# An Improved microRNA Annotation of the Canine Genome

**DOI:** 10.1371/journal.pone.0153453

**Published:** 2016-04-27

**Authors:** Luca Penso-Dolfin, Ross Swofford, Jeremy Johnson, Jessica Alföldi, Kerstin Lindblad-Toh, David Swarbreck, Simon Moxon, Federica Di Palma

**Affiliations:** 1 Vertebrate and Health Genomics, The Genome Analysis Centre, Norwich, United Kingdom; 2 Regulatory Genomics, The Genome Analysis Centre, Norwich, United Kingdom; 3 Vertebrate Genome Biology, Broad Institute of MIT and Harvard, Cambridge, Massachusetts, United States of America; 4 Science for Life Laboratory, Department of Medical Biochemistry and Microbiology, Uppsala University, Uppsala, Sweden; Kunming University of Science and Technology, CHINA

## Abstract

The domestic dog, *Canis familiaris*, is a valuable model for studying human diseases. The publication of the latest Canine genome build and annotation, *CanFam3*.*1* provides an opportunity to enhance our understanding of gene regulation across tissues in the dog model system. In this study, we used the latest dog genome assembly and small RNA sequencing data from 9 different dog tissues to predict novel miRNAs in the dog genome, as well as to annotate conserved miRNAs from the *miRBase* database that were missing from the current dog annotation. We used both *miRCat* and *miRDeep2* algorithms to computationally predict miRNA loci. The resulting, putative hairpin sequences were analysed in order to discard false positives, based on predicted secondary structures and patterns of small RNA read alignments. Results were further divided into high and low confidence miRNAs, using the same criteria. We generated tissue specific expression profiles for the resulting set of 811 loci: 720 conserved miRNAs, (207 of which had not been previously annotated in the dog genome) and 91 novel miRNA loci. Comparative analyses revealed 8 putative homologues of some novel miRNA in ferret, and one in microbat. All miRNAs were also classified into the genic and intergenic categories, based on the Ensembl RefSeq gene annotation for *CanFam3*.*1*. This additionally allowed us to identify four previously undescribed MiRtrons among our total set of miRNAs. We additionally annotated piRNAs, using *proTRAC* on the same input data. We thus identified 263 putative clusters, most of which (211 clusters) were found to be expressed in testis. Our results represent an important improvement of the dog genome annotation, paving the way to further research on the evolution of gene regulation, as well as on the contribution of post-transcriptional regulation to pathological conditions.

## Introduction

MicroRNAs (miRNAs) are small (~22 nt) non-coding RNAs found in both plants and animals. By binding to complementary RNA molecules, they can lead to translational repression or mRNA degradation [1–4] and have also been proposed as regulators of long non-coding RNA (lncRNA) expression [5]. MiRNAs typically derive from a primary transcript, the *pri-miRNA*, which is first transcribed inside the nucleus of a cell. The pri-miRNA contains one or more stem-loop structures which are processed by the enzyme Drosha, cleaving the double-stranded stem region. The resulting molecule, called the *pre-miRNA*, is then transported out of the nucleus, where a second enzyme, Dicer, excises the loop region and generates a ~22 bp, double stranded RNA molecule [6, 7]. One of these two strands (referred as 5p- and 3p-miRNA) will typically be degraded [7], while the other will be loaded into the miRNA-induced silencing complex and guide the targeting of mRNA molecules, by partial base-pairing [6].

First described in *C*. *elegans* two decades ago [8], miRNAs have increasingly attracted the interest of the scientific community, as their crucial implications in embryonic development, pathology [9, 10], and functional evolution [11] became clearer. Alteration of miRNA expression occurs in cancer cells [12–15] and represents a valid tool for the distinction of different tumor types [10]. Mutations in the miRNA seed region (typically positions 2–7 of the mature miRNA, crucial for target recognition) [16] are associated with diseases such as hereditary progressive hearing loss [17], or skeletal and growth defects [18]. Because of their wide implication in gene regulation, there is a great interest in identifying novel miRNAs in human and model animal systems, both for elucidating the biology of disease conditions and the evolution of phenotypic variation.

Recent advances in sequencing technologies and compute performance in the last decade means we can generate and analyse much larger amounts of data, with greater sensitivity and exponentially lowered costs. In particular, the advent of RNA-Seq technology [19] gives us a unique opportunity to study miRNA function and evolution. Parallel to such technological innovations came the development of computational tools for the prediction of novel miRNA loci [20–22]. Such tools utilise small RNA data and a genome assembly from a particular organism as the input information, returning a list of putative miRNA loci in that genome. These tools have allowed for an integrated experimental and *in silico* approach in the discovery of new miRNAs [21, 23, 24].

Other studies focused on the computational identification of conserved miRNAs between species, using a publicly available catalogue of annotated miRNAs, miRBase [25]. Buza *et al*. [26], for instance, identified a set of homologous counterparts of human disease-associated miRNAs in domestic animals. A similar approach was used by Sunkar *et al*. [27] for the identification of miRNA homologues in a variety of plant genomes. However, homology-based annotation will not find new species-specific miRNAs, as opposed to the tools described in [20–22], or provide information on tissue or stage specific expression.

Our study focuses on the domestic dog, *Canis familiaris*, the result of wolf (*Canis lupus*) domestication, which started around 10,000 years ago [28, 29]. Since then, hundreds of dog breeds have been artificially selected, leading to very high levels of morphological and behavioral variation. Having shared the environment with humans ever since its appearance, the dog has been exposed to similar pathogens, and thus represents an important model system for the study of human diseases [30].

The first high quality draft genome of the dog appeared in 2005 [31]; the authors also generated a map of single nucleotide polymorphisms (SNPs), revealed long-range haplotype structure and explored possible approaches for genome-wide association studies of trait and disease loci. Successful GWAS studies followed, providing new insights into the biology of selected traits [32–34], while other studies lead to the identification of important candidate genes for heritable diseases [35–38] improving our understanding of the corresponding human conditions.

The link between miRNAs and pathological phenotypes in dog has been suggested by many studies. Altered expression of some miRNAs was identified in canine melanomas, lymphomas and osteosarcomas, providing insights into human cancer treatment [39–42]. Other studies include the identification of miRNAs expression profiles in a canine heart failure model [43, 44], retinal degeneration [45] and in dogs infected by canine influenza virus [46].

MiRBase currently provides a catalogue of 502 miRNAs annotated in the dog genome (ftp://mirbase.org/pub/mirbase/CURRENT/genomes/cfa.gff3). However, the recent publication of an improved canine genome build, *canFam3*.*1* [30] provides a unique chance to further improve this annotation. In this study, we used the latest genome assembly and small RNA sequencing data to 1) validate all previously annotated dog miRNAs, 2) identify novel miRNA loci *in silico* and 3) strengthen our predictions by the comparison of multiple, independent tissue samples.

## Materials and Methods

### Library generation

Small RNA libraries for 9 different tissues (blood, brain, heart, kidney, lung, ovary, skin, smooth muscle and testis) were prepared using NEB's *Multiplex Small RNA Library Prep Set for Ilumina* (product E7300S, following the included protocol), and sequenced on an Illumina *HiSeq*2000 machine. Generated small RNA data was first processed by removing adaptor sequences, then combined into a superset of 126,263,963 reads.

### Annotation of known and novel miRNAs

We first aligned the complete set of all miRBase hairpin sequences to the canFam3.1 genomic sequence, using the command-line version of *BLASTN* (e-value ≤ 10^−6^). This gave us an initial set of putative miRNA homologues in the dog genome. We then mapped mature miRBase sequences to these putative pre-miRNA hairpins, and selected those for which at least one alignment with zero or 1 mismatch was observed.

In order to identify miRBase homologues, which were previously not annotated in dog, we downloaded the existing miRBase dog annotation (ftp://mirbase.org/pub/mirbase/CURRENT/genomes/cfa.gff3) and compared it to our complete set of predicted conserved miRNAs. We then excluded, using *Bedtools merge* [47], all of our miRNAs coordinates overlapping at least one entry in the existing miRBase annotation.

For the identification of novel loci in the combined set of *miRCat-miRDeep2* predictions, we similarly used *Bedtools merge* [47] and selected all predicted miRNAs whose coordinates do not overlap with any locus of our annotation of conserved miRNAs.

### Predictions of homologous miRNAs

In order to identify putative homologous loci, we used *BLASTN* [48] with an e-value cutoff of 10^−6^ to align our set of novel hairpin sequences to the latest genome assembly (downloaded from Ensembl) of the following species: Human, Pig, Mouse, Ferret, Elephant, Microbat, Lizard, Chicken, Zebrafish and Nile Tilapia. Genomic hits were then filtered, selecting hits with alignment length of at least 60 bps. The ferret genome had the highest number of hits (10), followed by microbat (2) and human and chicken with a single hit. In order to gain more confidence on the reliability of our inferences, we mapped the corresponding dog mature sequences of these conserved miRNAs to the putative homologous loci, using *PatMaN* [49] with parameters*–g 0 –e 1* (no gaps, 1 mismatch allowed). The corresponding dog mature sequences successfully aligned to 8 predicted ferret loci and one microbat locus (S7 Table), but not to the putative miRNA common to human and chicken from our predictions. We then decided to remove this locus from our final results.

### Annotating miRNAs by genomic location

We downloaded the GTF file of canFam3.1 from the Ensembl website (http://www.ensembl.org/info/data/ftp/index.html), converted it into a BED file of genomic coordinates and removed any row corresponding to a known miRBase miRNA. Using *Bedtools* [47], we found all overlaps between our hairpin coordinates and the entries from the GTF file. We selected overlaps implicating at least 15% of the total hairpin sequences. We were then able to classify our miRNAs into the categories UTR and EXON, according to the label of the overlapping entry in the GTF file. Using *Genometools* [50], we were also able to generate a gff file containing intronic coordinates for the canFam3.1 assembly. We then used an identical approach to identify miRNA loci located inside an intron (or overlapping one by 15% of their sequence). Finally, all unclassified loci were assigned to the INTERGENIC category.

We also looked for miRtrons, using the following approach: first we selected introns of length 50–120 bps [51]; we then used *Bedtools intersect* to find overlapping intronic and hairpin sequences, such that at least 95% of the intronic sequence was covered. Next, we aligned the full set of small RNA reads to these selected introns, using *PatMaN* [49] with parameters*–g 0 –e 0* (no gaps or mismatches allowed). When two clear peaks of read alignments at the 5’ and 3’ ends of the intronic hairpin sequence were observed (taking into account the possible presence of a short 3’ or 5’ tail [52], trimmed as part of the post-transcriptional modifications), the locus was considered a putative miRtron.

### Predicting piRNA clusters

Small RNA reads and CanFam3.1 annotation was used as input for the software *proTRAC-2*.*0*.*5* [53]. We used the following pipeline: initially we mapped our small RNA reads to the dog genome sequence; then we performed a hit counts normalization (based on uniquely mapping sequence reads) using the script *reallocate*.*pl* (part of *proTRAC* package). The resulting file of mapping reads with normalized counts was used as an input for the main *proTRAC* script, returning the piRNA predictions. For this step, we used the following command line parameters: *-map canine_miRNA*.*map*.*weighted -repeatmasker canFam3repeatmasker -geneset CanFam3*.*1*.*81*.*gtf -genome CanFam3*.*1*.*fa -clsize 1000 -1Tor10A 0*.*5 -distr 1–90 –nr–nh*. Finally, we merged overlapping coordinates from the combined set of predicted piRNA clusters across all tissues, using *Bedtools merge* [47].

### Gene target prediction

Target predictions of all novel miRNAs, as well as all newly discovered conserved miRNAs, were carried out using *TargetScan7* software [16]. We used the UTR sequences and the code available for download (http://www.targetscan.org/cgi-bin/targetscan/data_download.cgi?db=vert_70) together with the CanFam3.1 coding sequences obtained from Biomart (http://www.biomart.org), reformatted to meet the requirements of TargetScan7.

### Accession number

Small RNA data used for this project has been deposited at the National Center for Biotechnology Information (NCBI) under the BioProject accession PRJNA305157.

## Results

### miRCat and miRDeep2 identify known and novel miRNA loci

**Fig 1:**
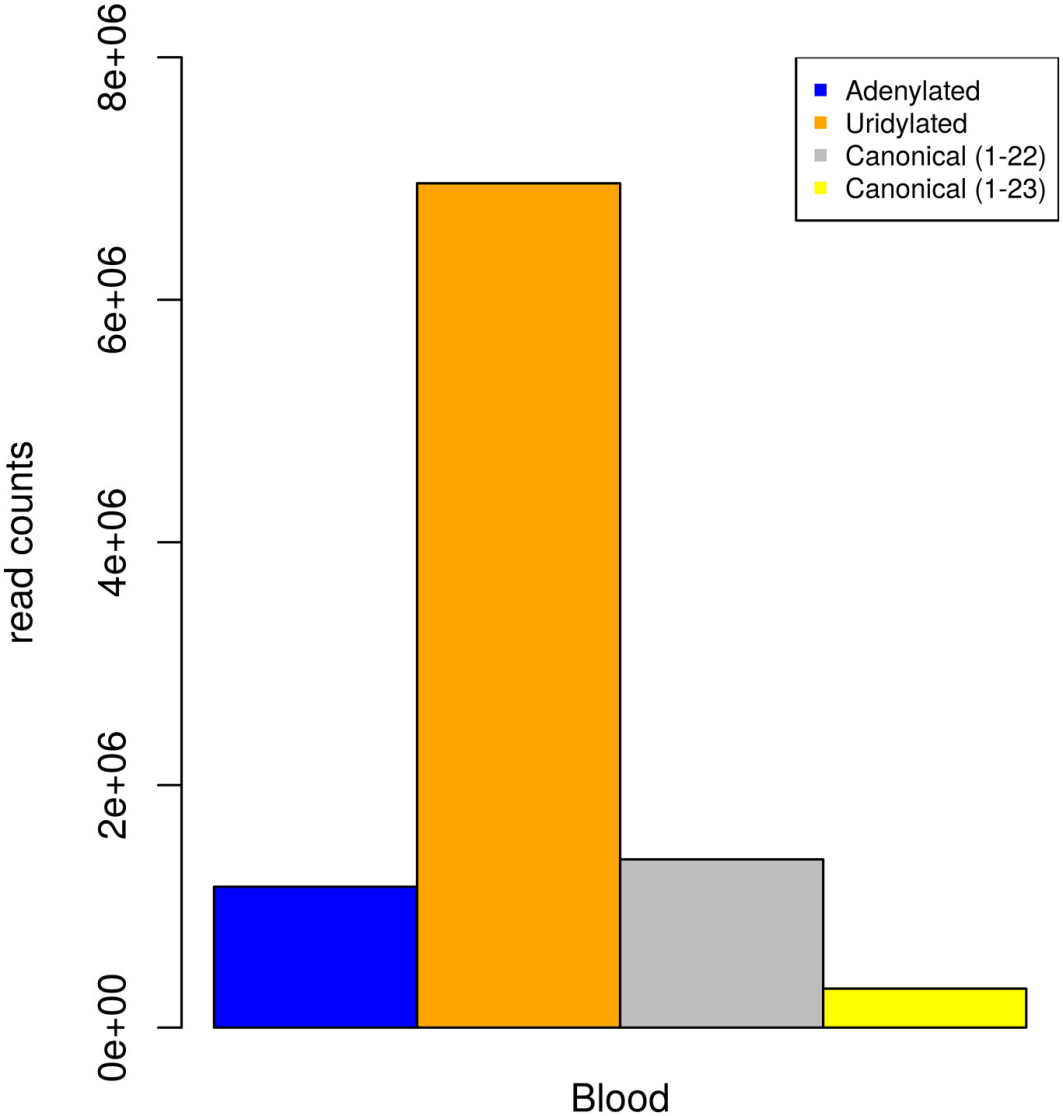
Counts of different isoforms of miRNA cfa-mir-486-5p in the blood sample.

**Fig 2:**
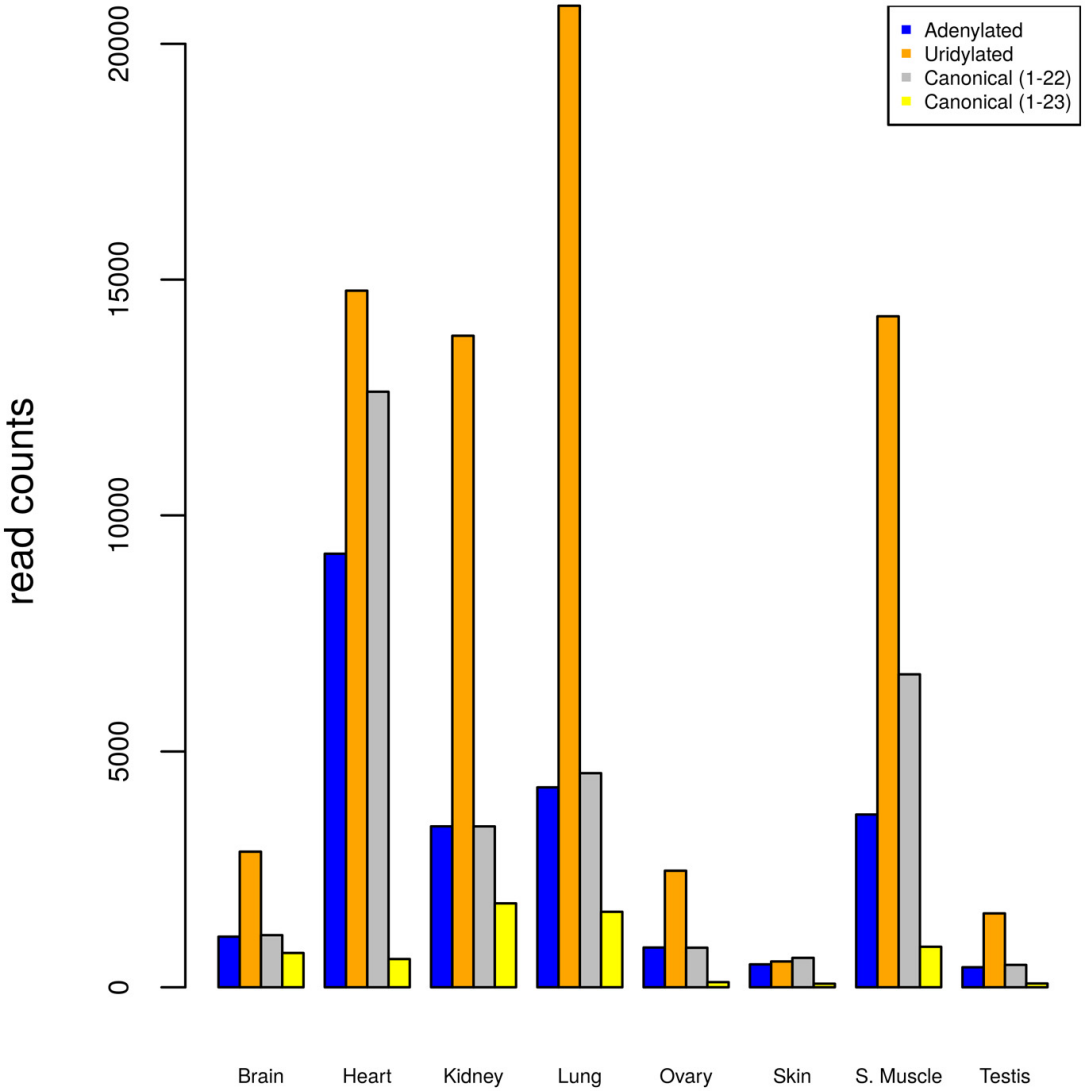
Counts of different isoforms of miRNA cfa-mir-486-5p in all samples except blood.

Size distribution (S1 and S2 Figs) and counts of genome matching reads for each tissue (S3 Fig) were generated as a preliminary quality control. We observed a clear enrichment for 22 nt long reads (57,642,938 or 45% of the total number), the typical size of a mature miRNA. Only the blood sample had a peak at size class 23. When we looked at the size distribution of genome matching reads (S3 Fig), however, the expected enrichment for 22 nt long reads was observed, although the count for 23 nt long reads was still high. Additional analyses showed how the peak at 23nt in the blood sample was mainly due to two highly abundant sequences that did not map to the reference genome. These sequences were searched against the miRBase database and returned perfect alignments to the first 22 nt of miR-486-5p (previously described as an important regulator of erythropoiesis) [54, 55]. The 3’ nucleotide of the two miRNA sequences (A and U respectively) were non-templated bases suggesting that this miRNA undergoes uridylation (6,960,721 reads in the blood sample) and adenylation (1,161,985 reads) [56] in the blood sample studied (Figs 1 and 2). These modifications are of great interest as they can affect the stability of the mature miRNA [57] which can in turn affect regulation of target genes. The abundance of the canonical 22-nt mature miRNA and 23-nt adenylated sequences appeared to be similar across all 9 tissues, while the 23-nt uridylated isoform was found to be consistently the most abundant (with the only exception of the skin sample). However, the counts for all isoforms were significantly higher in blood than in any other sample (Figs 1 and 2) leading to a peak at 23nt in the size distribution of total trimmed reads in this sample.

**Fig 3:**
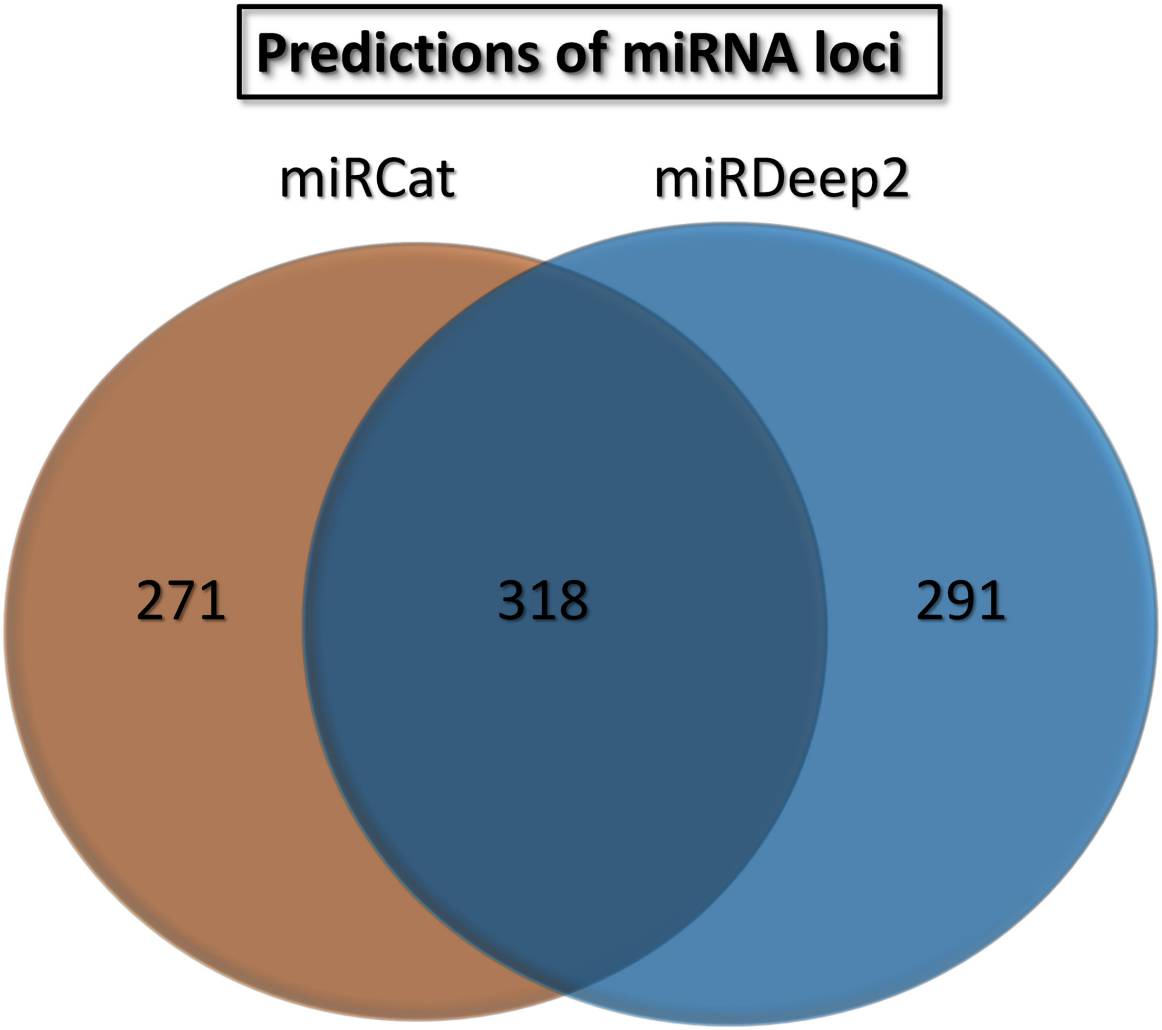
Results of the computational prediction of miRNA loci, using miRCat and miRDeep2.

Next, we ran *miRCat* [20] and *miRDeep2* [22] on the total set of 85,810,181 trimmed, genome matching reads, thus obtaining two distinct sets of miRNA predictions (Fig 3). Genomic coordinates of the 589 hairpin sequences predicted by miRCat, and the 609 loci predicted by *miRDeep2* were merged using *Bedtools merge* [47], thus obtaining a non-overlapping combined set of 880 coordinates.

### Re-annotation of the canFam3.1 assembly separates high and low confidence miRNAs

**Fig 4:**
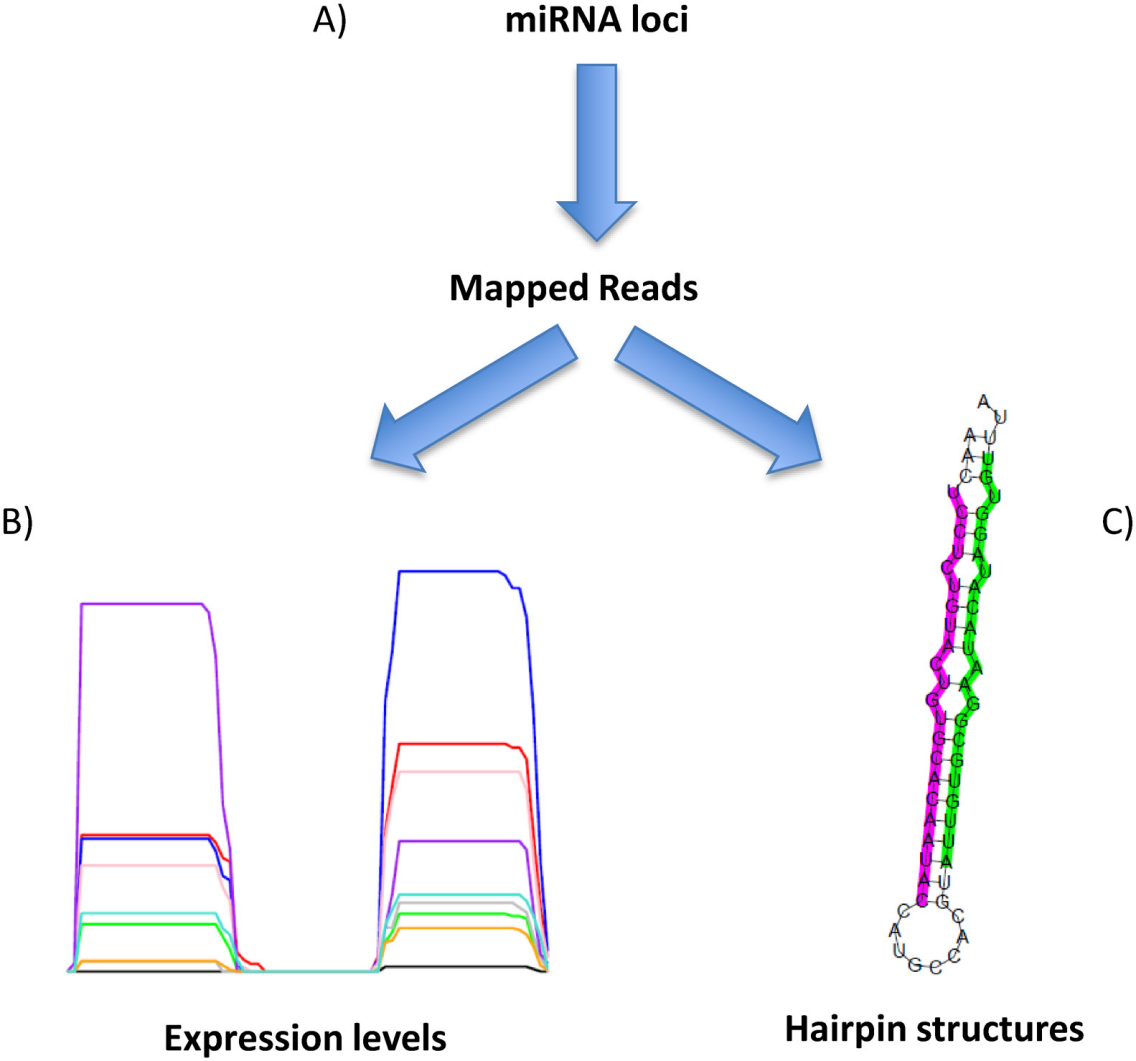
Workflow for the generation of miRNA expression profiles and secondary structures. Small RNA reads are first mapped (A) to the hairpin sequences, looking for two peak alignment patterns as shown in B (see S4 and S5 Figs). Expression levels across each hairpin (B) and secondary structures (C) are then derived (S6 and S7 Figs).

**Fig 5:**
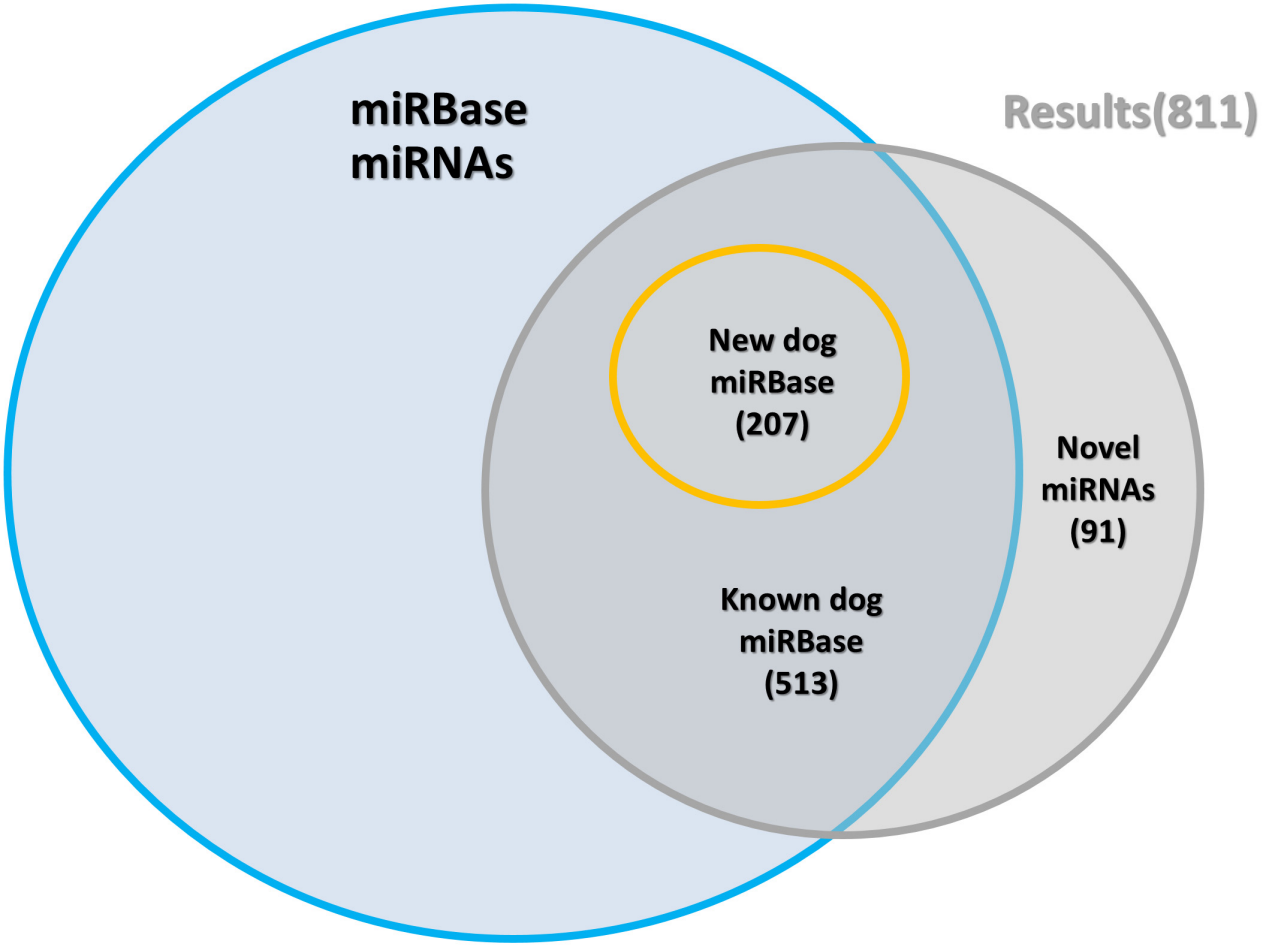
Identification of known and novel miRNA loci.

Hairpin sequences for both novel and conserved miRNA candidates were derived from their genomic coordinates. Small RNA reads were then aligned to these sequences, and we identified patterns of alignment which were consistent with Dicer and Drosha processing (typically, a precise “peak” at both the 5' end and 3' end of the sequence) [25, 58]. Where the presence of 3p-miRNA and 5p-miRNA sequences (S3 and S4 Tables) was supported by alignments (S5 and S6 Tables), hairpin secondary structures were generated (see Fig 4) using the Vienna-RNA package [59]. This software determines the most likely folding structure based on the minimum free-energy criterion. Based on the consistency of both the alignments and predicted secondary structure, 720 conserved miRNAs (S1 Table) and 91 high confidence novel loci (S2 Table) were identified (see also Fig 5). Each conserved miRNA was either classified as “high confidence” (consistent alignment pattern and secondary structure, 417 loci) or “low confidence” (where any of the above criteria was not met, 303 loci). The complete set of conserved and novel miRNA predicted structures can be found in S6 and S8 Figs, respectively. Small RNA reads provided evidence of expression of both the 3p- and 5p-miRNA of all newly identified loci but only 467 of the 720 conserved miRNAs (whose structures can be found in S7 Fig) received the same level of support. As we only sequenced nine different tissues it is entirely possible that the miRNAs we have annotated as low confidence could be supported by expression data in other tissues and therefore we have annotated them solely on similarity to miRNAs currently annotated in miRBase and conservation of the annotated mature miRNA.

**Fig 6:**
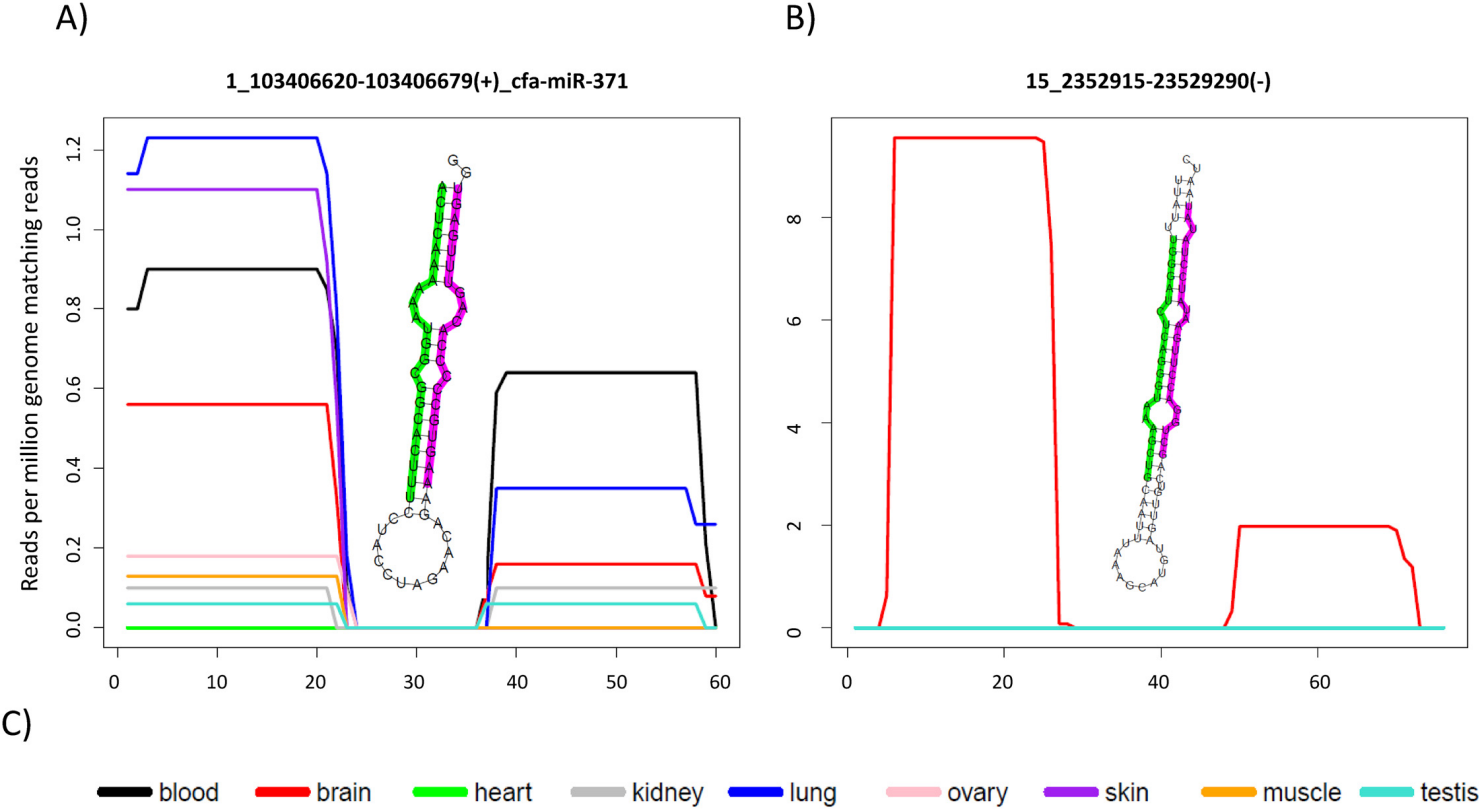
MiRNA expression plots and predicted secondary structures. Different colours in the plot correspond to different tissues as described in C). Green and purple-labelled bases in the hairpin figure indicate the most abundant read mapping to the 5' and 3' ends; conserved miRNA cfa-miR-371 (A) appears to be absent in heart and shows variable expression levels in the other tissues. B) Plots for a novel miRNA located in chromosome 15, supported by small RNA reads from the brain sample.

By excluding all of our miRNAs coordinates overlapping at least one entry in the existing miRBase annotation (see Materials and Methods), we were also able to identify, from our set of 720 conserved loci, 207 newly annotated miRNAs, 65 of which were classified as “high confidence”. Tissue specific expression plots for all novel (S4 Fig) and conserved miRNAs (S5 Fig) were also generated allowing visualization of expression data across each of the tissues sampled and demonstrating that predictions are expressed in multiple independent datasets (Fig 6B).

The complete annotation of both conserved and novel miRNAs is available online, as part of the UCSC genome browser “Broad Institute Canine Annotation” Track Hub (https://genome.ucsc.edu/cgi-bin/hgGateway?hgsid=432322929\_FBTCtBIVIPVoA0nQXPzwAguagWPr).

### Read abundance plots indicate arm switching across tissues

Generating expression plots as shown in Fig 6 allowed for the visualization of a very important phenomenon: the switching in abundance across tissues of the 5’ and 3’ mature sequences. These changes can be associated with important functional consequences, as different mature strands might target different mRNAs [60]. In order to gain a better understanding of the type of variation in our data, we generated abundance plots, using *Rstudio* (https://www.rstudio.com/), where the read abundance at the 5’ end for each tissue is plotted against the corresponding abundance at the 3’ (S10 Fig). We observed cases of arm switching, possibly indicating functional diversification across tissues. For example, novel miRNA *18/50102465-50102527(+)* shows prevalence of the 3p-miRNA in blood and heart, but prevalence of 5p-miRNA in brain, lung and testis (and no evidence of expression in the remaining tissues). Similarly, *cfa-mir-18a* shows a clear prevalence of 3p-miRNA in blood, while in all other tissues the 5p-miRNA is more abundant. Interestingly, the 3p-miRNA counts in blood are much higher than those of the 5p-miRNA in the other tissues.

We also generated histograms for the read counts across the hairpin sequences (S9 Fig). Unsurprisingly, we observed highly 3’ or 5’ biased distributions of reads, suggesting that in most cases only one strand of the mature miRNA is retained and plays a role in gene regulation.

### Comparative analyses of novel miRNAs reveal 9 putative homologues

Next we addressed the question whether some of our newly identified dog miRNAs are present in other vertebrates. We selected the following species: Human, Pig, Mouse, Ferret, Elephant, Microbat, Lizard, Chicken, Zebrafish and Nile Tilapia. In order to identify putative homologous loci in this diverse set of organisms, we used *BLASTN* to align our set of novel hairpin sequences to the latest genome assembly (downloaded from Ensembl) of each species. Selected hits (see Materials and Methods for more details) were further analysed by mapping the corresponding dog mature sequences to these putative homologous hairpins, allowing for 1 mismatch. This lead to the identification of 8 miRNA loci in ferret and one in microbat (S7 Table). We also compared the genomic annotation of these homologous miRNAs: we found perfect correspondence intron to intron or exon to exon for all putative homologues, giving further support to our predictions (Table 1). Our results thus indicate that these novel miRNAs are not conserved across long evolutionary timescales; however, when we look at the two evolutionary most closely related species (ferret, with an estimated divergence from dog of about 46 MYA, and microbat, about ~82 MYA) (http://www.timetree.org) we find some putative conserved miRNAs.

**Table 1:** Putative miRNA loci, homologous to some novel dog miRNAs, identified in ferret and microbat.

Species	Chromosome	Start	End	CanFam3.1 locus	Strand	Dog annotation	Species annotation
Ferret	GL896934.1	8105928	8105988	12/37307857-37307917(+)	+	INTRON	INTRON
Ferret	GL897283.1	41870	41948	15/23529215-23529290(-)	+	INTRON	INTRON
Ferret	GL896969.1	5729349	5729420	18/49182272-49182343(+)	-	INTRON	INTRON
Ferret	GL896988.1	2200068	2200144	3/84489968-84490044(-)	-	INTRON	INTRON
Ferret	GL896988.1	2218967	2219043	3/84489968-84490044(-)	+	INTRON	INTRON
Ferret	GL896900.1	21231318	21231382	8/42665000-42665064(-)	-	CDS	CDS
Ferret	GL896965.1	8980331	8980407	X/15639699-15639775(-)	-	INTRON	INTRON
Ferret	GL896945.1	1693147	1693222	X/32945413-32945490(-)	-	INTRON	INTRON
Microbat	GL429826	6144016	6144076	12/37307857-37307917(+)	+	INTRON	INTRON

### Canfam3.1 gene annotation reveals 4 possible MiRtrons in our dataset

**Fig 7:**
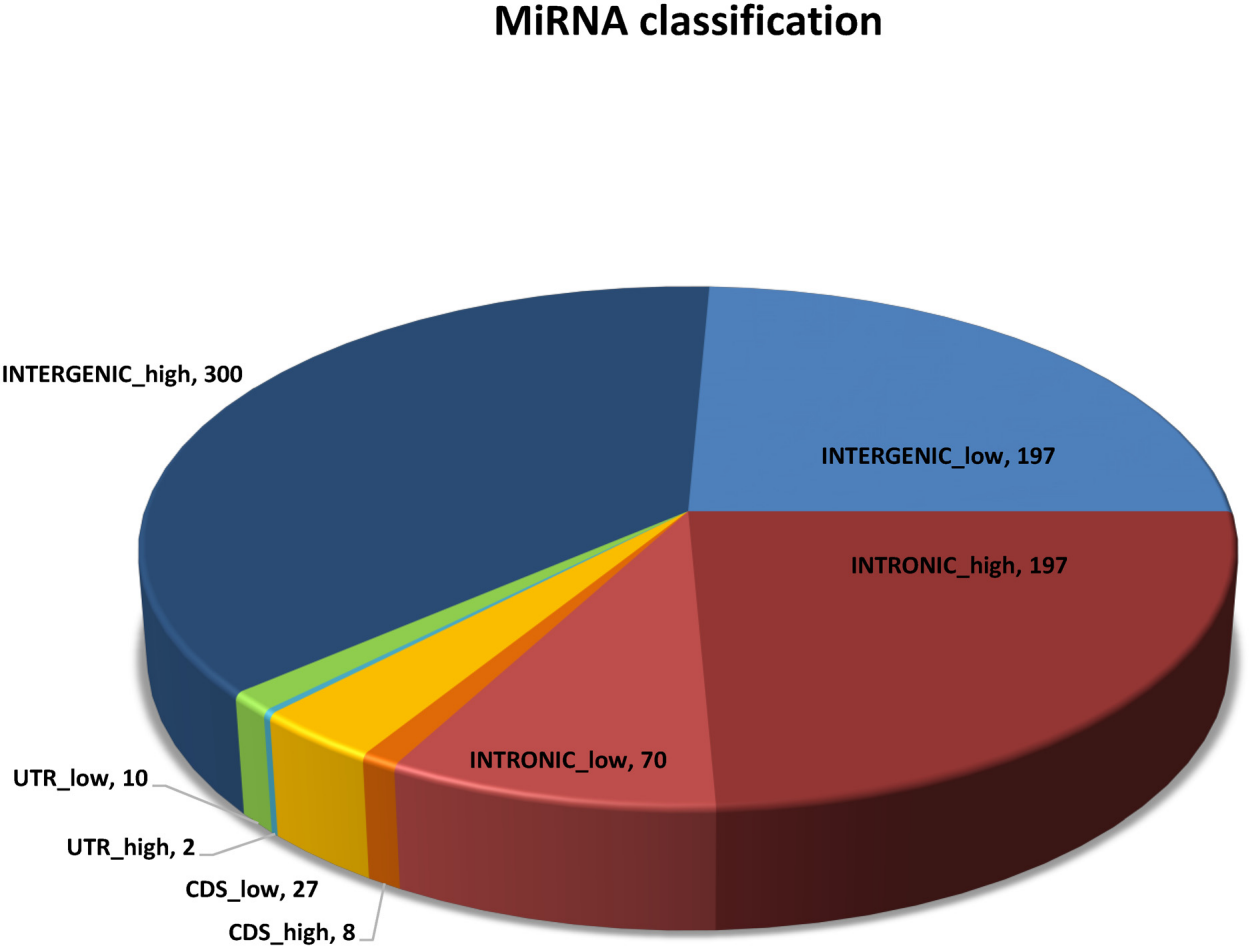
Annotation of the complete set of miRNAs, based on genomic location.

Next, we used the latest dog genome annotation (available from the Ensembl website, http://www.ensembl.org/info/data/ftp/index.html) to classify our complete set of miRNAs, based on their genomic coordinates. As expected, the vast majority of our loci fell into the intronic or intergenic (all unclassified miRNAs) categories, with fewer located in UTR or CDS regions (see Fig 7). By comparing the genomic locations of conserved and novel miRNAs (S11 Fig) we observed much higher proportions of intronic loci in the novel set (66%) compared to the conserved miRNAs (25%). This is in agreement with what Meunier *et al*. [11] observed for recently evolved mammalian miRNAs. The overrepresentation of novel loci in intronic sequences is unsurprising if we consider that the transcription of the host coding gene will favor the evolution of a novel miRNA [6]. Moreover, the interaction between Drosha and the spliceosome can lead to the formation of an intronic miRNA precursor, excised from the primary mRNA transcript [61].

A particularly interesting class of intronic miRNAs is represented by miRtrons, whose pre-miRNA is generated by mRNA splicing, rather than by Drosha cleavage. Given the observed high number of intronic loci in our dataset, we addressed the question whether some of them might represent miRtrons. We would expect these pre-miRNAs to cover the majority of the short intronic sequences they are processed from, with the 3p-miRNA and 5p-miRNA mapping to the corresponding ends of the intron. By aligning the full set of small RNA reads to these selected introns (as previously done with all predicted miRNA loci), we could identify 4 putative miRtrons: *12/406899-406968(+)_mir-877* (whose human homologue has been proposed as a miRtron) and novel miRNAs *4/35856564-35856639(+)*, *9/22412453-22412517* and *13/37816081-37816136*. These results represent the first description of putative miRtrons in the dog genome, and give further relevance to the novel predictions of this study.

### Annotation of piRNAs

**Fig 8:**
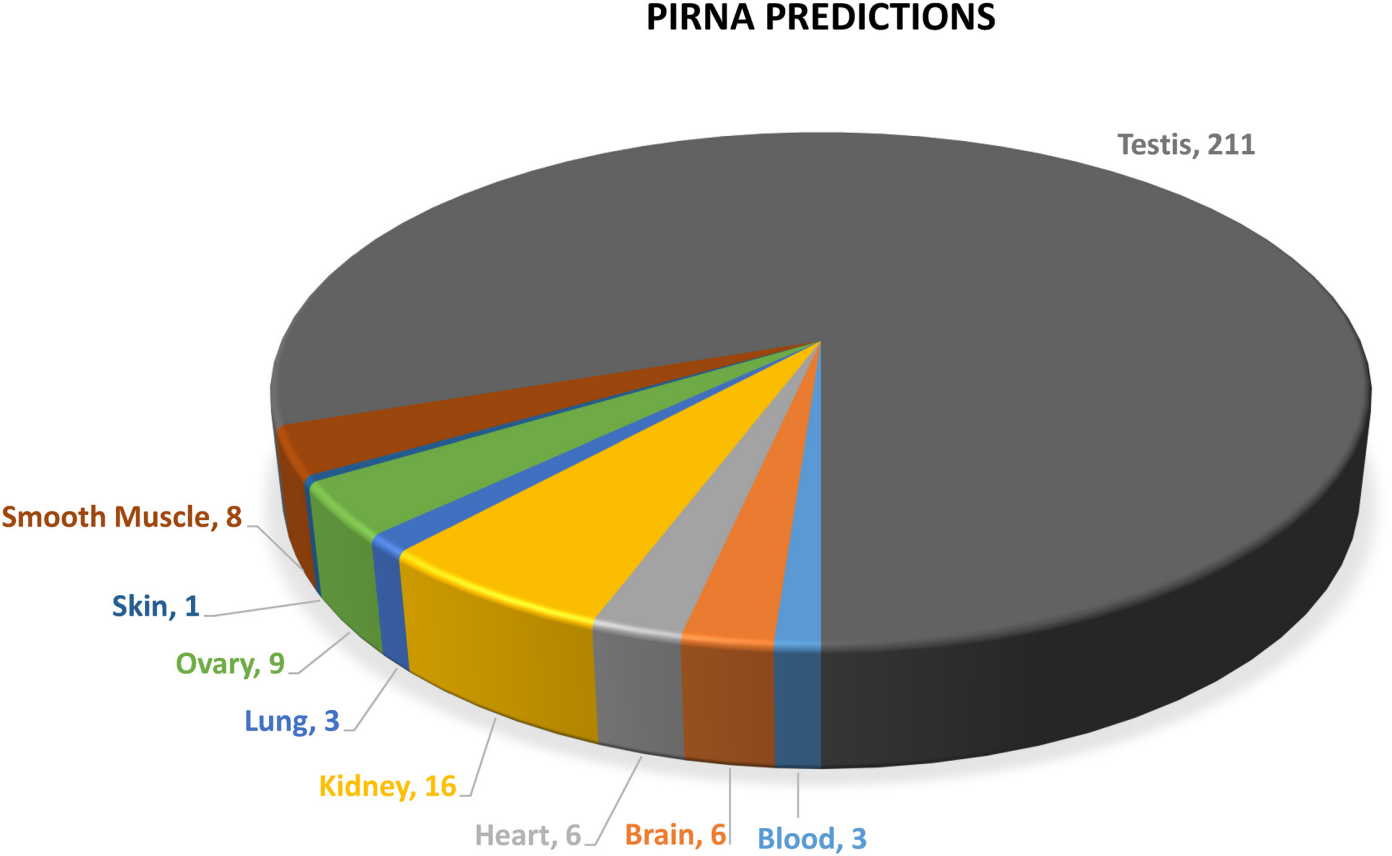
Counts of proTRAC piRNA predictions across tissues.

The size distribution of our combined small RNA dataset suggested not only the presence of miRNAs, but also longer, 24–30 bp sequences likely to represent piRNAs. These are an additional class of non-coding RNA molecules involved in the silencing of transposons and other repetitive elements [62–64]. We were thus able to identify a total of 263 predicted clusters (S8 Table). Unsurprisingly [65], we observed a clear enrichment in testis (211 clusters) compared to all other tissues (see Fig 8). We then selected the 231 mono-strand predicted clusters and used *Bedtools merge* [47] to combine overlapping coordinates. This returned a set of 205 merged coordinates, which are listed in S9 Table.

### Target prediction analysis

We performed target predictions of all new miRNAs (S10 Table), as well as all newly annotated conserved loci (S11 Table), using *TargetScan7* software [16]. Results provide an overview of possible gene targets, as well as information about the predicted strength of the miRNA-mRNA interaction (expressed by the context++ score). For a detailed description of the analyses, see section Materials and Methods.

## Discussion

Our study provides an important improvement of the dog miRNA annotation, summarized by the classification of 720 conserved miRNAs into the high and low confidence categories, along with the identification of 91, previously undescribed loci. Analysis of 9 tissues gives a broad overview of miRNA expression levels (S4 and S5 Figs), providing support across independent samples for a subset of 417 conserved miRNAs and all 91 novel miRNA predictions. Our improved microRNA annotation (available online as part of the UCSC genome browser “Broad Institute Canine Annotation” Track Hub) represents a valuable resource for the community, as it provides a possible starting point to both biomedical and evolutionary studies on the dog model system. Many polymorphisms in miRNAs target sites have already been identified which are associated with pathological conditions [66]. Investigating the functional role of these novel miRNAs has the potential of further elucidating the biology of several human diseases, and the evolution of artificially selected phenotypic traits in dog.

Our data has also highlighted an example of highly abundant adenylated and uridylated mature miRNA in the blood sample (Fig 1). This is in agreement with a study by Koppers-Lalic *et al*. [67], who observed high proportions of non-canonical, monoadenylated mir-486-5p sequences. The total count for the two modified cfa-mir-486-5p sequences in blood (8,122,706) represents as much as 43% of all size selected, trimmed reads in the sample. These modifications appear to be associated with increased stability of the mature miRNA [57] and hence can have an effect on target genes expression. Given the implication of mir-486 in erythropoiesis [54, 55], the observed modifications are of great interest, as they are likely to be linked to important regulatory mechanisms in blood.

An additional source of variation in our data is represented by a switching in the ratio of abundance between 5p- and 3p- miRNA sequences across samples. These results support the idea that distinct mature miRNA strands can be retained, and thus be functionally active, in different tissues. Such variation can be associated with differences in gene regulation, as a result of differential targeting or changes in targeting efficacy. Investigating the contribution of this variation in tissue specific regulation represents an important and interesting challenge.

miRNAs appear to be a key component in the evolution of life [6, 68]. Differential expression, target switching across tissues and organisms, together with the birth and death of miRNA loci in an evolutionary lineage, provide a wide spectrum of possibilities for phenotypic diversification. As opposed to the emergence of novel gene functions, functional miRNA genes represent a much easier way to explore new evolutionary pathways. Subtle modifications in how every gene is regulated in each tissue may have well represented, in some cases, the necessary base of a wider phenotypic diversity, or additional organismal complexity. Further research is needed in order to better elucidate the link between functional evolution and the observed diversity of miRNA expression, appearance and loss across taxa.

## Supporting Information

S1 Fig Read length distribution of trimmed, size filtered (≥16 bps) reads of each sample. (PDF)

S2 Fig Read length distribution of trimmed, size filtered (≥16 bps) genome matching reads for each sample. (PDF)

S3 Fig Counts of all reads, trimmed ≥16 bps and trimmed genome matching reads across all samples. (PDF)

S4 Fig Line plots of expression levels across the hairpin for all novel miRNAs. The base position along the hairpin (x axis) is plotted against the normalized read count (reads per million genome matching reads). Different colors in the plot correspond to different tissues, as described by the legend on the bottom of each plot.
(PDF)

S5 Fig Line plots of expression levels across the hairpin for all conserved miRNA candidates. The base position along the hairpin (x axis) is plotted against the normalized read count (reads per million genome matching reads). Different colors in the plot correspond to different tissues, as described by the legend on the bottom of each plot.
(PDF)

S6 Fig Predicted secondary structures for all conserved miRNA candidates. (PDF)

S7 Fig Predicted secondary structures for a subset of 467 conserved miRNA candidates, supported by evidence of expression of both the 5p- and 3p miRNAs. Purple and green labelled regions respectively correspond to the 5p- and 3p miRNA sequences, as predicted by small RNA reads alignments to the hairpin sequence.
(PDF)

S8 Fig Predicted secondary structures for all novel miRNAs. Purple and green labelled regions respectively correspond to the 5p- and 3p miRNA sequences, as predicted by small RNA reads alignments to the hairpin sequence.
(PDF)

S9 Fig Sample specific histograms of the proportion of 5’ reads (considering all 811 miRNAs), divided into 10 intervals. High counts for the first and tenth bin indicate the expected extreme values for the 3’/5’ proportions.
(PDF)

S10 Fig Abundance plots of all miRNAs across different tissues. Counts of reads at the 5’ end are plotted against the counts at the 3’ end. Different symbols indicate different tissues, as described by the legend.
(PDF)

S11 Fig Counts of novel and conserved miRNAs, classified by genomic location. (PDF)

S1 Table Genomic coordinates and hairpin sequences of all conserved miRNAs. (XLSX)

S2 Table Genomic coordinates and hairpin sequences of all novel miRNAs. (XLSX)

S3 Table Genomic coordinates and sequences of all conserved mature miRNAs. (XLSX)

S4 Table Genomic coordinates and sequences of all novel mature miRNAs. (TXT)

S5 Table Alignments of small RNA reads to the hairpin sequences of conserved miRNAs. (ZIP)

S6 Table Alignments of small RNA reads to the hairpin sequences of novel miRNAs. (ZIP)

S7 Table Genomic coordinates for 9 predicted miRNAs (homologous to some novel miRNA) in ferret and microbat. (TXT)

S8 Table Genomic coordinates of all piRNA clusters predicted by the software proTRAC. (TXT)

S9 Table Merged genomic coordinates of all piRNA clusters predicted by the software proTRAC. (ZIP)

S10 Table List of all novel miRNAs target predictions, as returned by the software TargetScan7. (BZ2)

S11 Table List of target predictions for all newly annotated conserved miRNAs, as returned by the software TargetScan7. (BZ2)
