# Supplementary figures and images for: An Improved microRNA Annotation of the Canine Genome
Source: PLoS One. 2016 Apr 27;11(4):e0153453. doi: 10.1371/journal.pone.0153453 (PMC4847789; doi:10.1371/journal.pone.0153453)

# Blood

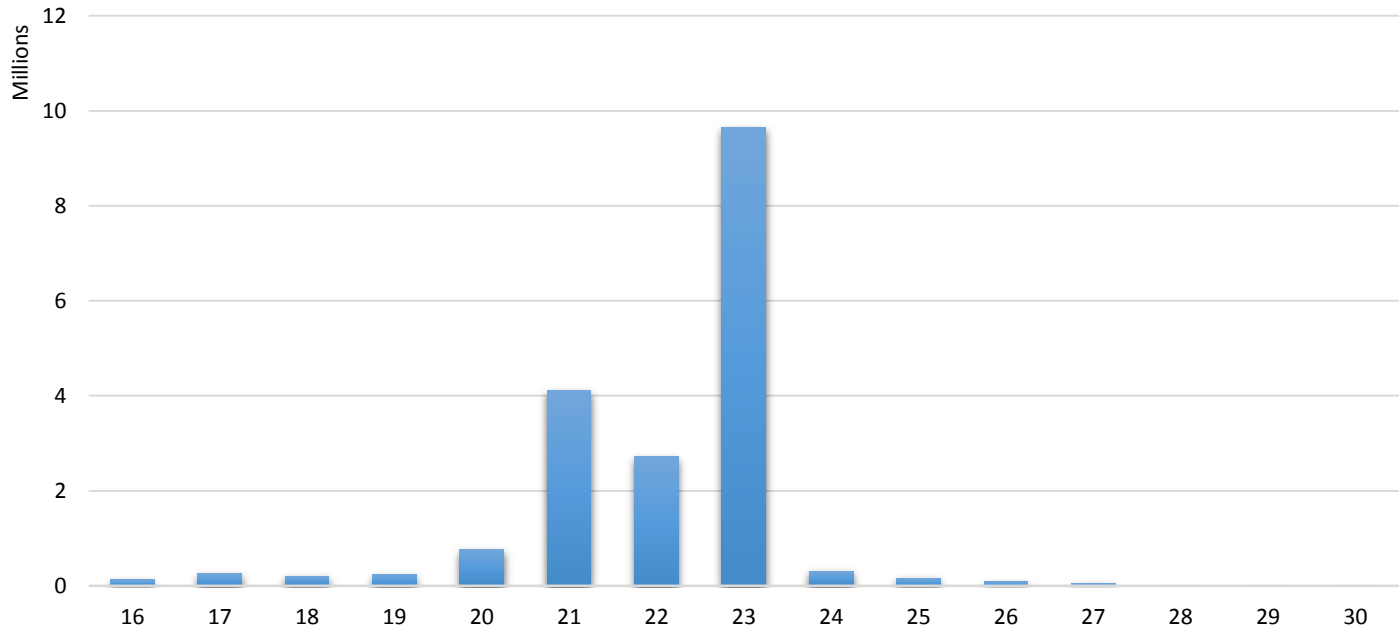

# Brain

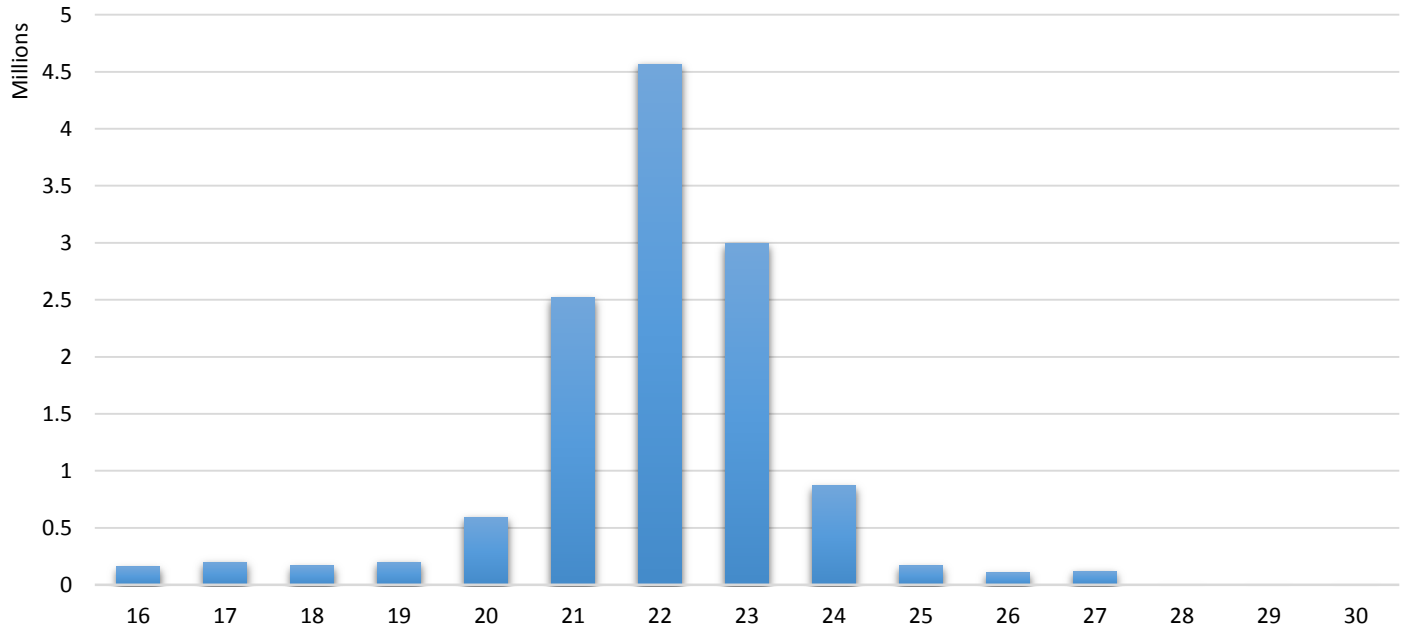

# Heart

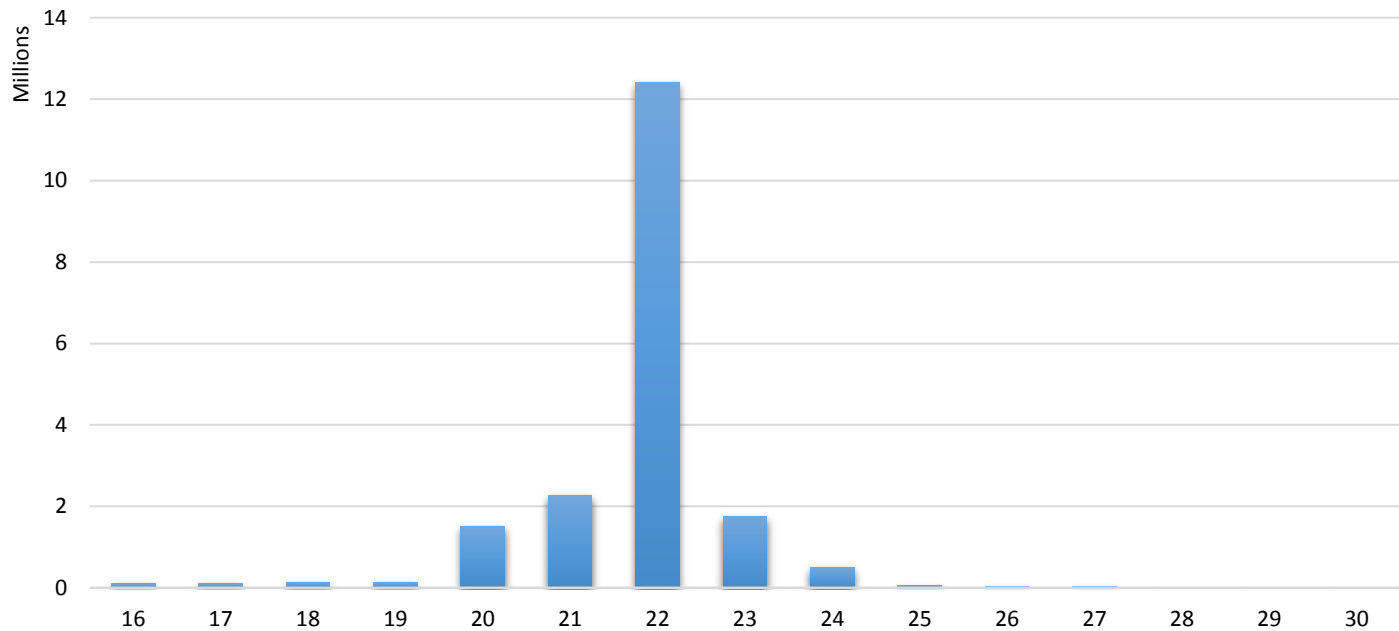

# Kidney

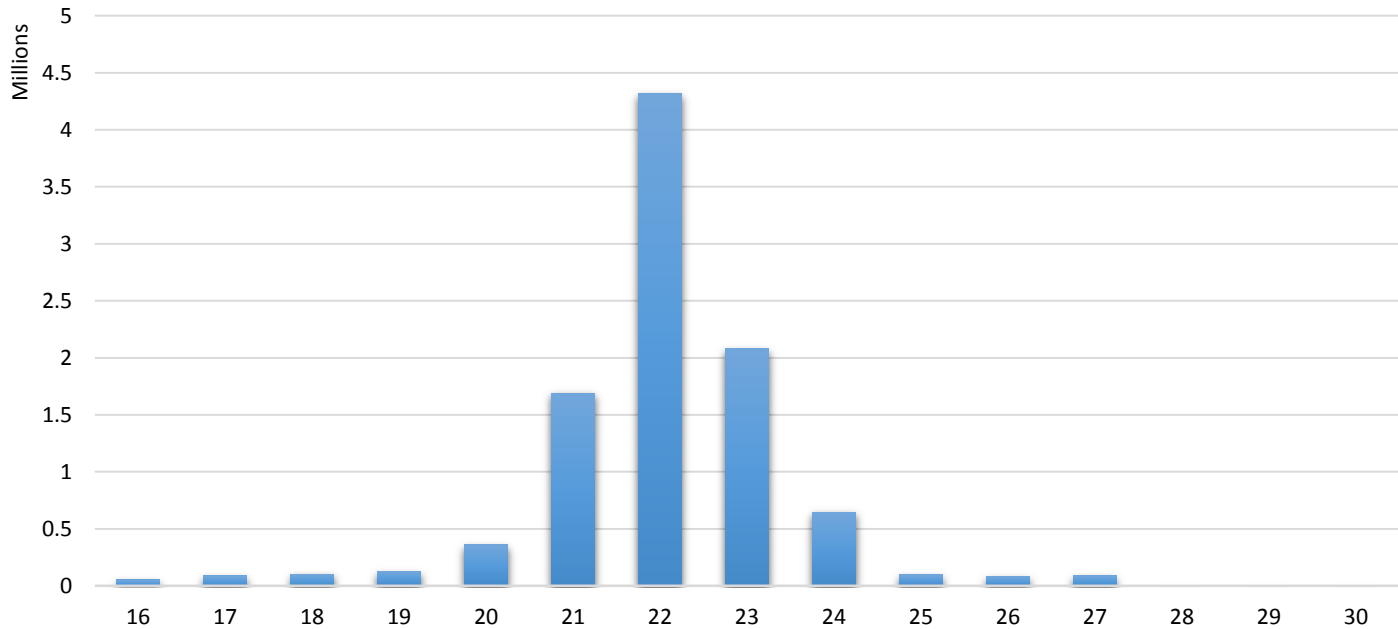

# Lung

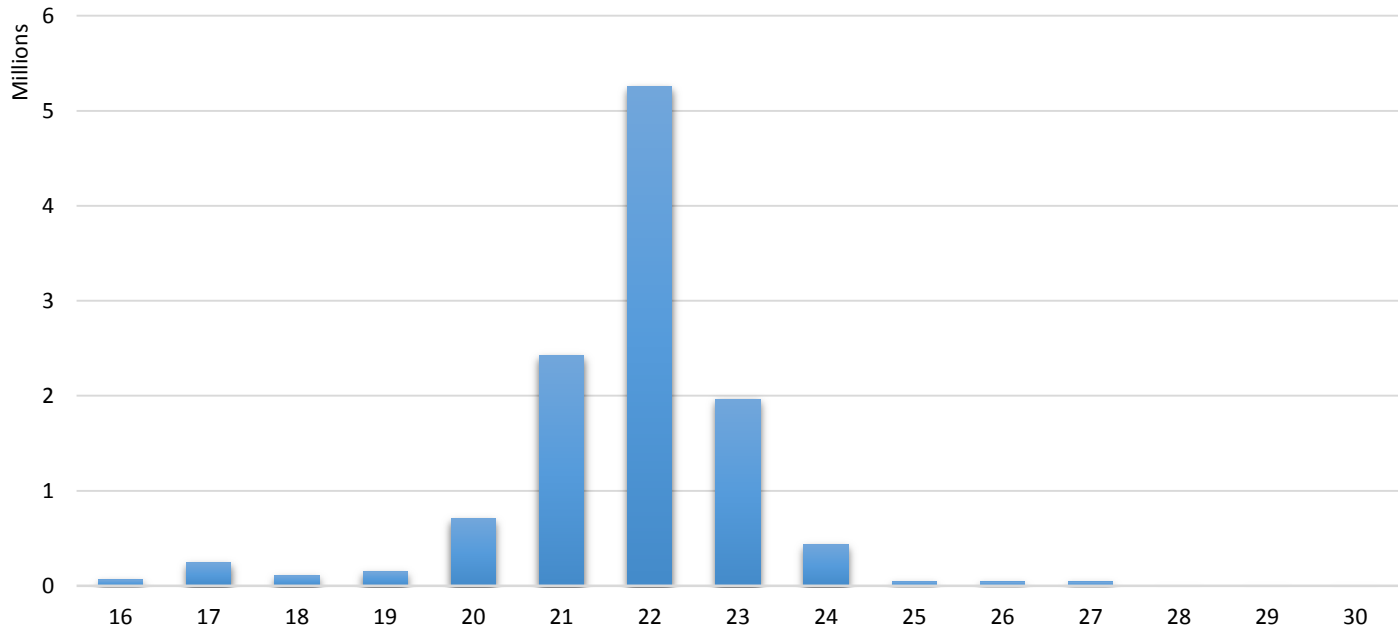

# Ovary

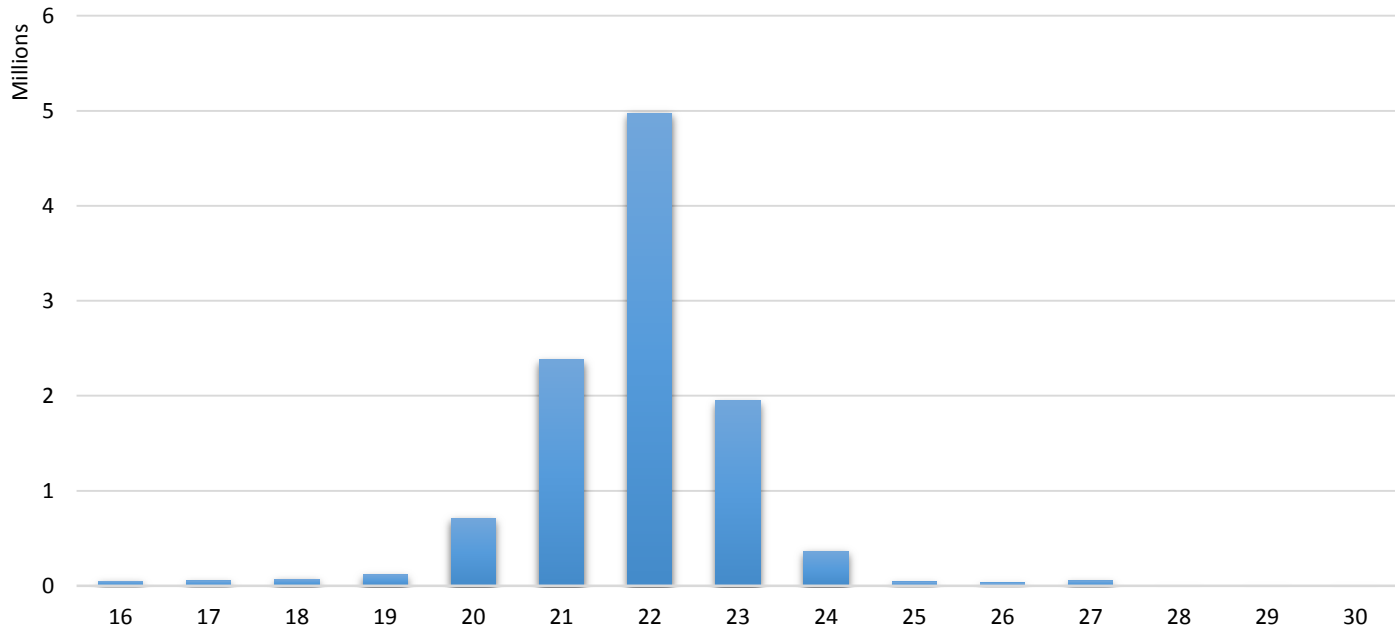

# Skin

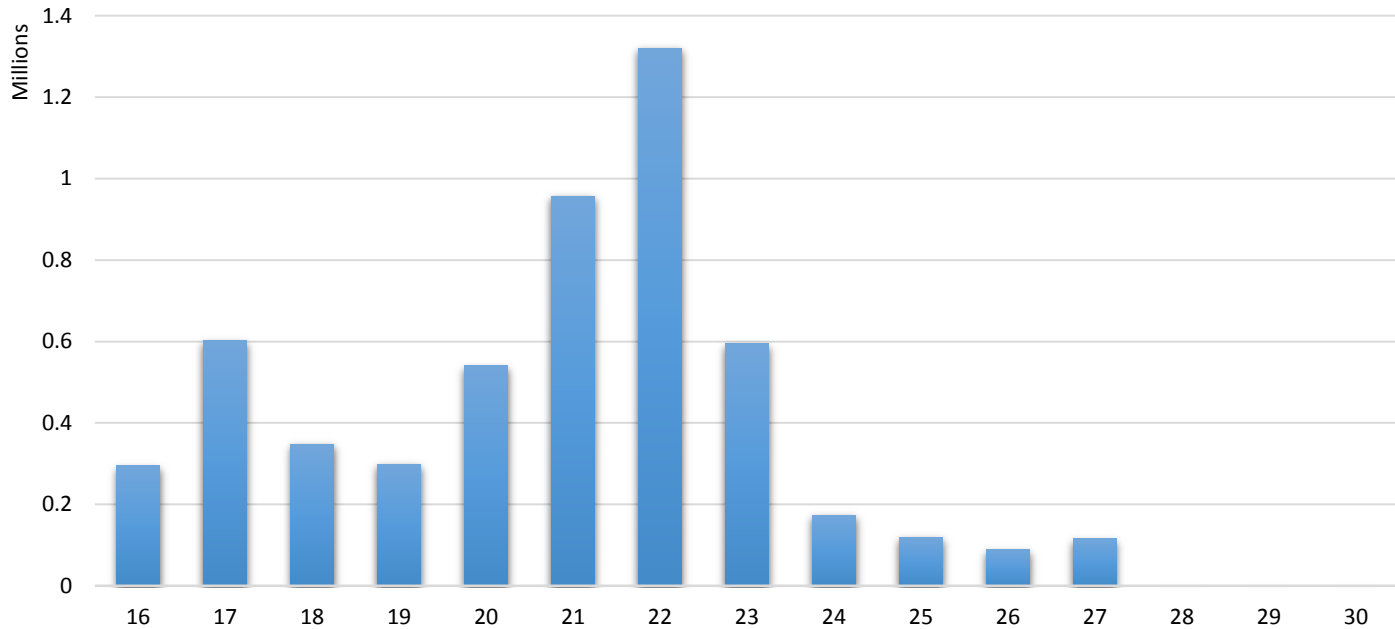

# Smooth Muscle

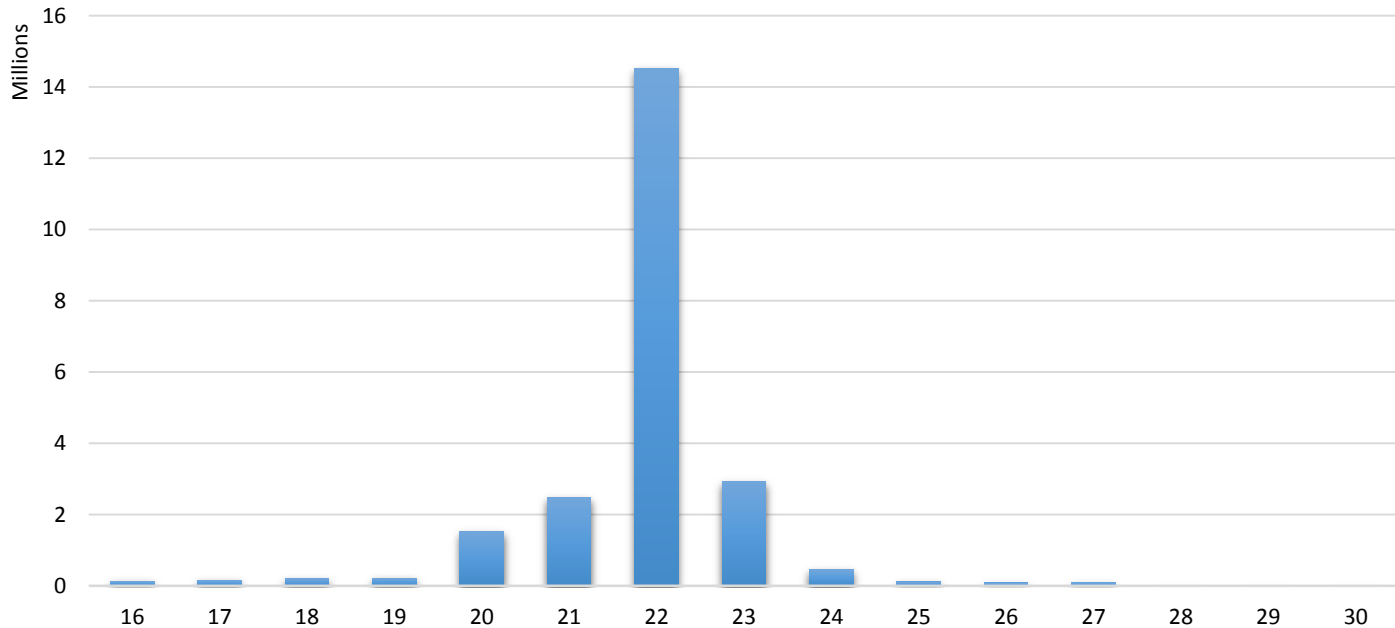

# Testis

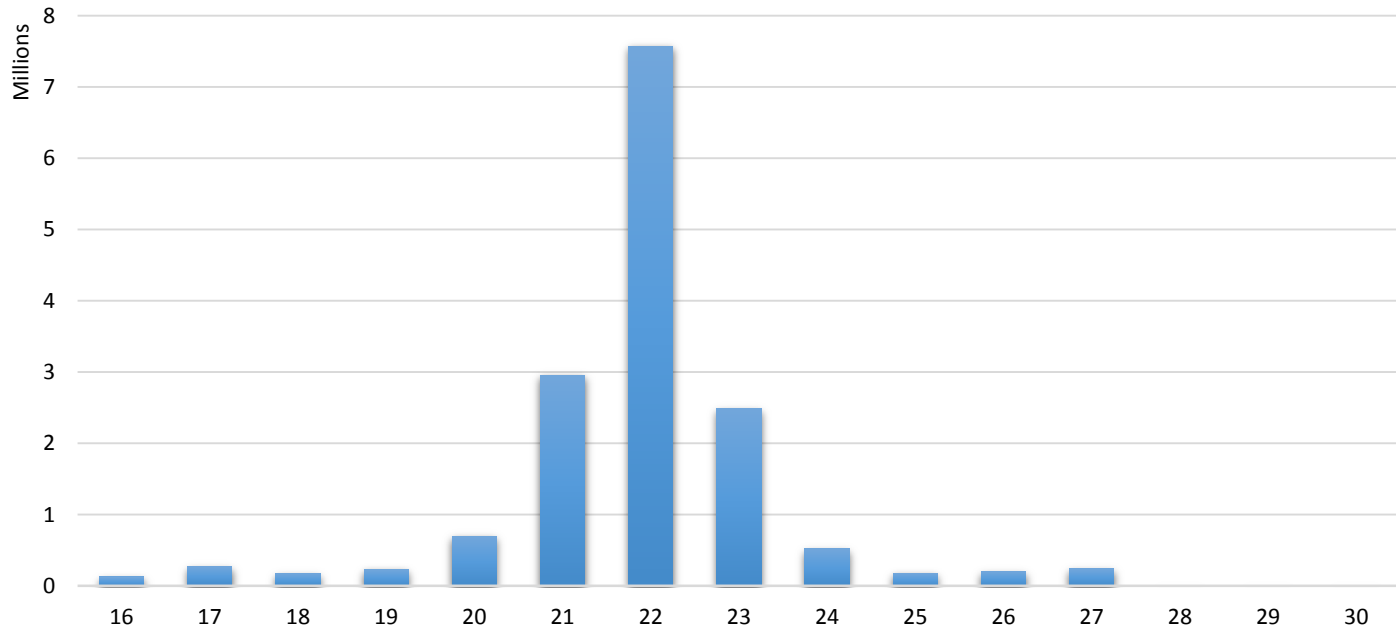

Supplement: S1 Fig — (PDF) [file pone.0153453.s001.pdf]

## Blood

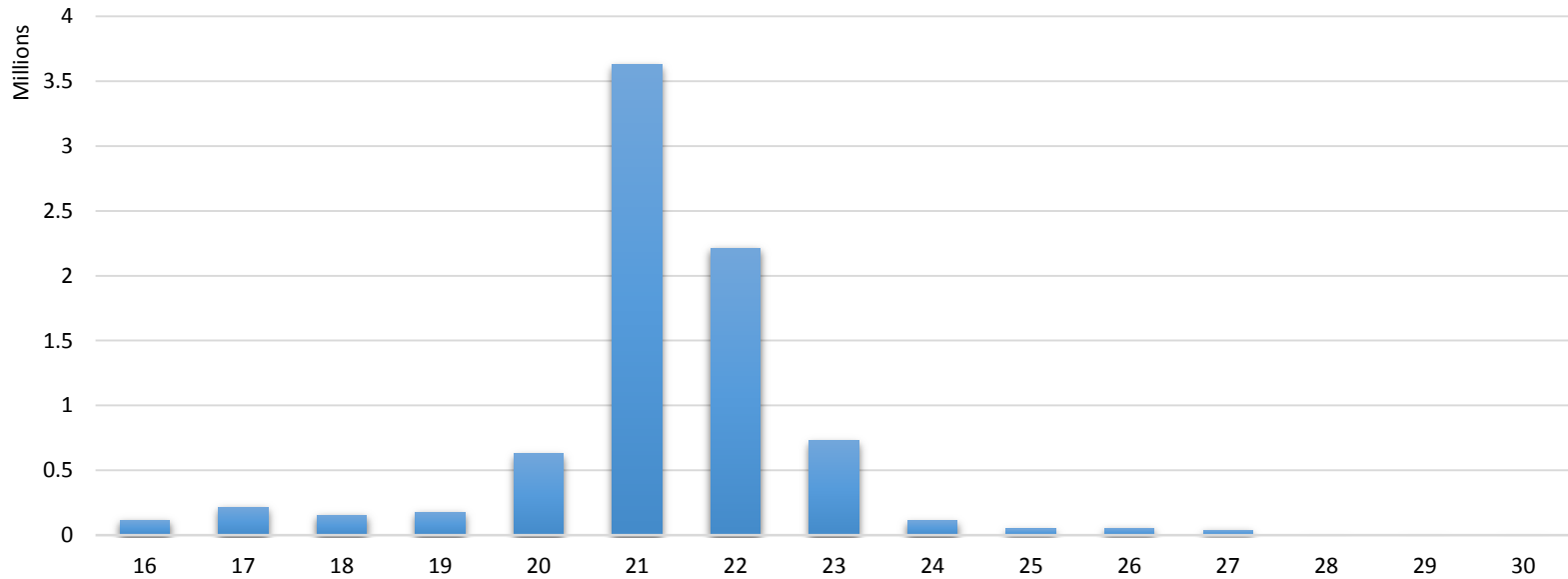

## Brain

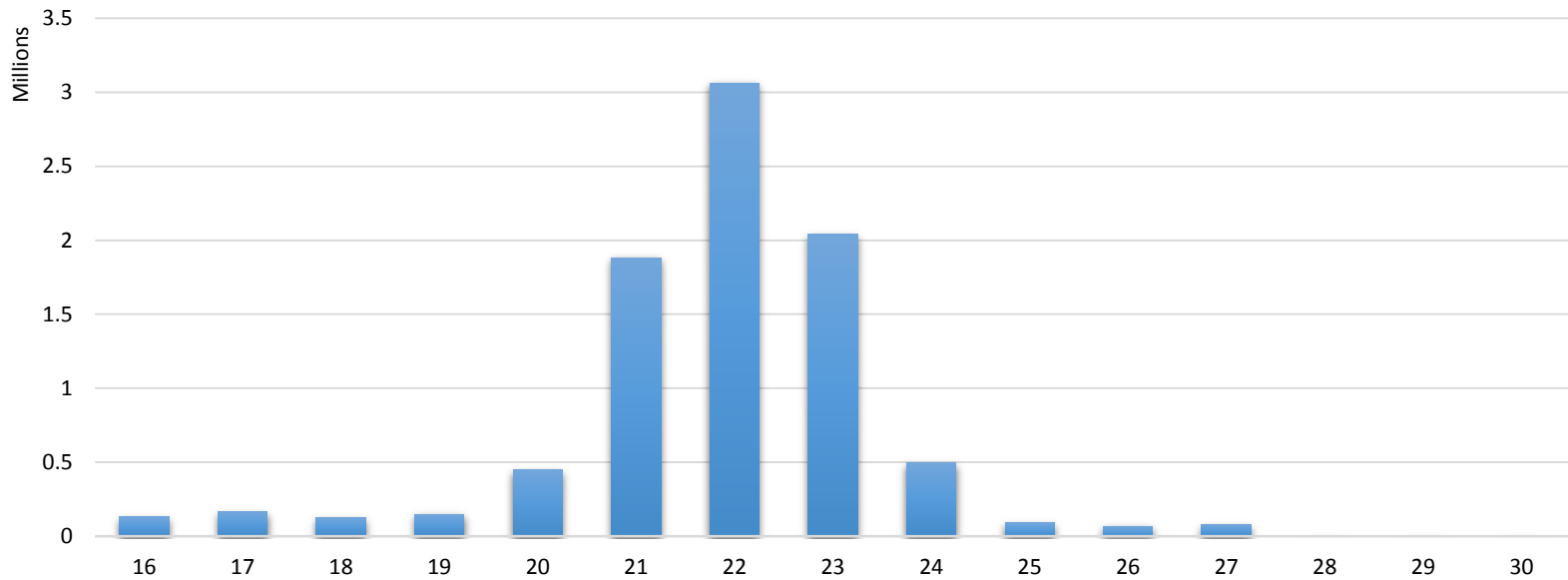

# Heart

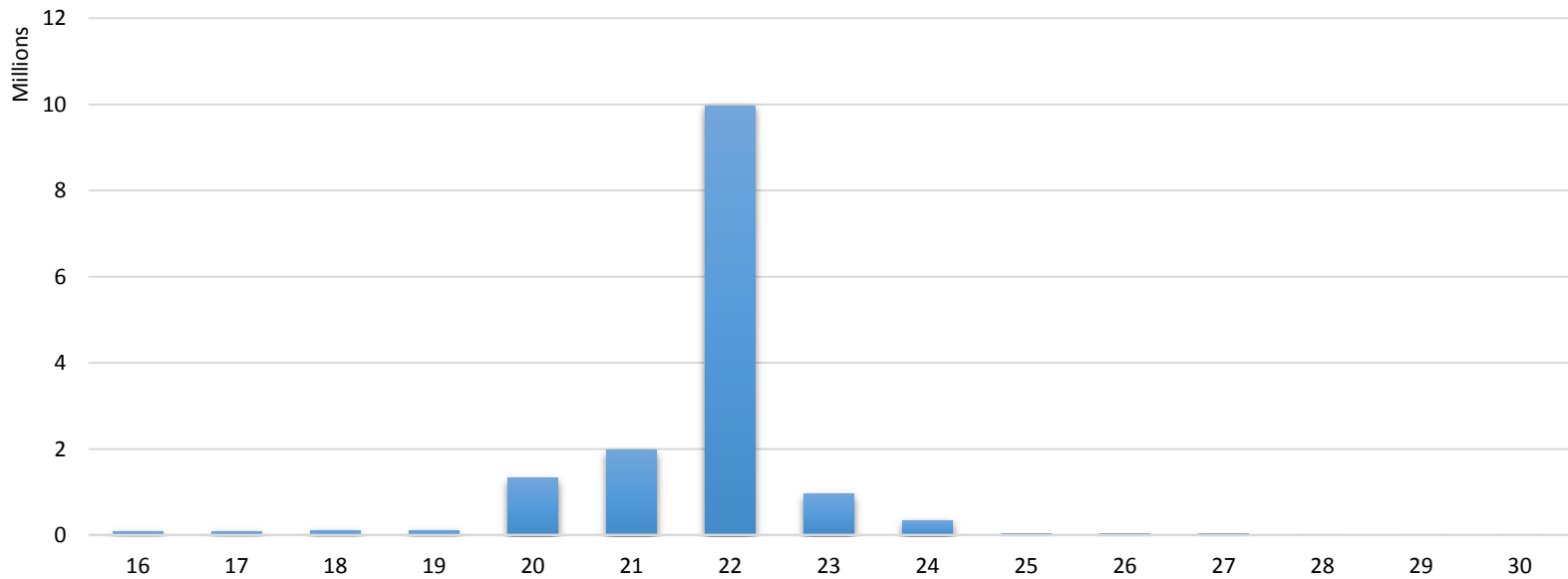

## Kidney

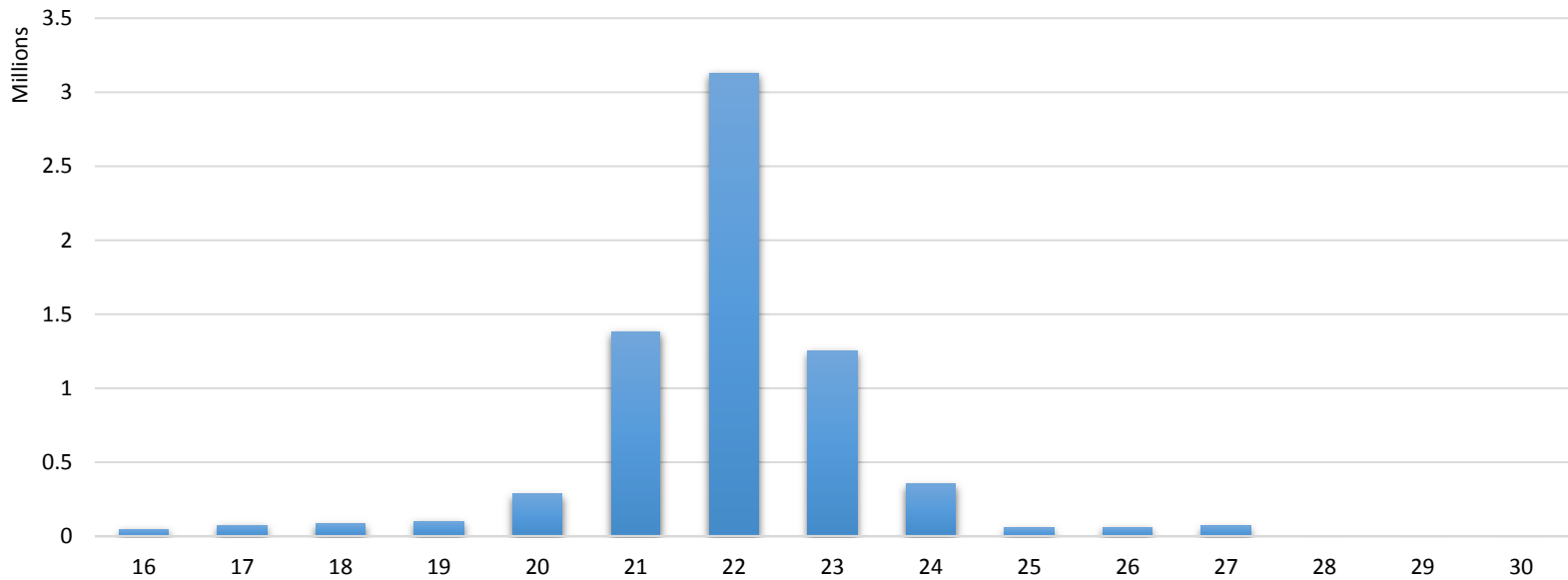

# Lung

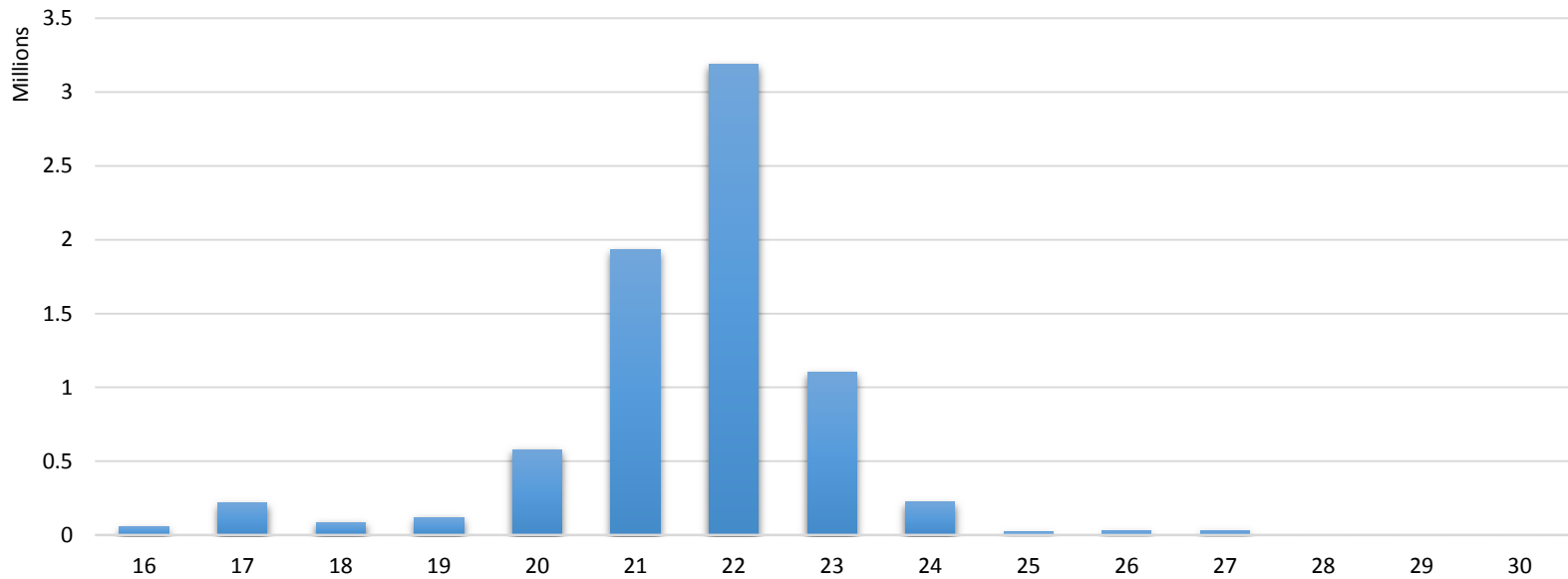

## Ovary

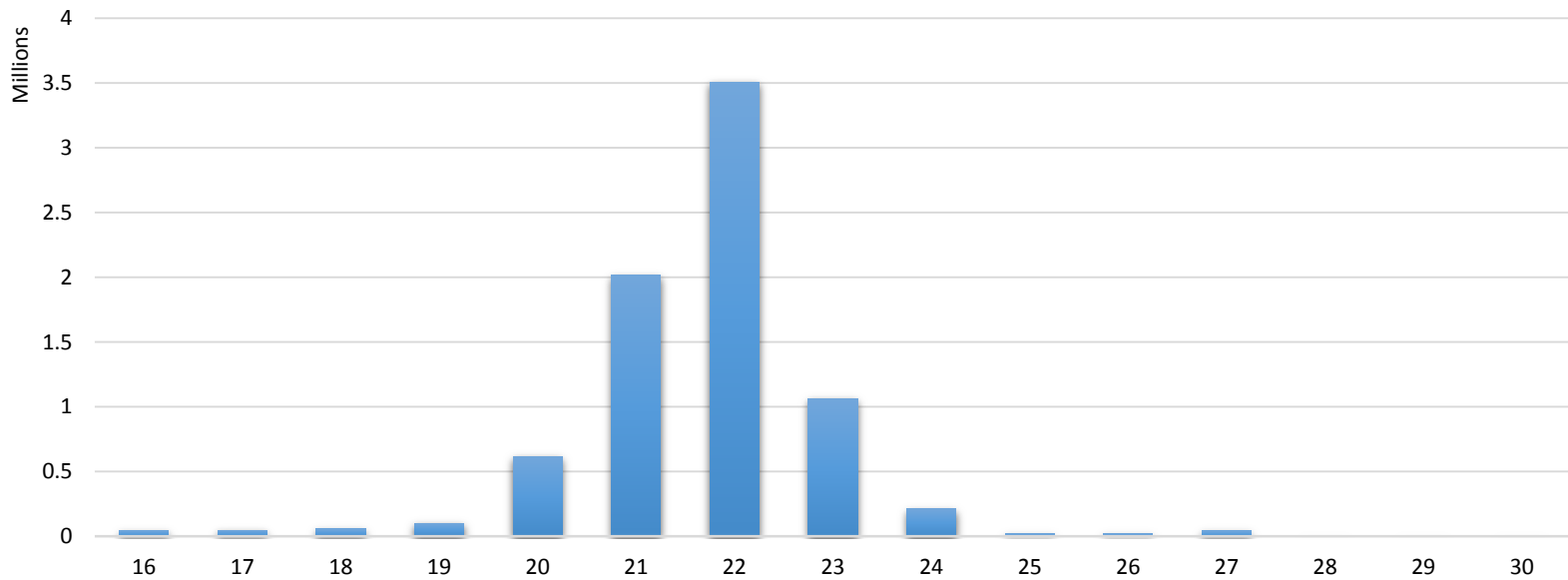

## Skin

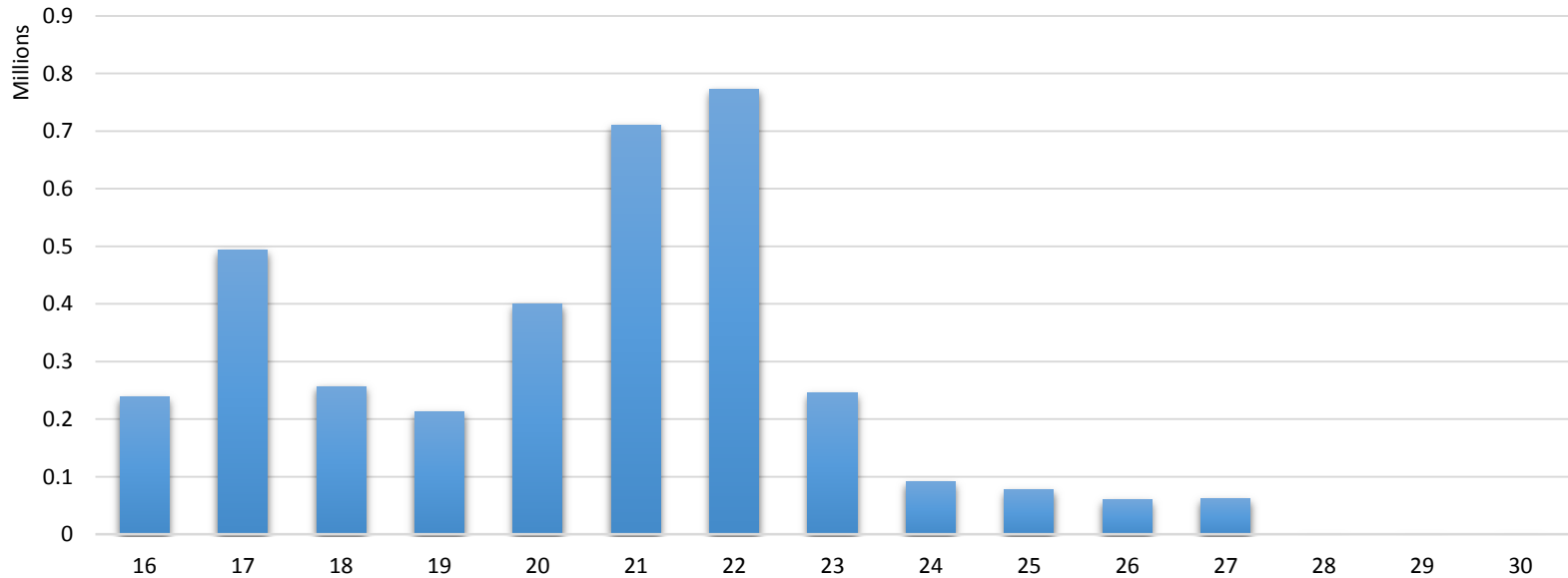

## Smooth Muscle

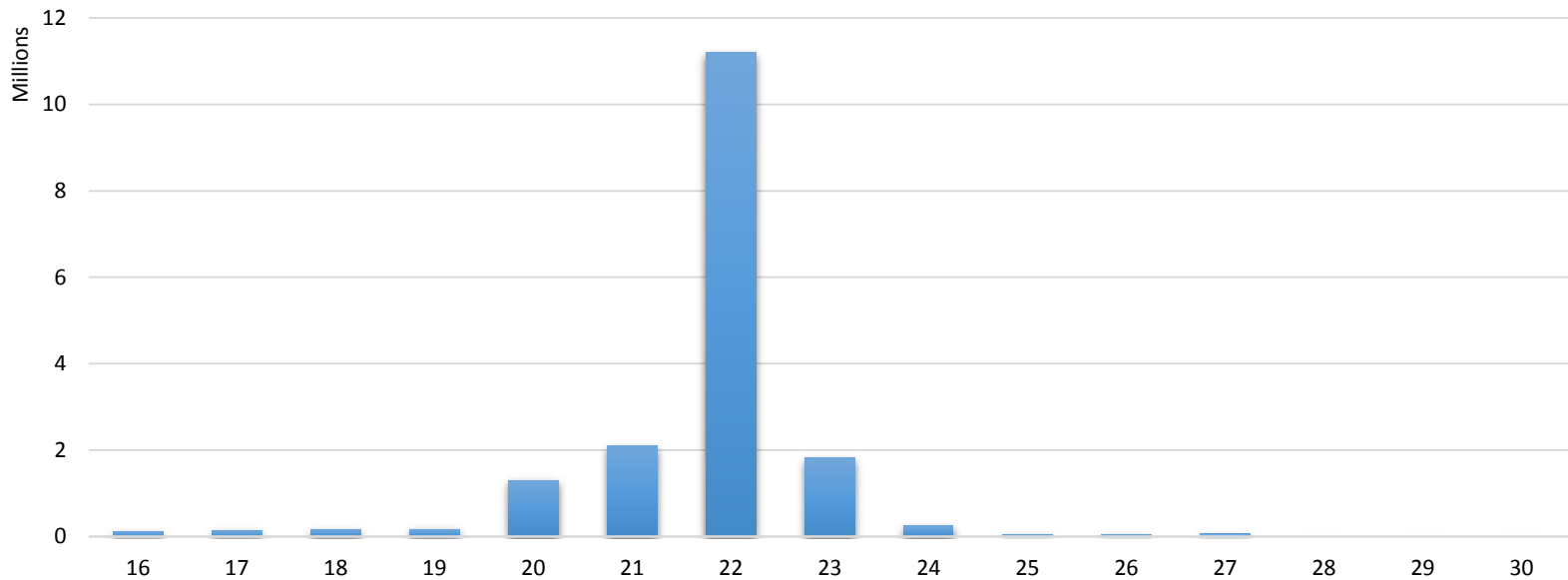

## Testis

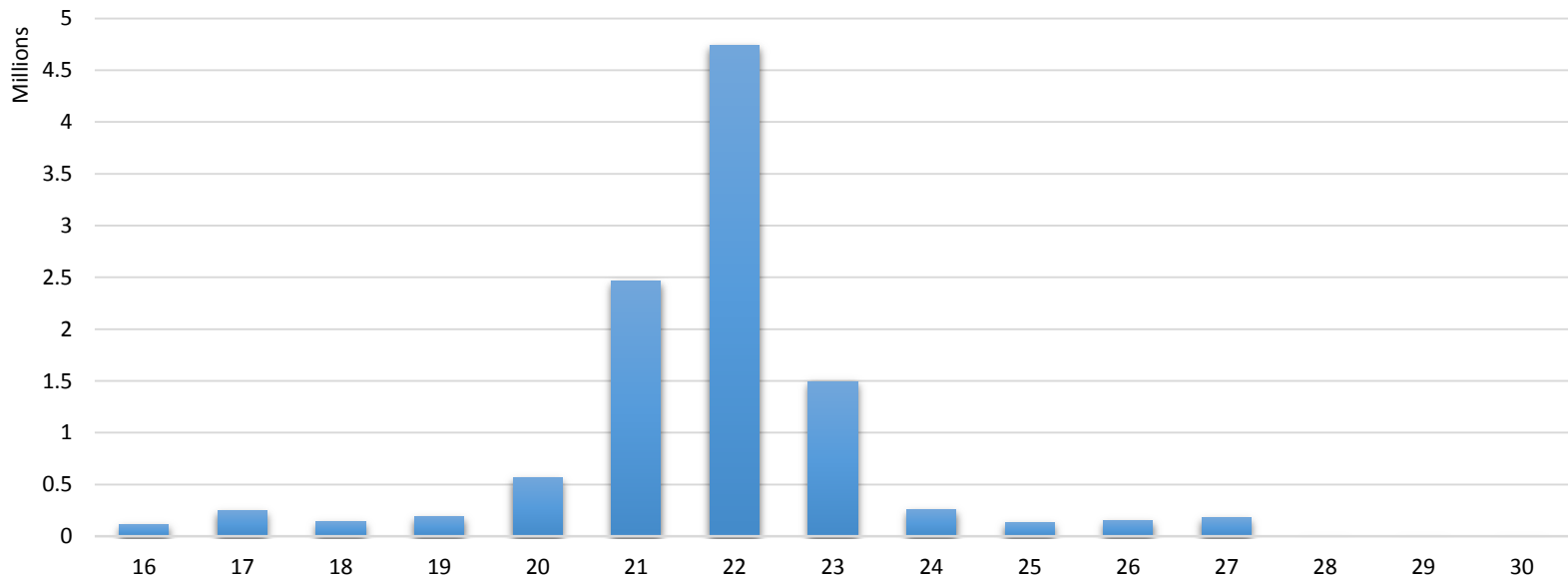

Supplement: S2 Fig — (PDF) [file pone.0153453.s002.pdf]

## Counts of trimmed genome matching reads

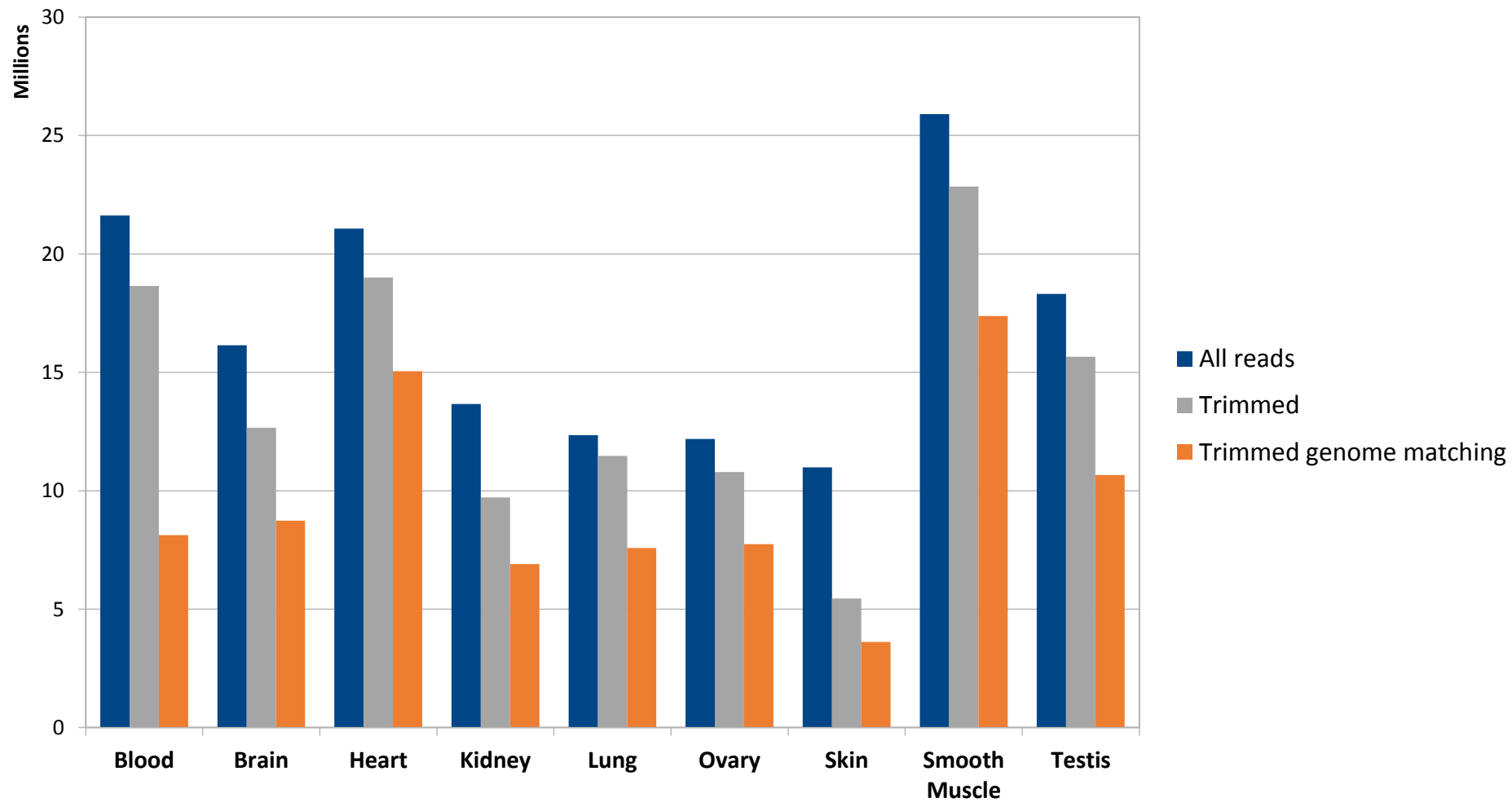

Supplement: S3 Fig — (PDF) [file pone.0153453.s003.pdf]

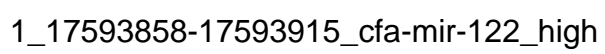

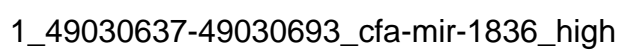

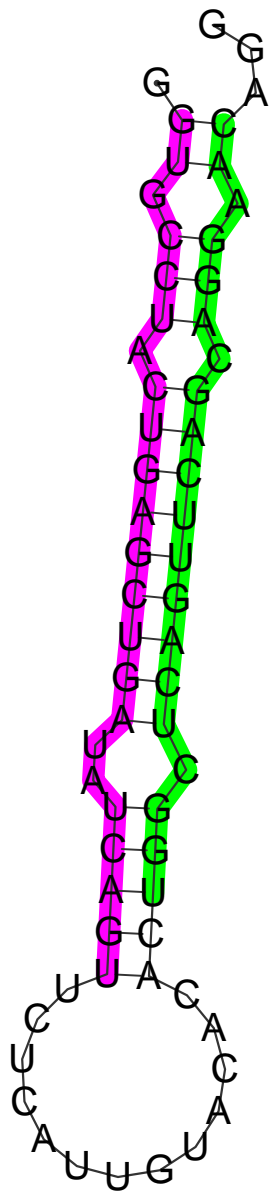

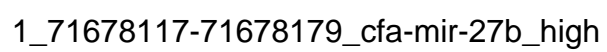

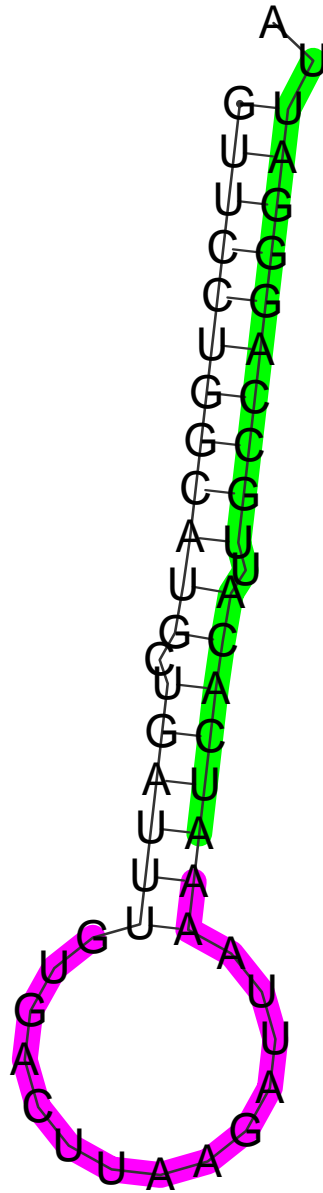

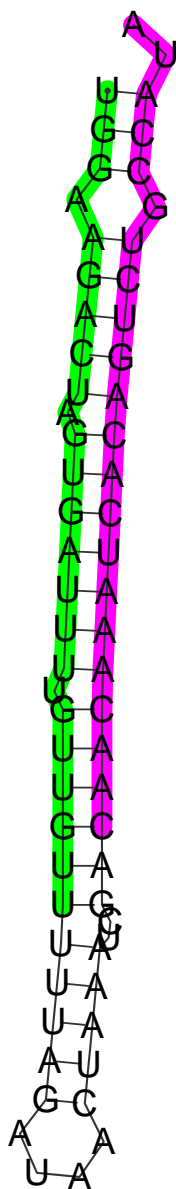

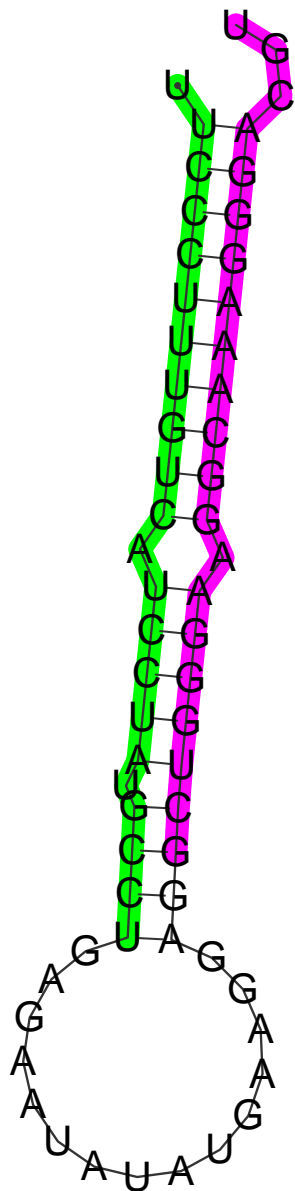

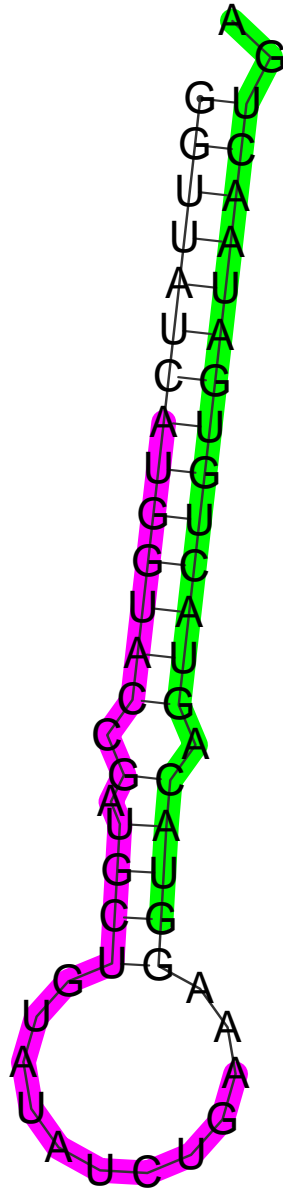

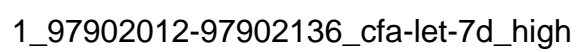

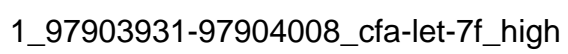

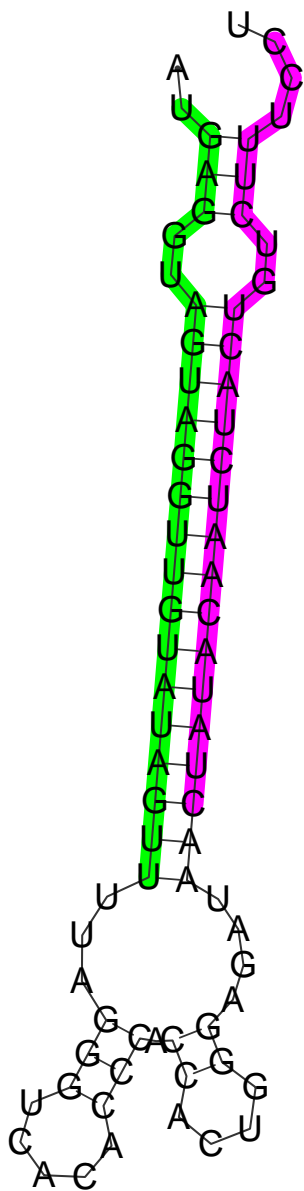

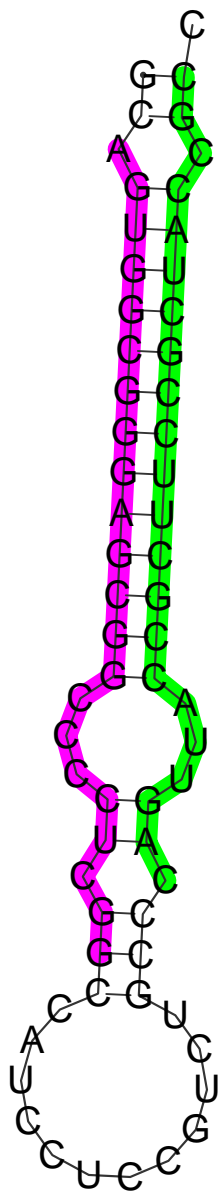

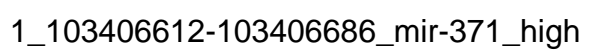



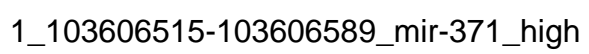

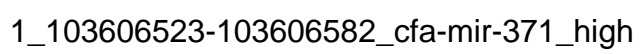

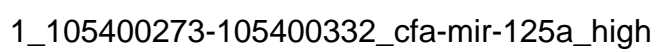

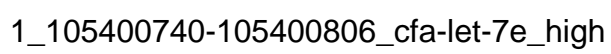

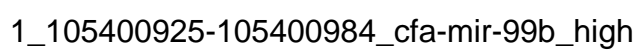

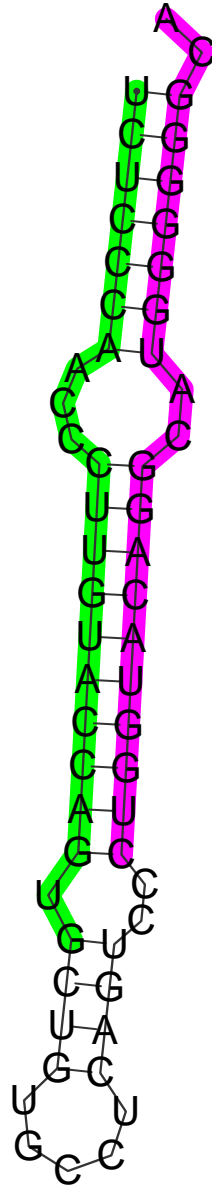

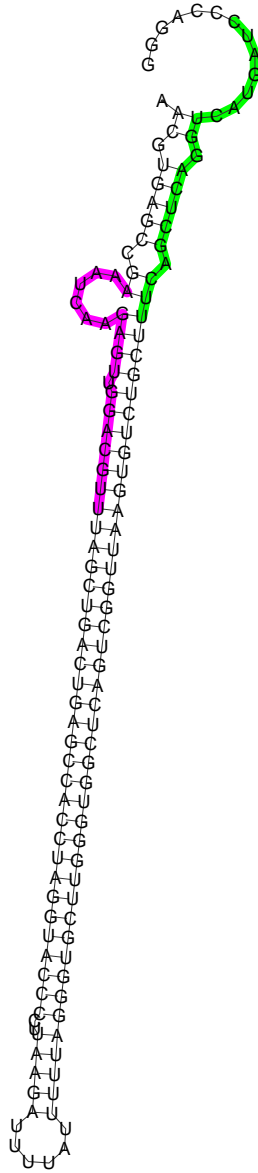

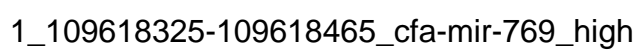

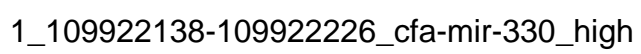

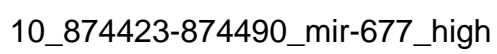

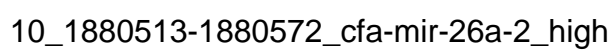

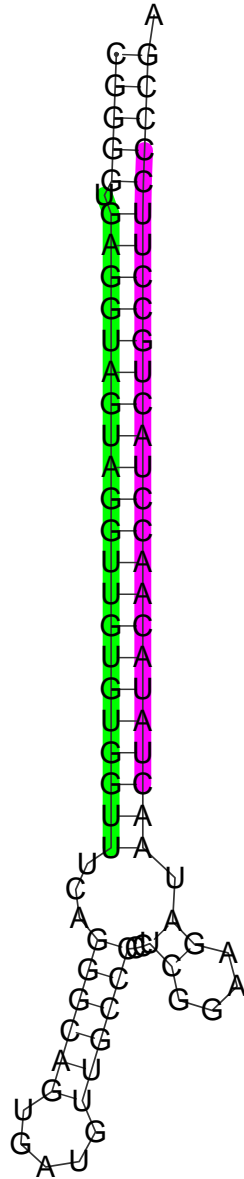



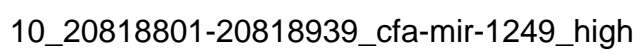

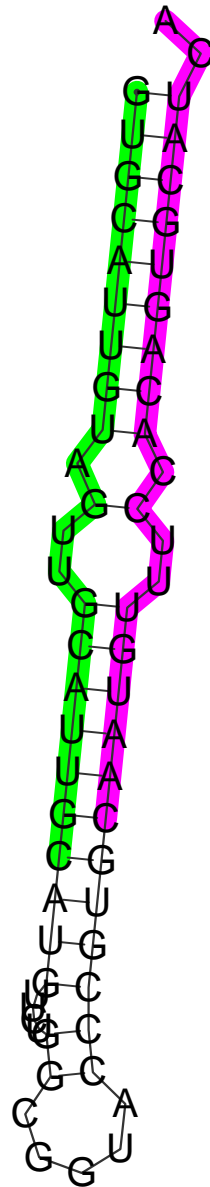



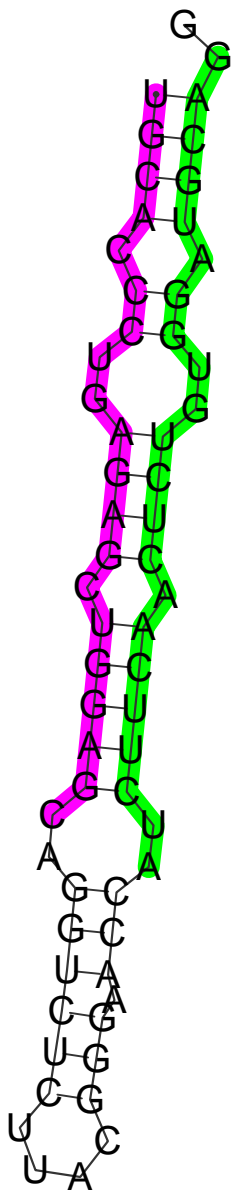

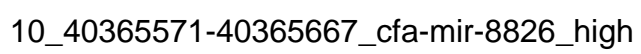

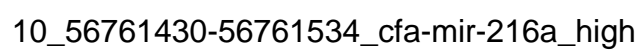

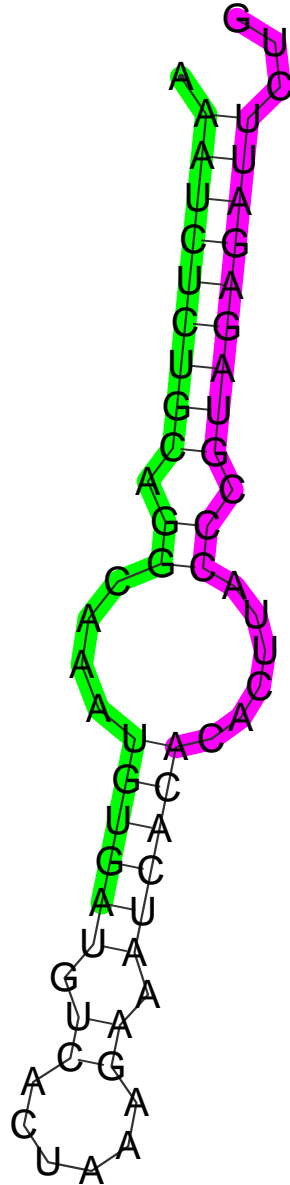

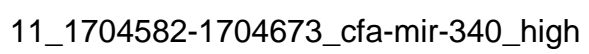

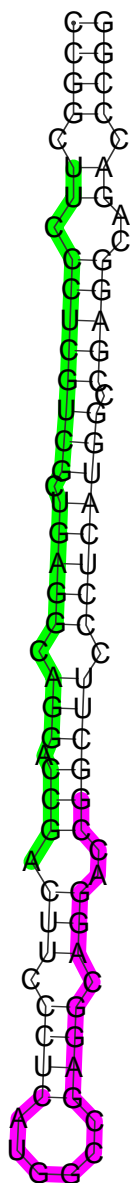

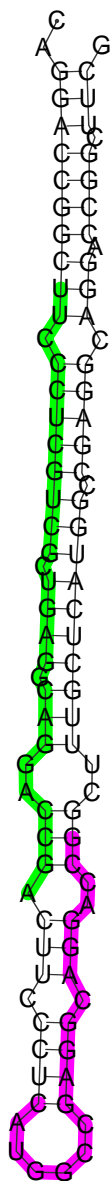

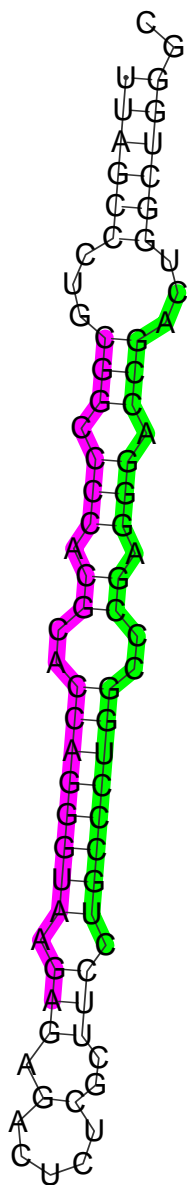

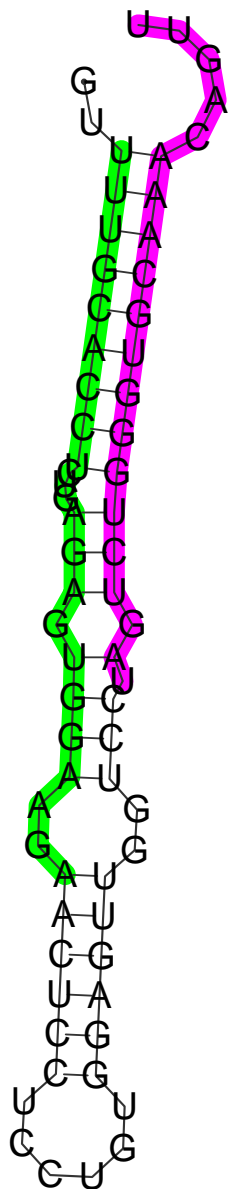

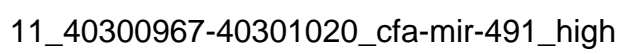

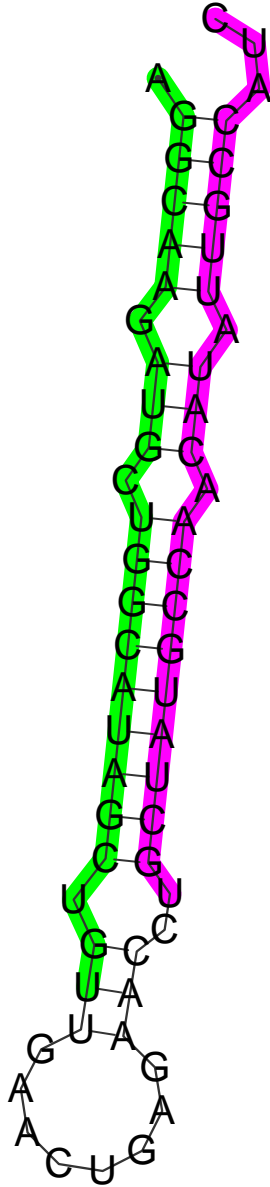

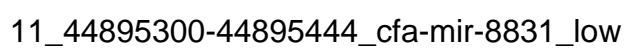

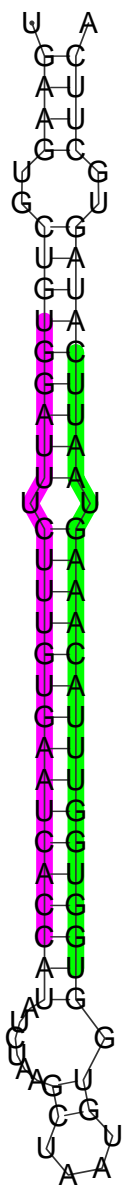

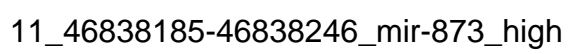

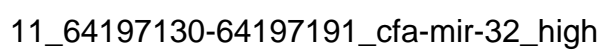



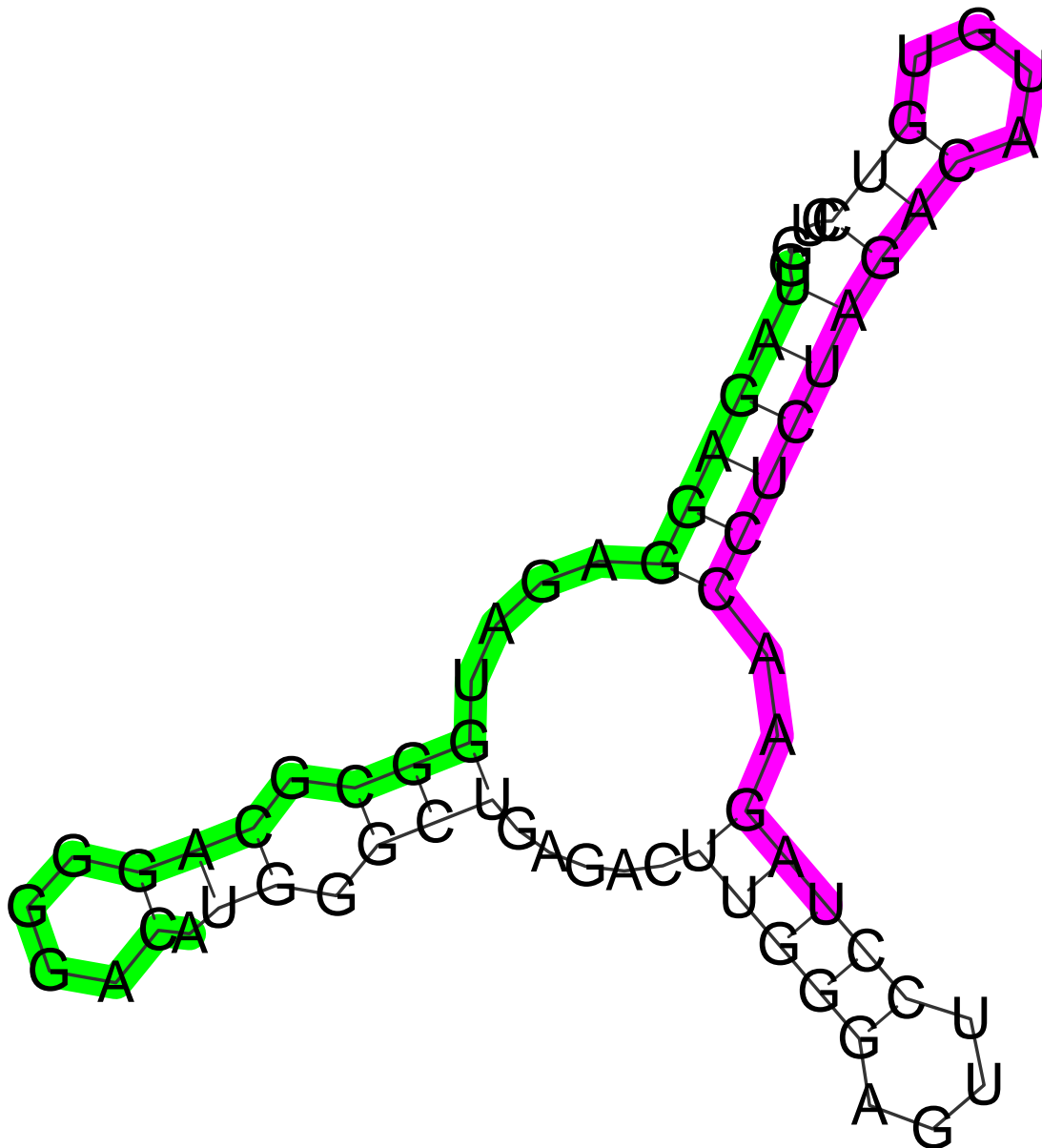

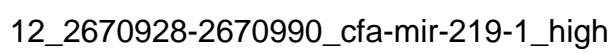



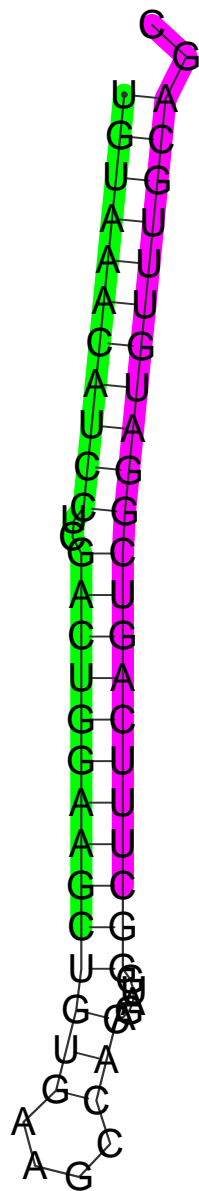

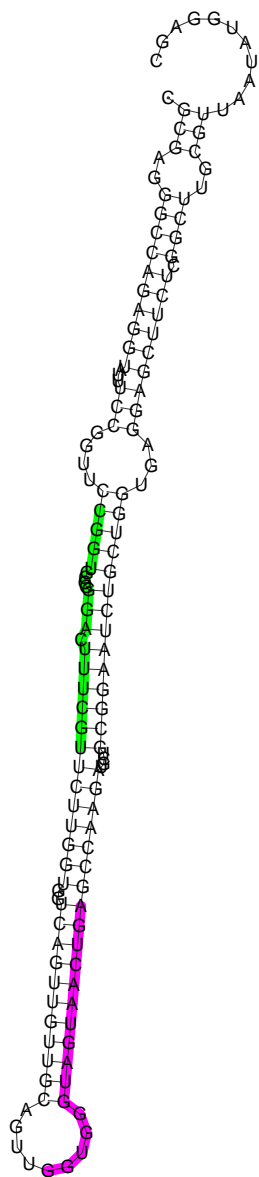

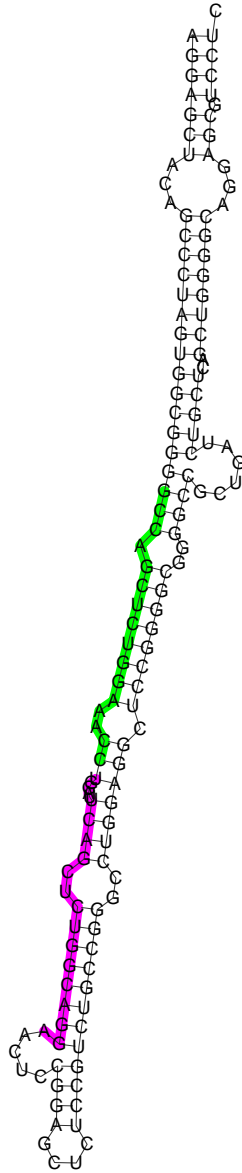

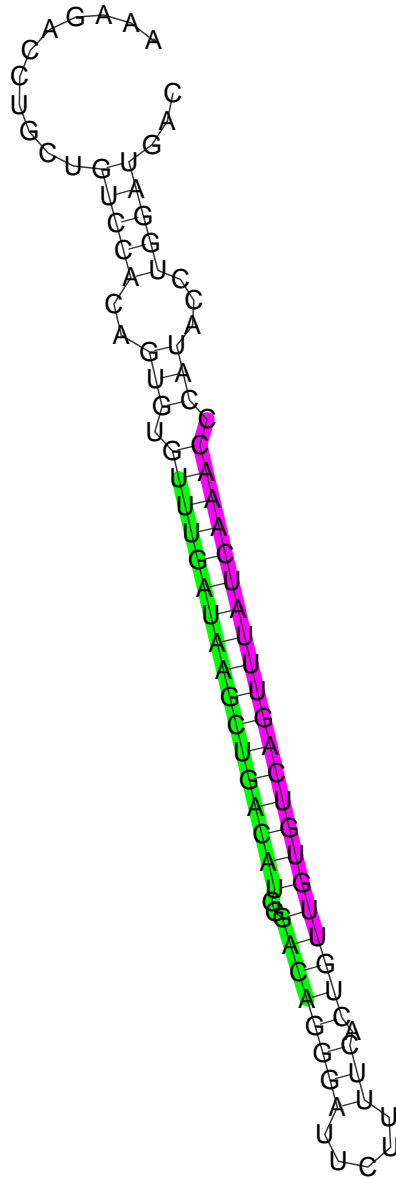

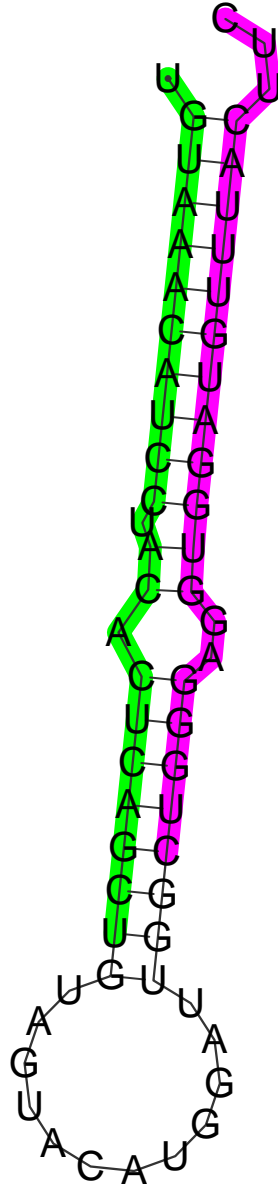

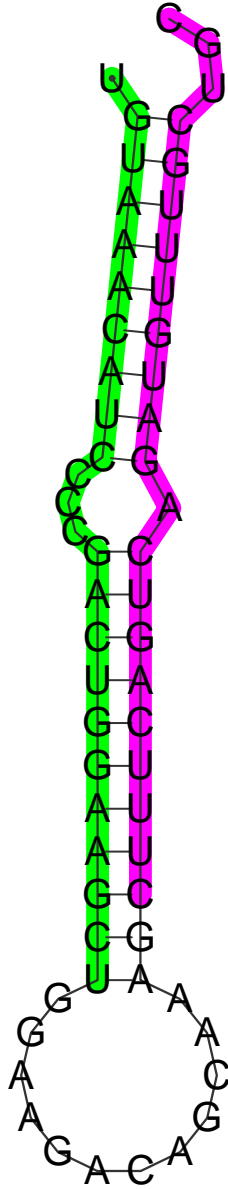

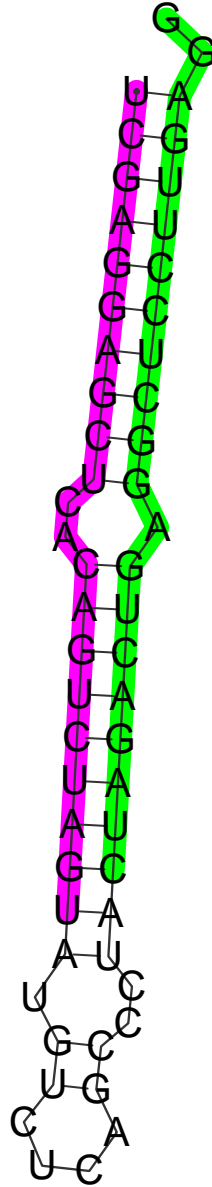

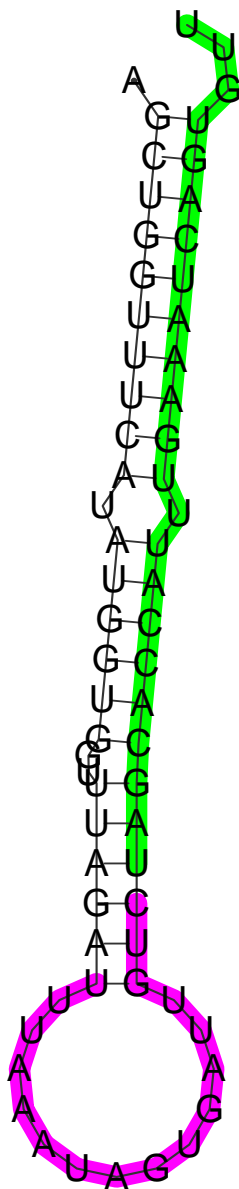

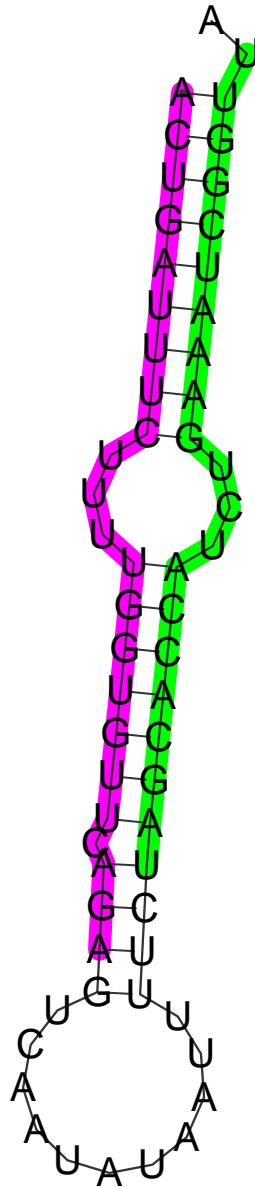

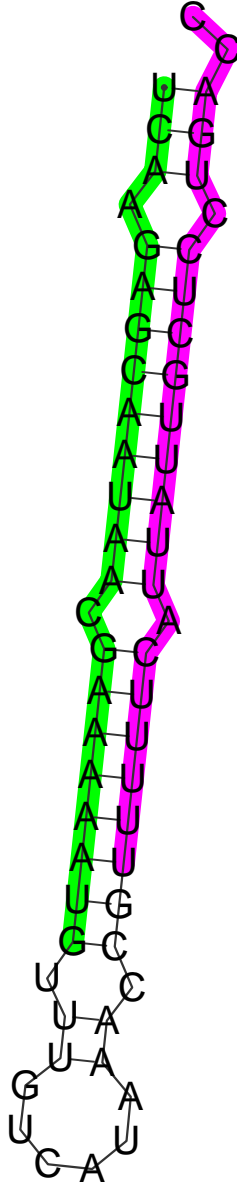

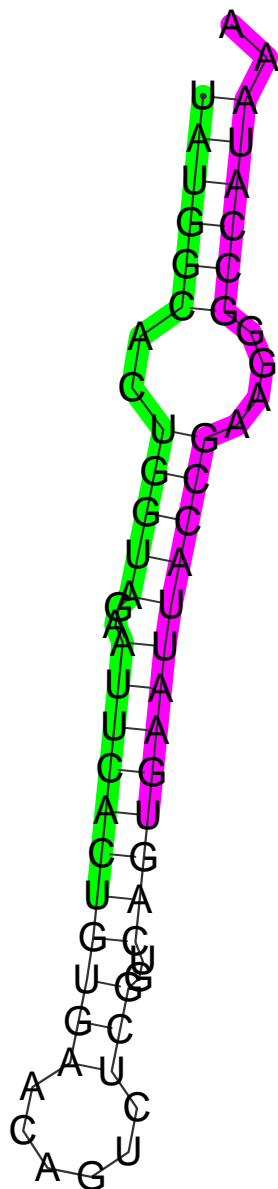

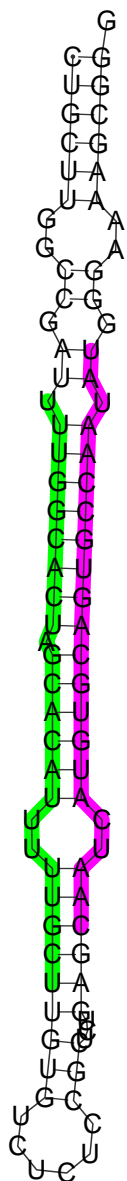

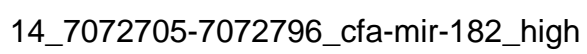

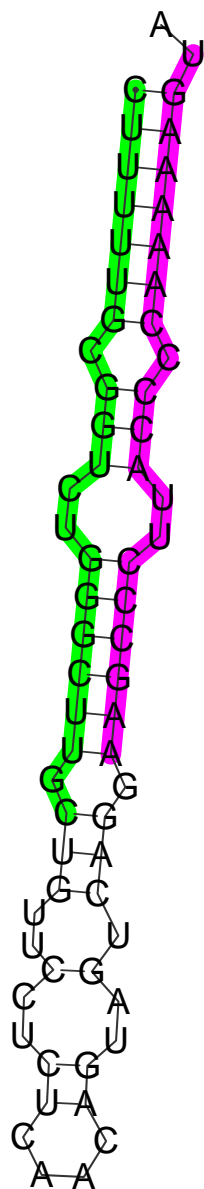

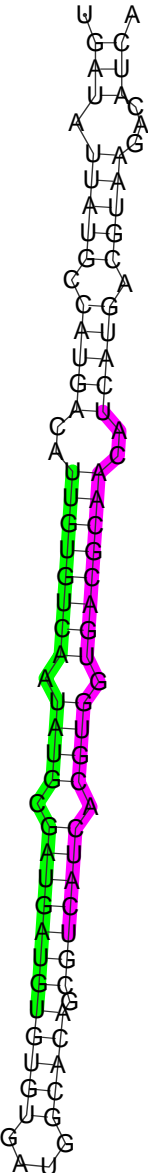

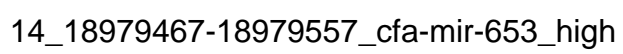

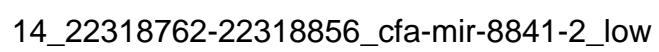

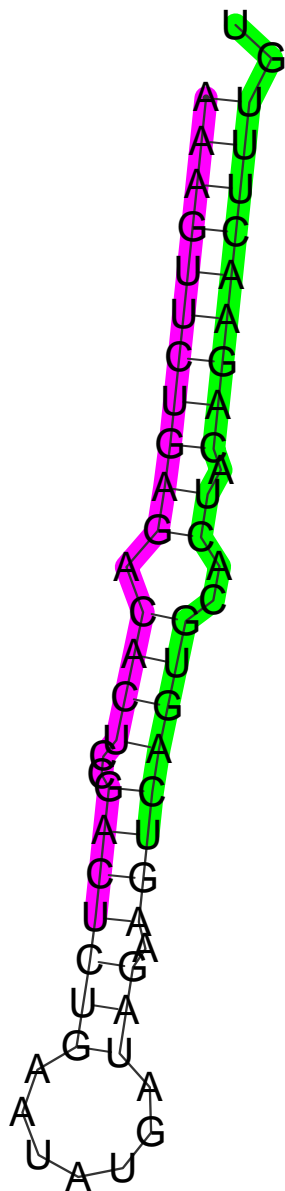

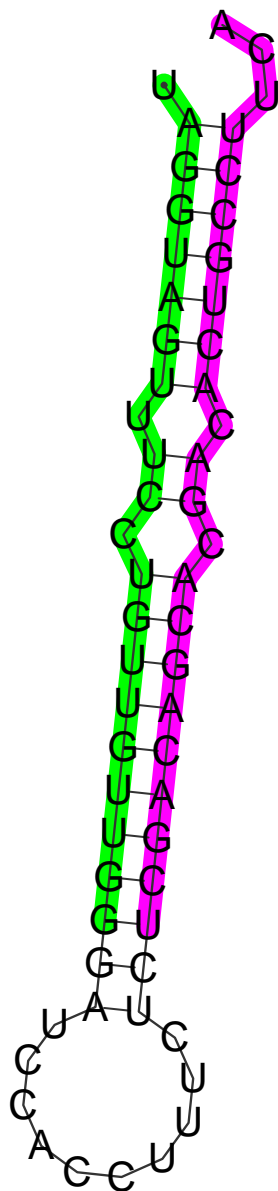

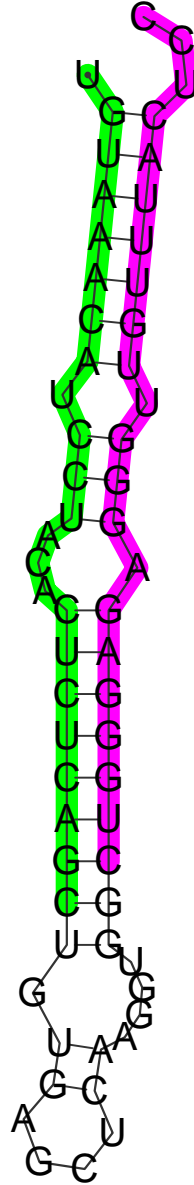

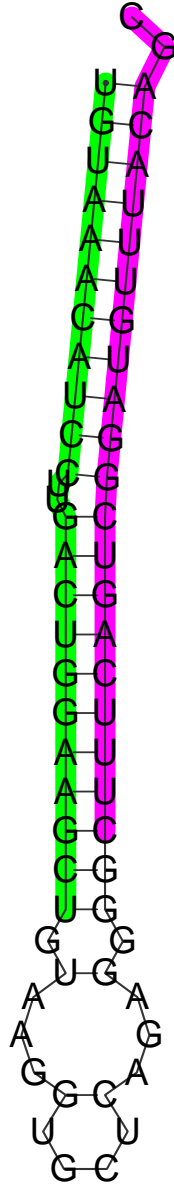

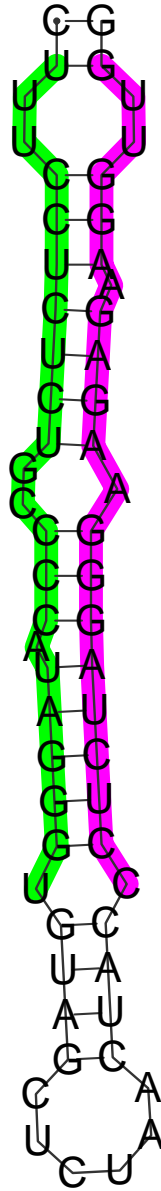

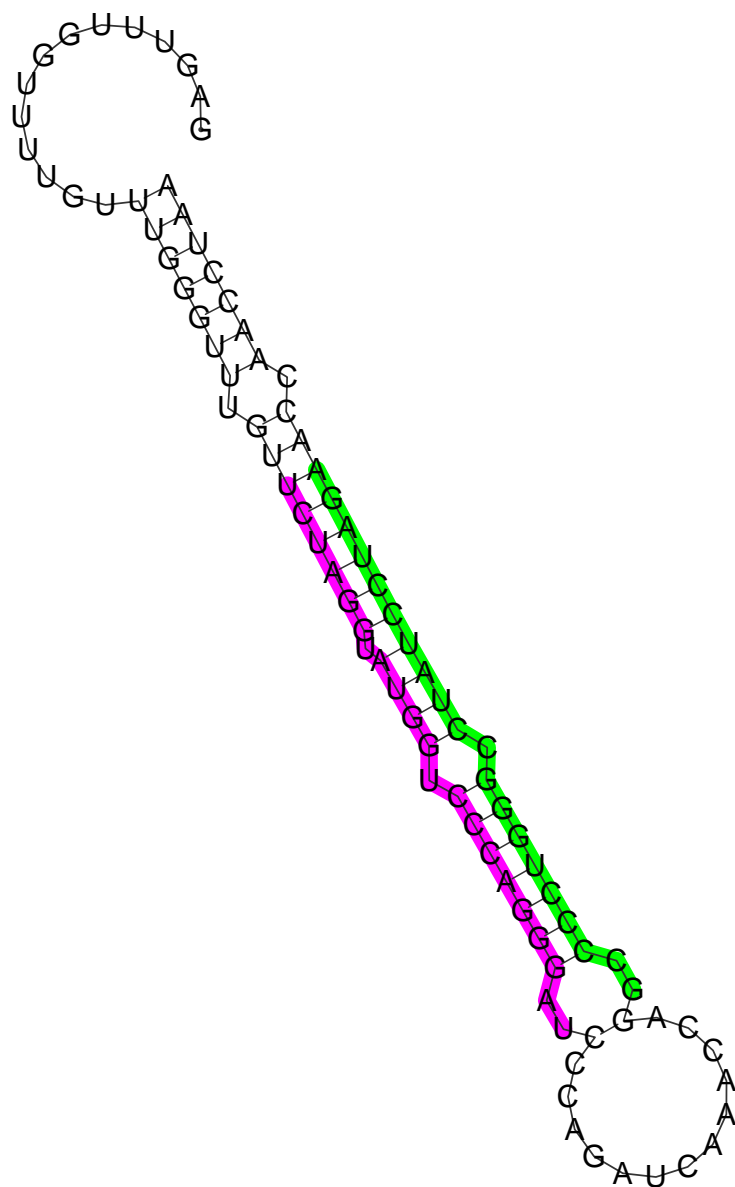



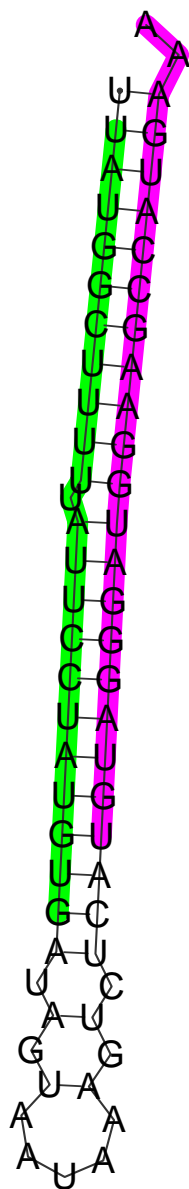

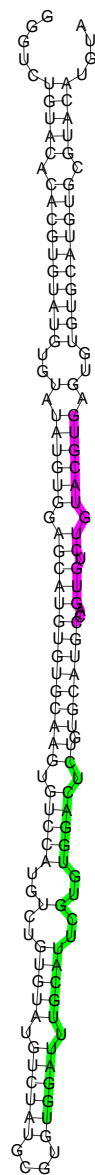

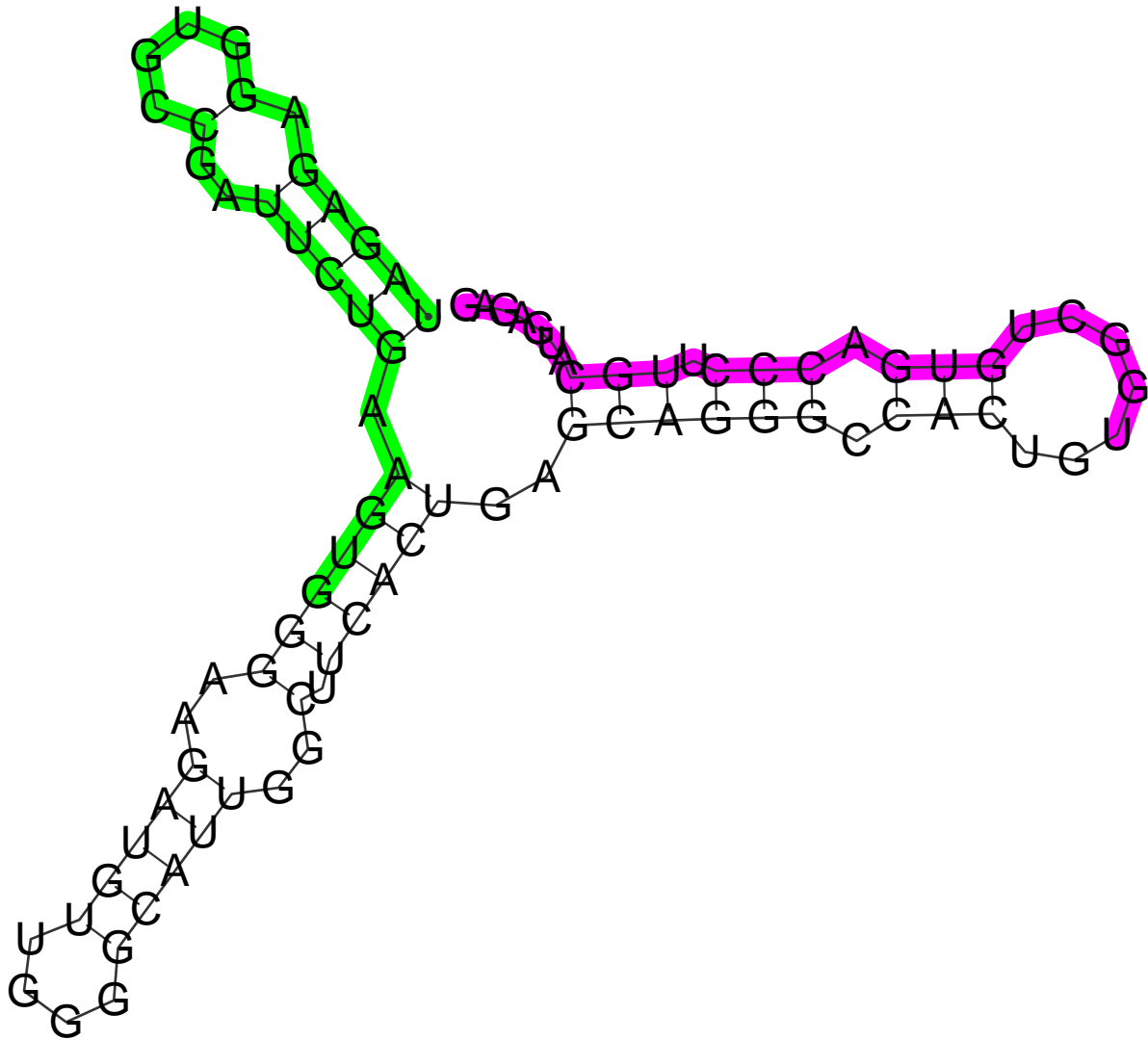

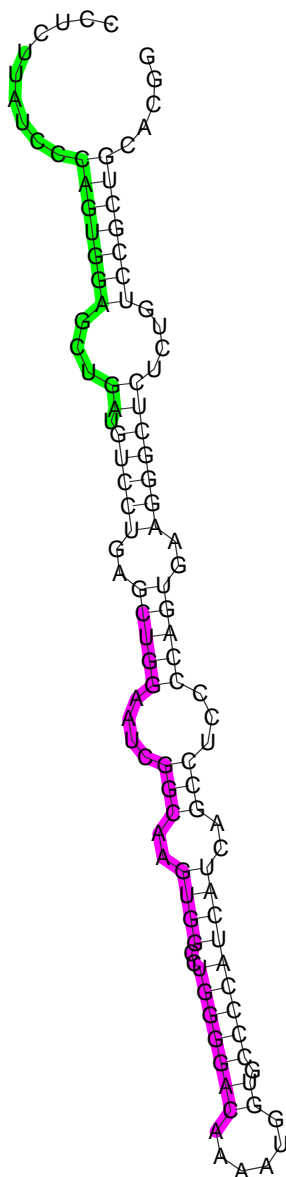

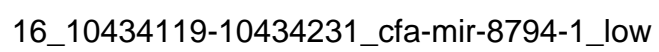



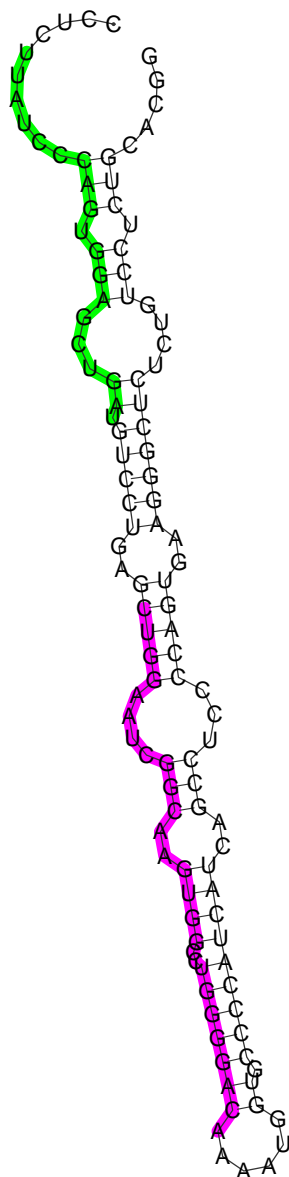

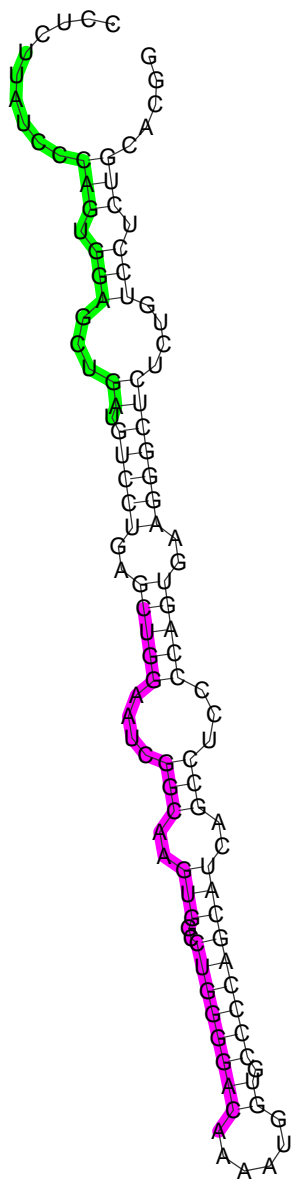

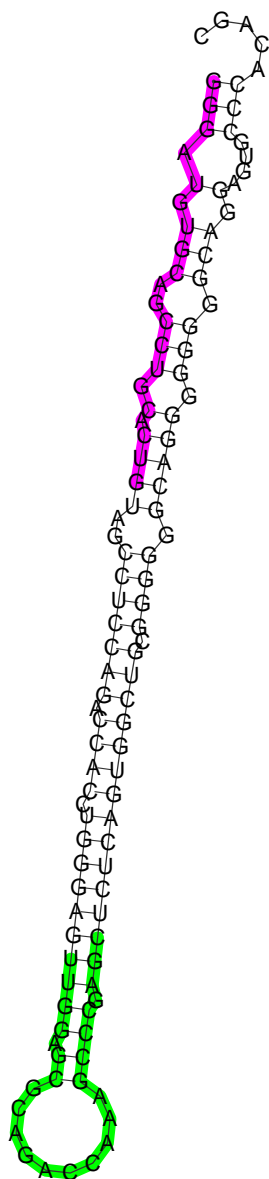

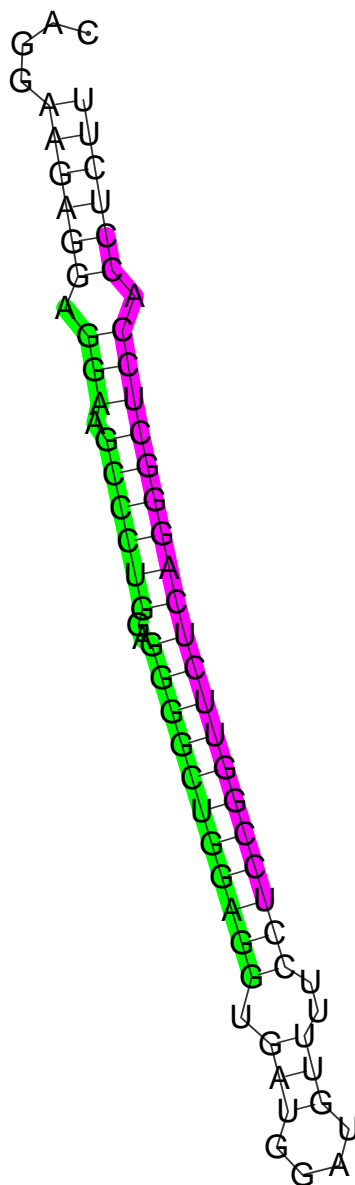

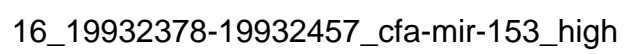

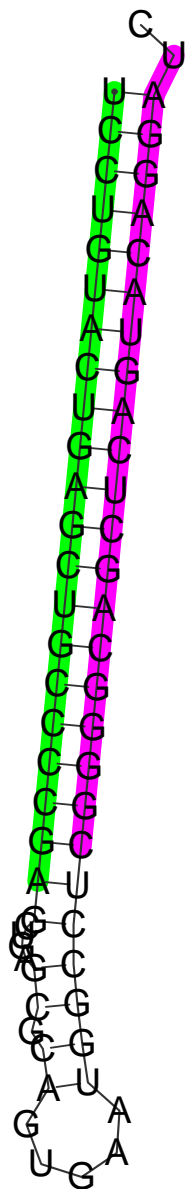

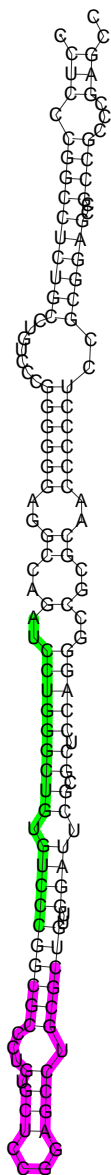

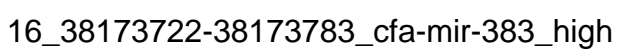

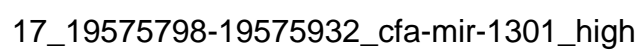

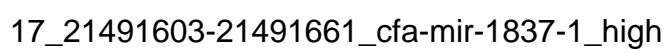

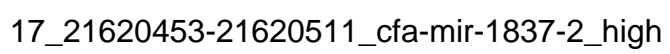

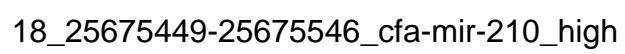

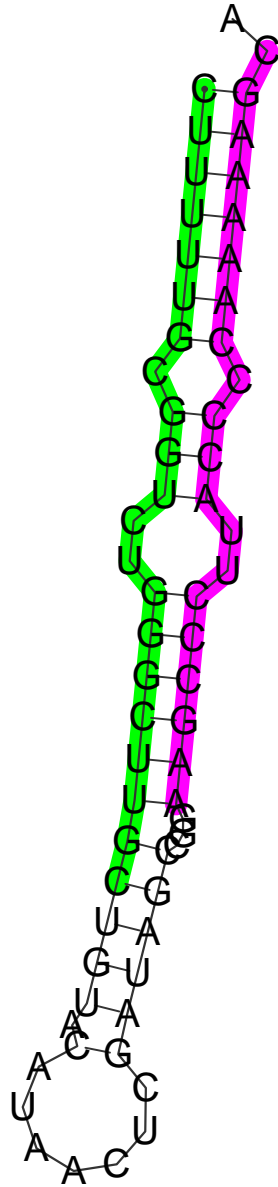

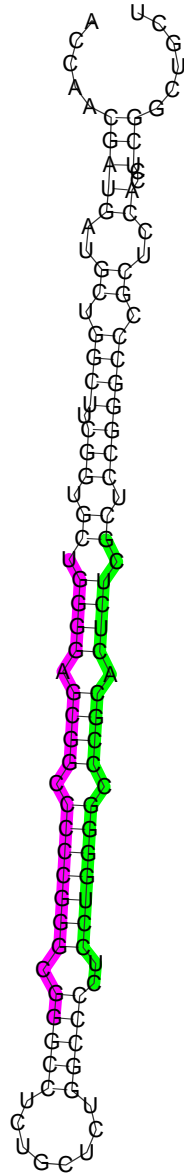

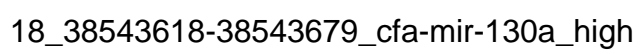

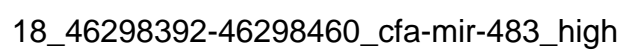

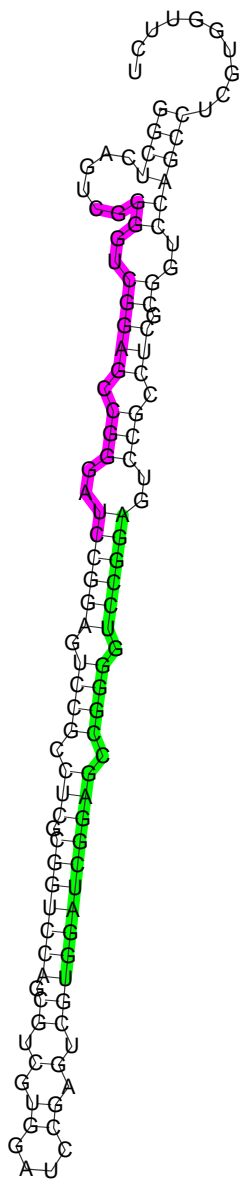

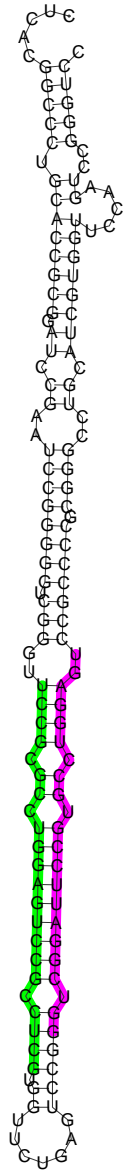

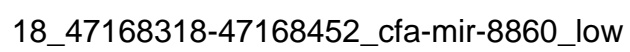

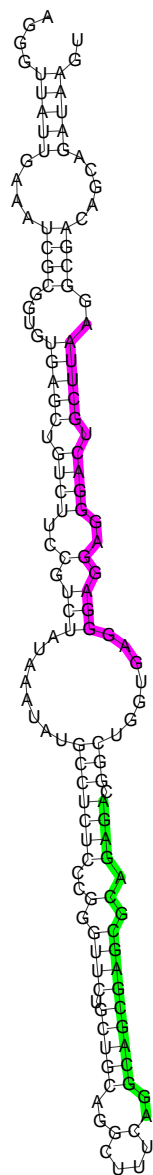

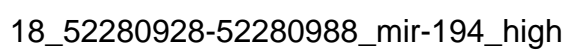

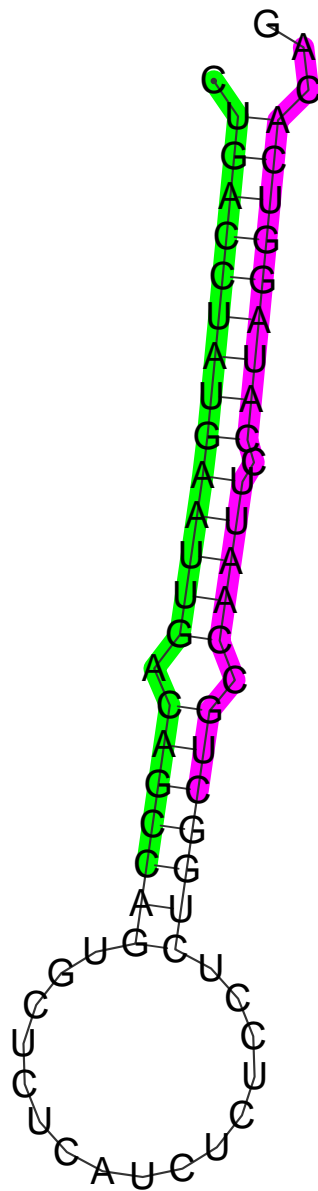

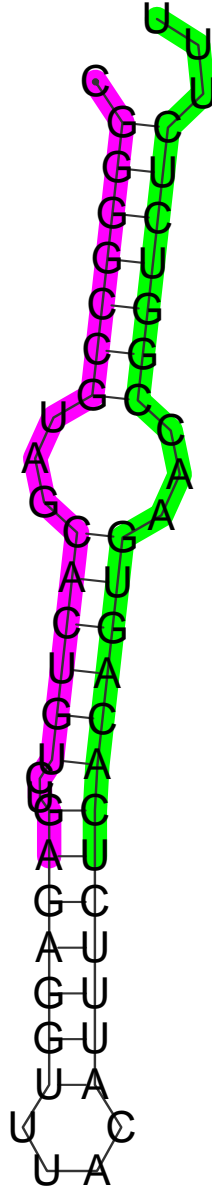

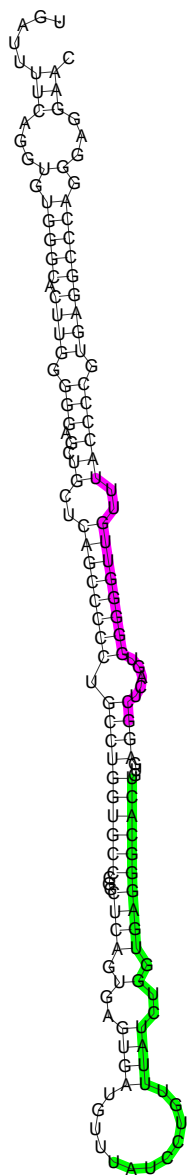

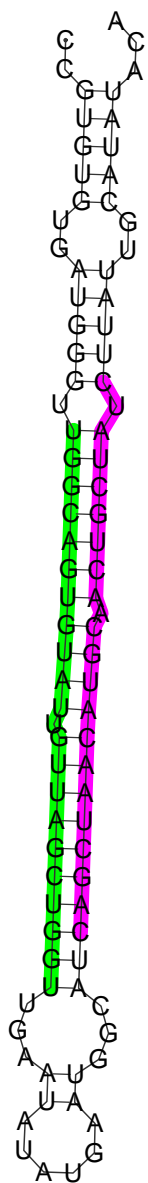

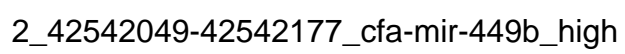

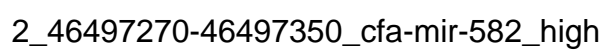

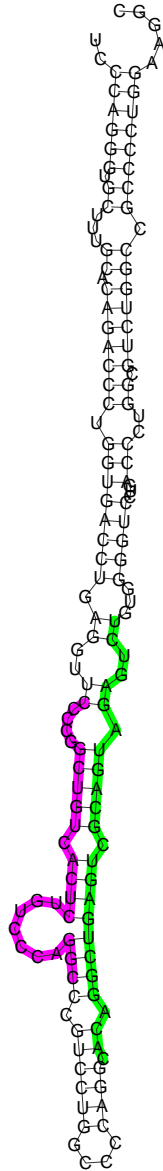

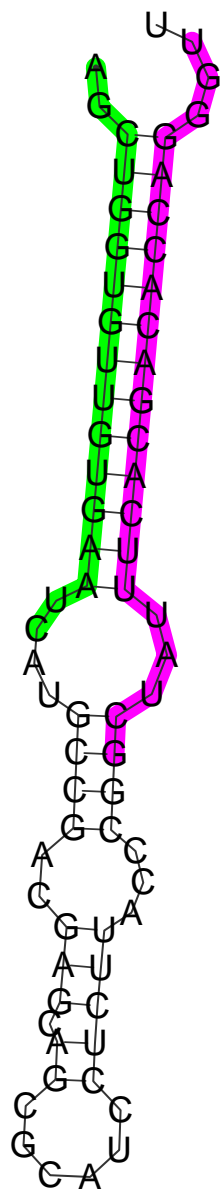

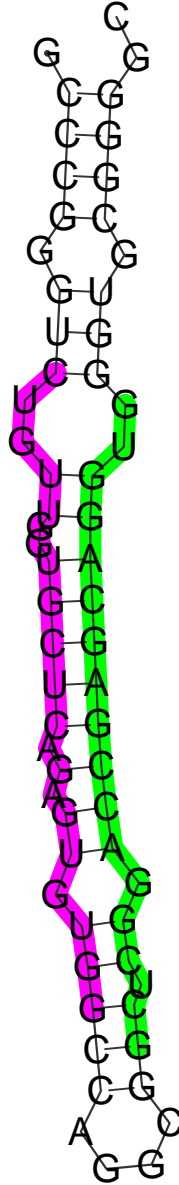

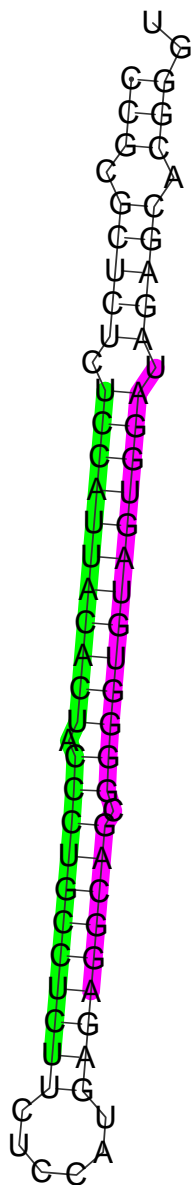

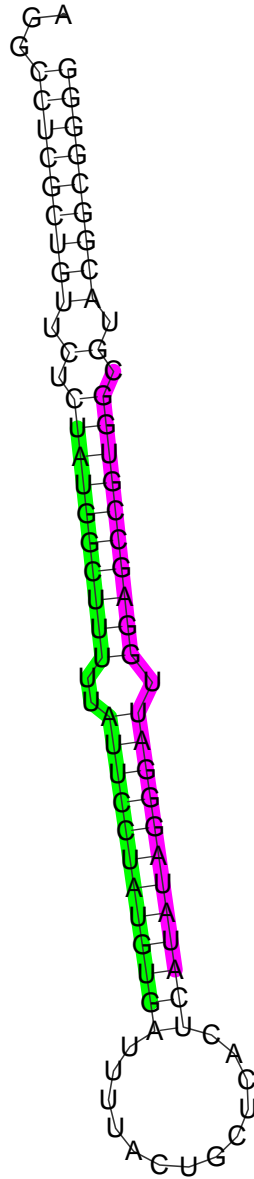

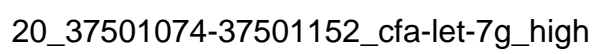

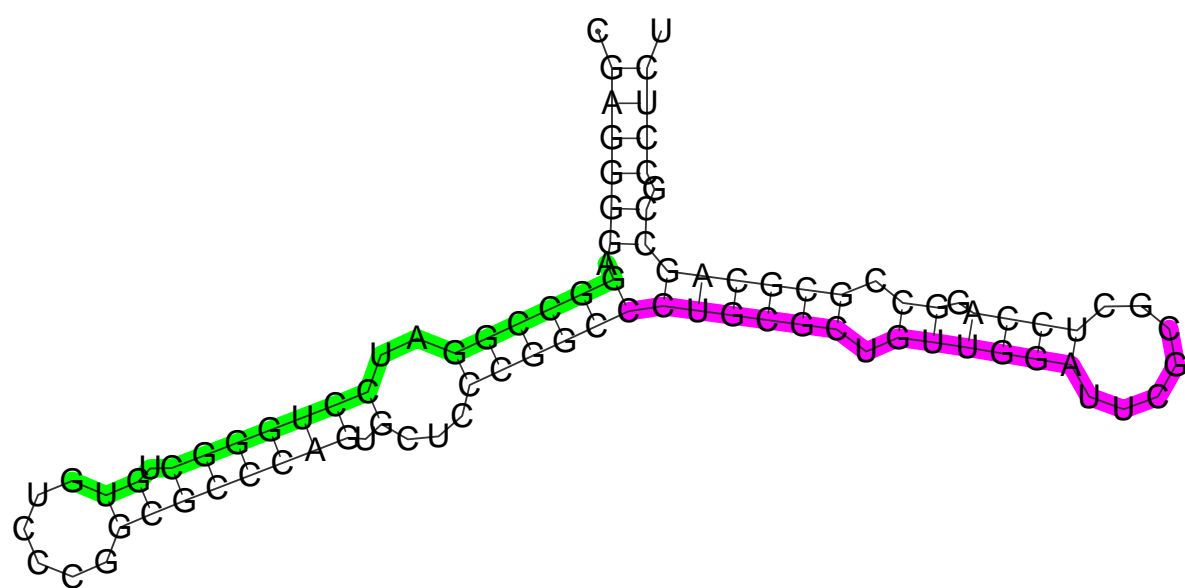

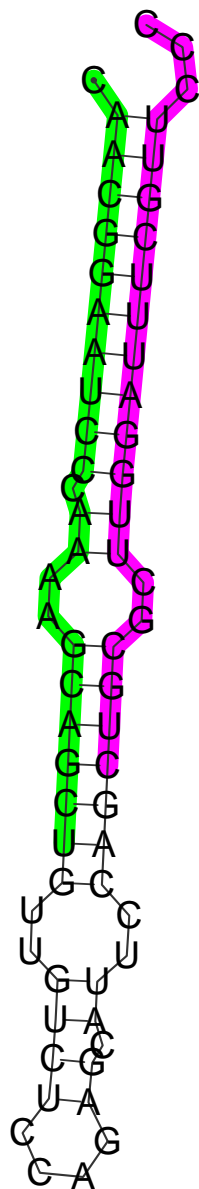

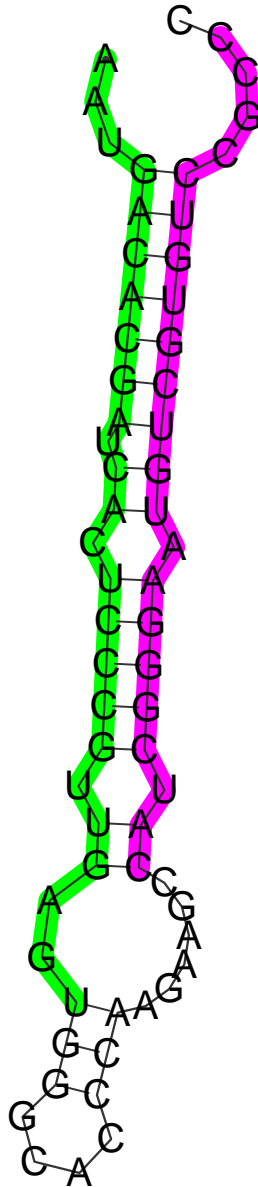

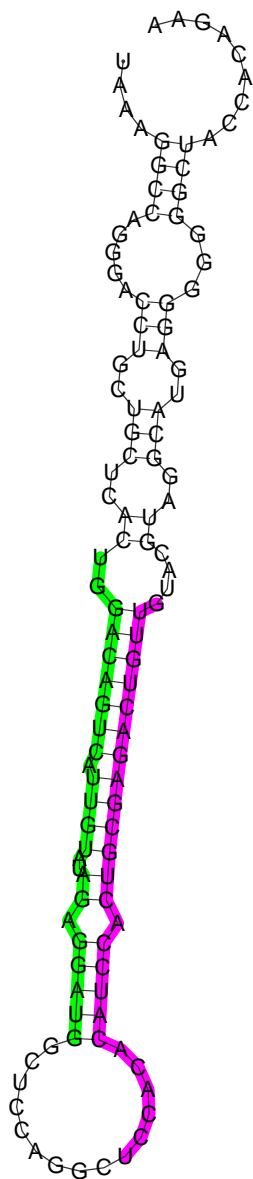

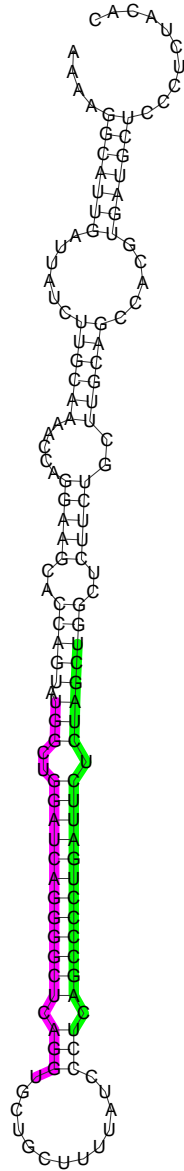

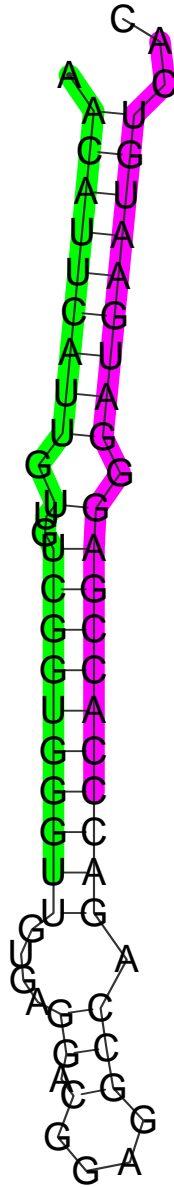

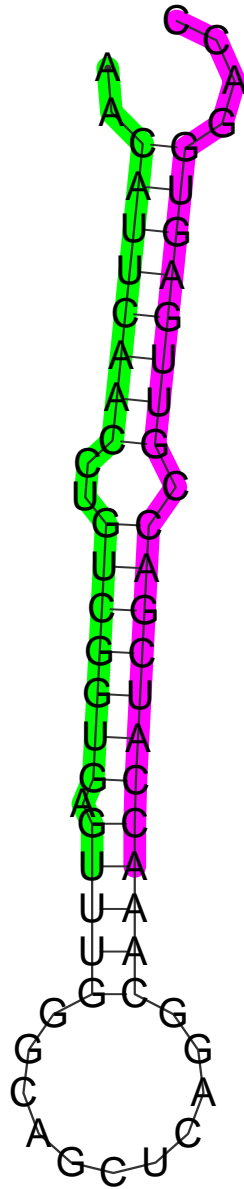

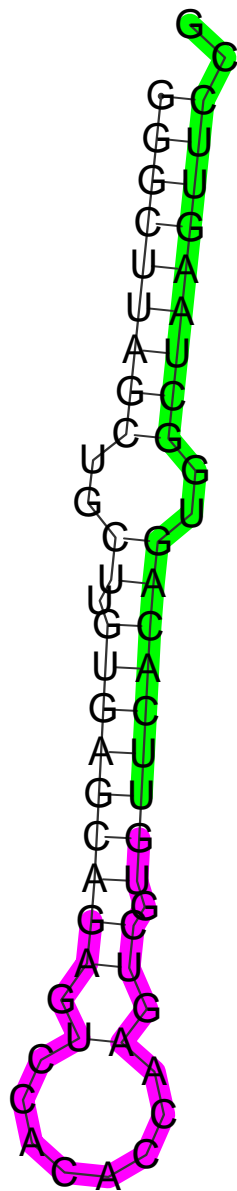

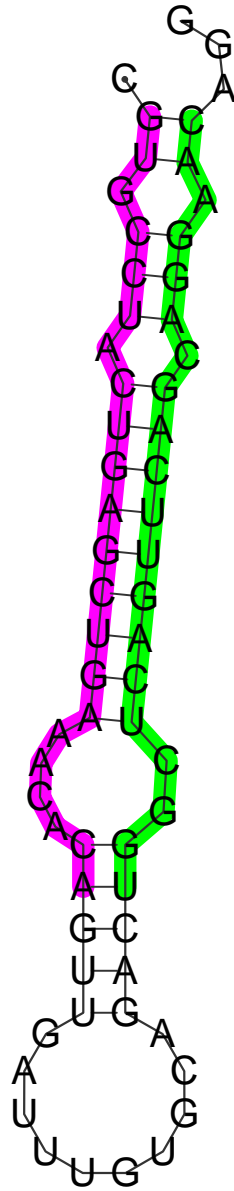

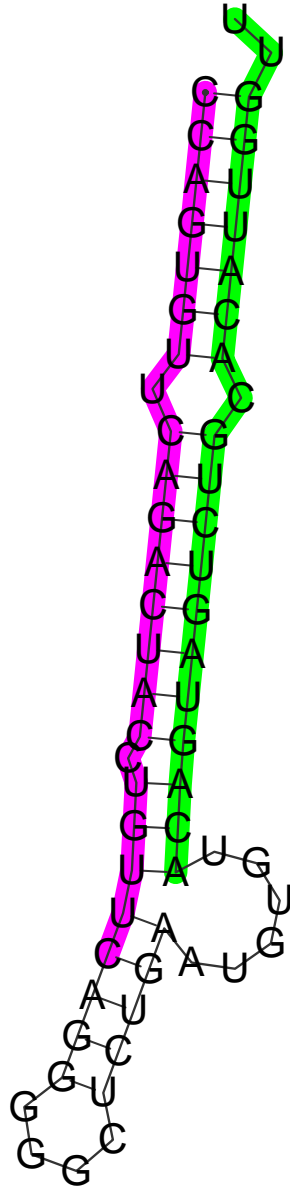

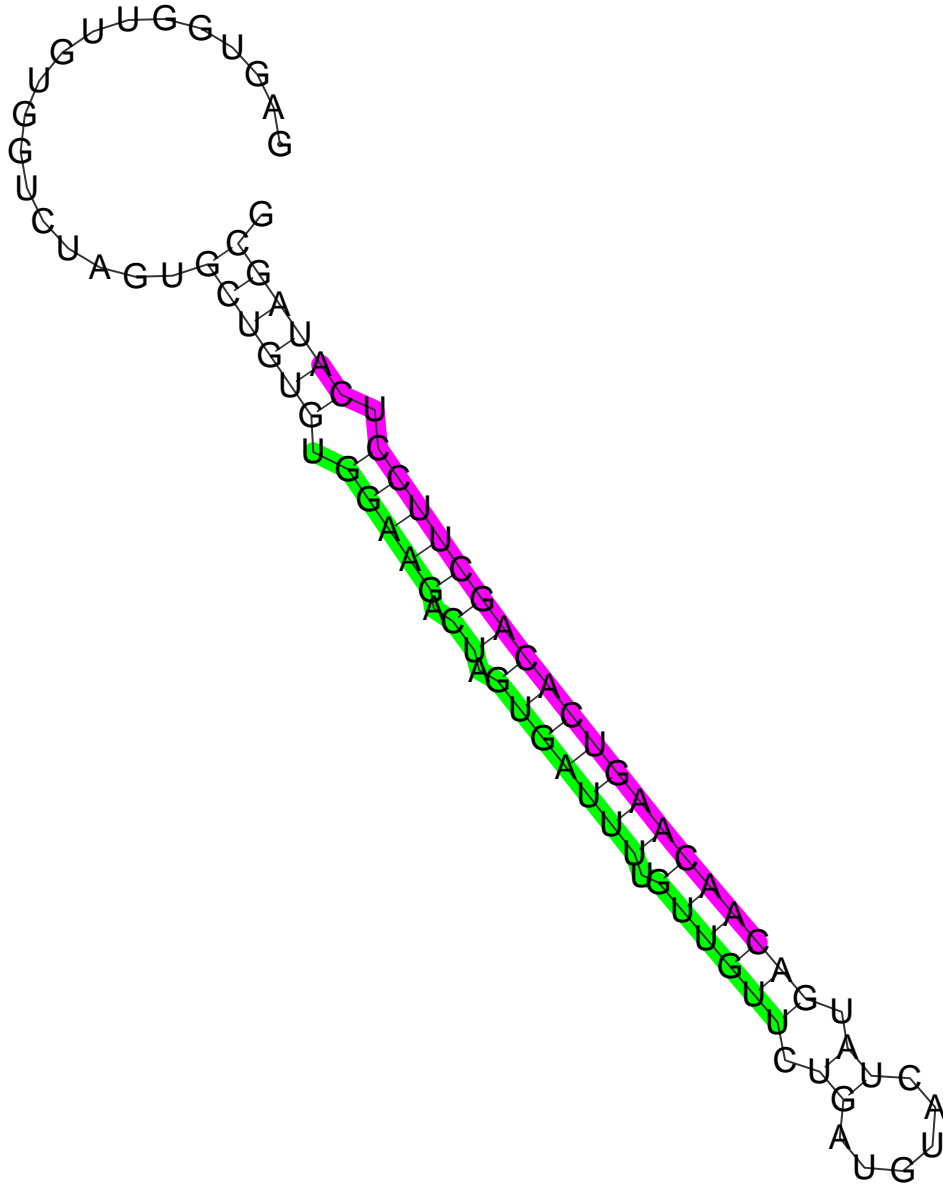



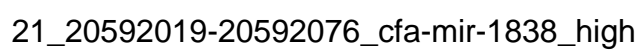

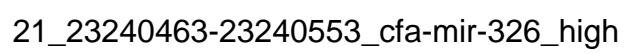

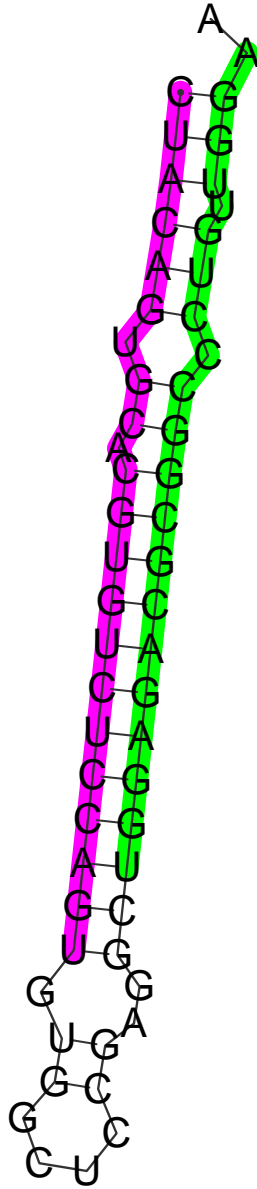

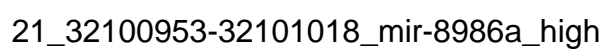

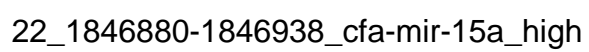

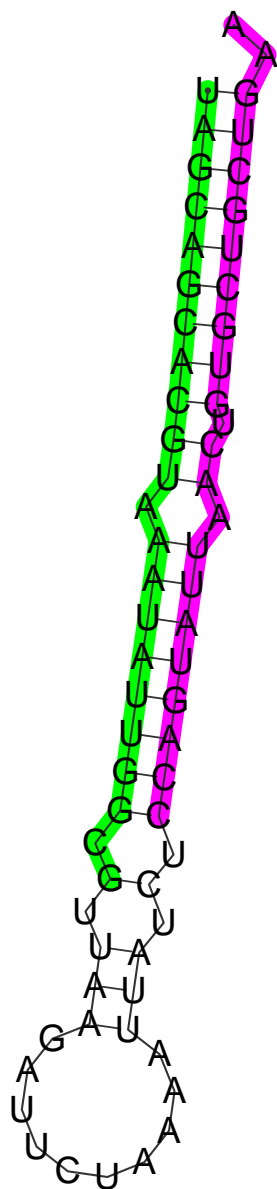

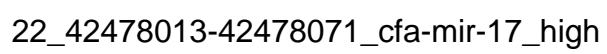

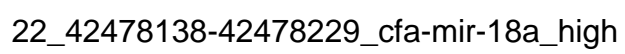

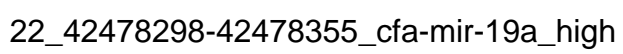

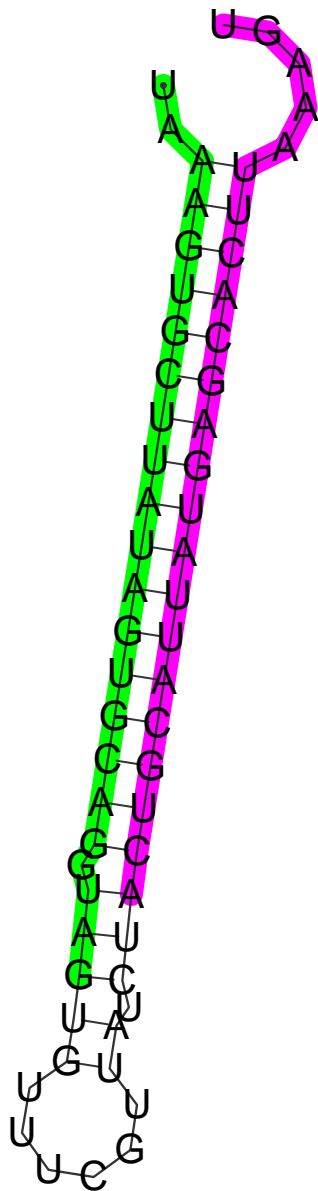

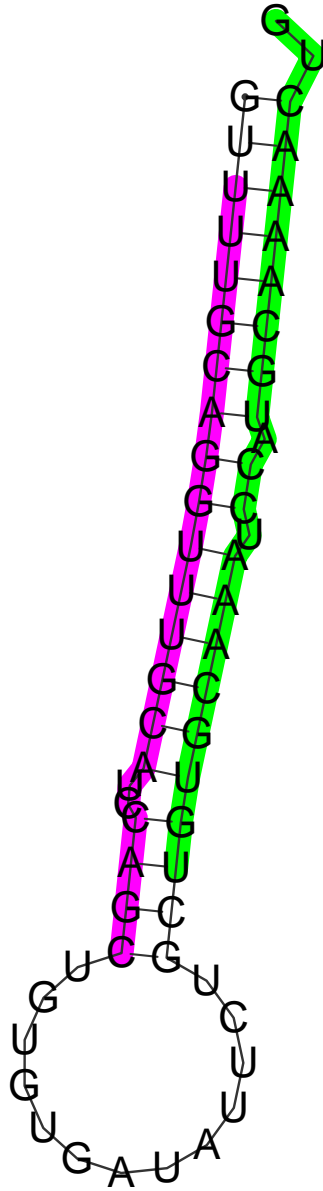

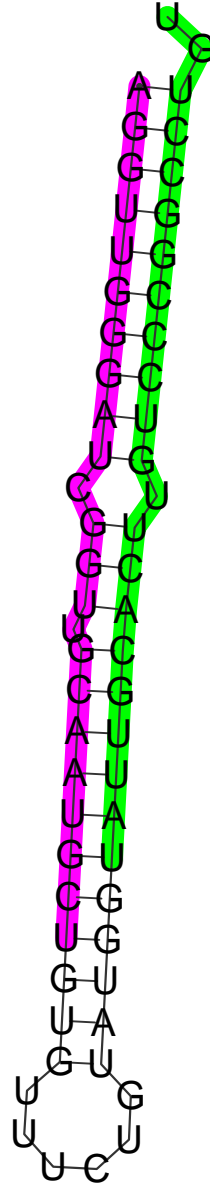



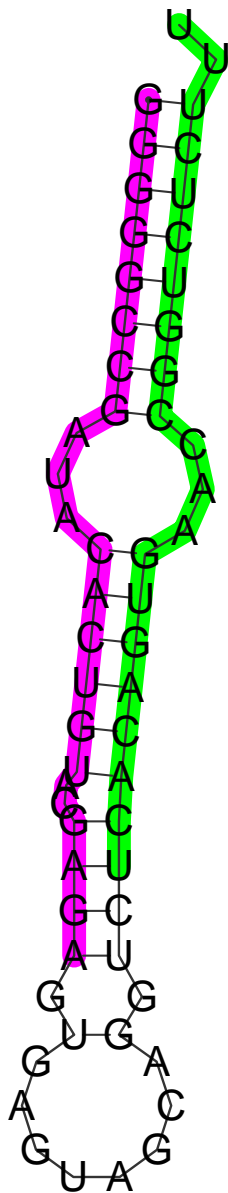

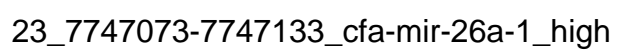

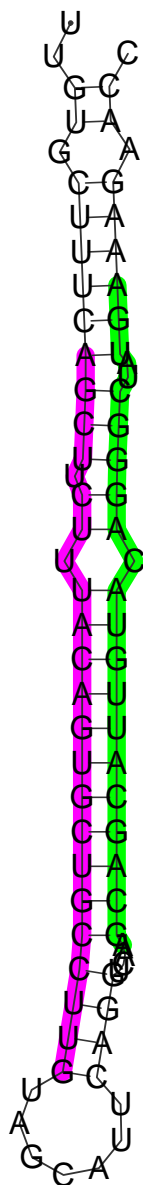

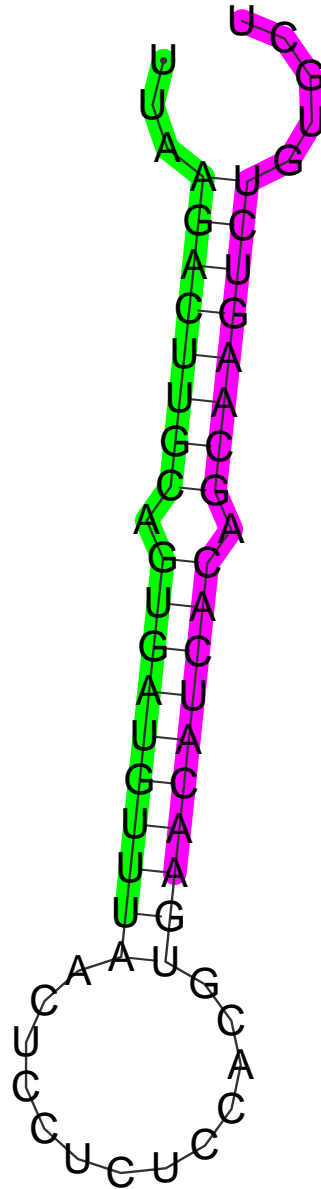

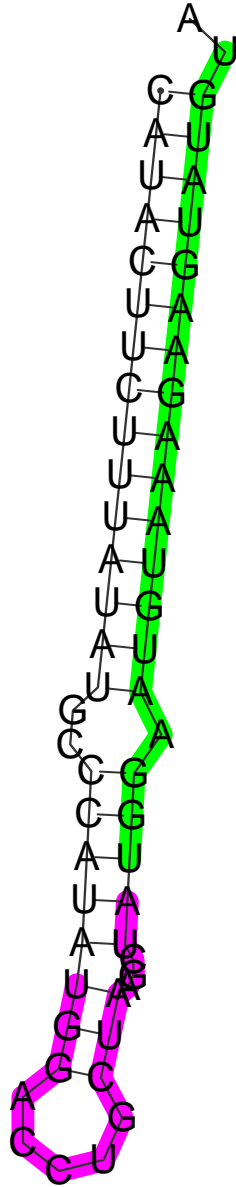

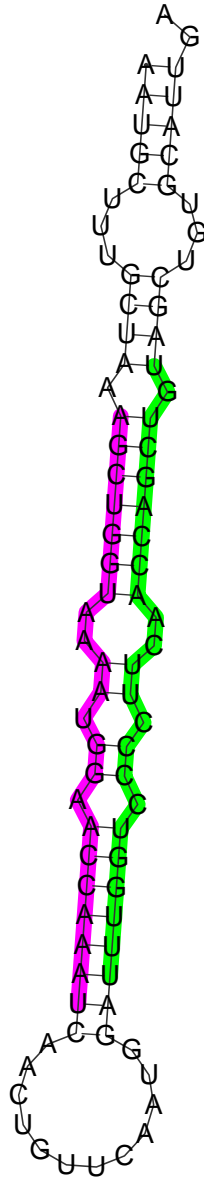

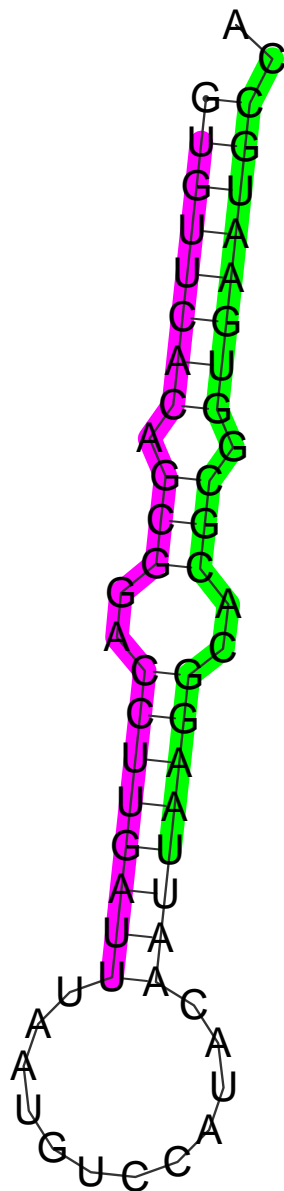

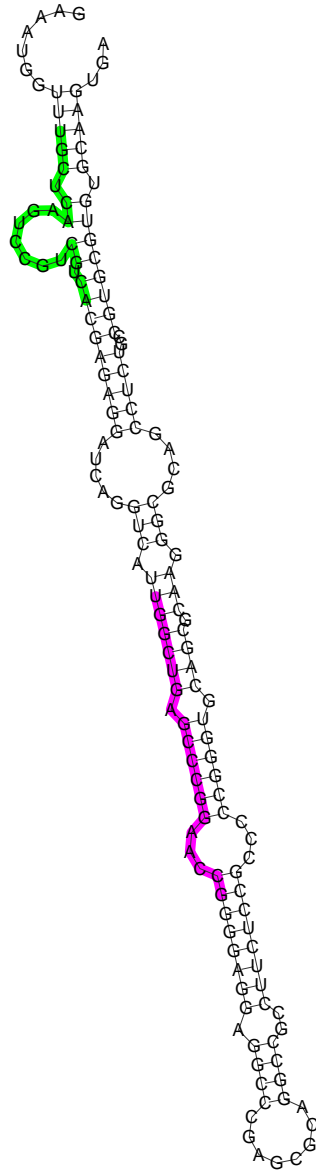

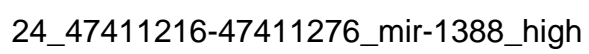

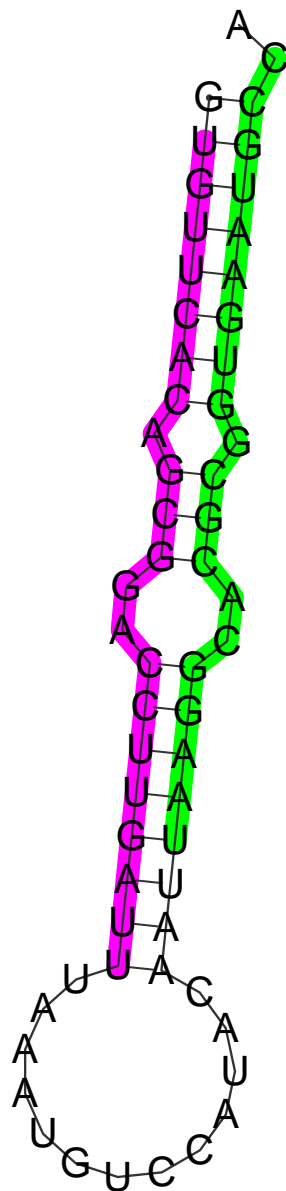

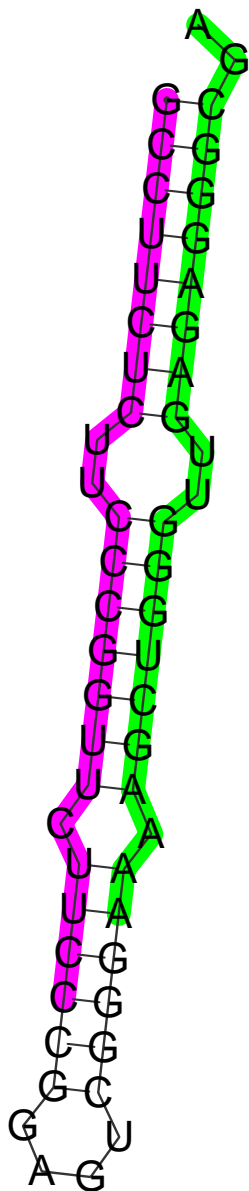

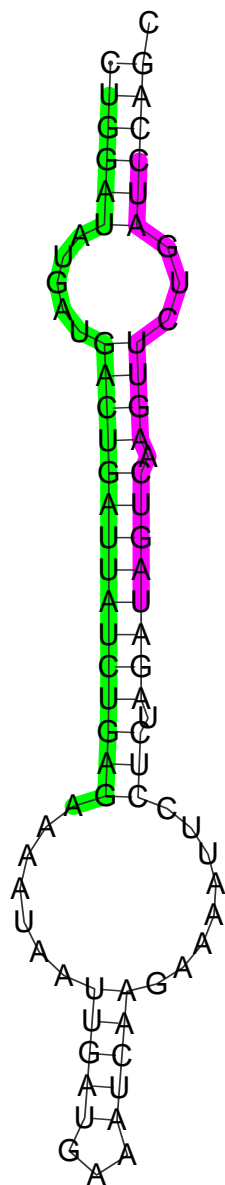

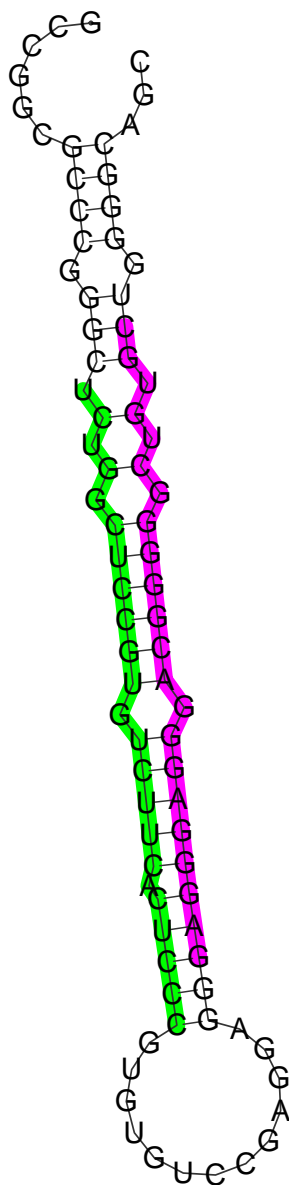

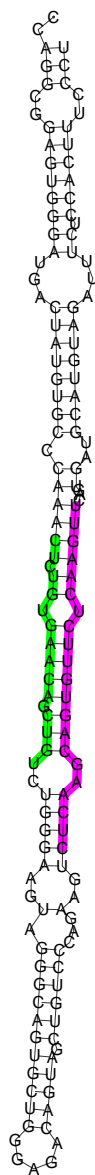

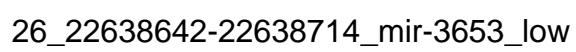

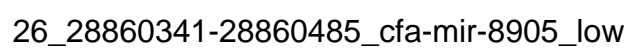

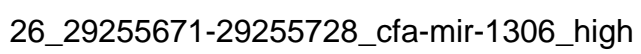

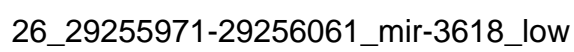

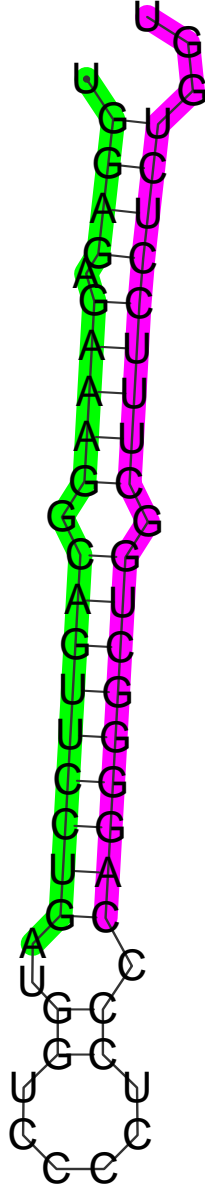

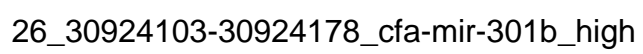

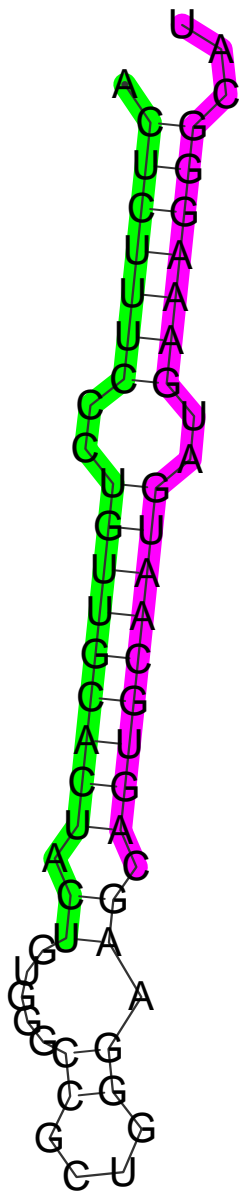

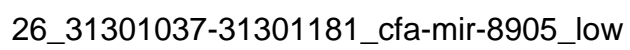

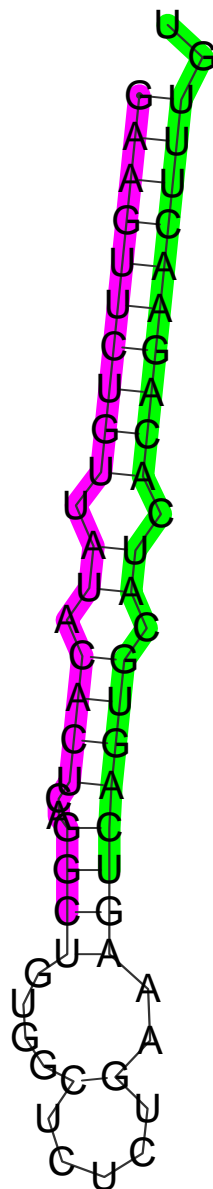

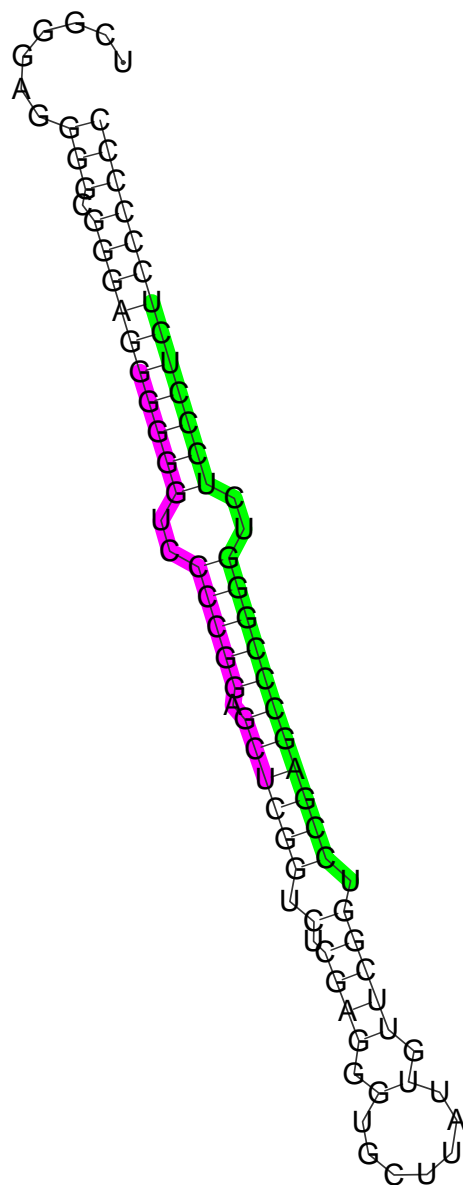

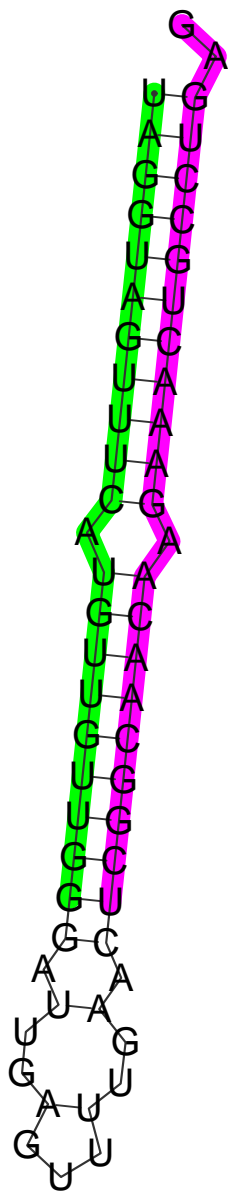

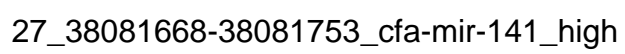

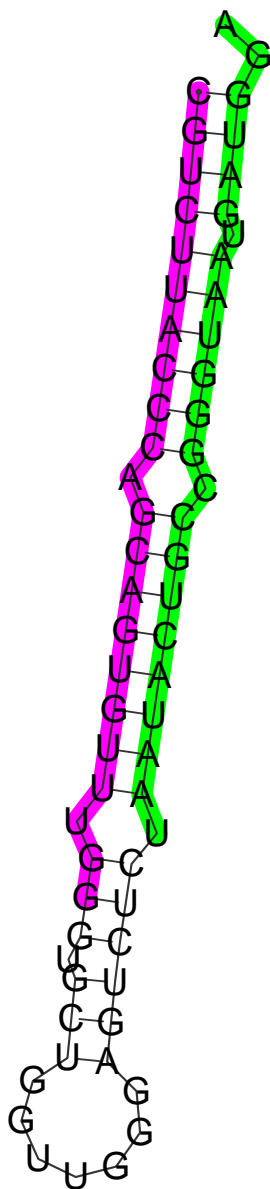

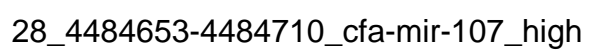

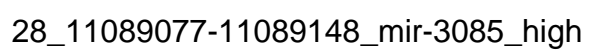

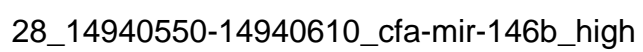

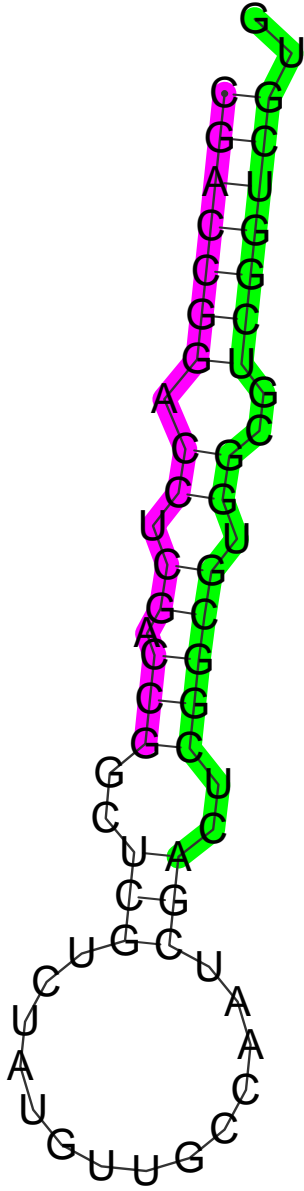

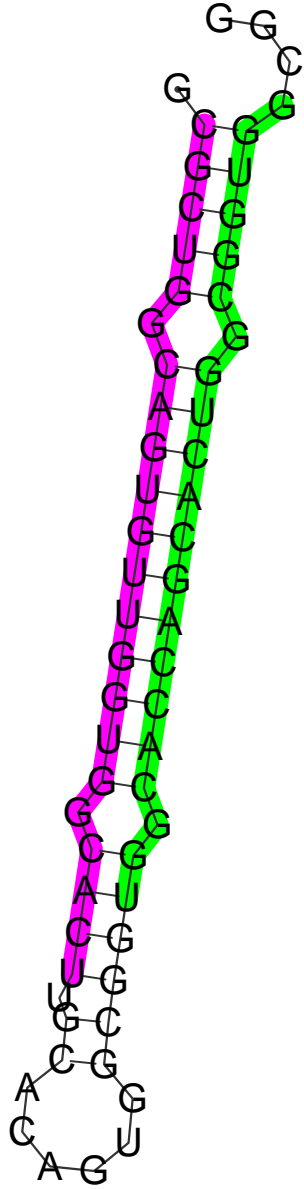

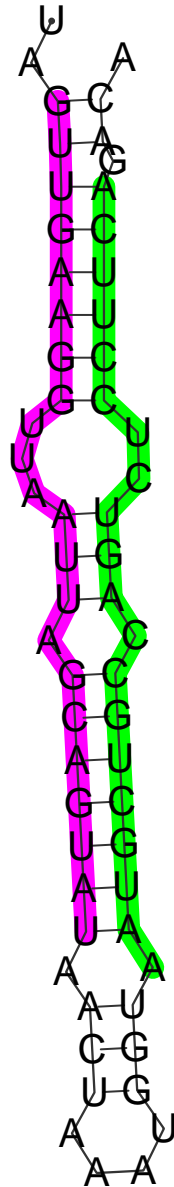

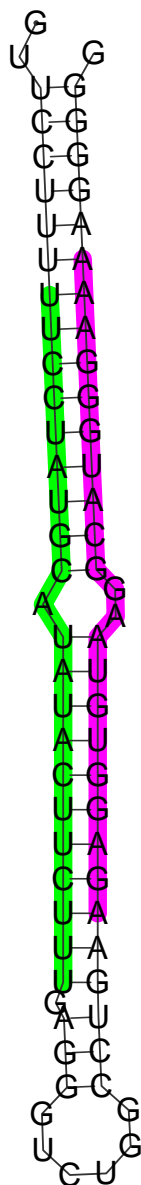

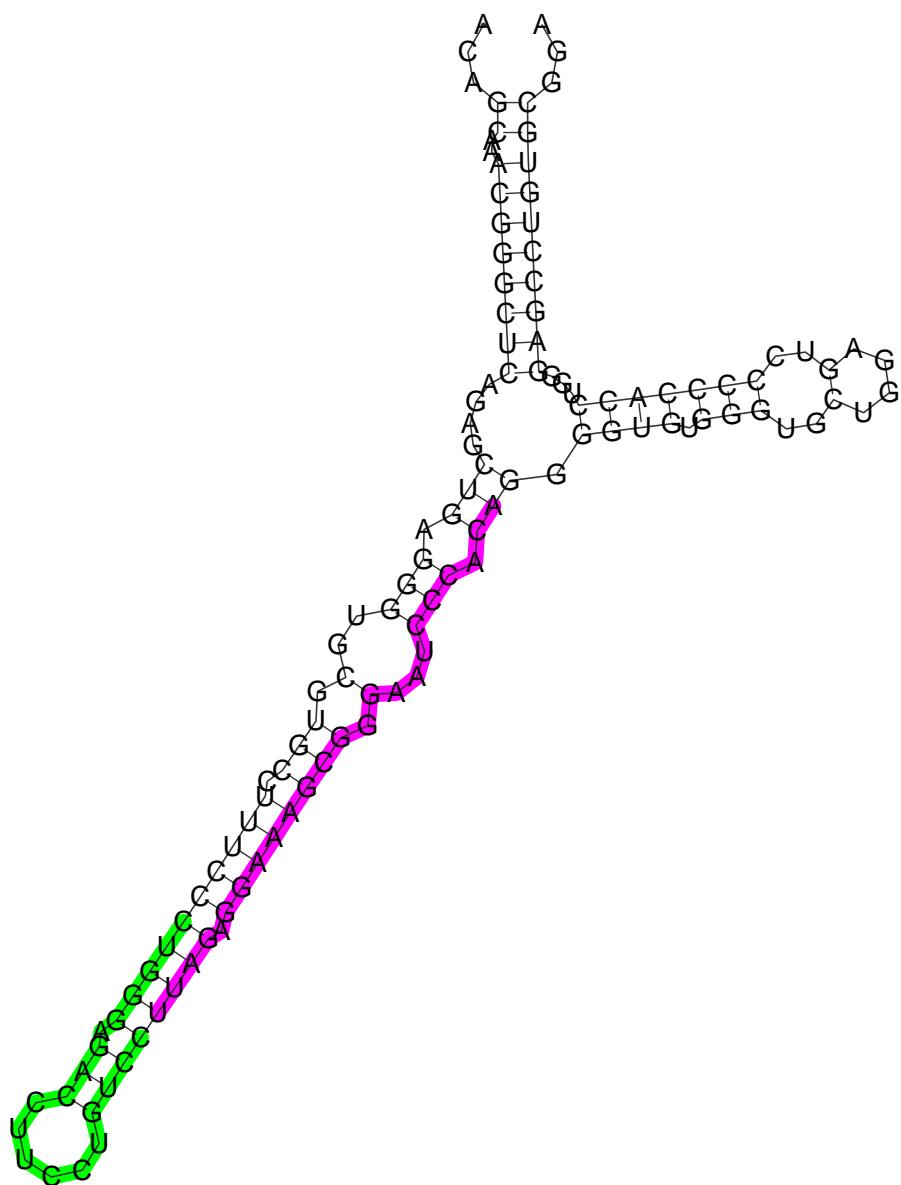

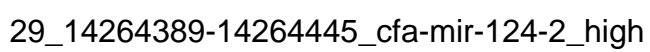

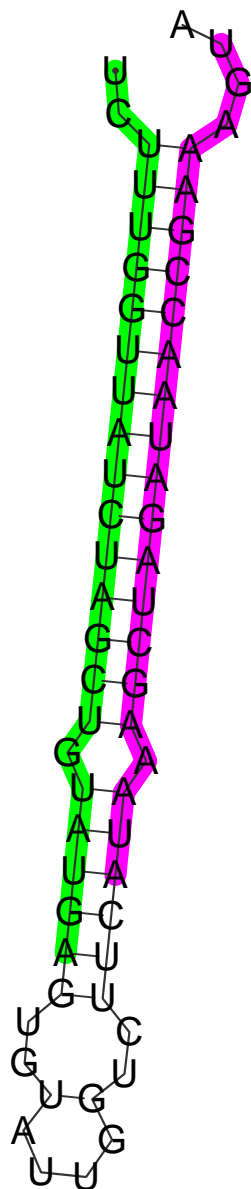

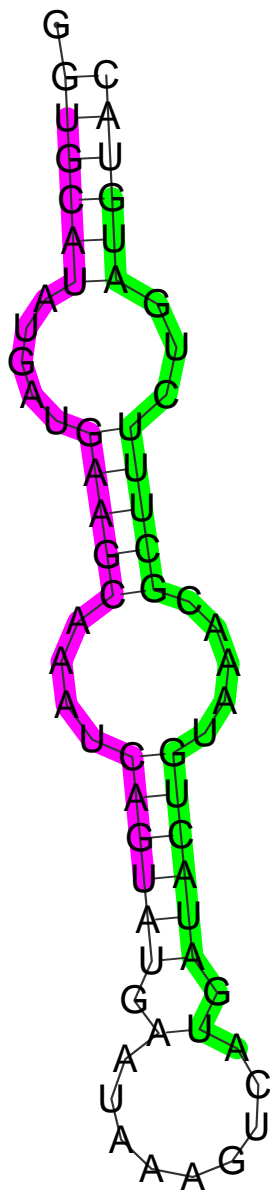

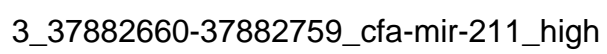

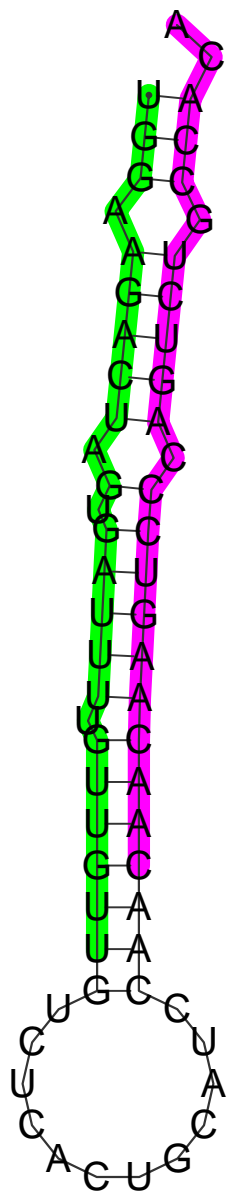

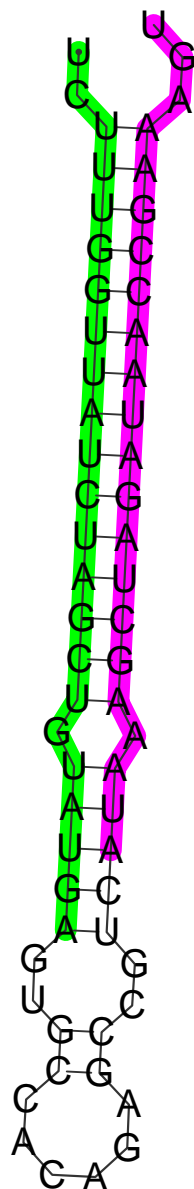

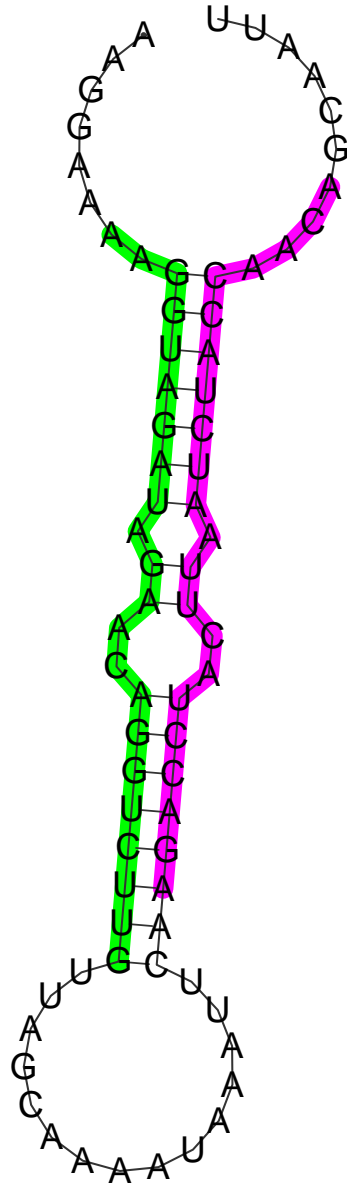

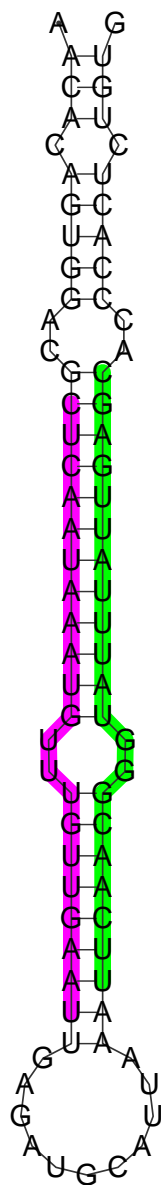

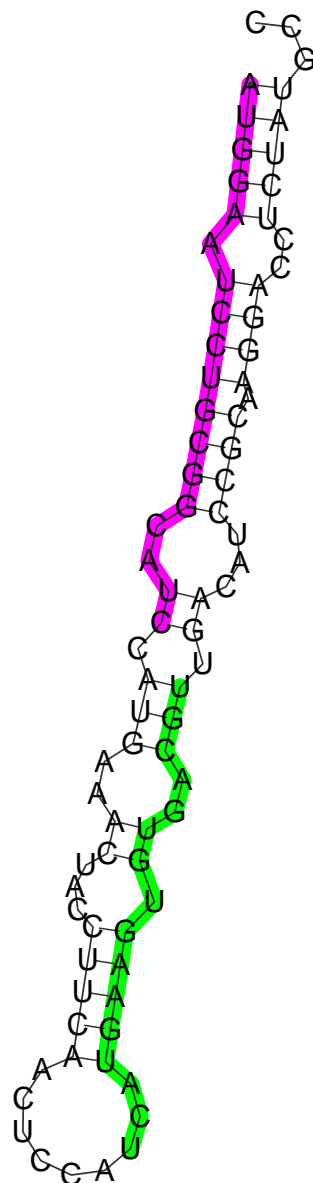

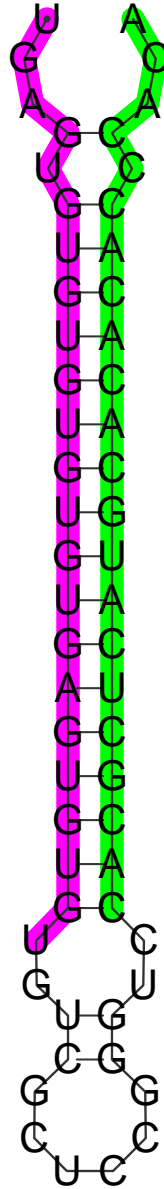

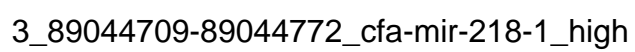

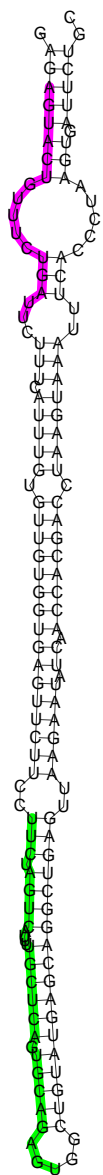

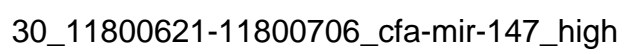



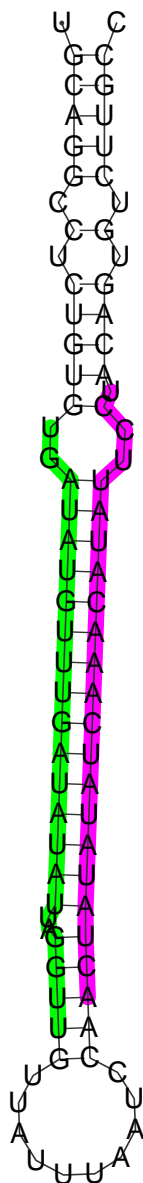



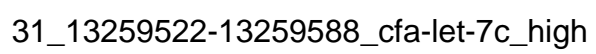

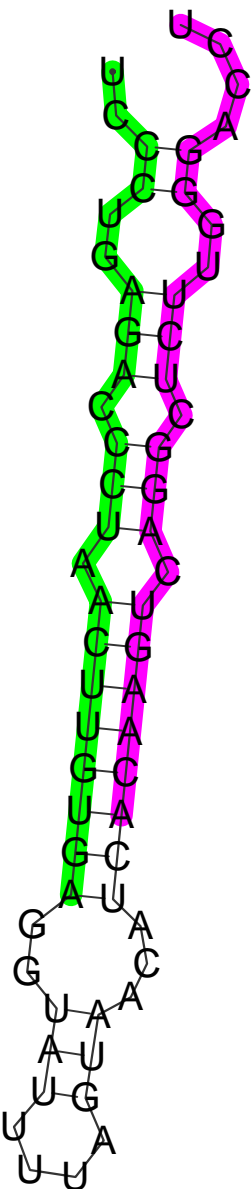

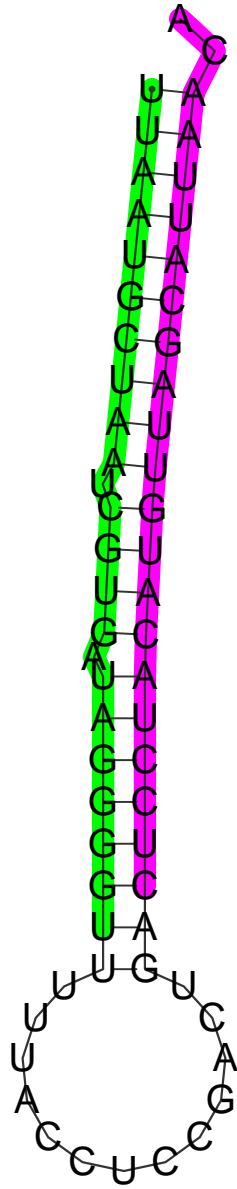

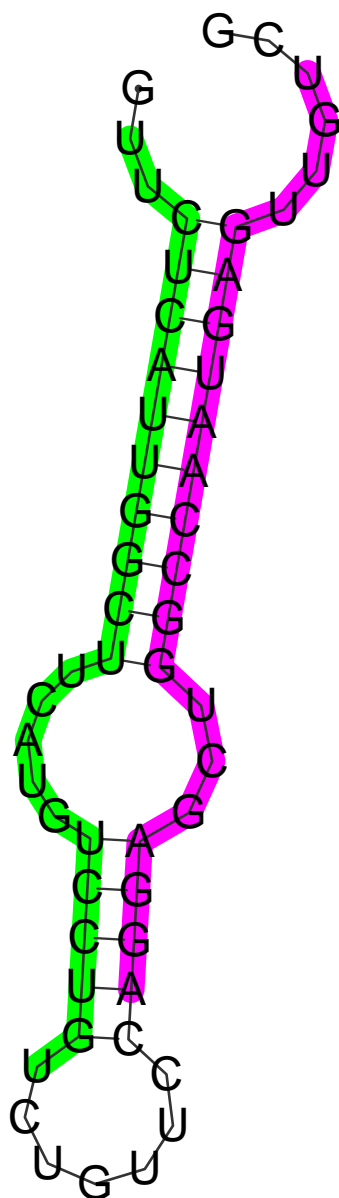

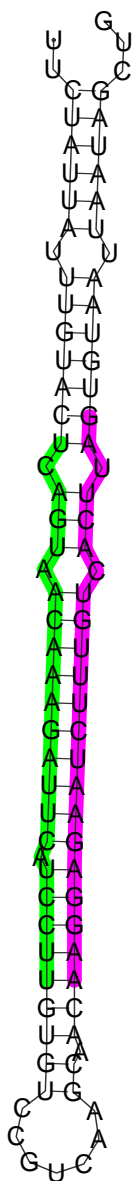

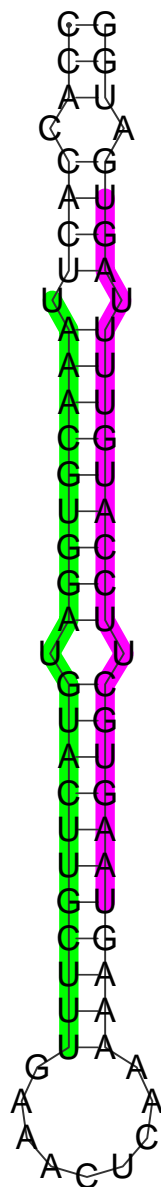

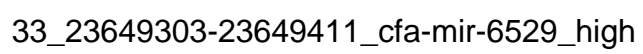

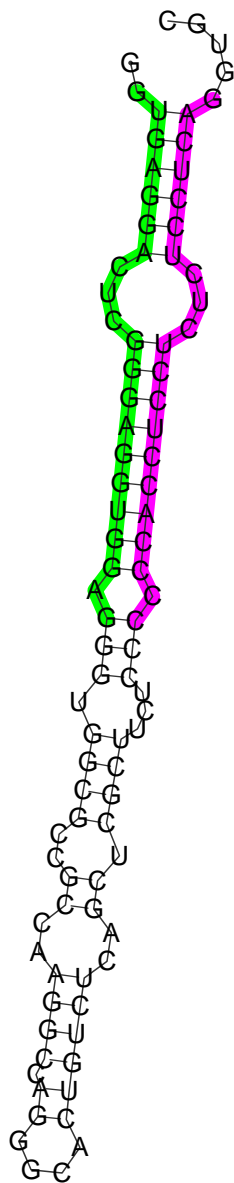

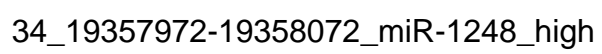

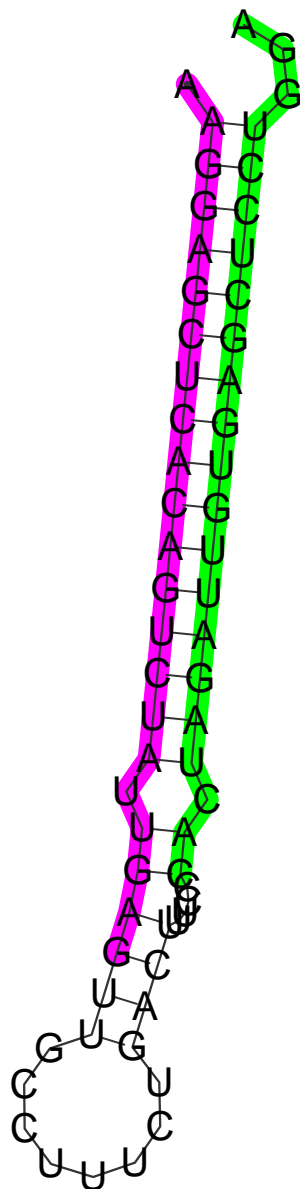

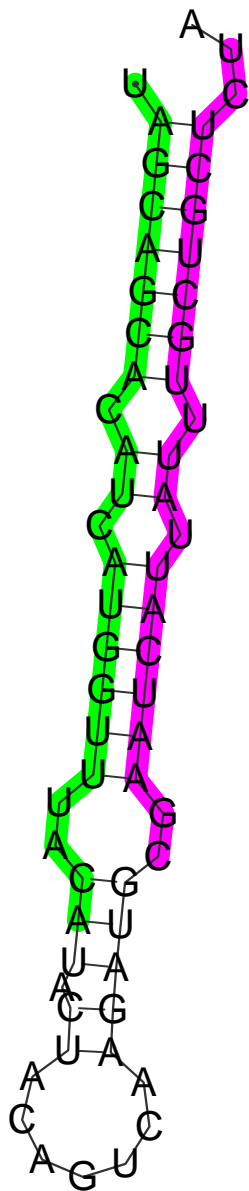

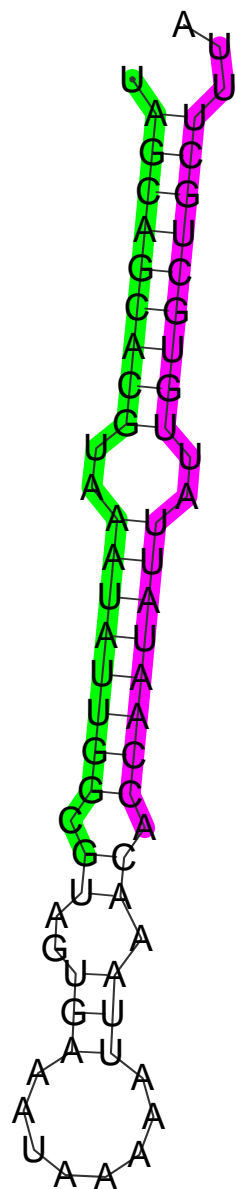

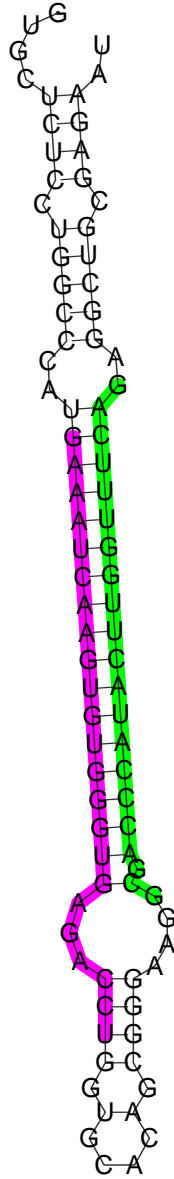

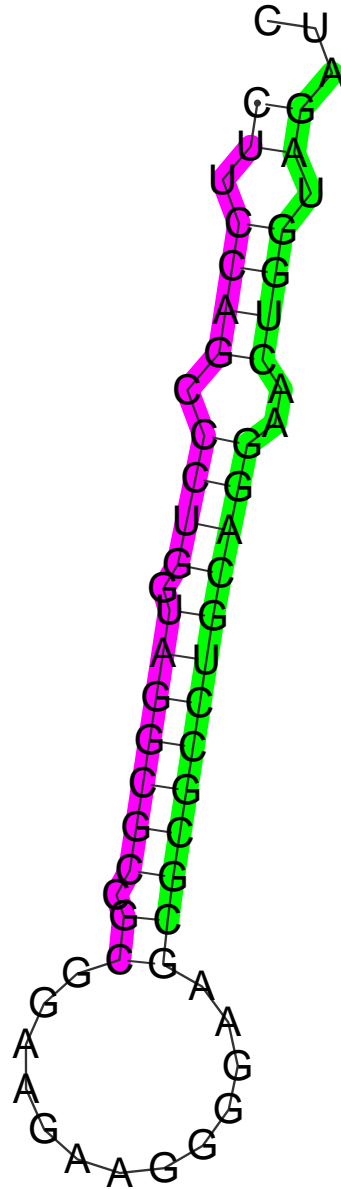

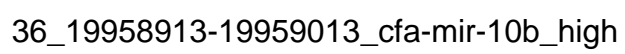

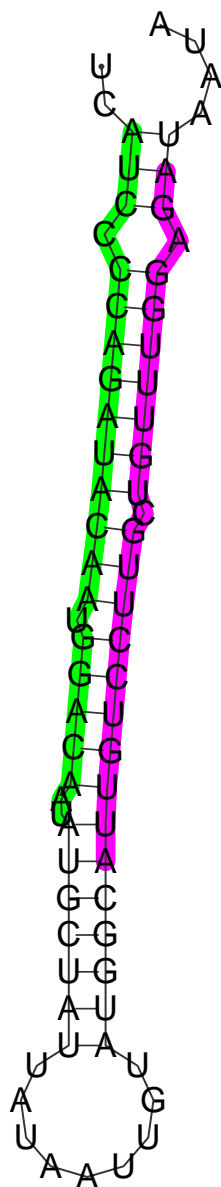

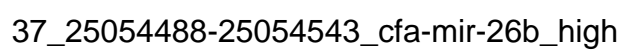

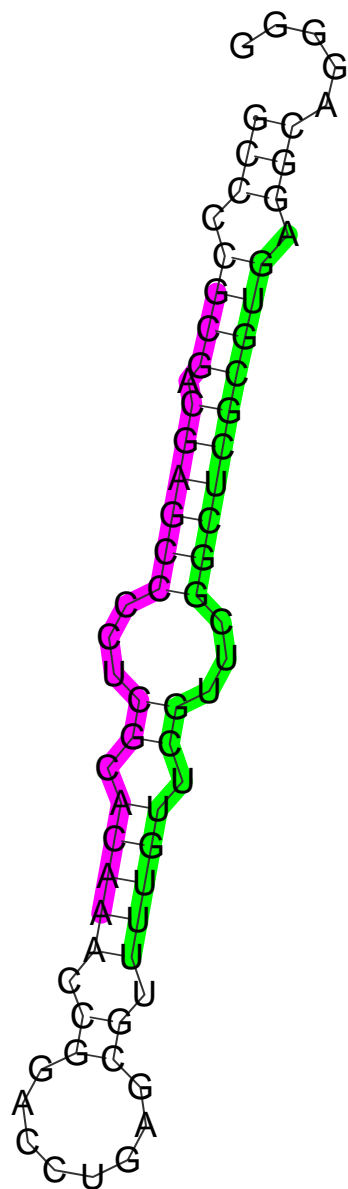



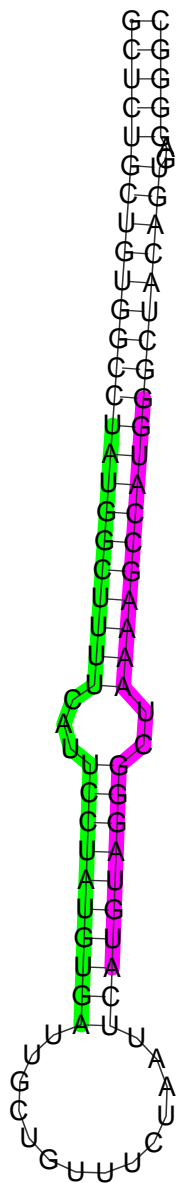

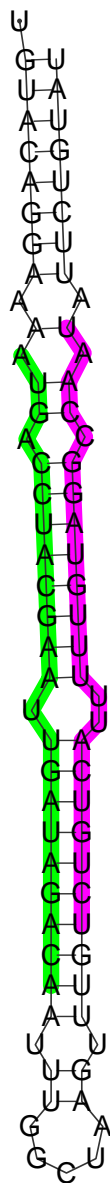

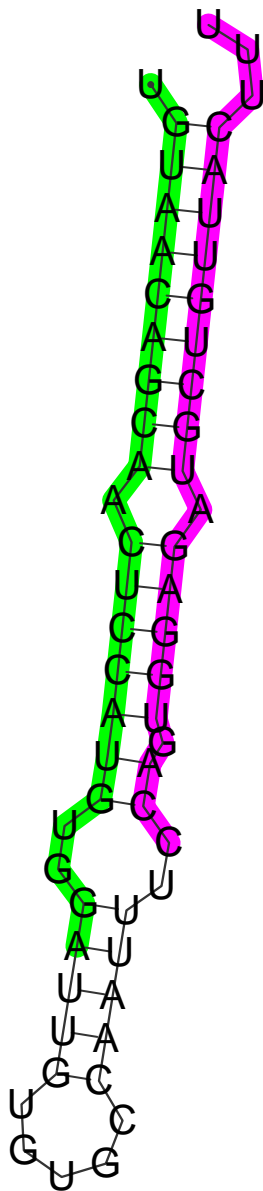

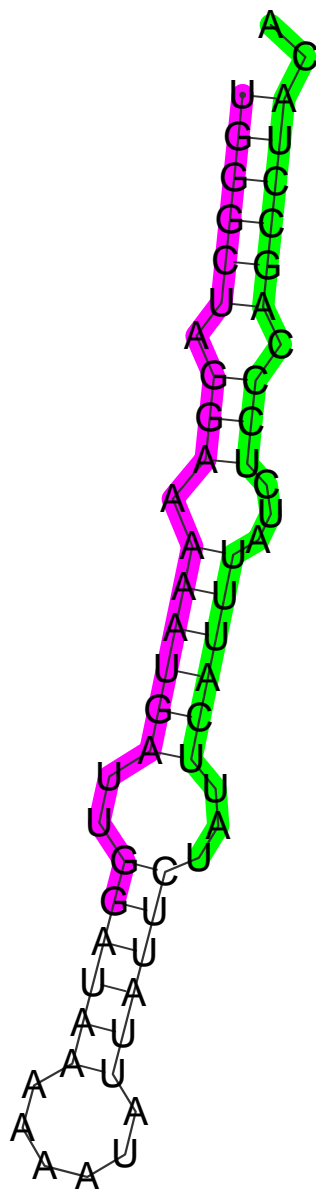

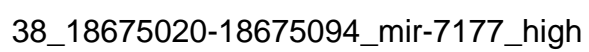

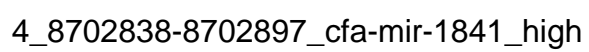

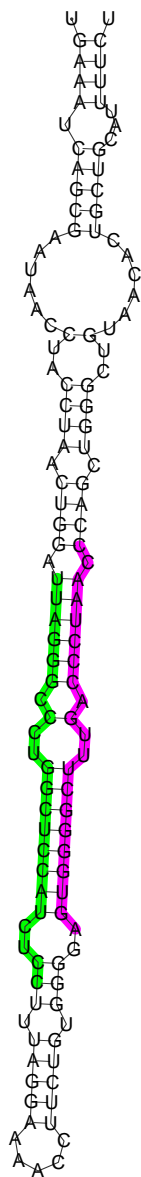

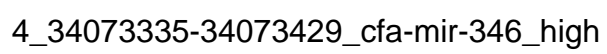

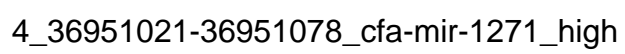

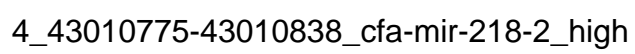

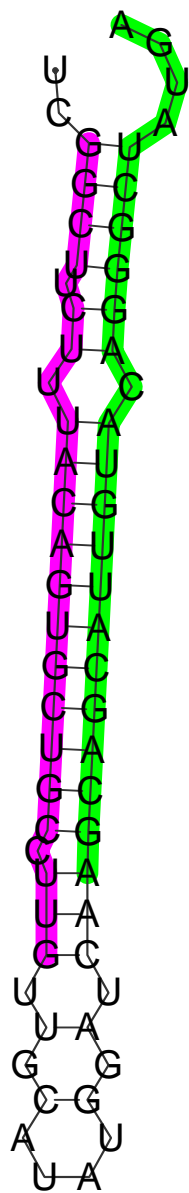

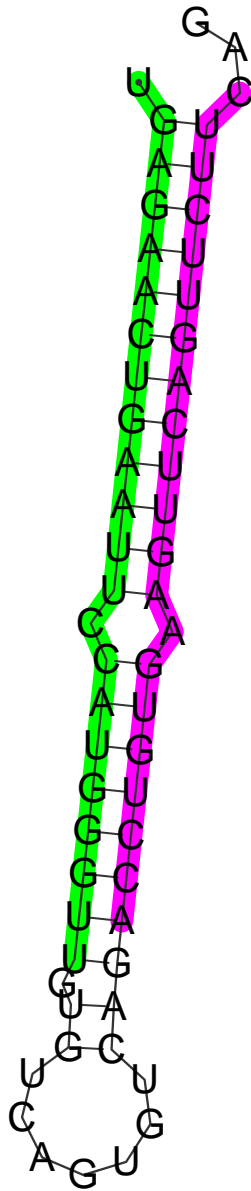

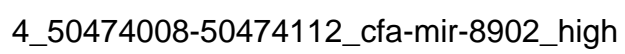

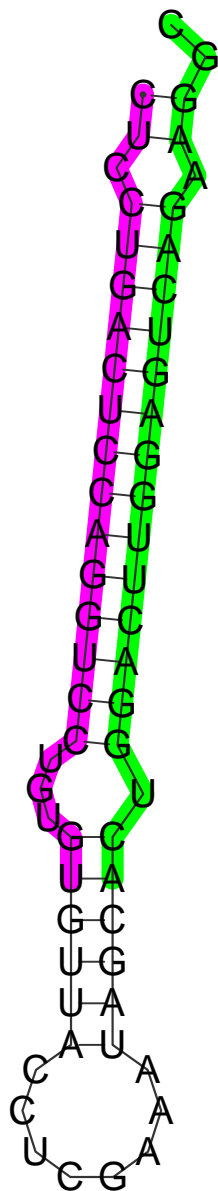

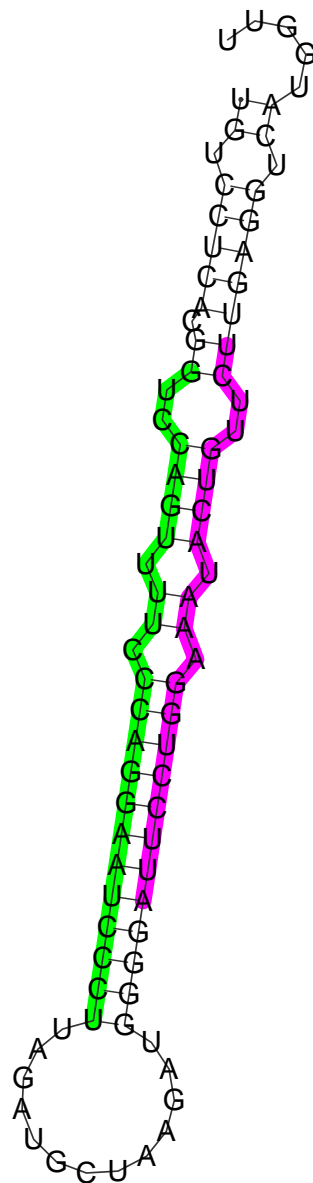



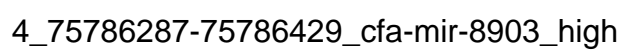

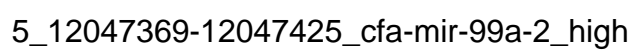

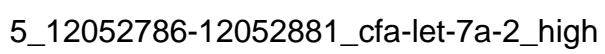

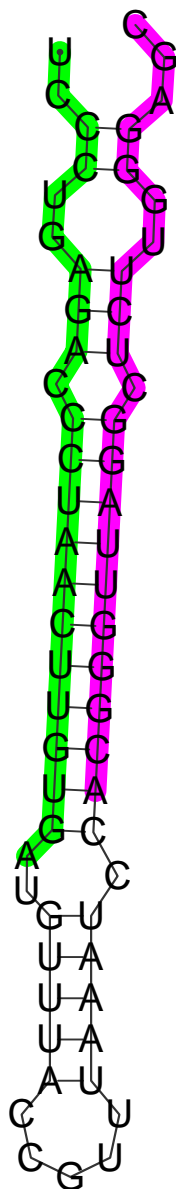

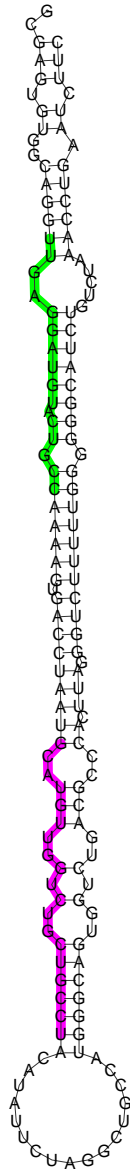

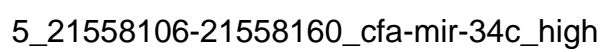

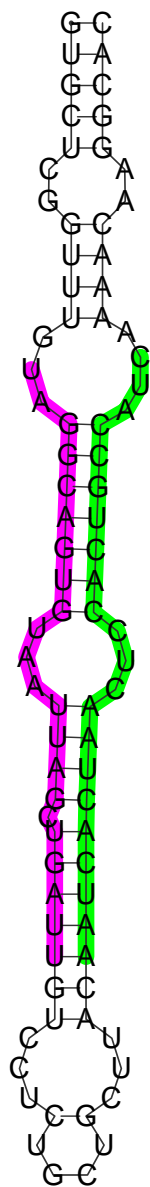

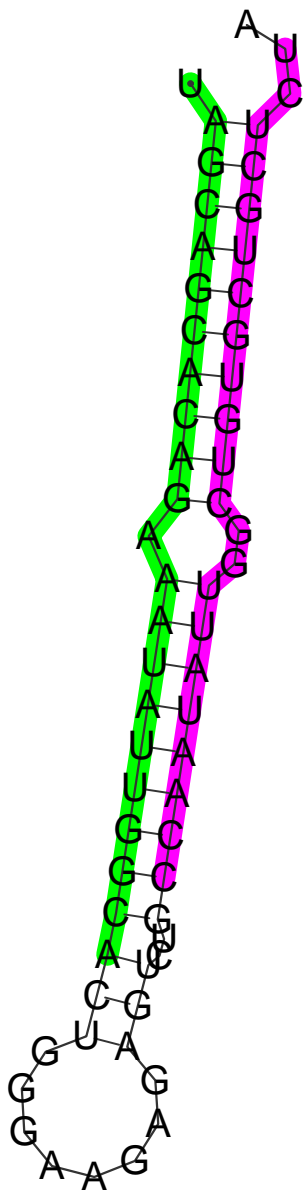

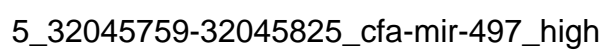

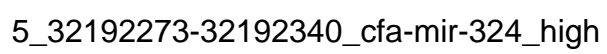

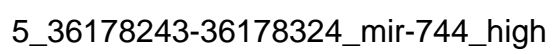





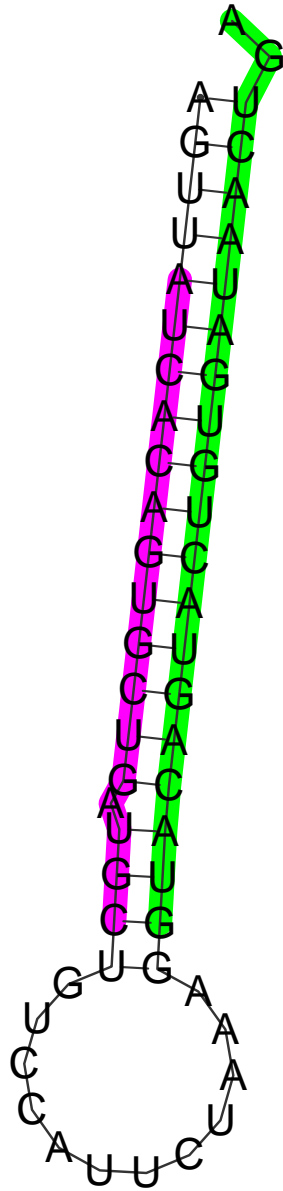

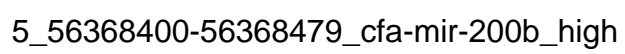

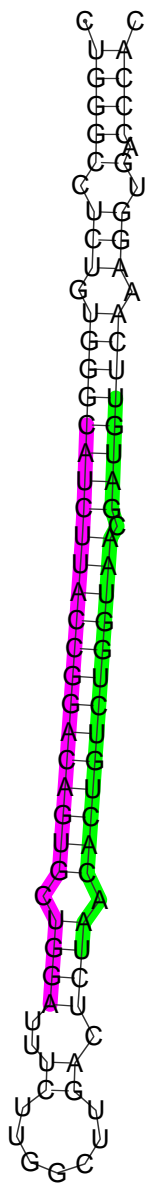

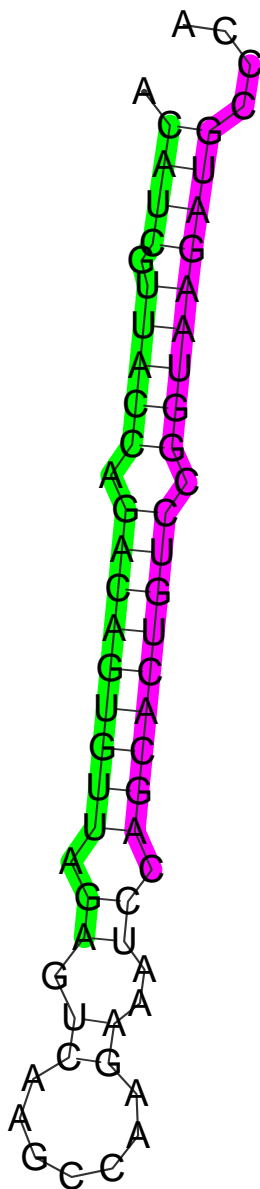

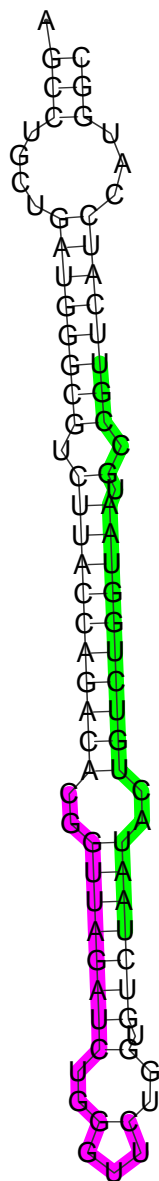



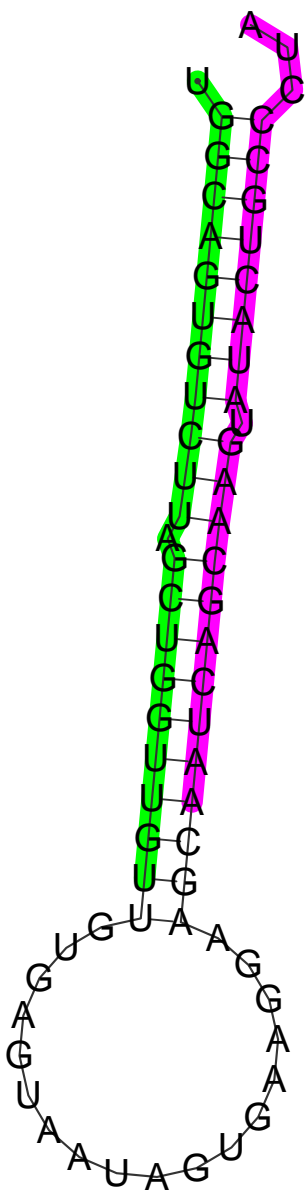

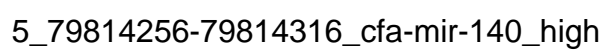

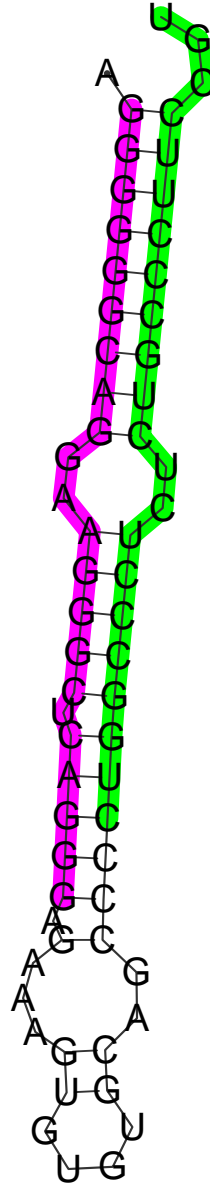

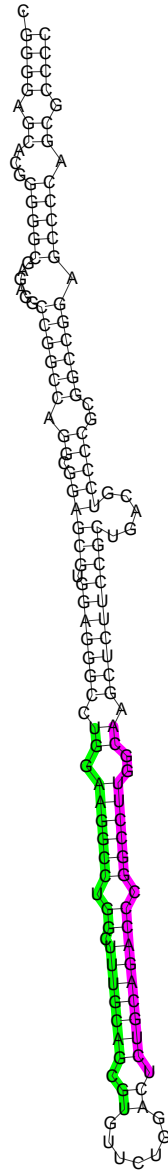

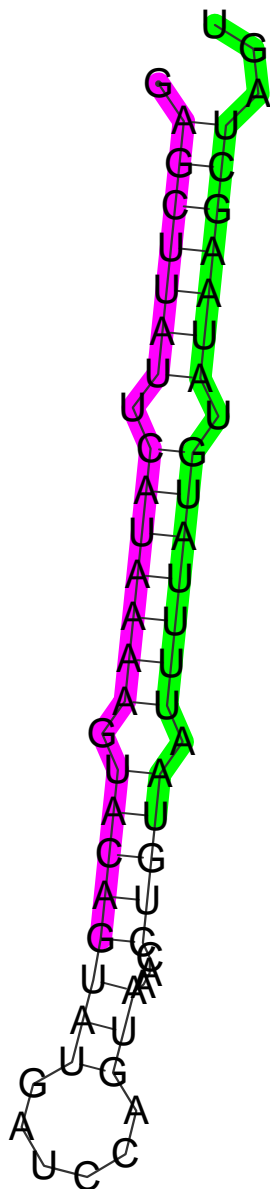

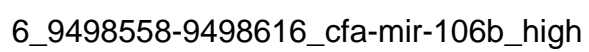

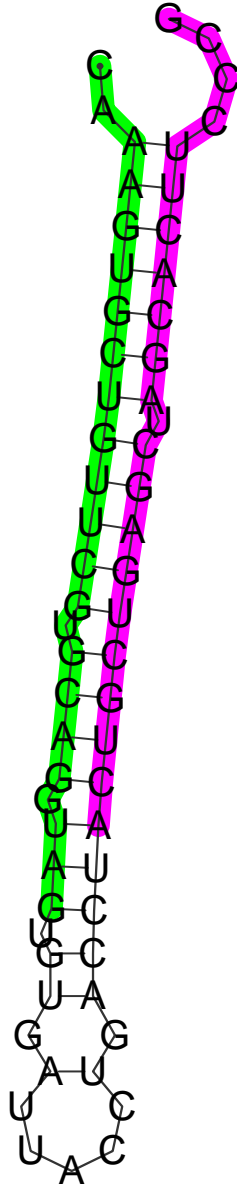

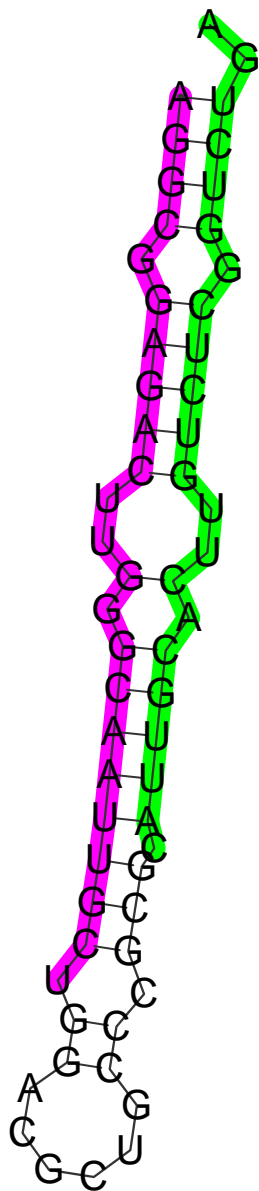

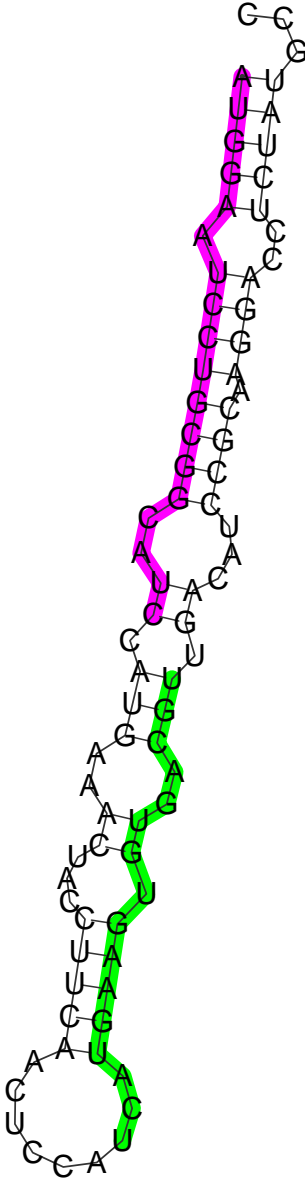

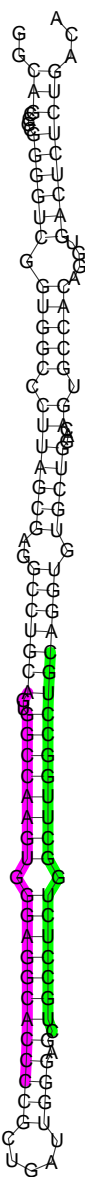

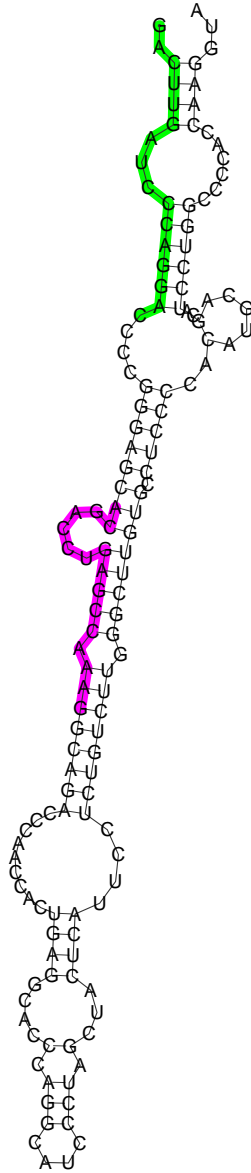

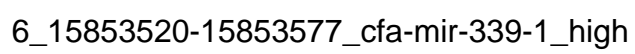



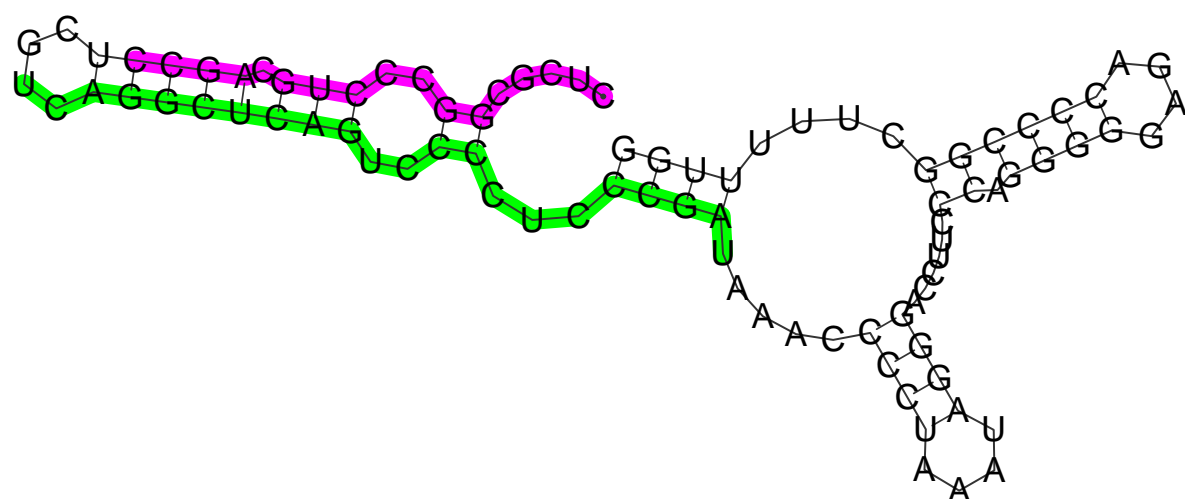

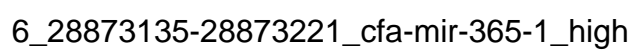

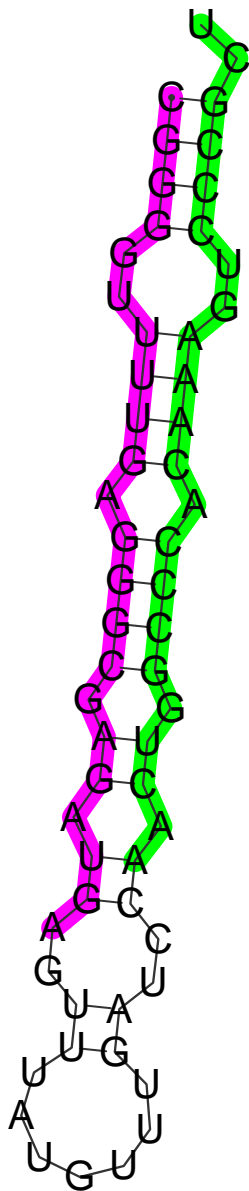

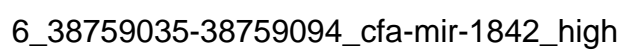

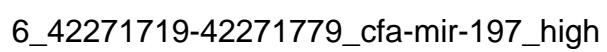

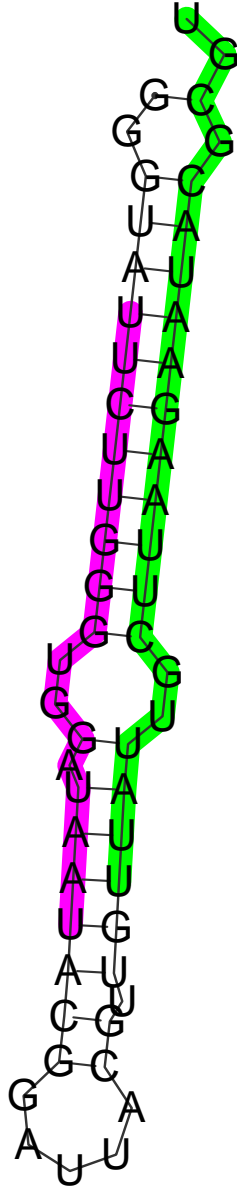

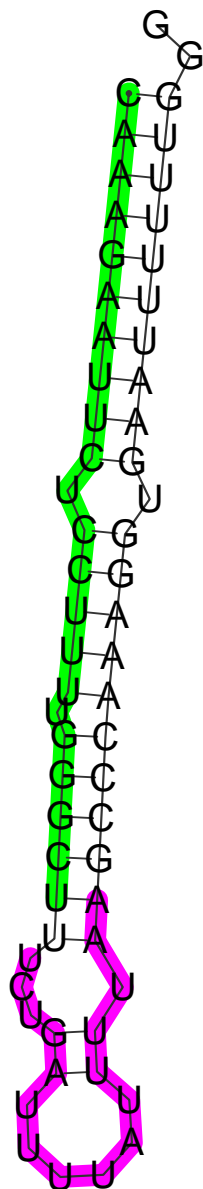

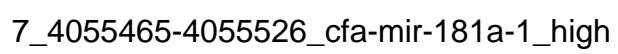

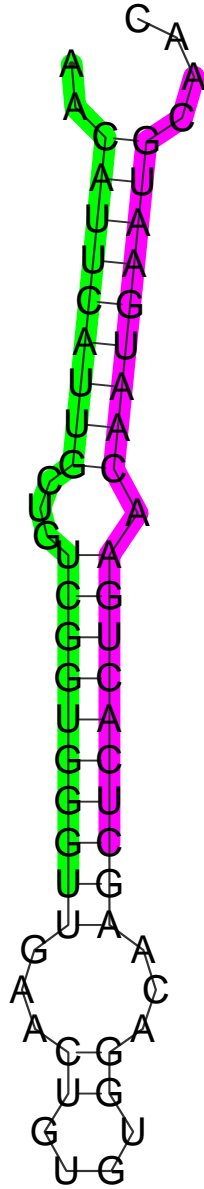





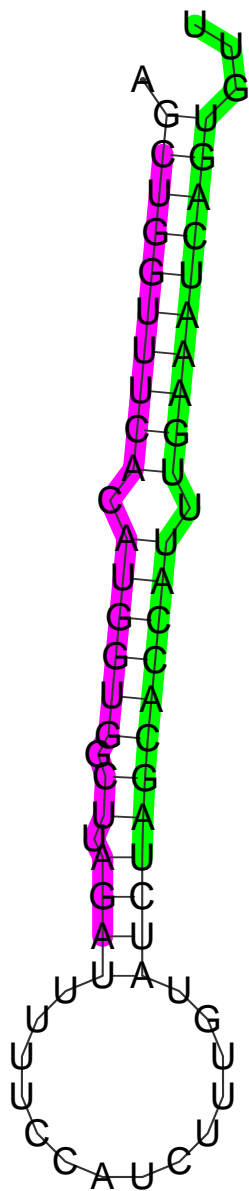

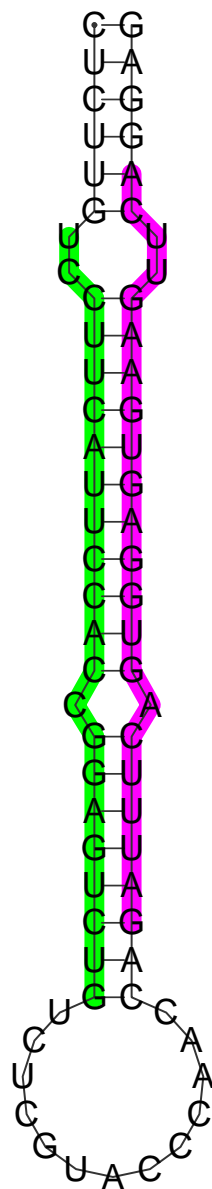

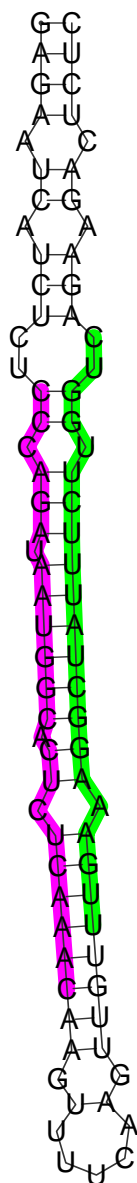

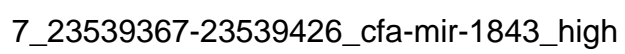

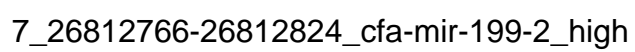

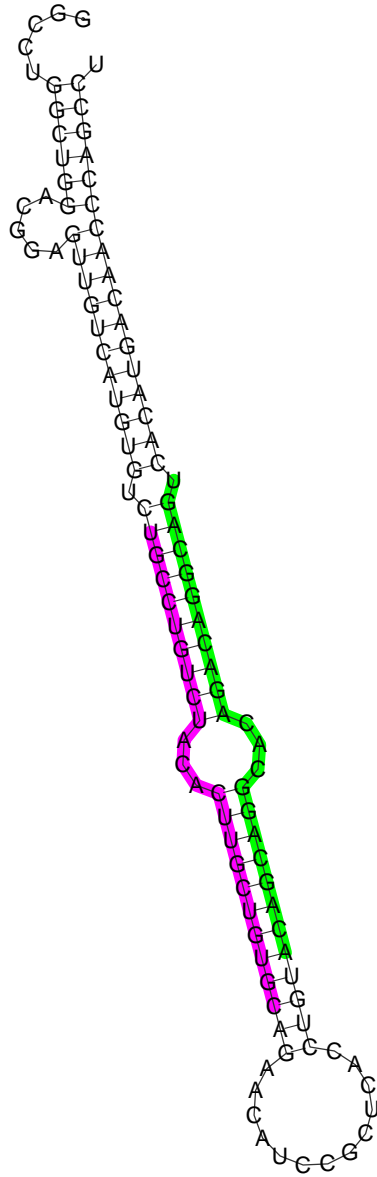

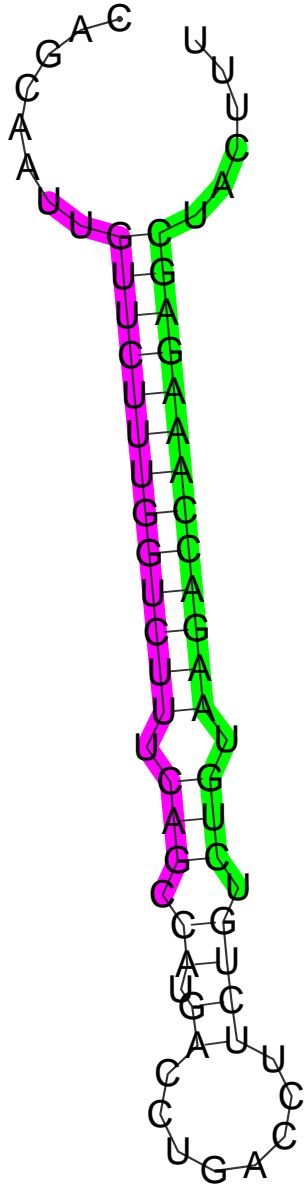

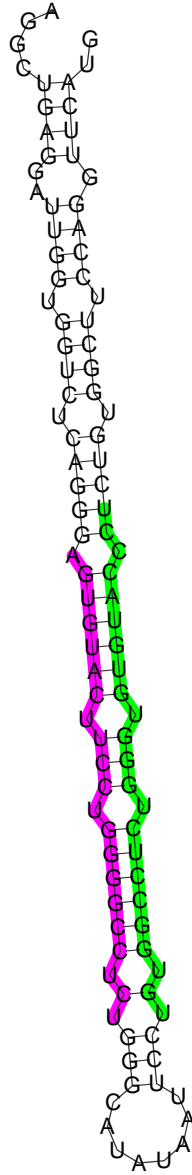

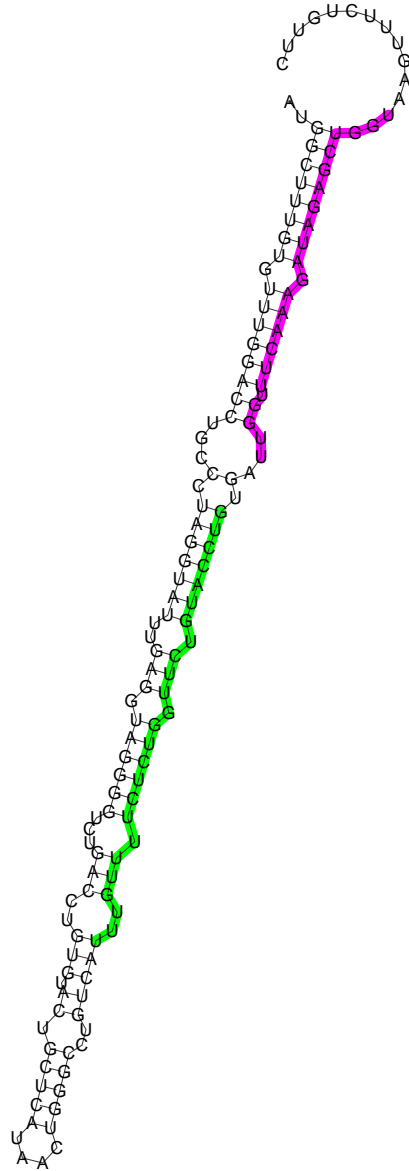

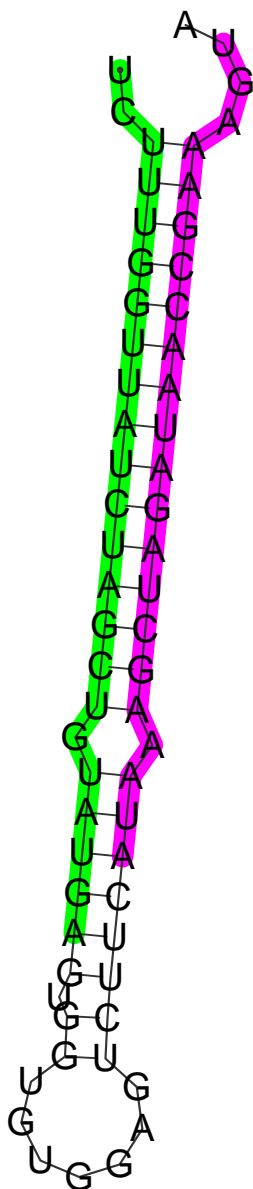

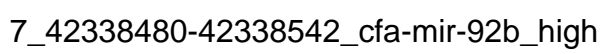

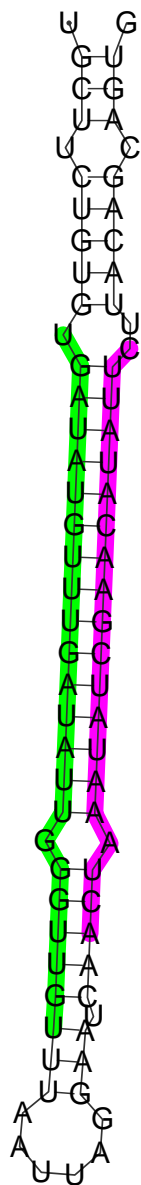

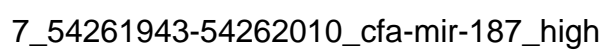

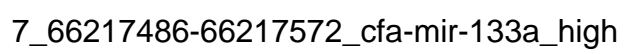

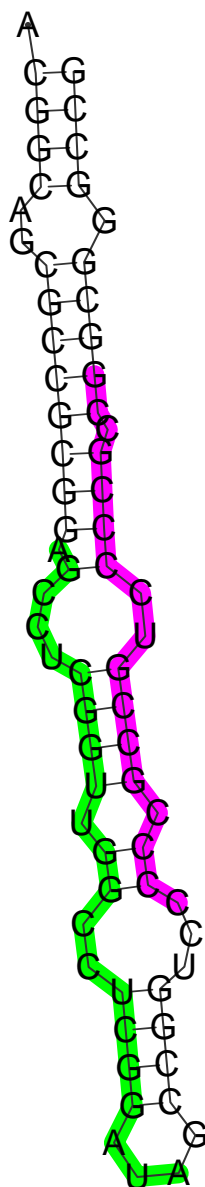

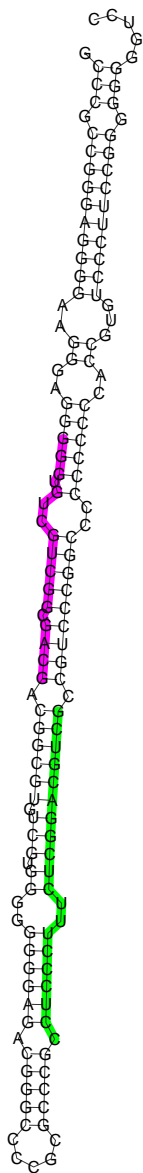

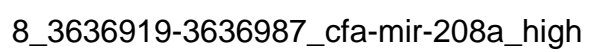

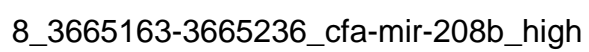

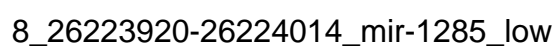

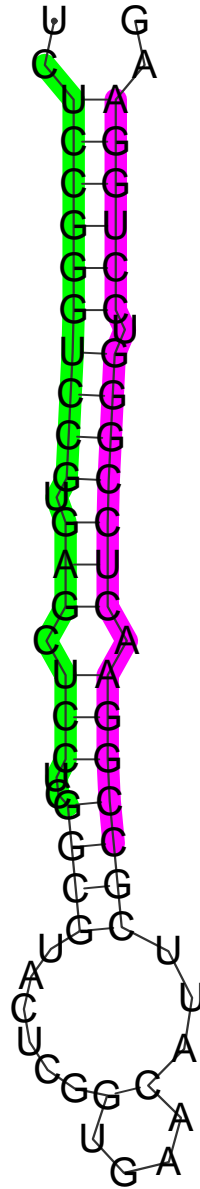

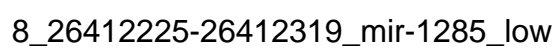

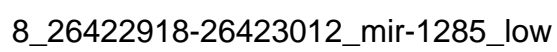

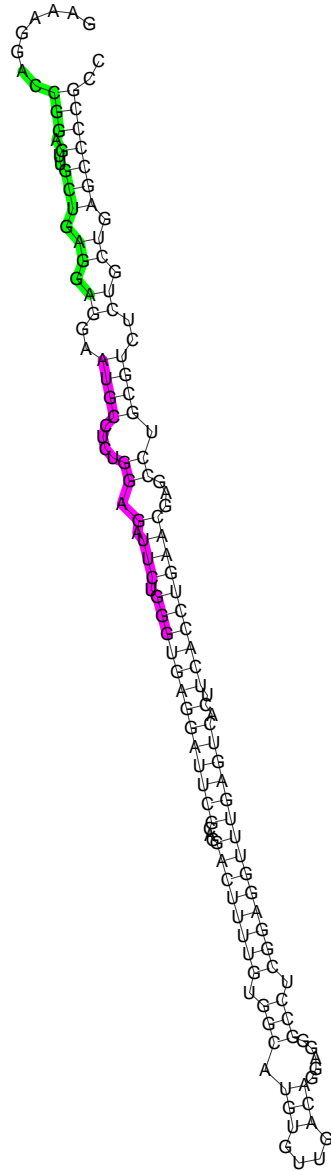

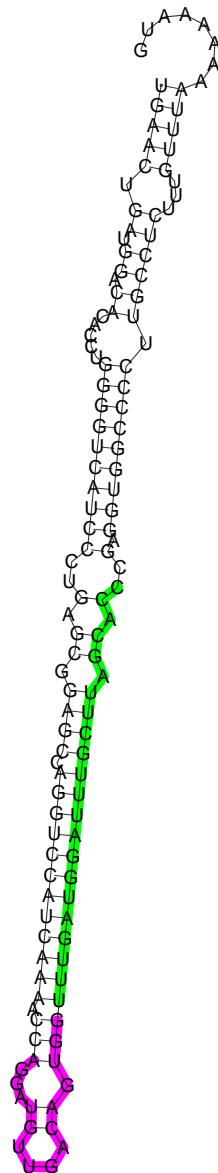

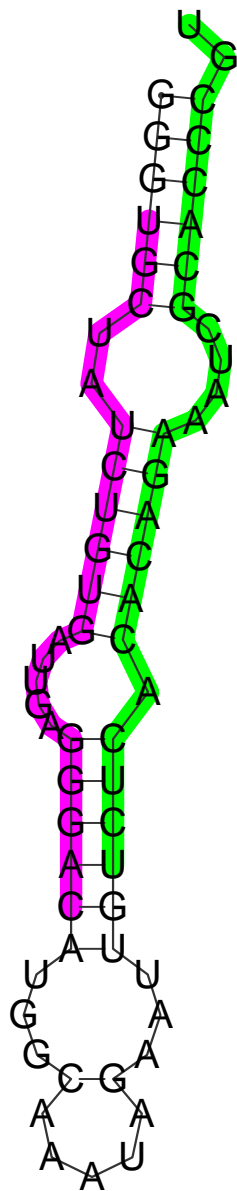



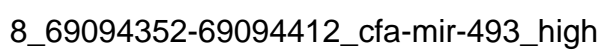

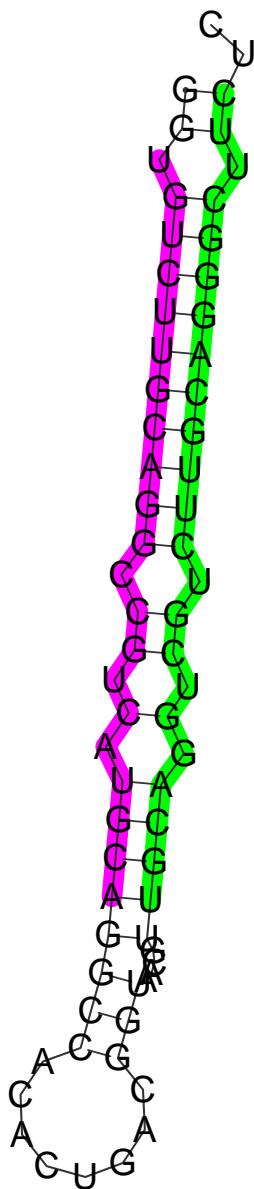

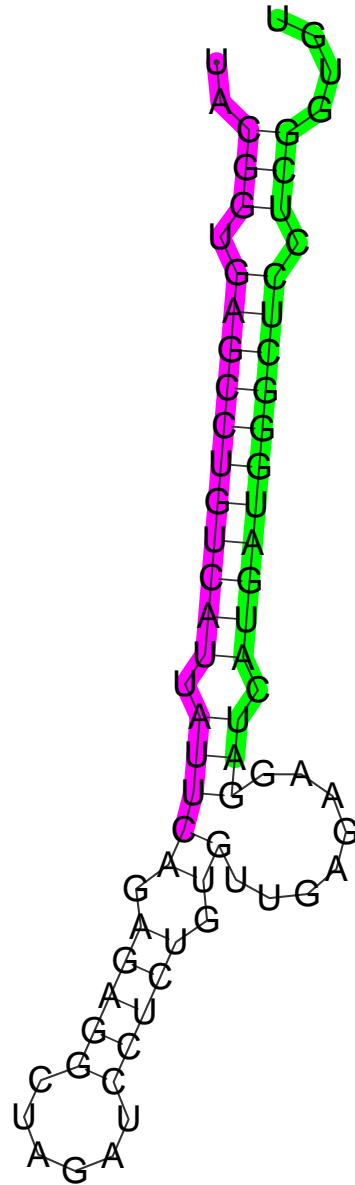

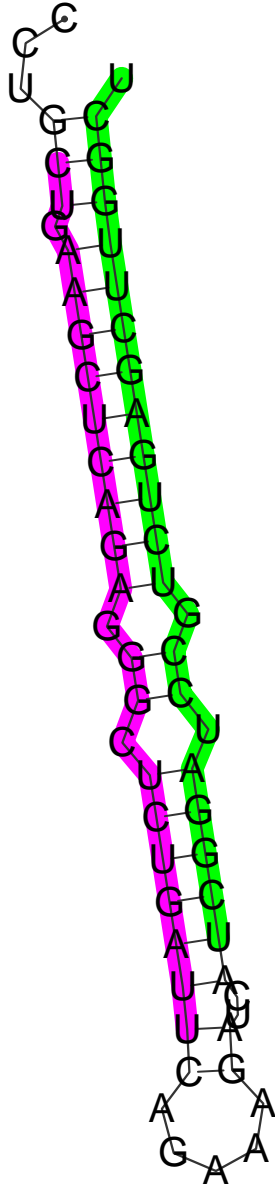

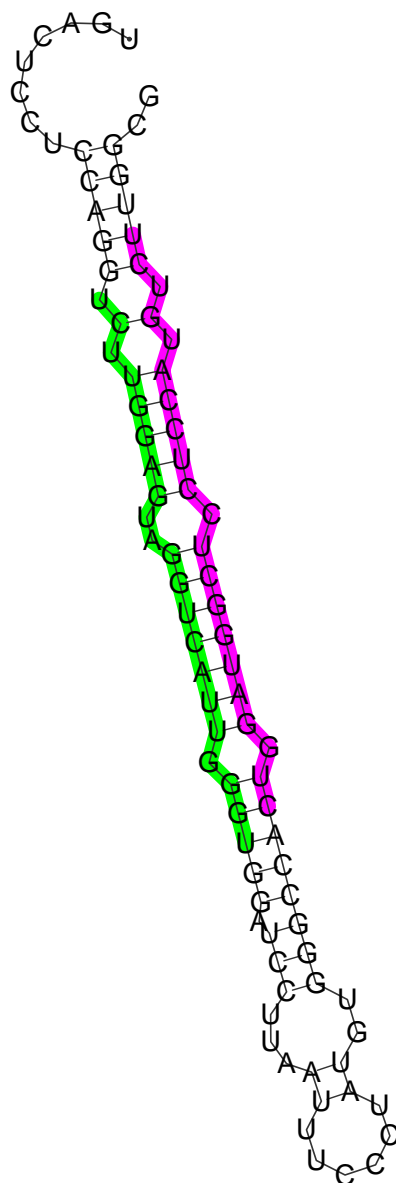



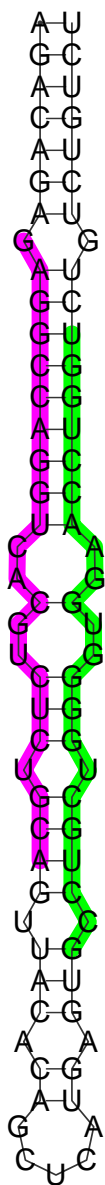

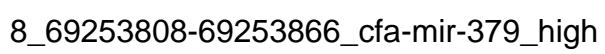

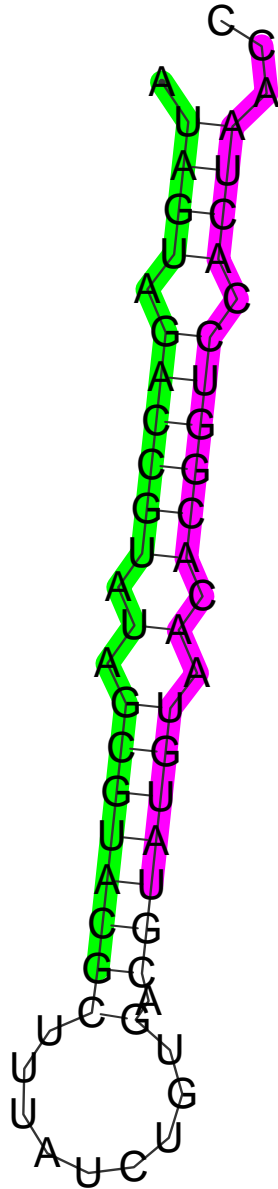

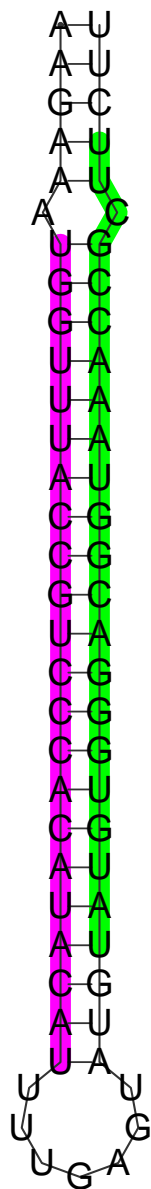

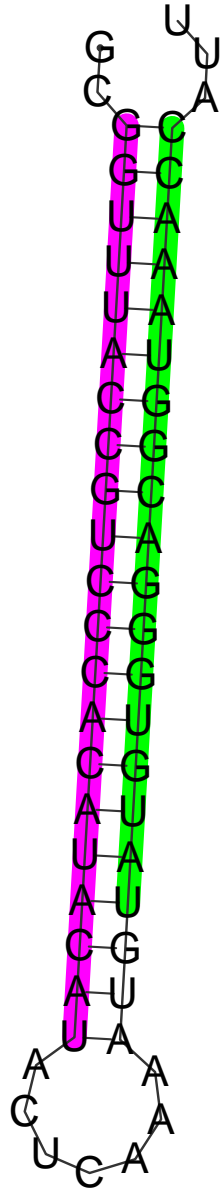



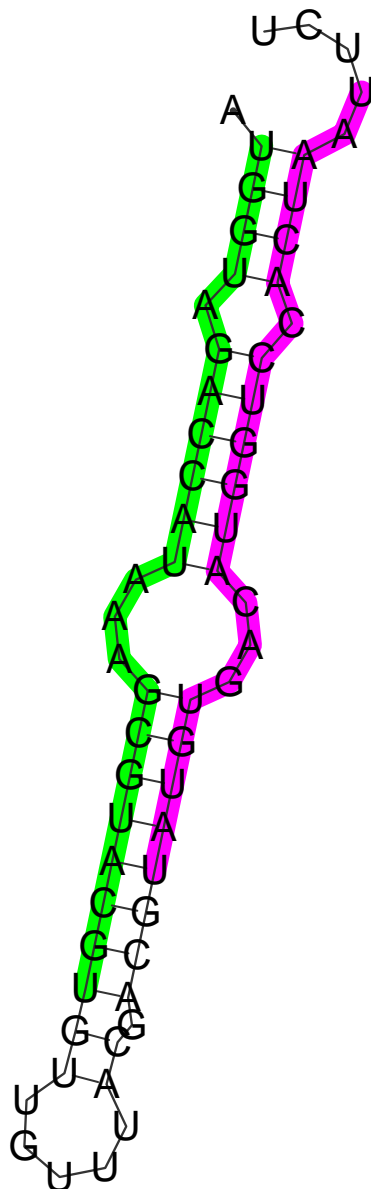

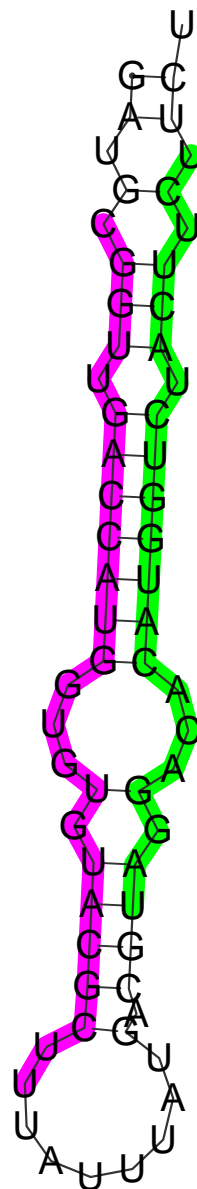



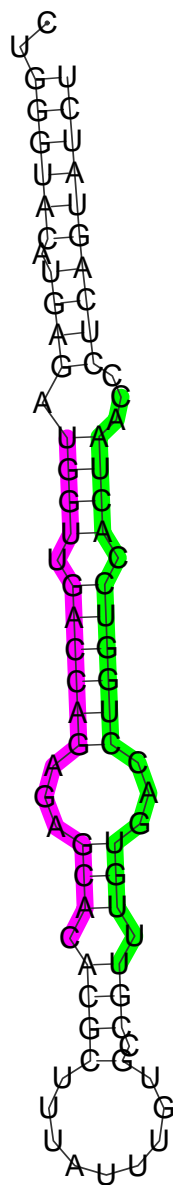

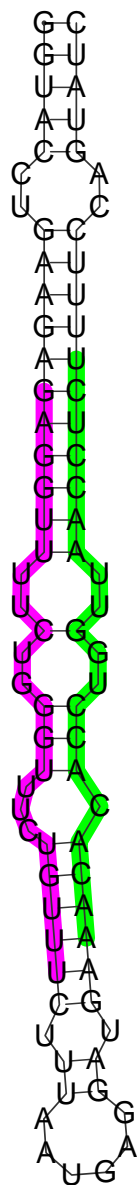

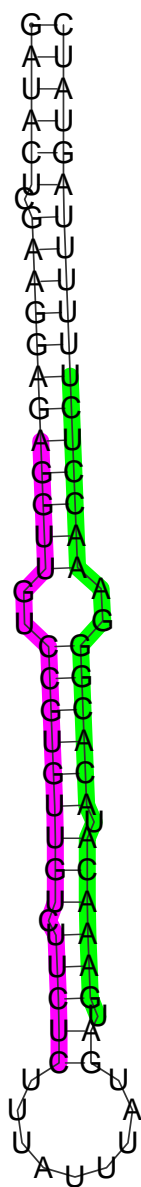



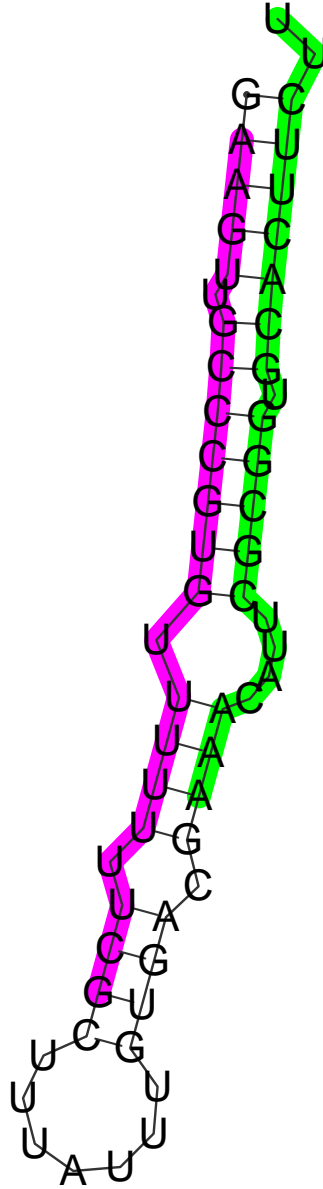



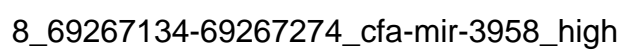

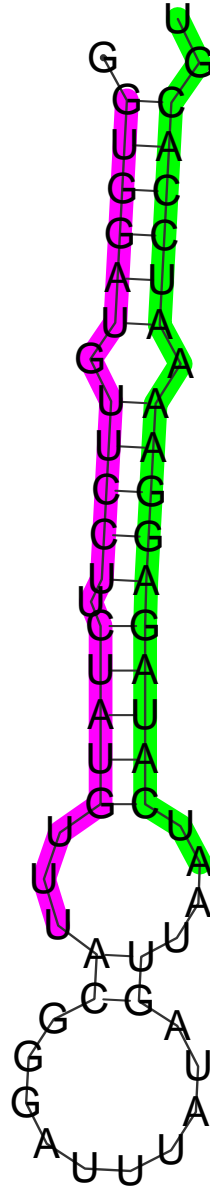

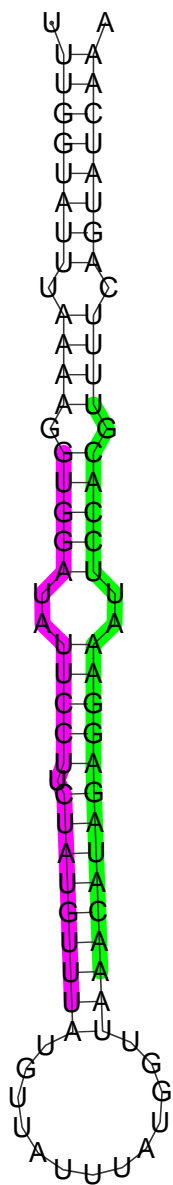

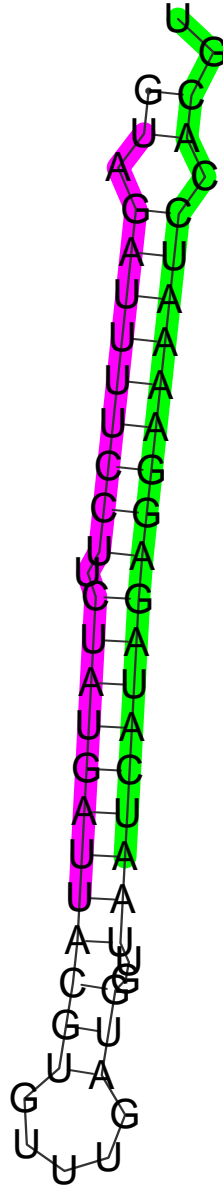

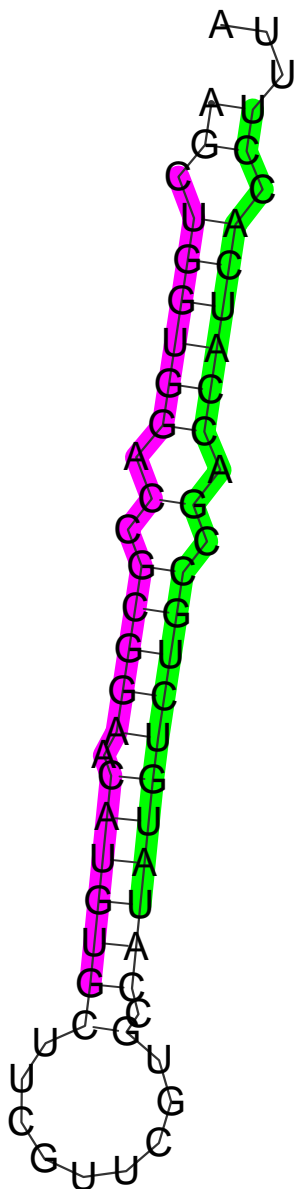

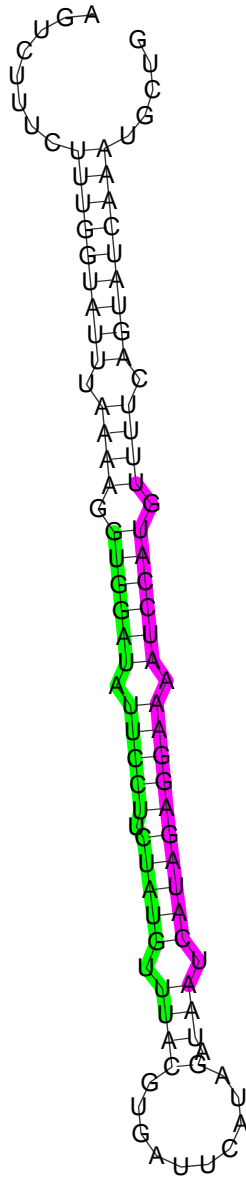

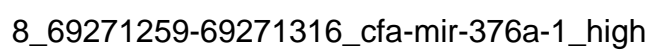

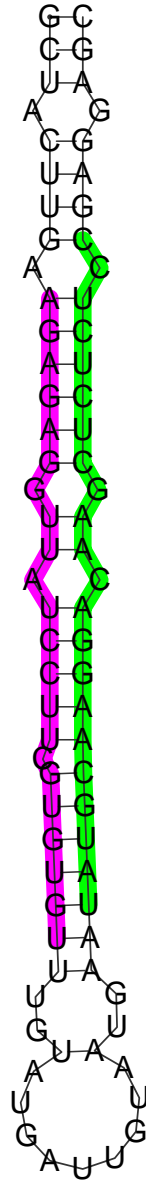

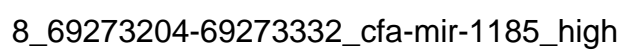

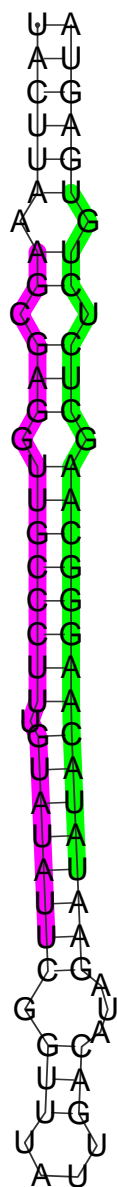

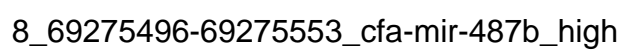

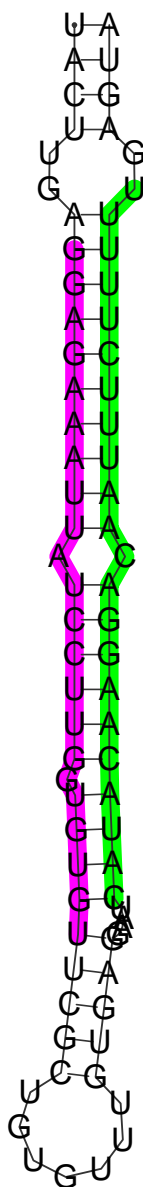

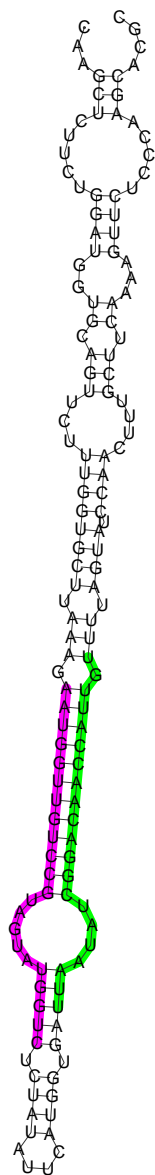

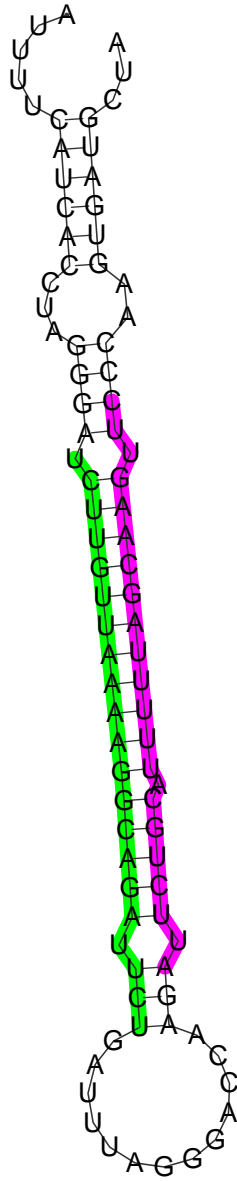

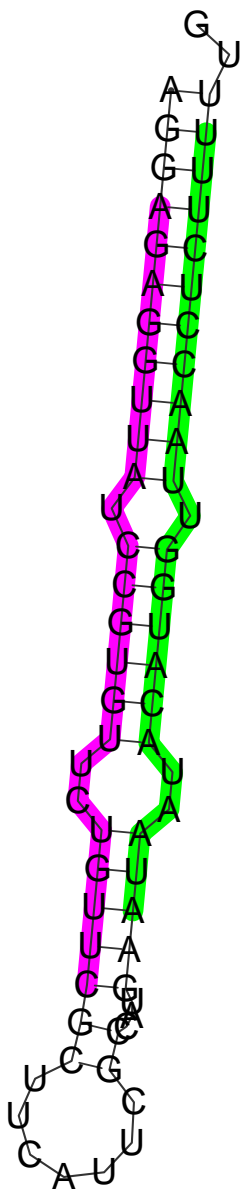

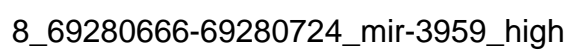

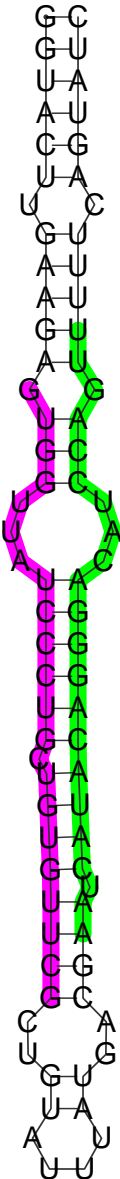



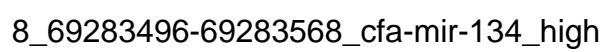

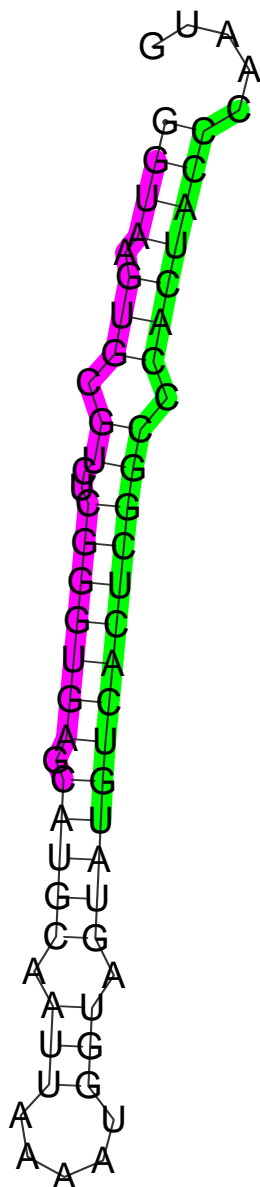

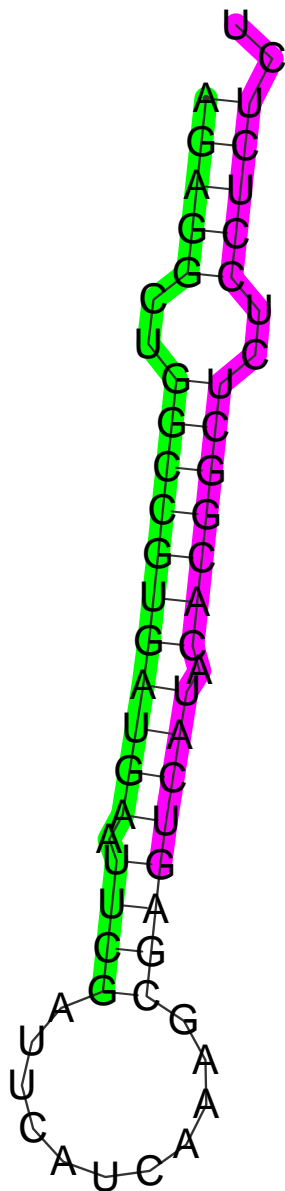

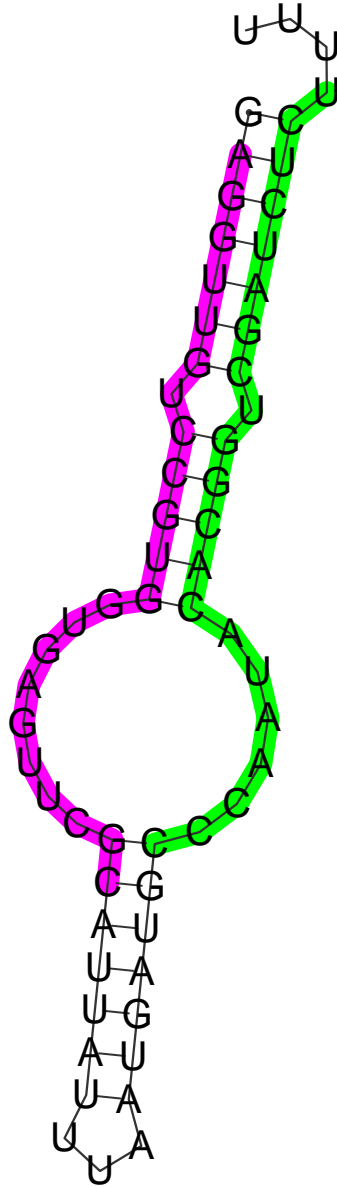

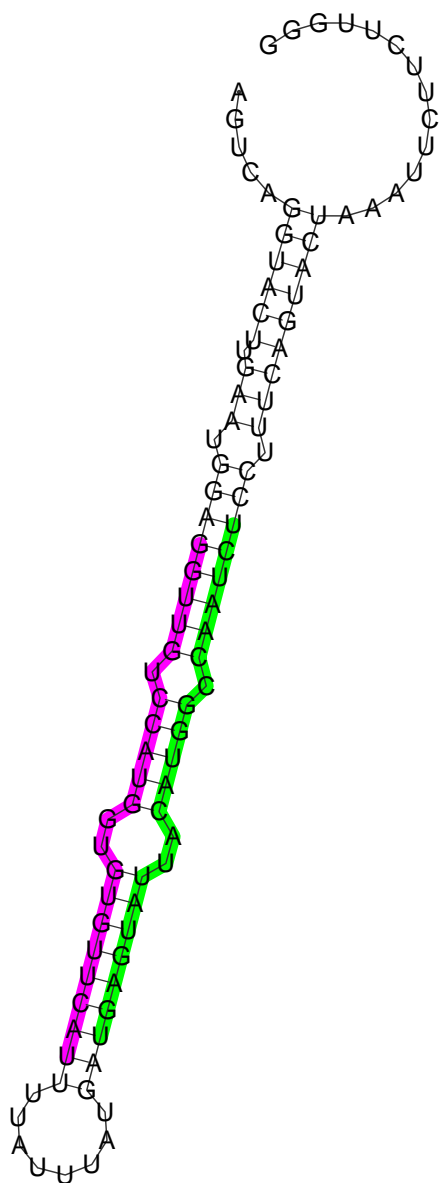





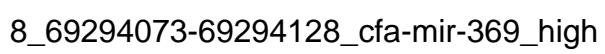





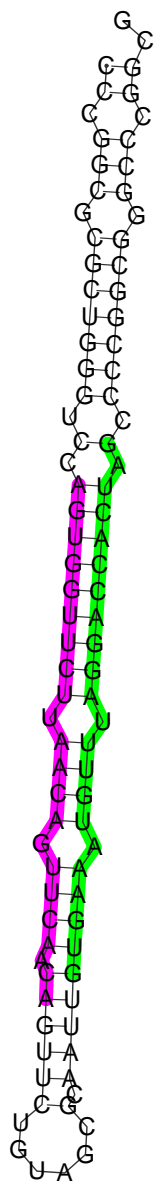





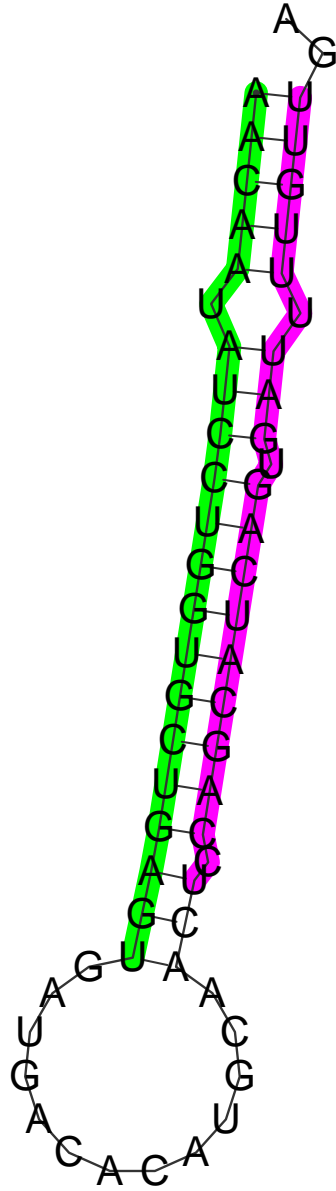



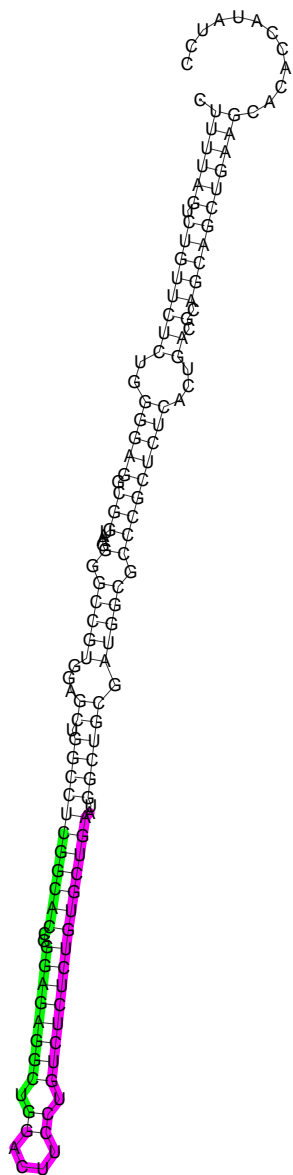

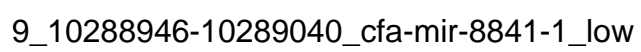

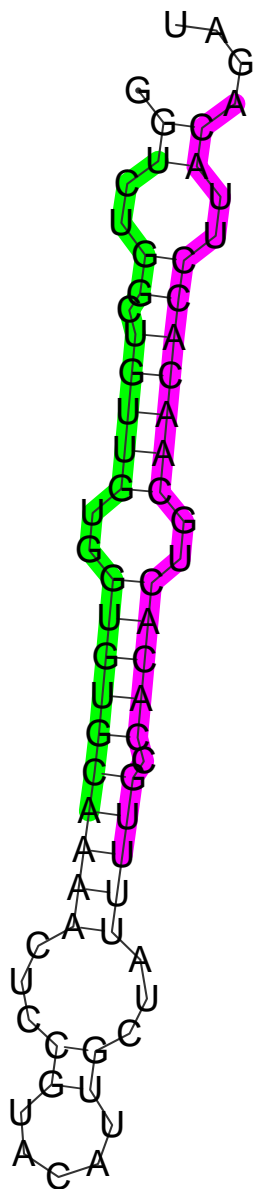

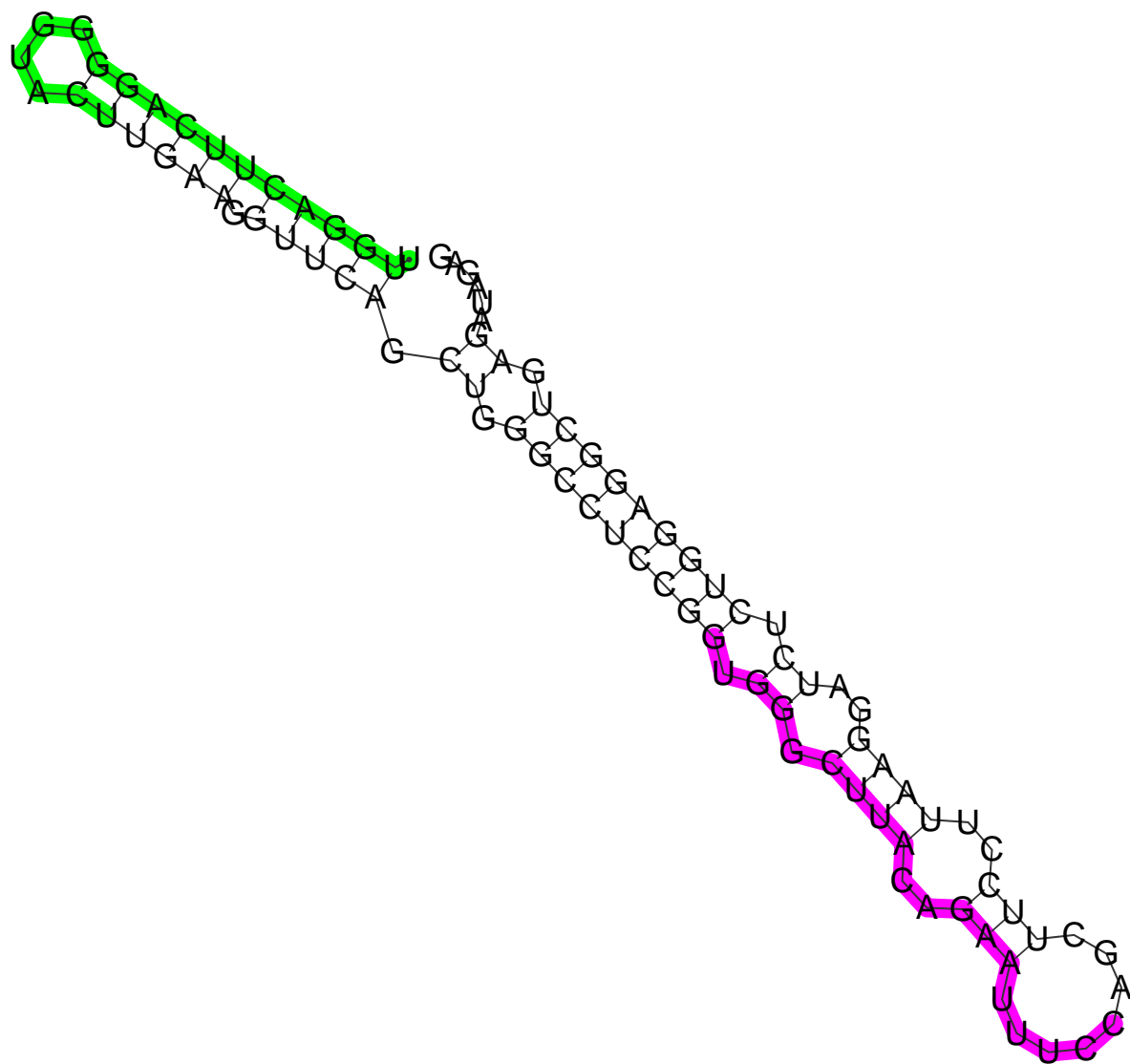

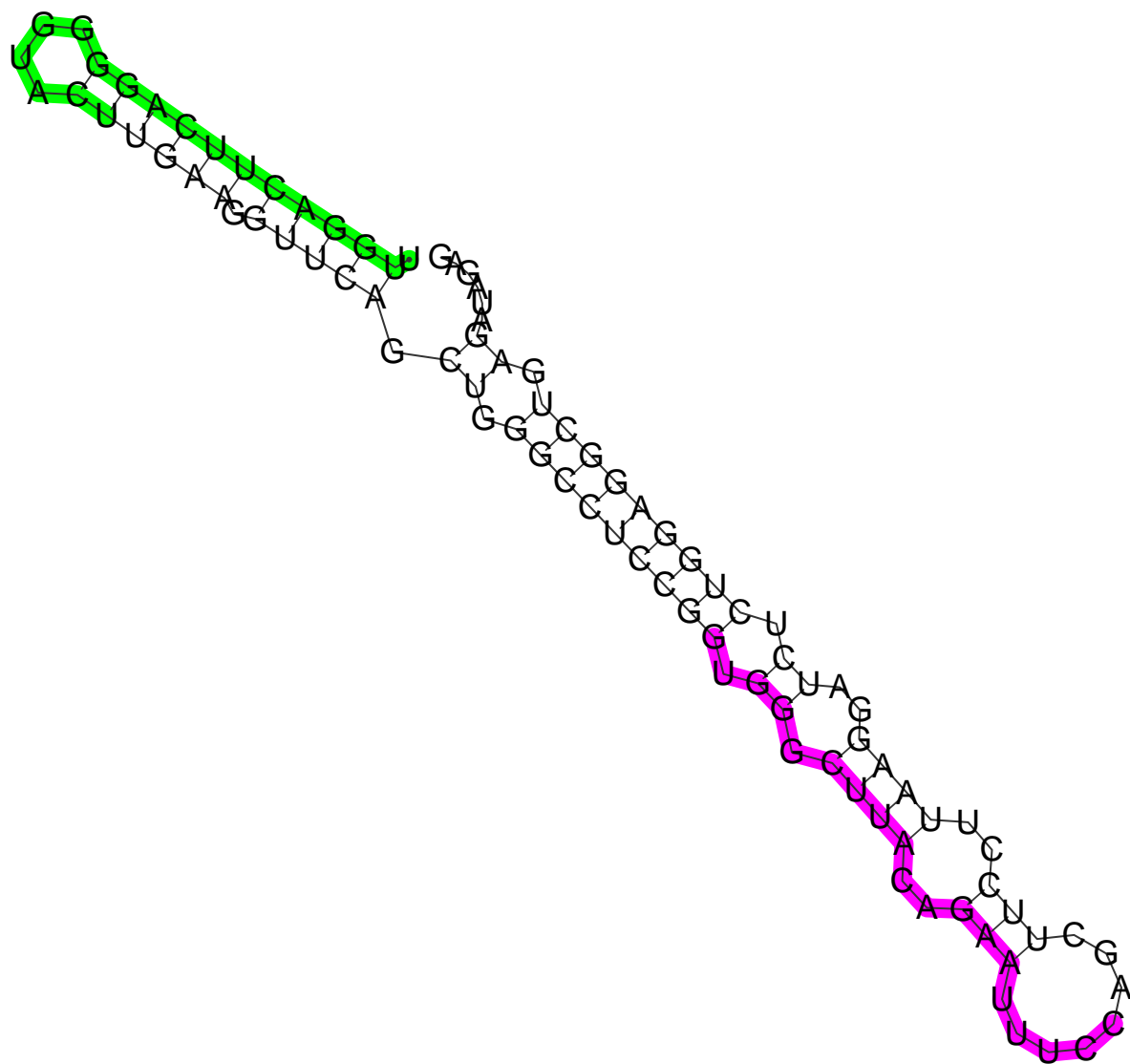

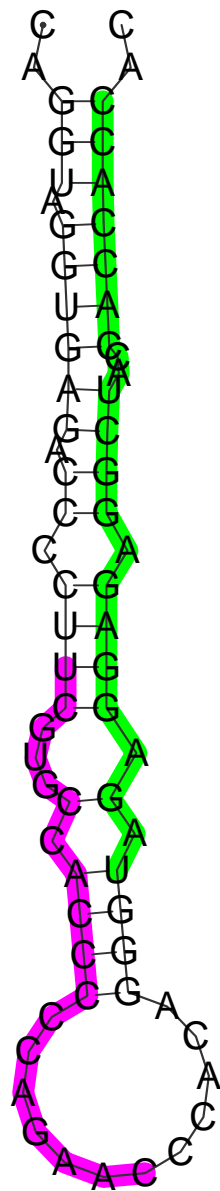



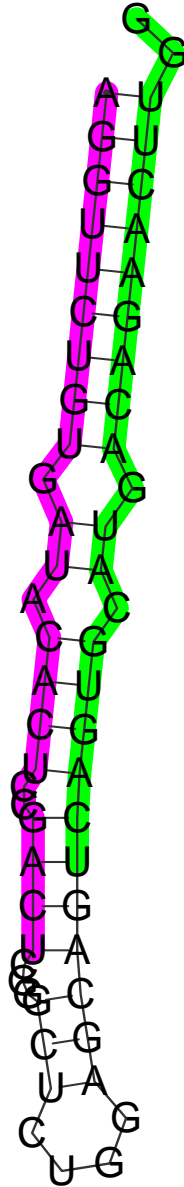

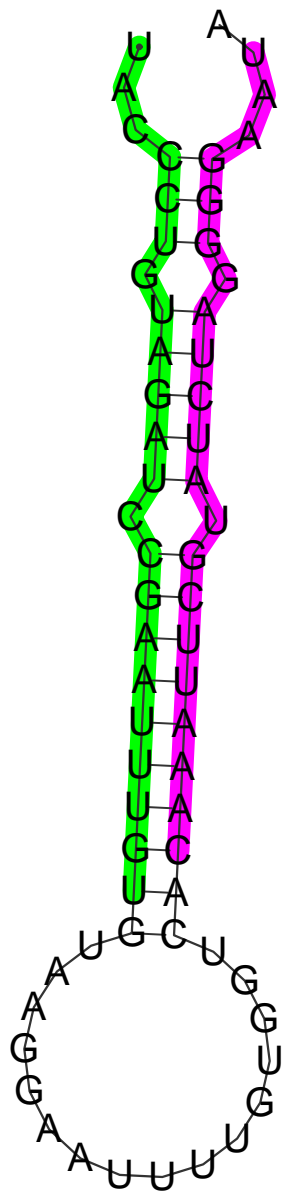

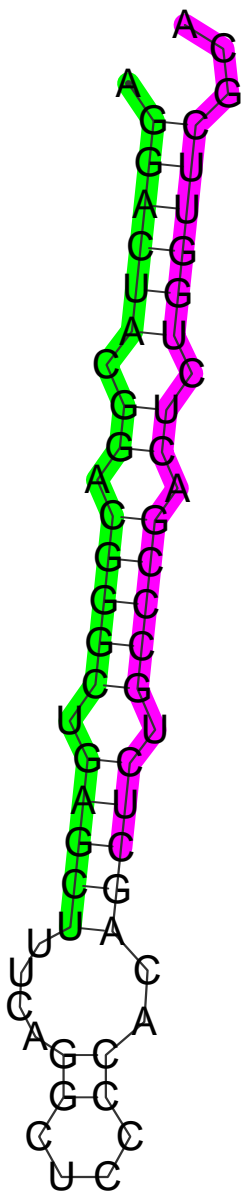

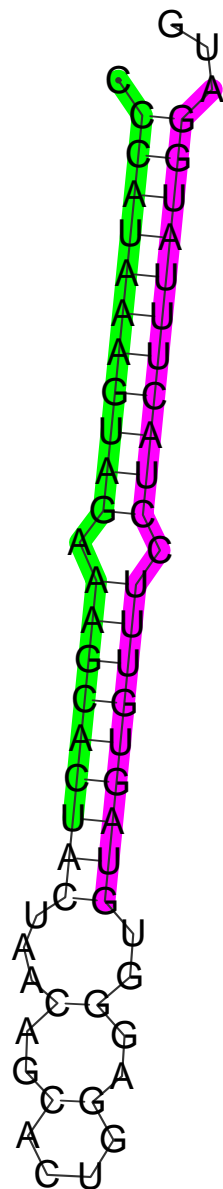

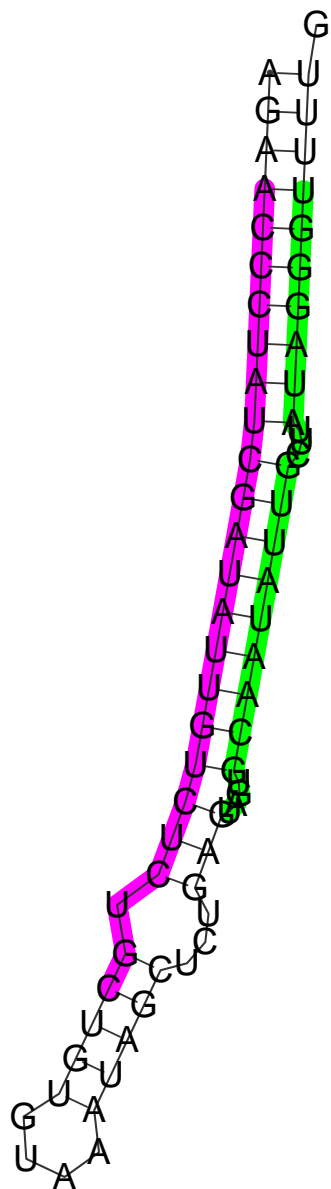



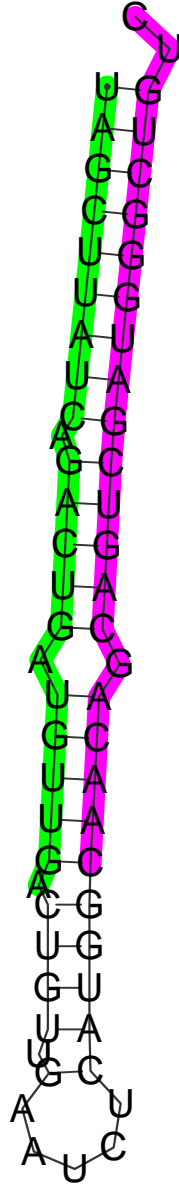

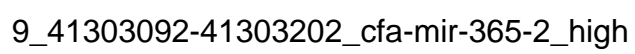

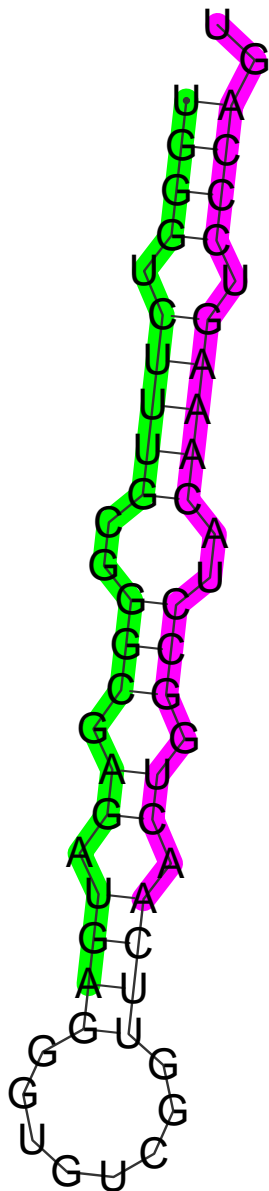

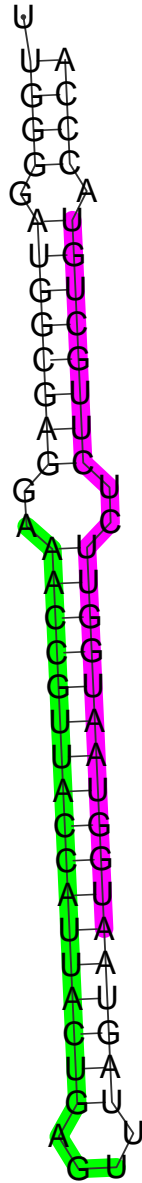

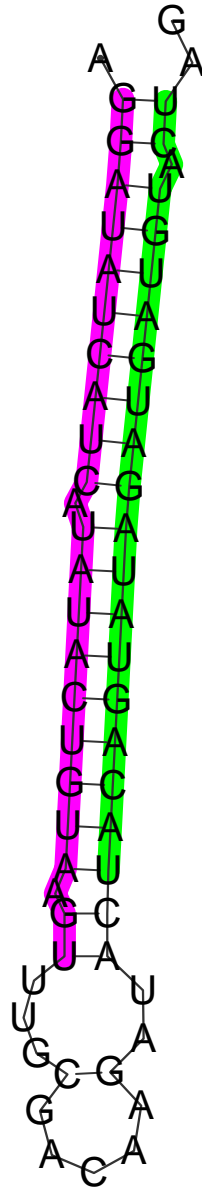

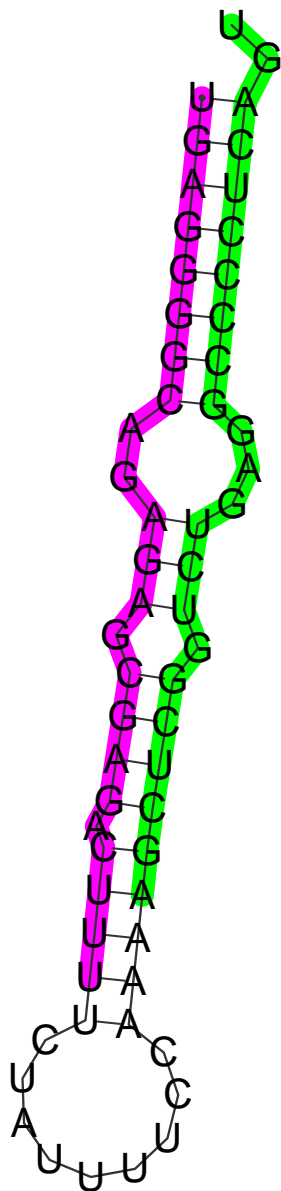

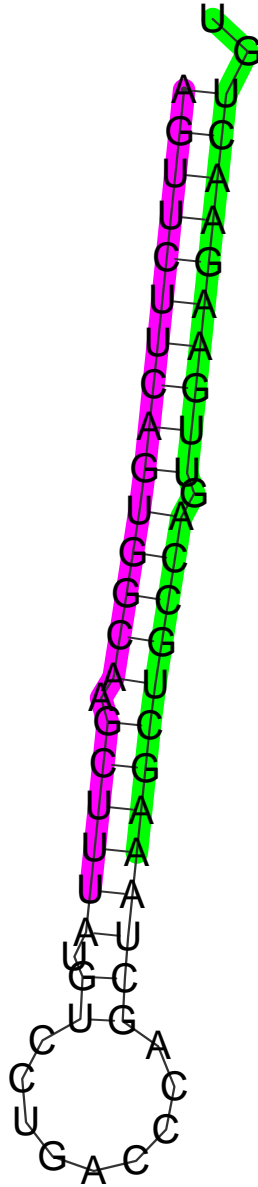

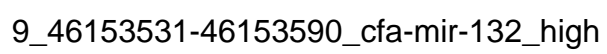

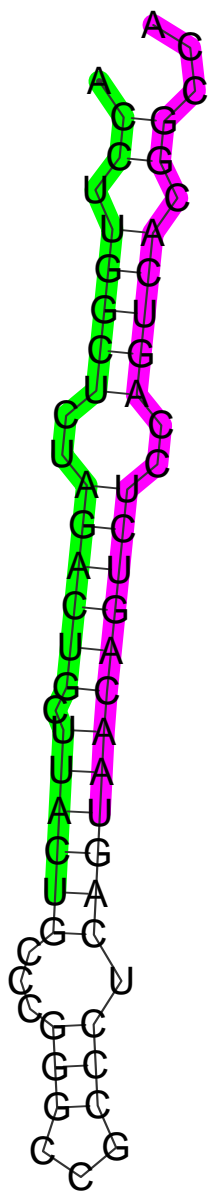

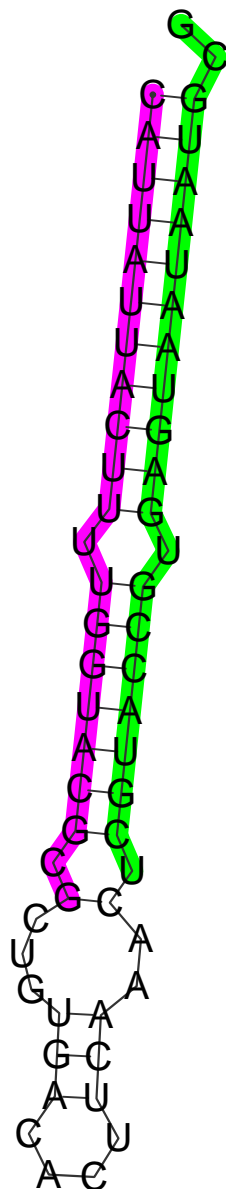

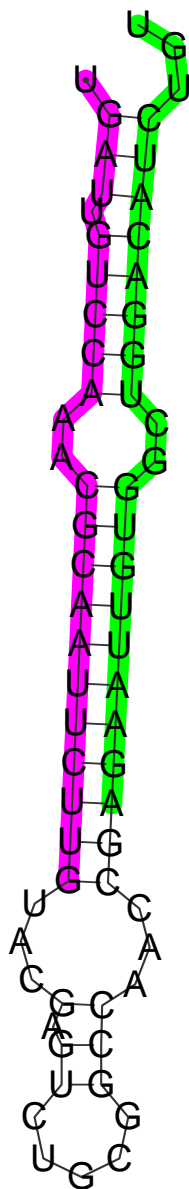

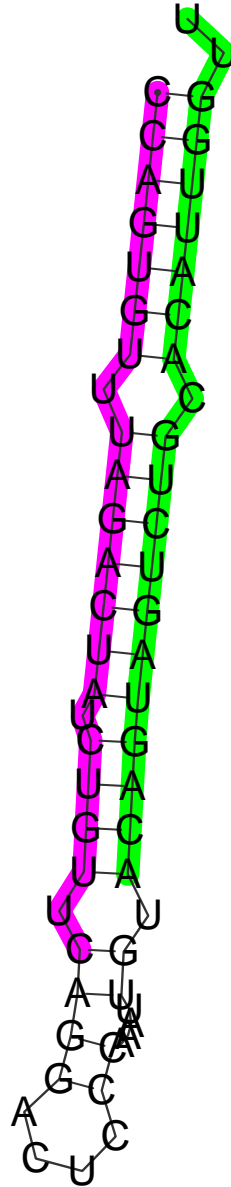

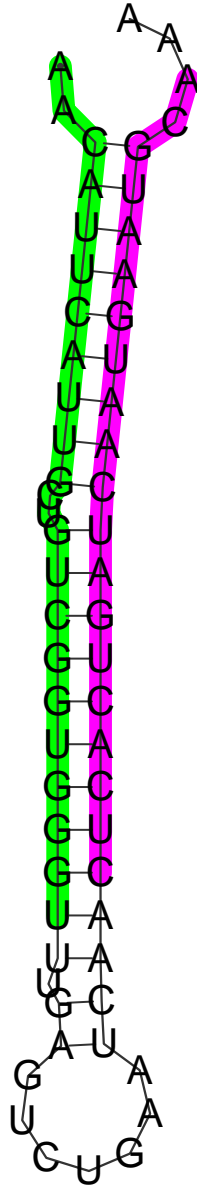

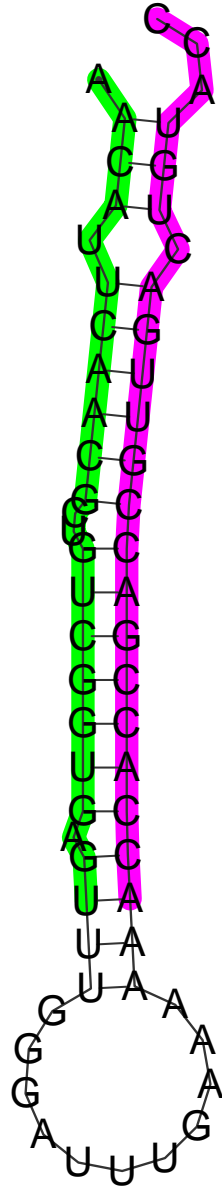

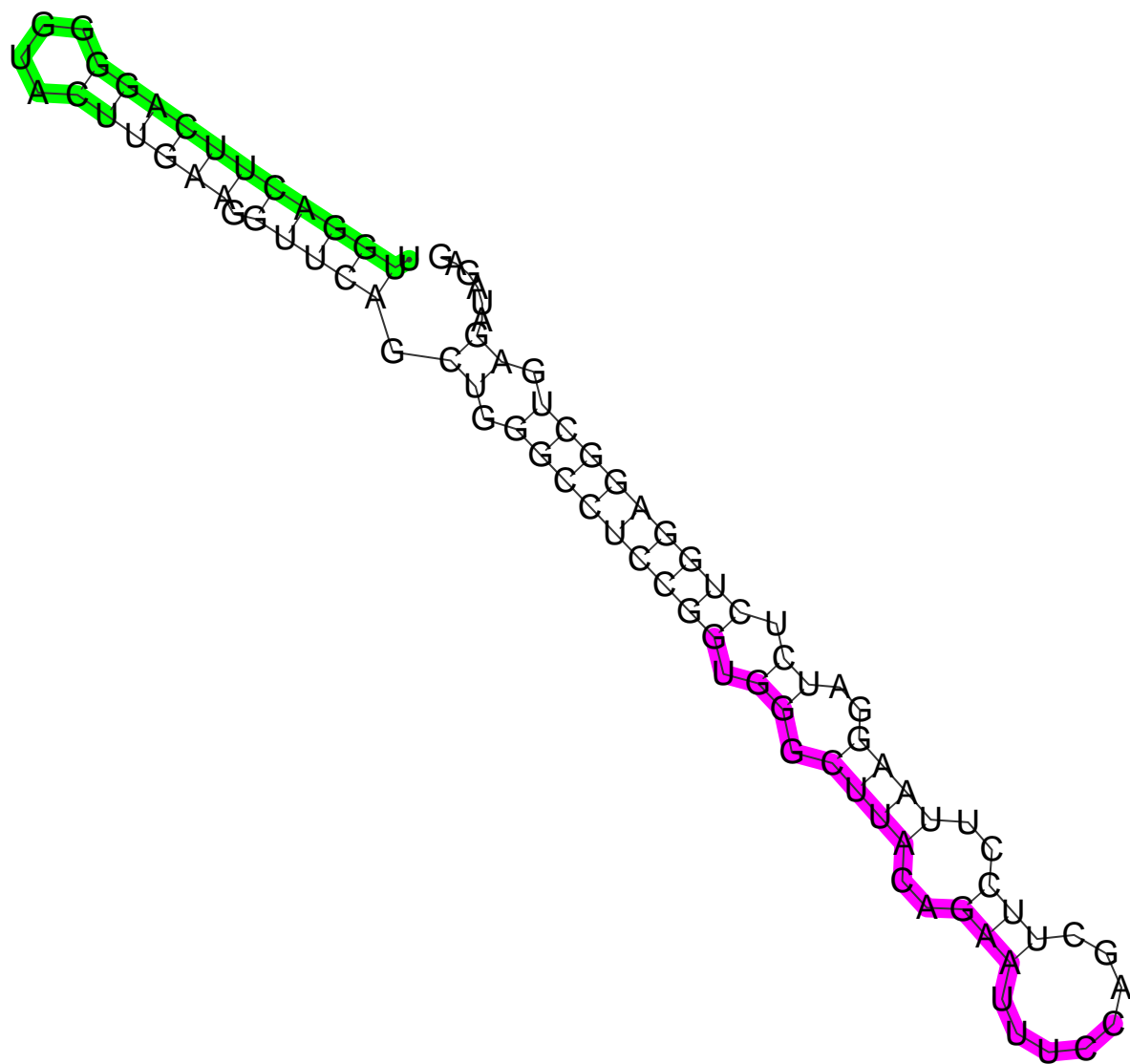

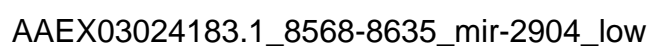

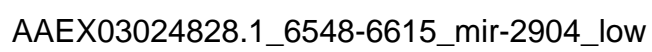



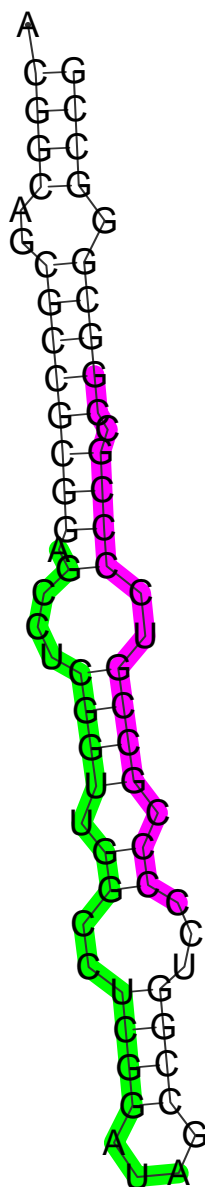

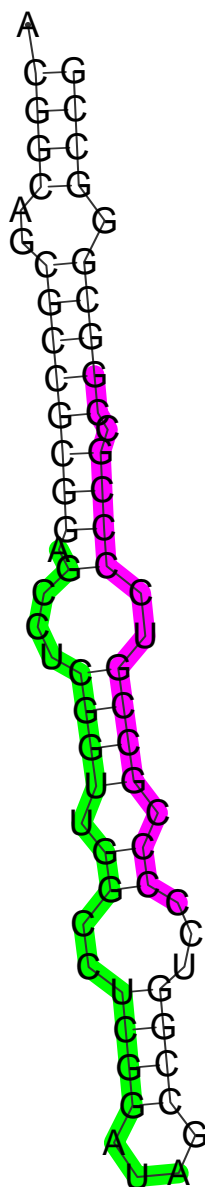

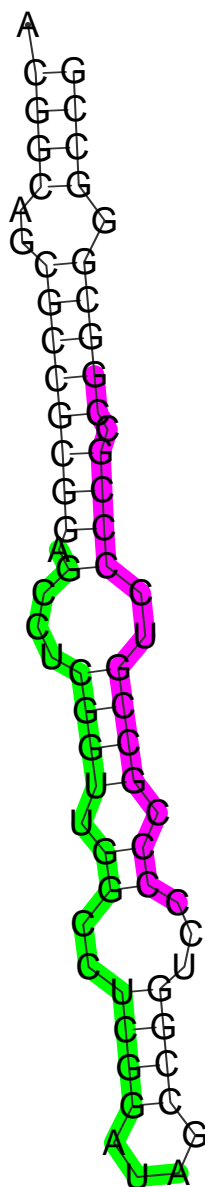



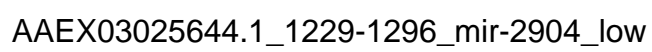

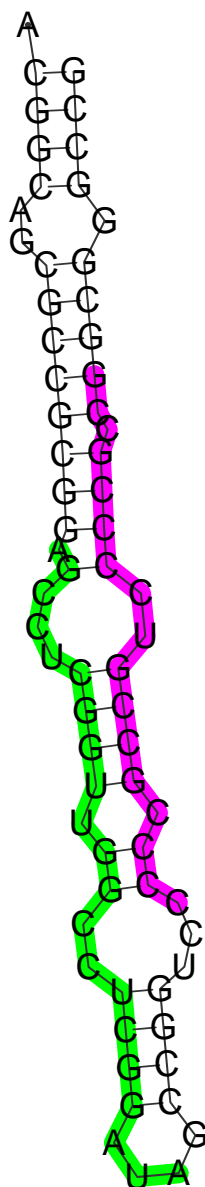

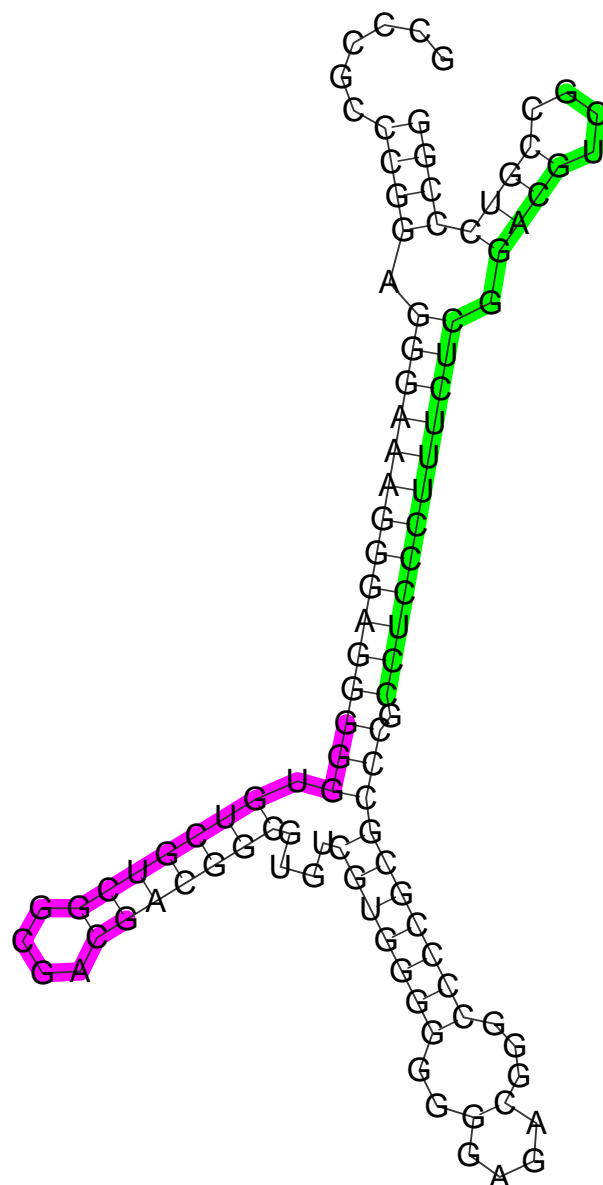

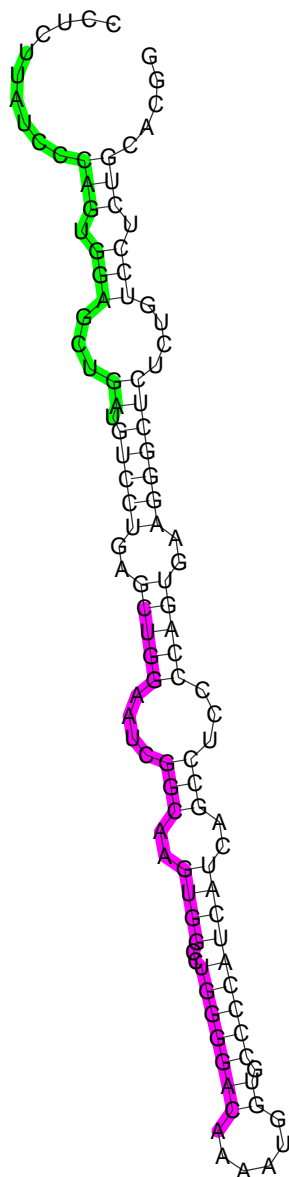

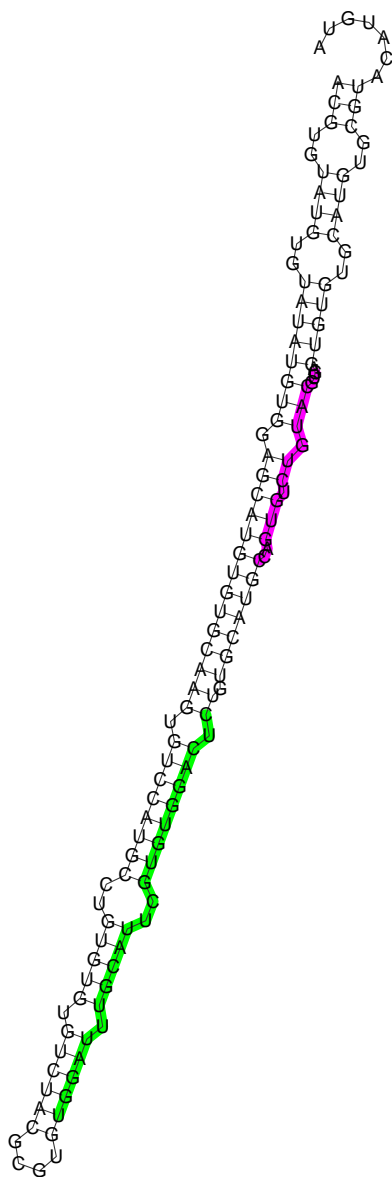

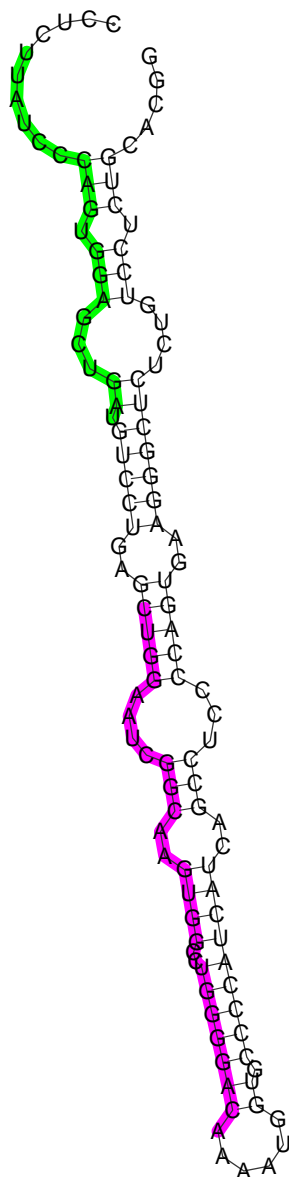



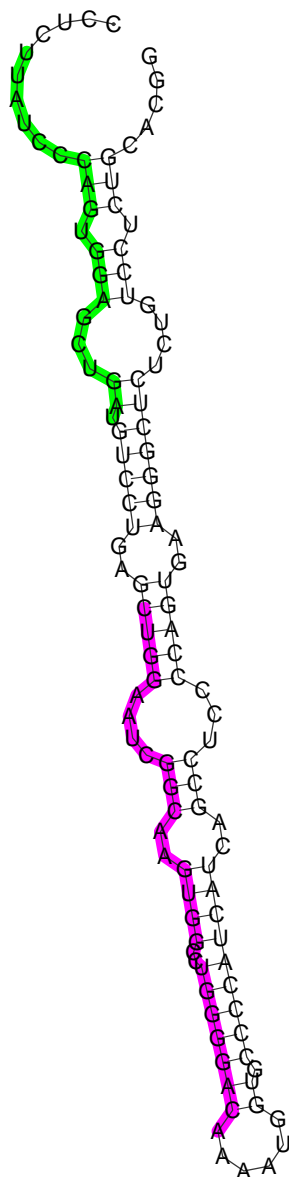

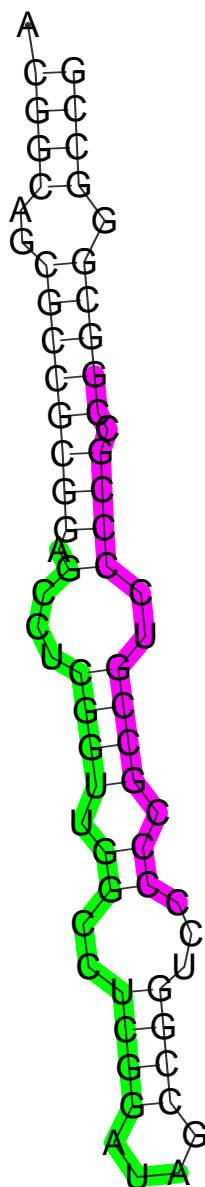

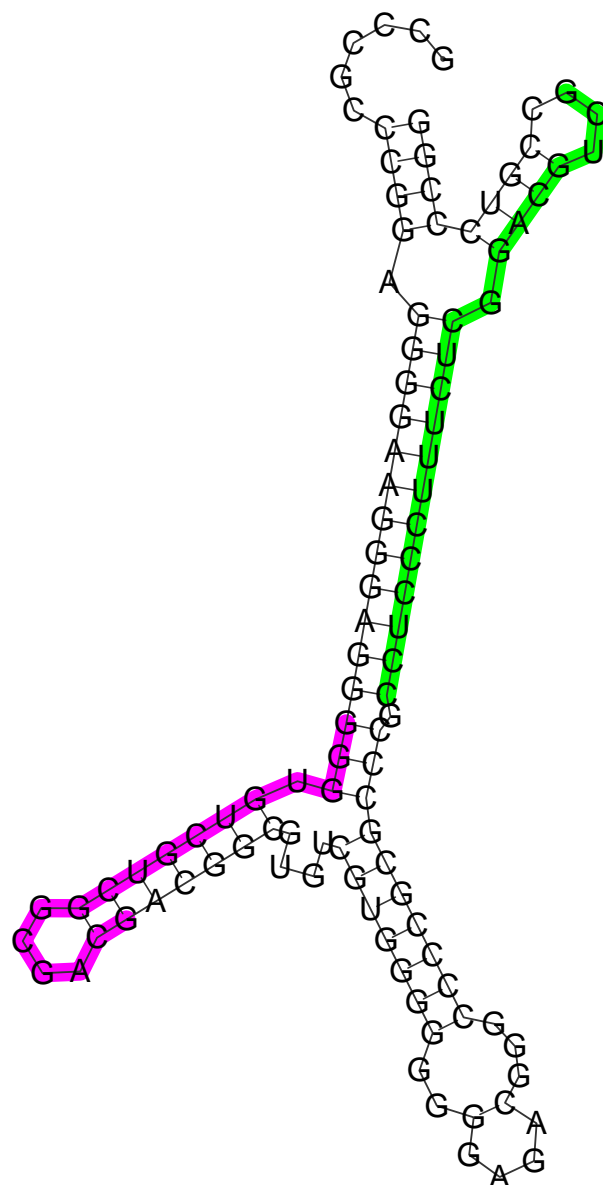

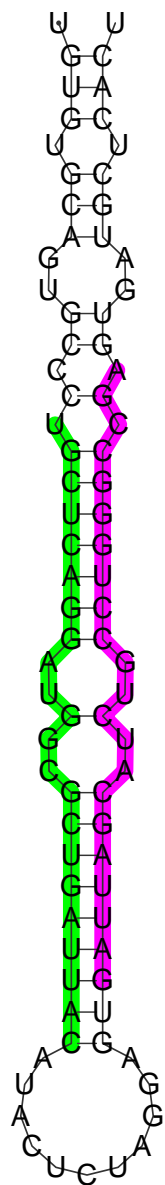

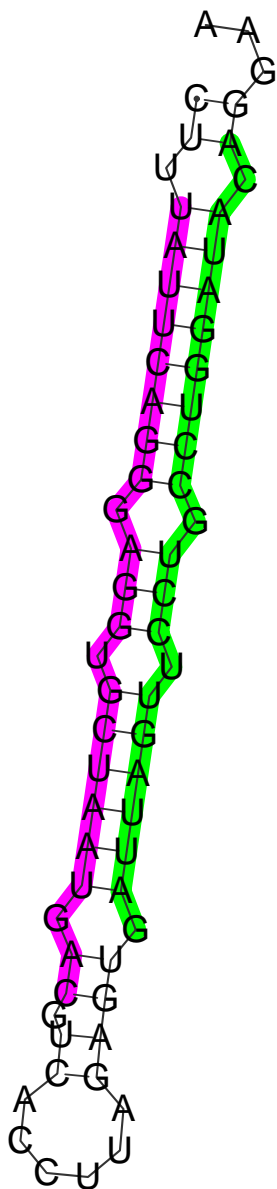

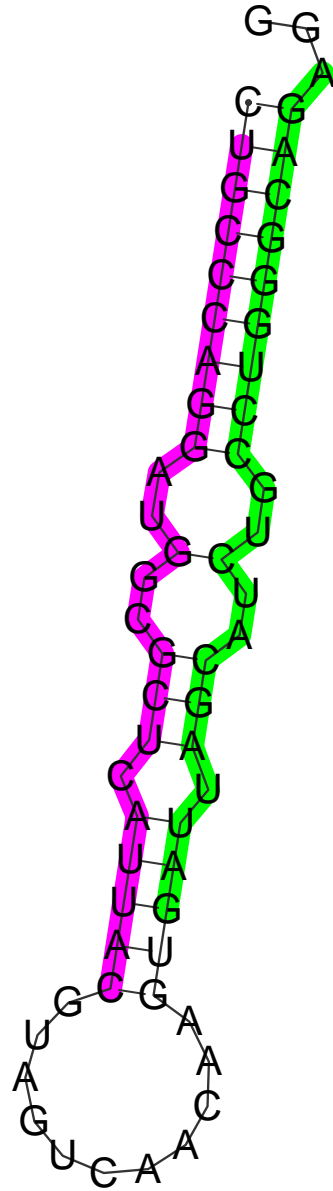

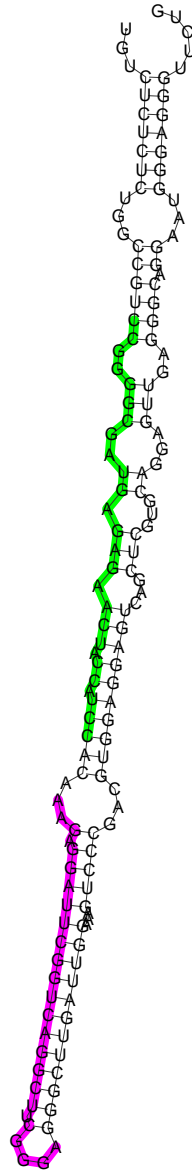

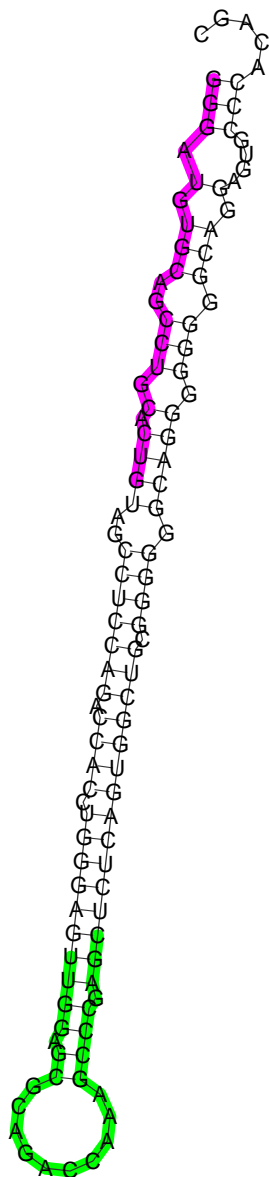

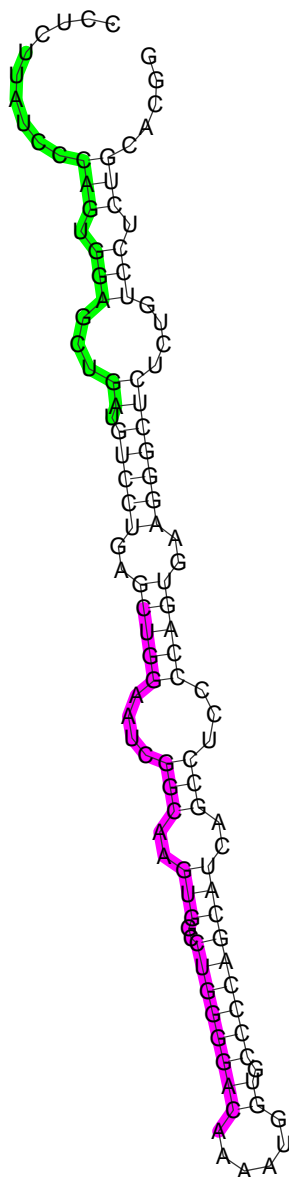

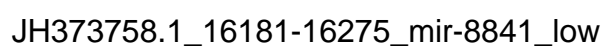

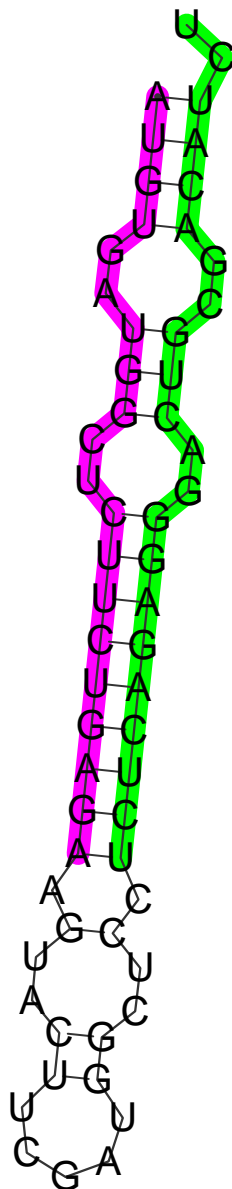

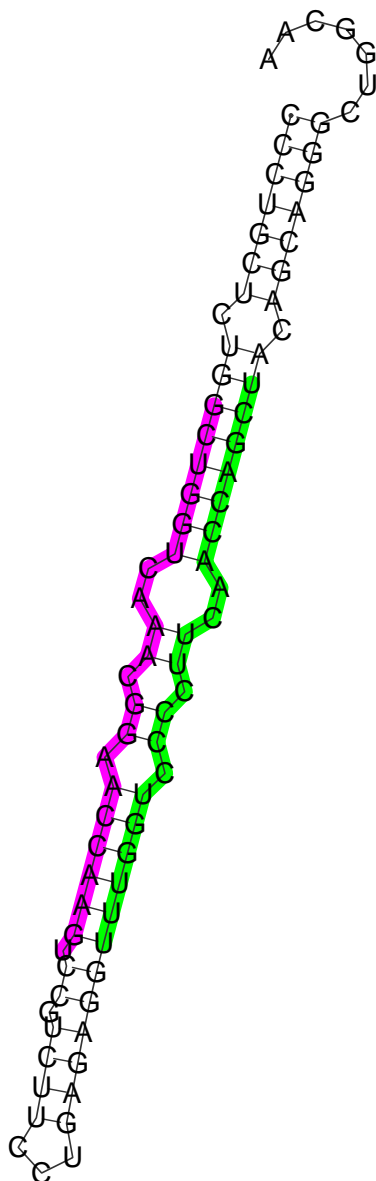

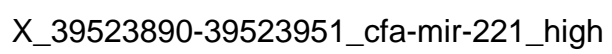

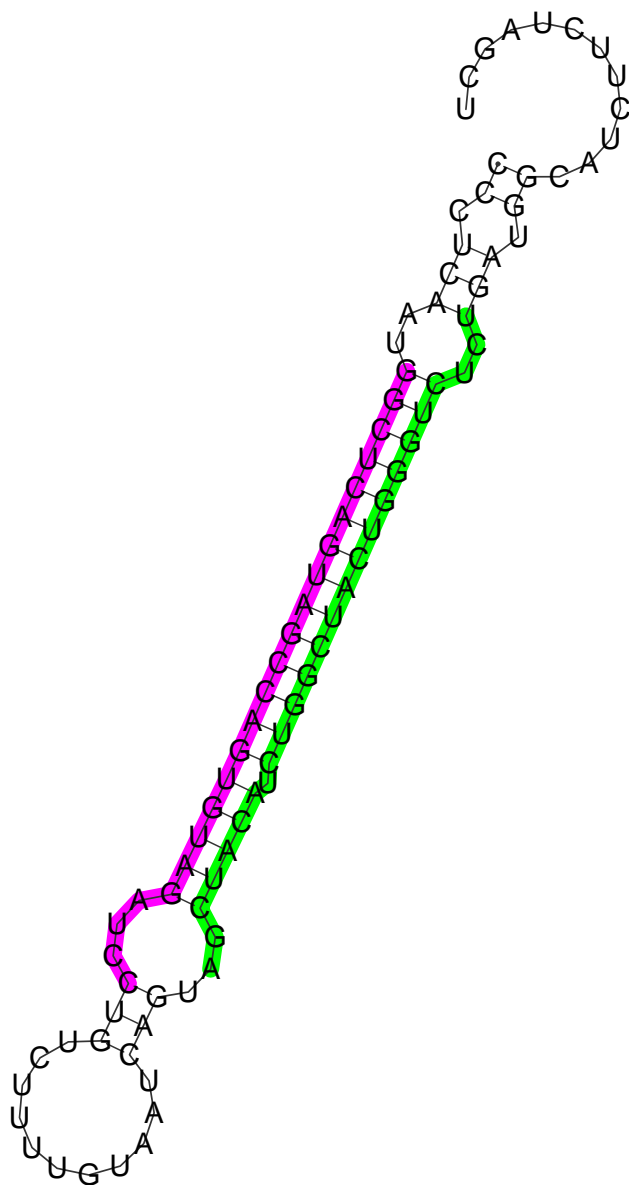

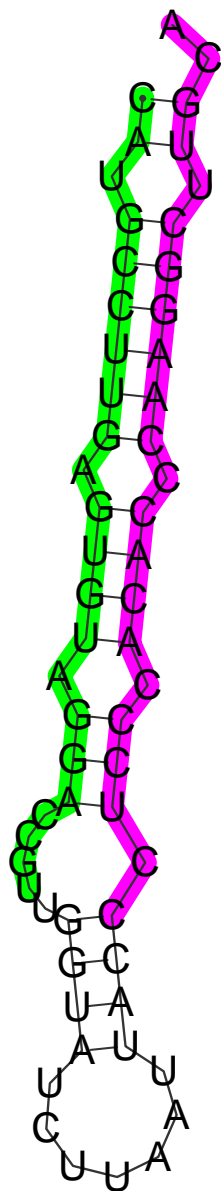

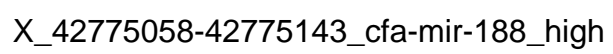

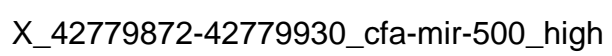

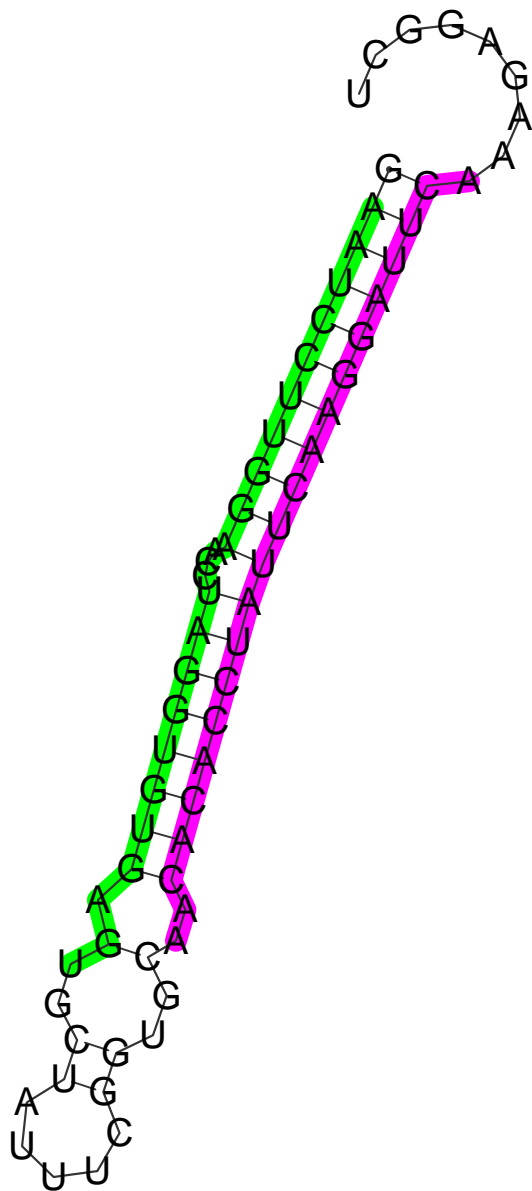

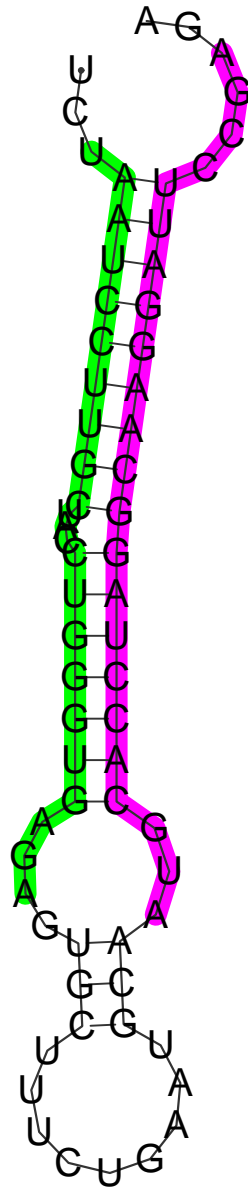

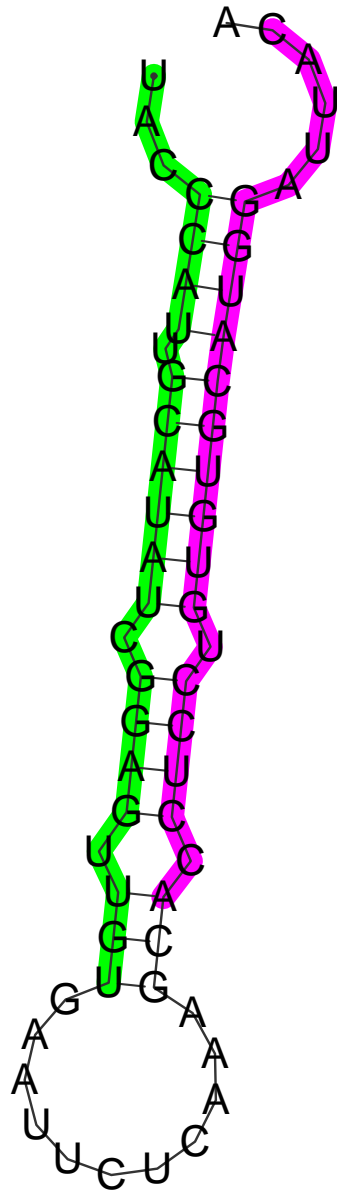

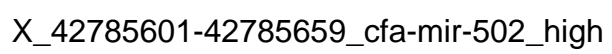

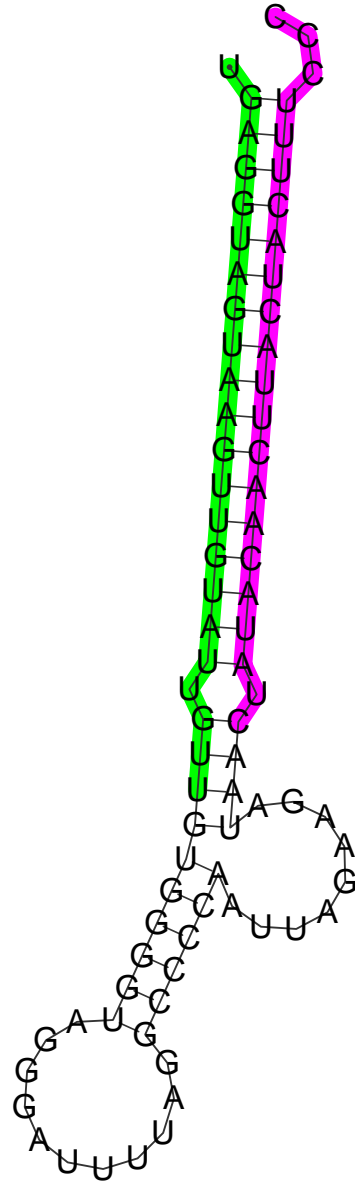

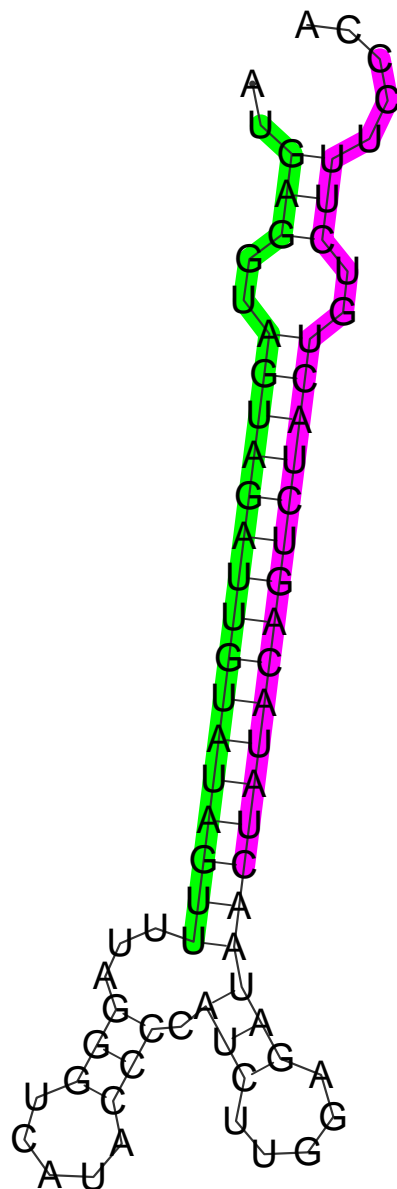

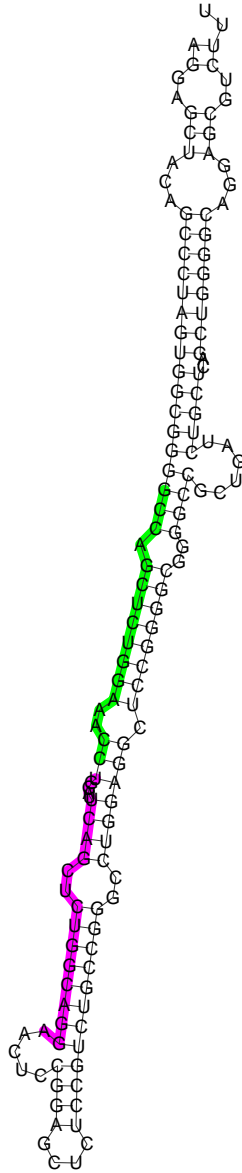

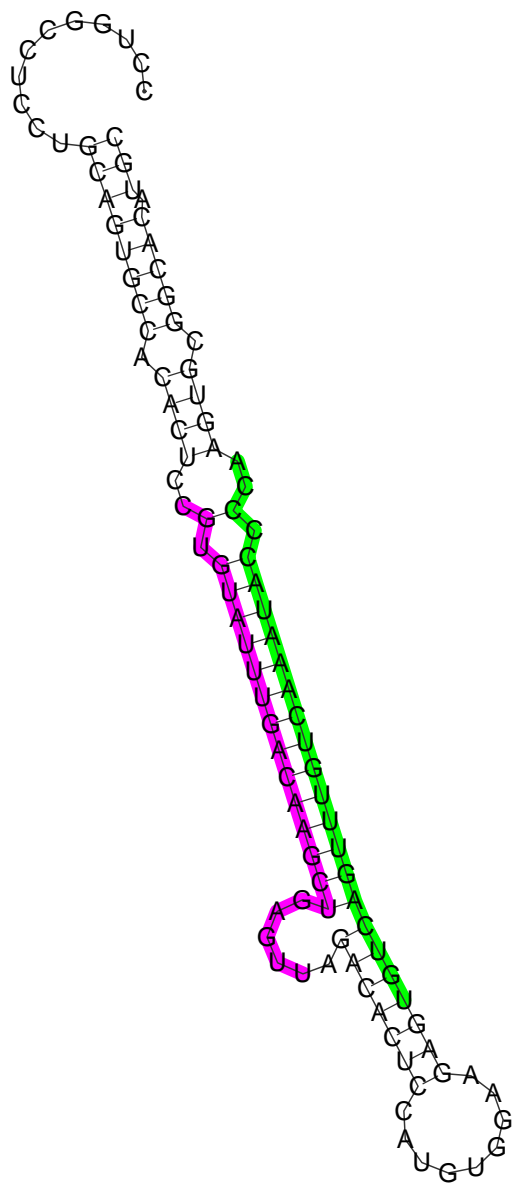

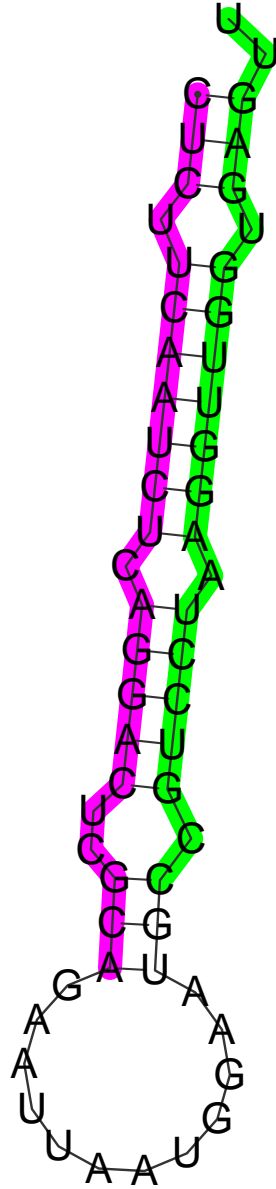

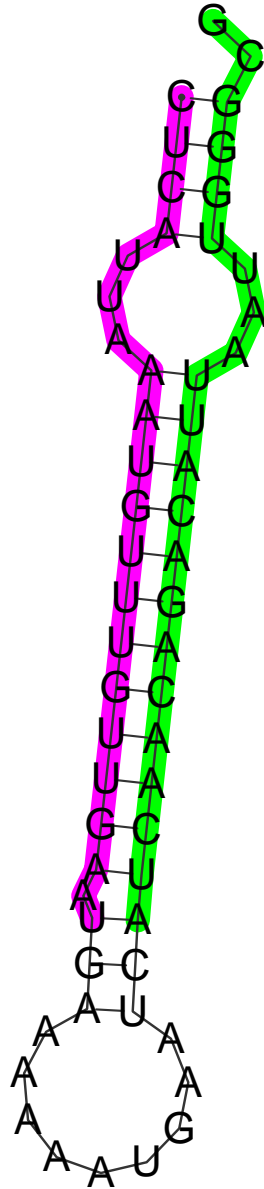

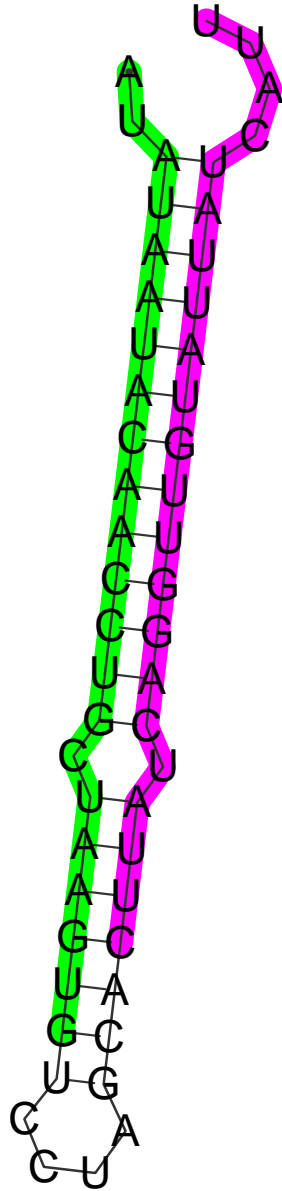

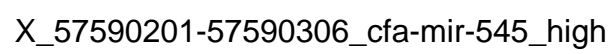

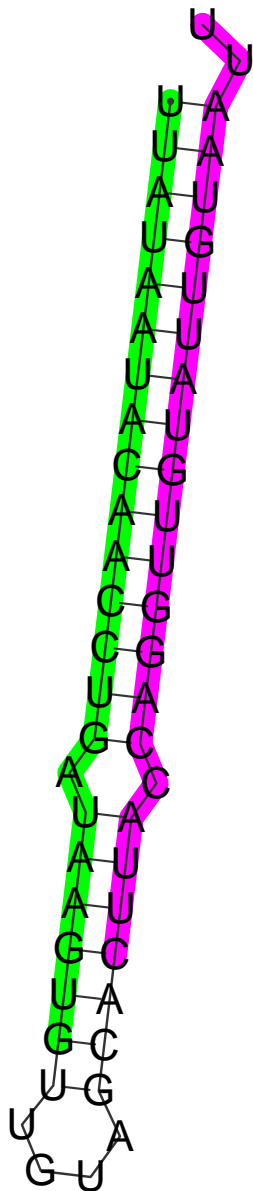

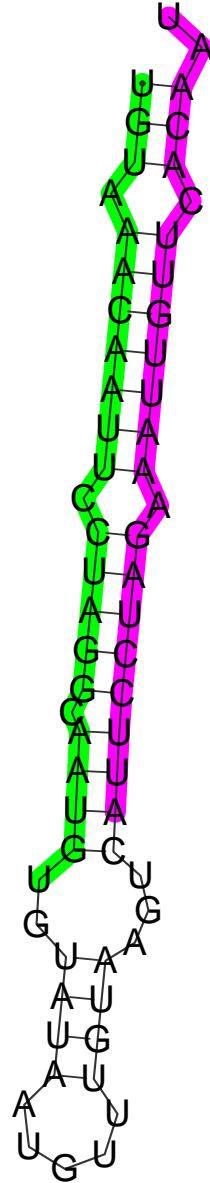

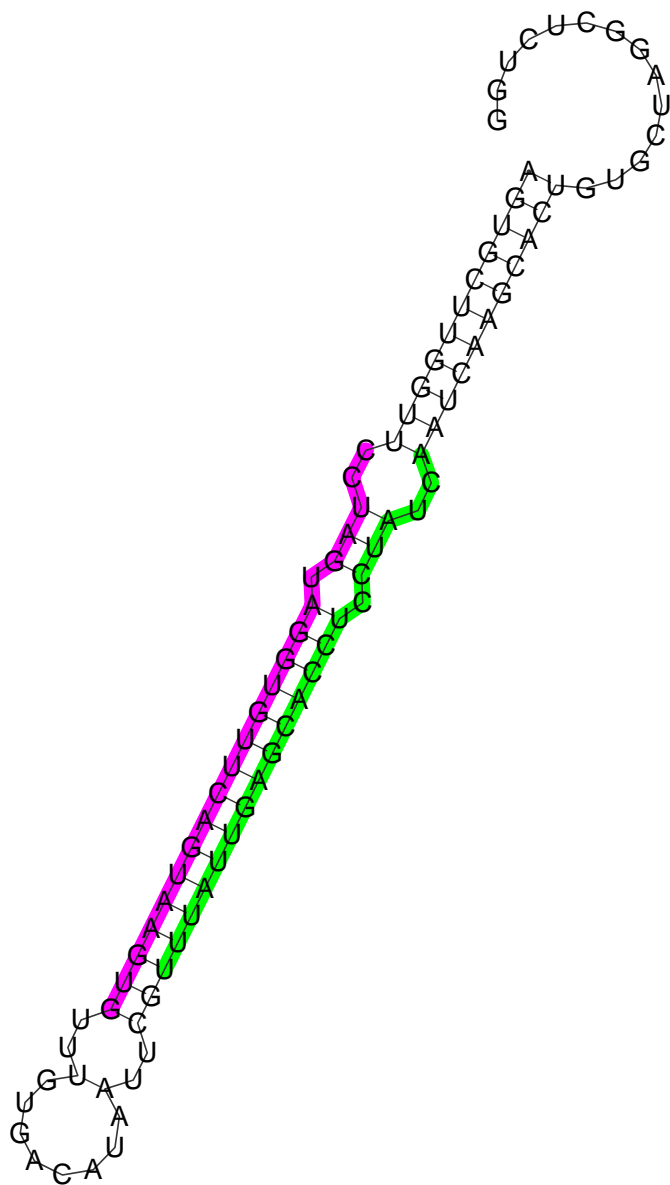

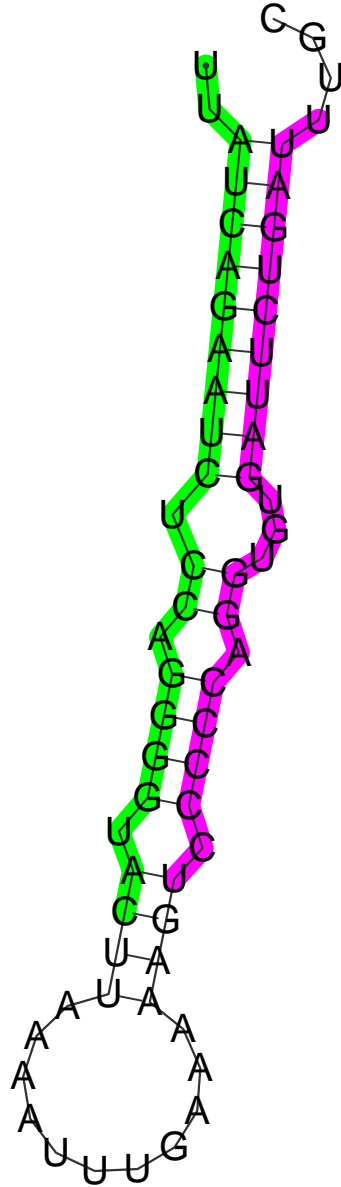

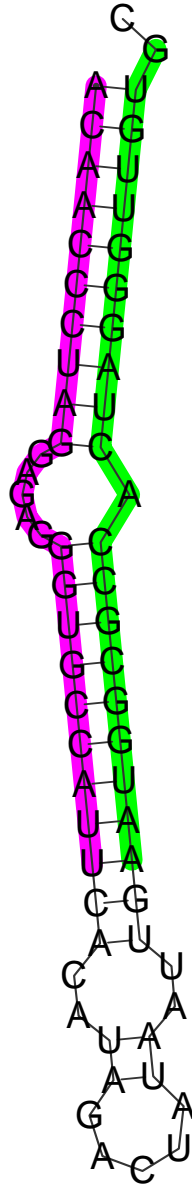

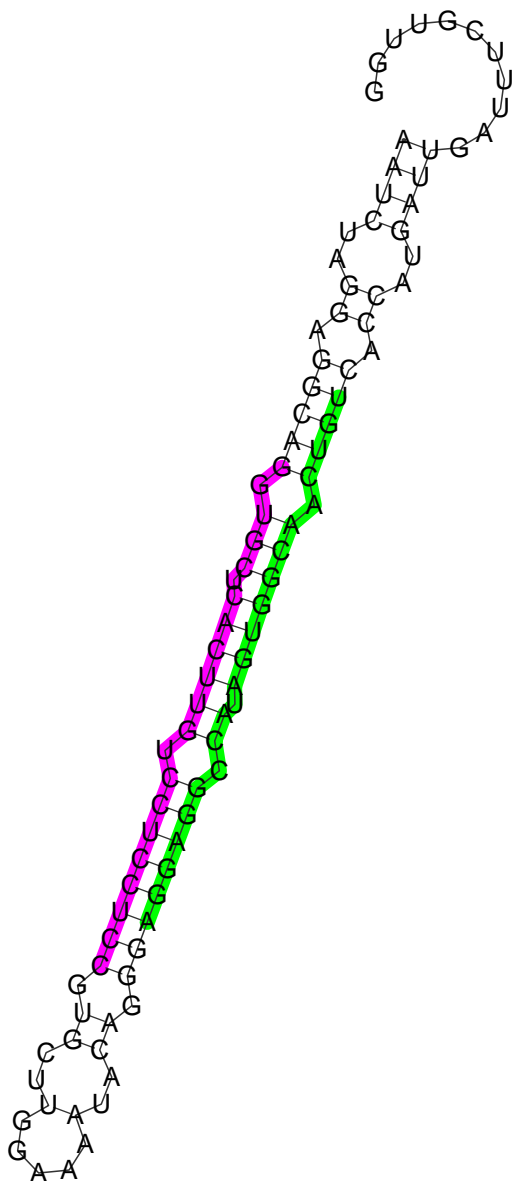

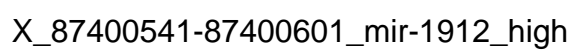

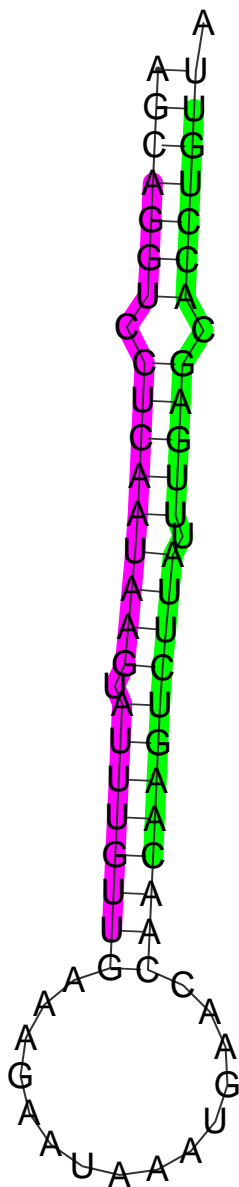

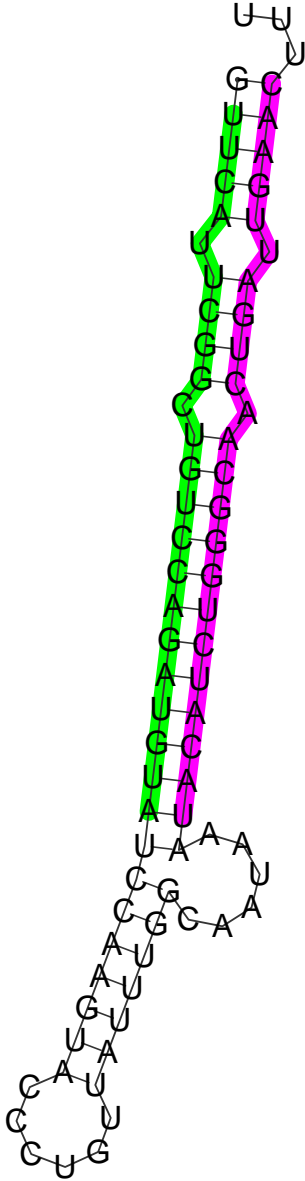

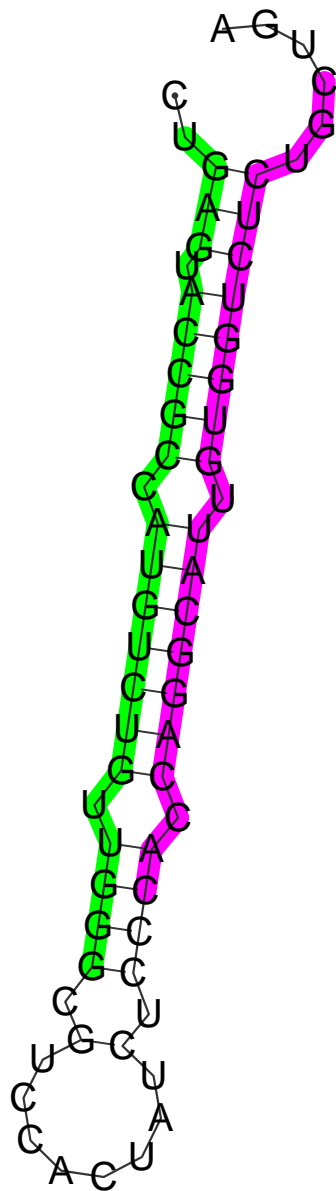

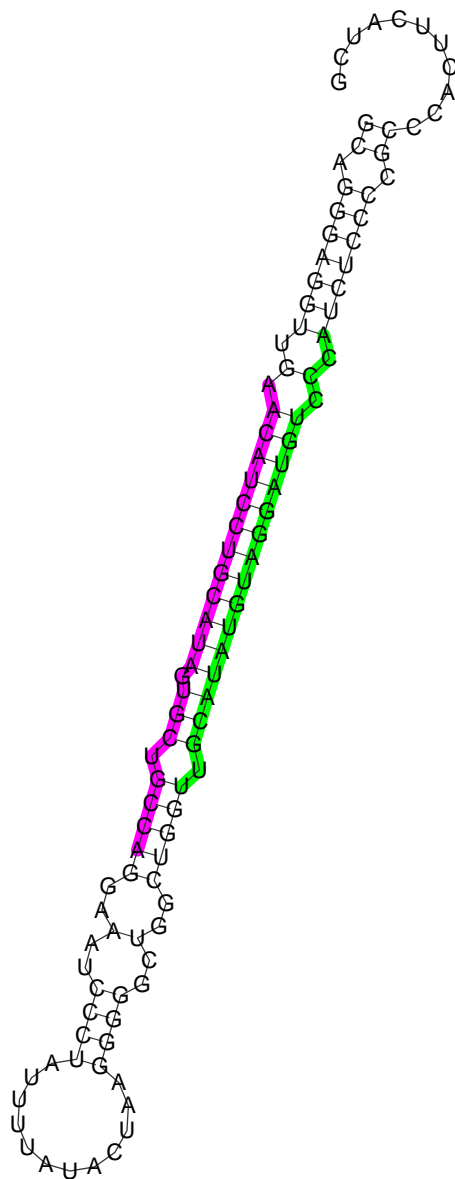

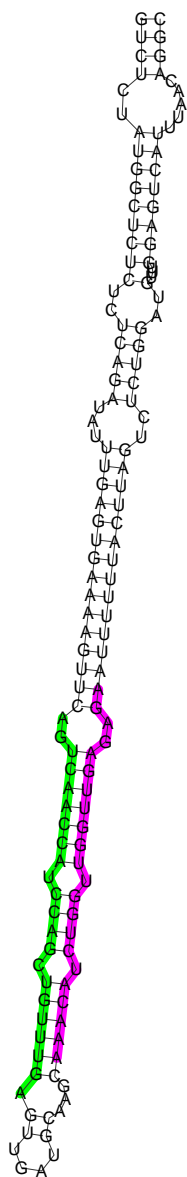

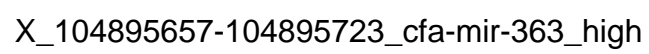

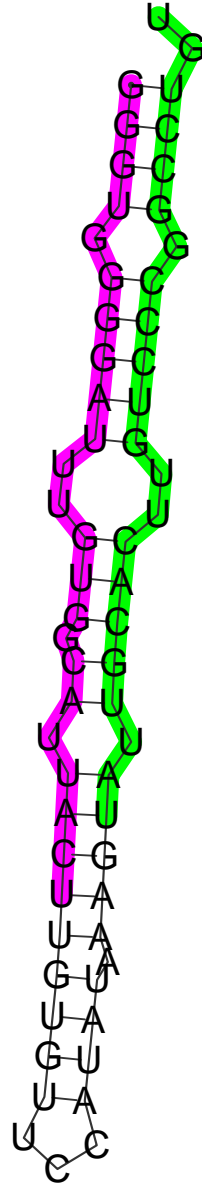

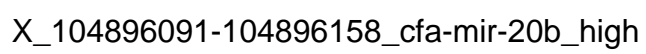

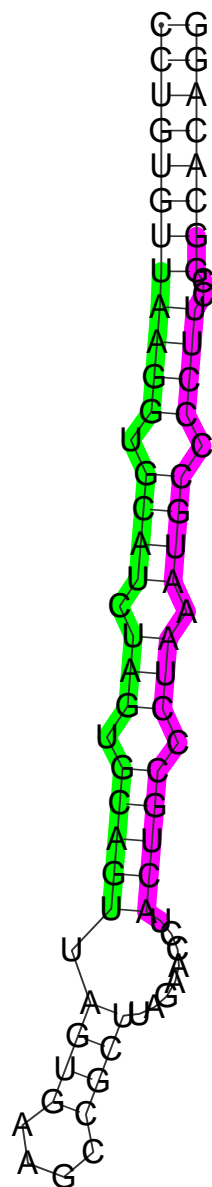

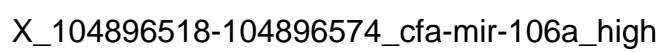

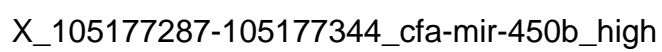



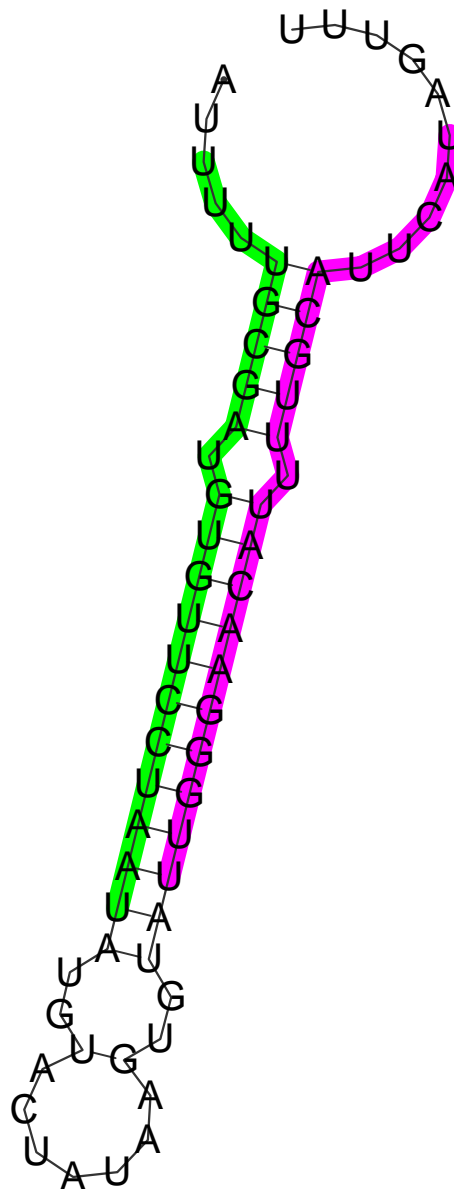

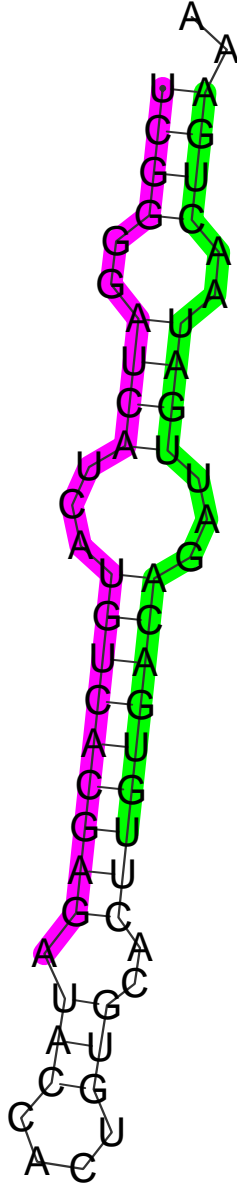

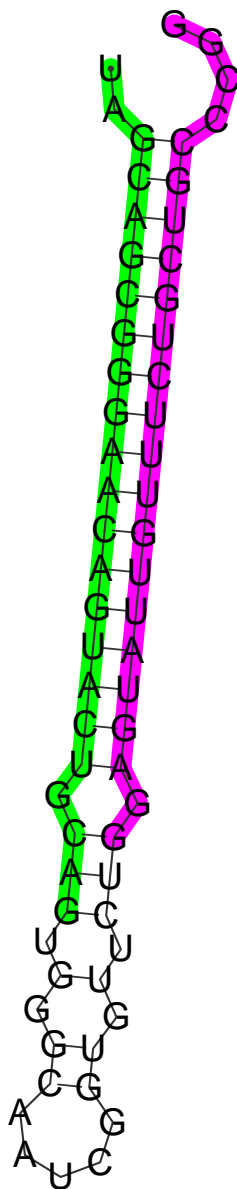

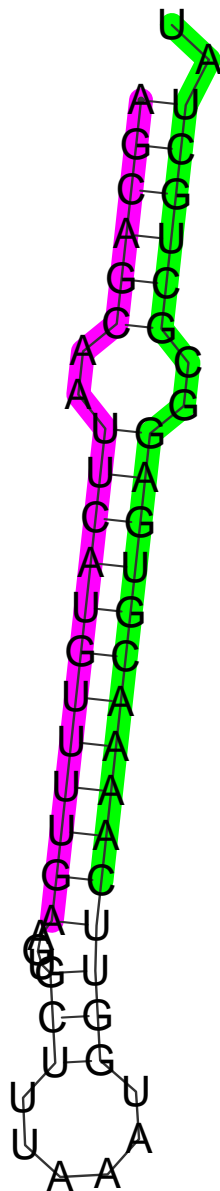

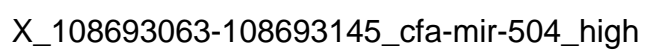

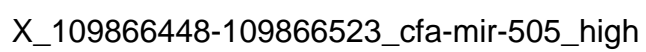

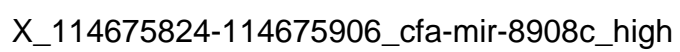

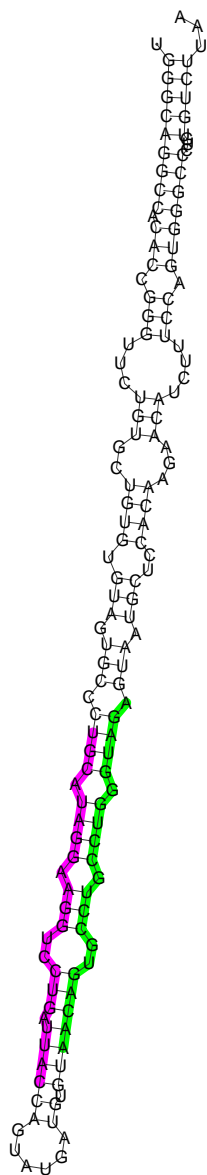

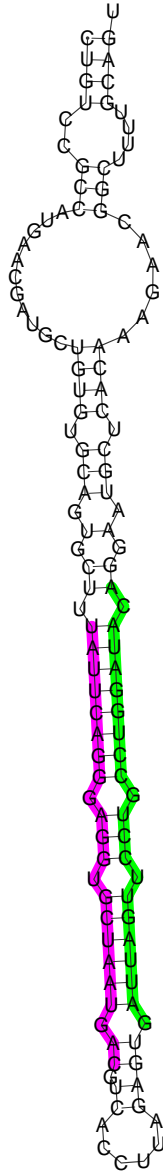

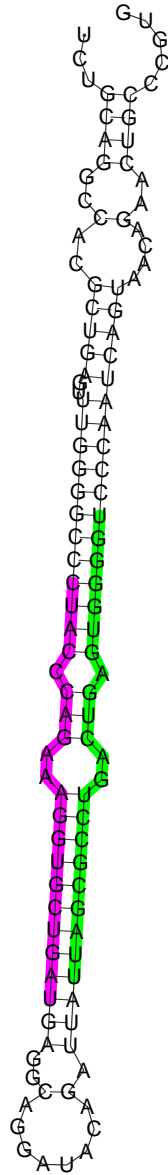

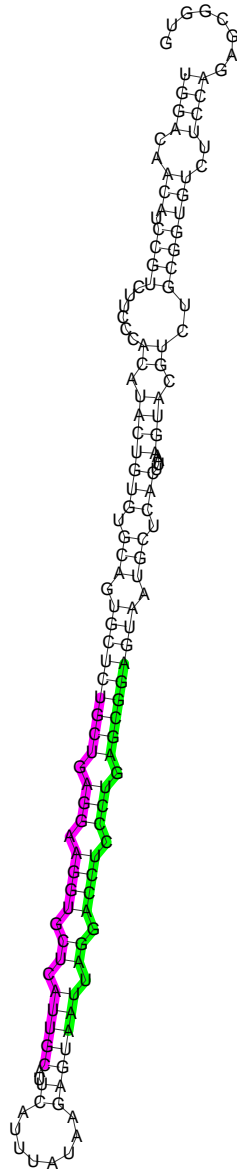

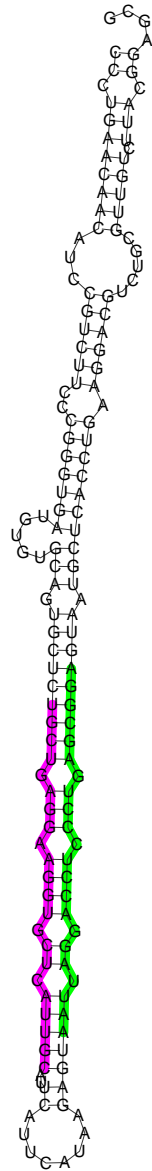

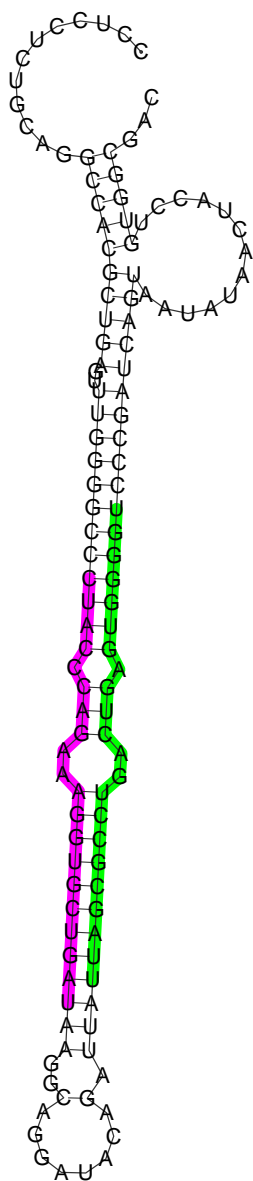

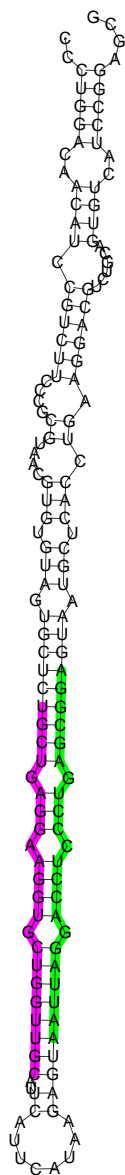

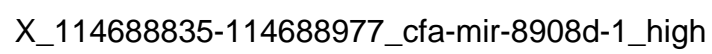

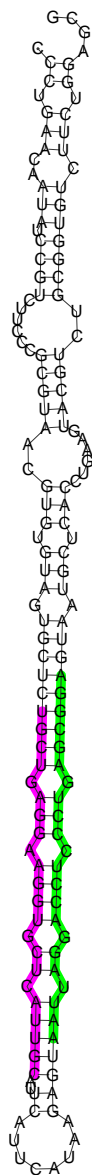

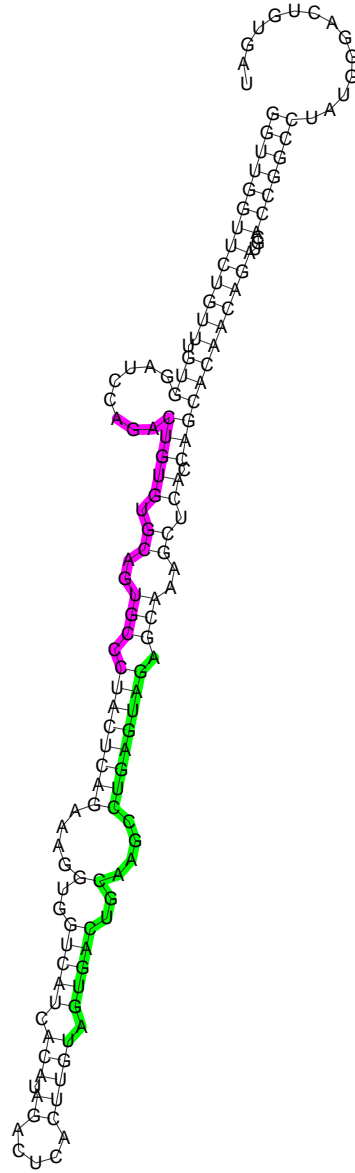

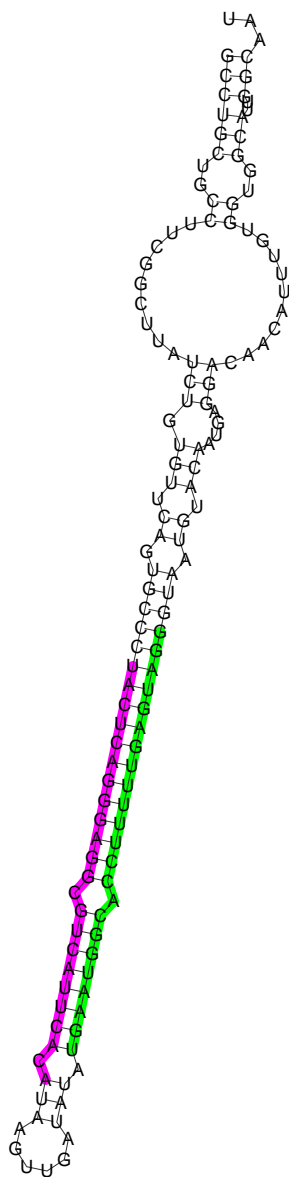

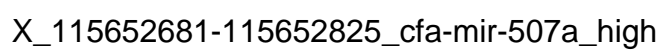

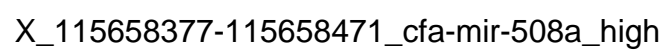

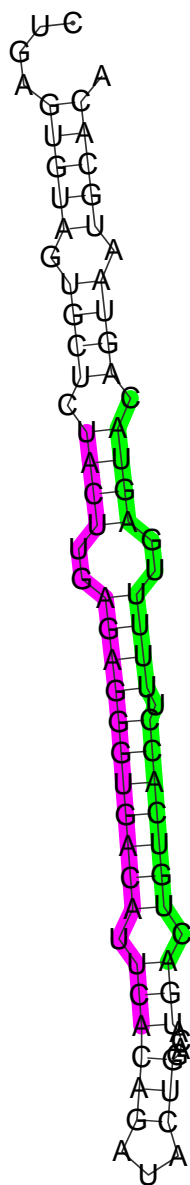

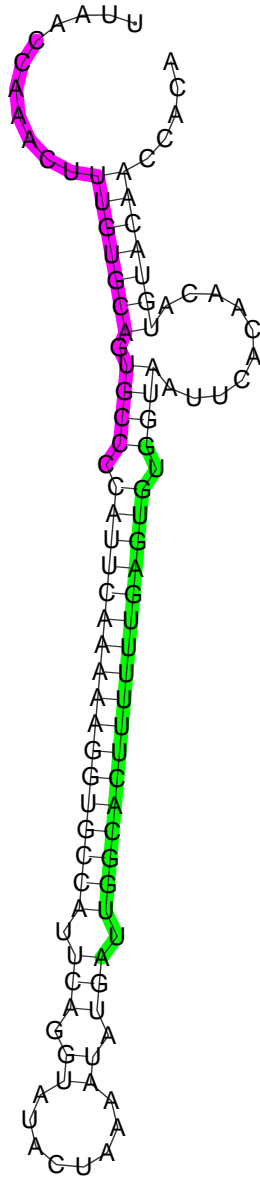

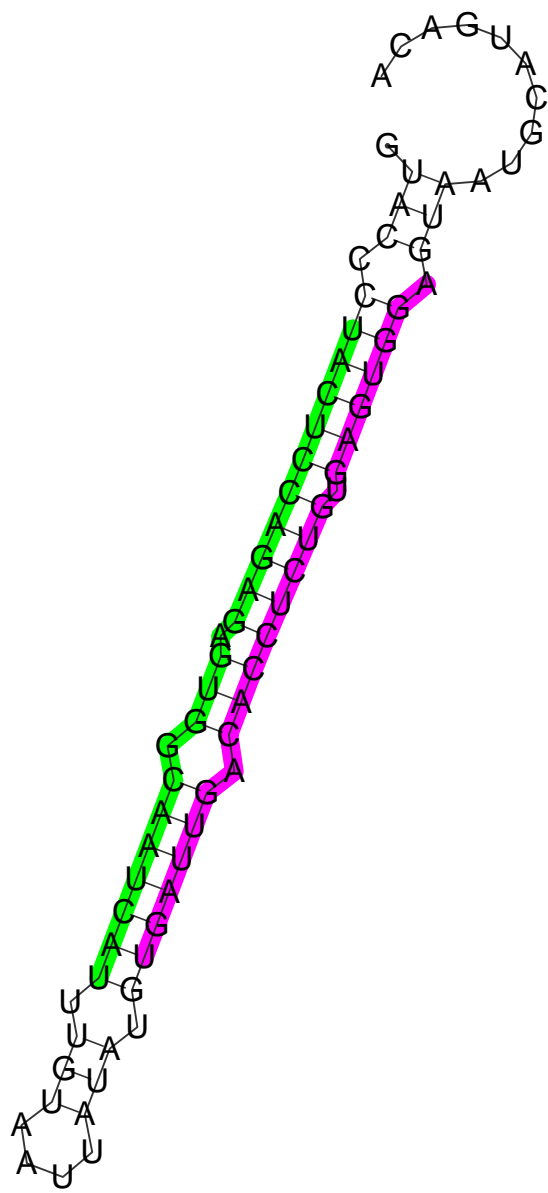

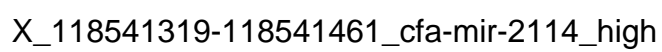

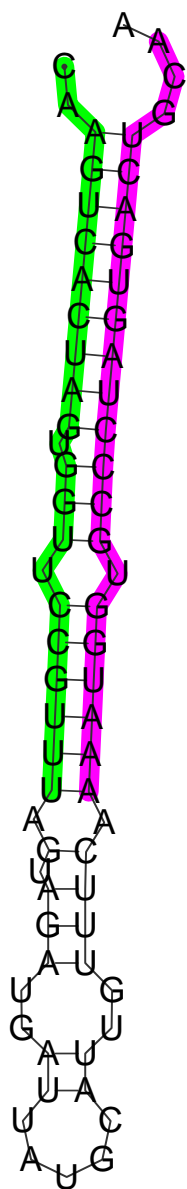

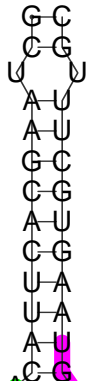

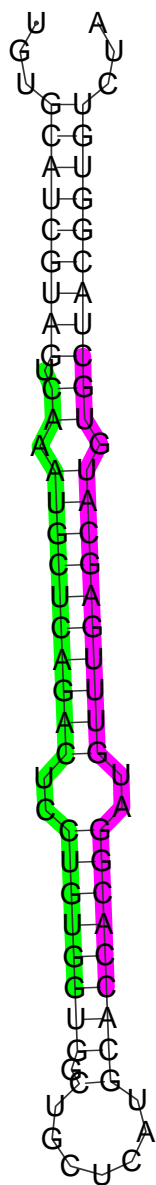

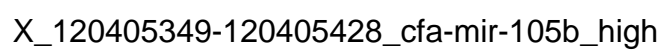

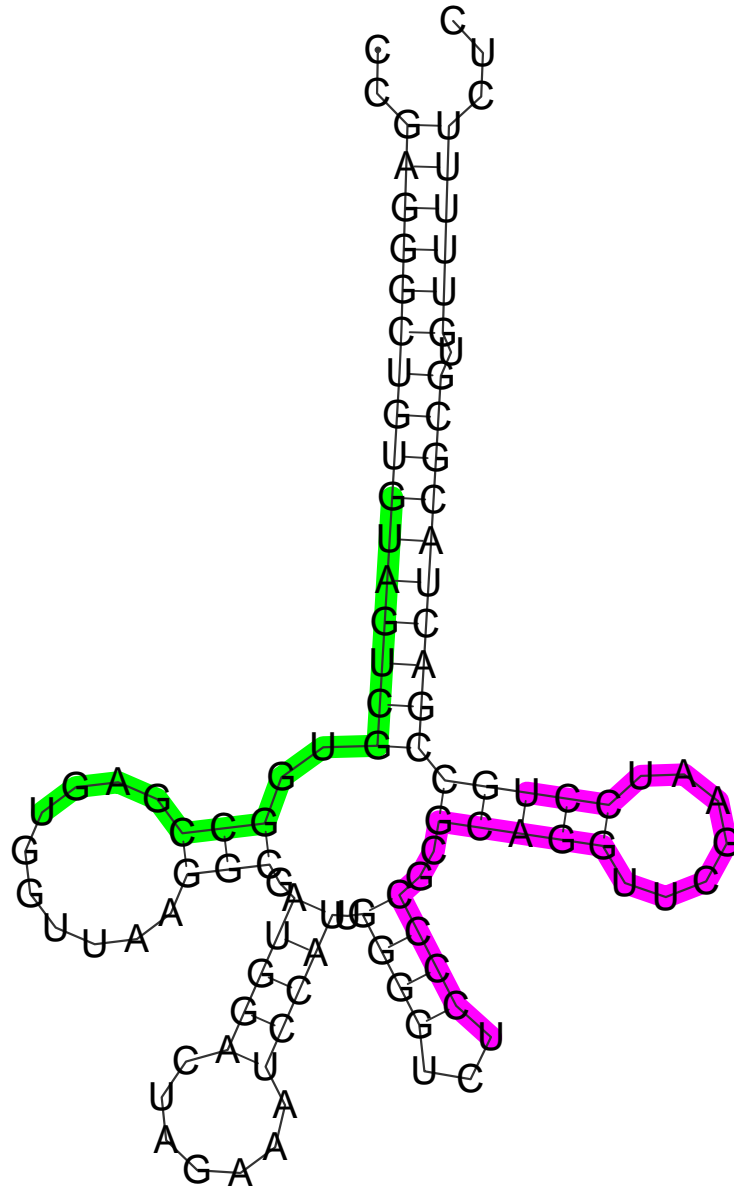

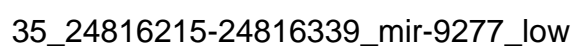

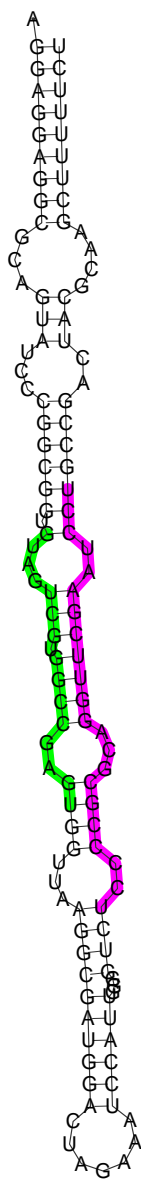

Supplement: S7 Fig — Purple and green labelled regions respectively correspond to the 5p- and 3p miRNA sequences, as predicted by small RNA reads alignments to the hairpin sequence. (PDF) [file pone.0153453.s007.pdf]

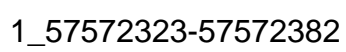

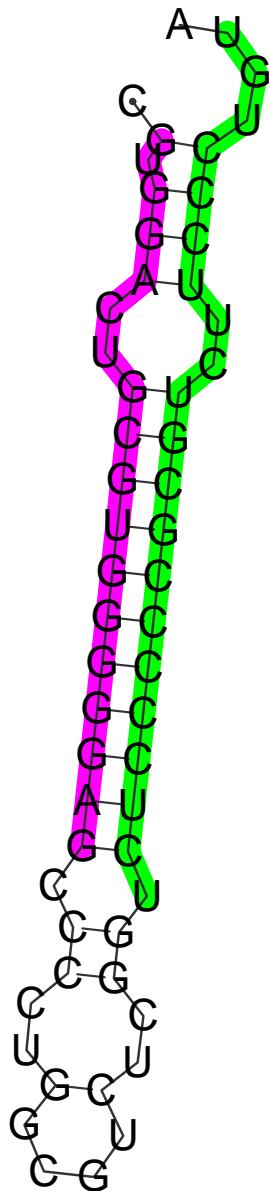

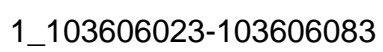

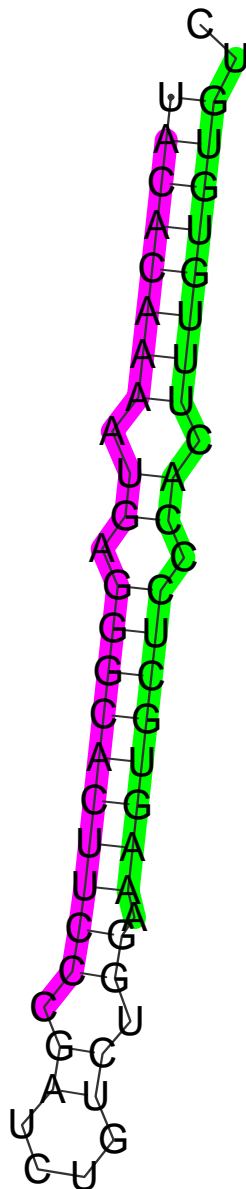

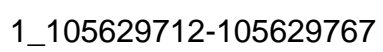

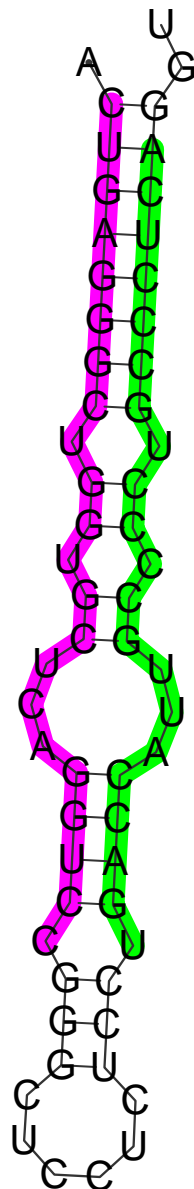

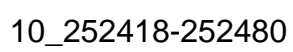

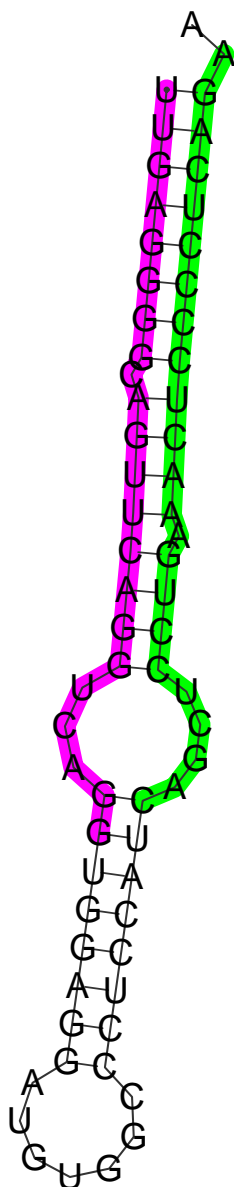

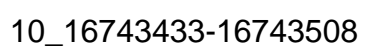

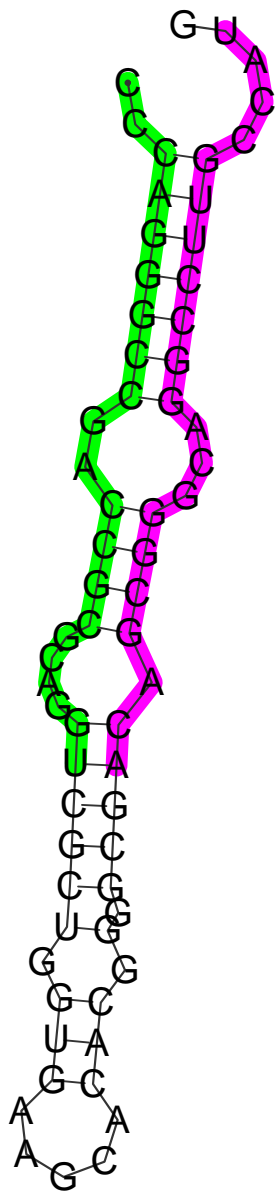

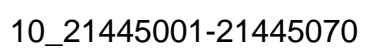

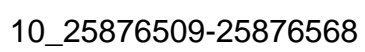

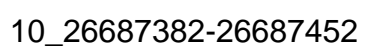

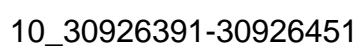

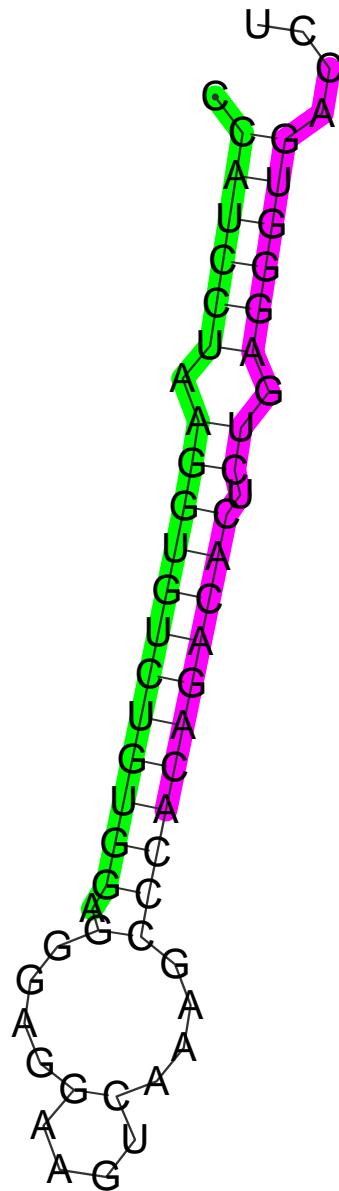

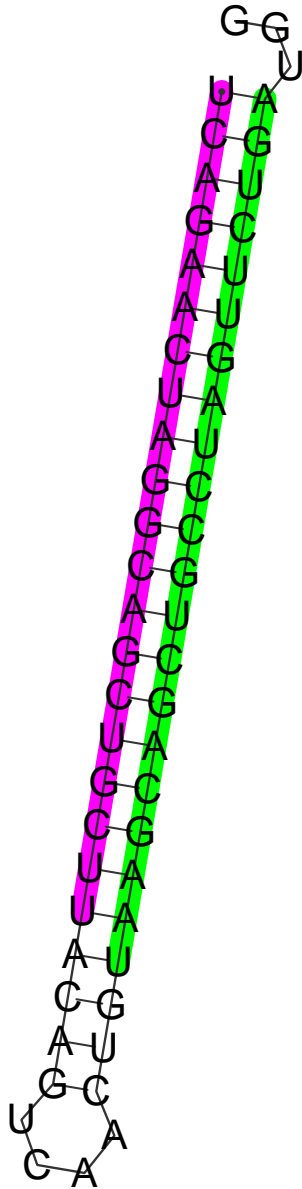

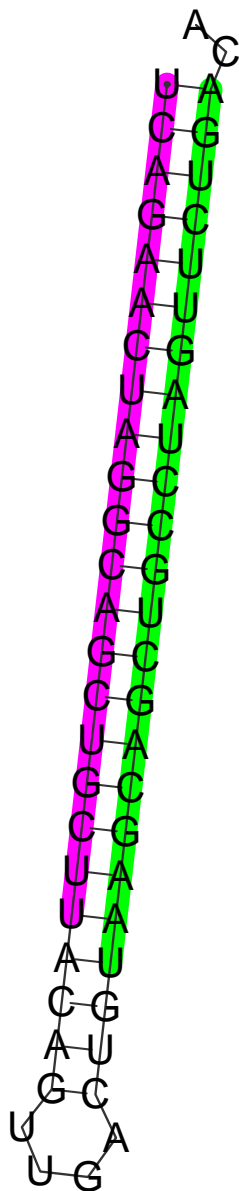

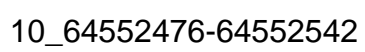

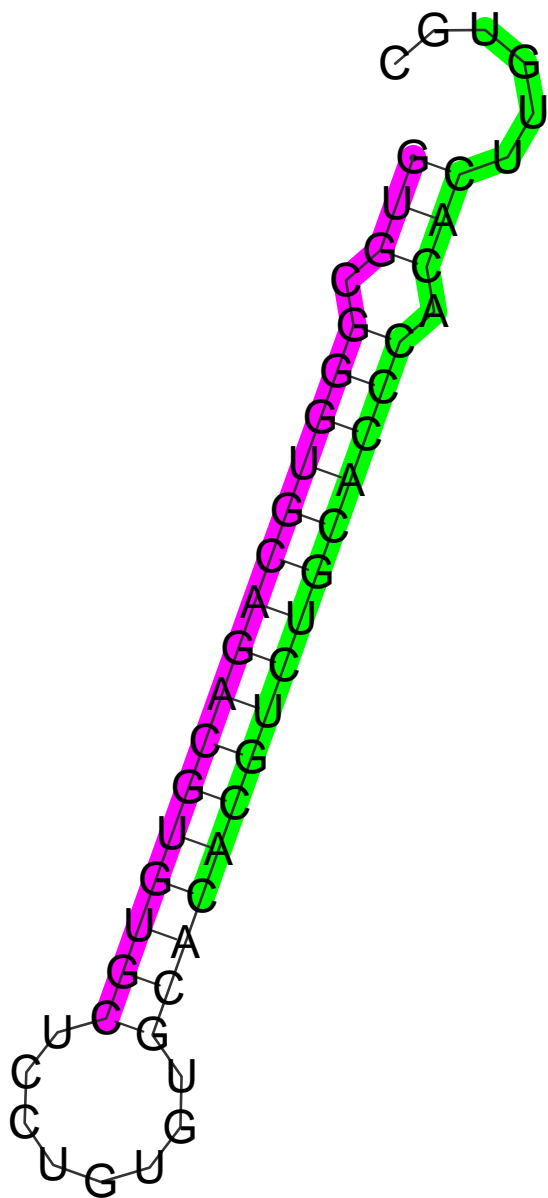

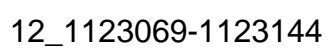

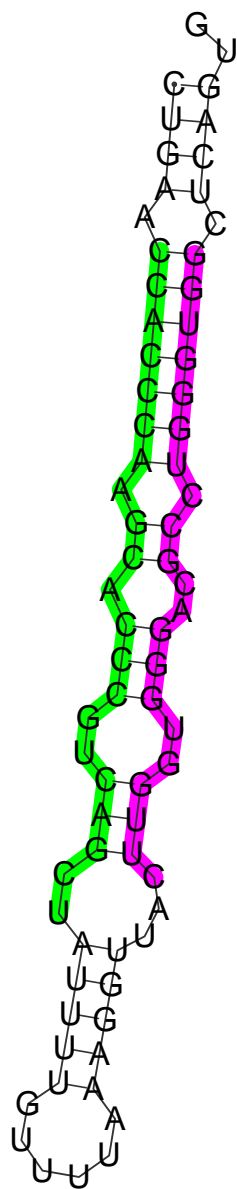

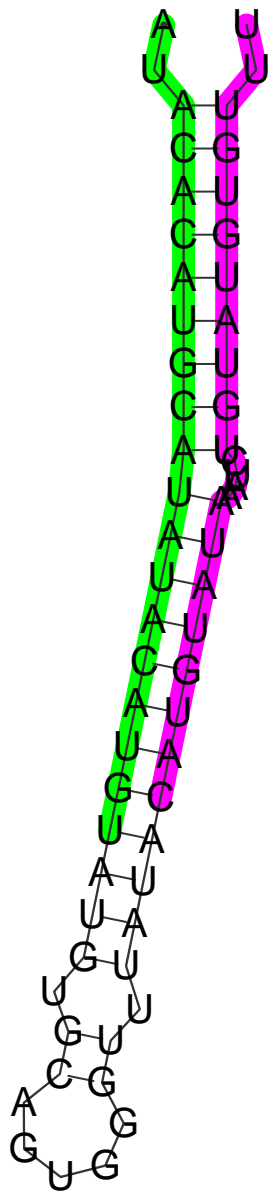

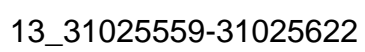

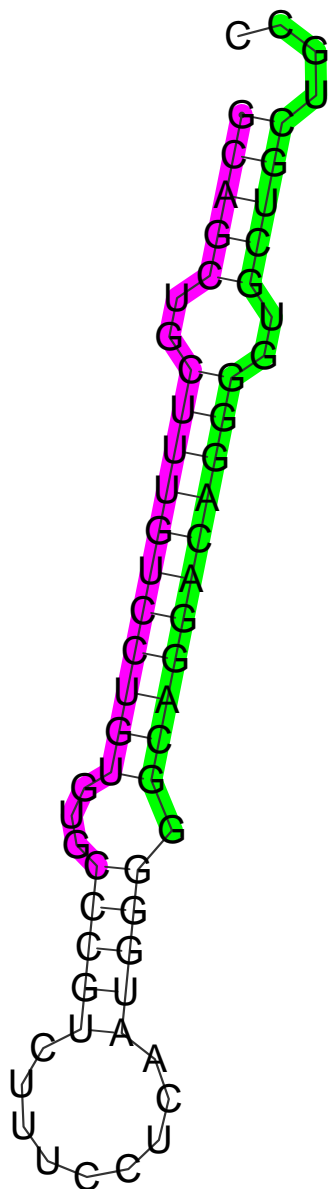

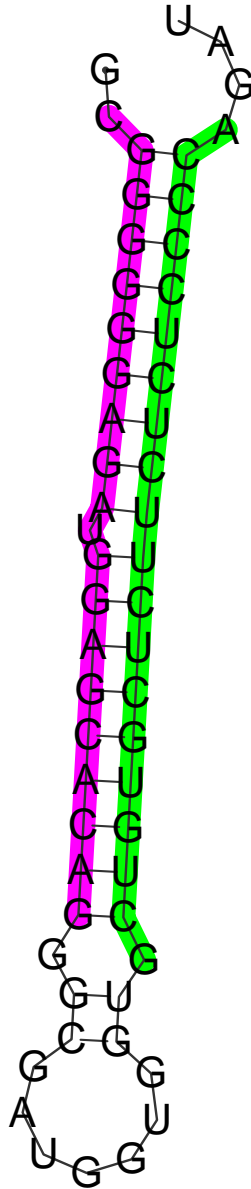

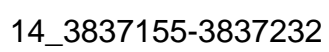

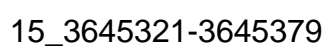

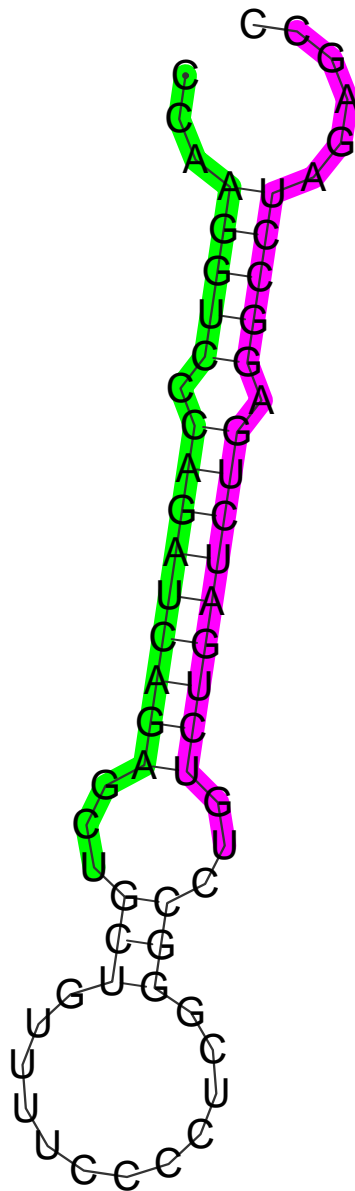

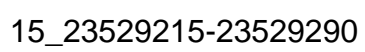

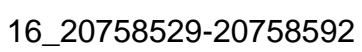

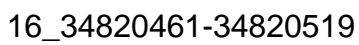

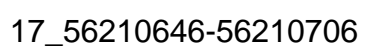

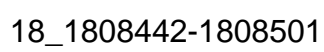



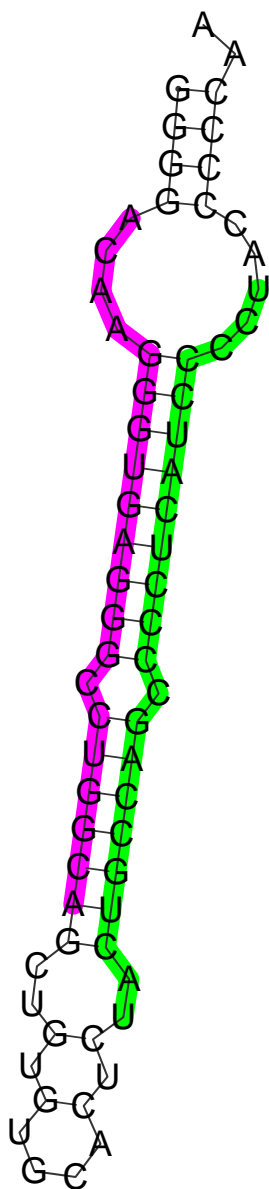



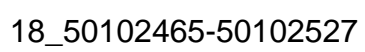

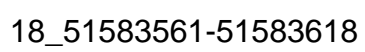

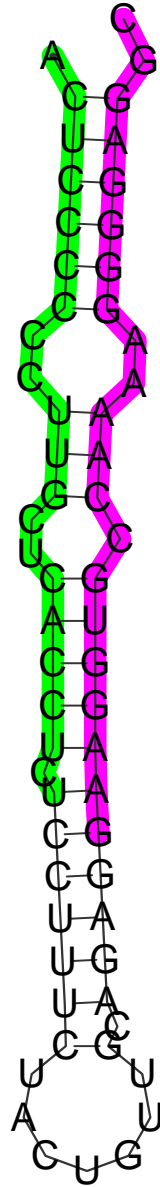

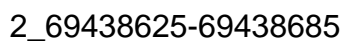

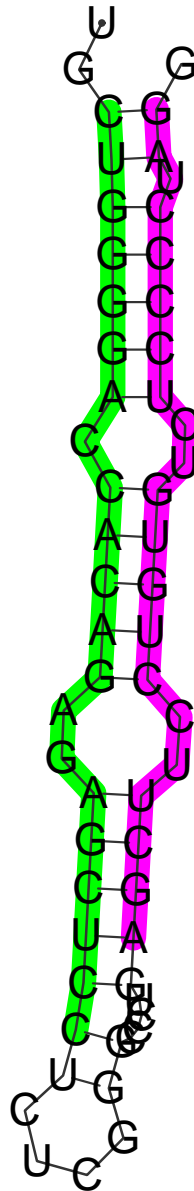

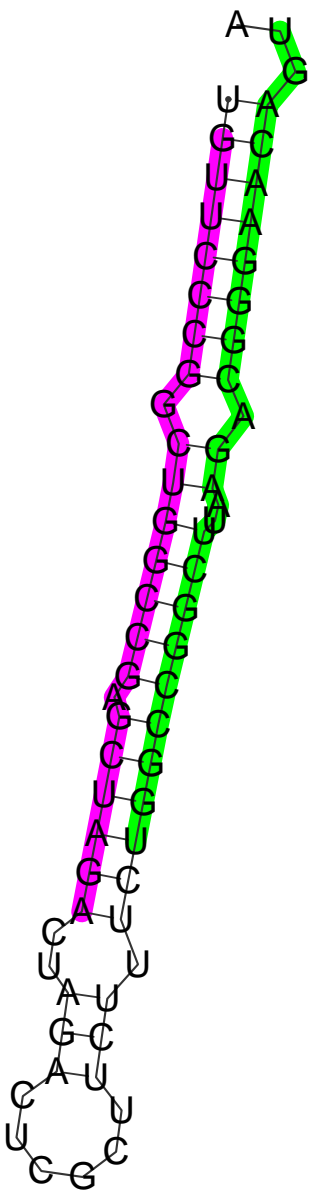

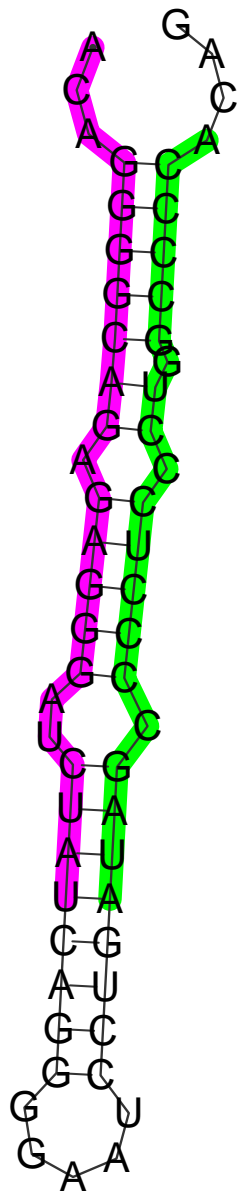

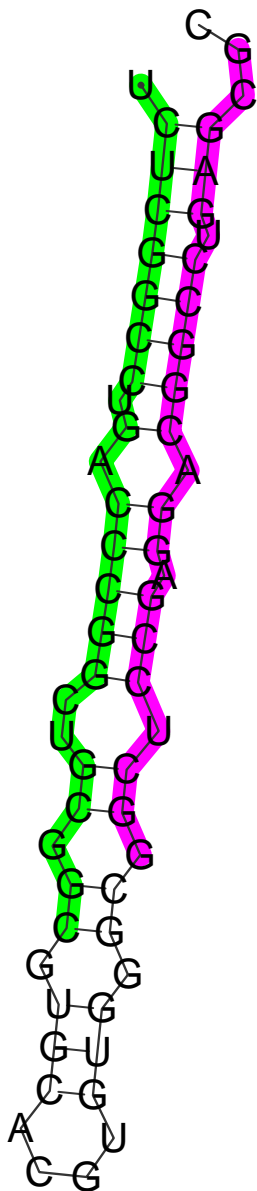

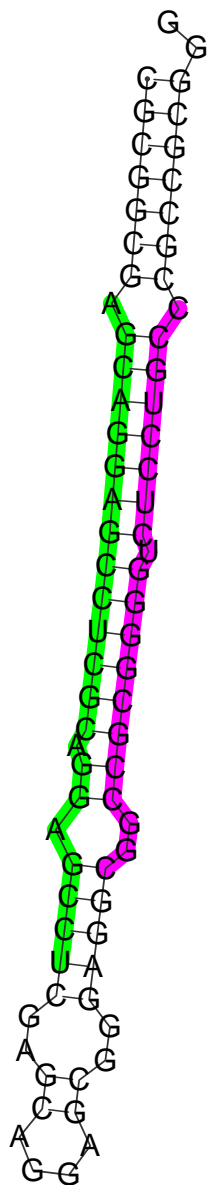

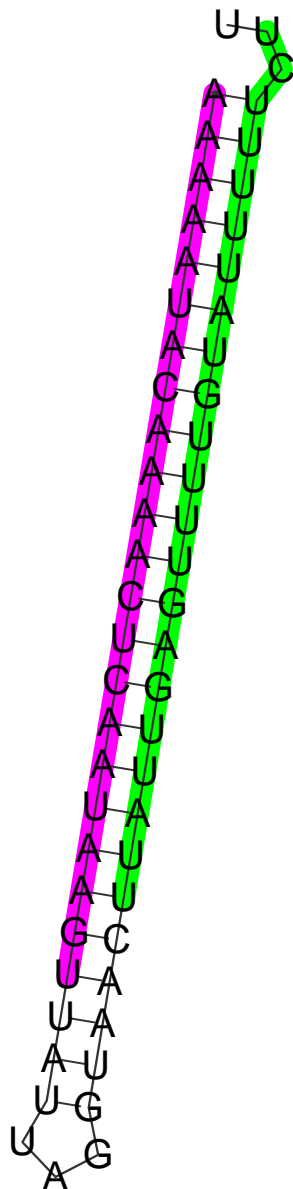

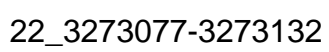

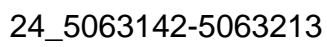

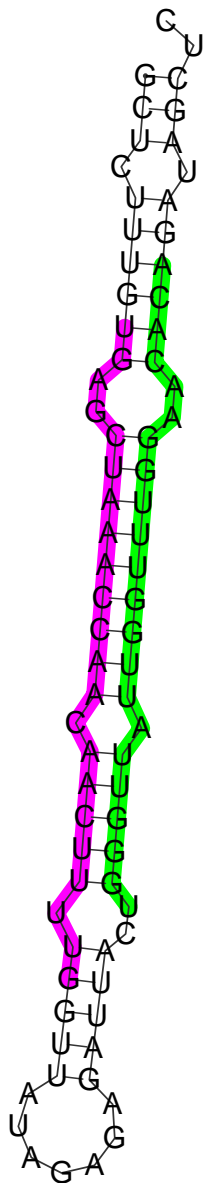



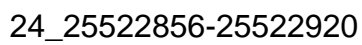

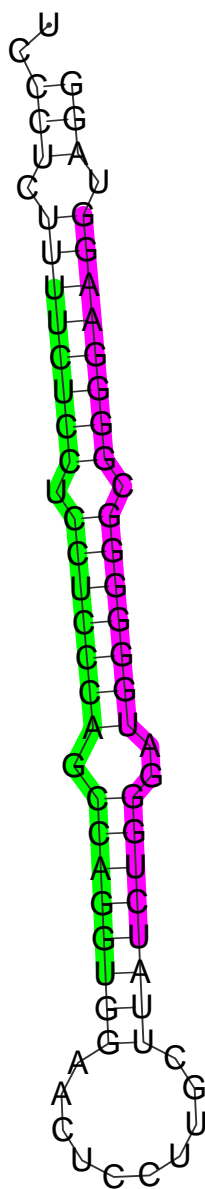

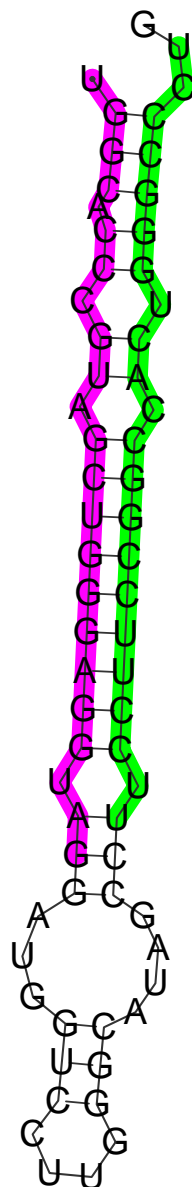

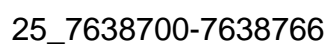

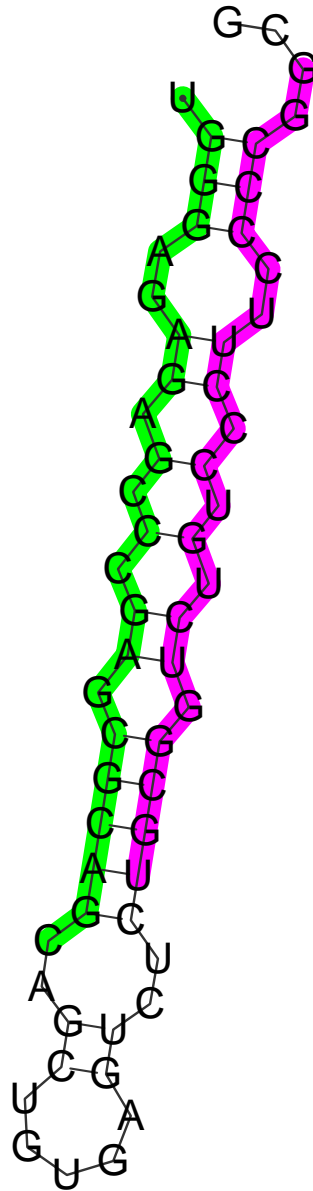

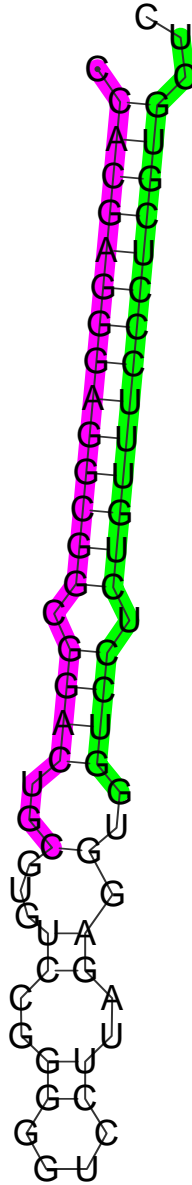



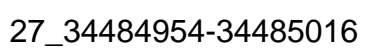

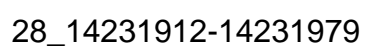

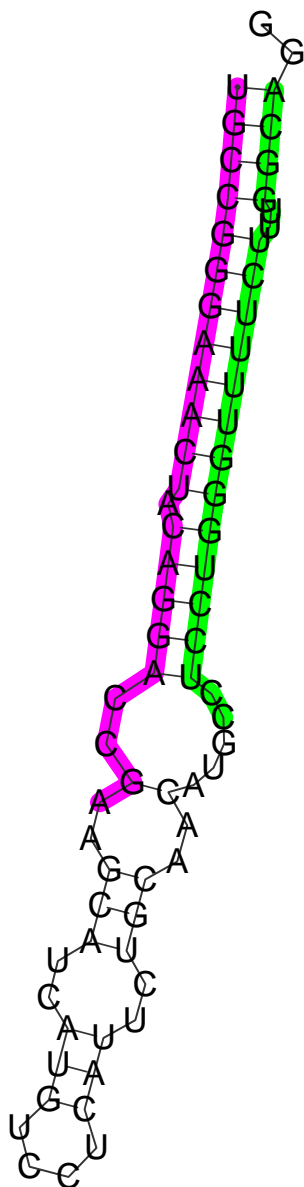

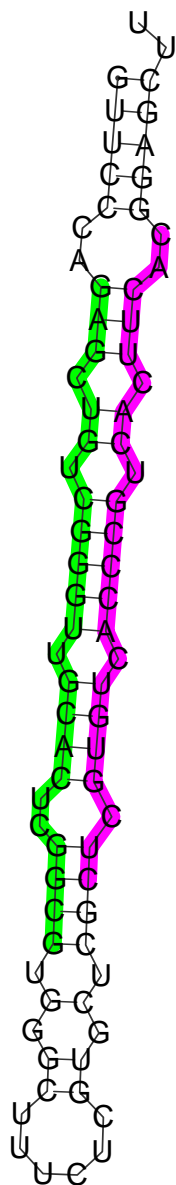



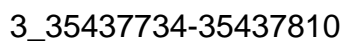

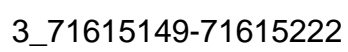

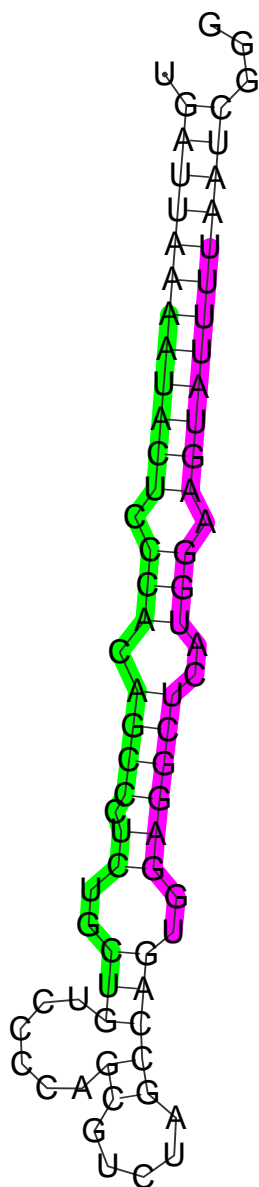

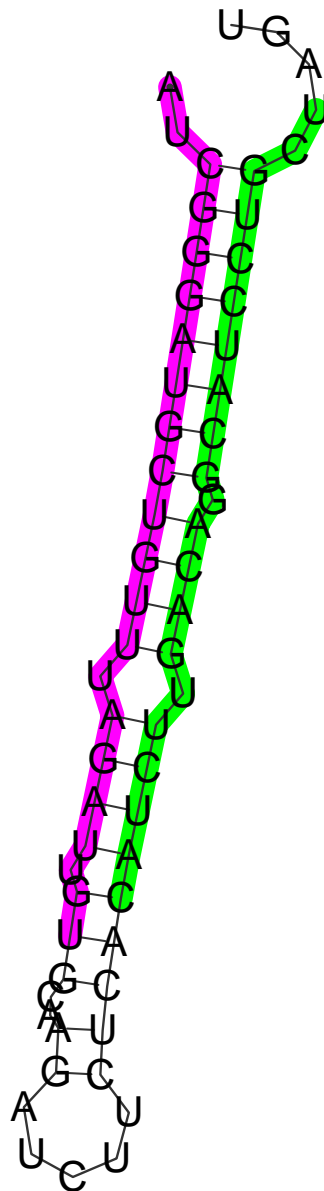



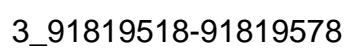

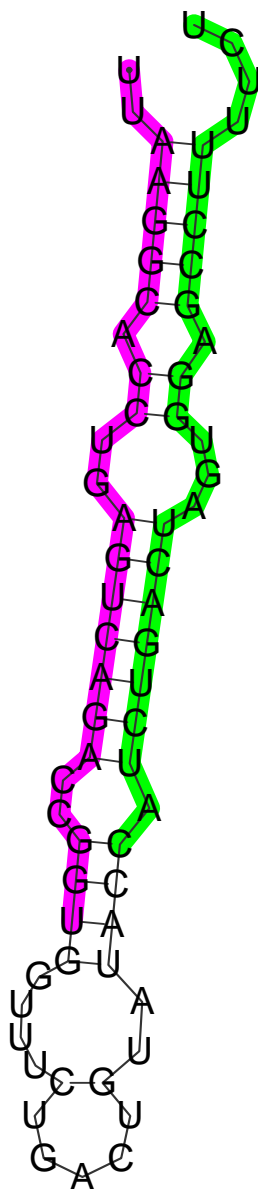

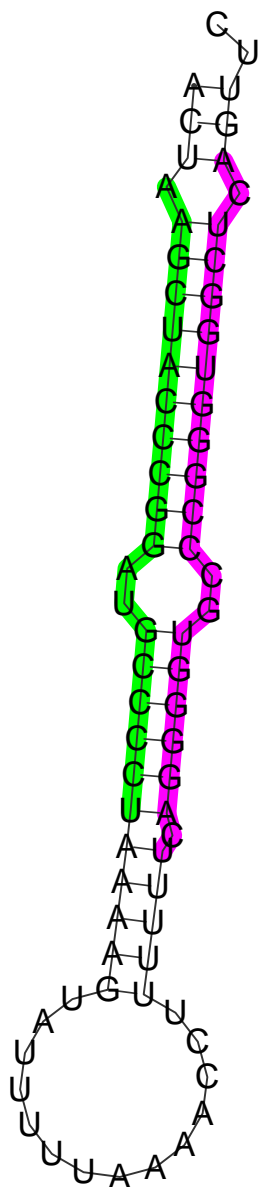

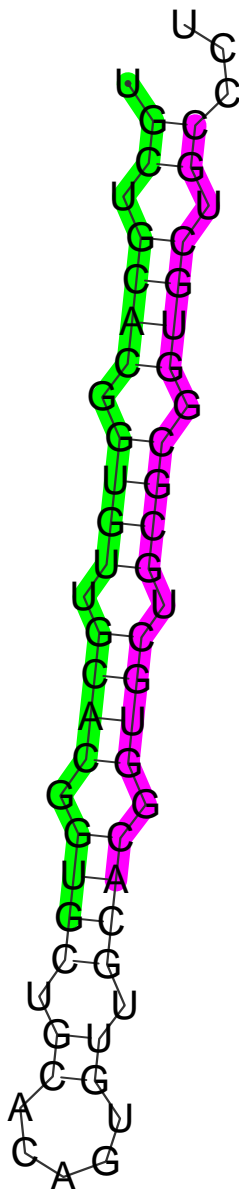

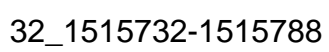

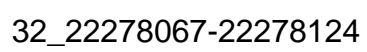

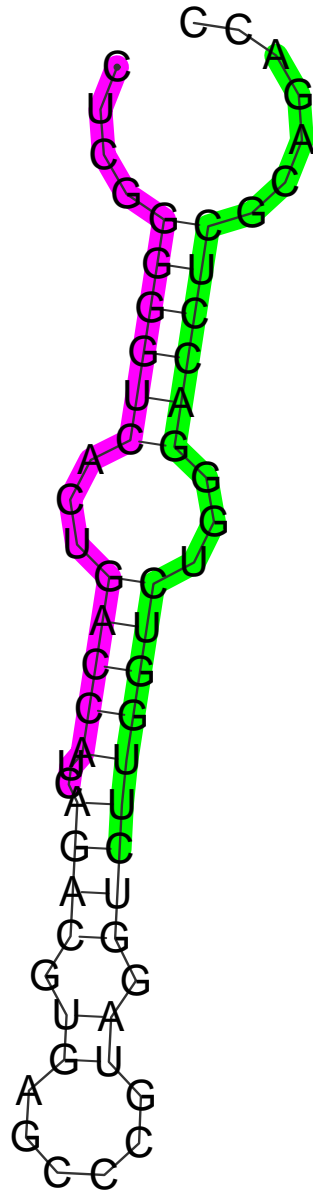

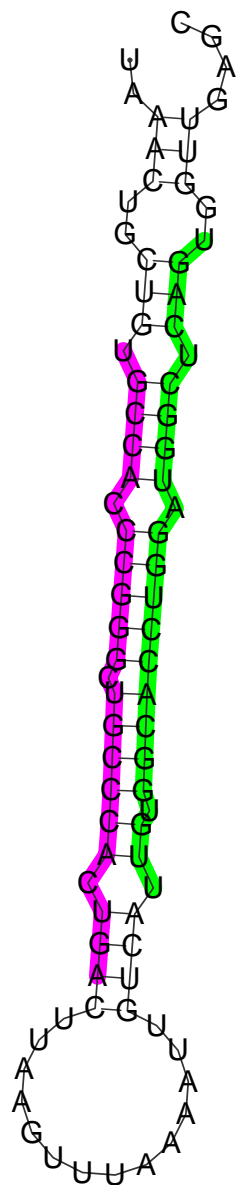

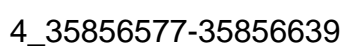

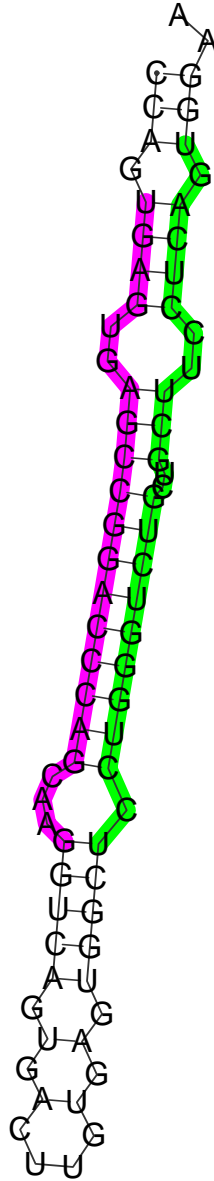

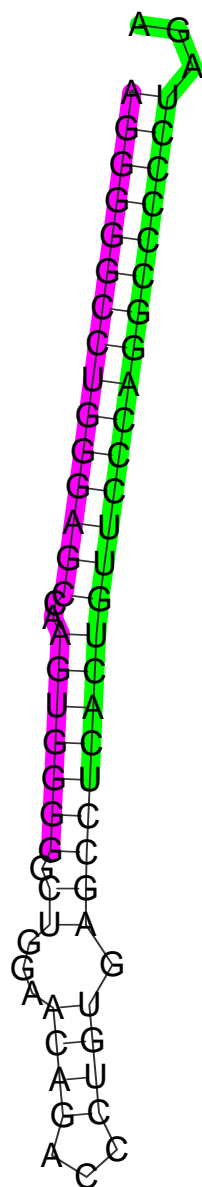

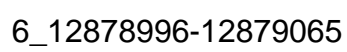

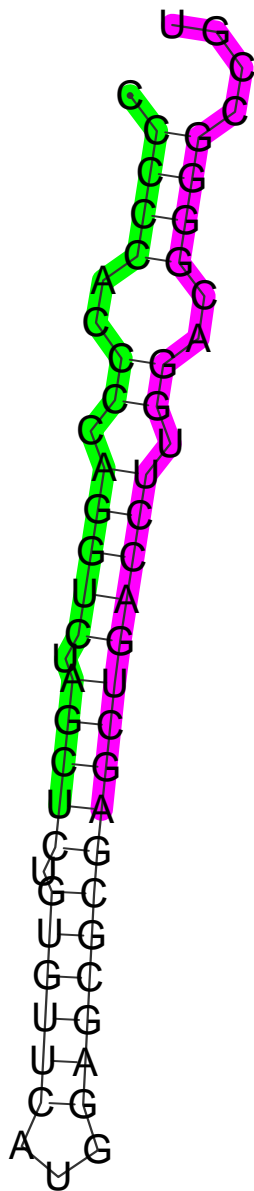

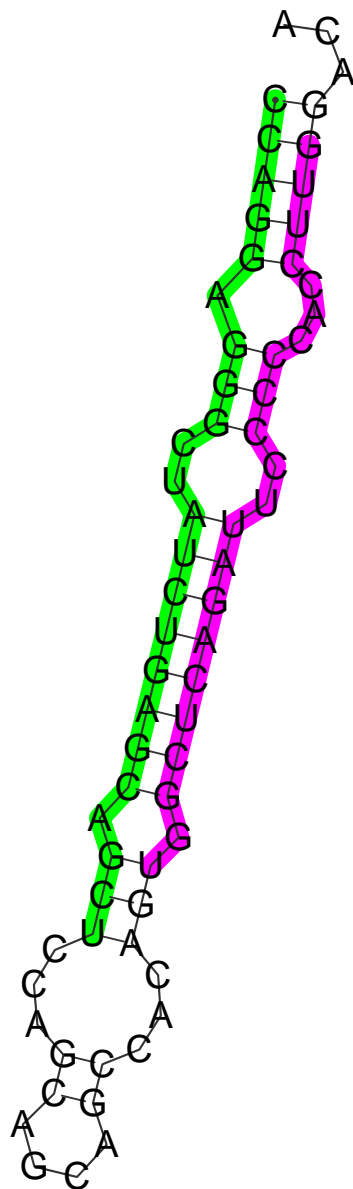

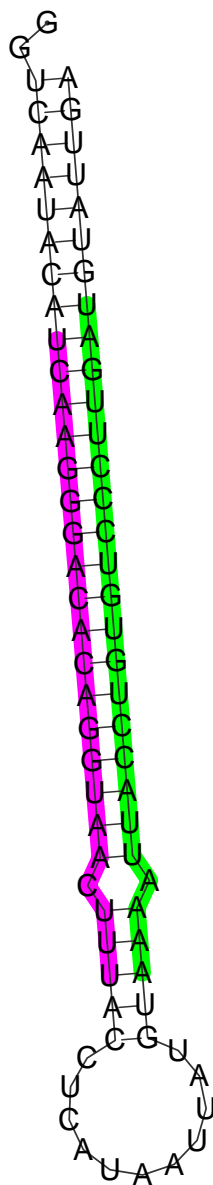

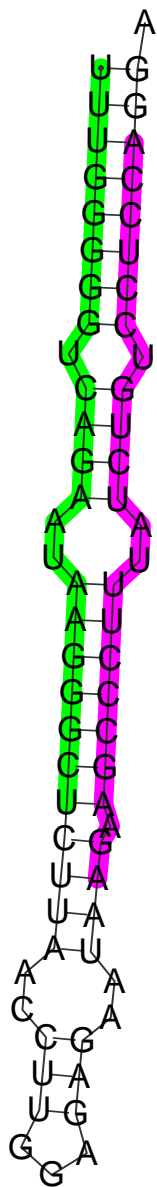

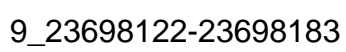

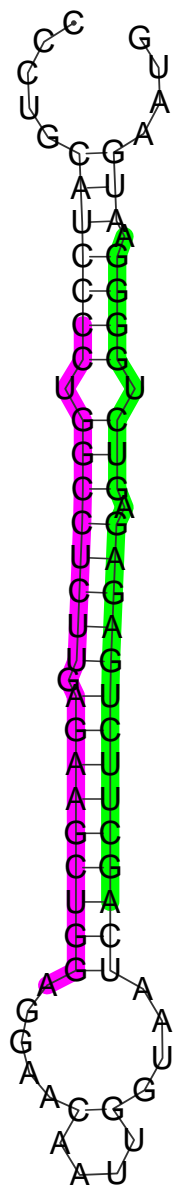



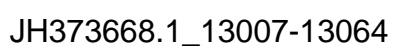

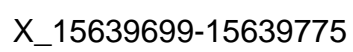

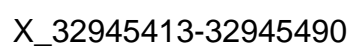

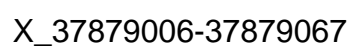

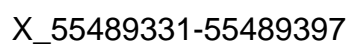

Supplement: S8 Fig — Purple and green labelled regions respectively correspond to the 5p- and 3p miRNA sequences, as predicted by small RNA reads alignments to the hairpin sequence. (PDF) [file pone.0153453.s008.pdf]

# GENOMIC LOCATION OF NOVEL AND CONSERVED MIRNAS

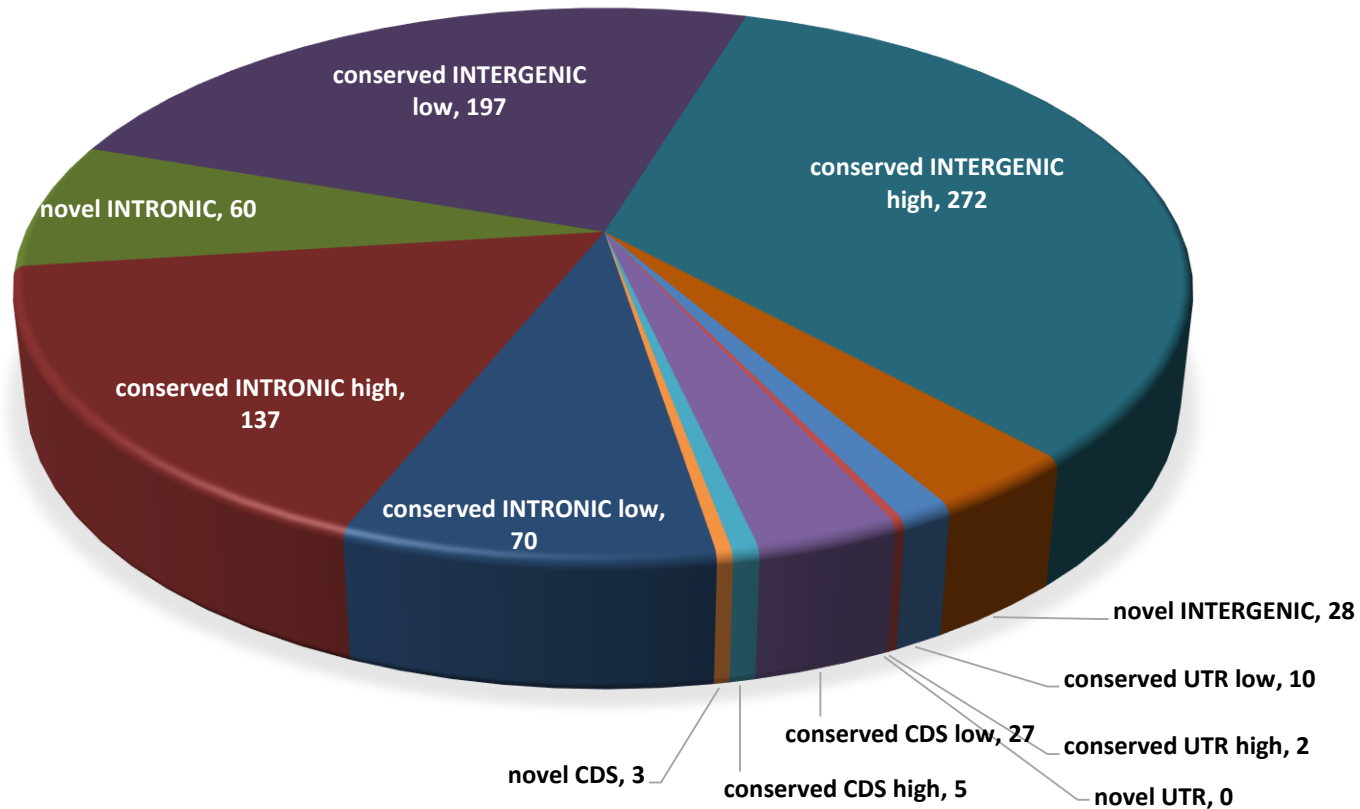

Supplement: S11 Fig — (PDF) [file pone.0153453.s011.pdf]
