# Supplementary material for: An Improved microRNA Annotation of the Canine Genome
Source: PLoS One. 2016 Apr 27;11(4):e0153453. doi: 10.1371/journal.pone.0153453 (PMC4847789; doi:10.1371/journal.pone.0153453)
Supplement: S4 Fig — The base position along the hairpin (x axis) is plotted against the normalized read count (reads per million genome matching reads). Different colors in the plot correspond to different tissues, as described by the legend on the bottom of each plot. (PDF) [file pone.0153453.s004.pdf]

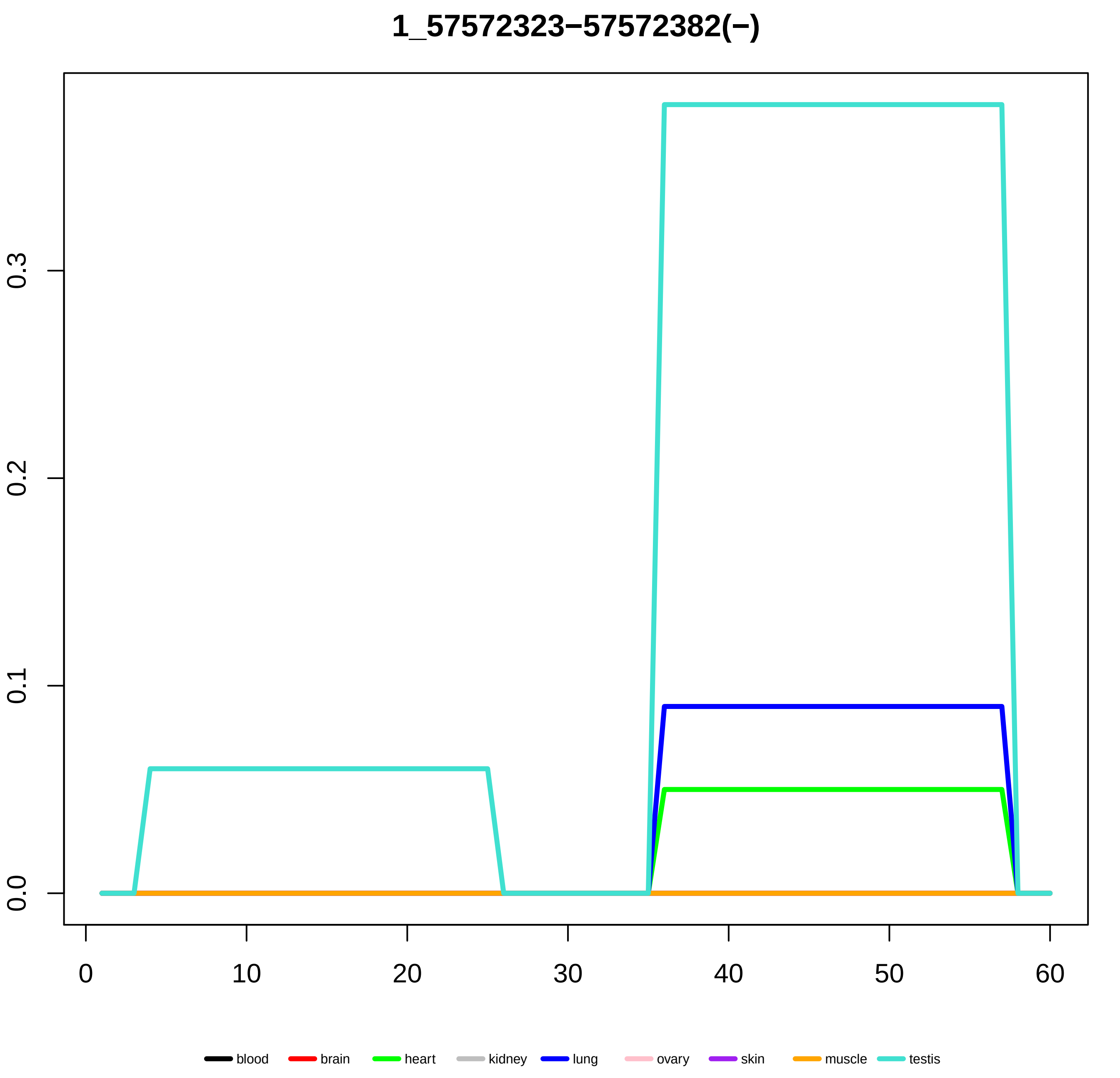

1_57572323−57572382(−)
0.0
0.1
0.2
0.3
0
10
20
30
40
50
60
blood
brain
heart
kidney
lung
ovary
skin
muscle
testis

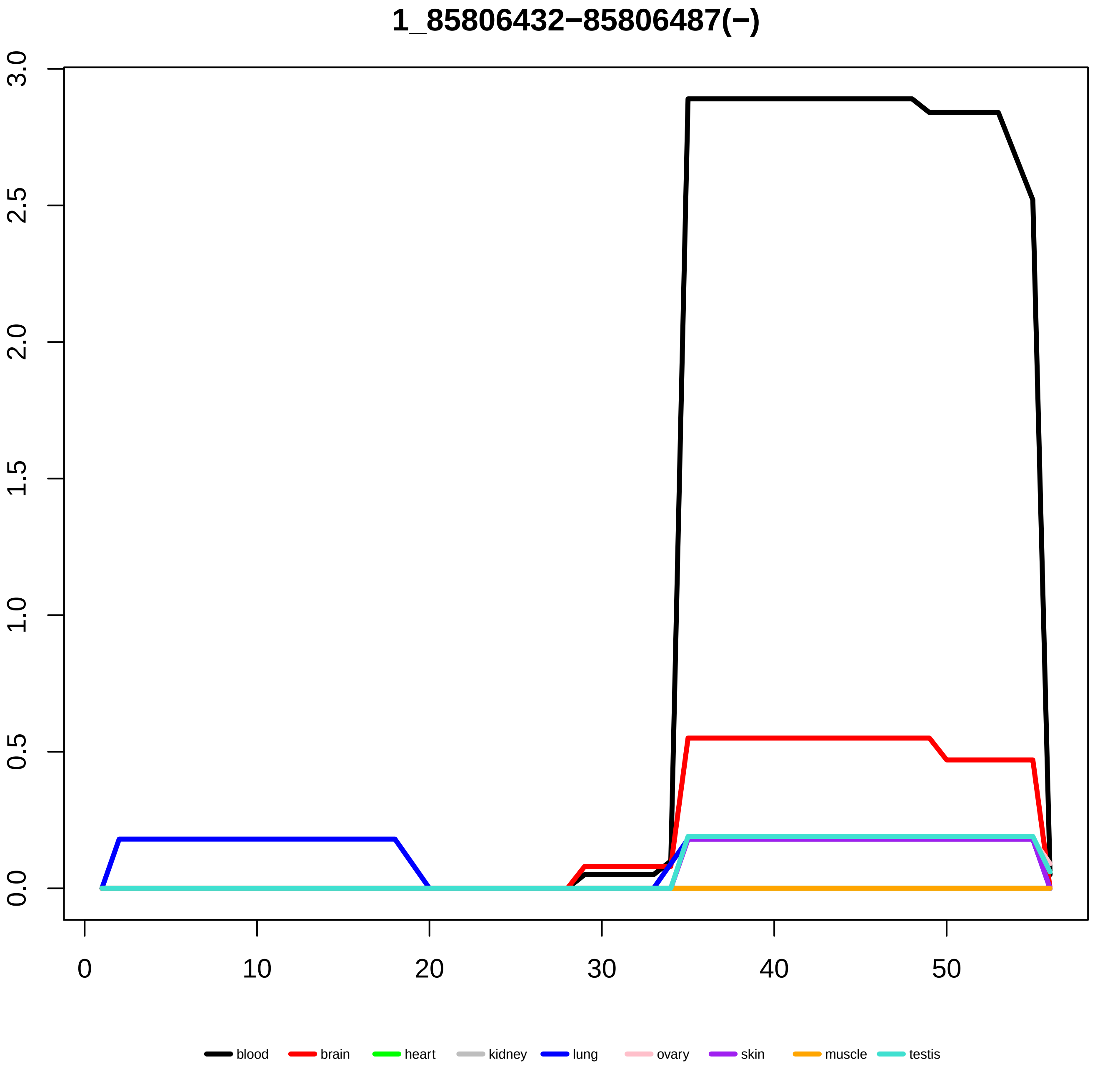
1_85806432−85806487(−)
0.0
0.5
1.0
1.5
2.0
2.5
3.0
0
10
20
30
40
50
blood
brain
heart
kidney
lung
ovary
skin
muscle
testis

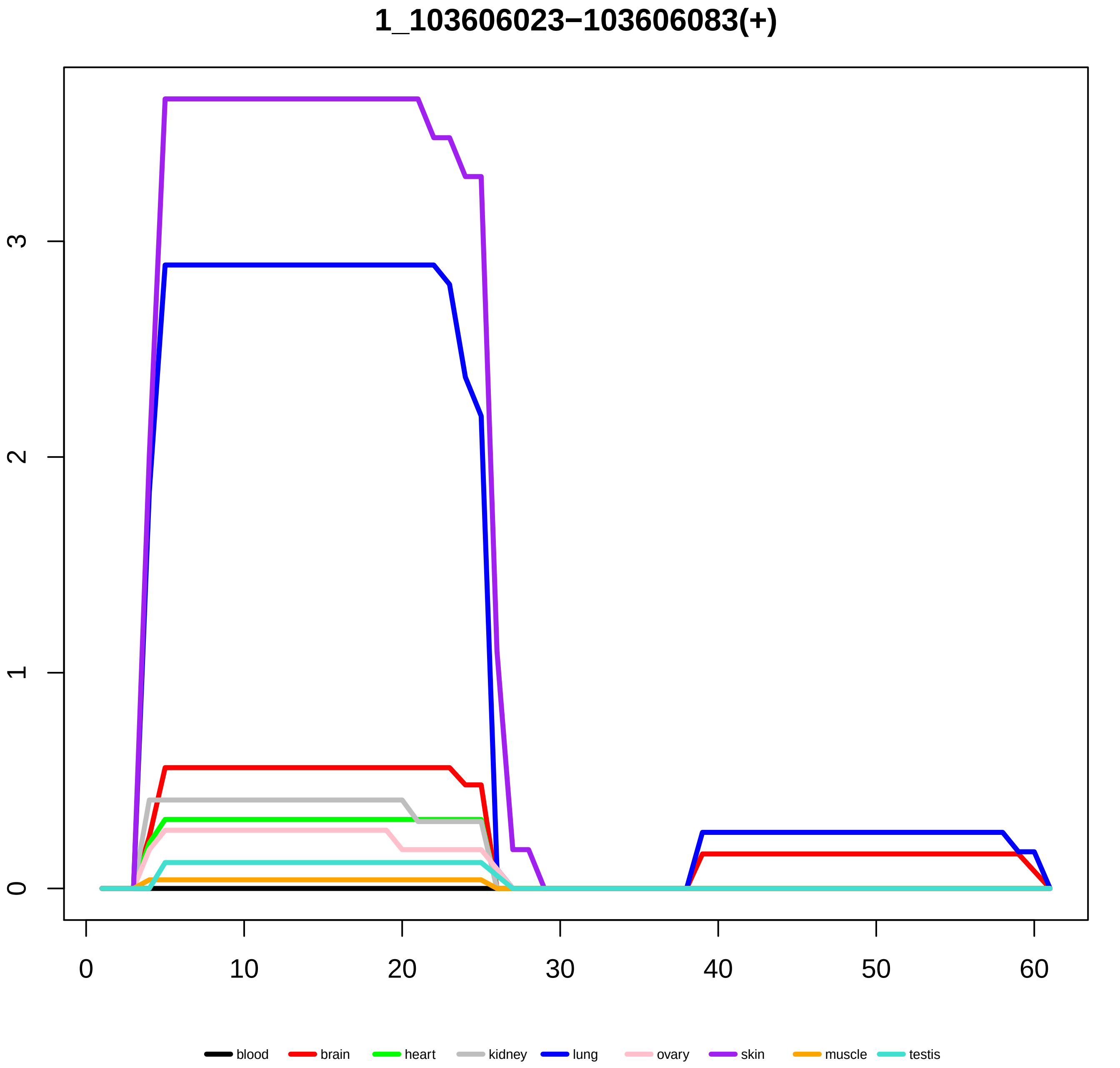

1_103606023−103606083(+)
0
1
2
3
0
10
20
30
40
50
60
blood
brain
heart
kidney
lung
ovary
skin
muscle
testis

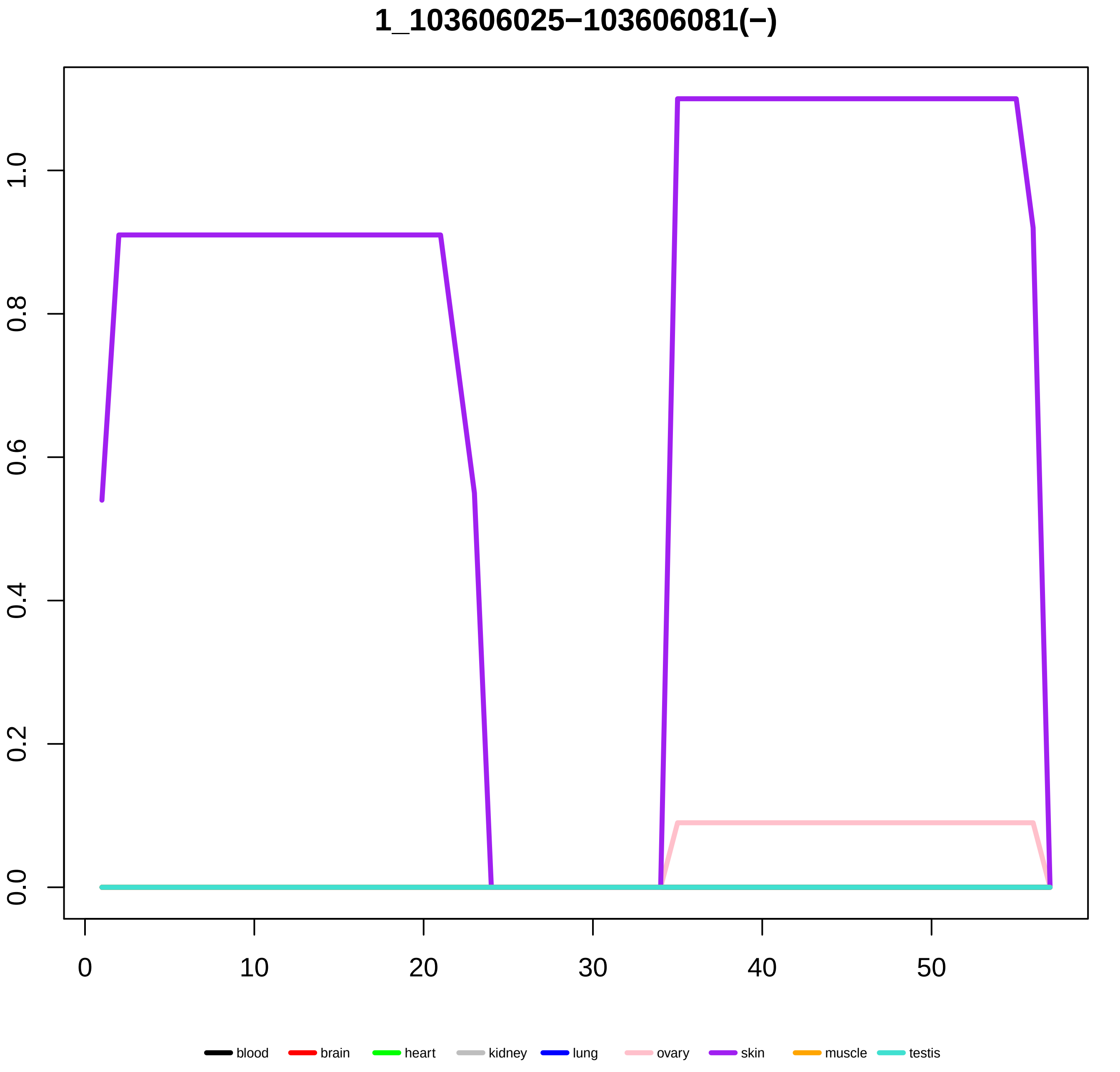

1_103606025−103606081(−)
0.0
0.2
0.4
0.6
0.8
1.0
0
10
20
30
40
50
blood
brain
heart
kidney
lung
ovary
skin
muscle
testis

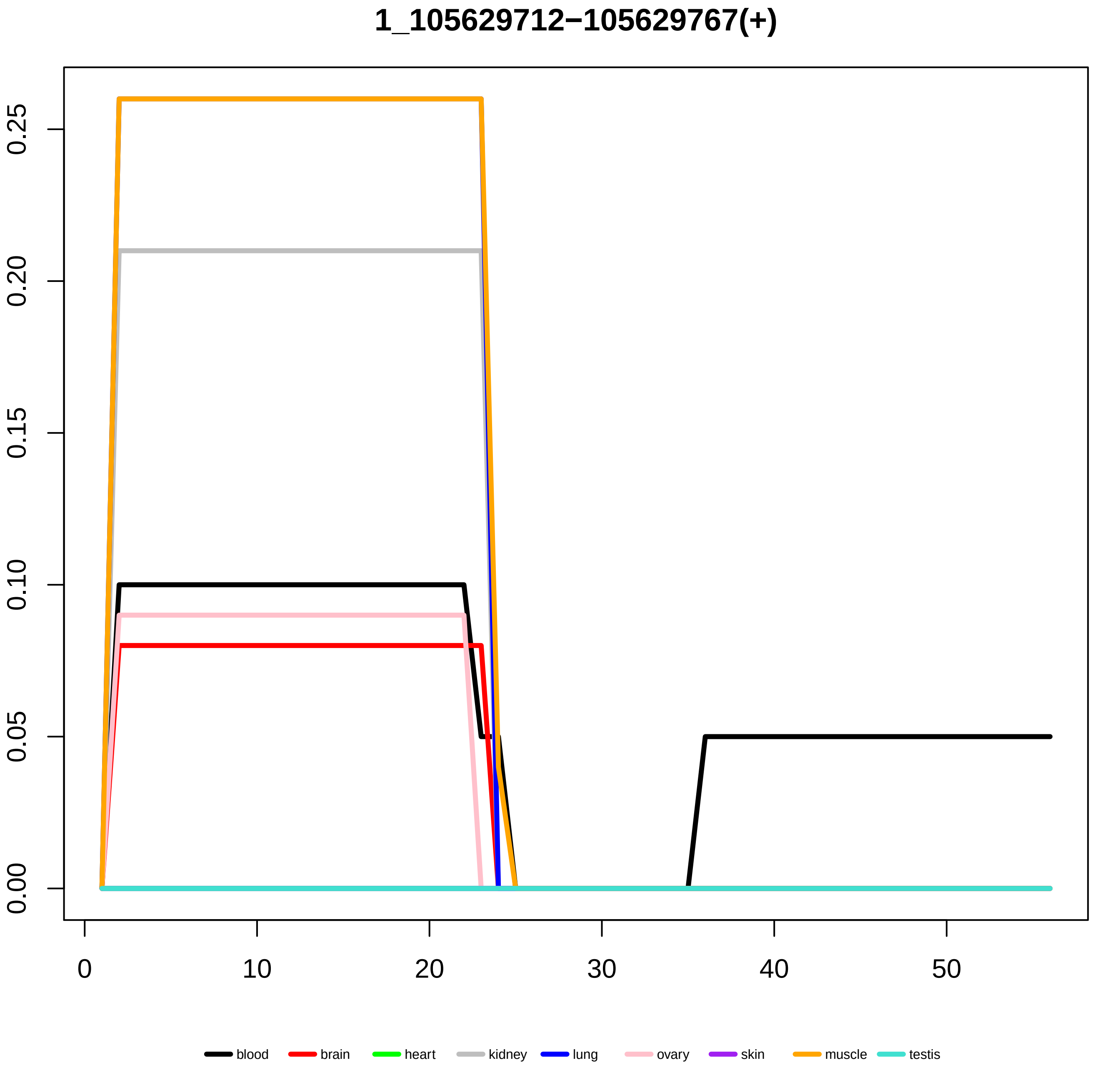
1_105629712−105629767(+)
0.00
0.05
0.10
0.15
0.20
0.25
0
10
20
30
40
50
blood
brain
heart
kidney
lung
ovary
skin
muscle
testis

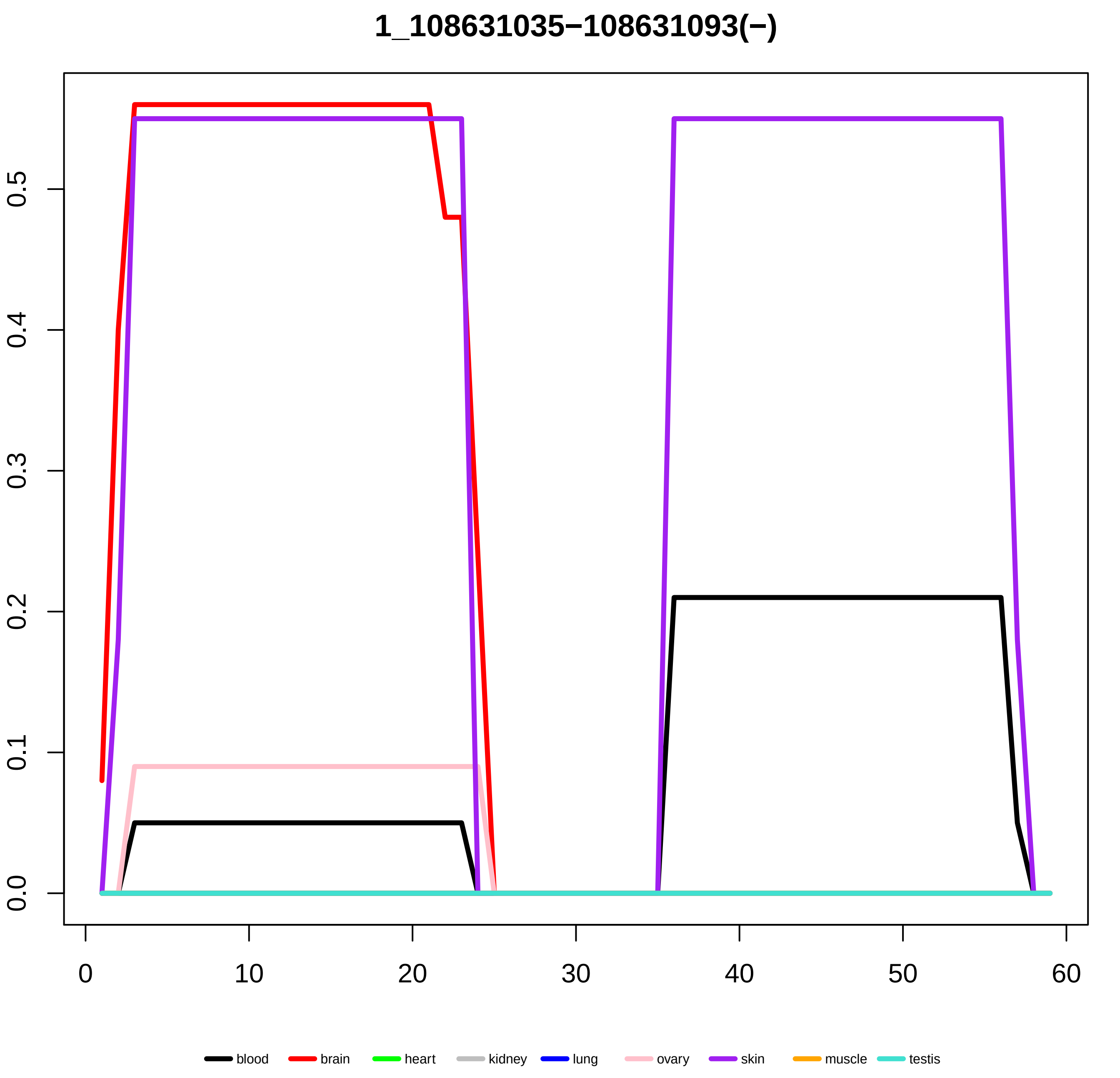

1_108631035−108631093(−)
0.0
0.1
0.2
0.3
0.4
0.5
0
10
20
30
40
50
60
blood
brain
heart
kidney
lung
ovary
skin
muscle
testis

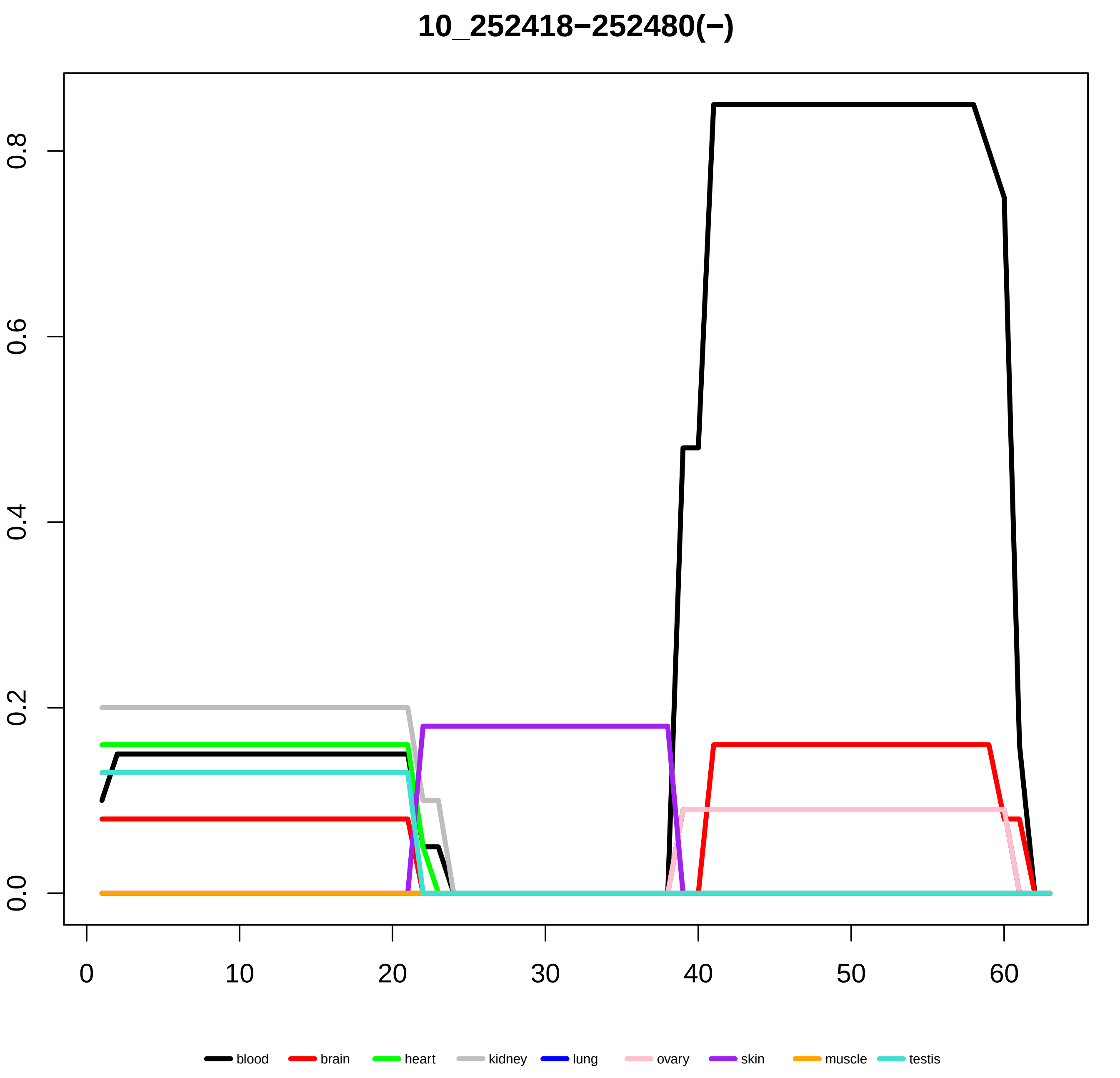
10_252418−252480(−)
0.0
0.2
0.4
0.6
0.8
0
10
20
30
40
50
60
blood
brain
heart
kidney
lung
ovary
skin
muscle
testis

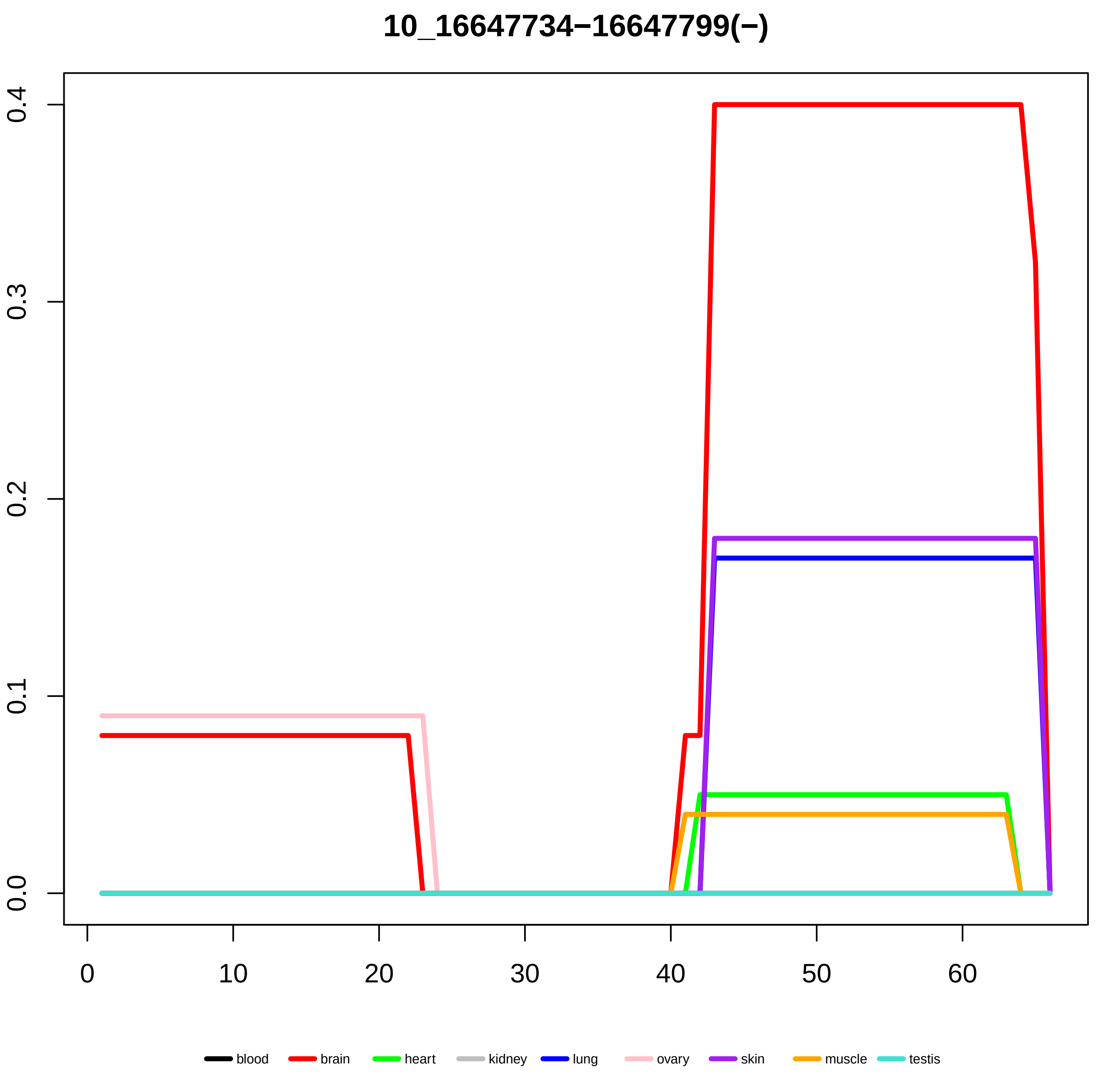

10_16647734−16647799(−)
0.0
0.1
0.2
0.3
0.4
0
10
20
30
40
50
60
blood
brain
heart
kidney
lung
ovary
skin
muscle
testis

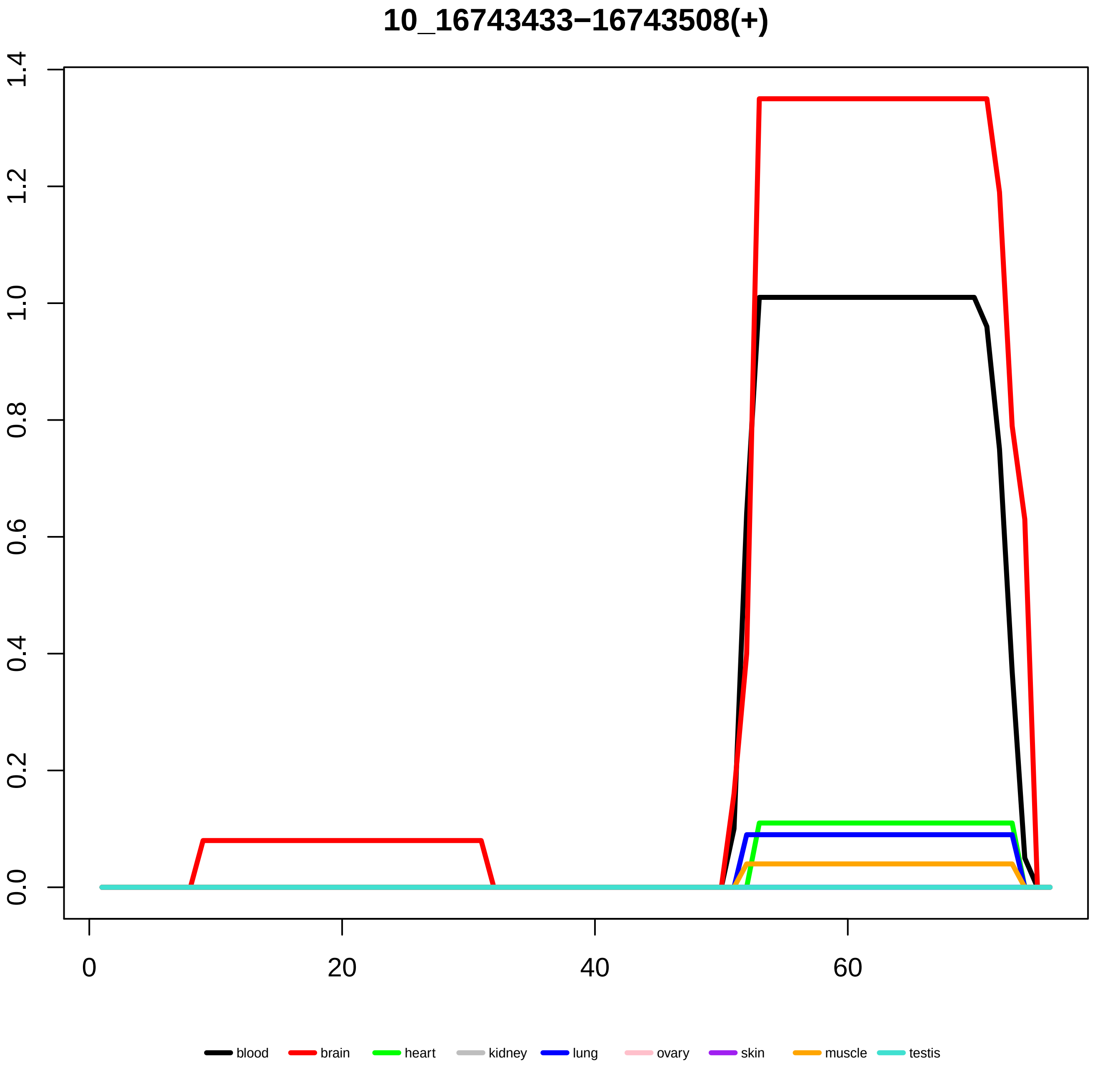
10_16743433−16743508(+)
0.0
0.2
0.4
0.6
0.8
1.0
1.2
1.4
0
20
40
60
blood
brain
heart
kidney
lung
ovary
skin
muscle
testis

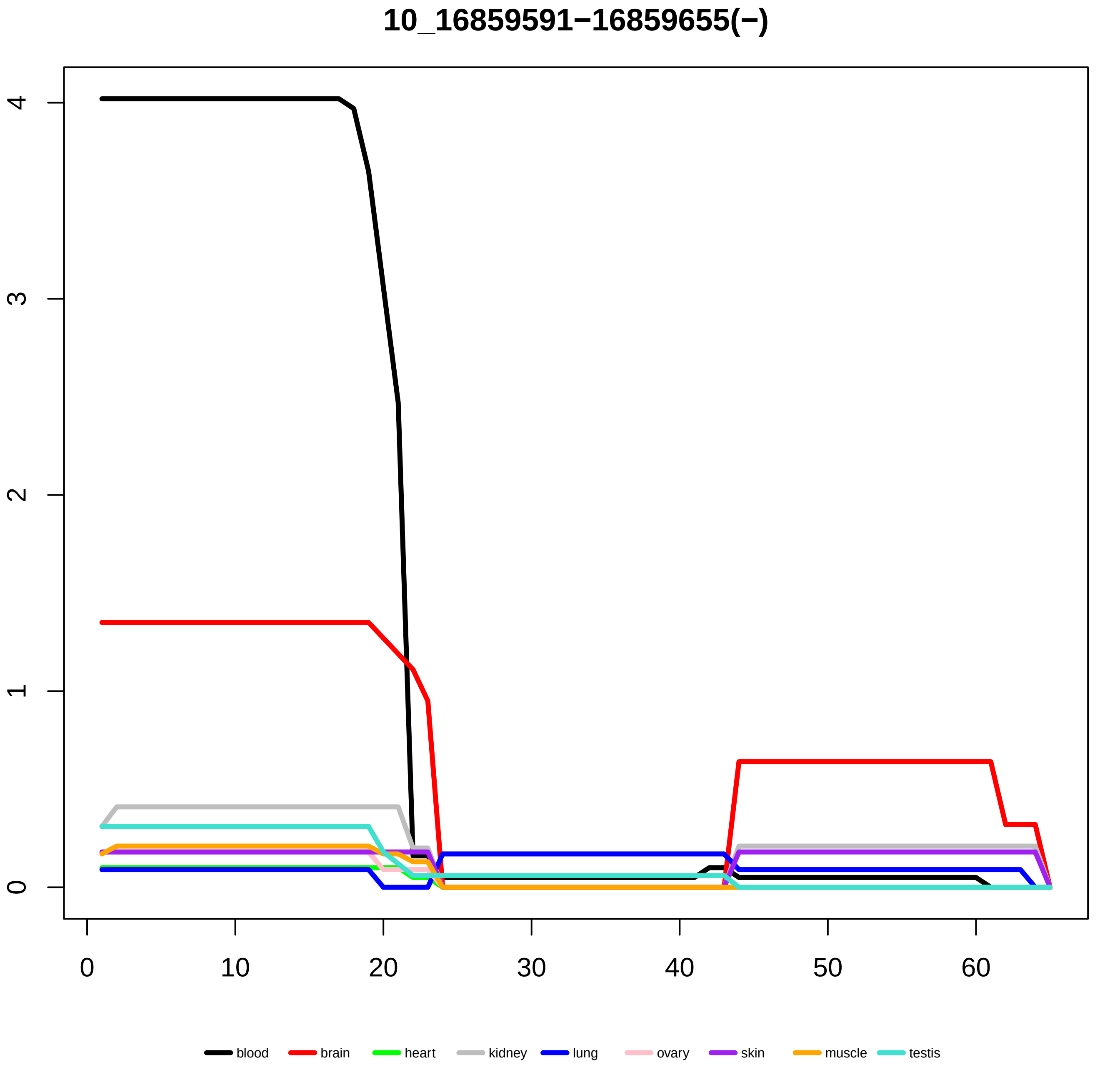
10_16859591−16859655(−)
0
1
2
3
4
0
10
20
30
40
50
60
blood
brain
heart
kidney
lung
ovary
skin
muscle
testis

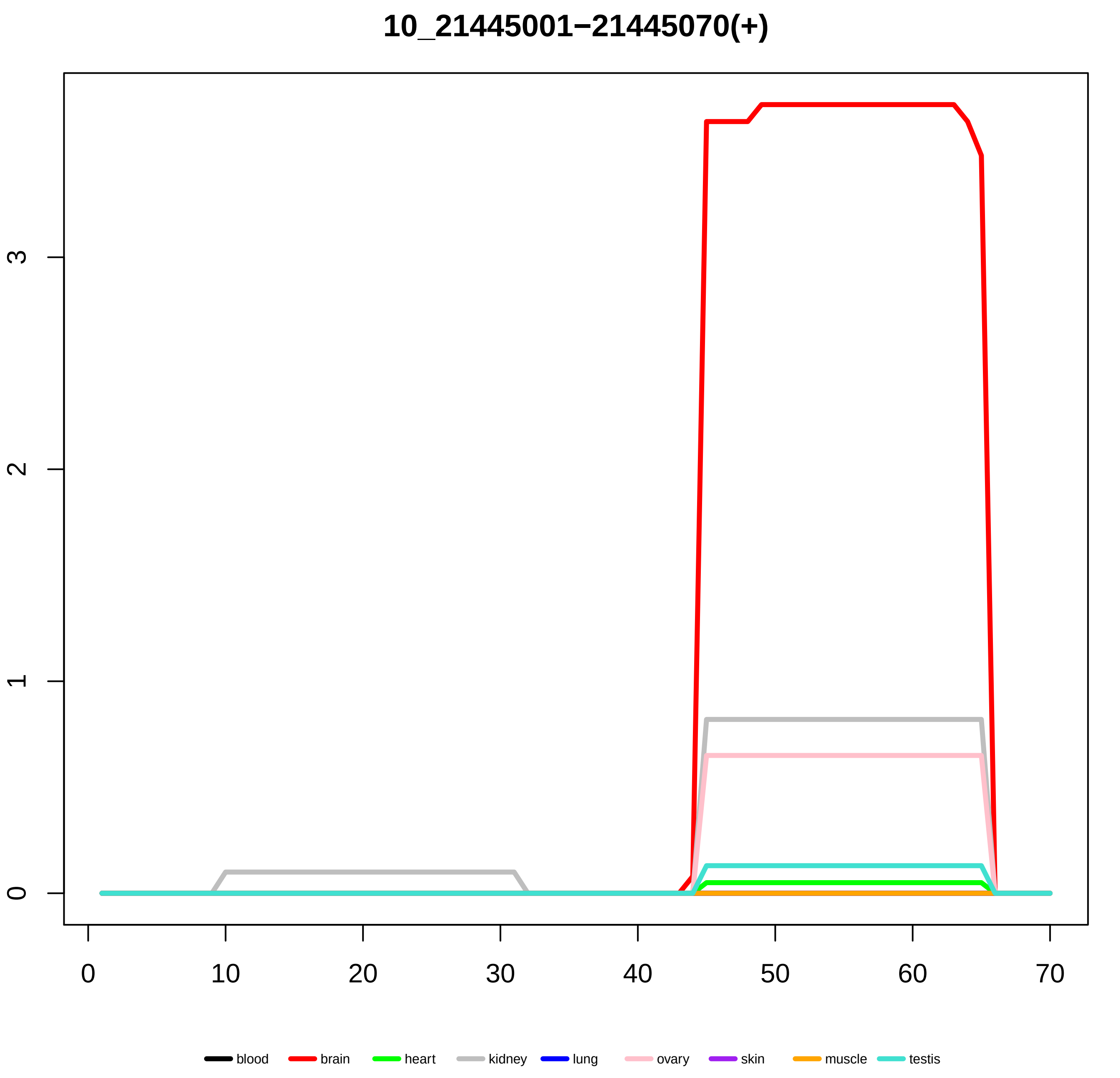
10_21445001−21445070(+)
0
1
2
3
0
10
20
30
40
50
60
70
blood
brain
heart
kidney
lung
ovary
skin
muscle
testis

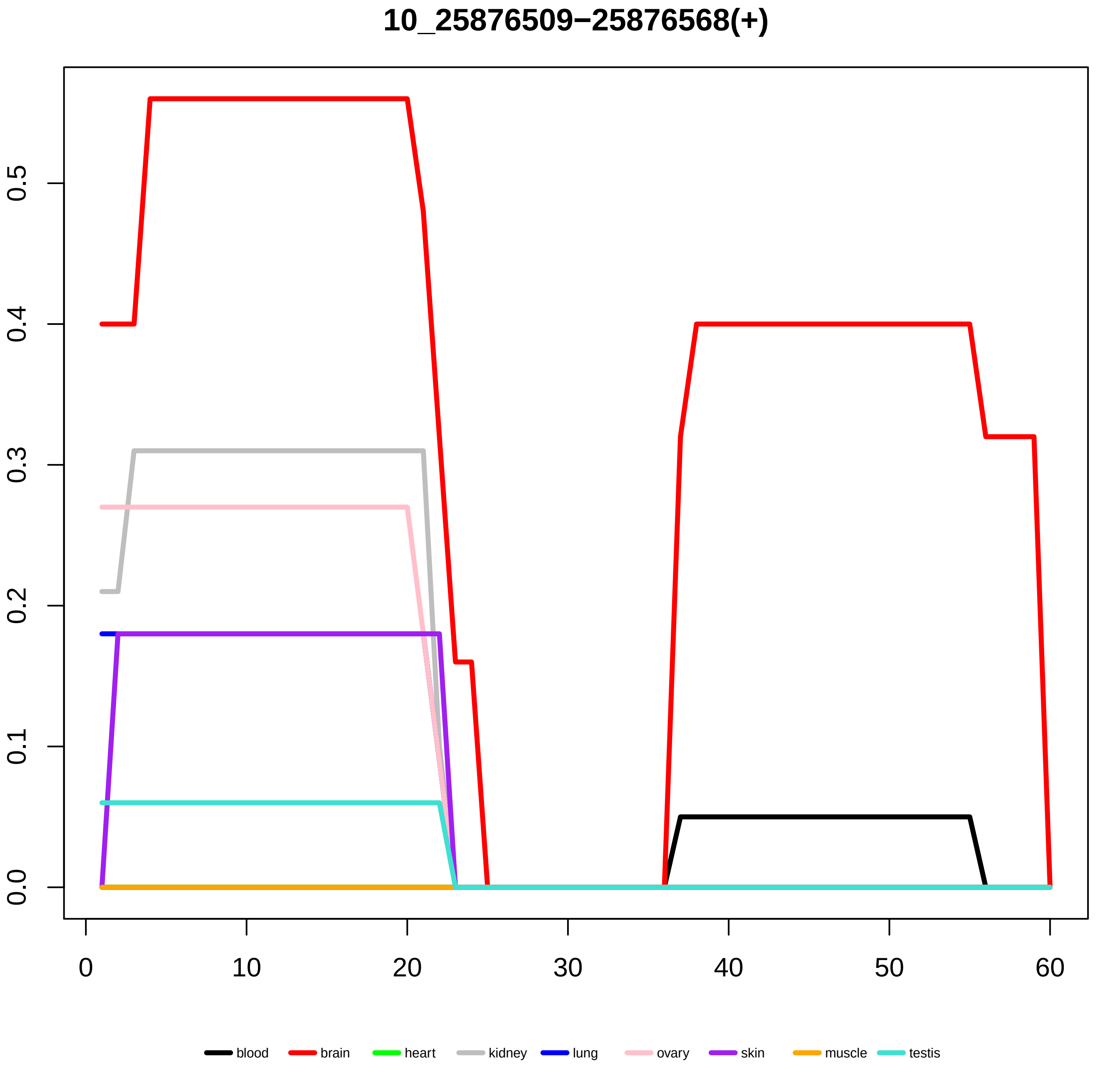
10_25876509−25876568(+)
0.0
0.1
0.2
0.3
0.4
0.5
0
10
20
30
40
50
60
blood
brain
heart
kidney
lung
ovary
skin
muscle
testis

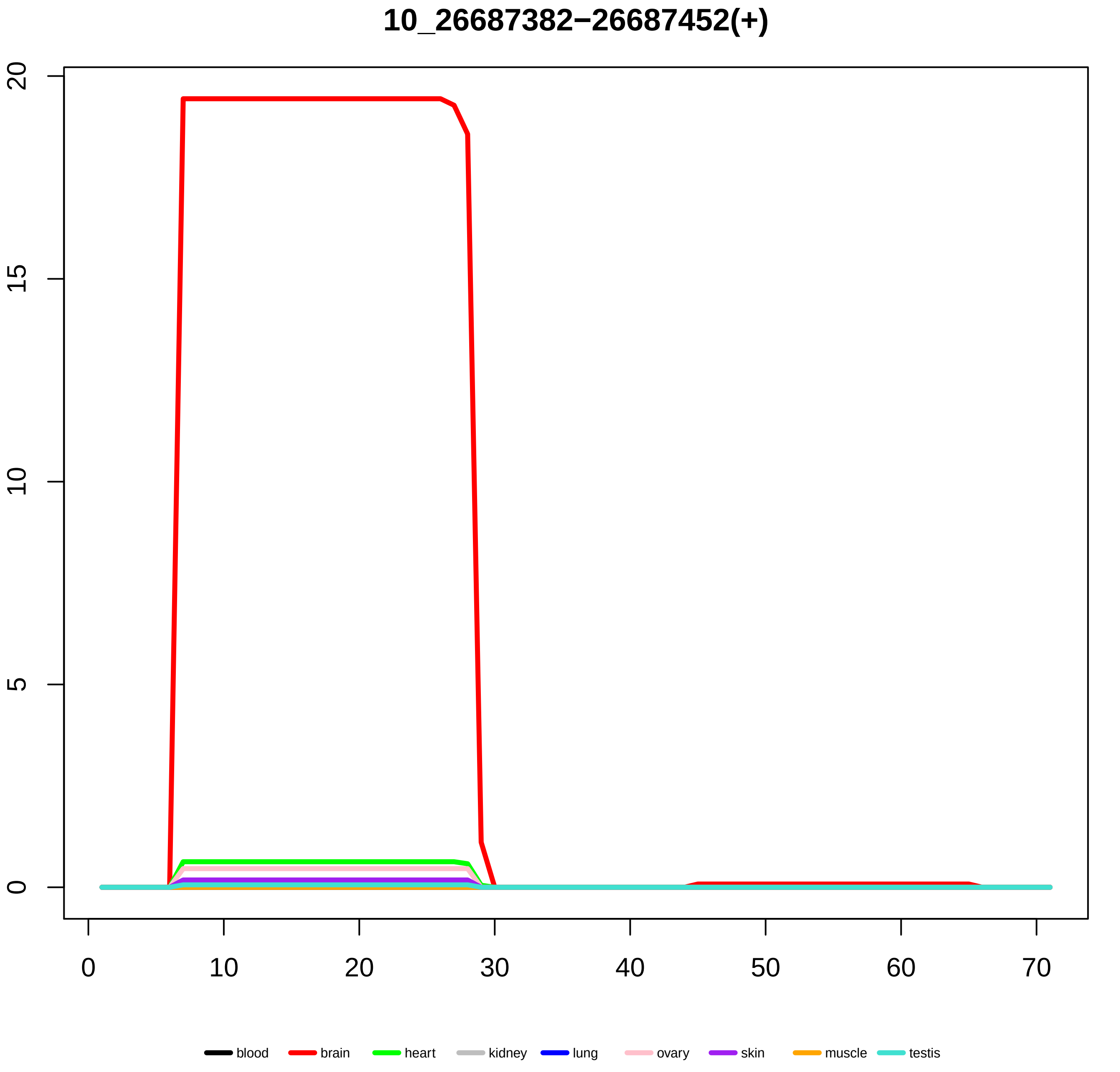
10_26687382−26687452(+)
0
5
10
15
20
0
10
20
30
40
50
60
70
blood
brain
heart
kidney
lung
ovary
skin
muscle
testis

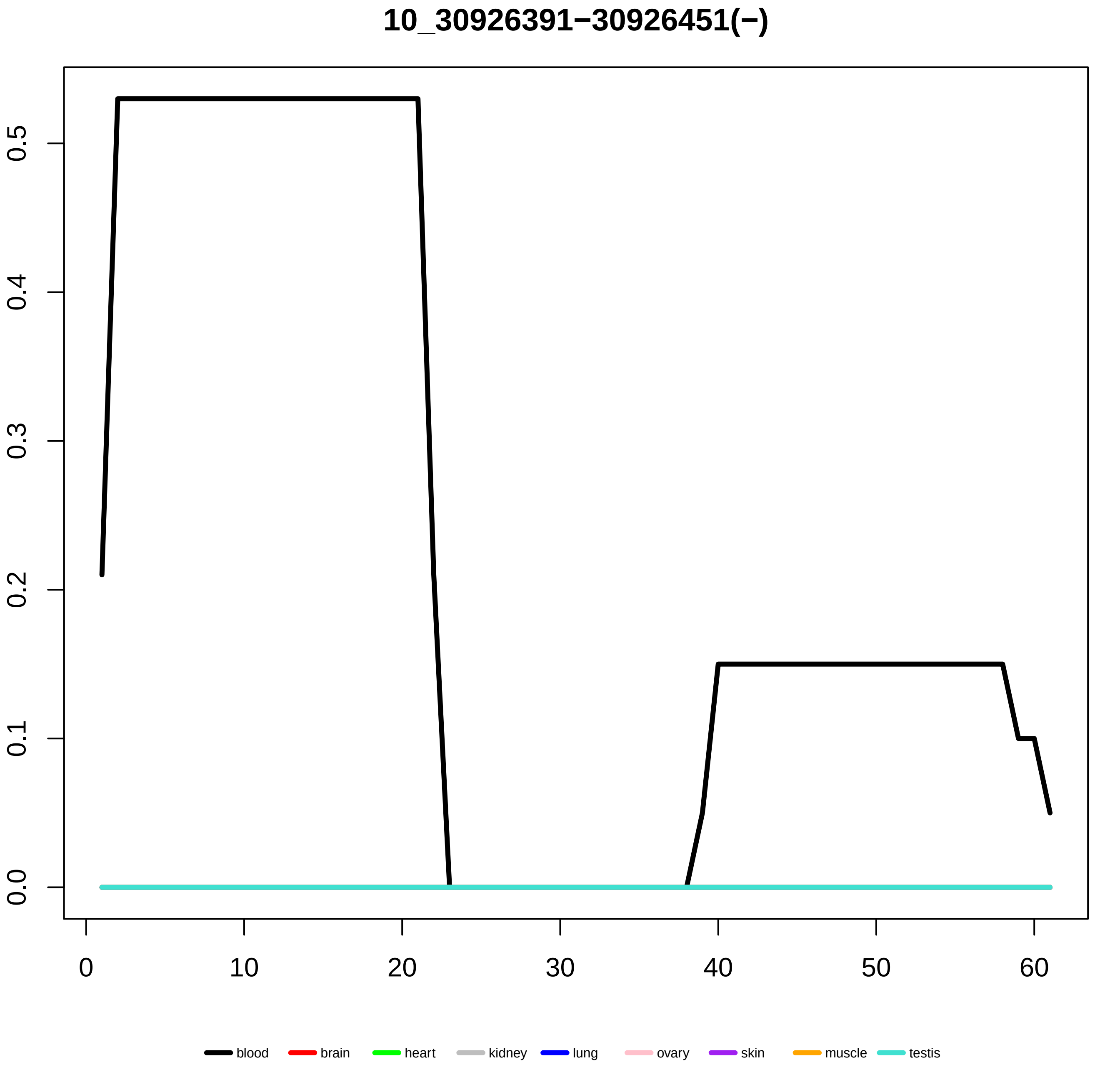
10_30926391−30926451(−)
0.0
0.1
0.2
0.3
0.4
0.5
0
10
20
30
40
50
60
blood
brain
heart
kidney
lung
ovary
skin
muscle
testis

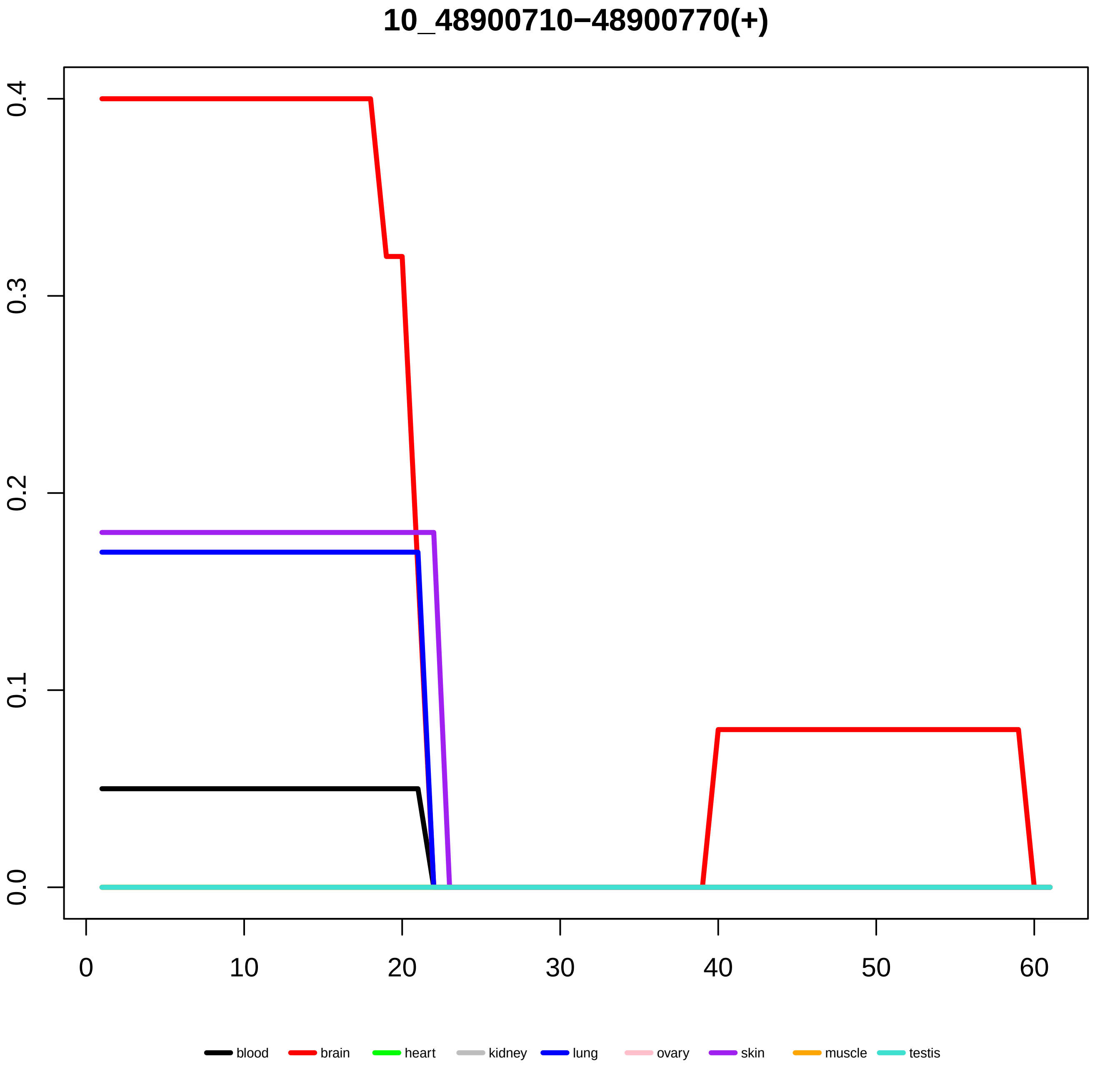

10_48900710−48900770(+)
0.0
0.1
0.2
0.3
0.4
0
10
20
30
40
50
60
blood
brain
heart
kidney
lung
ovary
skin
muscle
testis

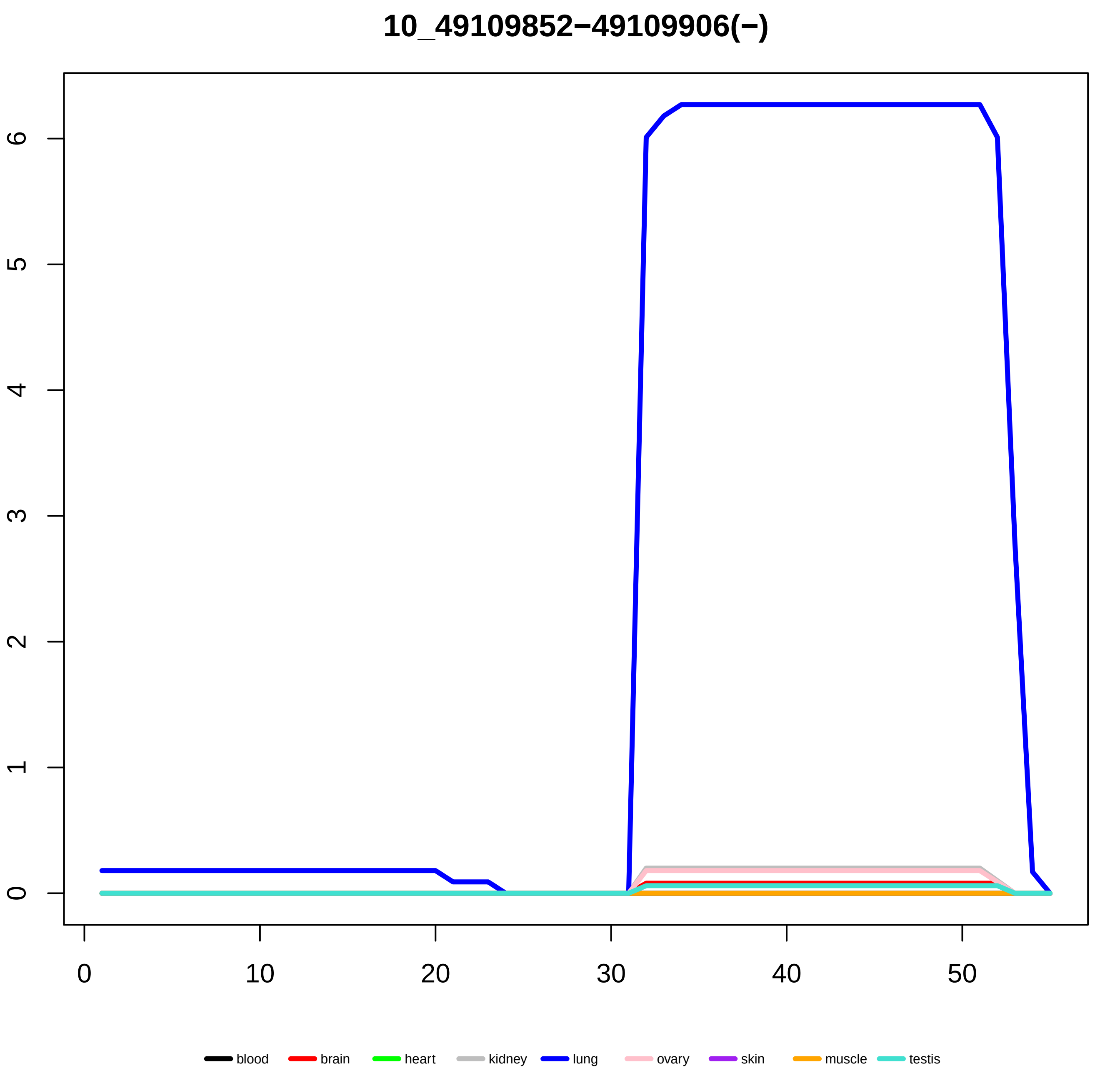
10_49109852−49109906(−)
0
1
2
3
4
5
6
0
10
20
30
40
50
blood
brain
heart
kidney
lung
ovary
skin
muscle
testis

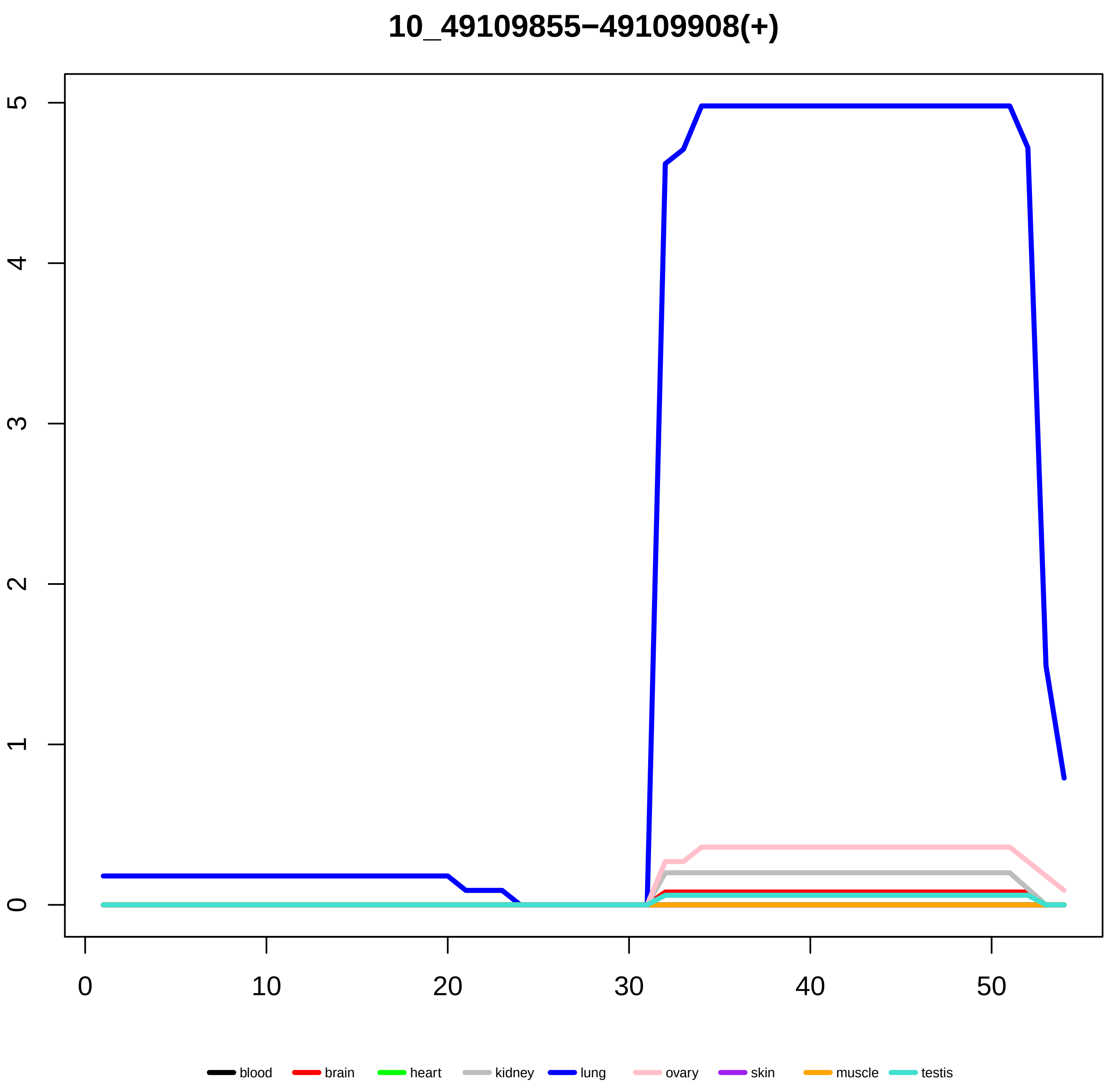

10_49109855−49109908(+)
0
1
2
3
4
5
0
10
20
30
40
50
blood
brain
heart
kidney
lung
ovary
skin
muscle
testis

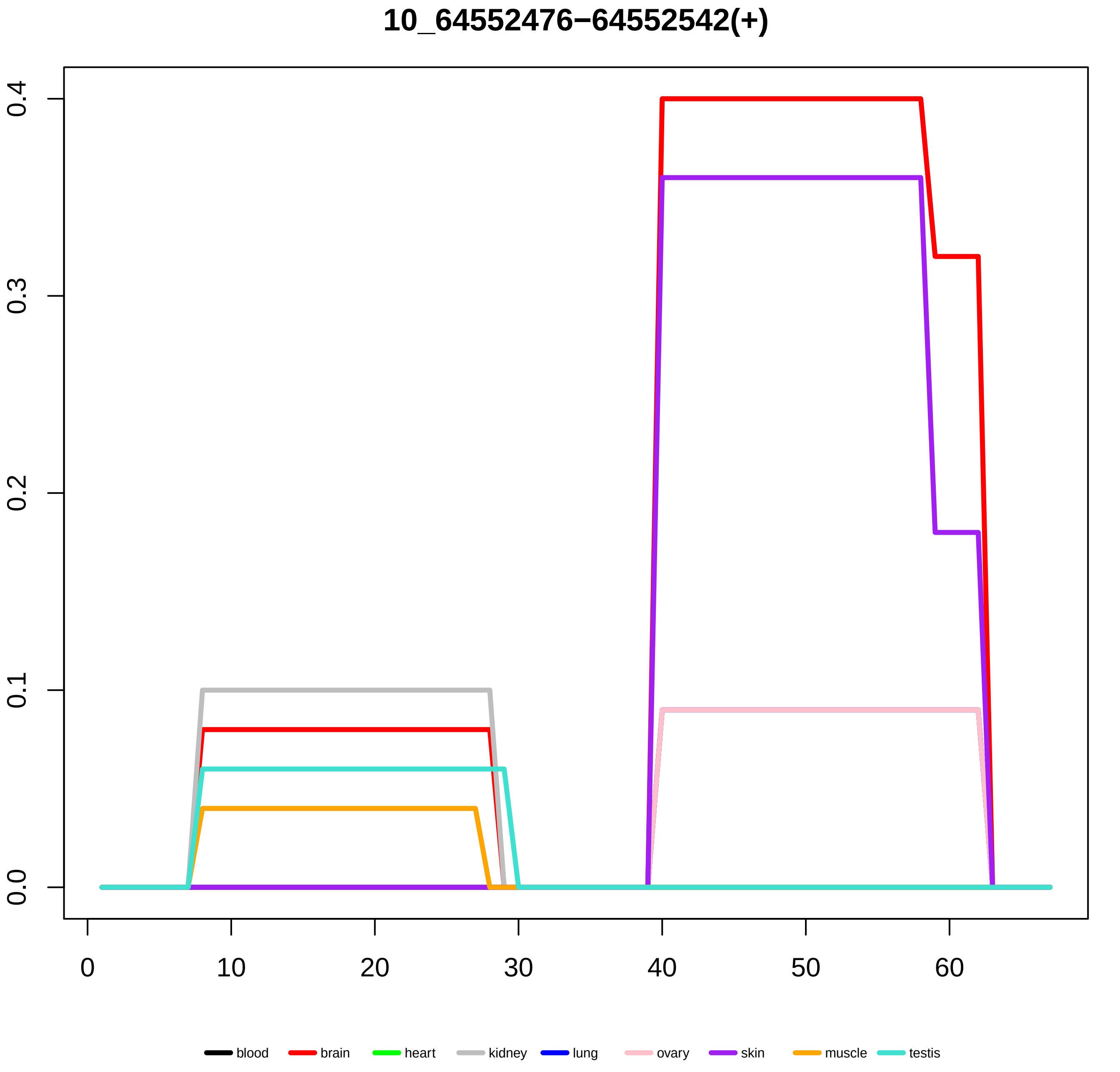

10_64552476−64552542(+)
0.0
0.1
0.2
0.3
0.4
0
10
20
30
40
50
60
blood
brain
heart
kidney
lung
ovary
skin
muscle
testis

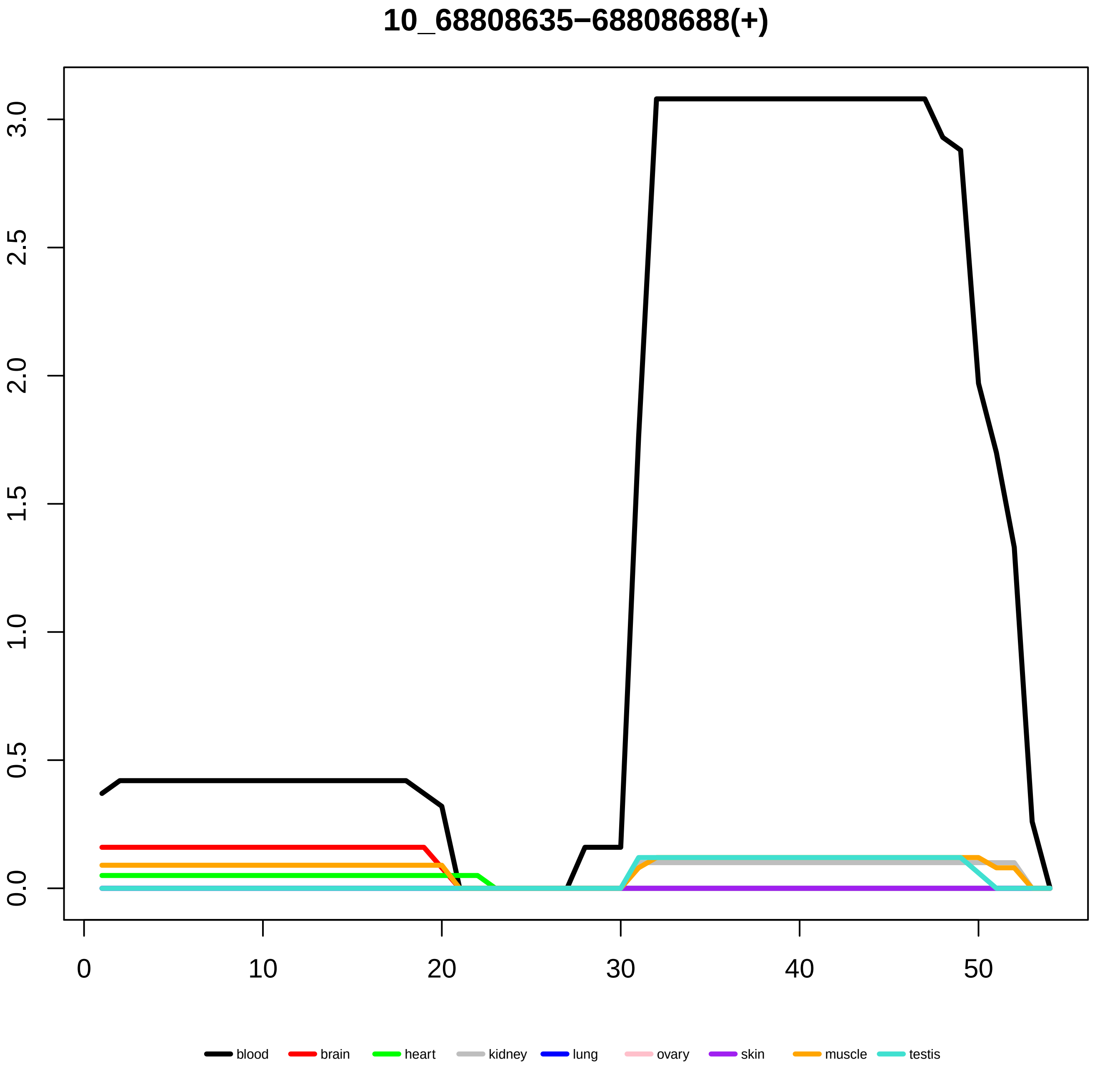

10_68808635−68808688(+)
0.0
0.5
1.0
1.5
2.0
2.5
3.0
0
10
20
30
40
50
blood
brain
heart
kidney
lung
ovary
skin
muscle
testis

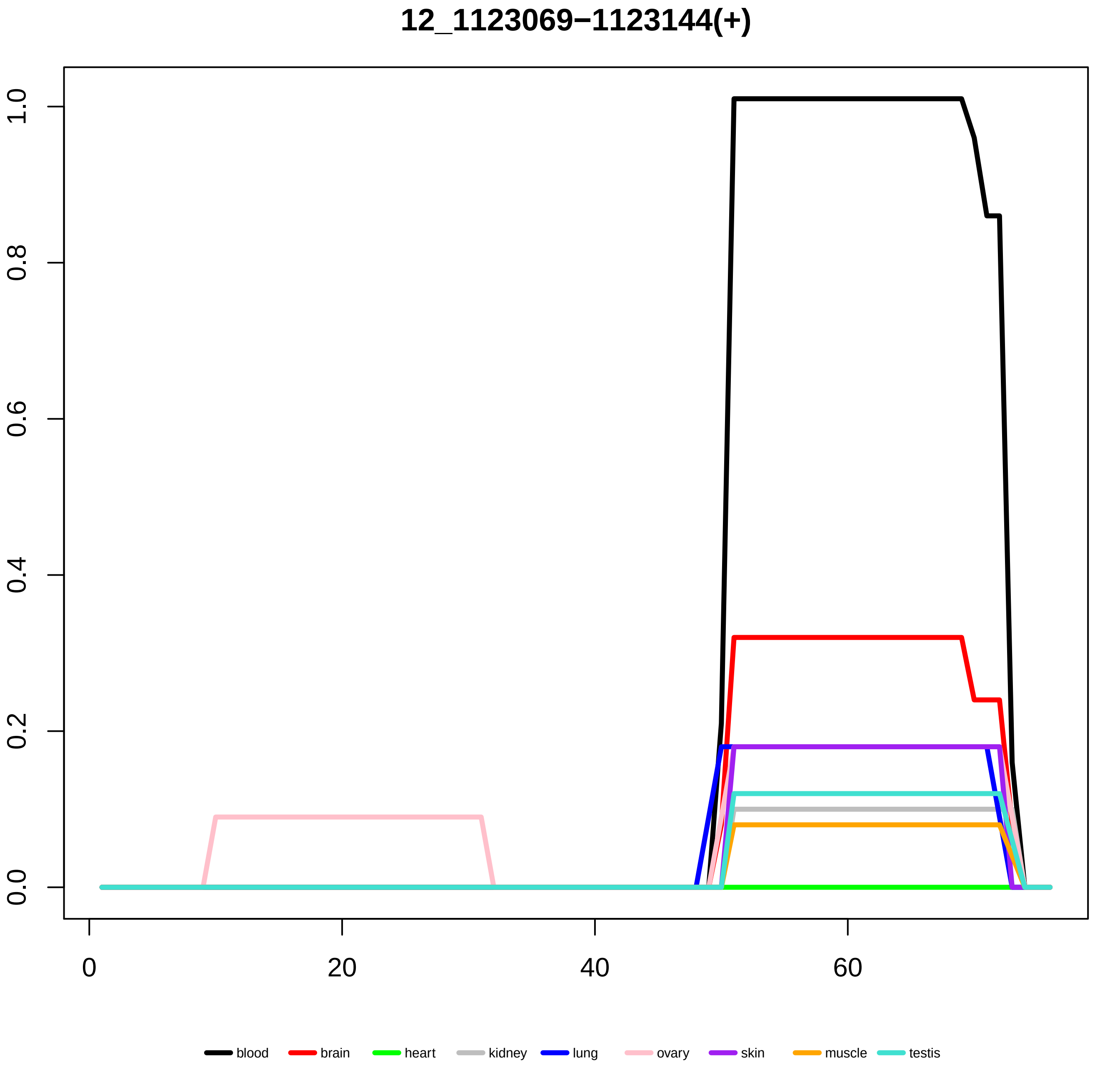

12_1123069−1123144(+)
0.0
0.2
0.4
0.6
0.8
1.0
0
20
40
60
blood
brain
heart
kidney
lung
ovary
skin
muscle
testis

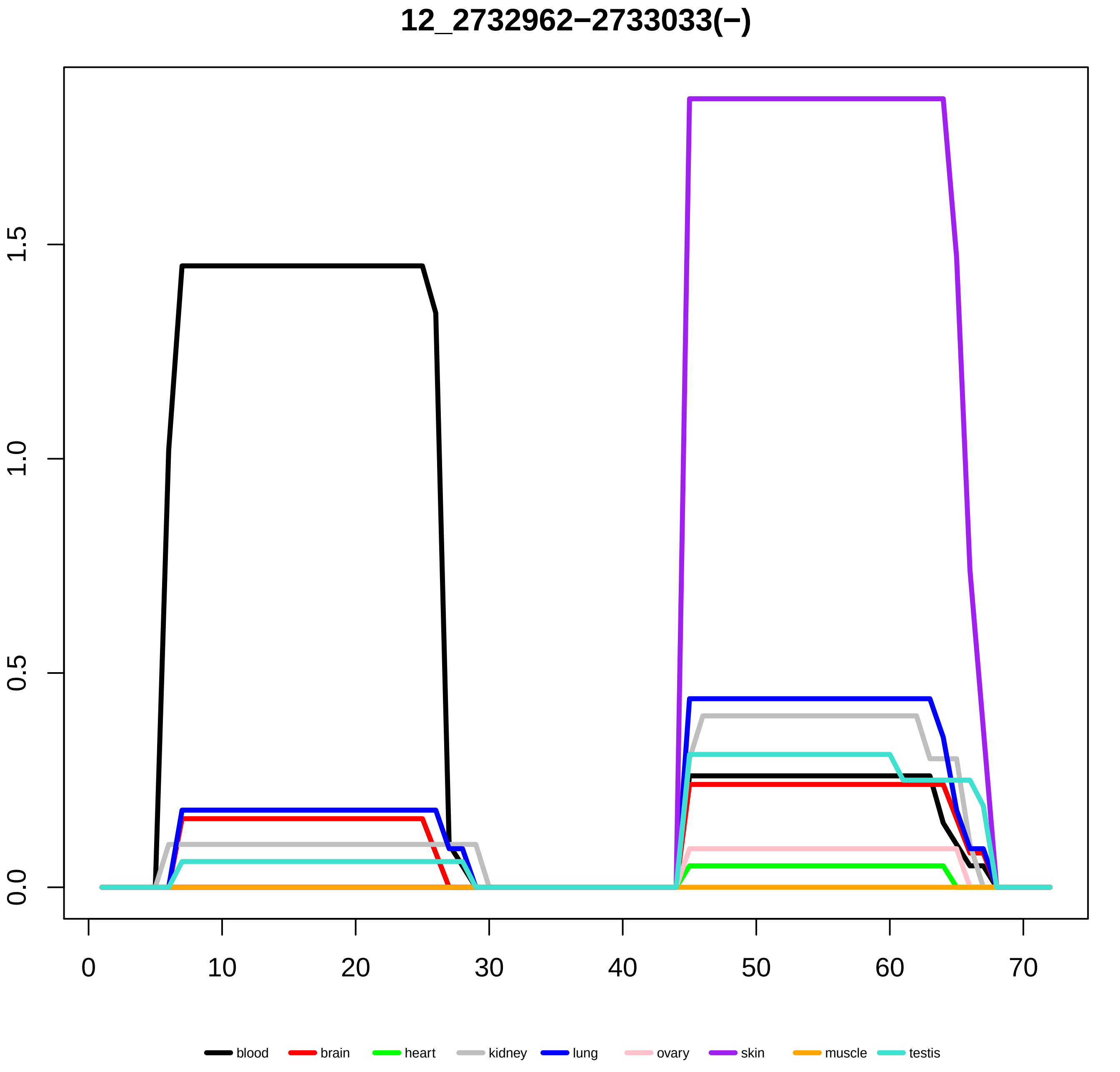

12_2732962−2733033(−)
0.0
0.5
1.0
1.5
0
10
20
30
40
50
60
70
blood
brain
heart
kidney
lung
ovary
skin
muscle
testis

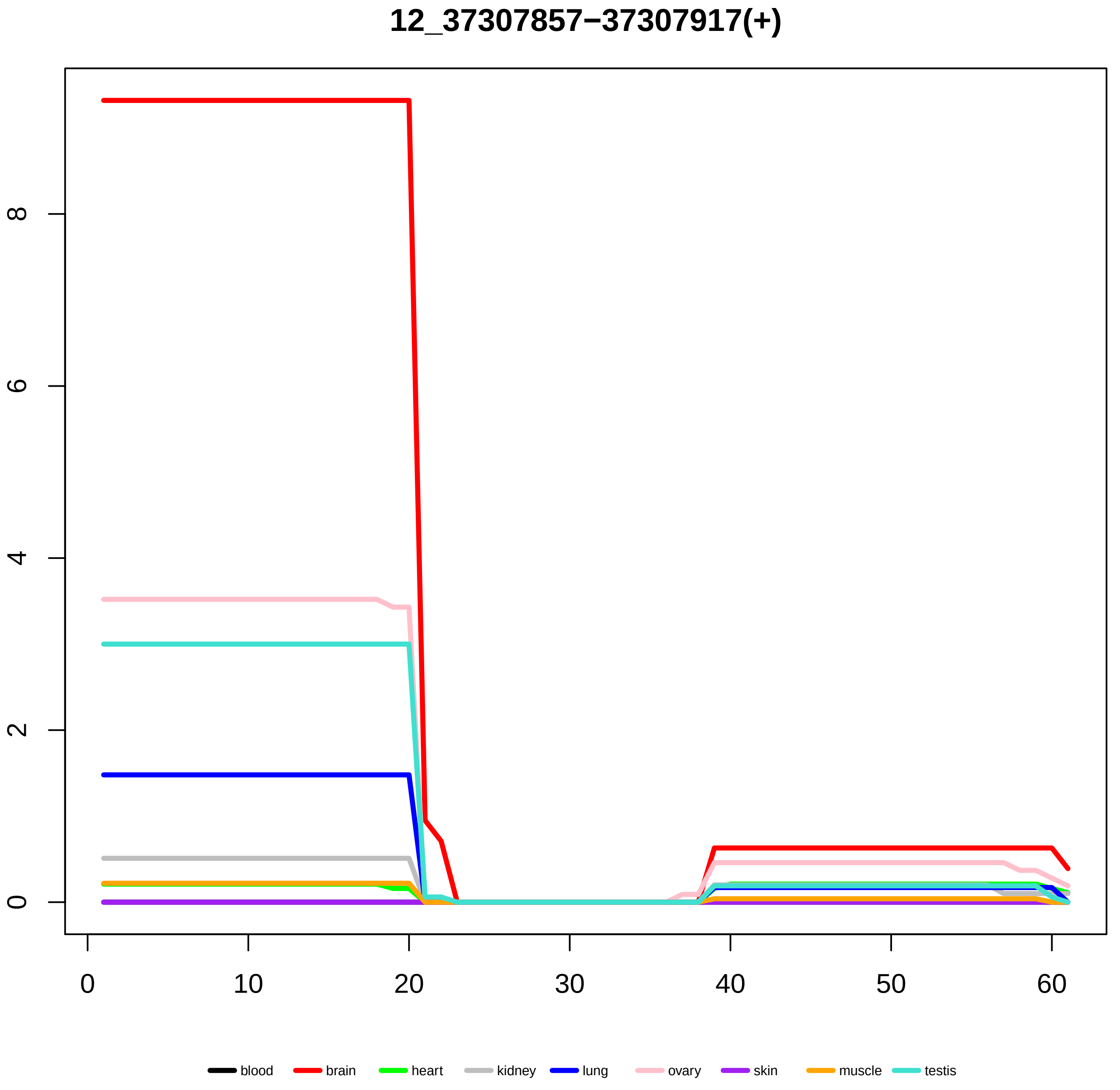

12_37307857−37307917(+)
0
2
4
6
8
0
10
20
30
40
50
60
blood
brain
heart
kidney
lung
ovary
skin
muscle
testis

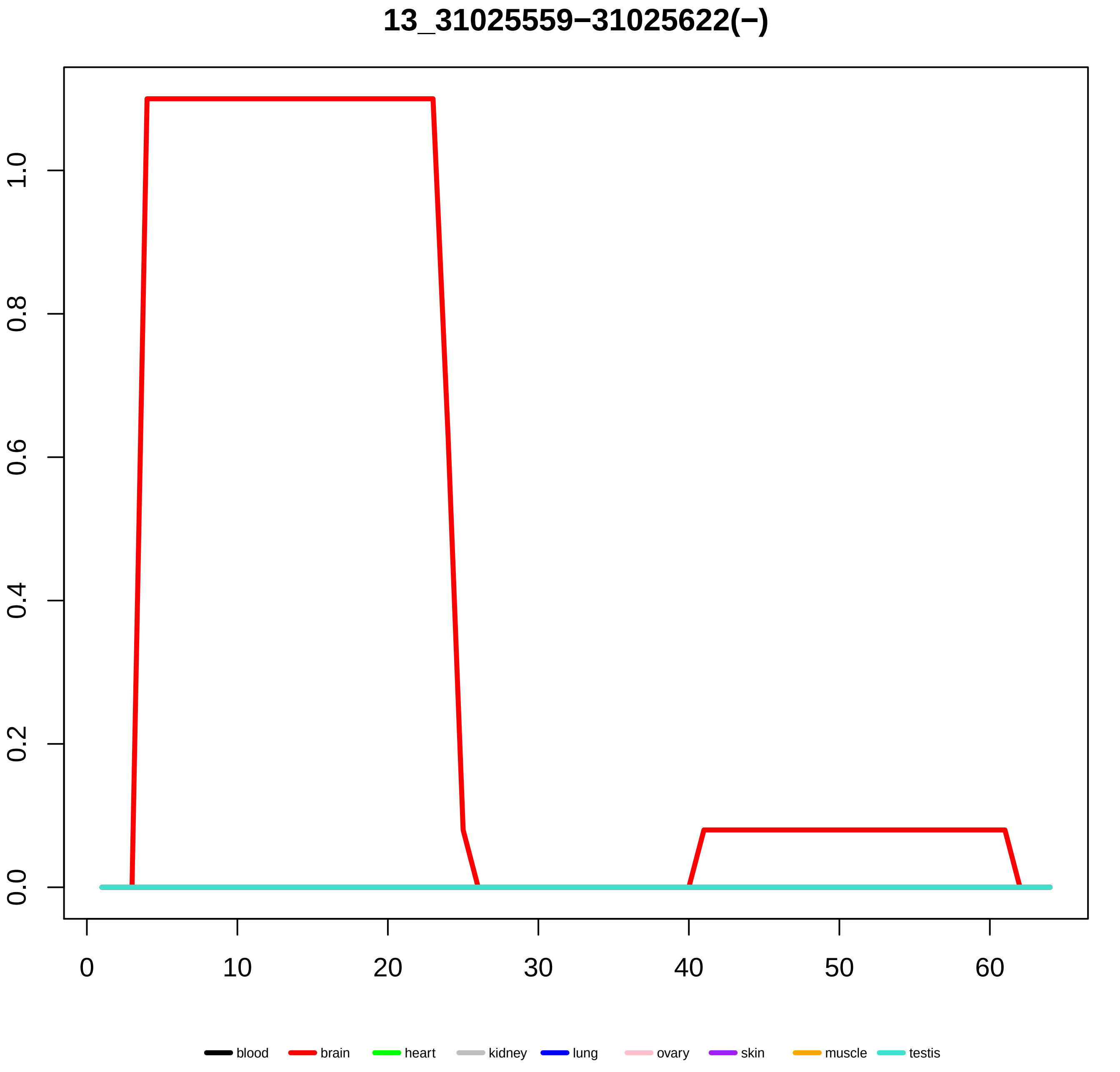
13_31025559−31025622(−)
0.0
0.2
0.4
0.6
0.8
1.0
0
10
20
30
40
50
60
blood
brain
heart
kidney
lung
ovary
skin
muscle
testis

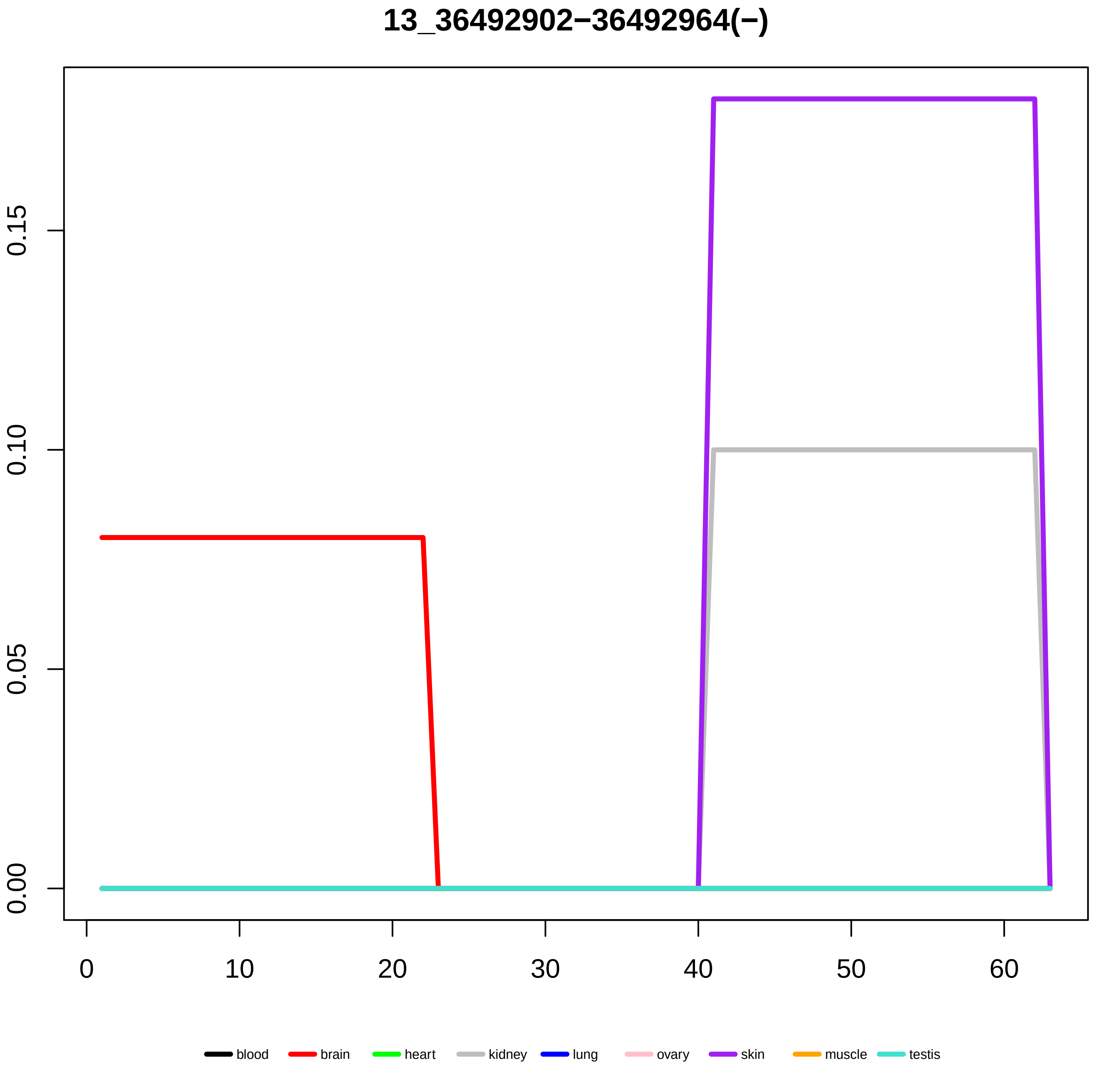
13_36492902−36492964(−)
0.00
0.05
0.10
0.15
0
10
20
30
40
50
60
blood
brain
heart
kidney
lung
ovary
skin
muscle
testis

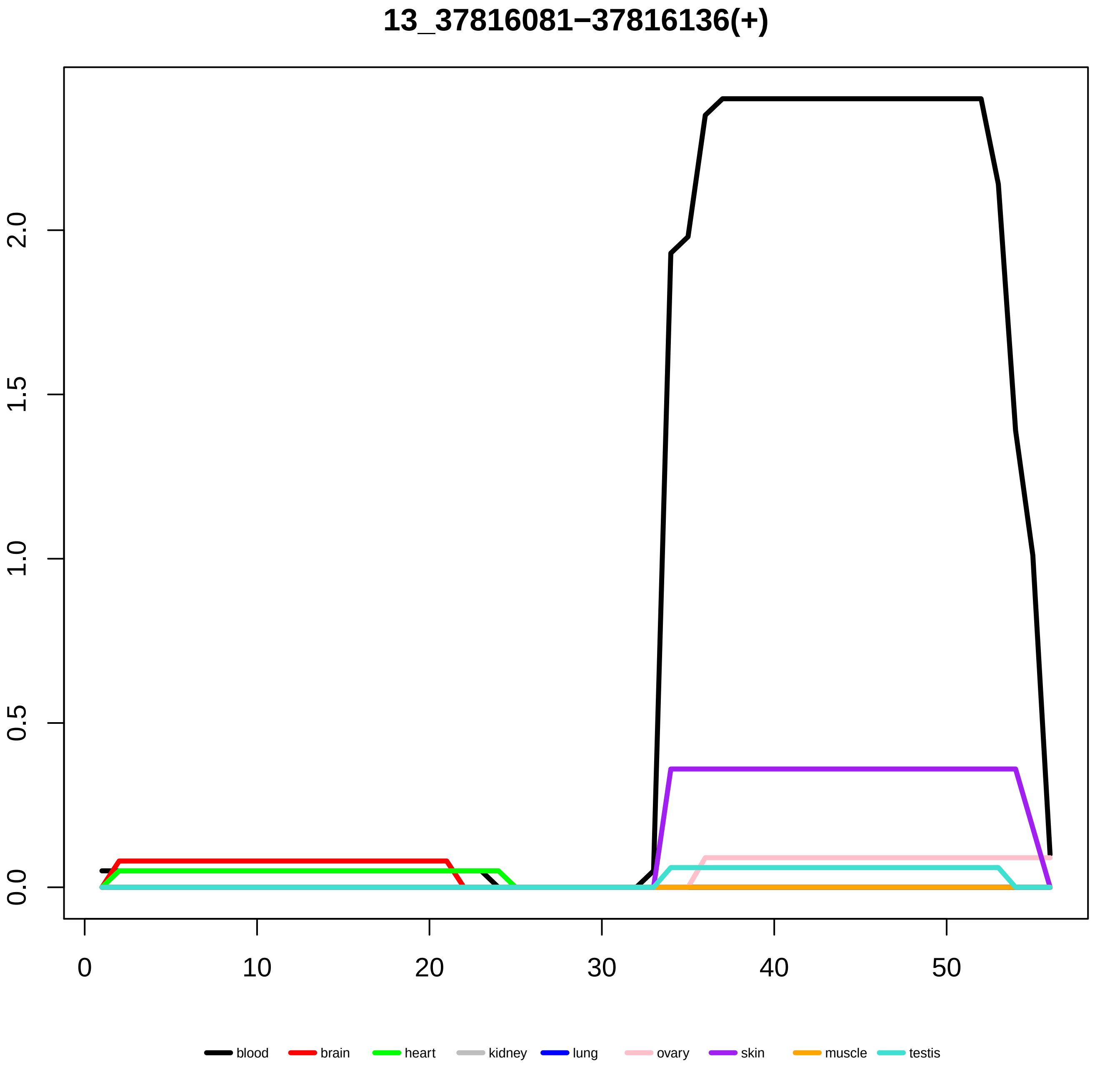
13_37816081−37816136(+)
0.0
0.5
1.0
1.5
2.0
0
10
20
30
40
50
blood
brain
heart
kidney
lung
ovary
skin
muscle
testis

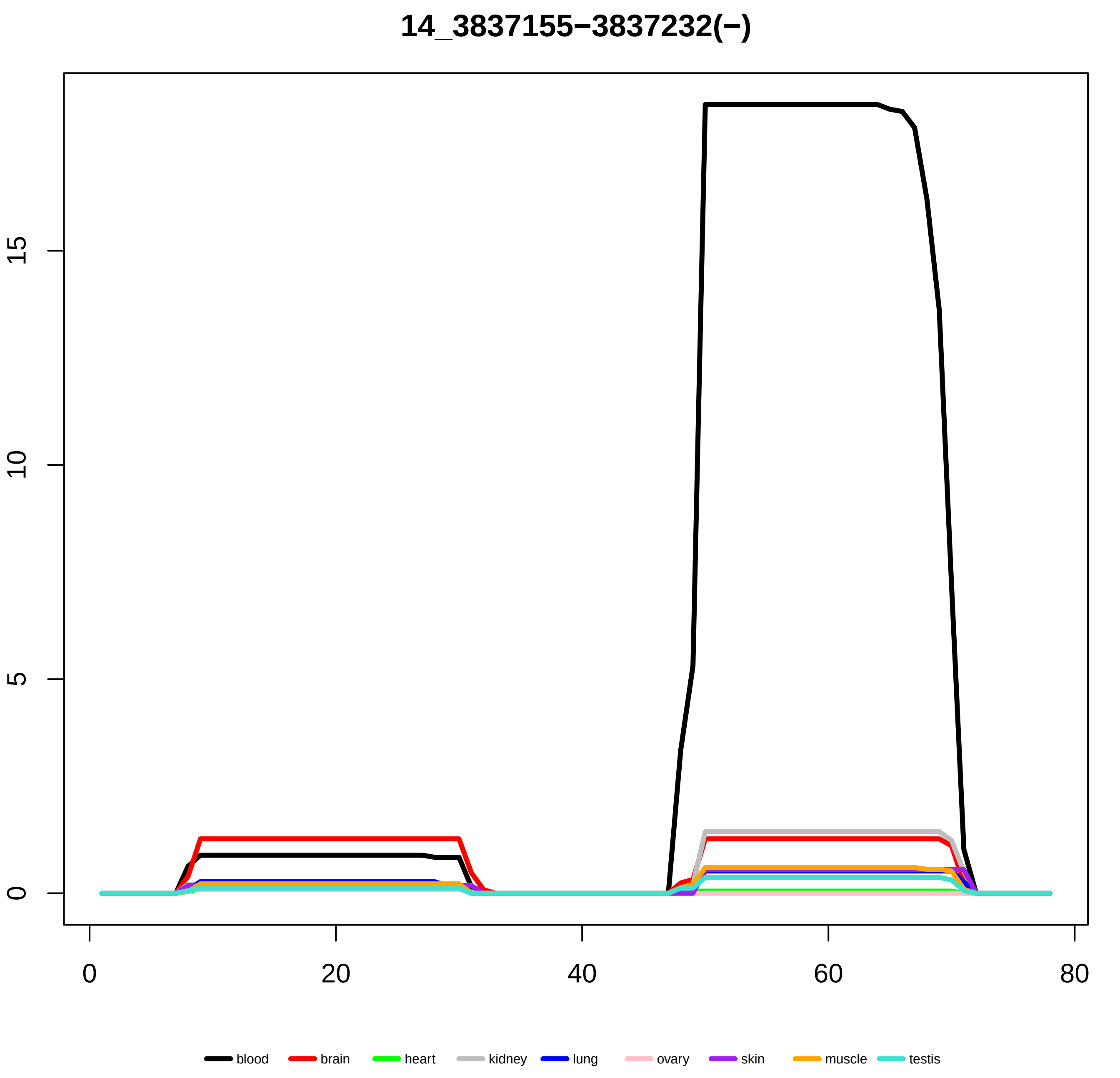

14_3837155−3837232(−)
0
5
10
15
0
20
40
60
80
blood
brain
heart
kidney
lung
ovary
skin
muscle
testis

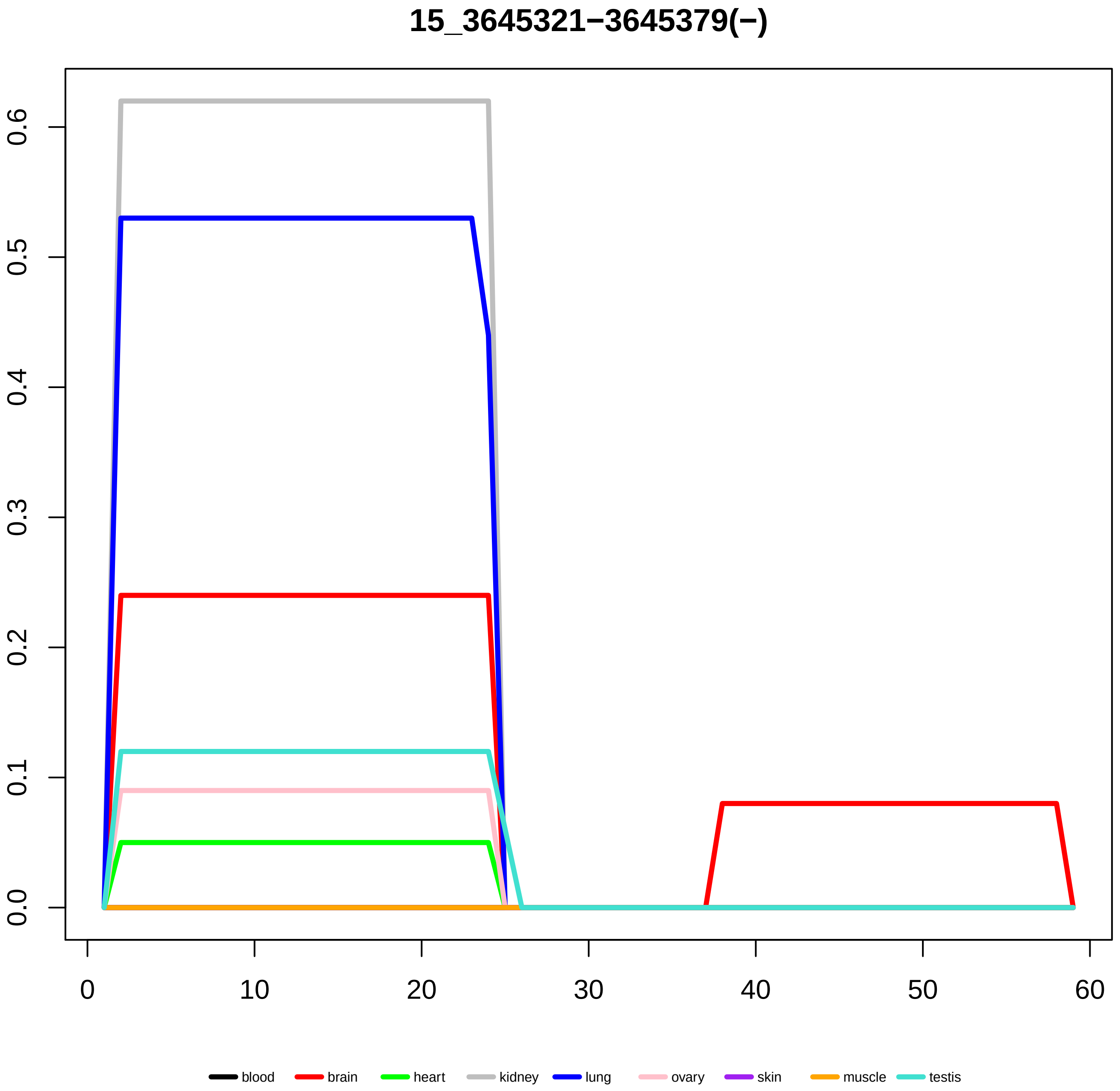
15_3645321−3645379(−)
0.0
0.1
0.2
0.3
0.4
0.5
0.6
0
10
20
30
40
50
60
blood
brain
heart
kidney
lung
ovary
skin
muscle
testis

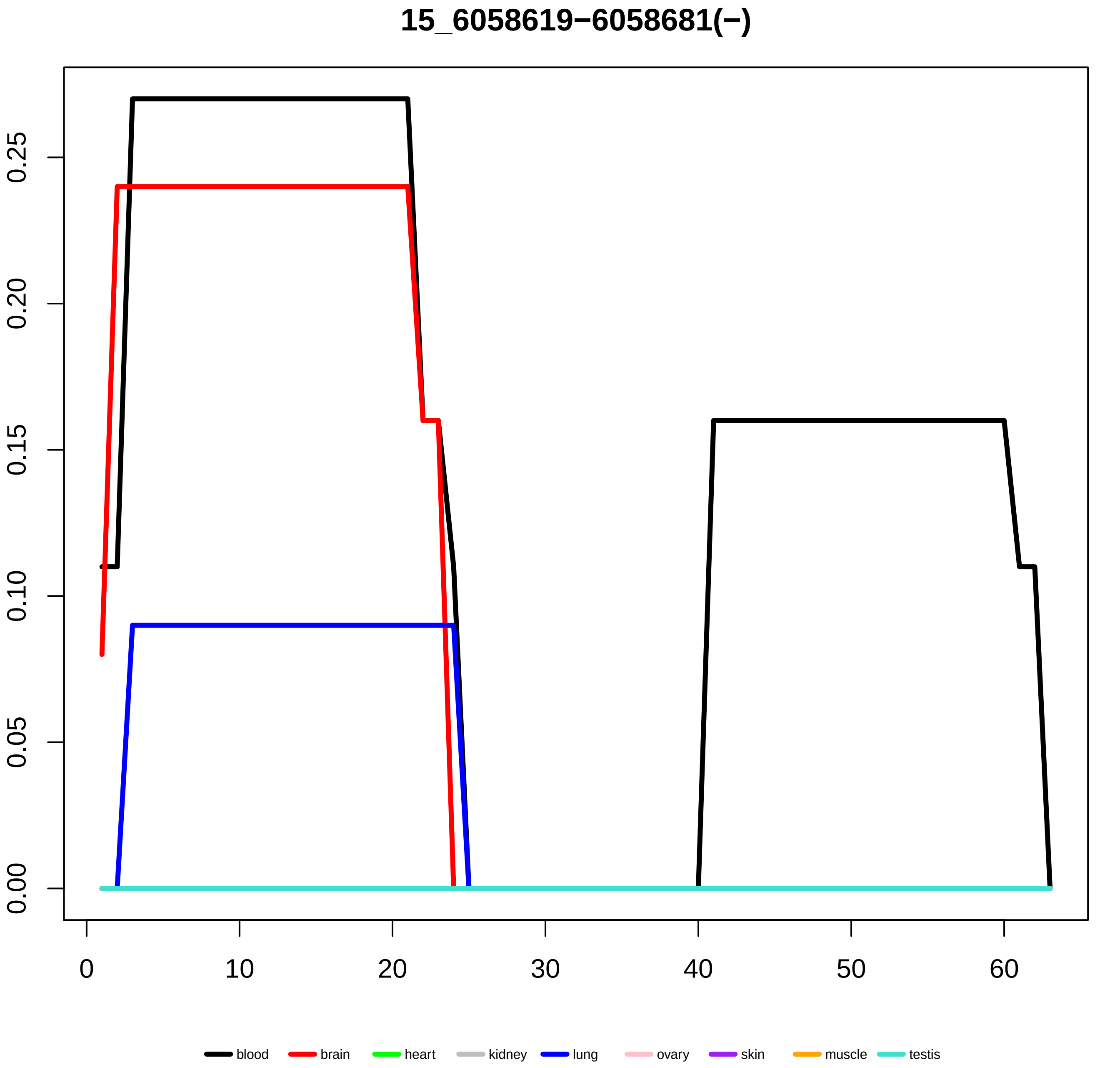

15_6058619−6058681(−)
0.00
0.05
0.10
0.15
0.20
0.25
0
10
20
30
40
50
60
blood
brain
heart
kidney
lung
ovary
skin
muscle
testis

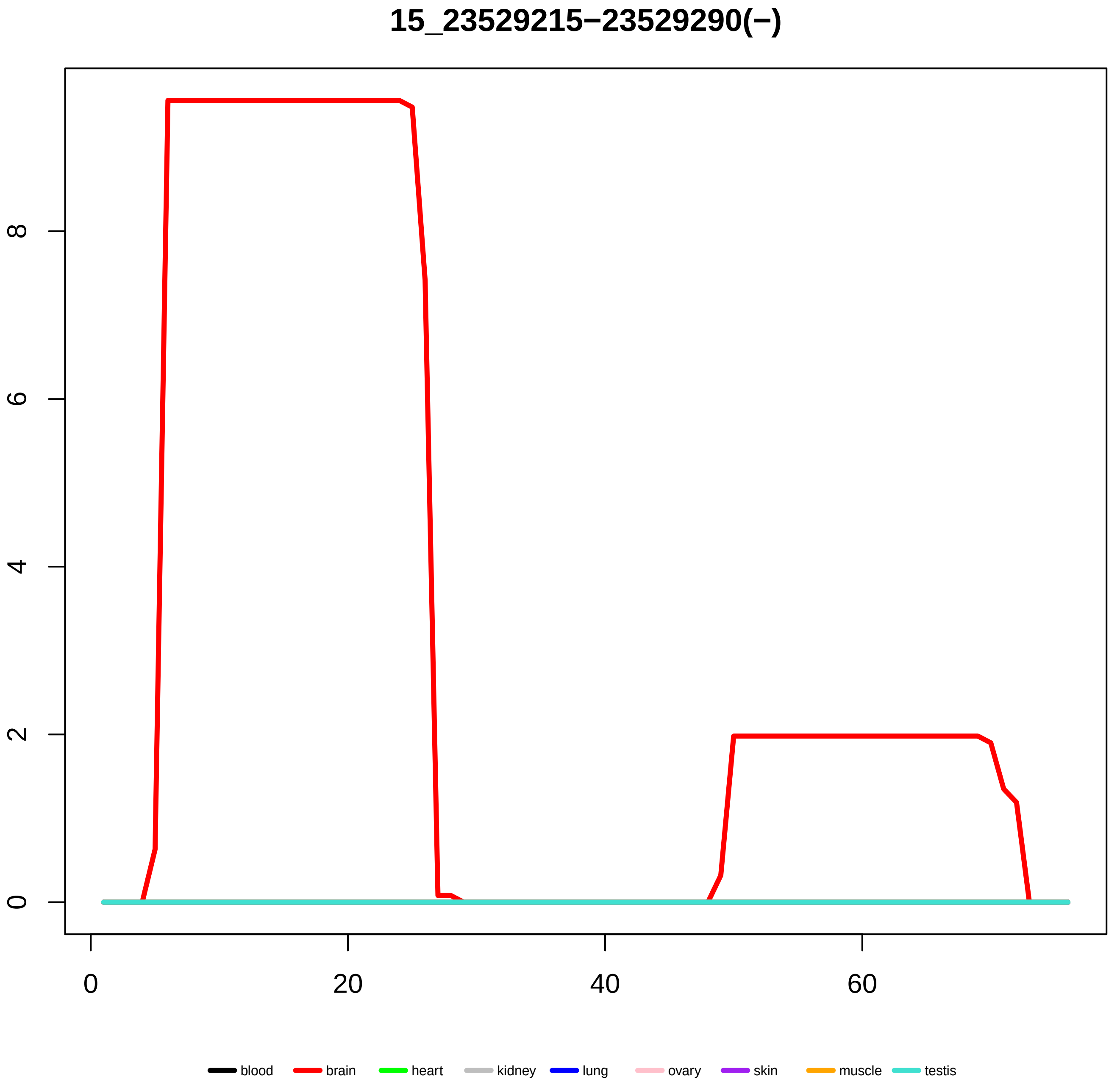
15_23529215−23529290(−)
0
2
4
6
8
0
20
40
60
blood
brain
heart
kidney
lung
ovary
skin
muscle
testis

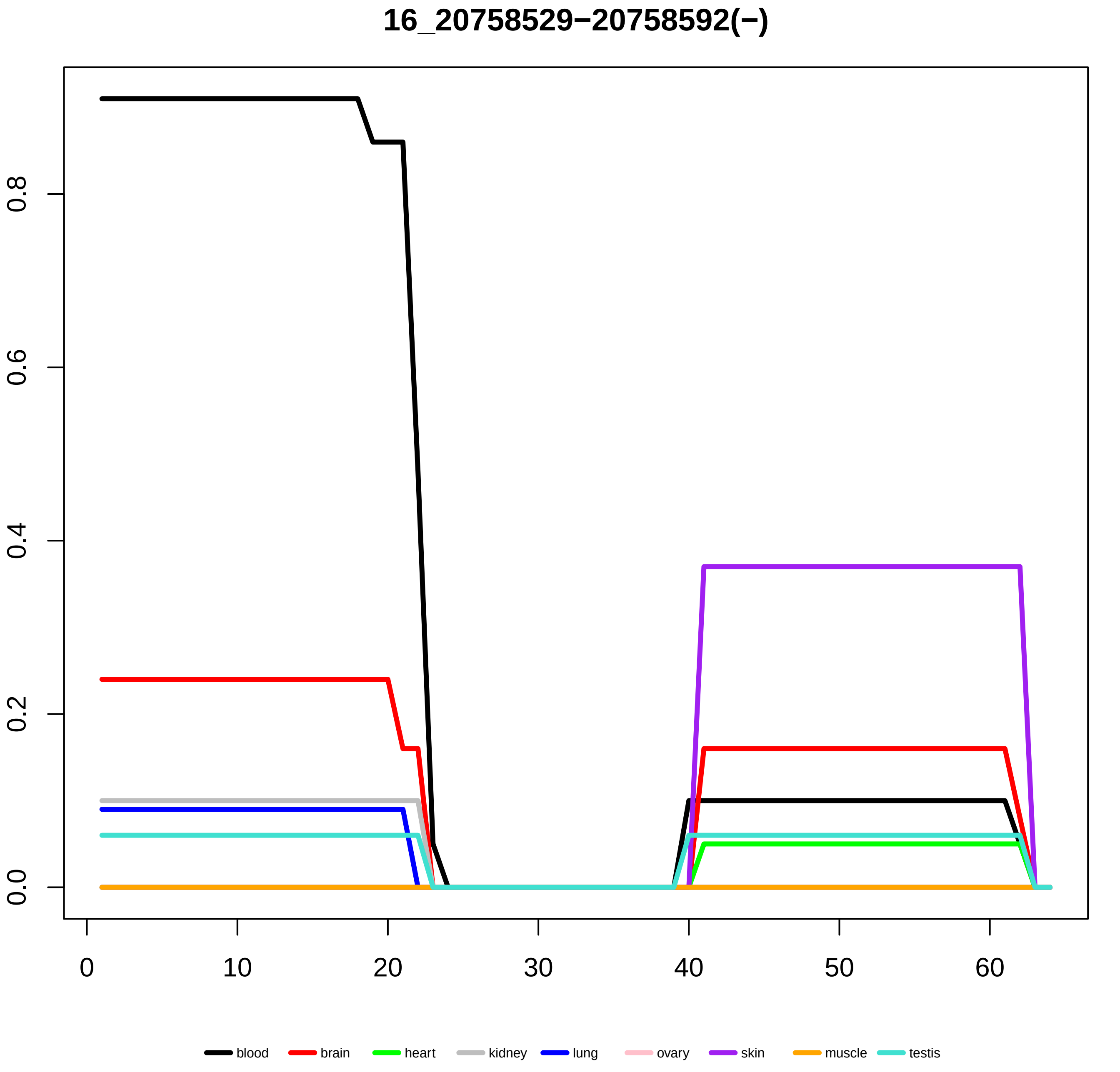
16_20758529−20758592(−)
0.0
0.2
0.4
0.6
0.8
0
10
20
30
40
50
60
blood
brain
heart
kidney
lung
ovary
skin
muscle
testis

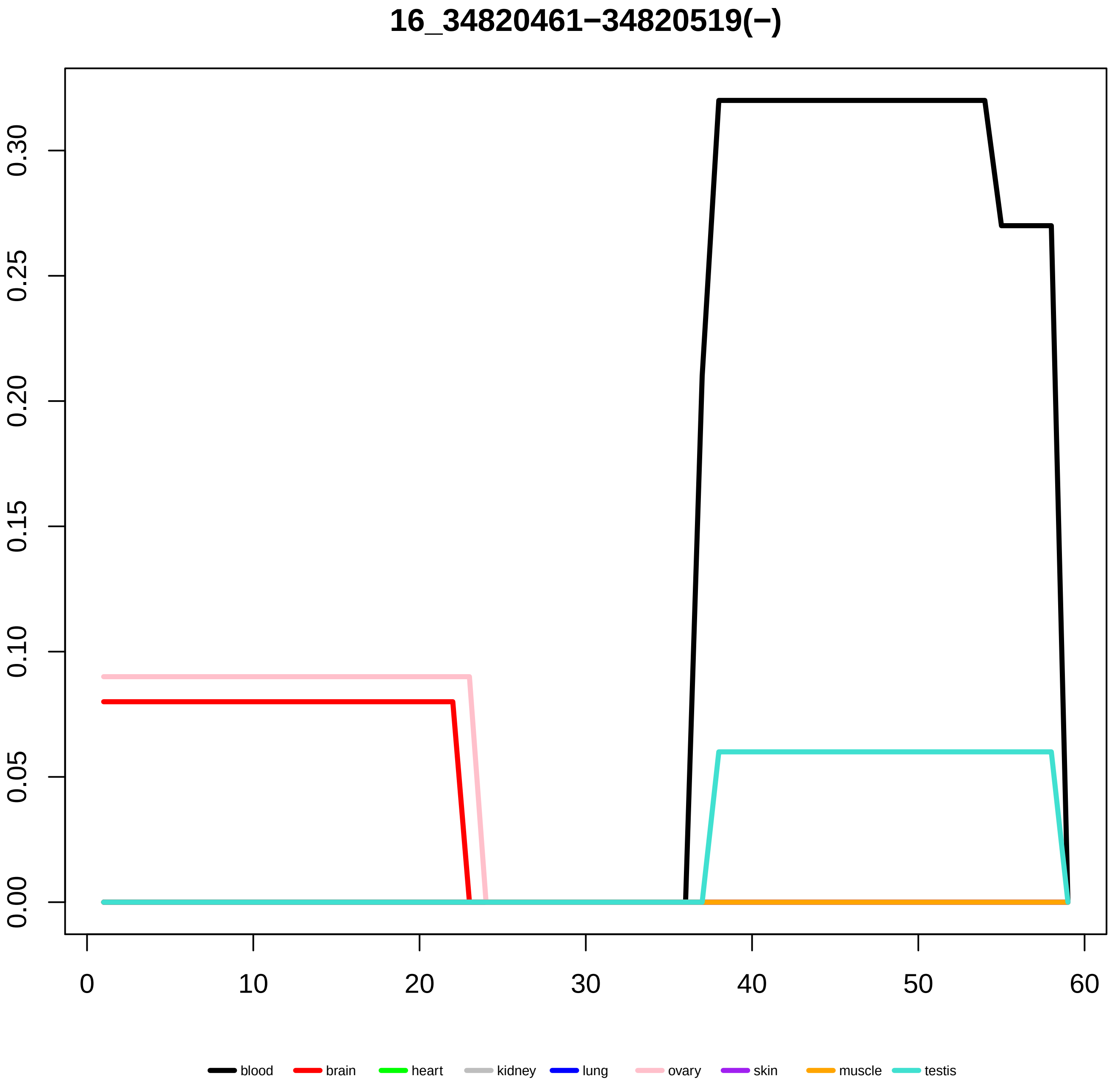

16_34820461−34820519(−)
0.00
0.05
0.10
0.15
0.20
0.25
0.30
0
10
20
30
40
50
60
blood
brain
heart
kidney
lung
ovary
skin
muscle
testis

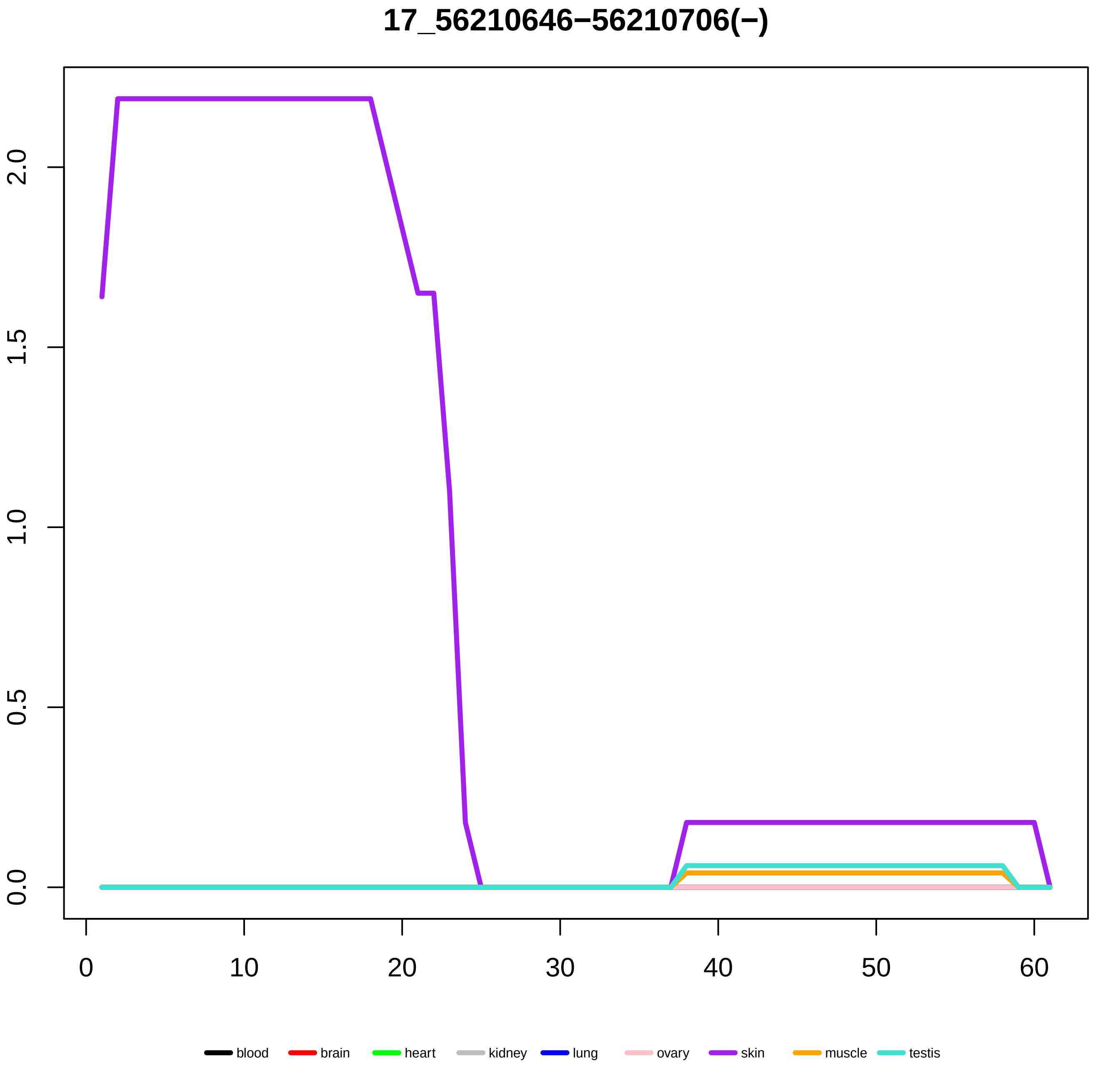

17_56210646−56210706(−)
0.0
0.5
1.0
1.5
2.0
0
10
20
30
40
50
60
blood
brain
heart
kidney
lung
ovary
skin
muscle
testis

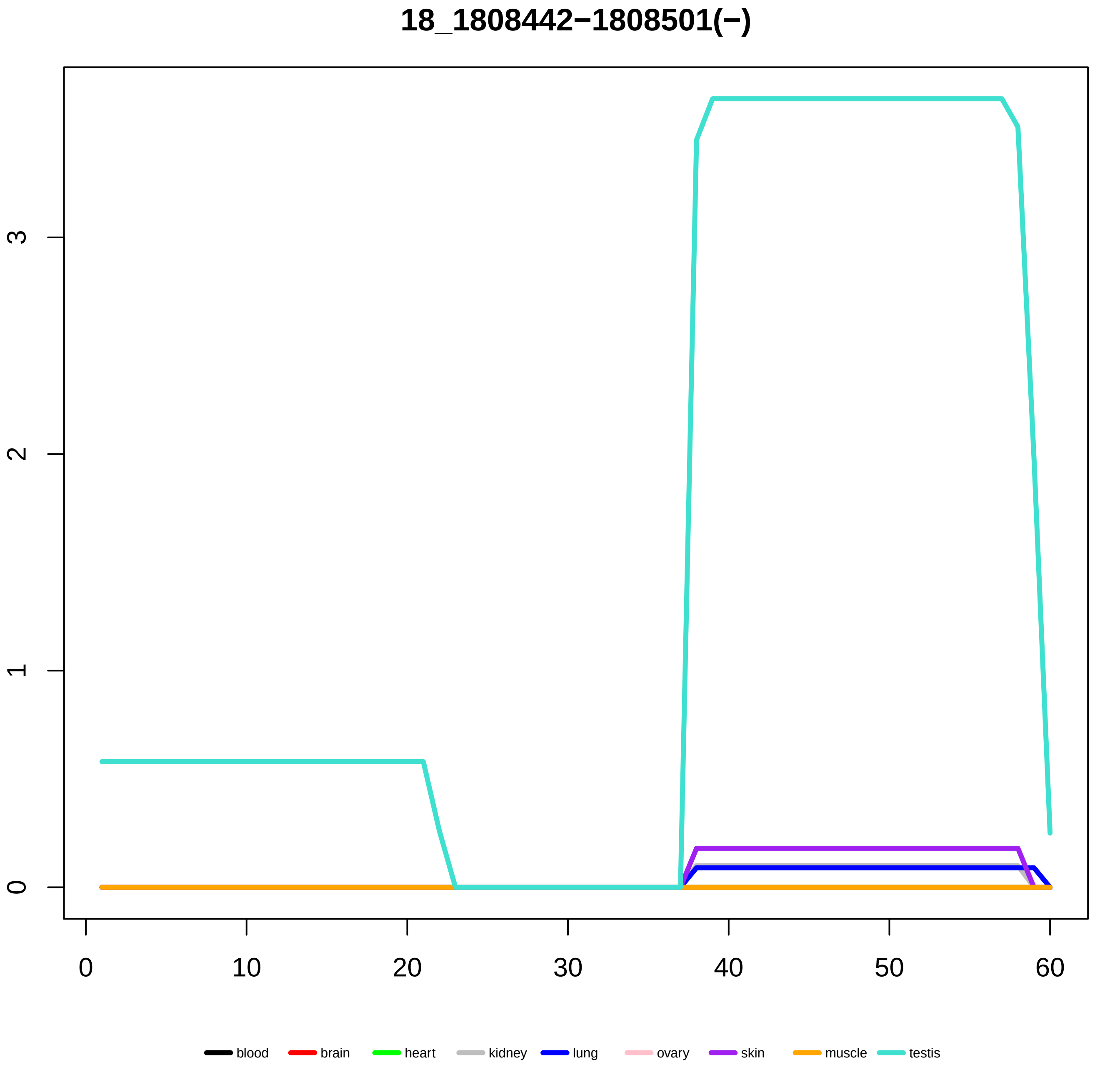
18_1808442−1808501(−)
0
1
2
3
0
10
20
30
40
50
60
blood
brain
heart
kidney
lung
ovary
skin
muscle
testis

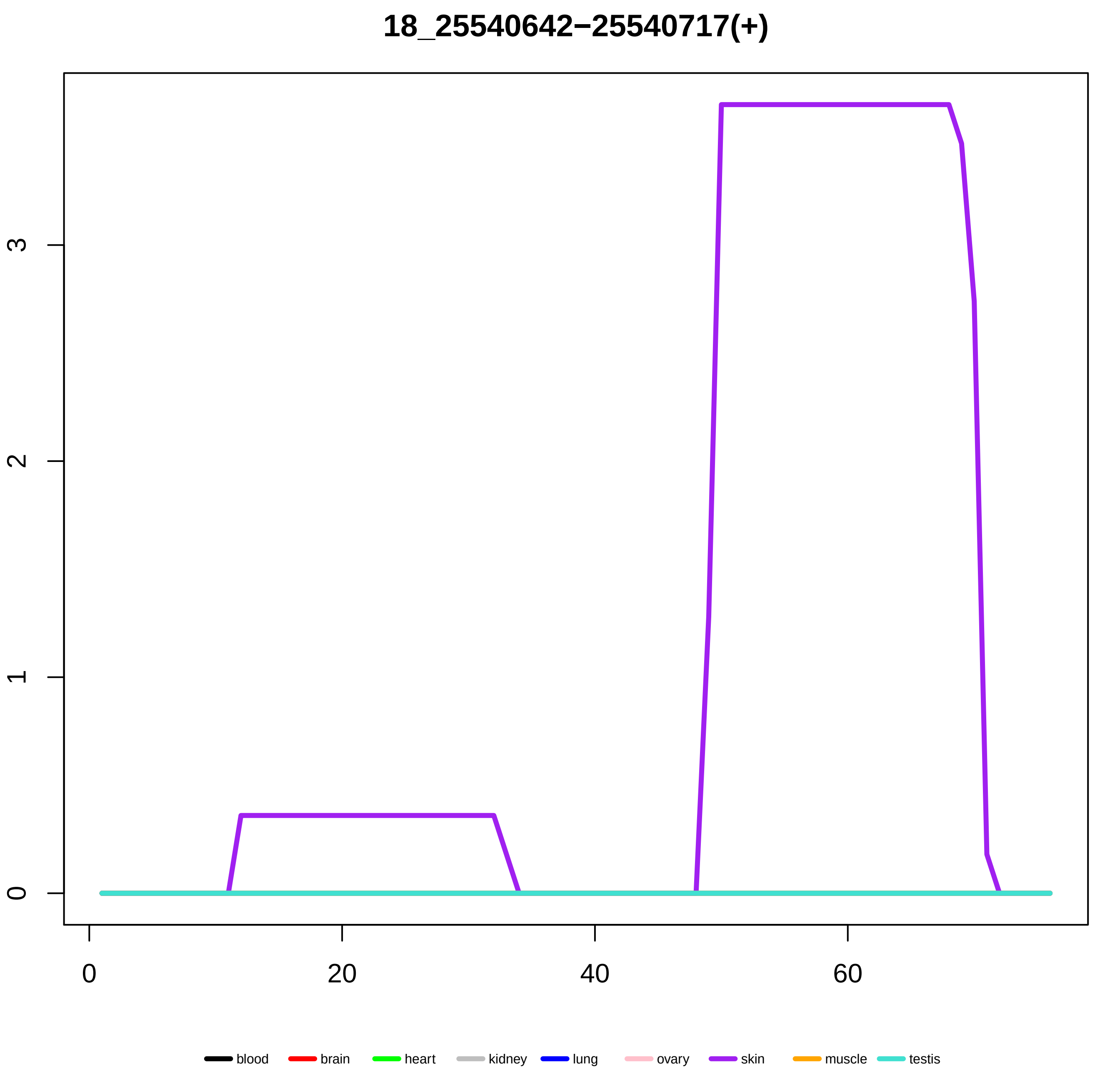

18_25540642−25540717(+)
0
1
2
3
0
20
40
60
blood
brain
heart
kidney
lung
ovary
skin
muscle
testis

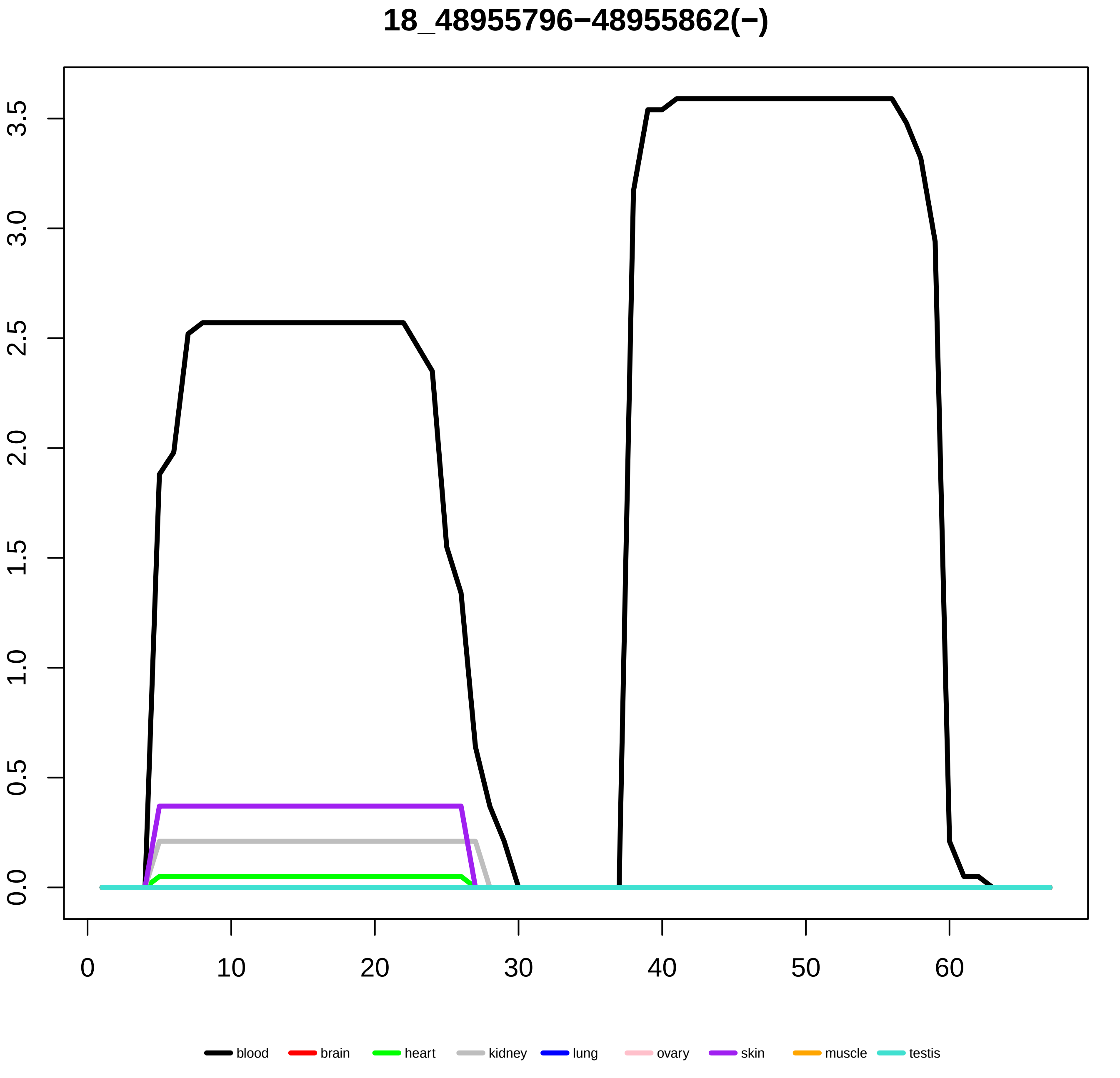
18_48955796−48955862(−)
0.0
0.5
1.0
1.5
2.0
2.5
3.0
3.5
0
10
20
30
40
50
60
blood
brain
heart
kidney
lung
ovary
skin
muscle
testis

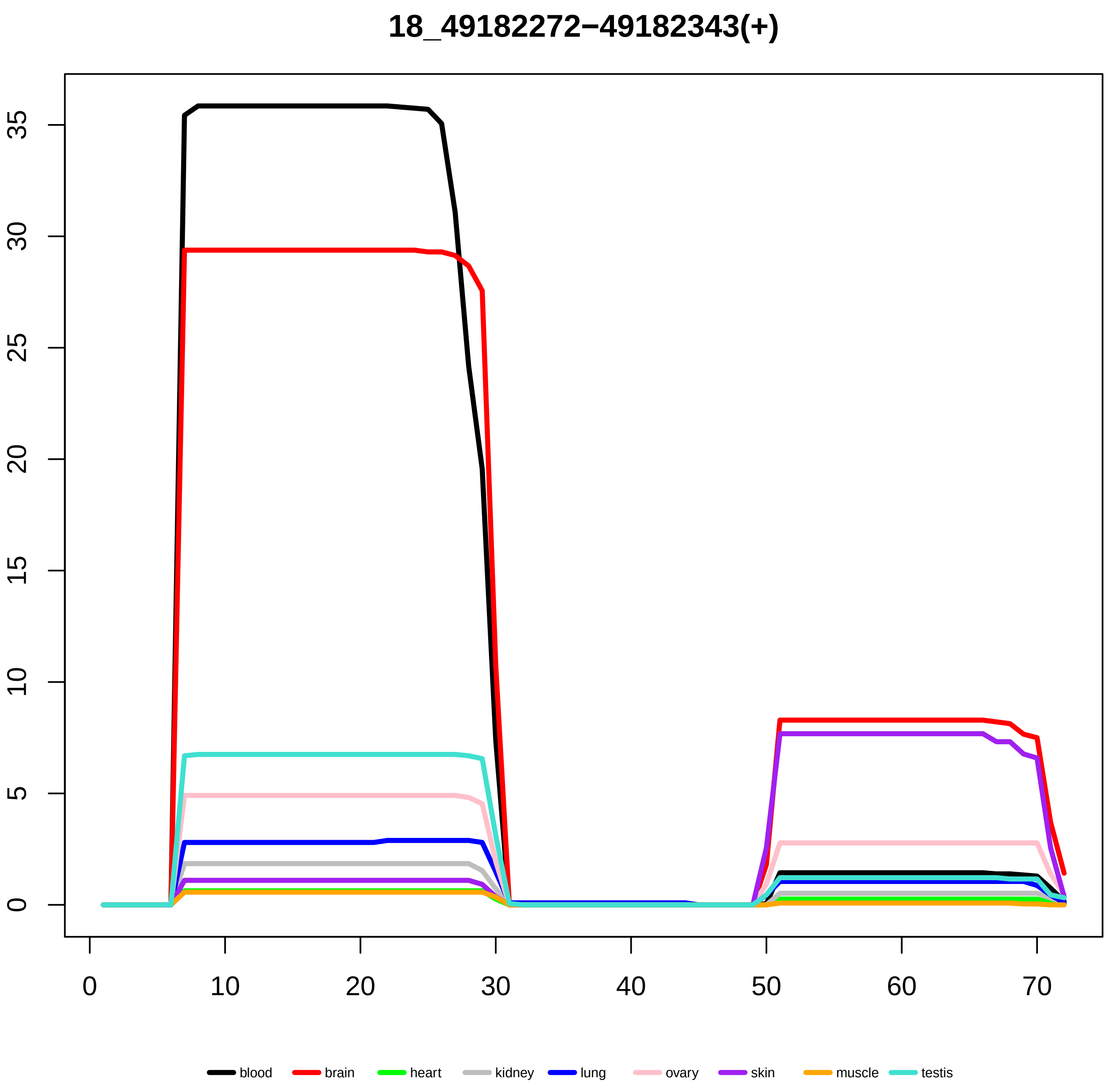
18_49182272−49182343(+)
0
5
10
15
20
25
30
35
0
10
20
30
40
50
60
70
blood
brain
heart
kidney
lung
ovary
skin
muscle
testis

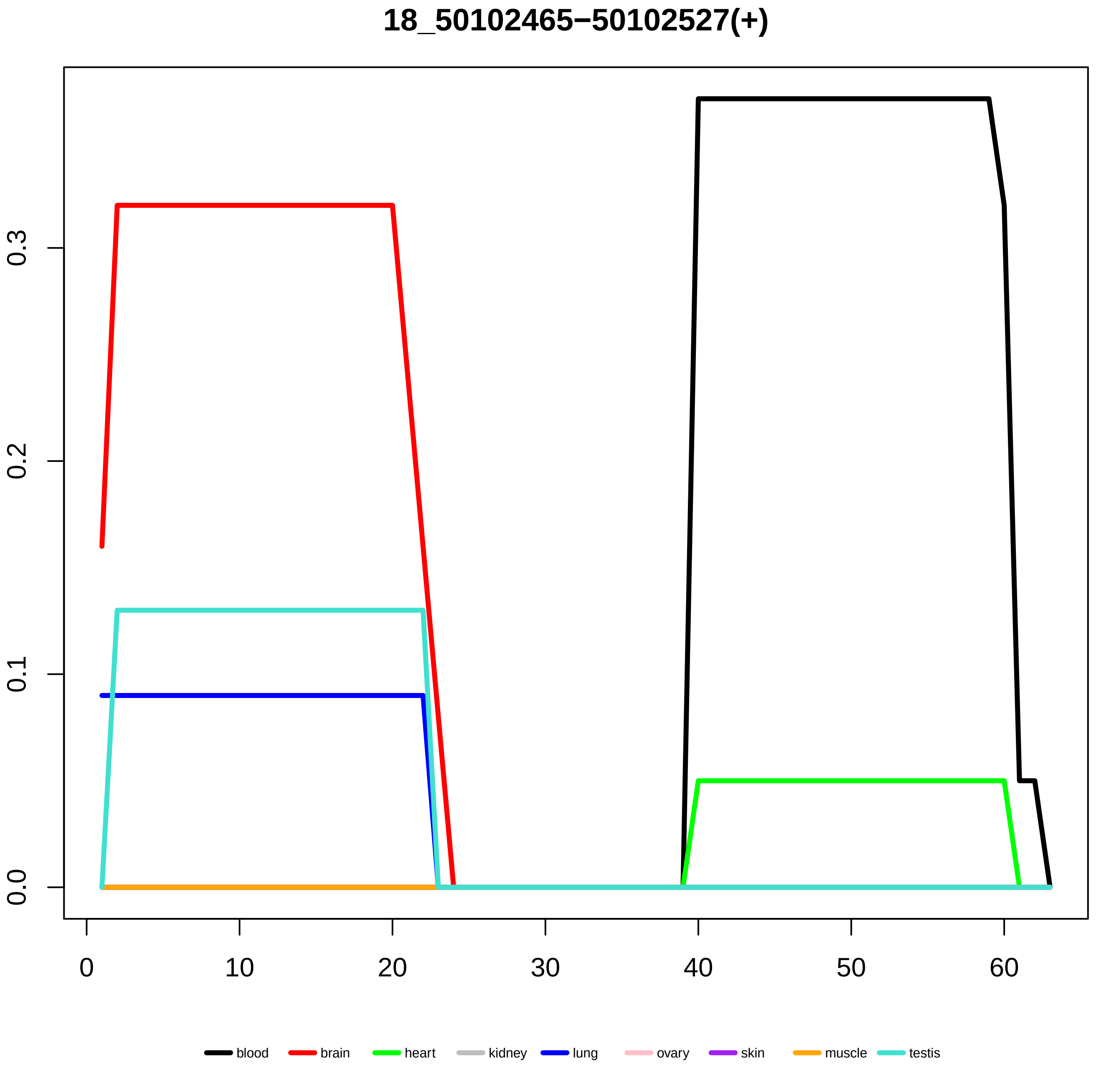

18_50102465−50102527(+)
0.0
0.1
0.2
0.3
0
10
20
30
40
50
60
blood
brain
heart
kidney
lung
ovary
skin
muscle
testis

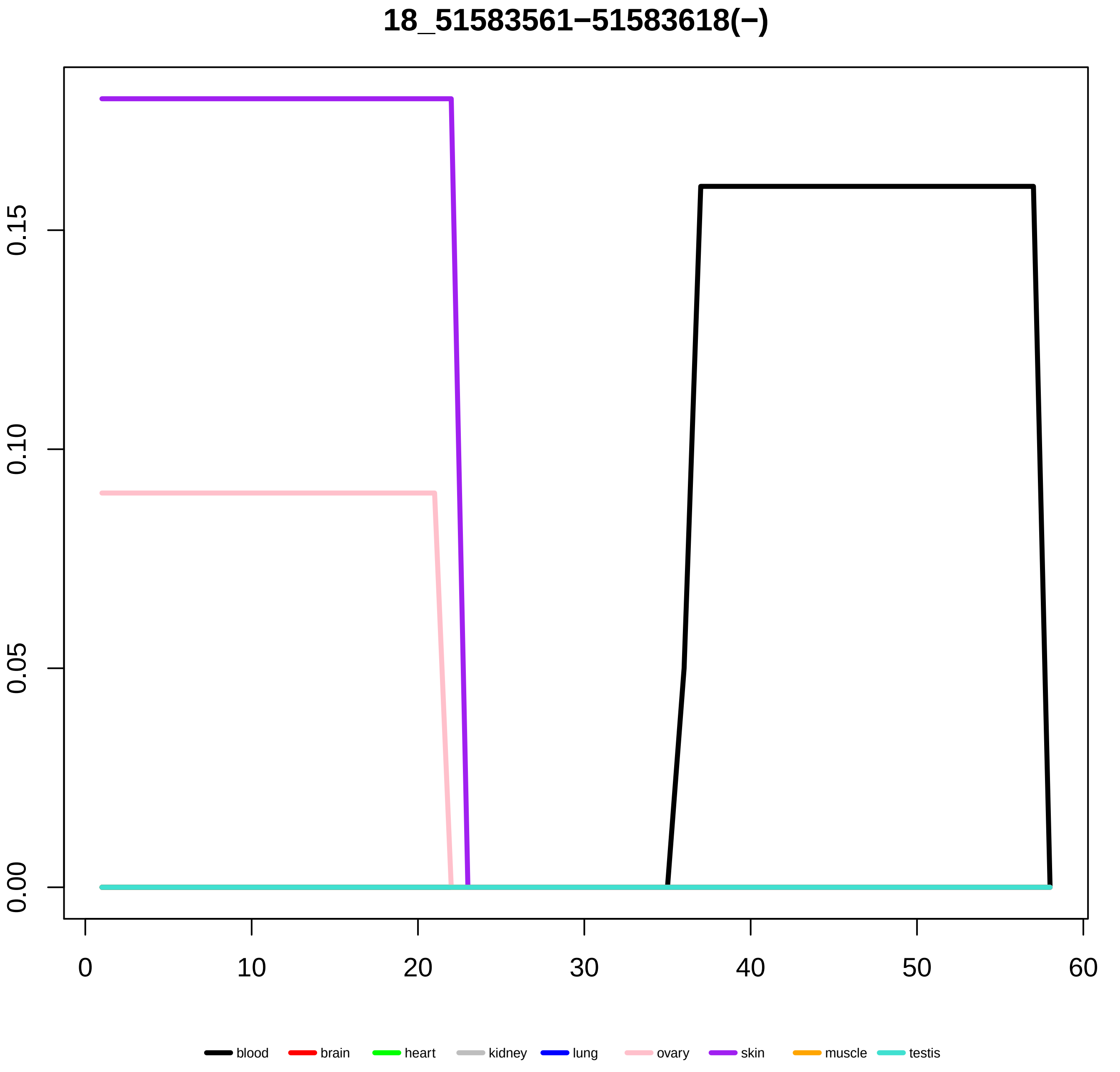

18_51583561−51583618(−)
0.00
0.05
0.10
0.15
0
10
20
30
40
50
60
blood
brain
heart
kidney
lung
ovary
skin
muscle
testis

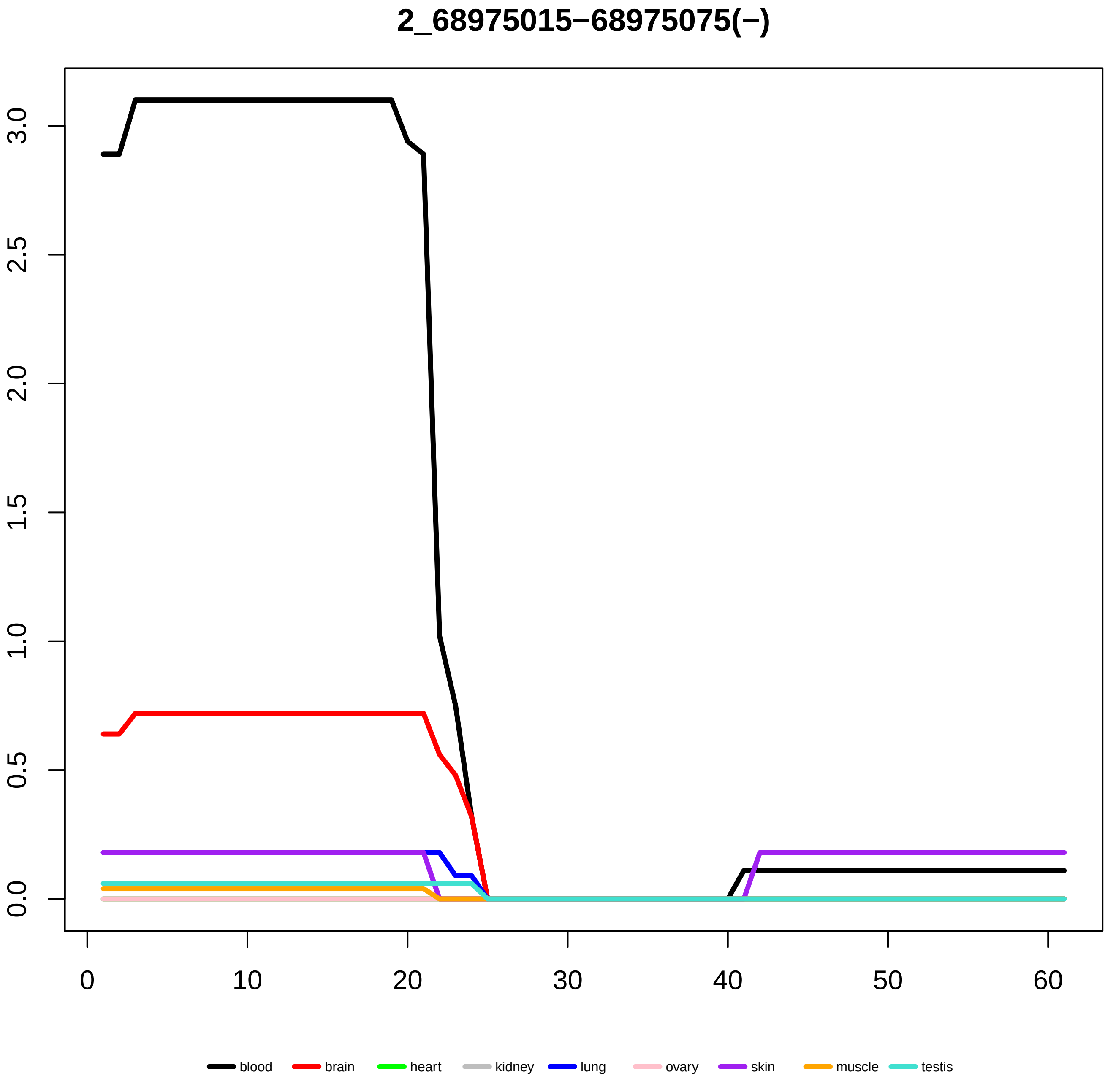
2_68975015−68975075(−)
3.0
2.5
2.0
1.5
1.0
0.5
0.0
0
10
20
30
40
50
60
blood
brain
heart
kidney
lung
ovary
skin
muscle
testis

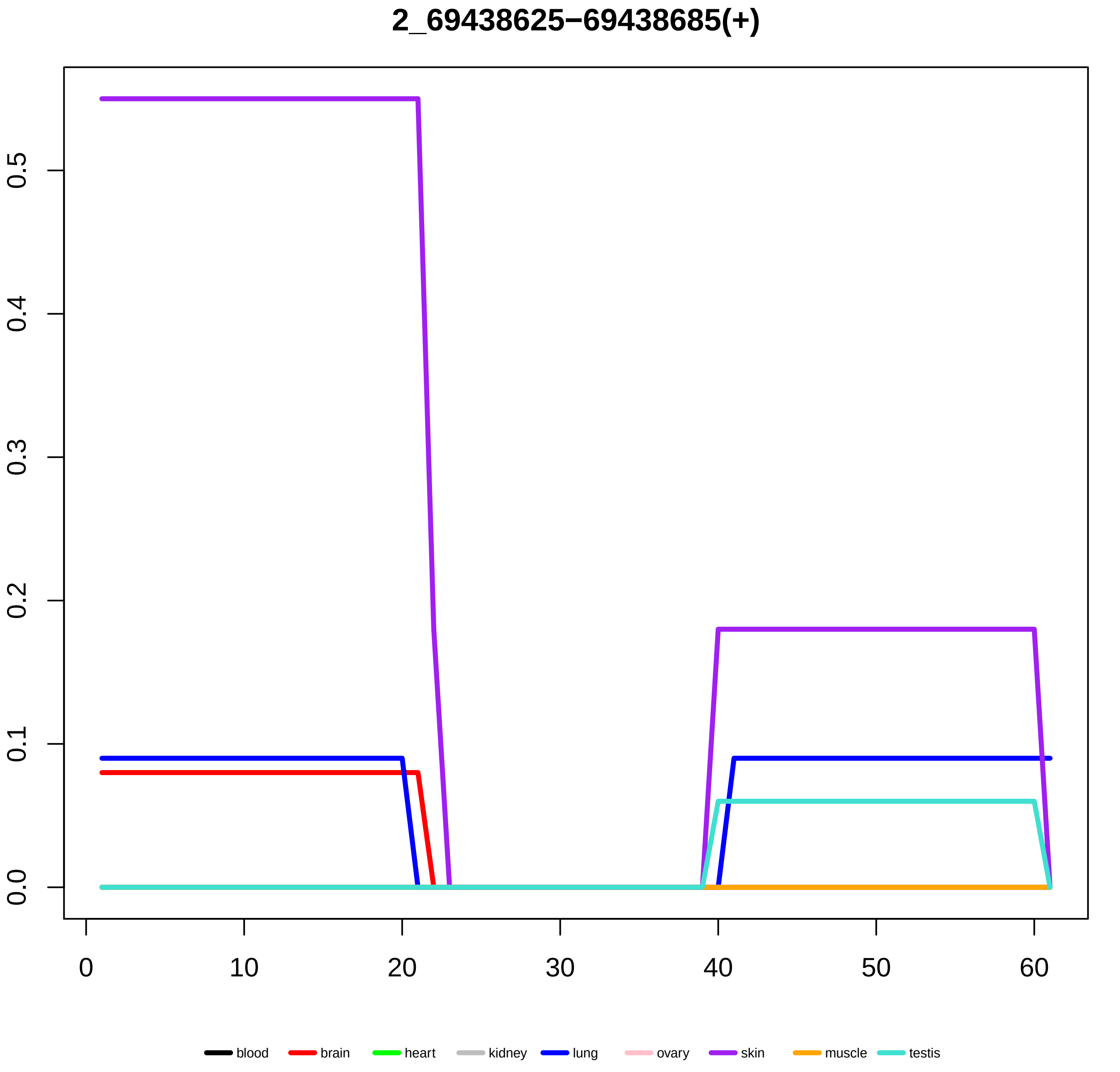
2_69438625−69438685(+)
0.0
0.1
0.2
0.3
0.4
0.5
0
10
20
30
40
50
60
blood
brain
heart
kidney
lung
ovary
skin
muscle
testis

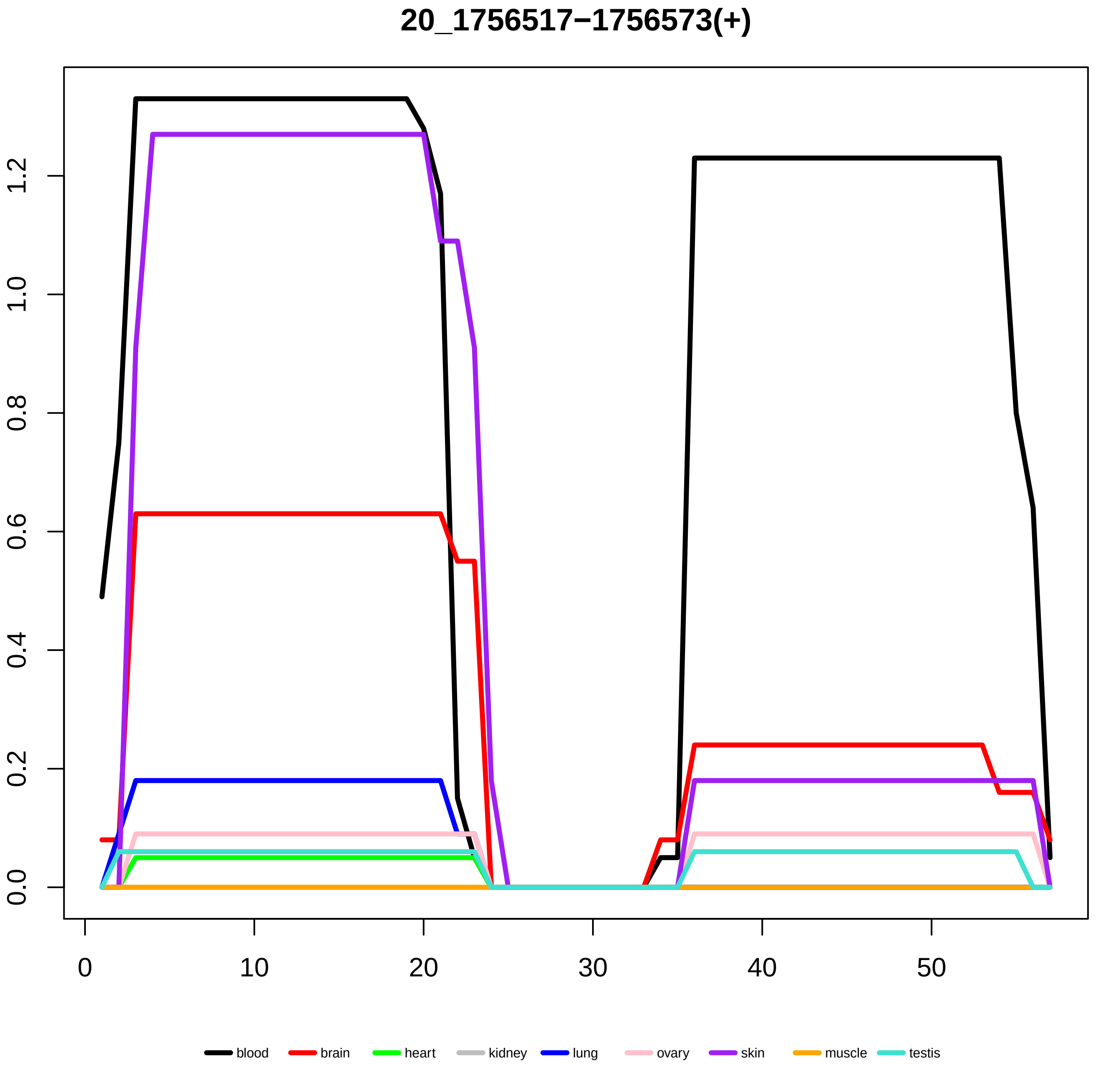

20_1756517−1756573(+)
0.0
0.2
0.4
0.6
0.8
1.0
1.2
0
10
20
30
40
50
blood
brain
heart
kidney
lung
ovary
skin
muscle
testis

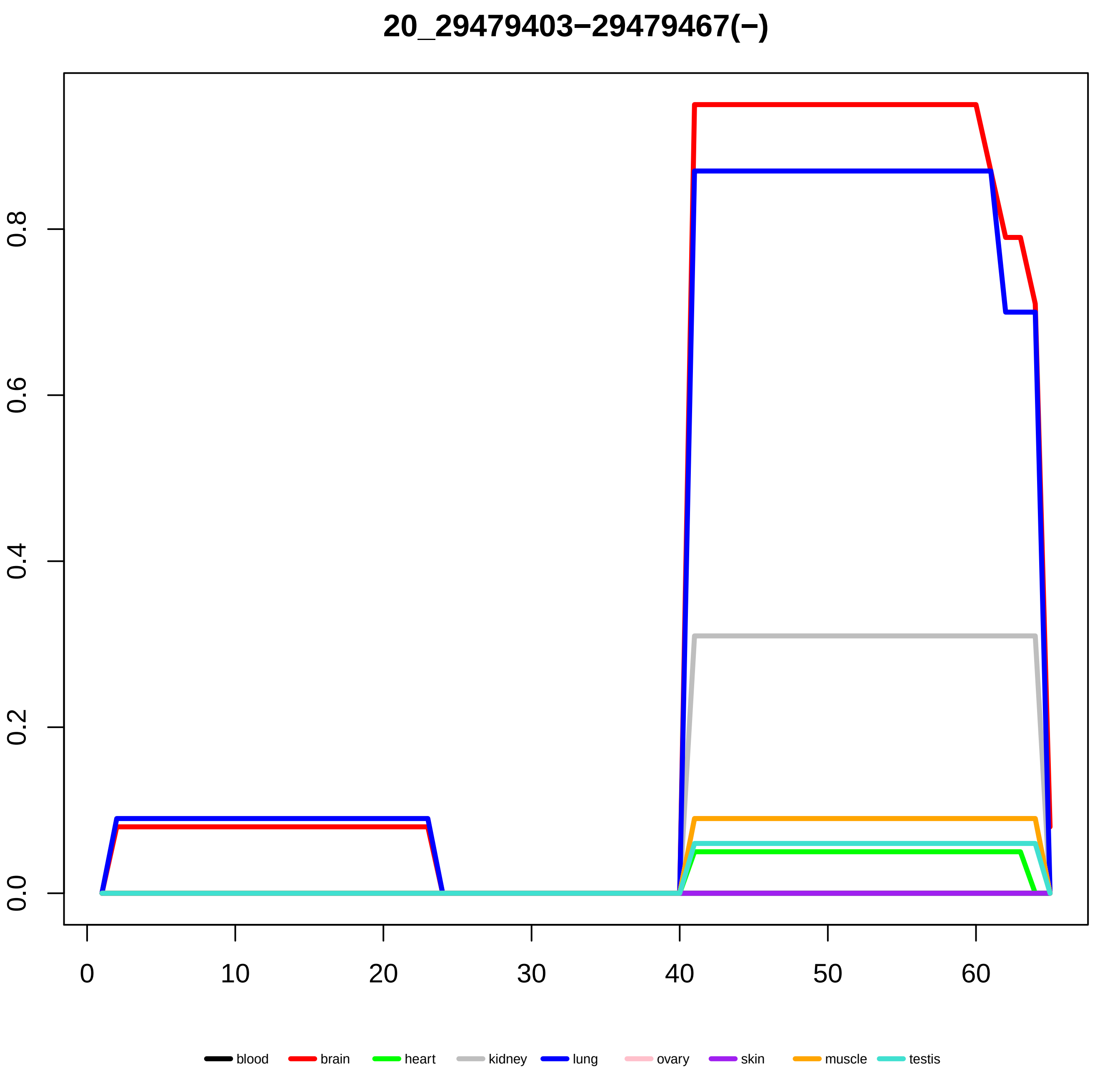
20_29479403−29479467(−)
0.0
0.2
0.4
0.6
0.8
0
10
20
30
40
50
60
blood
brain
heart
kidney
lung
ovary
skin
muscle
testis

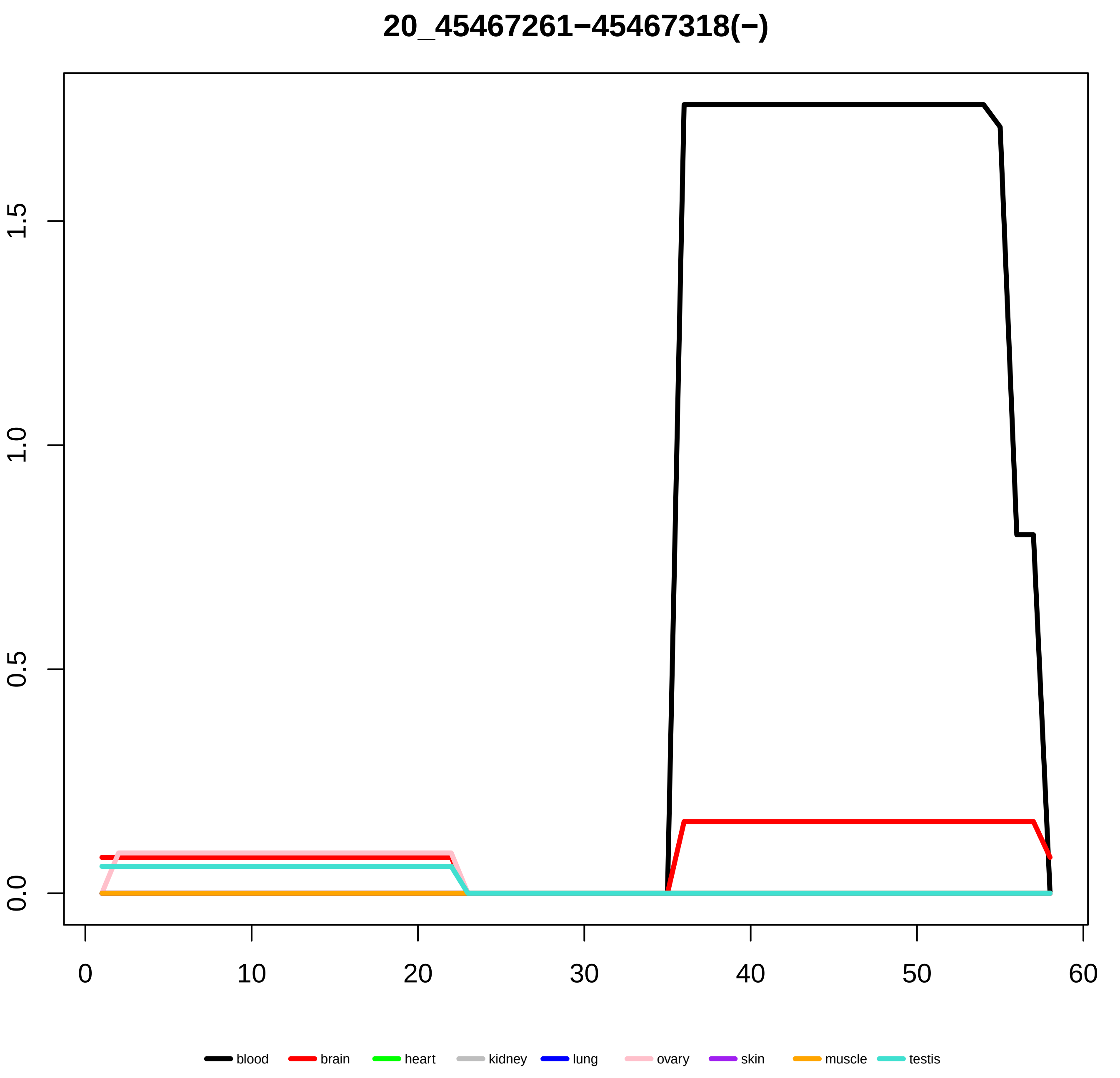
20_45467261−45467318(−)
0.0
0.5
1.0
1.5
0
10
20
30
40
50
60
blood
brain
heart
kidney
lung
ovary
skin
muscle
testis

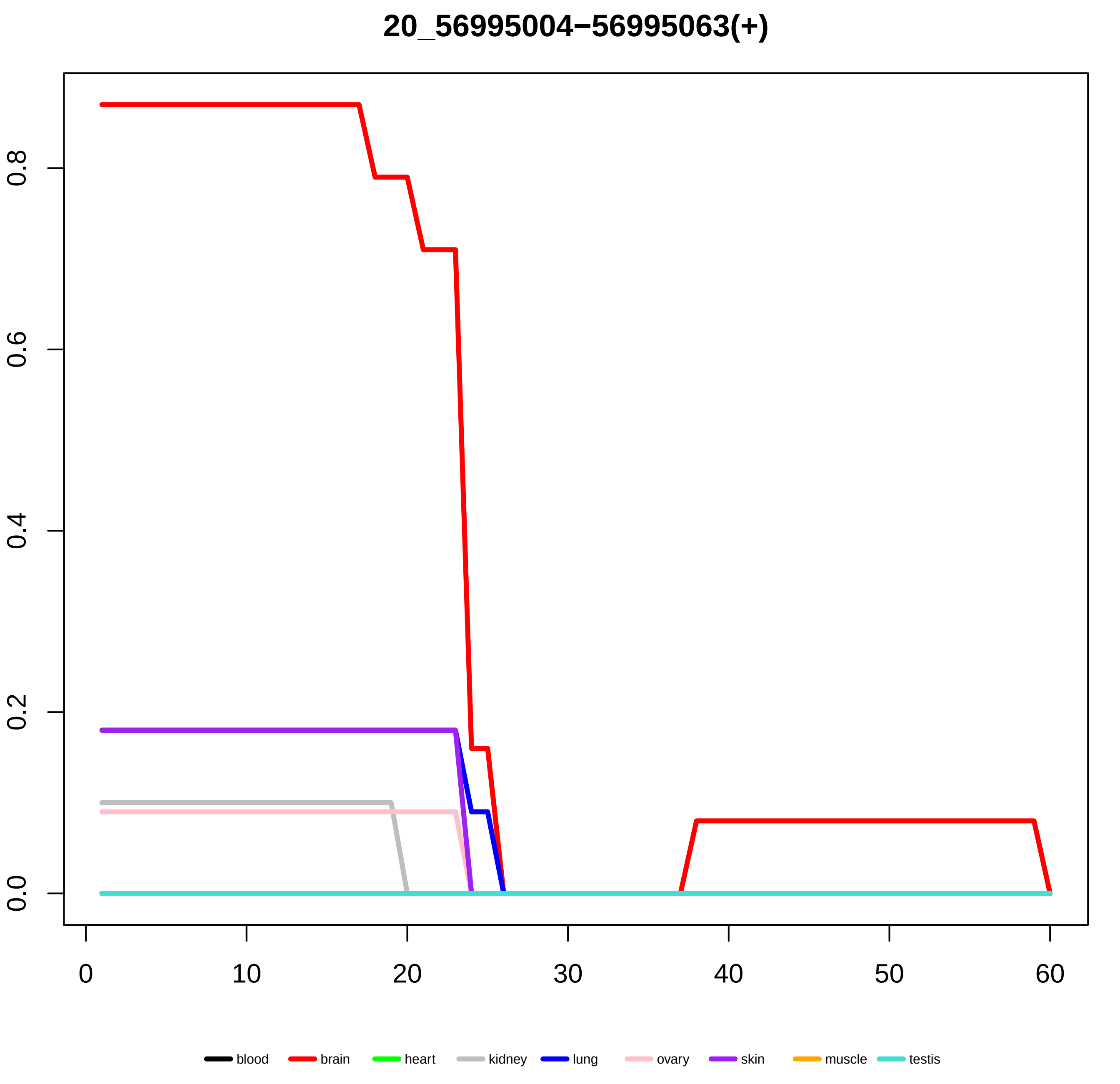

20_56995004−56995063(+)
0.0
0.2
0.4
0.6
0.8
0
10
20
30
40
50
60
blood
brain
heart
kidney
lung
ovary
skin
muscle
testis

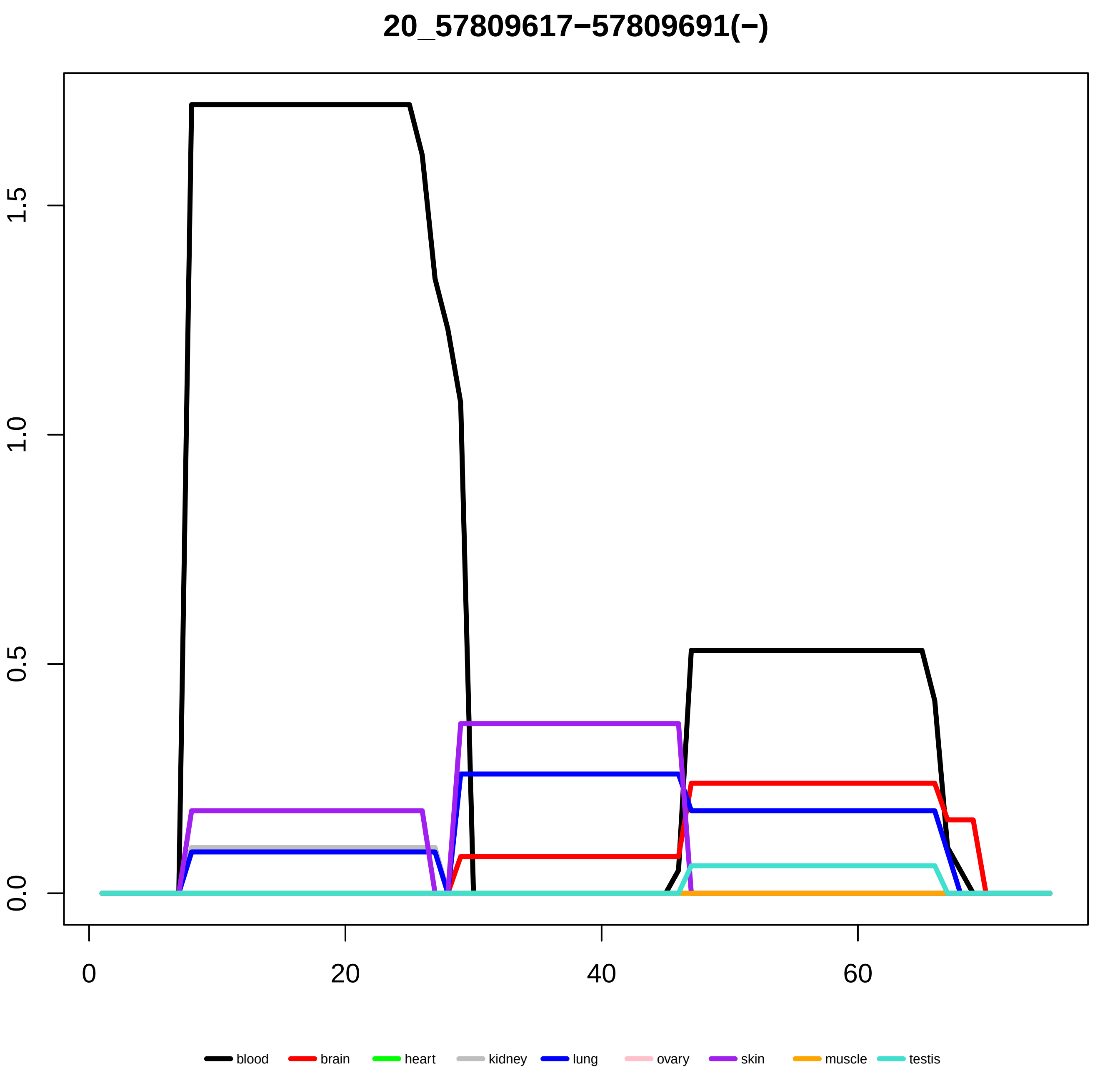
20_57809617−57809691(−)
0.0
0.5
1.0
1.5
0
20
40
60
blood
brain
heart
kidney
lung
ovary
skin
muscle
testis

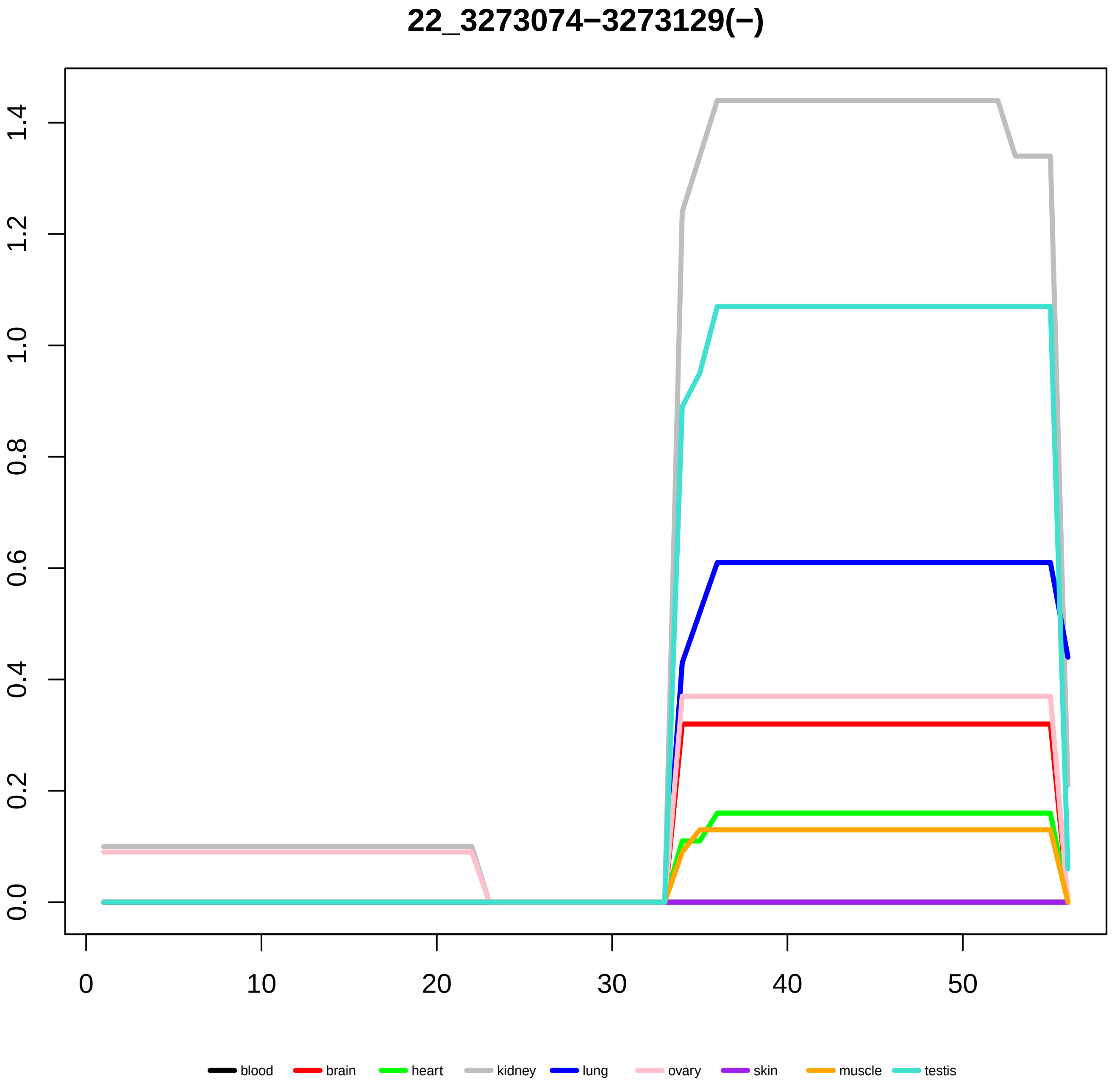

22_3273074−3273129(−)
0.0
0.2
0.4
0.6
0.8
1.0
1.2
1.4
0
10
20
30
40
50
blood
brain
heart
kidney
lung
ovary
skin
muscle
testis

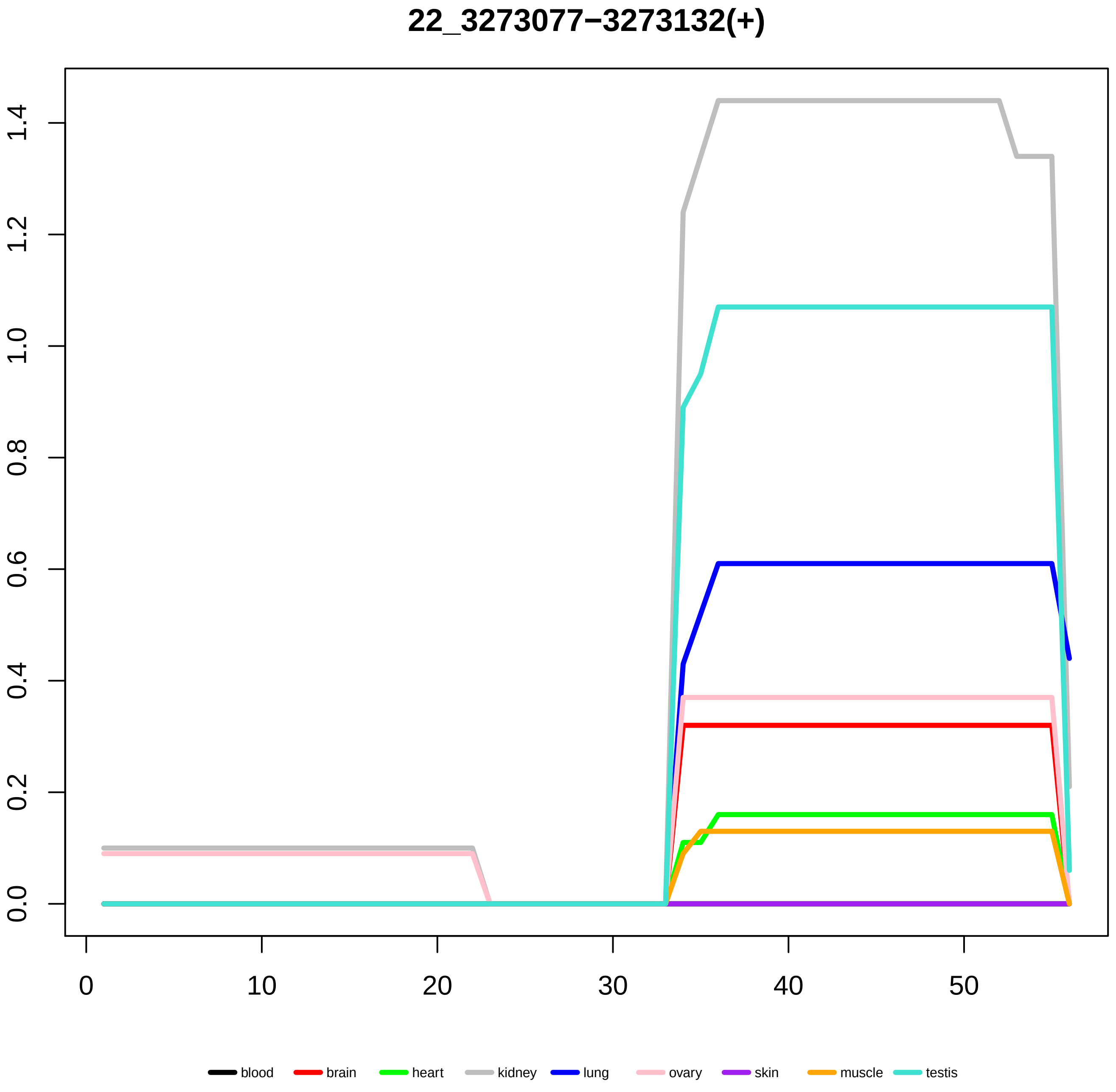

22_3273077−3273132(+)
1.4
1.2
1.0
0.8
0.6
0.4
0.2
0.0
0
10
20
30
40
50
blood
brain
heart
kidney
lung
ovary
skin
muscle
testis

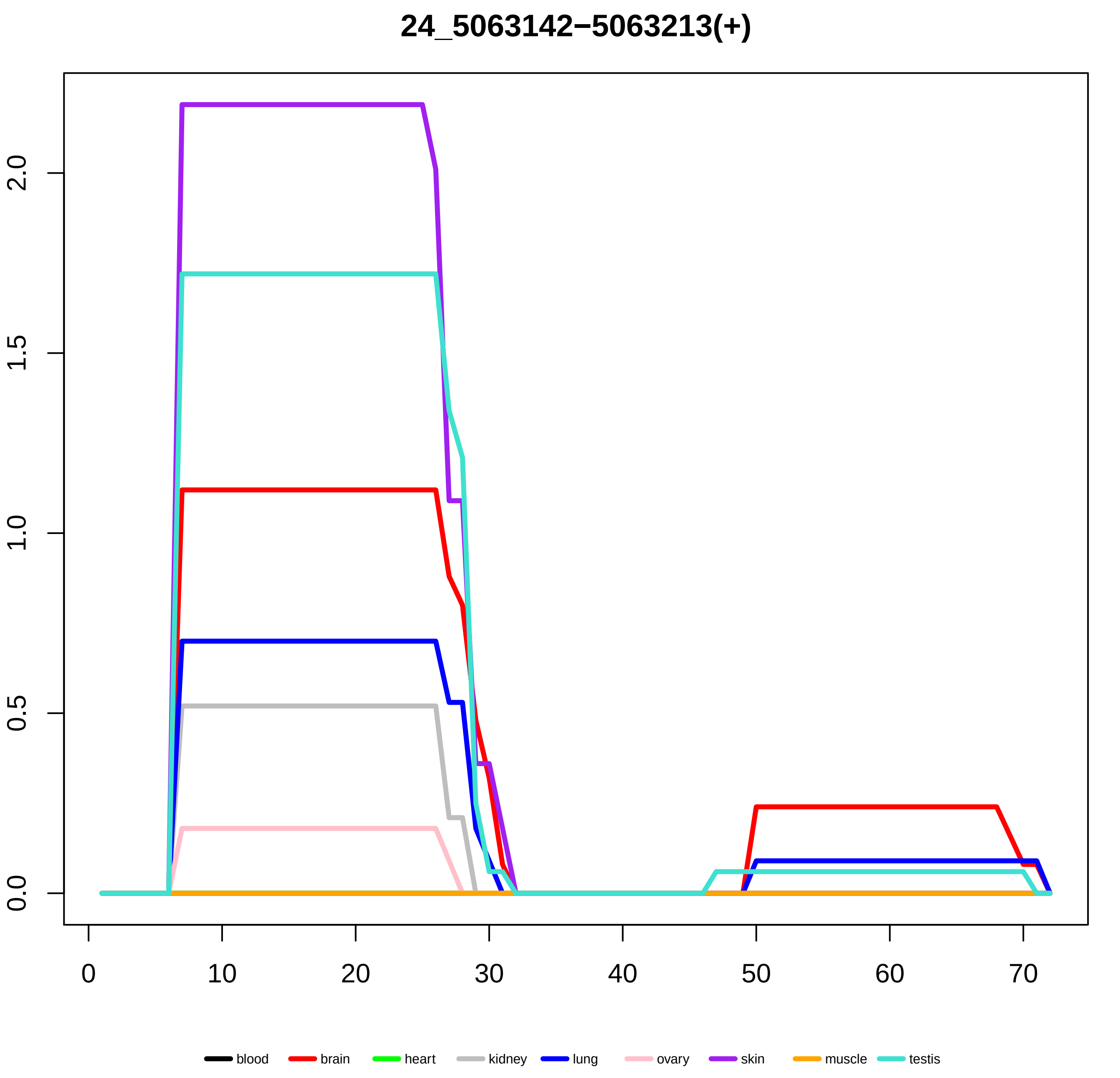

24_5063142−5063213(+)
0.0
0.5
1.0
1.5
2.0
0
10
20
30
40
50
60
70
blood
brain
heart
kidney
lung
ovary
skin
muscle
testis

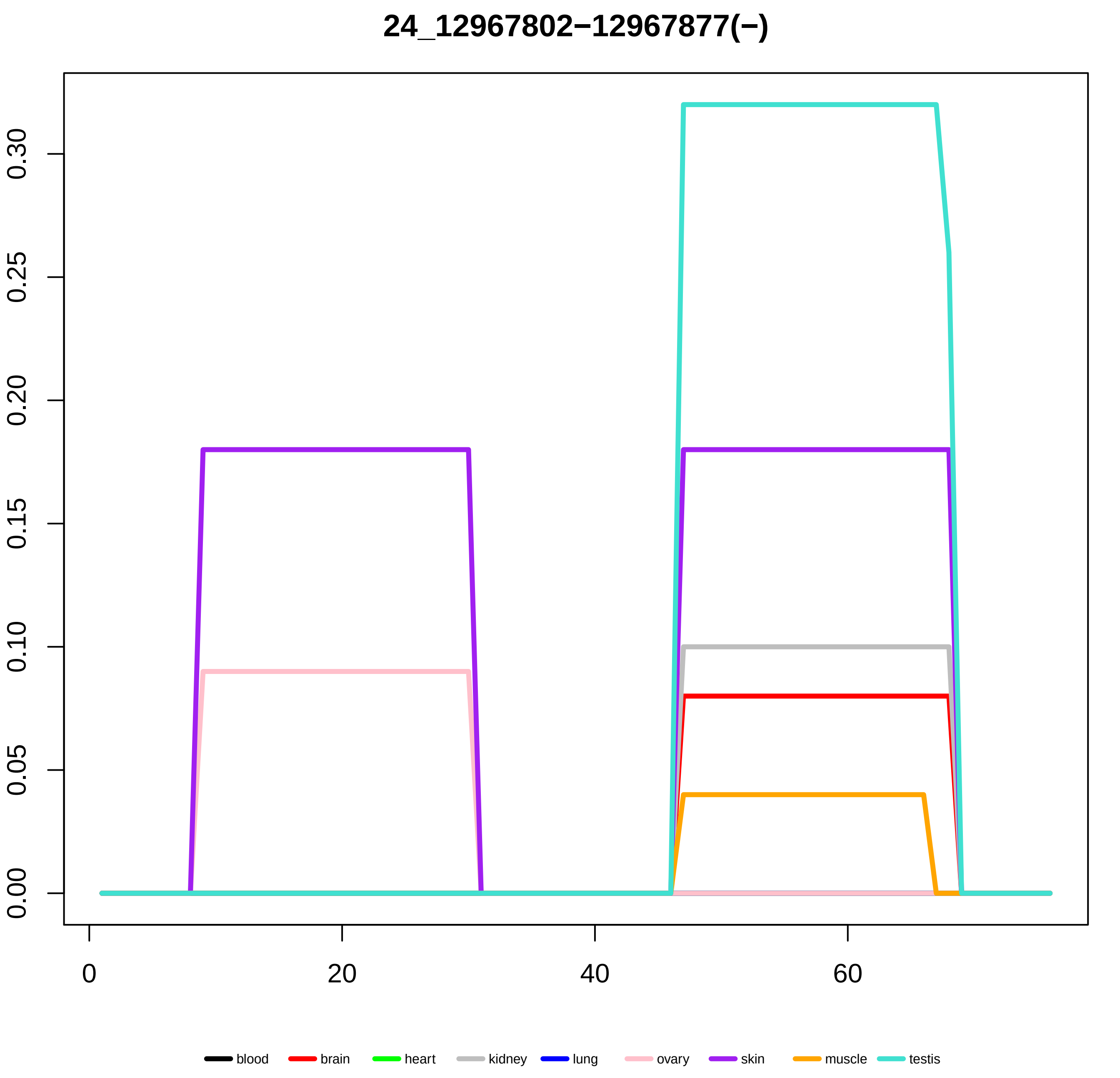

24_12967802−12967877(−)
0.00
0.05
0.10
0.15
0.20
0.25
0.30
0
20
40
60
blood
brain
heart
kidney
lung
ovary
skin
muscle
testis

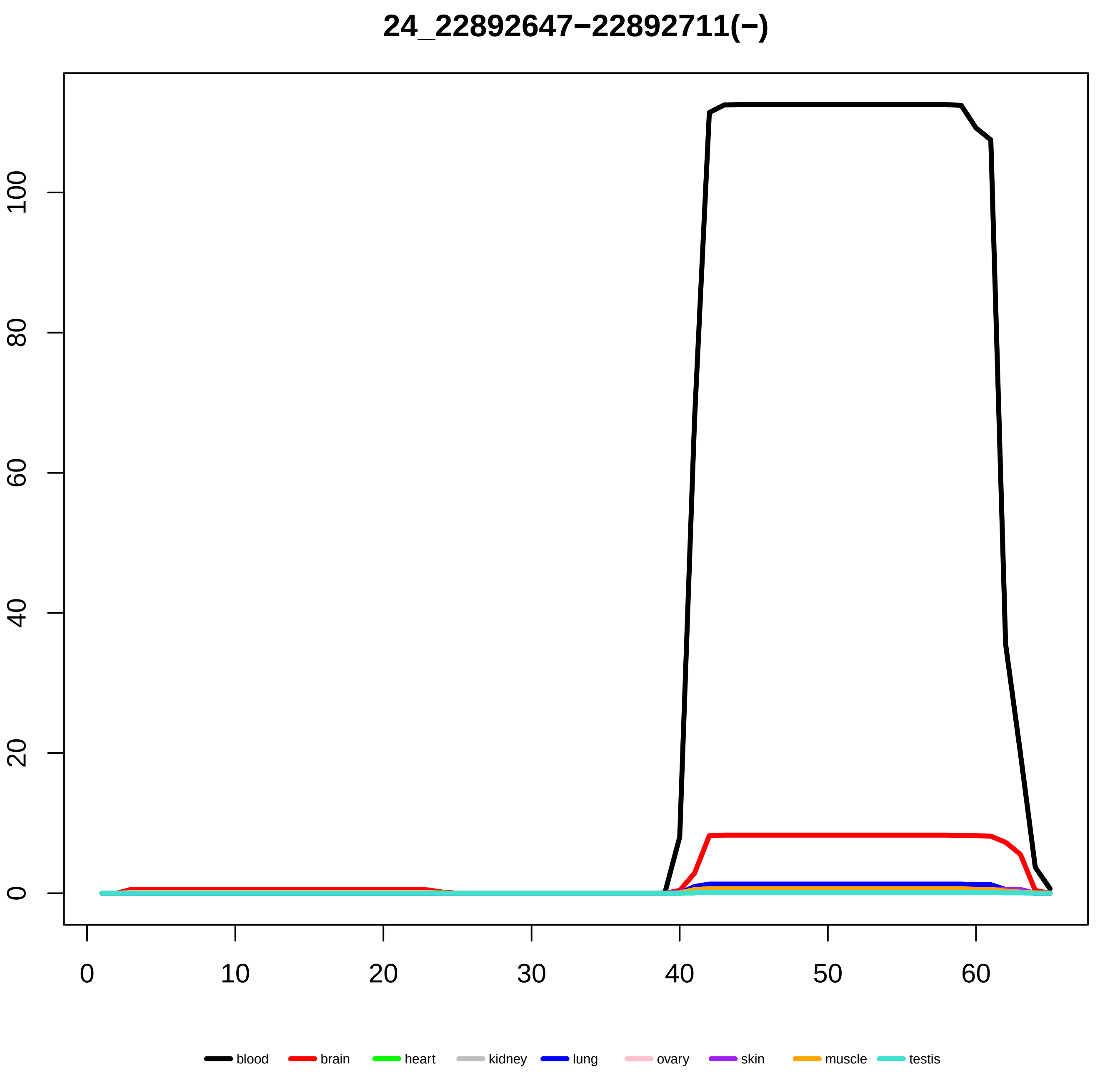

24_22892647−22892711(−)
0
20
40
60
80
100
0
10
20
30
40
50
60
blood
brain
heart
kidney
lung
ovary
skin
muscle
testis

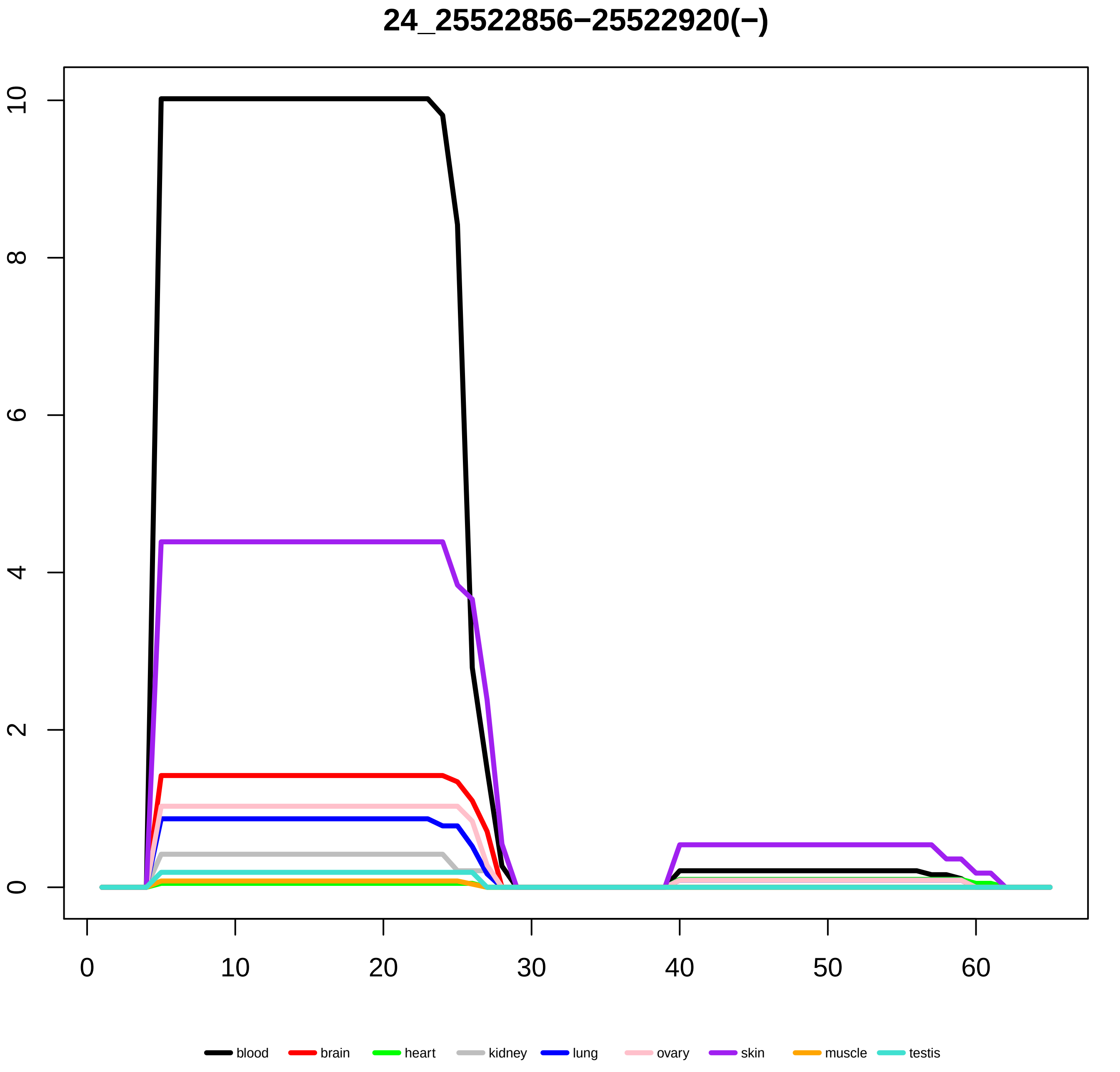
24_25522856−25522920(−)
0
2
4
6
8
10
0
10
20
30
40
50
60
blood
brain
heart
kidney
lung
ovary
skin
muscle
testis

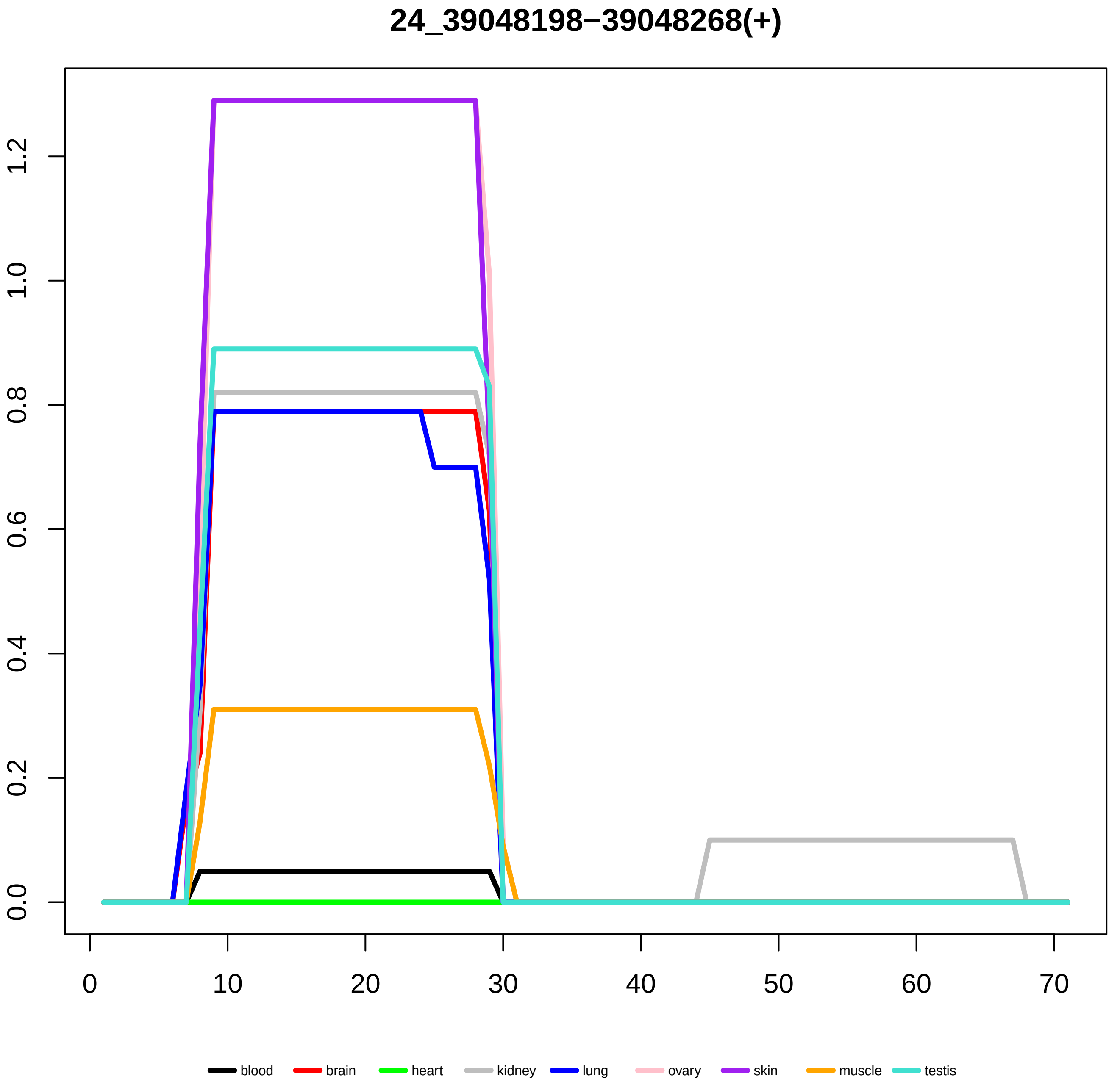
24_39048198−39048268(+)
0.0
0.2
0.4
0.6
0.8
1.0
1.2
0
10
20
30
40
50
60
70
blood
brain
heart
kidney
lung
ovary
skin
muscle
testis

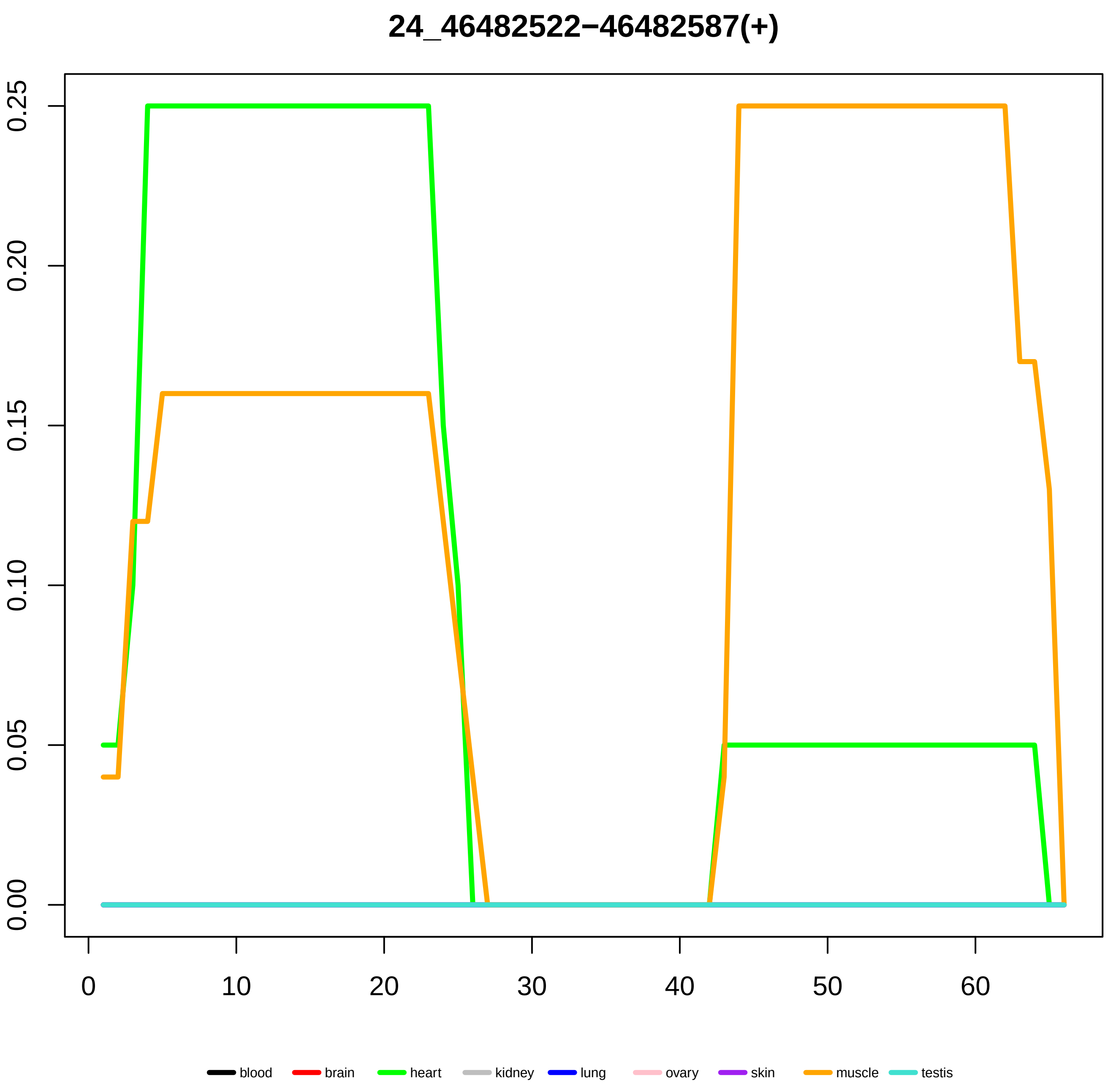

24_46482522−46482587(+)
0.00
0.05
0.10
0.15
0.20
0.25
0
10
20
30
40
50
60
blood
brain
heart
kidney
lung
ovary
skin
muscle
testis

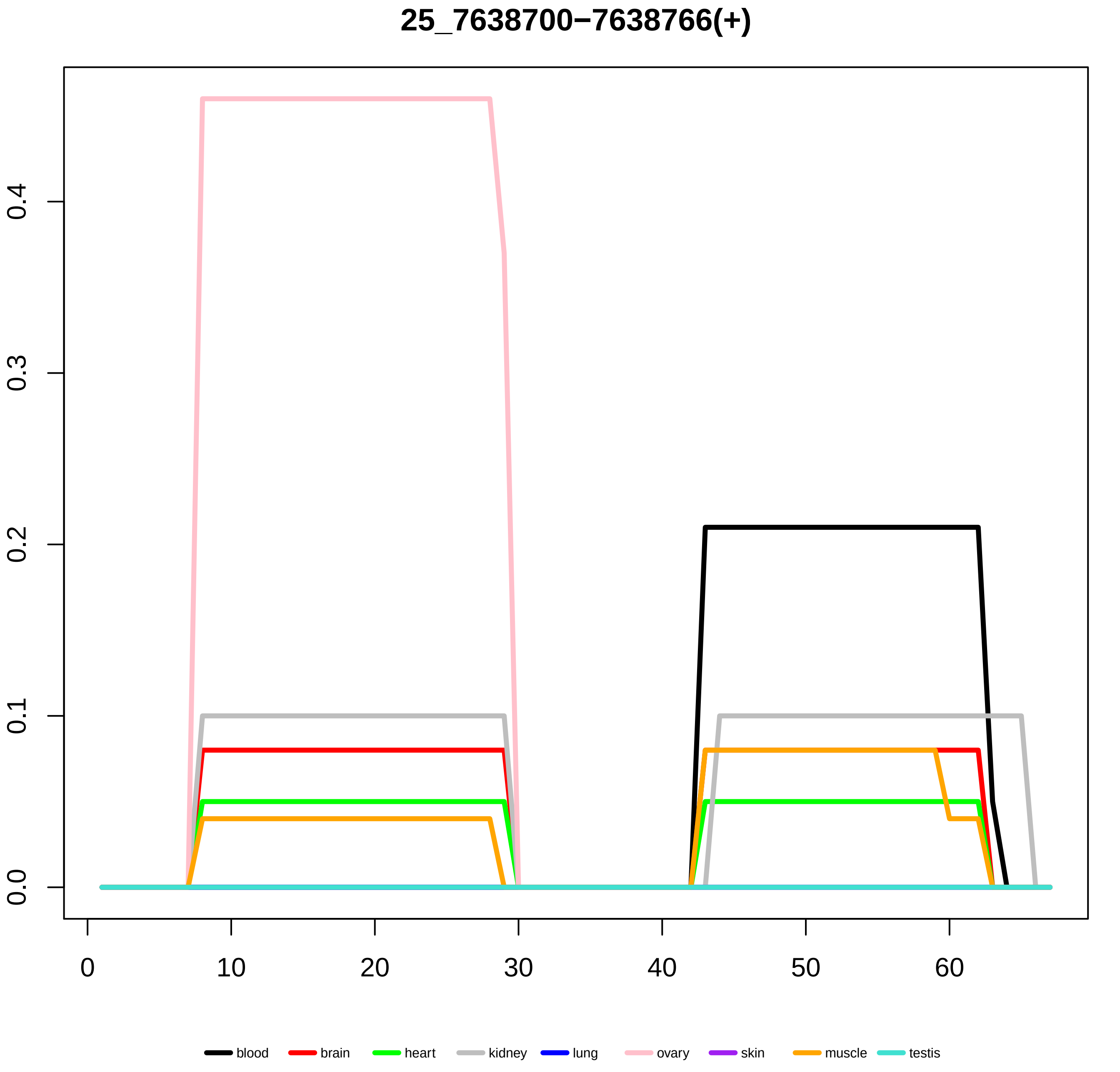

25_7638700−7638766(+)
0.0
0.1
0.2
0.3
0.4
0
10
20
30
40
50
60
blood
brain
heart
kidney
lung
ovary
skin
muscle
testis

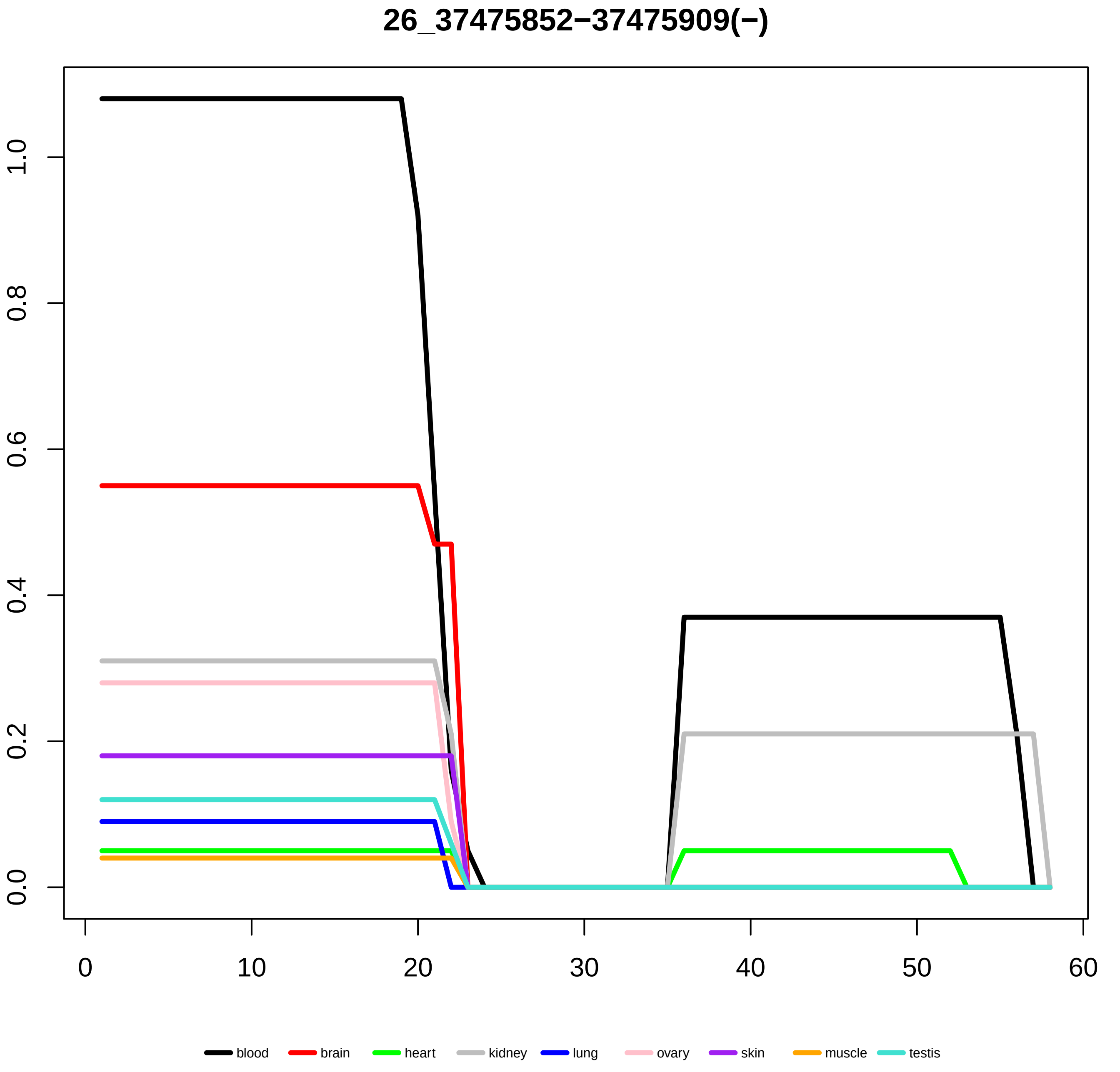
26_37475852−37475909(−)
0.0
0.2
0.4
0.6
0.8
1.0
0
10
20
30
40
50
60
blood
brain
heart
kidney
lung
ovary
skin
muscle
testis

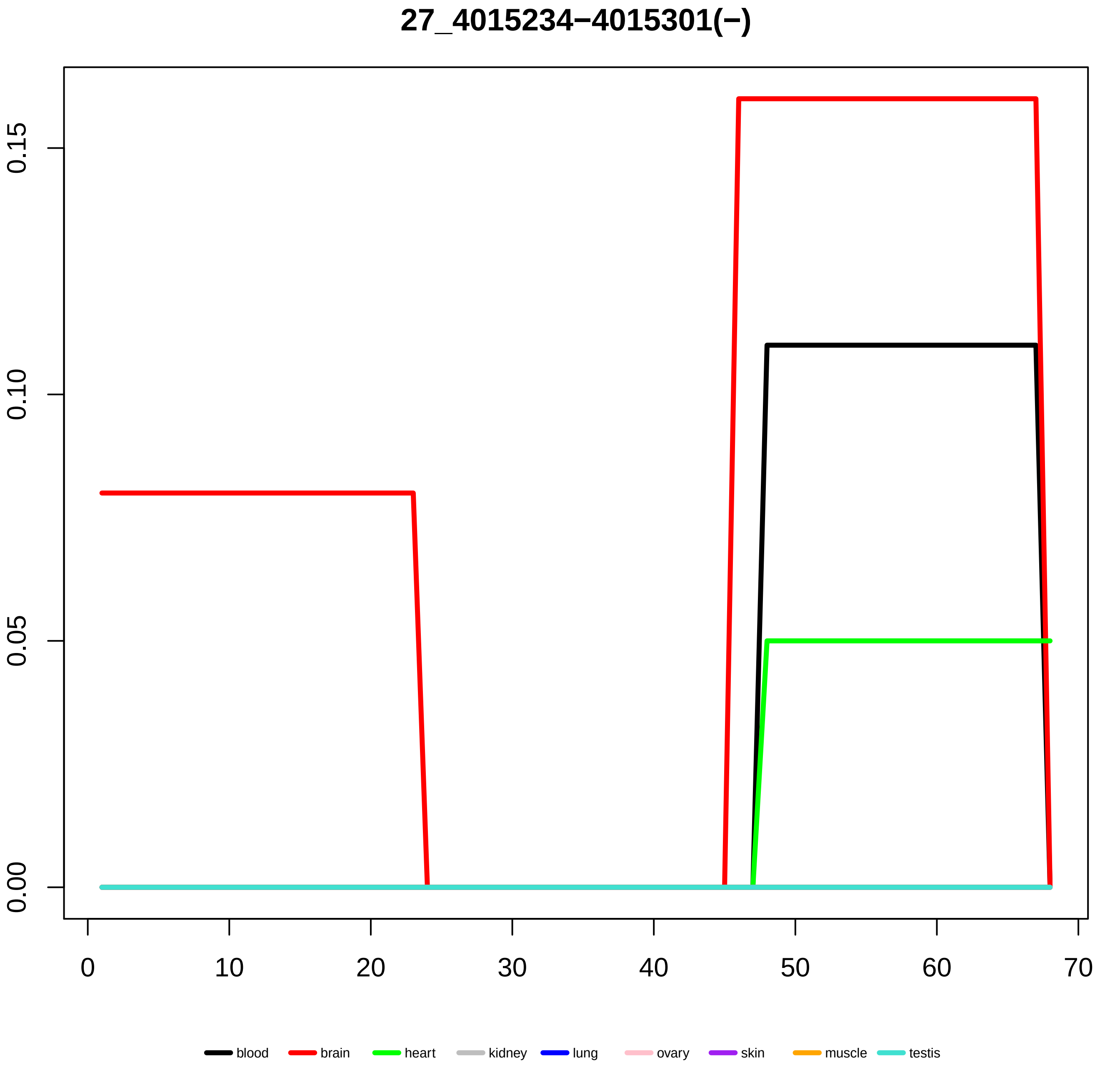
27_4015234−4015301(−)
0.00
0.05
0.10
0.15
0
10
20
30
40
50
60
70
blood
brain
heart
kidney
lung
ovary
skin
muscle
testis

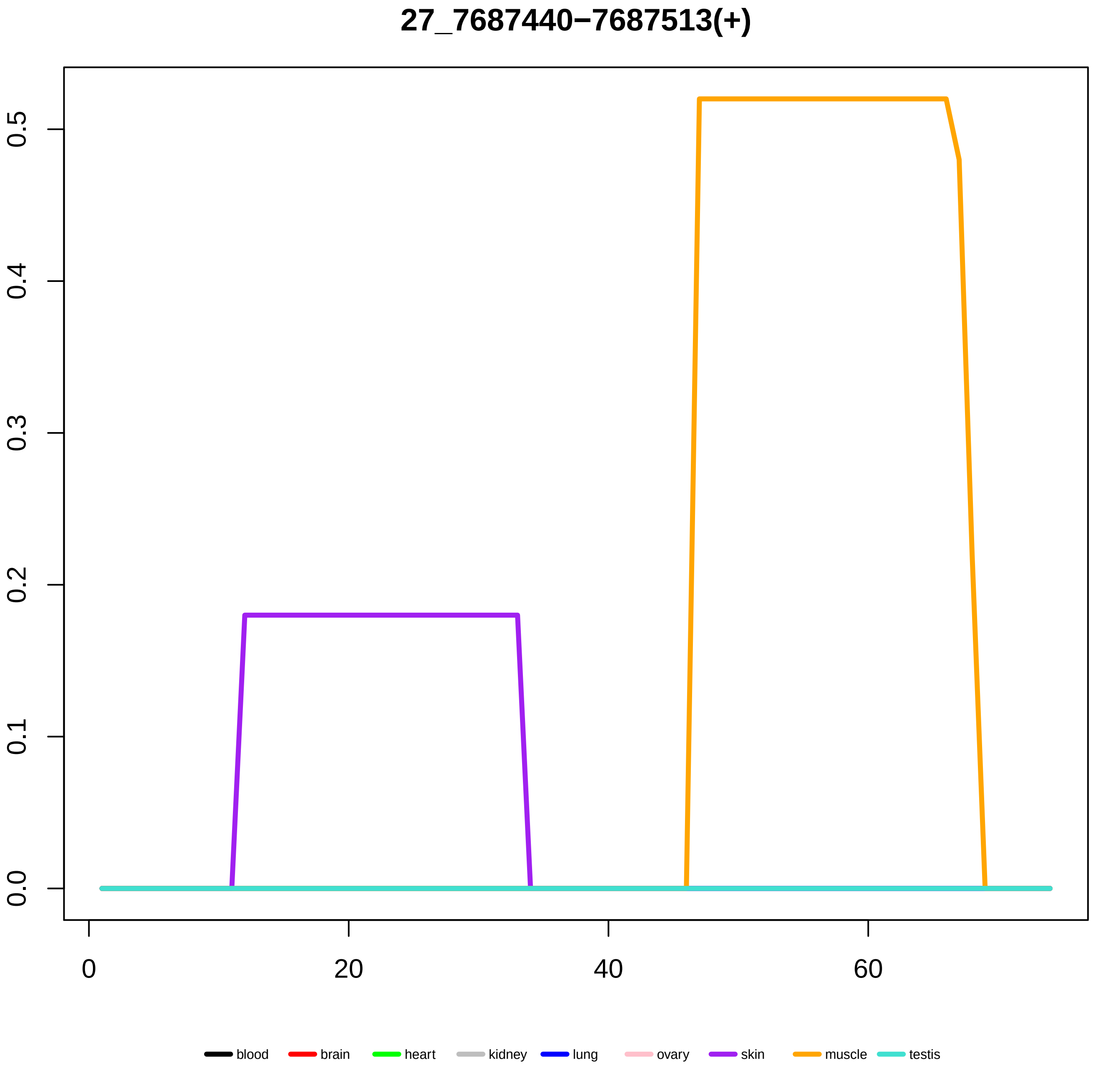

27_7687440−7687513(+)
0.0
0.1
0.2
0.3
0.4
0.5
0
20
40
60
blood
brain
heart
kidney
lung
ovary
skin
muscle
testis

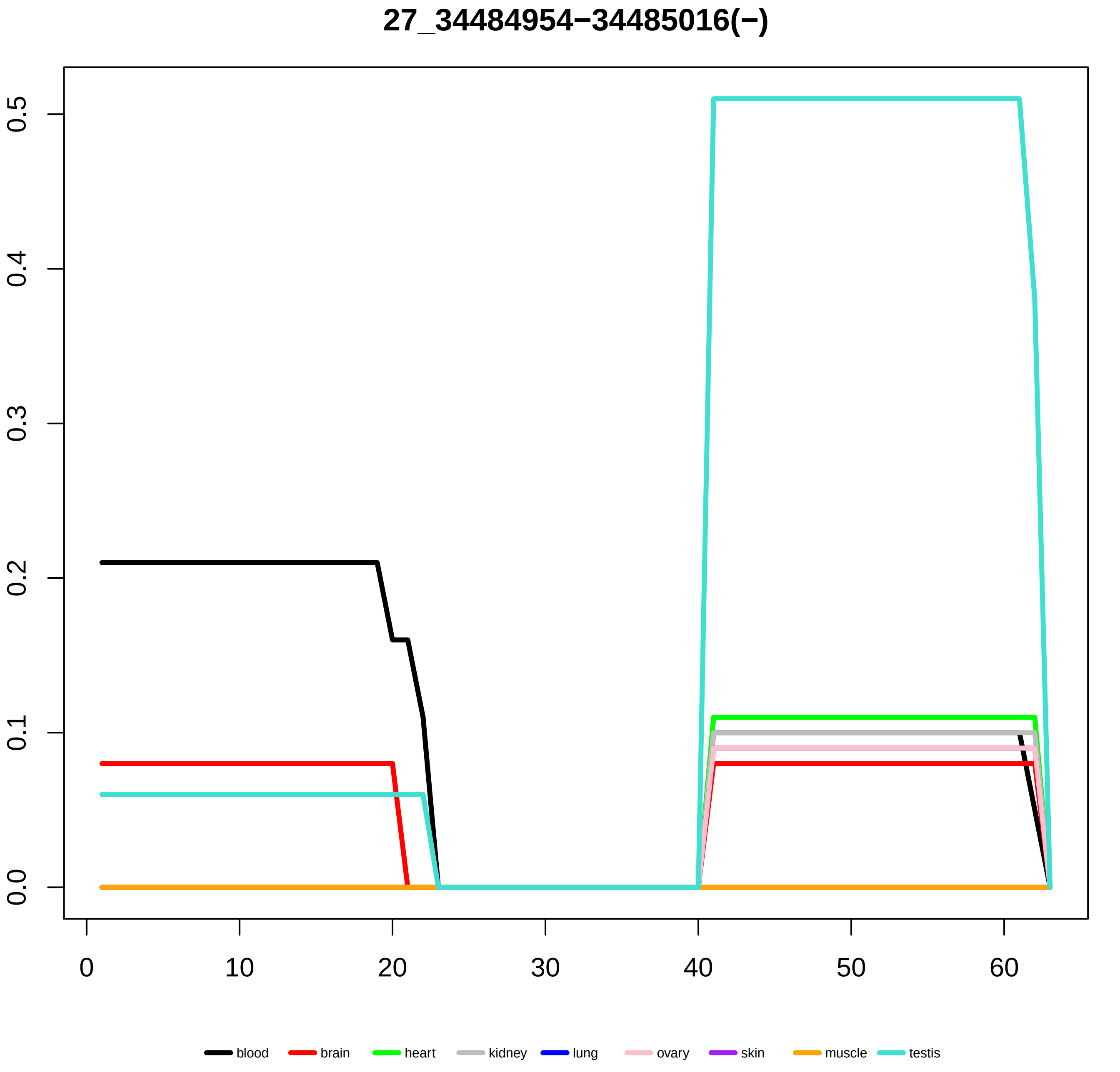
27_34484954−34485016(−)
0.0
0.1
0.2
0.3
0.4
0.5
0
10
20
30
40
50
60
blood
brain
heart
kidney
lung
ovary
skin
muscle
testis

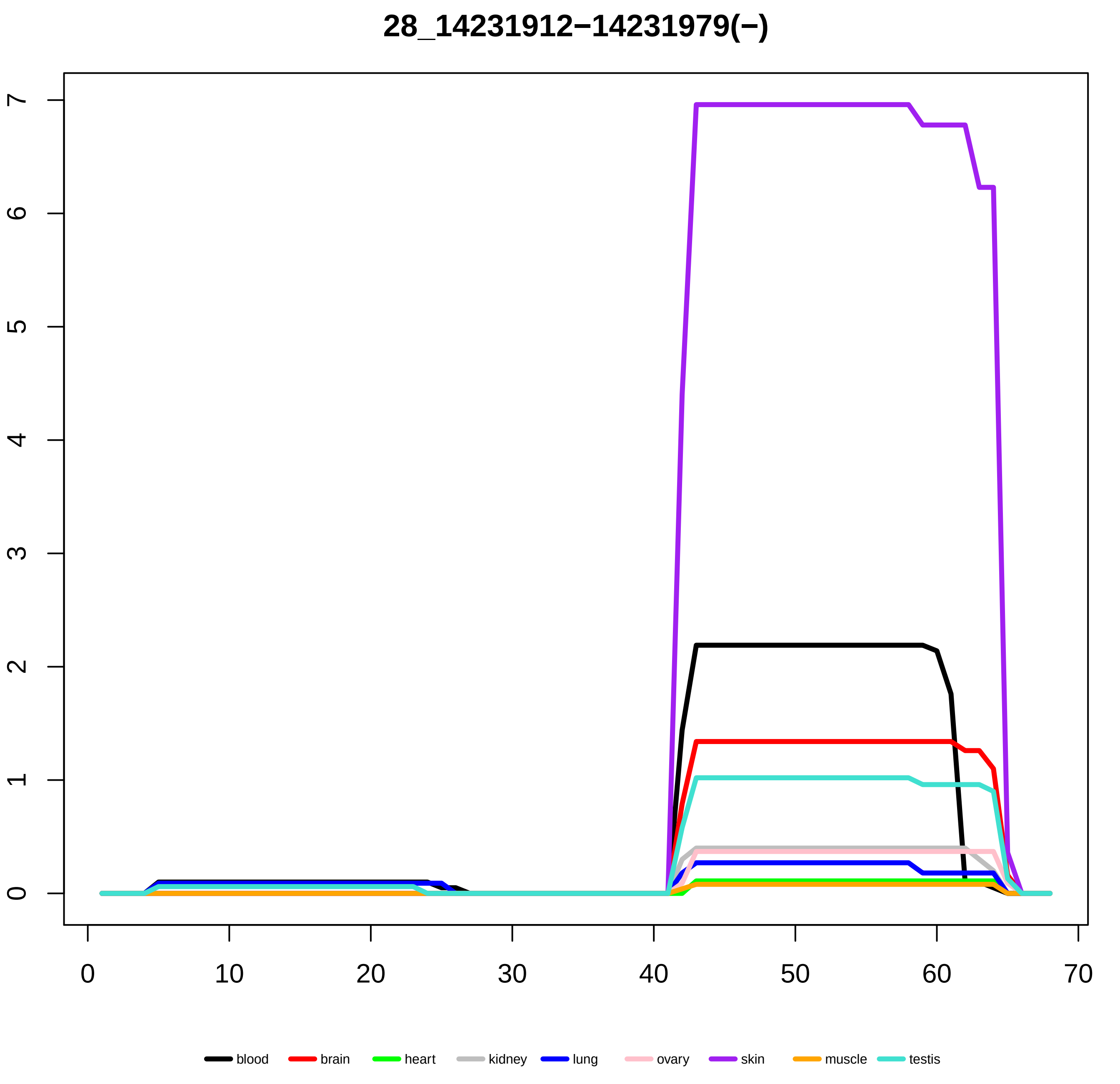
28_14231912−14231979(−)
0
1
2
3
4
5
6
7
0
10
20
30
40
50
60
70
blood
brain
heart
kidney
lung
ovary
skin
muscle
testis

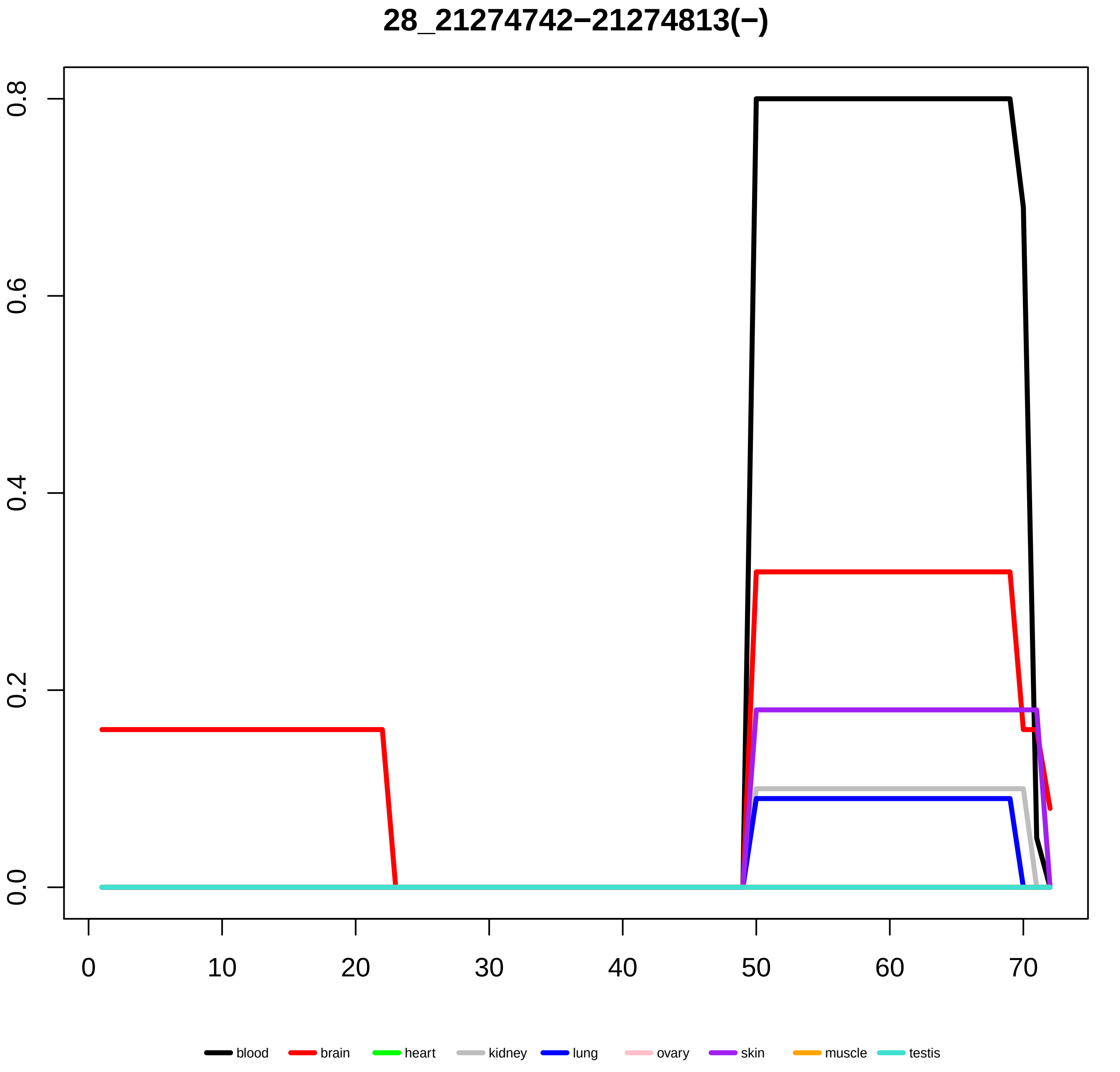
28_21274742−21274813(−)
0.0
0.2
0.4
0.6
0.8
0
10
20
30
40
50
60
70
blood
brain
heart
kidney
lung
ovary
skin
muscle
testis

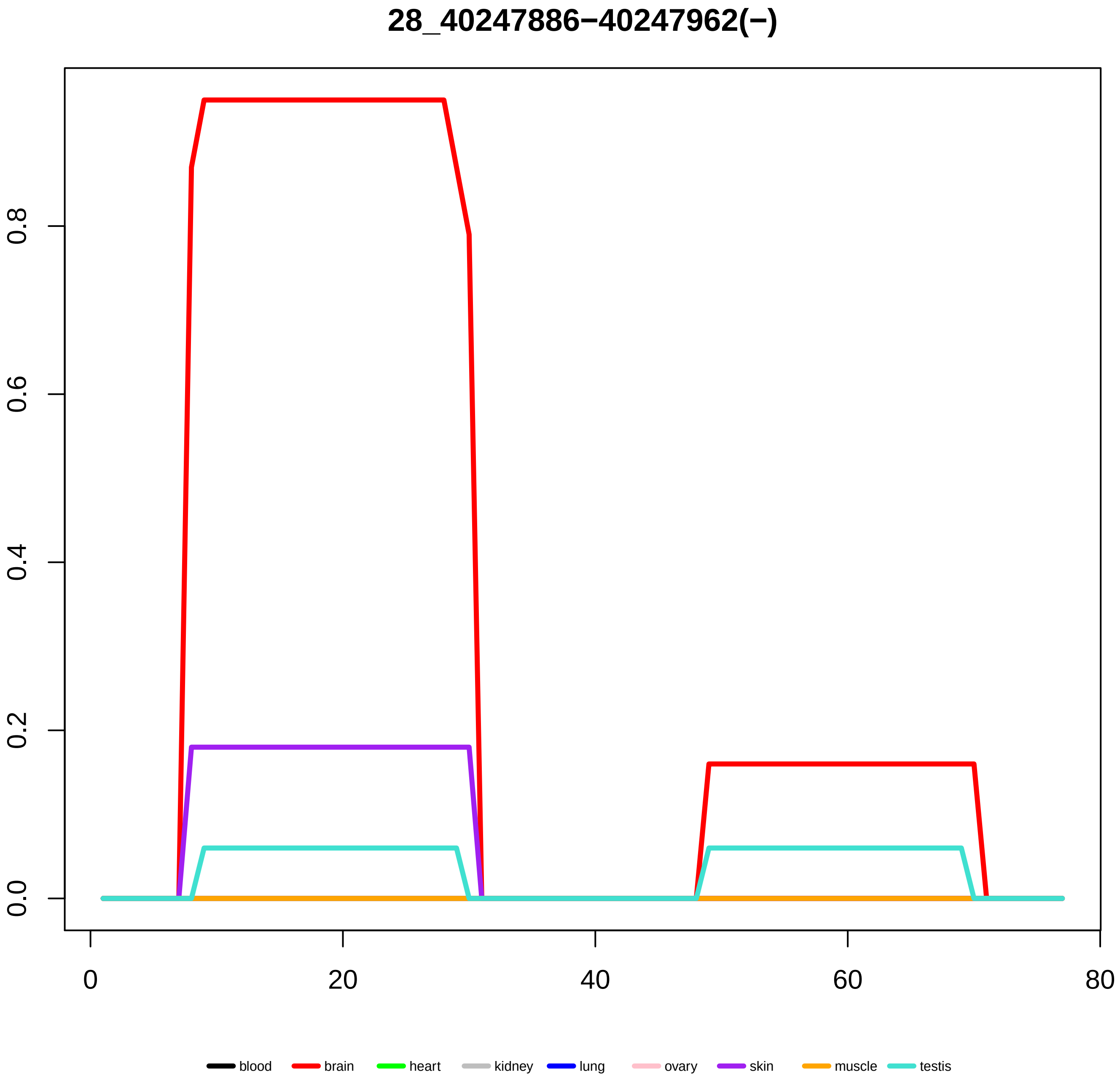
28_40247886−40247962(−)
0.0
0.2
0.4
0.6
0.8
0
20
40
60
80
blood
brain
heart
kidney
lung
ovary
skin
muscle
testis

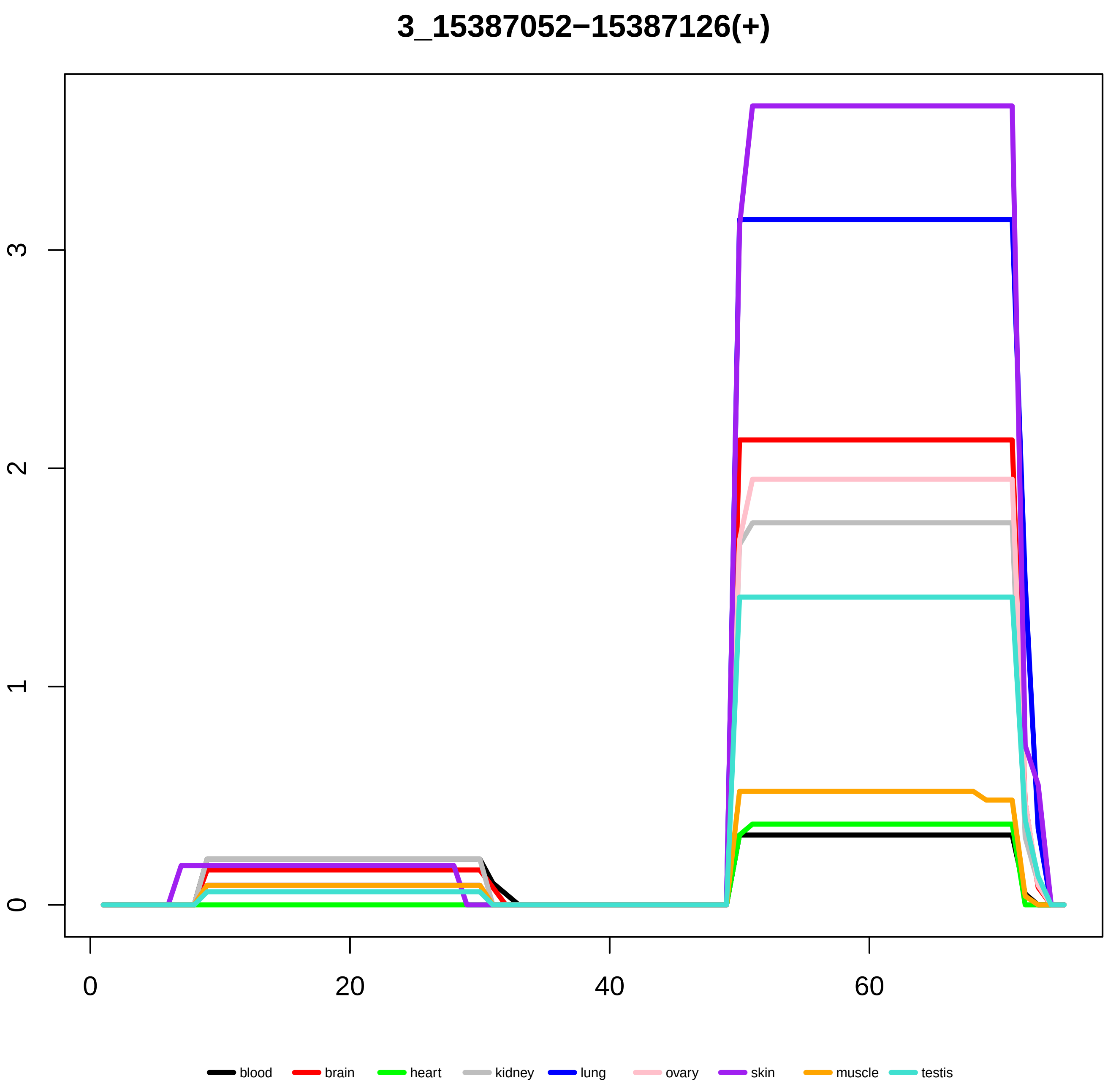
3_15387052−15387126(+)
0
1
2
3
0
20
40
60
blood
brain
heart
kidney
lung
ovary
skin
muscle
testis

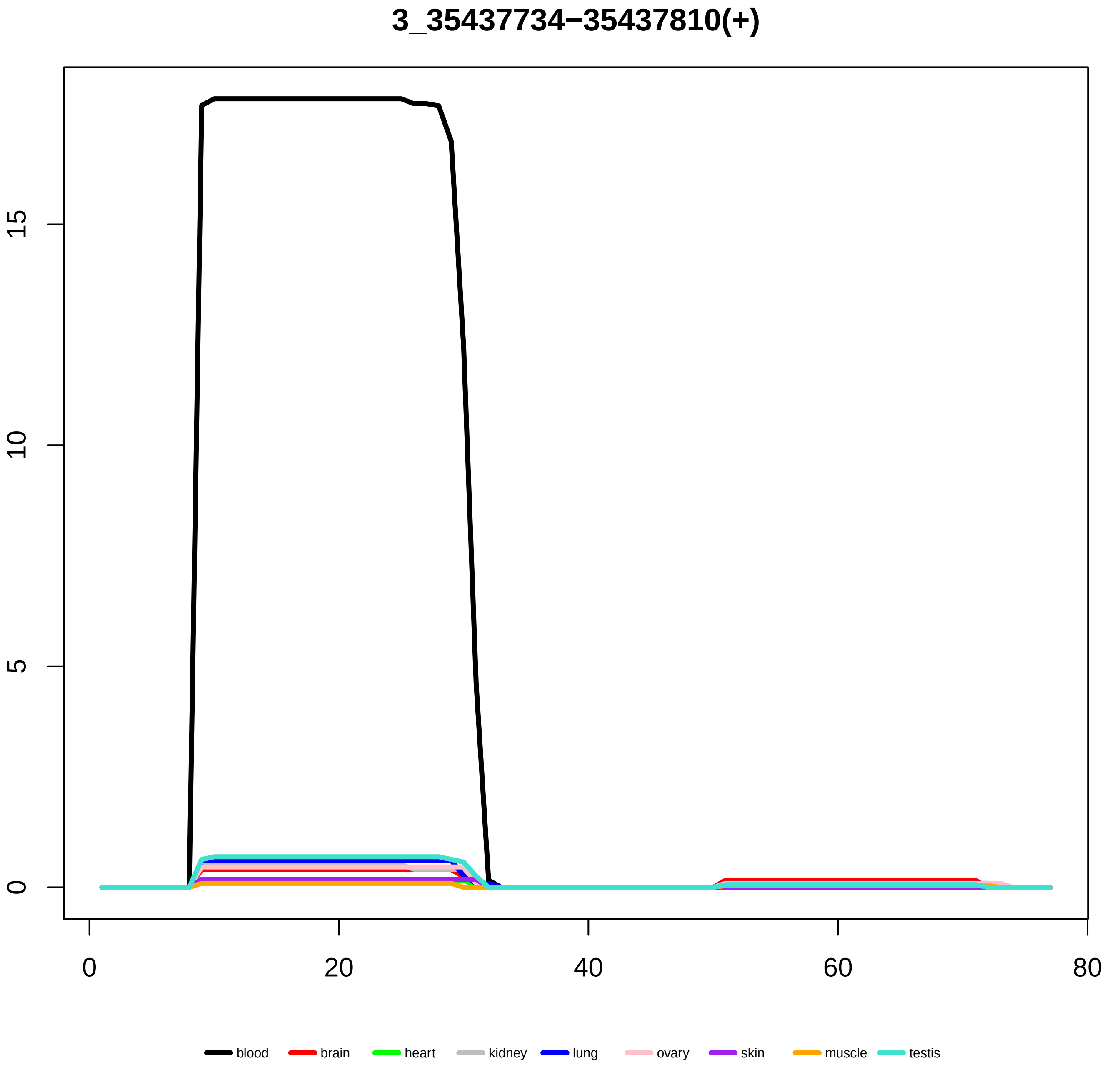
3_35437734−35437810(+)
0
5
10
15
0
20
40
60
80
blood
brain
heart
kidney
lung
ovary
skin
muscle
testis

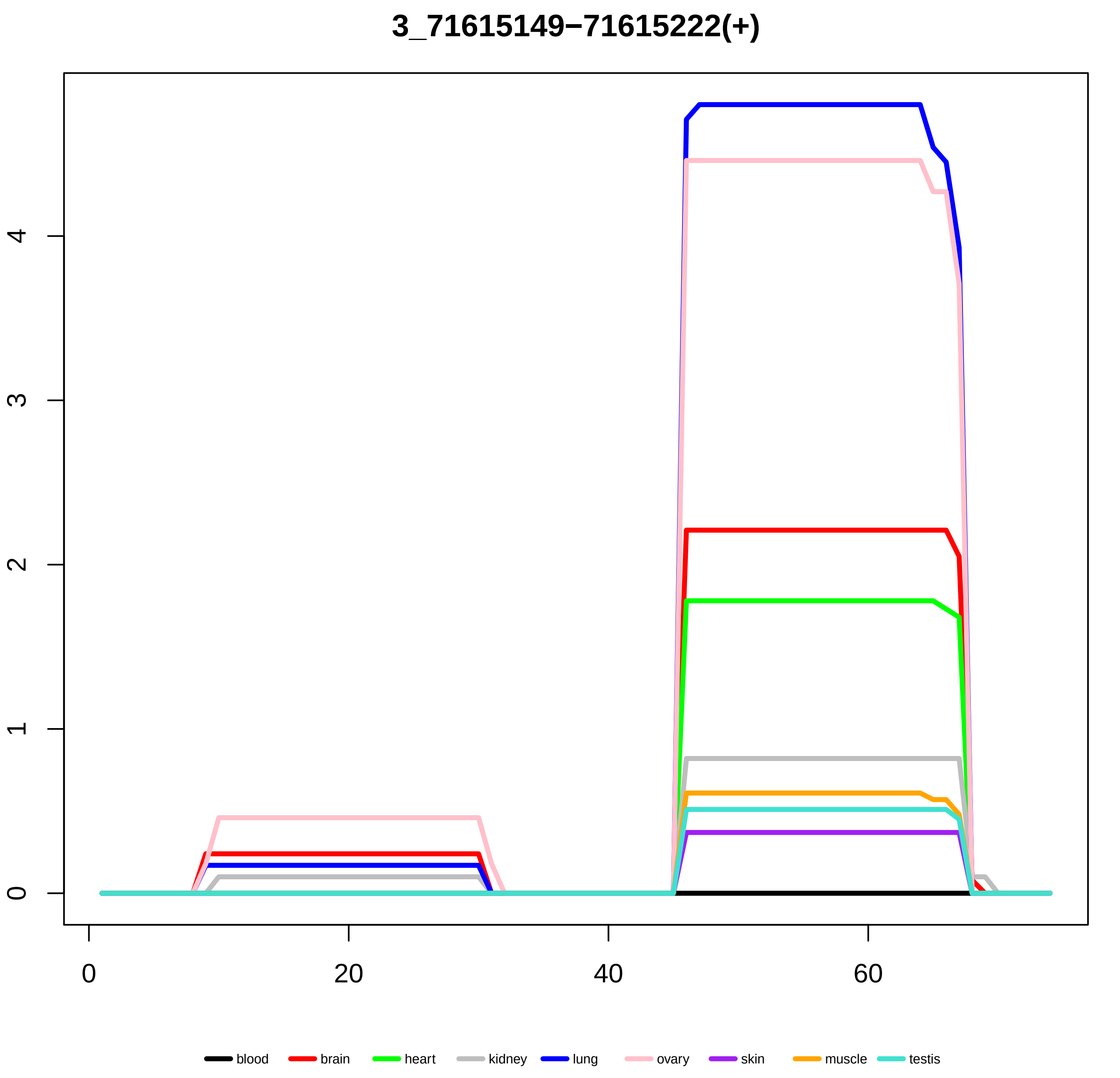
3_71615149−71615222(+)
0
1
2
3
4
0
20
40
60
blood
brain
heart
kidney
lung
ovary
skin
muscle
testis

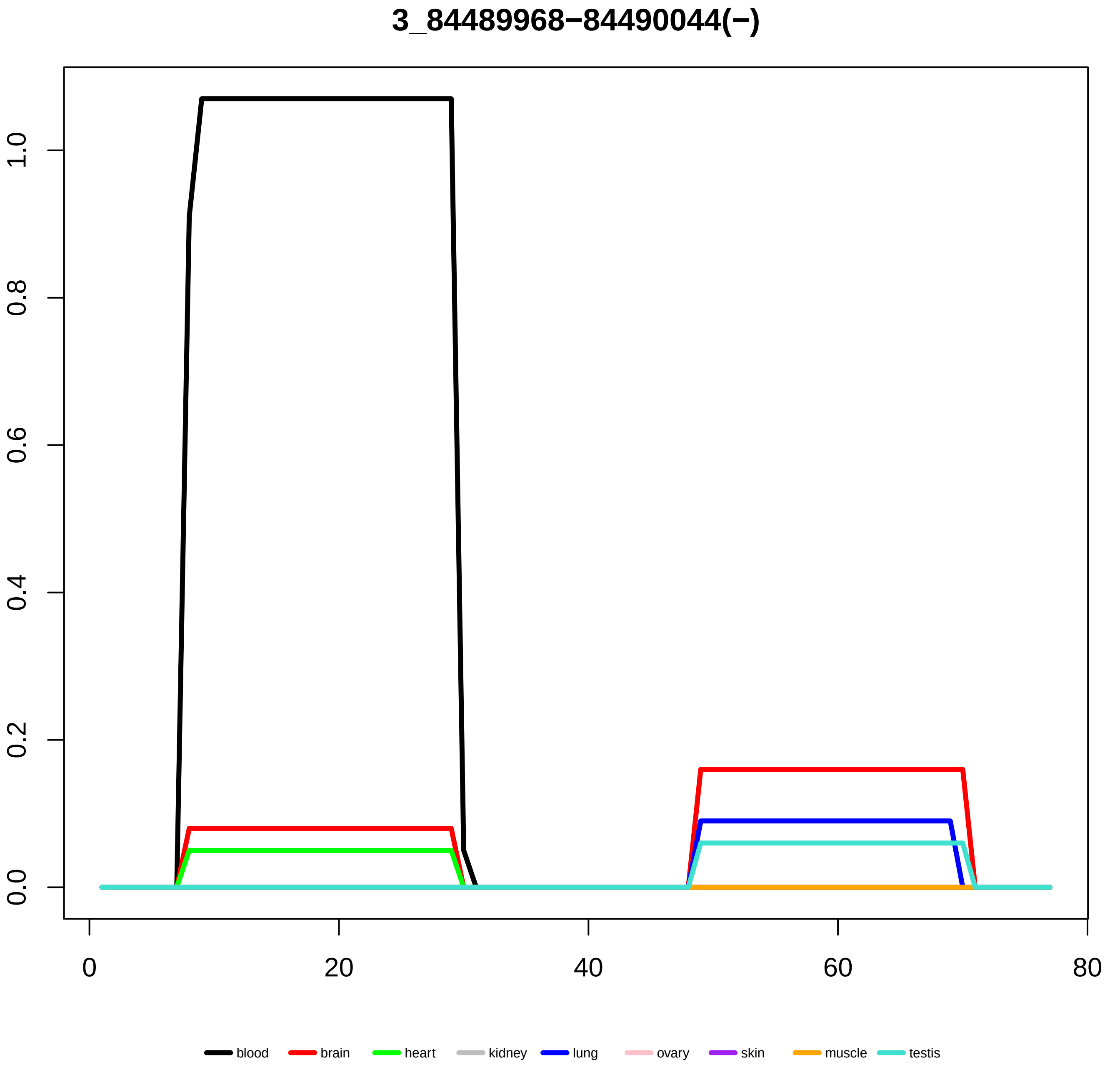
3_84489968−84490044(−)
0.0
0.2
0.4
0.6
0.8
1.0
0
20
40
60
80
blood
brain
heart
kidney
lung
ovary
skin
muscle
testis

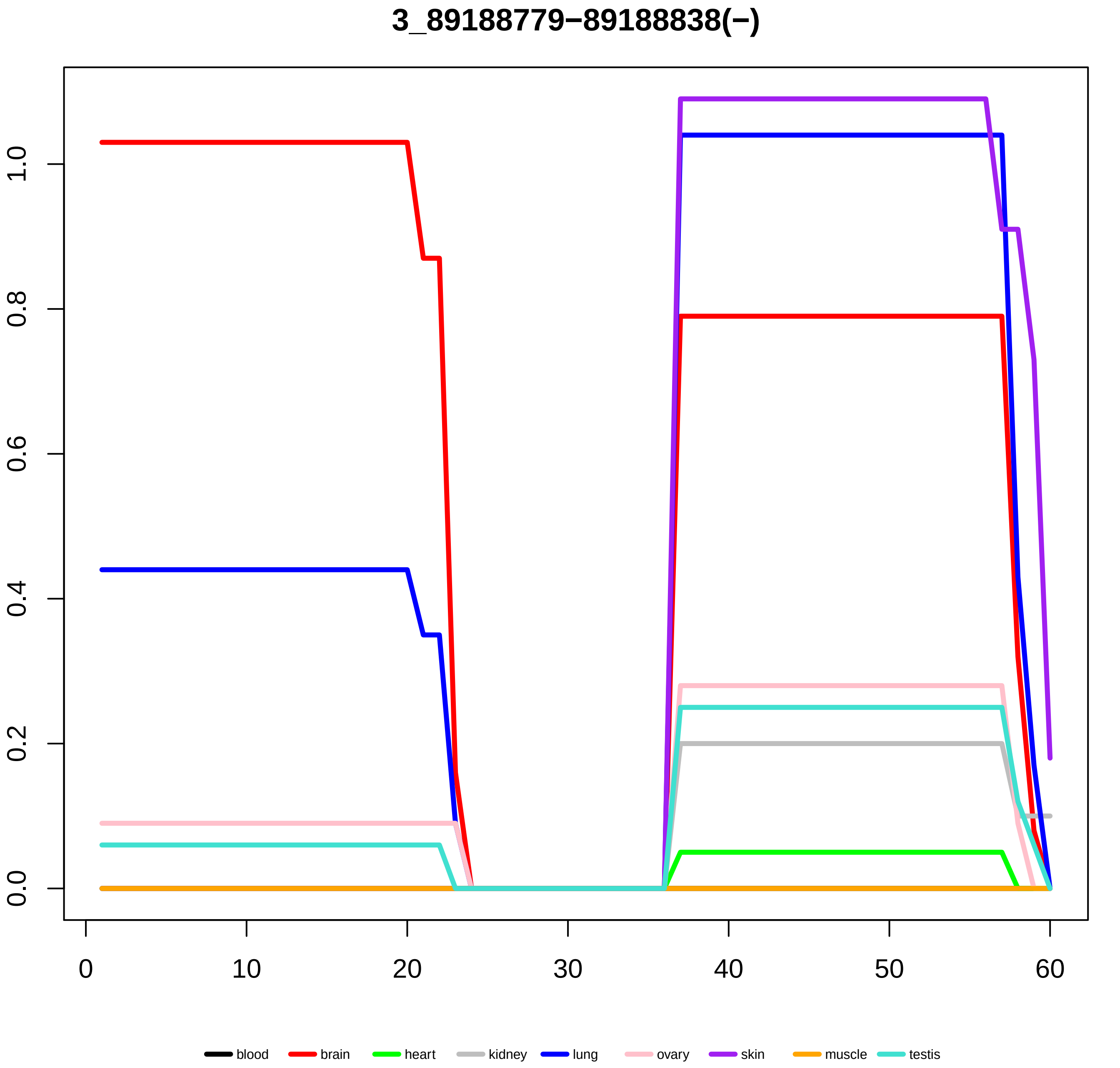

3_89188779−89188838(−)
0.0
0.2
0.4
0.6
0.8
1.0
0
10
20
30
40
50
60
blood
brain
heart
kidney
lung
ovary
skin
muscle
testis

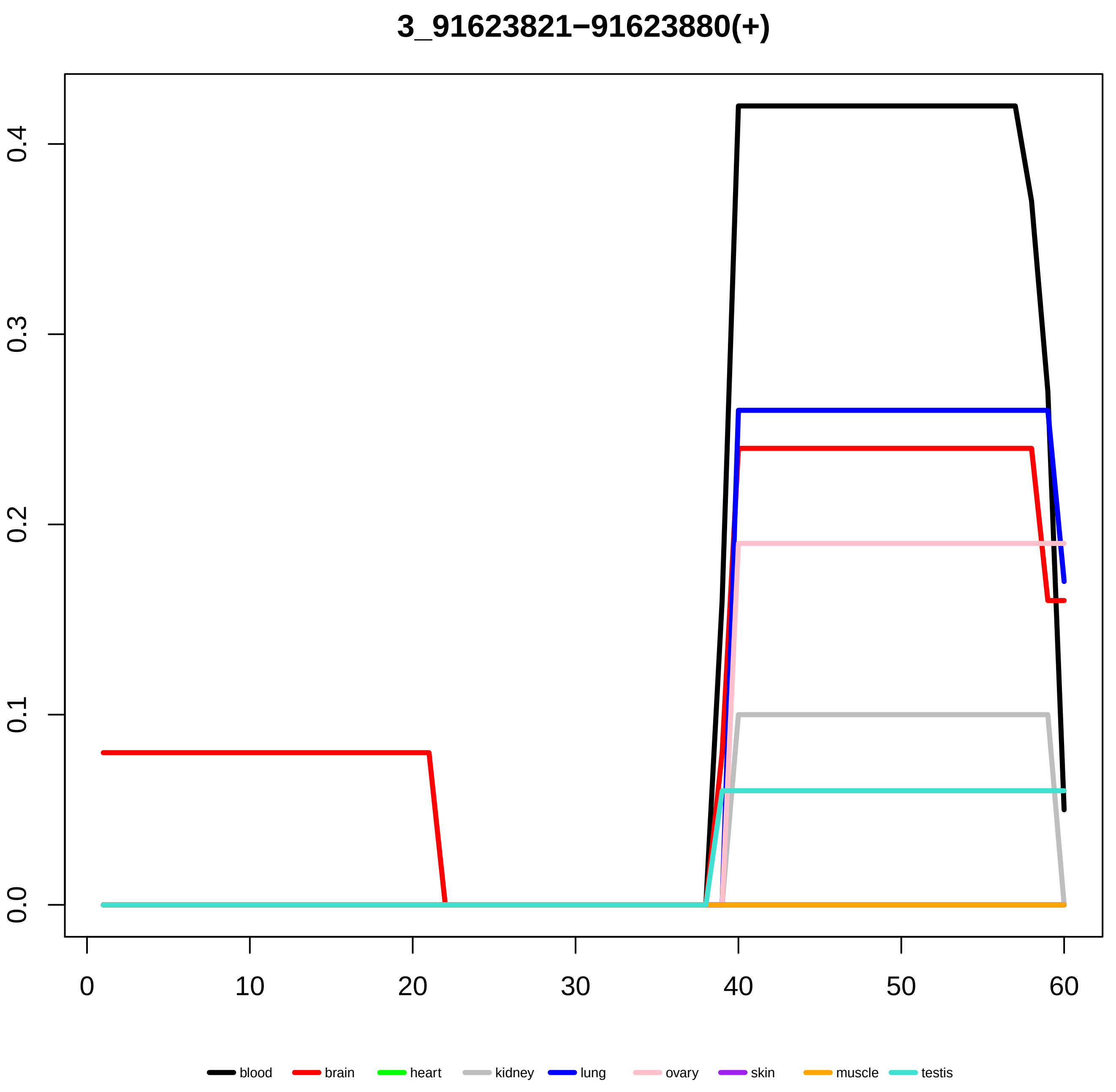

3_91623821−91623880(+)
0.0
0.1
0.2
0.3
0.4
0
10
20
30
40
50
60
blood
brain
heart
kidney
lung
ovary
skin
muscle
testis

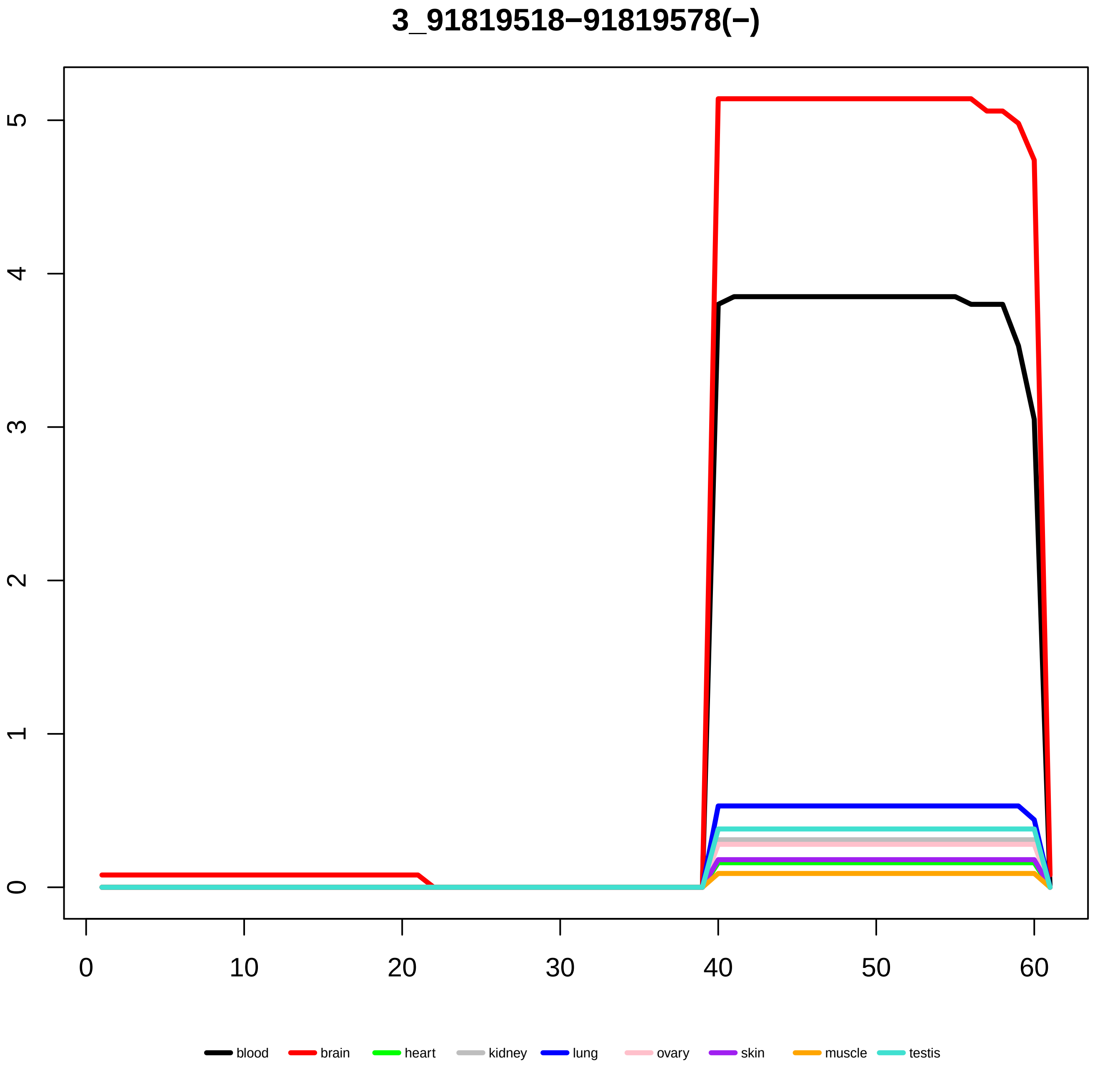

3_91819518−91819578(−)
0
1
2
3
4
5
0
10
20
30
40
50
60
blood
brain
heart
kidney
lung
ovary
skin
muscle
testis

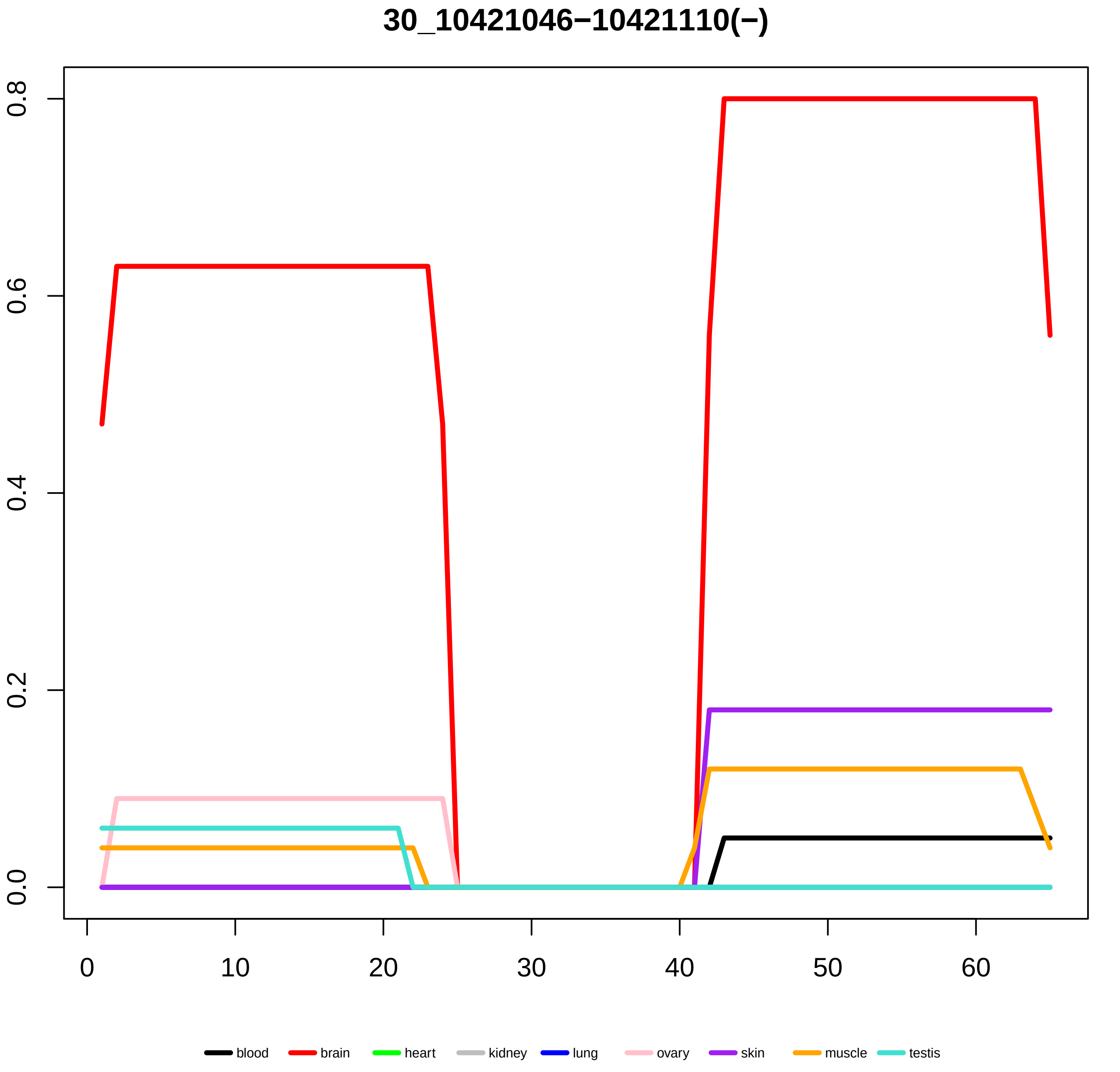

30_10421046−10421110(−)
0.0
0.2
0.4
0.6
0.8
0
10
20
30
40
50
60
blood
brain
heart
kidney
lung
ovary
skin
muscle
testis

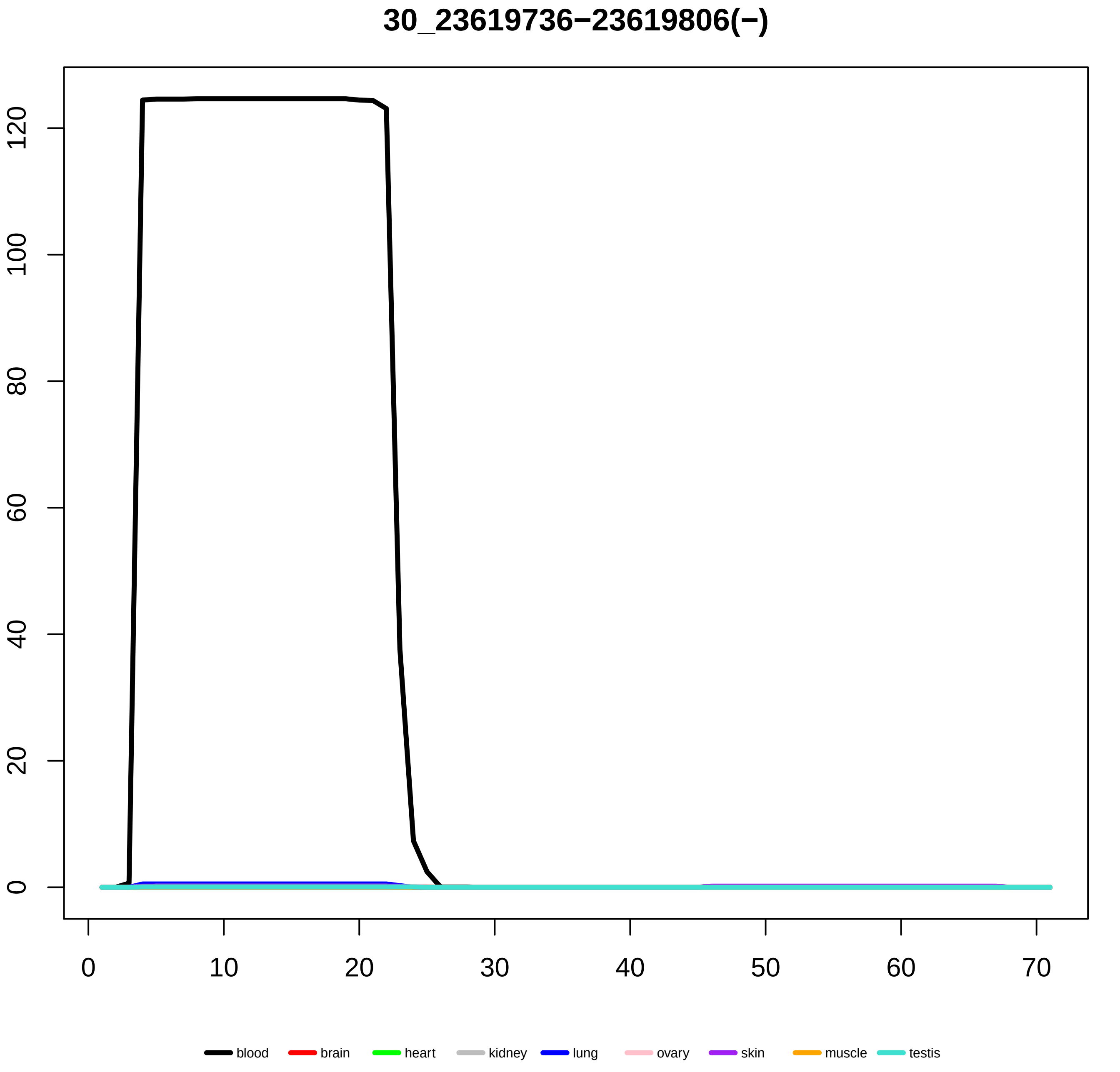
30_23619736−23619806(−)
0
20
40
60
80
100
120
0
10
20
30
40
50
60
70
blood
brain
heart
kidney
lung
ovary
skin
muscle
testis

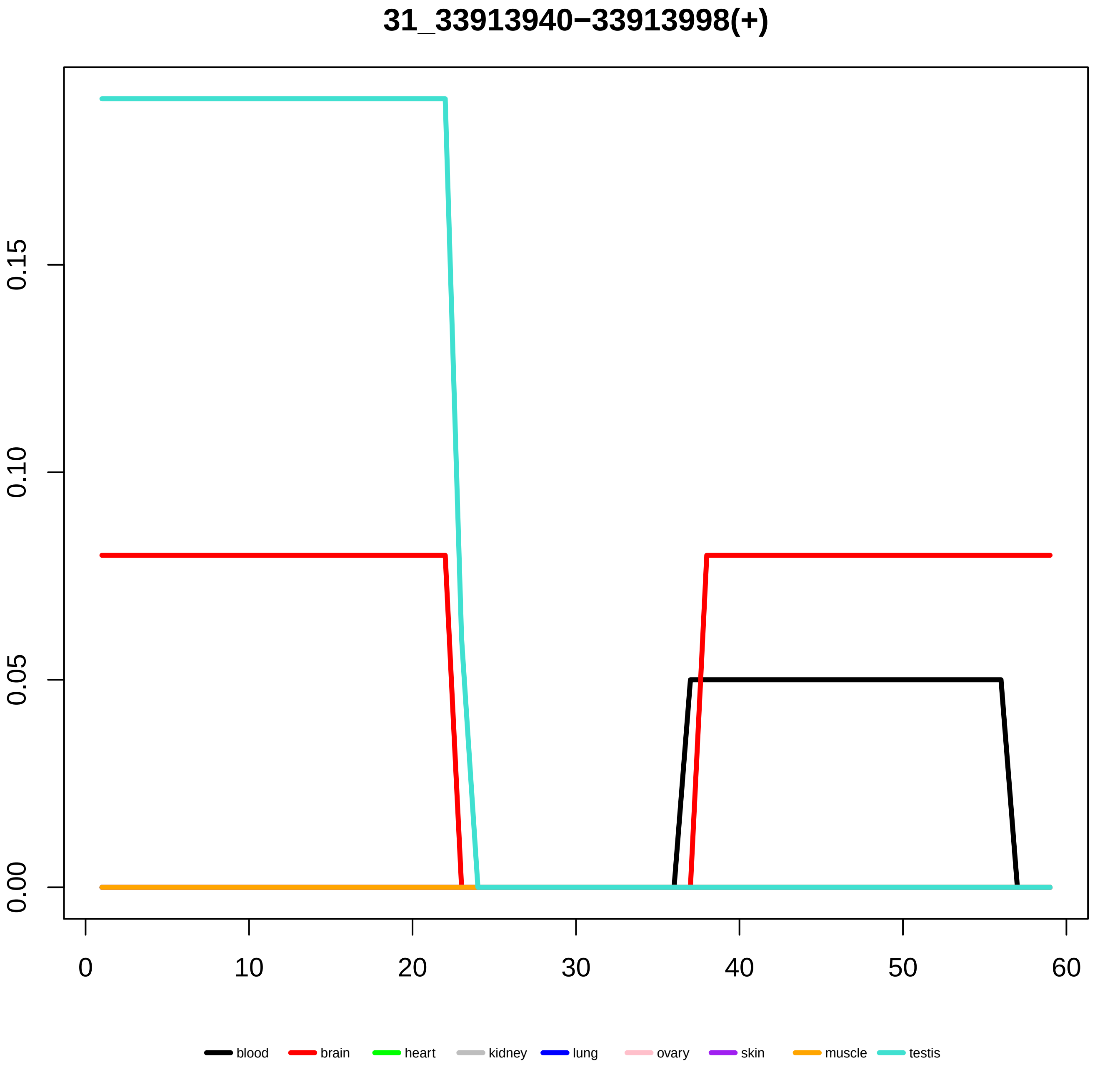

31_33913940−33913998(+)
0.00
0.05
0.10
0.15
0
10
20
30
40
50
60
blood
brain
heart
kidney
lung
ovary
skin
muscle
testis

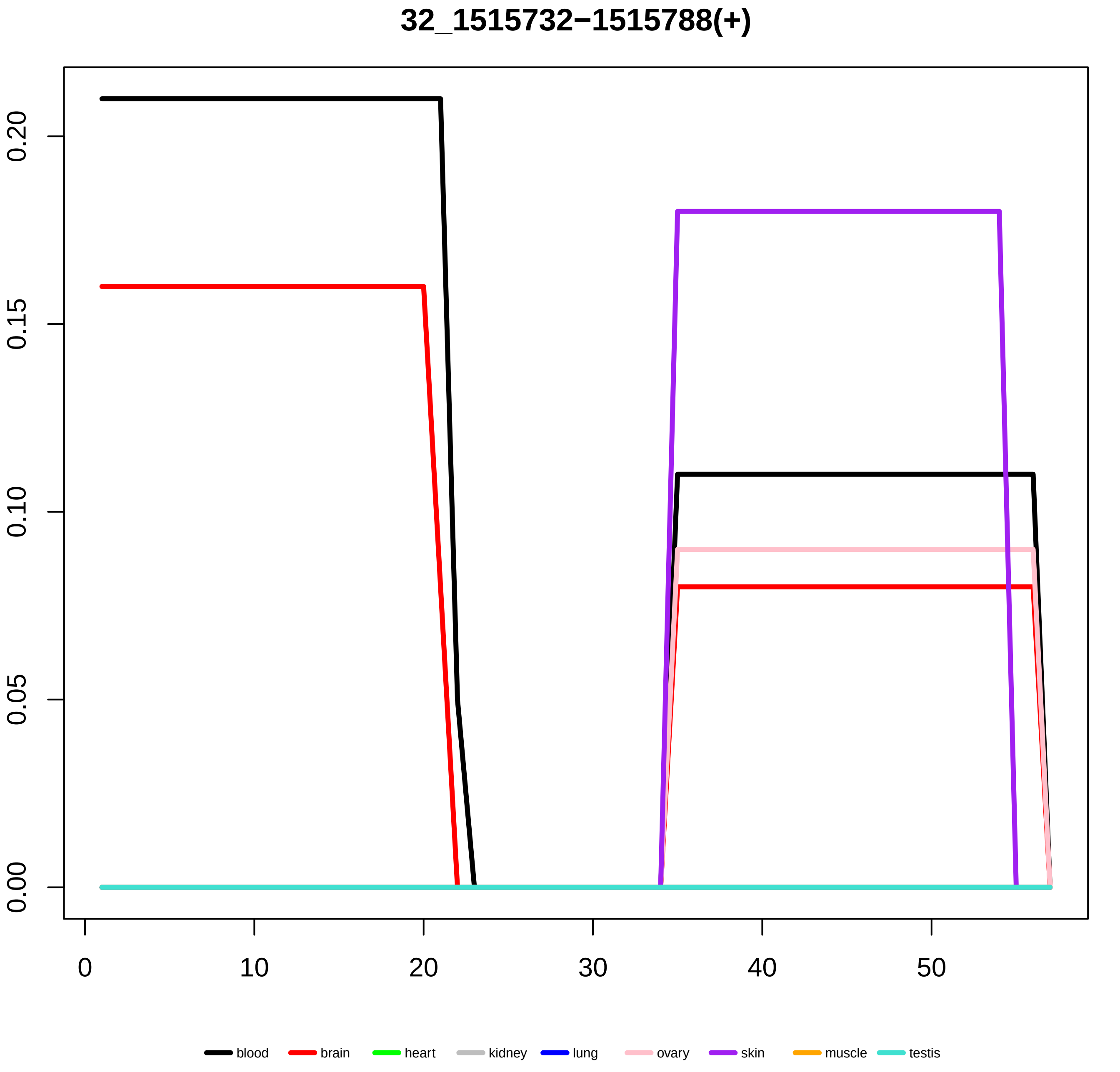

32_1515732−1515788(+)
0.00
0.05
0.10
0.15
0.20
0
10
20
30
40
50
blood
brain
heart
kidney
lung
ovary
skin
muscle
testis

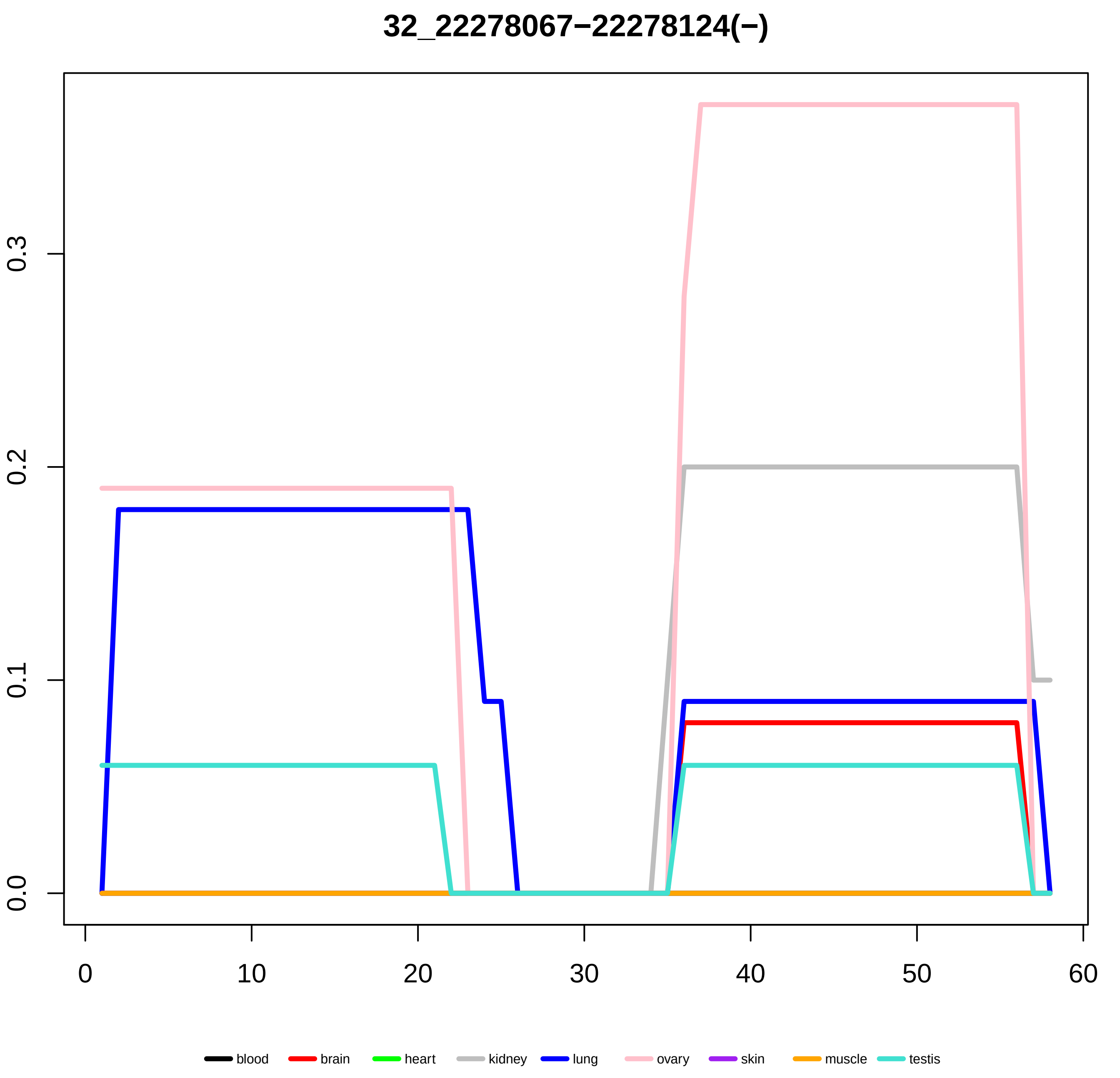

32_22278067−22278124(−)
0.0
0.1
0.2
0.3
0
10
20
30
40
50
60
blood
brain
heart
kidney
lung
ovary
skin
muscle
testis

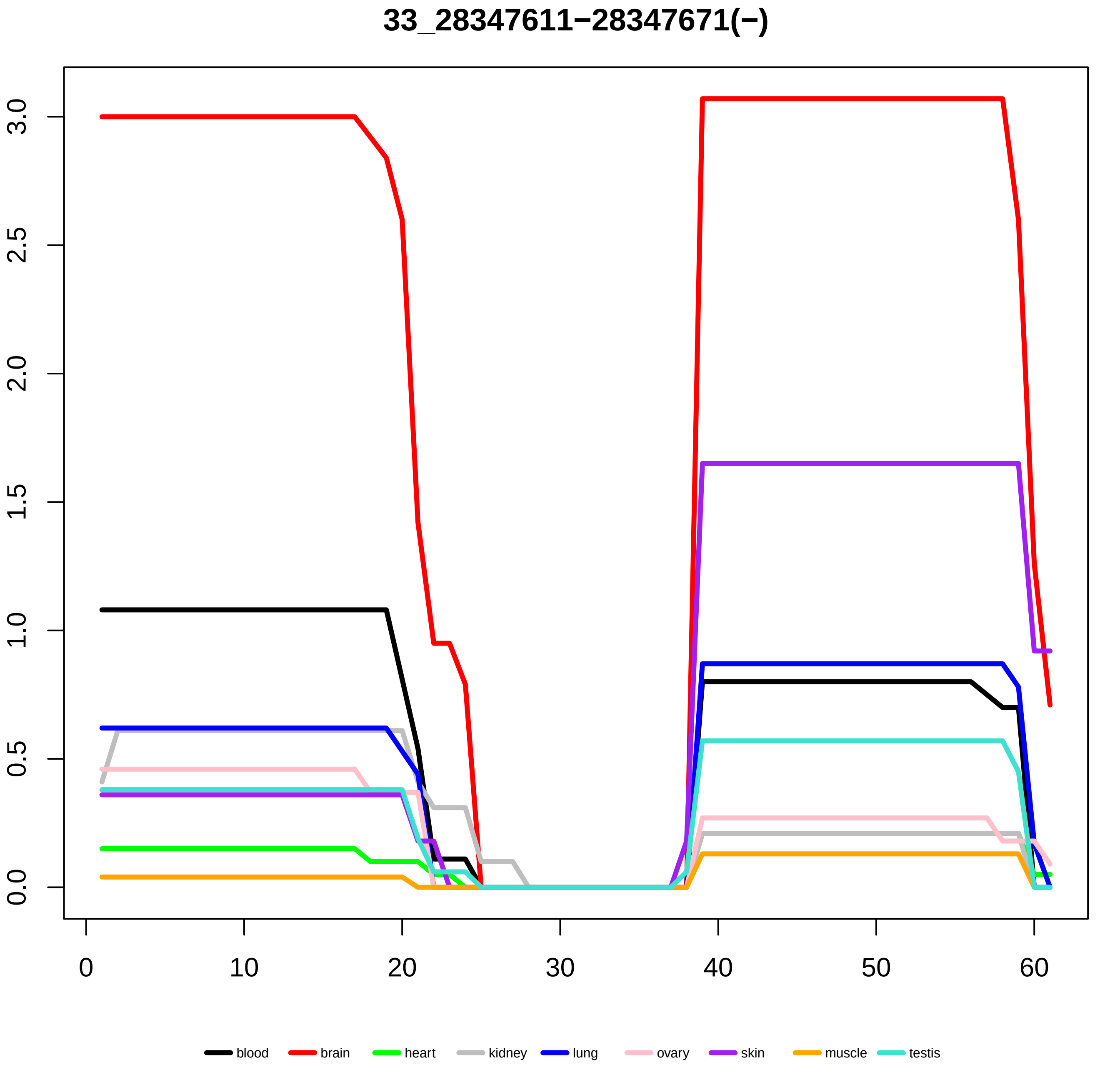
33_28347611−28347671(−)
0.0
0.5
1.0
1.5
2.0
2.5
3.0
0
10
20
30
40
50
60
blood
brain
heart
kidney
lung
ovary
skin
muscle
testis

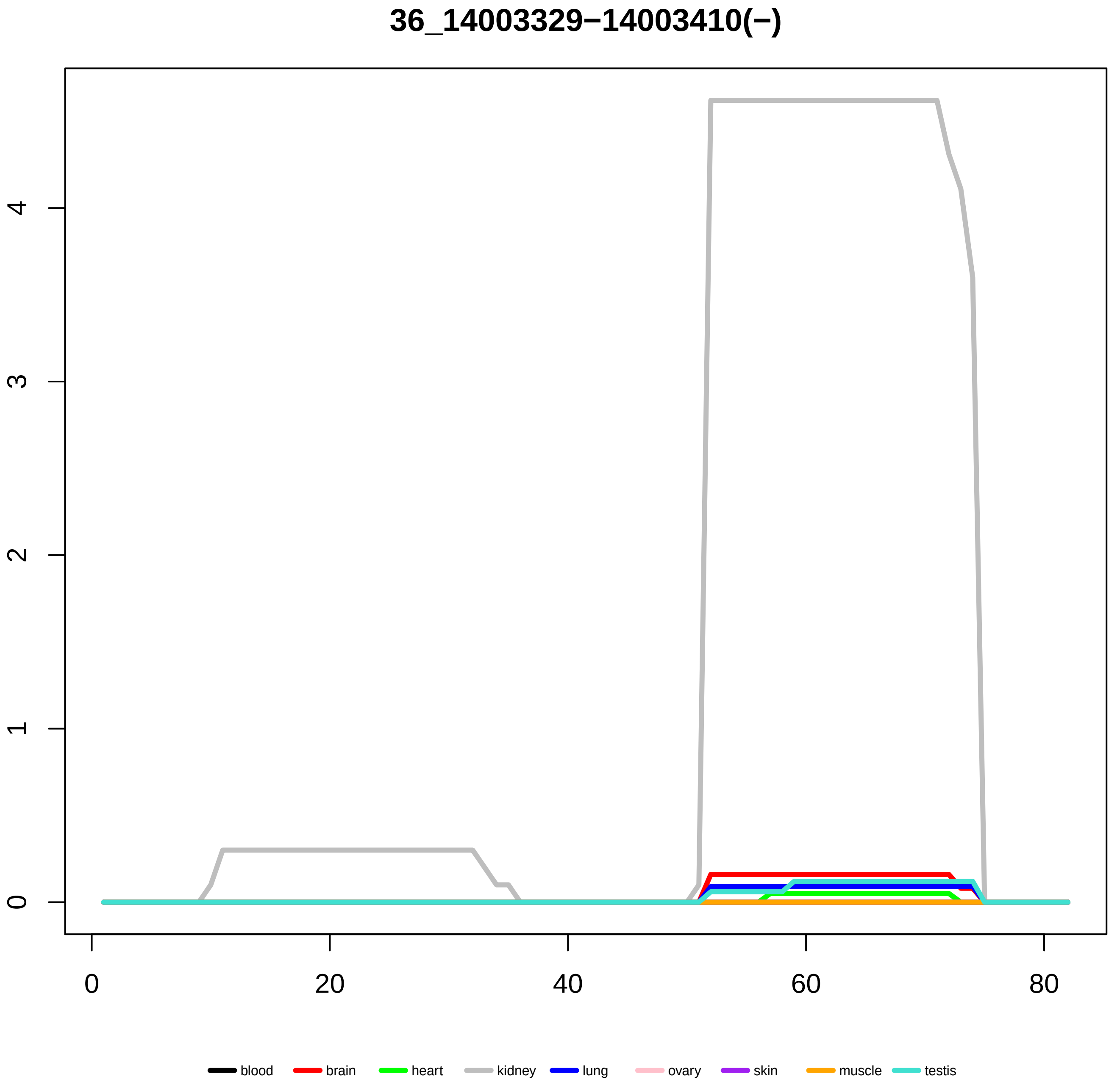

36_14003329−14003410(−)
0
1
2
3
4
0
20
40
60
80
blood
brain
heart
kidney
lung
ovary
skin
muscle
testis

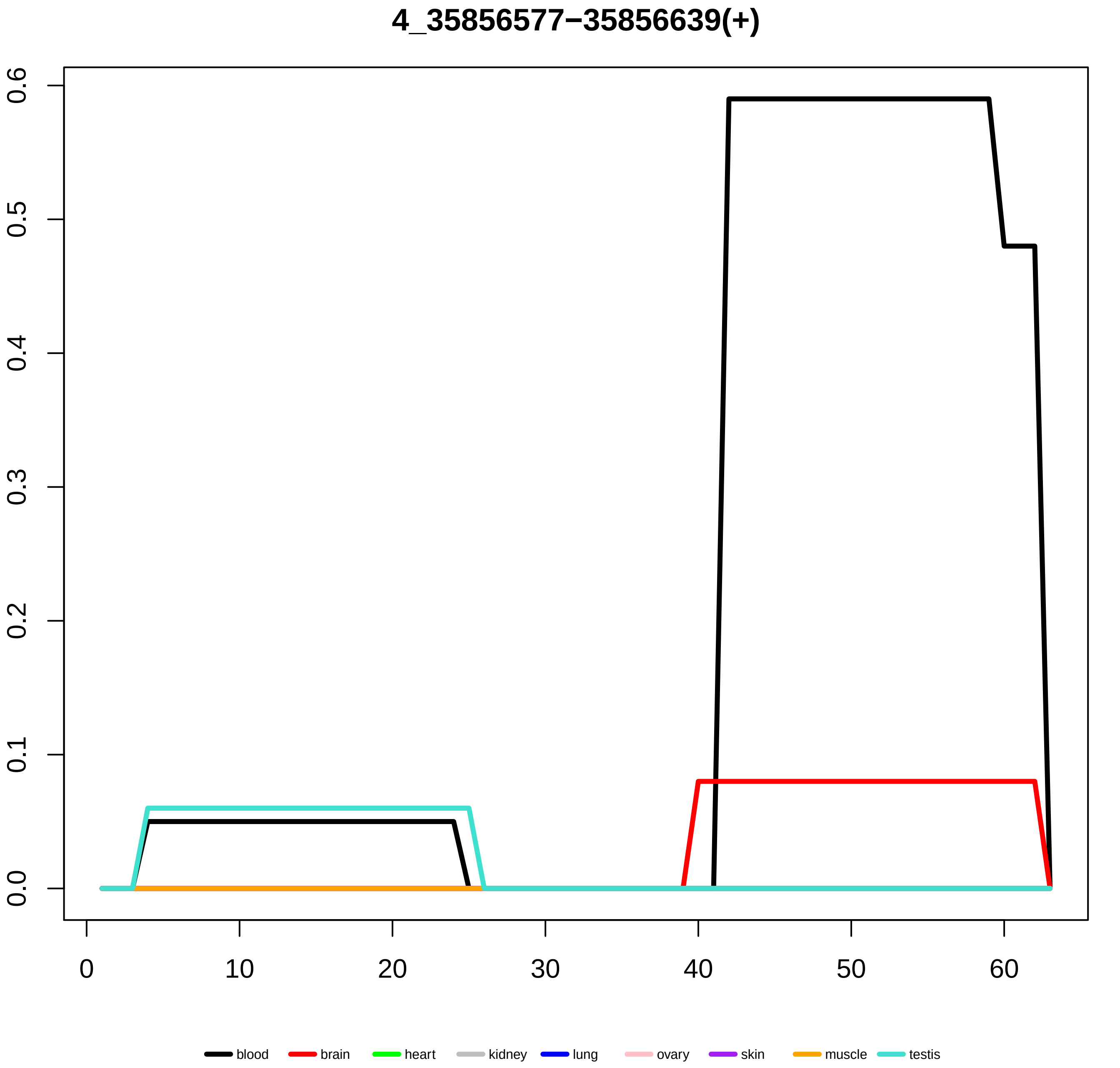
4_35856577−35856639(+)
0.0
0.1
0.2
0.3
0.4
0.5
0.6
0
10
20
30
40
50
60
blood
brain
heart
kidney
lung
ovary
skin
muscle
testis

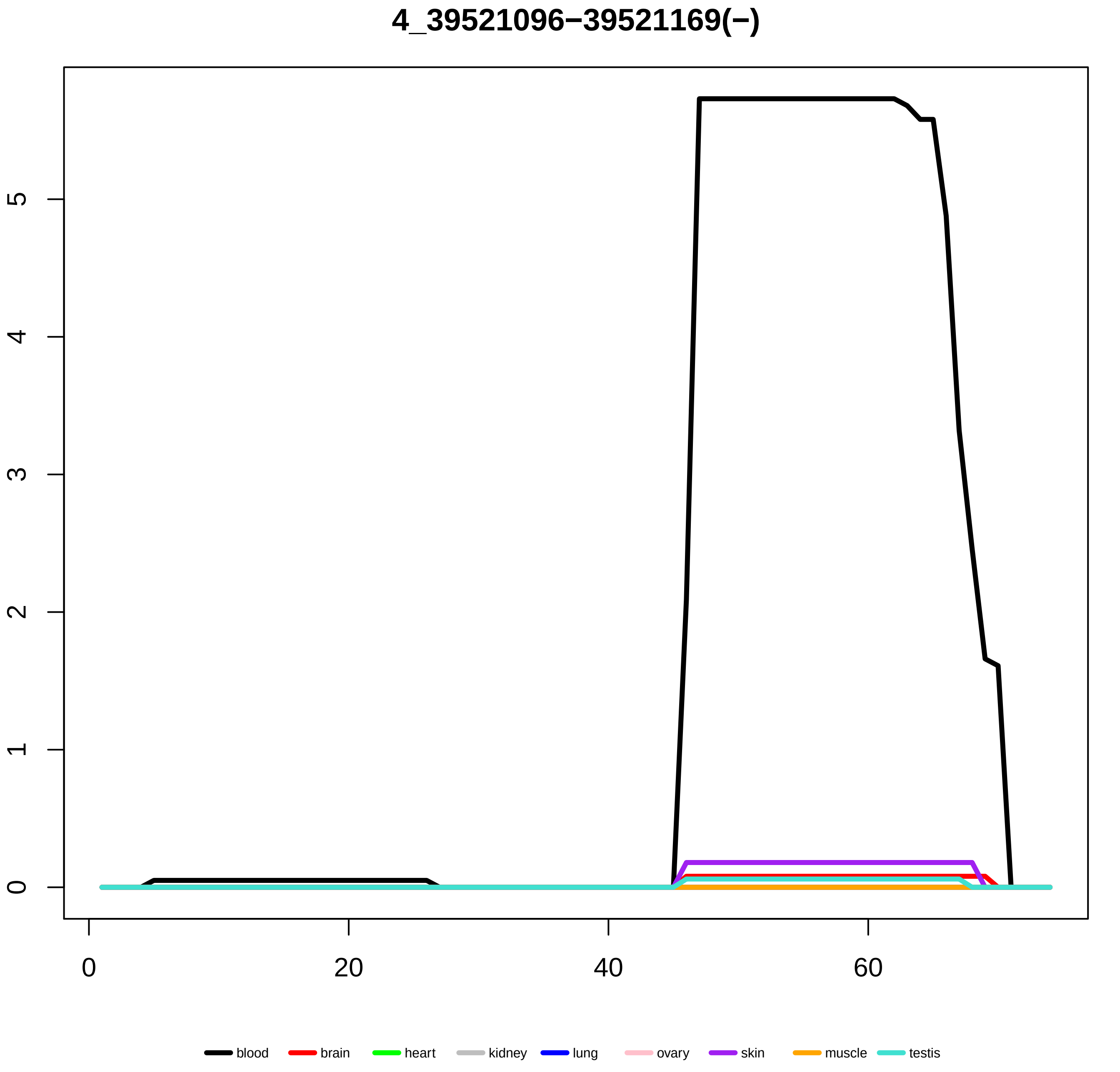
4_39521096−39521169(−)
0
1
2
3
4
5
0
20
40
60
blood
brain
heart
kidney
lung
ovary
skin
muscle
testis

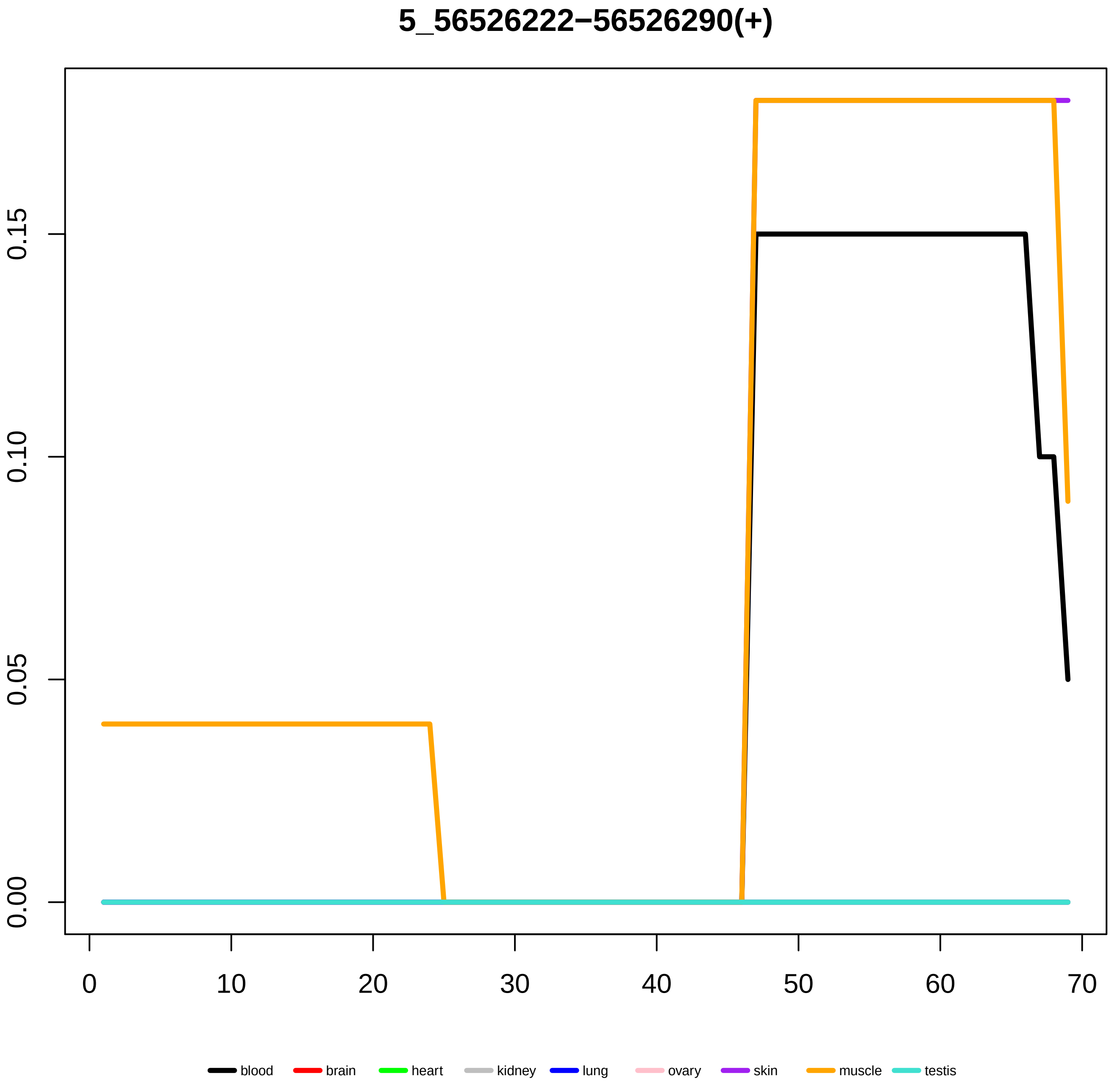
5_56526222−56526290(+)
0.00
0.05
0.10
0.15
0
10
20
30
40
50
60
70
blood
brain
heart
kidney
lung
ovary
skin
muscle
testis

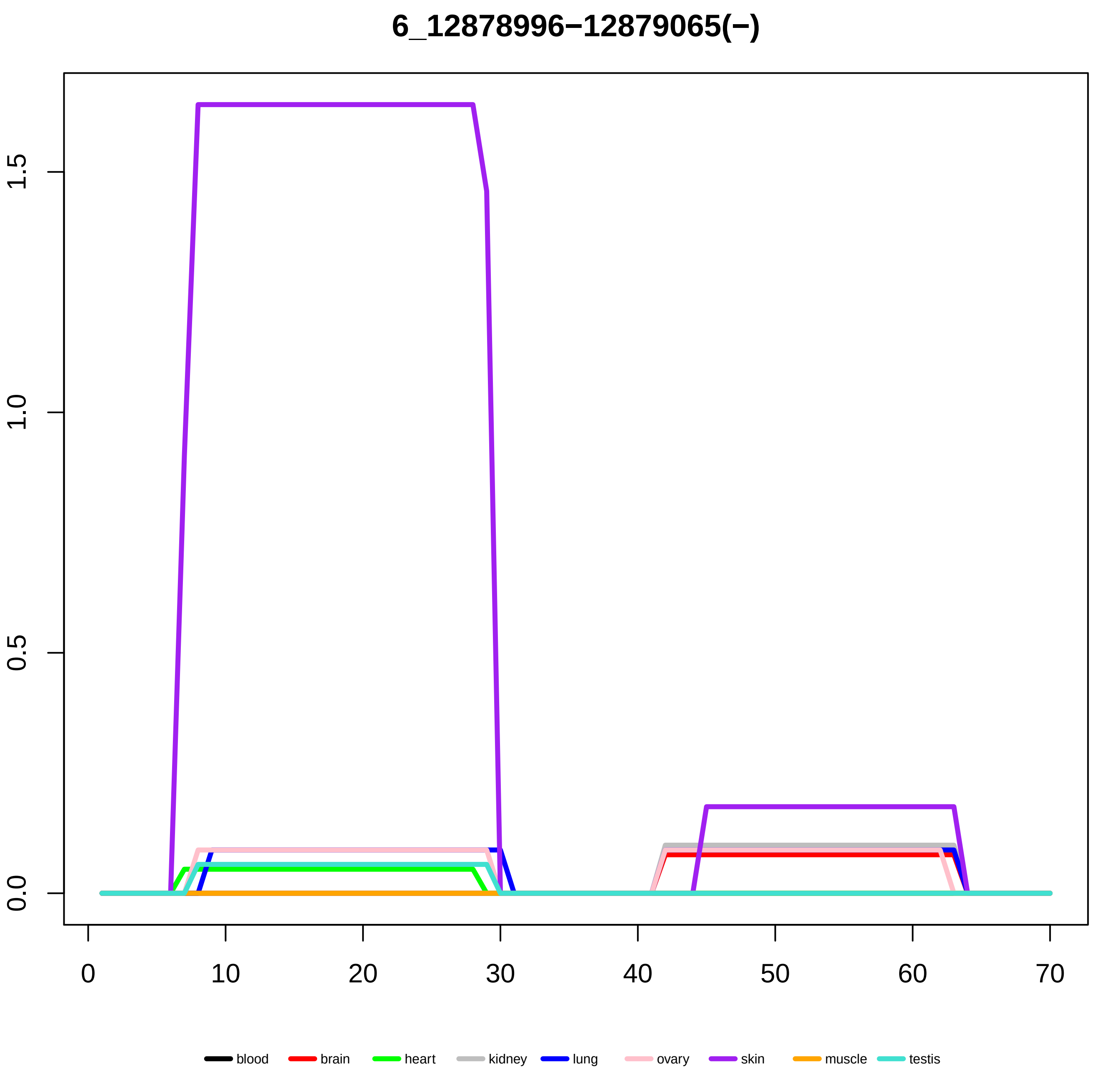
6_12878996−12879065(−)
0.0
0.5
1.0
1.5
0
10
20
30
40
50
60
70
blood
brain
heart
kidney
lung
ovary
skin
muscle
testis

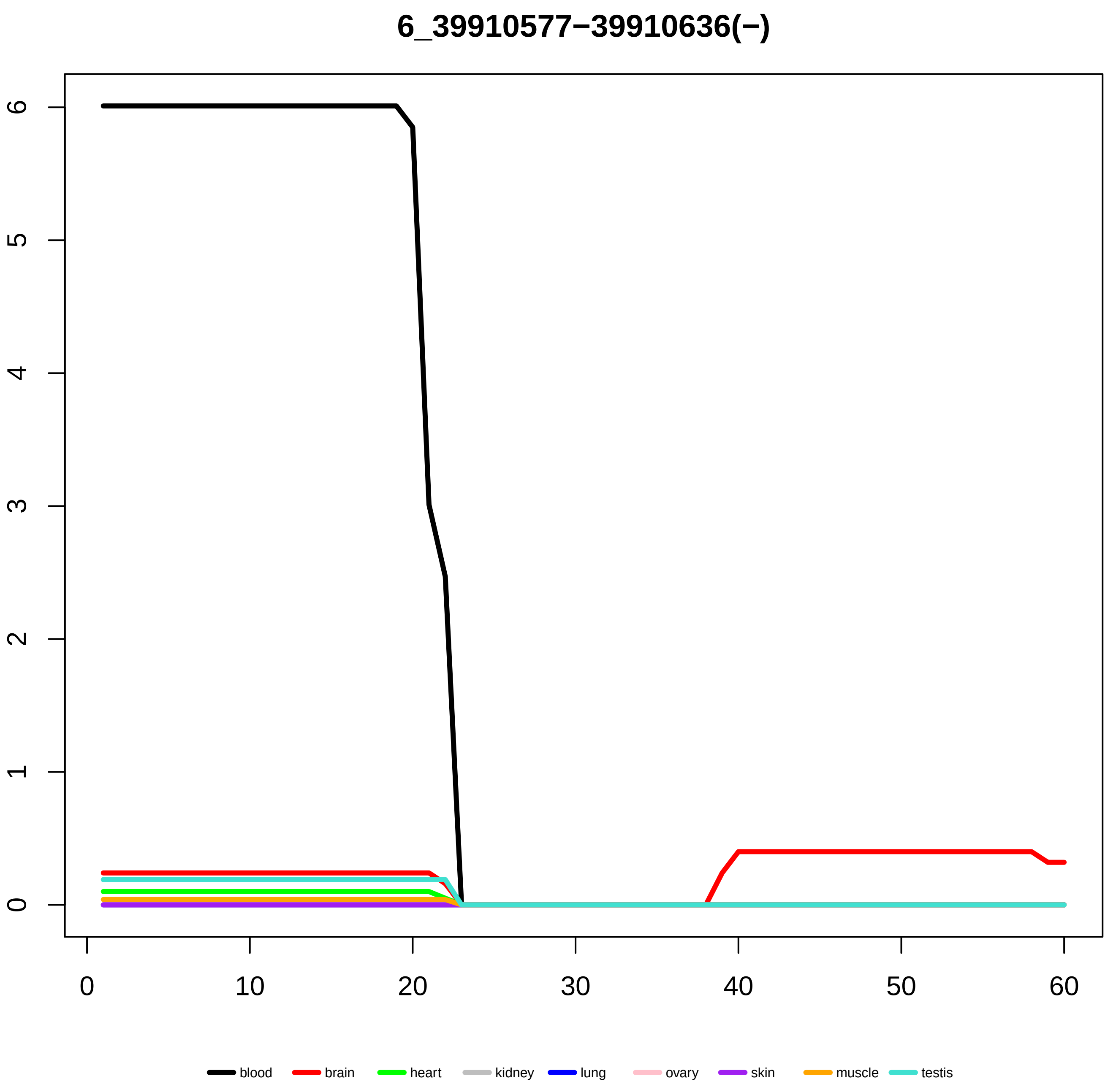

6_39910577−39910636(−)
0
1
2
3
4
5
6
0
10
20
30
40
50
60
blood
brain
heart
kidney
lung
ovary
skin
muscle
testis

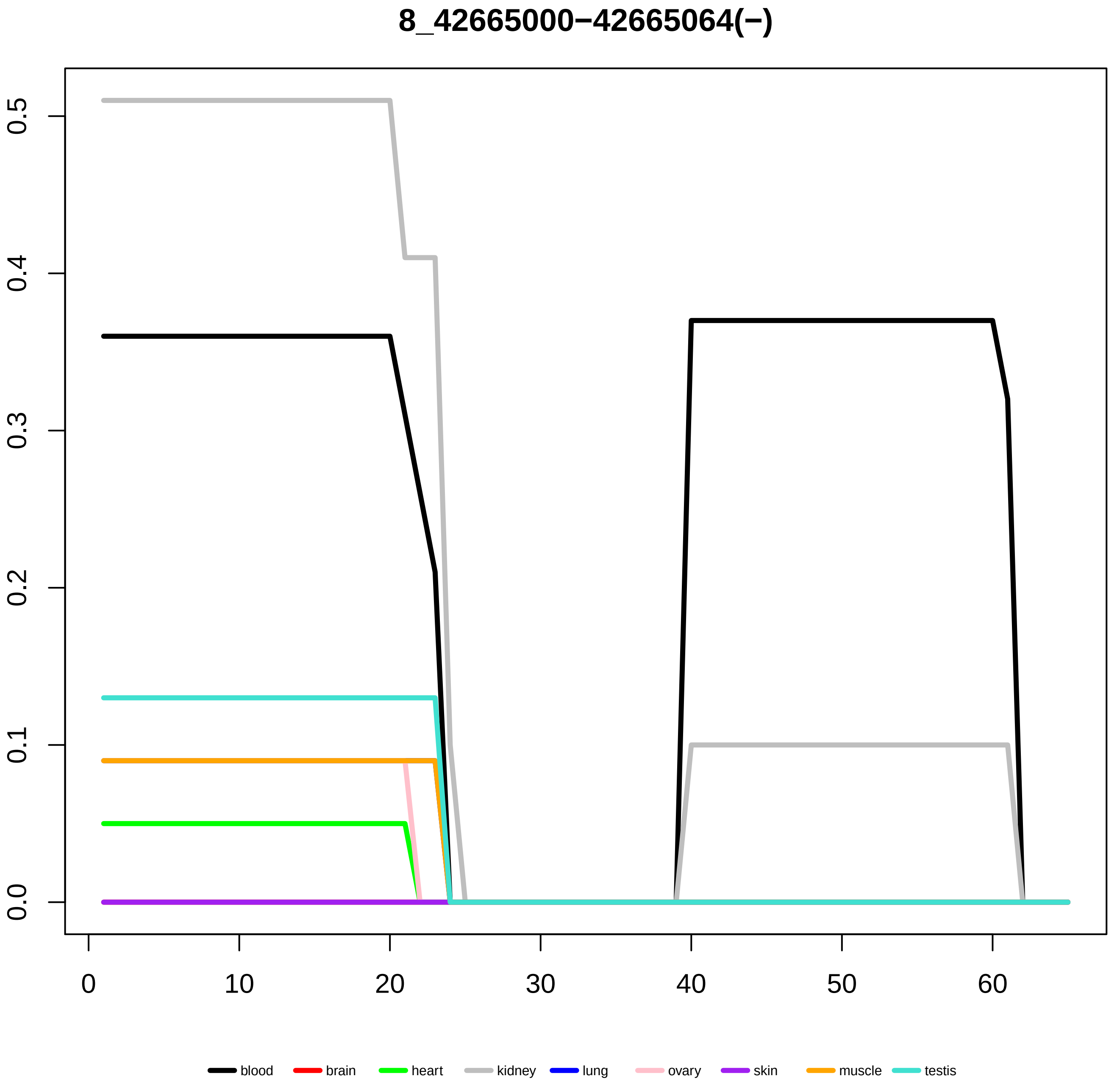
8_42665000−42665064(−)
0.0
0.1
0.2
0.3
0.4
0.5
0
10
20
30
40
50
60
blood
brain
heart
kidney
lung
ovary
skin
muscle
testis

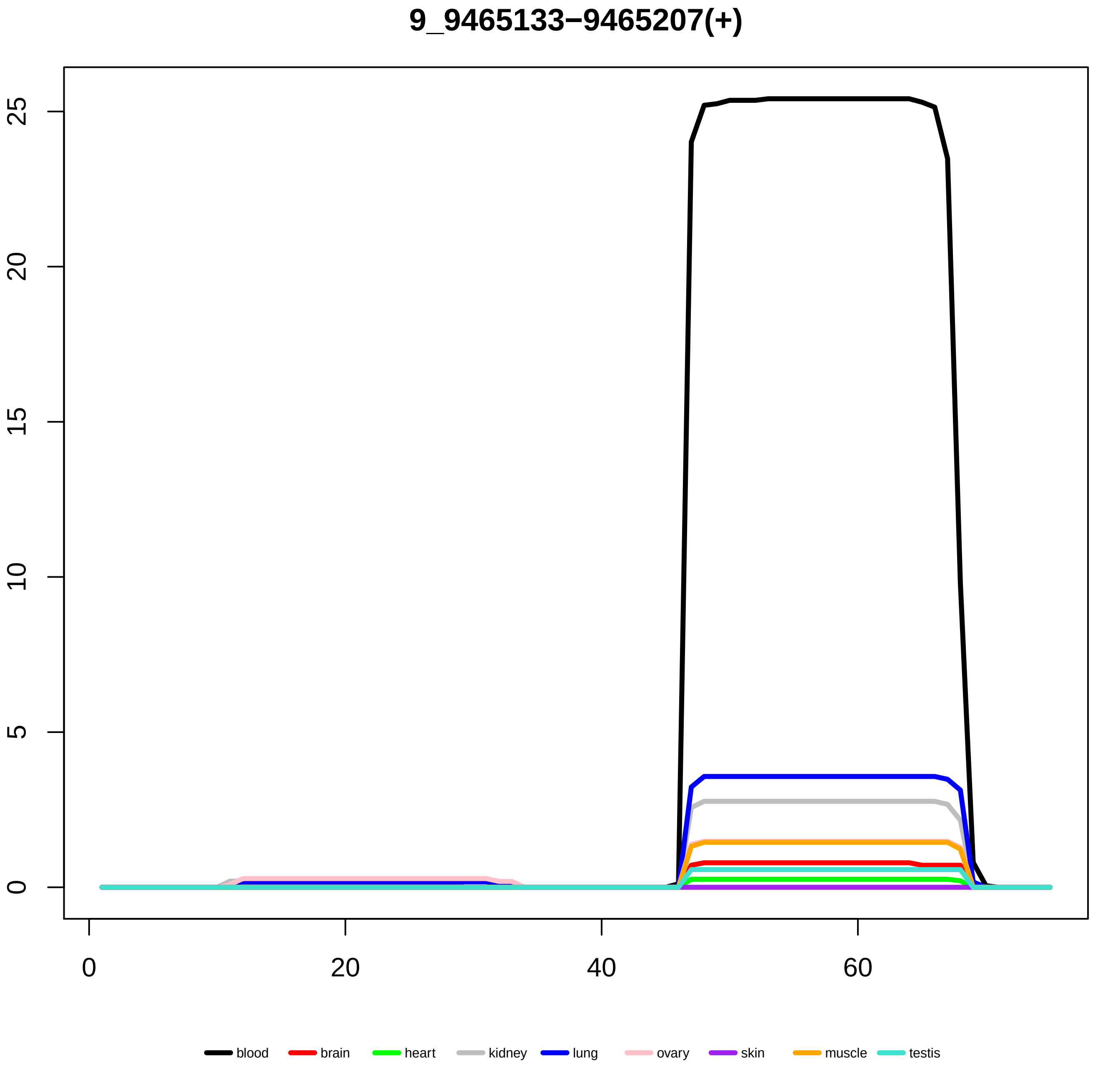

9_9465133−9465207(+)
0
5
10
15
20
25
0
20
40
60
blood
brain
heart
kidney
lung
ovary
skin
muscle
testis

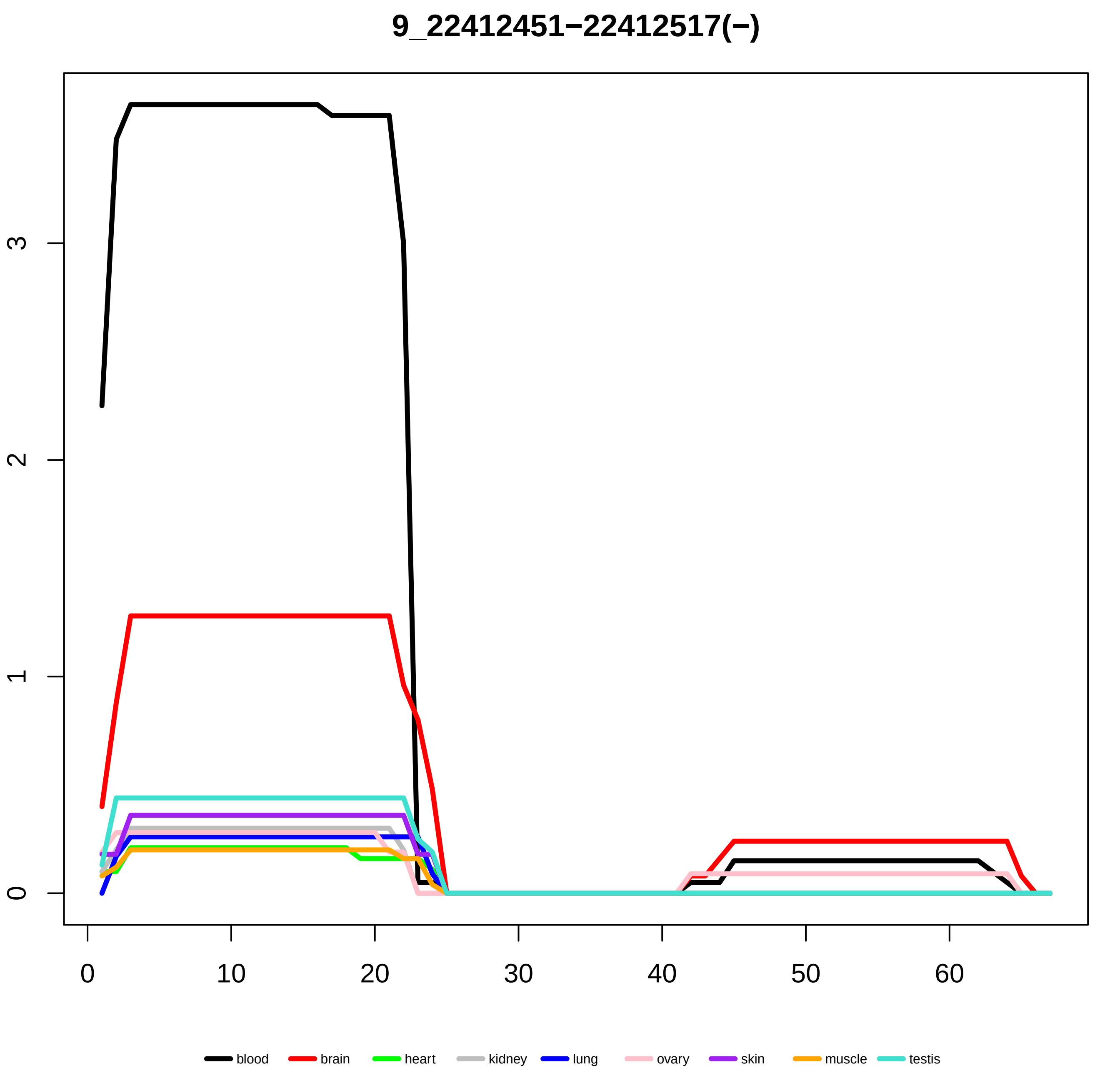
9_22412451−22412517(−)
0
1
2
3
0
10
20
30
40
50
60
blood
brain
heart
kidney
lung
ovary
skin
muscle
testis

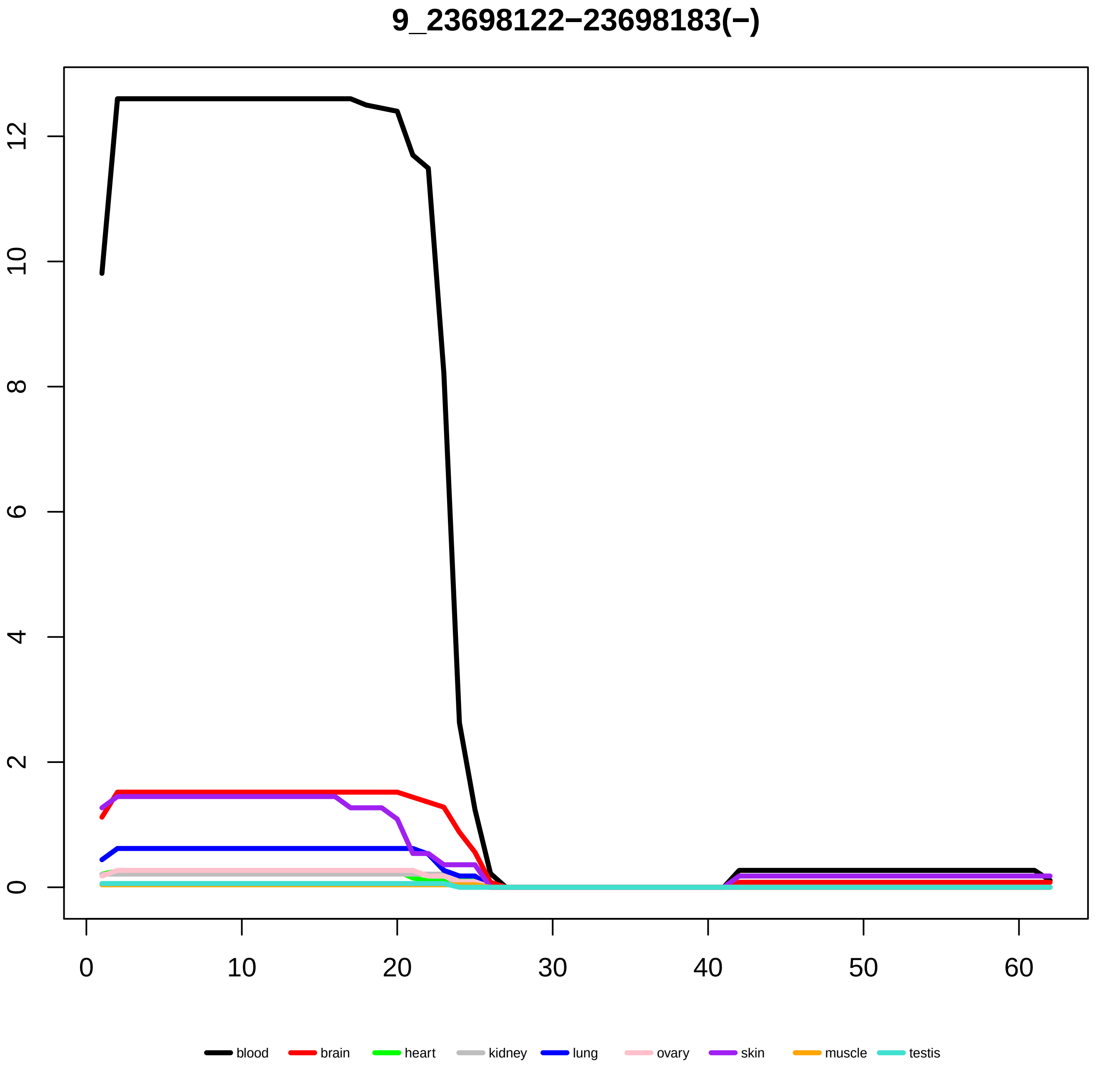

9_23698122−23698183(−)
0
2
4
6
8
10
12
0
10
20
30
40
50
60
blood
brain
heart
kidney
lung
ovary
skin
muscle
testis

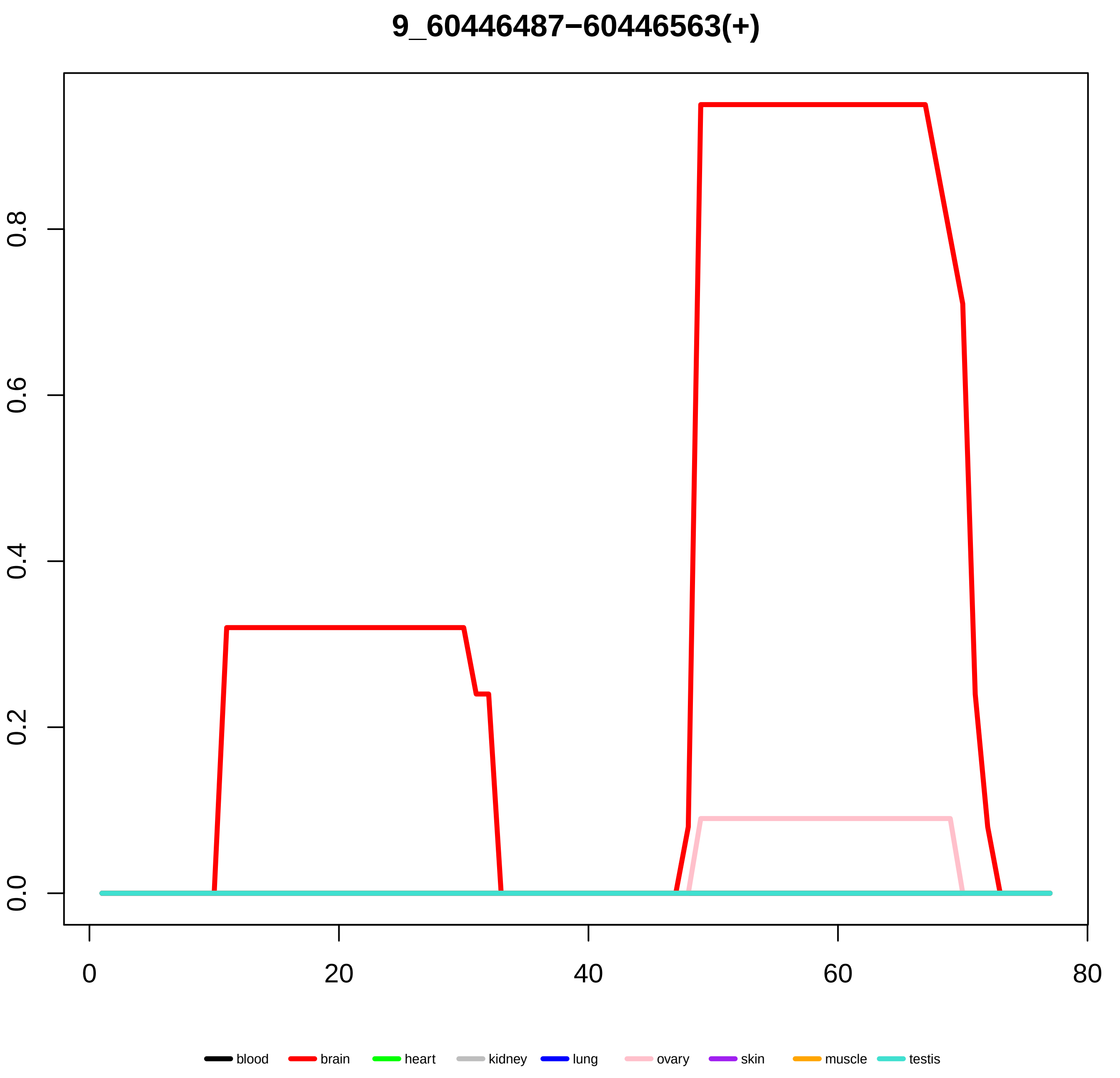

9_60446487−60446563(+)
0.0
0.2
0.4
0.6
0.8
0
20
40
60
80
blood
brain
heart
kidney
lung
ovary
skin
muscle
testis

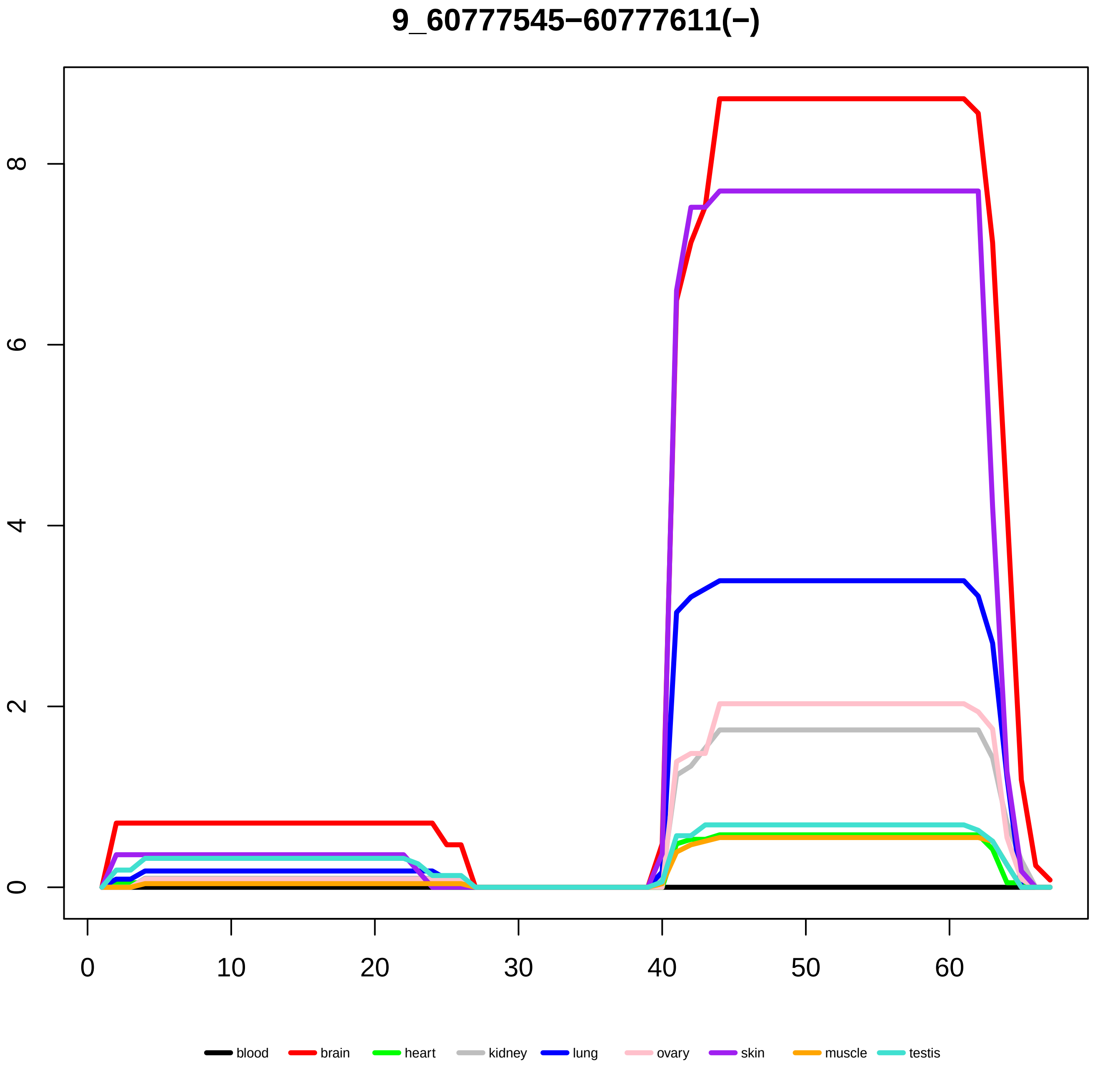
9_60777545−60777611(−)
0
2
4
6
8
0
10
20
30
40
50
60
blood
brain
heart
kidney
lung
ovary
skin
muscle
testis

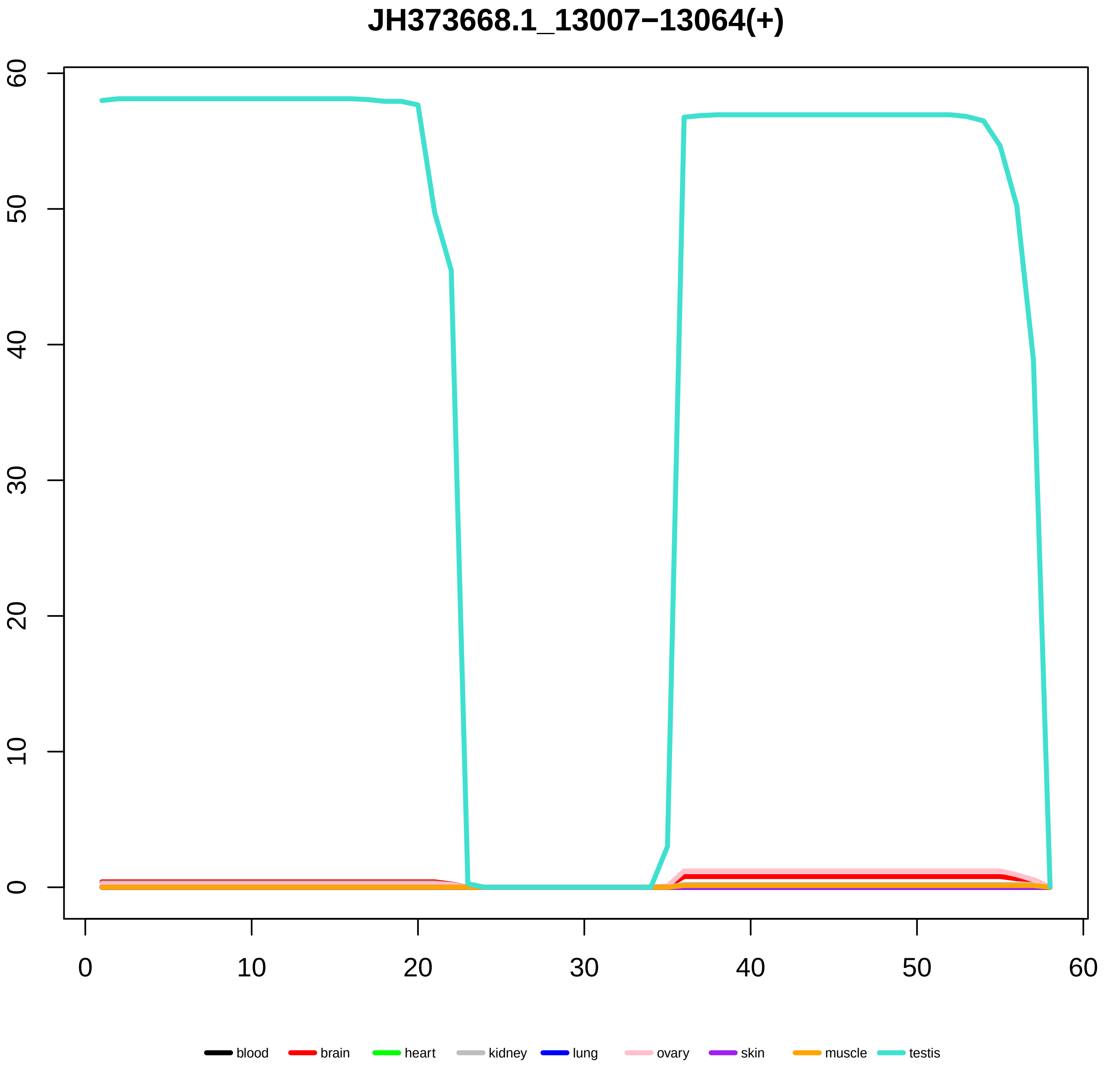
JH373668.1_13007−13064(+)
0
10
20
30
40
50
60
0
10
20
30
40
50
60
blood
brain
heart
kidney
lung
ovary
skin
muscle
testis

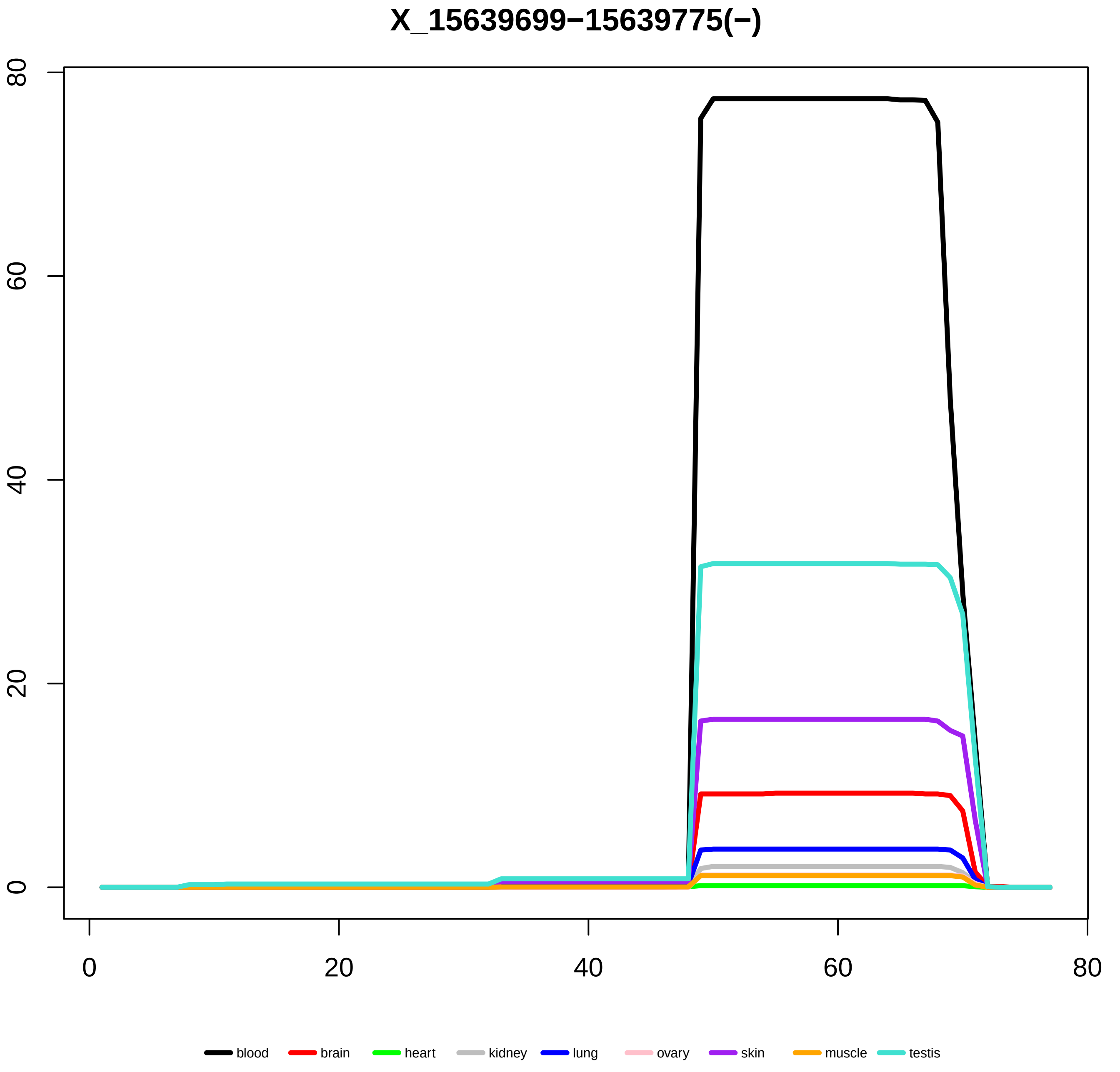
X_15639699−15639775(−)
0
20
40
60
80
0
20
40
60
80
blood
brain
heart
kidney
lung
ovary
skin
muscle
testis

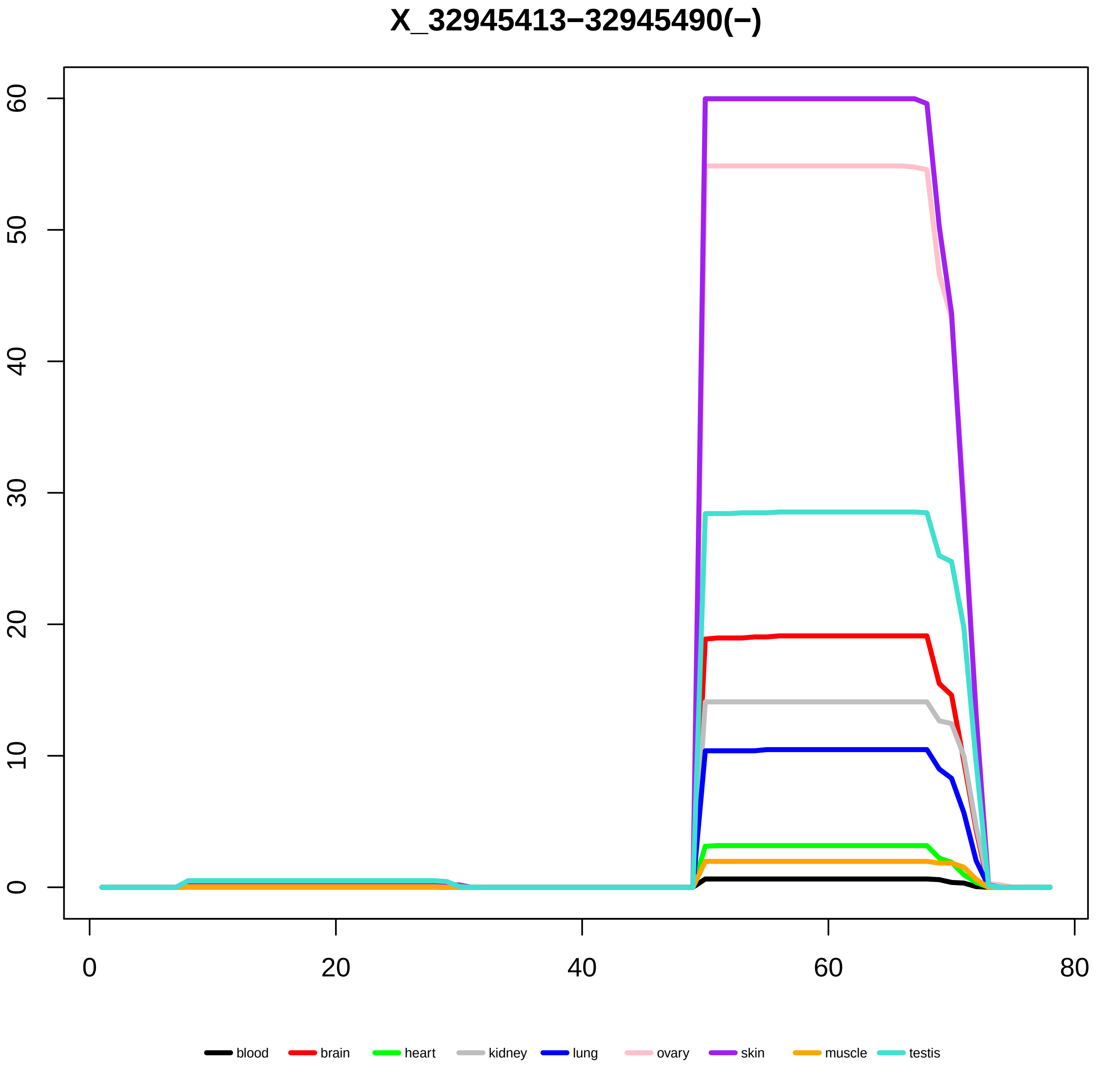
X_32945413−32945490(−)
0
10
20
30
40
50
60
0
20
40
60
80
blood
brain
heart
kidney
lung
ovary
skin
muscle
testis

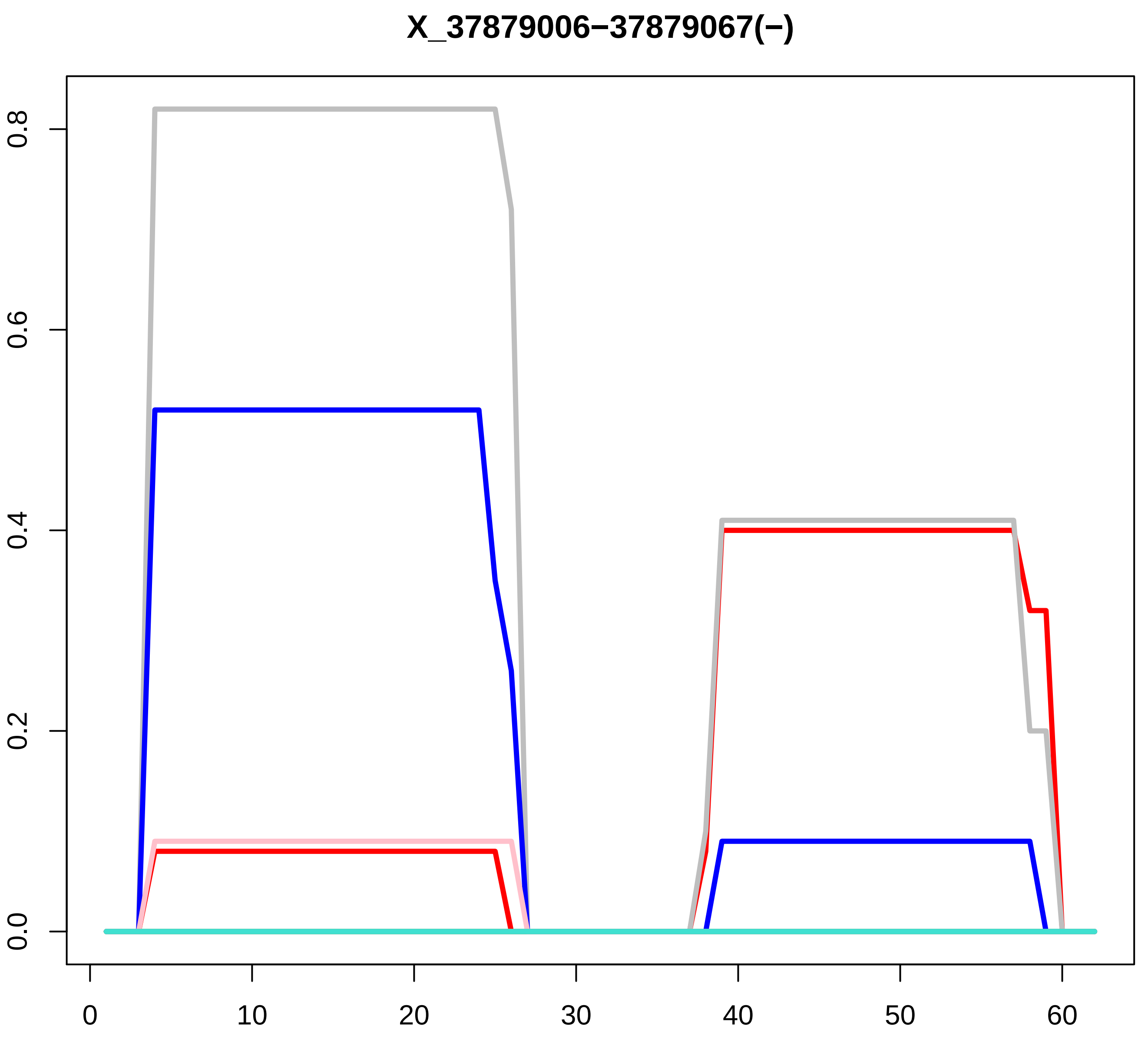
X_37879006−37879067(−)
0.0
0.2
0.4
0.6
0.8
0
10
20
30
40
50
60
