# Supplementary material for: An Improved microRNA Annotation of the Canine Genome
Source: PLoS One. 2016 Apr 27;11(4):e0153453. doi: 10.1371/journal.pone.0153453 (PMC4847789; doi:10.1371/journal.pone.0153453)
Supplement: S5 Fig — The base position along the hairpin (x axis) is plotted against the normalized read count (reads per million genome matching reads). Different colors in the plot correspond to different tissues, as described by the legend on the bottom of each plot. (PDF) [file pone.0153453.s005.pdf]

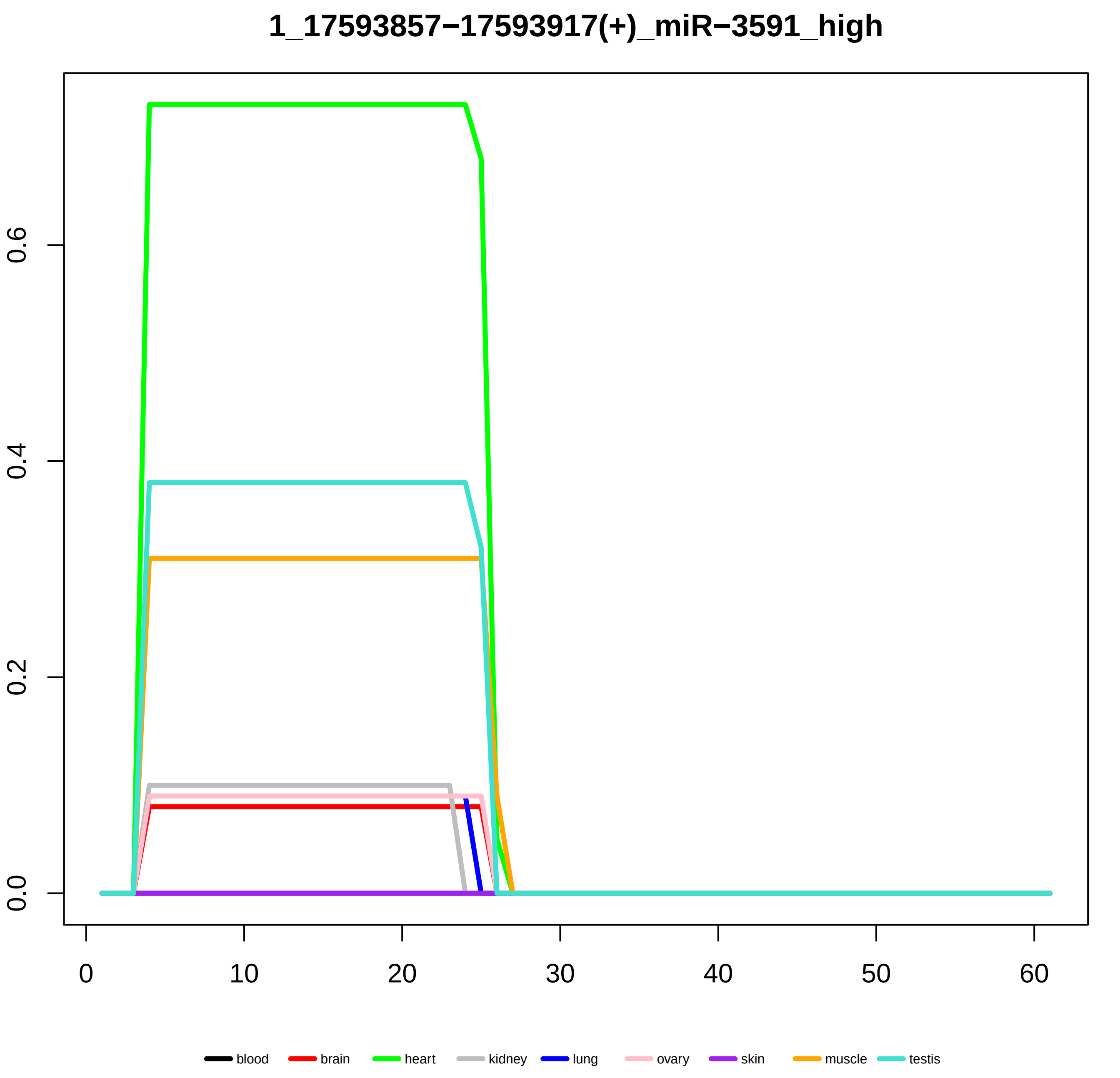
1_17593857−17593917(+)_miR−3591_high
0.0
0.2
0.4
0.6
0
10
20
30
40
50
60
blood
brain
heart
kidney
lung
ovary
skin
muscle
testis

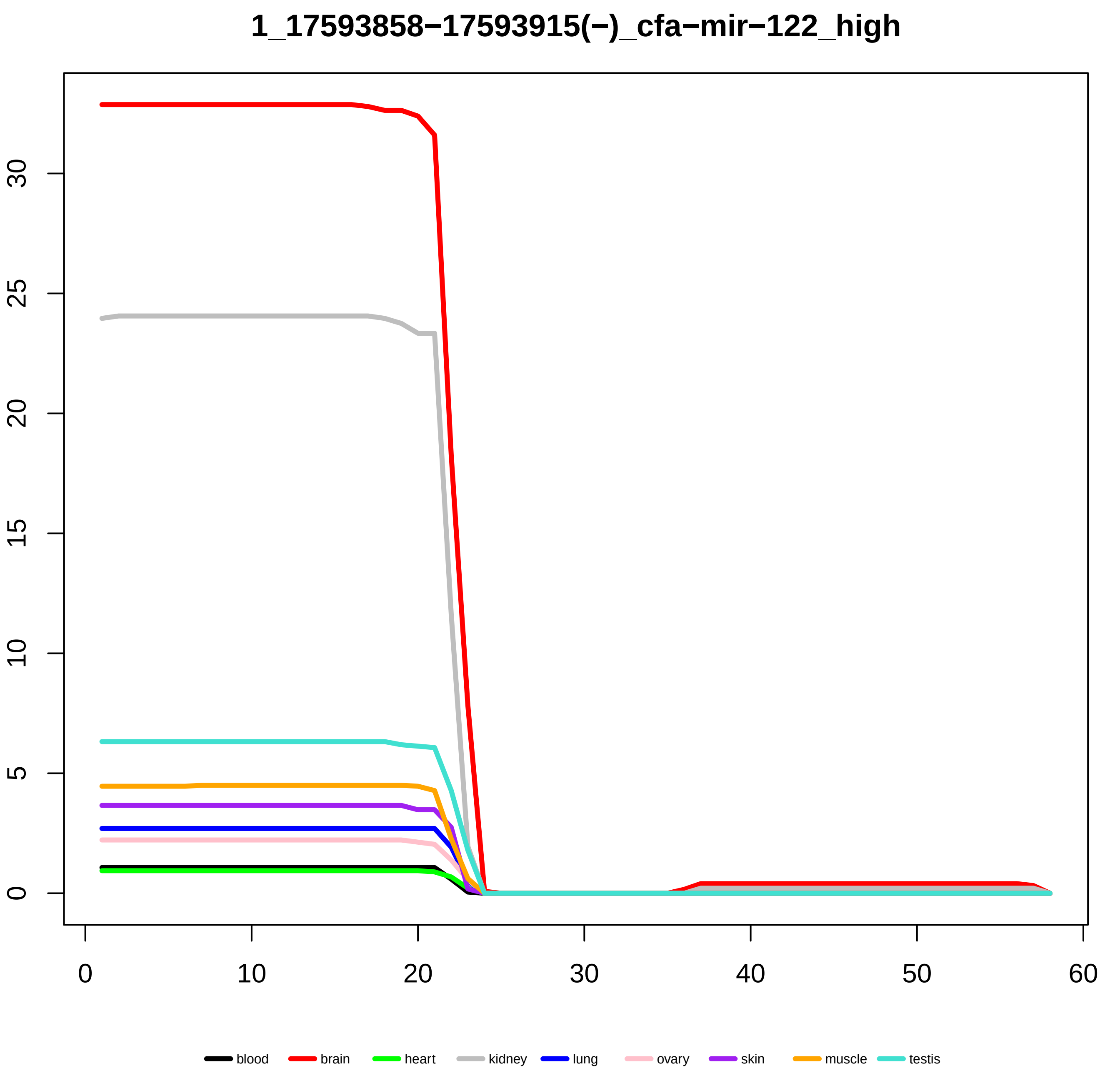
1_17593858−17593915(−)_cfa−mir−122_high
0
5
10
15
20
25
30
0
10
20
30
40
50
60
blood
brain
heart
kidney
lung
ovary
skin
muscle
testis

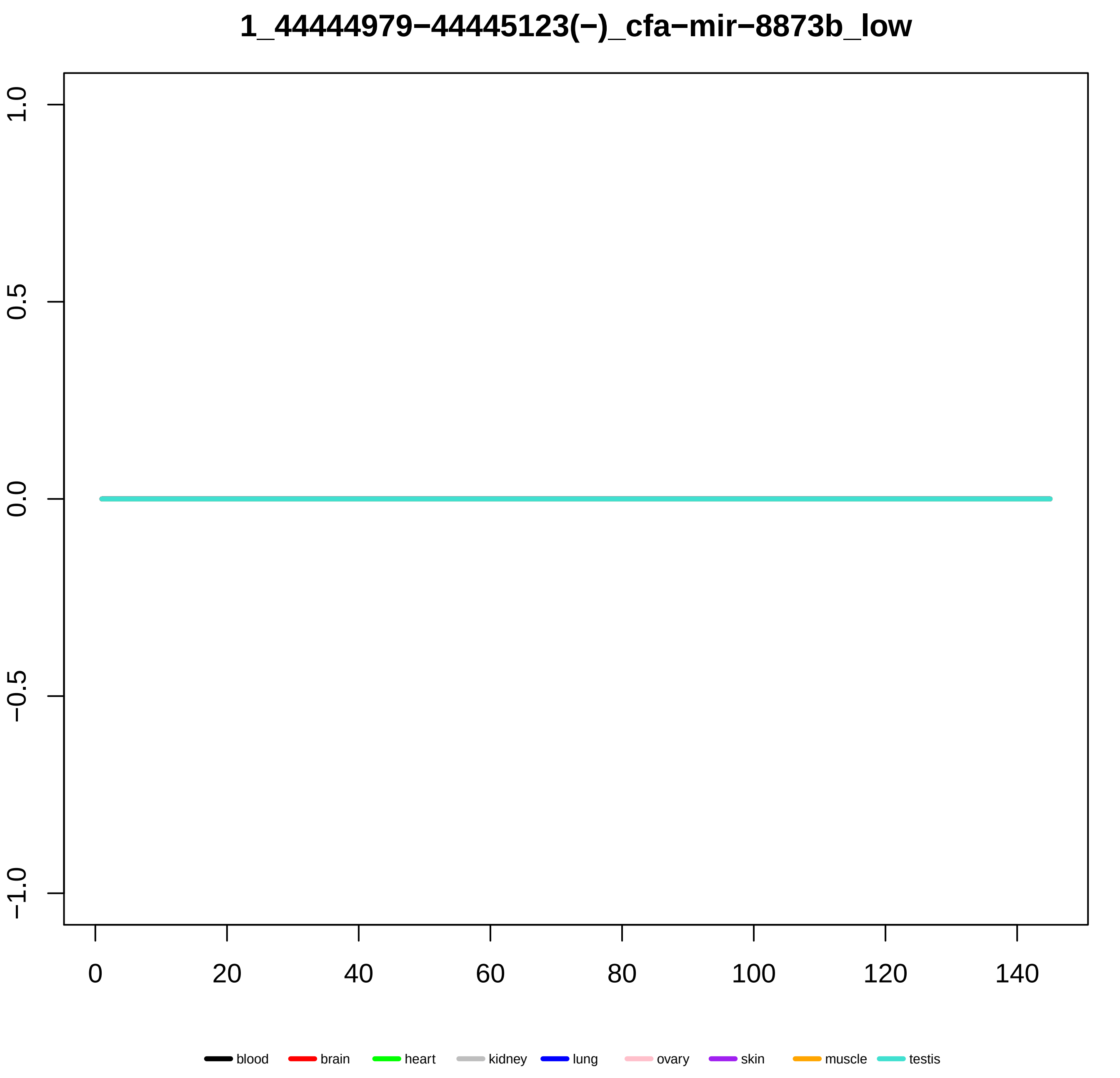

1_44444979−44445123(−)_cfa−mir−8873b_low
1.0
0.5
0.0
−0.5
−1.0
0
20
40
60
80
100
120
140
blood
brain
heart
kidney
lung
ovary
skin
muscle
testis

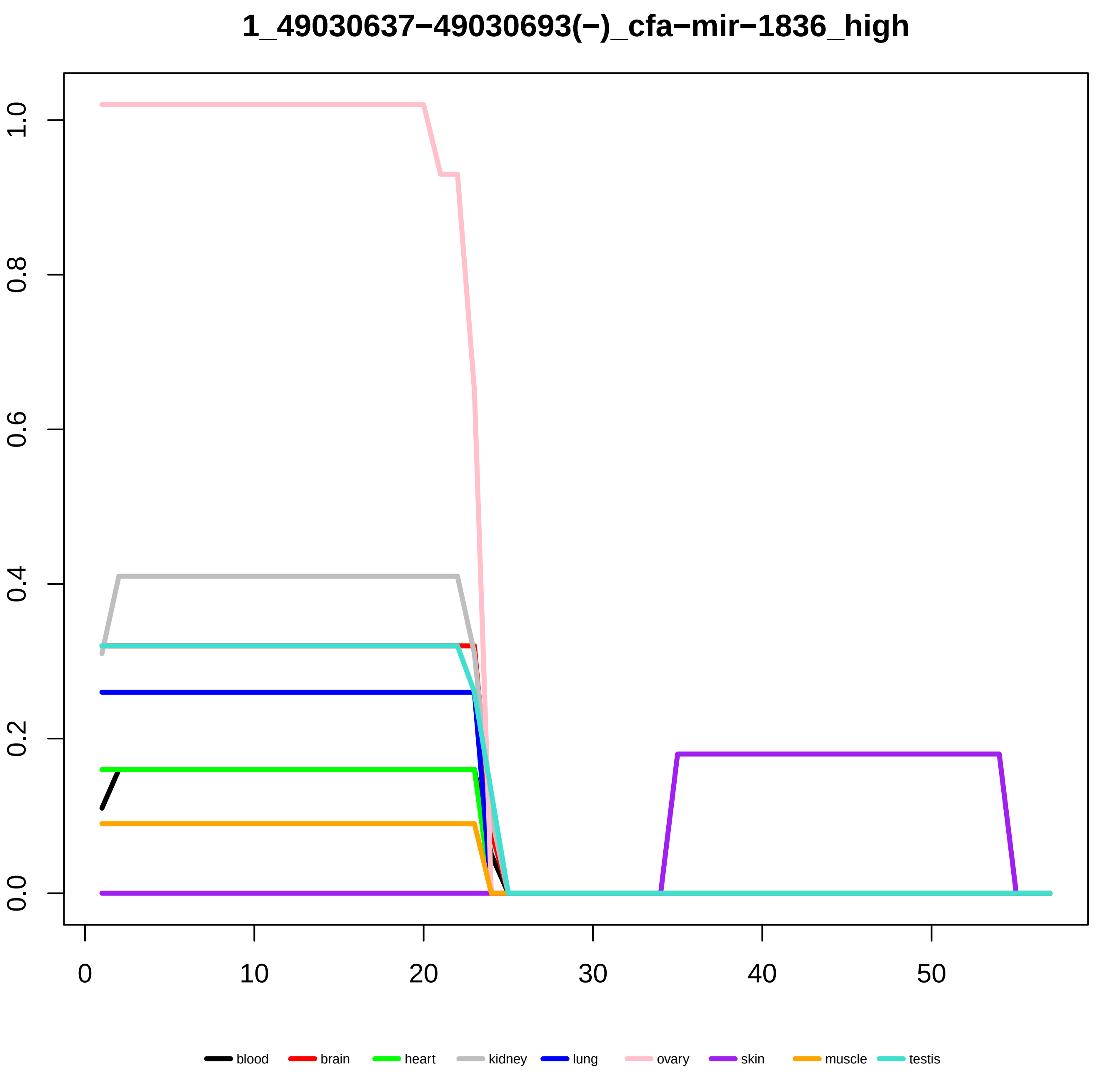
1_49030637−49030693(−)_cfa−mir−1836_high
0.0
0.2
0.4
0.6
0.8
1.0
0
10
20
30
40
50
blood
brain
heart
kidney
lung
ovary
skin
muscle
testis

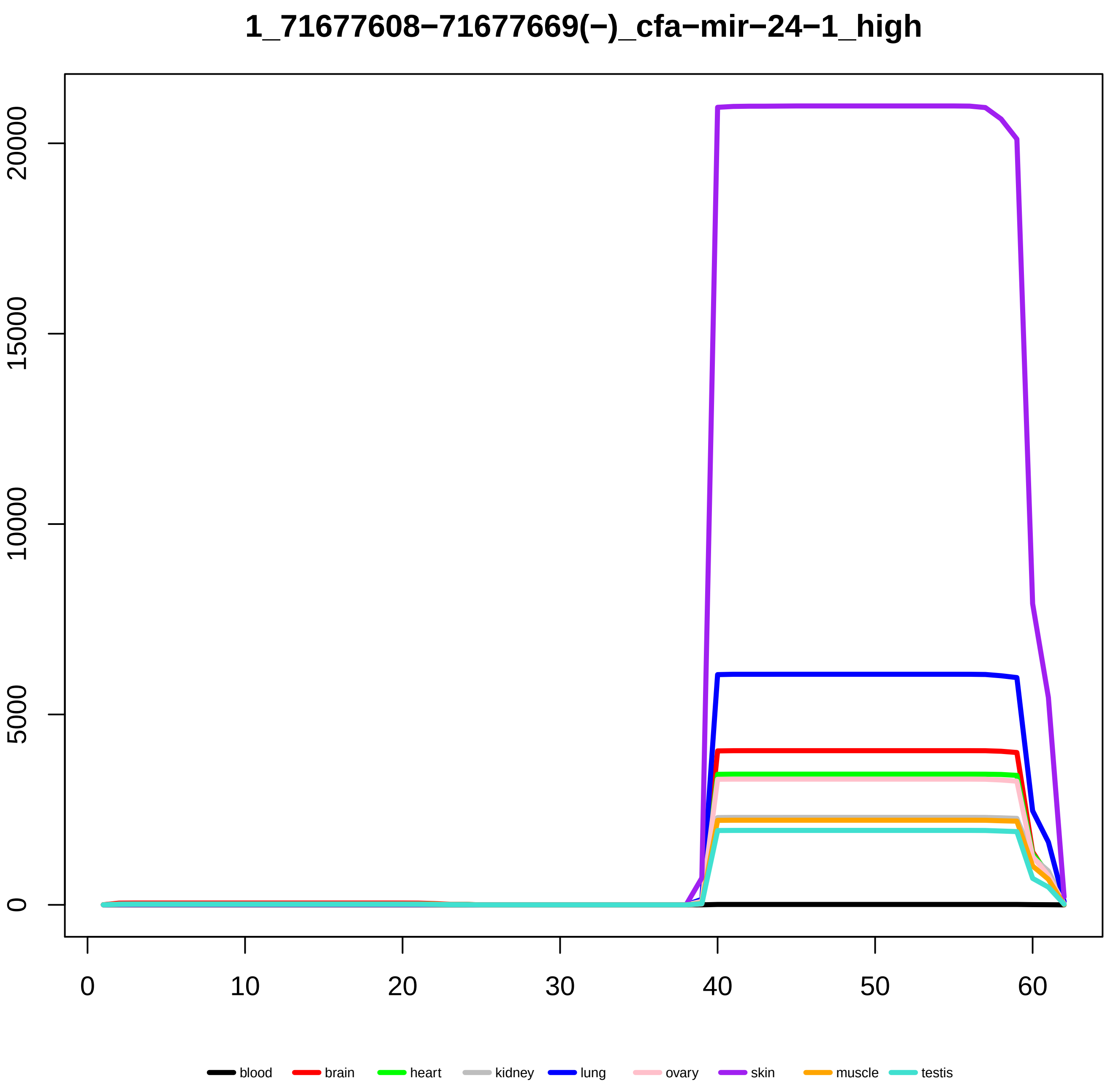

1_71677608−71677669(−)_cfa−mir−24−1_high
0
5000
10000
15000
20000
0
10
20
30
40
50
60
blood
brain
heart
kidney
lung
ovary
skin
muscle
testis

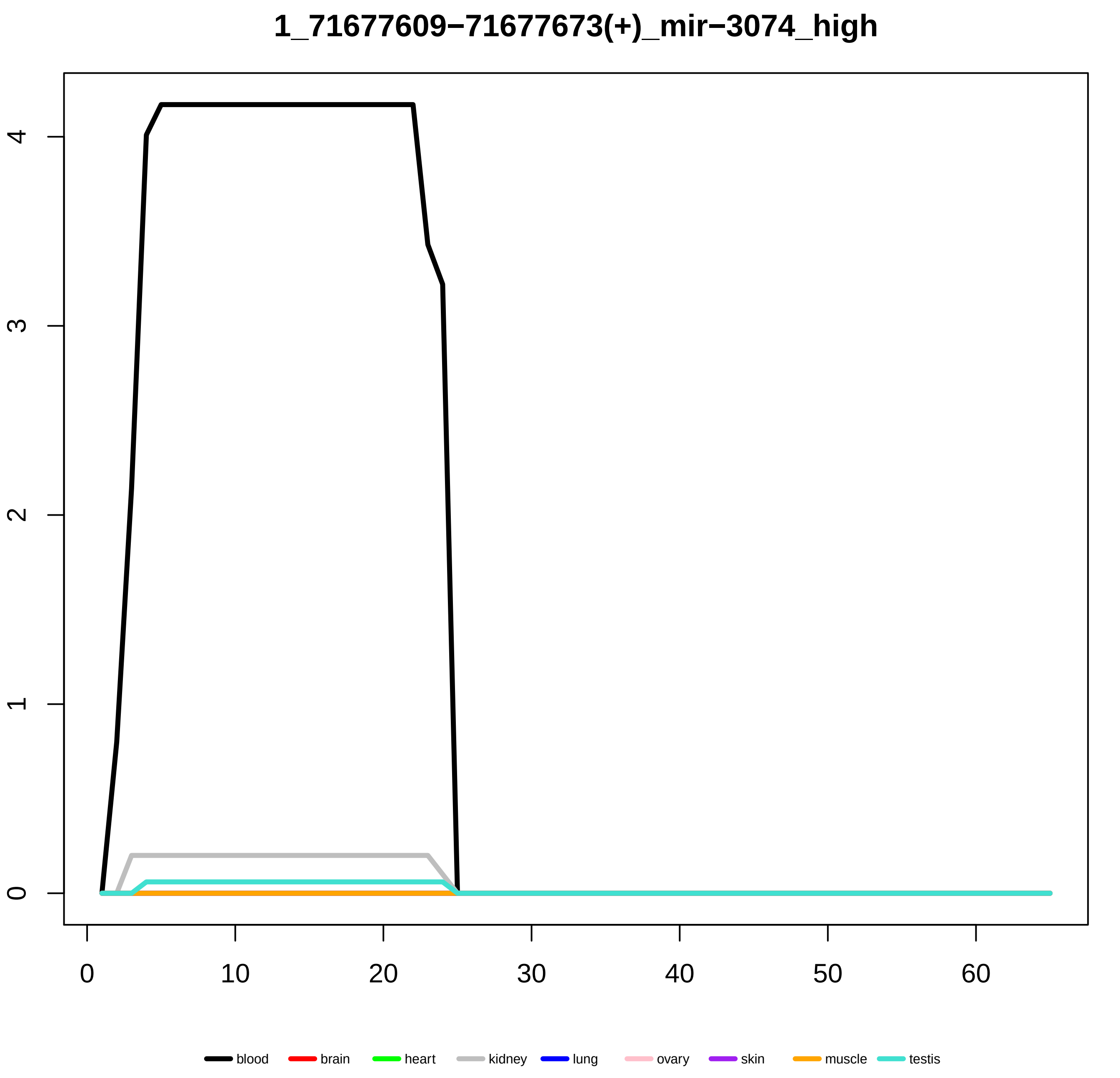

1_71677609−71677673(+)_mir−3074_high
0
1
2
3
4
0
10
20
30
40
50
60
blood
brain
heart
kidney
lung
ovary
skin
muscle
testis

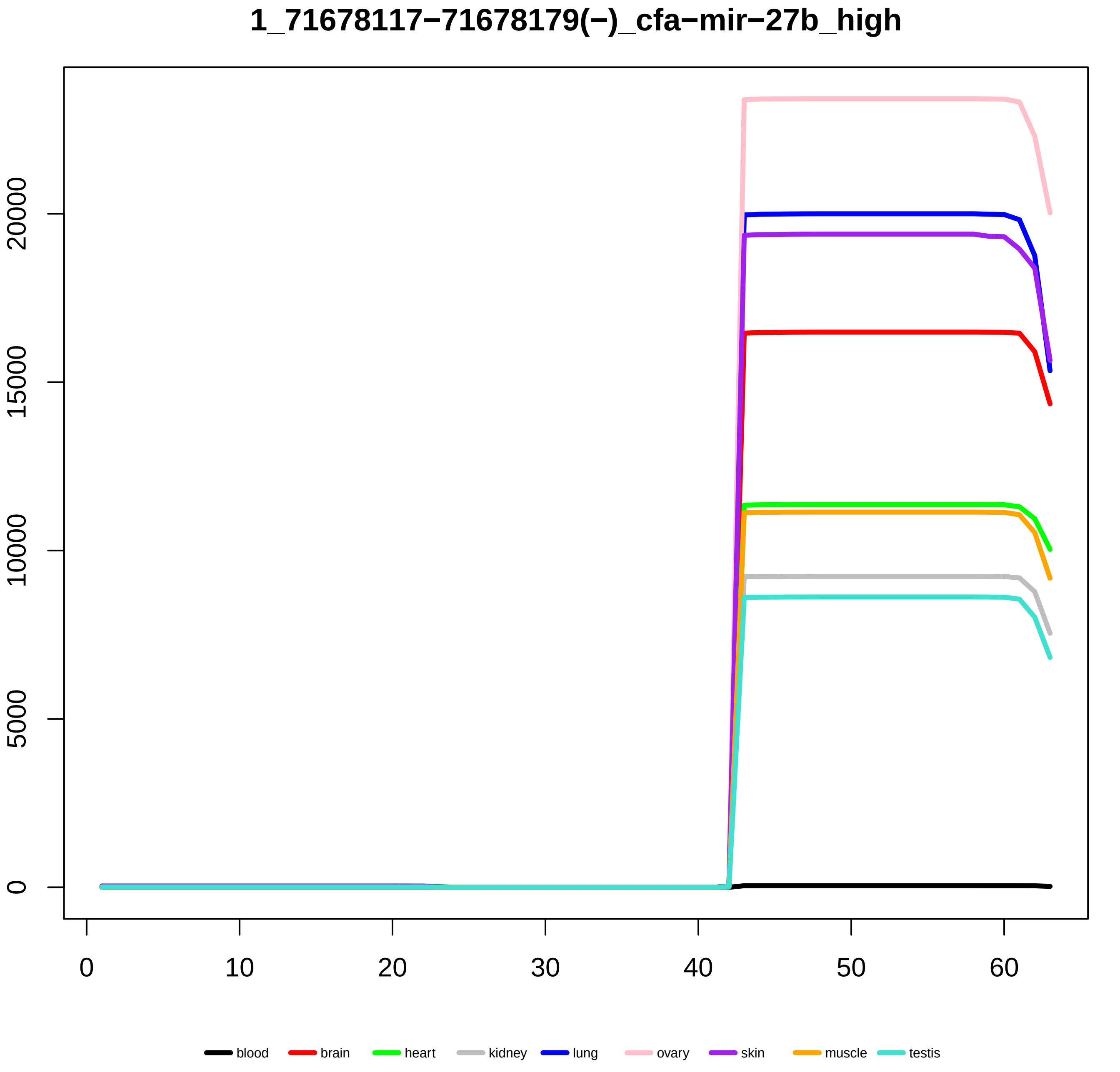

1_71678117−71678179(−)_cfa−mir−27b_high
0
5000
10000
15000
20000
0
10
20
30
40
50
60
blood
brain
heart
kidney
lung
ovary
skin
muscle
testis

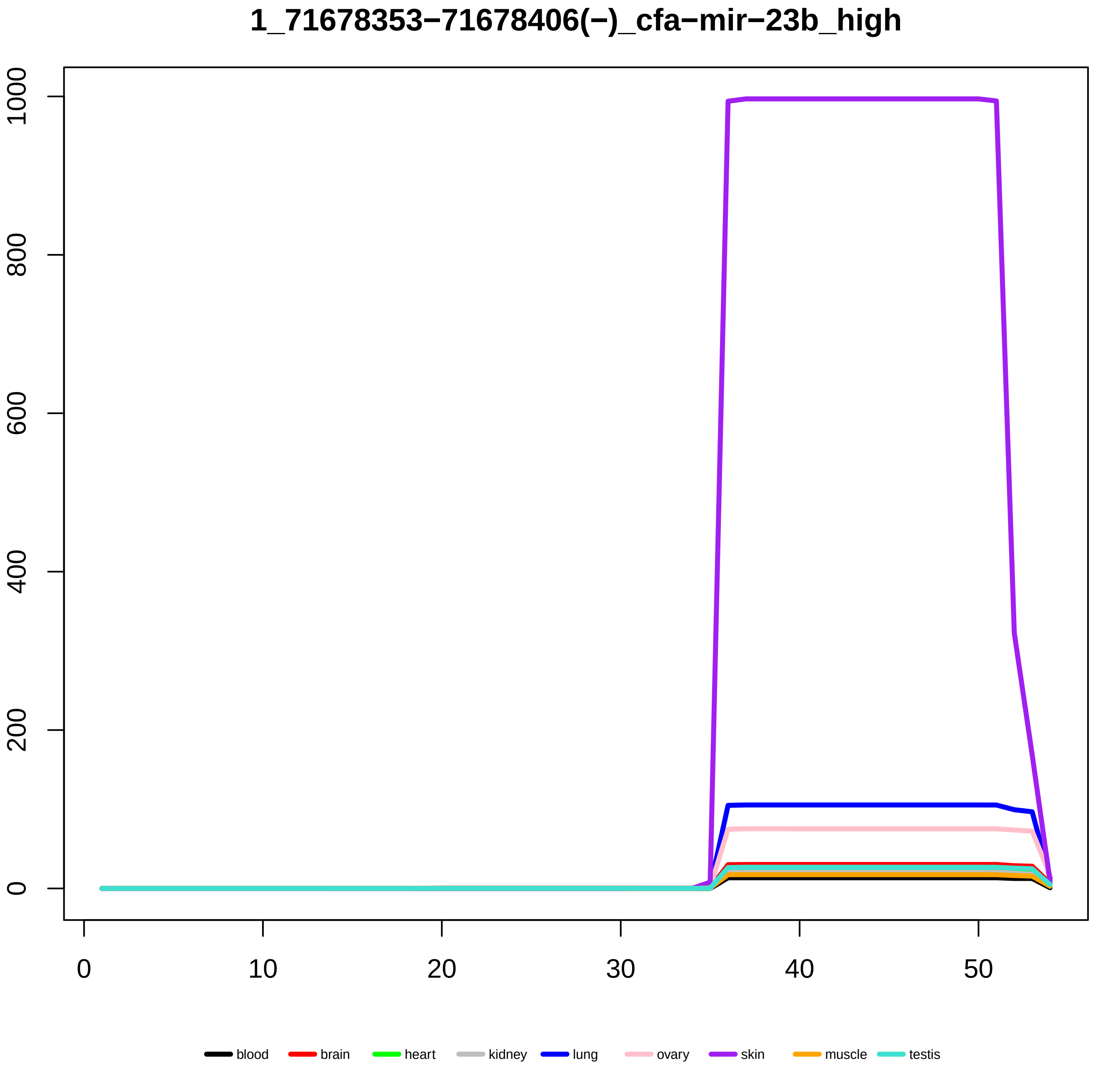
1_71678353−71678406(−)_cfa−mir−23b_high
0
200
400
600
800
1000
0
10
20
30
40
50
blood
brain
heart
kidney
lung
ovary
skin
muscle
testis

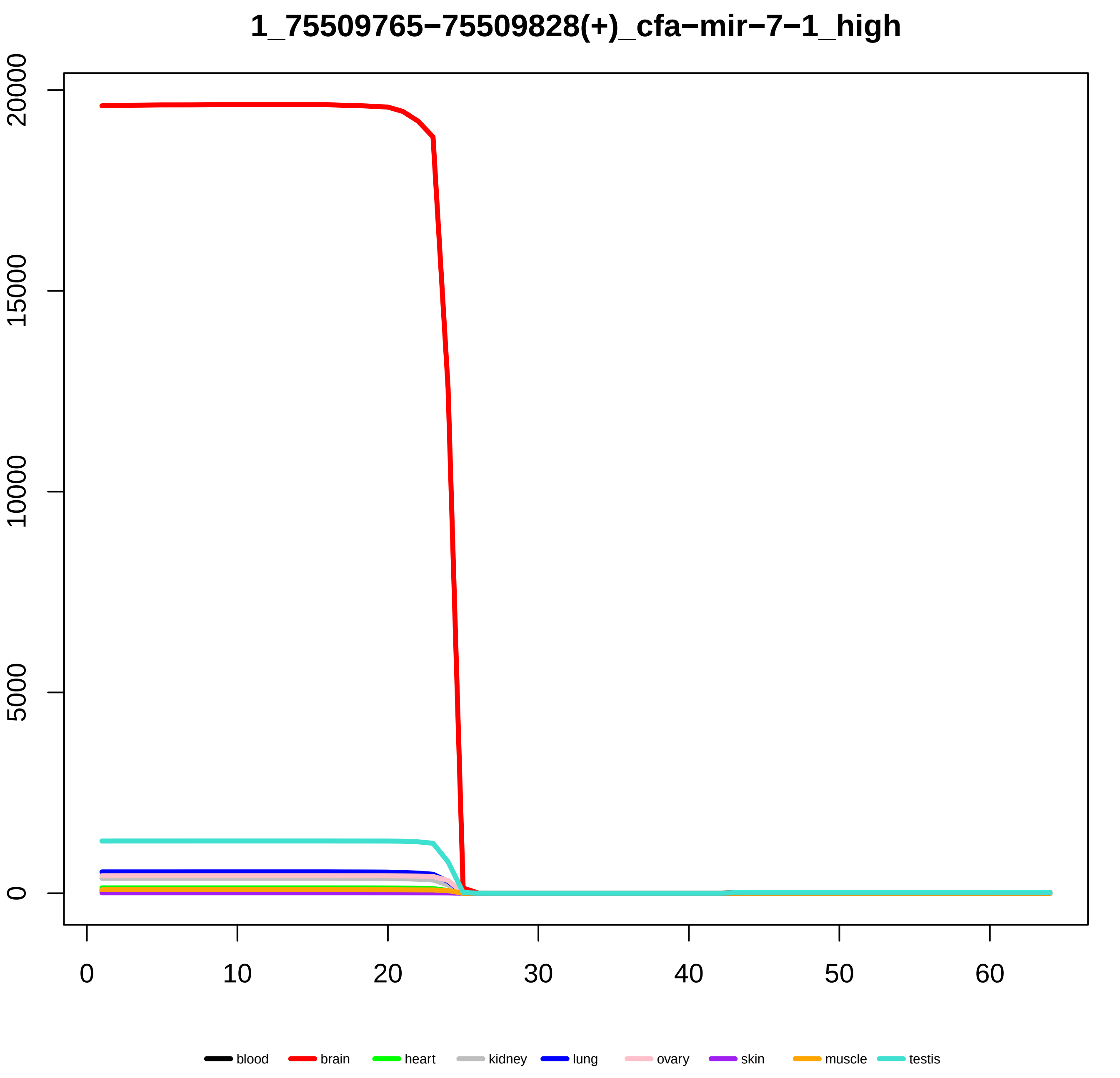

1_75509765−75509828(+)_cfa−mir−7−1_high
0
5000
10000
15000
20000
0
10
20
30
40
50
60
blood
brain
heart
kidney
lung
ovary
skin
muscle
testis

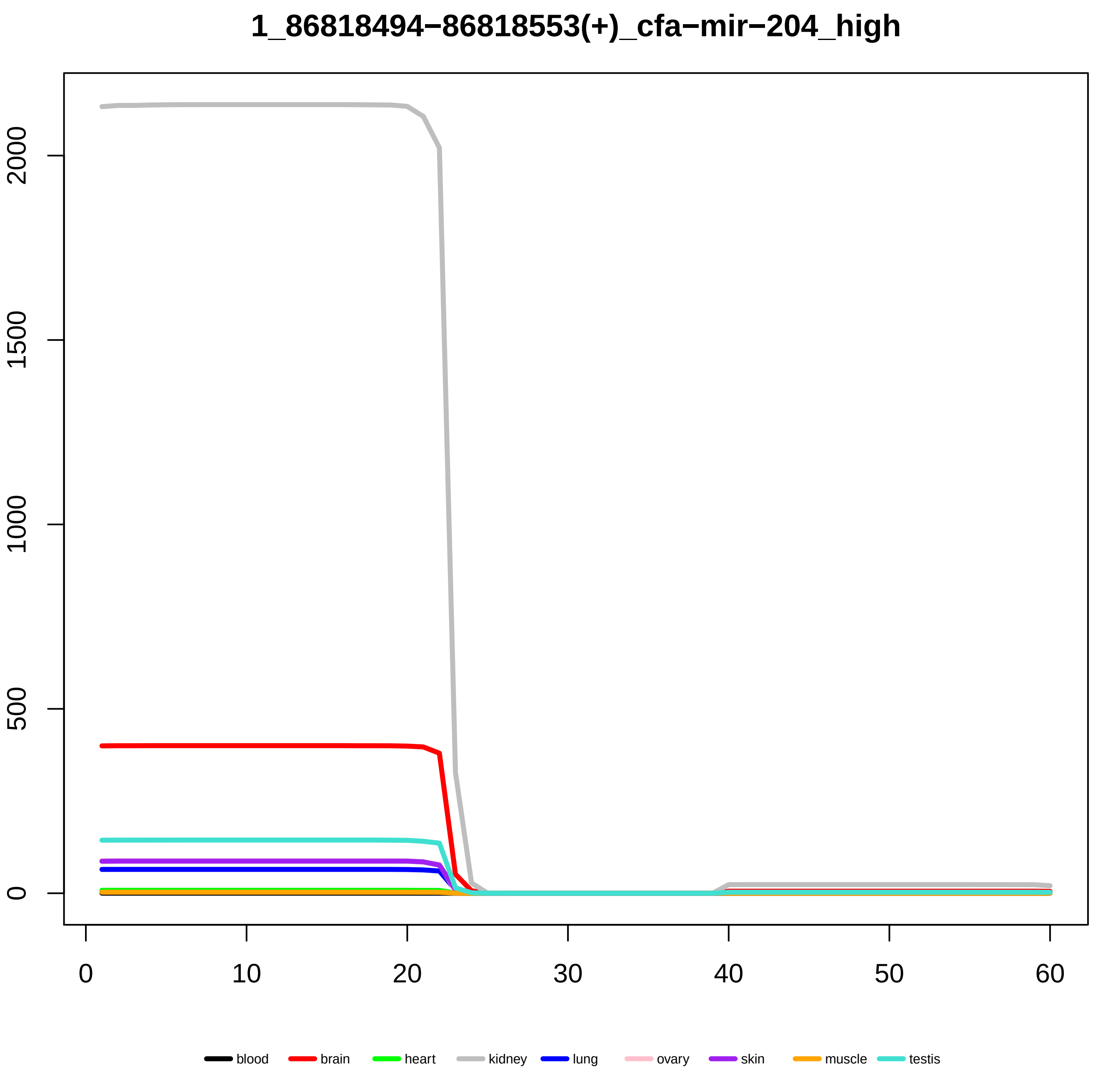

1_86818494−86818553(+)_cfa−mir−204_high
0
500
1000
1500
2000
0
10
20
30
40
50
60
blood
brain
heart
kidney
lung
ovary
skin
muscle
testis

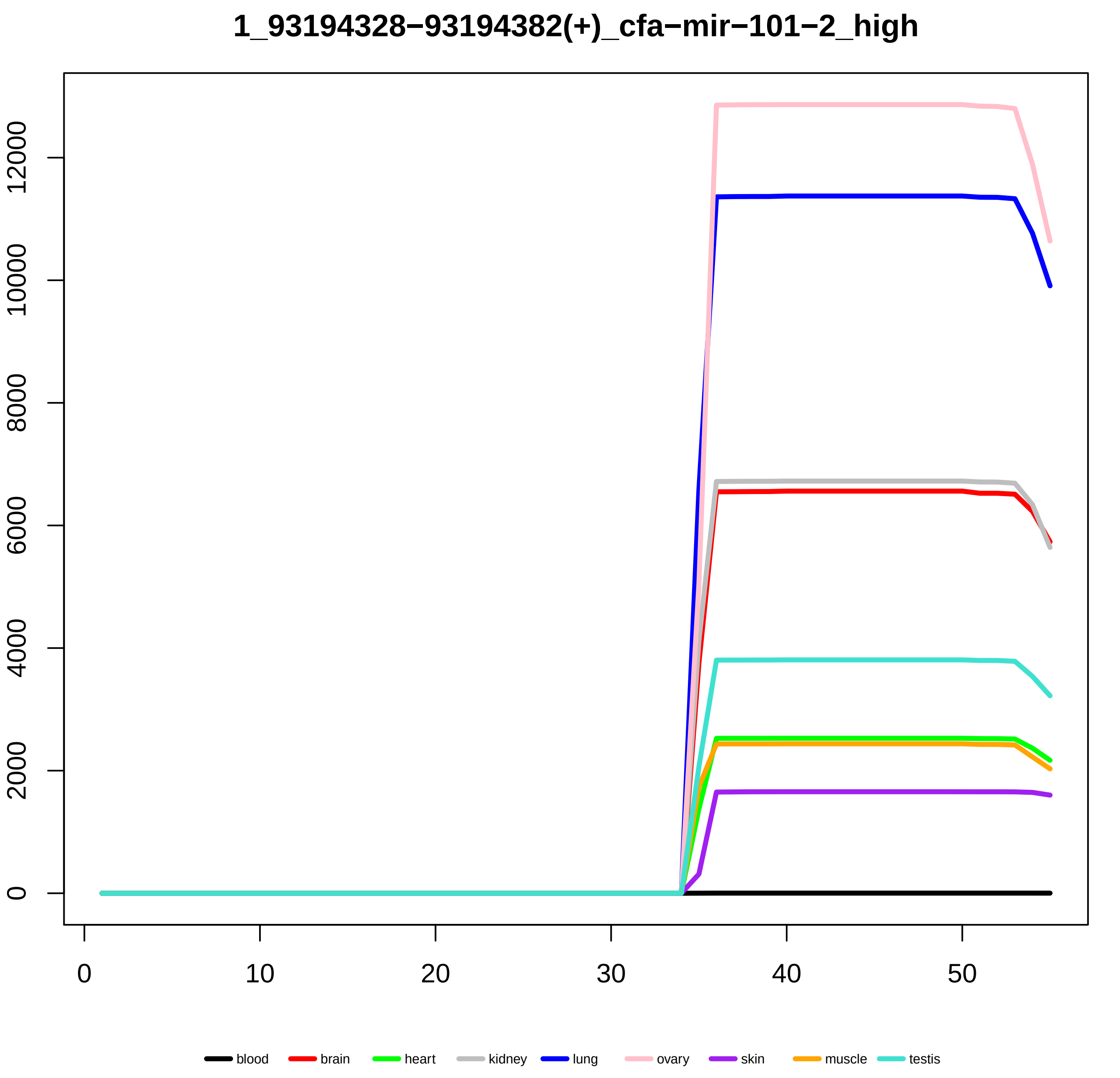

1_93194328−93194382(+)_cfa−mir−101−2_high
0
2000
4000
6000
8000
10000
12000
0
10
20
30
40
50
blood
brain
heart
kidney
lung
ovary
skin
muscle
testis

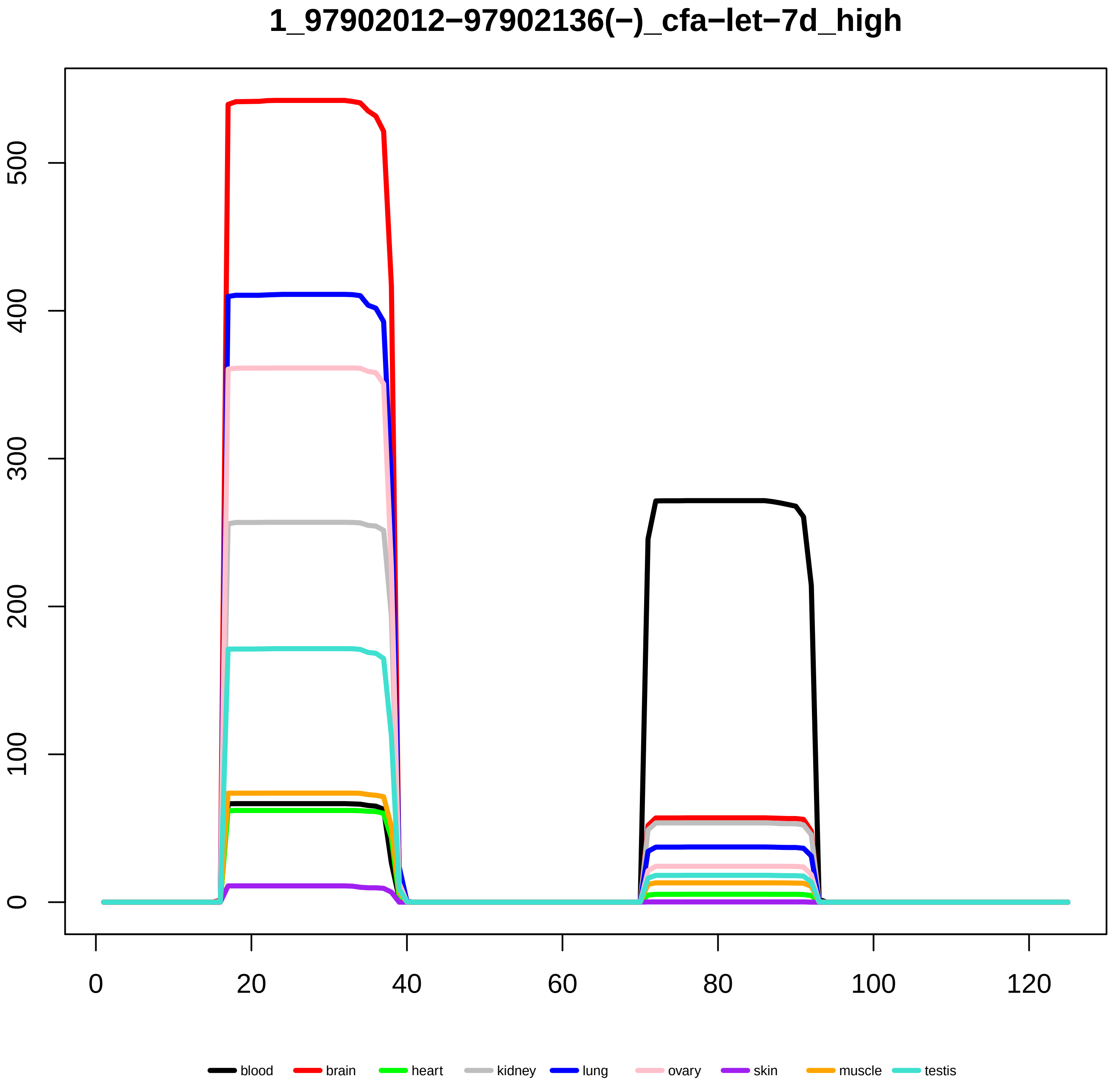

1_97902012−97902136(−)_cfa−let−7d_high
0
100
200
300
400
500
0
20
40
60
80
100
120
blood
brain
heart
kidney
lung
ovary
skin
muscle
testis

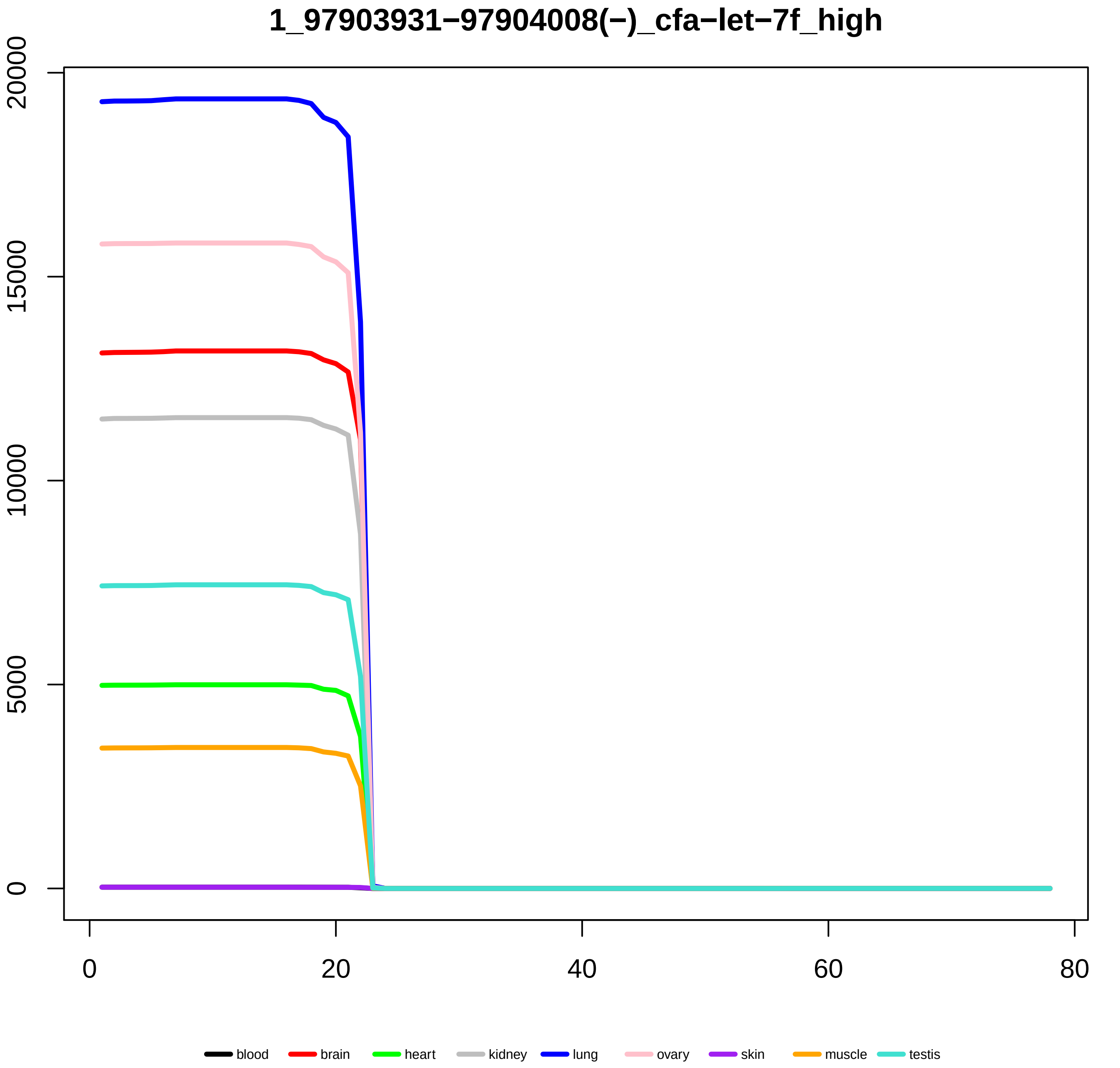
1_97903931−97904008(−)_cfa−let−7f_high
20000
15000
10000
5000
0
0
20
40
60
80
blood
brain
heart
kidney
lung
ovary
skin
muscle
testis

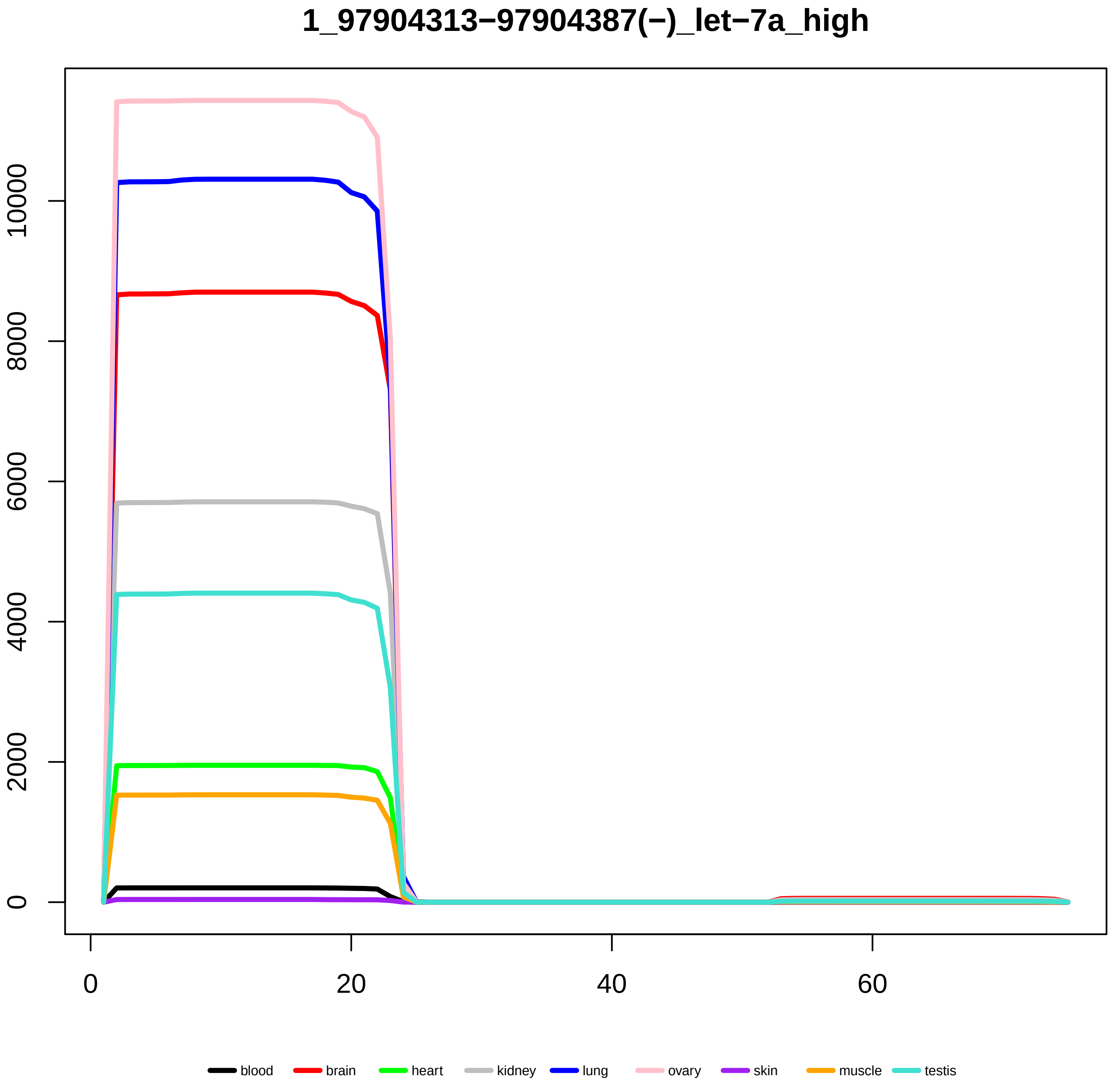
1_97904313−97904387(−)_let−7a_high
0
2000
4000
6000
8000
10000
0
20
40
60
blood
brain
heart
kidney
lung
ovary
skin
muscle
testis

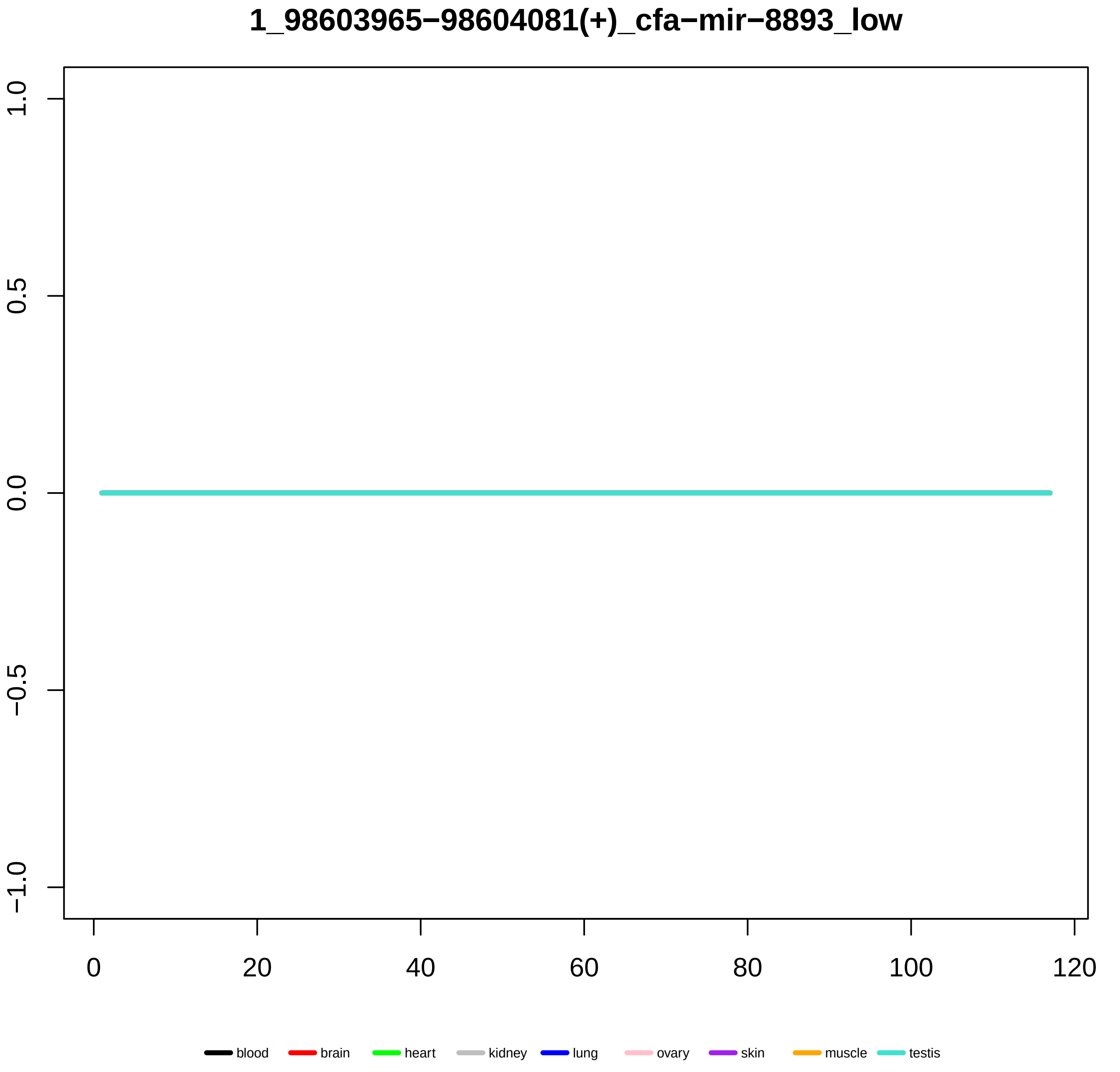
1_98603965−98604081(+)_cfa−mir−8893_low
1.0
0.5
0.0
−0.5
−1.0
0
20
40
60
80
100
120
blood
brain
heart
kidney
lung
ovary
skin
muscle
testis

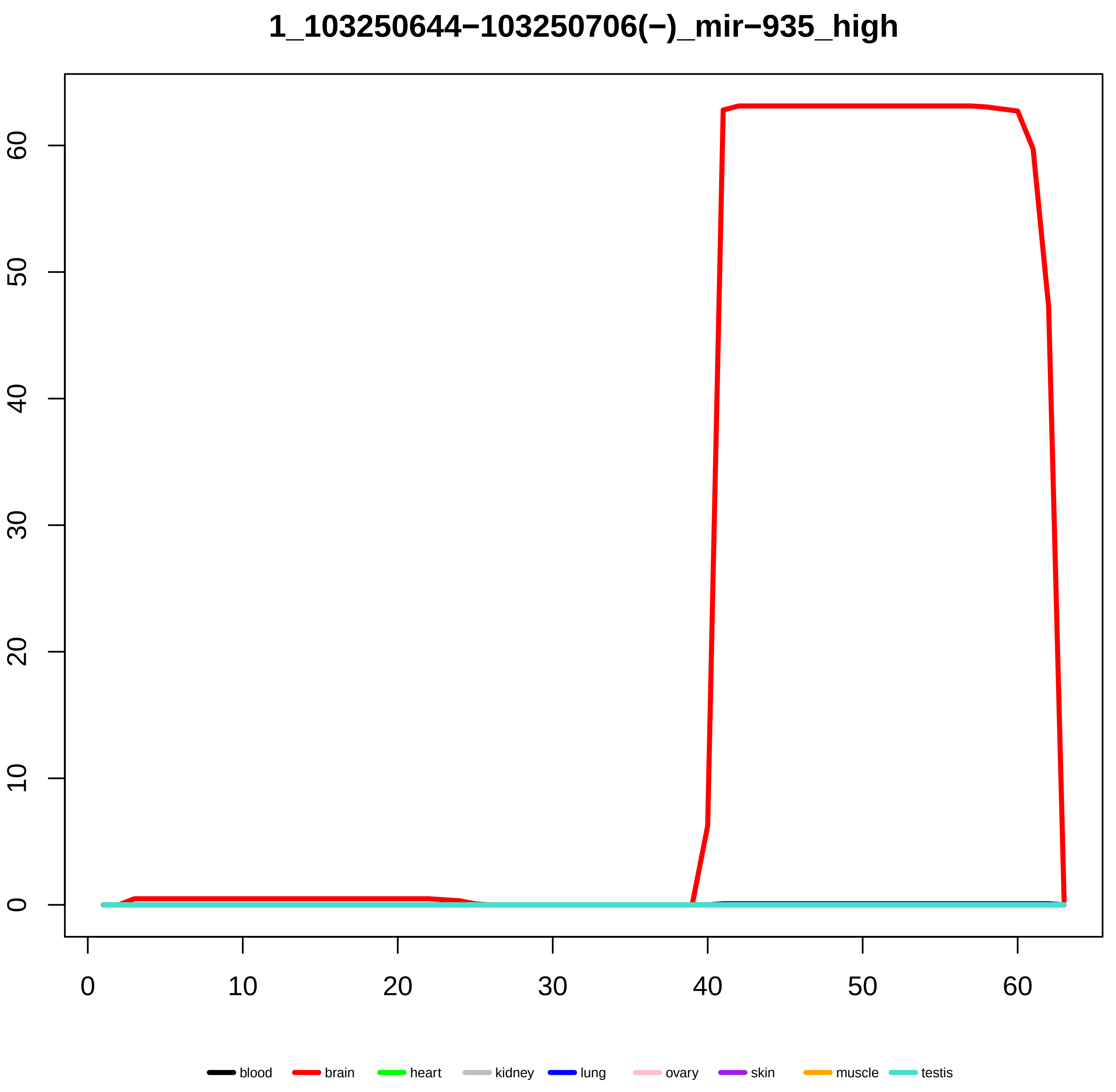
1_103250644−103250706(−)_mir−935_high
0
10
20
30
40
50
60
0
10
20
30
40
50
60
blood
brain
heart
kidney
lung
ovary
skin
muscle
testis

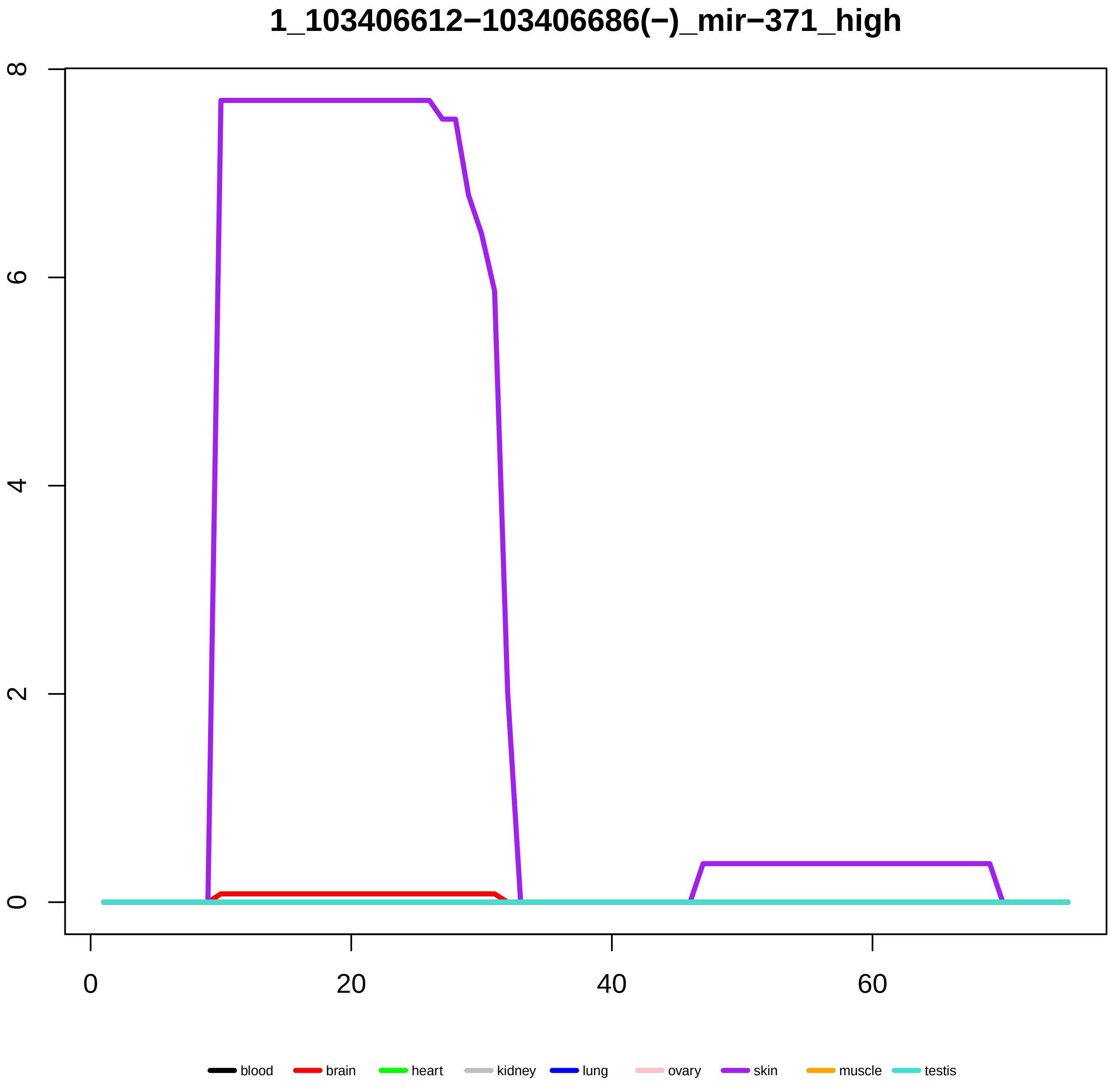

1_103406612−103406686(−)_mir−371_high
0
2
4
6
8
0
20
40
60
blood
brain
heart
kidney
lung
ovary
skin
muscle
testis

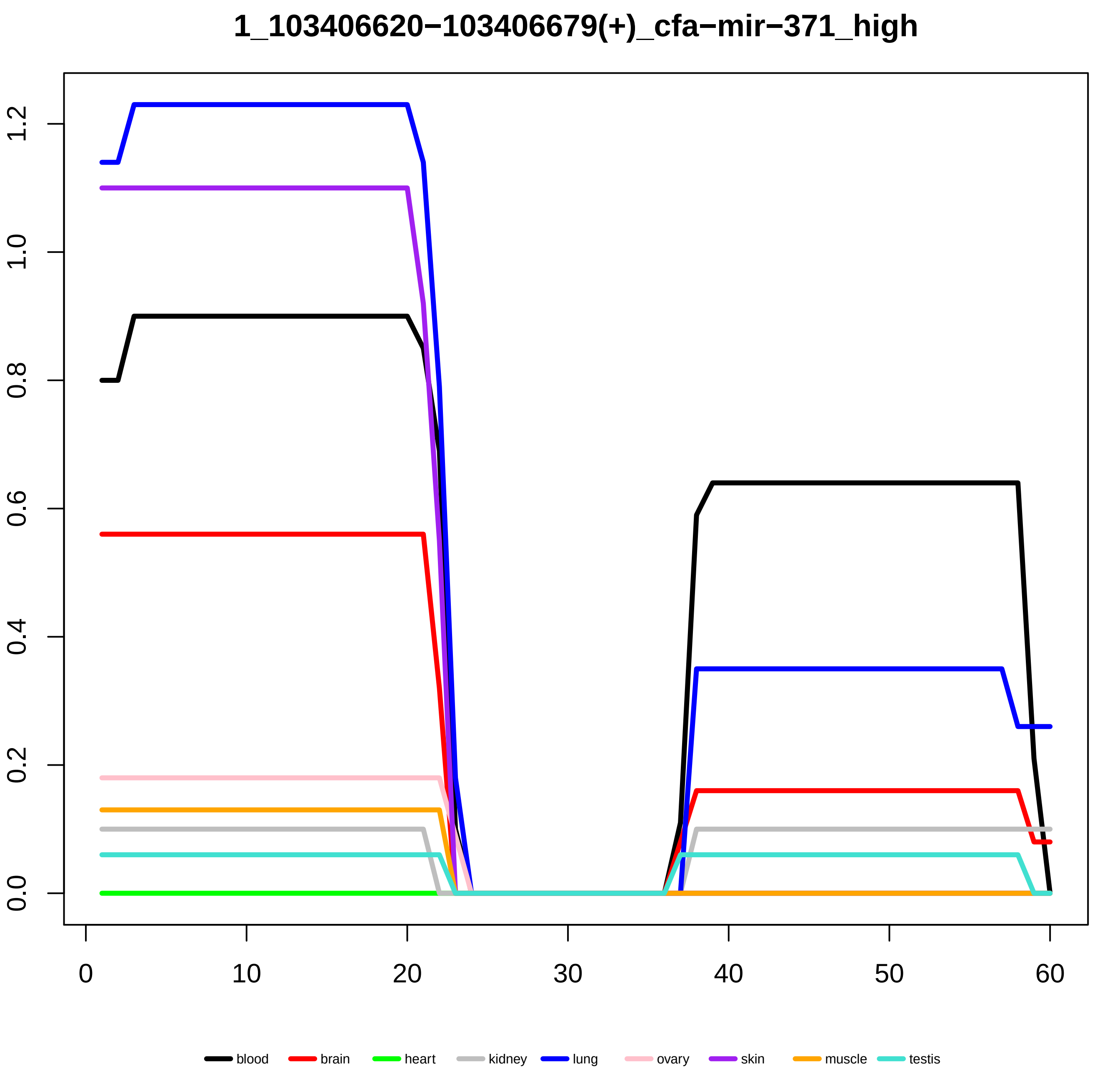
1_103406620−103406679(+)_cfa−mir−371_high
0.0
0.2
0.4
0.6
0.8
1.0
1.2
0
10
20
30
40
50
60
blood
brain
heart
kidney
lung
ovary
skin
muscle
testis

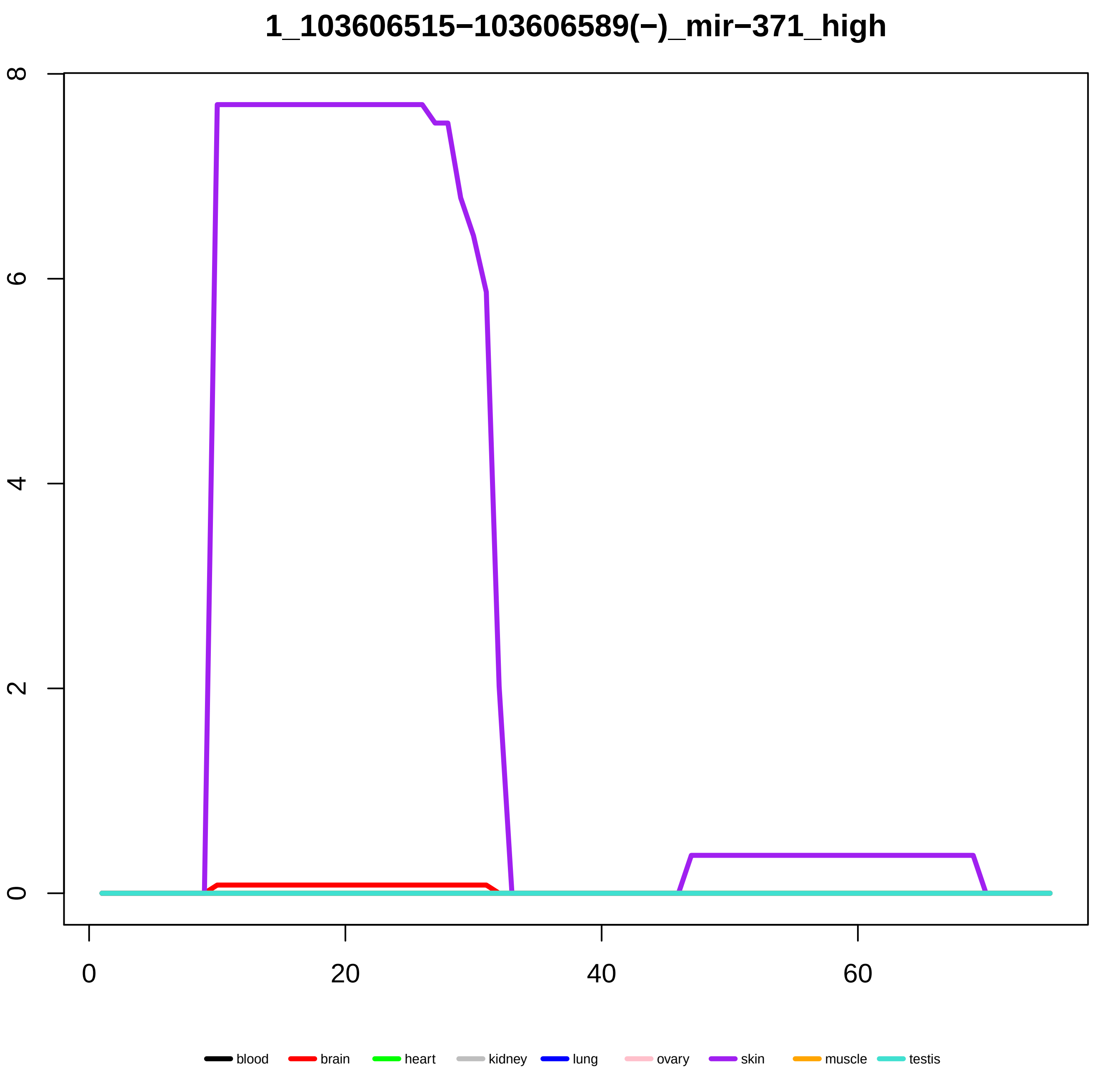
1_103606515−103606589(−)_mir−371_high
0
2
4
6
8
0
20
40
60
blood
brain
heart
kidney
lung
ovary
skin
muscle
testis

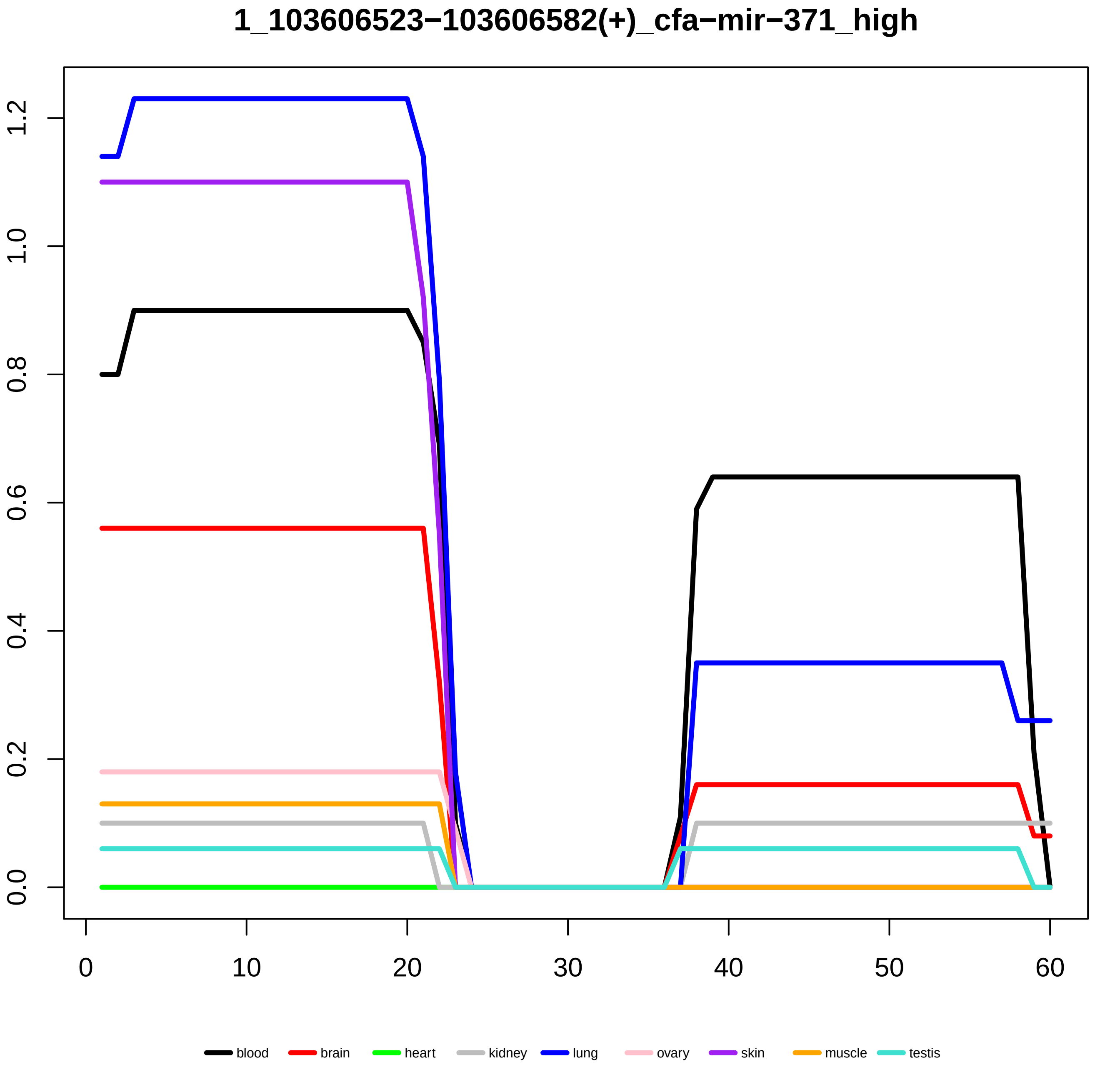

1_103606523−103606582(+)_cfa−mir−371_high
0.0
0.2
0.4
0.6
0.8
1.0
1.2
0
10
20
30
40
50
60
blood
brain
heart
kidney
lung
ovary
skin
muscle
testis

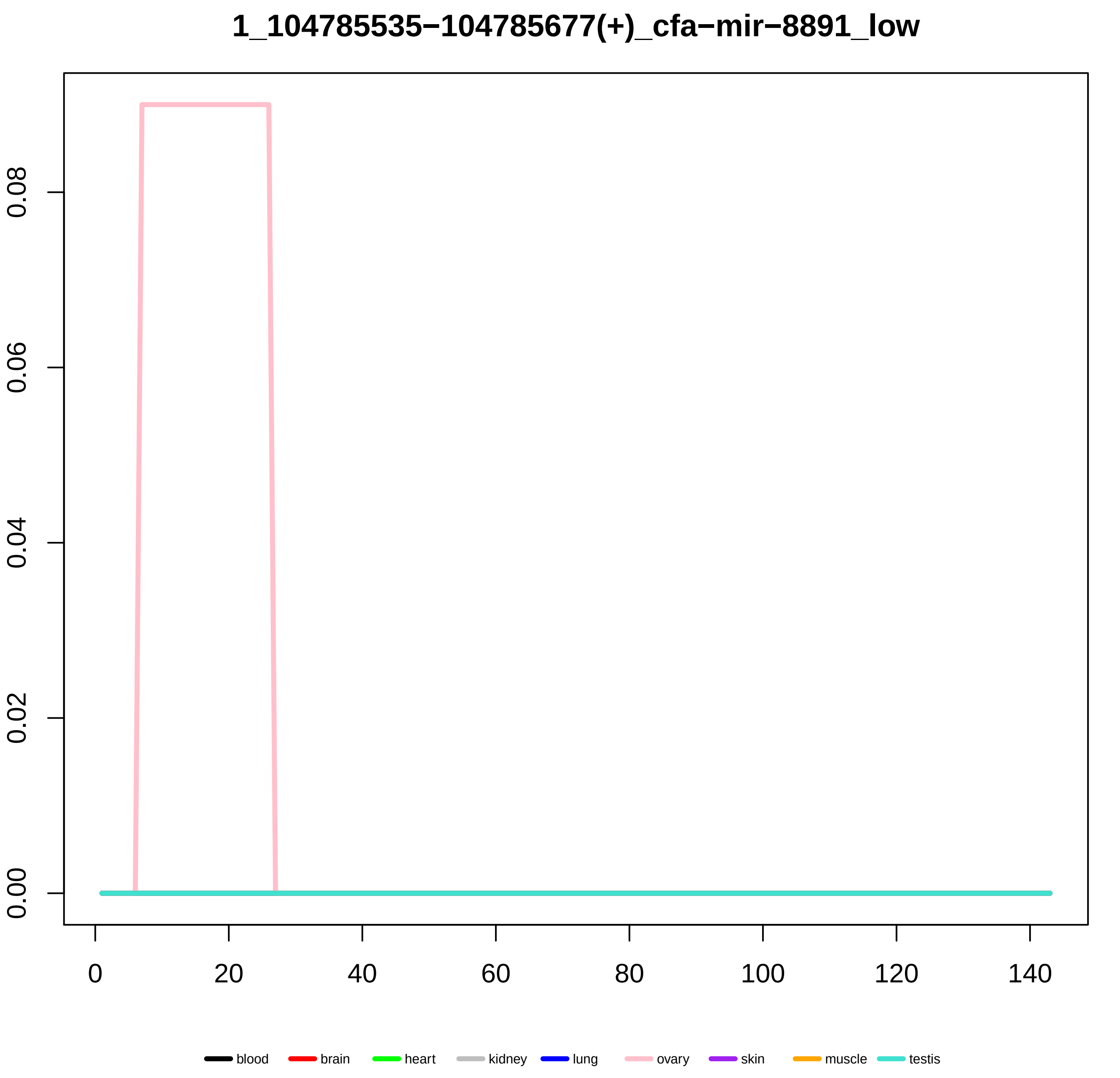

1_104785535−104785677(+)_cfa−mir−8891_low
0.00
0.02
0.04
0.06
0.08
0
20
40
60
80
100
120
140
blood
brain
heart
kidney
lung
ovary
skin
muscle
testis

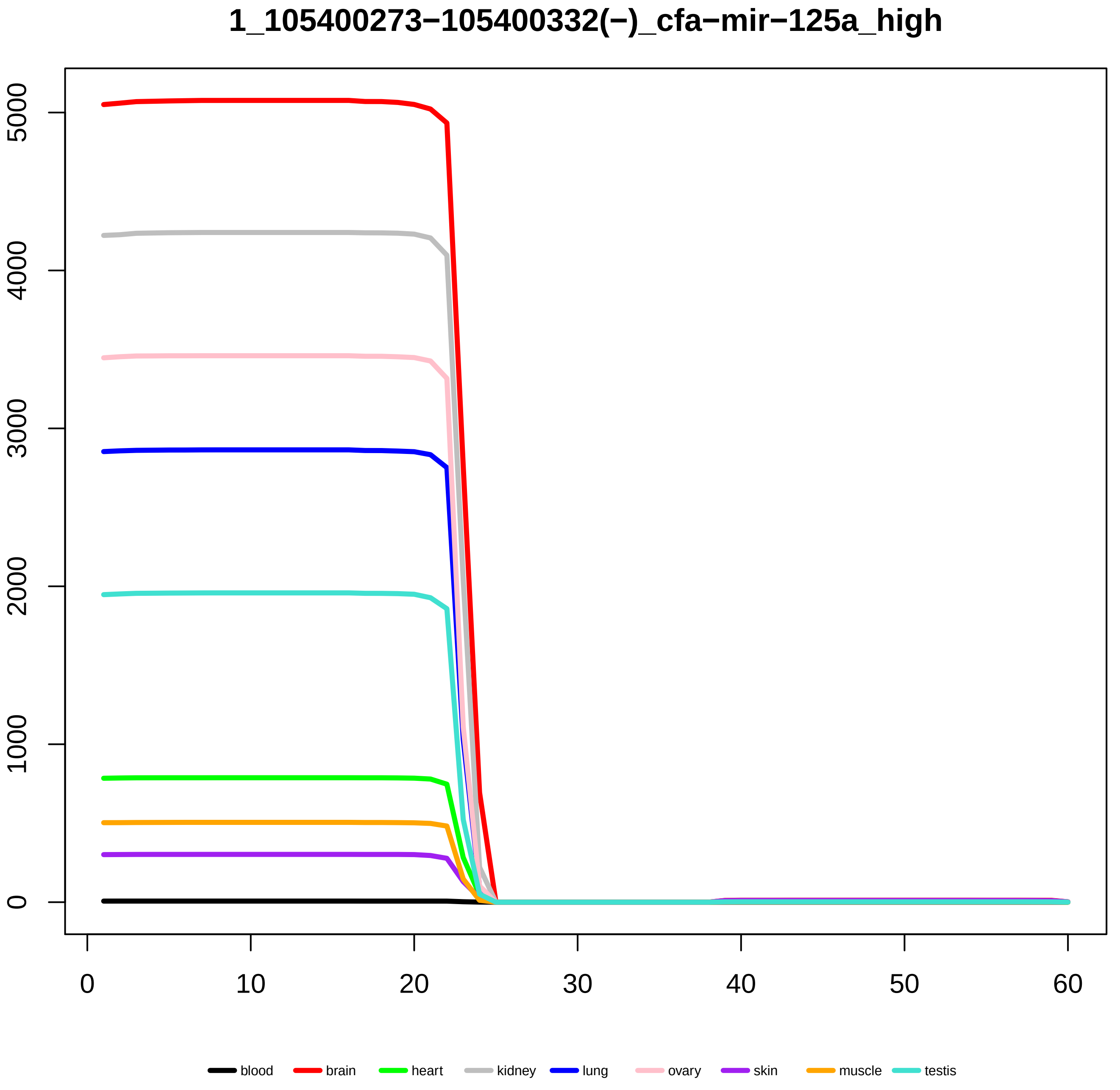

1_105400273−105400332(−)_cfa−mir−125a_high
0
1000
2000
3000
4000
5000
0
10
20
30
40
50
60
blood
brain
heart
kidney
lung
ovary
skin
muscle
testis

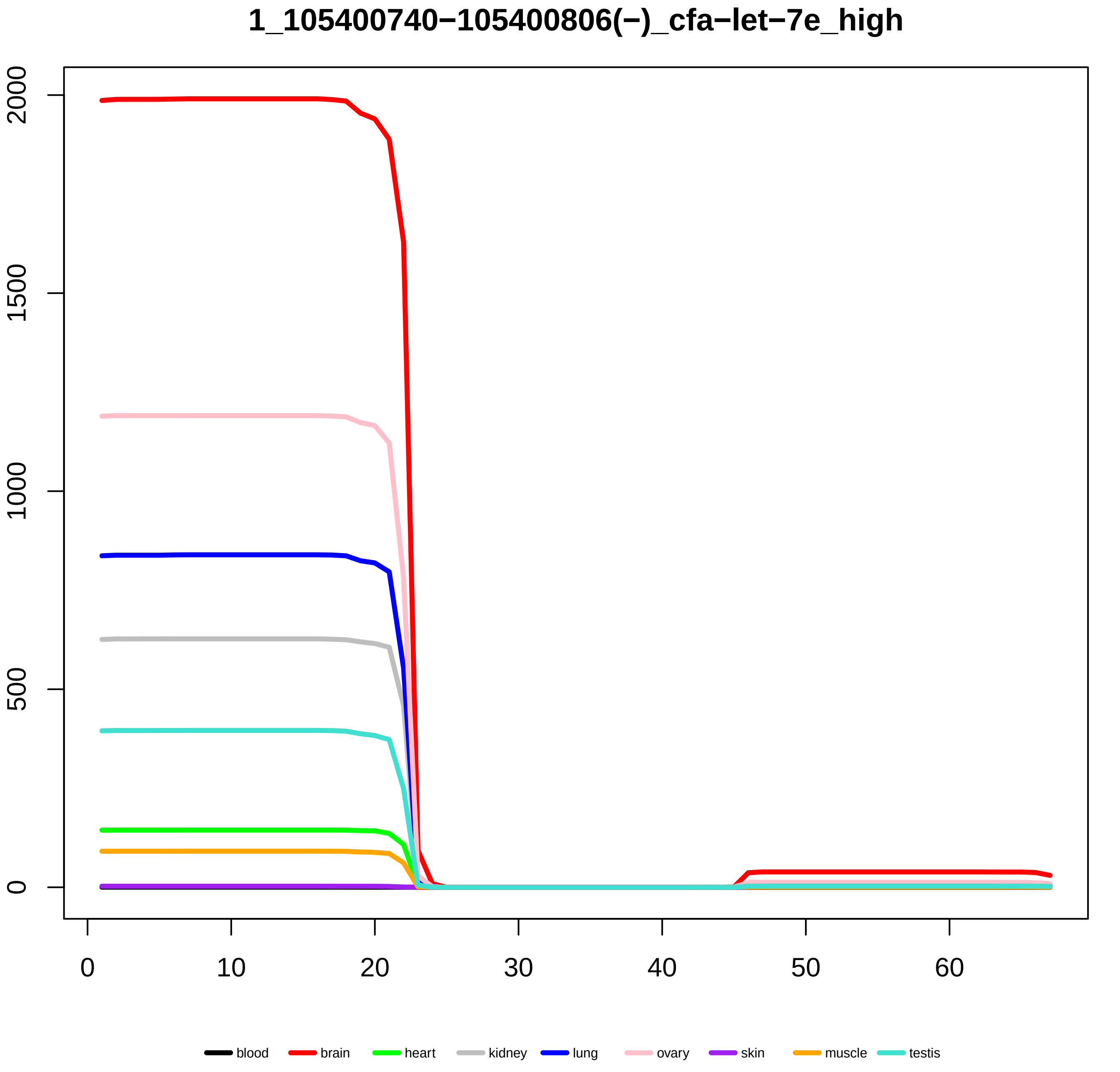

1_105400740−105400806(−)_cfa−let−7e_high
0
500
1000
1500
2000
0
10
20
30
40
50
60
blood
brain
heart
kidney
lung
ovary
skin
muscle
testis

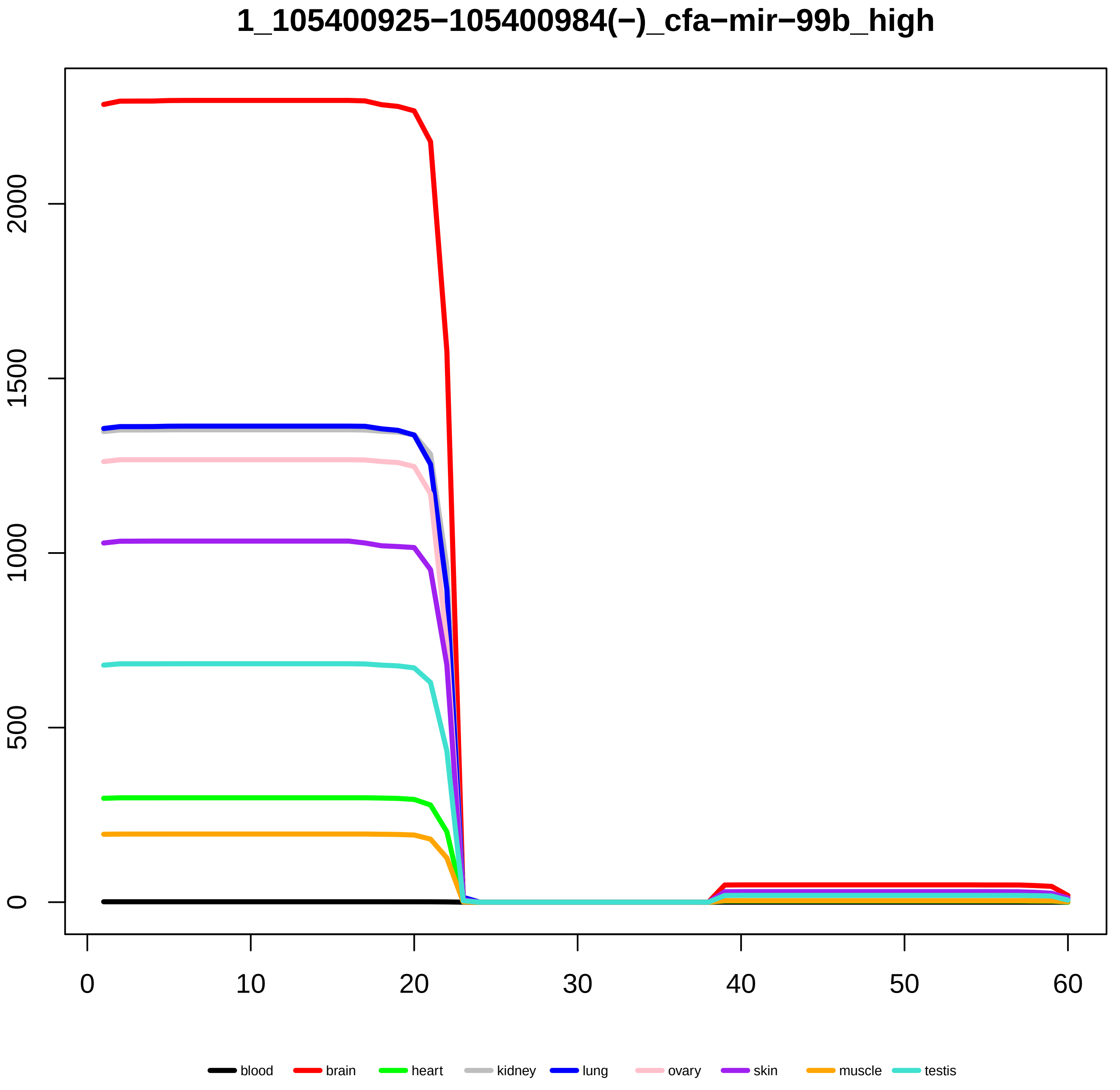

1_105400925−105400984(−)_cfa−mir−99b_high
0
500
1000
1500
2000
0
10
20
30
40
50
60
blood
brain
heart
kidney
lung
ovary
skin
muscle
testis

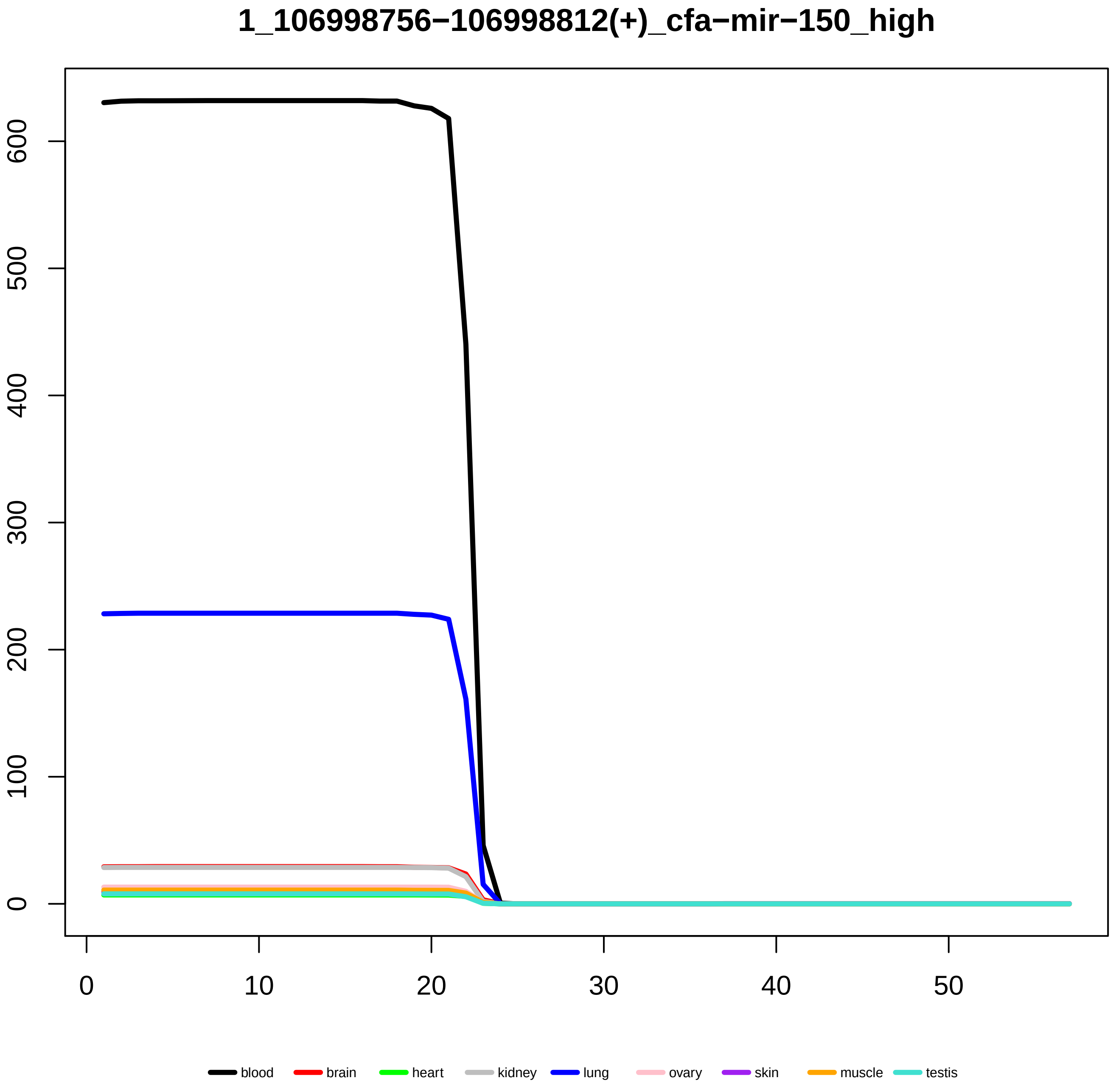
1_106998756−106998812(+)_cfa−mir−150_high
0
100
200
300
400
500
600
0
10
20
30
40
50
blood
brain
heart
kidney
lung
ovary
skin
muscle
testis

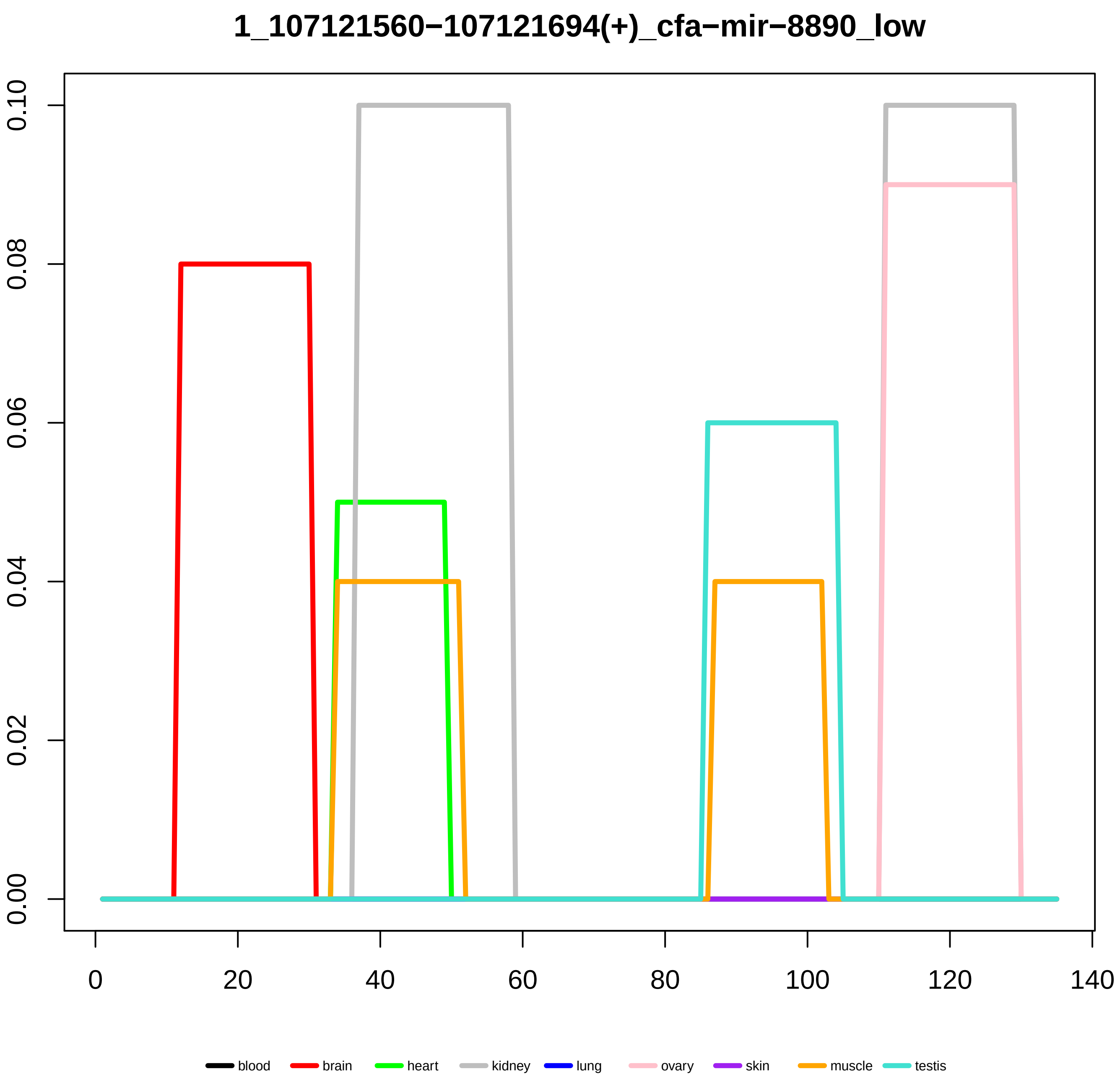

1_107121560−107121694(+)_cfa−mir−8890_low
0.00
0.02
0.04
0.06
0.08
0.10
0
20
40
60
80
100
120
140
blood
brain
heart
kidney
lung
ovary
skin
muscle
testis

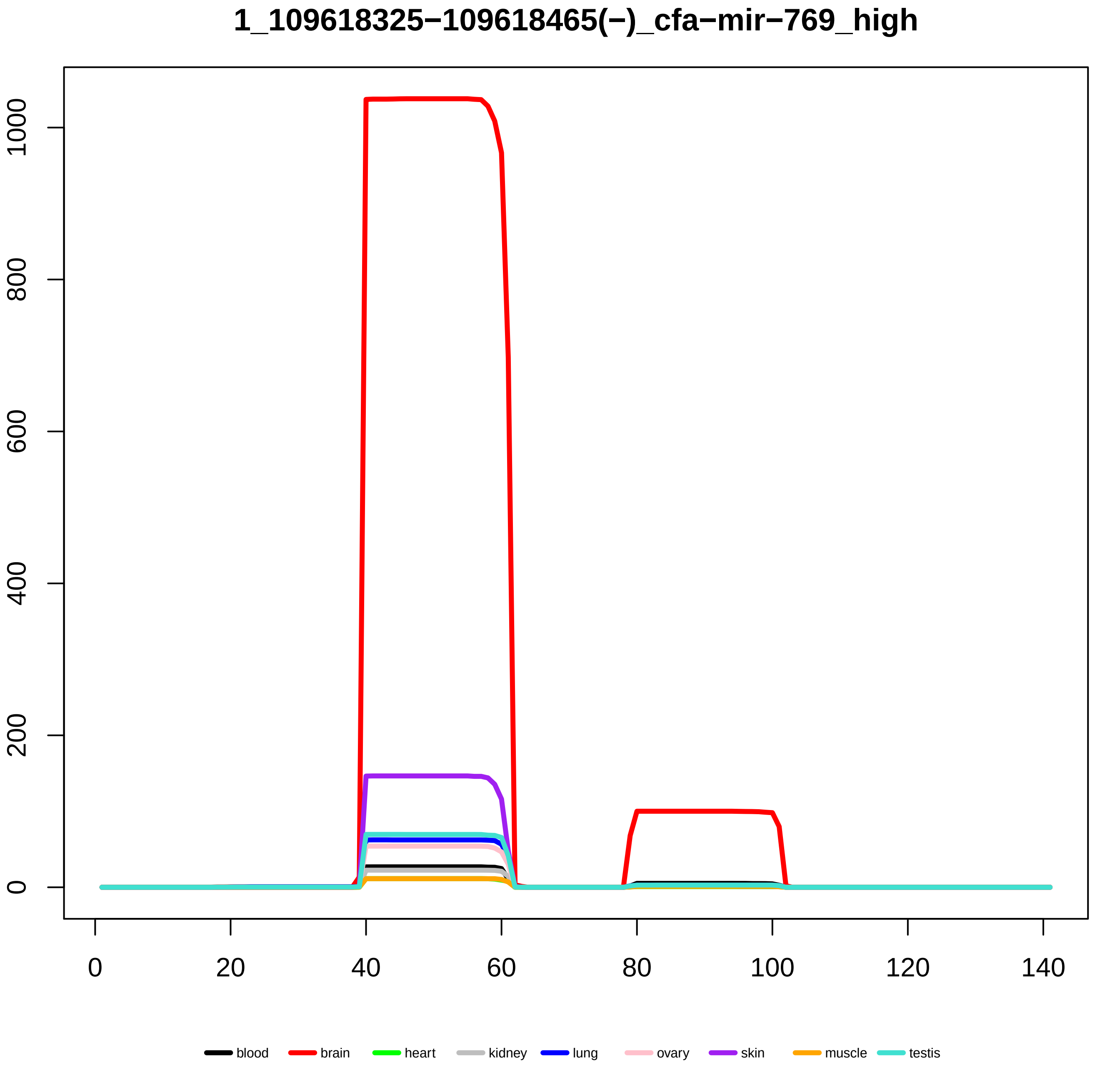
1_109618325−109618465(−)_cfa−mir−769_high
0
200
400
600
800
1000
0
20
40
60
80
100
120
140
blood
brain
heart
kidney
lung
ovary
skin
muscle
testis

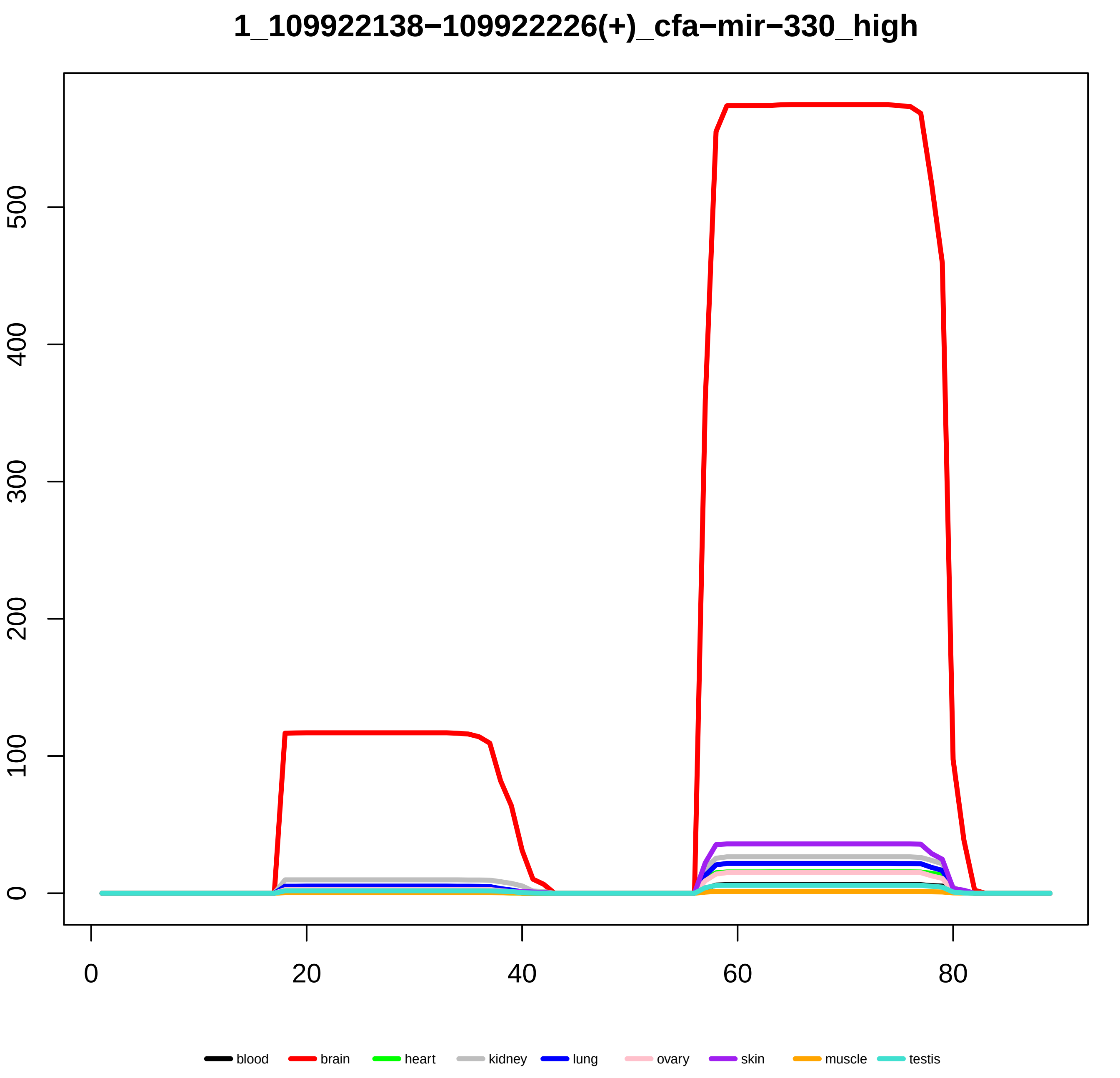

1_109922138−109922226(+)_cfa−mir−330_high
0
100
200
300
400
500
0
20
40
60
80
blood
brain
heart
kidney
lung
ovary
skin
muscle
testis

1_110690638−110690711(+)_mir−6516_low

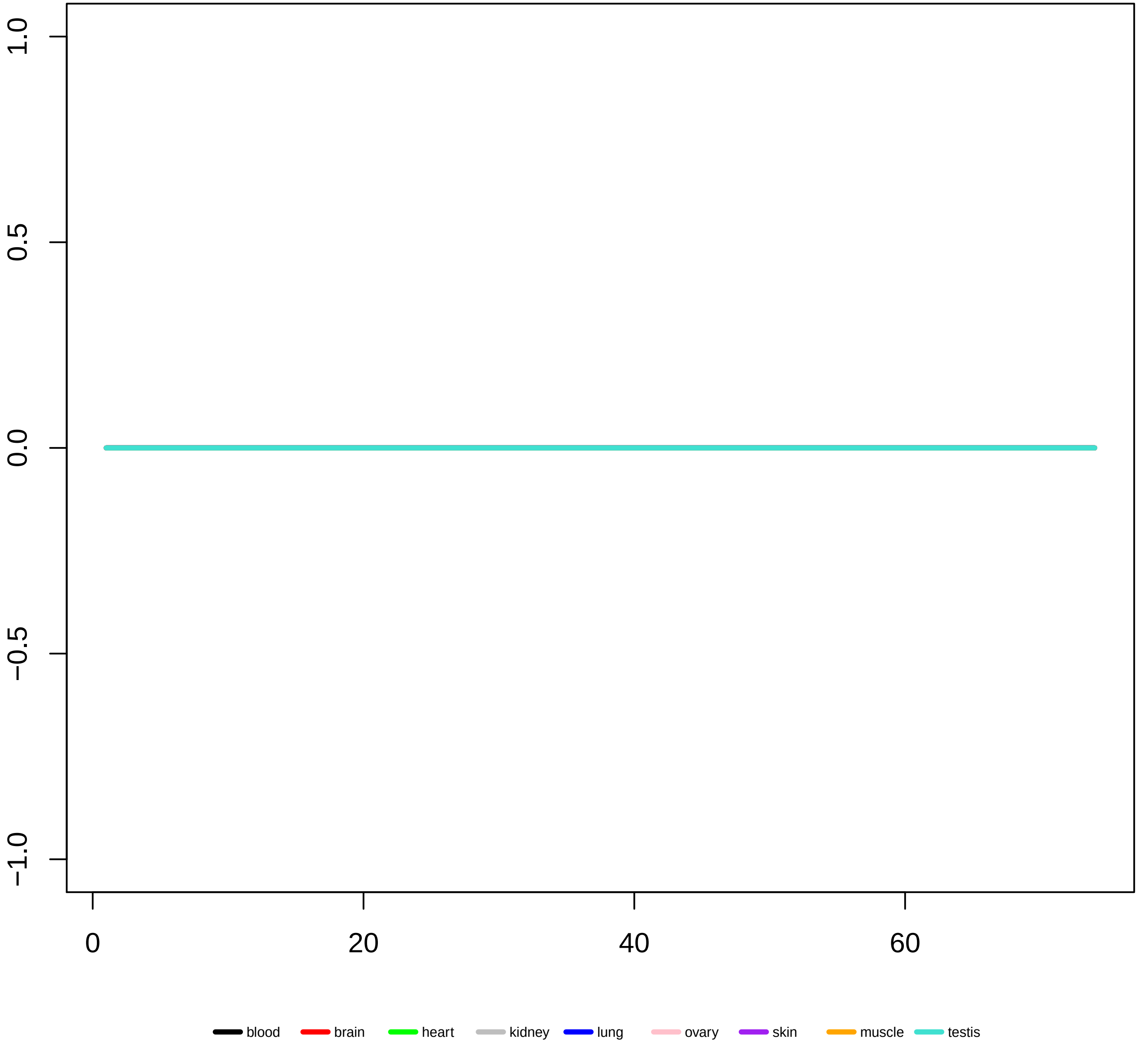

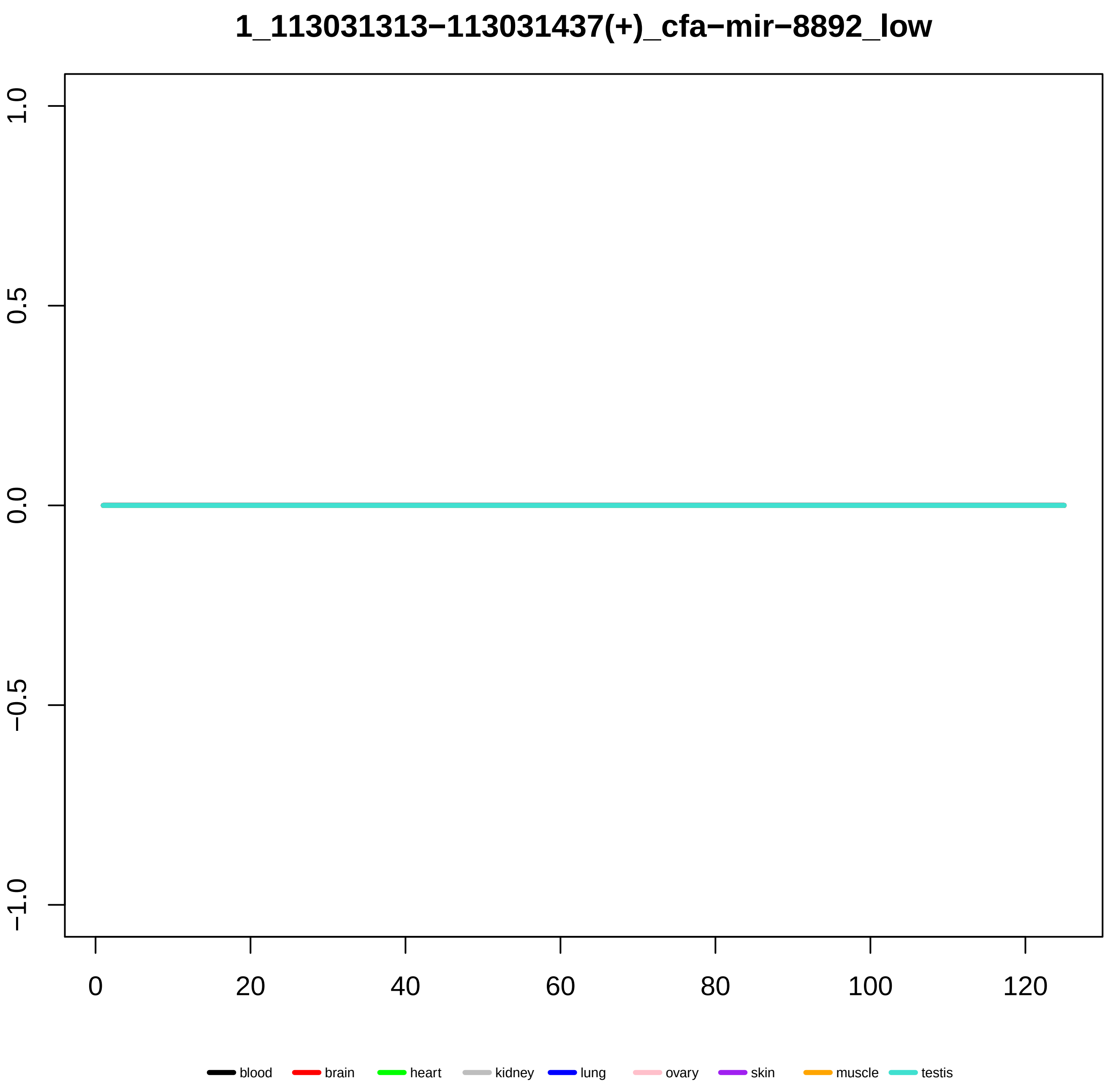

1_113031313−113031437(+)_cfa−mir−8892_low
1.0
0.5
0.0
−0.5
−1.0
0
20
40
60
80
100
120
blood
brain
heart
kidney
lung
ovary
skin
muscle
testis

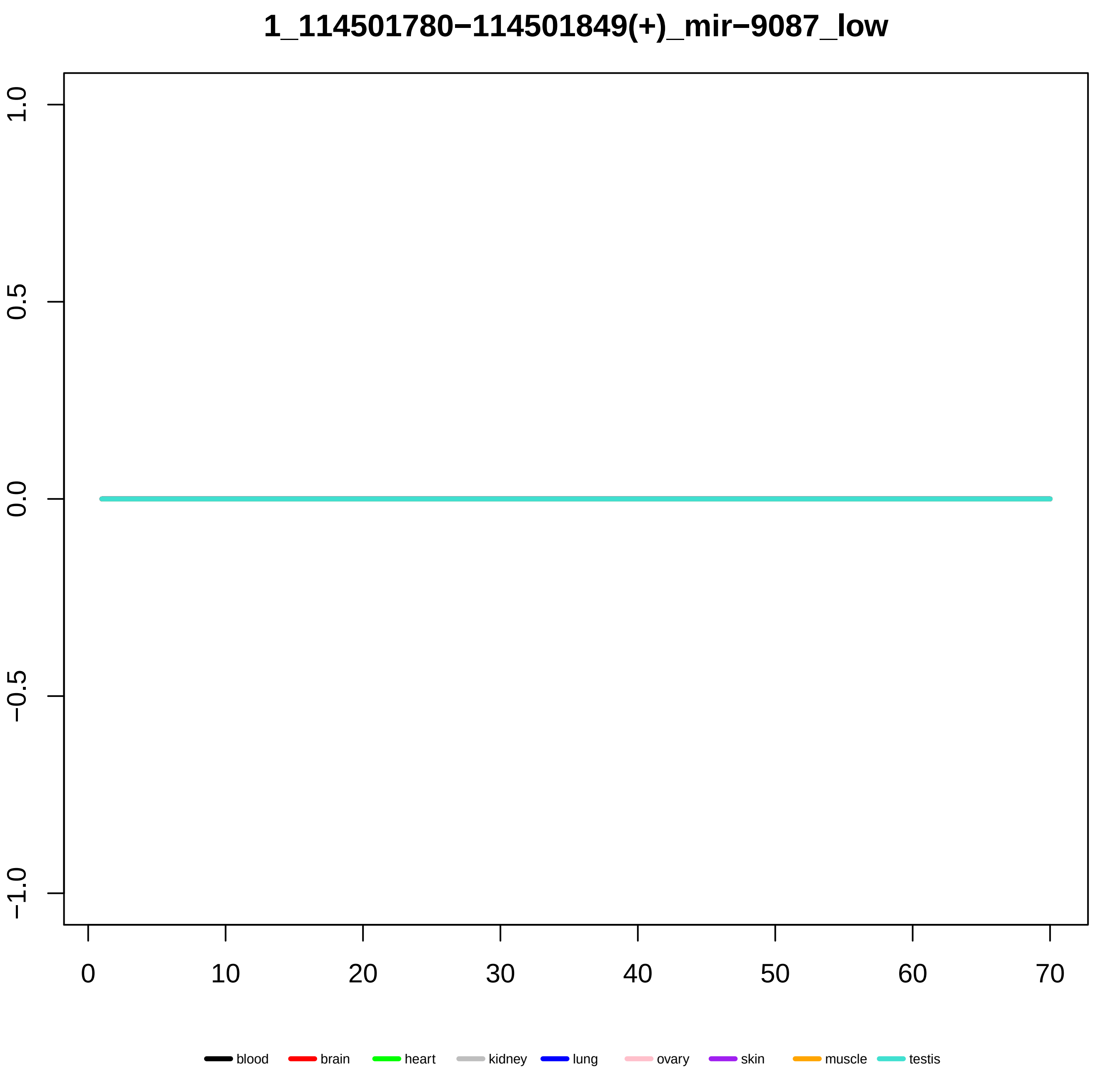

1_114501780−114501849(+)_mir−9087_low
1.0
0.5
0.0
−0.5
−1.0
0
10
20
30
40
50
60
70
blood
brain
heart
kidney
lung
ovary
skin
muscle
testis

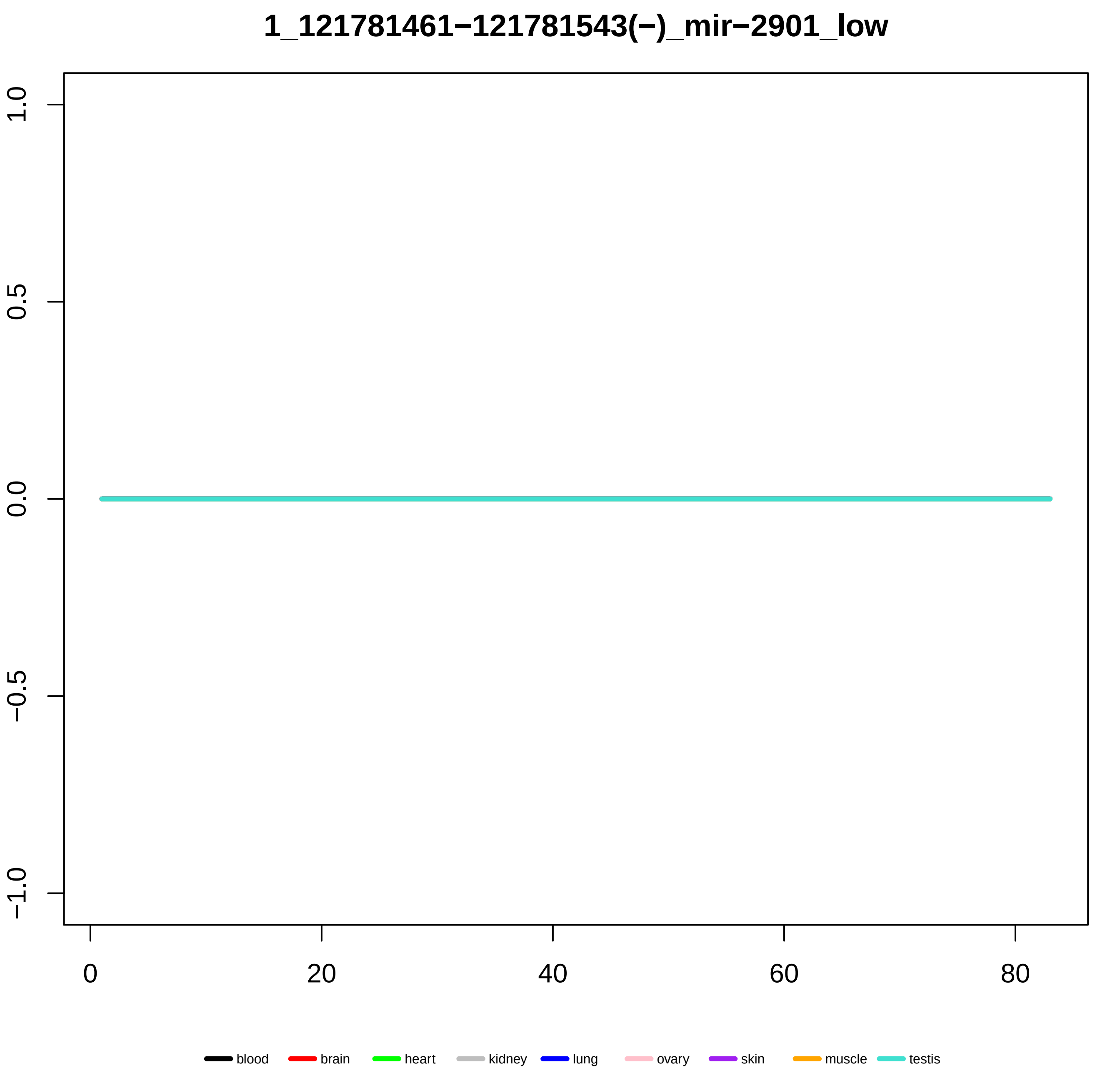
1_121781461−121781543(−)_mir−2901_low
1.0
0.5
0.0
−0.5
−1.0
0
20
40
60
80
blood
brain
heart
kidney
lung
ovary
skin
muscle
testis

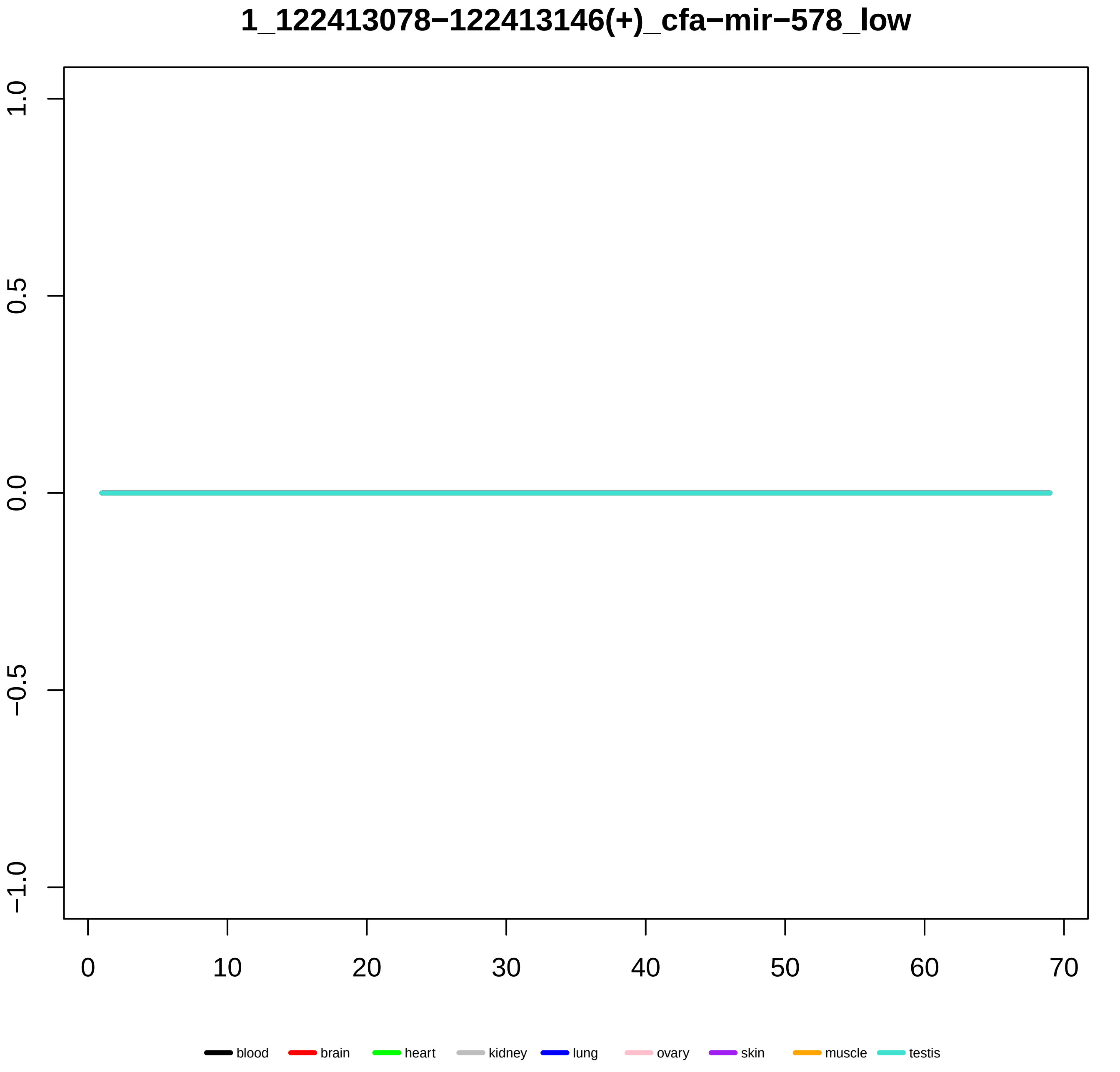

1_122413078−122413146(+)_cfa−mir−578_low
1.0
0.5
0.0
−0.5
−1.0
0
10
20
30
40
50
60
70
blood
brain
heart
kidney
lung
ovary
skin
muscle
testis

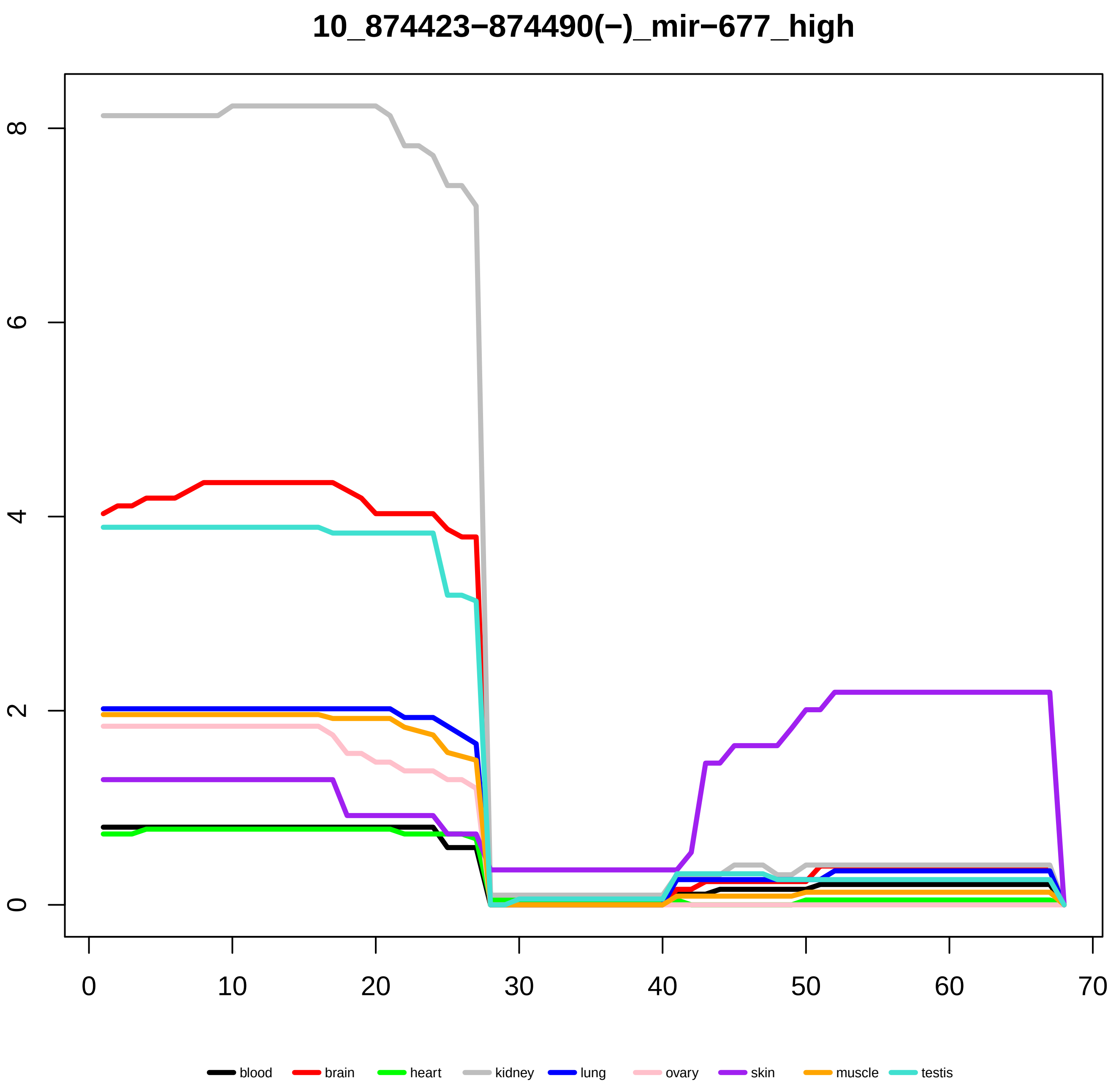

10_874423−874490(−)_mir−677_high
0
2
4
6
8
0
10
20
30
40
50
60
70
blood
brain
heart
kidney
lung
ovary
skin
muscle
testis

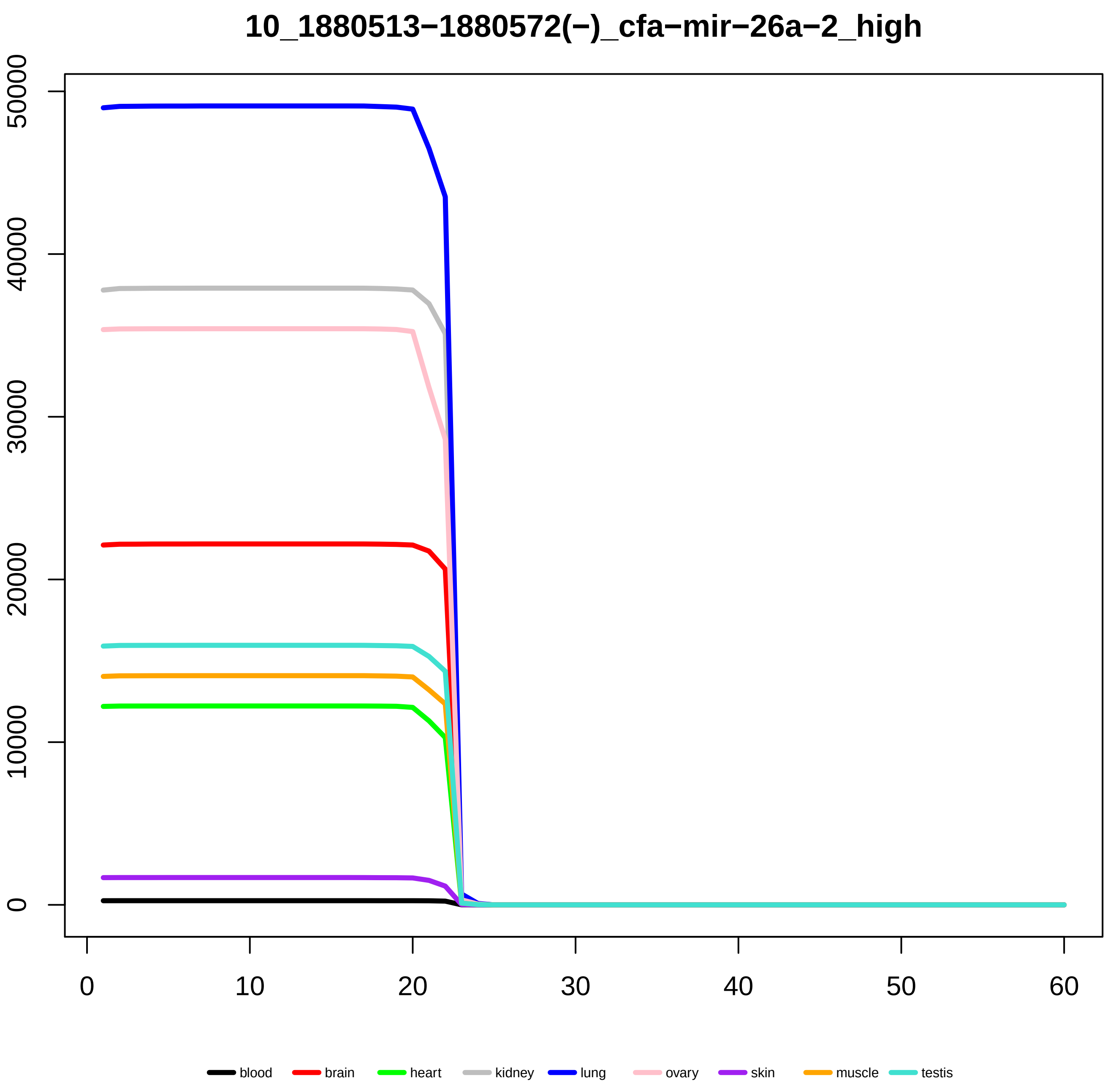

10_1880513−1880572(−)_cfa−mir−26a−2_high
0
10000
20000
30000
40000
50000
0
10
20
30
40
50
60
blood
brain
heart
kidney
lung
ovary
skin
muscle
testis

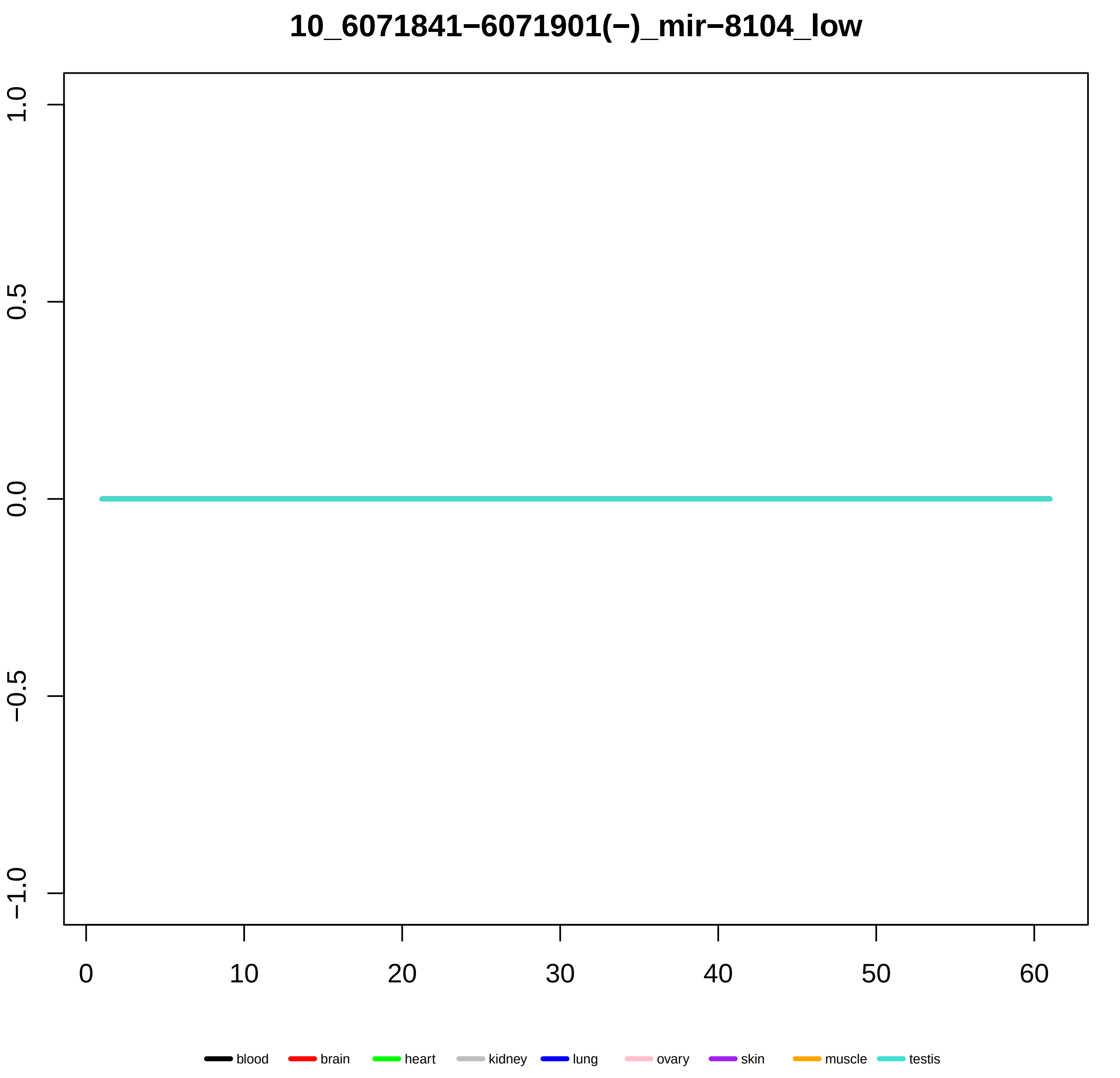
10_6071841−6071901(−)_mir−8104_low
1.0
0.5
0.0
−0.5
−1.0
0
10
20
30
40
50
60
blood
brain
heart
kidney
lung
ovary
skin
muscle
testis

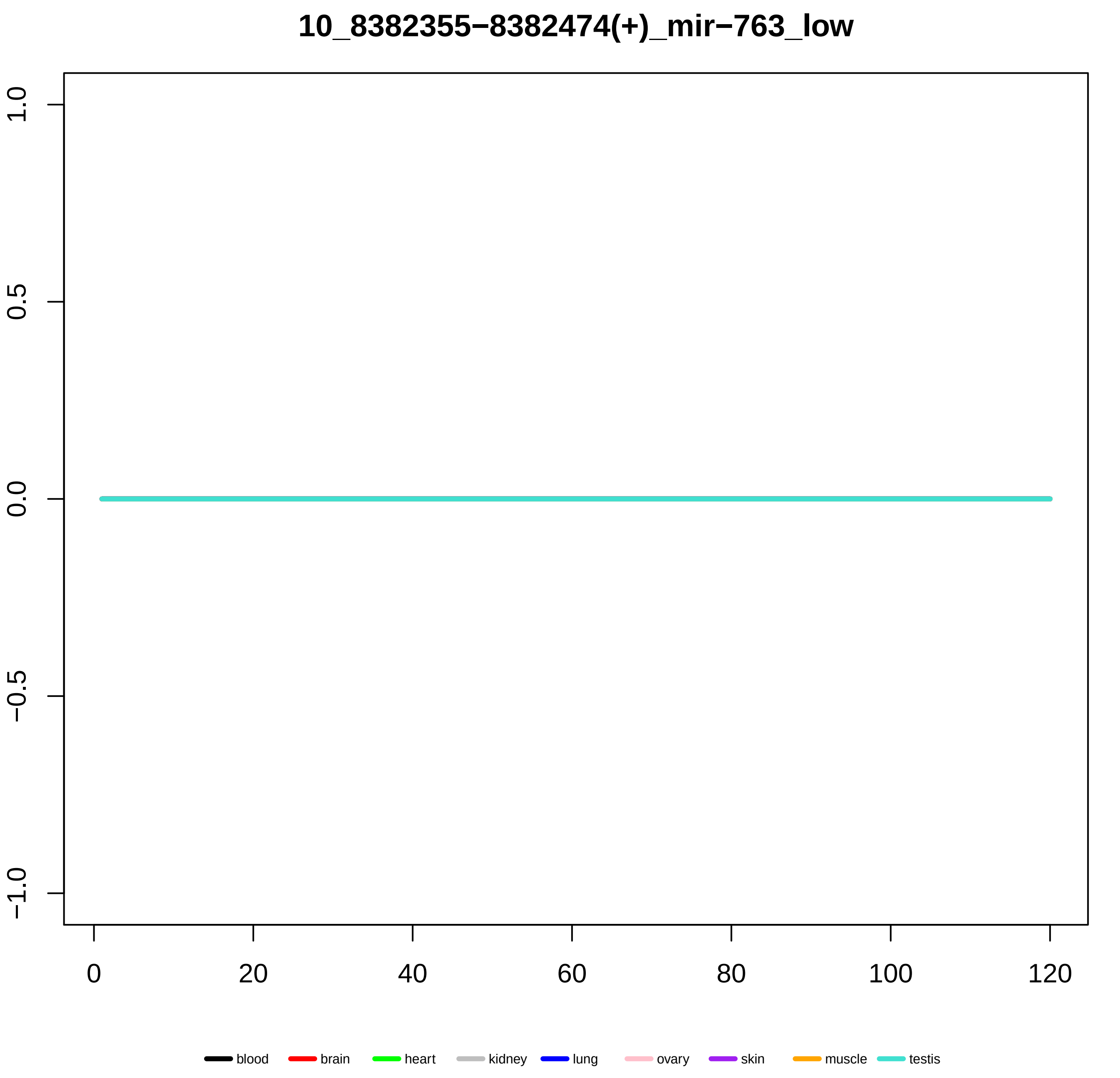

10_8382355−8382474(+)_mir−763_low
1.0
0.5
0.0
−0.5
−1.0
0
20
40
60
80
100
120
blood
brain
heart
kidney
lung
ovary
skin
muscle
testis

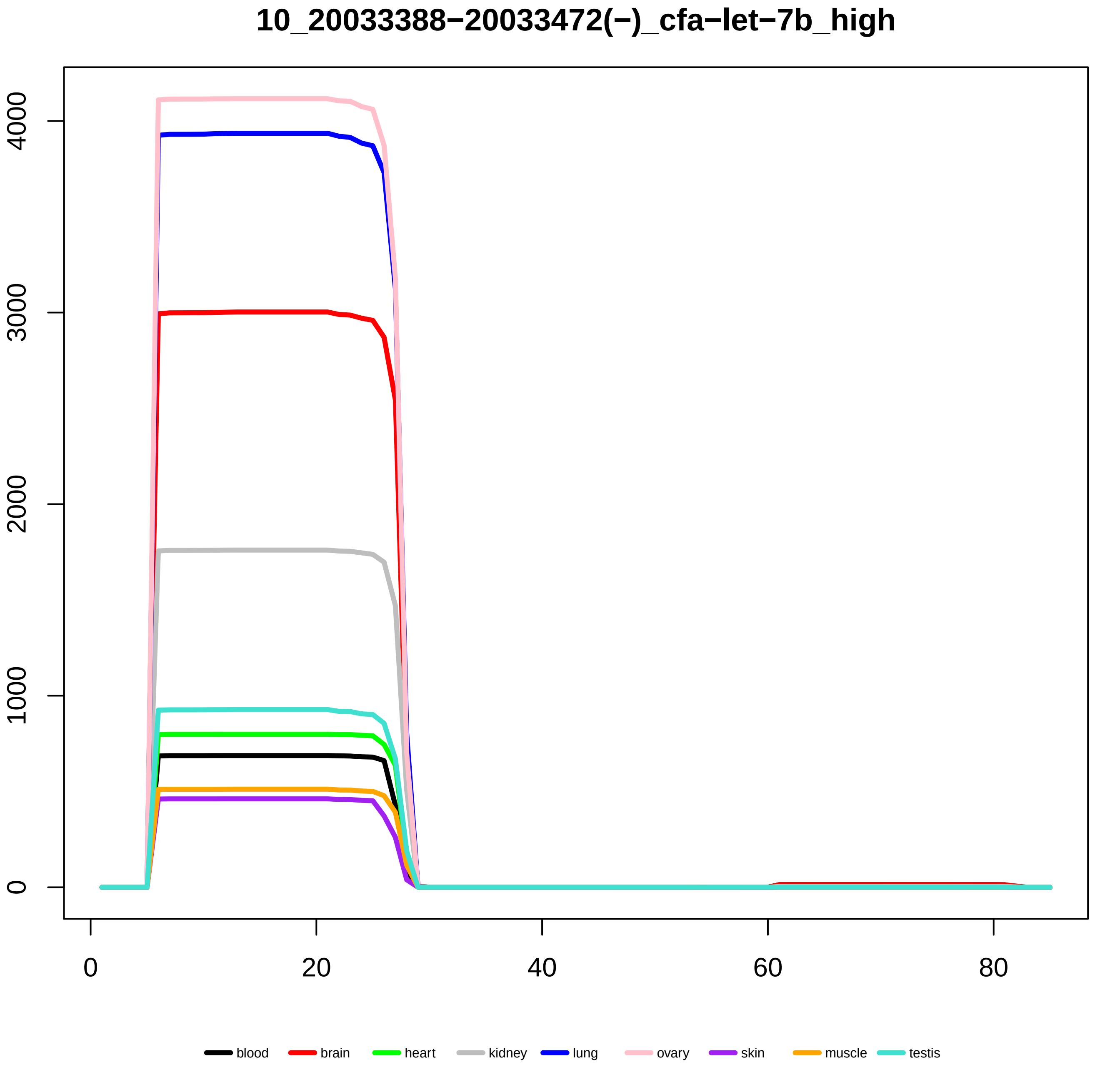

10_20033388−20033472(−)_cfa−let−7b_high
0
1000
2000
3000
4000
0
20
40
60
80
blood
brain
heart
kidney
lung
ovary
skin
muscle
testis

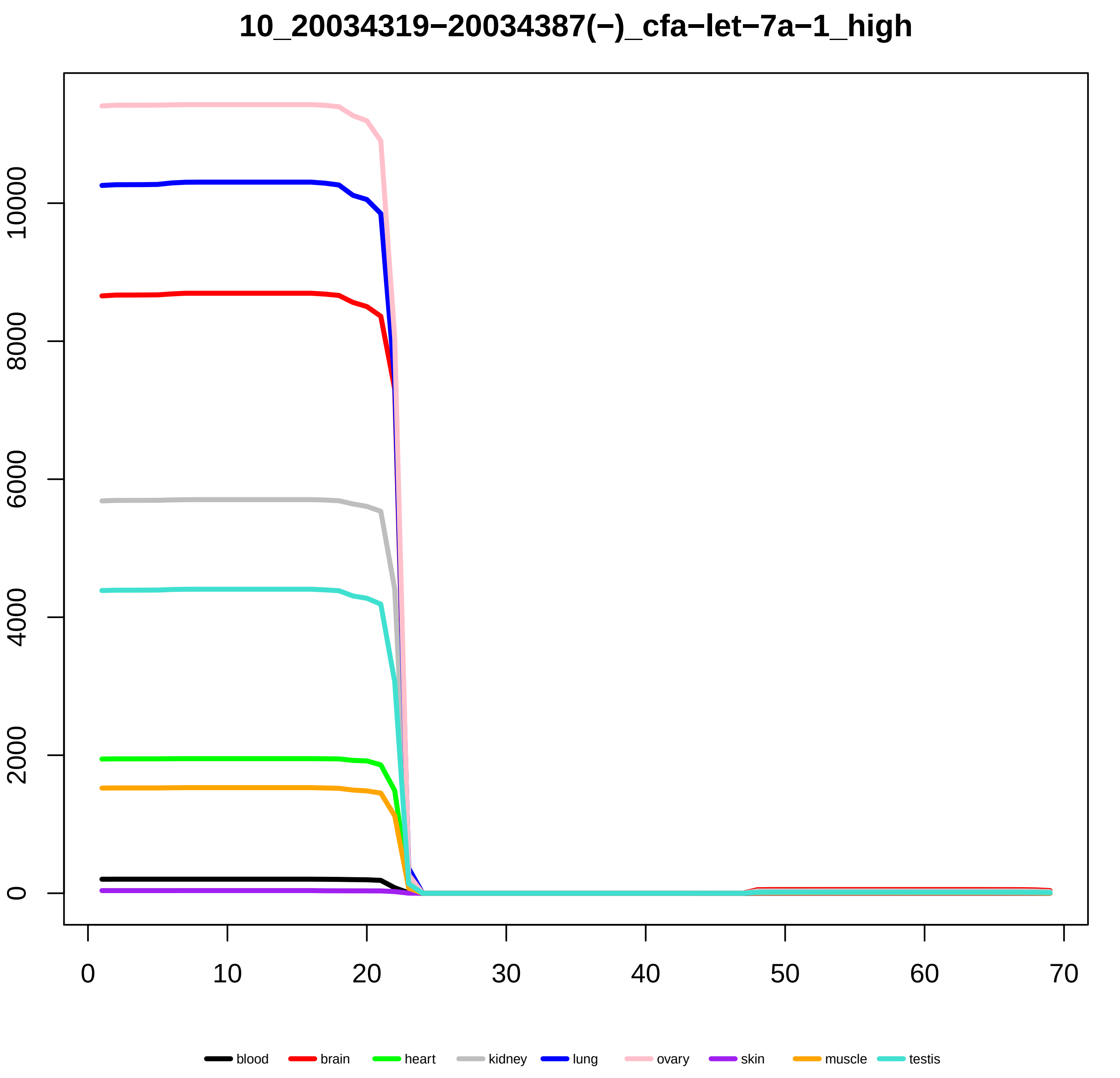
10_20034319−20034387(−)_cfa−let−7a−1_high
0
2000
4000
6000
8000
10000
0
10
20
30
40
50
60
70
blood
brain
heart
kidney
lung
ovary
skin
muscle
testis

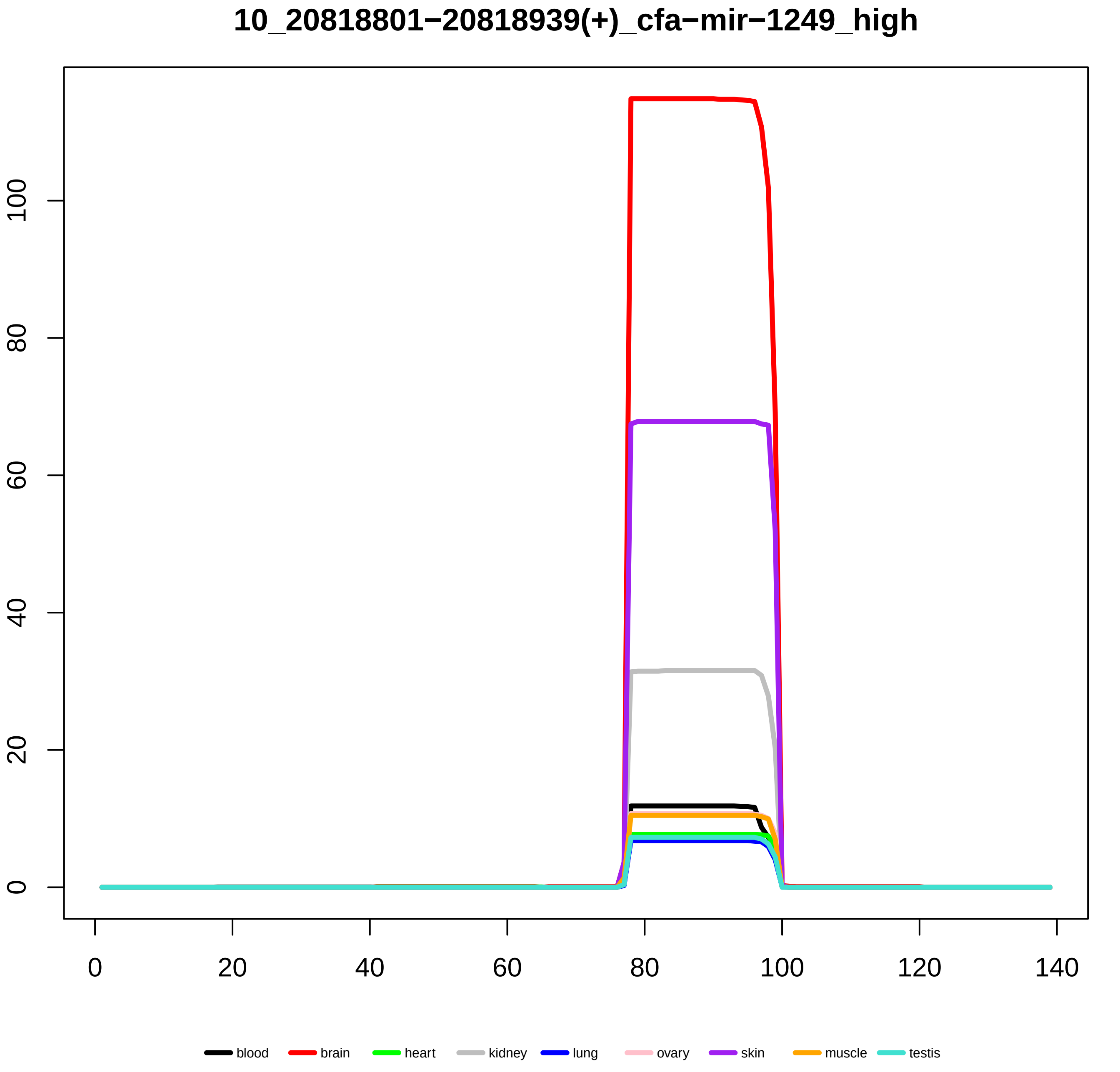

10_20818801−20818939(+)_cfa−mir−1249_high
0
20
40
60
80
100
120
140
blood
brain
heart
kidney
lung
ovary
skin
muscle
testis

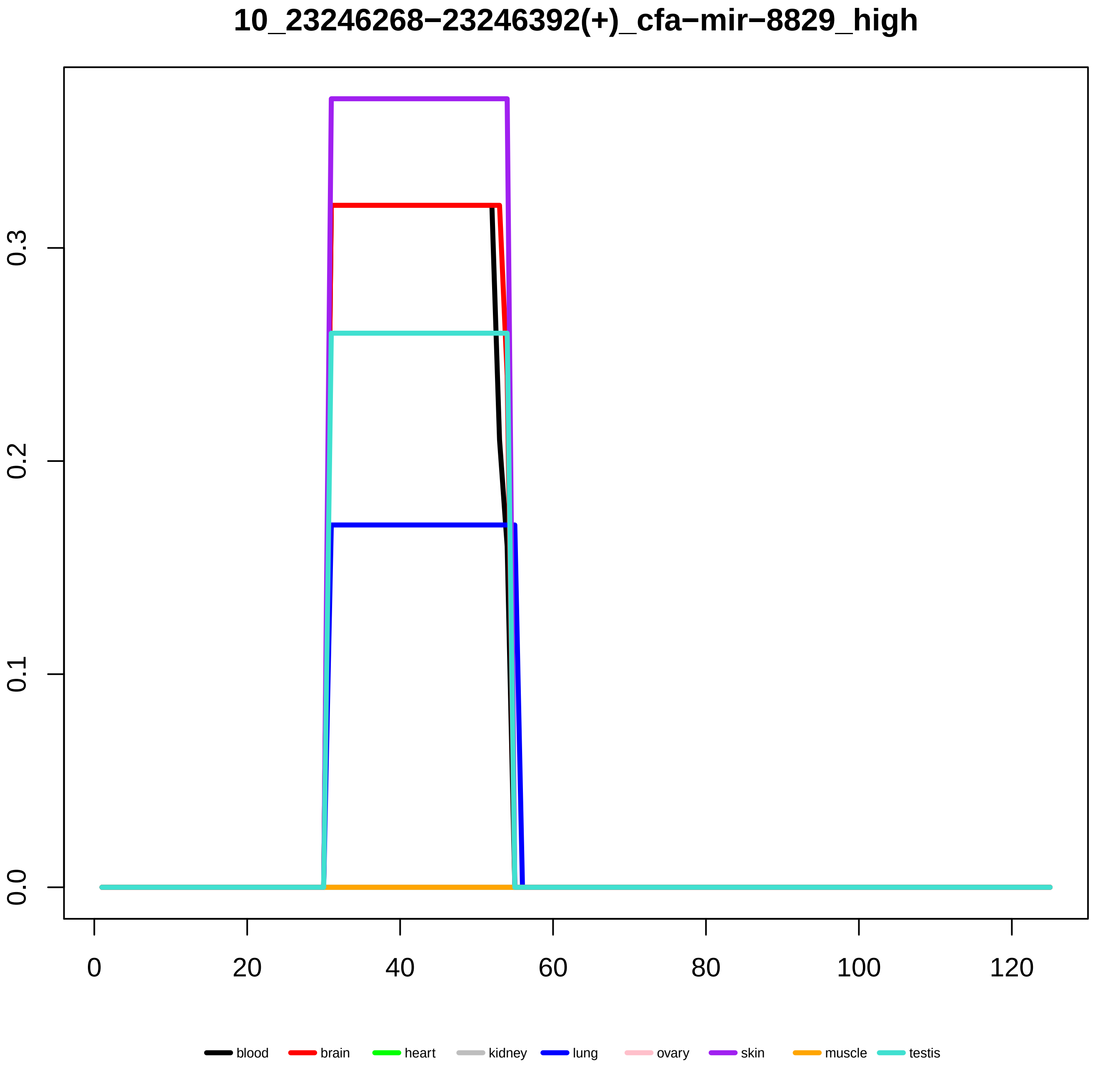

10_23246268−23246392(+)_cfa−mir−8829_high
0.0
0.1
0.2
0.3
0
20
40
60
80
100
120
blood
brain
heart
kidney
lung
ovary
skin
muscle
testis

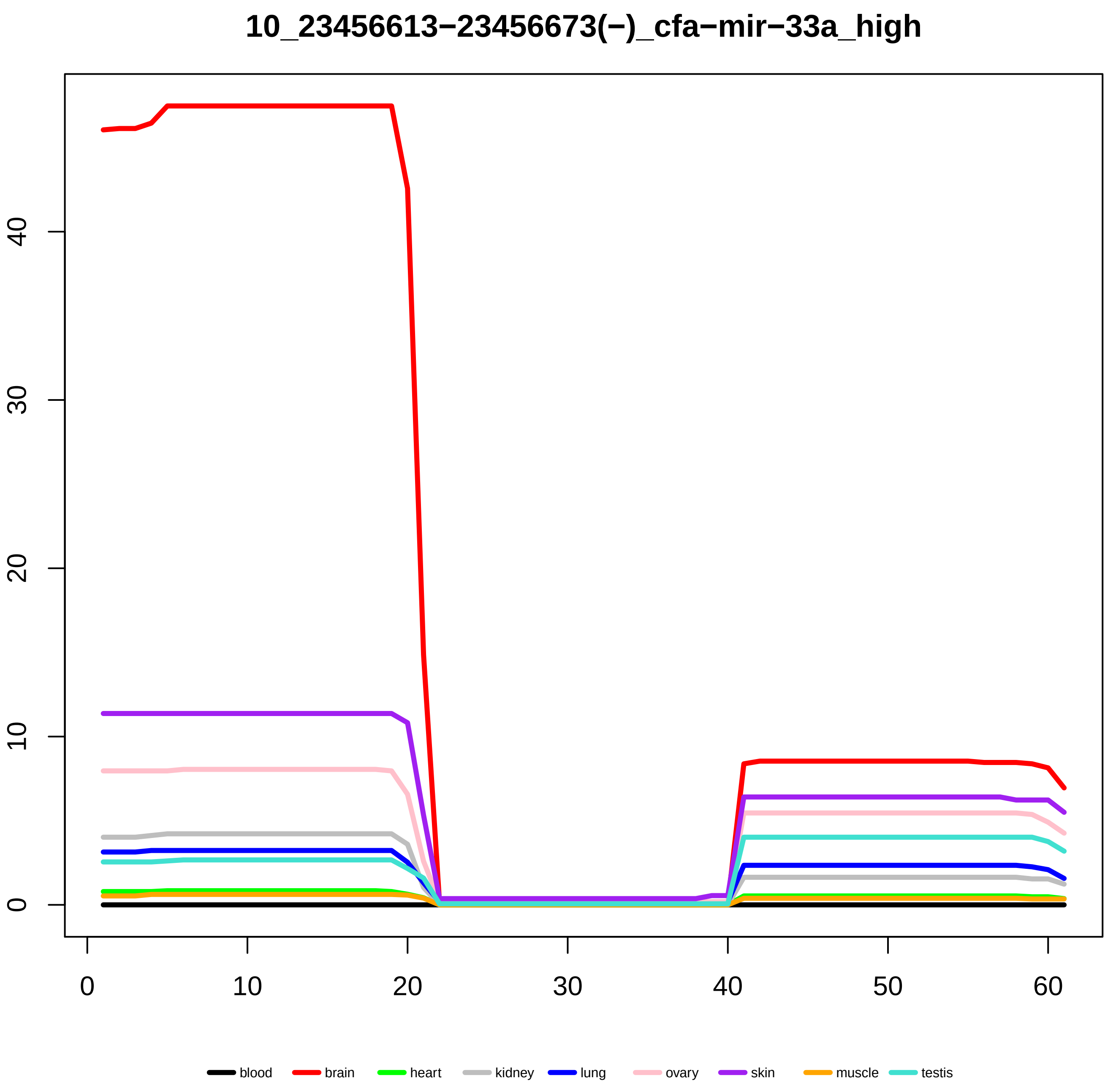

10_23456613−23456673(−)_cfa−mir−33a_high
0
10
20
30
40
50
60
blood
brain
heart
kidney
lung
ovary
skin
muscle
testis

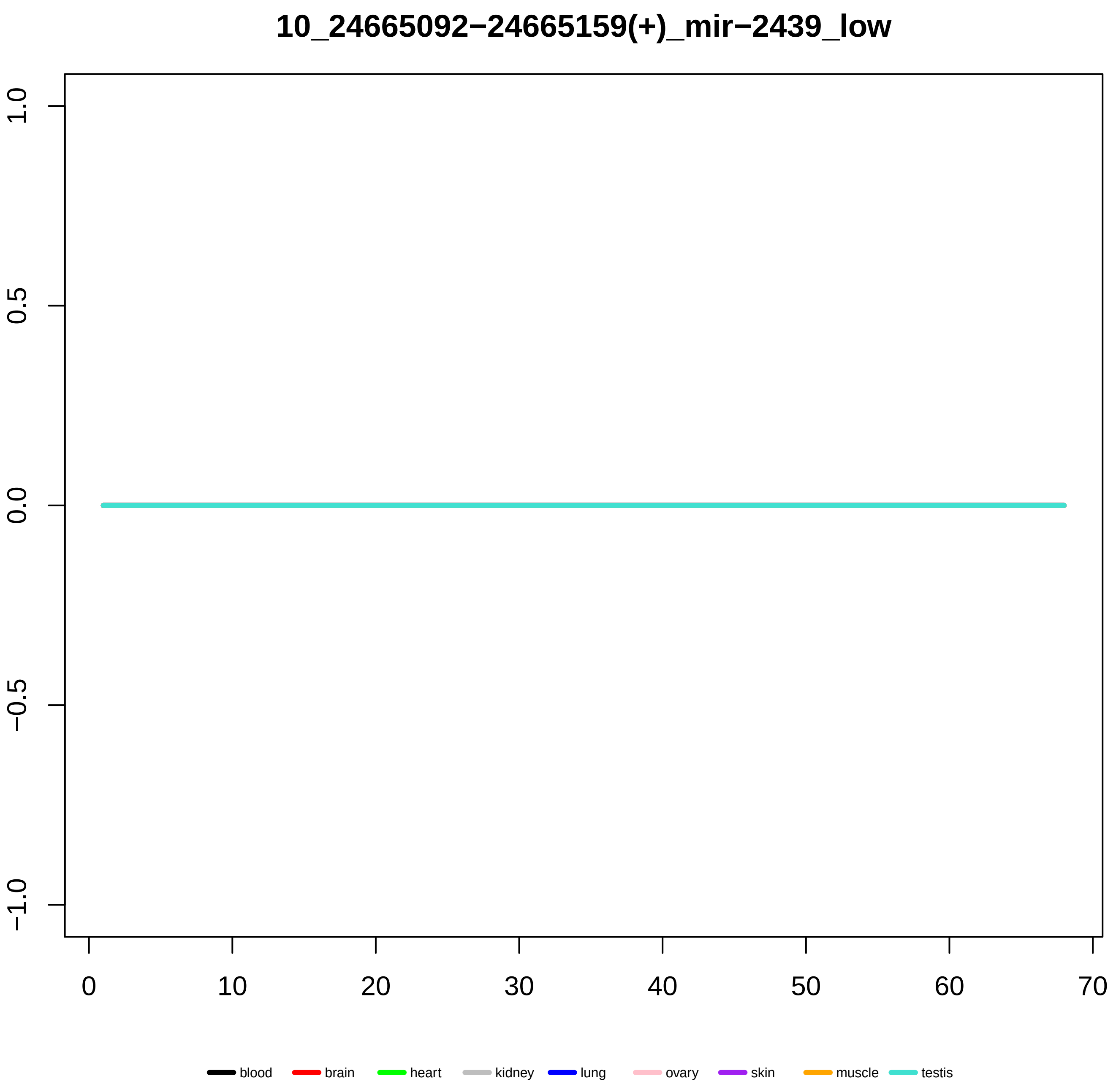

10_24665092−24665159(+)_mir−2439_low
1.0
0.5
0.0
−0.5
−1.0
0
10
20
30
40
50
60
70
blood
brain
heart
kidney
lung
ovary
skin
muscle
testis

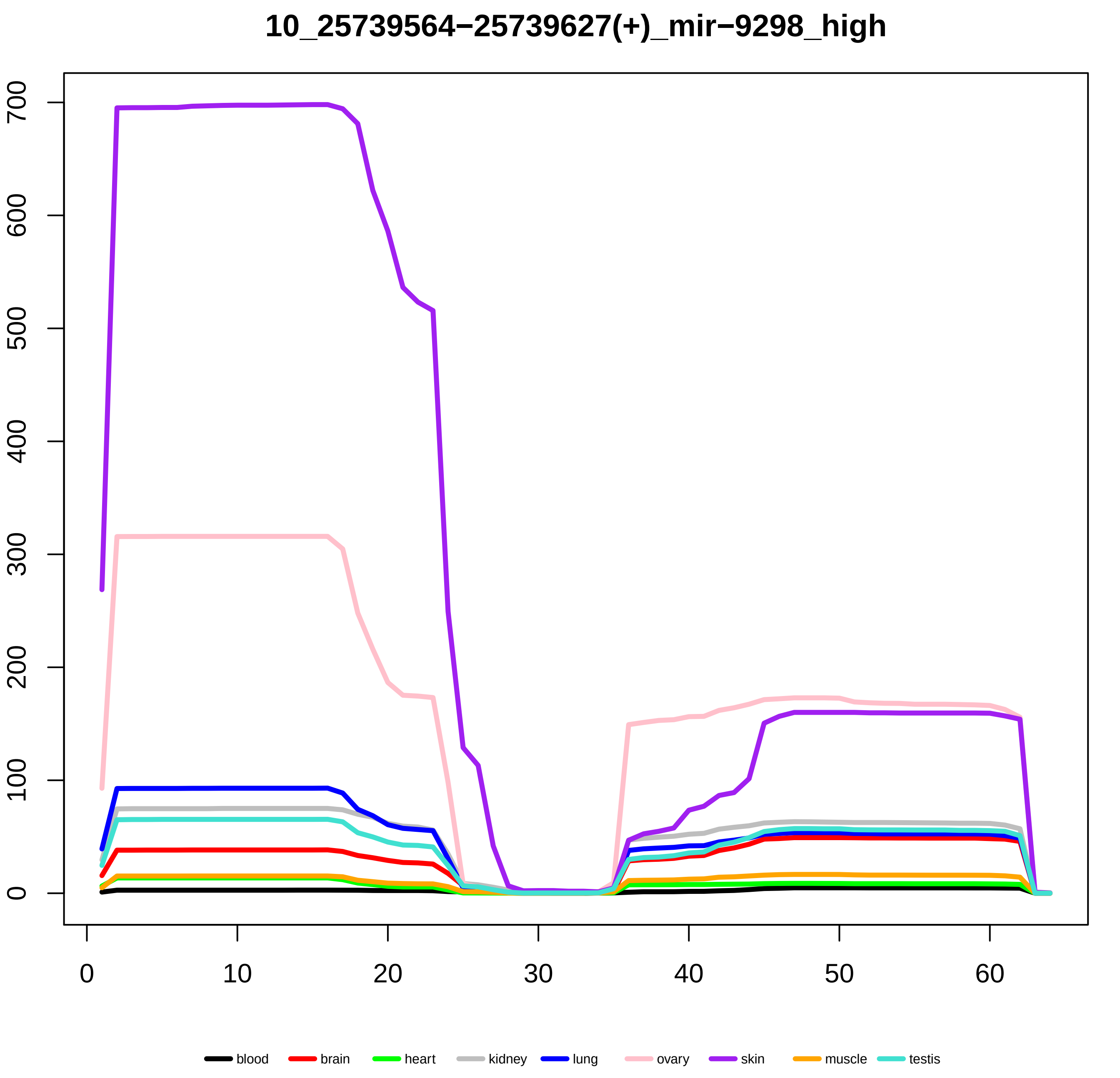

10_25739564−25739627(+)_mir−9298_high
0
100
200
300
400
500
600
700
0
10
20
30
40
50
60
blood
brain
heart
kidney
lung
ovary
skin
muscle
testis

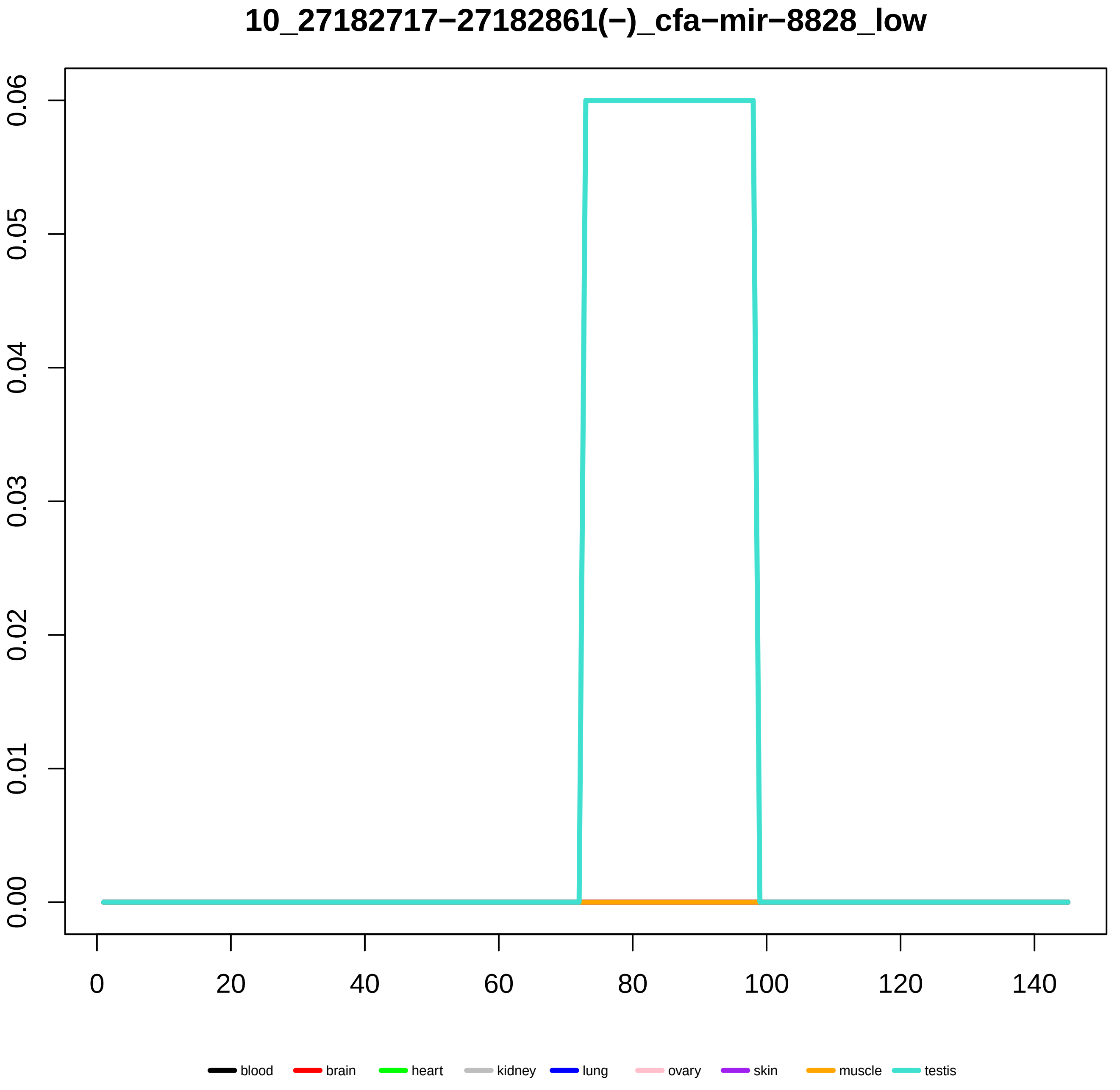
10_27182717−27182861(−)_cfa−mir−8828_low
0.00
0.01
0.02
0.03
0.04
0.05
0.06
0
20
40
60
80
100
120
140
blood
brain
heart
kidney
lung
ovary
skin
muscle
testis

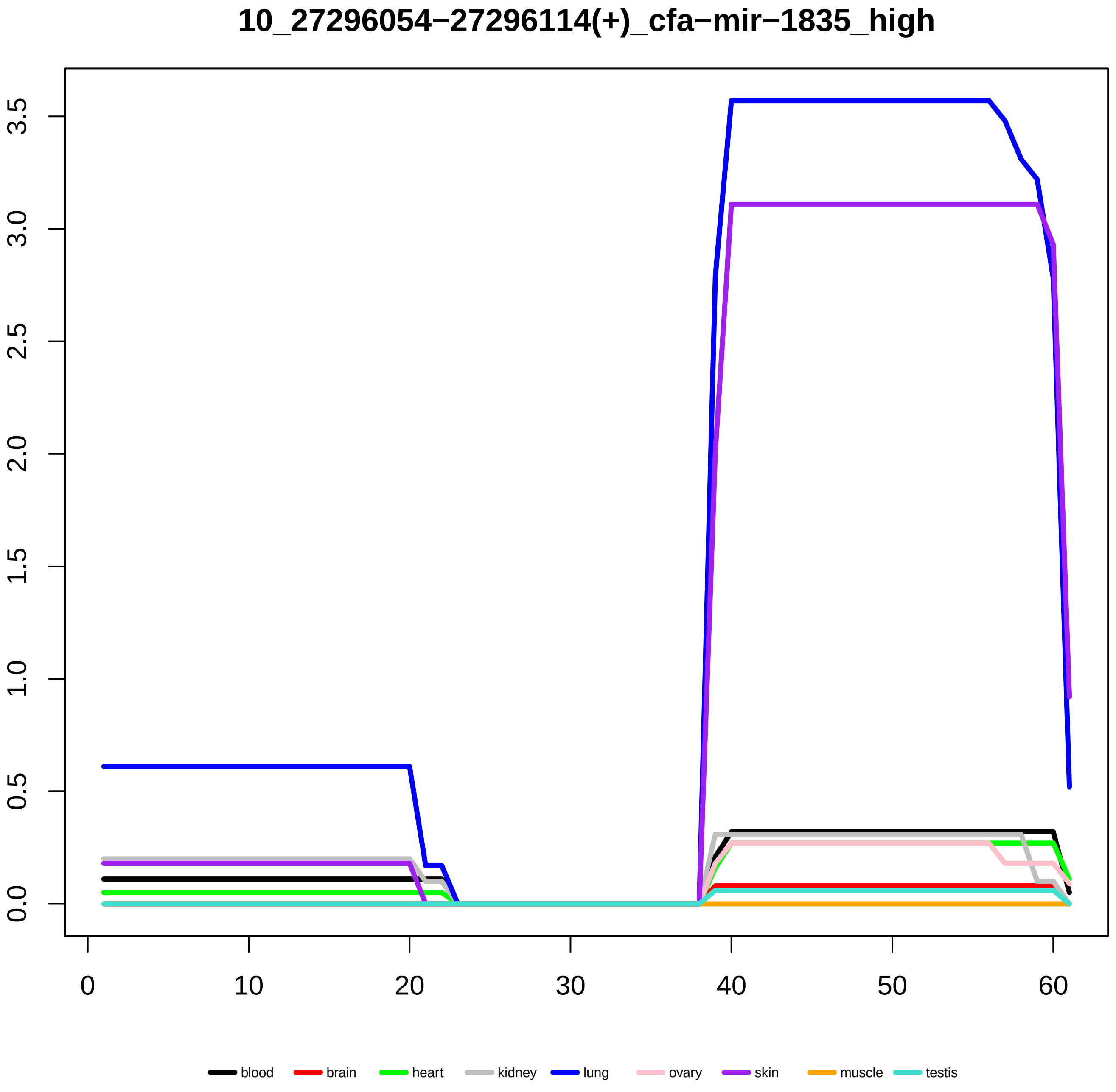
10_27296054−27296114(+)_cfa−mir−1835_high
0.0
0.5
1.0
1.5
2.0
2.5
3.0
3.5
0
10
20
30
40
50
60
blood
brain
heart
kidney
lung
ovary
skin
muscle
testis

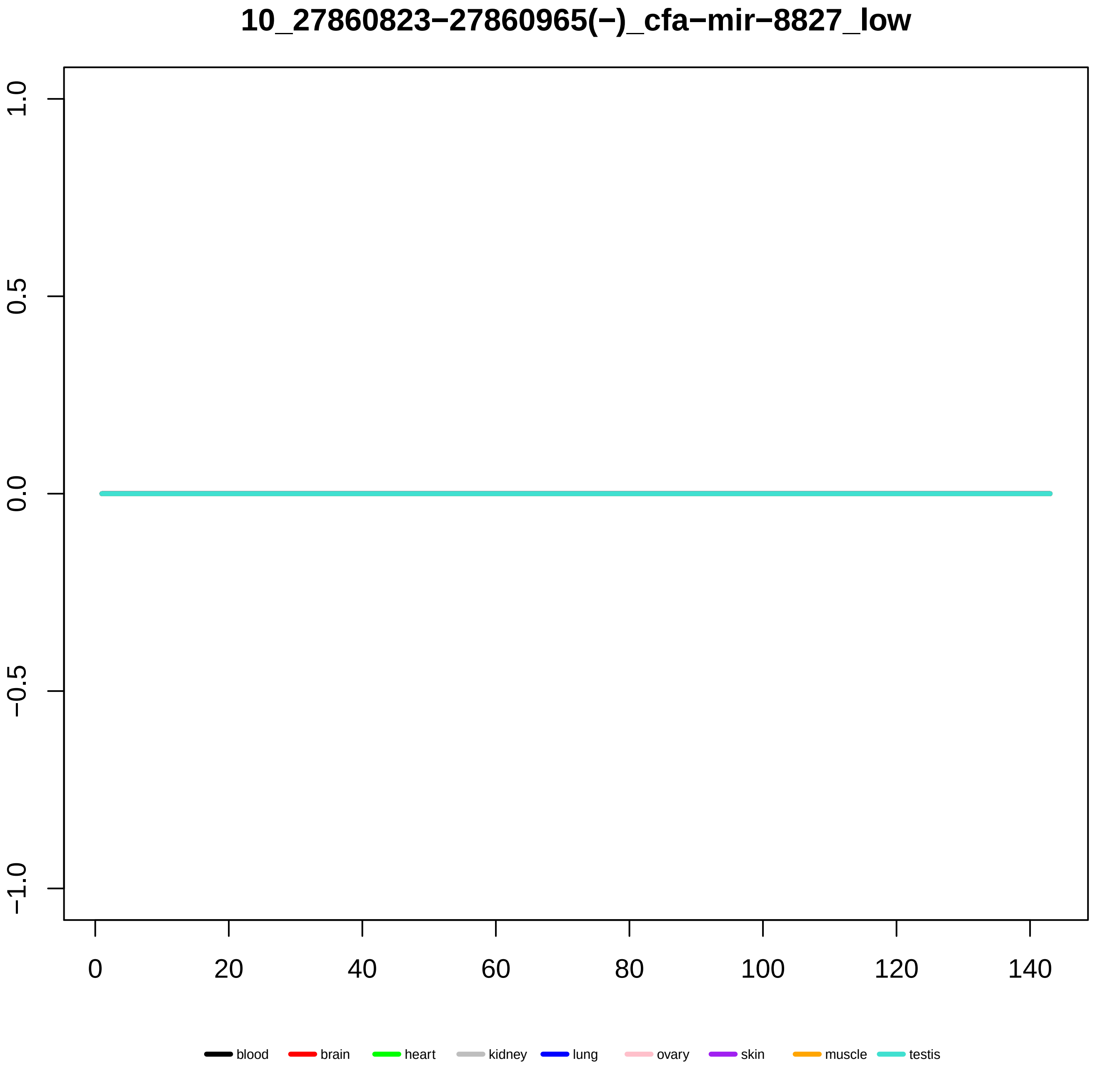
10_27860823−27860965(−)_cfa−mir−8827_low
1.0
0.5
0.0
−0.5
−1.0
0
20
40
60
80
100
120
140
blood
brain
heart
kidney
lung
ovary
skin
muscle
testis

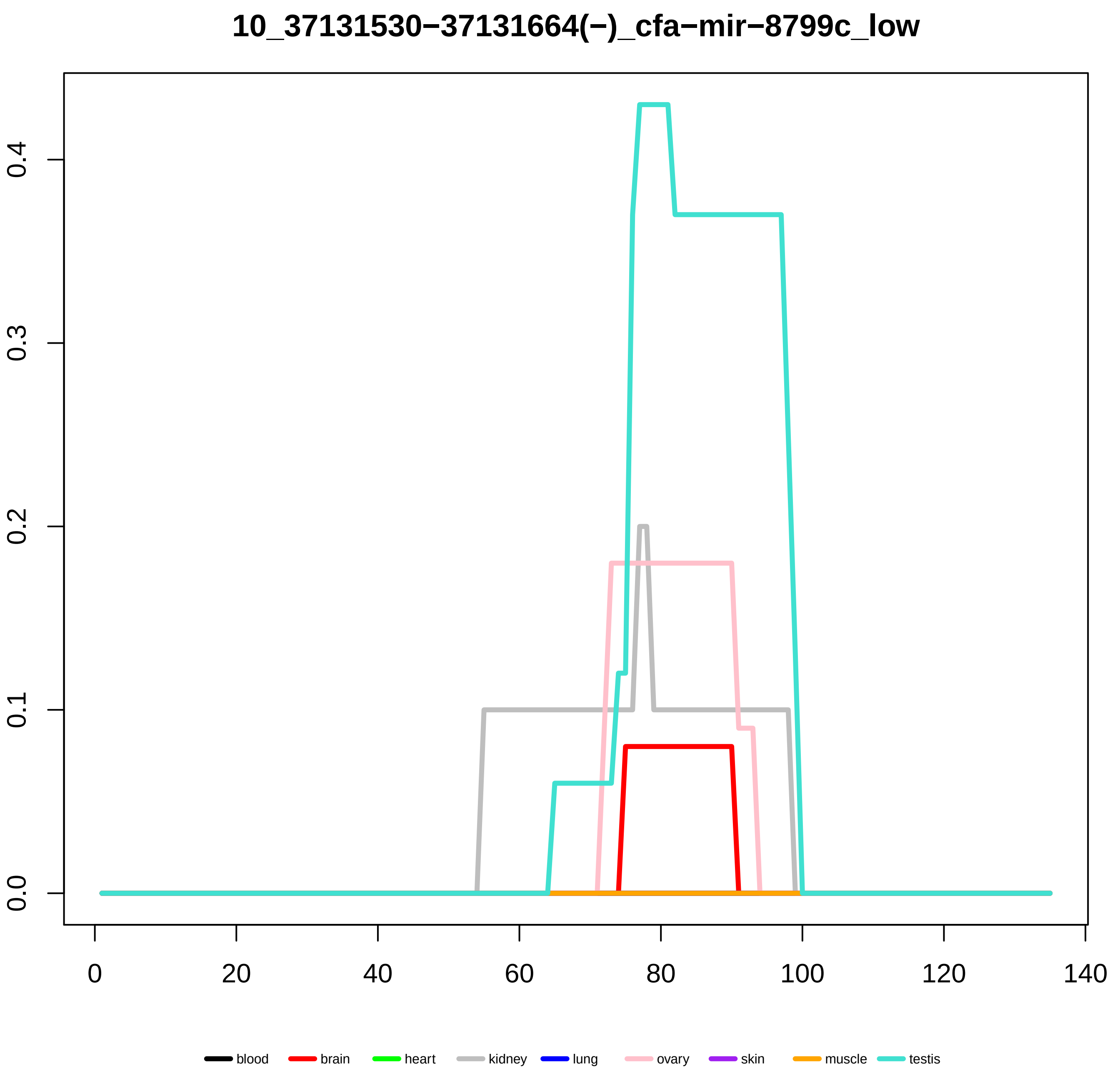

10_37131530−37131664(−)_cfa−mir−8799c_low
0.0
0.1
0.2
0.3
0.4
0
20
40
60
80
100
120
140
blood
brain
heart
kidney
lung
ovary
skin
muscle
testis

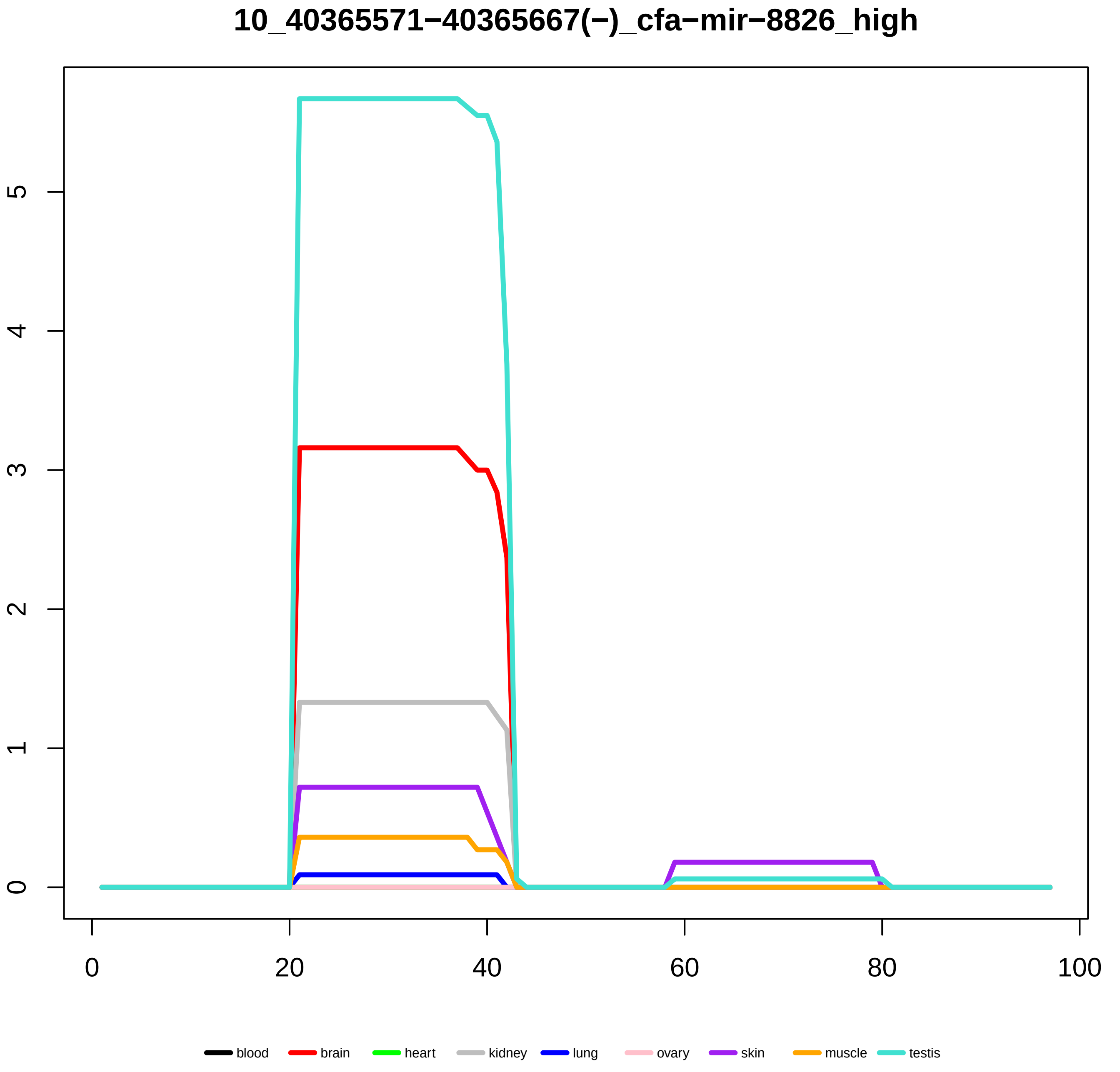

10_40365571−40365667(−)_cfa−mir−8826_high
0
1
2
3
4
5
0
20
40
60
80
100
blood
brain
heart
kidney
lung
ovary
skin
muscle
testis

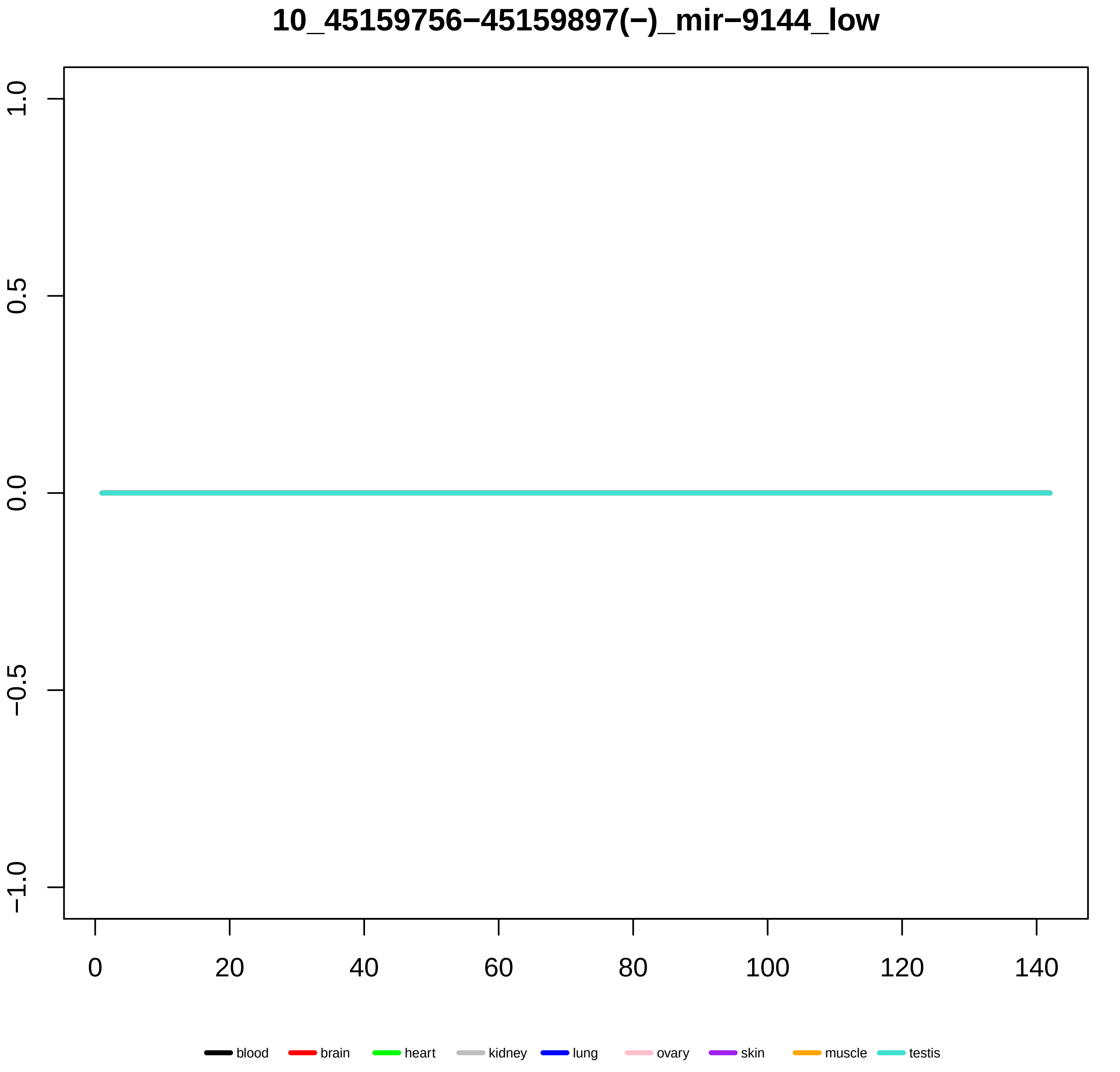

10_45159756−45159897(−)_mir−9144_low
1.0
0.5
0.0
−0.5
−1.0
0
20
40
60
80
100
120
140
blood
brain
heart
kidney
lung
ovary
skin
muscle
testis

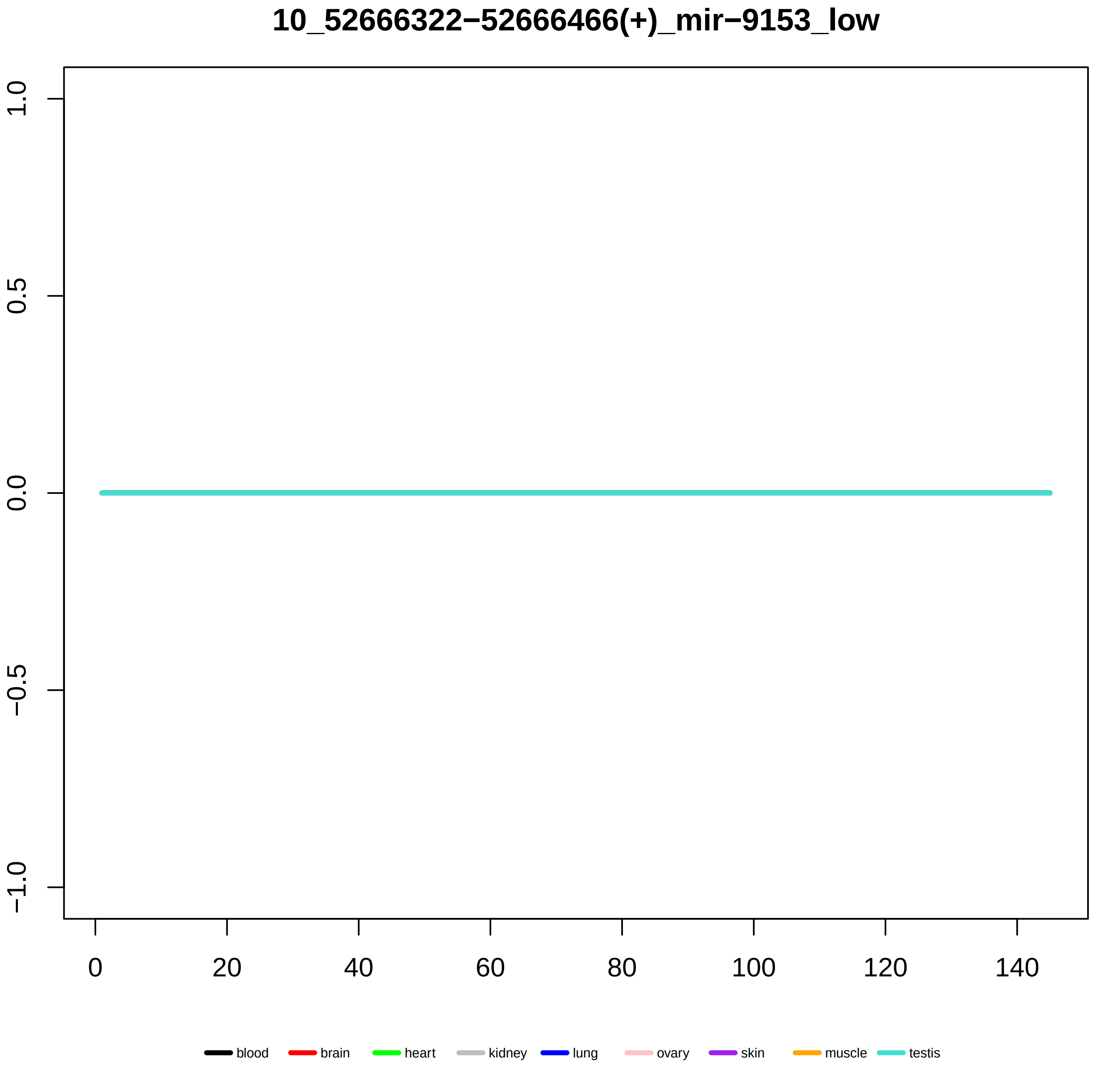
10_52666322−52666466(+)_mir−9153_low
1.0
0.5
0.0
−0.5
−1.0
0
20
40
60
80
100
120
140
blood
brain
heart
kidney
lung
ovary
skin
muscle
testis

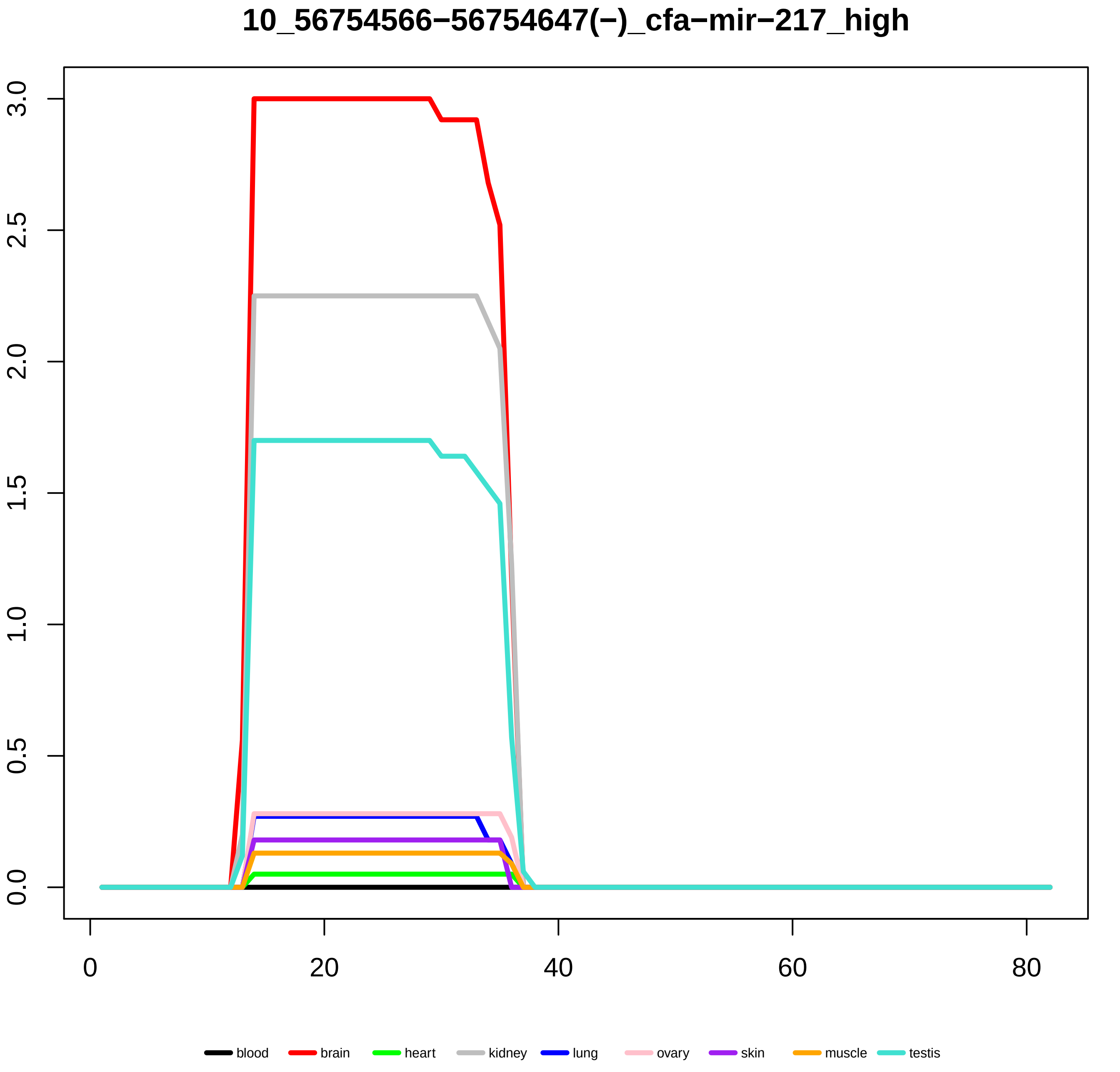

10_56754566−56754647(−)_cfa−mir−217_high
0.0
0.5
1.0
1.5
2.0
2.5
3.0
0
20
40
60
80
blood
brain
heart
kidney
lung
ovary
skin
muscle
testis

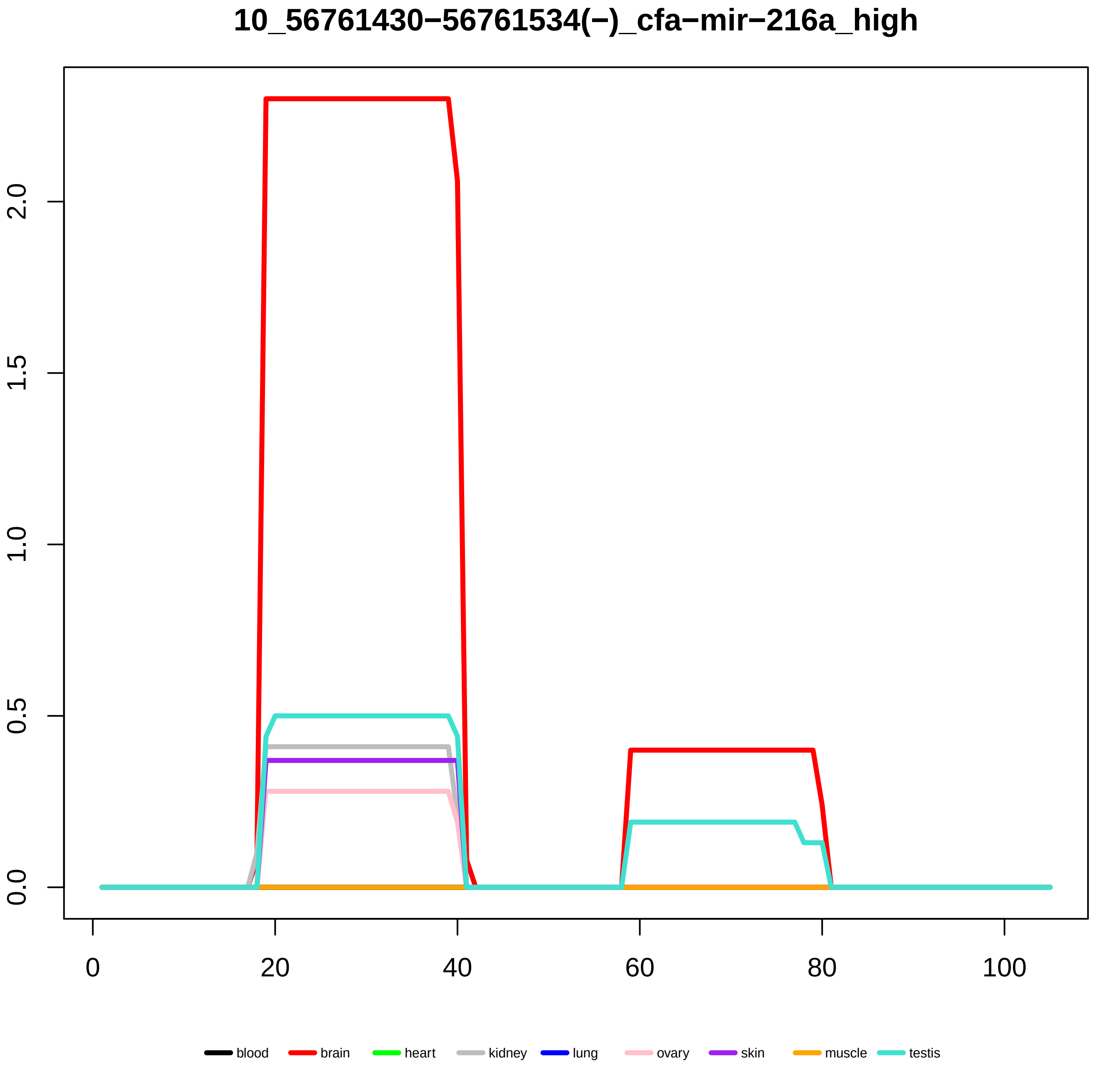
10_56761430−56761534(−)_cfa−mir−216a_high
0.0
0.5
1.0
1.5
2.0
0
20
40
60
80
100
blood
brain
heart
kidney
lung
ovary
skin
muscle
testis

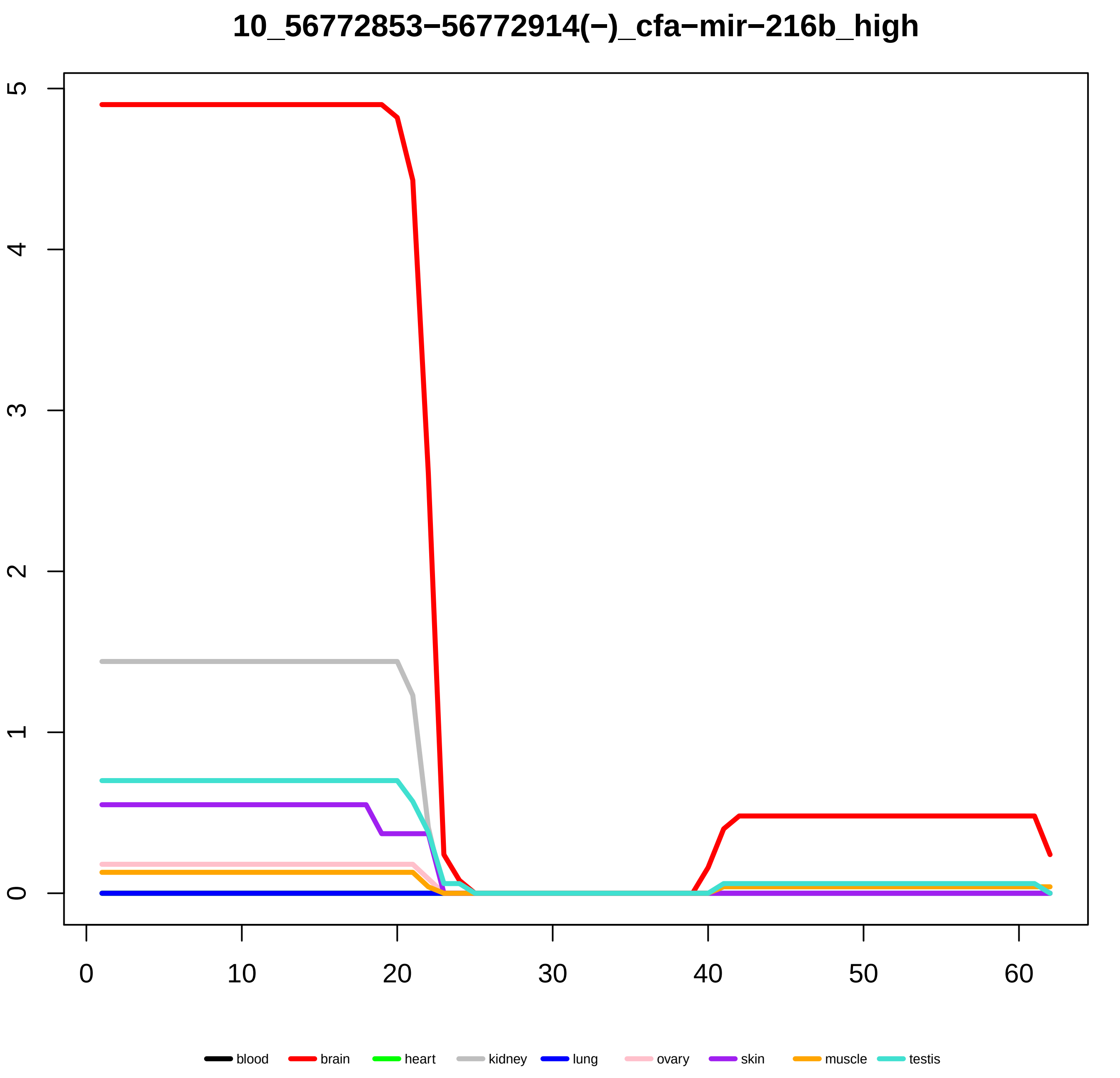
10_56772853−56772914(−)_cfa−mir−216b_high
0
1
2
3
4
5
0
10
20
30
40
50
60
blood
brain
heart
kidney
lung
ovary
skin
muscle
testis

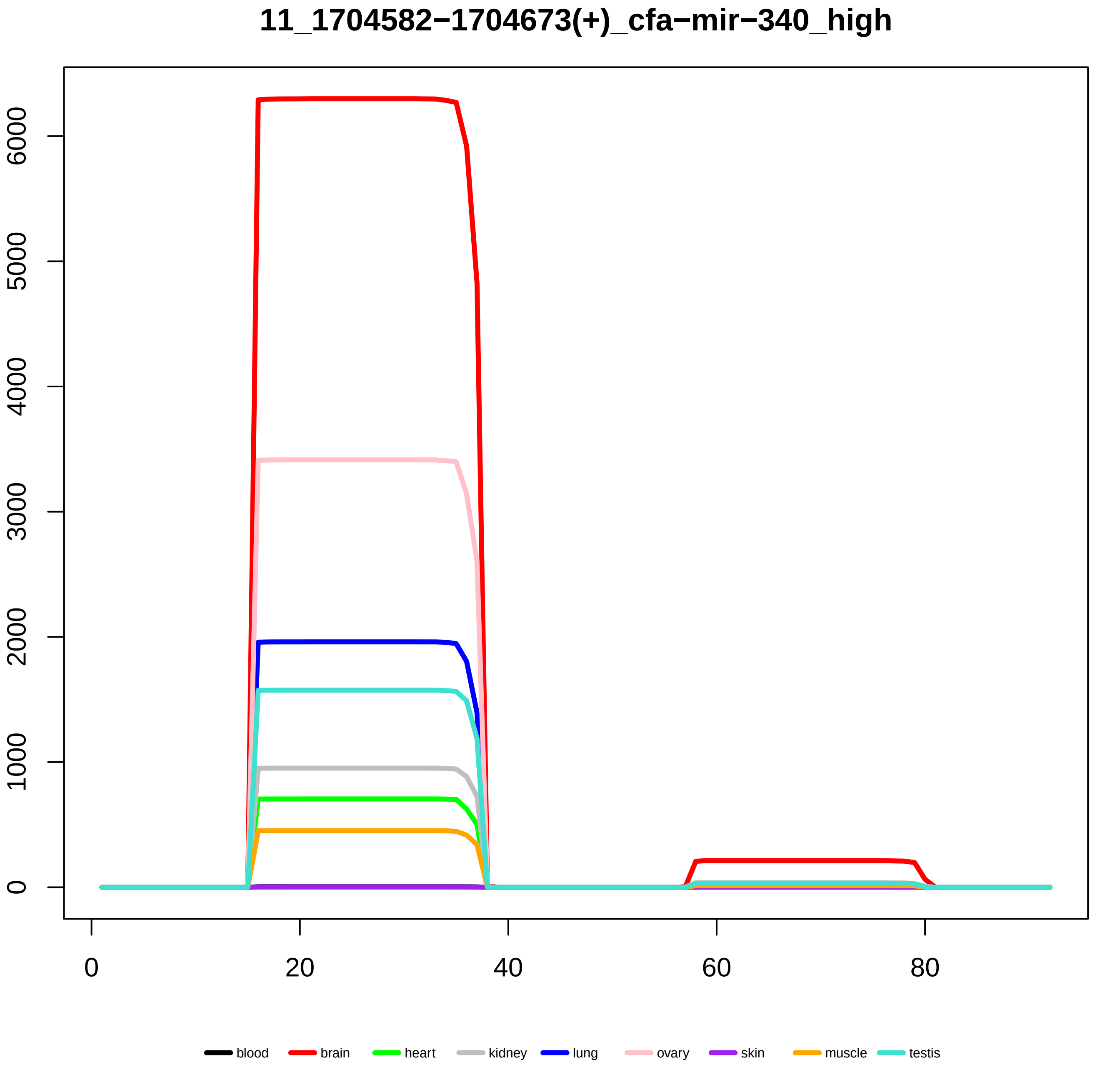
11_1704582−1704673(+)_cfa−mir−340_high
0
1000
2000
3000
4000
5000
6000
0
20
40
60
80
blood
brain
heart
kidney
lung
ovary
skin
muscle
testis

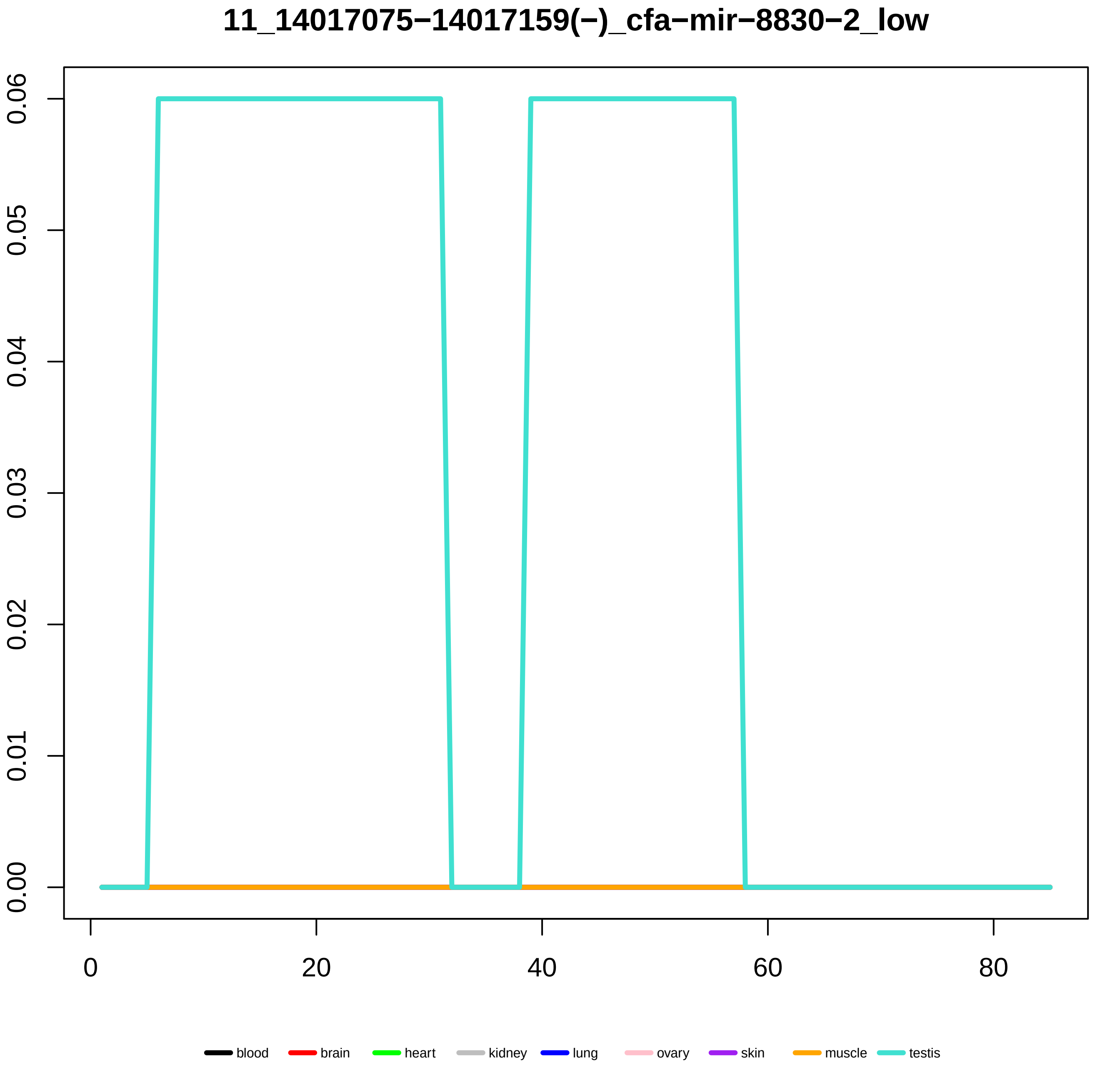

11_14017075−14017159(−)_cfa−mir−8830−2_low
0.00
0.01
0.02
0.03
0.04
0.05
0.06
0
20
40
60
80
blood
brain
heart
kidney
lung
ovary
skin
muscle
testis

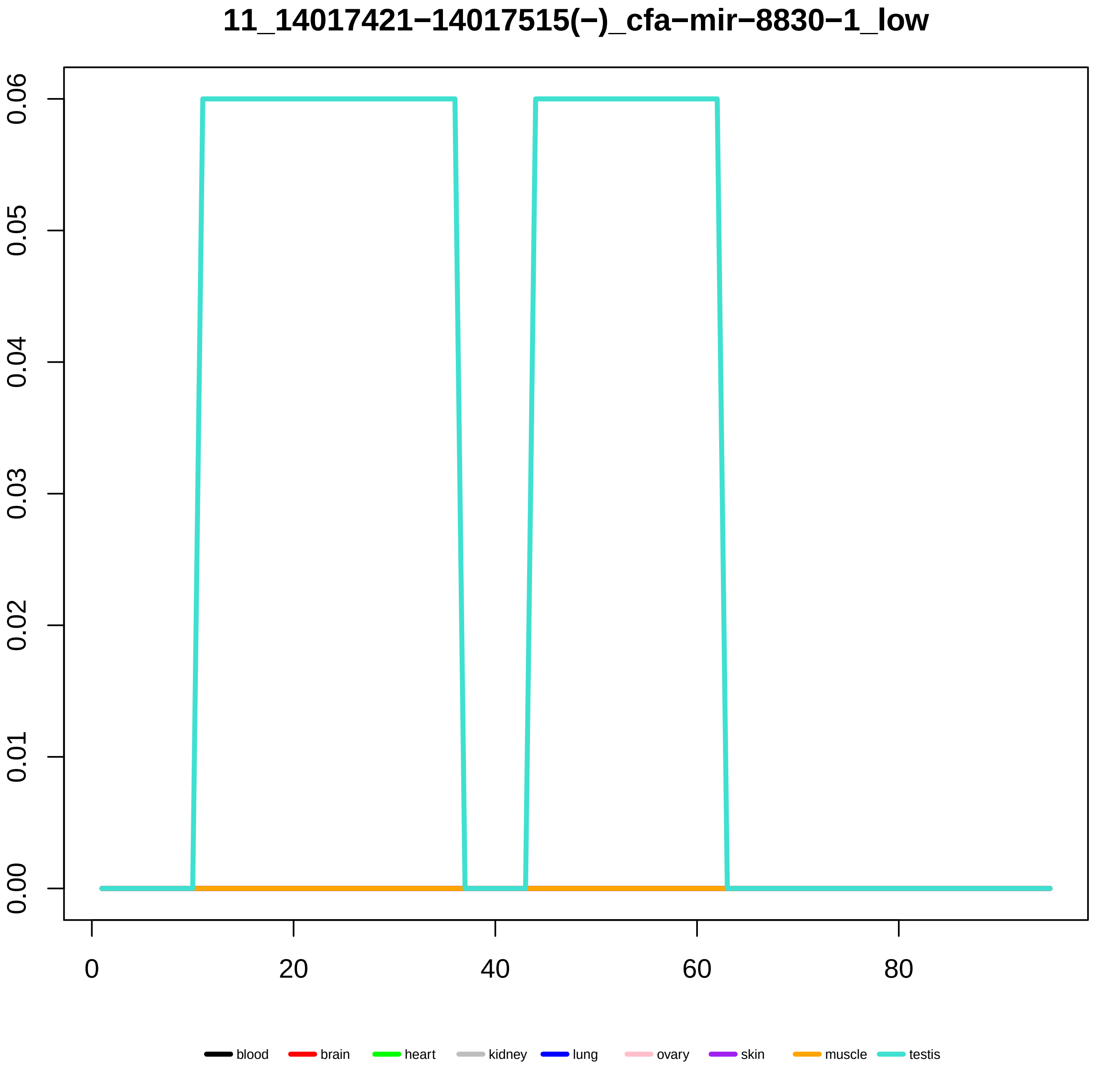

11_14017421−14017515(−)_cfa−mir−8830−1_low
0.06
0.05
0.04
0.03
0.02
0.01
0.00
0
20
40
60
80
blood
brain
heart
kidney
lung
ovary
skin
muscle
testis

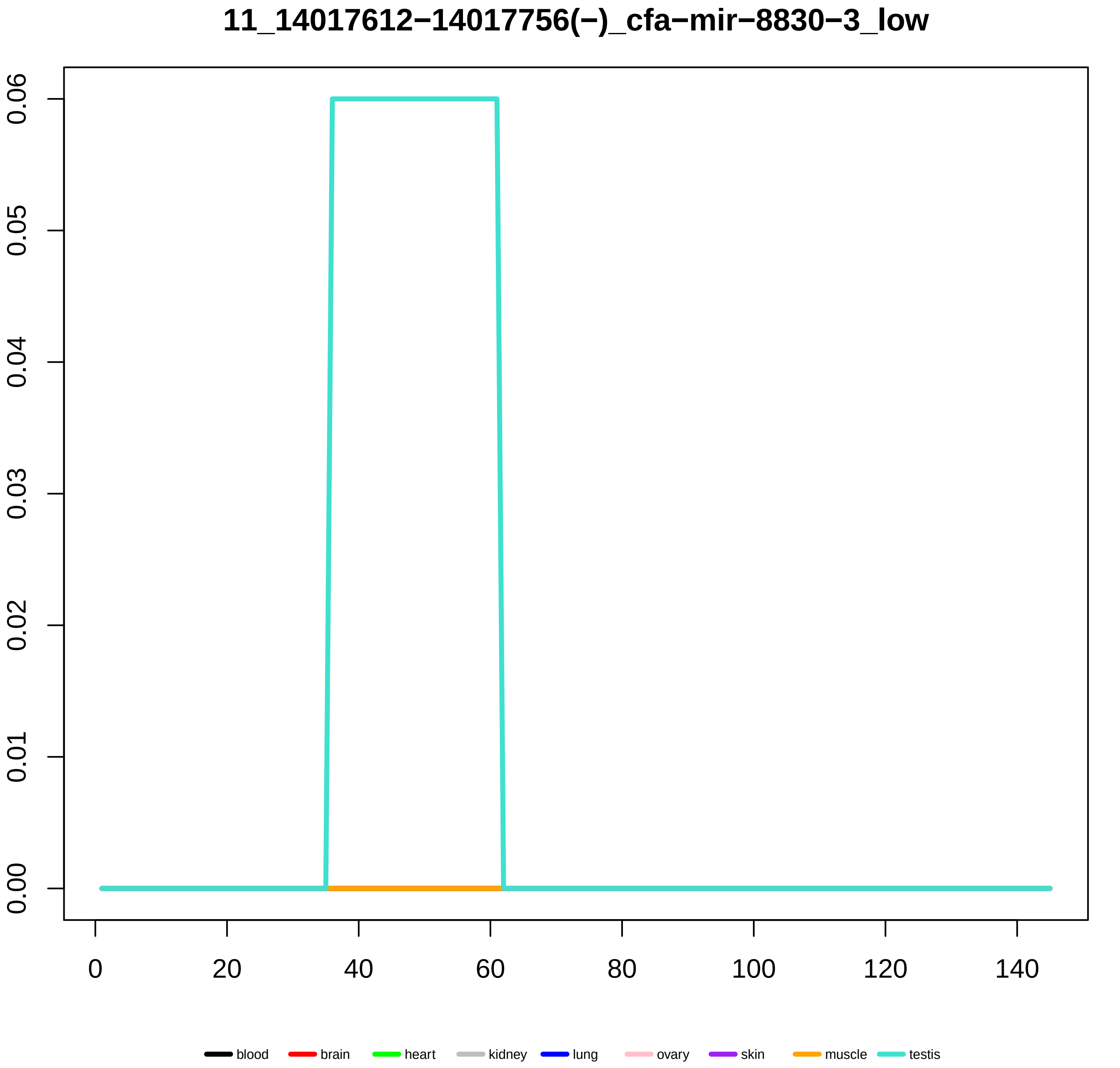

11_14017612−14017756(−)_cfa−mir−8830−3_low
0.00
0.01
0.02
0.03
0.04
0.05
0.06
0
20
40
60
80
100
120
140
blood
brain
heart
kidney
lung
ovary
skin
muscle
testis

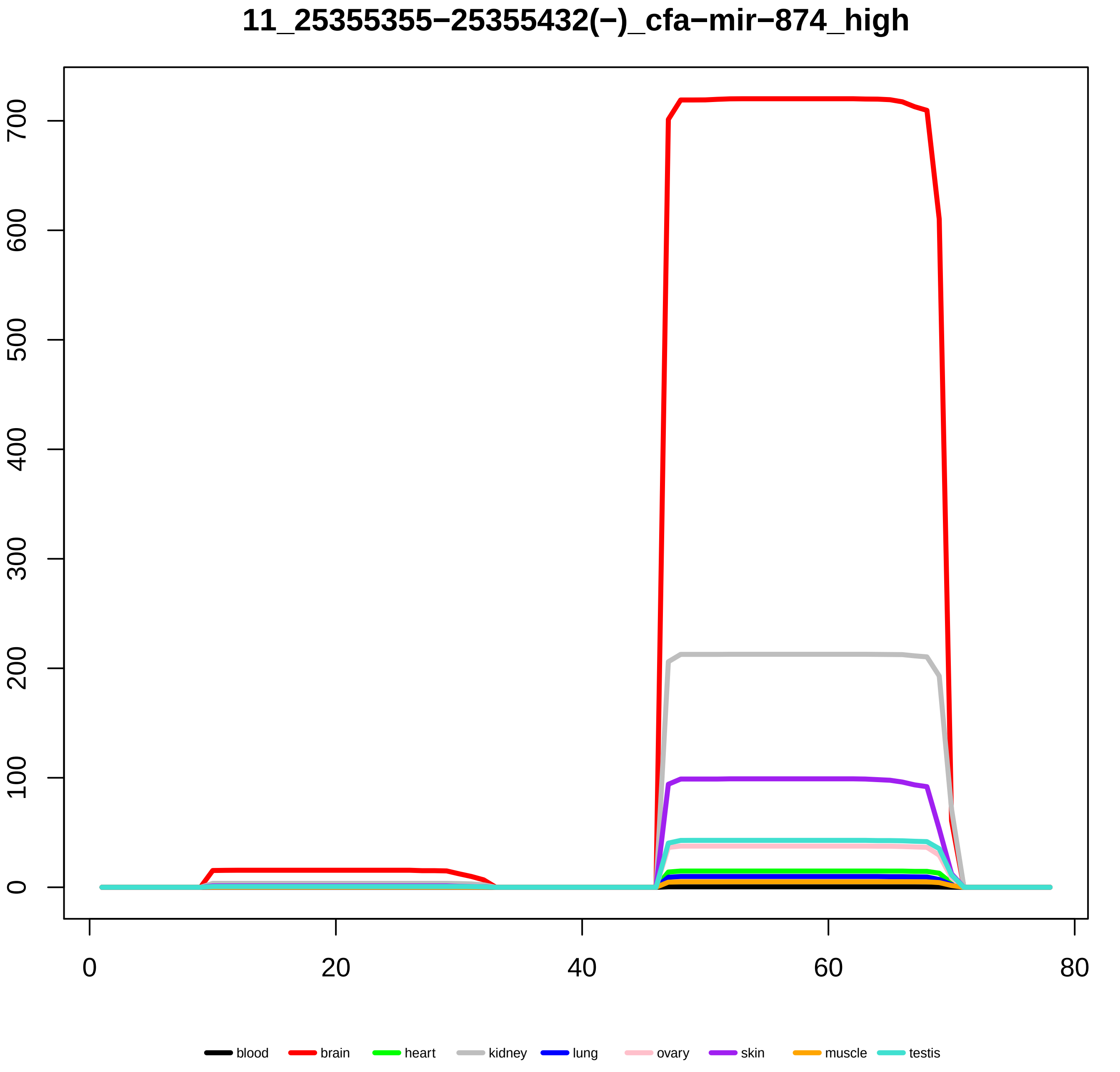
11_25355355−25355432(−)_cfa−mir−874_high
0
100
200
300
400
500
600
700
0
20
40
60
80
blood
brain
heart
kidney
lung
ovary
skin
muscle
testis

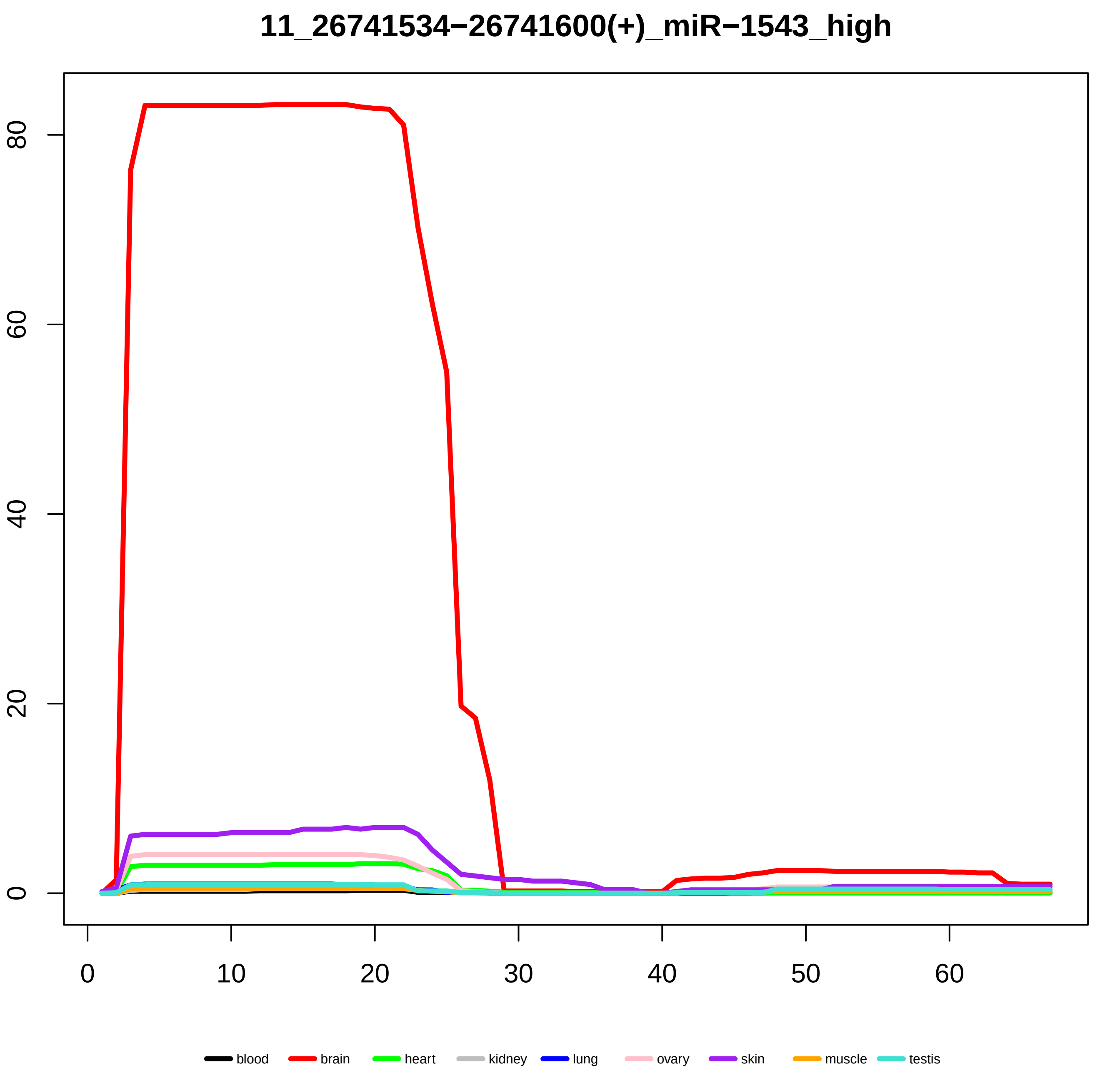

11_26741534−26741600(+)_miR−1543_high
0
20
40
60
80
0
10
20
30
40
50
60
blood
brain
heart
kidney
lung
ovary
skin
muscle
testis

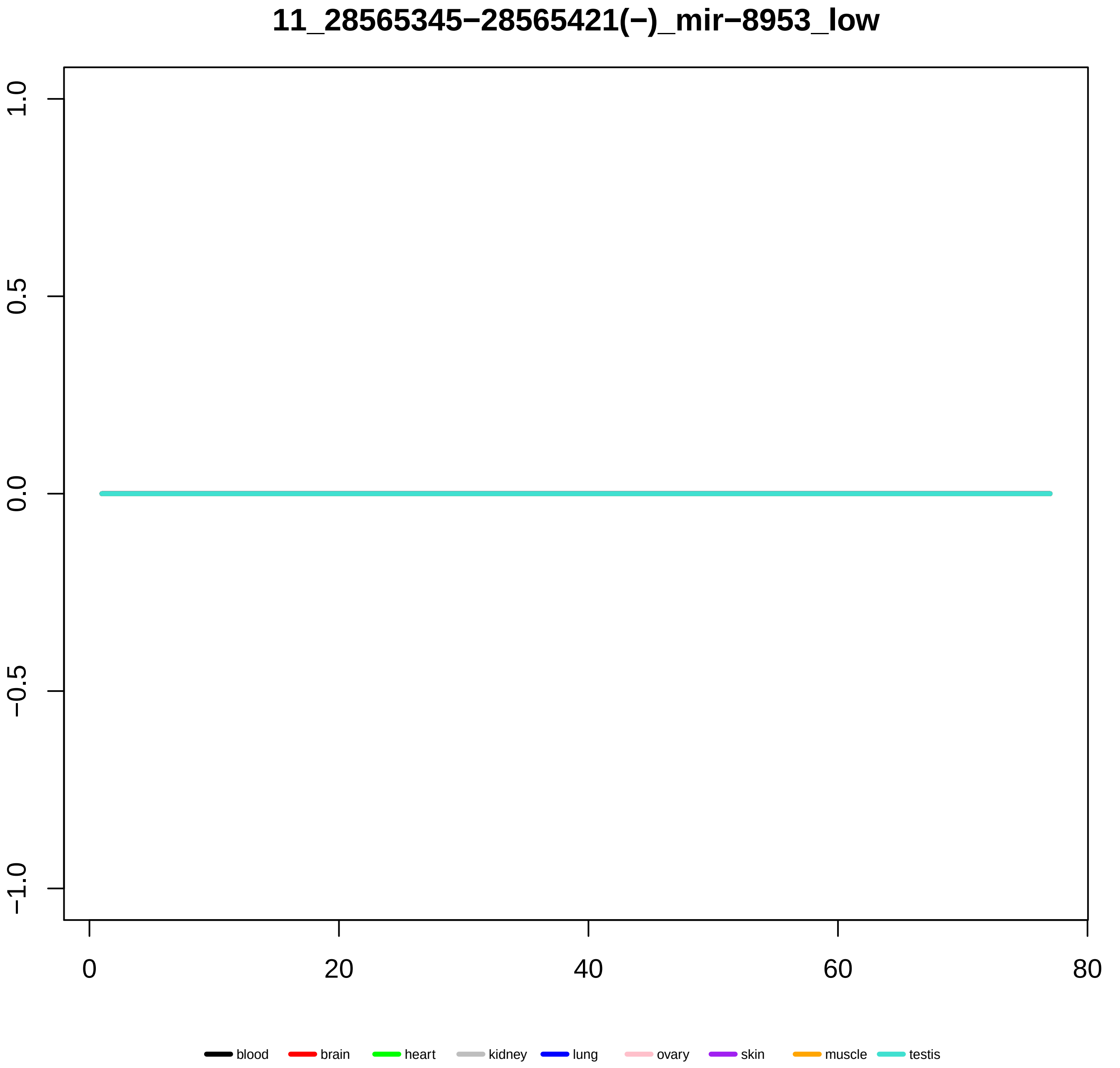
11_28565345−28565421(−)_mir−8953_low
1.0
0.5
0.0
−0.5
−1.0
0
20
40
60
80
blood
brain
heart
kidney
lung
ovary
skin
muscle
testis

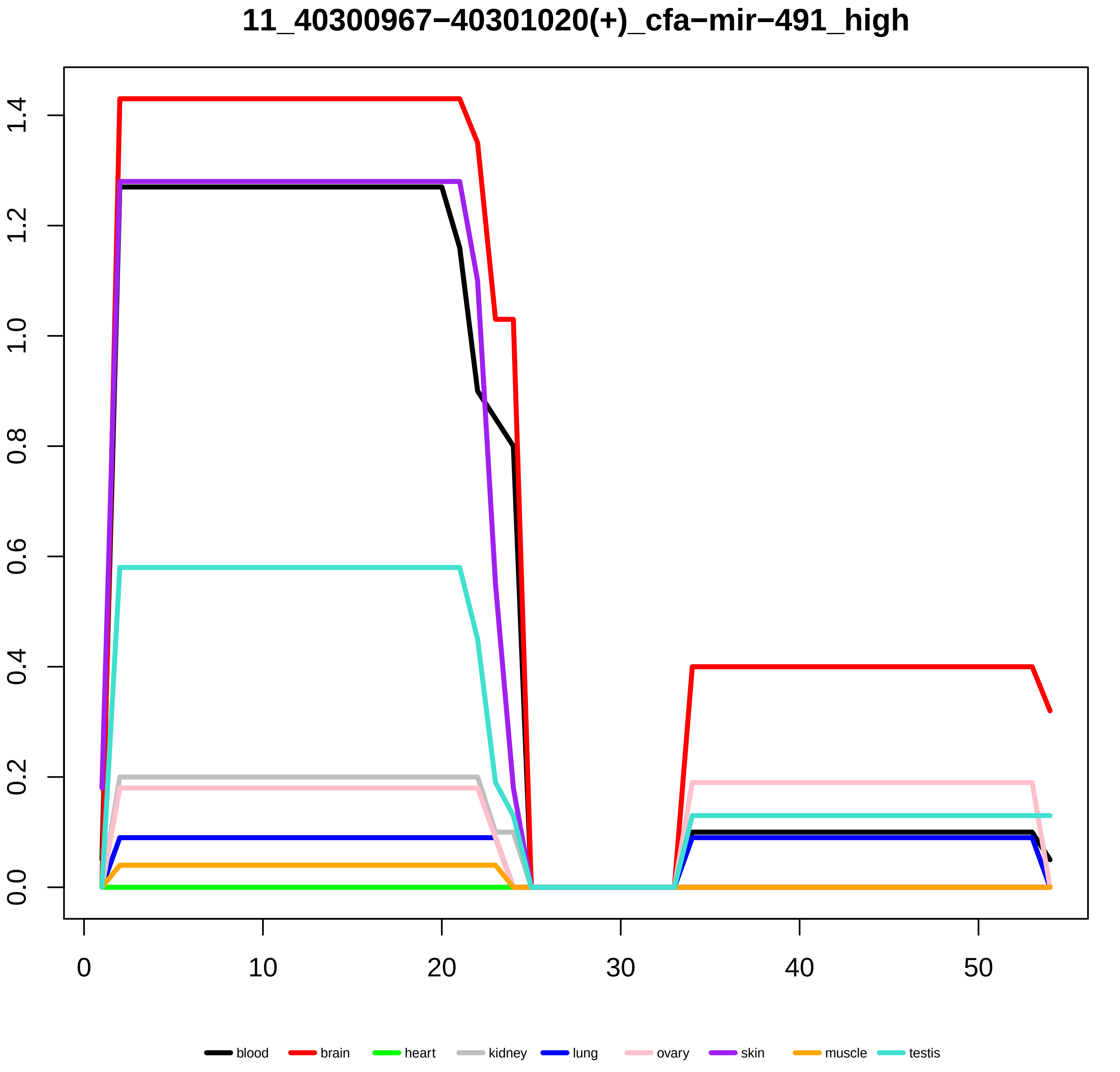

11_40300967−40301020(+)_cfa−mir−491_high
0.0
0.2
0.4
0.6
0.8
1.0
1.2
1.4
0
10
20
30
40
50
blood
brain
heart
kidney
lung
ovary
skin
muscle
testis

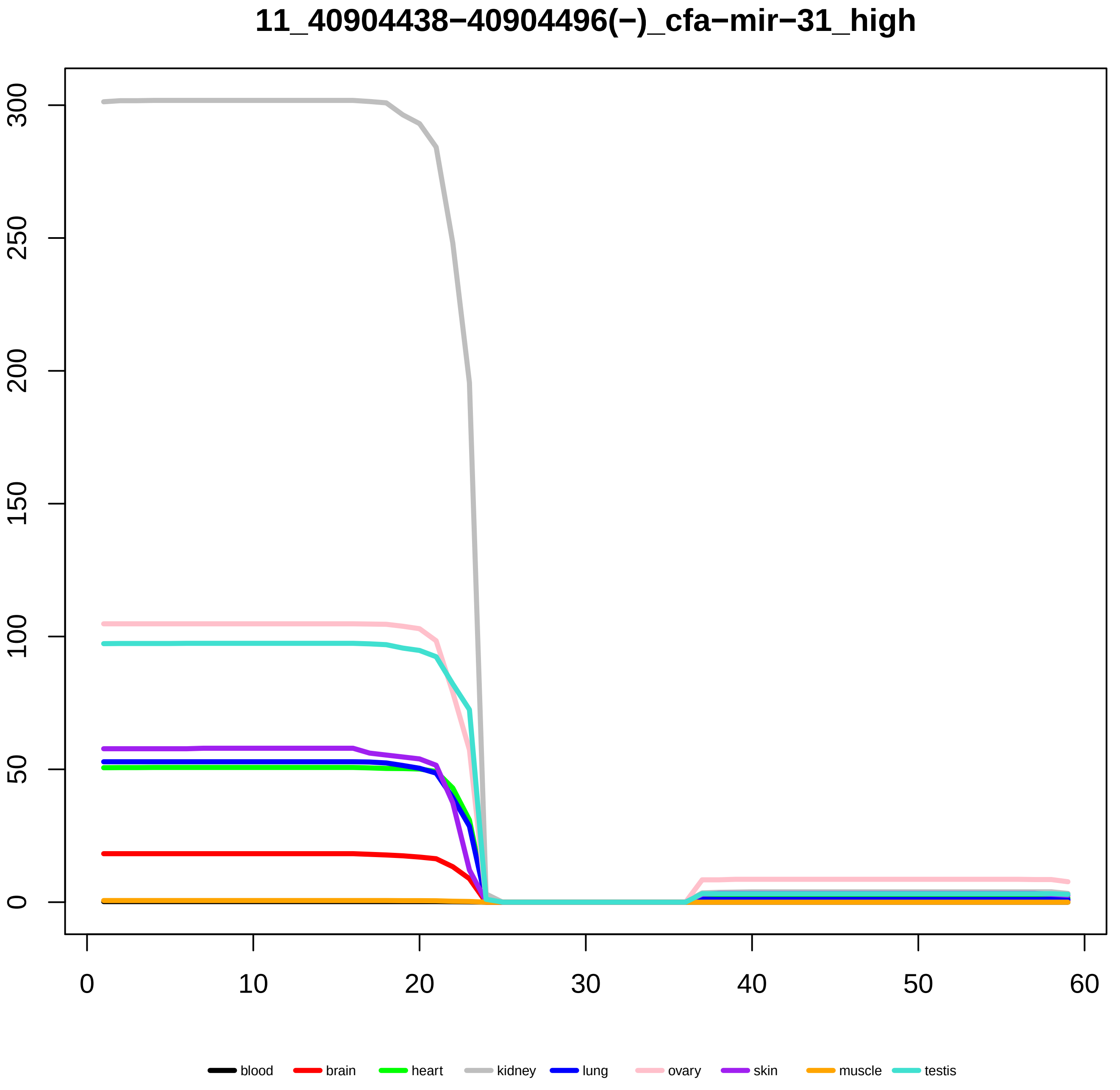
11_40904438−40904496(−)_cfa−mir−31_high
0
50
100
150
200
250
300
0
10
20
30
40
50
60
blood
brain
heart
kidney
lung
ovary
skin
muscle
testis

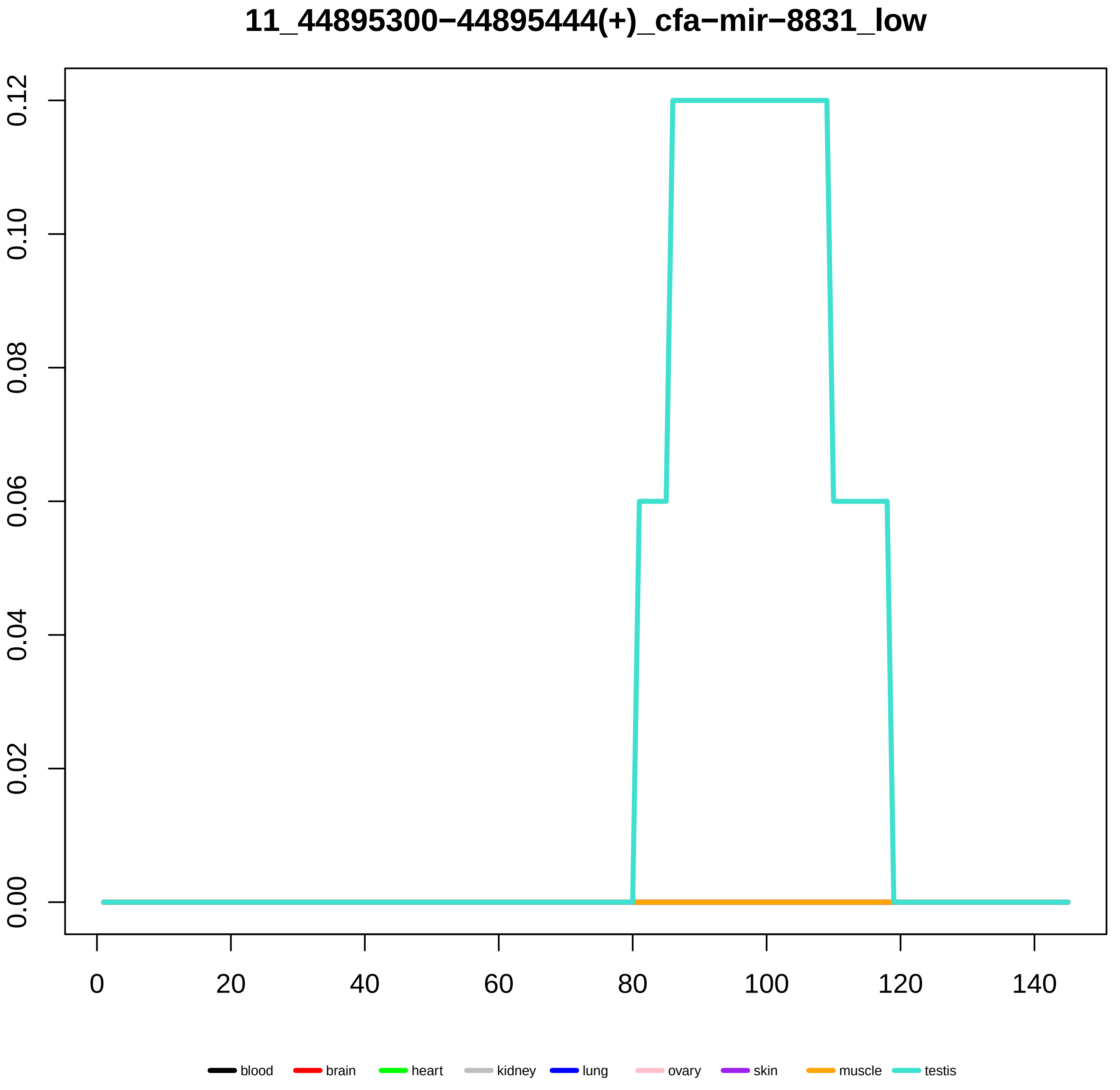

11_44895300−44895444(+)_cfa−mir−8831_low
0.00
0.02
0.04
0.06
0.08
0.10
0.12
0
20
40
60
80
100
120
140
blood
brain
heart
kidney
lung
ovary
skin
muscle
testis

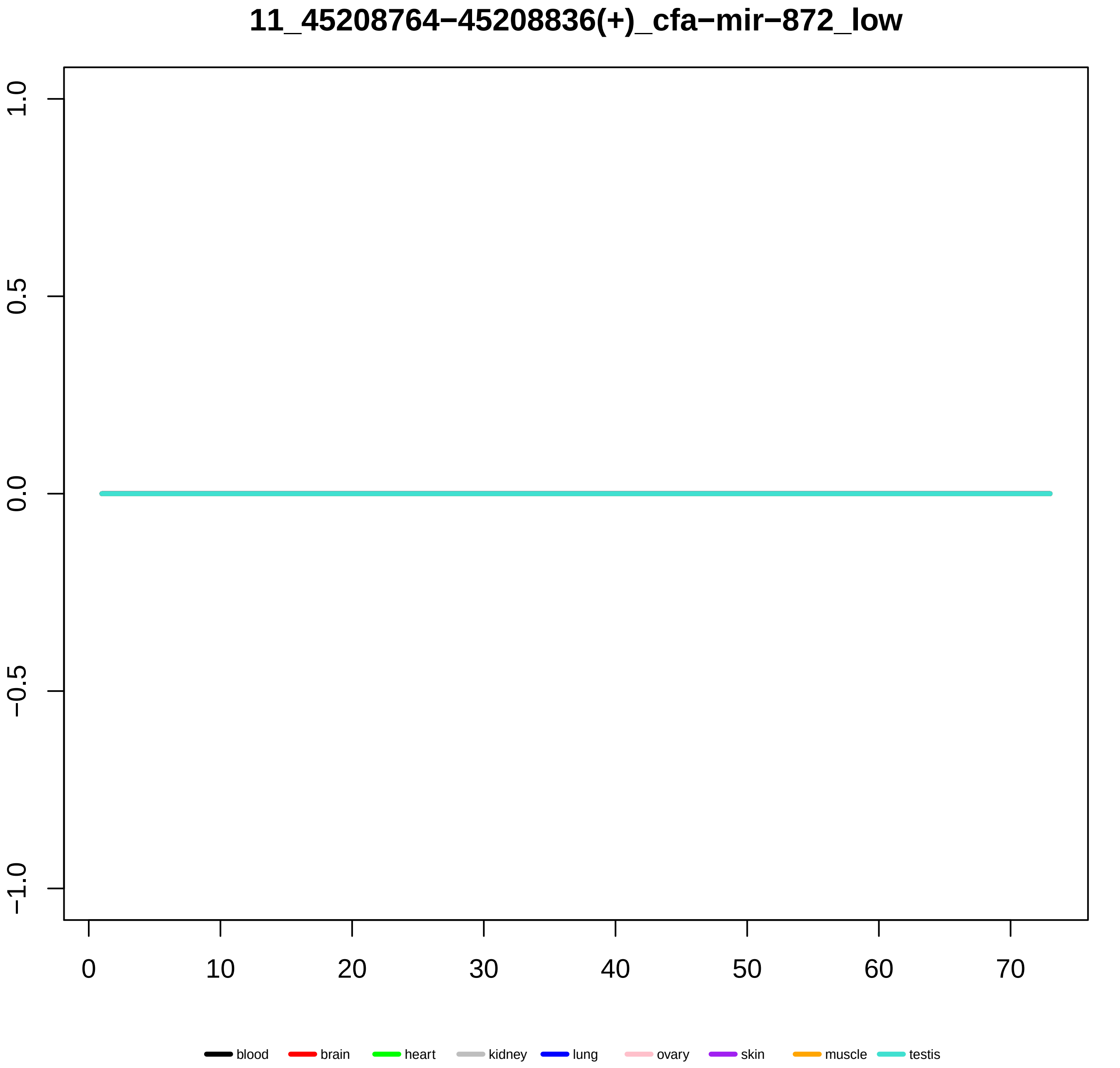

11_45208764−45208836(+)_cfa−mir−872_low
1.0
0.5
0.0
−0.5
−1.0
0
10
20
30
40
50
60
70
blood
brain
heart
kidney
lung
ovary
skin
muscle
testis

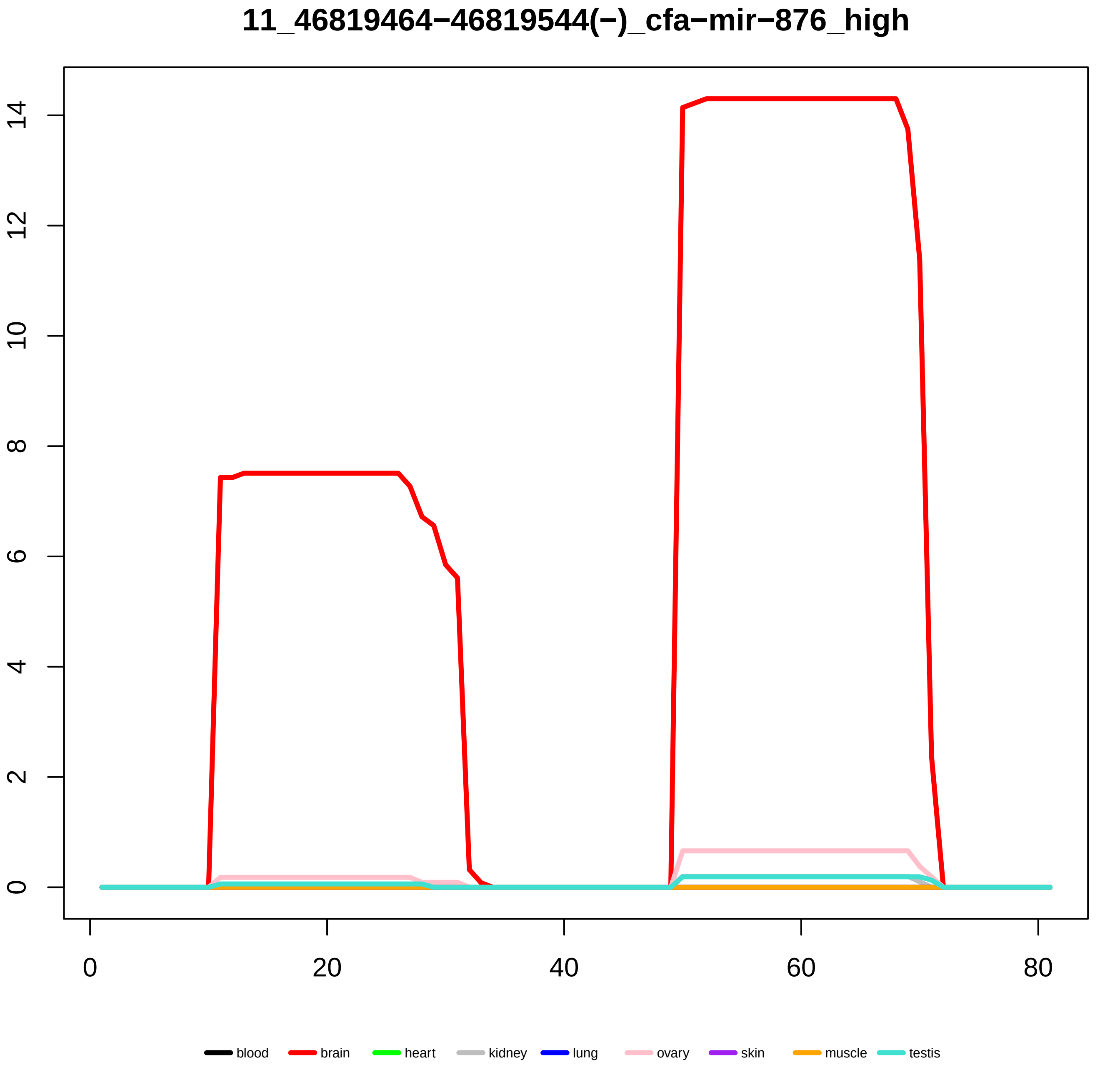
11_46819464−46819544(−)_cfa−mir−876_high
0
2
4
6
8
10
12
14
0
20
40
60
80
blood
brain
heart
kidney
lung
ovary
skin
muscle
testis

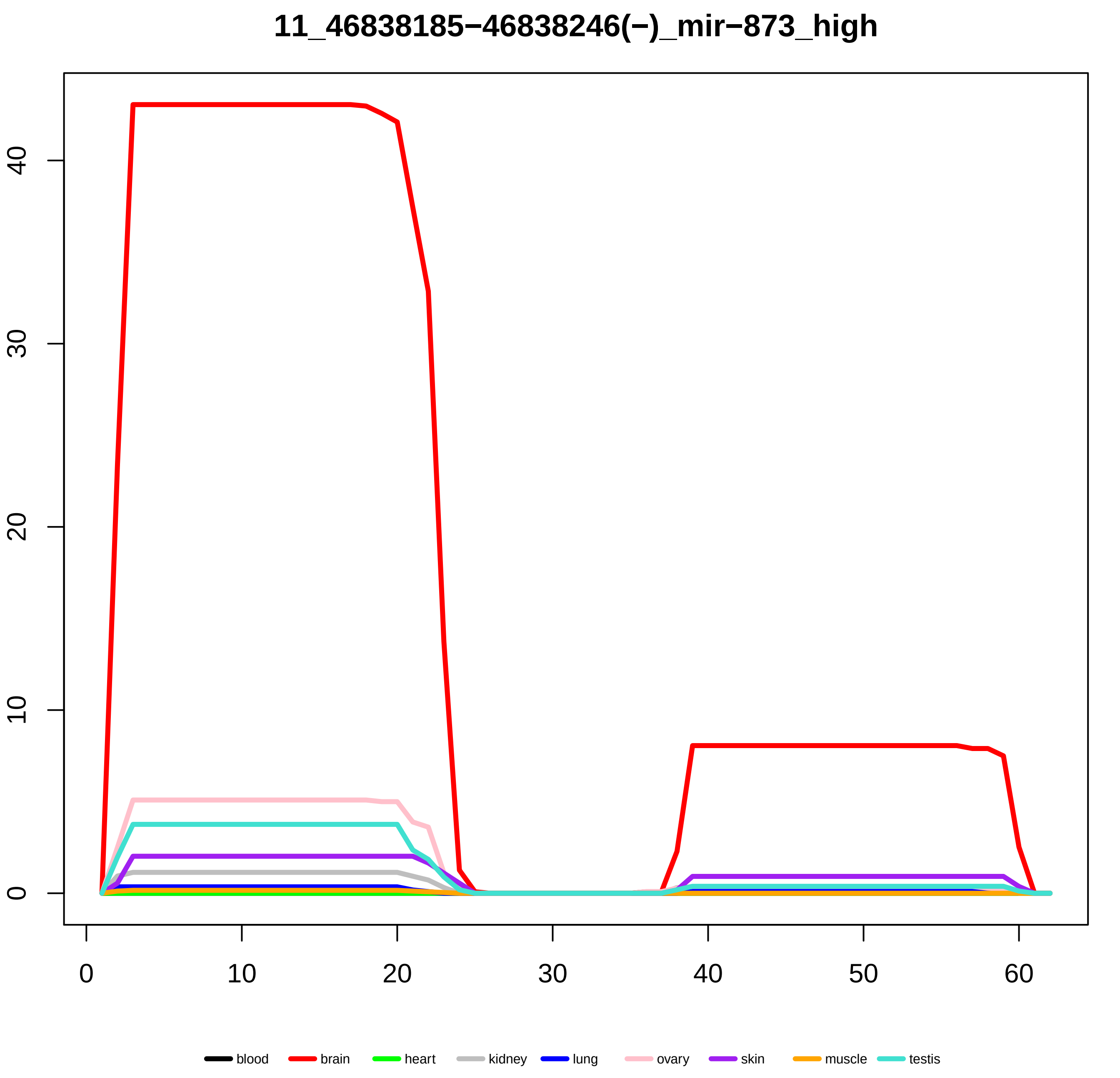

11_46838185−46838246(−)_mir−873_high
0
10
20
30
40
0
10
20
30
40
50
60
blood
brain
heart
kidney
lung
ovary
skin
muscle
testis

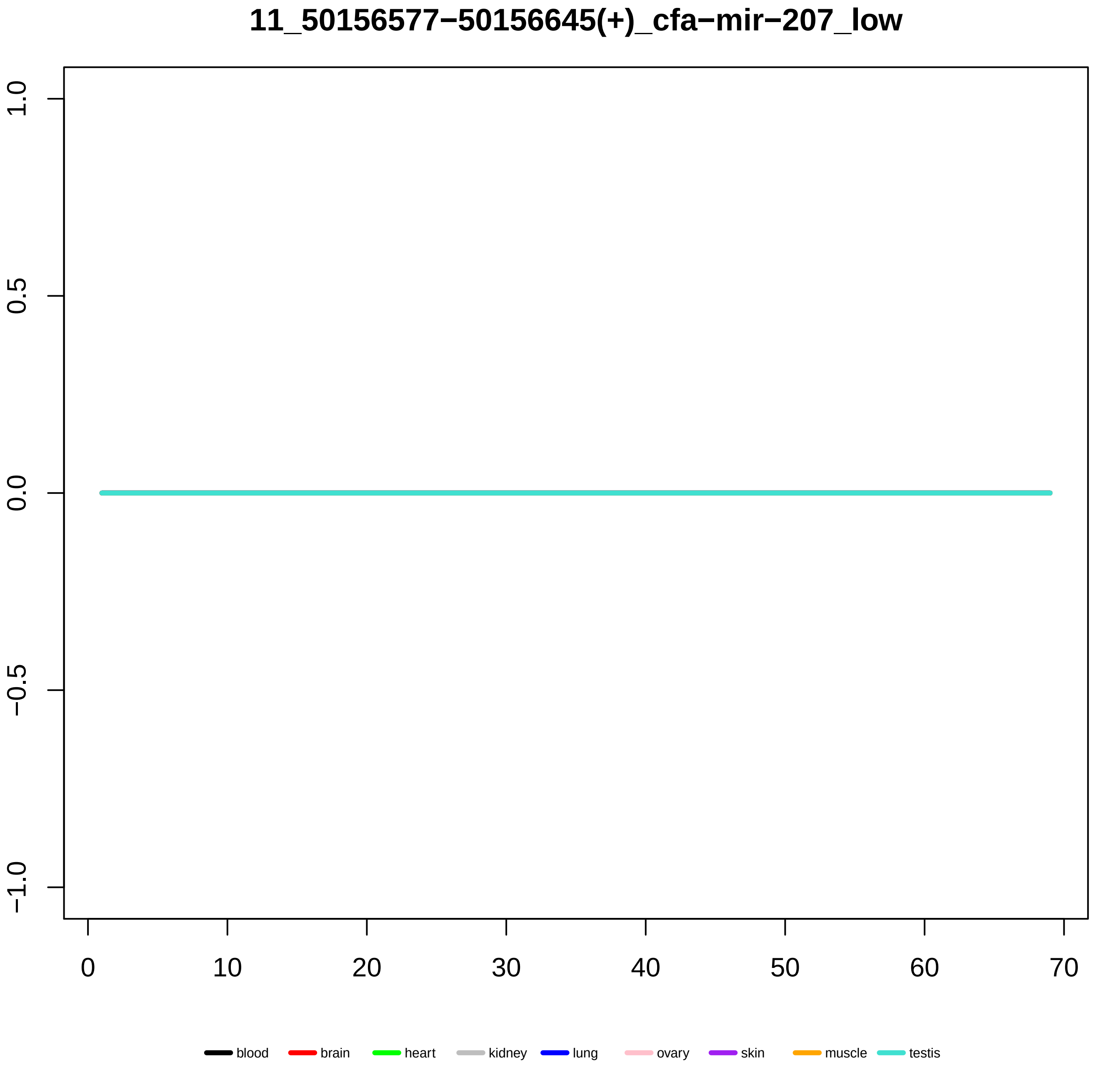
11_50156577−50156645(+)_cfa−mir−207_low
1.0
0.5
0.0
−0.5
−1.0
0
10
20
30
40
50
60
70
blood
brain
heart
kidney
lung
ovary
skin
muscle
testis

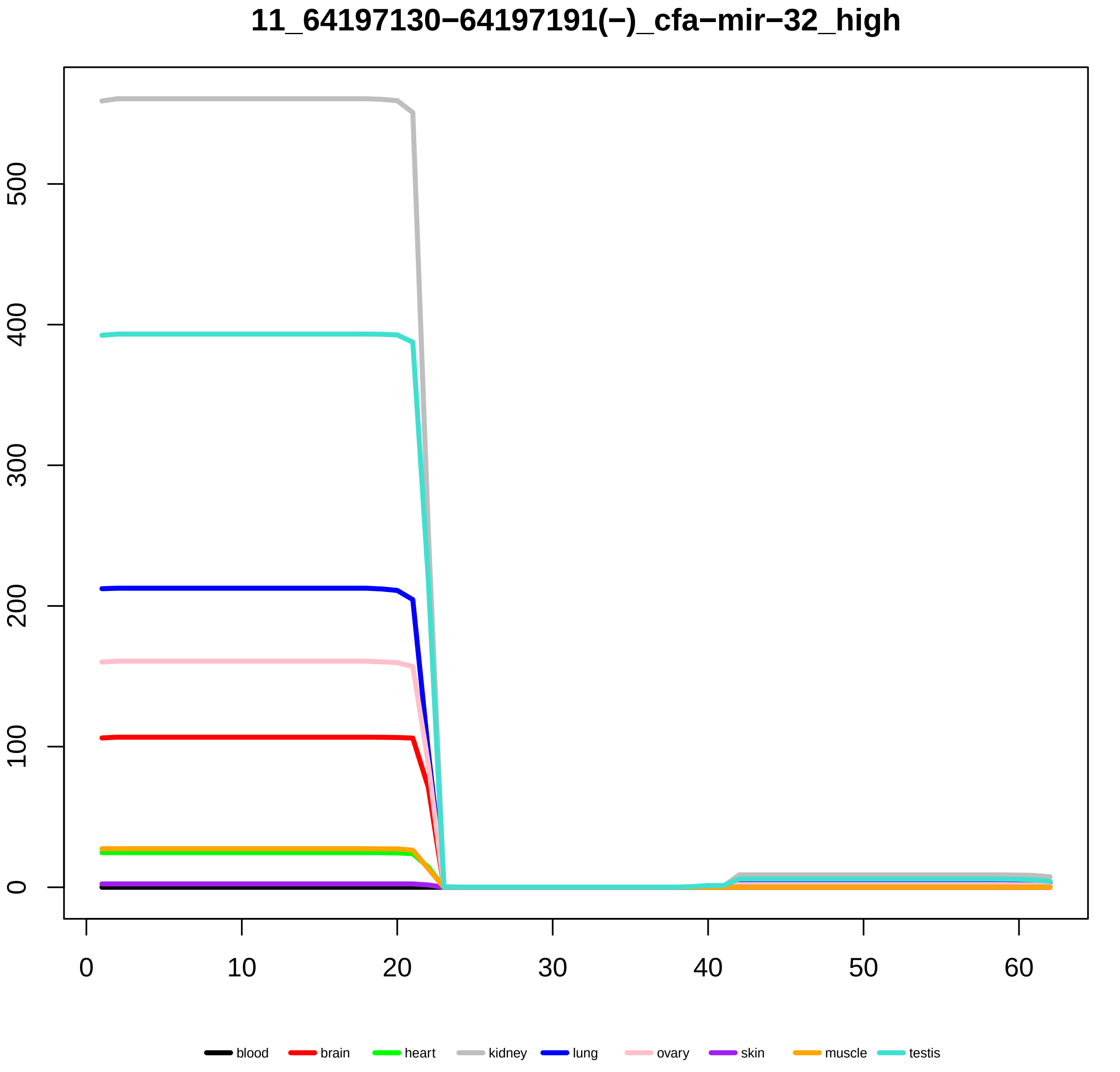
11_64197130−64197191(−)_cfa−mir−32_high
0
100
200
300
400
500
0
10
20
30
40
50
60
blood
brain
heart
kidney
lung
ovary
skin
muscle
testis

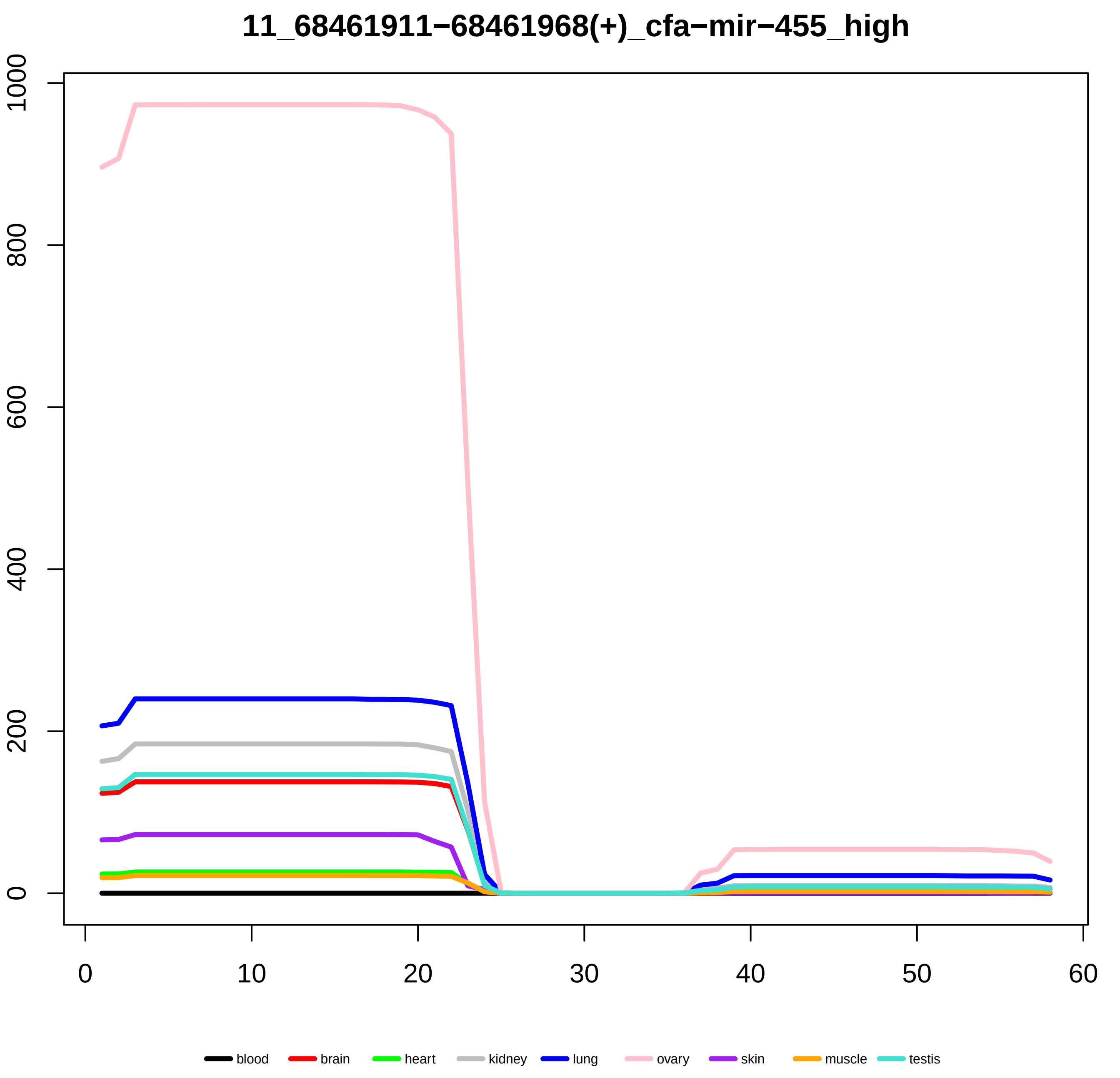

11_68461911−68461968(+)_cfa−mir−455_high
0
200
400
600
800
1000
0
10
20
30
40
50
60
blood
brain
heart
kidney
lung
ovary
skin
muscle
testis

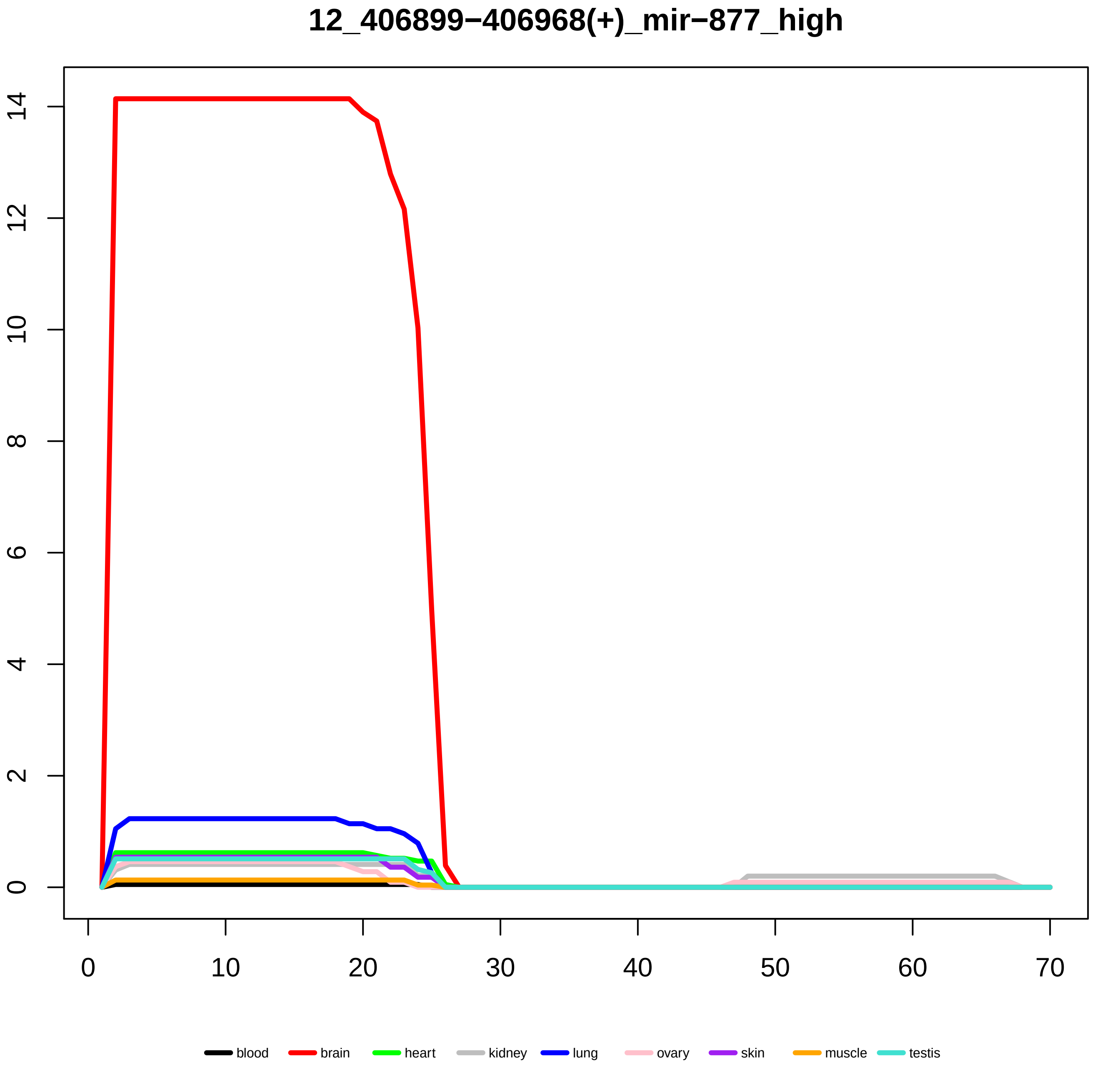

12_406899−406968(+)_mir−877_high
0
2
4
6
8
10
12
14
0
10
20
30
40
50
60
70
blood
brain
heart
kidney
lung
ovary
skin
muscle
testis

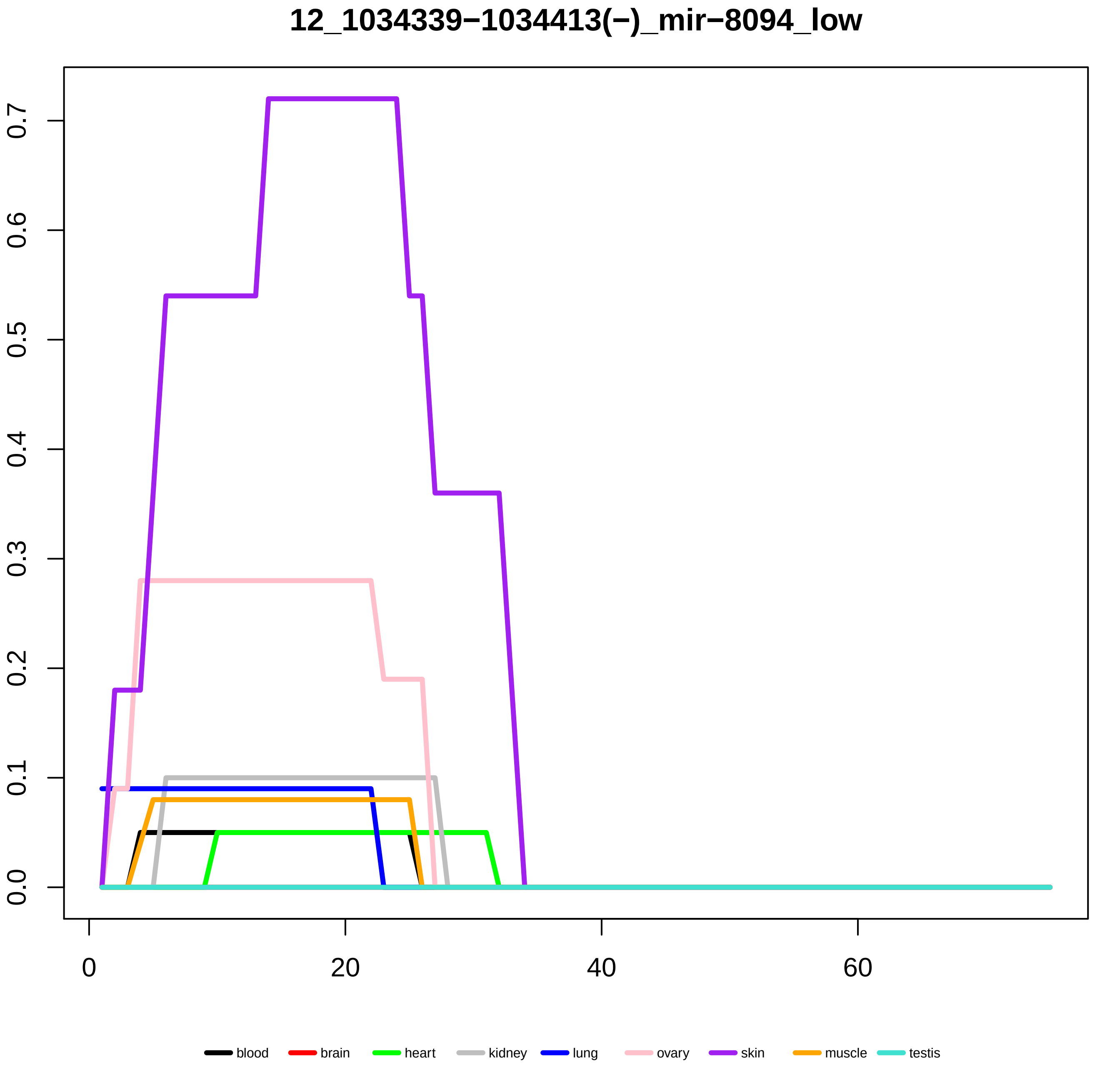

12_1034339−1034413(−)_mir−8094_low
0.0
0.1
0.2
0.3
0.4
0.5
0.6
0.7
0
20
40
60
blood
brain
heart
kidney
lung
ovary
skin
muscle
testis

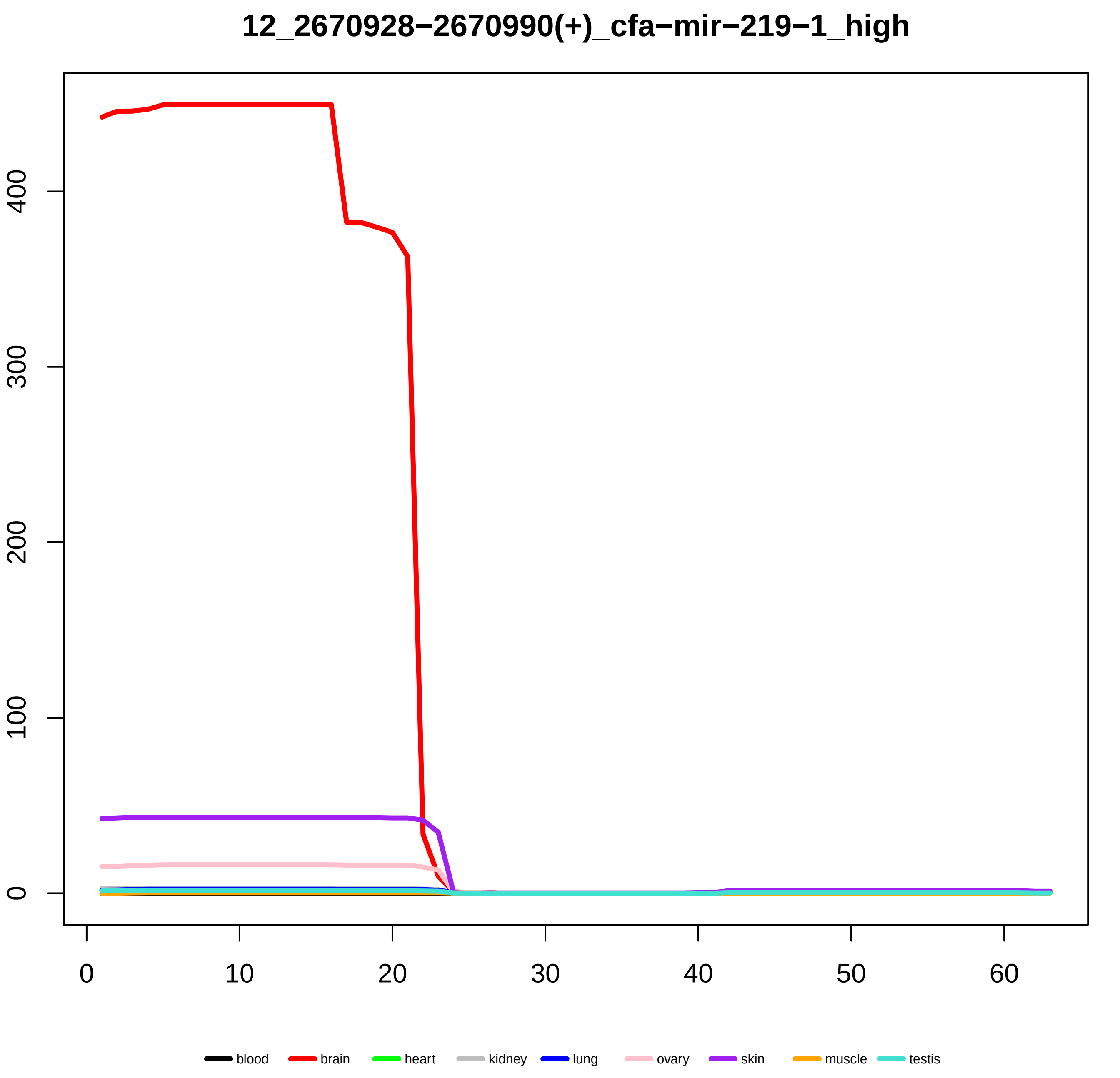

12_2670928−2670990(+)_cfa−mir−219−1_high
0
100
200
300
400
0
10
20
30
40
50
60
blood
brain
heart
kidney
lung
ovary
skin
muscle
testis

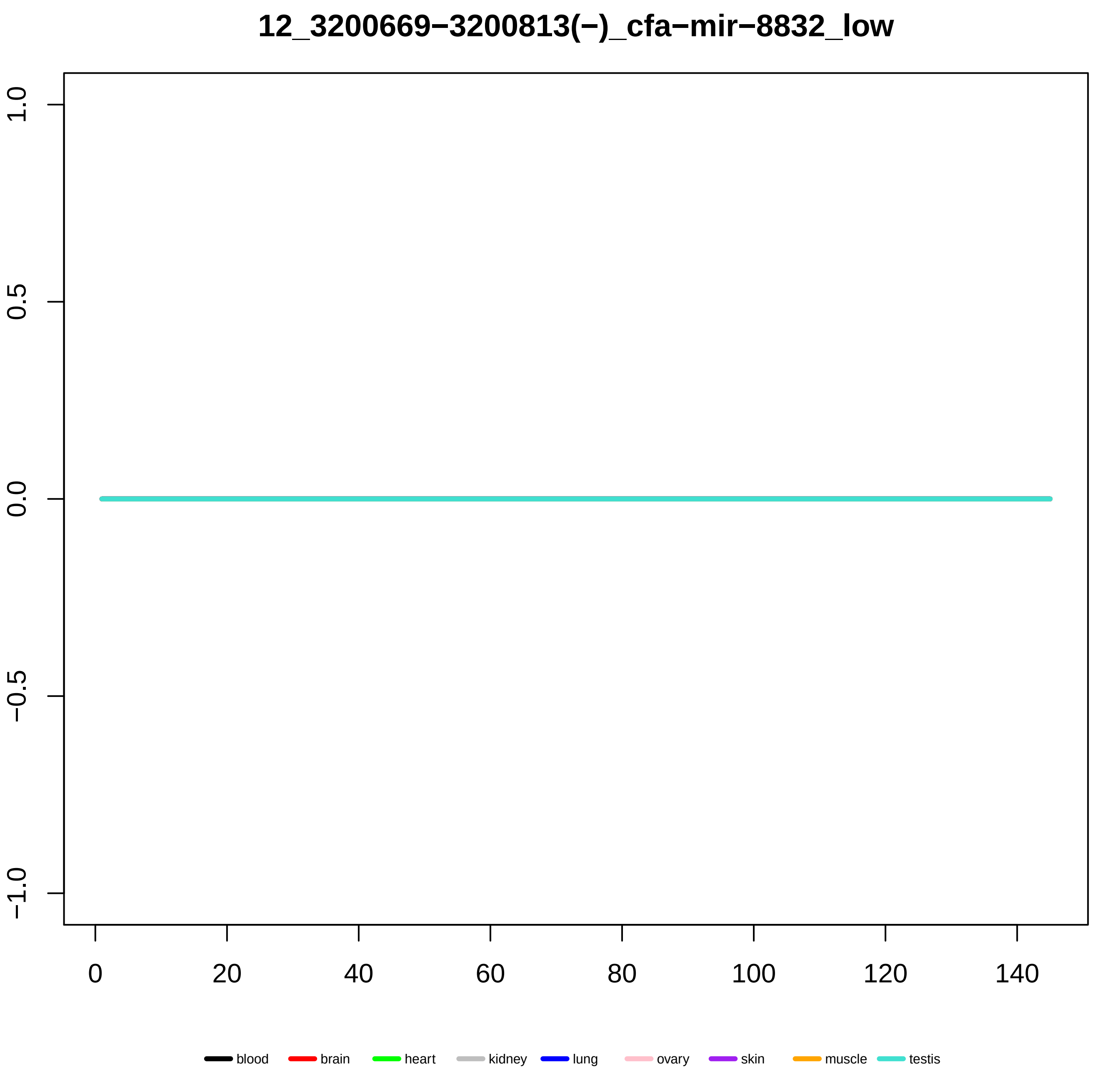
12_3200669−3200813(−)_cfa−mir−8832_low
1.0
0.5
0.0
−0.5
−1.0
0
20
40
60
80
100
120
140
blood
brain
heart
kidney
lung
ovary
skin
muscle
testis

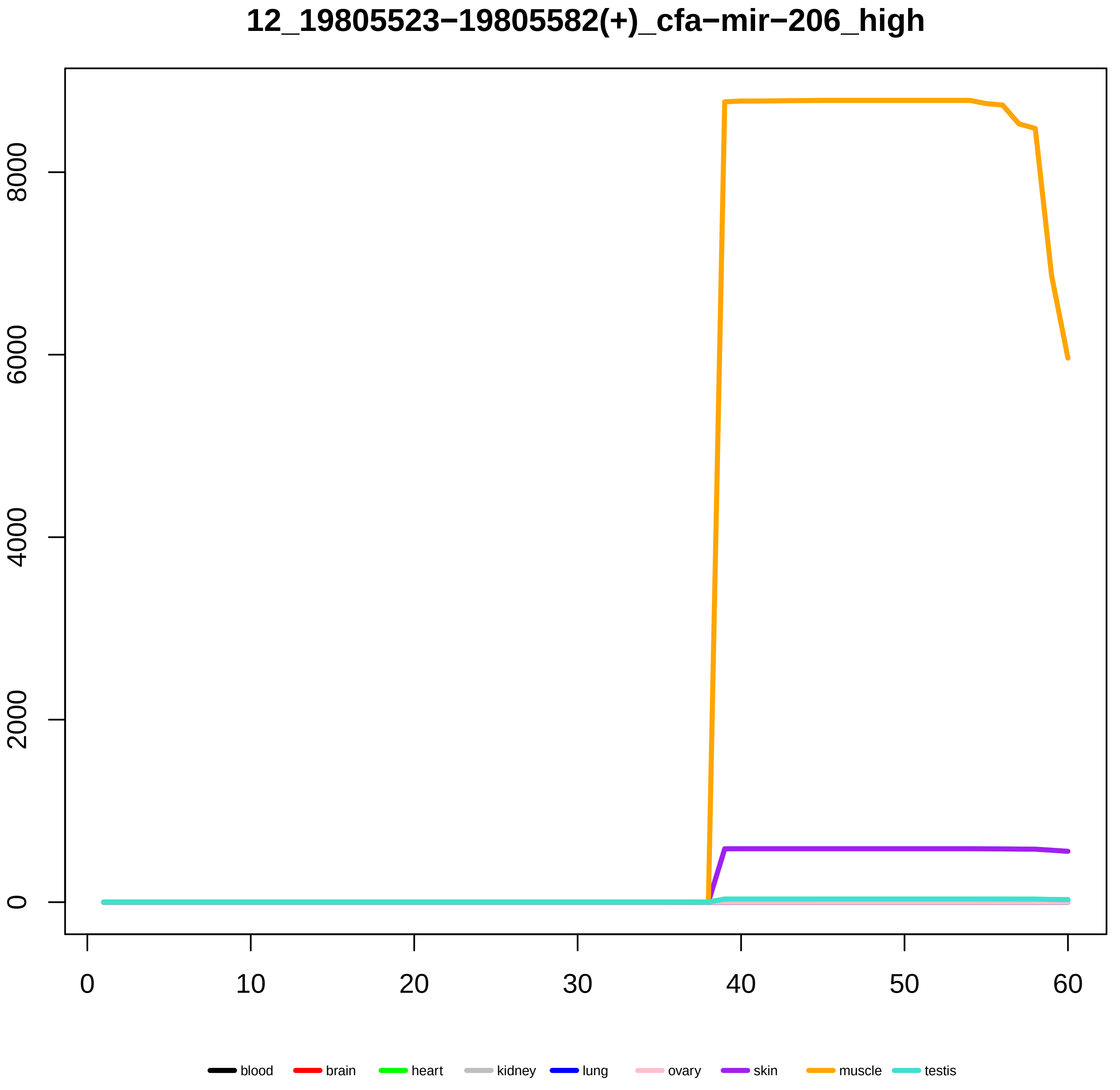

12_19805523−19805582(+)_cfa−mir−206_high
0
2000
4000
6000
8000
0
10
20
30
40
50
60
blood
brain
heart
kidney
lung
ovary
skin
muscle
testis

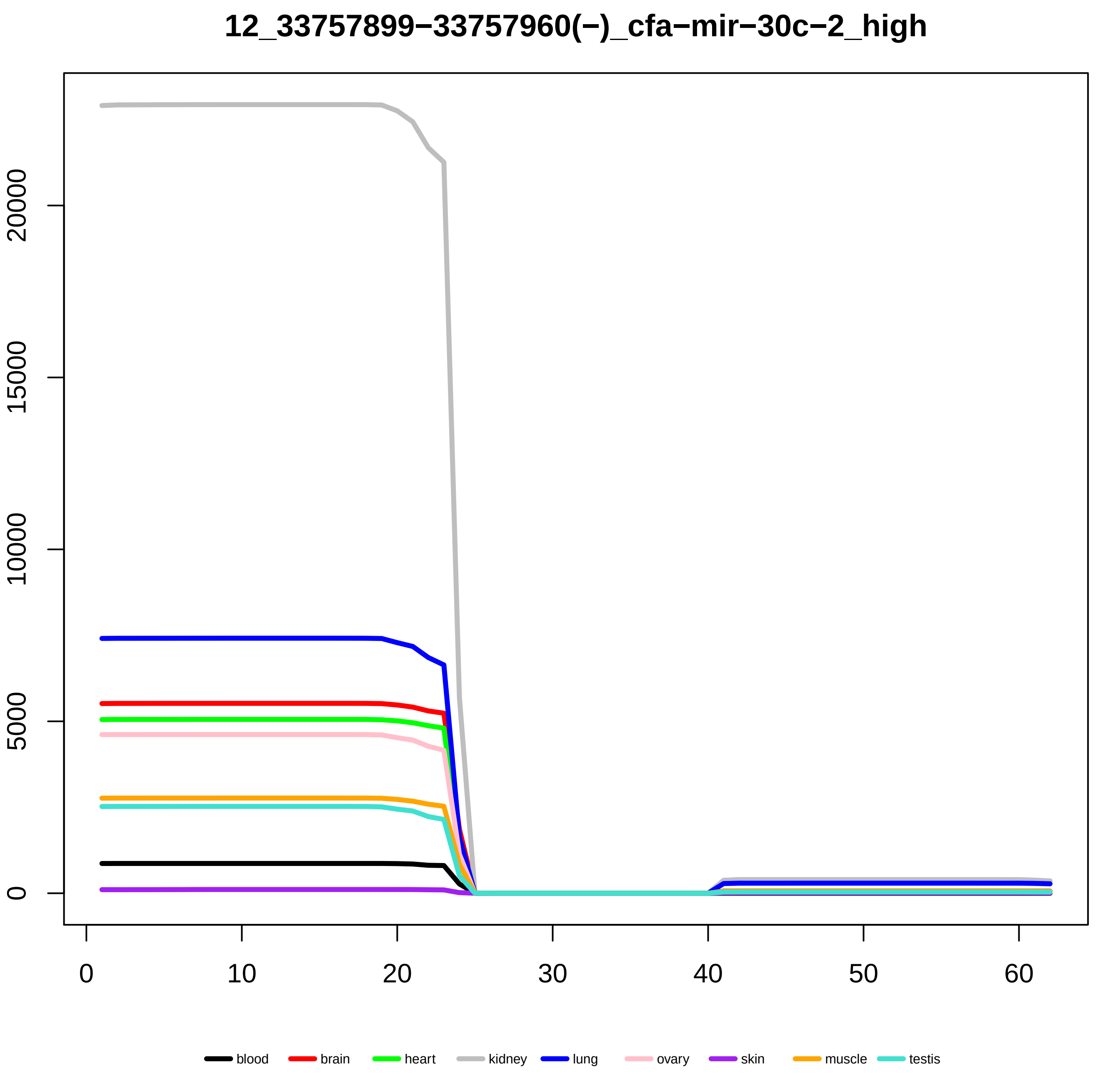

12_33757899−33757960(−)_cfa−mir−30c−2_high
0
5000
10000
15000
20000
0
10
20
30
40
50
60
blood
brain
heart
kidney
lung
ovary
skin
muscle
testis

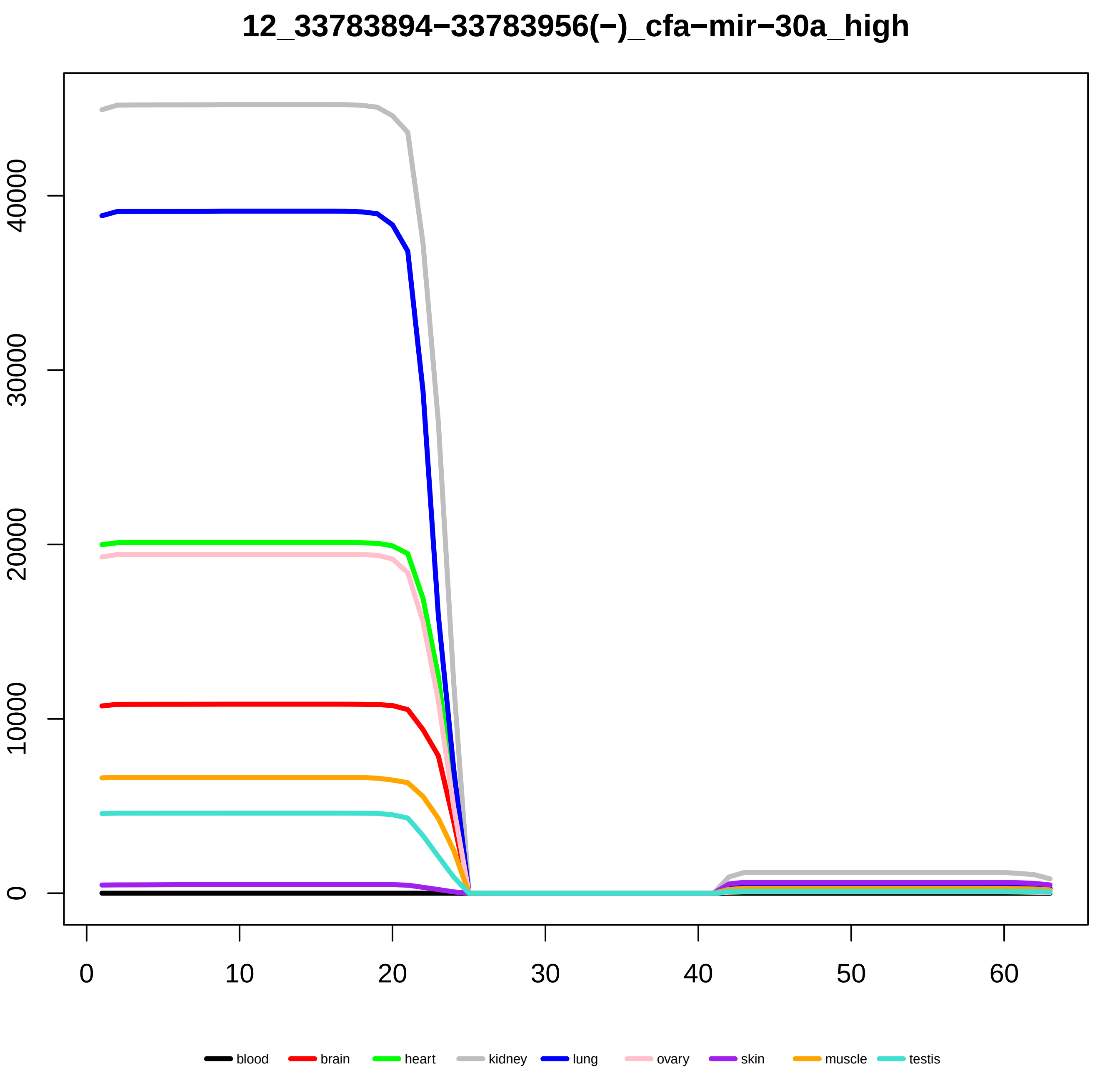
12_33783894−33783956(−)_cfa−mir−30a_high
0
10000
20000
30000
40000
0
10
20
30
40
50
60
blood
brain
heart
kidney
lung
ovary
skin
muscle
testis

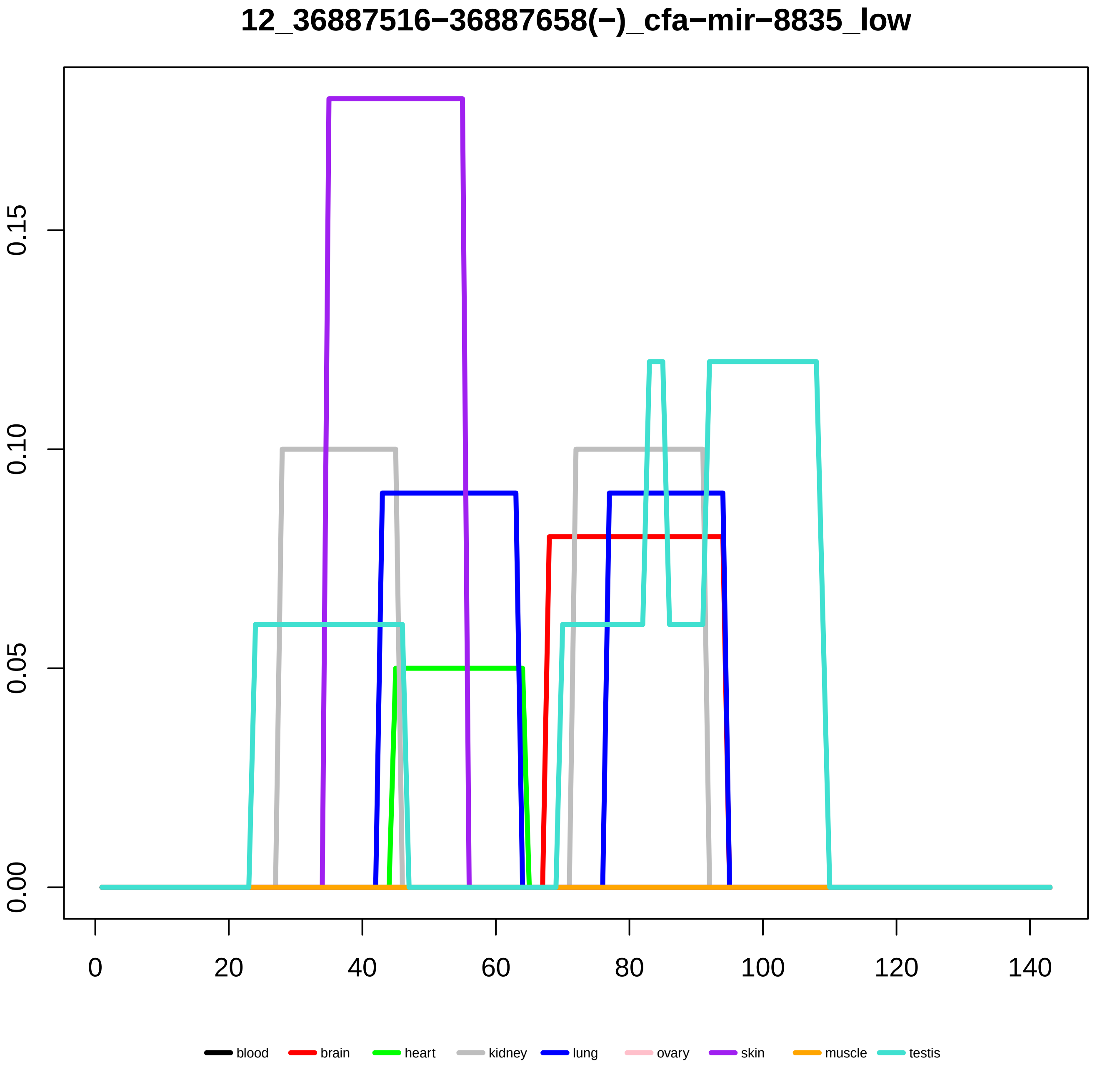

12_36887516−36887658(−)_cfa−mir−8835_low
0.00
0.05
0.10
0.15
0
20
40
60
80
100
120
140
blood
brain
heart
kidney
lung
ovary
skin
muscle
testis

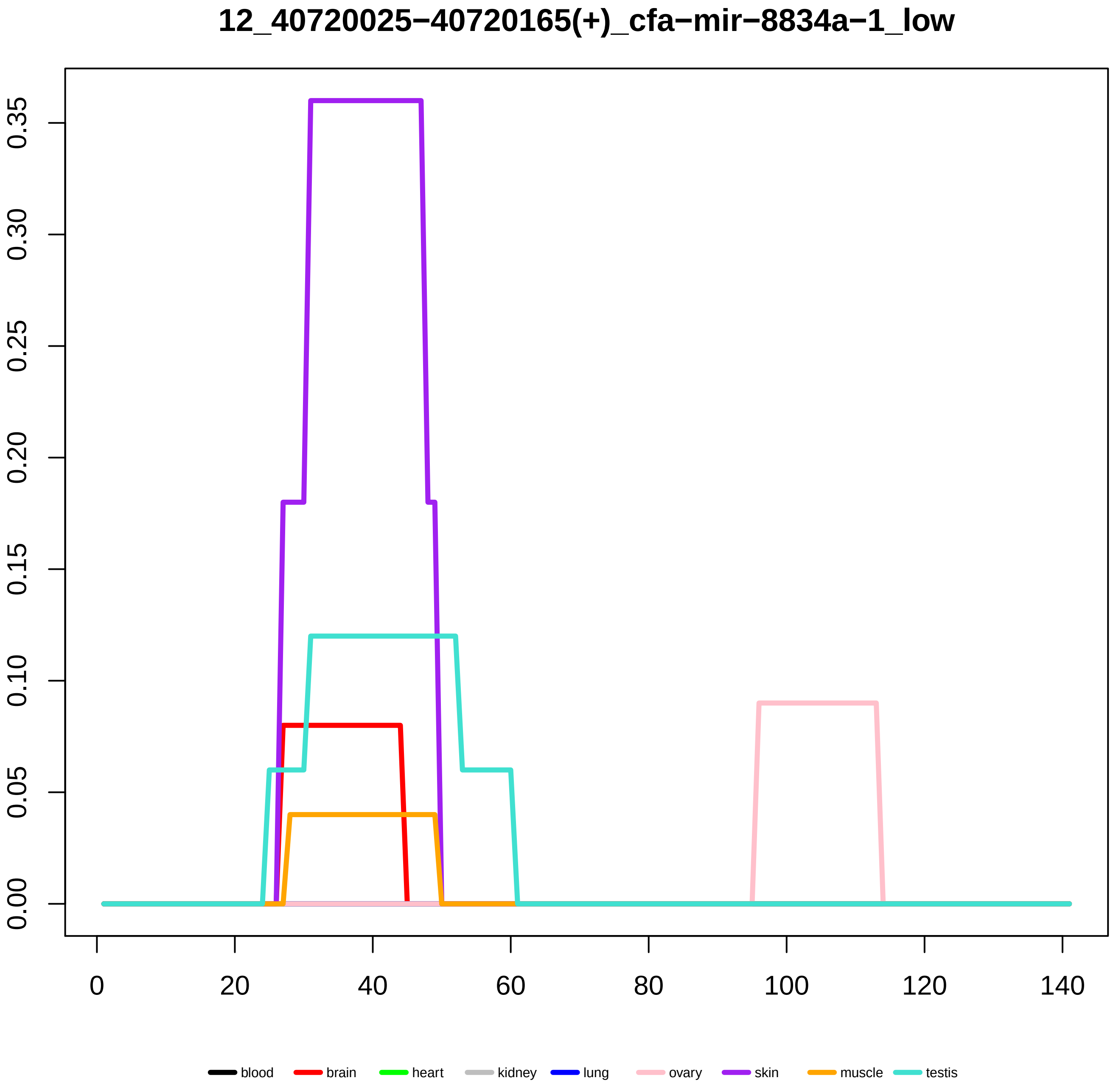
12_40720025−40720165(+)_cfa−mir−8834a−1_low
0.00
0.05
0.10
0.15
0.20
0.25
0.30
0.35
0
20
40
60
80
100
120
140
blood
brain
heart
kidney
lung
ovary
skin
muscle
testis

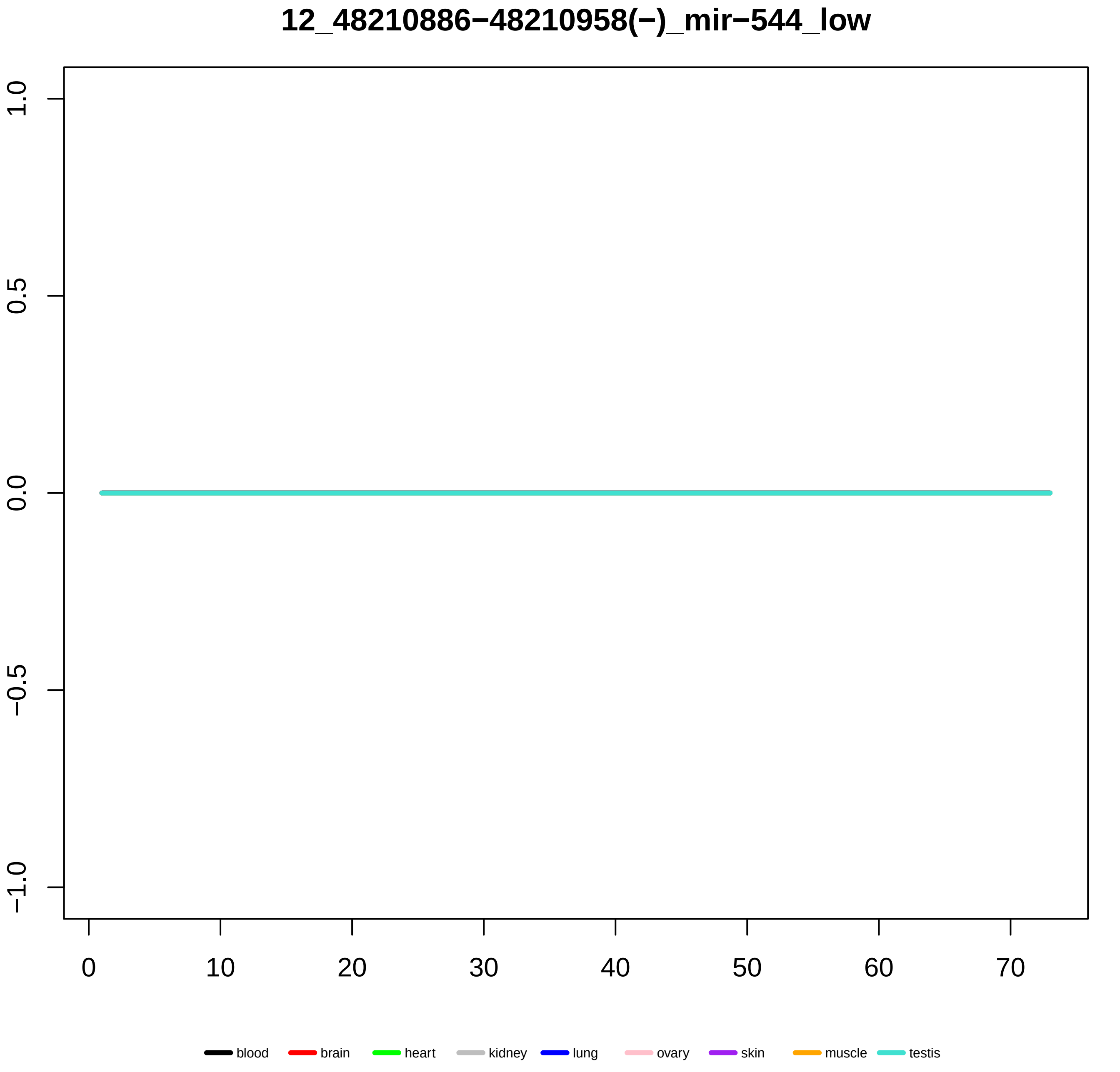

12_48210886−48210958(−)_mir−544_low
1.0
0.5
0.0
−0.5
−1.0
0
10
20
30
40
50
60
70
blood
brain
heart
kidney
lung
ovary
skin
muscle
testis

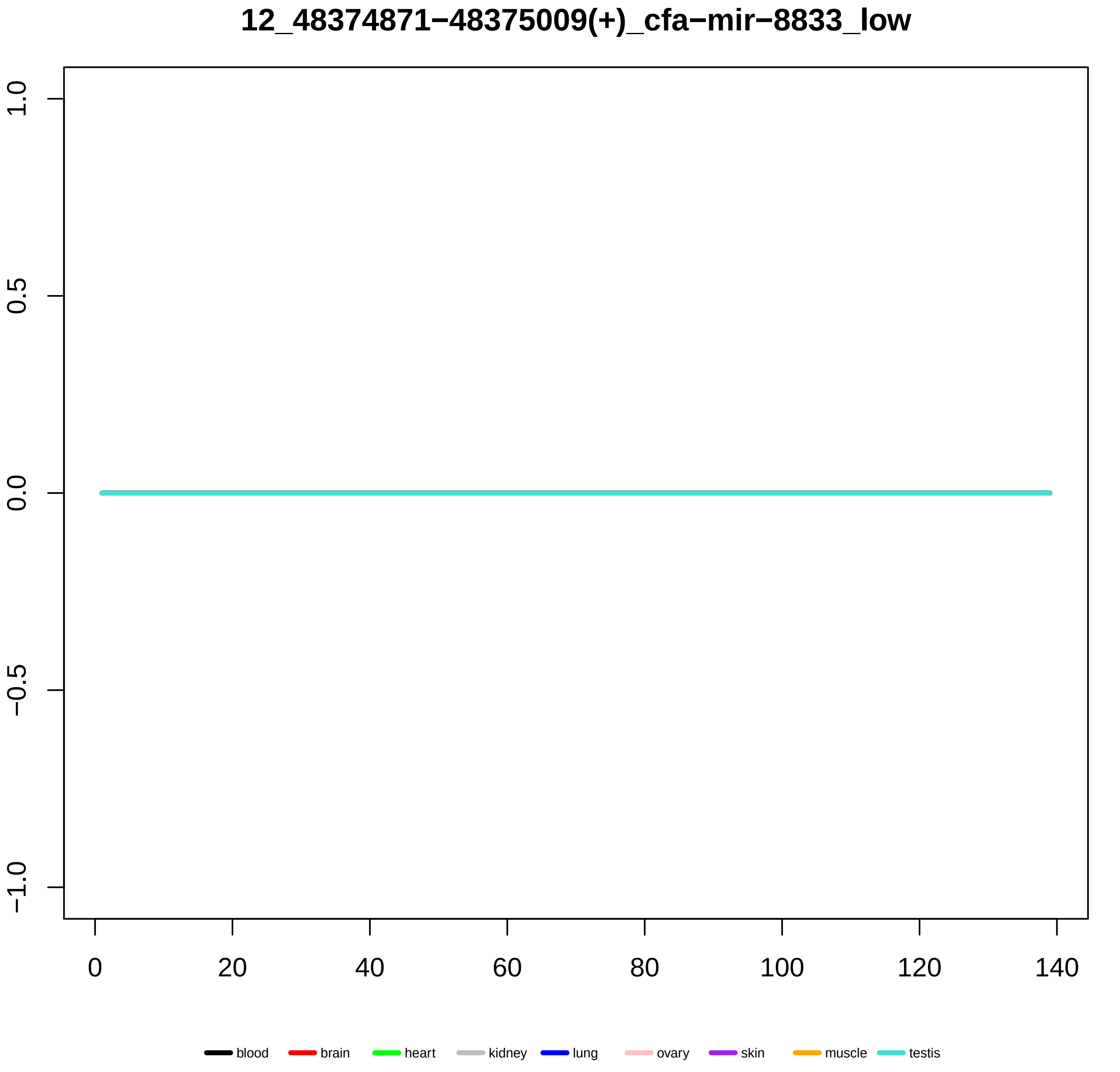

12_48374871−48375009(+)_cfa−mir−8833_low
1.0
0.5
0.0
−0.5
−1.0
0
20
40
60
80
100
120
140
blood
brain
heart
kidney
lung
ovary
skin
muscle
testis

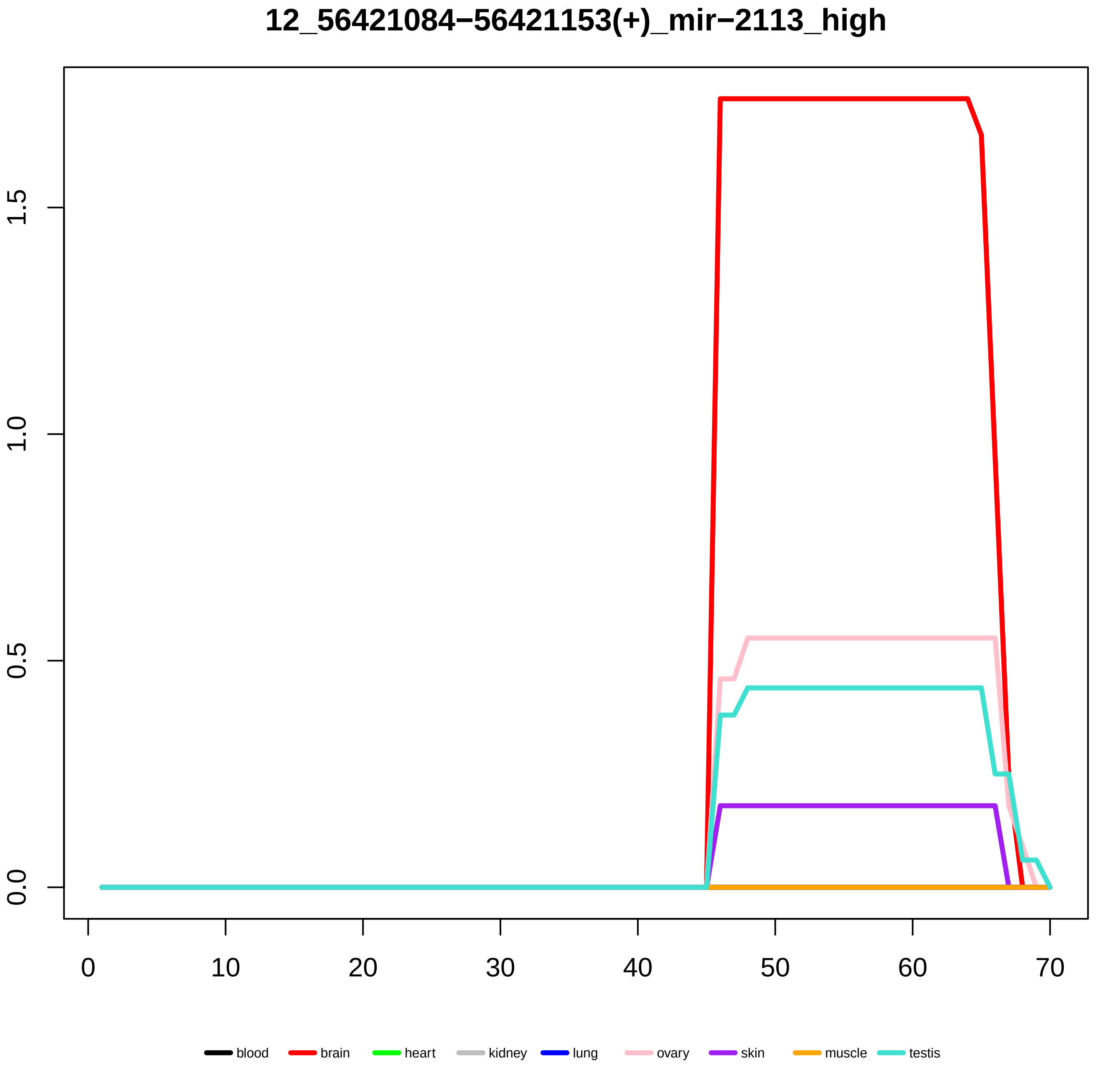
12_56421084−56421153(+)_mir−2113_high
0.0
0.5
1.0
1.5
0
10
20
30
40
50
60
70
blood
brain
heart
kidney
lung
ovary
skin
muscle
testis

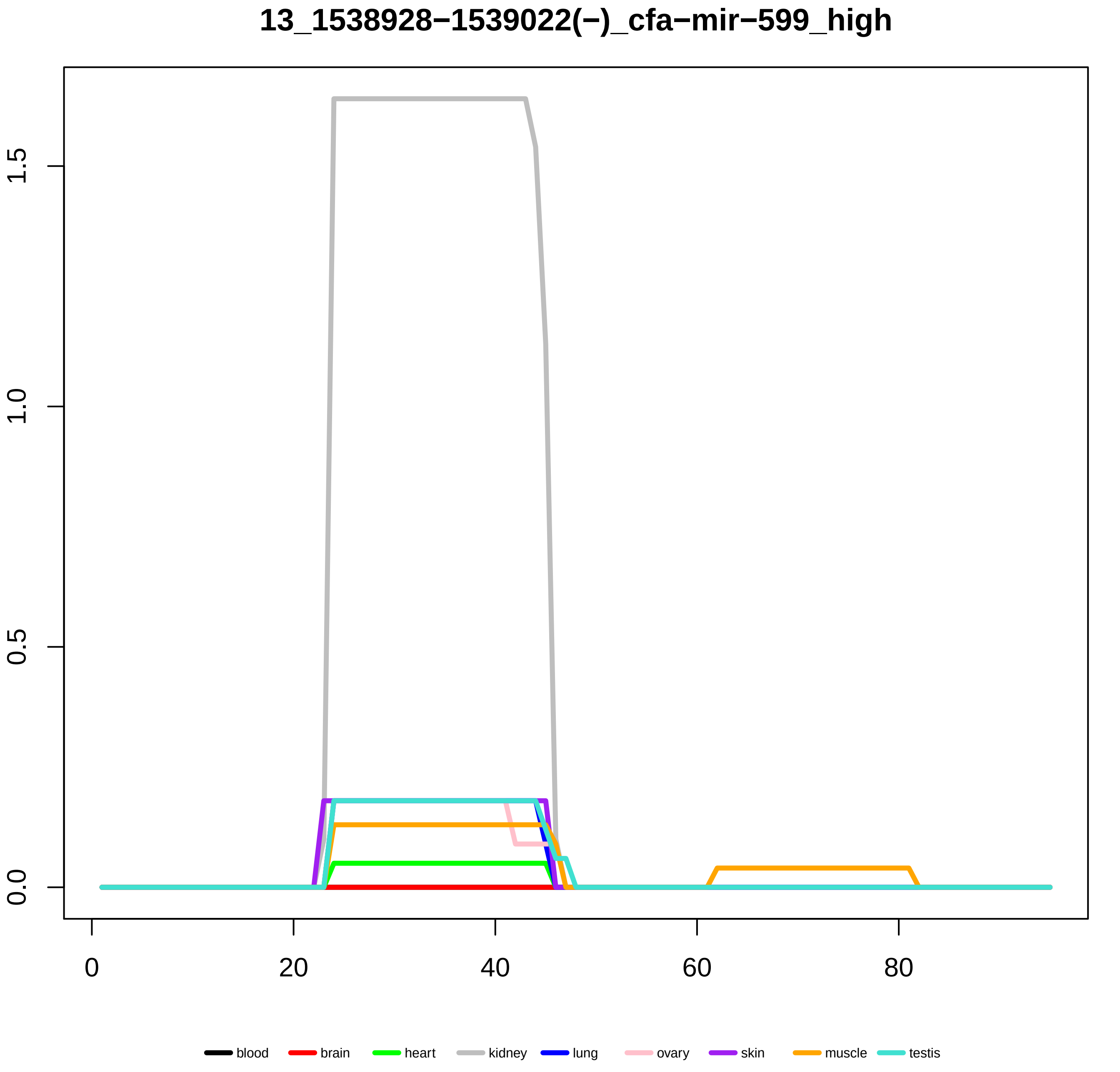

13_1538928−1539022(−)_cfa−mir−599_high
0.0
0.5
1.0
1.5
0
20
40
60
80
blood
brain
heart
kidney
lung
ovary
skin
muscle
testis

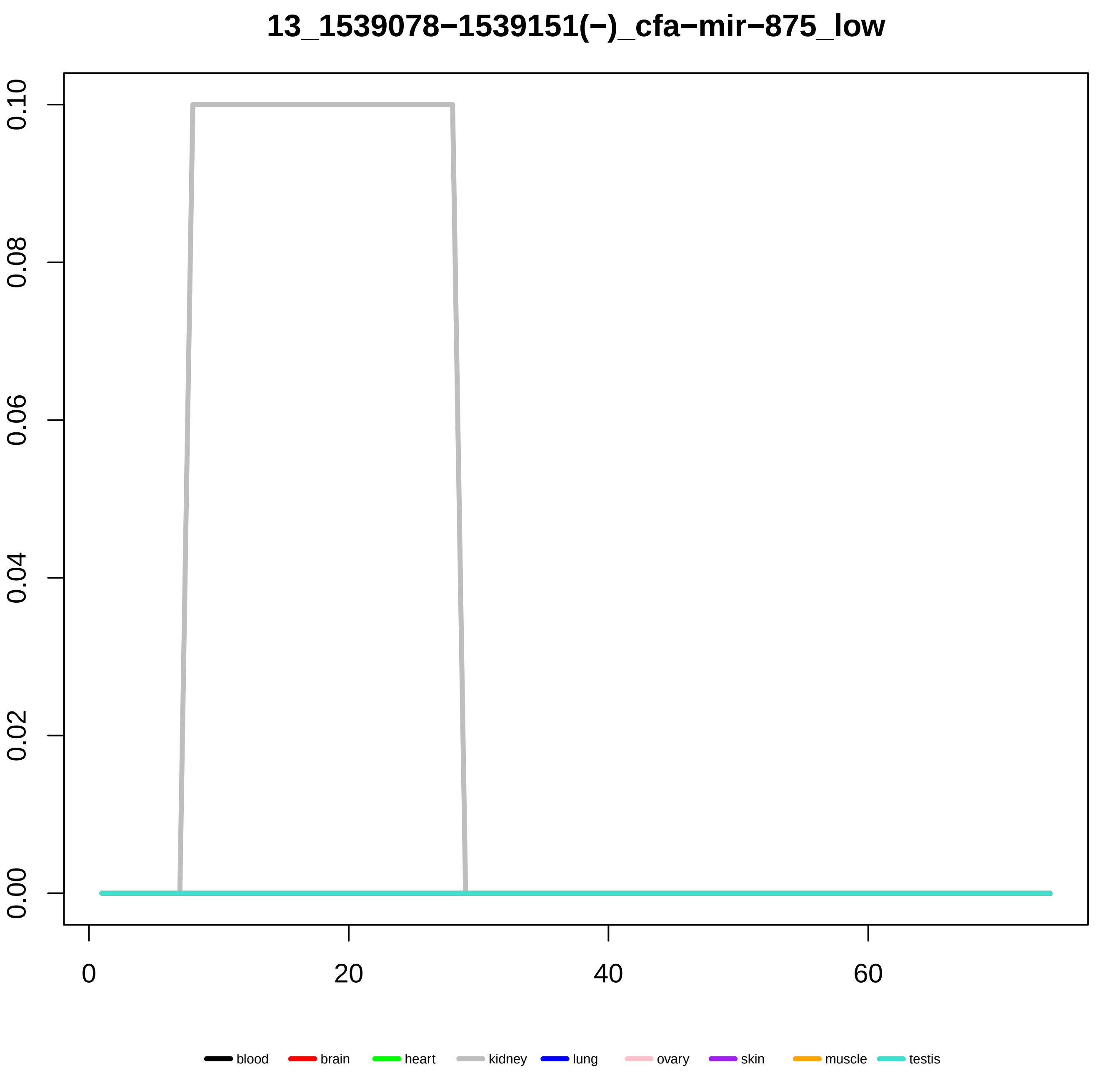
13_1539078−1539151(−)_cfa−mir−875_low
0.00
0.02
0.04
0.06
0.08
0.10
0
20
40
60
blood
brain
heart
kidney
lung
ovary
skin
muscle
testis

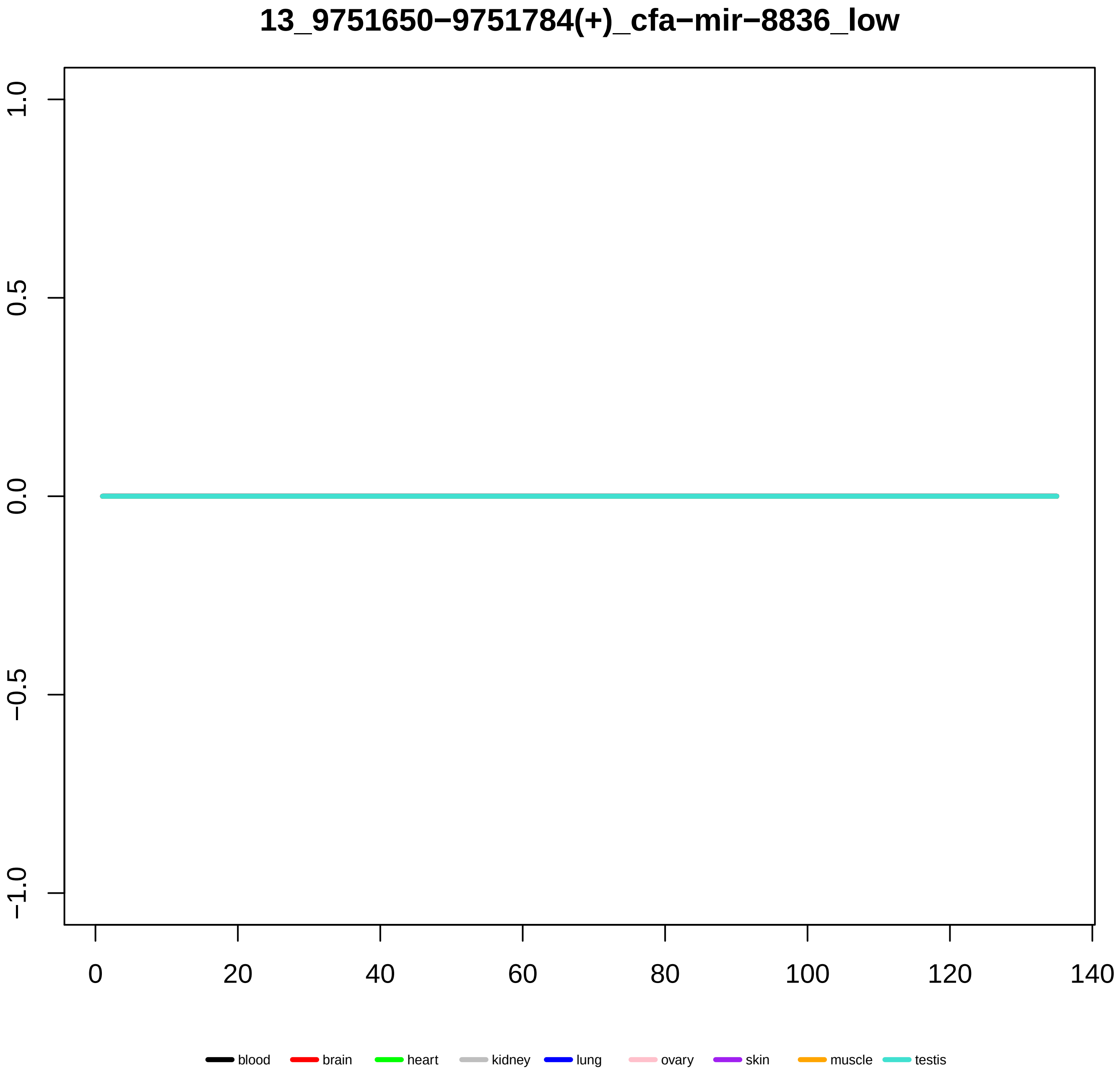
13_9751650−9751784(+)_cfa−mir−8836_low
1.0
0.5
0.0
−0.5
−1.0
0
20
40
60
80
100
120
140
blood
brain
heart
kidney
lung
ovary
skin
muscle
testis

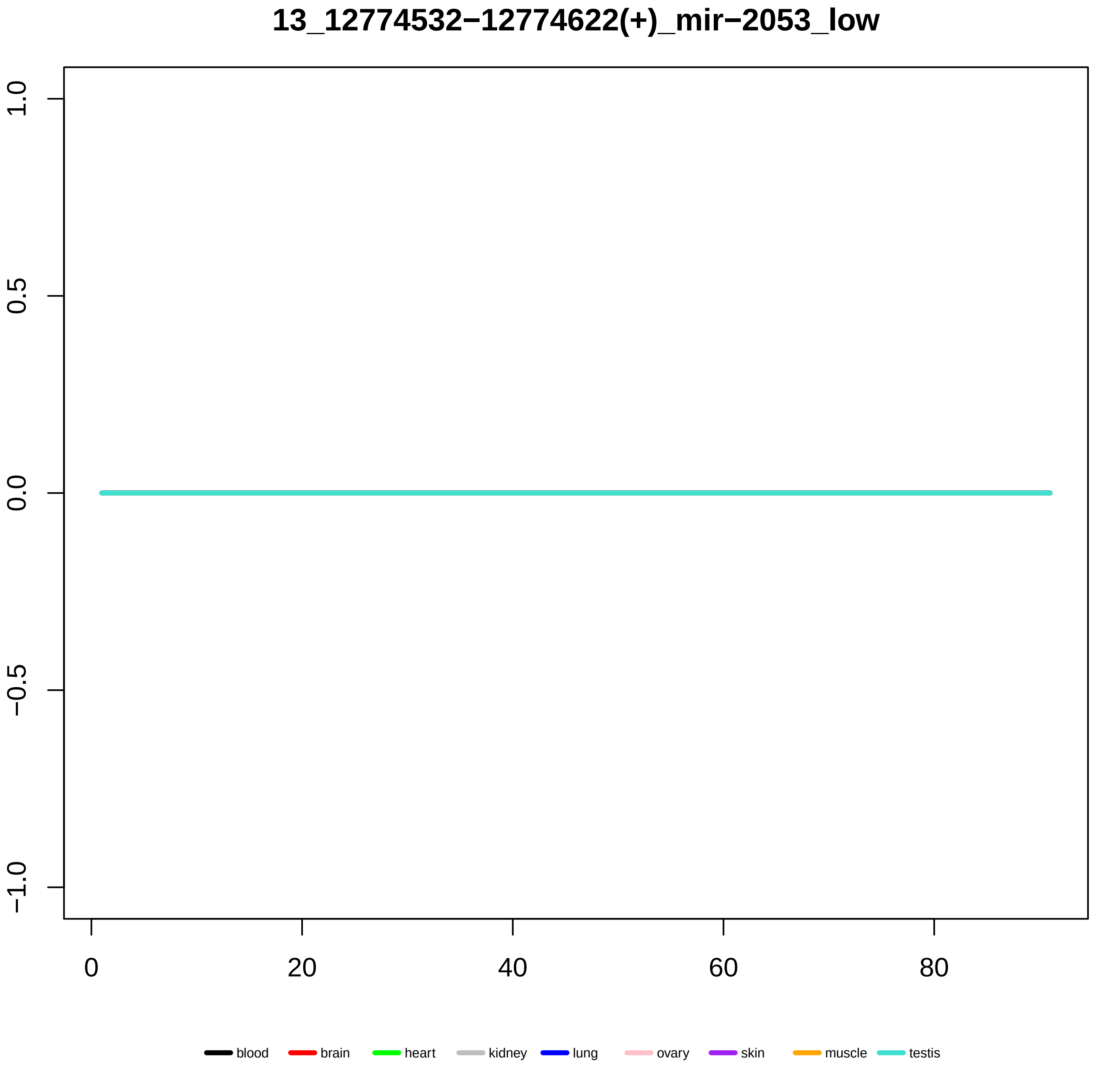

13_12774532−12774622(+)_mir−2053_low
1.0
0.5
0.0
−0.5
−1.0
0
20
40
60
80
blood
brain
heart
kidney
lung
ovary
skin
muscle
testis

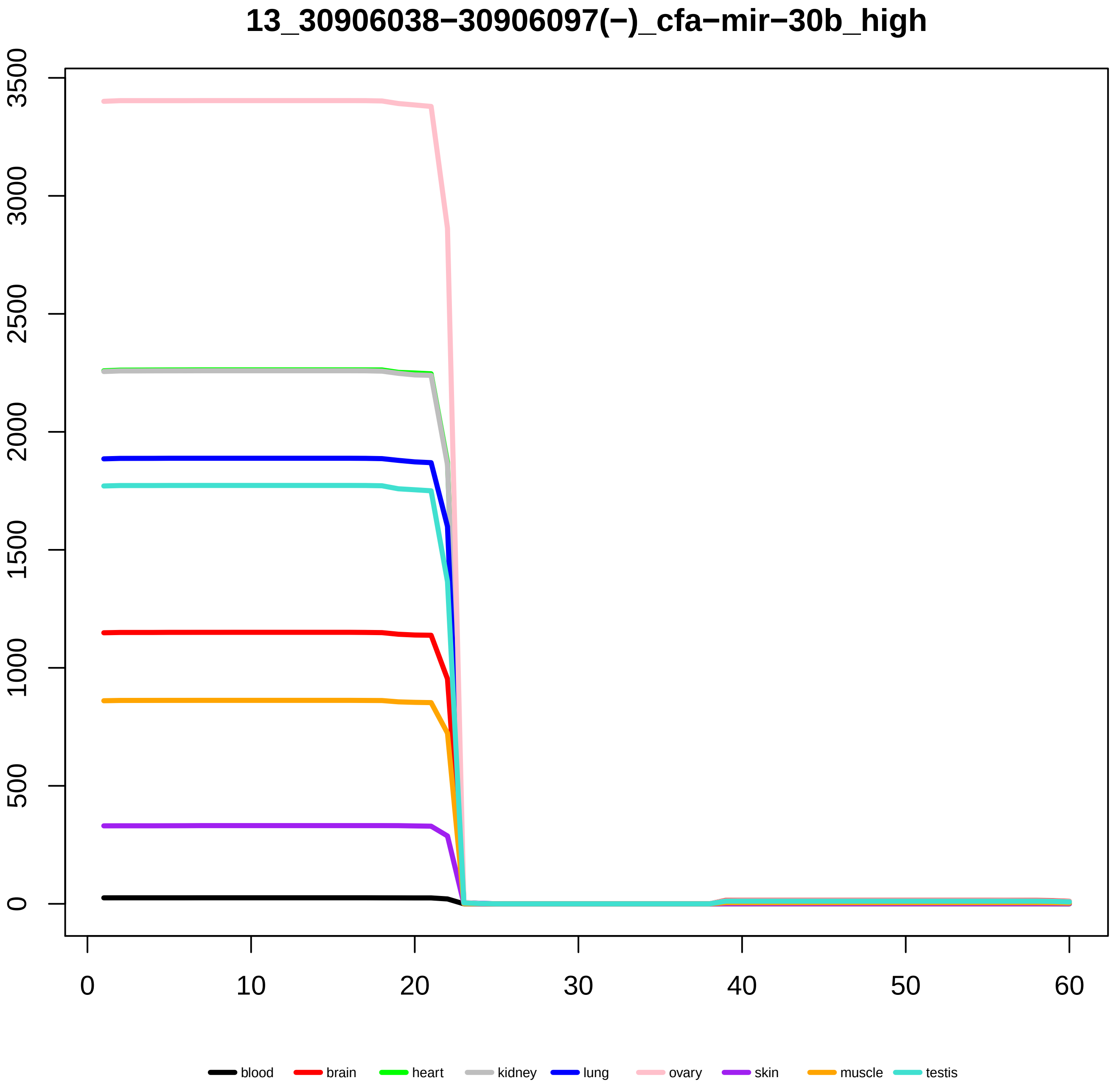

13_30906038−30906097(−)_cfa−mir−30b_high
0
500
1000
1500
2000
2500
3000
3500
0
10
20
30
40
50
60
blood
brain
heart
kidney
lung
ovary
skin
muscle
testis

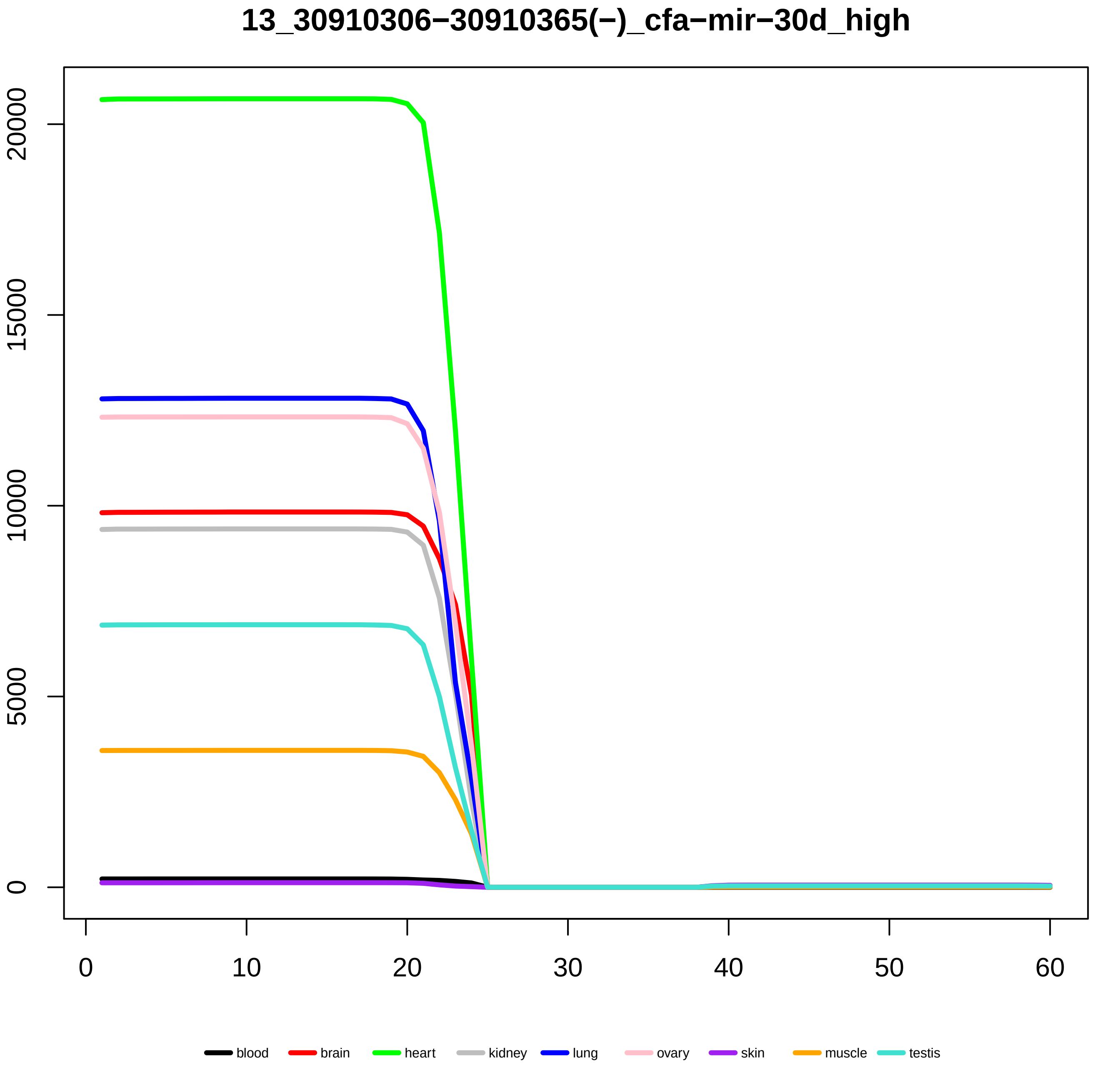
13_30910306−30910365(−)_cfa−mir−30d_high
20000
15000
10000
5000
0
0
10
20
30
40
50
60
blood
brain
heart
kidney
lung
ovary
skin
muscle
testis

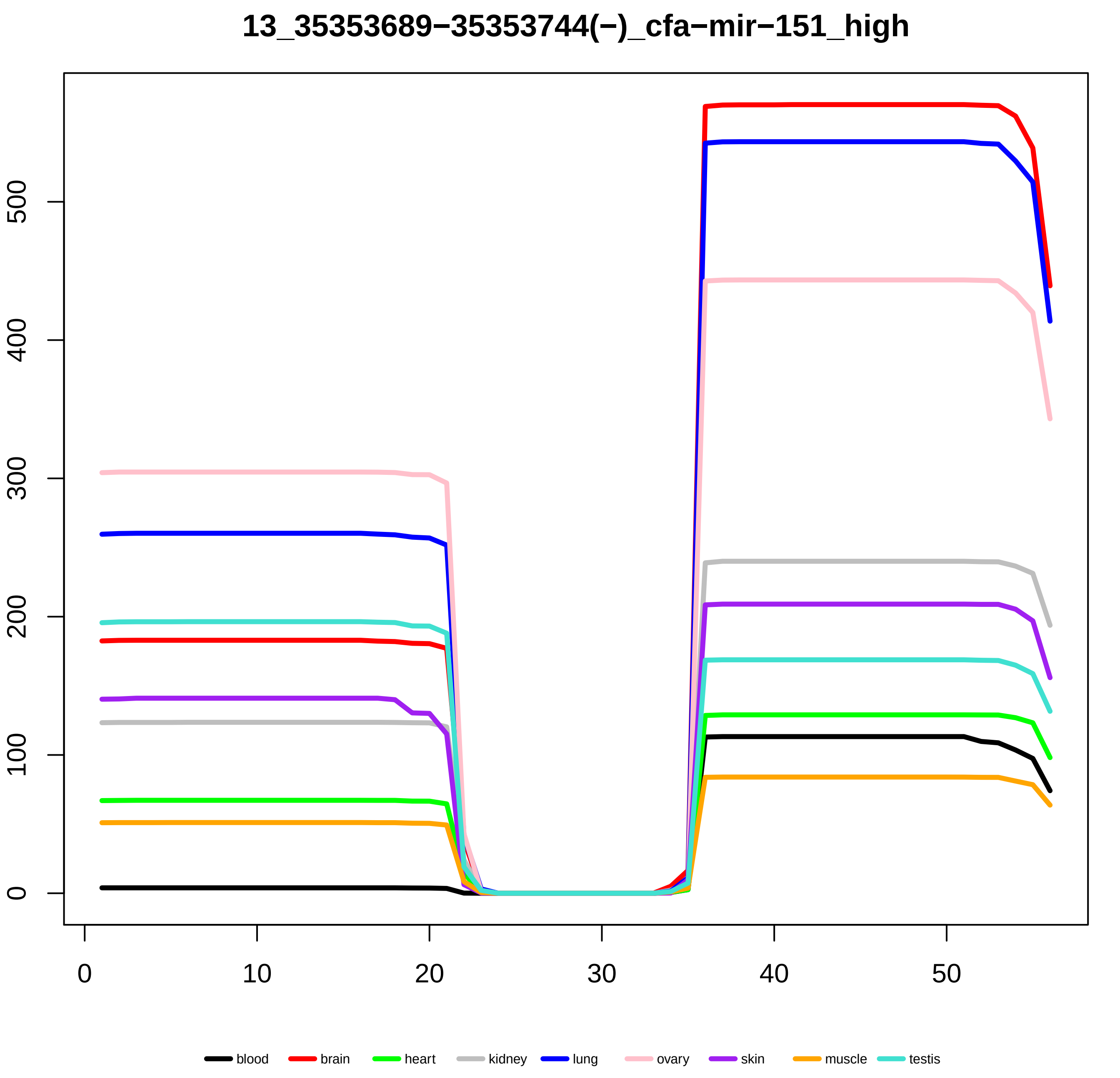
13_35353689−35353744(−)_cfa−mir−151_high
0
100
200
300
400
500
0
10
20
30
40
50
blood
brain
heart
kidney
lung
ovary
skin
muscle
testis

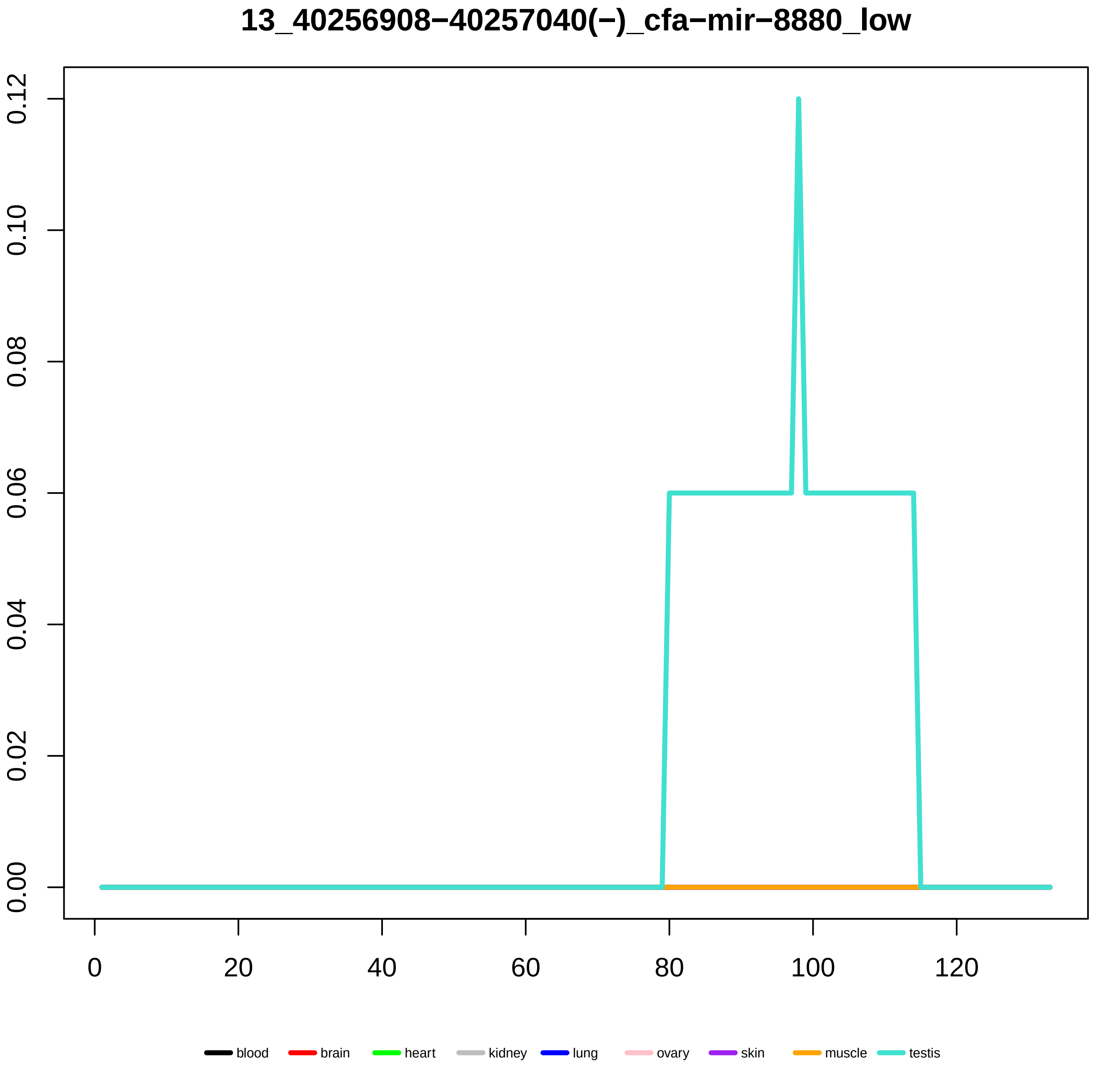

13_40256908−40257040(−)_cfa−mir−8880_low
0.00
0.02
0.04
0.06
0.08
0.10
0.12
0
20
40
60
80
100
120
blood
brain
heart
kidney
lung
ovary
skin
muscle
testis

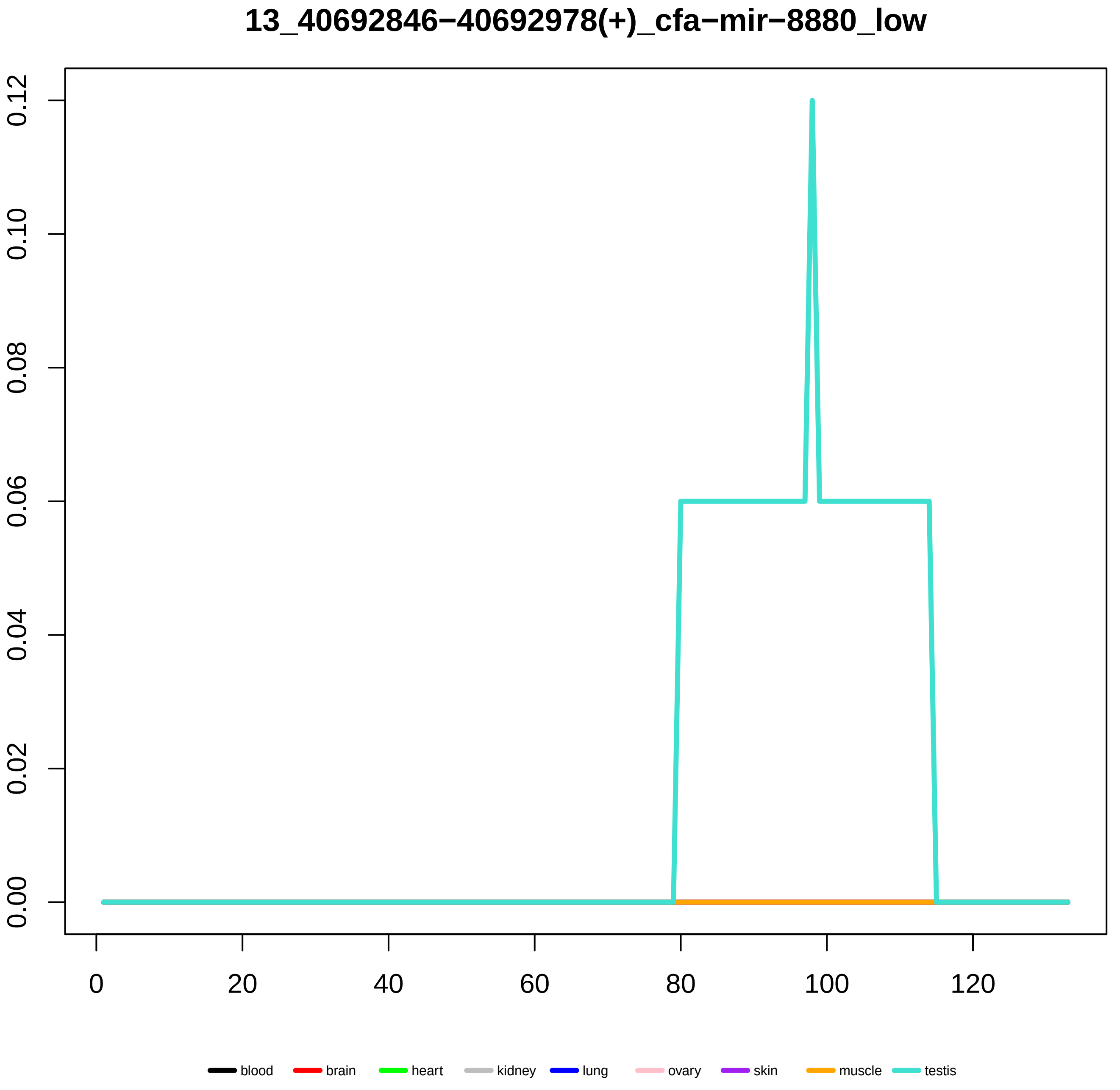
13_40692846−40692978(+)_cfa−mir−8880_low
0.00
0.02
0.04
0.06
0.08
0.10
0.12
0
20
40
60
80
100
120
blood
brain
heart
kidney
lung
ovary
skin
muscle
testis

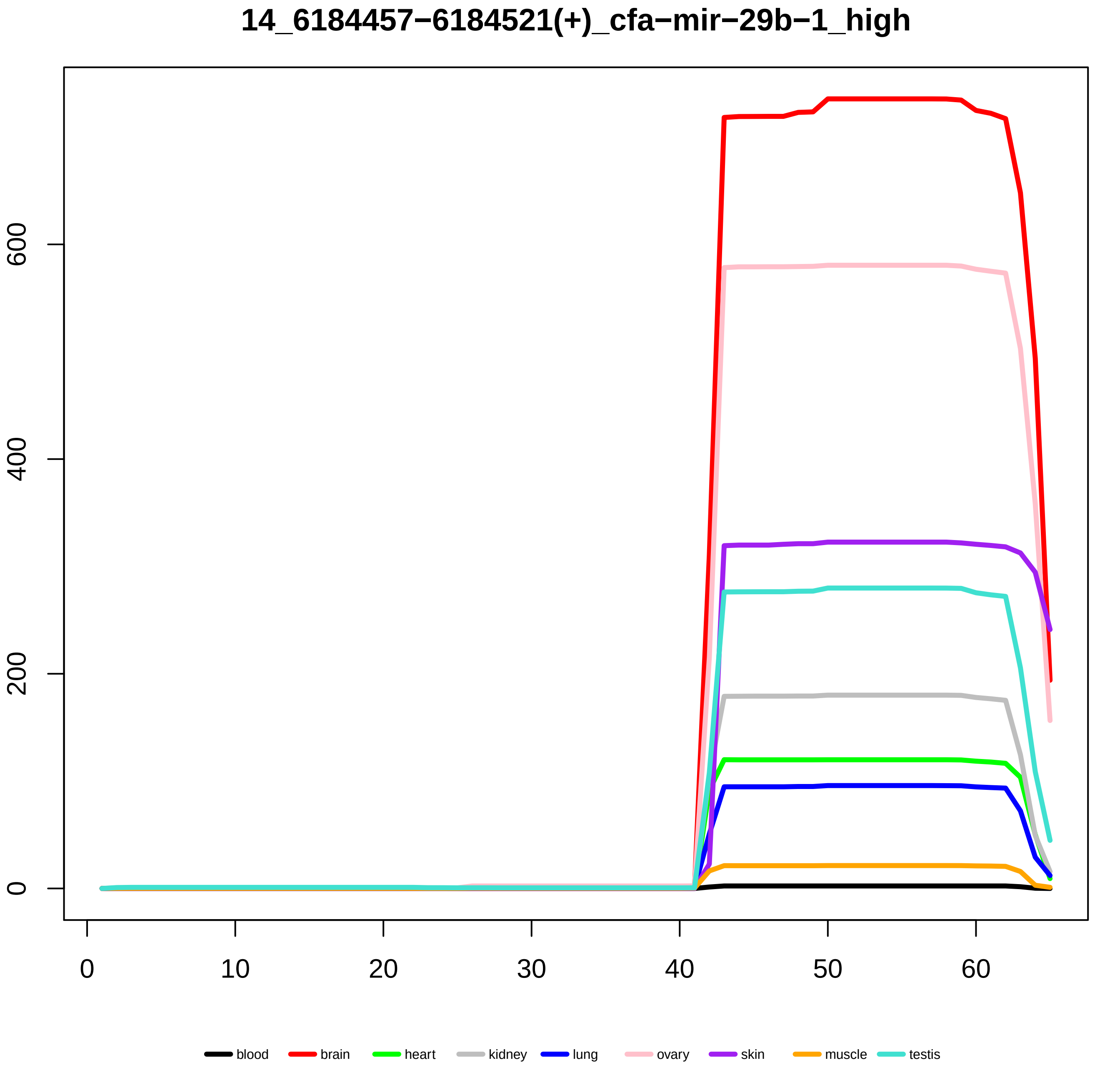

14_6184457−6184521(+)_cfa−mir−29b−1_high
0
200
400
600
0
10
20
30
40
50
60
blood
brain
heart
kidney
lung
ovary
skin
muscle
testis

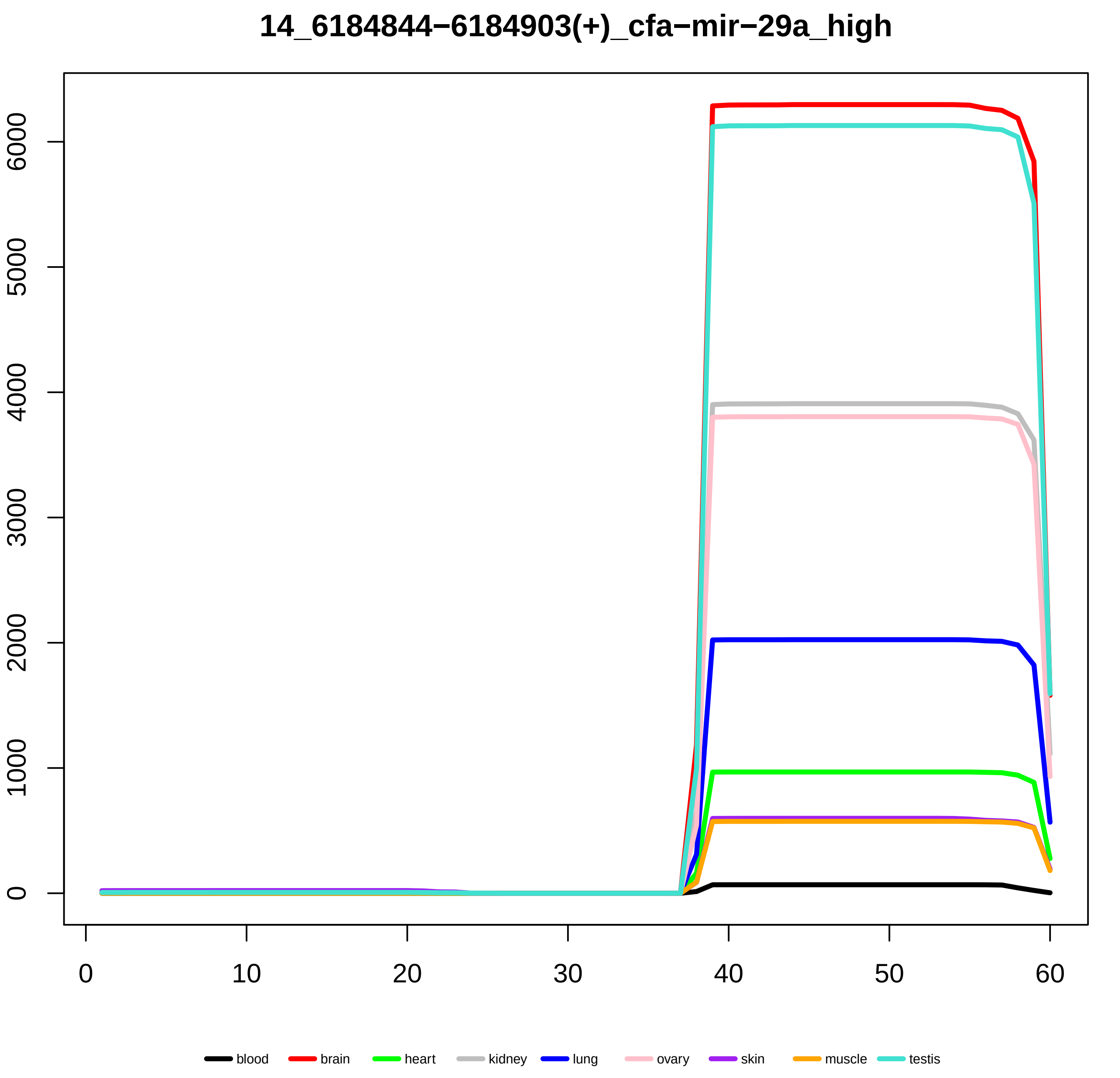

14_6184844−6184903(+)_cfa−mir−29a_high
0
1000
2000
3000
4000
5000
6000
0
10
20
30
40
50
60
blood
brain
heart
kidney
lung
ovary
skin
muscle
testis

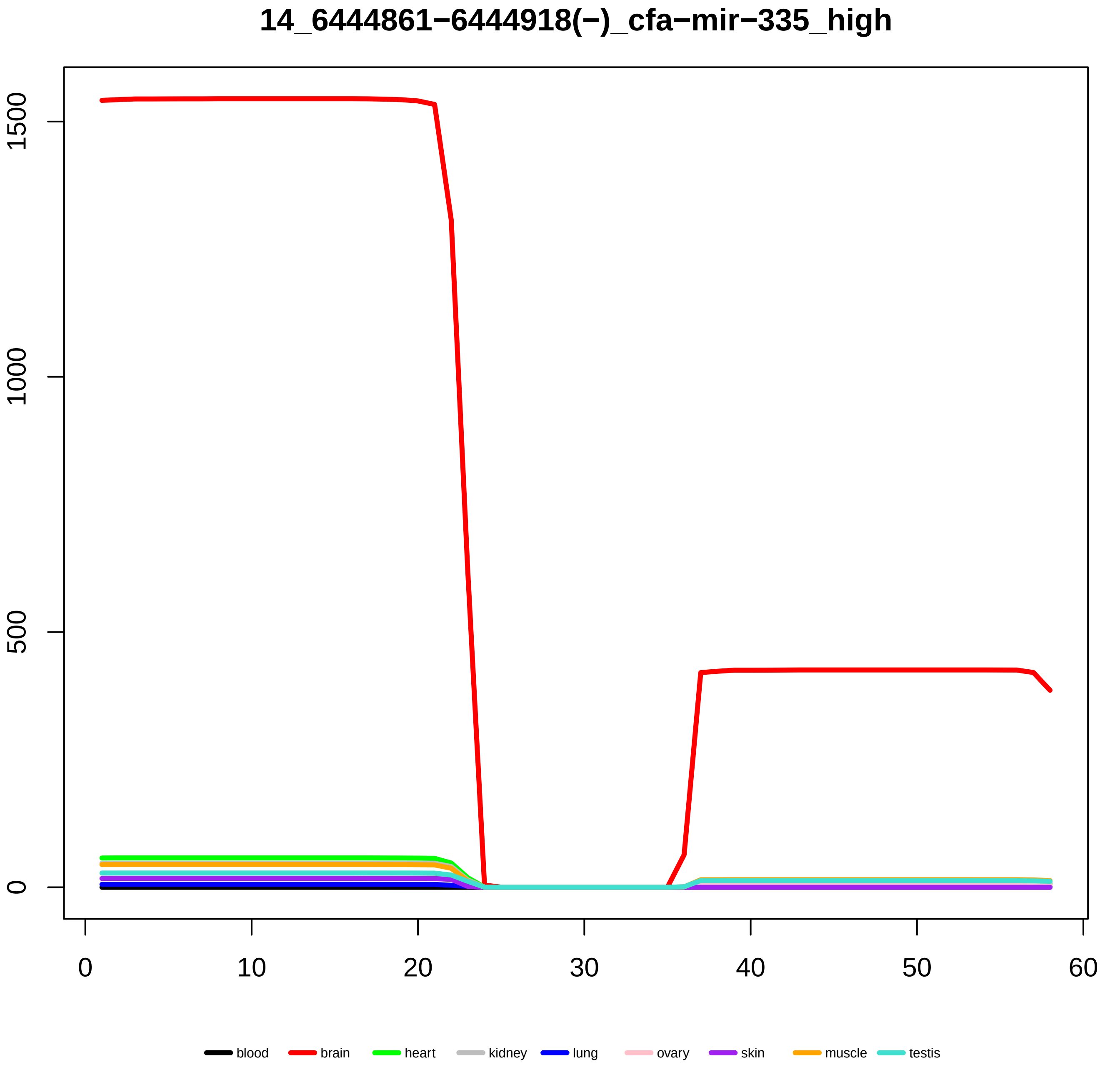
14_6444861−6444918(−)_cfa−mir−335_high
0
500
1000
1500
0
10
20
30
40
50
60
blood
brain
heart
kidney
lung
ovary
skin
muscle
testis

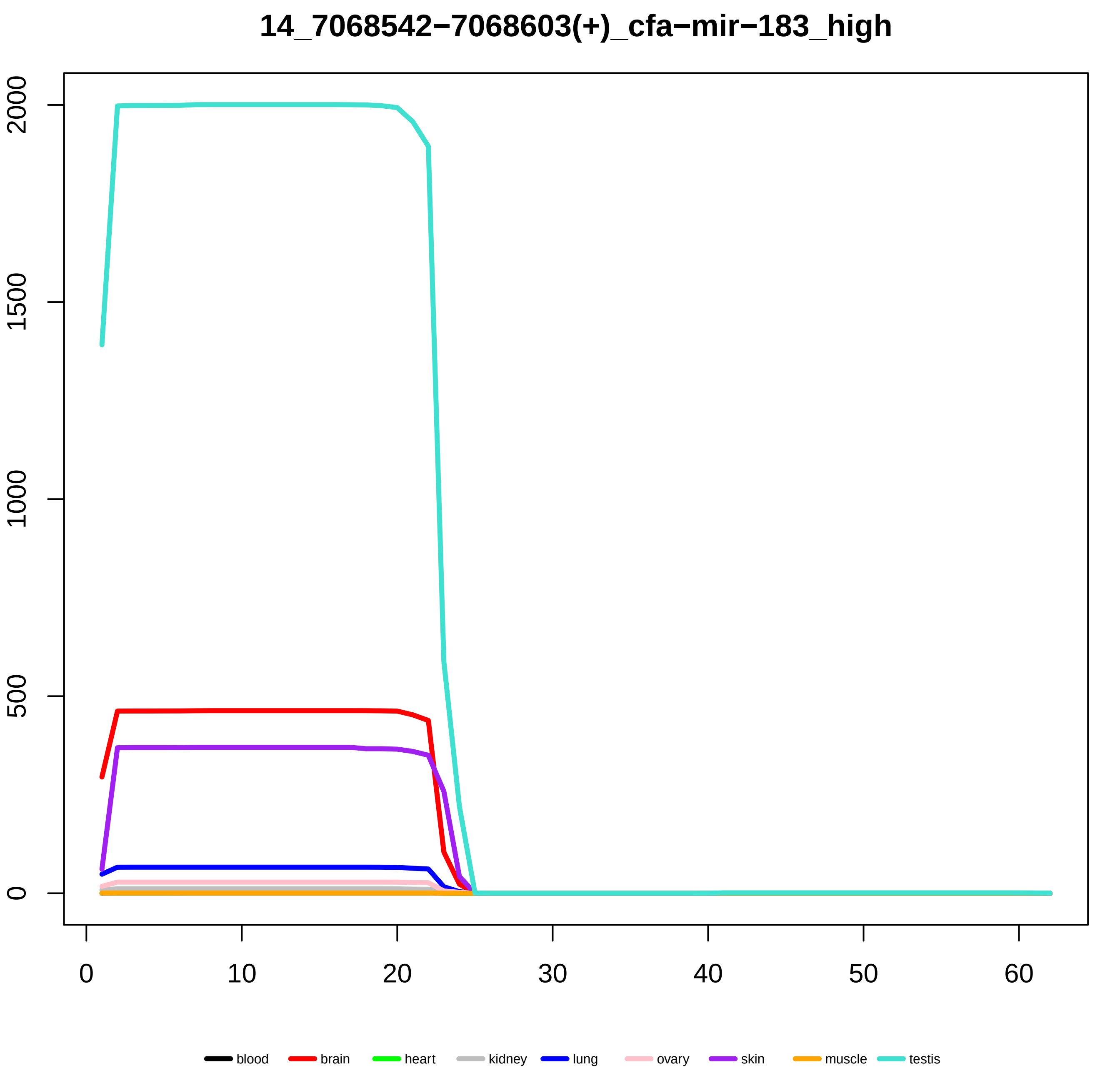

14_7068542−7068603(+)_cfa−mir−183_high
0
500
1000
1500
2000
0
10
20
30
40
50
60
blood
brain
heart
kidney
lung
ovary
skin
muscle
testis

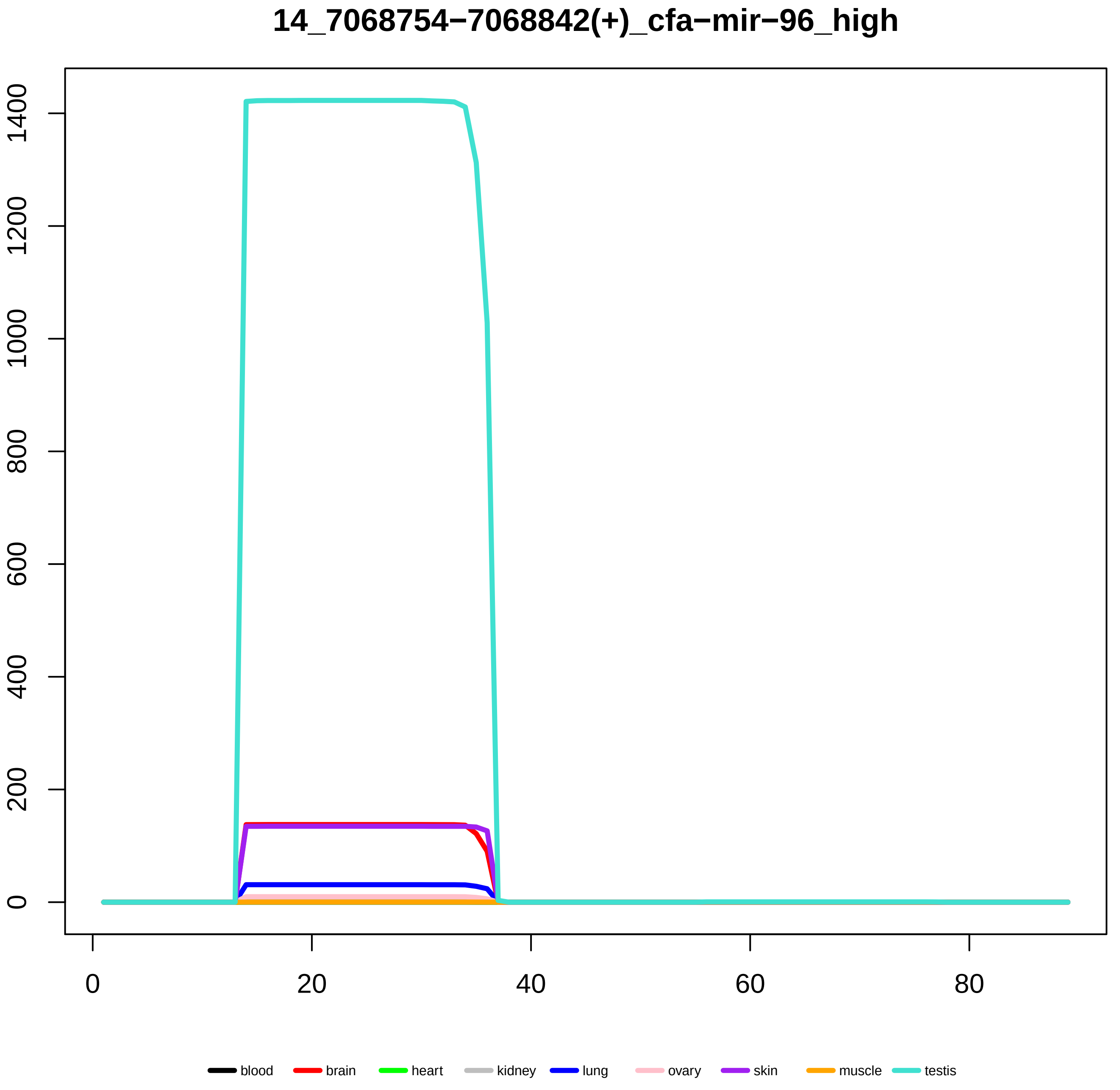

14_7068754−7068842(+)_cfa−mir−96_high
0
200
400
600
800
1000
1200
1400
0
20
40
60
80
blood
brain
heart
kidney
lung
ovary
skin
muscle
testis

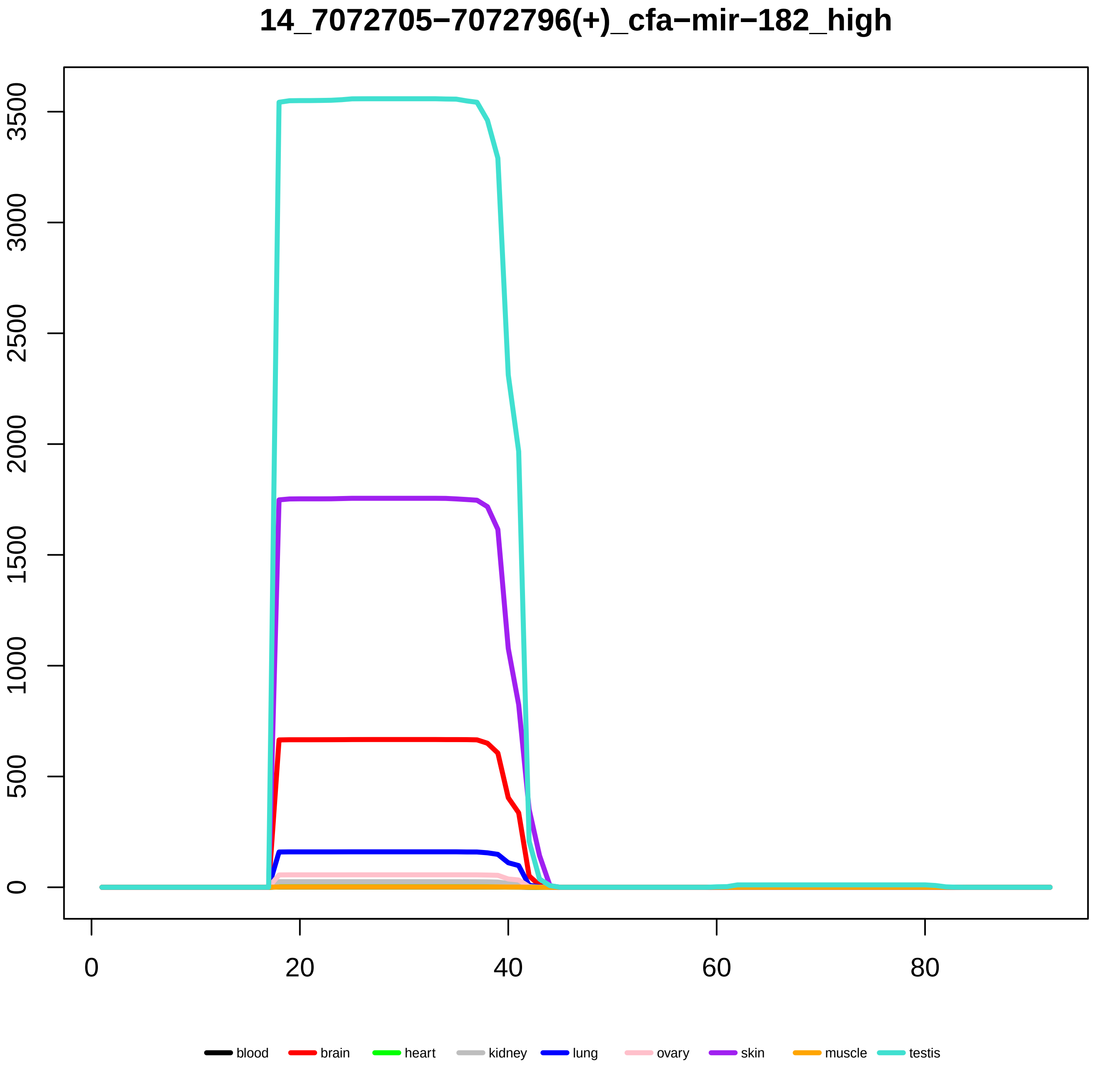

14_7072705−7072796(+)_cfa−mir−182_high
0
500
1000
1500
2000
2500
3000
3500
0
20
40
60
80
blood
brain
heart
kidney
lung
ovary
skin
muscle
testis

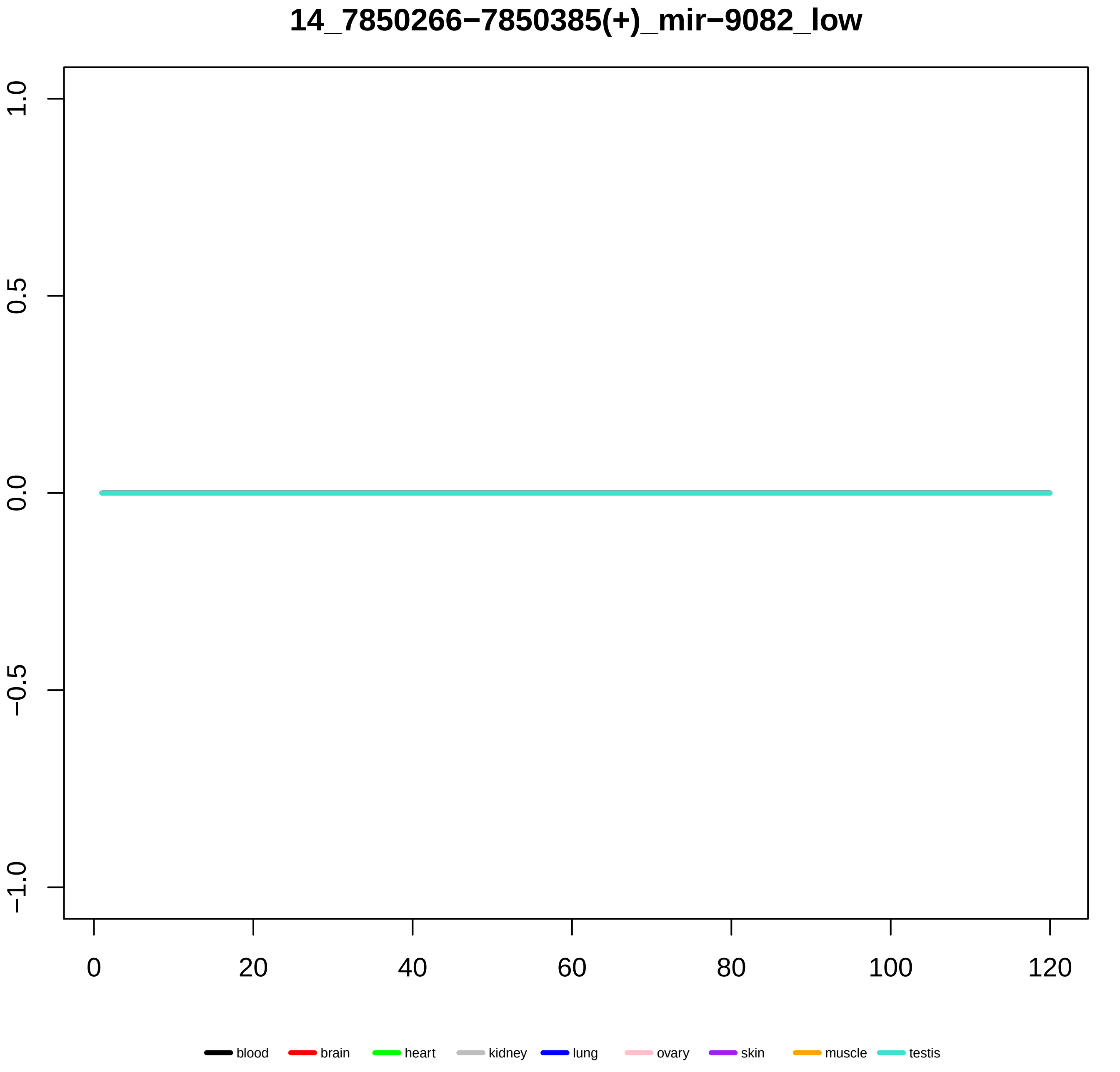

14_7850266−7850385(+)_mir−9082_low
1.0
0.5
0.0
−0.5
−1.0
0
20
40
60
80
100
120
blood
brain
heart
kidney
lung
ovary
skin
muscle
testis

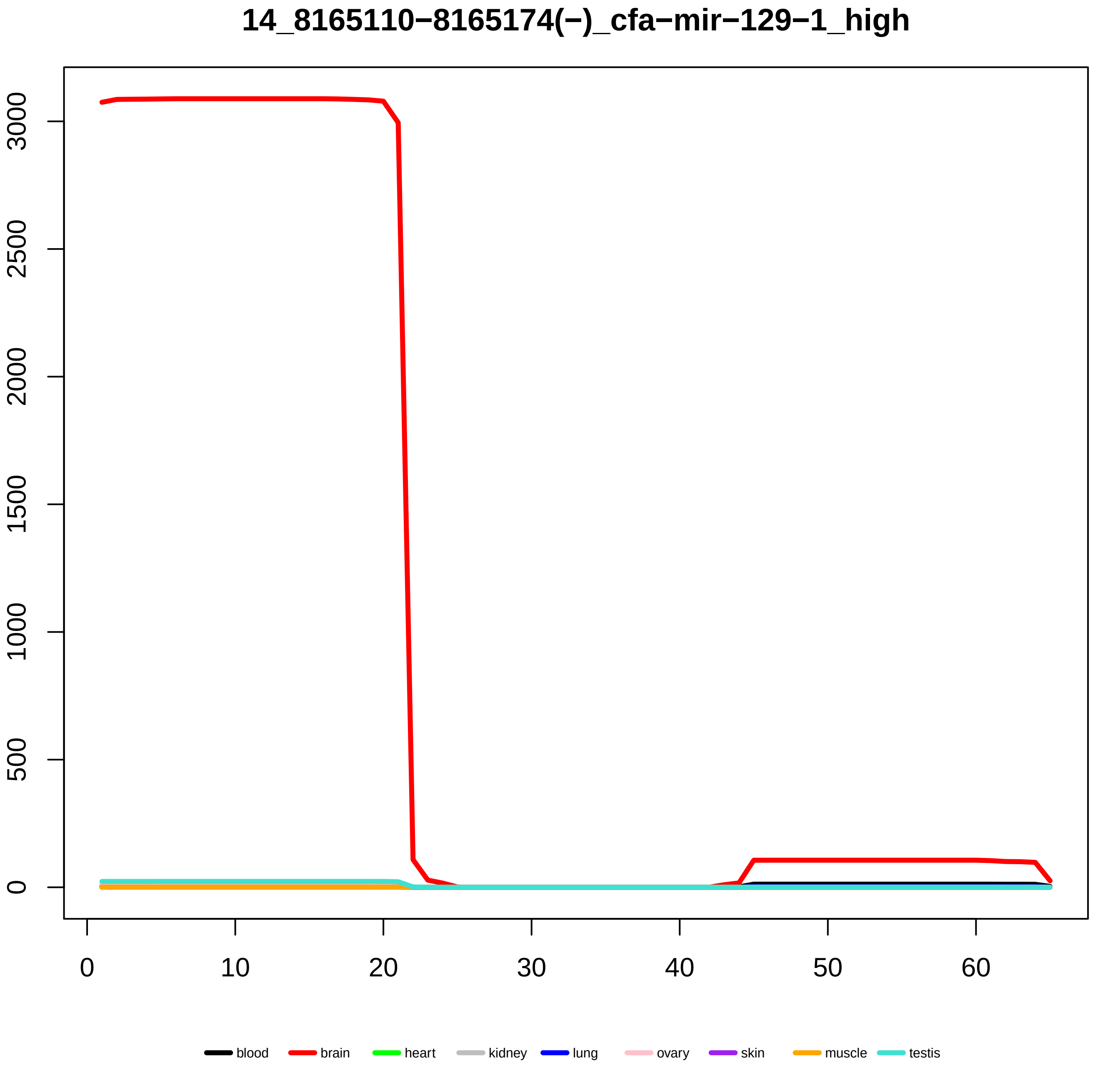
14_8165110−8165174(−)_cfa−mir−129−1_high
0
500
1000
1500
2000
2500
3000
0
10
20
30
40
50
60
blood
brain
heart
kidney
lung
ovary
skin
muscle
testis

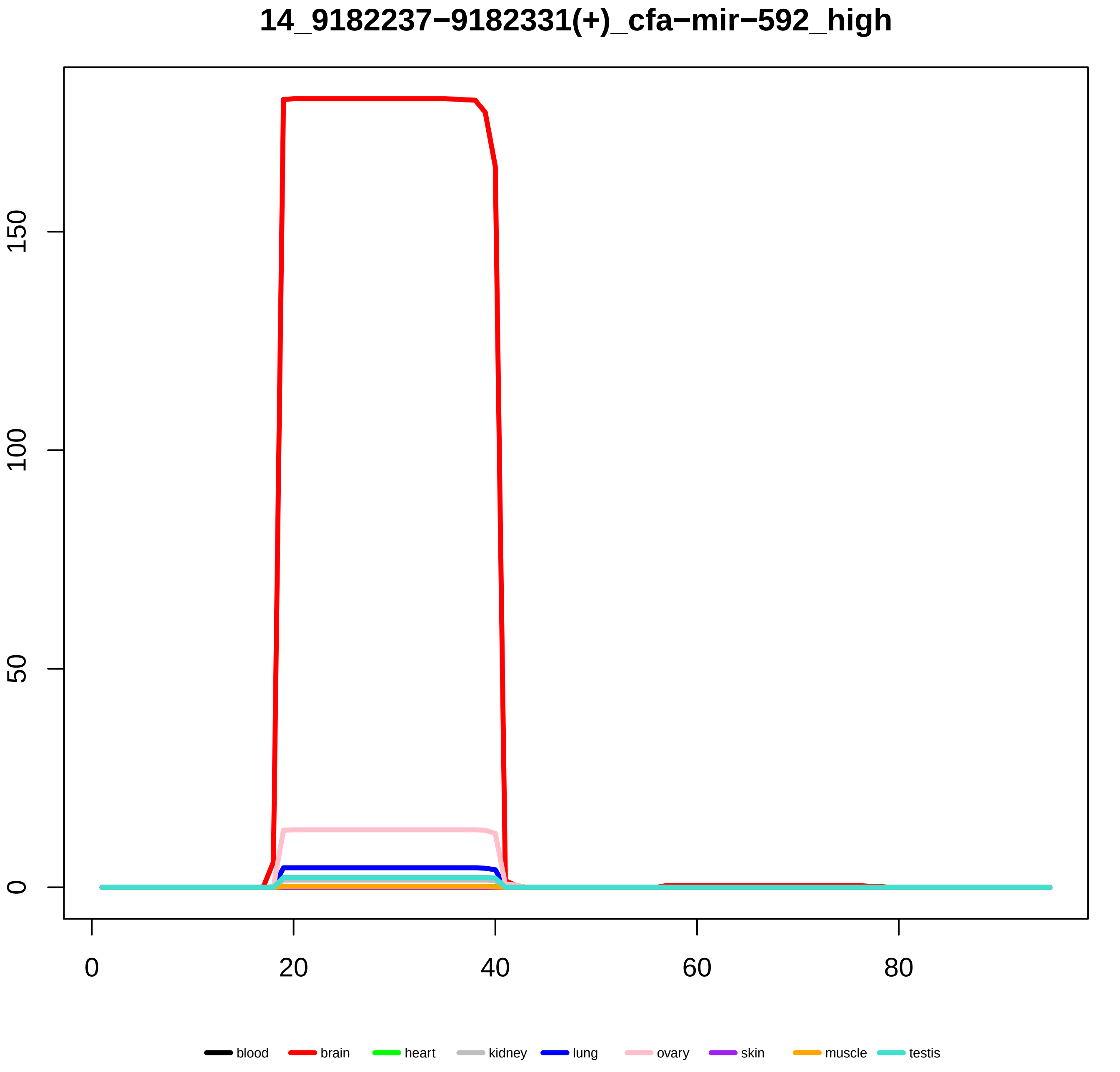

14_9182237−9182331(+)_cfa−mir−592_high
0
50
100
150
0
20
40
60
80
blood
brain
heart
kidney
lung
ovary
skin
muscle
testis

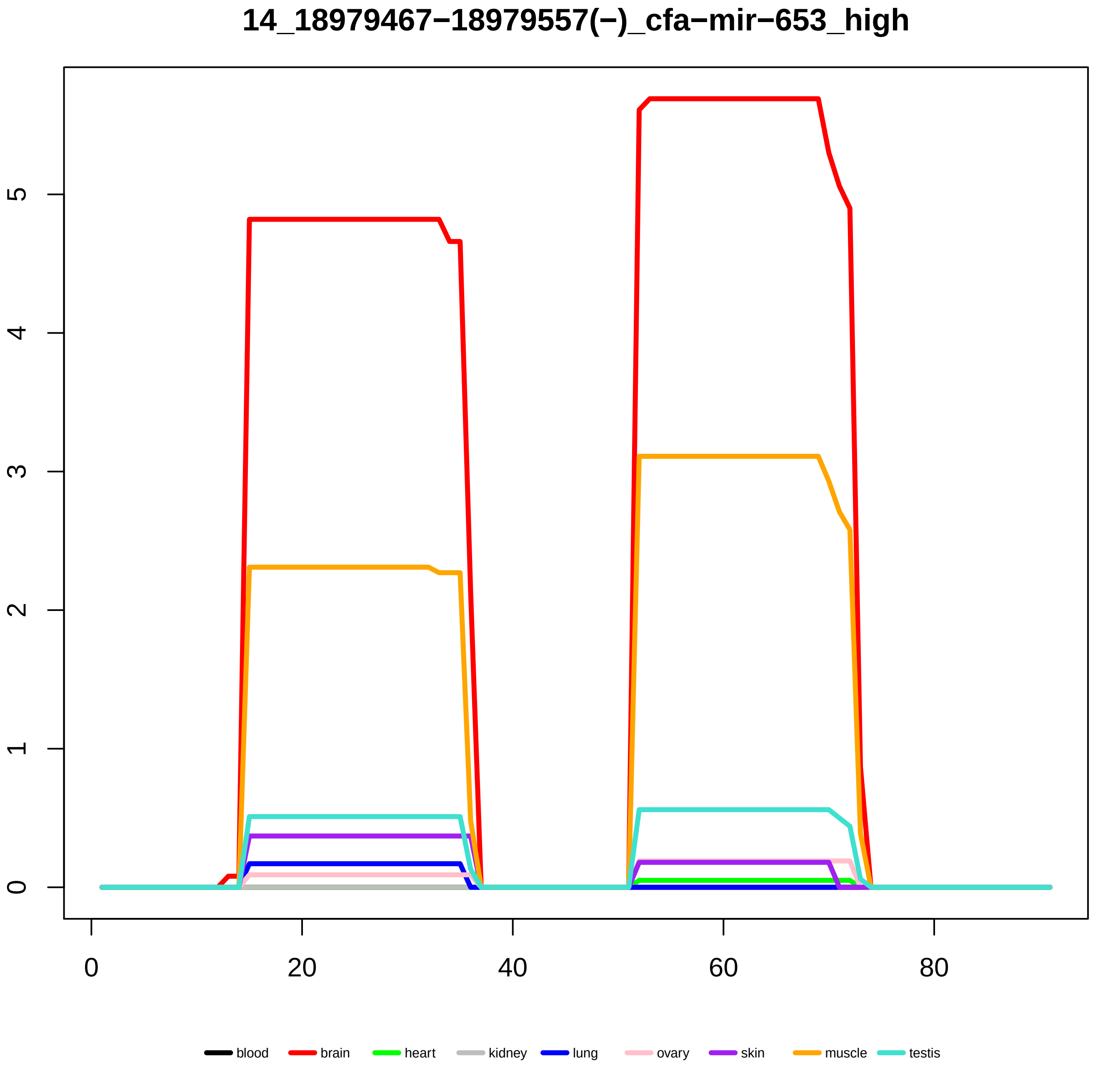

14_18979467−18979557(−)_cfa−mir−653_high
0
1
2
3
4
5
0
20
40
60
80
blood
brain
heart
kidney
lung
ovary
skin
muscle
testis

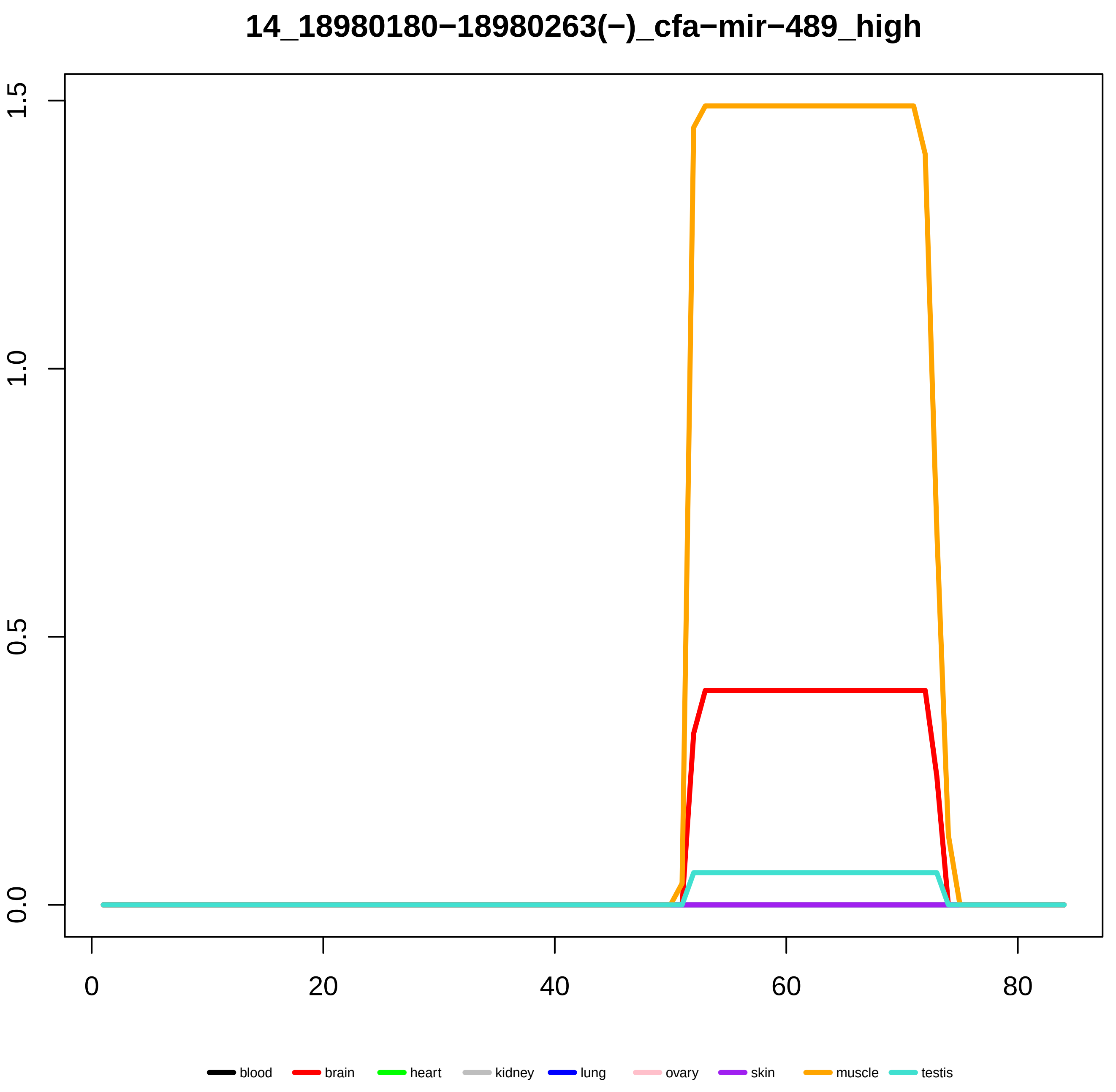

14_18980180−18980263(−)_cfa−mir−489_high
0.0
0.5
1.0
1.5
0
20
40
60
80
blood
brain
heart
kidney
lung
ovary
skin
muscle
testis

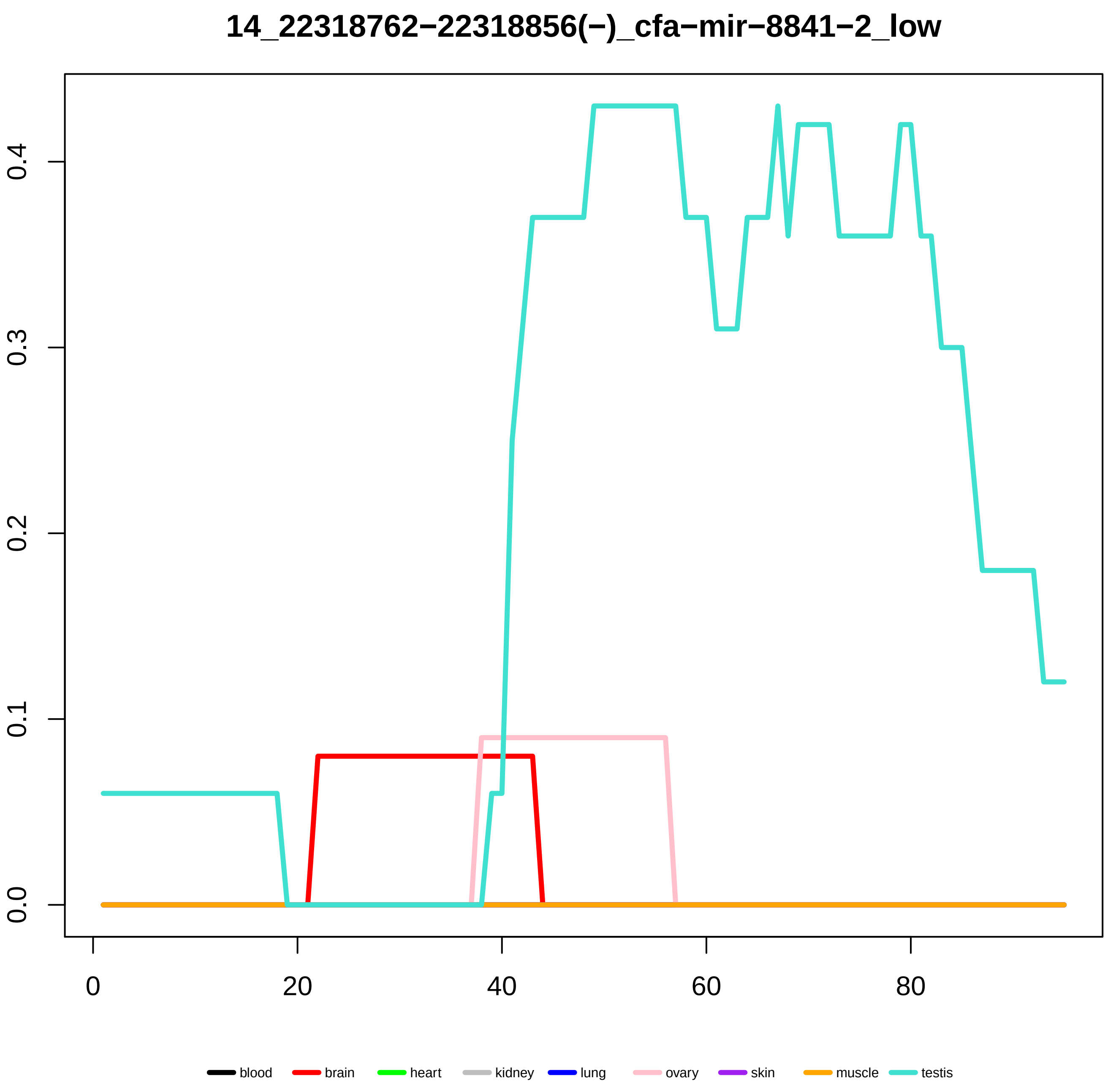

14_22318762−22318856(−)_cfa−mir−8841−2_low
0.0
0.1
0.2
0.3
0.4
0
20
40
60
80
blood
brain
heart
kidney
lung
ovary
skin
muscle
testis

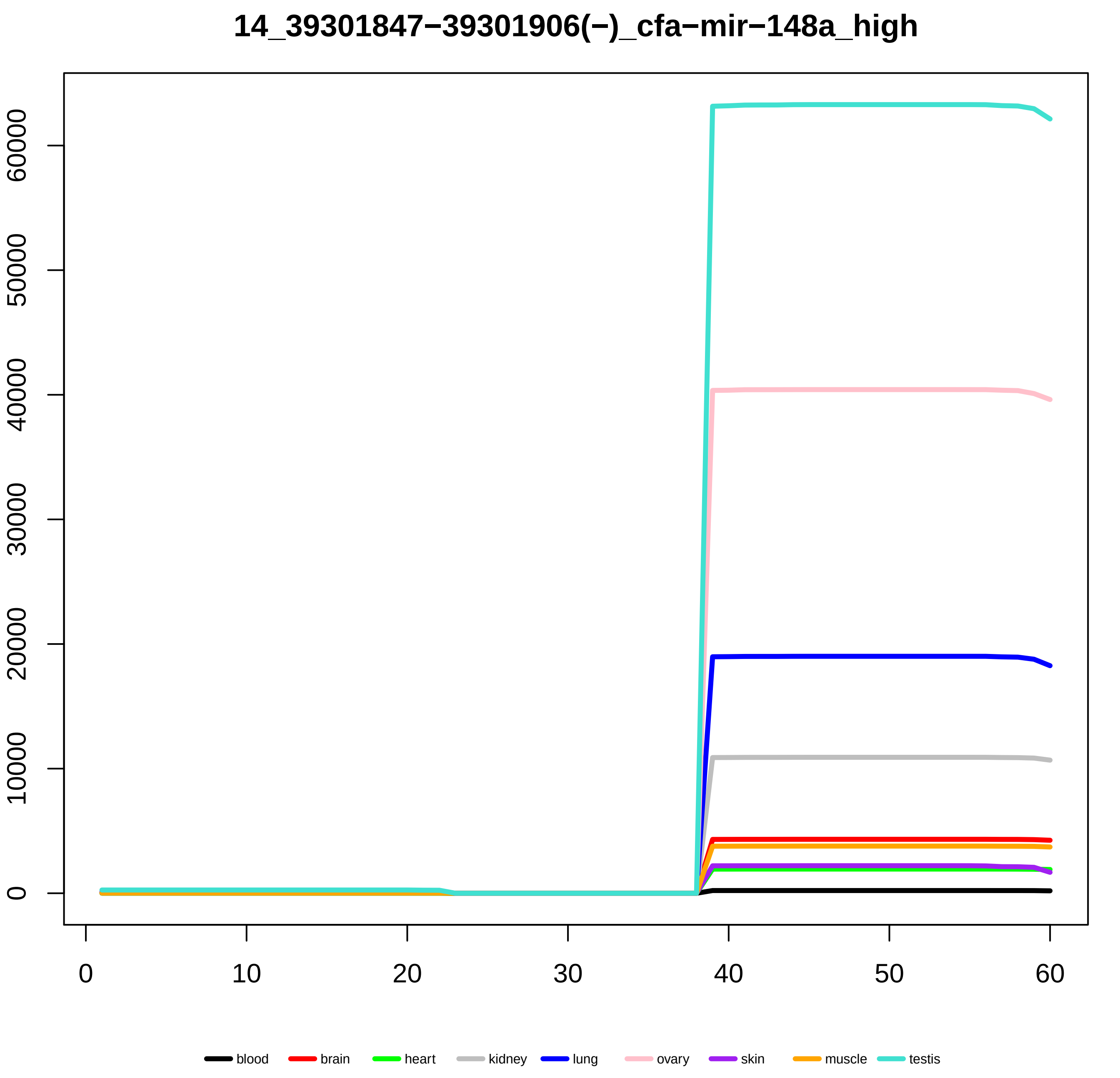
14_39301847−39301906(−)_cfa−mir−148a_high
0
10000
20000
30000
40000
50000
60000
0
10
20
30
40
50
60
blood
brain
heart
kidney
lung
ovary
skin
muscle
testis

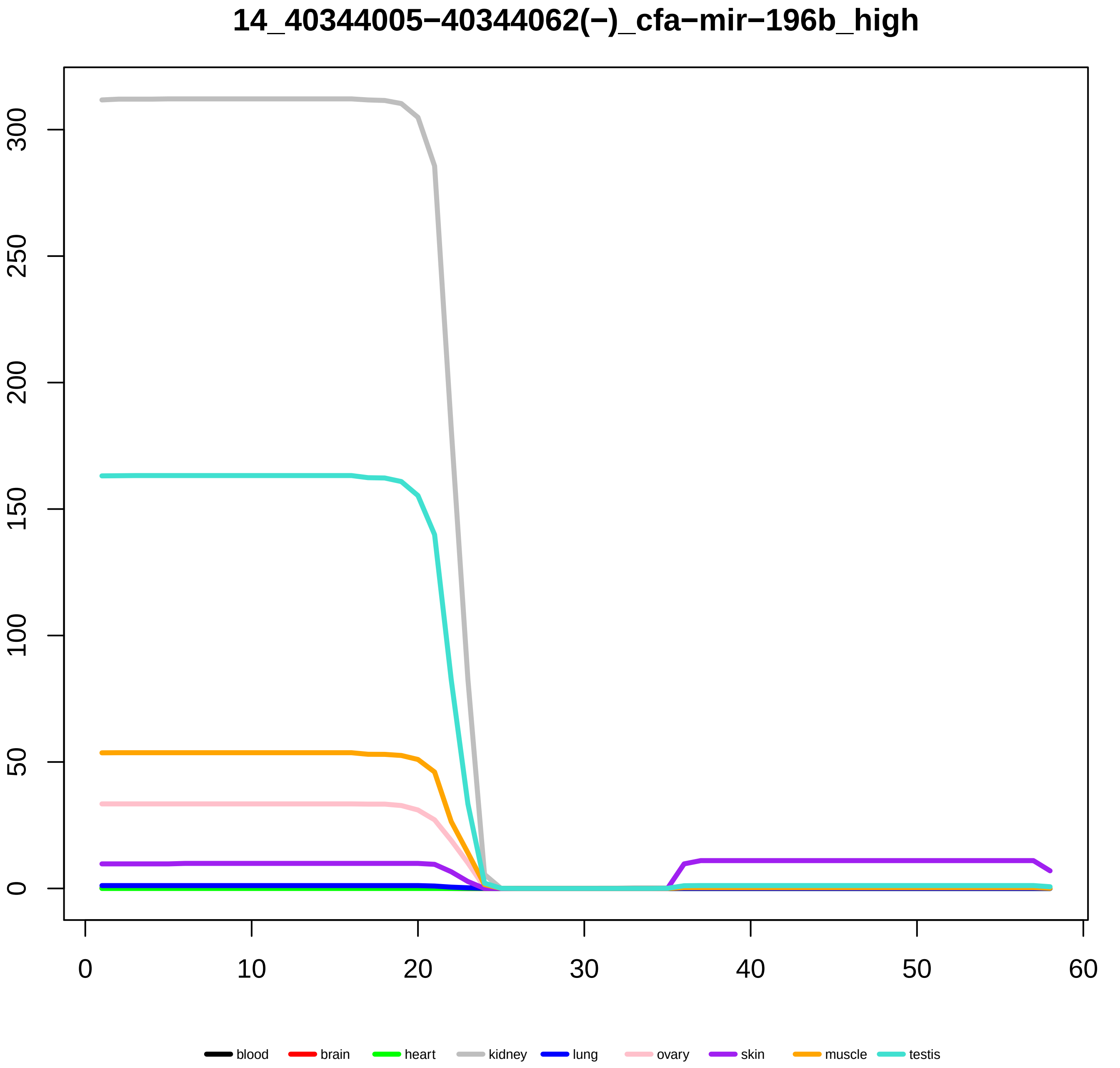
14_40344005−40344062(−)_cfa−mir−196b_high
0
50
100
150
200
250
300
0
10
20
30
40
50
60
blood
brain
heart
kidney
lung
ovary
skin
muscle
testis

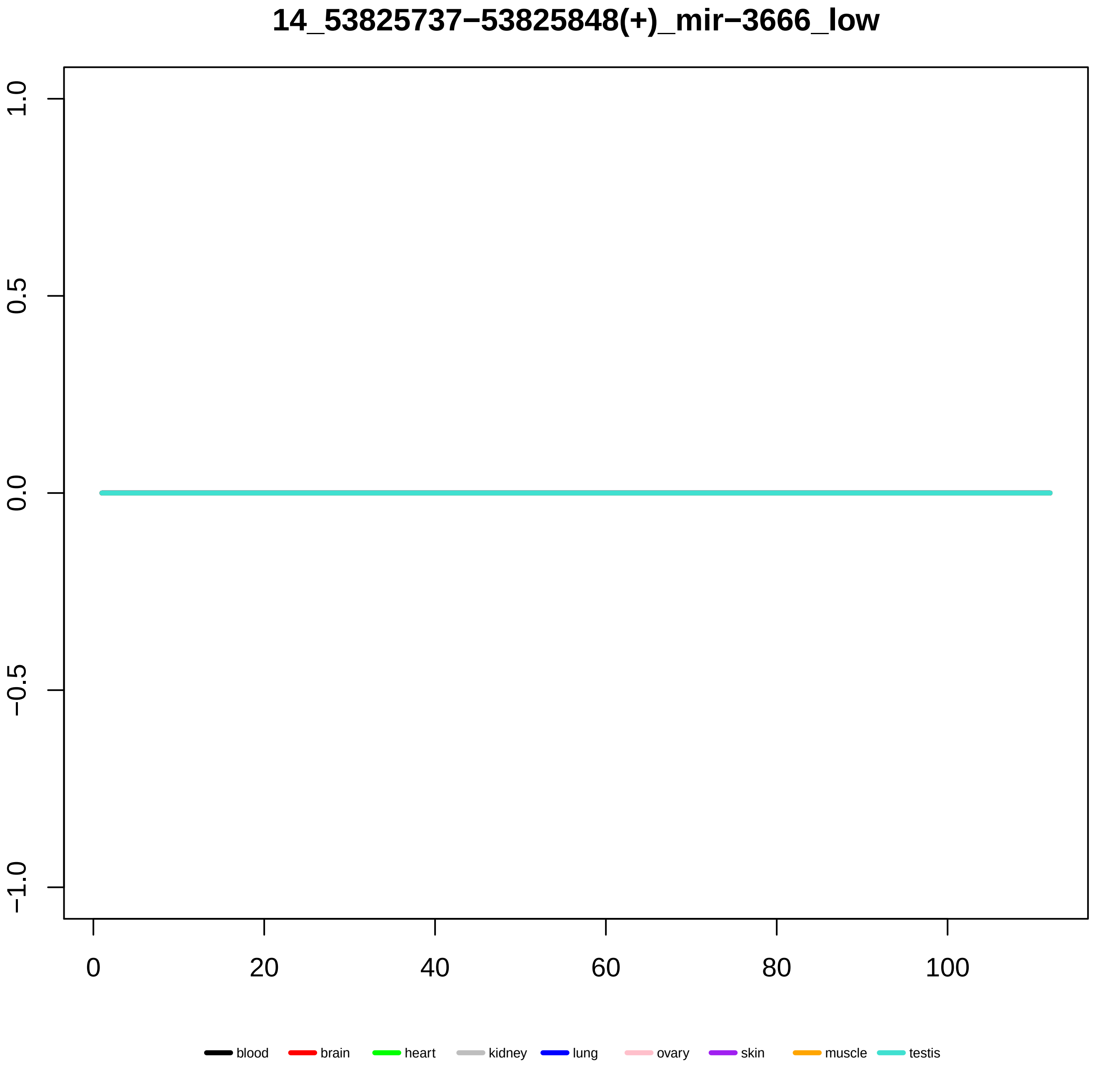

14_53825737−53825848(+)_mir−3666_low
1.0
0.5
0.0
−0.5
−1.0
0
20
40
60
80
100
blood
brain
heart
kidney
lung
ovary
skin
muscle
testis

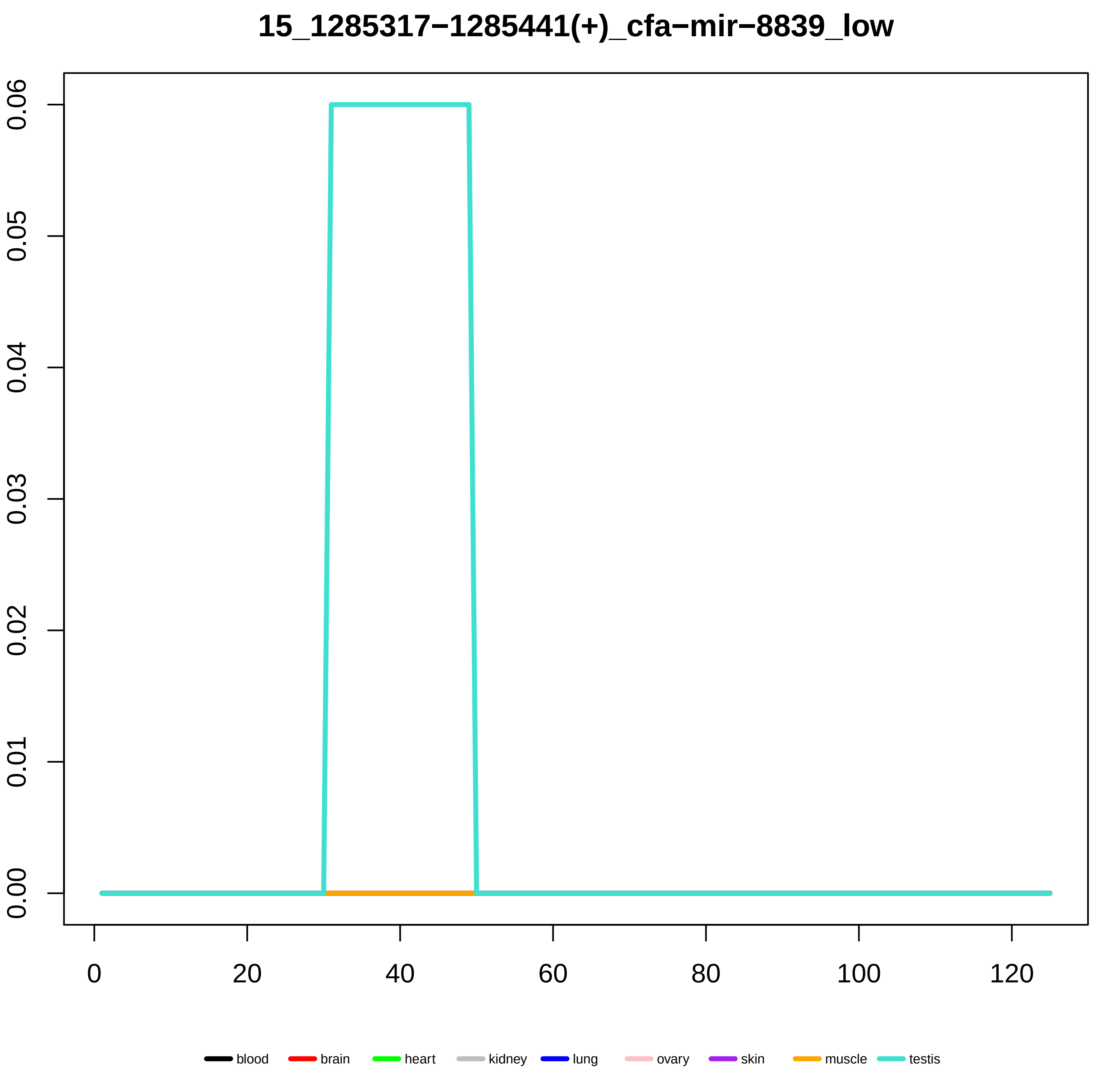

15_1285317−1285441(+)_cfa−mir−8839_low
0.00
0.01
0.02
0.03
0.04
0.05
0.06
0
20
40
60
80
100
120
blood
brain
heart
kidney
lung
ovary
skin
muscle
testis

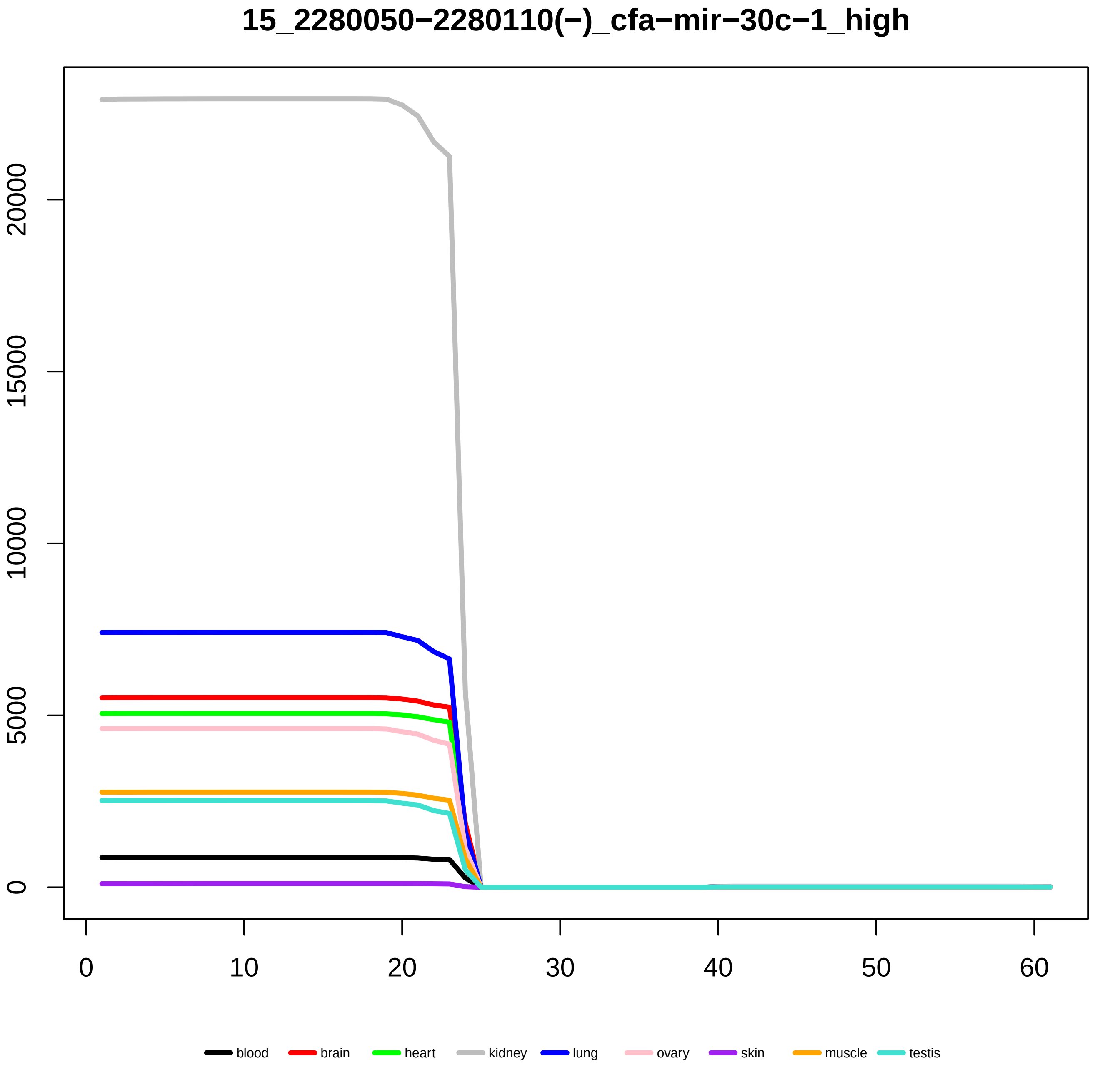
15_2280050−2280110(−)_cfa−mir−30c−1_high
0
5000
10000
15000
20000
0
10
20
30
40
50
60
blood
brain
heart
kidney
lung
ovary
skin
muscle
testis

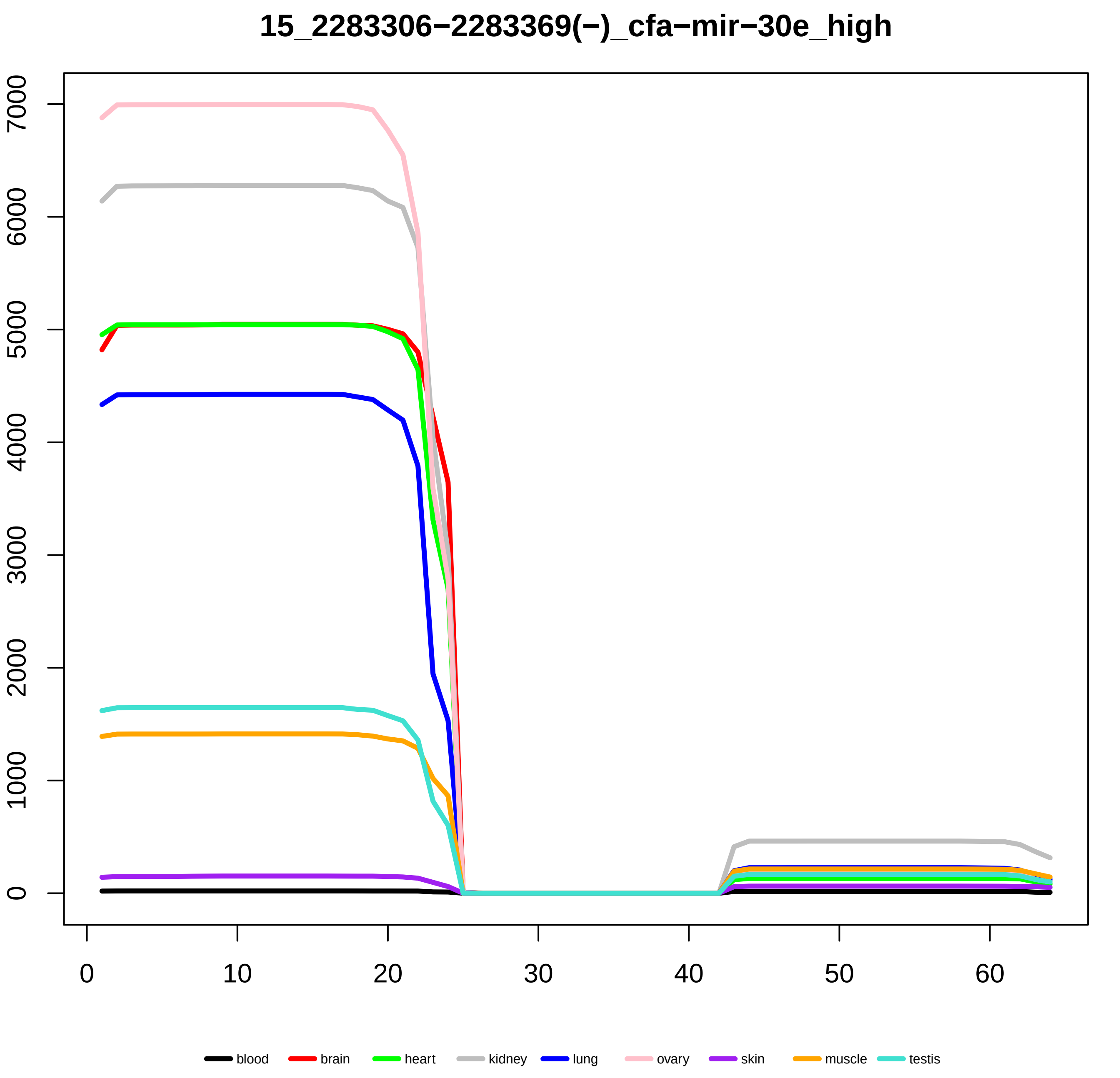

15_2283306−2283369(−)_cfa−mir−30e_high
0
1000
2000
3000
4000
5000
6000
7000
0
10
20
30
40
50
60
blood
brain
heart
kidney
lung
ovary
skin
muscle
testis

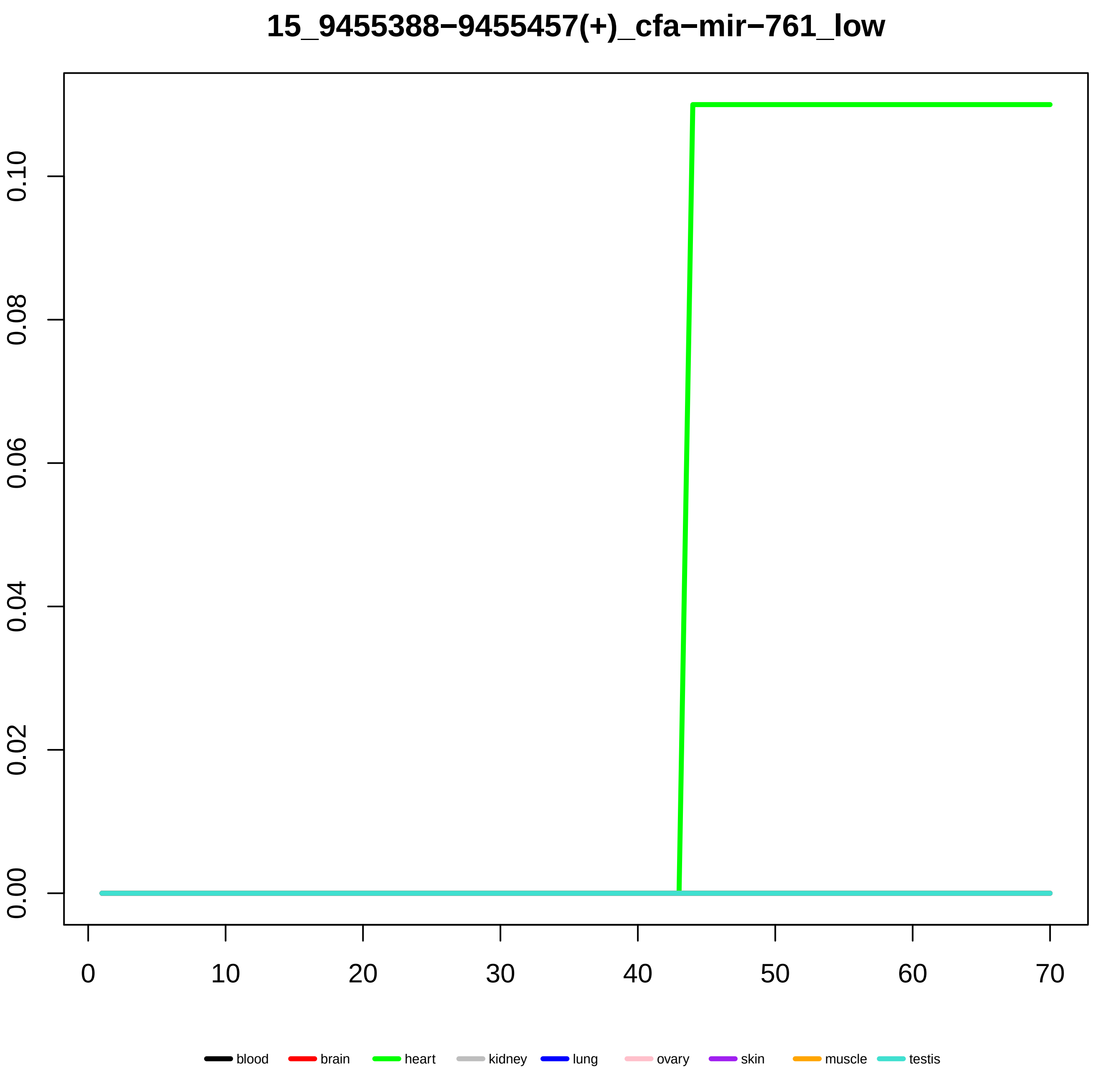
15_9455388−9455457(+)_cfa−mir−761_low
0.00
0.02
0.04
0.06
0.08
0.10
0
10
20
30
40
50
60
70
blood
brain
heart
kidney
lung
ovary
skin
muscle
testis

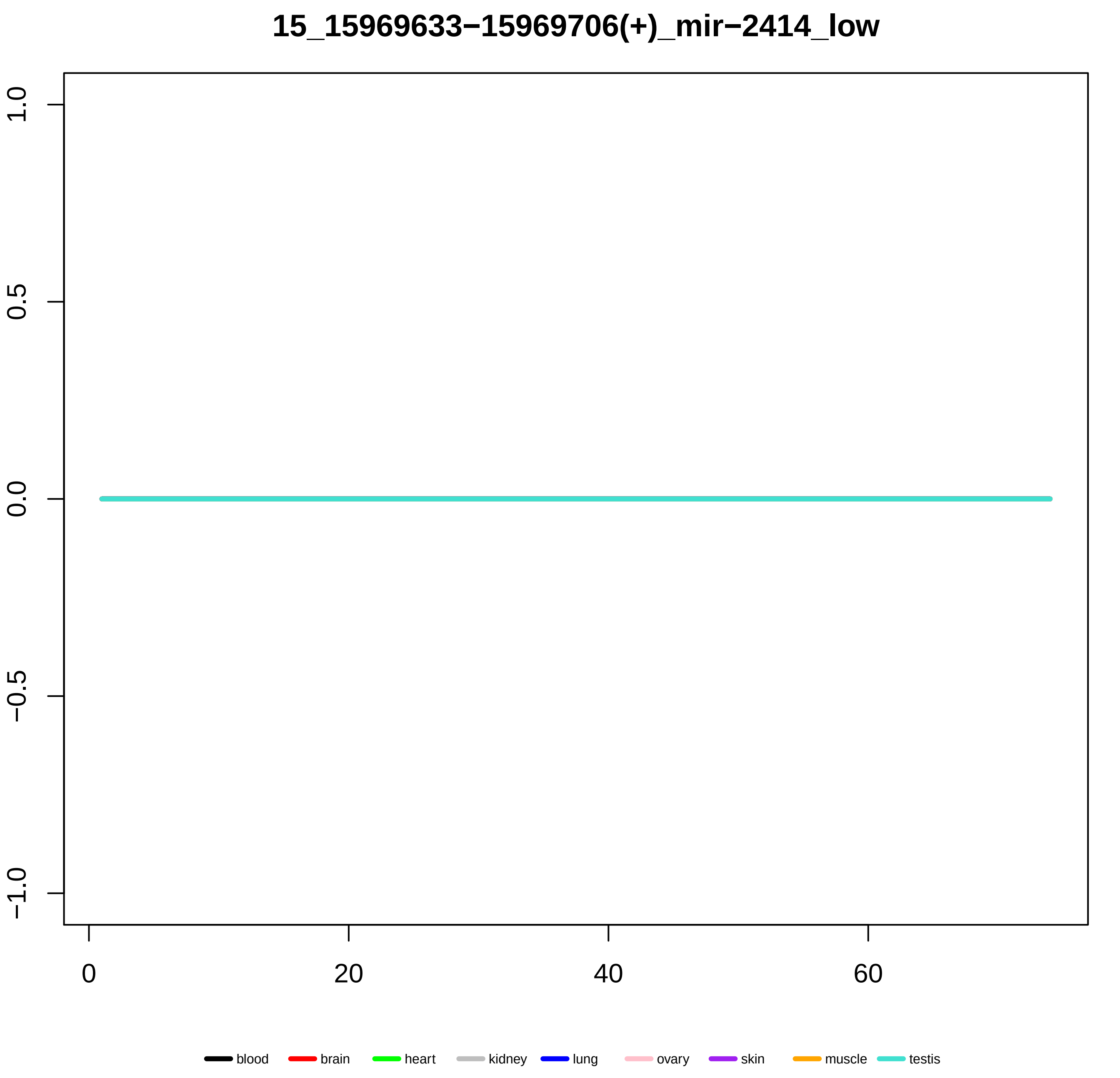
15_15969633−15969706(+)_mir−2414_low
1.0
0.5
0.0
−0.5
−1.0
0
20
40
60
blood
brain
heart
kidney
lung
ovary
skin
muscle
testis

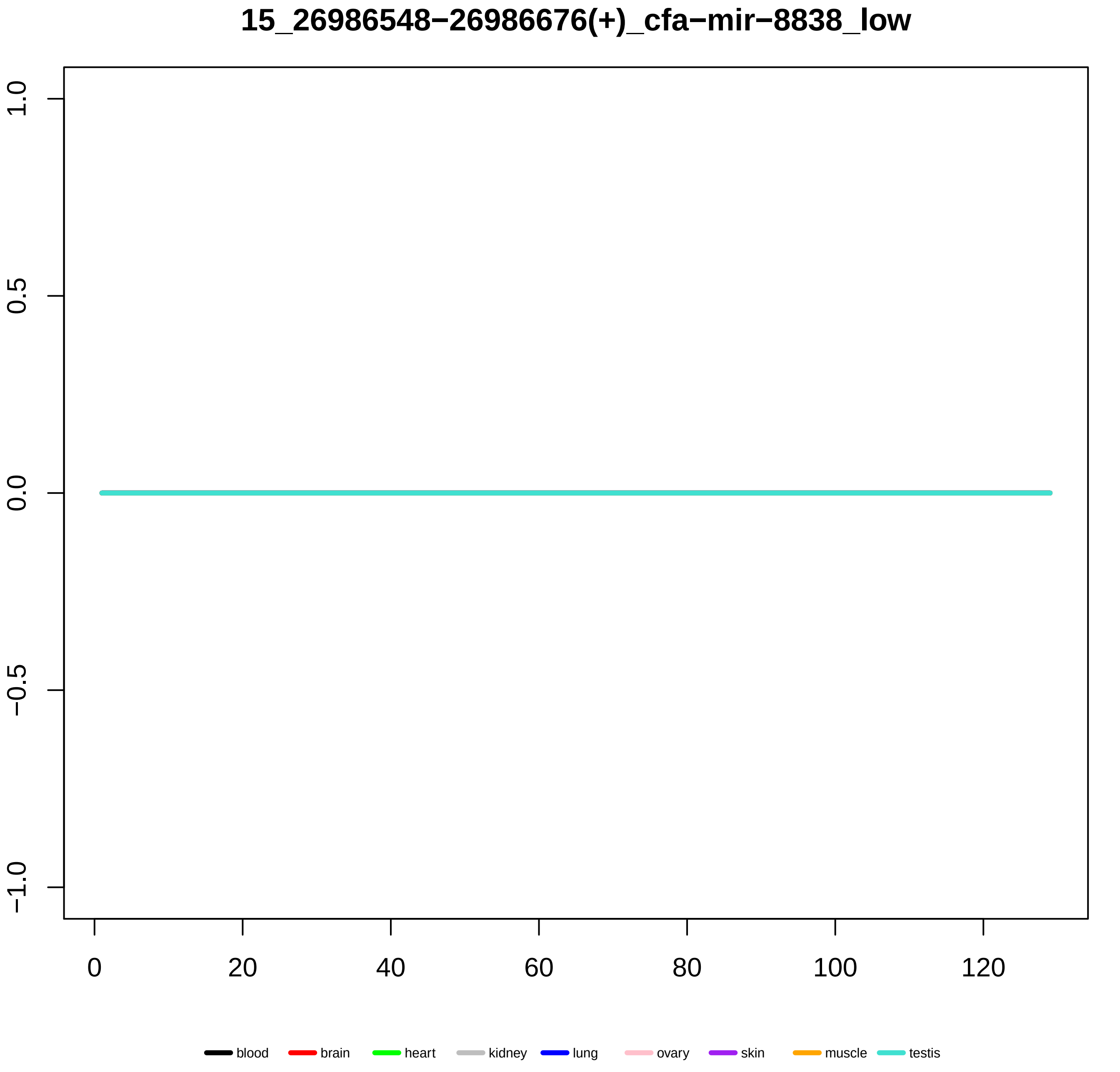

15_26986548−26986676(+)_cfa−mir−8838_low
1.0
0.5
0.0
−0.5
−1.0
0
20
40
60
80
100
120
blood
brain
heart
kidney
lung
ovary
skin
muscle
testis

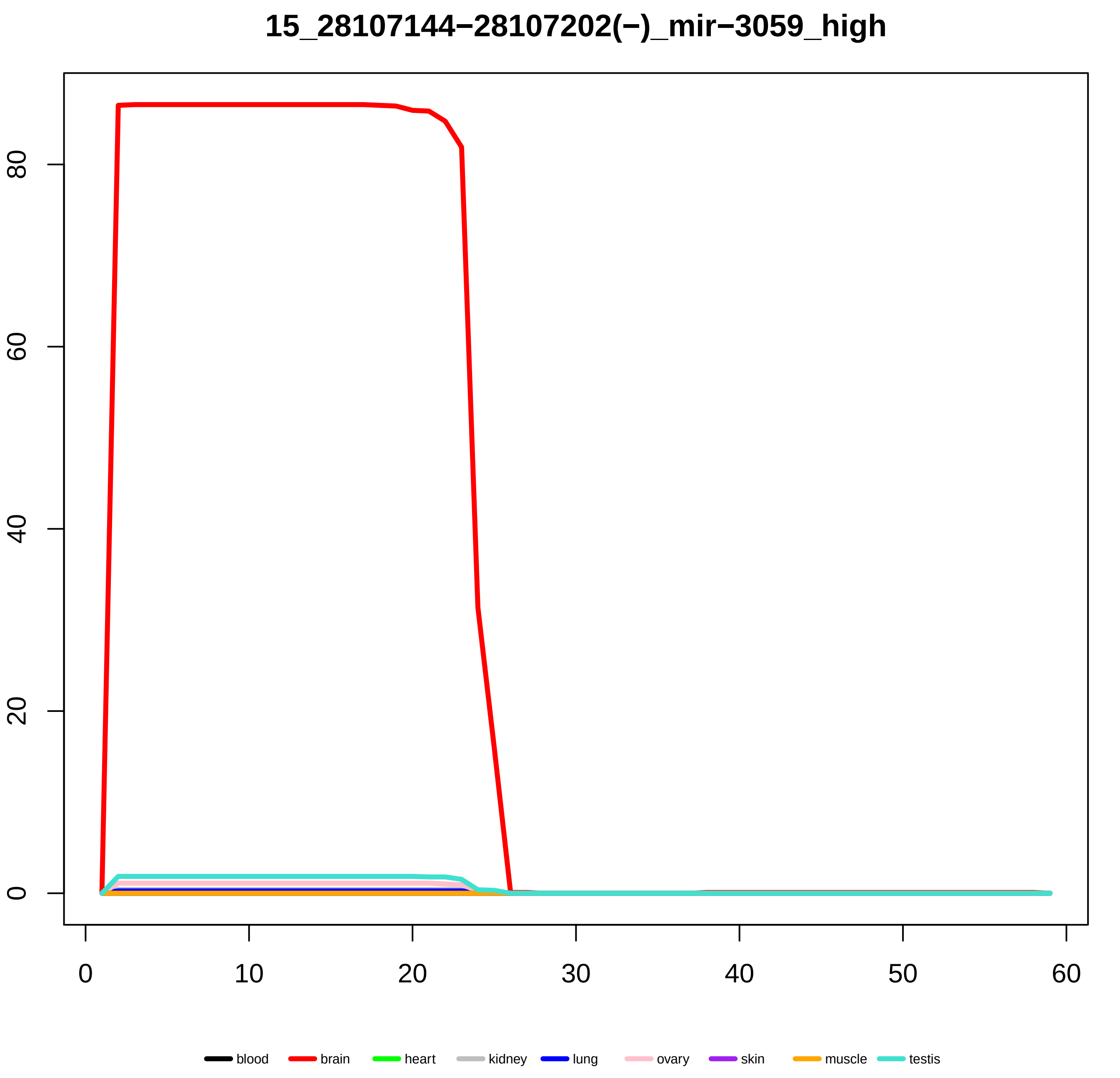

15_28107144−28107202(−)_mir−3059_high
0
20
40
60
80
0
10
20
30
40
50
60
blood
brain
heart
kidney
lung
ovary
skin
muscle
testis

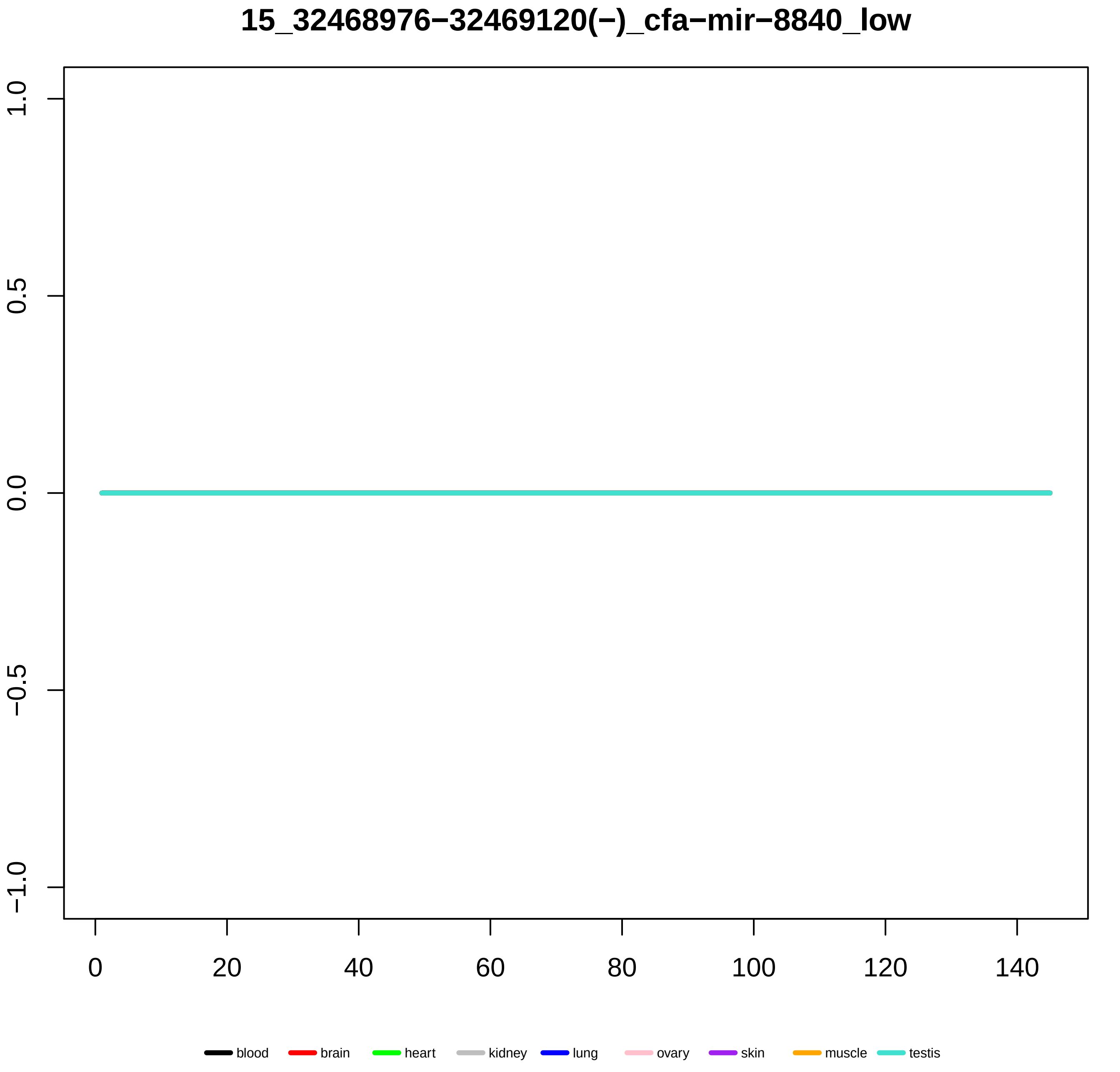
15_32468976−32469120(−)_cfa−mir−8840_low
1.0
0.5
0.0
−0.5
−1.0
0
20
40
60
80
100
120
140
blood
brain
heart
kidney
lung
ovary
skin
muscle
testis

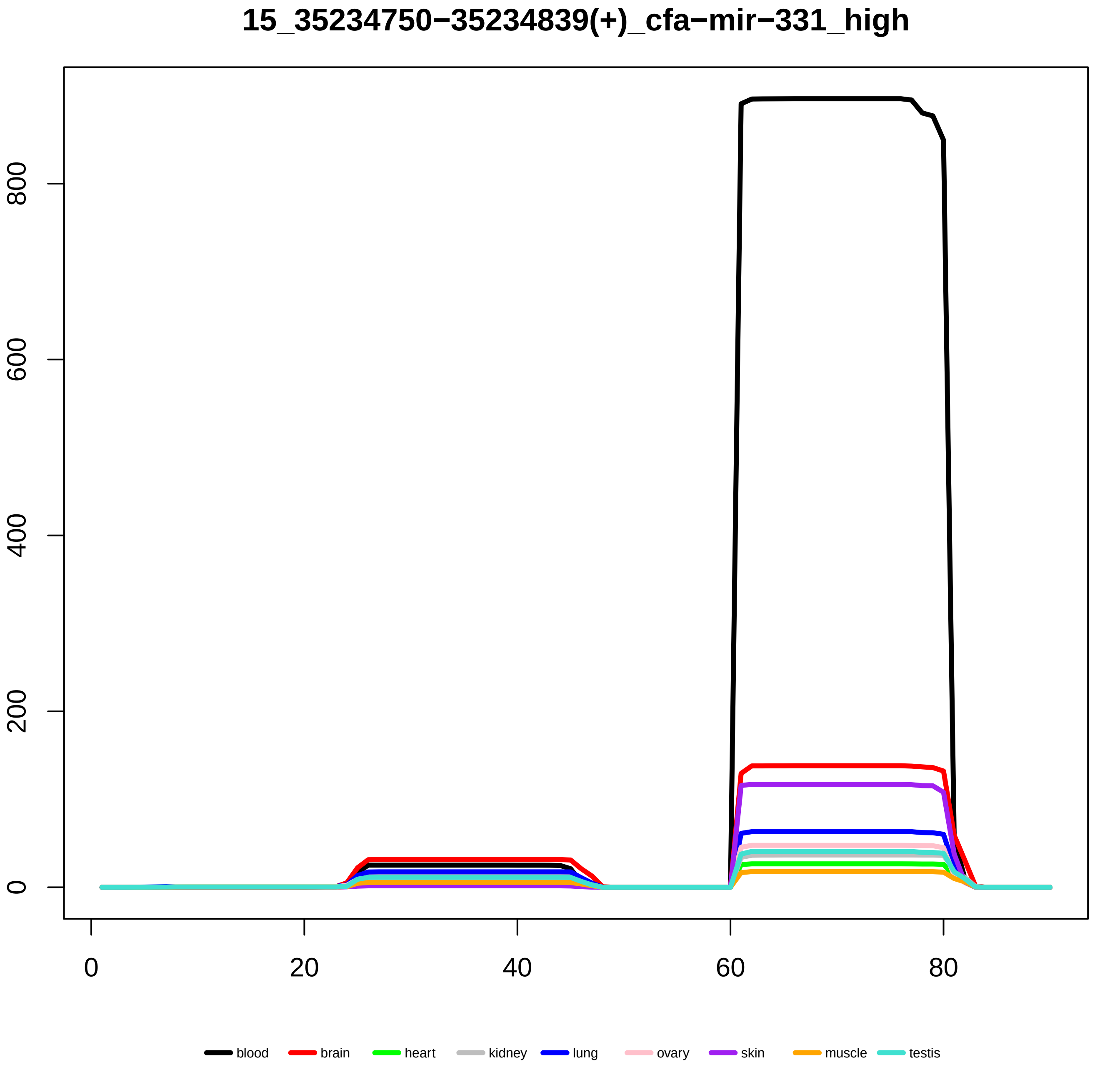

15_35234750−35234839(+)_cfa−mir−331_high
0
200
400
600
800
0
20
40
60
80
blood
brain
heart
kidney
lung
ovary
skin
muscle
testis

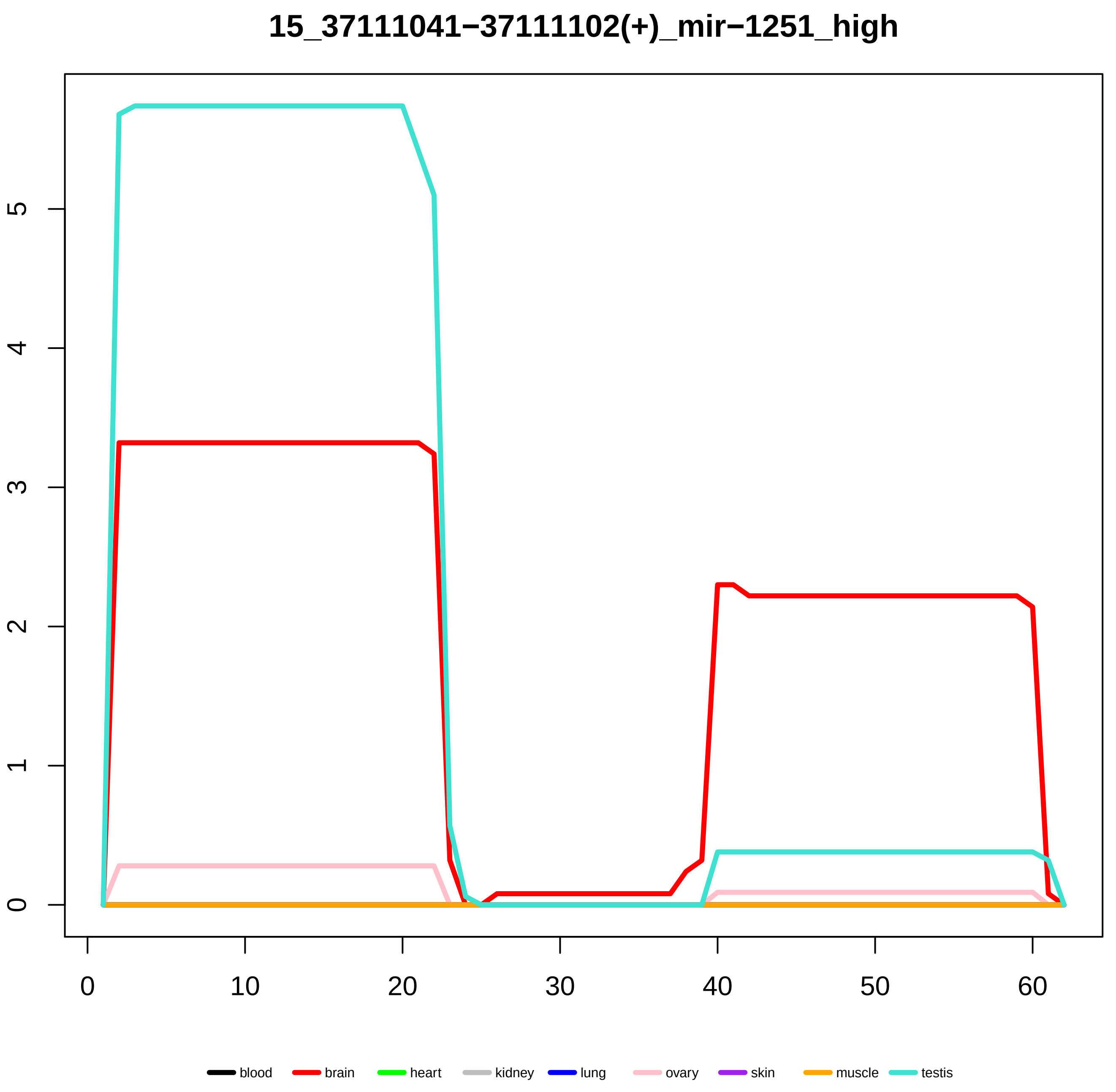
15_37111041−37111102(+)_mir−1251_high
0
1
2
3
4
5
0
10
20
30
40
50
60
blood
brain
heart
kidney
lung
ovary
skin
muscle
testis

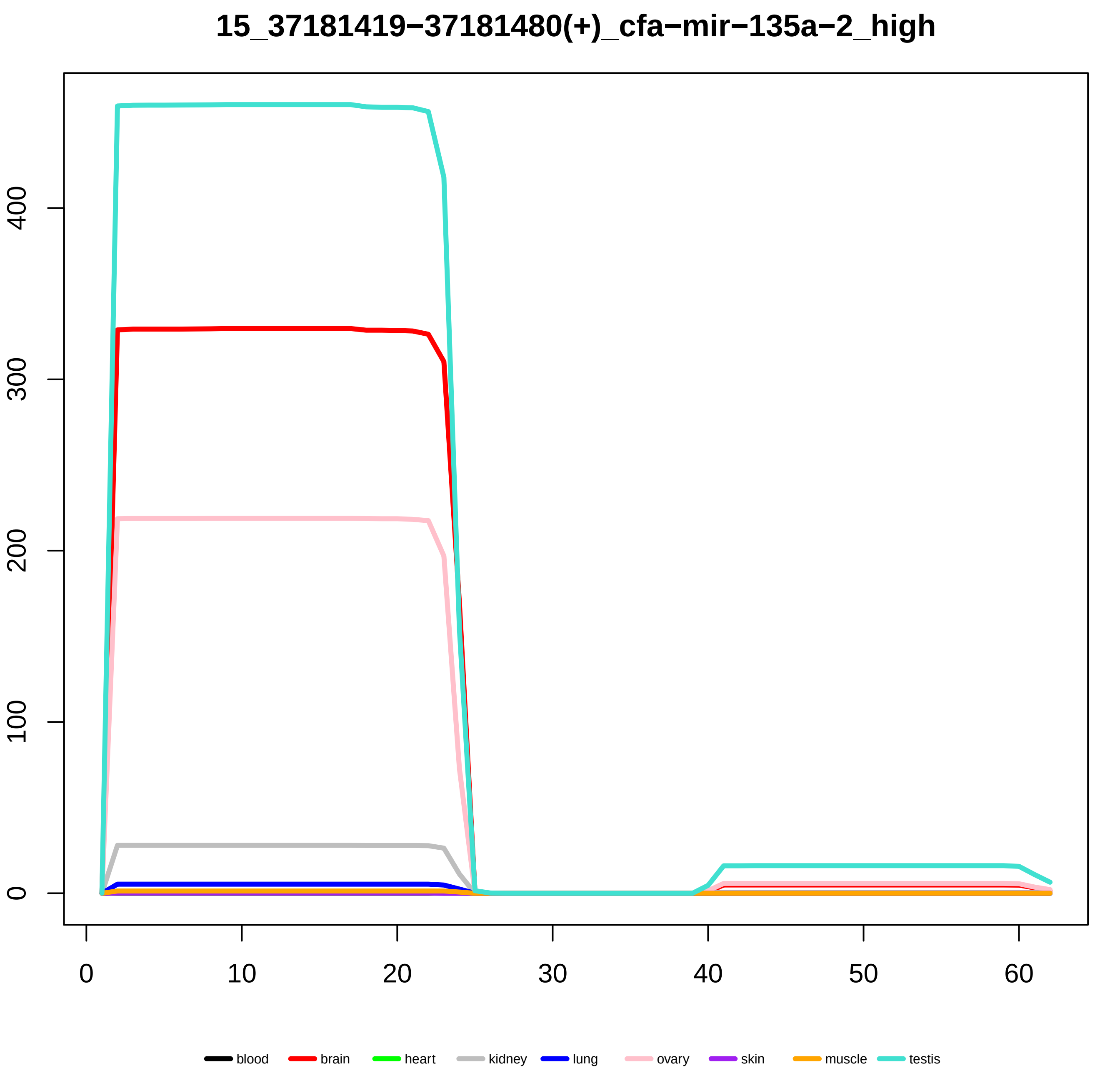
15_37181419−37181480(+)_cfa−mir−135a−2_high
0
100
200
300
400
0
10
20
30
40
50
60
blood
brain
heart
kidney
lung
ovary
skin
muscle
testis

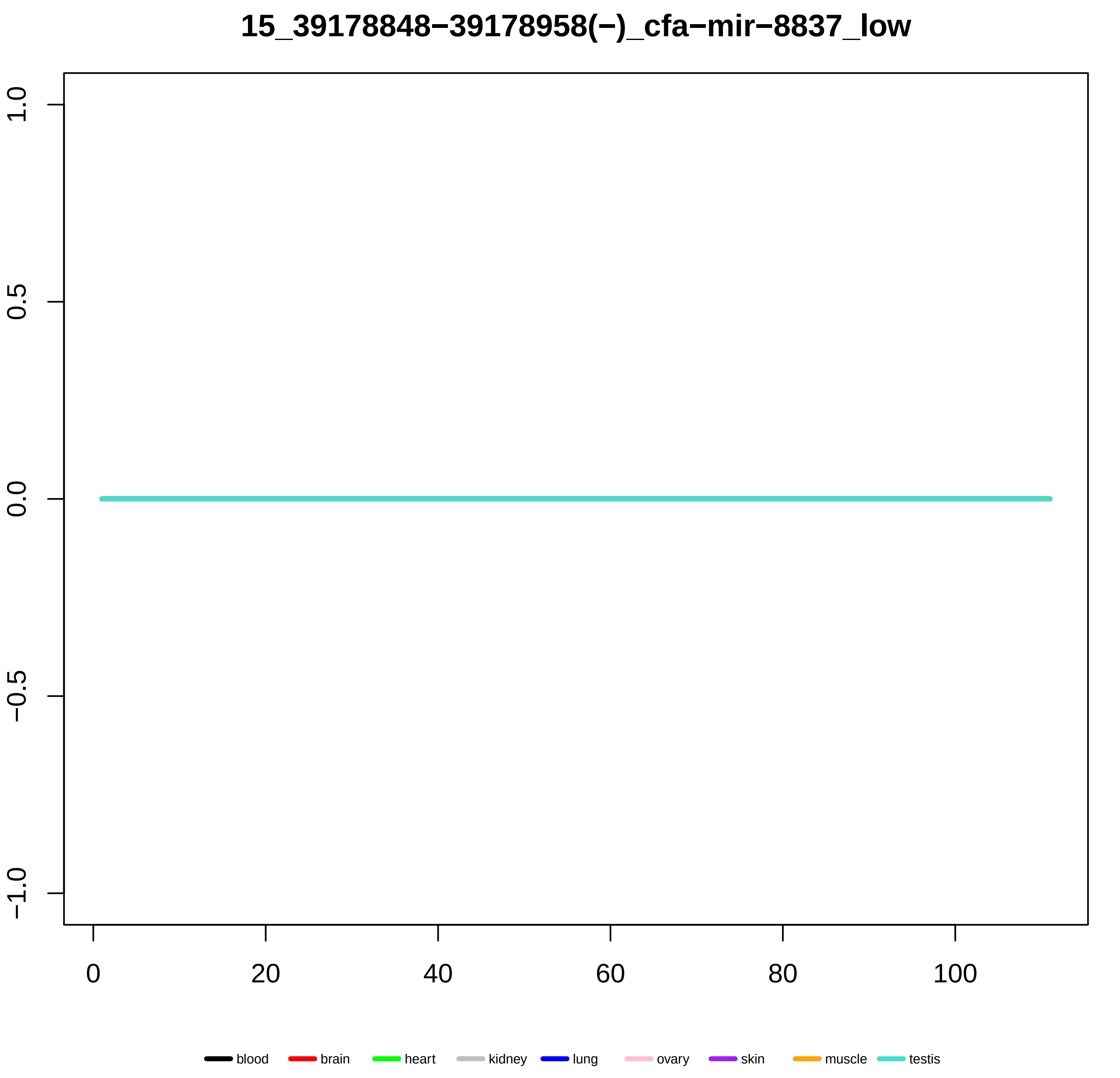

15_39178848−39178958(−)_cfa−mir−8837_low
1.0
0.5
0.0
−0.5
−1.0
0
20
40
60
80
100
blood
brain
heart
kidney
lung
ovary
skin
muscle
testis

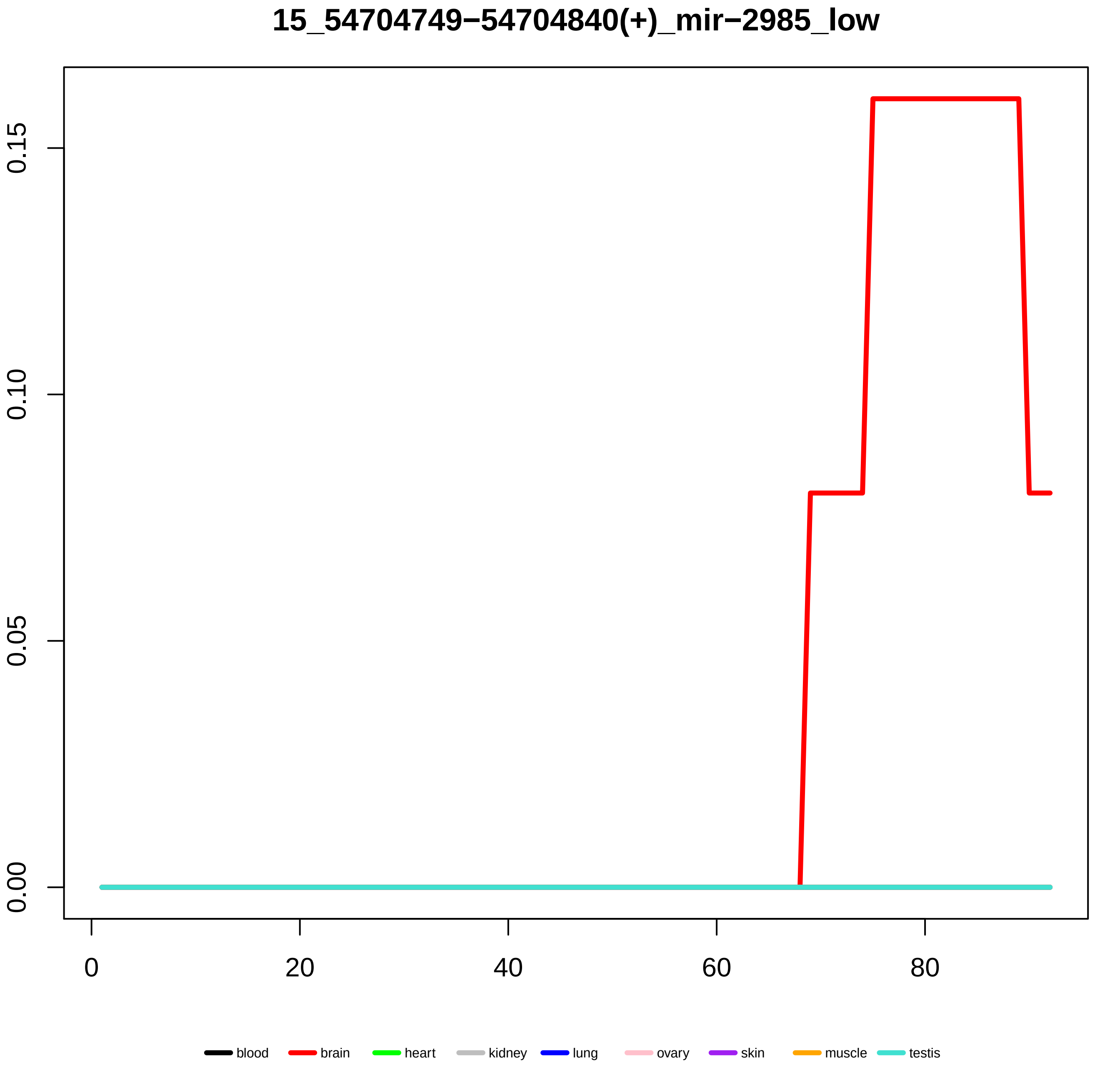
15_54704749−54704840(+)_mir−2985_low
0.00
0.05
0.10
0.15
0
20
40
60
80
blood
brain
heart
kidney
lung
ovary
skin
muscle
testis

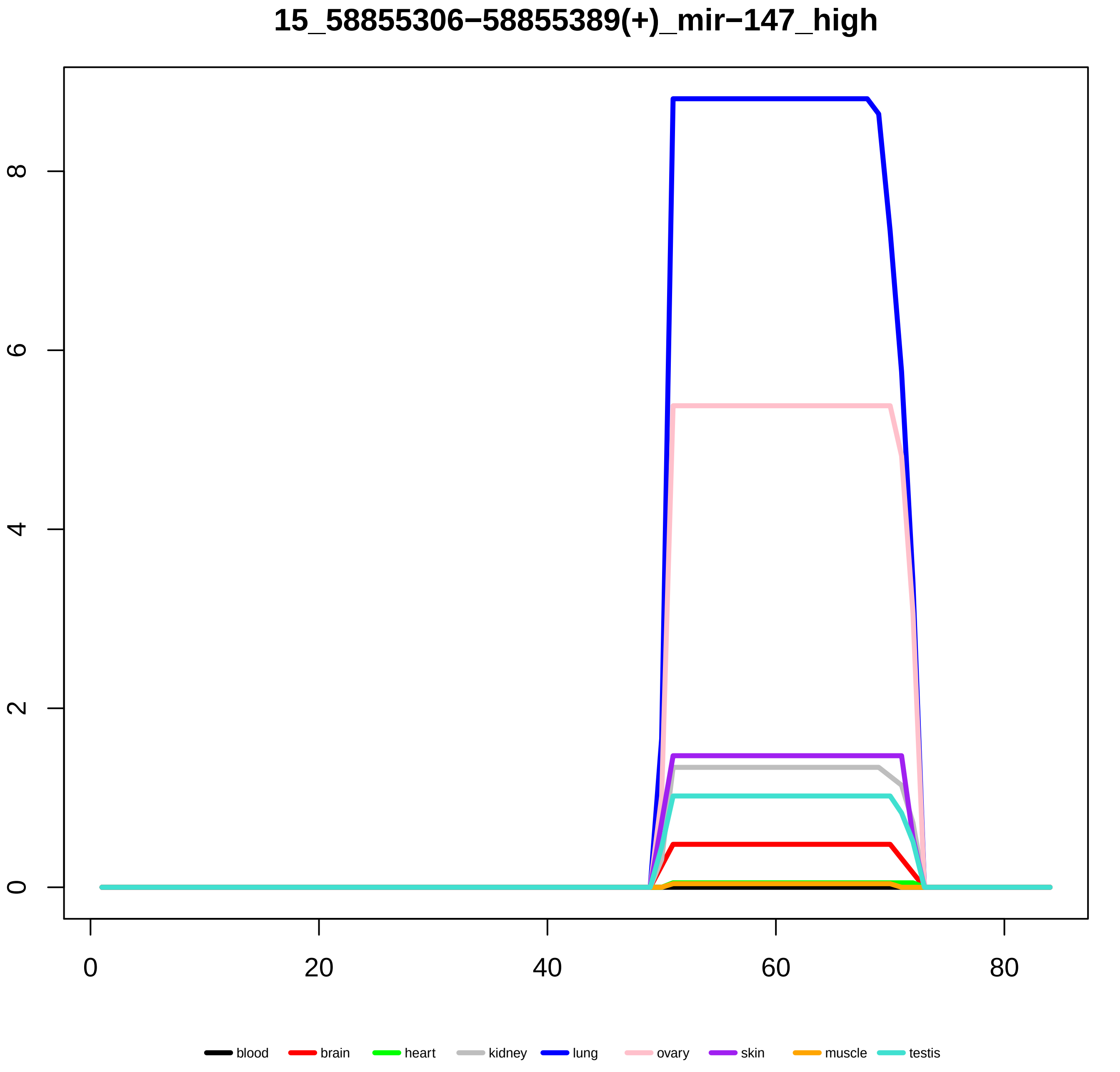

15_58855306−58855389(+)_mir−147_high
0
2
4
6
8
0
20
40
60
80
blood
brain
heart
kidney
lung
ovary
skin
muscle
testis

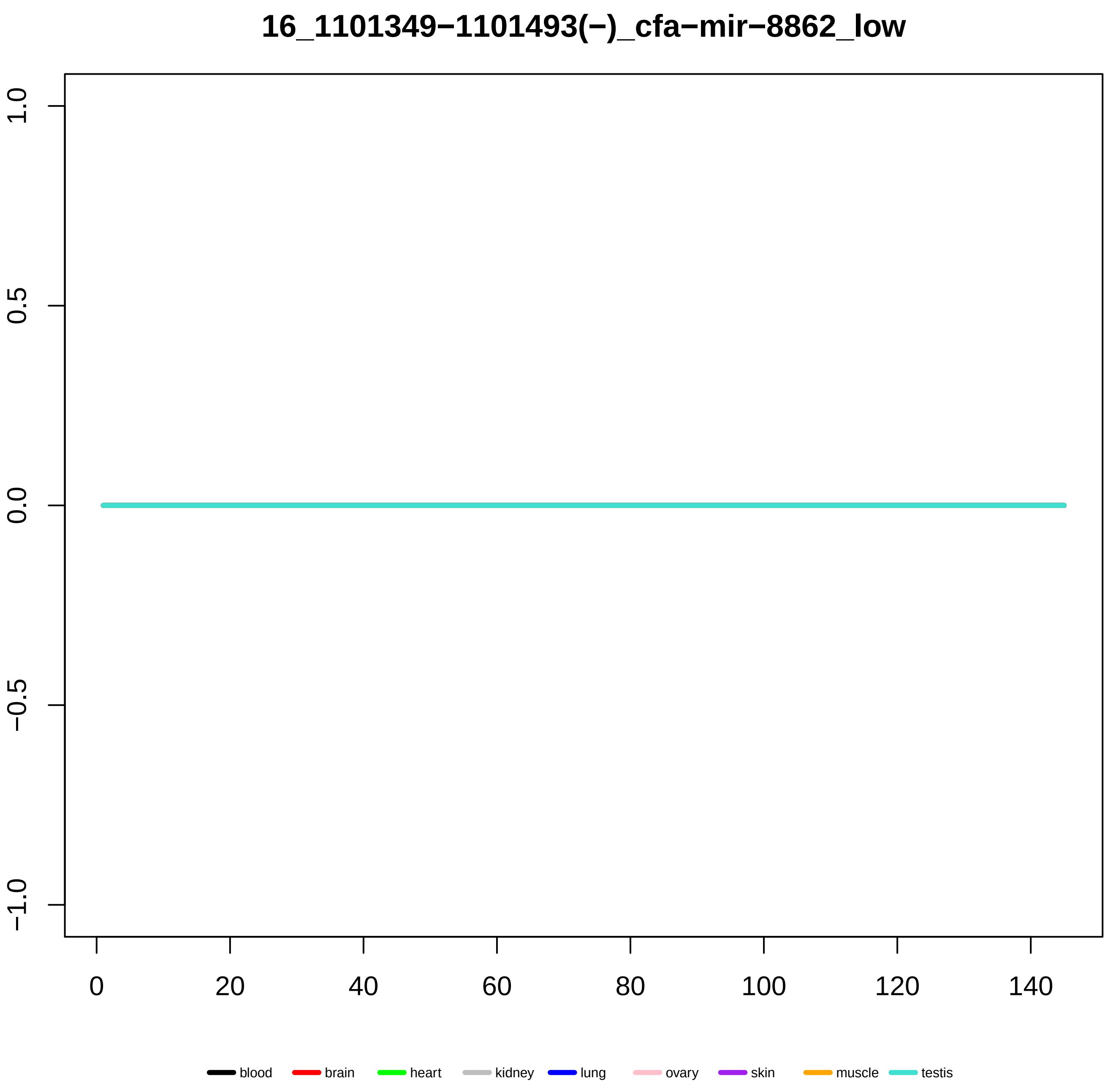
16_1101349−1101493(−)_cfa−mir−8862_low
1.0
0.5
0.0
−0.5
−1.0
0
20
40
60
80
100
120
140
blood
brain
heart
kidney
lung
ovary
skin
muscle
testis

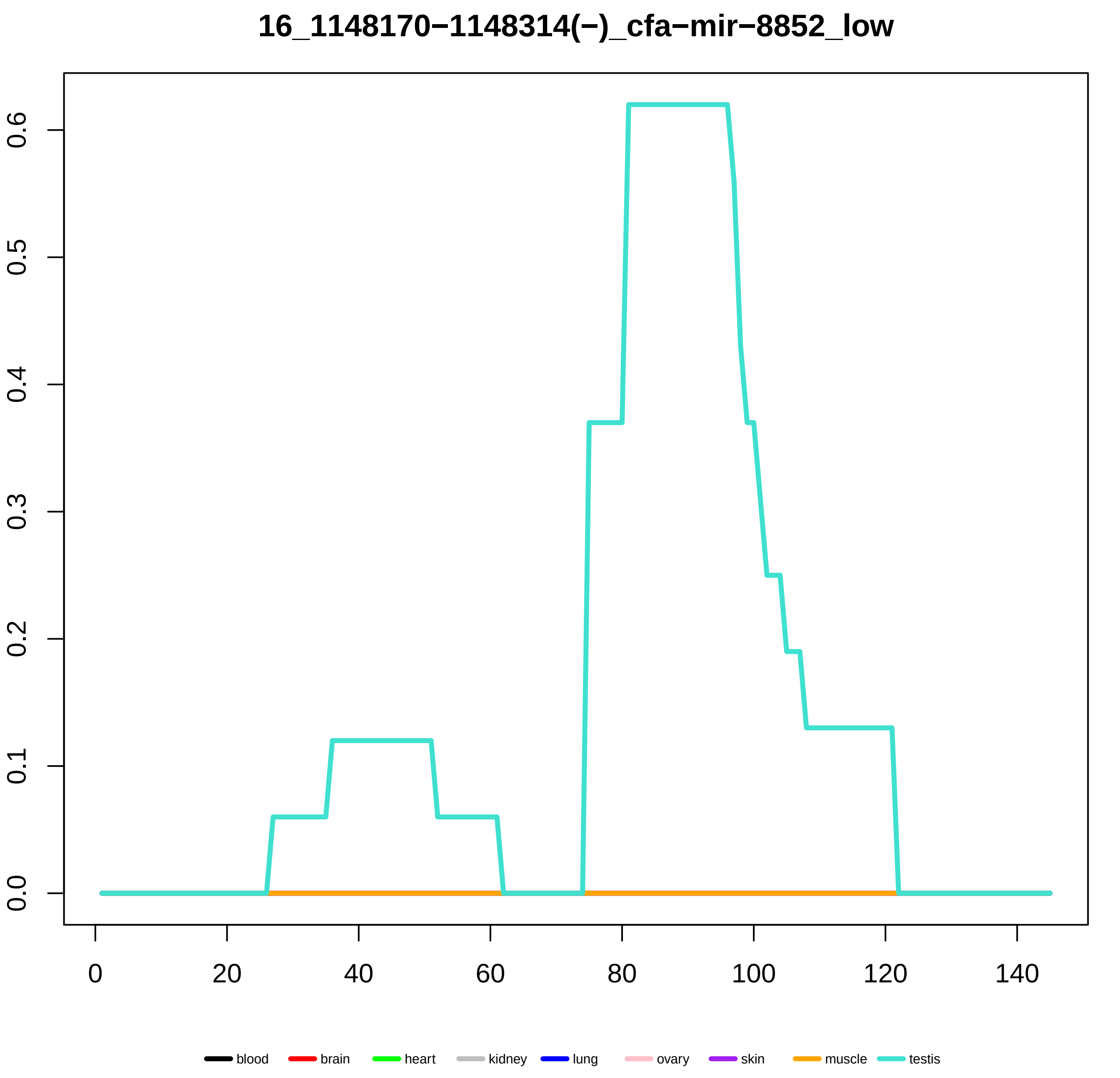
16_1148170−1148314(−)_cfa−mir−8852_low
0.0
0.1
0.2
0.3
0.4
0.5
0.6
0
20
40
60
80
100
120
140
blood
brain
heart
kidney
lung
ovary
skin
muscle
testis

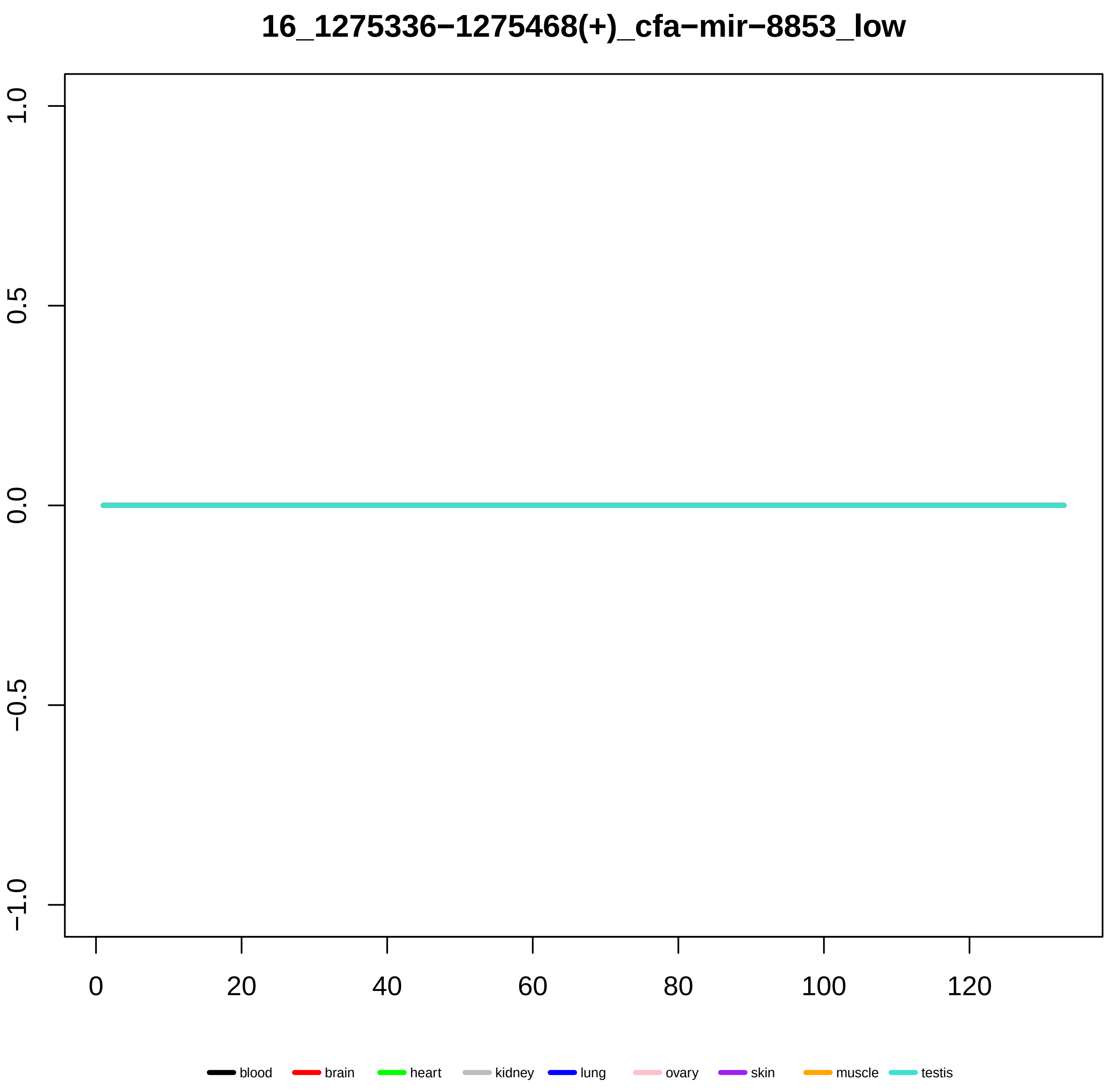
16_1275336−1275468(+)_cfa−mir−8853_low
1.0
0.5
0.0
−0.5
−1.0
0
20
40
60
80
100
120
blood
brain
heart
kidney
lung
ovary
skin
muscle
testis

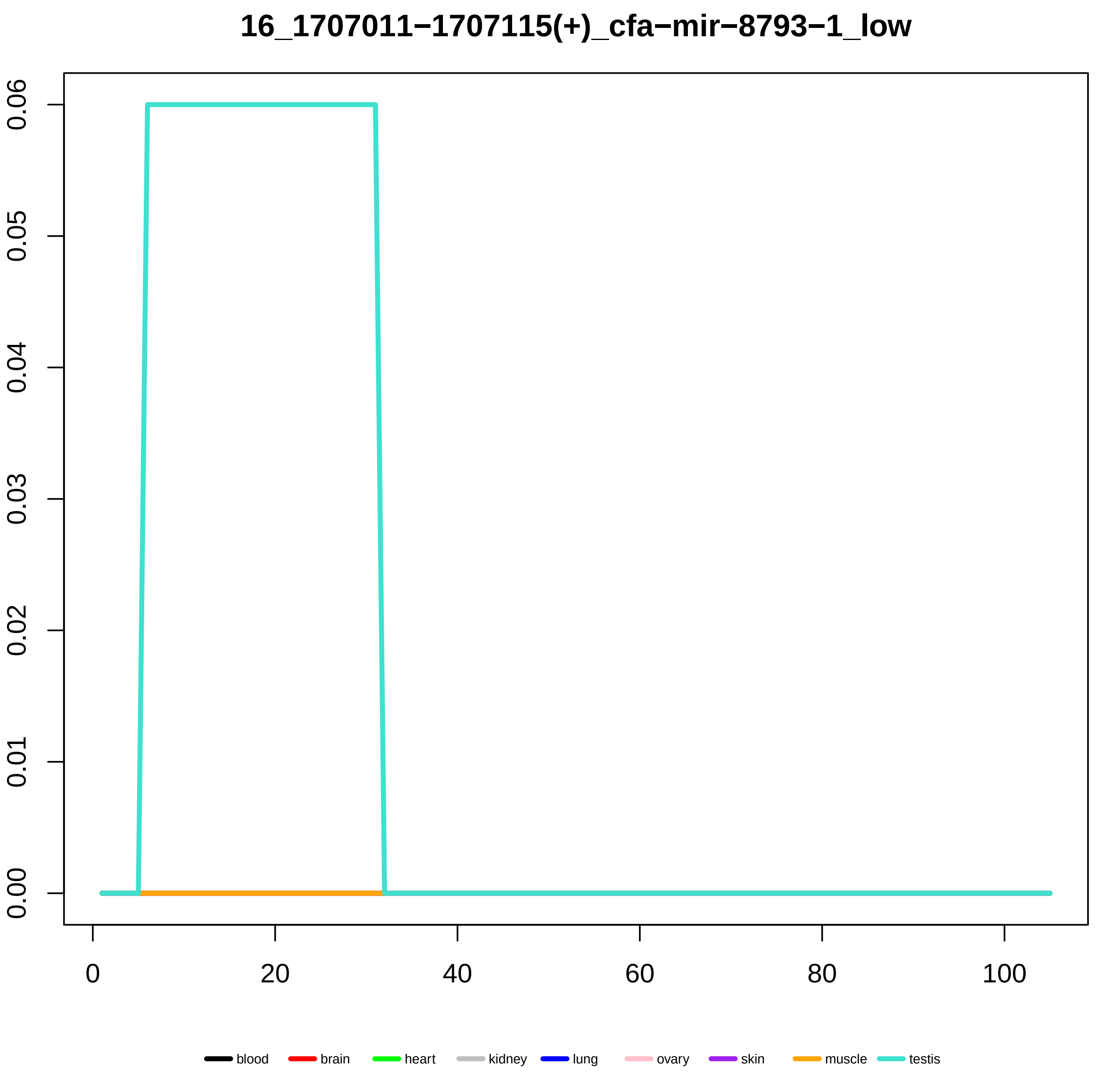
16_1707011−1707115(+)_cfa−mir−8793−1_low
0.00
0.01
0.02
0.03
0.04
0.05
0.06
0
20
40
60
80
100
blood
brain
heart
kidney
lung
ovary
skin
muscle
testis

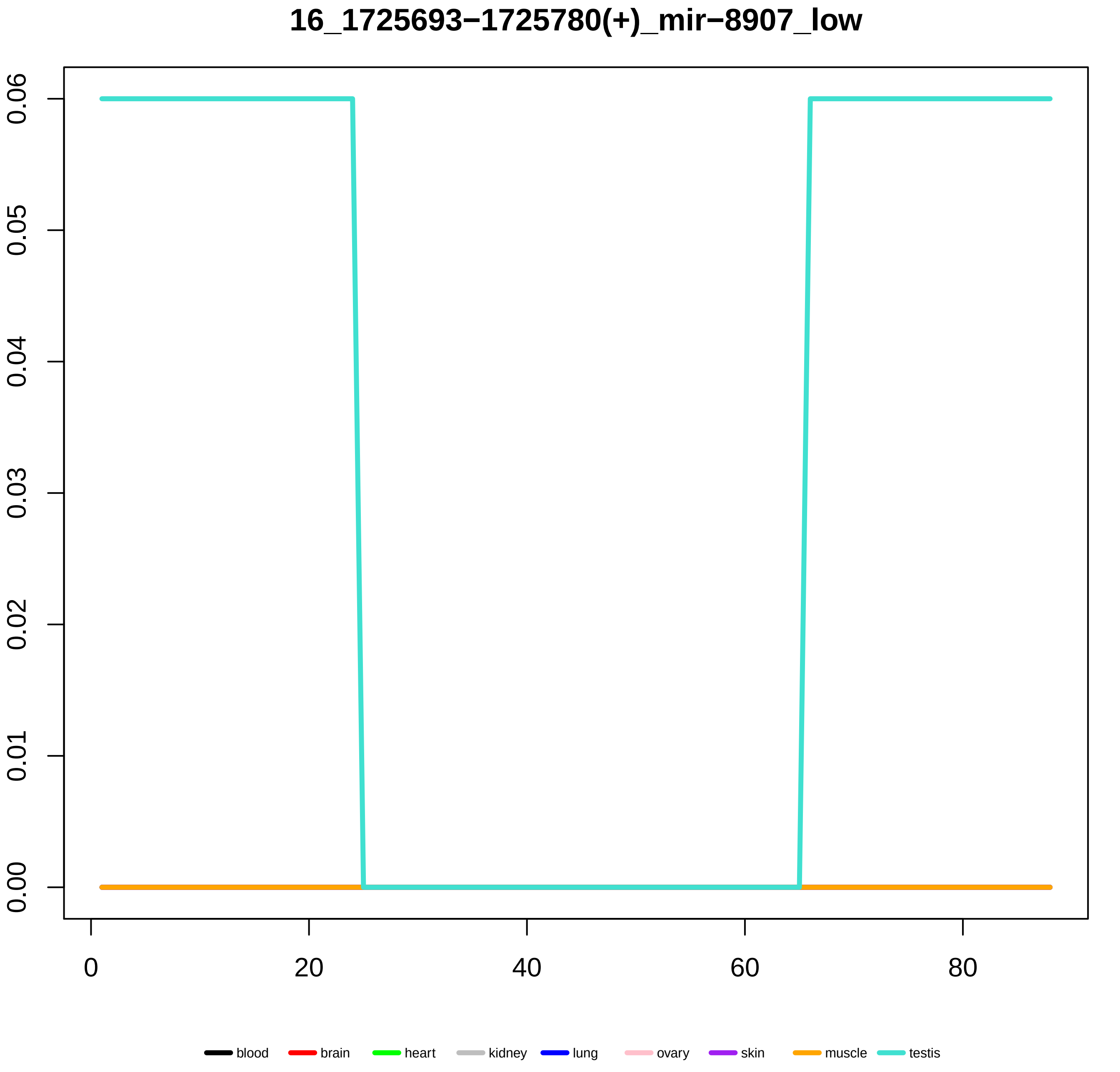

16_1725693−1725780(+)_mir−8907_low
0.00
0.01
0.02
0.03
0.04
0.05
0.06
0
20
40
60
80
blood
brain
heart
kidney
lung
ovary
skin
muscle
testis

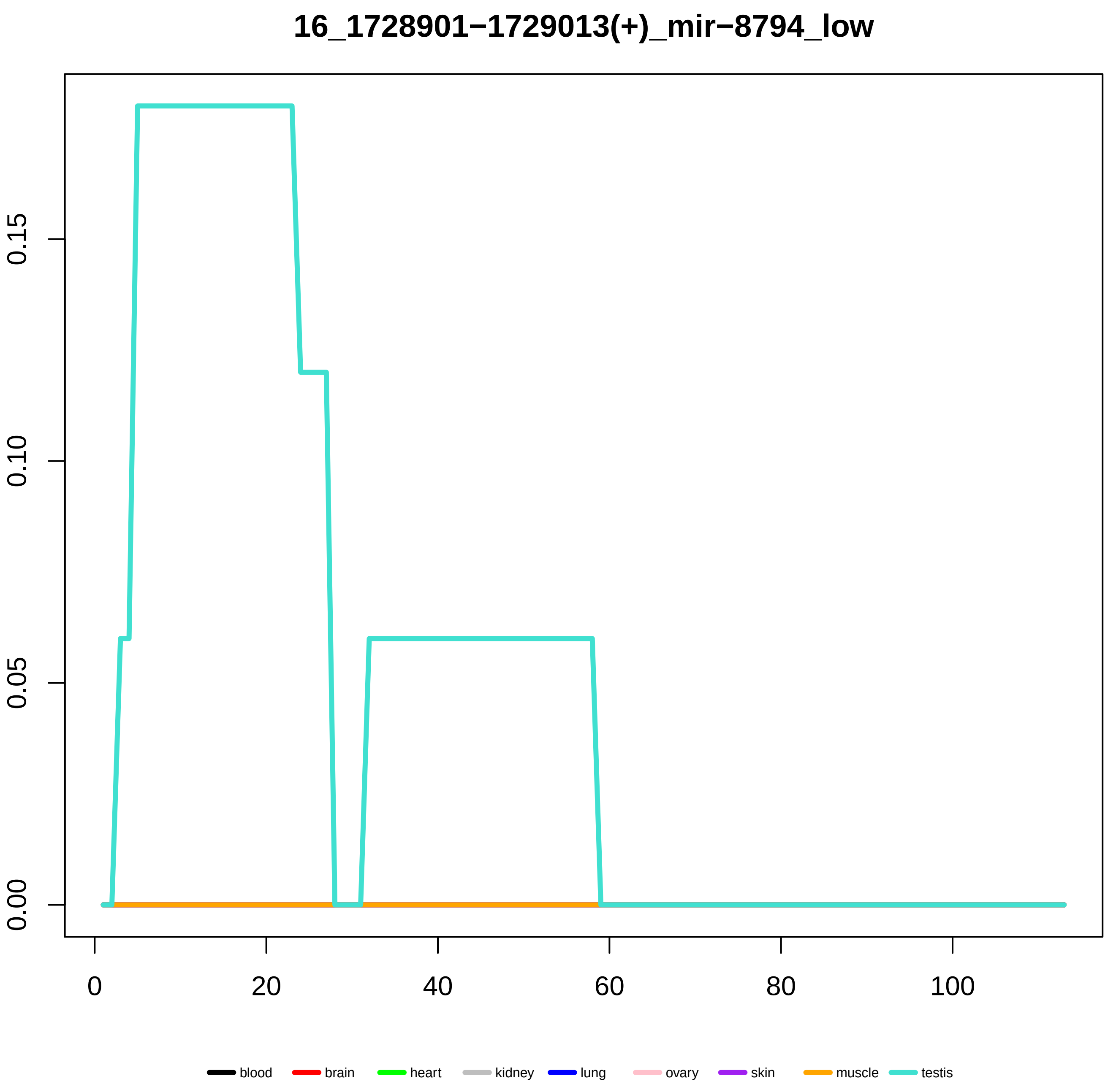
16_1728901−1729013(+)_mir−8794_low
0.00
0.05
0.10
0.15
0
20
40
60
80
100
blood
brain
heart
kidney
lung
ovary
skin
muscle
testis

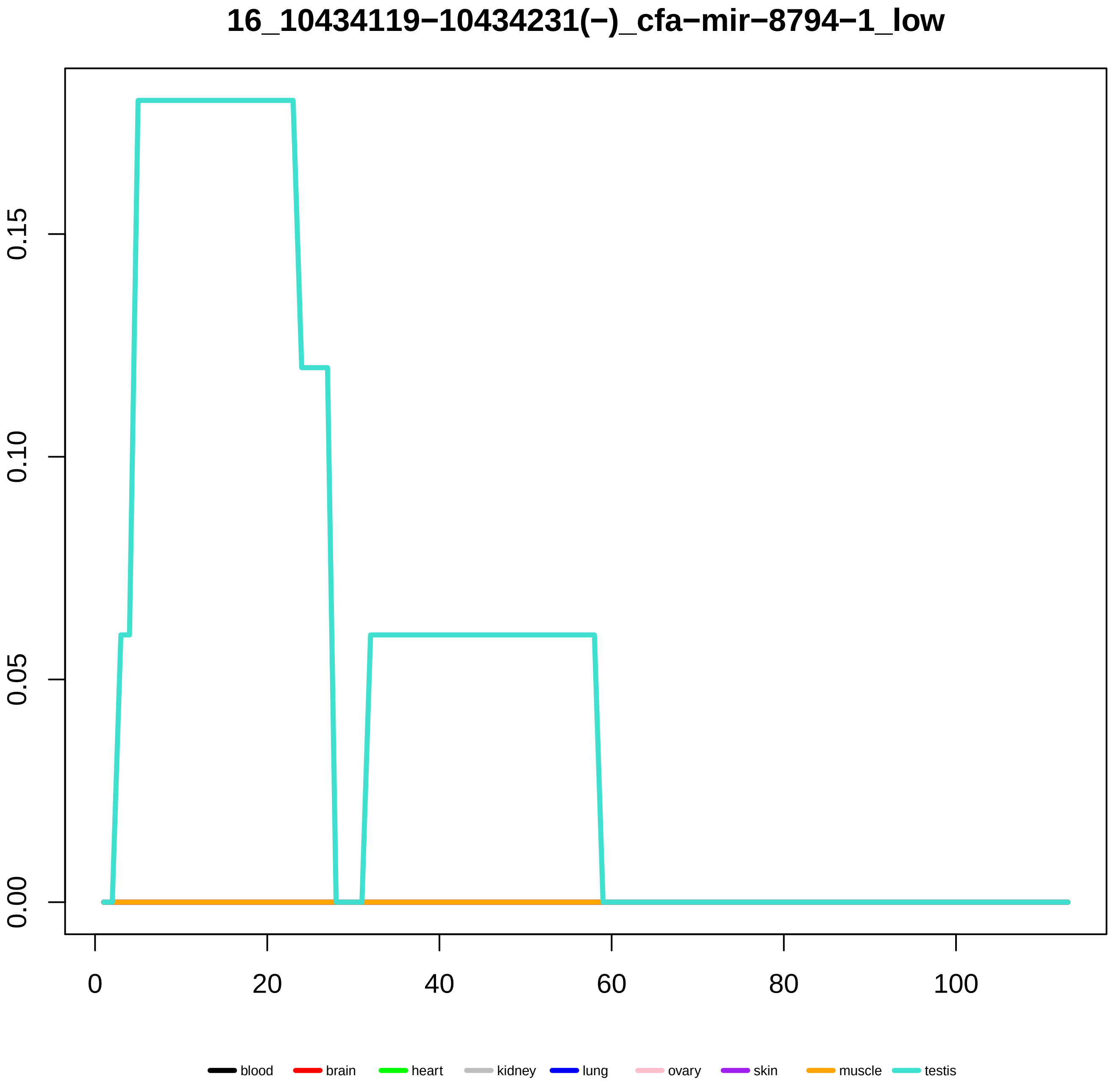
16_10434119−10434231(−)_cfa−mir−8794−1_low
0.00
0.05
0.10
0.15
0
20
40
60
80
100
blood
brain
heart
kidney
lung
ovary
skin
muscle
testis

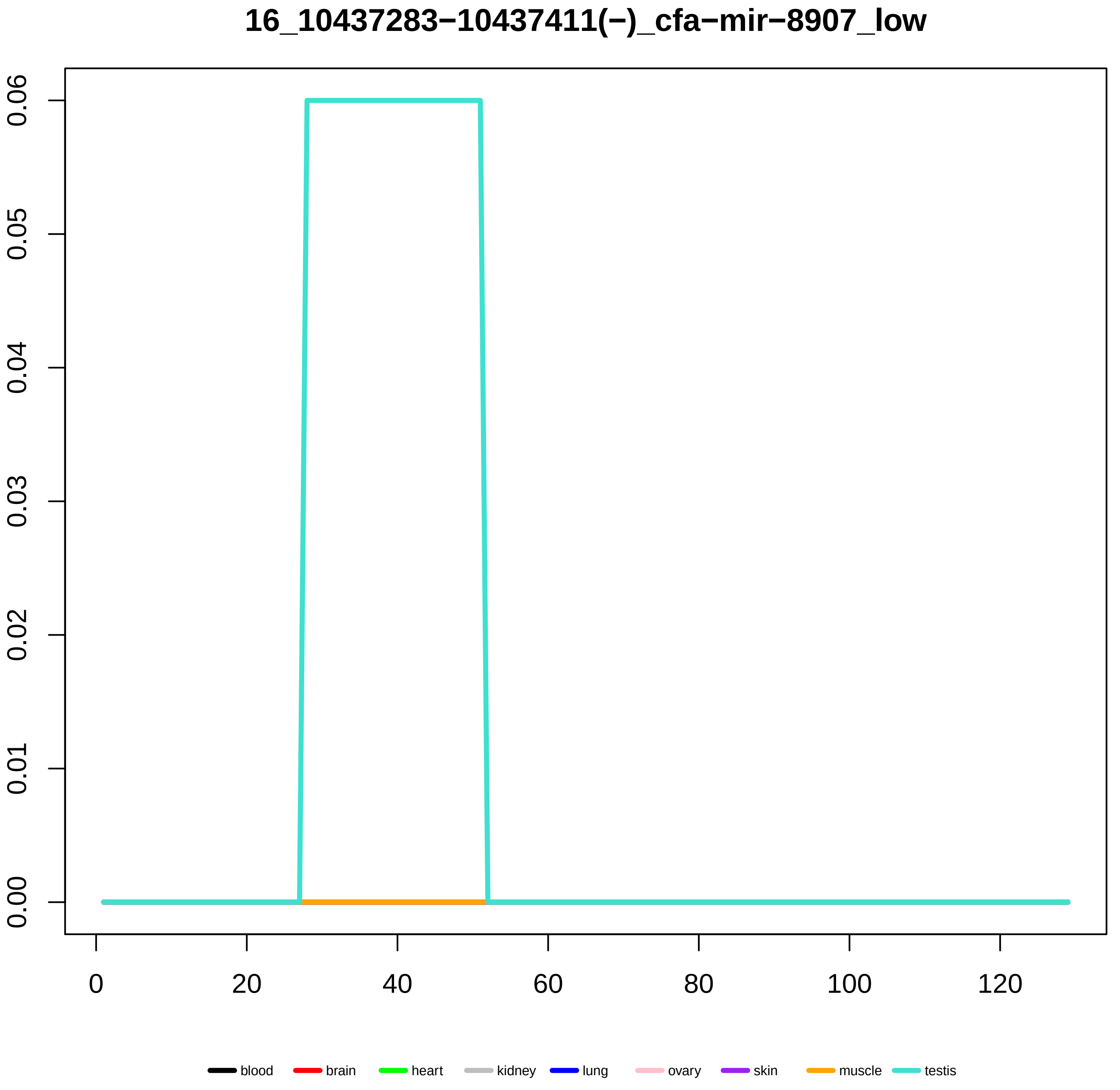

16_10437283−10437411(−)_cfa−mir−8907_low
0.00
0.01
0.02
0.03
0.04
0.05
0.06
0
20
40
60
80
100
120
blood
brain
heart
kidney
lung
ovary
skin
muscle
testis

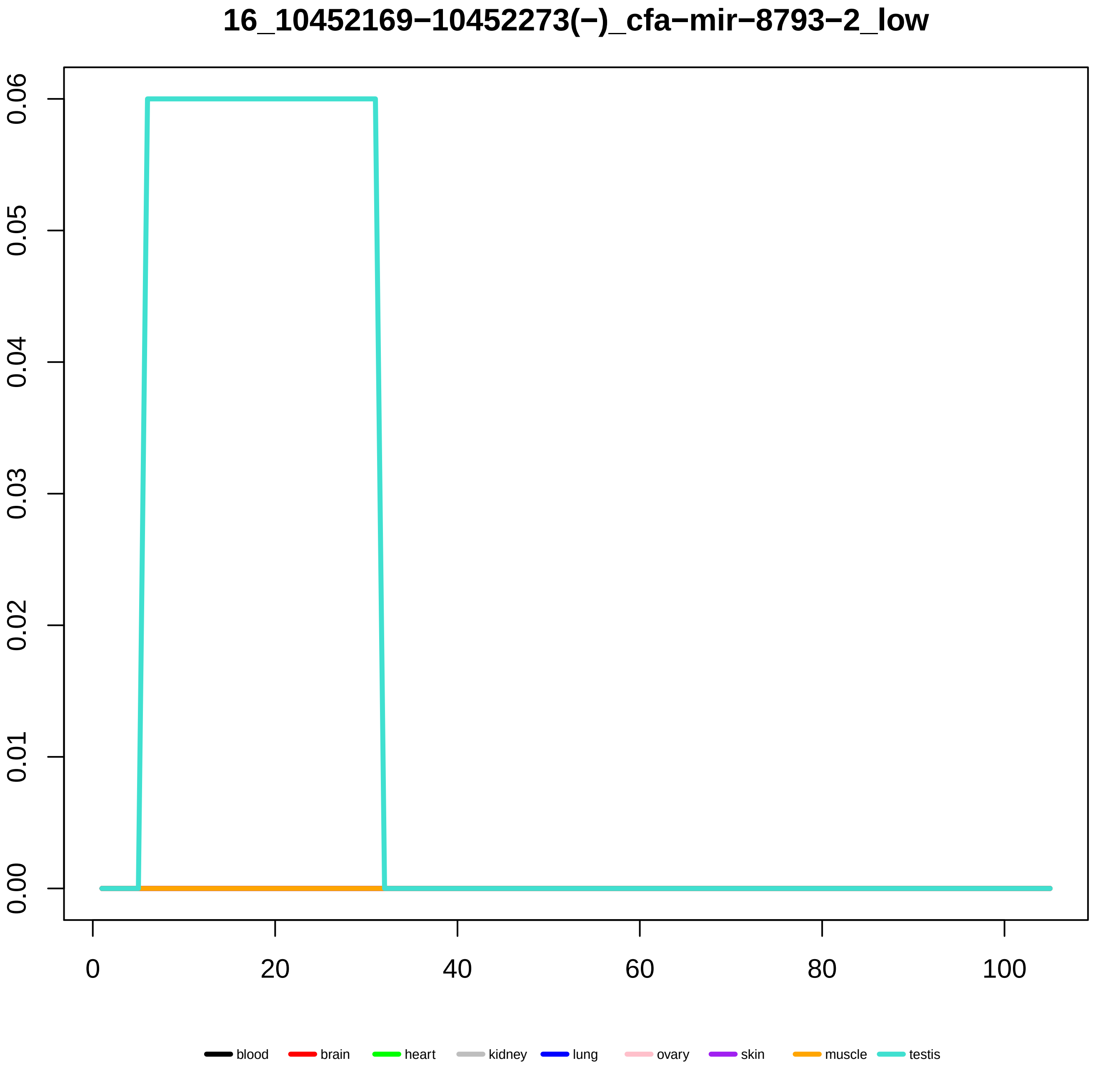
16_10452169−10452273(−)_cfa−mir−8793−2_low
0.00
0.01
0.02
0.03
0.04
0.05
0.06
0
20
40
60
80
100
blood
brain
heart
kidney
lung
ovary
skin
muscle
testis

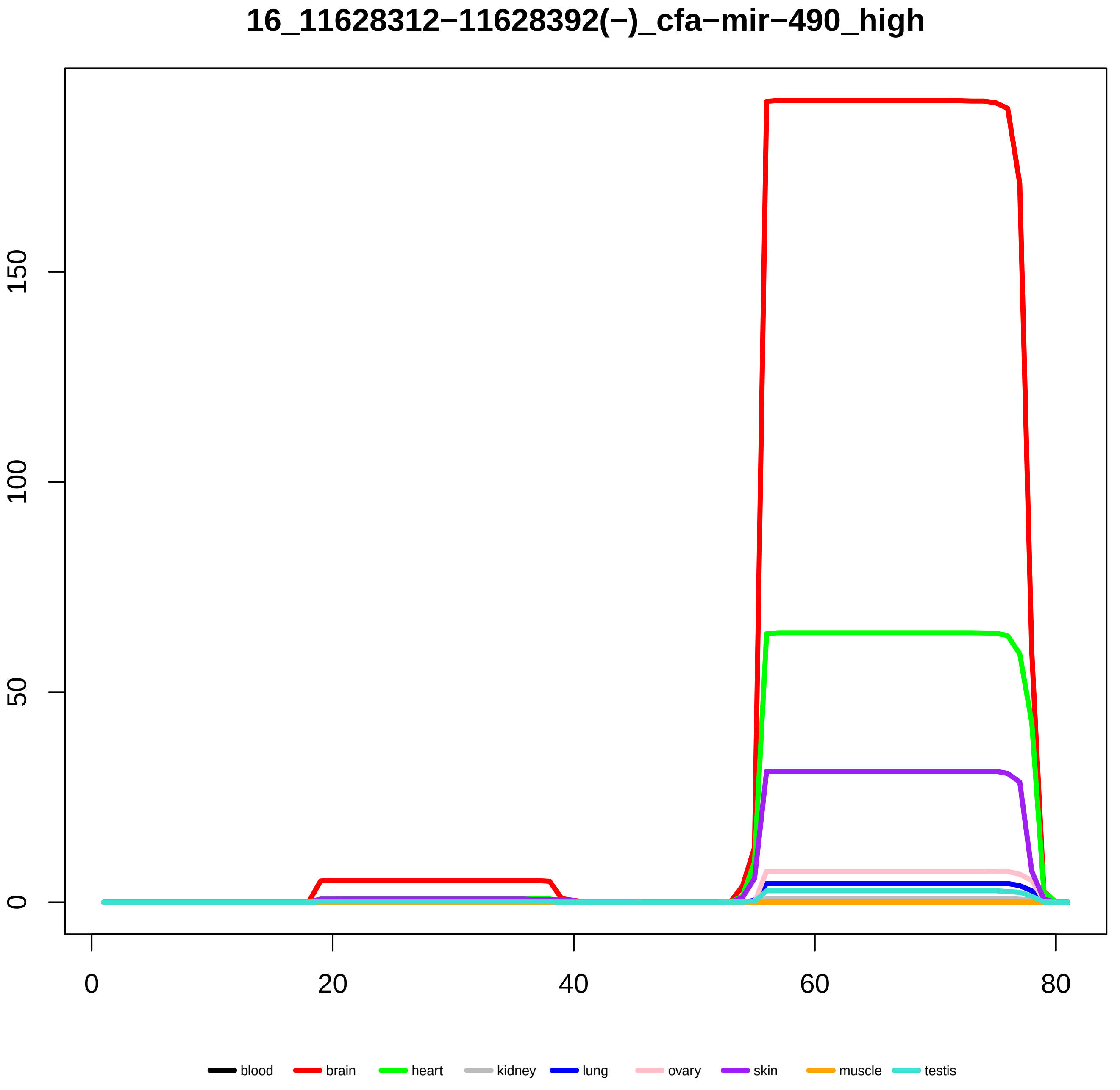
16_11628312−11628392(−)_cfa−mir−490_high
0
50
100
150
0
20
40
60
80
blood
brain
heart
kidney
lung
ovary
skin
muscle
testis

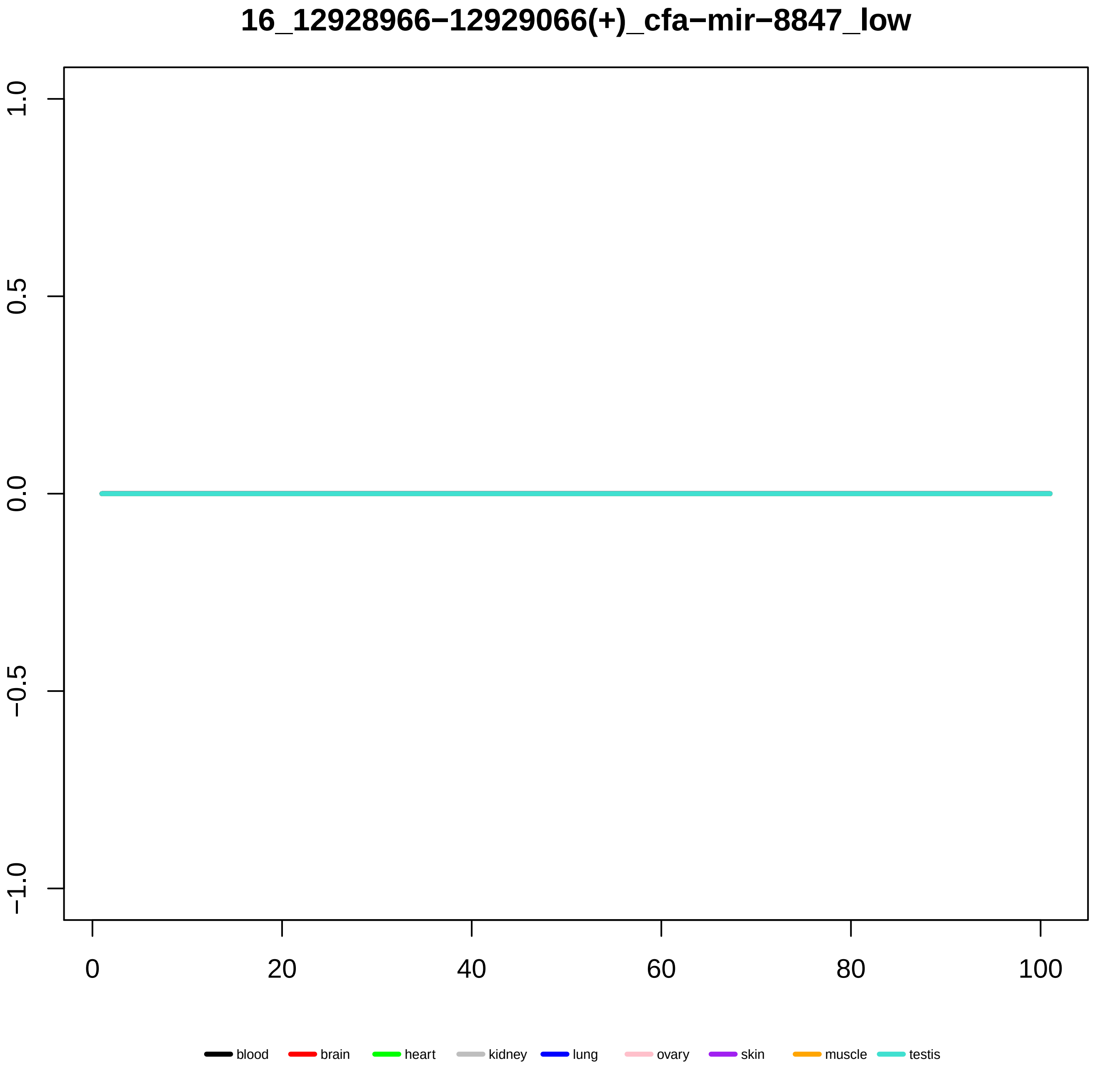
16_12928966−12929066(+)_cfa−mir−8847_low
1.0
0.5
0.0
−0.5
−1.0
0
20
40
60
80
100
blood
brain
heart
kidney
lung
ovary
skin
muscle
testis

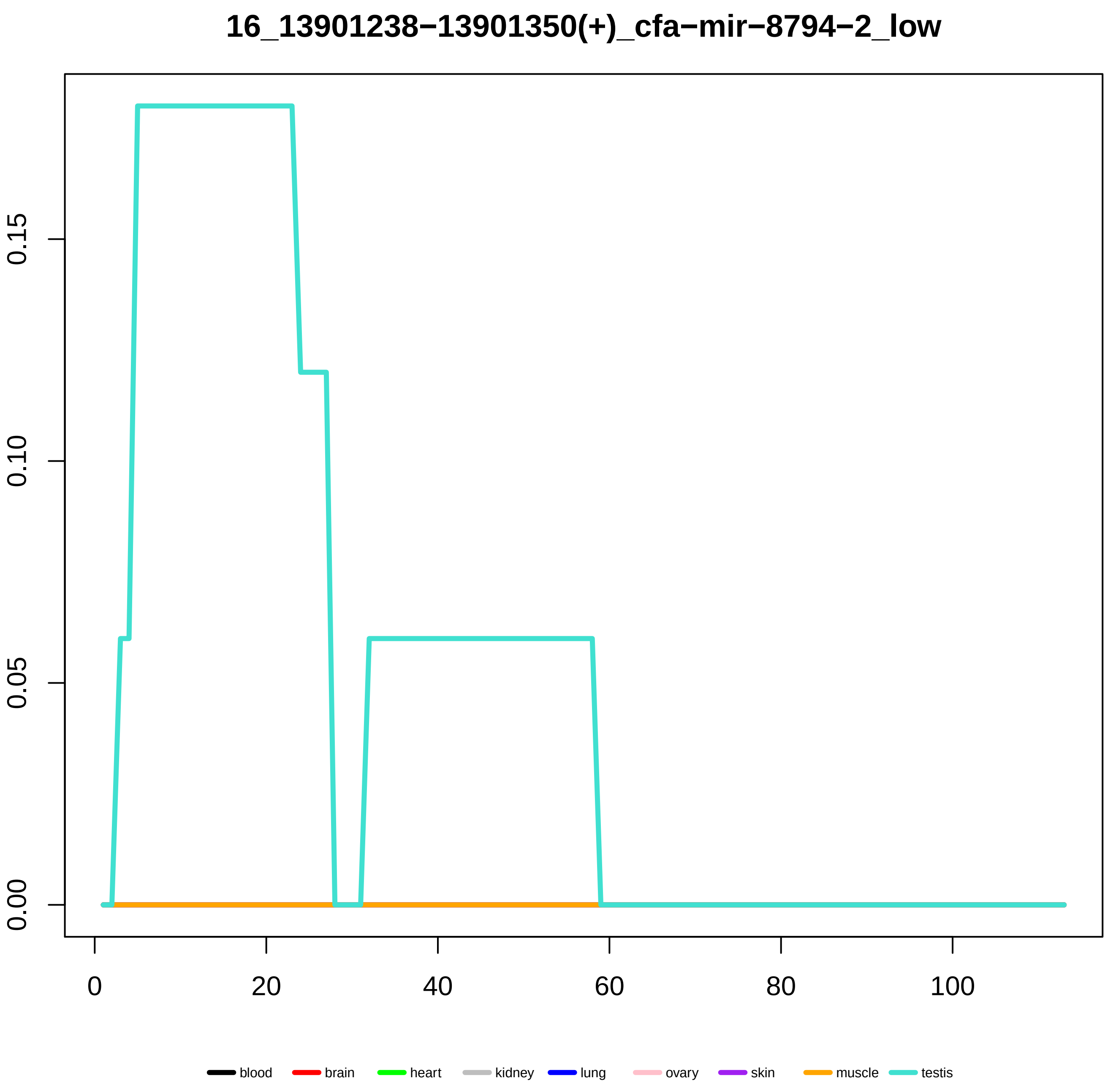
16_13901238−13901350(+)_cfa−mir−8794−2_low
0.00
0.05
0.10
0.15
0
20
40
60
80
100
blood
brain
heart
kidney
lung
ovary
skin
muscle
testis

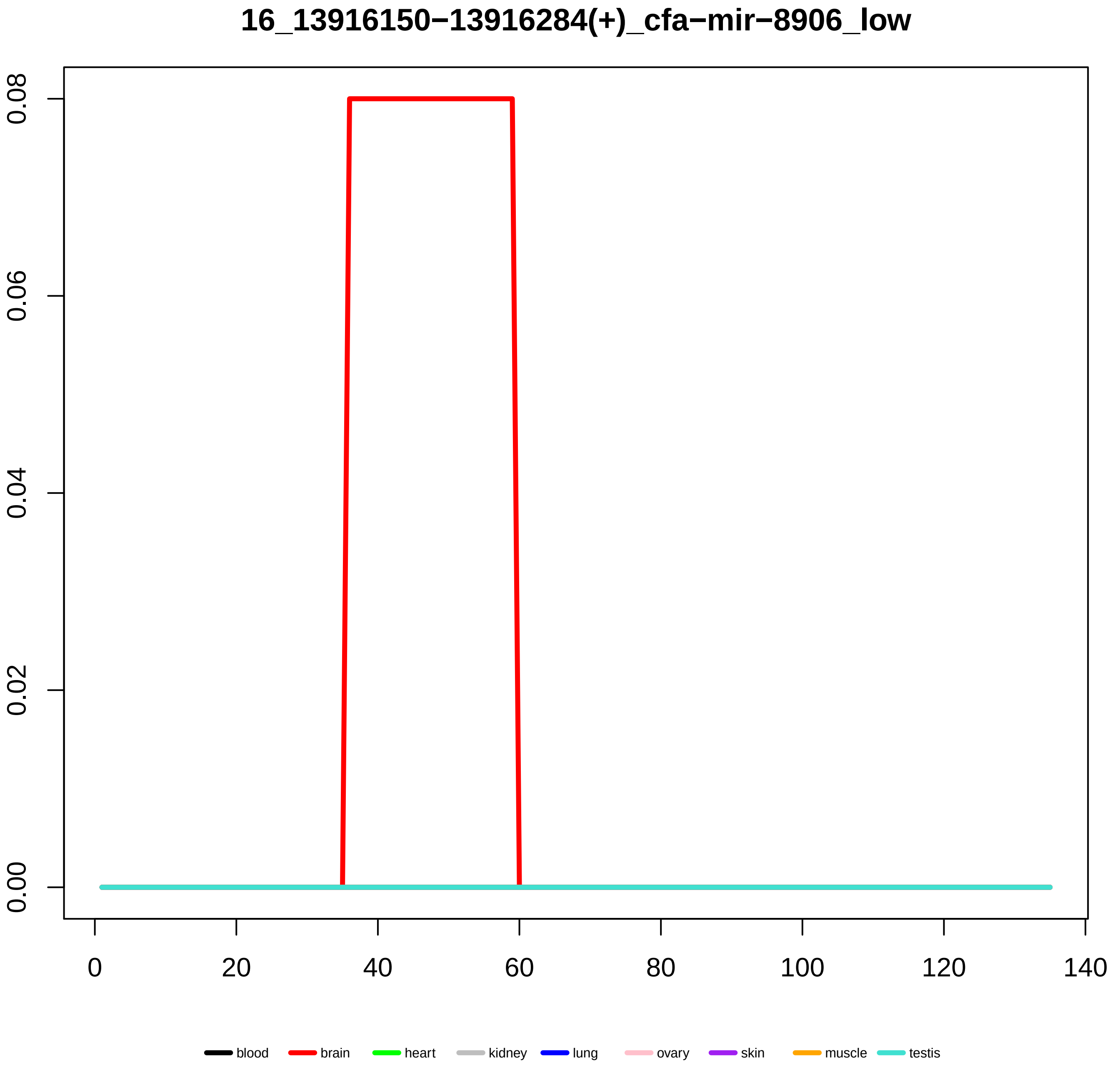
16_13916150−13916284(+)_cfa−mir−8906_low
0.00
0.02
0.04
0.06
0.08
0
20
40
60
80
100
120
140
blood
brain
heart
kidney
lung
ovary
skin
muscle
testis

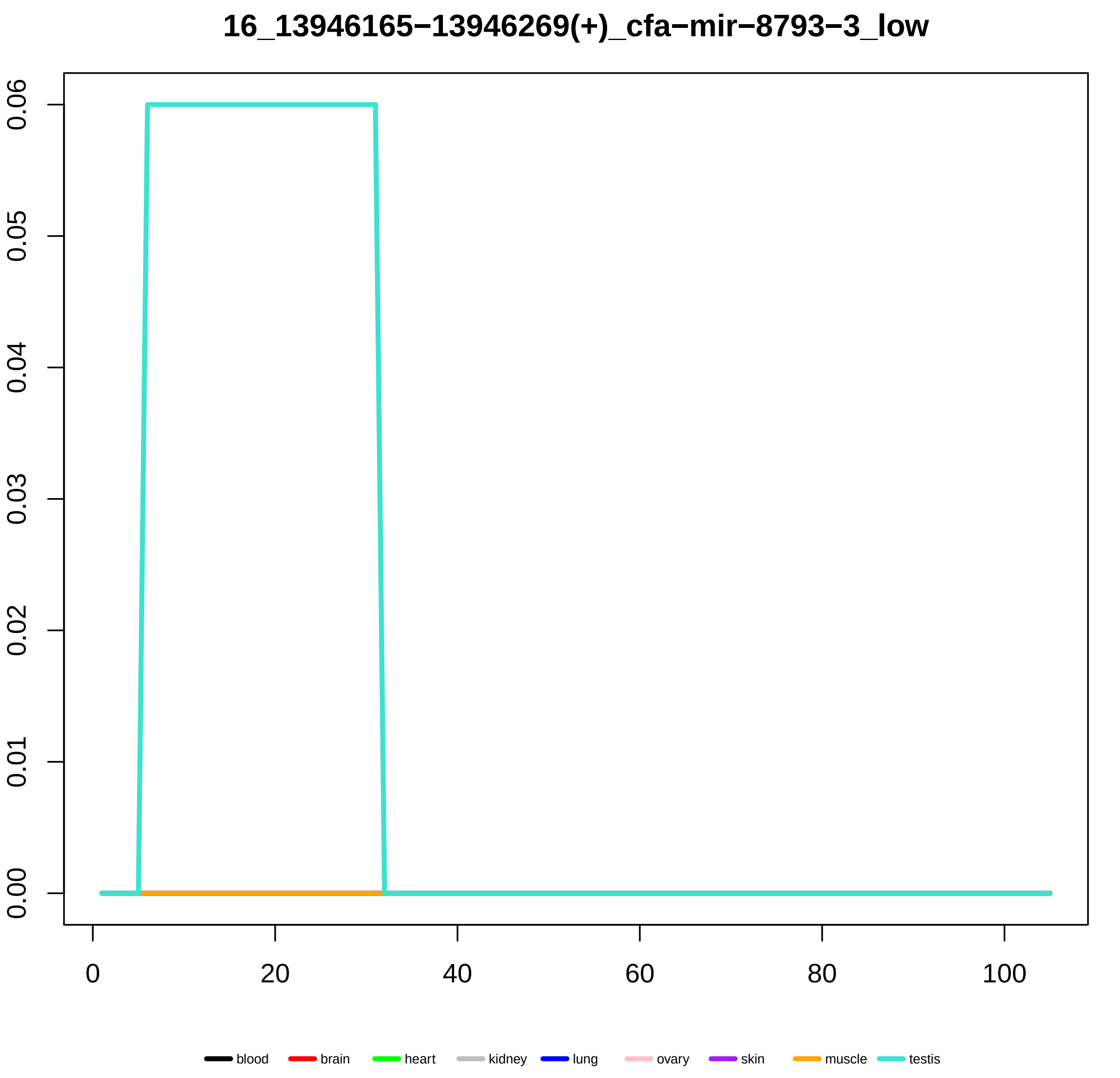
16_13946165−13946269(+)_cfa−mir−8793−3_low
0.00
0.01
0.02
0.03
0.04
0.05
0.06
0
20
40
60
80
100
blood
brain
heart
kidney
lung
ovary
skin
muscle
testis

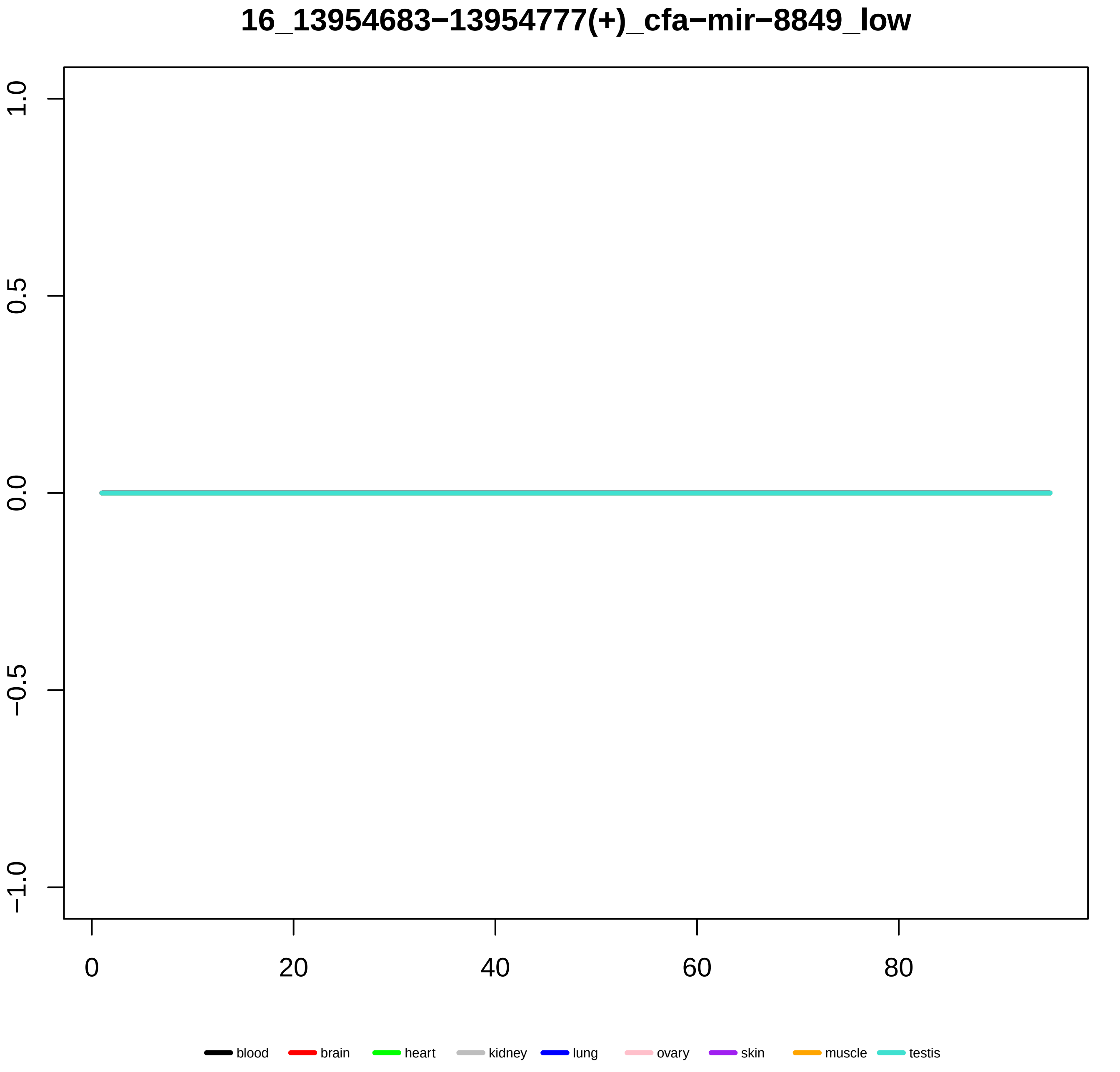

16_13954683−13954777(+)_cfa−mir−8849_low
1.0
0.5
0.0
−0.5
−1.0
0
20
40
60
80
blood
brain
heart
kidney
lung
ovary
skin
muscle
testis

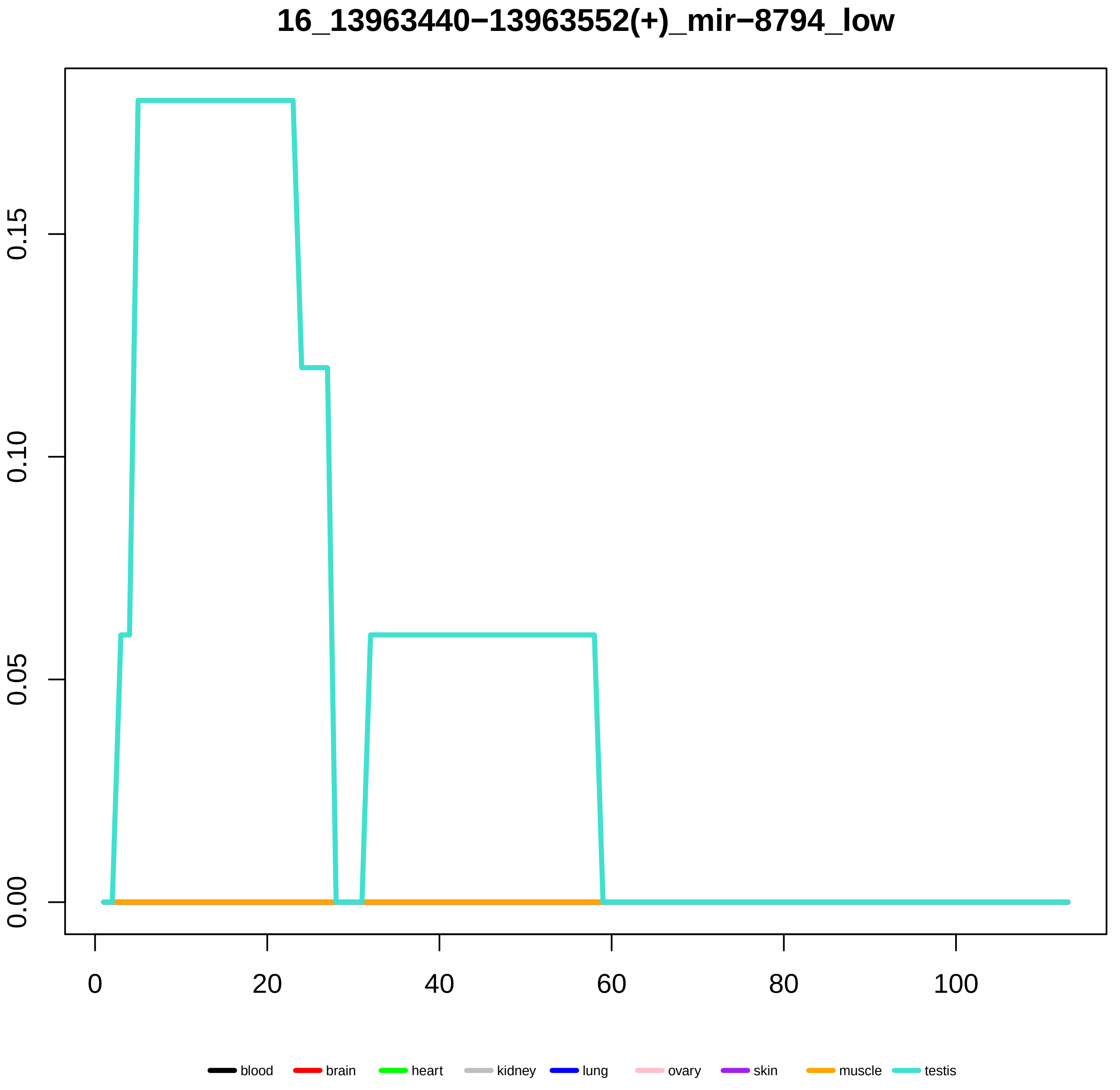
16_13963440−13963552(+)_mir−8794_low
0.00
0.05
0.10
0.15
0
20
40
60
80
100
blood
brain
heart
kidney
lung
ovary
skin
muscle
testis

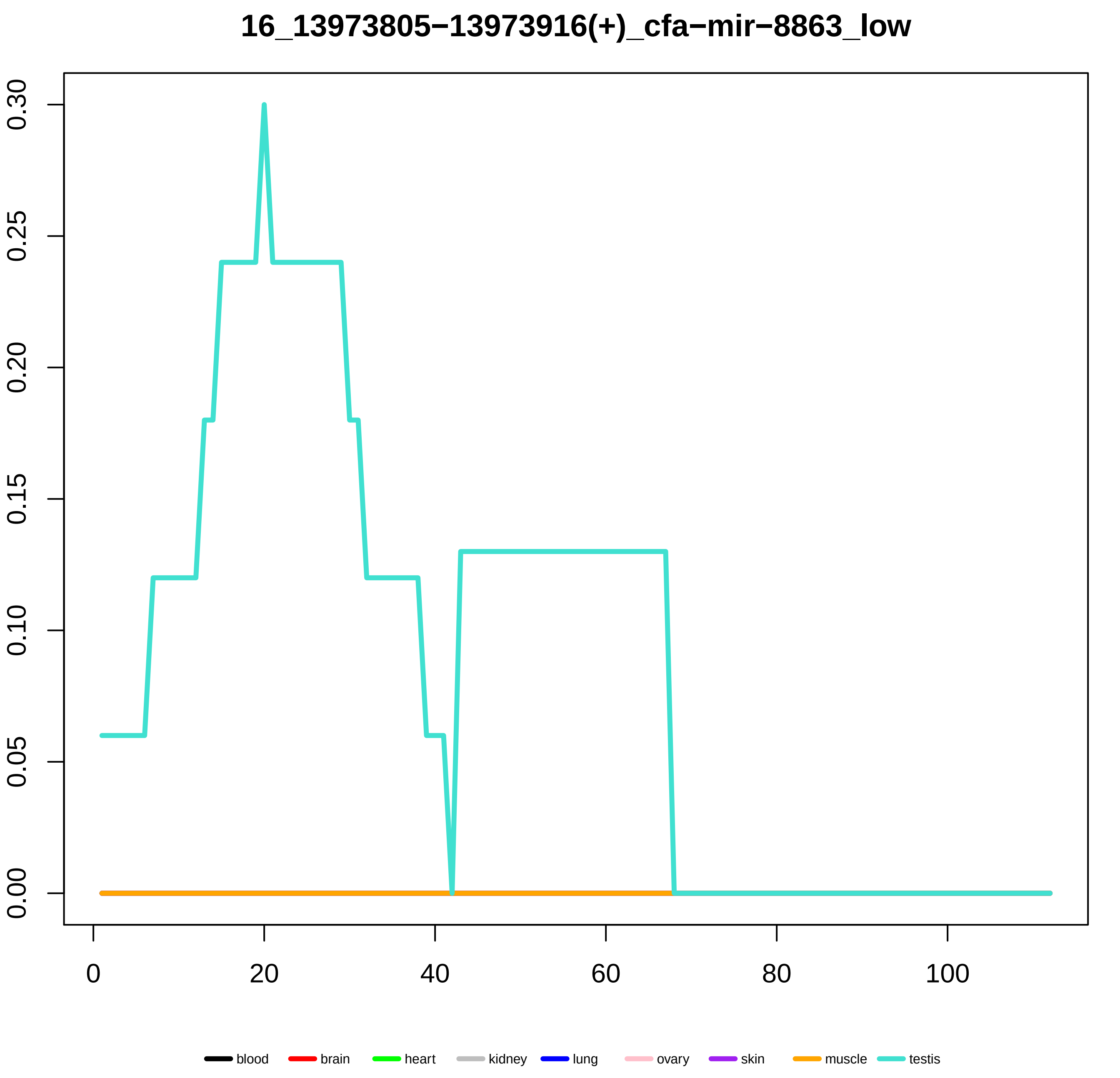

16_13973805−13973916(+)_cfa−mir−8863_low
0.00
0.05
0.10
0.15
0.20
0.25
0.30
0
20
40
60
80
100
blood
brain
heart
kidney
lung
ovary
skin
muscle
testis

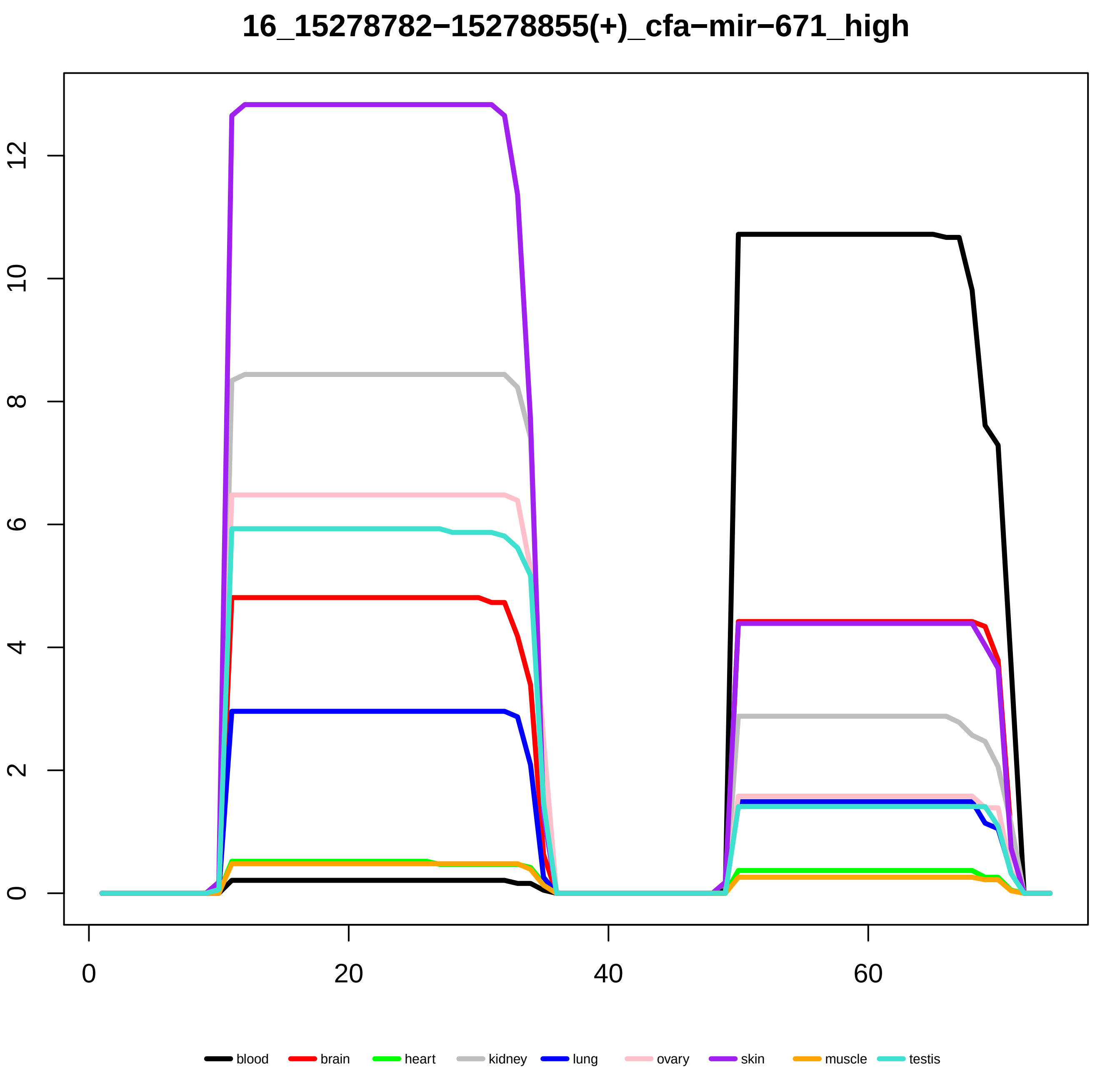
16_15278782−15278855(+)_cfa−mir−671_high
0
2
4
6
8
10
12
0
20
40
60
blood
brain
heart
kidney
lung
ovary
skin
muscle
testis

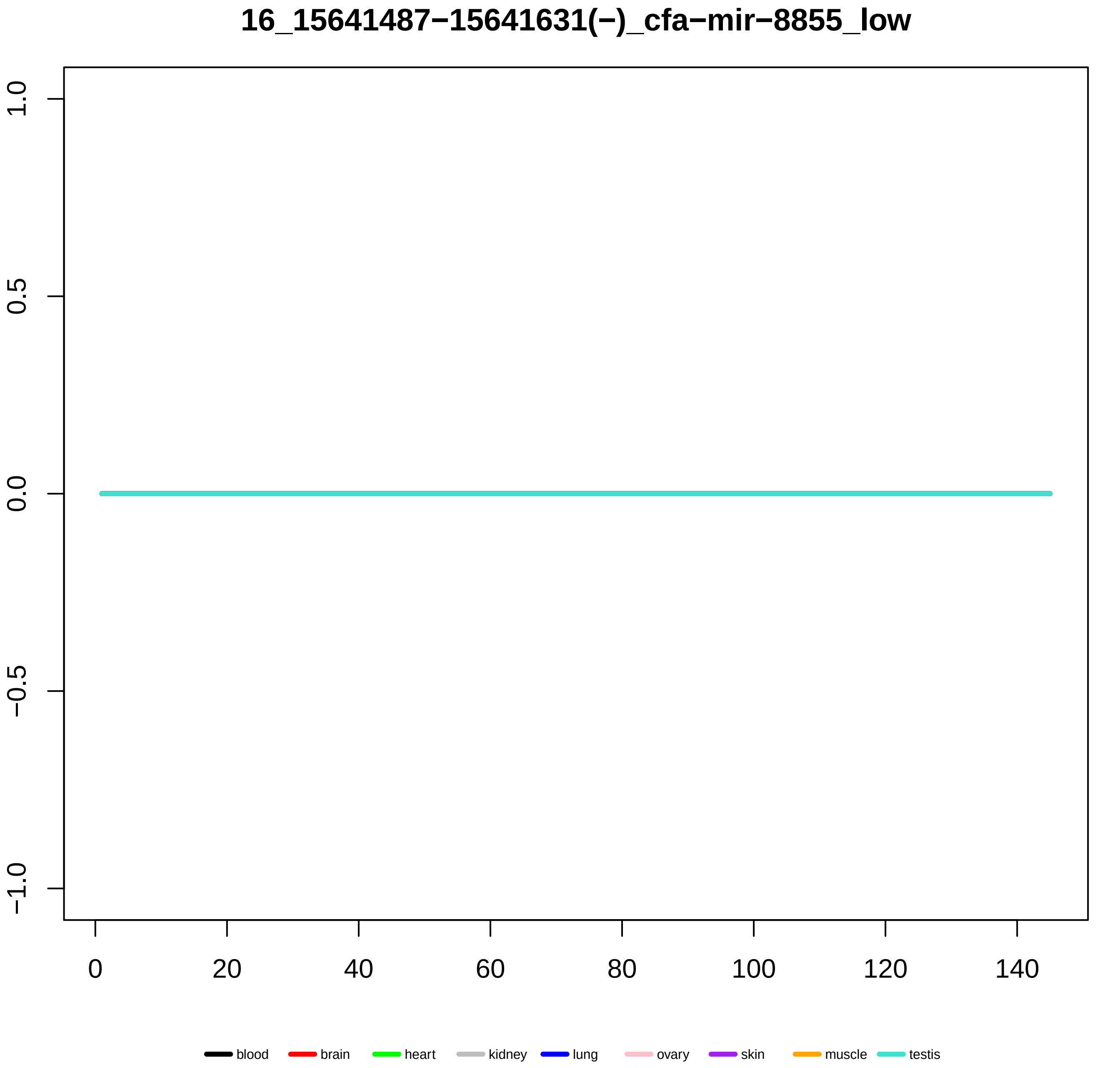

16_15641487−15641631(−)_cfa−mir−8855_low
1.0
0.5
0.0
−0.5
−1.0
0
20
40
60
80
100
120
140
blood
brain
heart
kidney
lung
ovary
skin
muscle
testis

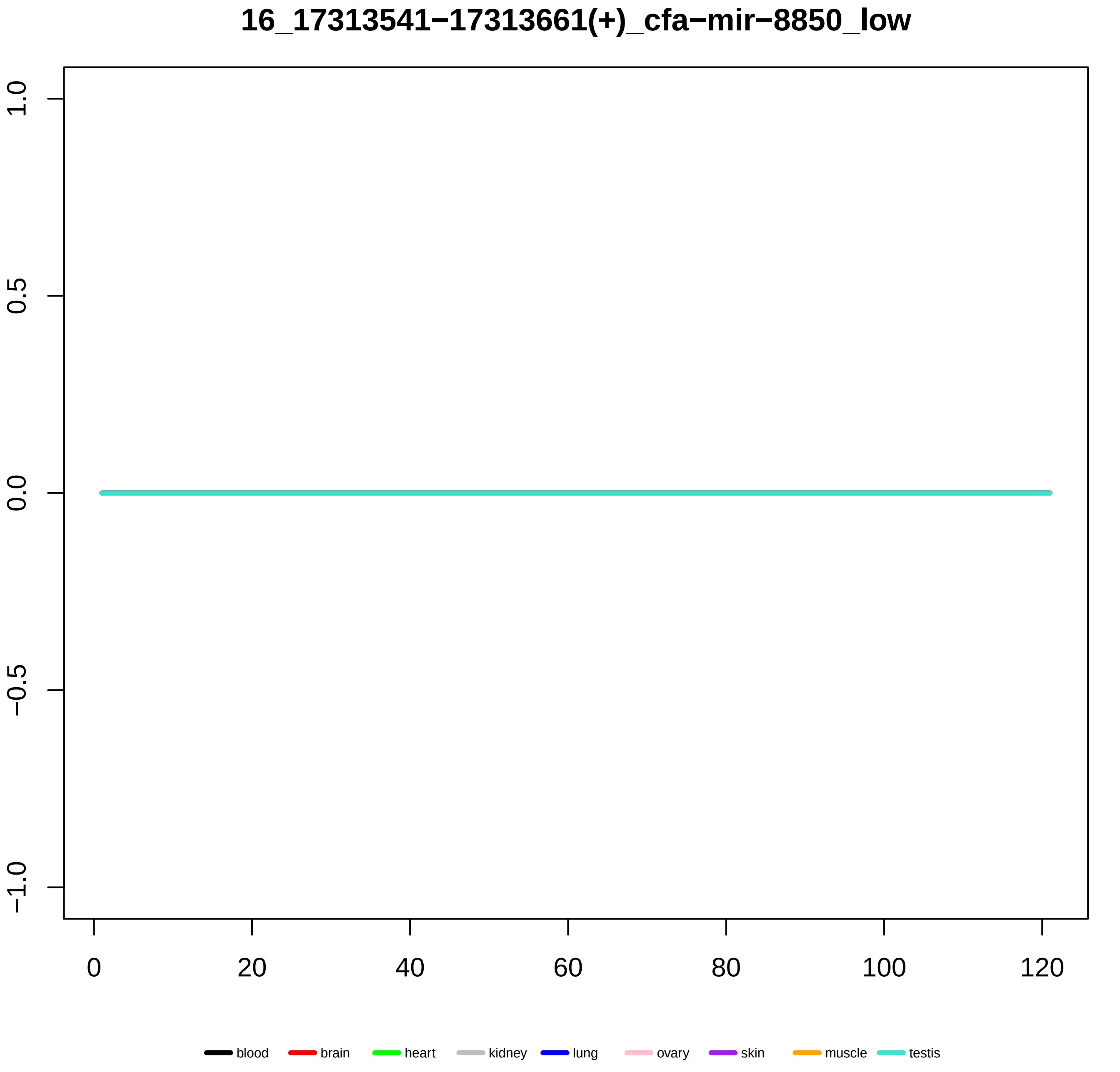
16_17313541−17313661(+)_cfa−mir−8850_low
1.0
0.5
0.0
−0.5
−1.0
0
20
40
60
80
100
120
blood
brain
heart
kidney
lung
ovary
skin
muscle
testis

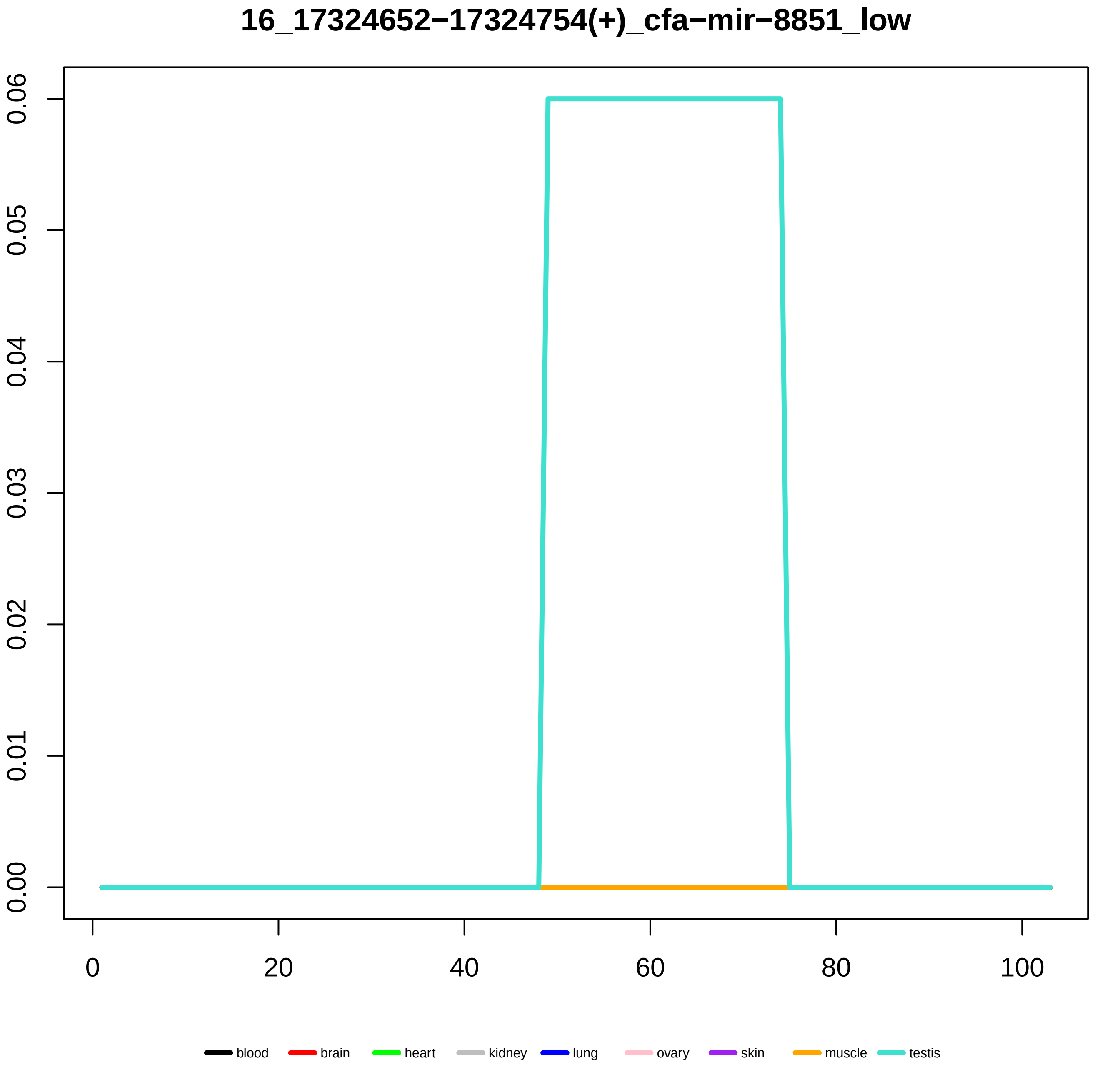

16_17324652−17324754(+)_cfa−mir−8851_low
0.00
0.01
0.02
0.03
0.04
0.05
0.06
0
20
40
60
80
100
blood
brain
heart
kidney
lung
ovary
skin
muscle
testis

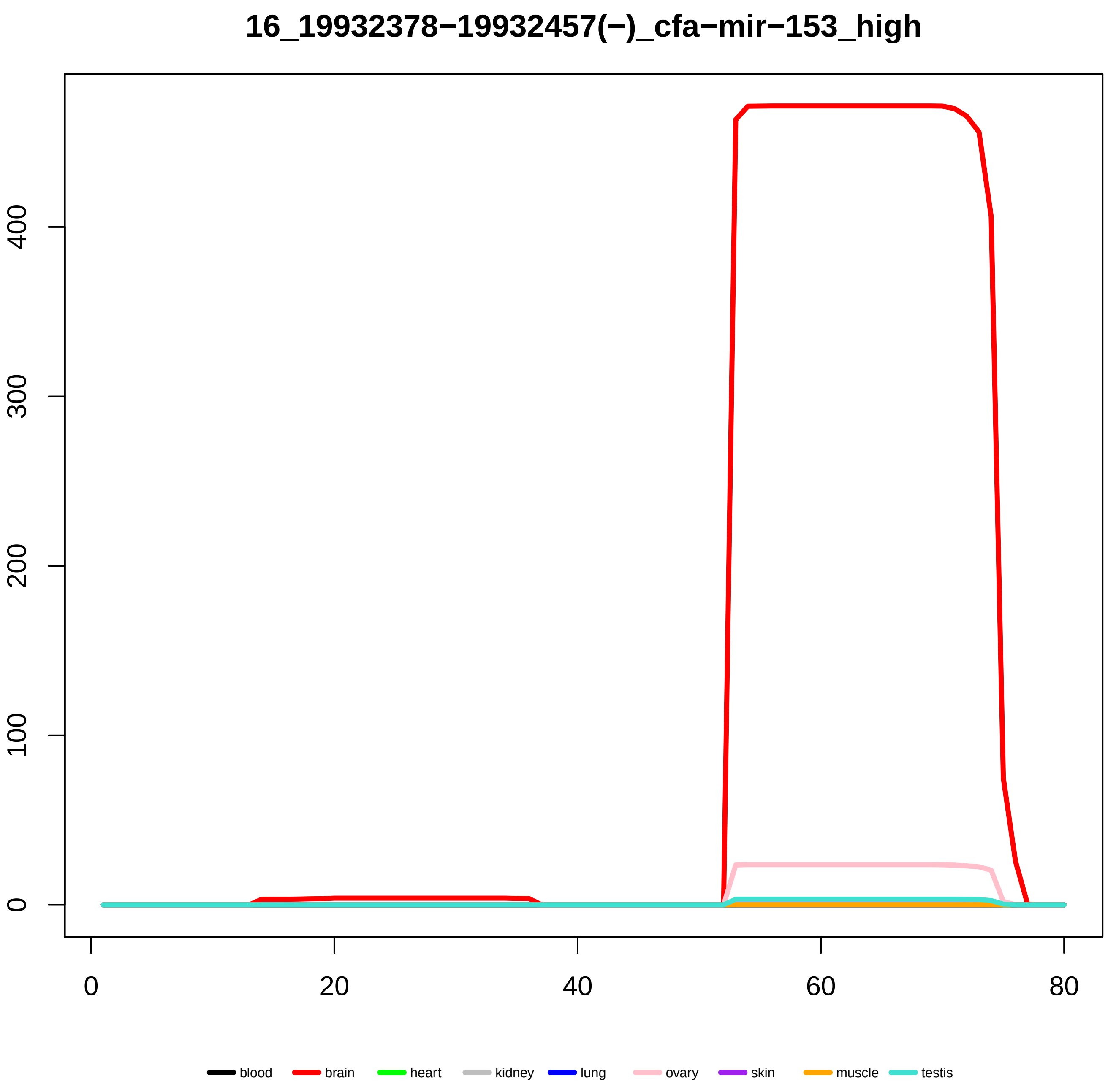

16_19932378−19932457(−)_cfa−mir−153_high
0
100
200
300
400
0
20
40
60
80
blood
brain
heart
kidney
lung
ovary
skin
muscle
testis

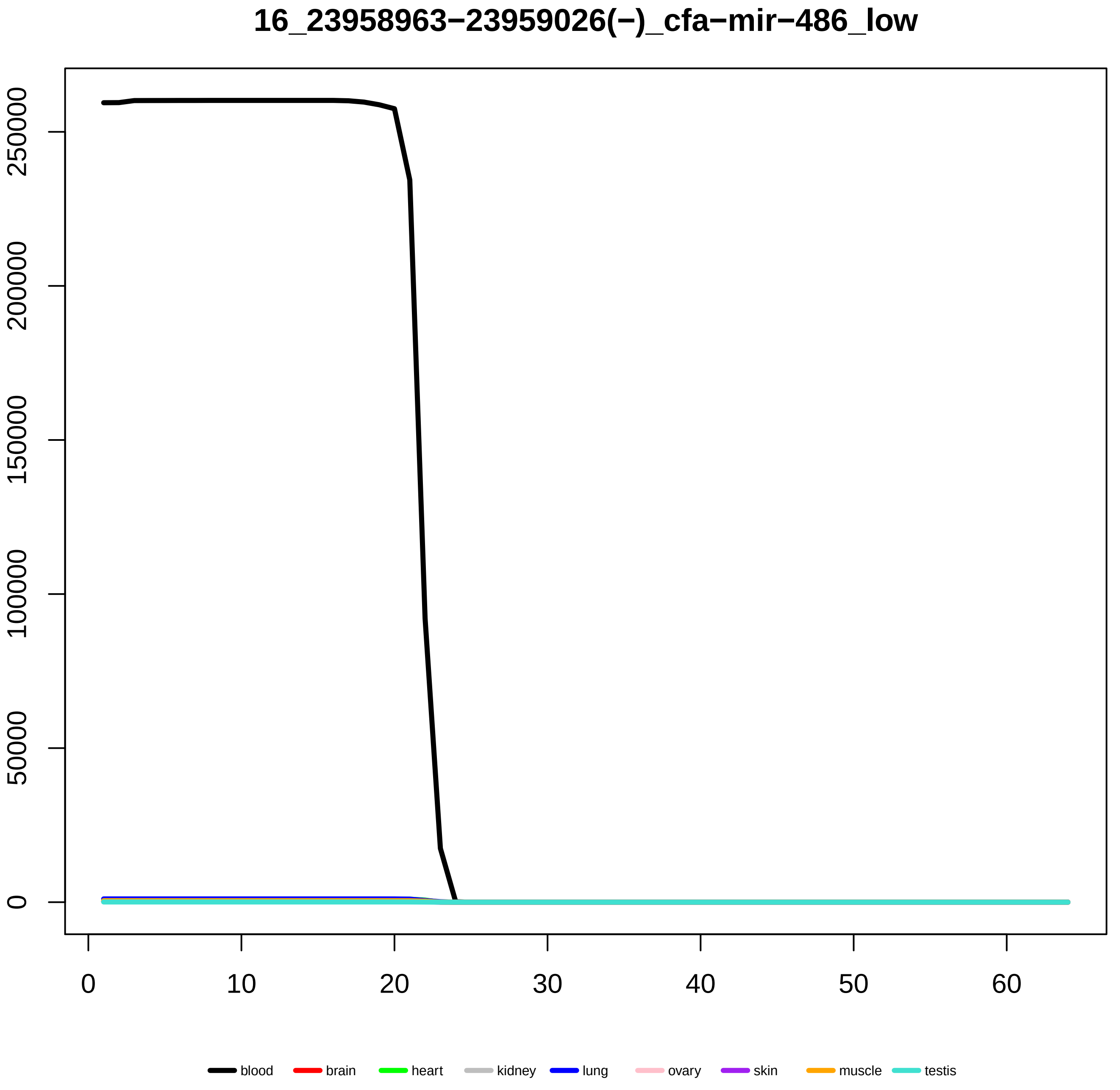
16_23958963−23959026(−)_cfa−mir−486_low
0
50000
100000
150000
200000
250000
0
10
20
30
40
50
60
blood
brain
heart
kidney
lung
ovary
skin
muscle
testis

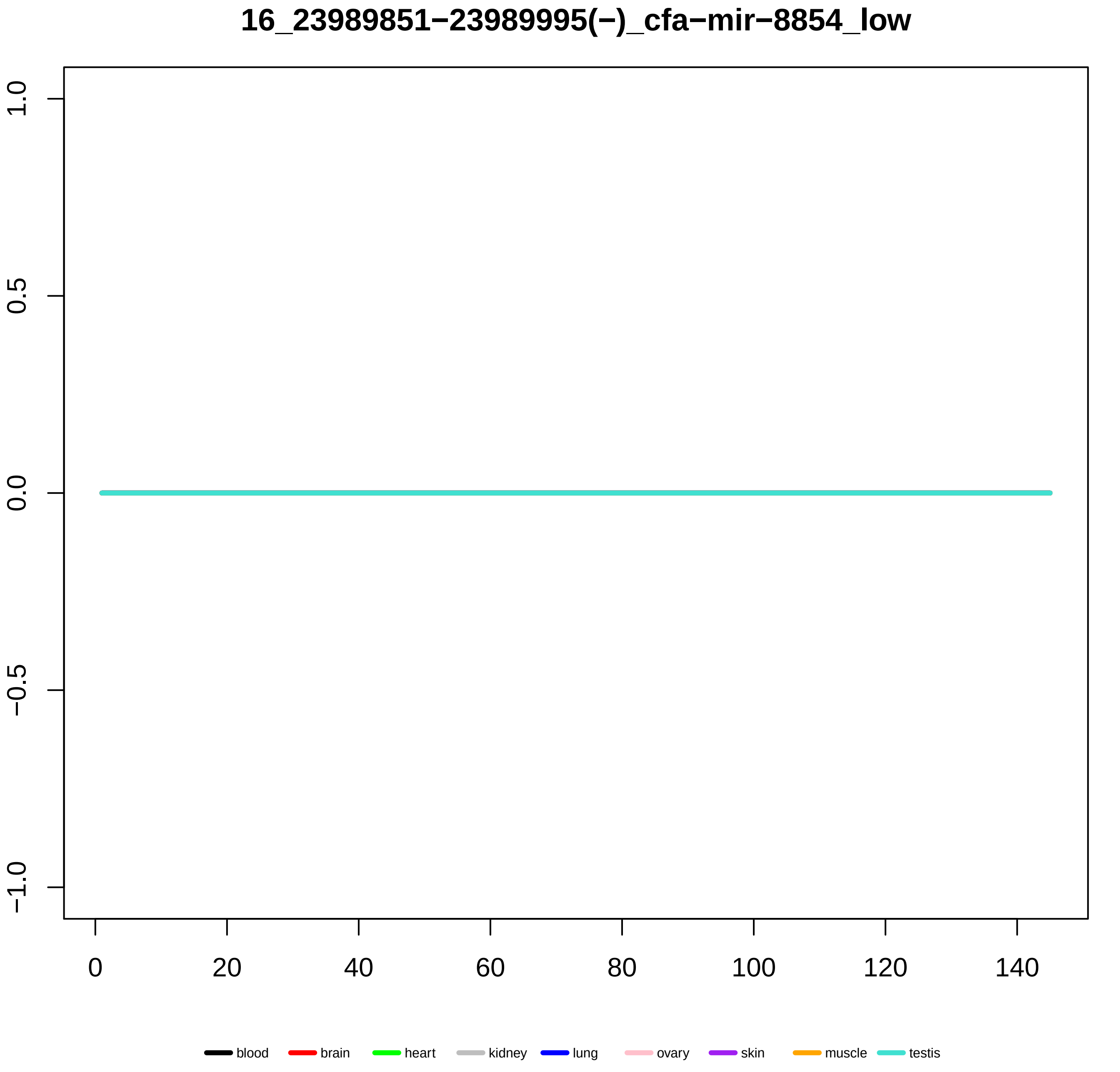

16_23989851−23989995(−)_cfa−mir−8854_low
1.0
0.5
0.0
−0.5
−1.0
0
20
40
60
80
100
120
140
blood
brain
heart
kidney
lung
ovary
skin
muscle
testis

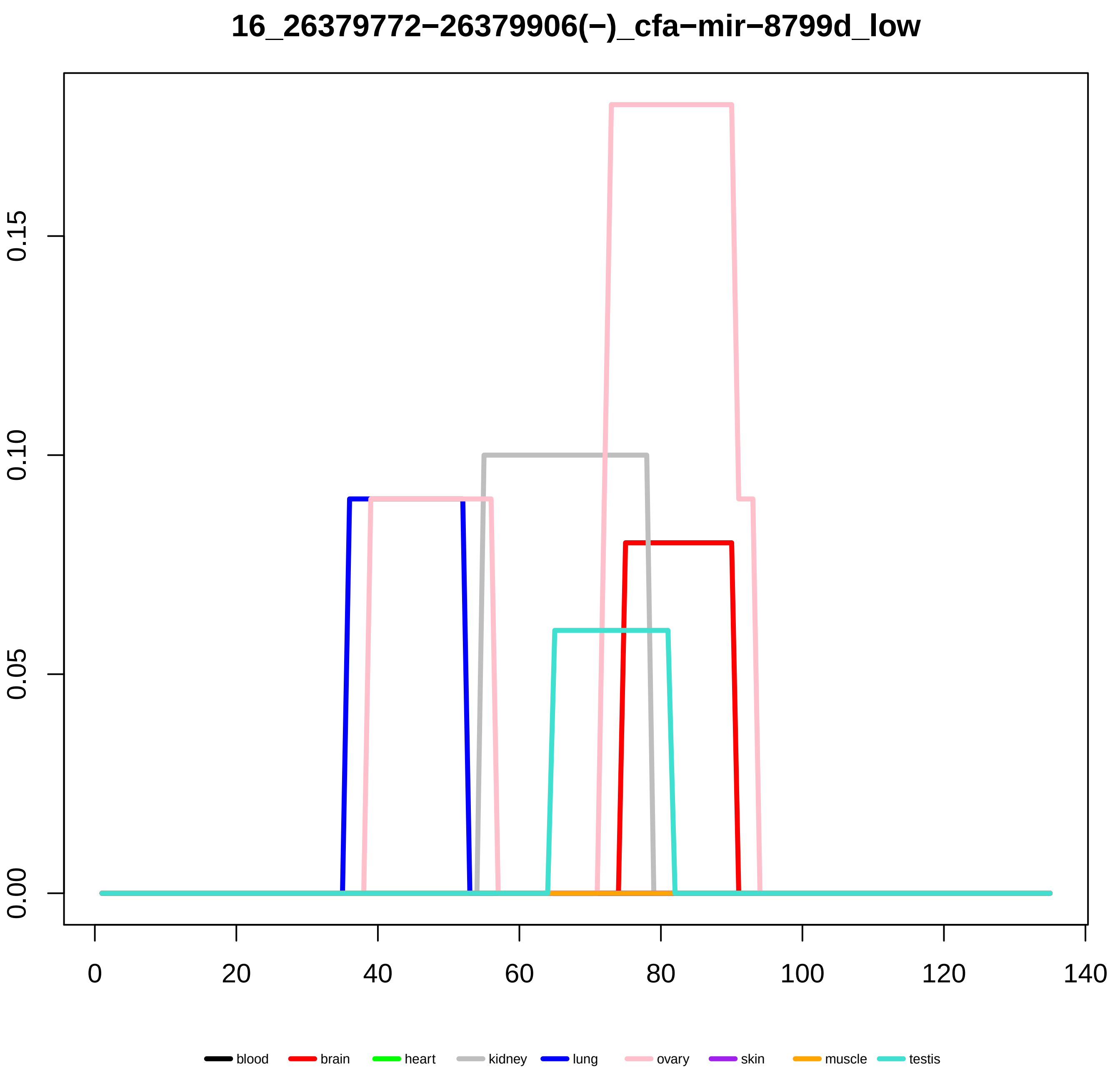
16_26379772−26379906(−)_cfa−mir−8799d_low
0.00
0.05
0.10
0.15
0
20
40
60
80
100
120
140
blood
brain
heart
kidney
lung
ovary
skin
muscle
testis

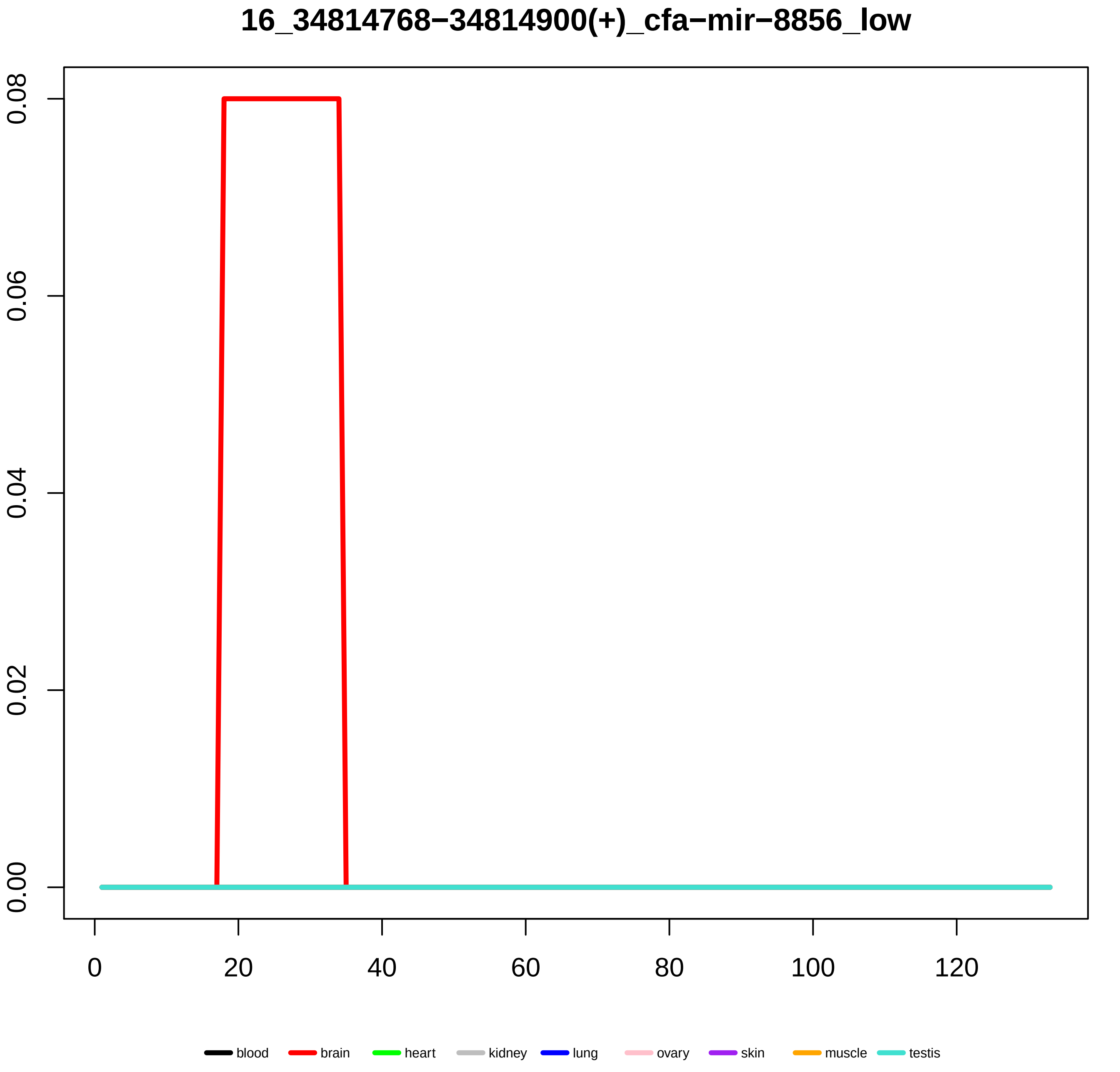
16_34814768−34814900(+)_cfa−mir−8856_low
0.00
0.02
0.04
0.06
0.08
0
20
40
60
80
100
120
blood
brain
heart
kidney
lung
ovary
skin
muscle
testis

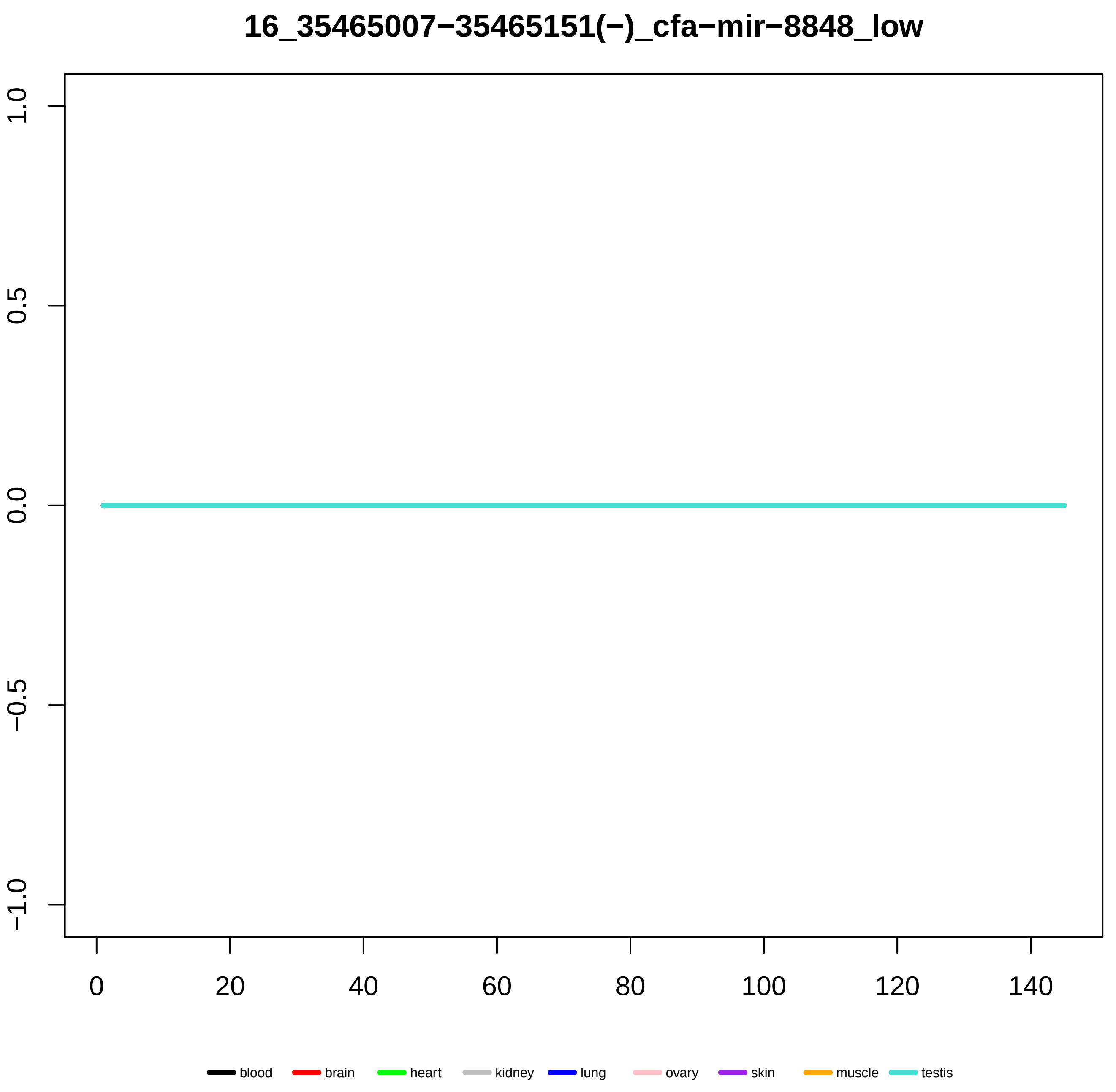
16_35465007−35465151(−)_cfa−mir−8848_low
1.0
0.5
0.0
−0.5
−1.0
0
20
40
60
80
100
120
140
blood
brain
heart
kidney
lung
ovary
skin
muscle
testis

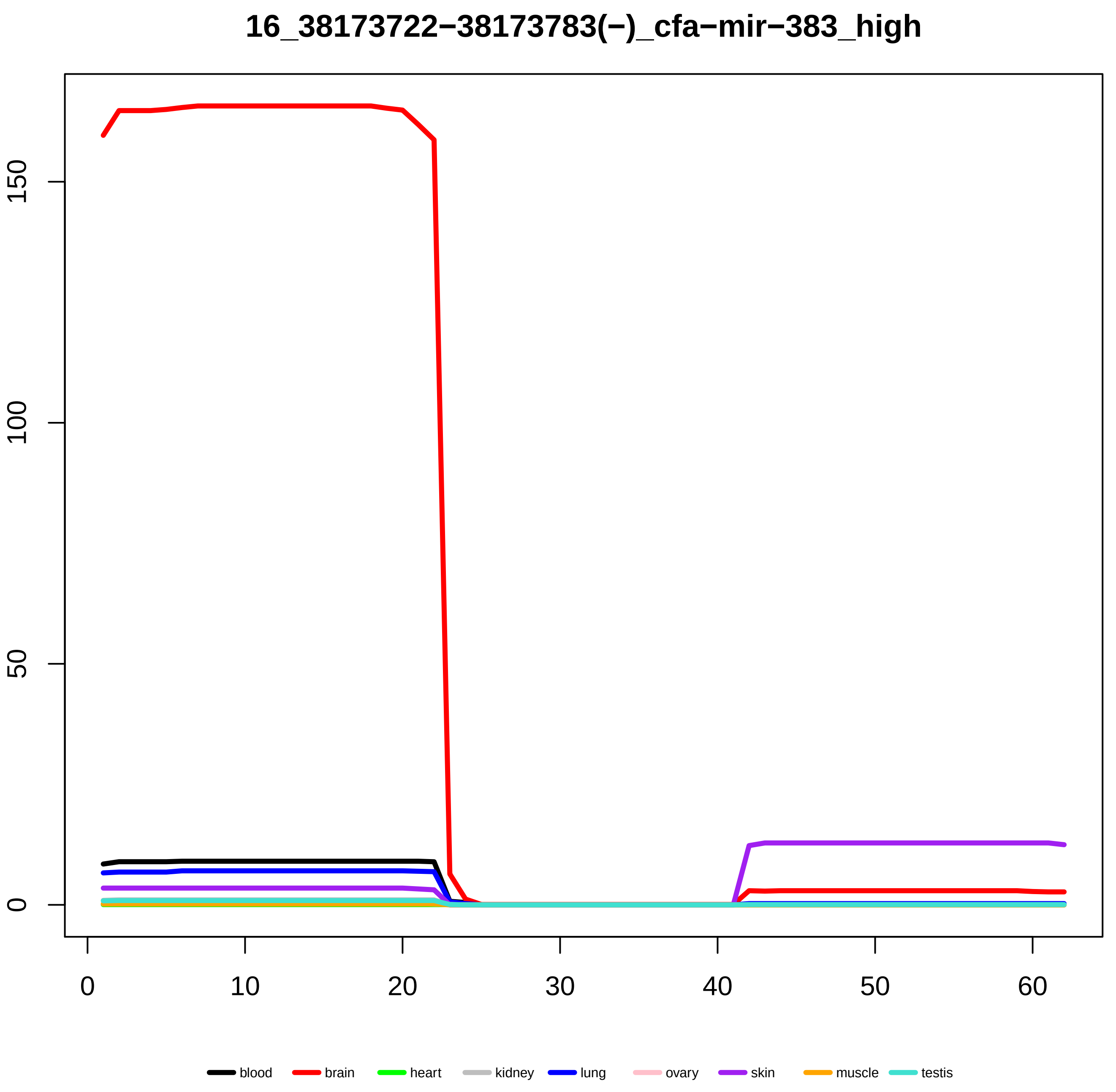

16_38173722−38173783(−)_cfa−mir−383_high
0
50
100
150
0
10
20
30
40
50
60
blood
brain
heart
kidney
lung
ovary
skin
muscle
testis

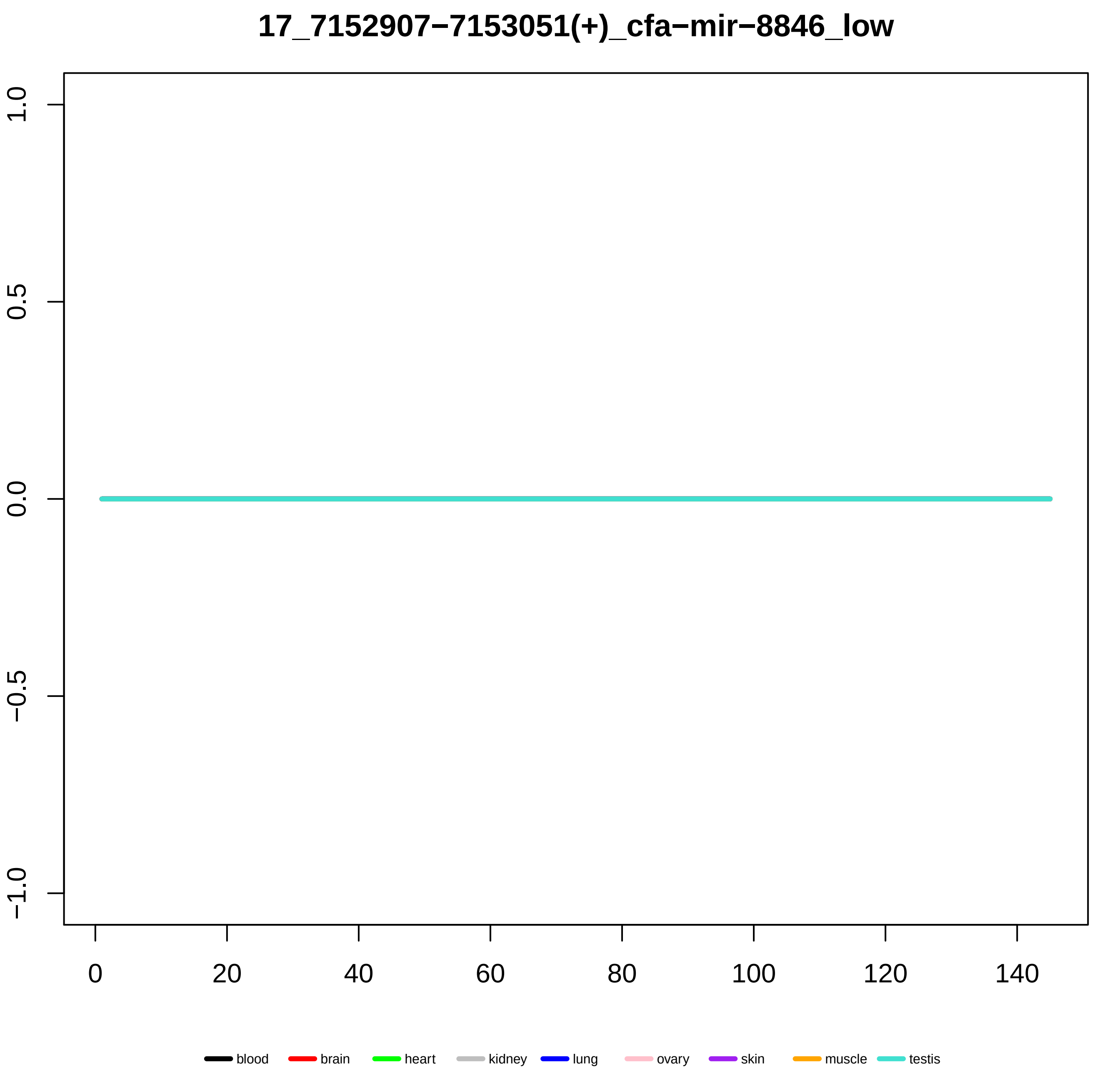

17_7152907−7153051(+)_cfa−mir−8846_low
1.0
0.5
0.0
−0.5
−1.0
0
20
40
60
80
100
120
140
blood
brain
heart
kidney
lung
ovary
skin
muscle
testis

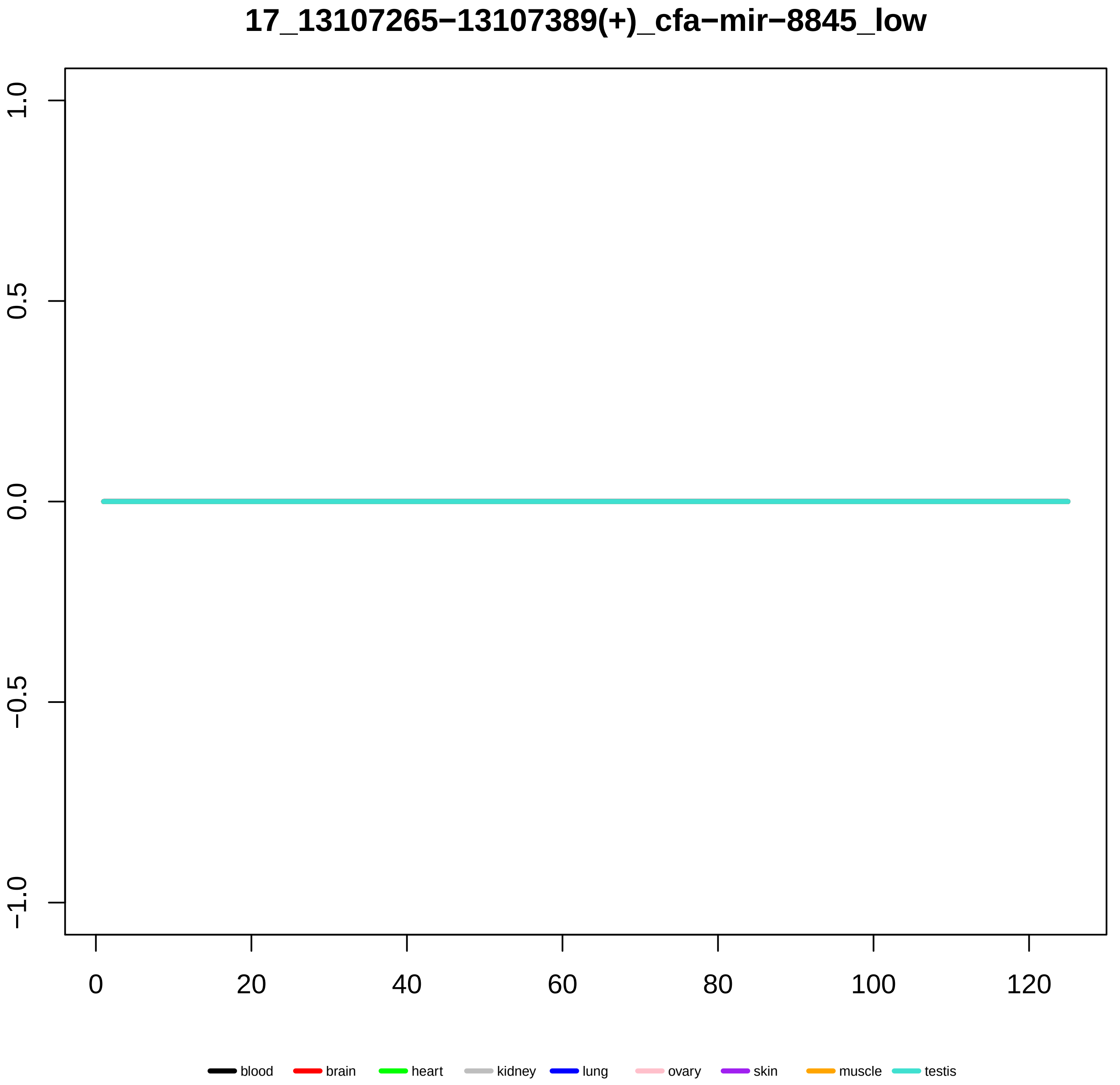

17_13107265−13107389(+)_cfa−mir−8845_low
1.0
0.5
0.0
−0.5
−1.0
0
20
40
60
80
100
120
blood
brain
heart
kidney
lung
ovary
skin
muscle
testis

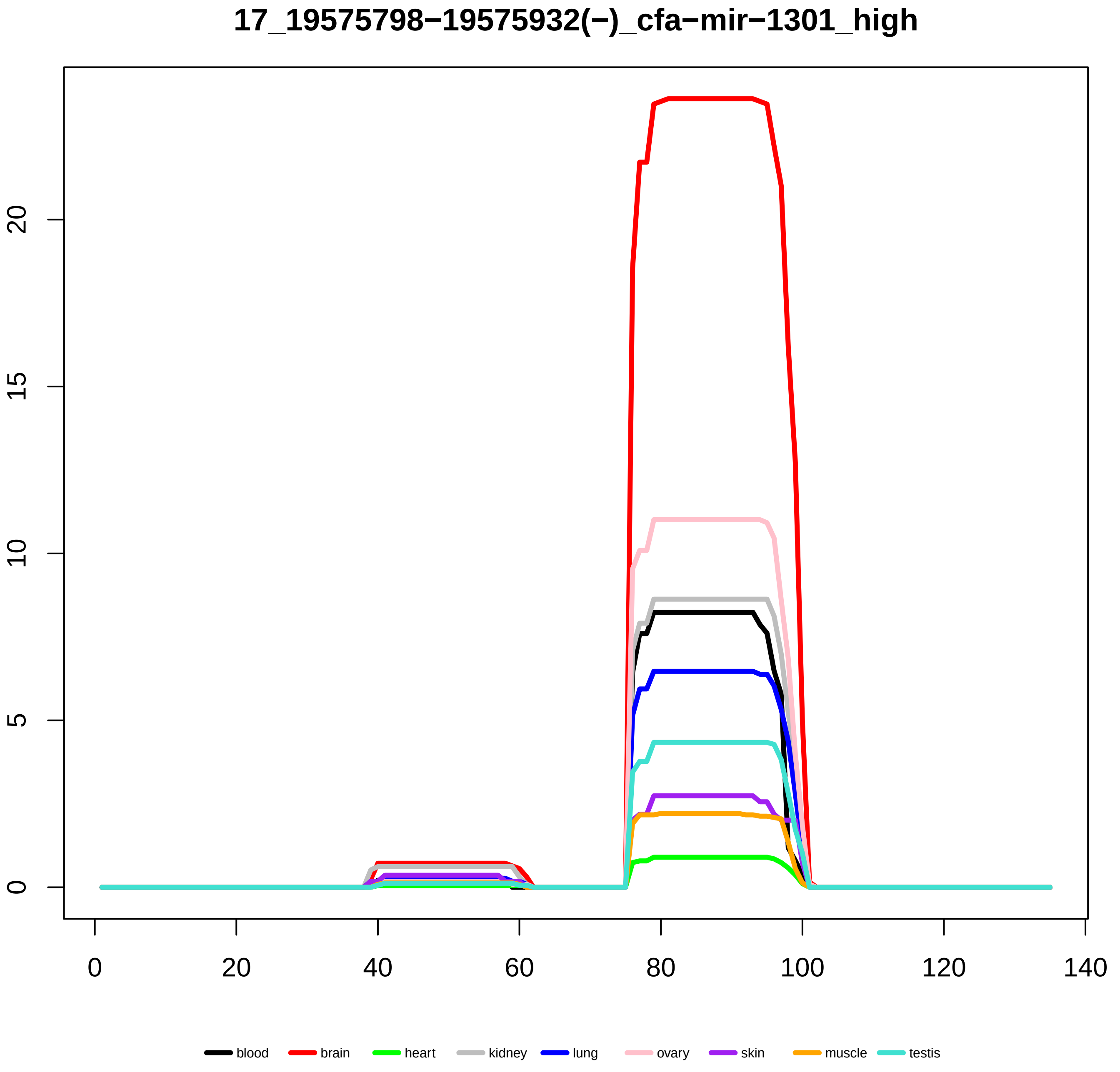

17_19575798−19575932(−)_cfa−mir−1301_high
0
5
10
15
20
0
20
40
60
80
100
120
140
blood
brain
heart
kidney
lung
ovary
skin
muscle
testis

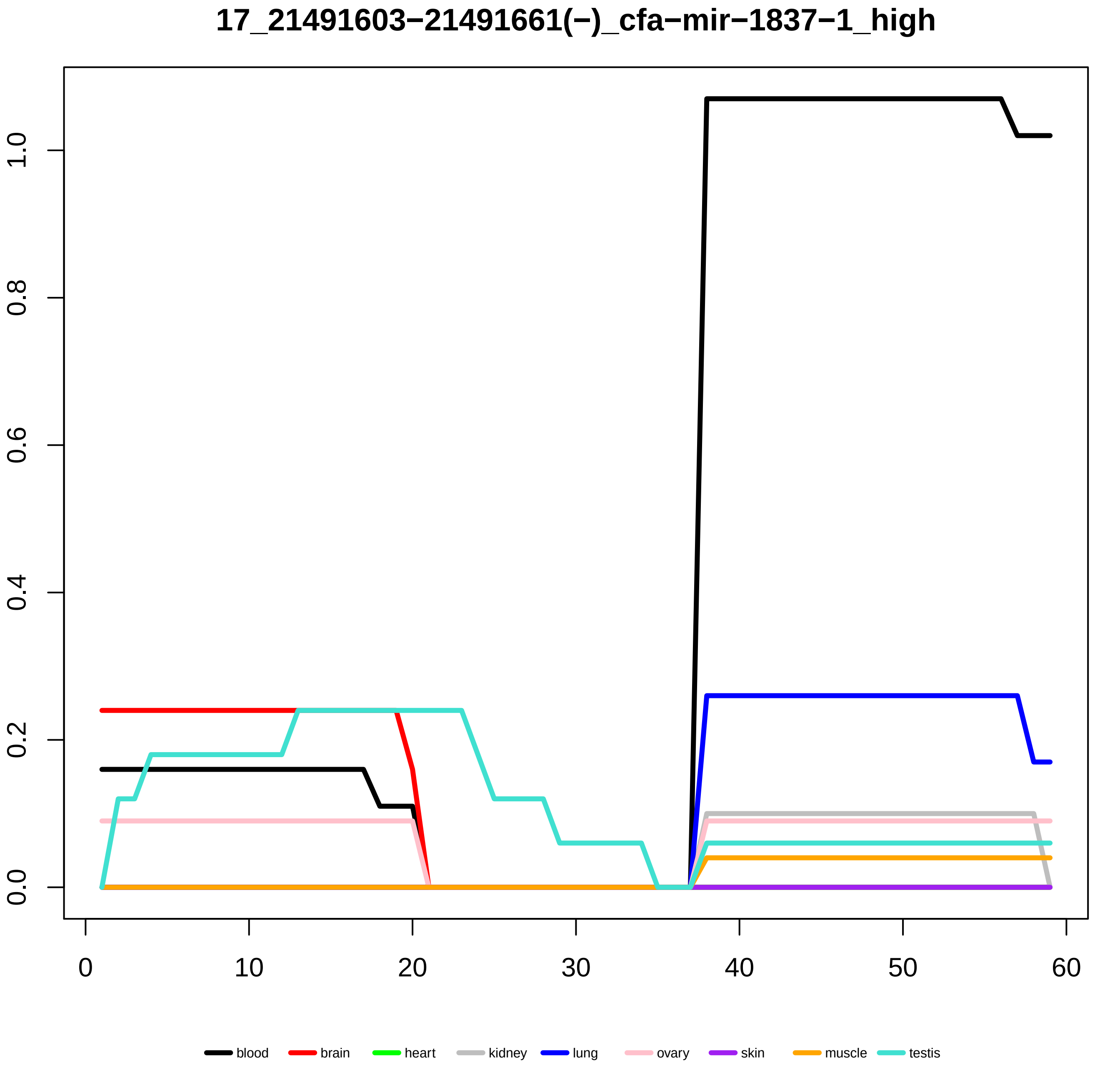

17_21491603−21491661(−)_cfa−mir−1837−1_high
0.0
0.2
0.4
0.6
0.8
1.0
0
10
20
30
40
50
60
blood
brain
heart
kidney
lung
ovary
skin
muscle
testis

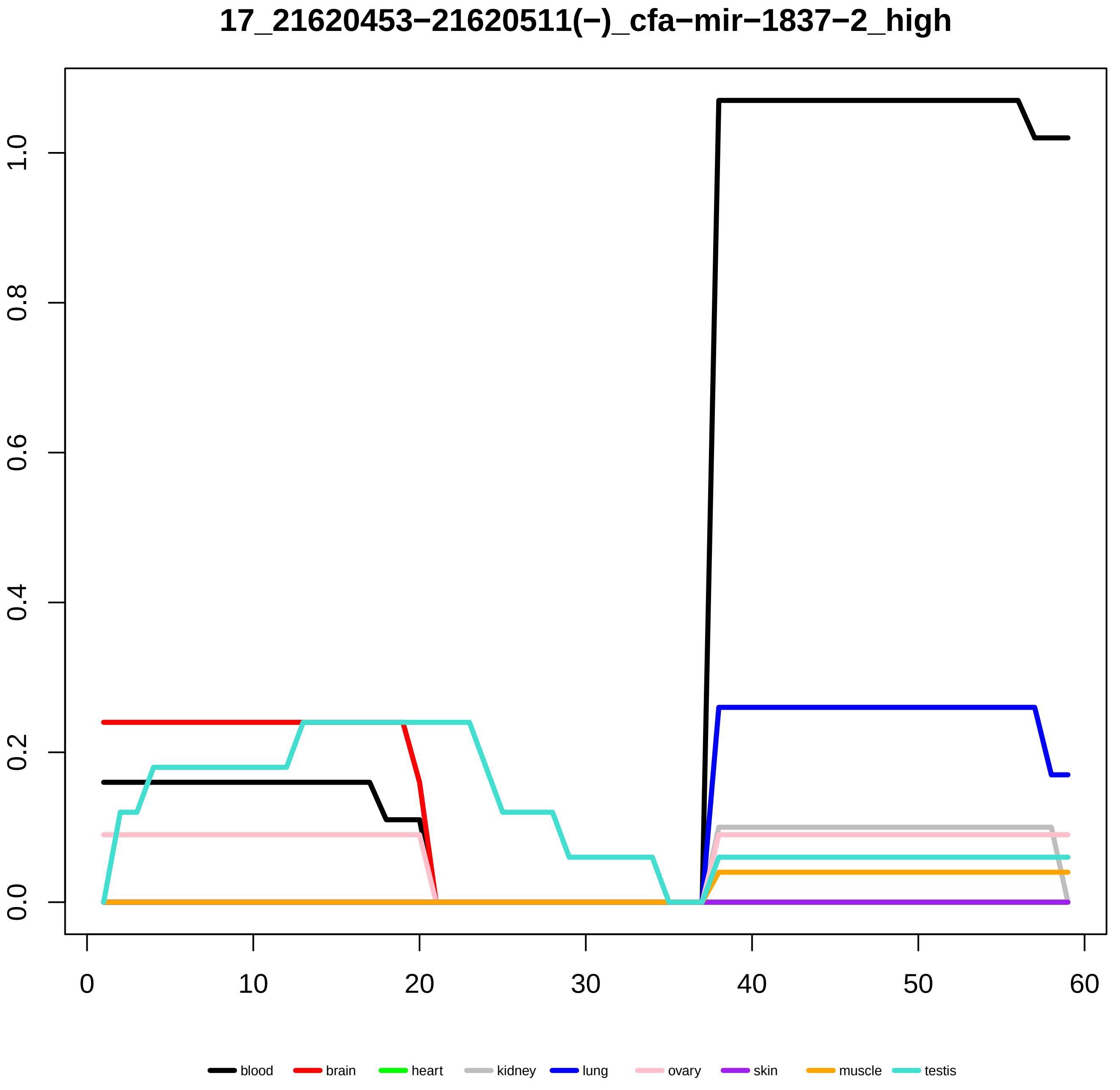
17_21620453−21620511(−)_cfa−mir−1837−2_high
0.0
0.2
0.4
0.6
0.8
1.0
0
10
20
30
40
50
60
blood
brain
heart
kidney
lung
ovary
skin
muscle
testis

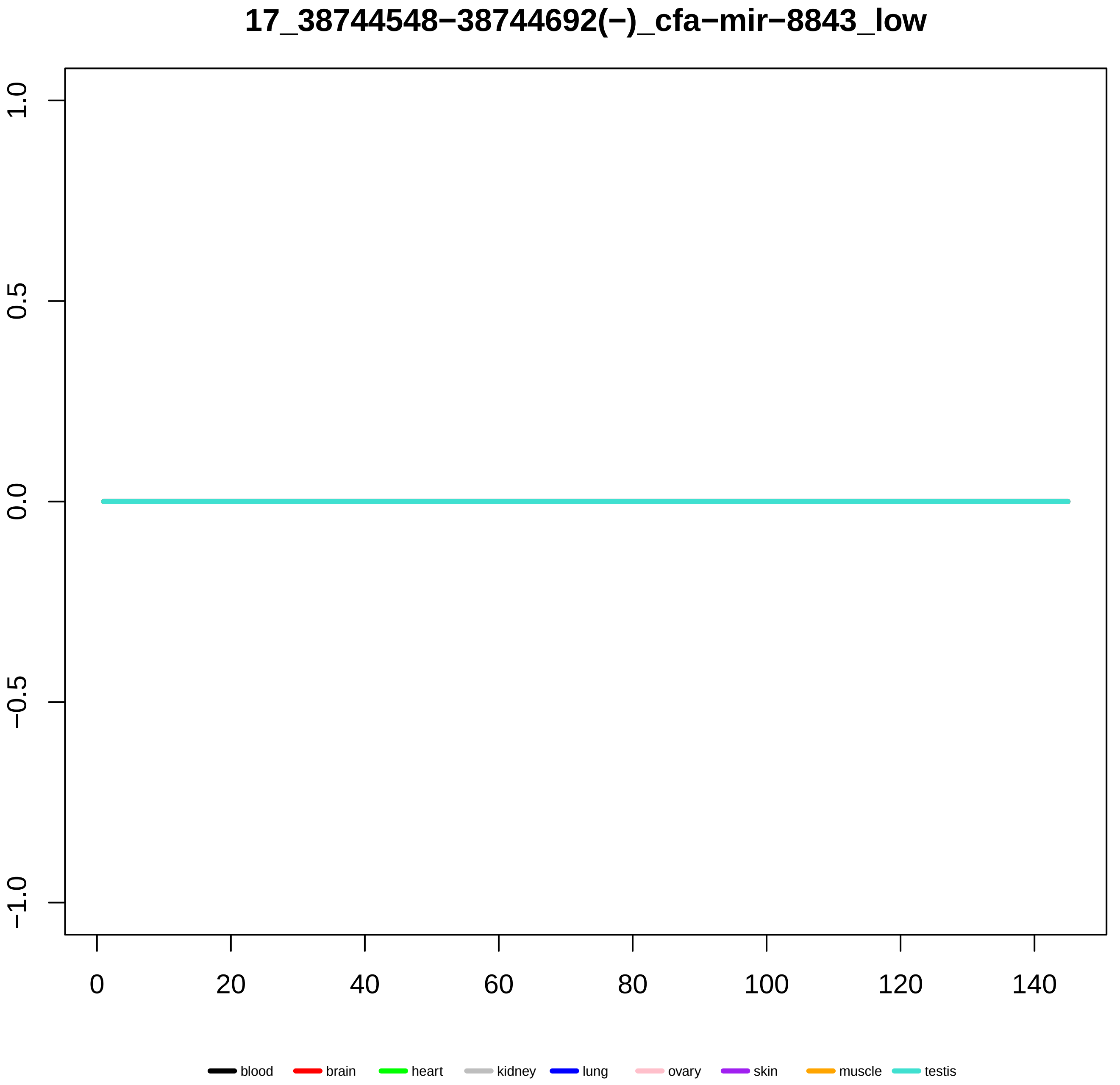
17_38744548−38744692(−)_cfa−mir−8843_low
1.0
0.5
0.0
−0.5
−1.0
0
20
40
60
80
100
120
140
blood
brain
heart
kidney
lung
ovary
skin
muscle
testis

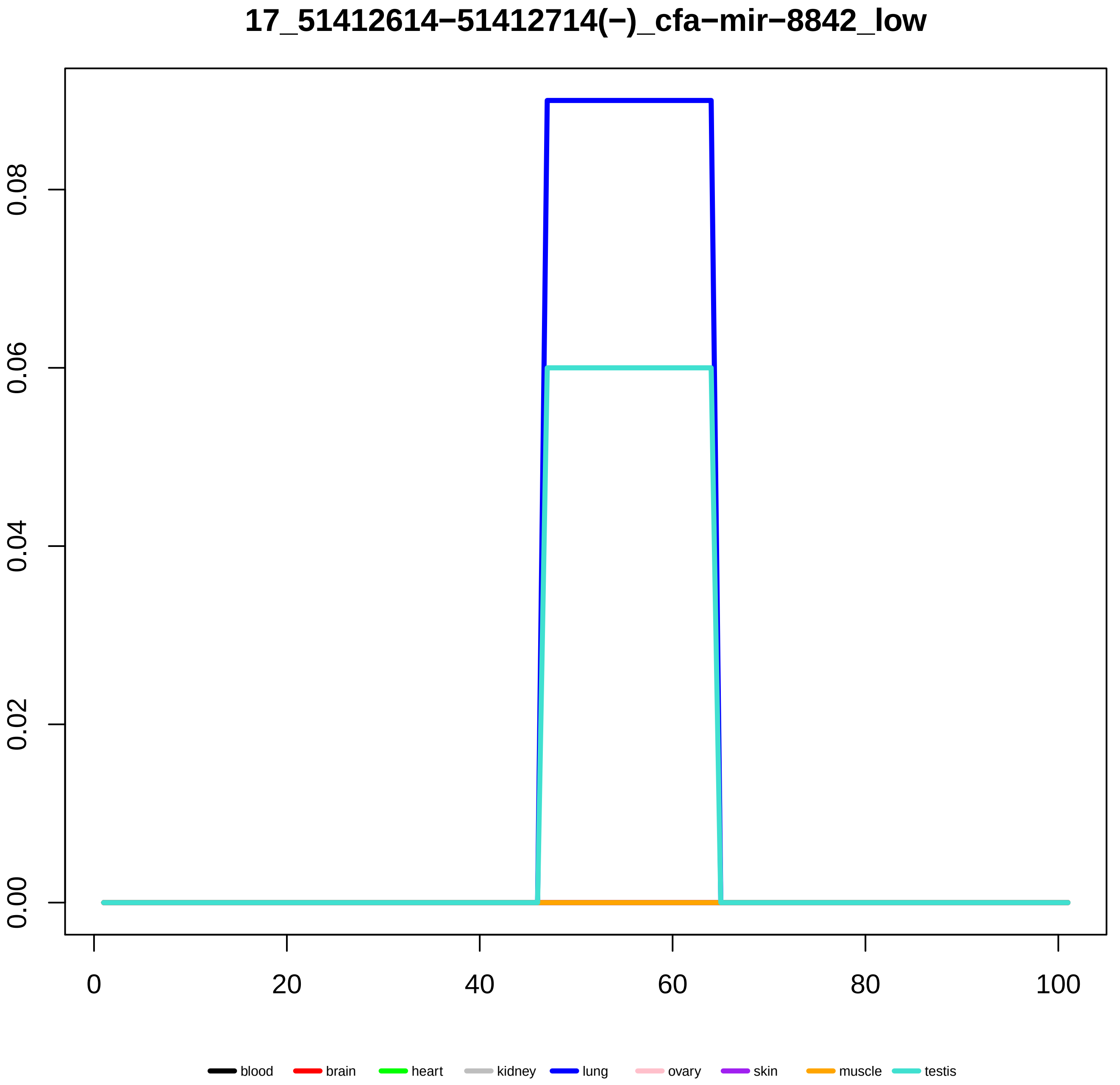

17_51412614−51412714(−)_cfa−mir−8842_low
0.00
0.02
0.04
0.06
0.08
0
20
40
60
80
100
blood
brain
heart
kidney
lung
ovary
skin
muscle
testis

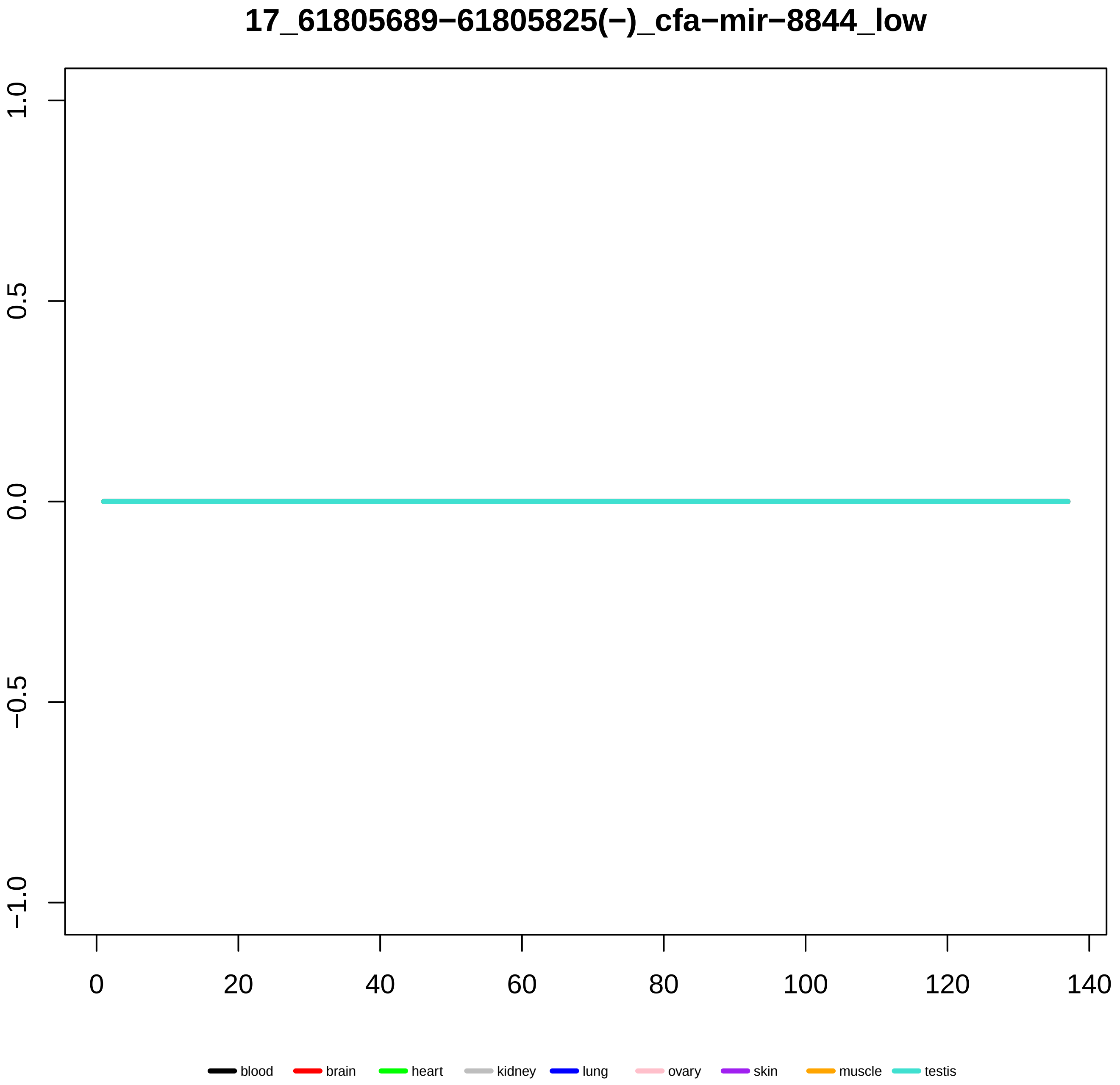
17_61805689−61805825(−)_cfa−mir−8844_low
1.0
0.5
0.0
−0.5
−1.0
0
20
40
60
80
100
120
140
blood
brain
heart
kidney
lung
ovary
skin
muscle
testis

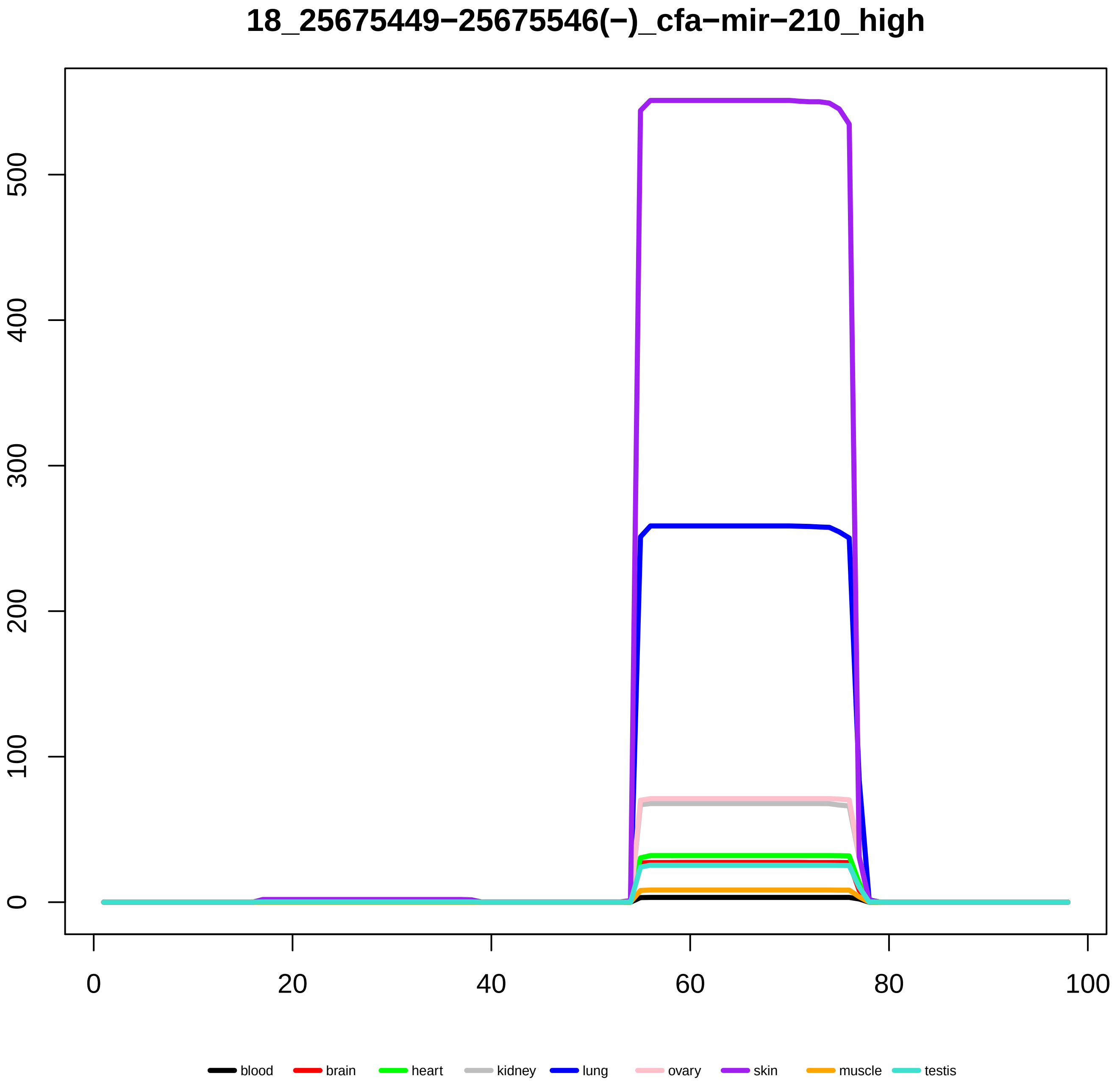
18_25675449−25675546(−)_cfa−mir−210_high
0
100
200
300
400
500
0
20
40
60
80
100
blood
brain
heart
kidney
lung
ovary
skin
muscle
testis

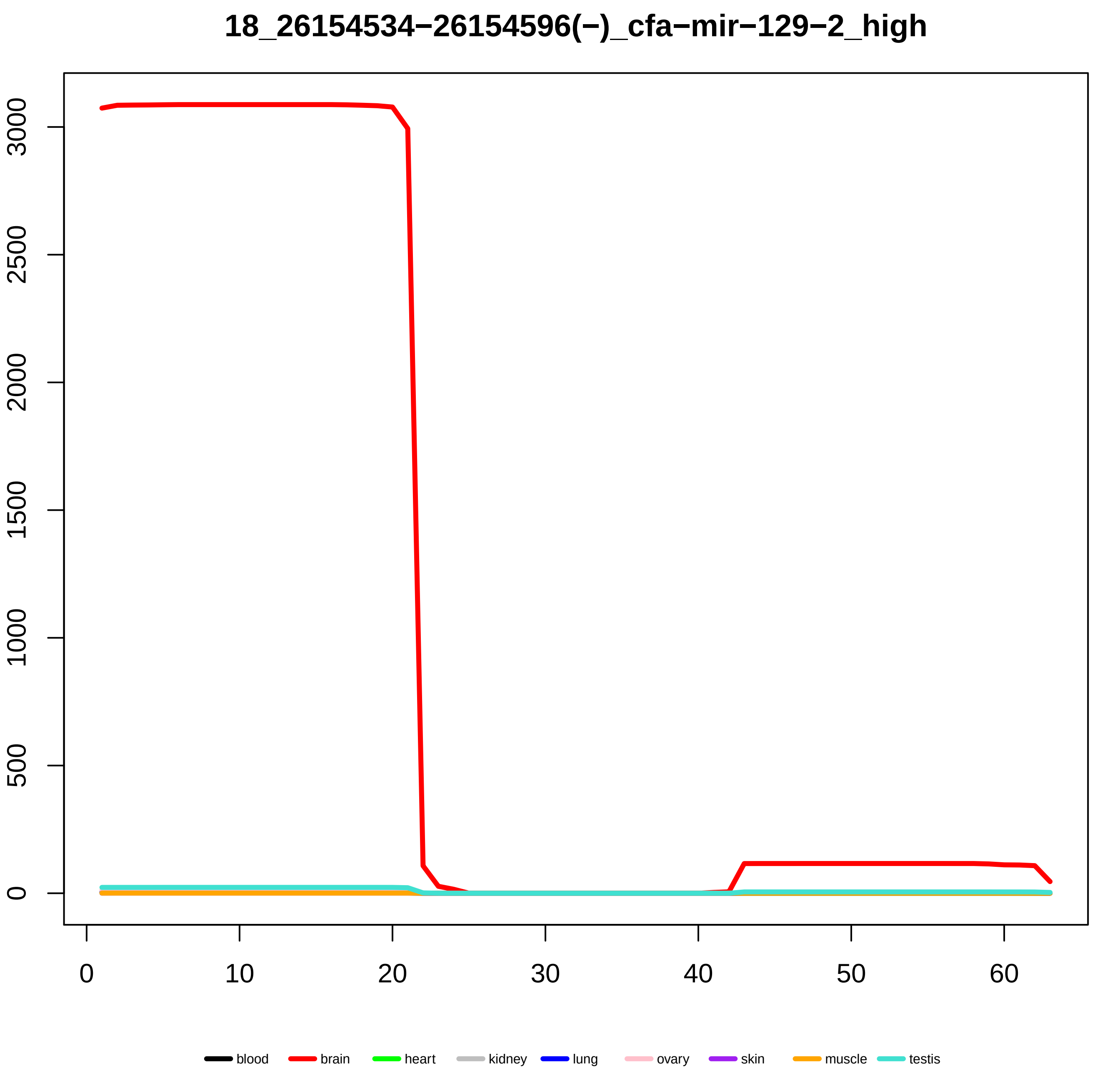

18_26154534−26154596(−)_cfa−mir−129−2_high
0
500
1000
1500
2000
2500
3000
0
10
20
30
40
50
60
blood
brain
heart
kidney
lung
ovary
skin
muscle
testis

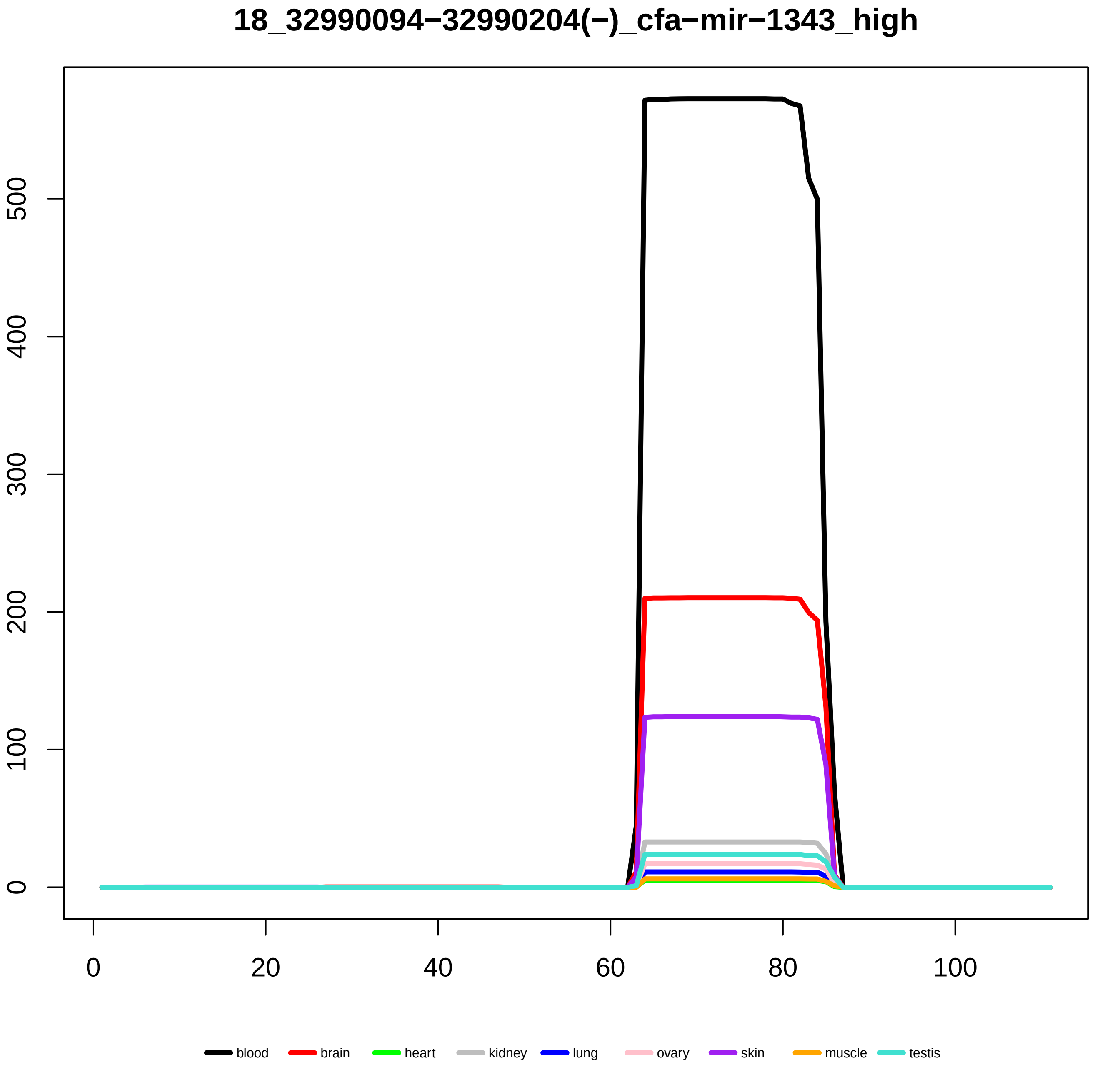

18_32990094−32990204(−)_cfa−mir−1343_high
0
100
200
300
400
500
0
20
40
60
80
100
blood
brain
heart
kidney
lung
ovary
skin
muscle
testis

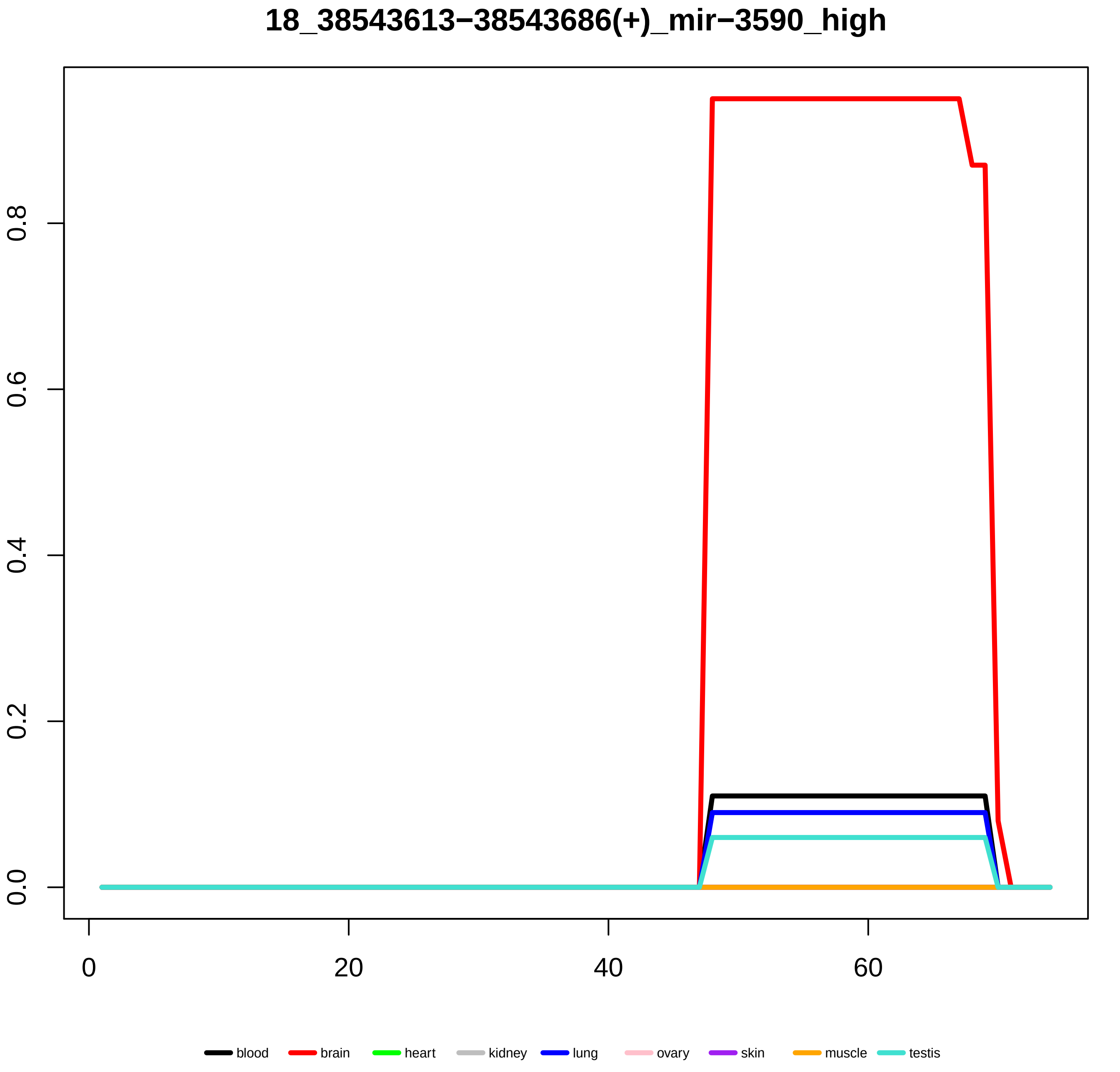

18_38543613−38543686(+)_mir−3590_high
0.0
0.2
0.4
0.6
0.8
0
20
40
60
blood
brain
heart
kidney
lung
ovary
skin
muscle
testis

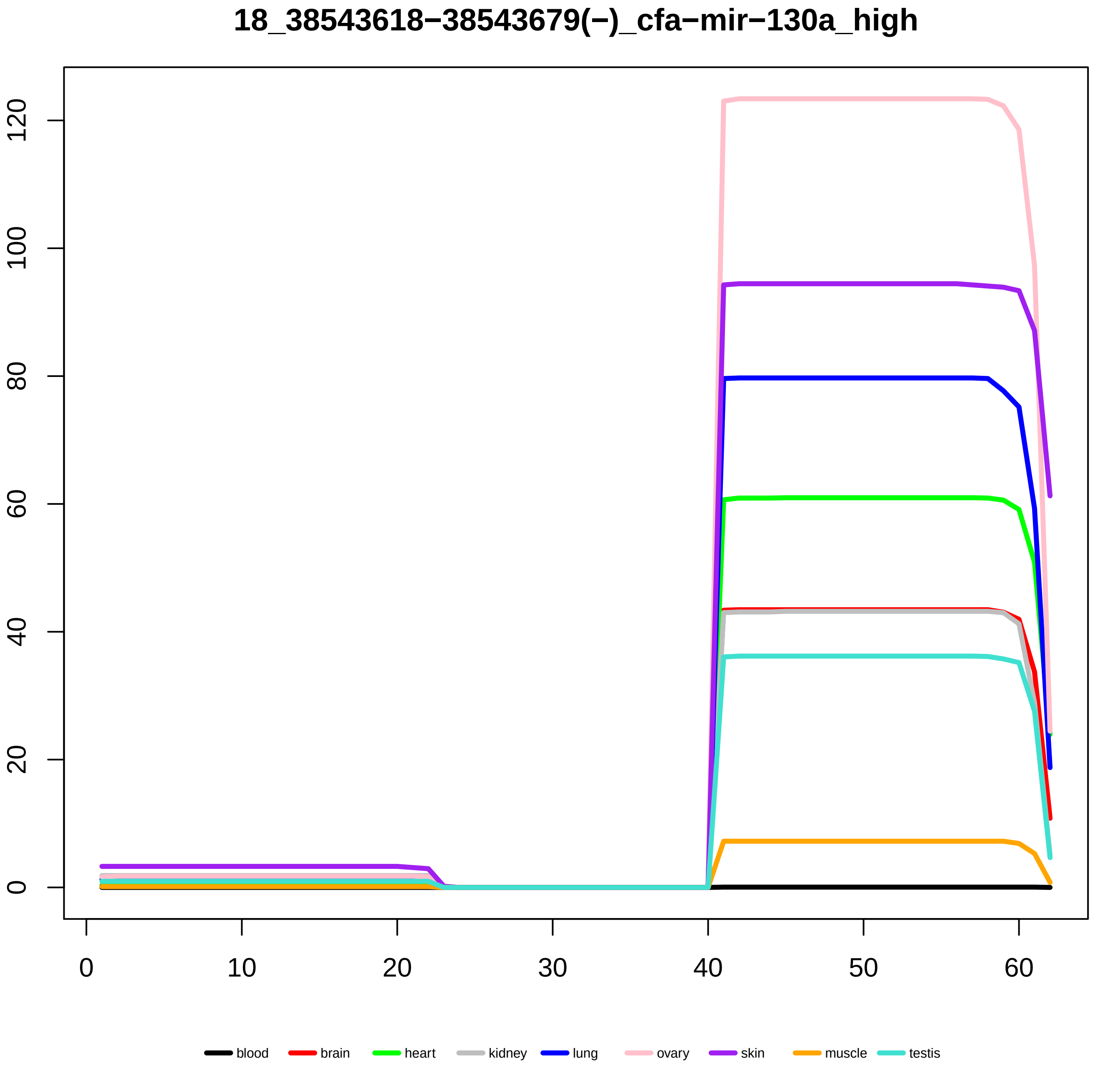
18_38543618−38543679(−)_cfa−mir−130a_high
0
20
40
60
80
100
120
0
10
20
30
40
50
60
blood
brain
heart
kidney
lung
ovary
skin
muscle
testis

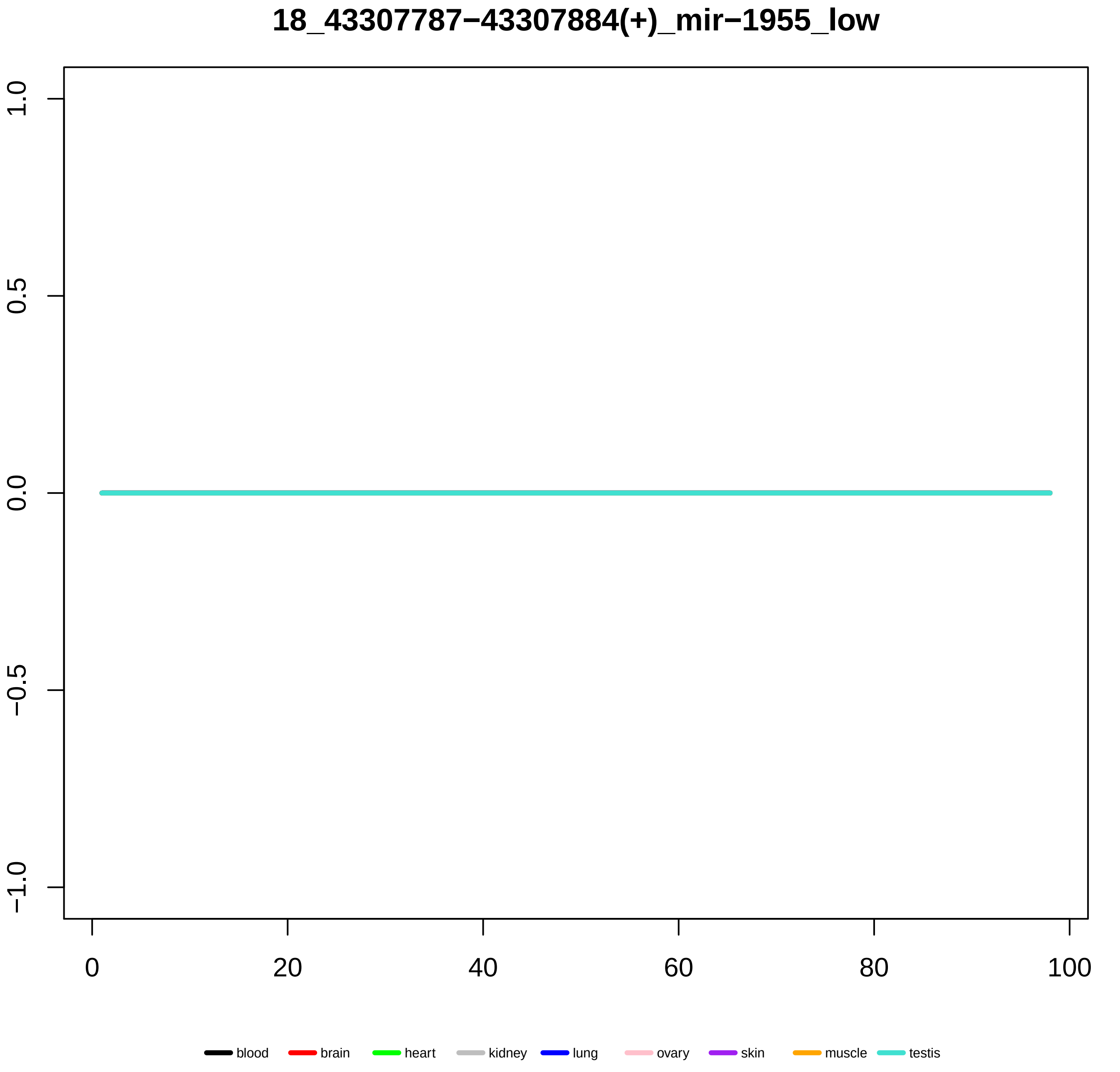

18_43307787−43307884(+)_mir−1955_low
1.0
0.5
0.0
−0.5
−1.0
0
20
40
60
80
100
blood
brain
heart
kidney
lung
ovary
skin
muscle
testis

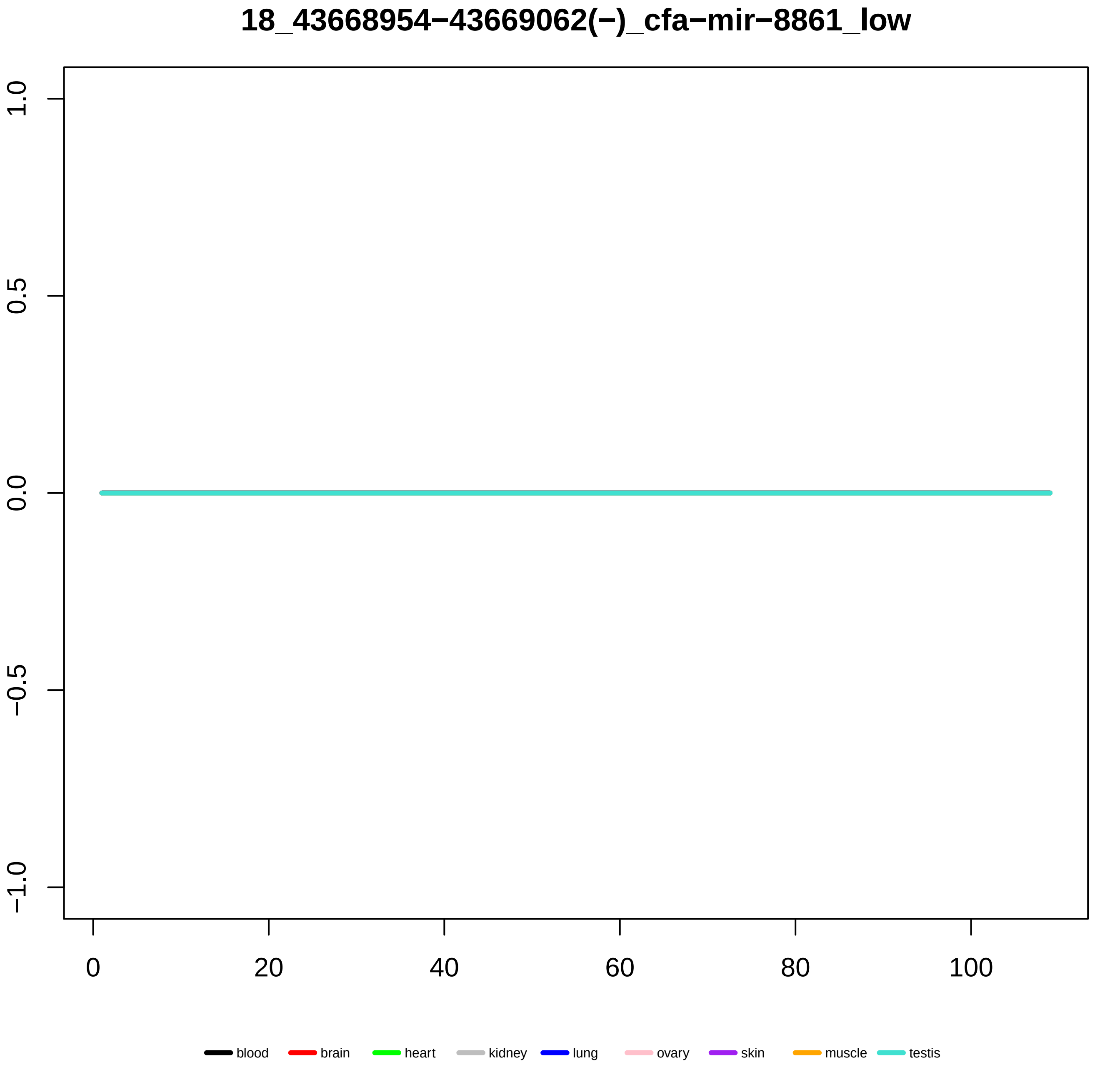

18_43668954−43669062(−)_cfa−mir−8861_low
1.0
0.5
0.0
−0.5
−1.0
0
20
40
60
80
100
blood
brain
heart
kidney
lung
ovary
skin
muscle
testis

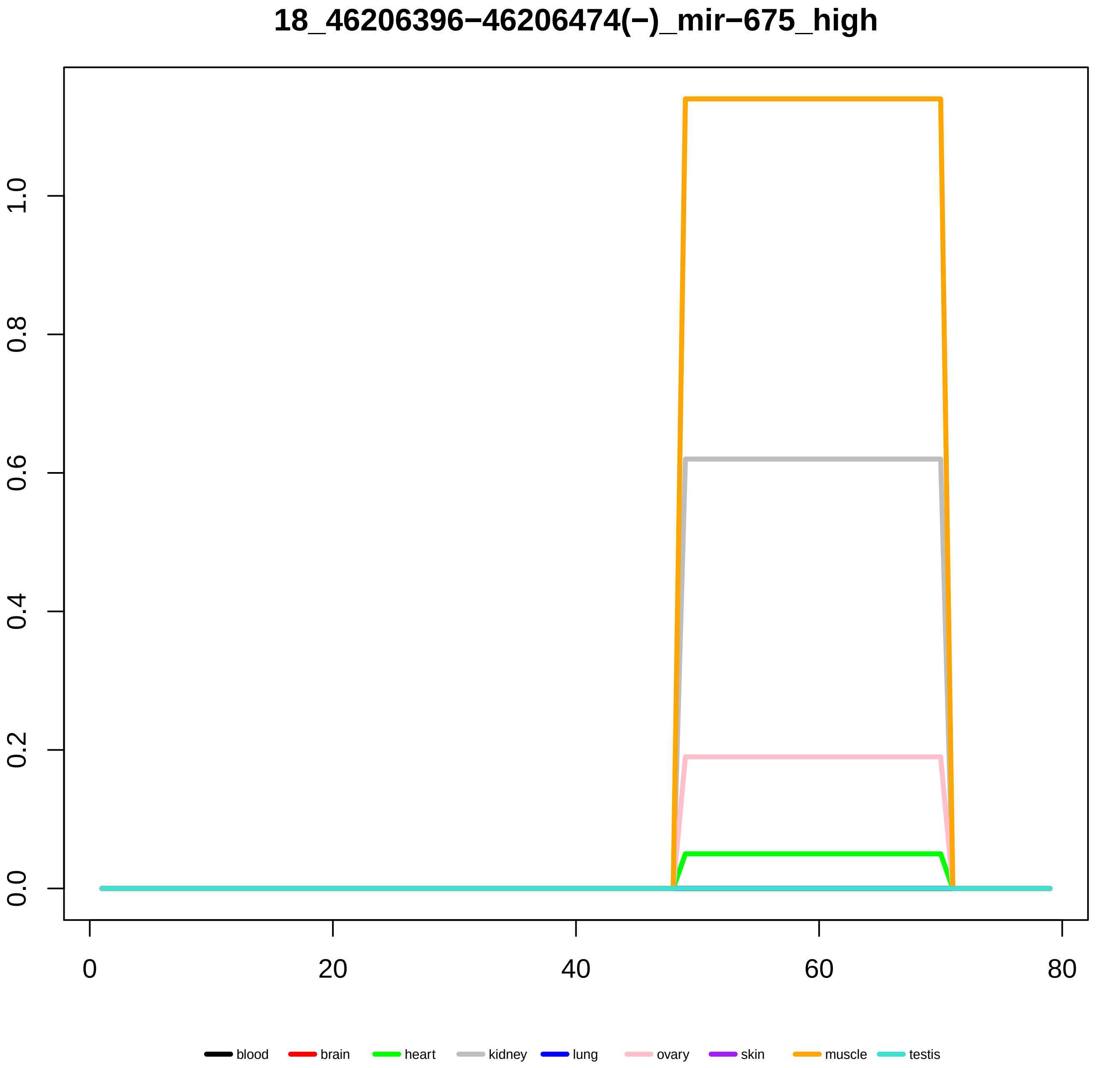
18_46206396−46206474(−)_mir−675_high
0.0
0.2
0.4
0.6
0.8
1.0
0
20
40
60
80
blood
brain
heart
kidney
lung
ovary
skin
muscle
testis

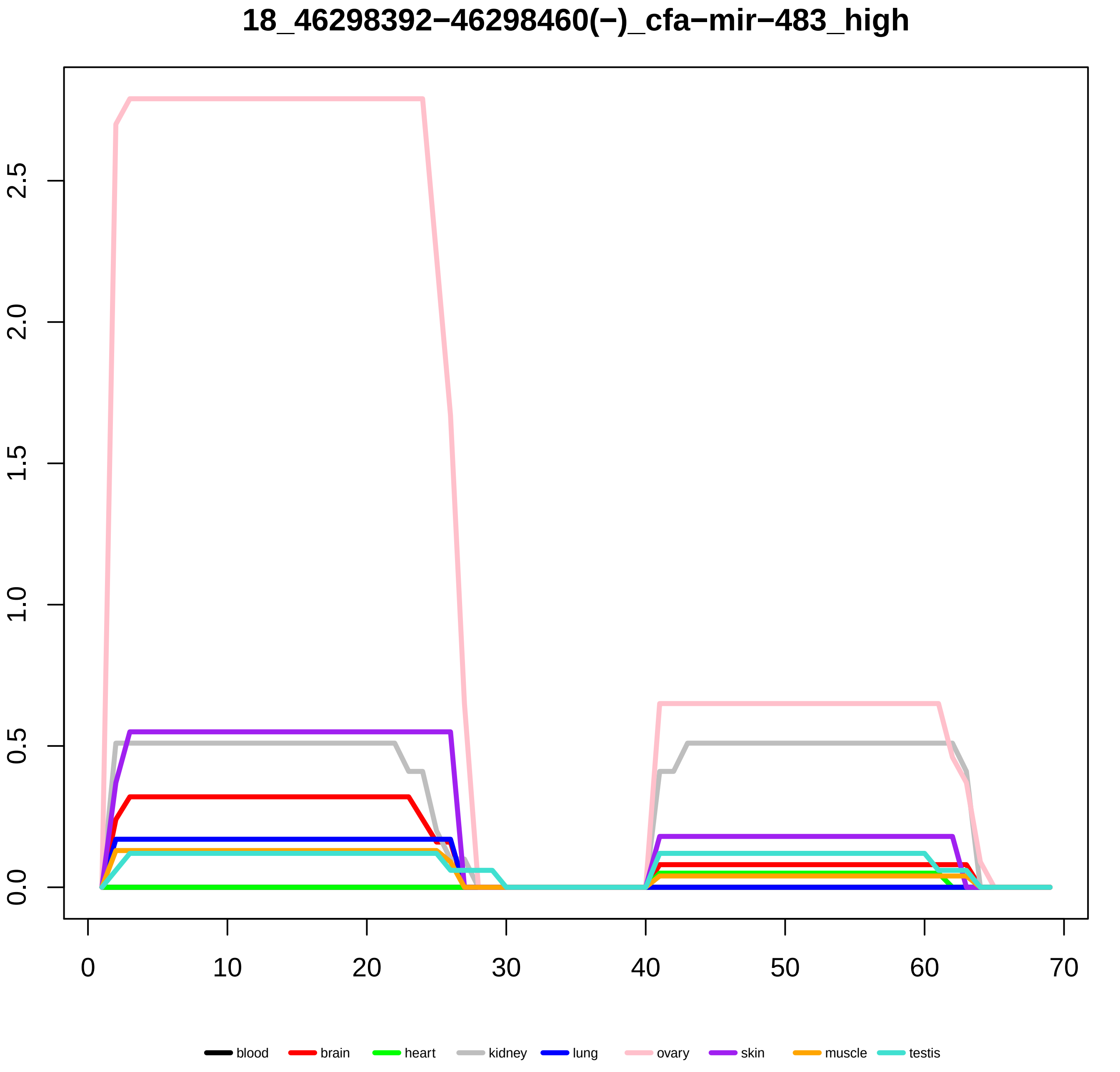

18_46298392−46298460(−)_cfa−mir−483_high
0.0
0.5
1.0
1.5
2.0
2.5
0
10
20
30
40
50
60
70
blood
brain
heart
kidney
lung
ovary
skin
muscle
testis

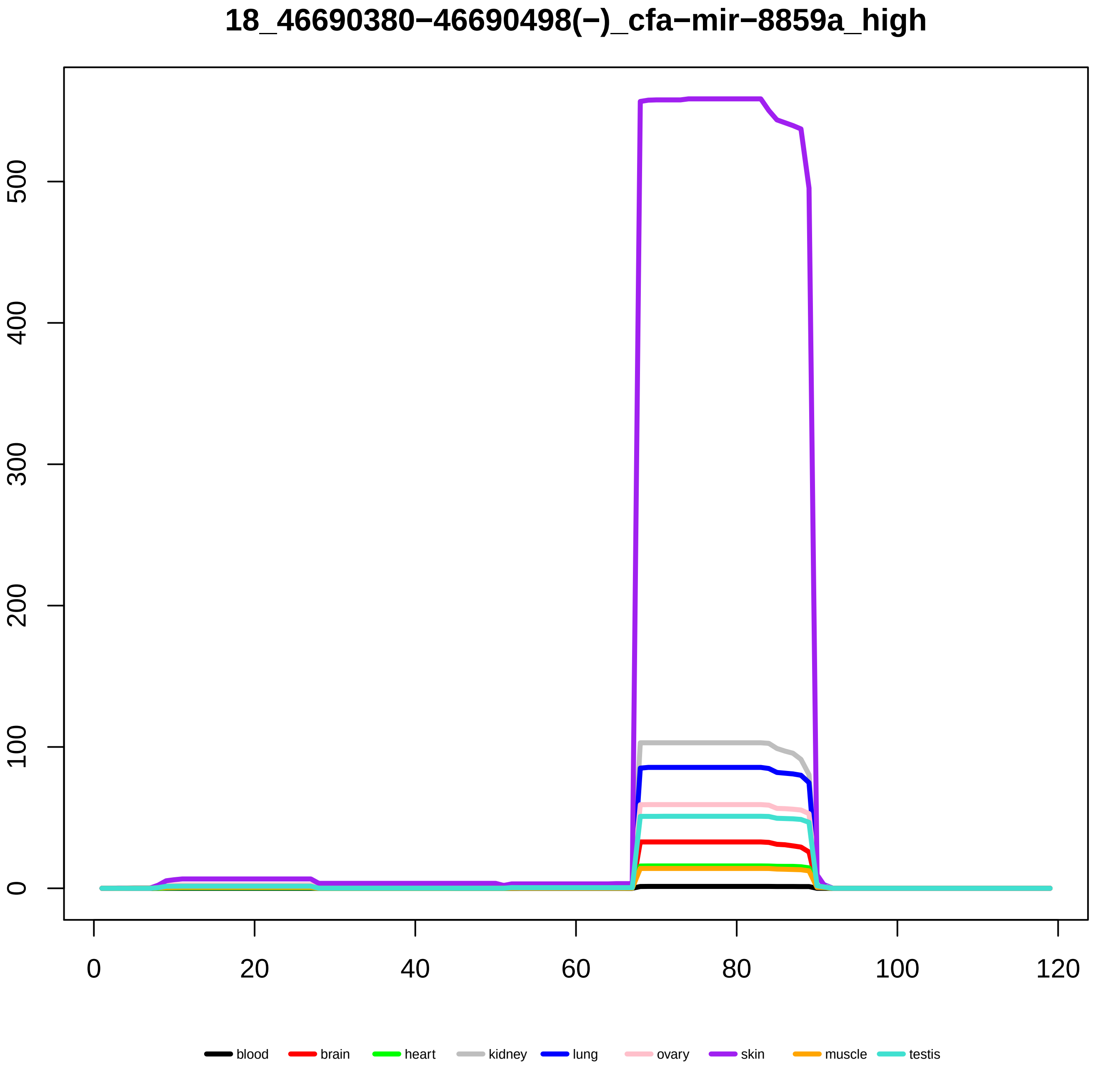

18_46690380−46690498(−)_cfa−mir−8859a_high
0
100
200
300
400
500
0
20
40
60
80
100
120
blood
brain
heart
kidney
lung
ovary
skin
muscle
testis

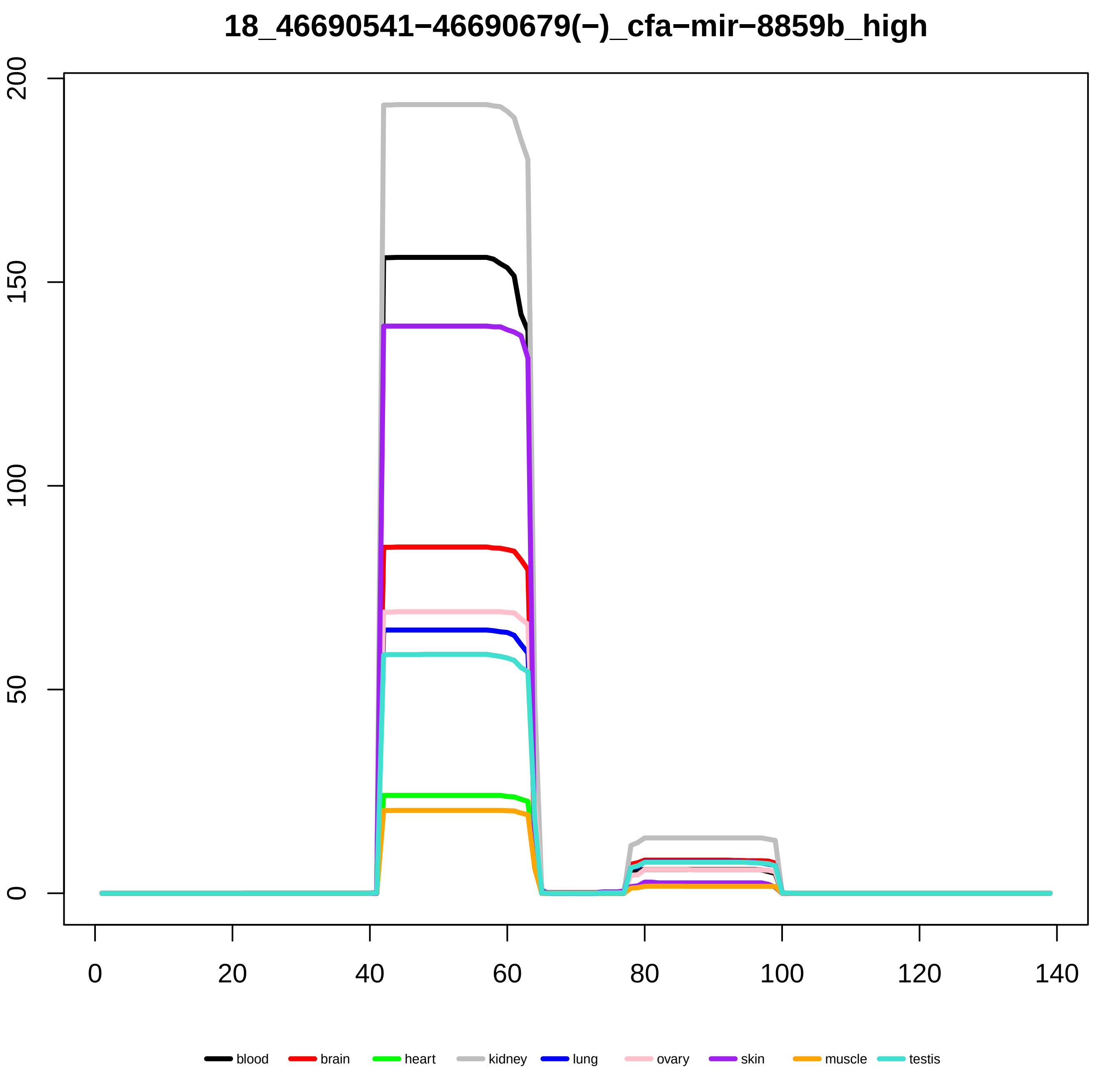

18_46690541−46690679(−)_cfa−mir−8859b_high
0
50
100
150
200
0
20
40
60
80
100
120
140
blood
brain
heart
kidney
lung
ovary
skin
muscle
testis

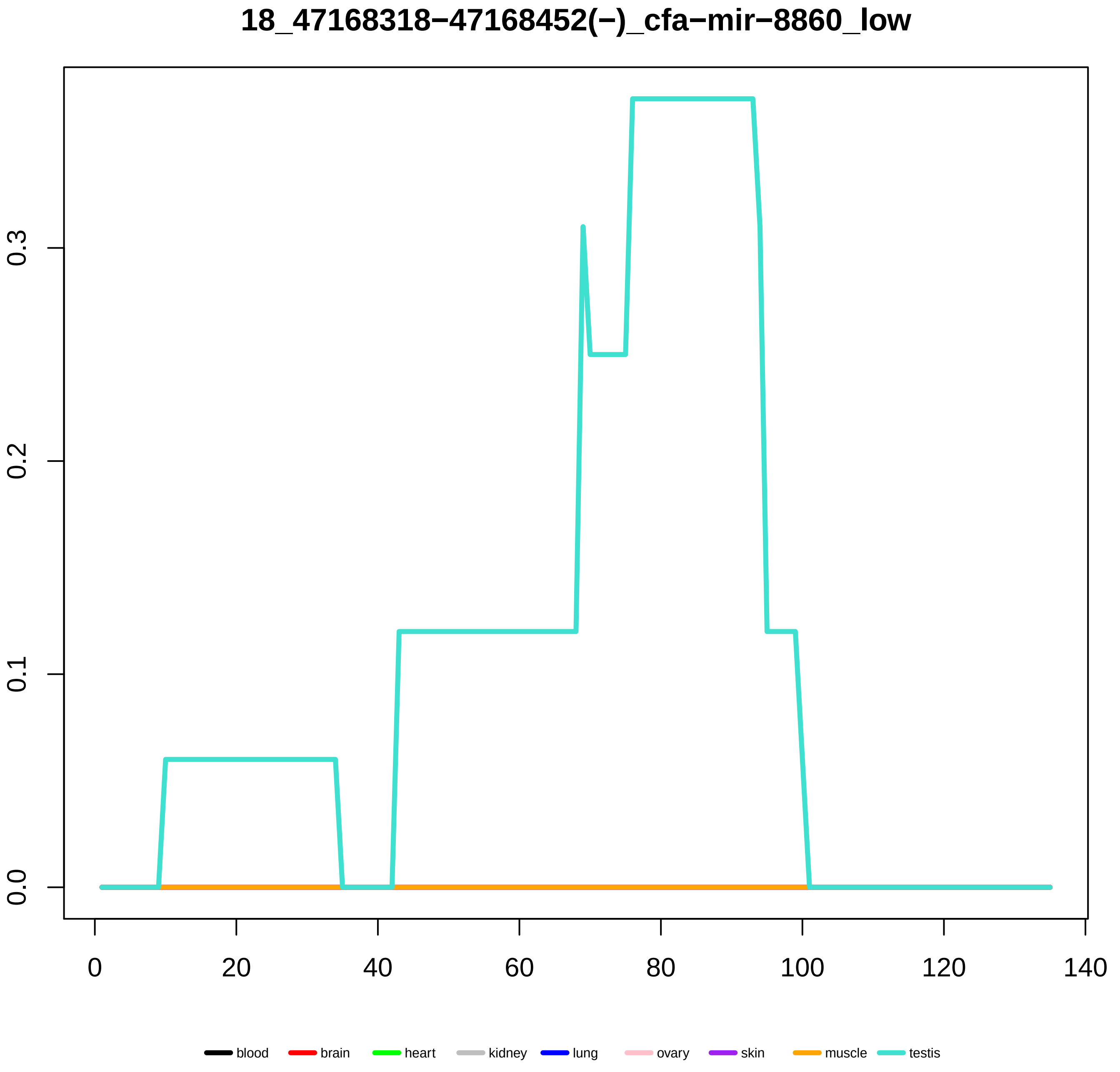

18_47168318−47168452(−)_cfa−mir−8860_low
0.0
0.1
0.2
0.3
0
20
40
60
80
100
120
140
blood
brain
heart
kidney
lung
ovary
skin
muscle
testis

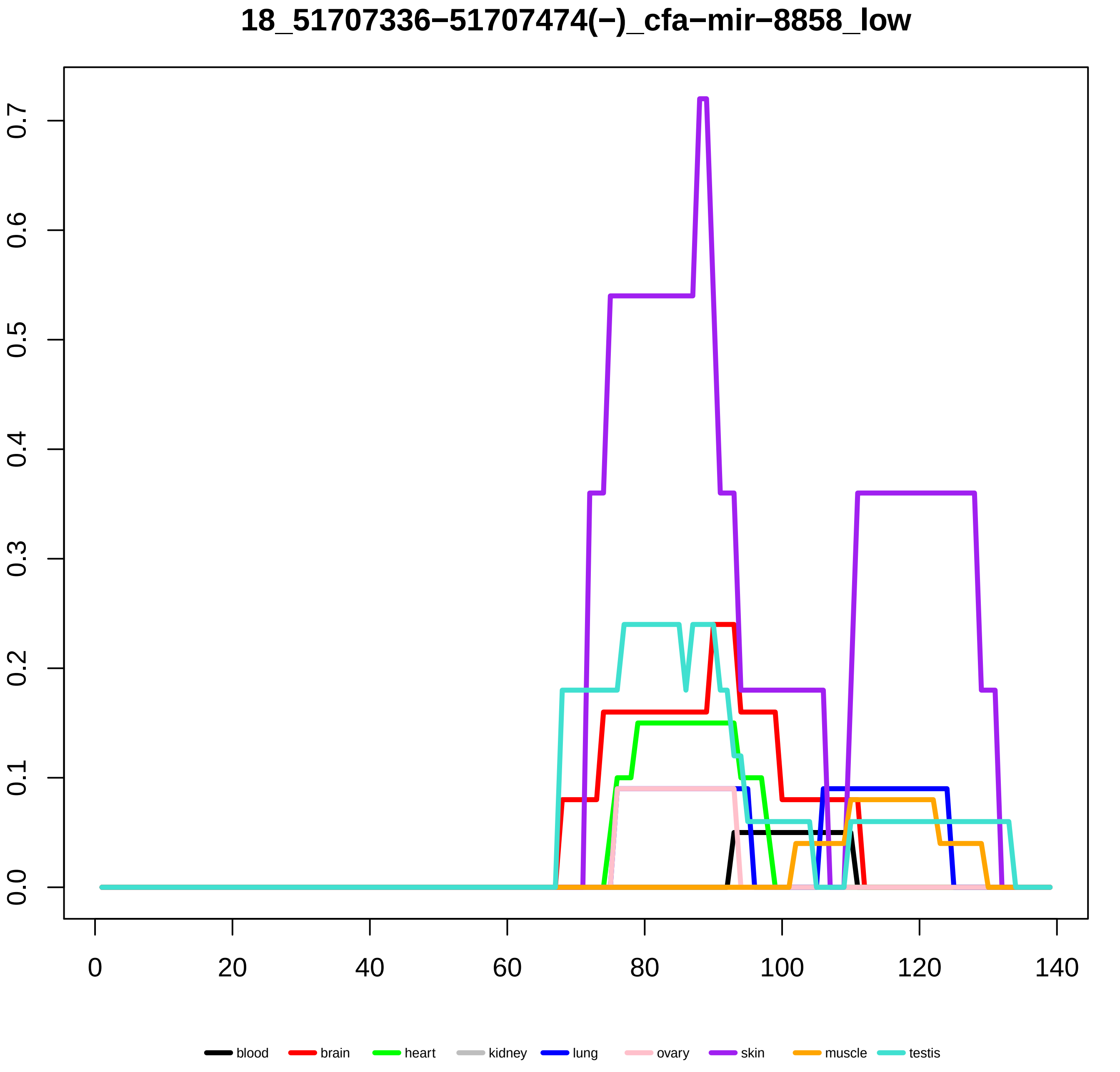
18_51707336−51707474(−)_cfa−mir−8858_low
0.0
0.1
0.2
0.3
0.4
0.5
0.6
0.7
0
20
40
60
80
100
120
140
blood
brain
heart
kidney
lung
ovary
skin
muscle
testis

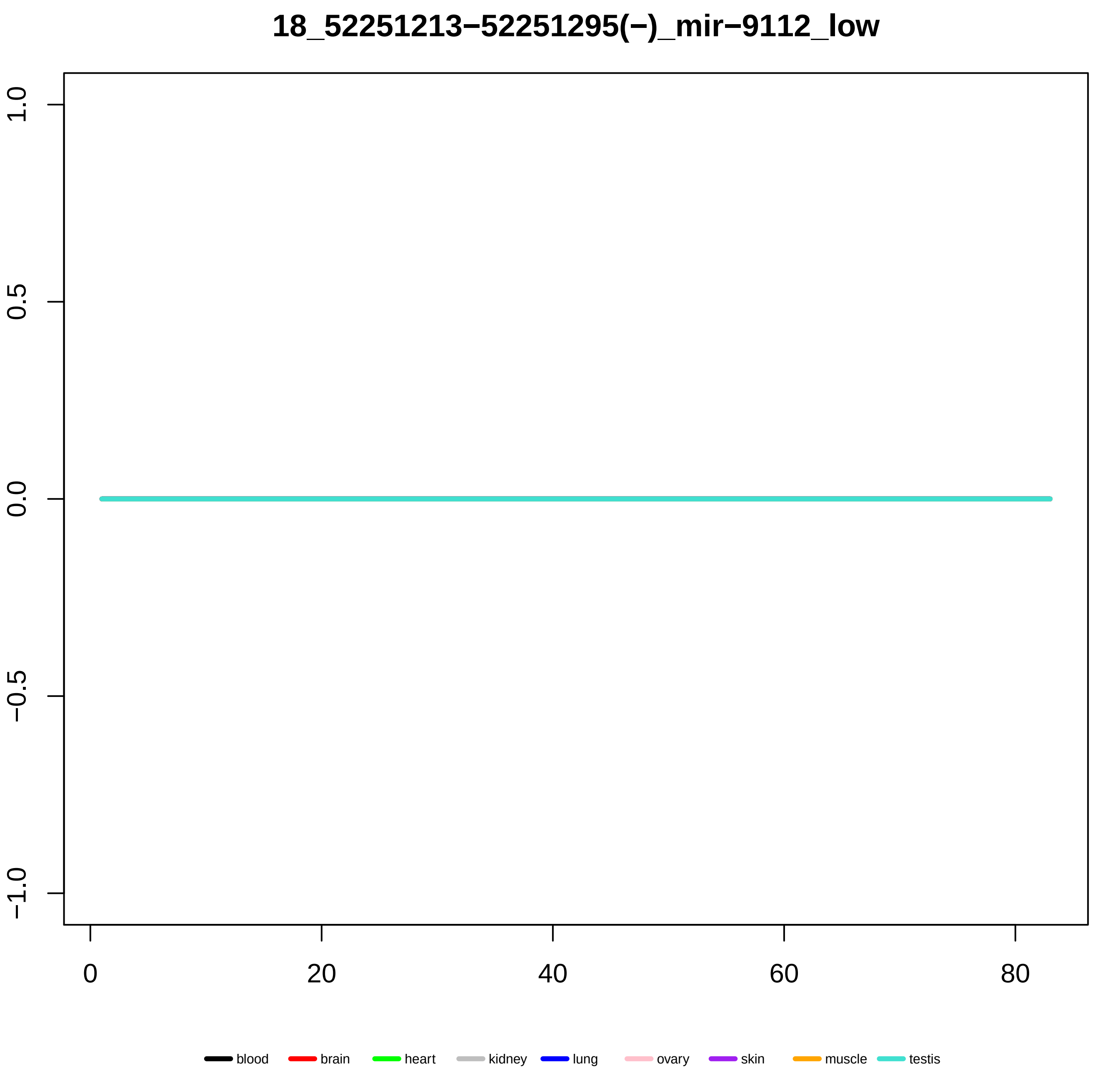

18_52251213−52251295(−)_mir−9112_low
1.0
0.5
0.0
−0.5
−1.0
0
20
40
60
80
blood
brain
heart
kidney
lung
ovary
skin
muscle
testis

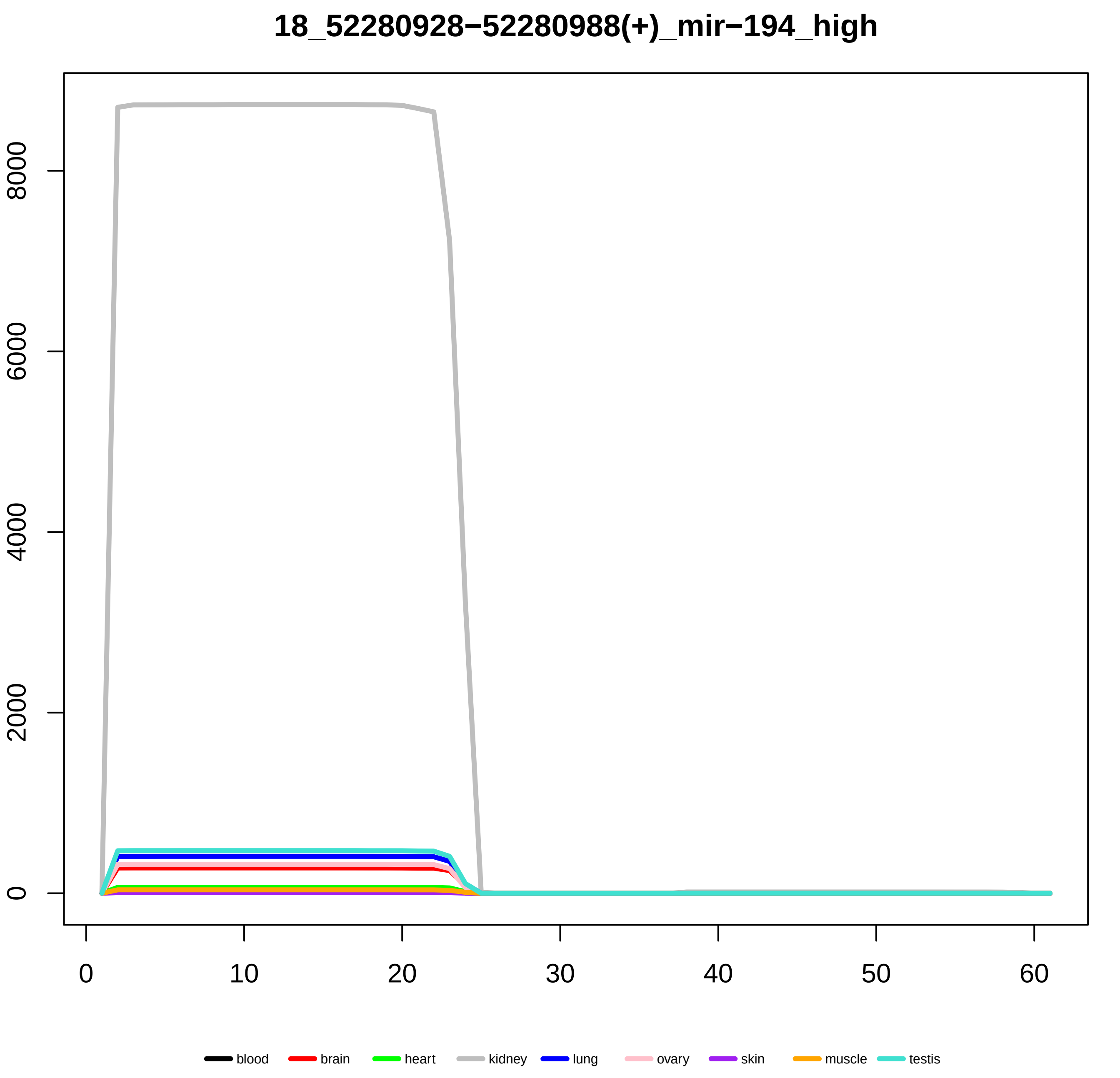

18_52280928−52280988(+)_mir−194_high
0
2000
4000
6000
8000
0
10
20
30
40
50
60
blood
brain
heart
kidney
lung
ovary
skin
muscle
testis

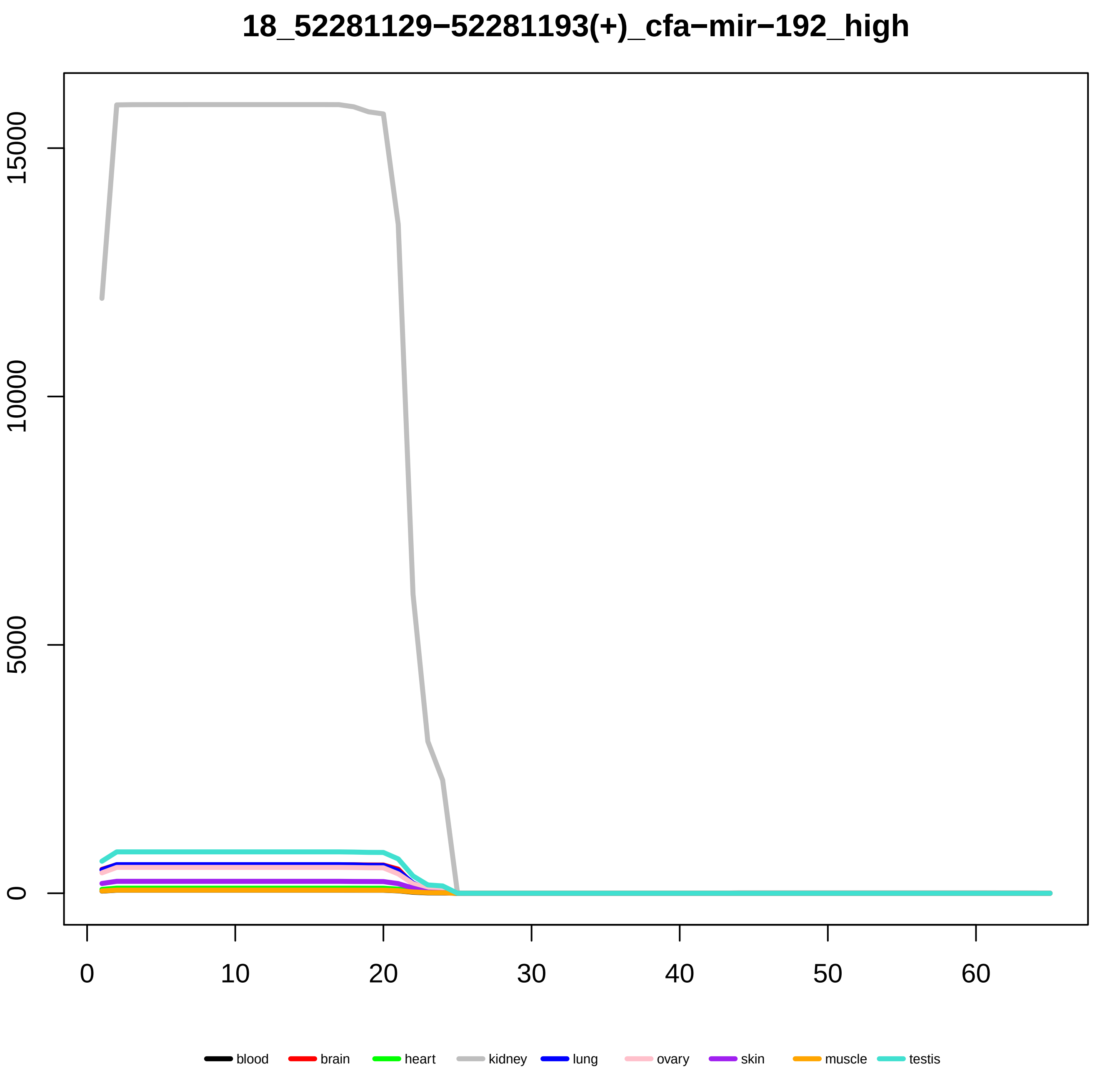
18_52281129−52281193(+)_cfa−mir−192_high
15000
10000
5000
0
0
10
20
30
40
50
60
blood
brain
heart
kidney
lung
ovary
skin
muscle
testis

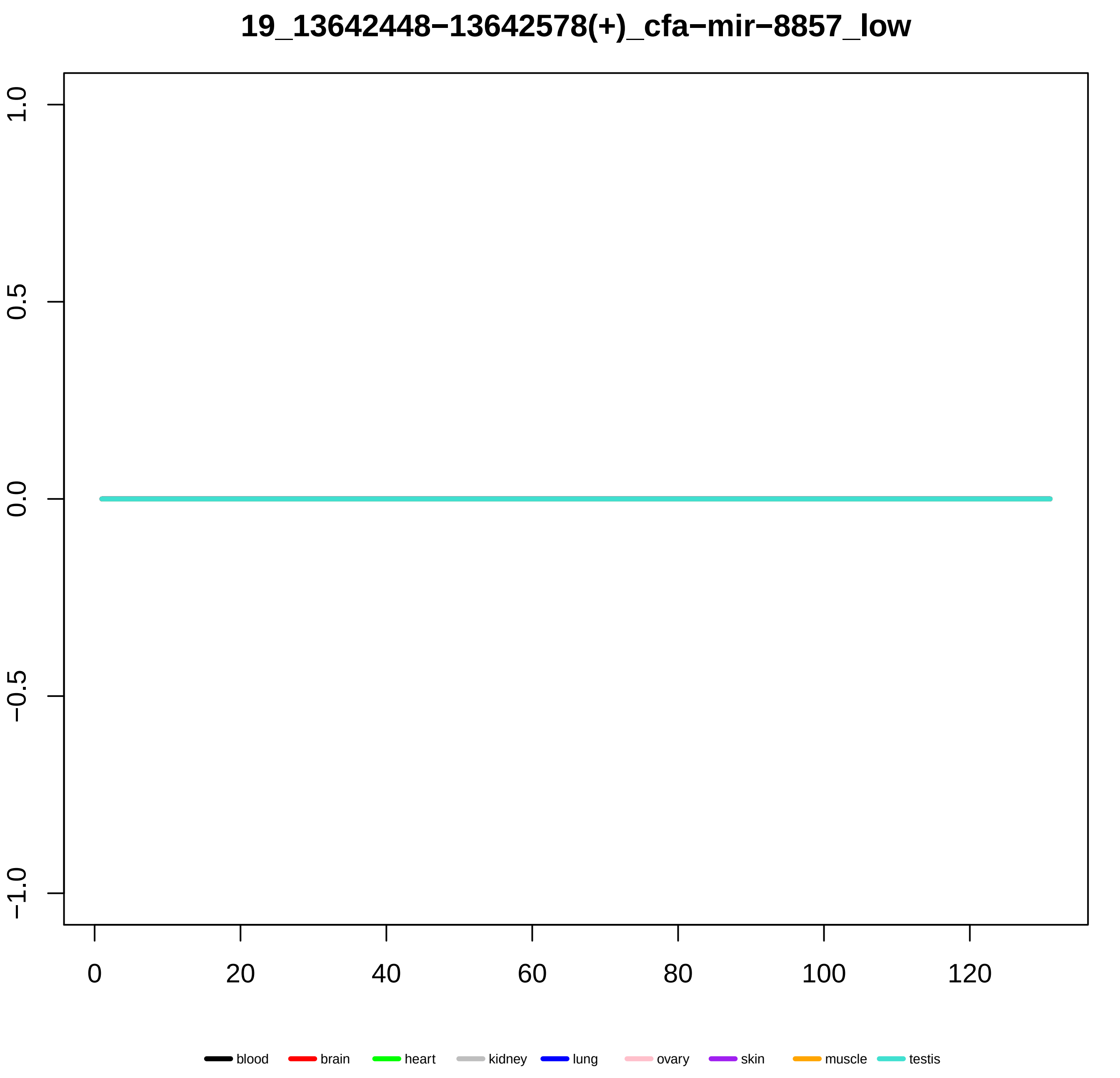

19_13642448−13642578(+)_cfa−mir−8857_low
1.0
0.5
0.0
−0.5
−1.0
0
20
40
60
80
100
120
blood
brain
heart
kidney
lung
ovary
skin
muscle
testis

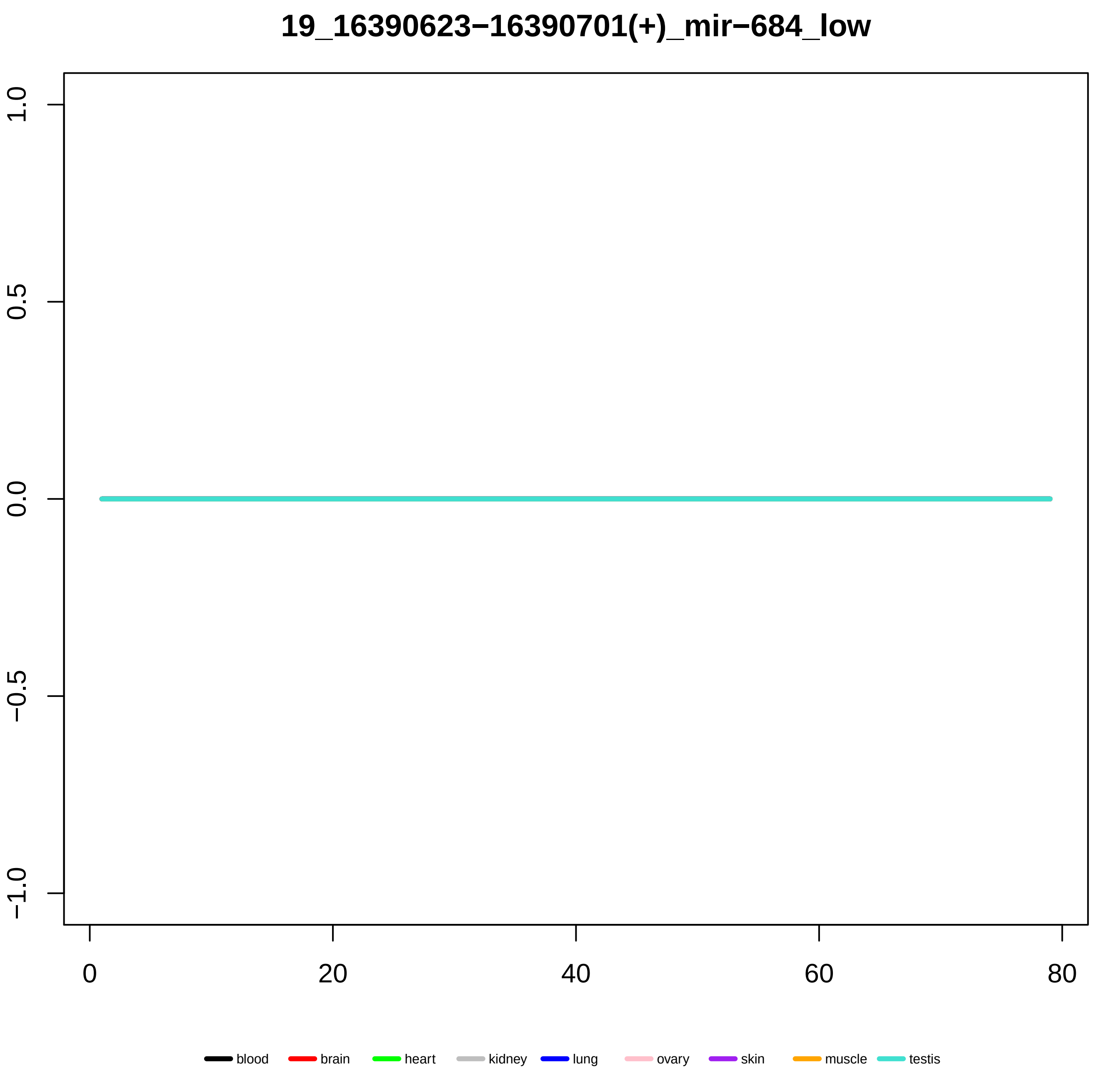

19_16390623−16390701(+)_mir−684_low
1.0
0.5
0.0
−0.5
−1.0
0
20
40
60
80
blood
brain
heart
kidney
lung
ovary
skin
muscle
testis

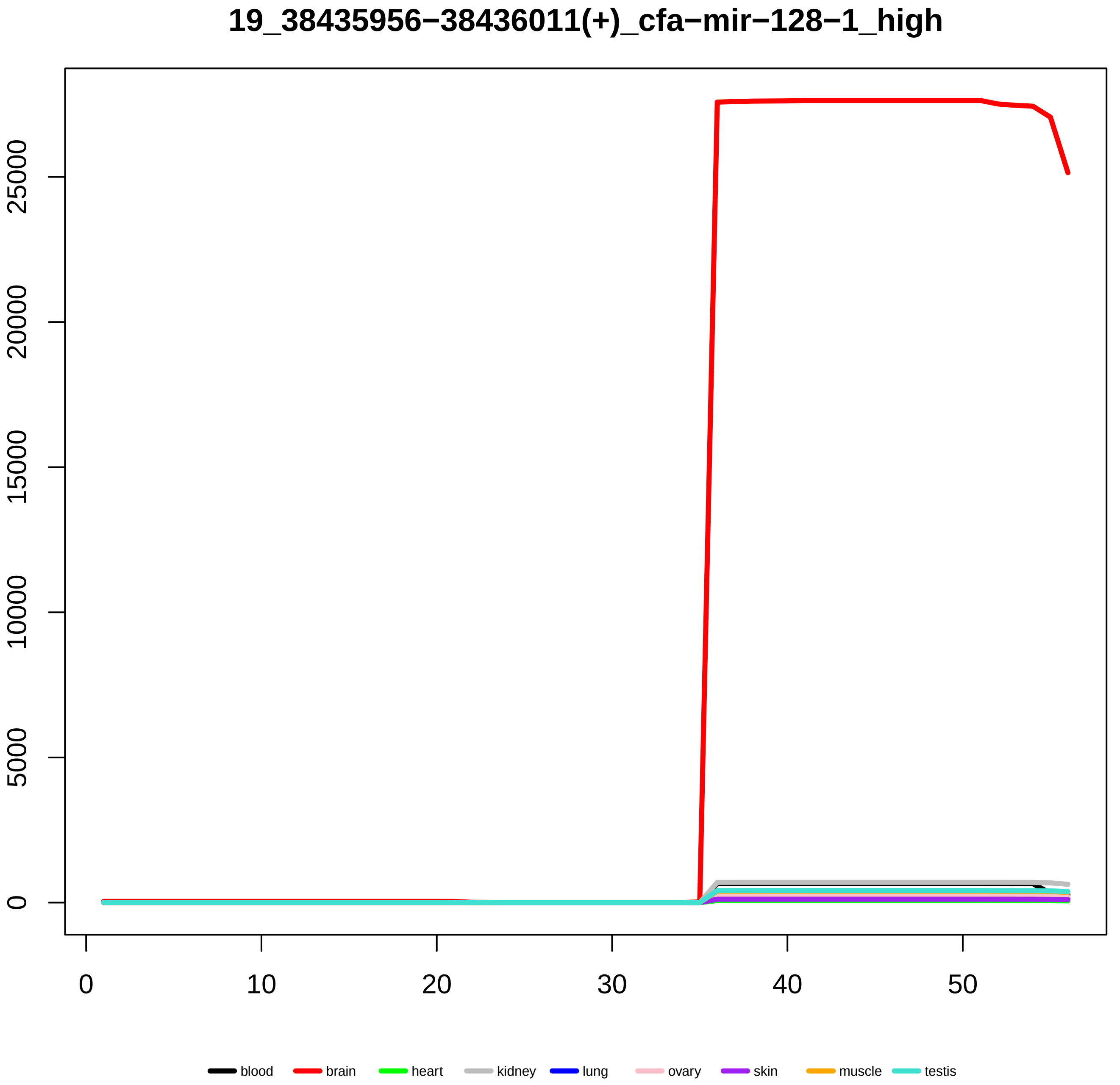

19_38435956−38436011(+)_cfa−mir−128−1_high
0
5000
10000
15000
20000
25000
0
10
20
30
40
50
blood
brain
heart
kidney
lung
ovary
skin
muscle
testis

2_1319449−1319518(+)_mir−4683_high

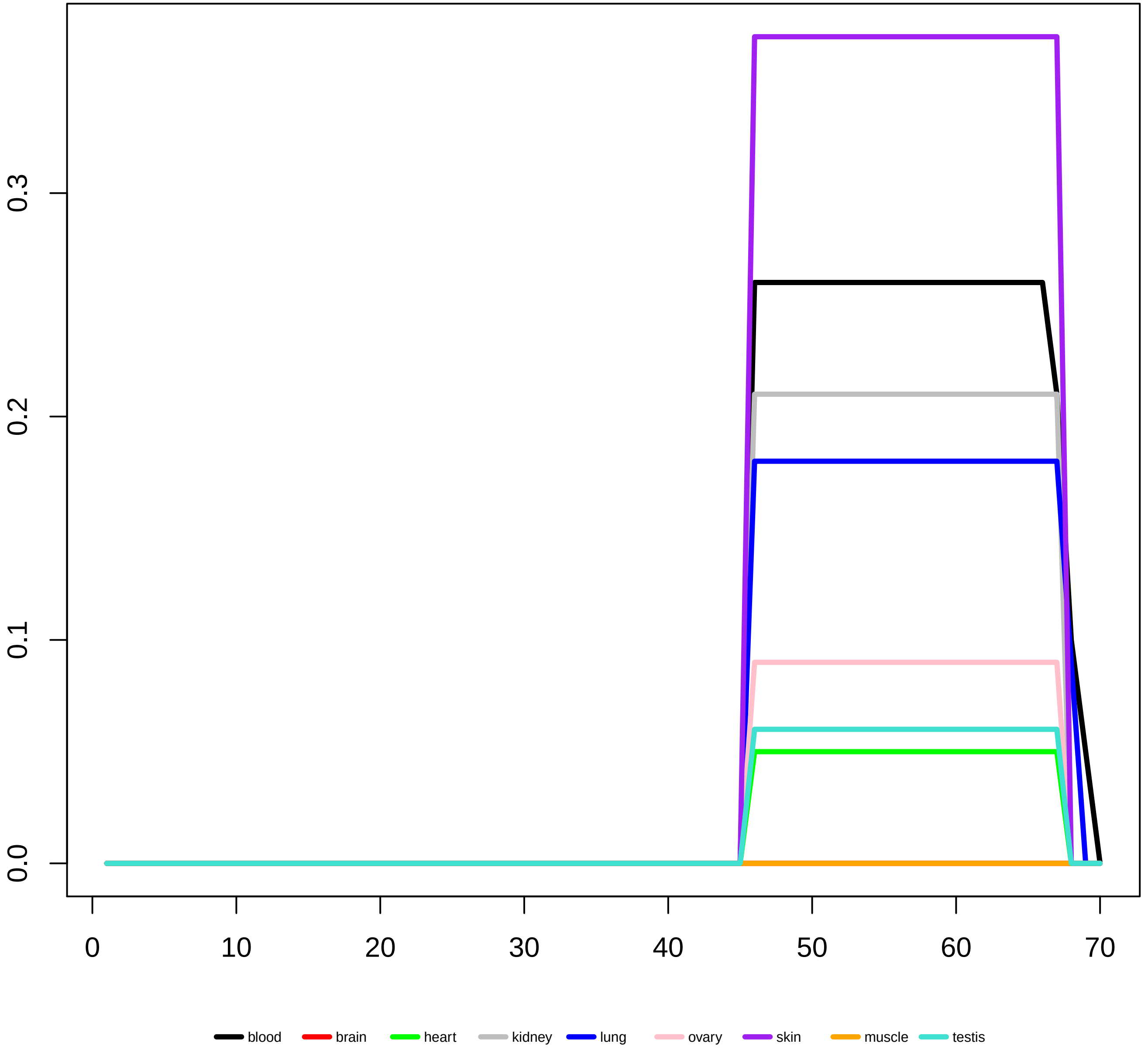

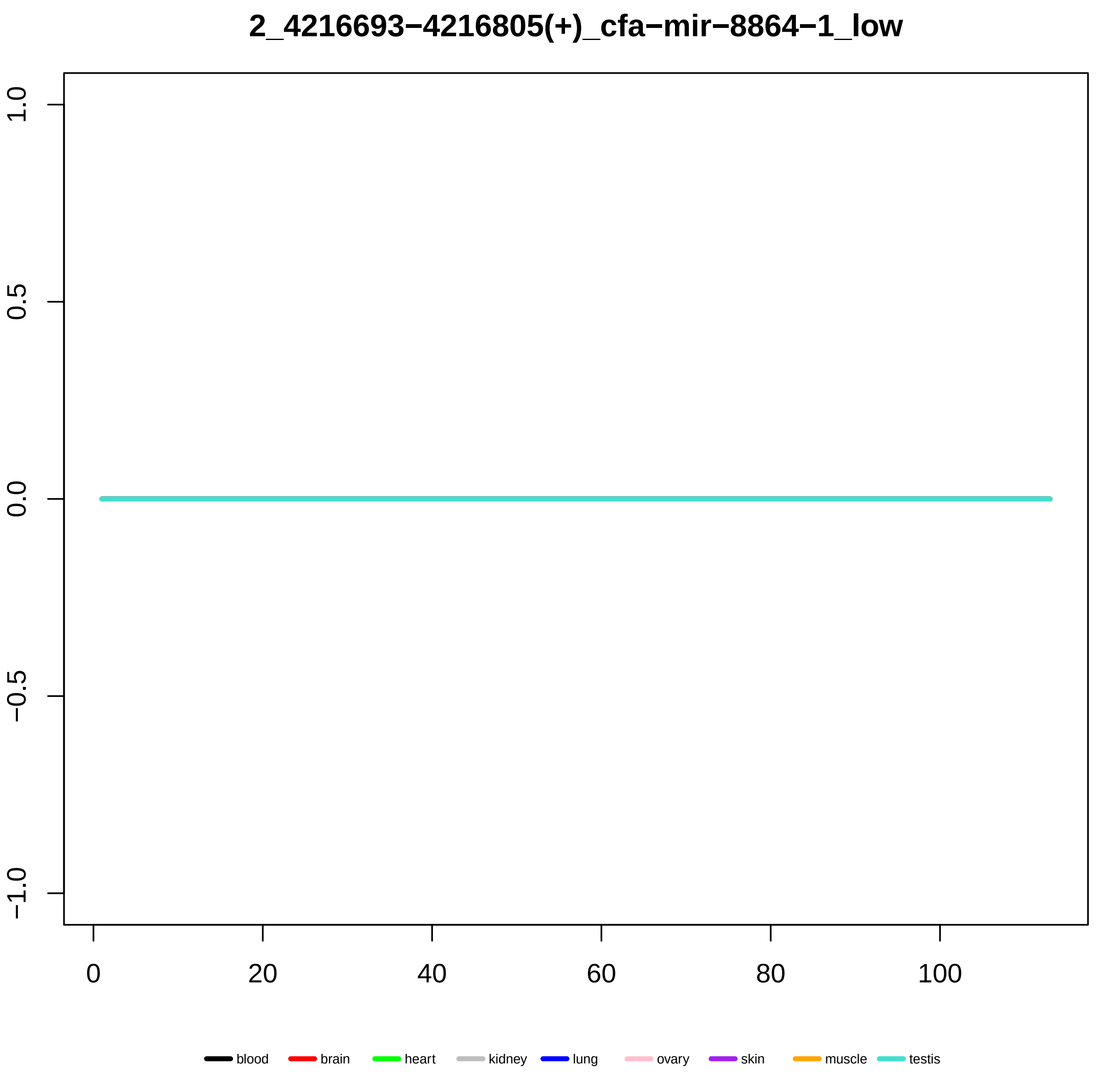

2_4216693−4216805(+)_cfa−mir−8864−1_low
1.0
0.5
0.0
−0.5
−1.0
0
20
40
60
80
100
blood
brain
heart
kidney
lung
ovary
skin
muscle
testis

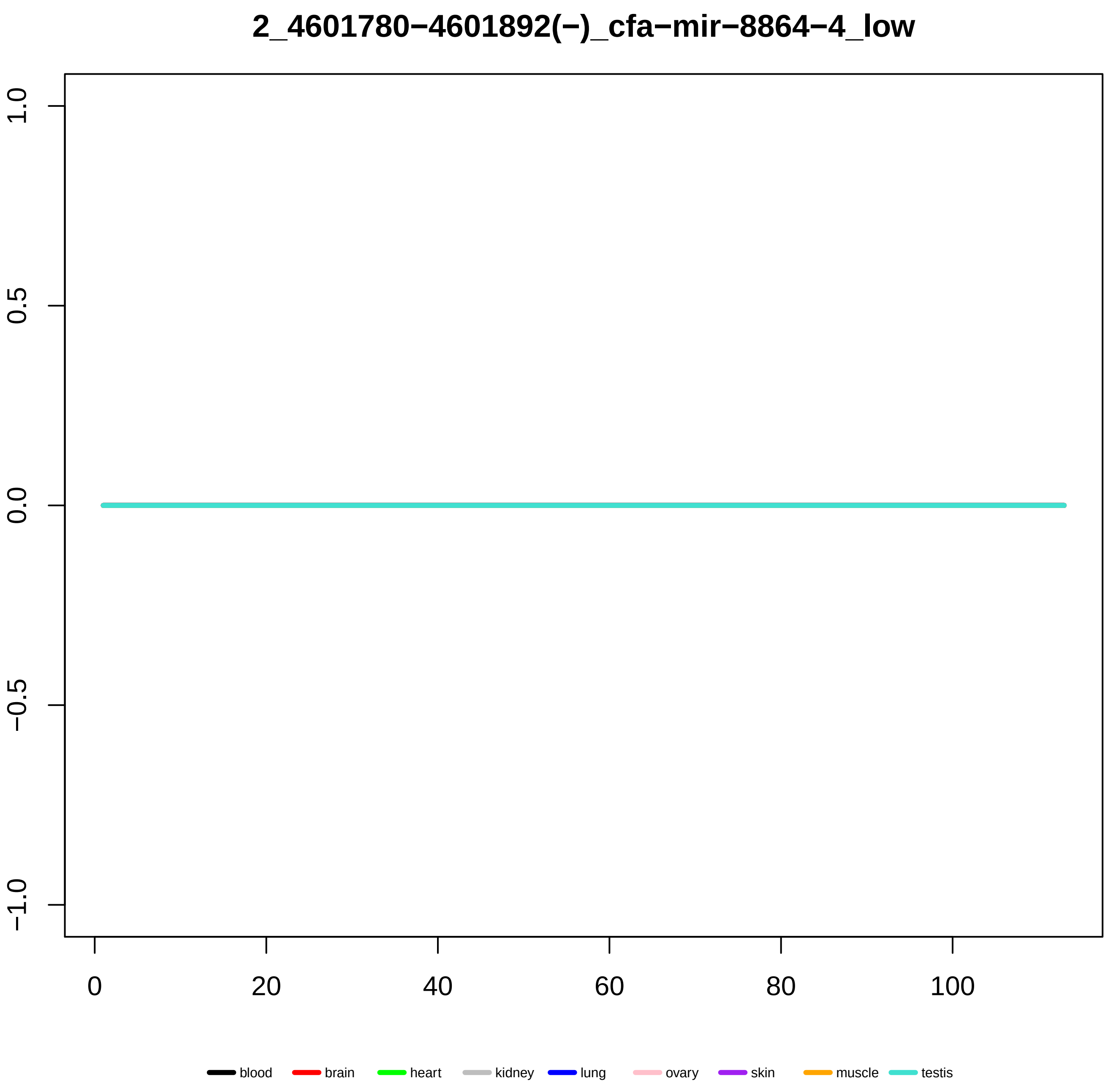

2_4601780−4601892(−)_cfa−mir−8864−4_low
1.0
0.5
0.0
−0.5
−1.0
0
20
40
60
80
100
blood
brain
heart
kidney
lung
ovary
skin
muscle
testis

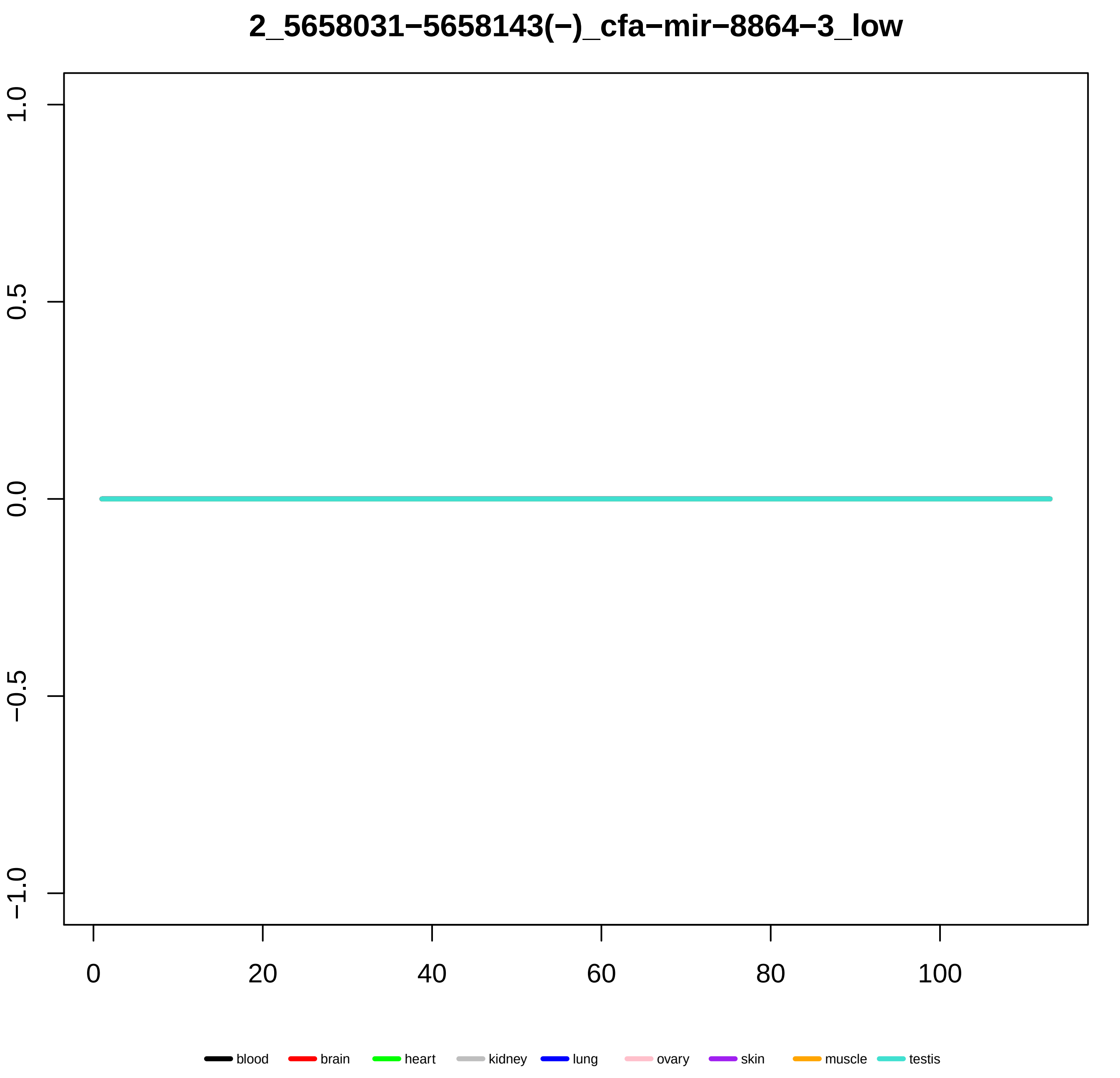
2_5658031−5658143(−)_cfa−mir−8864−3_low
1.0
0.5
0.0
−0.5
−1.0
0
20
40
60
80
100
blood
brain
heart
kidney
lung
ovary
skin
muscle
testis

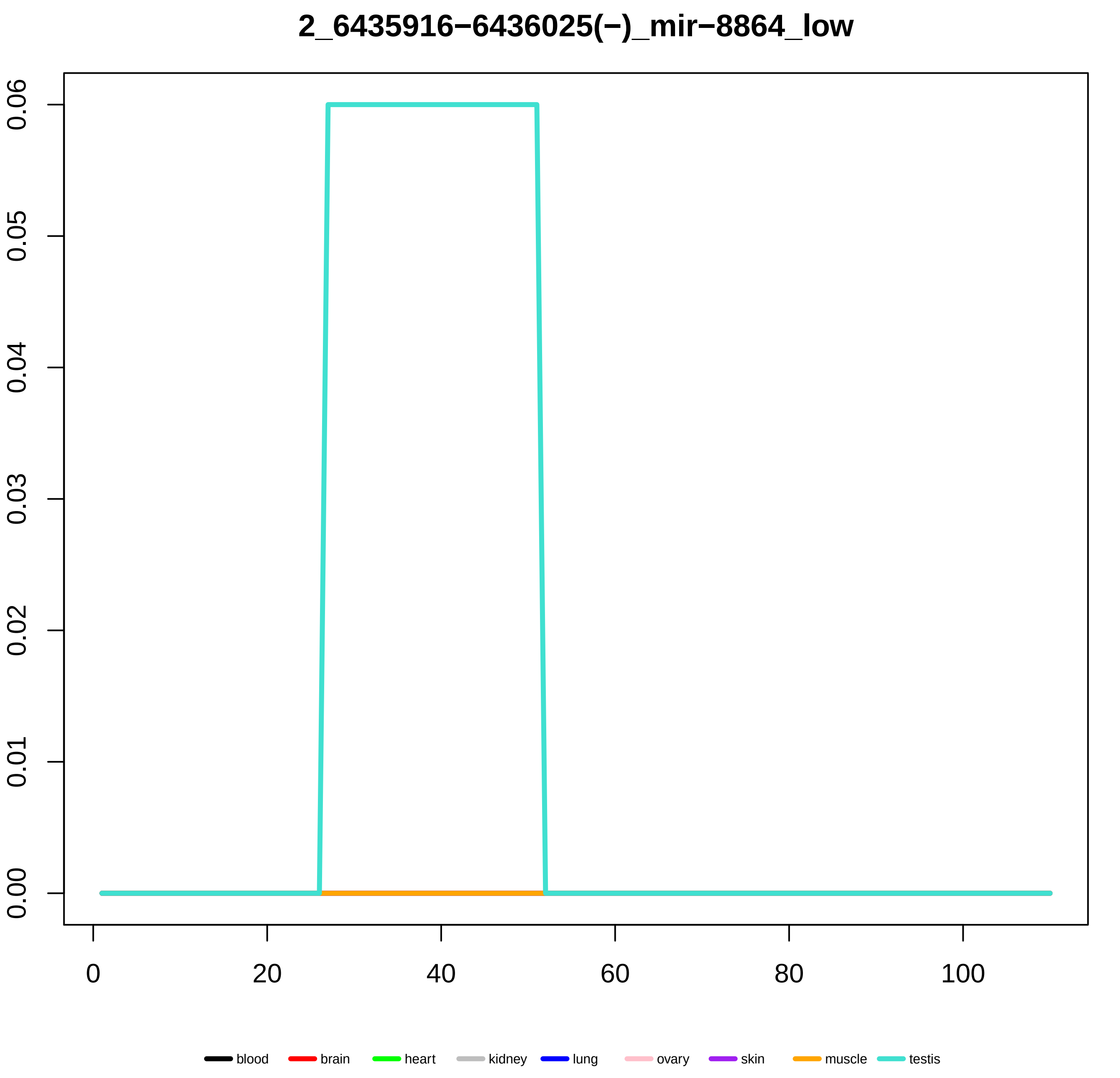
2_6435916−6436025(−)_mir−8864_low
0.00
0.01
0.02
0.03
0.04
0.05
0.06
0
20
40
60
80
100
blood
brain
heart
kidney
lung
ovary
skin
muscle
testis

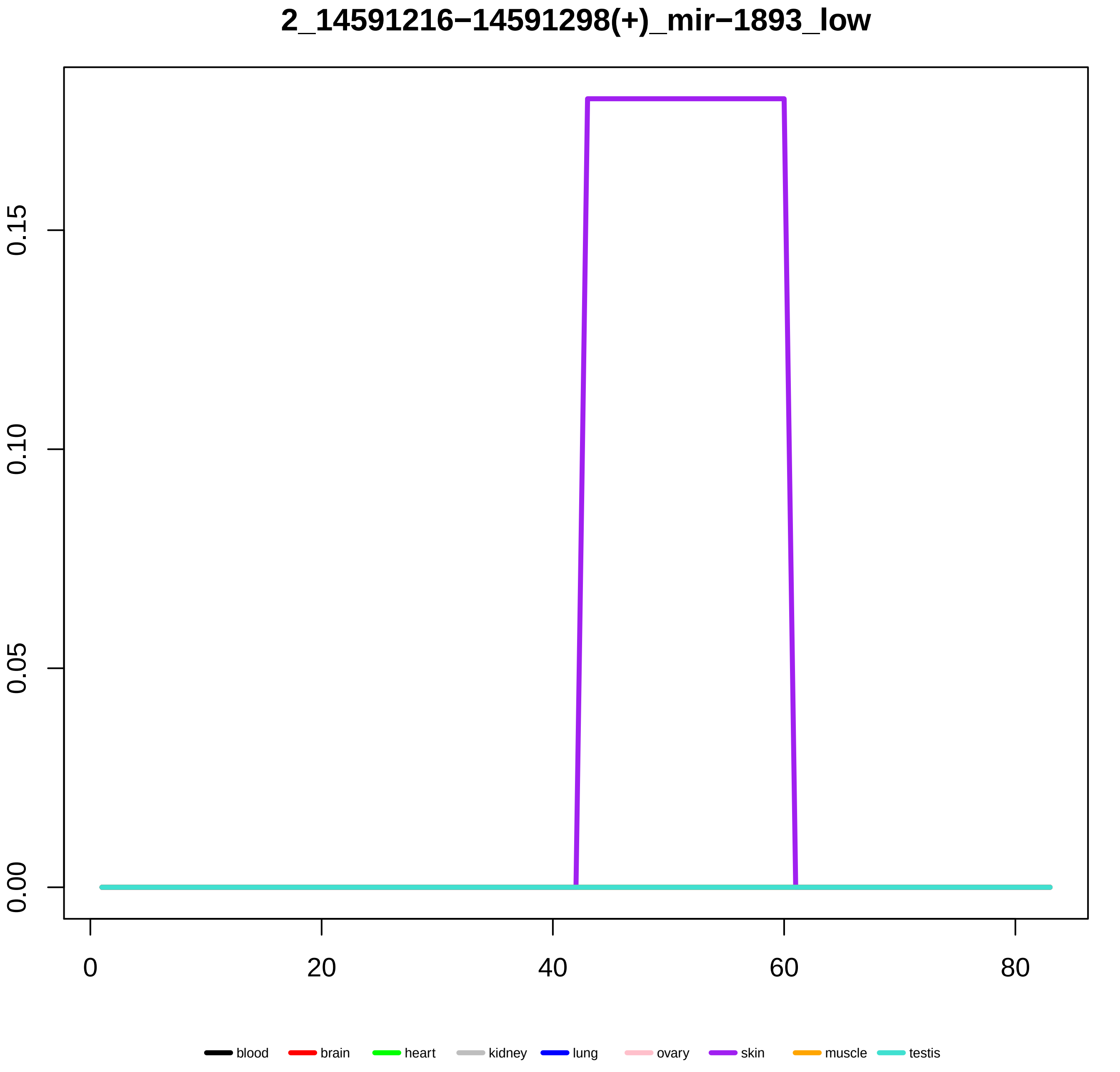

2_14591216−14591298(+)_mir−1893_low
0.00
0.05
0.10
0.15
0
20
40
60
80
blood
brain
heart
kidney
lung
ovary
skin
muscle
testis

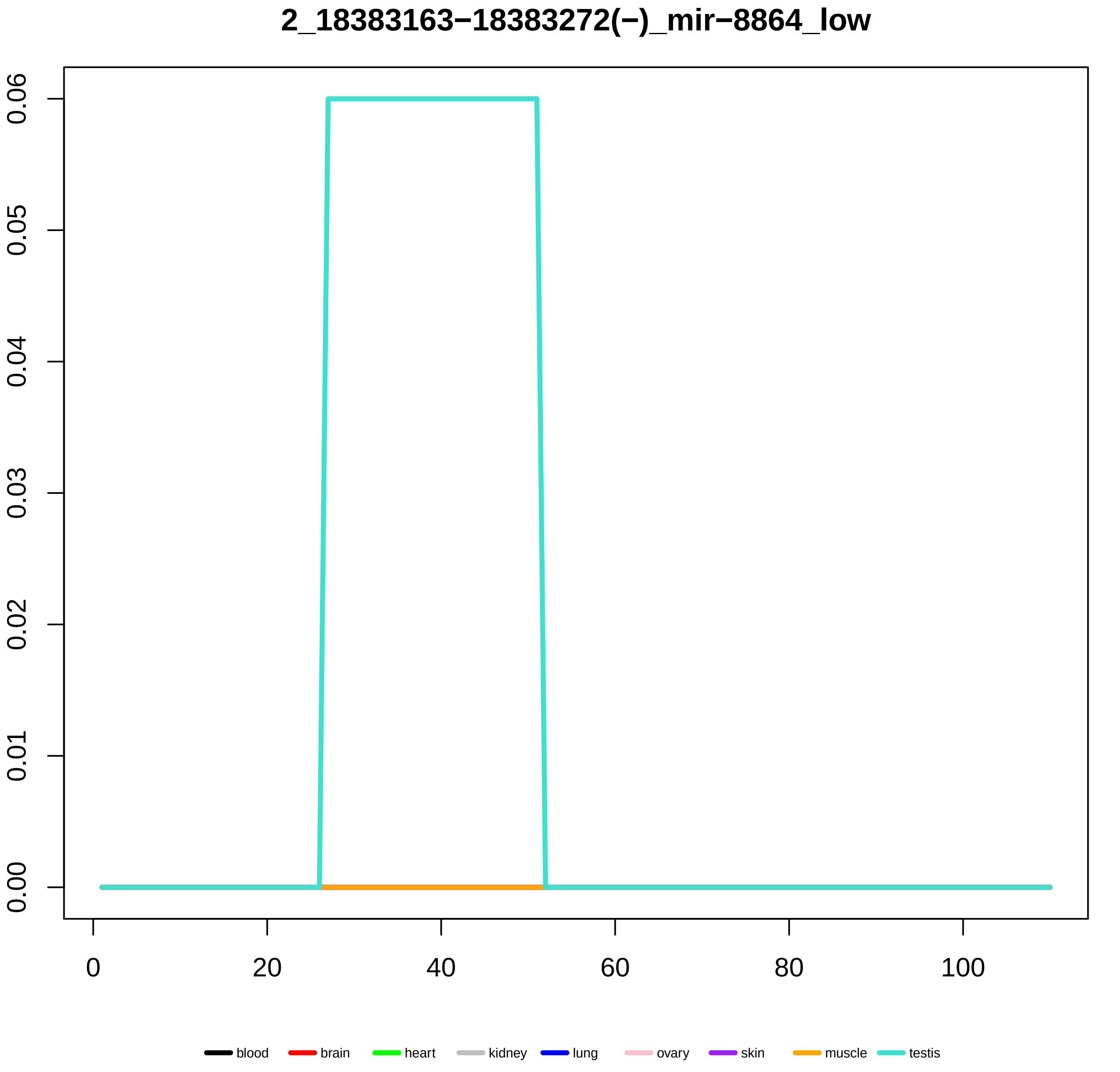
2_18383163−18383272(−)_mir−8864_low
0.00
0.01
0.02
0.03
0.04
0.05
0.06
0
20
40
60
80
100
blood
brain
heart
kidney
lung
ovary
skin
muscle
testis

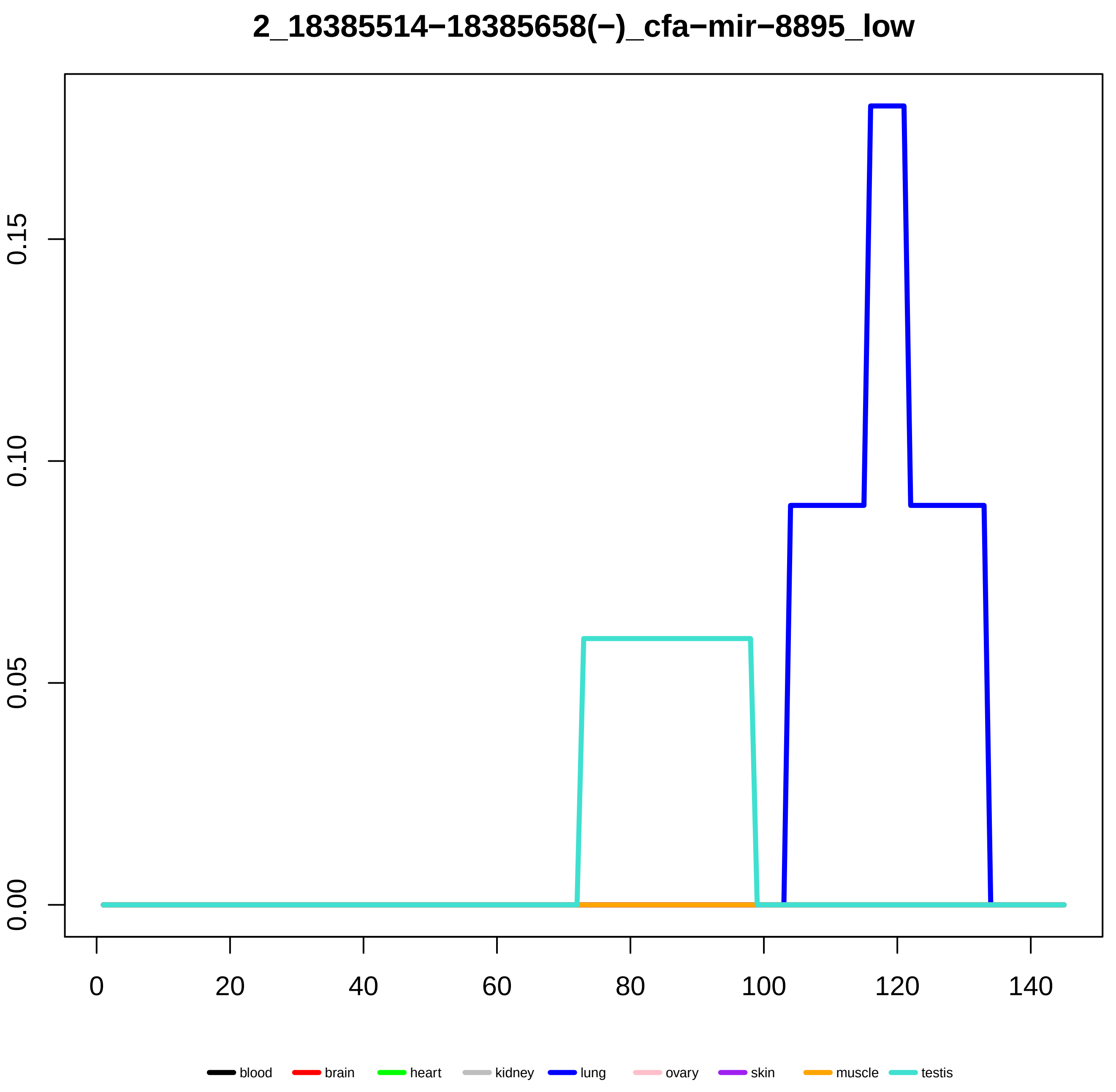

2_18385514−18385658(−)_cfa−mir−8895_low
0.00
0.05
0.10
0.15
0
20
40
60
80
100
120
140
blood
brain
heart
kidney
lung
ovary
skin
muscle
testis

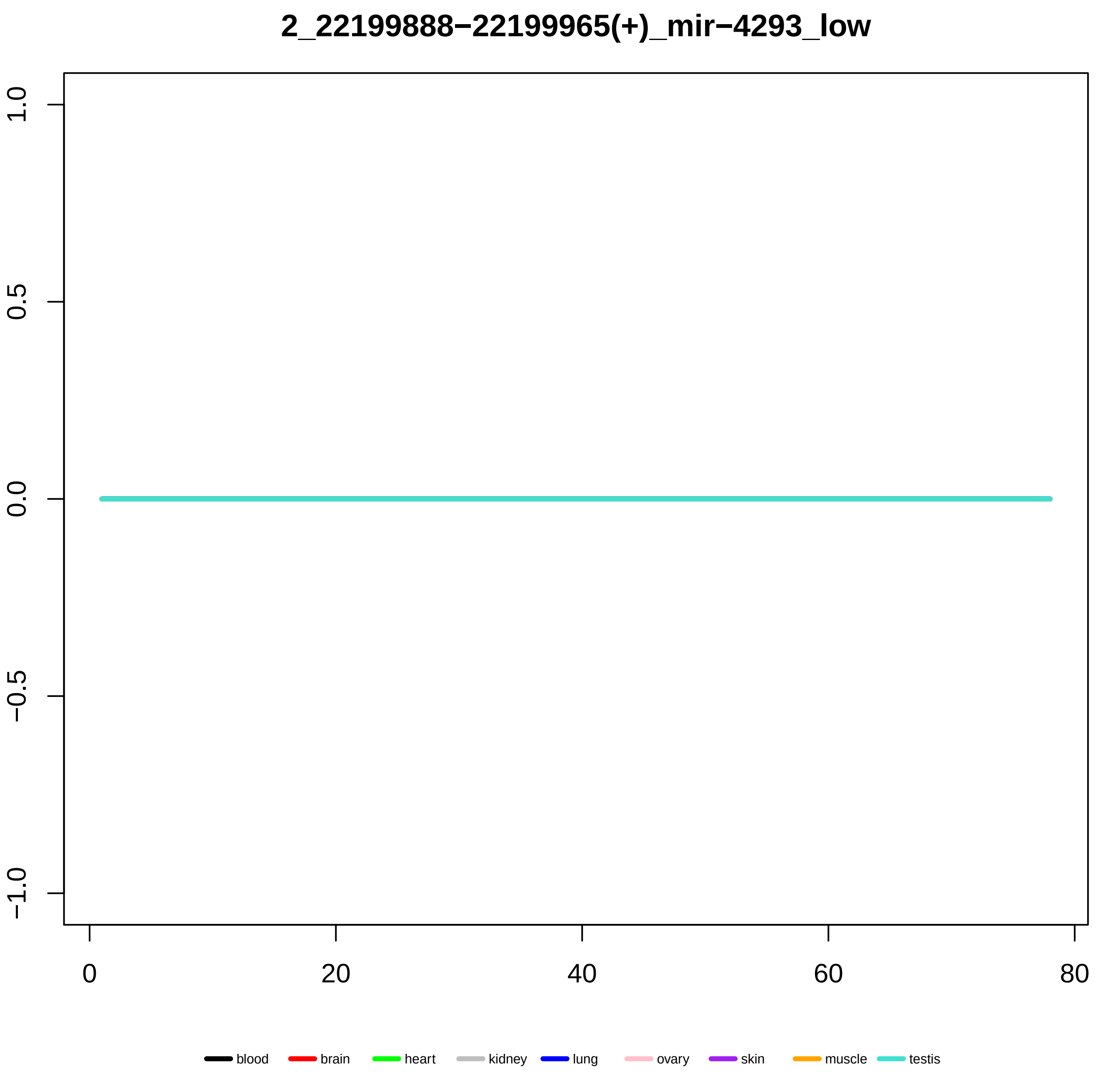
2_22199888−22199965(+)_mir−4293_low
1.0
0.5
0.0
−0.5
−1.0
0
20
40
60
80
blood
brain
heart
kidney
lung
ovary
skin
muscle
testis

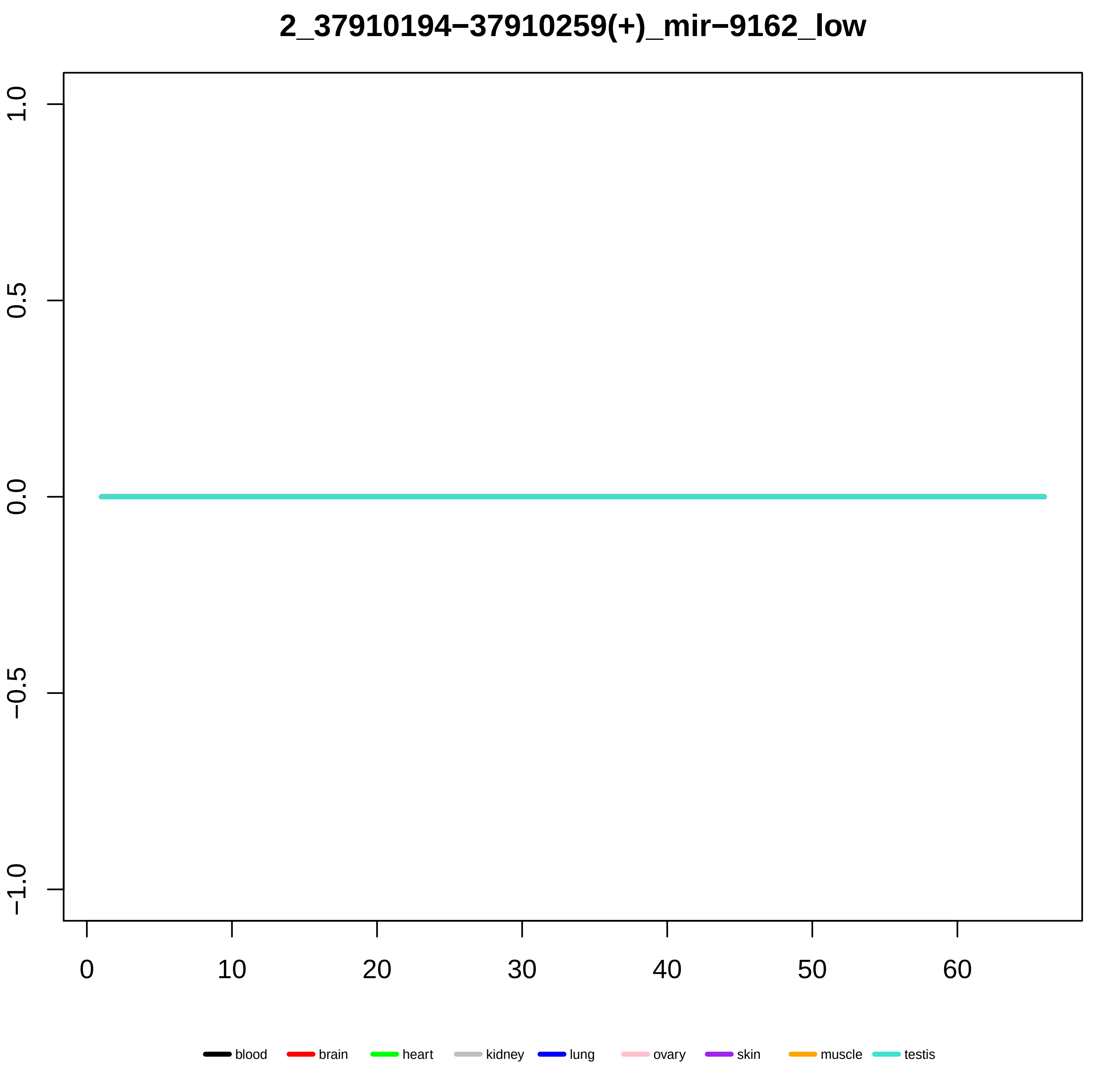
2_37910194−37910259(+)_mir−9162_low
1.0
0.5
0.0
−0.5
−1.0
0
10
20
30
40
50
60
blood
brain
heart
kidney
lung
ovary
skin
muscle
testis

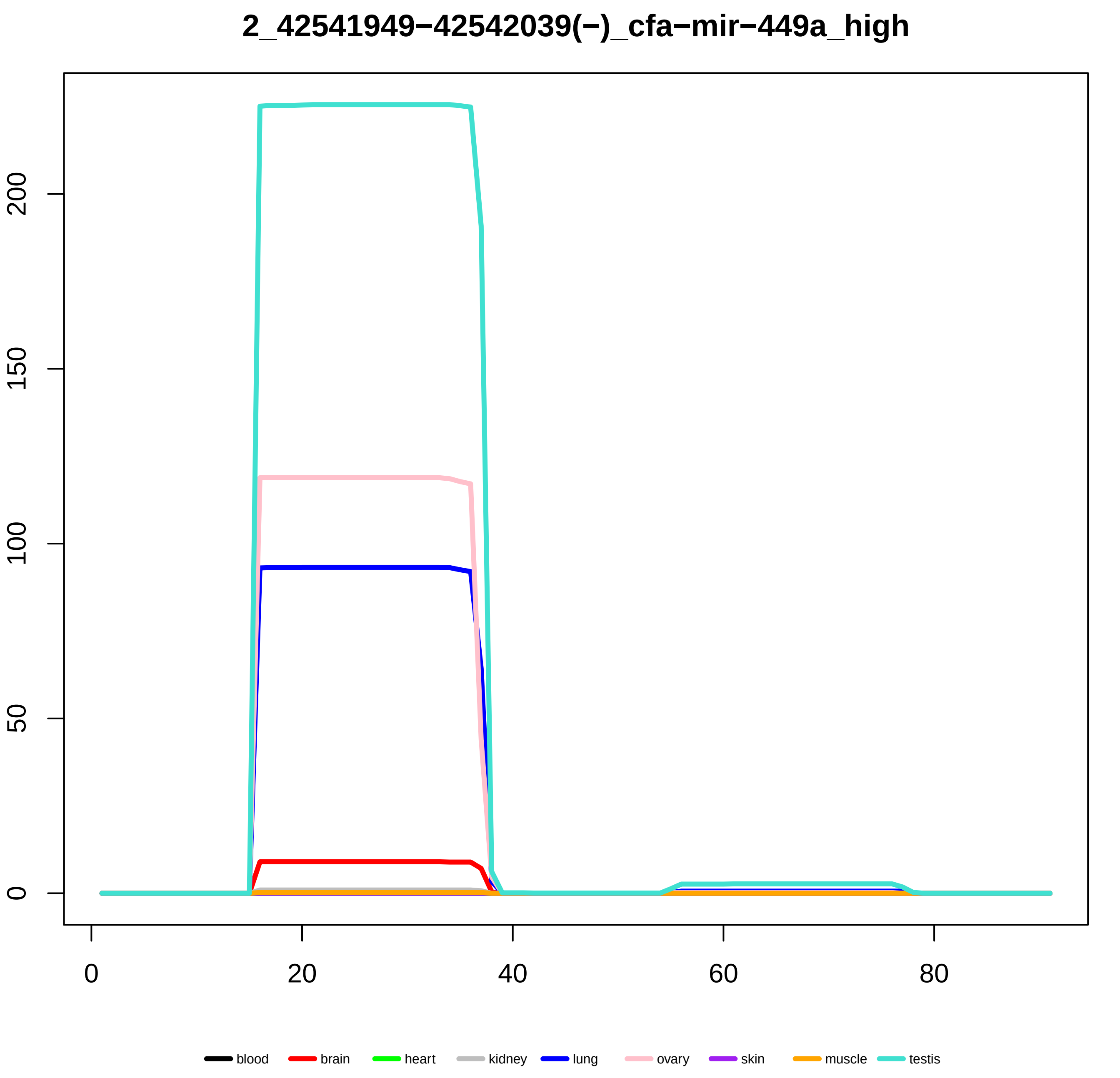

2_42541949−42542039(−)_cfa−mir−449a_high
0
50
100
150
200
0
20
40
60
80
blood
brain
heart
kidney
lung
ovary
skin
muscle
testis

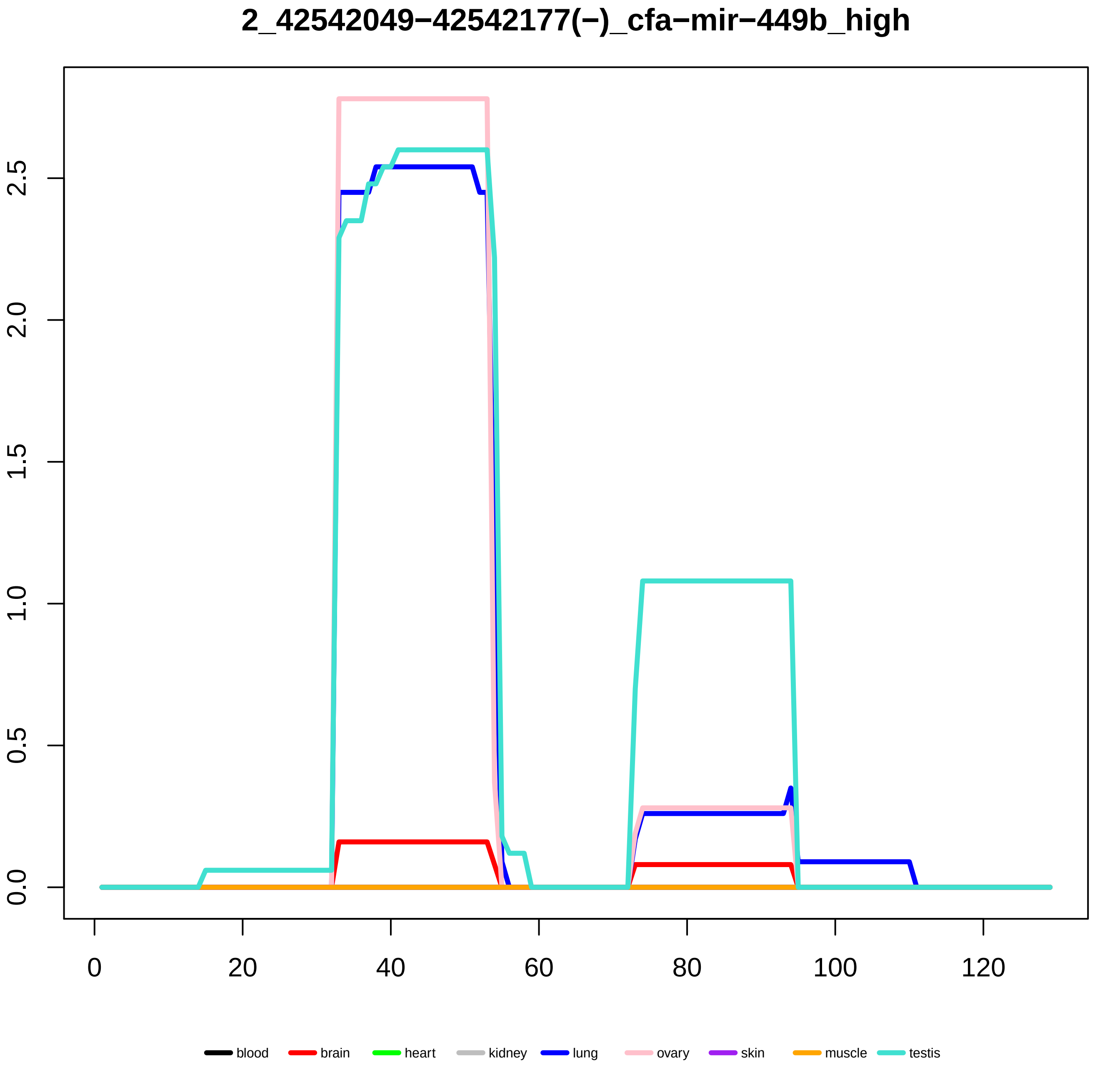
2_42542049−42542177(−)_cfa−mir−449b_high
0.0
0.5
1.0
1.5
2.0
2.5
0
20
40
60
80
100
120
blood
brain
heart
kidney
lung
ovary
skin
muscle
testis

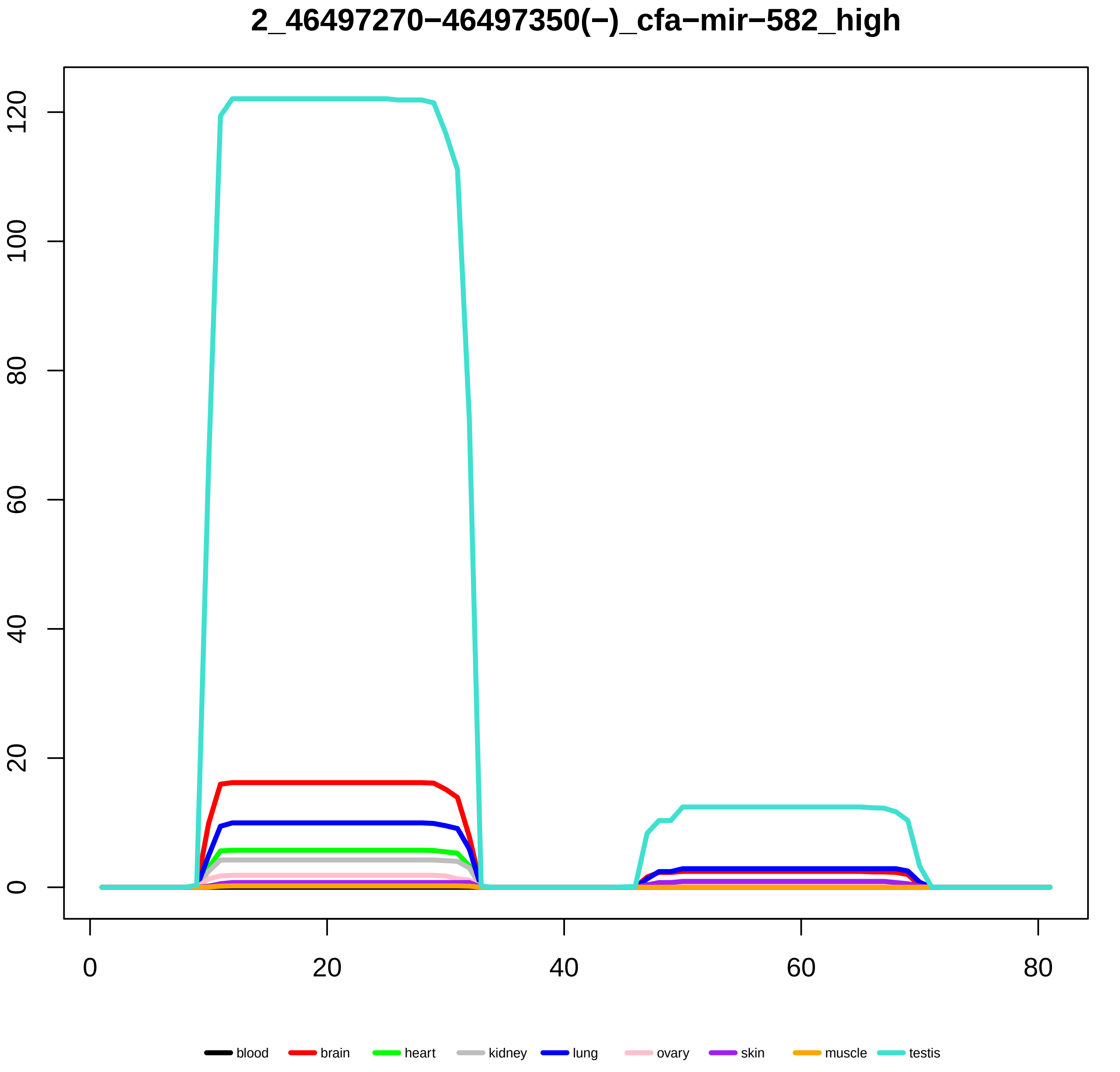

2_46497270−46497350(−)_cfa−mir−582_high
0
20
40
60
80
100
120
0
20
40
60
80
blood
brain
heart
kidney
lung
ovary
skin
muscle
testis

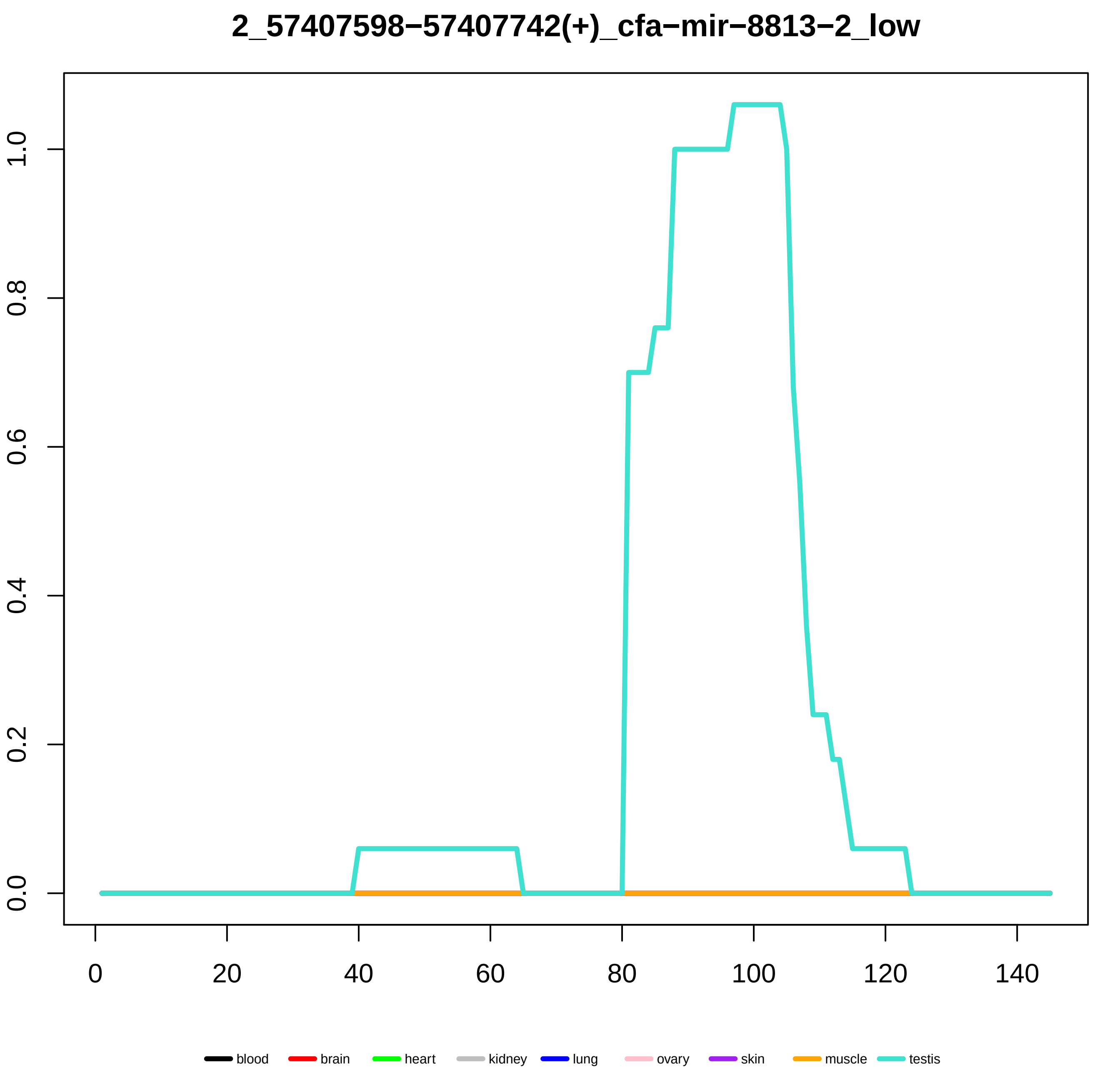
2_57407598−57407742(+)_cfa−mir−8813−2_low
0.0
0.2
0.4
0.6
0.8
1.0
0
20
40
60
80
100
120
140
blood
brain
heart
kidney
lung
ovary
skin
muscle
testis

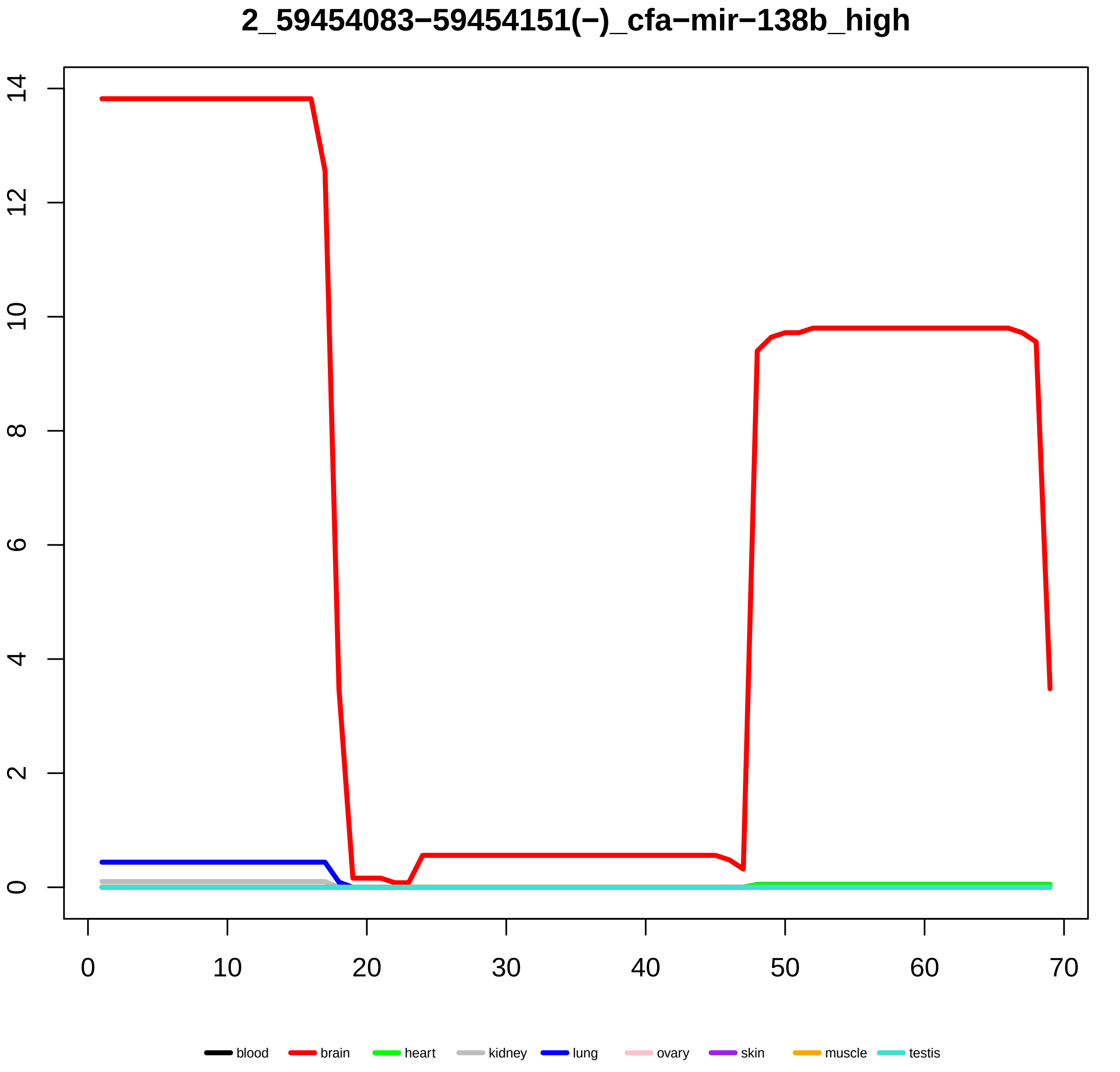
2_59454083−59454151(−)_cfa−mir−138b_high
0
2
4
6
8
10
12
14
0
10
20
30
40
50
60
70
blood
brain
heart
kidney
lung
ovary
skin
muscle
testis

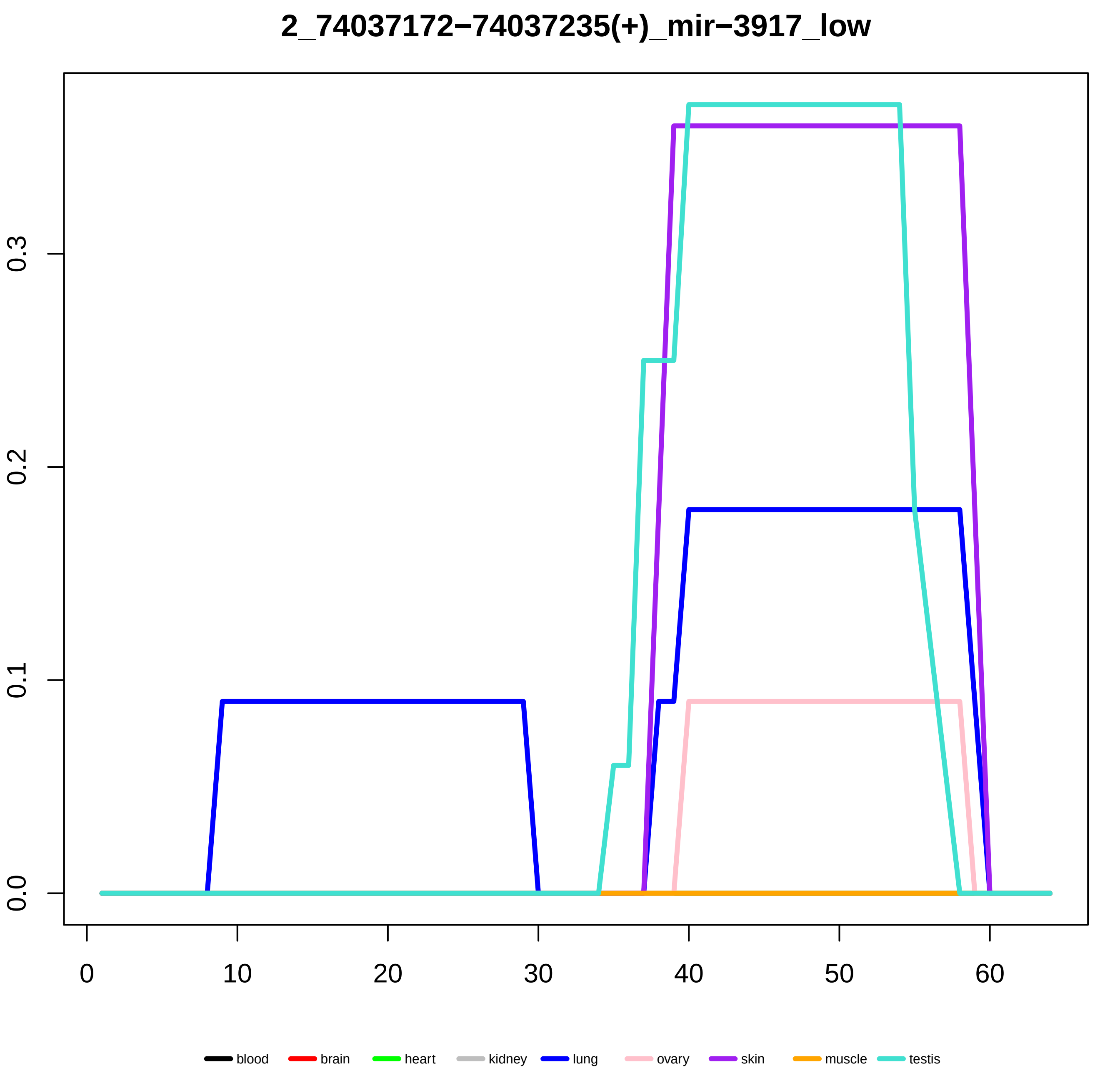

2_74037172−74037235(+)_mir−3917_low
0.0
0.1
0.2
0.3
0
10
20
30
40
50
60
blood
brain
heart
kidney
lung
ovary
skin
muscle
testis

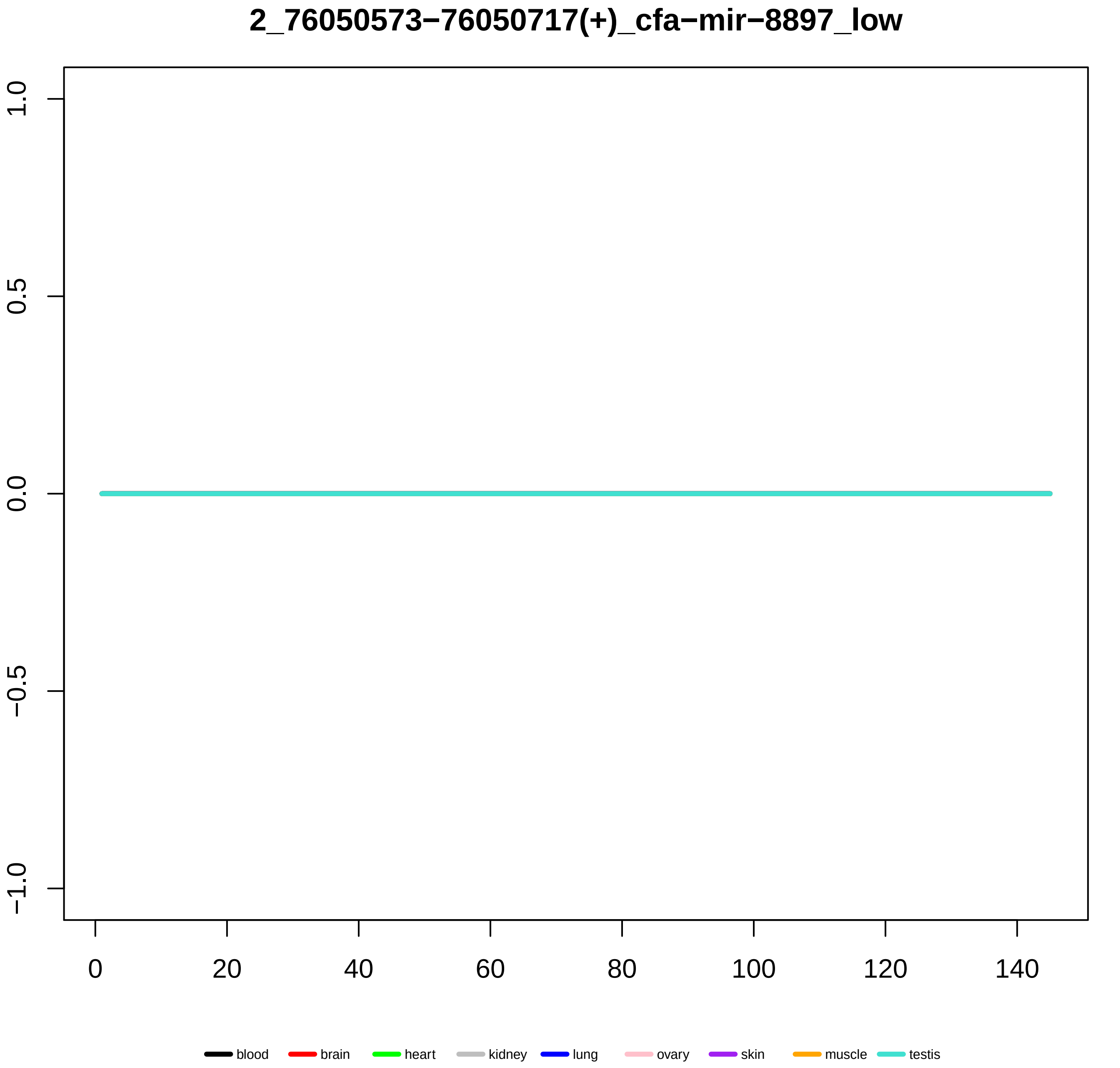

2_76050573−76050717(+)_cfa−mir−8897_low
1.0
0.5
0.0
−0.5
−1.0
0
20
40
60
80
100
120
140
blood
brain
heart
kidney
lung
ovary
skin
muscle
testis

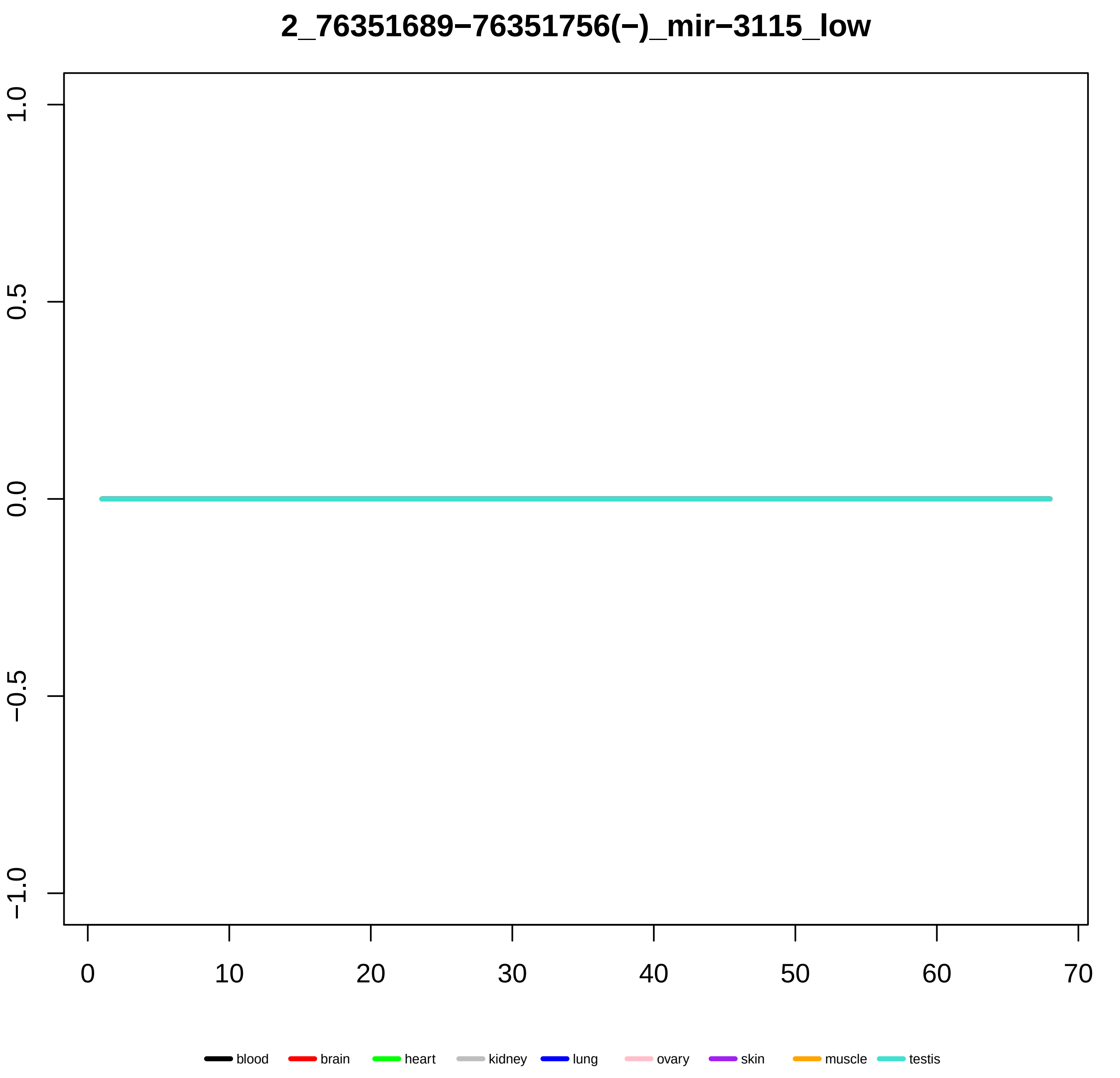
2_76351689−76351756(−)_mir−3115_low
1.0
0.5
0.0
−0.5
−1.0
0
10
20
30
40
50
60
70
blood
brain
heart
kidney
lung
ovary
skin
muscle
testis

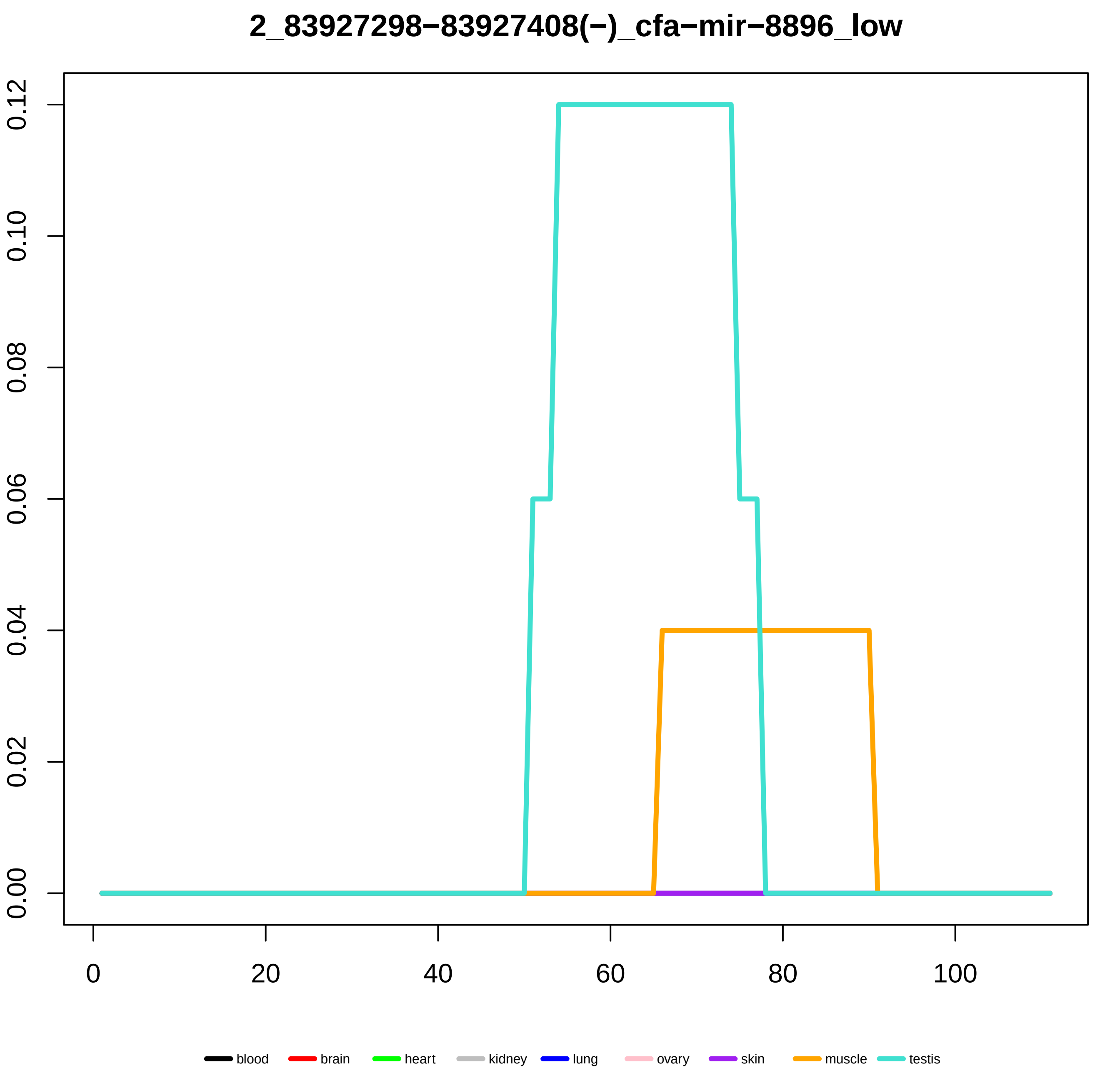

2_83927298−83927408(−)_cfa−mir−8896_low
0.00
0.02
0.04
0.06
0.08
0.10
0.12
0
20
40
60
80
100
blood
brain
heart
kidney
lung
ovary
skin
muscle
testis

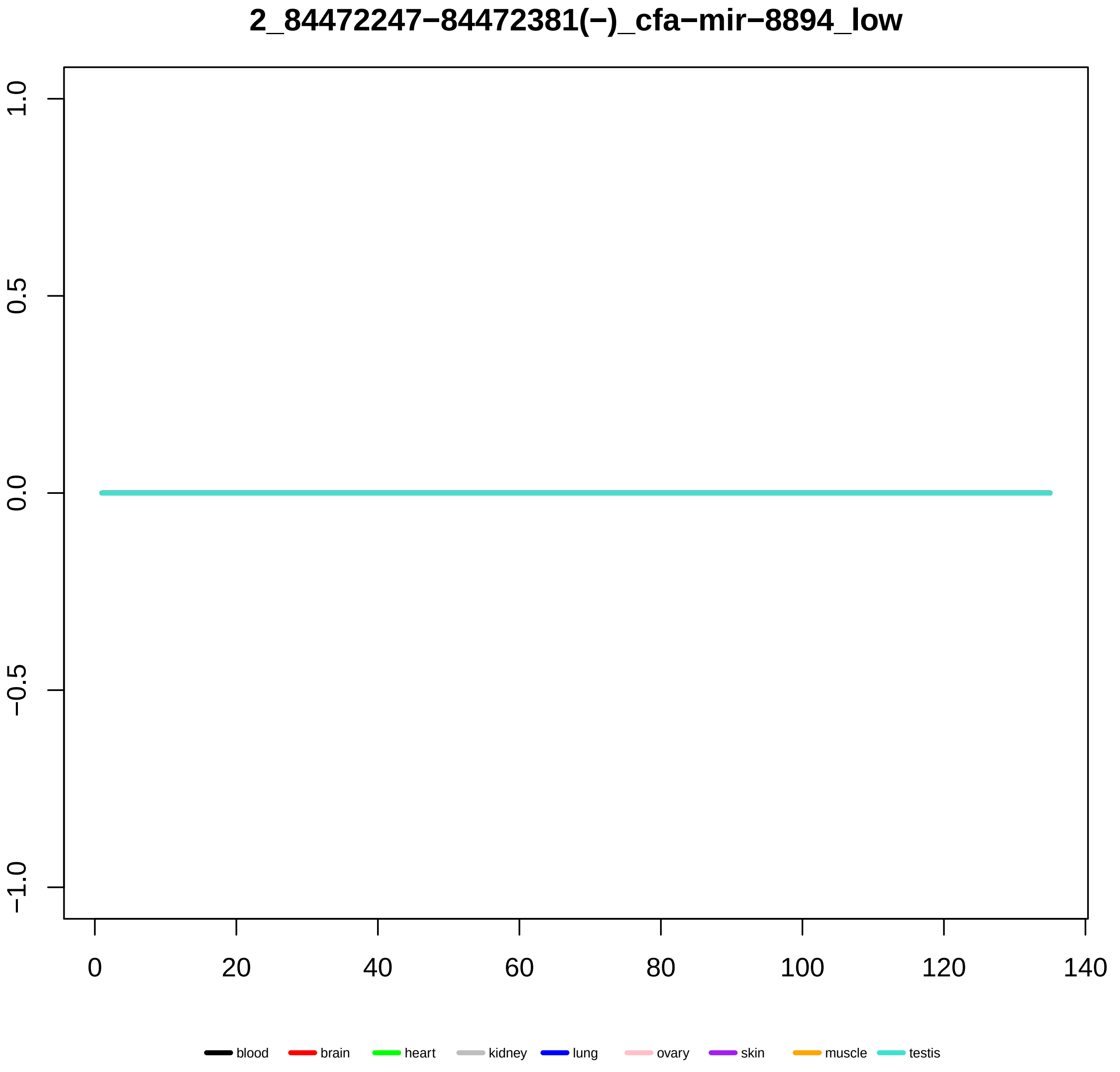

2_84472247−84472381(−)_cfa−mir−8894_low
1.0
0.5
0.0
−0.5
−1.0
0
20
40
60
80
100
120
140
blood
brain
heart
kidney
lung
ovary
skin
muscle
testis

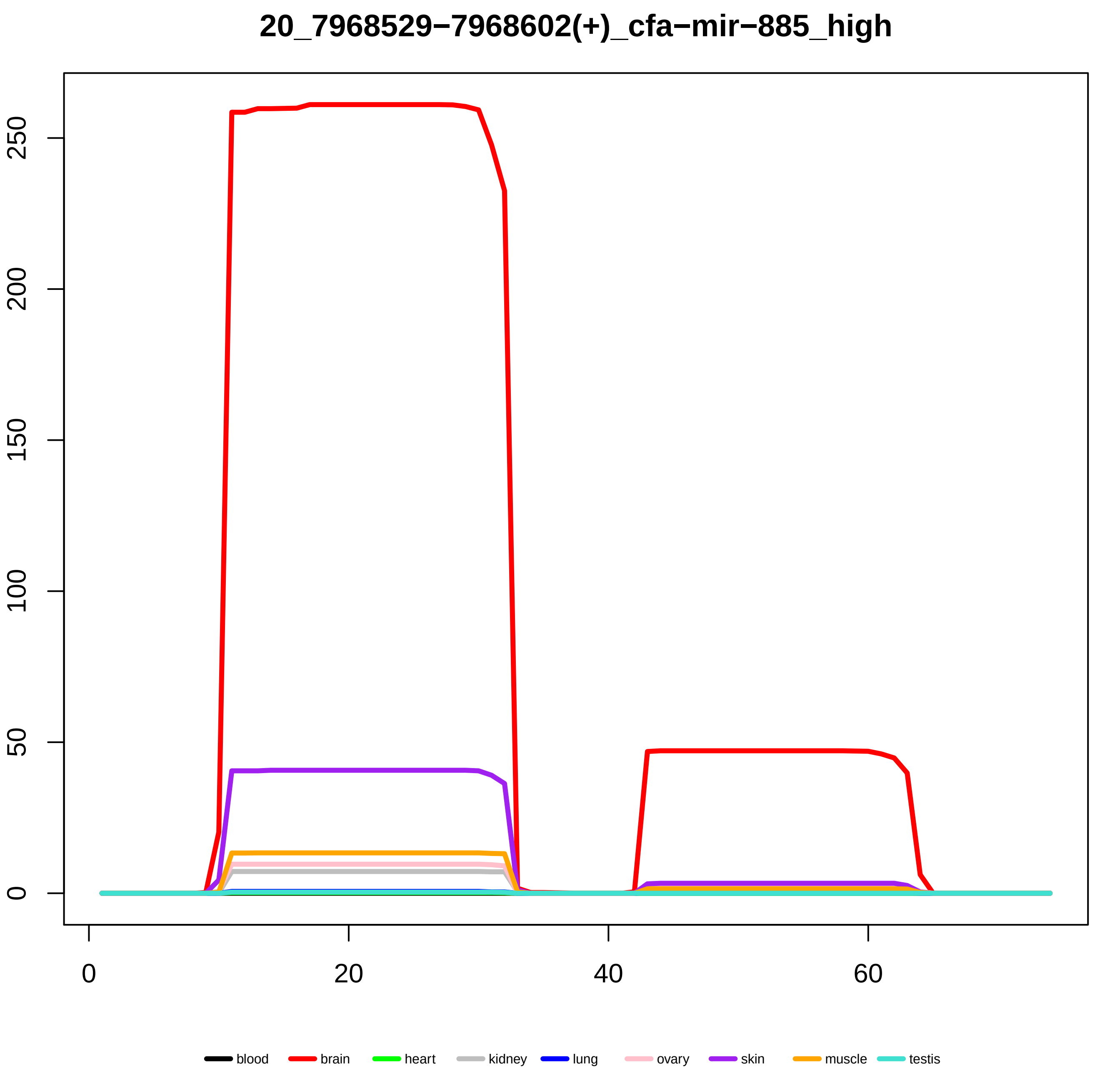
20_7968529−7968602(+)_cfa−mir−885_high
0
50
100
150
200
250
0
20
40
60
blood
brain
heart
kidney
lung
ovary
skin
muscle
testis

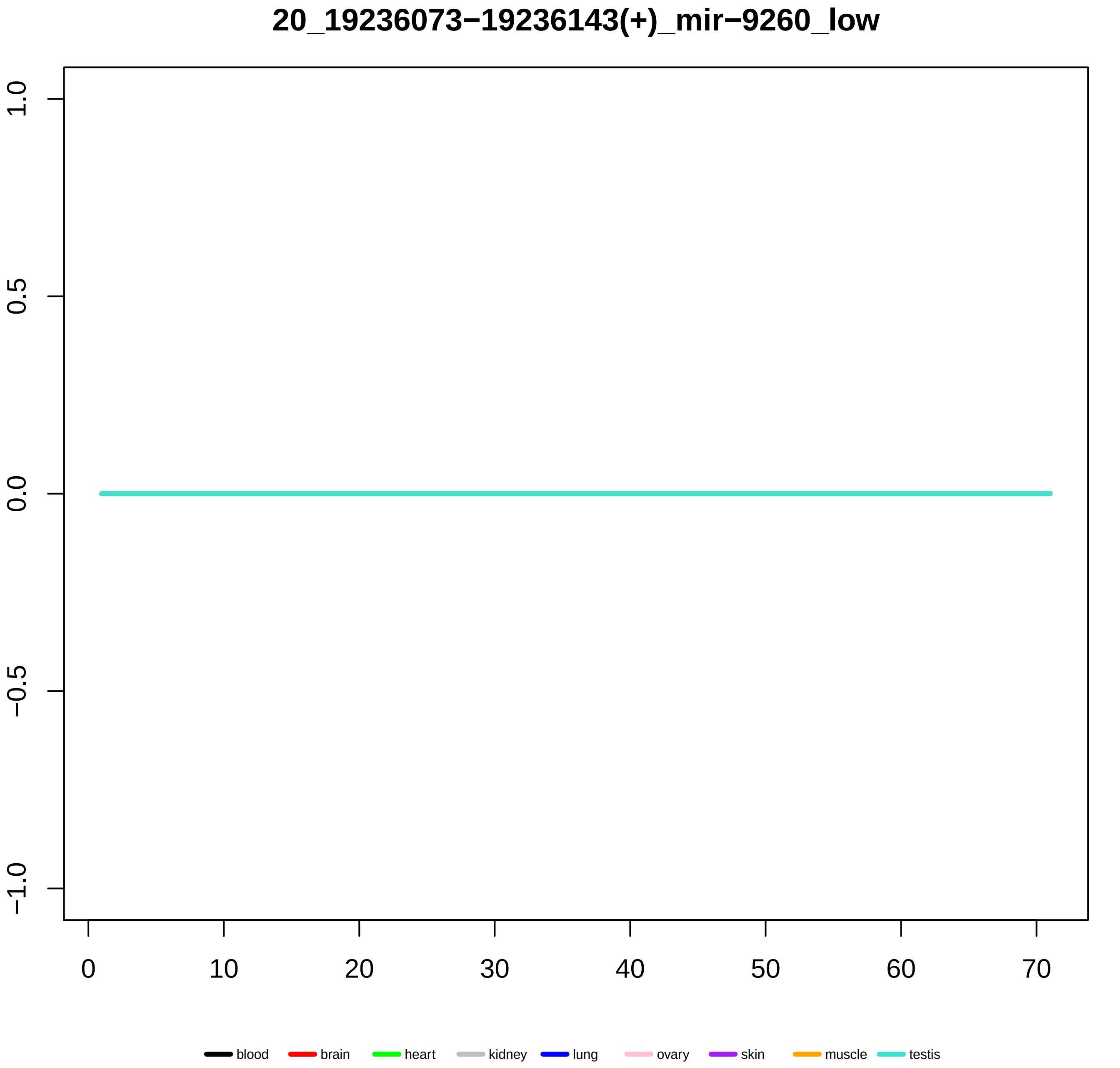

20_19236073−19236143(+)_mir−9260_low
1.0
0.5
0.0
−0.5
−1.0
0
10
20
30
40
50
60
70
blood
brain
heart
kidney
lung
ovary
skin
muscle
testis

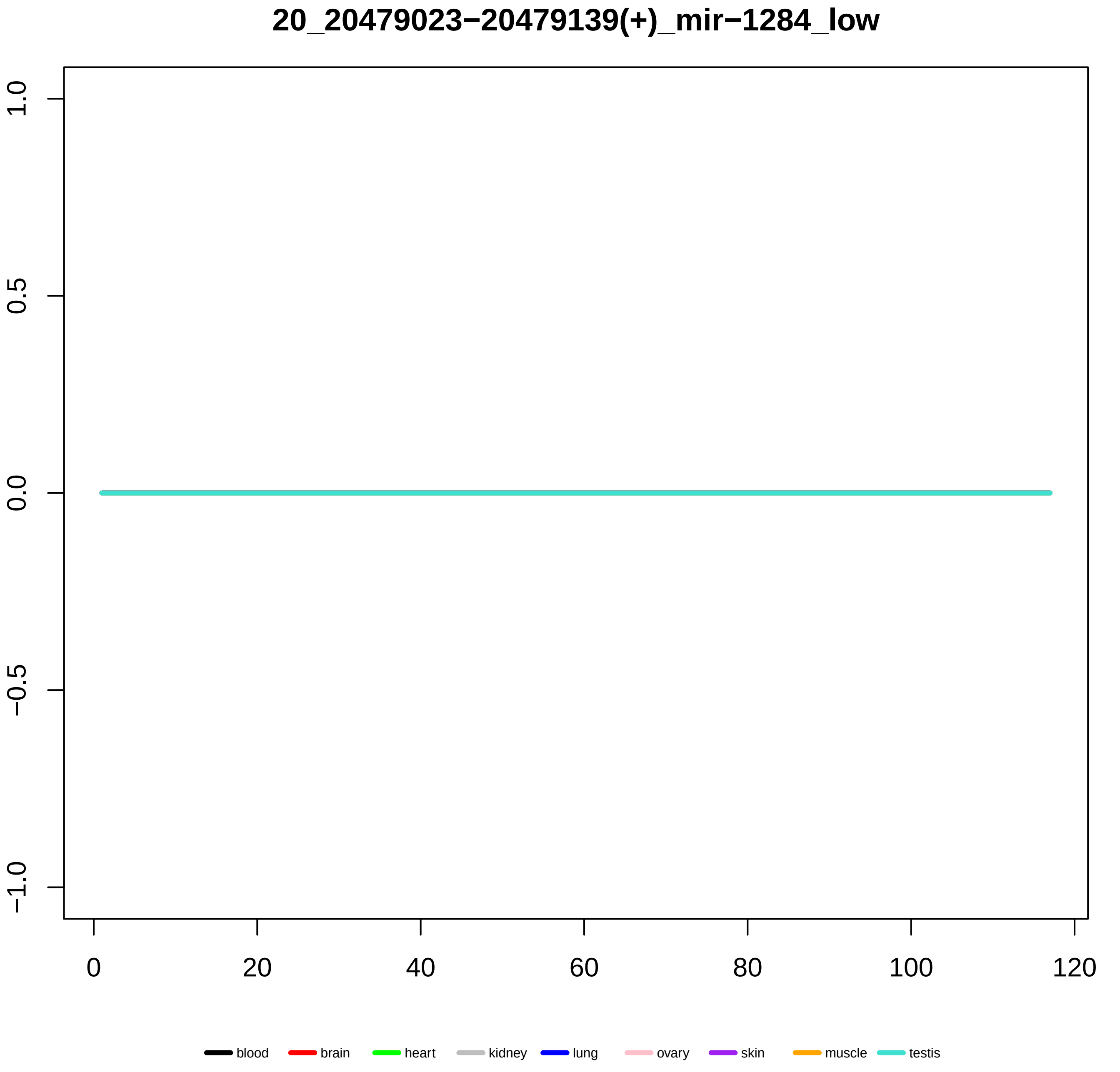

20_20479023−20479139(+)_mir−1284_low
1.0
0.5
0.0
−0.5
−1.0
0
20
40
60
80
100
120
blood
brain
heart
kidney
lung
ovary
skin
muscle
testis

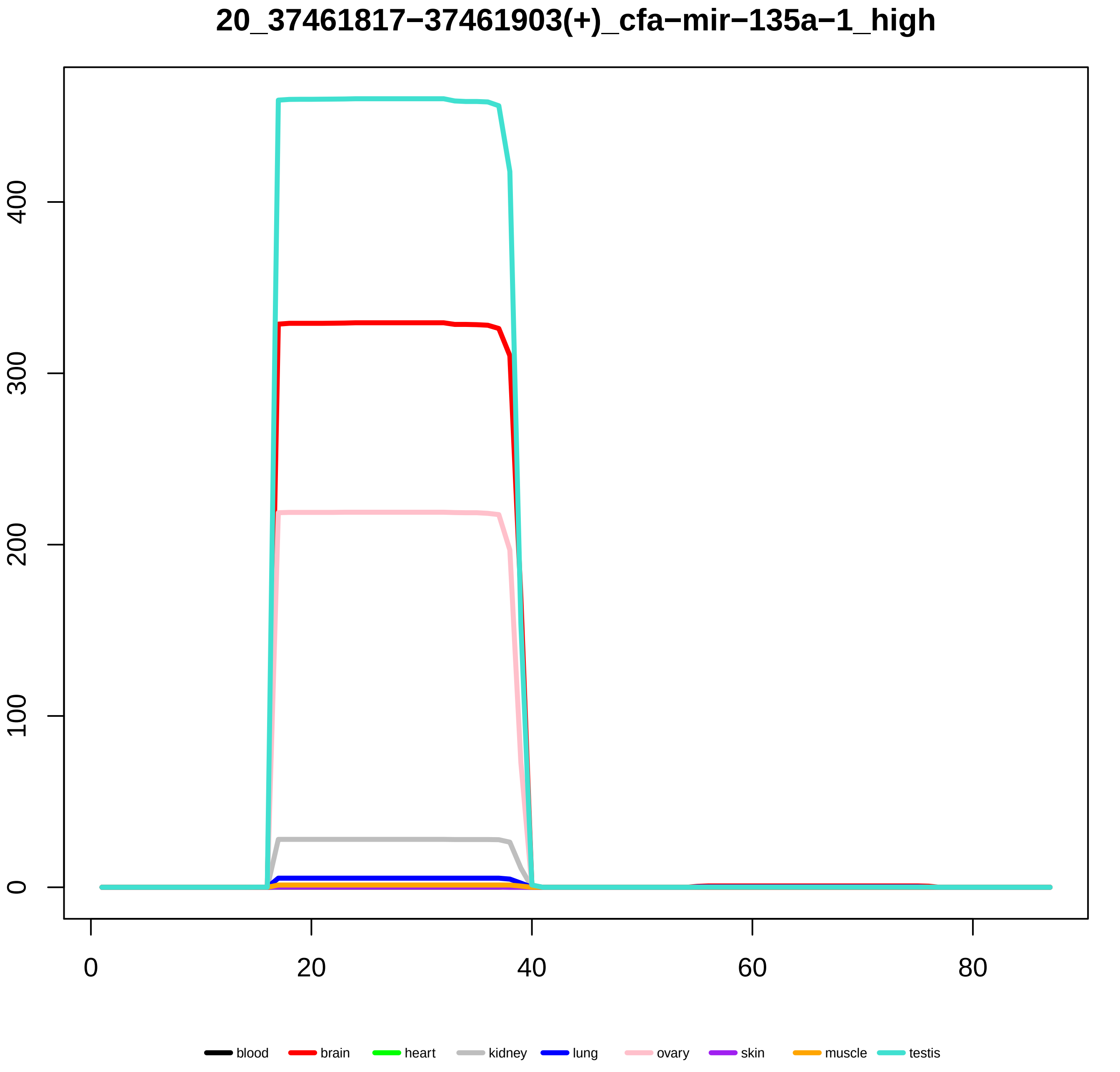

20_37461817−37461903(+)_cfa−mir−135a−1_high
0
100
200
300
400
0
20
40
60
80
blood
brain
heart
kidney
lung
ovary
skin
muscle
testis

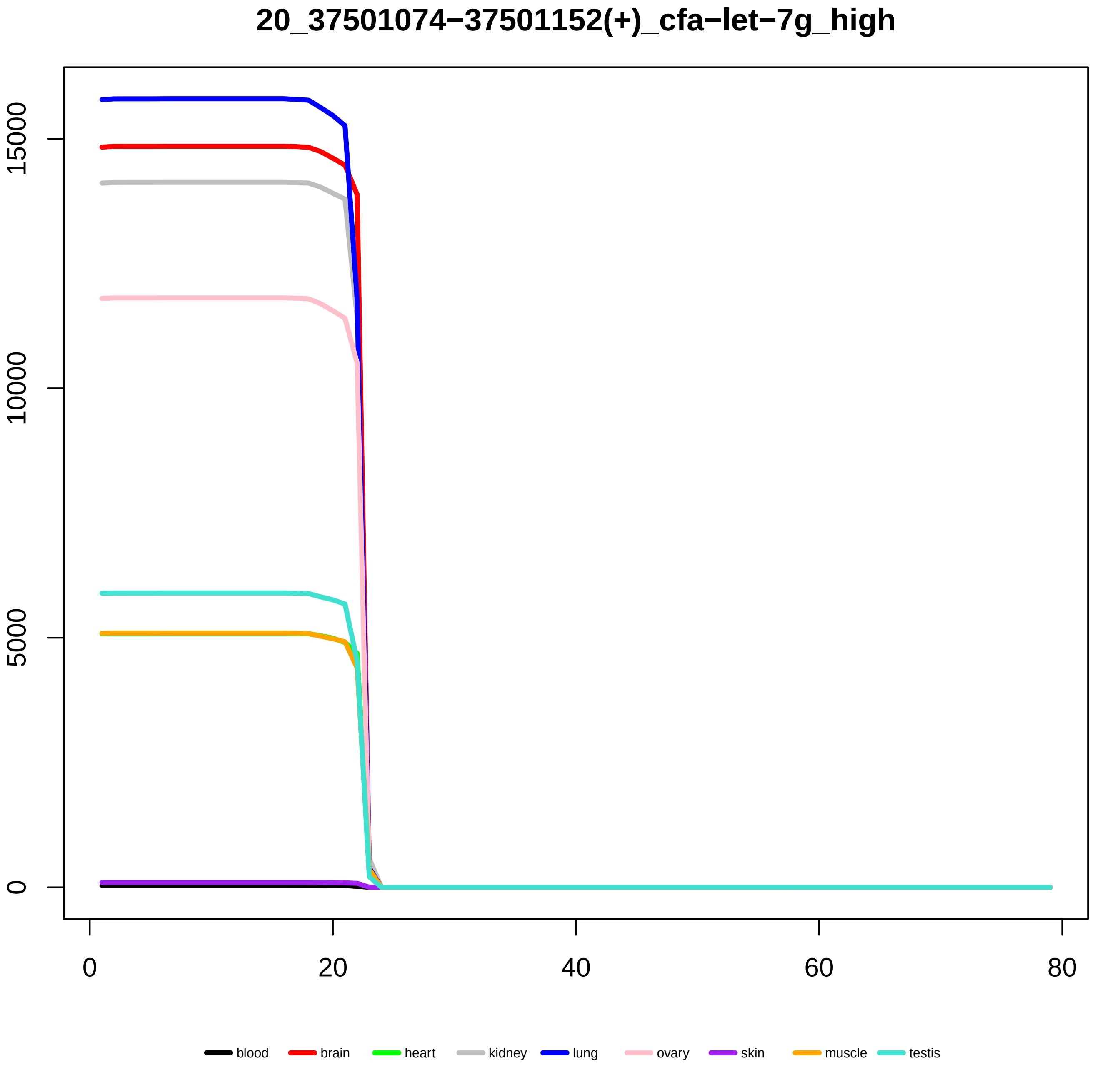

20_37501074−37501152(+)_cfa−let−7g_high
0
5000
10000
15000
0
20
40
60
80
blood
brain
heart
kidney
lung
ovary
skin
muscle
testis

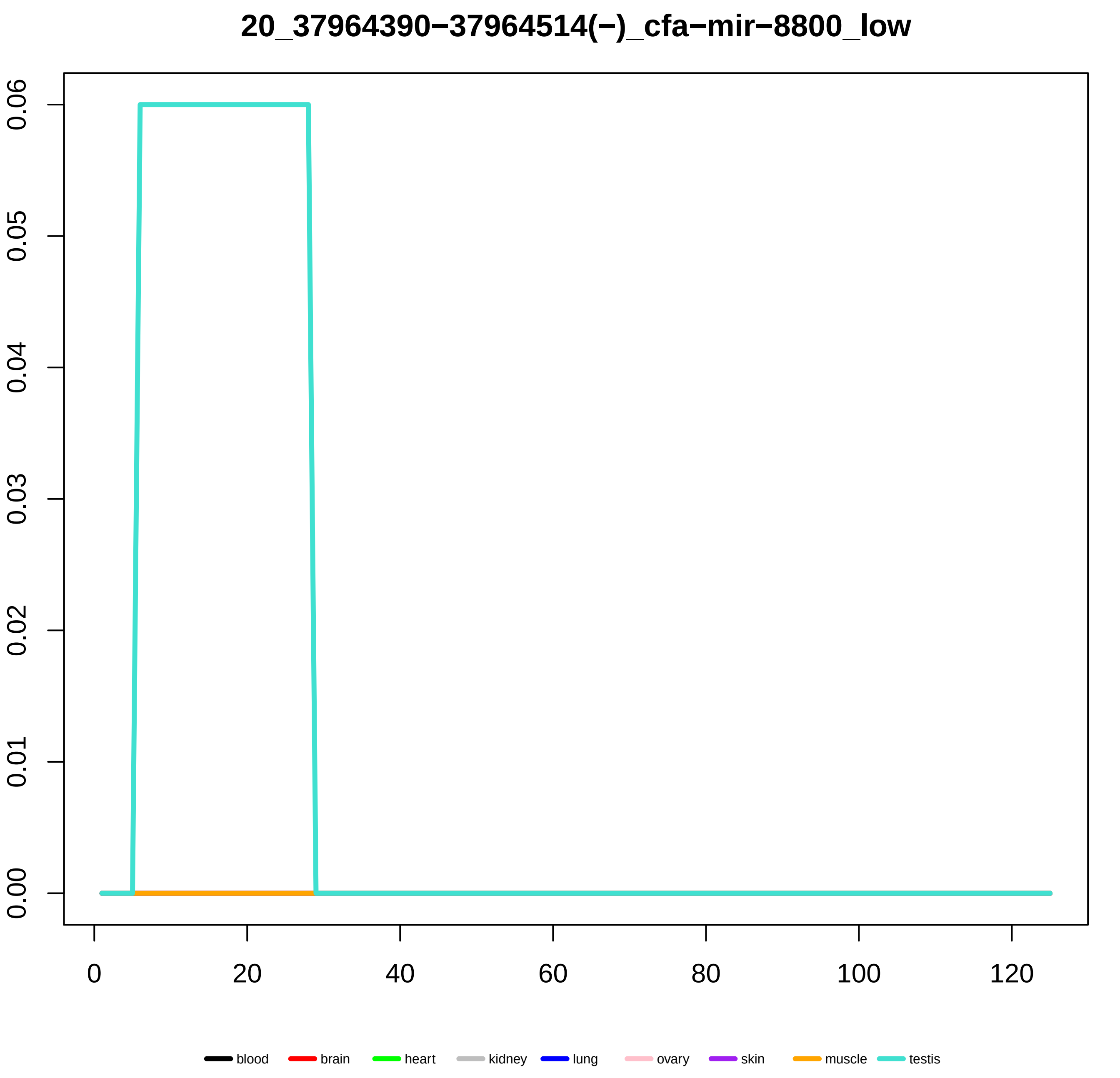
20_37964390−37964514(−)_cfa−mir−8800_low
0.00
0.01
0.02
0.03
0.04
0.05
0.06
0
20
40
60
80
100
120
blood
brain
heart
kidney
lung
ovary
skin
muscle
testis

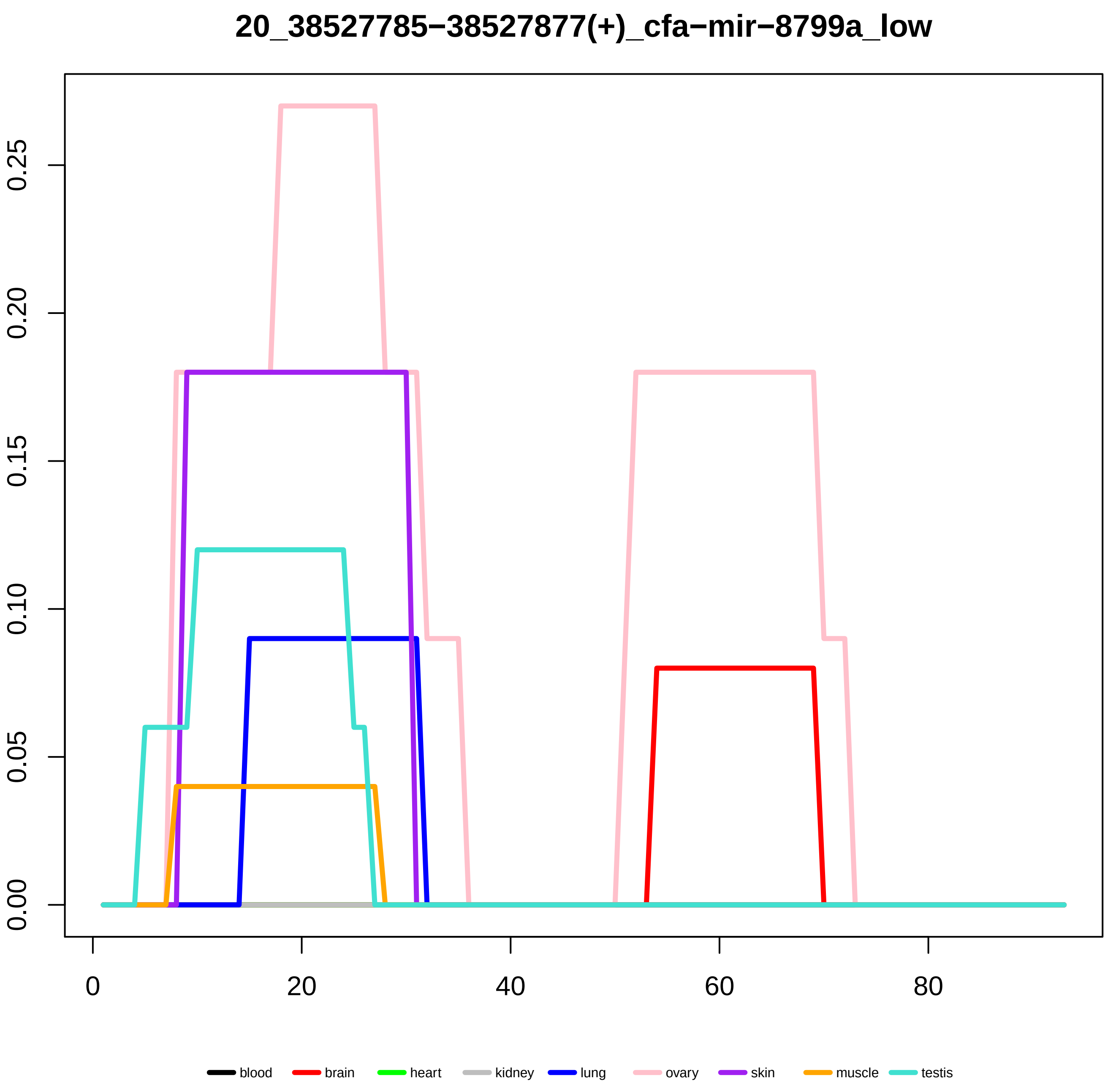
20_38527785−38527877(+)_cfa−mir−8799a_low
0.00
0.05
0.10
0.15
0.20
0.25
0
20
40
60
80
blood
brain
heart
kidney
lung
ovary
skin
muscle
testis

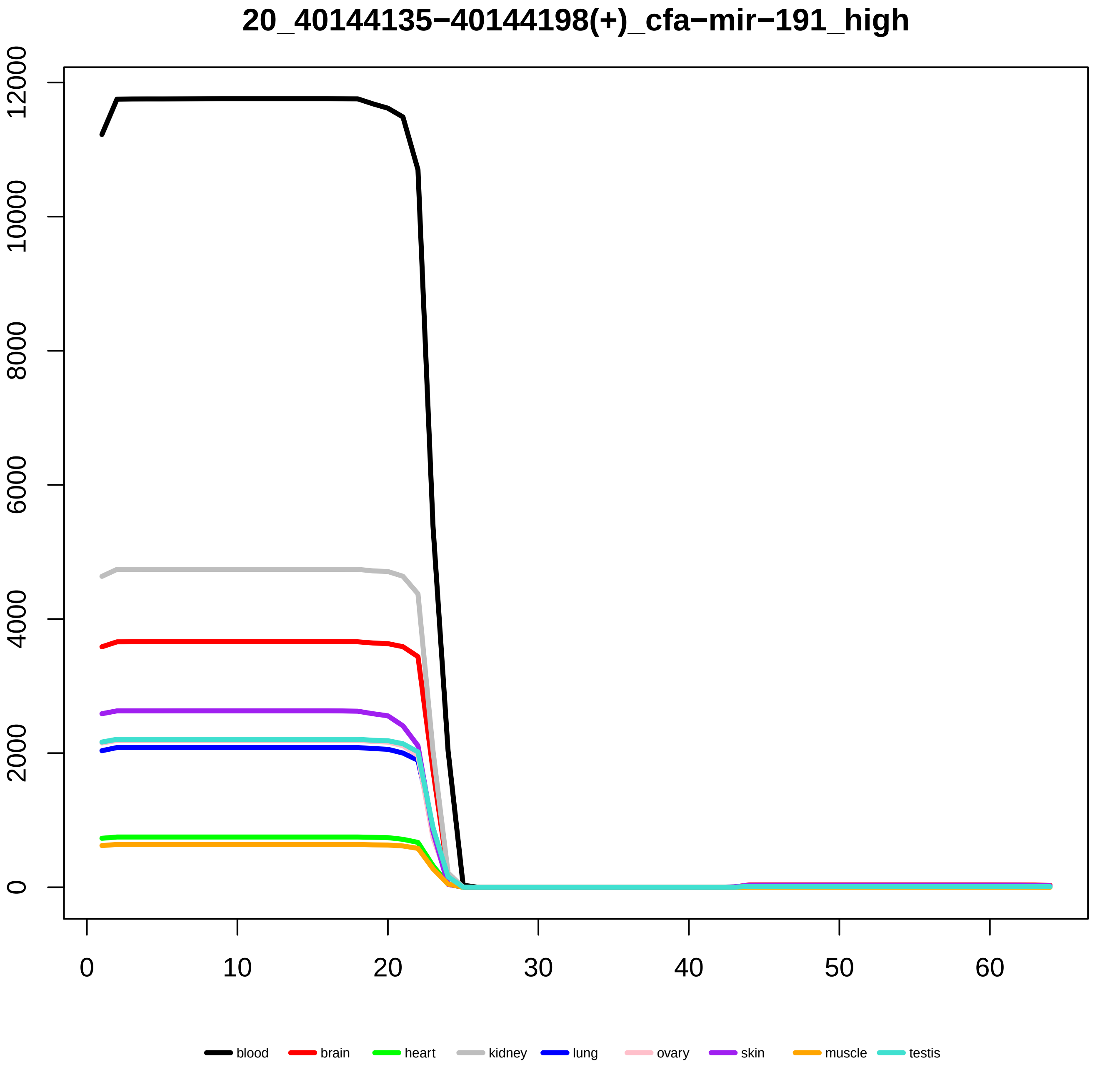

20_40144135−40144198(+)_cfa−mir−191_high
0
2000
4000
6000
8000
10000
12000
0
10
20
30
40
50
60
blood
brain
heart
kidney
lung
ovary
skin
muscle
testis

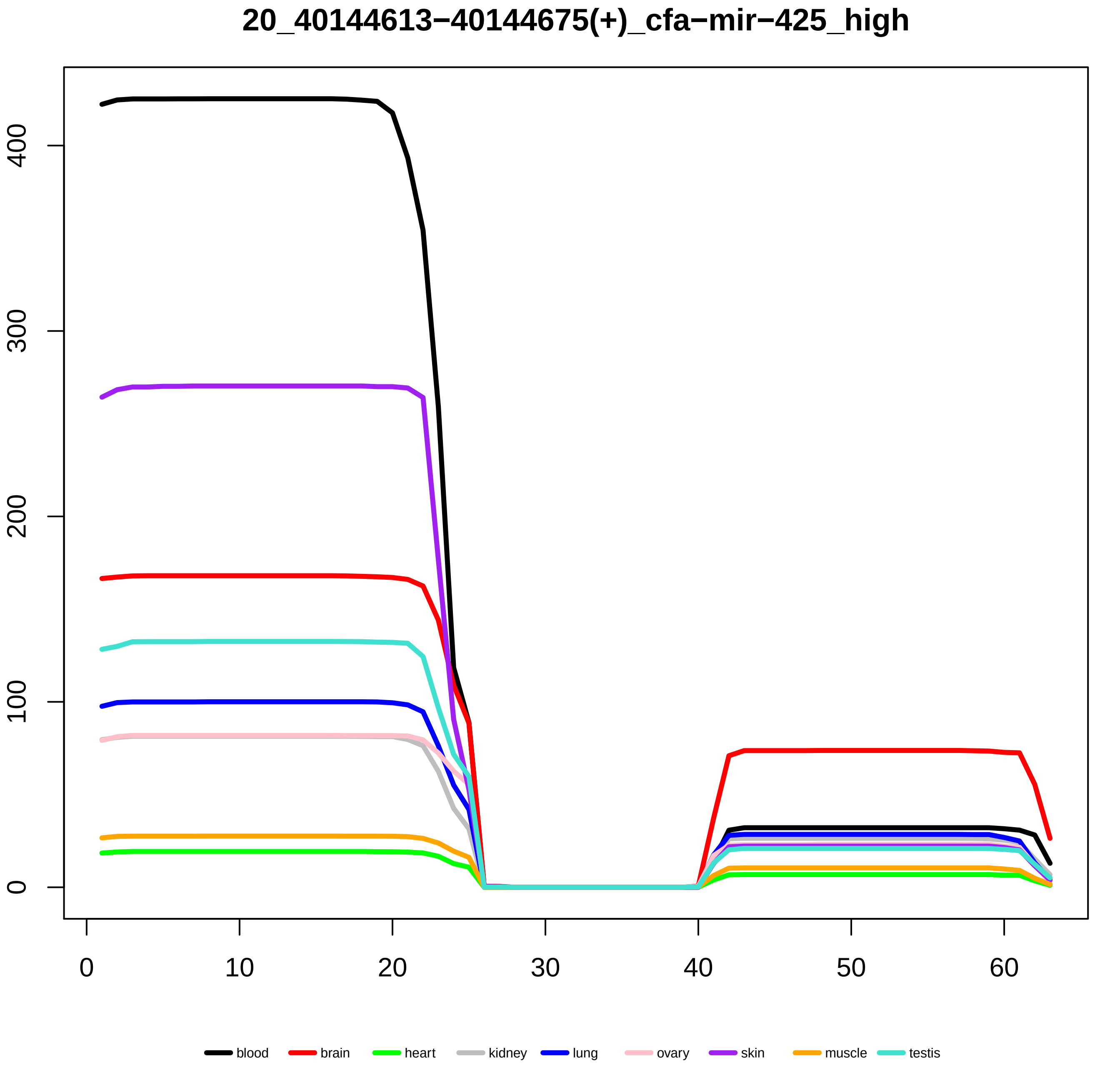
20_40144613−40144675(+)_cfa−mir−425_high
0
100
200
300
400
0
10
20
30
40
50
60
blood
brain
heart
kidney
lung
ovary
skin
muscle
testis

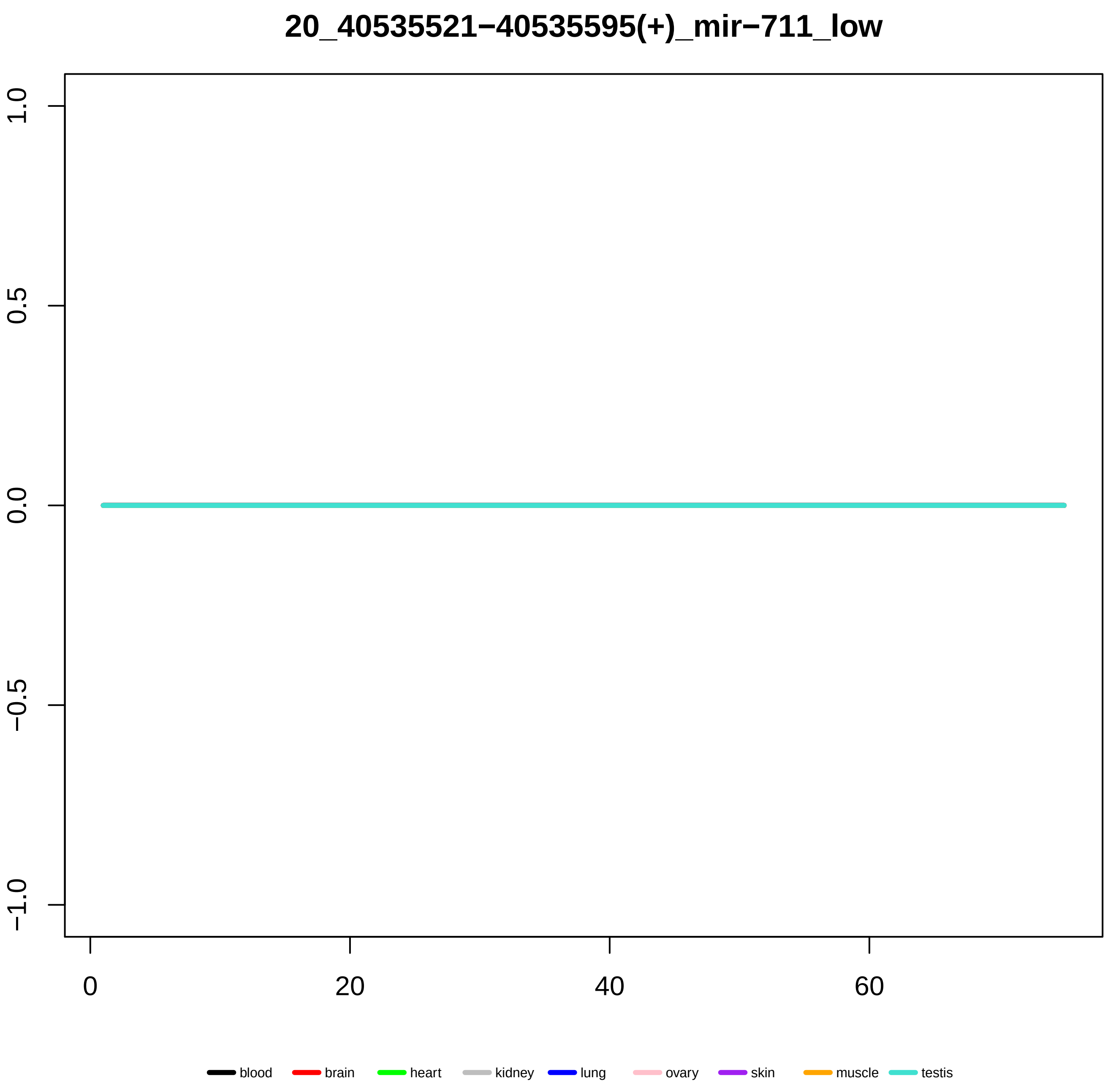

20_40535521−40535595(+)_mir−711_low
1.0
0.5
0.0
−0.5
−1.0
0
20
40
60
blood
brain
heart
kidney
lung
ovary
skin
muscle
testis

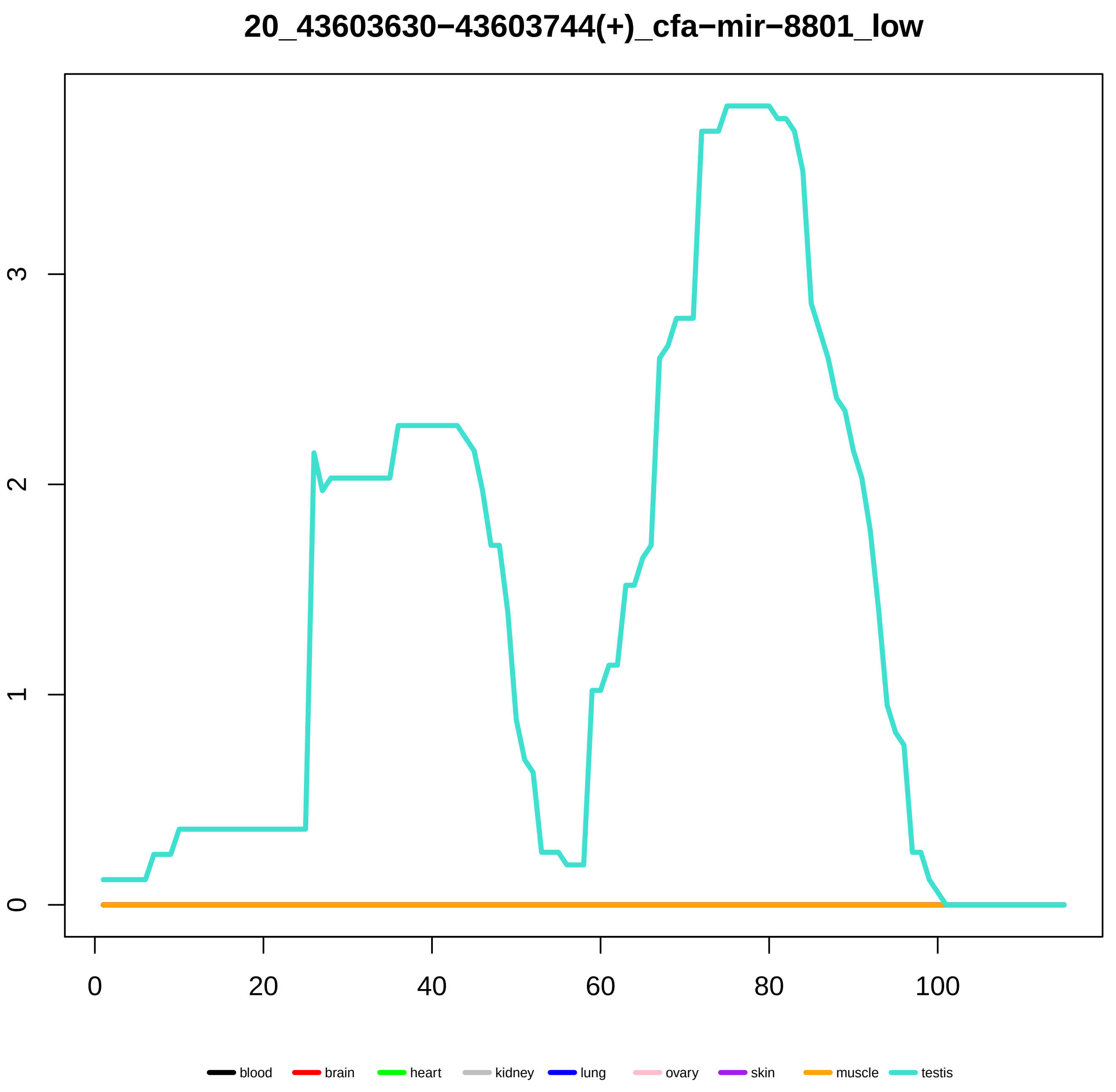
20_43603630−43603744(+)_cfa−mir−8801_low
0
1
2
3
0
20
40
60
80
100
blood
brain
heart
kidney
lung
ovary
skin
muscle
testis

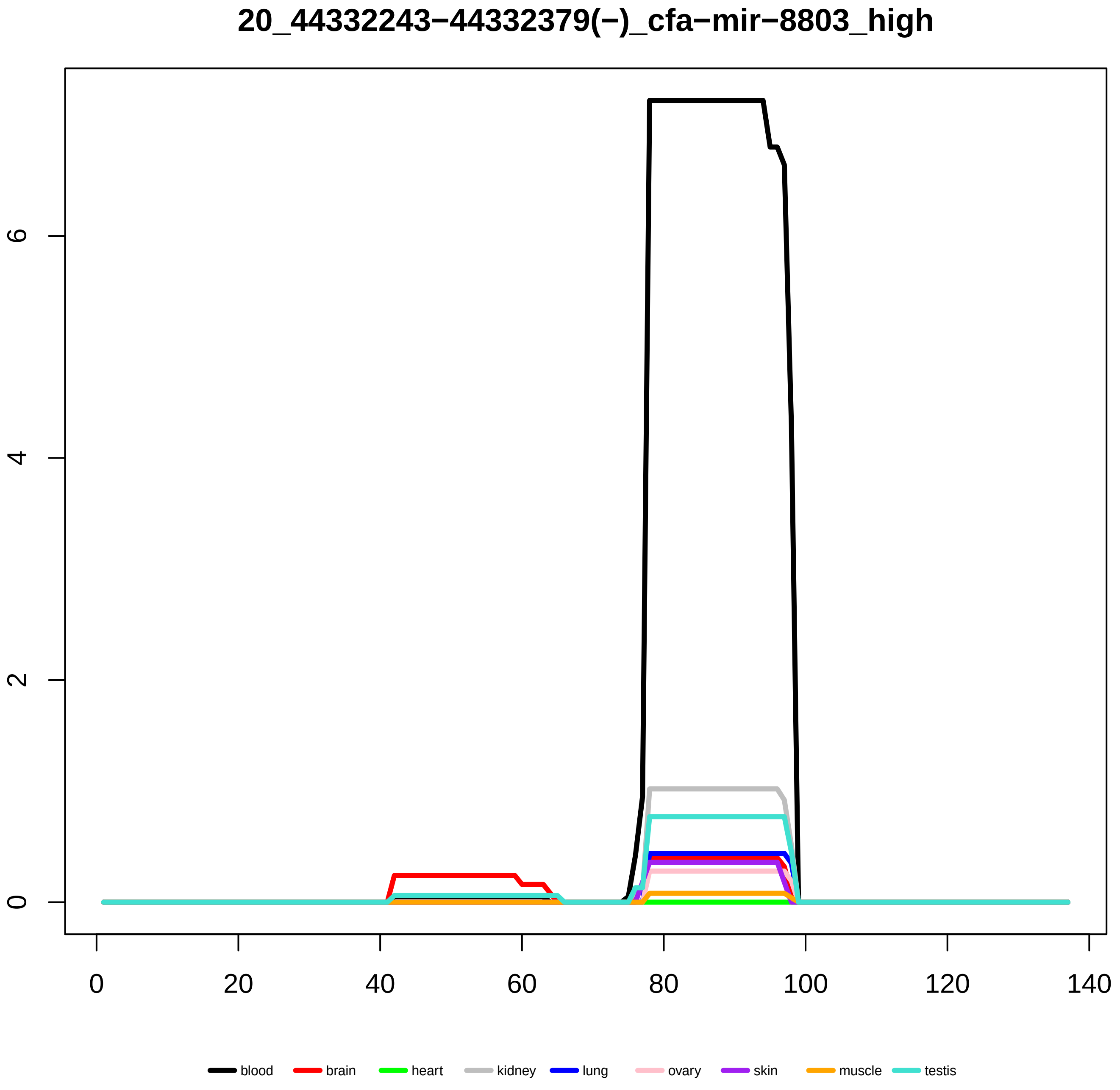

20_44332243−44332379(−)_cfa−mir−8803_high
0
2
4
6
0
20
40
60
80
100
120
140
blood
brain
heart
kidney
lung
ovary
skin
muscle
testis

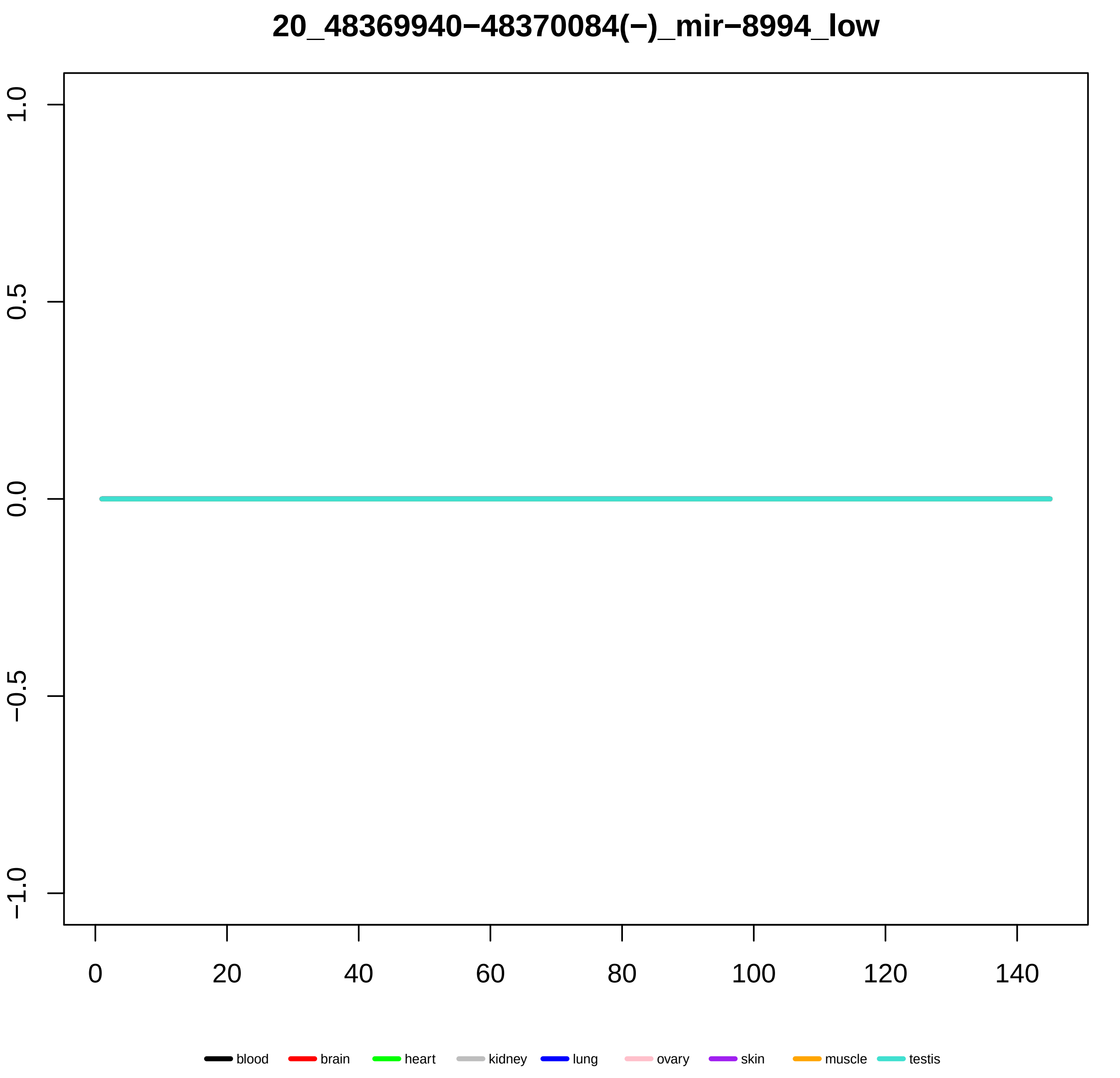
20_48369940−48370084(−)_mir−8994_low
1.0
0.5
0.0
−0.5
−1.0
0
20
40
60
80
100
120
140
blood
brain
heart
kidney
lung
ovary
skin
muscle
testis

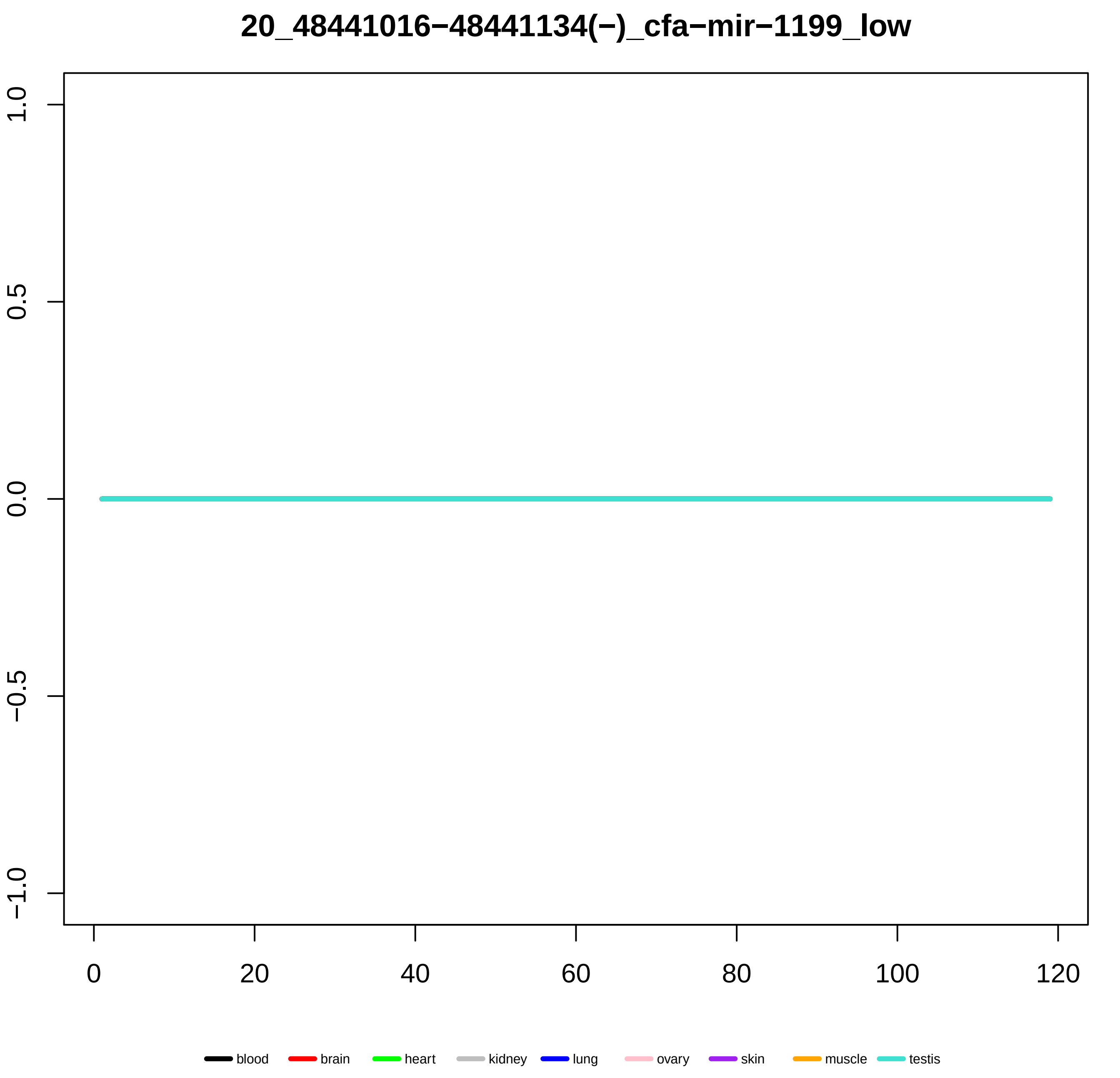
20_48441016−48441134(−)_cfa−mir−1199_low
1.0
0.5
0.0
−0.5
−1.0
0
20
40
60
80
100
120
blood
brain
heart
kidney
lung
ovary
skin
muscle
testis

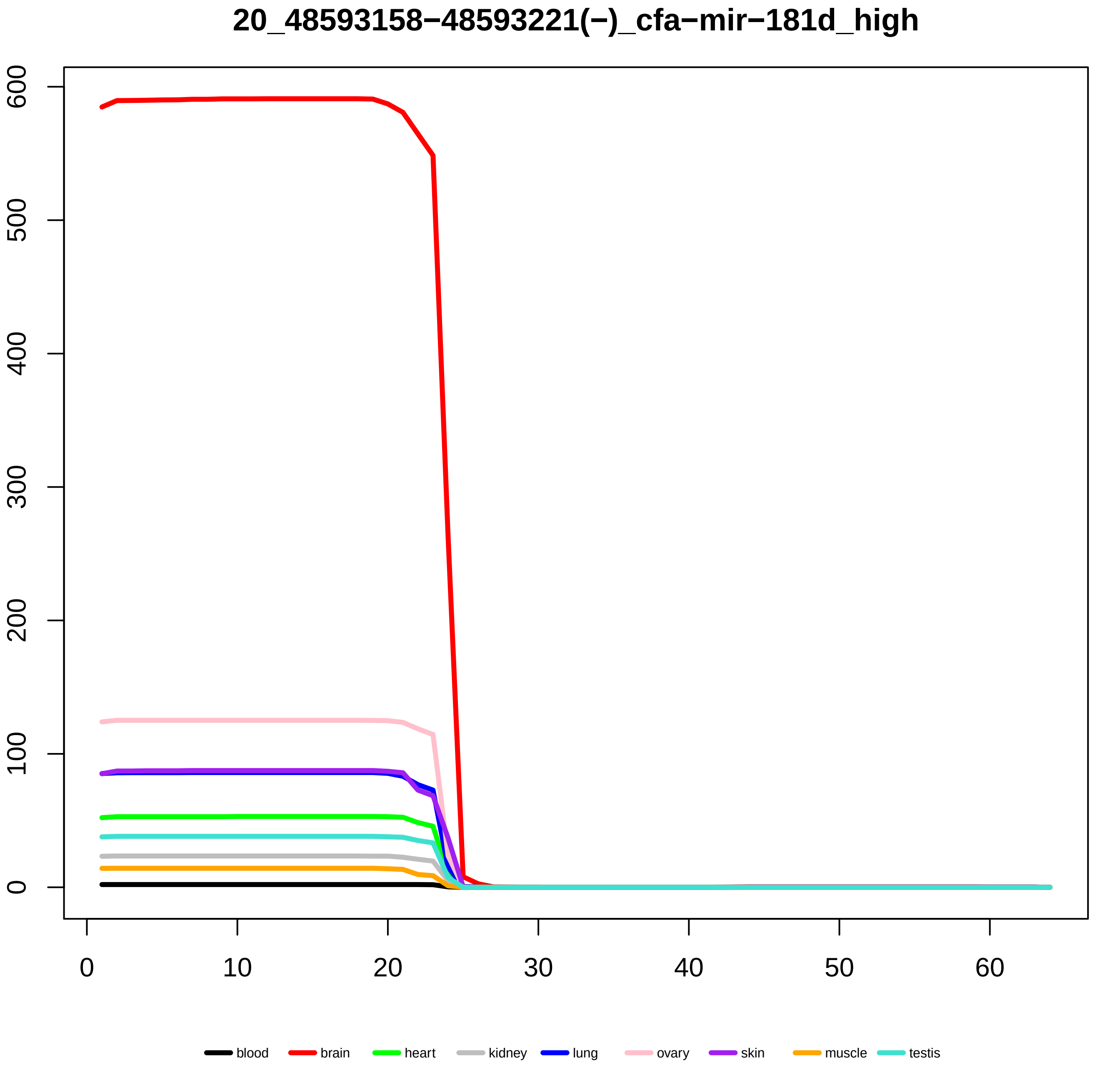
20_48593158−48593221(−)_cfa−mir−181d_high
0
100
200
300
400
500
600
0
10
20
30
40
50
60
blood
brain
heart
kidney
lung
ovary
skin
muscle
testis

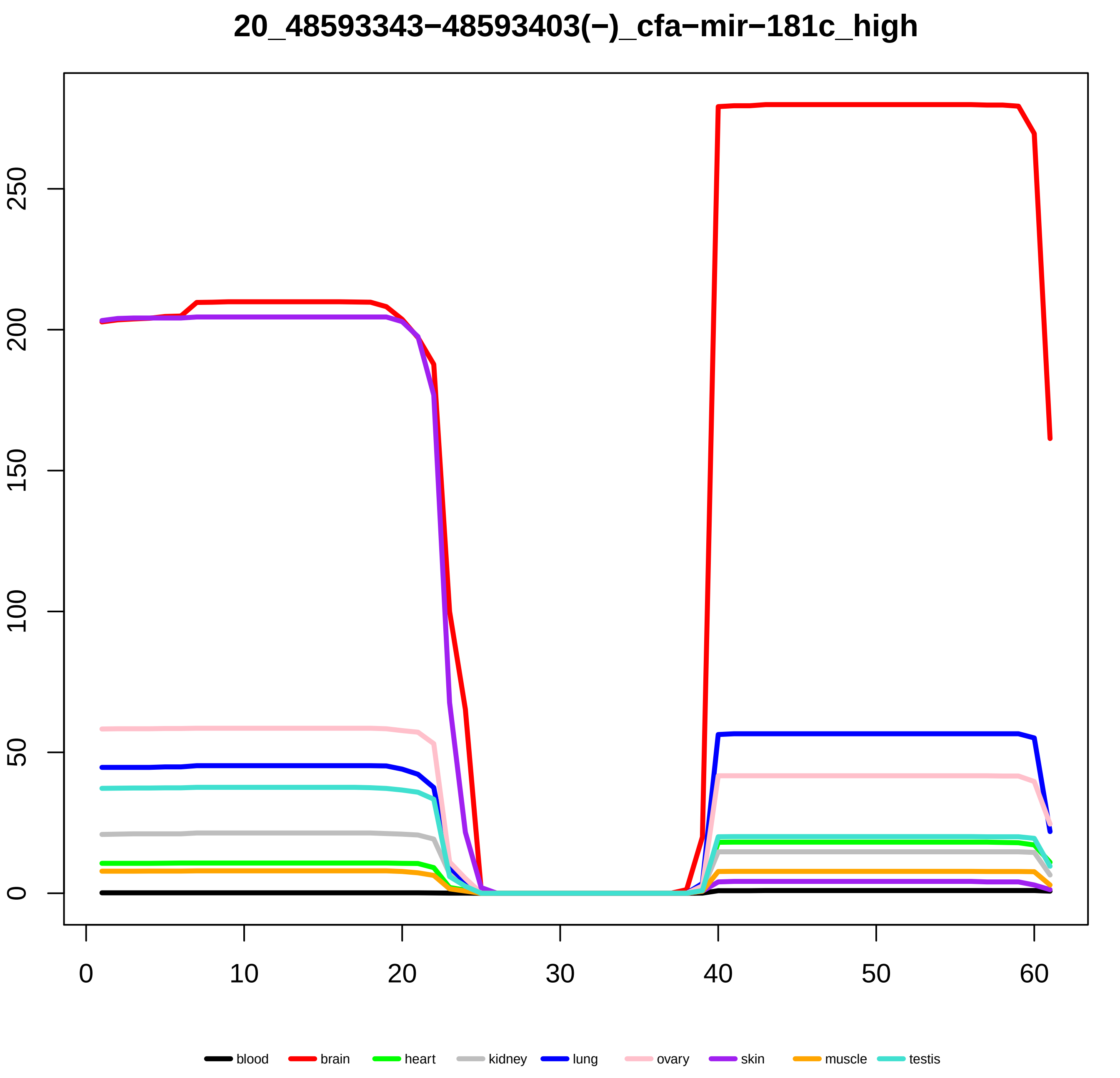
20_48593343−48593403(−)_cfa−mir−181c_high
0
50
100
150
200
250
0
10
20
30
40
50
60
blood
brain
heart
kidney
lung
ovary
skin
muscle
testis

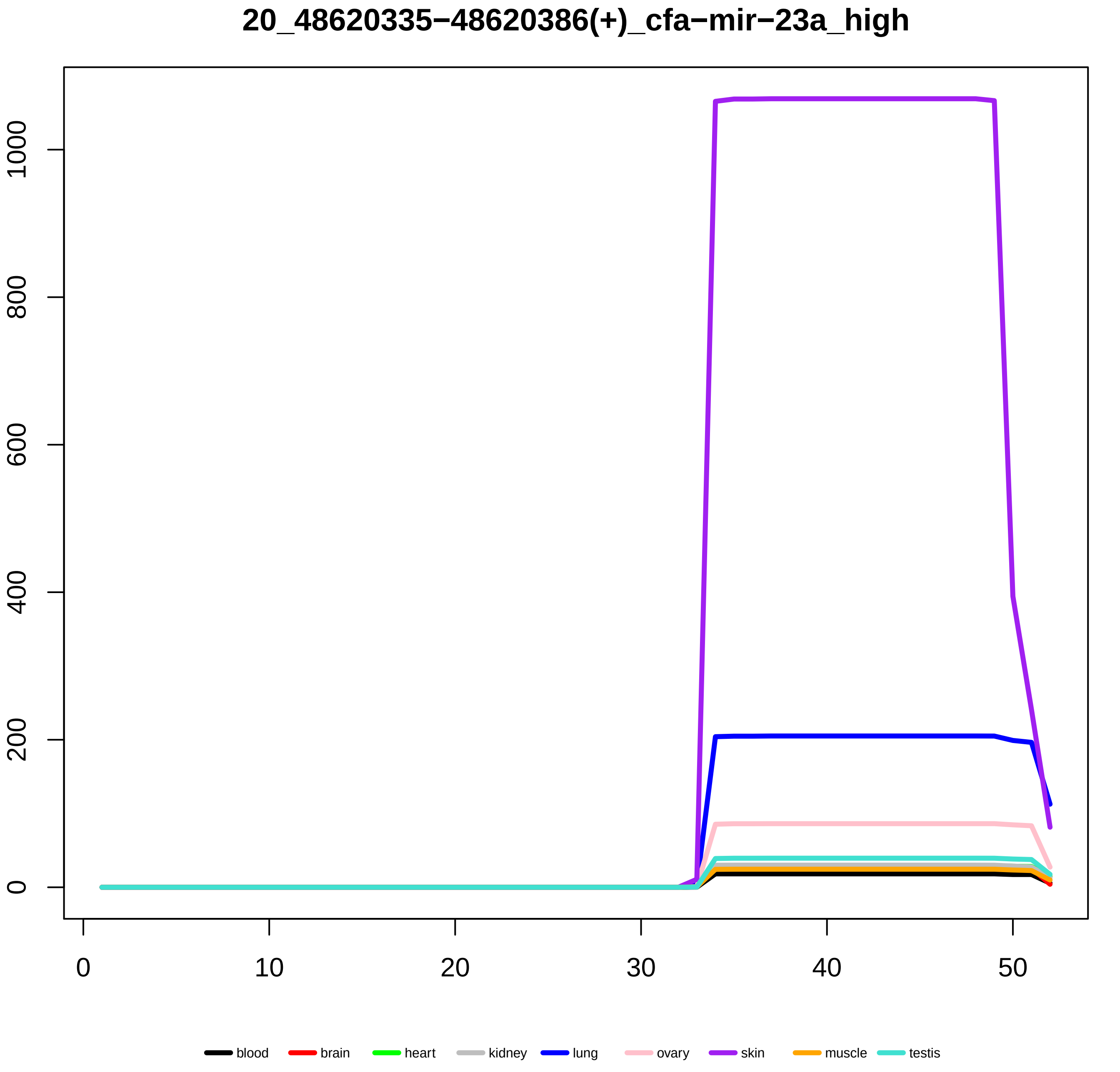

20_48620335−48620386(+)_cfa−mir−23a_high
0
200
400
600
800
1000
0
10
20
30
40
50
blood
brain
heart
kidney
lung
ovary
skin
muscle
testis

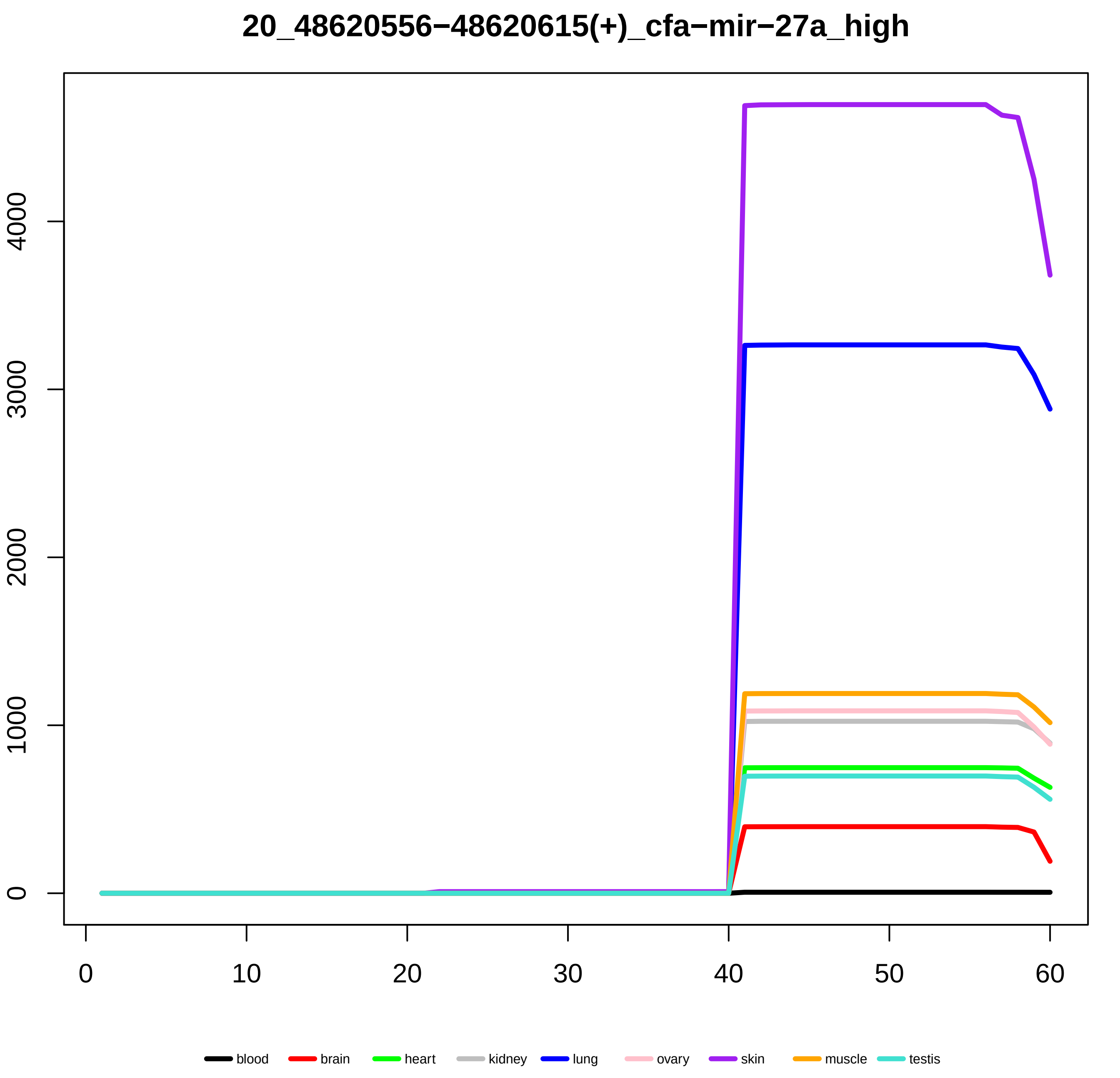

20_48620556−48620615(+)_cfa−mir−27a_high
0
1000
2000
3000
4000
0
10
20
30
40
50
60
blood
brain
heart
kidney
lung
ovary
skin
muscle
testis

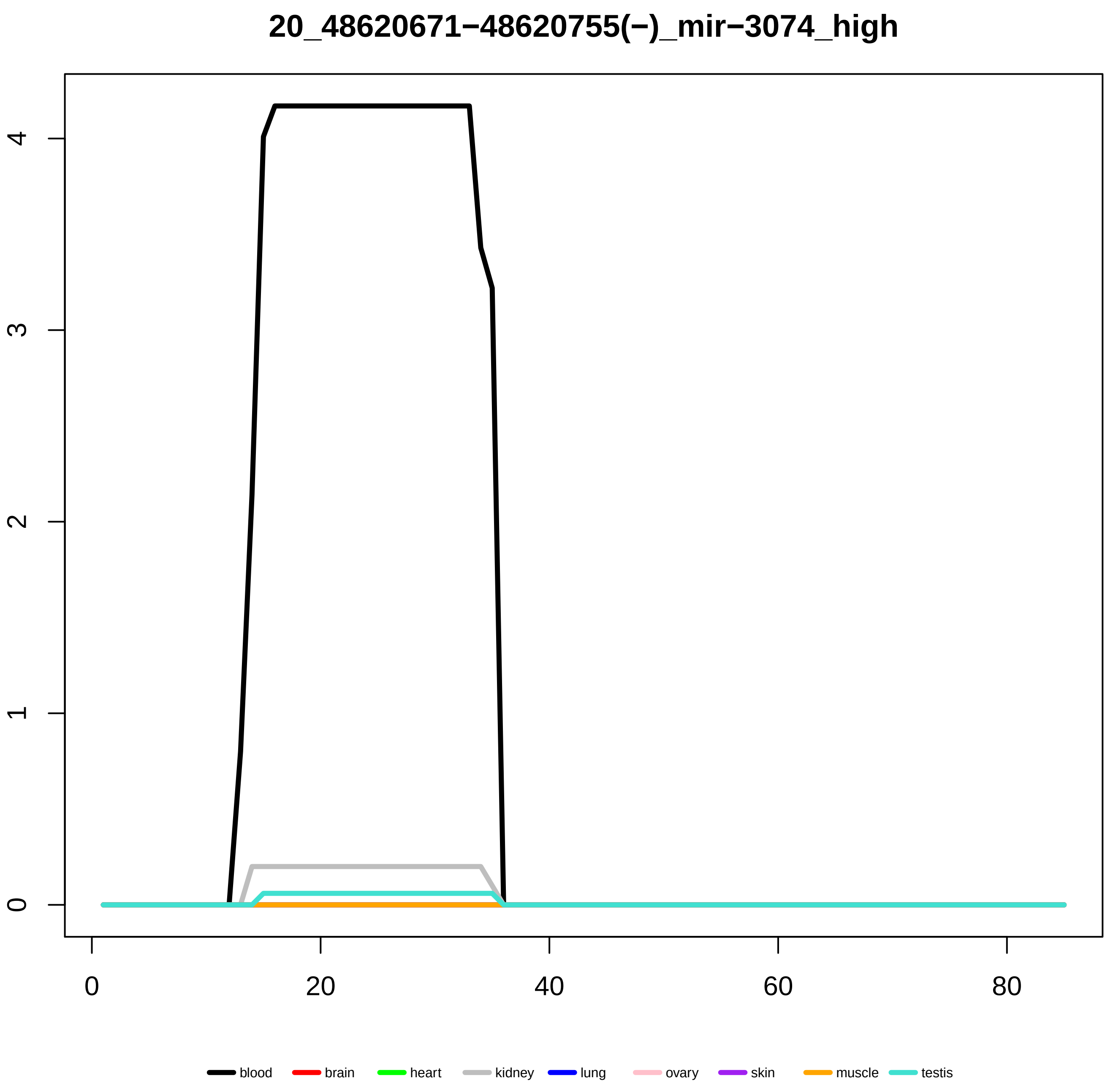

20_48620671−48620755(−)_mir−3074_high
0
1
2
3
4
0
20
40
60
80
blood
brain
heart
kidney
lung
ovary
skin
muscle
testis

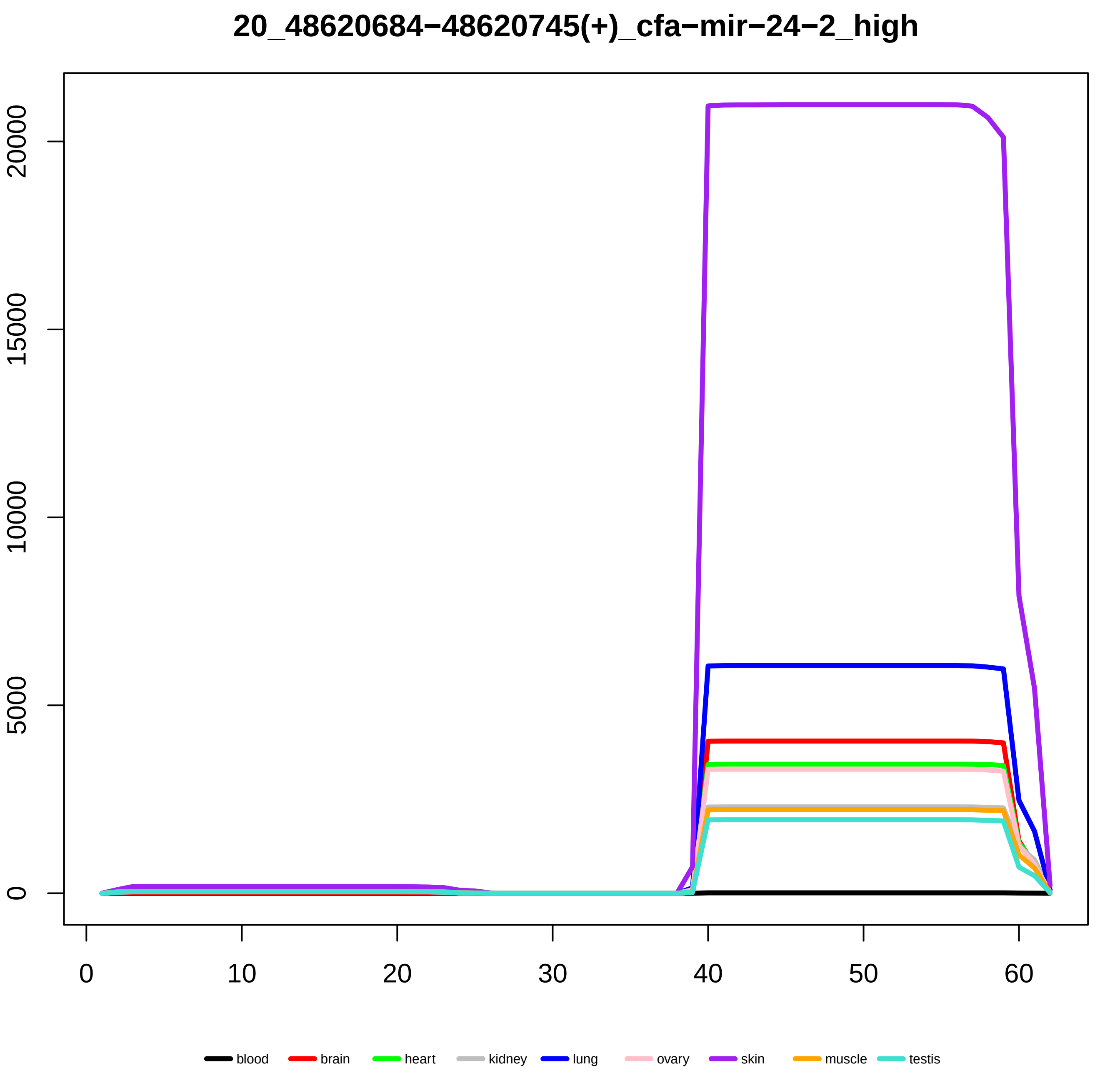
20_48620684−48620745(+)_cfa−mir−24−2_high
0
5000
10000
15000
20000
0
10
20
30
40
50
60
blood
brain
heart
kidney
lung
ovary
skin
muscle
testis

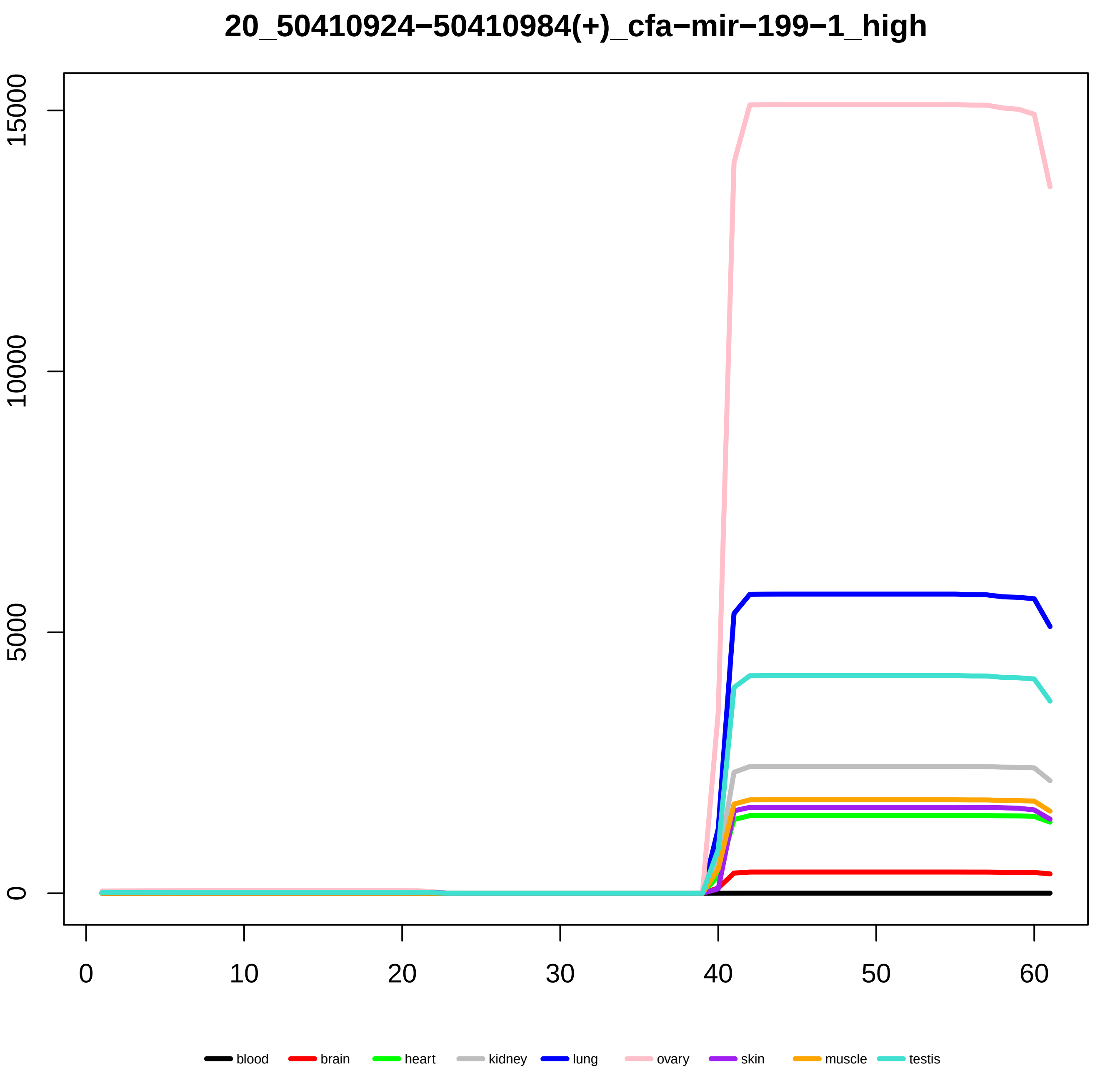
20_50410924−50410984(+)_cfa−mir−199−1_high
0
5000
10000
15000
0
10
20
30
40
50
60
blood
brain
heart
kidney
lung
ovary
skin
muscle
testis

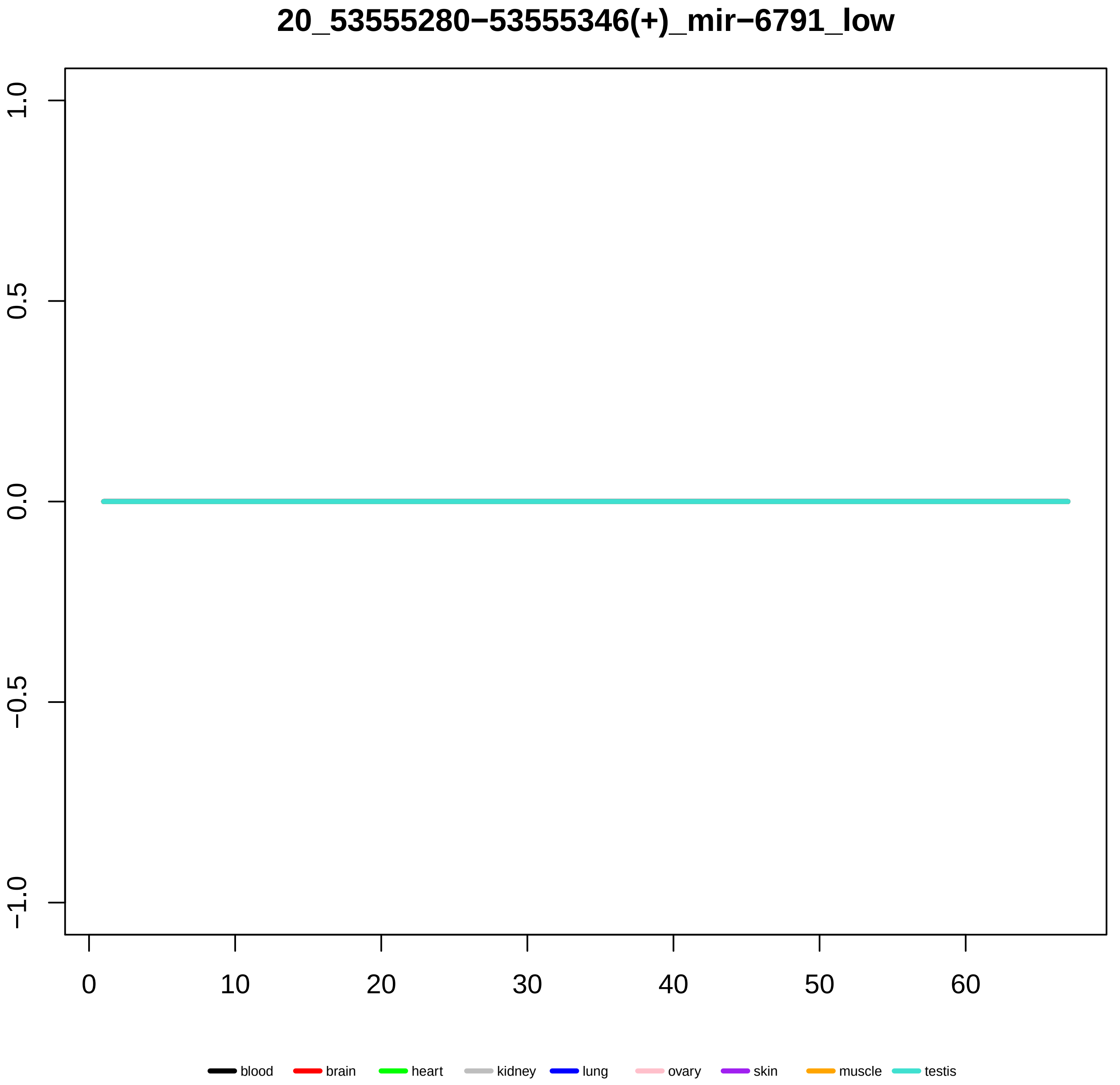

20_53555280−53555346(+)_mir−6791_low
1.0
0.5
0.0
−0.5
−1.0
0
10
20
30
40
50
60
blood
brain
heart
kidney
lung
ovary
skin
muscle
testis

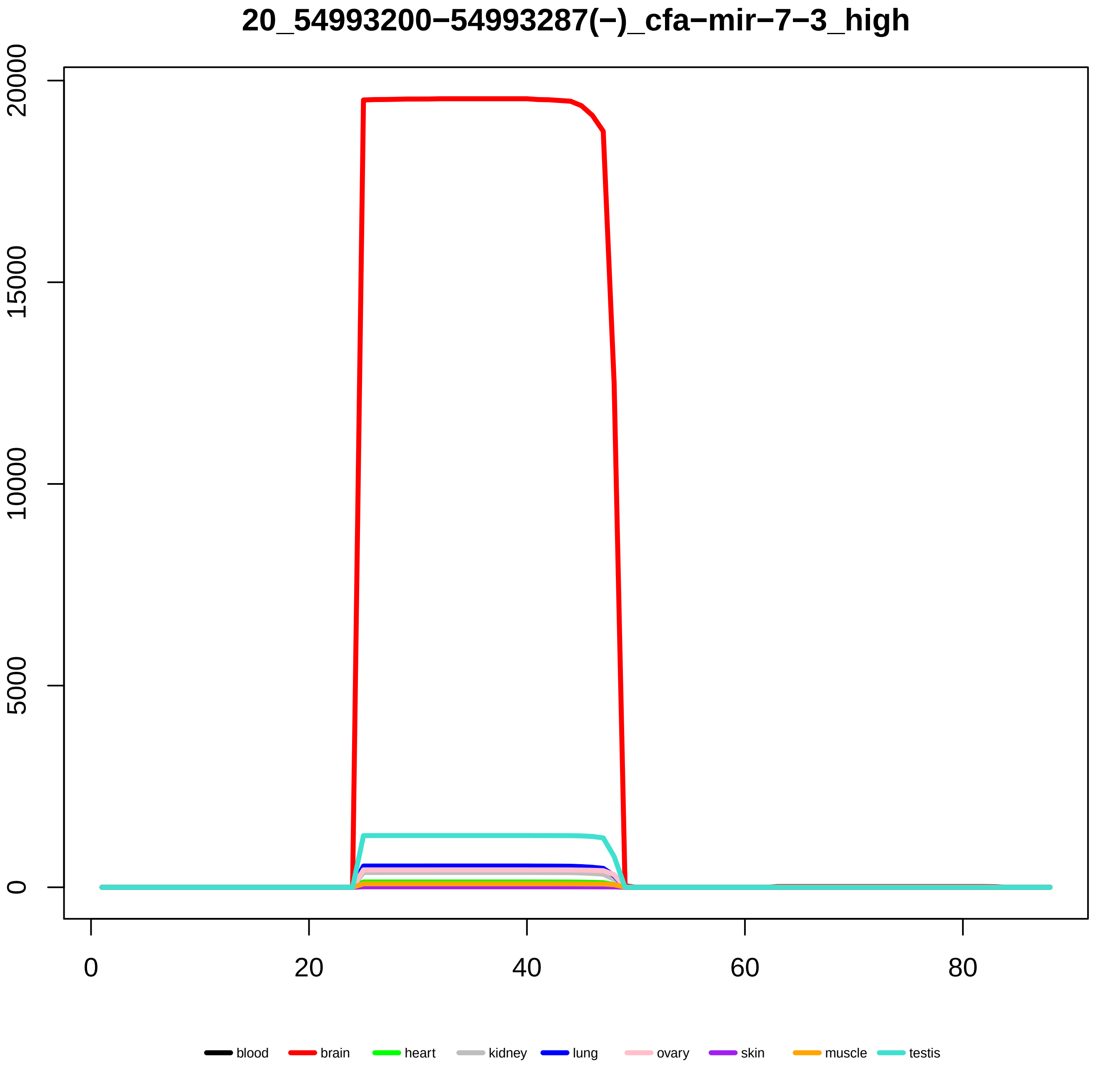

20_54993200−54993287(−)_cfa−mir−7−3_high
0
5000
10000
15000
20000
0
20
40
60
80
blood
brain
heart
kidney
lung
ovary
skin
muscle
testis

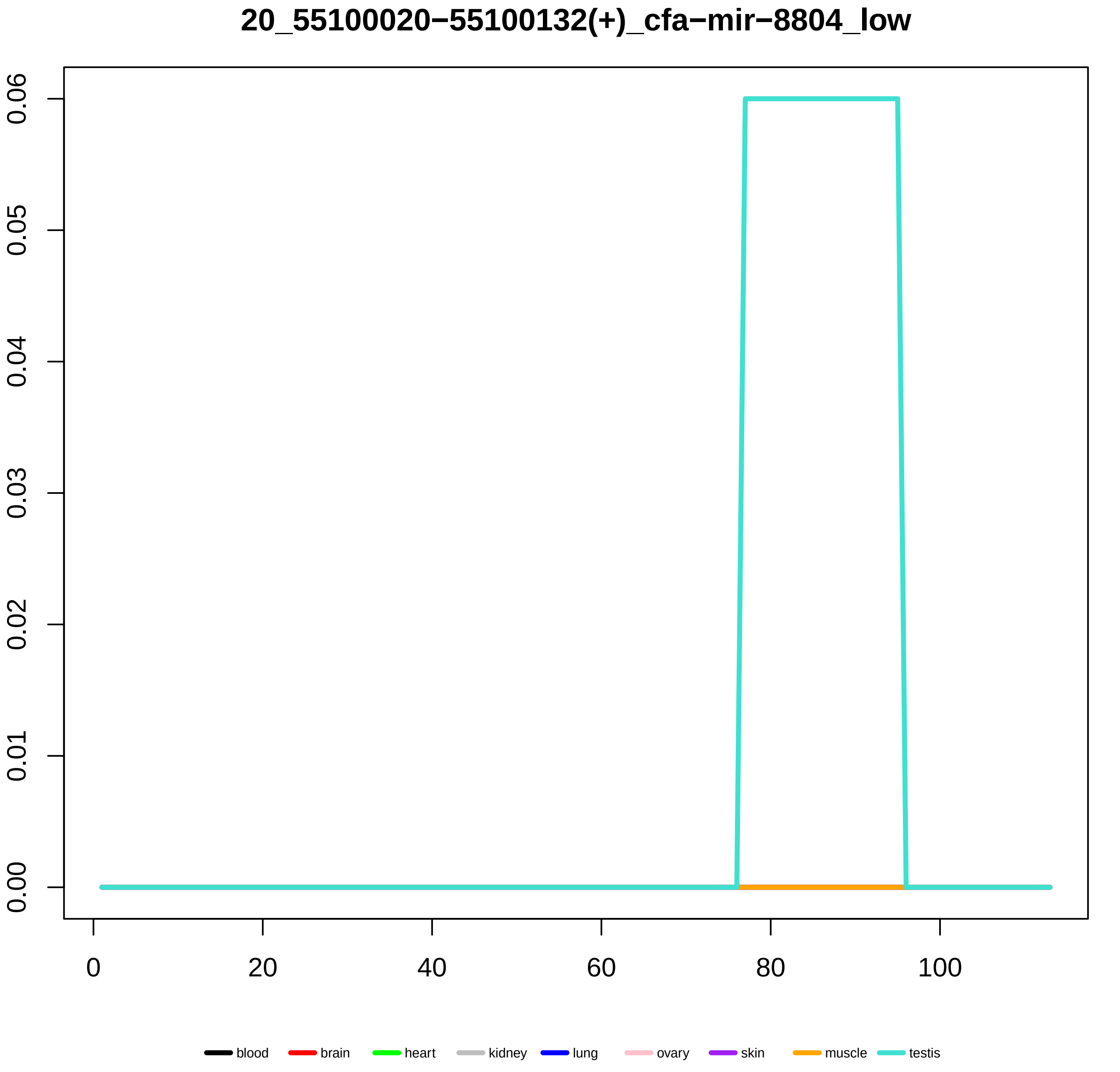

20_55100020−55100132(+)_cfa−mir−8804_low
0.00
0.01
0.02
0.03
0.04
0.05
0.06
0
20
40
60
80
100
blood
brain
heart
kidney
lung
ovary
skin
muscle
testis

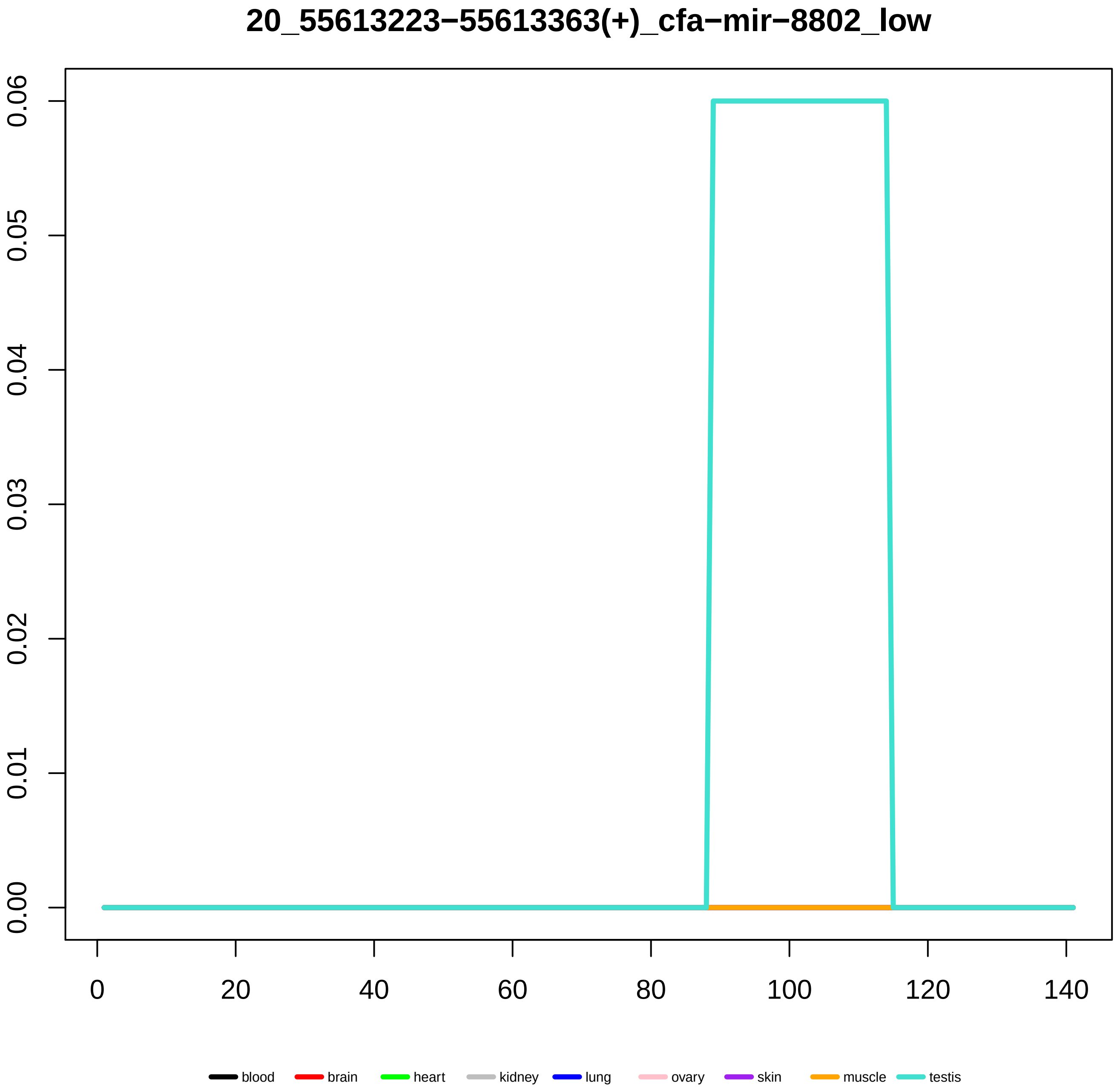
20_55613223−55613363(+)_cfa−mir−8802_low
0.00
0.01
0.02
0.03
0.04
0.05
0.06
0
20
40
60
80
100
120
140
blood
brain
heart
kidney
lung
ovary
skin
muscle
testis

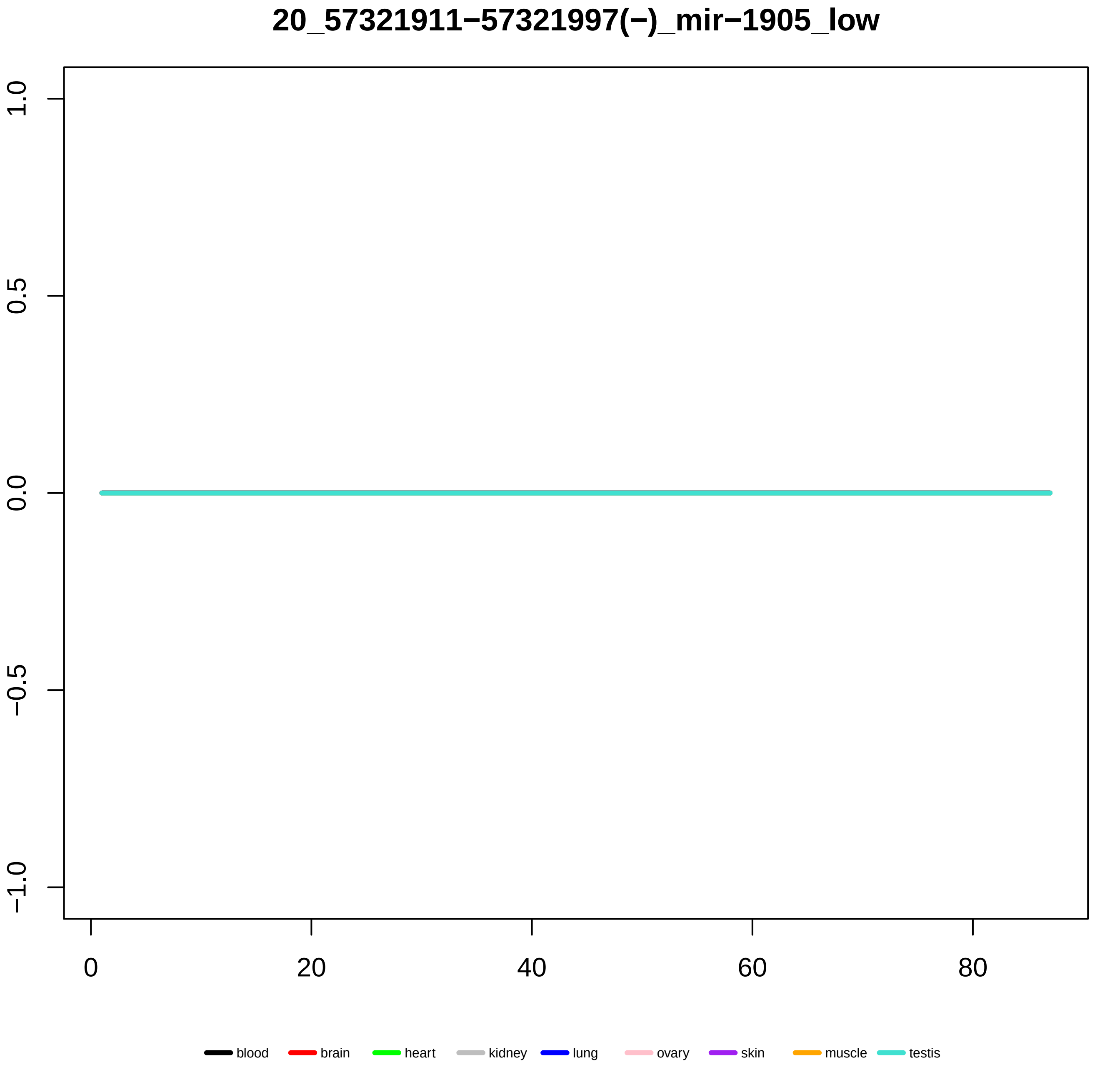
20_57321911−57321997(−)_mir−1905_low
1.0
0.5
0.0
−0.5
−1.0
0
20
40
60
80
blood
brain
heart
kidney
lung
ovary
skin
muscle
testis

20_57346963−57347107(−)_cfa−mir−8805_low

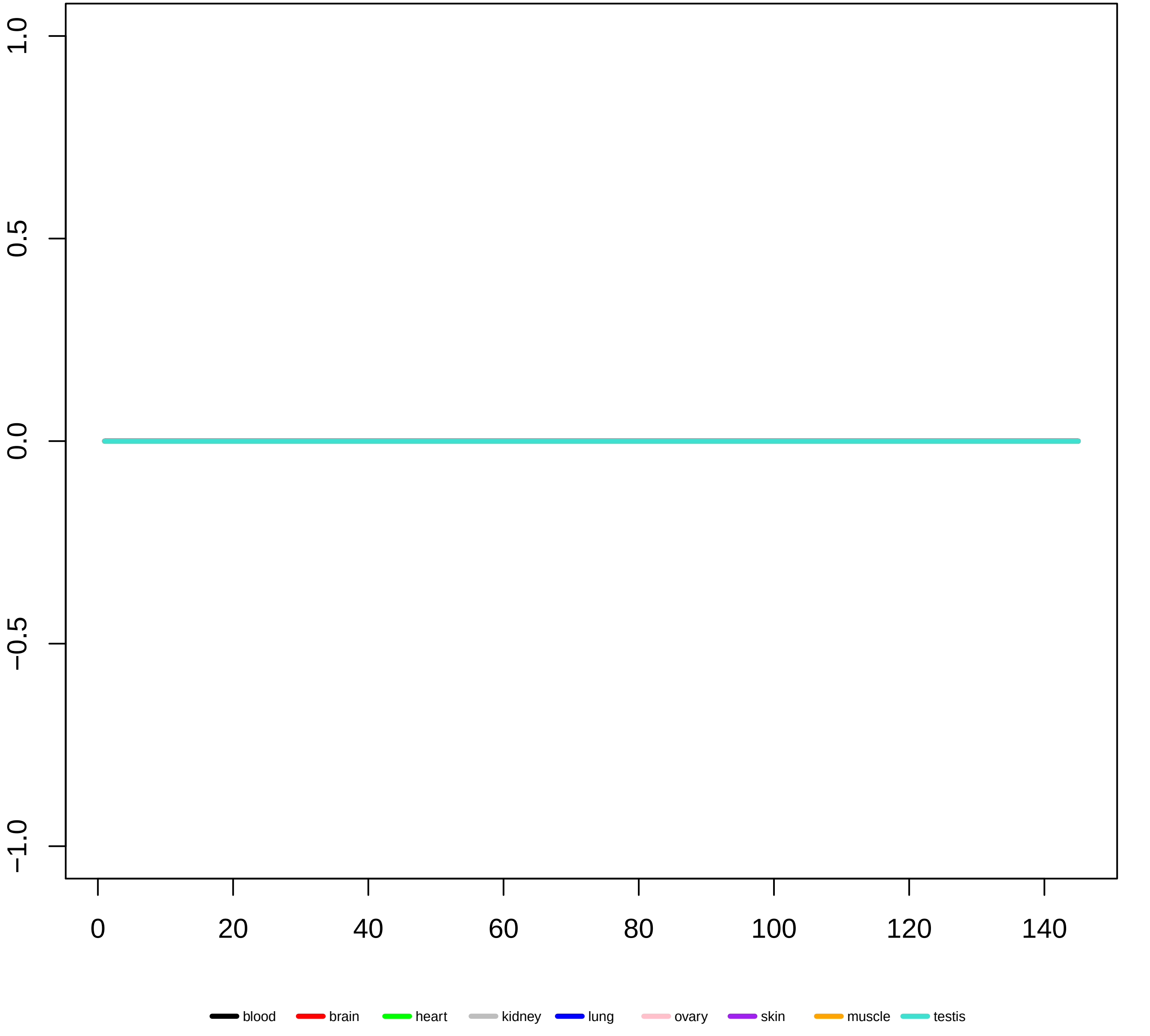

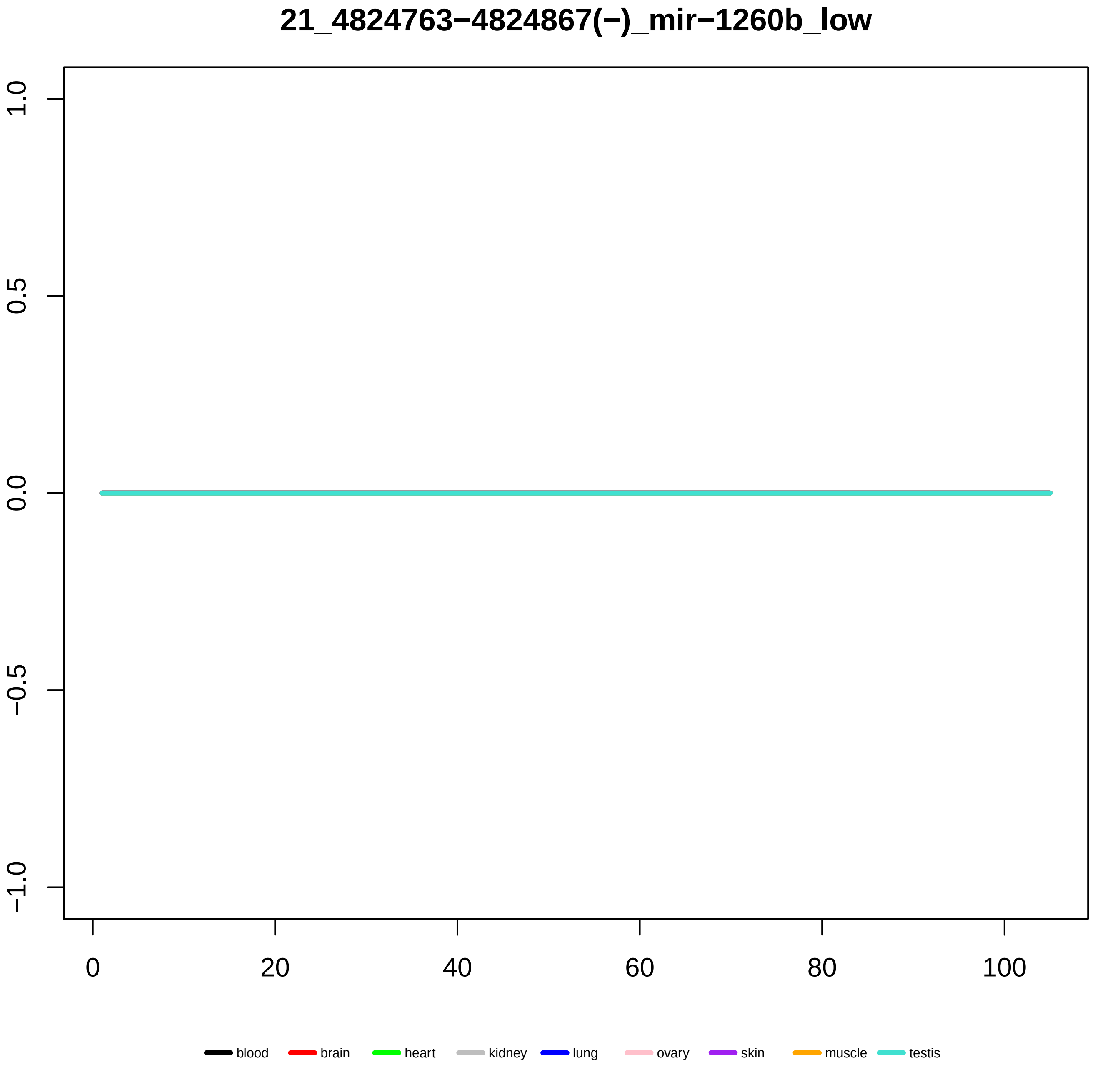

21_4824763−4824867(−)_mir−1260b_low
1.0
0.5
0.0
−0.5
−1.0
0
20
40
60
80
100
blood
brain
heart
kidney
lung
ovary
skin
muscle
testis

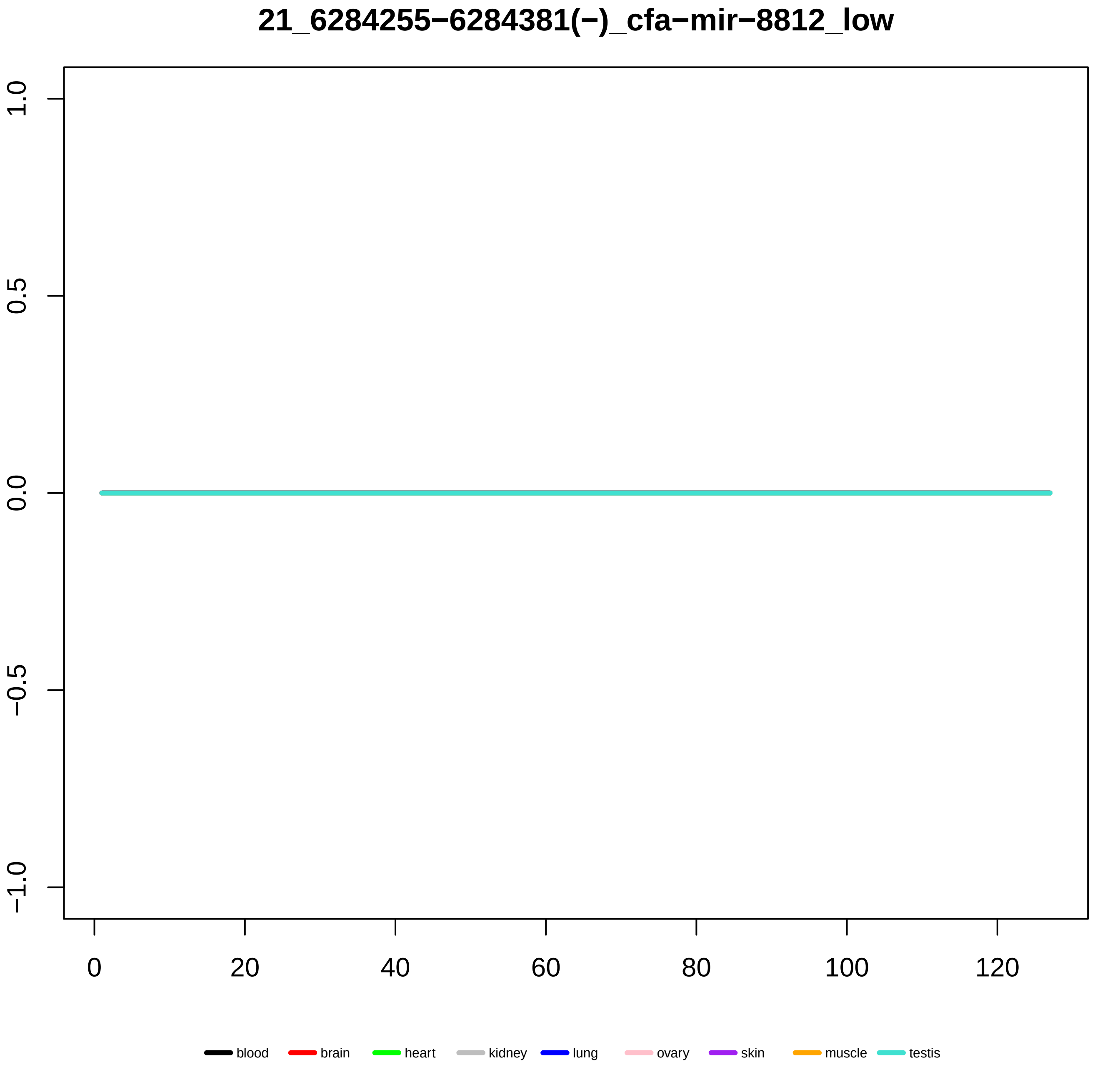
21_6284255−6284381(−)_cfa−mir−8812_low
1.0
0.5
0.0
−0.5
−1.0
0
20
40
60
80
100
120
blood
brain
heart
kidney
lung
ovary
skin
muscle
testis

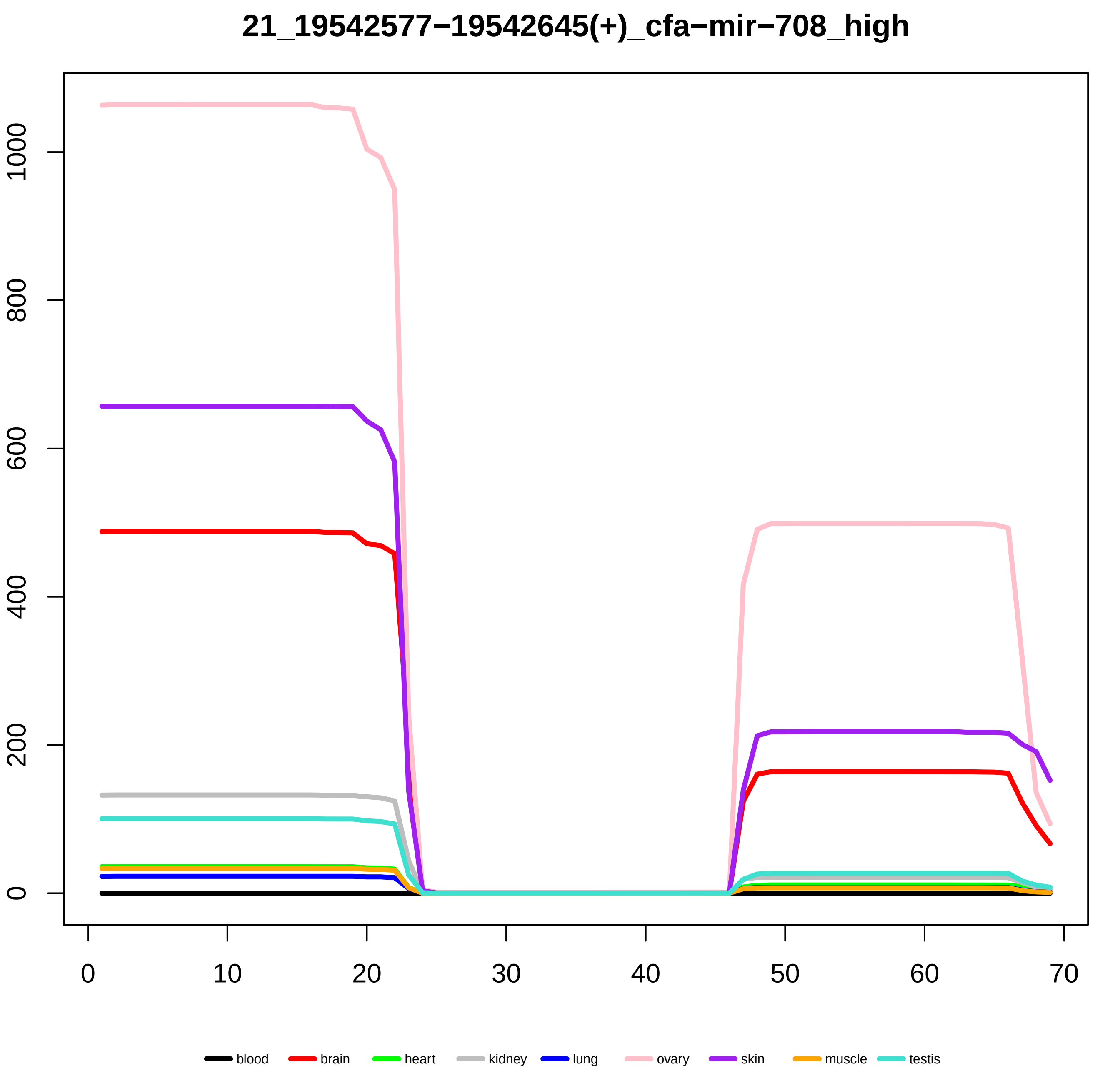

21_19542577−19542645(+)_cfa−mir−708_high
0
200
400
600
800
1000
0
10
20
30
40
50
60
70
blood
brain
heart
kidney
lung
ovary
skin
muscle
testis

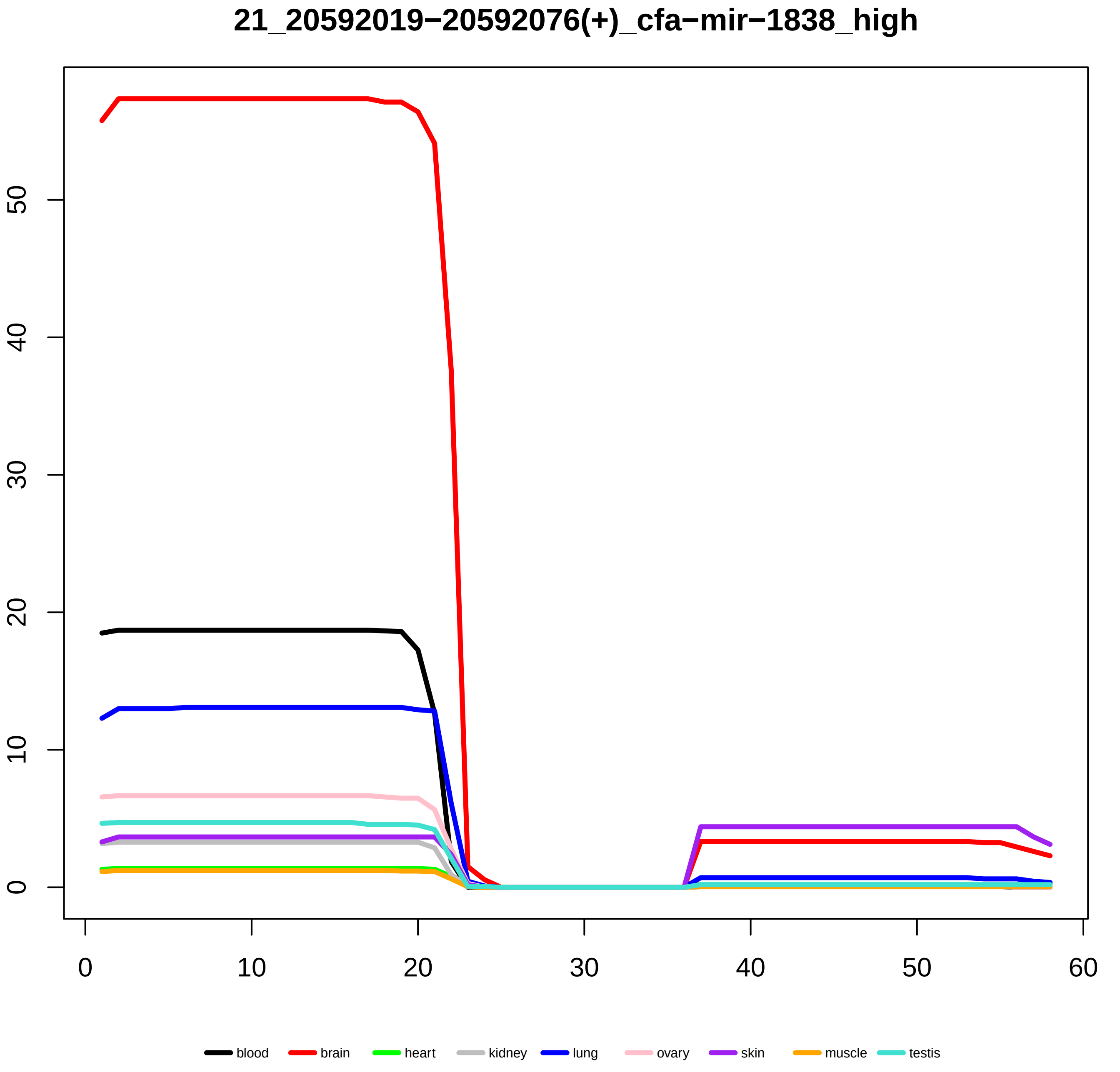

21_20592019−20592076(+)_cfa−mir−1838_high
0
10
20
30
40
50
0
10
20
30
40
50
60
blood
brain
heart
kidney
lung
ovary
skin
muscle
testis

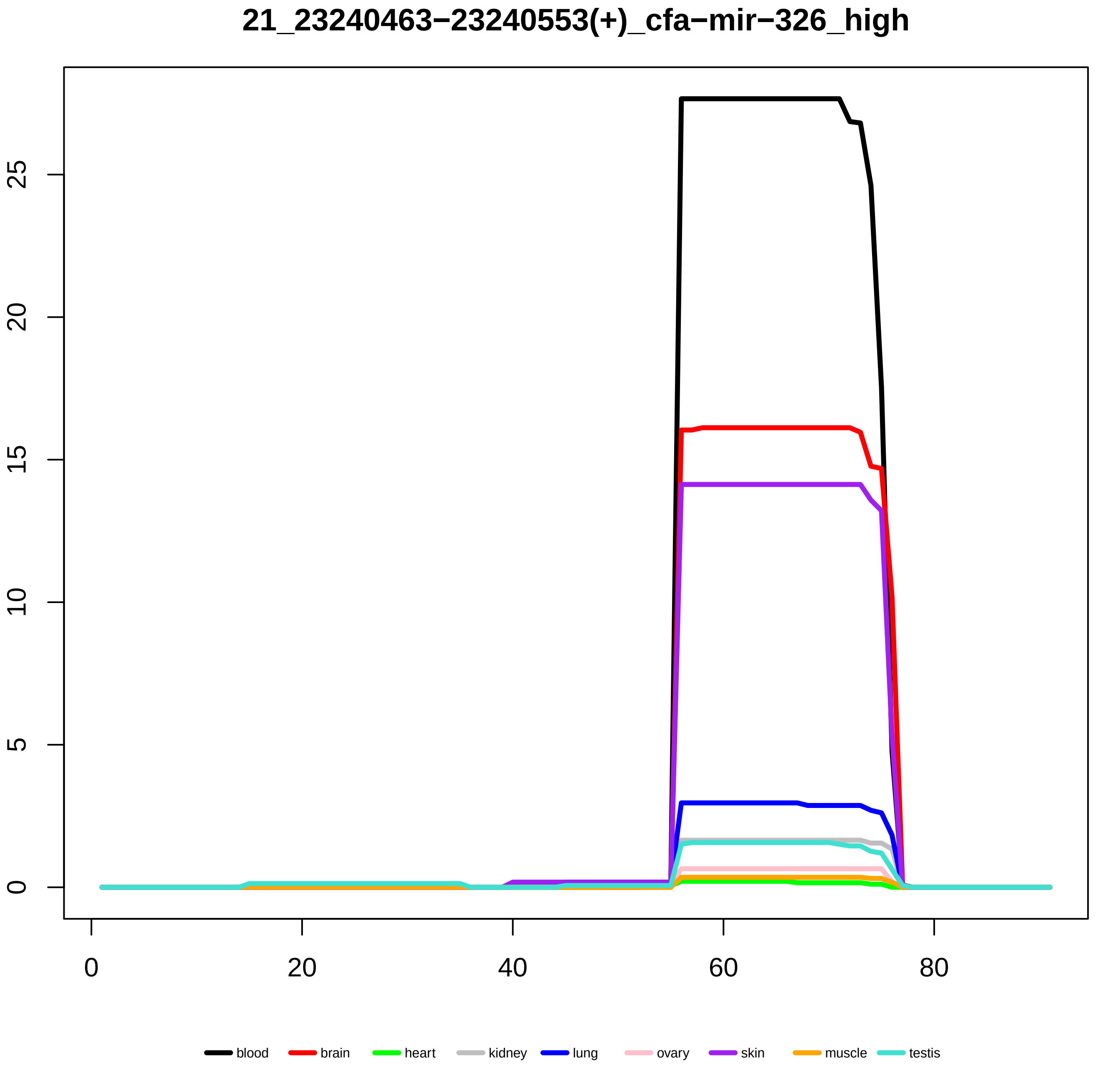

21_23240463−23240553(+)_cfa−mir−326_high
0
5
10
15
20
25
0
20
40
60
80
blood
brain
heart
kidney
lung
ovary
skin
muscle
testis

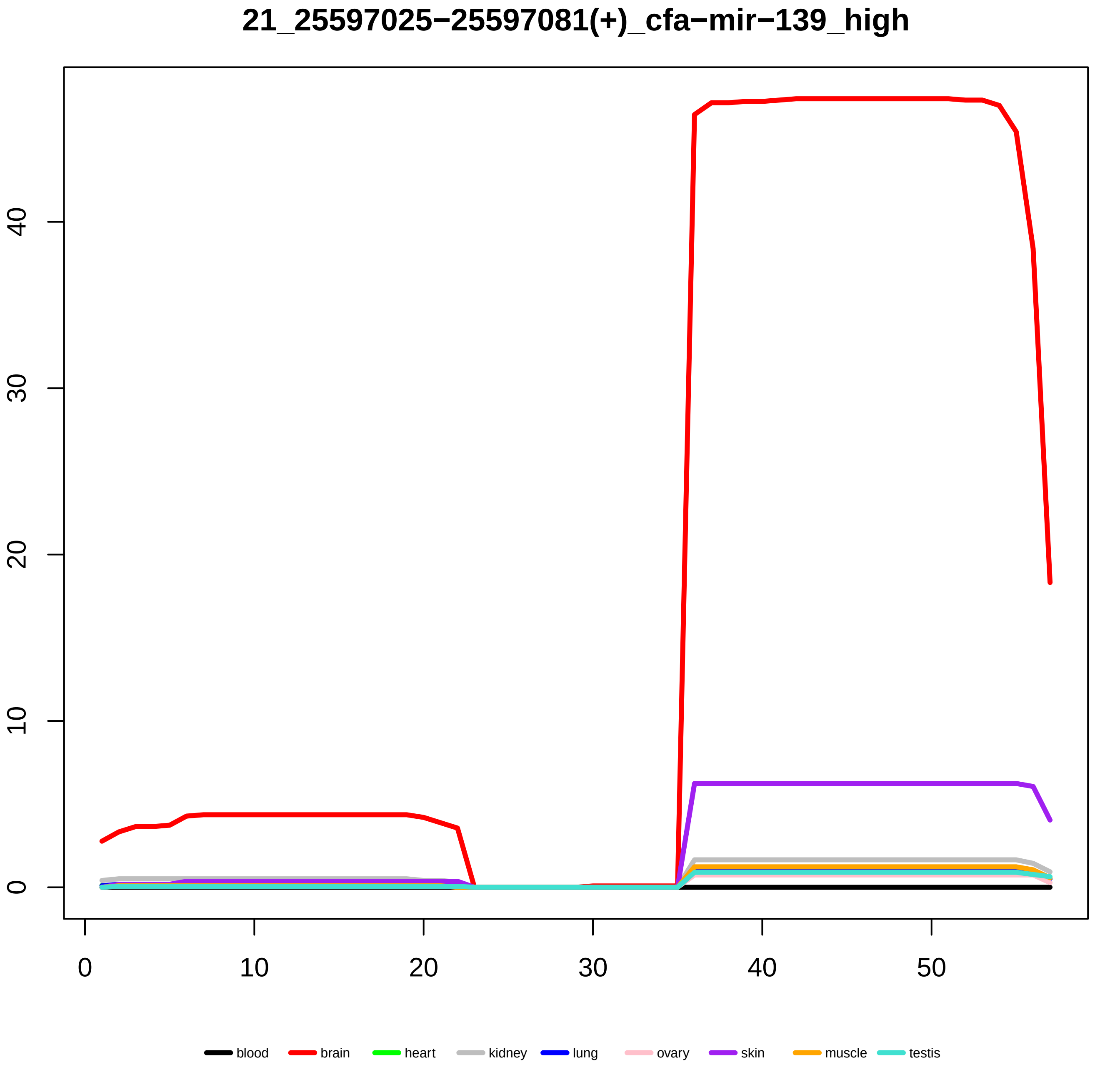

21_25597025−25597081(+)_cfa−mir−139_high
0
10
20
30
40
0
10
20
30
40
50
blood
brain
heart
kidney
lung
ovary
skin
muscle
testis

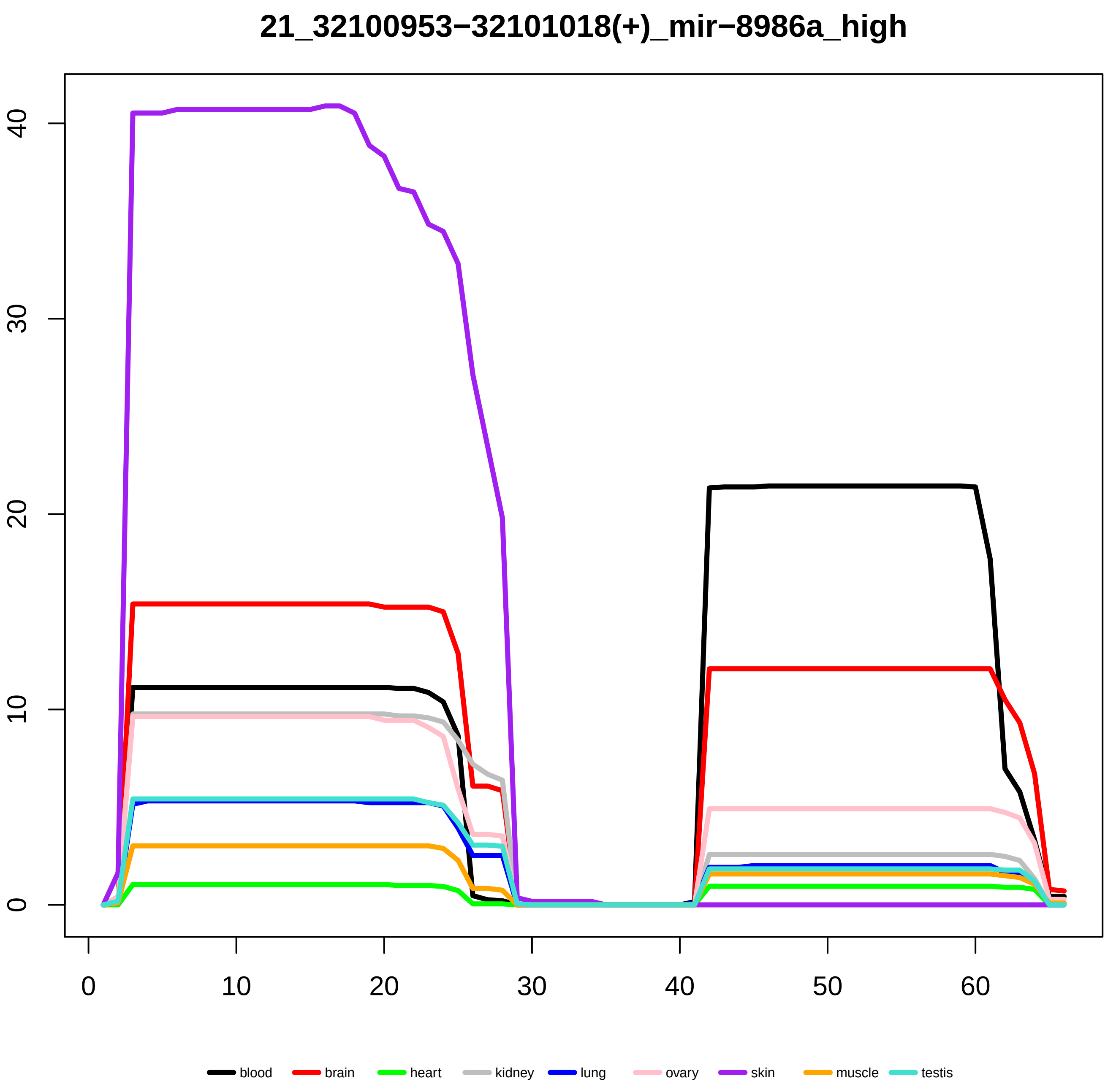

21_32100953−32101018(+)_mir−8986a_high
0
10
20
30
40
0
10
20
30
40
50
60
blood
brain
heart
kidney
lung
ovary
skin
muscle
testis

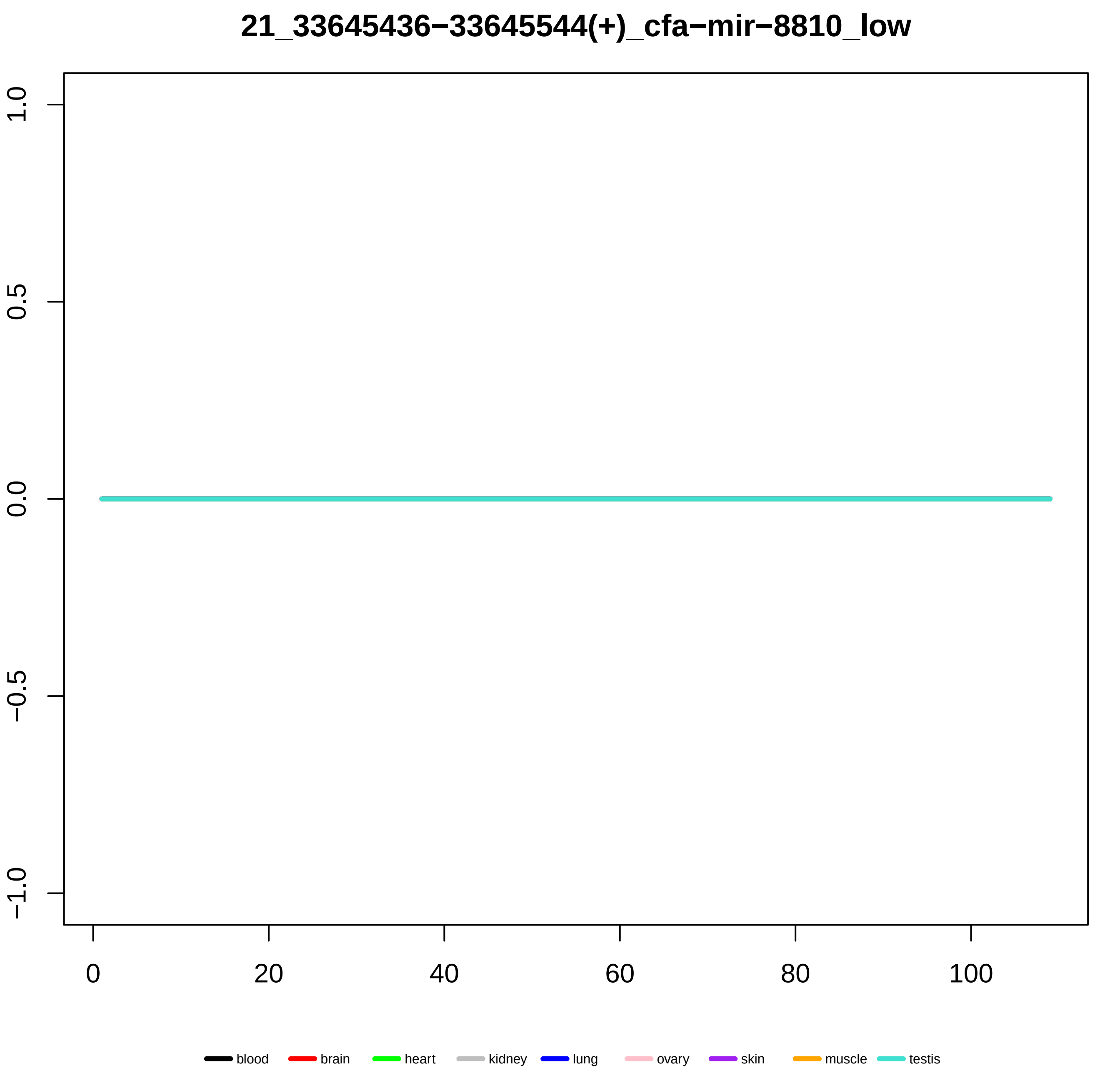
21_33645436−33645544(+)_cfa−mir−8810_low
1.0
0.5
0.0
−0.5
−1.0
0
20
40
60
80
100
blood
brain
heart
kidney
lung
ovary
skin
muscle
testis

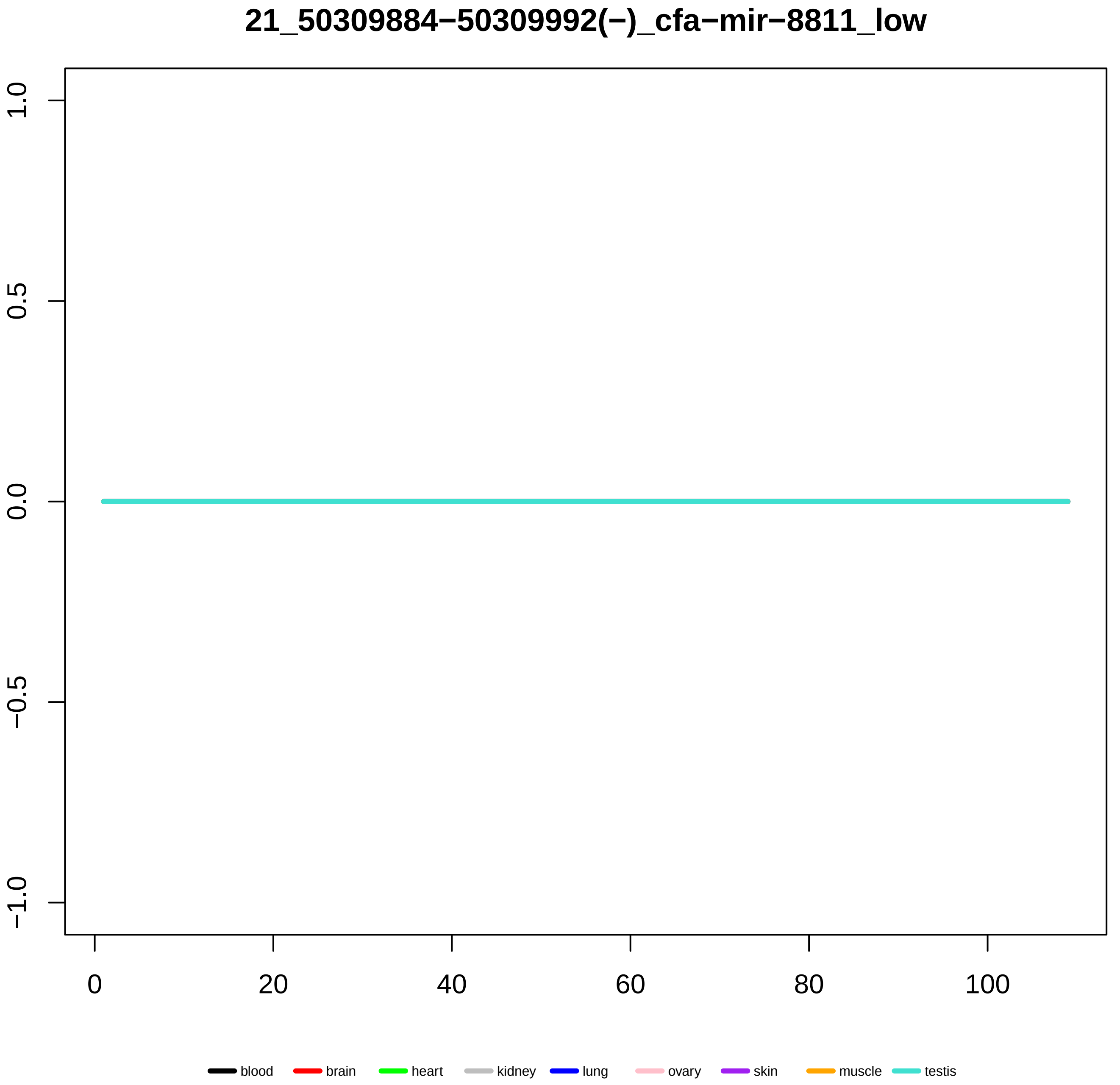

21_50309884−50309992(−)_cfa−mir−8811_low
1.0
0.5
0.0
−0.5
−1.0
0
20
40
60
80
100
blood
brain
heart
kidney
lung
ovary
skin
muscle
testis

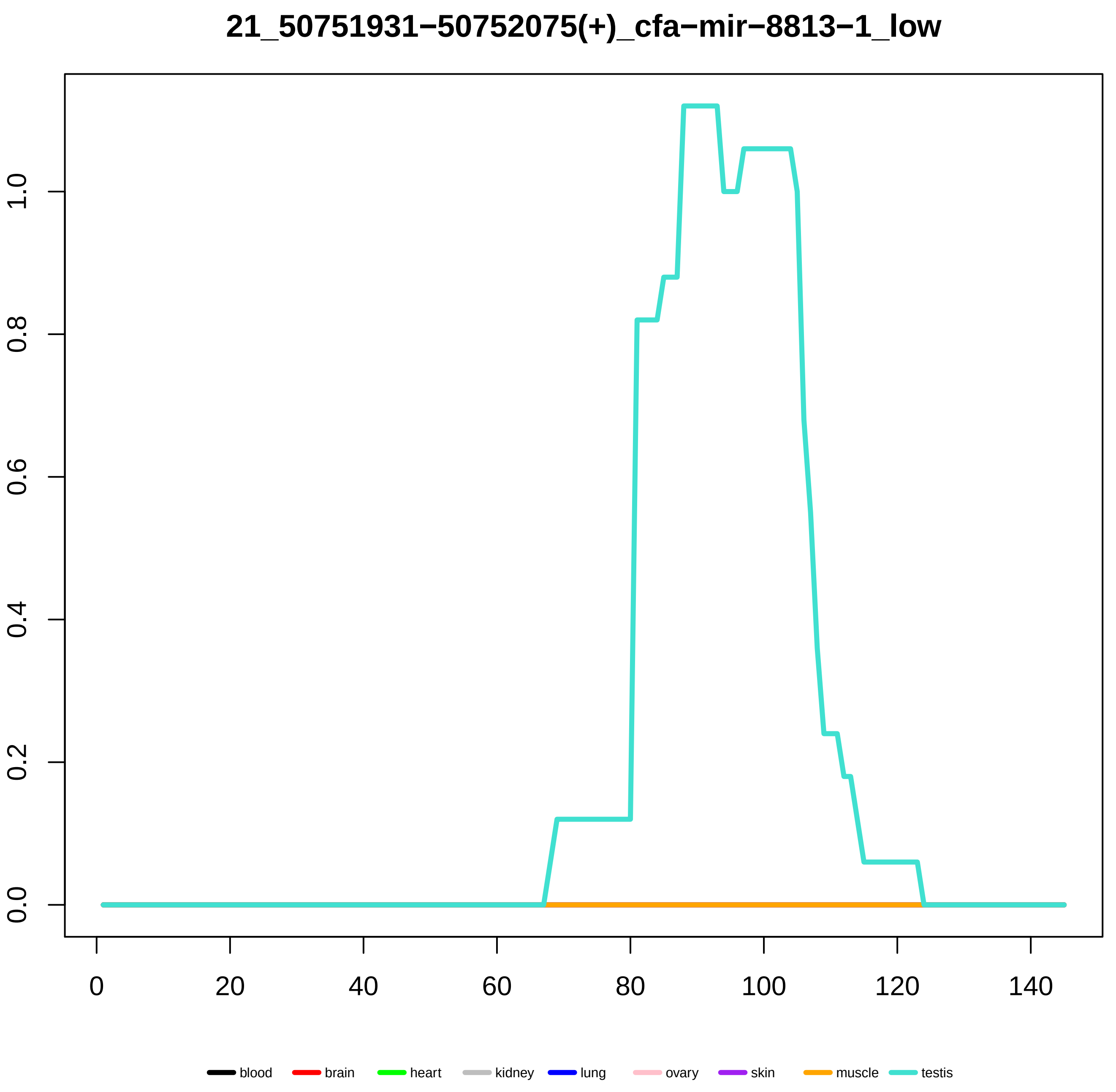

21_50751931−50752075(+)_cfa−mir−8813−1_low
0.0
0.2
0.4
0.6
0.8
1.0
0
20
40
60
80
100
120
140
blood
brain
heart
kidney
lung
ovary
skin
muscle
testis

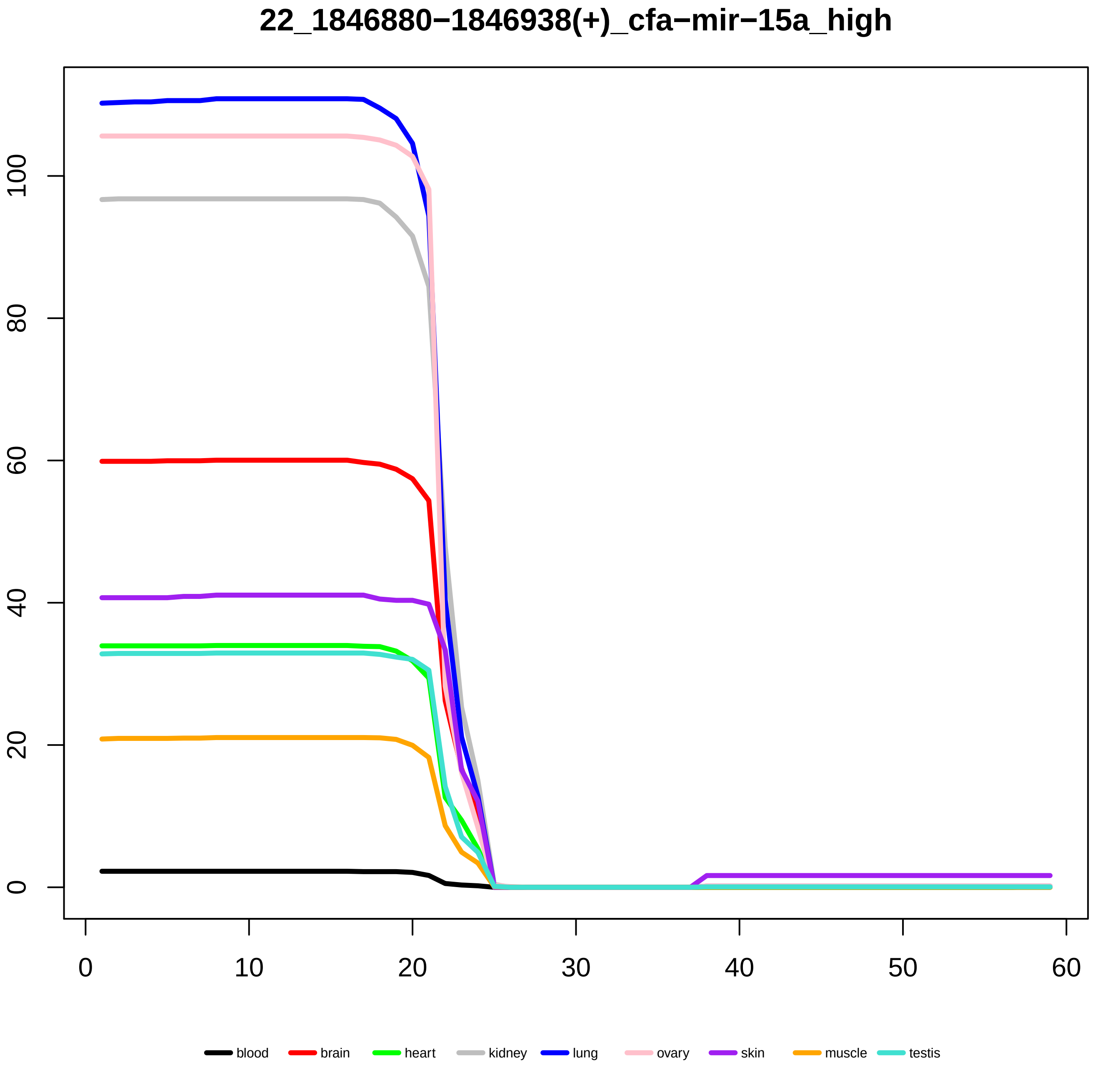

22_1846880−1846938(+)_cfa−mir−15a_high
0
20
40
60
80
100
0
10
20
30
40
50
60
blood
brain
heart
kidney
lung
ovary
skin
muscle
testis

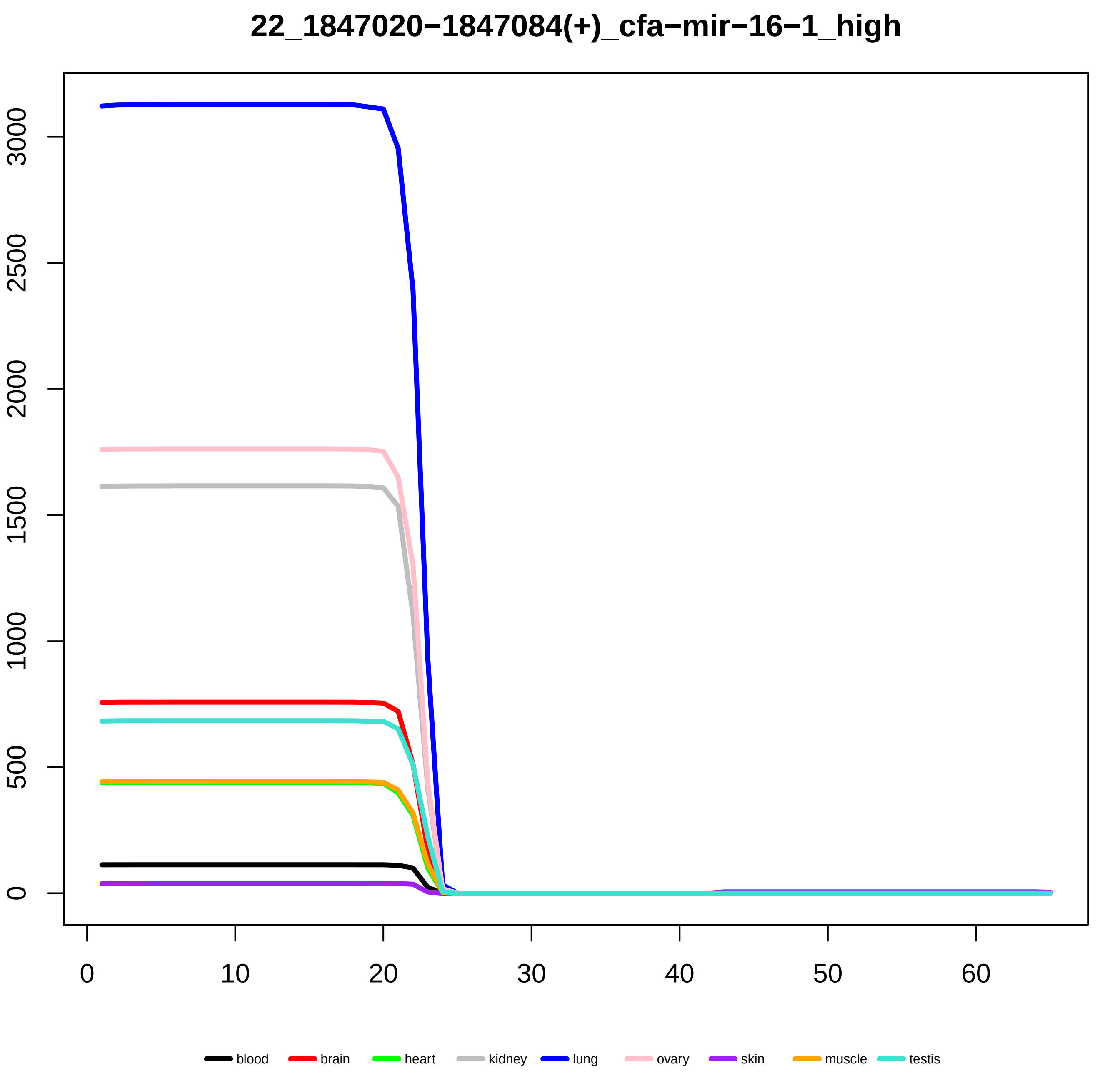
22_1847020−1847084(+)_cfa−mir−16−1_high
0
500
1000
1500
2000
2500
3000
0
10
20
30
40
50
60
blood
brain
heart
kidney
lung
ovary
skin
muscle
testis

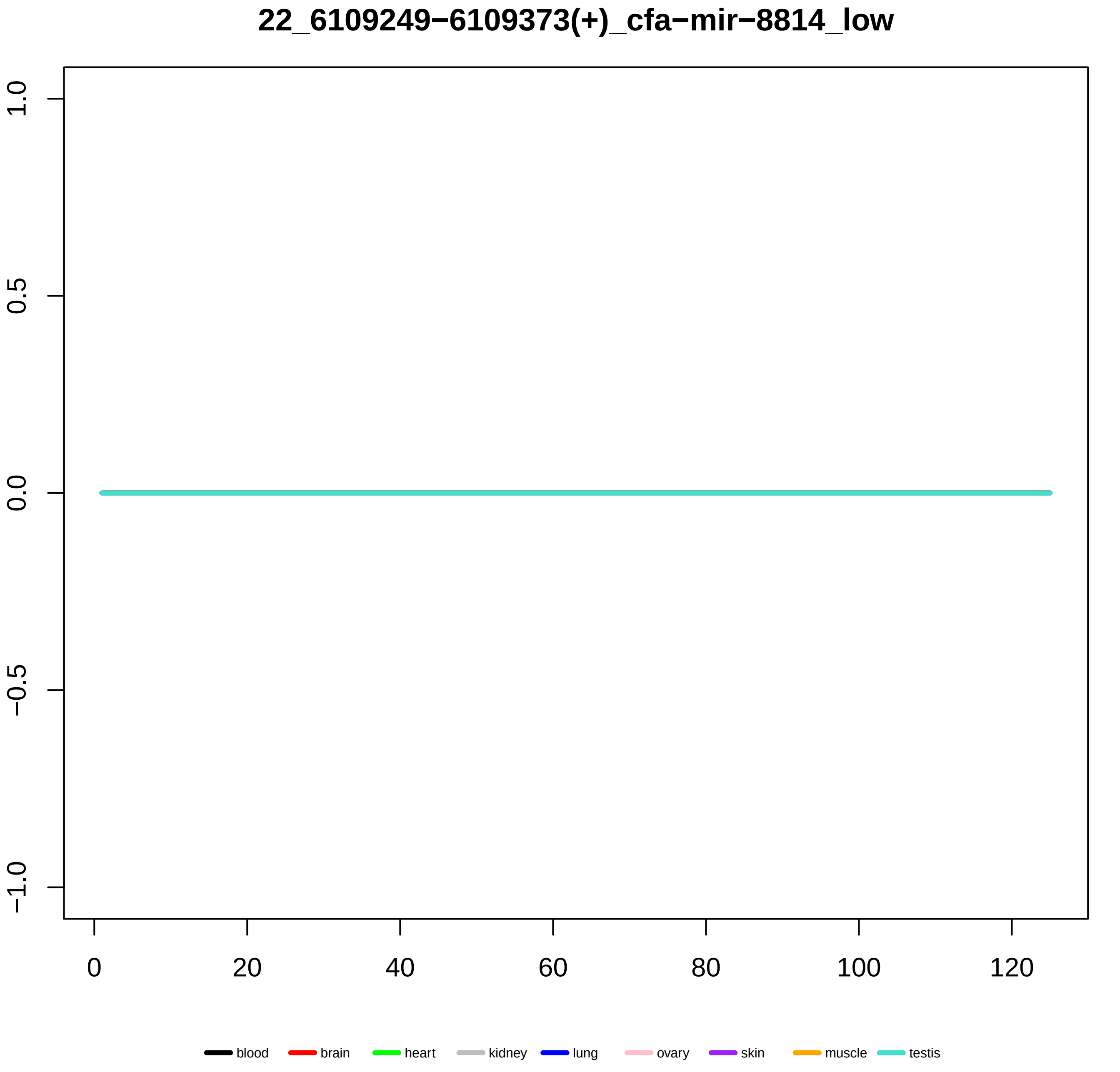

22_6109249−6109373(+)_cfa−mir−8814_low
1.0
0.5
0.0
−0.5
−1.0
0
20
40
60
80
100
120
blood
brain
heart
kidney
lung
ovary
skin
muscle
testis

22_9147830−9147922(+)_mir−5006_low

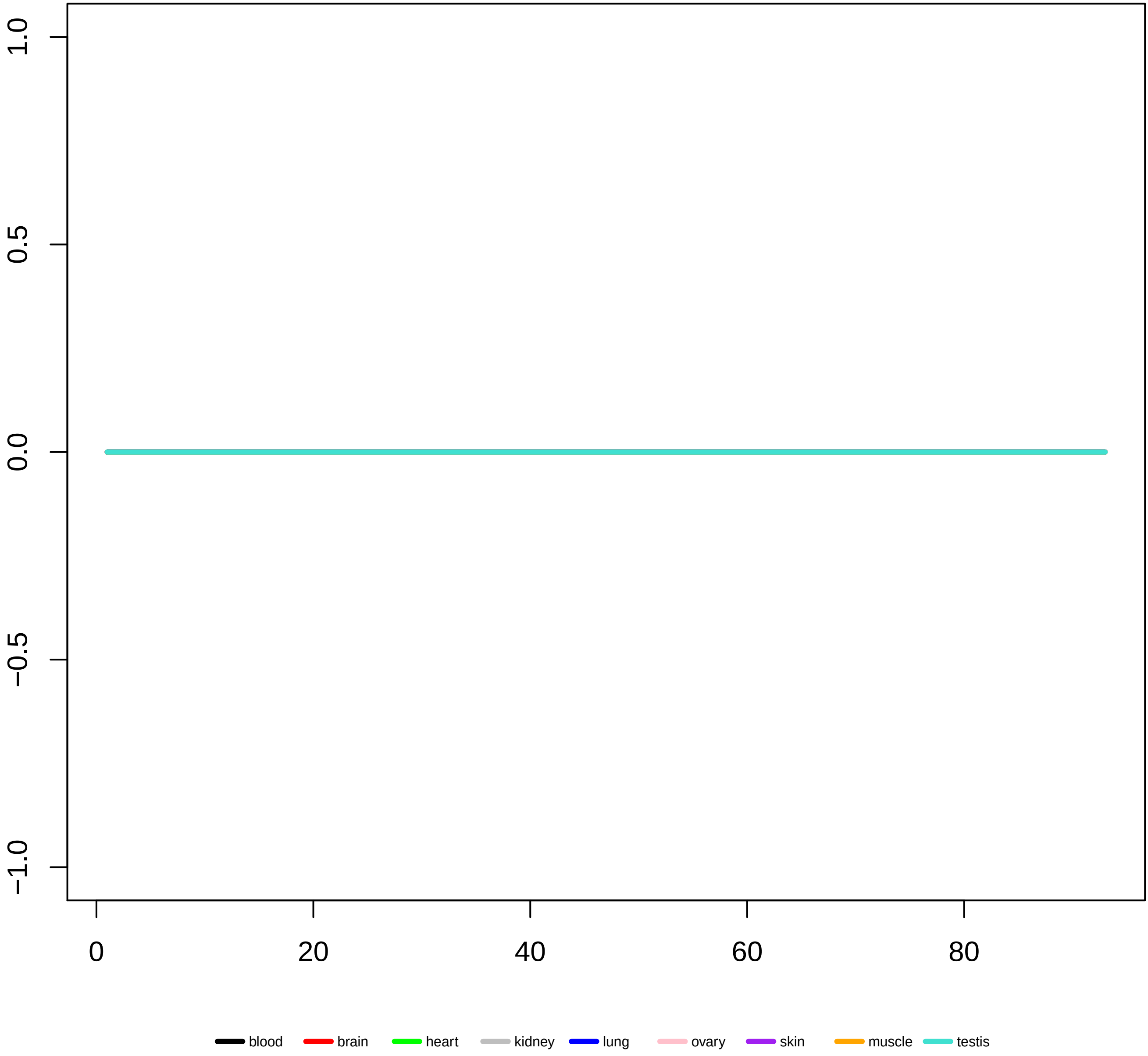

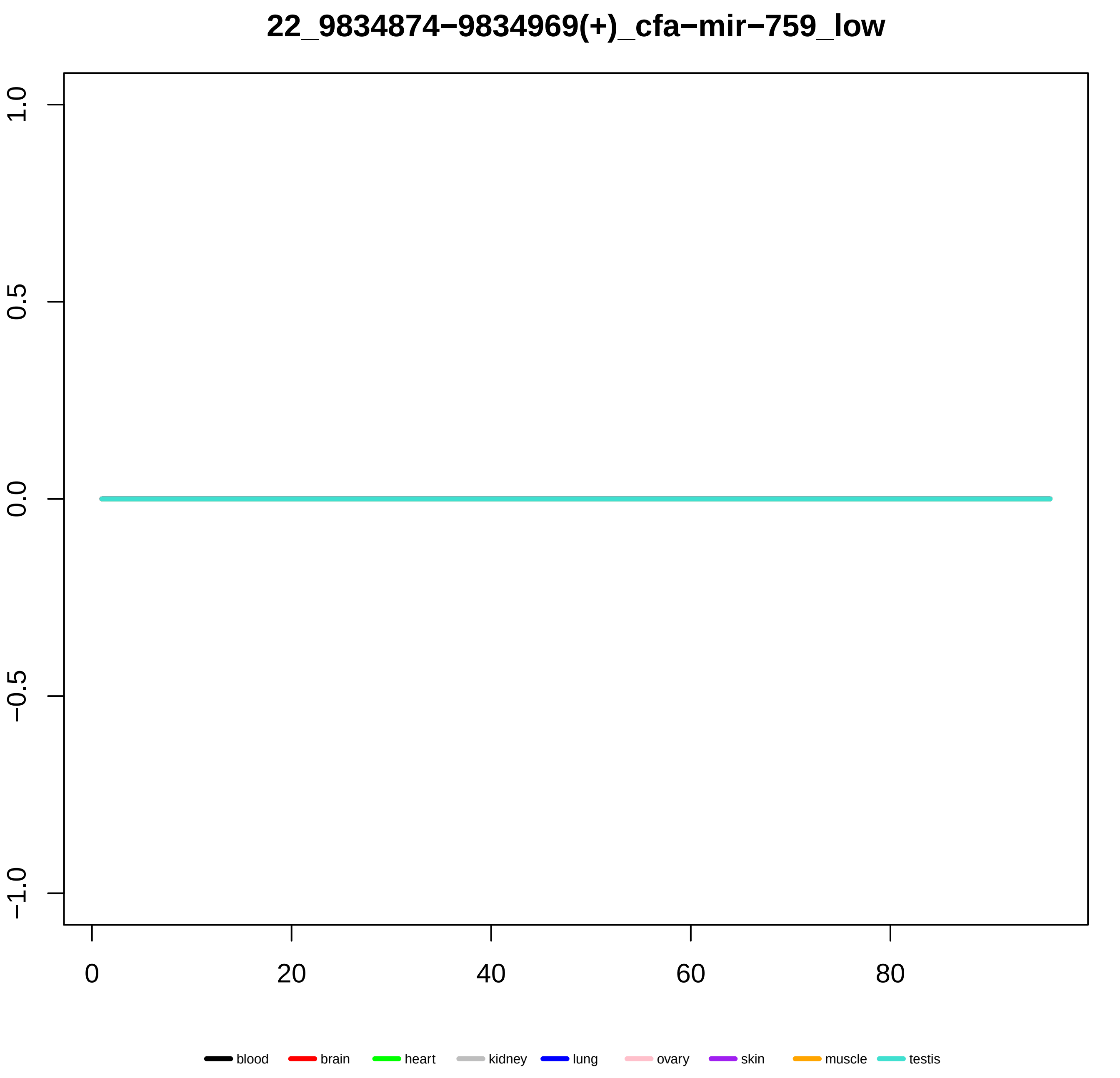

22_9834874−9834969(+)_cfa−mir−759_low
1.0
0.5
0.0
−0.5
−1.0
0
20
40
60
80
blood
brain
heart
kidney
lung
ovary
skin
muscle
testis

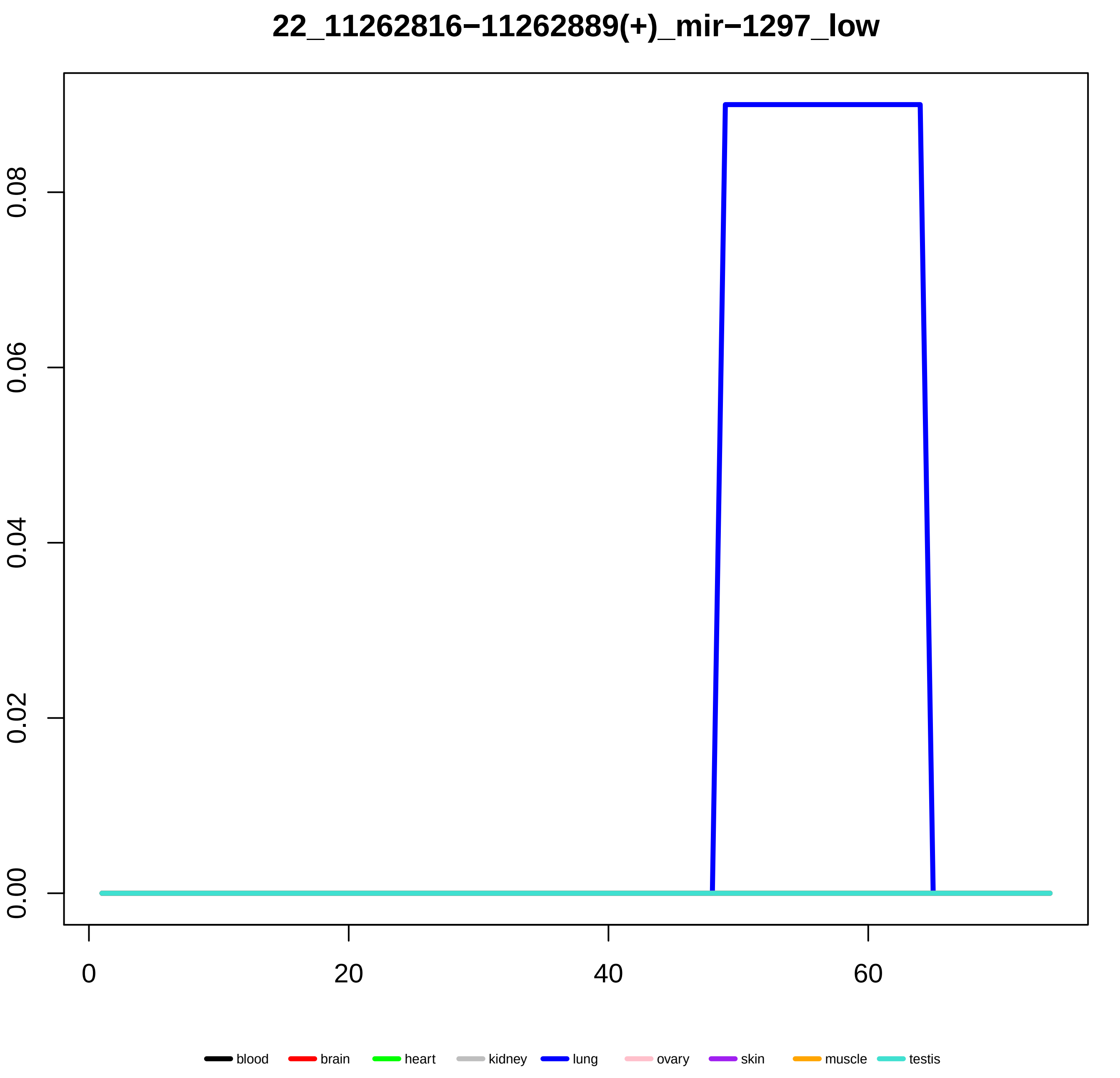
22_11262816−11262889(+)_mir−1297_low
0.00
0.02
0.04
0.06
0.08
0
20
40
60
blood
brain
heart
kidney
lung
ovary
skin
muscle
testis

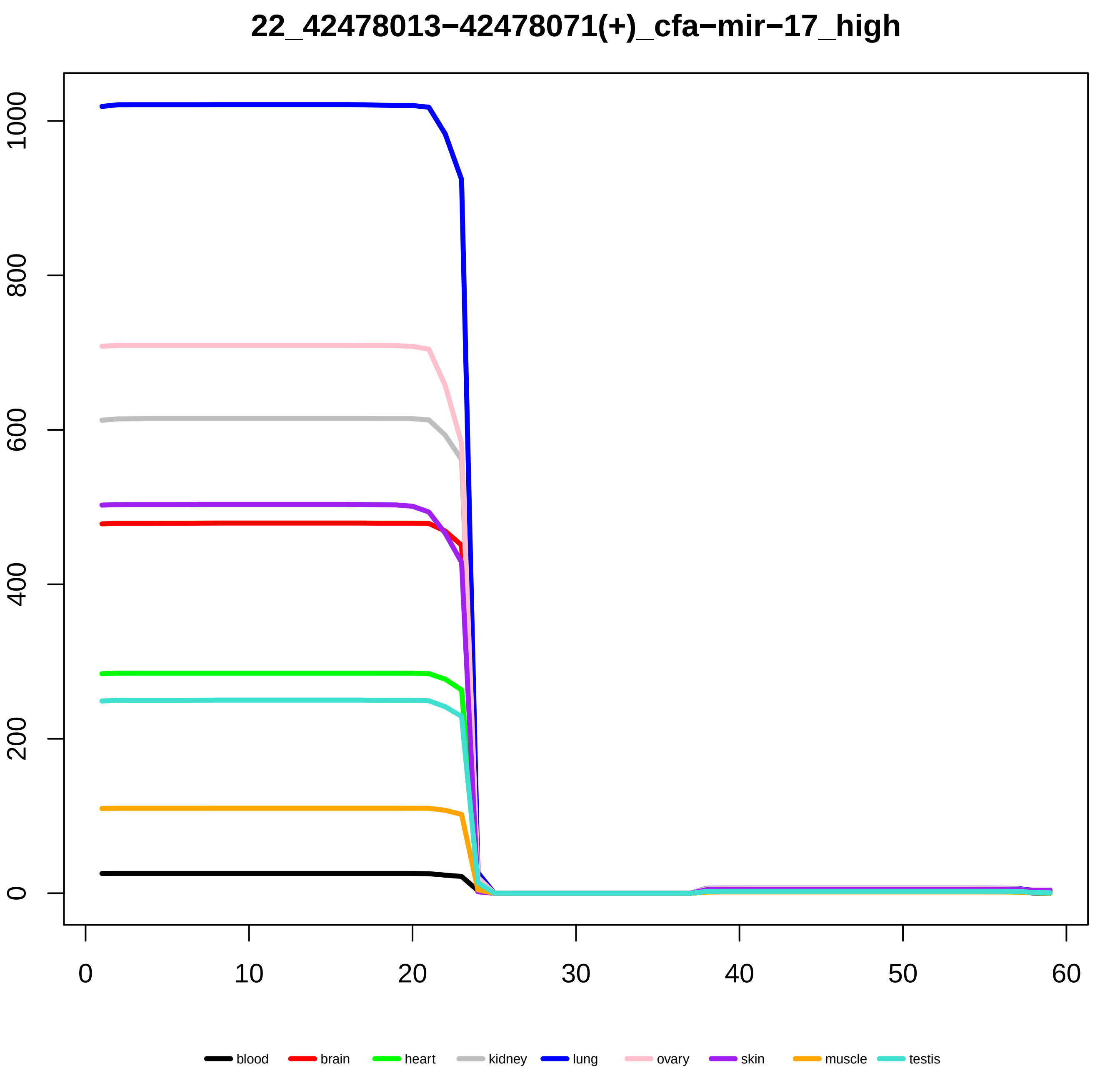
22_42478013−42478071(+)_cfa−mir−17_high
0
200
400
600
800
1000
0
10
20
30
40
50
60
blood
brain
heart
kidney
lung
ovary
skin
muscle
testis

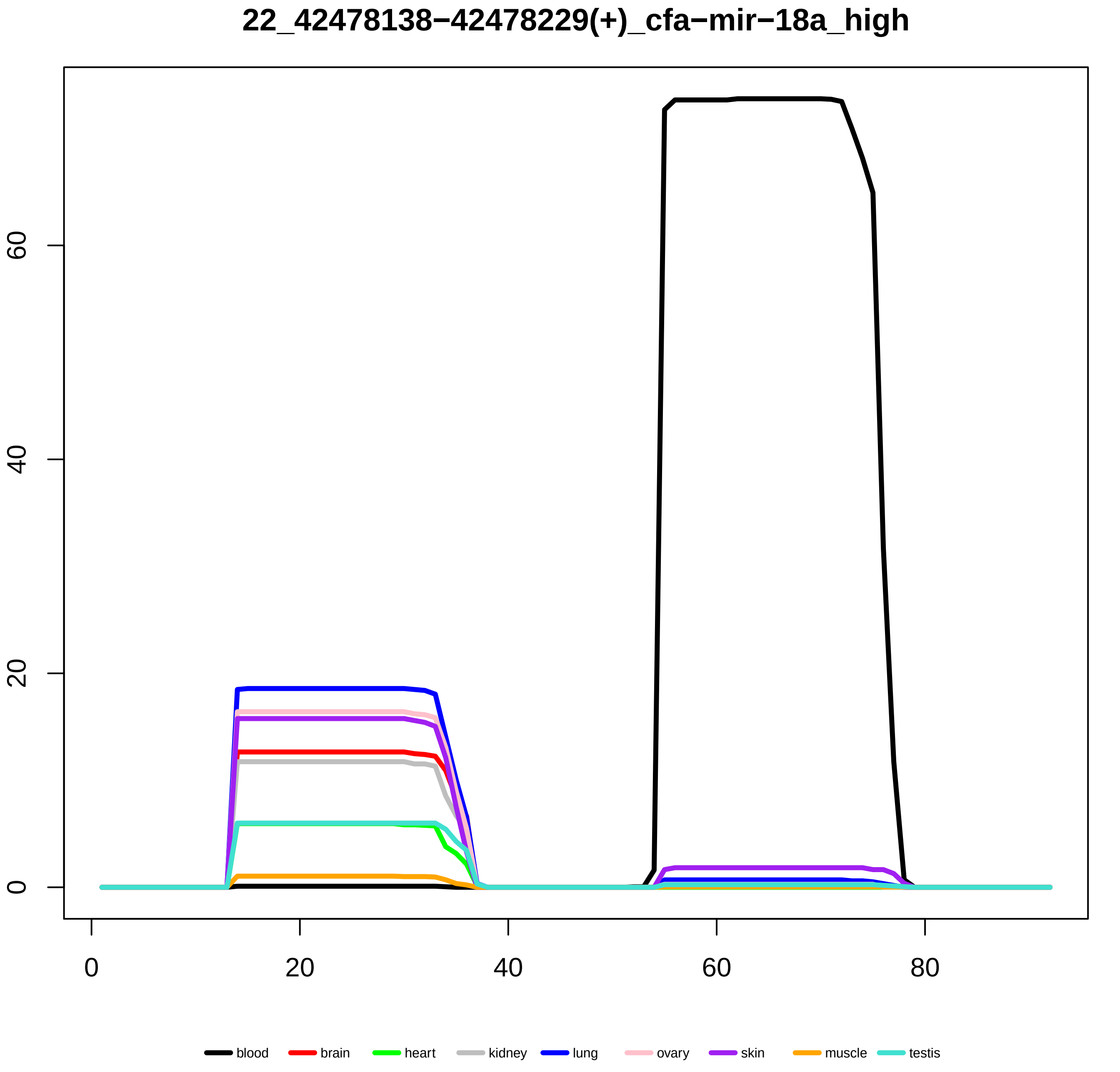

22_42478138−42478229(+)_cfa−mir−18a_high
0
20
40
60
0
20
40
60
80
blood
brain
heart
kidney
lung
ovary
skin
muscle
testis

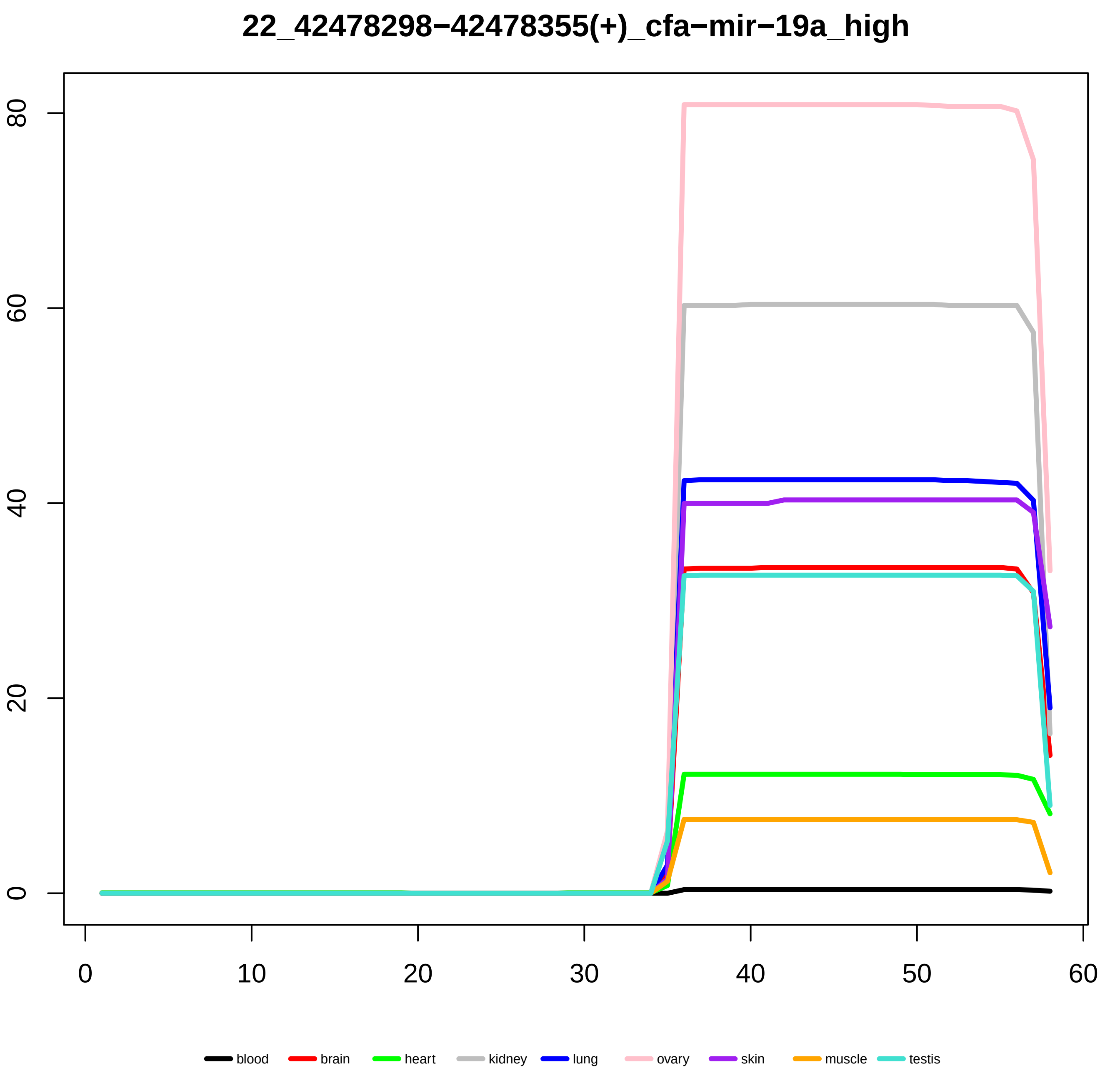

22_42478298−42478355(+)_cfa−mir−19a_high
0
20
40
60
80
0
10
20
30
40
50
60
blood
brain
heart
kidney
lung
ovary
skin
muscle
testis

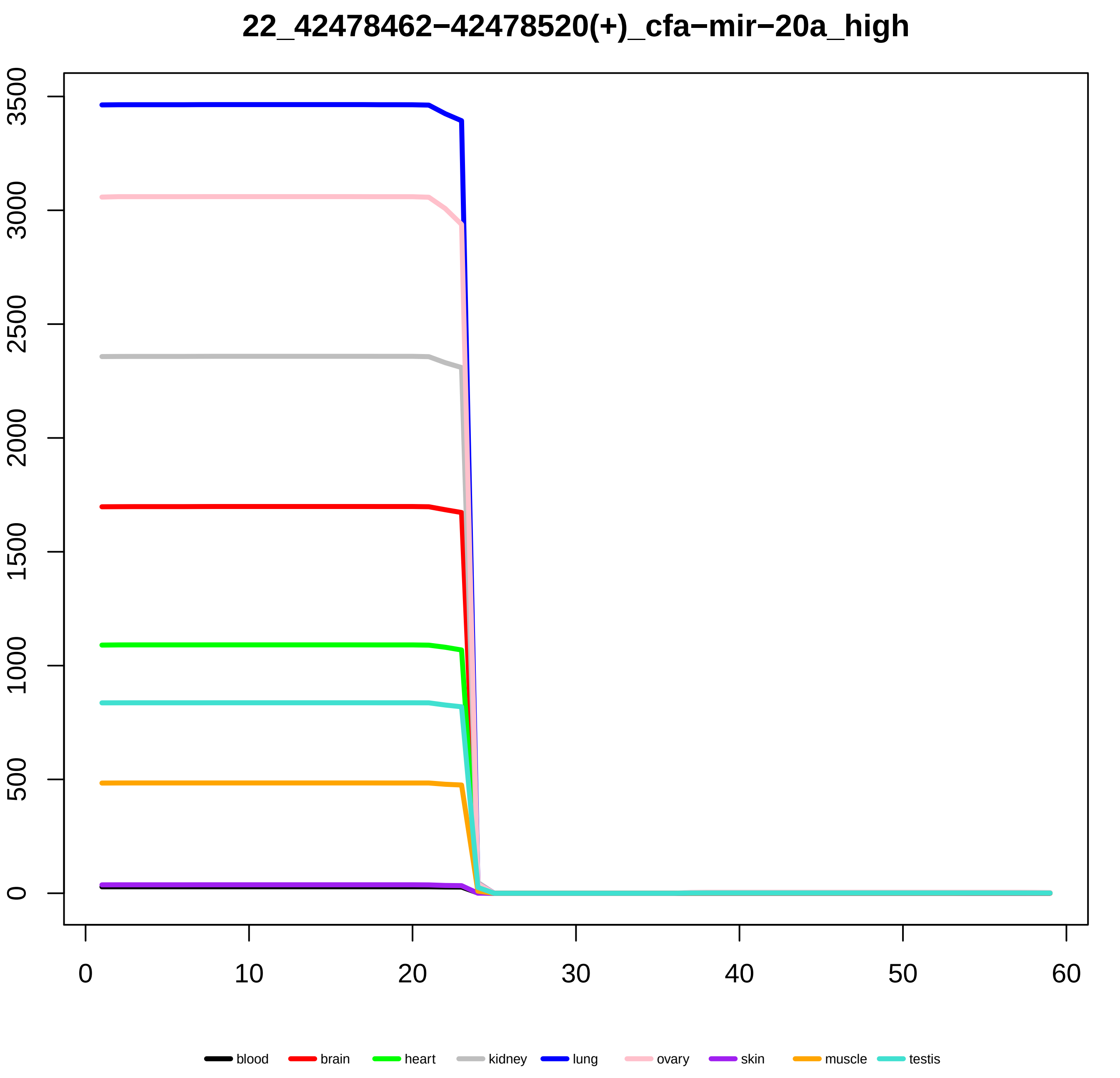

22_42478462−42478520(+)_cfa−mir−20a_high
0
500
1000
1500
2000
2500
3000
3500
0
10
20
30
40
50
60
blood
brain
heart
kidney
lung
ovary
skin
muscle
testis

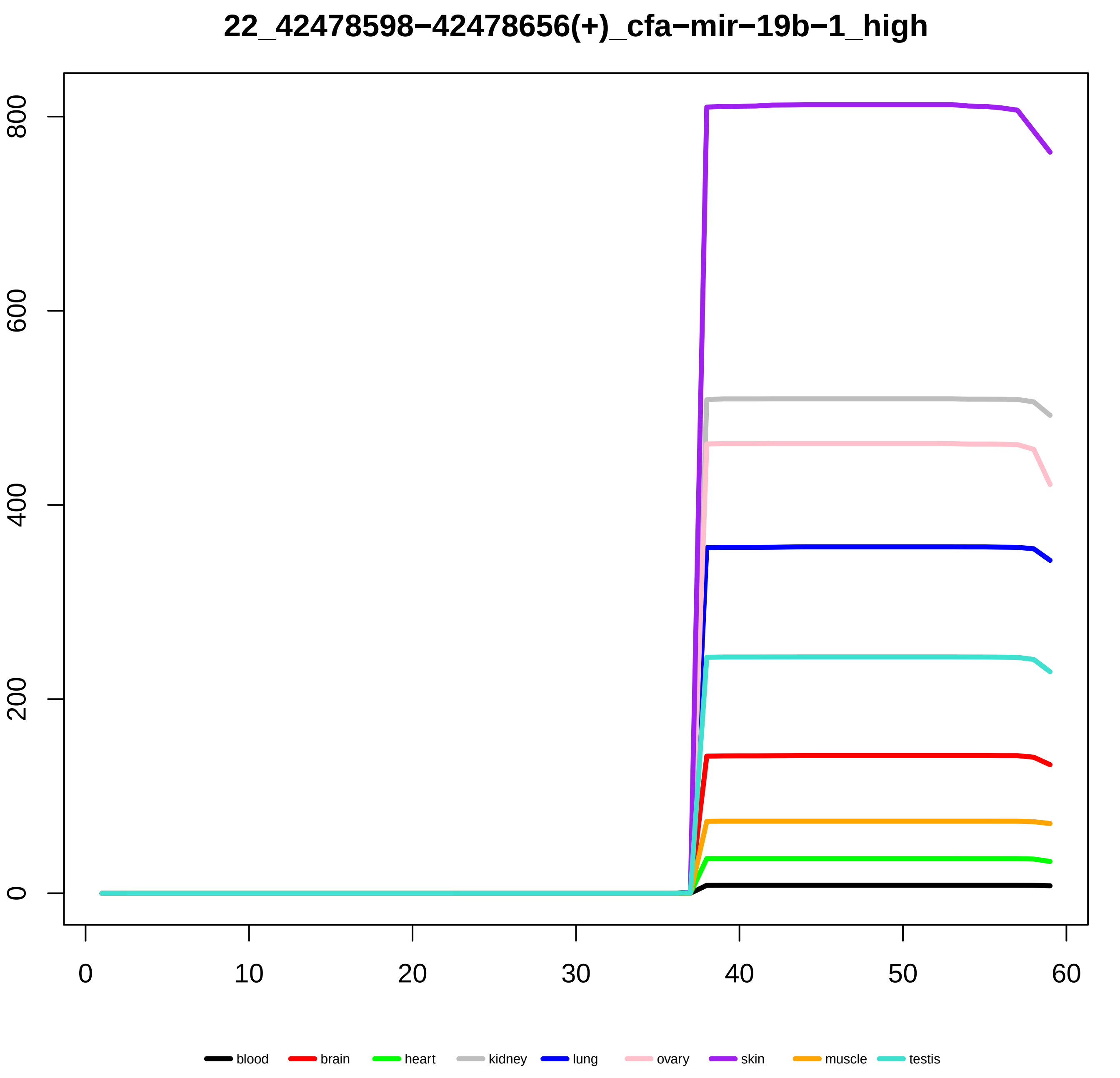

22_42478598−42478656(+)_cfa−mir−19b−1_high
0
200
400
600
800
0
10
20
30
40
50
60
blood
brain
heart
kidney
lung
ovary
skin
muscle
testis

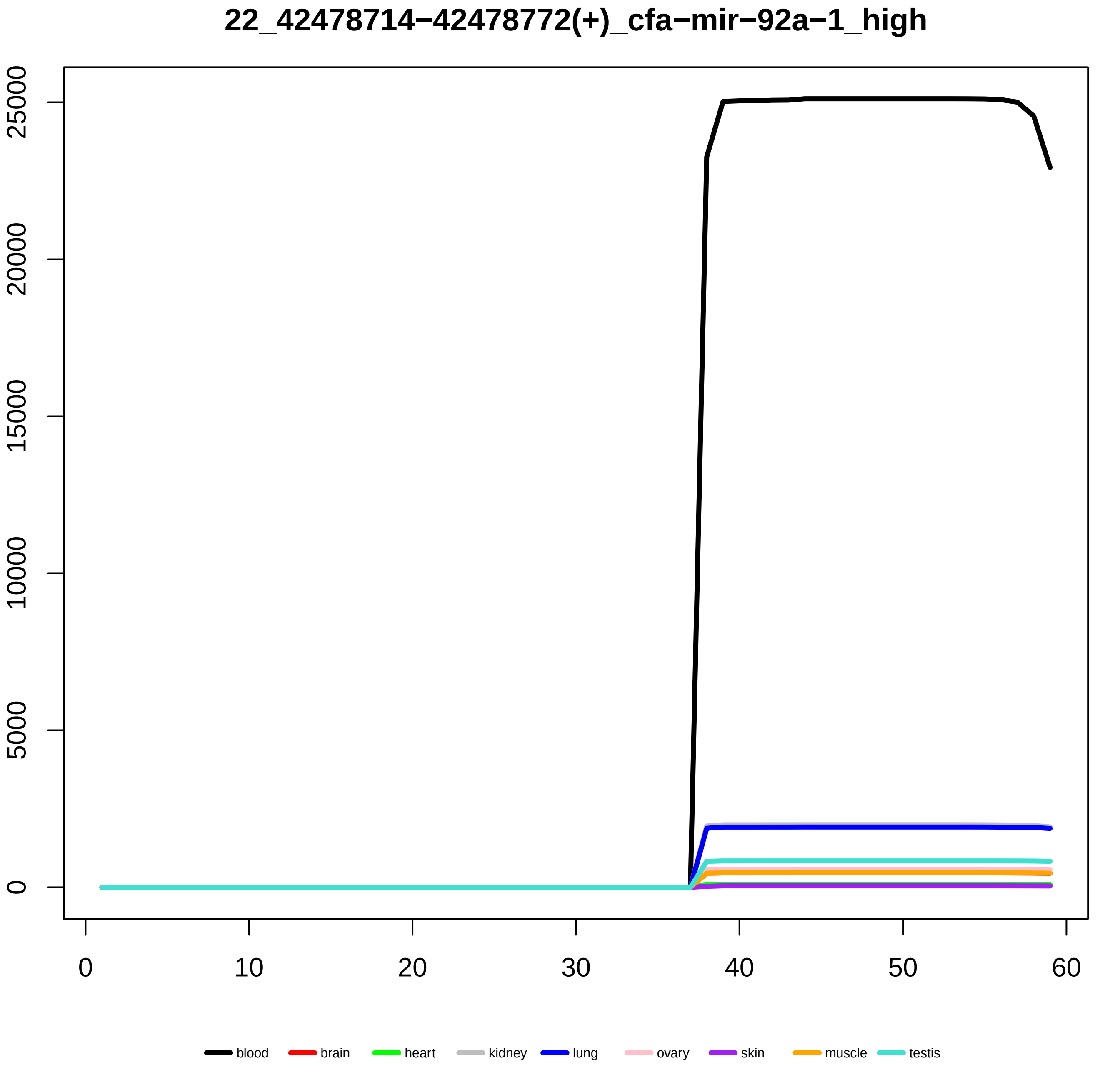

22_42478714−42478772(+)_cfa−mir−92a−1_high
0
5000
10000
15000
20000
25000
0
10
20
30
40
50
60
blood
brain
heart
kidney
lung
ovary
skin
muscle
testis

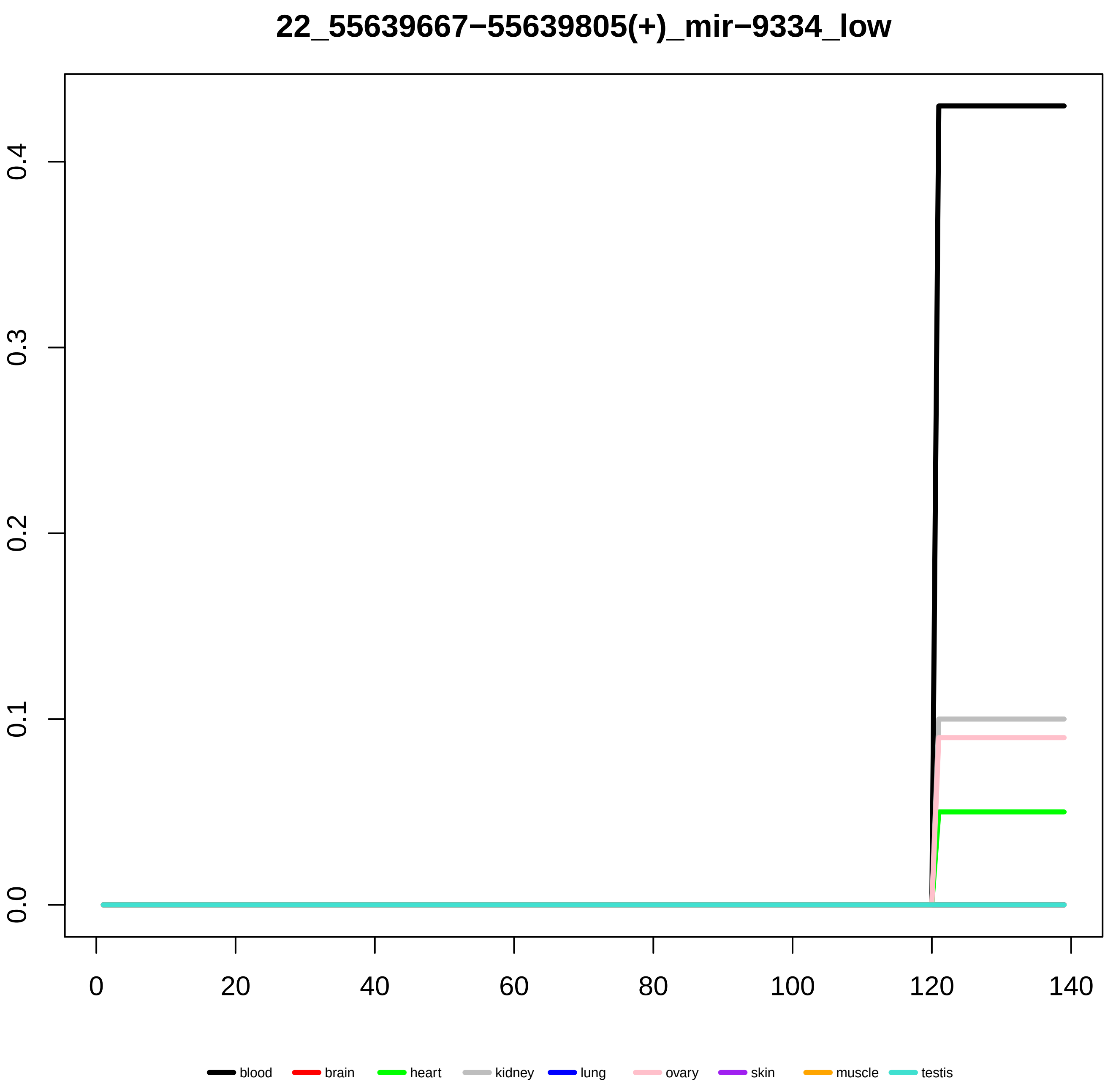
22_55639667−55639805(+)_mir−9334_low
0.0
0.1
0.2
0.3
0.4
0
20
40
60
80
100
120
140
blood
brain
heart
kidney
lung
ovary
skin
muscle
testis

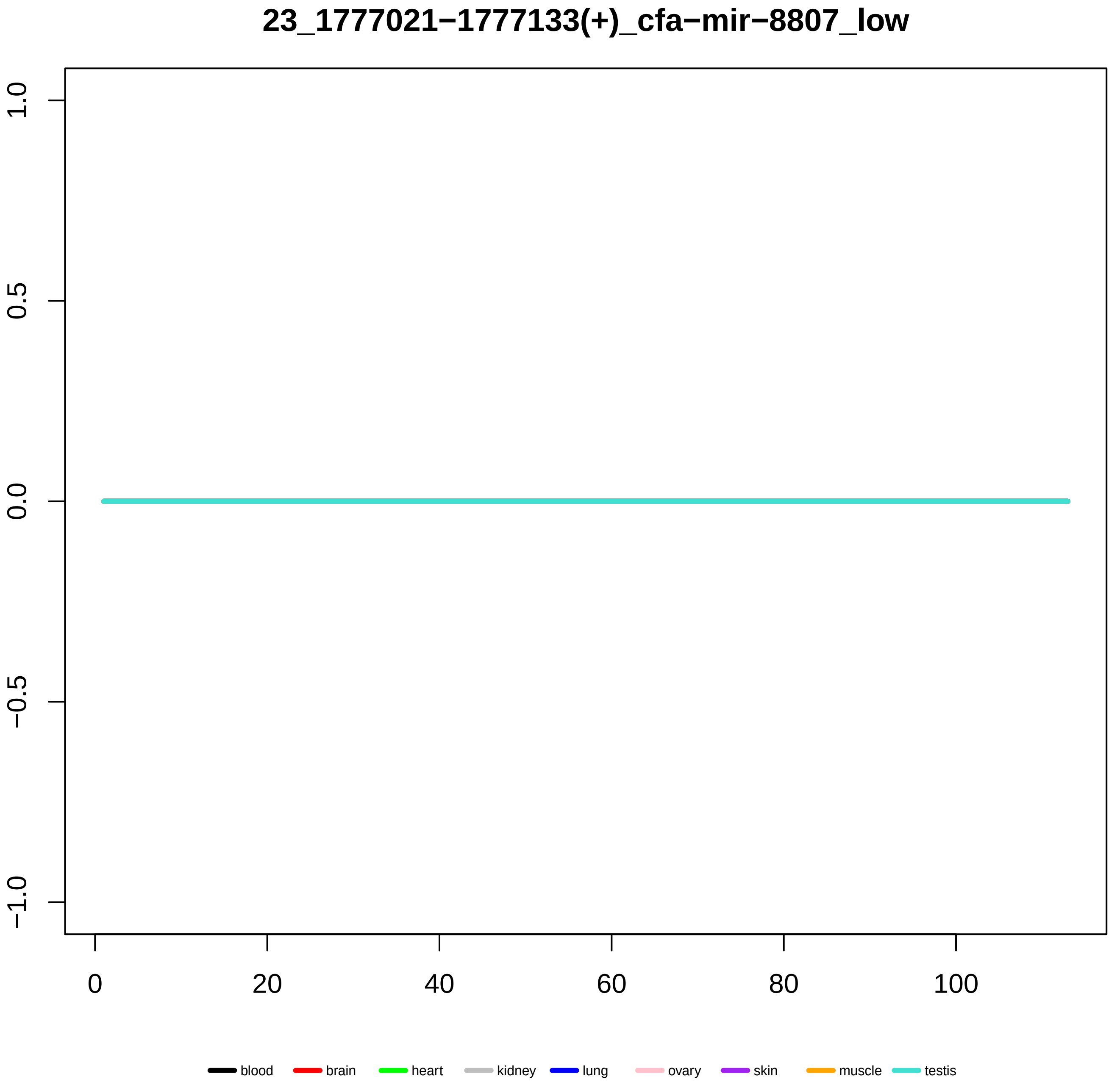

23_1777021−1777133(+)_cfa−mir−8807_low
1.0
0.5
0.0
−0.5
−1.0
0
20
40
60
80
100
blood
brain
heart
kidney
lung
ovary
skin
muscle
testis

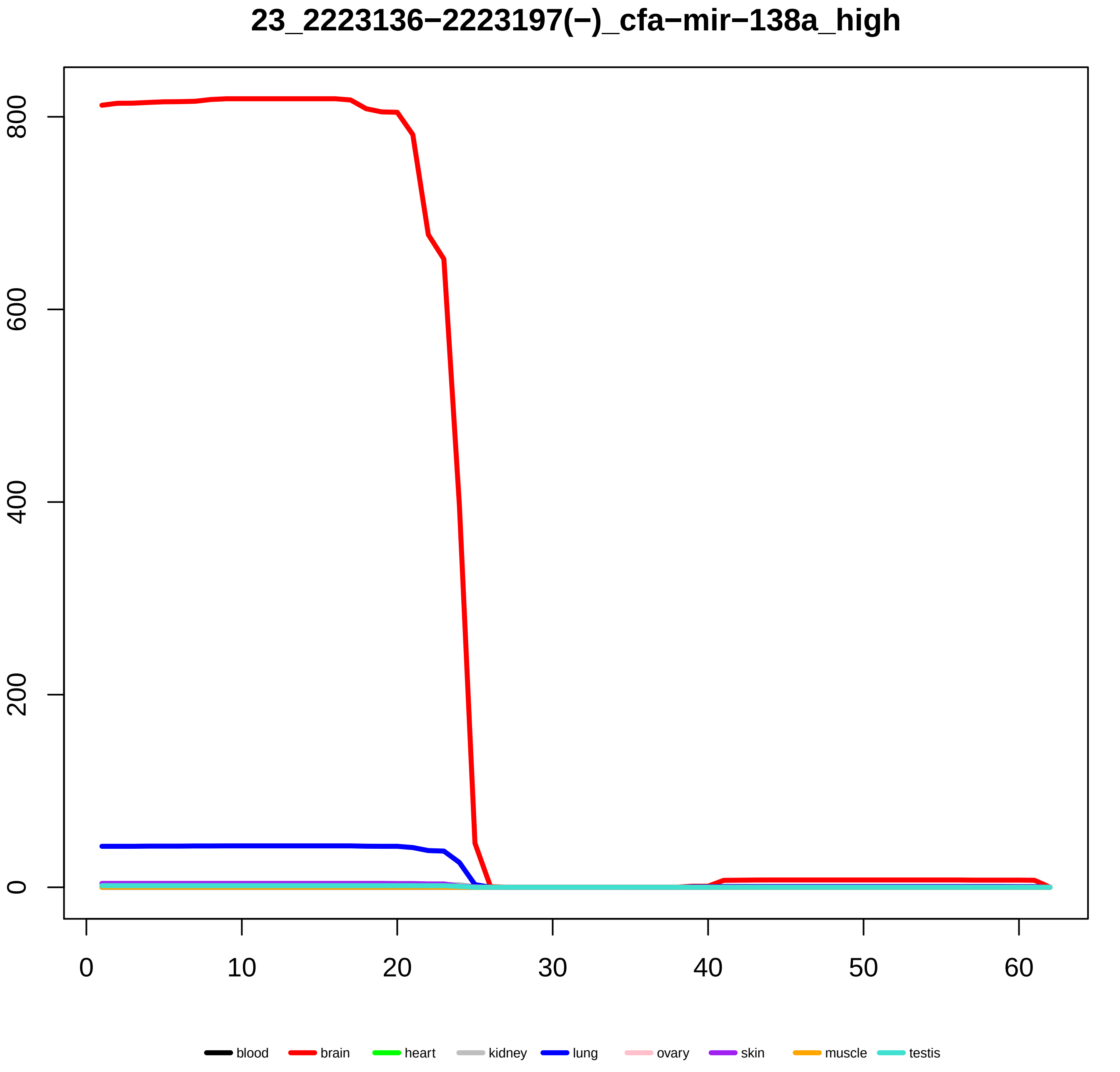
23_2223136−2223197(−)_cfa−mir−138a_high
0
200
400
600
800
0
10
20
30
40
50
60
blood
brain
heart
kidney
lung
ovary
skin
muscle
testis

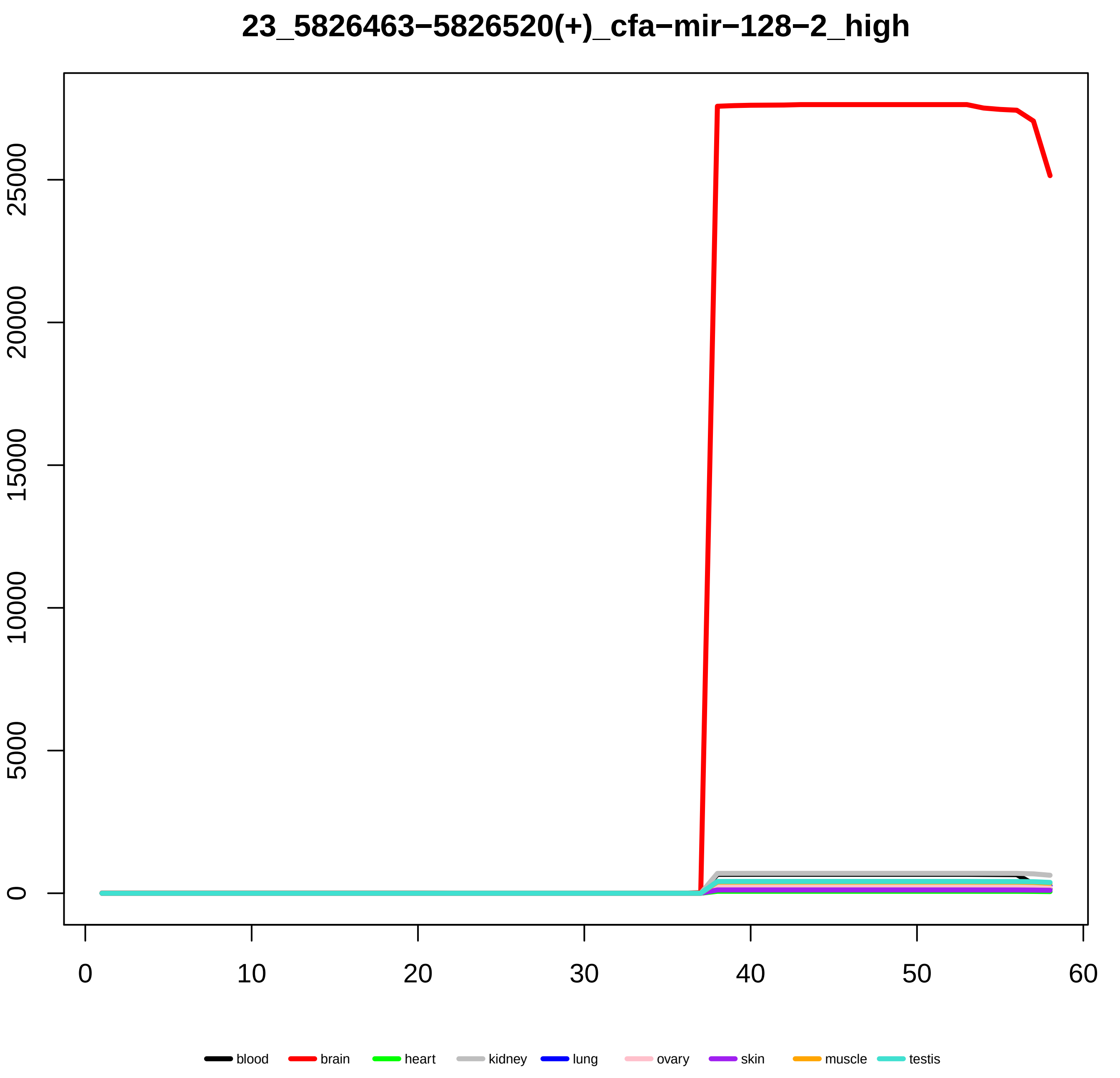
23_5826463−5826520(+)_cfa−mir−128−2_high
0
5000
10000
15000
20000
25000
0
10
20
30
40
50
60
blood
brain
heart
kidney
lung
ovary
skin
muscle
testis

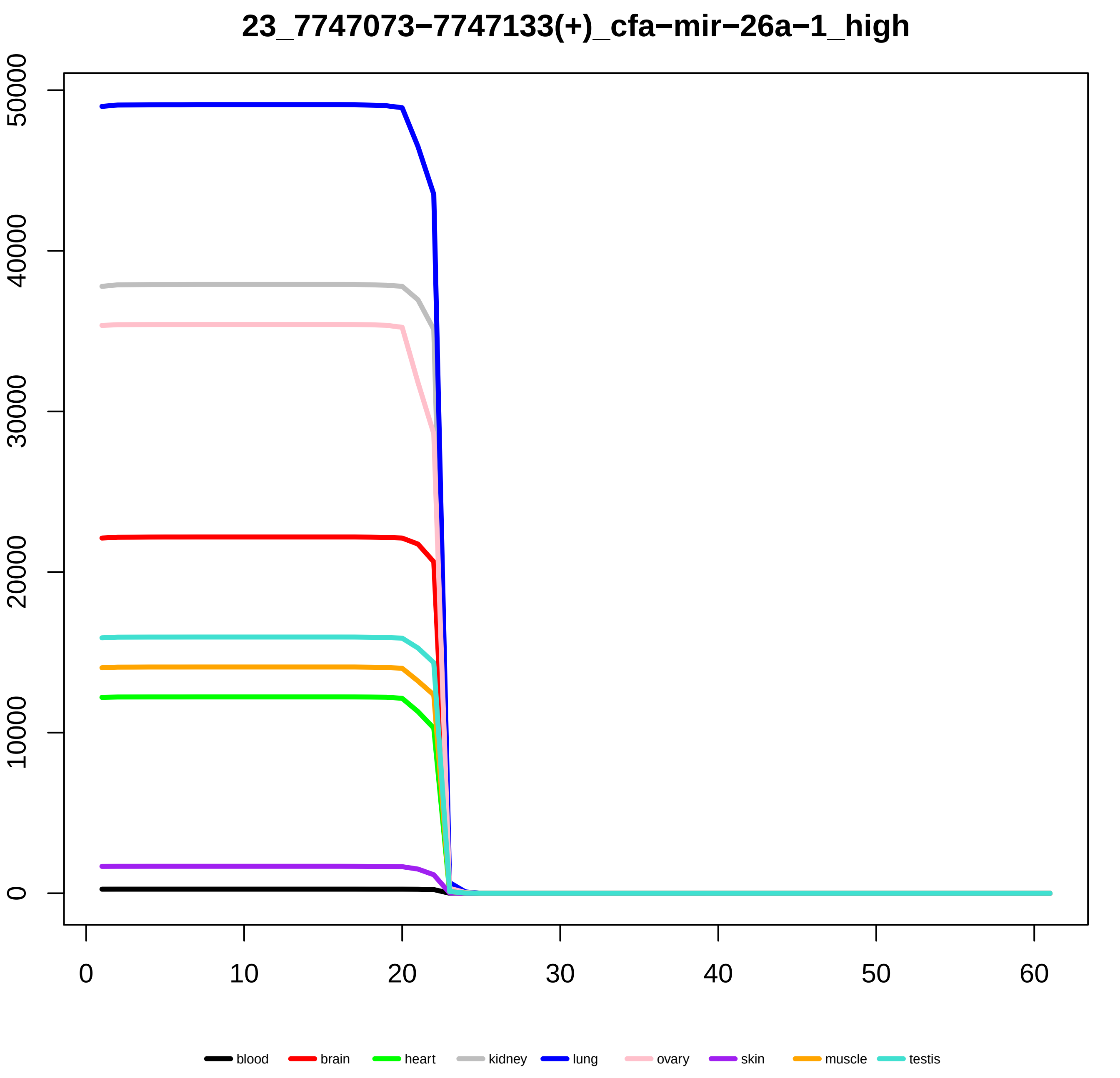

23_7747073−7747133(+)_cfa−mir−26a−1_high
0
10000
20000
30000
40000
50000
0
10
20
30
40
50
60
blood
brain
heart
kidney
lung
ovary
skin
muscle
testis

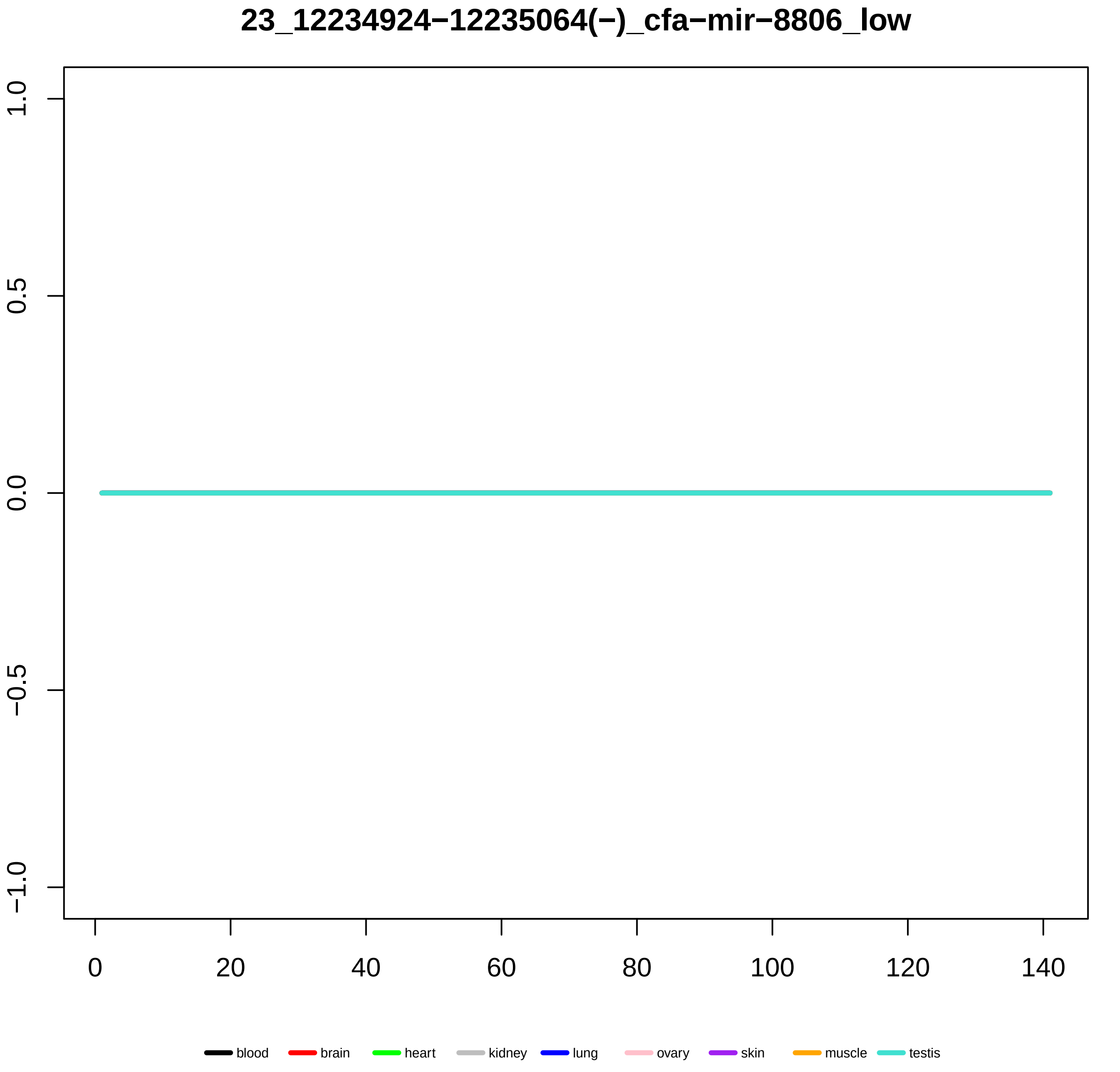
23_12234924−12235064(−)_cfa−mir−8806_low
1.0
0.5
0.0
−0.5
−1.0
0
20
40
60
80
100
120
140
blood
brain
heart
kidney
lung
ovary
skin
muscle
testis

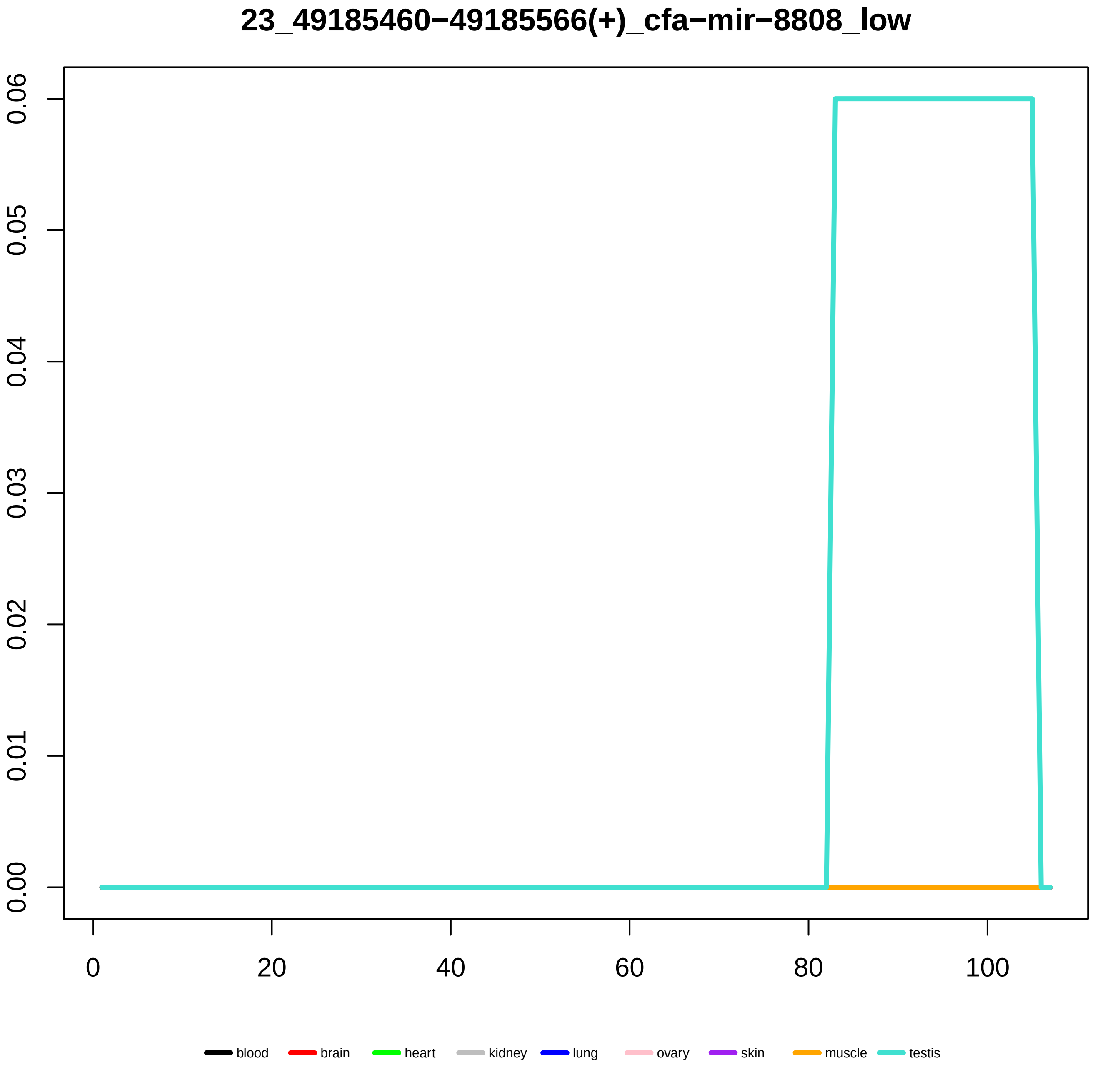
23_49185460−49185566(+)_cfa−mir−8808_low
0.00
0.01
0.02
0.03
0.04
0.05
0.06
0
20
40
60
80
100
blood
brain
heart
kidney
lung
ovary
skin
muscle
testis

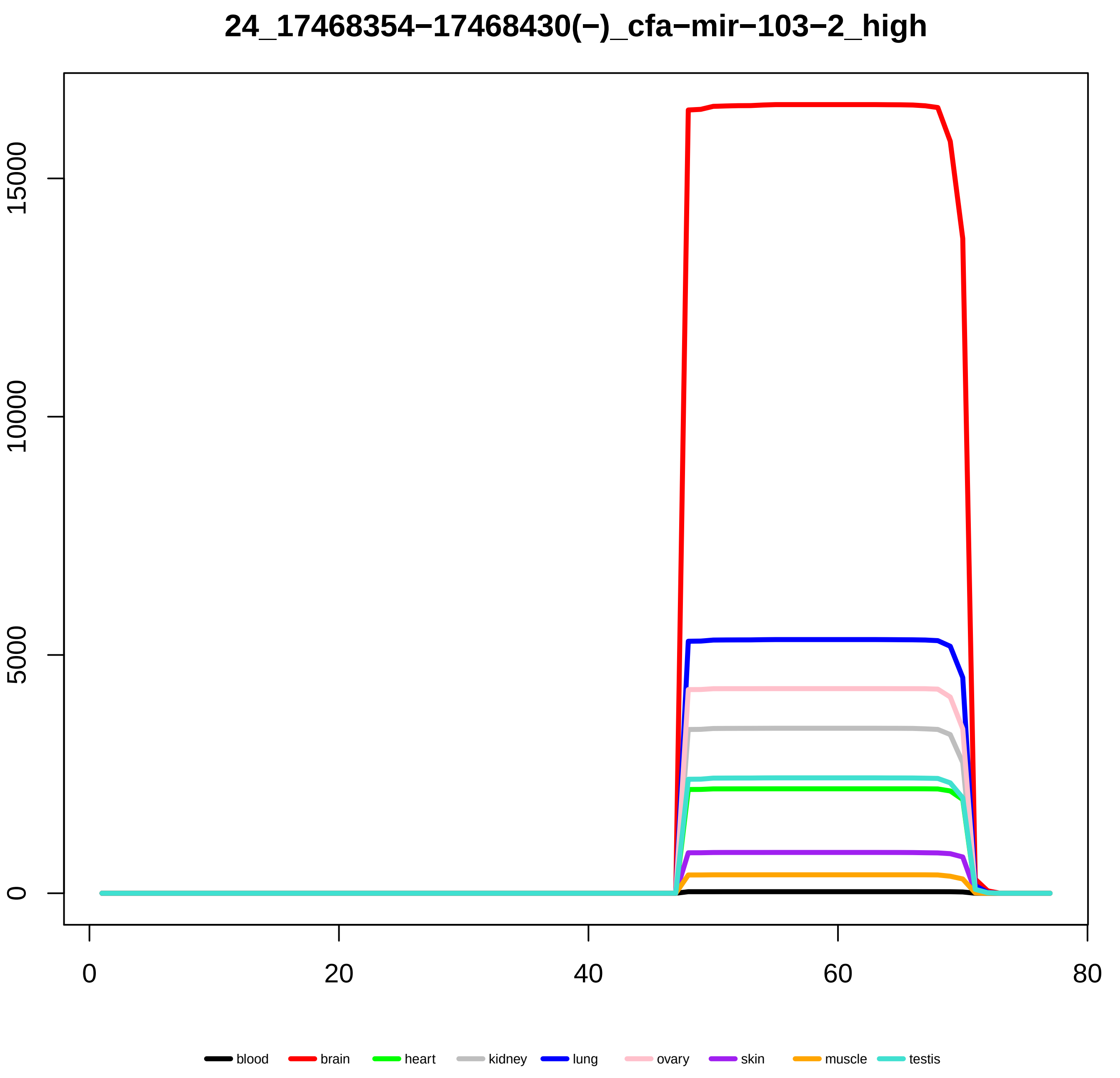
24_17468354−17468430(−)_cfa−mir−103−2_high
0
5000
10000
15000
0
20
40
60
80
blood
brain
heart
kidney
lung
ovary
skin
muscle
testis

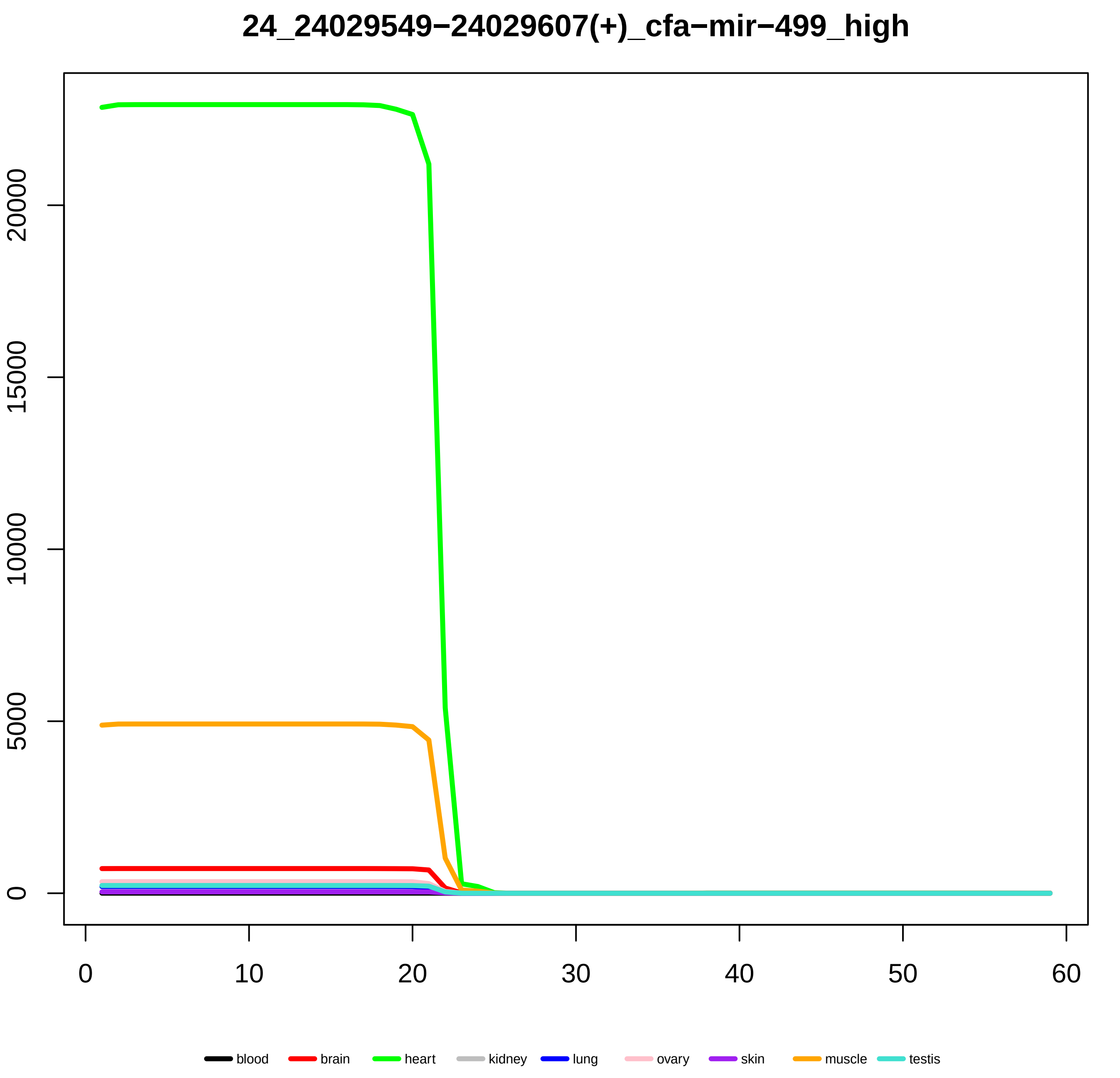
24_24029549−24029607(+)_cfa−mir−499_high
0
5000
10000
15000
20000
0
10
20
30
40
50
60
blood
brain
heart
kidney
lung
ovary
skin
muscle
testis

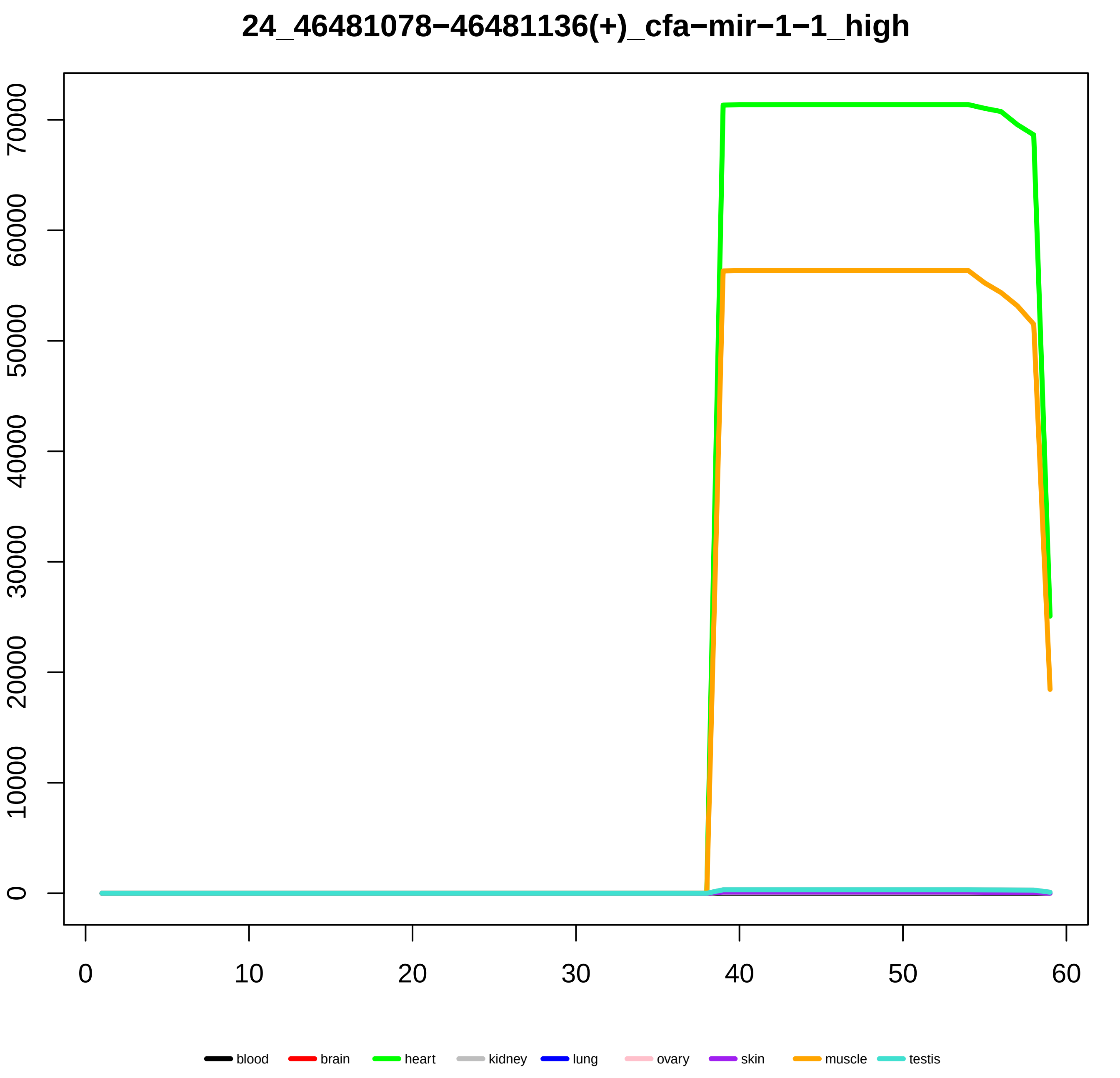
24_46481078−46481136(+)_cfa−mir−1−1_high
0
10000
20000
30000
40000
50000
60000
70000
0
10
20
30
40
50
60
blood
brain
heart
kidney
lung
ovary
skin
muscle
testis

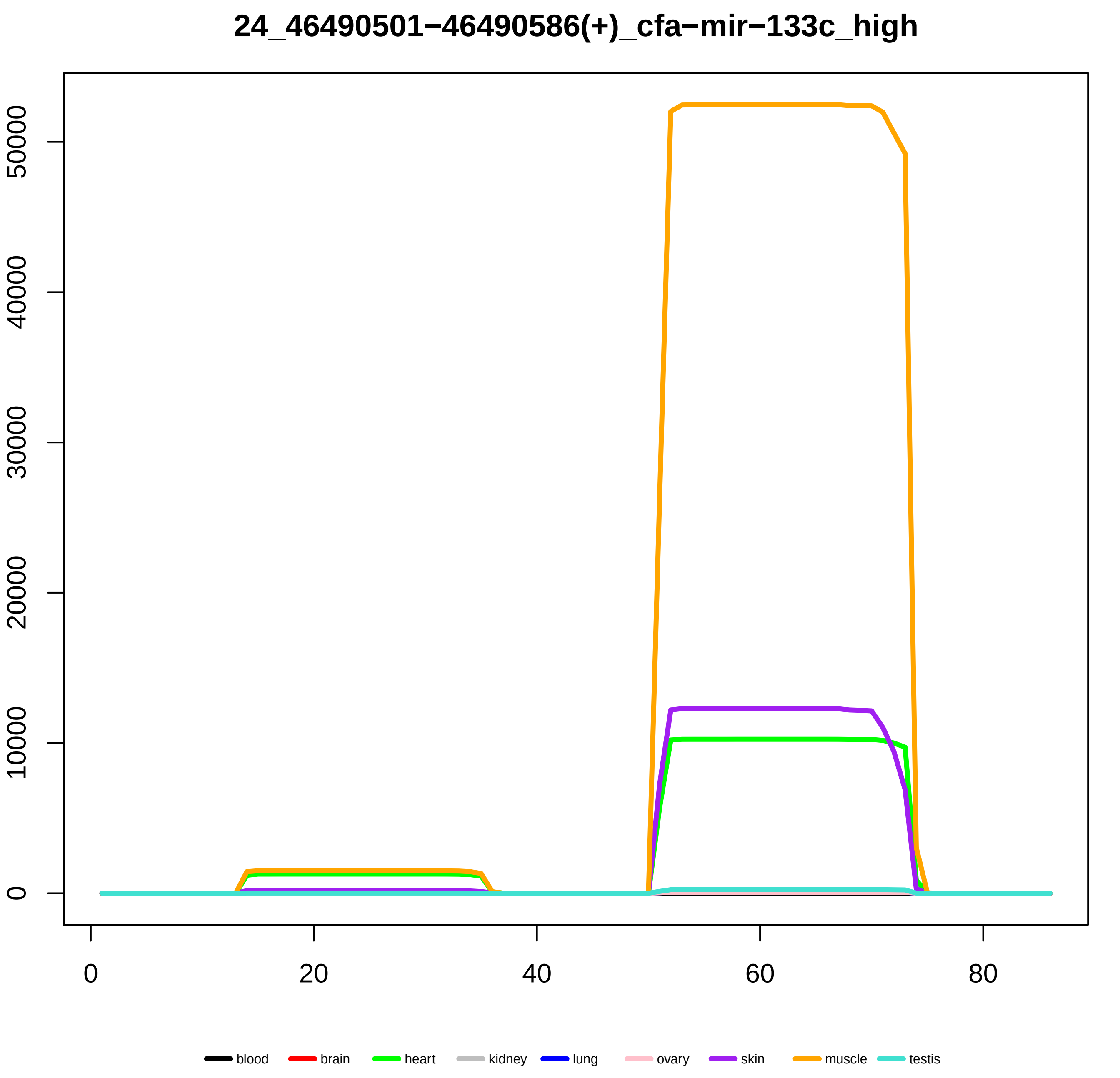

24_46490501−46490586(+)_cfa−mir−133c_high
0
10000
20000
30000
40000
50000
0
20
40
60
80
blood
brain
heart
kidney
lung
ovary
skin
muscle
testis

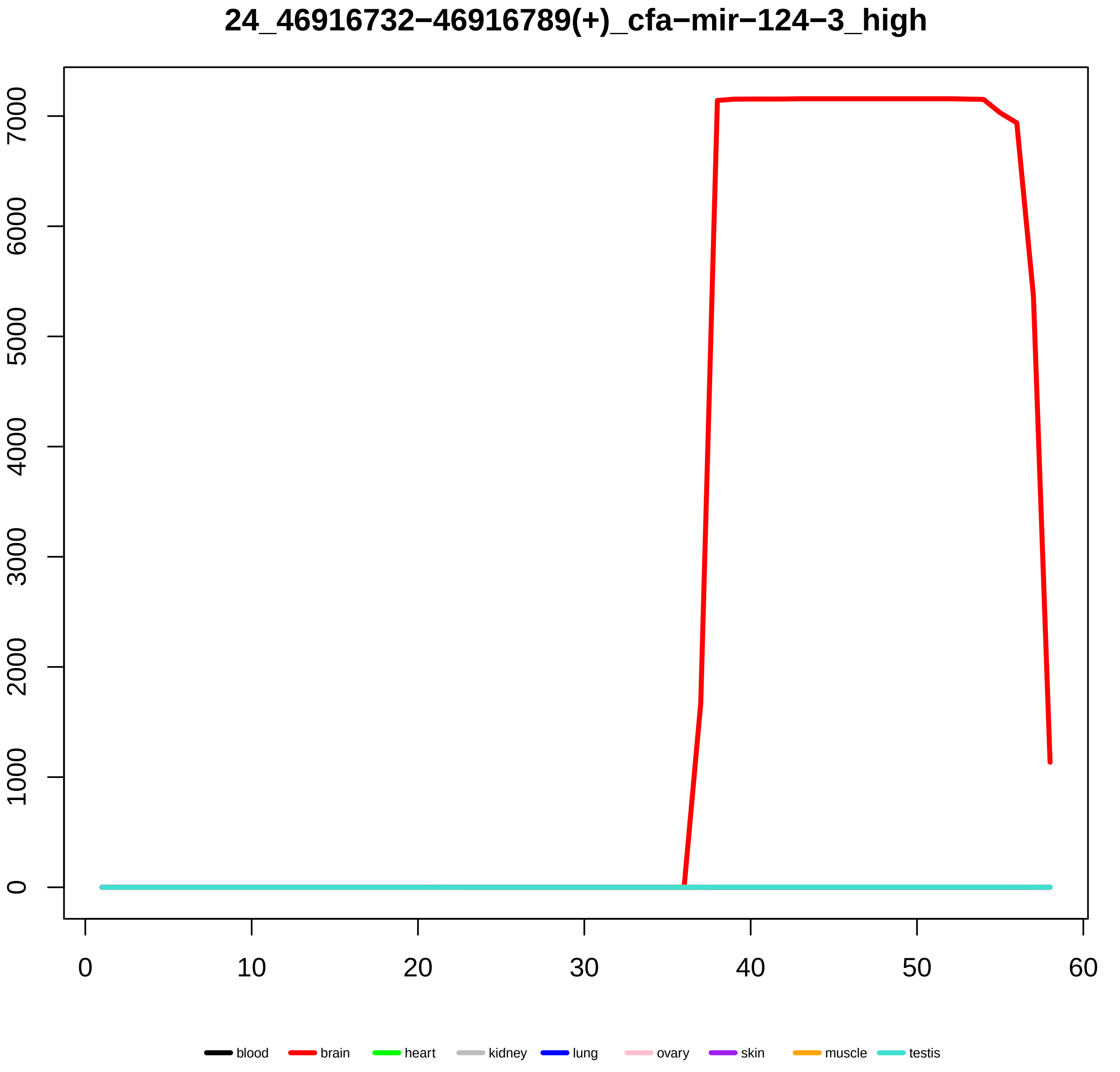
24_46916732−46916789(+)_cfa−mir−124−3_high
0
1000
2000
3000
4000
5000
6000
7000
0
10
20
30
40
50
60
blood
brain
heart
kidney
lung
ovary
skin
muscle
testis

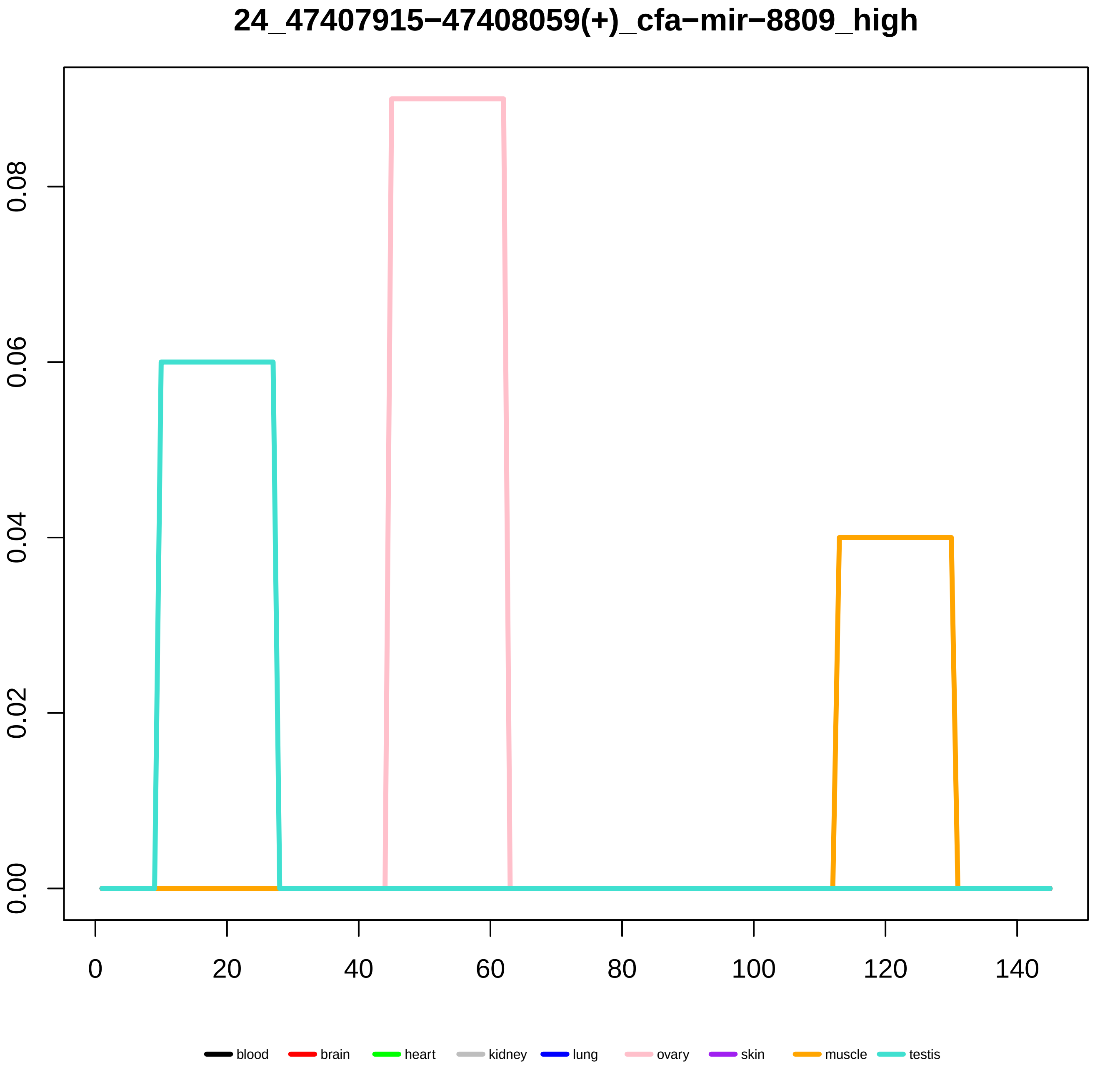
24_47407915−47408059(+)_cfa−mir−8809_high
0.00
0.02
0.04
0.06
0.08
0
20
40
60
80
100
120
140
blood
brain
heart
kidney
lung
ovary
skin
muscle
testis

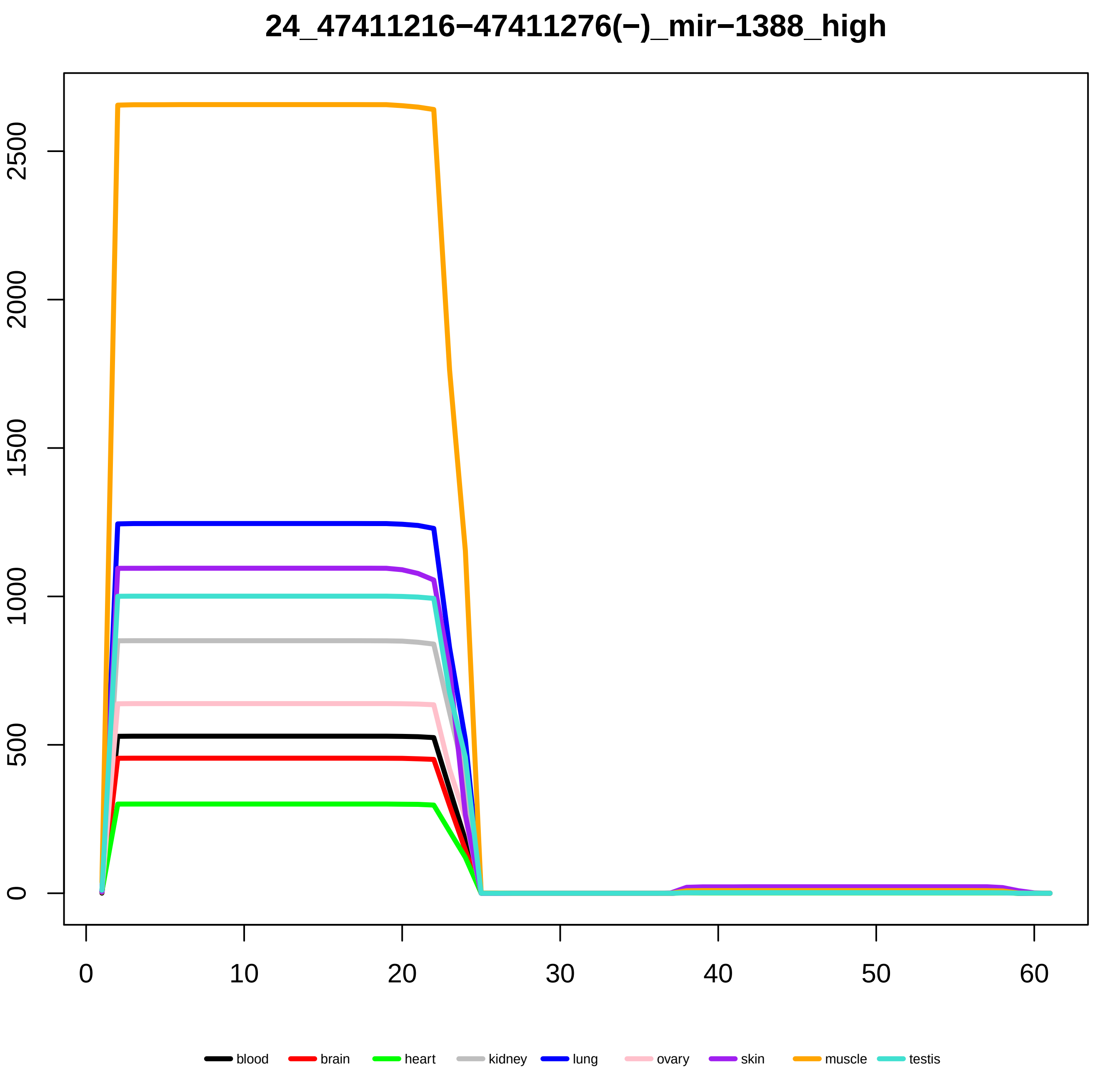

24_47411216−47411276(−)_mir−1388_high
0
500
1000
1500
2000
2500
0
10
20
30
40
50
60
blood
brain
heart
kidney
lung
ovary
skin
muscle
testis

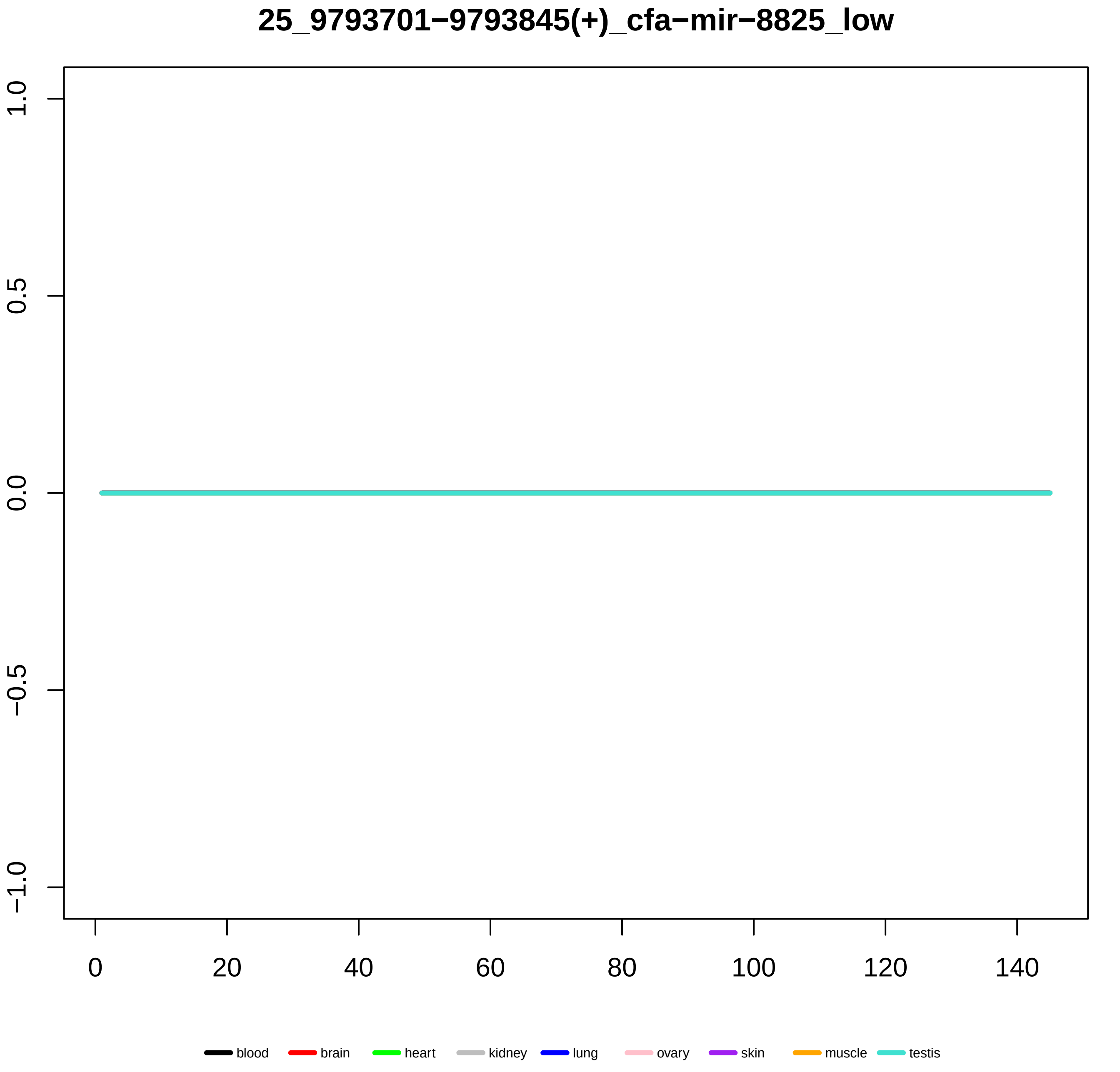

25_9793701−9793845(+)_cfa−mir−8825_low
1.0
0.5
0.0
−0.5
−1.0
0
20
40
60
80
100
120
140
blood
brain
heart
kidney
lung
ovary
skin
muscle
testis

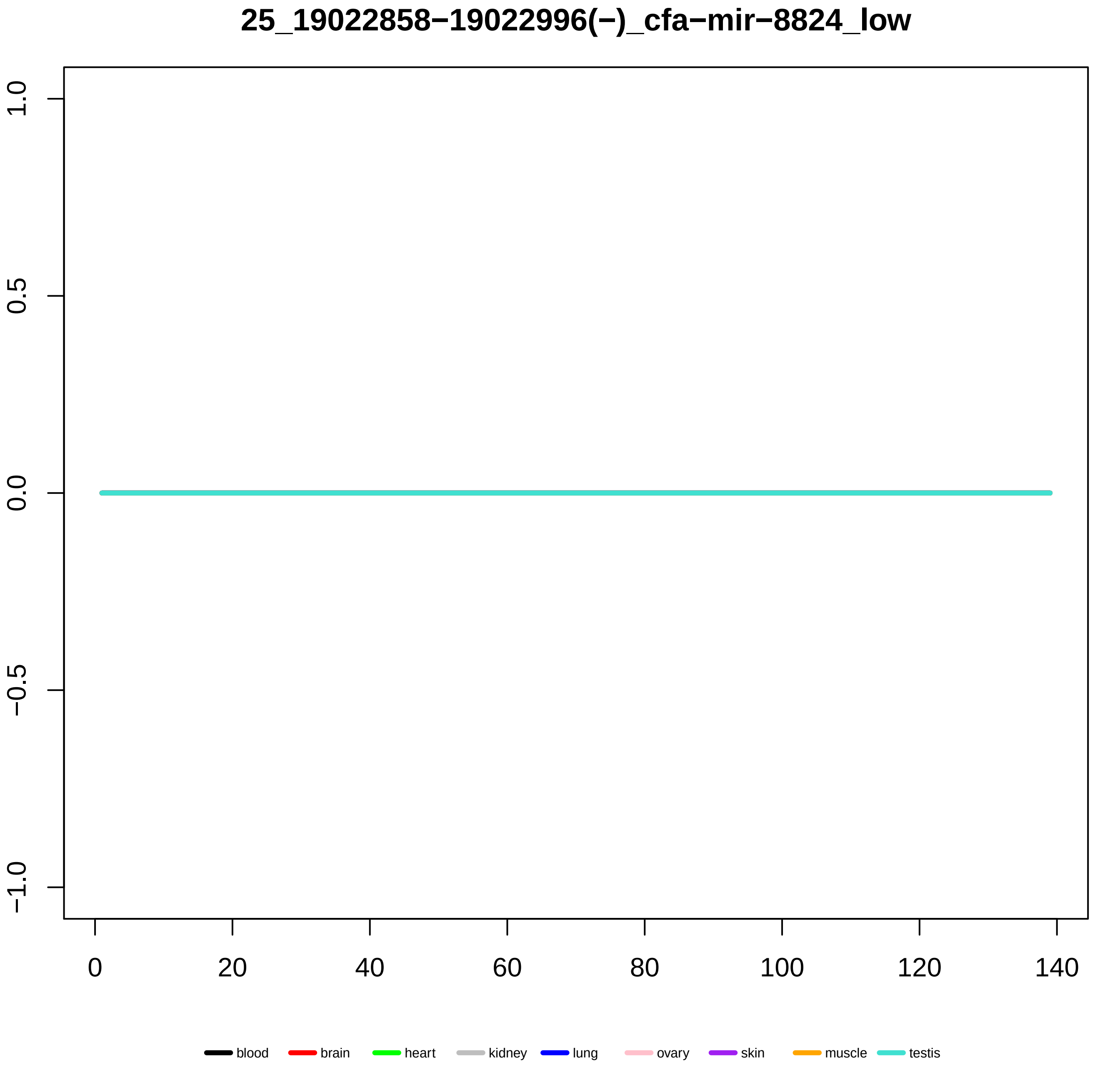
25_19022858−19022996(−)_cfa−mir−8824_low
1.0
0.5
0.0
−0.5
−1.0
0
20
40
60
80
100
120
140
blood
brain
heart
kidney
lung
ovary
skin
muscle
testis

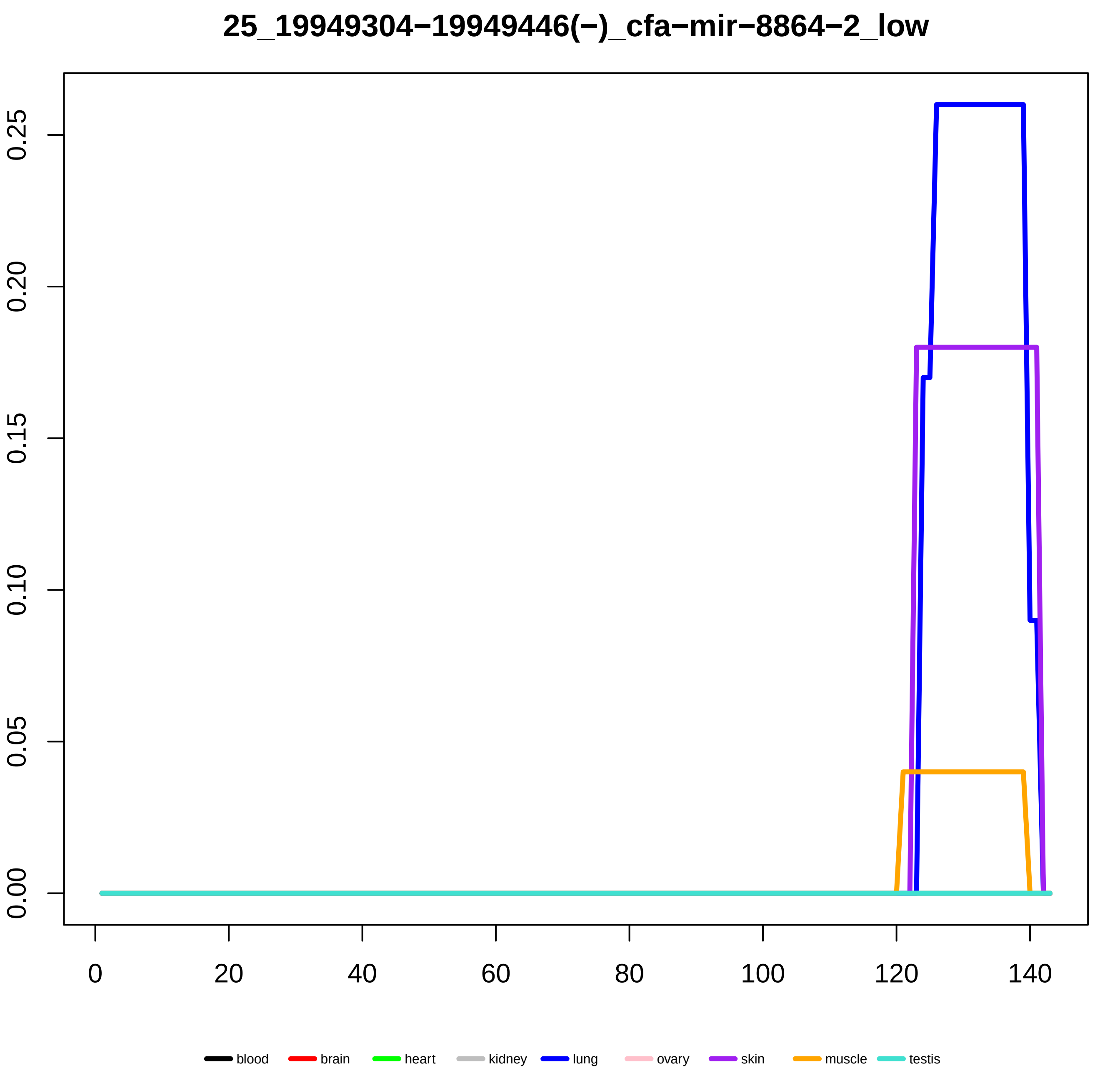

25_19949304−19949446(−)_cfa−mir−8864−2_low
0.00
0.05
0.10
0.15
0.20
0.25
0
20
40
60
80
100
120
140
blood
brain
heart
kidney
lung
ovary
skin
muscle
testis

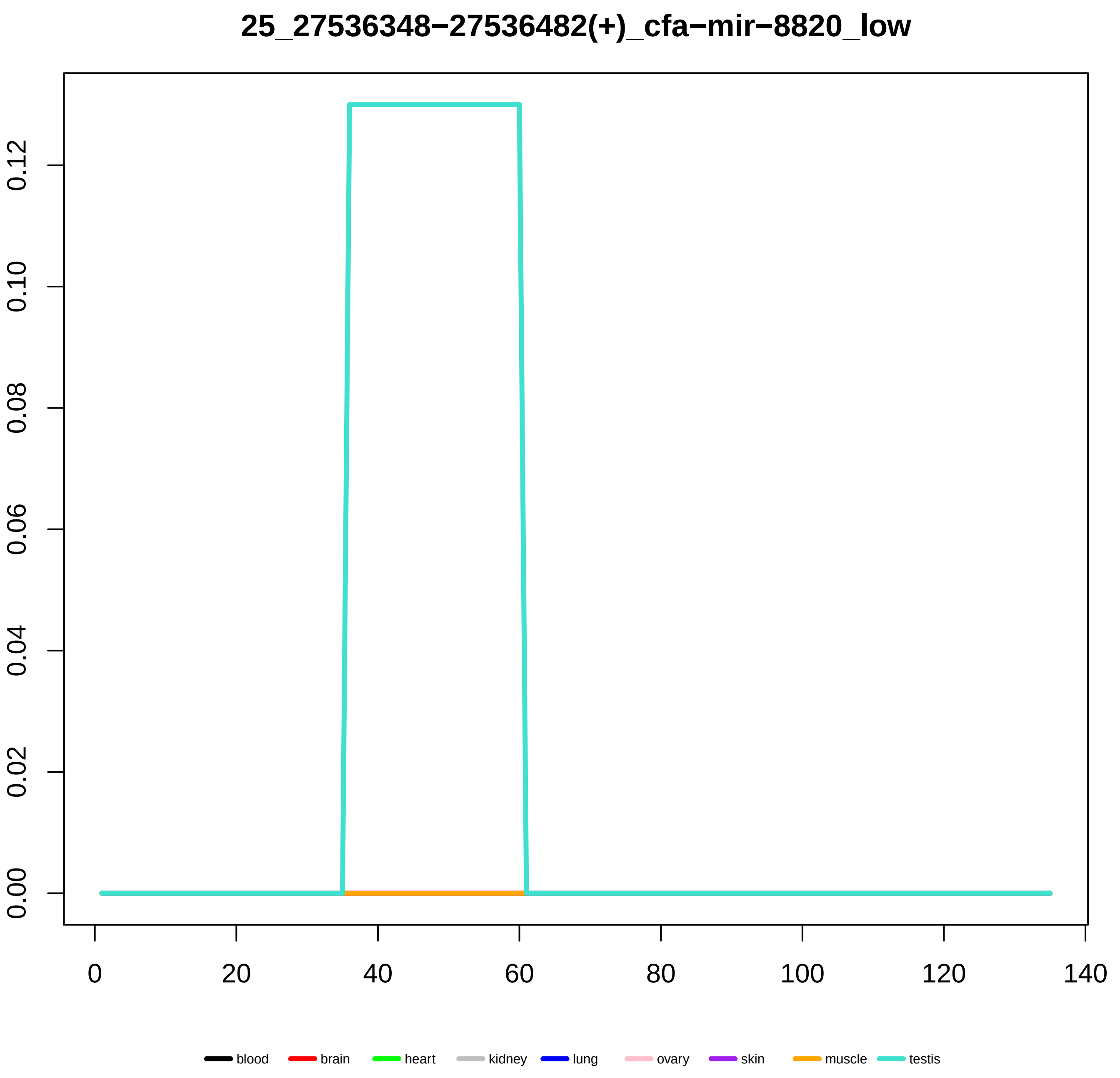
25_27536348−27536482(+)_cfa−mir−8820_low
0.00
0.02
0.04
0.06
0.08
0.10
0.12
0
20
40
60
80
100
120
140
blood
brain
heart
kidney
lung
ovary
skin
muscle
testis

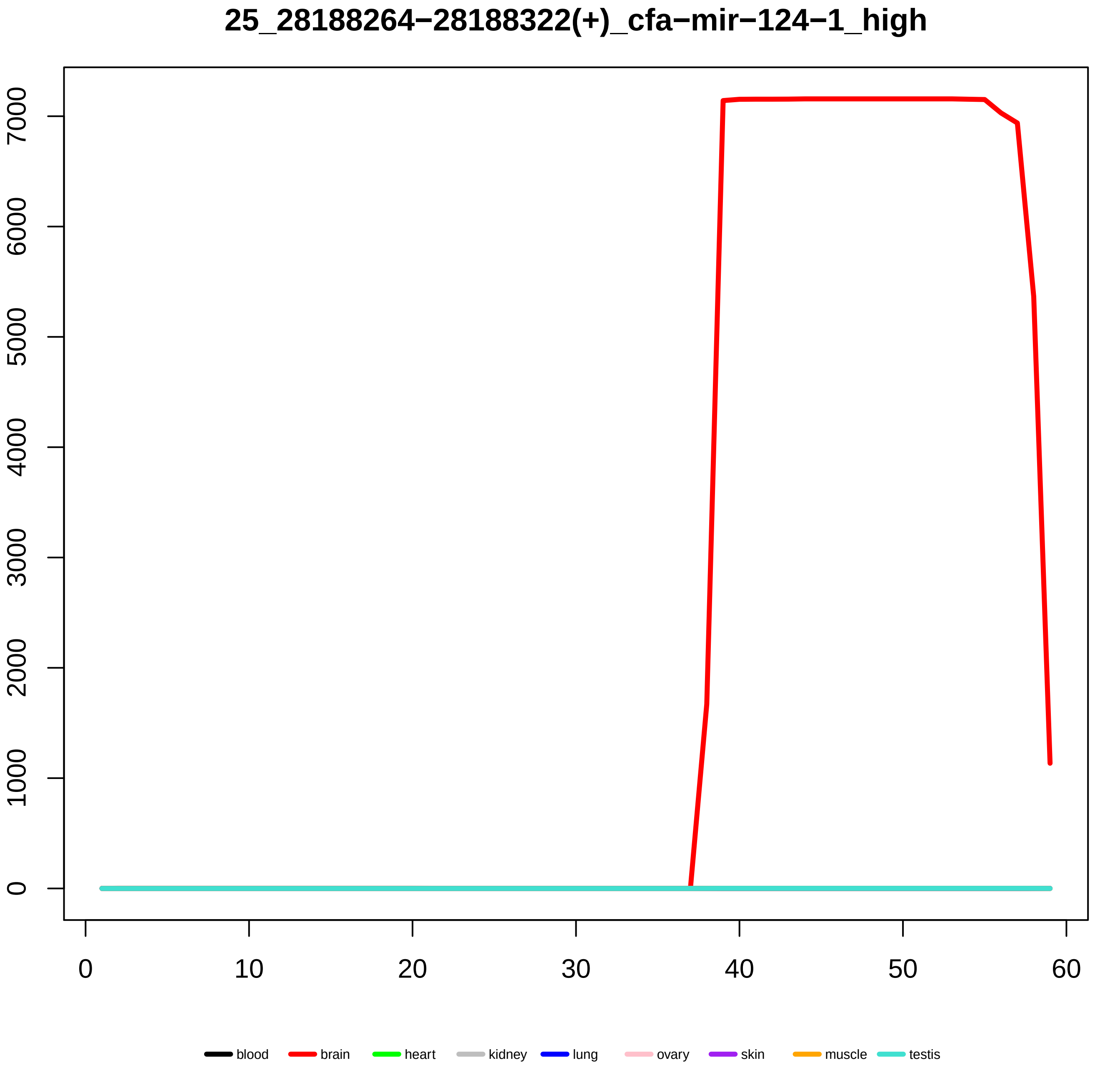

25_28188264−28188322(+)_cfa−mir−124−1_high
0
1000
2000
3000
4000
5000
6000
7000
0
10
20
30
40
50
60
blood
brain
heart
kidney
lung
ovary
skin
muscle
testis

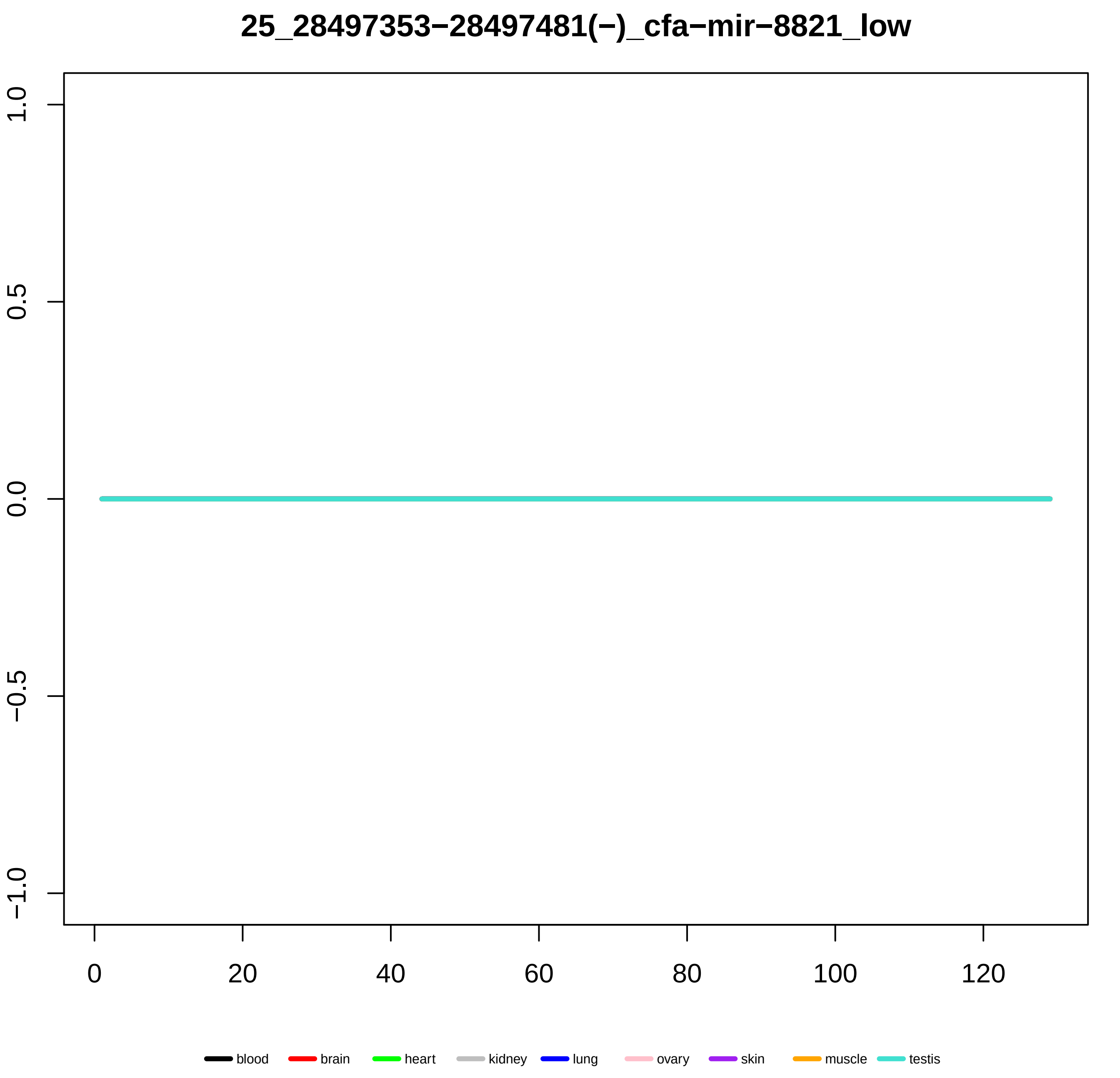
25_28497353−28497481(−)_cfa−mir−8821_low
1.0
0.5
0.0
−0.5
−1.0
0
20
40
60
80
100
120
blood
brain
heart
kidney
lung
ovary
skin
muscle
testis

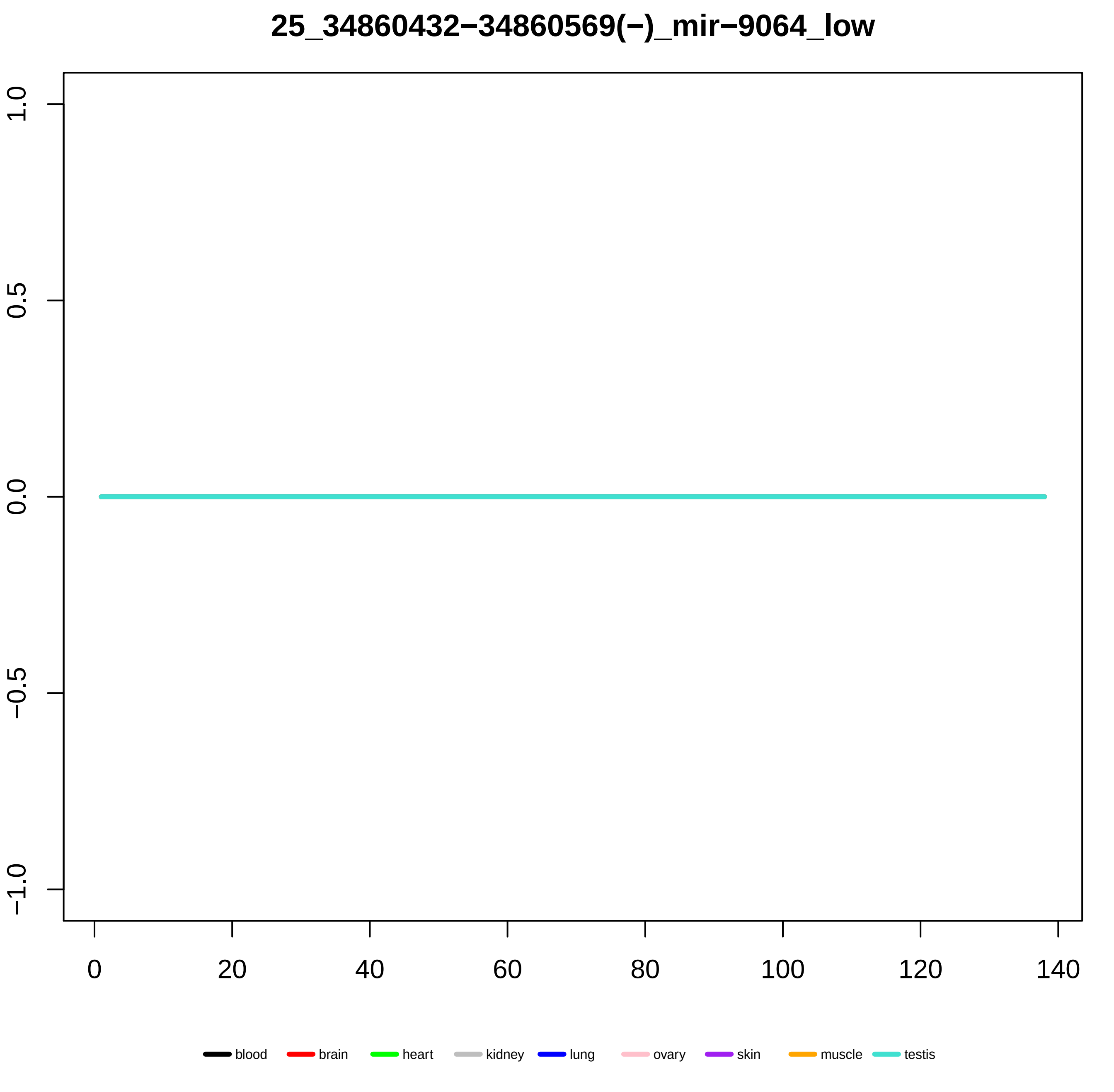

25_34860432−34860569(−)_mir−9064_low
1.0
0.5
0.0
−0.5
−1.0
0
20
40
60
80
100
120
140
blood
brain
heart
kidney
lung
ovary
skin
muscle
testis

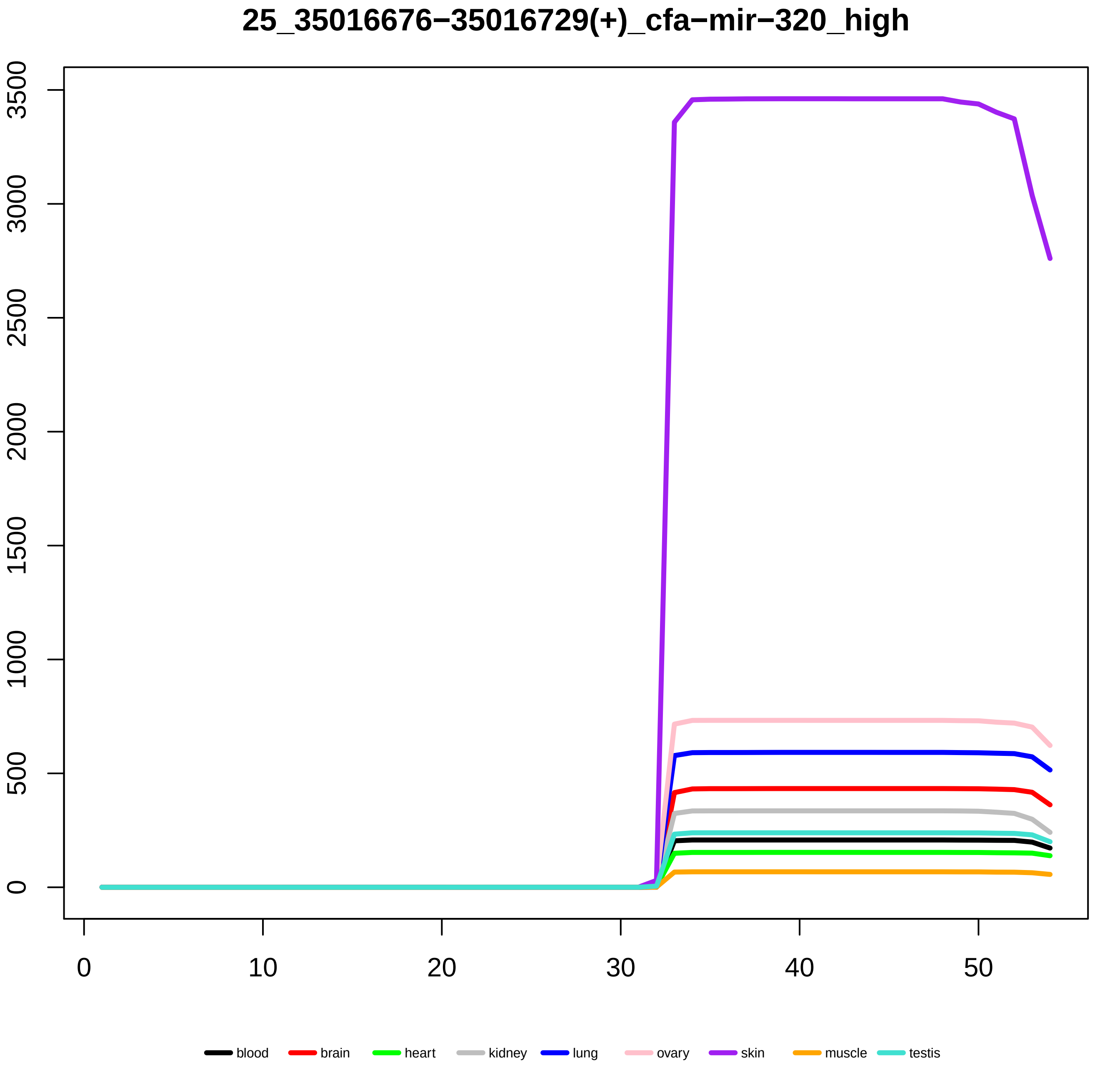

25_35016676−35016729(+)_cfa−mir−320_high
0
500
1000
1500
2000
2500
3000
3500
0
10
20
30
40
50
blood
brain
heart
kidney
lung
ovary
skin
muscle
testis

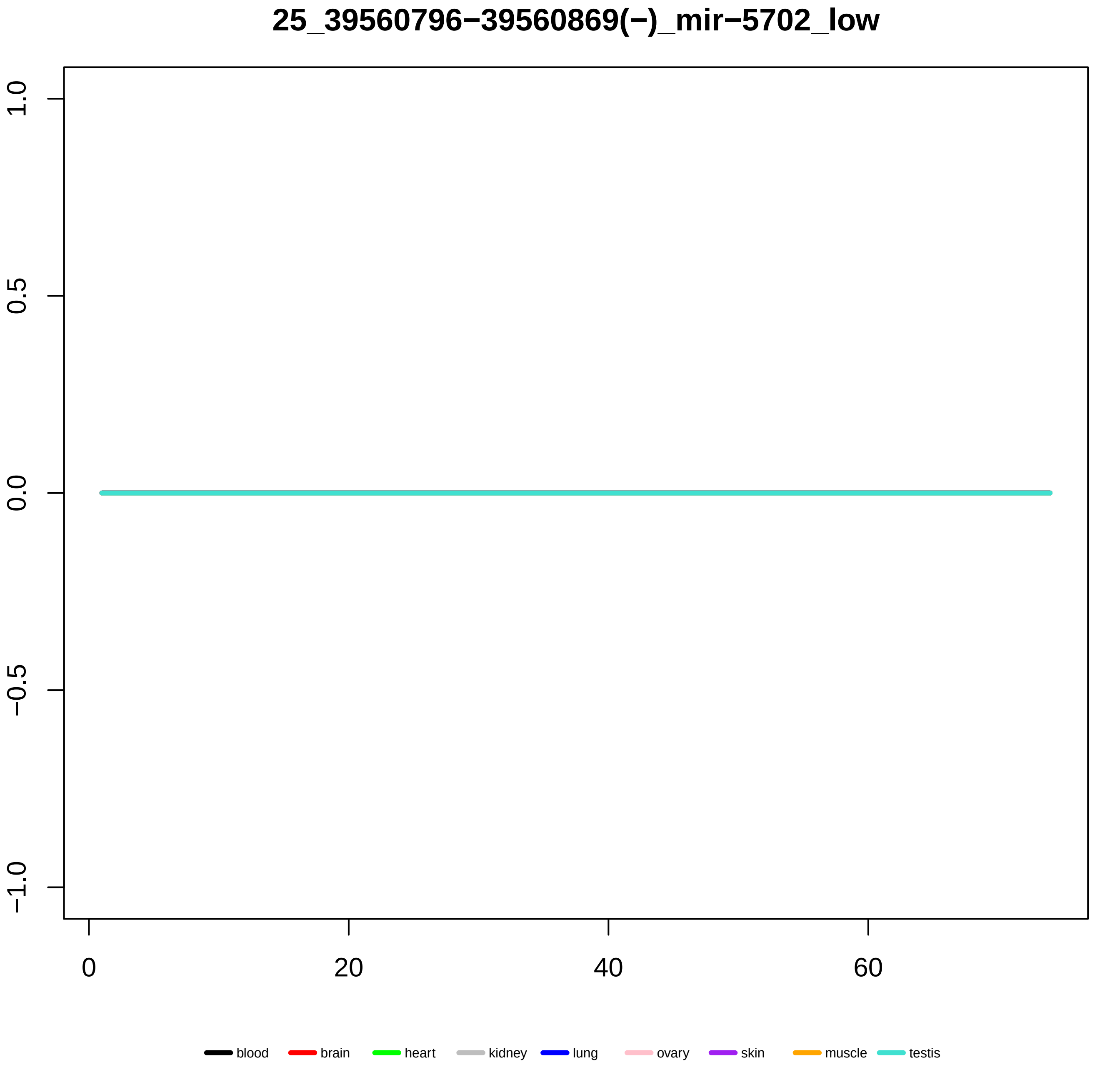

25_39560796−39560869(−)_mir−5702_low
1.0
0.5
0.0
−0.5
−1.0
0
20
40
60
blood
brain
heart
kidney
lung
ovary
skin
muscle
testis

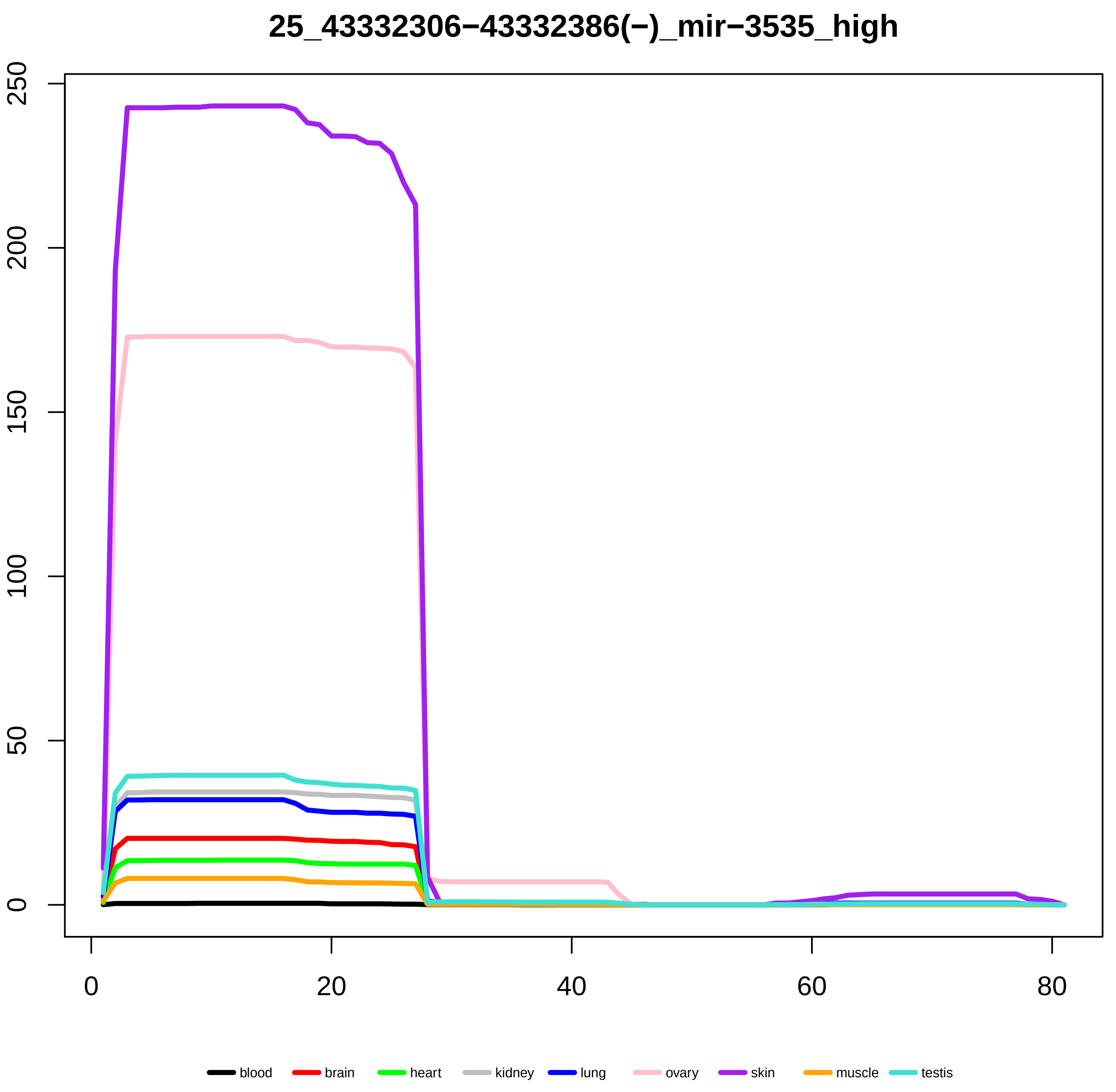

25_43332306−43332386(−)_mir−3535_high
0
50
100
150
200
250
0
20
40
60
80
blood
brain
heart
kidney
lung
ovary
skin
muscle
testis

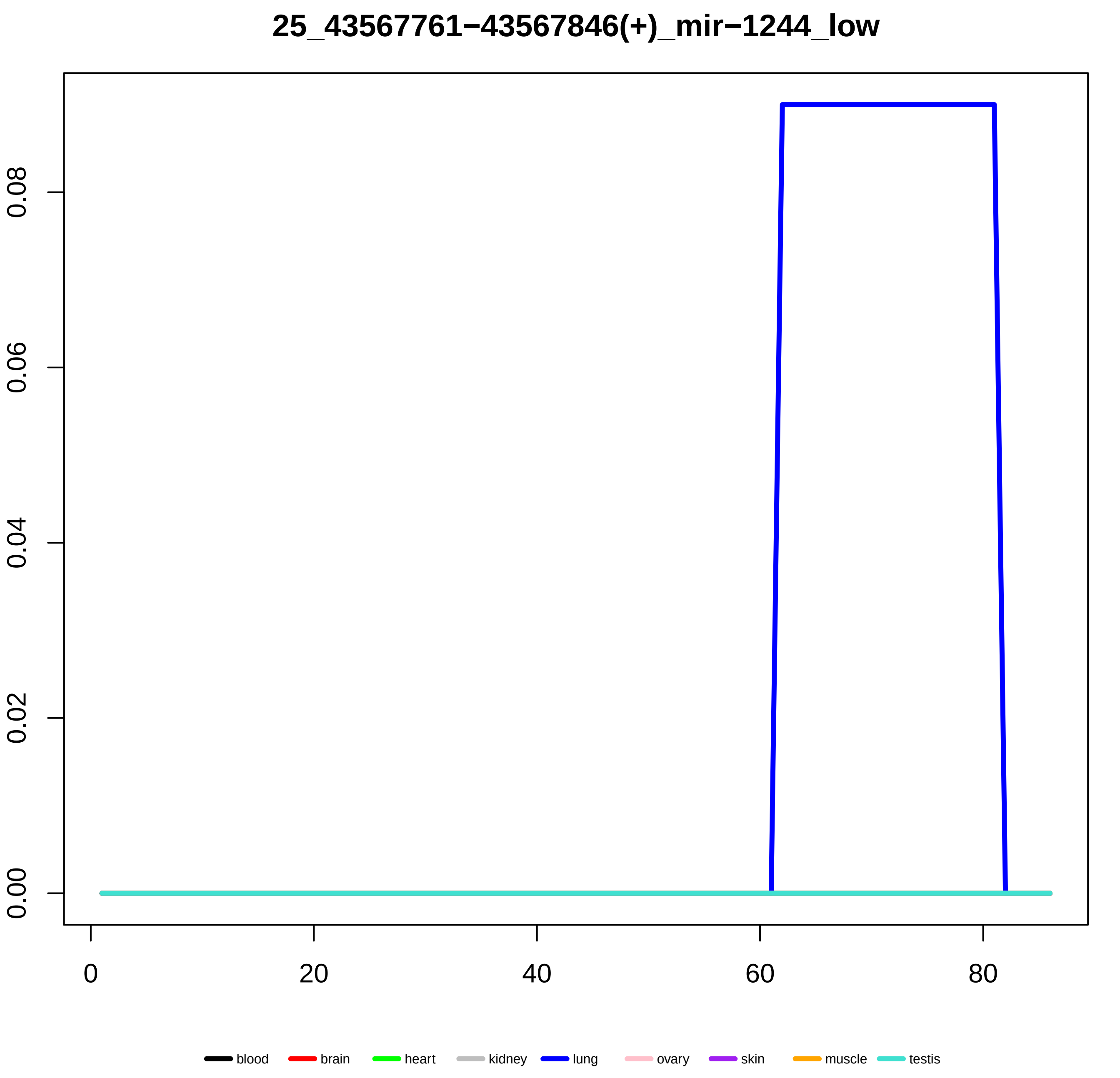

25_43567761−43567846(+)_mir−1244_low
0.00
0.02
0.04
0.06
0.08
0
20
40
60
80
blood
brain
heart
kidney
lung
ovary
skin
muscle
testis

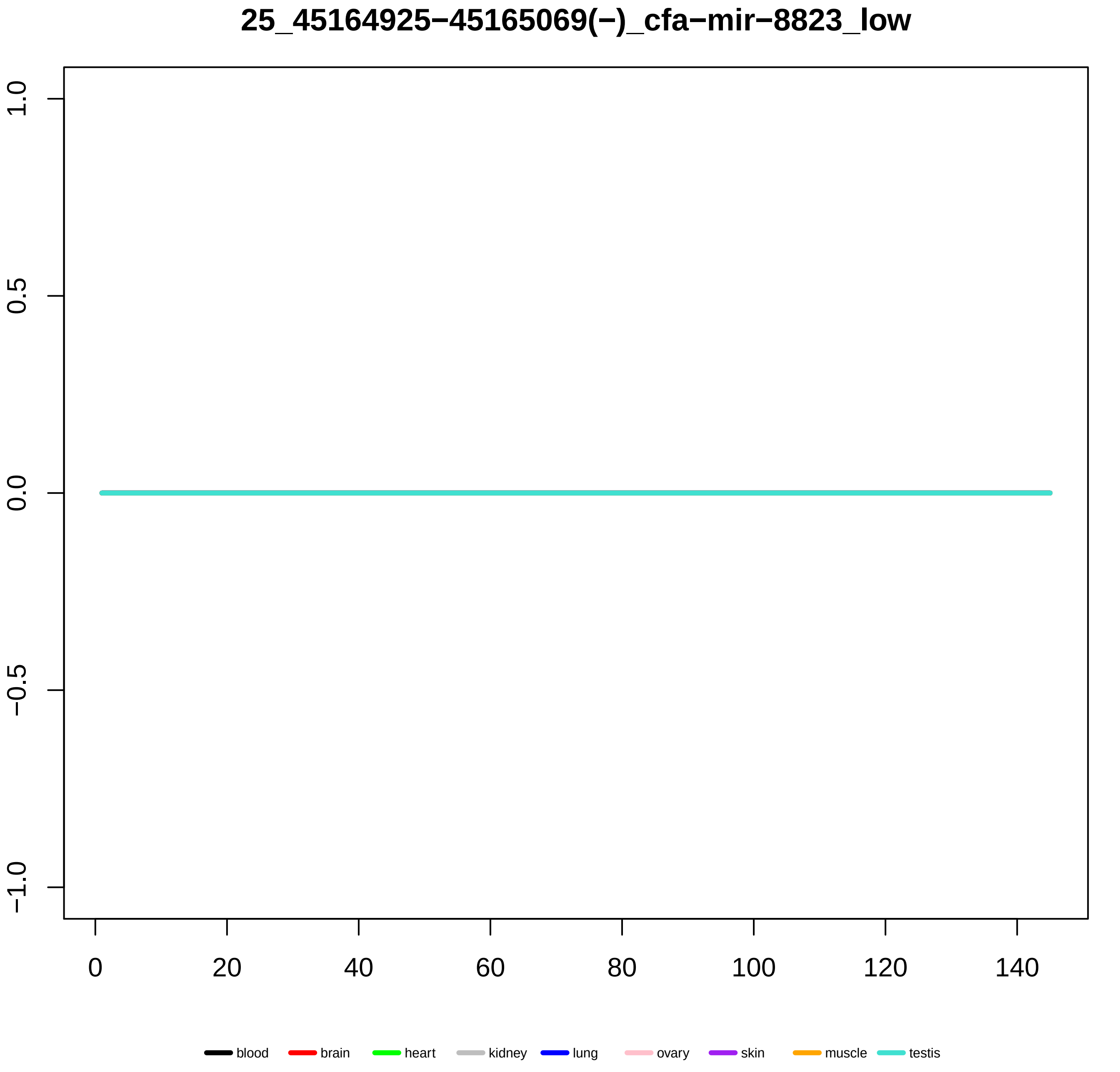
25_45164925−45165069(−)_cfa−mir−8823_low
1.0
0.5
0.0
−0.5
−1.0
0
20
40
60
80
100
120
140
blood
brain
heart
kidney
lung
ovary
skin
muscle
testis

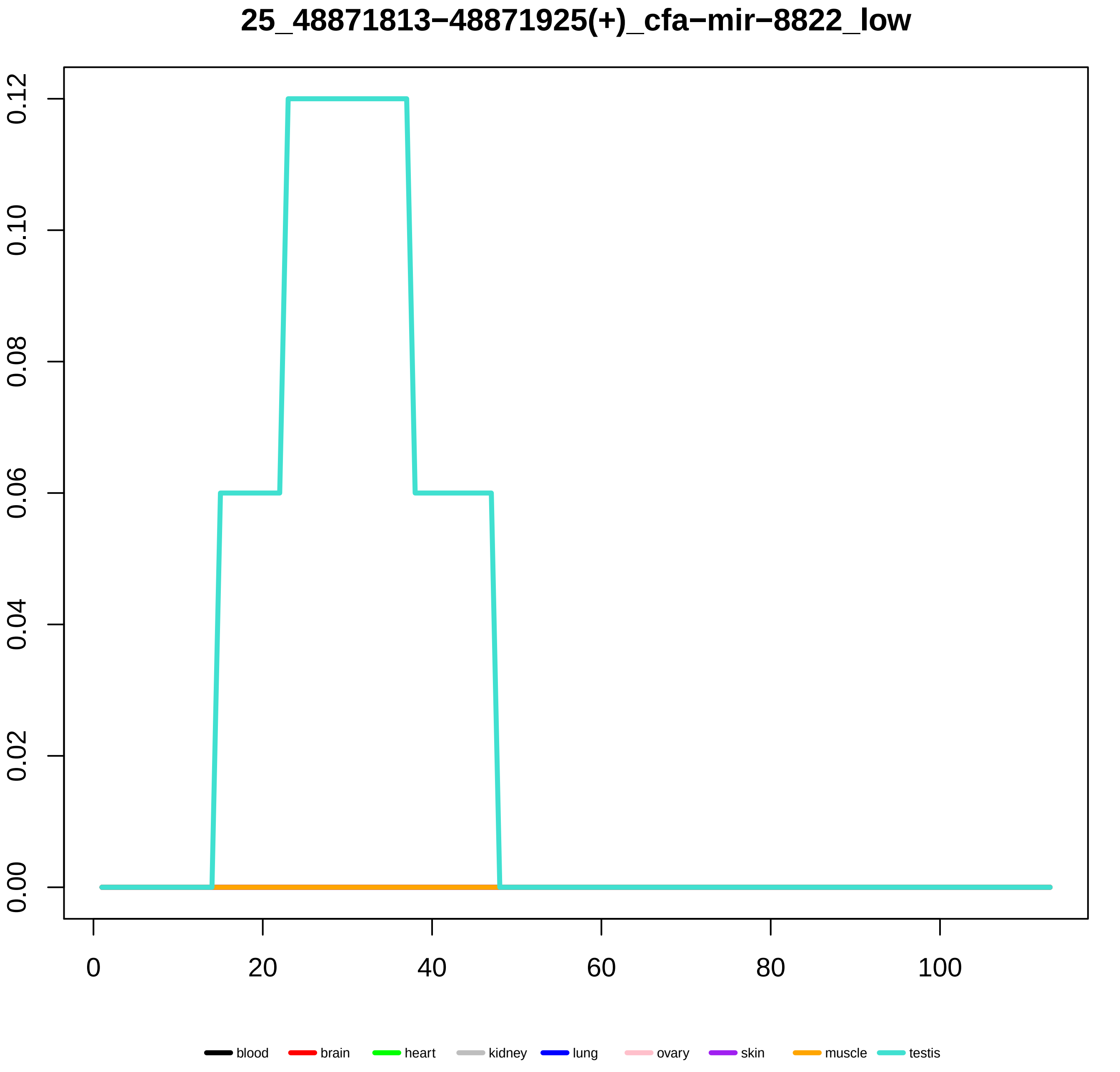

25_48871813−48871925(+)_cfa−mir−8822_low
0.00
0.02
0.04
0.06
0.08
0.10
0.12
0
20
40
60
80
100
blood
brain
heart
kidney
lung
ovary
skin
muscle
testis

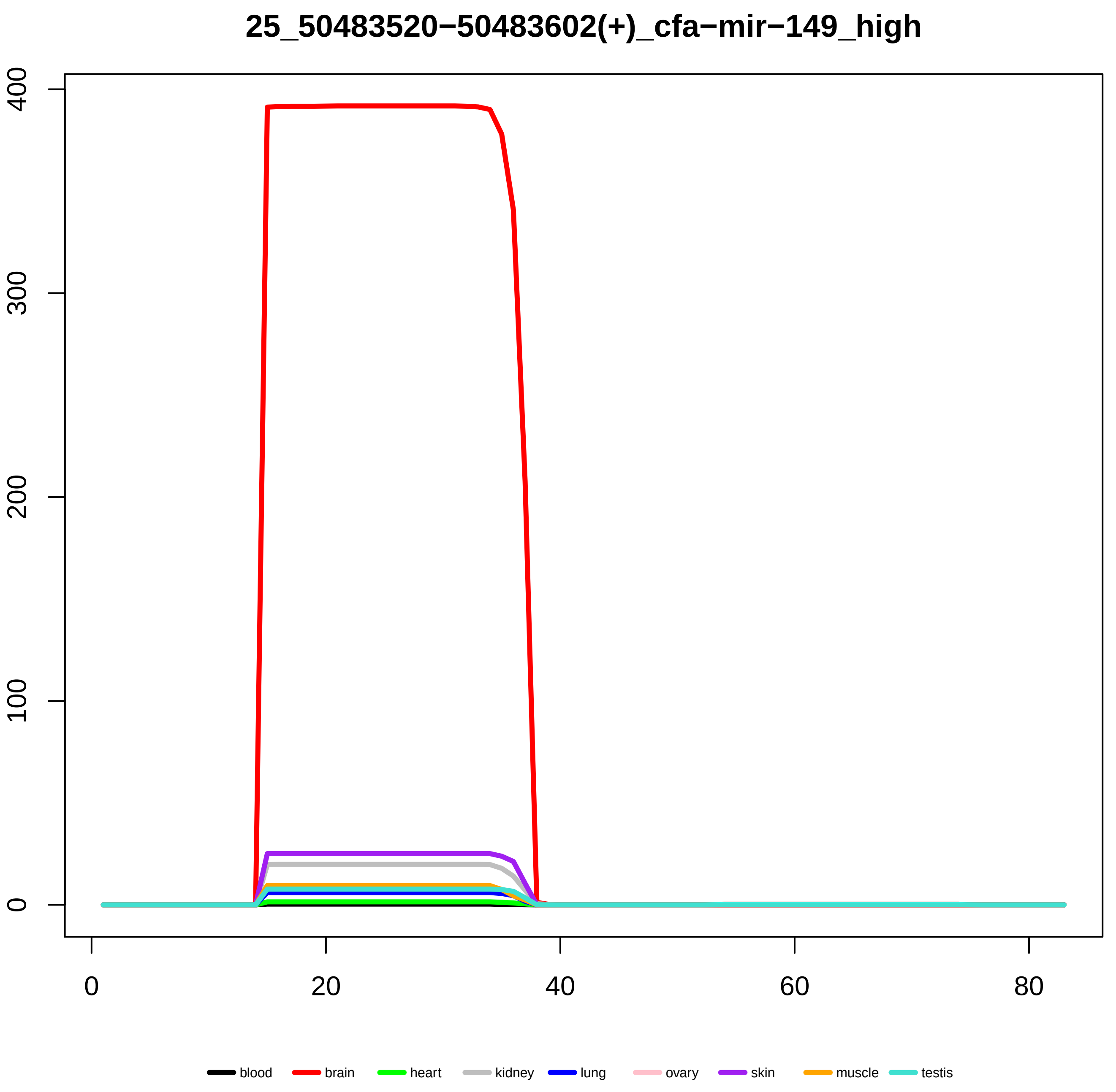

25_50483520−50483602(+)_cfa−mir−149_high
0
100
200
300
400
0
20
40
60
80
blood
brain
heart
kidney
lung
ovary
skin
muscle
testis

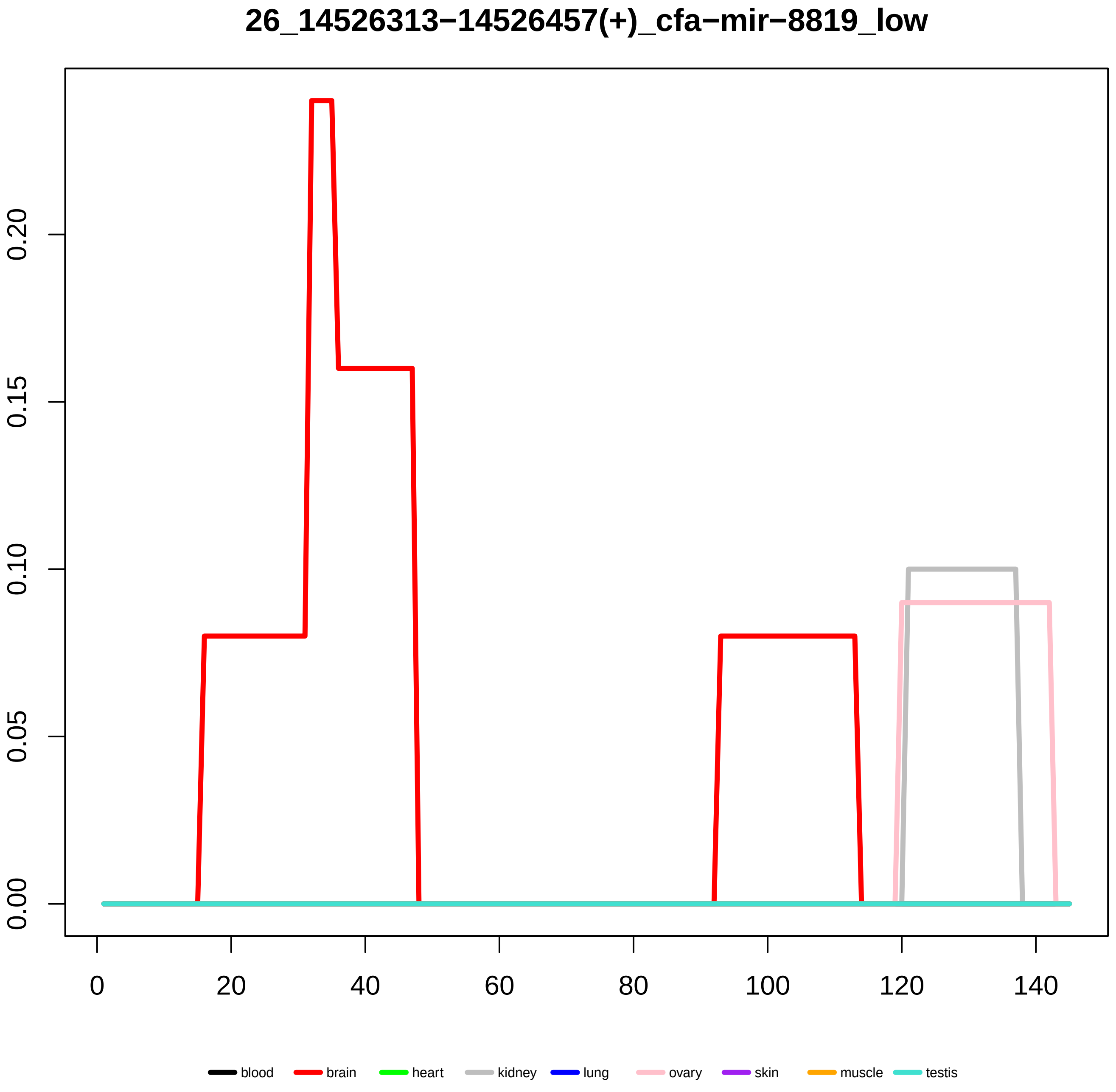
26_14526313−14526457(+)_cfa−mir−8819_low
0.00
0.05
0.10
0.15
0.20
0
20
40
60
80
100
120
140
blood
brain
heart
kidney
lung
ovary
skin
muscle
testis

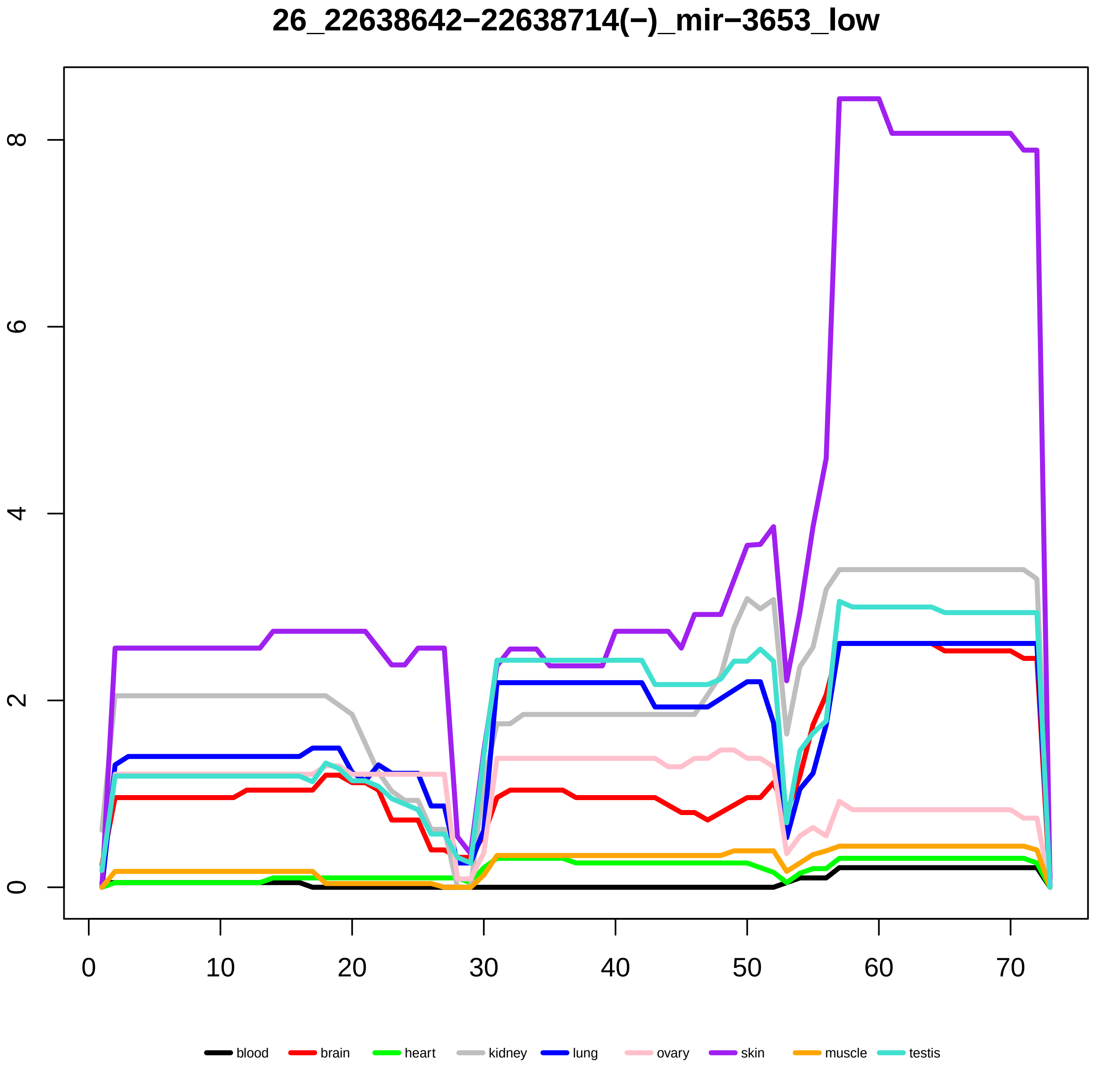

26_22638642−22638714(−)_mir−3653_low
0
2
4
6
8
0
10
20
30
40
50
60
70
blood
brain
heart
kidney
lung
ovary
skin
muscle
testis

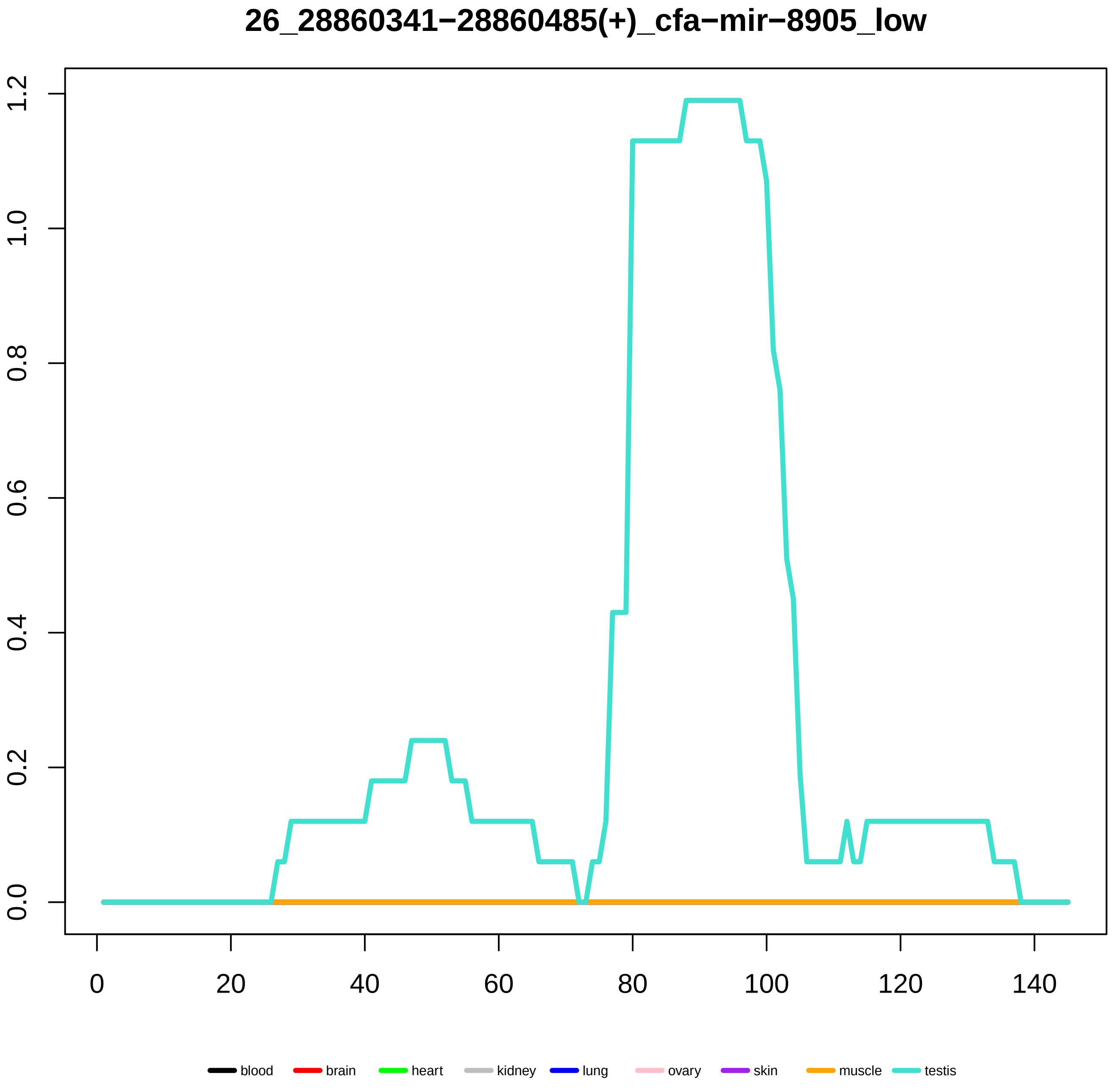
26_28860341−28860485(+)_cfa−mir−8905_low
0.0
0.2
0.4
0.6
0.8
1.0
1.2
0
20
40
60
80
100
120
140
blood
brain
heart
kidney
lung
ovary
skin
muscle
testis

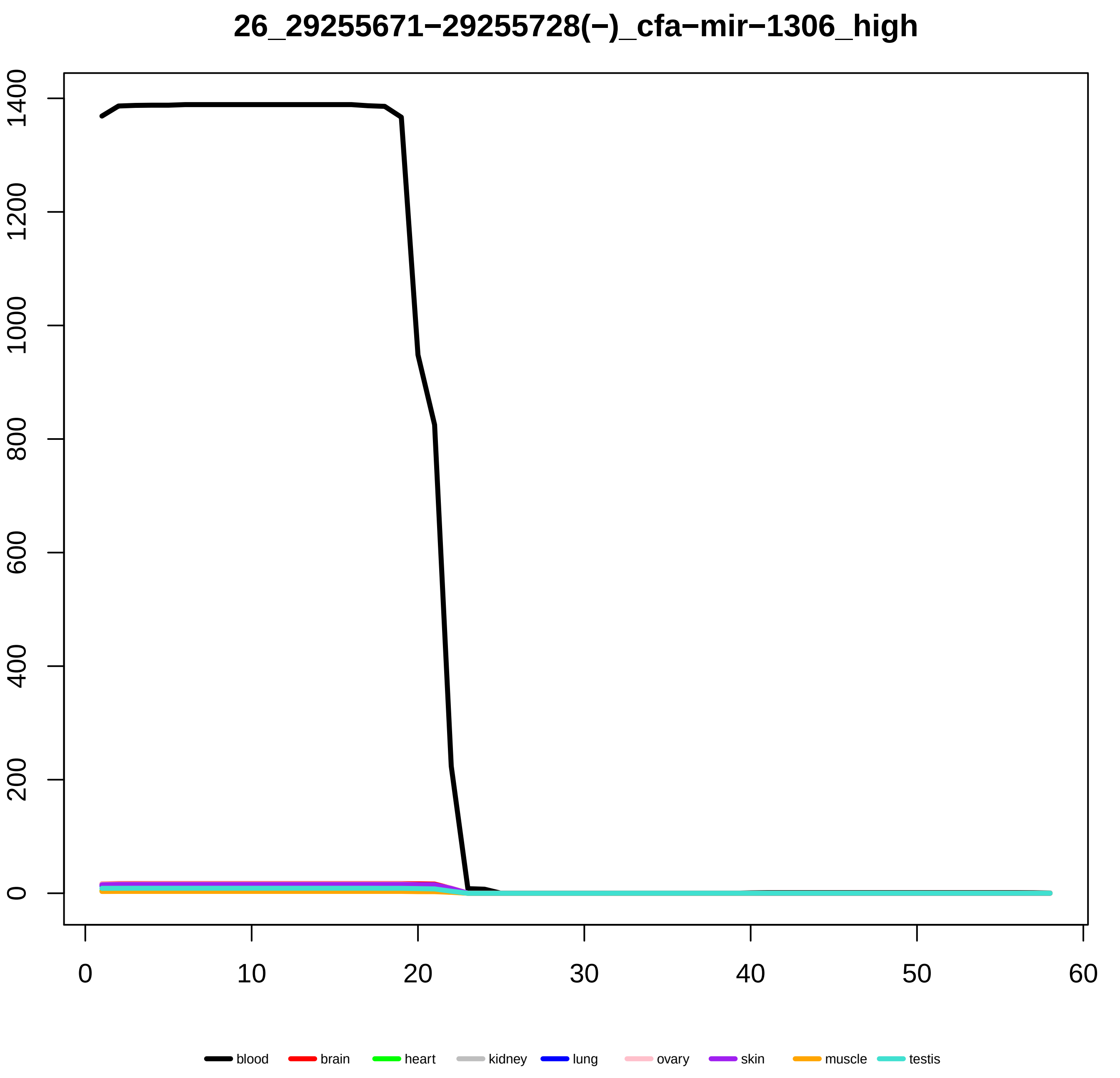

26_29255671−29255728(−)_cfa−mir−1306_high
0
200
400
600
800
1000
1200
1400
0
10
20
30
40
50
60
blood
brain
heart
kidney
lung
ovary
skin
muscle
testis

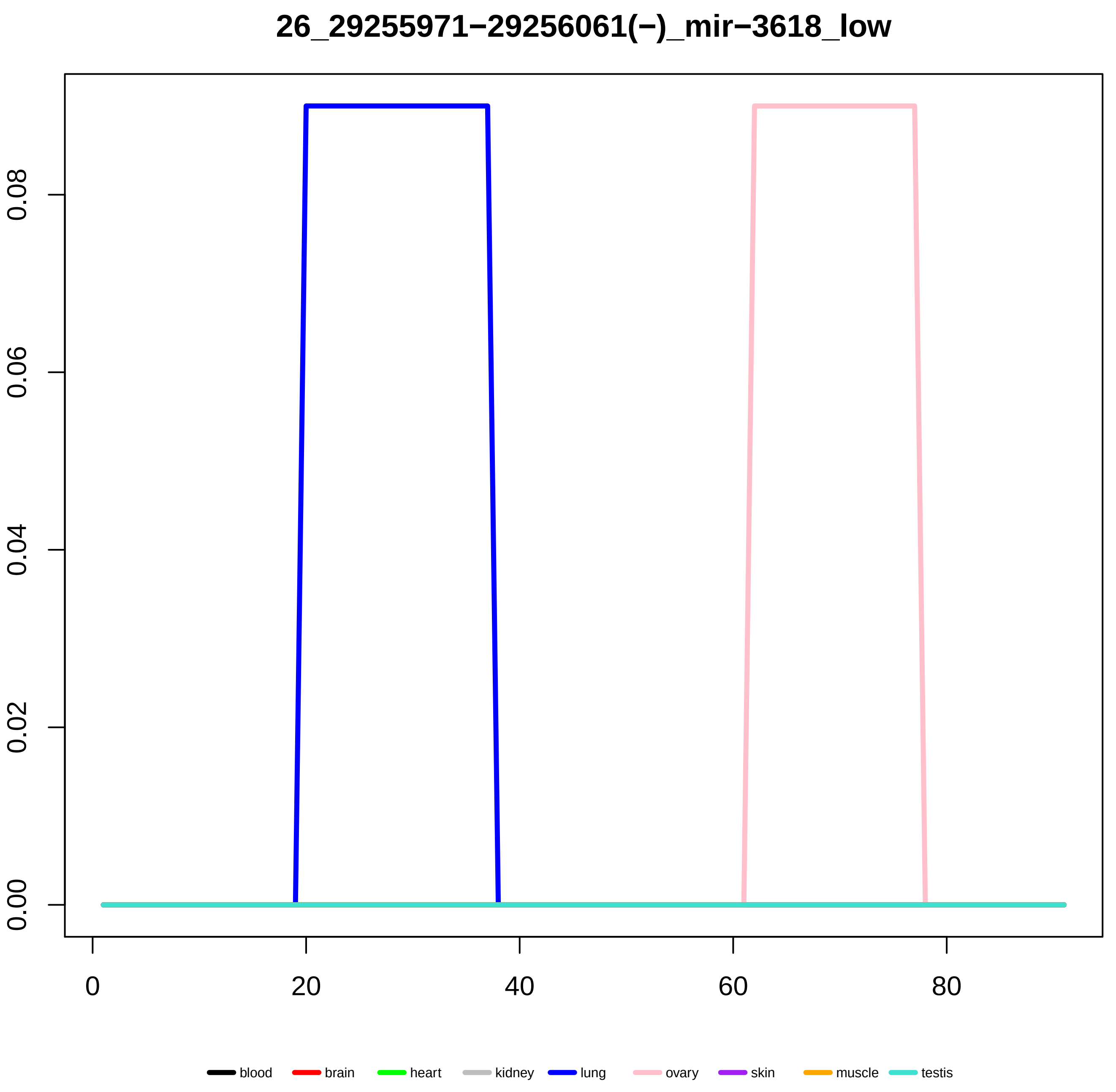
26_29255971−29256061(−)_mir−3618_low
0.00
0.02
0.04
0.06
0.08
0
20
40
60
80
blood
brain
heart
kidney
lung
ovary
skin
muscle
testis

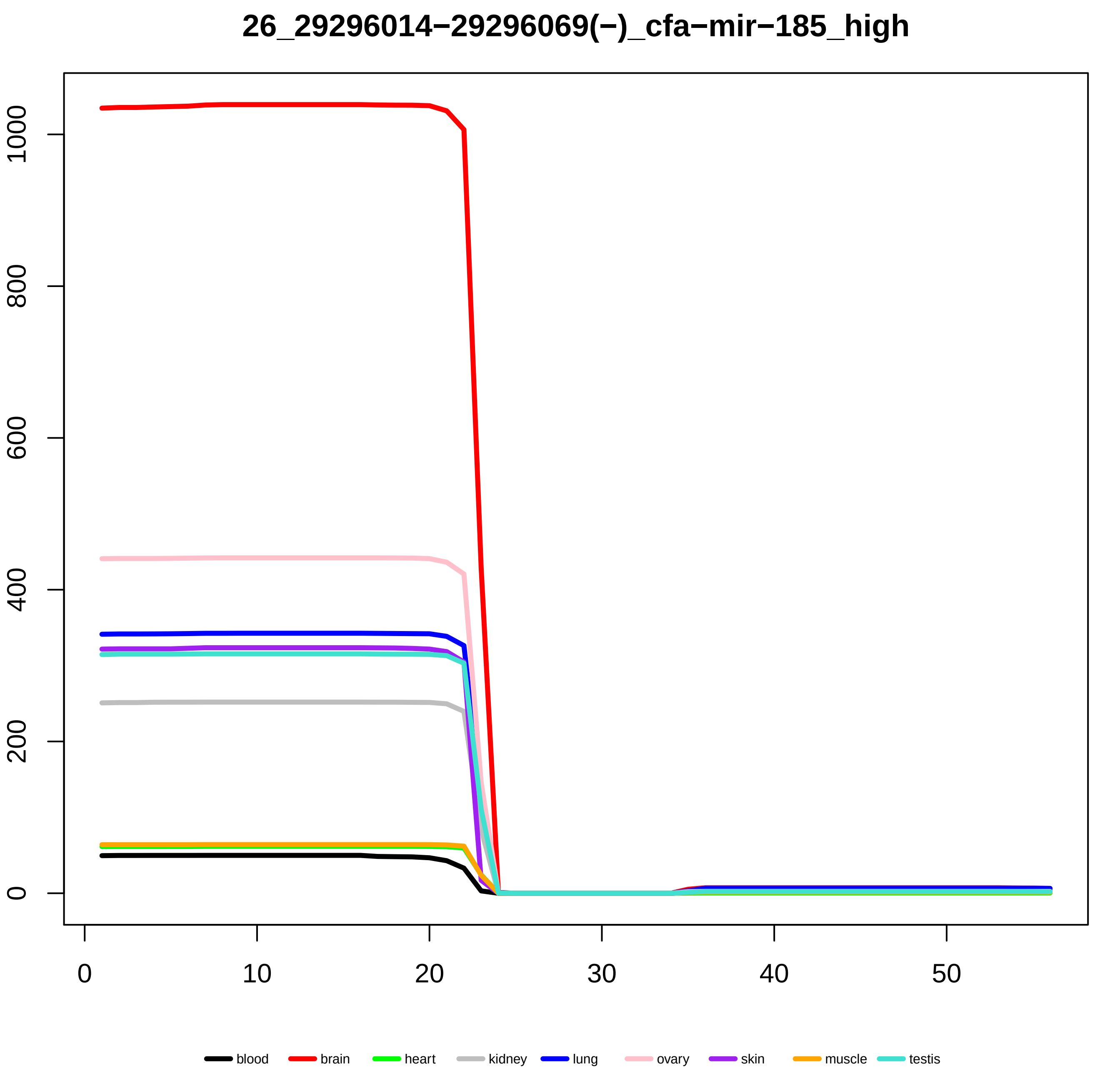
26_29296014−29296069(−)_cfa−mir−185_high
0
200
400
600
800
1000
0
10
20
30
40
50
blood
brain
heart
kidney
lung
ovary
skin
muscle
testis

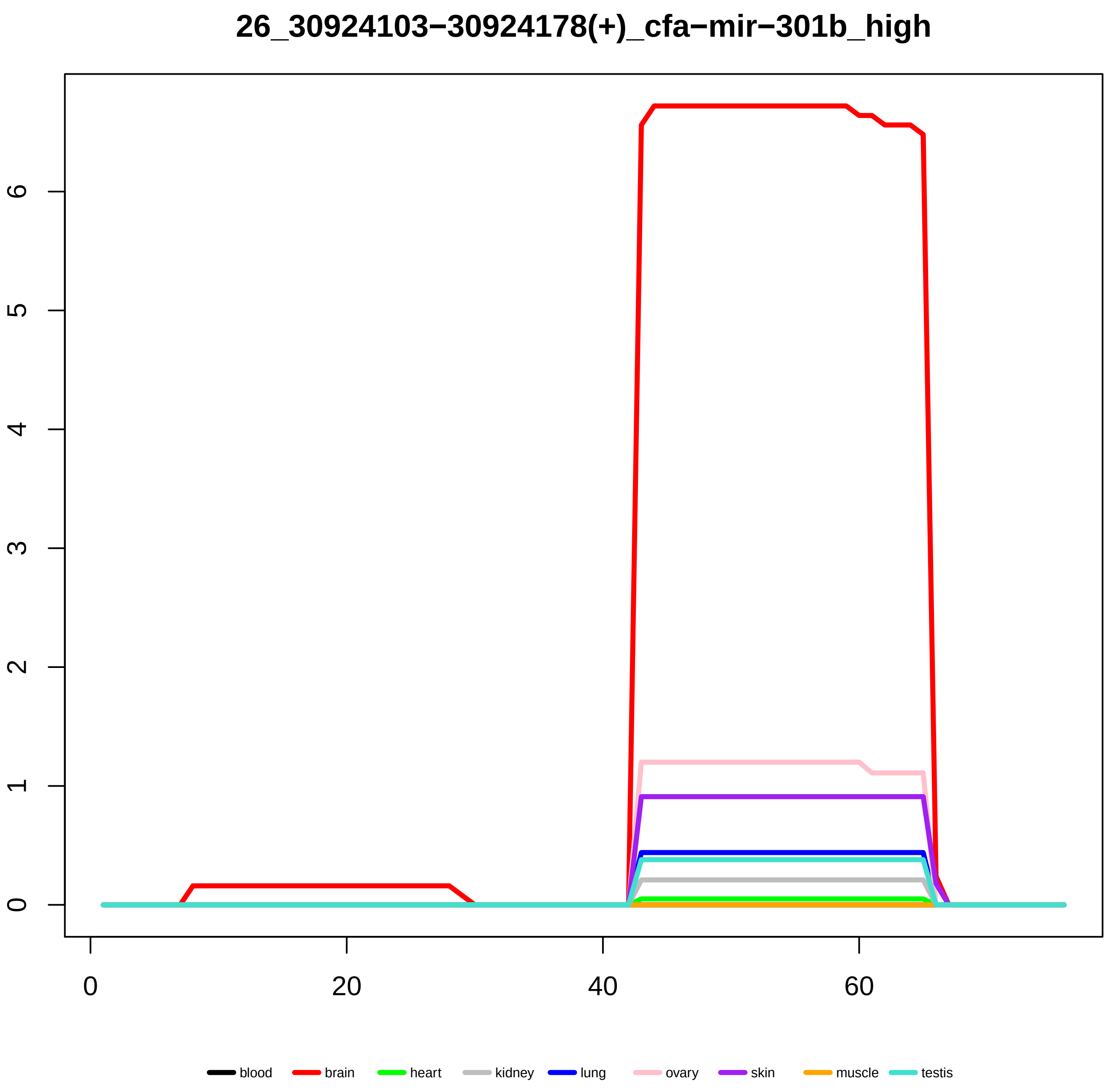

26_30924103−30924178(+)_cfa−mir−301b_high
0
1
2
3
4
5
6
0
20
40
60
blood
brain
heart
kidney
lung
ovary
skin
muscle
testis

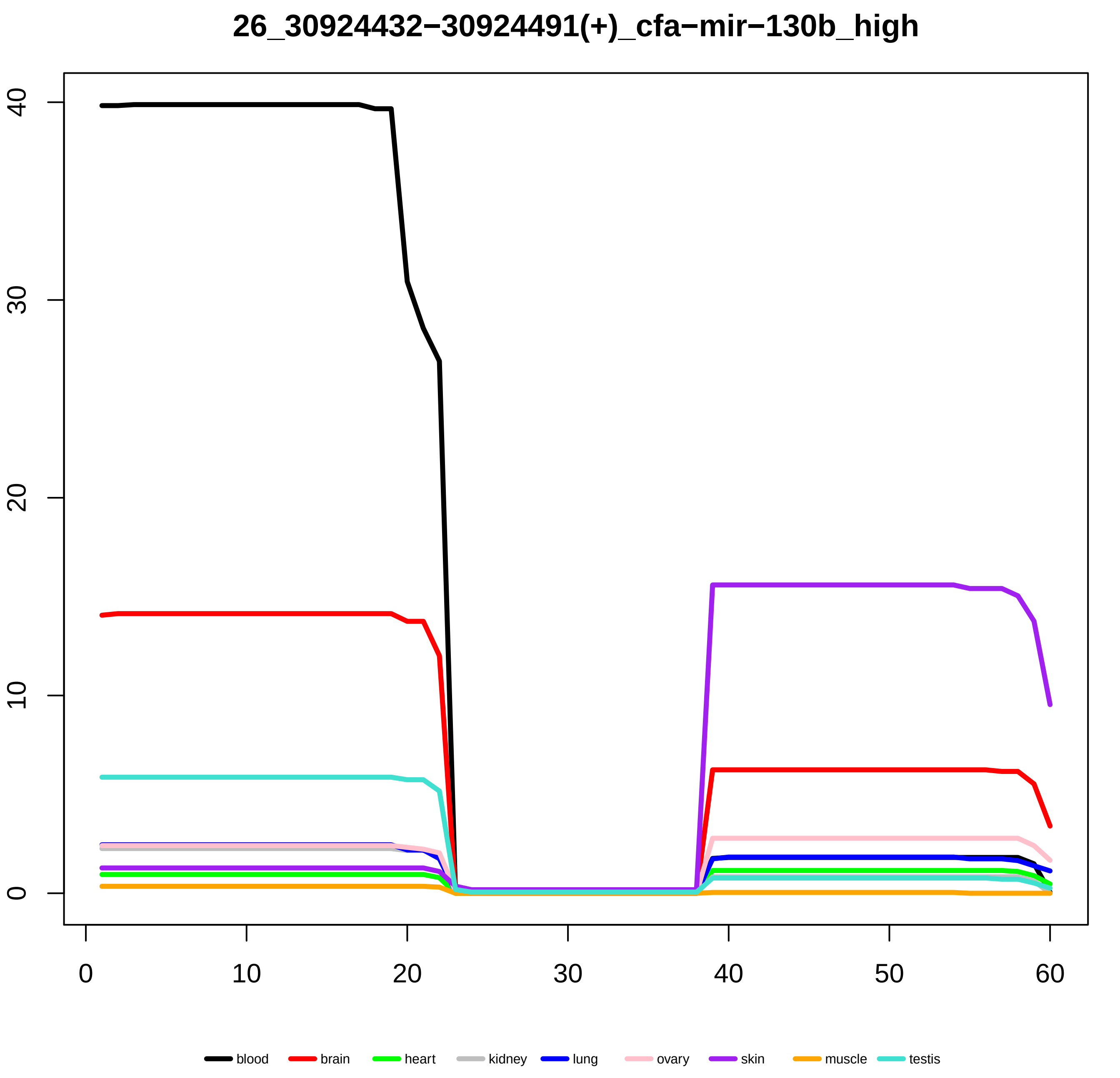

26_30924432−30924491(+)_cfa−mir−130b_high
0
10
20
30
40
0
10
20
30
40
50
60
blood
brain
heart
kidney
lung
ovary
skin
muscle
testis

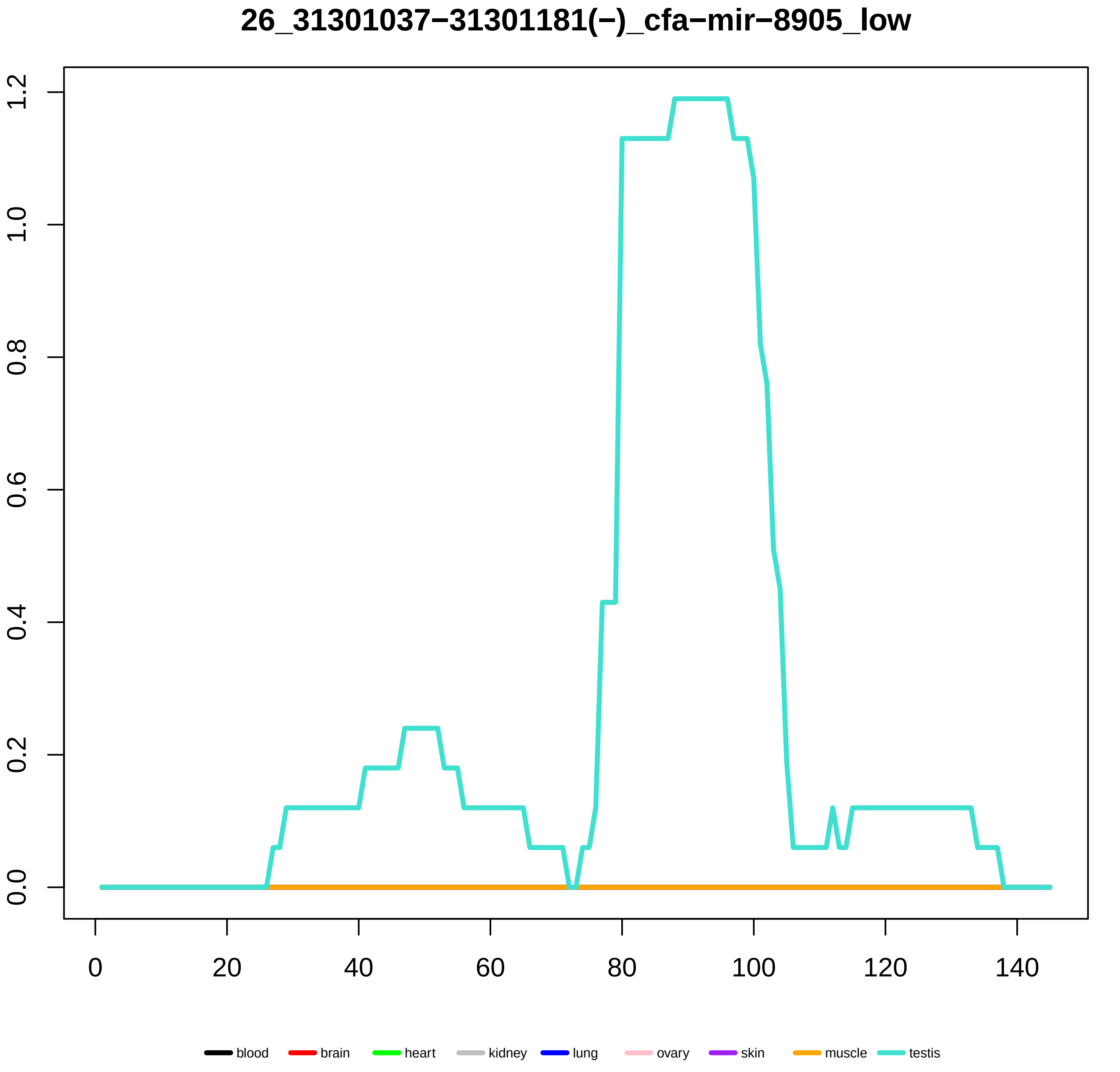

26_31301037−31301181(−)_cfa−mir−8905_low
0.0
0.2
0.4
0.6
0.8
1.0
1.2
0
20
40
60
80
100
120
140
blood
brain
heart
kidney
lung
ovary
skin
muscle
testis

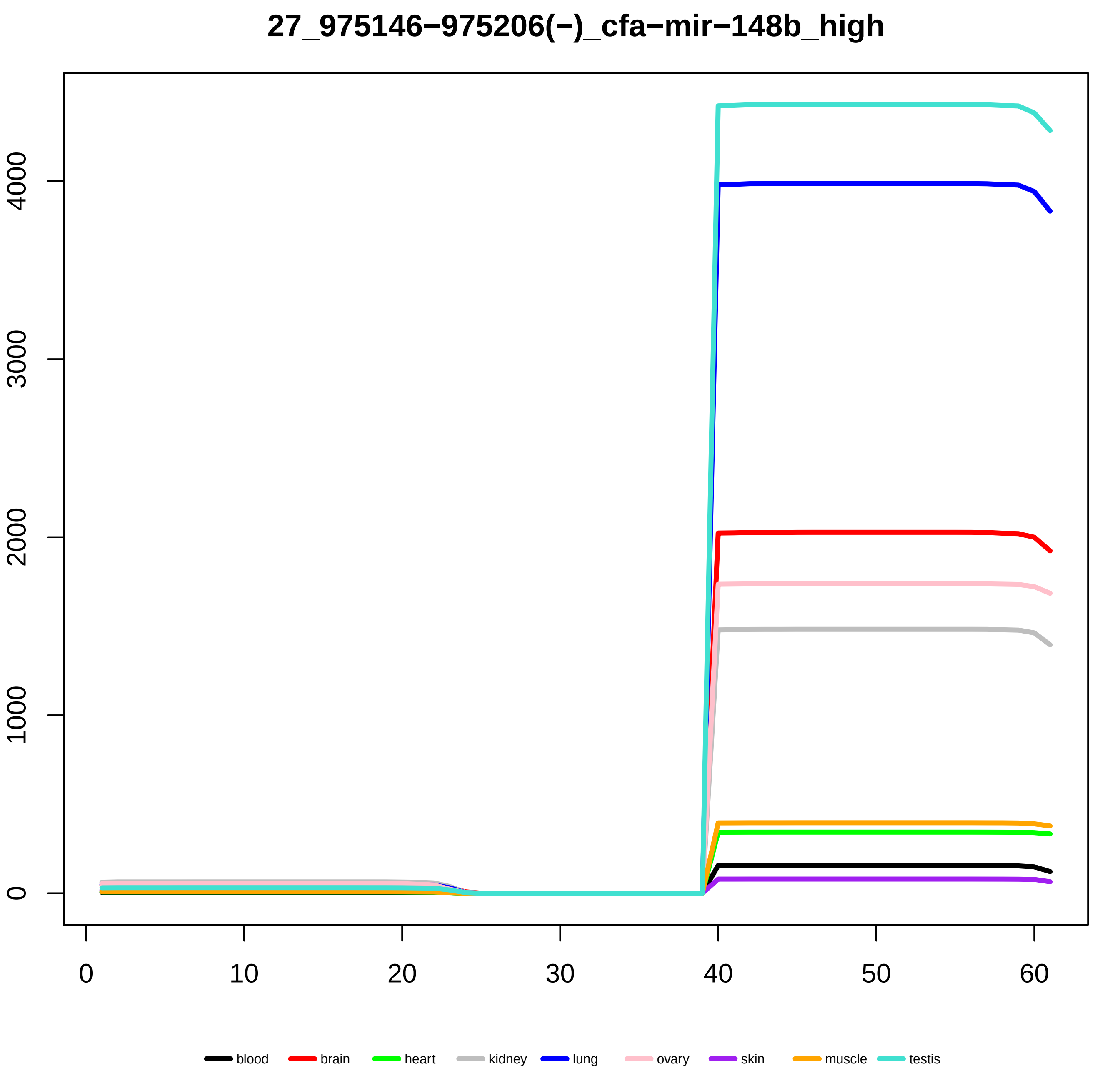
27_975146−975206(−)_cfa−mir−148b_high
0
1000
2000
3000
4000
0
10
20
30
40
50
60
blood
brain
heart
kidney
lung
ovary
skin
muscle
testis

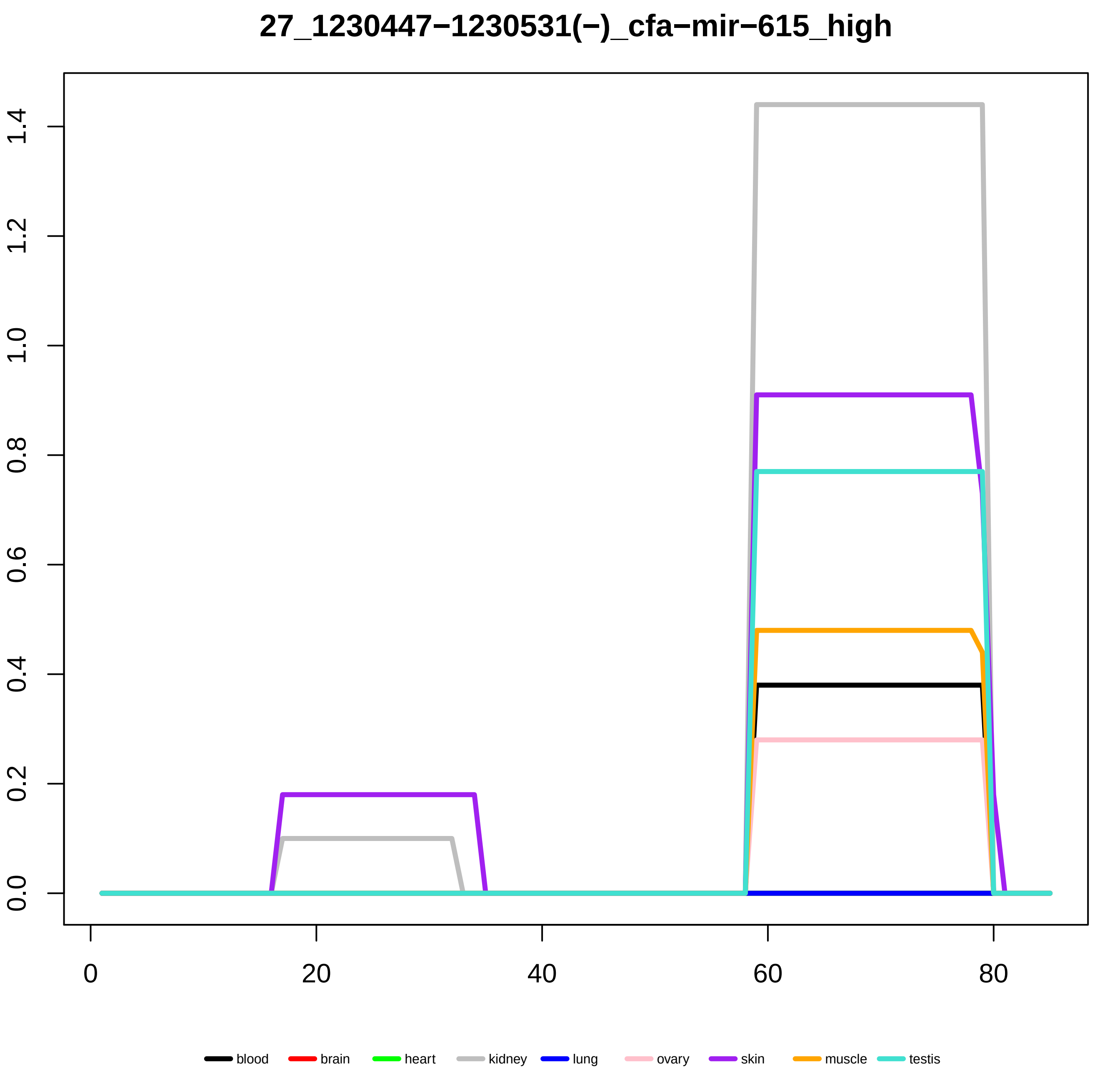

27_1230447−1230531(−)_cfa−mir−615_high
0.0
0.2
0.4
0.6
0.8
1.0
1.2
1.4
0
20
40
60
80
blood
brain
heart
kidney
lung
ovary
skin
muscle
testis

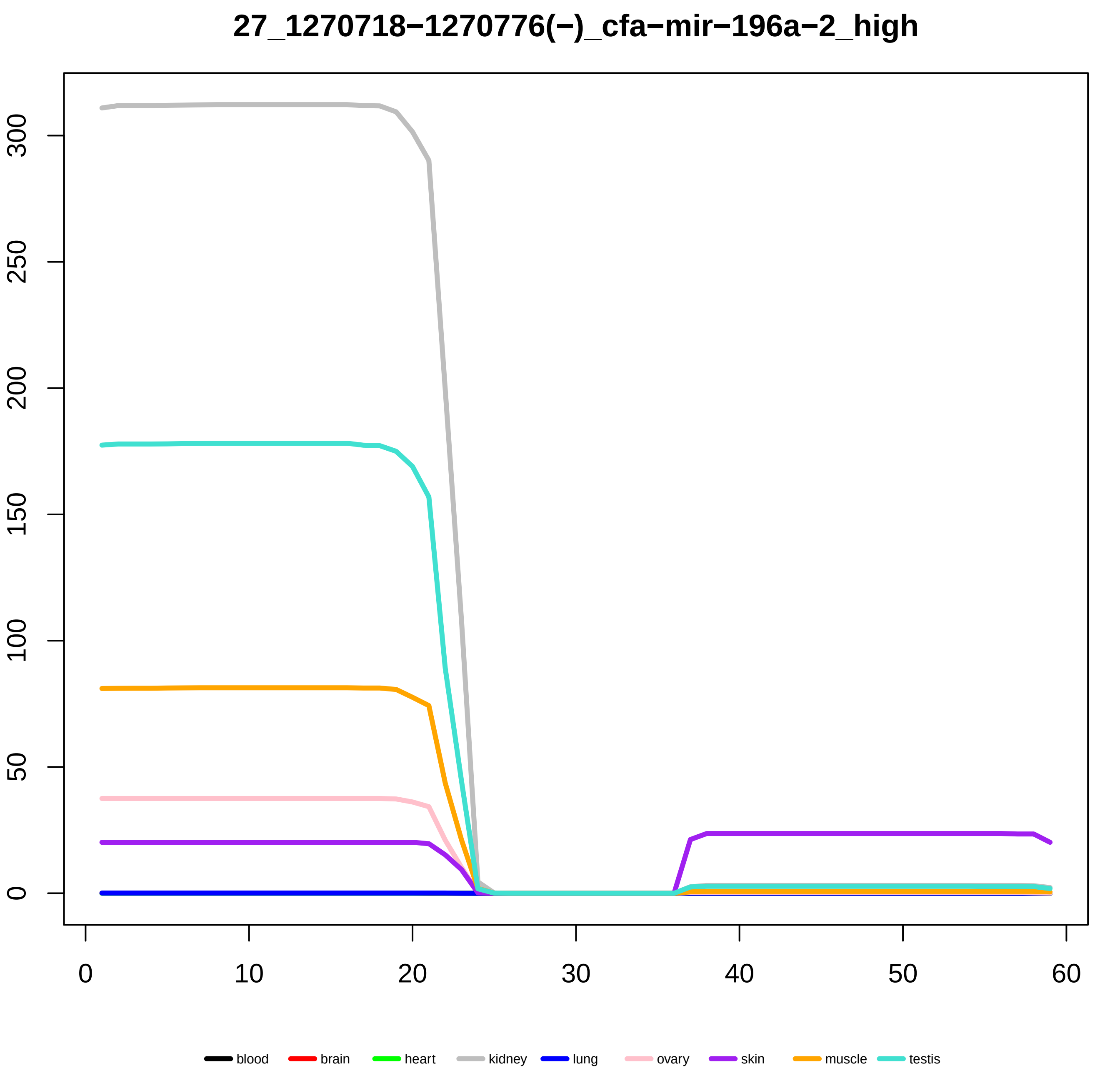

27_1270718−1270776(−)_cfa−mir−196a−2_high
0
50
100
150
200
250
300
0
10
20
30
40
50
60
blood
brain
heart
kidney
lung
ovary
skin
muscle
testis

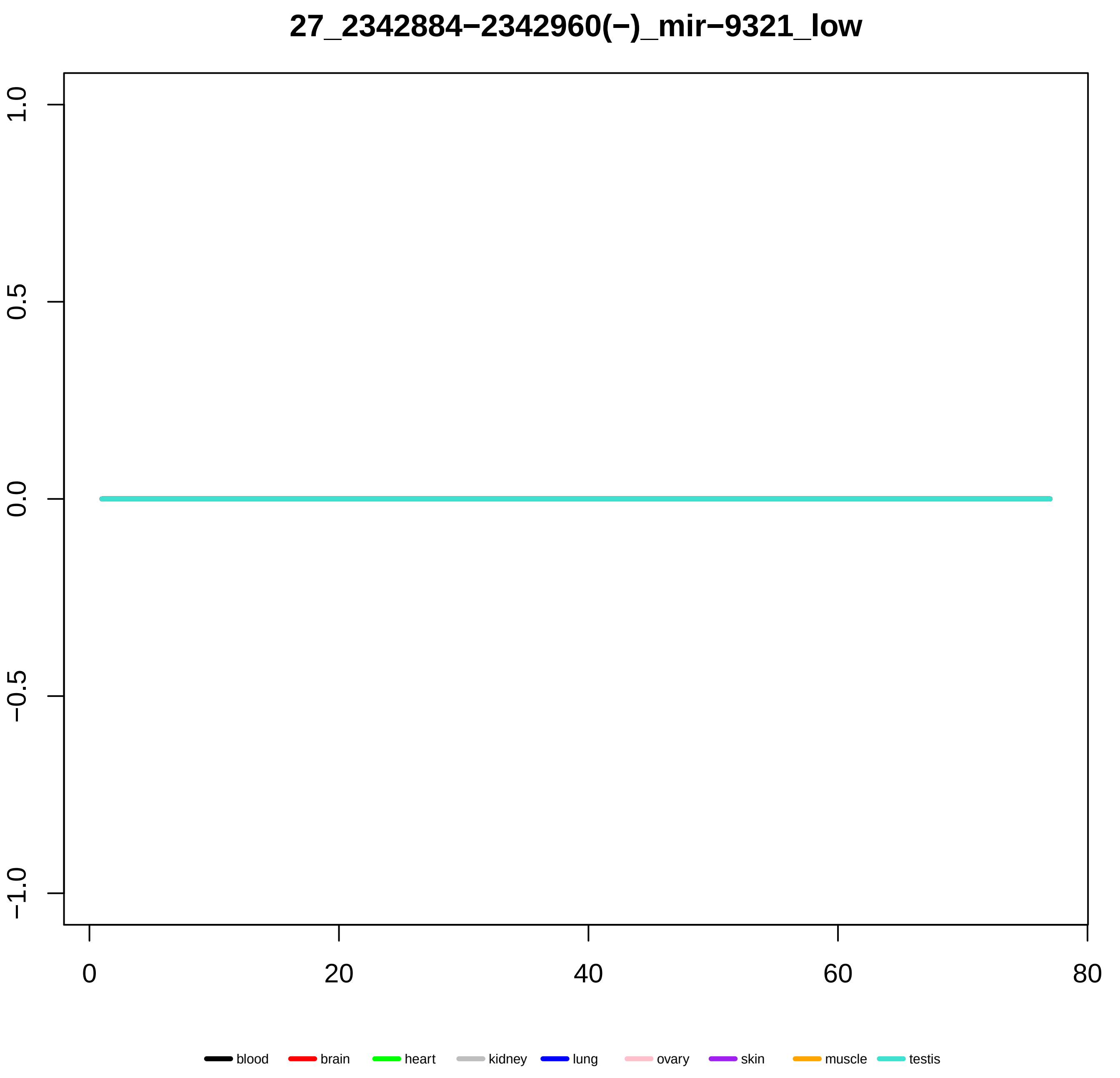

27_2342884−2342960(−)_mir−9321_low
1.0
0.5
0.0
−0.5
−1.0
0
20
40
60
80
blood
brain
heart
kidney
lung
ovary
skin
muscle
testis

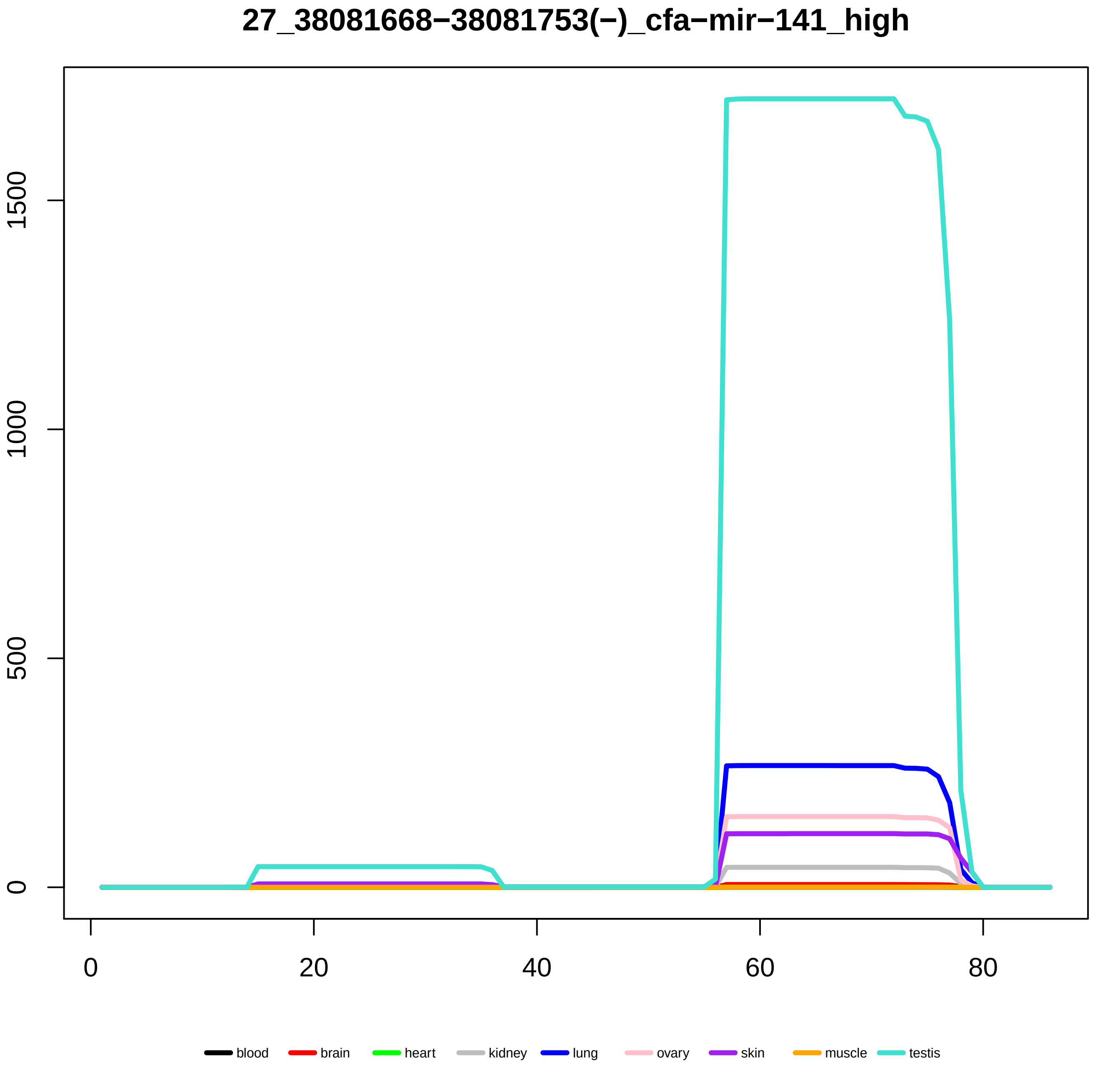
27_38081668−38081753(−)_cfa−mir−141_high
0
500
1000
1500
0
20
40
60
80
blood
brain
heart
kidney
lung
ovary
skin
muscle
testis

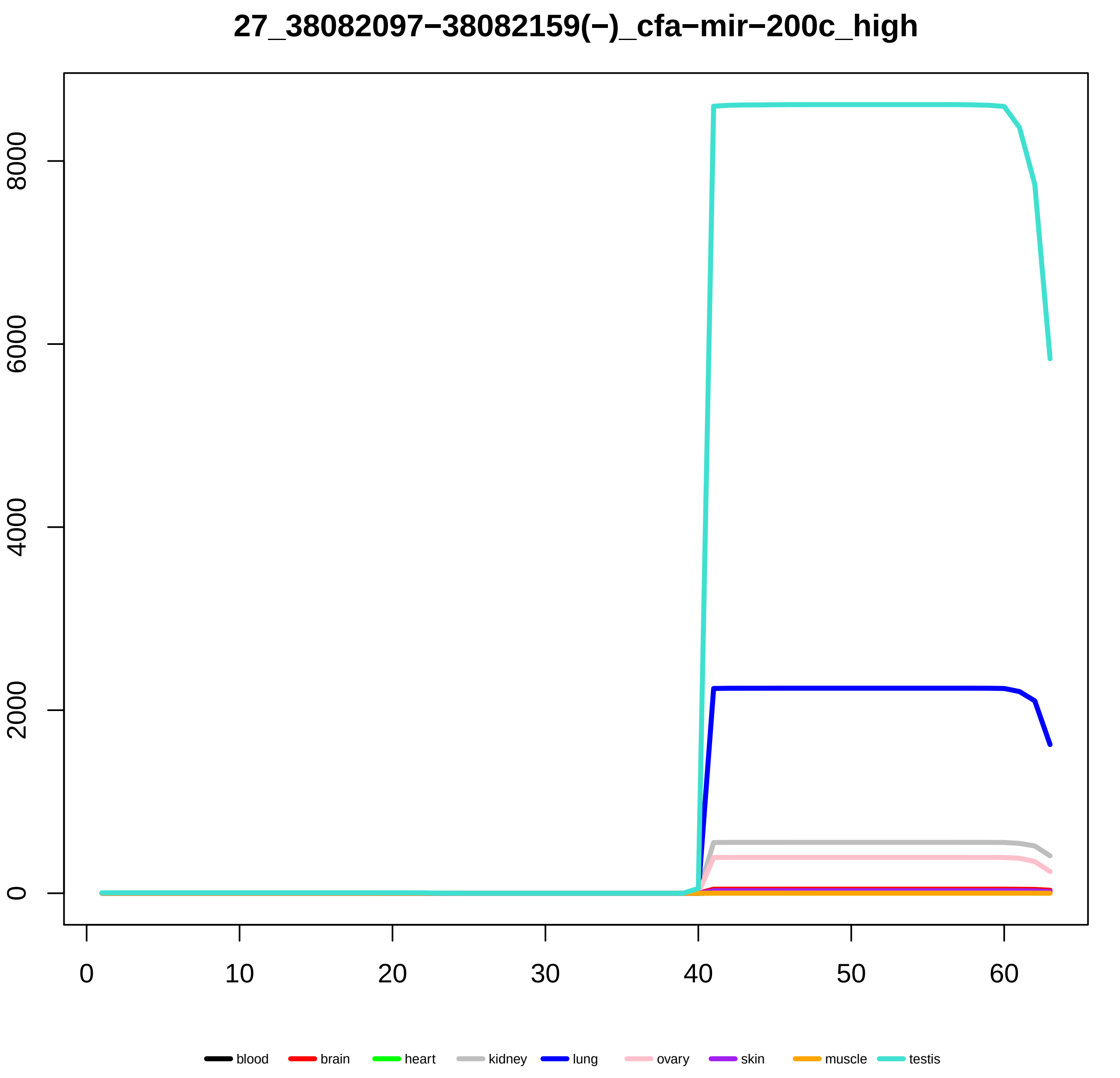

27_38082097−38082159(−)_cfa−mir−200c_high
0
2000
4000
6000
8000
0
10
20
30
40
50
60
blood
brain
heart
kidney
lung
ovary
skin
muscle
testis

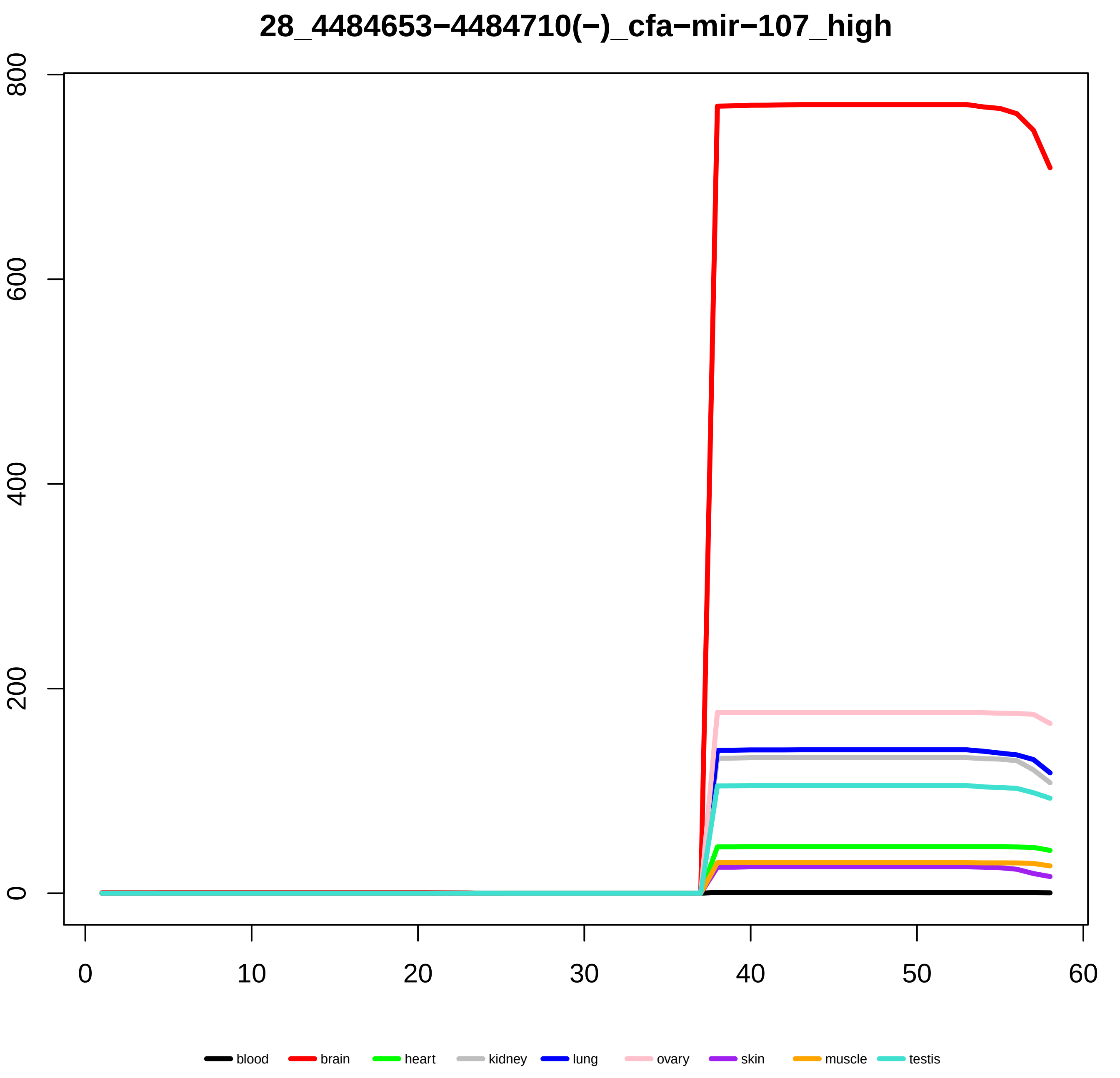

28_4484653−4484710(−)_cfa−mir−107_high
0
200
400
600
800
0
10
20
30
40
50
60
blood
brain
heart
kidney
lung
ovary
skin
muscle
testis

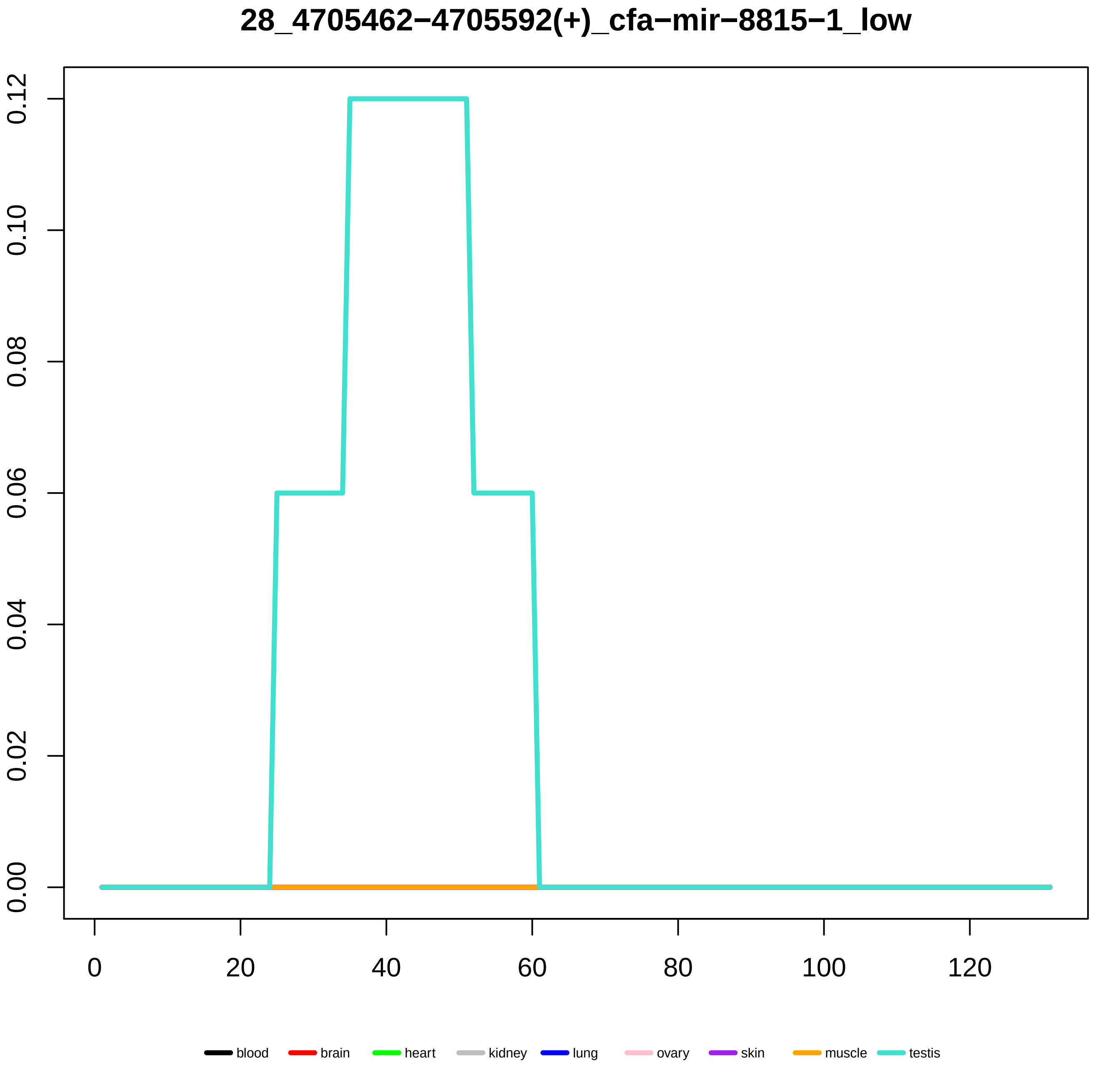

28_4705462−4705592(+)_cfa−mir−8815−1_low
0.00
0.02
0.04
0.06
0.08
0.10
0.12
0
20
40
60
80
100
120
blood
brain
heart
kidney
lung
ovary
skin
muscle
testis

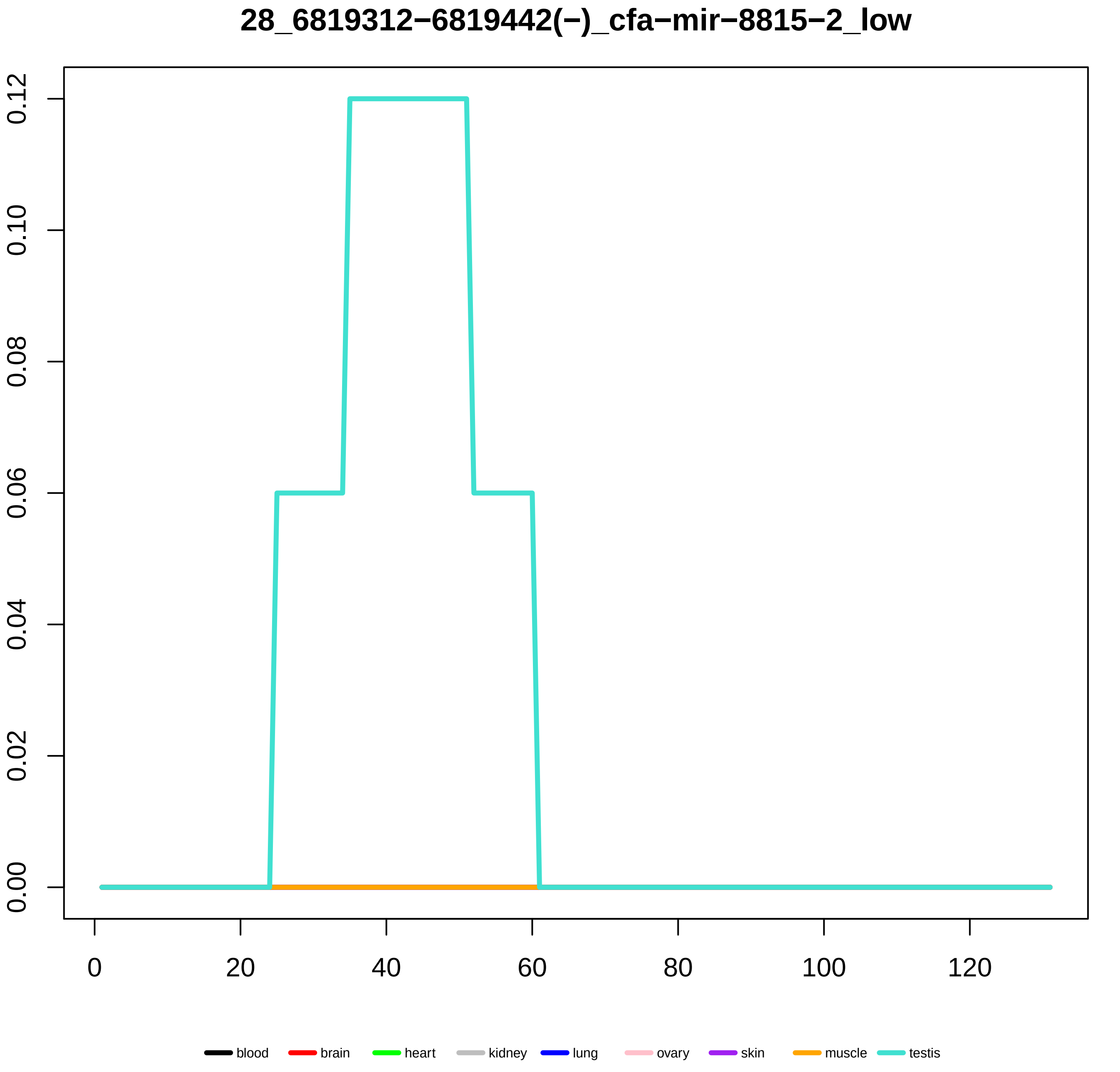

28_6819312−6819442(−)_cfa−mir−8815−2_low
0.00
0.02
0.04
0.06
0.08
0.10
0.12
0
20
40
60
80
100
120
blood
brain
heart
kidney
lung
ovary
skin
muscle
testis

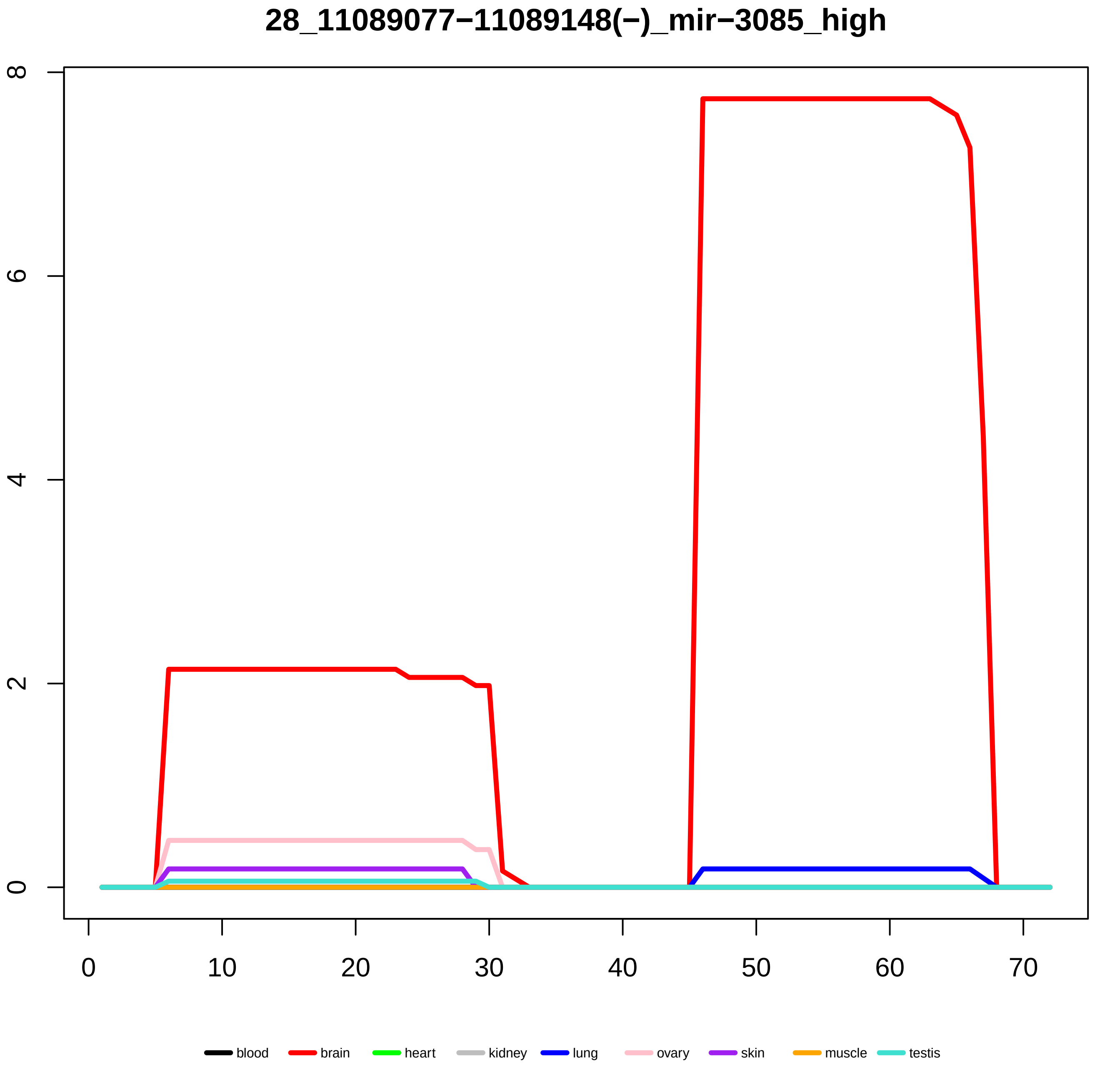

28_11089077−11089148(−)_mir−3085_high
0
2
4
6
8
0
10
20
30
40
50
60
70
blood
brain
heart
kidney
lung
ovary
skin
muscle
testis

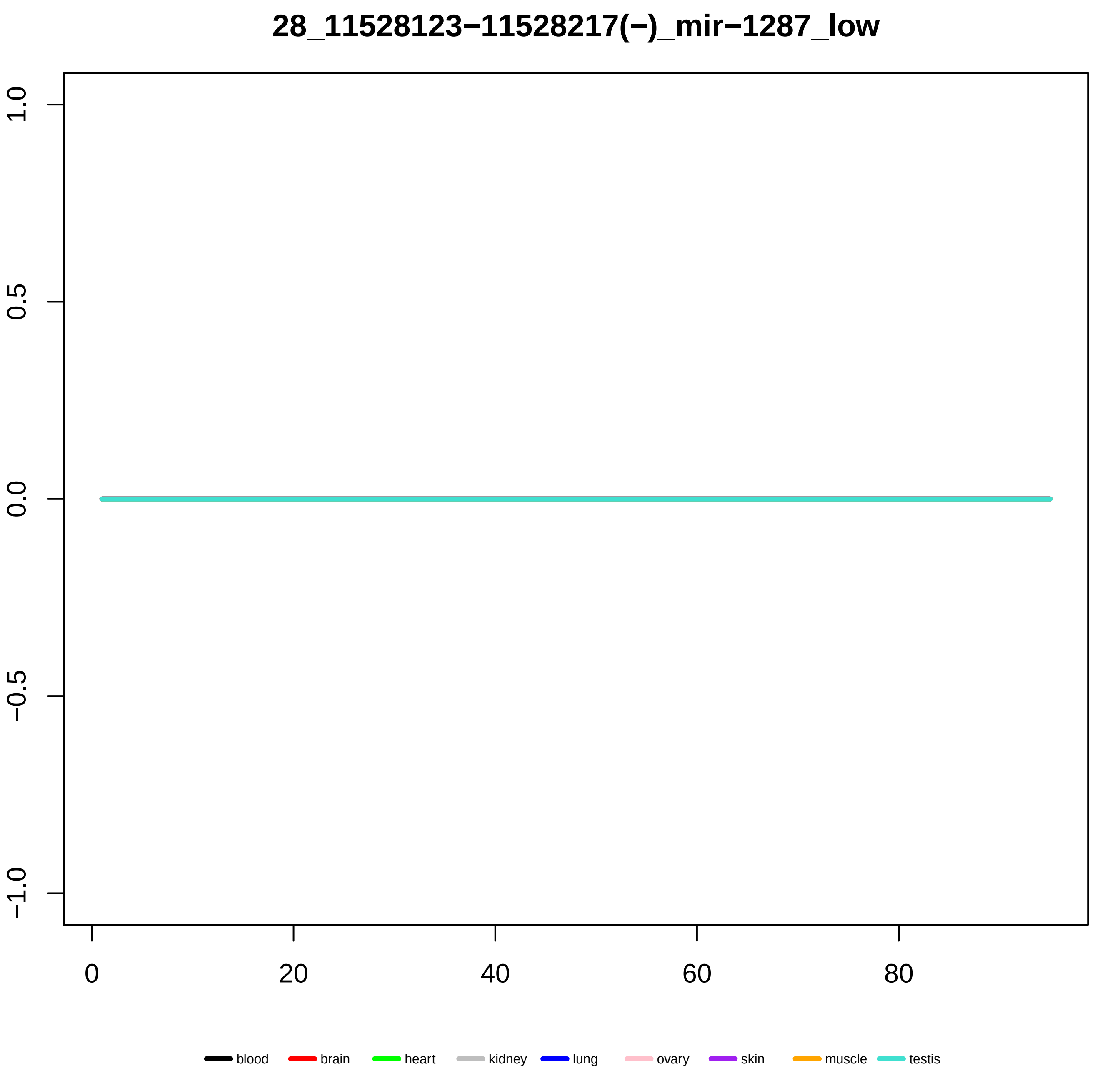

28_11528123−11528217(−)_mir−1287_low
1.0
0.5
0.0
−0.5
−1.0
0
20
40
60
80
blood
brain
heart
kidney
lung
ovary
skin
muscle
testis

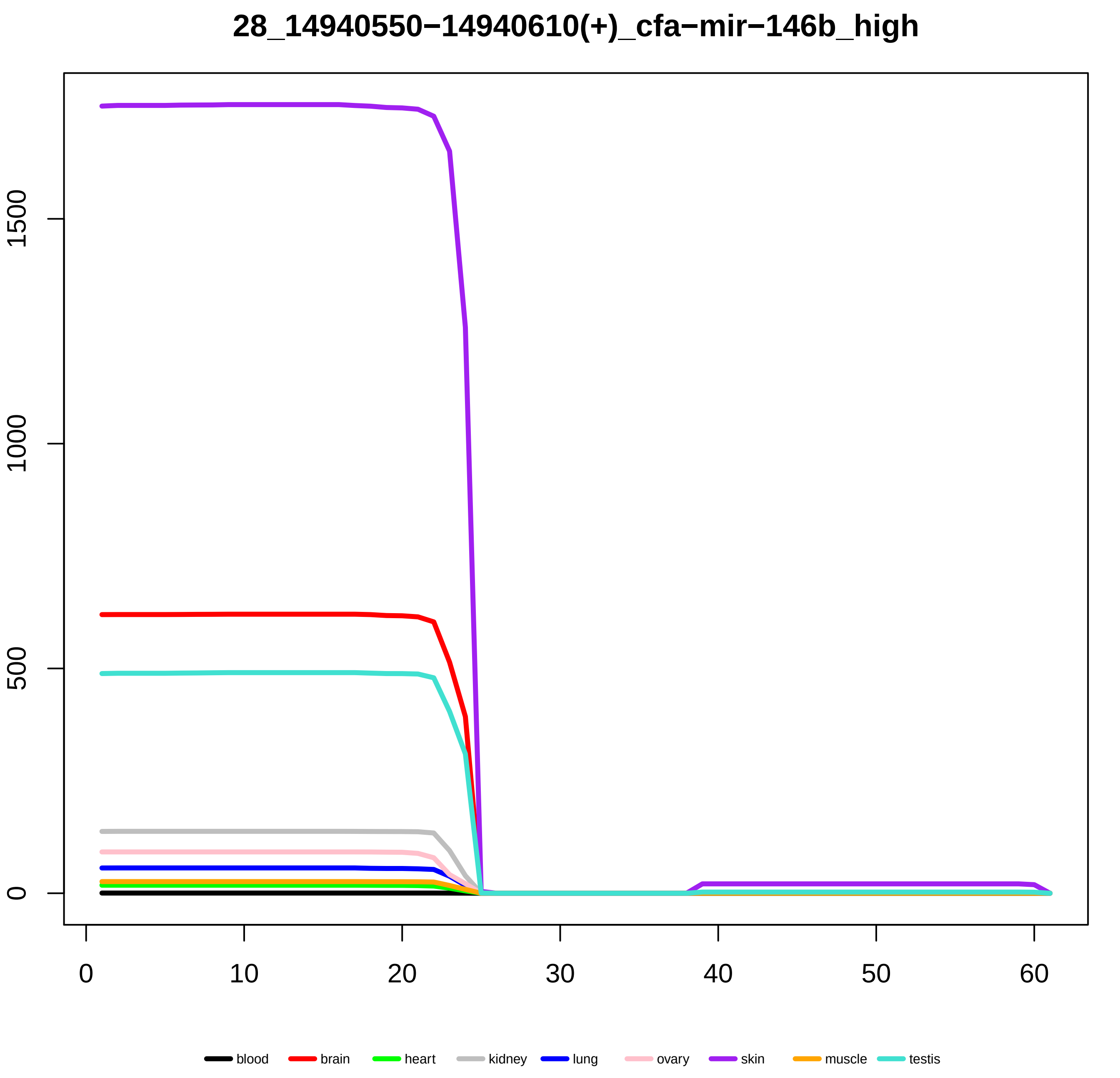
28_14940550−14940610(+)_cfa−mir−146b_high
0
500
1000
1500
0
10
20
30
40
50
60
blood
brain
heart
kidney
lung
ovary
skin
muscle
testis

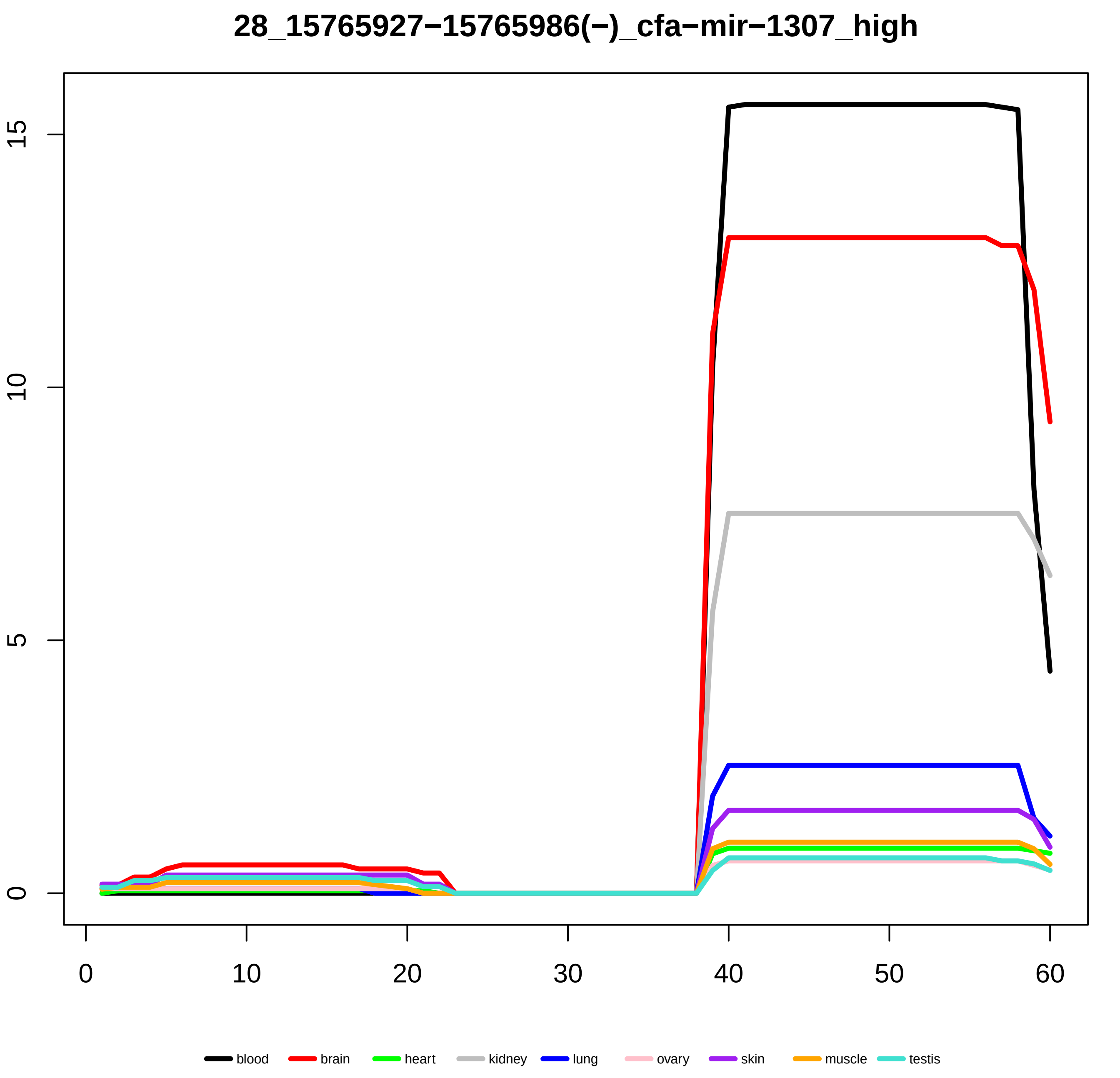

28_15765927−15765986(−)_cfa−mir−1307_high
0
5
10
15
0
10
20
30
40
50
60
blood
brain
heart
kidney
lung
ovary
skin
muscle
testis

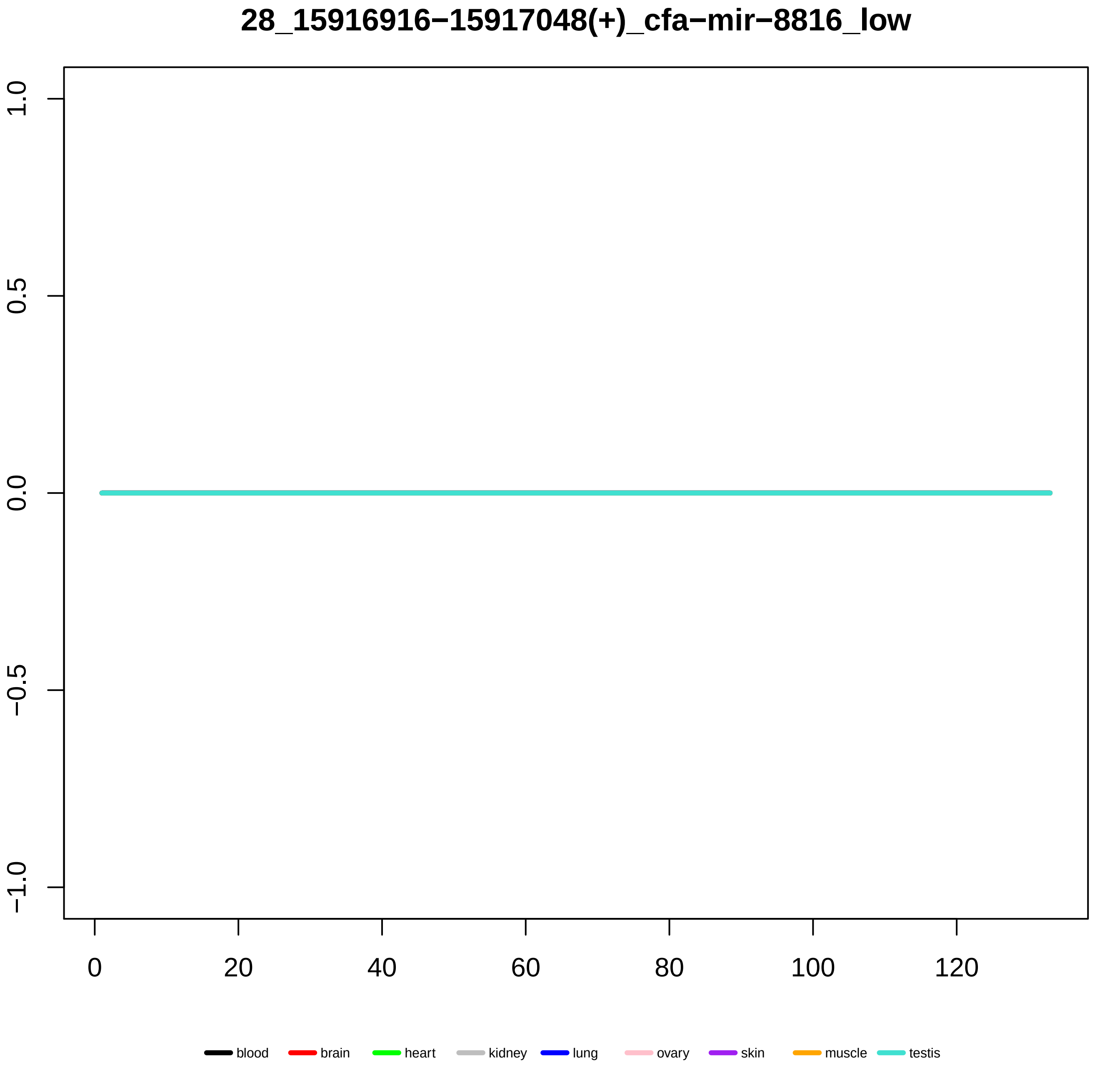

28_15916916−15917048(+)_cfa−mir−8816_low
1.0
0.5
0.0
−0.5
−1.0
0
20
40
60
80
100
120
blood
brain
heart
kidney
lung
ovary
skin
muscle
testis

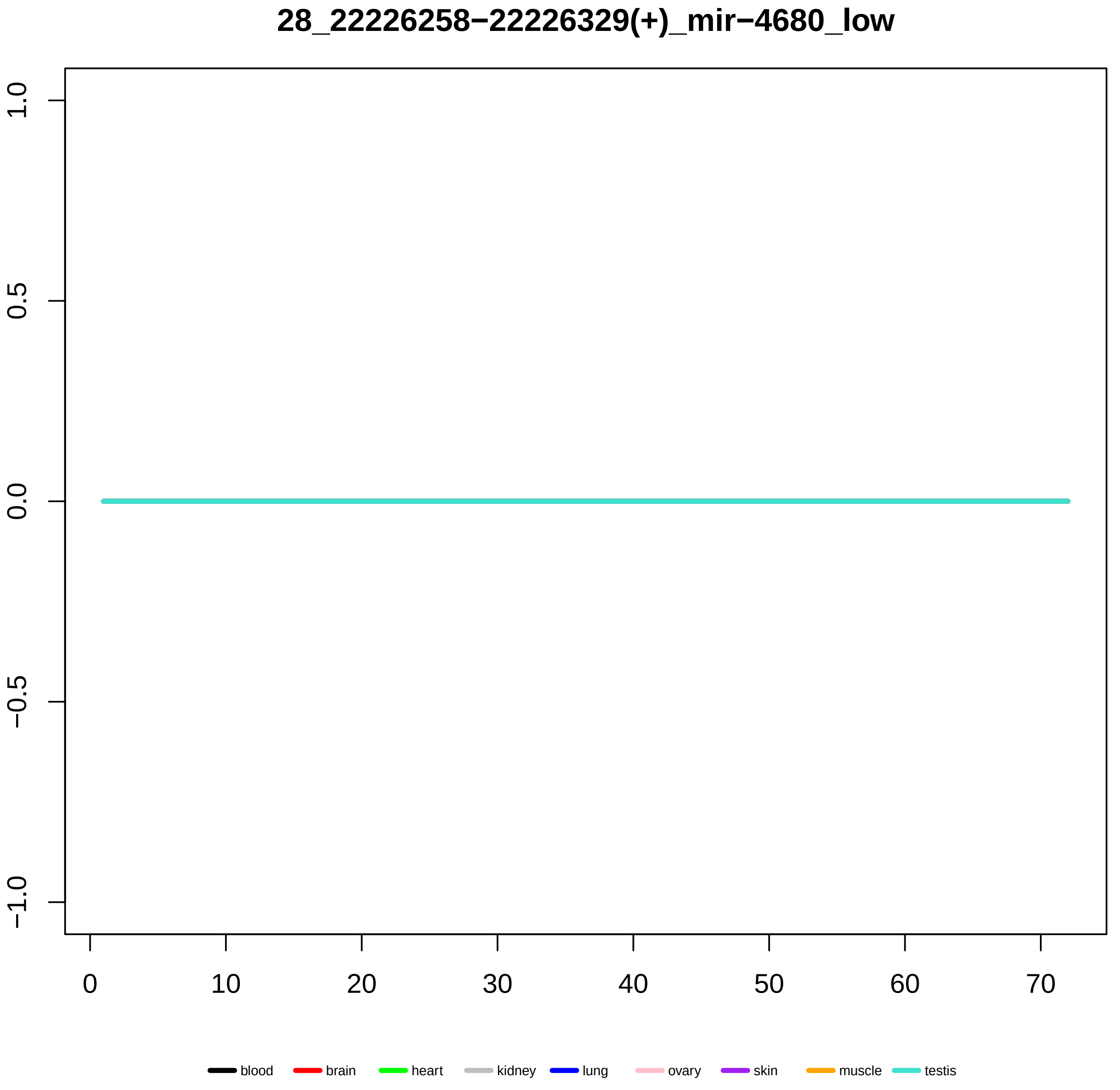

28_22226258−22226329(+)_mir−4680_low
1.0
0.5
0.0
−0.5
−1.0
0
10
20
30
40
50
60
70
blood
brain
heart
kidney
lung
ovary
skin
muscle
testis

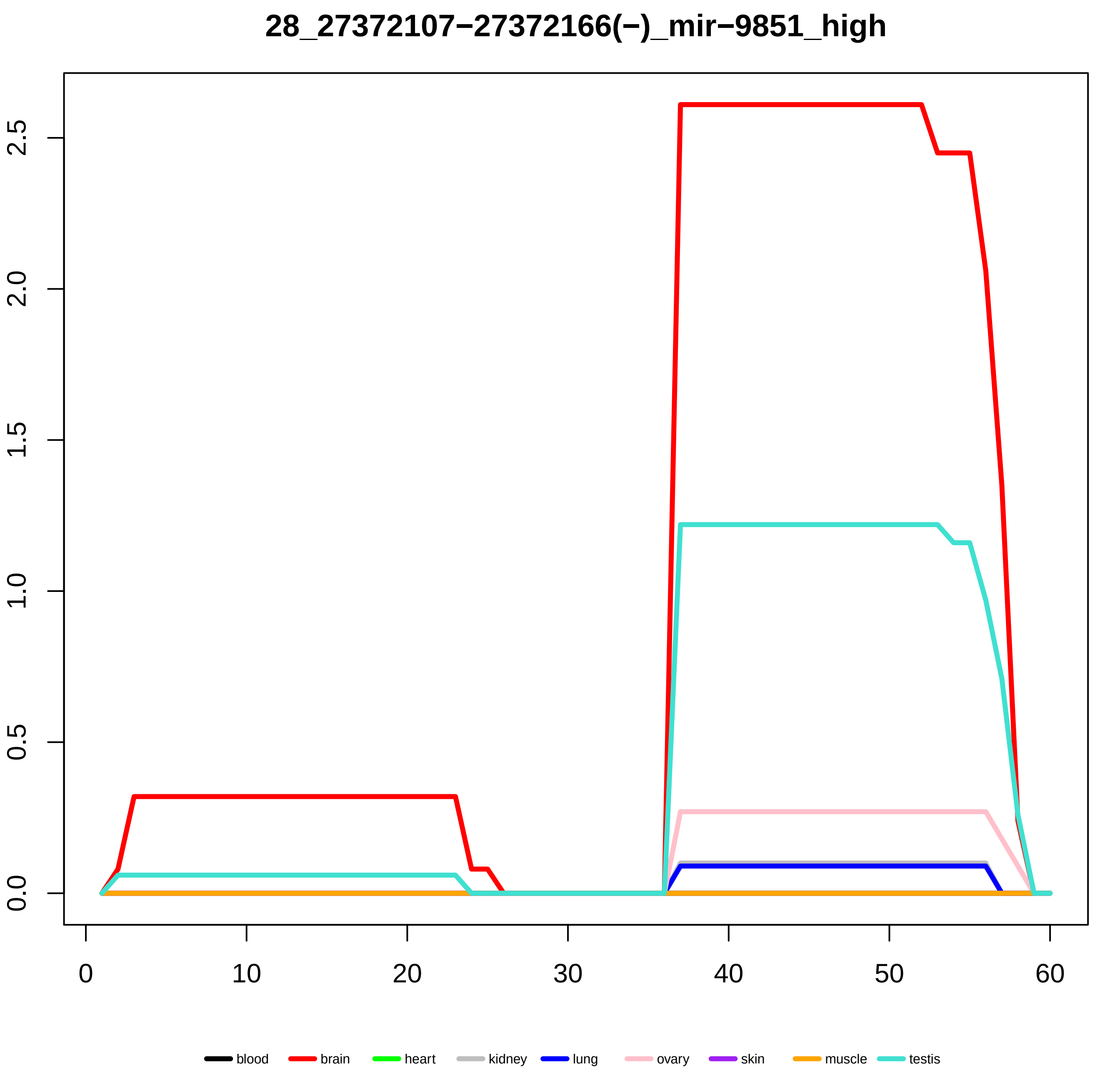

28_27372107−27372166(−)_mir−9851_high
0.0
0.5
1.0
1.5
2.0
2.5
0
10
20
30
40
50
60
blood
brain
heart
kidney
lung
ovary
skin
muscle
testis

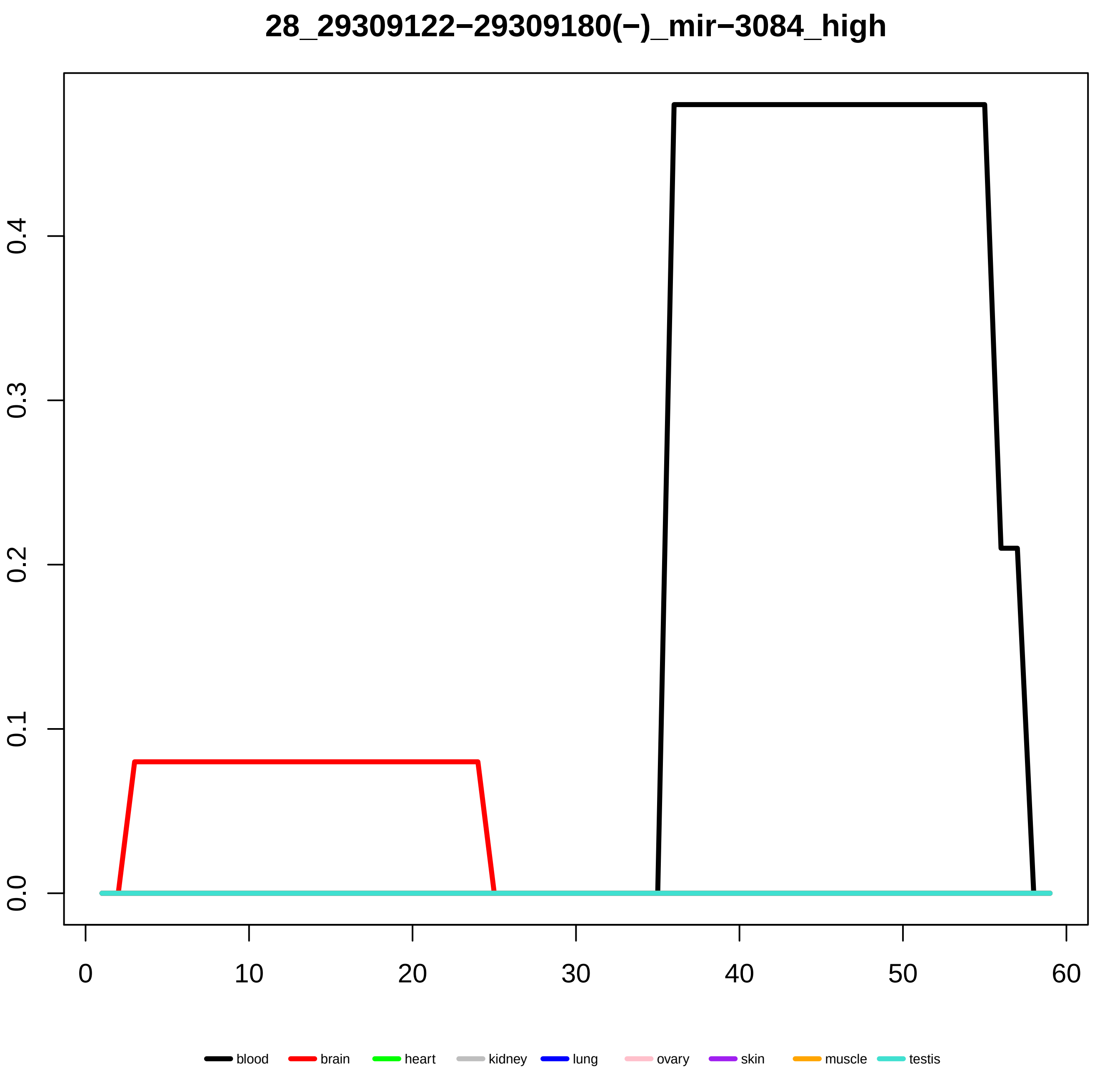

28_29309122−29309180(−)_mir−3084_high
0.0
0.1
0.2
0.3
0.4
0
10
20
30
40
50
60
blood
brain
heart
kidney
lung
ovary
skin
muscle
testis

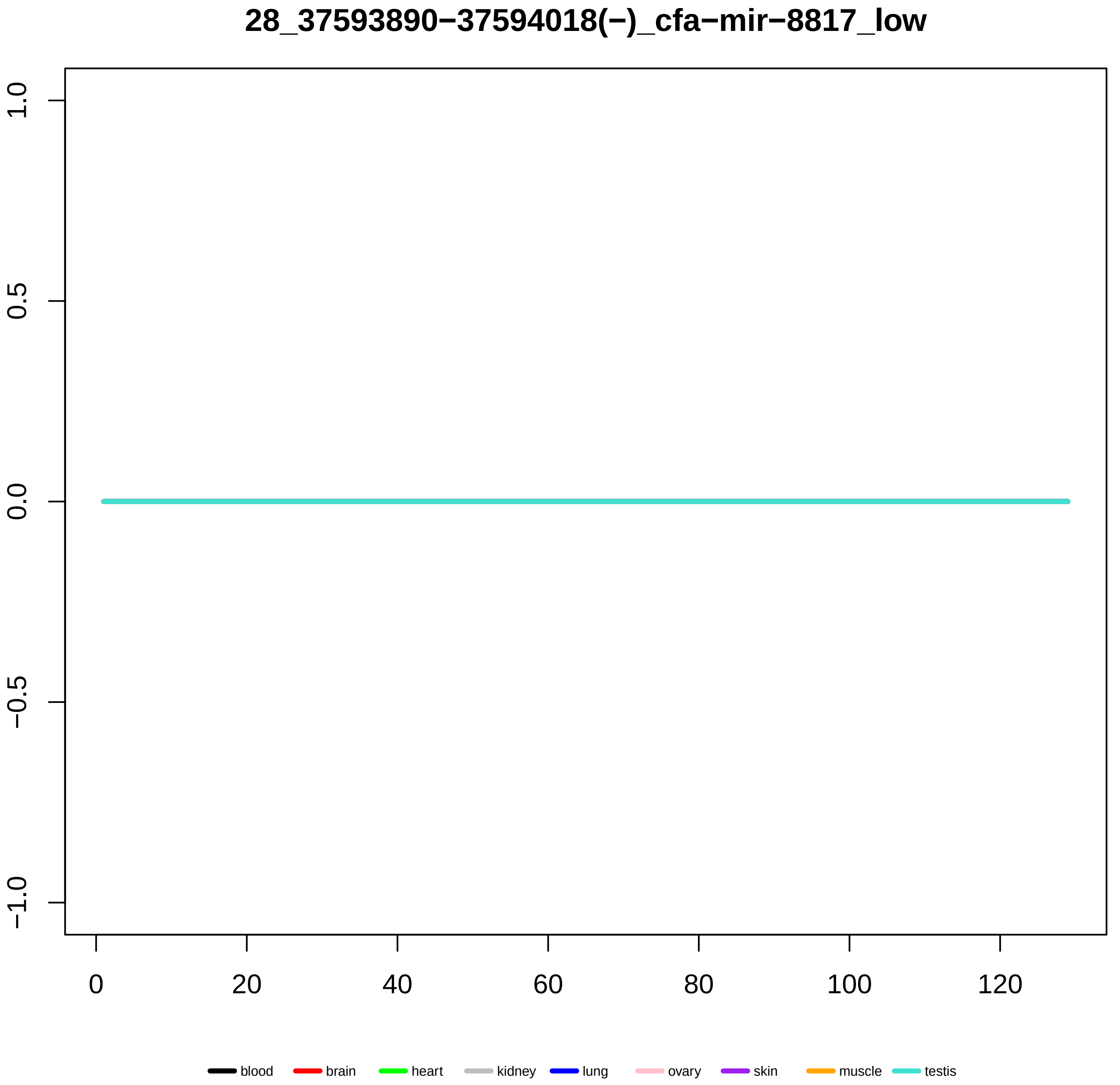
28_37593890−37594018(−)_cfa−mir−8817_low
1.0
0.5
0.0
−0.5
−1.0
0
20
40
60
80
100
120
blood
brain
heart
kidney
lung
ovary
skin
muscle
testis

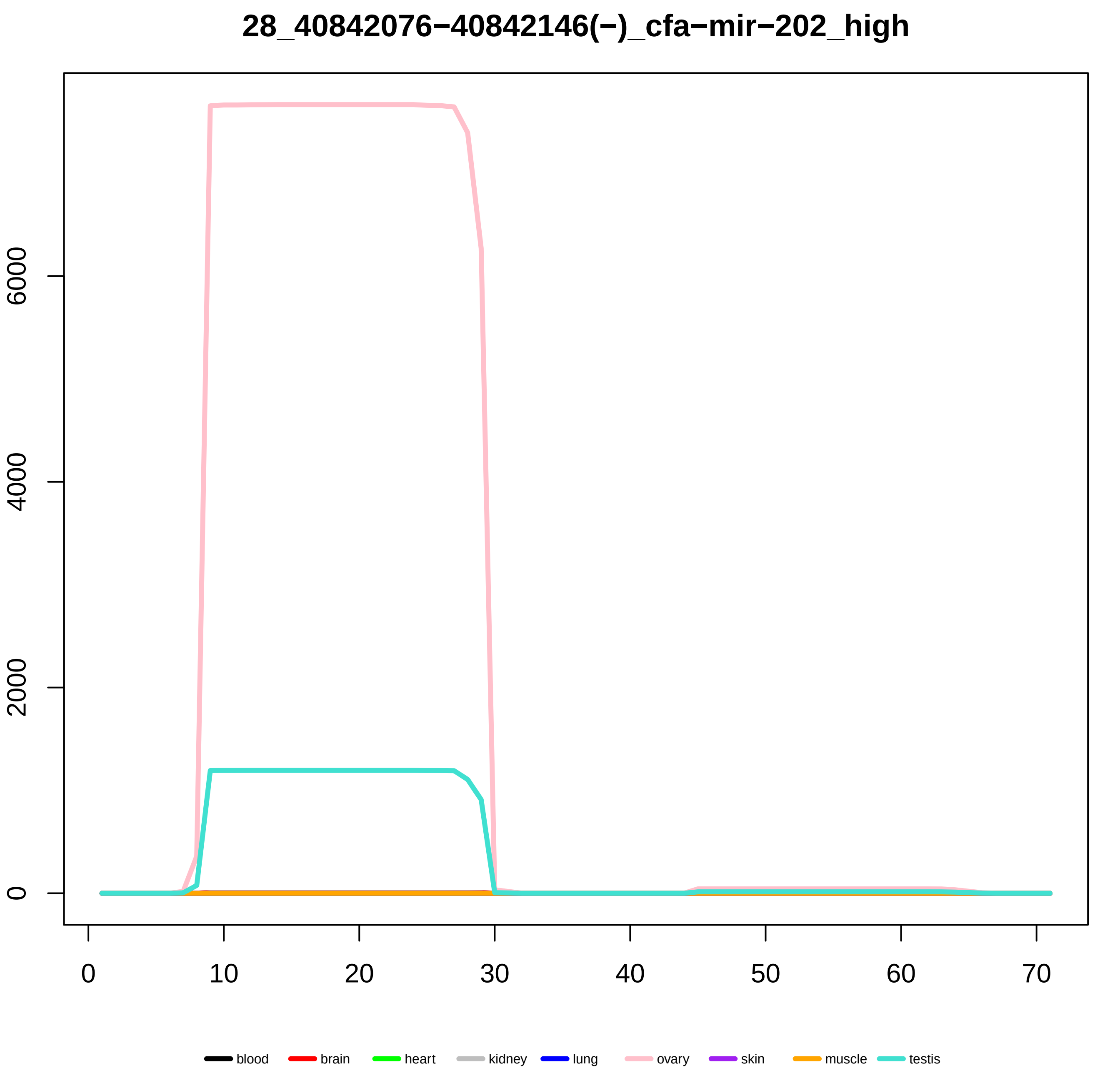

28_40842076−40842146(−)_cfa−mir−202_high
0
2000
4000
6000
0
10
20
30
40
50
60
70
blood
brain
heart
kidney
lung
ovary
skin
muscle
testis

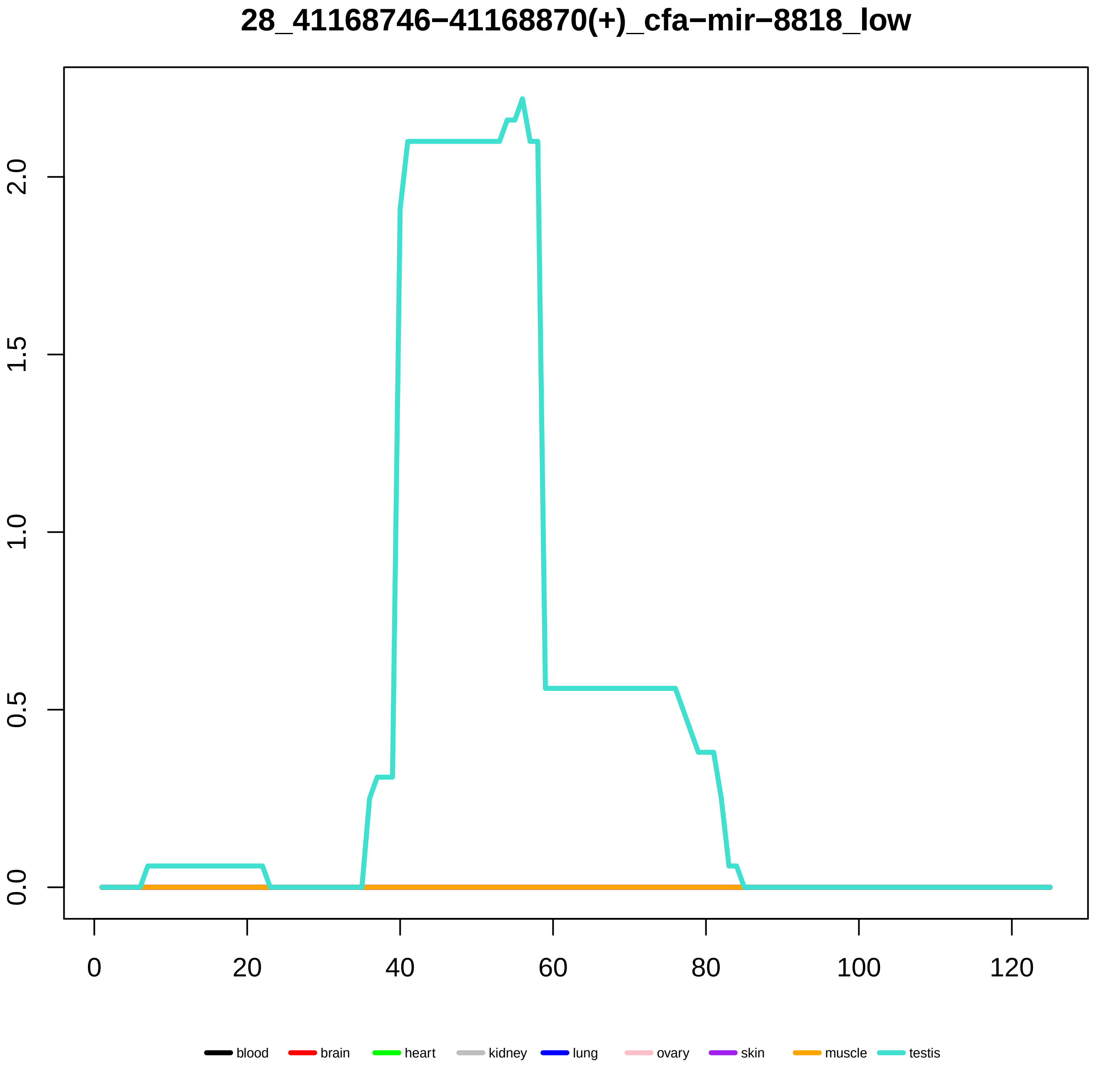
28_41168746−41168870(+)_cfa−mir−8818_low
0.0
0.5
1.0
1.5
2.0
0
20
40
60
80
100
120
blood
brain
heart
kidney
lung
ovary
skin
muscle
testis

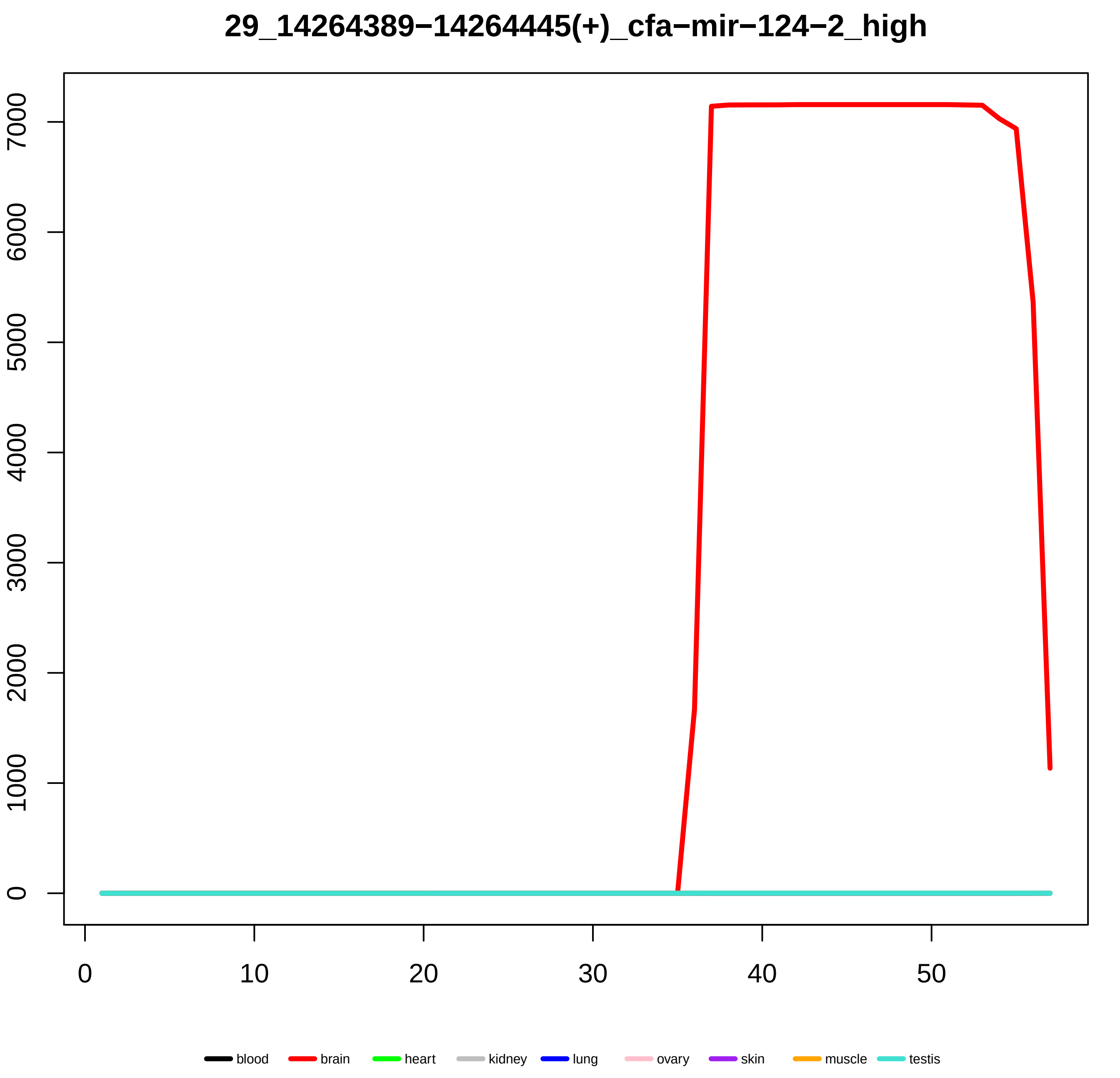

29_14264389−14264445(+)_cfa−mir−124−2_high
0
1000
2000
3000
4000
5000
6000
7000
0
10
20
30
40
50
blood
brain
heart
kidney
lung
ovary
skin
muscle
testis

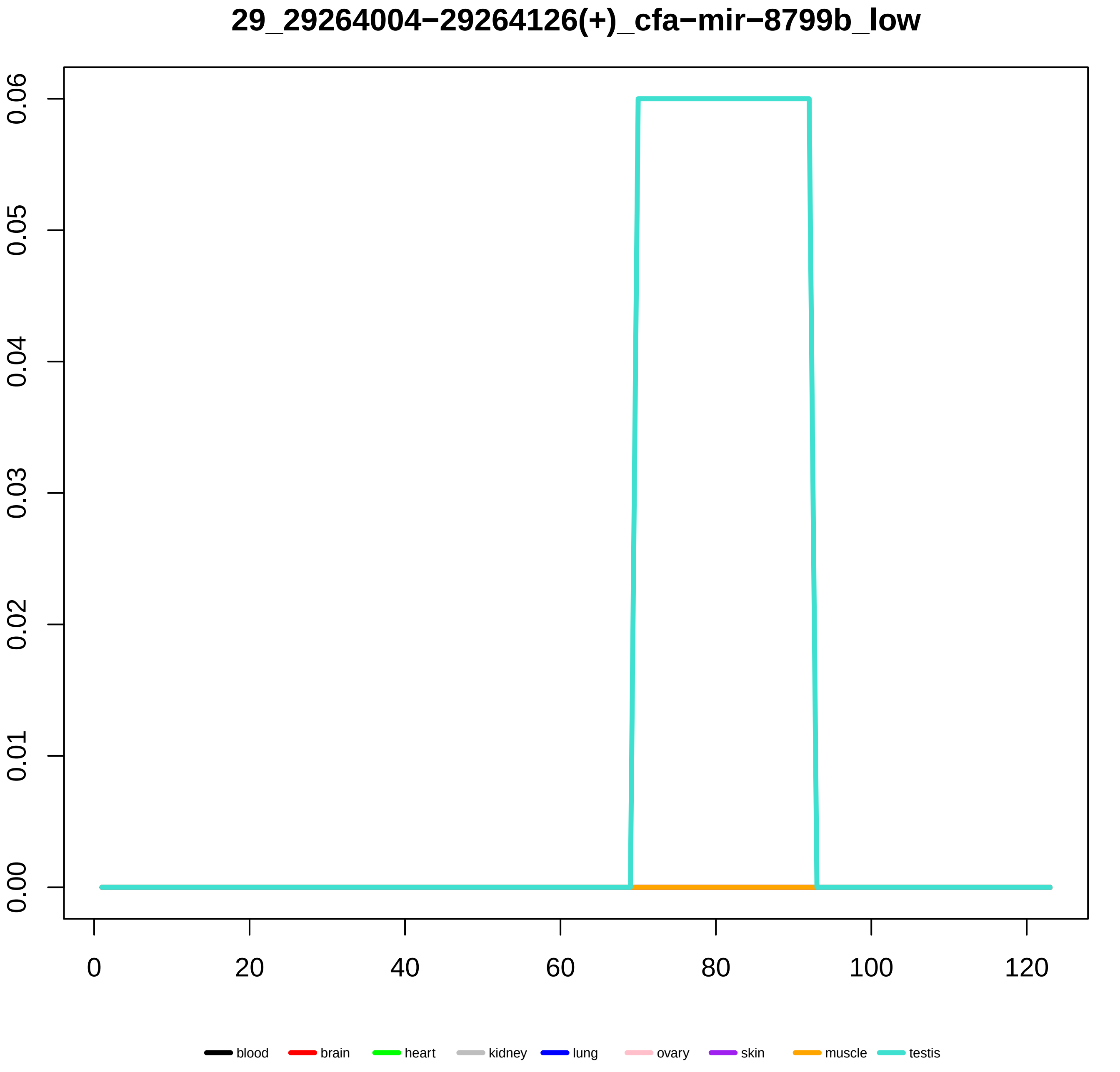
29_29264004−29264126(+)_cfa−mir−8799b_low
0.00
0.01
0.02
0.03
0.04
0.05
0.06
0
20
40
60
80
100
120
blood
brain
heart
kidney
lung
ovary
skin
muscle
testis

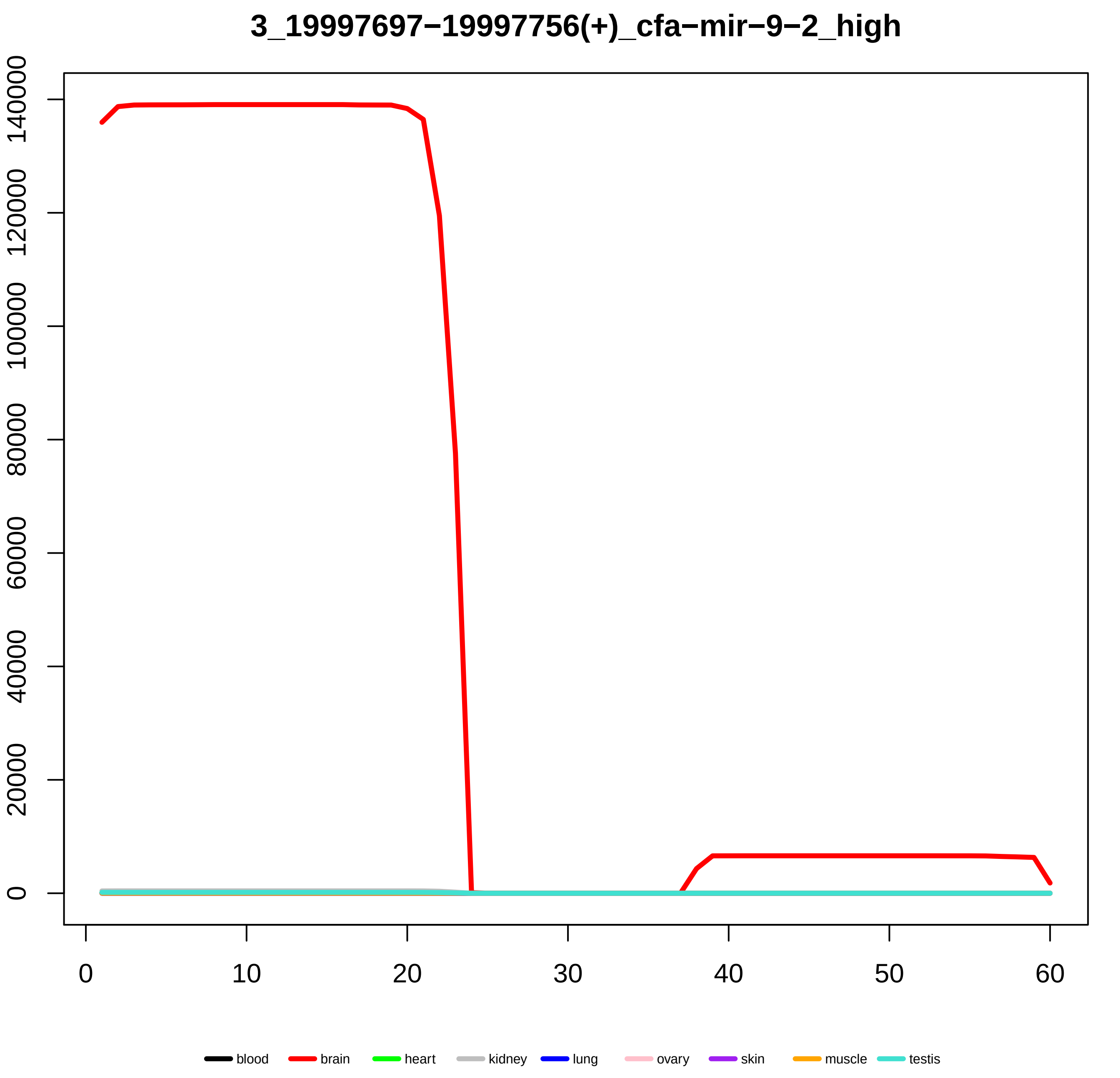
3_19997697−19997756(+)_cfa−mir−9−2_high
0
20000
40000
60000
80000
100000
120000
140000
0
10
20
30
40
50
60
blood
brain
heart
kidney
lung
ovary
skin
muscle
testis

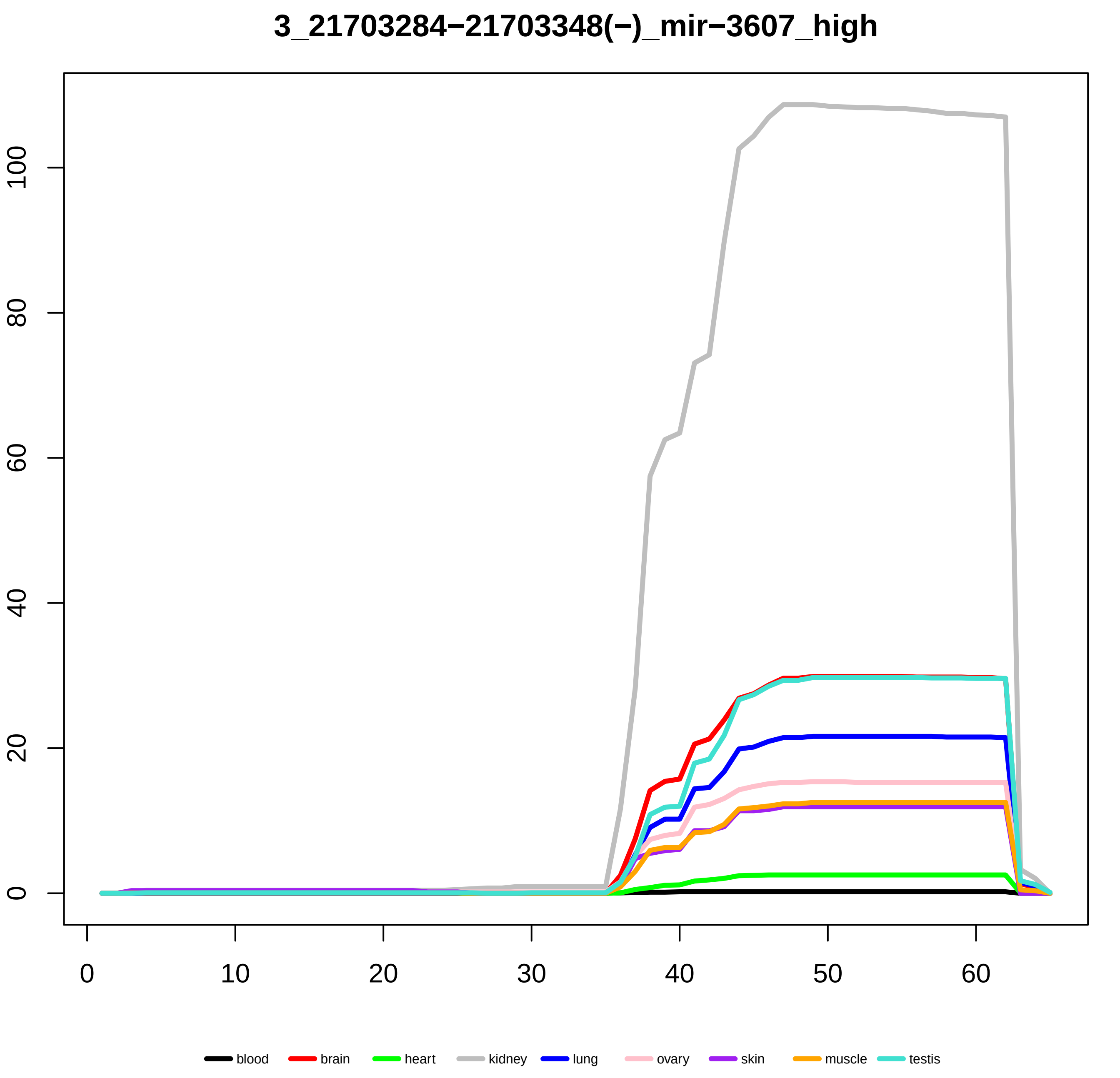

3_21703284−21703348(−)_mir−3607_high
0
20
40
60
80
100
0
10
20
30
40
50
60
blood
brain
heart
kidney
lung
ovary
skin
muscle
testis

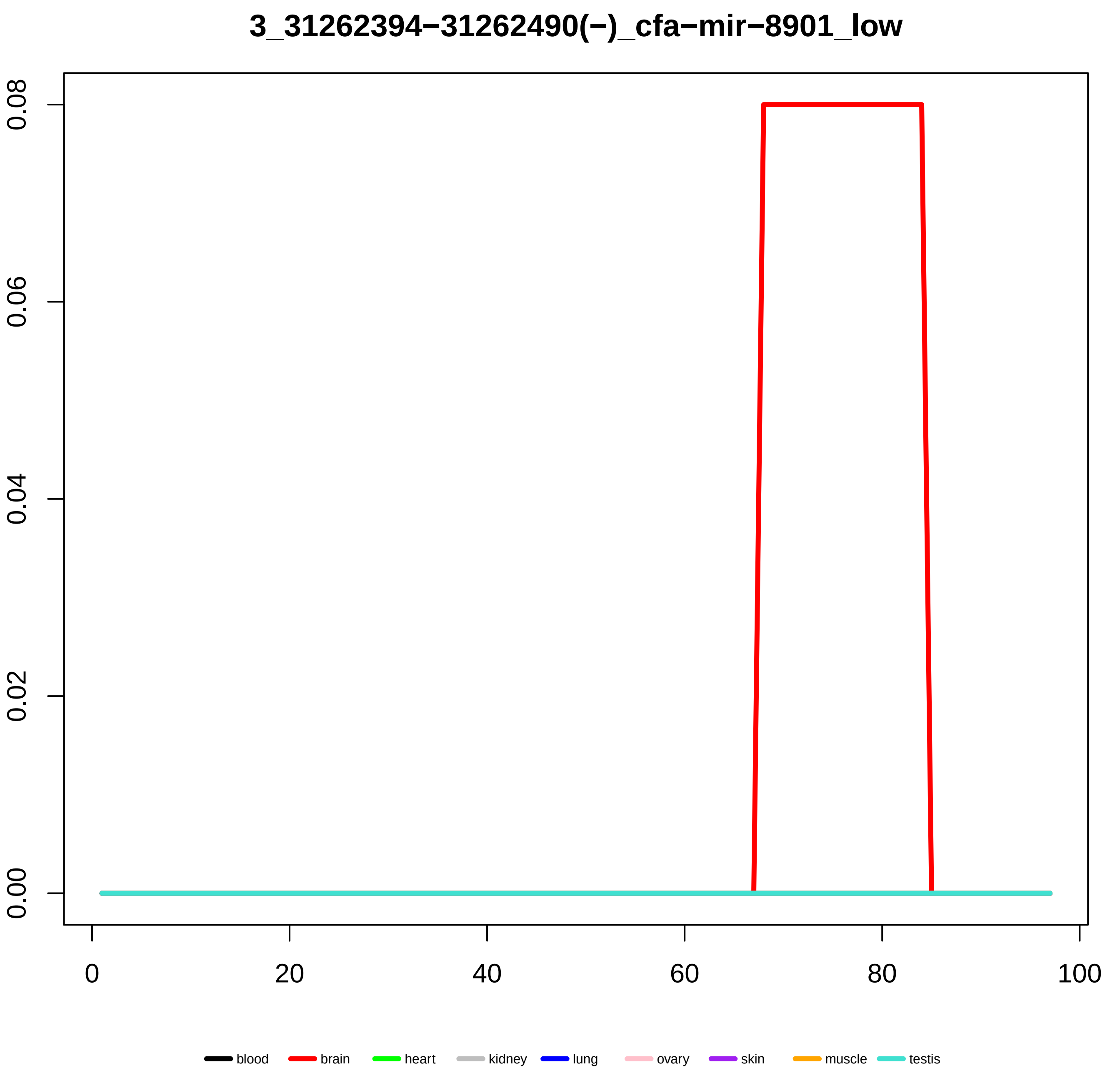
3_31262394−31262490(−)_cfa−mir−8901_low
0.00
0.02
0.04
0.06
0.08
0
20
40
60
80
100
blood
brain
heart
kidney
lung
ovary
skin
muscle
testis

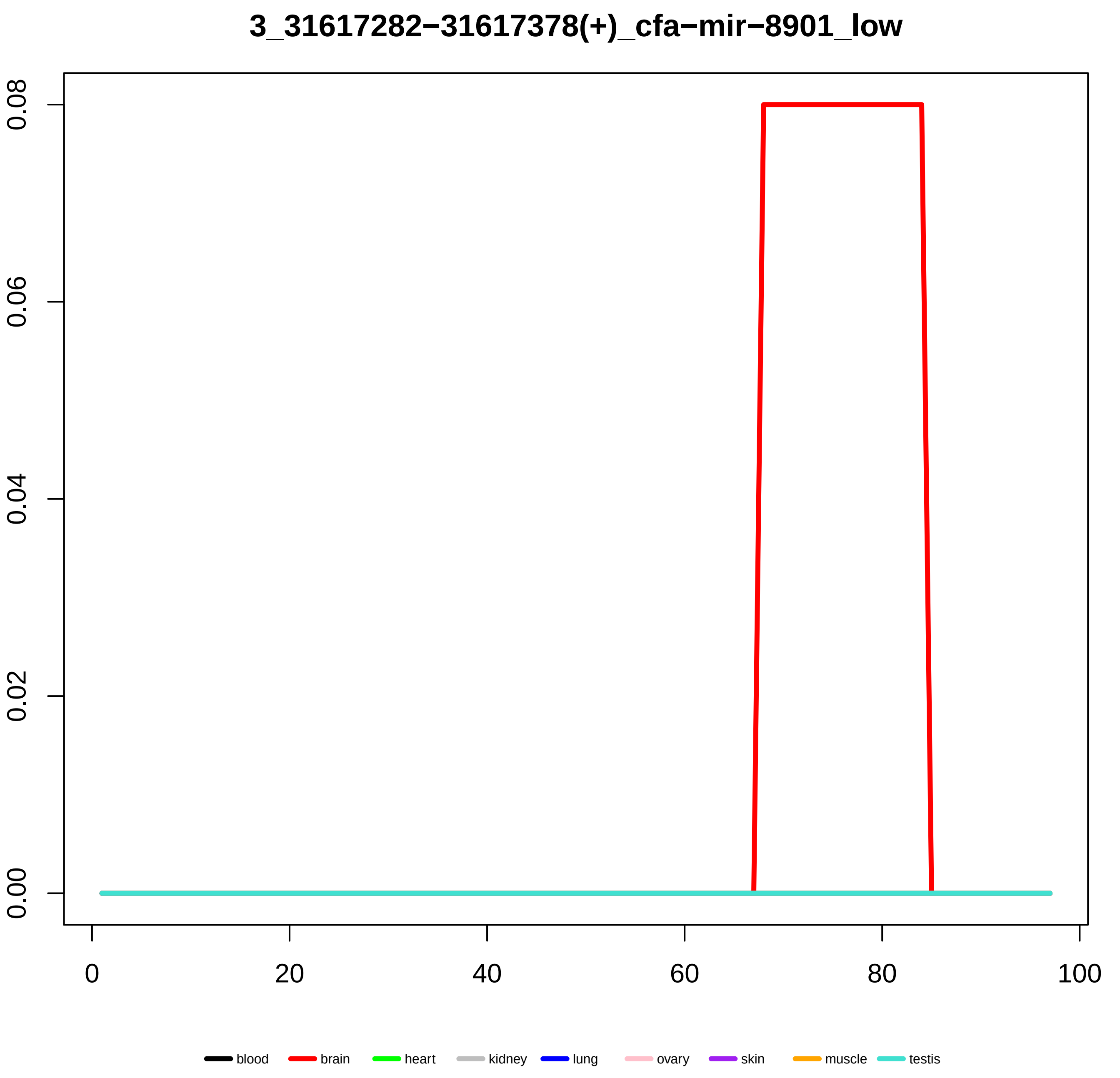

3_31617282−31617378(+)_cfa−mir−8901_low
0.00
0.02
0.04
0.06
0.08
0
20
40
60
80
100
blood
brain
heart
kidney
lung
ovary
skin
muscle
testis

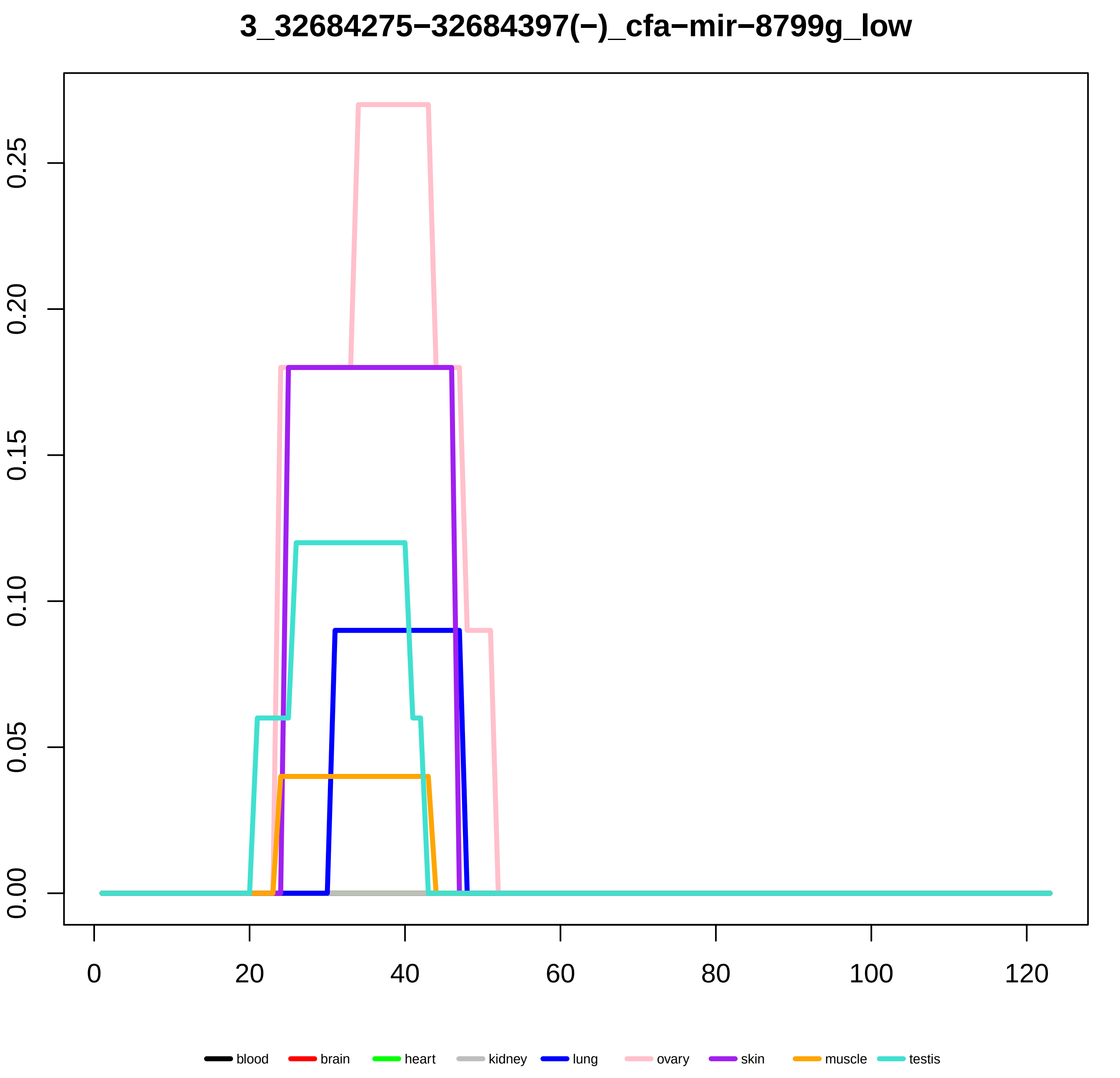
3_32684275−32684397(−)_cfa−mir−8799g_low
0.00
0.05
0.10
0.15
0.20
0.25
0
20
40
60
80
100
120
blood
brain
heart
kidney
lung
ovary
skin
muscle
testis

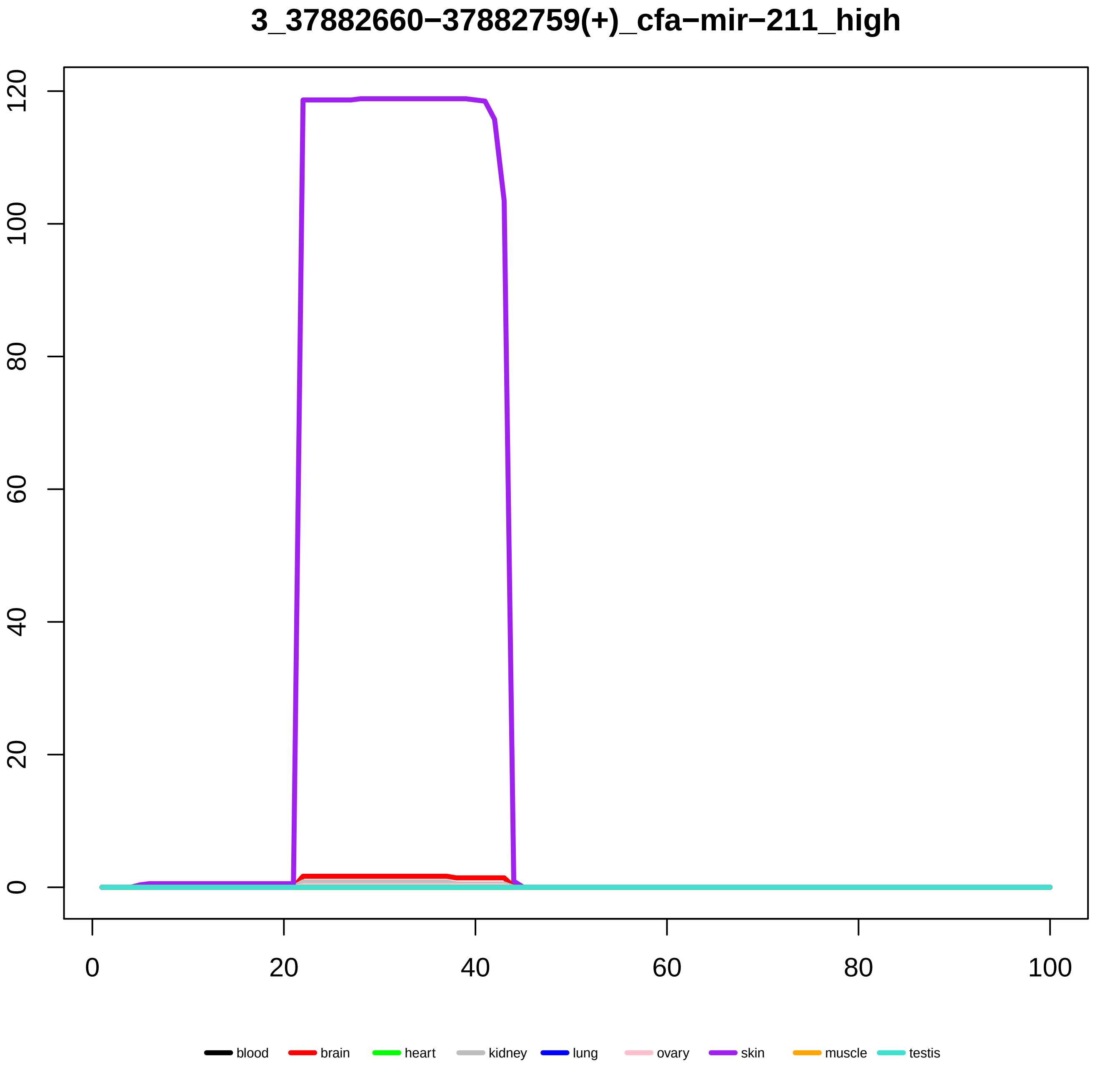

3_37882660−37882759(+)_cfa−mir−211_high
0
20
40
60
80
100
120
0
20
40
60
80
100
blood
brain
heart
kidney
lung
ovary
skin
muscle
testis

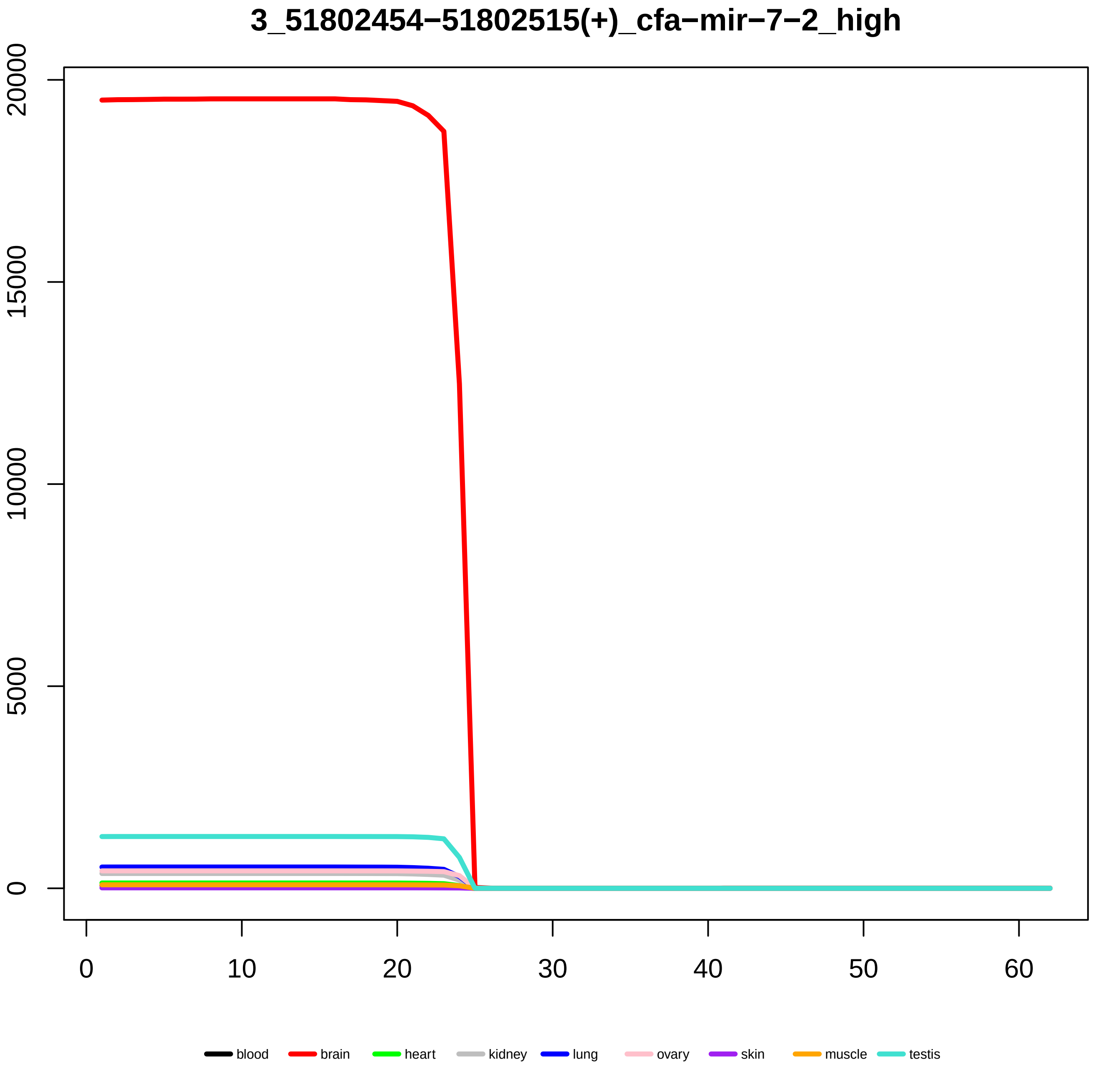

3_51802454−51802515(+)_cfa−mir−7−2_high
0
5000
10000
15000
20000
0
10
20
30
40
50
60
blood
brain
heart
kidney
lung
ovary
skin
muscle
testis

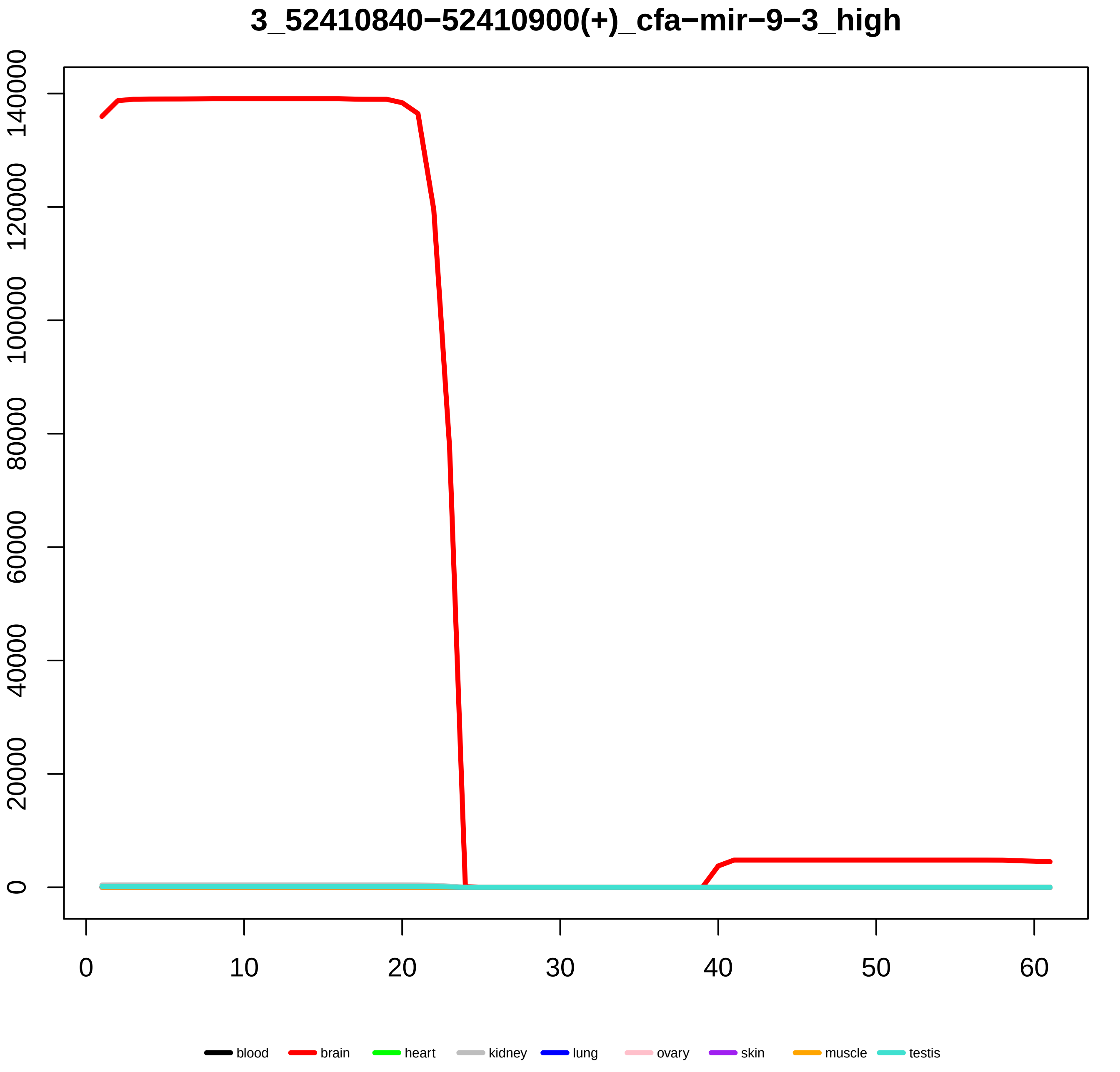
3_52410840−52410900(+)_cfa−mir−9−3_high
0
20000
40000
60000
80000
100000
120000
140000
0
10
20
30
40
50
60
blood
brain
heart
kidney
lung
ovary
skin
muscle
testis

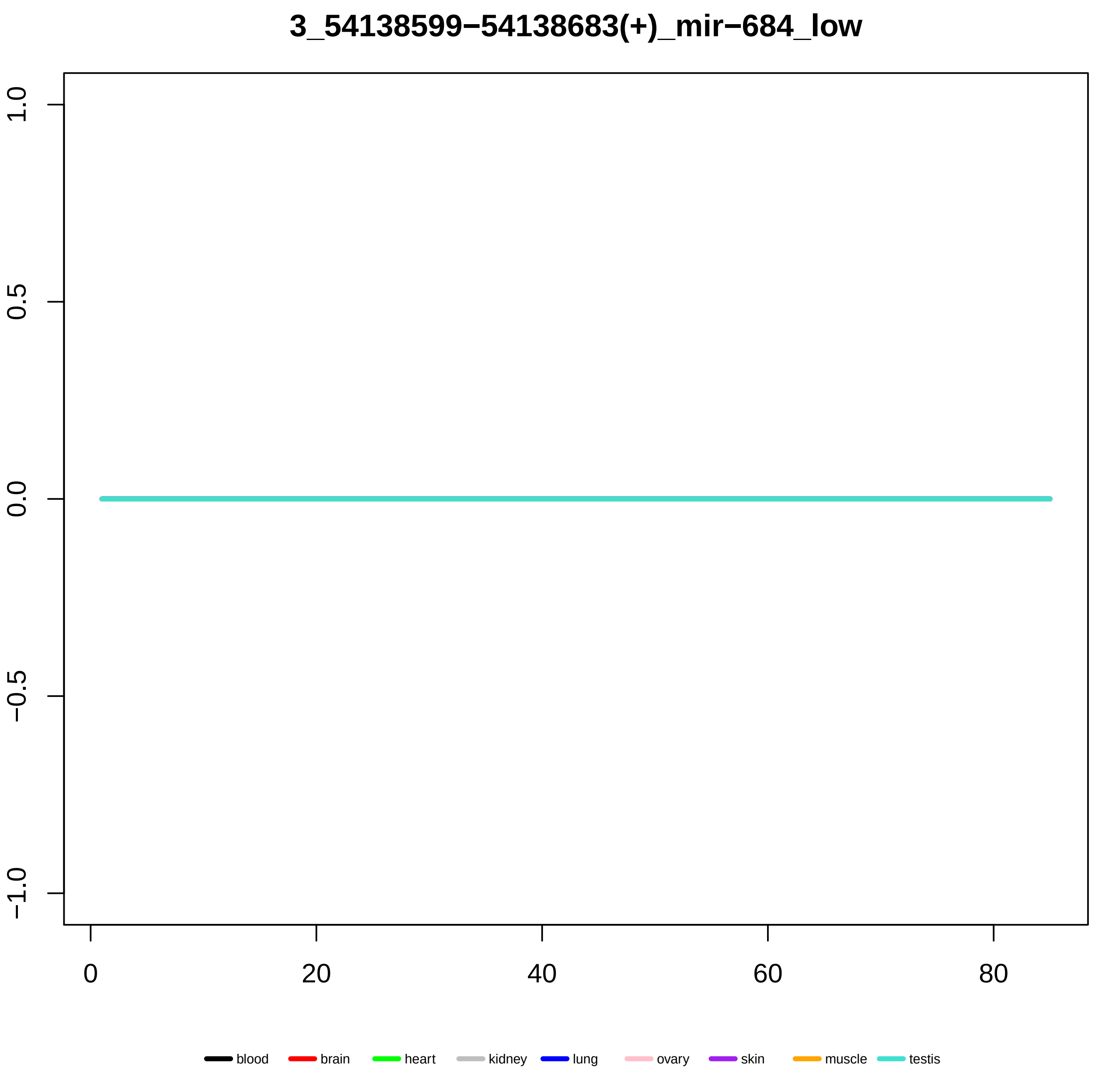
3_54138599−54138683(+)_mir−684_low
1.0
0.5
0.0
−0.5
−1.0
0
20
40
60
80
blood
brain
heart
kidney
lung
ovary
skin
muscle
testis

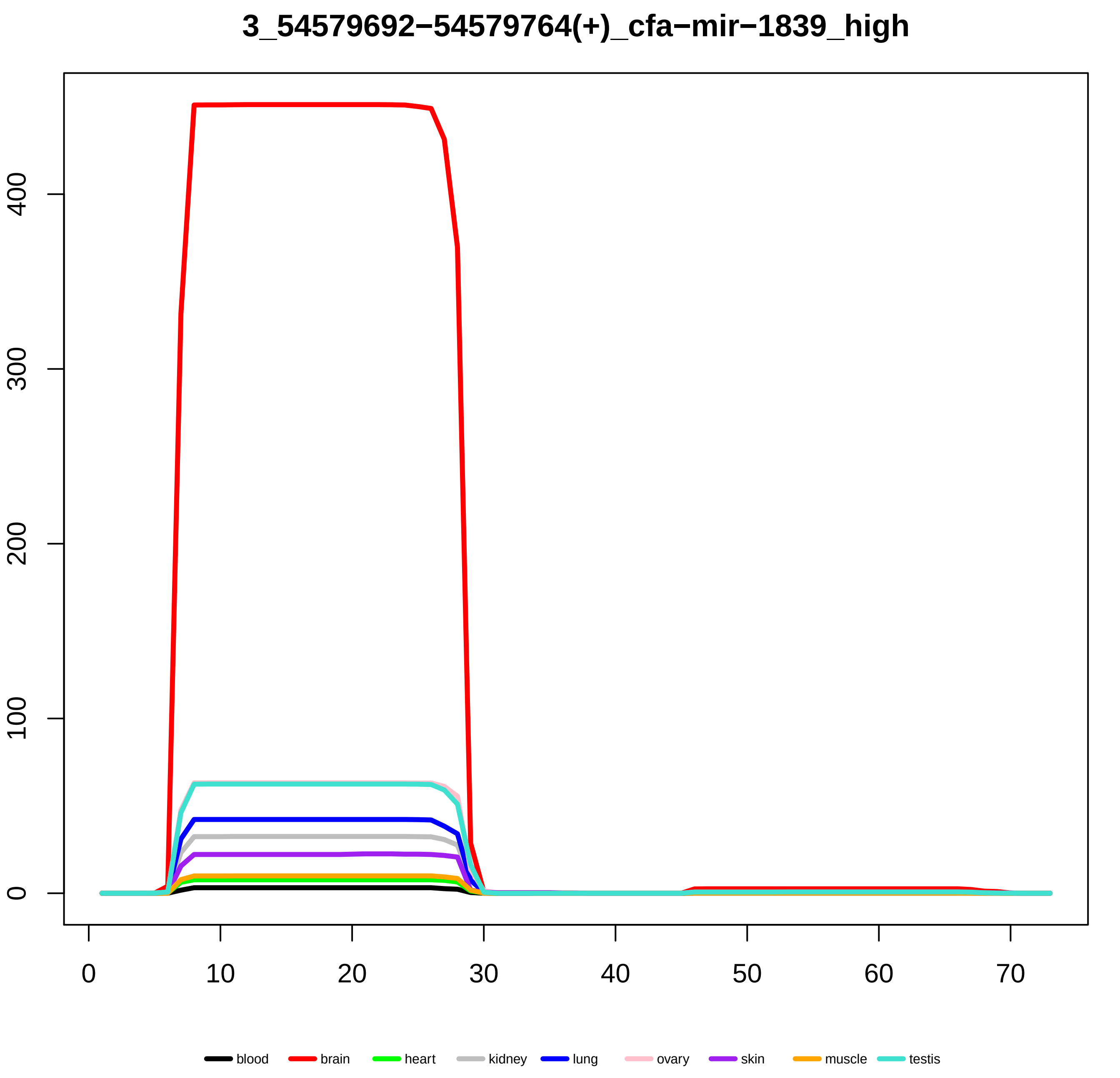
3_54579692−54579764(+)_cfa−mir−1839_high
0
100
200
300
400
0
10
20
30
40
50
60
70
blood
brain
heart
kidney
lung
ovary
skin
muscle
testis

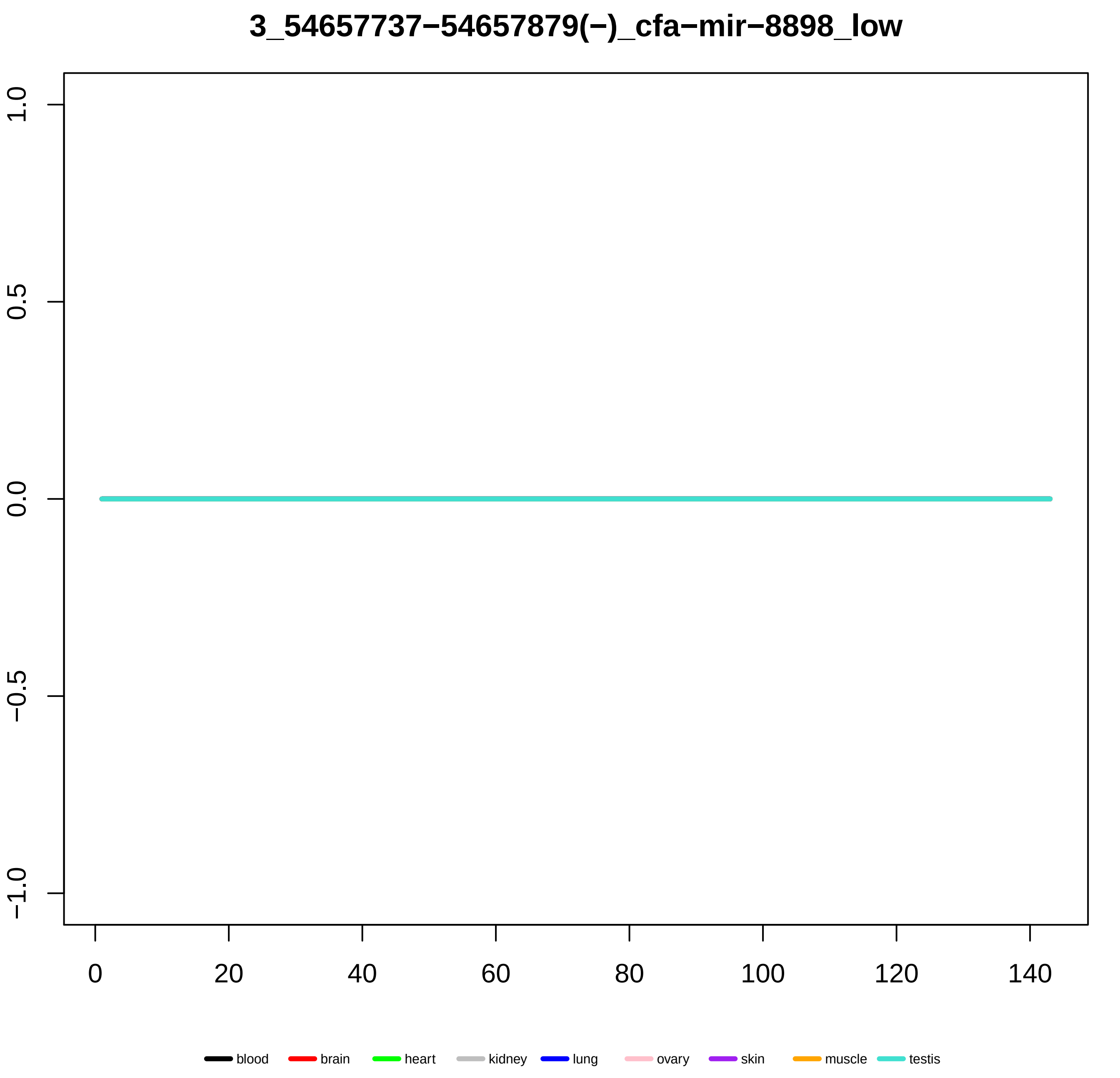
3_54657737−54657879(−)_cfa−mir−8898_low
1.0
0.5
0.0
−0.5
−1.0
0
20
40
60
80
100
120
140
blood
brain
heart
kidney
lung
ovary
skin
muscle
testis

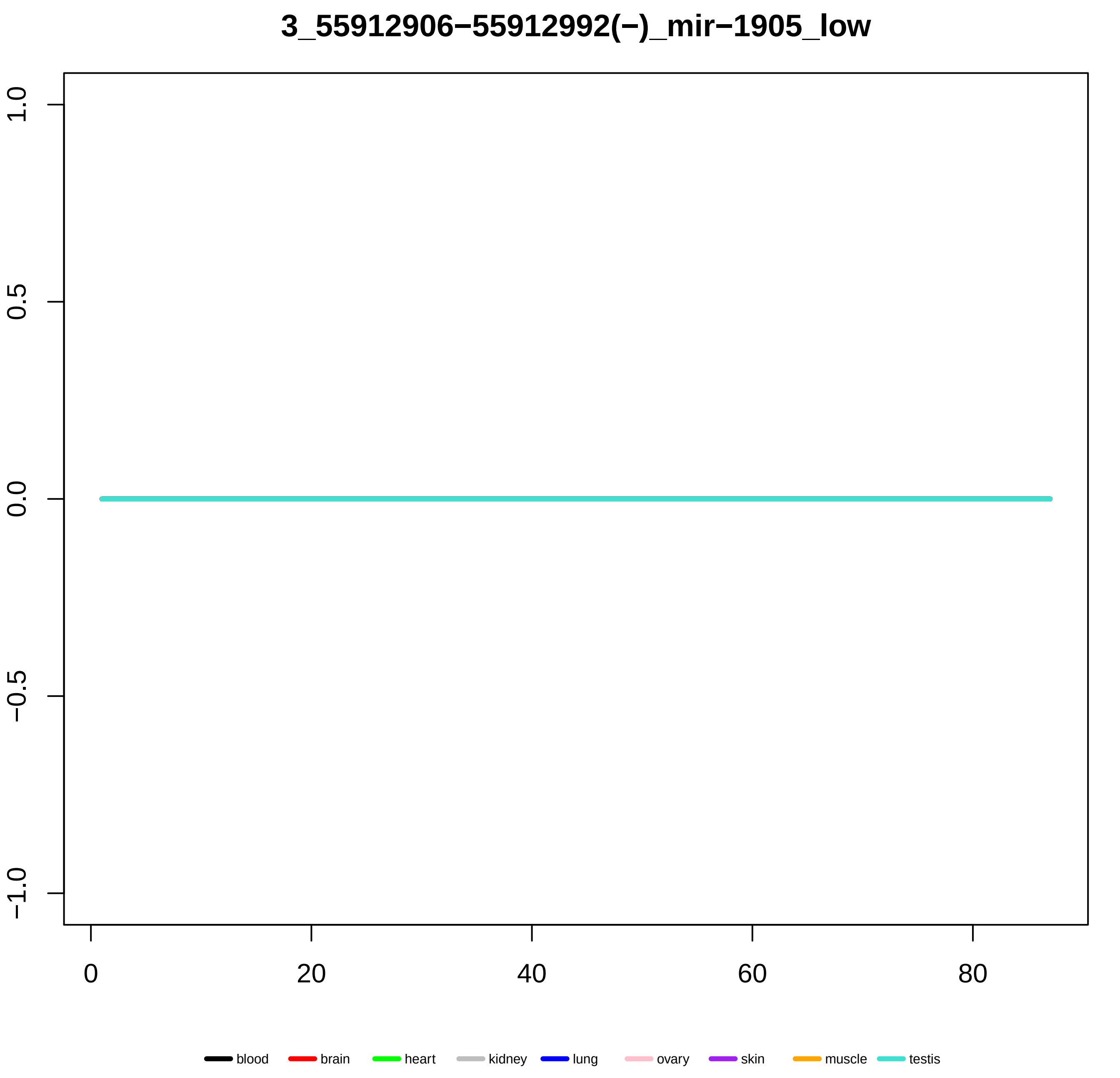

3_55912906−55912992(−)_mir−1905_low
1.0
0.5
0.0
−0.5
−1.0
0
20
40
60
80
blood
brain
heart
kidney
lung
ovary
skin
muscle
testis

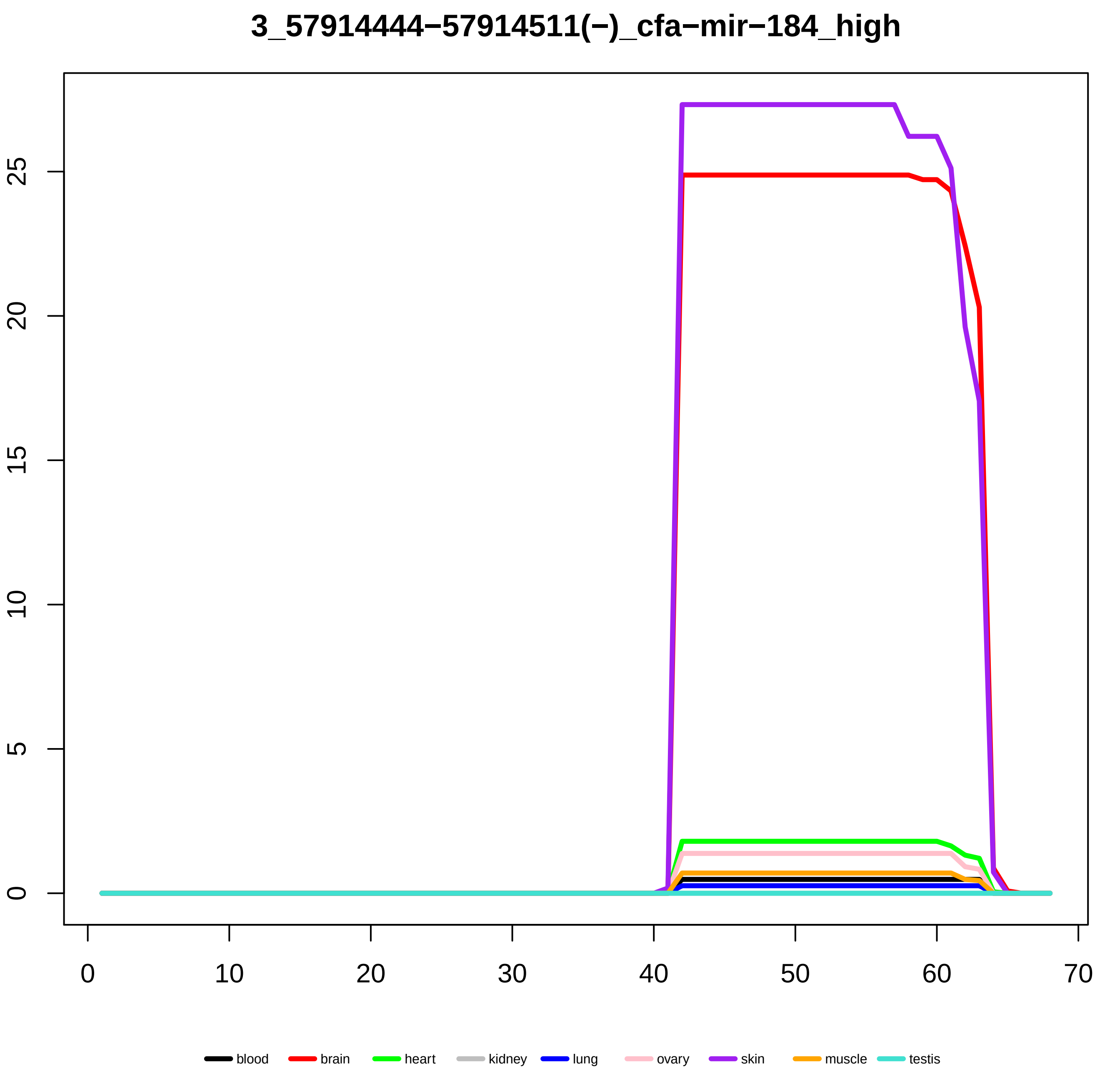
3_57914444−57914511(−)_cfa−mir−184_high
0
5
10
15
20
25
0
10
20
30
40
50
60
70
blood
brain
heart
kidney
lung
ovary
skin
muscle
testis

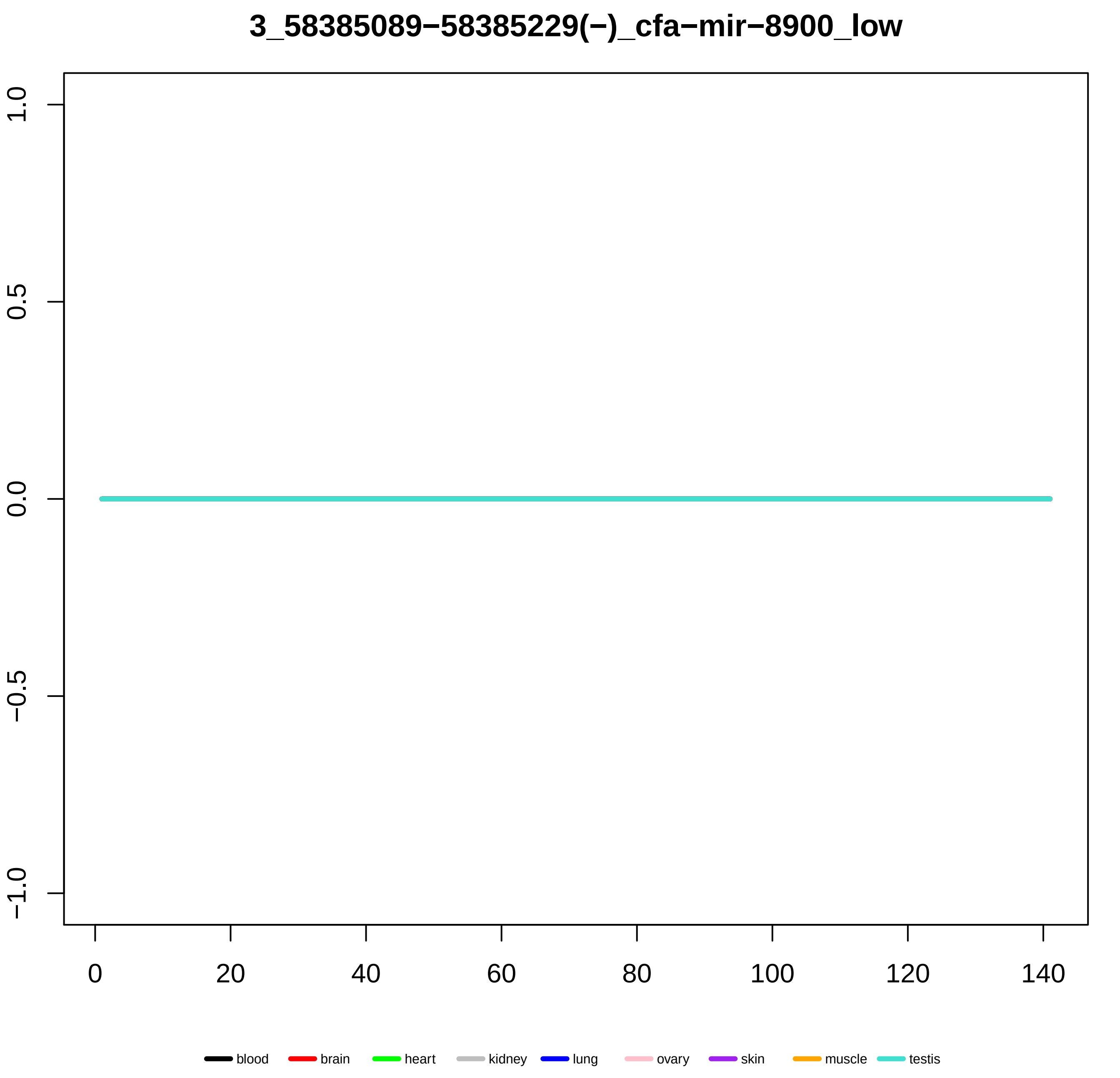

3_58385089−58385229(−)_cfa−mir−8900_low
1.0
0.5
0.0
−0.5
−1.0
0
20
40
60
80
100
120
140
blood
brain
heart
kidney
lung
ovary
skin
muscle
testis

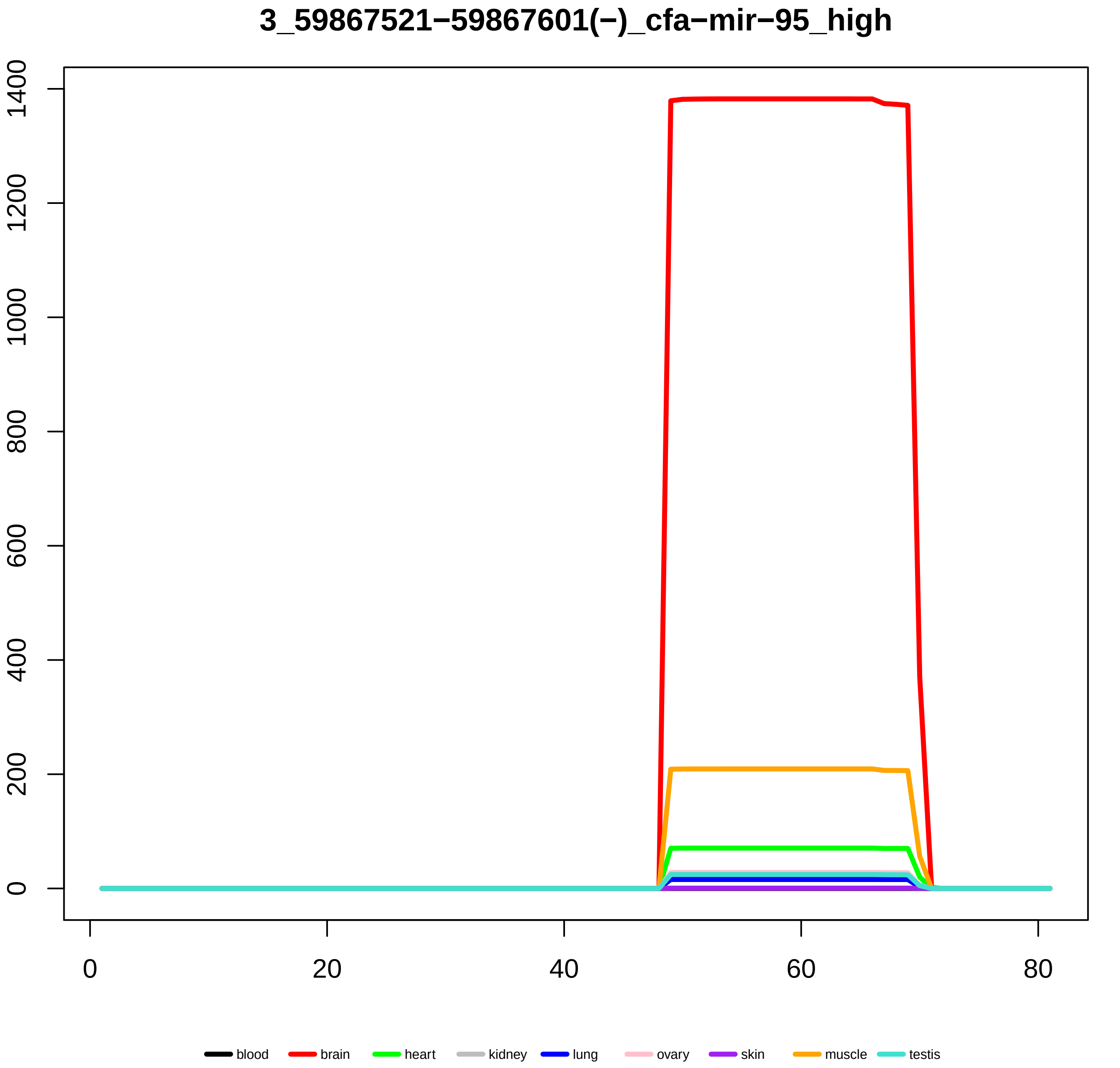

3_59867521−59867601(−)_cfa−mir−95_high
0
200
400
600
800
1000
1200
1400
0
20
40
60
80
blood
brain
heart
kidney
lung
ovary
skin
muscle
testis

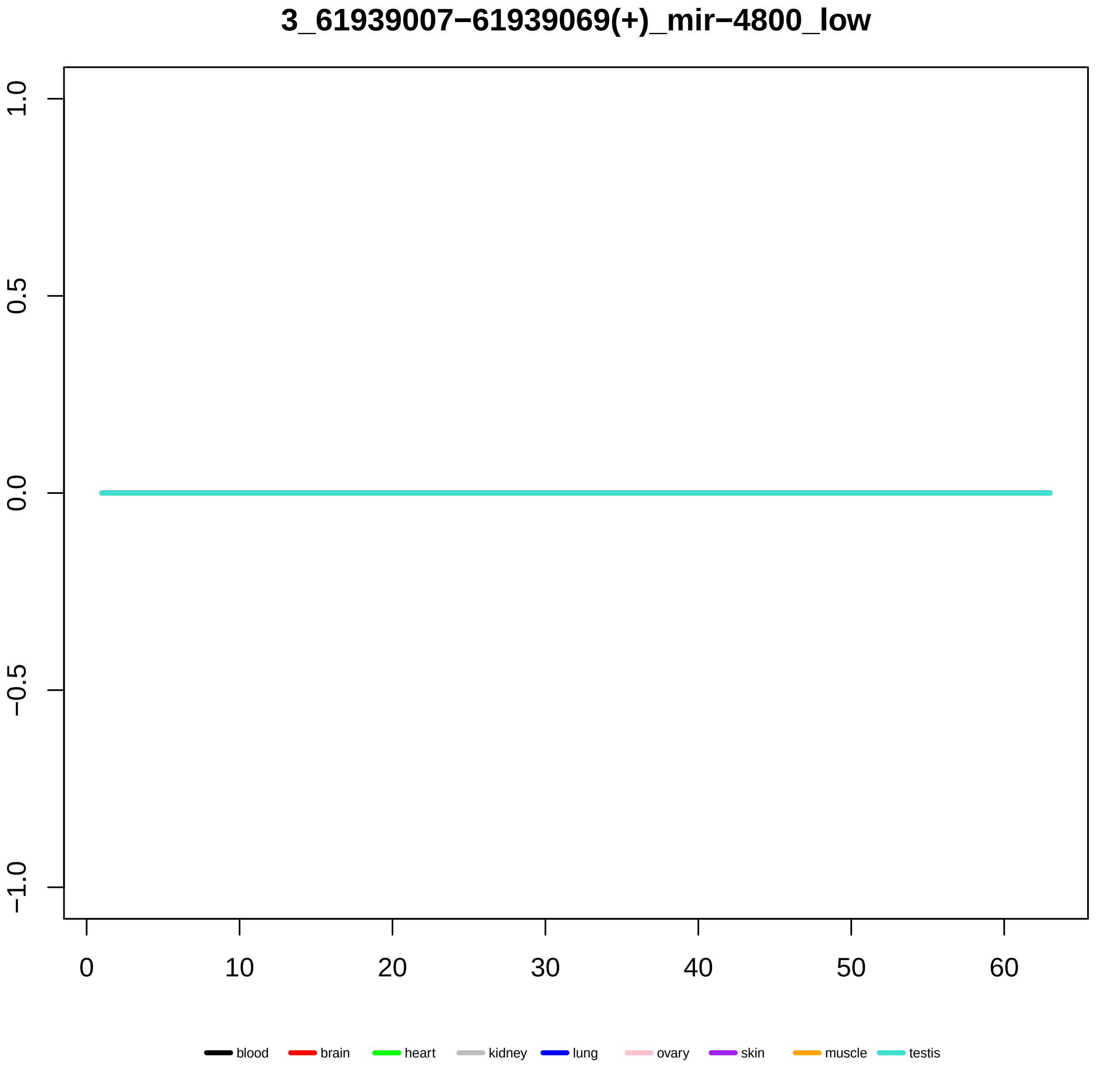

3_61939007−61939069(+)_mir−4800_low
1.0
0.5
0.0
−0.5
−1.0
0
10
20
30
40
50
60
blood
brain
heart
kidney
lung
ovary
skin
muscle
testis

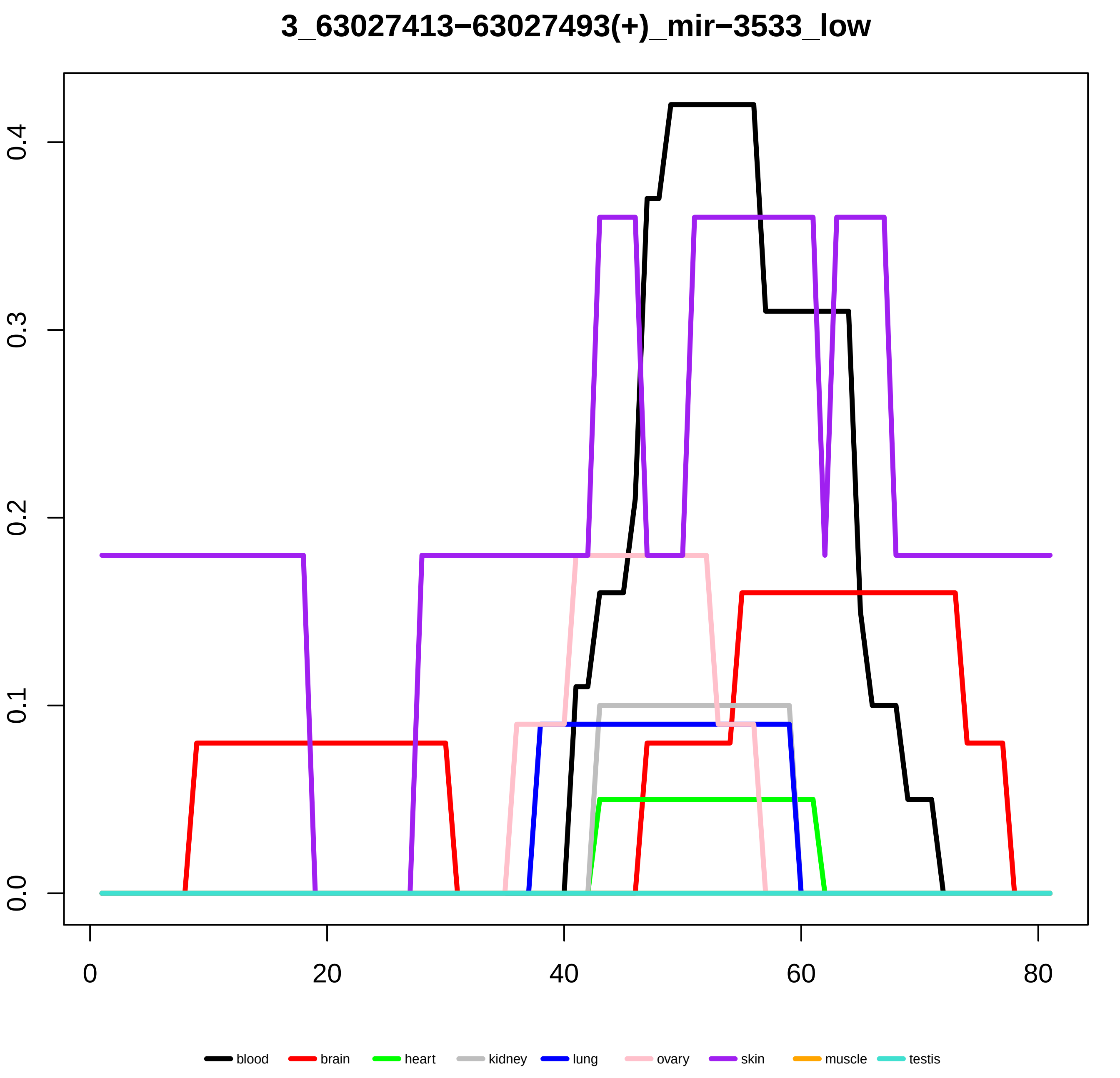

3_63027413−63027493(+)_mir−3533_low
0.0
0.1
0.2
0.3
0.4
0
20
40
60
80
blood
brain
heart
kidney
lung
ovary
skin
muscle
testis

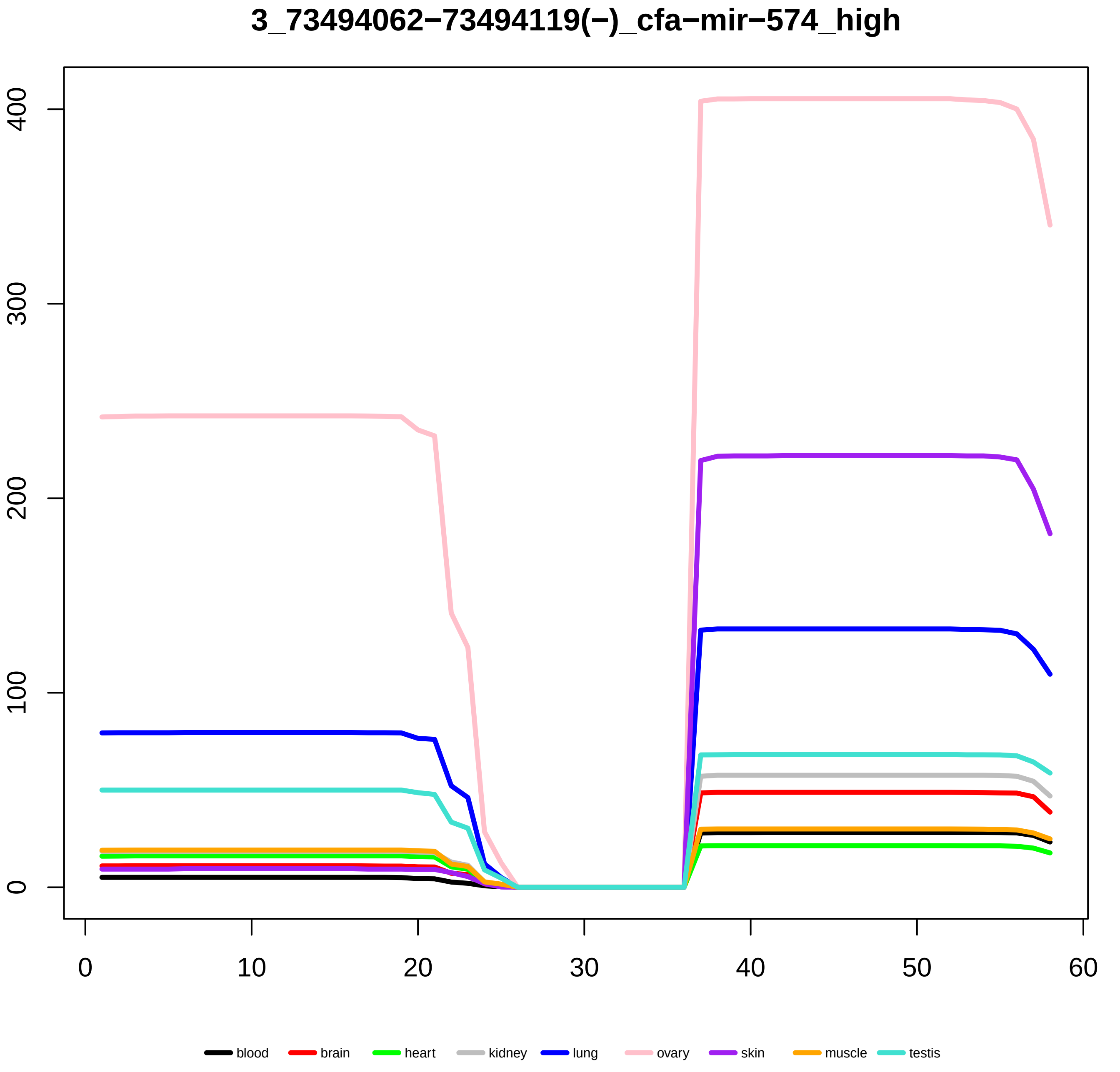

3_73494062−73494119(−)_cfa−mir−574_high
0
100
200
300
400
0
10
20
30
40
50
60
blood
brain
heart
kidney
lung
ovary
skin
muscle
testis

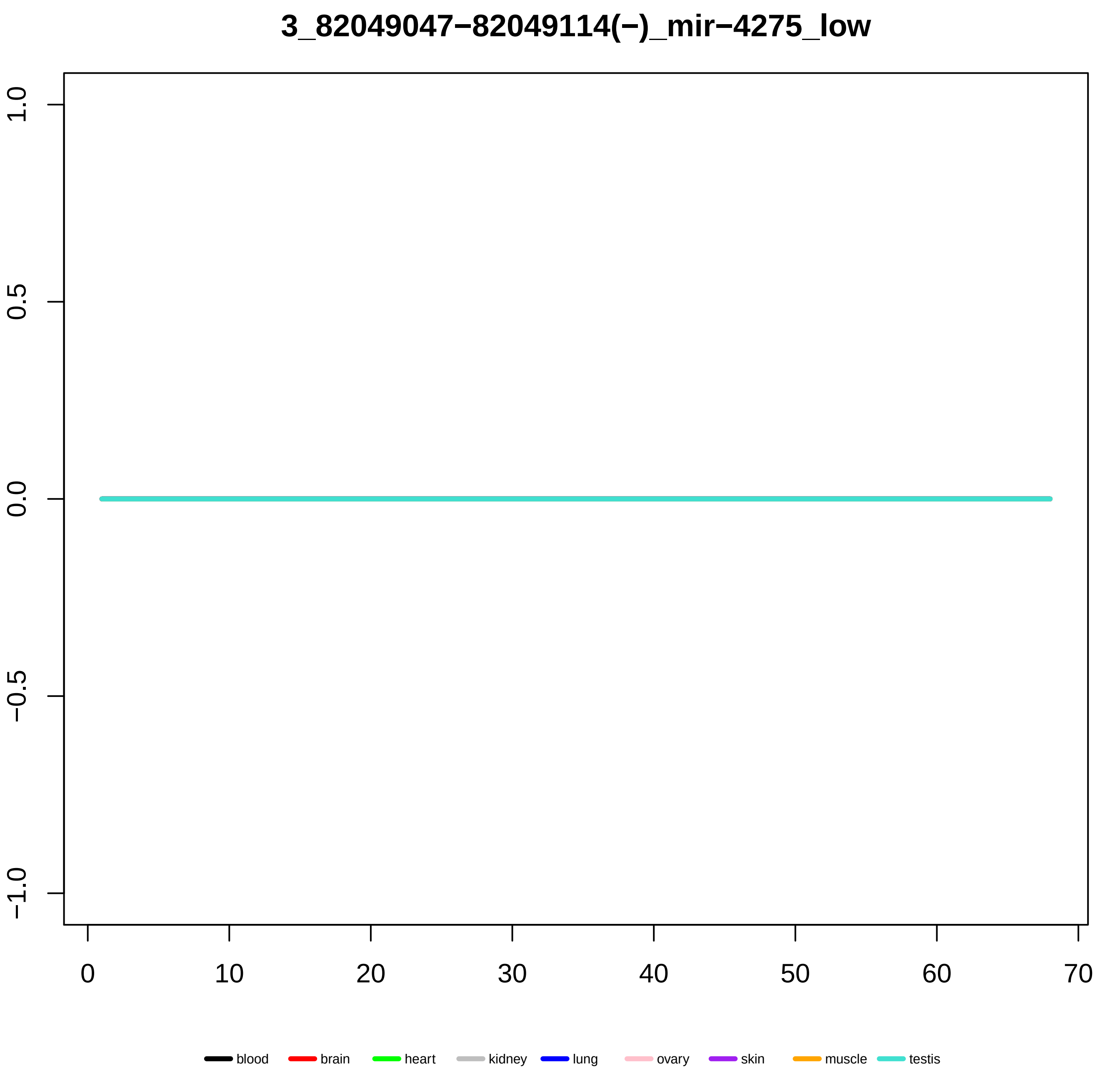
3_82049047−82049114(−)_mir−4275_low
1.0
0.5
0.0
−0.5
−1.0
0
10
20
30
40
50
60
70
blood
brain
heart
kidney
lung
ovary
skin
muscle
testis

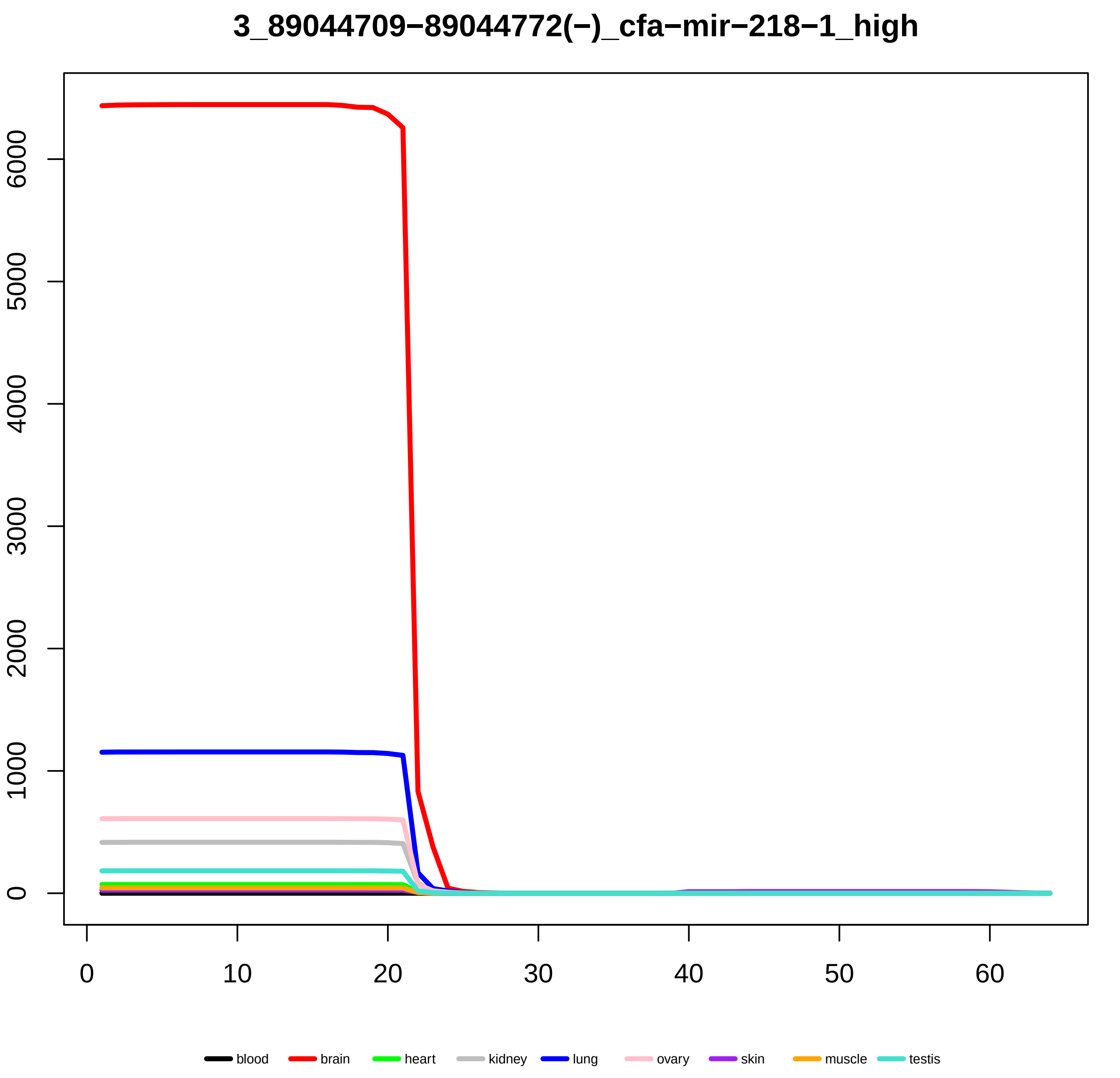

3_89044709−89044772(−)_cfa−mir−218−1_high
0
1000
2000
3000
4000
5000
6000
0
10
20
30
40
50
60
blood
brain
heart
kidney
lung
ovary
skin
muscle
testis

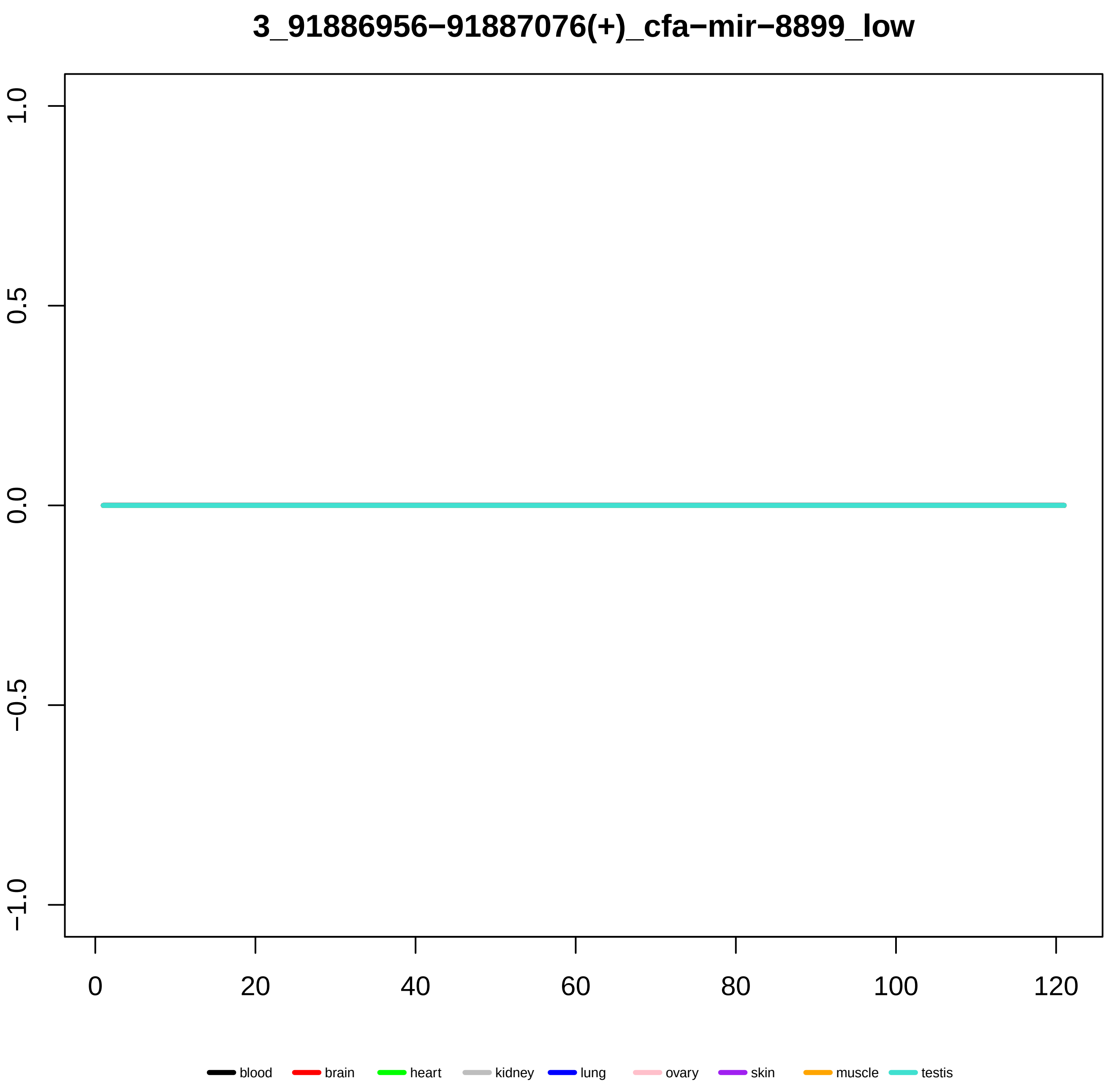

3_91886956−91887076(+)_cfa−mir−8899_low
1.0
0.5
0.0
−0.5
−1.0
0
20
40
60
80
100
120
blood
brain
heart
kidney
lung
ovary
skin
muscle
testis

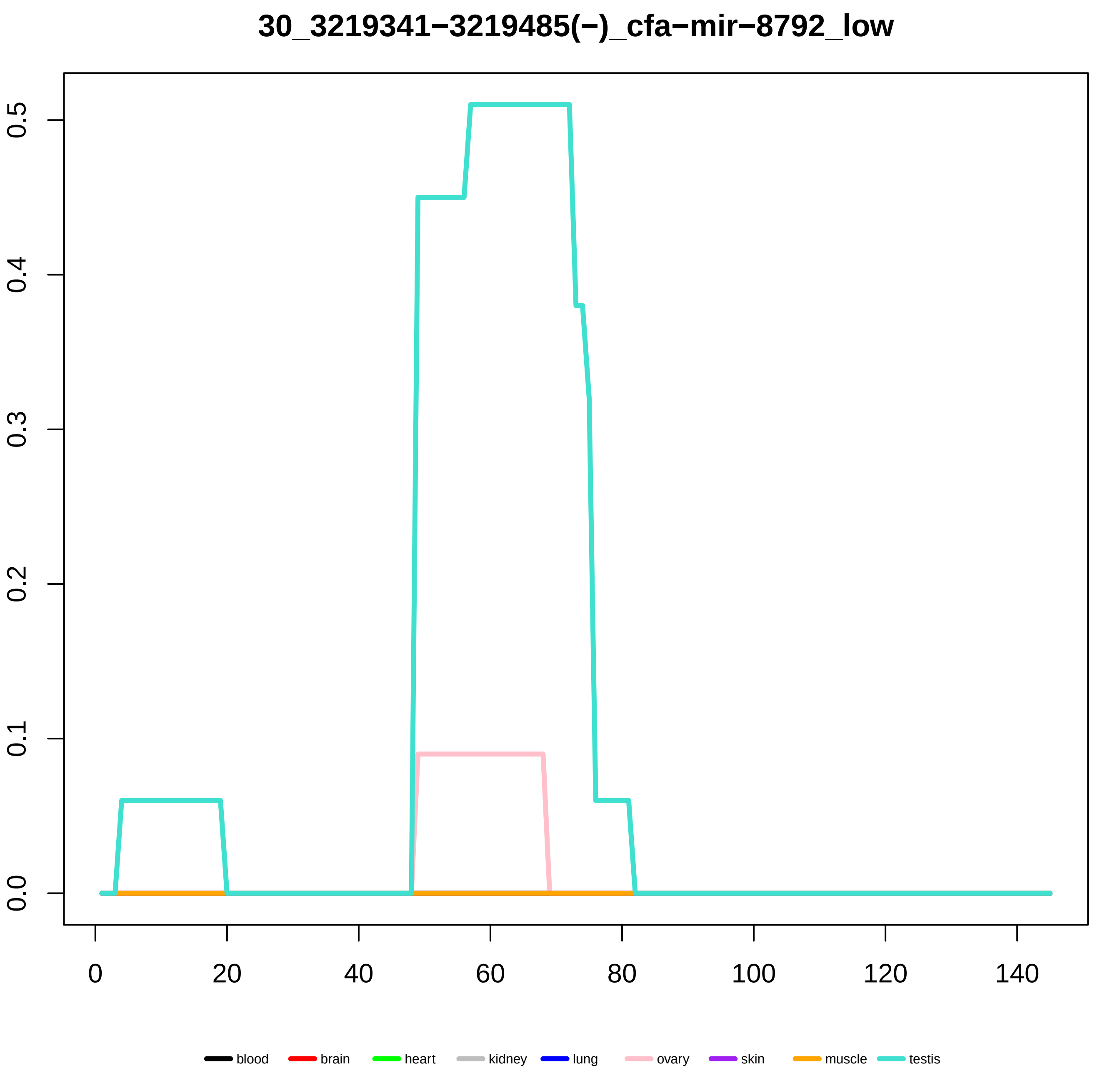
30_3219341−3219485(−)_cfa−mir−8792_low
0.0
0.1
0.2
0.3
0.4
0.5
0
20
40
60
80
100
120
140
blood
brain
heart
kidney
lung
ovary
skin
muscle
testis

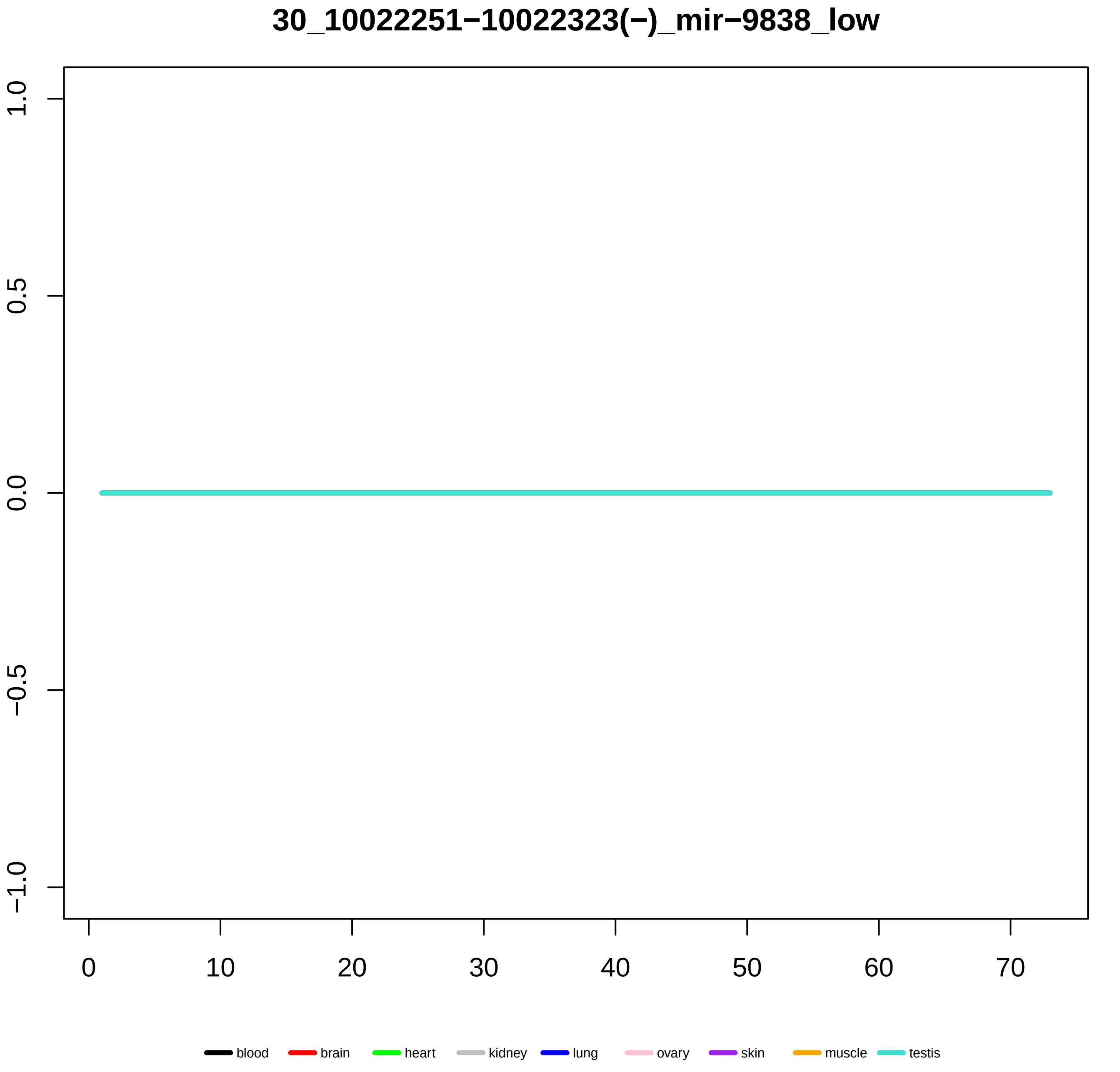

30_10022251−10022323(−)_mir−9838_low
1.0
0.5
0.0
−0.5
−1.0
0
10
20
30
40
50
60
70
blood
brain
heart
kidney
lung
ovary
skin
muscle
testis

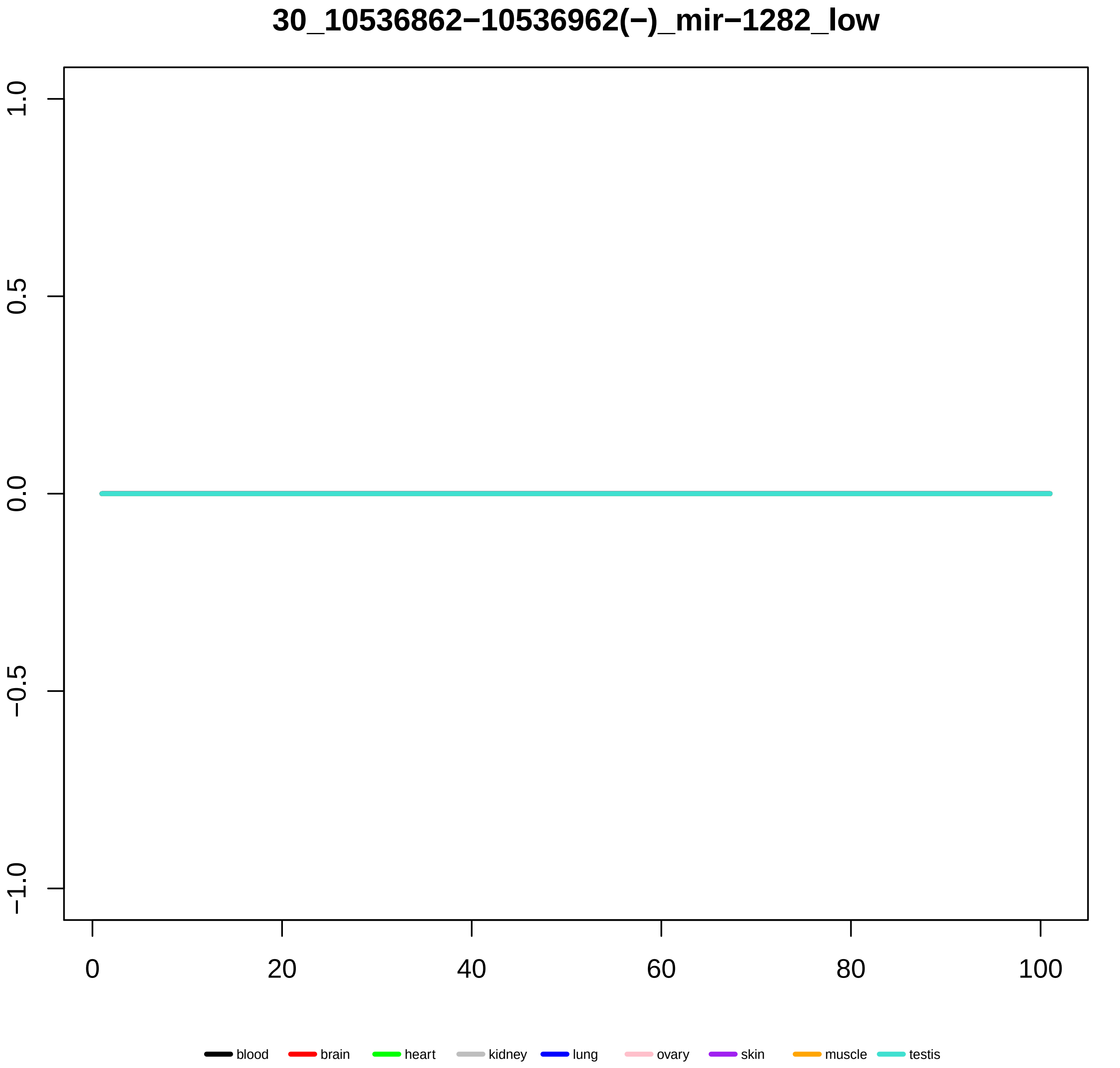

30_10536862−10536962(−)_mir−1282_low
1.0
0.5
0.0
−0.5
−1.0
0
20
40
60
80
100
blood
brain
heart
kidney
lung
ovary
skin
muscle
testis

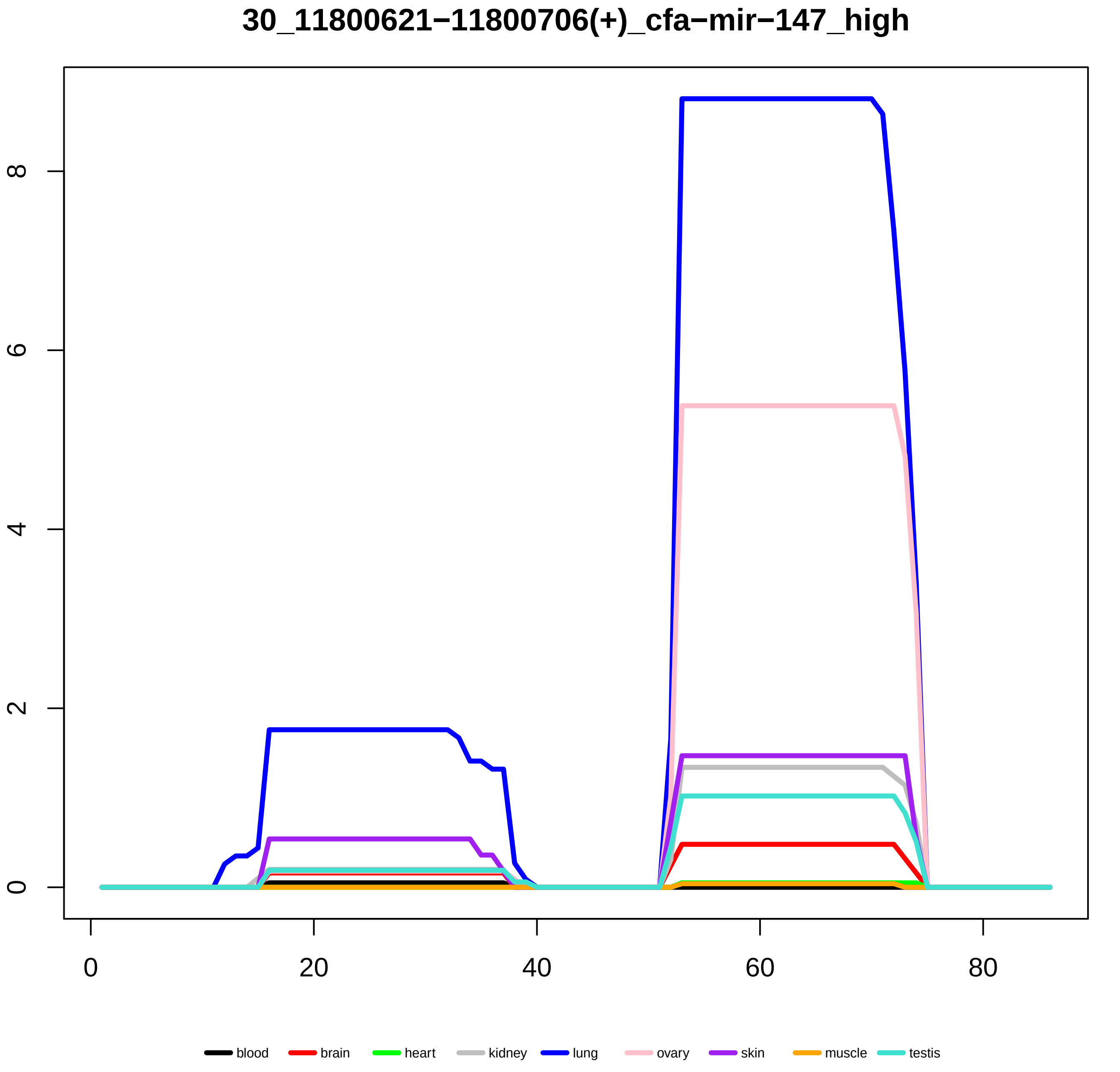

30_11800621−11800706(+)_cfa−mir−147_high
0
2
4
6
8
0
20
40
60
80
blood
brain
heart
kidney
lung
ovary
skin
muscle
testis

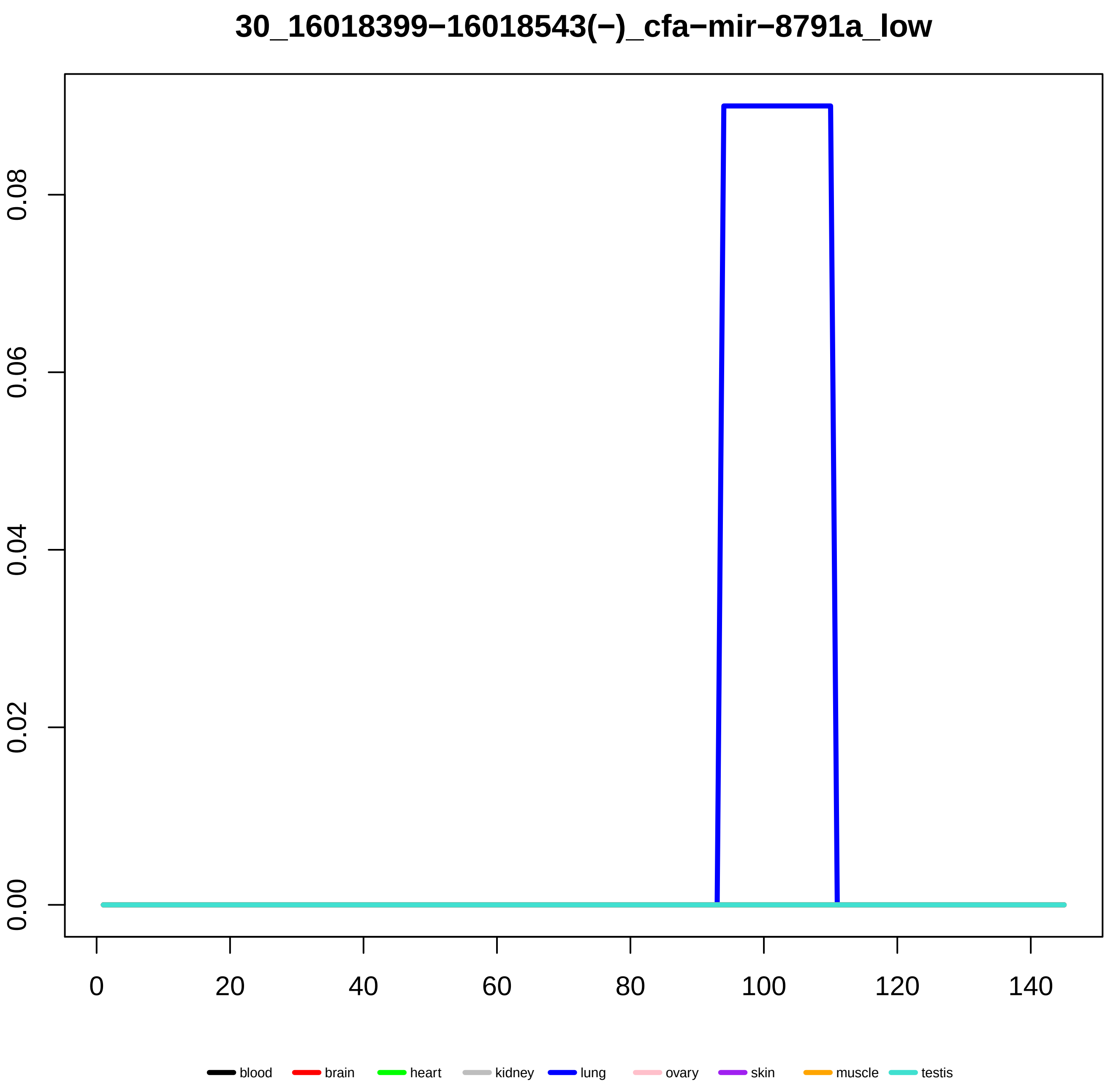

30_16018399−16018543(−)_cfa−mir−8791a_low
0.00
0.02
0.04
0.06
0.08
0
20
40
60
80
100
120
140
blood
brain
heart
kidney
lung
ovary
skin
muscle
testis

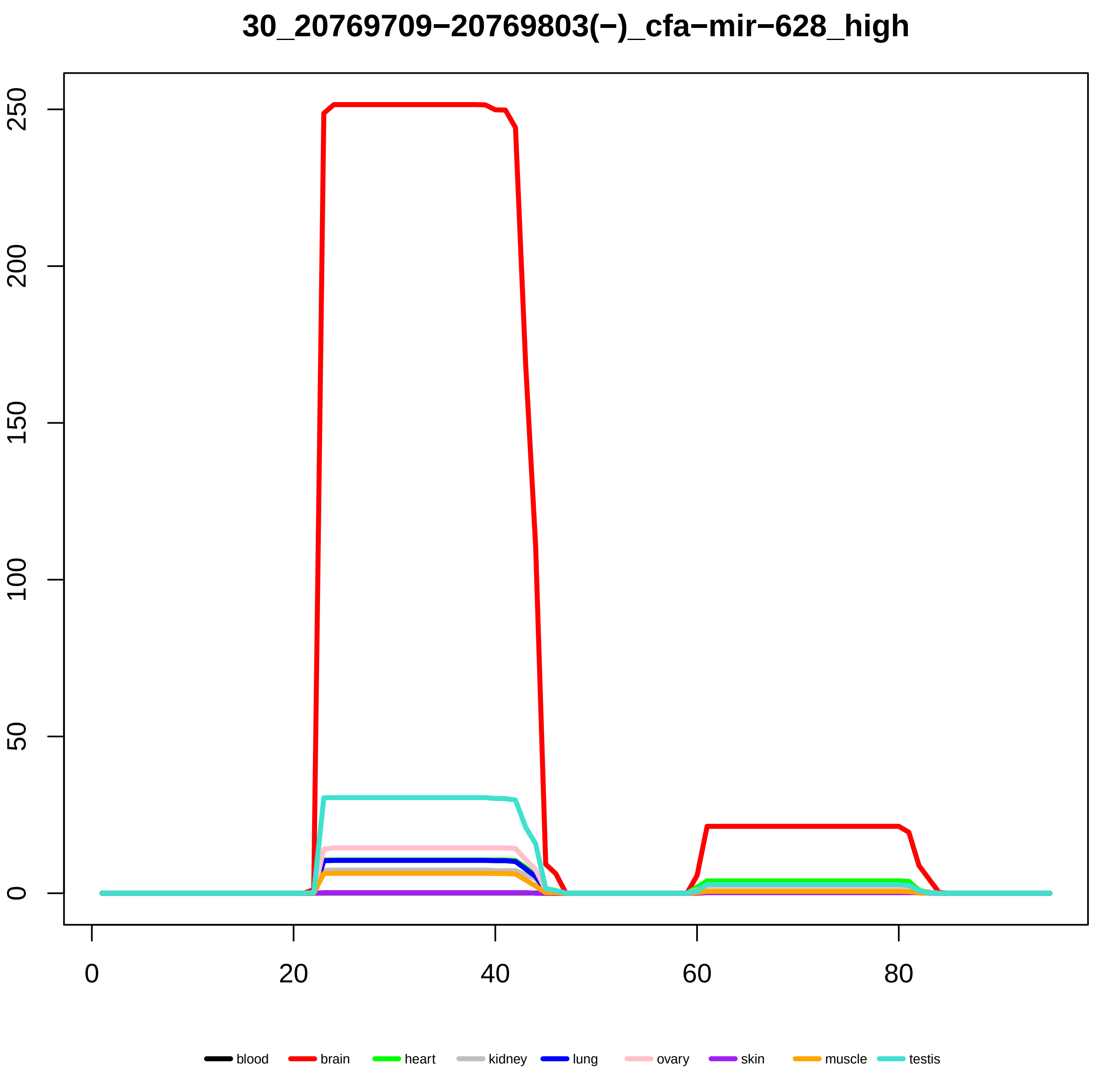

30_20769709−20769803(−)_cfa−mir−628_high
0
50
100
150
200
250
0
20
40
60
80
blood
brain
heart
kidney
lung
ovary
skin
muscle
testis

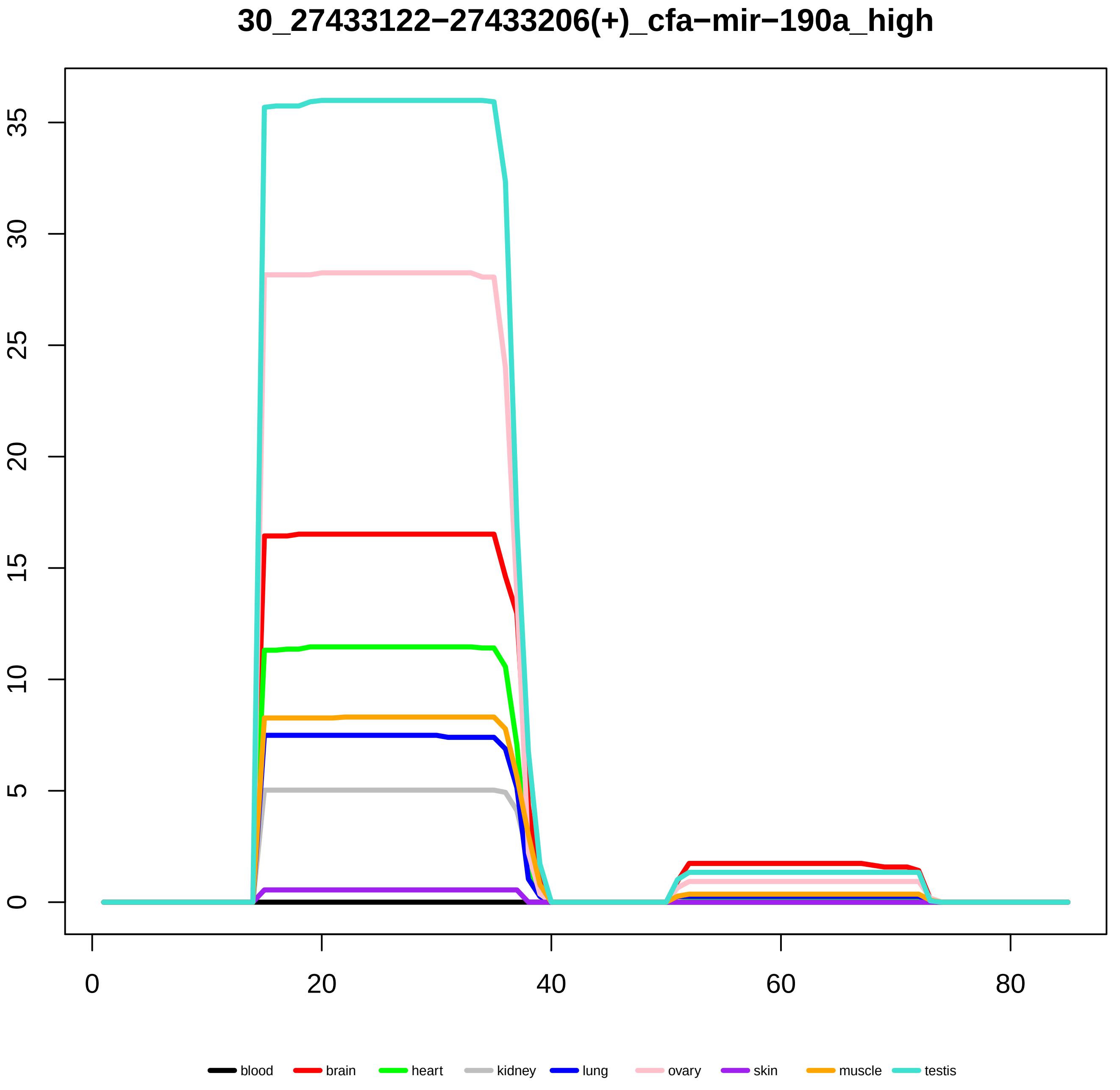

30_27433122−27433206(+)_cfa−mir−190a_high
0
5
10
15
20
25
30
35
0
20
40
60
80
blood
brain
heart
kidney
lung
ovary
skin
muscle
testis

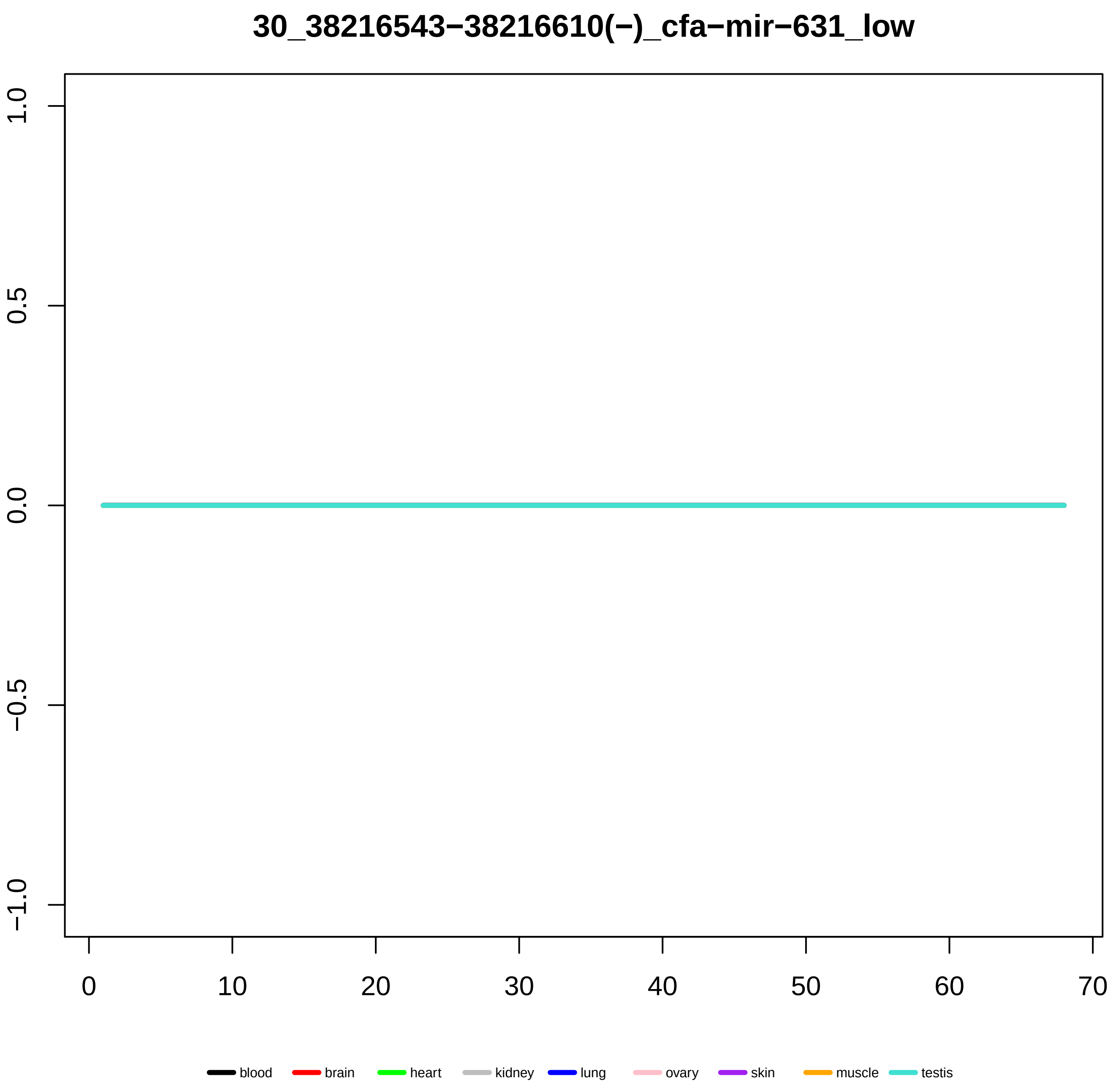

30_38216543−38216610(−)_cfa−mir−631_low
1.0
0.5
0.0
−0.5
−1.0
0
10
20
30
40
50
60
70
blood
brain
heart
kidney
lung
ovary
skin
muscle
testis

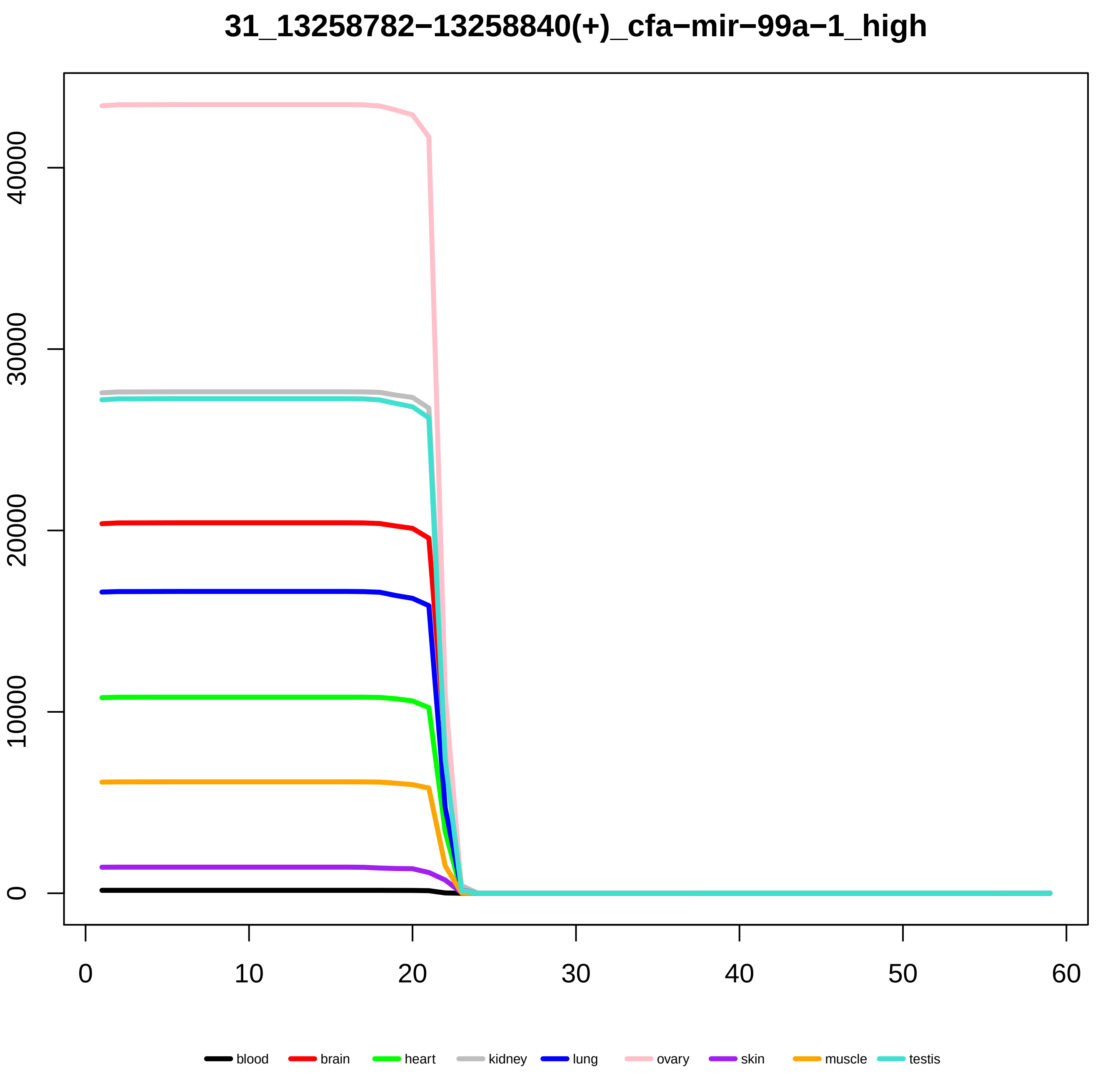

31_13258782−13258840(+)_cfa−mir−99a−1_high
0
10000
20000
30000
40000
0
10
20
30
40
50
60
blood
brain
heart
kidney
lung
ovary
skin
muscle
testis

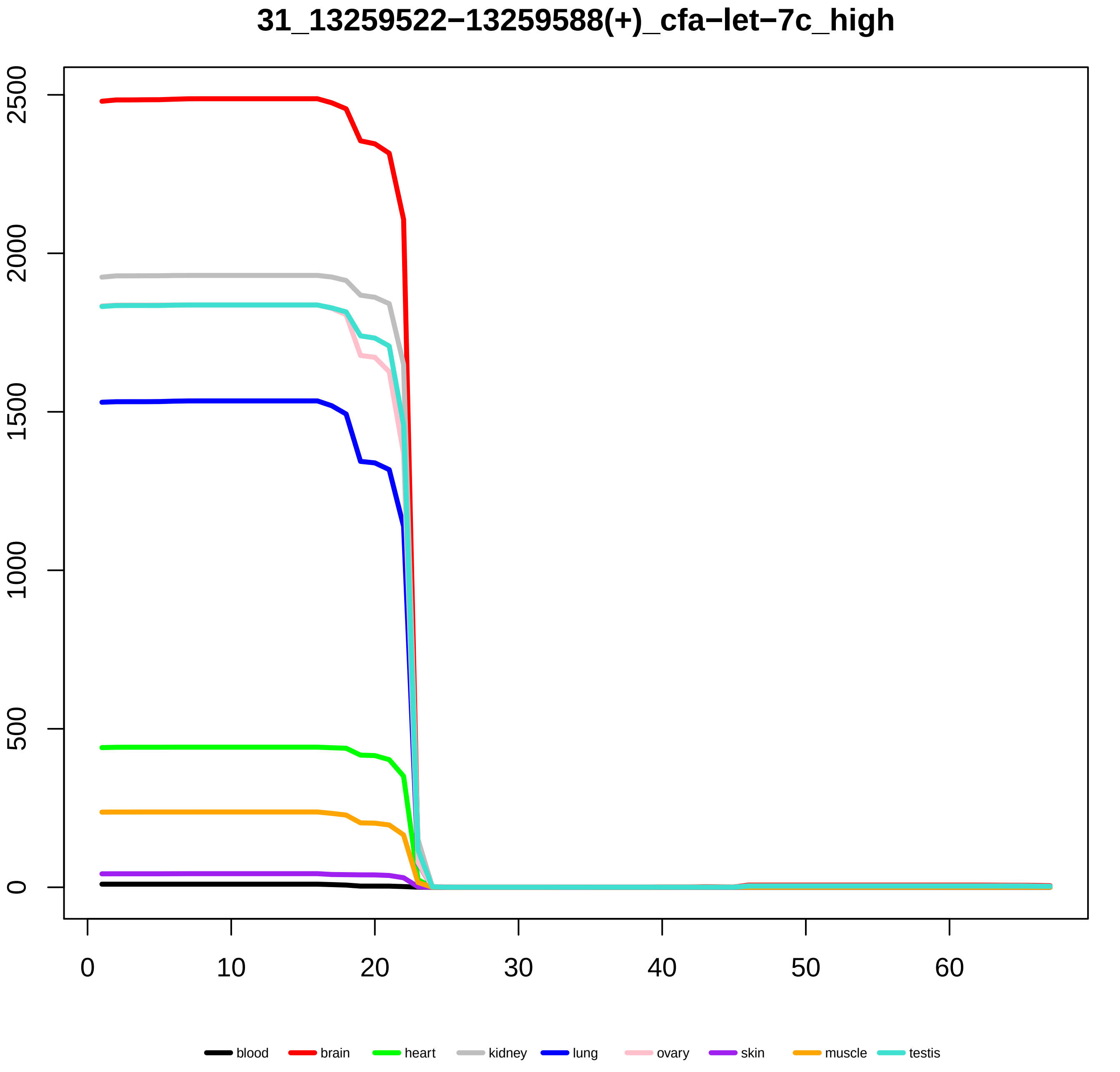

31_13259522−13259588(+)_cfa−let−7c_high
0
500
1000
1500
2000
2500
0
10
20
30
40
50
60
blood
brain
heart
kidney
lung
ovary
skin
muscle
testis

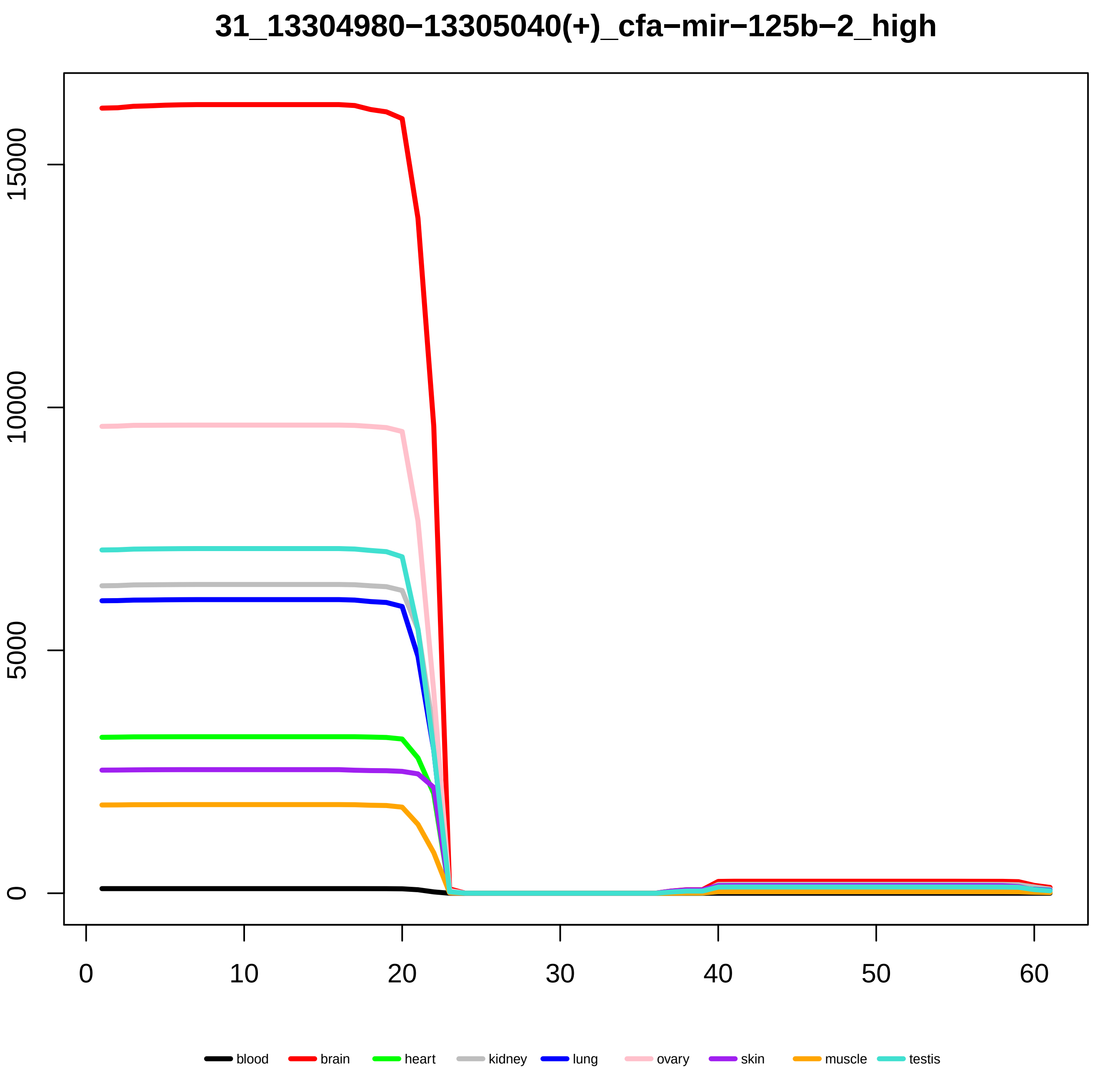
31_13304980−13305040(+)_cfa−mir−125b−2_high
0
5000
10000
15000
0
10
20
30
40
50
60
blood
brain
heart
kidney
lung
ovary
skin
muscle
testis

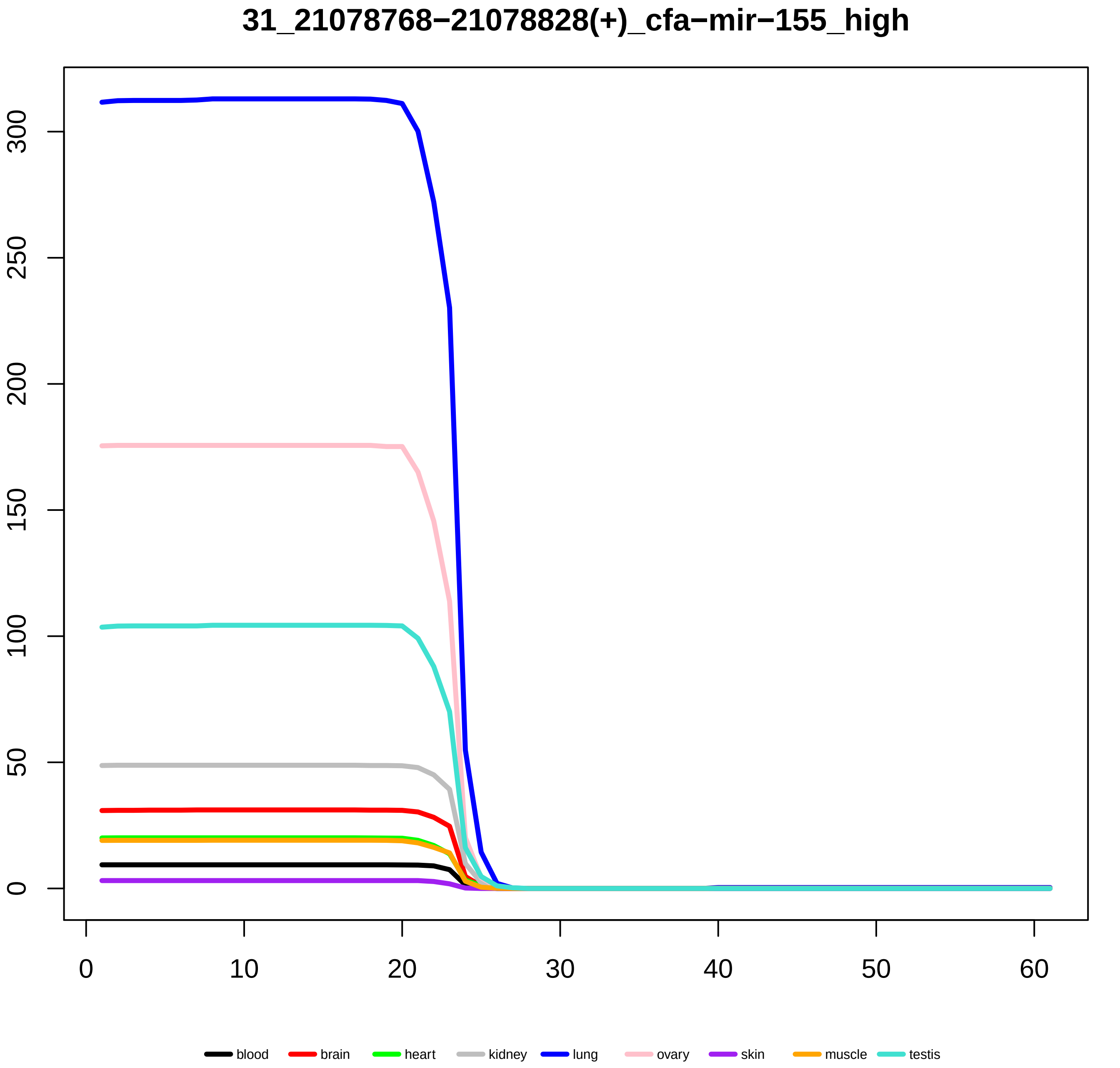

31_21078768−21078828(+)_cfa−mir−155_high
0
50
100
150
200
250
300
0
10
20
30
40
50
60
blood
brain
heart
kidney
lung
ovary
skin
muscle
testis

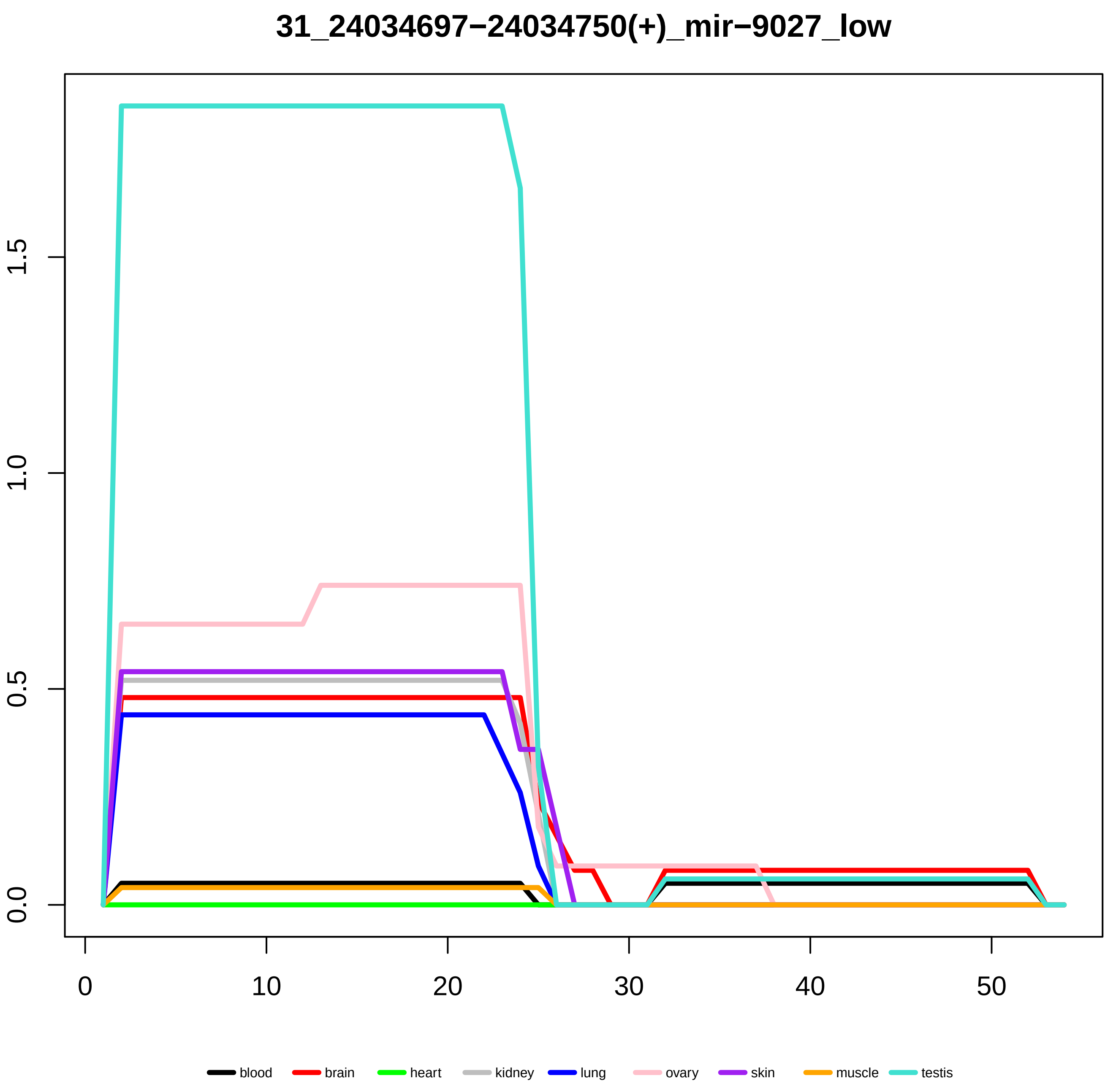

31_24034697−24034750(+)_mir−9027_low
0.0
0.5
1.0
1.5
0
10
20
30
40
50
blood
brain
heart
kidney
lung
ovary
skin
muscle
testis

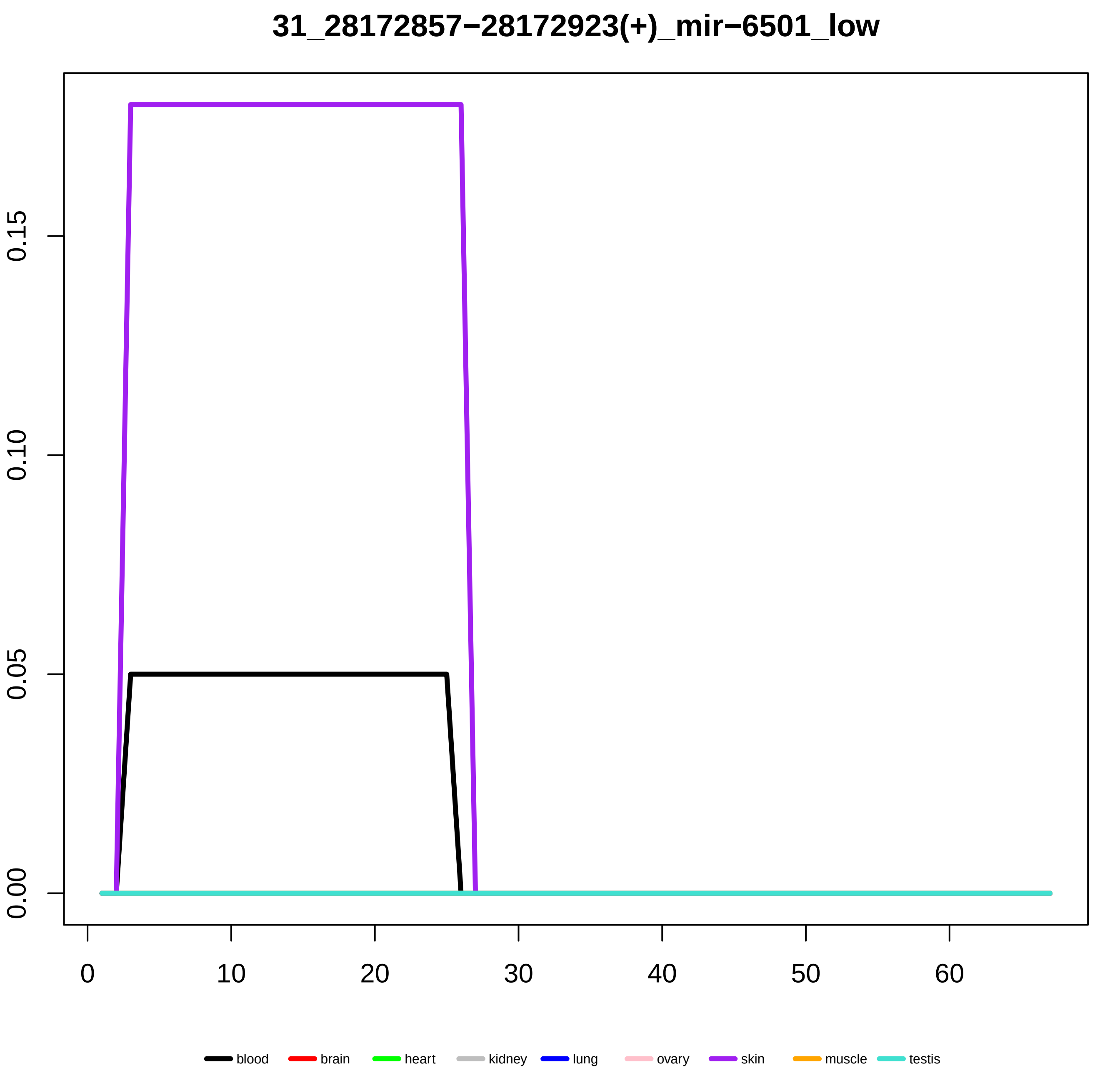

31_28172857−28172923(+)_mir−6501_low
0.00
0.05
0.10
0.15
0
10
20
30
40
50
60
blood
brain
heart
kidney
lung
ovary
skin
muscle
testis

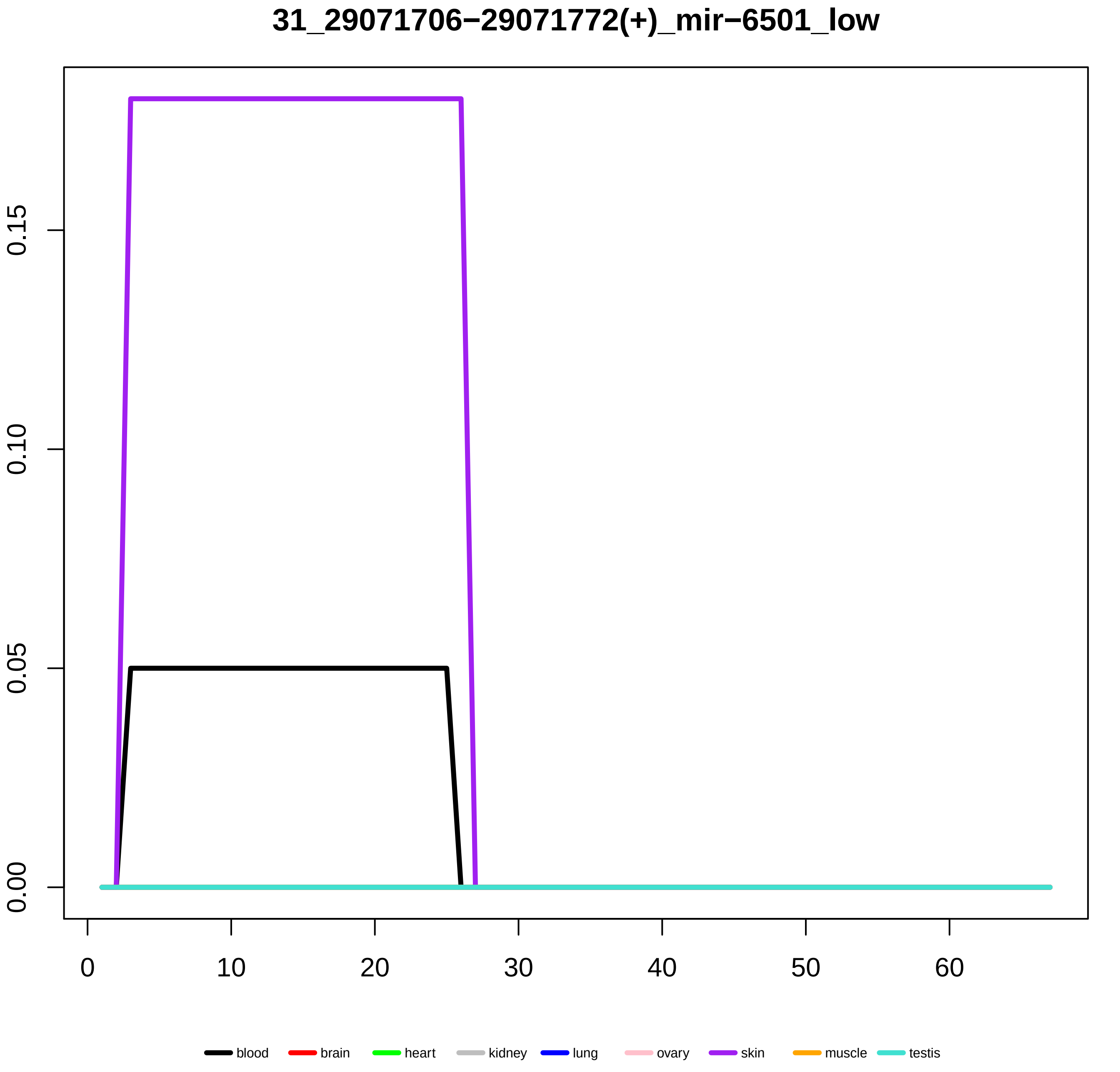

31_29071706−29071772(+)_mir−6501_low
0.00
0.05
0.10
0.15
0
10
20
30
40
50
60
blood
brain
heart
kidney
lung
ovary
skin
muscle
testis

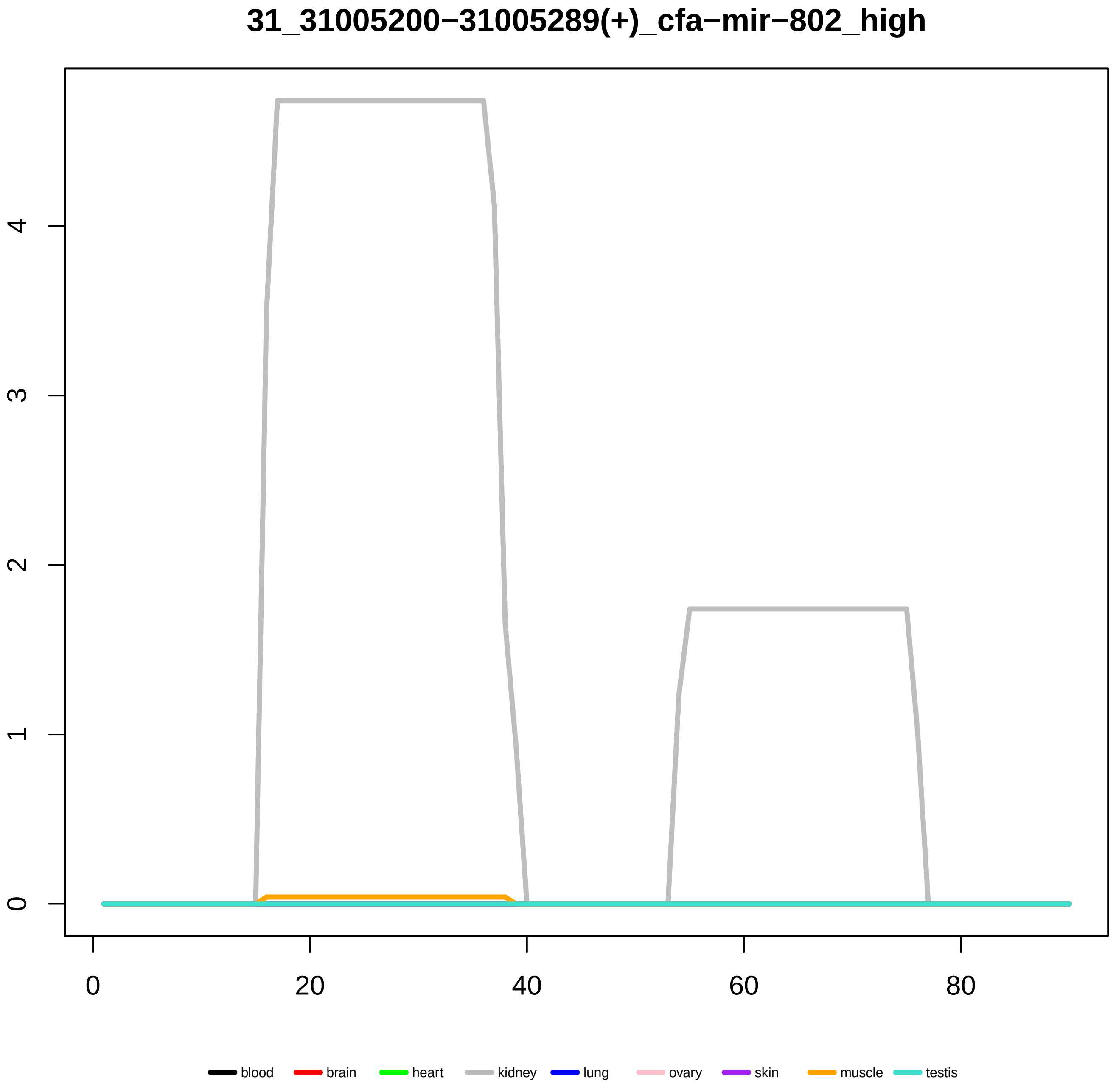
31_31005200−31005289(+)_cfa−mir−802_high
0
1
2
3
4
0
20
40
60
80
blood
brain
heart
kidney
lung
ovary
skin
muscle
testis

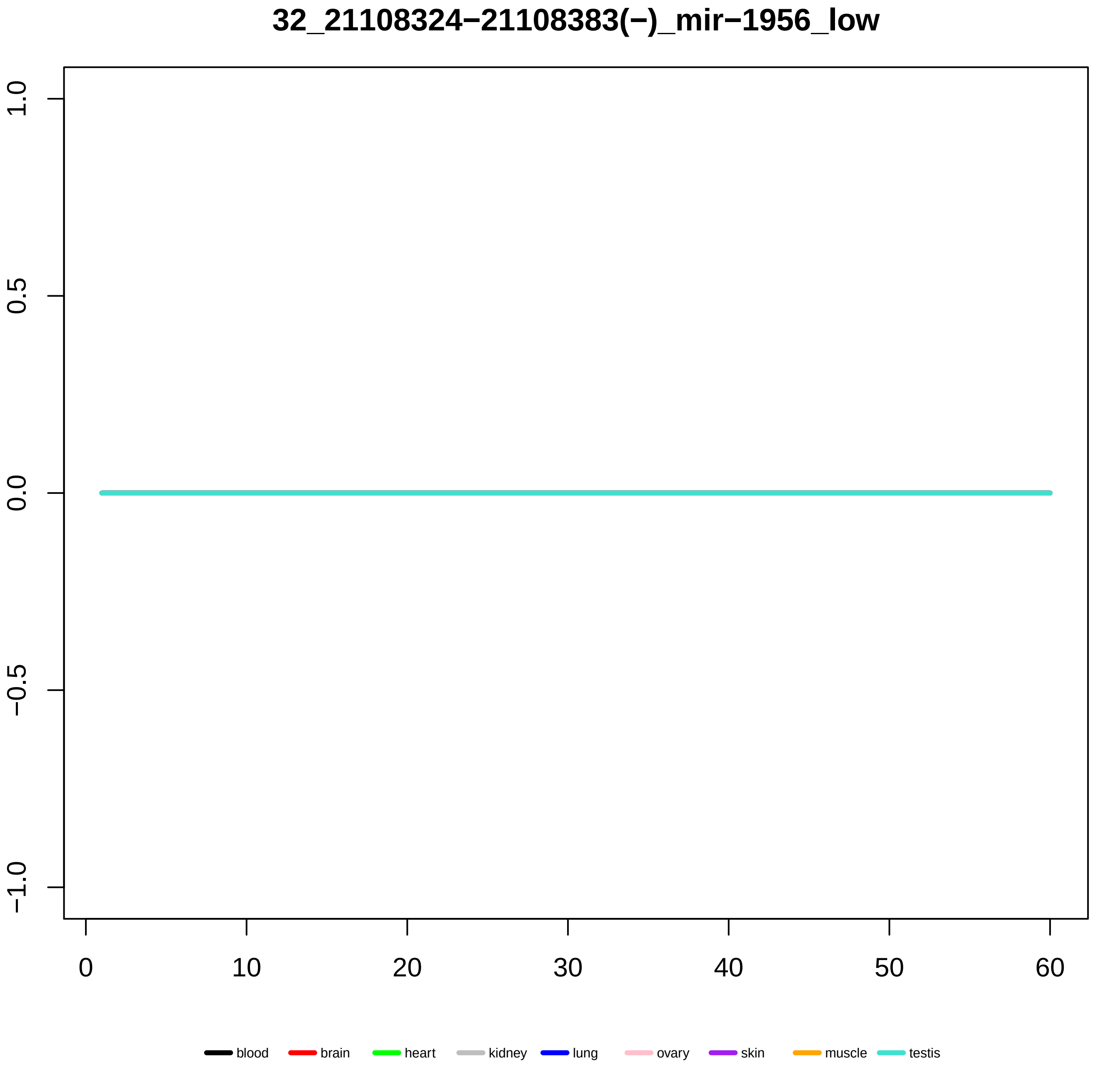
32_21108324−21108383(−)_mir−1956_low
1.0
0.5
0.0
−0.5
−1.0
0
10
20
30
40
50
60
blood
brain
heart
kidney
lung
ovary
skin
muscle
testis

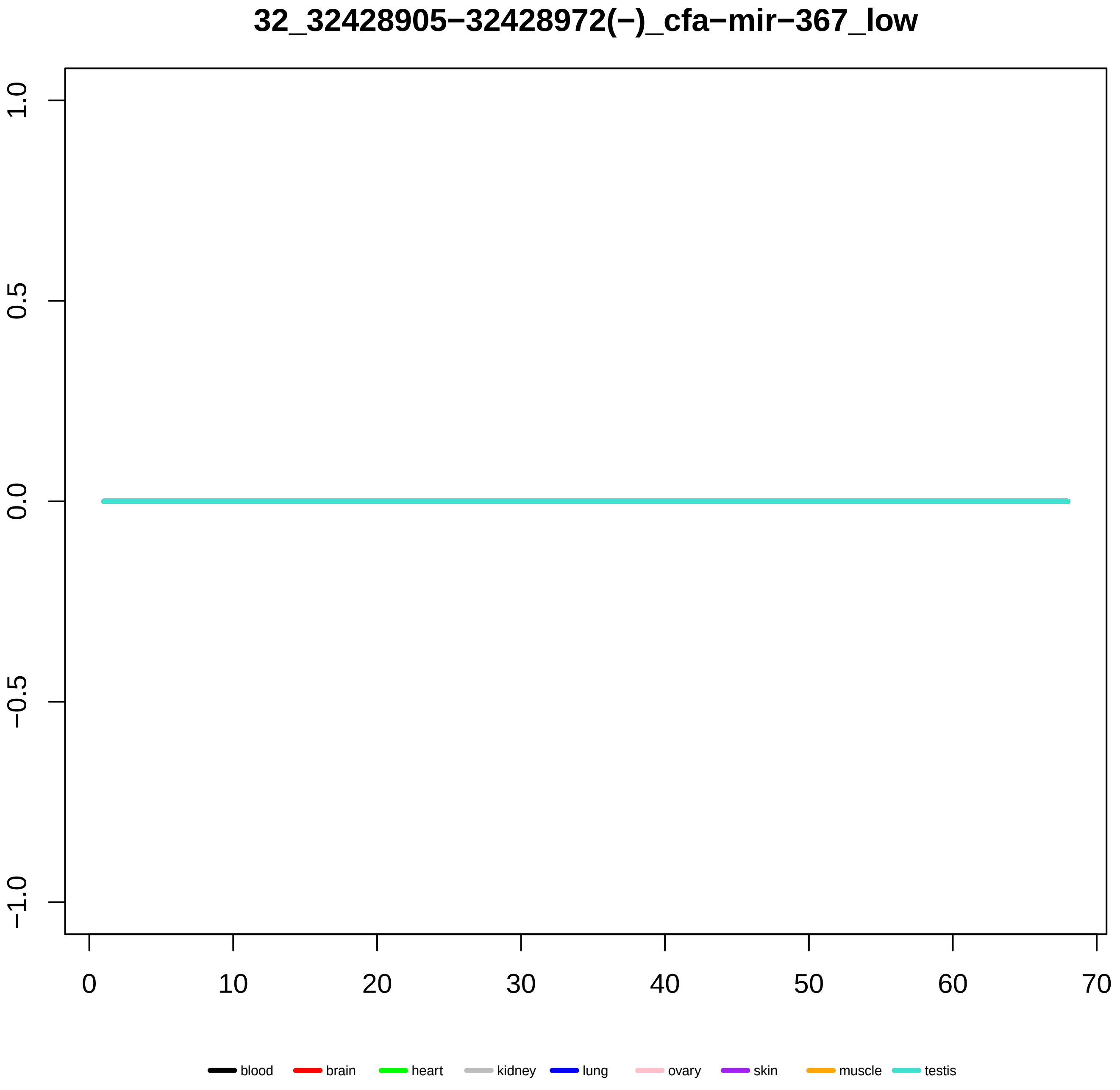

32_32428905−32428972(−)_cfa−mir−367_low
1.0
0.5
0.0
−0.5
−1.0
0
10
20
30
40
50
60
70
blood
brain
heart
kidney
lung
ovary
skin
muscle
testis

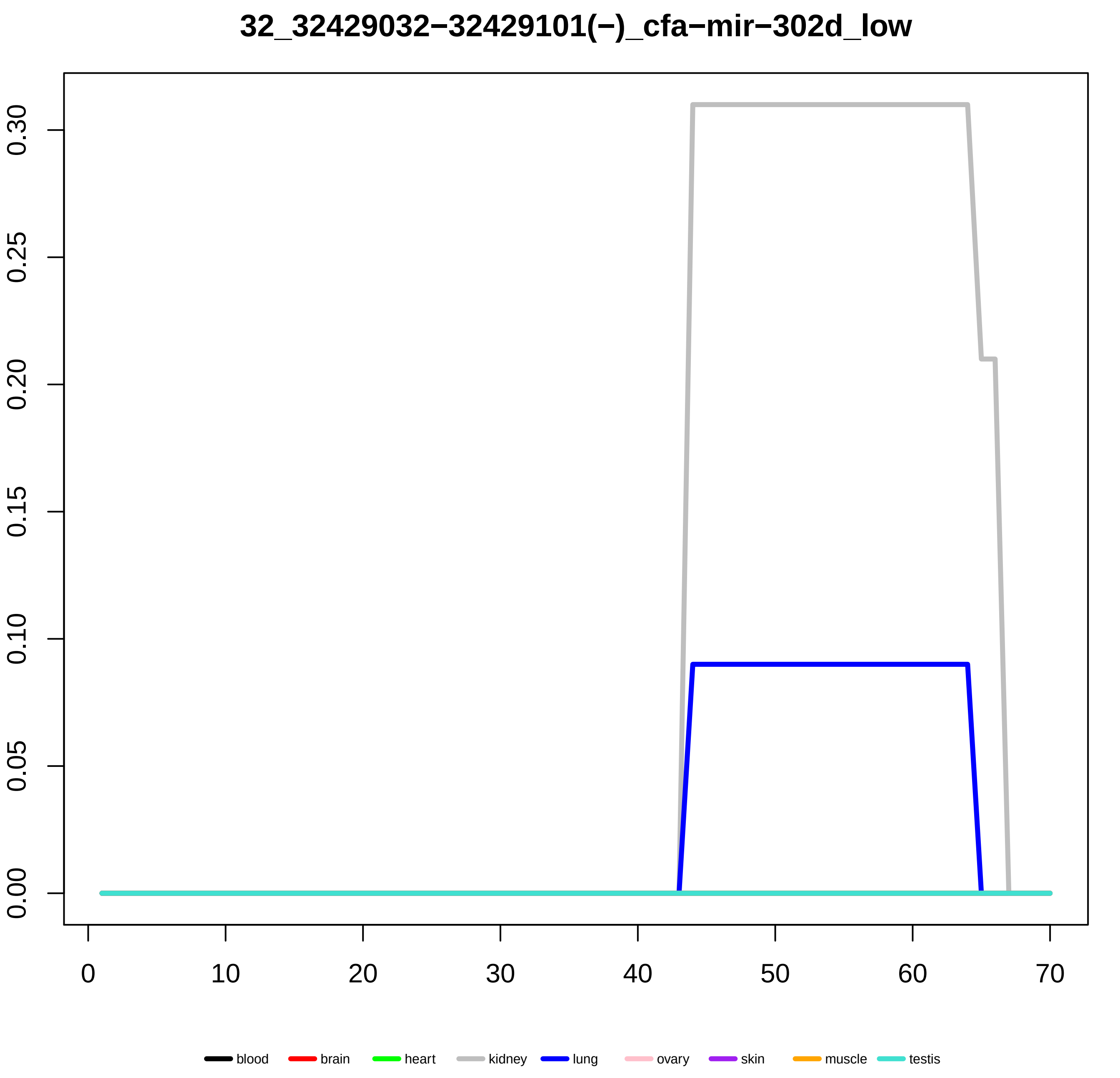

32_32429032−32429101(−)_cfa−mir−302d_low
0.00
0.05
0.10
0.15
0.20
0.25
0.30
0
10
20
30
40
50
60
70
blood
brain
heart
kidney
lung
ovary
skin
muscle
testis

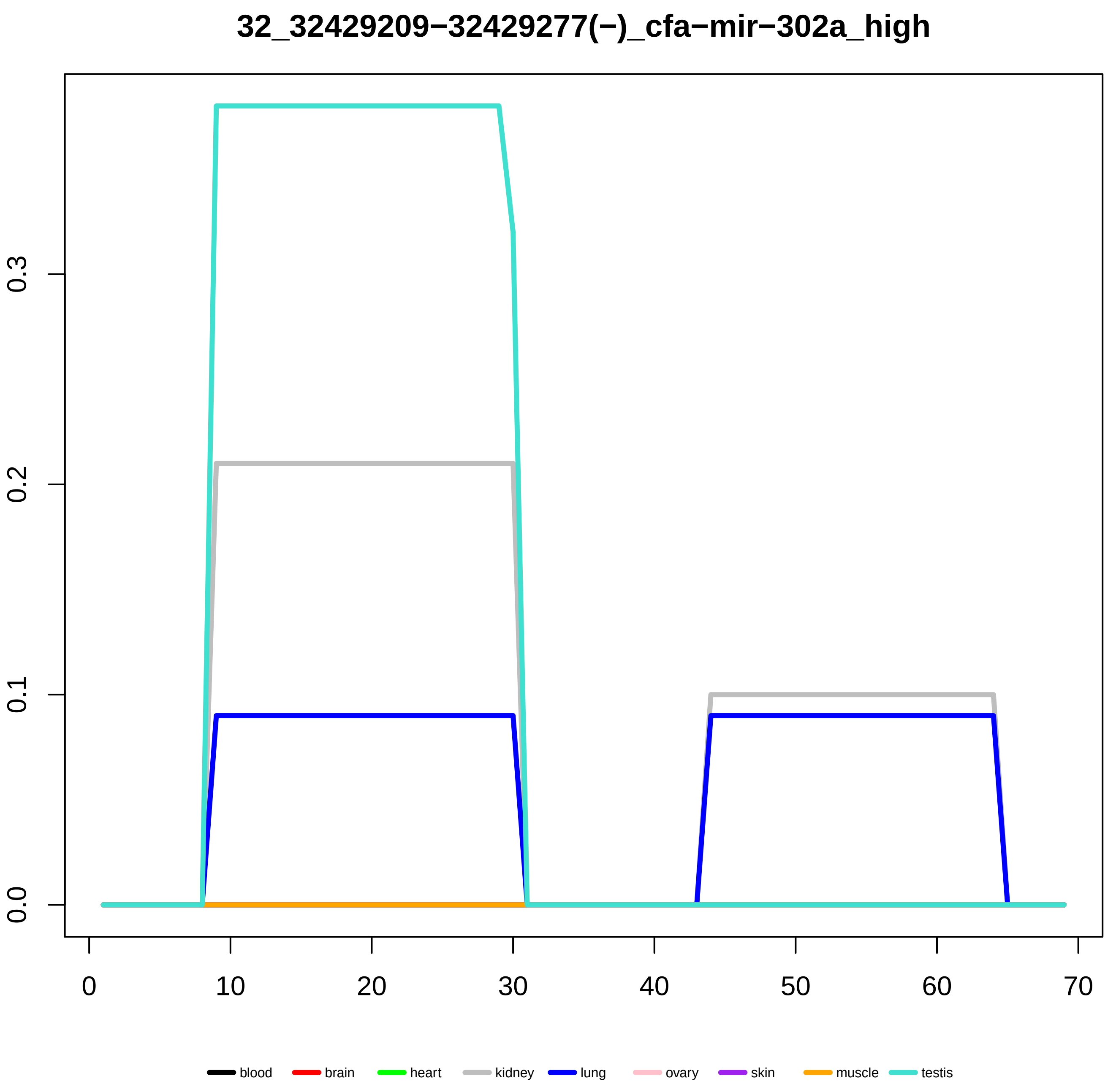

32_32429209−32429277(−)_cfa−mir−302a_high
0.0
0.1
0.2
0.3
0
10
20
30
40
50
60
70
blood
brain
heart
kidney
lung
ovary
skin
muscle
testis

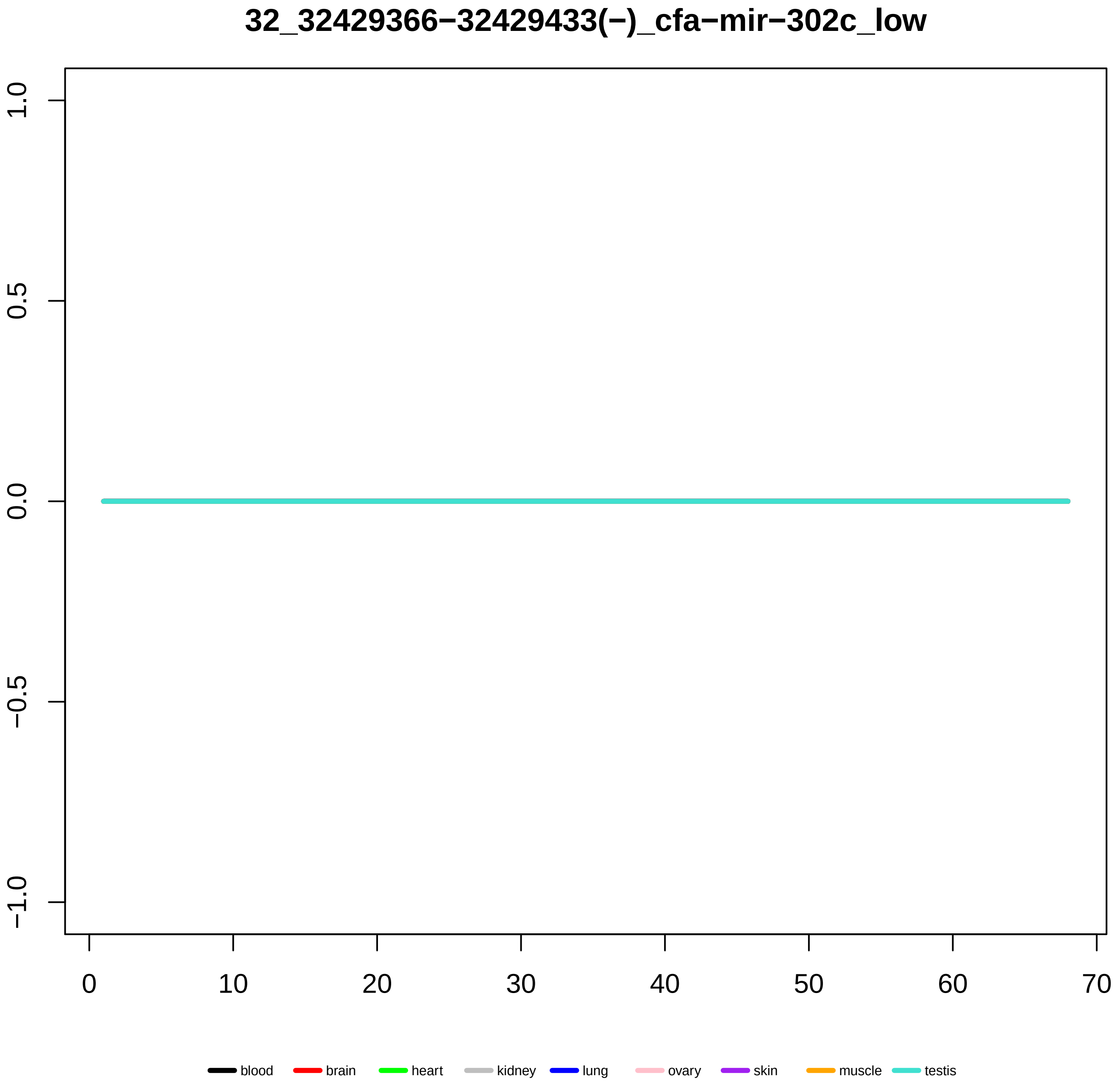

32_32429366−32429433(−)_cfa−mir−302c_low
1.0
0.5
0.0
−0.5
−1.0
0
10
20
30
40
50
60
70
blood
brain
heart
kidney
lung
ovary
skin
muscle
testis

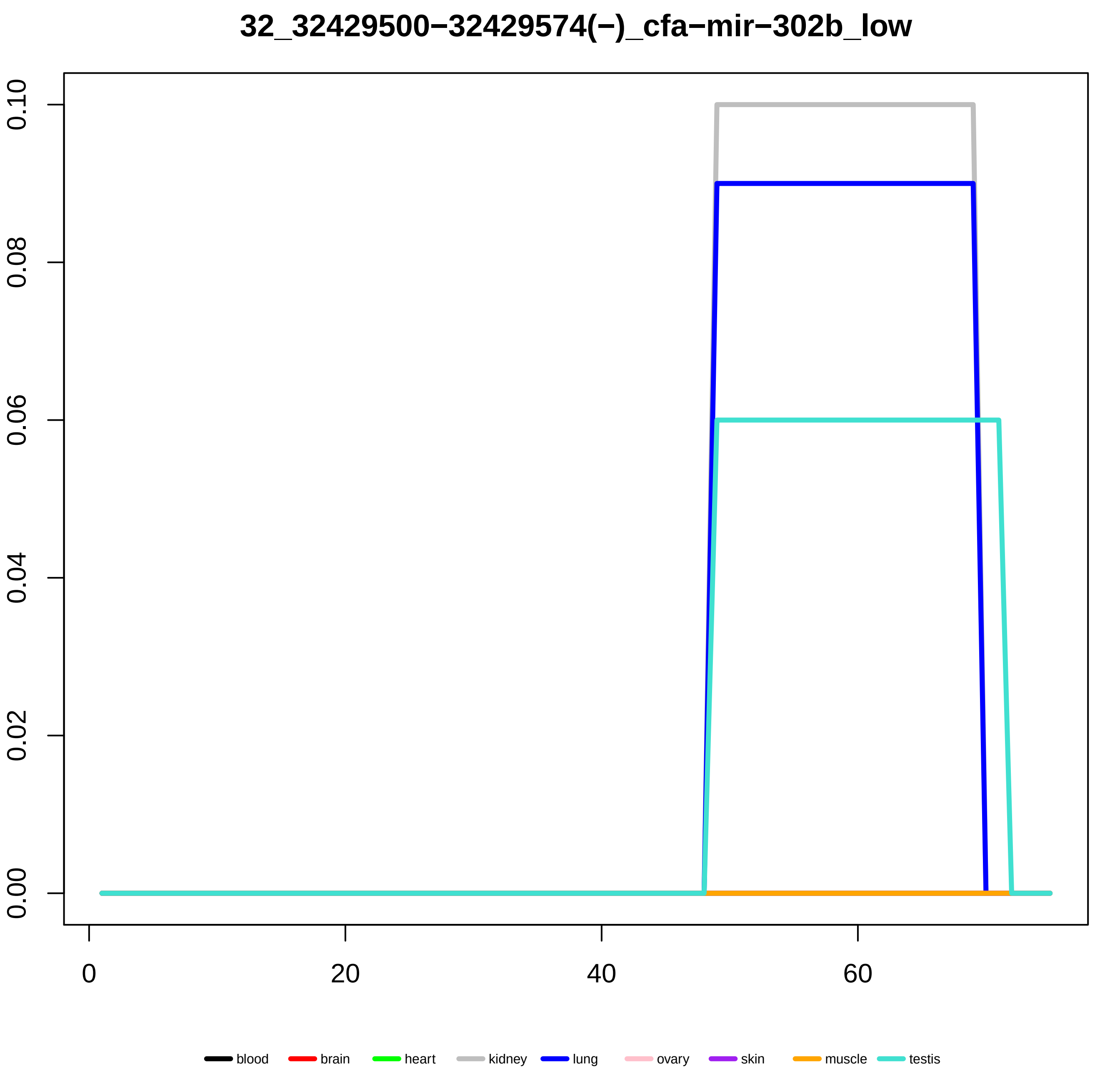
32_32429500−32429574(−)_cfa−mir−302b_low
0.00
0.02
0.04
0.06
0.08
0.10
0
20
40
60
blood
brain
heart
kidney
lung
ovary
skin
muscle
testis

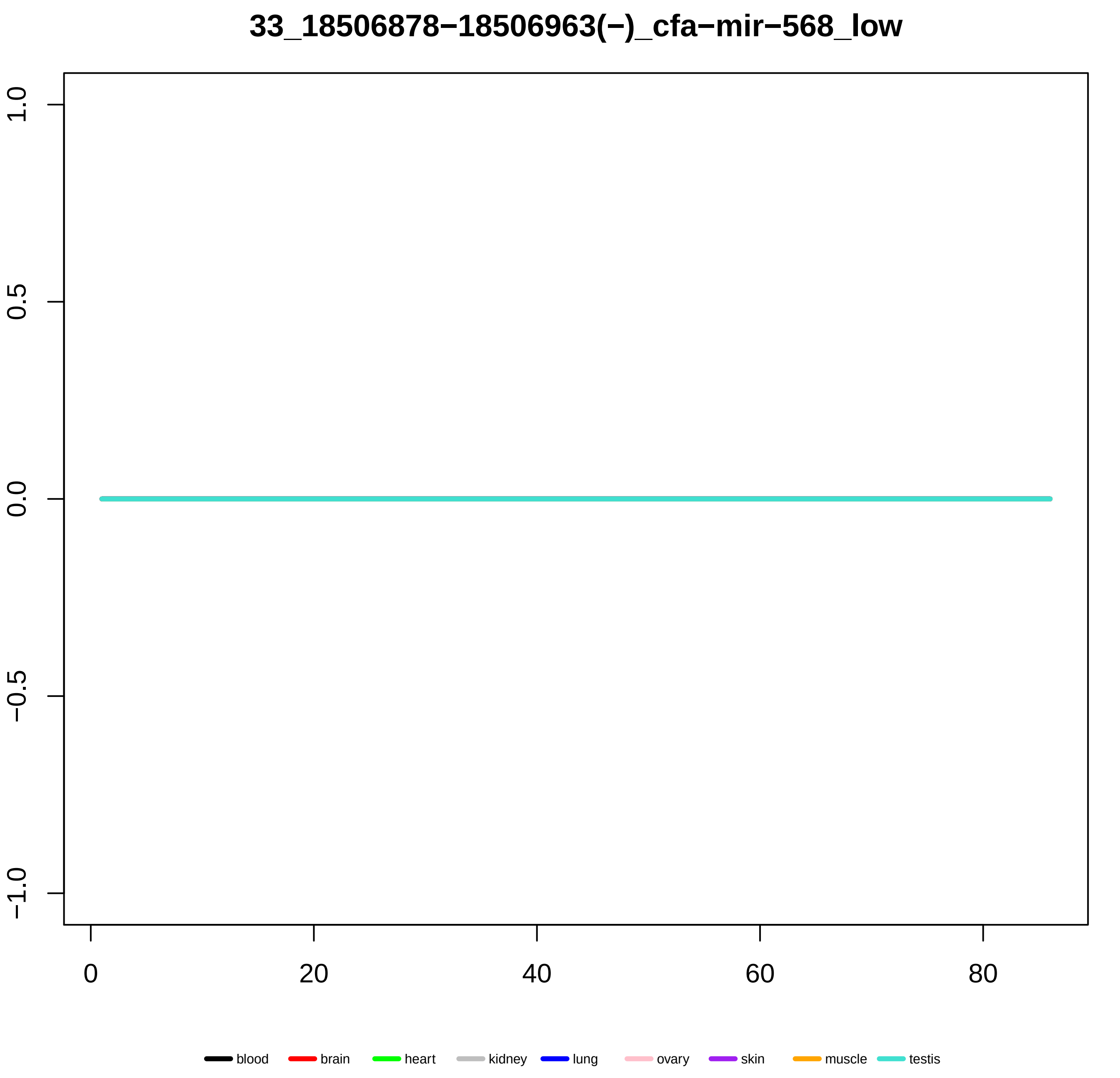
33_18506878−18506963(−)_cfa−mir−568_low
1.0
0.5
0.0
−0.5
−1.0
0
20
40
60
80
blood
brain
heart
kidney
lung
ovary
skin
muscle
testis

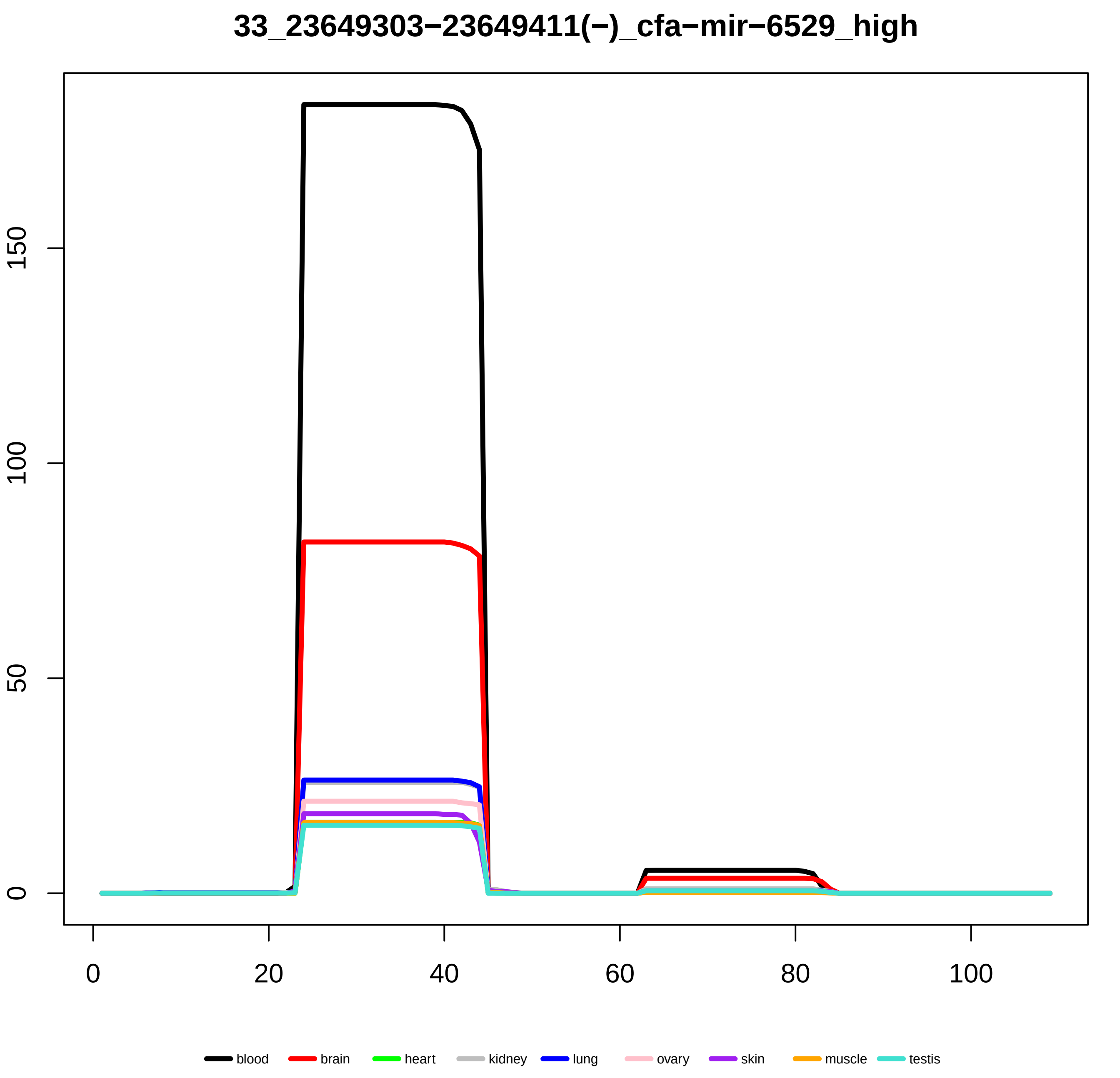

33_23649303−23649411(−)_cfa−mir−6529_high
0
50
100
150
0
20
40
60
80
100
blood
brain
heart
kidney
lung
ovary
skin
muscle
testis

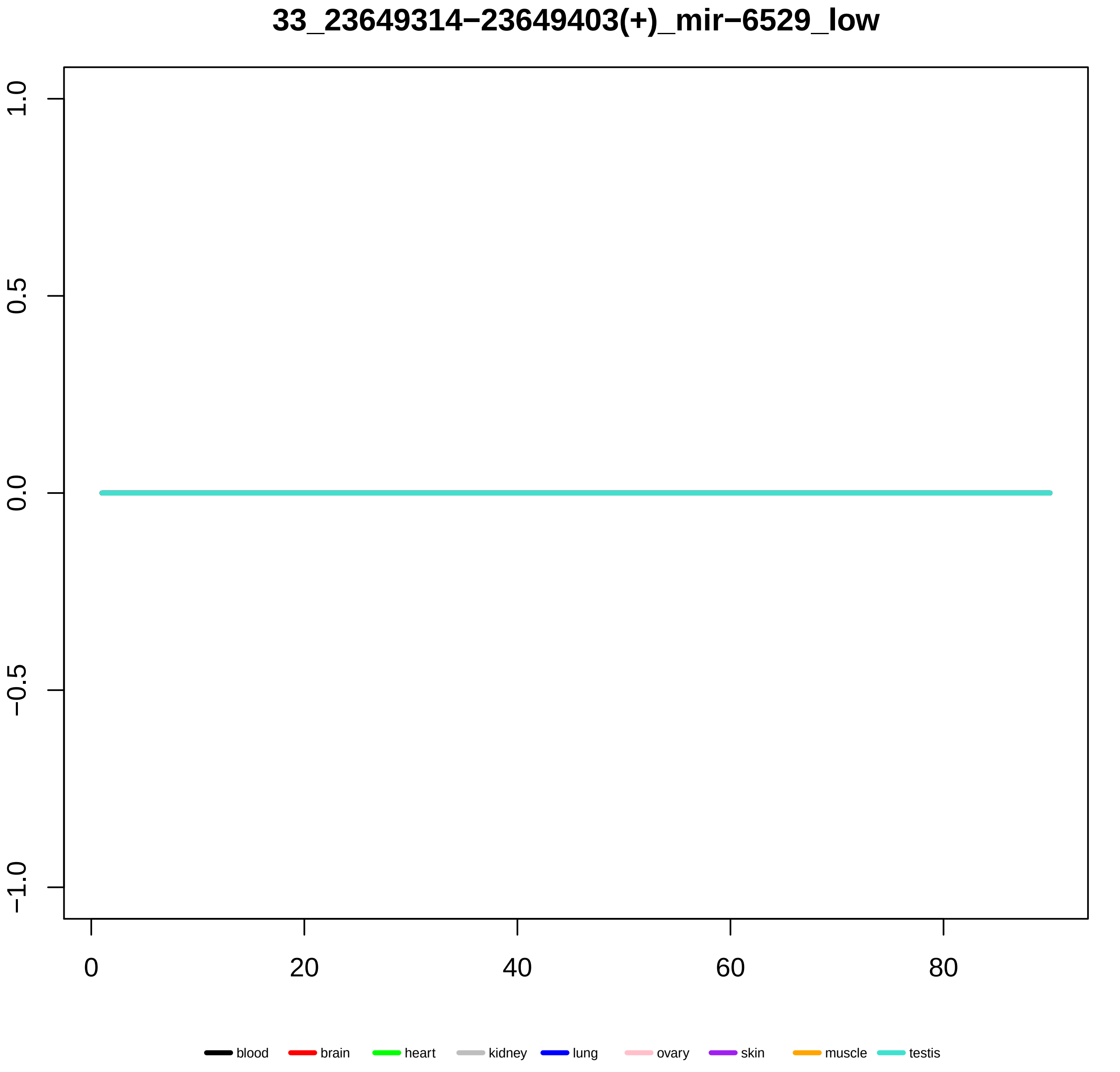
33_23649314−23649403(+)_mir−6529_low
1.0
0.5
0.0
−0.5
−1.0
0
20
40
60
80
blood
brain
heart
kidney
lung
ovary
skin
muscle
testis

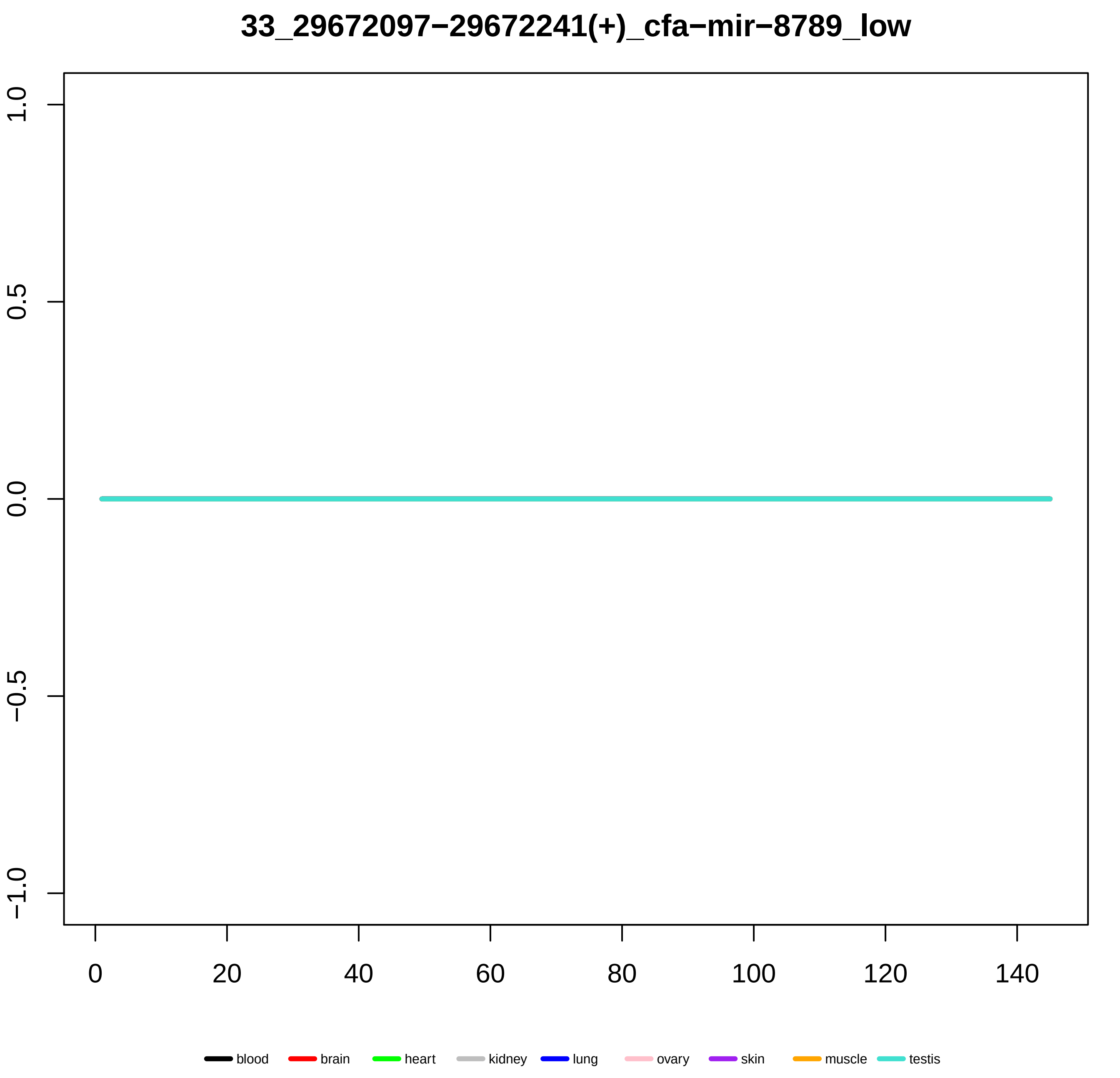

33_29672097−29672241(+)_cfa−mir−8789_low
1.0
0.5
0.0
−0.5
−1.0
0
20
40
60
80
100
120
140
blood
brain
heart
kidney
lung
ovary
skin
muscle
testis

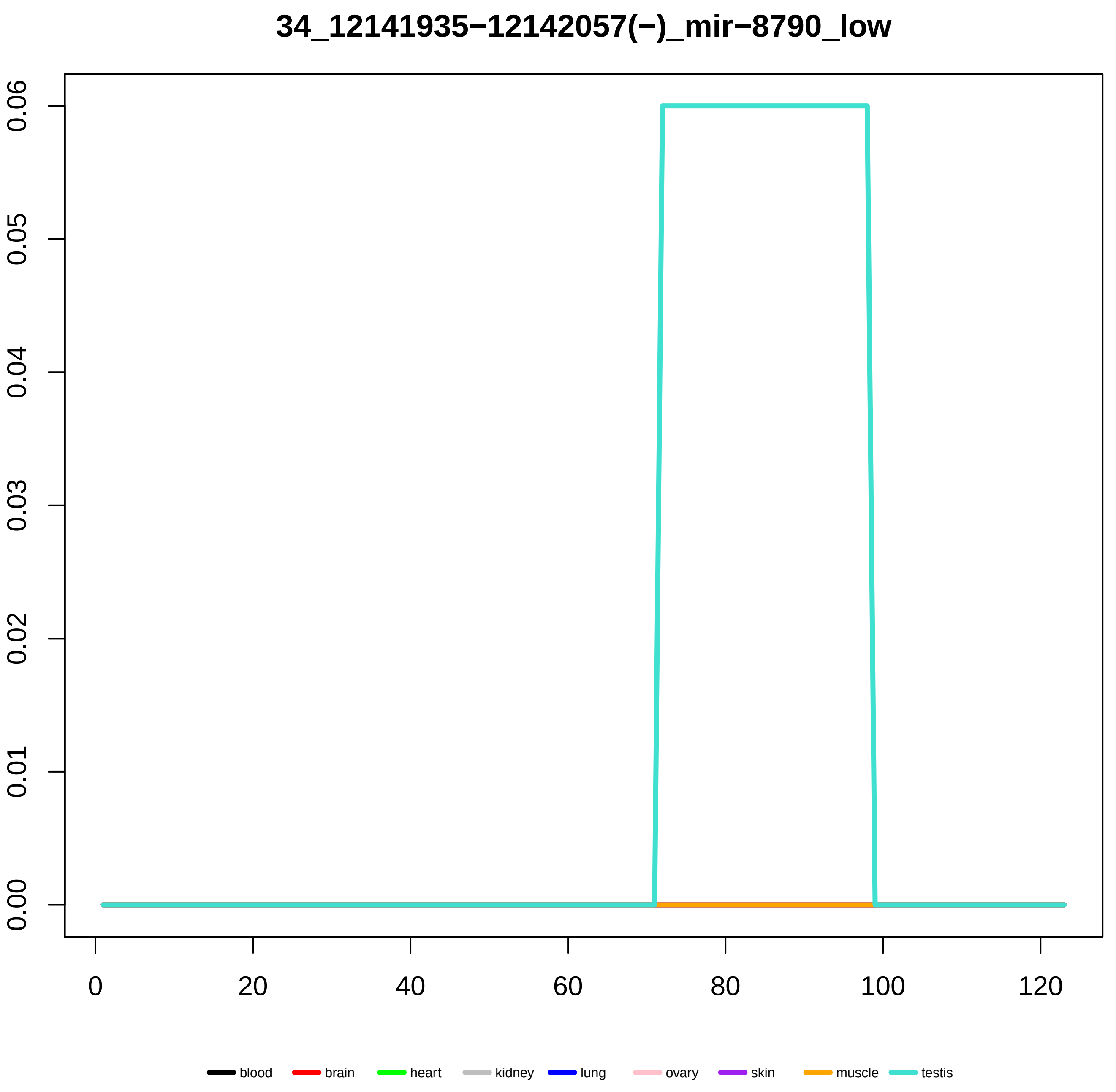
34_12141935−12142057(−)_mir−8790_low
0.00
0.01
0.02
0.03
0.04
0.05
0.06
0
20
40
60
80
100
120
blood
brain
heart
kidney
lung
ovary
skin
muscle
testis

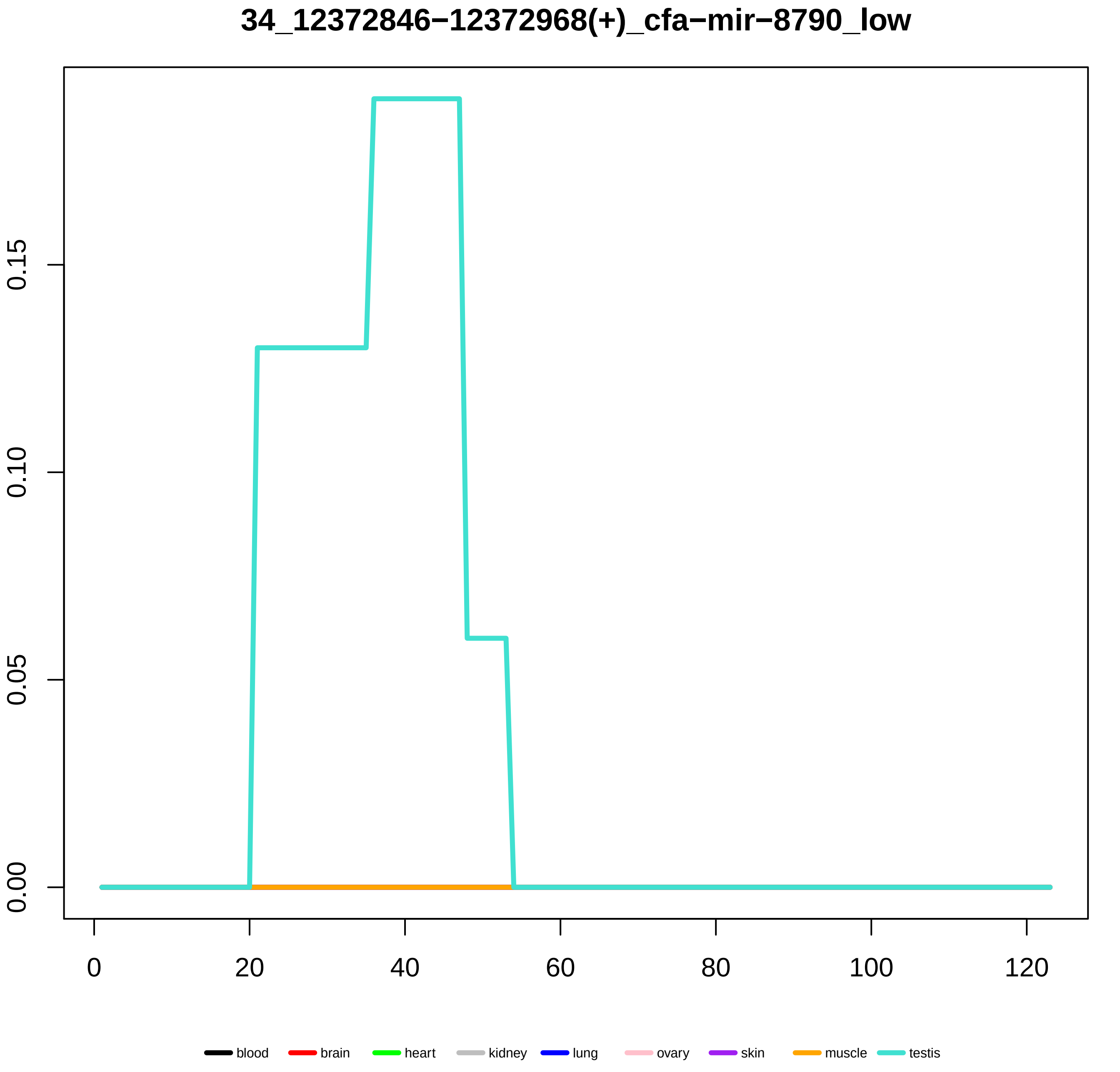
34_12372846−12372968(+)_cfa−mir−8790_low
0.00
0.05
0.10
0.15
0
20
40
60
80
100
120
blood
brain
heart
kidney
lung
ovary
skin
muscle
testis

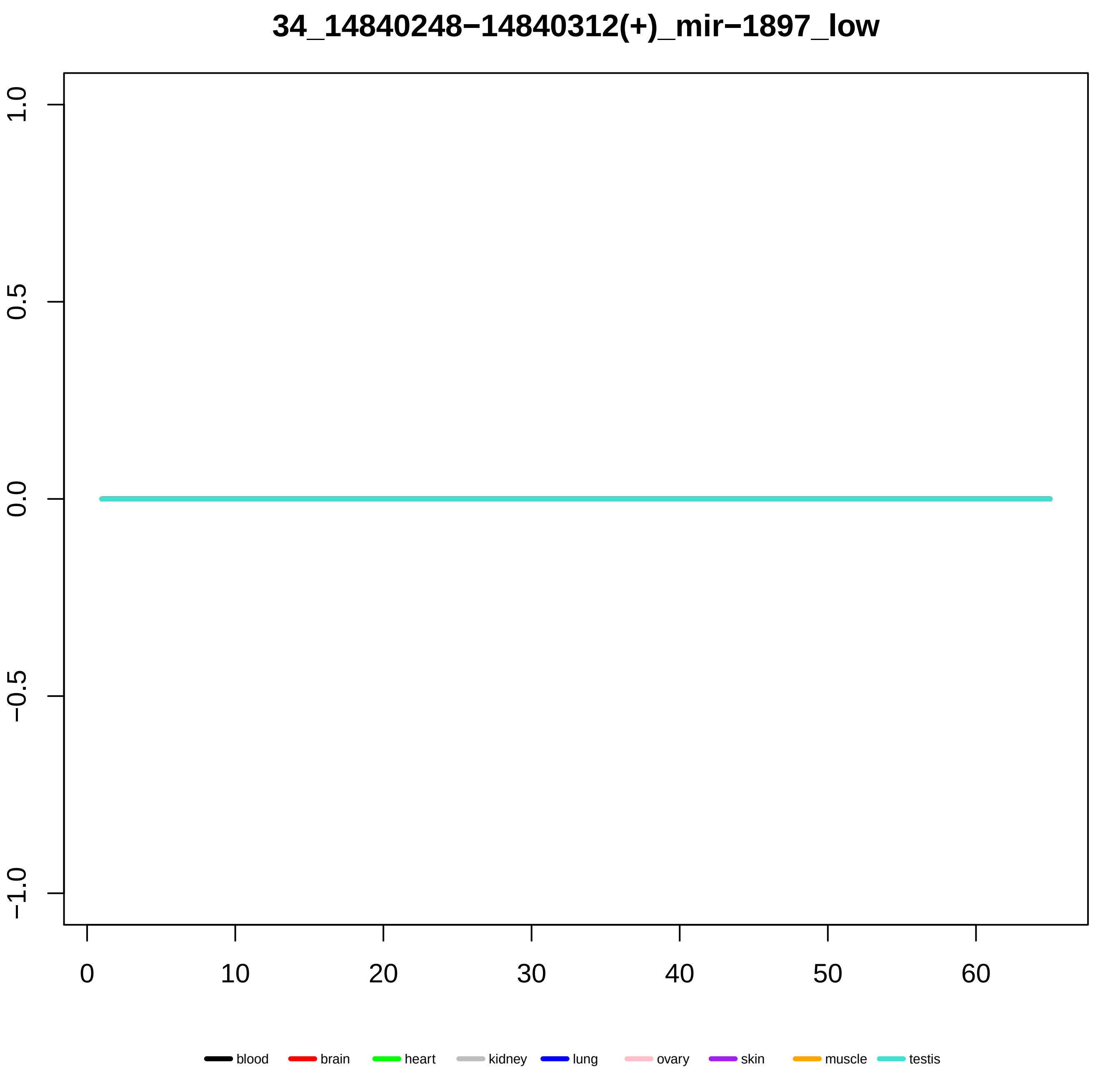
34_14840248−14840312(+)_mir−1897_low
1.0
0.5
0.0
−0.5
−1.0
0
10
20
30
40
50
60
blood
brain
heart
kidney
lung
ovary
skin
muscle
testis

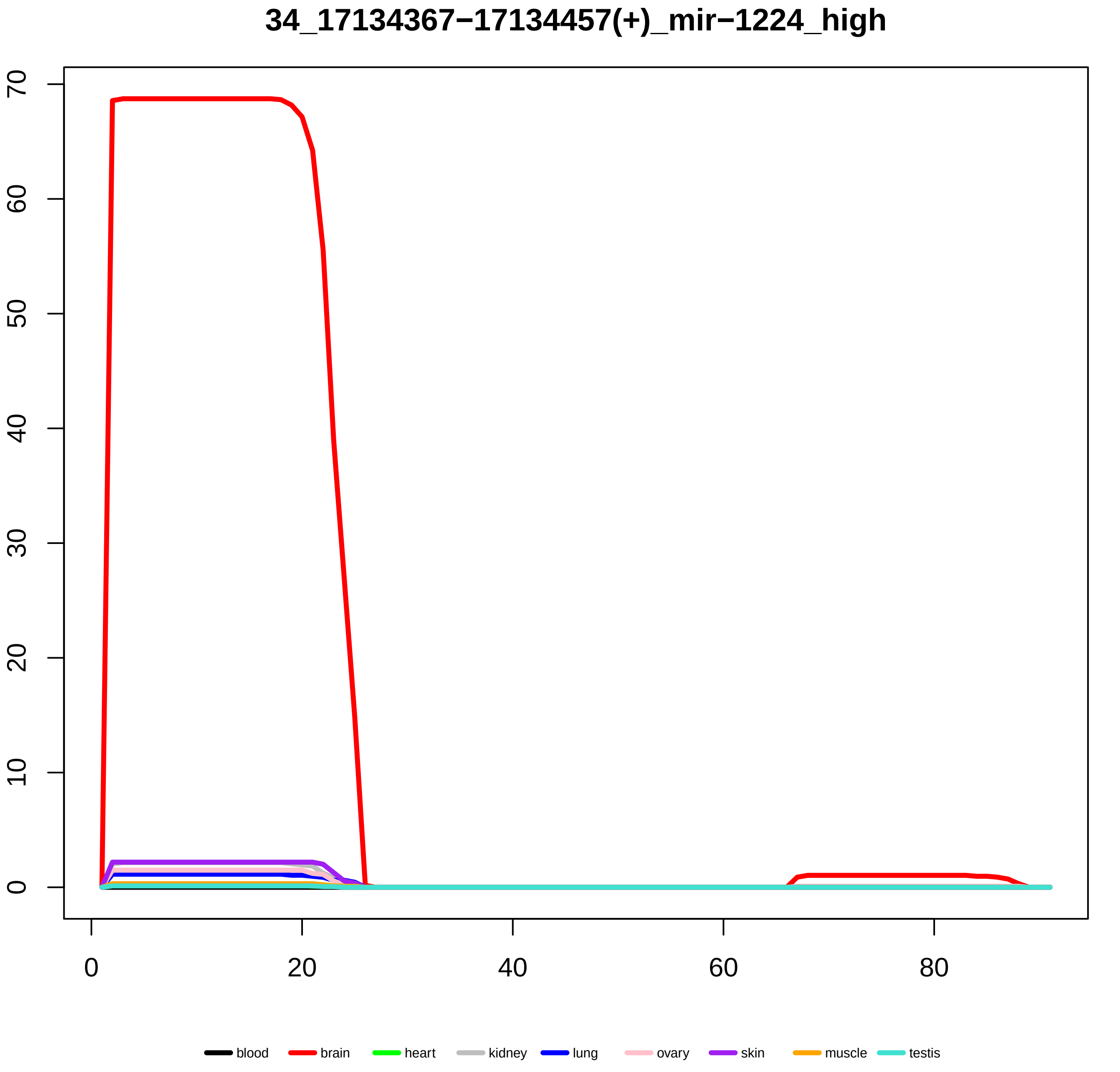
34_17134367−17134457(+)_mir−1224_high
70
60
50
40
30
20
10
0
0
20
40
60
80
blood
brain
heart
kidney
lung
ovary
skin
muscle
testis

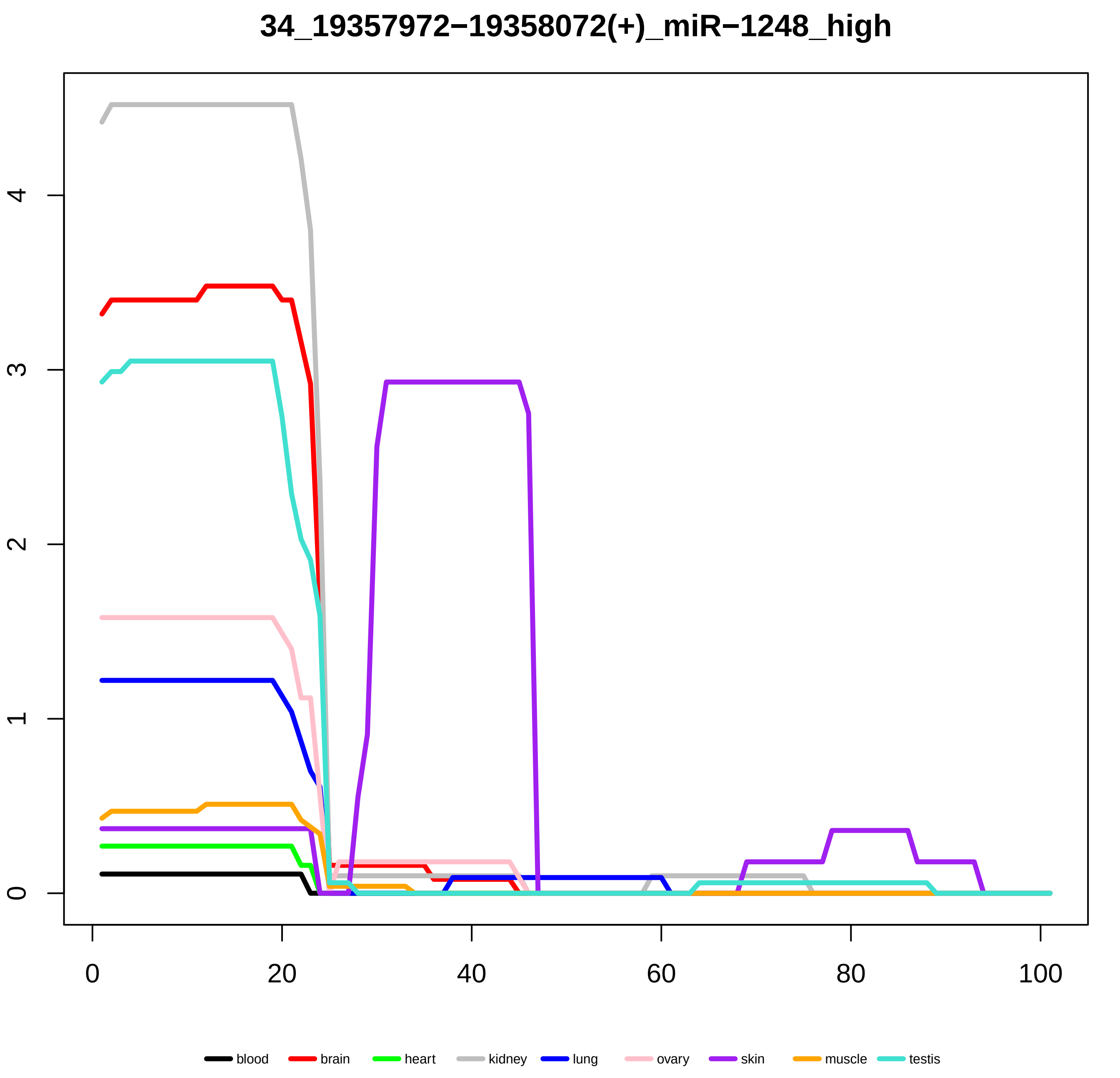
34_19357972−19358072(+)_miR−1248_high
0
1
2
3
4
0
20
40
60
80
100
blood
brain
heart
kidney
lung
ovary
skin
muscle
testis

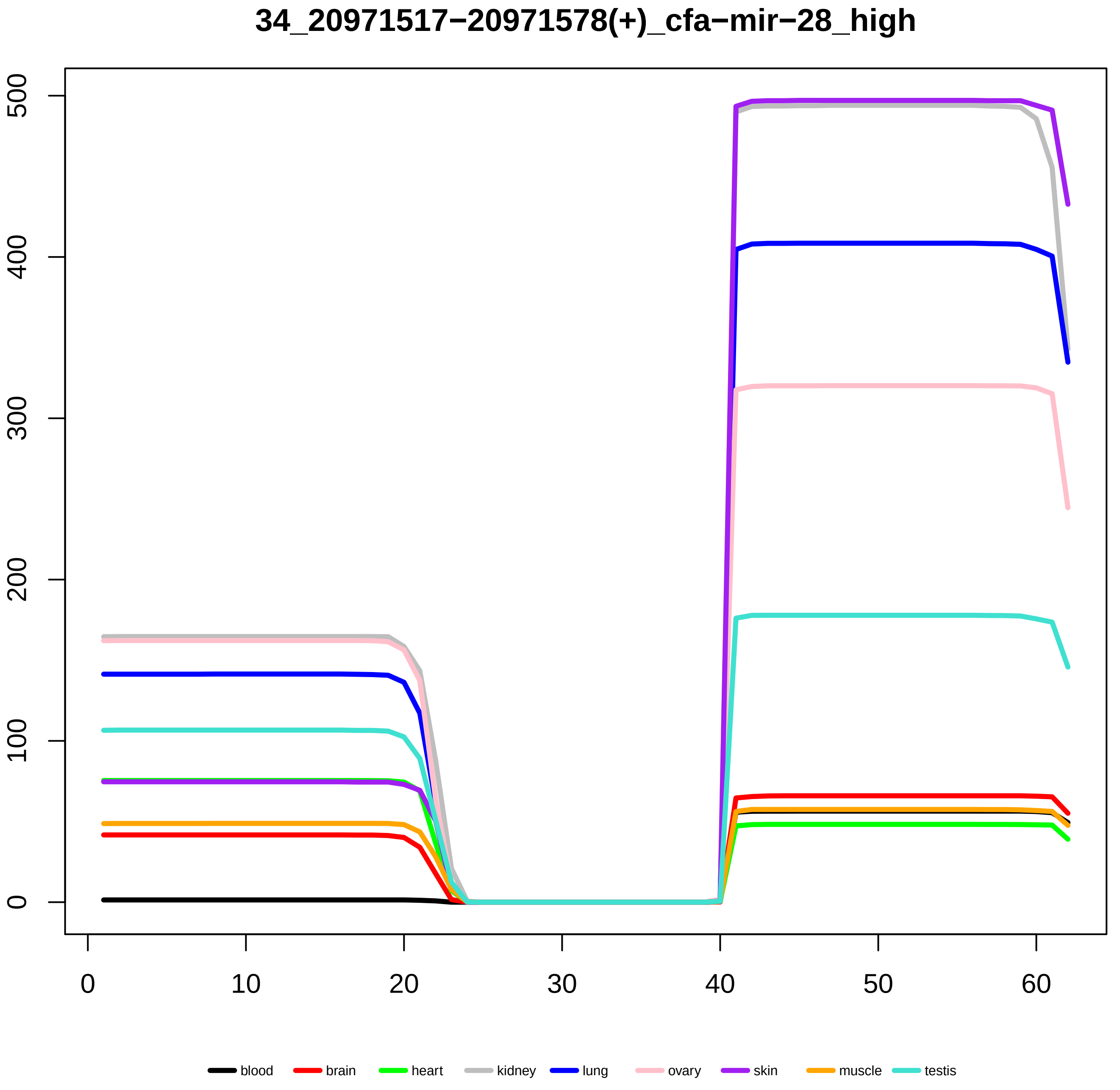

34_20971517−20971578(+)_cfa−mir−28_high
0
100
200
300
400
500
0
10
20
30
40
50
60
blood
brain
heart
kidney
lung
ovary
skin
muscle
testis

34_26460663−26460722(+)_cfa−mir−15b_high

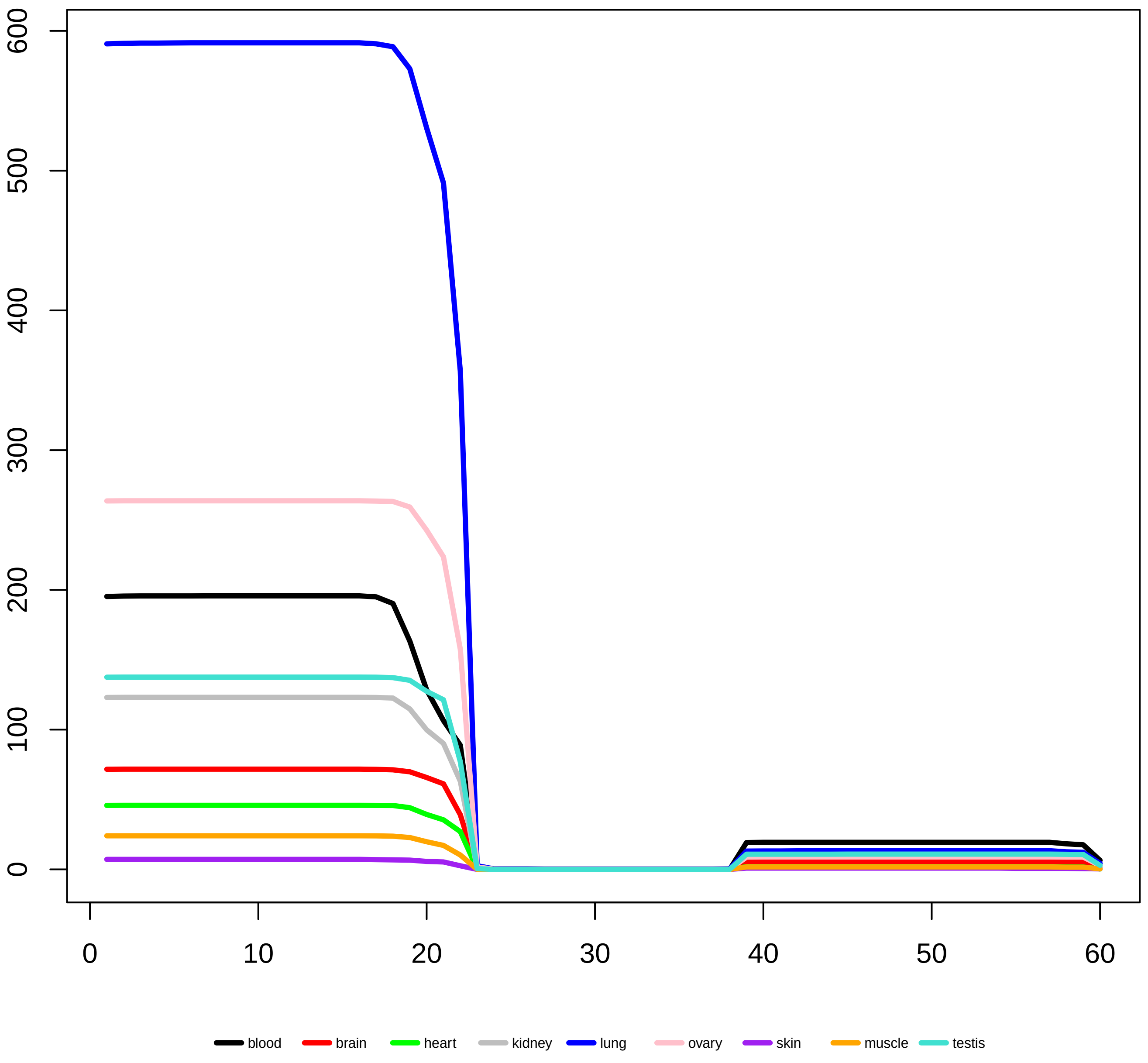

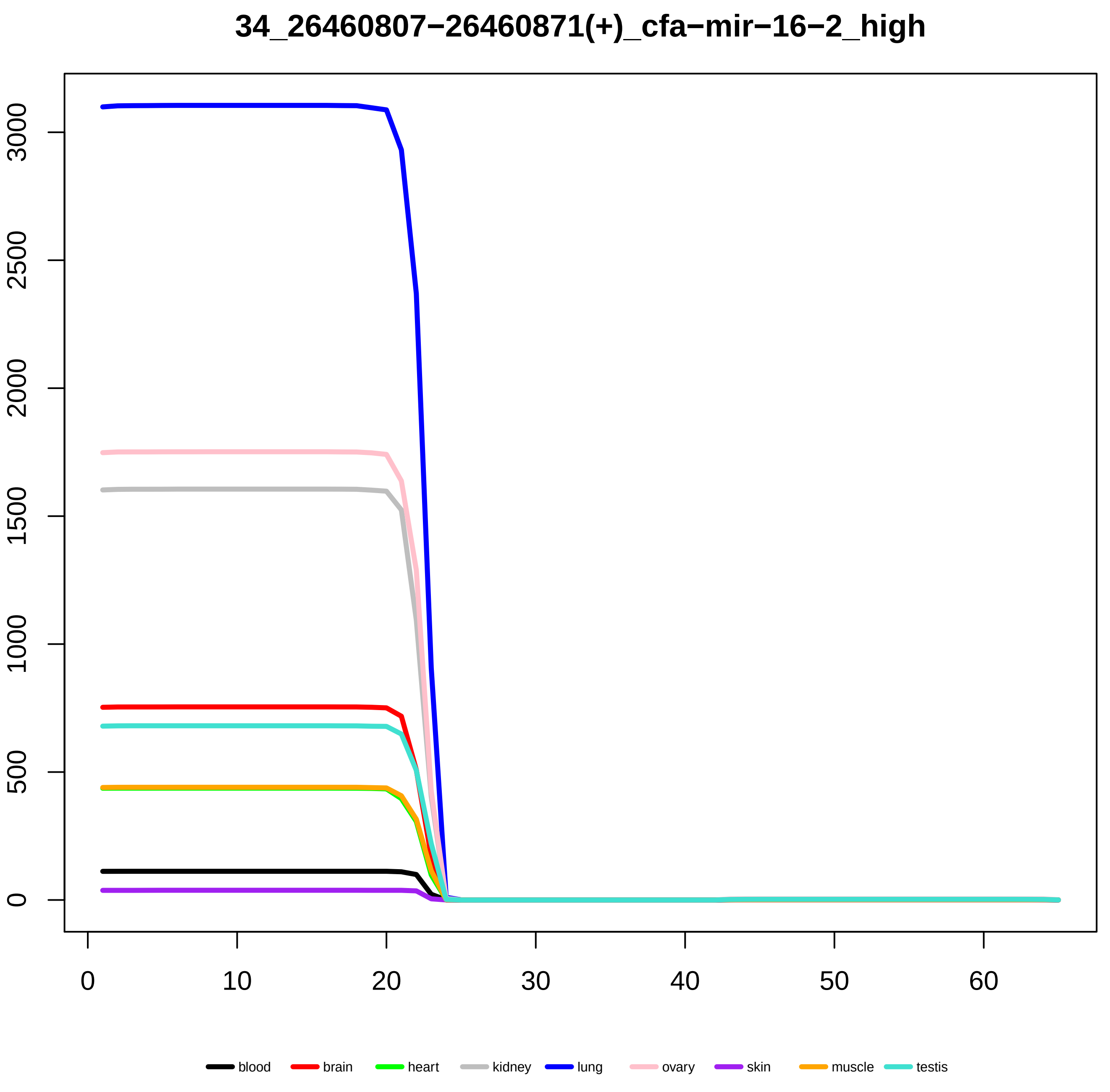

34_26460807−26460871(+)_cfa−mir−16−2_high
0
500
1000
1500
2000
2500
3000
0
10
20
30
40
50
60
blood
brain
heart
kidney
lung
ovary
skin
muscle
testis

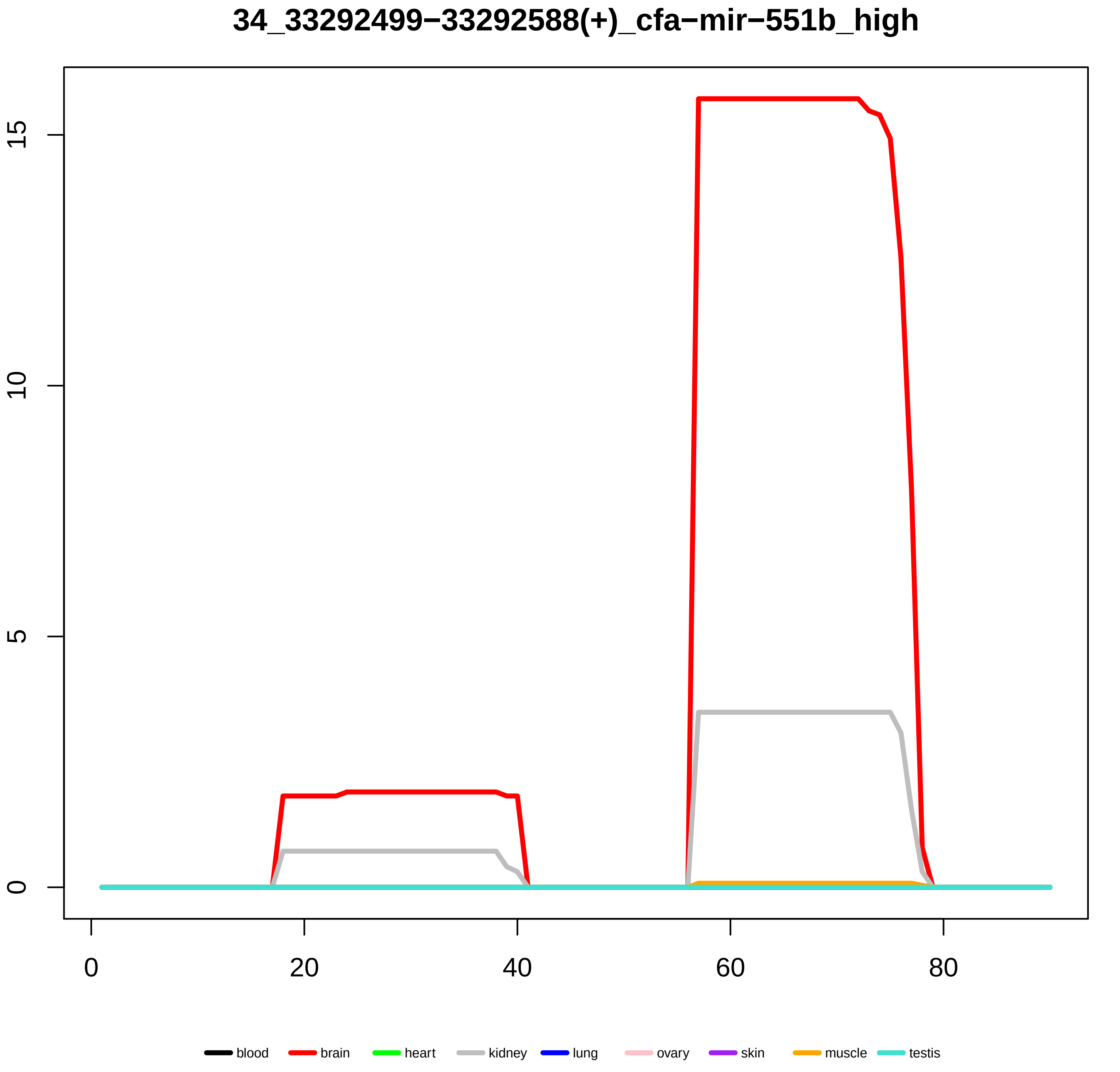
34_33292499−33292588(+)_cfa−mir−551b_high
0
5
10
15
0
20
40
60
80
blood
brain
heart
kidney
lung
ovary
skin
muscle
testis

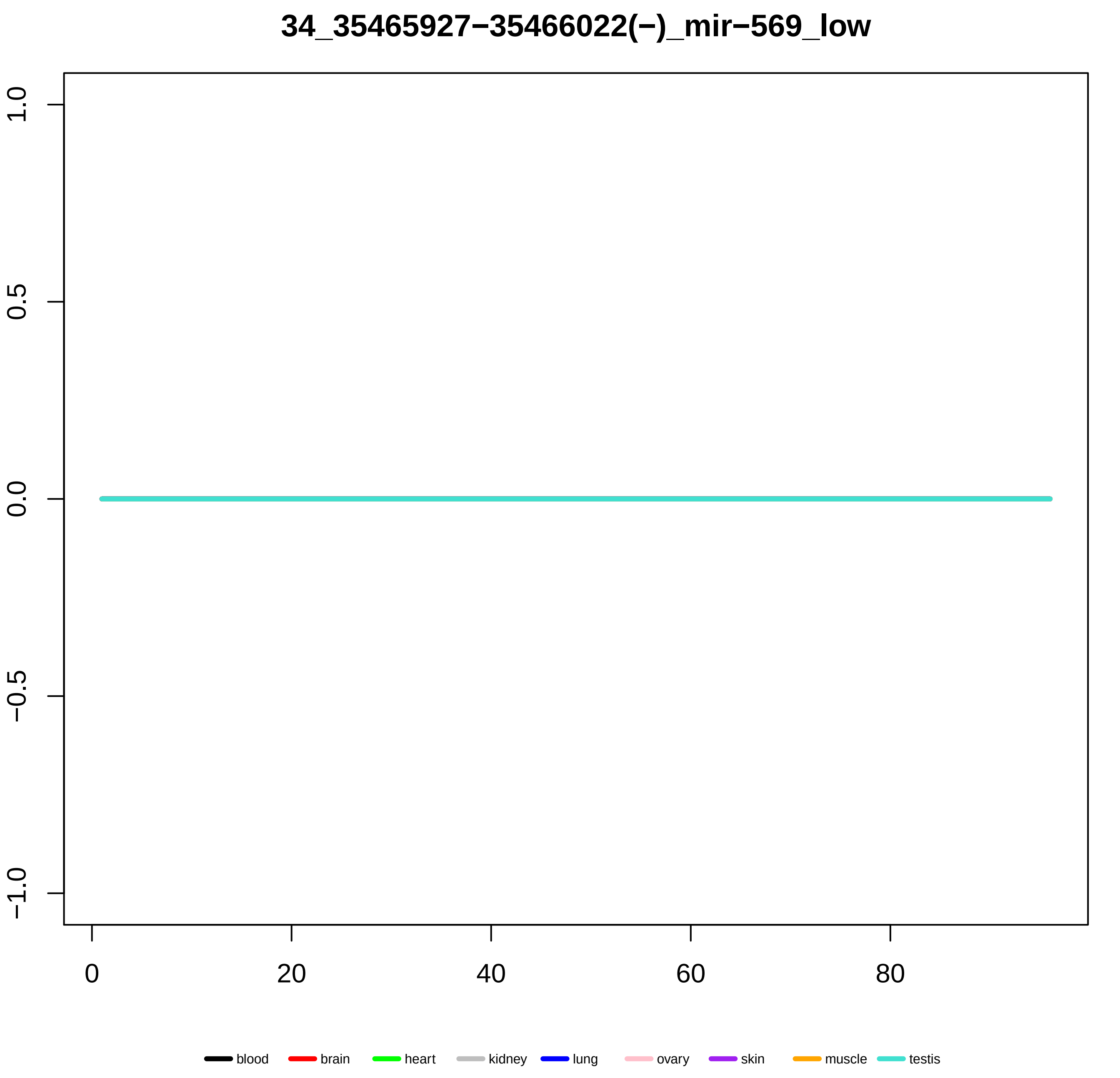
34_35465927−35466022(−)_mir−569_low
1.0
0.5
0.0
−0.5
−1.0
0
20
40
60
80
blood
brain
heart
kidney
lung
ovary
skin
muscle
testis

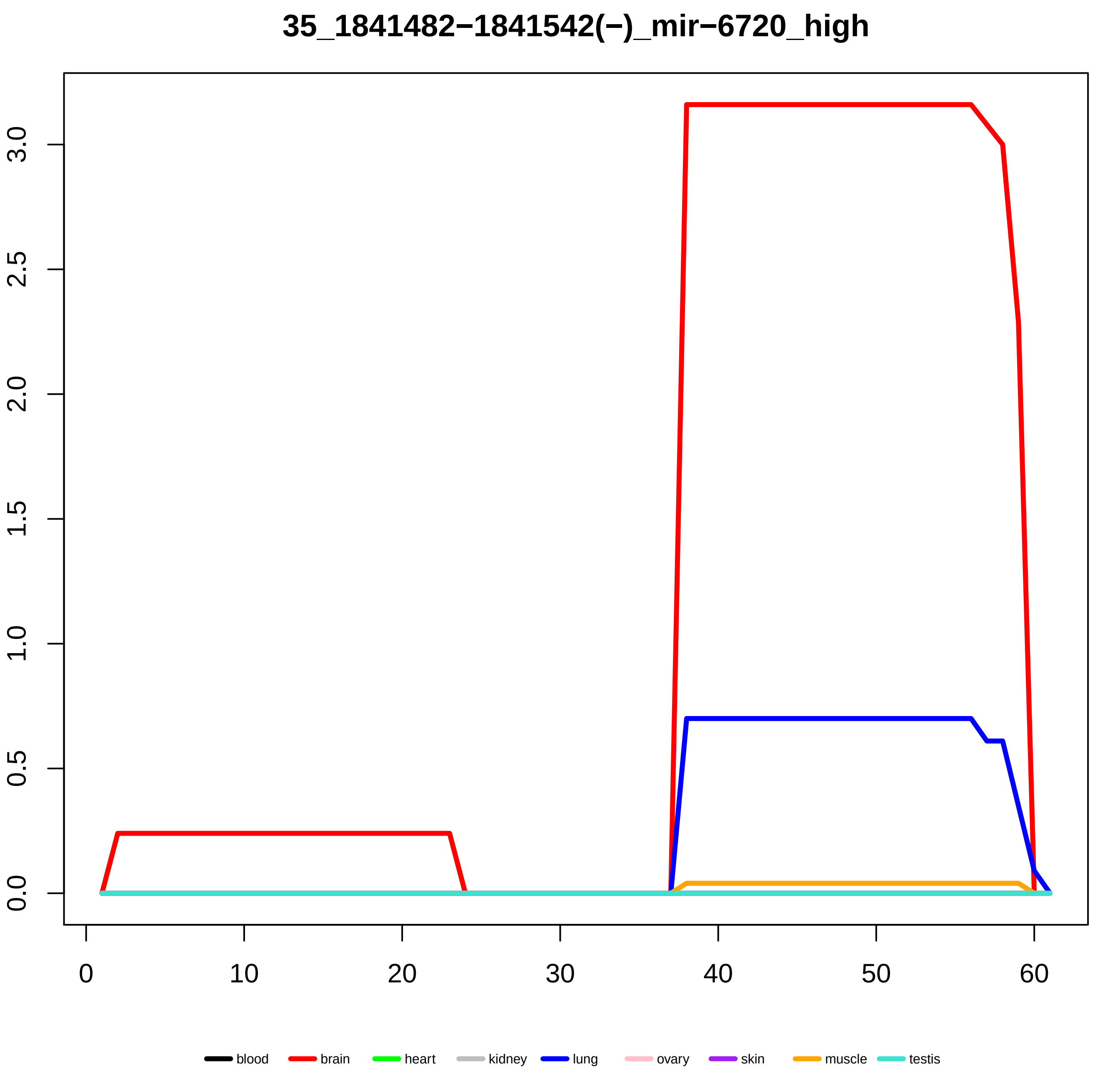

35_1841482−1841542(−)_mir−6720_high
0.0
0.5
1.0
1.5
2.0
2.5
3.0
0
10
20
30
40
50
60
blood
brain
heart
kidney
lung
ovary
skin
muscle
testis

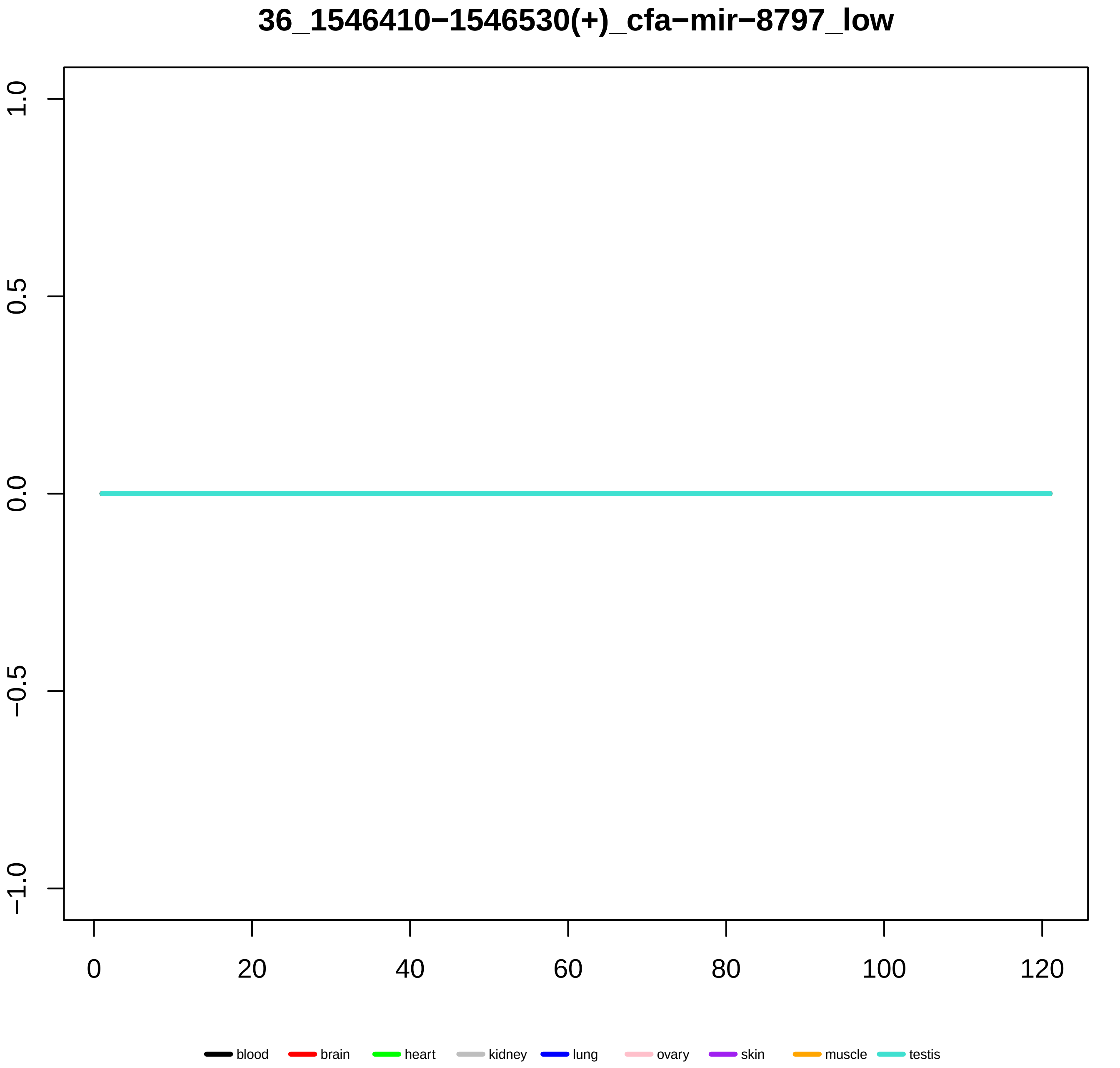

36_1546410−1546530(+)_cfa−mir−8797_low
1.0
0.5
0.0
−0.5
−1.0
0
20
40
60
80
100
120
blood
brain
heart
kidney
lung
ovary
skin
muscle
testis

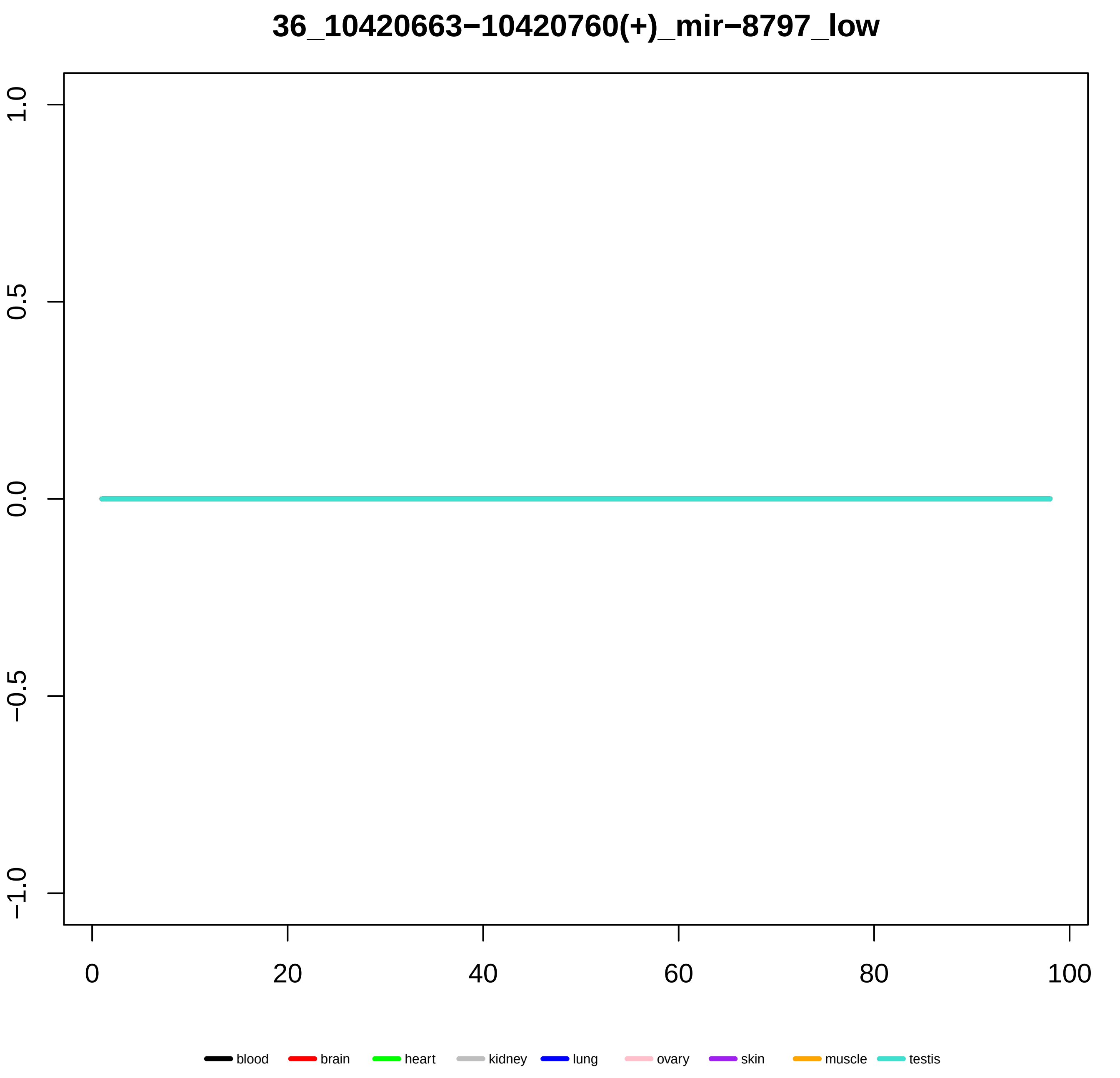

36_10420663−10420760(+)_mir−8797_low
1.0
0.5
0.0
−0.5
−1.0
0
20
40
60
80
100
blood
brain
heart
kidney
lung
ovary
skin
muscle
testis

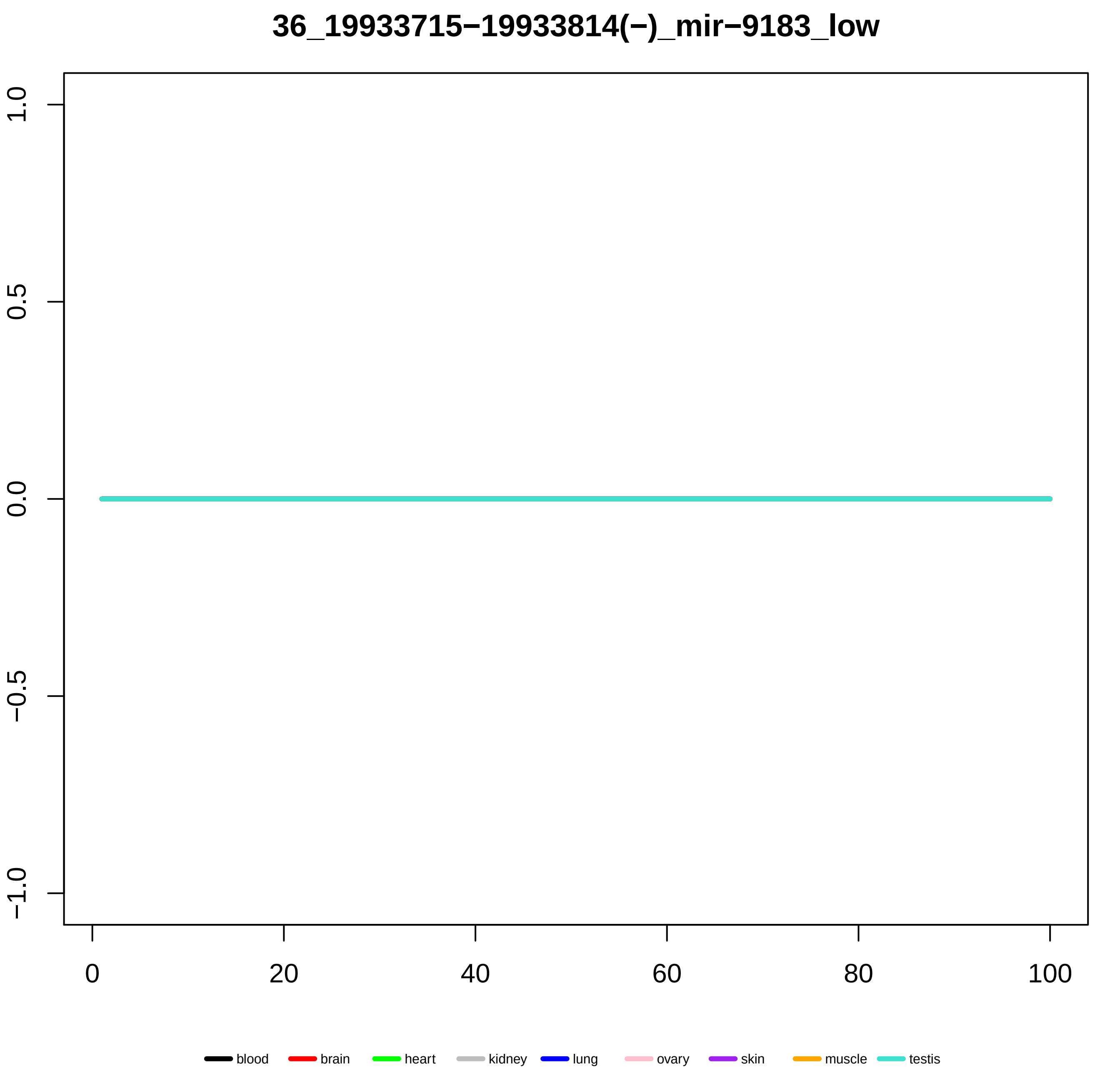

36_19933715−19933814(−)_mir−9183_low
1.0
0.5
0.0
−0.5
−1.0
0
20
40
60
80
100
blood
brain
heart
kidney
lung
ovary
skin
muscle
testis

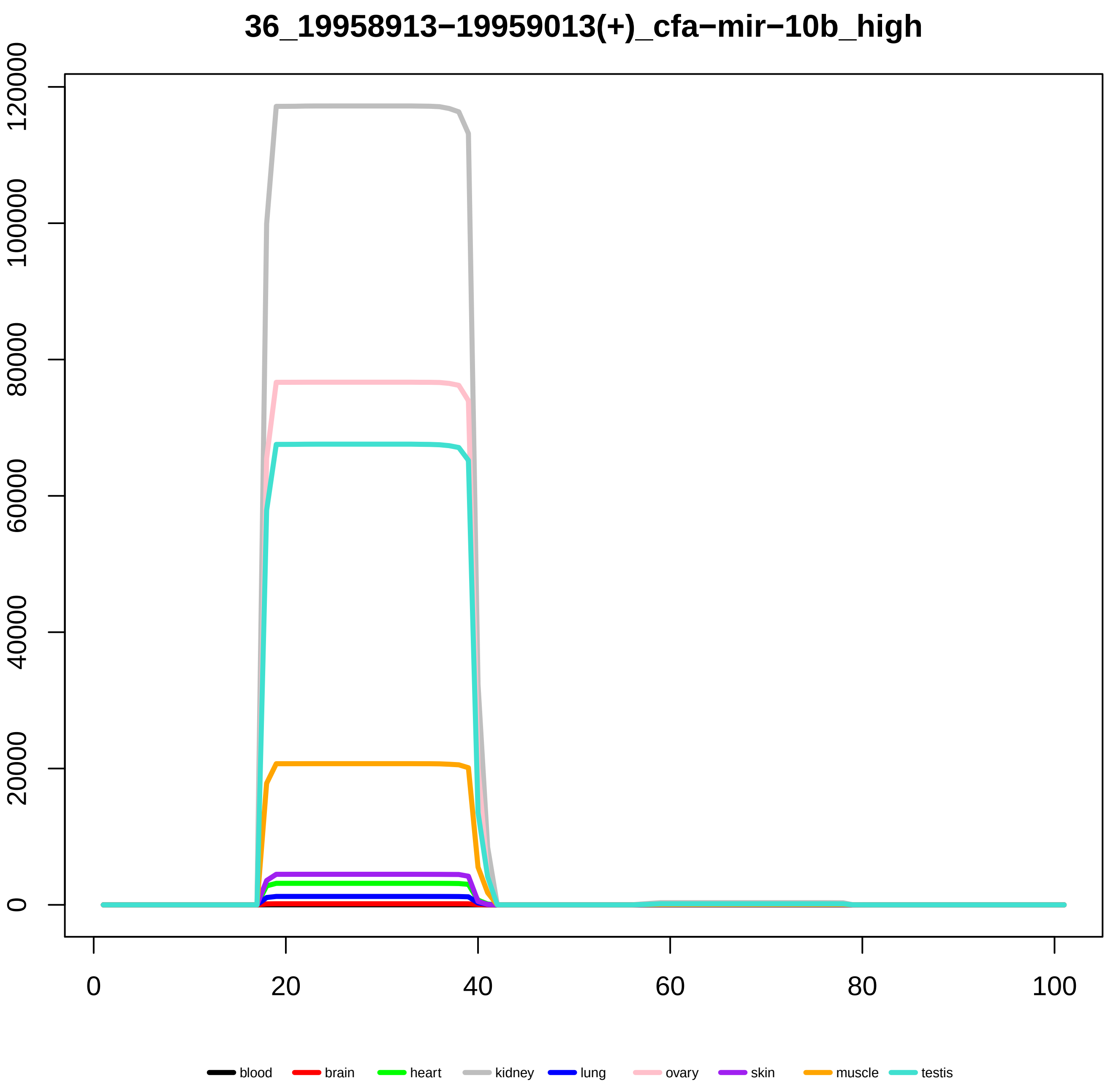
36_19958913−19959013(+)_cfa−mir−10b_high
0
20000
40000
60000
80000
100000
120000
0
20
40
60
80
100
blood
brain
heart
kidney
lung
ovary
skin
muscle
testis

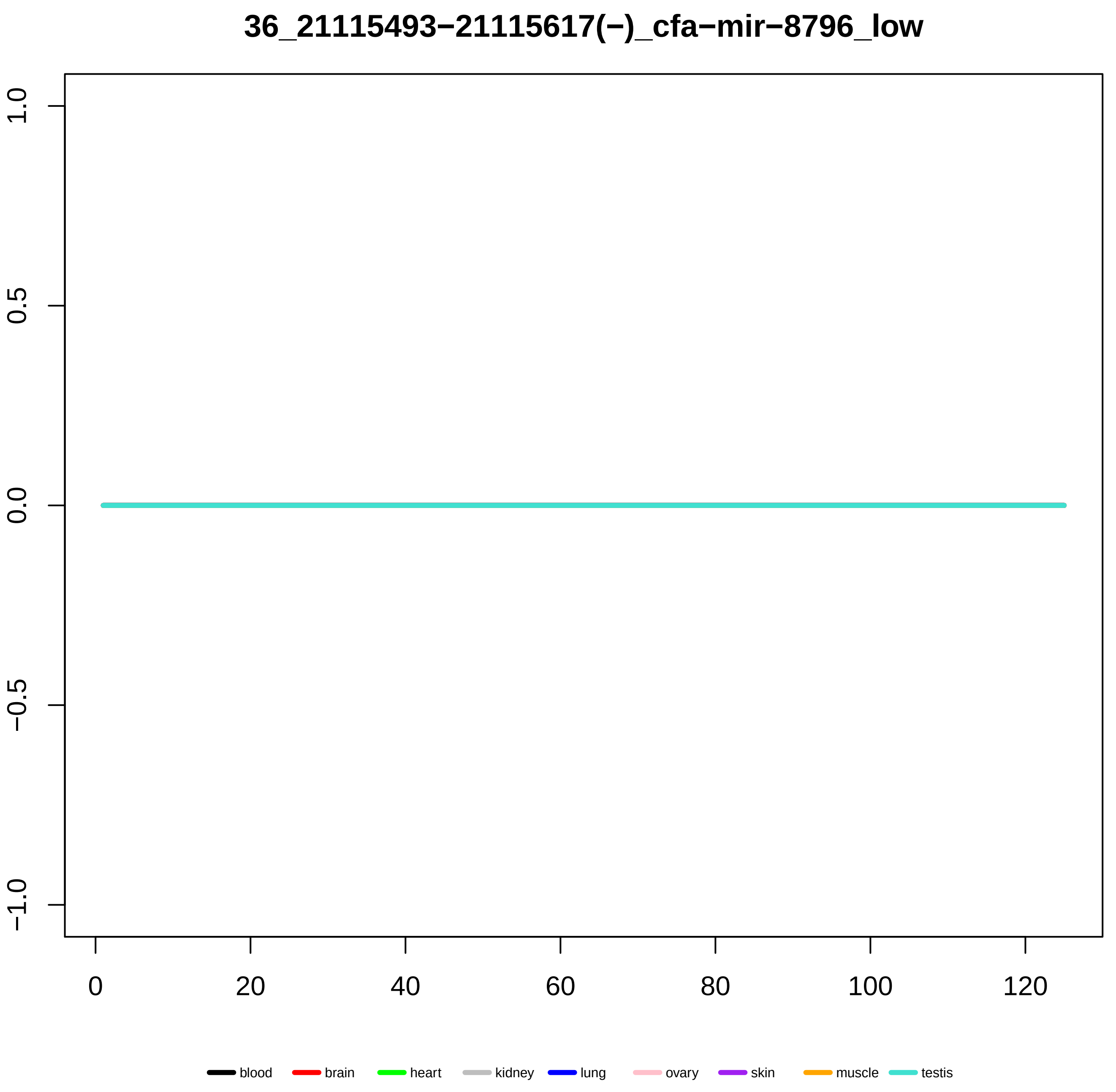
36_21115493−21115617(−)_cfa−mir−8796_low
1.0
0.5
0.0
−0.5
−1.0
0
20
40
60
80
100
120
blood
brain
heart
kidney
lung
ovary
skin
muscle
testis

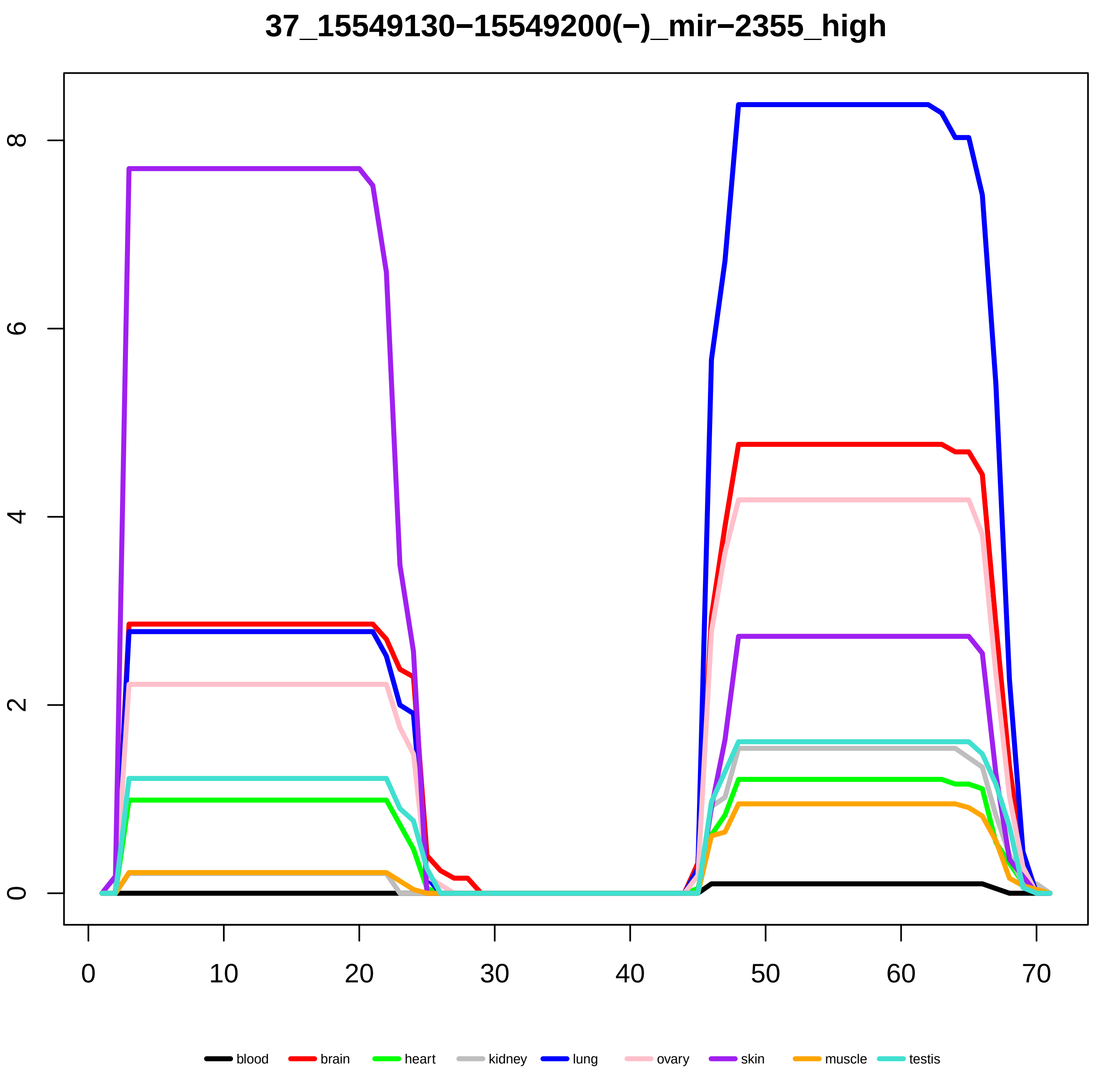
37_15549130−15549200(−)_mir−2355_high
0
2
4
6
8
0
10
20
30
40
50
60
70
blood
brain
heart
kidney
lung
ovary
skin
muscle
testis

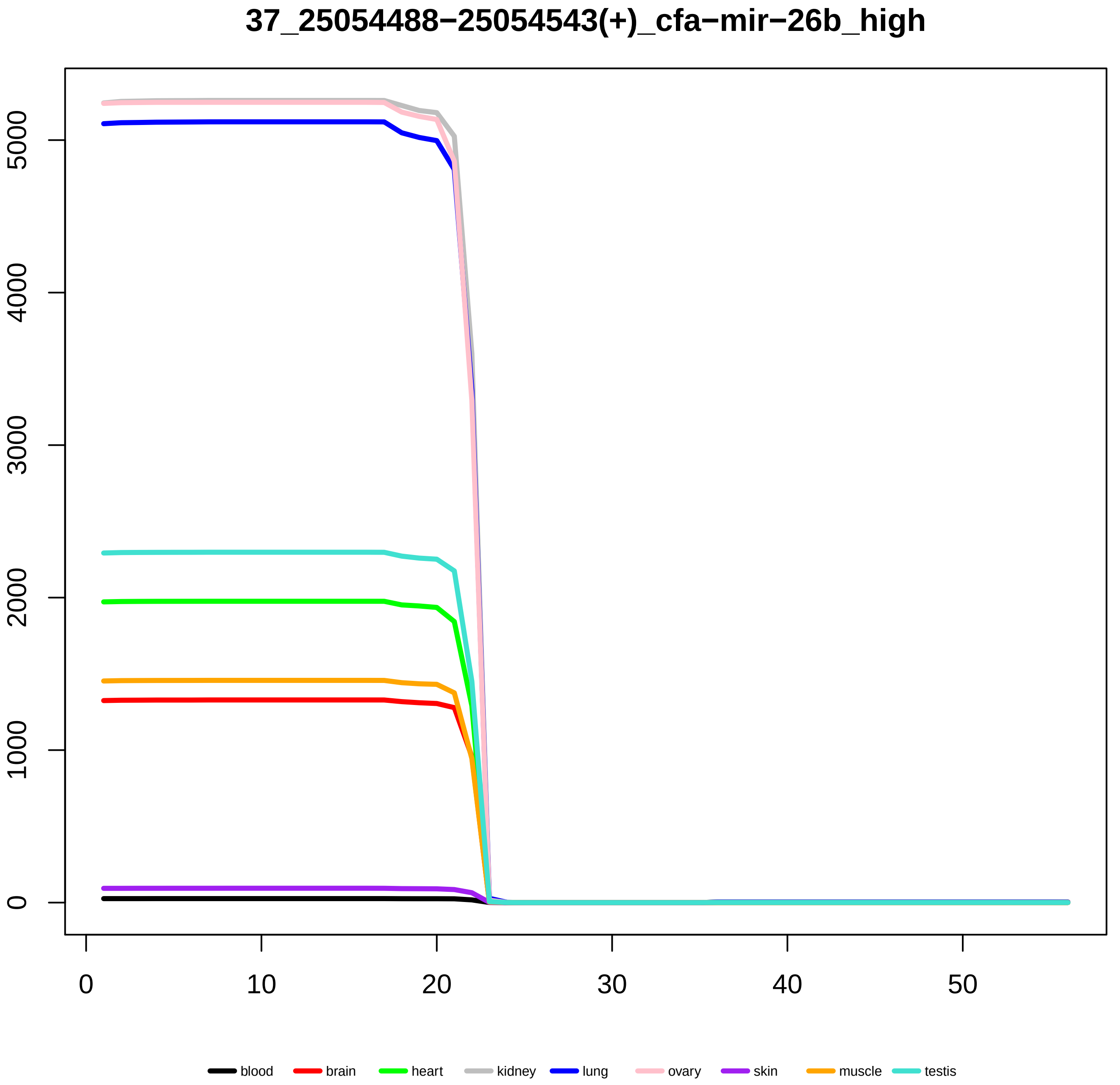

37_25054488−25054543(+)_cfa−mir−26b_high
0
1000
2000
3000
4000
5000
0
10
20
30
40
50
blood
brain
heart
kidney
lung
ovary
skin
muscle
testis

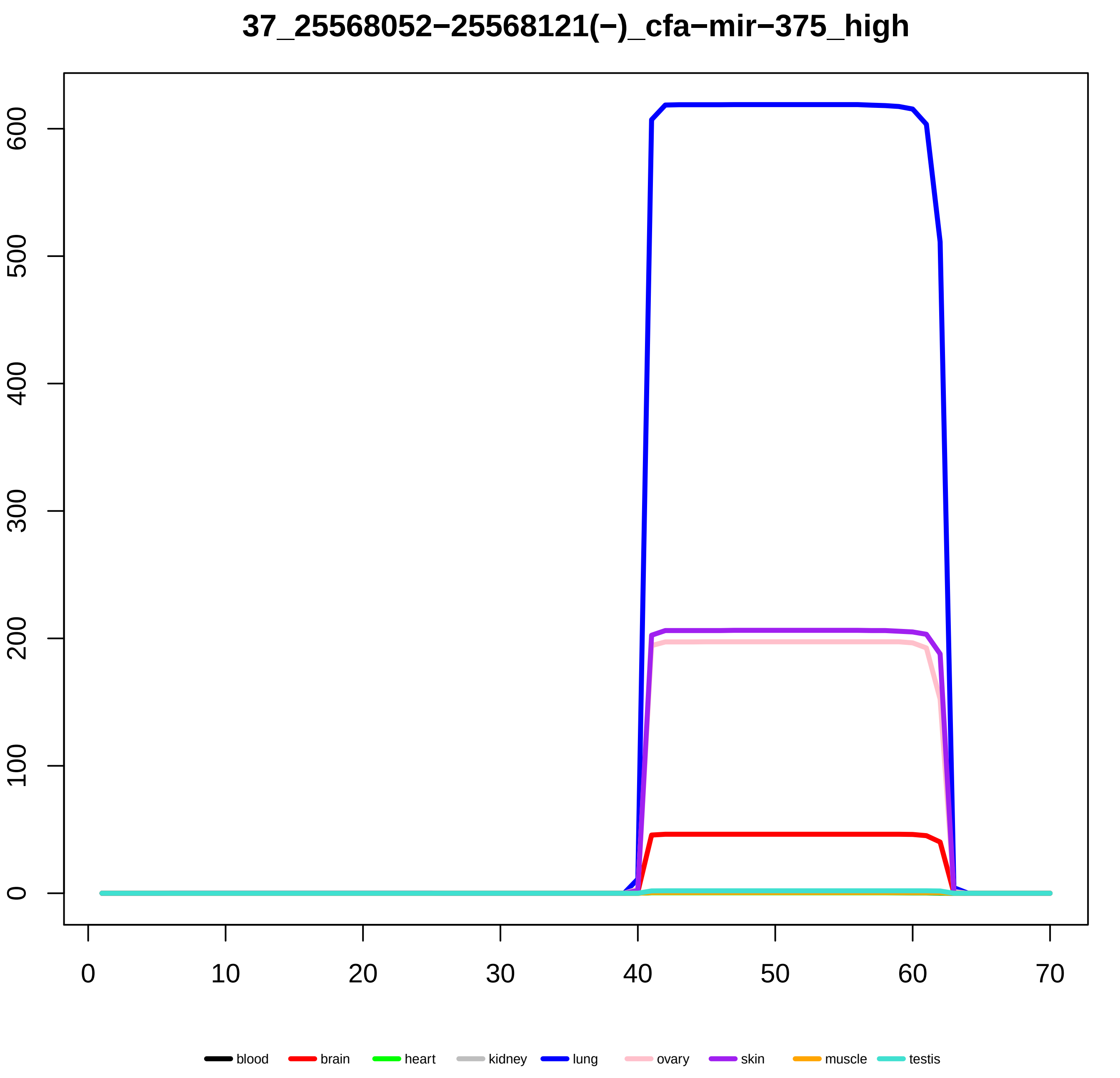

37_25568052−25568121(−)_cfa−mir−375_high
0
100
200
300
400
500
600
0
10
20
30
40
50
60
70
blood
brain
heart
kidney
lung
ovary
skin
muscle
testis

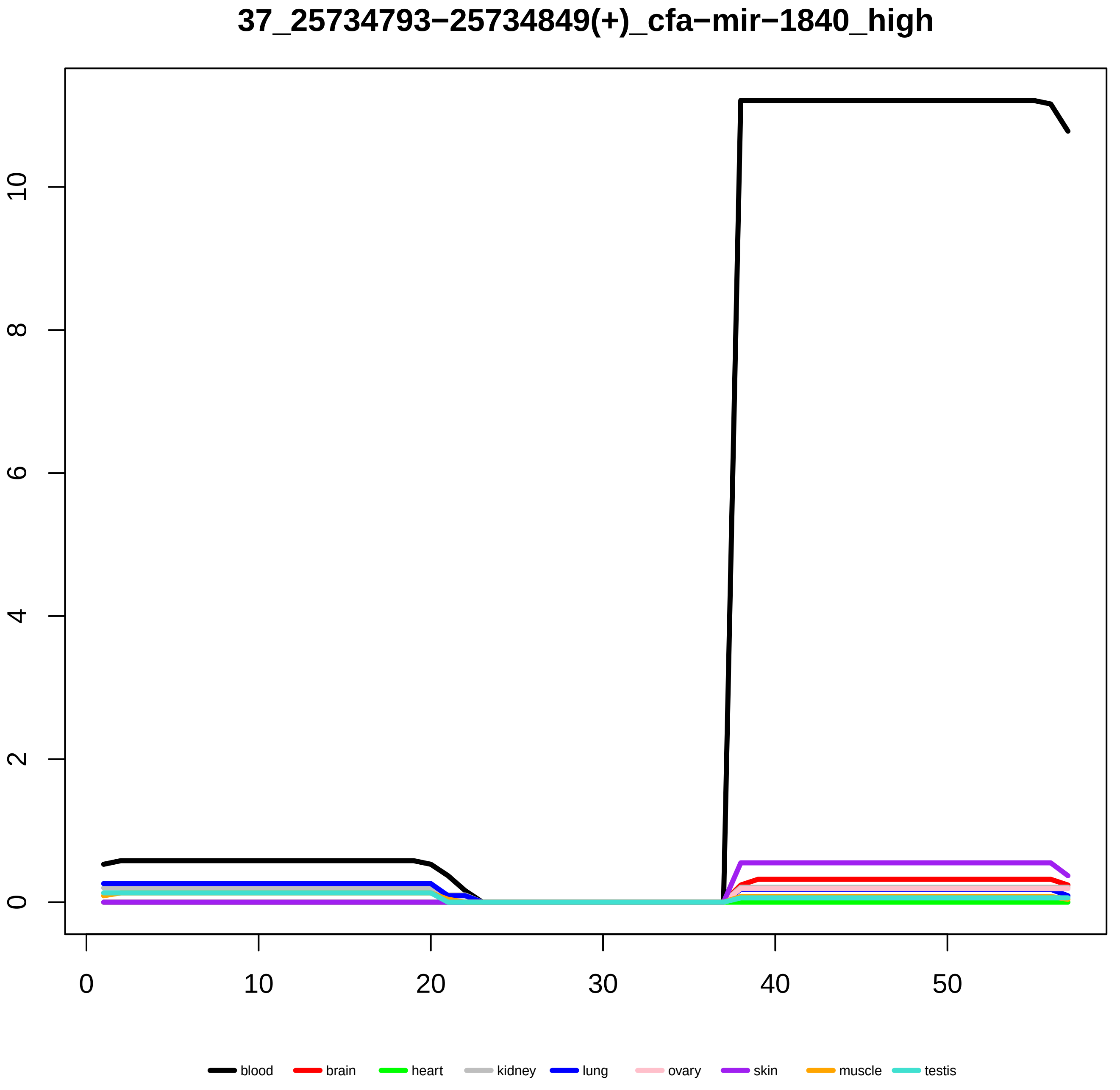

37_25734793−25734849(+)_cfa−mir−1840_high
0
2
4
6
8
10
0
10
20
30
40
50
blood
brain
heart
kidney
lung
ovary
skin
muscle
testis

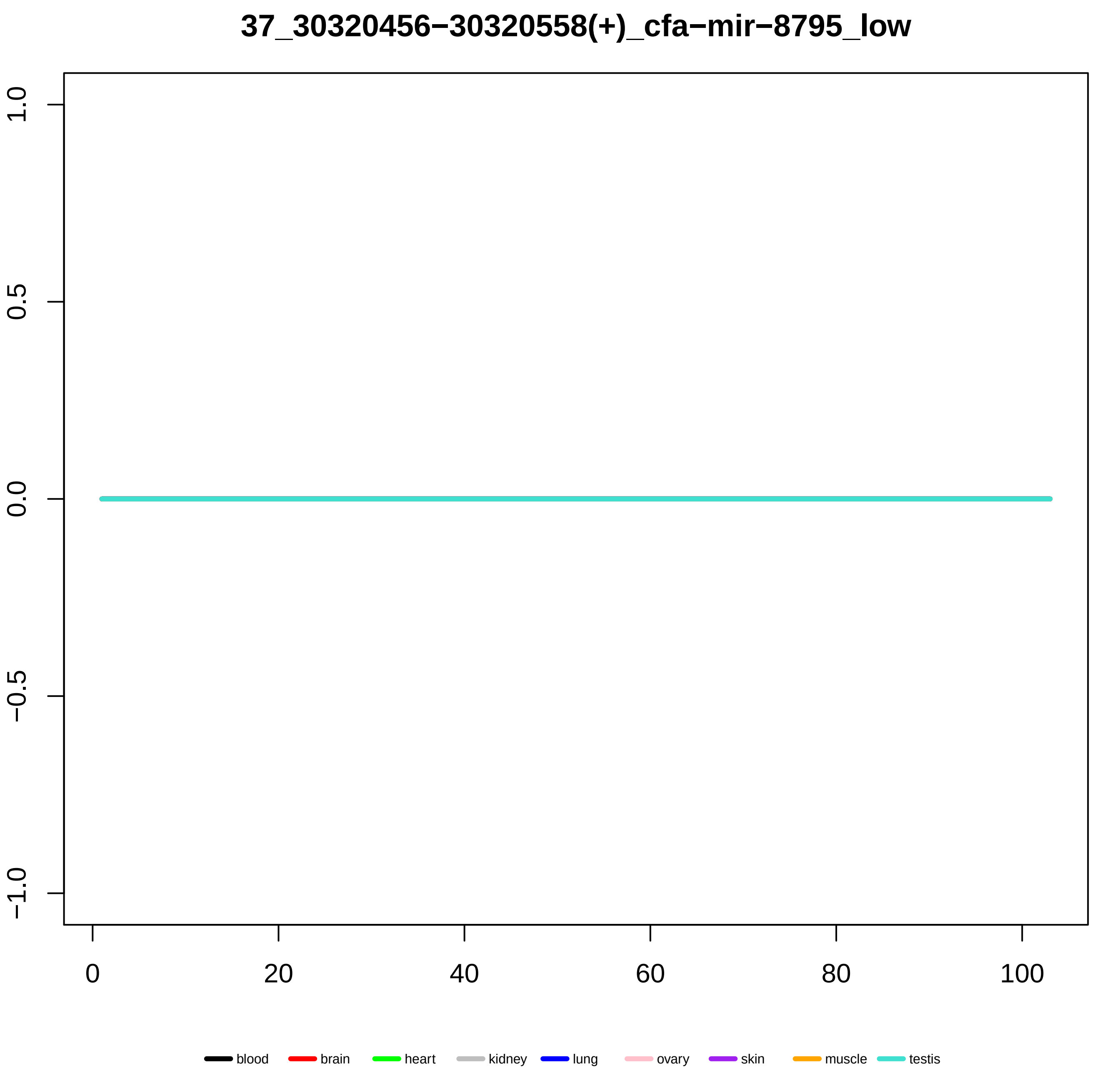

37_30320456−30320558(+)_cfa−mir−8795_low
1.0
0.5
0.0
−0.5
−1.0
0
20
40
60
80
100
blood
brain
heart
kidney
lung
ovary
skin
muscle
testis

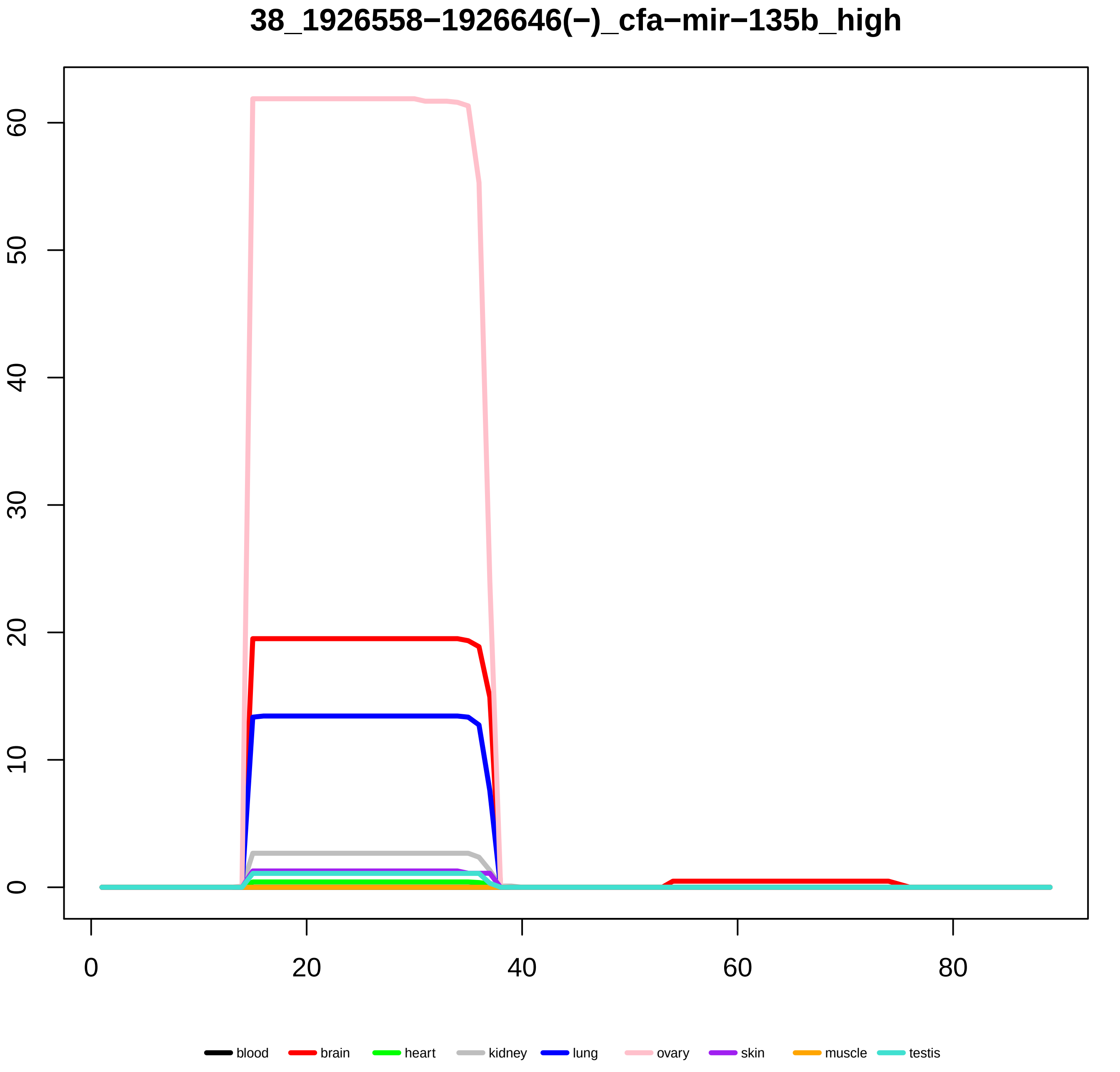

38_1926558−1926646(−)_cfa−mir−135b_high
0
10
20
30
40
50
60
0
20
40
60
80
blood
brain
heart
kidney
lung
ovary
skin
muscle
testis

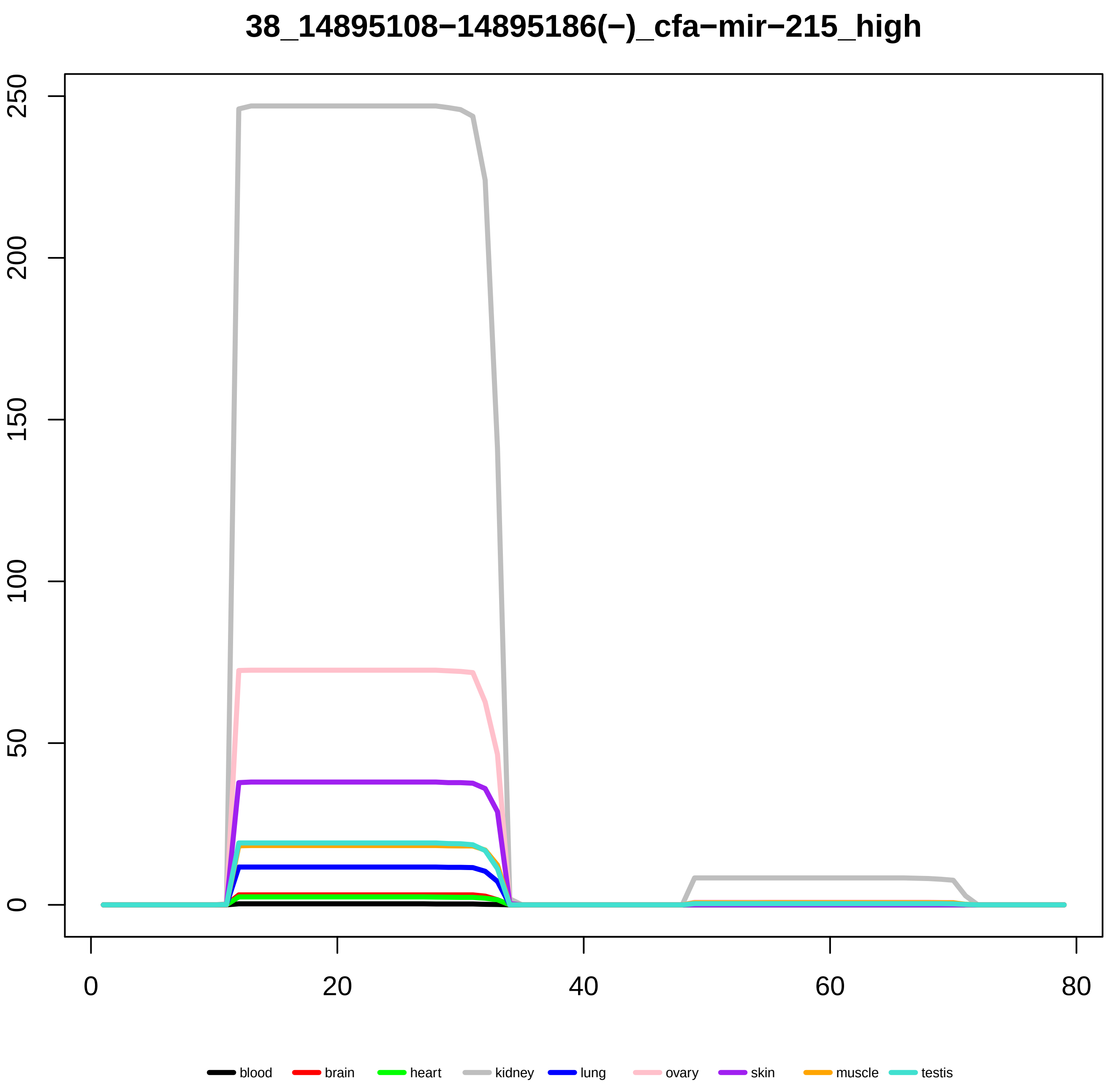

38_14895108−14895186(−)_cfa−mir−215_high
0
50
100
150
200
250
0
20
40
60
80
blood
brain
heart
kidney
lung
ovary
skin
muscle
testis

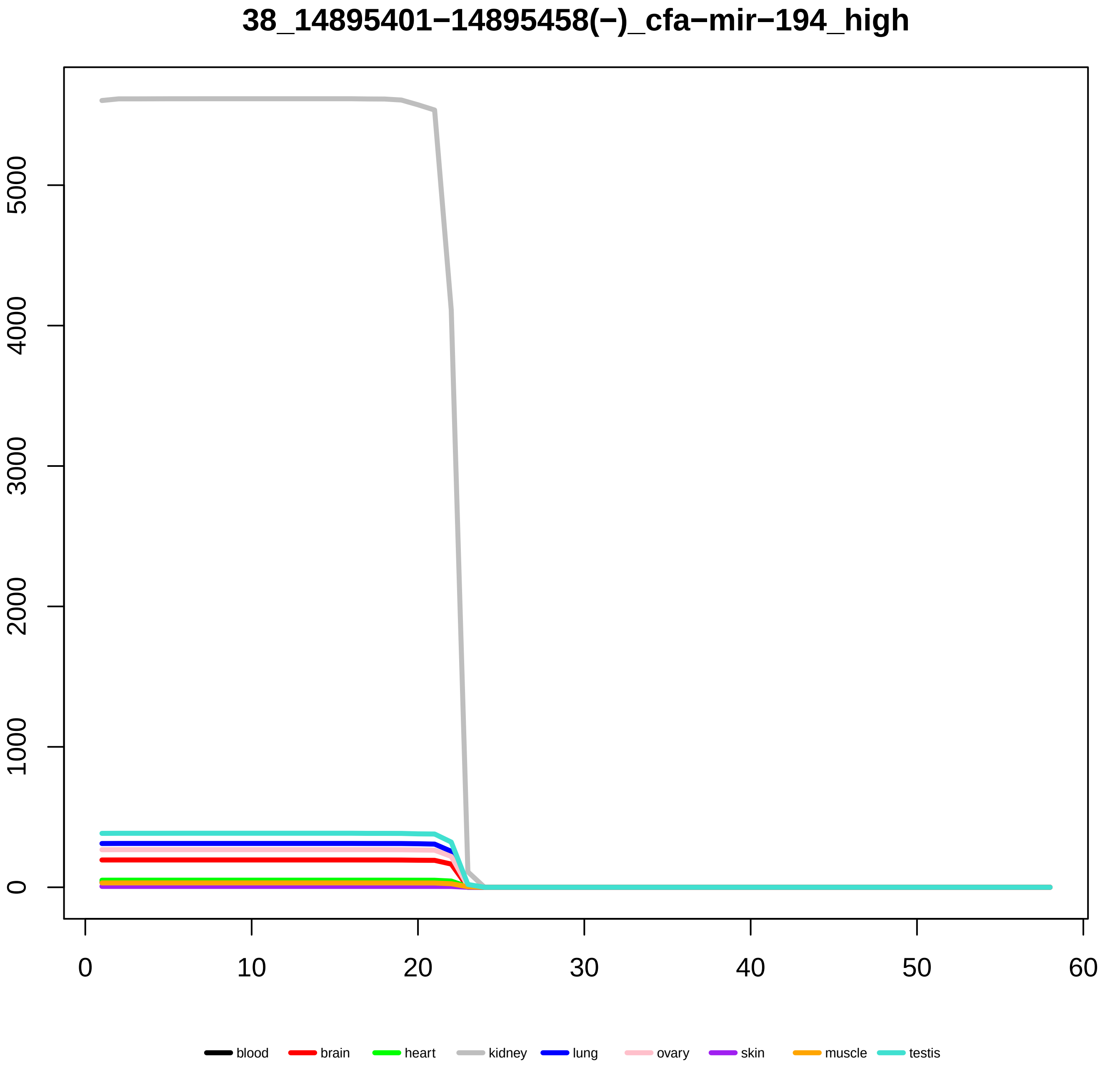

38_14895401−14895458(−)_cfa−mir−194_high
0
1000
2000
3000
4000
5000
0
10
20
30
40
50
60
blood
brain
heart
kidney
lung
ovary
skin
muscle
testis

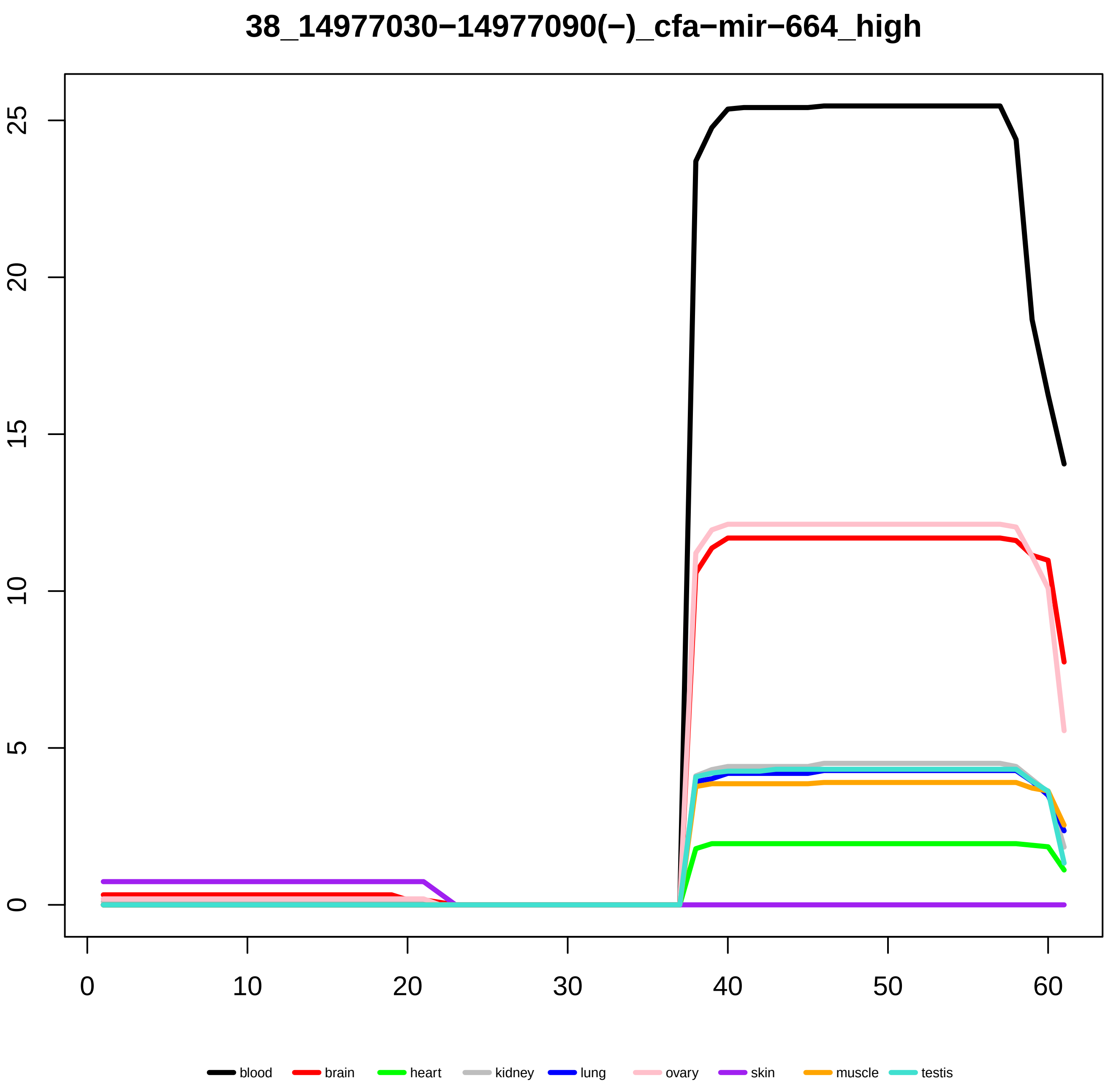

38_14977030−14977090(−)_cfa−mir−664_high
0
5
10
15
20
25
0
10
20
30
40
50
60
blood
brain
heart
kidney
lung
ovary
skin
muscle
testis

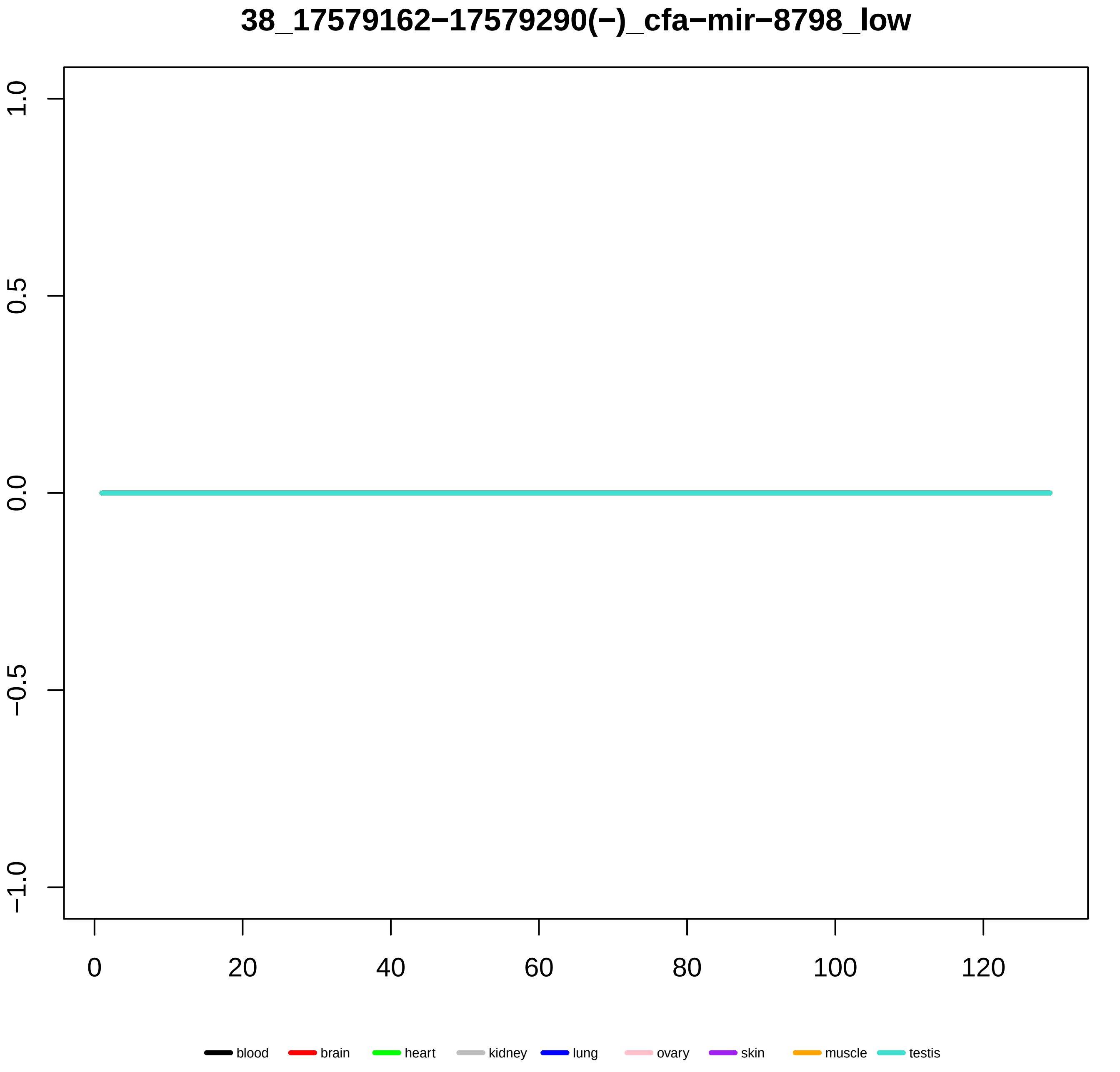

38_17579162−17579290(−)_cfa−mir−8798_low
1.0
0.5
0.0
−0.5
−1.0
0
20
40
60
80
100
120
blood
brain
heart
kidney
lung
ovary
skin
muscle
testis

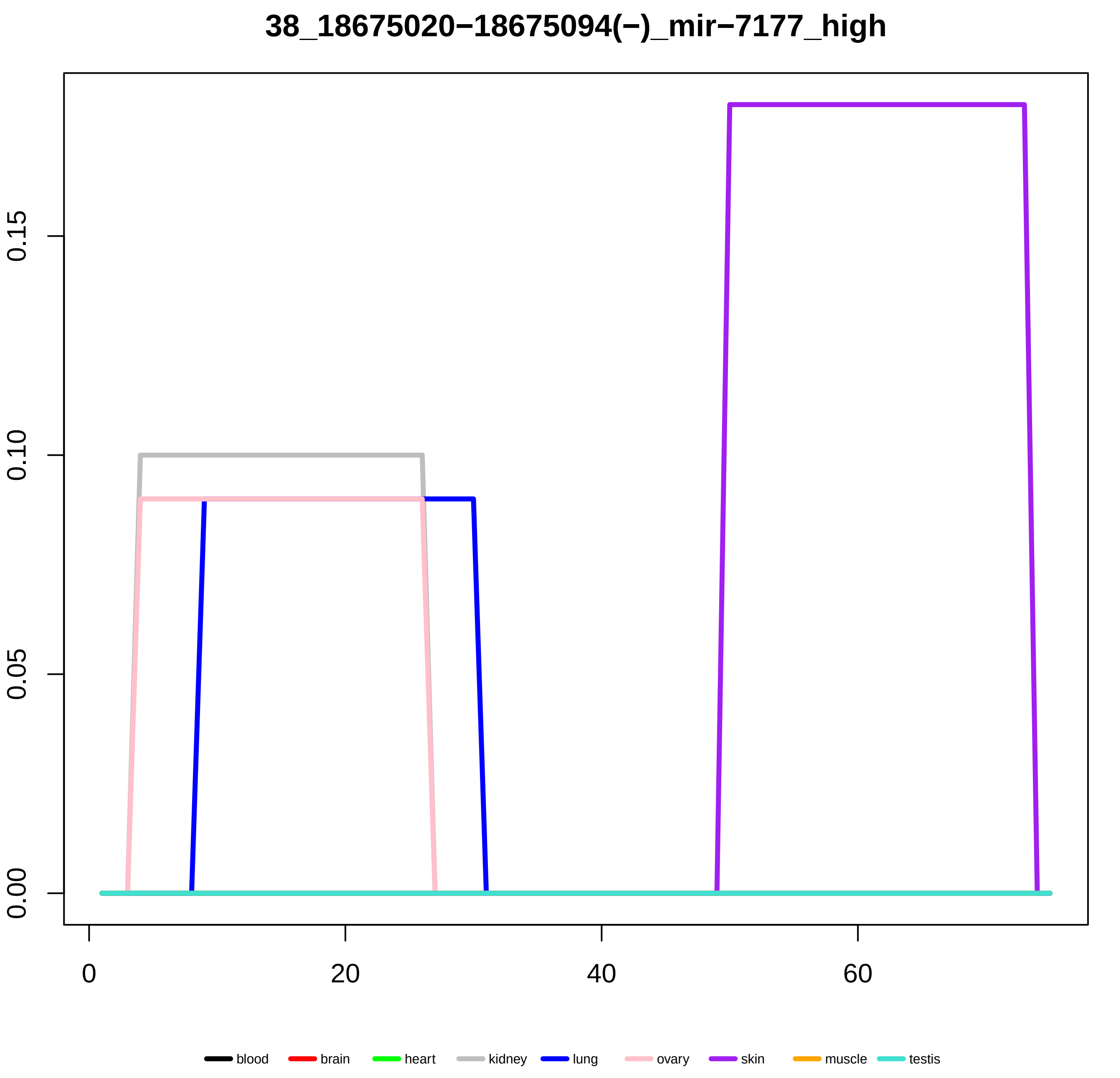

38_18675020−18675094(−)_mir−7177_high
0.00
0.05
0.10
0.15
0
20
40
60
blood
brain
heart
kidney
lung
ovary
skin
muscle
testis

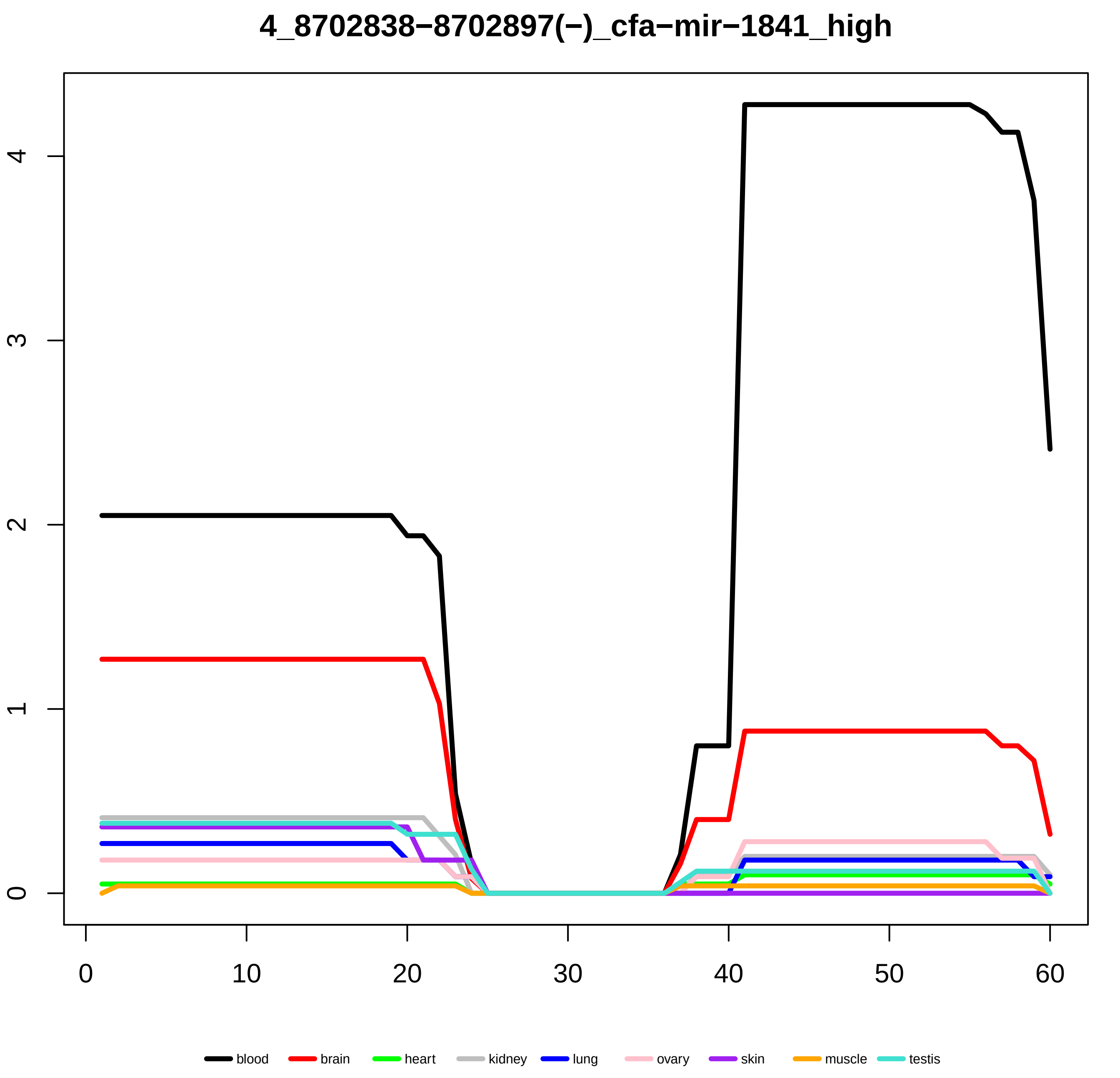

4_8702838−8702897(−)_cfa−mir−1841_high
0
1
2
3
4
0
10
20
30
40
50
60
blood
brain
heart
kidney
lung
ovary
skin
muscle
testis

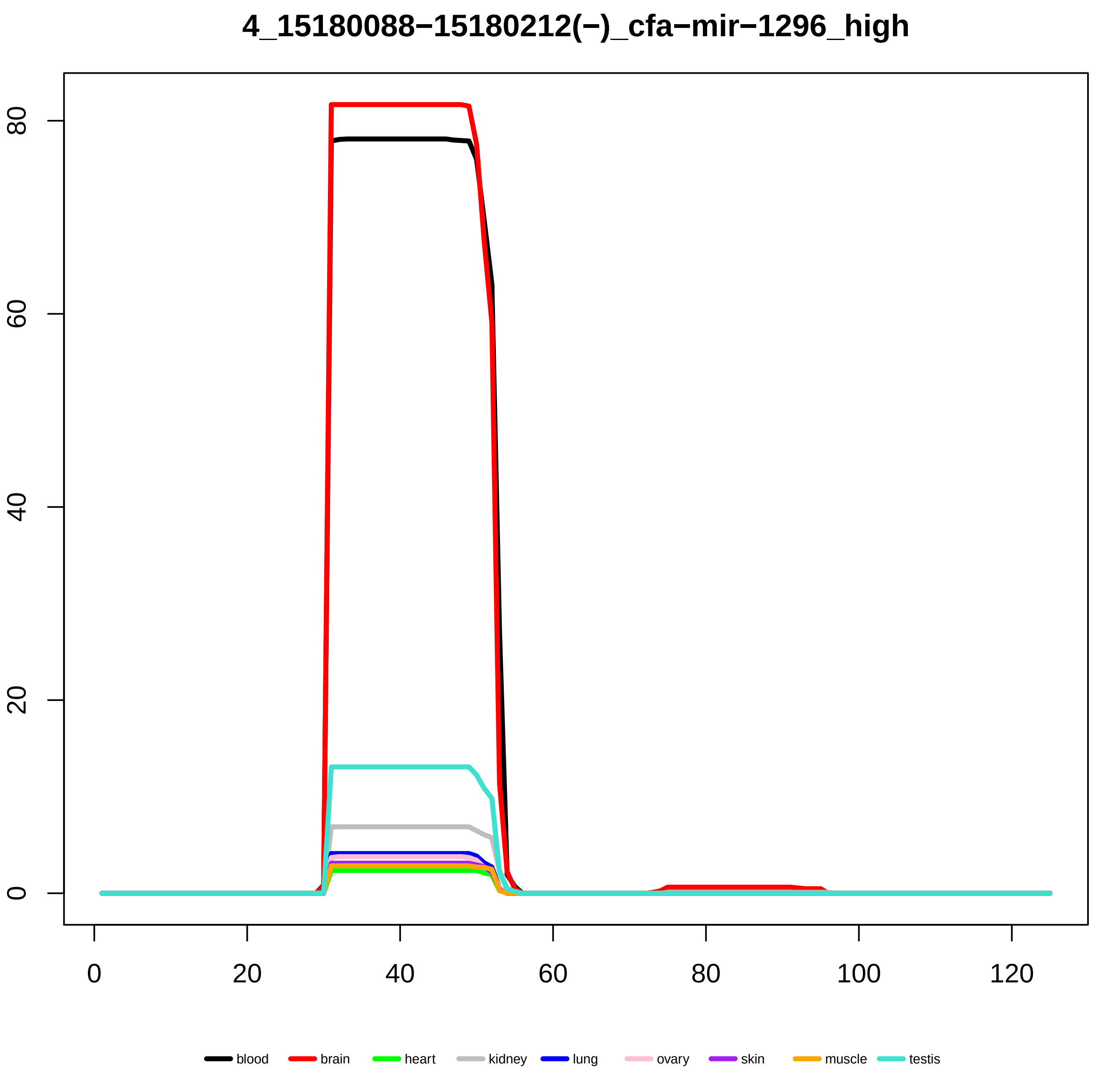

4_15180088−15180212(−)_cfa−mir−1296_high
0
20
40
60
80
0
20
40
60
80
100
120
blood
brain
heart
kidney
lung
ovary
skin
muscle
testis

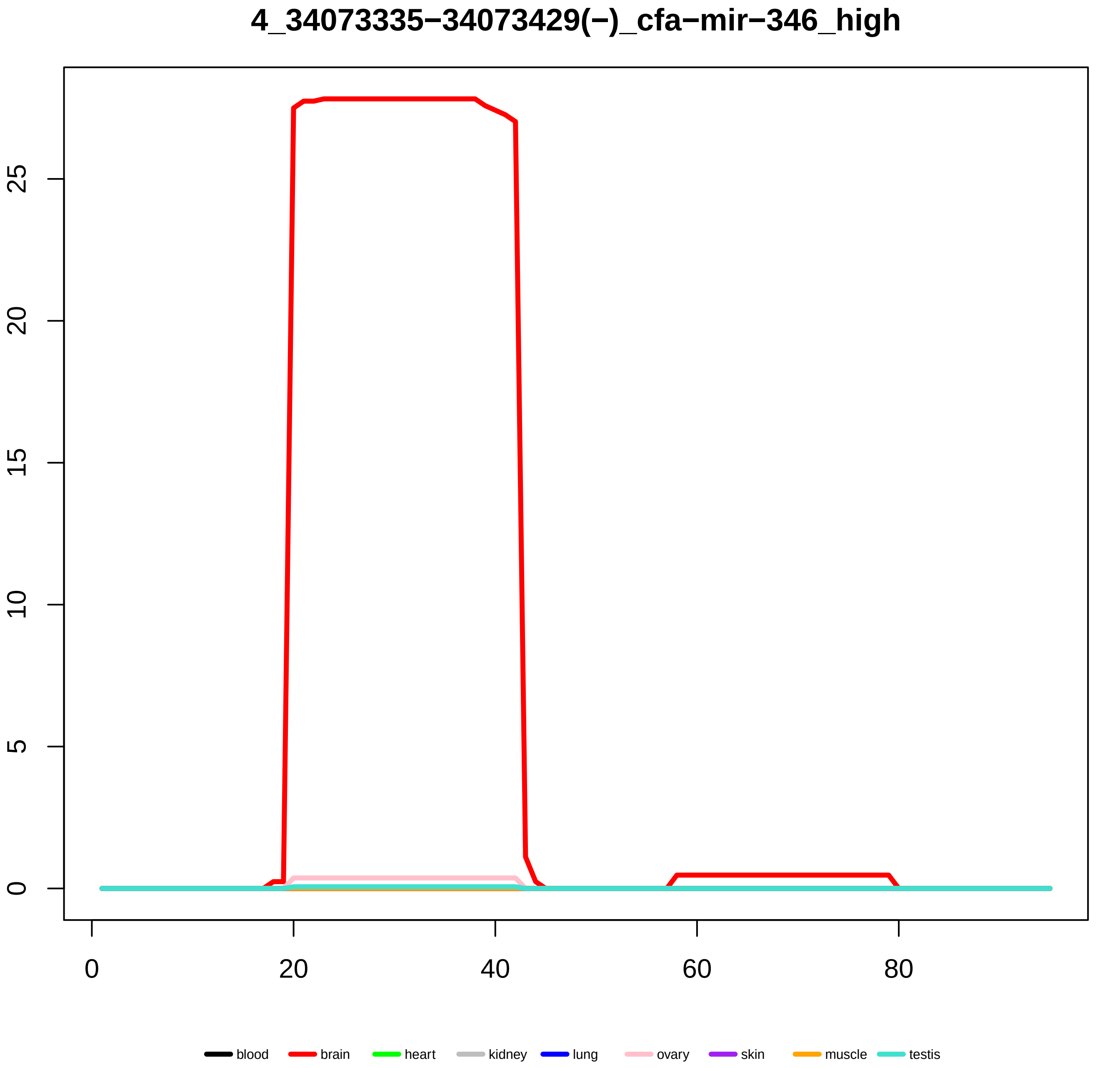

4_34073335−34073429(−)_cfa−mir−346_high
0
5
10
15
20
25
0
20
40
60
80
blood
brain
heart
kidney
lung
ovary
skin
muscle
testis

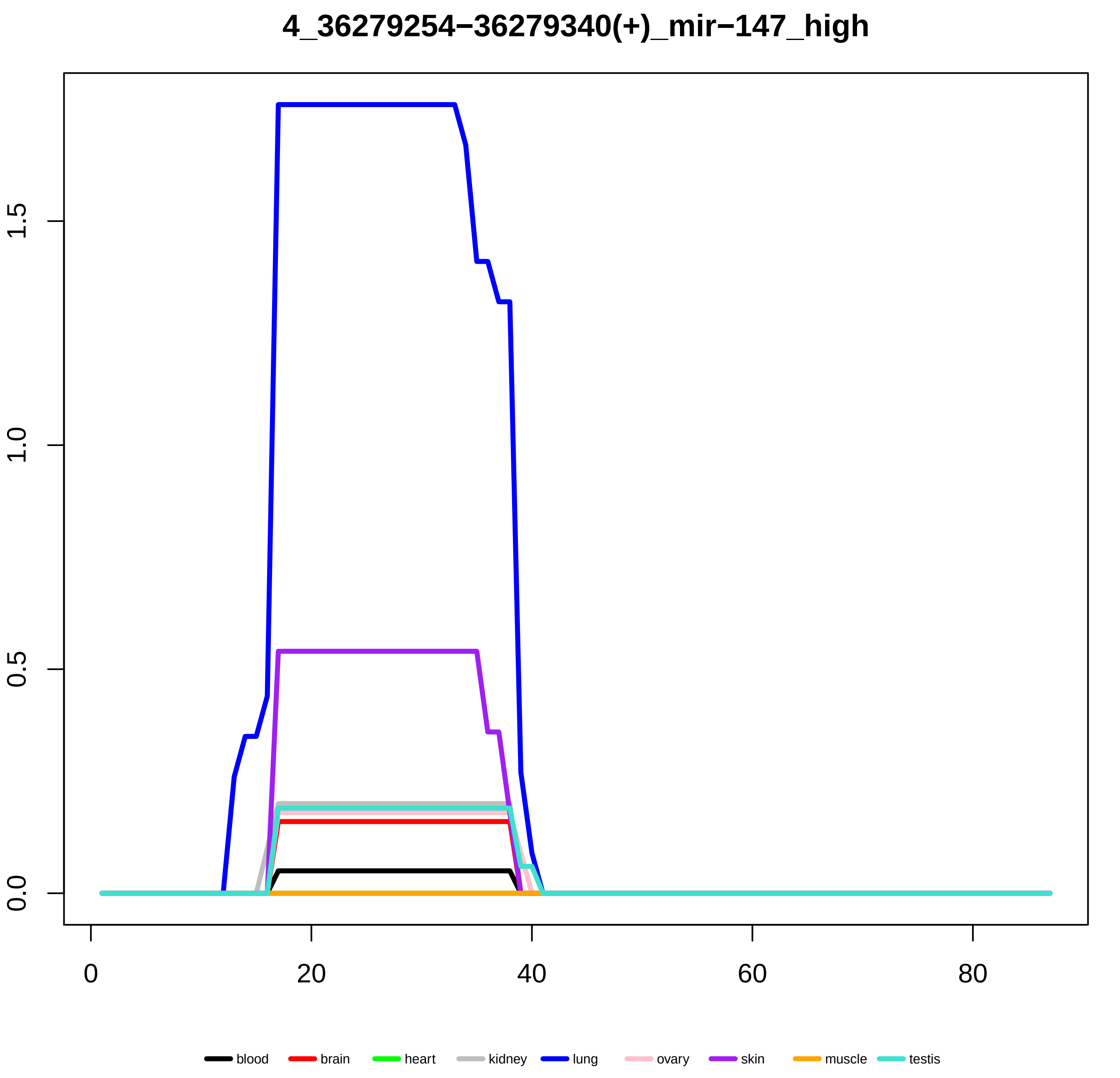

4_36279254−36279340(+)_mir−147_high
0.0
0.5
1.0
1.5
0
20
40
60
80
blood
brain
heart
kidney
lung
ovary
skin
muscle
testis

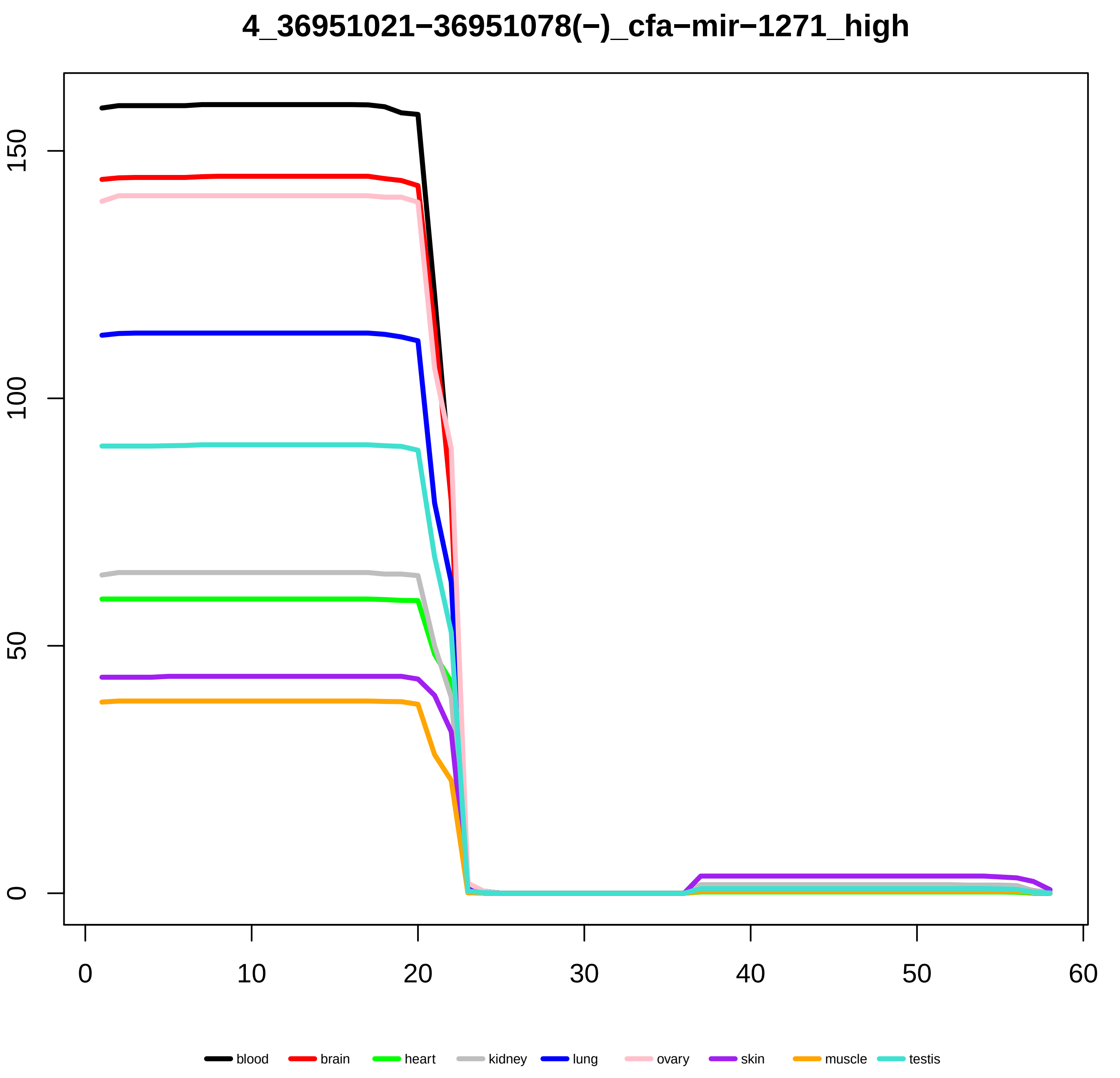
4_36951021−36951078(−)_cfa−mir−1271_high
0
50
100
150
0
10
20
30
40
50
60
blood
brain
heart
kidney
lung
ovary
skin
muscle
testis

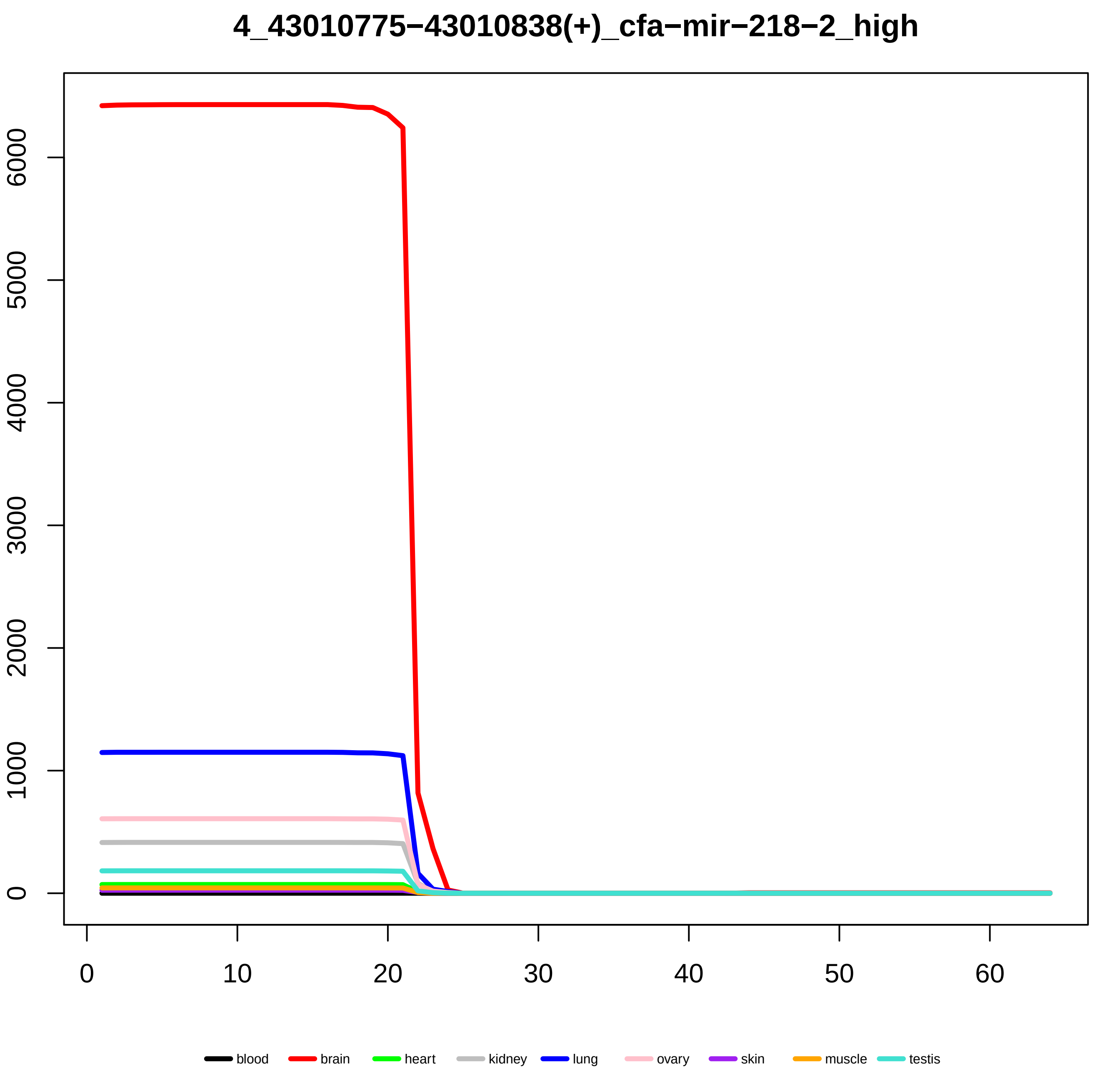
4_43010775−43010838(+)_cfa−mir−218−2_high
0
1000
2000
3000
4000
5000
6000
0
10
20
30
40
50
60
blood
brain
heart
kidney
lung
ovary
skin
muscle
testis

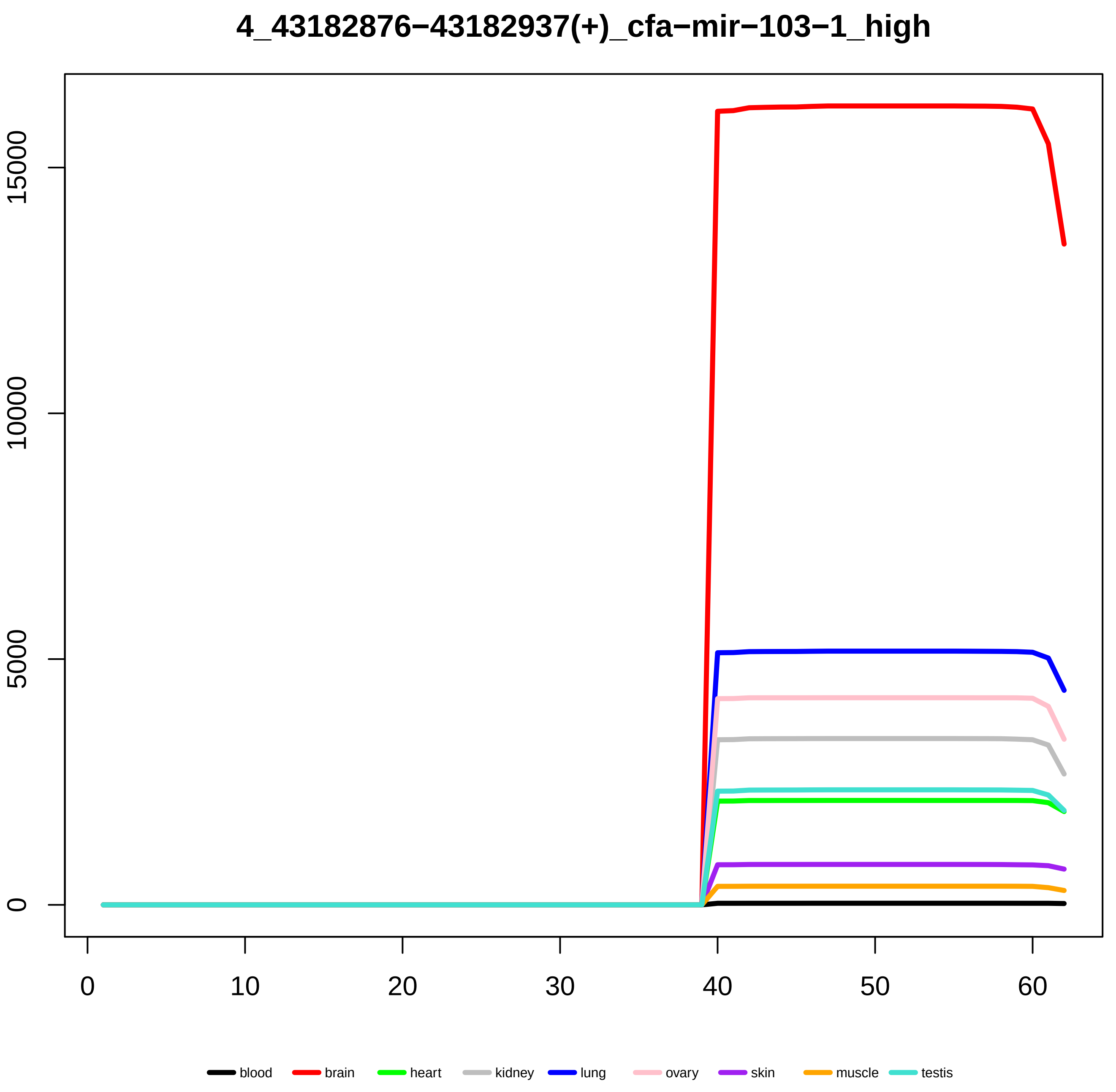
4_43182876−43182937(+)_cfa−mir−103−1_high
0
5000
10000
15000
0
10
20
30
40
50
60
blood
brain
heart
kidney
lung
ovary
skin
muscle
testis

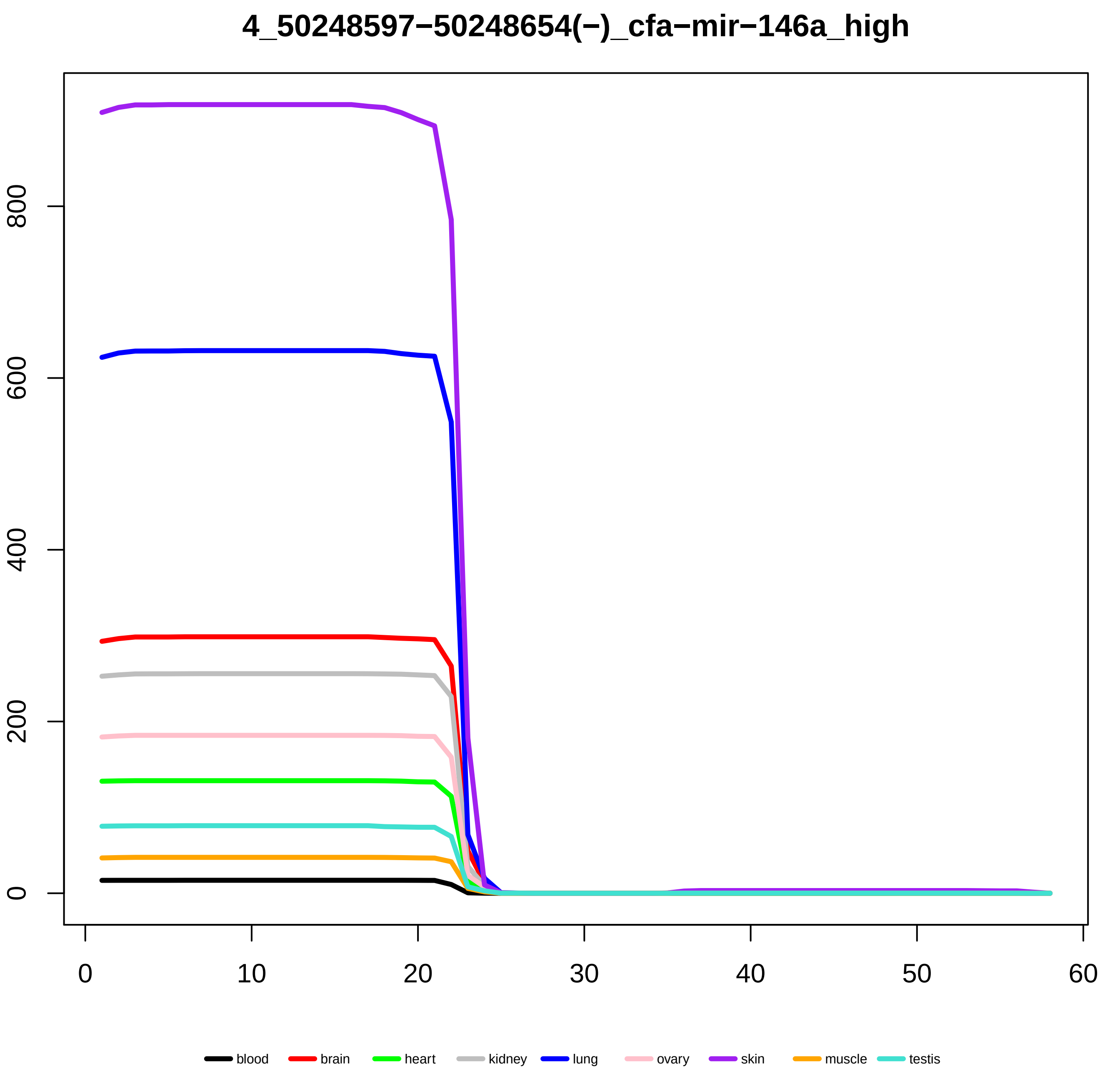
4_50248597−50248654(−)_cfa−mir−146a_high
0
200
400
600
800
0
10
20
30
40
50
60
blood
brain
heart
kidney
lung
ovary
skin
muscle
testis

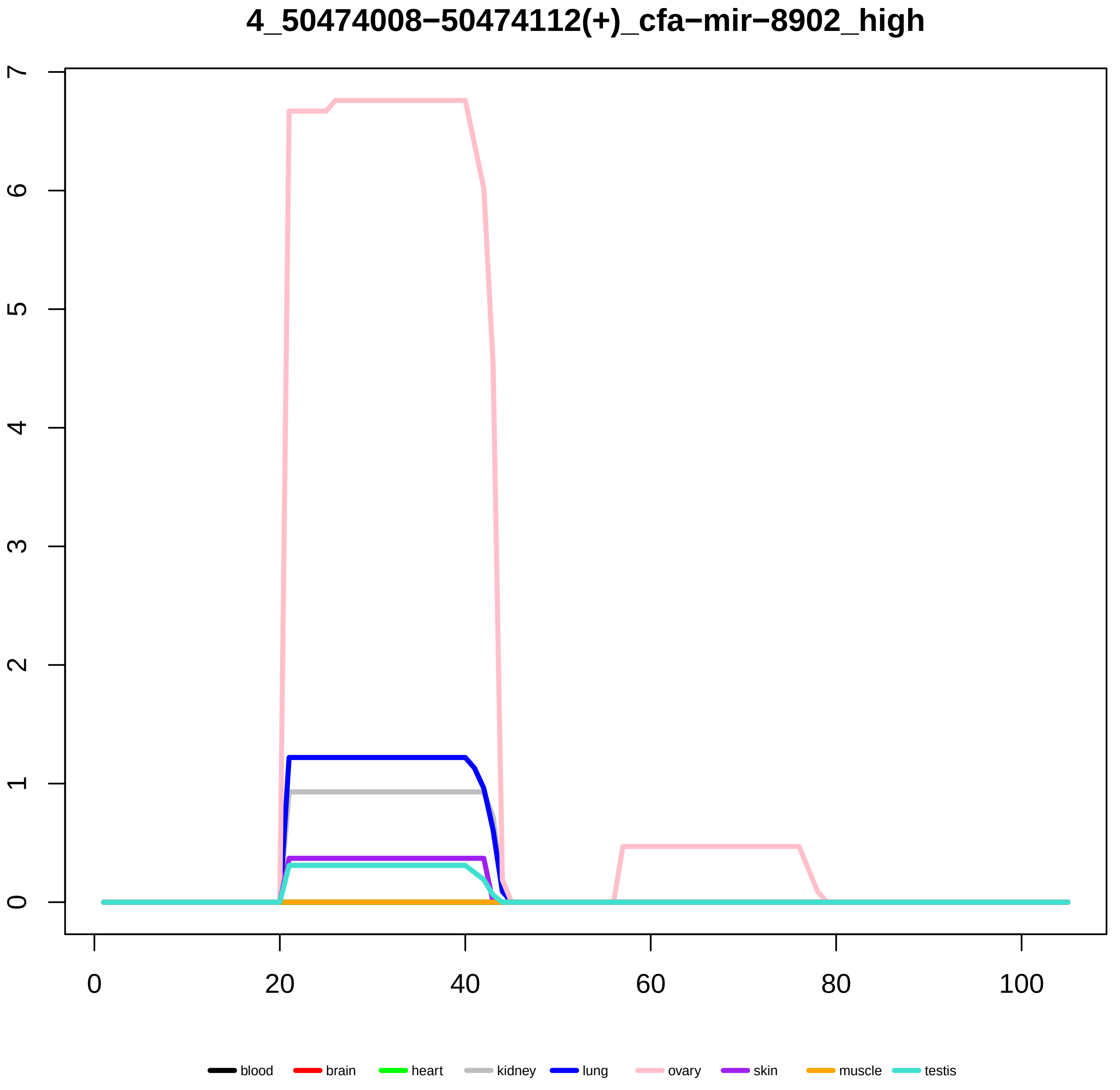
4_50474008−50474112(+)_cfa−mir−8902_high
0
1
2
3
4
5
6
7
0
20
40
60
80
100
blood
brain
heart
kidney
lung
ovary
skin
muscle
testis

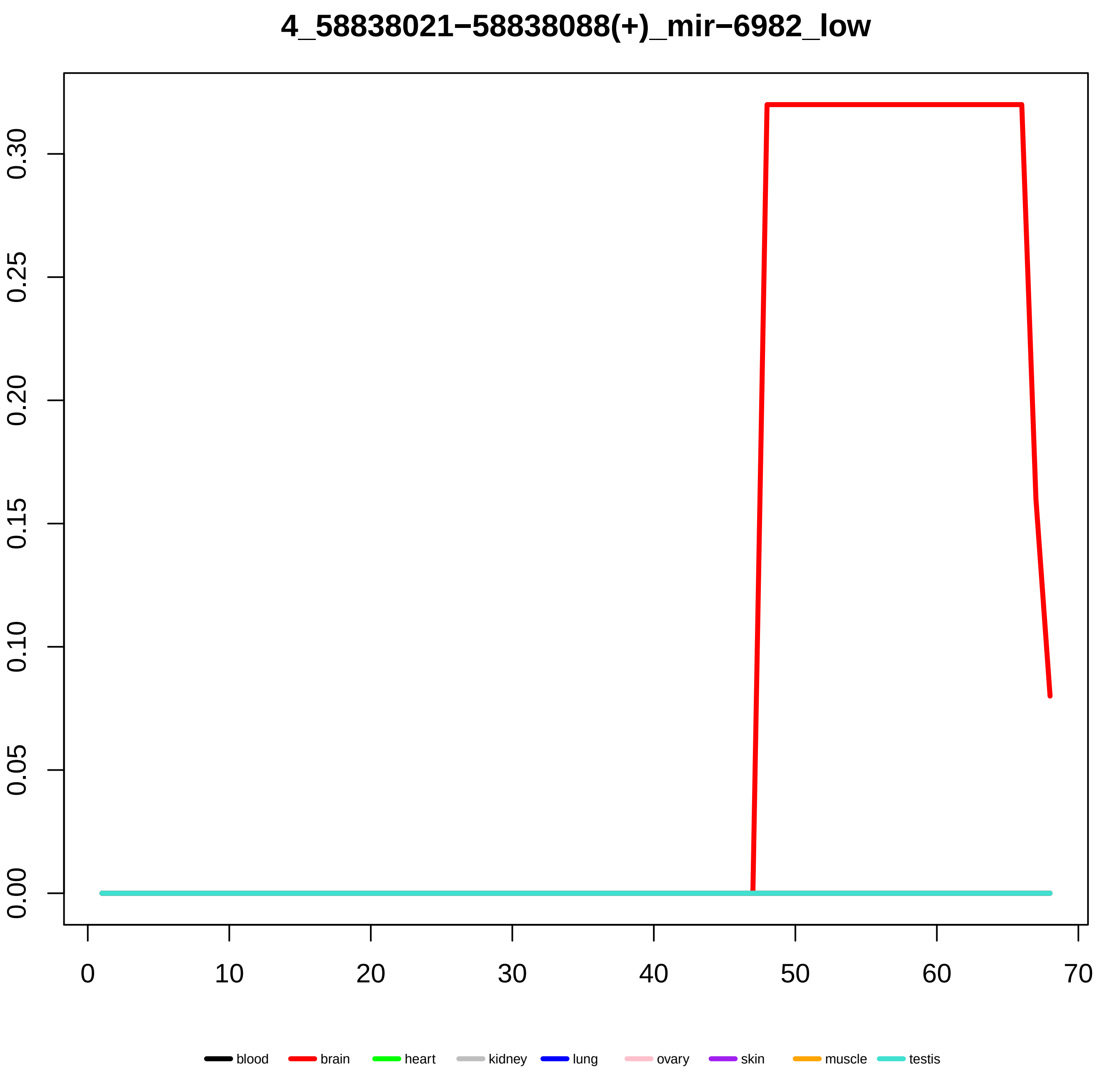

4_58838021−58838088(+)_mir−6982_low
0.00
0.05
0.10
0.15
0.20
0.25
0.30
0
10
20
30
40
50
60
70
blood
brain
heart
kidney
lung
ovary
skin
muscle
testis

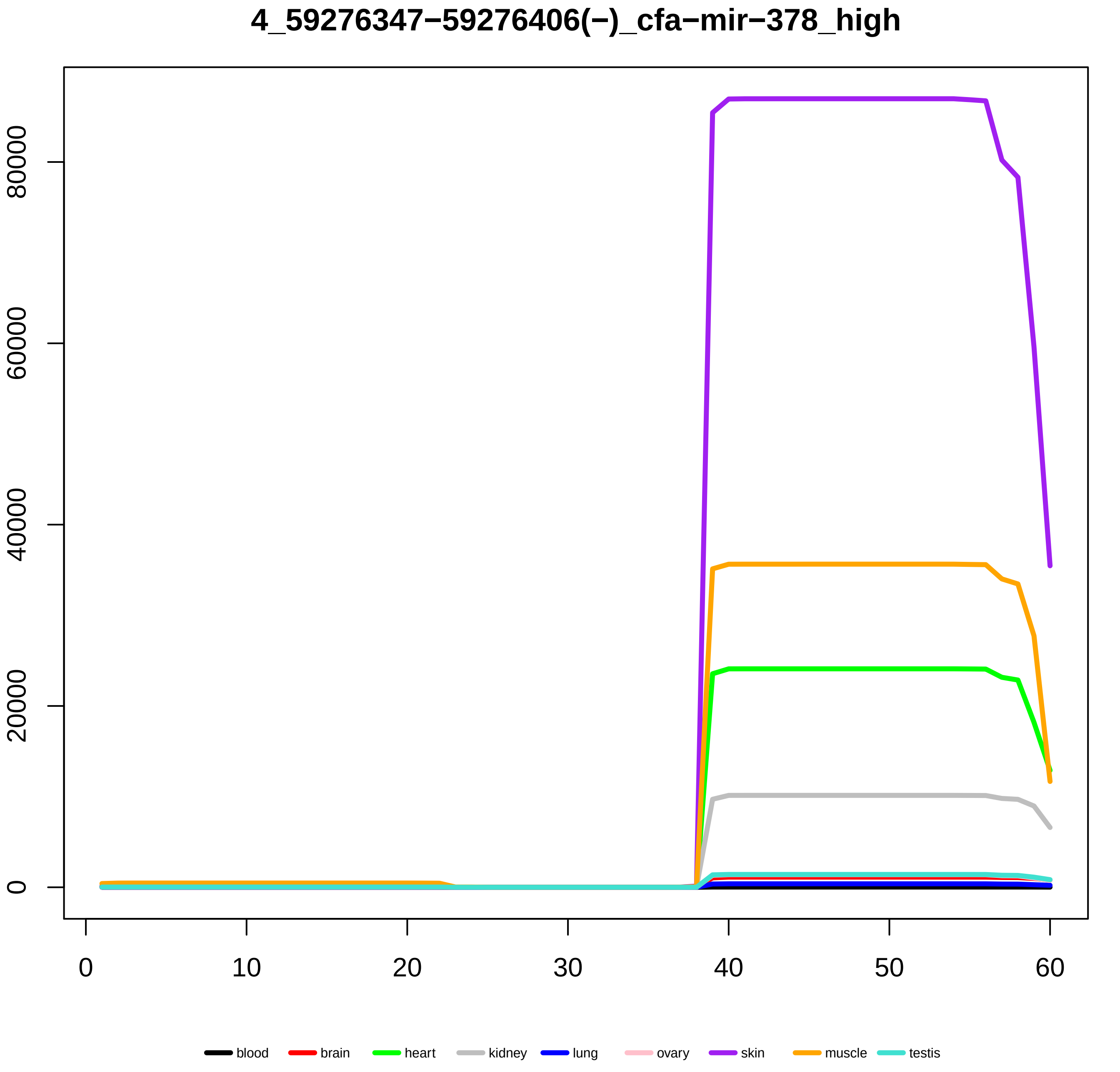

4_59276347−59276406(−)_cfa−mir−378_high
0
20000
40000
60000
80000
0
10
20
30
40
50
60
blood
brain
heart
kidney
lung
ovary
skin
muscle
testis

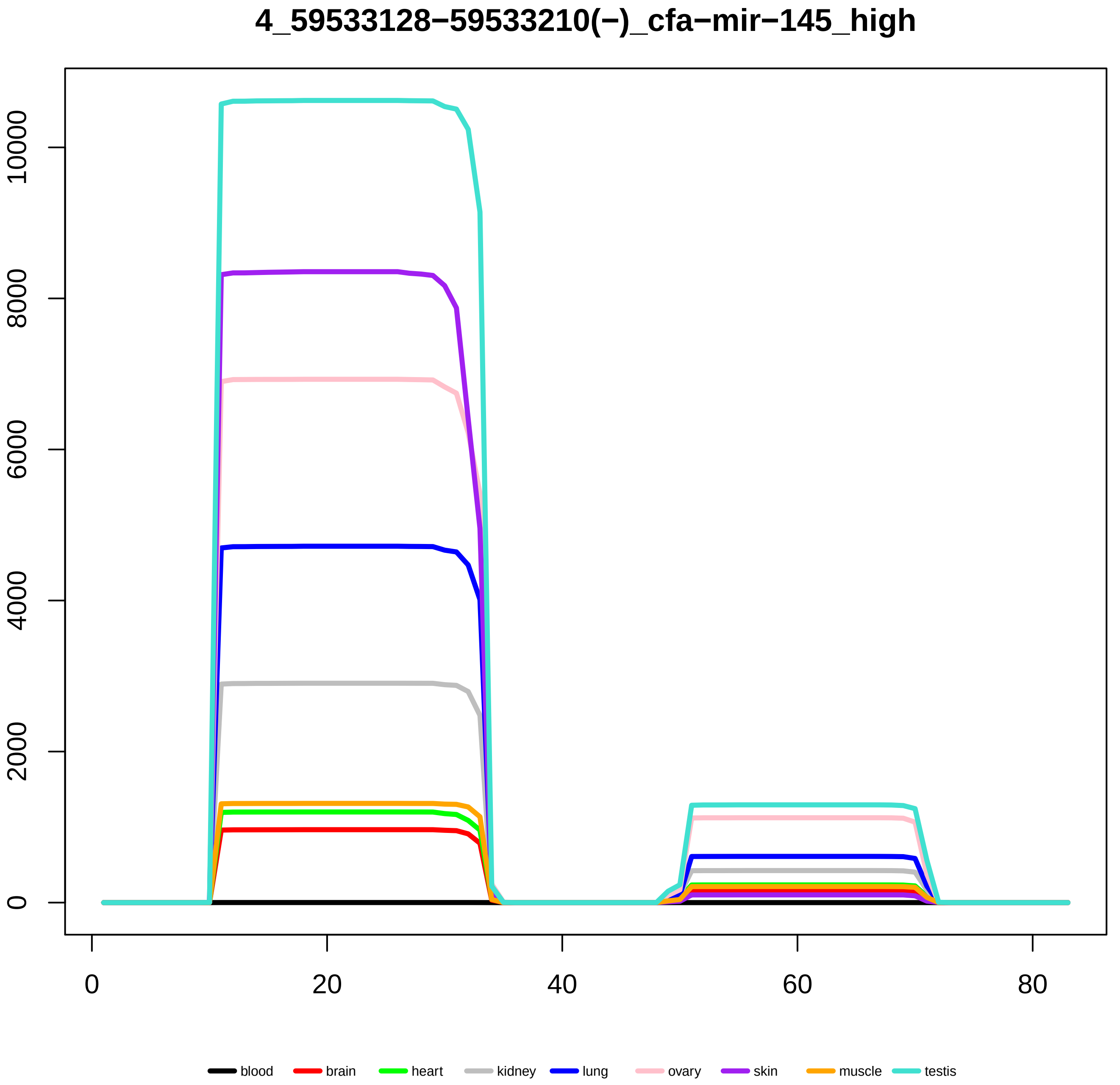

4_59533128−59533210(−)_cfa−mir−145_high
0
2000
4000
6000
8000
10000
0
20
40
60
80
blood
brain
heart
kidney
lung
ovary
skin
muscle
testis

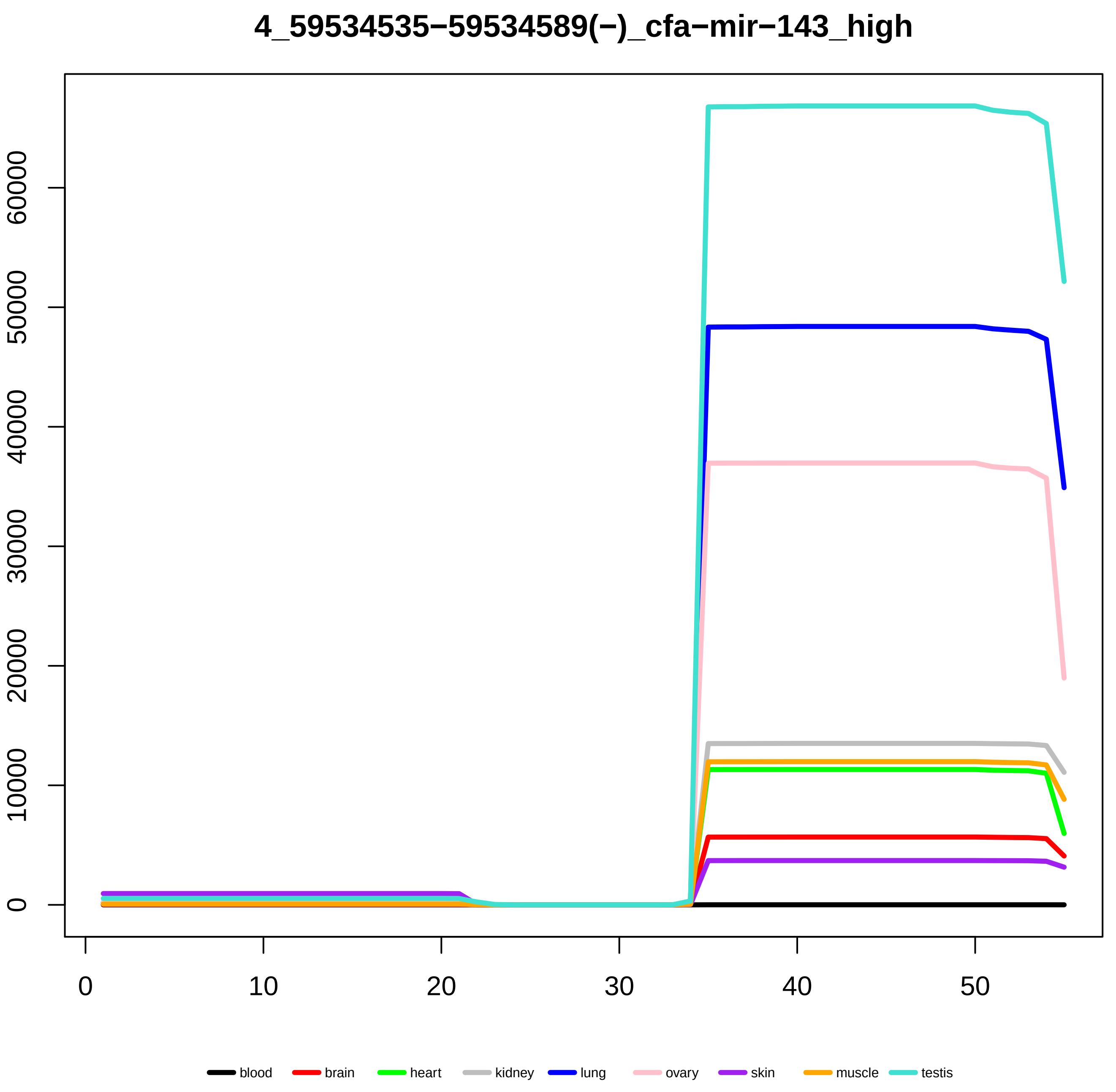
4_59534535−59534589(−)_cfa−mir−143_high
0
10000
20000
30000
40000
50000
60000
0
10
20
30
40
50
blood
brain
heart
kidney
lung
ovary
skin
muscle
testis

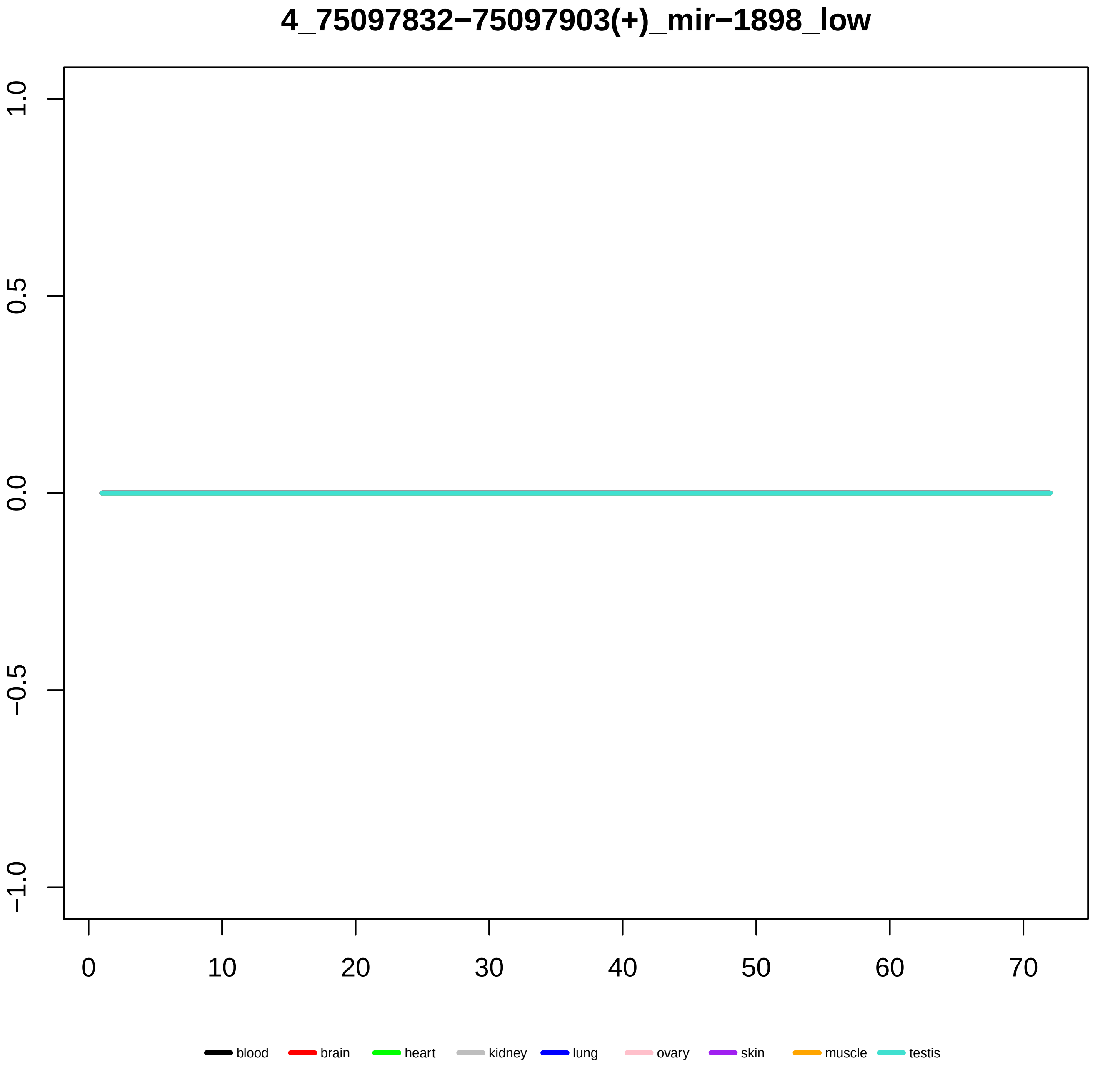

4_75097832−75097903(+)_mir−1898_low
1.0
0.5
0.0
−0.5
−1.0
0
10
20
30
40
50
60
70
blood
brain
heart
kidney
lung
ovary
skin
muscle
testis

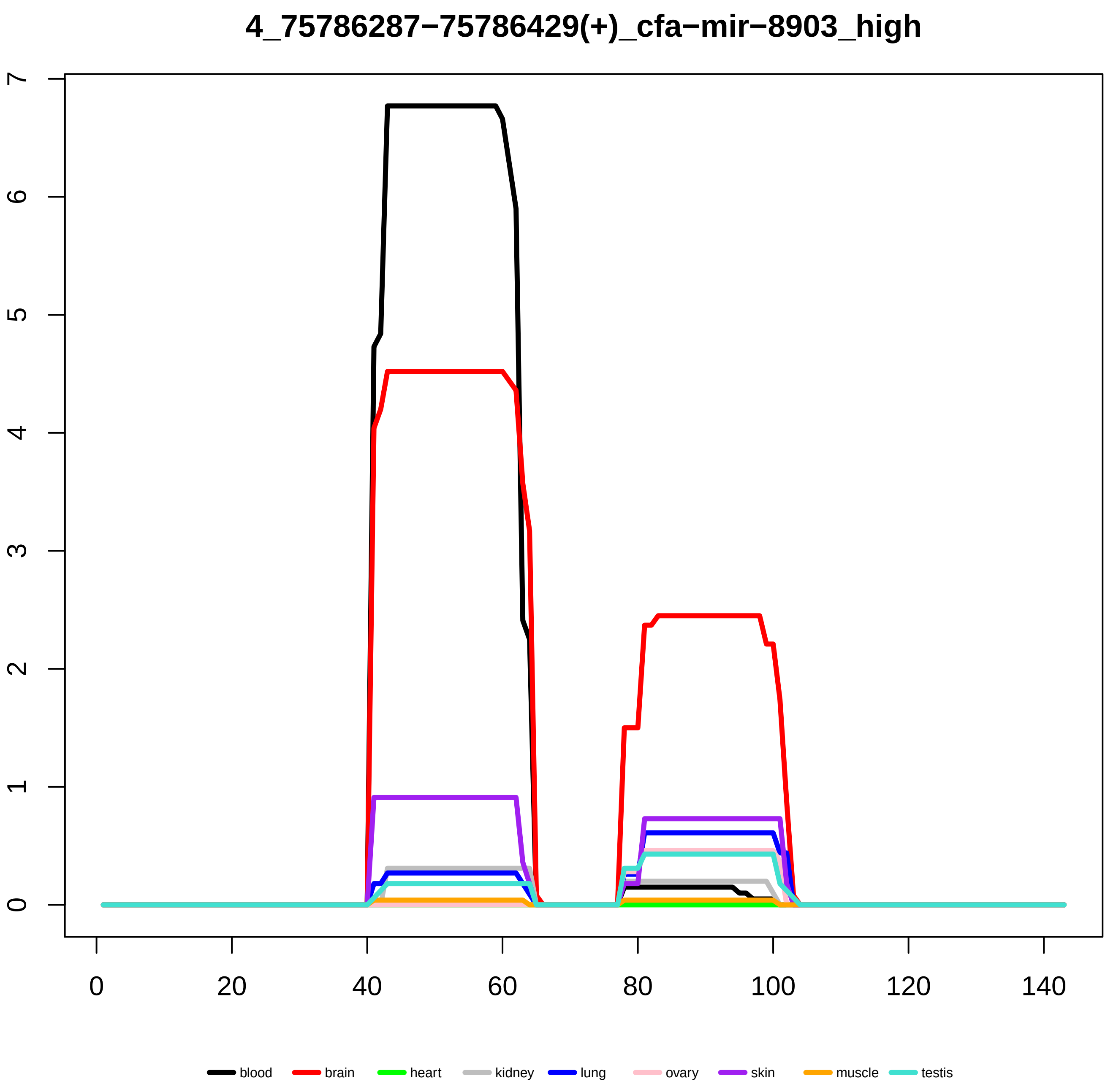

4_75786287−75786429(+)_cfa−mir−8903_high
0
1
2
3
4
5
6
7
0
20
40
60
80
100
120
140
blood
brain
heart
kidney
lung
ovary
skin
muscle
testis

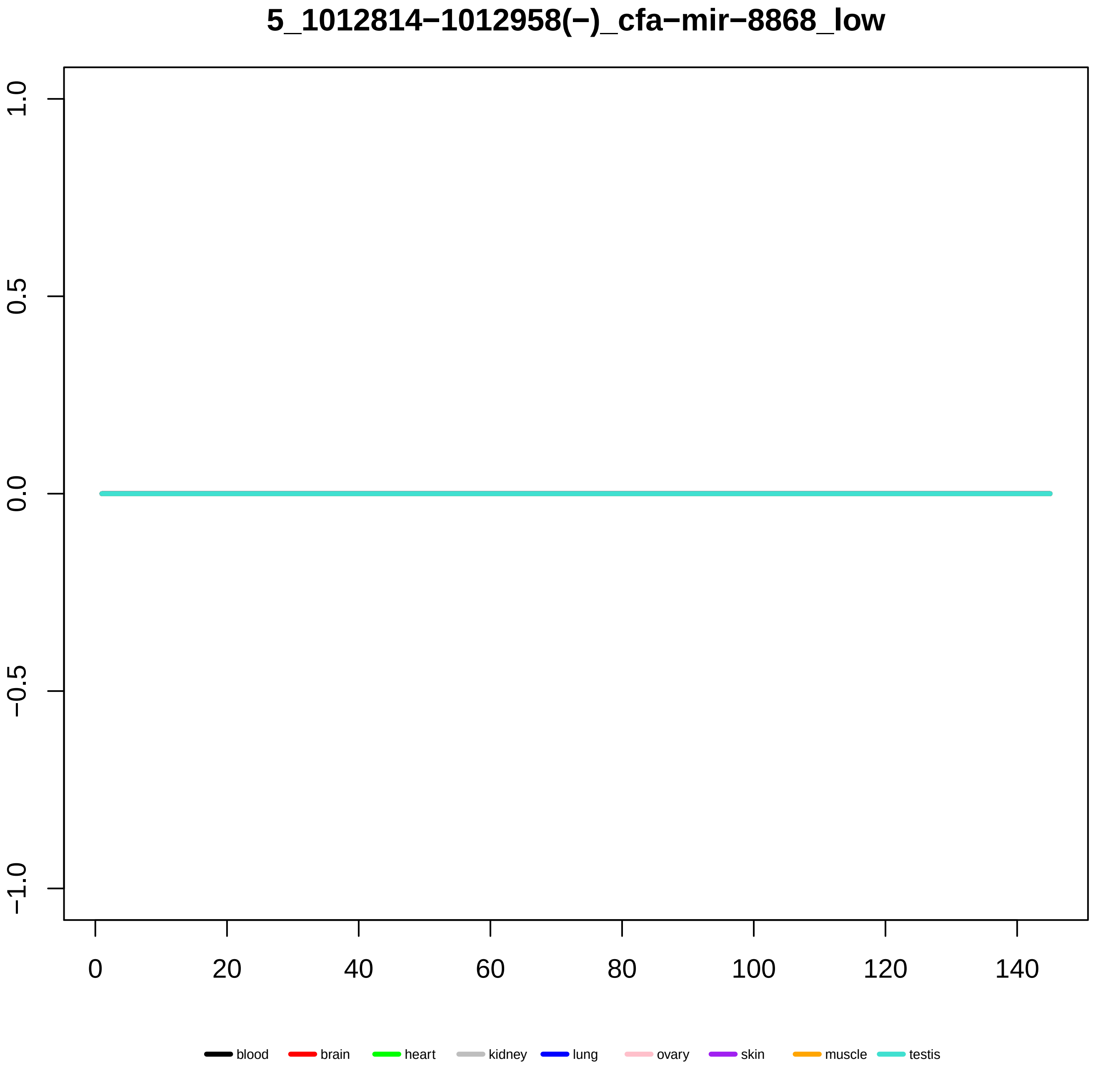

5_1012814−1012958(−)_cfa−mir−8868_low
1.0
0.5
0.0
−0.5
−1.0
0
20
40
60
80
100
120
140
blood
brain
heart
kidney
lung
ovary
skin
muscle
testis

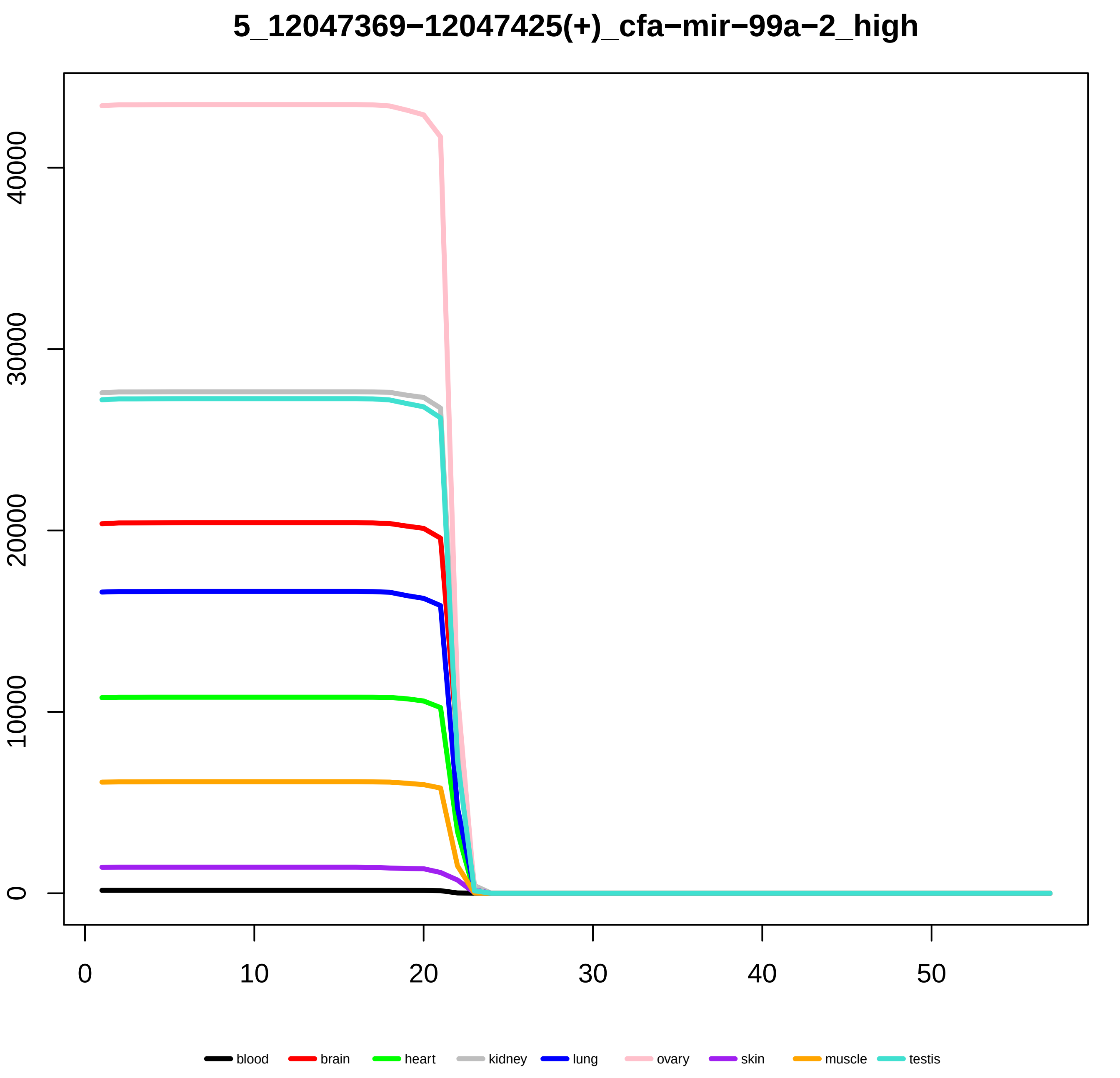

5_12047369−12047425(+)_cfa−mir−99a−2_high
0
10000
20000
30000
40000
0
10
20
30
40
50
blood
brain
heart
kidney
lung
ovary
skin
muscle
testis

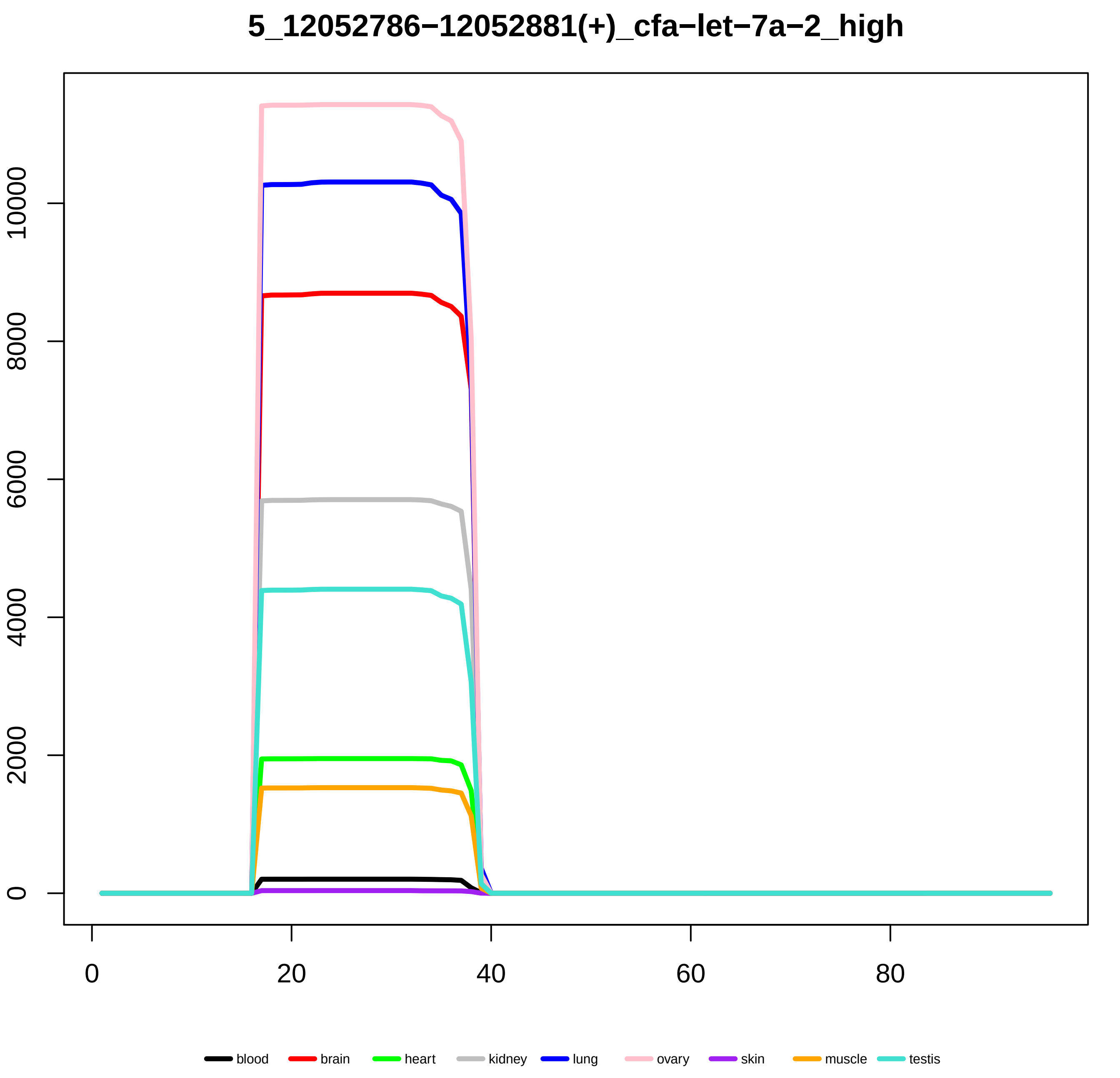

5_12052786−12052881(+)_cfa−let−7a−2_high
0
2000
4000
6000
8000
10000
0
20
40
60
80
blood
brain
heart
kidney
lung
ovary
skin
muscle
testis

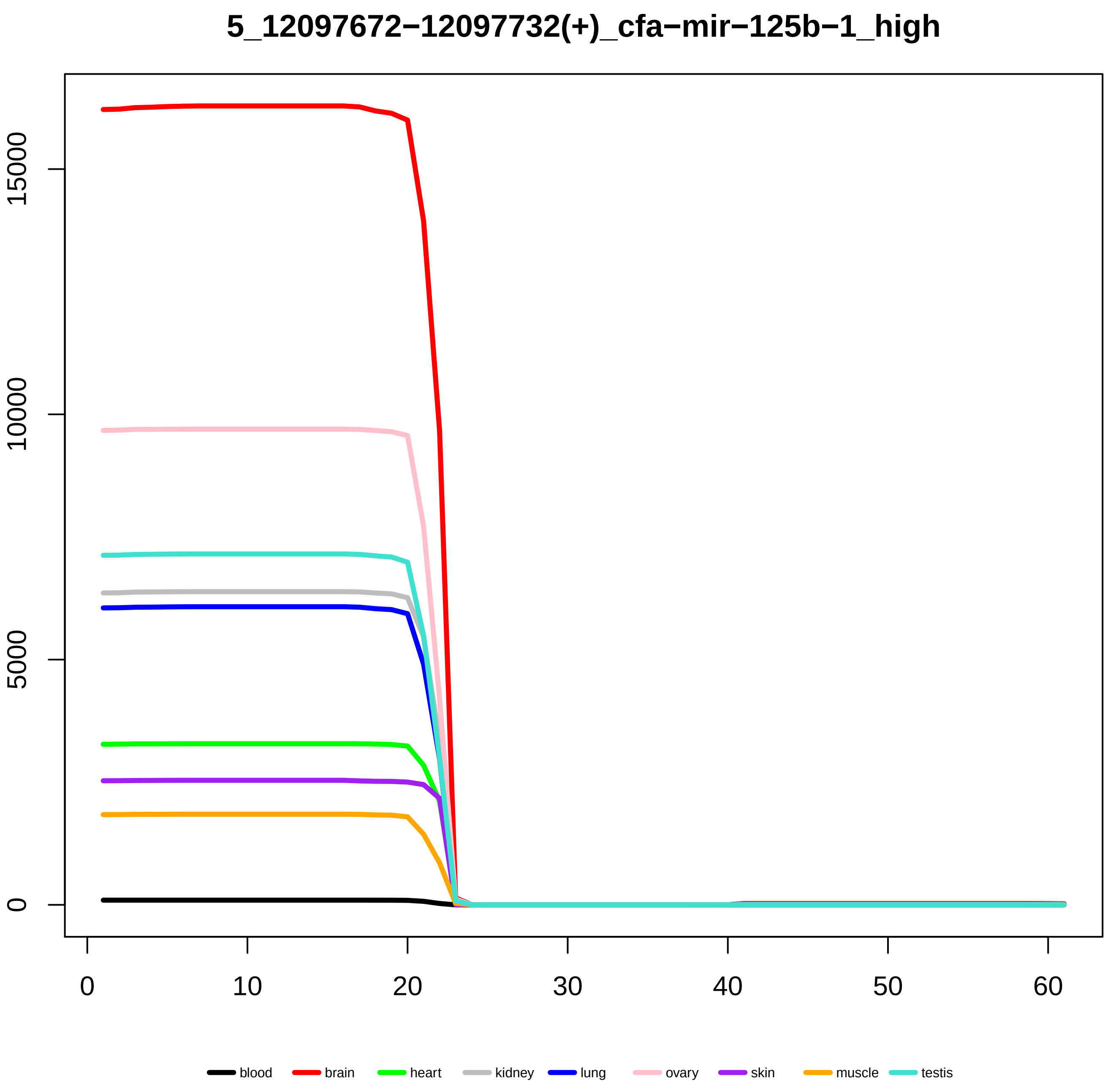

5_12097672−12097732(+)_cfa−mir−125b−1_high
15000
10000
5000
0
0
10
20
30
40
50
60
blood
brain
heart
kidney
lung
ovary
skin
muscle
testis

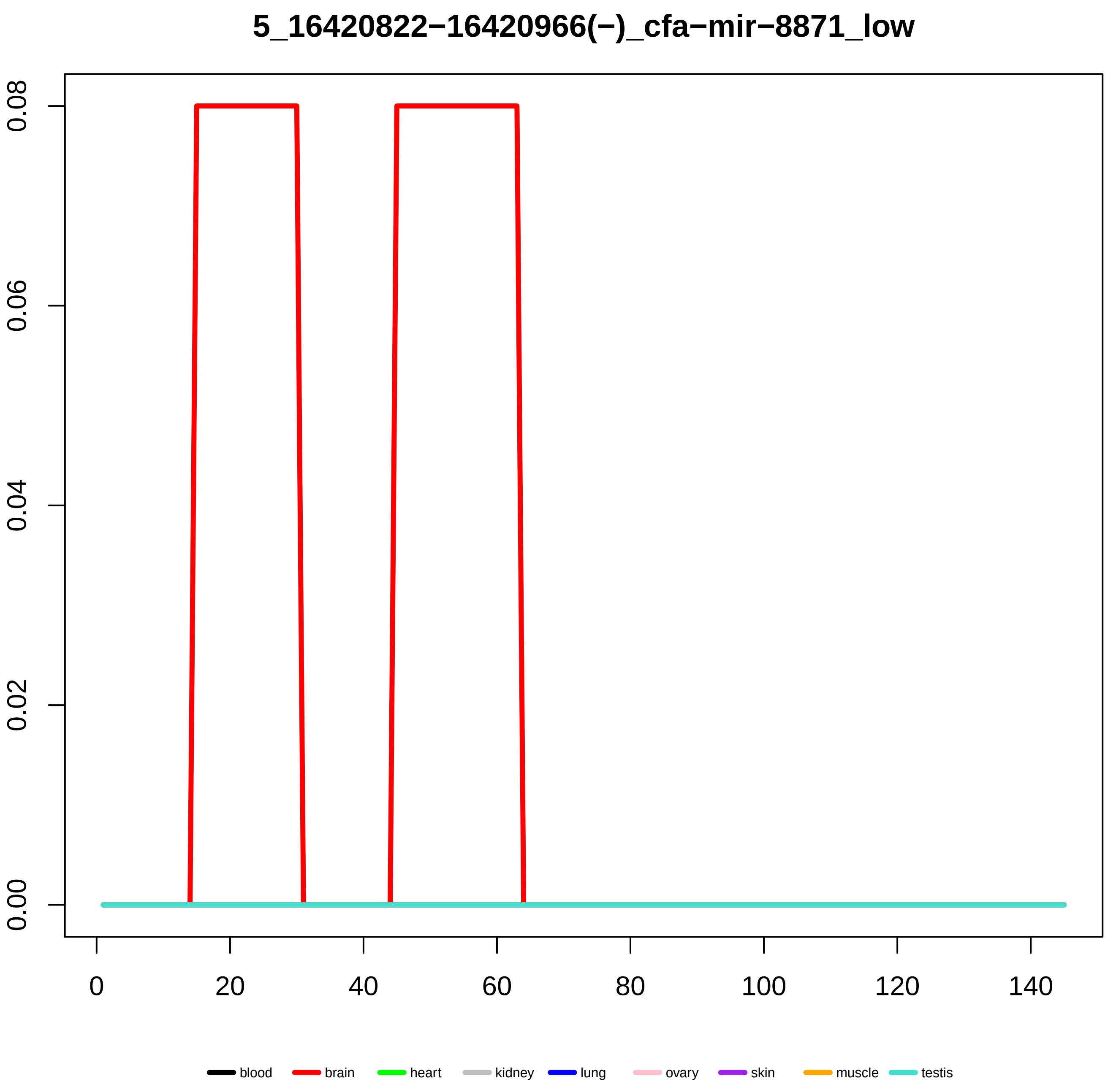

5_16420822−16420966(−)_cfa−mir−8871_low
0.00
0.02
0.04
0.06
0.08
0
20
40
60
80
100
120
140
blood
brain
heart
kidney
lung
ovary
skin
muscle
testis

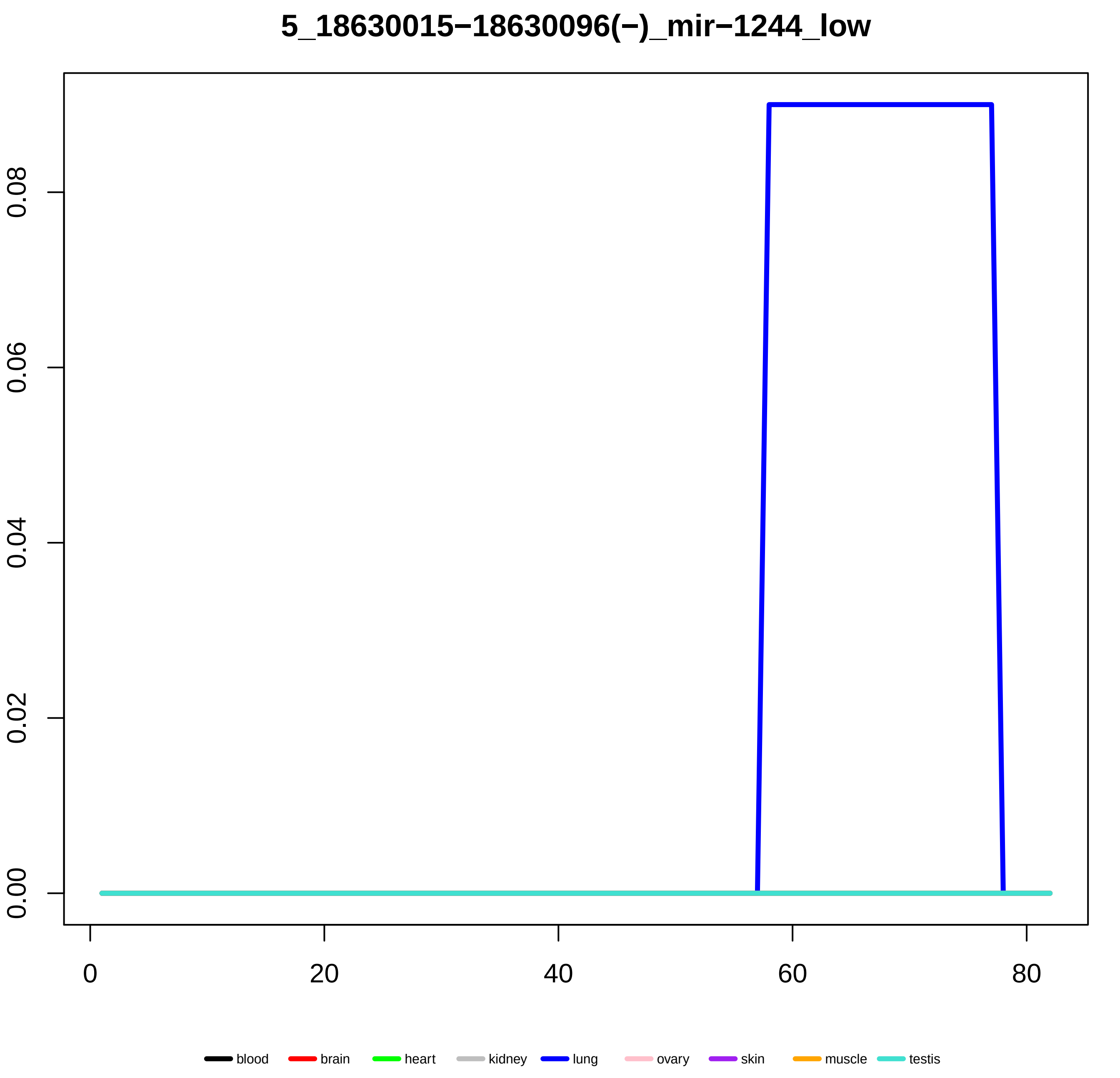

5_18630015−18630096(−)_mir−1244_low
0.00
0.02
0.04
0.06
0.08
0
20
40
60
80
blood
brain
heart
kidney
lung
ovary
skin
muscle
testis

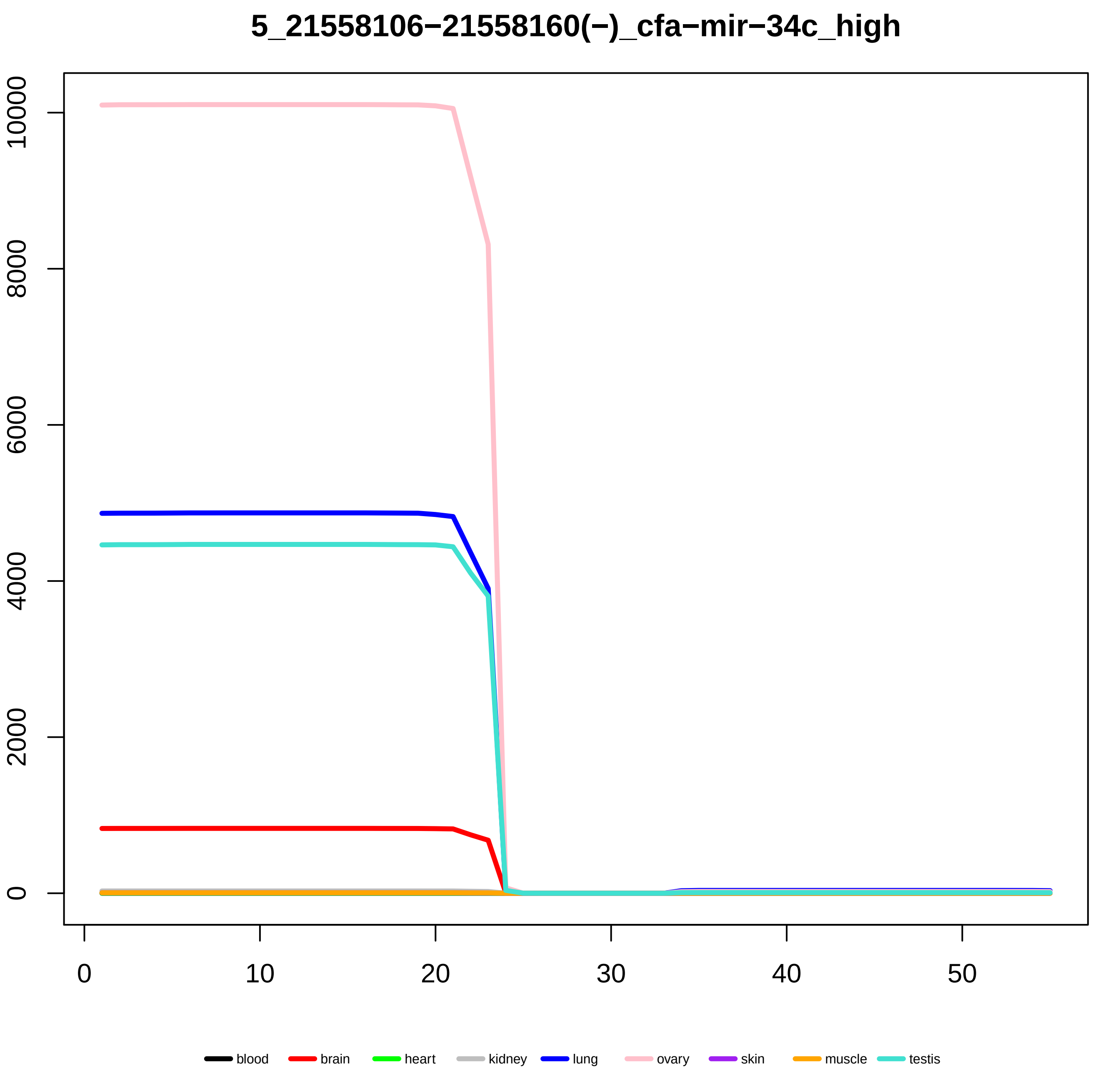

5_21558106−21558160(−)_cfa−mir−34c_high
0
2000
4000
6000
8000
10000
0
10
20
30
40
50
blood
brain
heart
kidney
lung
ovary
skin
muscle
testis

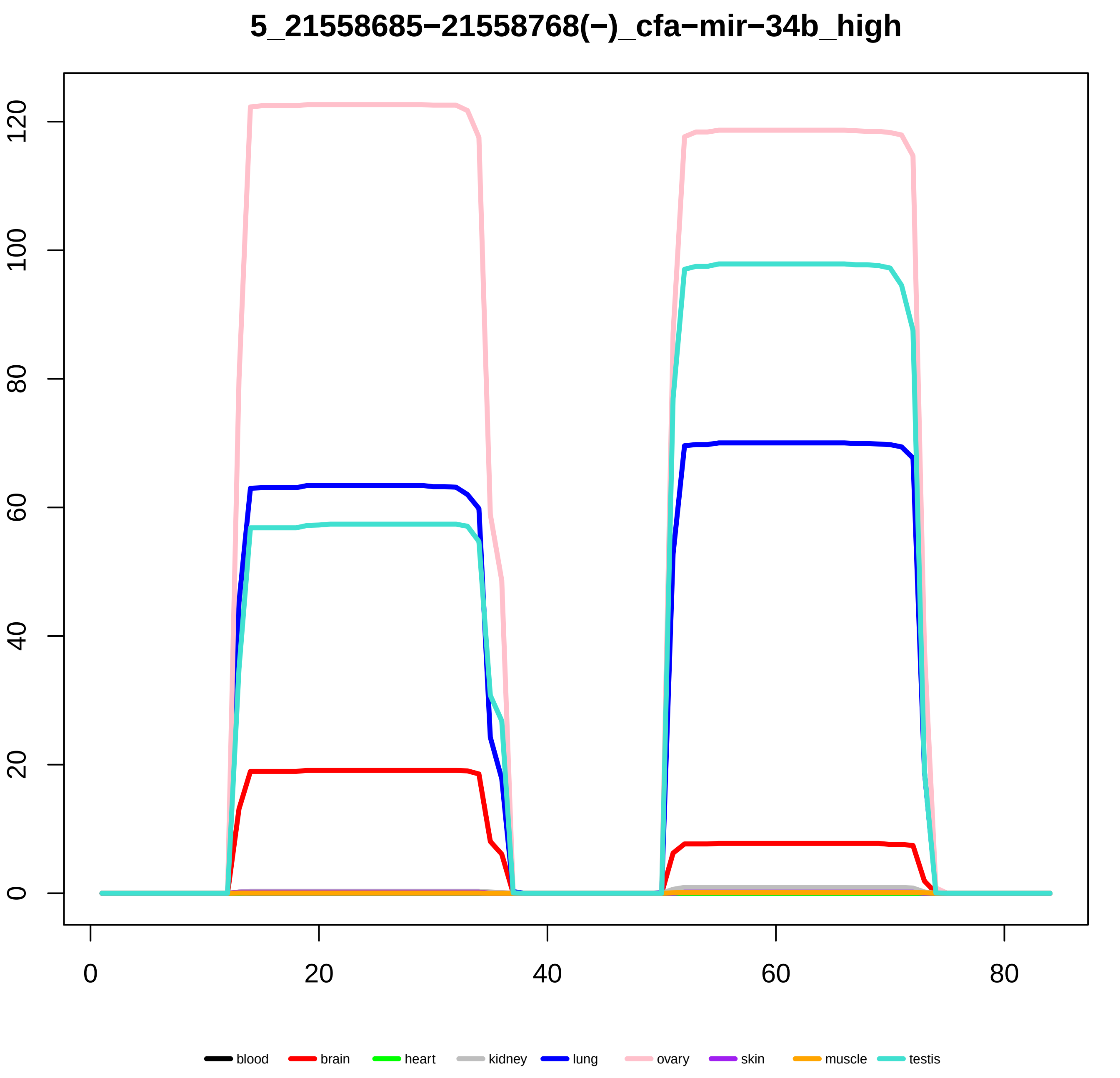

5_21558685−21558768(−)_cfa−mir−34b_high
0
20
40
60
80
100
120
0
20
40
60
80
blood
brain
heart
kidney
lung
ovary
skin
muscle
testis

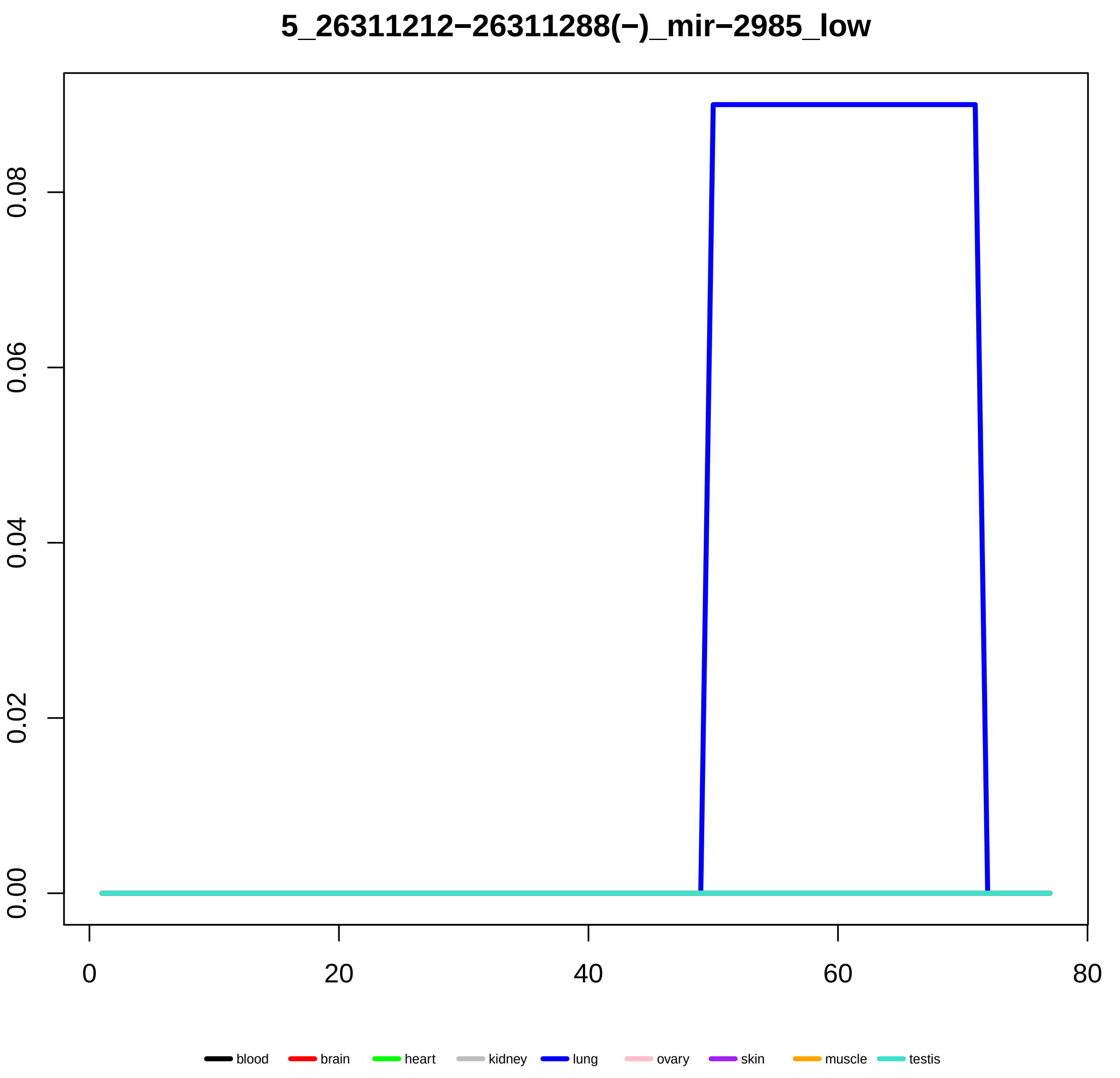

5_26311212−26311288(−)_mir−2985_low
0.00
0.02
0.04
0.06
0.08
0
20
40
60
80
blood
brain
heart
kidney
lung
ovary
skin
muscle
testis

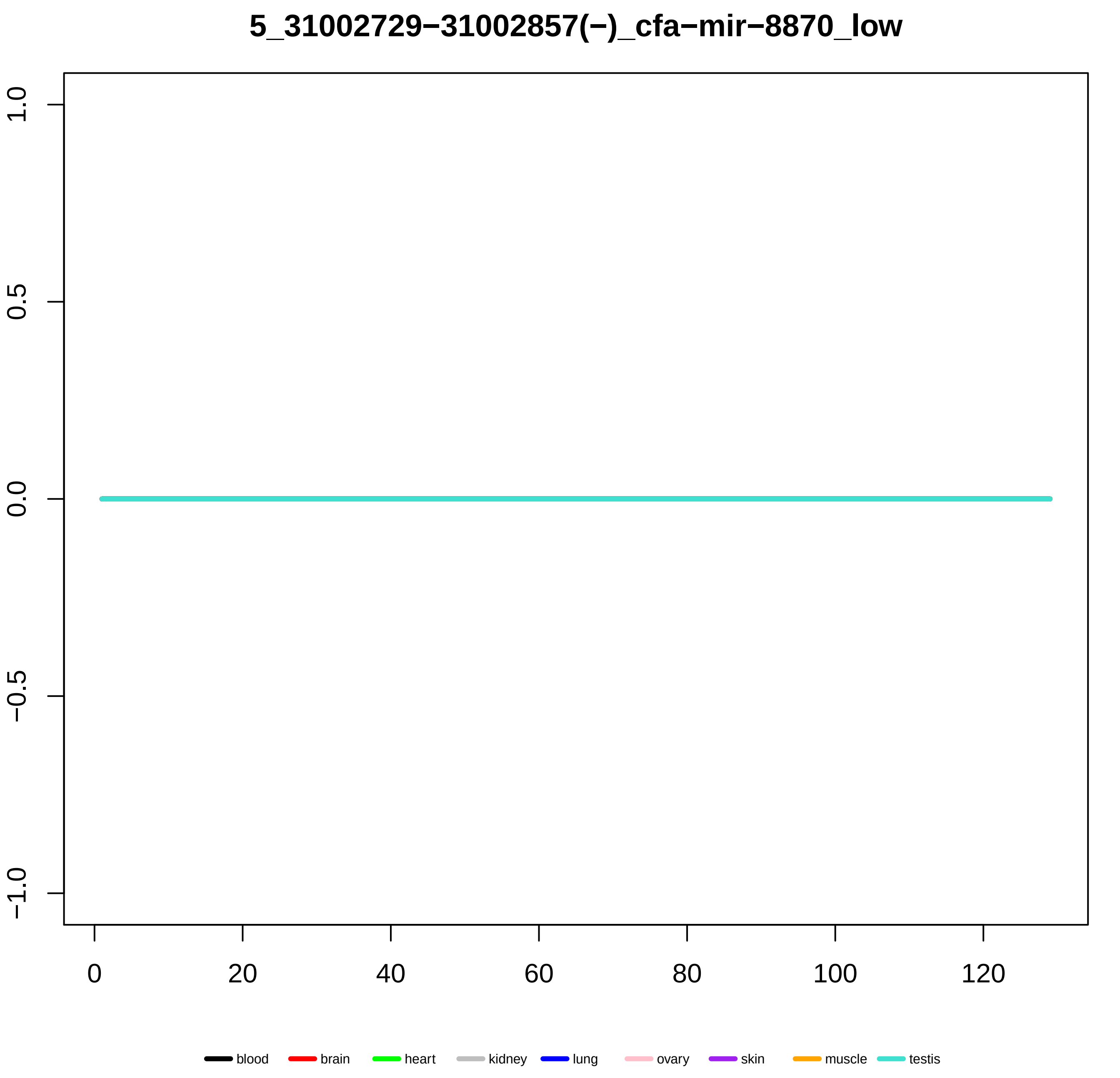
5_31002729−31002857(−)_cfa−mir−8870_low
1.0
0.5
0.0
−0.5
−1.0
0
20
40
60
80
100
120
blood
brain
heart
kidney
lung
ovary
skin
muscle
testis

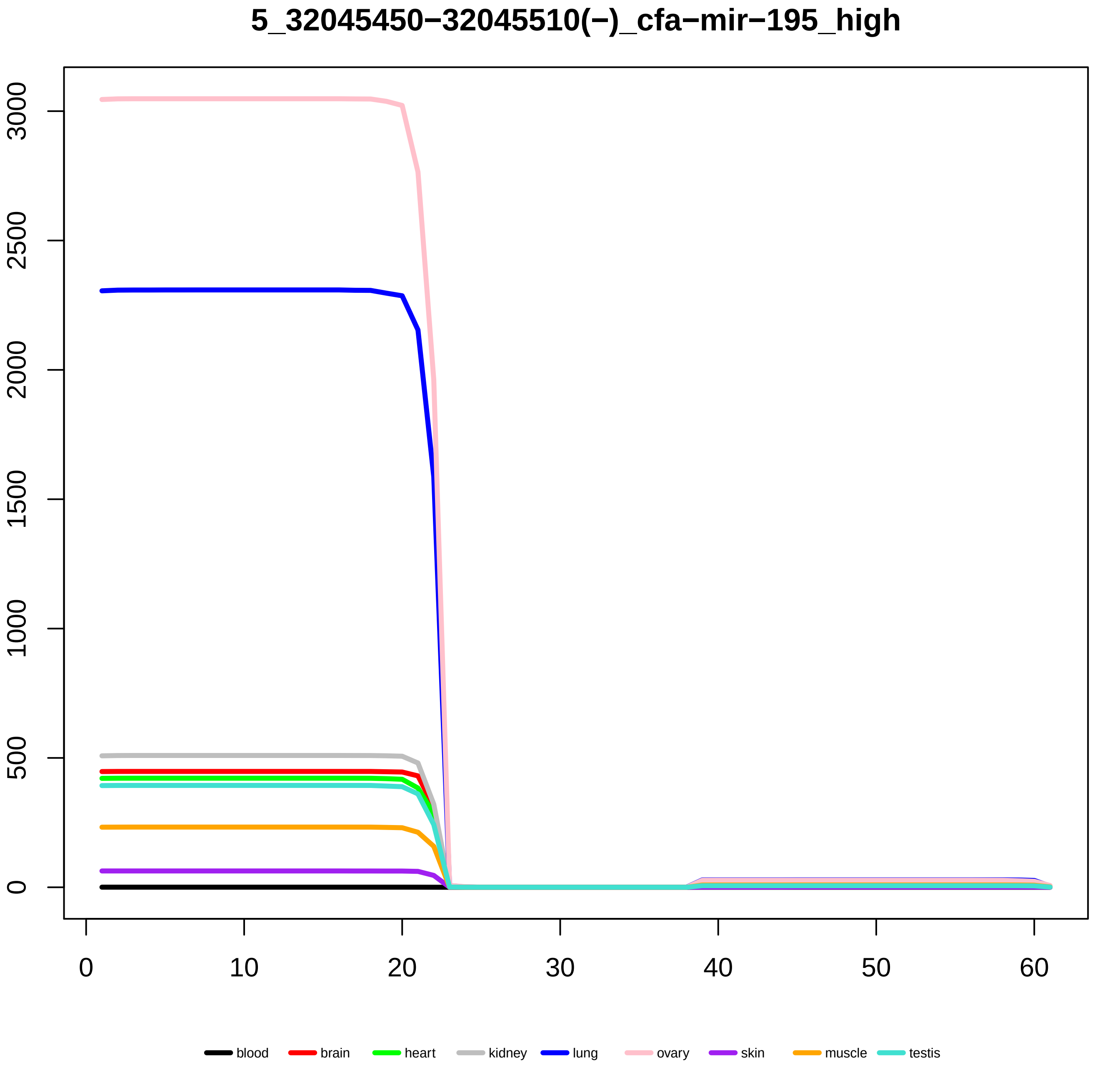

5_32045450−32045510(−)_cfa−mir−195_high
0
500
1000
1500
2000
2500
3000
0
10
20
30
40
50
60
blood
brain
heart
kidney
lung
ovary
skin
muscle
testis

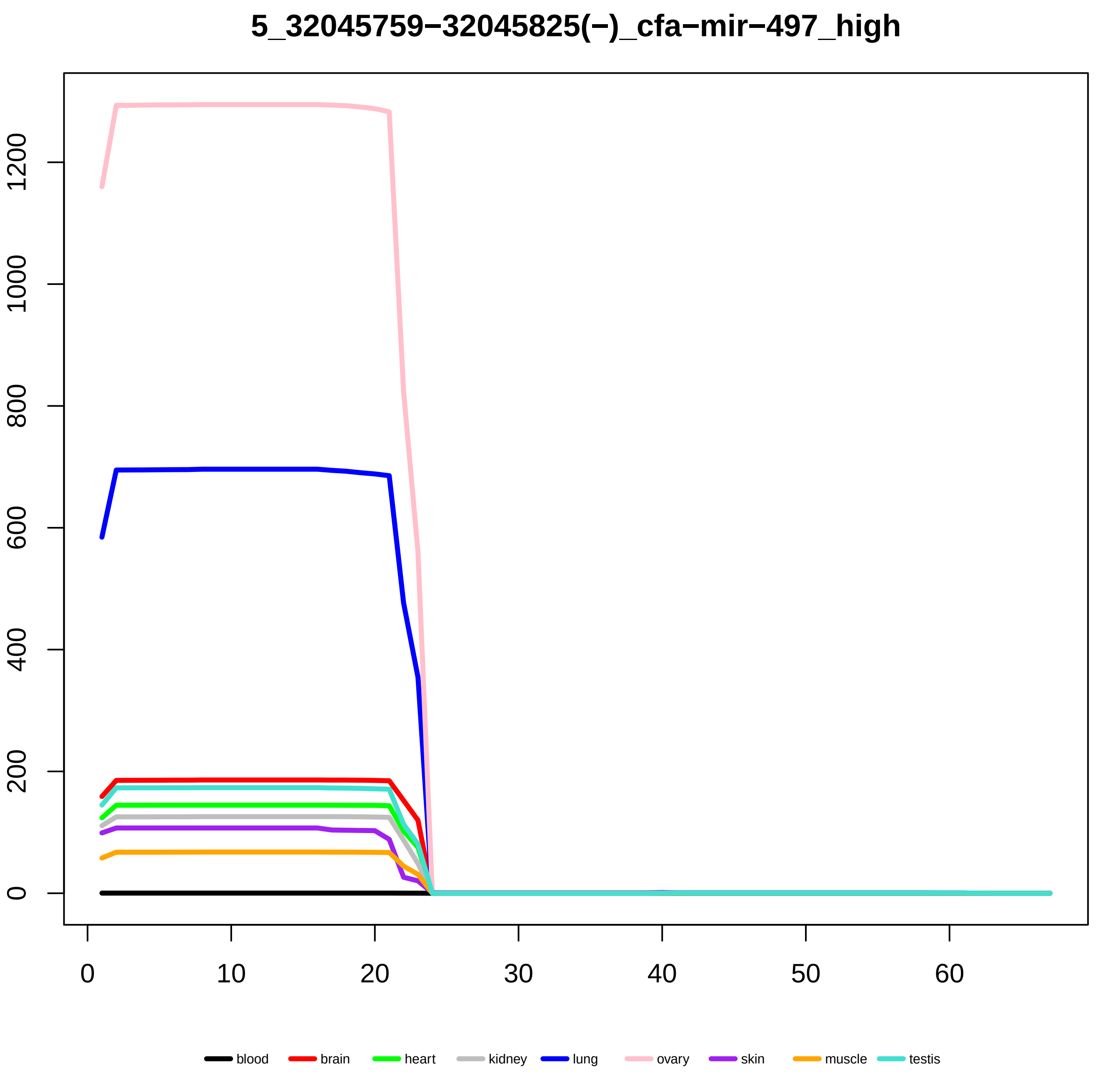

5_32045759−32045825(−)_cfa−mir−497_high
0
200
400
600
800
1000
1200
0
10
20
30
40
50
60
blood
brain
heart
kidney
lung
ovary
skin
muscle
testis

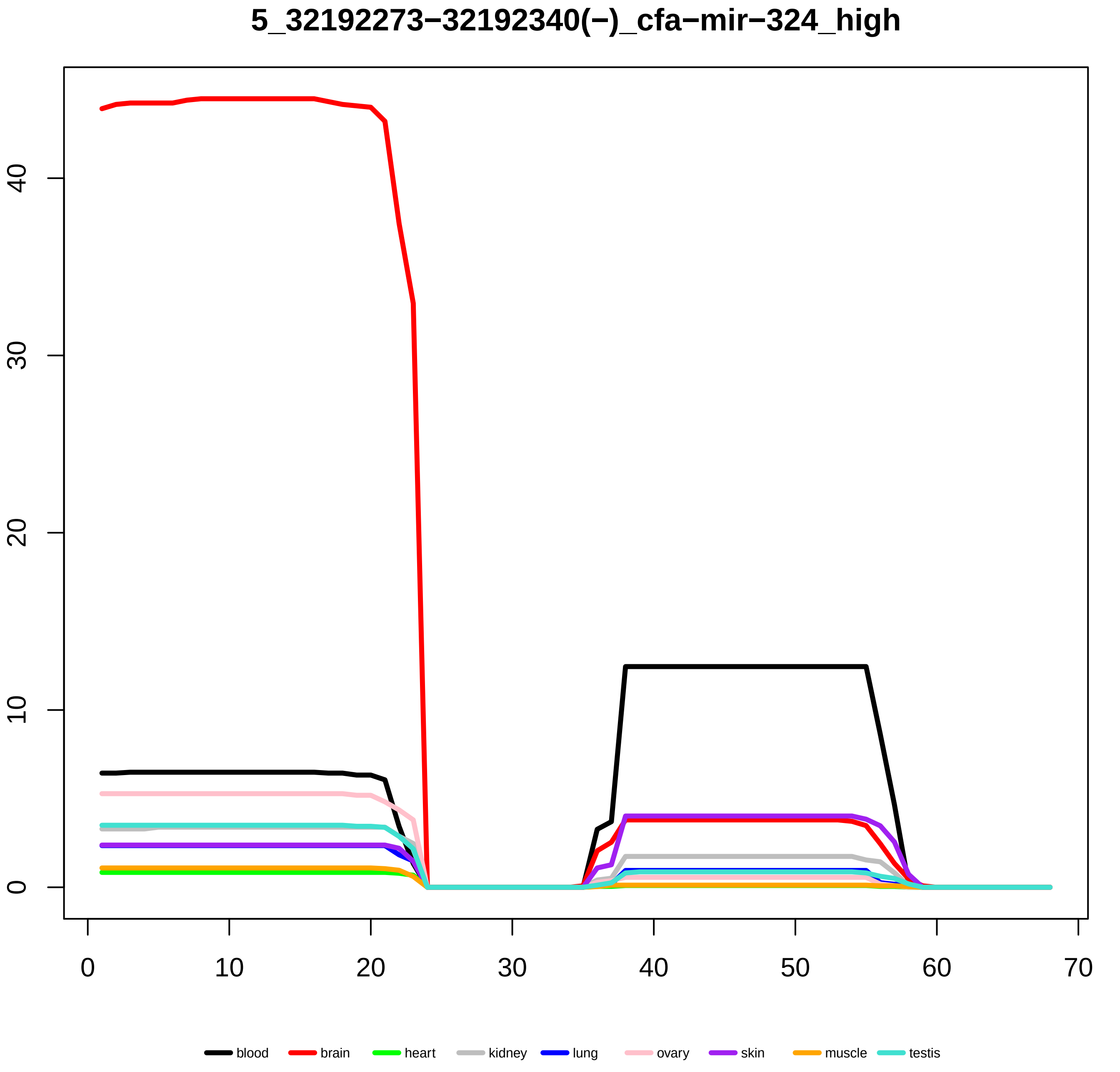

5_32192273−32192340(−)_cfa−mir−324_high
0
10
20
30
40
0
10
20
30
40
50
60
70
blood
brain
heart
kidney
lung
ovary
skin
muscle
testis

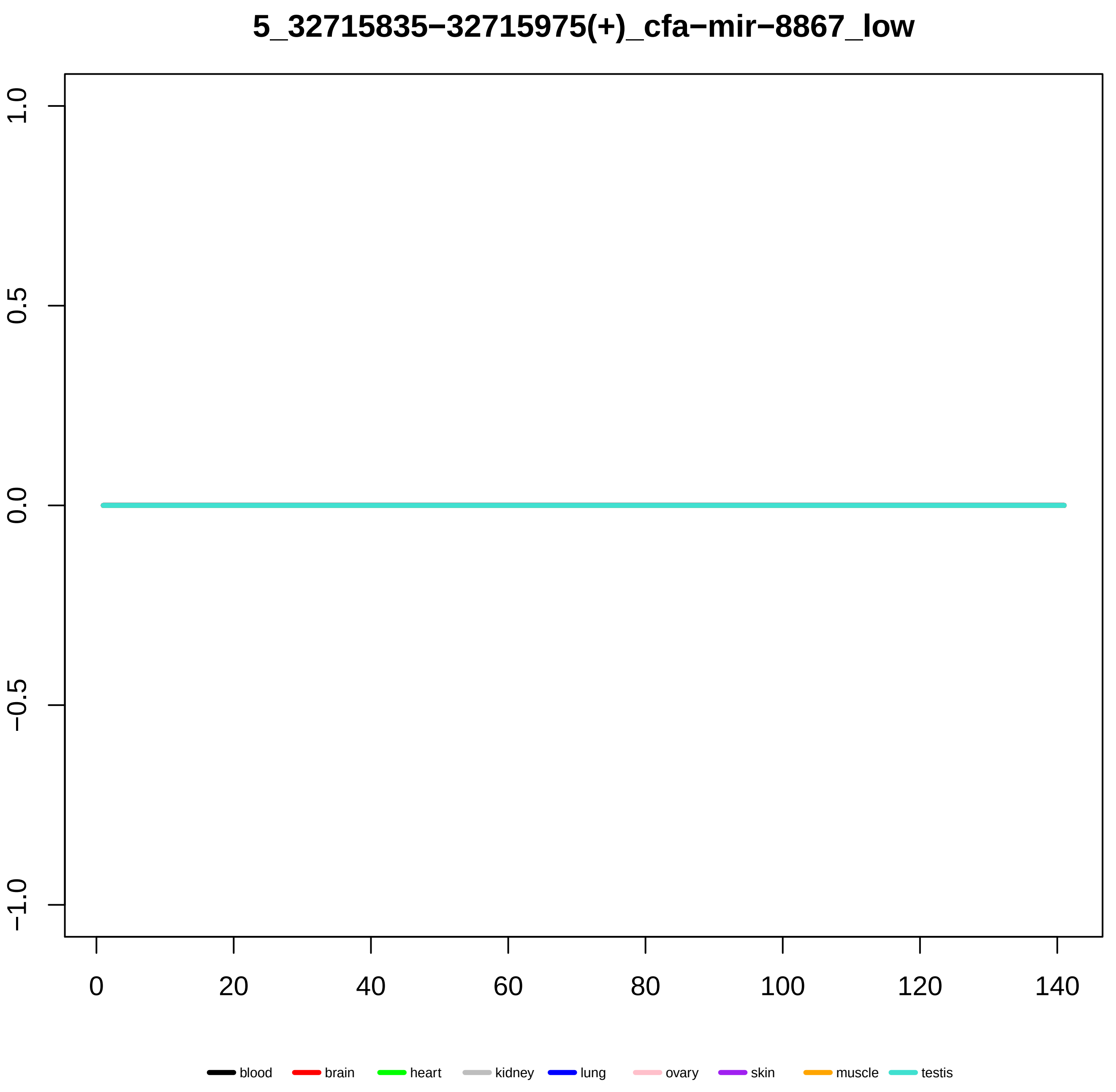

5_32715835−32715975(+)_cfa−mir−8867_low
1.0
0.5
0.0
−0.5
−1.0
0
20
40
60
80
100
120
140
blood
brain
heart
kidney
lung
ovary
skin
muscle
testis

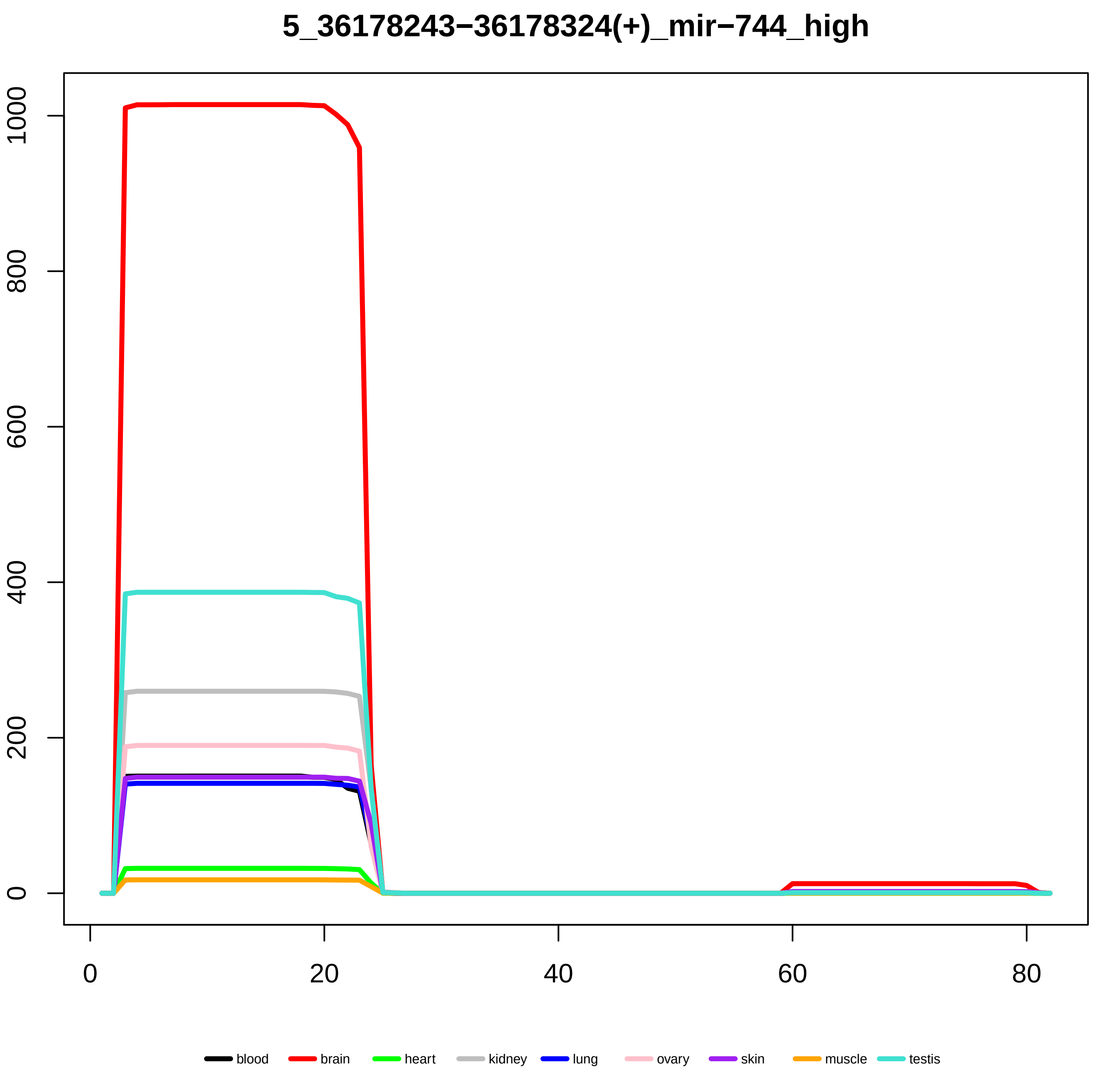

5_36178243−36178324(+)_mir−744_high
0
200
400
600
800
1000
0
20
40
60
80
blood
brain
heart
kidney
lung
ovary
skin
muscle
testis

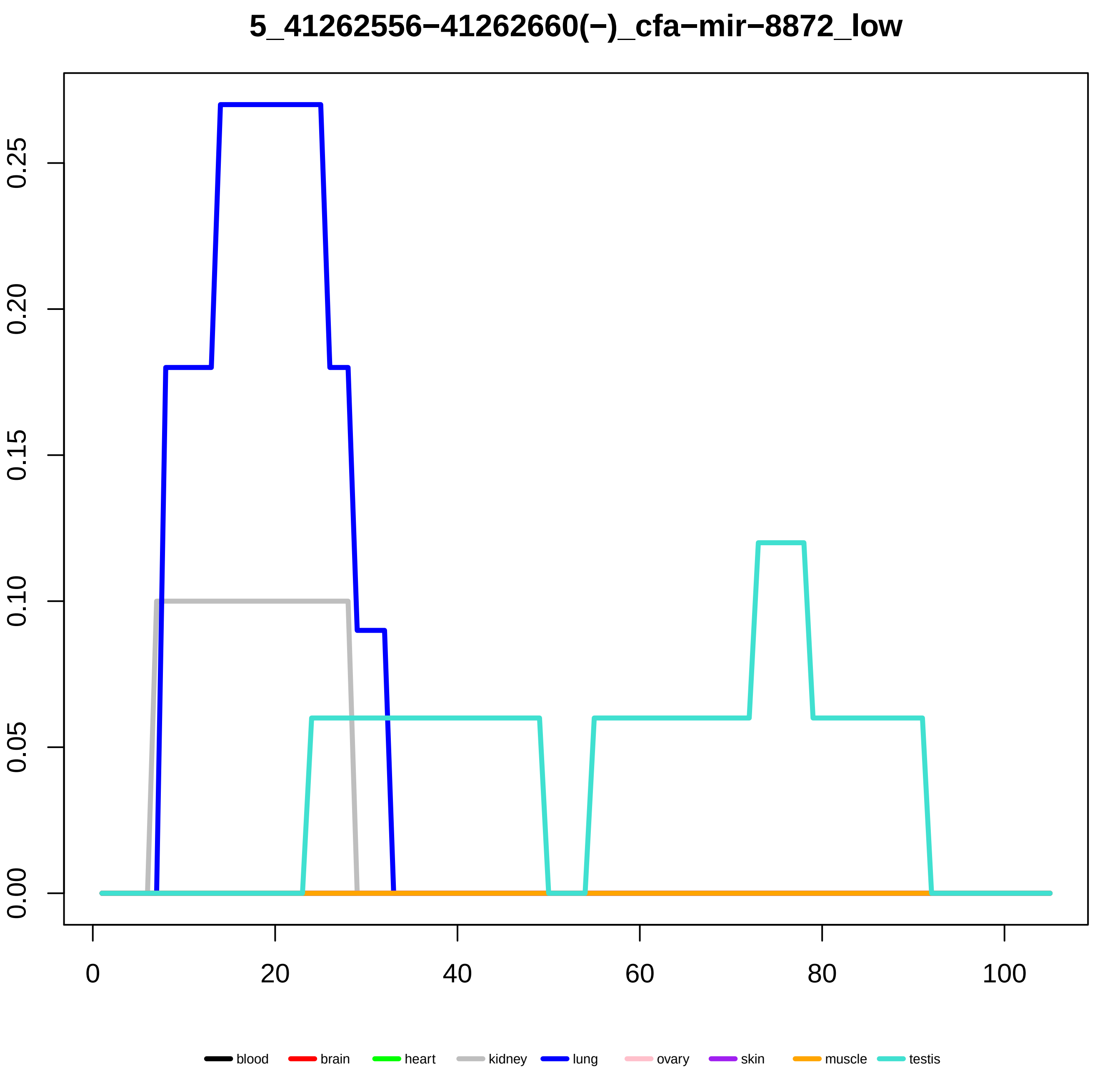

5_41262556−41262660(−)_cfa−mir−8872_low
0.00
0.05
0.10
0.15
0.20
0.25
0
20
40
60
80
100
blood
brain
heart
kidney
lung
ovary
skin
muscle
testis

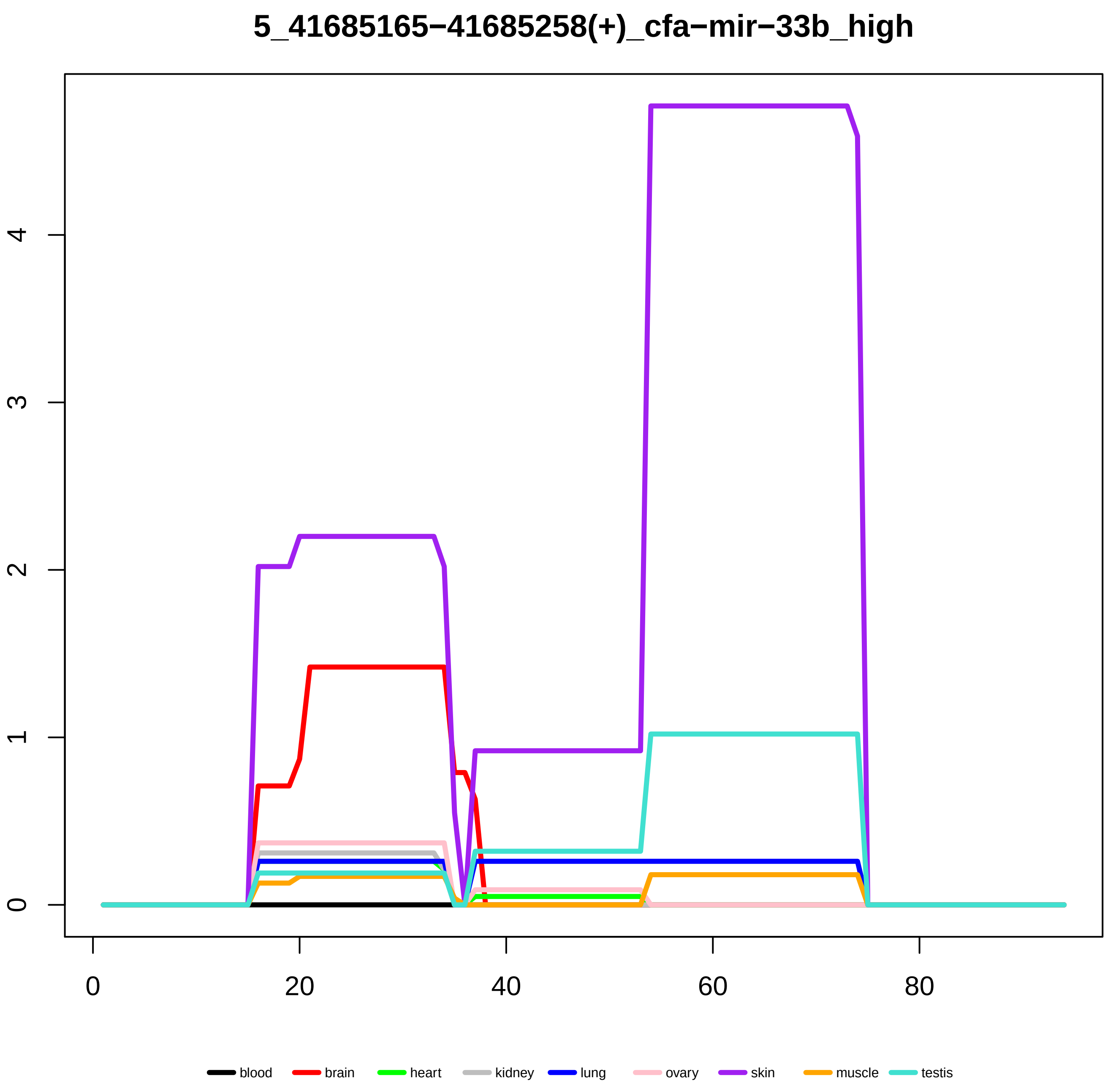
5_41685165−41685258(+)_cfa−mir−33b_high
0
1
2
3
4
0
20
40
60
80
blood
brain
heart
kidney
lung
ovary
skin
muscle
testis

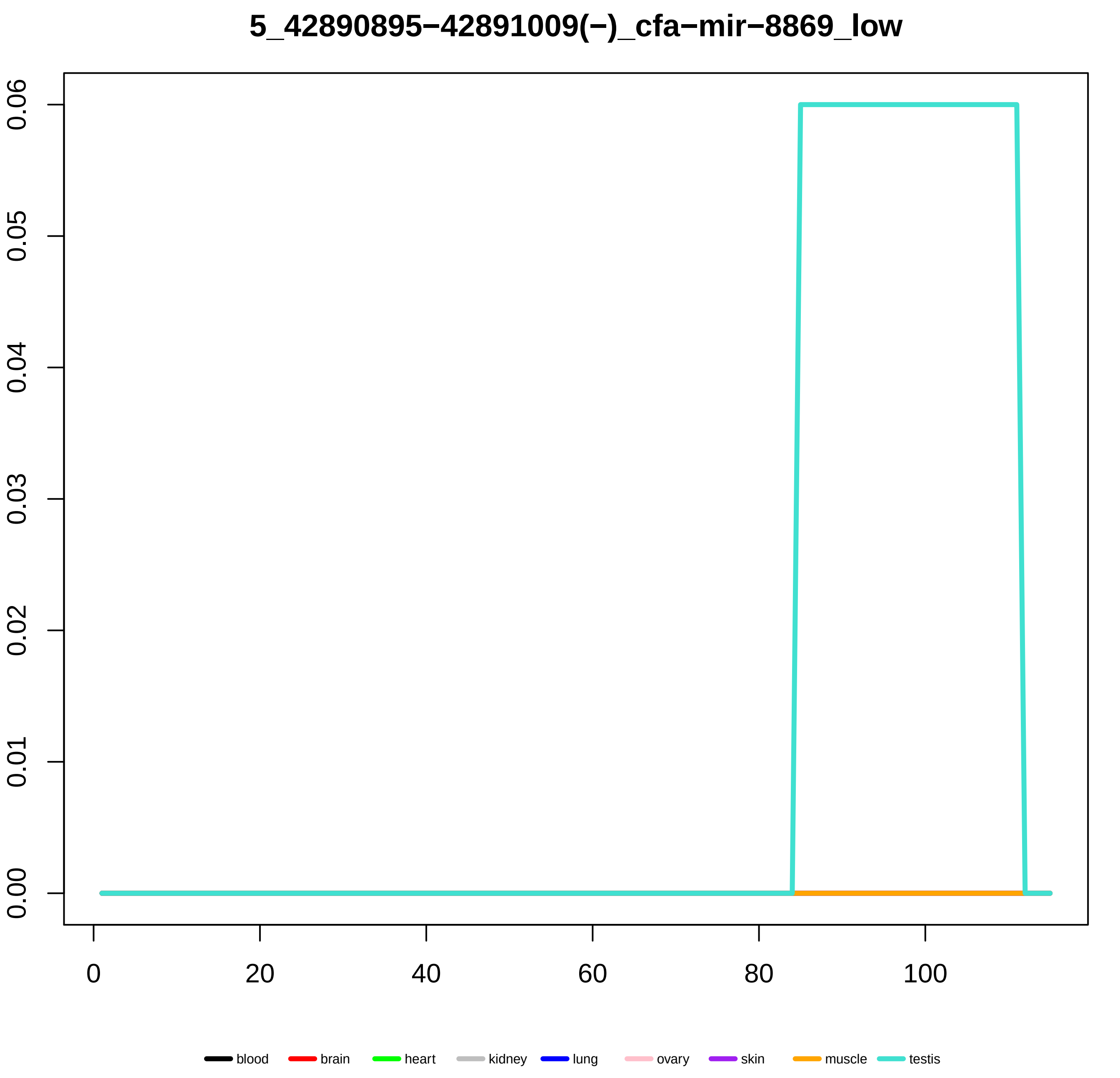

5_42890895−42891009(−)_cfa−mir−8869_low
0.00
0.01
0.02
0.03
0.04
0.05
0.06
0
20
40
60
80
100
blood
brain
heart
kidney
lung
ovary
skin
muscle
testis

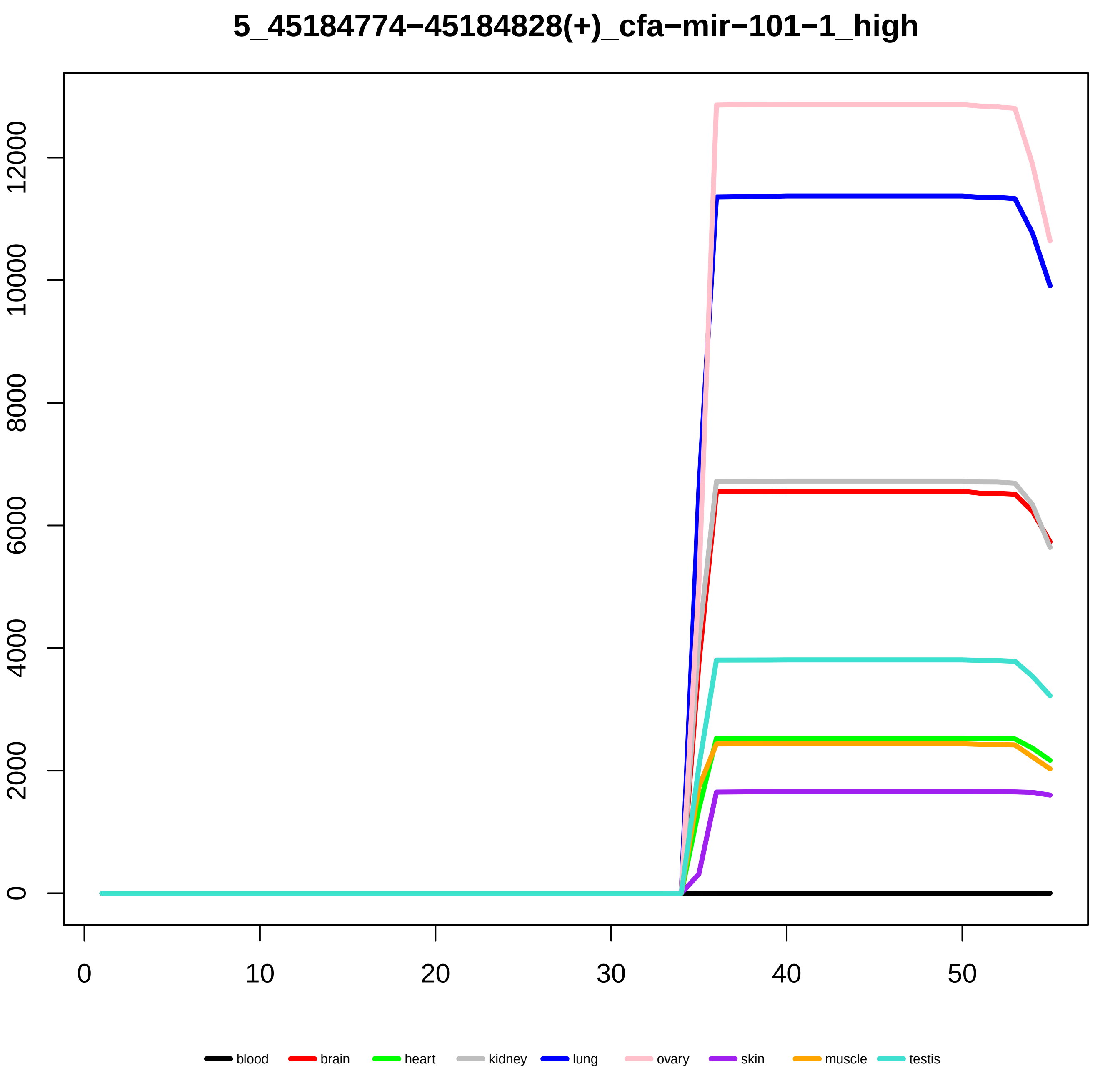

5_45184774−45184828(+)_cfa−mir−101−1_high
0
2000
4000
6000
8000
10000
12000
0
10
20
30
40
50
blood
brain
heart
kidney
lung
ovary
skin
muscle
testis

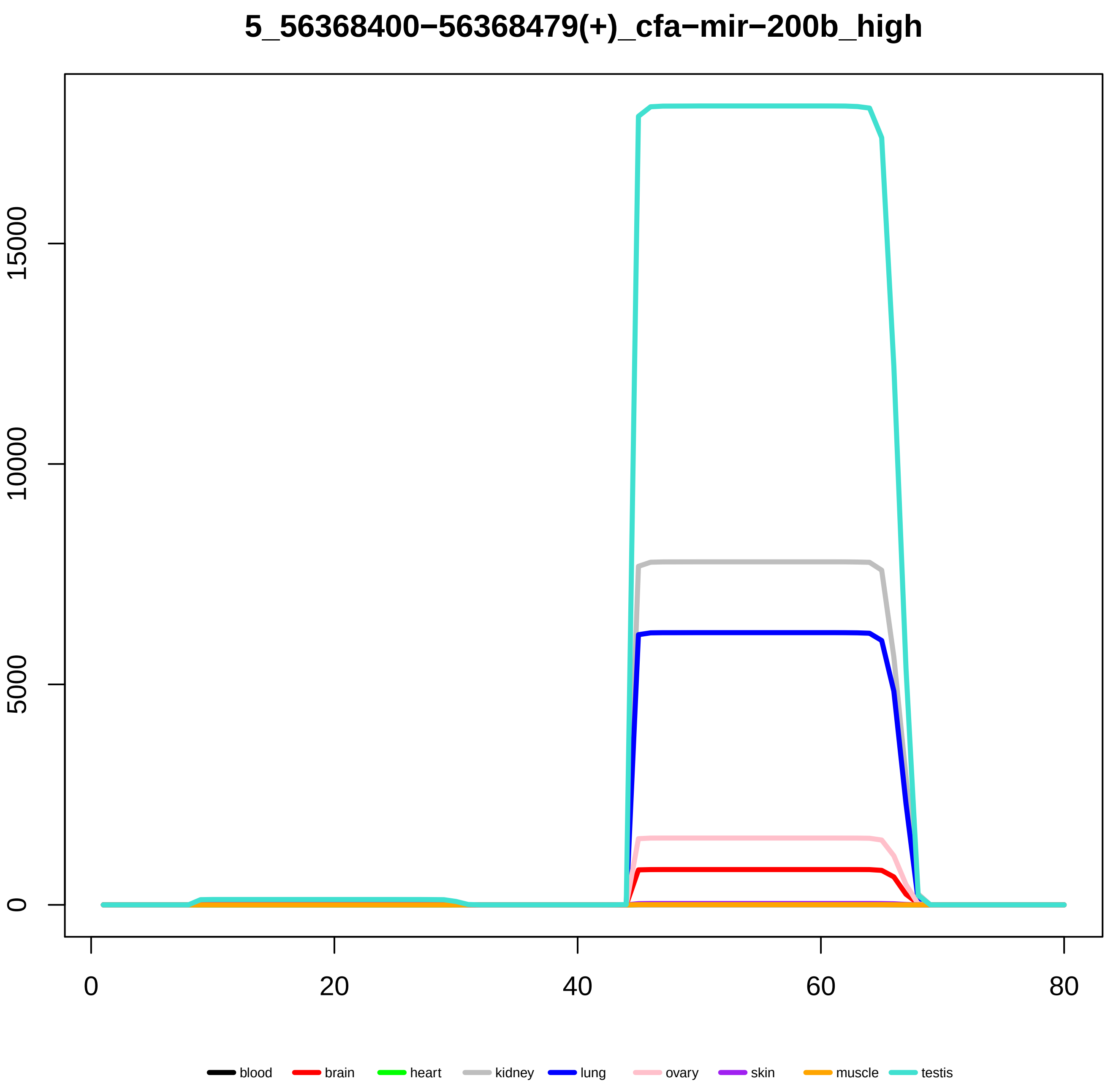

5_56368400−56368479(+)_cfa−mir−200b_high
0
5000
10000
15000
0
20
40
60
80
blood
brain
heart
kidney
lung
ovary
skin
muscle
testis

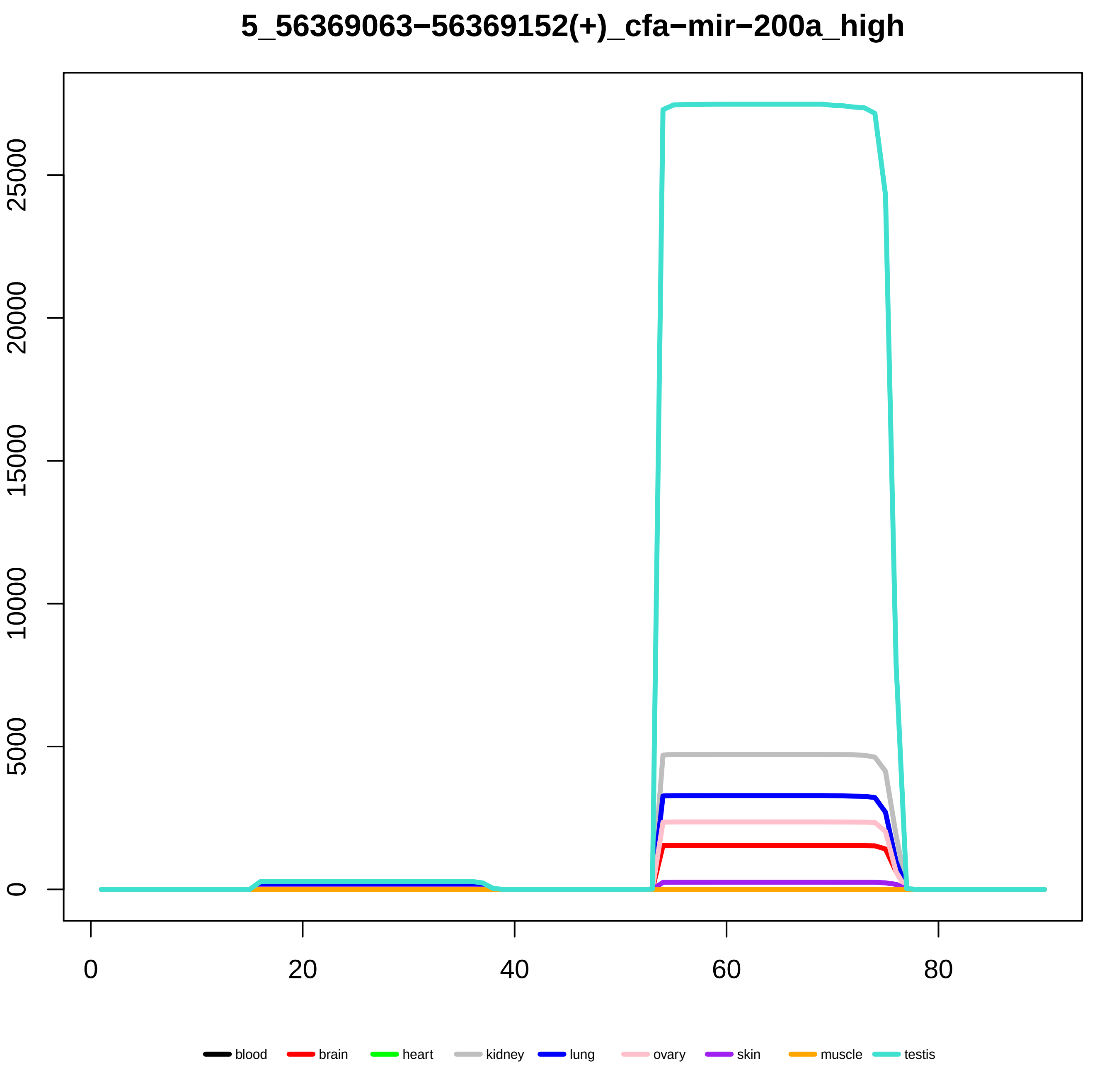

5_56369063−56369152(+)_cfa−mir−200a_high
0
5000
10000
15000
20000
25000
0
20
40
60
80
blood
brain
heart
kidney
lung
ovary
skin
muscle
testis

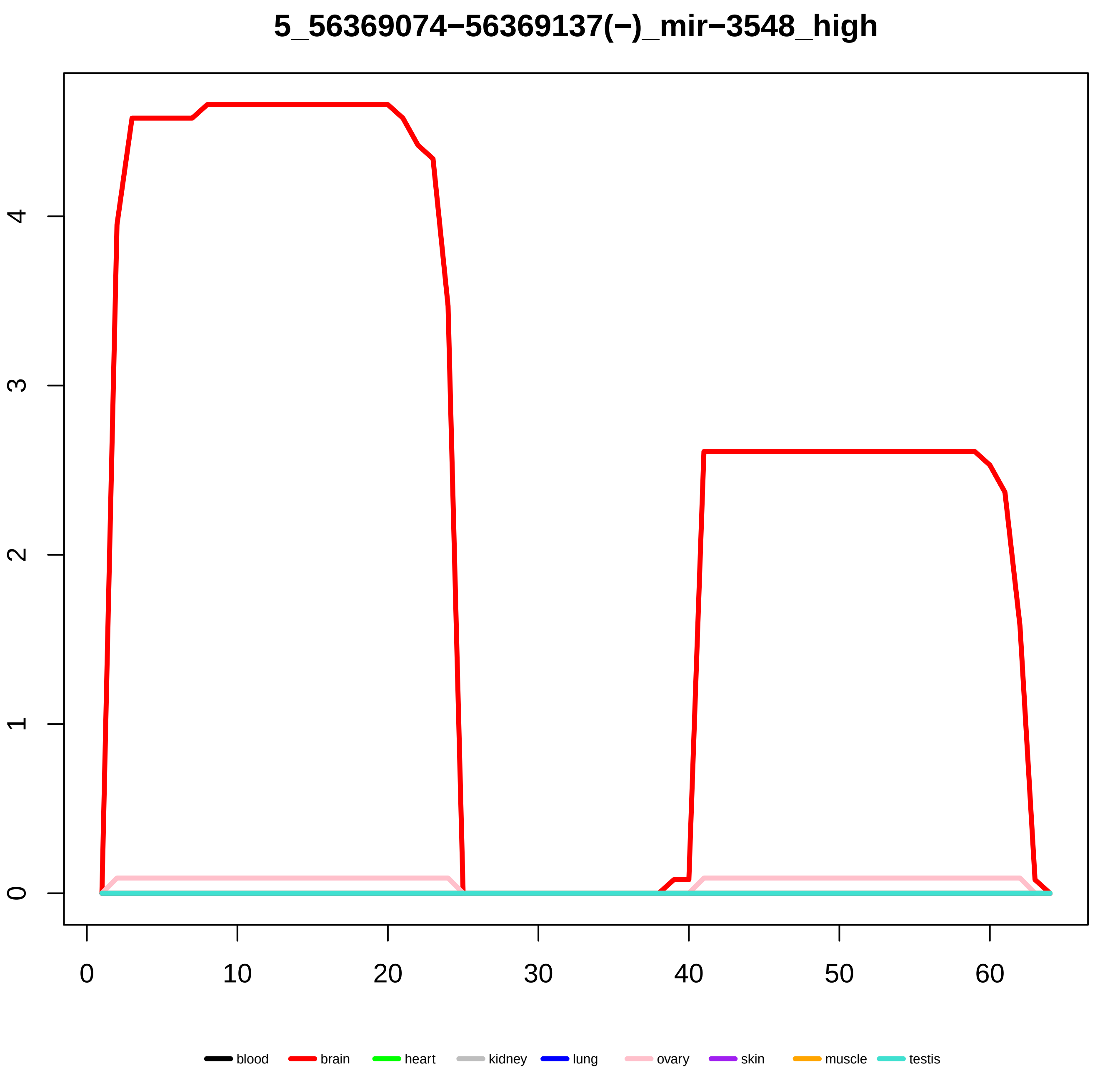

5_56369074−56369137(−)_mir−3548_high
0
1
2
3
4
0
10
20
30
40
50
60
blood
brain
heart
kidney
lung
ovary
skin
muscle
testis

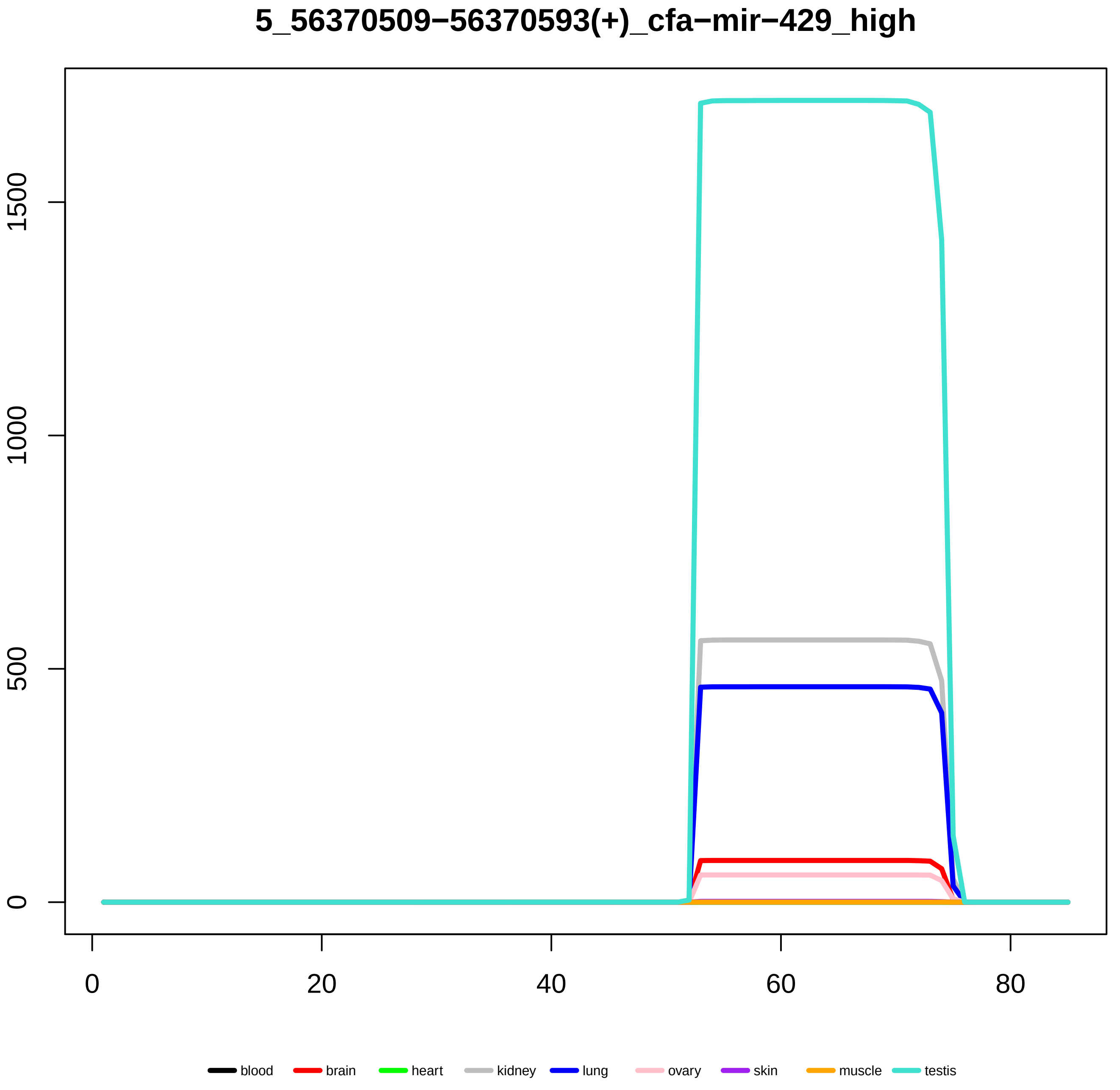
5_56370509−56370593(+)_cfa−mir−429_high
0
500
1000
1500
0
20
40
60
80
blood
brain
heart
kidney
lung
ovary
skin
muscle
testis

5_57395454−57395588(+)_cfa−mir−8866_low

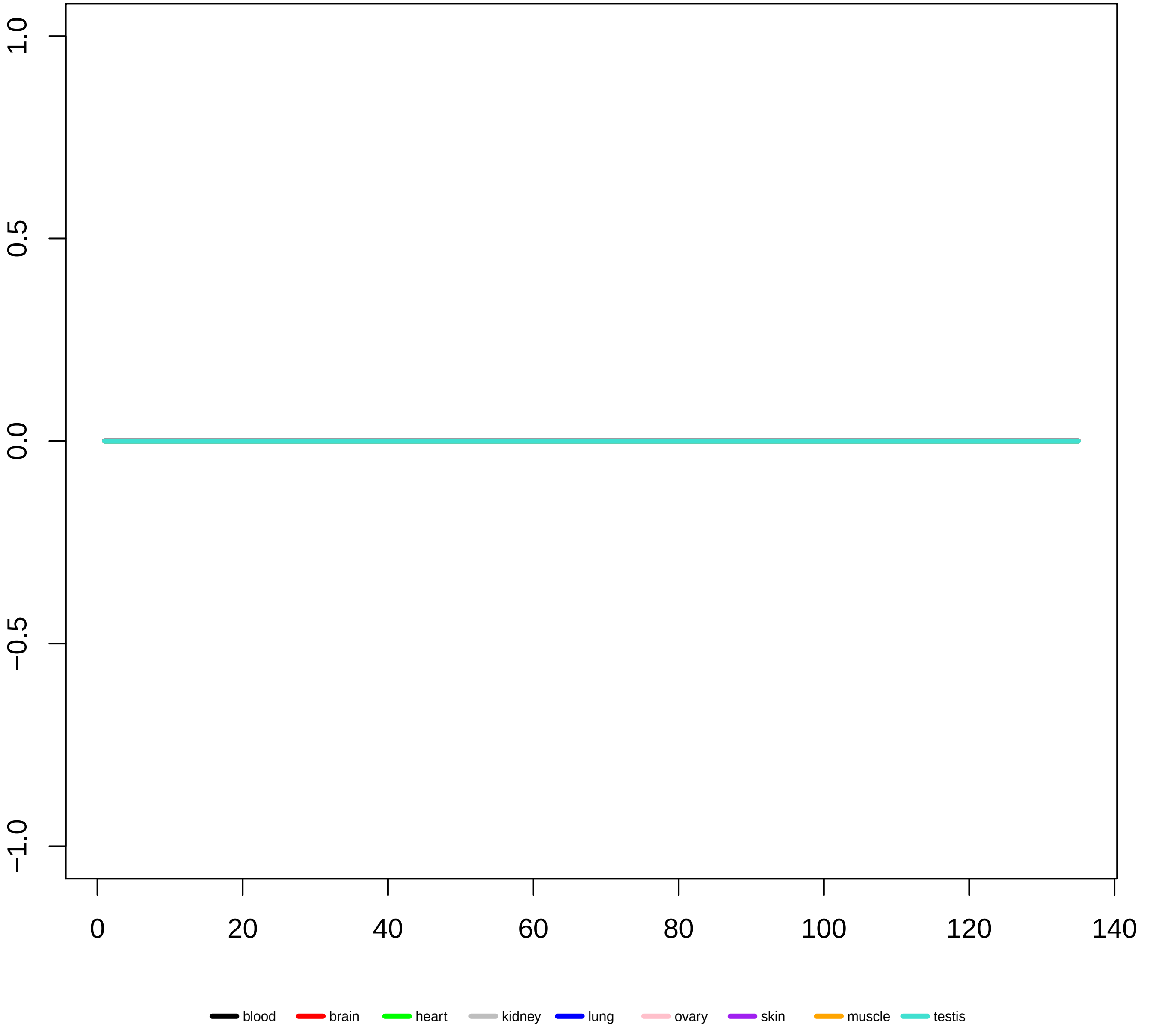

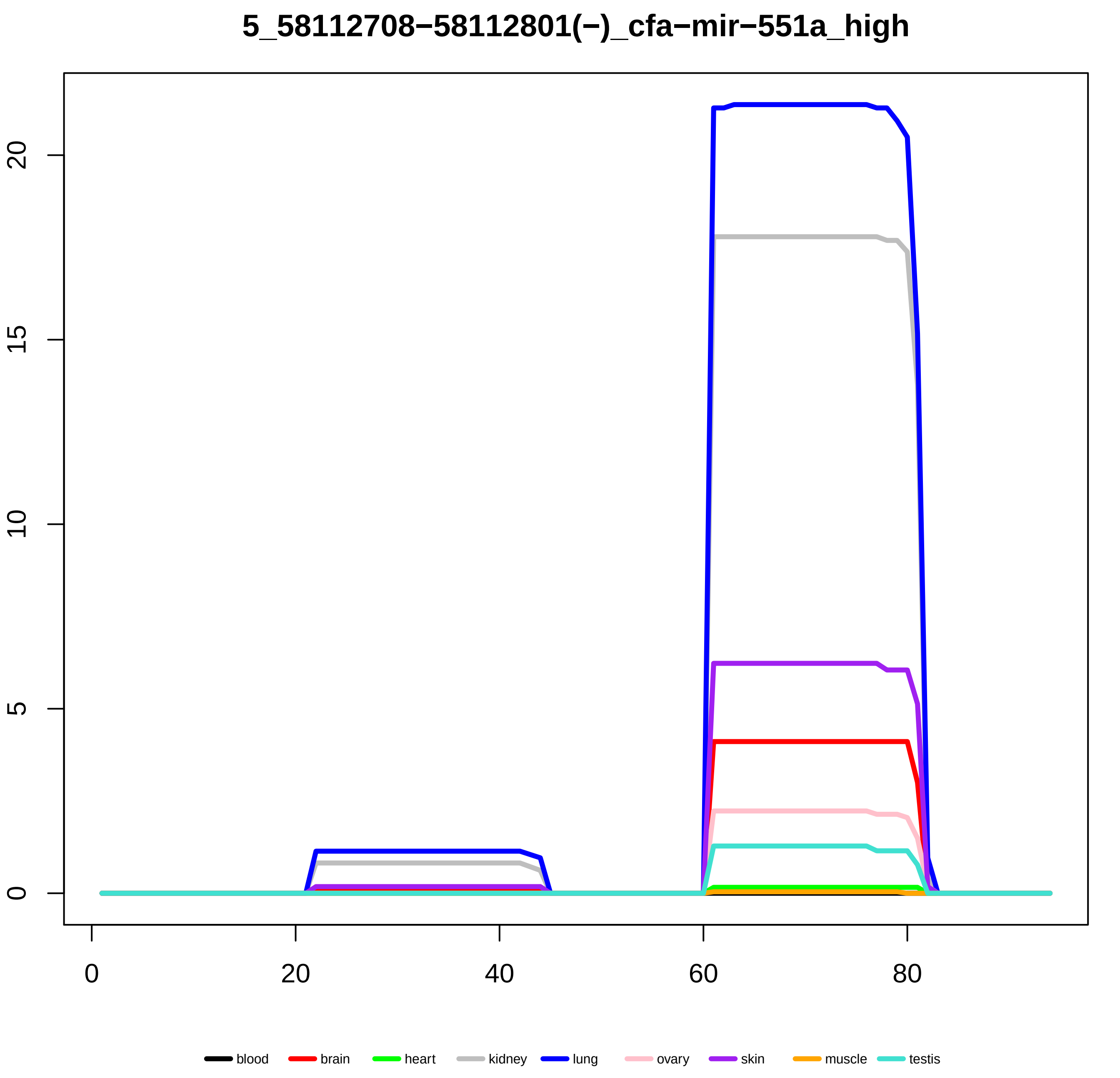

5_58112708−58112801(−)_cfa−mir−551a_high
0
5
10
15
20
0
20
40
60
80
blood
brain
heart
kidney
lung
ovary
skin
muscle
testis

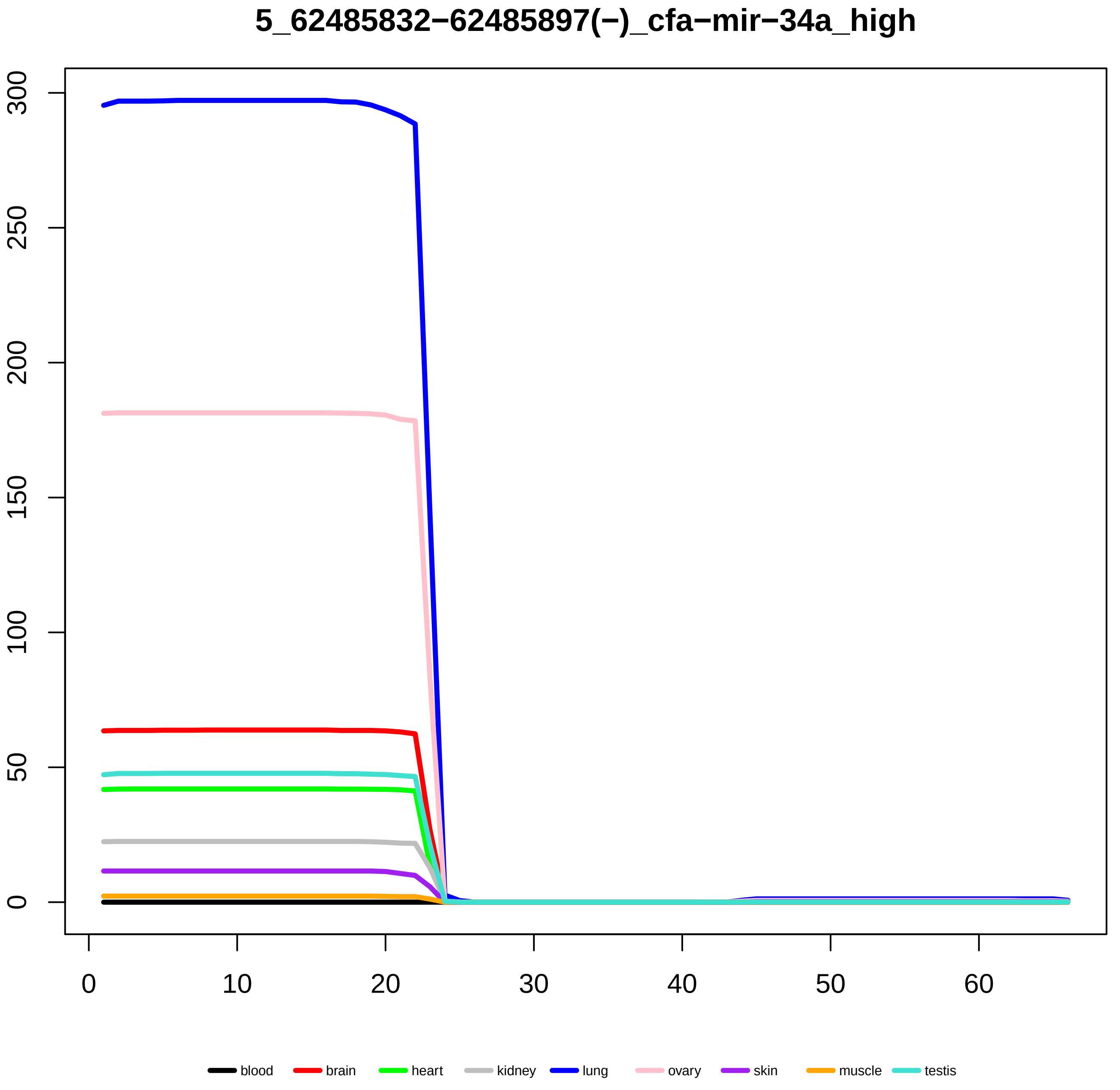

5_62485832−62485897(−)_cfa−mir−34a_high
0
50
100
150
200
250
300
0
10
20
30
40
50
60
blood
brain
heart
kidney
lung
ovary
skin
muscle
testis

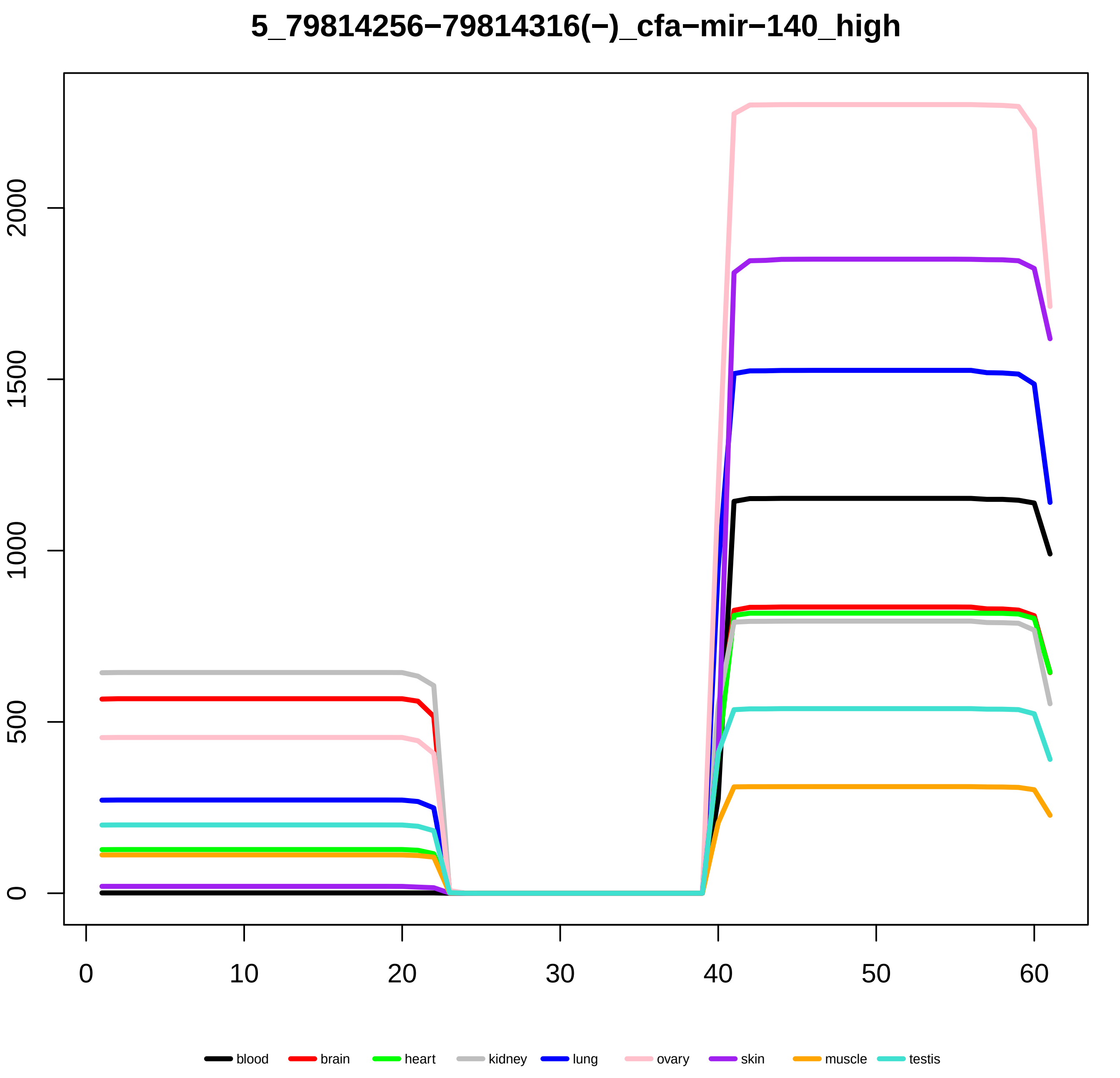

5_79814256−79814316(−)_cfa−mir−140_high
0
500
1000
1500
2000
0
10
20
30
40
50
60
blood
brain
heart
kidney
lung
ovary
skin
muscle
testis

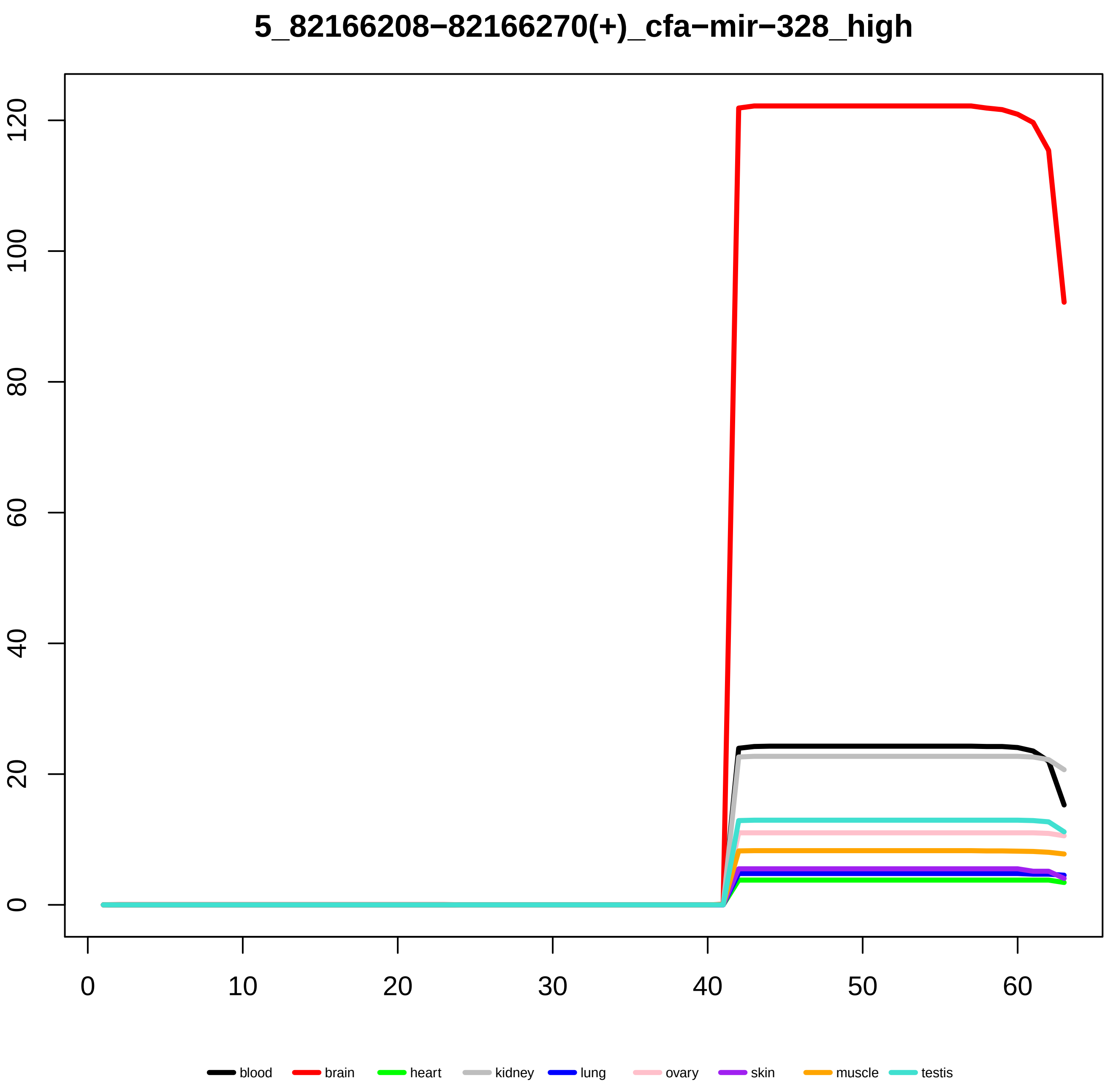

5_82166208−82166270(+)_cfa−mir−328_high
0
20
40
60
80
100
120
0
10
20
30
40
50
60
blood
brain
heart
kidney
lung
ovary
skin
muscle
testis

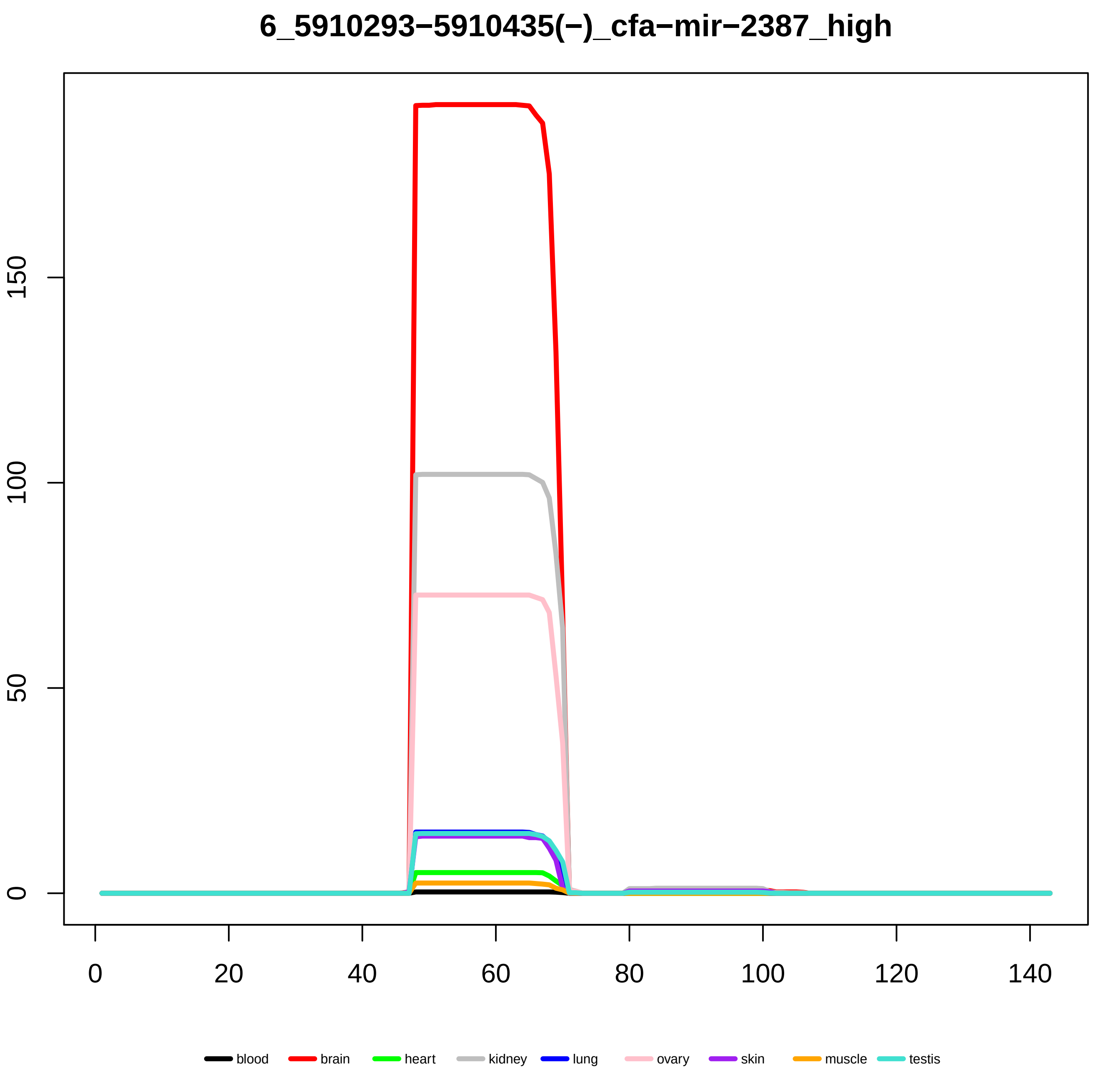

6_5910293−5910435(−)_cfa−mir−2387_high
0
50
100
150
0
20
40
60
80
100
120
140
blood
brain
heart
kidney
lung
ovary
skin
muscle
testis

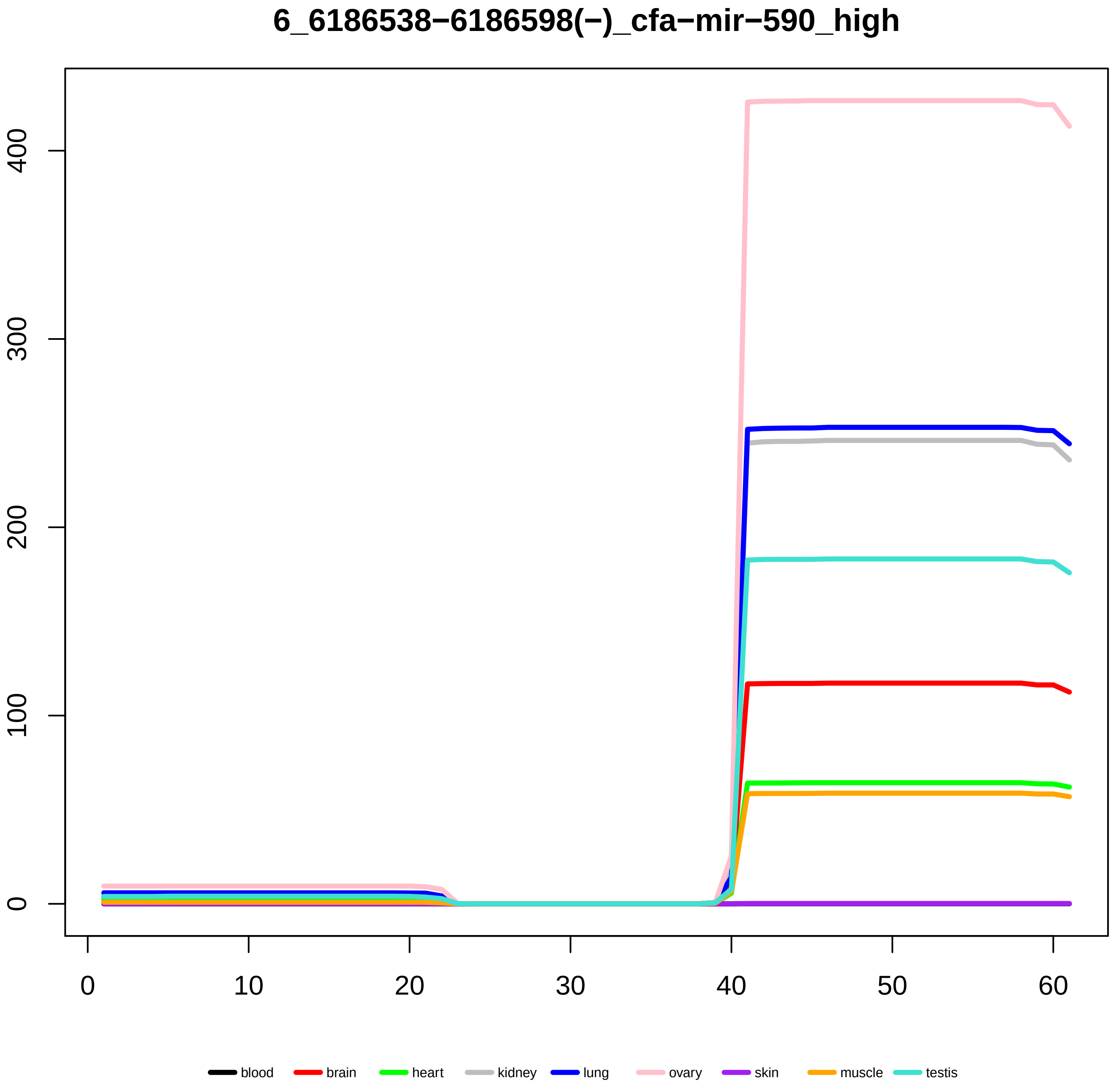

6_6186538−6186598(−)_cfa−mir−590_high
0
100
200
300
400
0
10
20
30
40
50
60
blood
brain
heart
kidney
lung
ovary
skin
muscle
testis

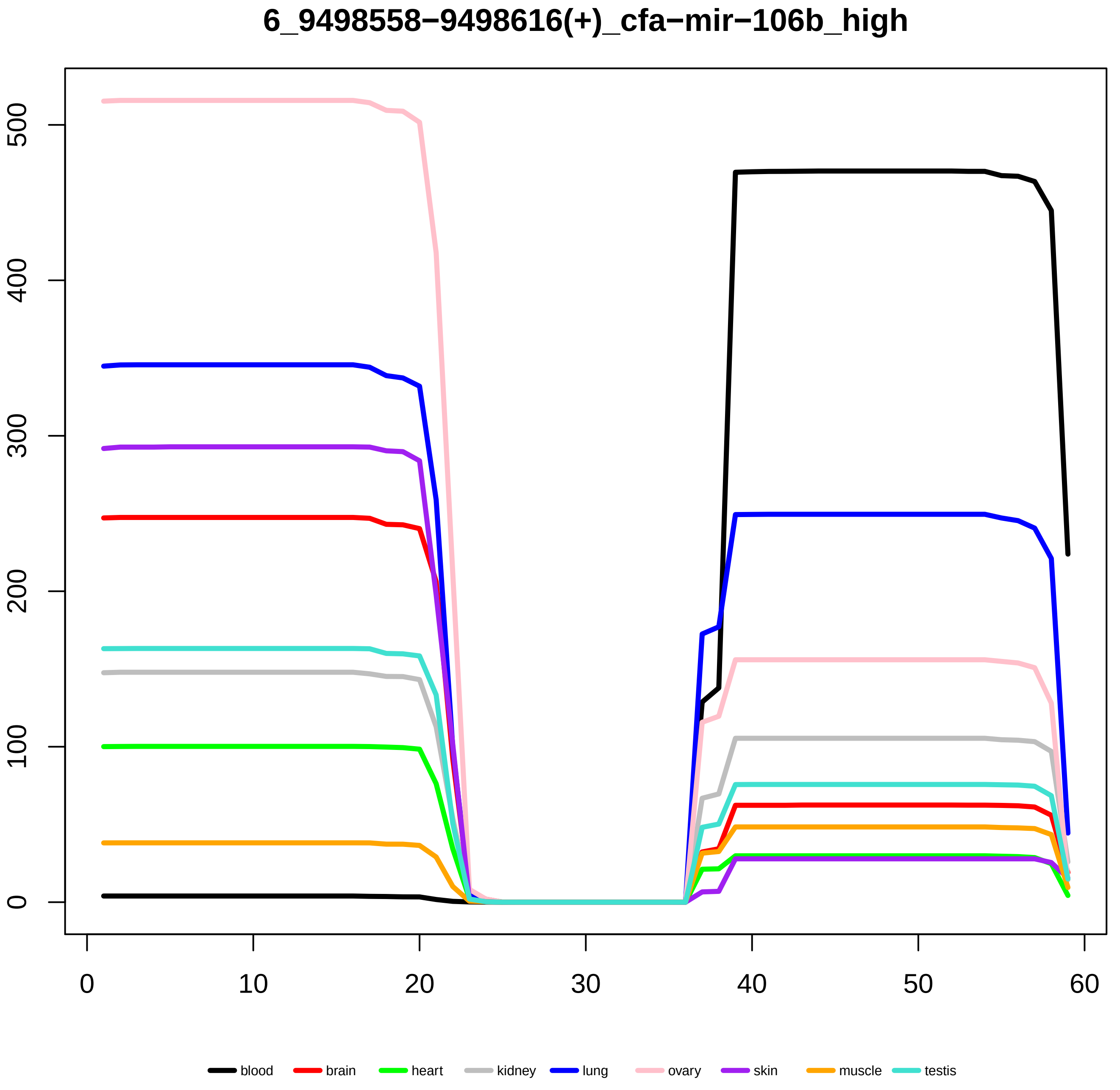
6_9498558−9498616(+)_cfa−mir−106b_high
0
100
200
300
400
500
0
10
20
30
40
50
60
blood
brain
heart
kidney
lung
ovary
skin
muscle
testis

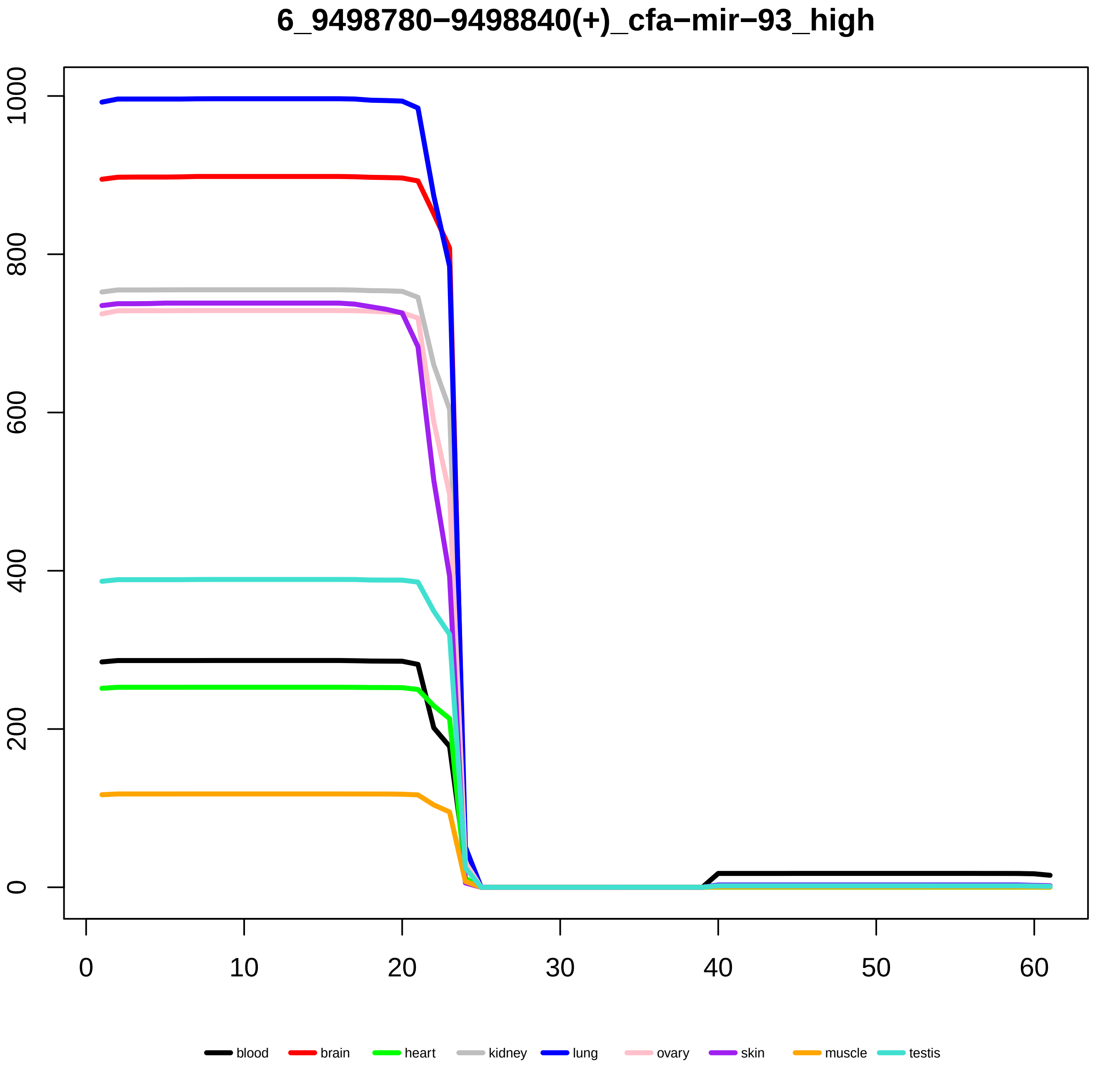
6_9498780−9498840(+)_cfa−mir−93_high
0
200
400
600
800
1000
0
10
20
30
40
50
60
blood
brain
heart
kidney
lung
ovary
skin
muscle
testis

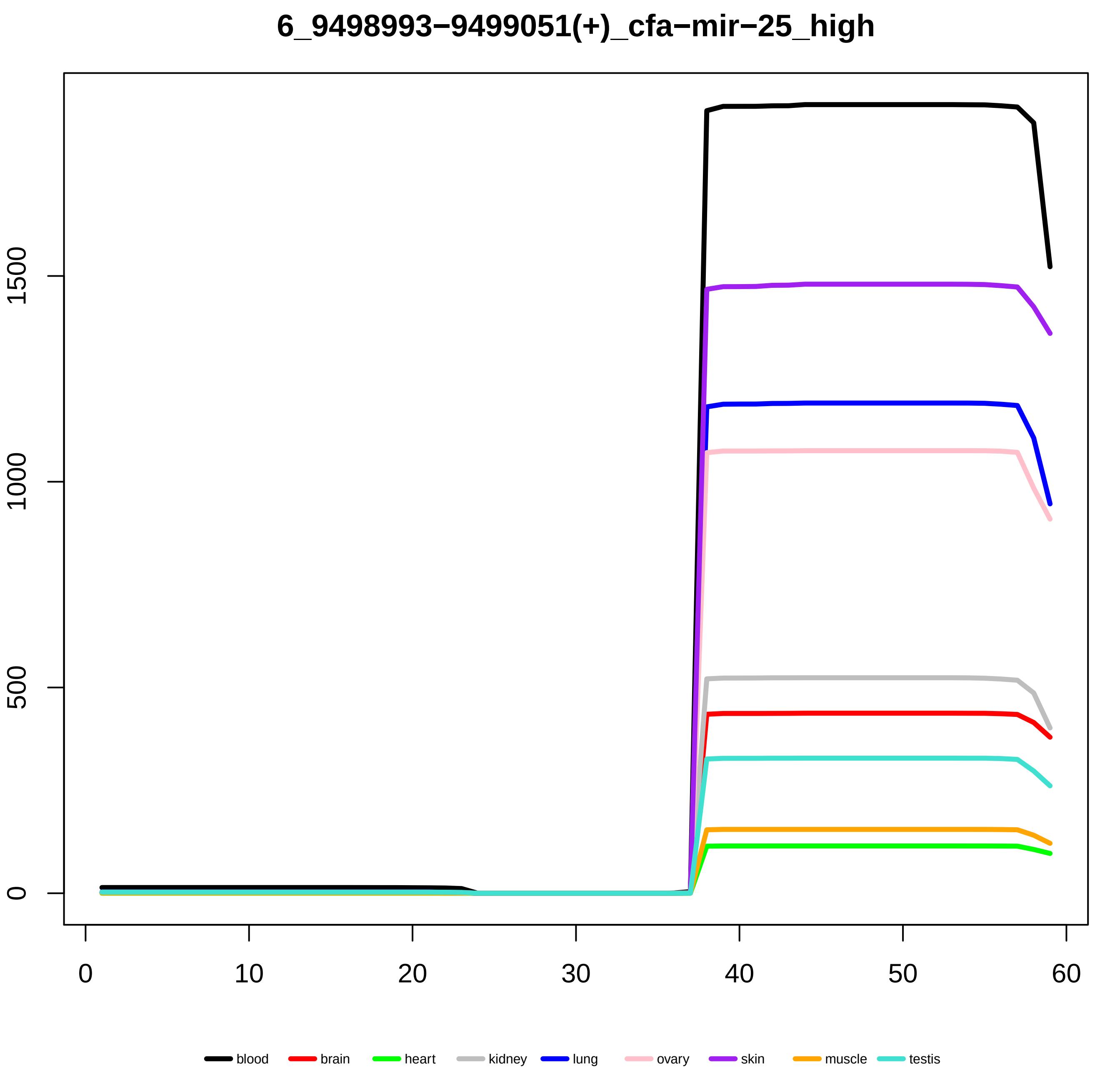
6_9498993−9499051(+)_cfa−mir−25_high
0
500
1000
1500
0
10
20
30
40
50
60
blood
brain
heart
kidney
lung
ovary
skin
muscle
testis

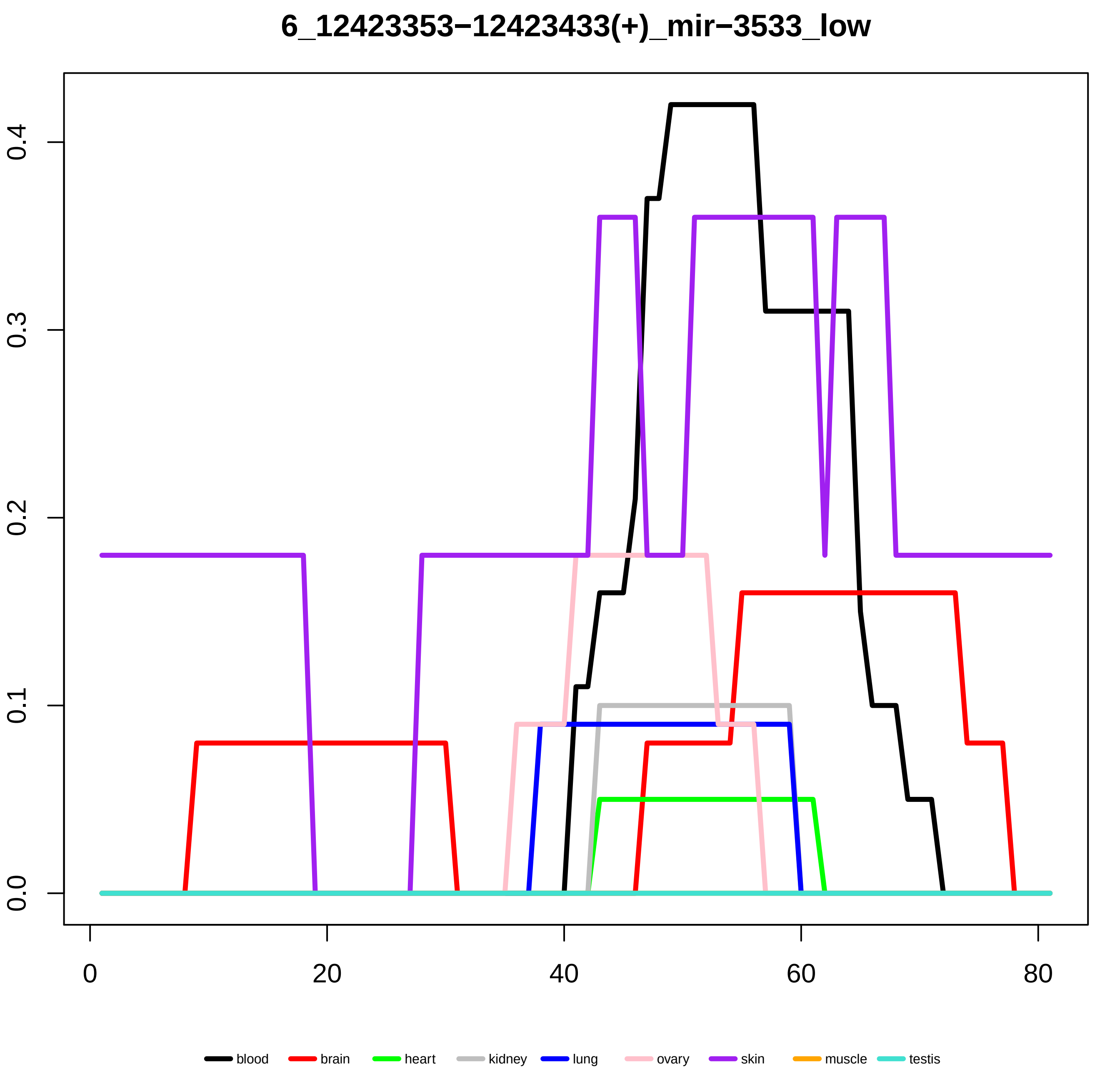

6_12423353−12423433(+)_mir−3533_low
0.0
0.1
0.2
0.3
0.4
0
20
40
60
80
blood
brain
heart
kidney
lung
ovary
skin
muscle
testis

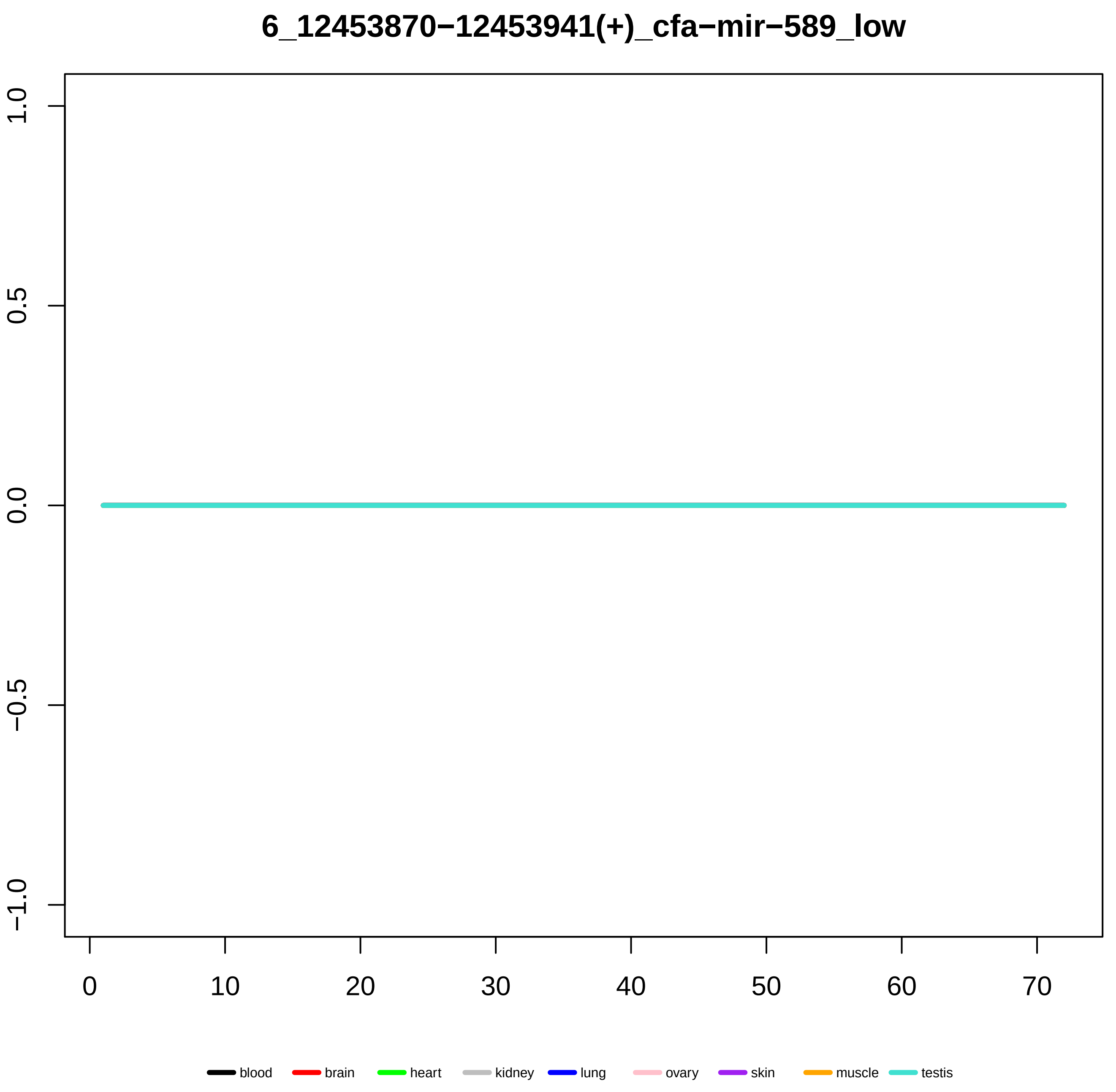
6_12453870−12453941(+)_cfa−mir−589_low
1.0
0.5
0.0
−0.5
−1.0
0
10
20
30
40
50
60
70
blood
brain
heart
kidney
lung
ovary
skin
muscle
testis

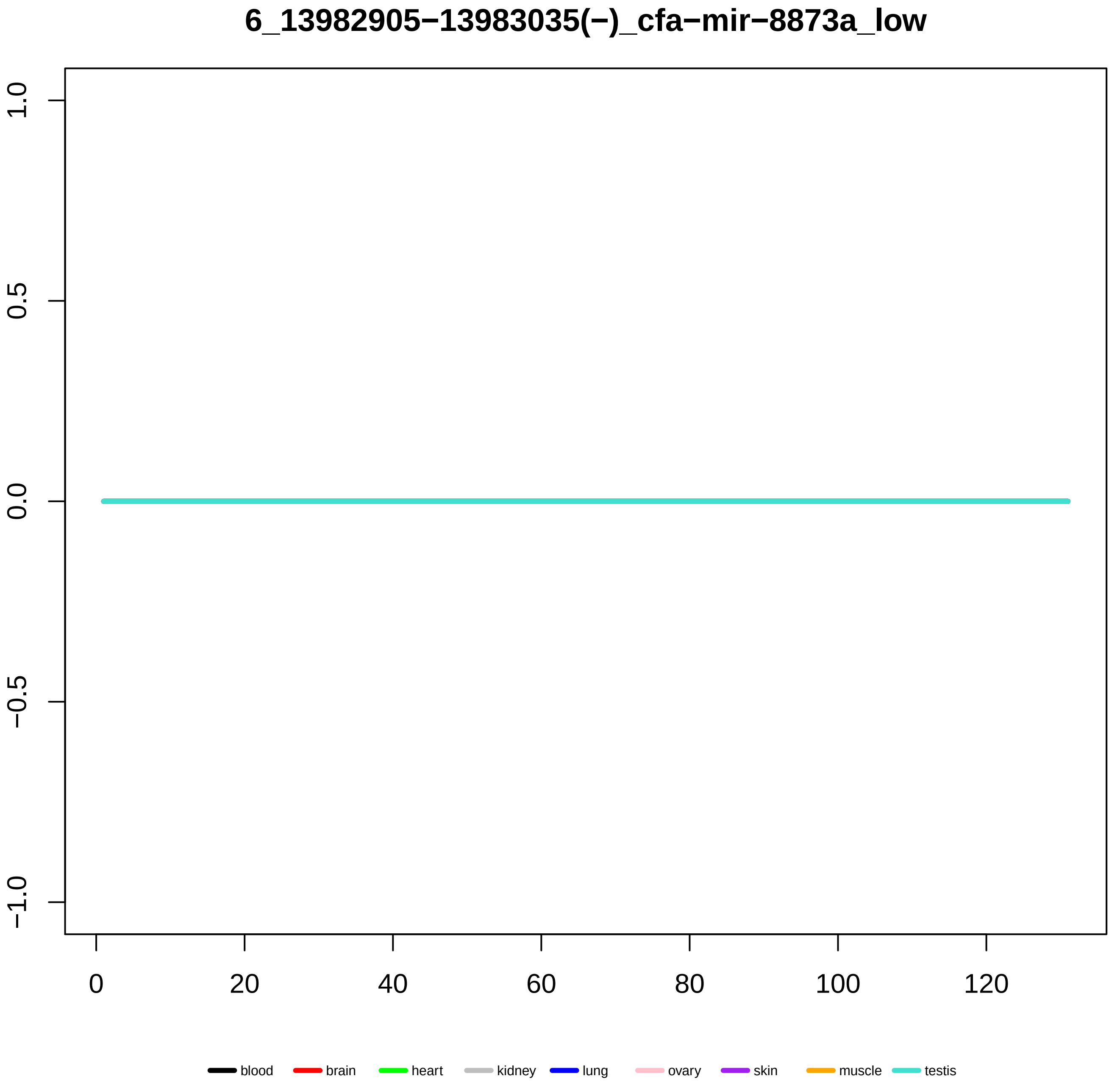

6_13982905−13983035(−)_cfa−mir−8873a_low
1.0
0.5
0.0
−0.5
−1.0
0
20
40
60
80
100
120
blood
brain
heart
kidney
lung
ovary
skin
muscle
testis

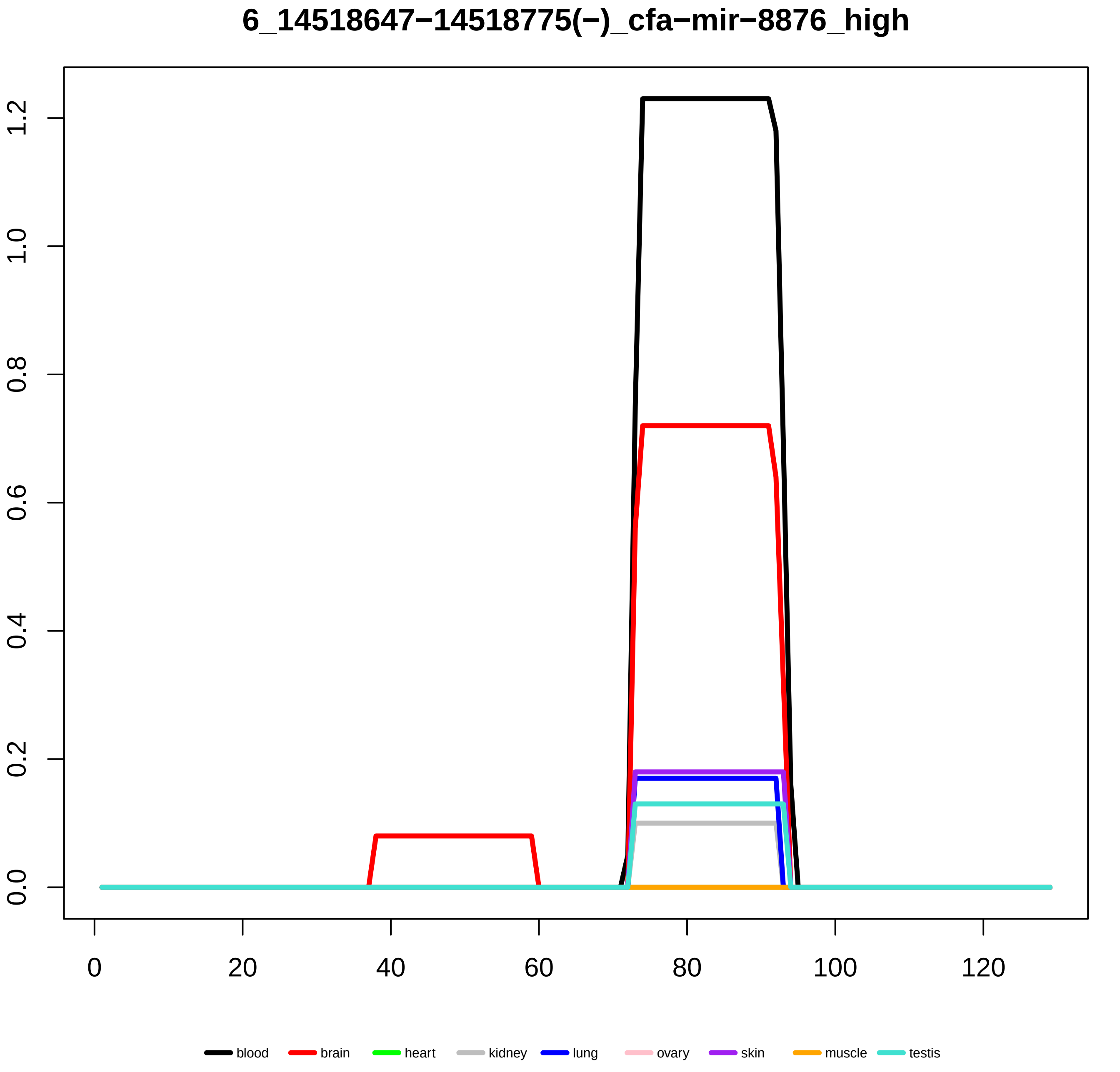

6_14518647−14518775(−)_cfa−mir−8876_high
0.0
0.2
0.4
0.6
0.8
1.0
1.2
0
20
40
60
80
100
120
blood
brain
heart
kidney
lung
ovary
skin
muscle
testis

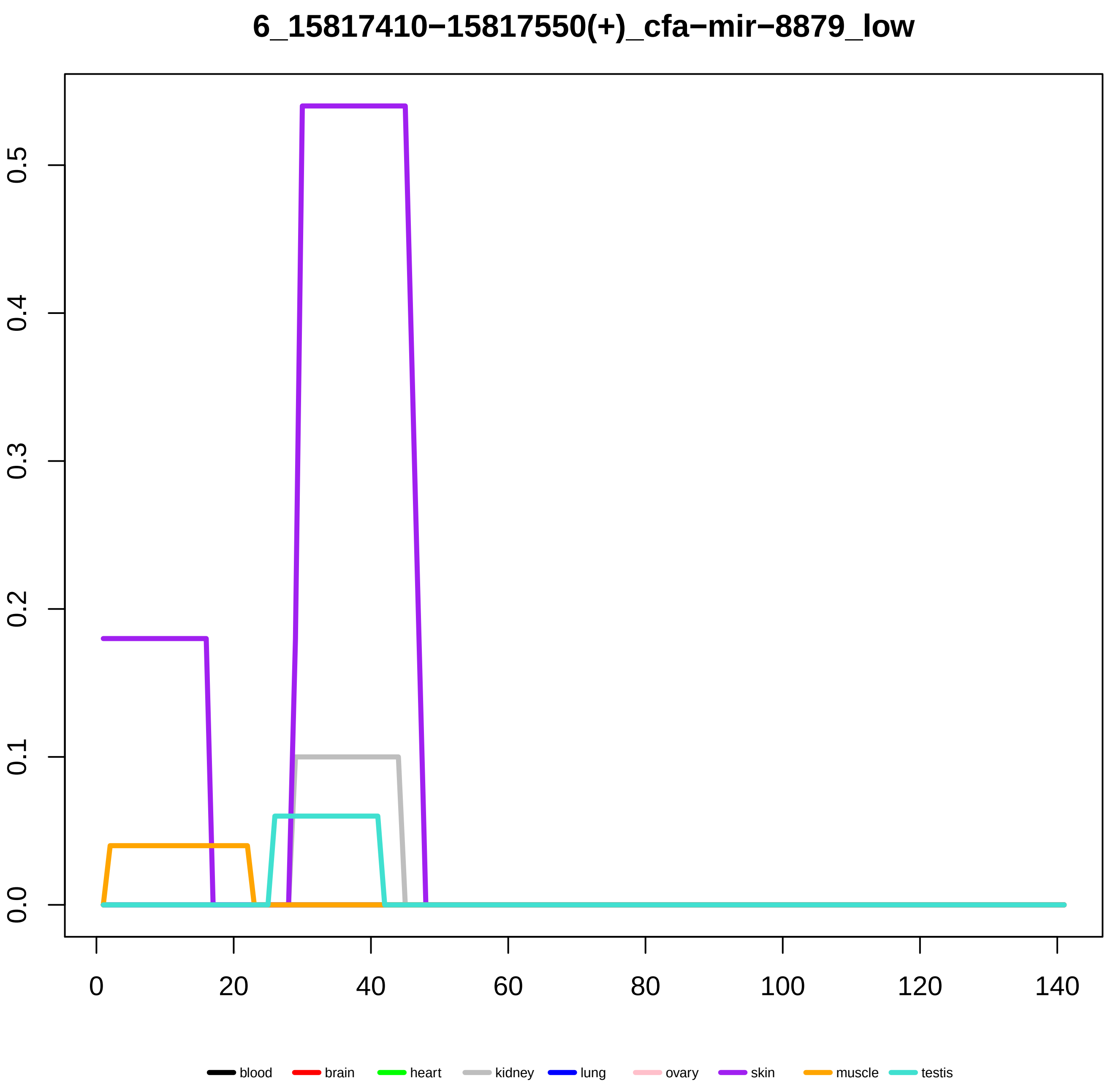
6_15817410−15817550(+)_cfa−mir−8879_low
0.0
0.1
0.2
0.3
0.4
0.5
0
20
40
60
80
100
120
140
blood
brain
heart
kidney
lung
ovary
skin
muscle
testis

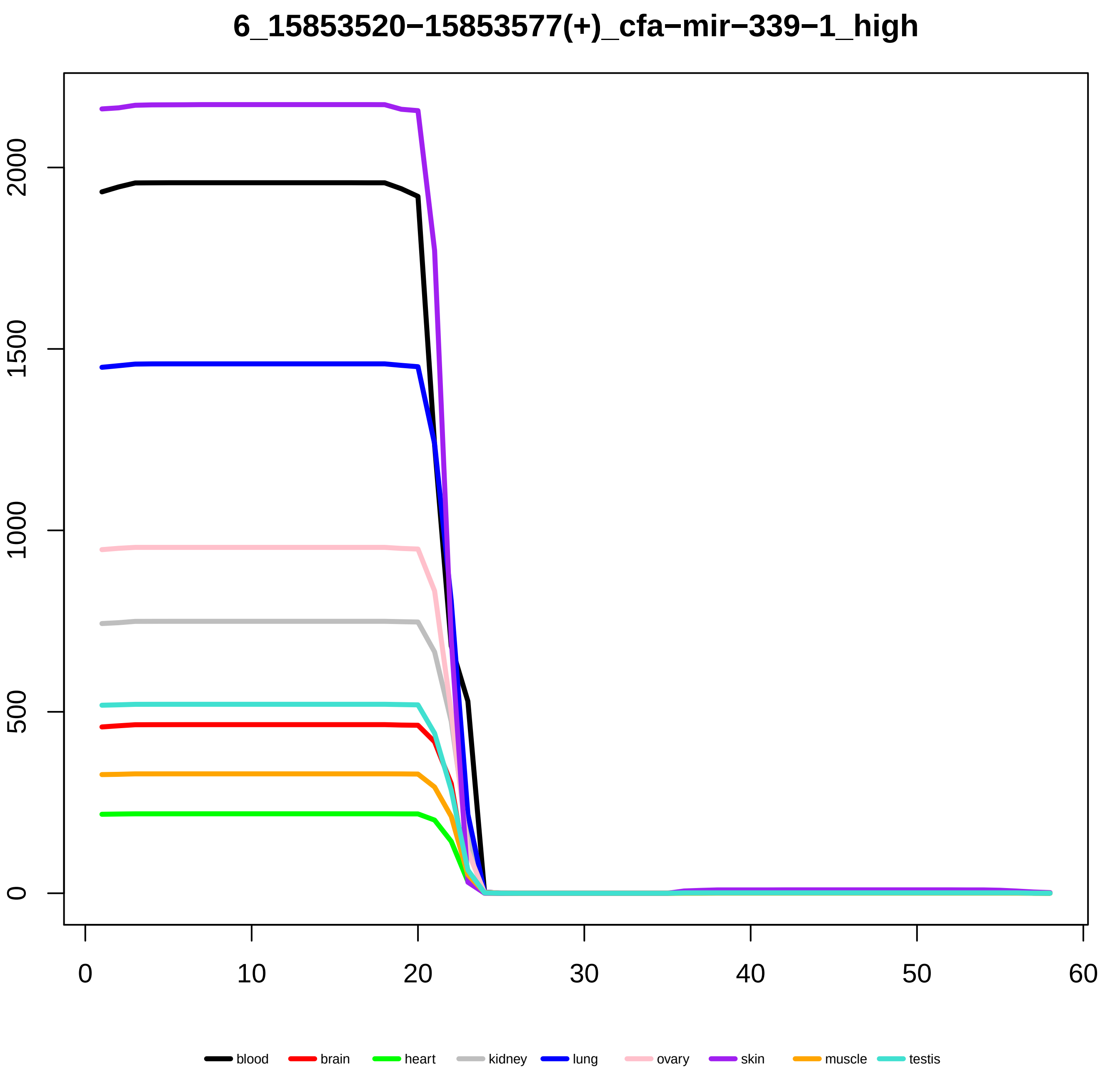

6_15853520−15853577(+)_cfa−mir−339−1_high
0
500
1000
1500
2000
0
10
20
30
40
50
60
blood
brain
heart
kidney
lung
ovary
skin
muscle
testis

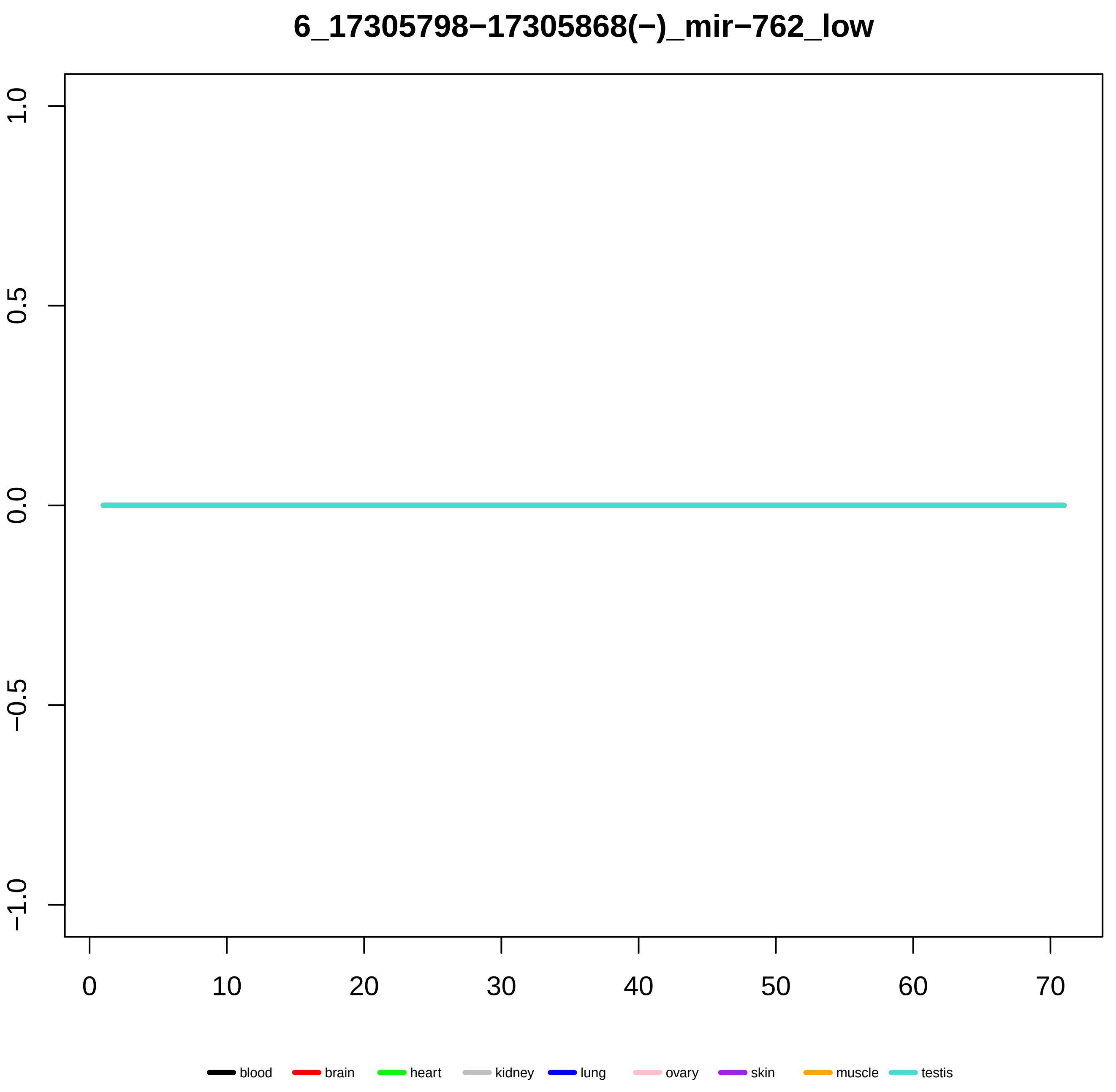
6_17305798−17305868(−)_mir−762_low
1.0
0.5
0.0
−0.5
−1.0
0
10
20
30
40
50
60
70
blood
brain
heart
kidney
lung
ovary
skin
muscle
testis

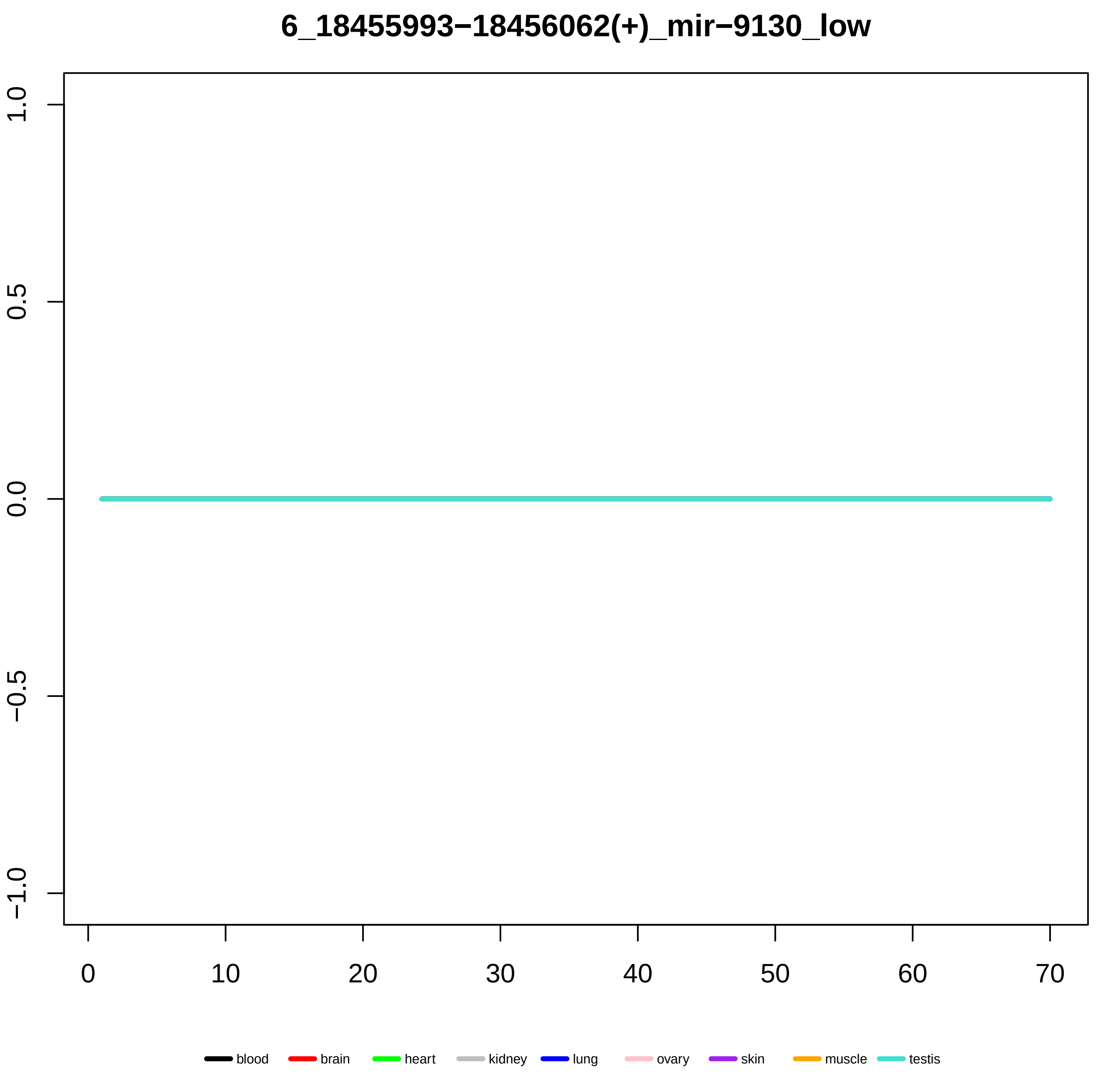

6_18455993−18456062(+)_mir−9130_low
1.0
0.5
0.0
−0.5
−1.0
0
10
20
30
40
50
60
70
blood
brain
heart
kidney
lung
ovary
skin
muscle
testis

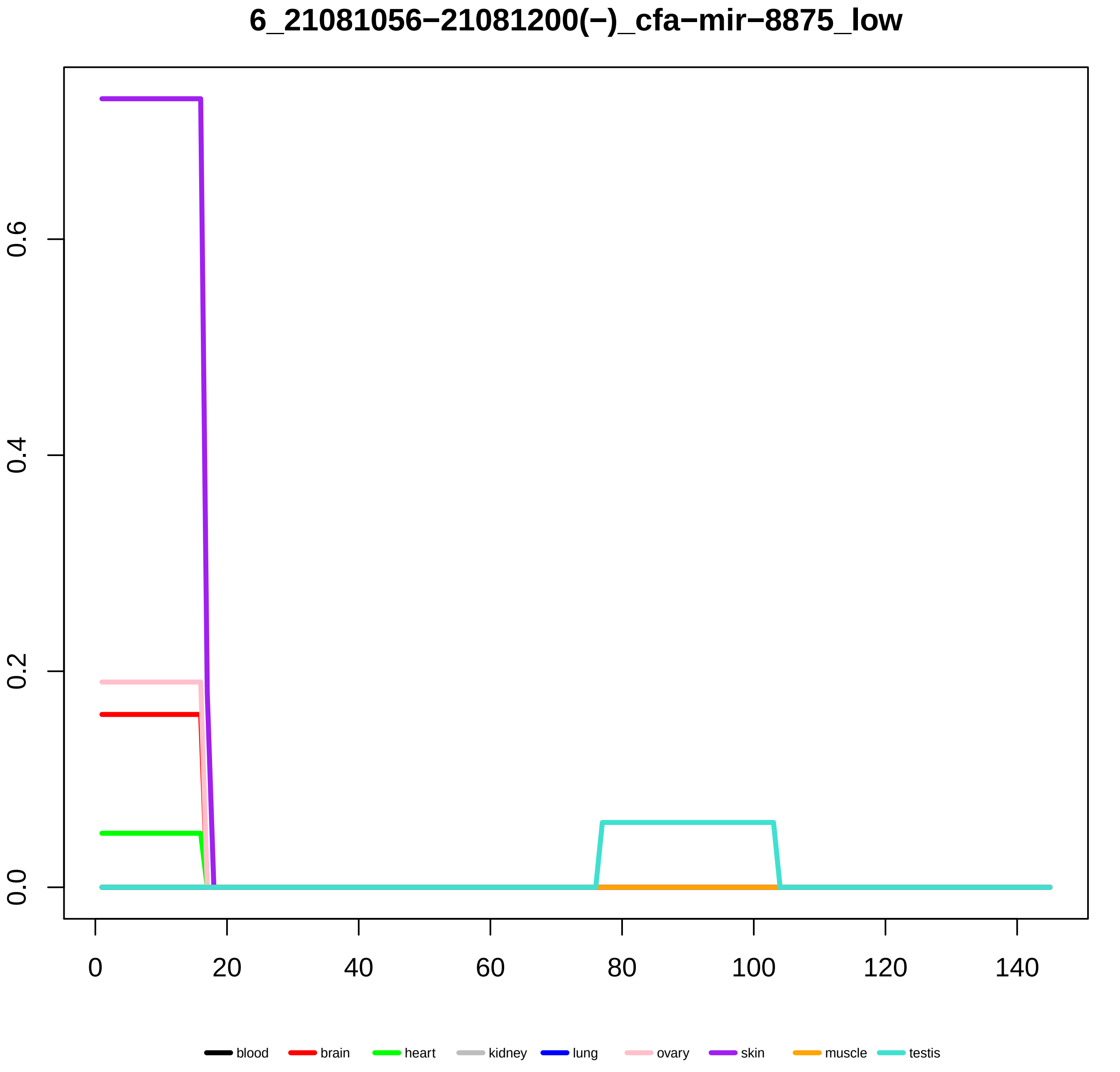

6_21081056−21081200(−)_cfa−mir−8875_low
0.0
0.2
0.4
0.6
0
20
40
60
80
100
120
140
blood
brain
heart
kidney
lung
ovary
skin
muscle
testis

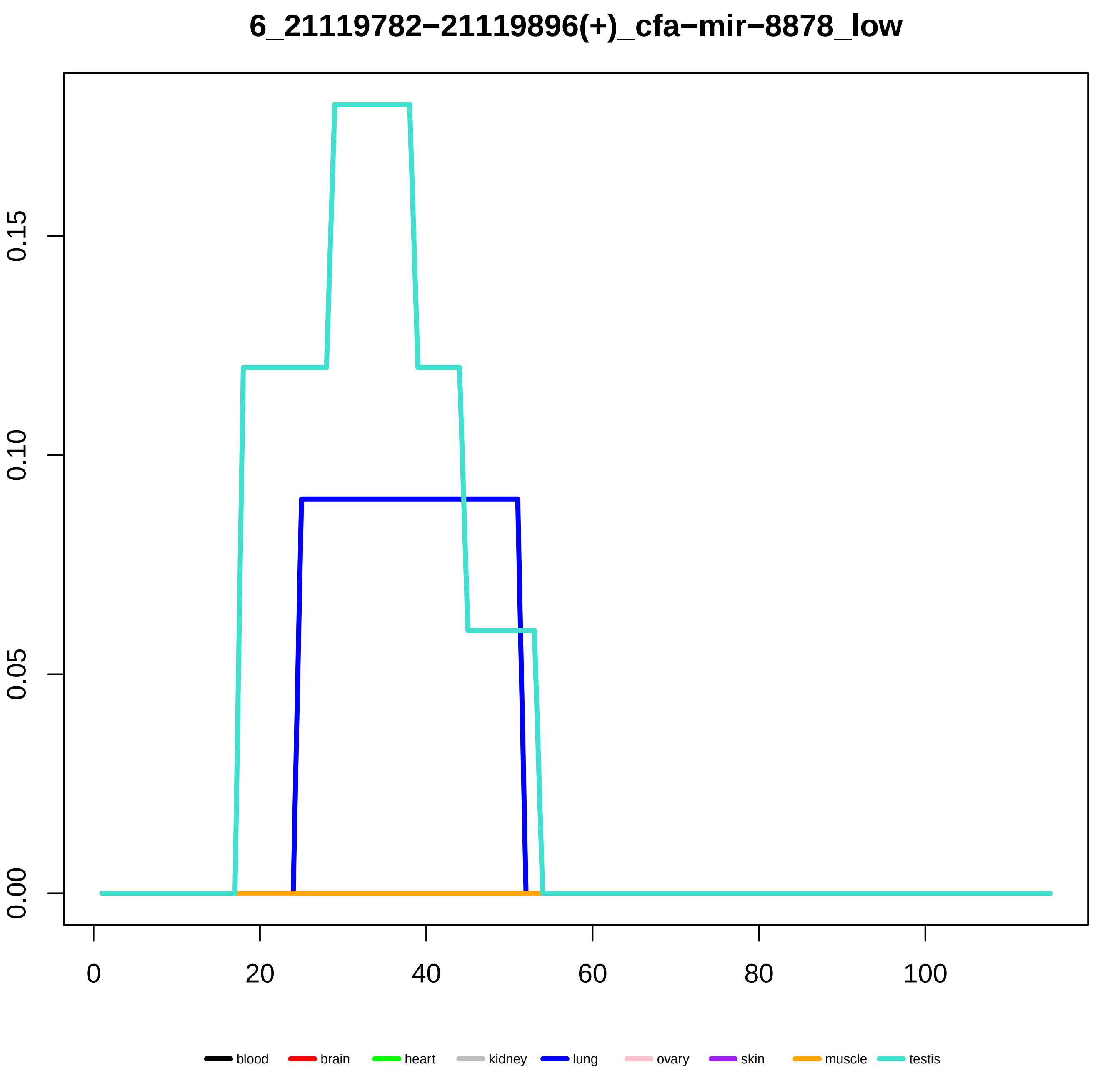

6_21119782−21119896(+)_cfa−mir−8878_low
0.00
0.05
0.10
0.15
0
20
40
60
80
100
blood
brain
heart
kidney
lung
ovary
skin
muscle
testis

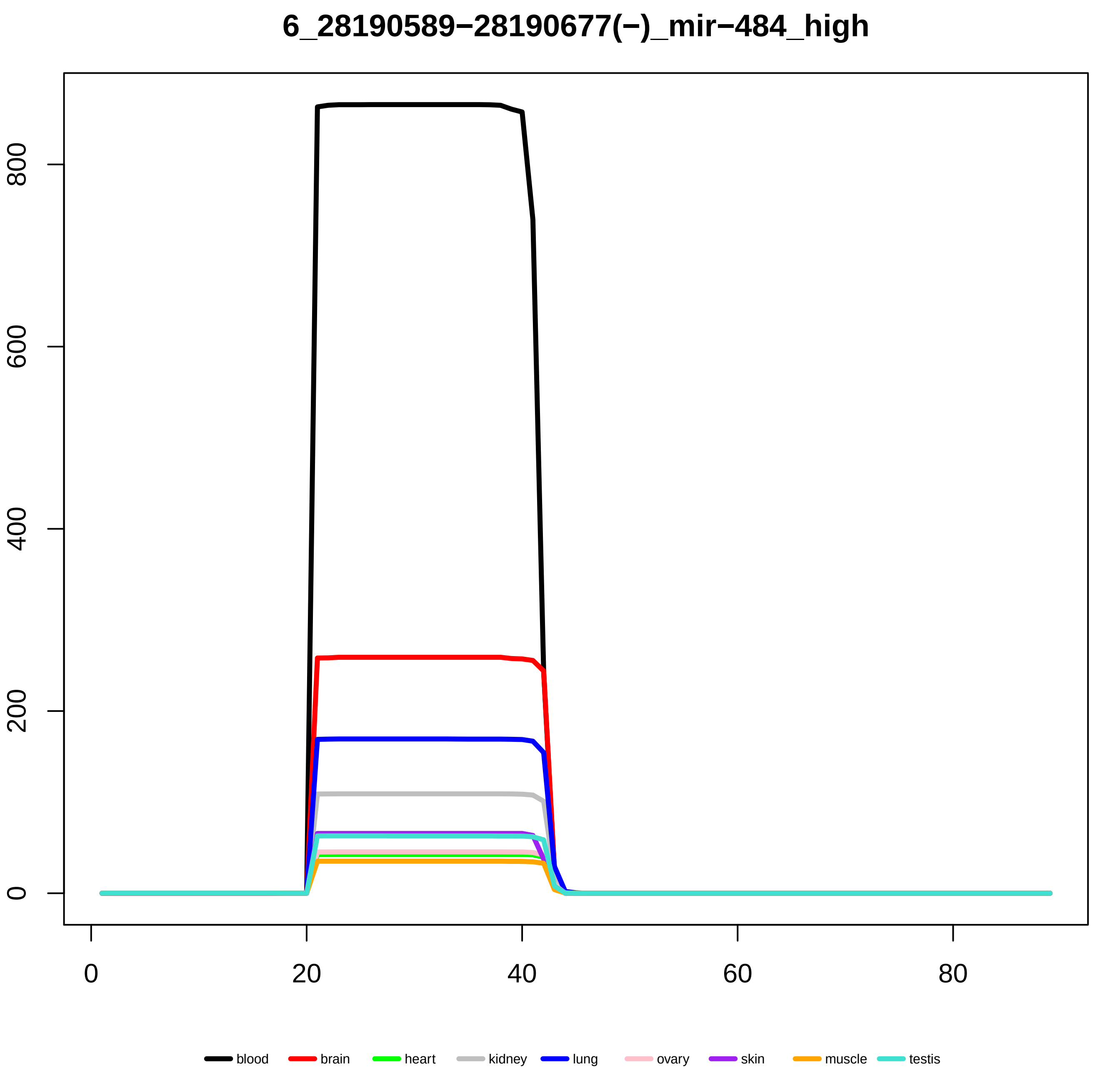
6_28190589−28190677(−)_mir−484_high
0
200
400
600
800
0
20
40
60
80
blood
brain
heart
kidney
lung
ovary
skin
muscle
testis

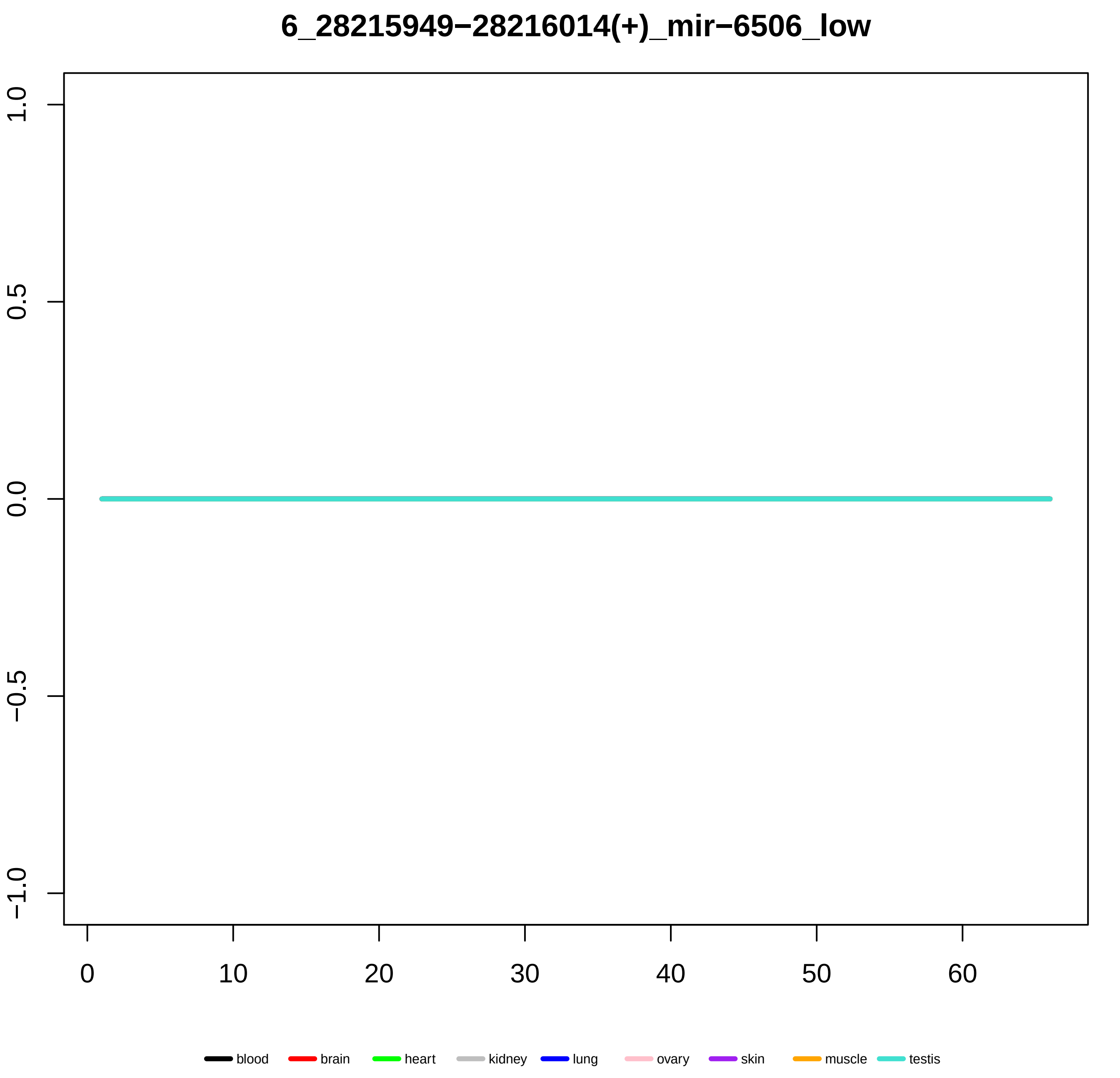

6_28215949−28216014(+)_mir−6506_low
1.0
0.5
0.0
−0.5
−1.0
0
10
20
30
40
50
60
blood
brain
heart
kidney
lung
ovary
skin
muscle
testis

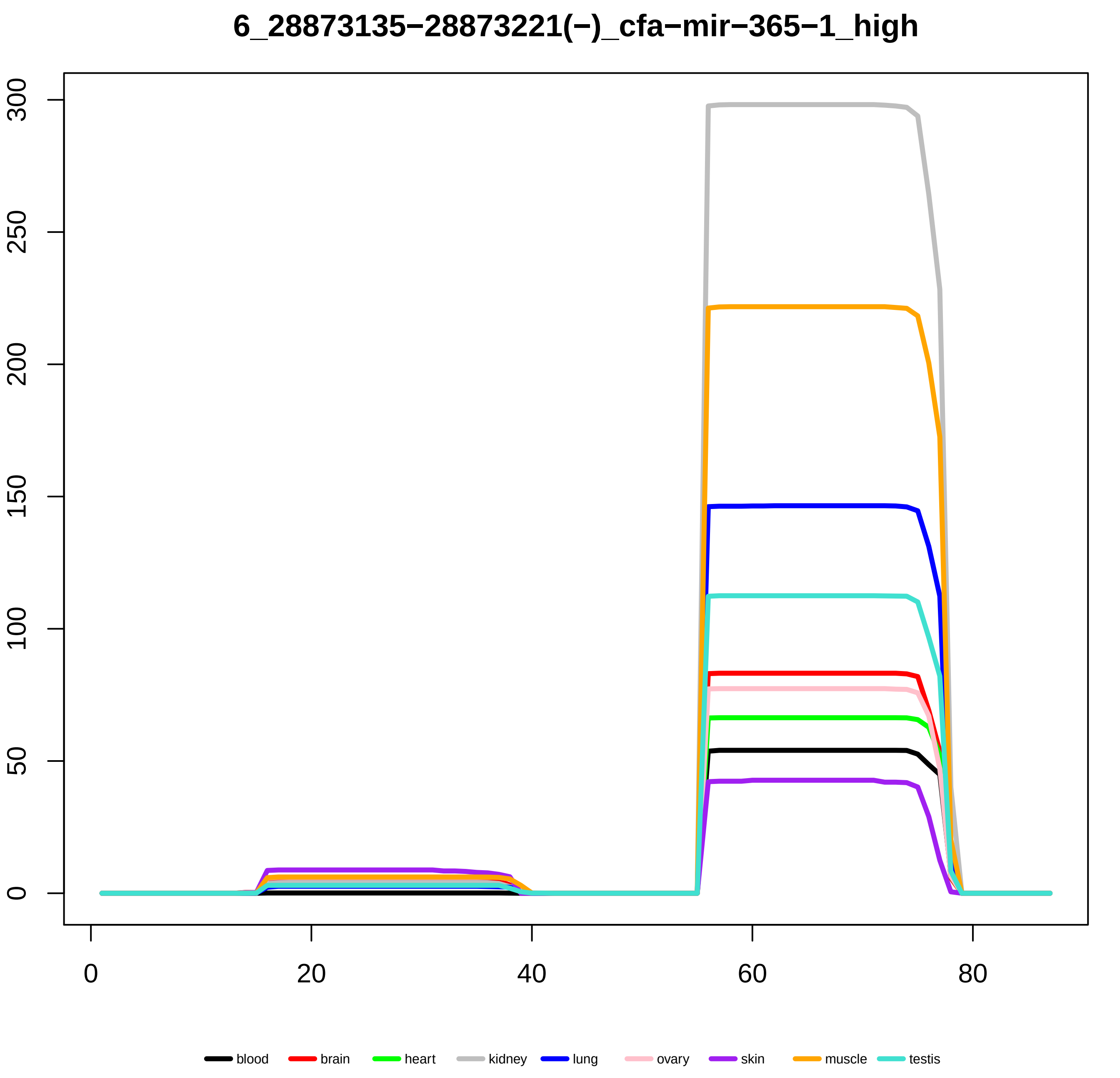

6_28873135−28873221(−)_cfa−mir−365−1_high
0
50
100
150
200
250
300
0
20
40
60
80
blood
brain
heart
kidney
lung
ovary
skin
muscle
testis

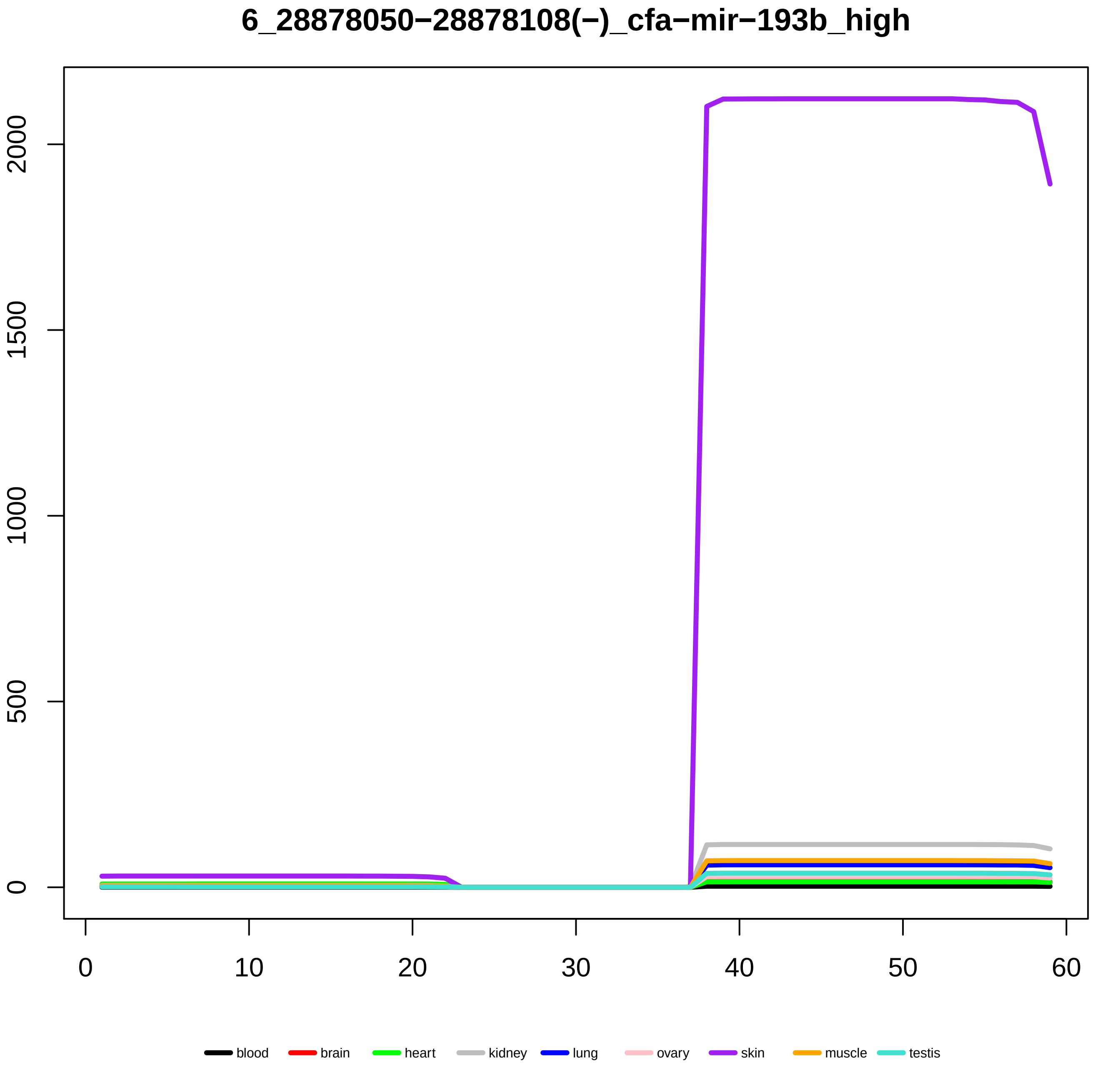

6_28878050−28878108(−)_cfa−mir−193b_high
0
500
1000
1500
2000
0
10
20
30
40
50
60
blood
brain
heart
kidney
lung
ovary
skin
muscle
testis

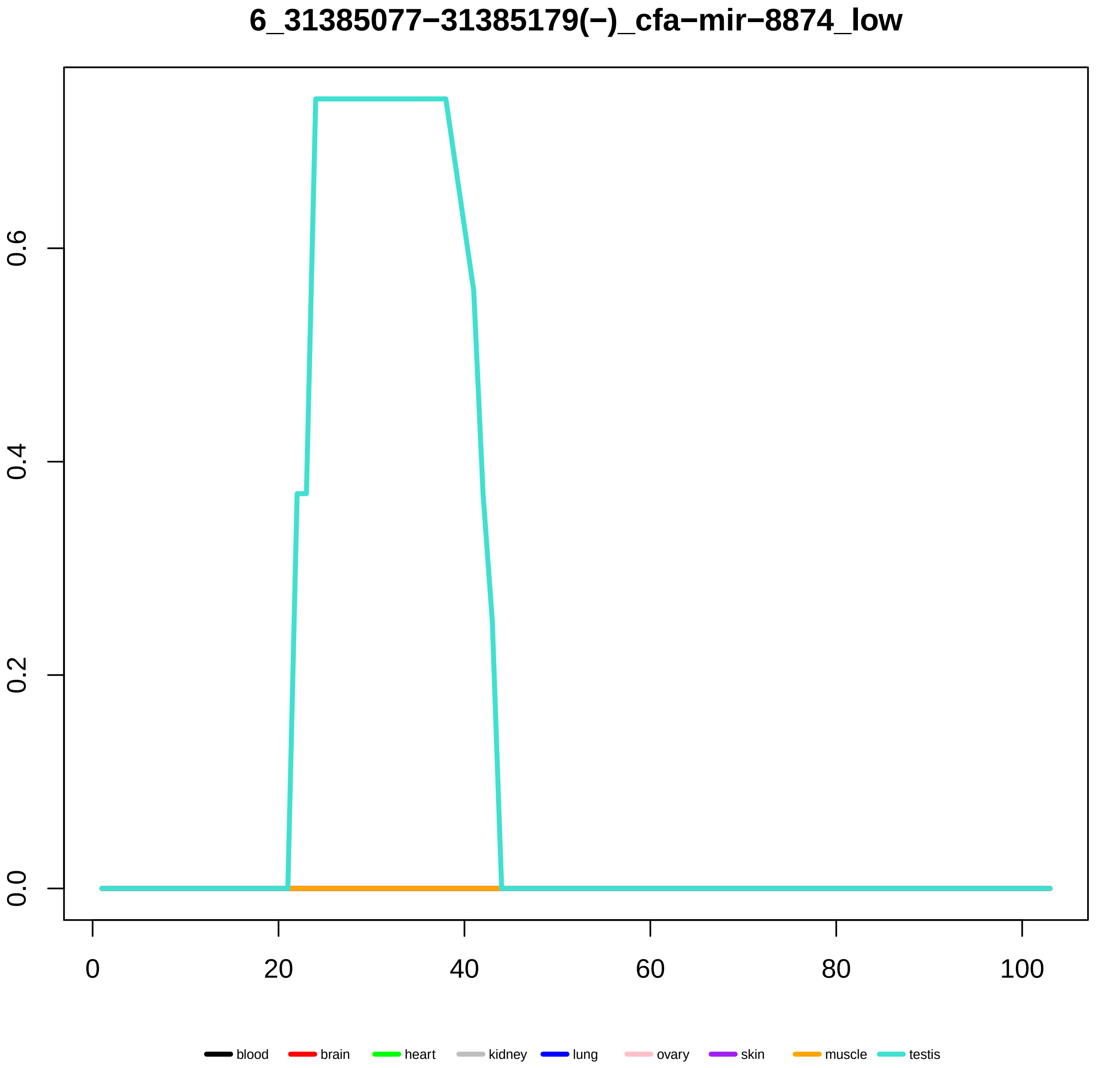

6_31385077−31385179(−)_cfa−mir−8874_low
0.0
0.2
0.4
0.6
0
20
40
60
80
100
blood
brain
heart
kidney
lung
ovary
skin
muscle
testis

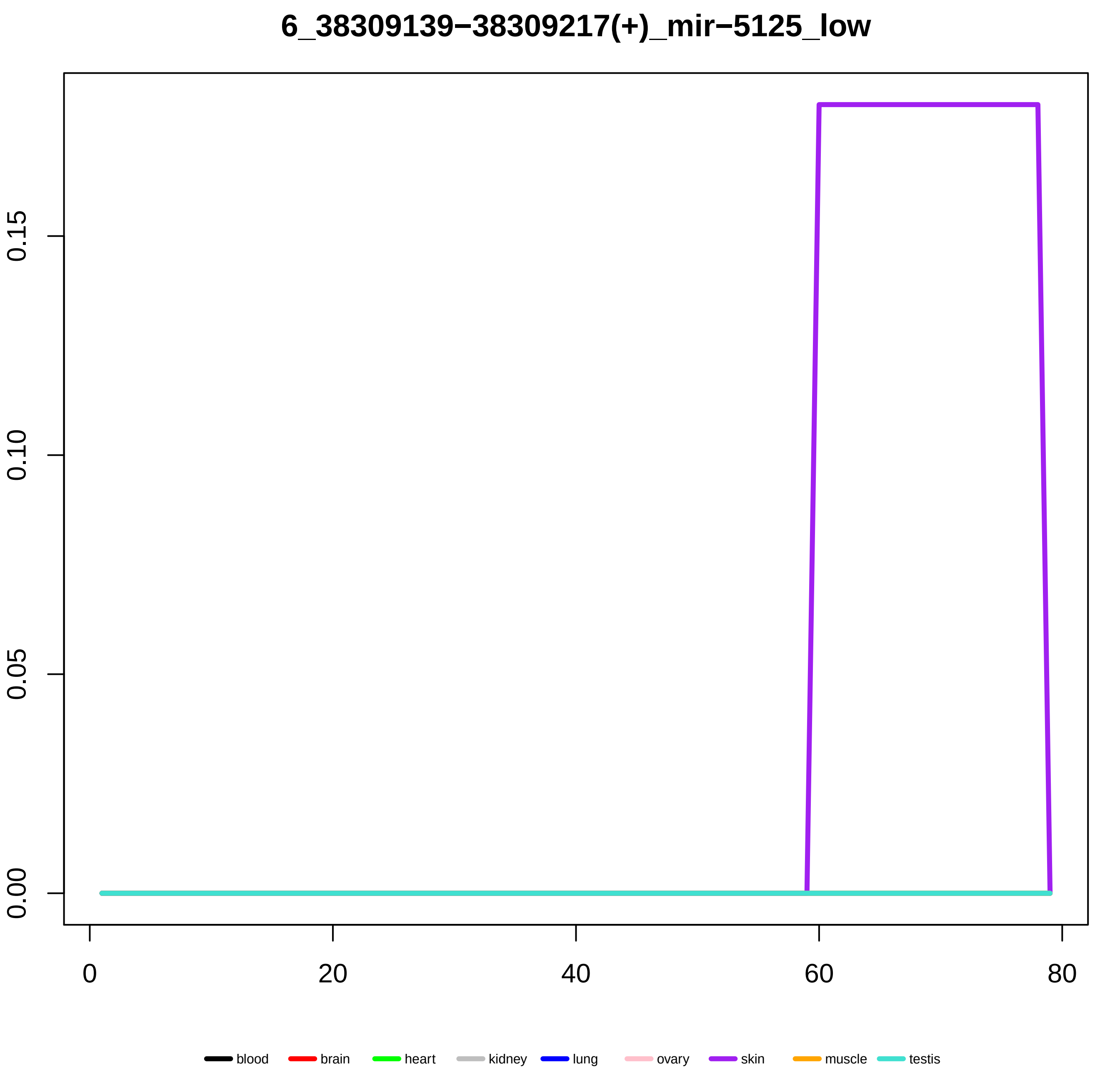

6_38309139−38309217(+)_mir−5125_low
0.00
0.05
0.10
0.15
0
20
40
60
80
blood
brain
heart
kidney
lung
ovary
skin
muscle
testis

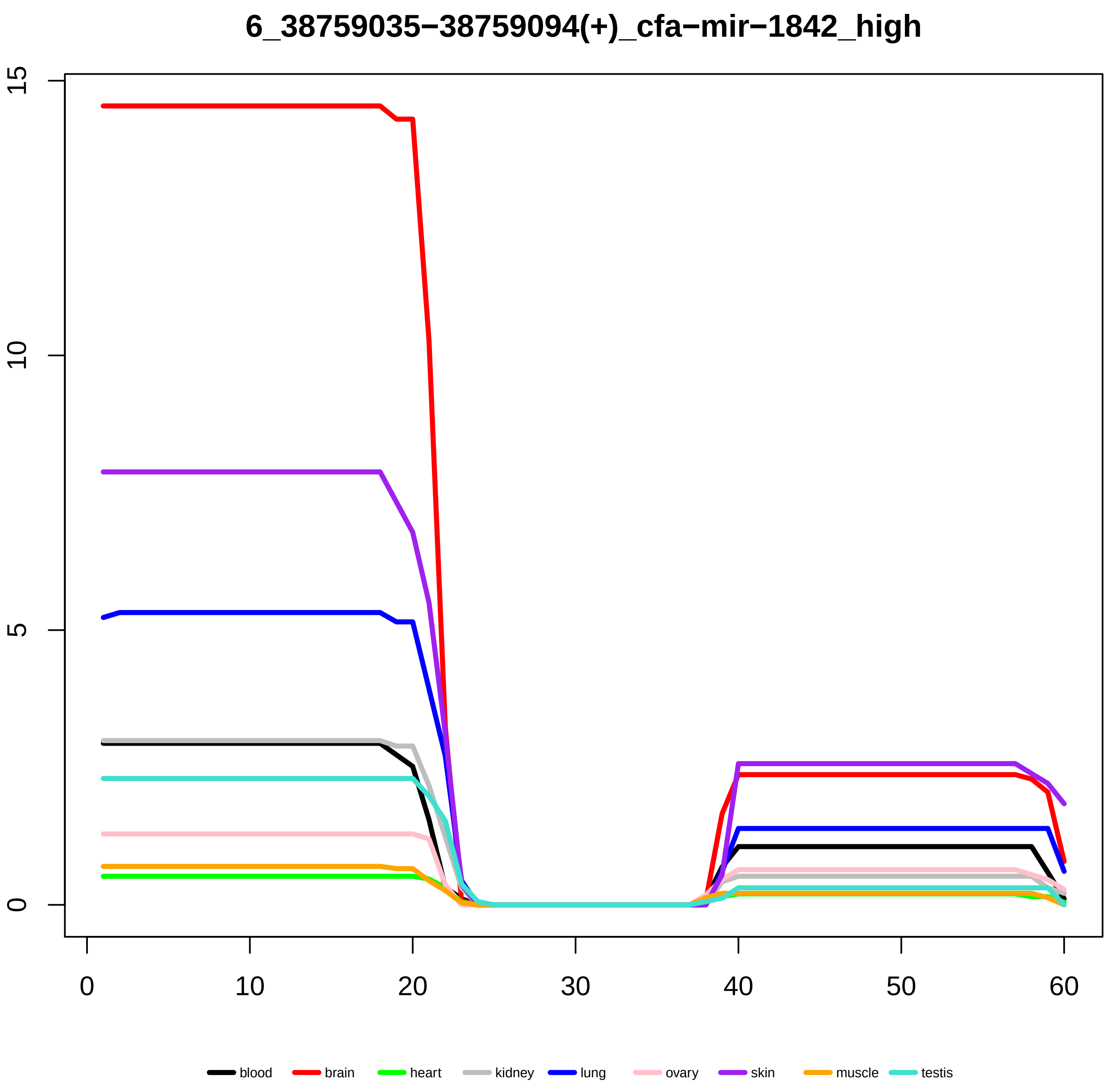
6_38759035−38759094(+)_cfa−mir−1842_high
0
5
10
15
0
10
20
30
40
50
60
blood
brain
heart
kidney
lung
ovary
skin
muscle
testis

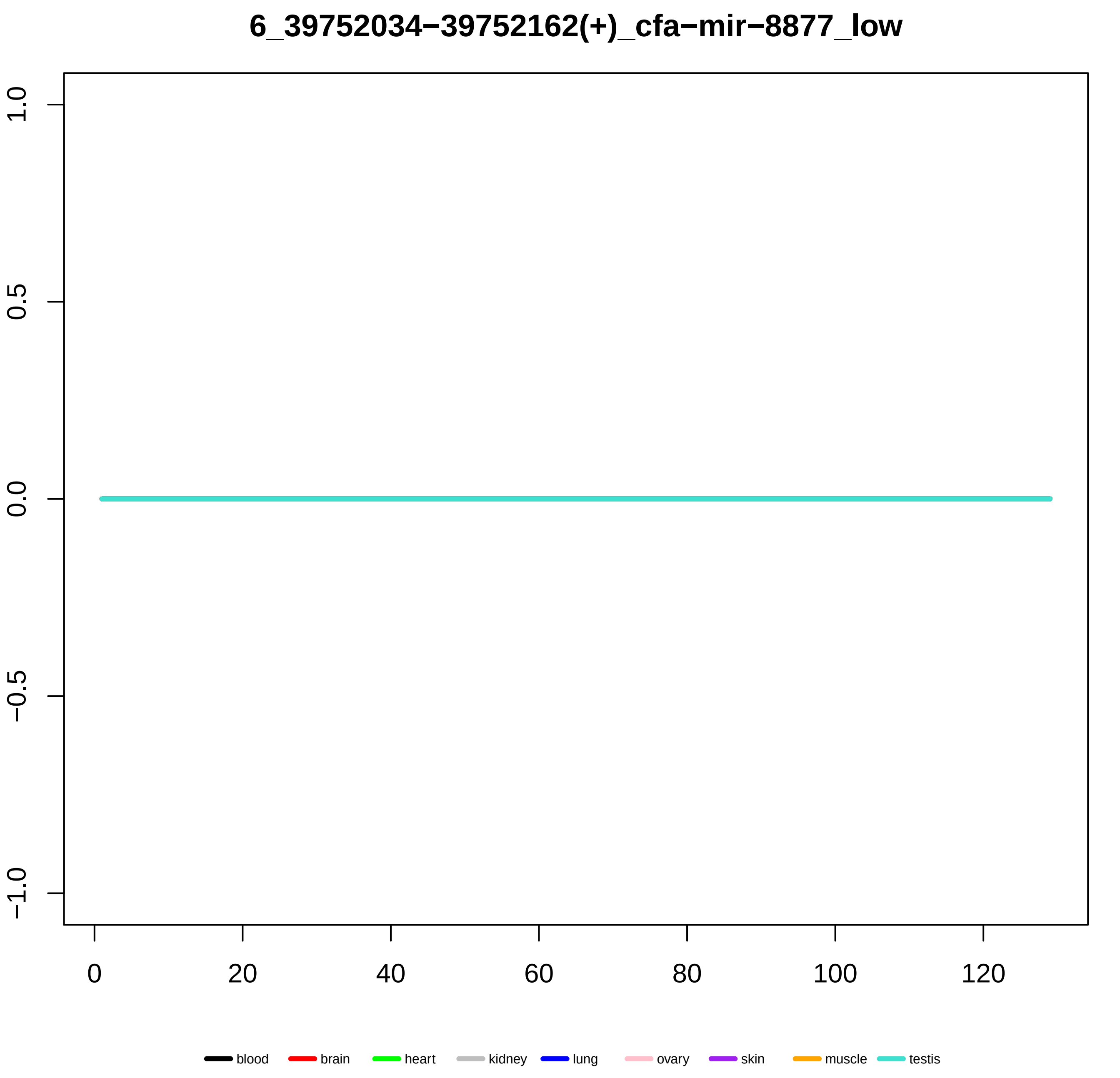
6_39752034−39752162(+)_cfa−mir−8877_low
1.0
0.5
0.0
−0.5
−1.0
0
20
40
60
80
100
120
blood
brain
heart
kidney
lung
ovary
skin
muscle
testis

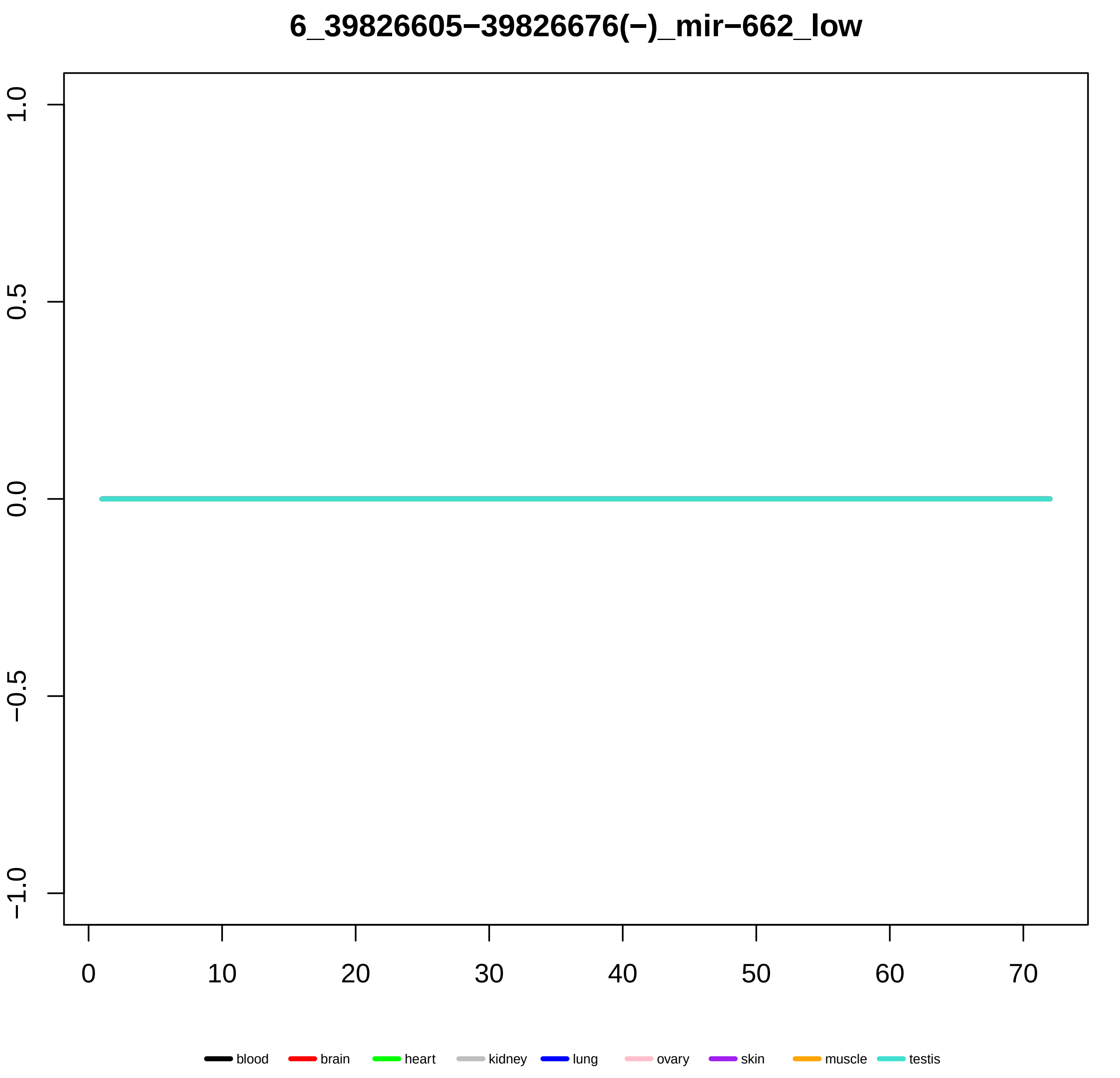

6_39826605−39826676(−)_mir−662_low
1.0
0.5
0.0
−0.5
−1.0
0
10
20
30
40
50
60
70
blood
brain
heart
kidney
lung
ovary
skin
muscle
testis

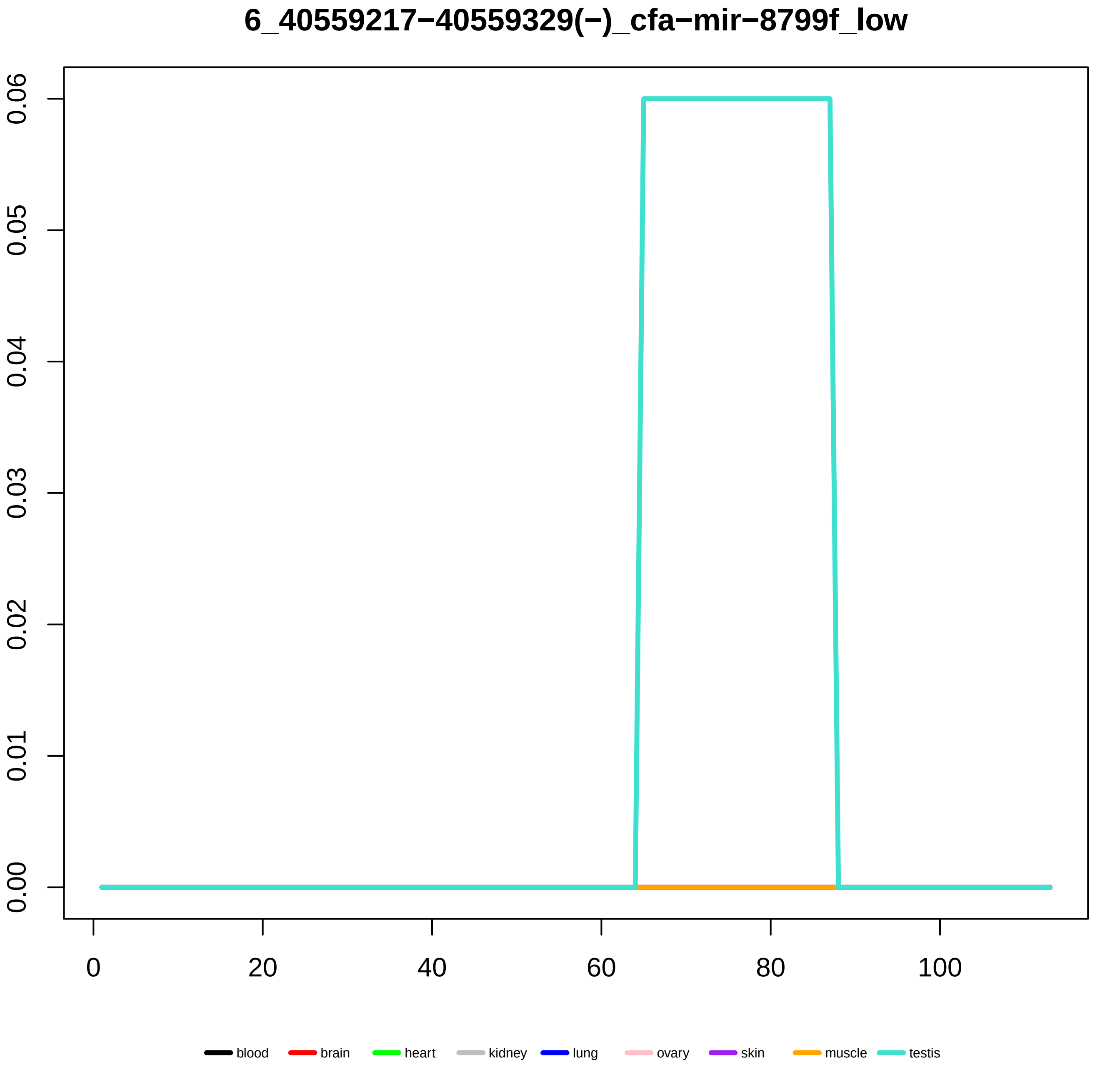
6_40559217−40559329(−)_cfa−mir−8799f_low
0.00
0.01
0.02
0.03
0.04
0.05
0.06
0
20
40
60
80
100
blood
brain
heart
kidney
lung
ovary
skin
muscle
testis

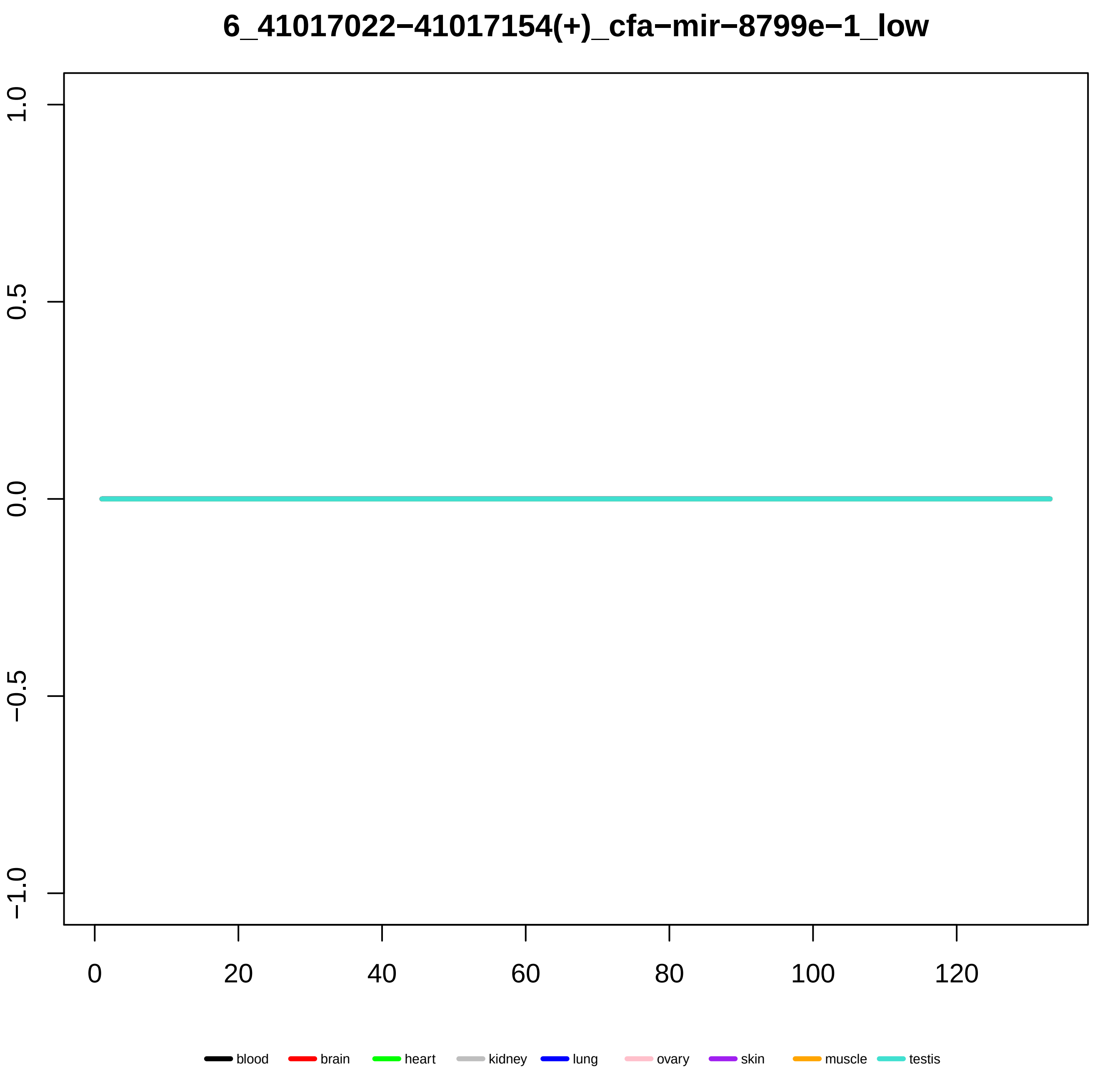

6_41017022−41017154(+)_cfa−mir−8799e−1_low
1.0
0.5
0.0
−0.5
−1.0
0
20
40
60
80
100
120
blood
brain
heart
kidney
lung
ovary
skin
muscle
testis

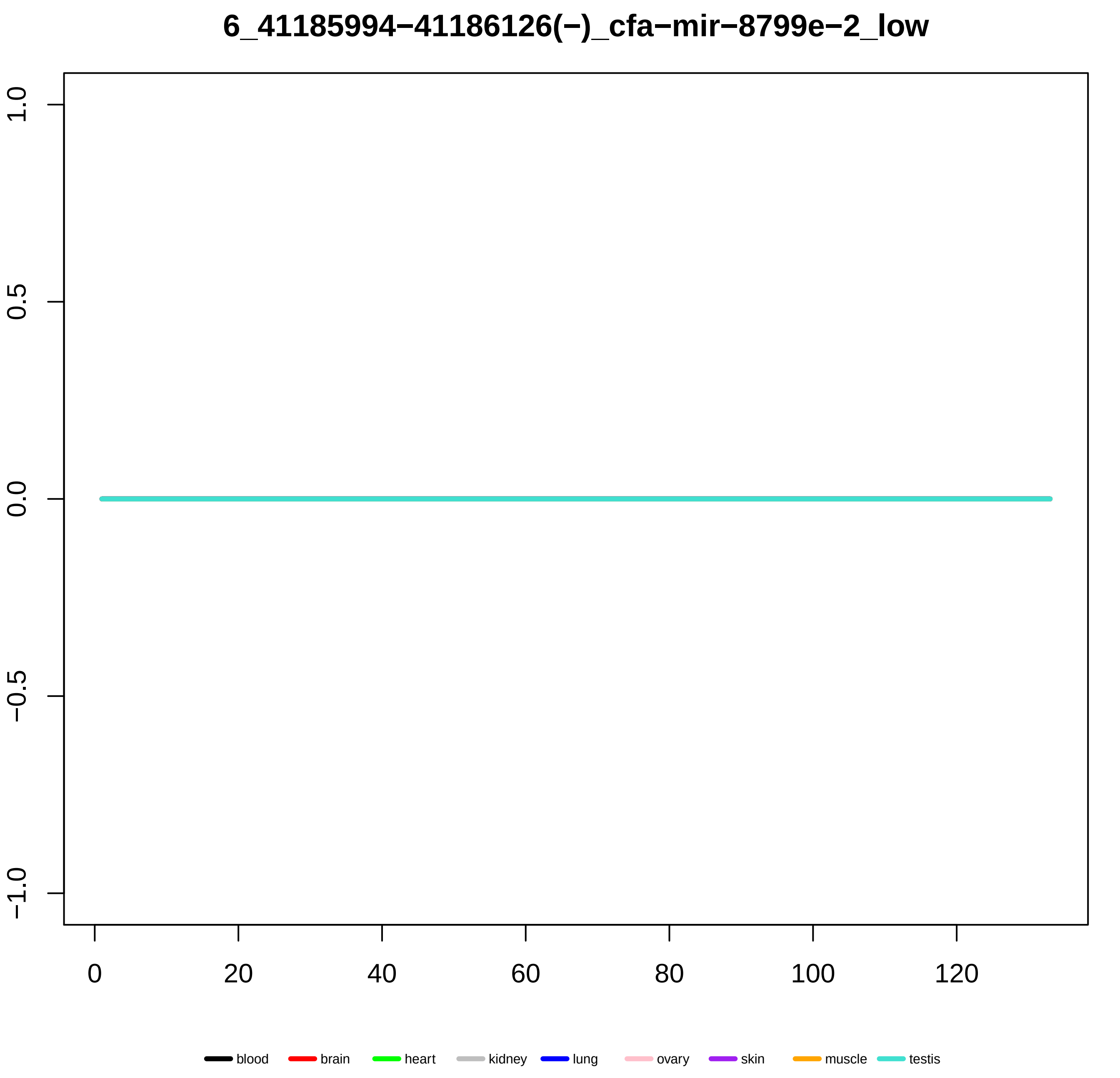

6_41185994−41186126(−)_cfa−mir−8799e−2_low
1.0
0.5
0.0
−0.5
−1.0
0
20
40
60
80
100
120
blood
brain
heart
kidney
lung
ovary
skin
muscle
testis

6_42271719−42271779(−)_cfa−mir−197_high

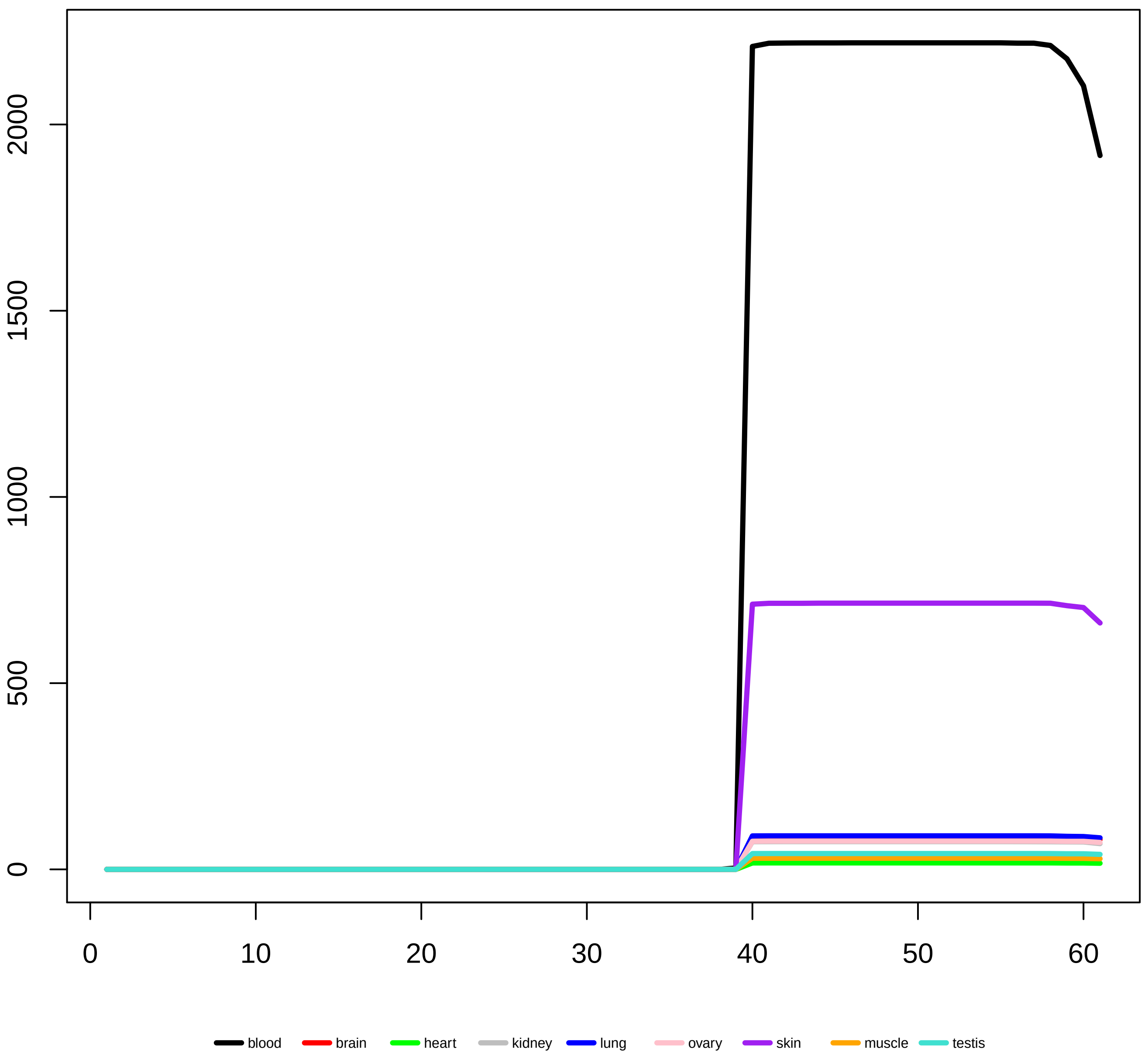

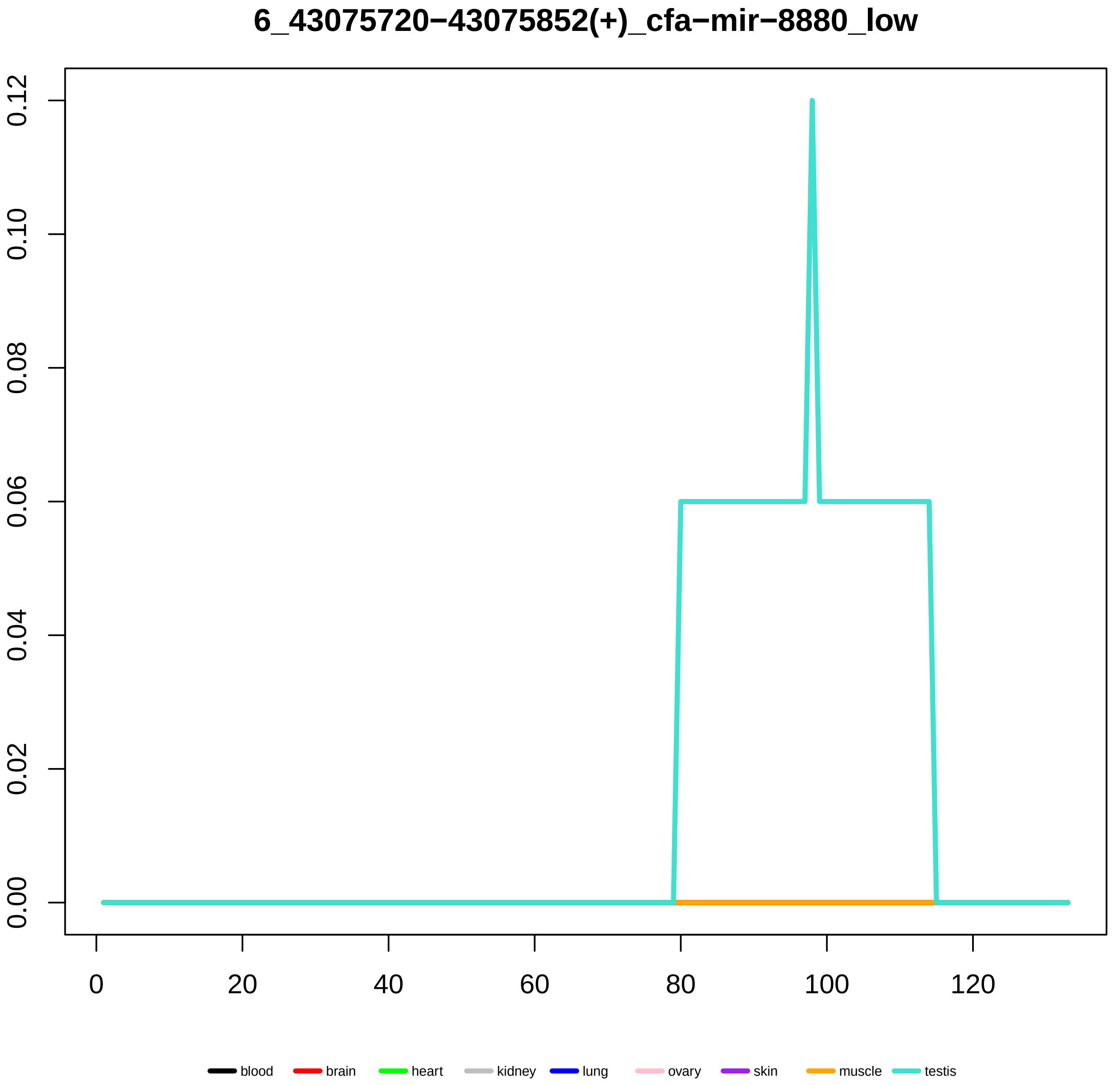
6_43075720−43075852(+)_cfa−mir−8880_low
0.00
0.02
0.04
0.06
0.08
0.10
0.12
0
20
40
60
80
100
120
blood
brain
heart
kidney
lung
ovary
skin
muscle
testis

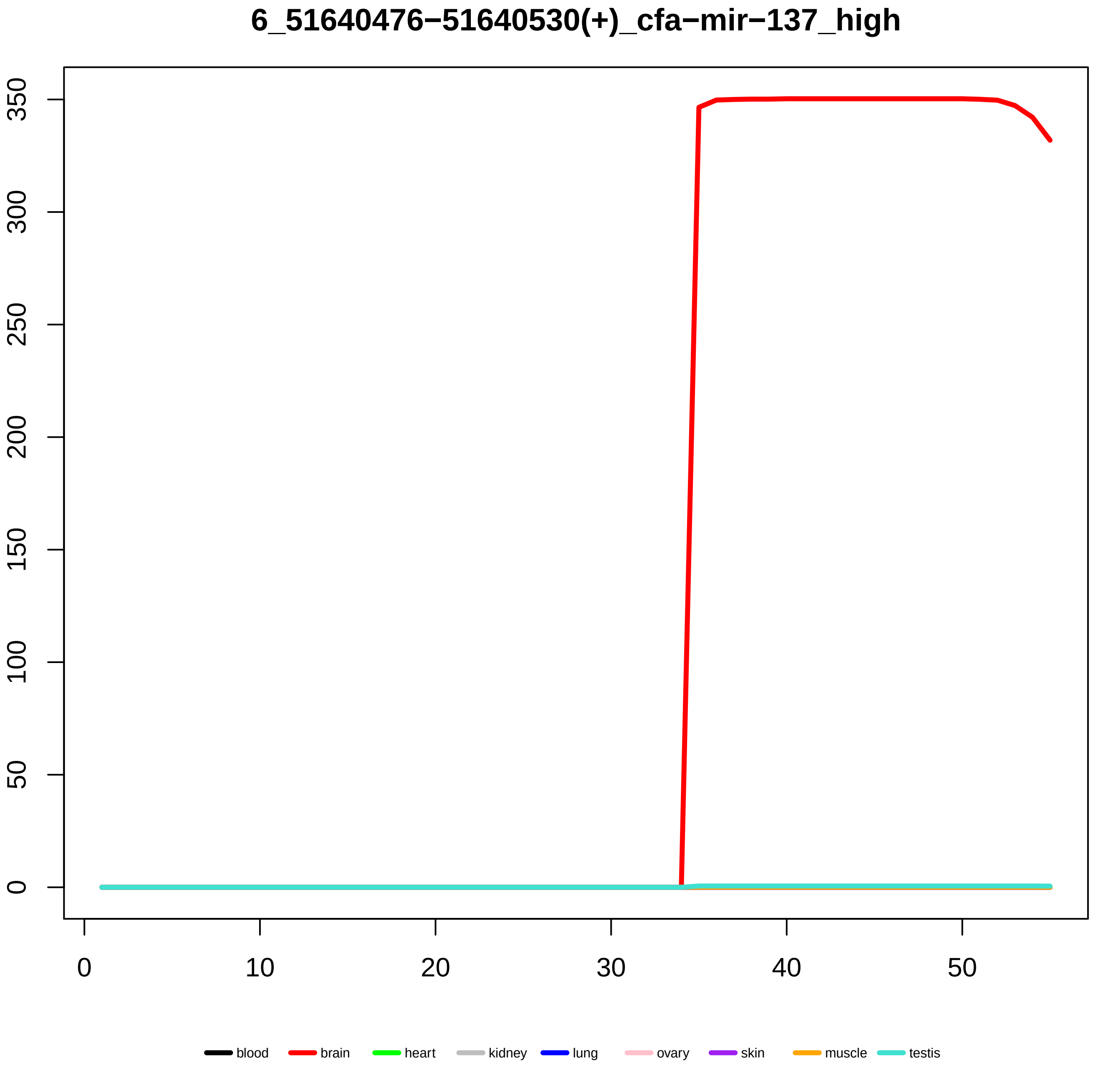
6_51640476−51640530(+)_cfa−mir−137_high
0
50
100
150
200
250
300
350
0
10
20
30
40
50
blood
brain
heart
kidney
lung
ovary
skin
muscle
testis

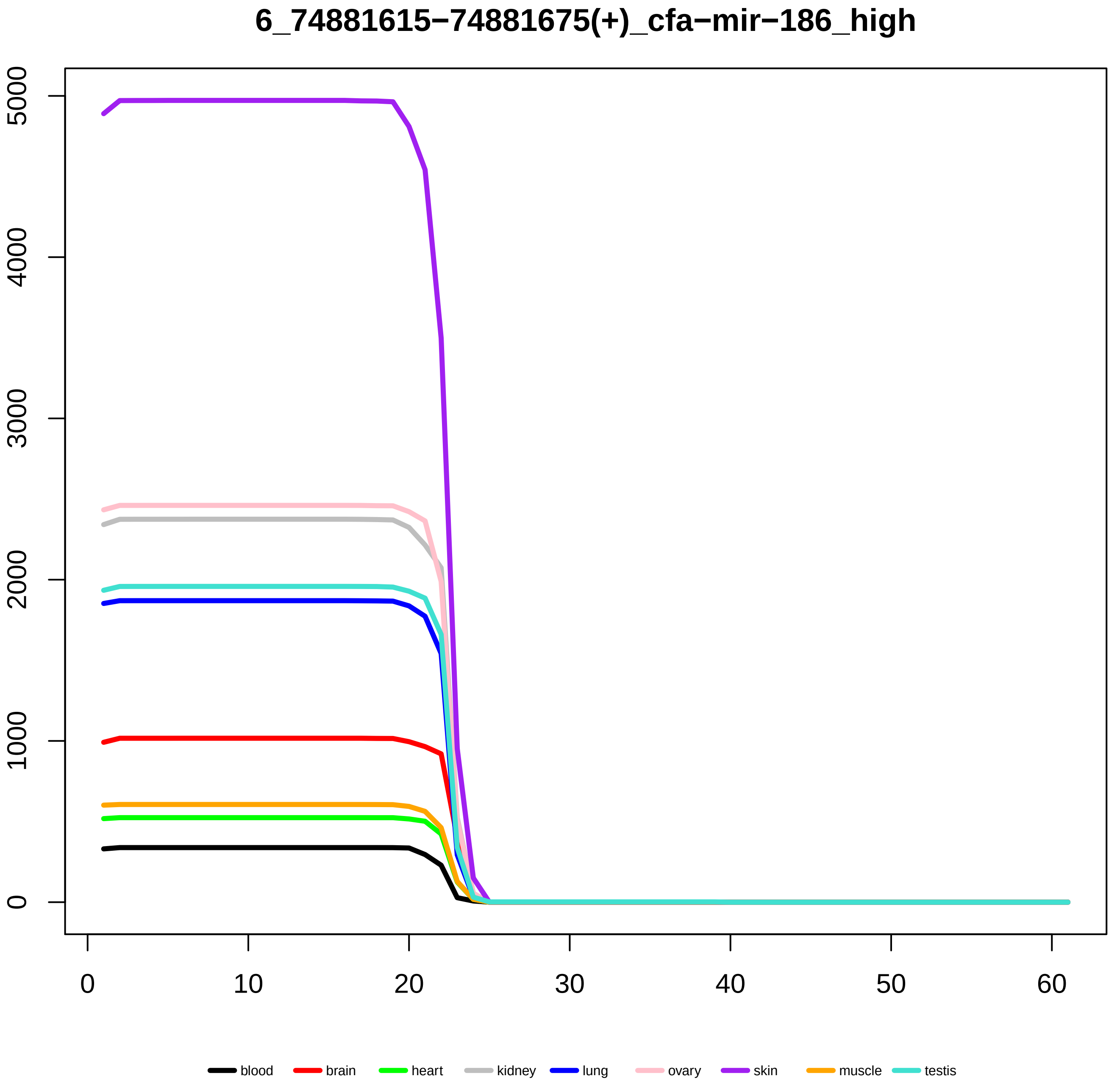

6_74881615−74881675(+)_cfa−mir−186_high
0
1000
2000
3000
4000
5000
0
10
20
30
40
50
60
blood
brain
heart
kidney
lung
ovary
skin
muscle
testis

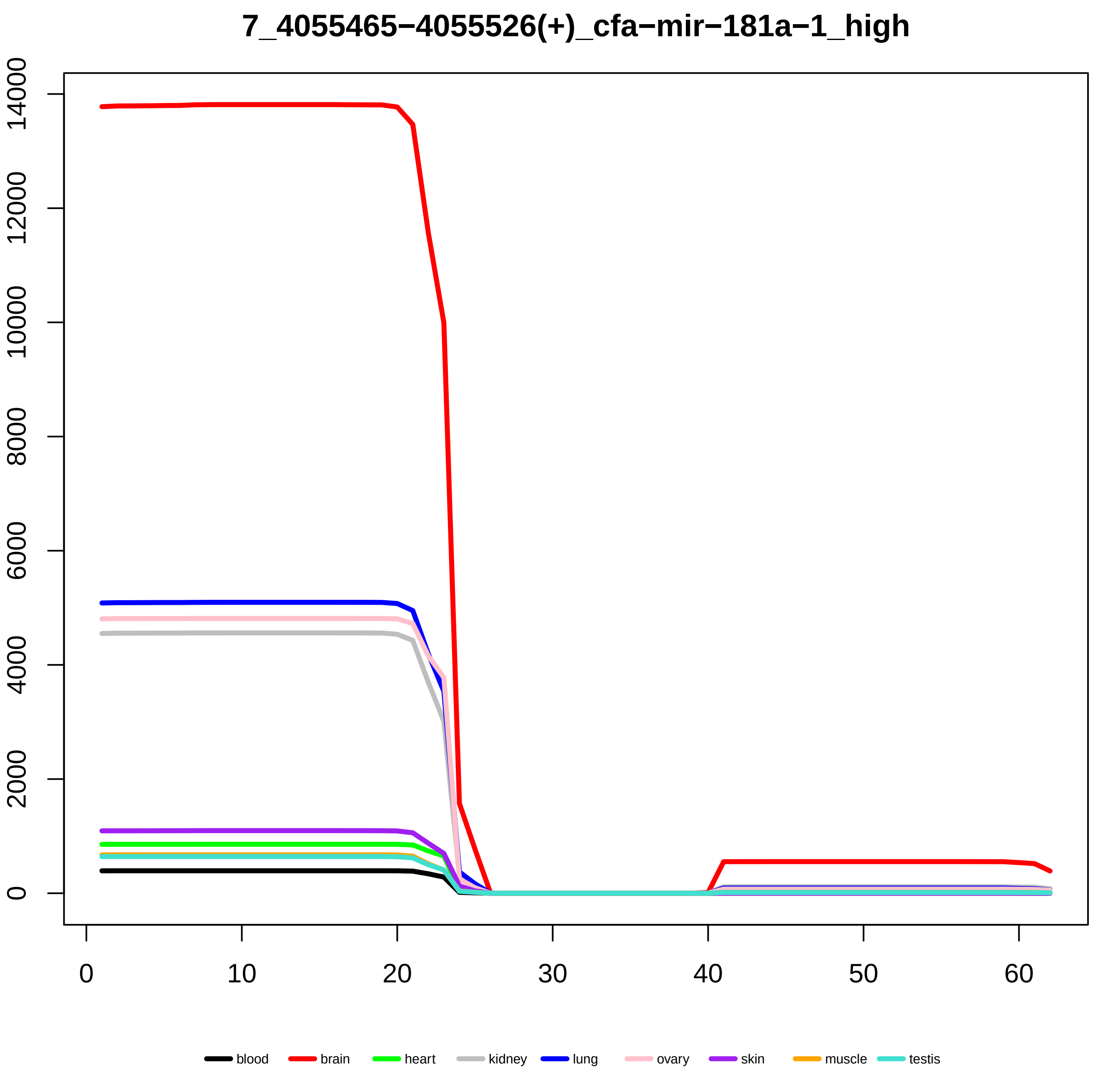

7_4055465−4055526(+)_cfa−mir−181a−1_high
0
2000
4000
6000
8000
10000
12000
14000
0
10
20
30
40
50
60
blood
brain
heart
kidney
lung
ovary
skin
muscle
testis

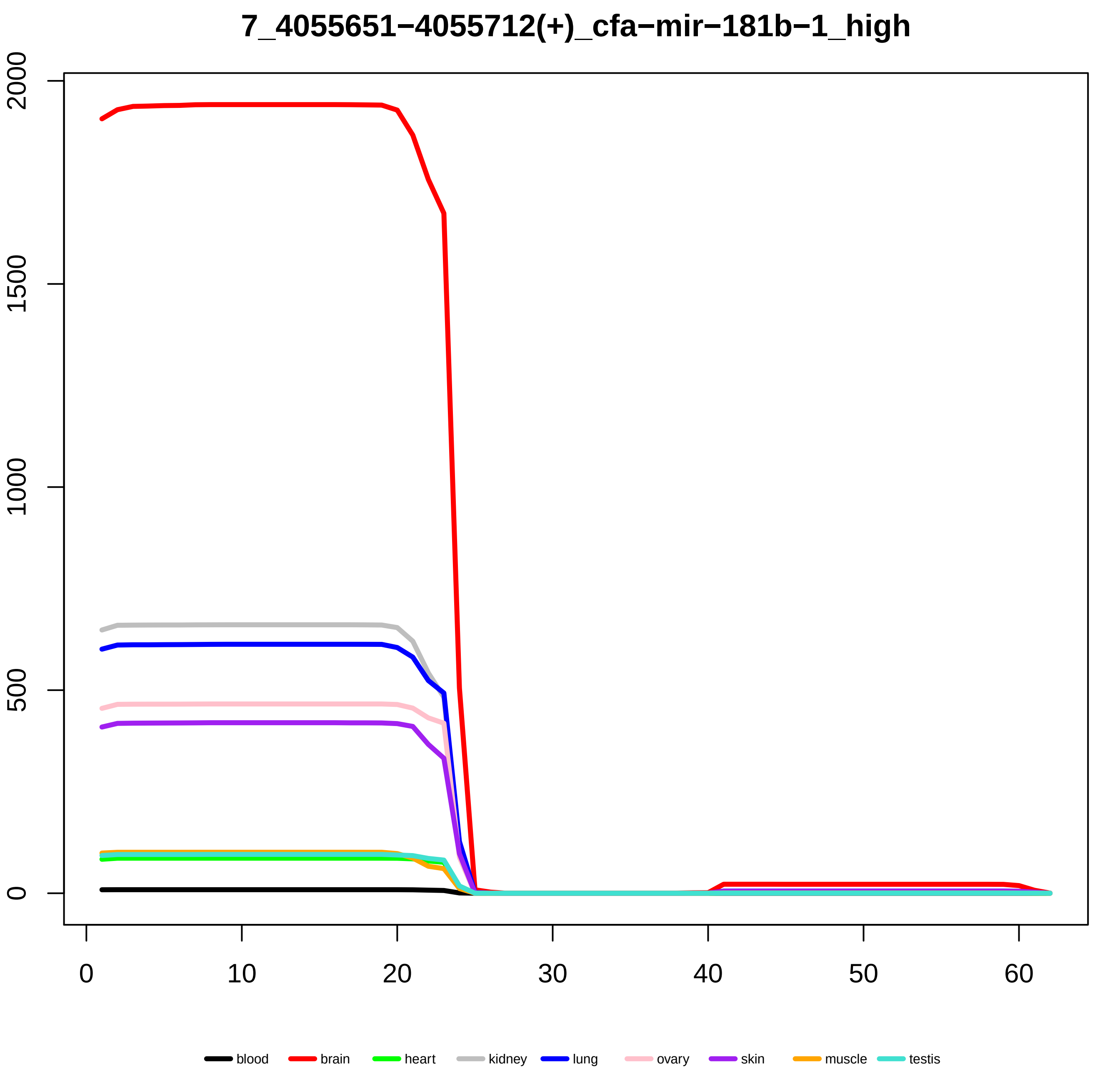

7_4055651−4055712(+)_cfa−mir−181b−1_high
0
500
1000
1500
2000
0
10
20
30
40
50
60
blood
brain
heart
kidney
lung
ovary
skin
muscle
testis

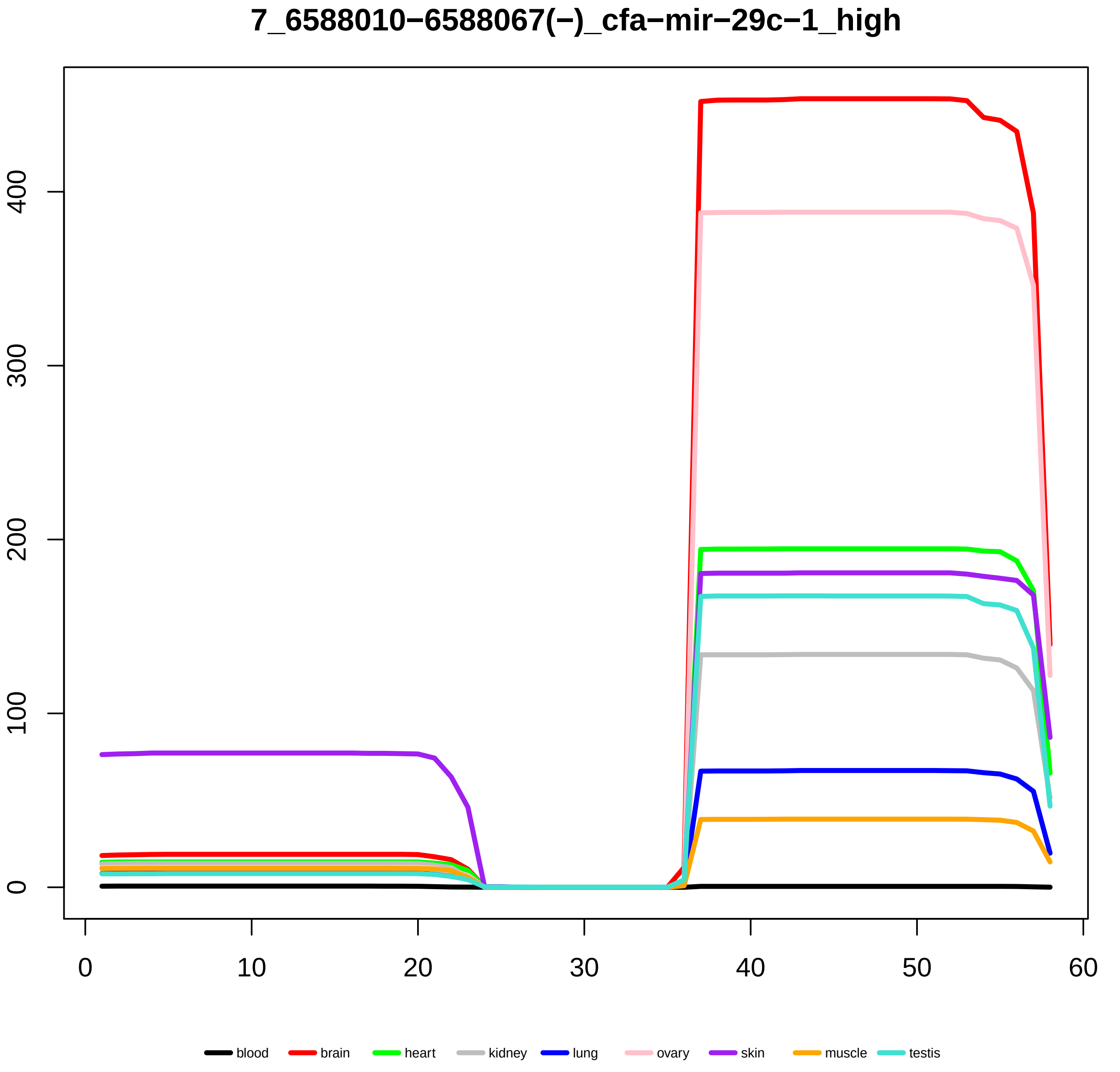
7_6588010−6588067(−)_cfa−mir−29c−1_high
0
100
200
300
400
0
10
20
30
40
50
60
blood
brain
heart
kidney
lung
ovary
skin
muscle
testis

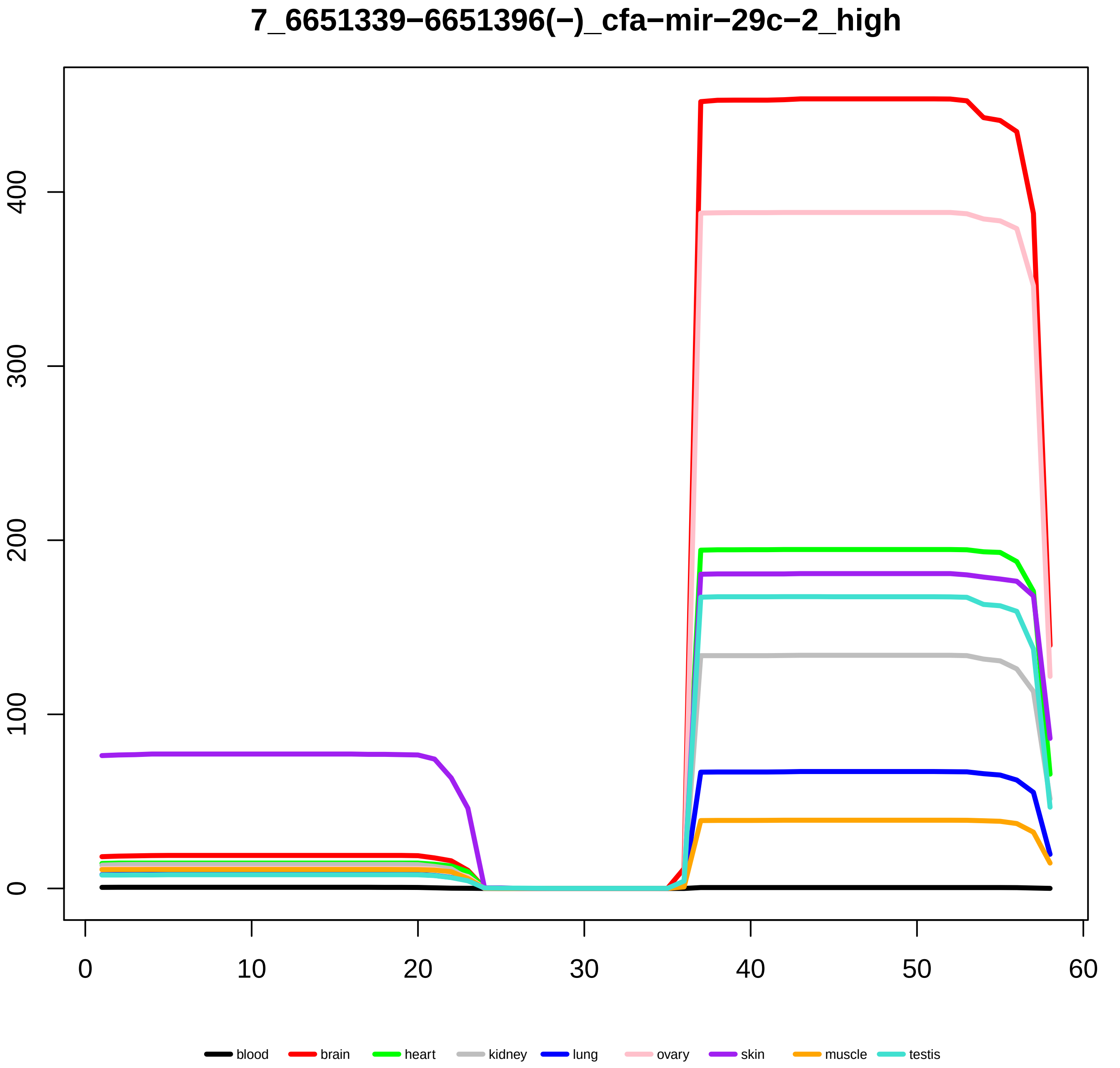
7_6651339−6651396(−)_cfa−mir−29c−2_high
0
100
200
300
400
0
10
20
30
40
50
60
blood
brain
heart
kidney
lung
ovary
skin
muscle
testis

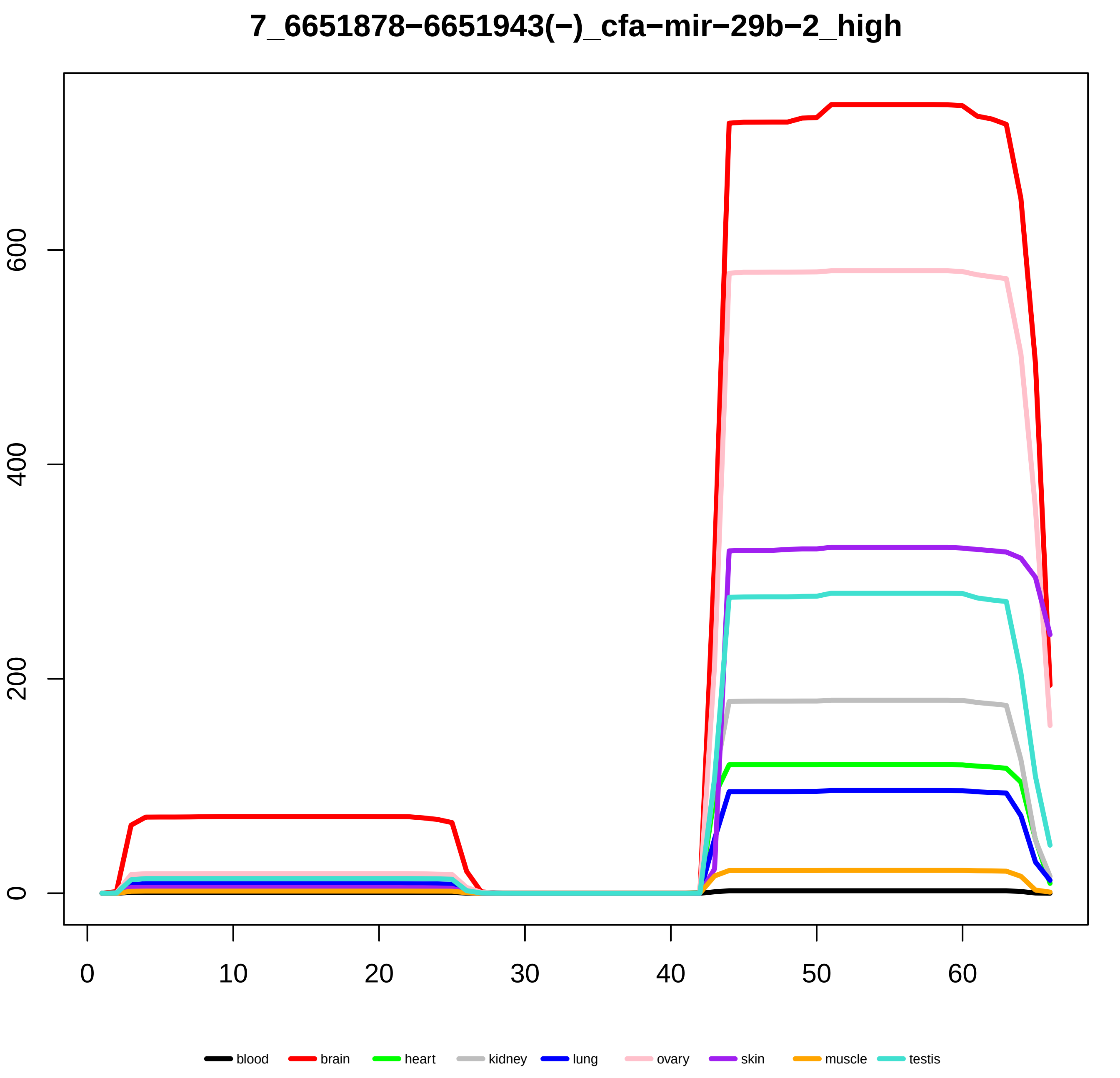

7_6651878−6651943(−)_cfa−mir−29b−2_high
0
200
400
600
0
10
20
30
40
50
60
blood
brain
heart
kidney
lung
ovary
skin
muscle
testis

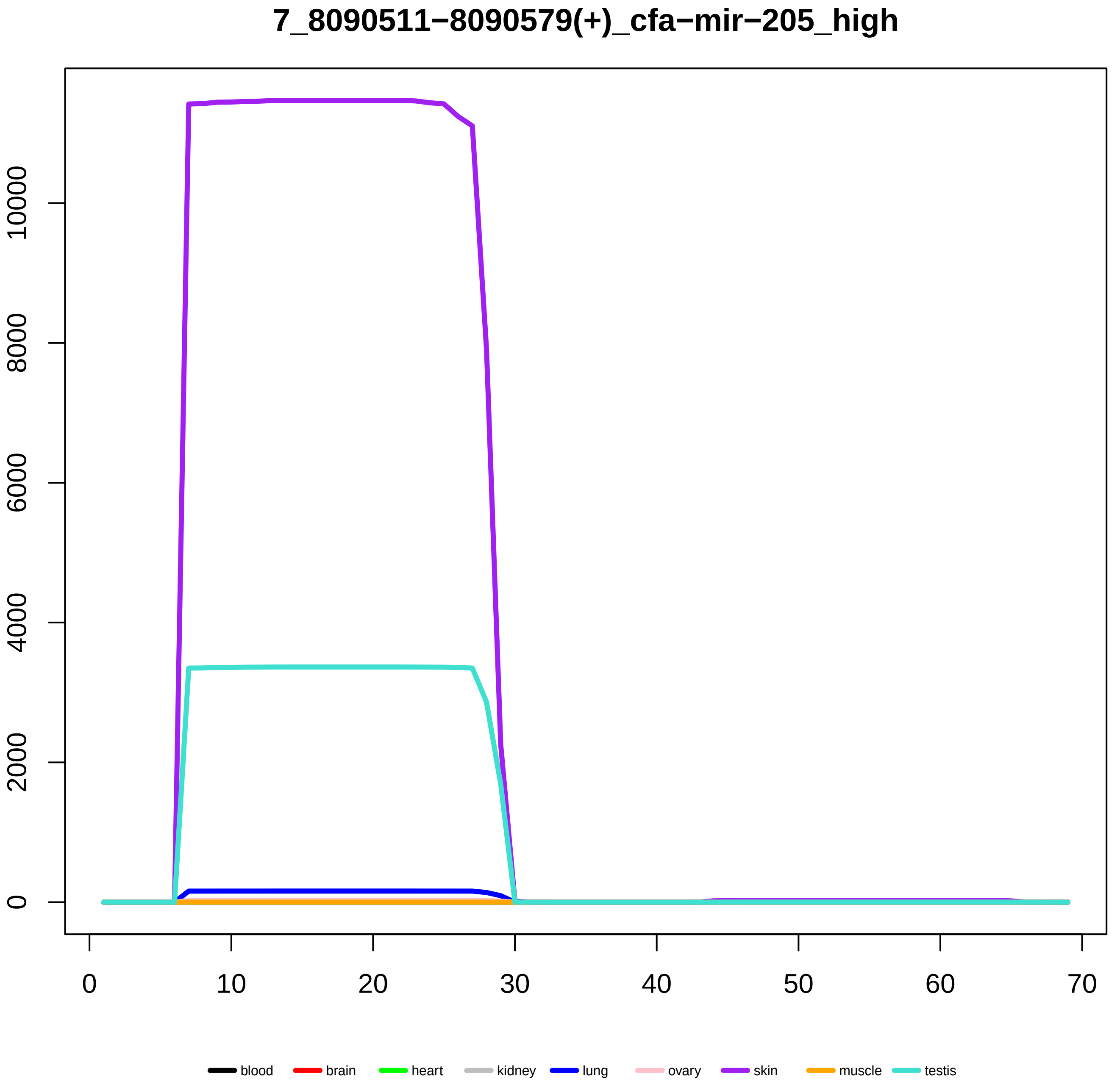
7_8090511−8090579(+)_cfa−mir−205_high
0
2000
4000
6000
8000
10000
0
10
20
30
40
50
60
70
blood
brain
heart
kidney
lung
ovary
skin
muscle
testis

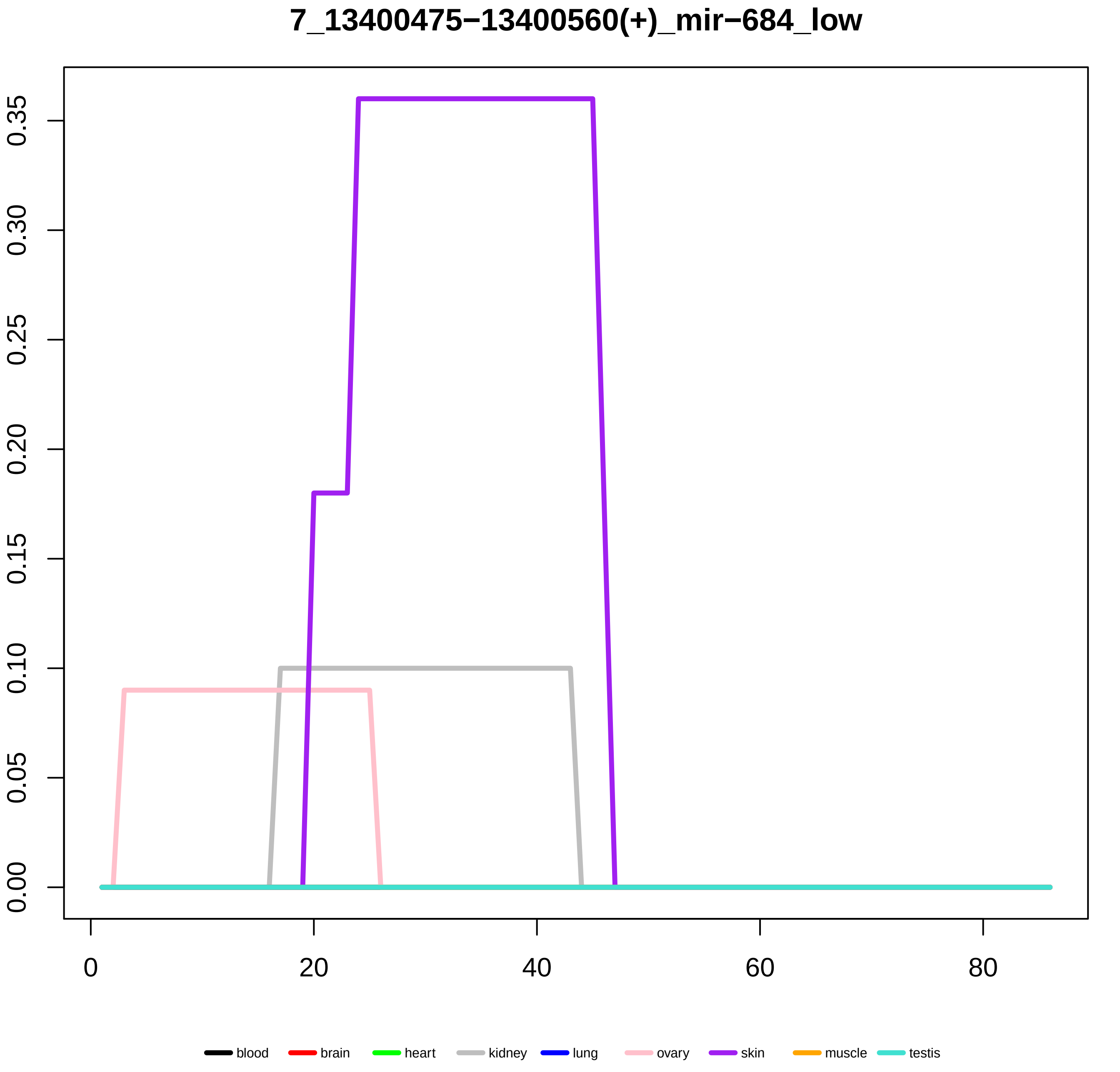

7_13400475−13400560(+)_mir−684_low
0.00
0.05
0.10
0.15
0.20
0.25
0.30
0.35
0
20
40
60
80
blood
brain
heart
kidney
lung
ovary
skin
muscle
testis

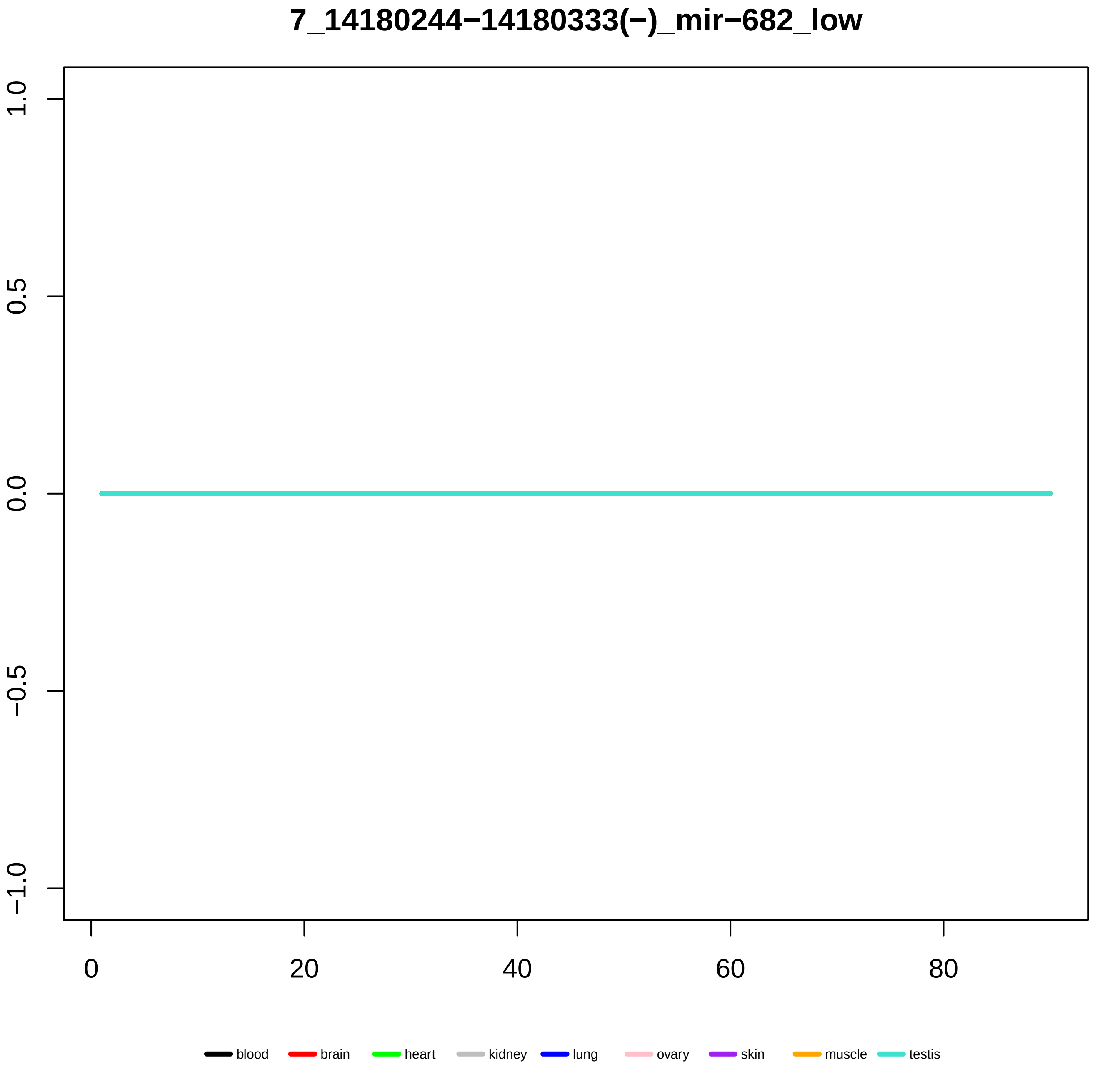

7_14180244−14180333(−)_mir−682_low
1.0
0.5
0.0
−0.5
−1.0
0
20
40
60
80
blood
brain
heart
kidney
lung
ovary
skin
muscle
testis

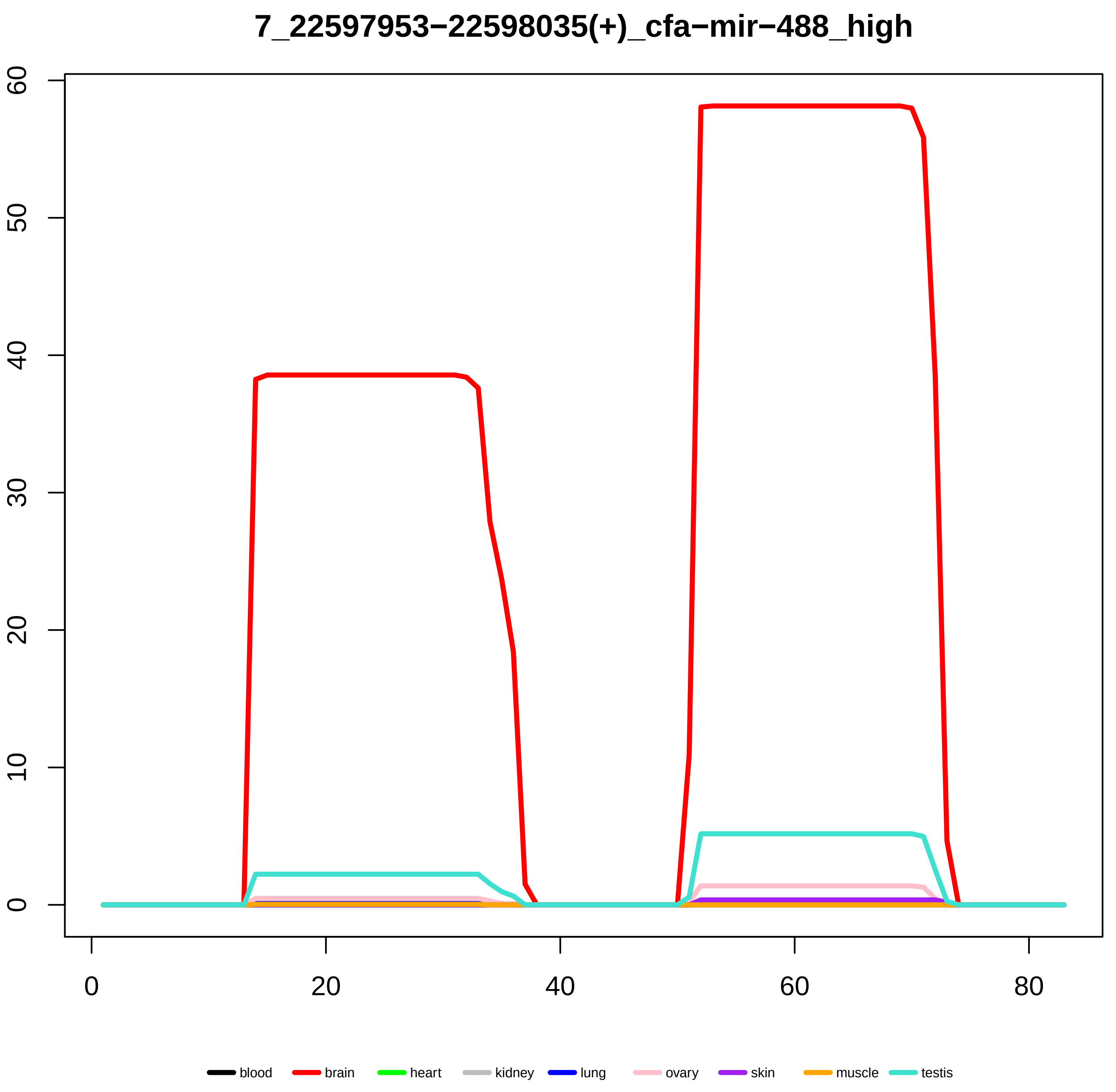

7_22597953−22598035(+)_cfa−mir−488_high
0
10
20
30
40
50
60
0
20
40
60
80
blood
brain
heart
kidney
lung
ovary
skin
muscle
testis

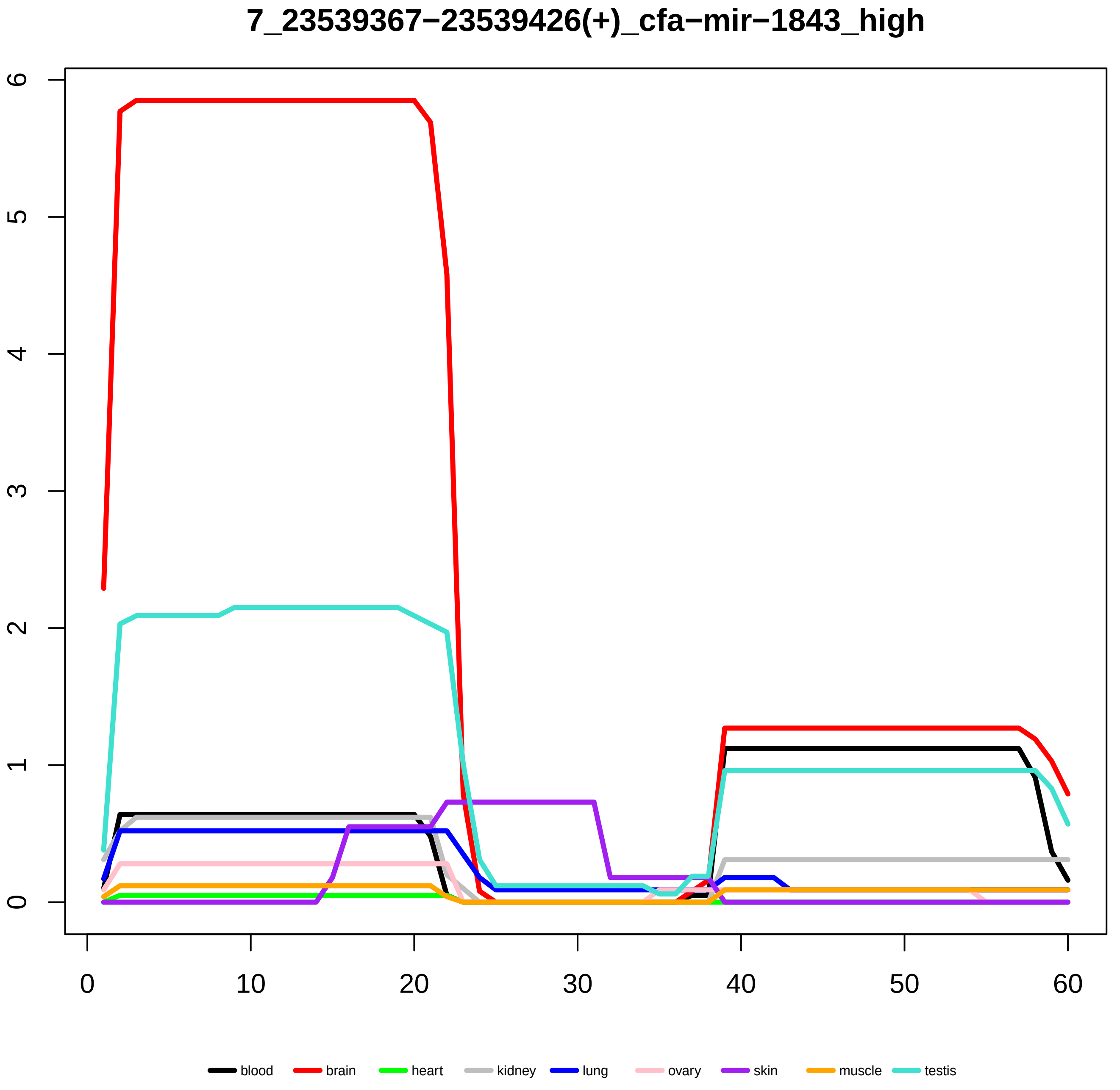

7_23539367−23539426(+)_cfa−mir−1843_high
0
1
2
3
4
5
6
0
10
20
30
40
50
60
blood
brain
heart
kidney
lung
ovary
skin
muscle
testis

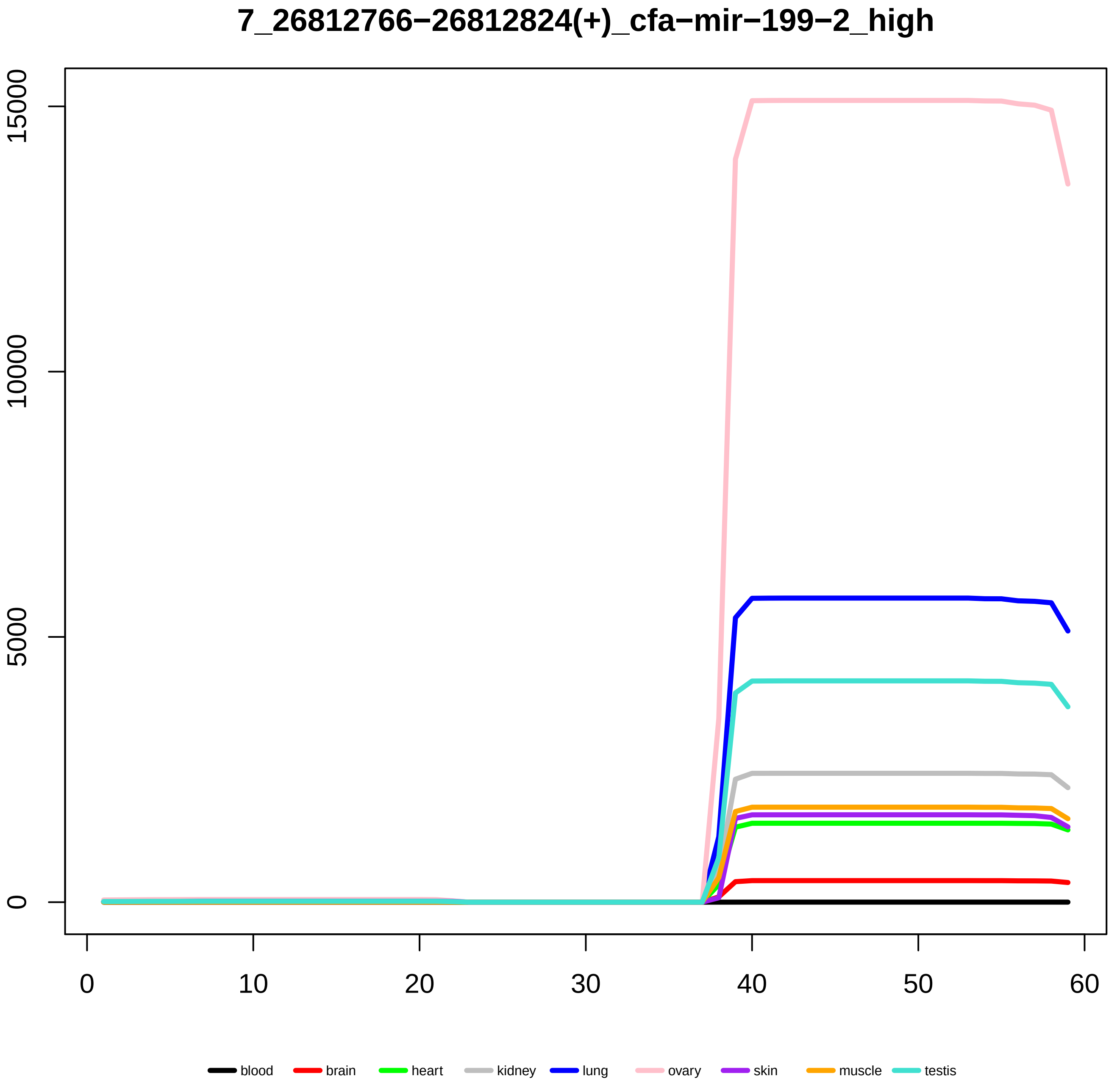
7_26812766−26812824(+)_cfa−mir−199−2_high
0
5000
10000
15000
0
10
20
30
40
50
60
blood
brain
heart
kidney
lung
ovary
skin
muscle
testis

7_26818559−26818668(+)_cfa−mir−214_high

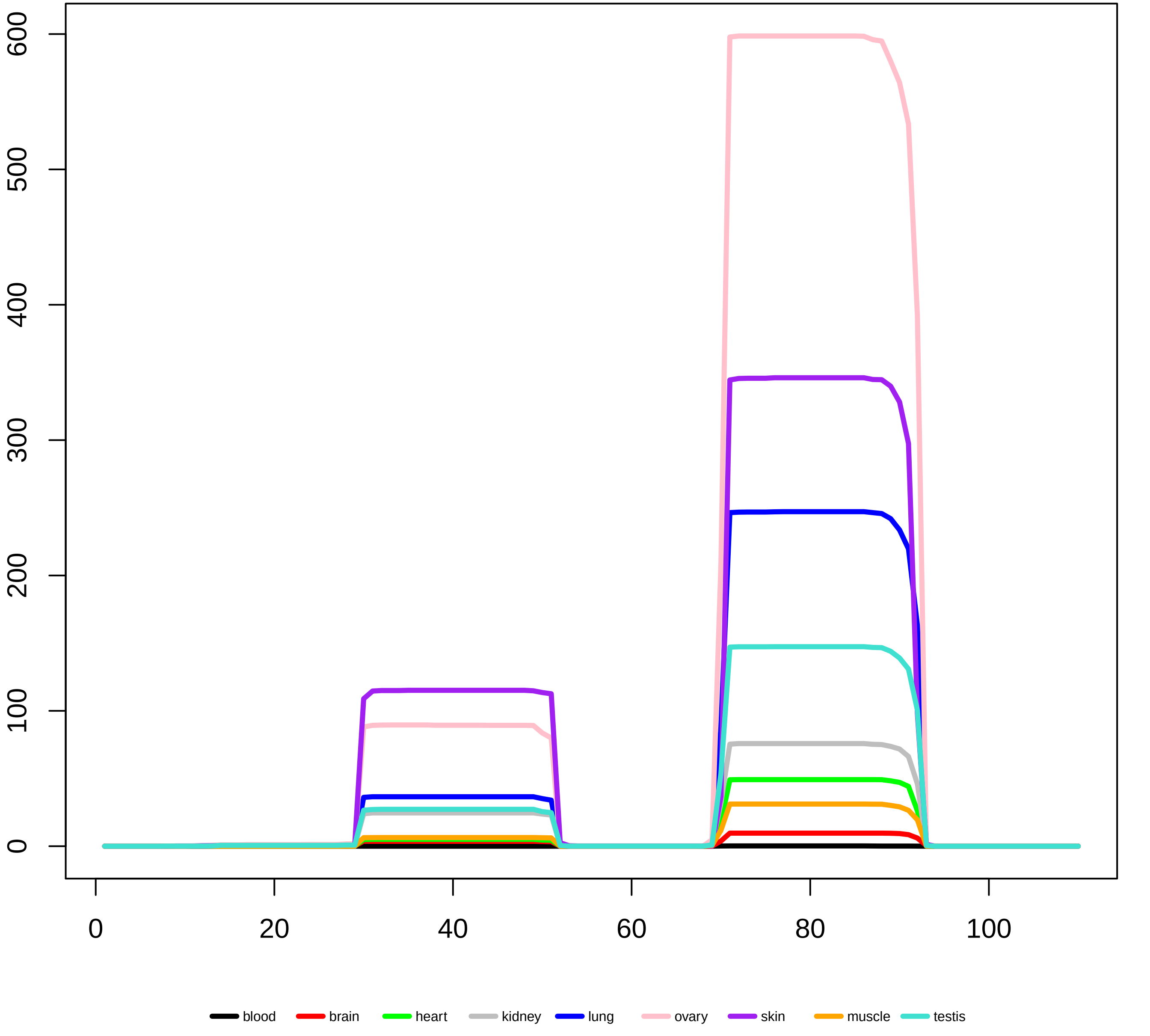

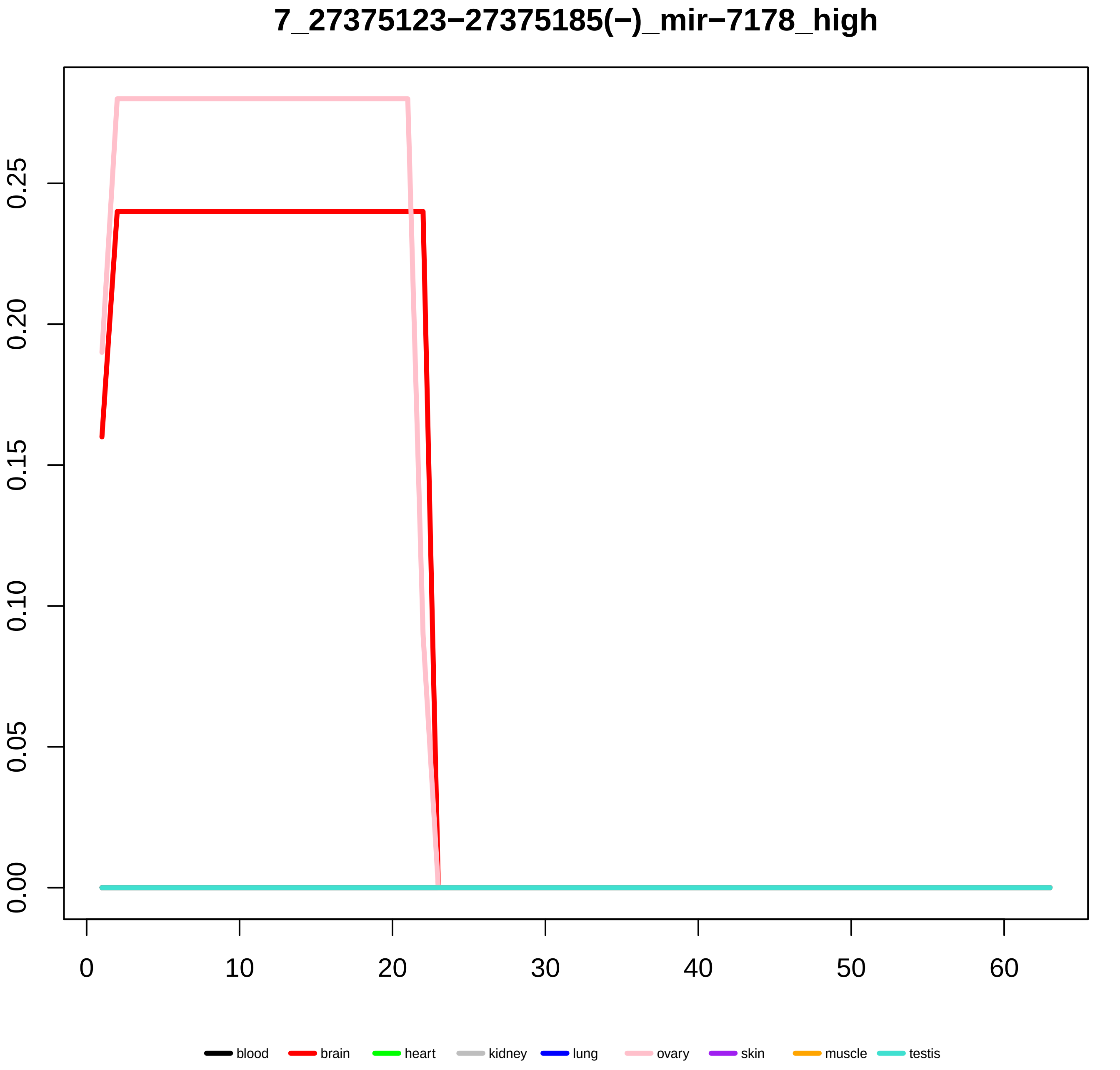

7_27375123−27375185(−)_mir−7178_high
0.00
0.05
0.10
0.15
0.20
0.25
0
10
20
30
40
50
60
blood
brain
heart
kidney
lung
ovary
skin
muscle
testis

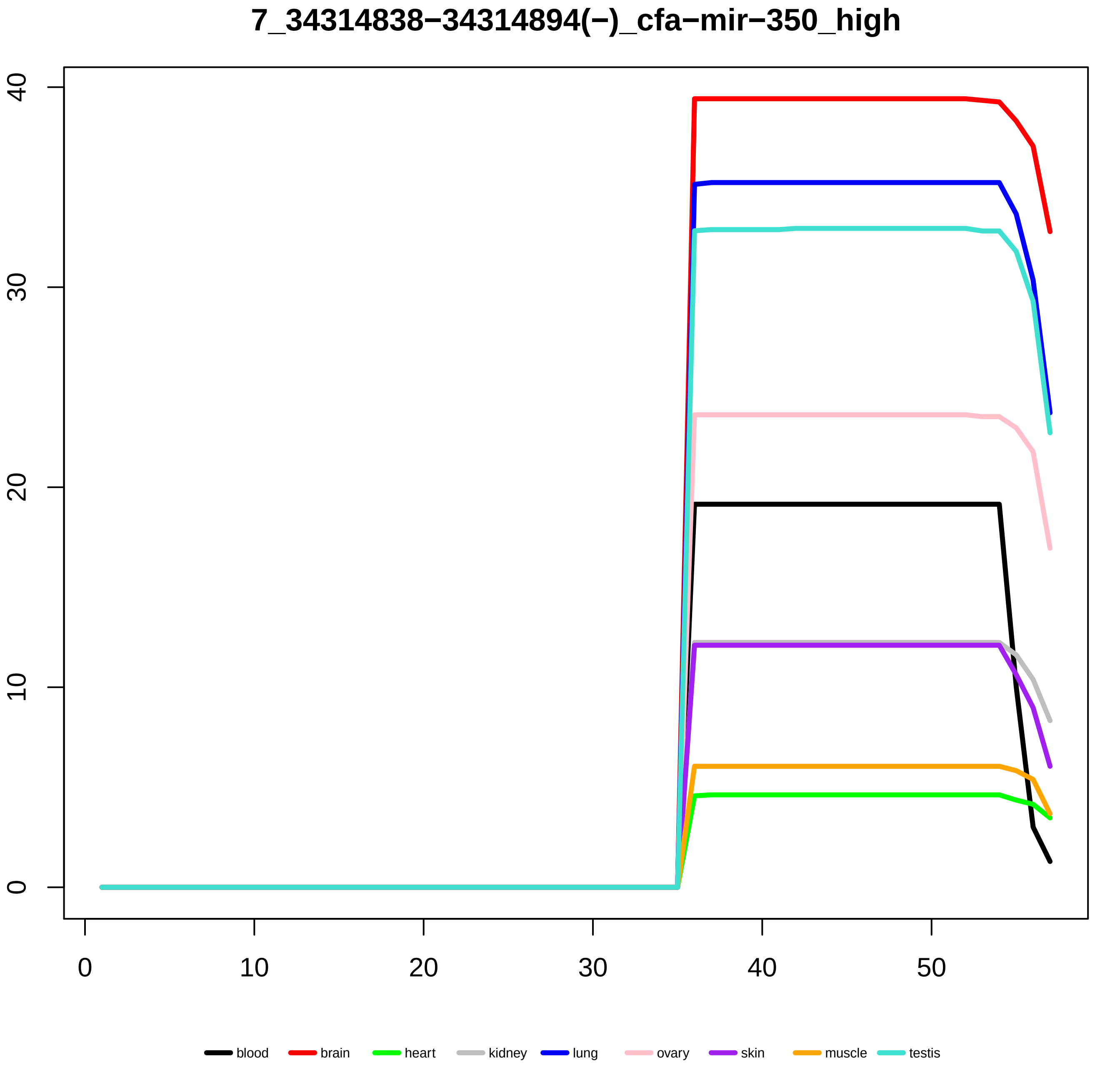

7_34314838−34314894(−)_cfa−mir−350_high
0
10
20
30
40
0
10
20
30
40
50
blood
brain
heart
kidney
lung
ovary
skin
muscle
testis

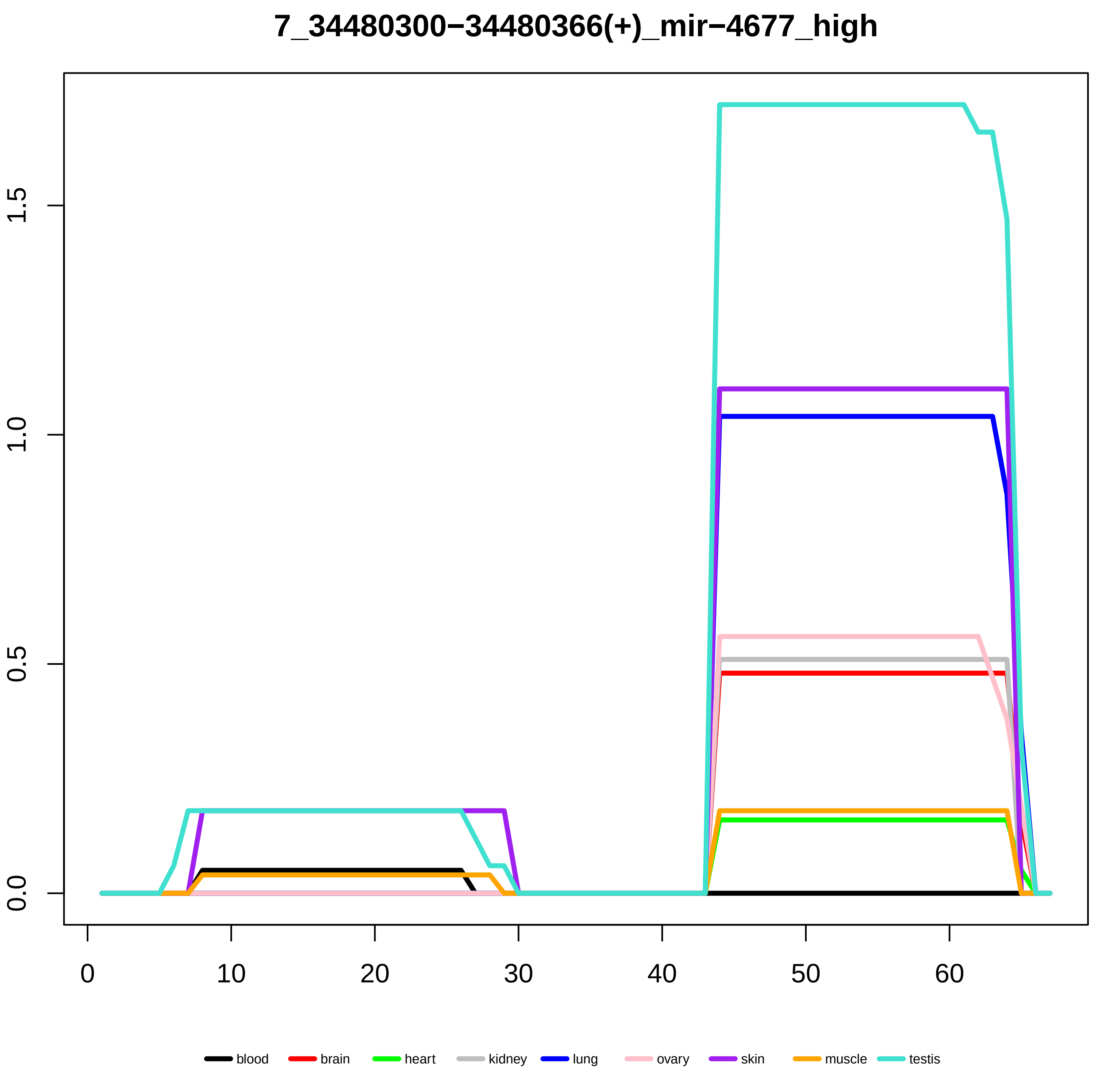

7_34480300−34480366(+)_mir−4677_high
0.0
0.5
1.0
1.5
0
10
20
30
40
50
60
blood
brain
heart
kidney
lung
ovary
skin
muscle
testis

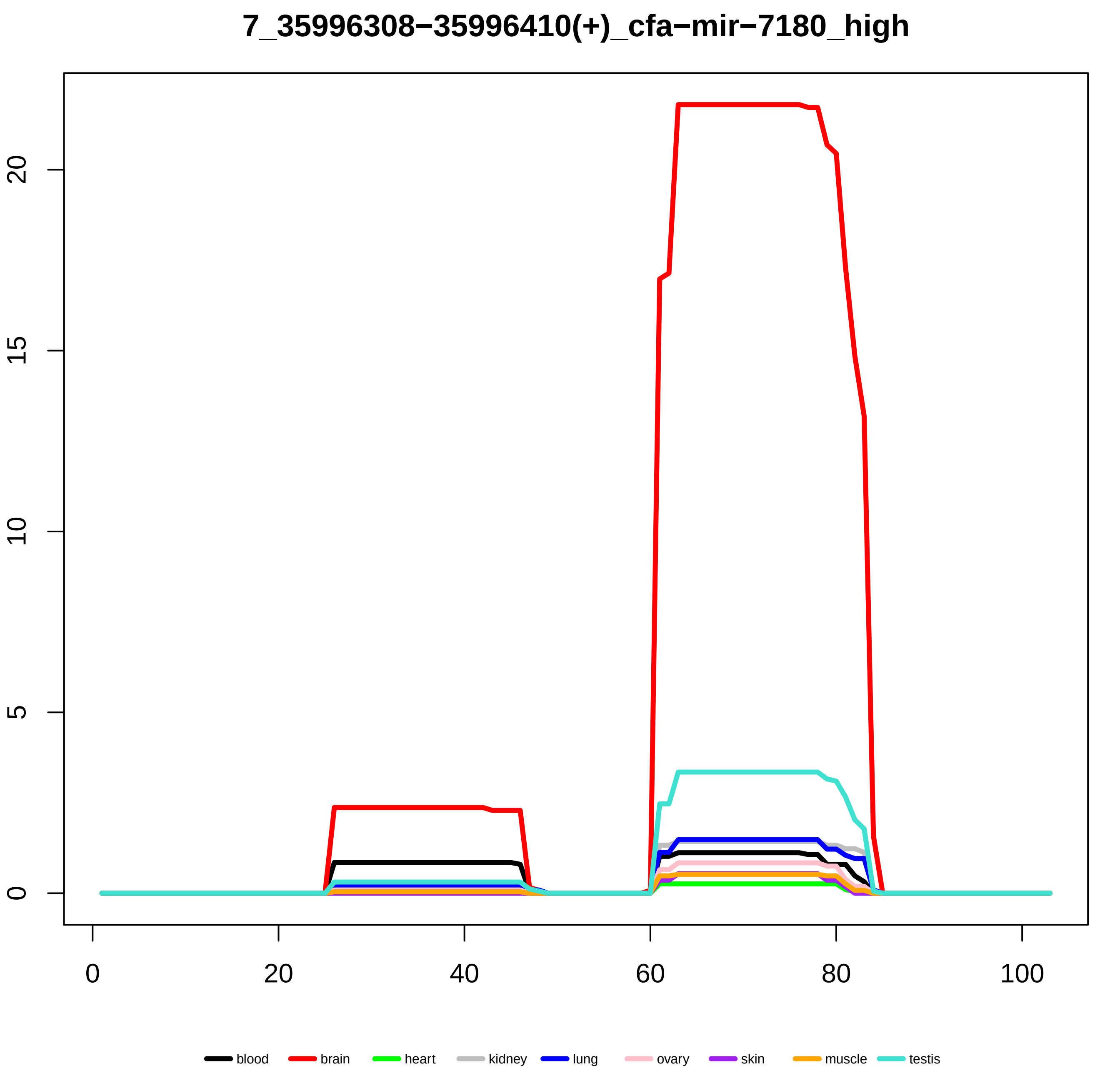

7_35996308−35996410(+)_cfa−mir−7180_high
0
5
10
15
20
0
20
40
60
80
100
blood
brain
heart
kidney
lung
ovary
skin
muscle
testis

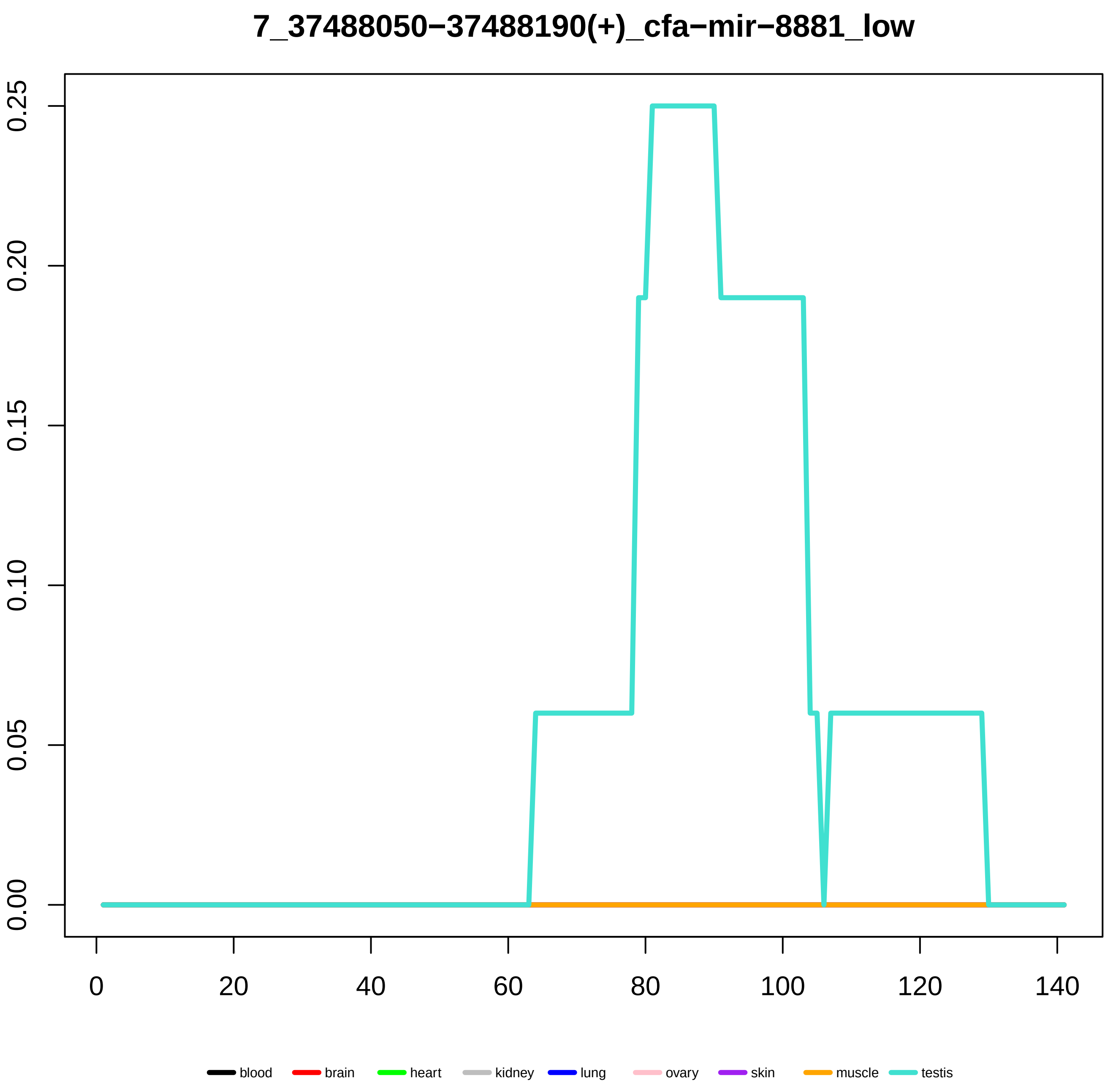

7_37488050−37488190(+)_cfa−mir−8881_low
0.00
0.05
0.10
0.15
0.20
0.25
0
20
40
60
80
100
120
140
blood
brain
heart
kidney
lung
ovary
skin
muscle
testis

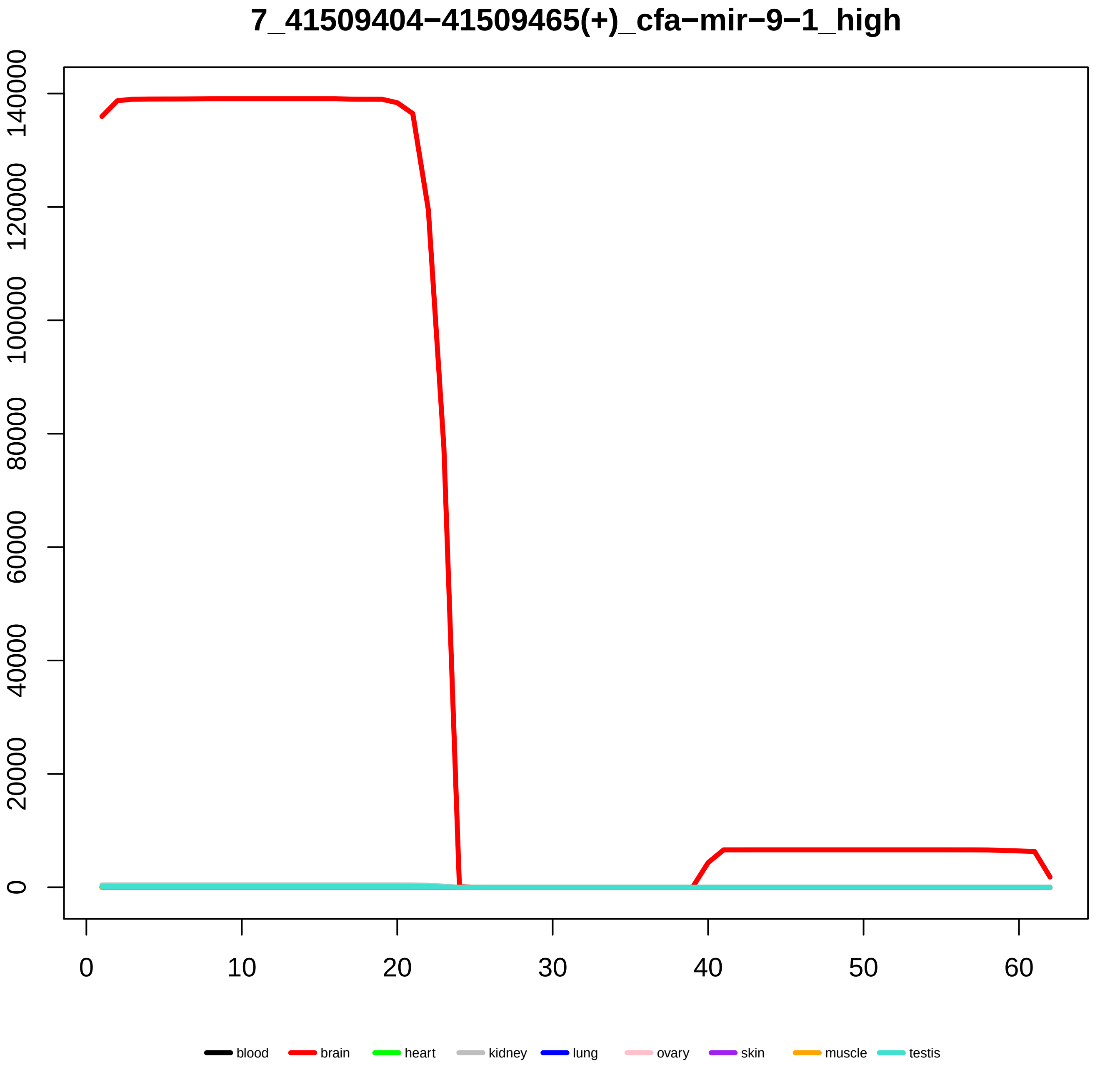

7_41509404−41509465(+)_cfa−mir−9−1_high
0
20000
40000
60000
80000
100000
120000
140000
0
10
20
30
40
50
60
blood
brain
heart
kidney
lung
ovary
skin
muscle
testis

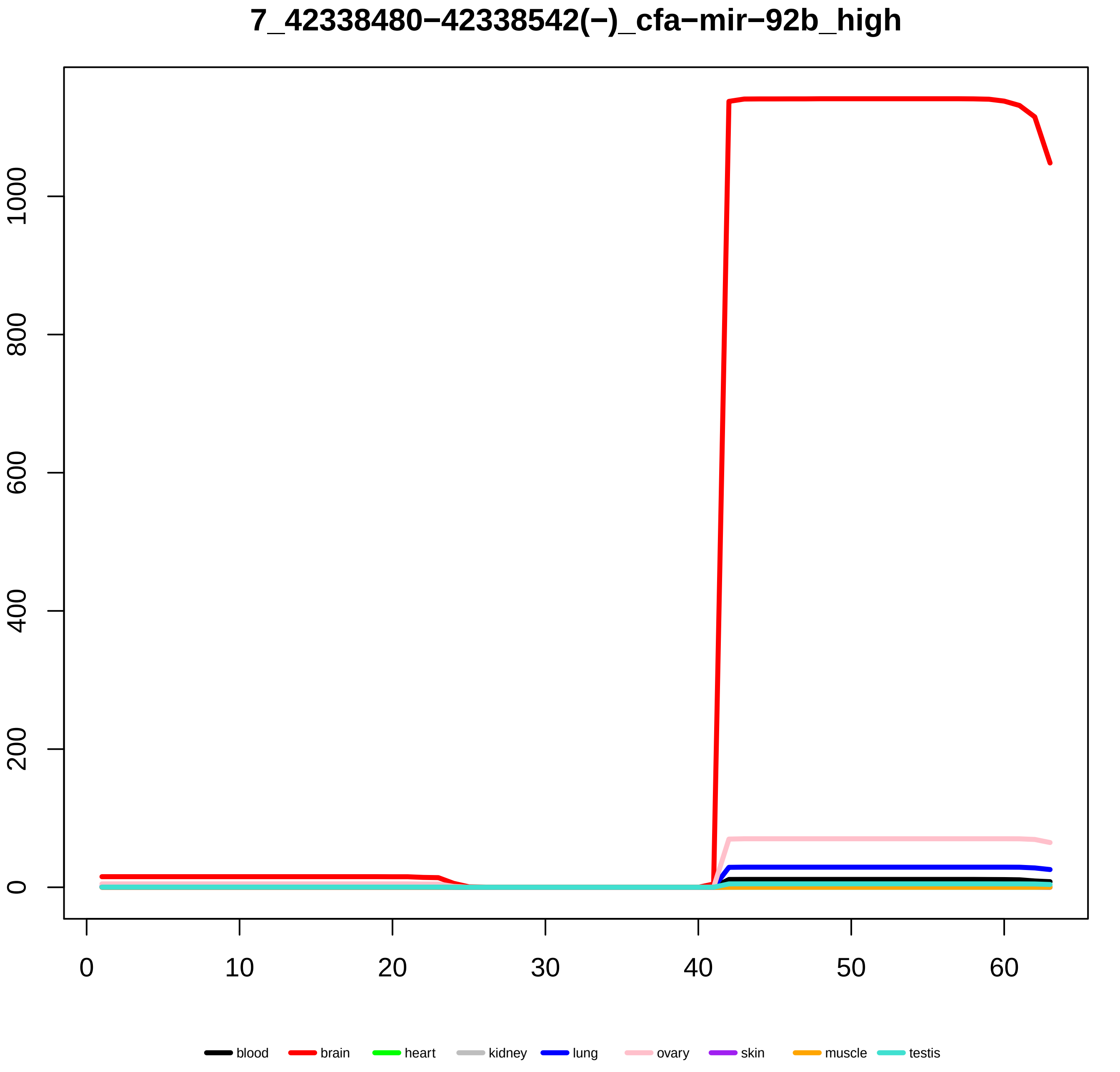

7_42338480−42338542(−)_cfa−mir−92b_high
0
200
400
600
800
1000
0
10
20
30
40
50
60
blood
brain
heart
kidney
lung
ovary
skin
muscle
testis

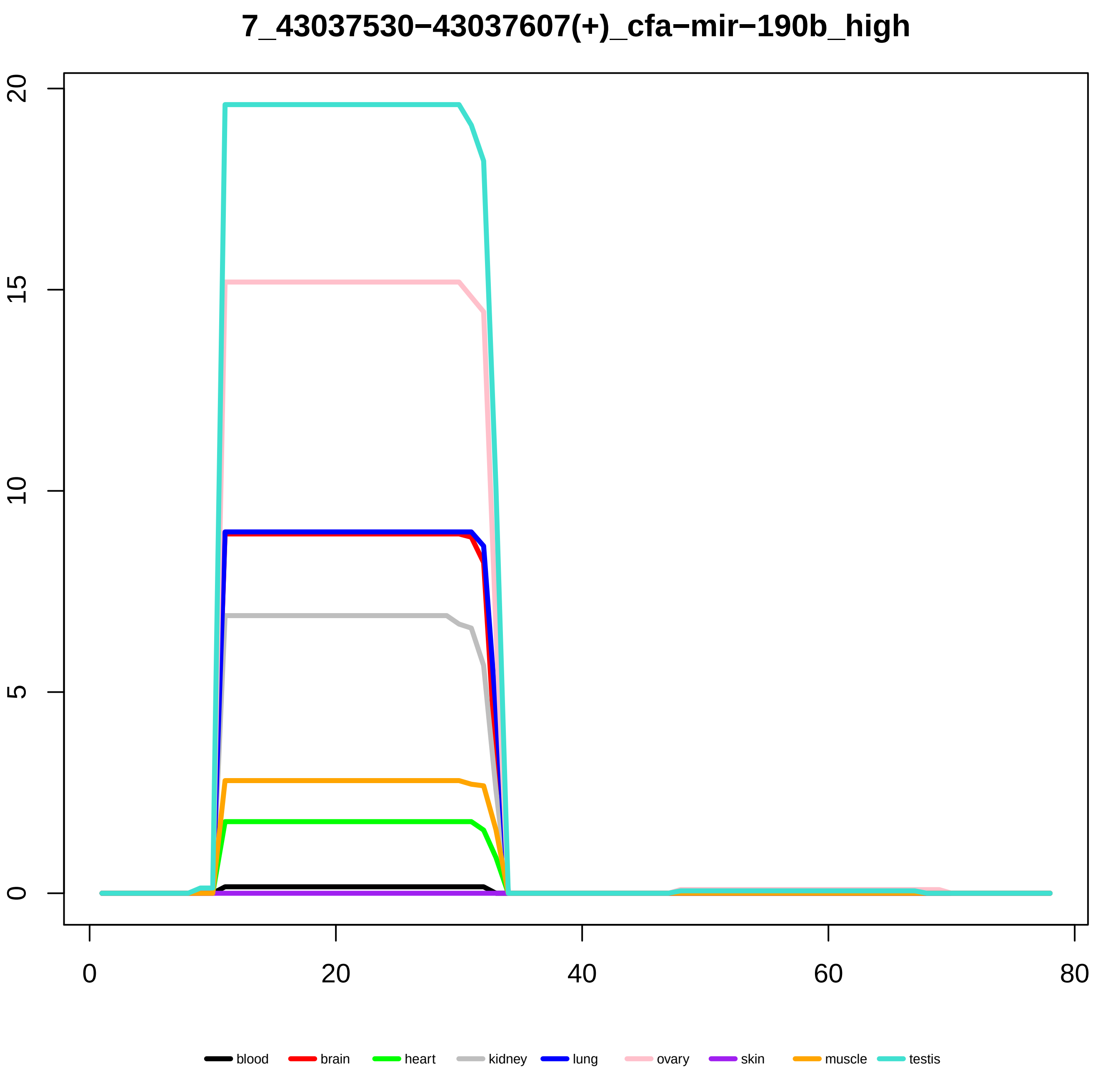

7_43037530−43037607(+)_cfa−mir−190b_high
20
15
10
5
0
0
20
40
60
80
blood
brain
heart
kidney
lung
ovary
skin
muscle
testis

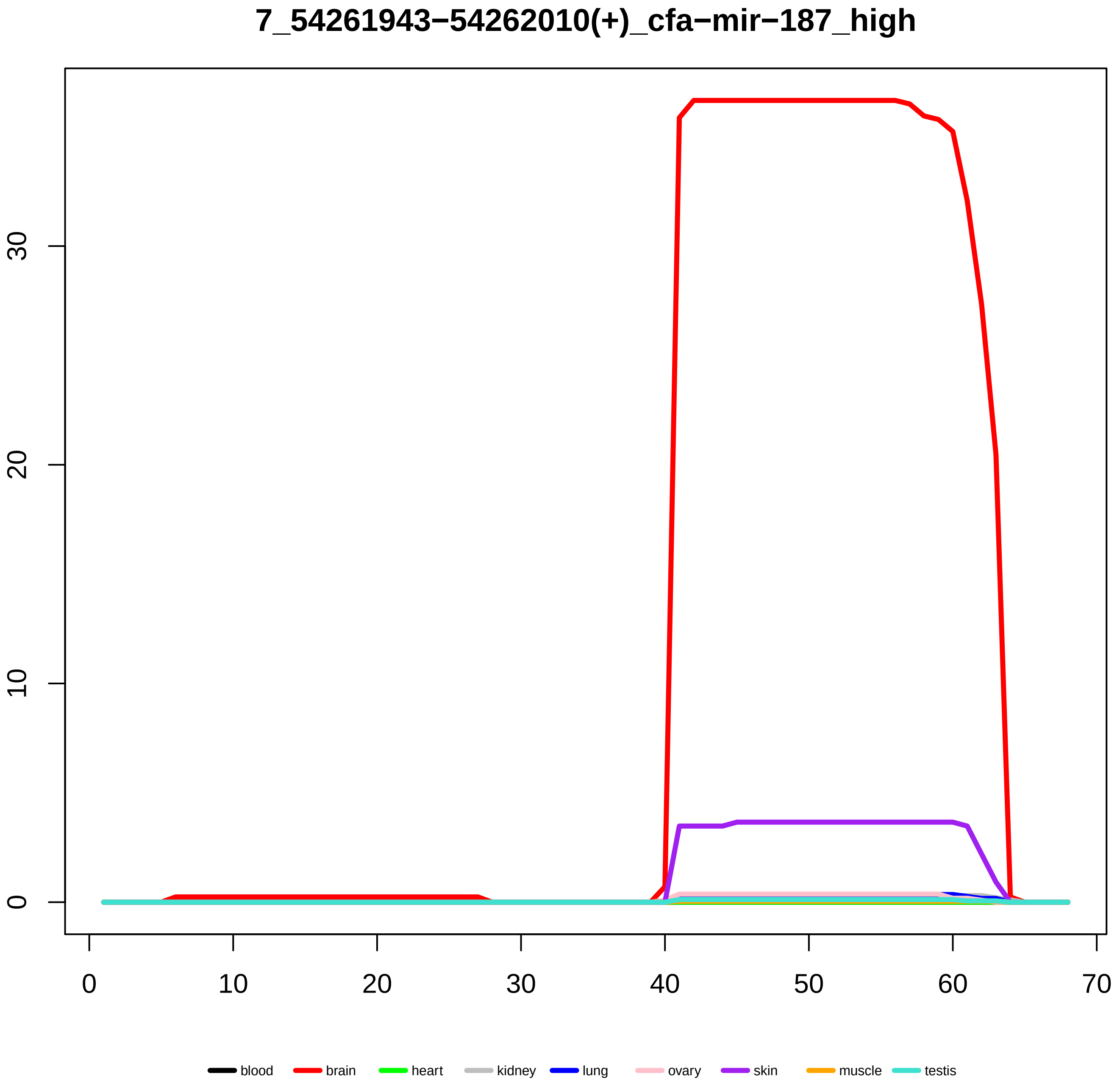
7_54261943−54262010(+)_cfa−mir−187_high
0
10
20
30
0
10
20
30
40
50
60
70
blood
brain
heart
kidney
lung
ovary
skin
muscle
testis

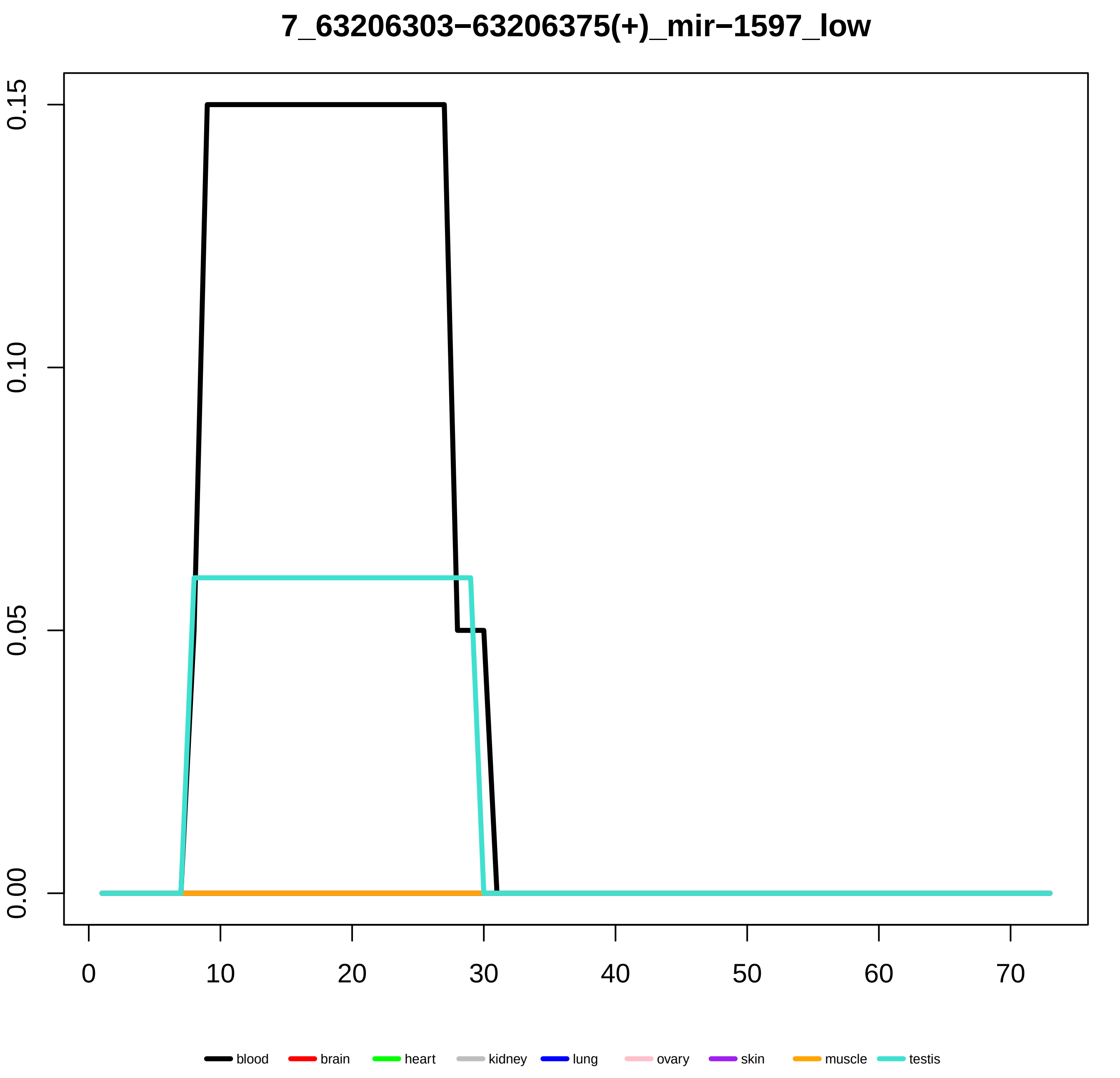

7_63206303−63206375(+)_mir−1597_low
0.00
0.05
0.10
0.15
0
10
20
30
40
50
60
70
blood
brain
heart
kidney
lung
ovary
skin
muscle
testis

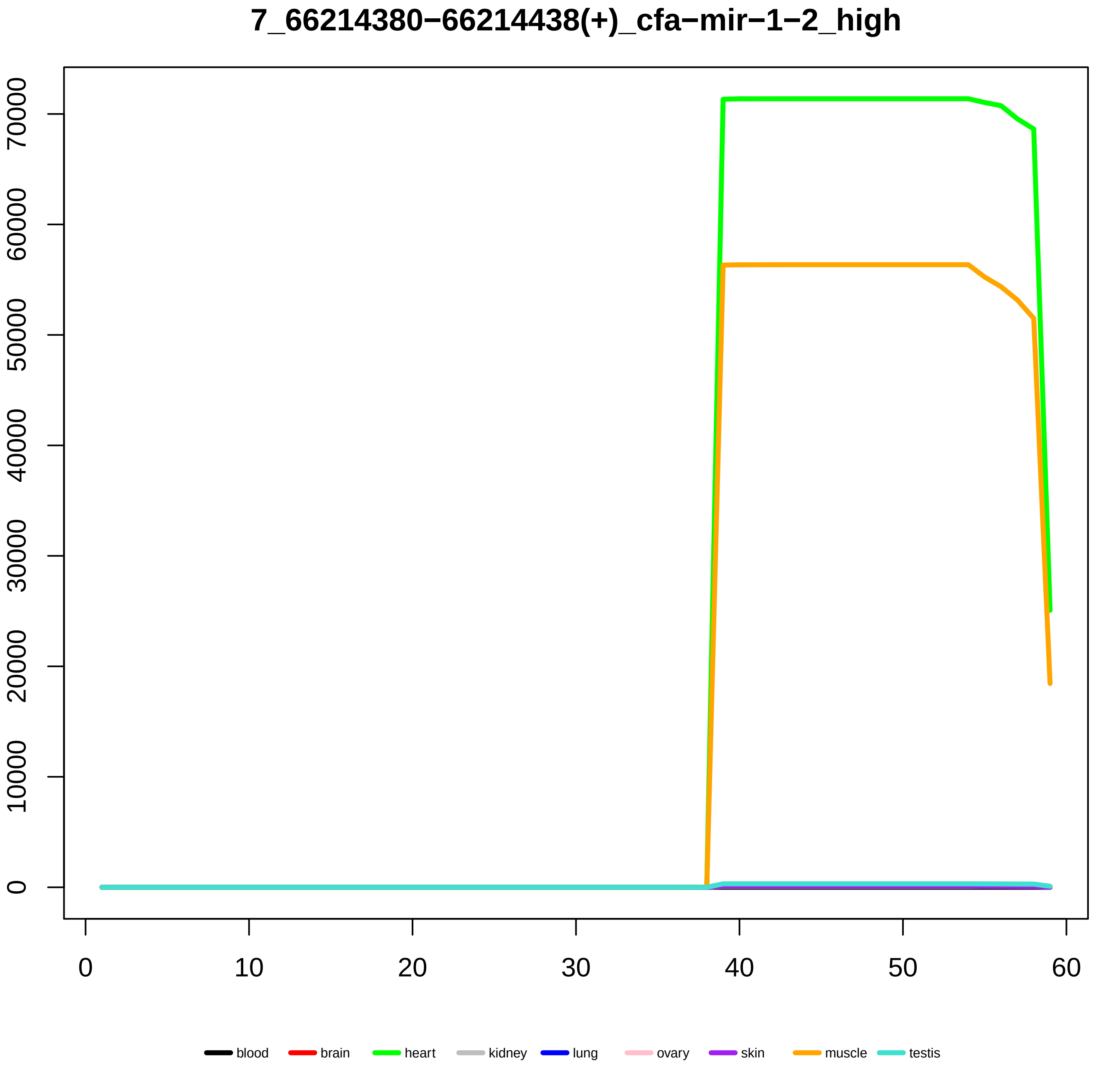
7_66214380−66214438(+)_cfa−mir−1−2_high
0
10000
20000
30000
40000
50000
60000
70000
0
10
20
30
40
50
60
blood
brain
heart
kidney
lung
ovary
skin
muscle
testis

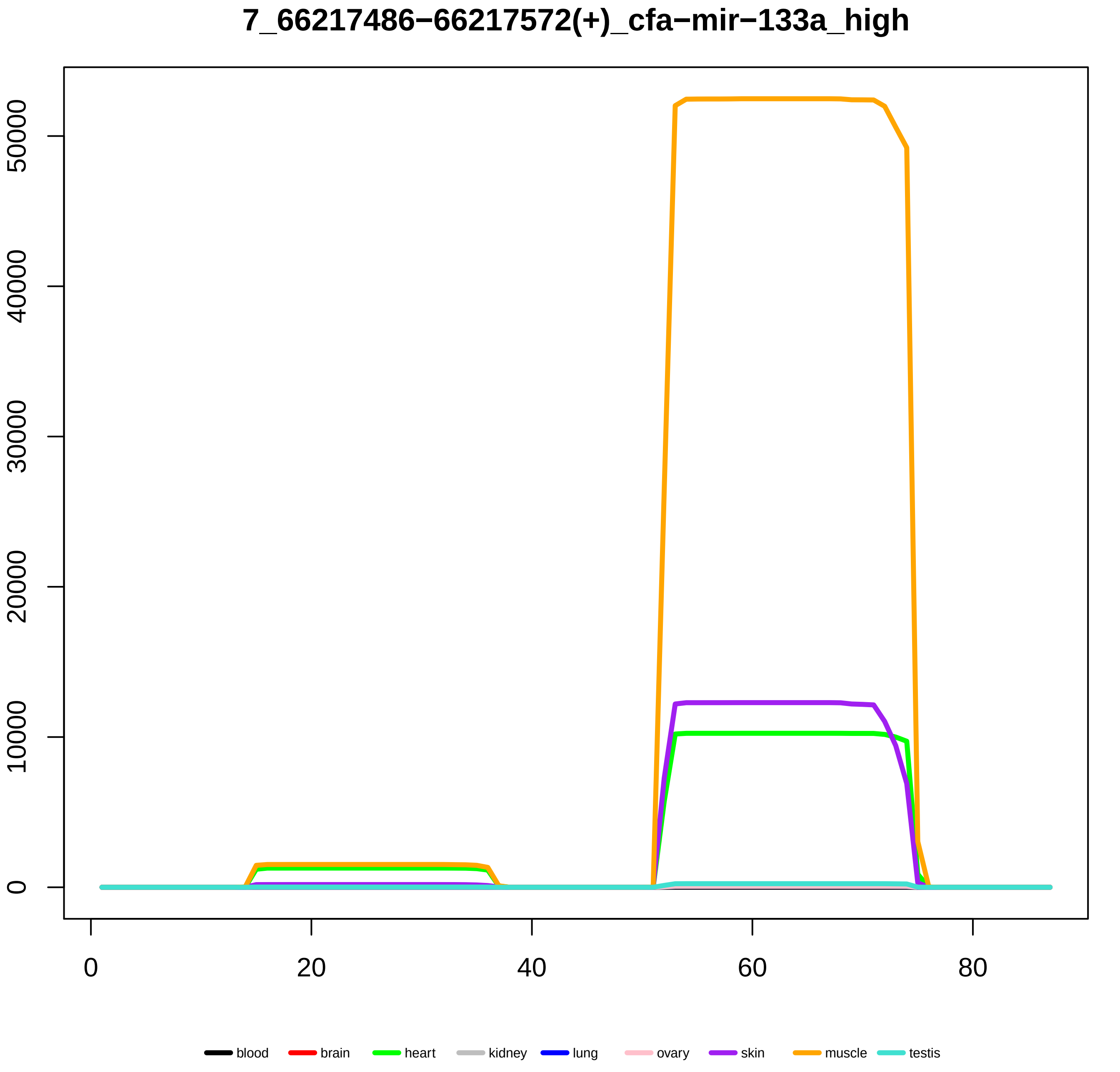
7_66217486−66217572(+)_cfa−mir−133a_high
0
10000
20000
30000
40000
50000
0
20
40
60
80
blood
brain
heart
kidney
lung
ovary
skin
muscle
testis

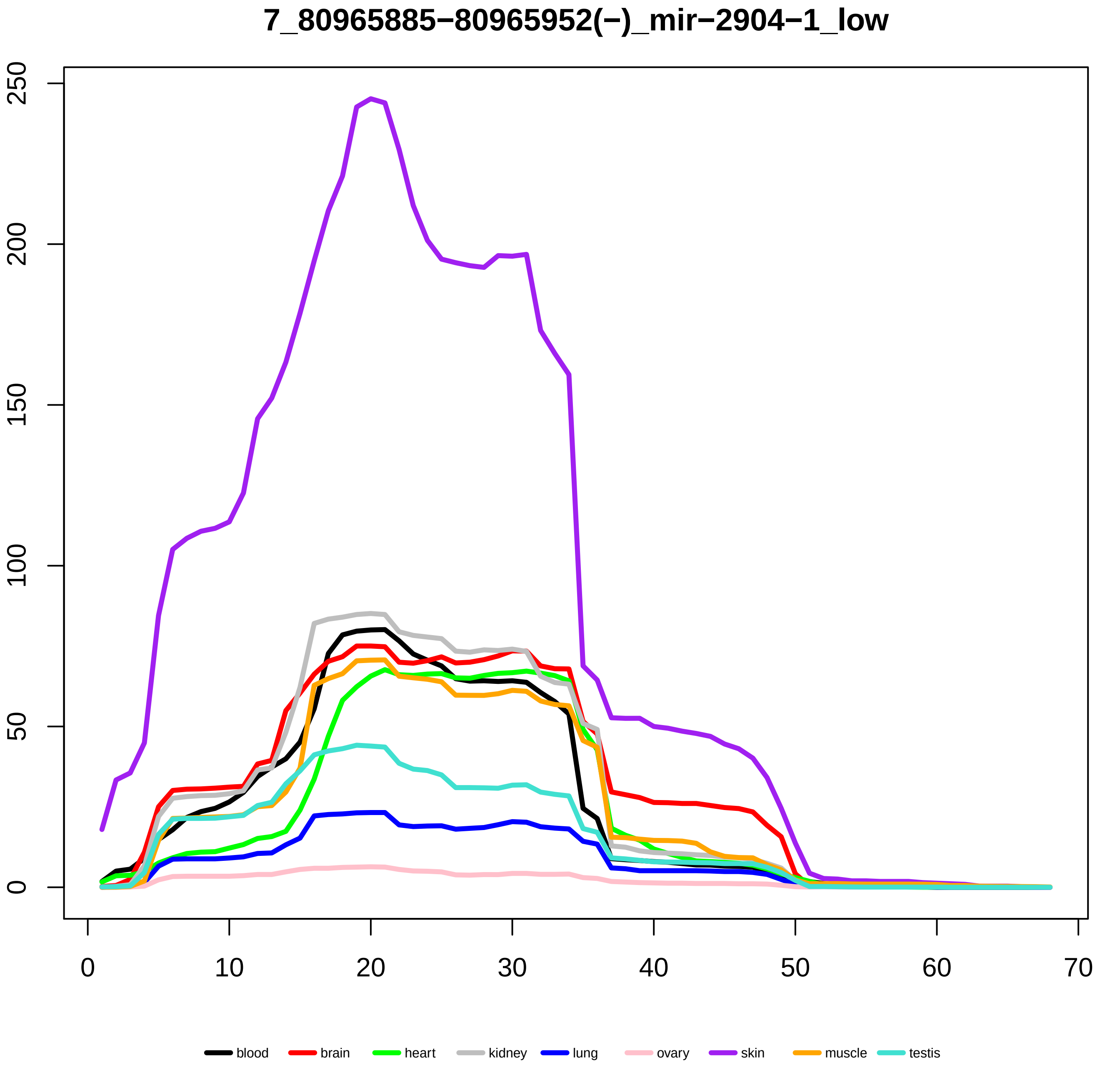
7_80965885−80965952(−)_mir−2904−1_low
0
50
100
150
200
250
0
10
20
30
40
50
60
70
blood
brain
heart
kidney
lung
ovary
skin
muscle
testis

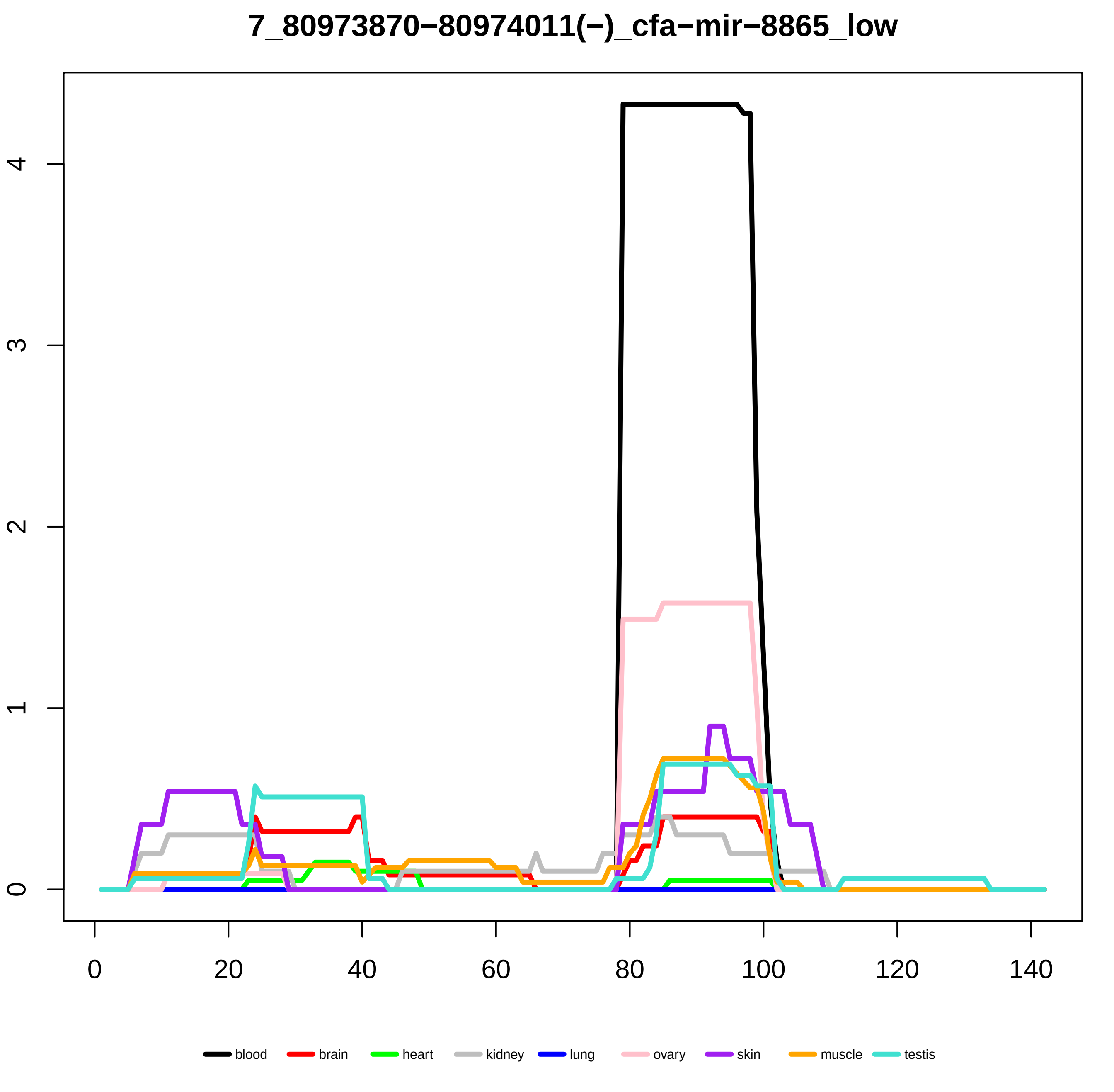

7_80973870−80974011(−)_cfa−mir−8865_low
0
1
2
3
4
0
20
40
60
80
100
120
140
blood
brain
heart
kidney
lung
ovary
skin
muscle
testis

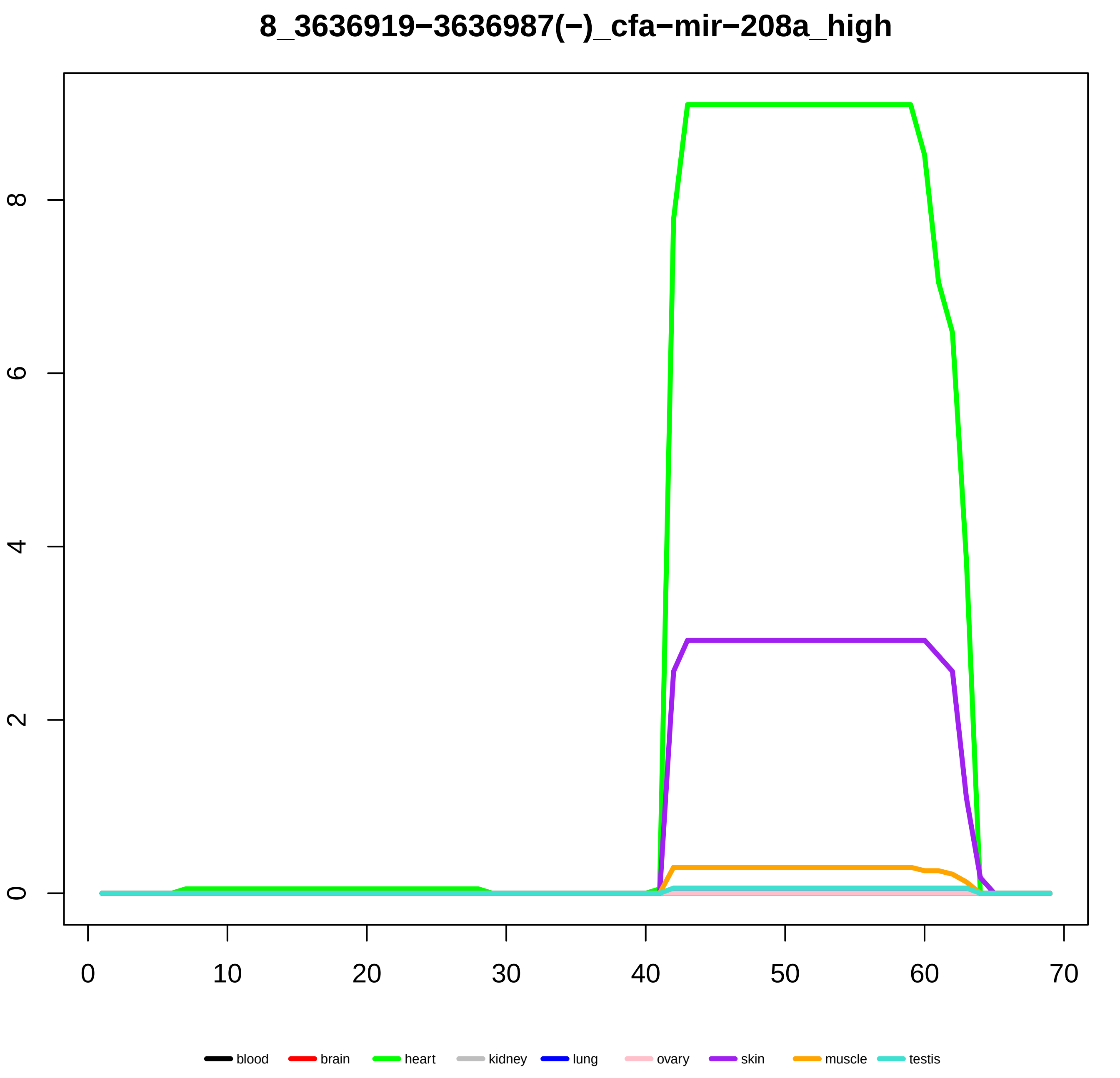

8_3636919−3636987(−)_cfa−mir−208a_high
0
2
4
6
8
0
10
20
30
40
50
60
70
blood
brain
heart
kidney
lung
ovary
skin
muscle
testis

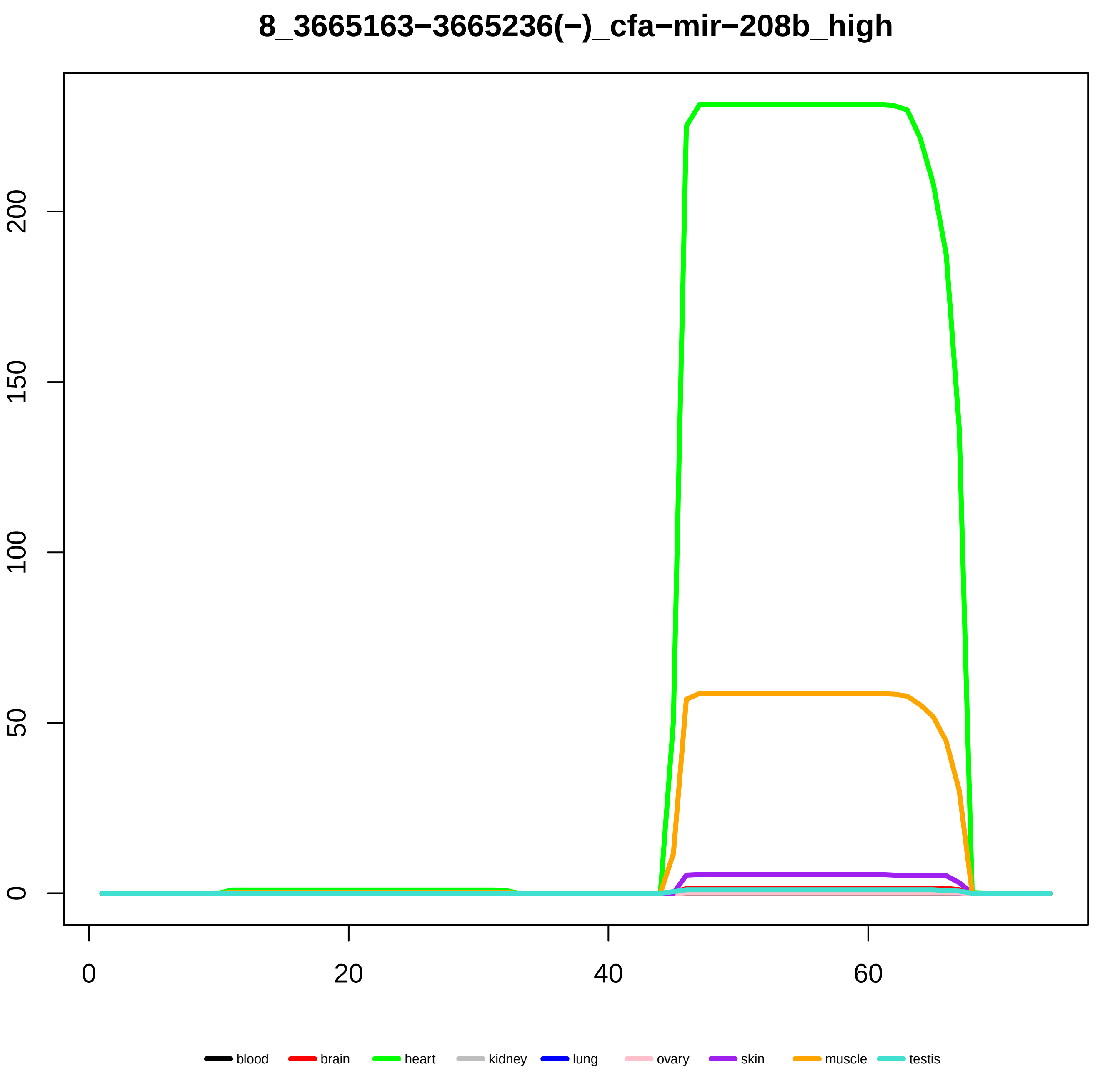
8_3665163−3665236(−)_cfa−mir−208b_high
0
50
100
150
200
0
20
40
60
blood
brain
heart
kidney
lung
ovary
skin
muscle
testis

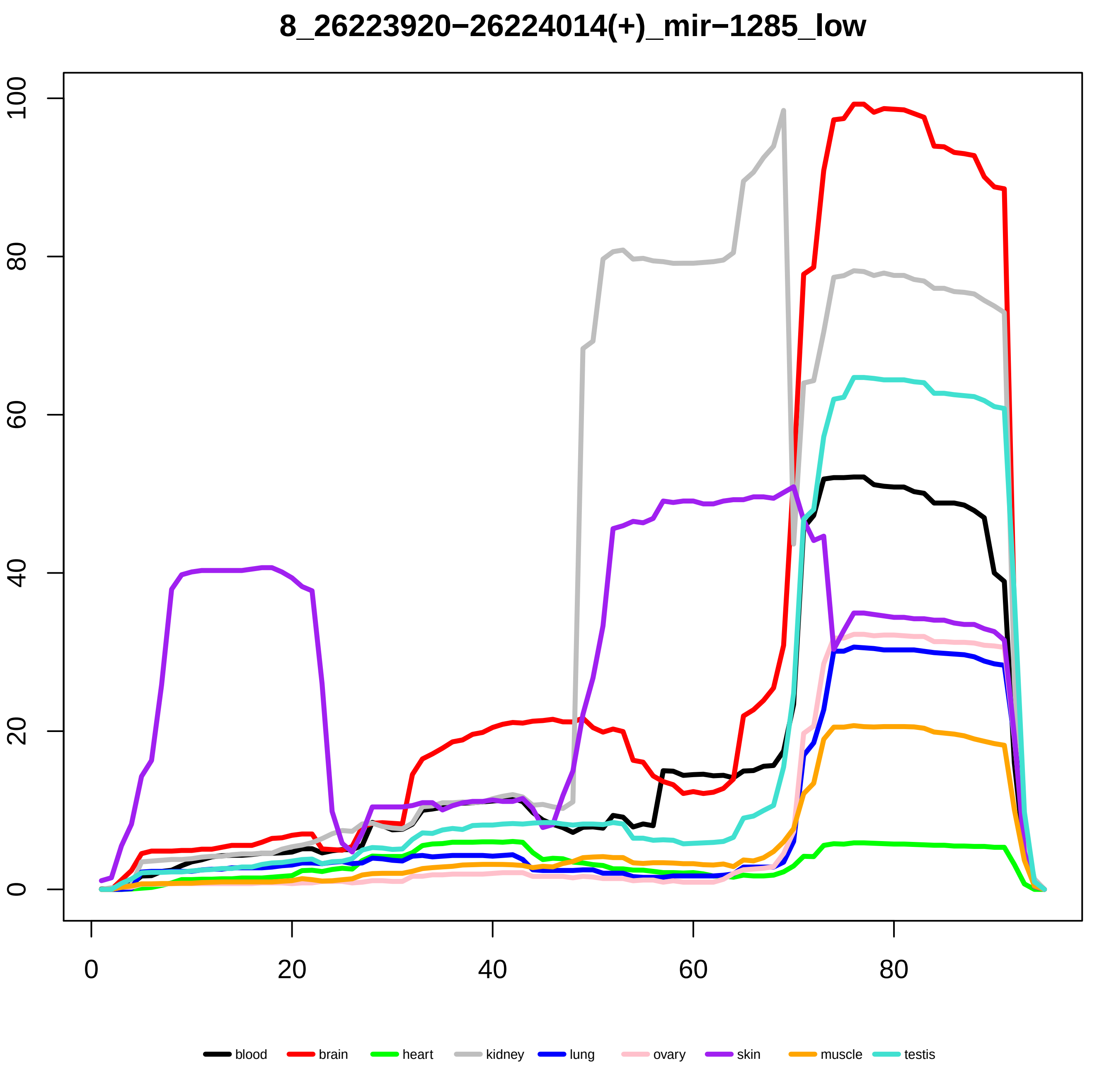
8_26223920−26224014(+)_mir−1285_low
0
20
40
60
80
100
0
20
40
60
80
blood
brain
heart
kidney
lung
ovary
skin
muscle
testis

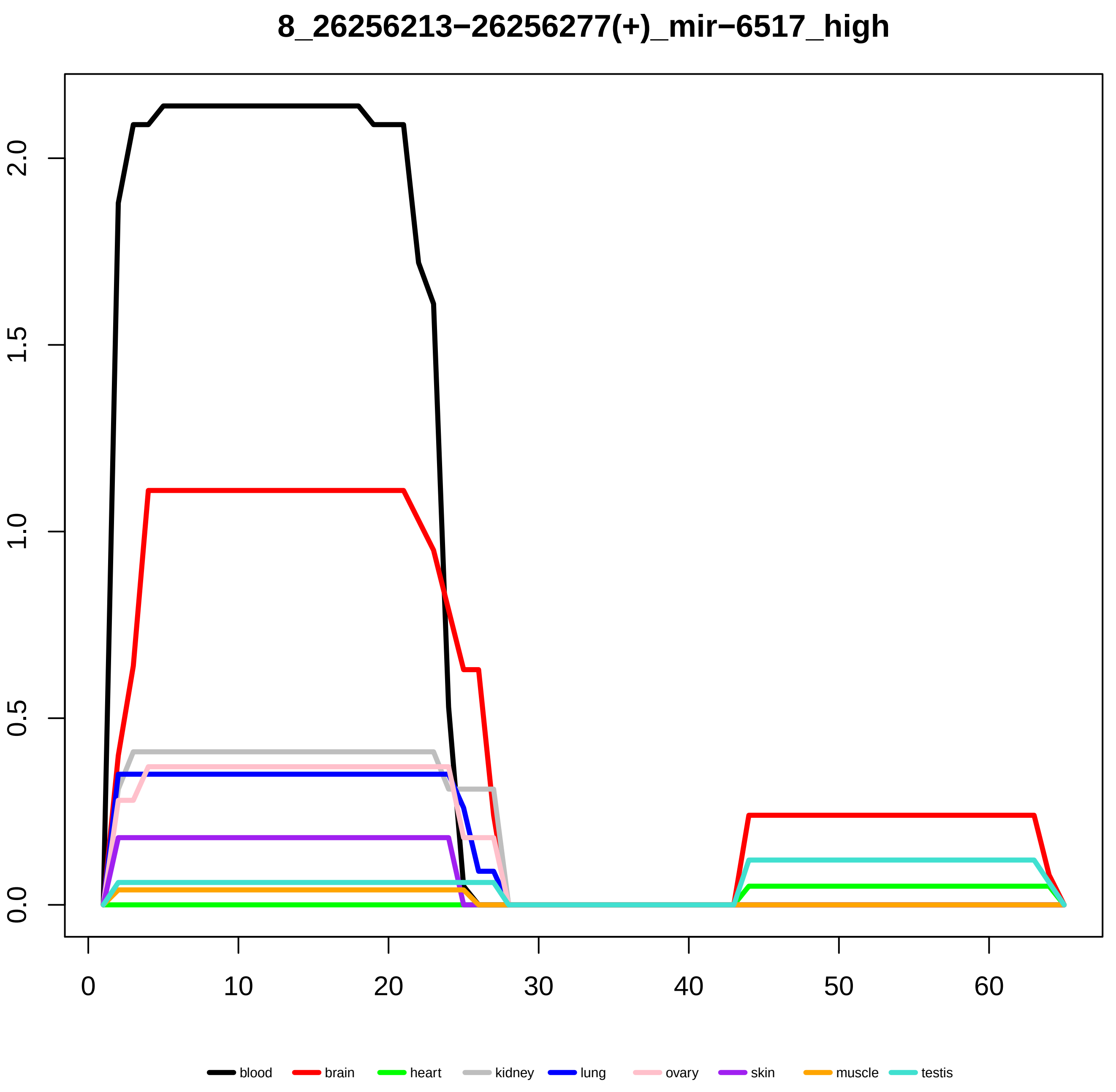

8_26256213−26256277(+)_mir−6517_high
0.0
0.5
1.0
1.5
2.0
0
10
20
30
40
50
60
blood
brain
heart
kidney
lung
ovary
skin
muscle
testis

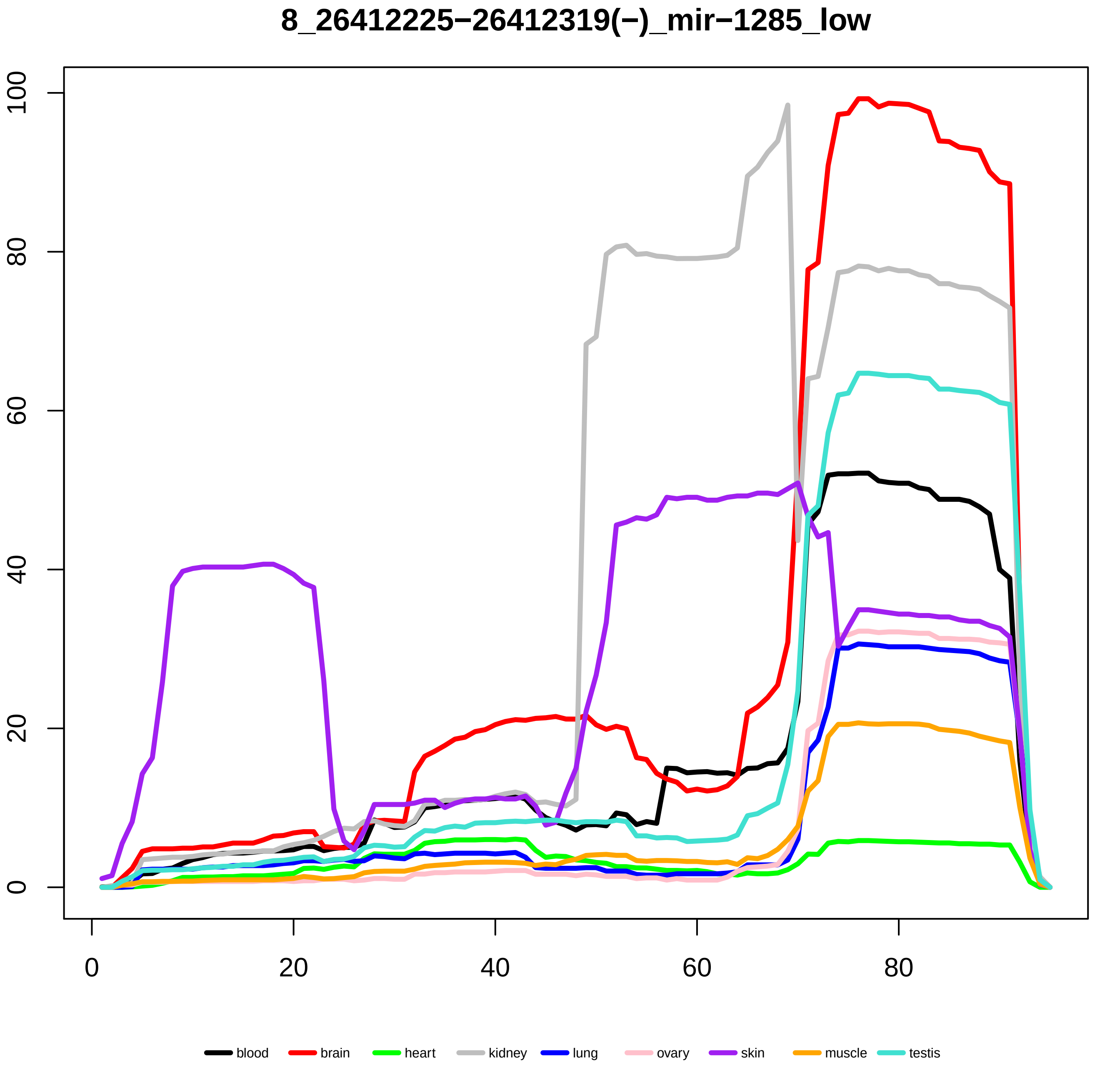

8_26412225−26412319(−)_mir−1285_low
0
20
40
60
80
100
0
20
40
60
80
blood
brain
heart
kidney
lung
ovary
skin
muscle
testis

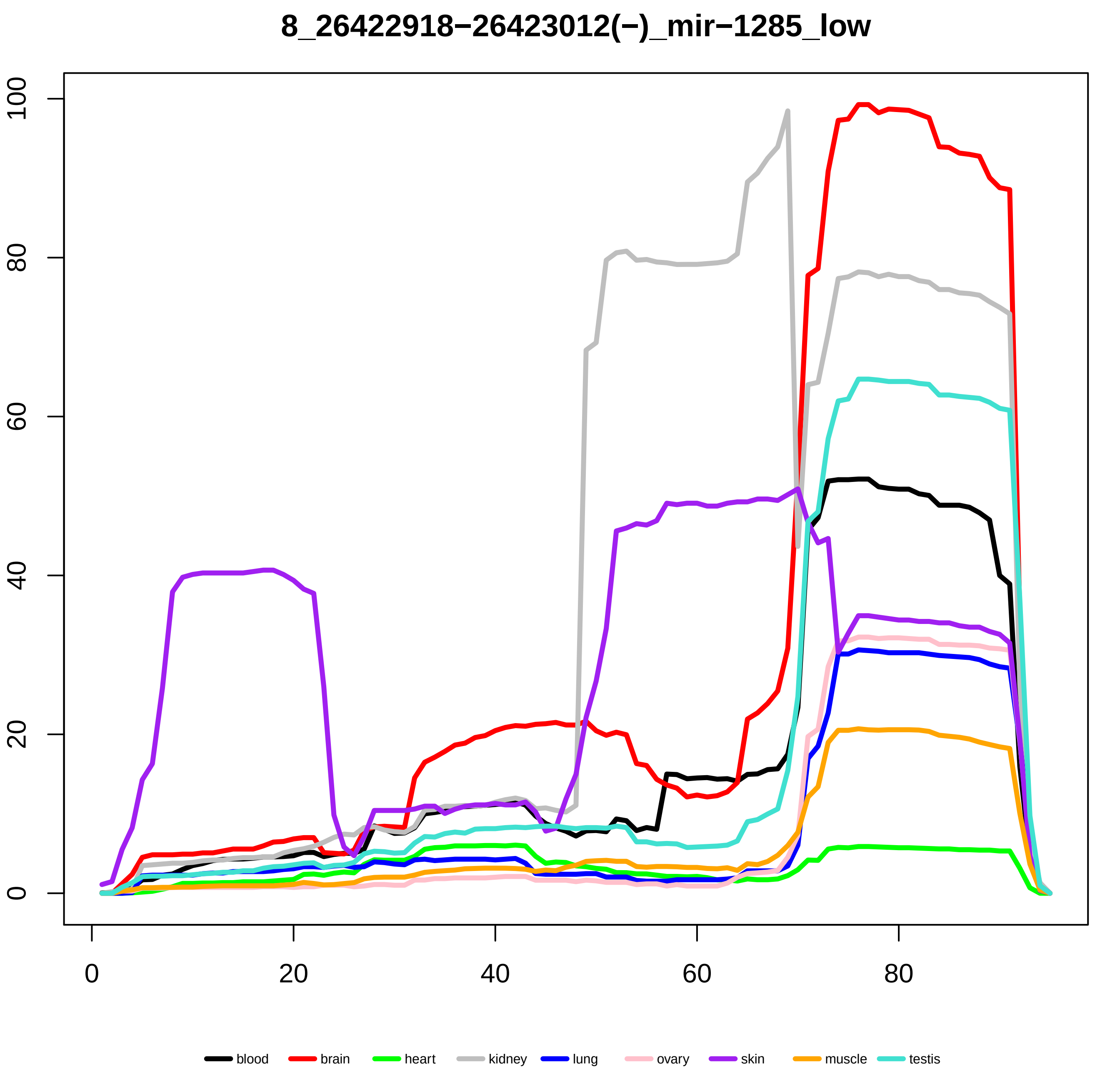
8_26422918−26423012(−)_mir−1285_low
0
20
40
60
80
100
0
20
40
60
80
blood
brain
heart
kidney
lung
ovary
skin
muscle
testis

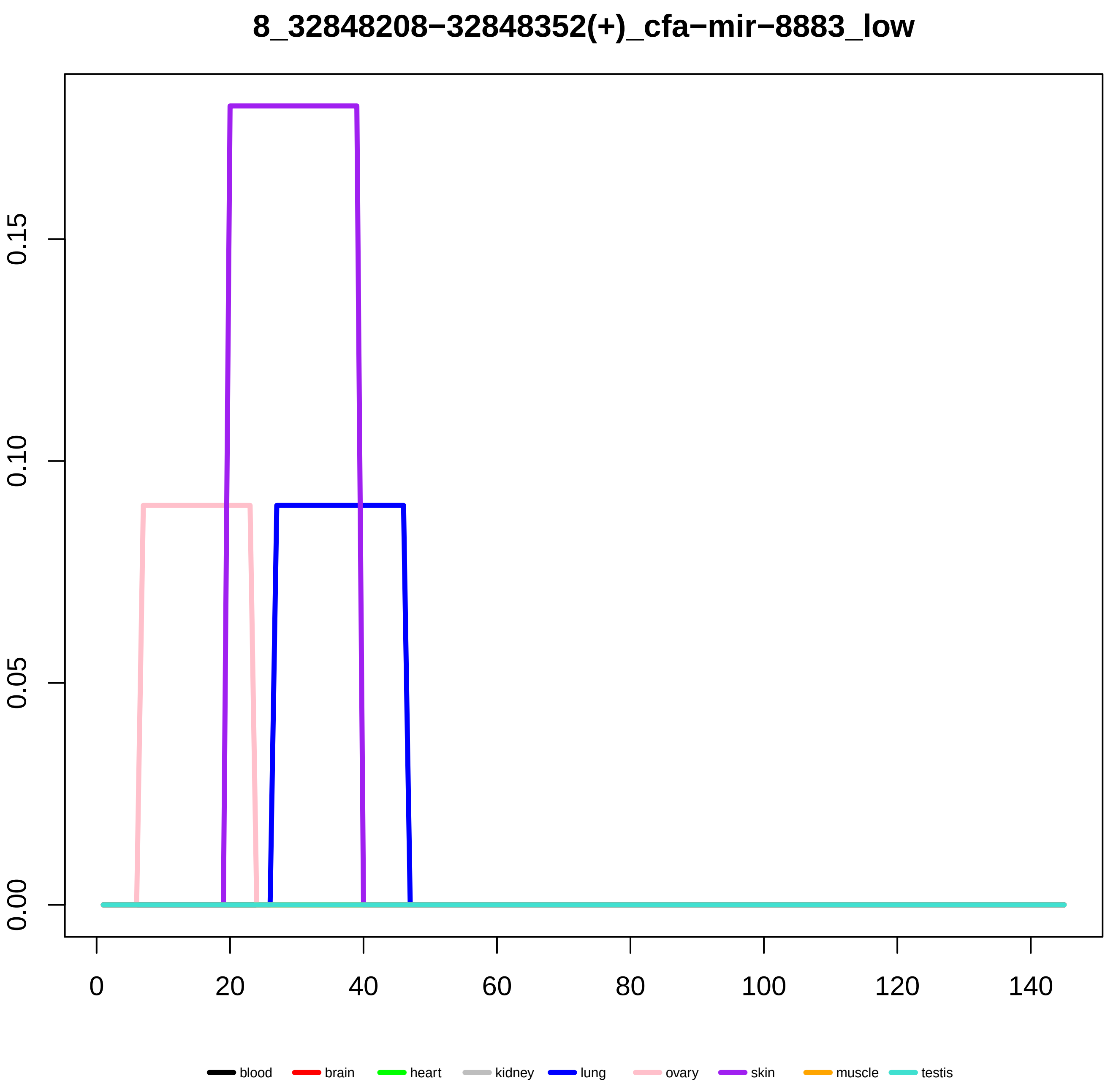
8_32848208−32848352(+)_cfa−mir−8883_low
0.00
0.05
0.10
0.15
0
20
40
60
80
100
120
140
blood
brain
heart
kidney
lung
ovary
skin
muscle
testis

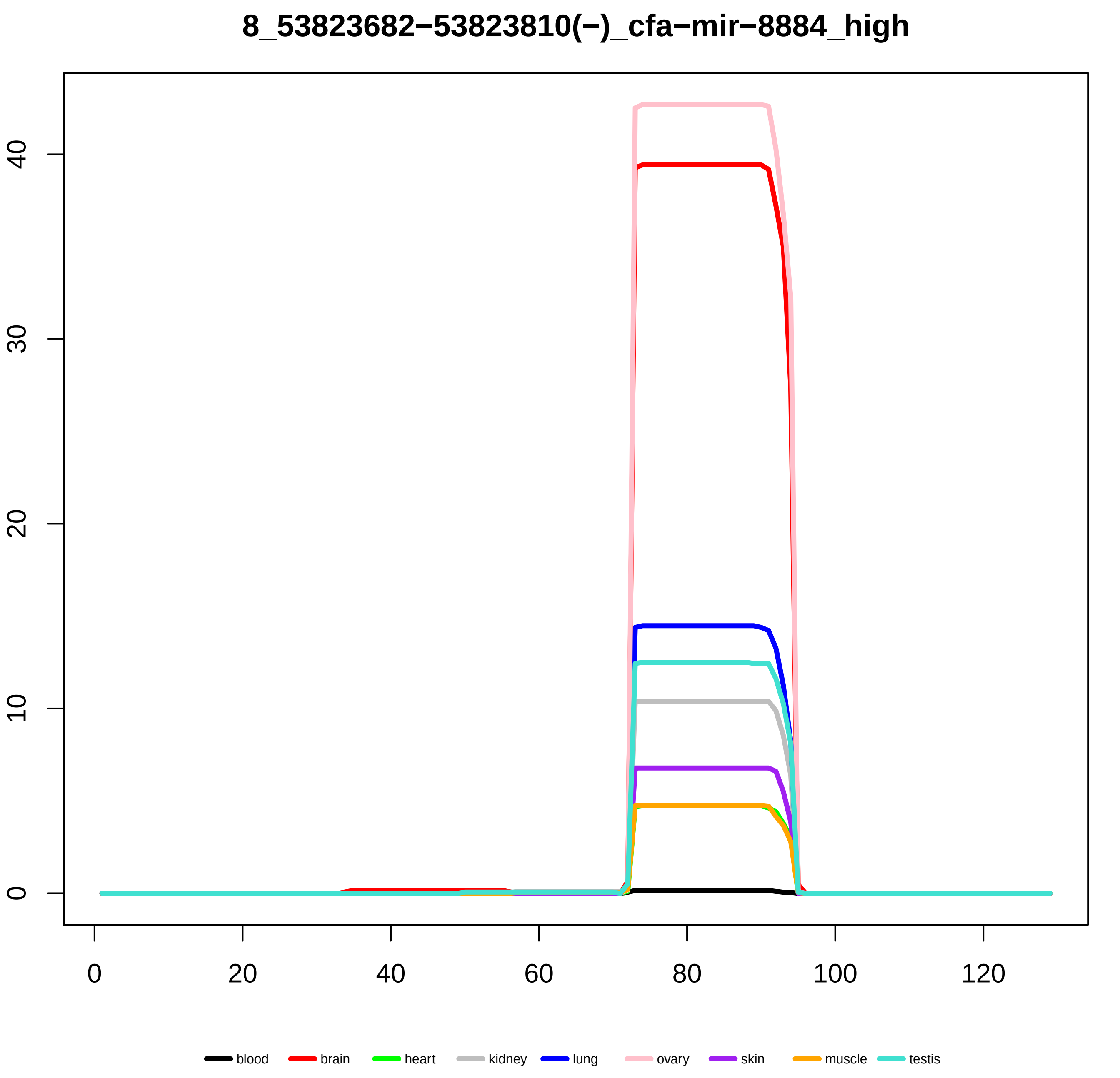
8_53823682−53823810(−)_cfa−mir−8884_high
0
10
20
30
40
0
20
40
60
80
100
120
blood
brain
heart
kidney
lung
ovary
skin
muscle
testis

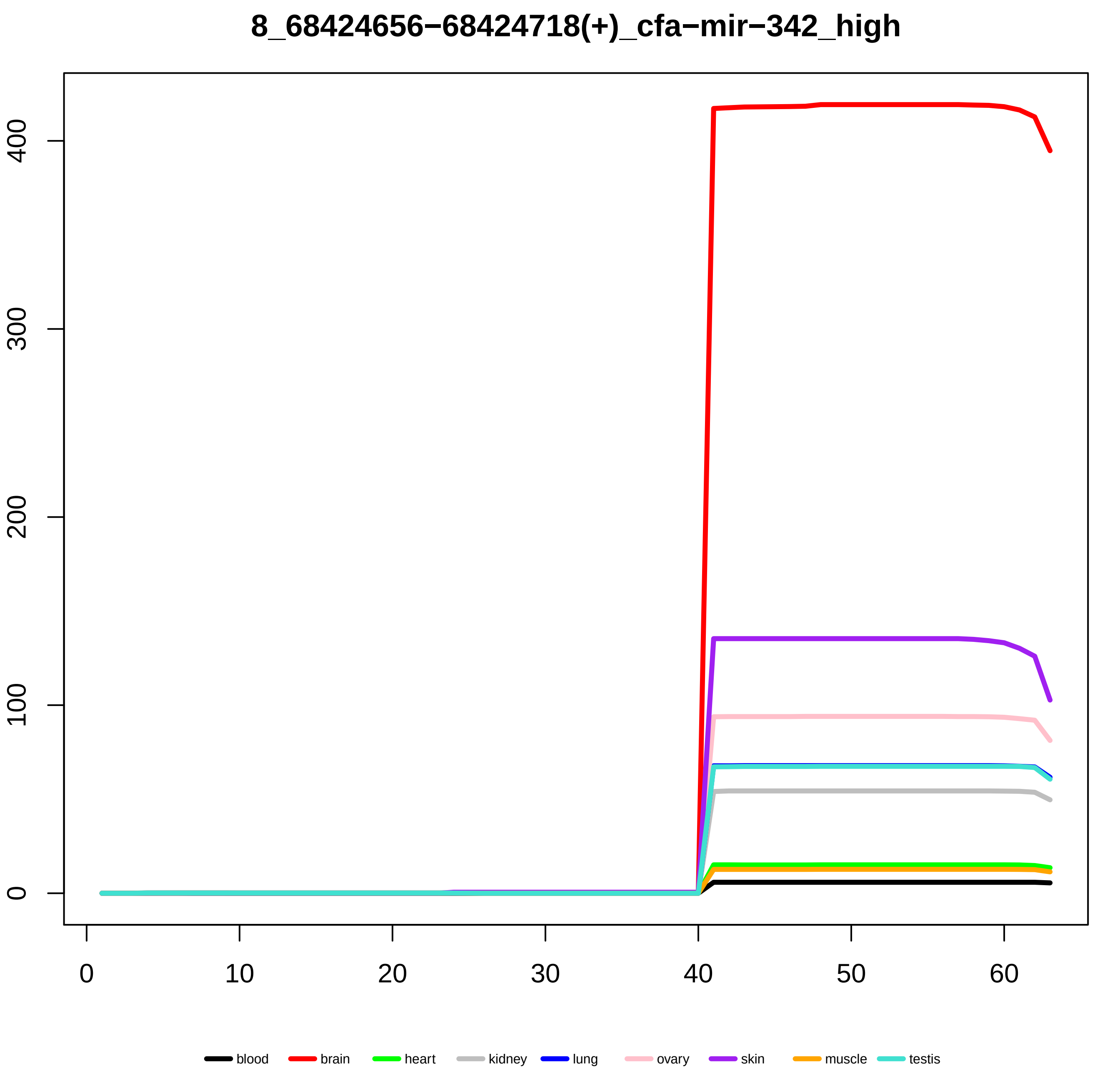

8_68424656−68424718(+)_cfa−mir−342_high
0
100
200
300
400
0
10
20
30
40
50
60
blood
brain
heart
kidney
lung
ovary
skin
muscle
testis

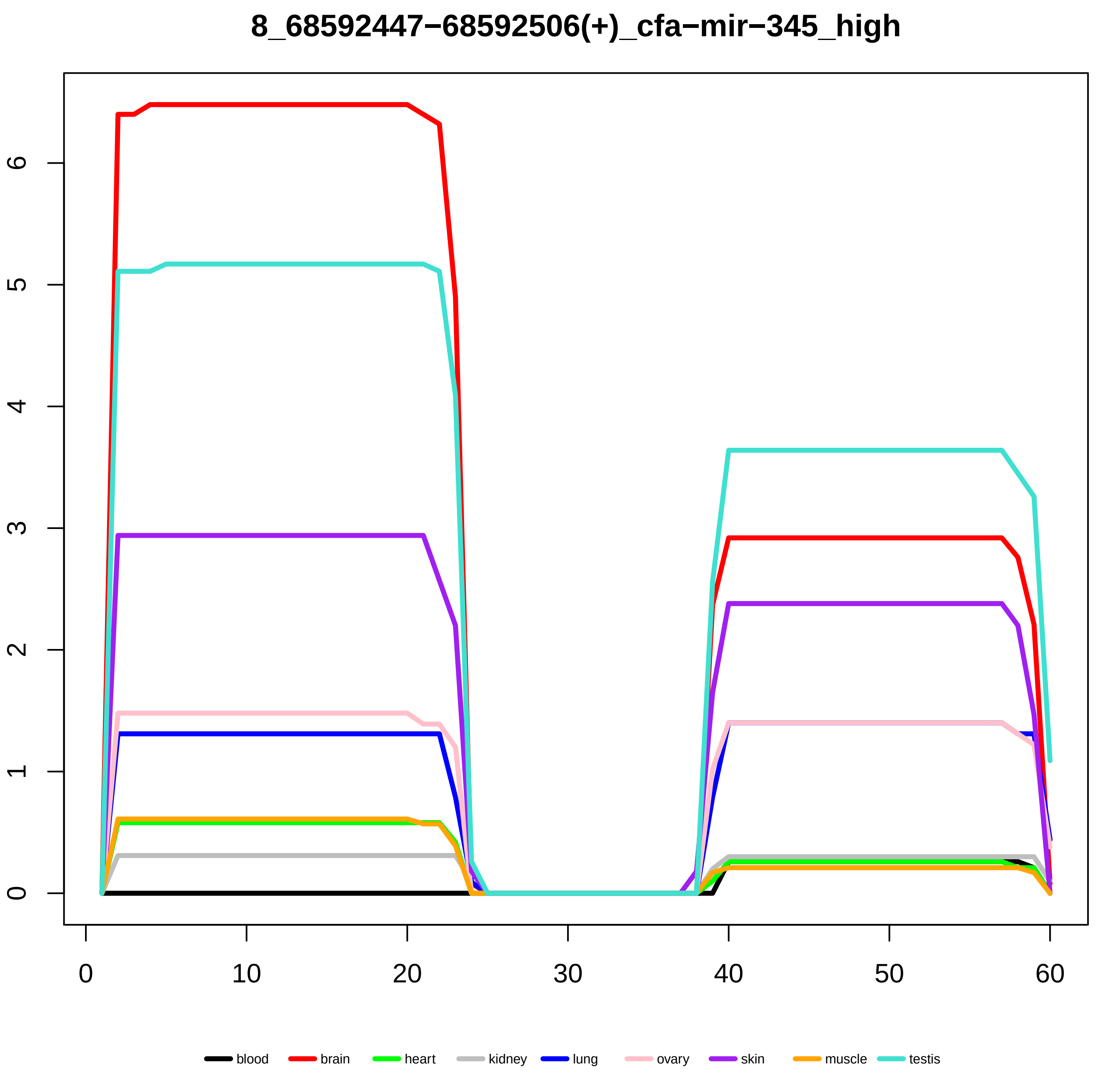
8_68592447−68592506(+)_cfa−mir−345_high
0
1
2
3
4
5
6
0
10
20
30
40
50
60
blood
brain
heart
kidney
lung
ovary
skin
muscle
testis

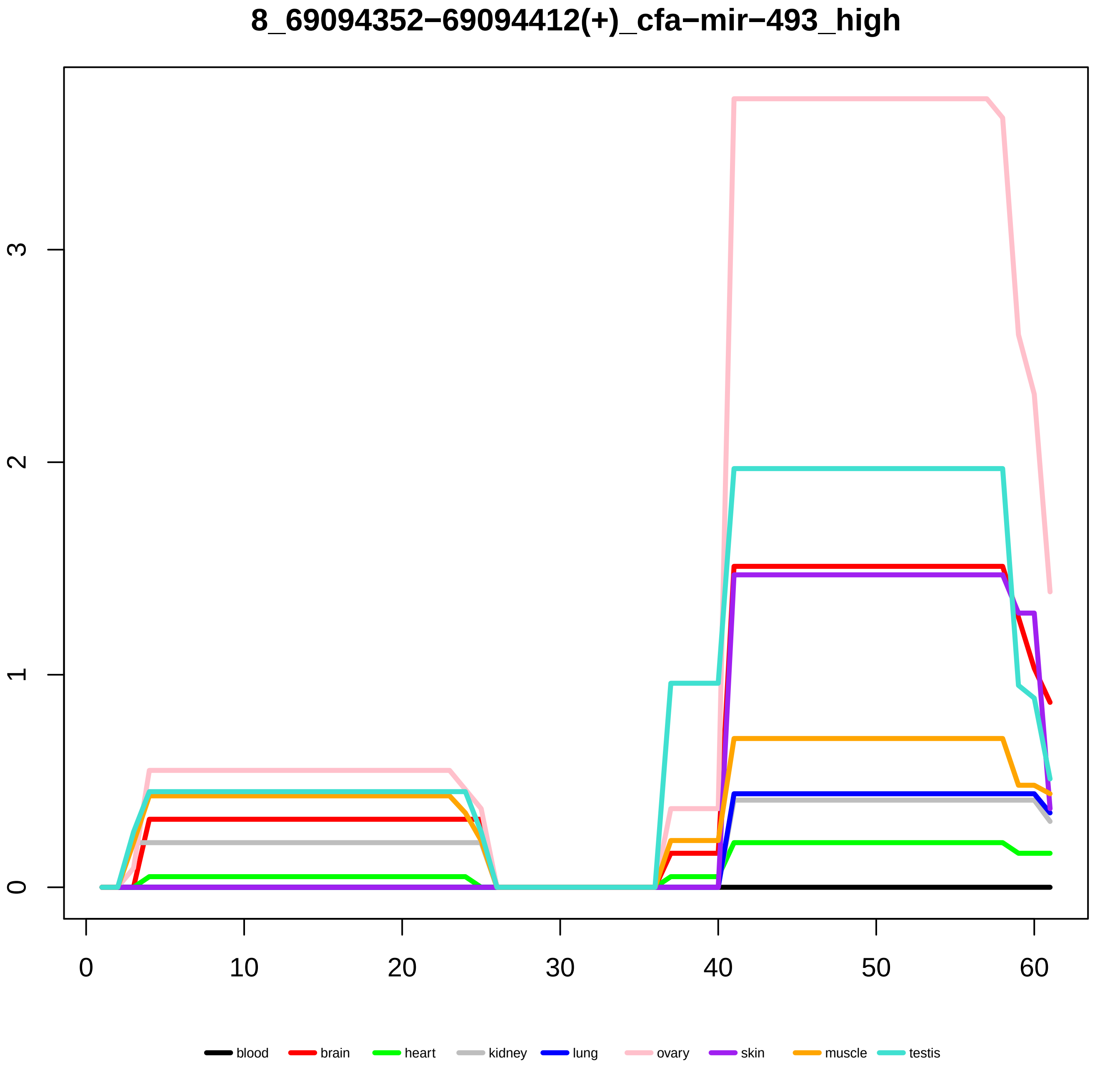

8_69094352−69094412(+)_cfa−mir−493_high
0
1
2
3
0
10
20
30
40
50
60
blood
brain
heart
kidney
lung
ovary
skin
muscle
testis

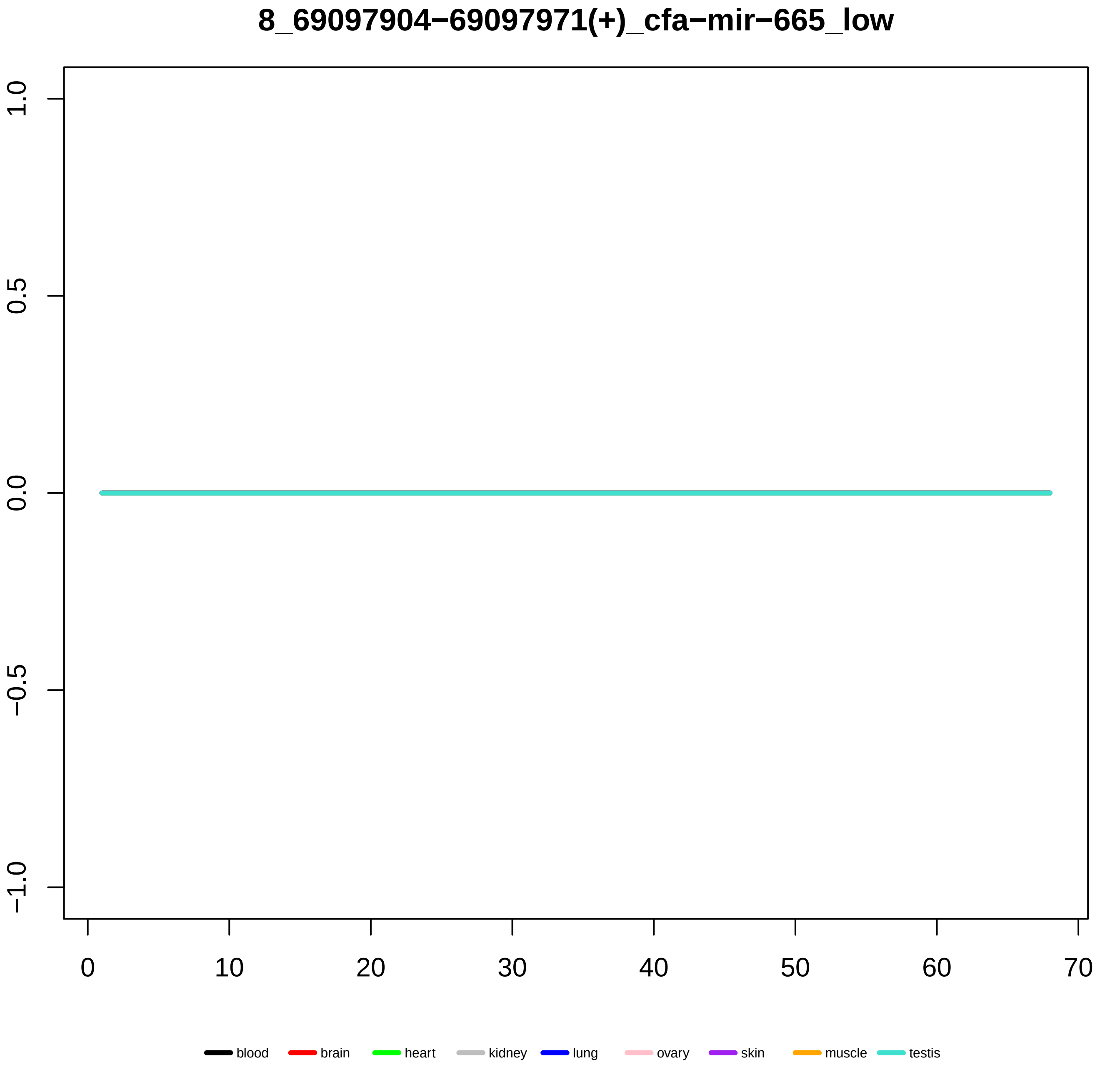
8_69097904−69097971(+)_cfa−mir−665_low
1.0
0.5
0.0
−0.5
−1.0
0
10
20
30
40
50
60
70
blood
brain
heart
kidney
lung
ovary
skin
muscle
testis

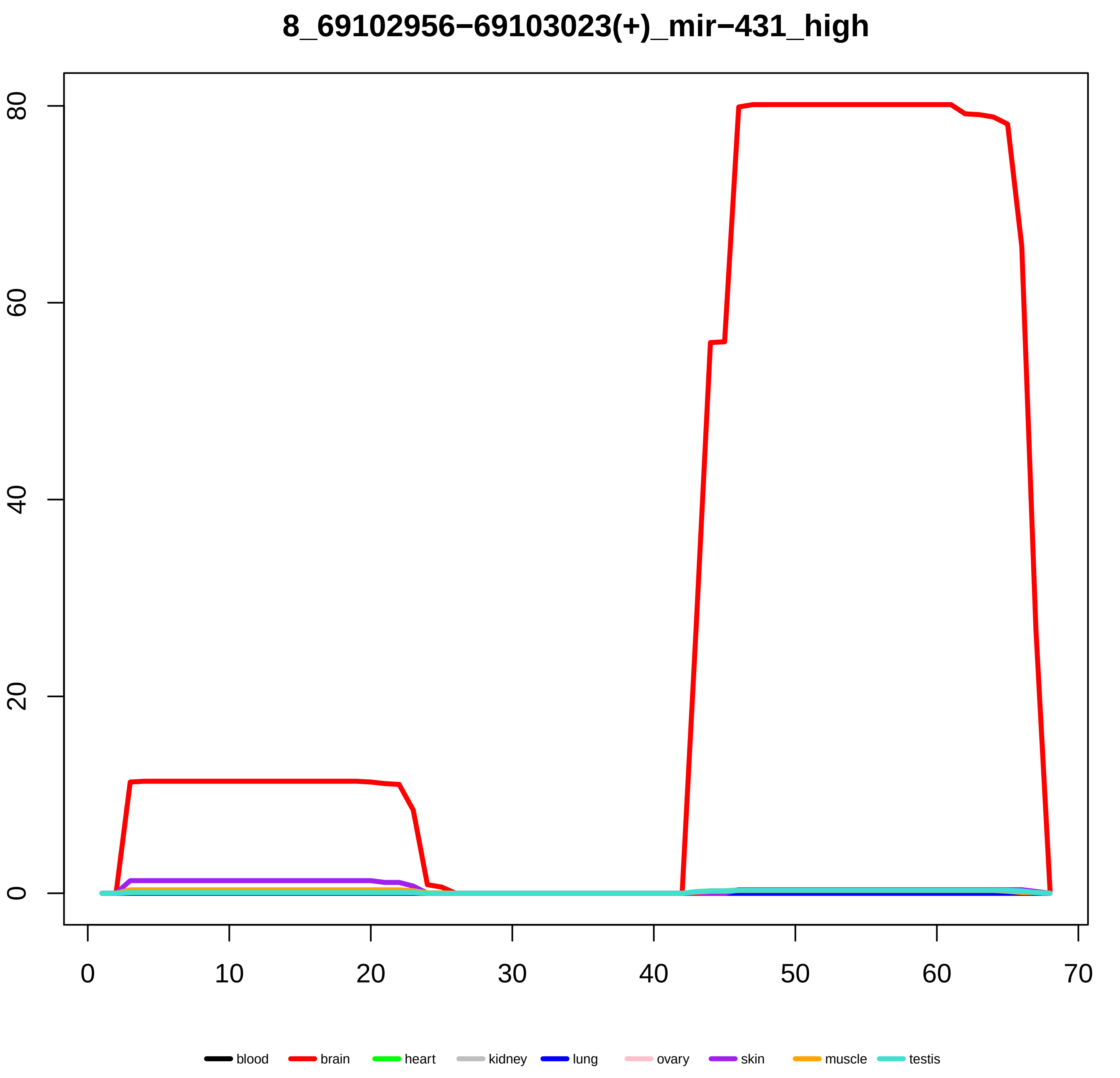
8_69102956−69103023(+)_mir−431_high
0
20
40
60
80
0
10
20
30
40
50
60
70
blood
brain
heart
kidney
lung
ovary
skin
muscle
testis

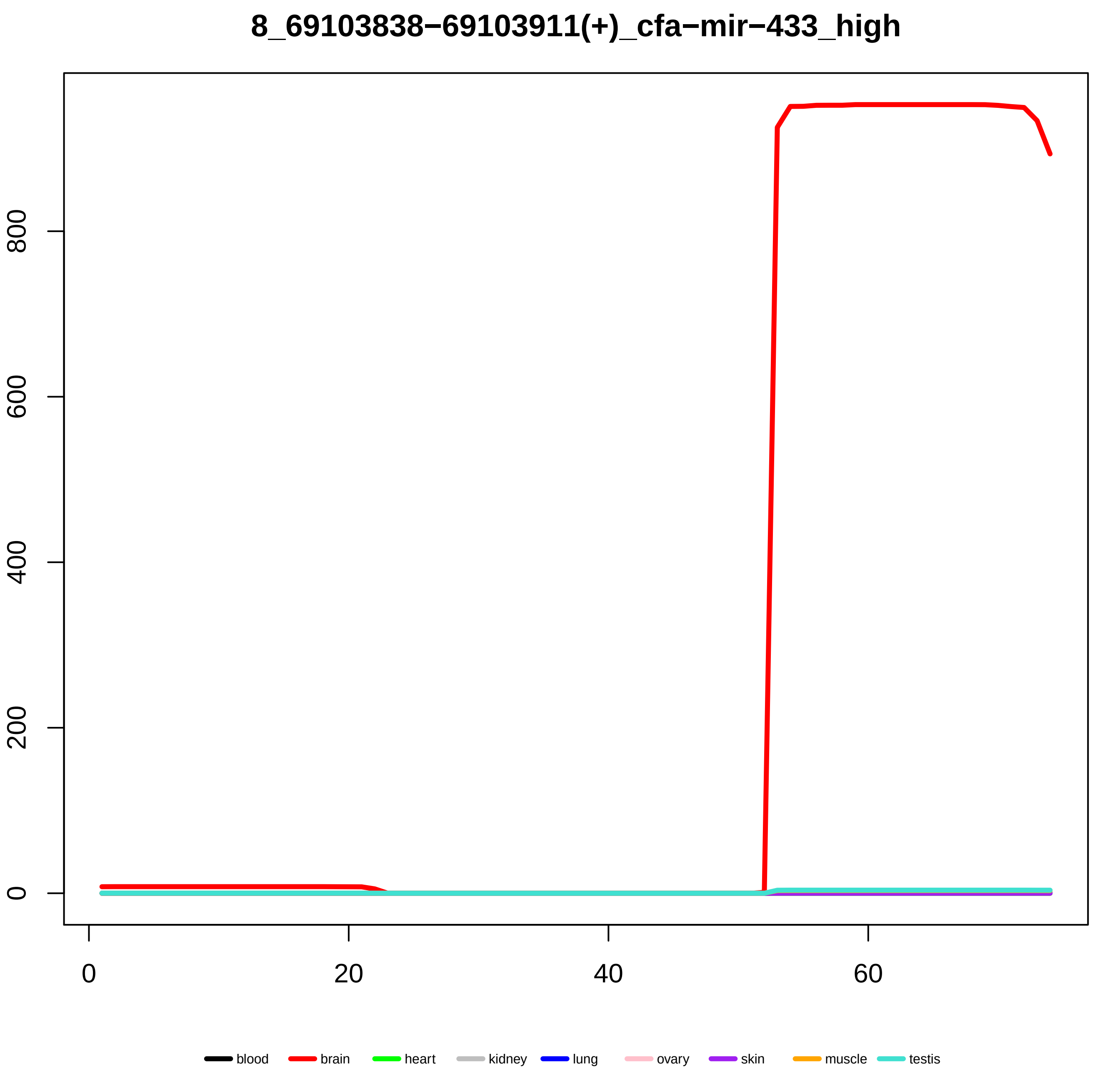
8_69103838−69103911(+)_cfa−mir−433_high
0
200
400
600
800
0
20
40
60
blood
brain
heart
kidney
lung
ovary
skin
muscle
testis

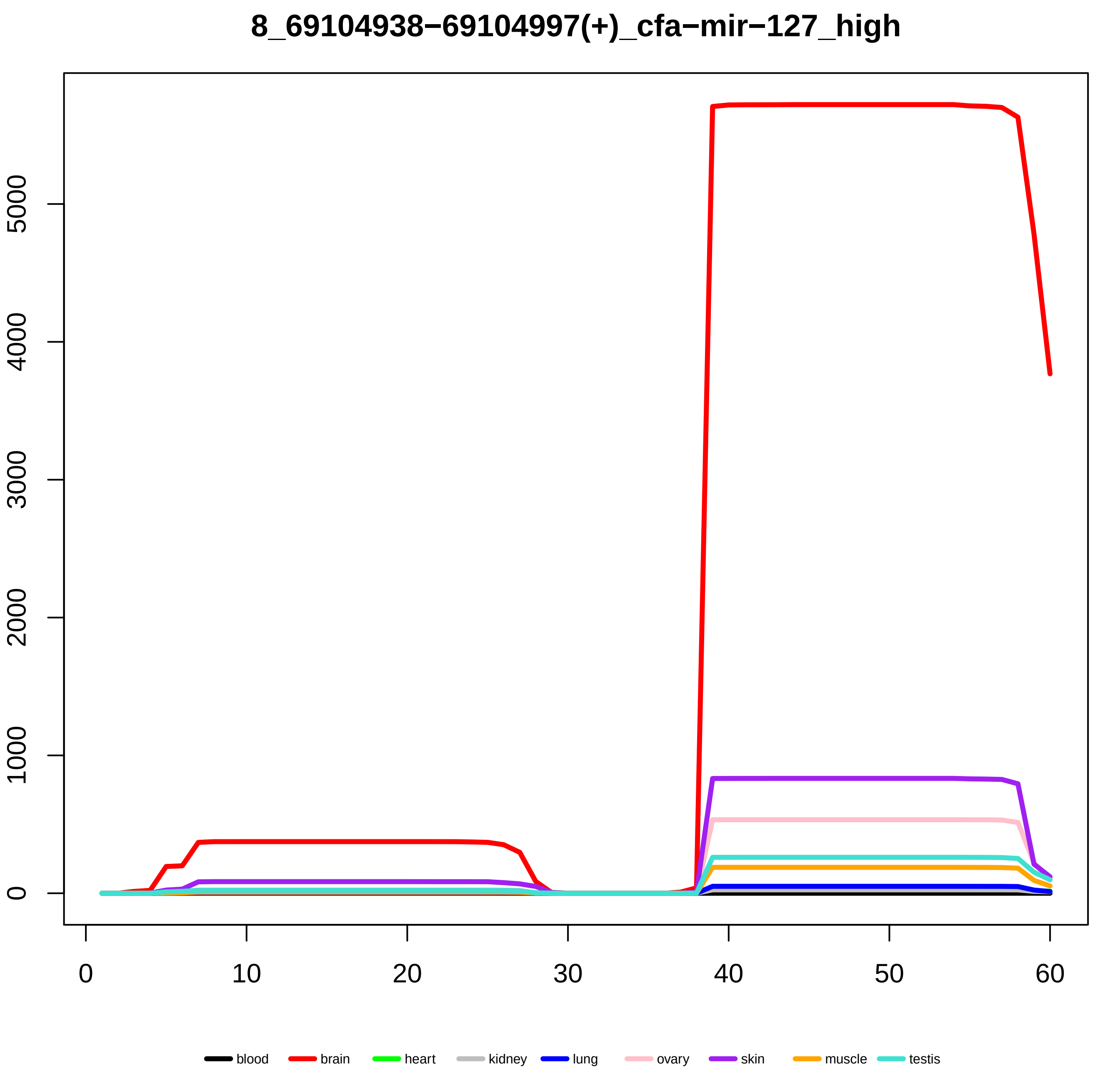
8_69104938−69104997(+)_cfa−mir−127_high
0
1000
2000
3000
4000
5000
0
10
20
30
40
50
60
blood
brain
heart
kidney
lung
ovary
skin
muscle
testis

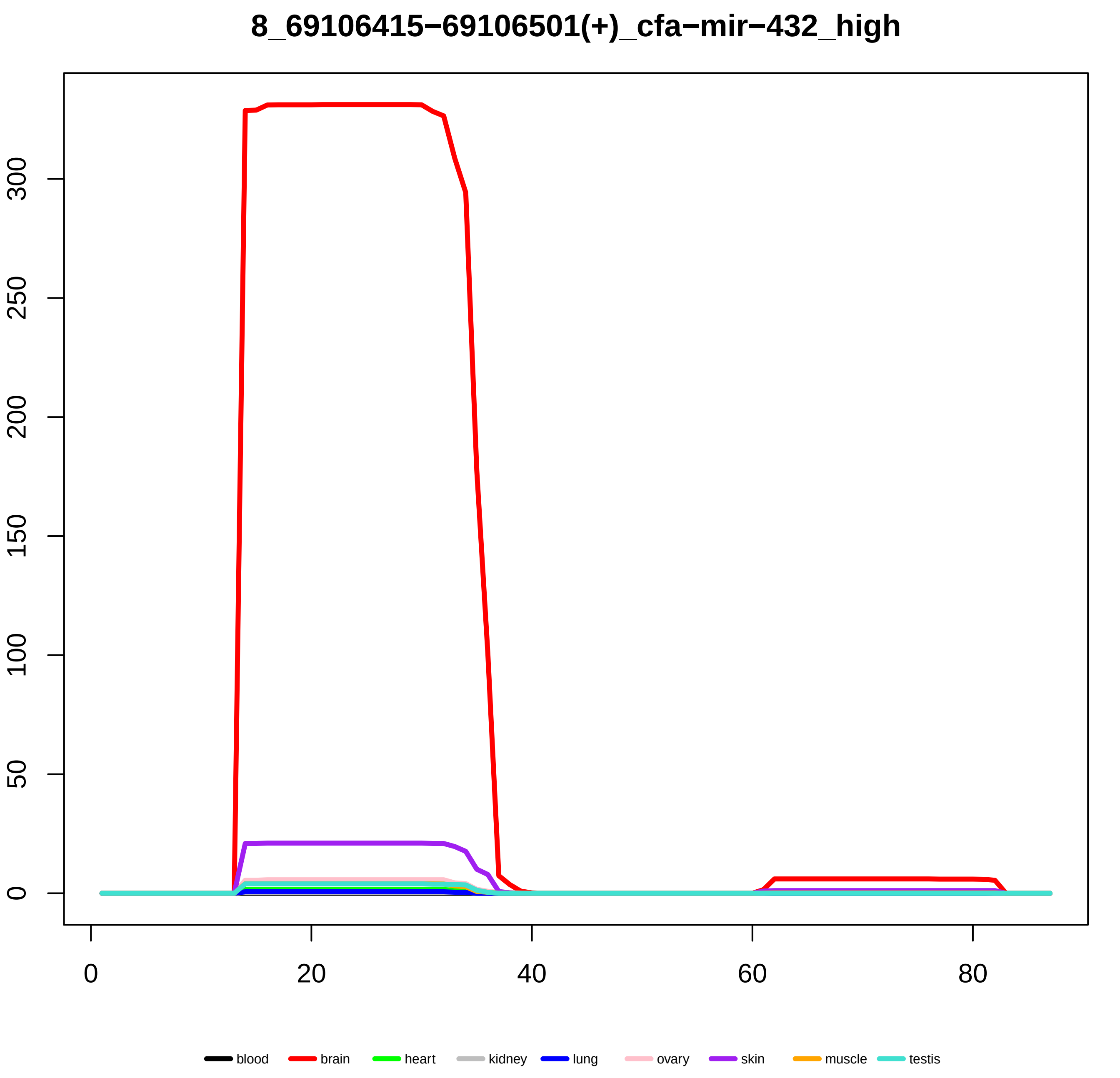
8_69106415−69106501(+)_cfa−mir−432_high
0
50
100
150
200
250
300
0
20
40
60
80
blood
brain
heart
kidney
lung
ovary
skin
muscle
testis

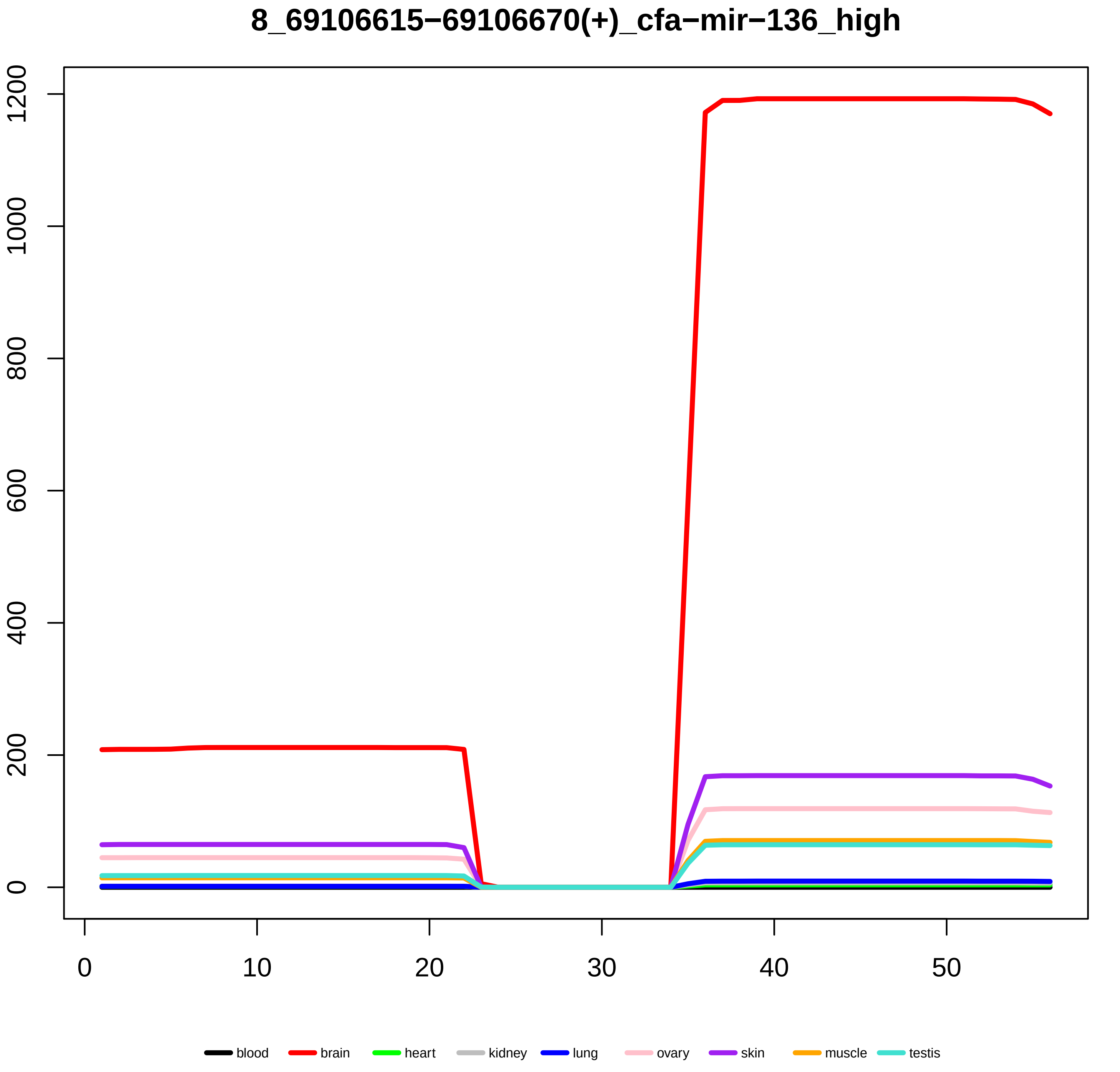

8_69106615−69106670(+)_cfa−mir−136_high
0
200
400
600
800
1000
1200
0
10
20
30
40
50
blood
brain
heart
kidney
lung
ovary
skin
muscle
testis

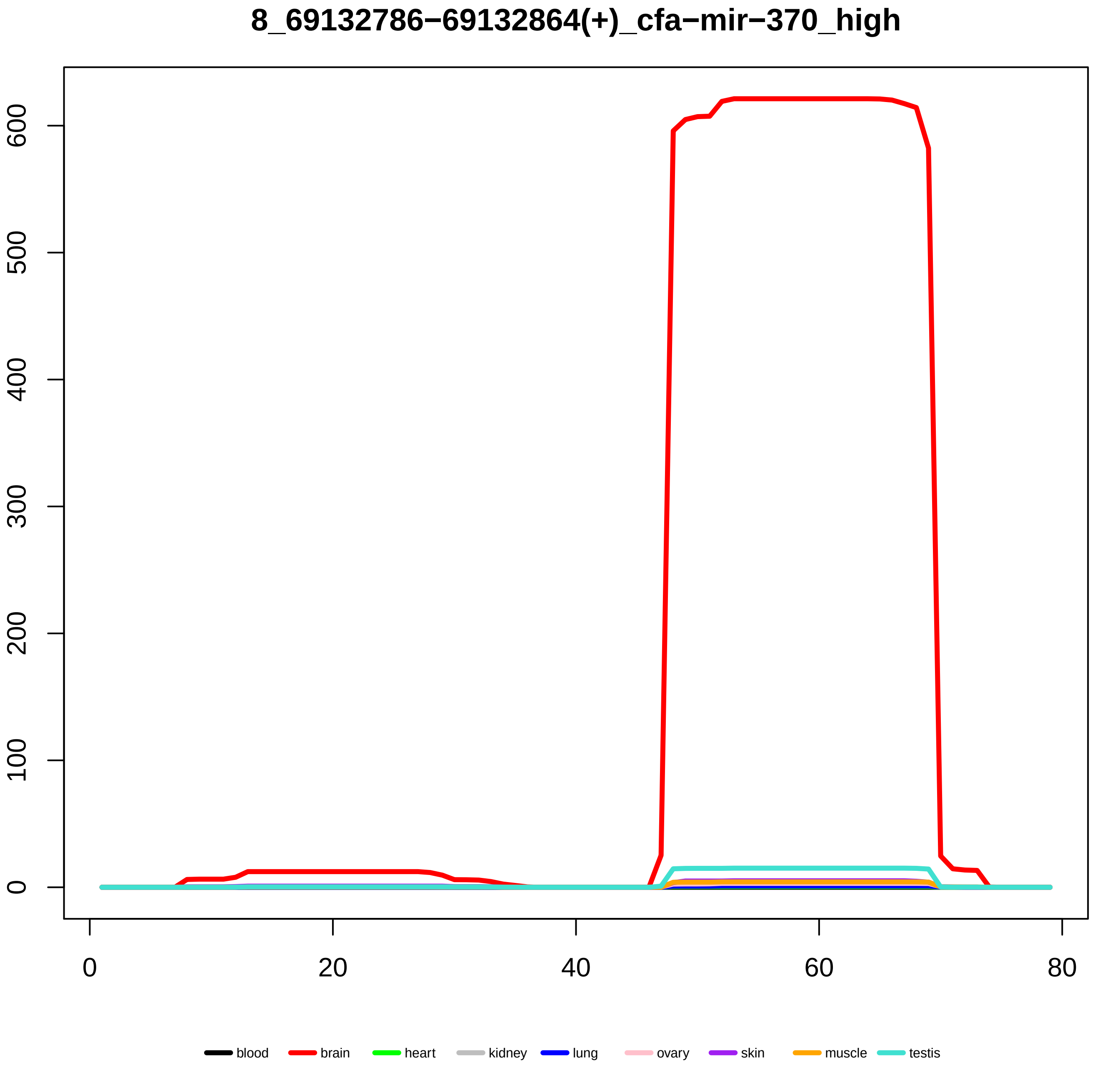
8_69132786−69132864(+)_cfa−mir−370_high
0
100
200
300
400
500
600
0
20
40
60
80
blood
brain
heart
kidney
lung
ovary
skin
muscle
testis

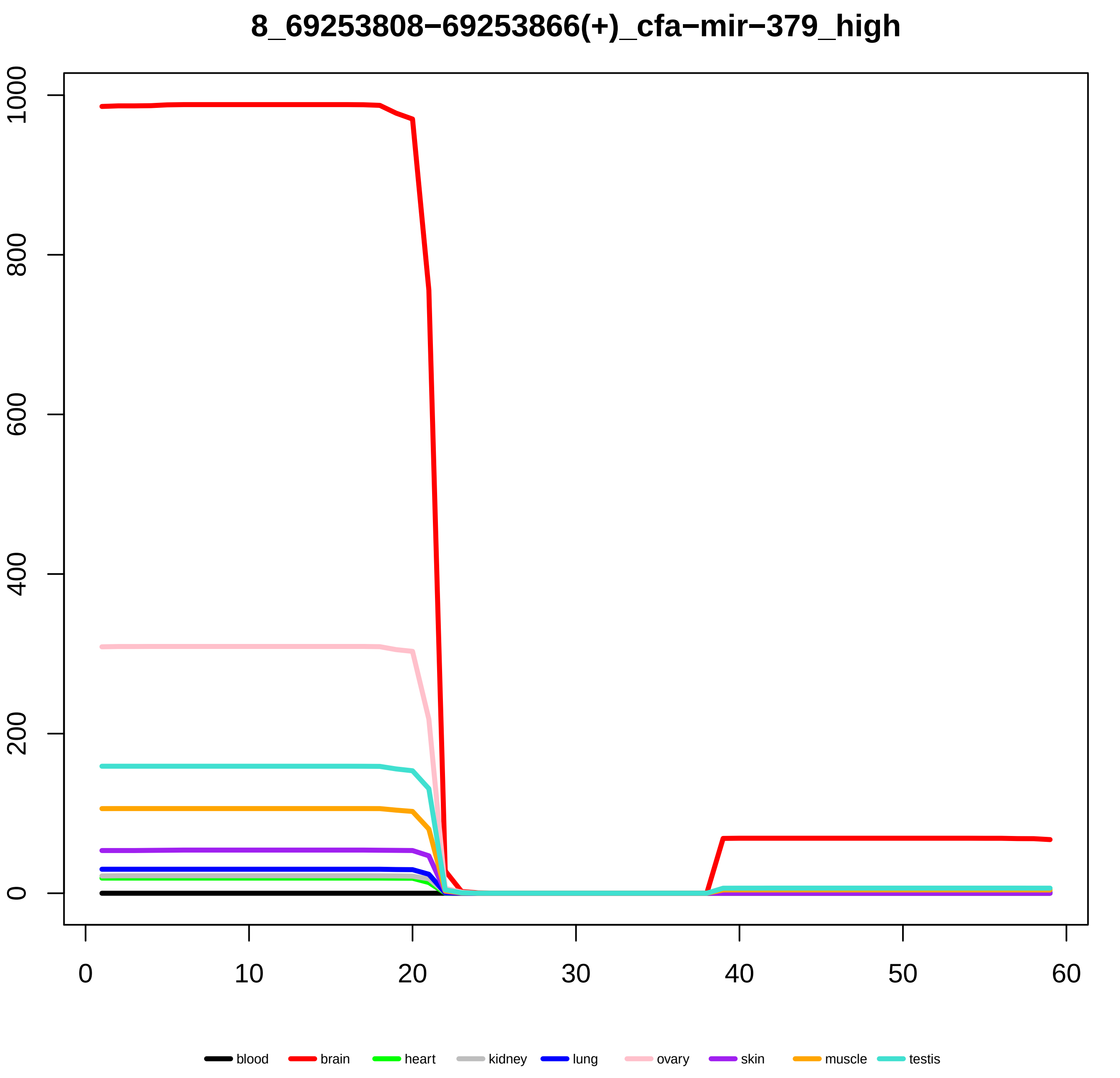

8_69253808−69253866(+)_cfa−mir−379_high
0
200
400
600
800
1000
0
10
20
30
40
50
60
blood
brain
heart
kidney
lung
ovary
skin
muscle
testis

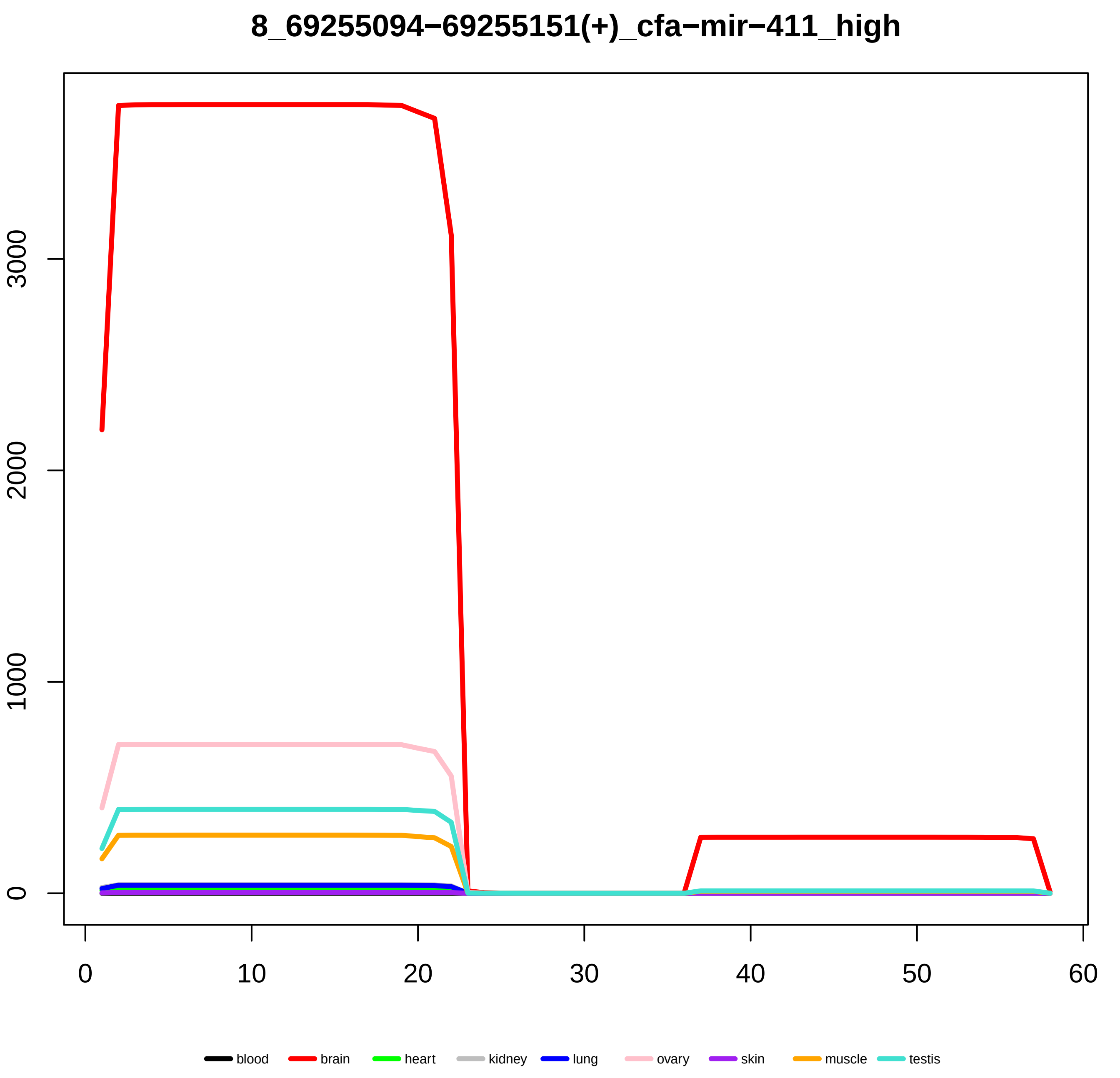

8_69255094−69255151(+)_cfa−mir−411_high
0
1000
2000
3000
0
10
20
30
40
50
60
blood
brain
heart
kidney
lung
ovary
skin
muscle
testis

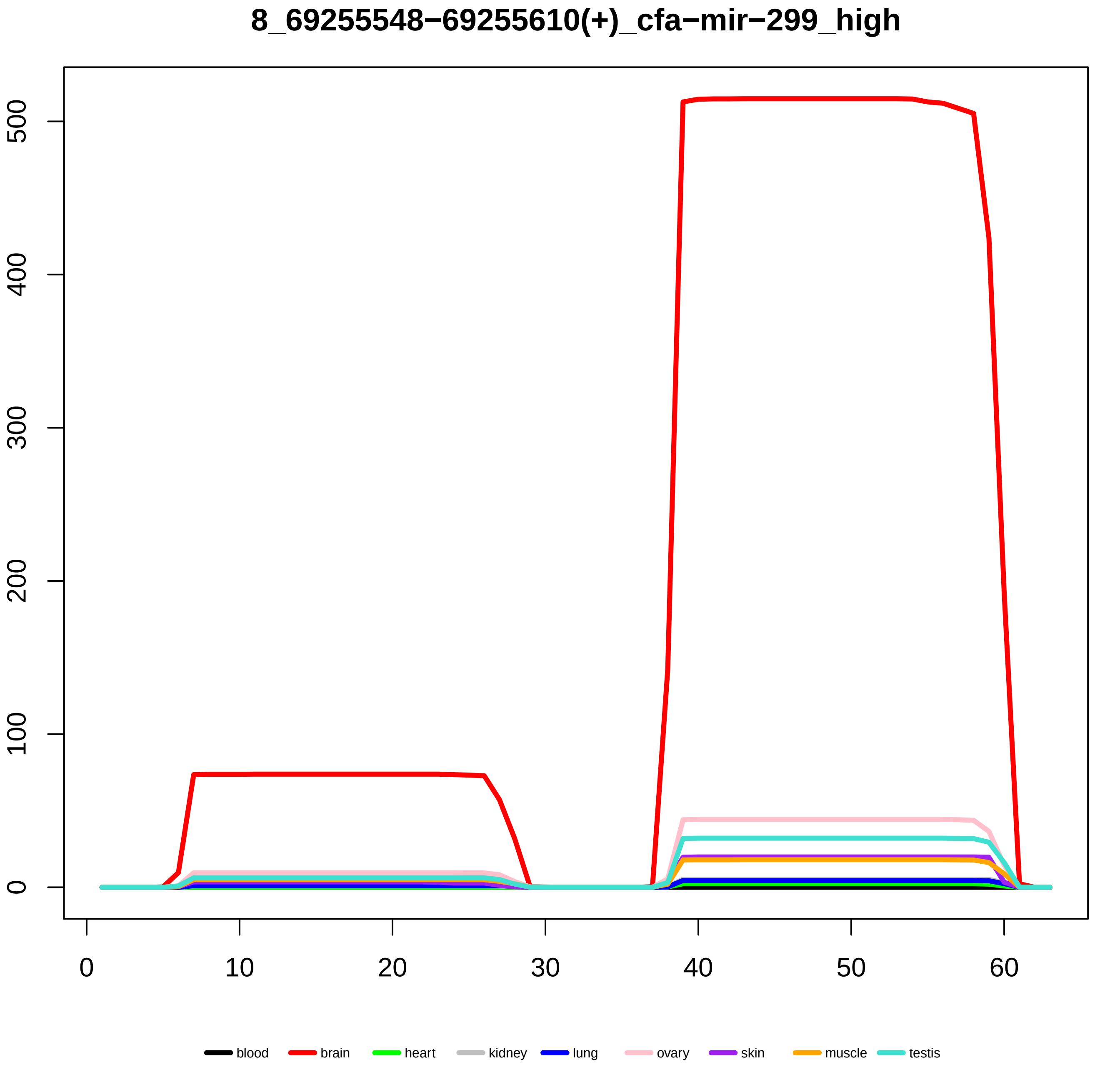
8_69255548−69255610(+)_cfa−mir−299_high
0
100
200
300
400
500
0
10
20
30
40
50
60
blood
brain
heart
kidney
lung
ovary
skin
muscle
testis

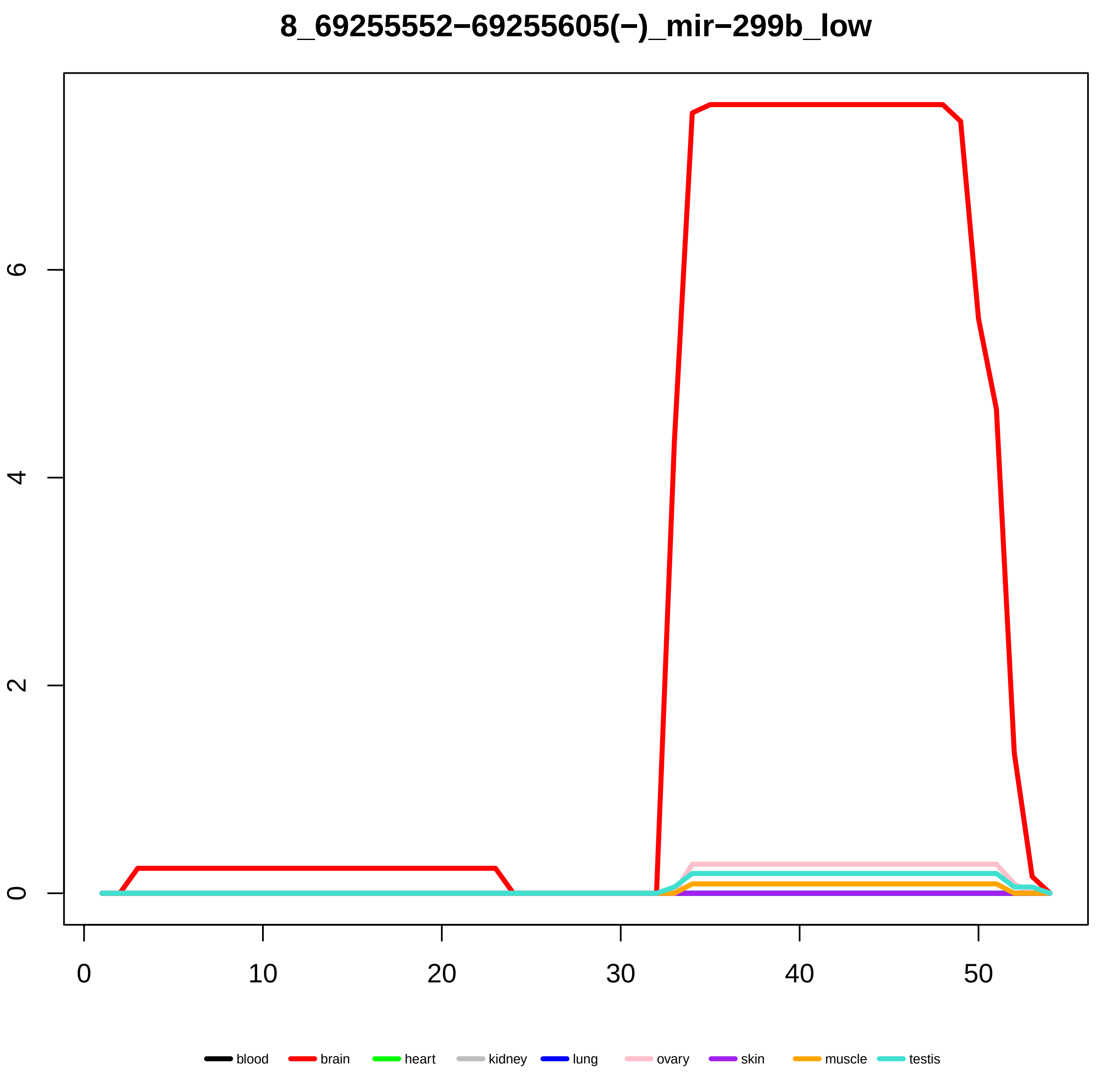
8_69255552−69255605(−)_mir−299b_low
0
2
4
6
0
10
20
30
40
50
blood
brain
heart
kidney
lung
ovary
skin
muscle
testis

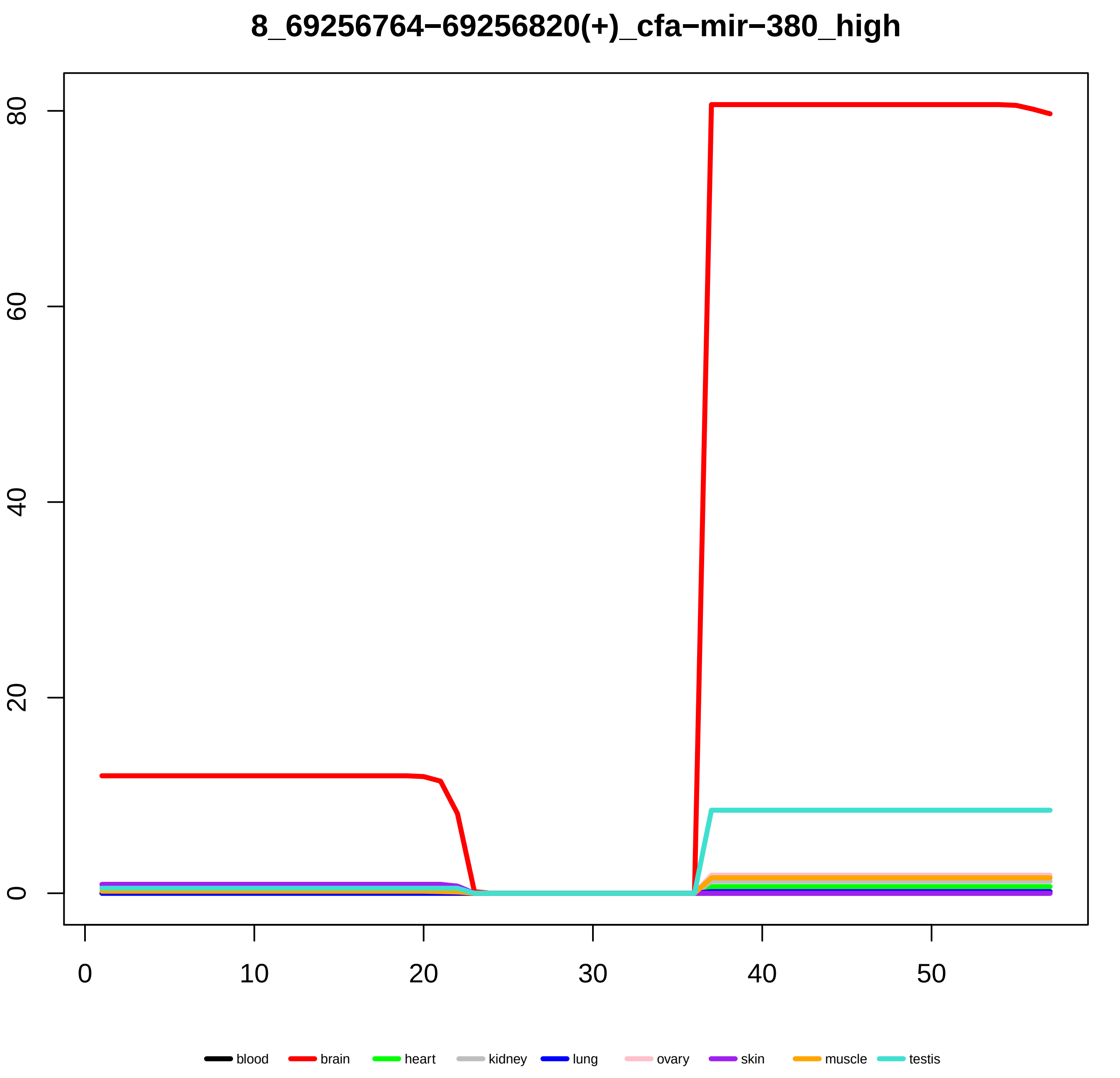

8_69256764−69256820(+)_cfa−mir−380_high
0
20
40
60
80
0
10
20
30
40
50
blood
brain
heart
kidney
lung
ovary
skin
muscle
testis

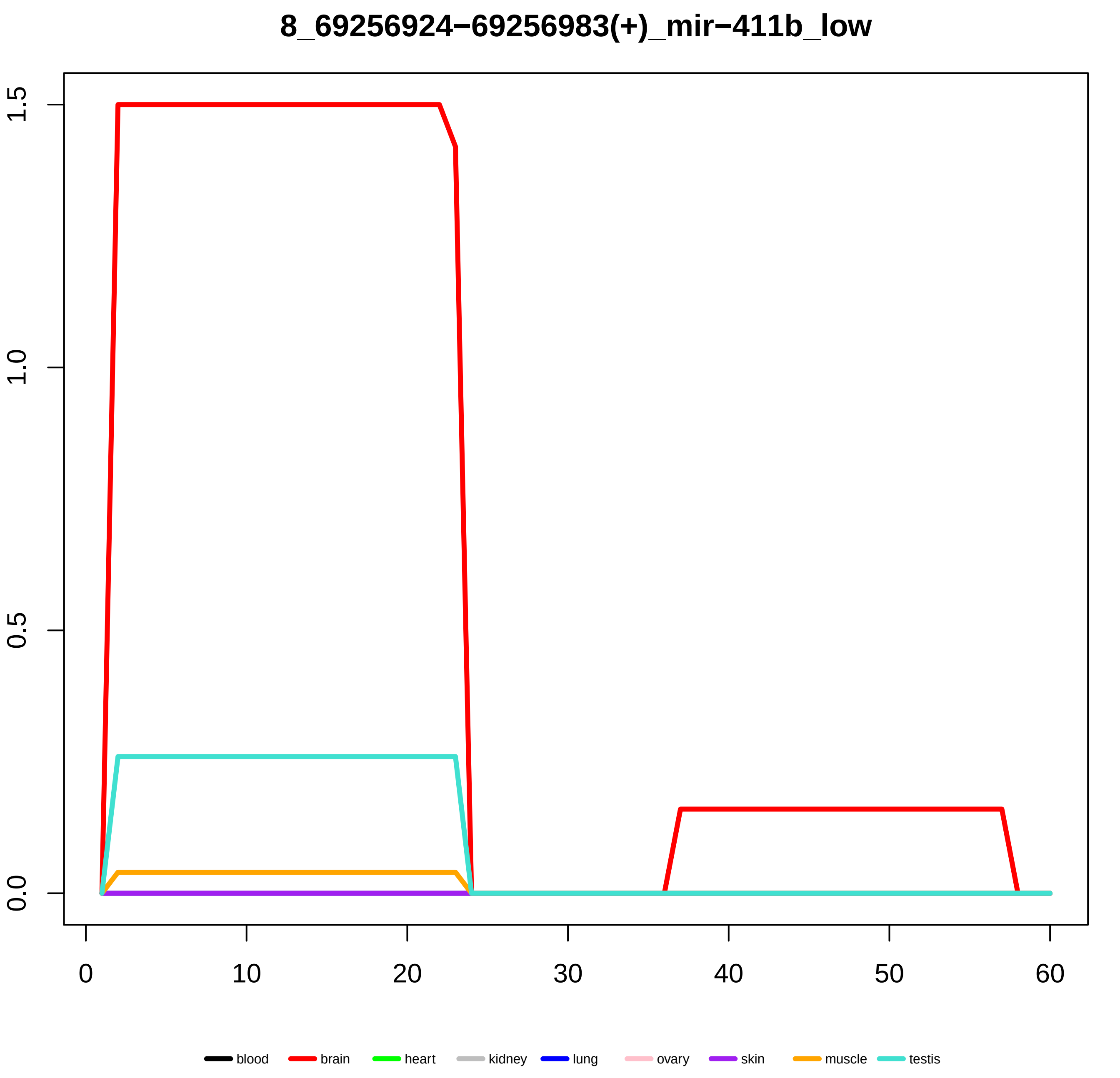

8_69256924−69256983(+)_mir−411b_low
0.0
0.5
1.0
1.5
0
10
20
30
40
50
60
blood
brain
heart
kidney
lung
ovary
skin
muscle
testis

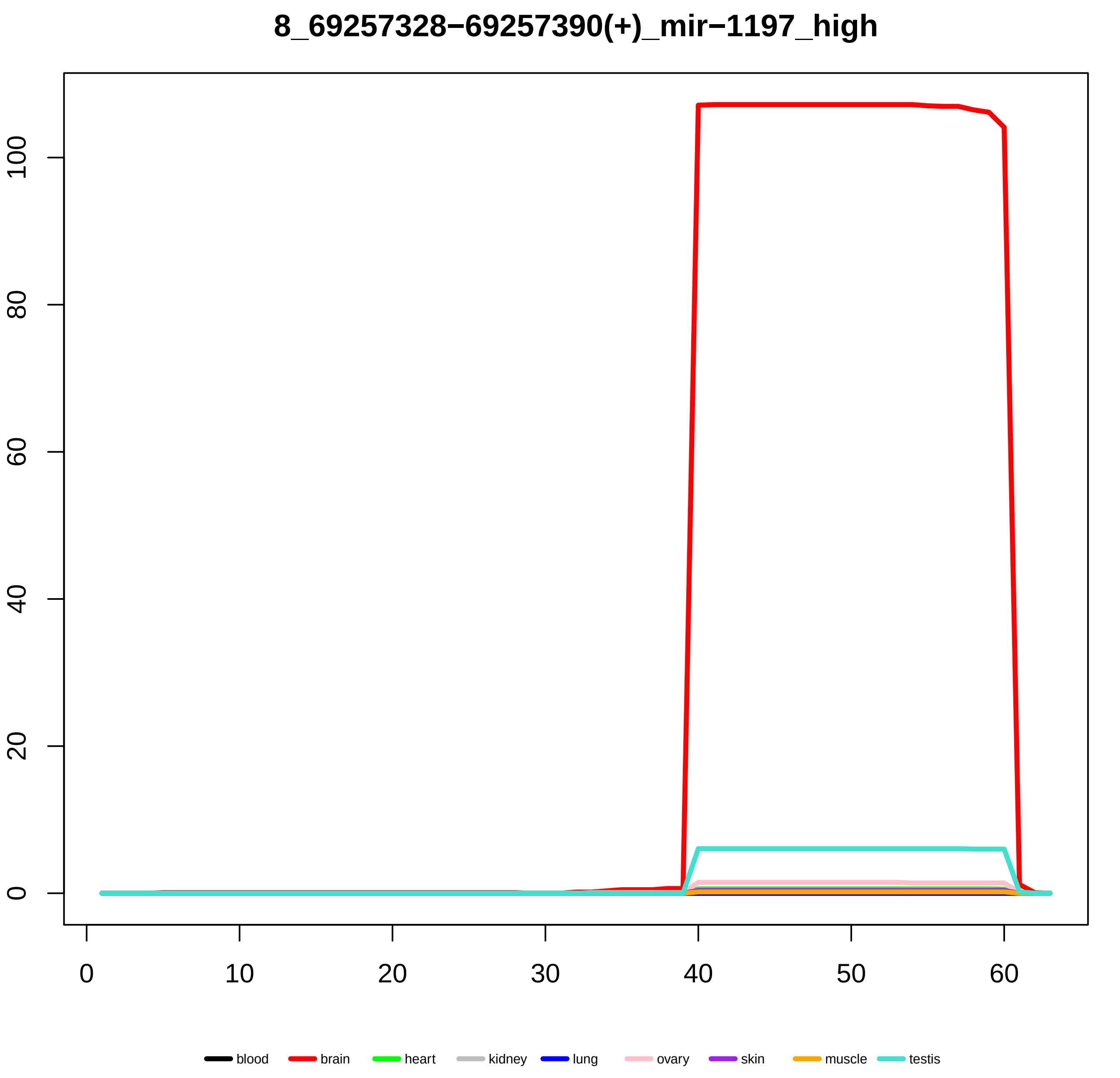
8_69257328−69257390(+)_mir−1197_high
0
20
40
60
80
100
0
10
20
30
40
50
60
blood
brain
heart
kidney
lung
ovary
skin
muscle
testis

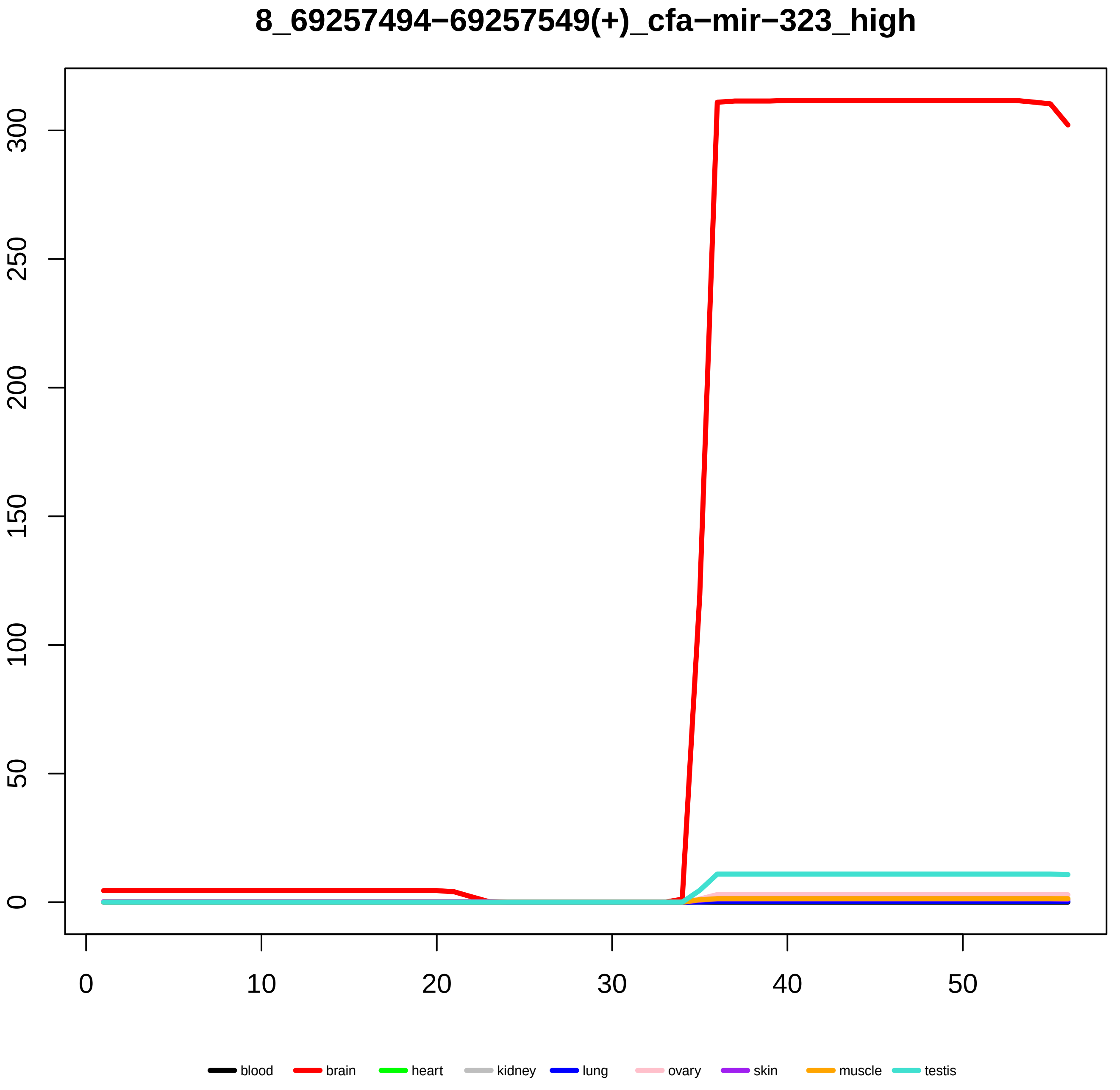
8_69257494−69257549(+)_cfa−mir−323_high
0
50
100
150
200
250
300
0
10
20
30
40
50
blood
brain
heart
kidney
lung
ovary
skin
muscle
testis

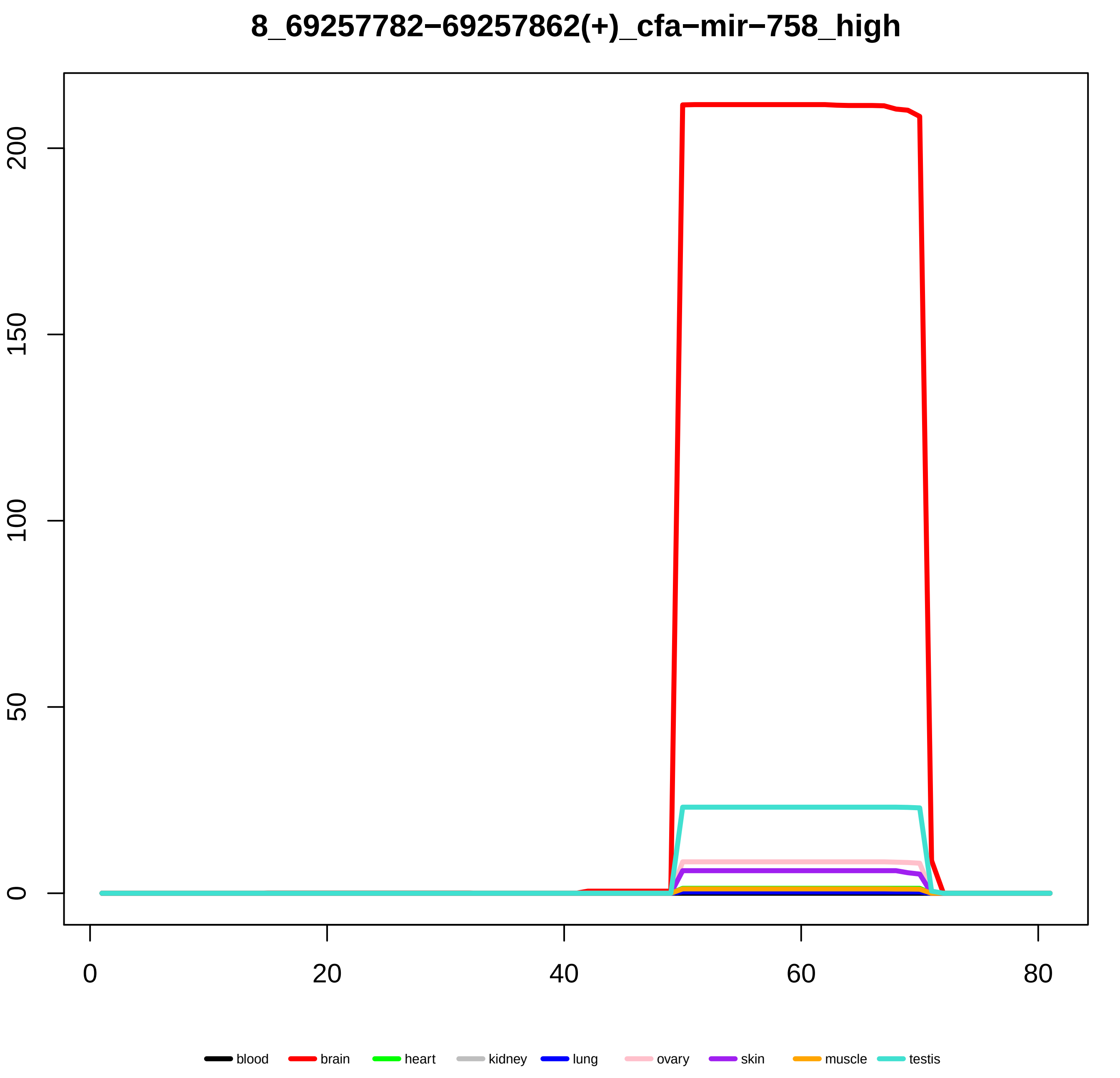
8_69257782−69257862(+)_cfa−mir−758_high
0
50
100
150
200
0
20
40
60
80
blood
brain
heart
kidney
lung
ovary
skin
muscle
testis

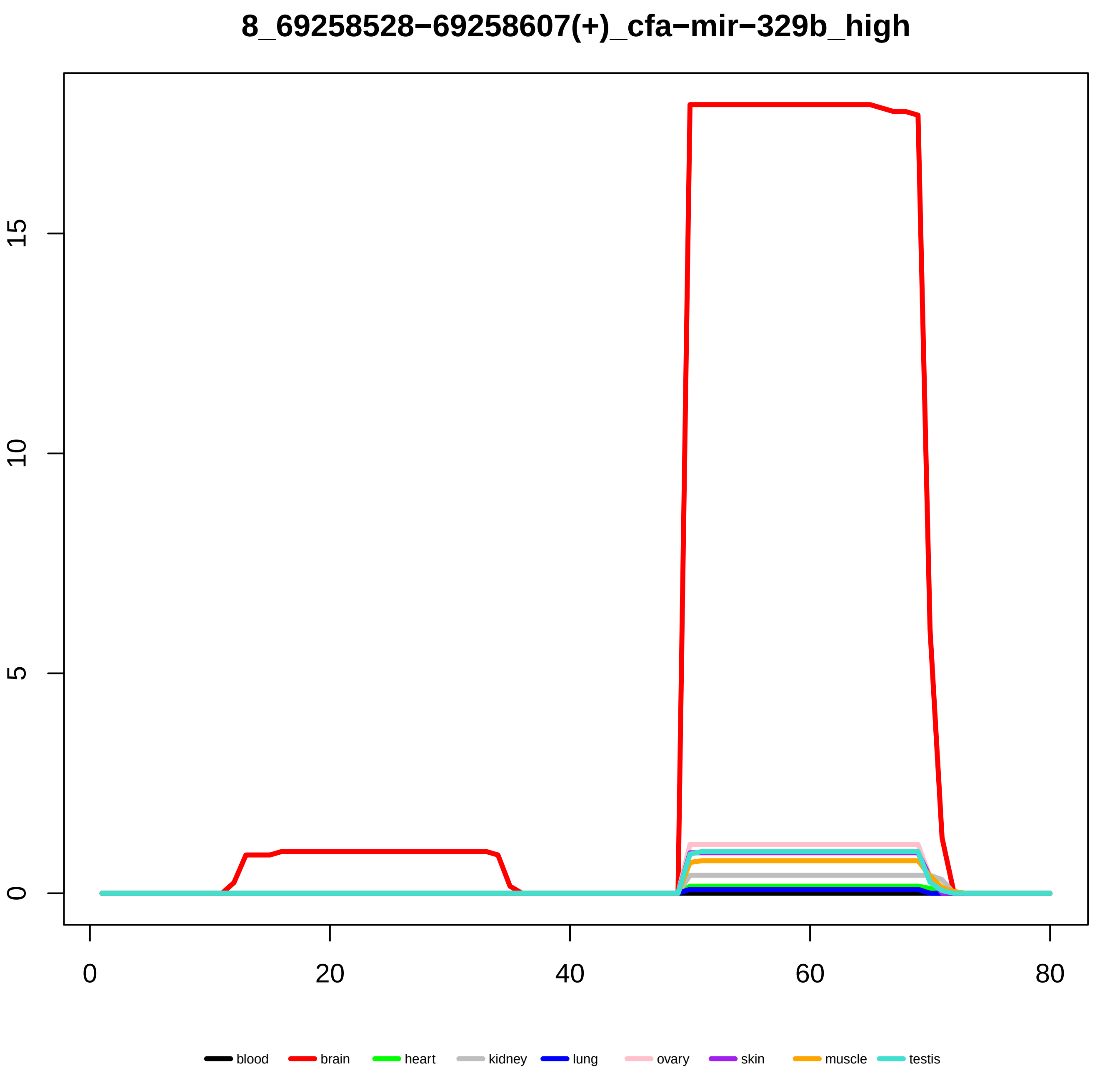
8_69258528−69258607(+)_cfa−mir−329b_high
0
5
10
15
0
20
40
60
80
blood
brain
heart
kidney
lung
ovary
skin
muscle
testis

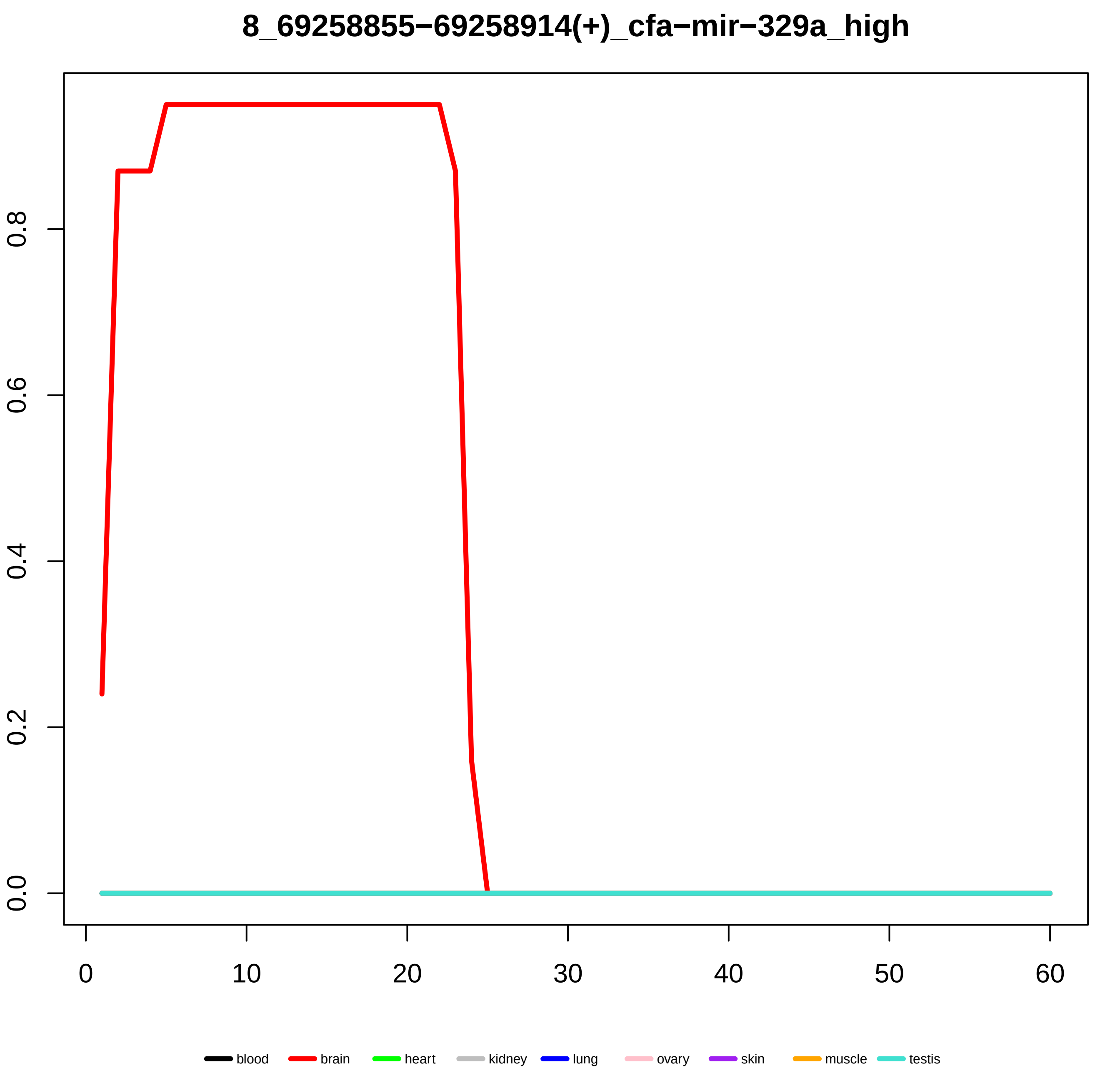

8_69258855−69258914(+)_cfa−mir−329a_high
0.0
0.2
0.4
0.6
0.8
0
10
20
30
40
50
60
blood
brain
heart
kidney
lung
ovary
skin
muscle
testis

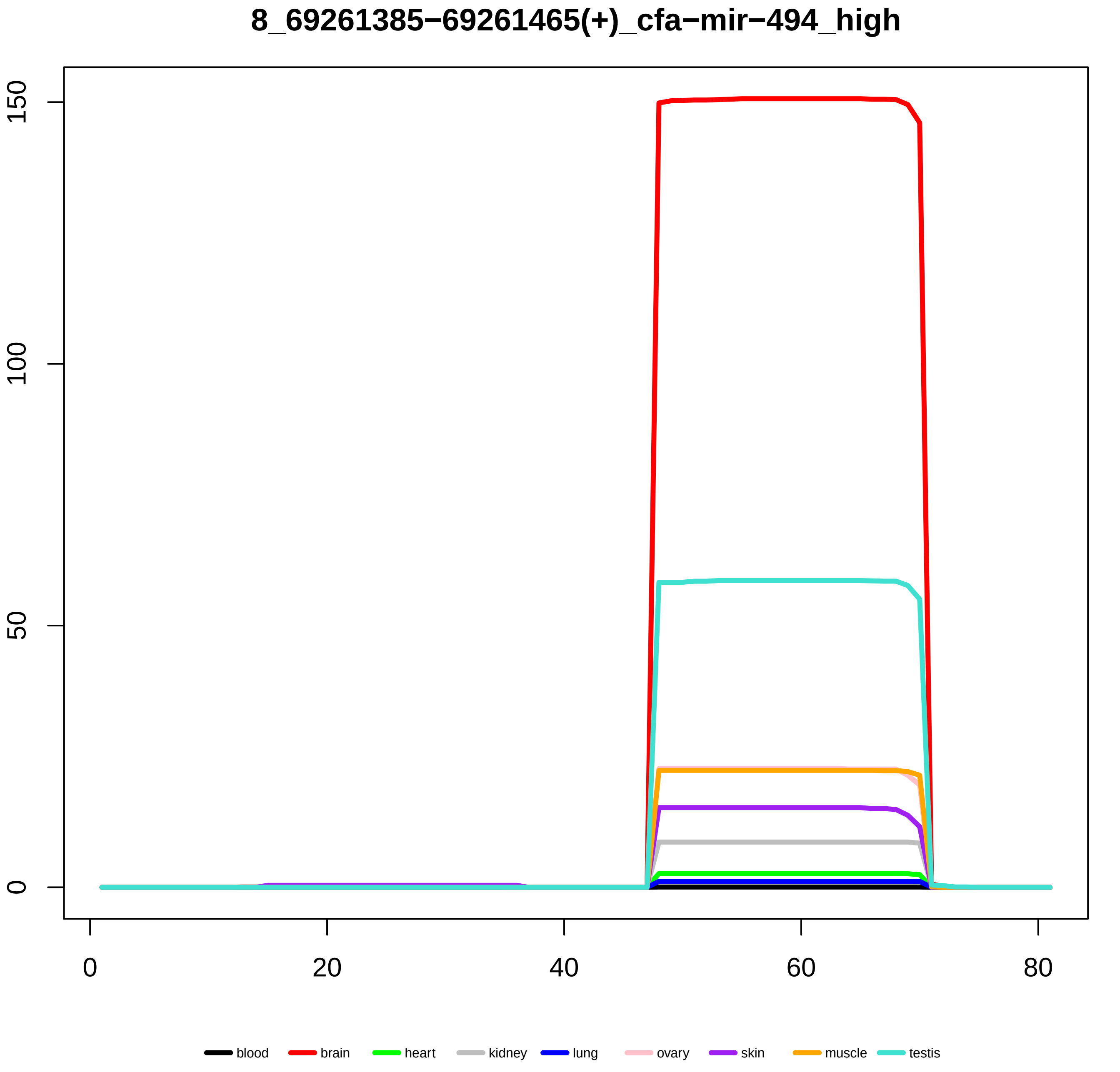
8_69261385−69261465(+)_cfa−mir−494_high
0
50
100
150
0
20
40
60
80
blood
brain
heart
kidney
lung
ovary
skin
muscle
testis

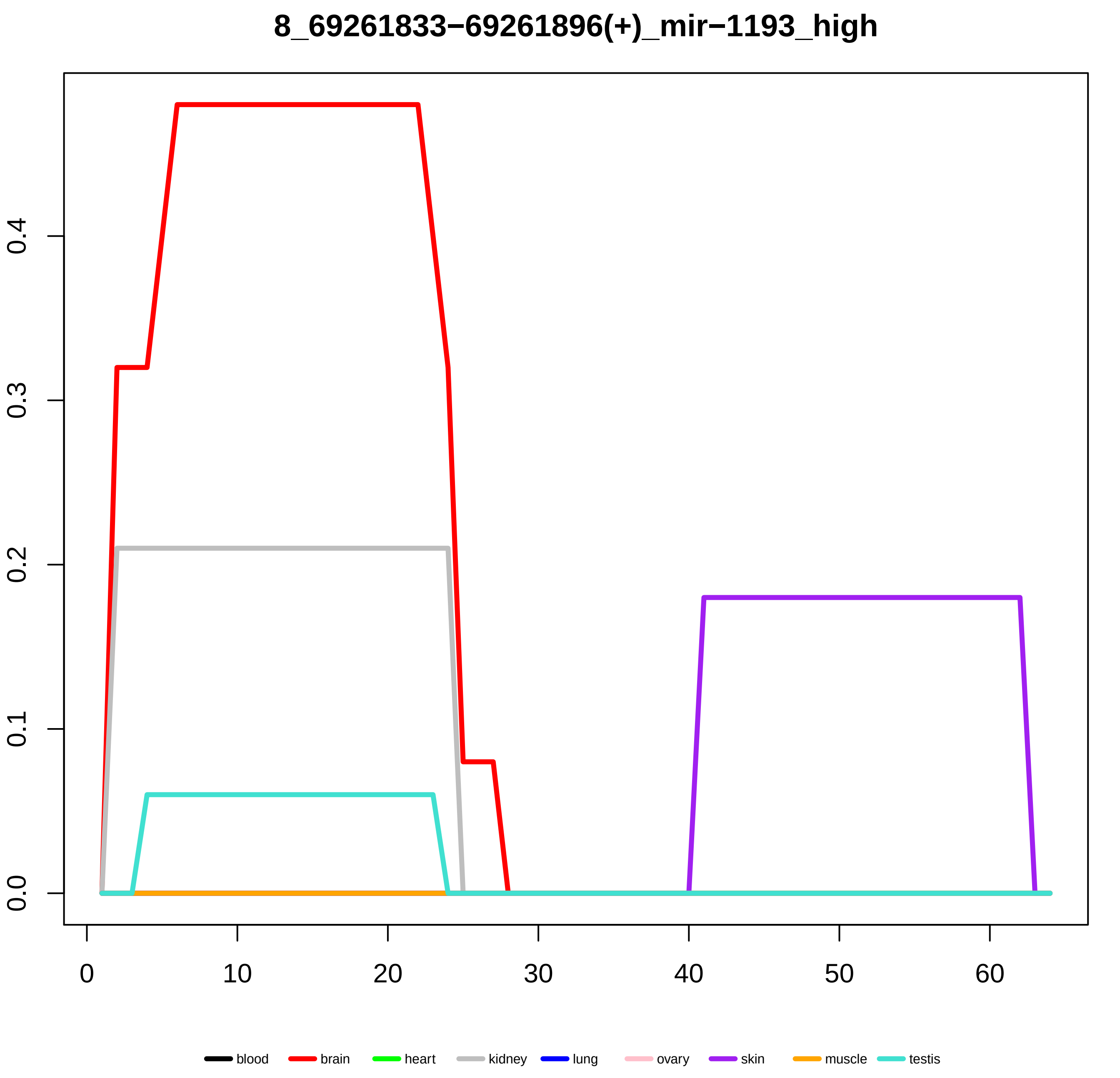

8_69261833−69261896(+)_mir−1193_high
0.0
0.1
0.2
0.3
0.4
0
10
20
30
40
50
60
blood
brain
heart
kidney
lung
ovary
skin
muscle
testis

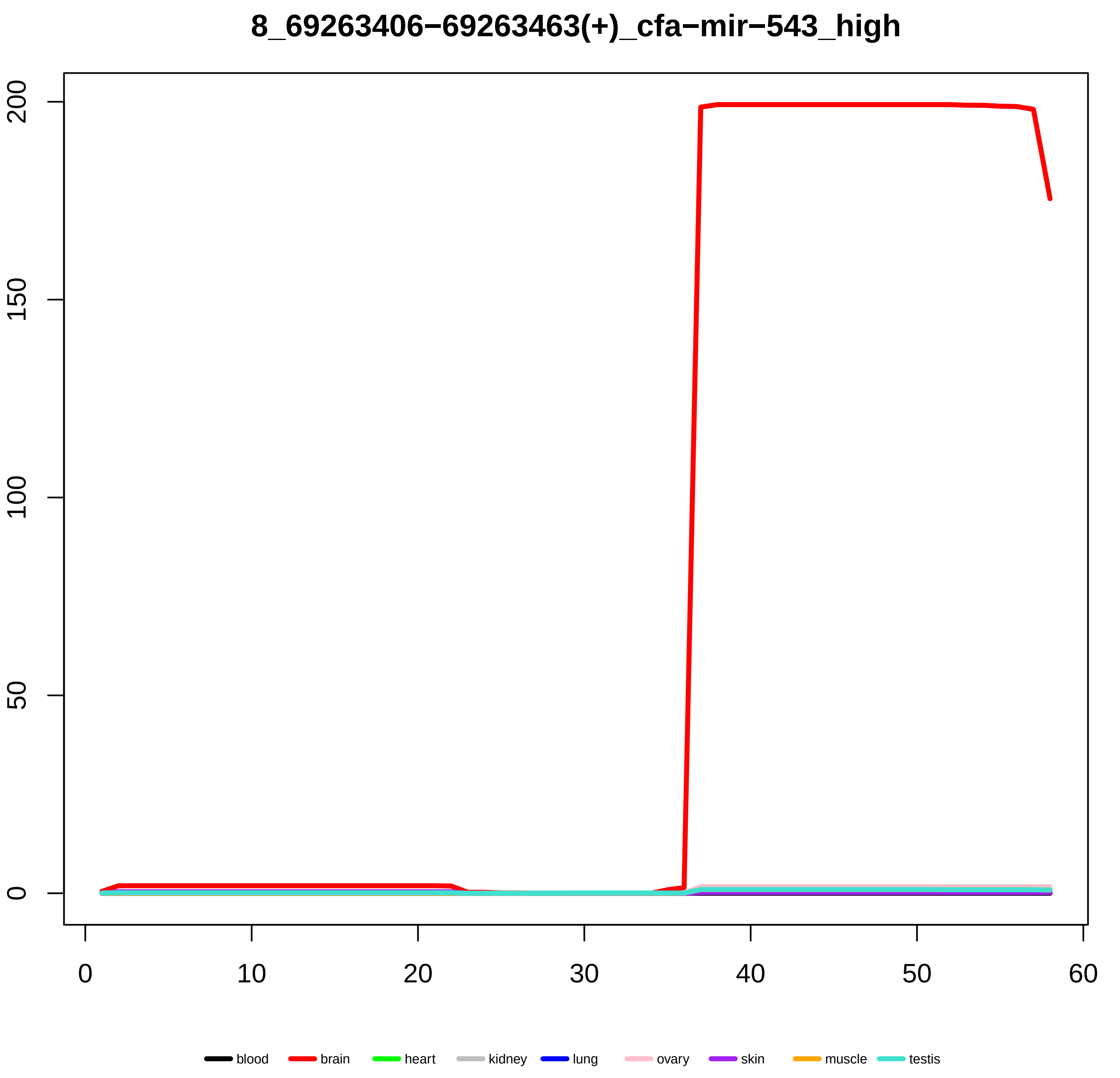

8_69263406−69263463(+)_cfa−mir−543_high
0
50
100
150
200
0
10
20
30
40
50
60
blood
brain
heart
kidney
lung
ovary
skin
muscle
testis

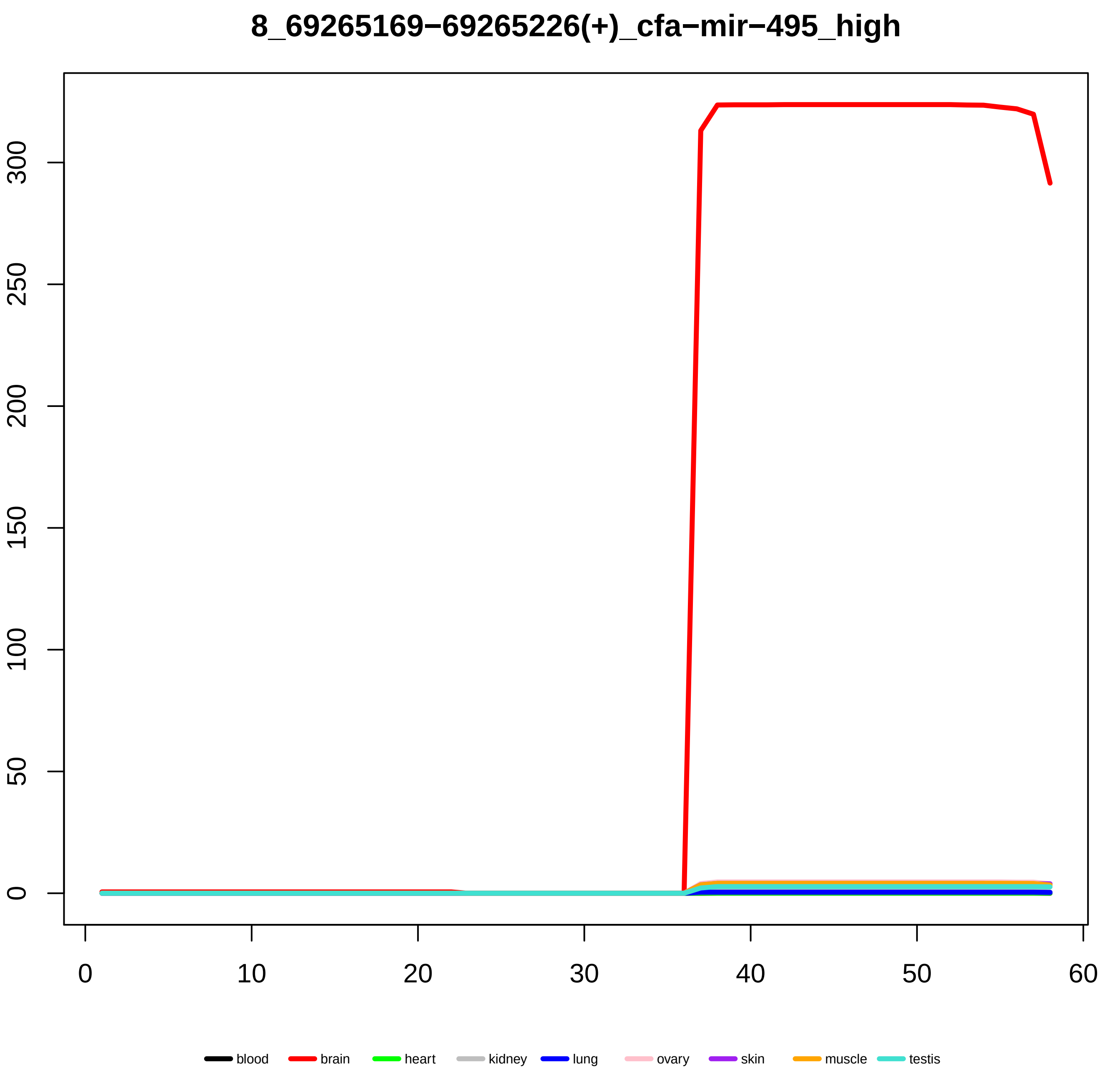
8_69265169−69265226(+)_cfa−mir−495_high
0
50
100
150
200
250
300
0
10
20
30
40
50
60
blood
brain
heart
kidney
lung
ovary
skin
muscle
testis

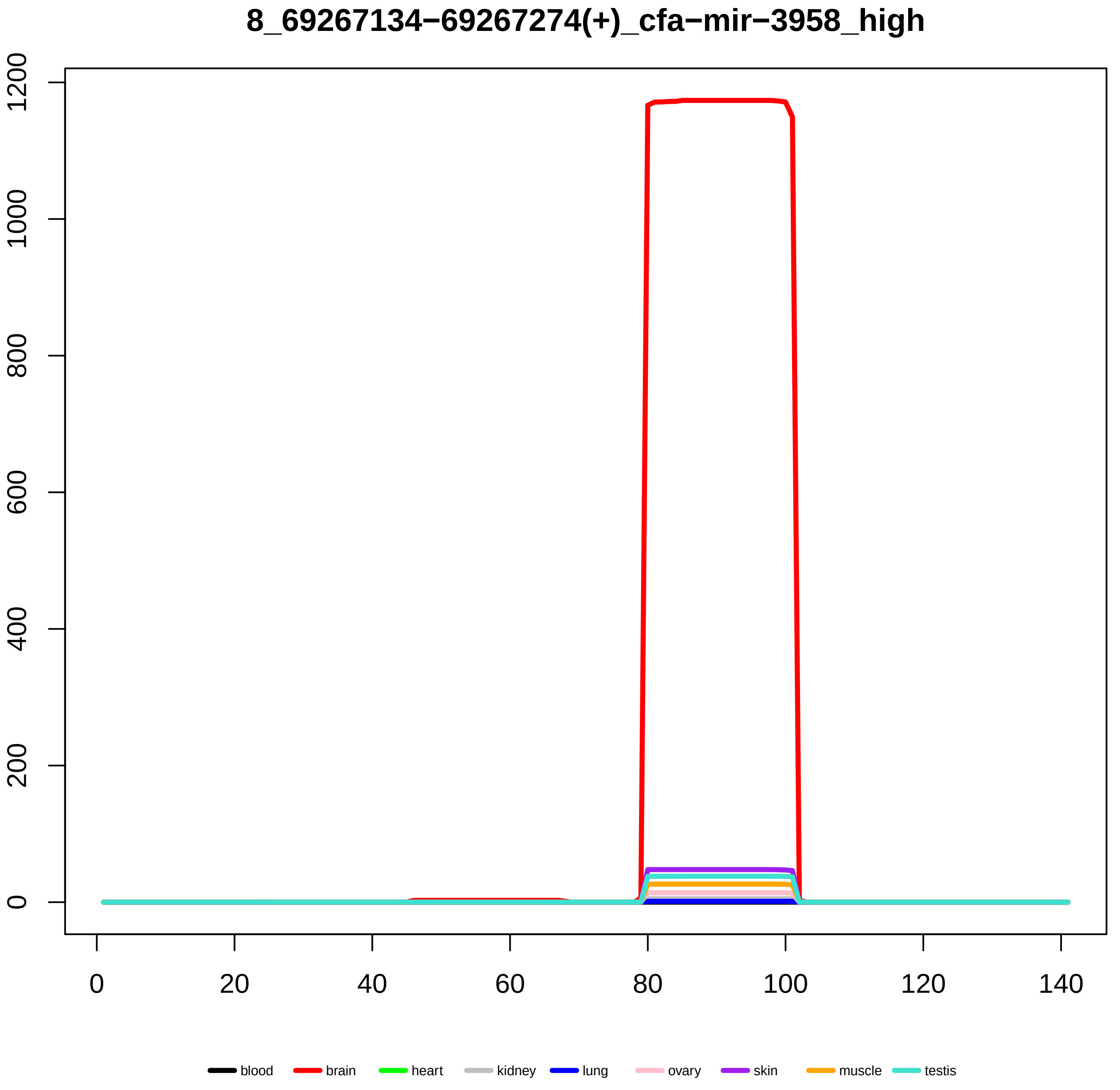

8_69267134−69267274(+)_cfa−mir−3958_high
0
200
400
600
800
1000
1200
0
20
40
60
80
100
120
140
blood
brain
heart
kidney
lung
ovary
skin
muscle
testis

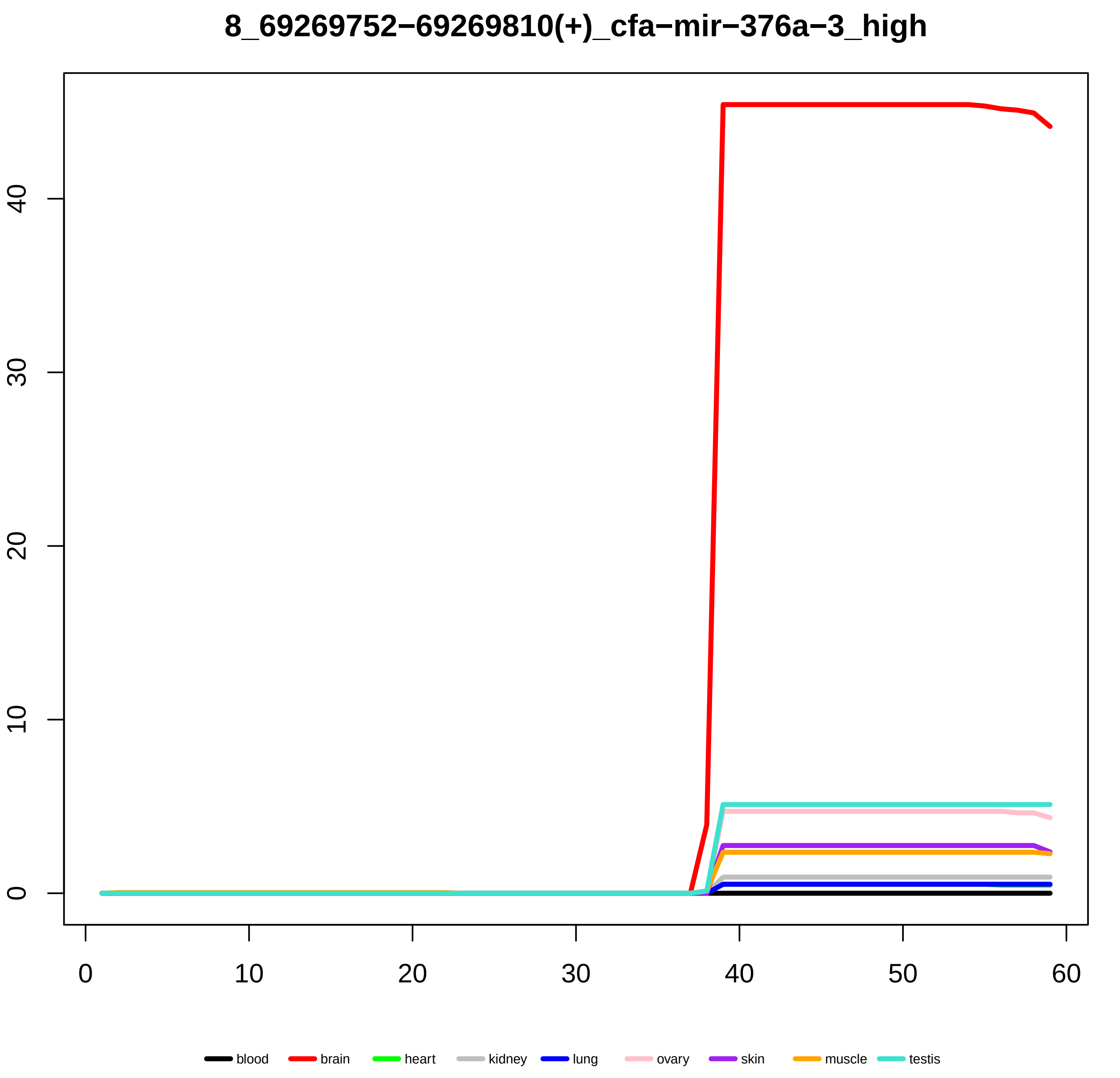

8_69269752−69269810(+)_cfa−mir−376a−3_high
0
10
20
30
40
0
10
20
30
40
50
60
blood
brain
heart
kidney
lung
ovary
skin
muscle
testis

8_69270100−69270185(+)_cfa−mir−376c_high

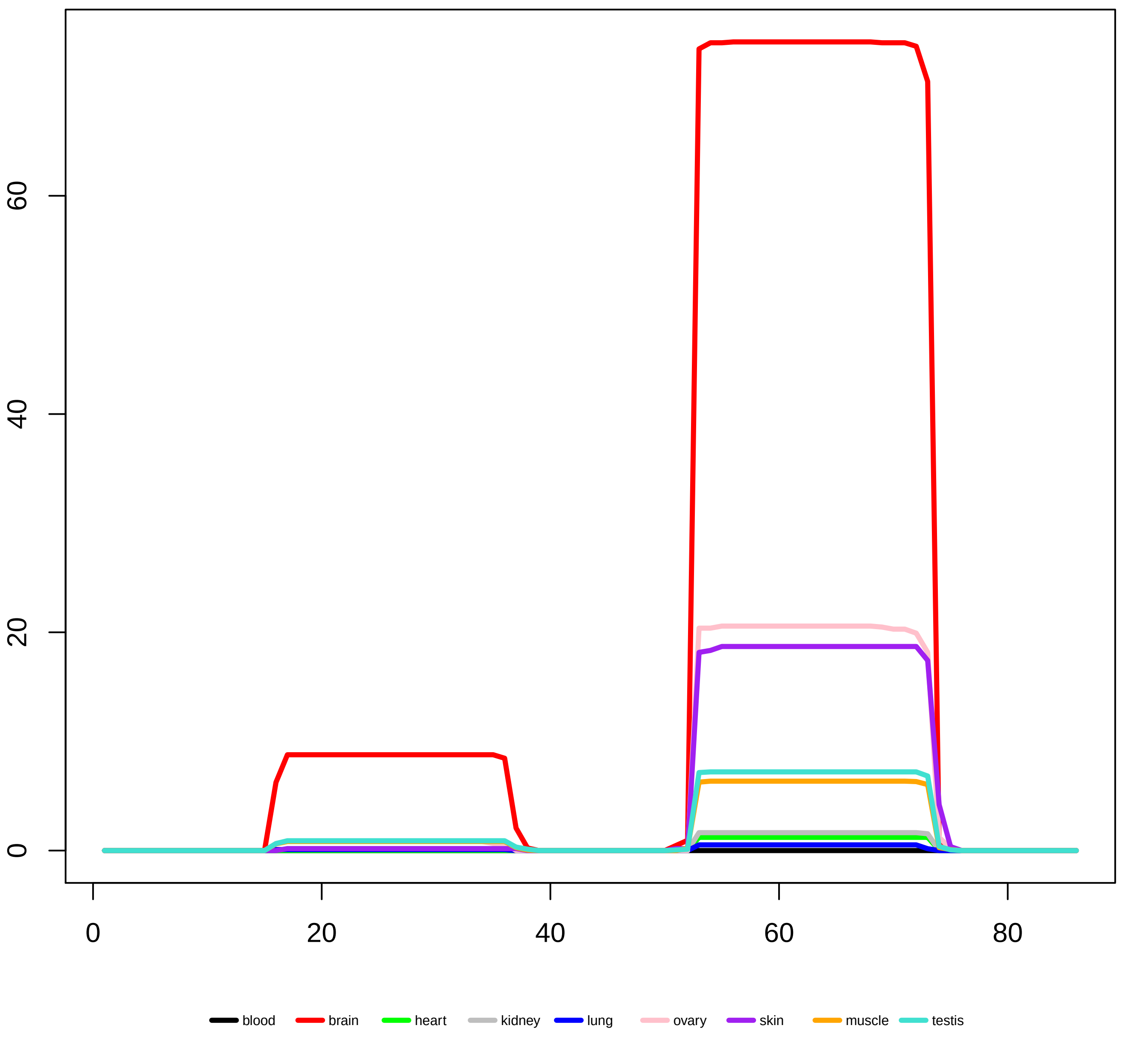

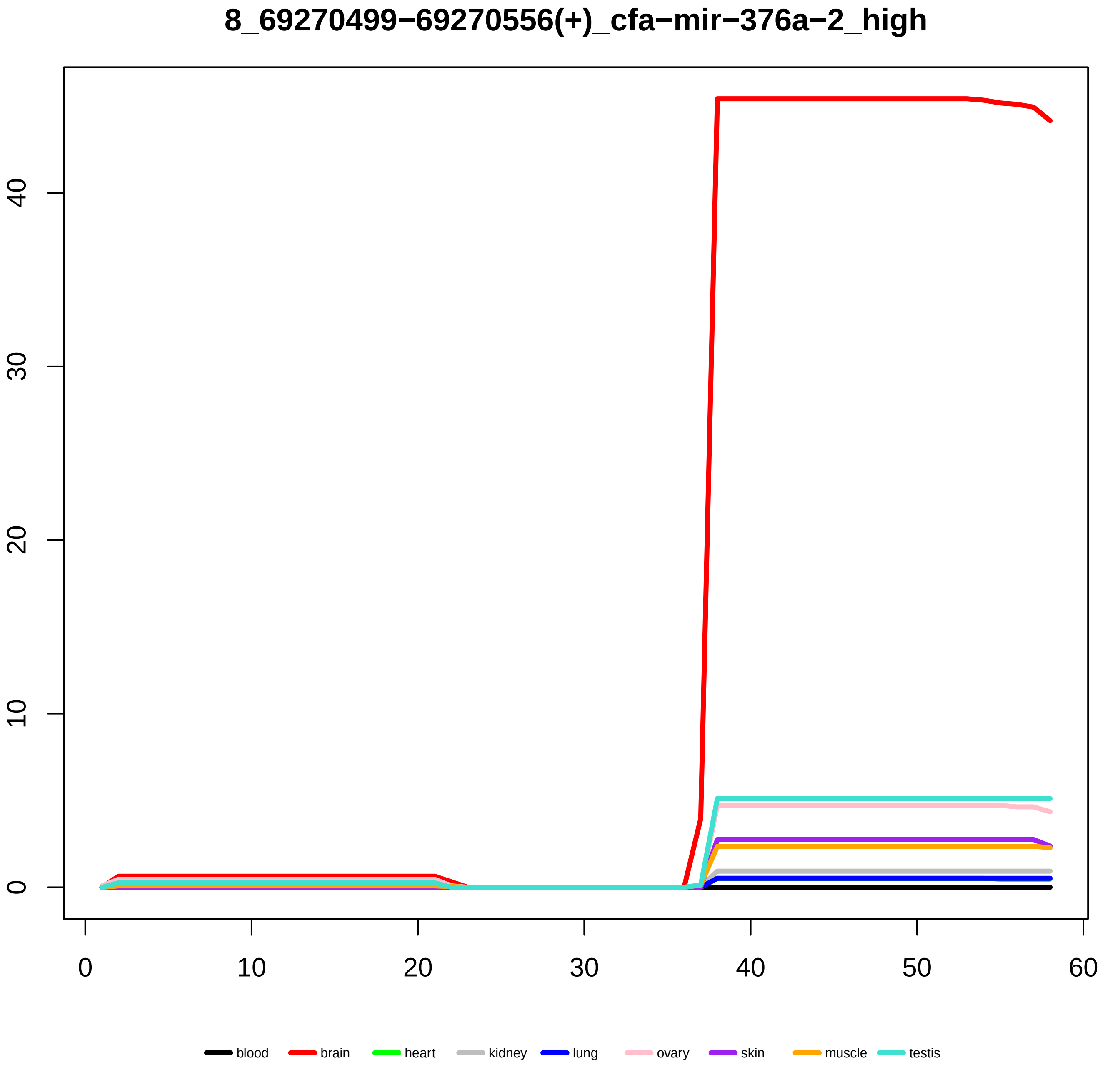

8_69270499−69270556(+)_cfa−mir−376a−2_high
0
10
20
30
40
0
10
20
30
40
50
60
blood
brain
heart
kidney
lung
ovary
skin
muscle
testis

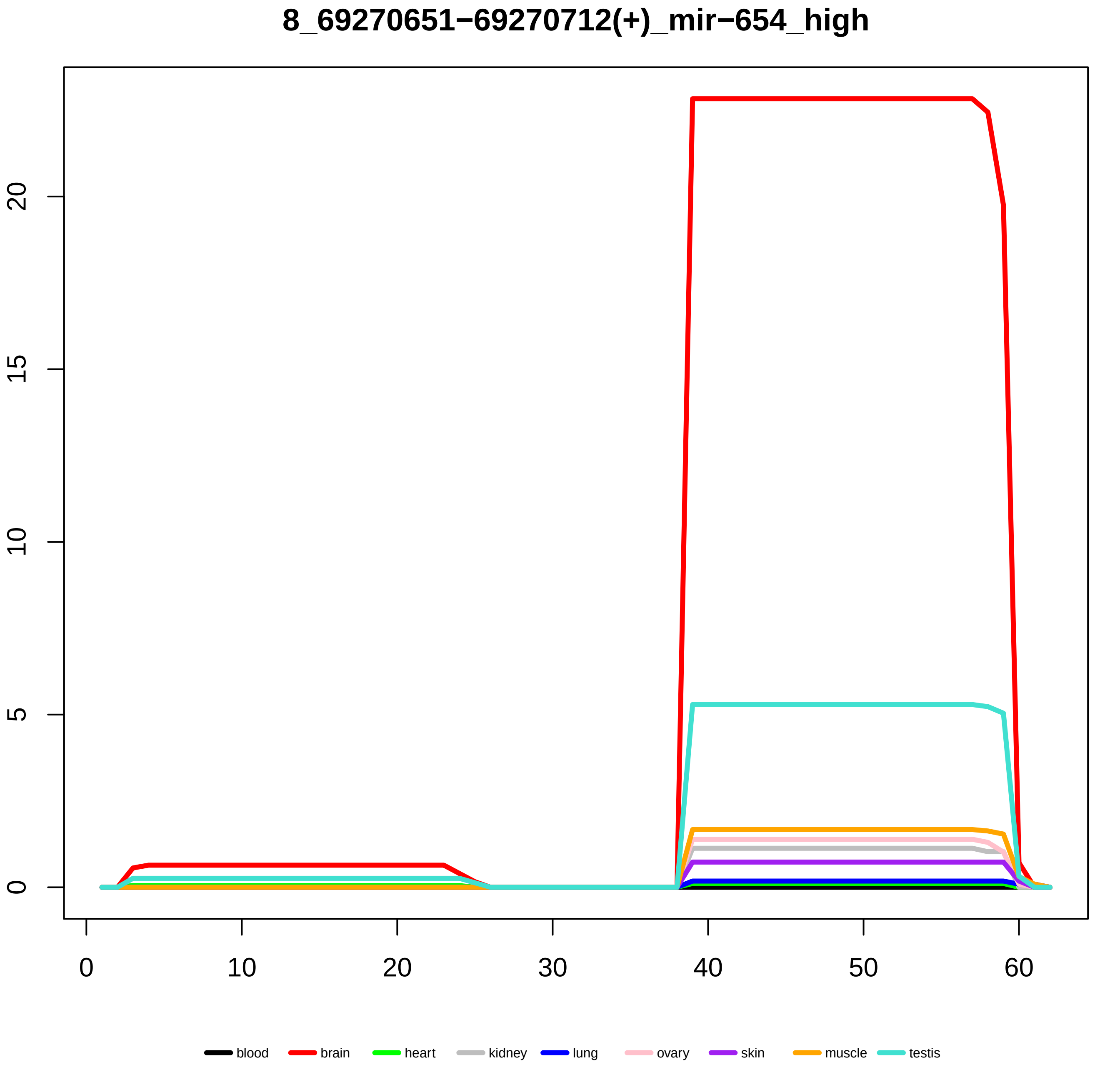

8_69270651−69270712(+)_mir−654_high
0
5
10
15
20
0
10
20
30
40
50
60
blood
brain
heart
kidney
lung
ovary
skin
muscle
testis

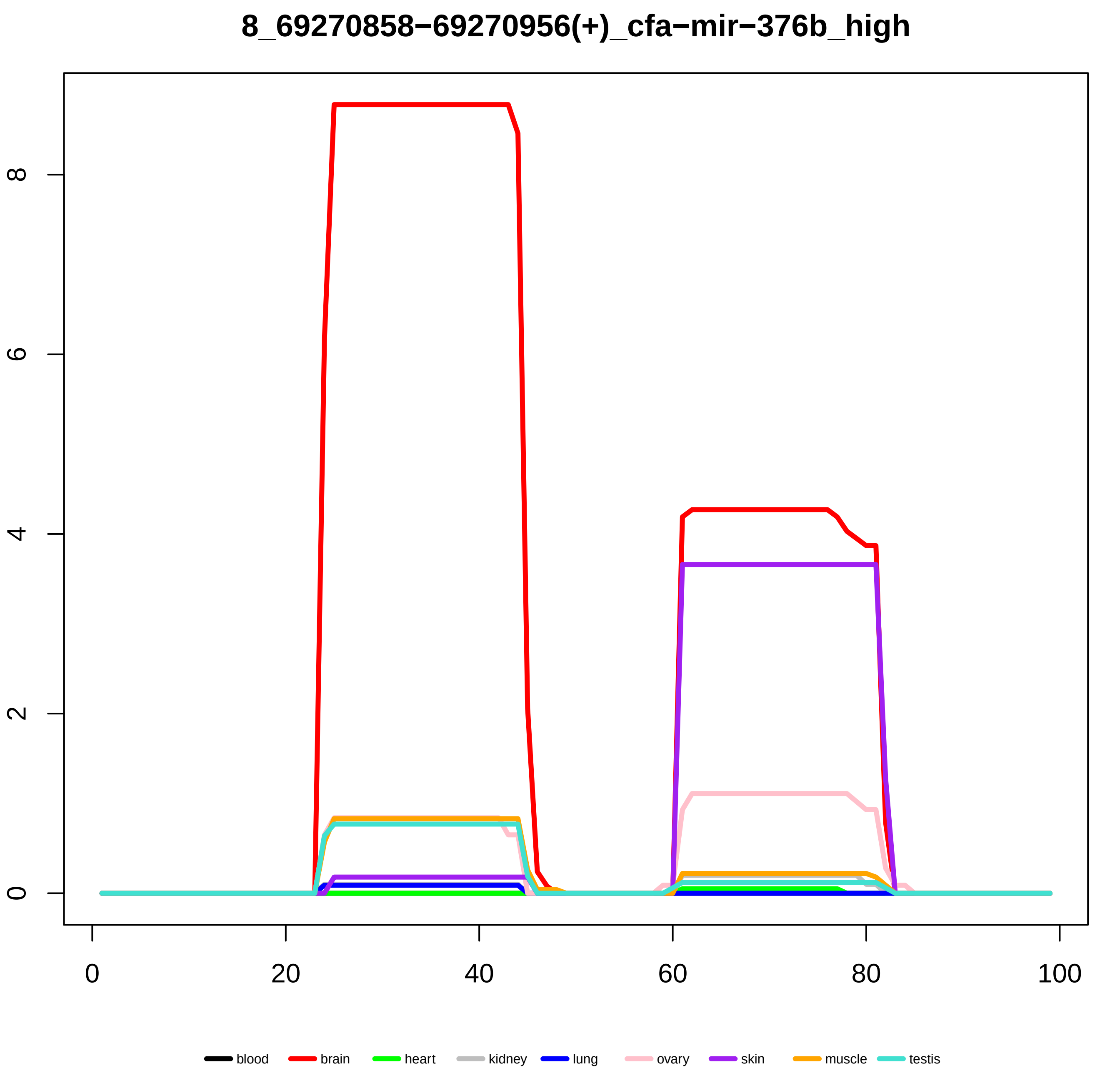

8_69270858−69270956(+)_cfa−mir−376b_high
0
2
4
6
8
0
20
40
60
80
100
blood
brain
heart
kidney
lung
ovary
skin
muscle
testis

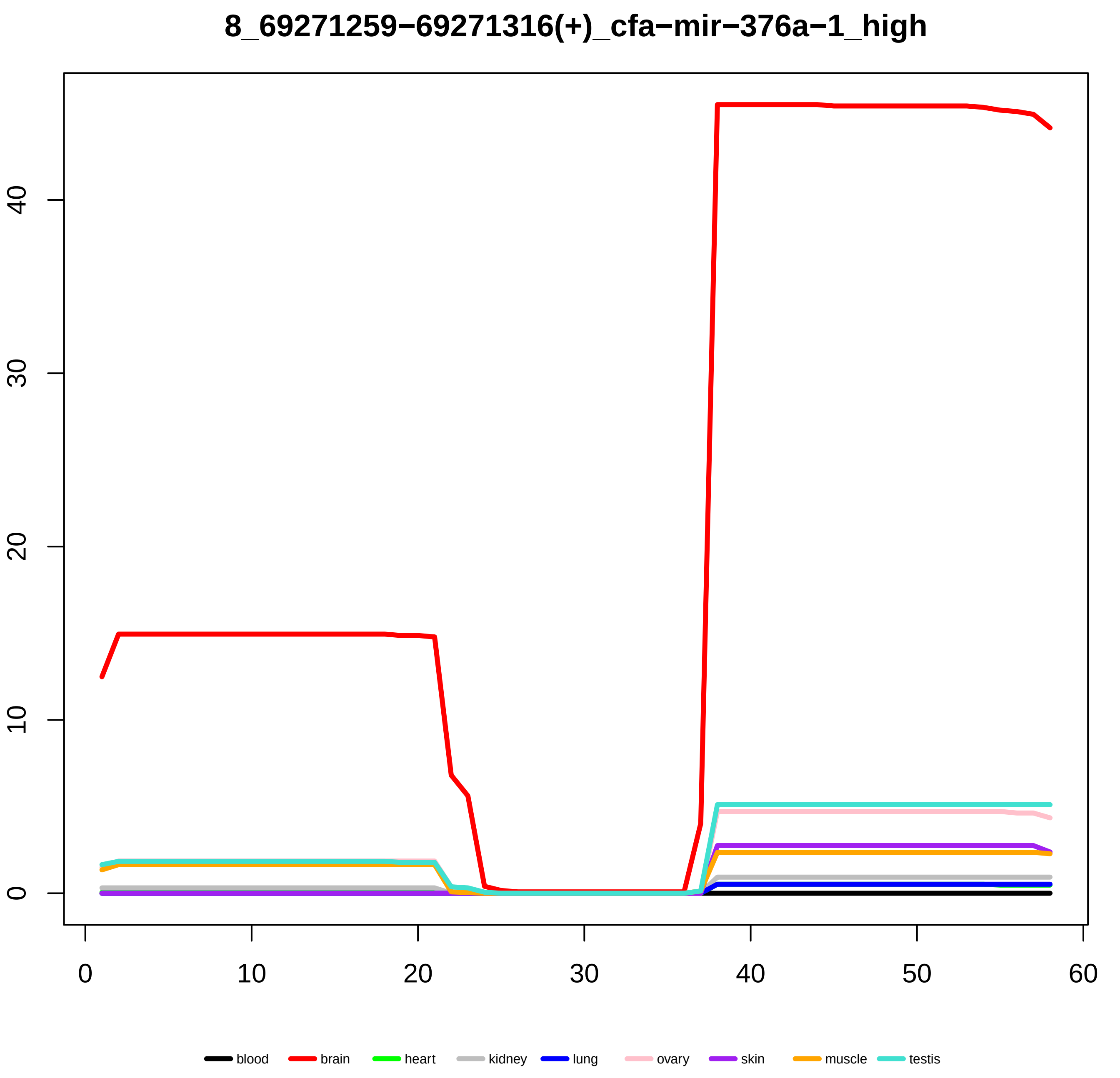

8_69271259−69271316(+)_cfa−mir−376a−1_high
0
10
20
30
40
0
10
20
30
40
50
60
blood
brain
heart
kidney
lung
ovary
skin
muscle
testis

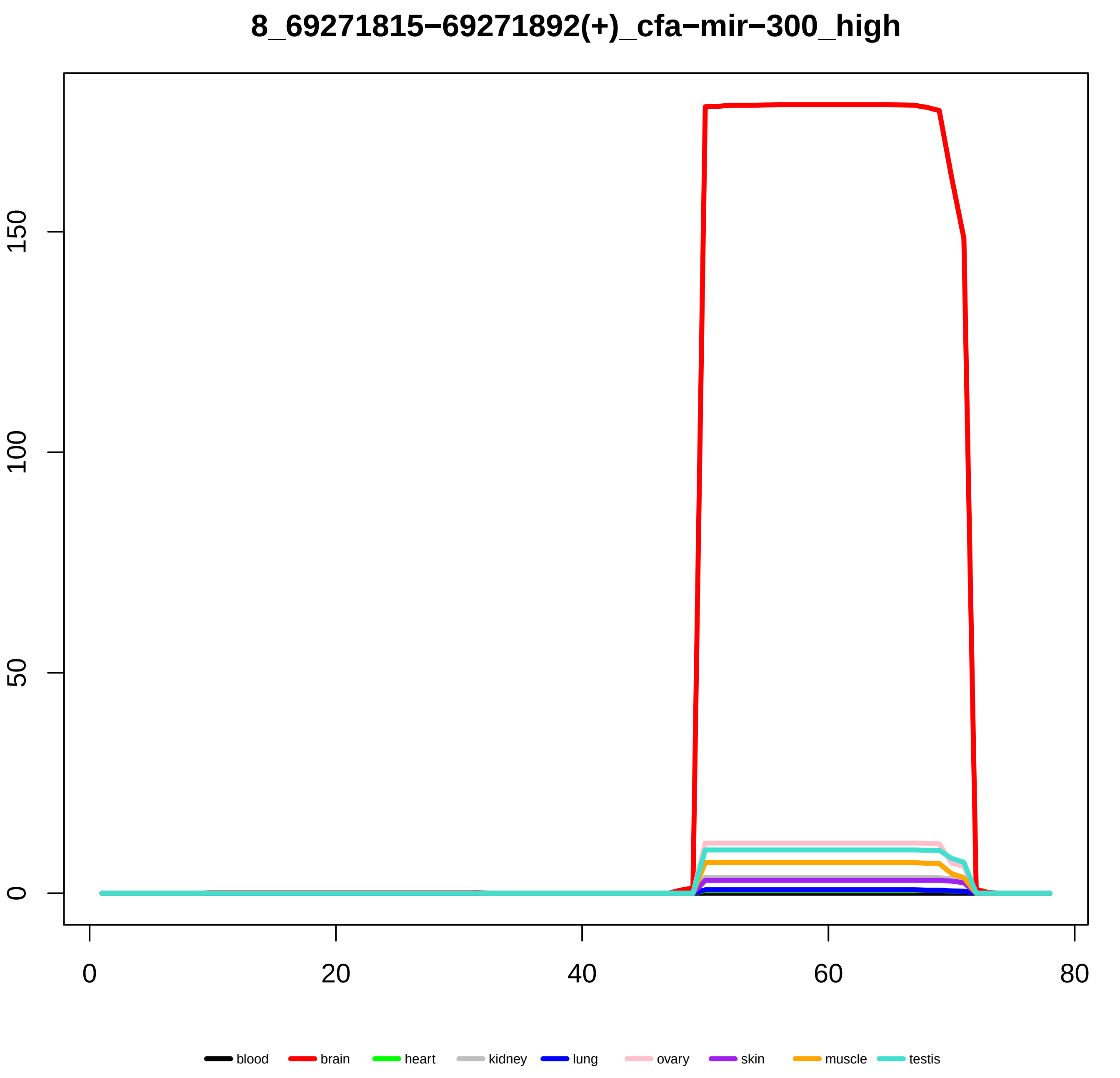

8_69271815−69271892(+)_cfa−mir−300_high
0
50
100
150
0
20
40
60
80
blood
brain
heart
kidney
lung
ovary
skin
muscle
testis

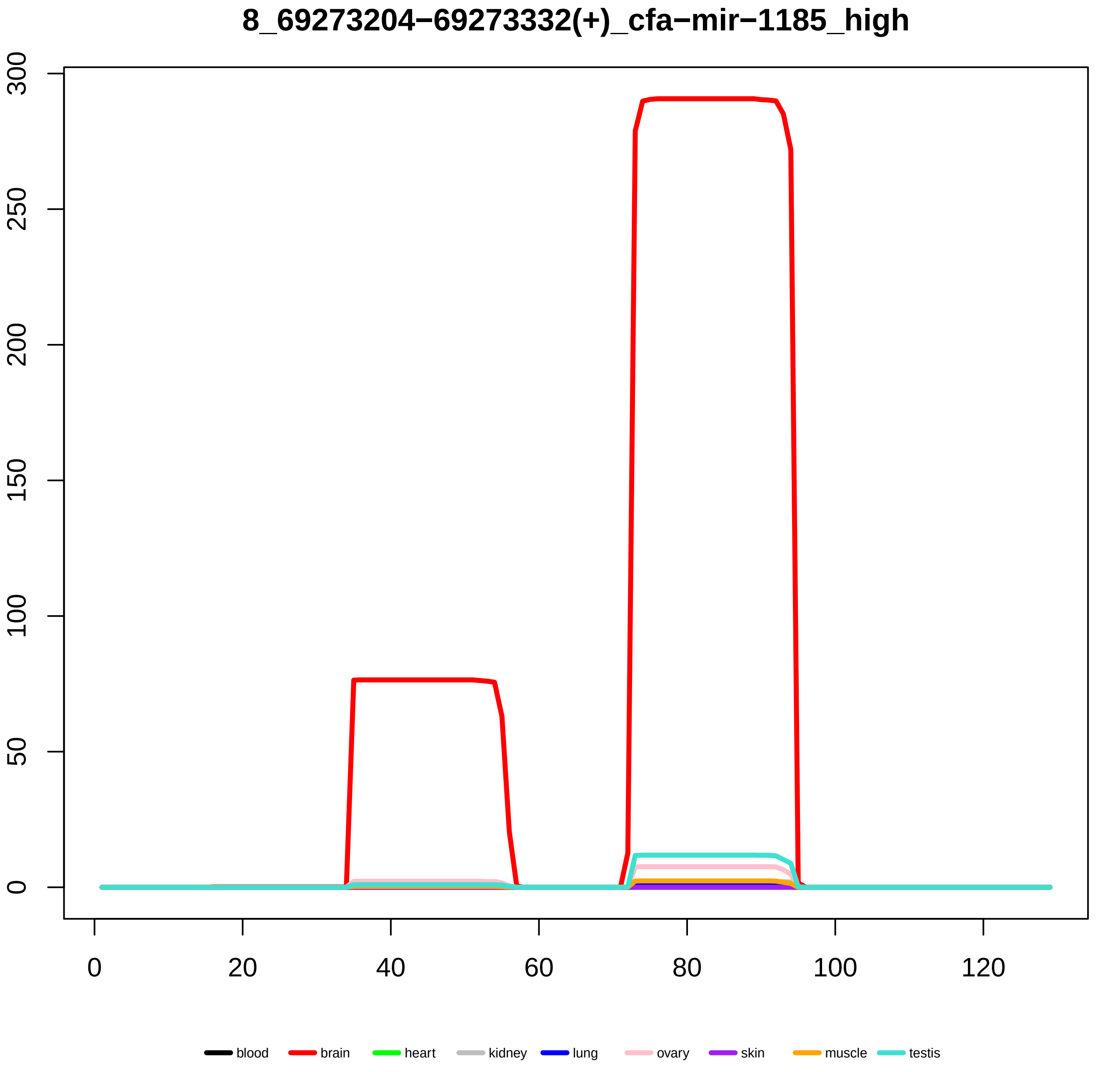
8_69273204−69273332(+)_cfa−mir−1185_high
0
50
100
150
200
250
300
0
20
40
60
80
100
120
blood
brain
heart
kidney
lung
ovary
skin
muscle
testis

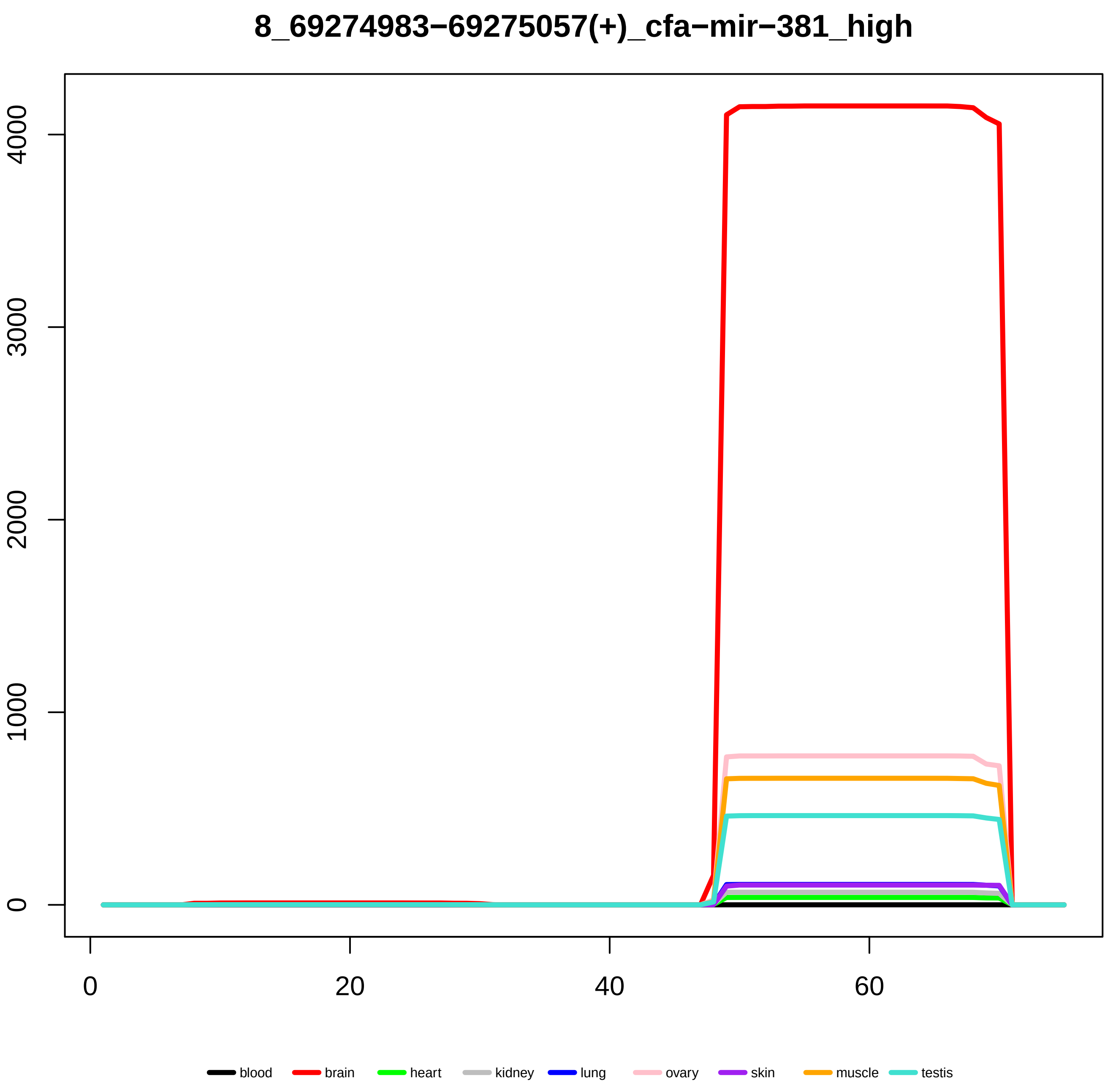

8_69274983−69275057(+)_cfa−mir−381_high
0
1000
2000
3000
4000
0
20
40
60
blood
brain
heart
kidney
lung
ovary
skin
muscle
testis

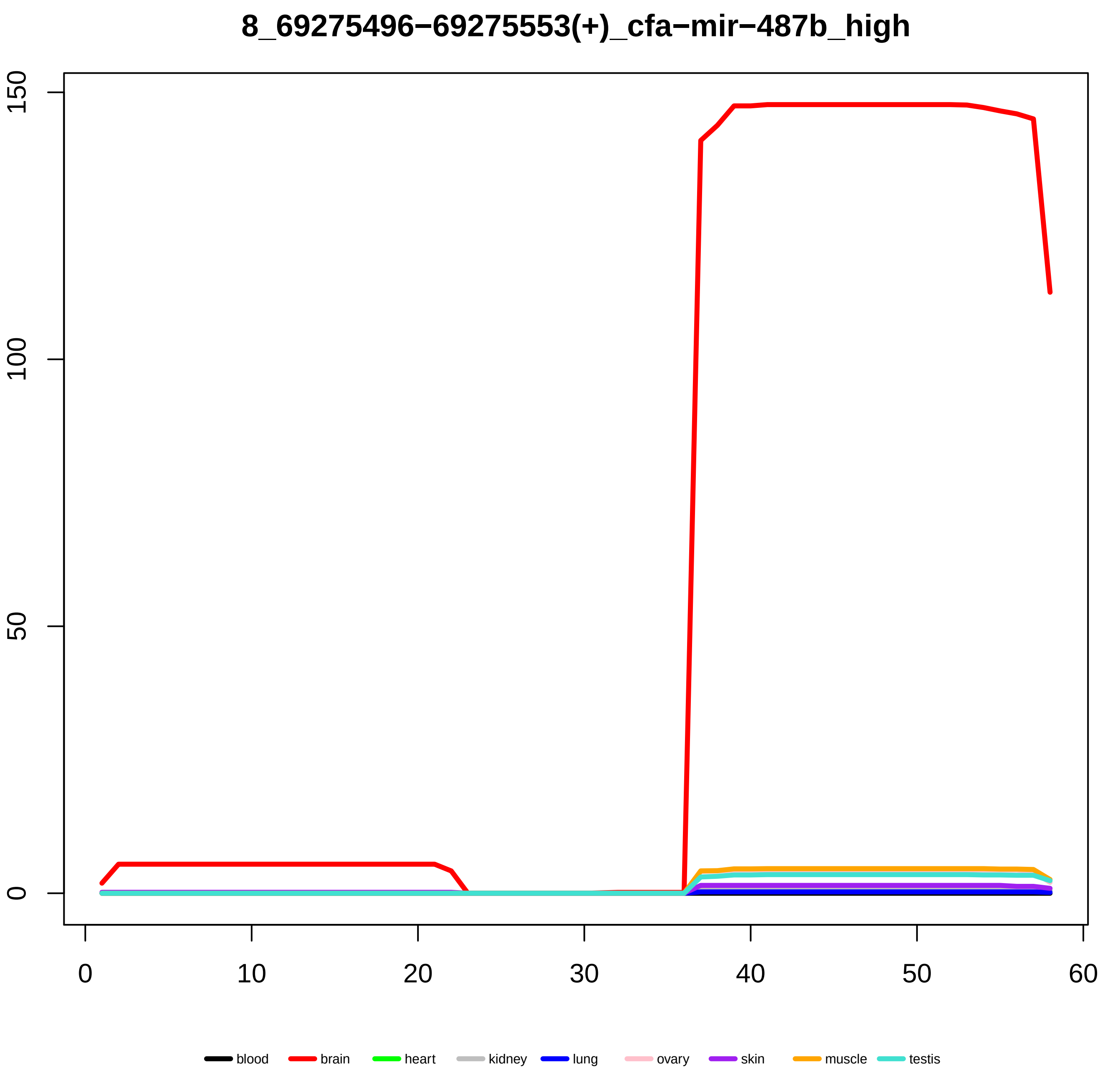
8_69275496−69275553(+)_cfa−mir−487b_high
0
50
100
150
0
10
20
30
40
50
60
blood
brain
heart
kidney
lung
ovary
skin
muscle
testis

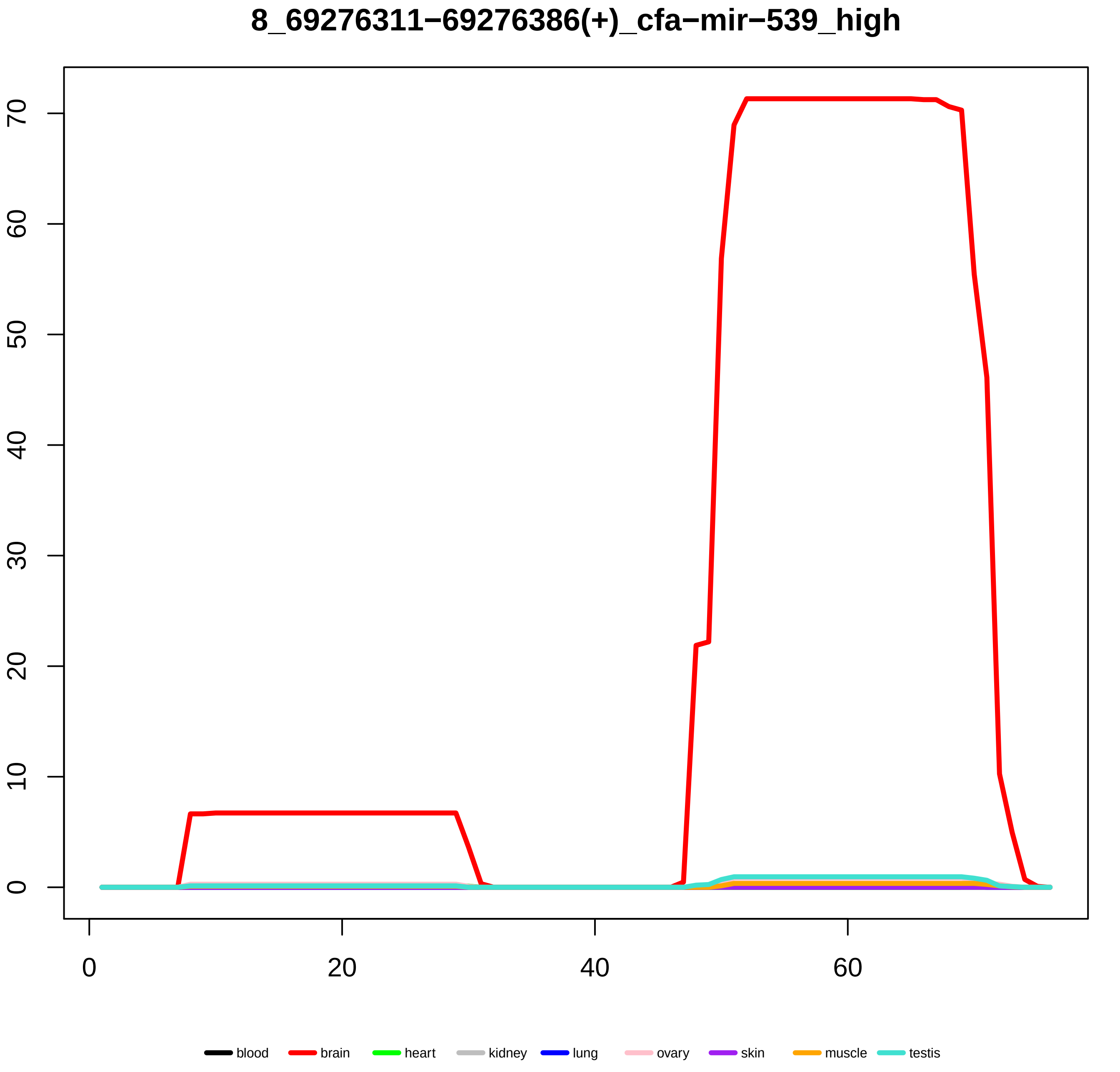

8_69276311−69276386(+)_cfa−mir−539_high
0
10
20
30
40
50
60
70
0
20
40
60
blood
brain
heart
kidney
lung
ovary
skin
muscle
testis

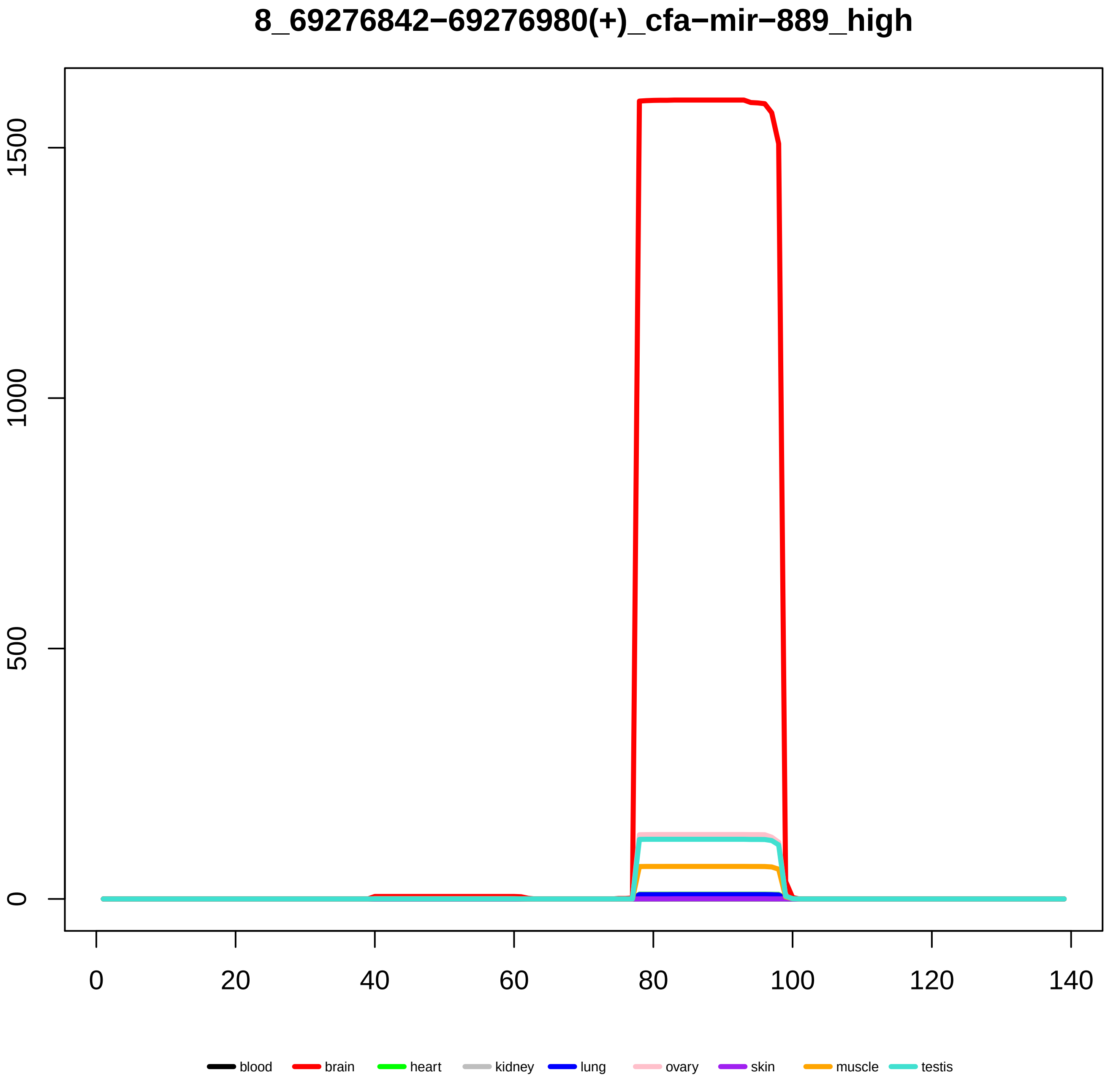

8_69276842−69276980(+)_cfa−mir−889_high
0
500
1000
1500
0
20
40
60
80
100
120
140
blood
brain
heart
kidney
lung
ovary
skin
muscle
testis

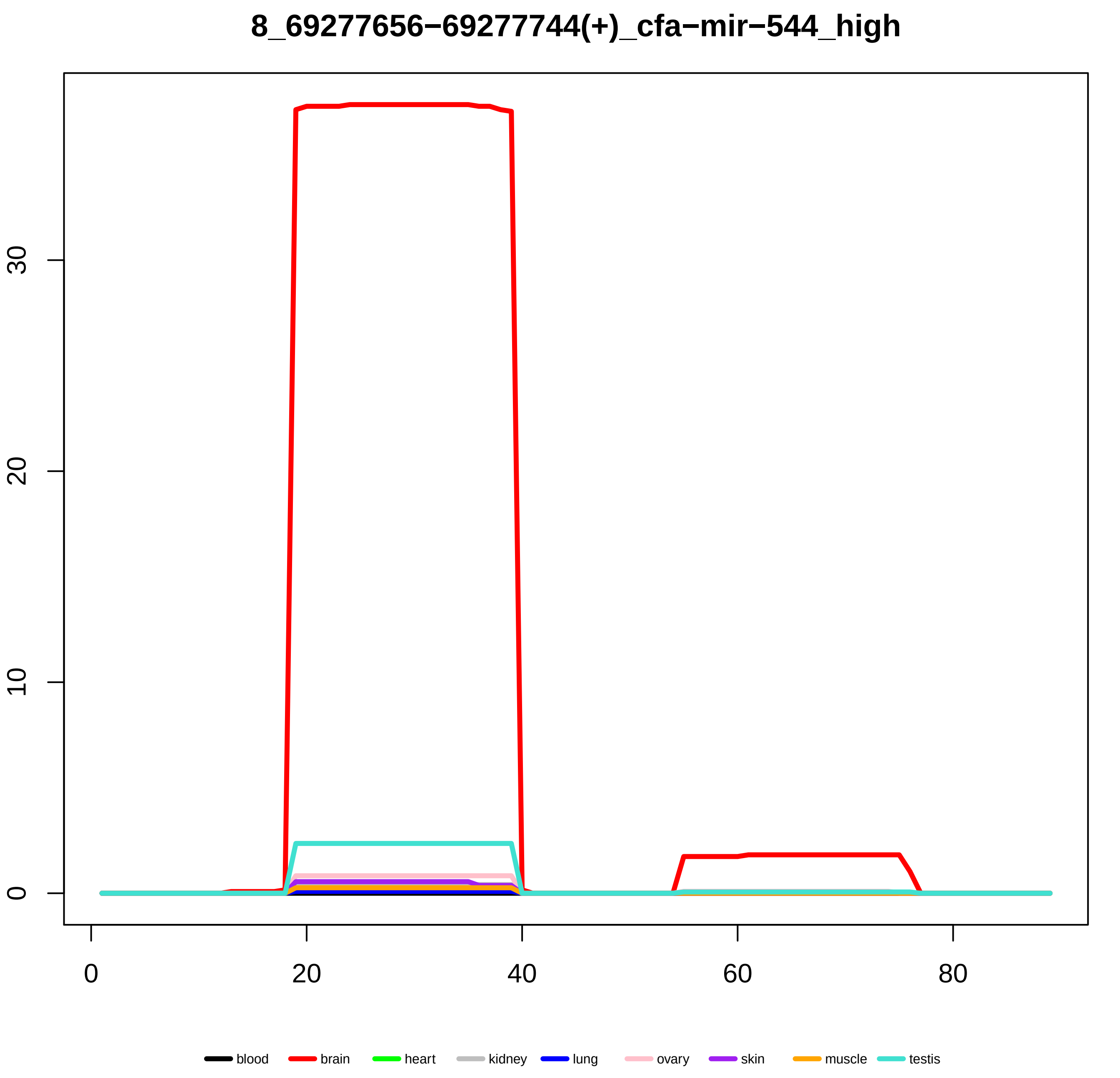
8_69277656−69277744(+)_cfa−mir−544_high
0
10
20
30
0
20
40
60
80
blood
brain
heart
kidney
lung
ovary
skin
muscle
testis

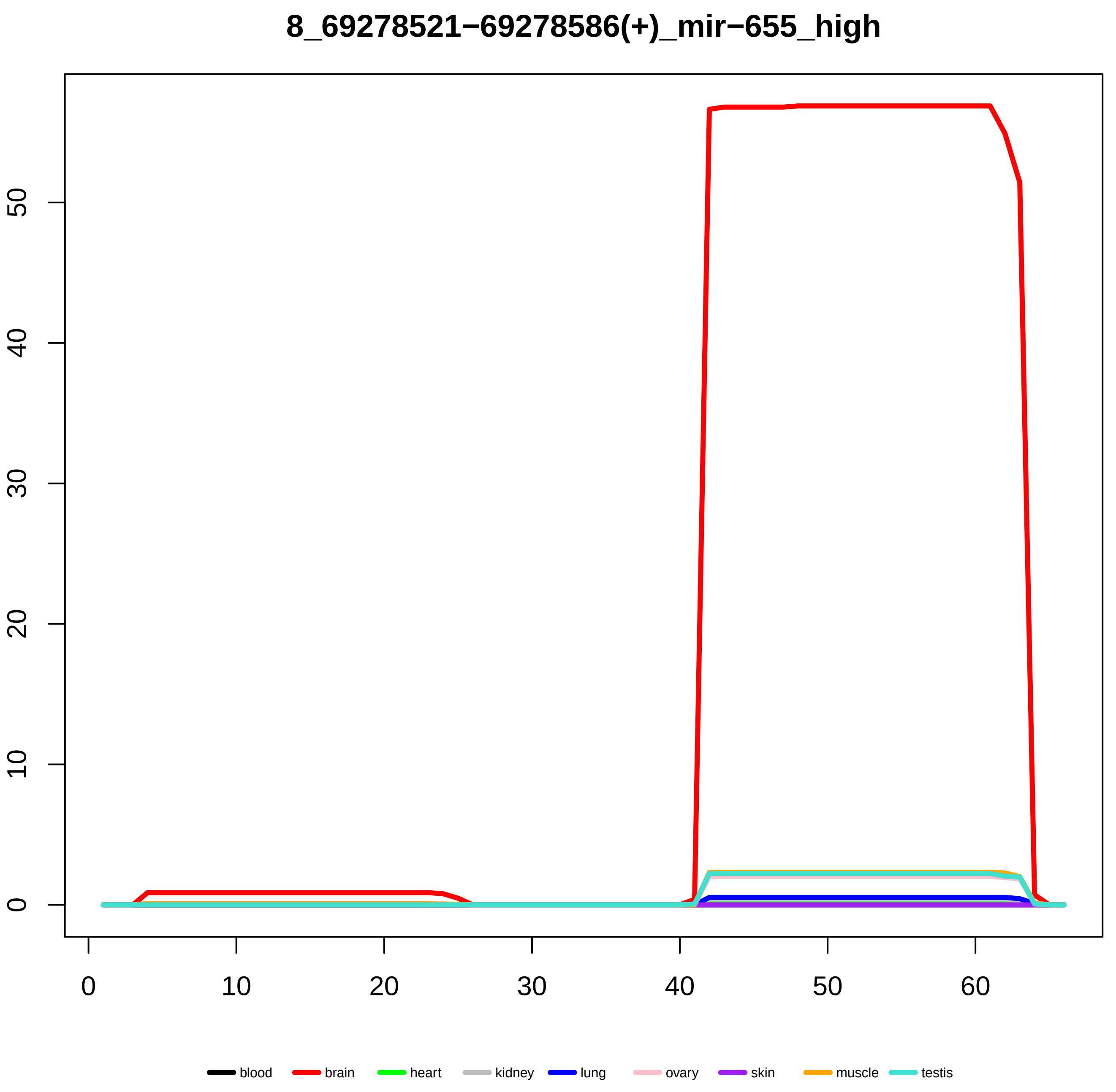
8_69278521−69278586(+)_mir−655_high
0
10
20
30
40
50
0
10
20
30
40
50
60
blood
brain
heart
kidney
lung
ovary
skin
muscle
testis

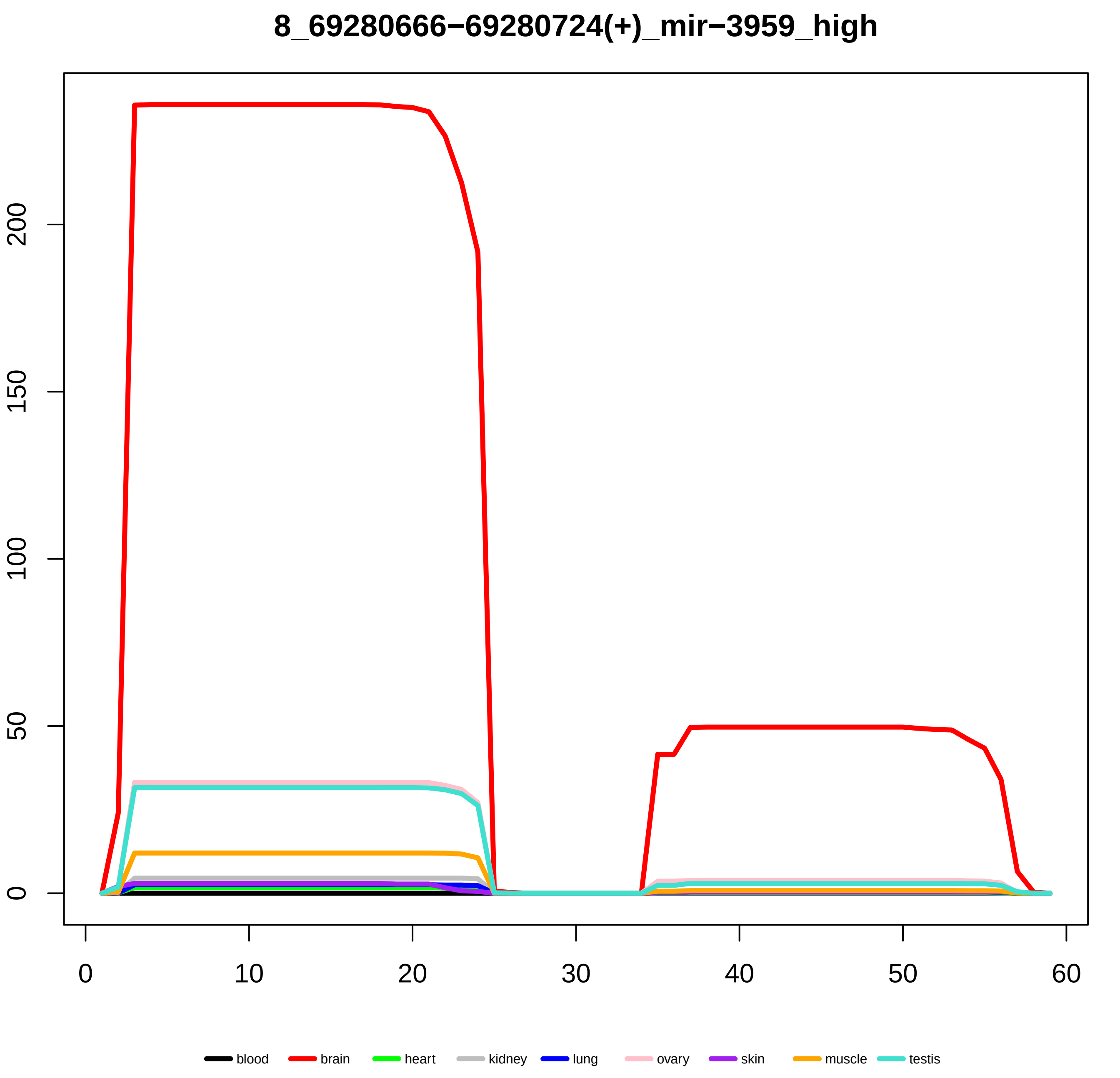
8_69280666−69280724(+)_mir−3959_high
0
50
100
150
200
0
10
20
30
40
50
60
blood
brain
heart
kidney
lung
ovary
skin
muscle
testis

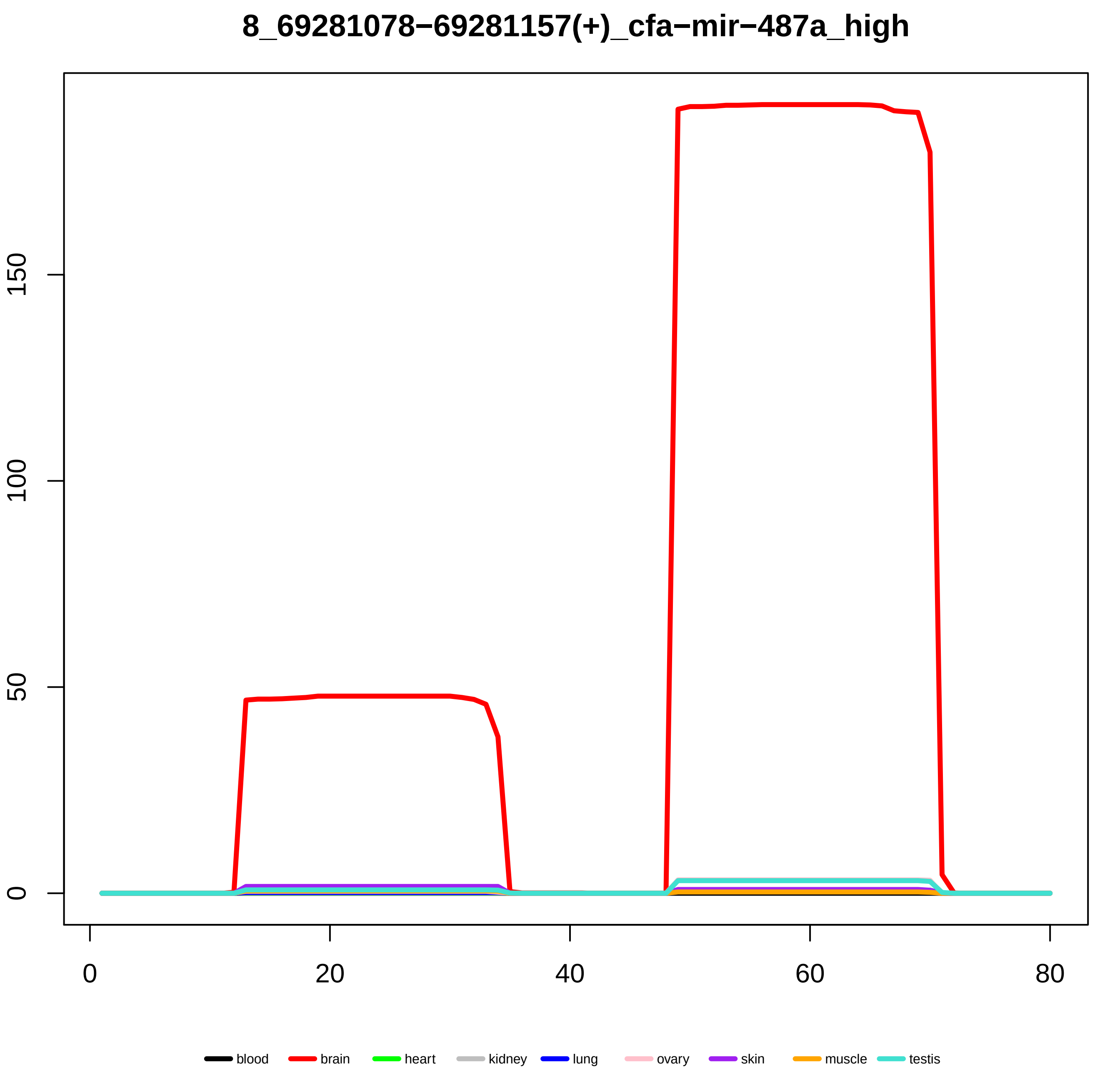
8_69281078−69281157(+)_cfa−mir−487a_high
0
50
100
150
0
20
40
60
80
blood
brain
heart
kidney
lung
ovary
skin
muscle
testis

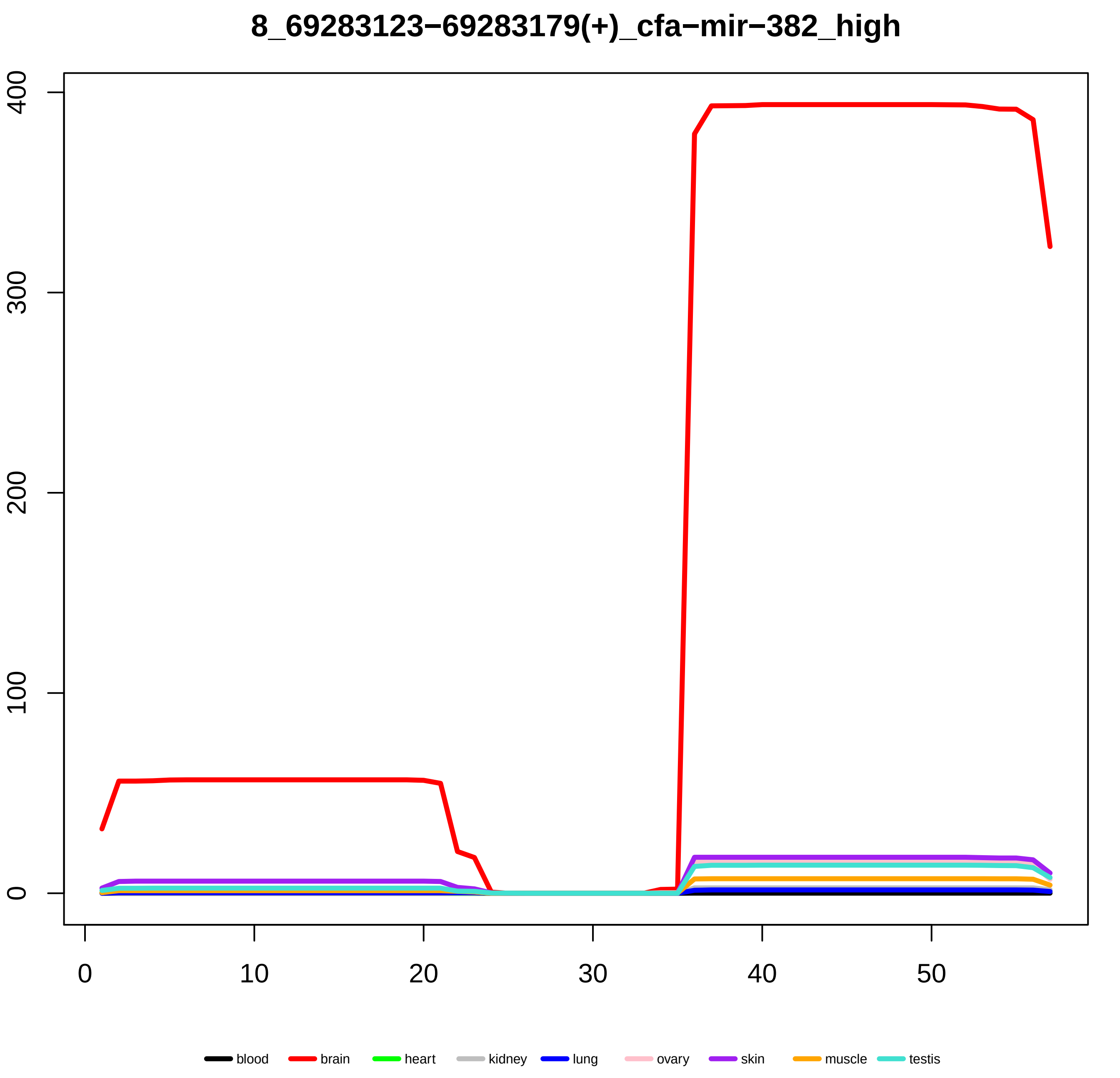

8_69283123−69283179(+)_cfa−mir−382_high
0
100
200
300
400
0
10
20
30
40
50
blood
brain
heart
kidney
lung
ovary
skin
muscle
testis

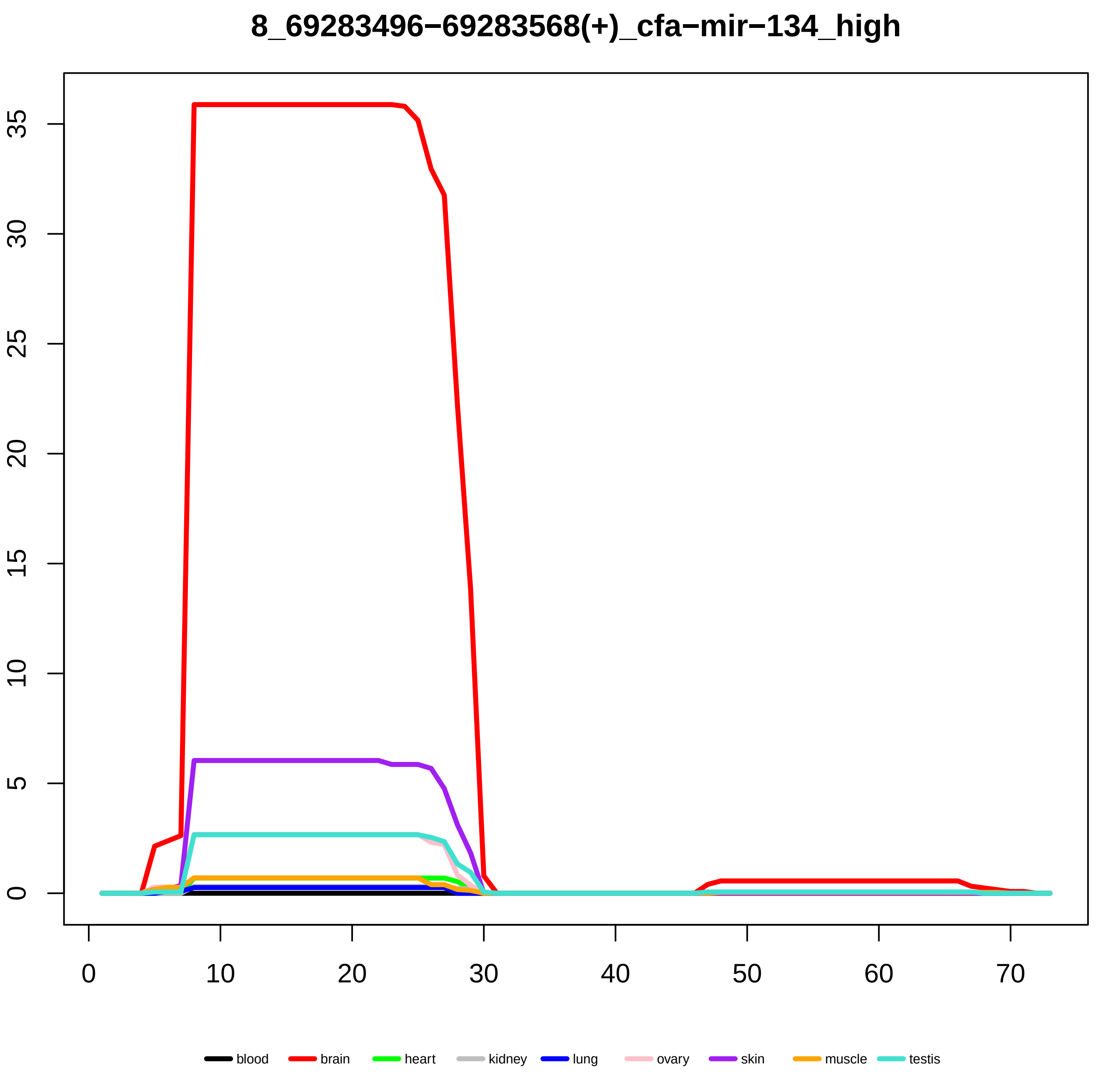

8_69283496−69283568(+)_cfa−mir−134_high
0
5
10
15
20
25
30
35
0
10
20
30
40
50
60
70
blood
brain
heart
kidney
lung
ovary
skin
muscle
testis

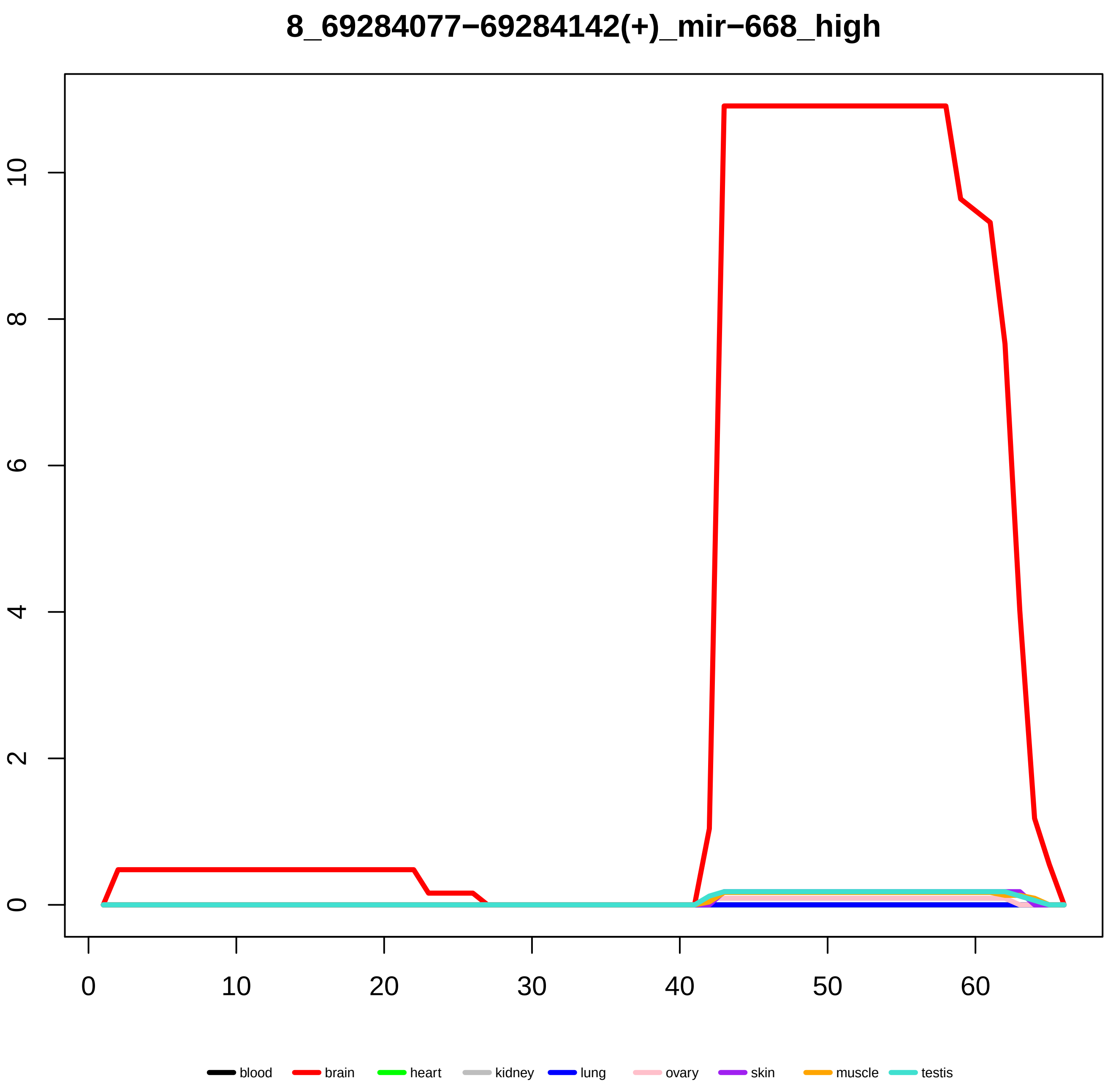

8_69284077−69284142(+)_mir−668_high
0
2
4
6
8
10
0
10
20
30
40
50
60
blood
brain
heart
kidney
lung
ovary
skin
muscle
testis

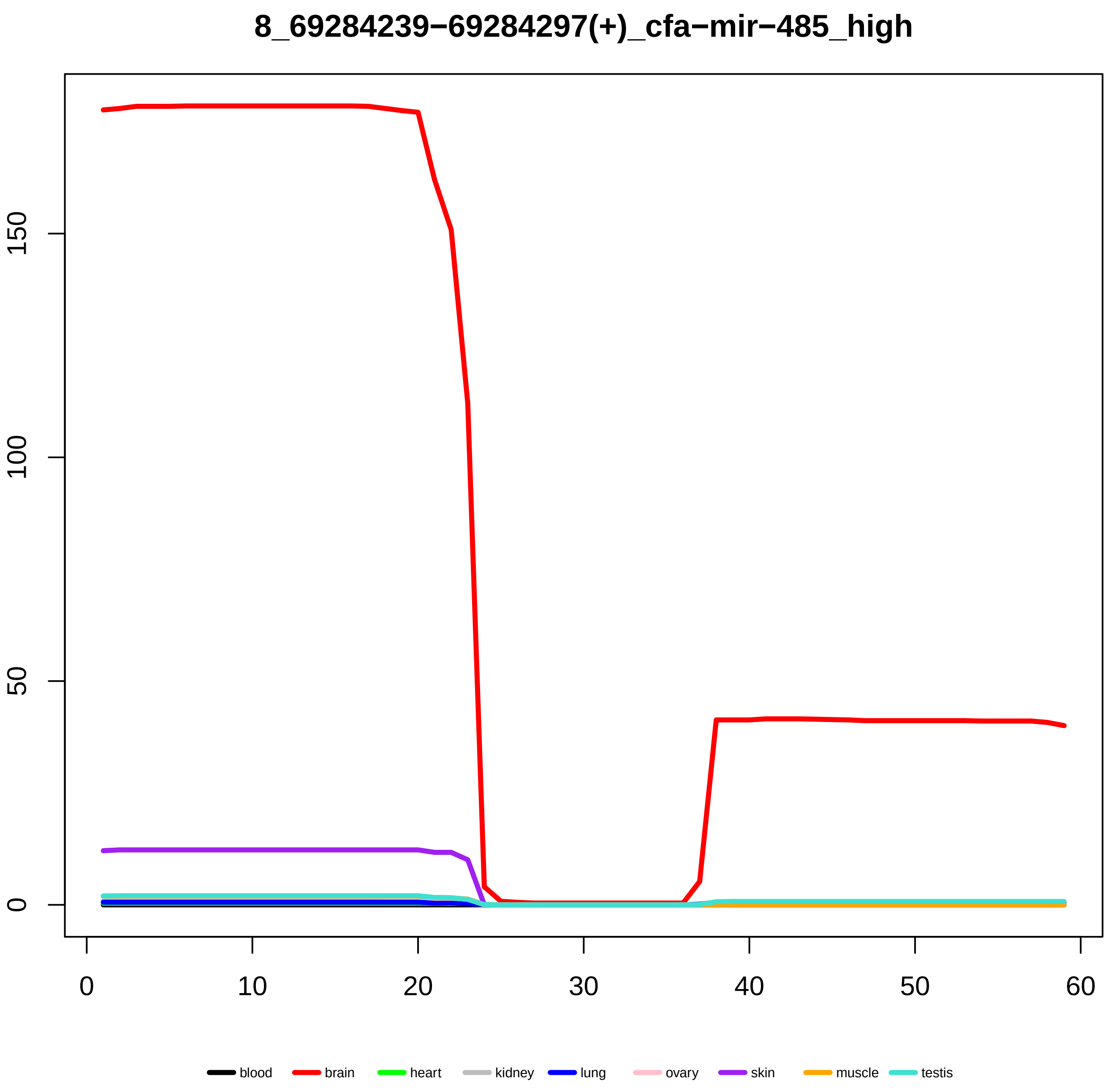

8_69284239−69284297(+)_cfa−mir−485_high
0
50
100
150
0
10
20
30
40
50
60
blood
brain
heart
kidney
lung
ovary
skin
muscle
testis

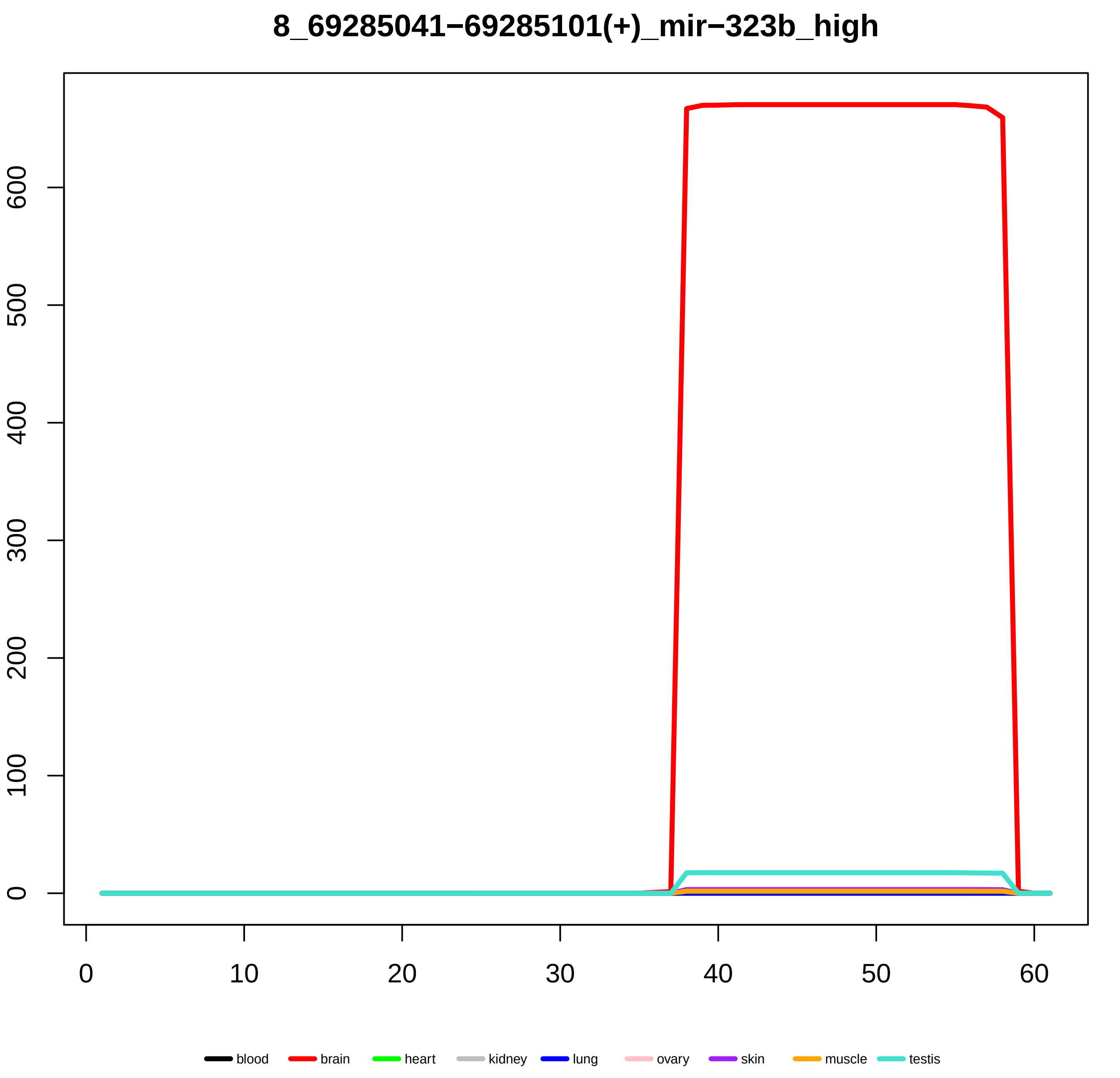
8_69285041−69285101(+)_mir−323b_high
0
100
200
300
400
500
600
0
10
20
30
40
50
60
blood
brain
heart
kidney
lung
ovary
skin
muscle
testis

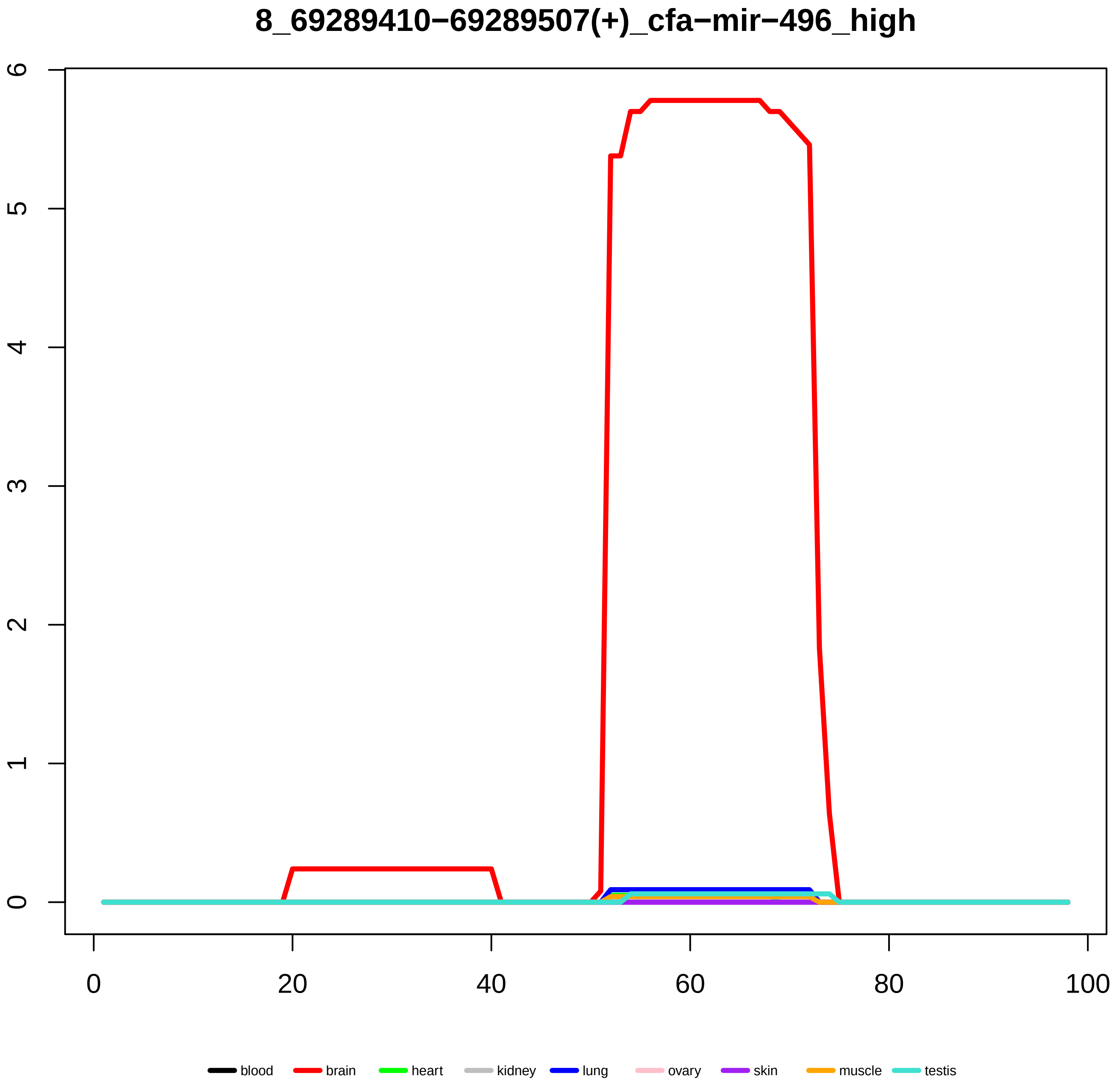

8_69289410−69289507(+)_cfa−mir−496_high
0
1
2
3
4
5
6
0
20
40
60
80
100
blood
brain
heart
kidney
lung
ovary
skin
muscle
testis

8_69290937−69291005(+)_cfa−mir−377_high

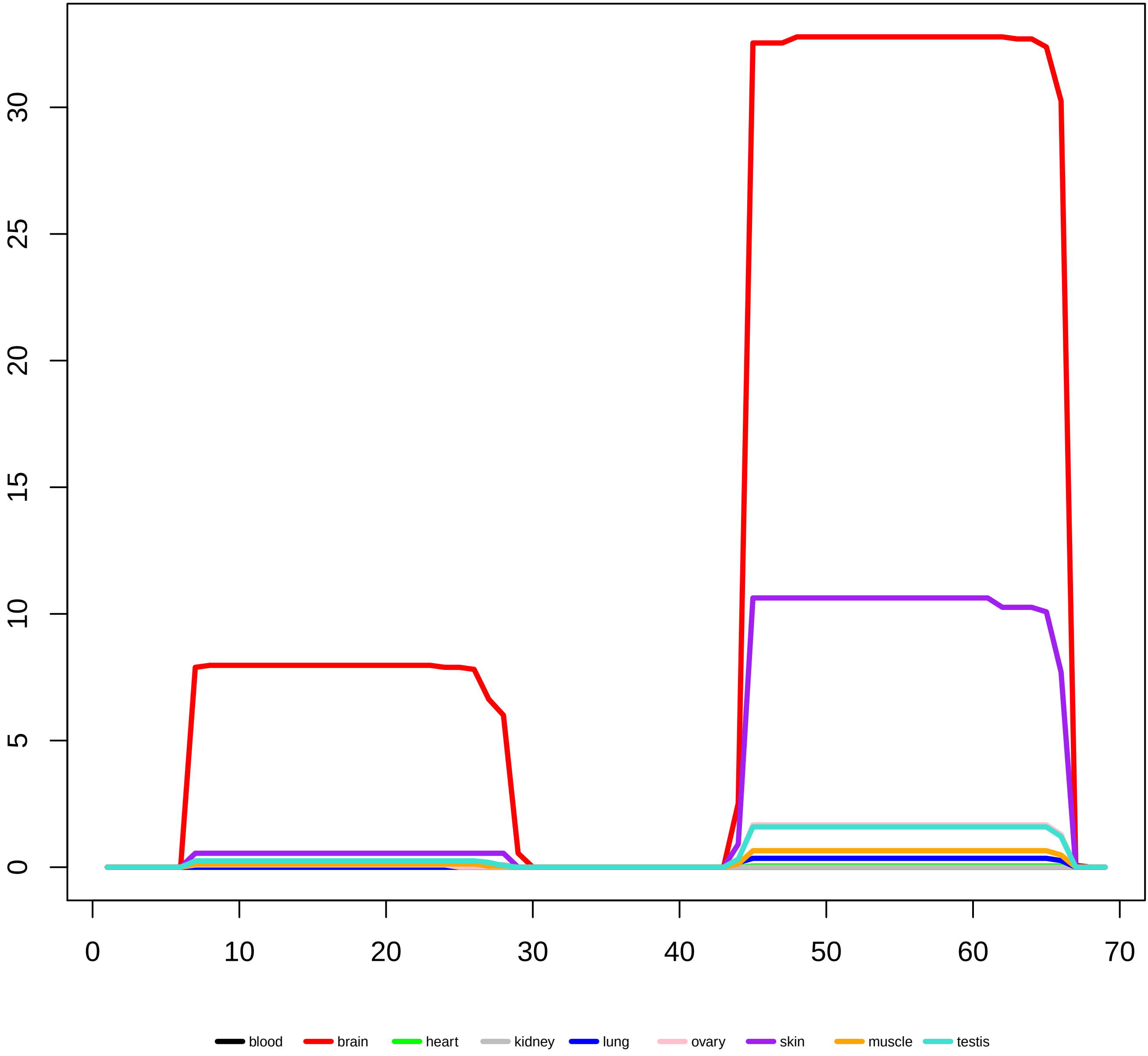

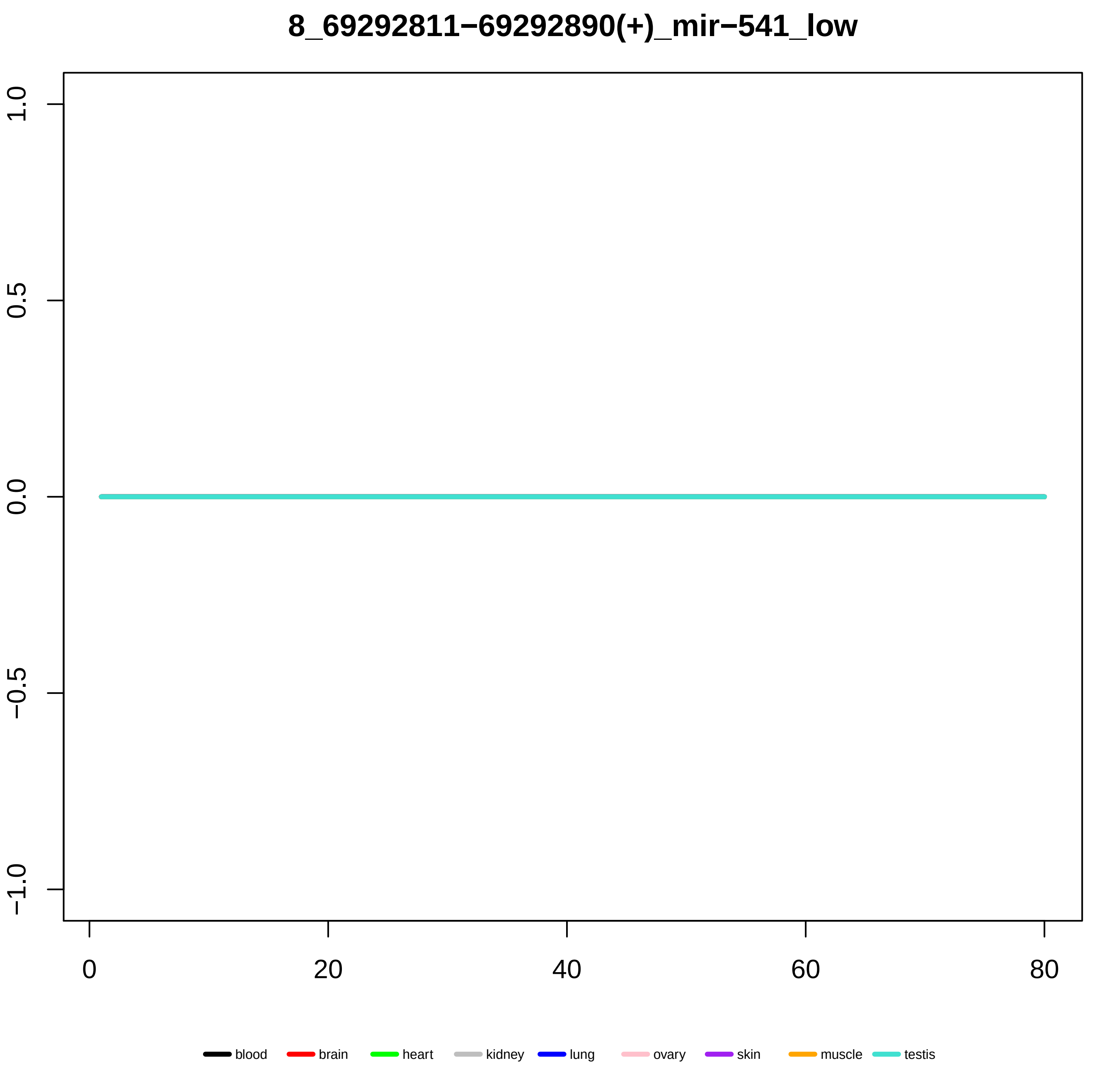
8_69292811−69292890(+)_mir−541_low
1.0
0.5
0.0
−0.5
−1.0
0
20
40
60
80
blood
brain
heart
kidney
lung
ovary
skin
muscle
testis

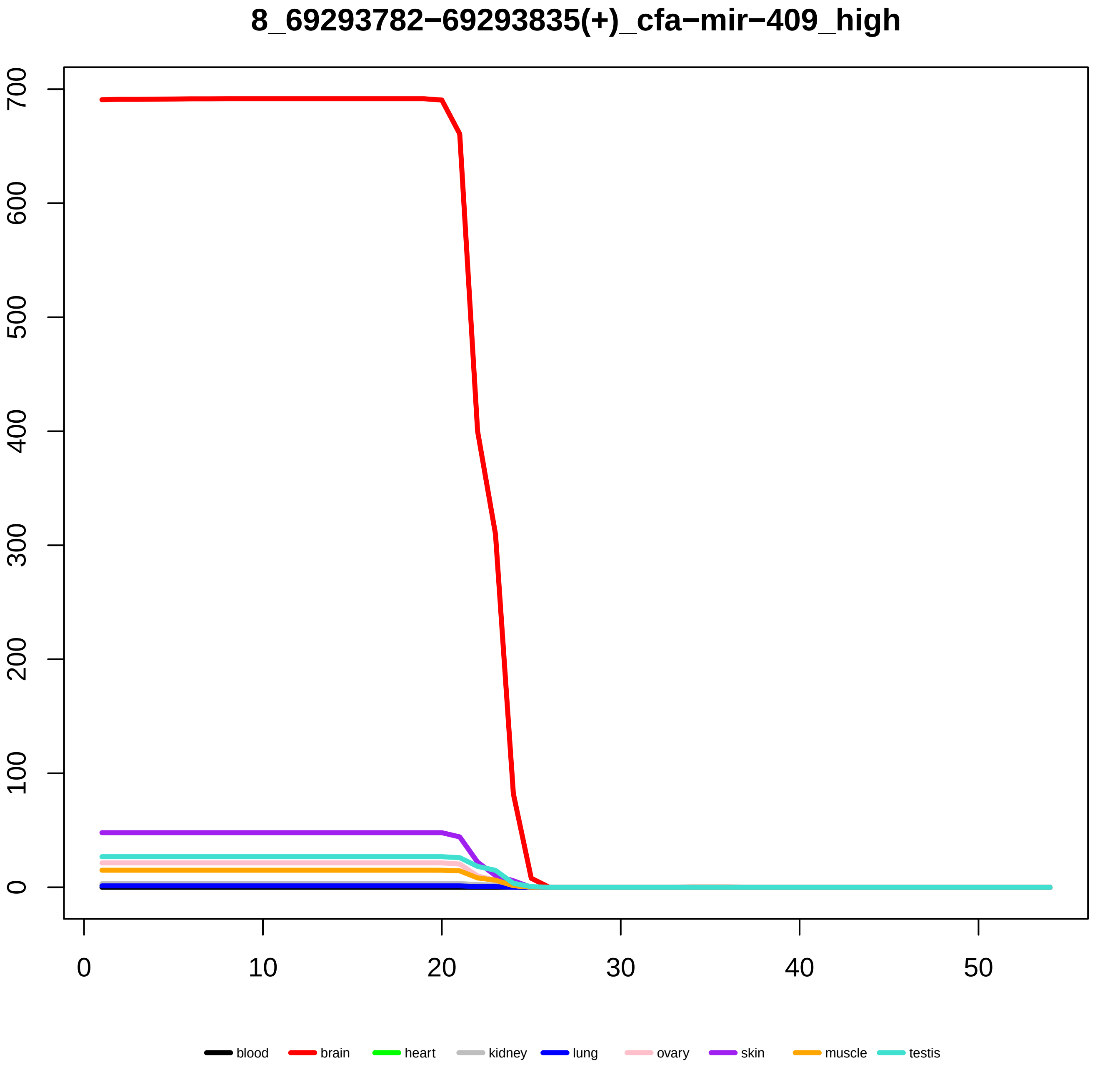

8_69293782−69293835(+)_cfa−mir−409_high
0
100
200
300
400
500
600
700
0
10
20
30
40
50
blood
brain
heart
kidney
lung
ovary
skin
muscle
testis

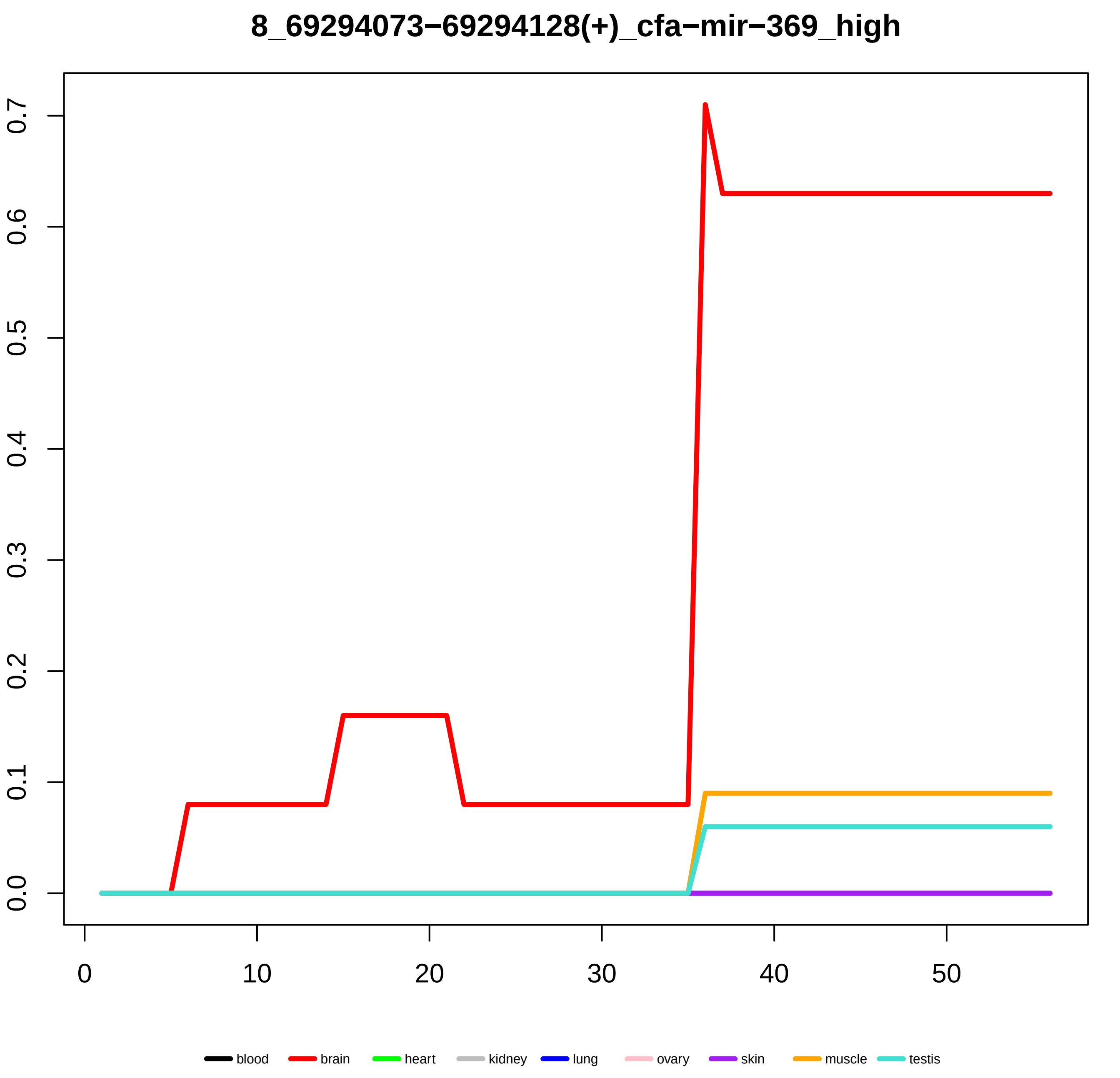

8_69294073−69294128(+)_cfa−mir−369_high
0.0
0.1
0.2
0.3
0.4
0.5
0.6
0.7
0
10
20
30
40
50
blood
brain
heart
kidney
lung
ovary
skin
muscle
testis

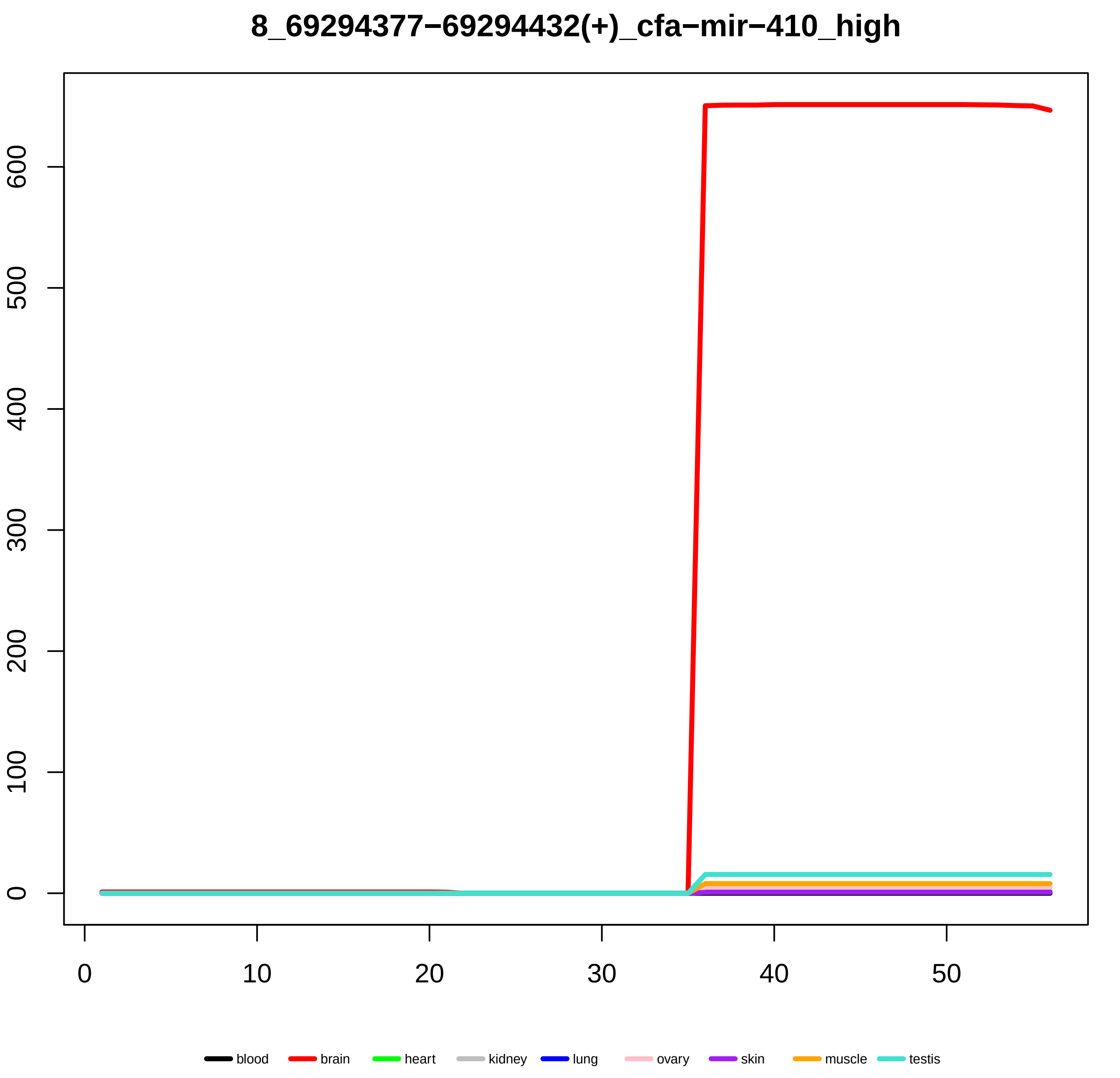
8_69294377−69294432(+)_cfa−mir−410_high
0
100
200
300
400
500
600
0
10
20
30
40
50
blood
brain
heart
kidney
lung
ovary
skin
muscle
testis

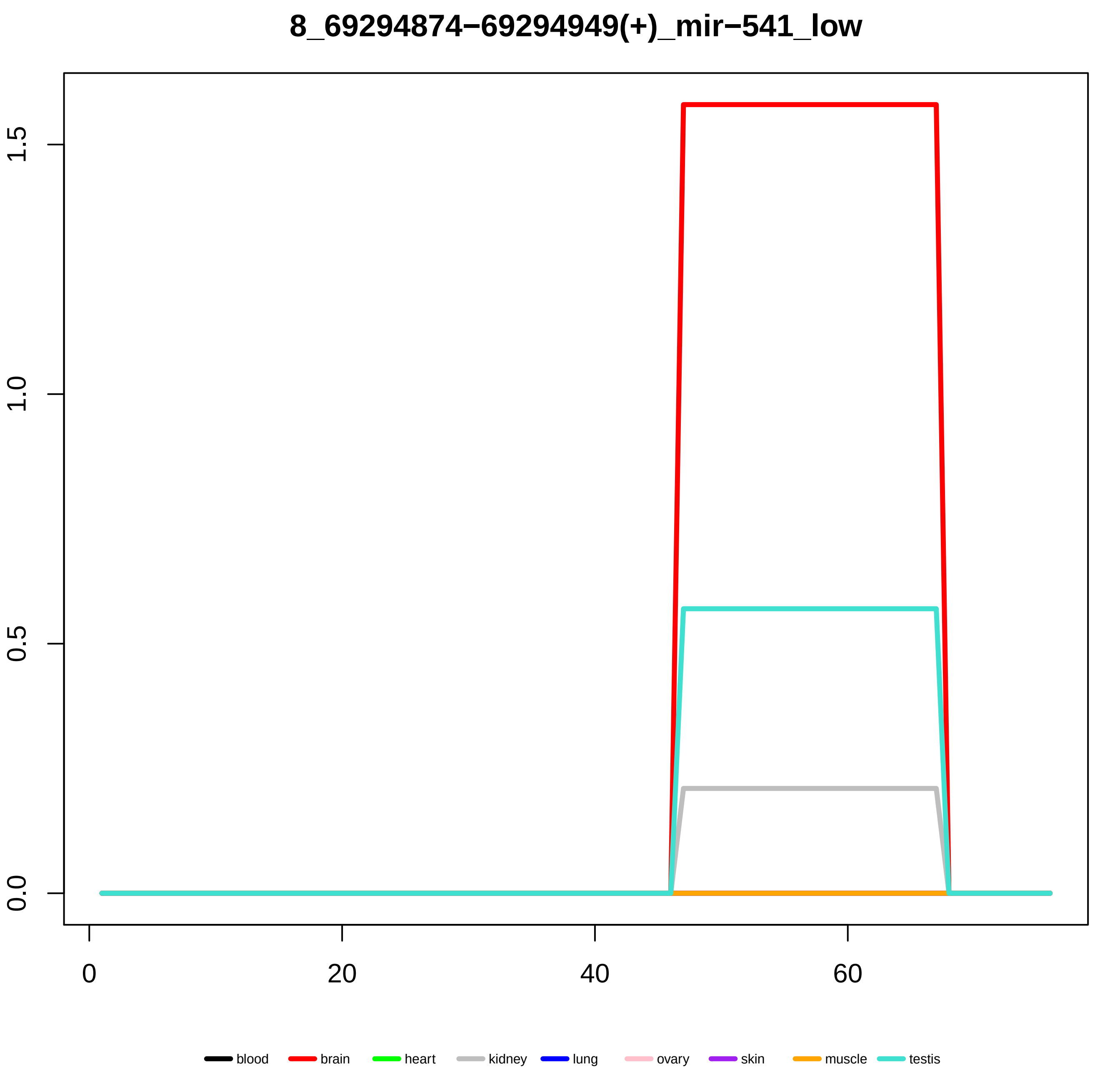
8_69294874−69294949(+)_mir−541_low
0.0
0.5
1.0
1.5
0
20
40
60
blood
brain
heart
kidney
lung
ovary
skin
muscle
testis

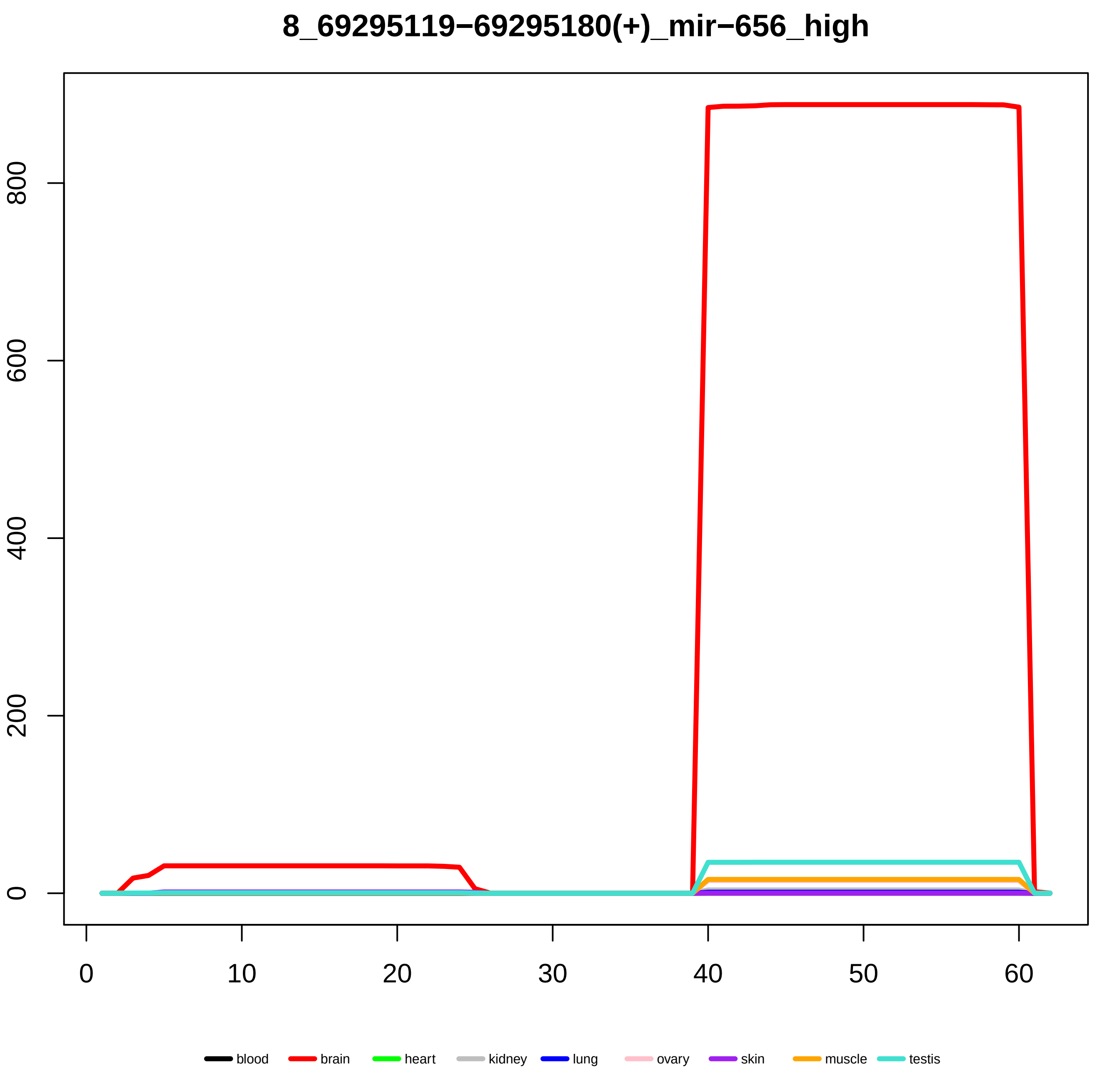

8_69295119−69295180(+)_mir−656_high
0
200
400
600
800
0
10
20
30
40
50
60
blood
brain
heart
kidney
lung
ovary
skin
muscle
testis

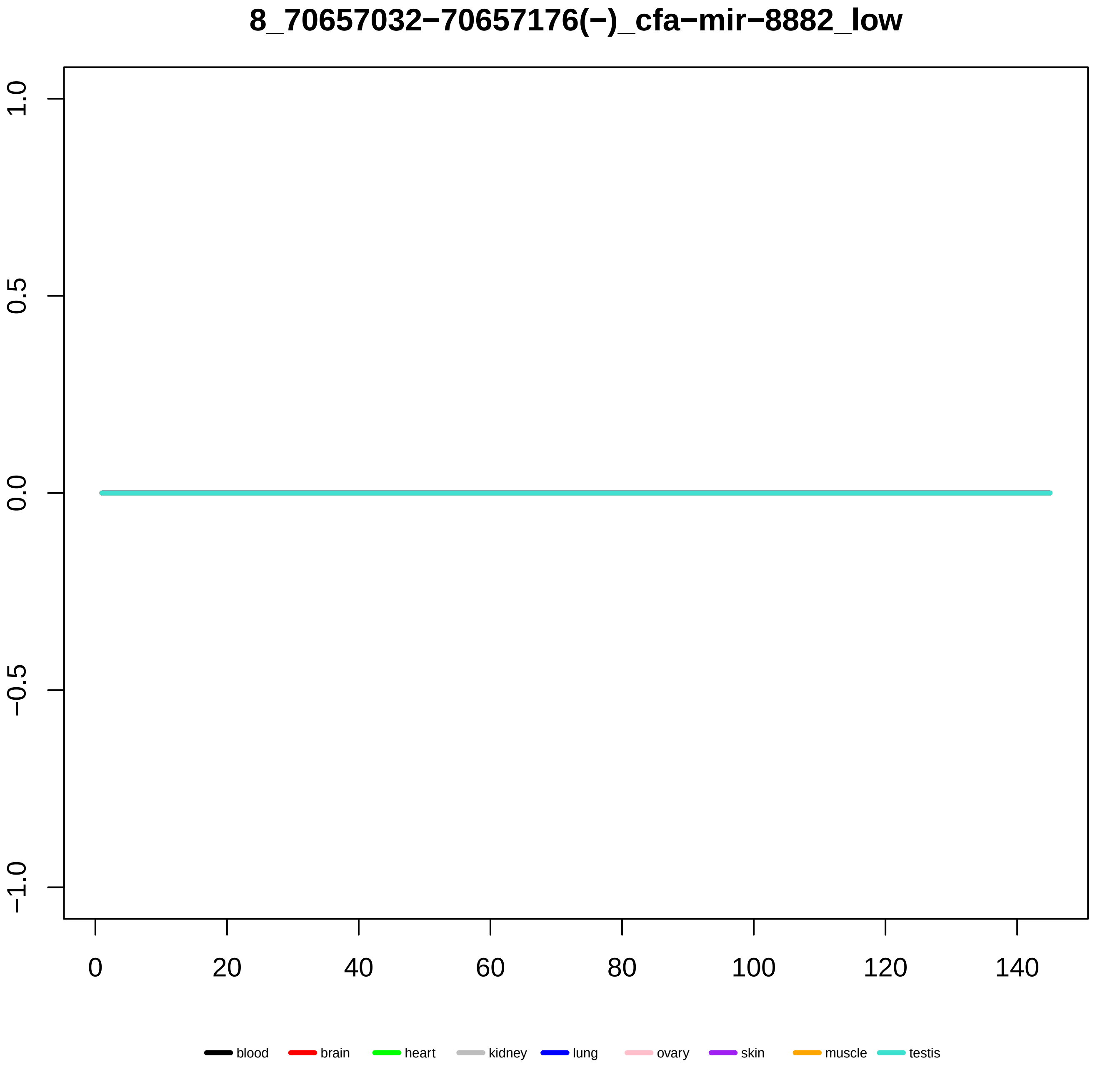
8_70657032−70657176(−)_cfa−mir−8882_low
1.0
0.5
0.0
−0.5
−1.0
0
20
40
60
80
100
120
140
blood
brain
heart
kidney
lung
ovary
skin
muscle
testis

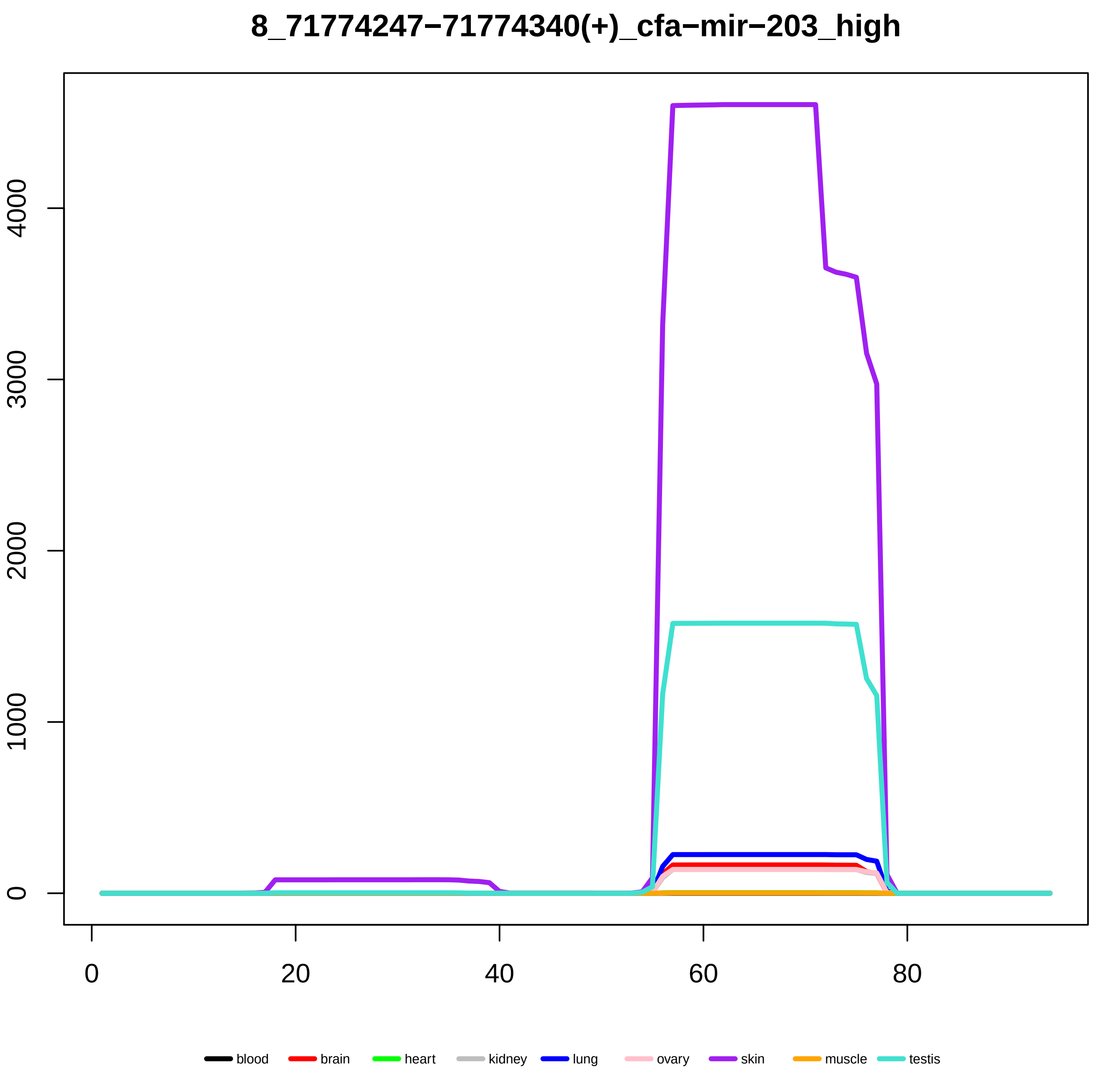
8_71774247−71774340(+)_cfa−mir−203_high
0
1000
2000
3000
4000
0
20
40
60
80
blood
brain
heart
kidney
lung
ovary
skin
muscle
testis

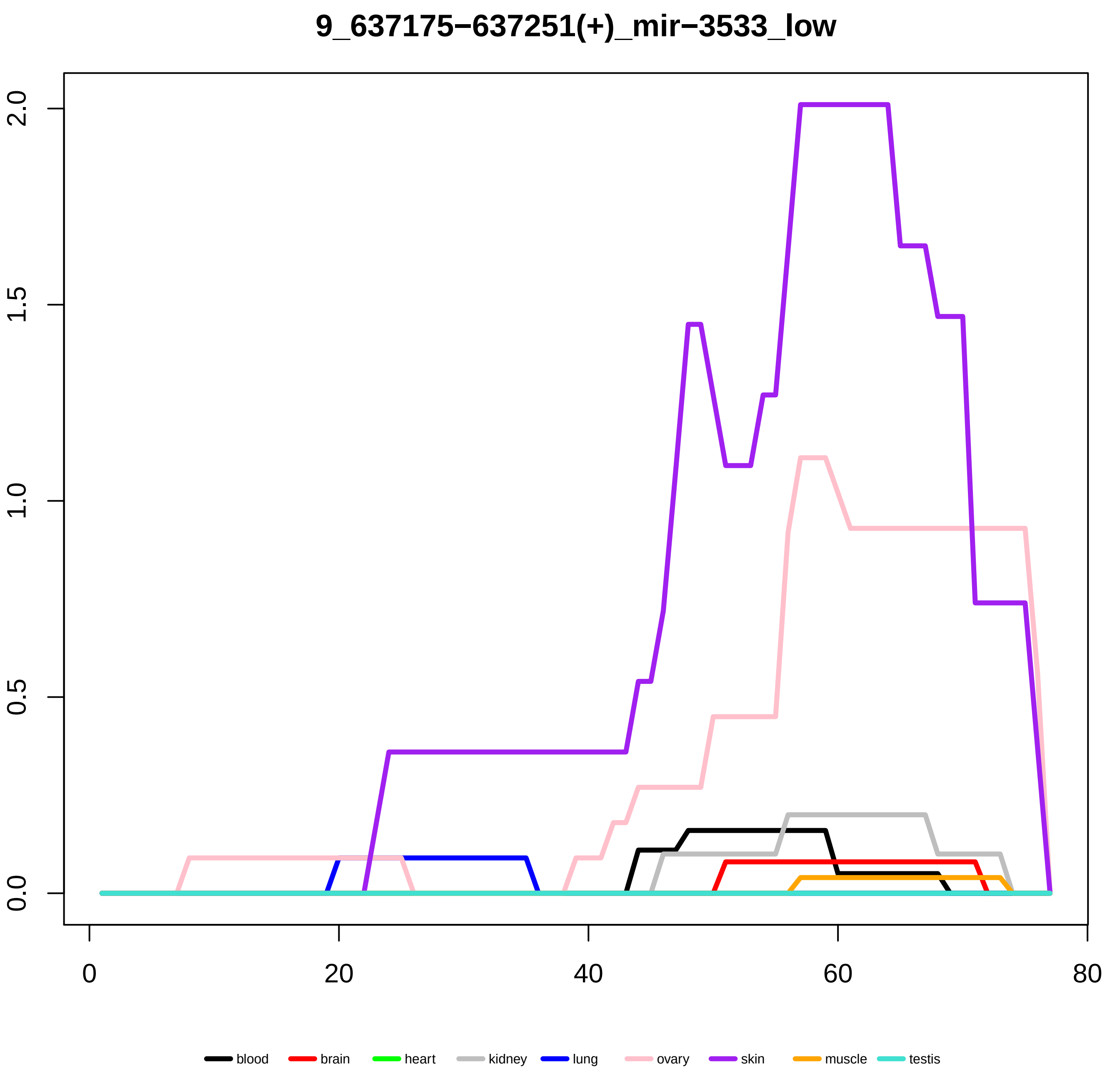
9_637175−637251(+)_mir−3533_low
0.0
0.5
1.0
1.5
2.0
0
20
40
60
80
blood
brain
heart
kidney
lung
ovary
skin
muscle
testis

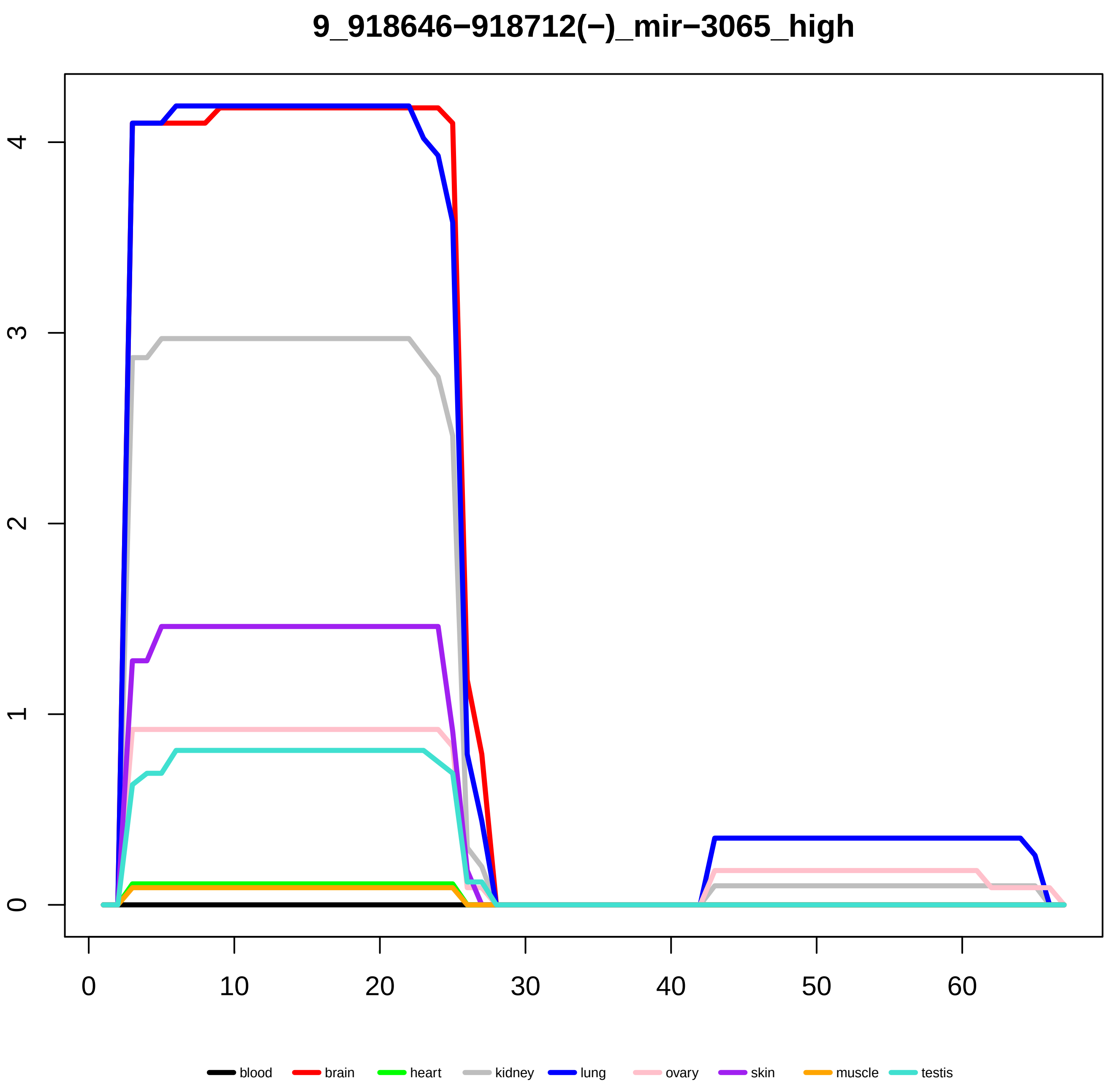

9_918646−918712(−)_mir−3065_high
0
1
2
3
4
0
10
20
30
40
50
60
blood
brain
heart
kidney
lung
ovary
skin
muscle
testis

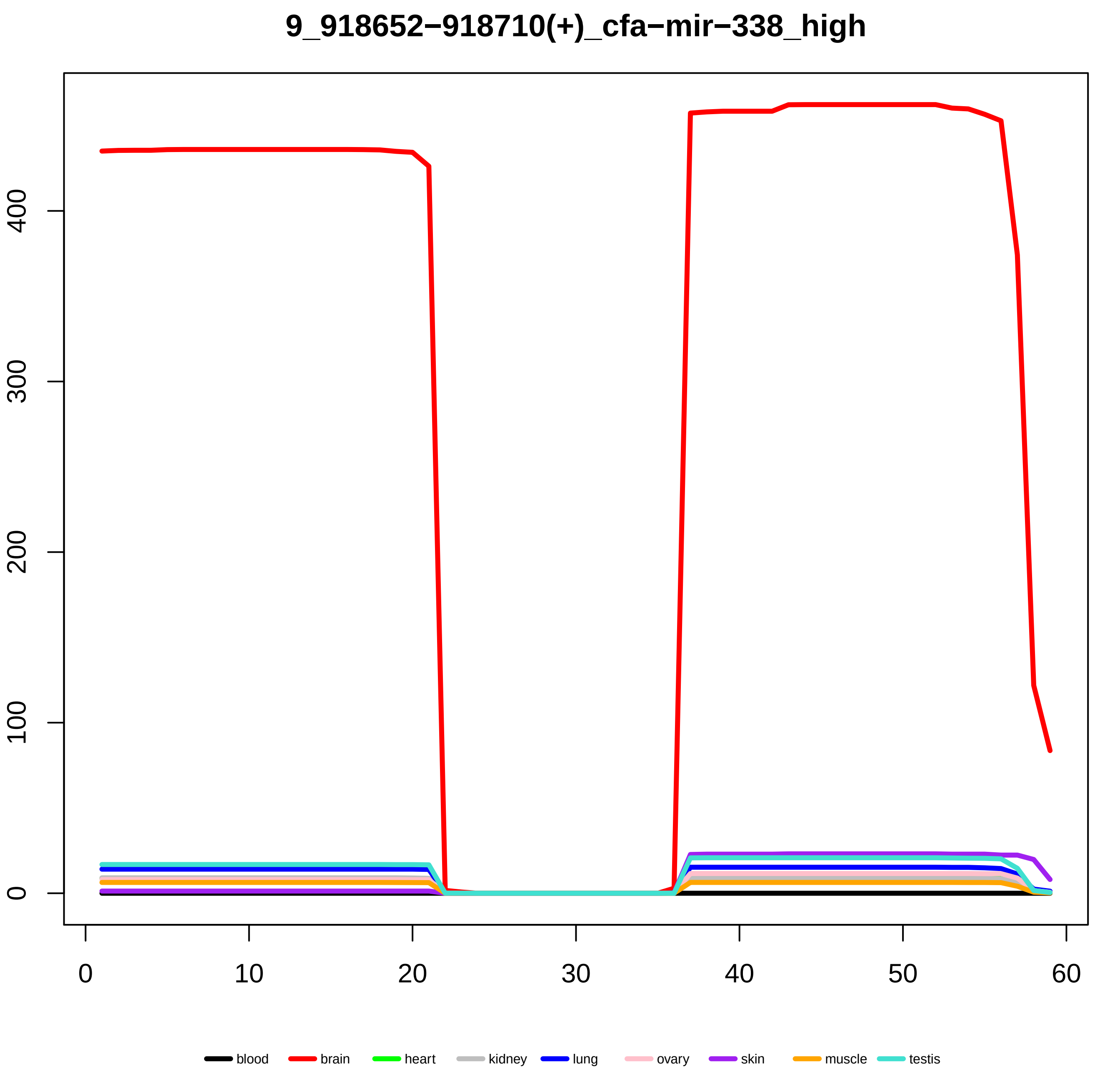
9_918652−918710(+)_cfa−mir−338_high
0
100
200
300
400
0
10
20
30
40
50
60
blood
brain
heart
kidney
lung
ovary
skin
muscle
testis

9_3776763−3776877(−)_cfa−mir−6516_high

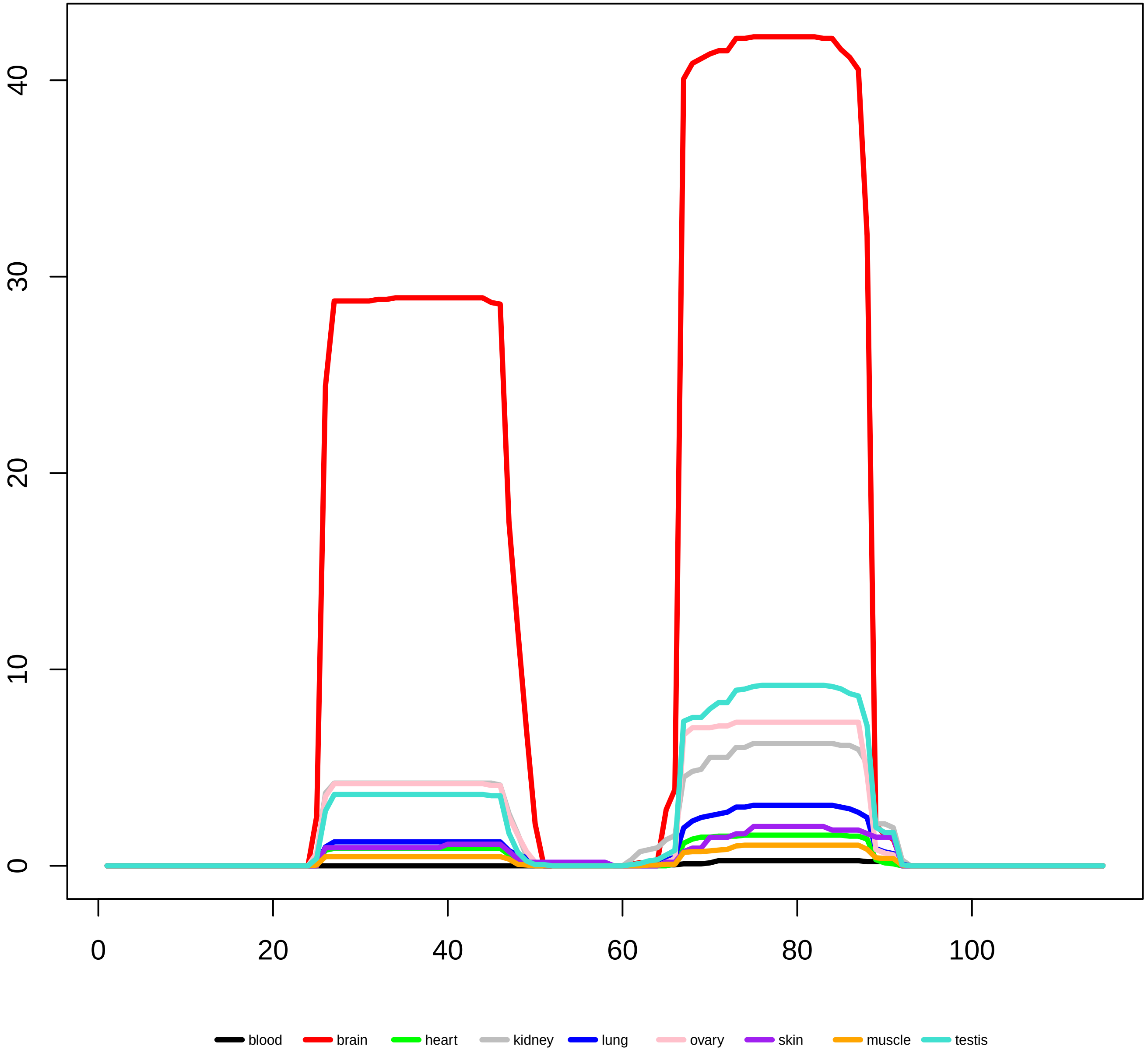

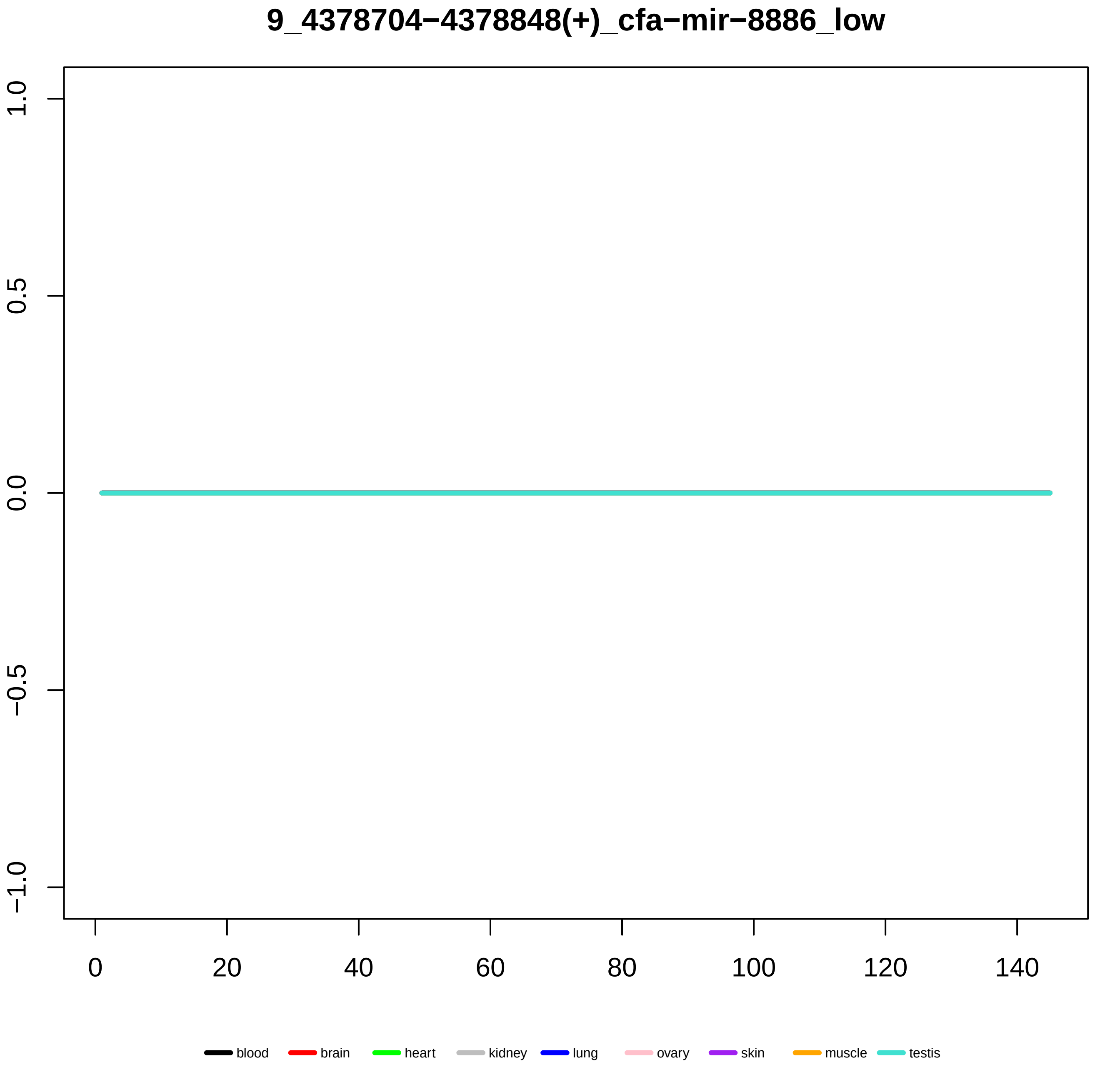
9_4378704−4378848(+)_cfa−mir−8886_low
1.0
0.5
0.0
−0.5
−1.0
0
20
40
60
80
100
120
140
blood
brain
heart
kidney
lung
ovary
skin
muscle
testis

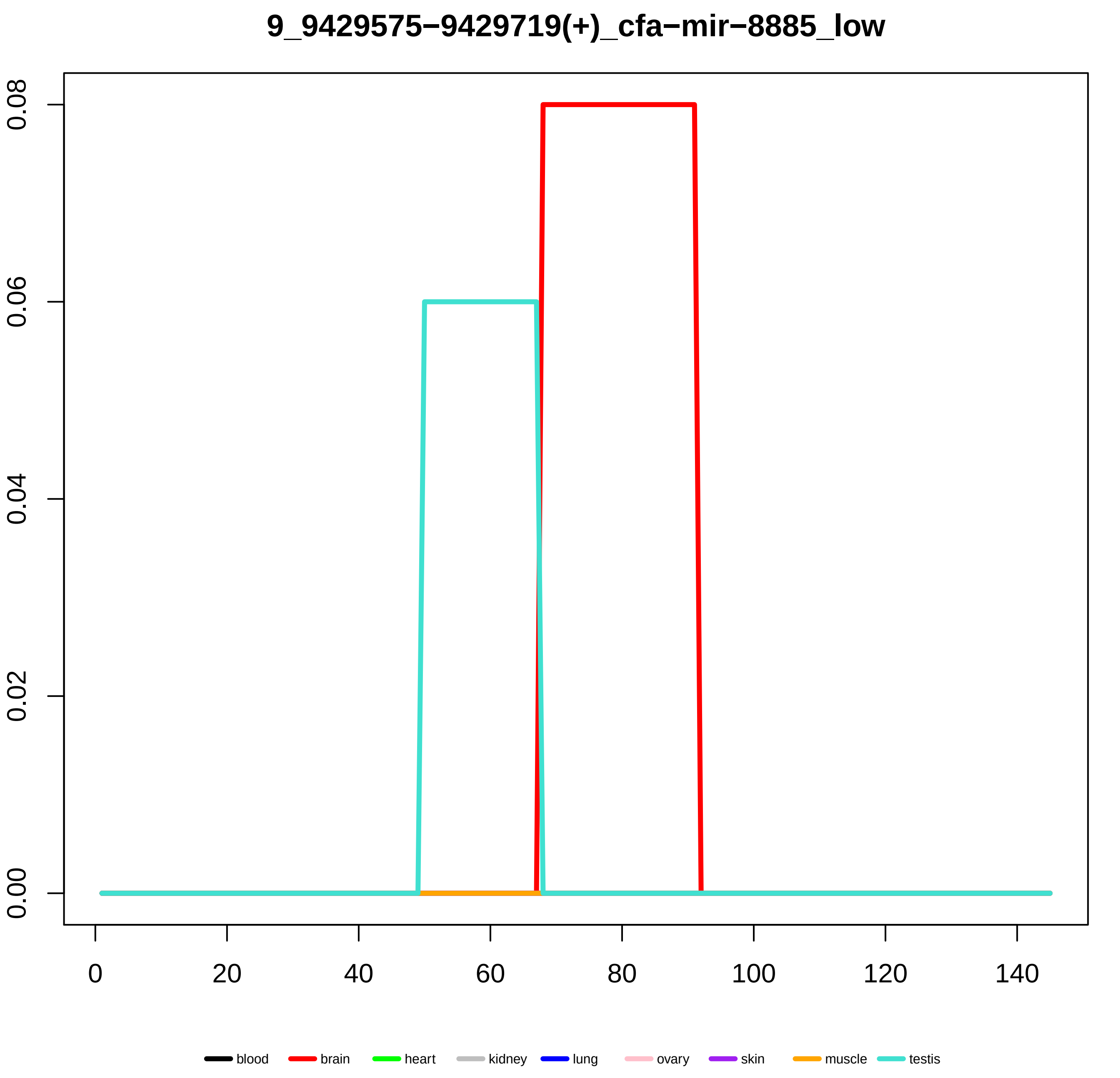

9_9429575−9429719(+)_cfa−mir−8885_low
0.00
0.02
0.04
0.06
0.08
0
20
40
60
80
100
120
140
blood
brain
heart
kidney
lung
ovary
skin
muscle
testis

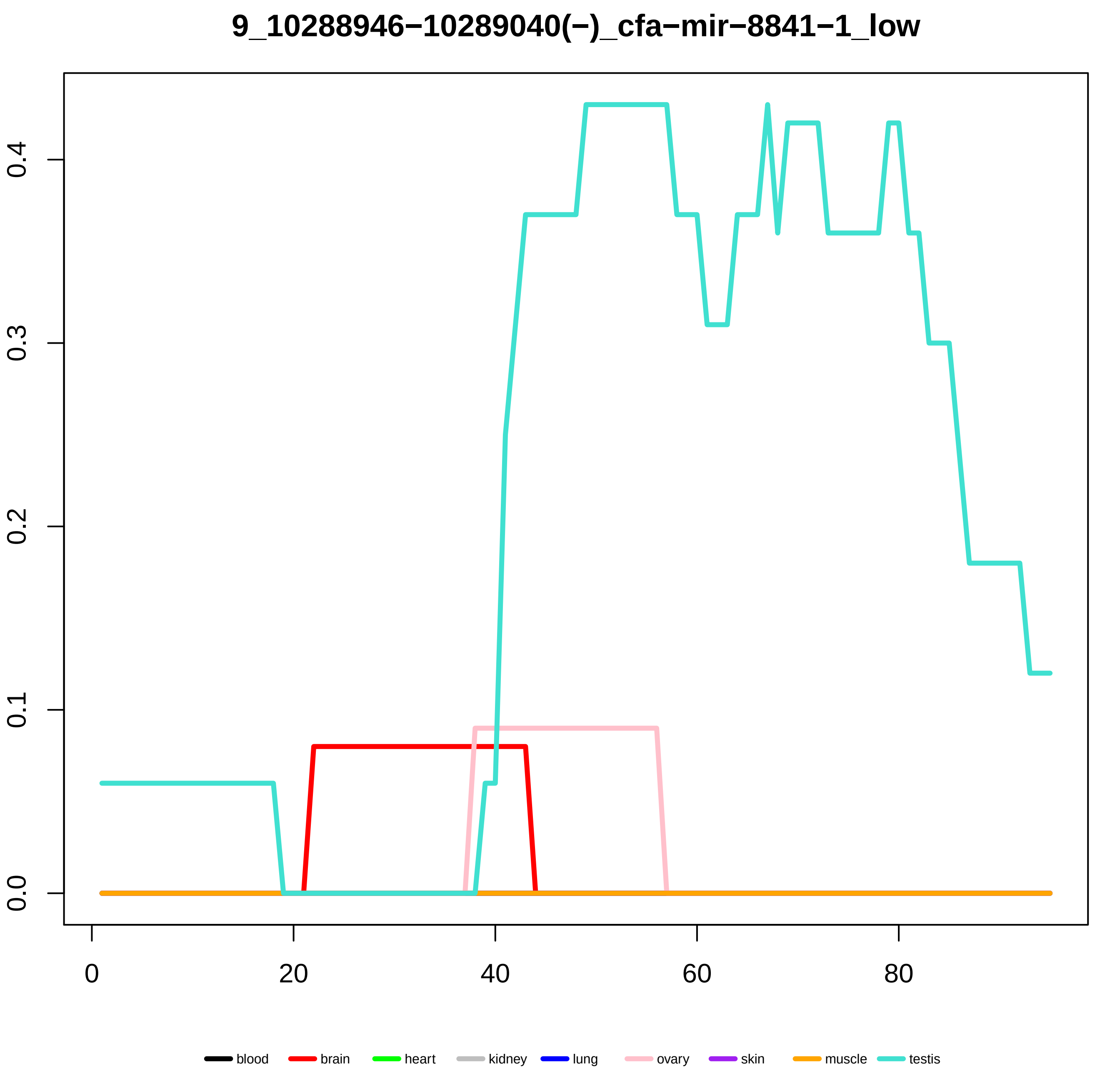
9_10288946−10289040(−)_cfa−mir−8841−1_low
0.0
0.1
0.2
0.3
0.4
0
20
40
60
80
blood
brain
heart
kidney
lung
ovary
skin
muscle
testis

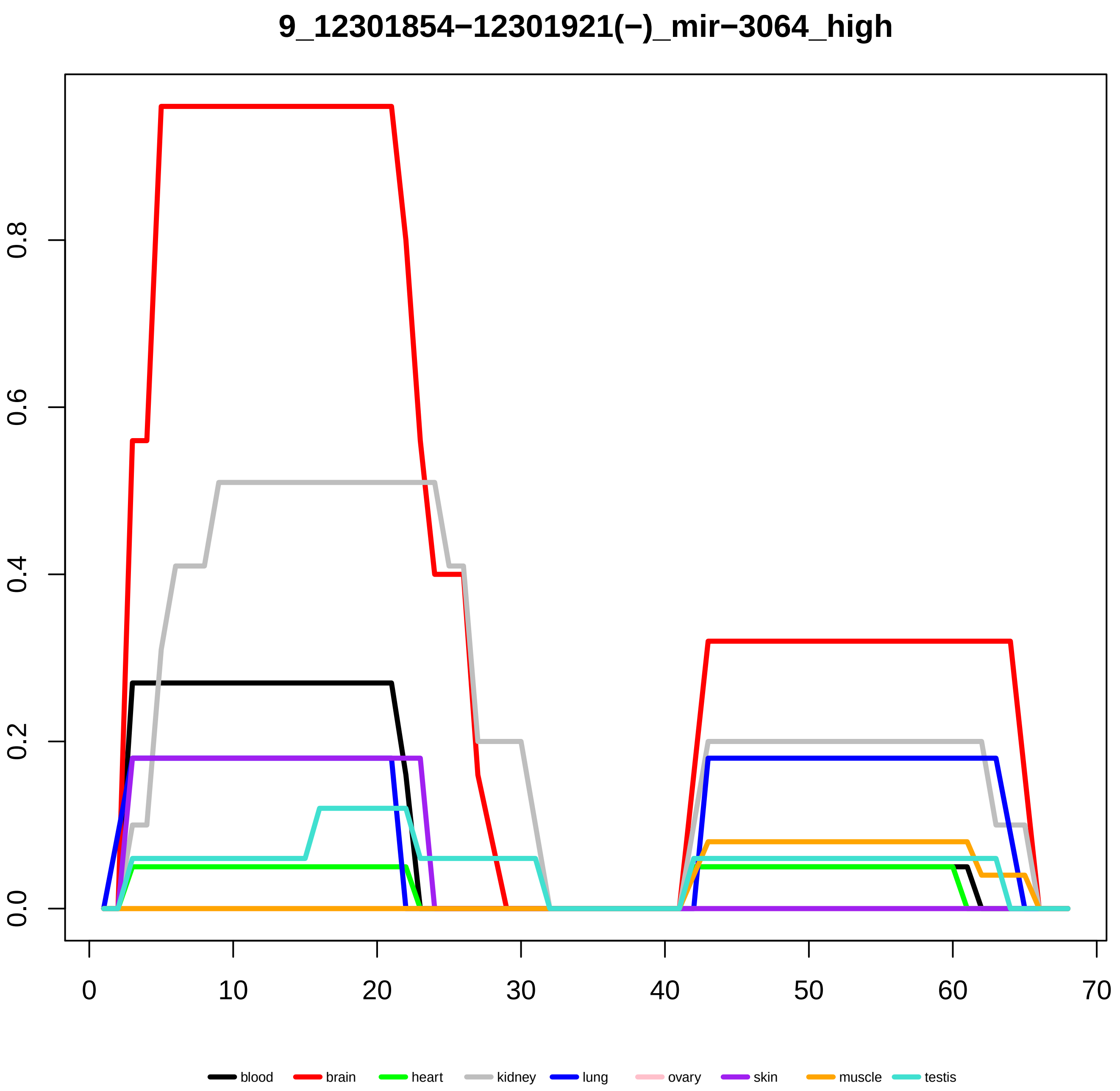

9_12301854−12301921(−)_mir−3064_high
0.0
0.2
0.4
0.6
0.8
0
10
20
30
40
50
60
70
blood
brain
heart
kidney
lung
ovary
skin
muscle
testis

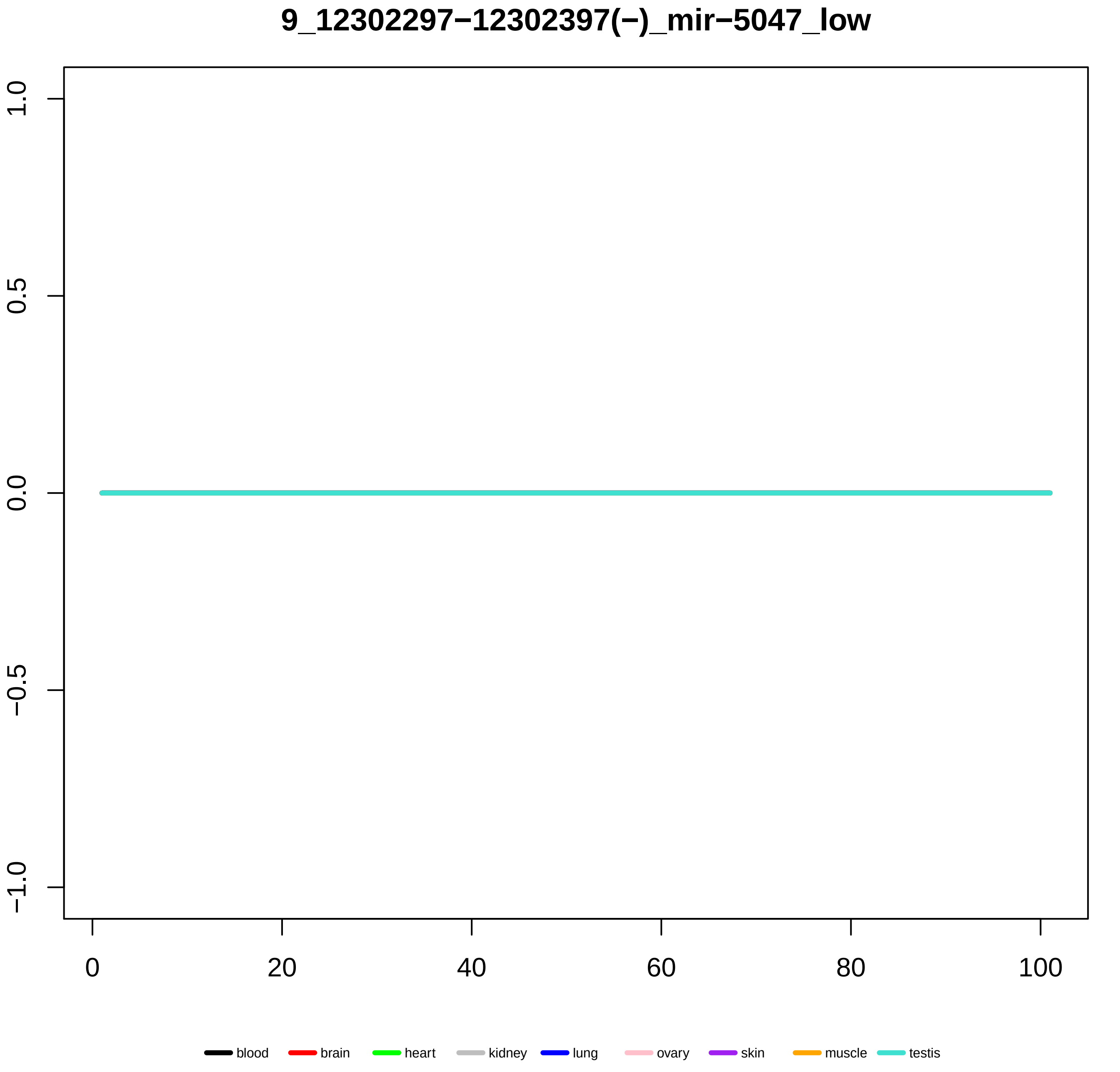
9_12302297−12302397(−)_mir−5047_low
1.0
0.5
0.0
−0.5
−1.0
0
20
40
60
80
100
blood
brain
heart
kidney
lung
ovary
skin
muscle
testis

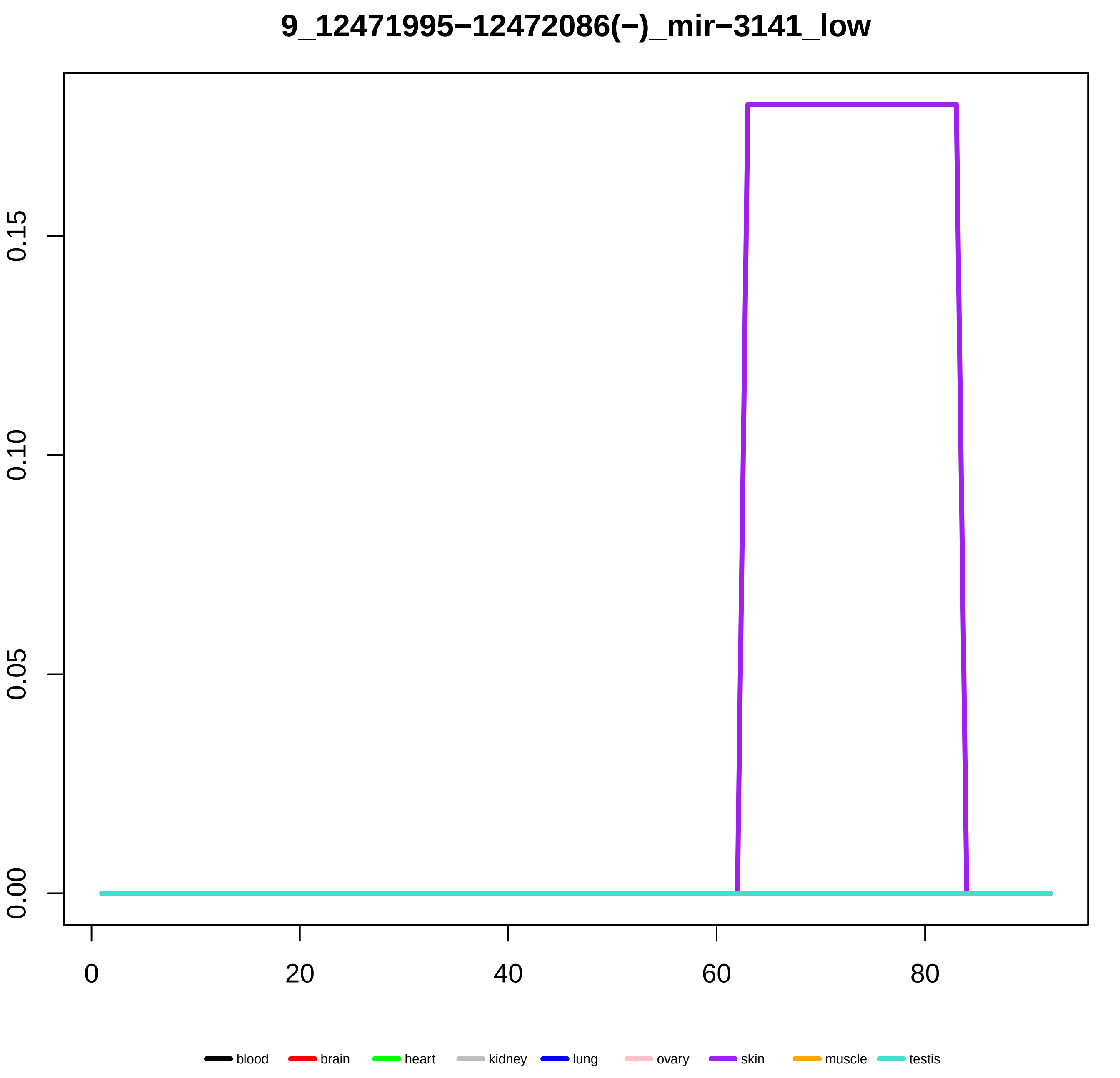

9_12471995−12472086(−)_mir−3141_low
0.00
0.05
0.10
0.15
0
20
40
60
80
blood
brain
heart
kidney
lung
ovary
skin
muscle
testis

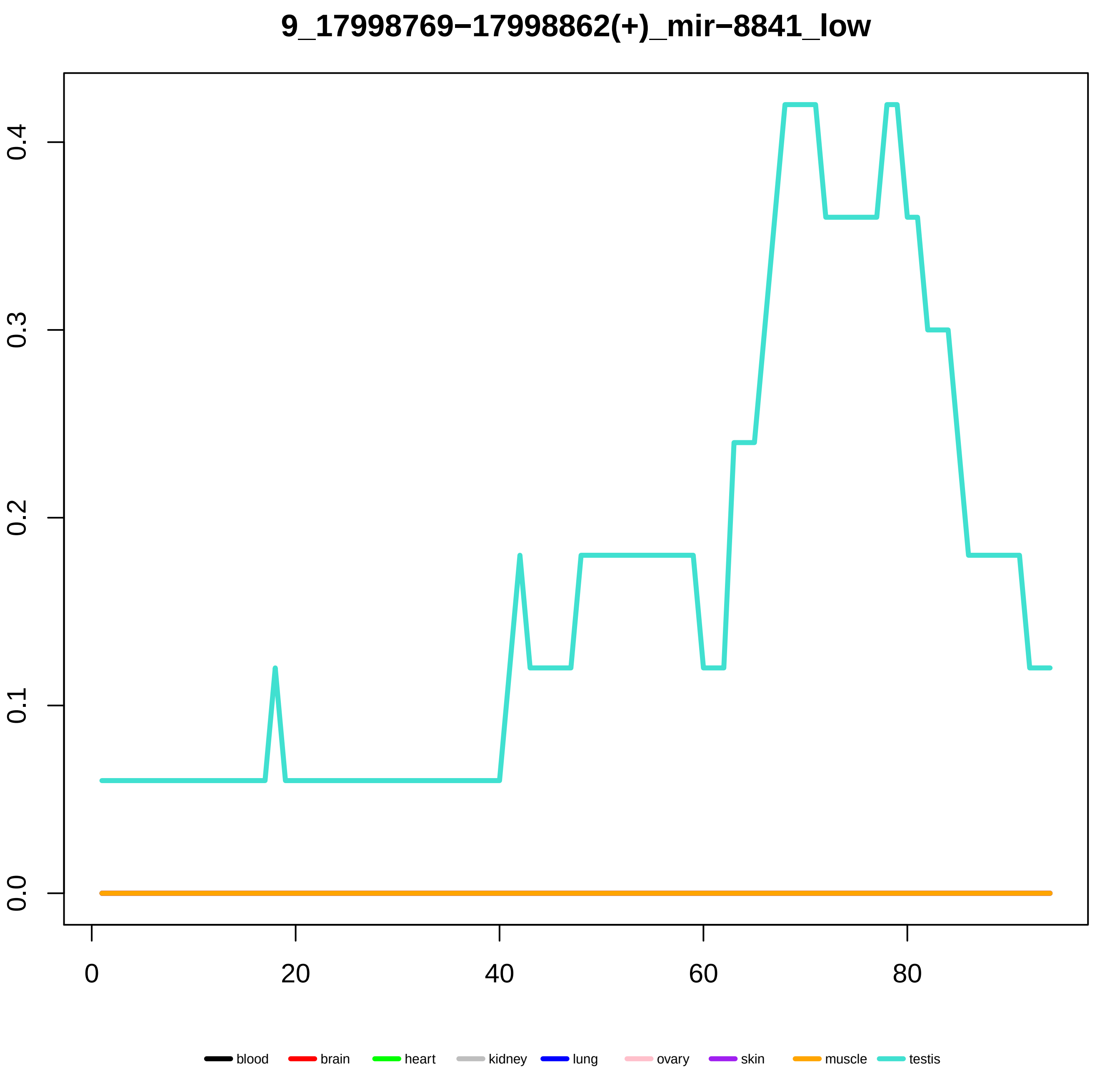
9_17998769−17998862(+)_mir−8841_low
0.0
0.1
0.2
0.3
0.4
0
20
40
60
80
blood
brain
heart
kidney
lung
ovary
skin
muscle
testis

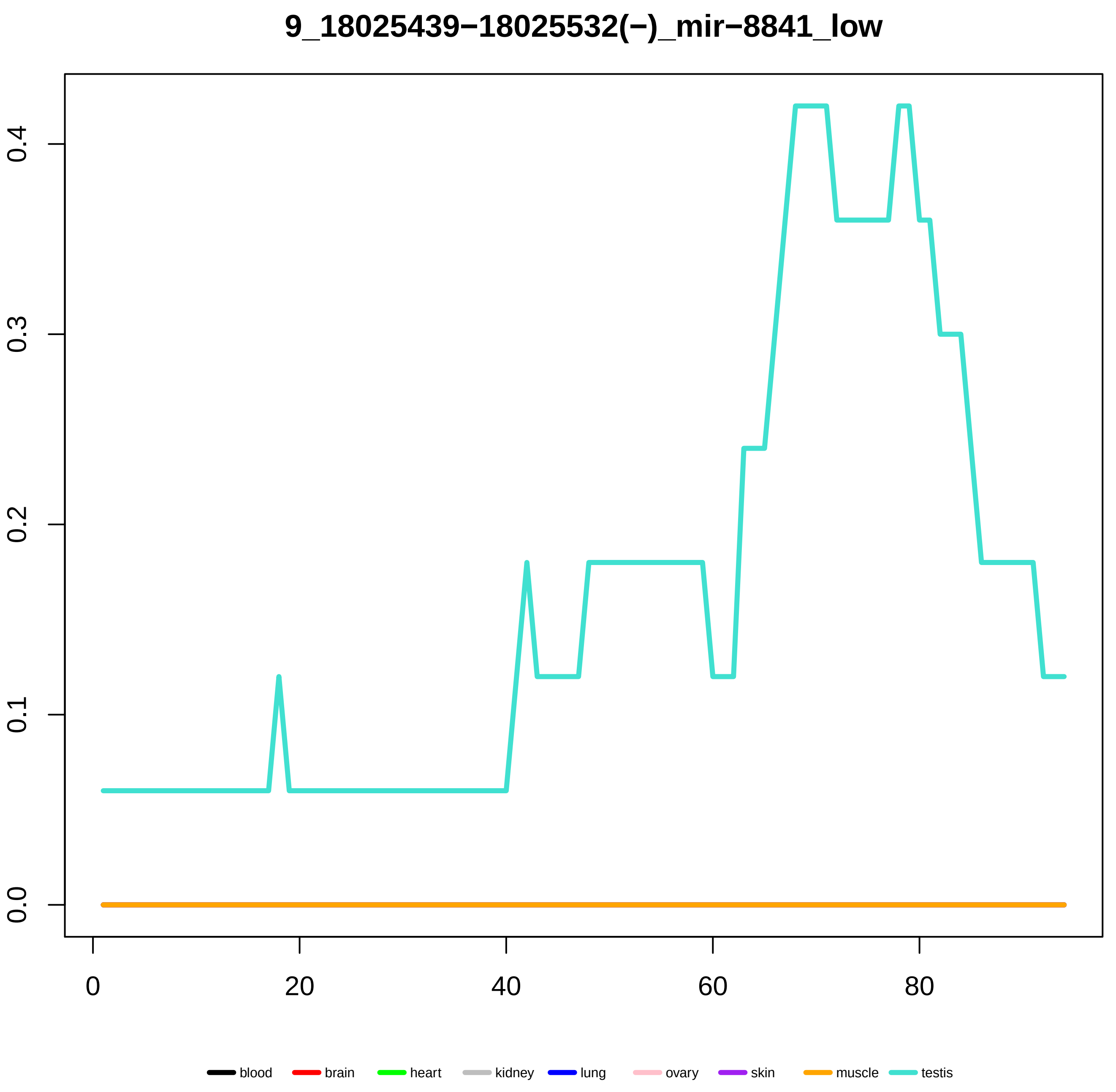
9_18025439−18025532(−)_mir−8841_low
0.0
0.1
0.2
0.3
0.4
0
20
40
60
80
blood
brain
heart
kidney
lung
ovary
skin
muscle
testis

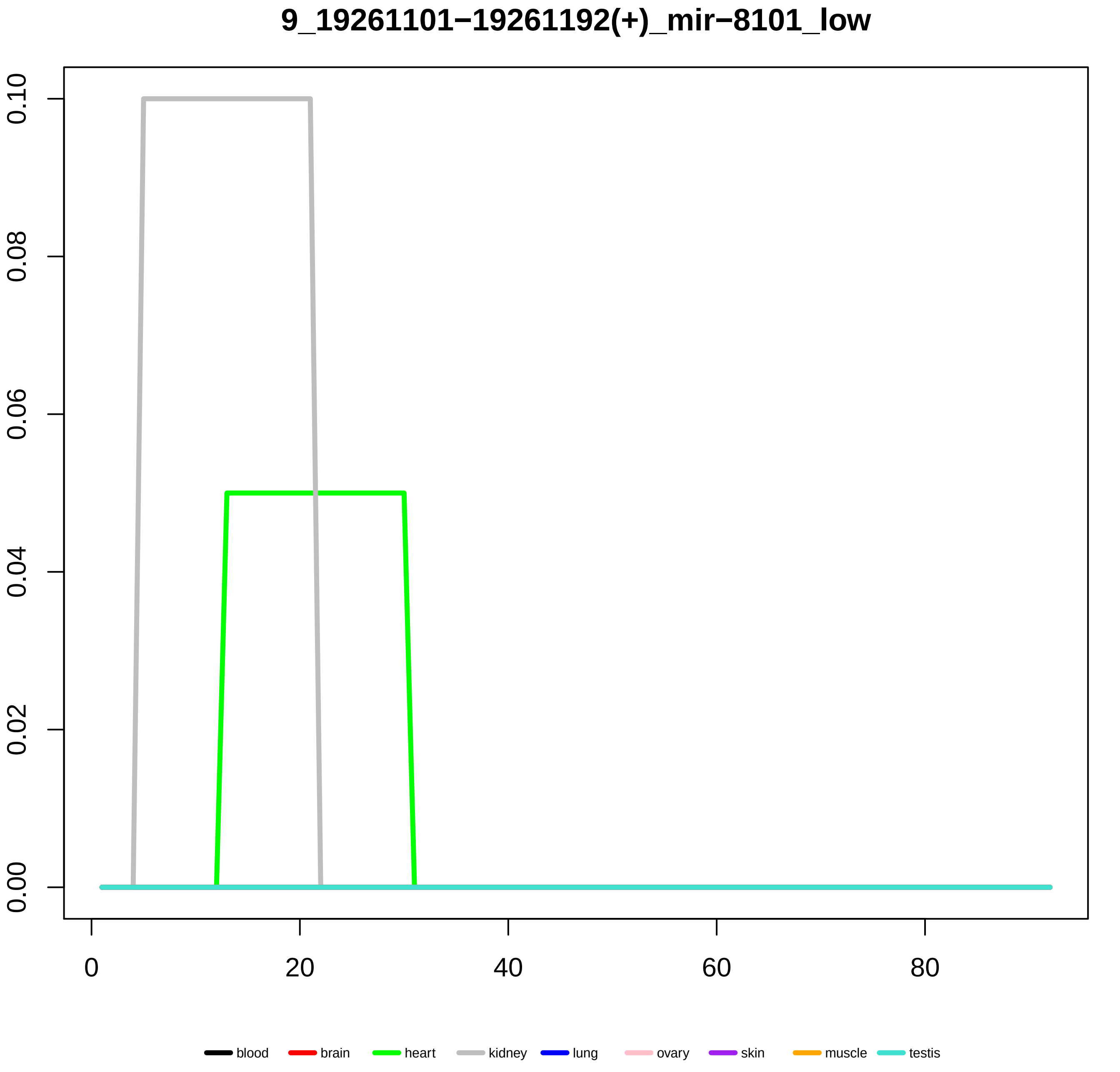

9_19261101−19261192(+)_mir−8101_low
0.00
0.02
0.04
0.06
0.08
0.10
0
20
40
60
80
blood
brain
heart
kidney
lung
ovary
skin
muscle
testis

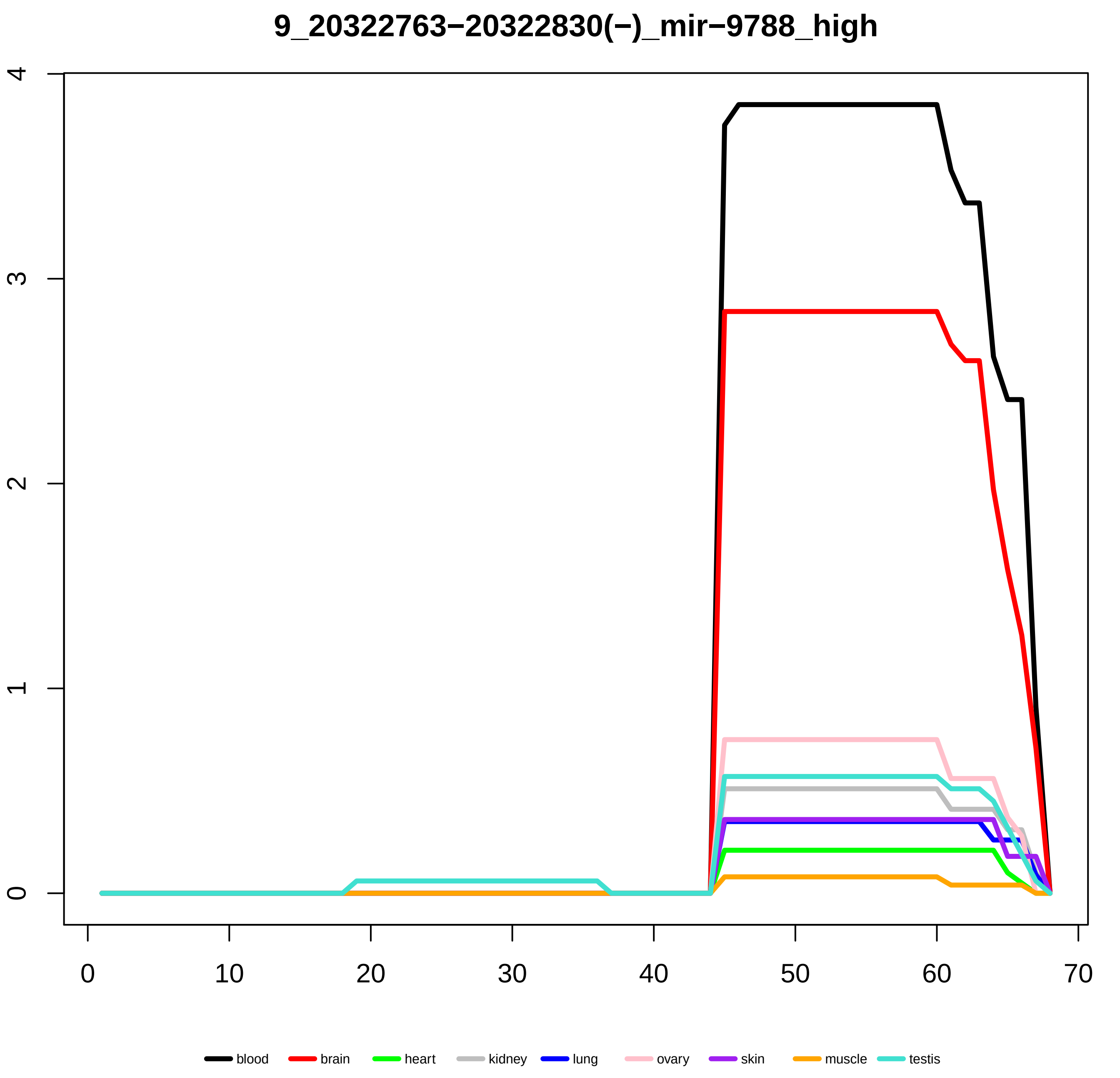
9_20322763−20322830(−)_mir−9788_high
0
1
2
3
4
0
10
20
30
40
50
60
70
blood
brain
heart
kidney
lung
ovary
skin
muscle
testis

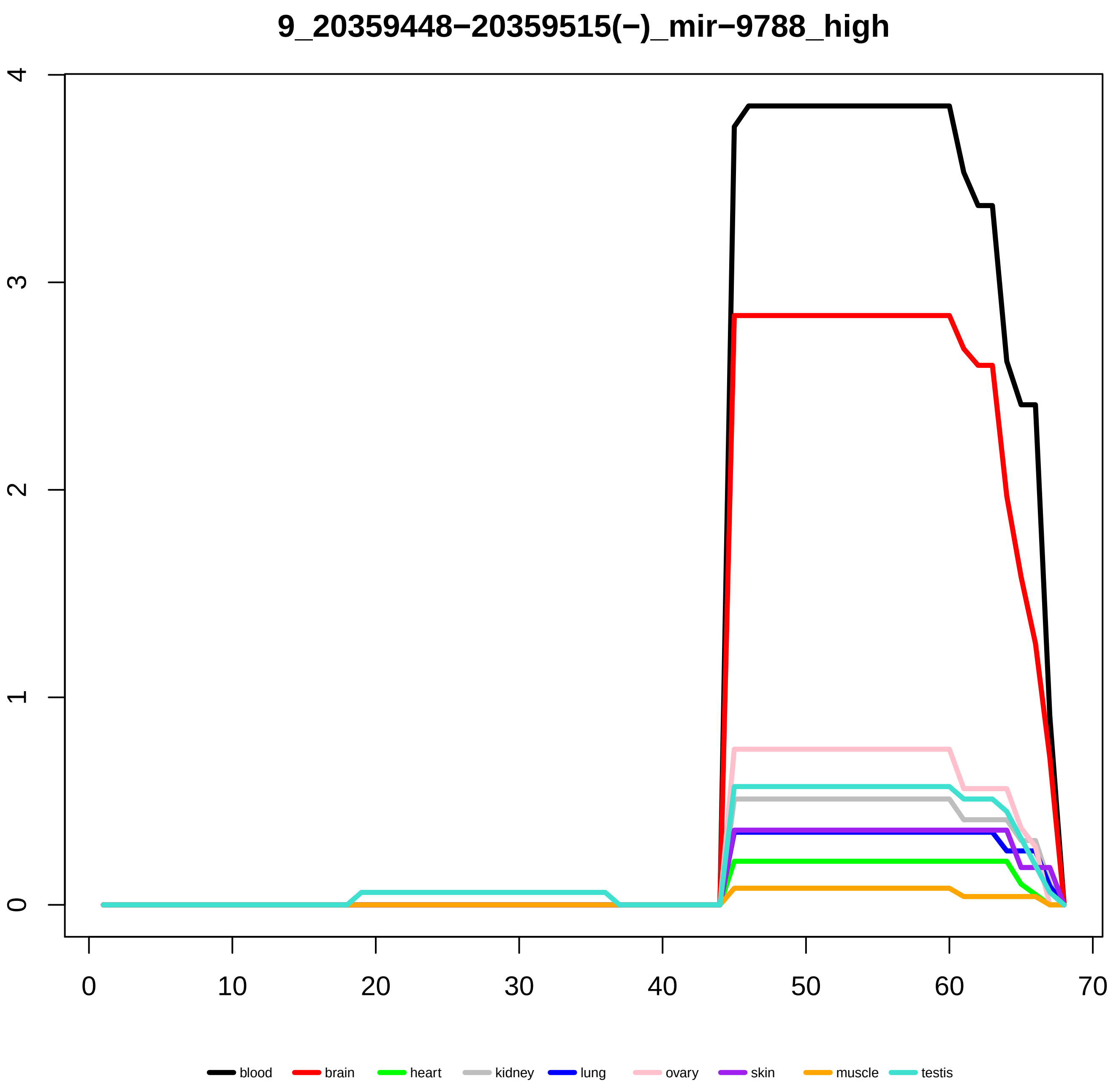

9_20359448−20359515(−)_mir−9788_high
0
1
2
3
4
0
10
20
30
40
50
60
70
blood
brain
heart
kidney
lung
ovary
skin
muscle
testis

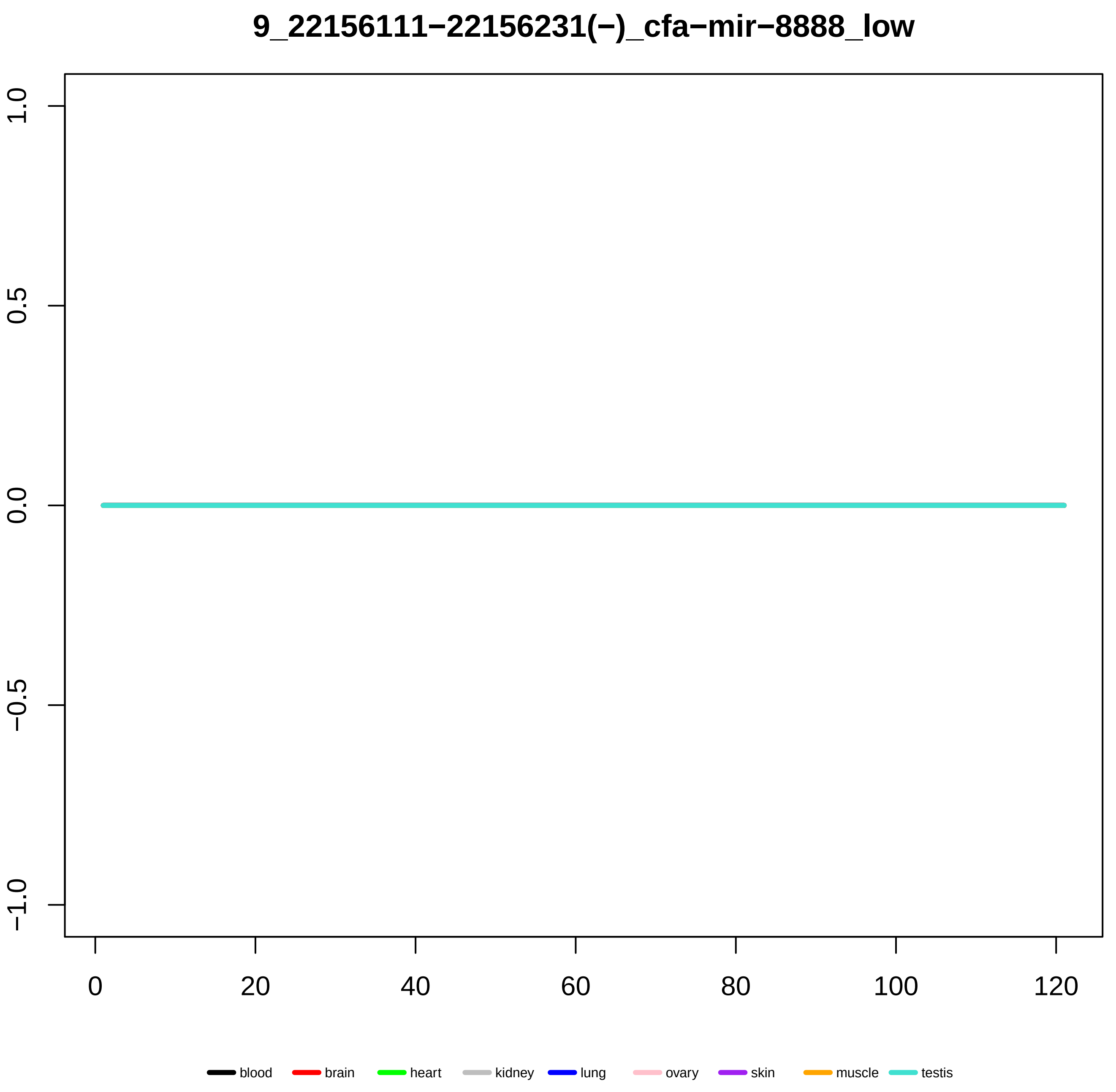

9_22156111−22156231(−)_cfa−mir−8888_low
1.0
0.5
0.0
−0.5
−1.0
0
20
40
60
80
100
120
blood
brain
heart
kidney
lung
ovary
skin
muscle
testis

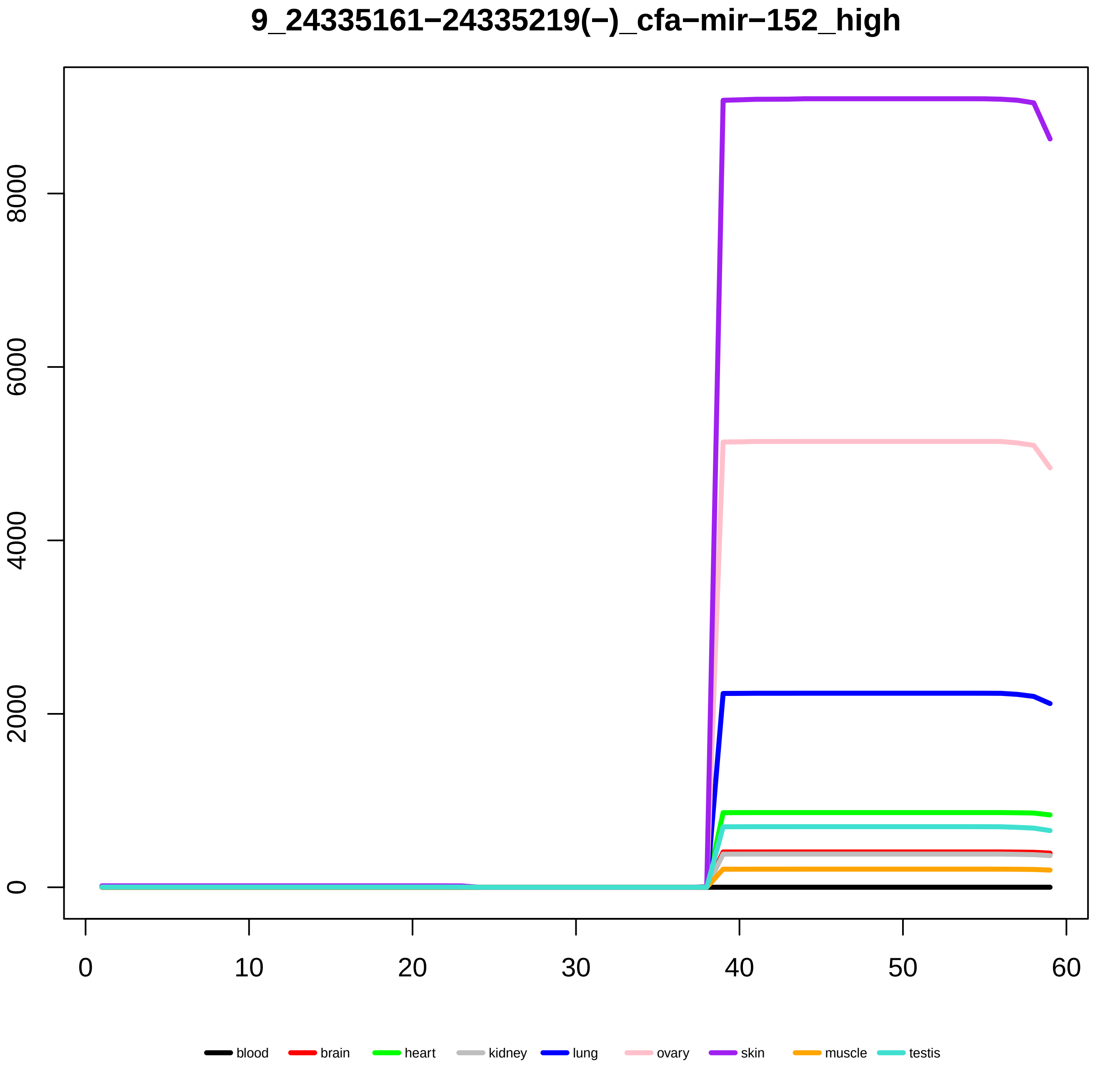

9_24335161−24335219(−)_cfa−mir−152_high
0
2000
4000
6000
8000
0
10
20
30
40
50
60
blood
brain
heart
kidney
lung
ovary
skin
muscle
testis

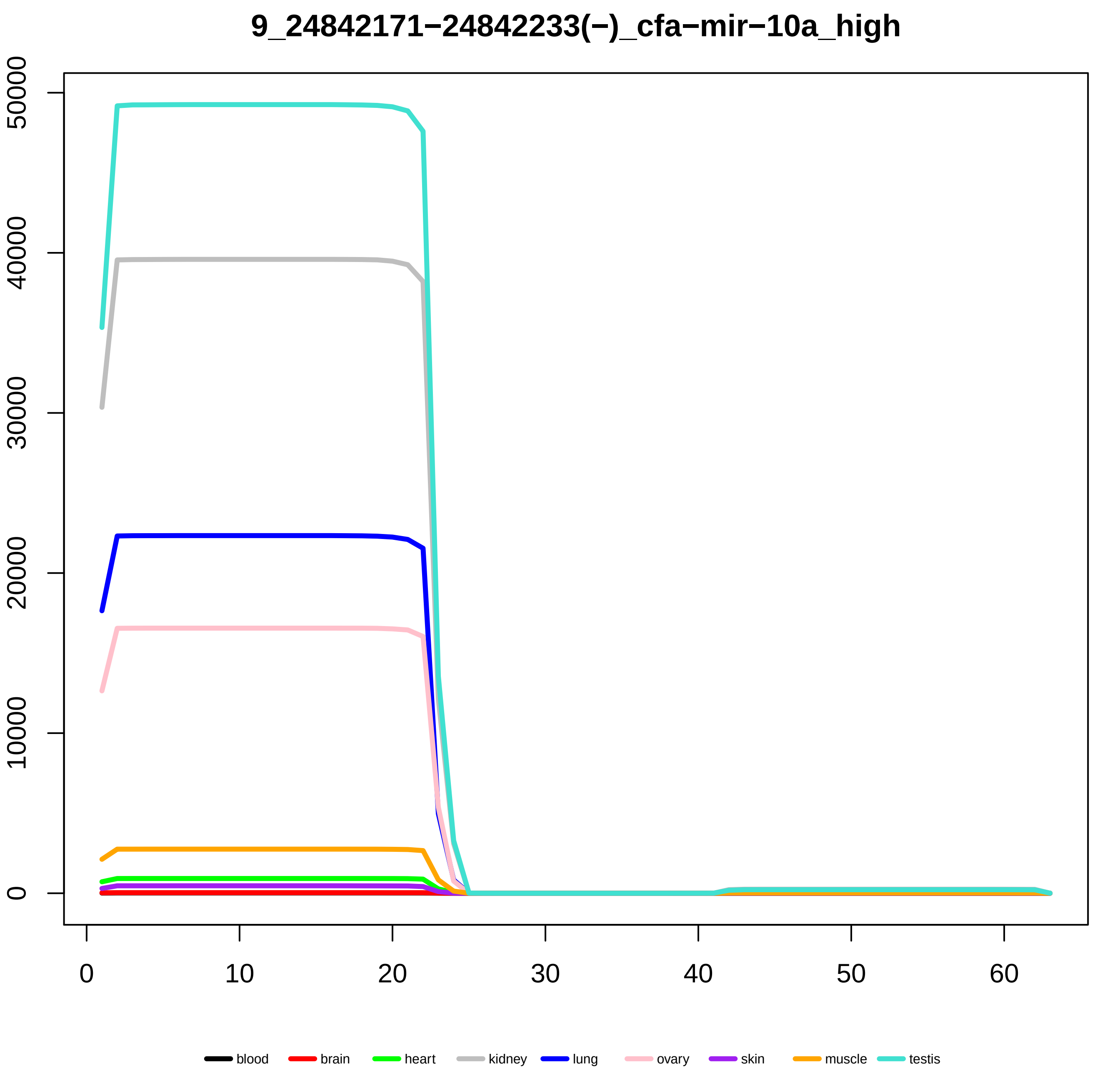
9_24842171−24842233(−)_cfa−mir−10a_high
0
10000
20000
30000
40000
50000
0
10
20
30
40
50
60
blood
brain
heart
kidney
lung
ovary
skin
muscle
testis

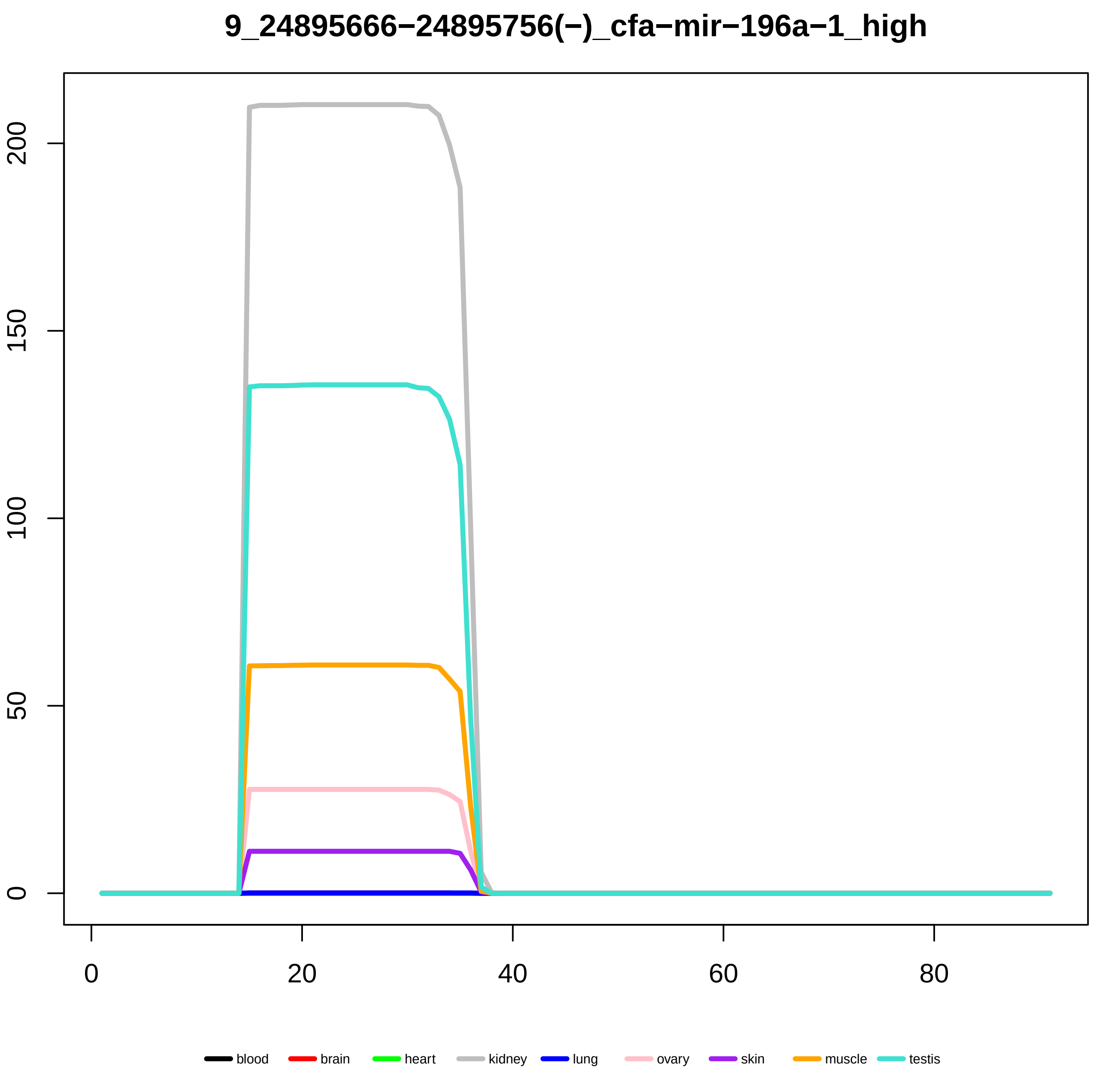
9_24895666−24895756(−)_cfa−mir−196a−1_high
0
50
100
150
200
0
20
40
60
80
blood
brain
heart
kidney
lung
ovary
skin
muscle
testis

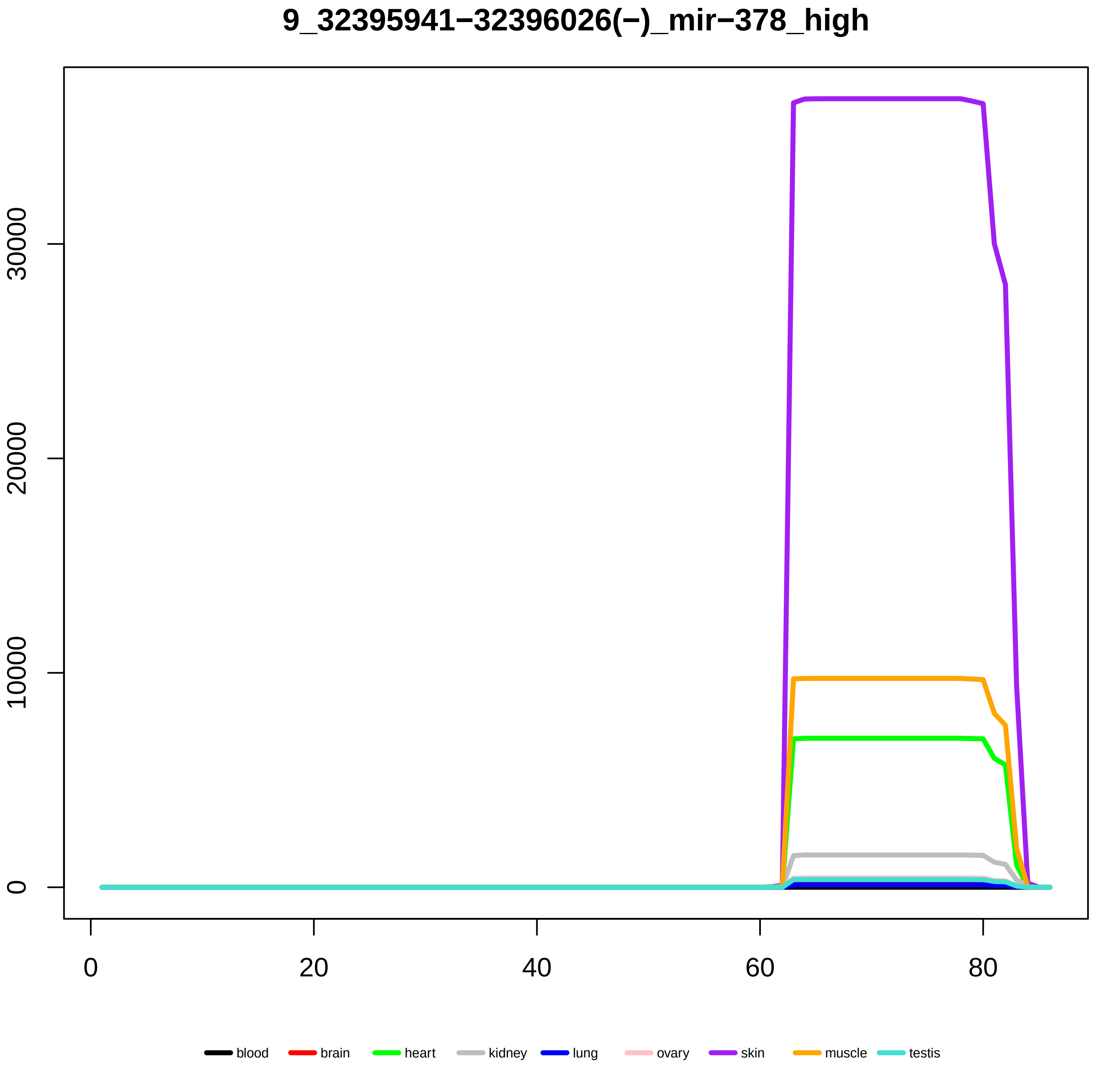
9_32395941−32396026(−)_mir−378_high
0
10000
20000
30000
0
20
40
60
80
blood
brain
heart
kidney
lung
ovary
skin
muscle
testis

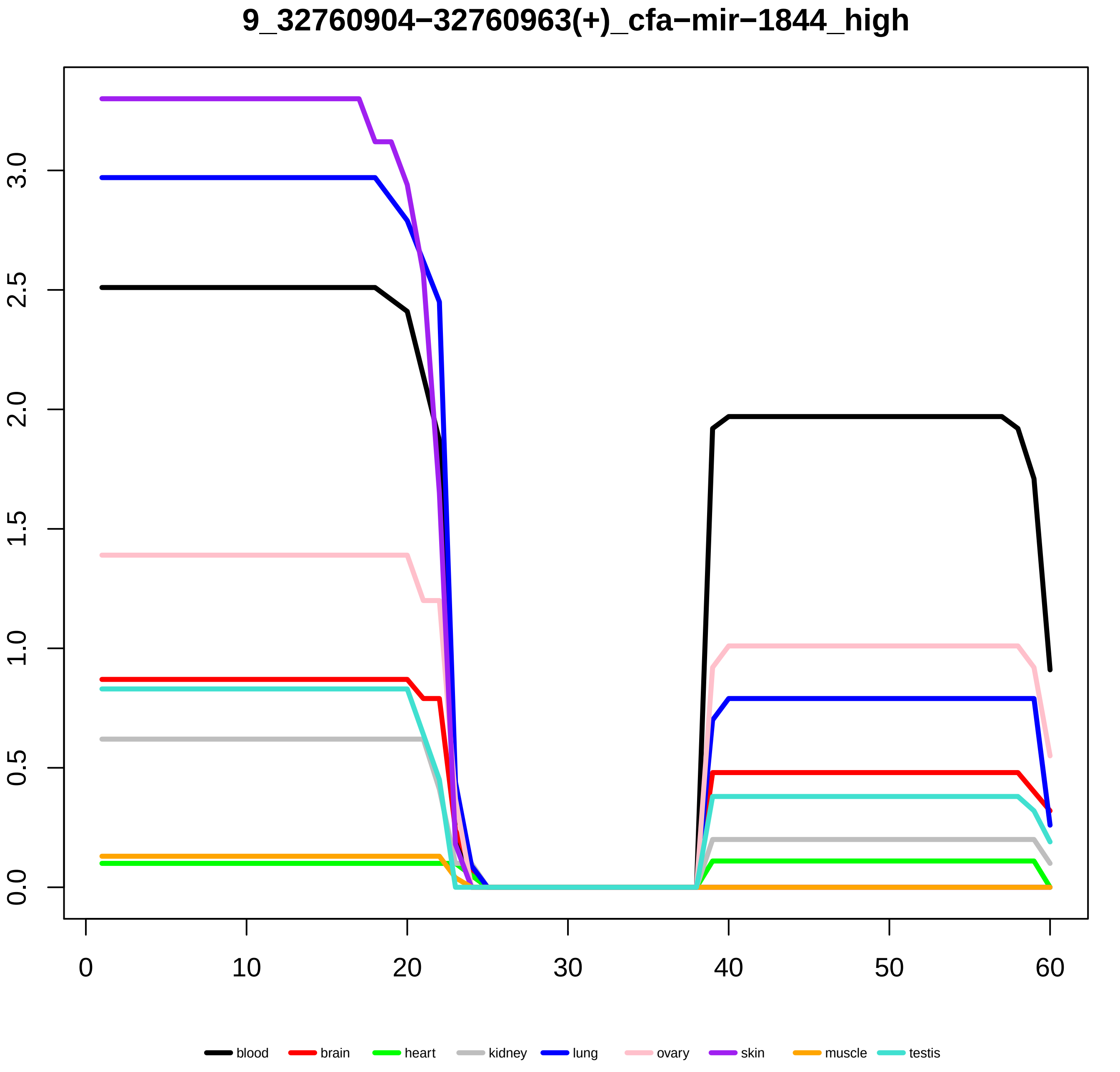
9_32760904−32760963(+)_cfa−mir−1844_high
0.0
0.5
1.0
1.5
2.0
2.5
3.0
0
10
20
30
40
50
60
blood
brain
heart
kidney
lung
ovary
skin
muscle
testis

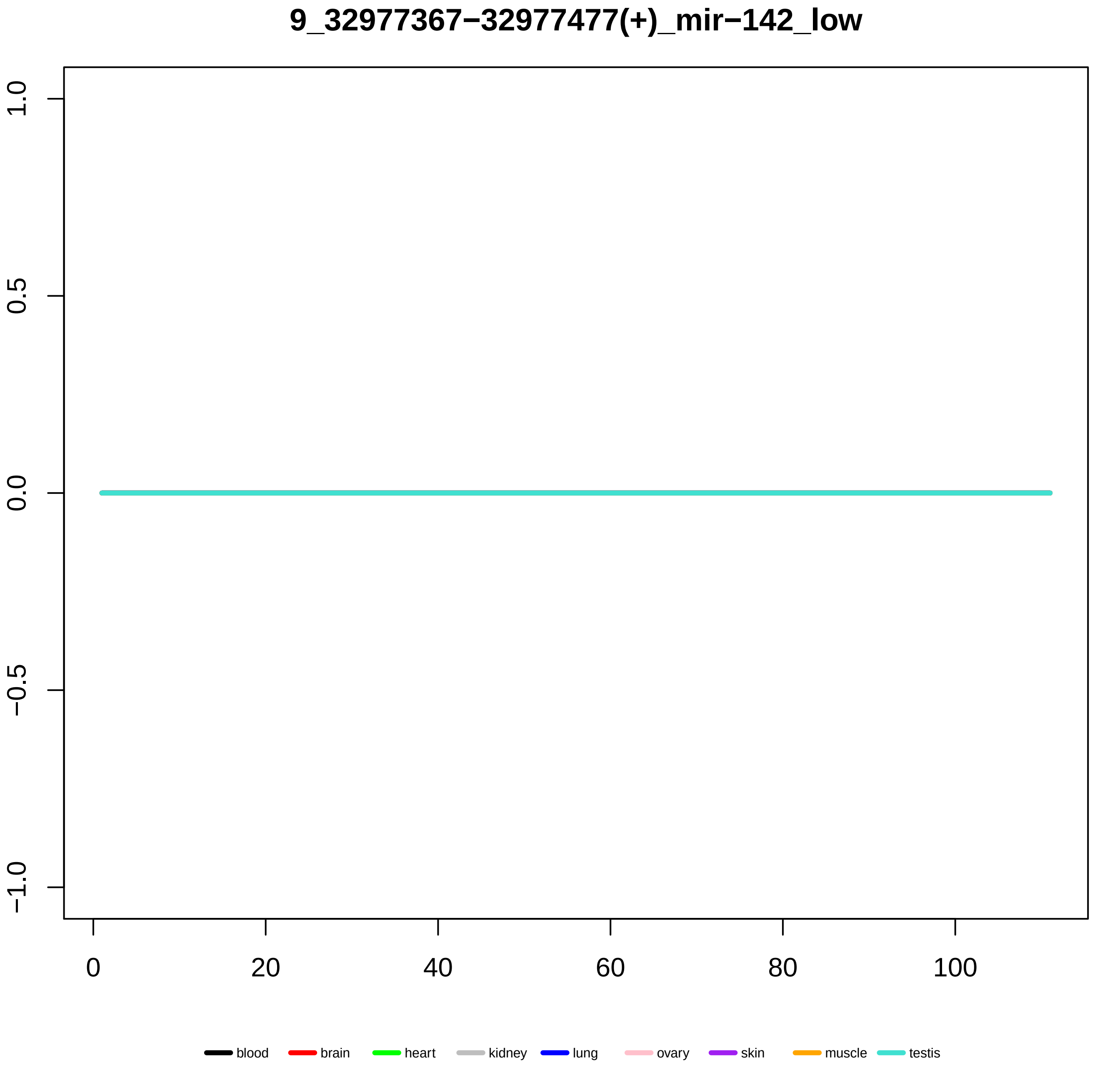

9_32977367−32977477(+)_mir−142_low
1.0
0.5
0.0
−0.5
−1.0
0
20
40
60
80
100
blood
brain
heart
kidney
lung
ovary
skin
muscle
testis

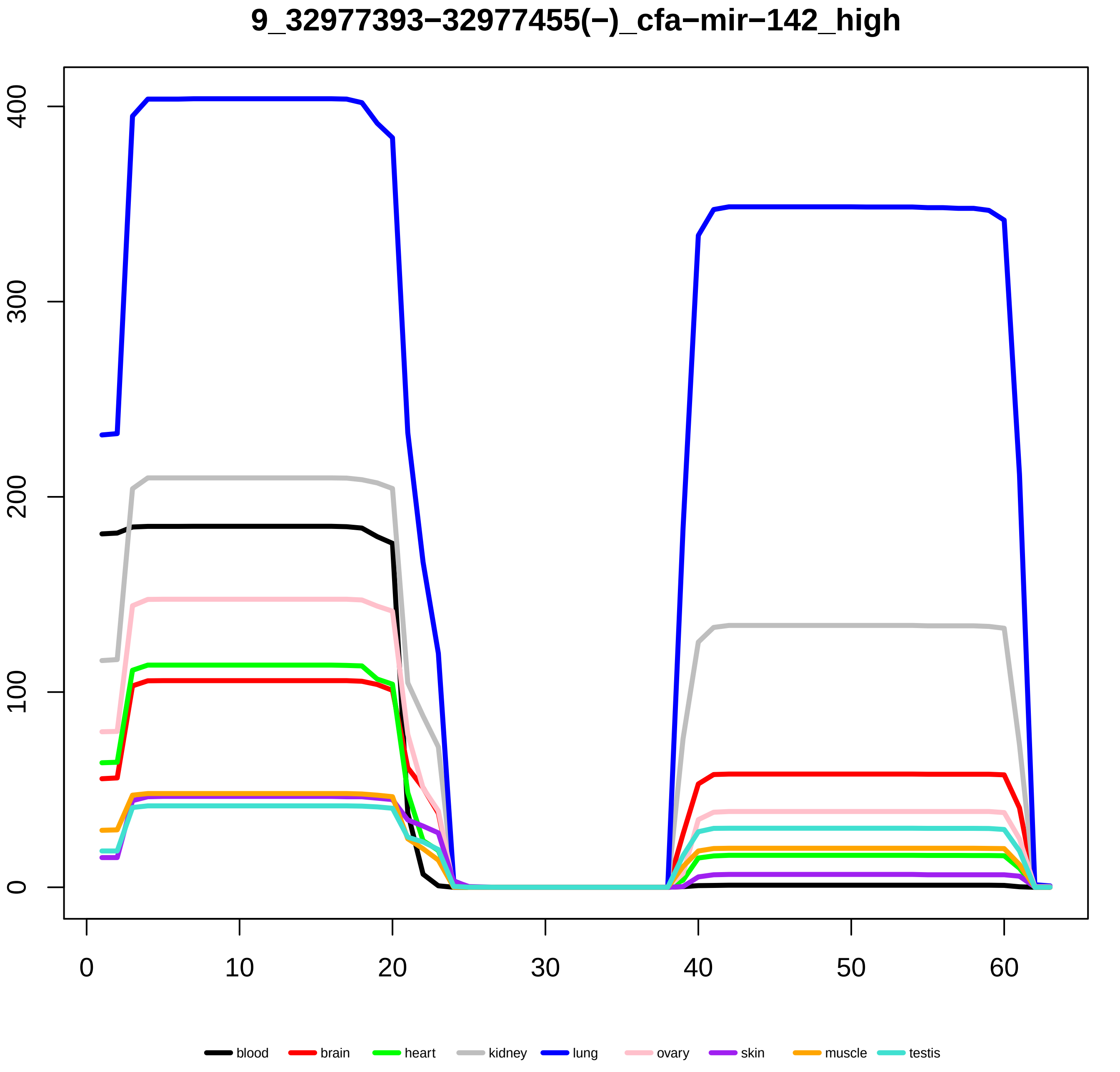
9_32977393−32977455(−)_cfa−mir−142_high
0
100
200
300
400
0
10
20
30
40
50
60
blood
brain
heart
kidney
lung
ovary
skin
muscle
testis

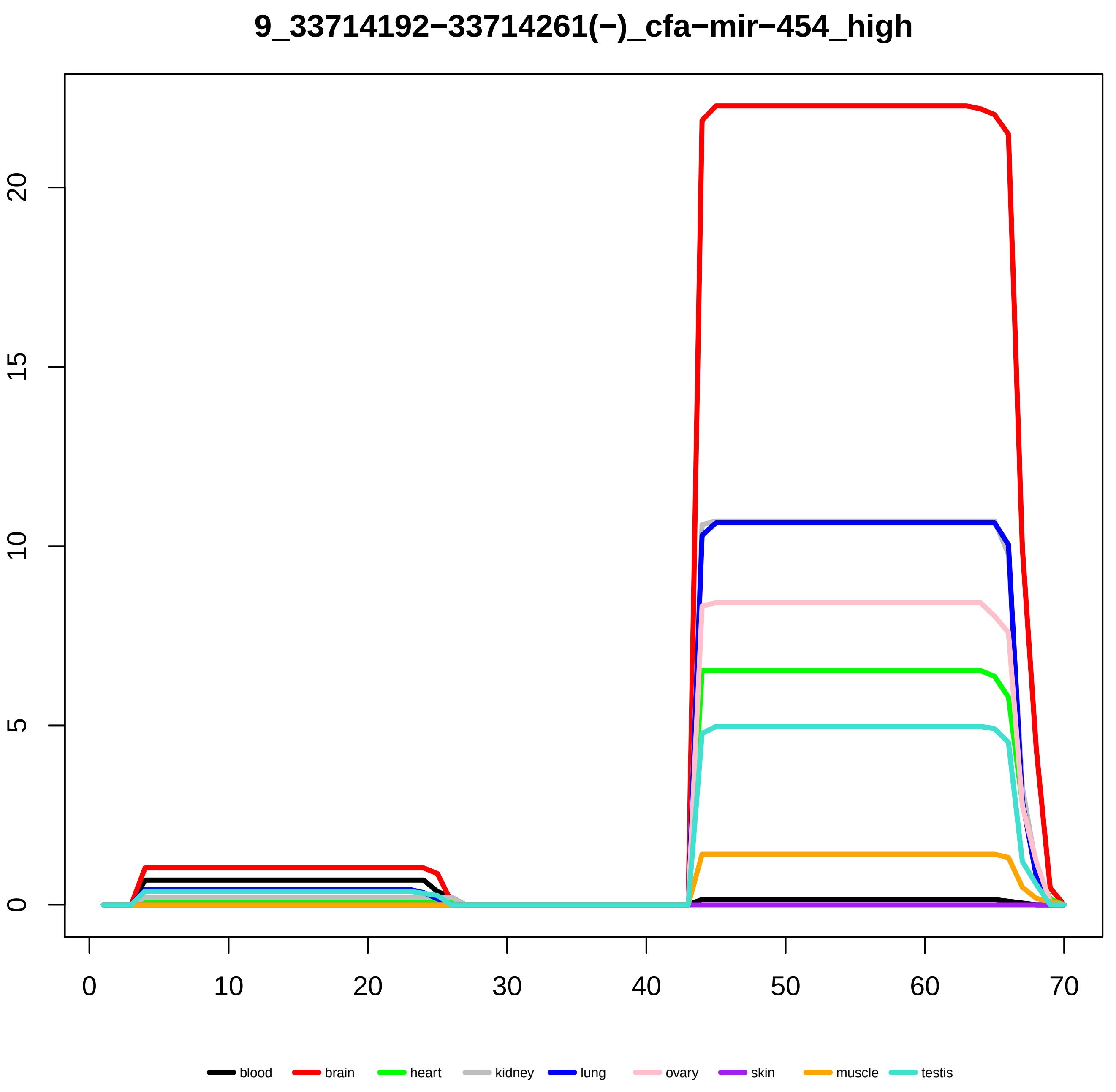
9_33714192−33714261(−)_cfa−mir−454_high
0
5
10
15
20
0
10
20
30
40
50
60
70
blood
brain
heart
kidney
lung
ovary
skin
muscle
testis

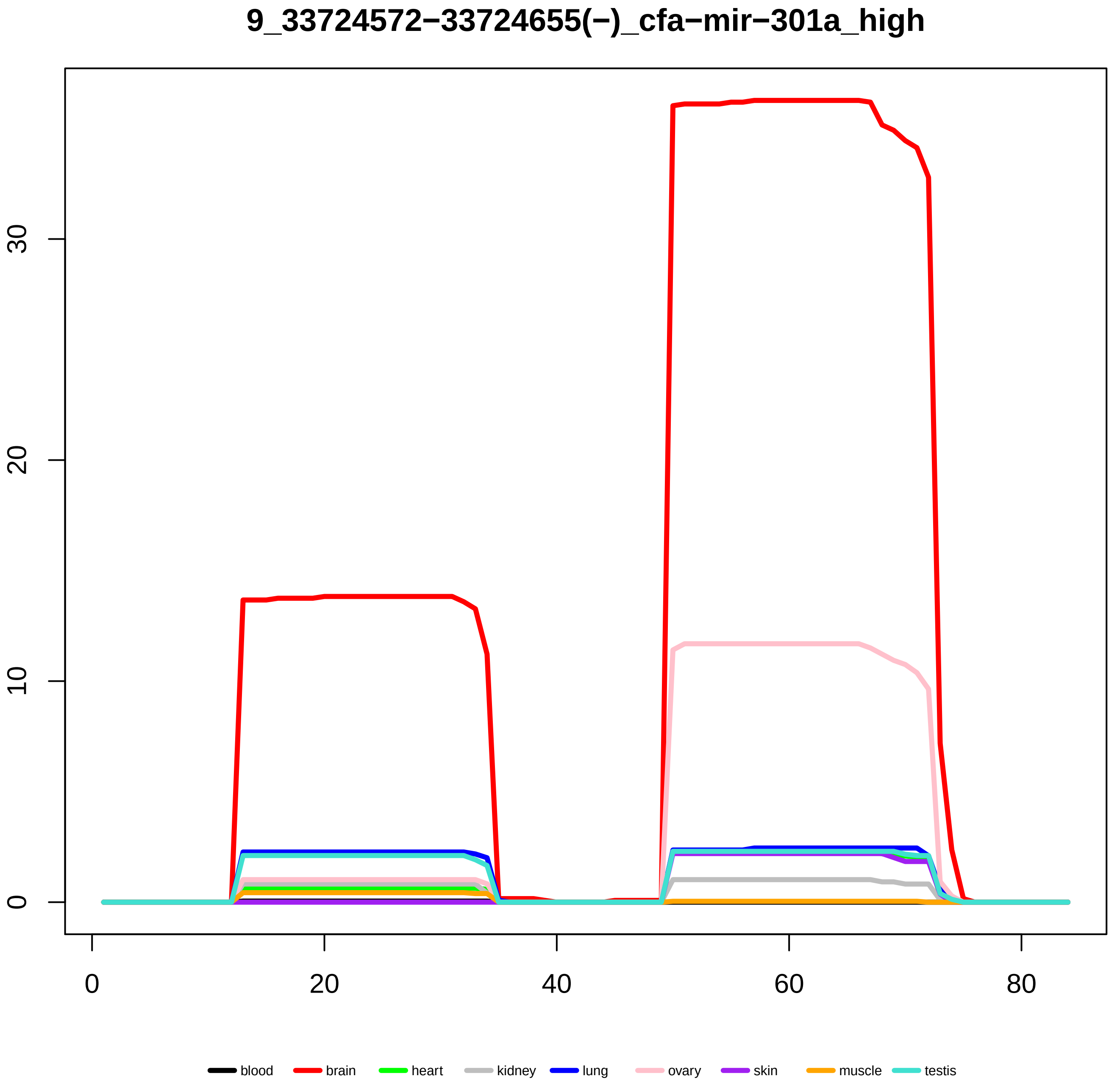

9_33724572−33724655(−)_cfa−mir−301a_high
0
10
20
30
0
20
40
60
80
blood
brain
heart
kidney
lung
ovary
skin
muscle
testis

9_34340550−34340609(+)_cfa−mir−21_high

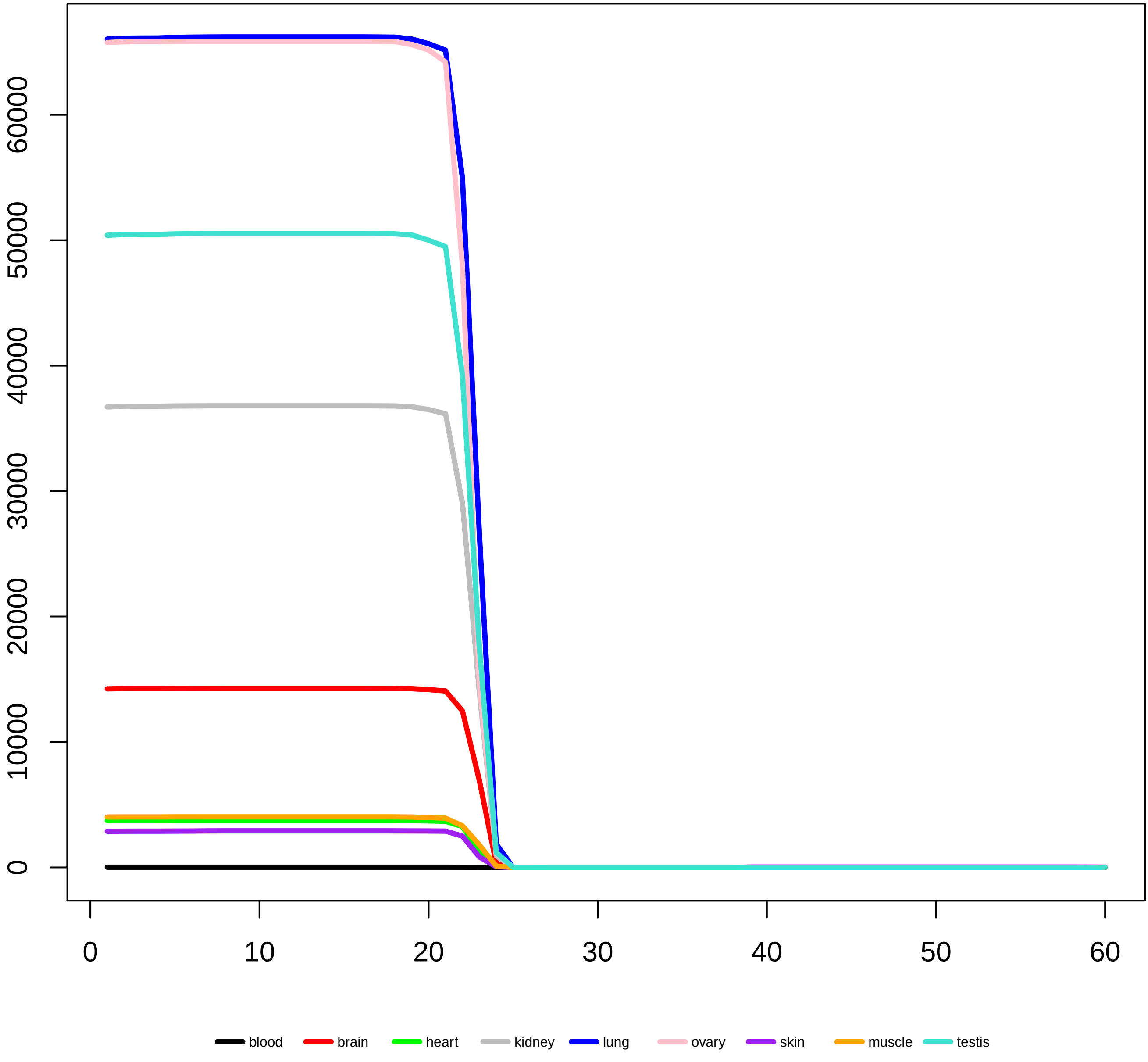

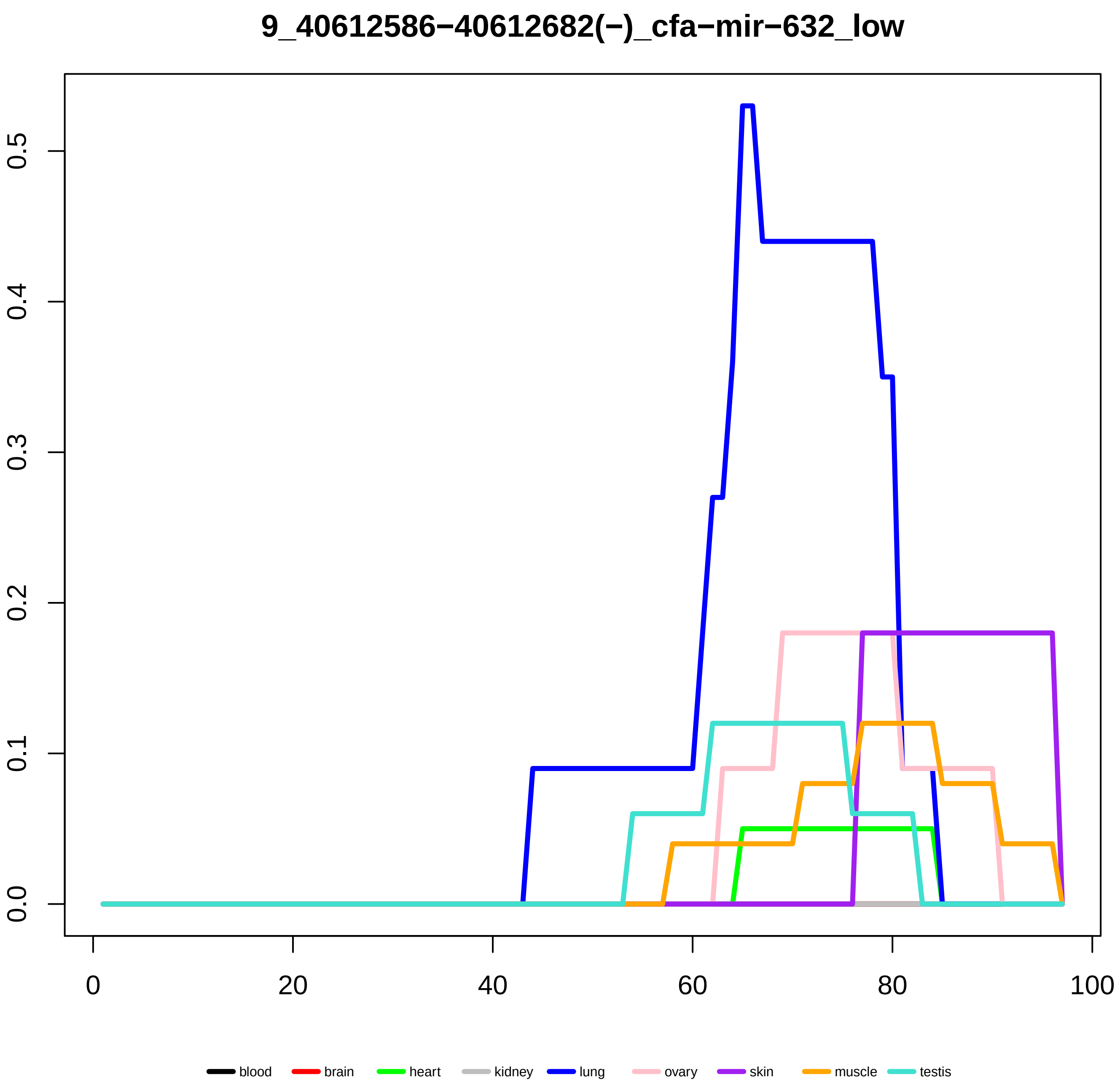

9_40612586−40612682(−)_cfa−mir−632_low
0.0
0.1
0.2
0.3
0.4
0.5
0
20
40
60
80
100
blood
brain
heart
kidney
lung
ovary
skin
muscle
testis

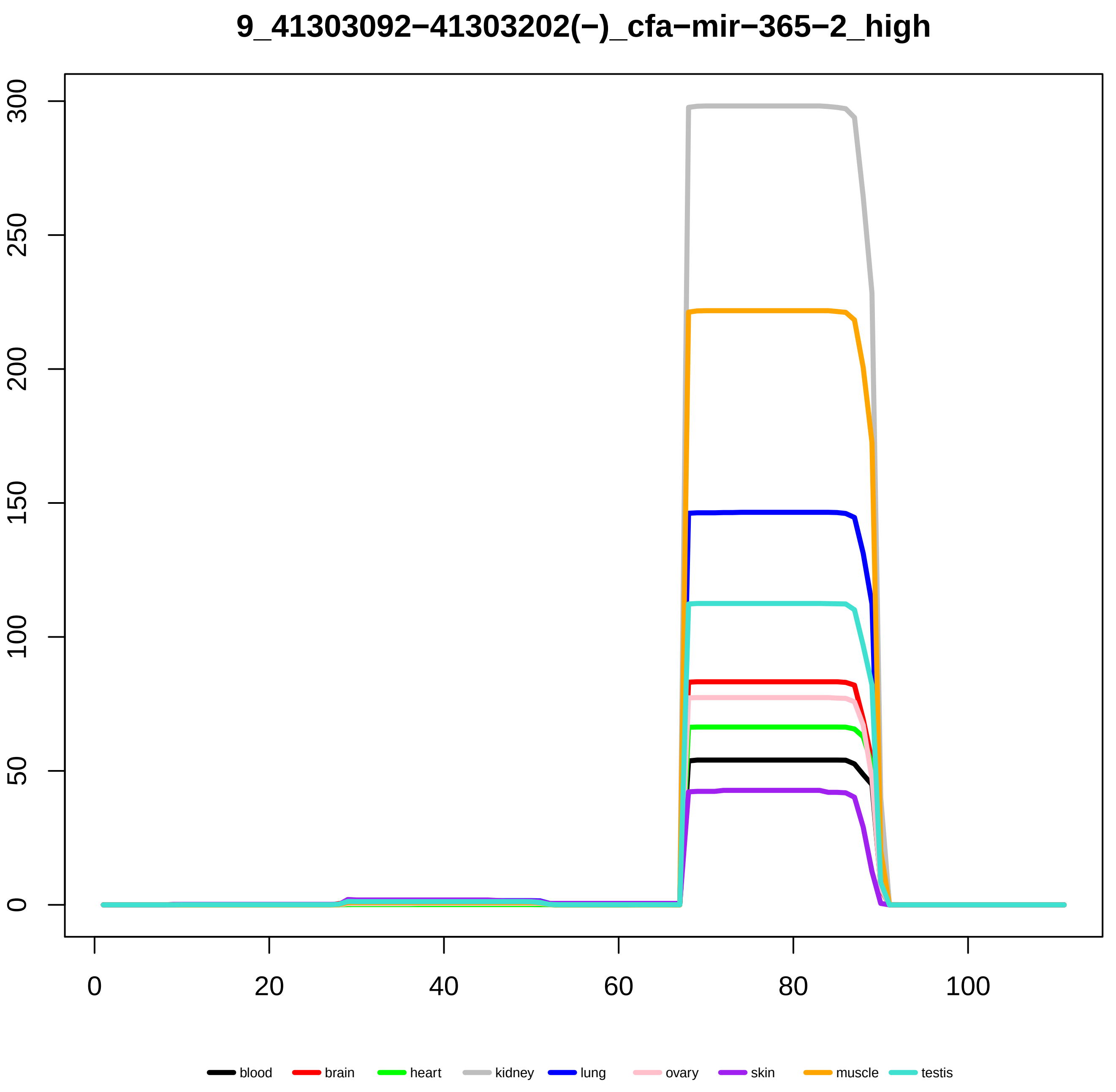

9_41303092−41303202(−)_cfa−mir−365−2_high
300
250
200
150
100
50
0
0
20
40
60
80
100
blood
brain
heart
kidney
lung
ovary
skin
muscle
testis

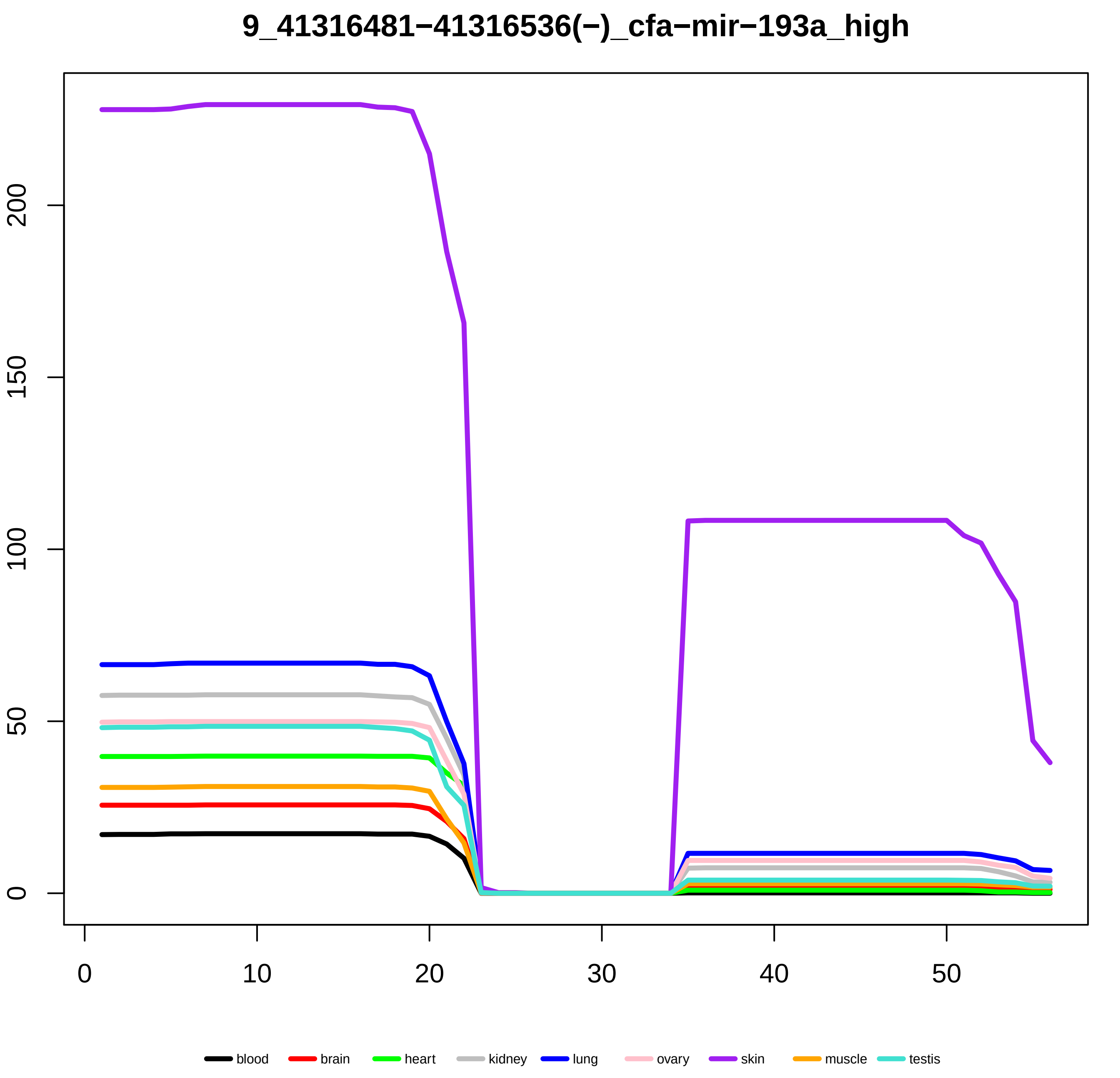

9_41316481−41316536(−)_cfa−mir−193a_high
0
50
100
150
200
0
10
20
30
40
50
blood
brain
heart
kidney
lung
ovary
skin
muscle
testis

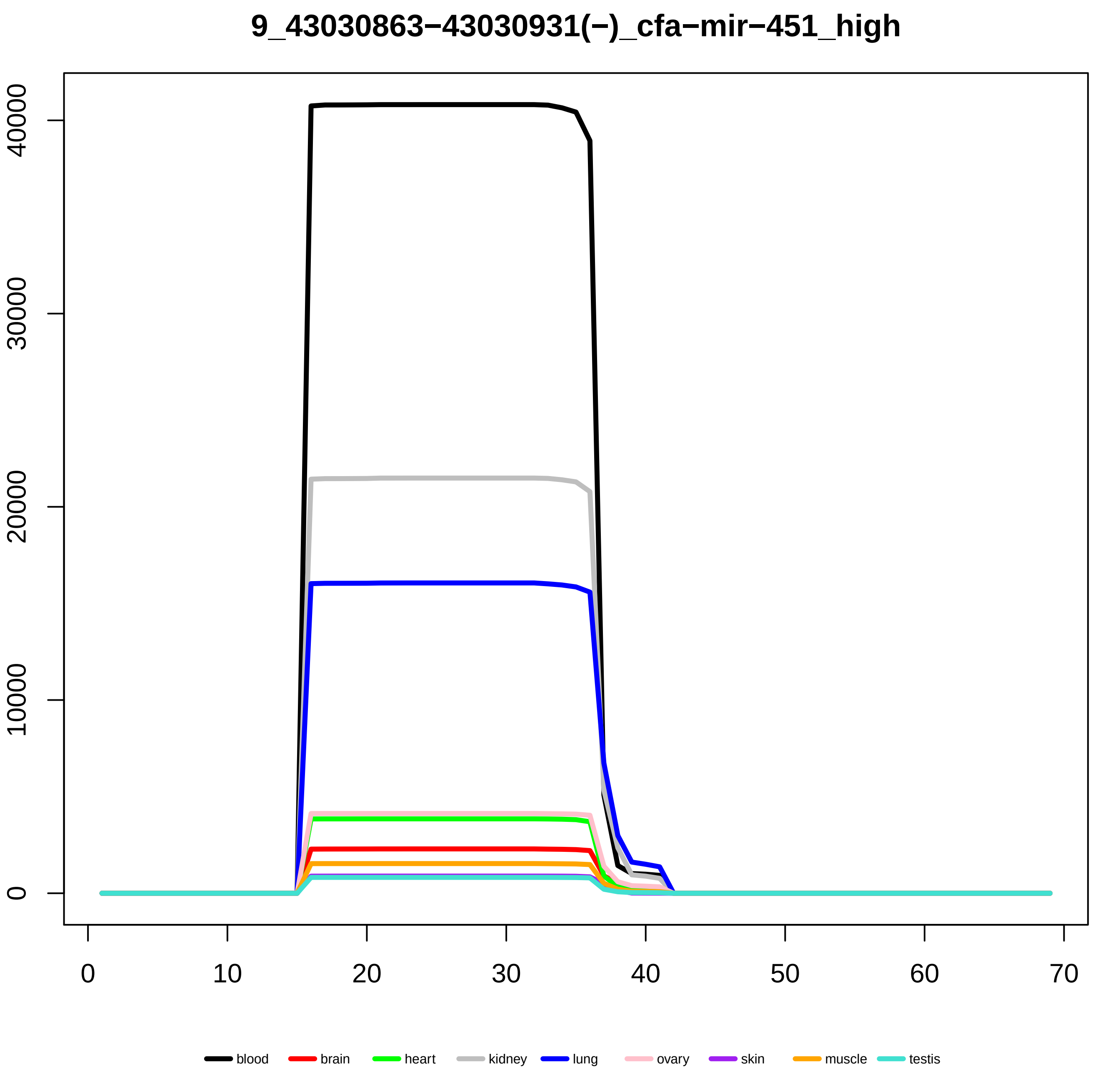

9_43030863−43030931(−)_cfa−mir−451_high
0
10000
20000
30000
40000
0
10
20
30
40
50
60
70
blood
brain
heart
kidney
lung
ovary
skin
muscle
testis

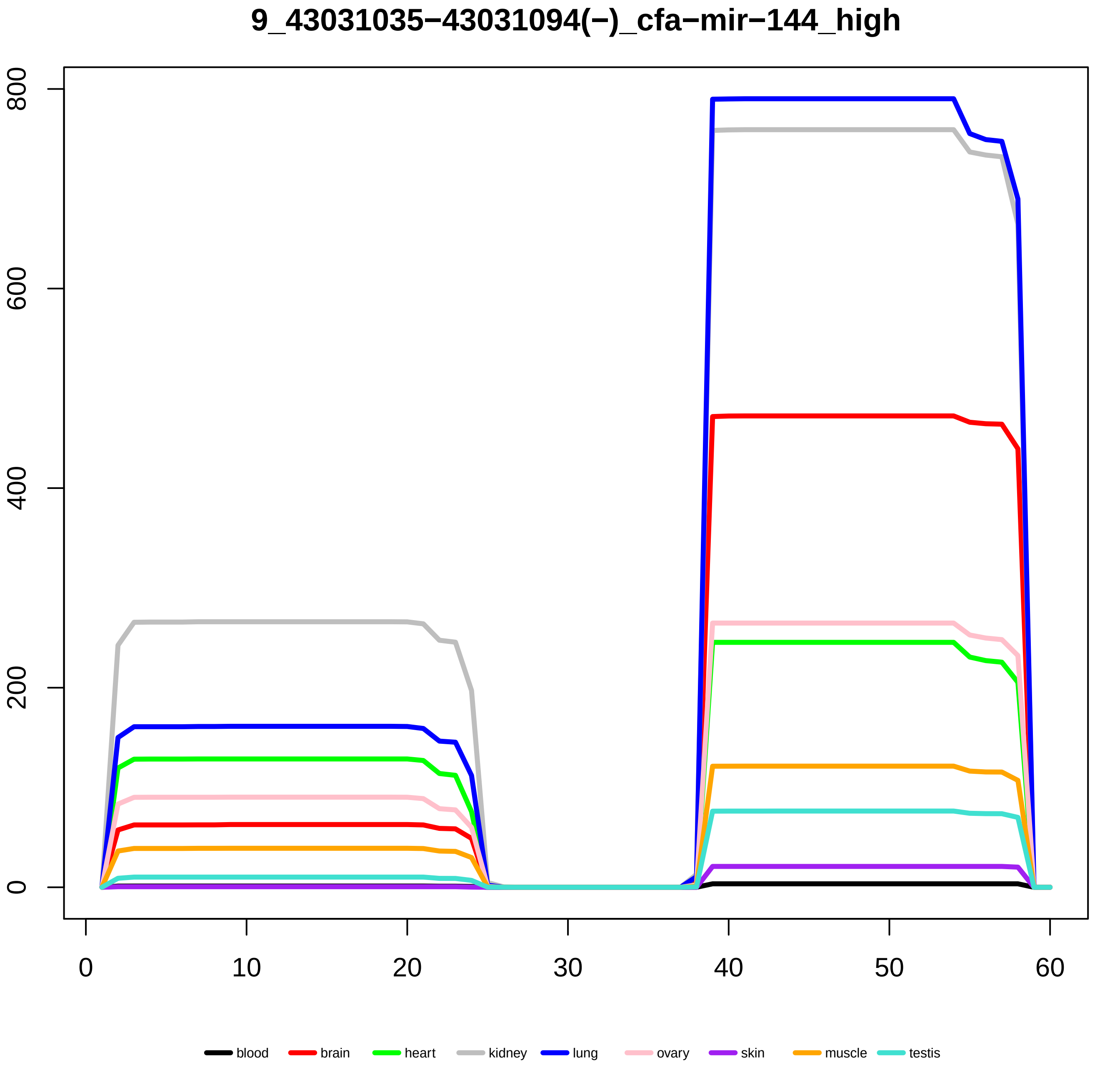
9_43031035−43031094(−)_cfa−mir−144_high
0
200
400
600
800
0
10
20
30
40
50
60
blood
brain
heart
kidney
lung
ovary
skin
muscle
testis

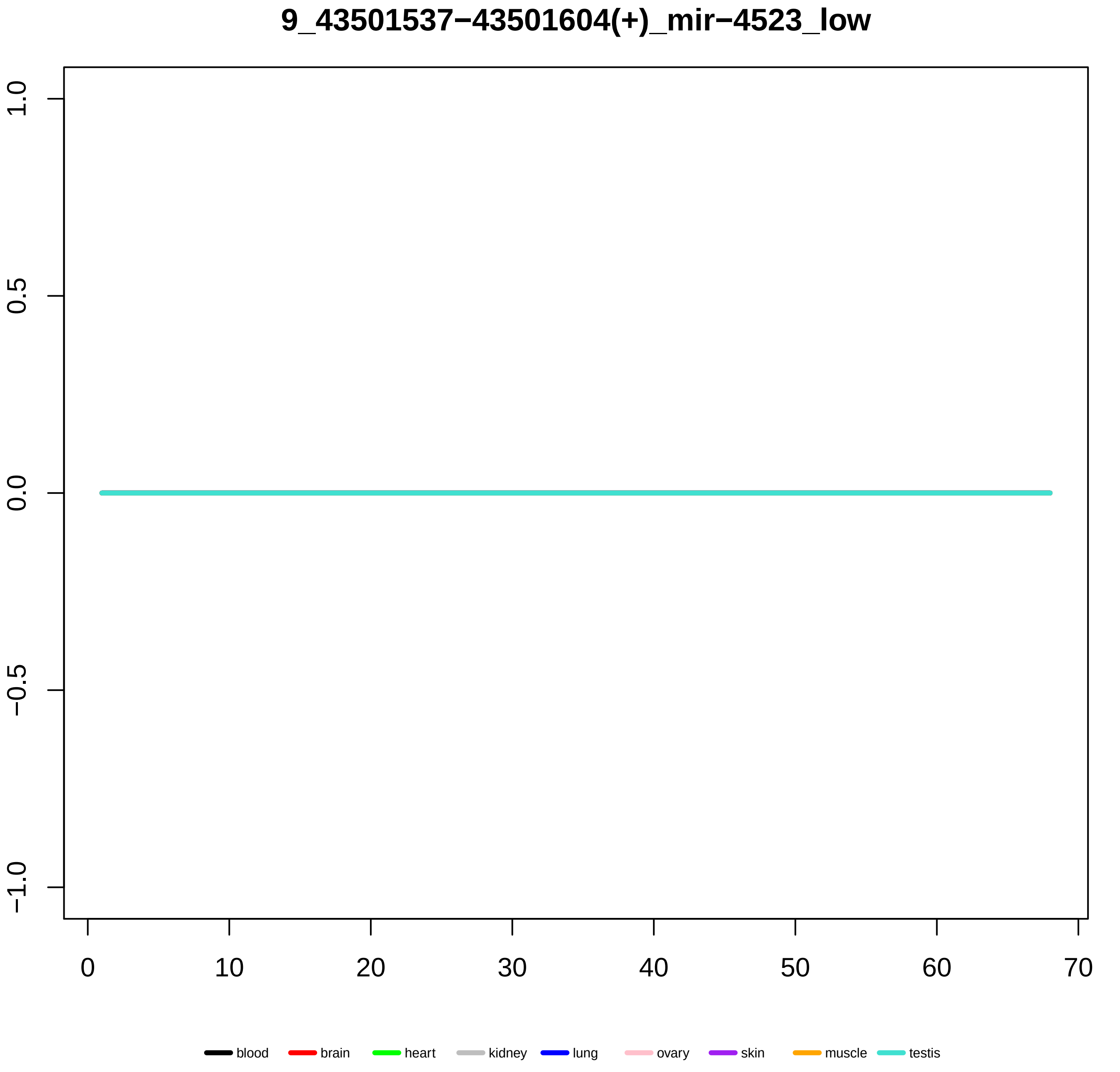

9_43501537−43501604(+)_mir−4523_low
1.0
0.5
0.0
−0.5
−1.0
0
10
20
30
40
50
60
70
blood
brain
heart
kidney
lung
ovary
skin
muscle
testis

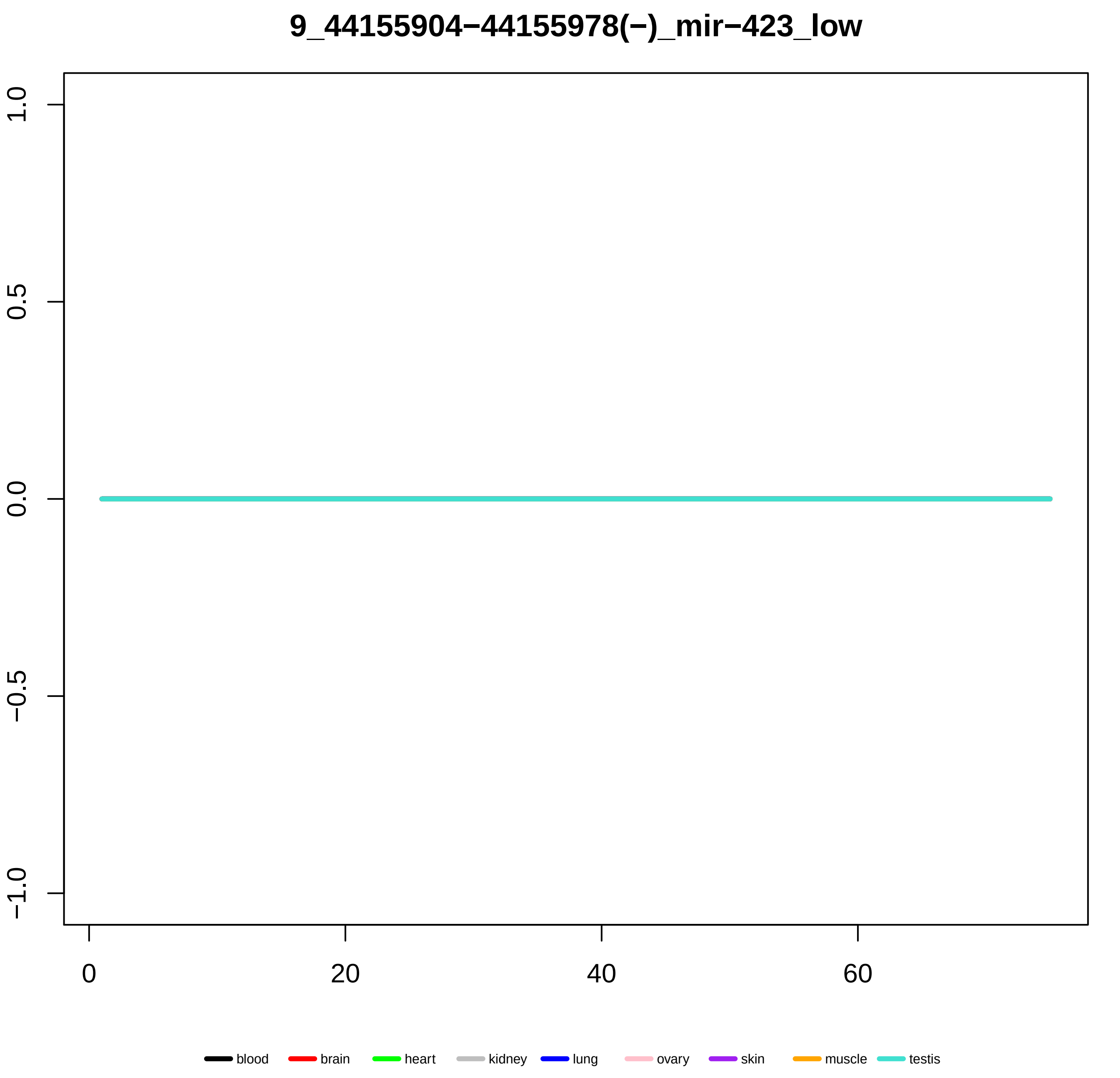
9_44155904−44155978(−)_mir−423_low
1.0
0.5
0.0
−0.5
−1.0
0
20
40
60
blood
brain
heart
kidney
lung
ovary
skin
muscle
testis

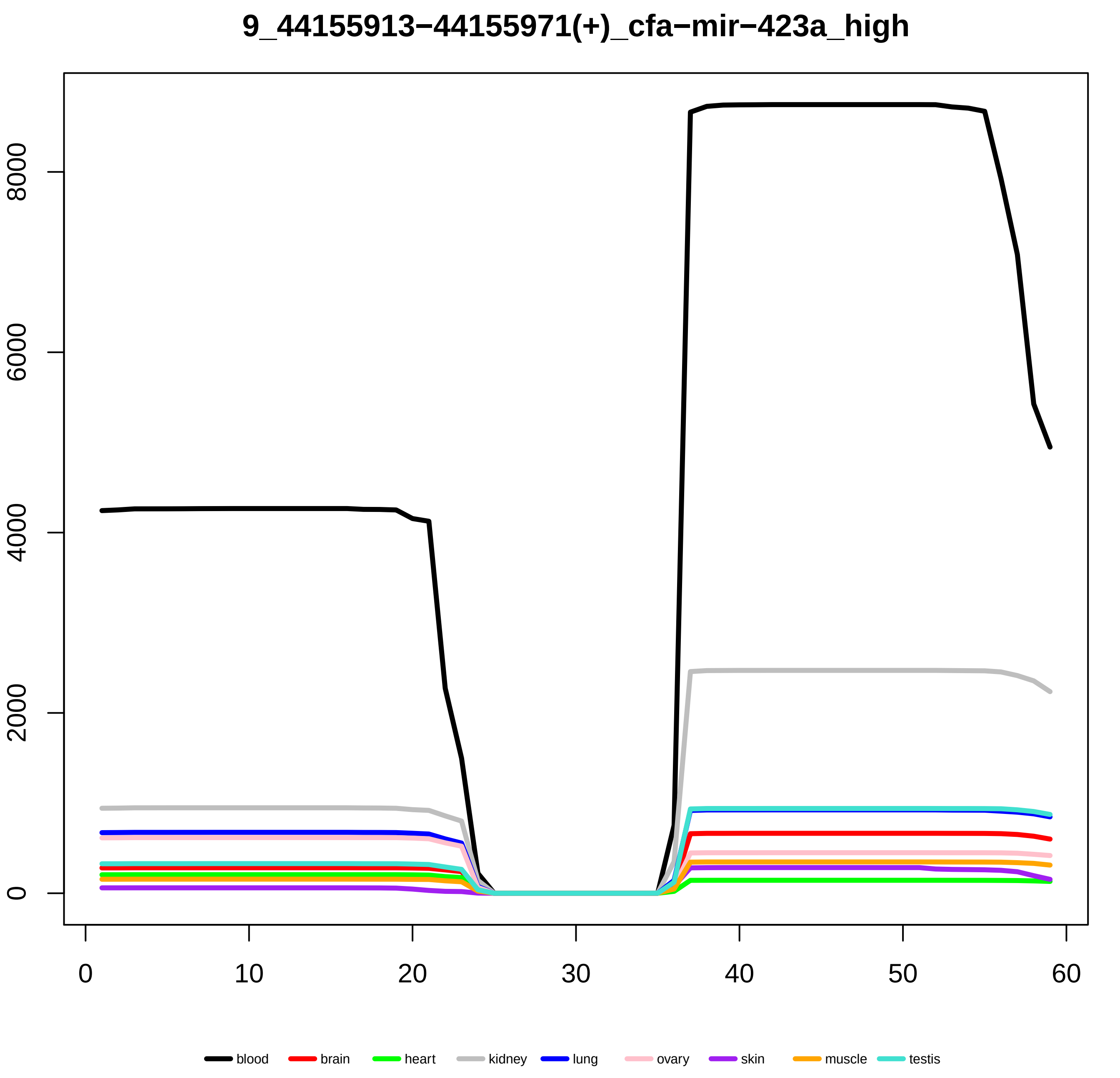

9_44155913−44155971(+)_cfa−mir−423a_high
0
2000
4000
6000
8000
0
10
20
30
40
50
60
blood
brain
heart
kidney
lung
ovary
skin
muscle
testis

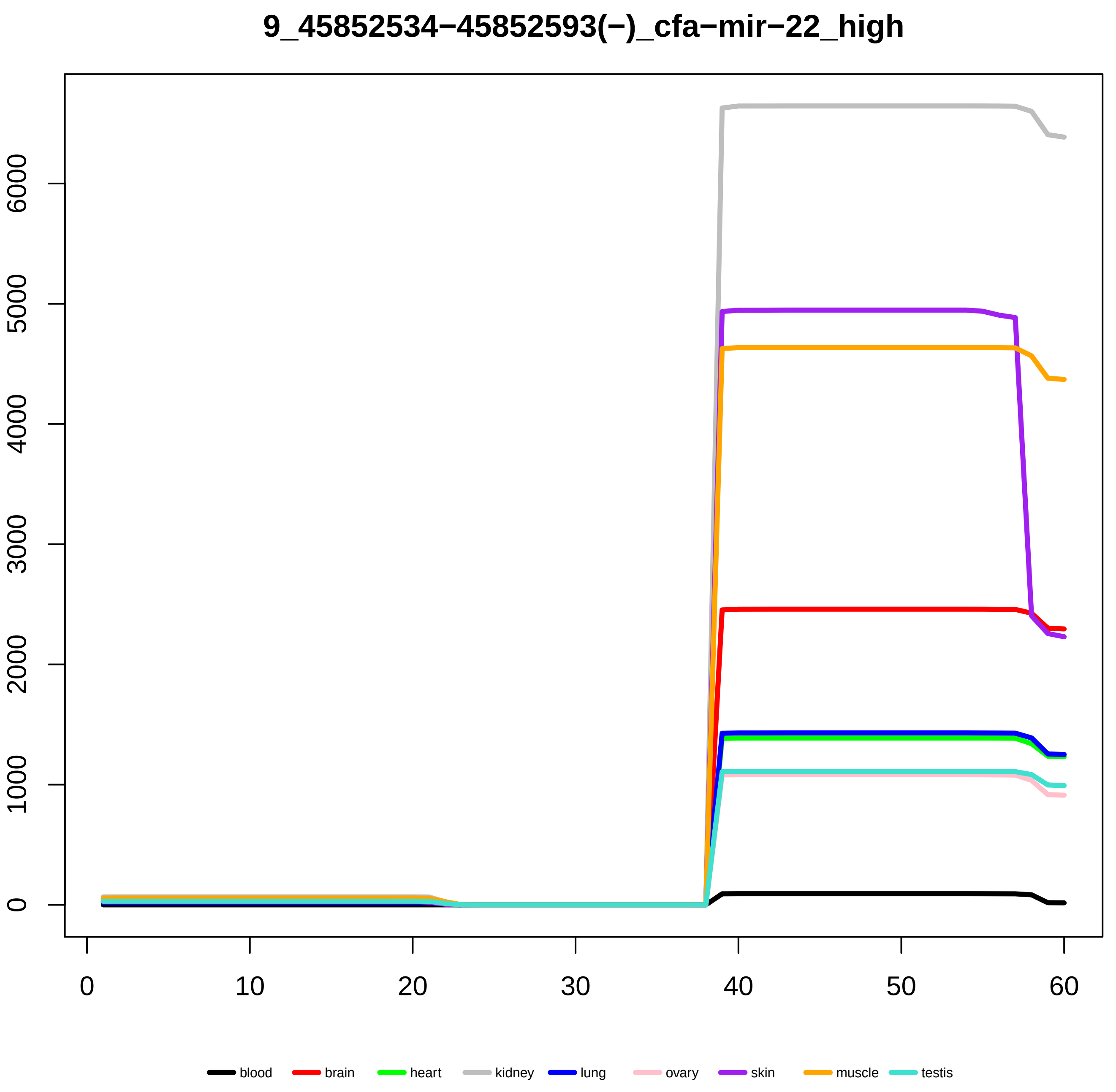

9_45852534−45852593(−)_cfa−mir−22_high
0
1000
2000
3000
4000
5000
6000
0
10
20
30
40
50
60
blood
brain
heart
kidney
lung
ovary
skin
muscle
testis

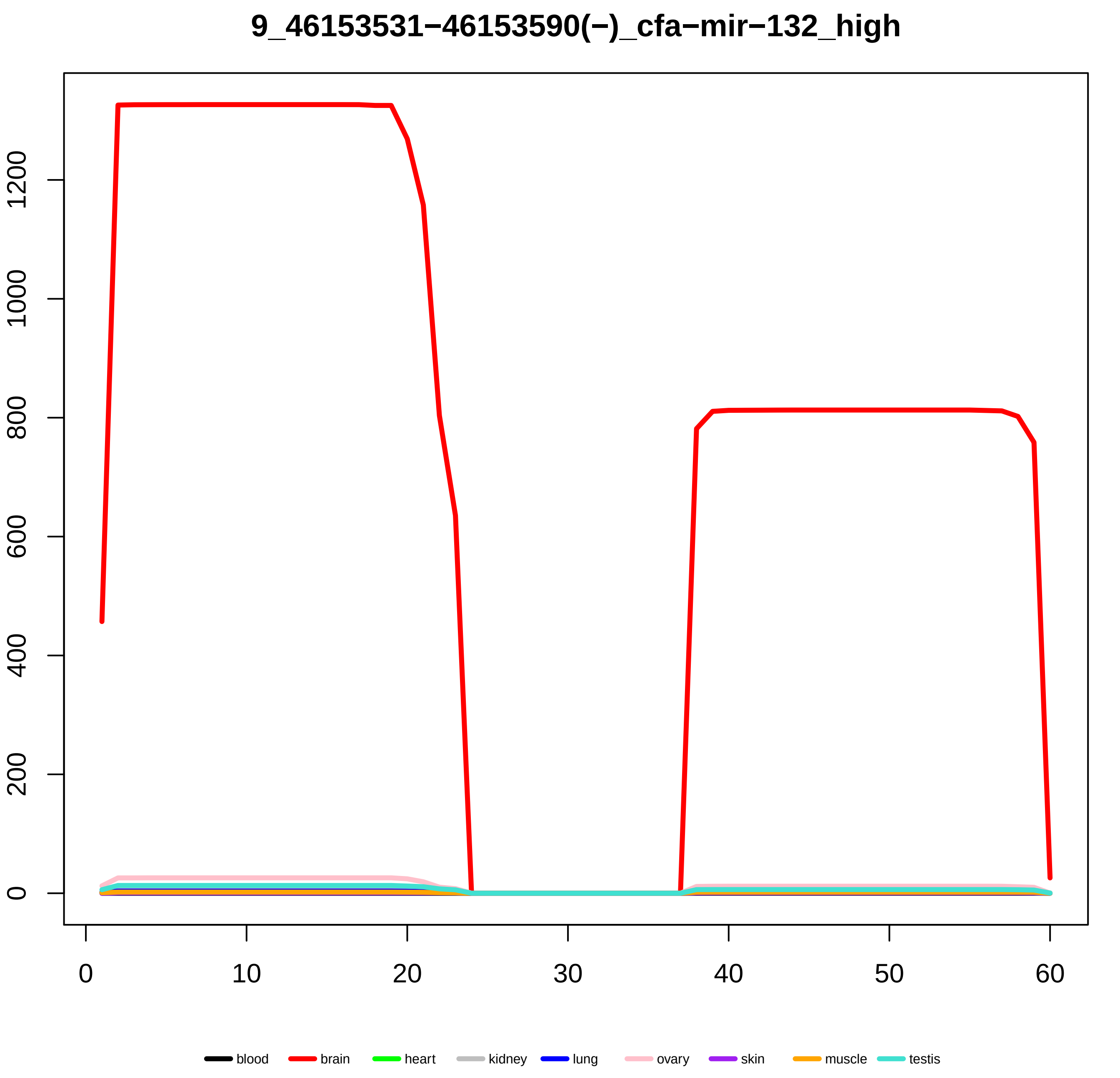

9_46153531−46153590(−)_cfa−mir−132_high
0
200
400
600
800
1000
1200
0
10
20
30
40
50
60
blood
brain
heart
kidney
lung
ovary
skin
muscle
testis

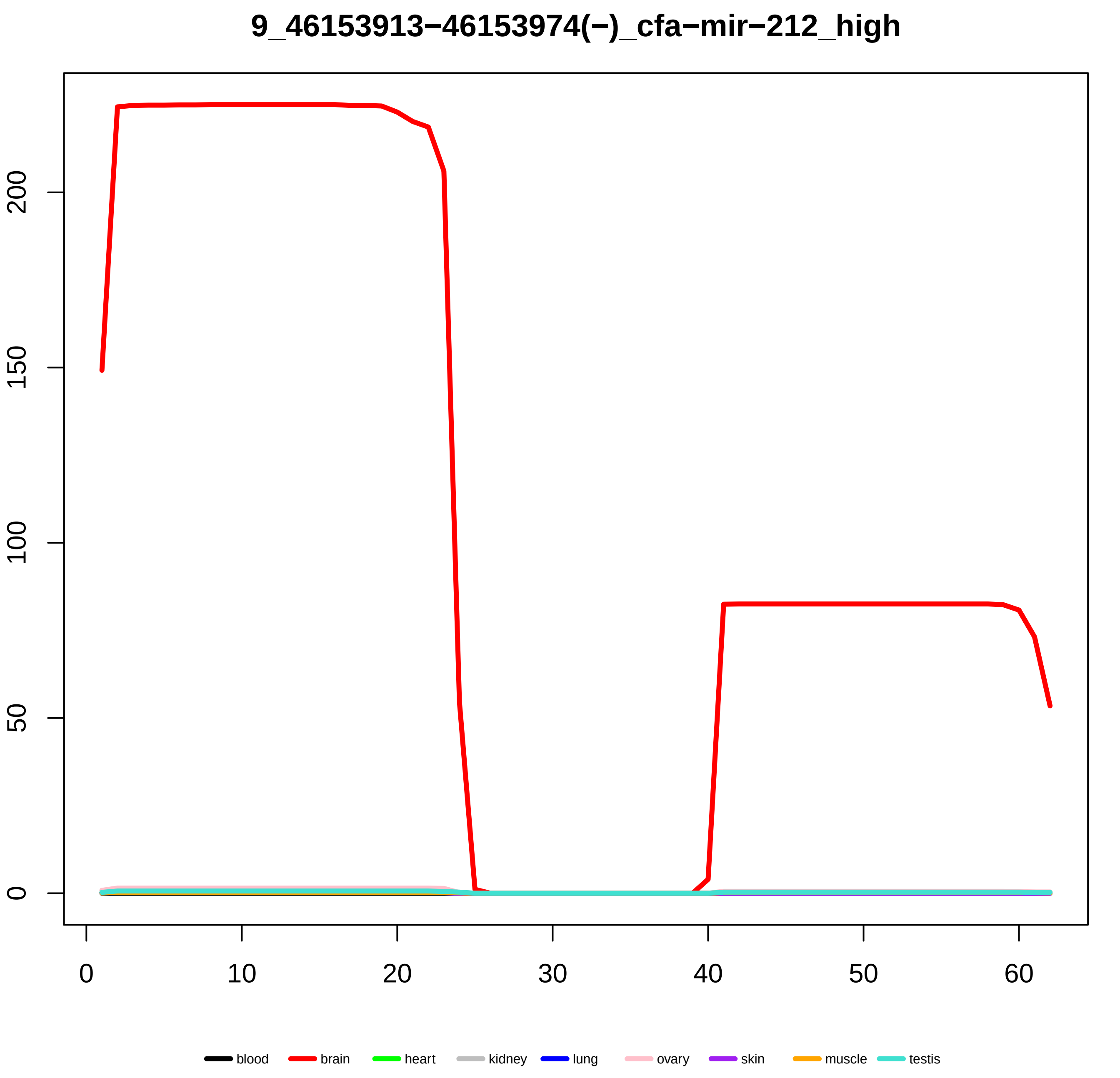

9_46153913−46153974(−)_cfa−mir−212_high
0
50
100
150
200
0
10
20
30
40
50
60
blood
brain
heart
kidney
lung
ovary
skin
muscle
testis

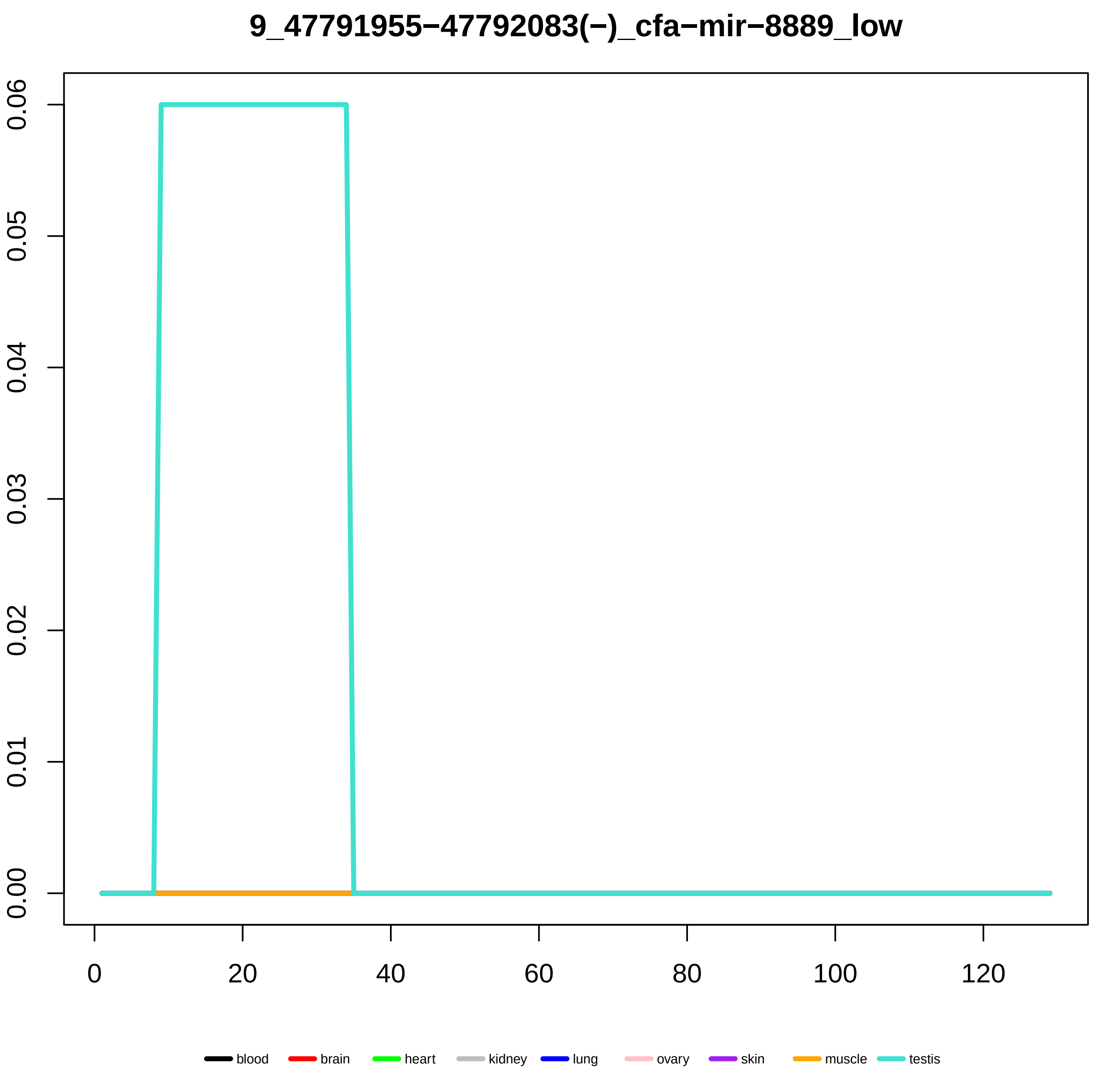

9_47791955−47792083(−)_cfa−mir−8889_low
0.00
0.01
0.02
0.03
0.04
0.05
0.06
0
20
40
60
80
100
120
blood
brain
heart
kidney
lung
ovary
skin
muscle
testis

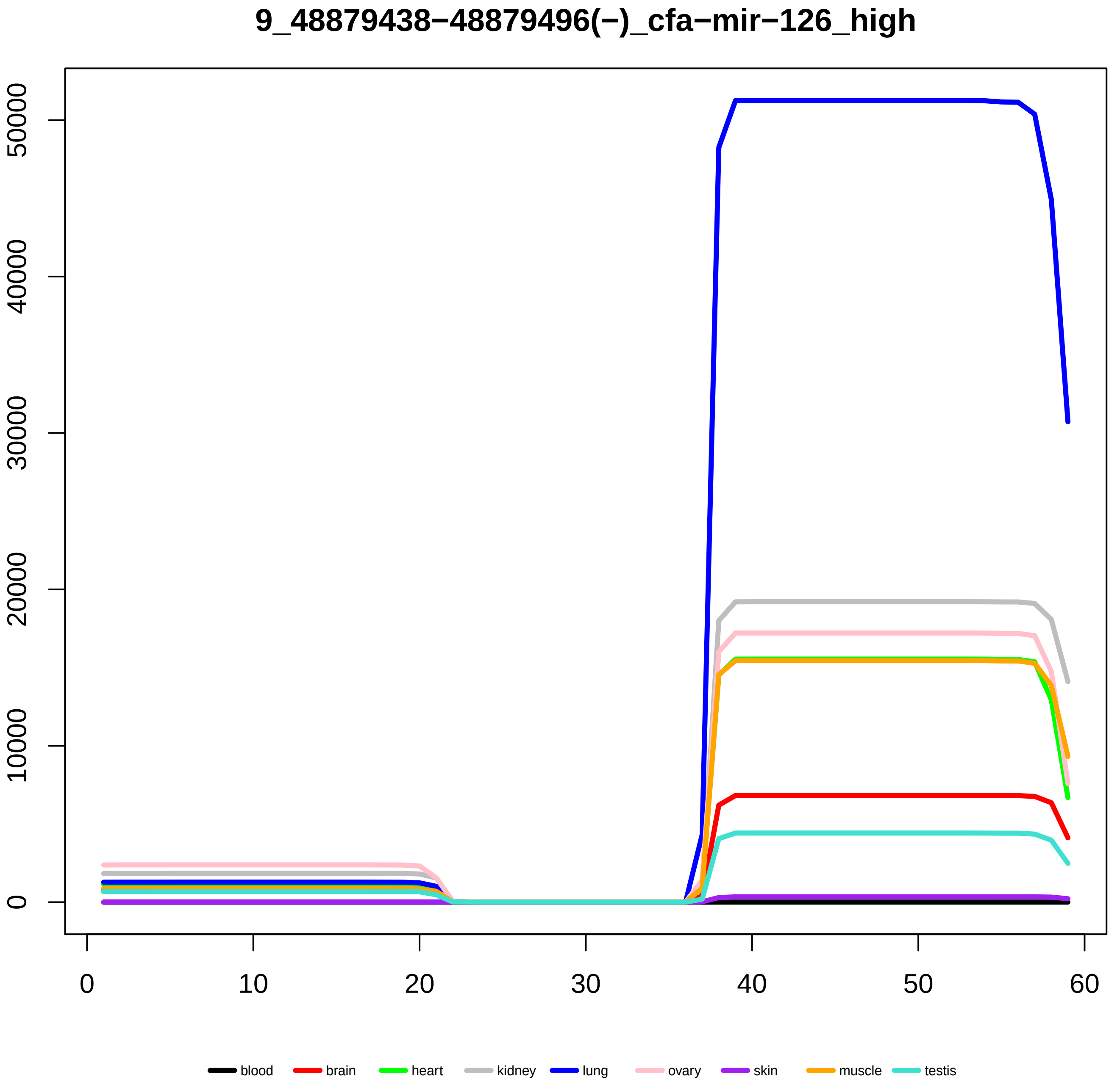
9_48879438−48879496(−)_cfa−mir−126_high
0
10000
20000
30000
40000
50000
0
10
20
30
40
50
60
blood
brain
heart
kidney
lung
ovary
skin
muscle
testis

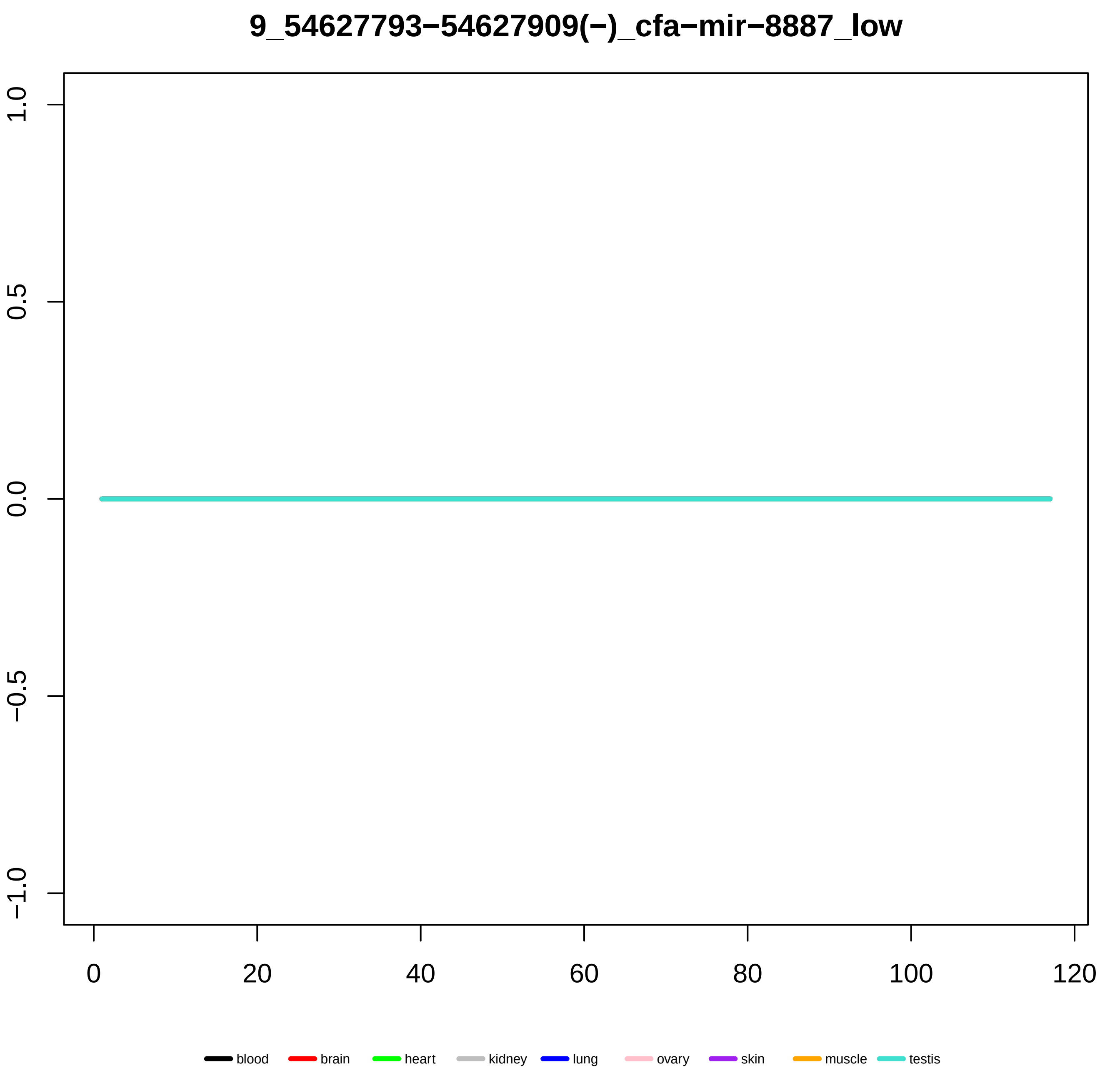
9_54627793−54627909(−)_cfa−mir−8887_low
1.0
0.5
0.0
−0.5
−1.0
0
20
40
60
80
100
120
blood
brain
heart
kidney
lung
ovary
skin
muscle
testis

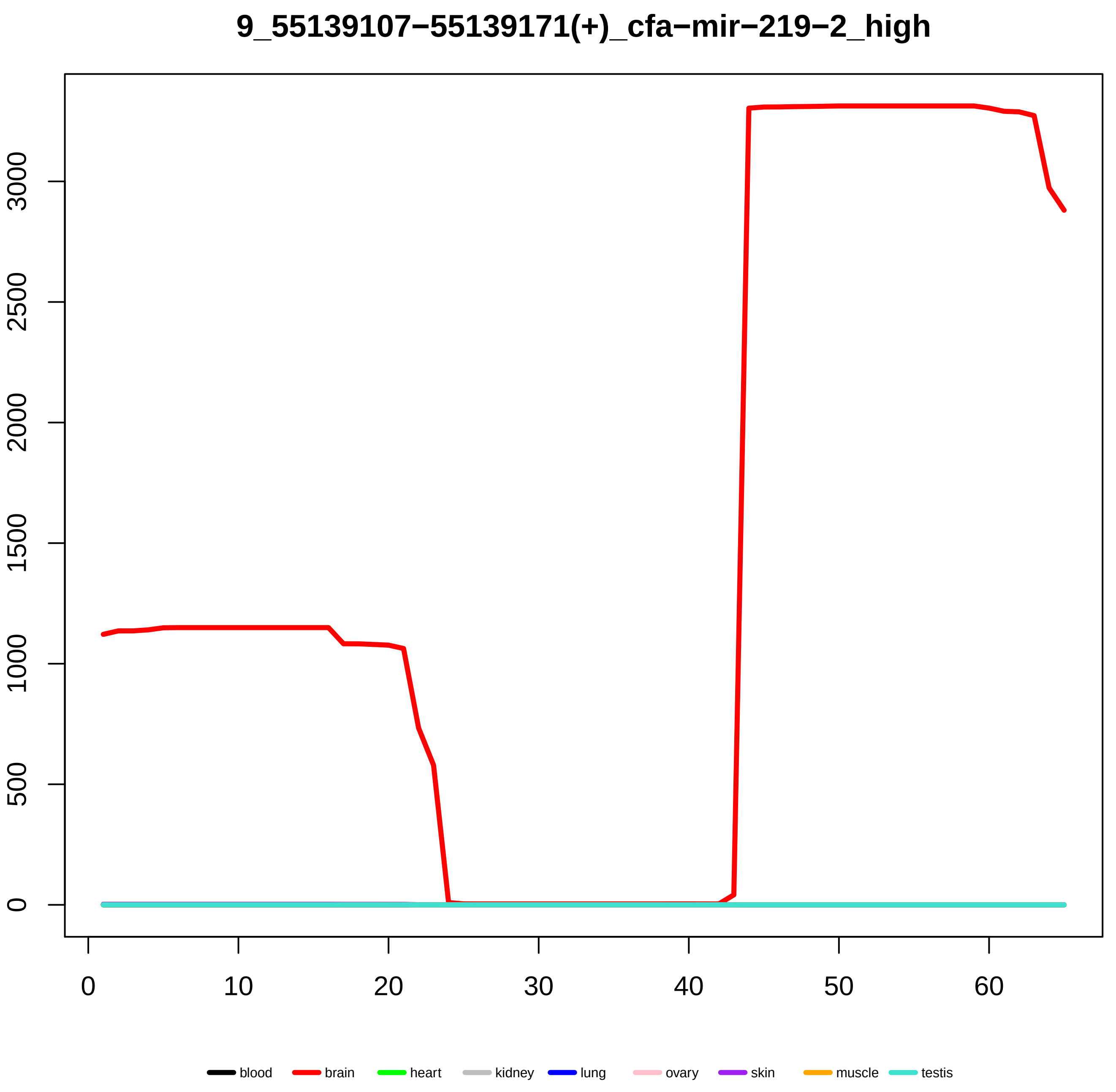

9_55139107−55139171(+)_cfa−mir−219−2_high
0
500
1000
1500
2000
2500
3000
0
10
20
30
40
50
60
blood
brain
heart
kidney
lung
ovary
skin
muscle
testis

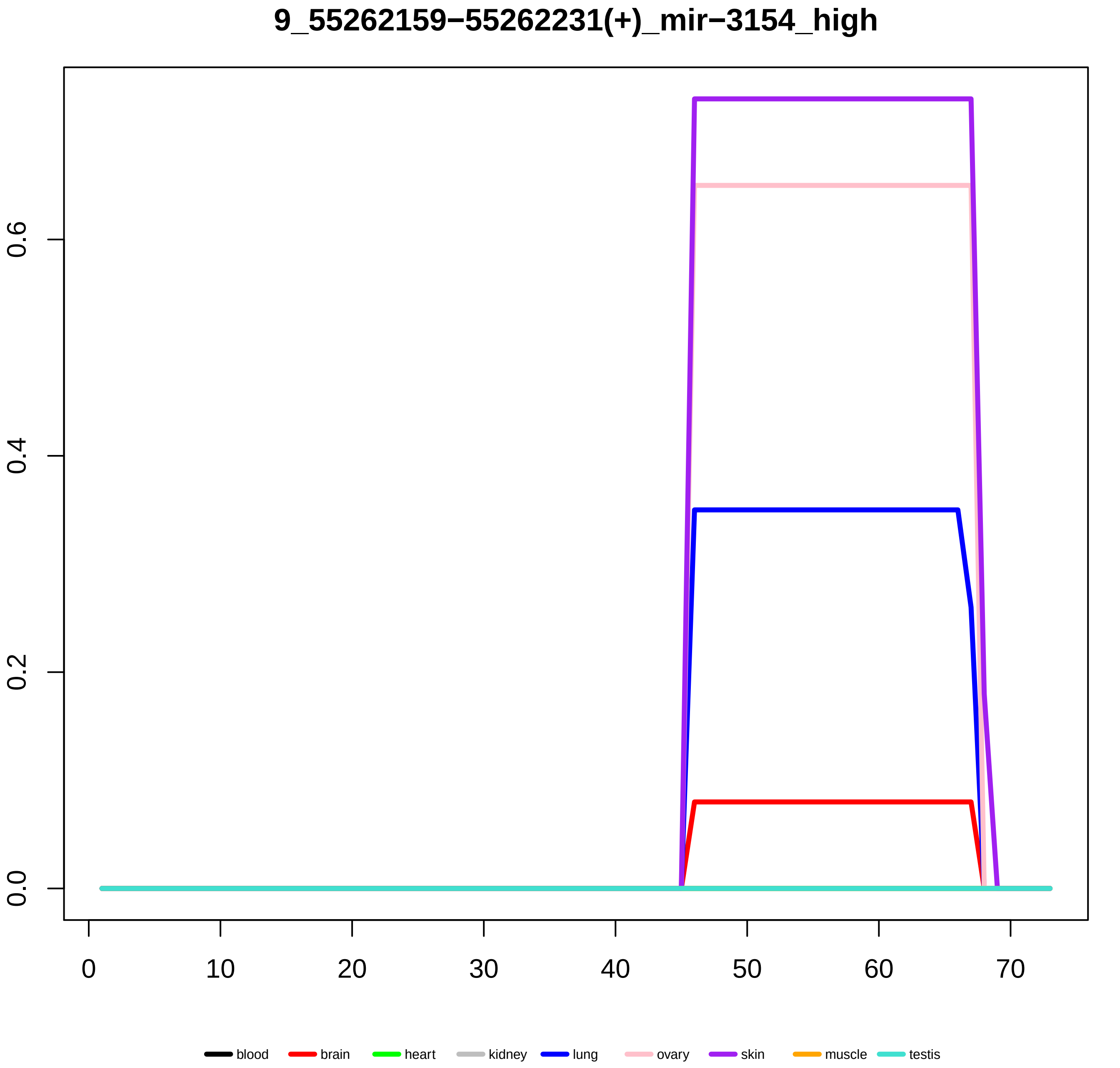

9_55262159−55262231(+)_mir−3154_high
0.0
0.2
0.4
0.6
0
10
20
30
40
50
60
70
blood
brain
heart
kidney
lung
ovary
skin
muscle
testis

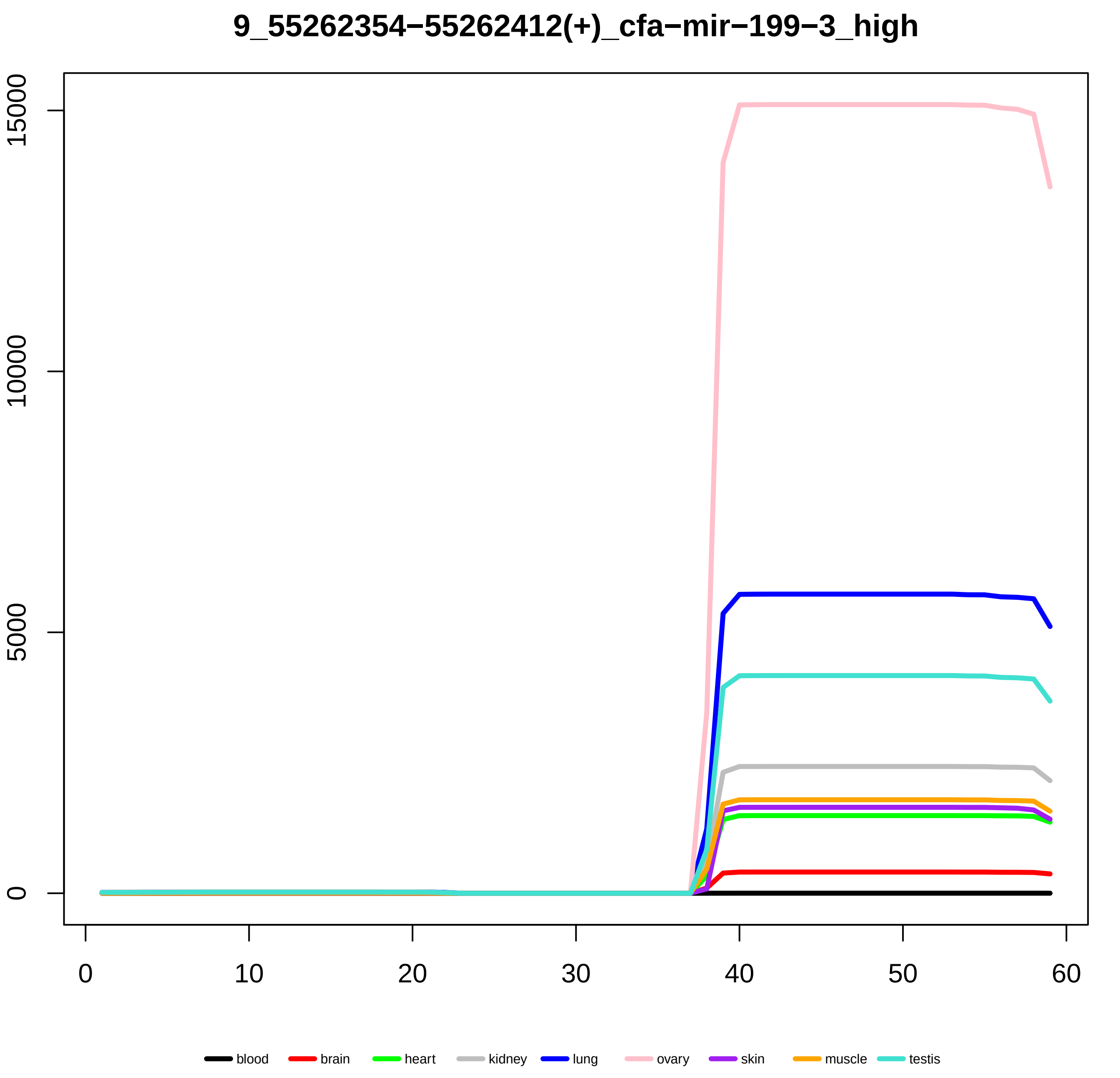
9_55262354−55262412(+)_cfa−mir−199−3_high
0
5000
10000
15000
0
10
20
30
40
50
60
blood
brain
heart
kidney
lung
ovary
skin
muscle
testis

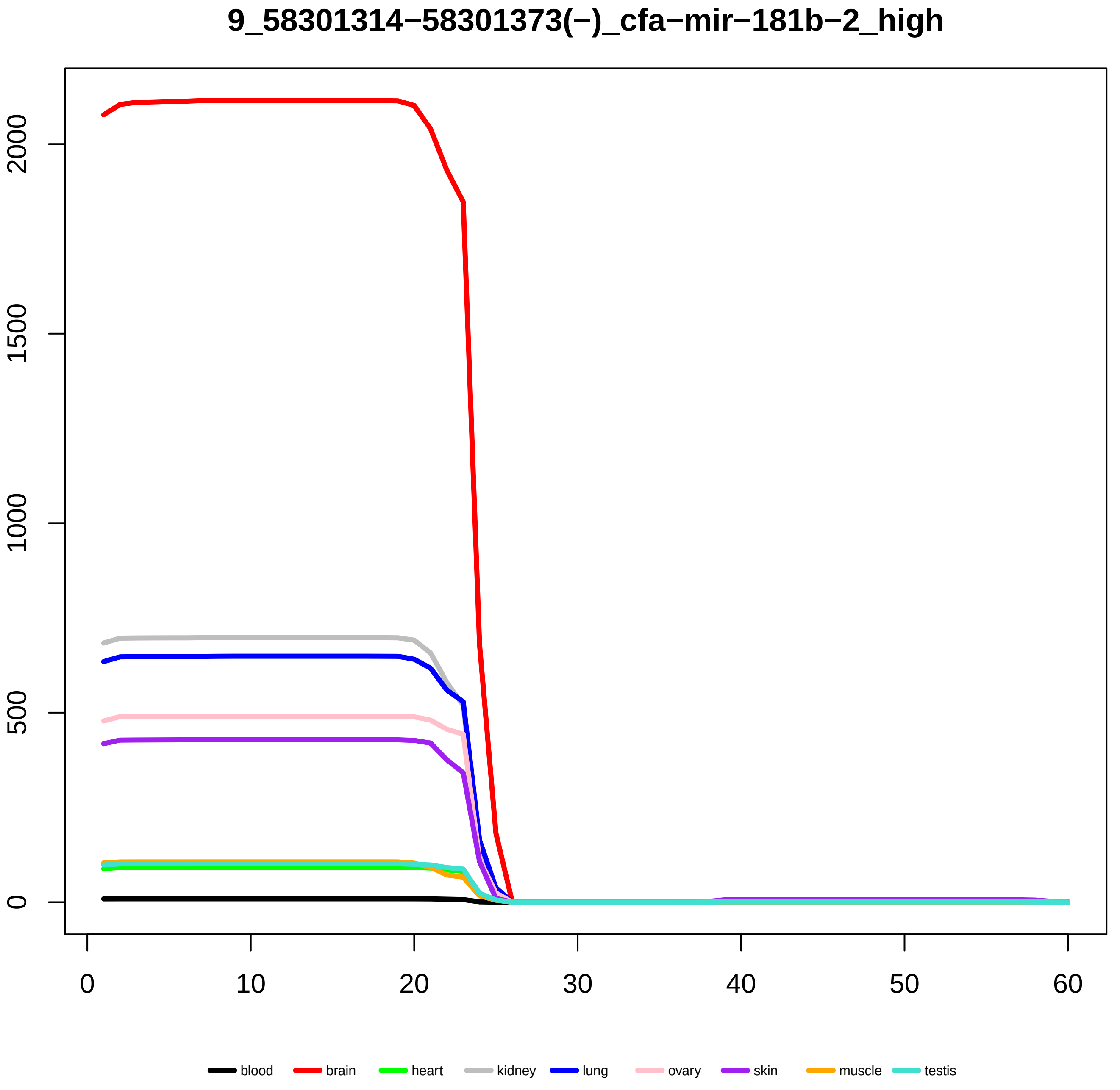

9_58301314−58301373(−)_cfa−mir−181b−2_high
0
500
1000
1500
2000
0
10
20
30
40
50
60
blood
brain
heart
kidney
lung
ovary
skin
muscle
testis

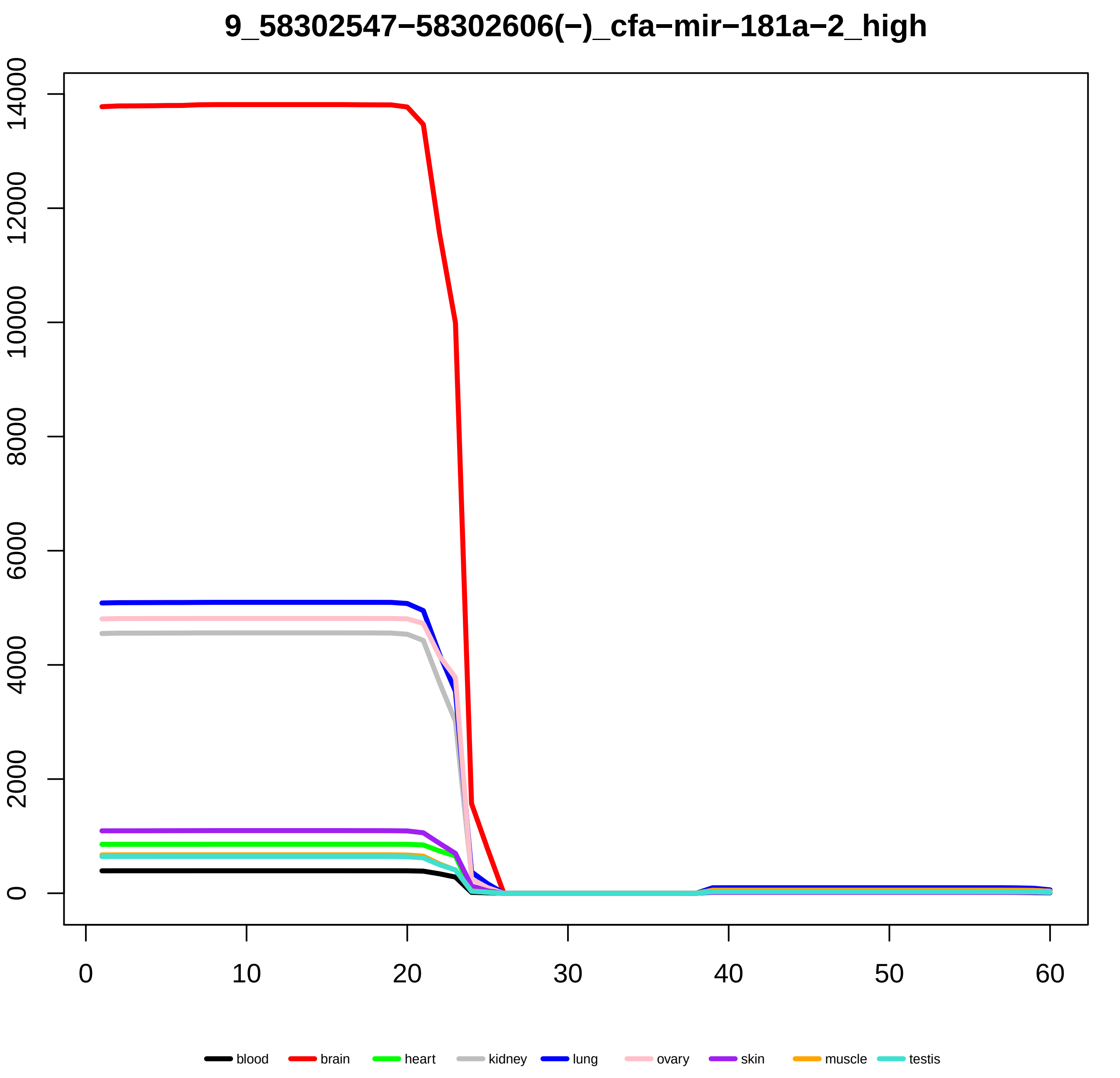
9_58302547−58302606(−)_cfa−mir−181a−2_high
0
2000
4000
6000
8000
10000
12000
14000
0
10
20
30
40
50
60
blood
brain
heart
kidney
lung
ovary
skin
muscle
testis

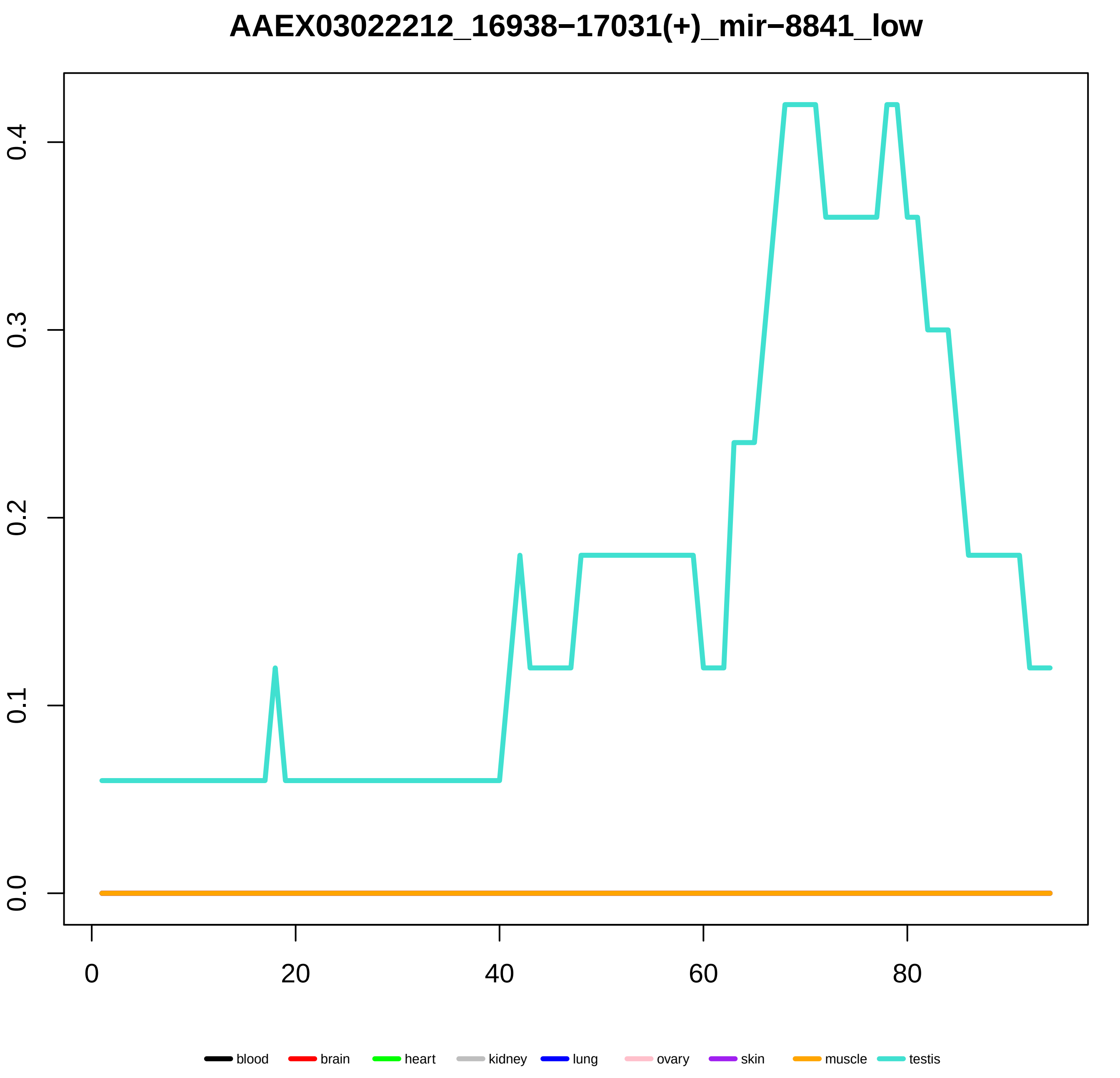

AAEX03022212_16938−17031(+)_mir−8841_low
0.0
0.1
0.2
0.3
0.4
0
20
40
60
80
blood
brain
heart
kidney
lung
ovary
skin
muscle
testis

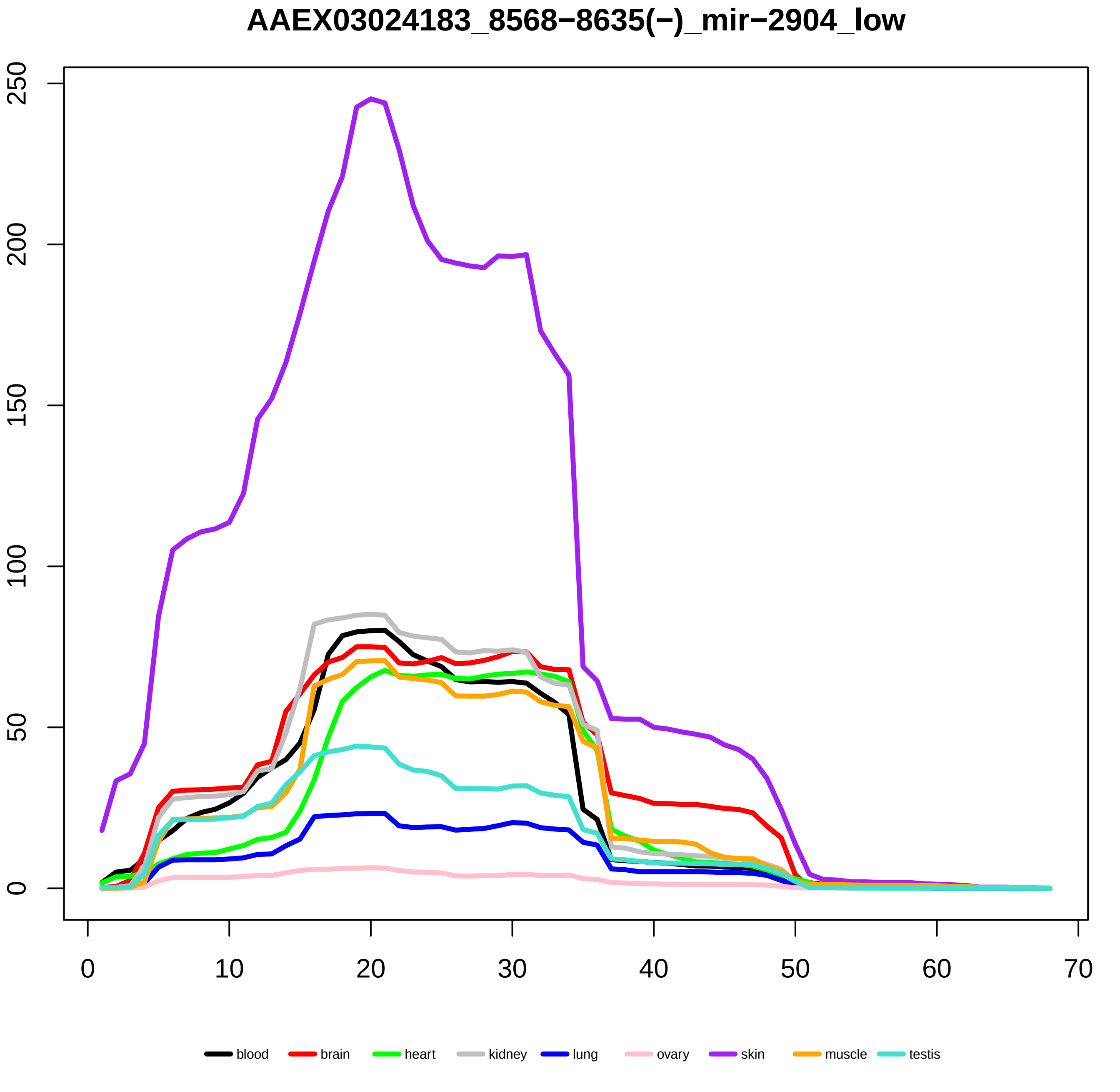

AAEX03024183_8568−8635(−)_mir−2904_low
0
50
100
150
200
250
0
10
20
30
40
50
60
70
blood
brain
heart
kidney
lung
ovary
skin
muscle
testis

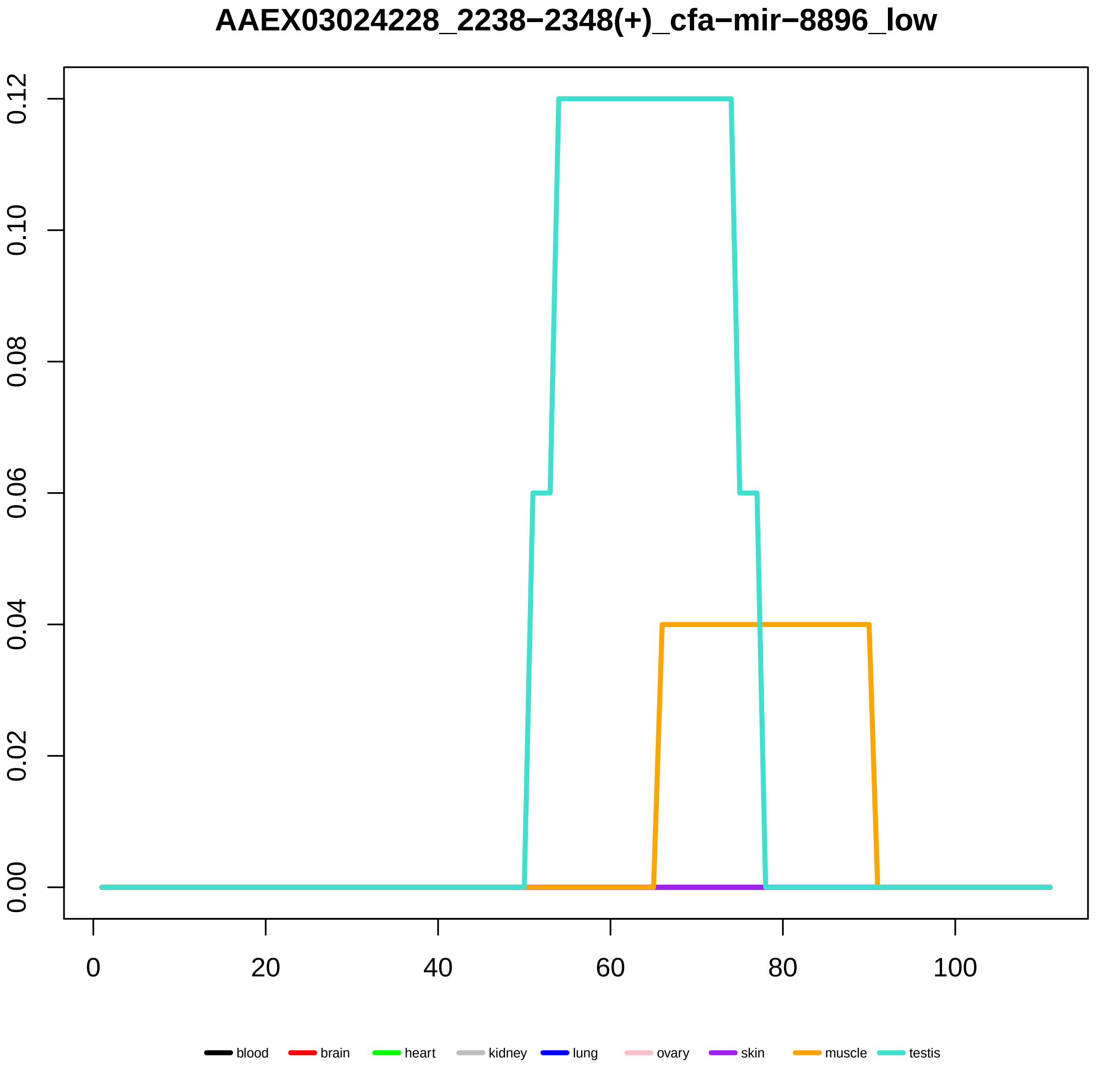

AAEX03024228_2238−2348(+)_cfa−mir−8896_low
0.00
0.02
0.04
0.06
0.08
0.10
0.12
0
20
40
60
80
100
blood
brain
heart
kidney
lung
ovary
skin
muscle
testis

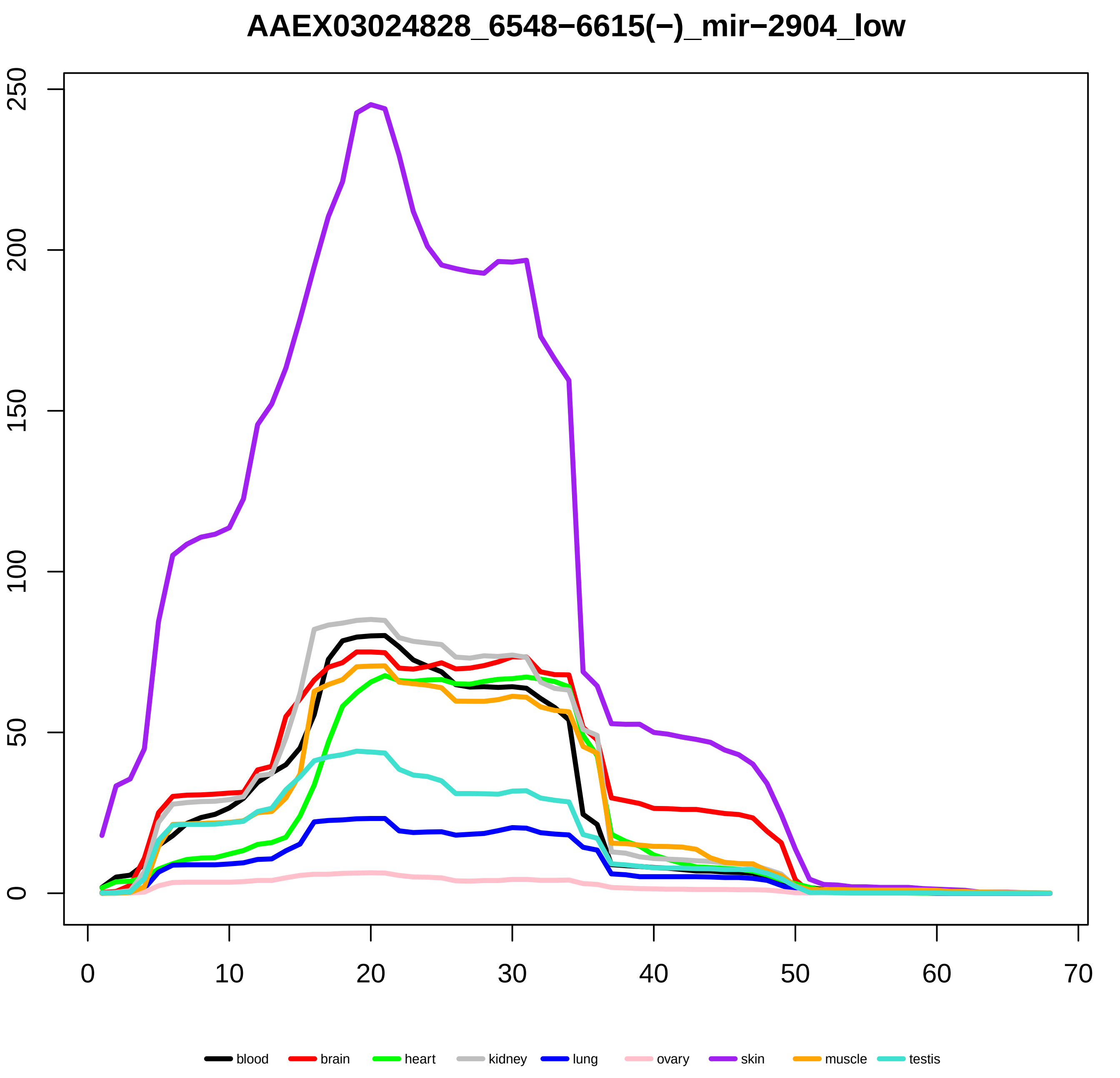
AAEX03024828_6548−6615(−)_mir−2904_low
0
50
100
150
200
250
0
10
20
30
40
50
60
70
blood
brain
heart
kidney
lung
ovary
skin
muscle
testis

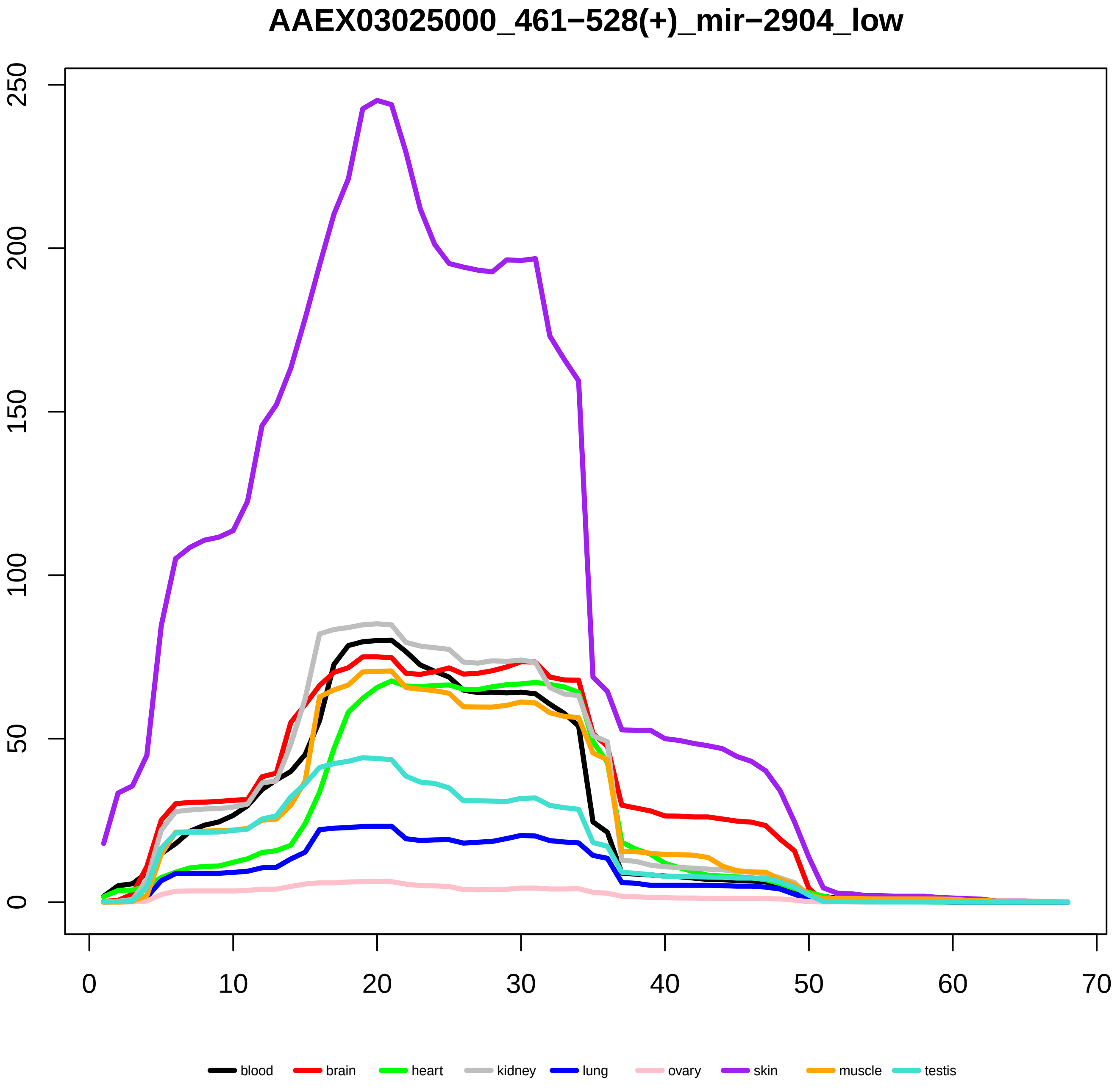

AAEX03025000_461−528(+)_mir−2904_low
0
50
100
150
200
250
0
10
20
30
40
50
60
70
blood
brain
heart
kidney
lung
ovary
skin
muscle
testis

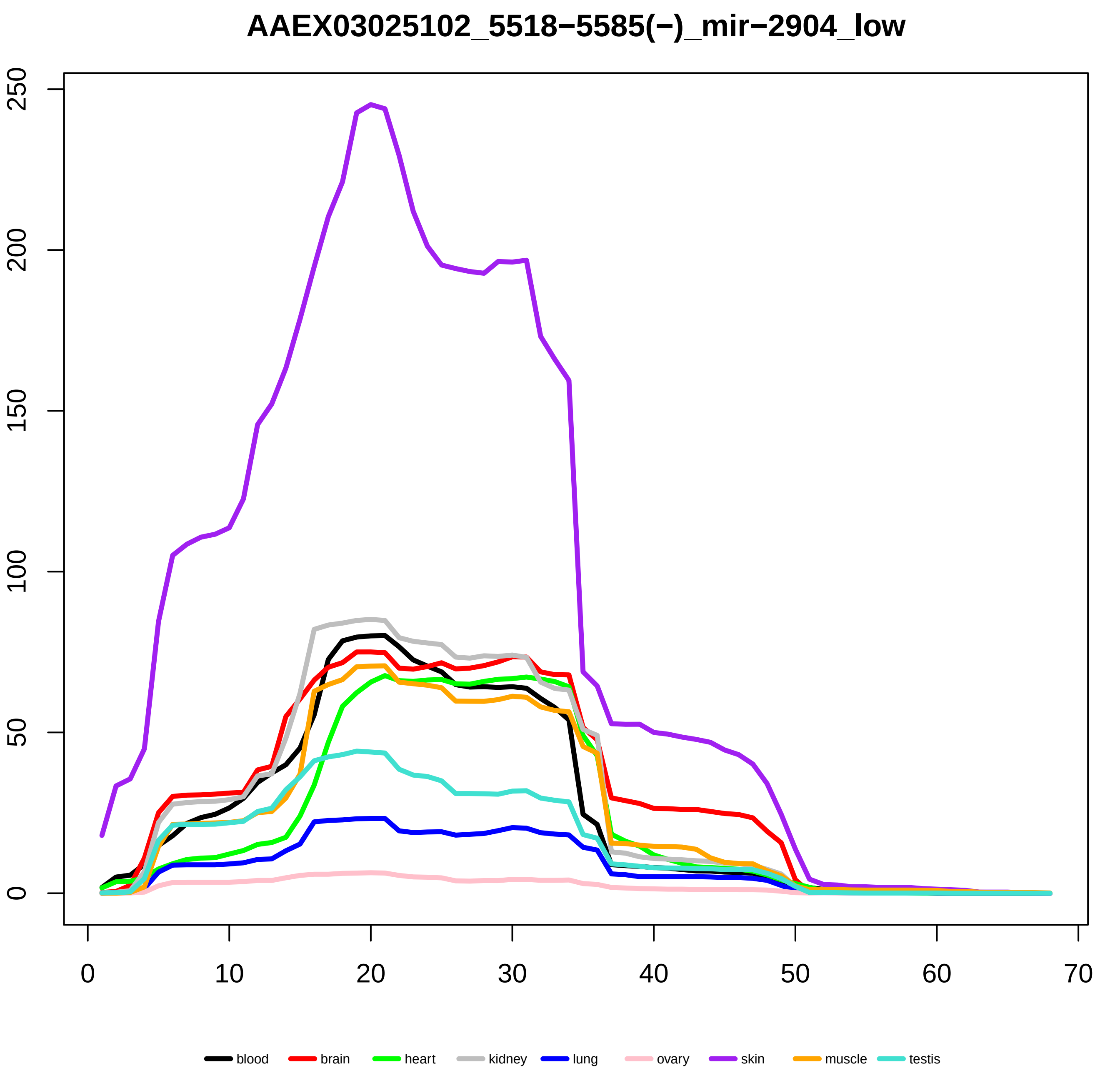
AAEX03025102_5518−5585(−)_mir−2904_low
0
50
100
150
200
250
0
10
20
30
40
50
60
70
blood
brain
heart
kidney
lung
ovary
skin
muscle
testis

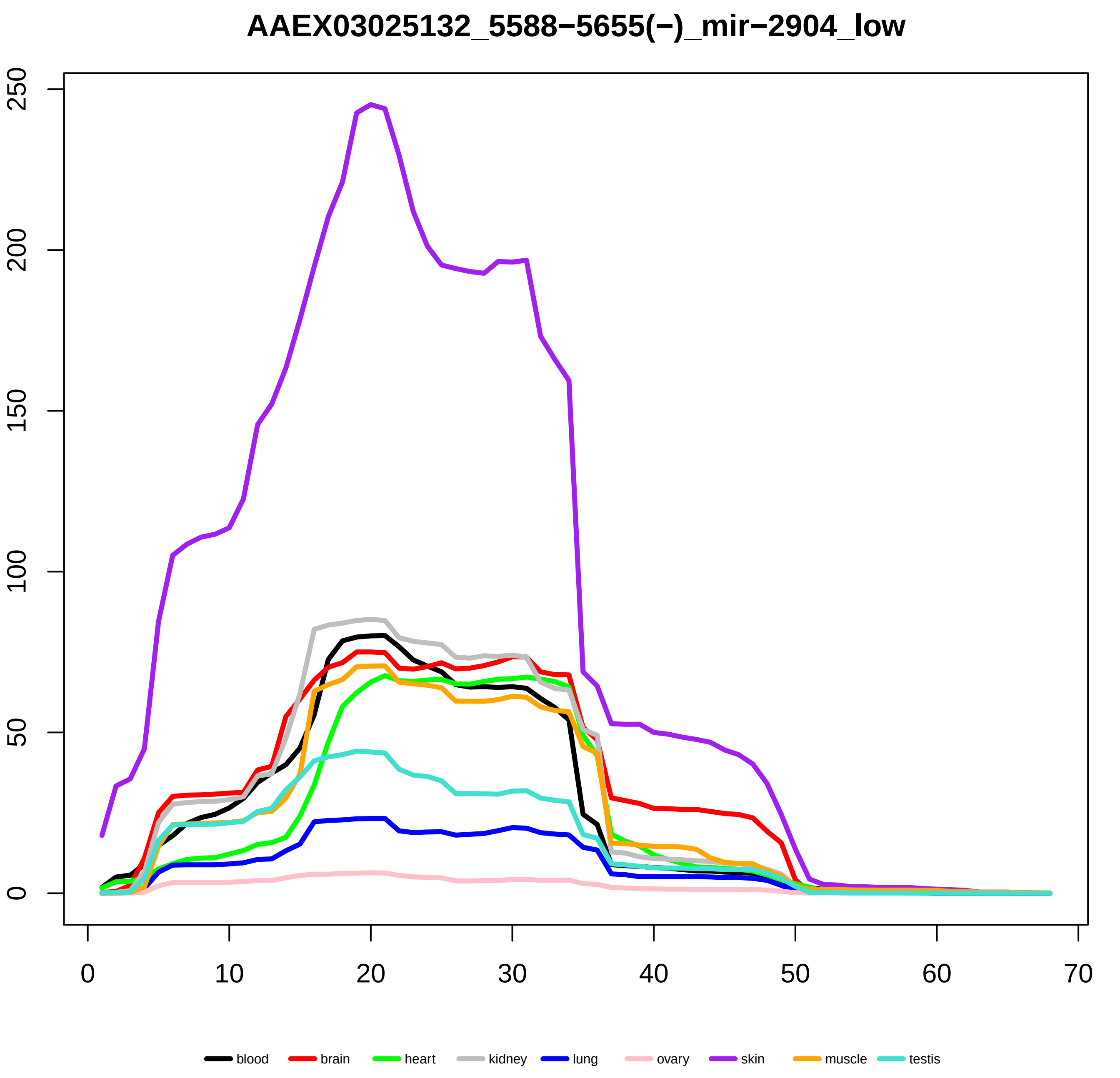

AAEX03025132_5588−5655(−)_mir−2904_low
0
50
100
150
200
250
0
10
20
30
40
50
60
70
blood
brain
heart
kidney
lung
ovary
skin
muscle
testis

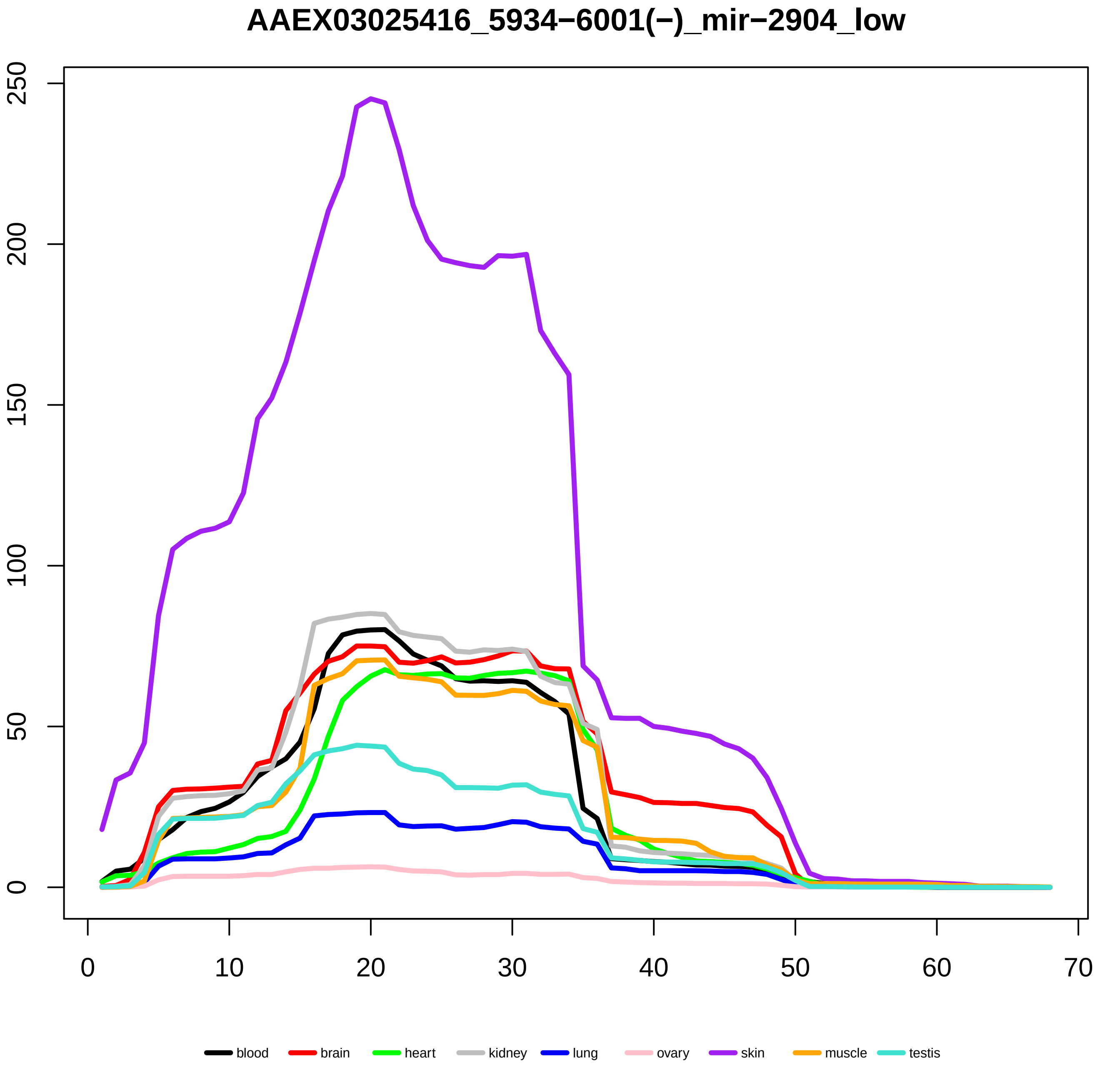

AAEX03025416_5934−6001(−)_mir−2904_low
0
50
100
150
200
250
0
10
20
30
40
50
60
70
blood
brain
heart
kidney
lung
ovary
skin
muscle
testis

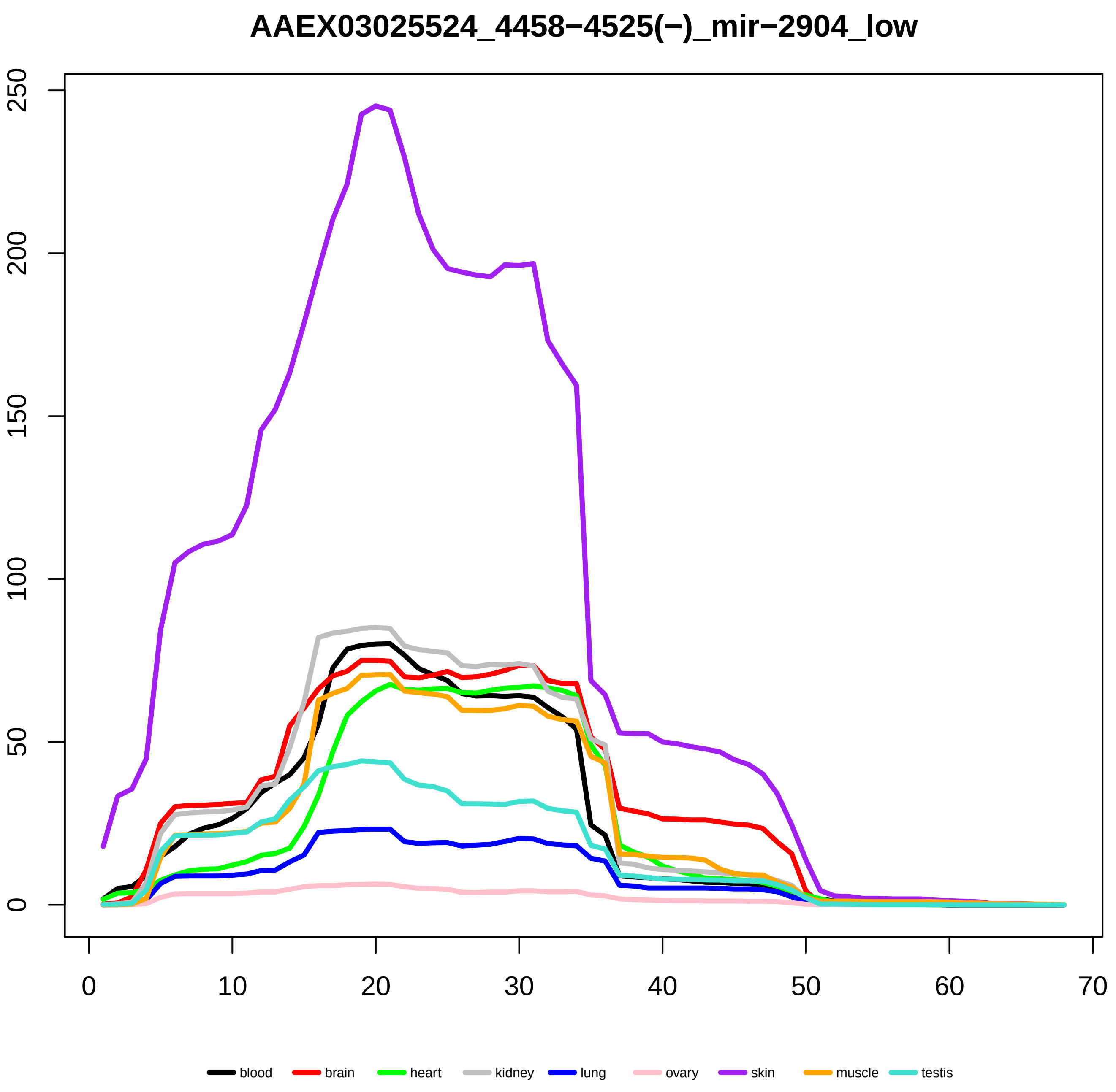

AAEX03025524_4458−4525(−)_mir−2904_low
0
50
100
150
200
250
0
10
20
30
40
50
60
70
blood
brain
heart
kidney
lung
ovary
skin
muscle
testis

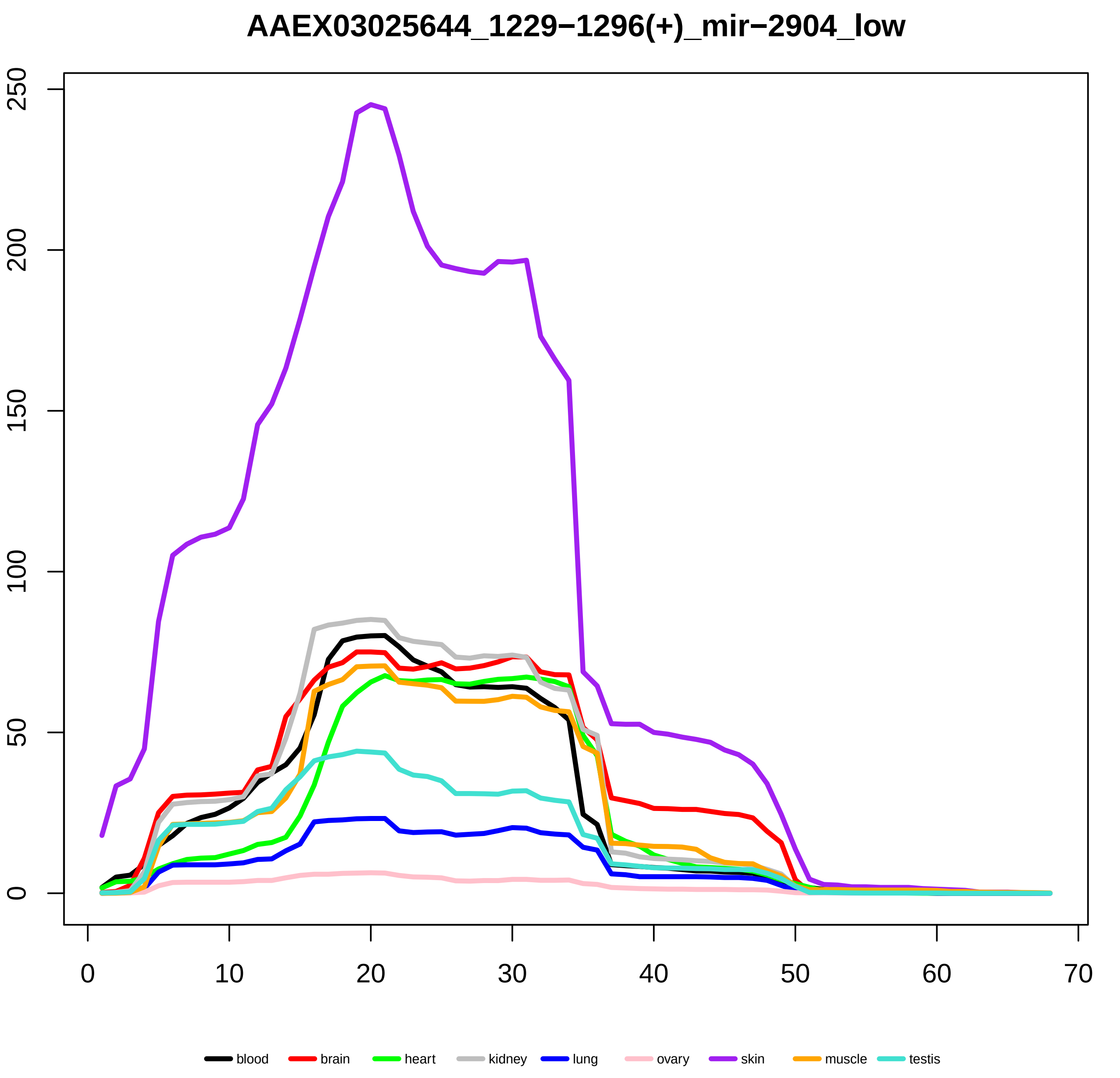

AAEX03025644_1229−1296(+)_mir−2904_low
0
50
100
150
200
250
0
10
20
30
40
50
60
70
blood
brain
heart
kidney
lung
ovary
skin
muscle
testis

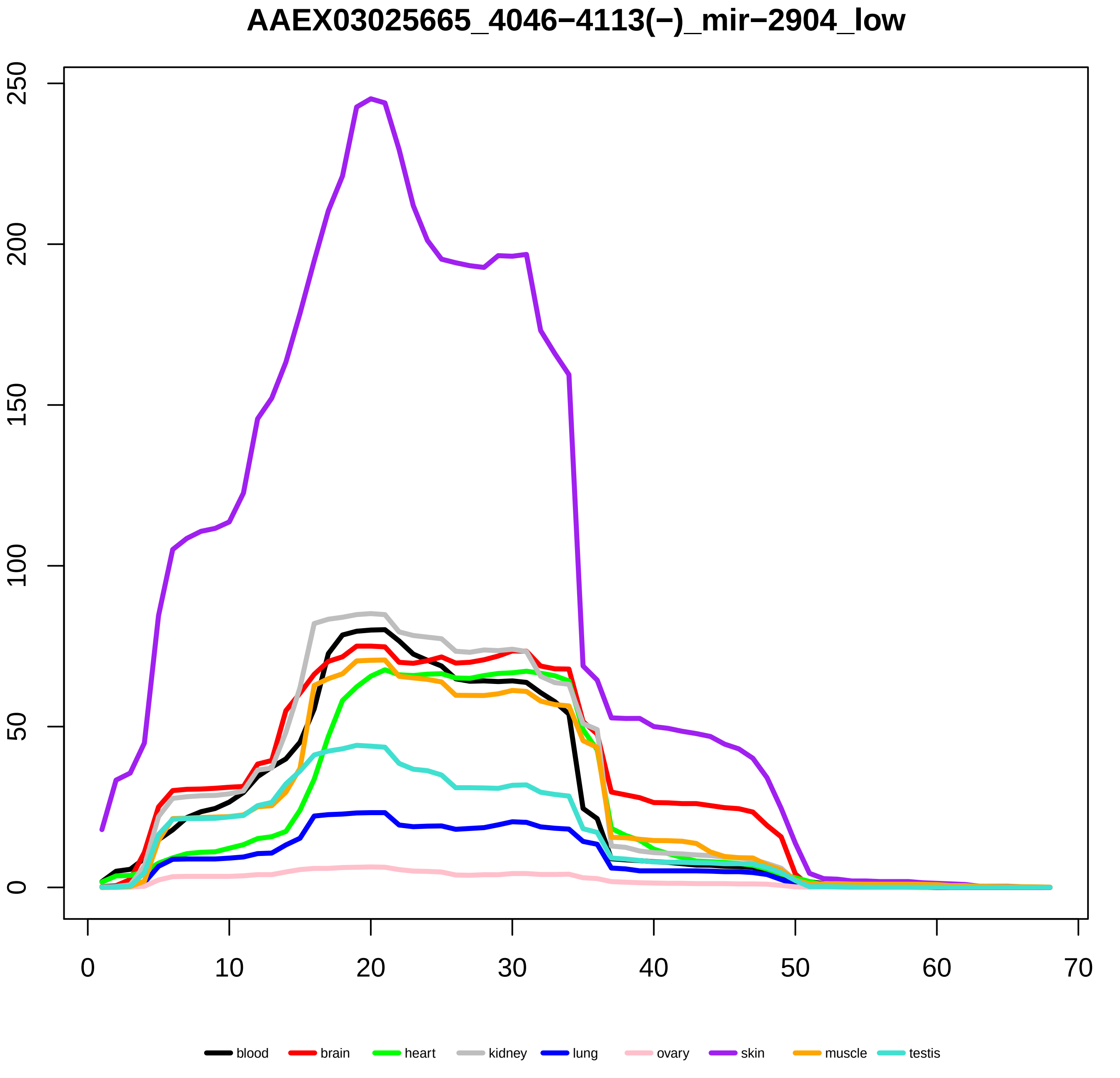

AAEX03025665_4046−4113(−)_mir−2904_low
0
50
100
150
200
250
0
10
20
30
40
50
60
70
blood
brain
heart
kidney
lung
ovary
skin
muscle
testis

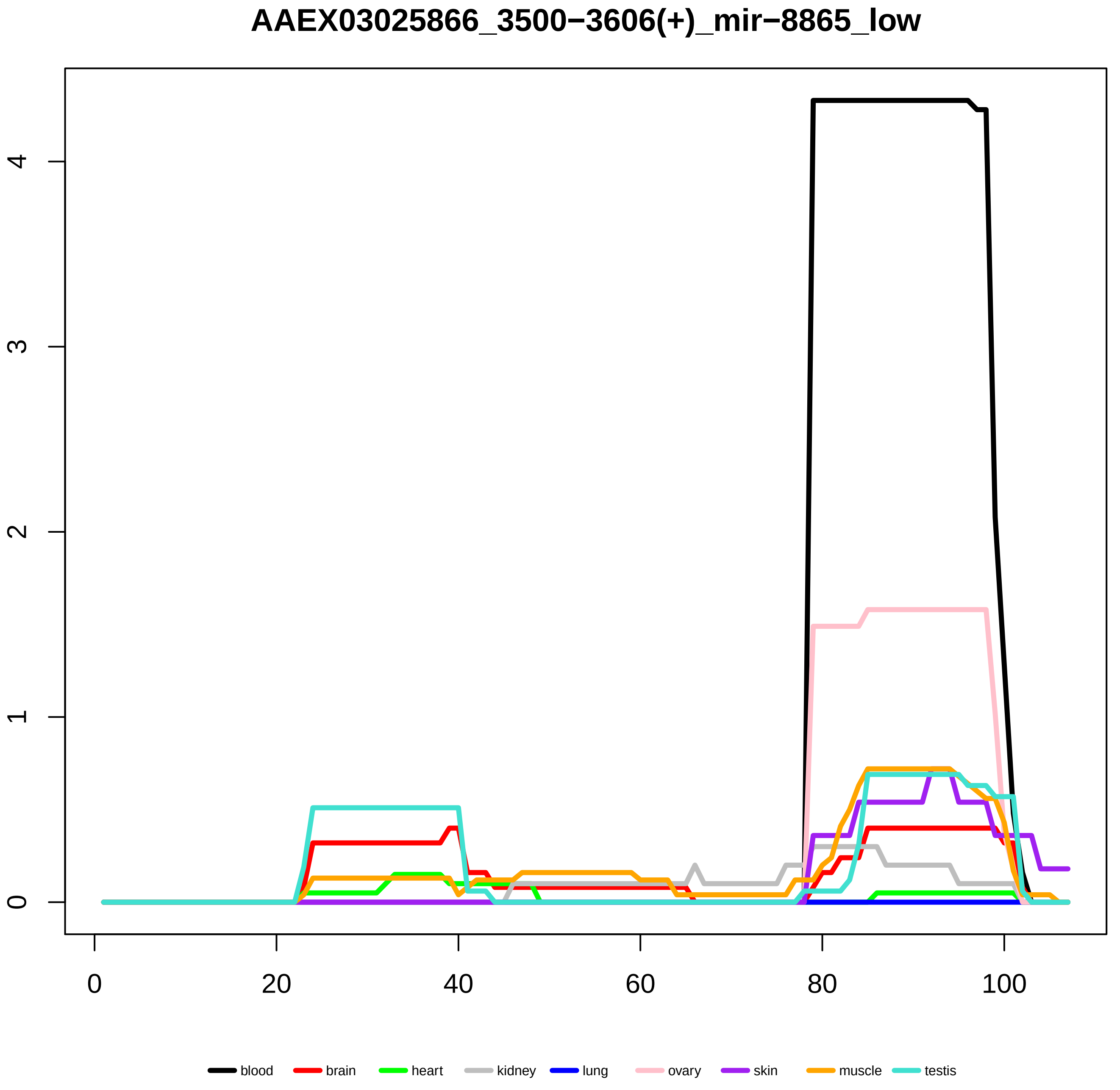

AAEX03025866_3500−3606(+)_mir−8865_low
0
1
2
3
4
0
20
40
60
80
100
blood
brain
heart
kidney
lung
ovary
skin
muscle
testis

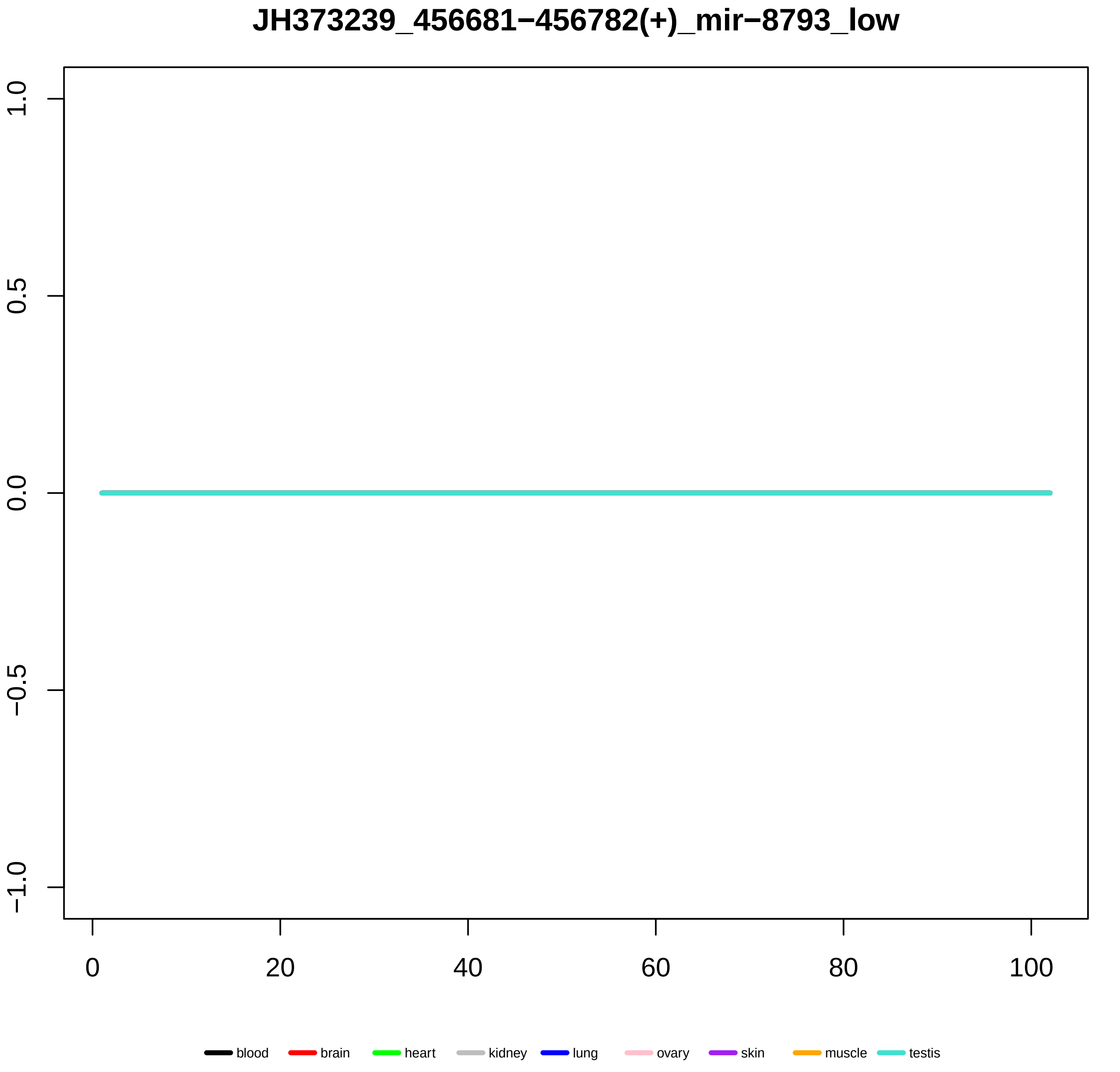
JH373239_456681−456782(+)_mir−8793_low
1.0
0.5
0.0
−0.5
−1.0
0
20
40
60
80
100
blood
brain
heart
kidney
lung
ovary
skin
muscle
testis

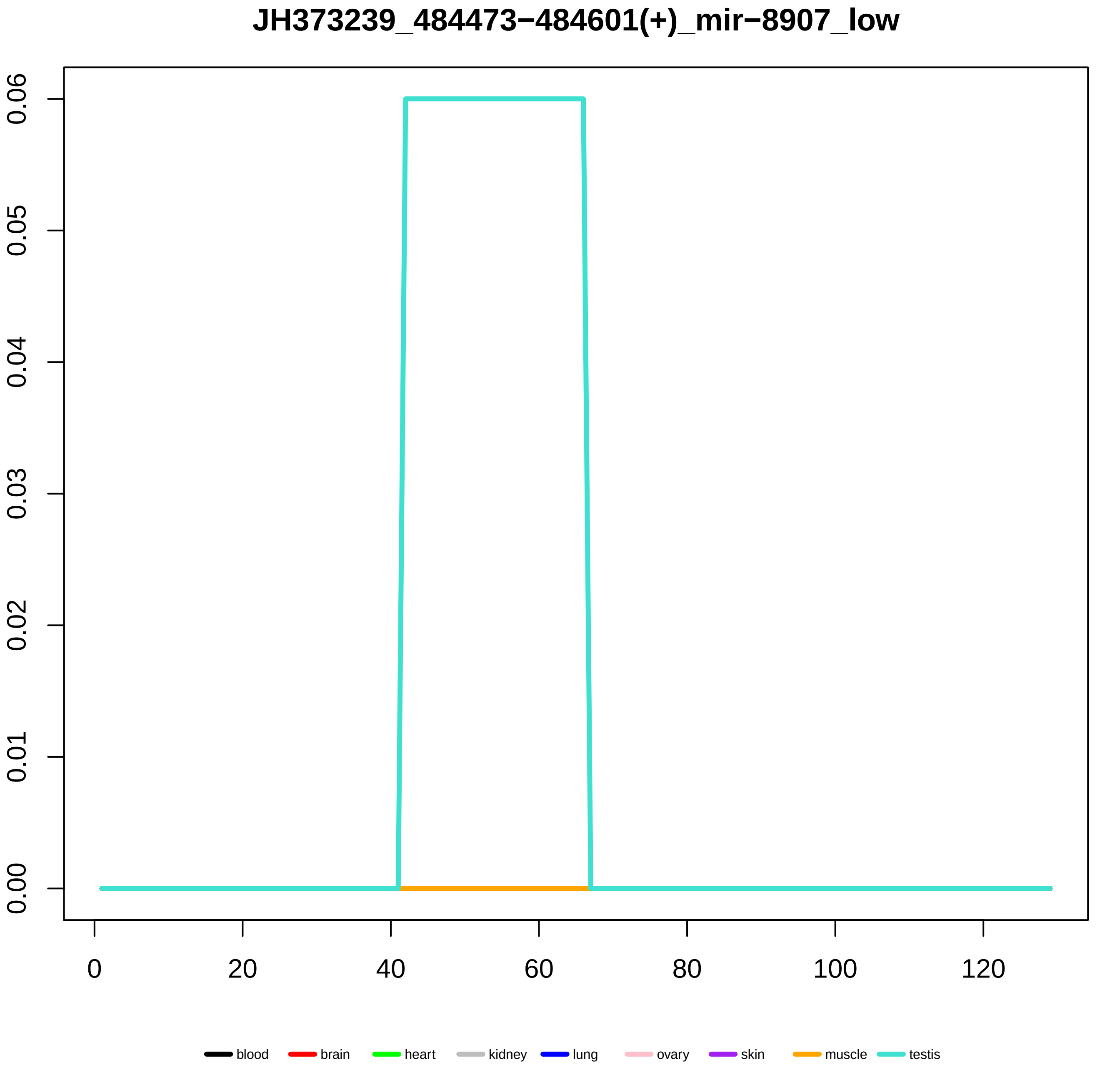
JH373239_484473−484601(+)_mir−8907_low
0.00
0.01
0.02
0.03
0.04
0.05
0.06
0
20
40
60
80
100
120
blood
brain
heart
kidney
lung
ovary
skin
muscle
testis

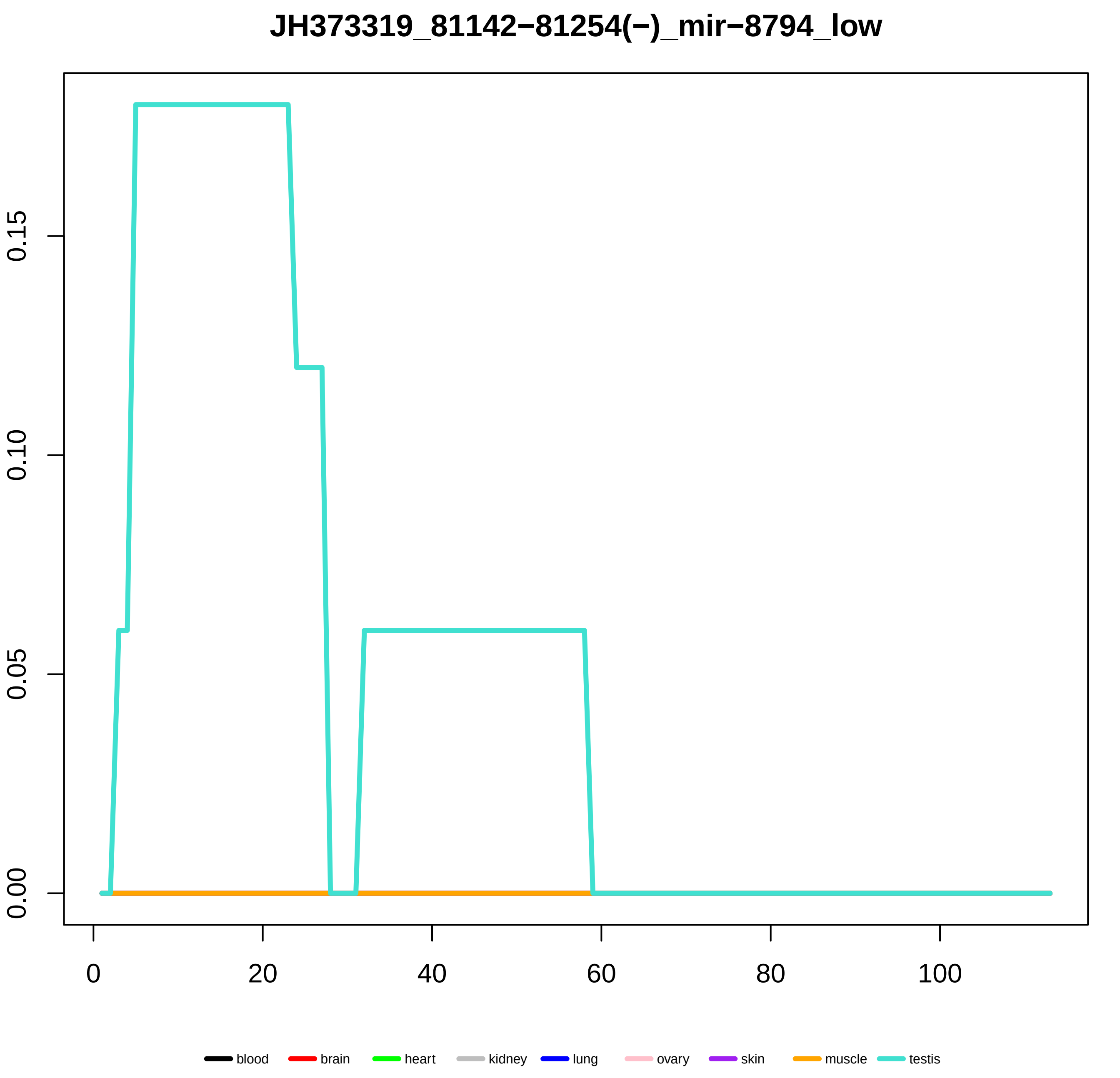

JH373319_81142−81254(−)_mir−8794_low
0.00
0.05
0.10
0.15
0
20
40
60
80
100
blood
brain
heart
kidney
lung
ovary
skin
muscle
testis

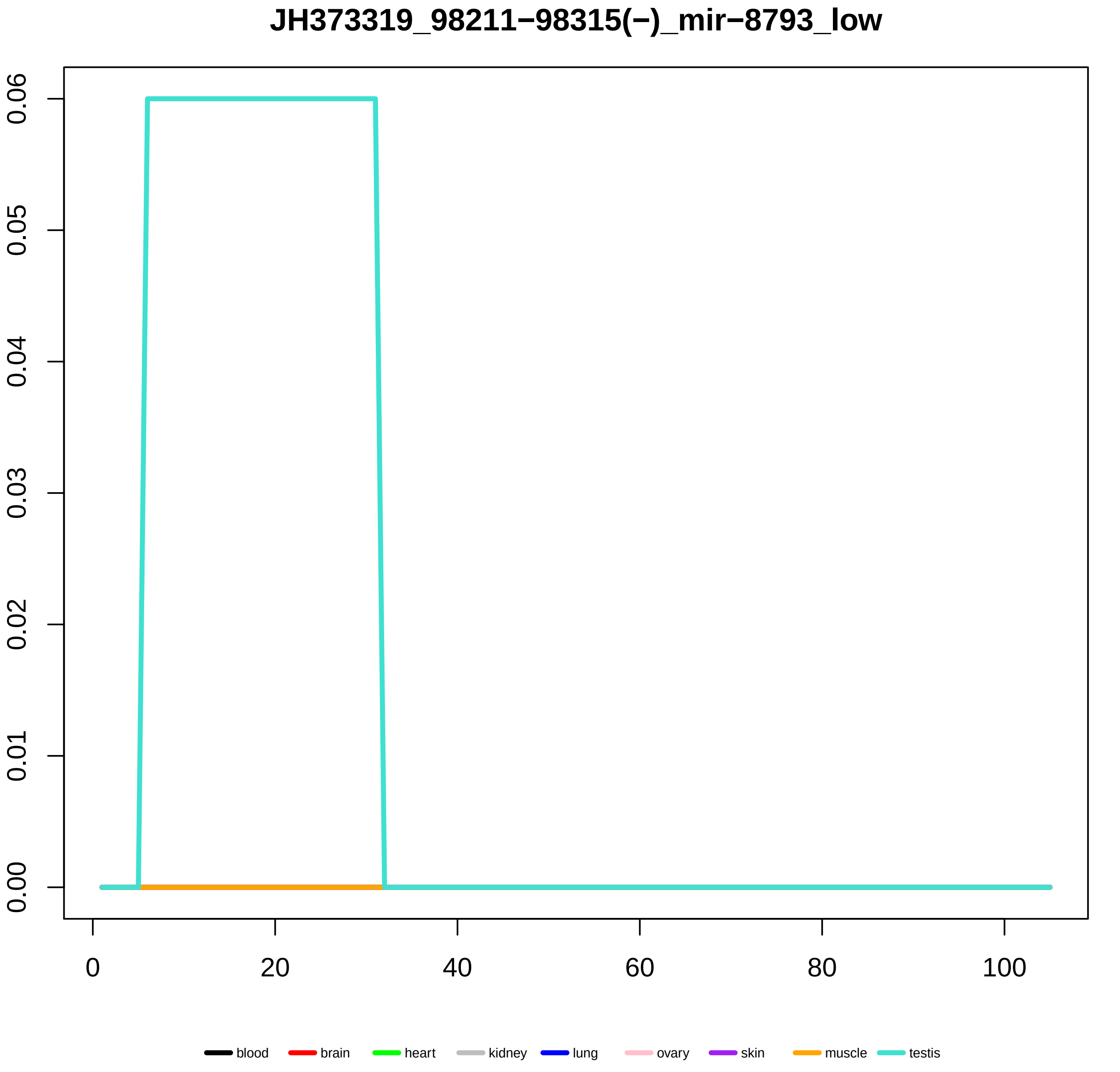
JH373319_98211−98315(−)_mir−8793_low
0.00
0.01
0.02
0.03
0.04
0.05
0.06
0
20
40
60
80
100
blood
brain
heart
kidney
lung
ovary
skin
muscle
testis

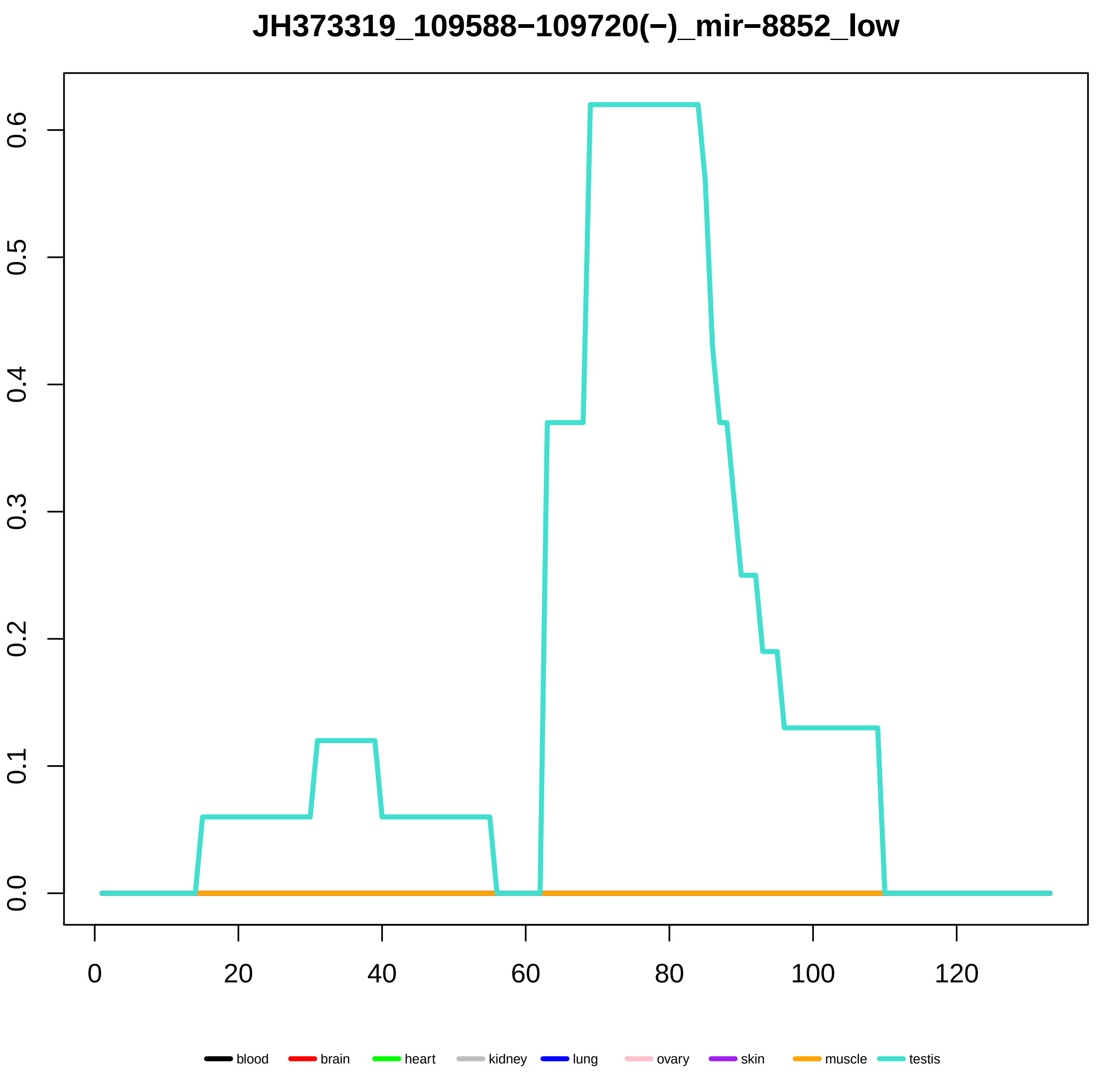

JH373319_109588−109720(−)_mir−8852_low
0.0
0.1
0.2
0.3
0.4
0.5
0.6
0
20
40
60
80
100
120
blood
brain
heart
kidney
lung
ovary
skin
muscle
testis

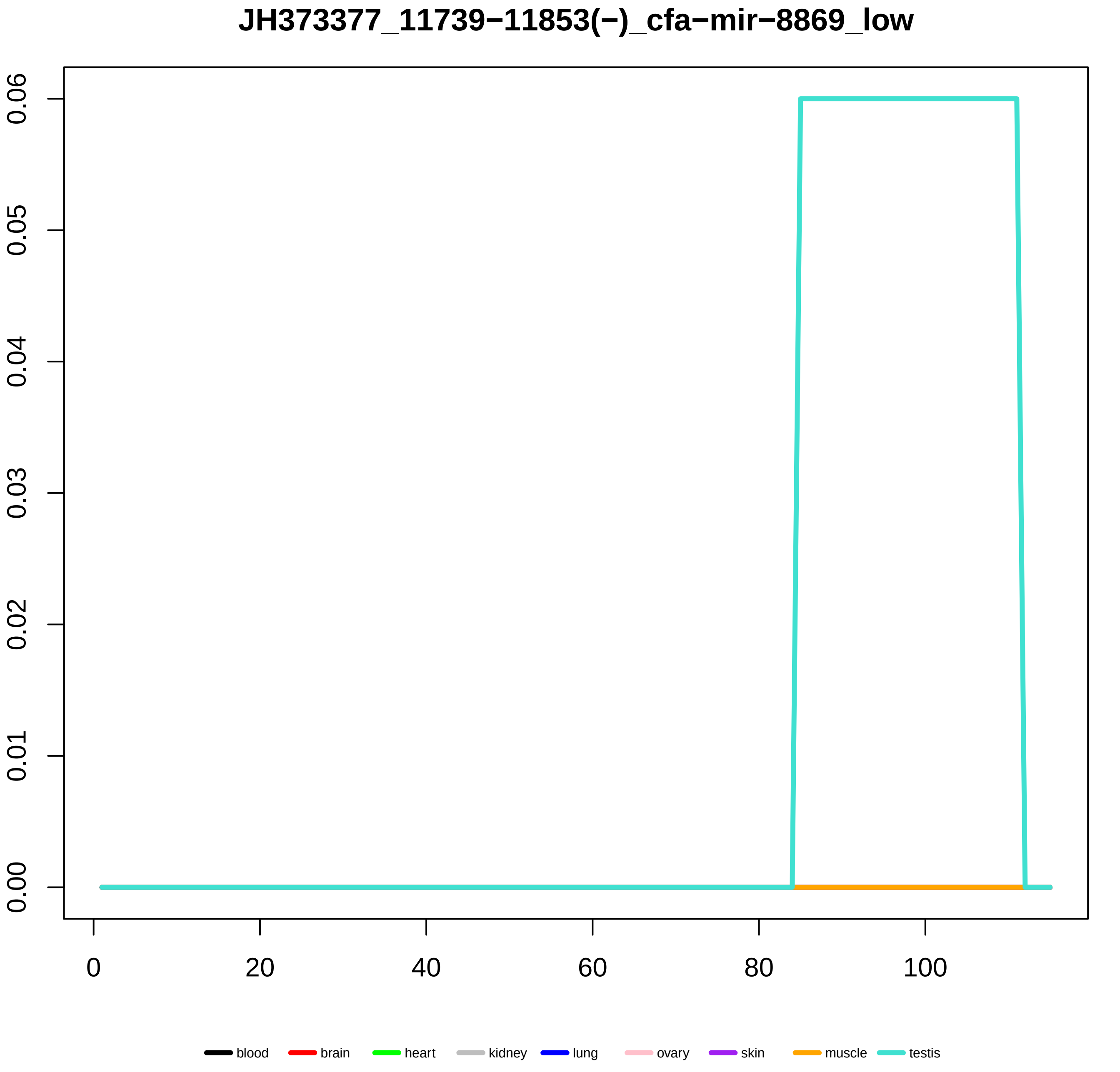

JH373377_11739−11853(−)_cfa−mir−8869_low
0.00
0.01
0.02
0.03
0.04
0.05
0.06
0
20
40
60
80
100
blood
brain
heart
kidney
lung
ovary
skin
muscle
testis

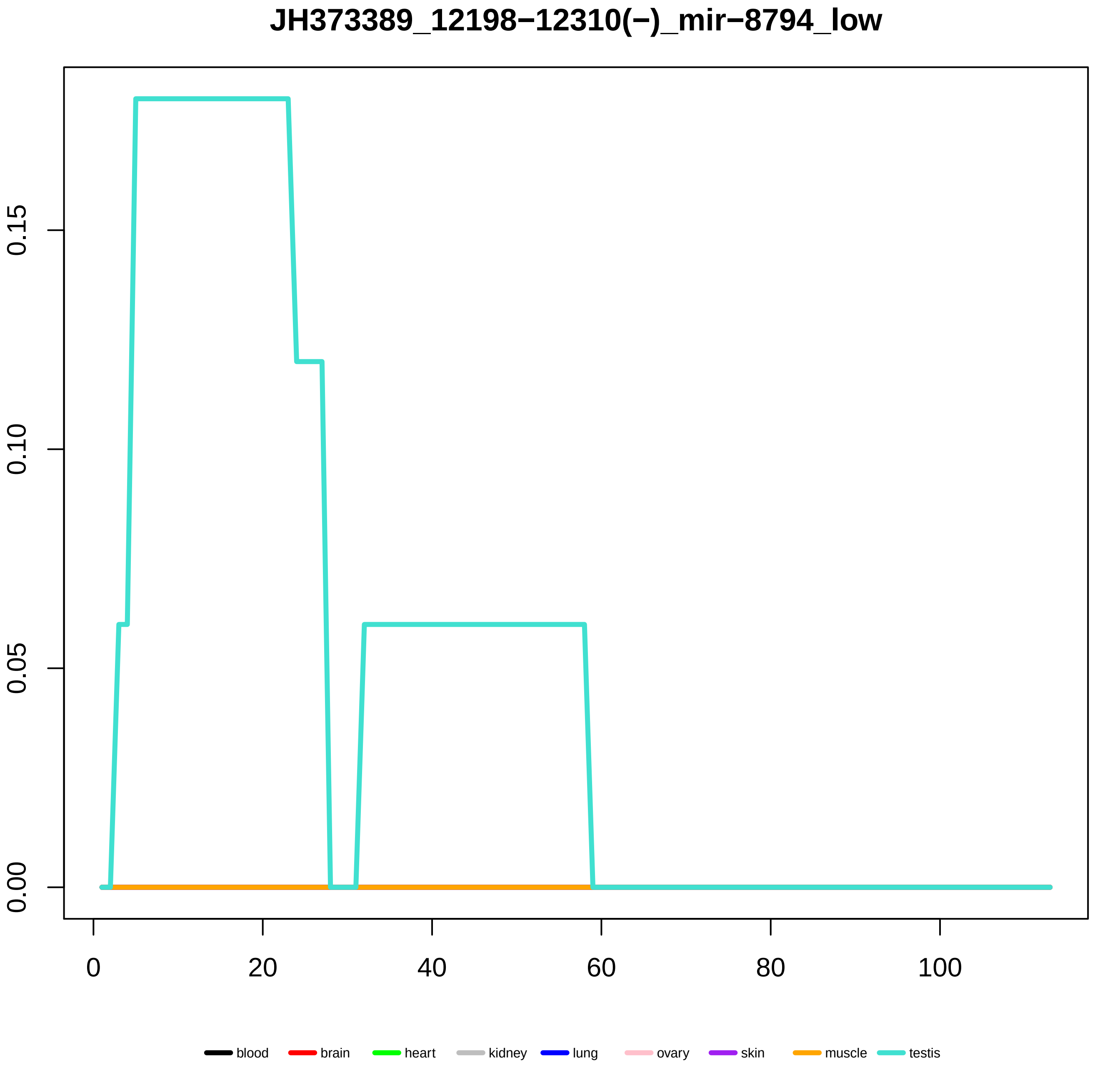
JH373389_12198−12310(−)_mir−8794_low
0.00
0.05
0.10
0.15
0
20
40
60
80
100
blood
brain
heart
kidney
lung
ovary
skin
muscle
testis

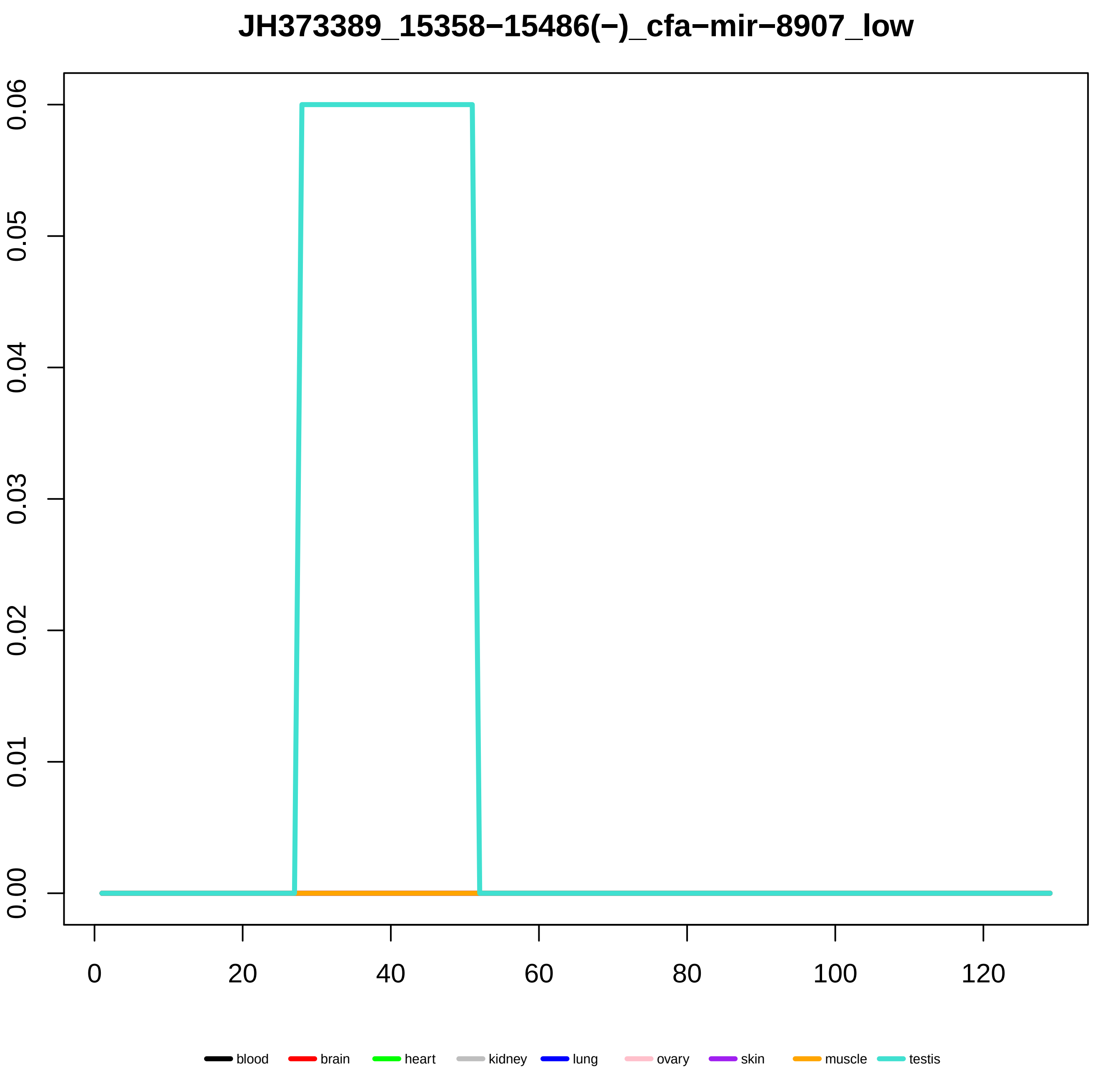
JH373389_15358−15486(−)_cfa−mir−8907_low
0.00
0.01
0.02
0.03
0.04
0.05
0.06
0
20
40
60
80
100
120
blood
brain
heart
kidney
lung
ovary
skin
muscle
testis

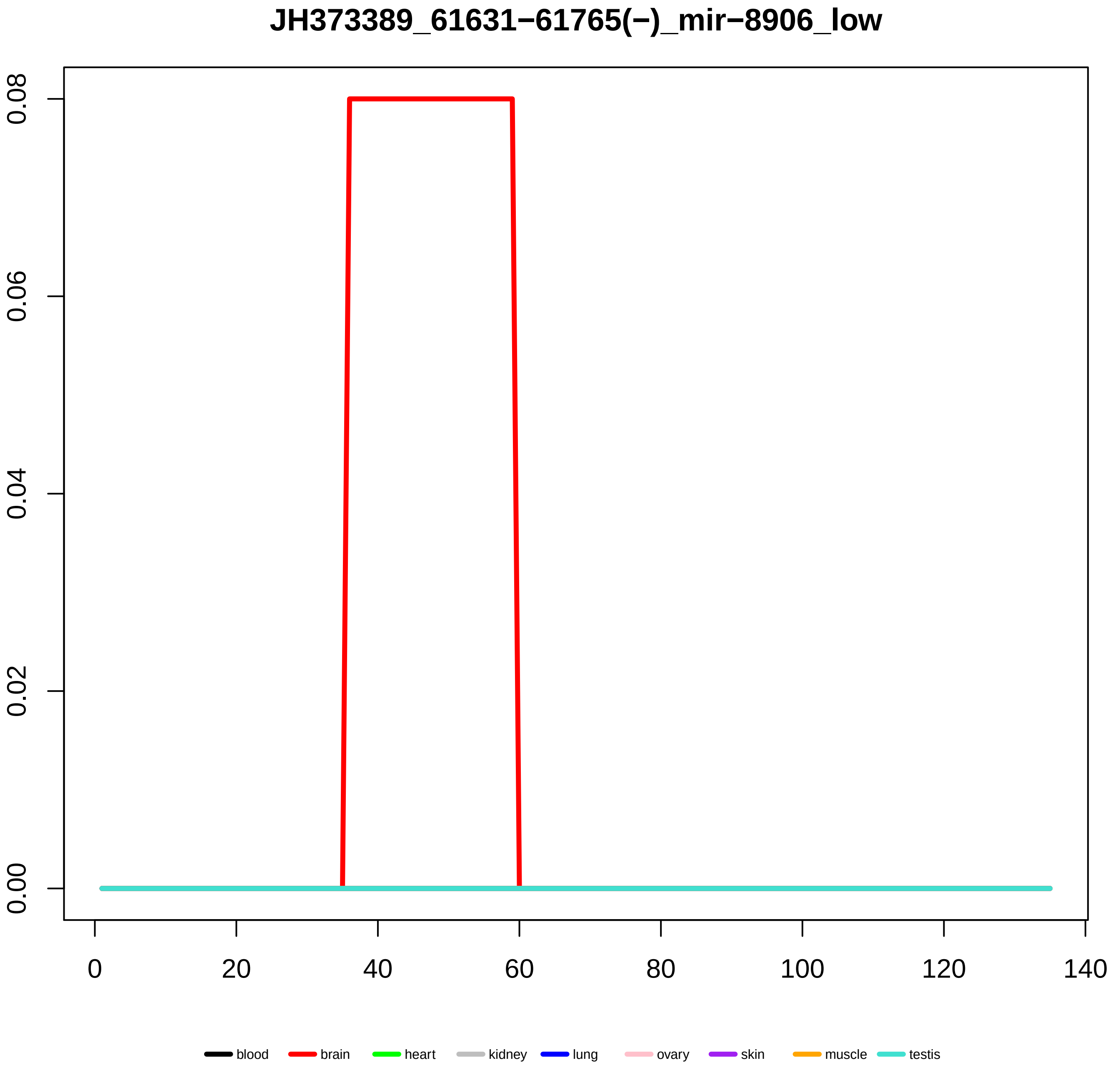

JH373389_61631−61765(−)_mir−8906_low
0.00
0.02
0.04
0.06
0.08
0
20
40
60
80
100
120
140
blood
brain
heart
kidney
lung
ovary
skin
muscle
testis

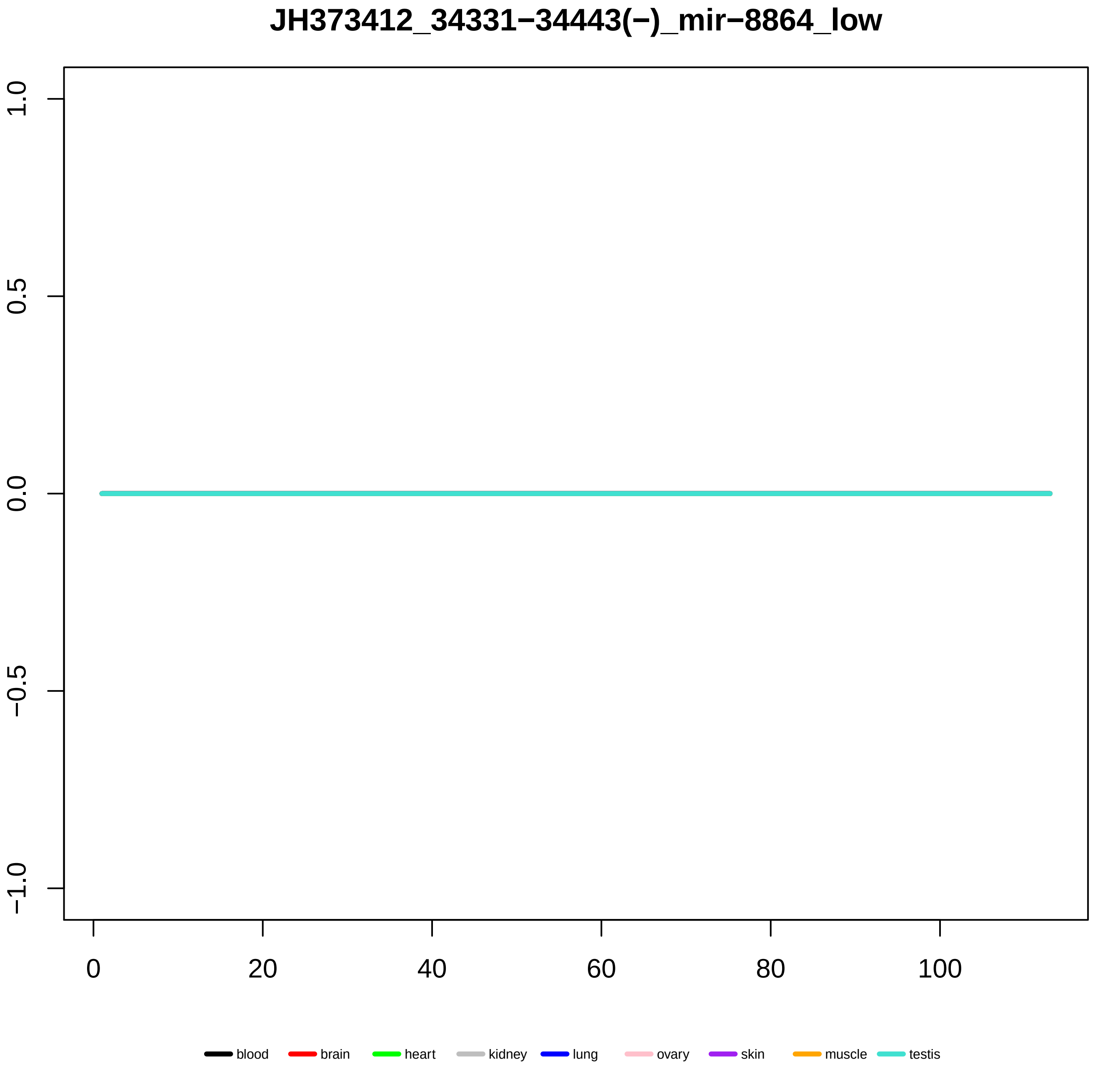
JH373412_34331−34443(−)_mir−8864_low
1.0
0.5
0.0
−0.5
−1.0
0
20
40
60
80
100
blood
brain
heart
kidney
lung
ovary
skin
muscle
testis

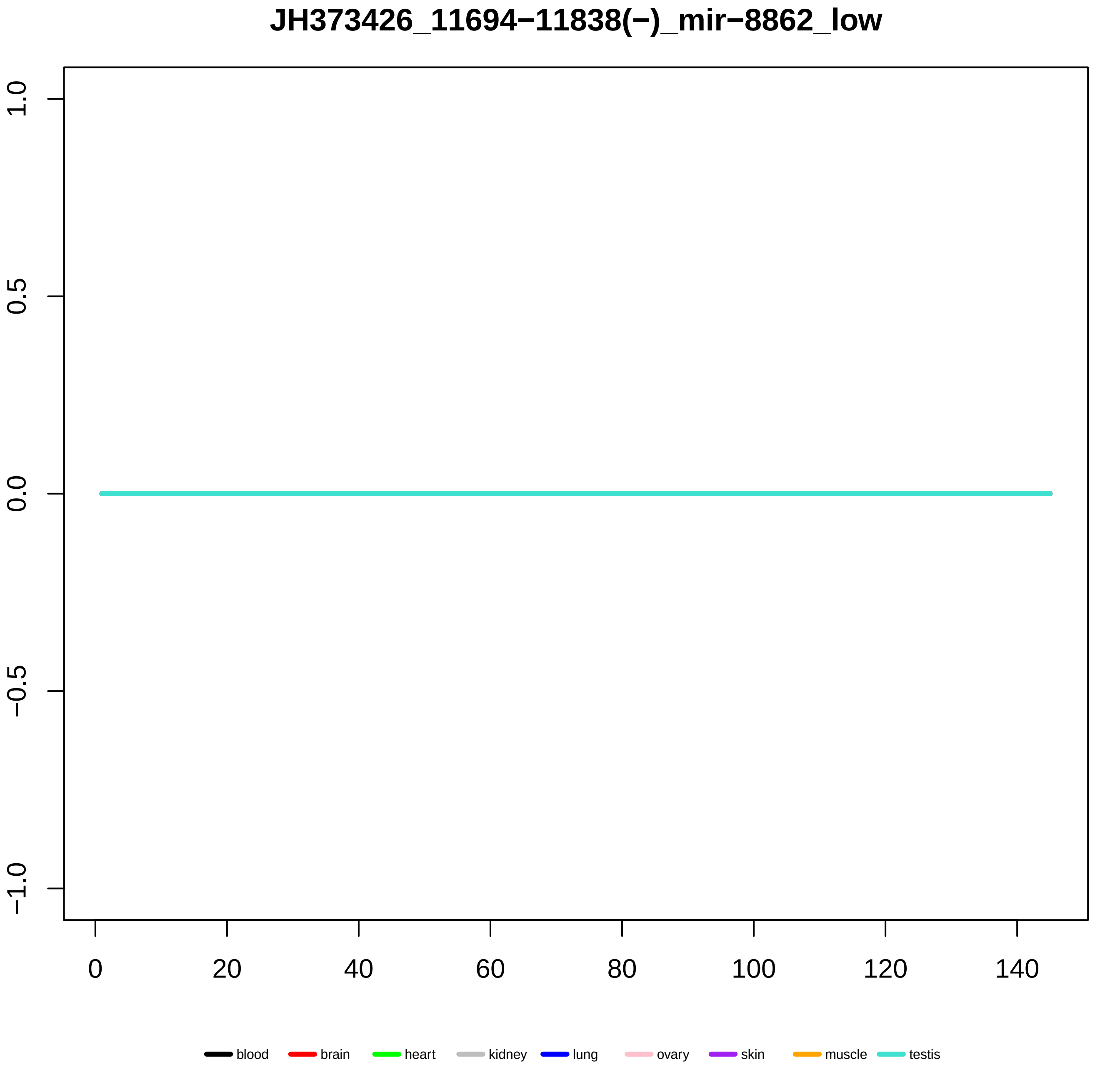
JH373426_11694−11838(−)_mir−8862_low
1.0
0.5
0.0
−0.5
−1.0
0
20
40
60
80
100
120
140
blood
brain
heart
kidney
lung
ovary
skin
muscle
testis

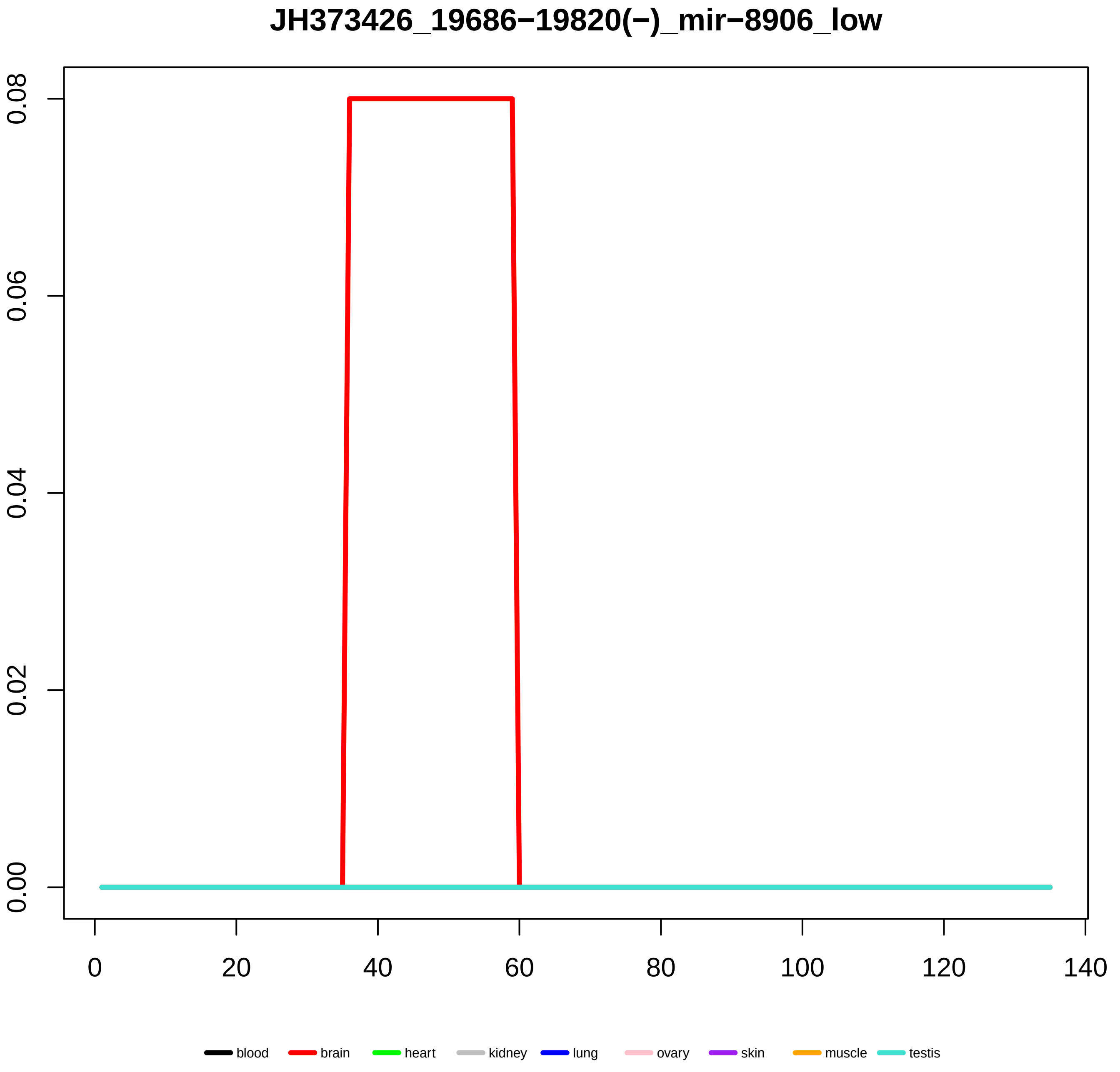

JH373426_19686−19820(−)_mir−8906_low
0.00
0.02
0.04
0.06
0.08
0
20
40
60
80
100
120
140
blood
brain
heart
kidney
lung
ovary
skin
muscle
testis

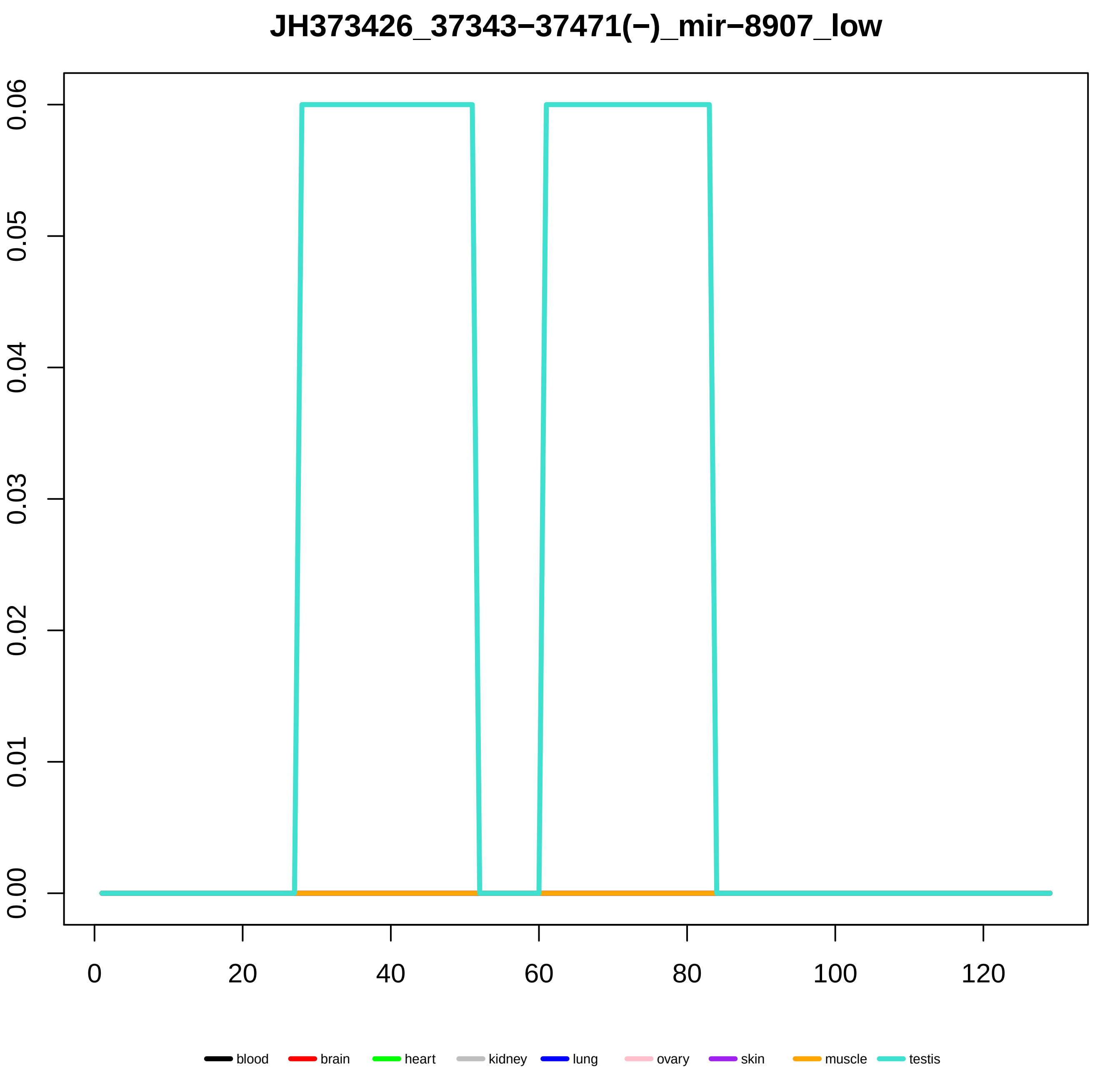

JH373426_37343−37471(−)_mir−8907_low
0.00
0.01
0.02
0.03
0.04
0.05
0.06
0
20
40
60
80
100
120
blood
brain
heart
kidney
lung
ovary
skin
muscle
testis

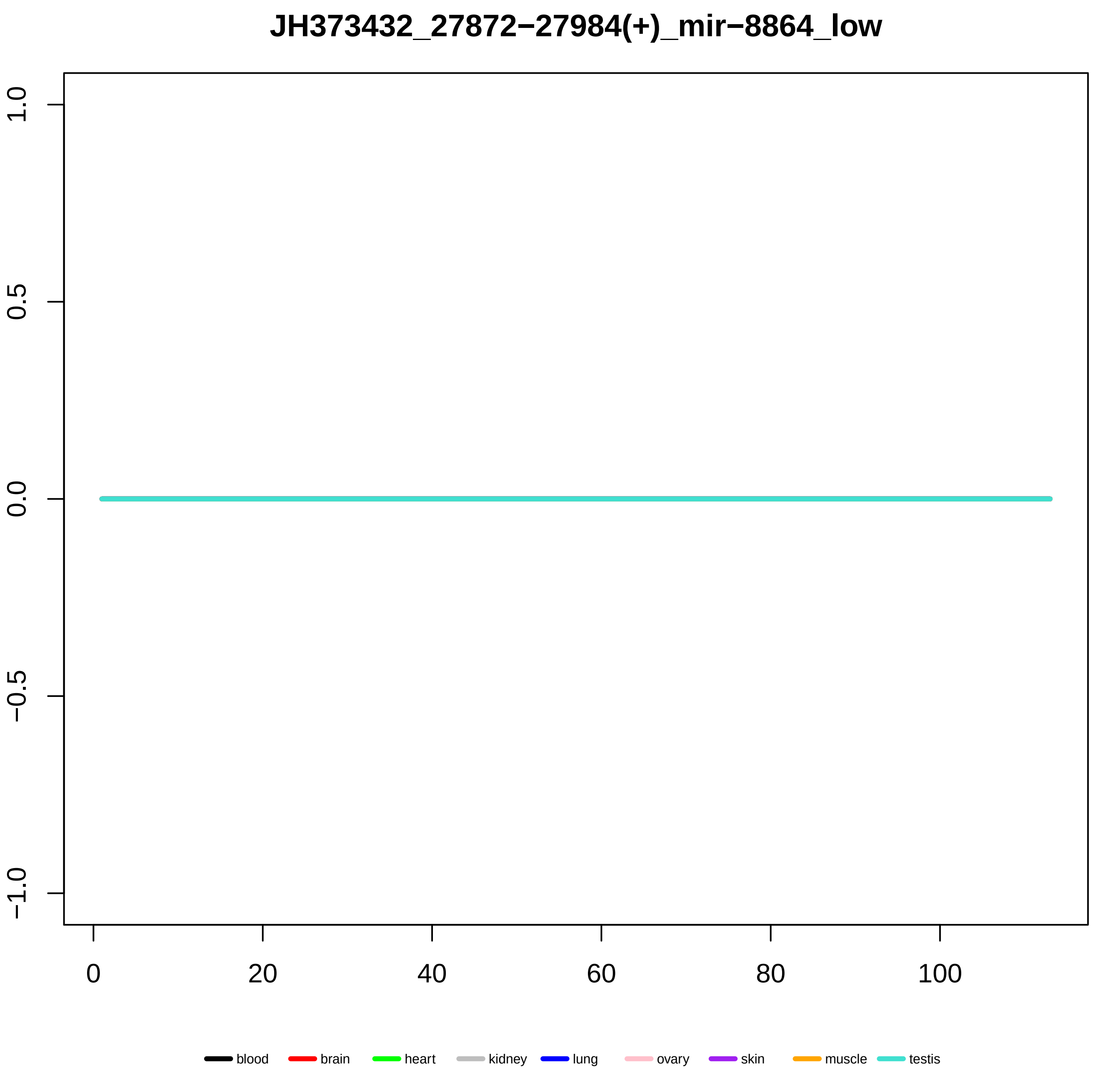
JH373432_27872−27984(+)_mir−8864_low
1.0
0.5
0.0
−0.5
−1.0
0
20
40
60
80
100
blood
brain
heart
kidney
lung
ovary
skin
muscle
testis

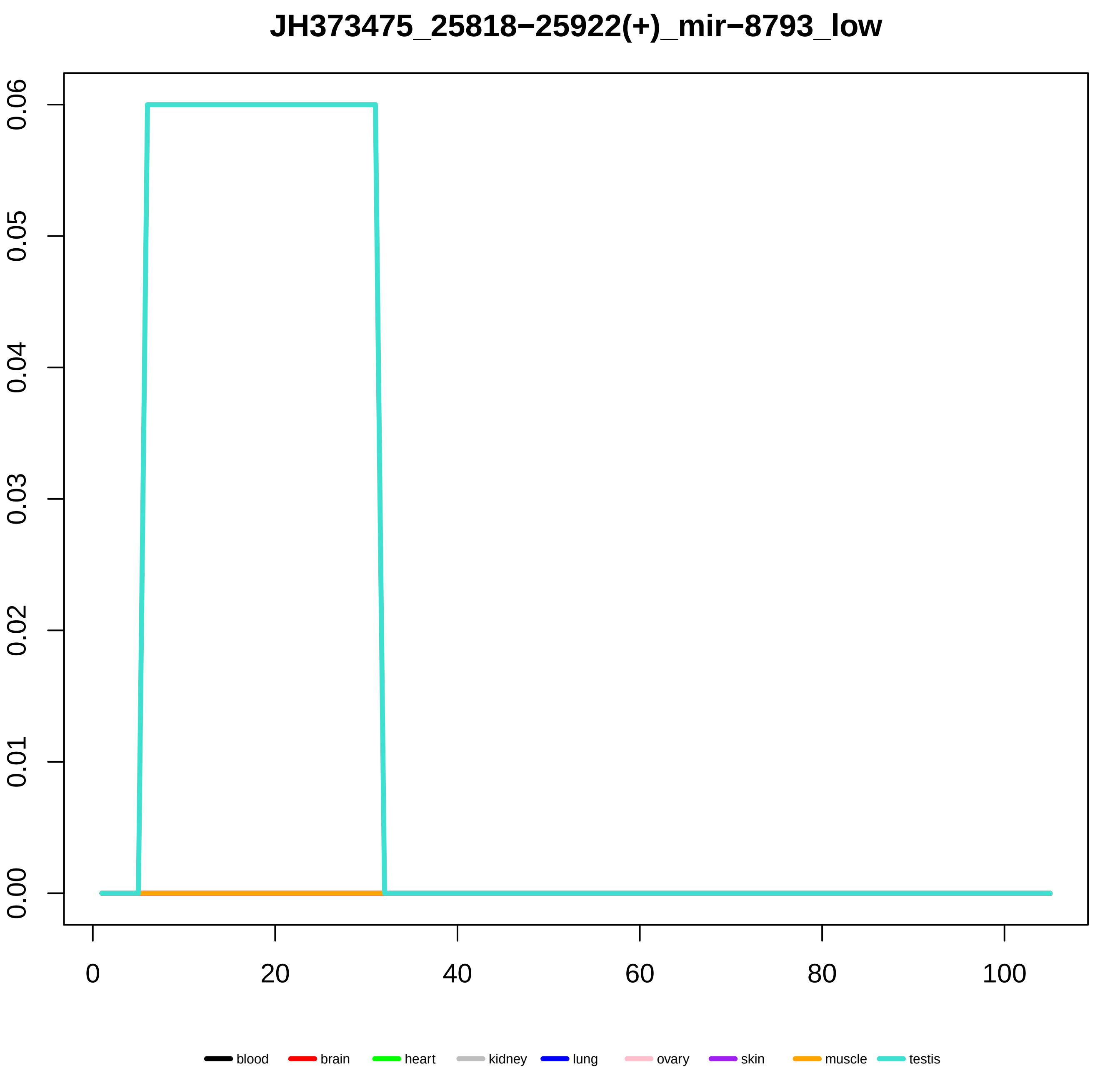

JH373475_25818−25922(+)_mir−8793_low
0.00
0.01
0.02
0.03
0.04
0.05
0.06
0
20
40
60
80
100
blood
brain
heart
kidney
lung
ovary
skin
muscle
testis

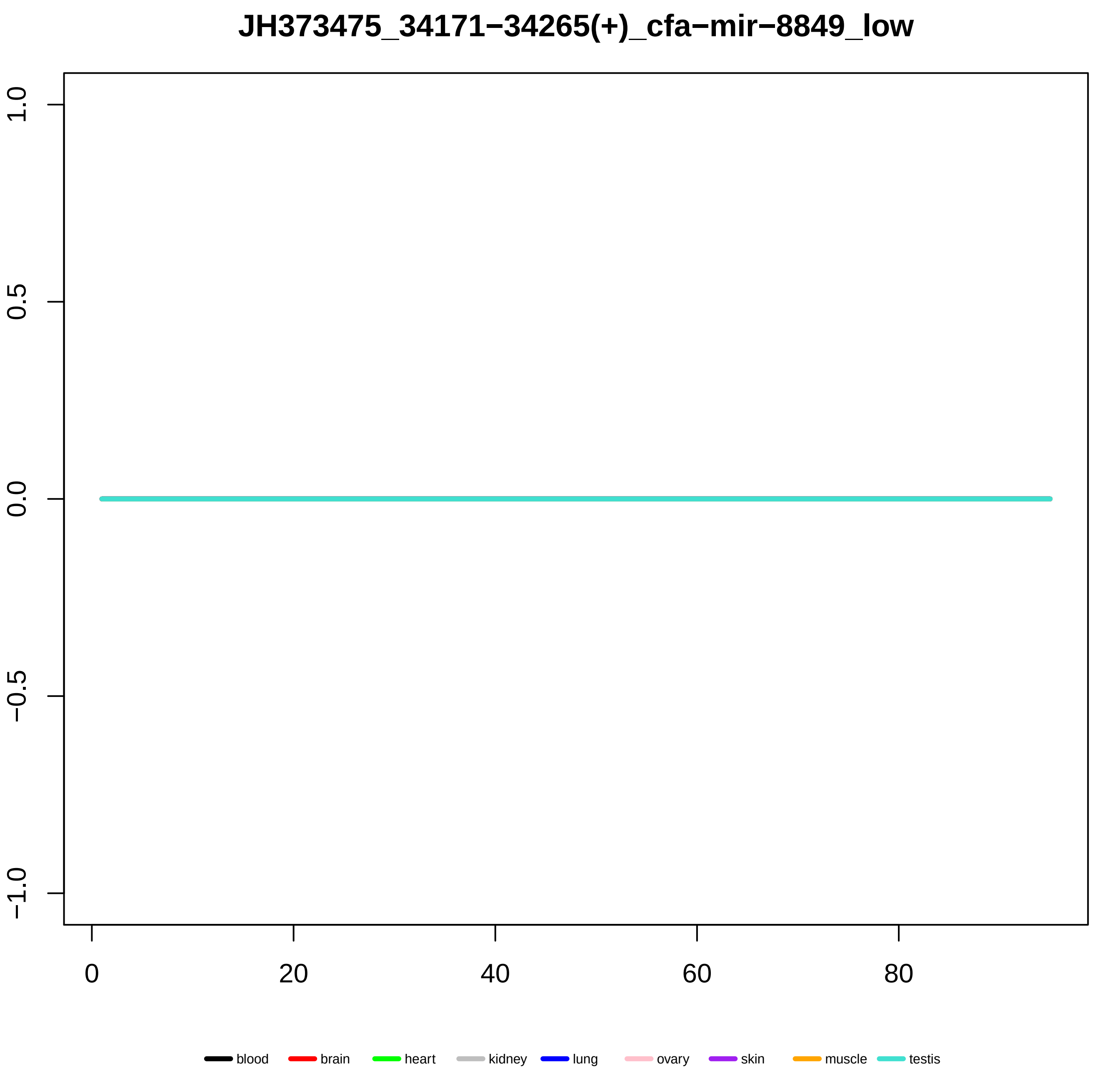

JH373475_34171−34265(+)_cfa−mir−8849_low
1.0
0.5
0.0
−0.5
−1.0
0
20
40
60
80
blood
brain
heart
kidney
lung
ovary
skin
muscle
testis

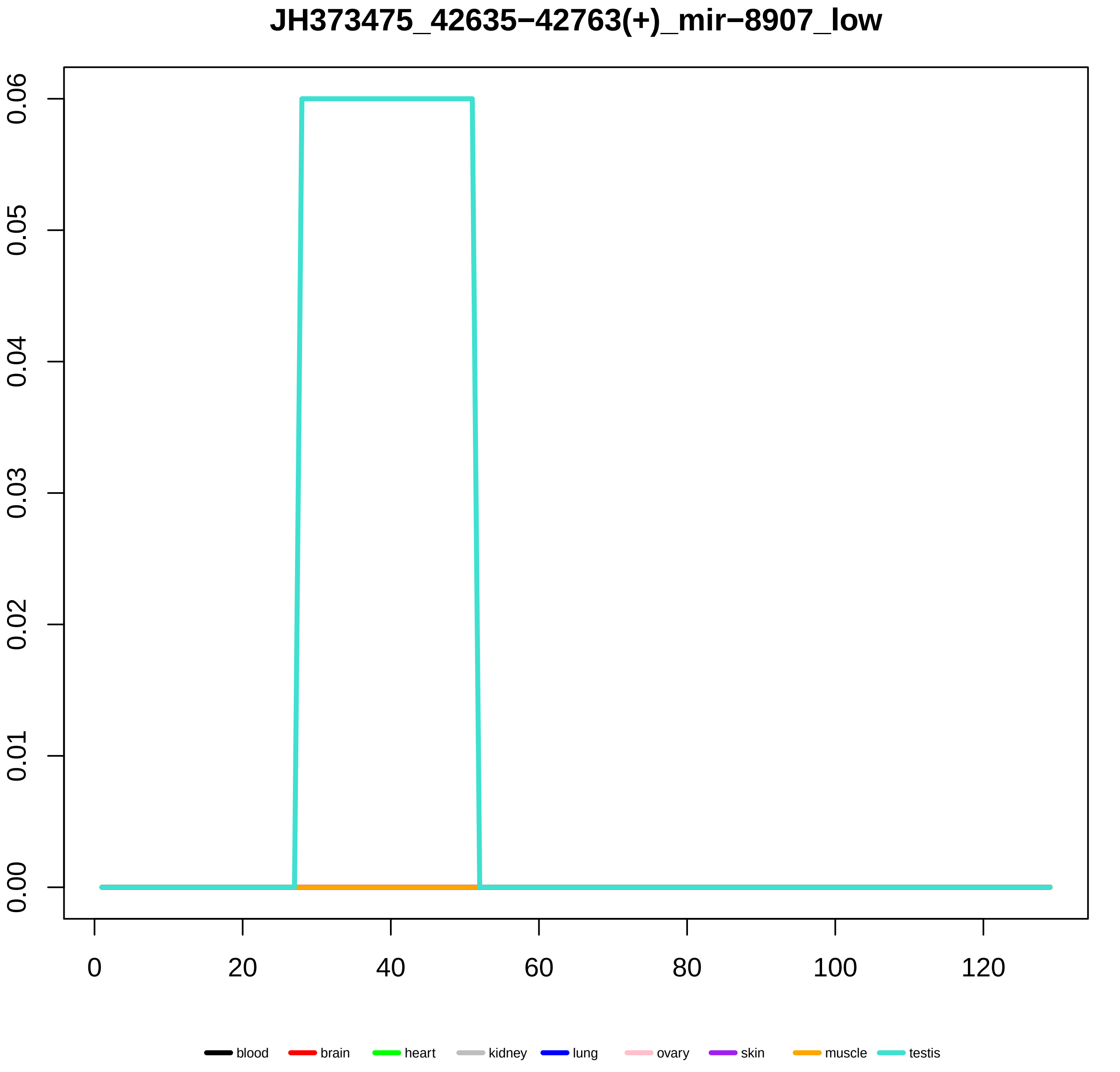

JH373475_42635−42763(+)_mir−8907_low
0.06
0.05
0.04
0.03
0.02
0.01
0.00
0
20
40
60
80
100
120
blood
brain
heart
kidney
lung
ovary
skin
muscle
testis

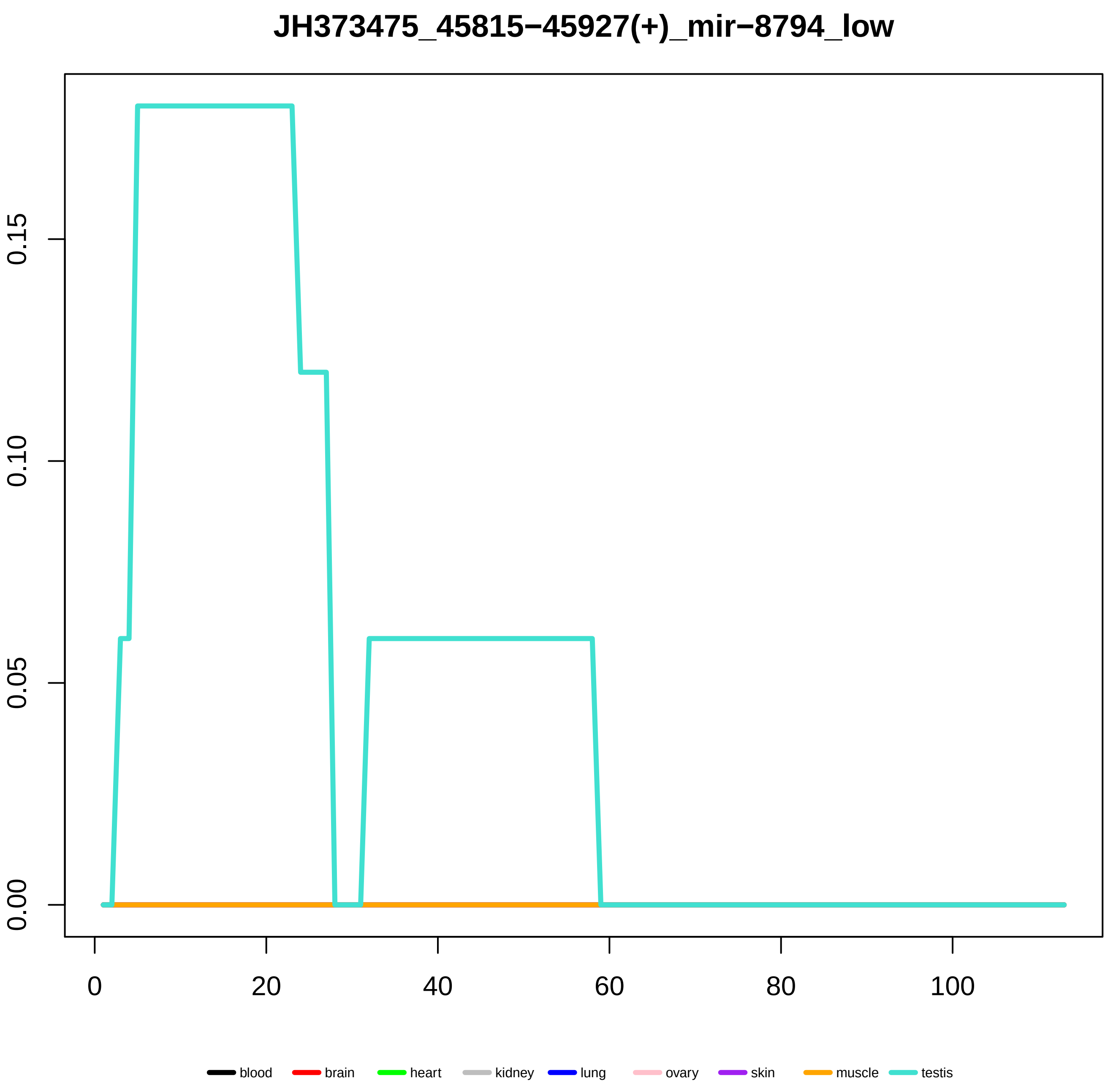

JH373475_45815−45927(+)_mir−8794_low
0.00
0.05
0.10
0.15
0
20
40
60
80
100
blood
brain
heart
kidney
lung
ovary
skin
muscle
testis

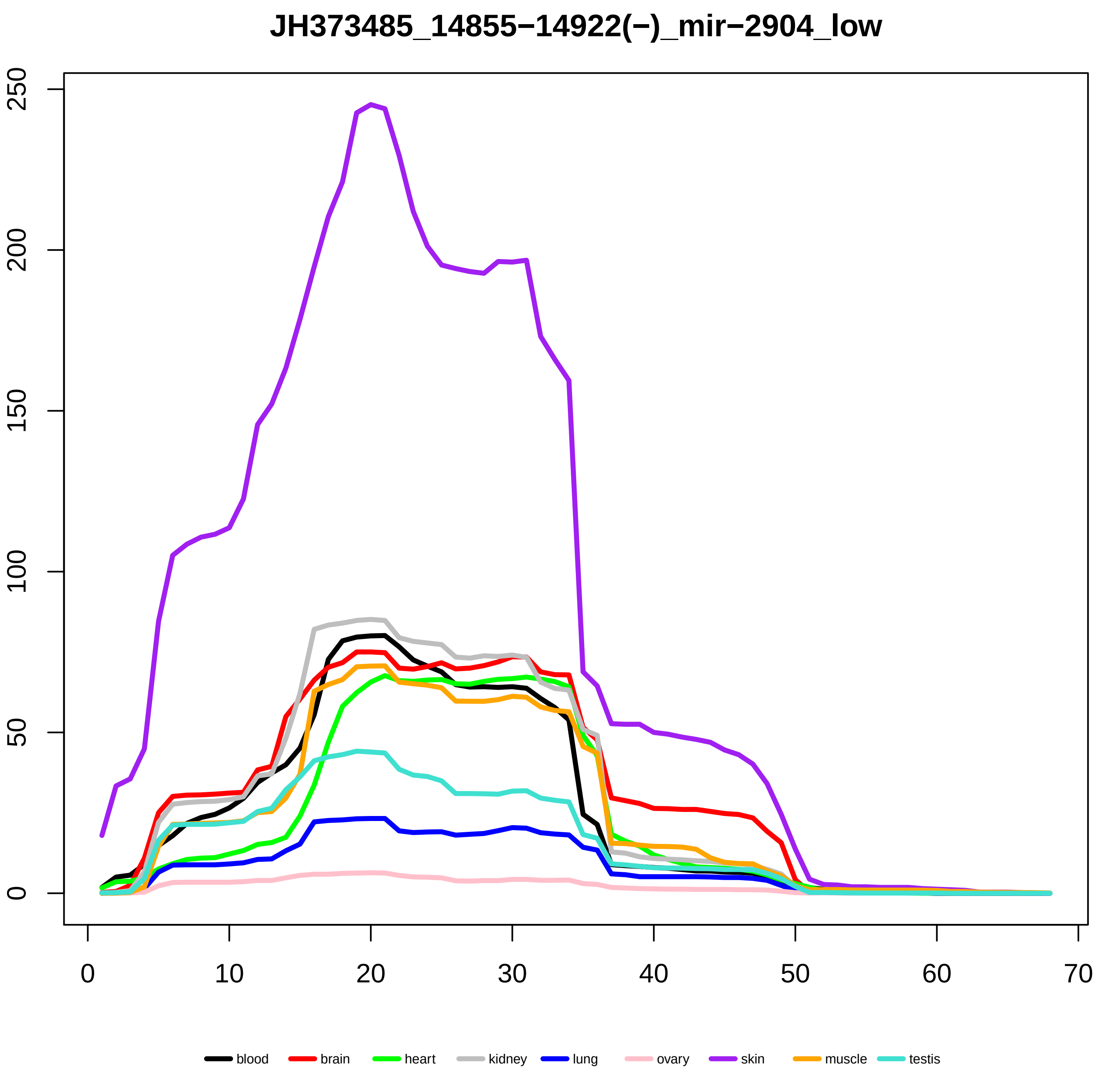

JH373485_14855−14922(−)_mir−2904_low
0
50
100
150
200
250
0
10
20
30
40
50
60
70
blood
brain
heart
kidney
lung
ovary
skin
muscle
testis

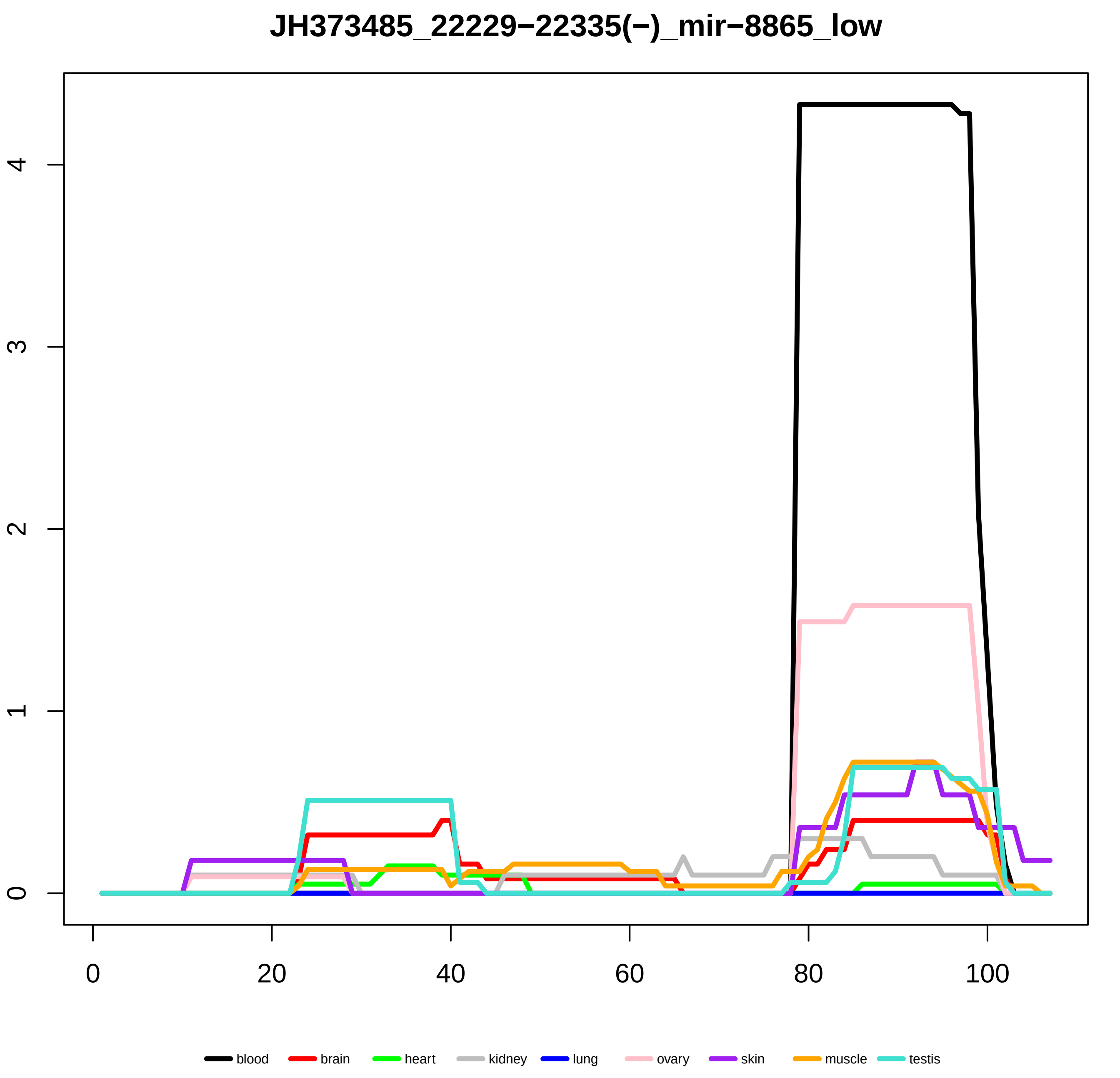
JH373485_22229−22335(−)_mir−8865_low
0
1
2
3
4
0
20
40
60
80
100
blood
brain
heart
kidney
lung
ovary
skin
muscle
testis

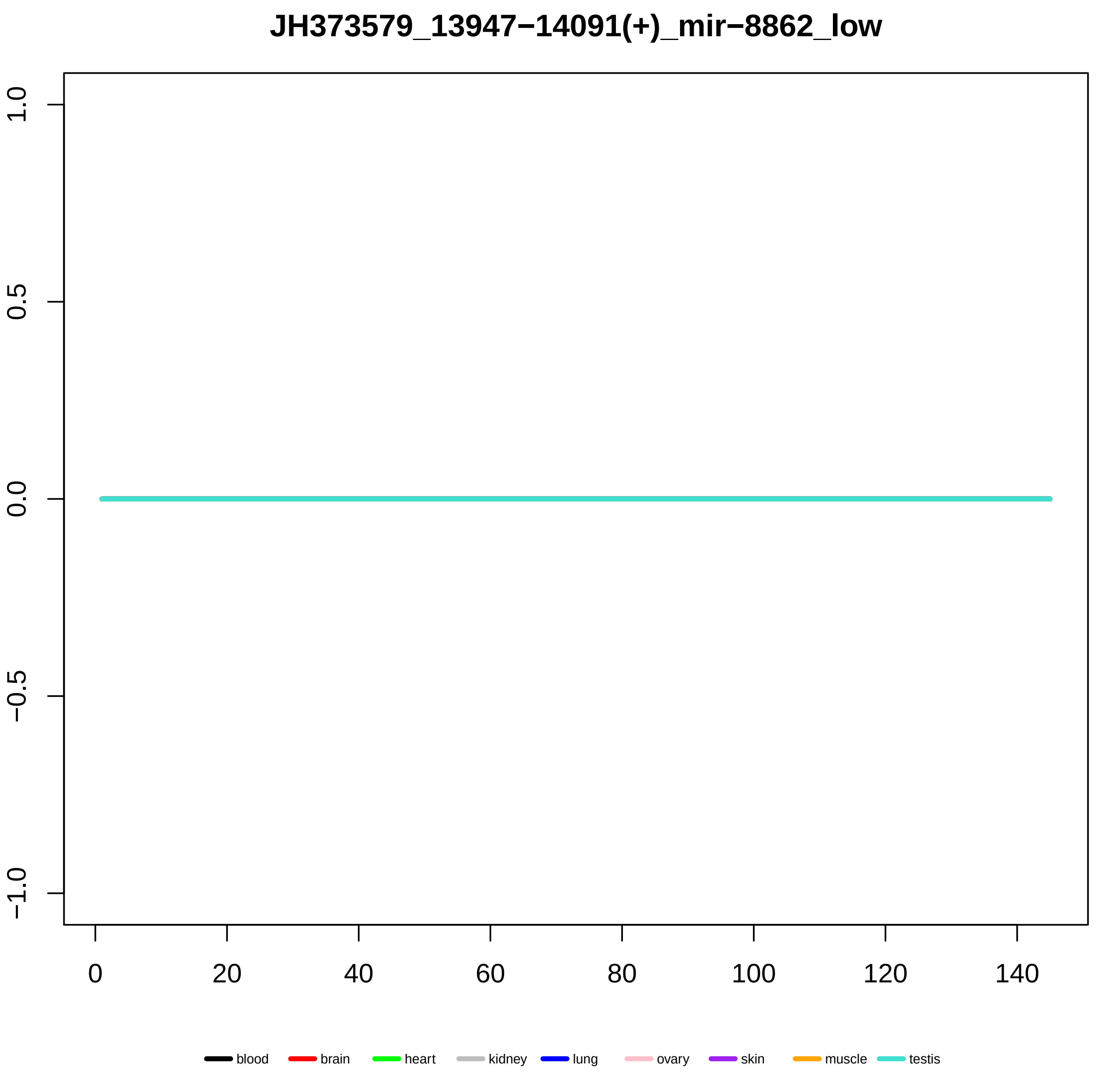
JH373579_13947−14091(+)_mir−8862_low
1.0
0.5
0.0
−0.5
−1.0
0
20
40
60
80
100
120
140
blood
brain
heart
kidney
lung
ovary
skin
muscle
testis

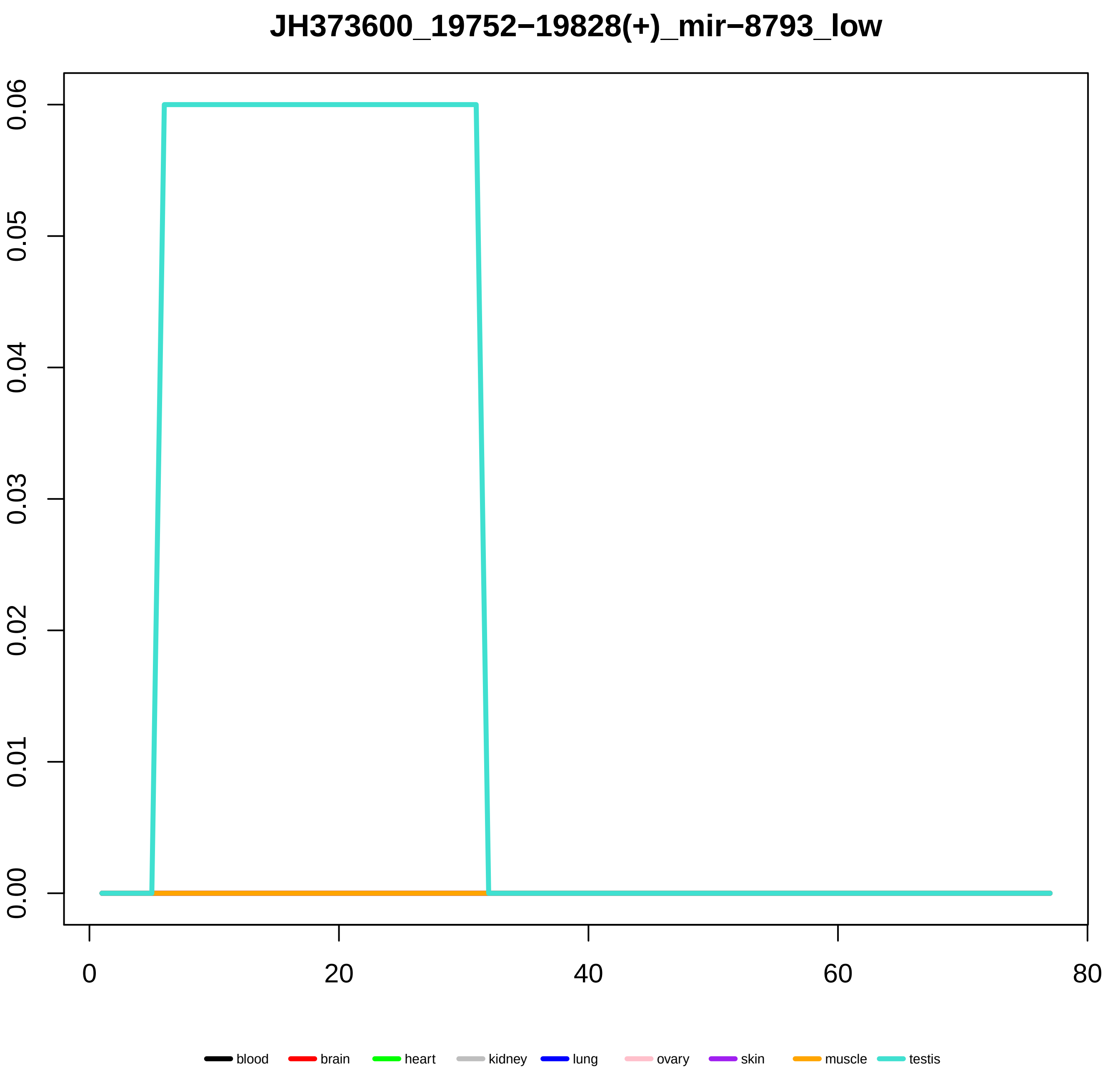

JH373600_19752−19828(+)_mir−8793_low
0.00
0.01
0.02
0.03
0.04
0.05
0.06
0
20
40
60
80
blood
brain
heart
kidney
lung
ovary
skin
muscle
testis

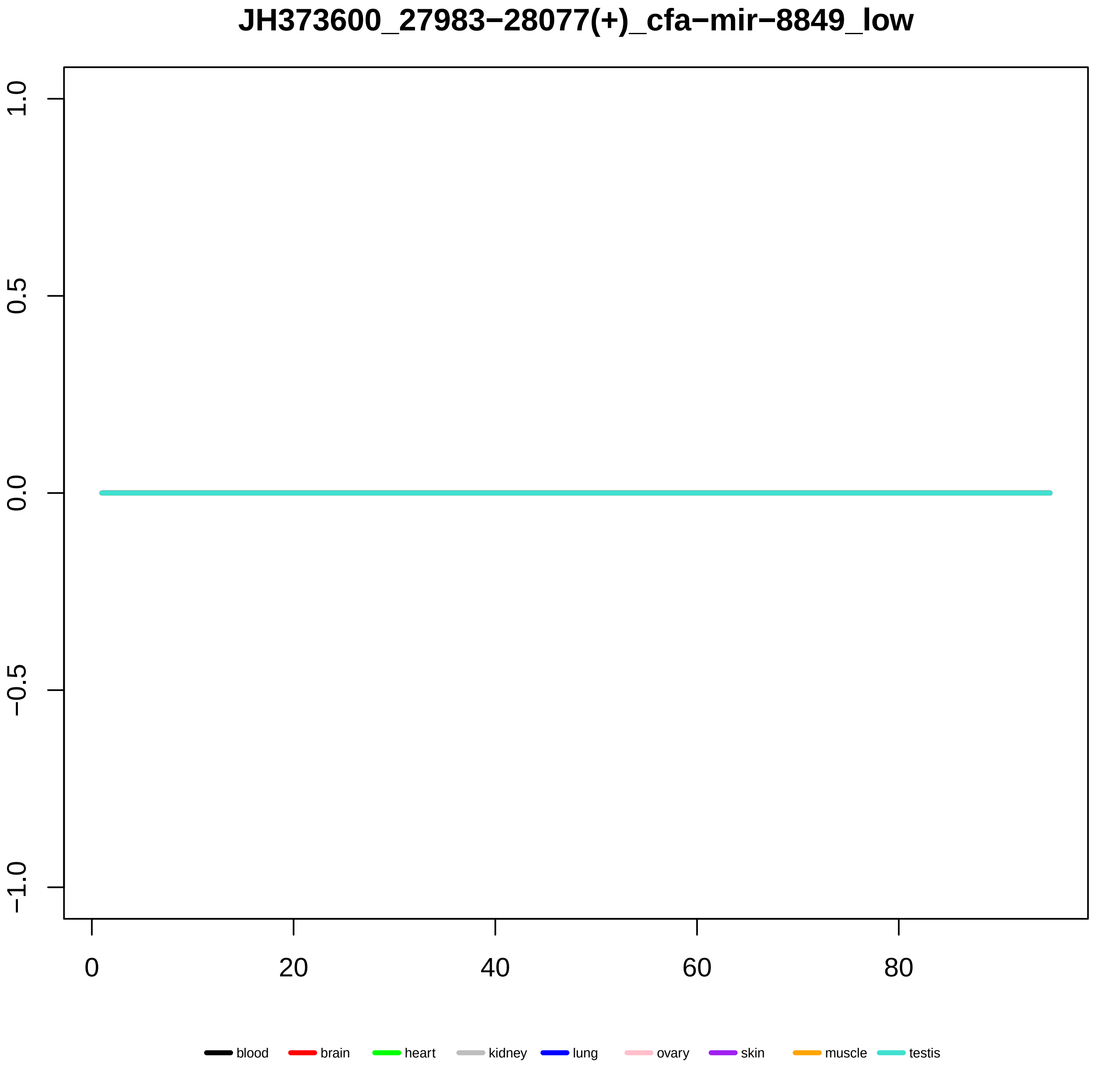

JH373600_27983−28077(+)_cfa−mir−8849_low
1.0
0.5
0.0
−0.5
−1.0
0
20
40
60
80
blood
brain
heart
kidney
lung
ovary
skin
muscle
testis

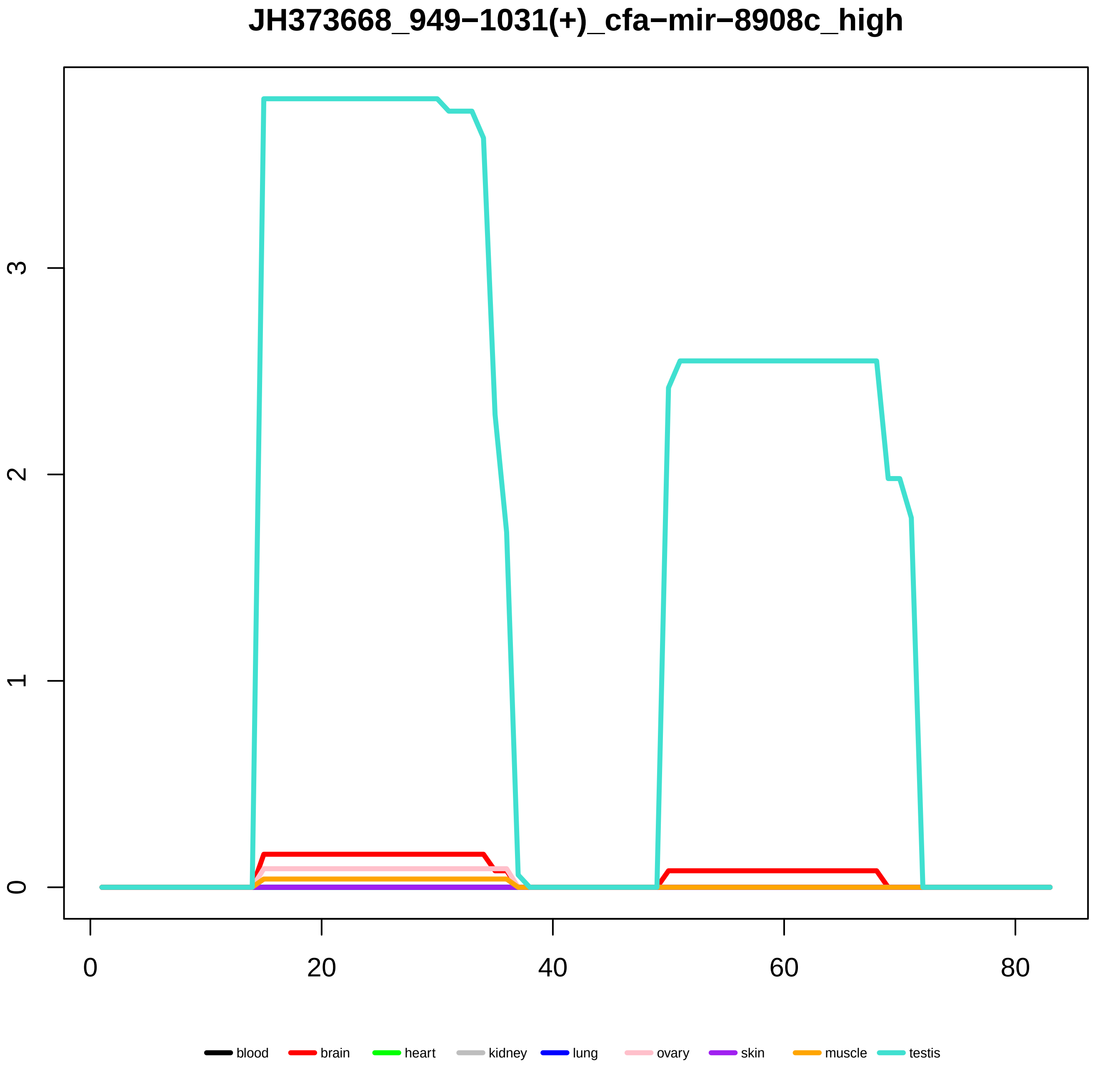

JH373668_949−1031(+)_cfa−mir−8908c_high
0
1
2
3
0
20
40
60
80
blood
brain
heart
kidney
lung
ovary
skin
muscle
testis

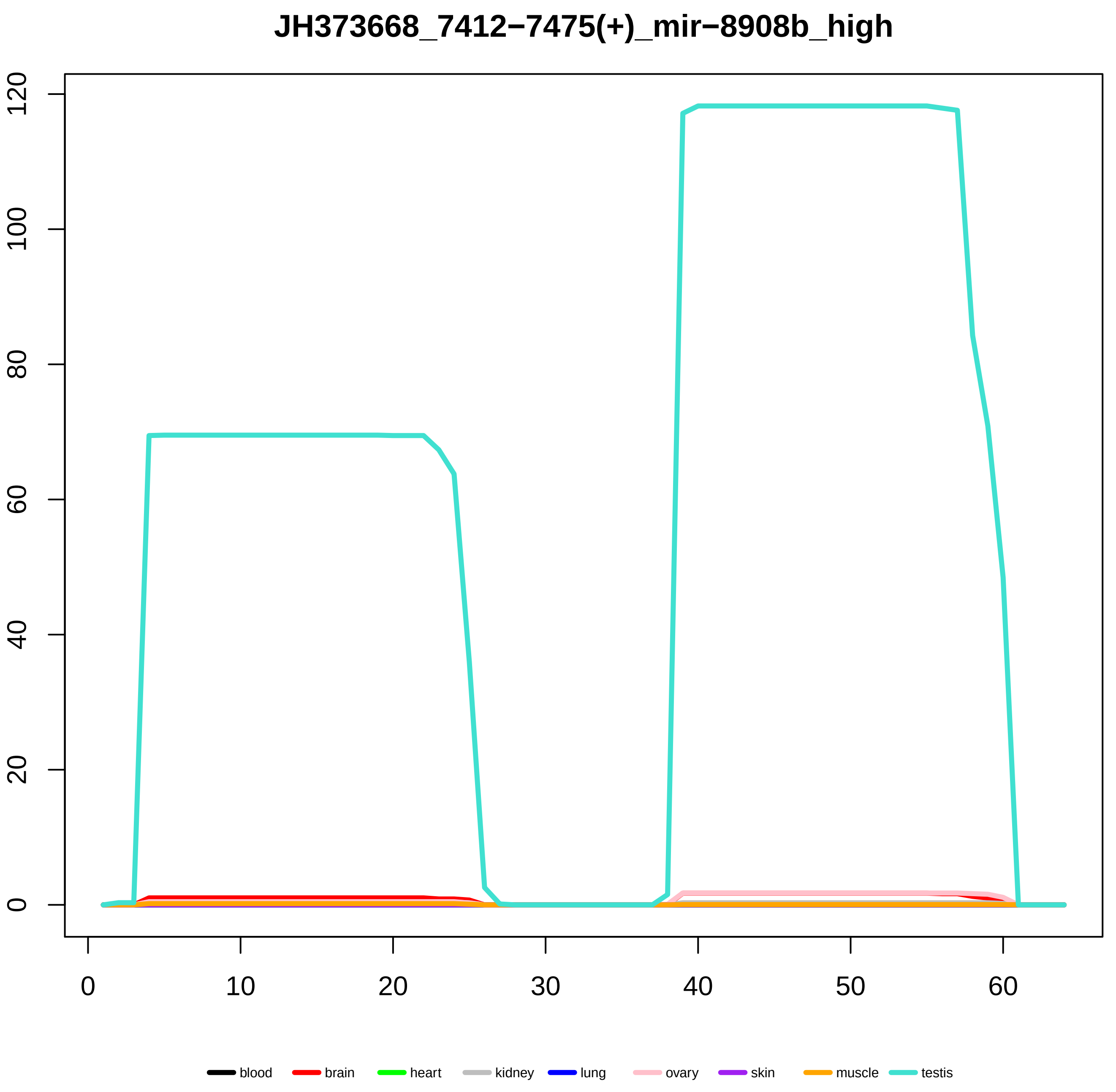
JH373668_7412−7475(+)_mir−8908b_high
0
20
40
60
80
100
120
0
10
20
30
40
50
60
blood
brain
heart
kidney
lung
ovary
skin
muscle
testis

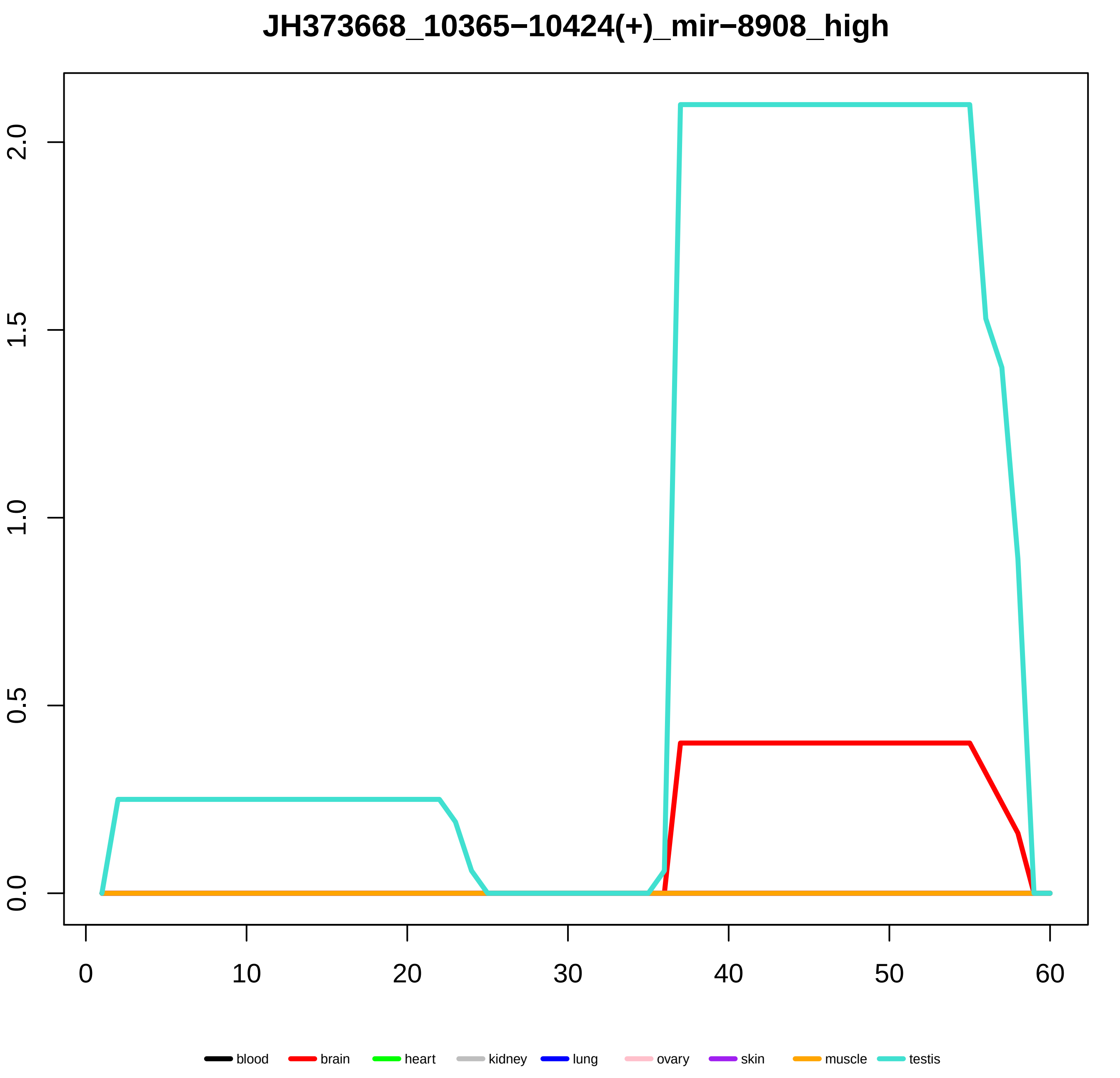

JH373668_10365−10424(+)_mir−8908_high
0.0
0.5
1.0
1.5
2.0
0
10
20
30
40
50
60
blood
brain
heart
kidney
lung
ovary
skin
muscle
testis

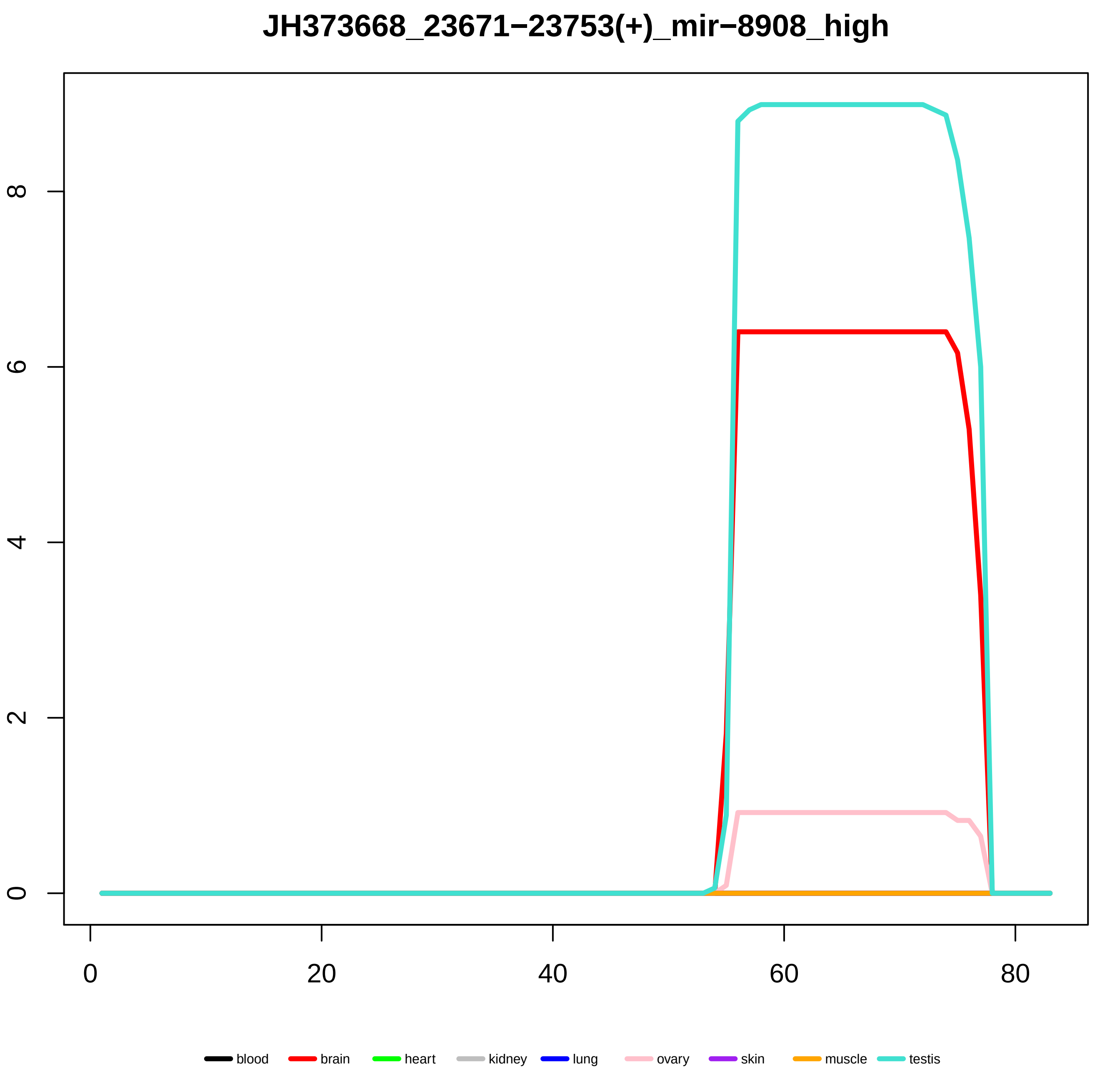

JH373668_23671−23753(+)_mir−8908_high
0
2
4
6
8
0
20
40
60
80
blood
brain
heart
kidney
lung
ovary
skin
muscle
testis

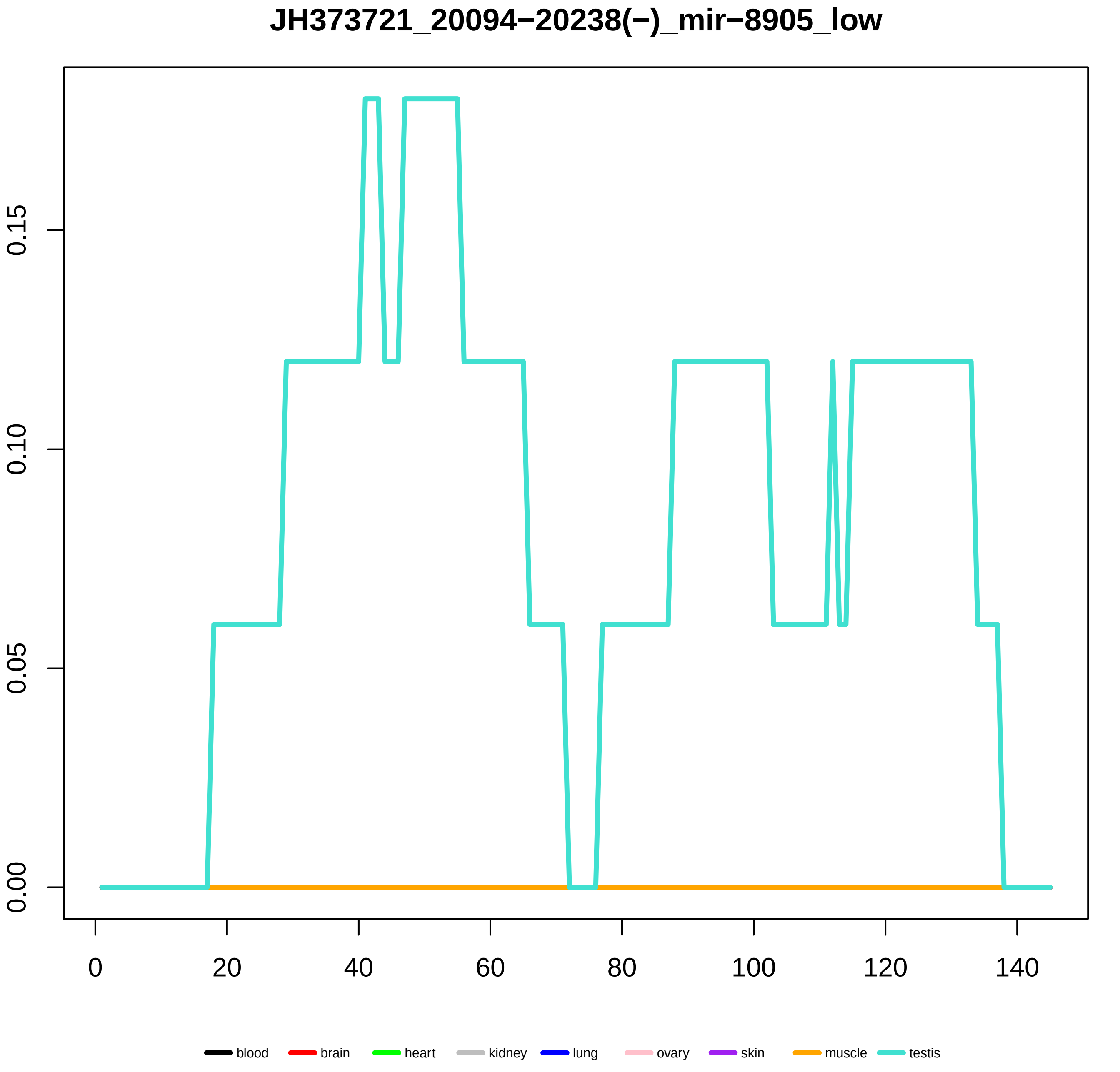

JH373721_20094−20238(−)_mir−8905_low
0.00
0.05
0.10
0.15
0
20
40
60
80
100
120
140
blood
brain
heart
kidney
lung
ovary
skin
muscle
testis

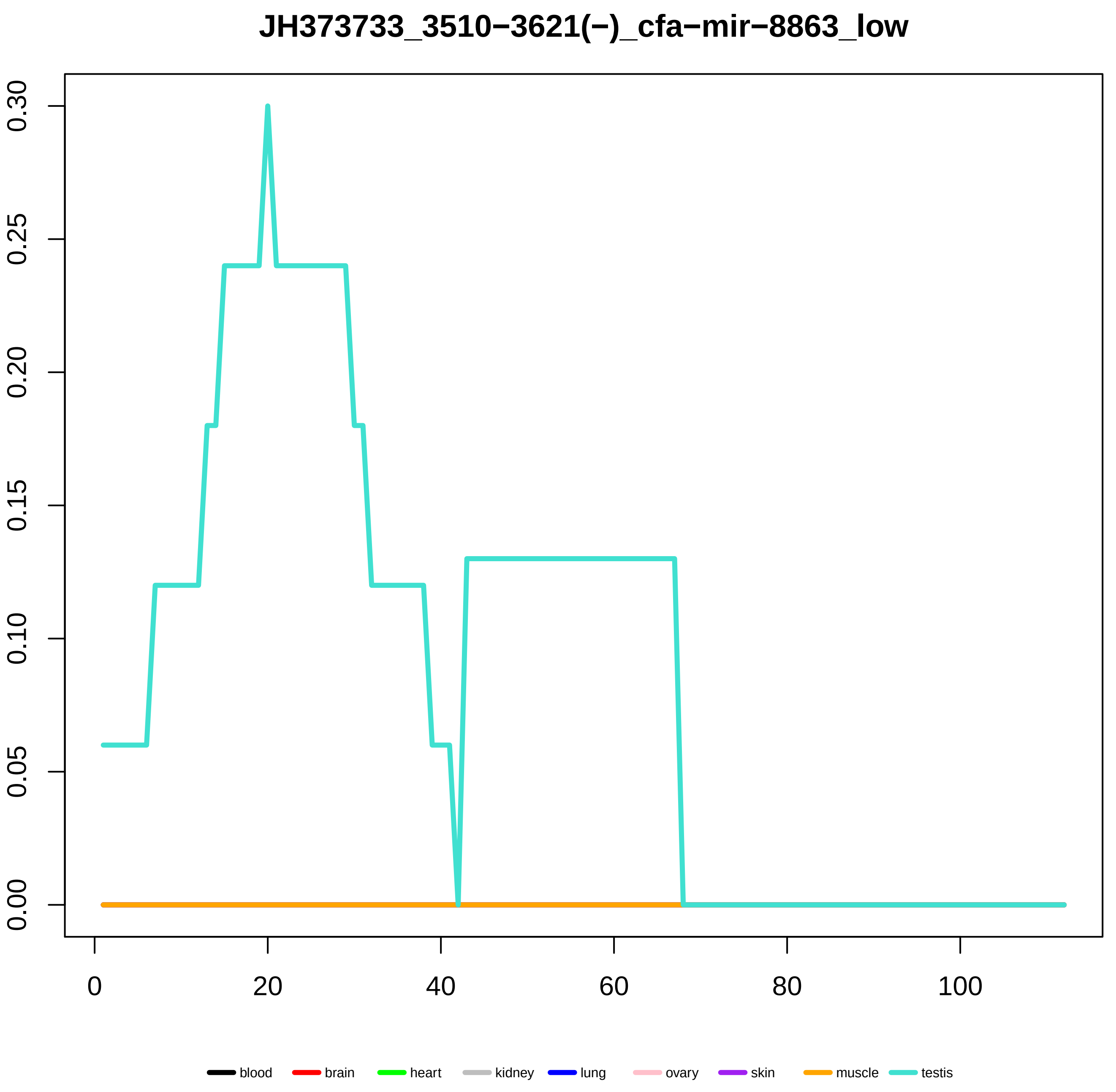

JH373733_3510−3621(−)_cfa−mir−8863_low
0.00
0.05
0.10
0.15
0.20
0.25
0.30
0
20
40
60
80
100
blood
brain
heart
kidney
lung
ovary
skin
muscle
testis

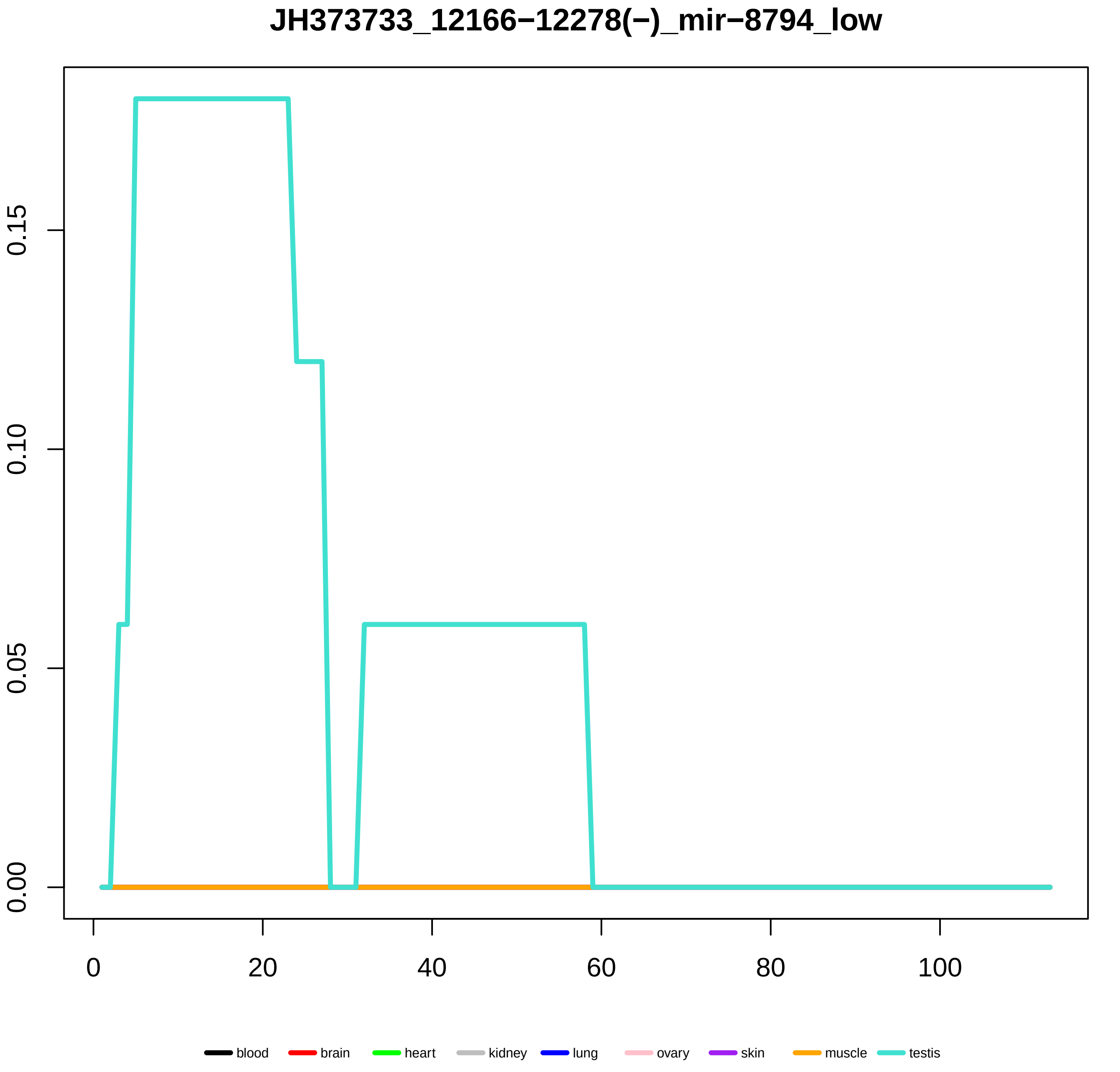
JH373733_12166−12278(−)_mir−8794_low
0.00
0.05
0.10
0.15
0
20
40
60
80
100
blood
brain
heart
kidney
lung
ovary
skin
muscle
testis

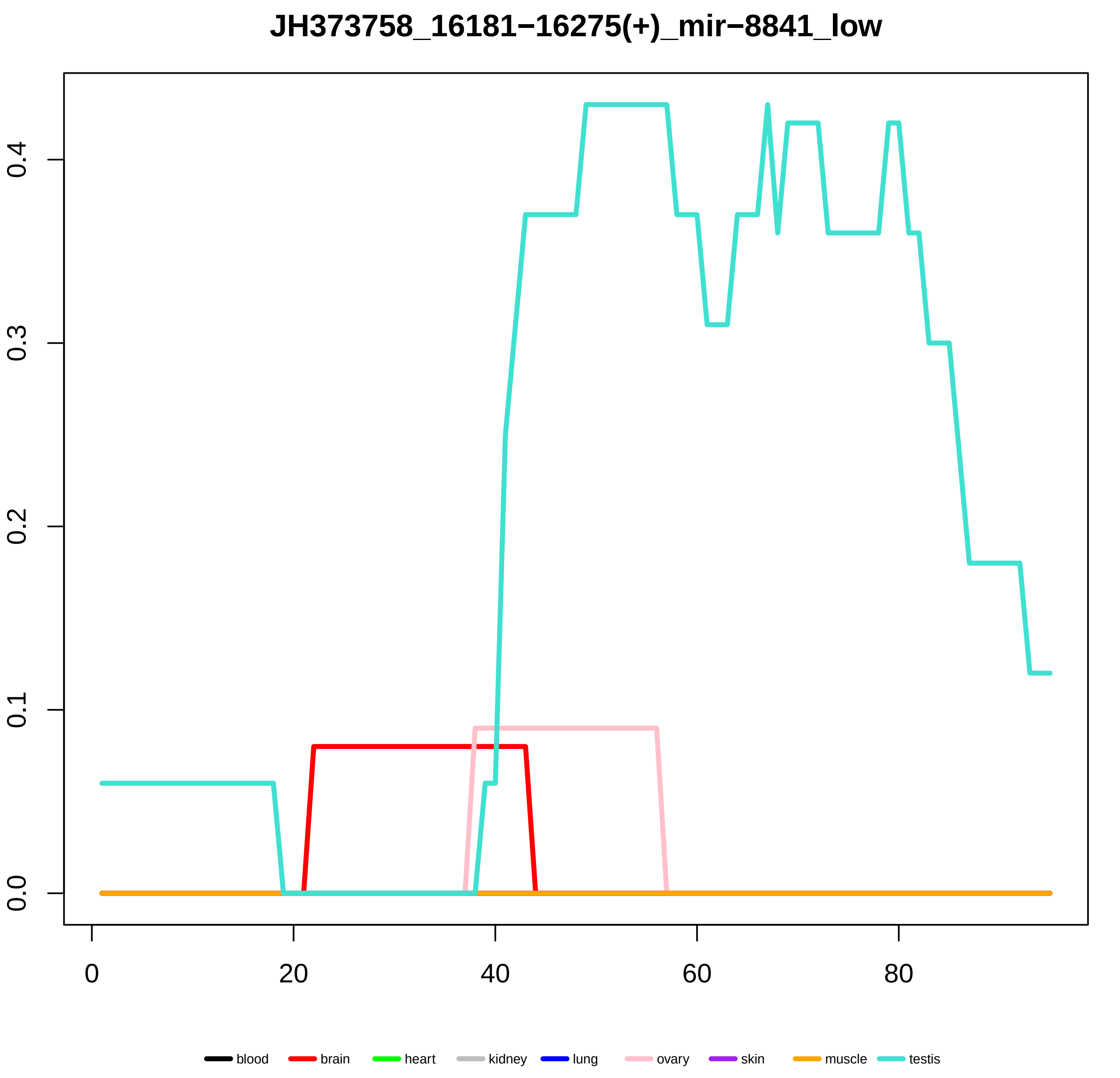
JH373758_16181−16275(+)_mir−8841_low
0.0
0.1
0.2
0.3
0.4
0
20
40
60
80
blood
brain
heart
kidney
lung
ovary
skin
muscle
testis

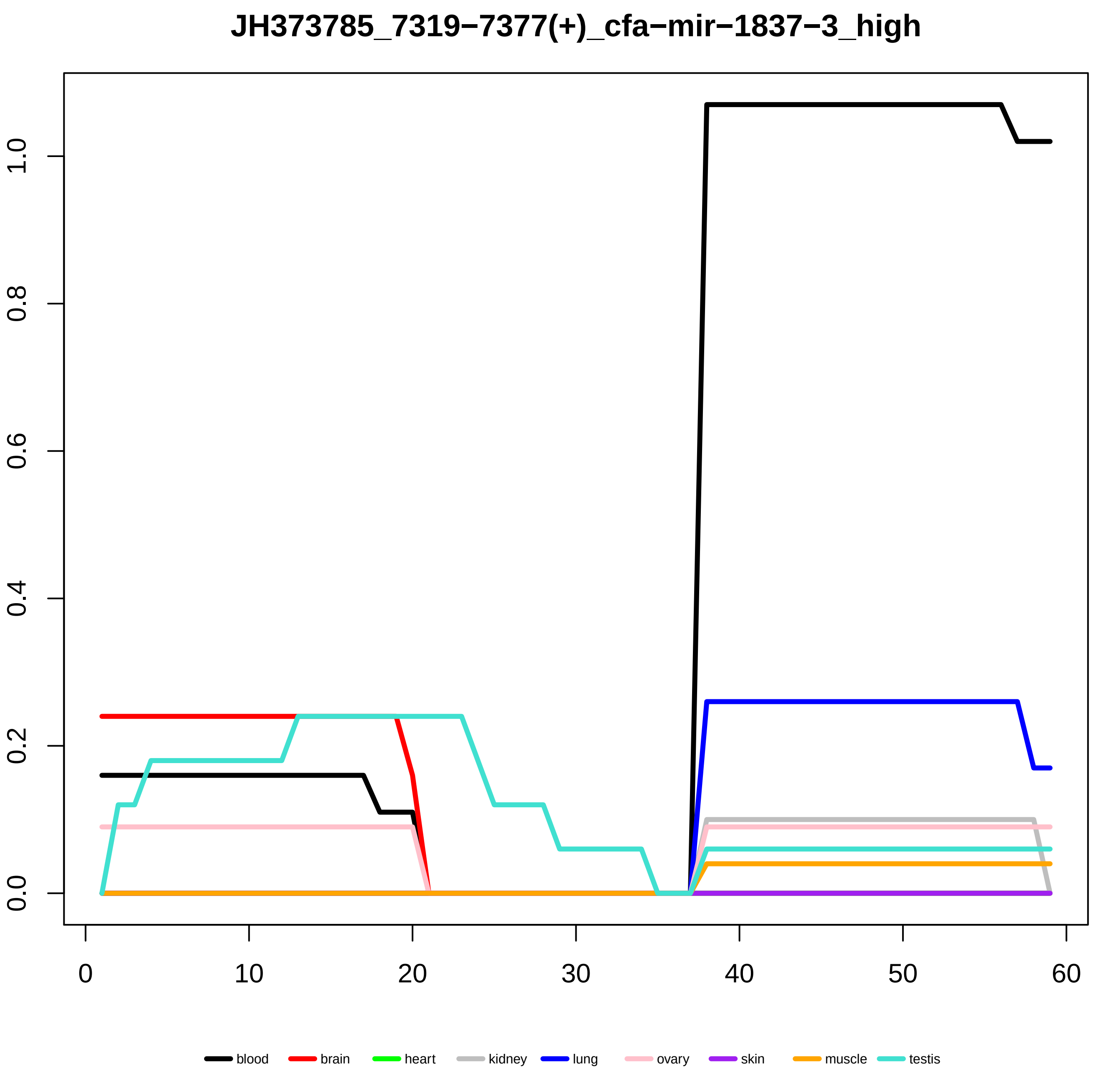

JH373785_7319−7377(+)_cfa−mir−1837−3_high
0.0
0.2
0.4
0.6
0.8
1.0
0
10
20
30
40
50
60
blood
brain
heart
kidney
lung
ovary
skin
muscle
testis

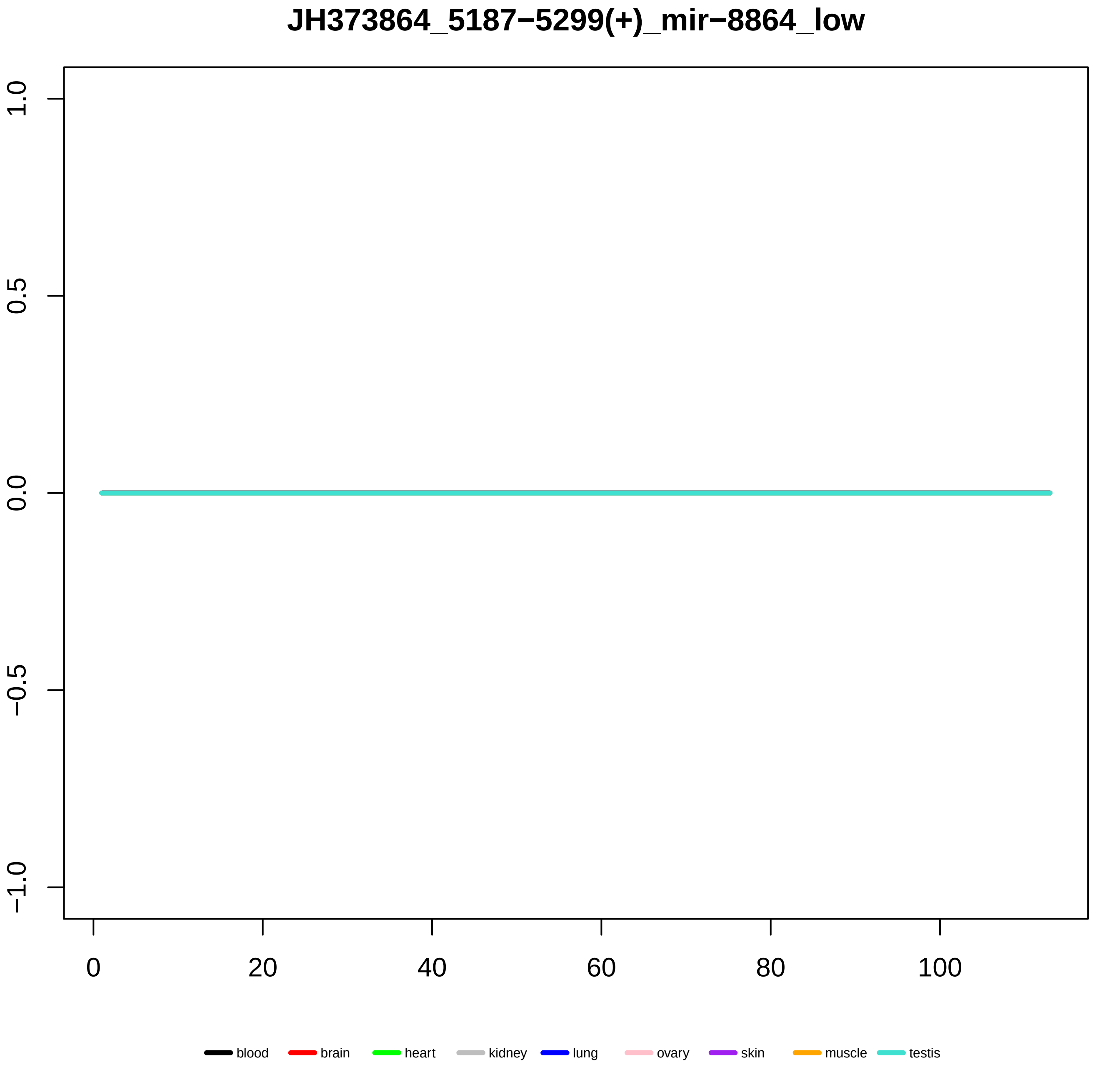
JH373864_5187−5299(+)_mir−8864_low
1.0
0.5
0.0
−0.5
−1.0
0
20
40
60
80
100
blood
brain
heart
kidney
lung
ovary
skin
muscle
testis

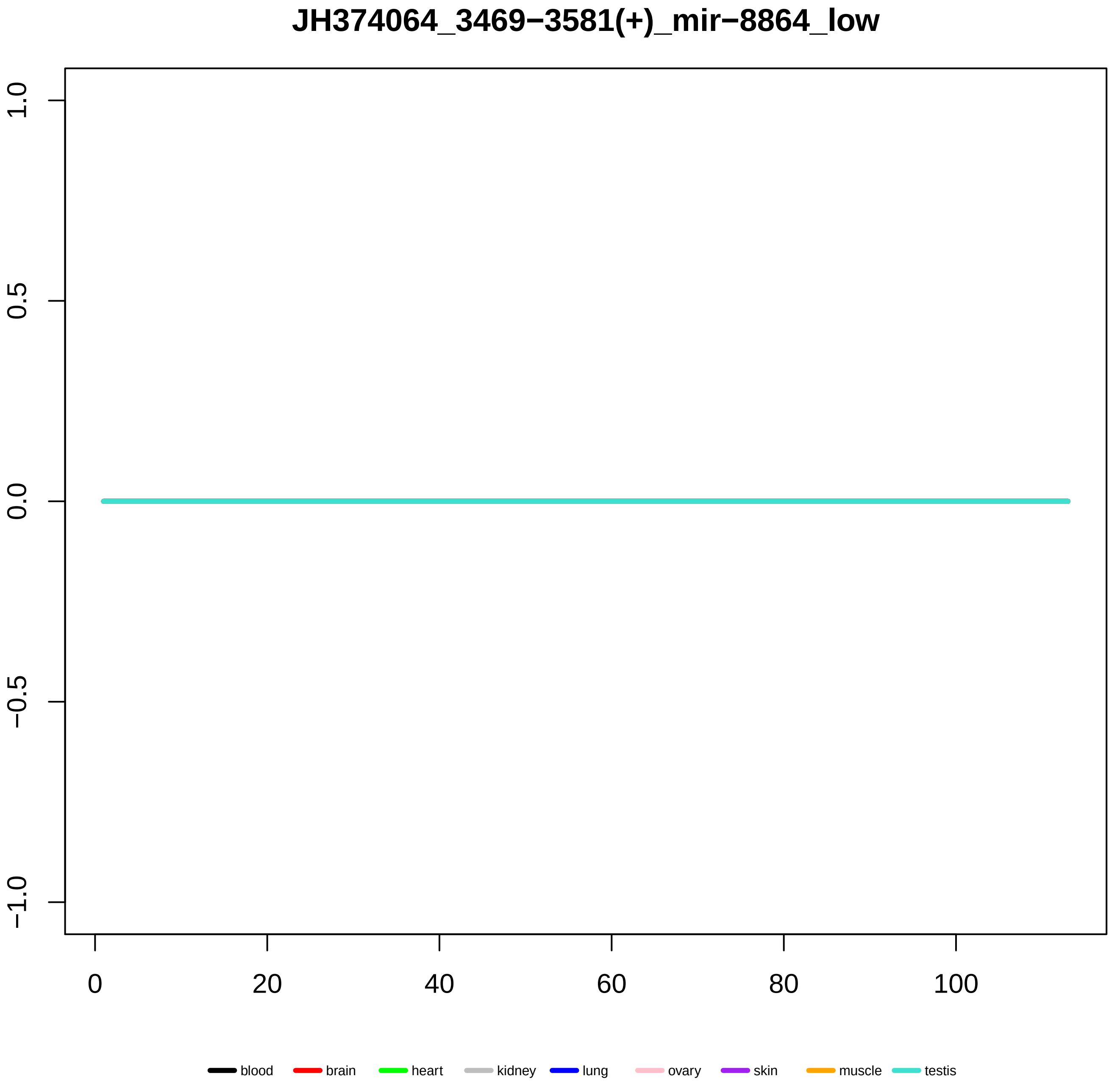
JH374064_3469−3581(+)_mir−8864_low
1.0
0.5
0.0
−0.5
−1.0
0
20
40
60
80
100
blood
brain
heart
kidney
lung
ovary
skin
muscle
testis

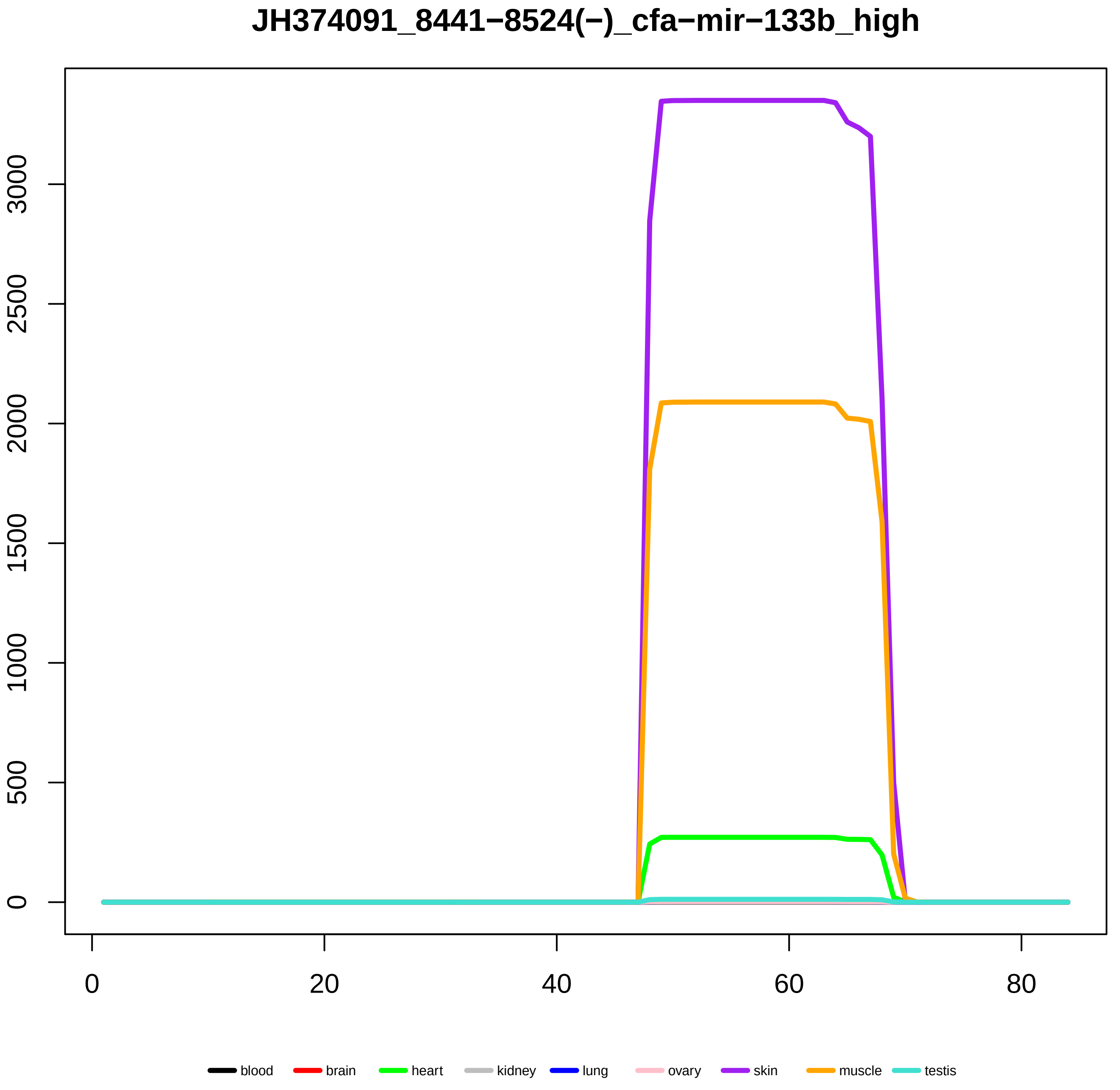
JH374091_8441−8524(−)_cfa−mir−133b_high
0
500
1000
1500
2000
2500
3000
0
20
40
60
80
blood
brain
heart
kidney
lung
ovary
skin
muscle
testis

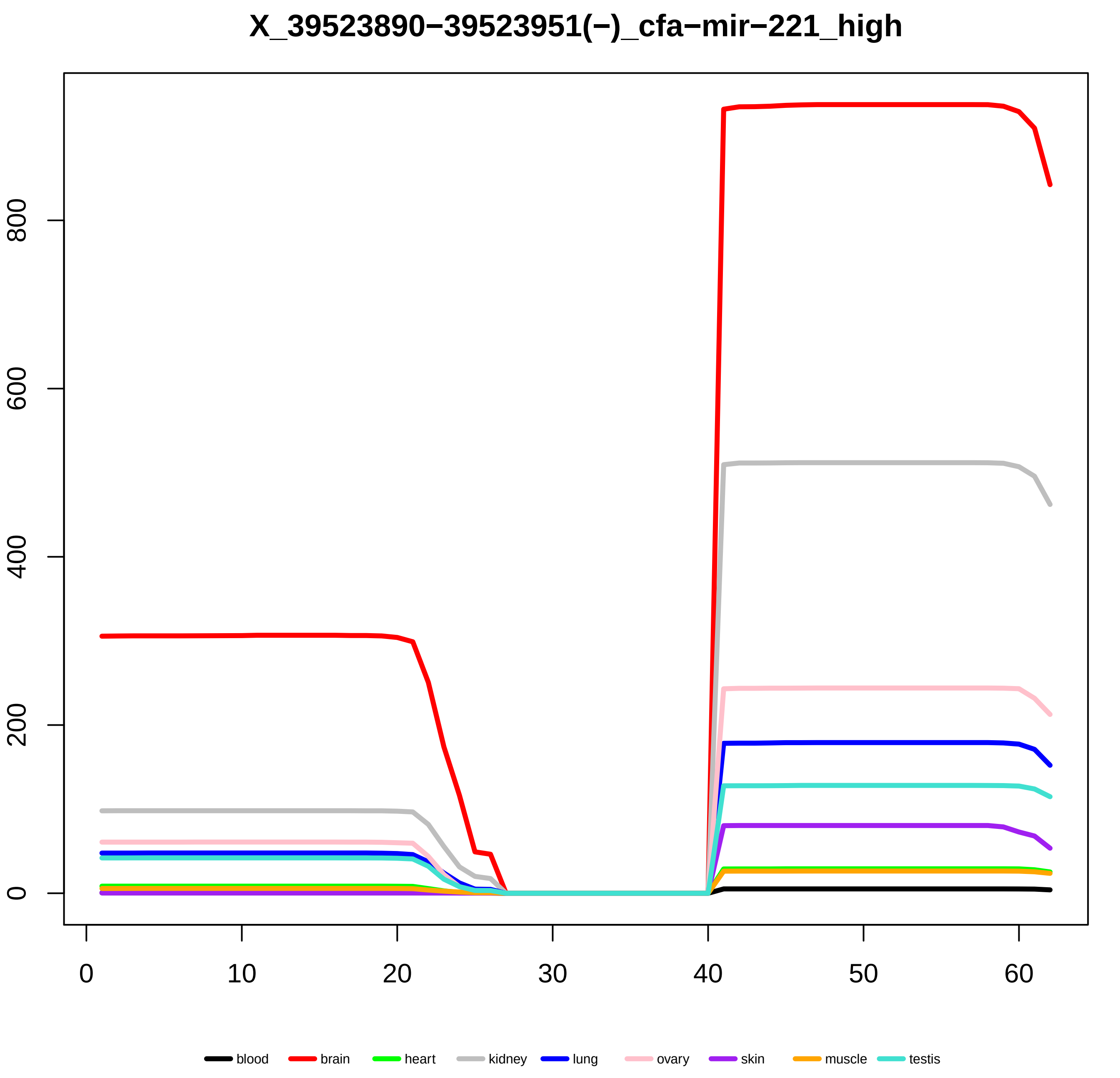
X_39523890−39523951(−)_cfa−mir−221_high
0
200
400
600
800
0
10
20
30
40
50
60
blood
brain
heart
kidney
lung
ovary
skin
muscle
testis

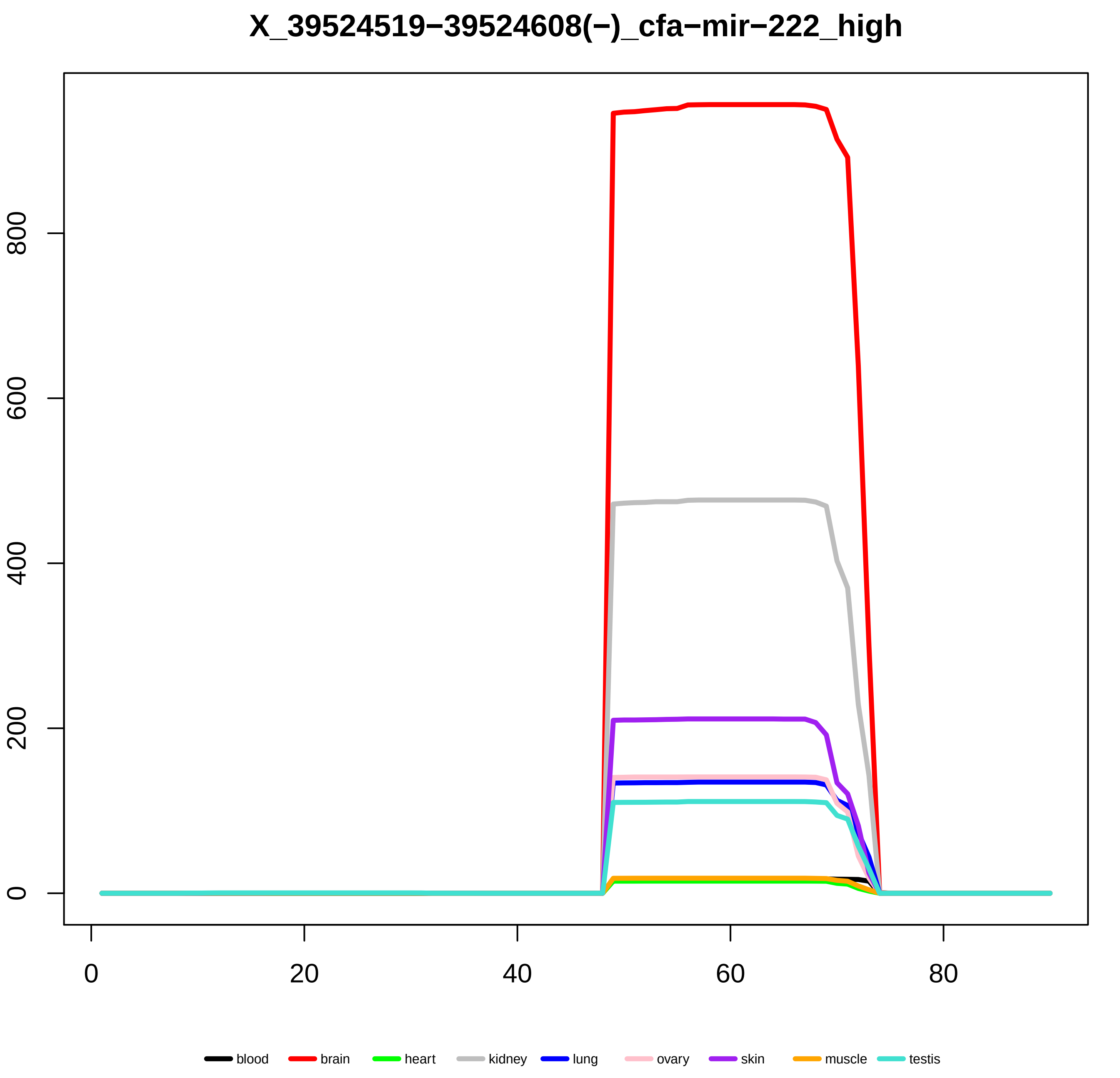
X_39524519−39524608(−)_cfa−mir−222_high
0
200
400
600
800
0
20
40
60
80
blood
brain
heart
kidney
lung
ovary
skin
muscle
testis

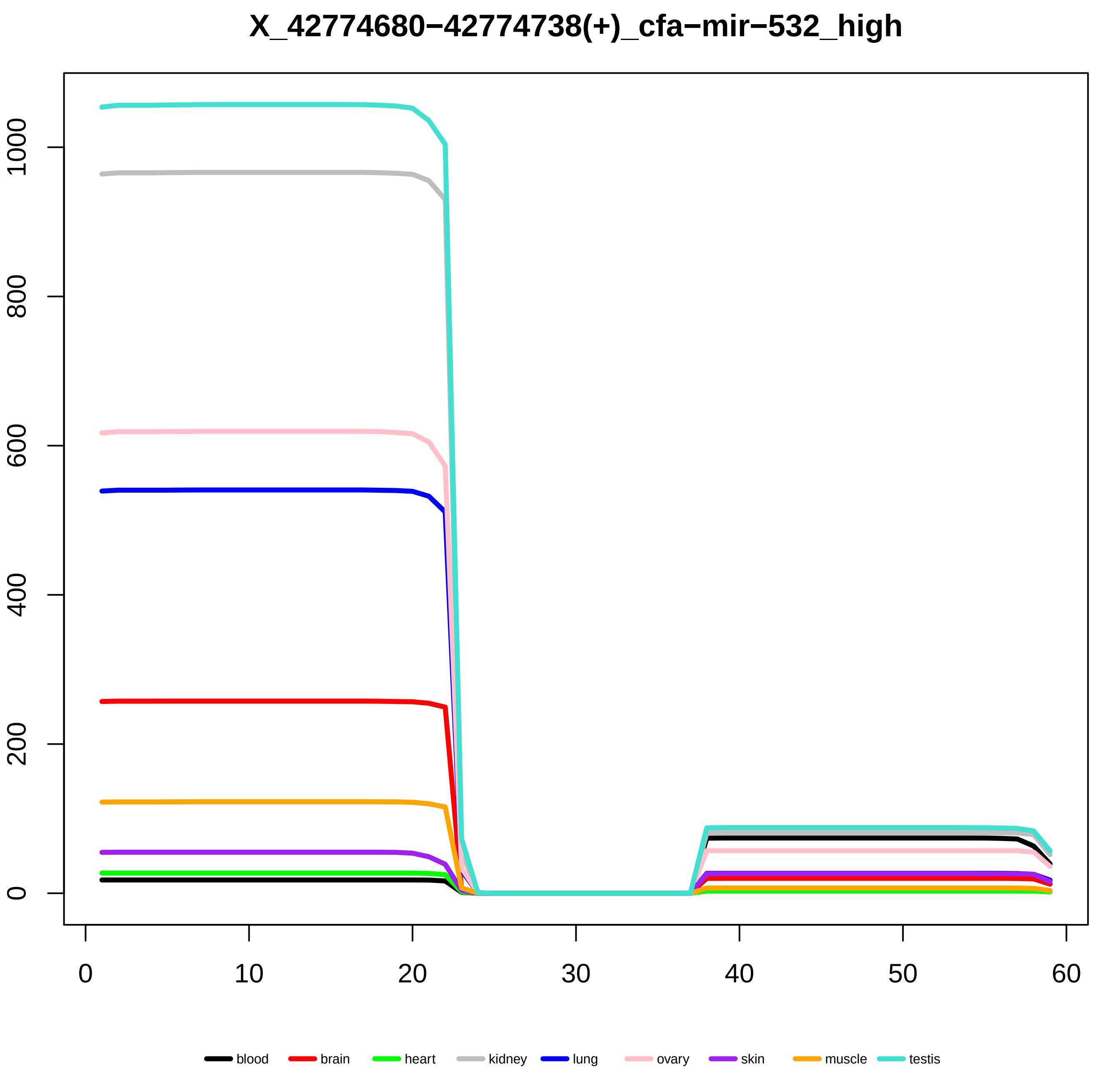

X_42774680−42774738(+)_cfa−mir−532_high
0
200
400
600
800
1000
0
10
20
30
40
50
60
blood
brain
heart
kidney
lung
ovary
skin
muscle
testis

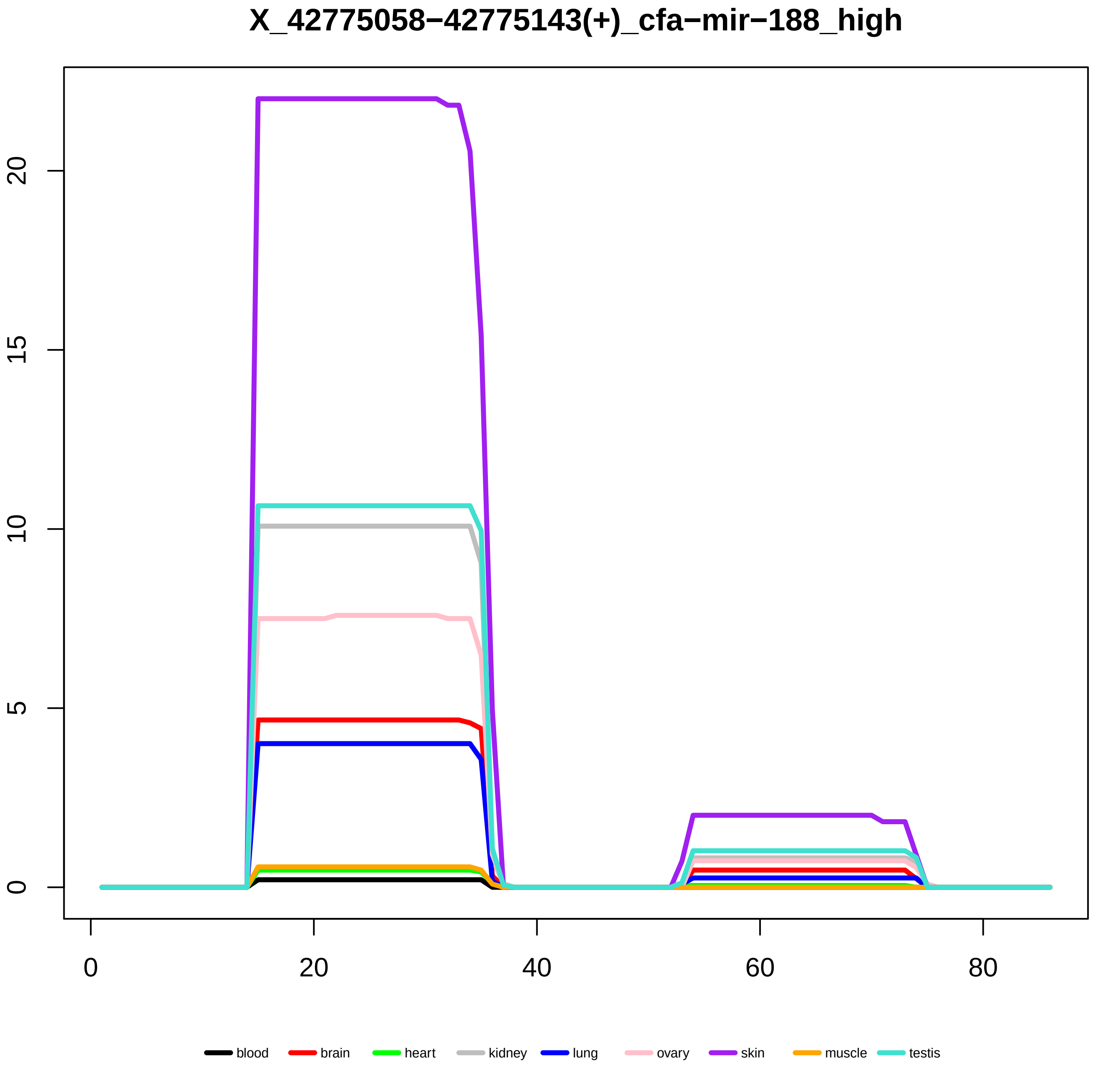
X_42775058−42775143(+)_cfa−mir−188_high
0
5
10
15
20
0
20
40
60
80
blood
brain
heart
kidney
lung
ovary
skin
muscle
testis

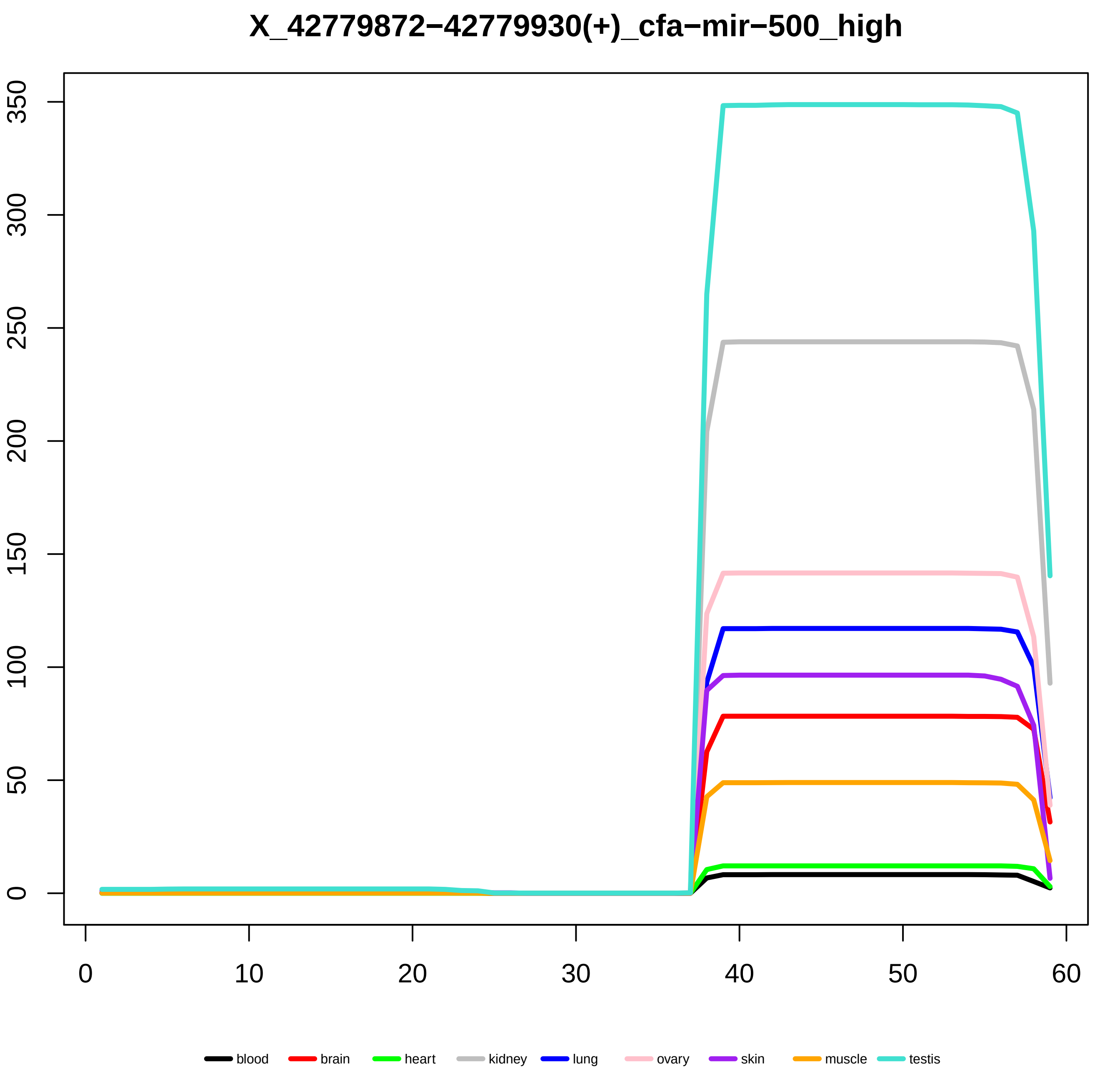

X_42779872−42779930(+)_cfa−mir−500_high
0
50
100
150
200
250
300
350
0
10
20
30
40
50
60
blood
brain
heart
kidney
lung
ovary
skin
muscle
testis

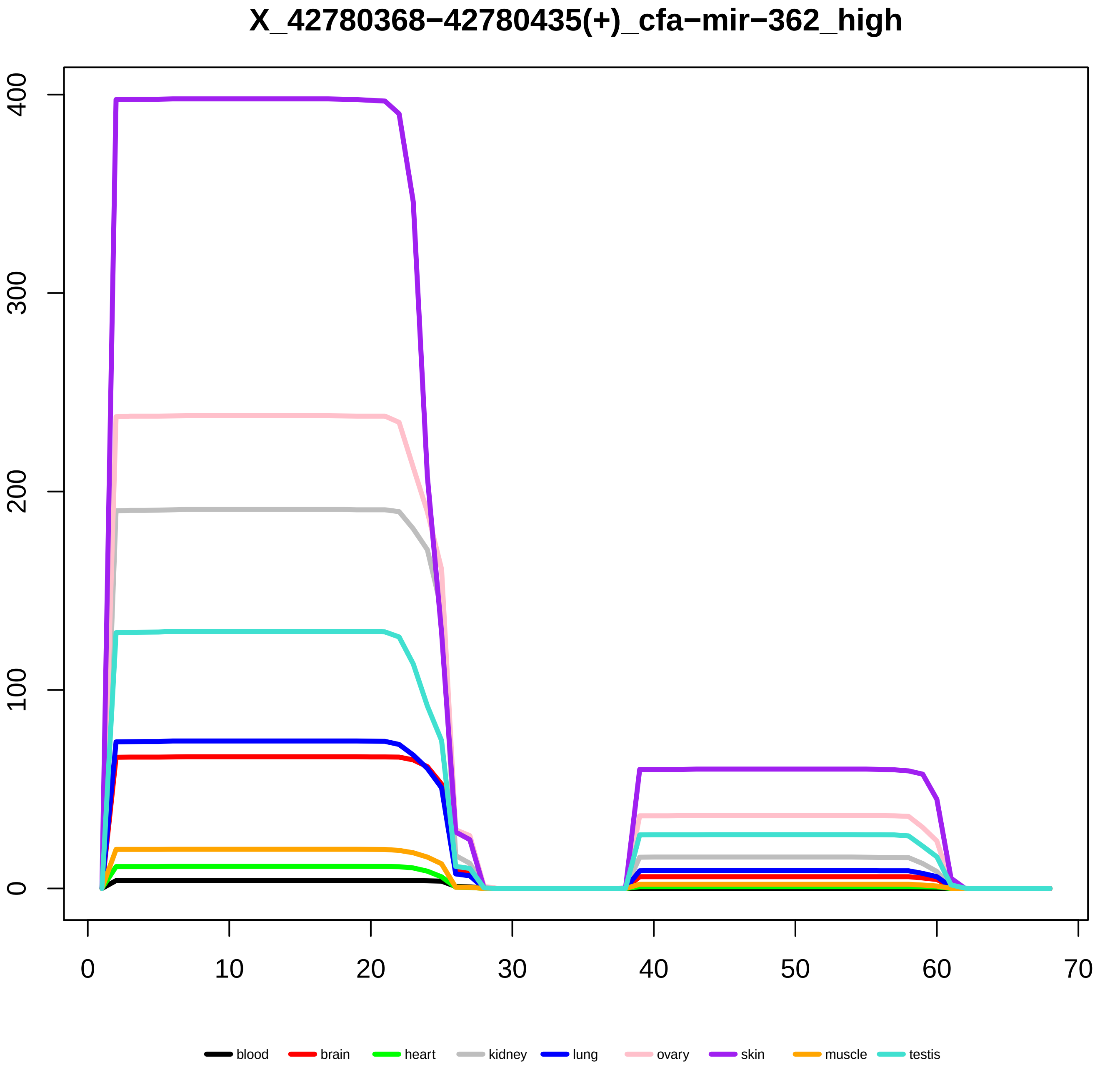

X_42780368−42780435(+)_cfa−mir−362_high
0
100
200
300
400
0
10
20
30
40
50
60
70
blood
brain
heart
kidney
lung
ovary
skin
muscle
testis

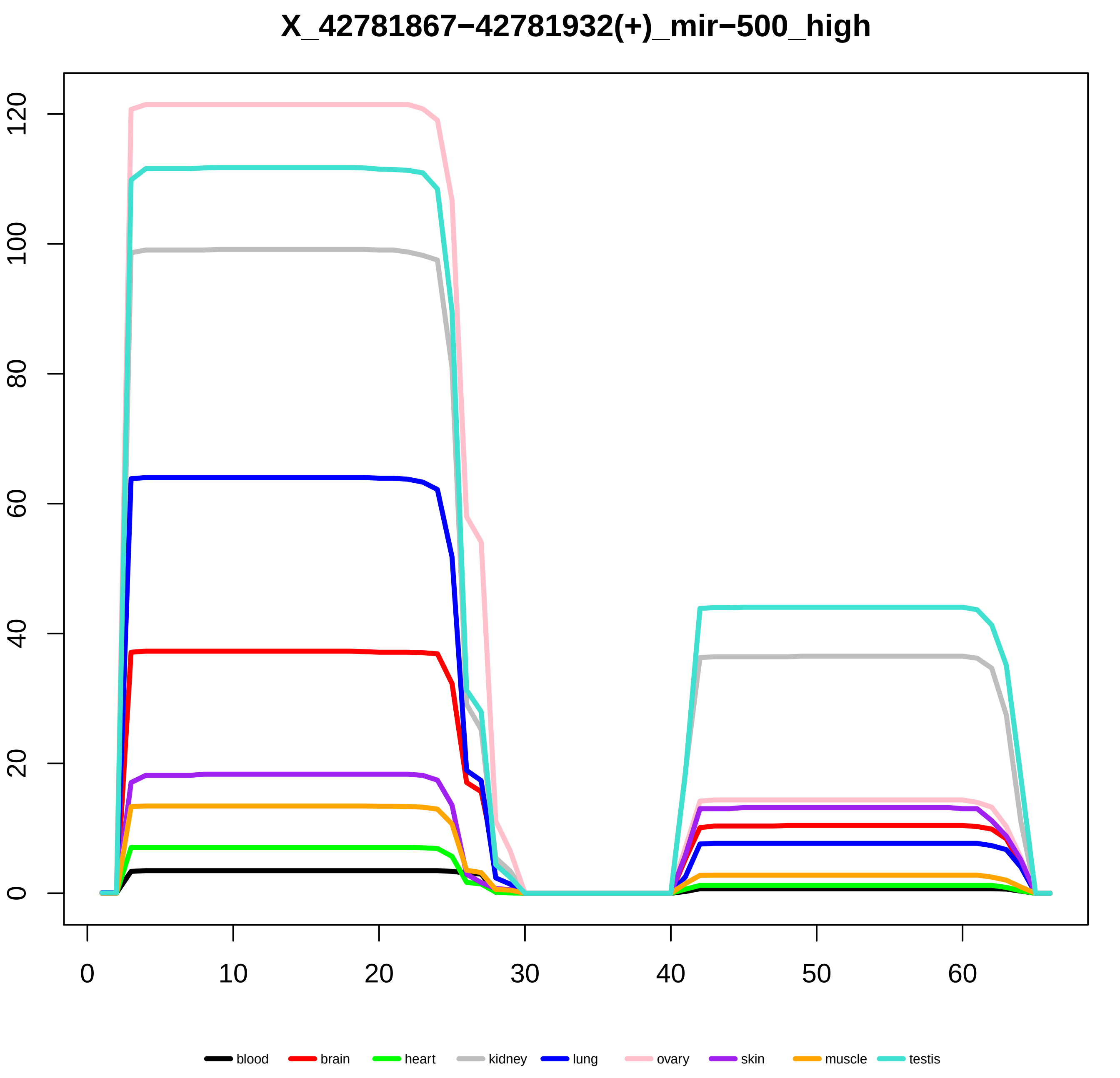

X_42781867−42781932(+)_mir−500_high
0
20
40
60
80
100
120
0
10
20
30
40
50
60
blood
brain
heart
kidney
lung
ovary
skin
muscle
testis

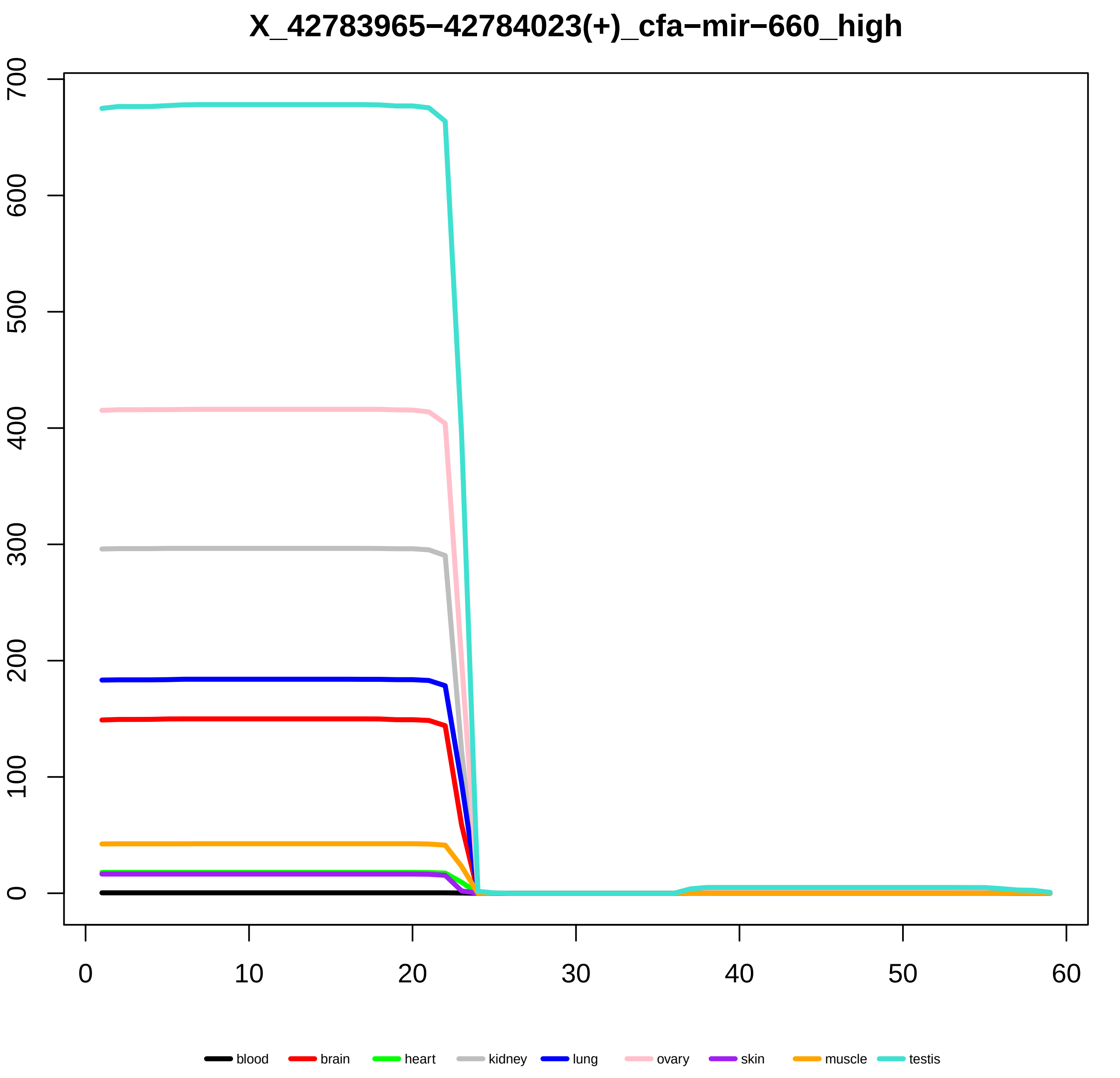

X_42783965−42784023(+)_cfa−mir−660_high
0
100
200
300
400
500
600
700
0
10
20
30
40
50
60
blood
brain
heart
kidney
lung
ovary
skin
muscle
testis

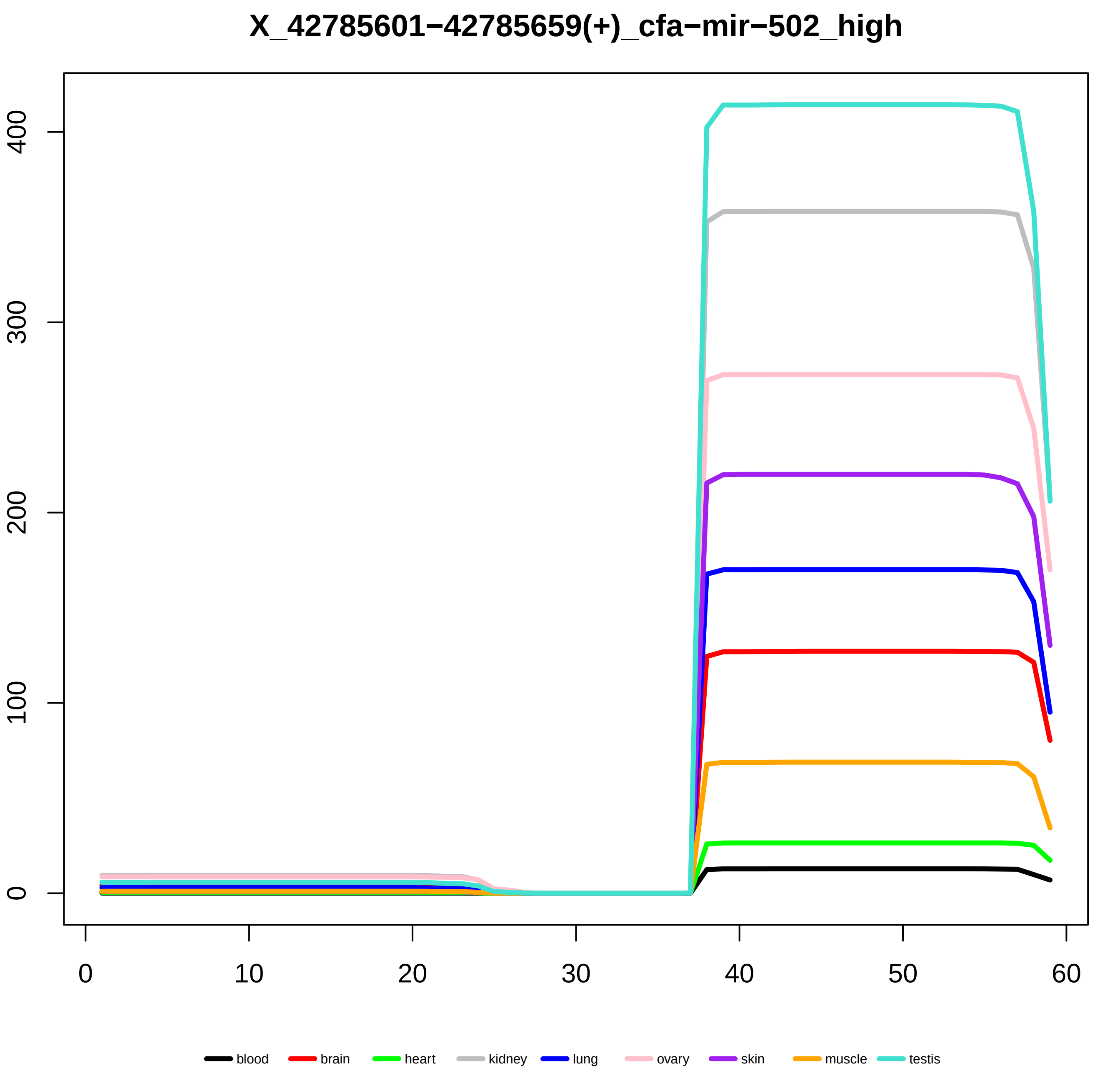
X_42785601−42785659(+)_cfa−mir−502_high
0
100
200
300
400
0
10
20
30
40
50
60
blood
brain
heart
kidney
lung
ovary
skin
muscle
testis

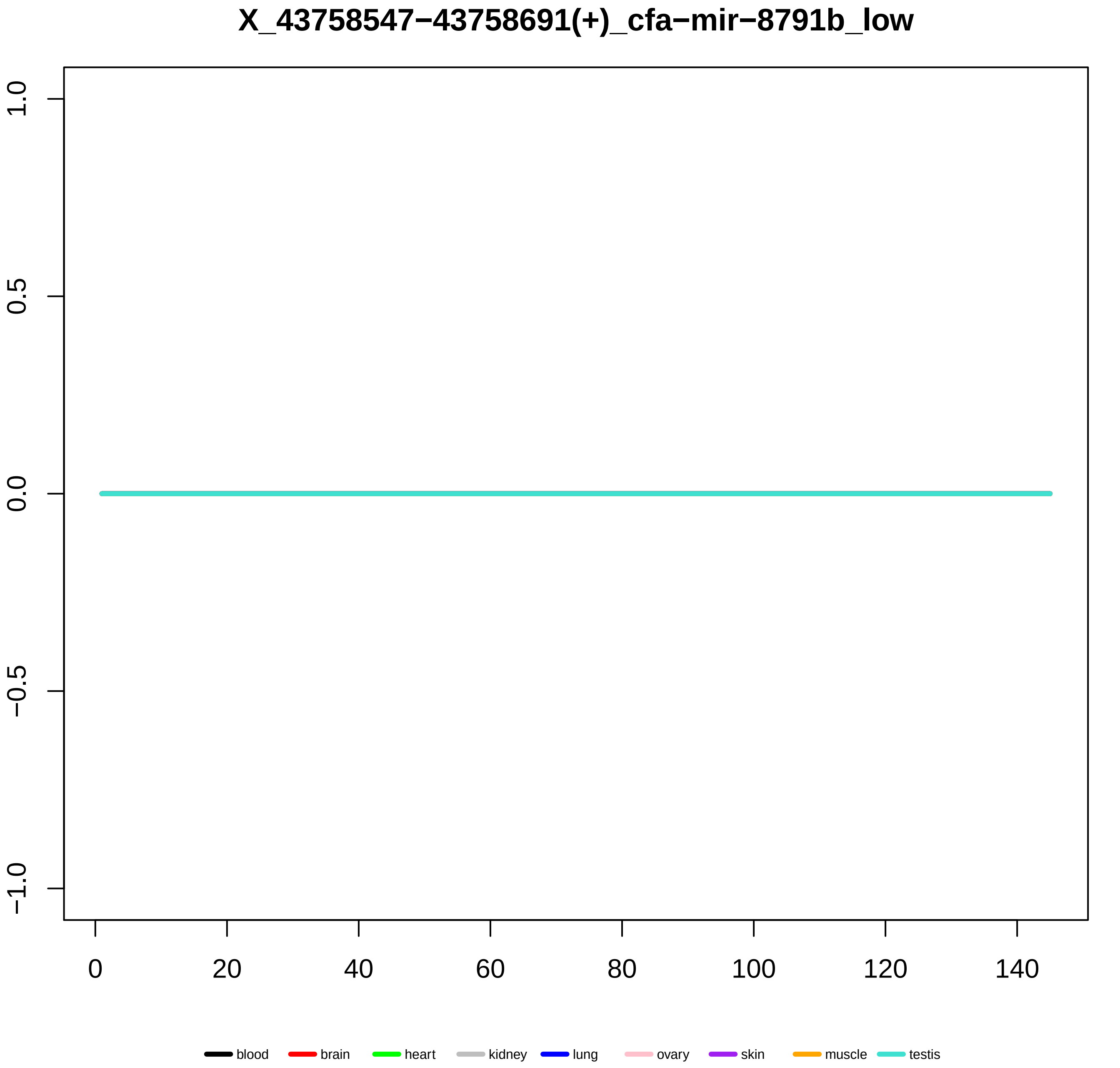
X_43758547−43758691(+)_cfa−mir−8791b_low
1.0
0.5
0.0
−0.5
−1.0
0
20
40
60
80
100
120
140
blood
brain
heart
kidney
lung
ovary
skin
muscle
testis

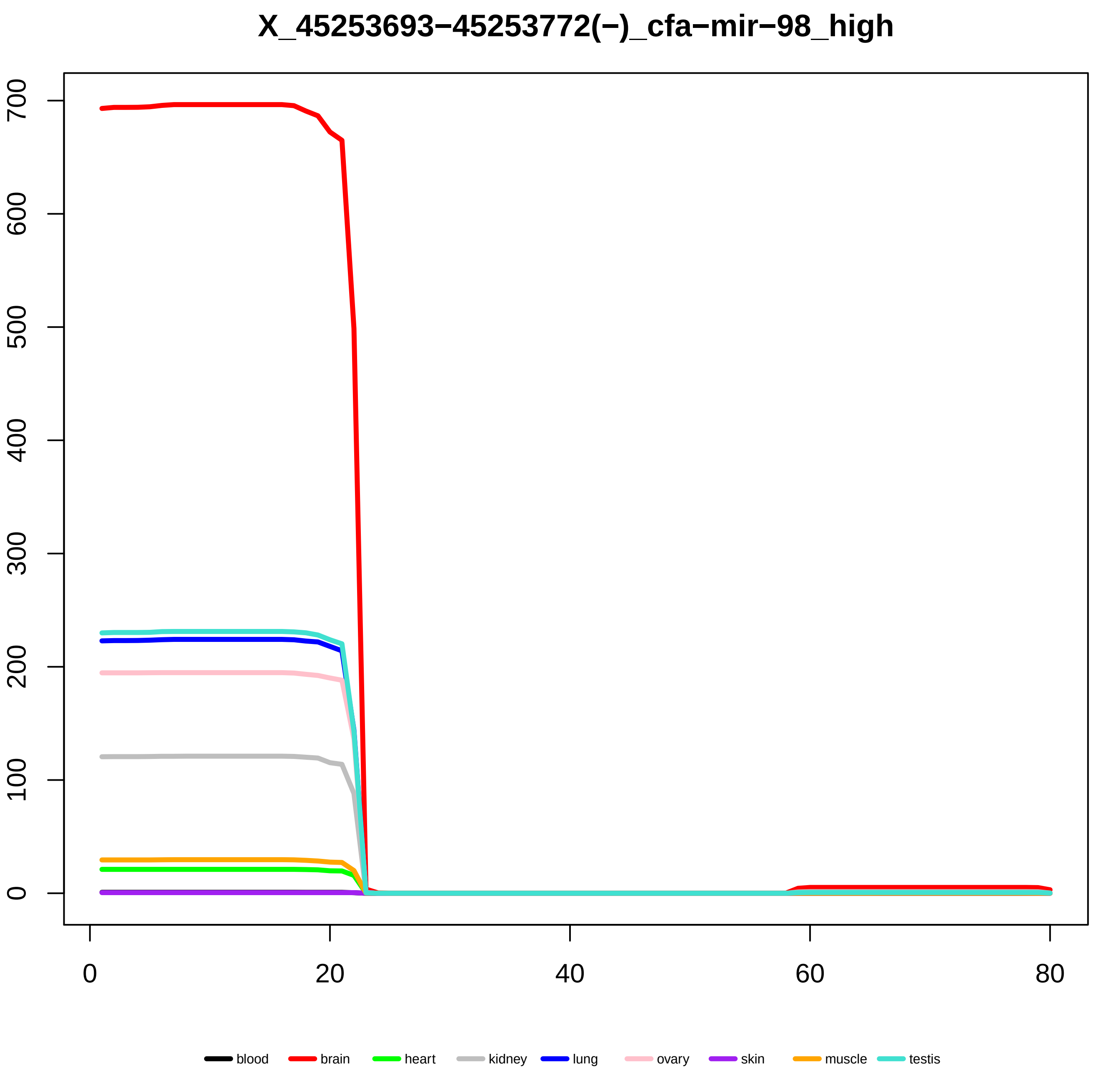
X_45253693−45253772(−)_cfa−mir−98_high
0
100
200
300
400
500
600
700
0
20
40
60
80
blood
brain
heart
kidney
lung
ovary
skin
muscle
testis

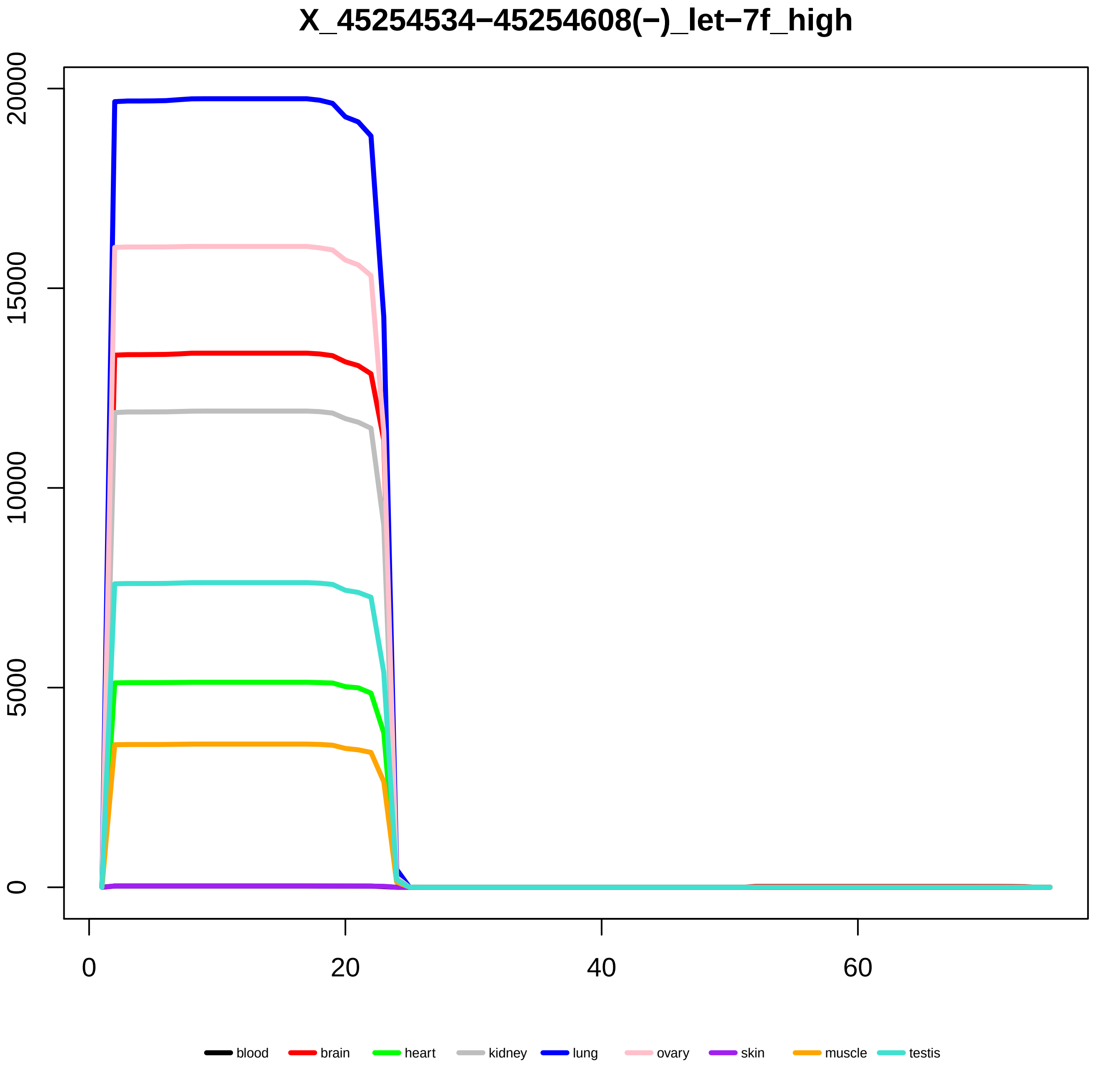

X_45254534−45254608(−)_let−7f_high
0
5000
10000
15000
20000
0
20
40
60
blood
brain
heart
kidney
lung
ovary
skin
muscle
testis

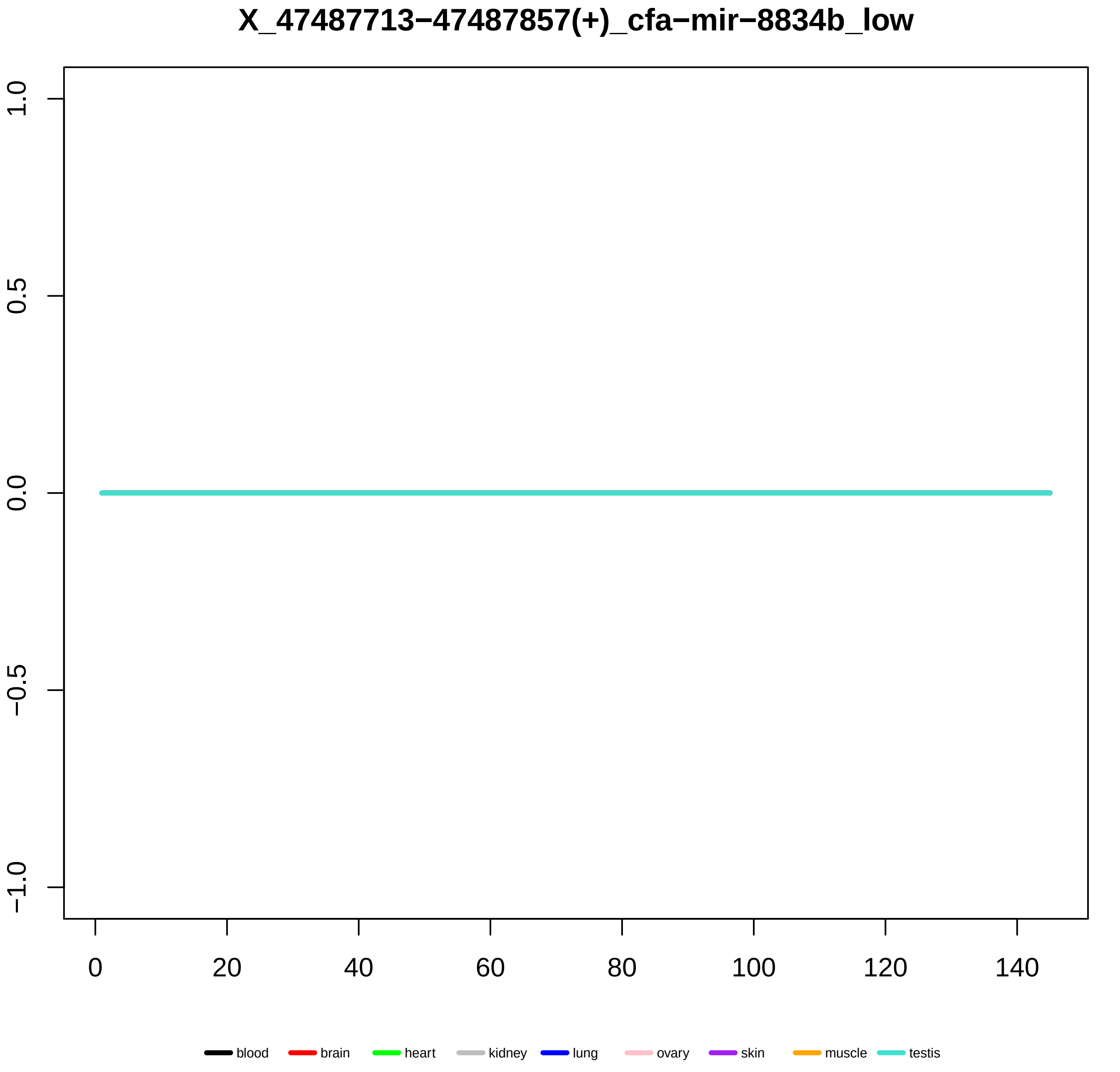

X_47487713−47487857(+)_cfa−mir−8834b_low
1.0
0.5
0.0
−0.5
−1.0
0
20
40
60
80
100
120
140
blood
brain
heart
kidney
lung
ovary
skin
muscle
testis

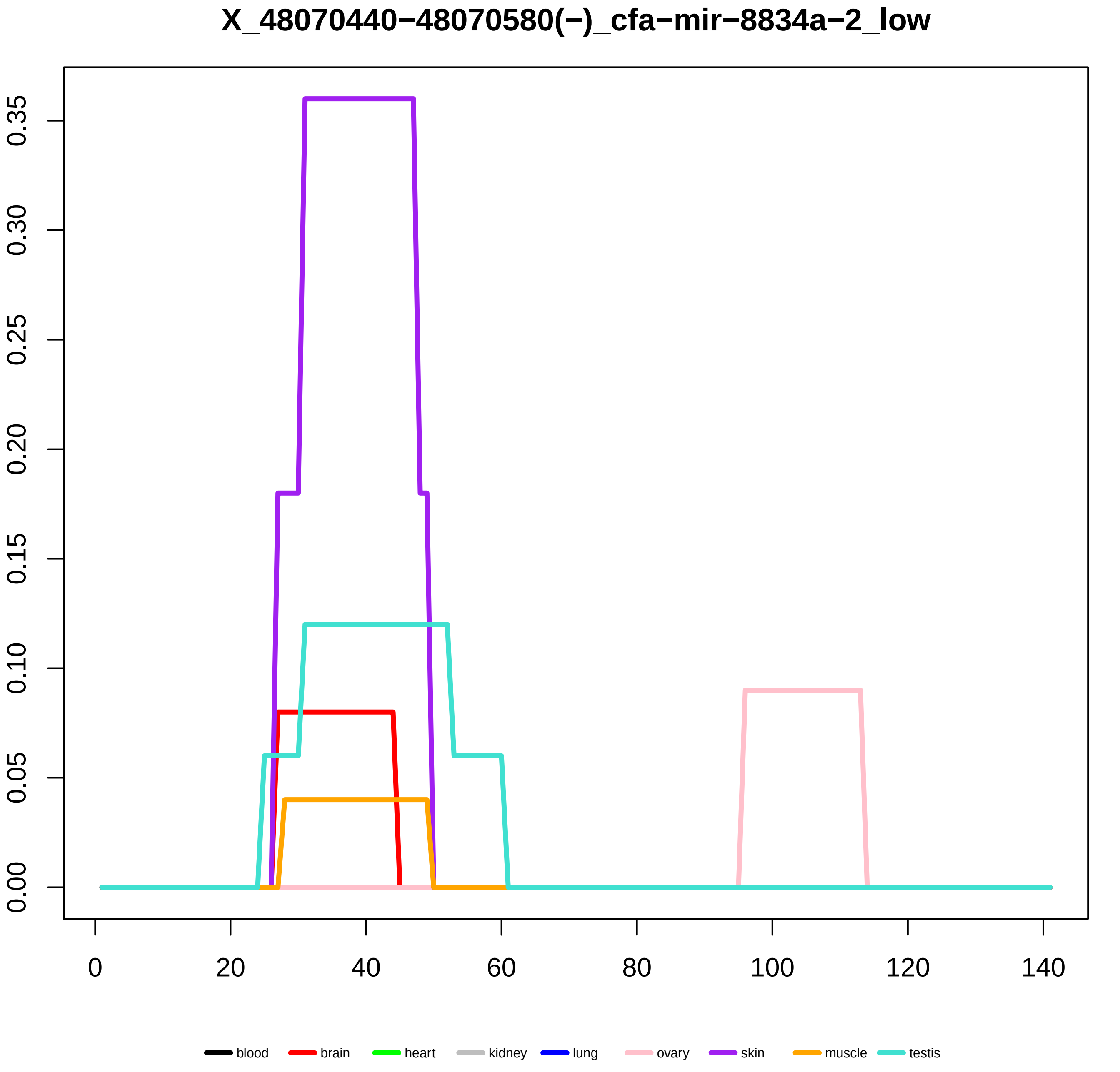

X_48070440−48070580(−)_cfa−mir−8834a−2_low
0.00
0.05
0.10
0.15
0.20
0.25
0.30
0.35
0
20
40
60
80
100
120
140
blood
brain
heart
kidney
lung
ovary
skin
muscle
testis

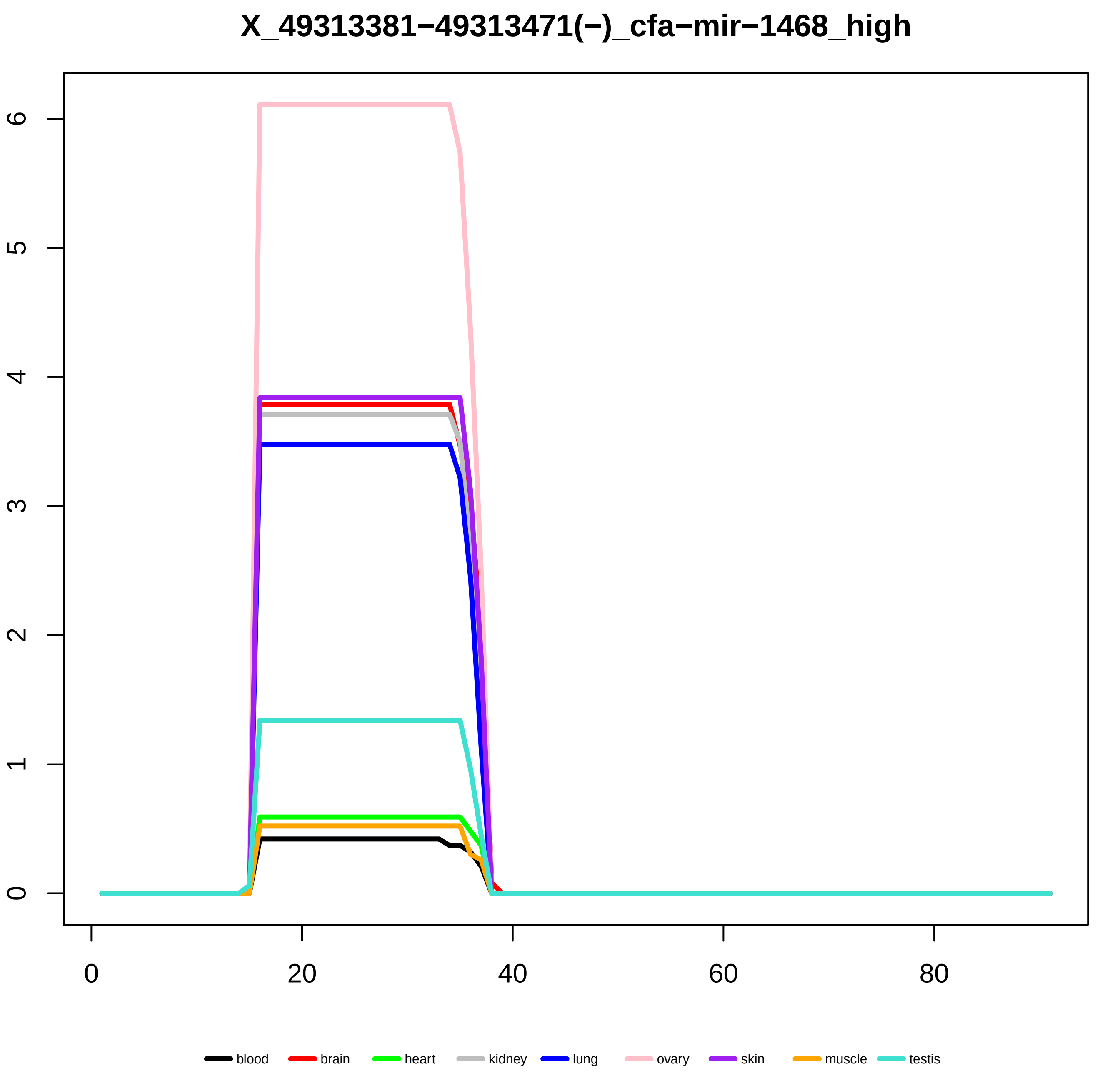

X_49313381−49313471(−)_cfa−mir−1468_high
0
1
2
3
4
5
6
0
20
40
60
80
blood
brain
heart
kidney
lung
ovary
skin
muscle
testis

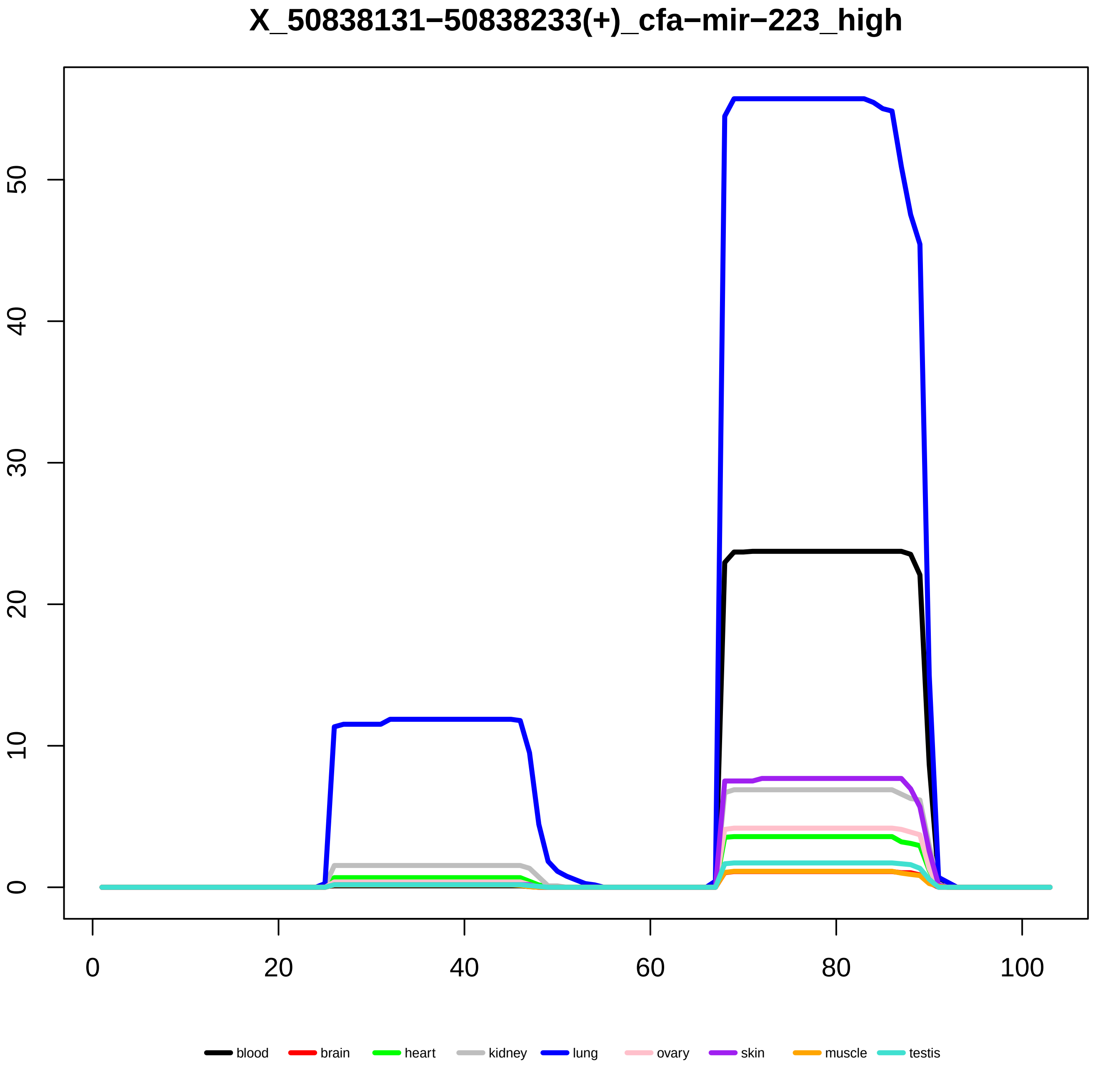

X_50838131−50838233(+)_cfa−mir−223_high
0
10
20
30
40
50
0
20
40
60
80
100
blood
brain
heart
kidney
lung
ovary
skin
muscle
testis

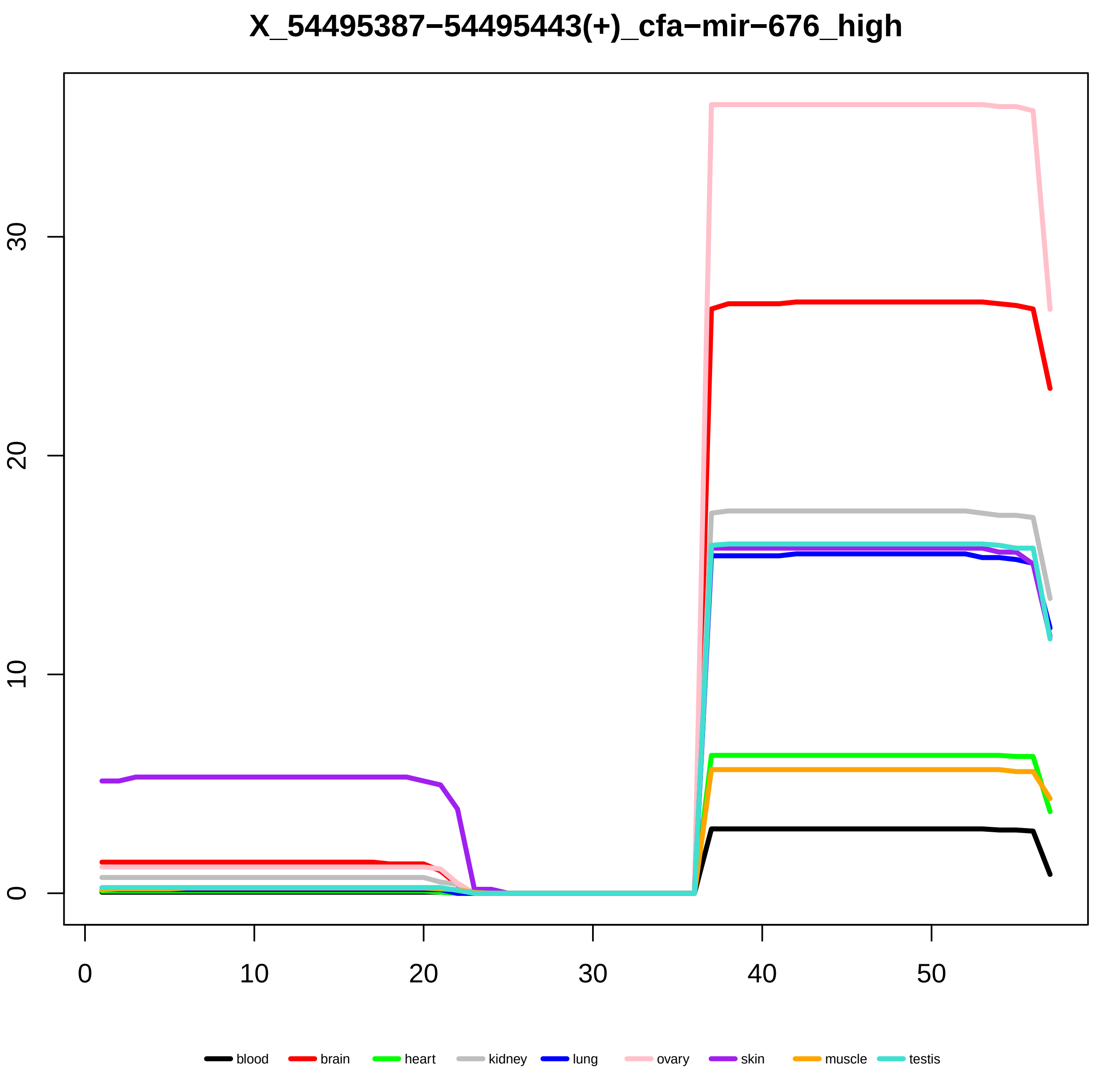

X_54495387−54495443(+)_cfa−mir−676_high
0
10
20
30
0
10
20
30
40
50
blood
brain
heart
kidney
lung
ovary
skin
muscle
testis

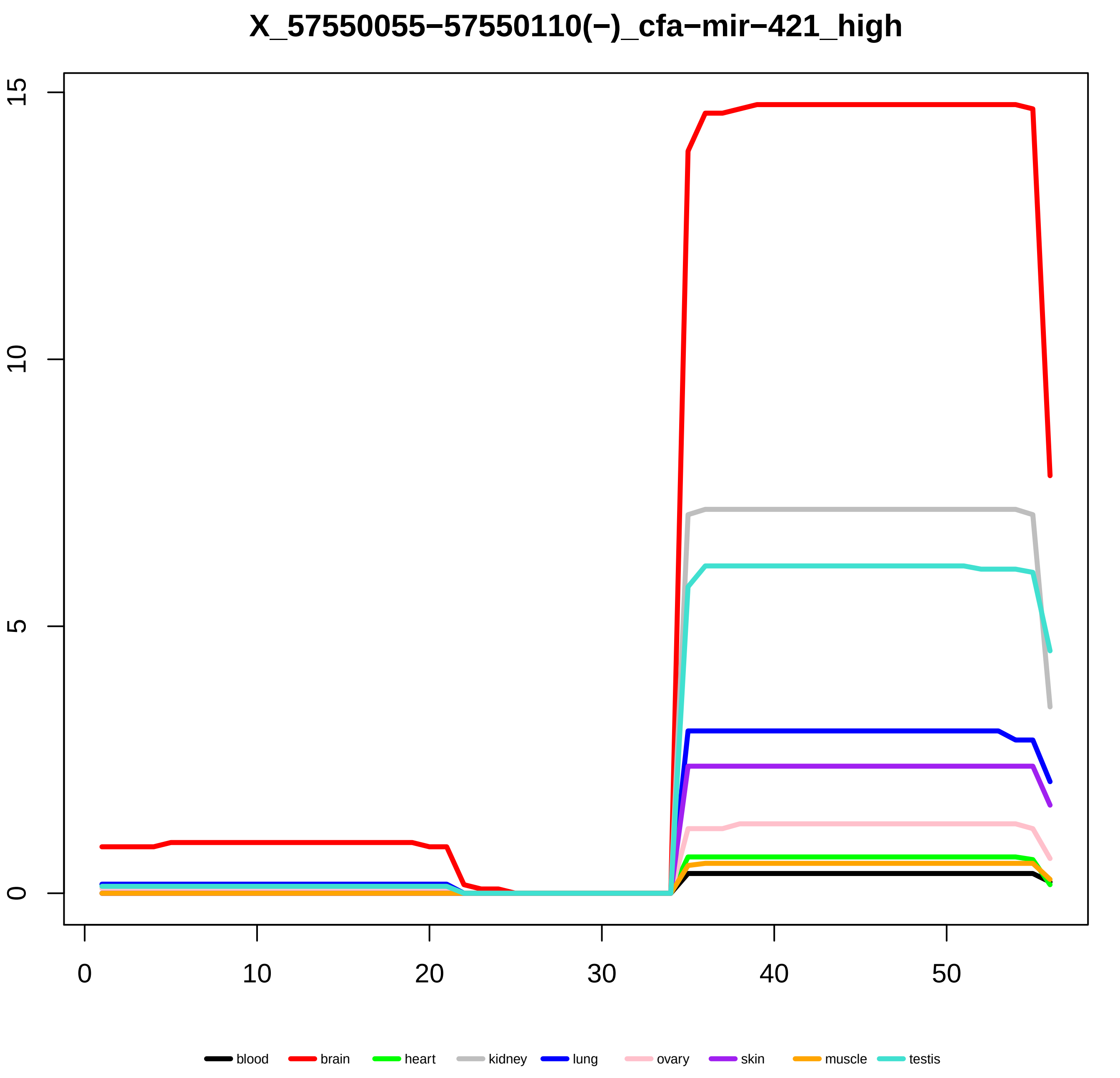
X_57550055−57550110(−)_cfa−mir−421_high
0
5
10
15
0
10
20
30
40
50
blood
brain
heart
kidney
lung
ovary
skin
muscle
testis

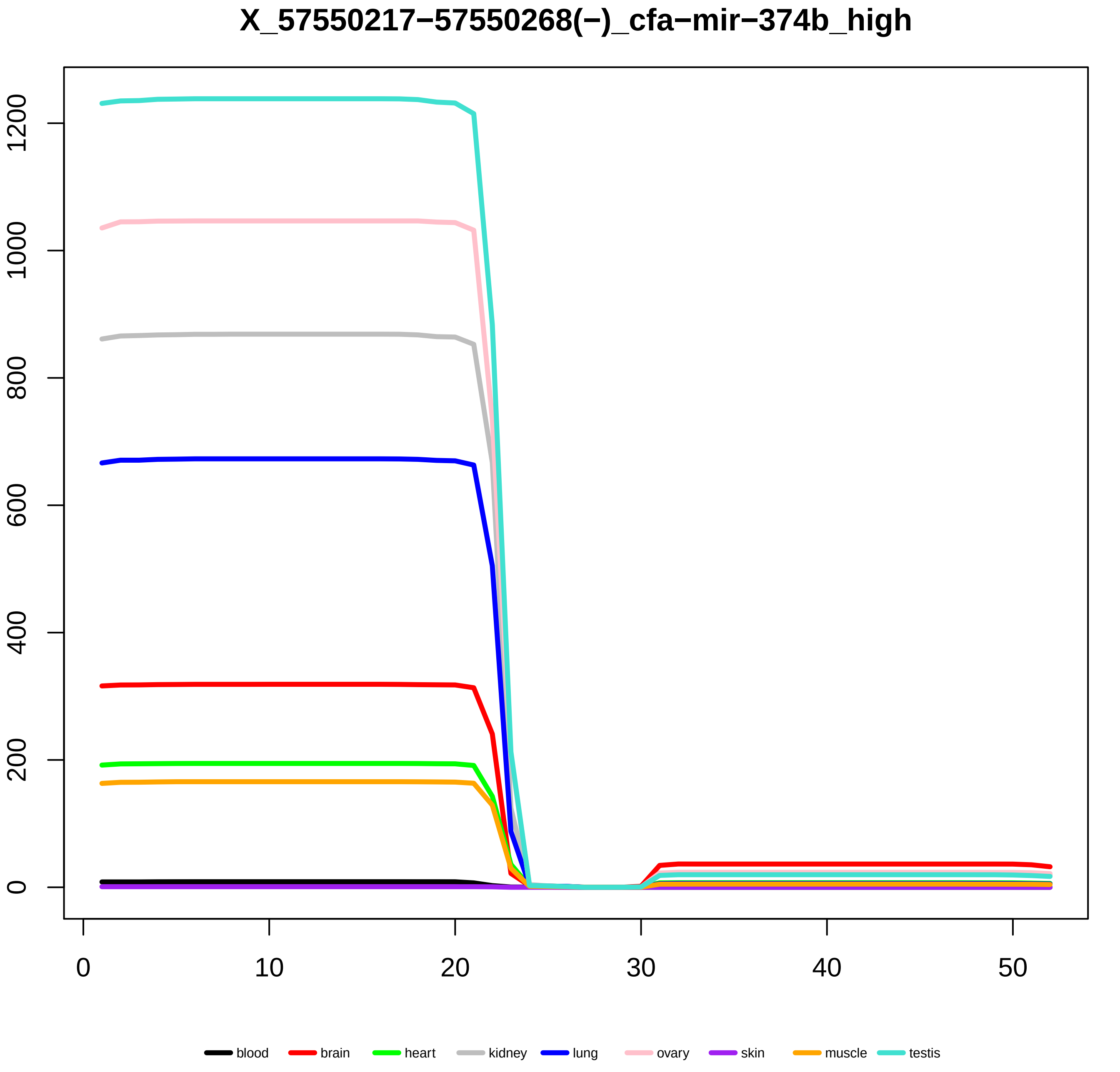

X_57550217−57550268(−)_cfa−mir−374b_high
0
200
400
600
800
1000
1200
0
10
20
30
40
50
blood
brain
heart
kidney
lung
ovary
skin
muscle
testis

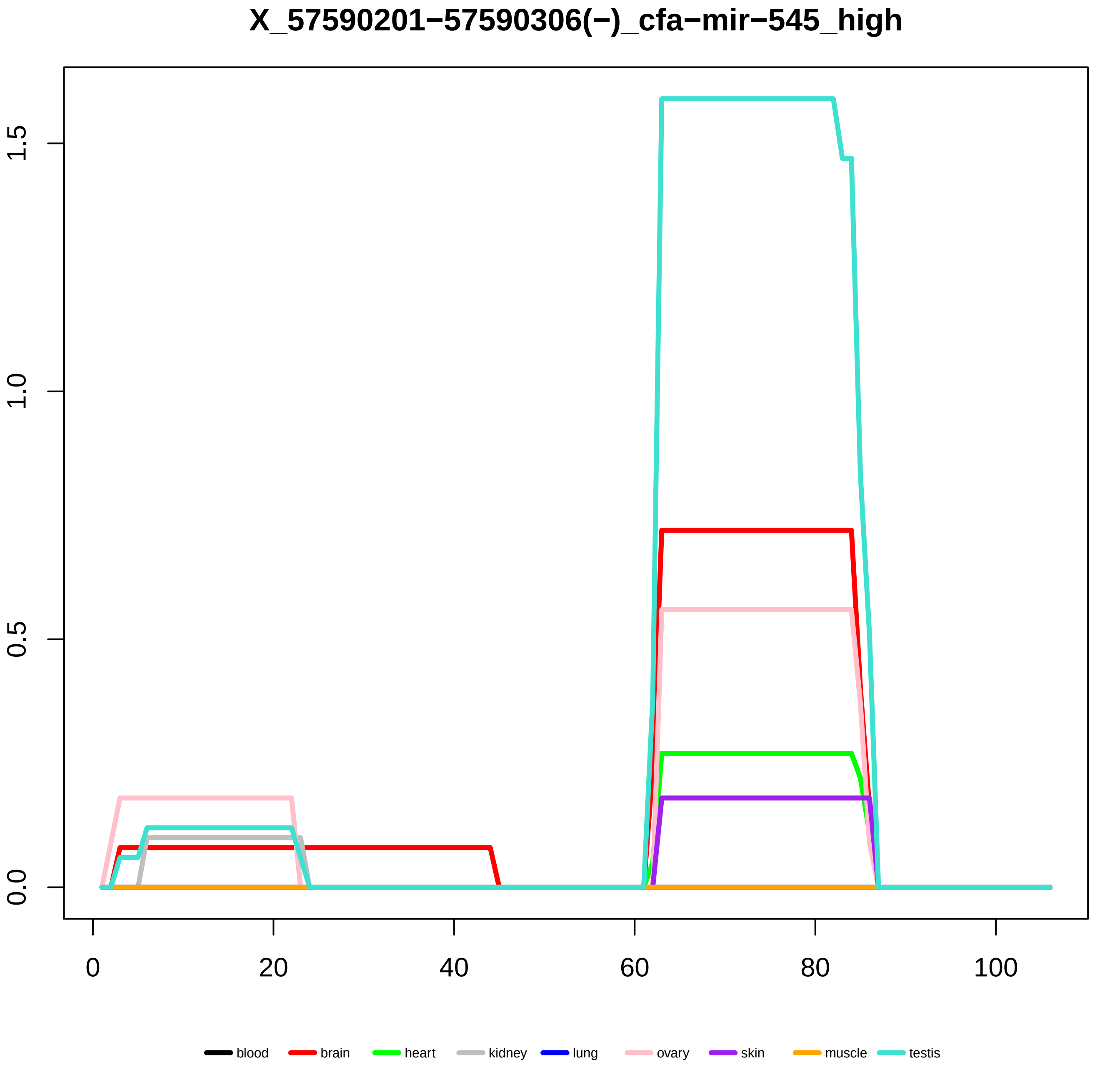

X_57590201−57590306(−)_cfa−mir−545_high
0.0
0.5
1.0
1.5
0
20
40
60
80
100
blood
brain
heart
kidney
lung
ovary
skin
muscle
testis

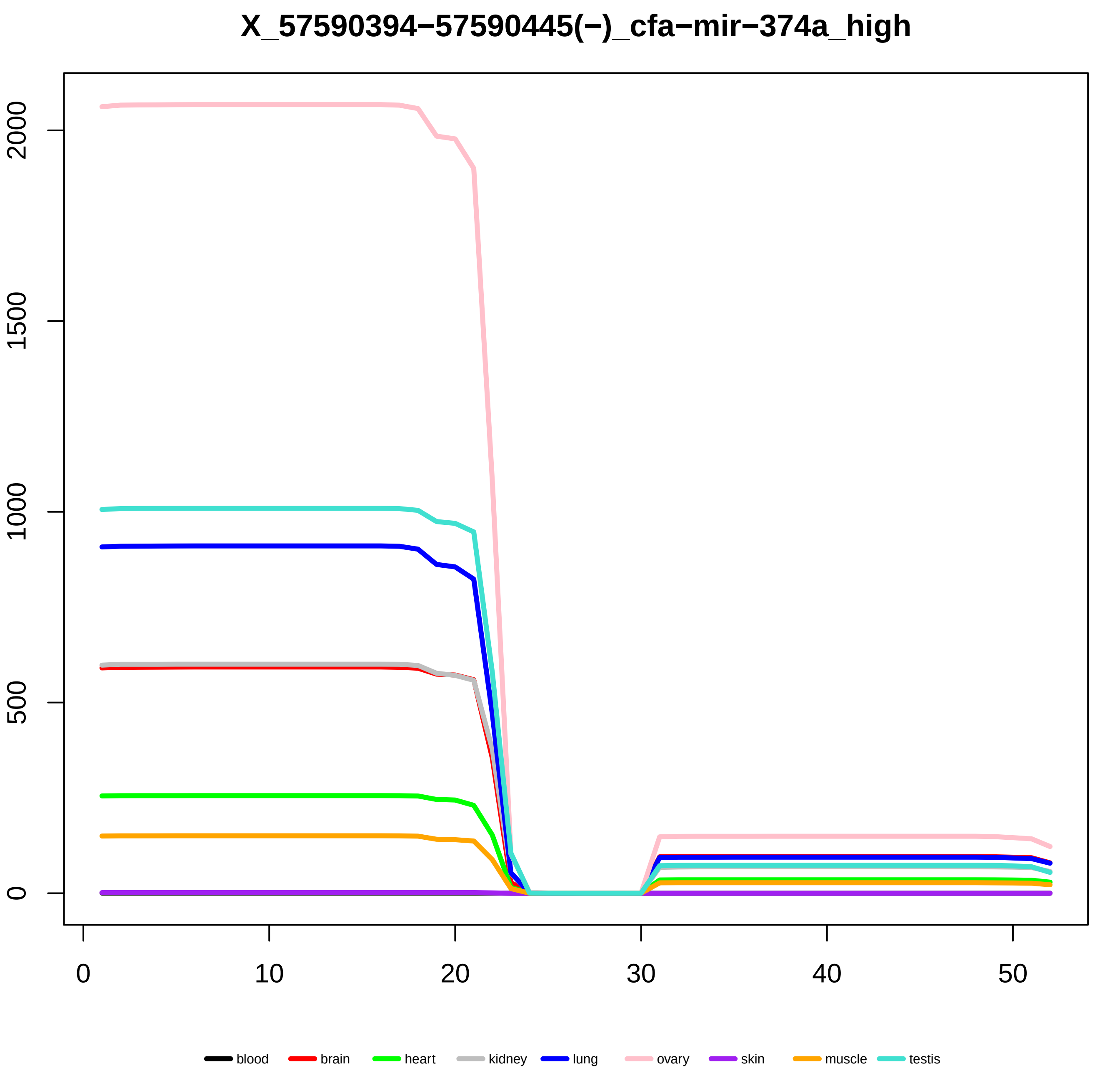

X_57590394−57590445(−)_cfa−mir−374a_high
0
500
1000
1500
2000
0
10
20
30
40
50
blood
brain
heart
kidney
lung
ovary
skin
muscle
testis

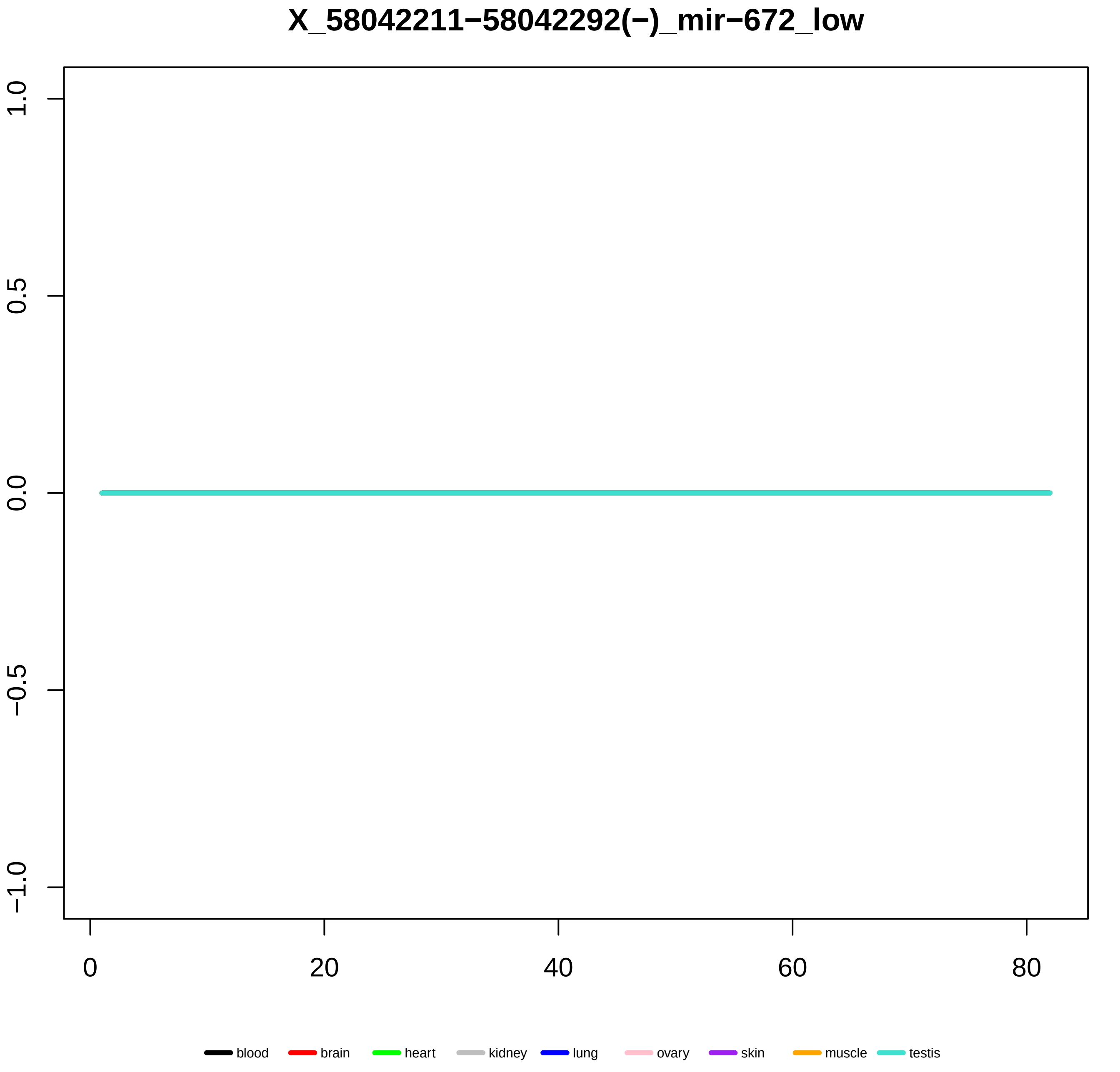

X_58042211−58042292(−)_mir−672_low
1.0
0.5
0.0
−0.5
−1.0
0
20
40
60
80
blood
brain
heart
kidney
lung
ovary
skin
muscle
testis

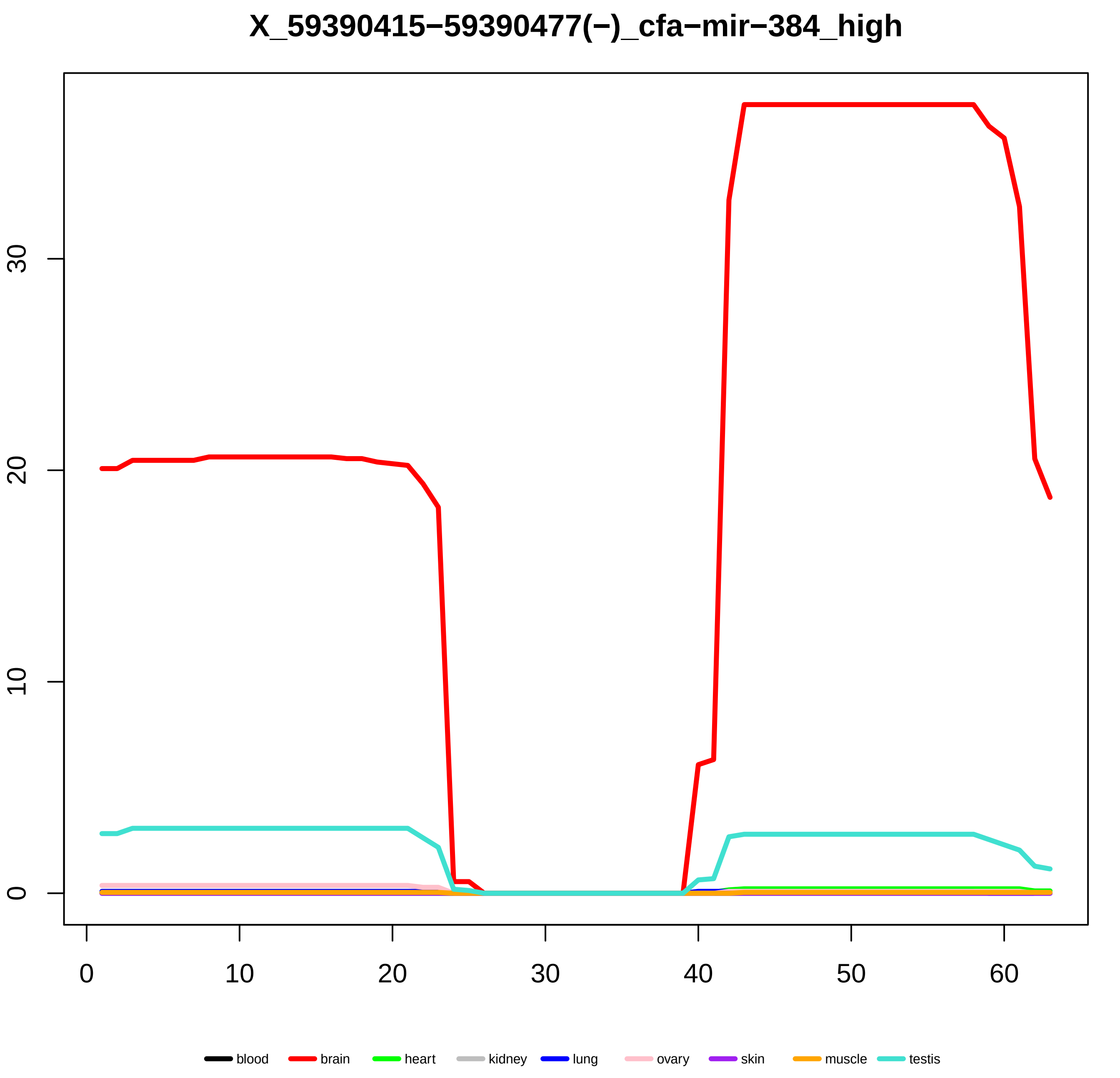

X_59390415−59390477(−)_cfa−mir−384_high
0
10
20
30
0
10
20
30
40
50
60
blood
brain
heart
kidney
lung
ovary
skin
muscle
testis

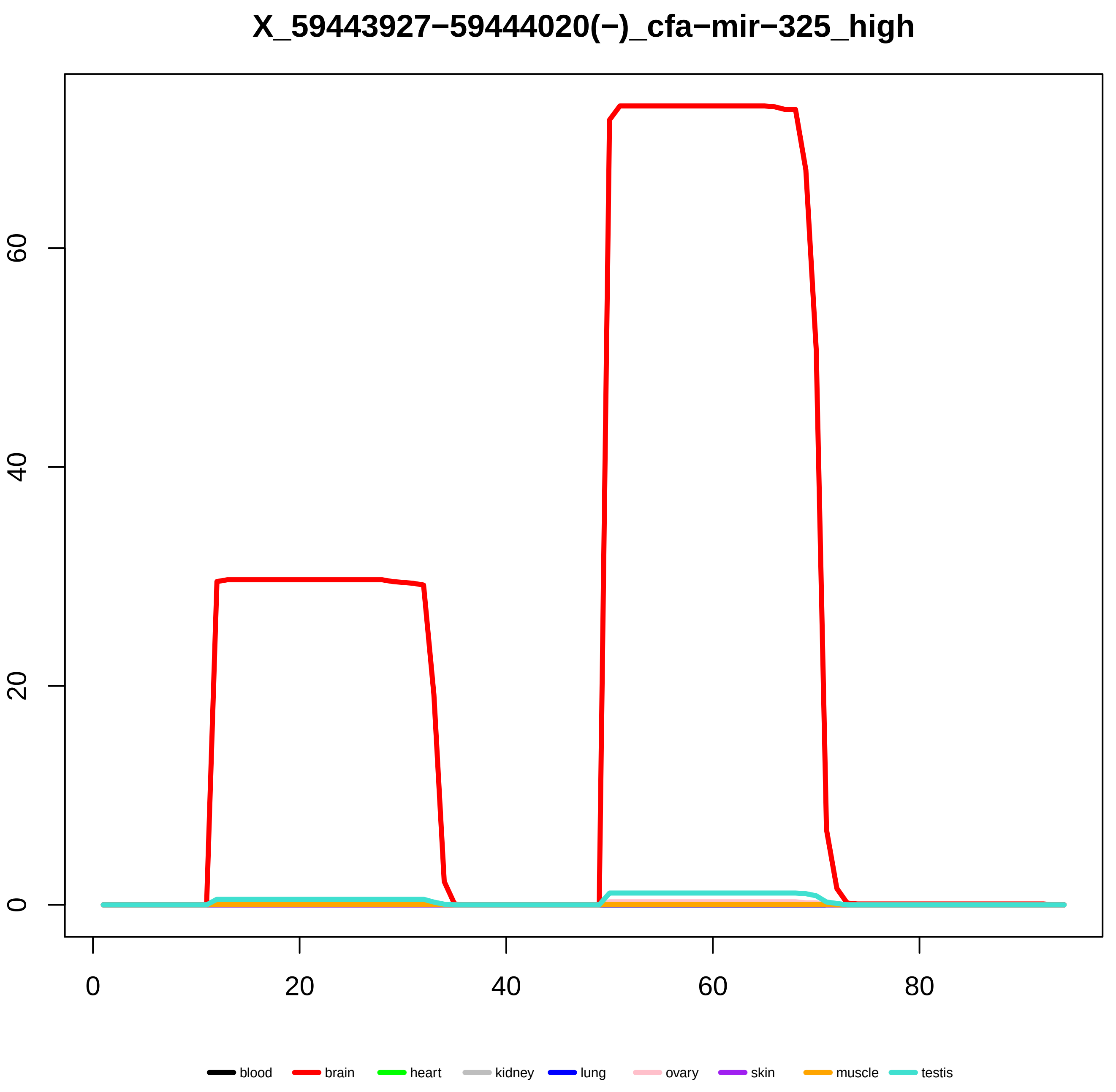

X_59443927−59444020(−)_cfa−mir−325_high
0
20
40
60
0
20
40
60
80
blood
brain
heart
kidney
lung
ovary
skin
muscle
testis

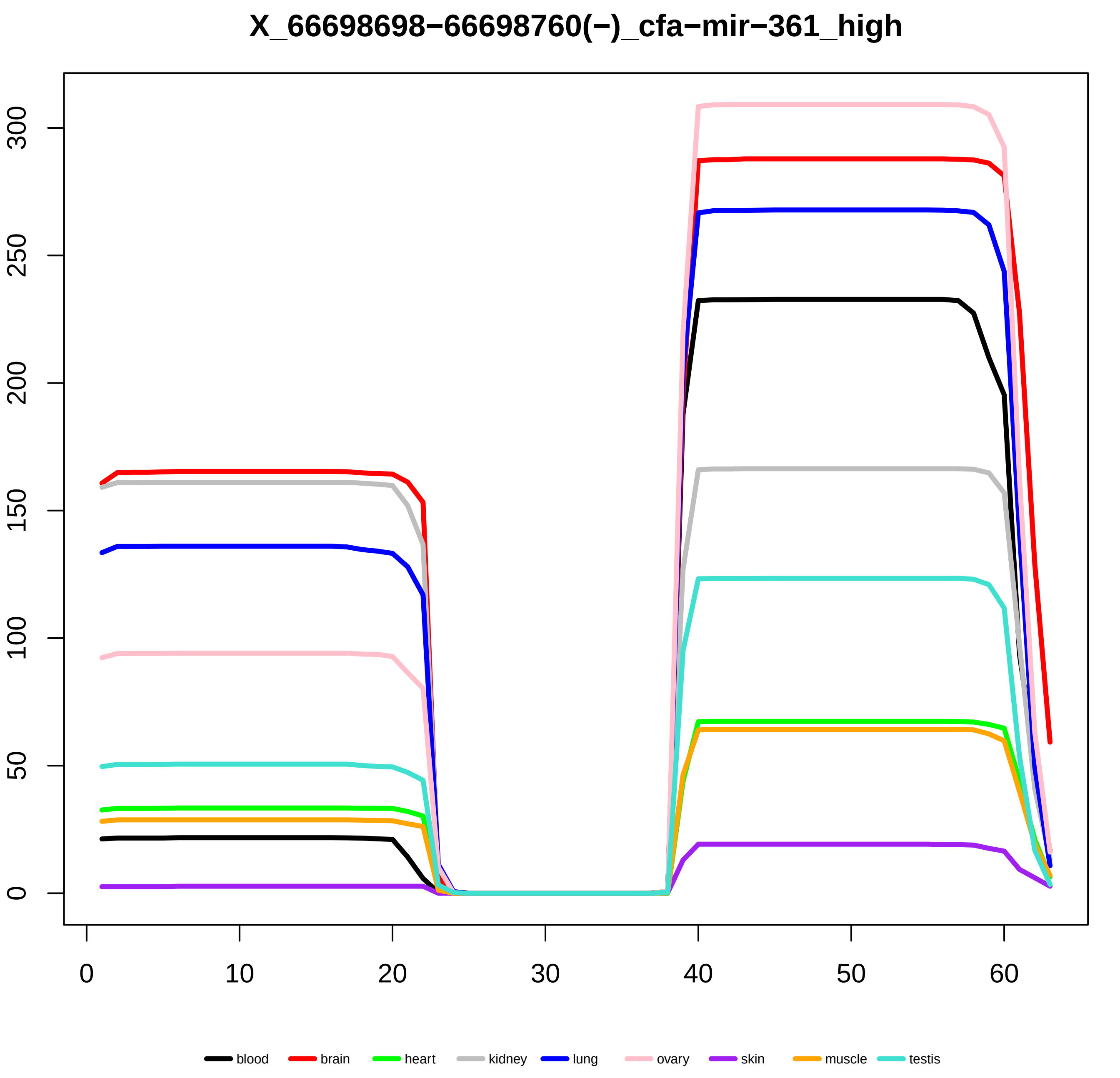

X_66698698−66698760(−)_cfa−mir−361_high
0
50
100
150
200
250
300
0
10
20
30
40
50
60
blood
brain
heart
kidney
lung
ovary
skin
muscle
testis

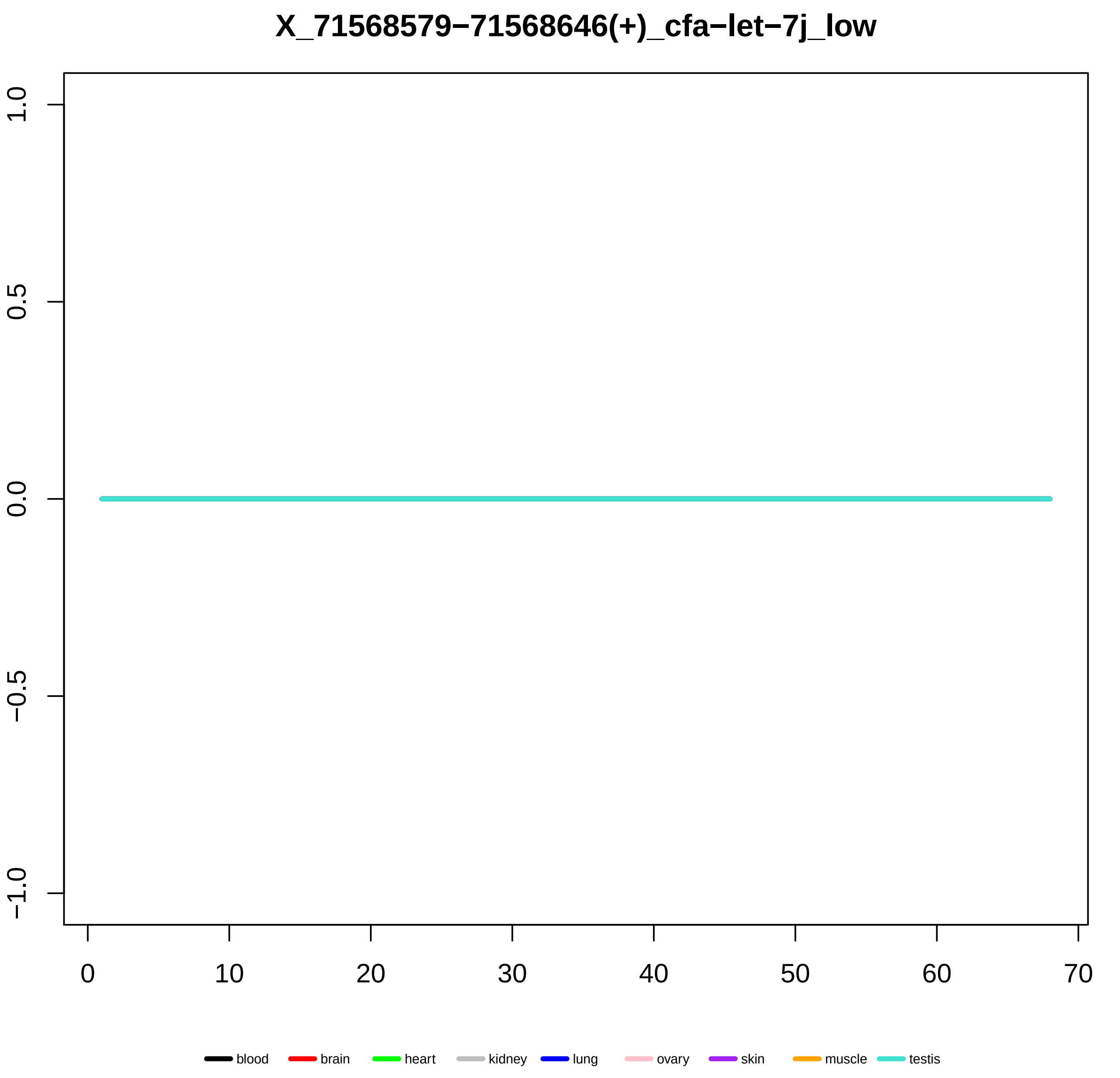
X_71568579−71568646(+)_cfa−let−7j_low
1.0
0.5
0.0
−0.5
−1.0
0
10
20
30
40
50
60
70
blood
brain
heart
kidney
lung
ovary
skin
muscle
testis

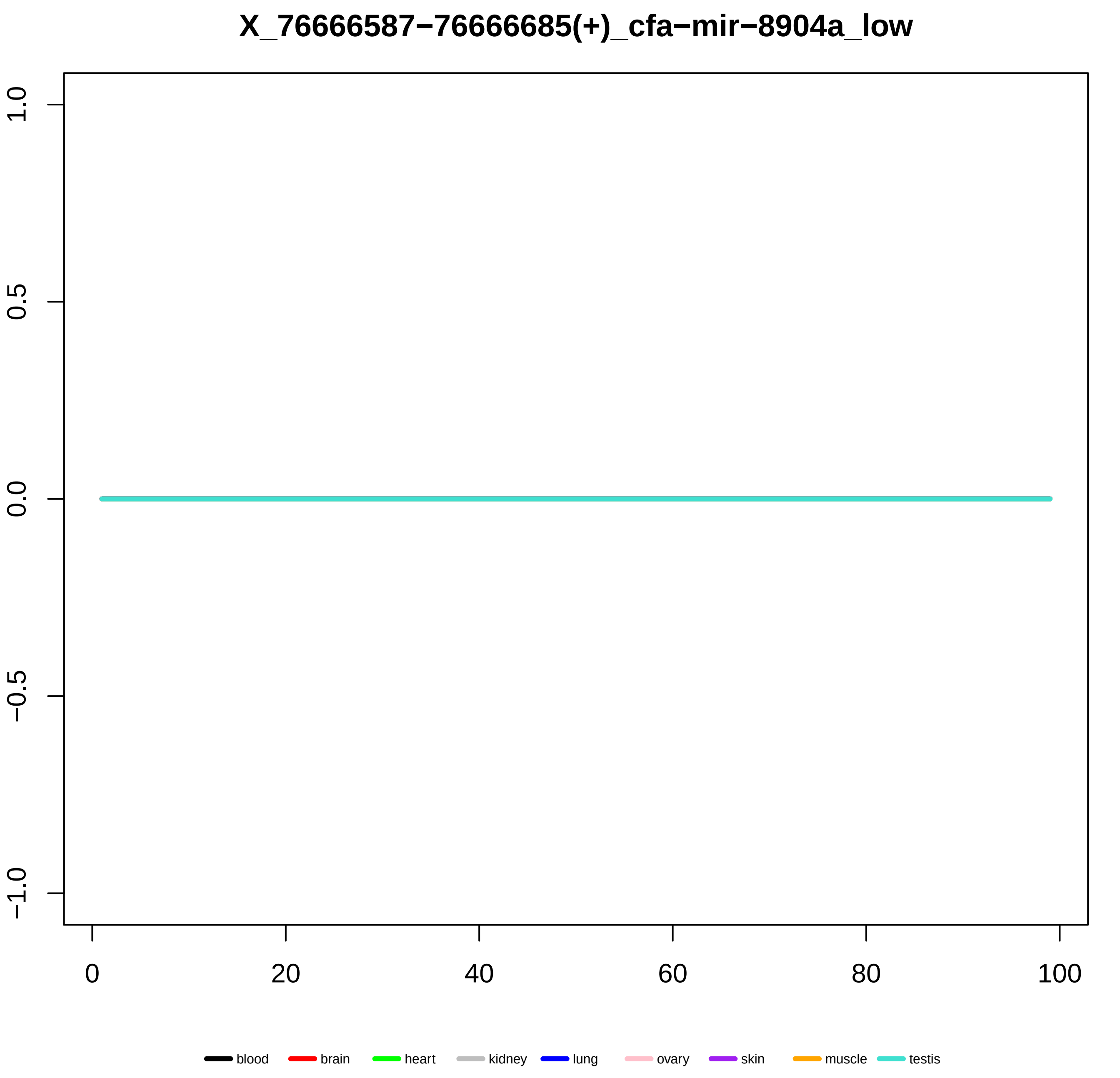

X_76666587−76666685(+)_cfa−mir−8904a_low
1.0
0.5
0.0
−0.5
−1.0
0
20
40
60
80
100
blood
brain
heart
kidney
lung
ovary
skin
muscle
testis

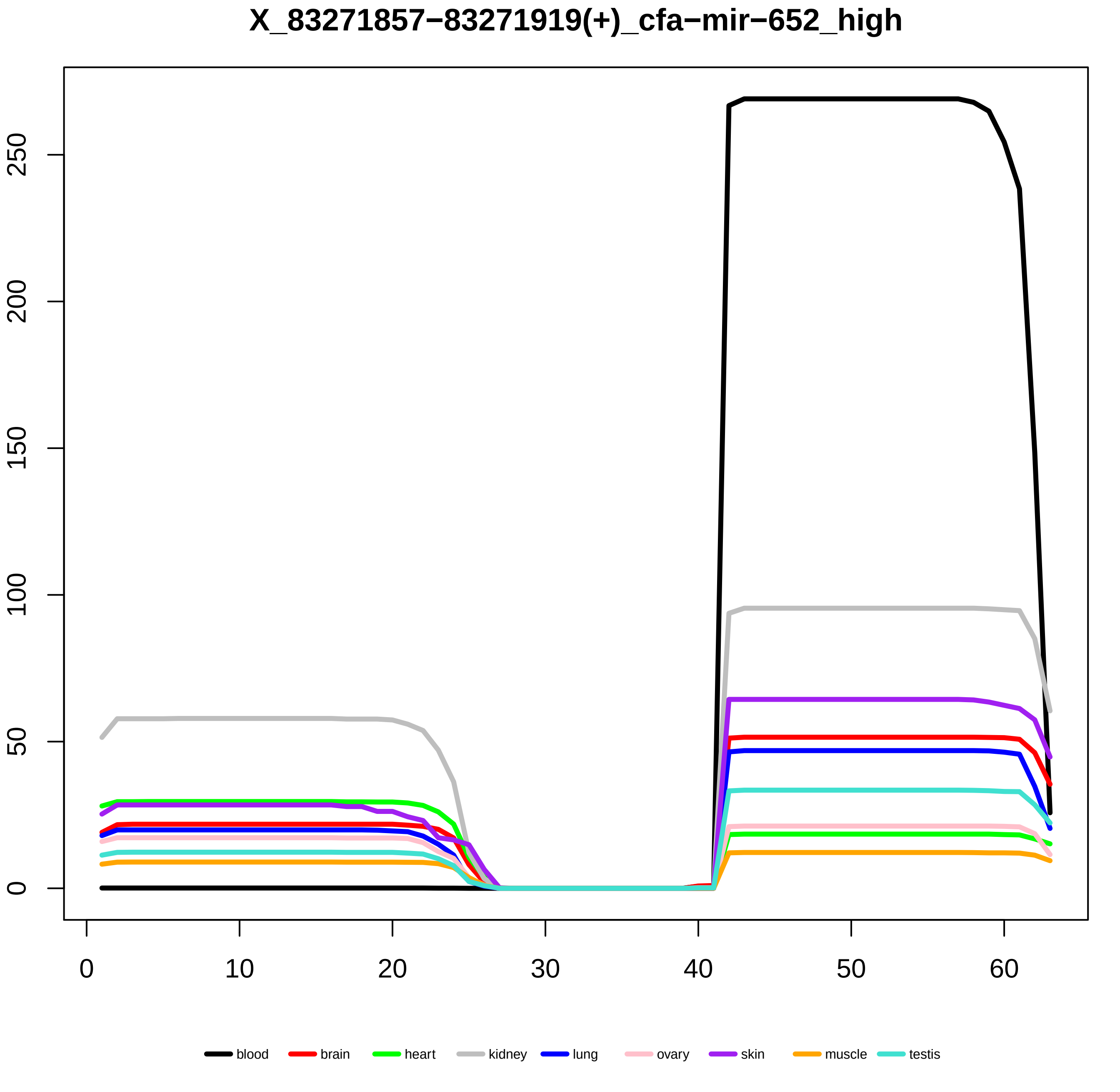
X_83271857−83271919(+)_cfa−mir−652_high
0
50
100
150
200
250
0
10
20
30
40
50
60
blood
brain
heart
kidney
lung
ovary
skin
muscle
testis

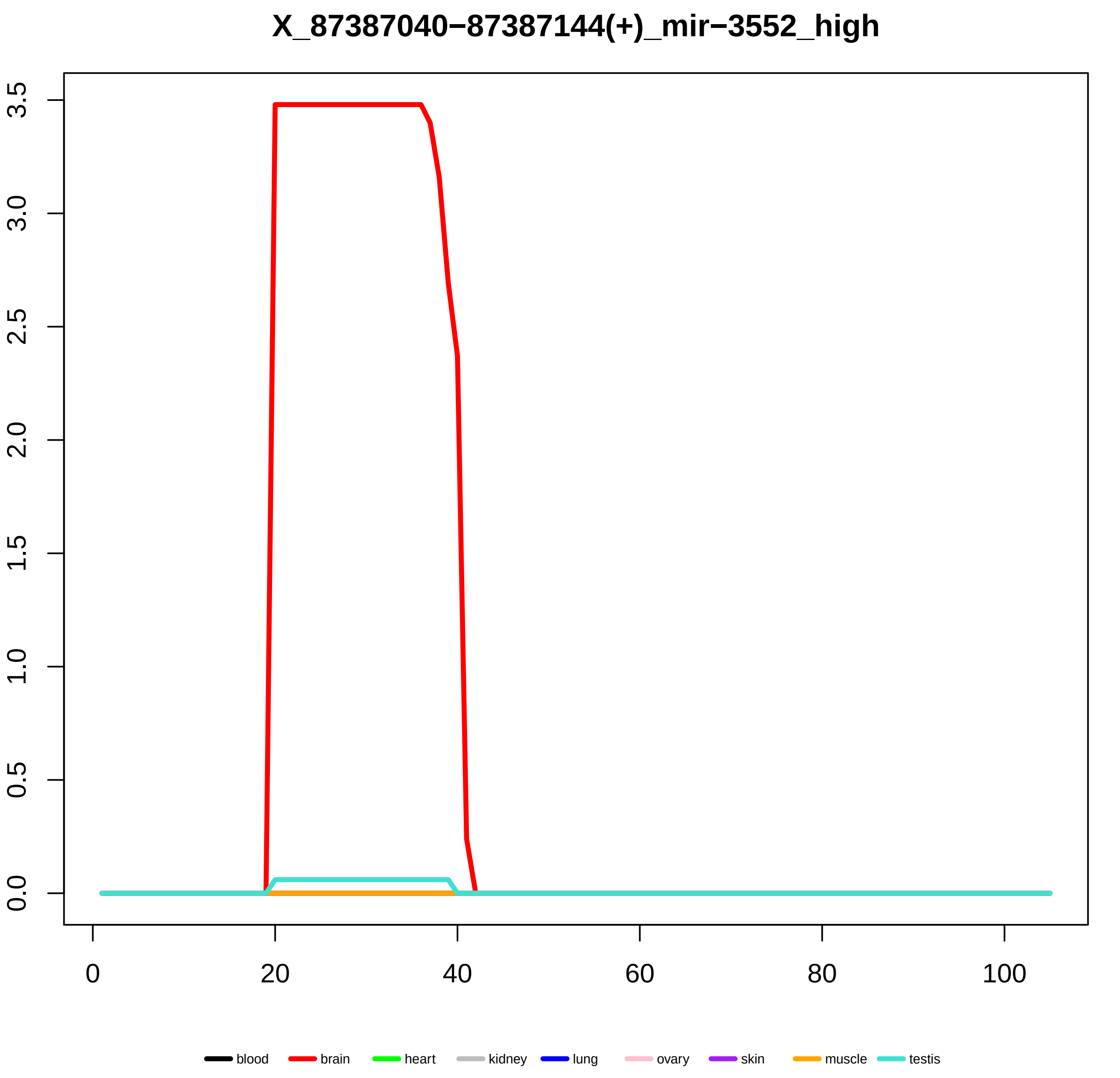
X_87387040−87387144(+)_mir−3552_high
0.0
0.5
1.0
1.5
2.0
2.5
3.0
3.5
0
20
40
60
80
100
blood
brain
heart
kidney
lung
ovary
skin
muscle
testis

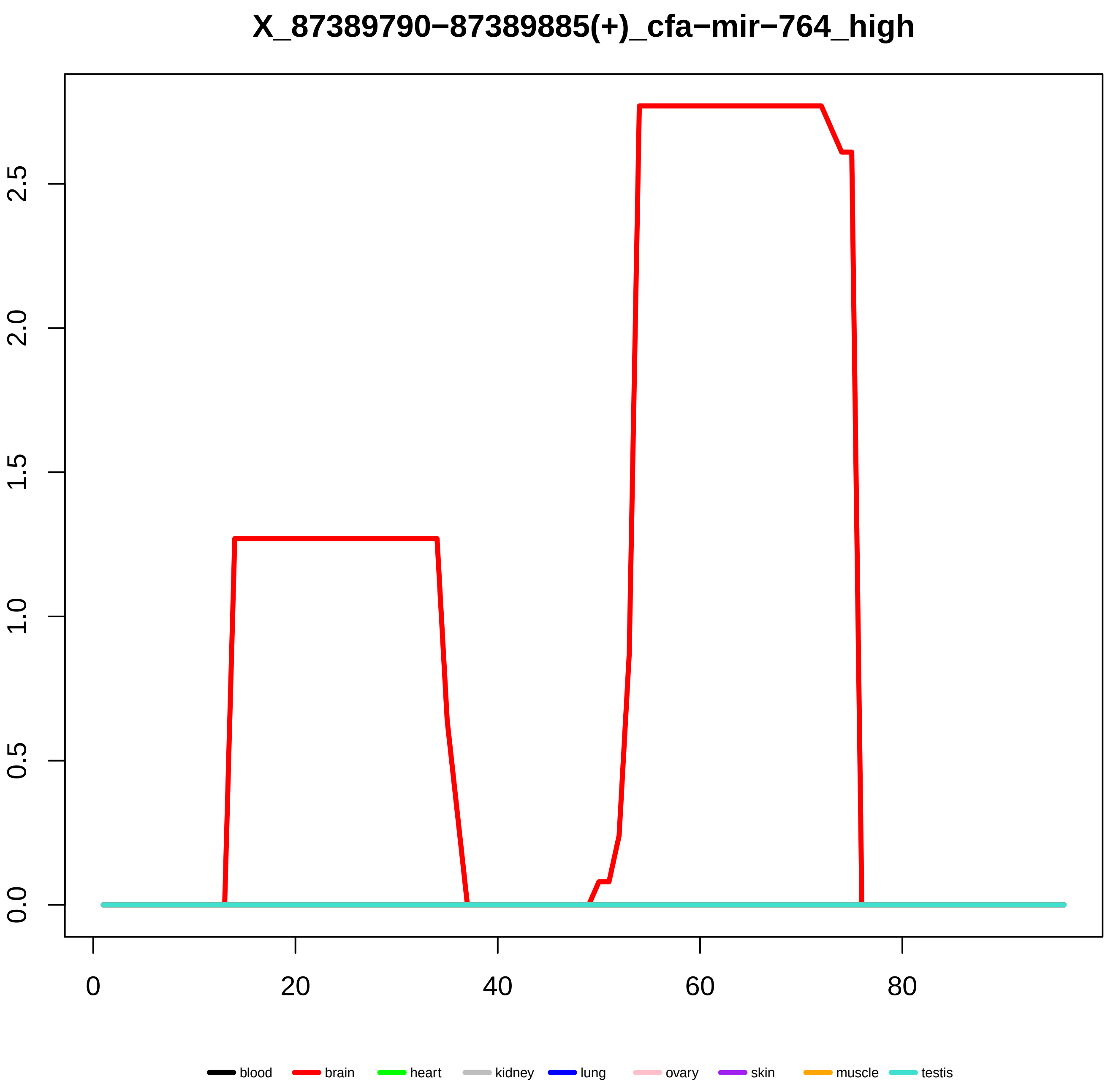
X_87389790−87389885(+)_cfa−mir−764_high
0.0
0.5
1.0
1.5
2.0
2.5
0
20
40
60
80
blood
brain
heart
kidney
lung
ovary
skin
muscle
testis

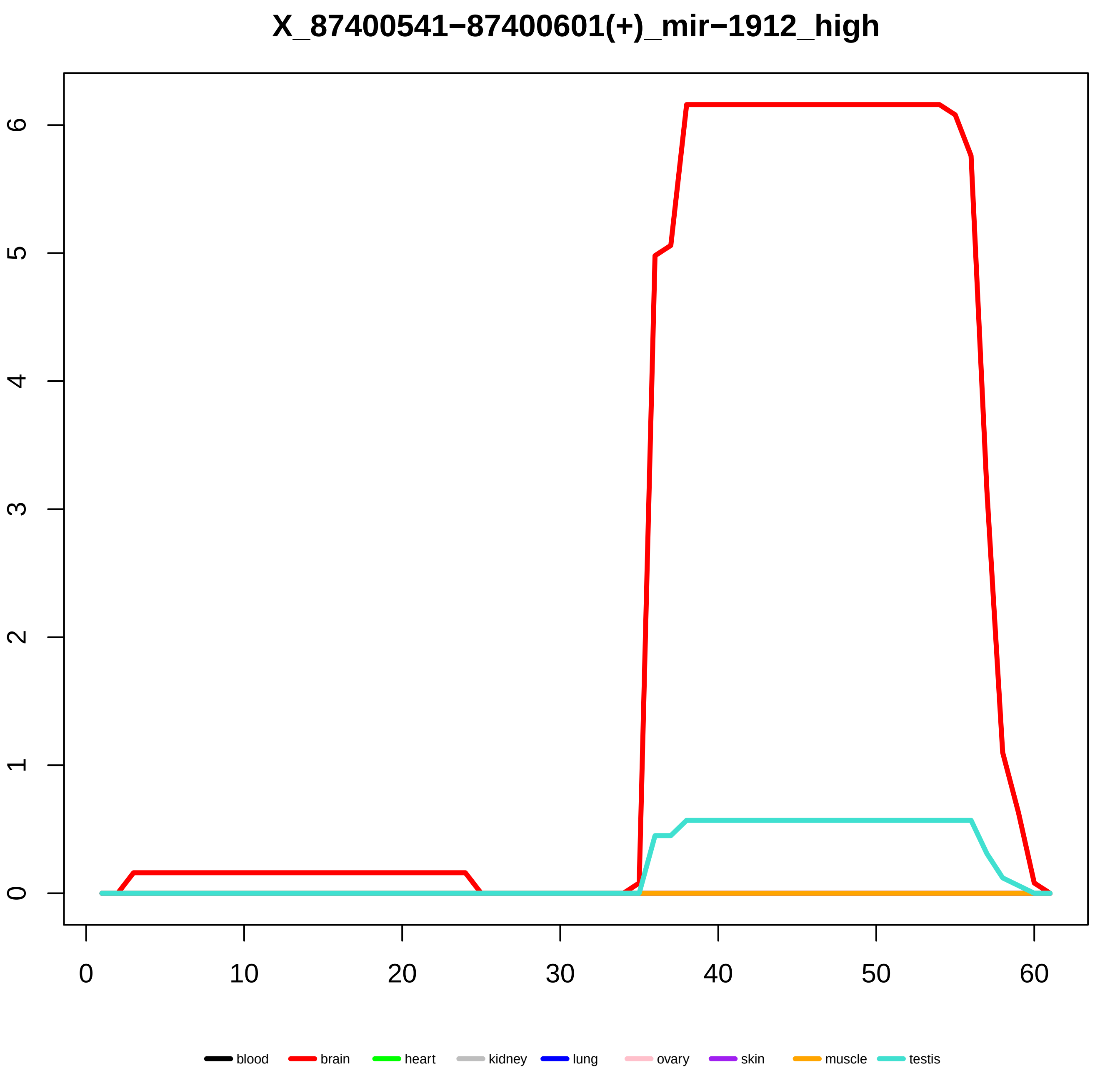
X_87400541−87400601(+)_mir−1912_high
0
1
2
3
4
5
6
0
10
20
30
40
50
60
blood
brain
heart
kidney
lung
ovary
skin
muscle
testis

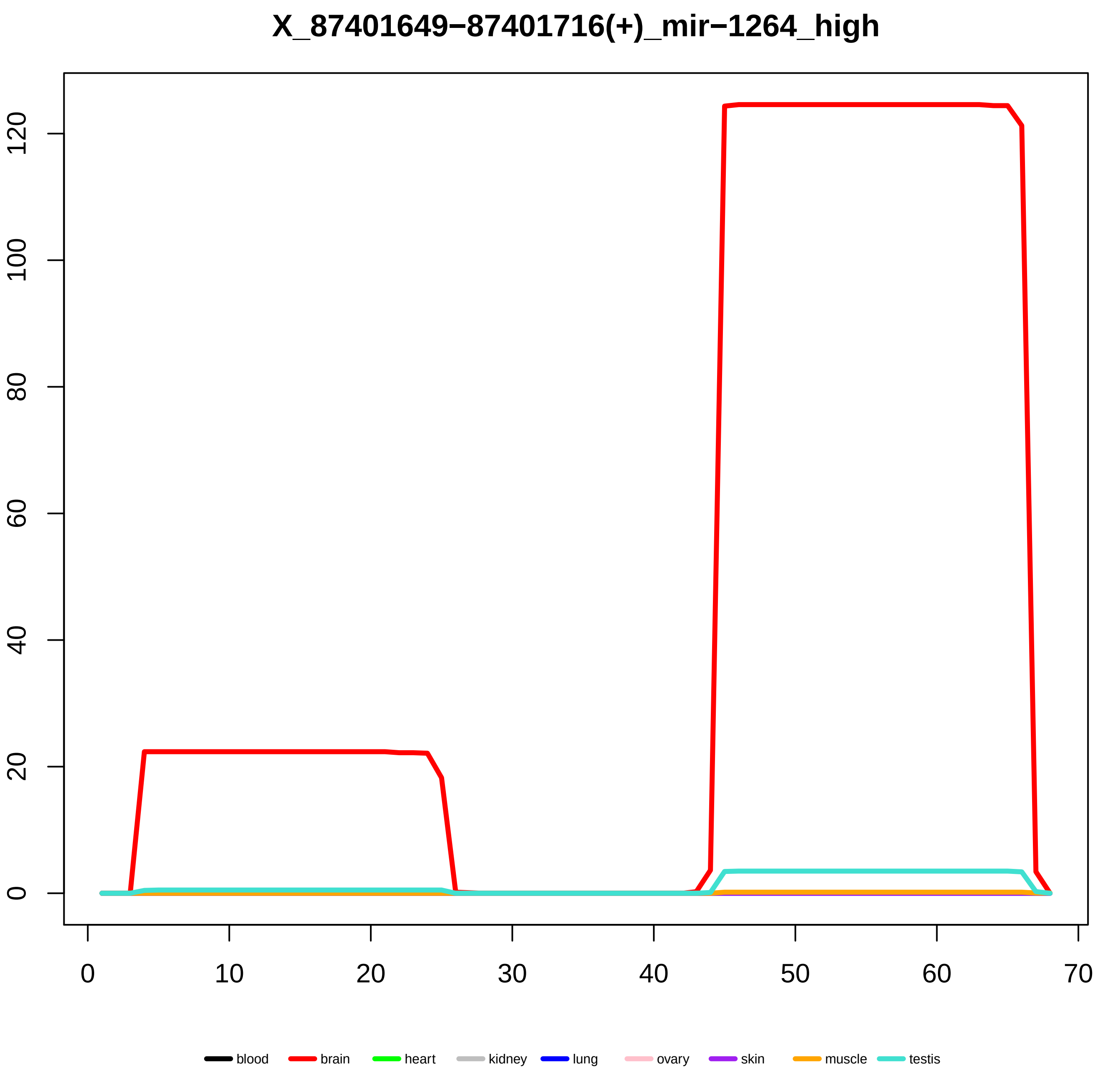
X_87401649−87401716(+)_mir−1264_high
0
20
40
60
80
100
120
0
10
20
30
40
50
60
70
blood
brain
heart
kidney
lung
ovary
skin
muscle
testis

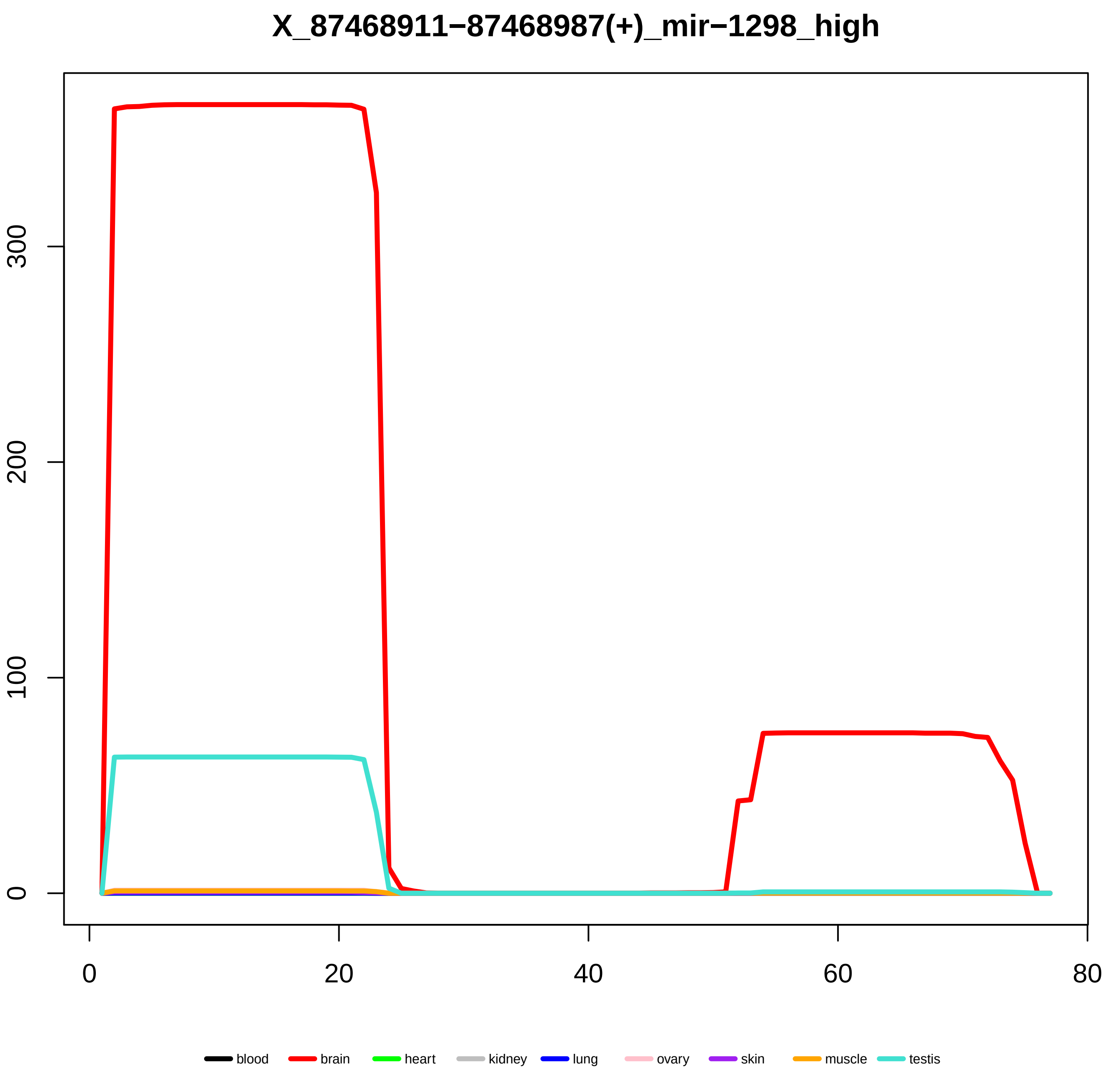
X_87468911−87468987(+)_mir−1298_high
0
100
200
300
0
20
40
60
80
blood
brain
heart
kidney
lung
ovary
skin
muscle
testis

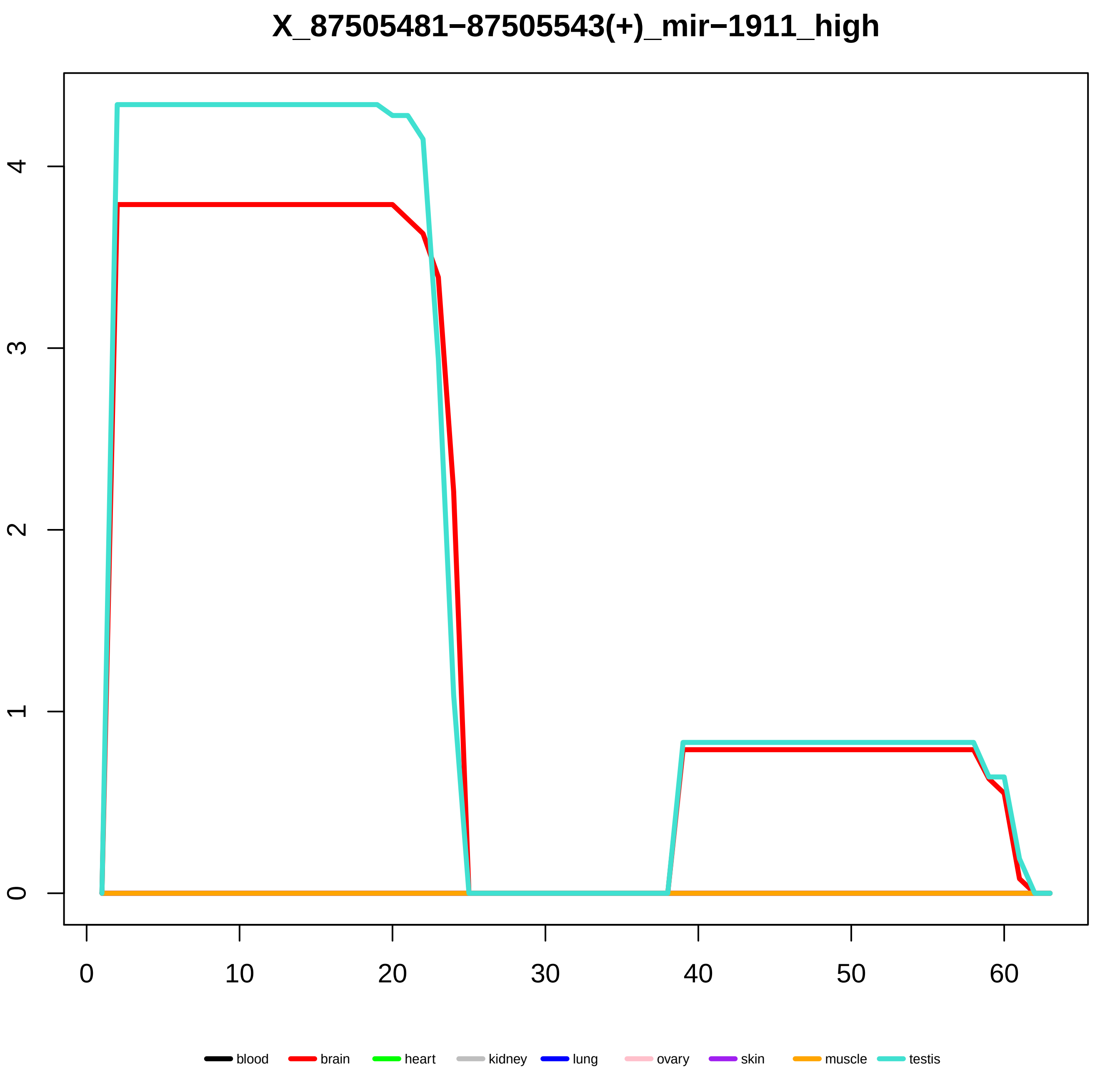
X_87505481−87505543(+)_mir−1911_high
0
1
2
3
4
0
10
20
30
40
50
60
blood
brain
heart
kidney
lung
ovary
skin
muscle
testis

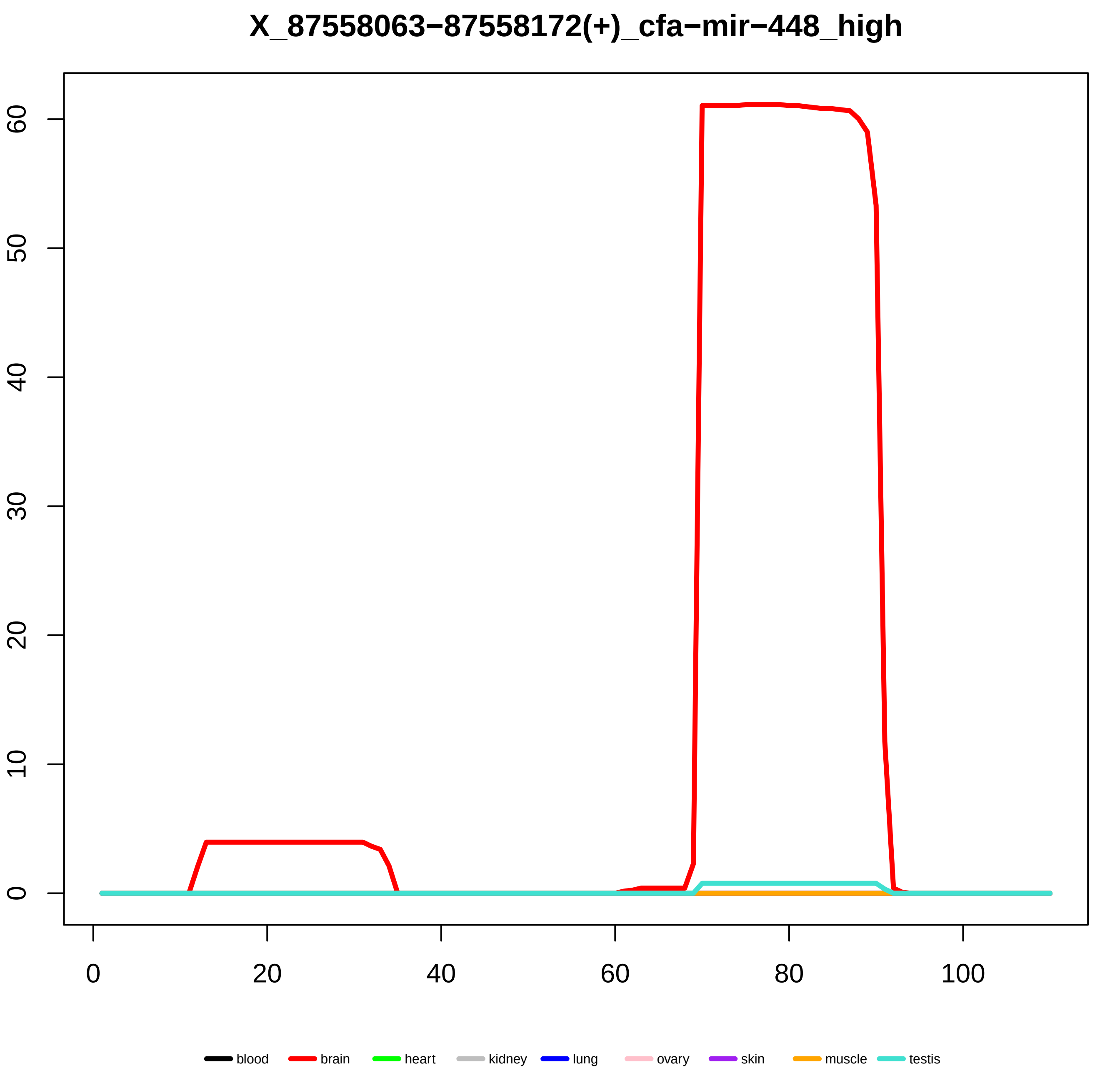

X_87558063−87558172(+)_cfa−mir−448_high
0
10
20
30
40
50
60
0
20
40
60
80
100
blood
brain
heart
kidney
lung
ovary
skin
muscle
testis

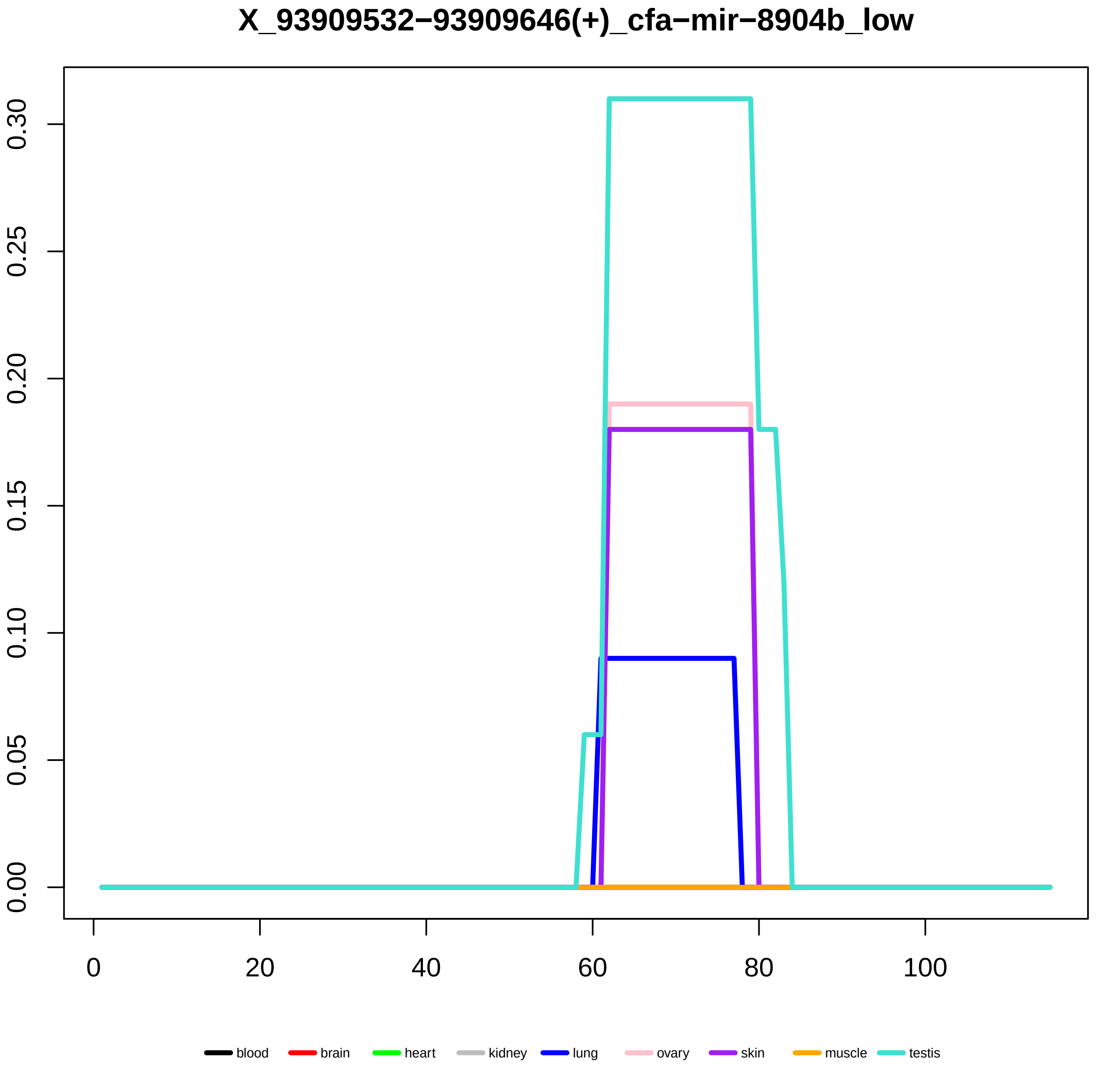

X_93909532−93909646(+)_cfa−mir−8904b_low
0.00
0.05
0.10
0.15
0.20
0.25
0.30
0
20
40
60
80
100
blood
brain
heart
kidney
lung
ovary
skin
muscle
testis

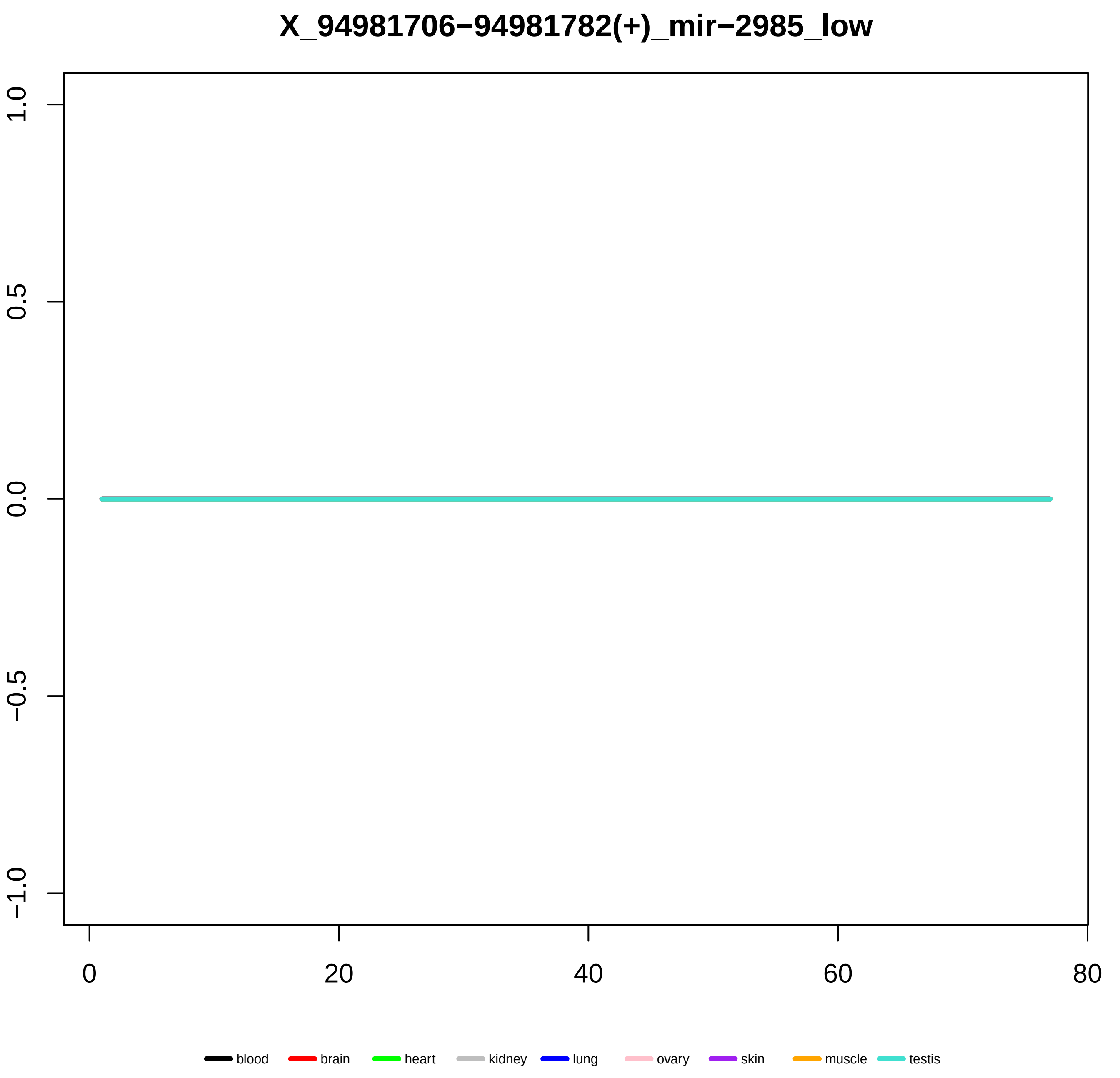

X_94981706−94981782(+)_mir−2985_low
1.0
0.5
0.0
−0.5
−1.0
0
20
40
60
80
blood
brain
heart
kidney
lung
ovary
skin
muscle
testis

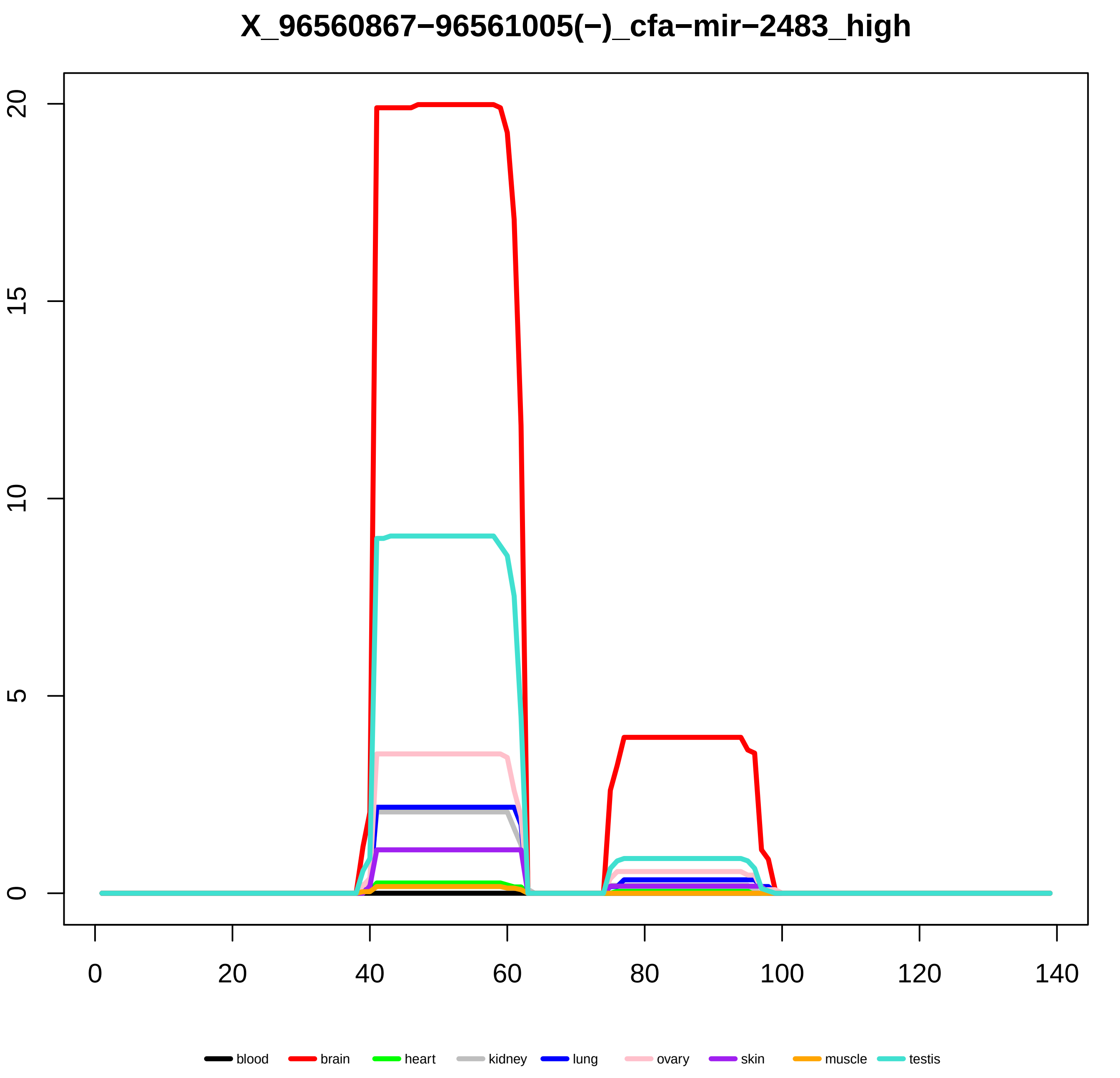
X_96560867−96561005(−)_cfa−mir−2483_high
0
5
10
15
20
0
20
40
60
80
100
120
140
blood
brain
heart
kidney
lung
ovary
skin
muscle
testis

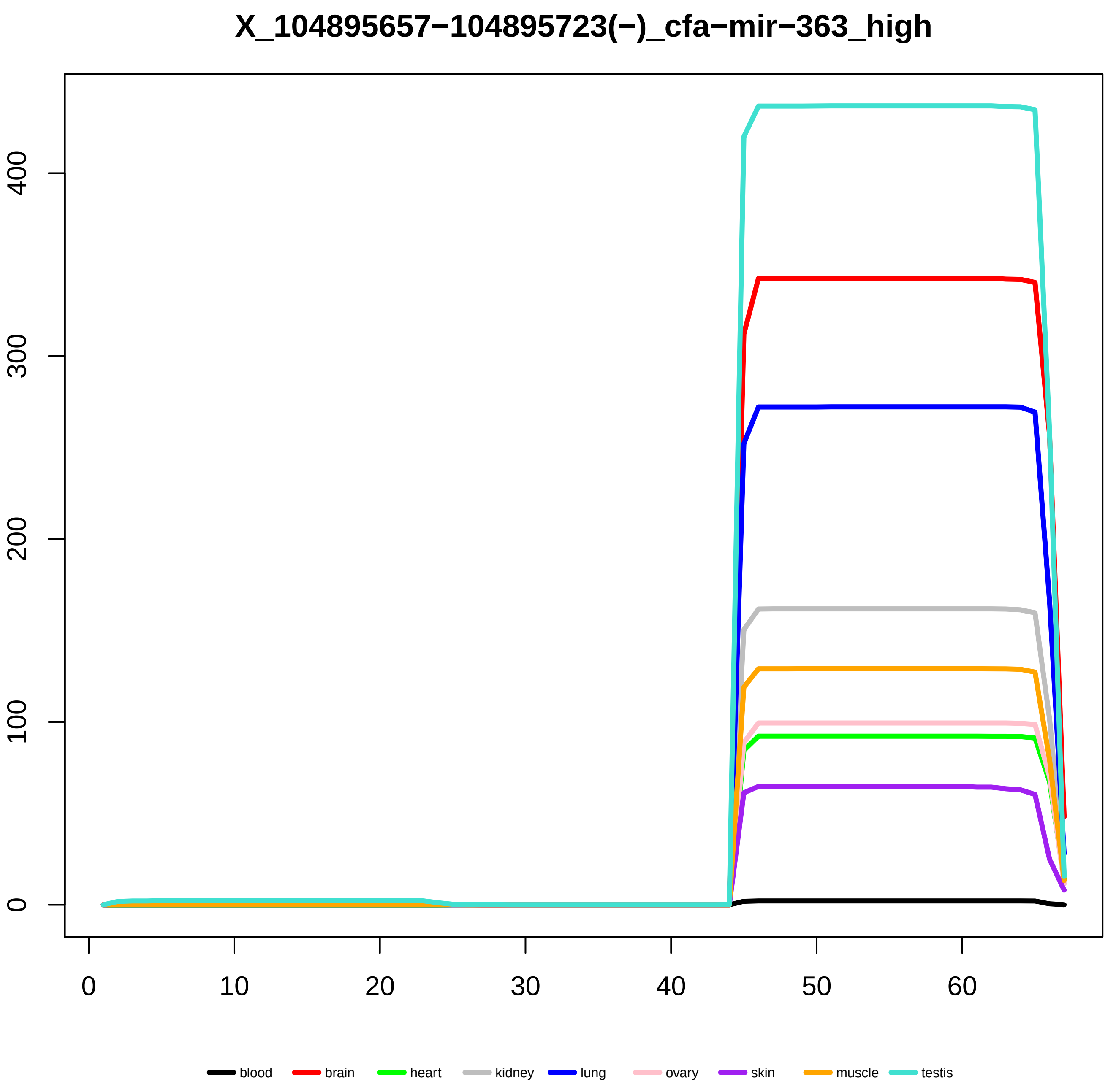
X_104895657−104895723(−)_cfa−mir−363_high
0
100
200
300
400
0
10
20
30
40
50
60
blood
brain
heart
kidney
lung
ovary
skin
muscle
testis

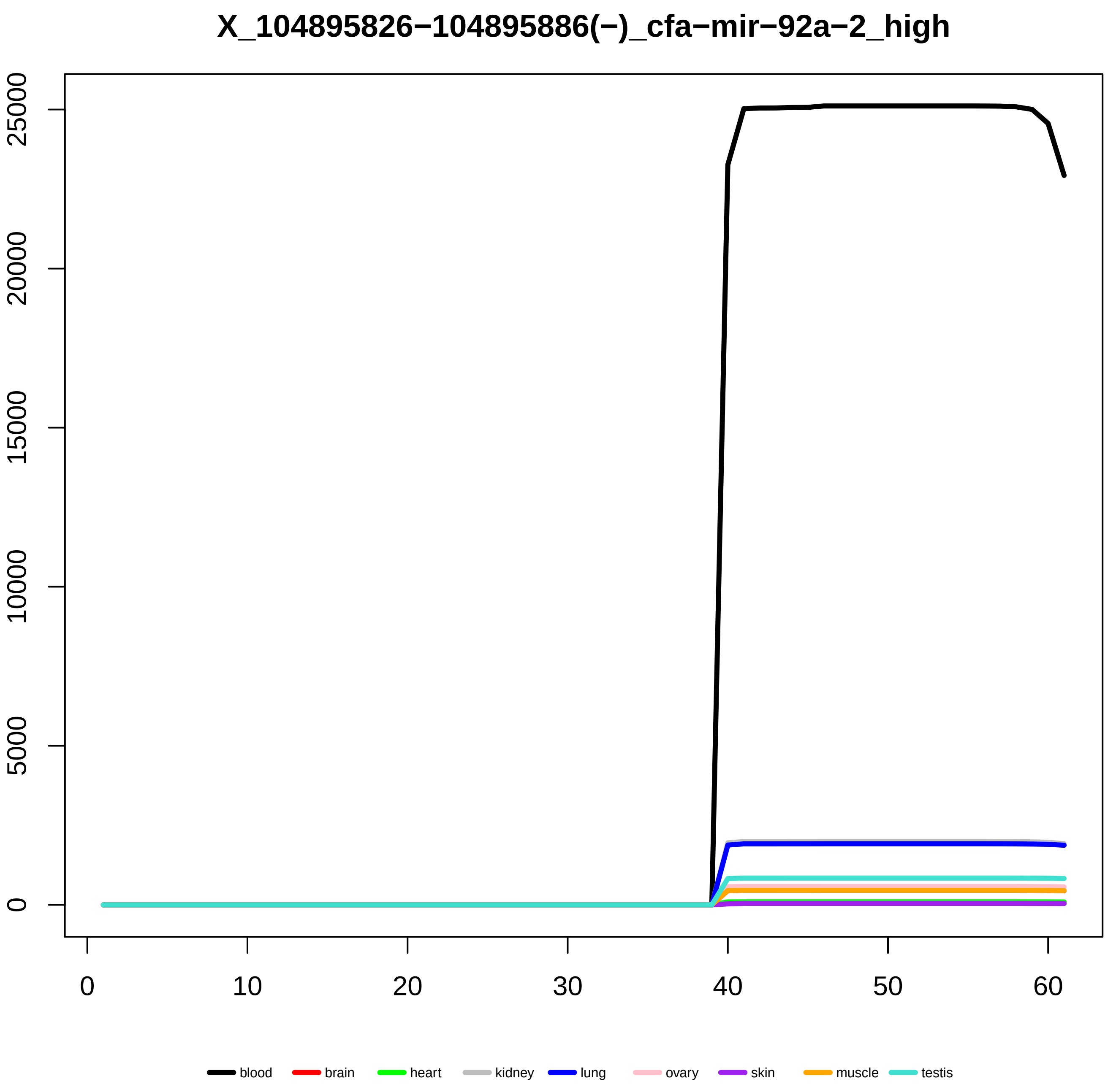
X_104895826−104895886(−)_cfa−mir−92a−2_high
0
5000
10000
15000
20000
25000
0
10
20
30
40
50
60
blood
brain
heart
kidney
lung
ovary
skin
muscle
testis

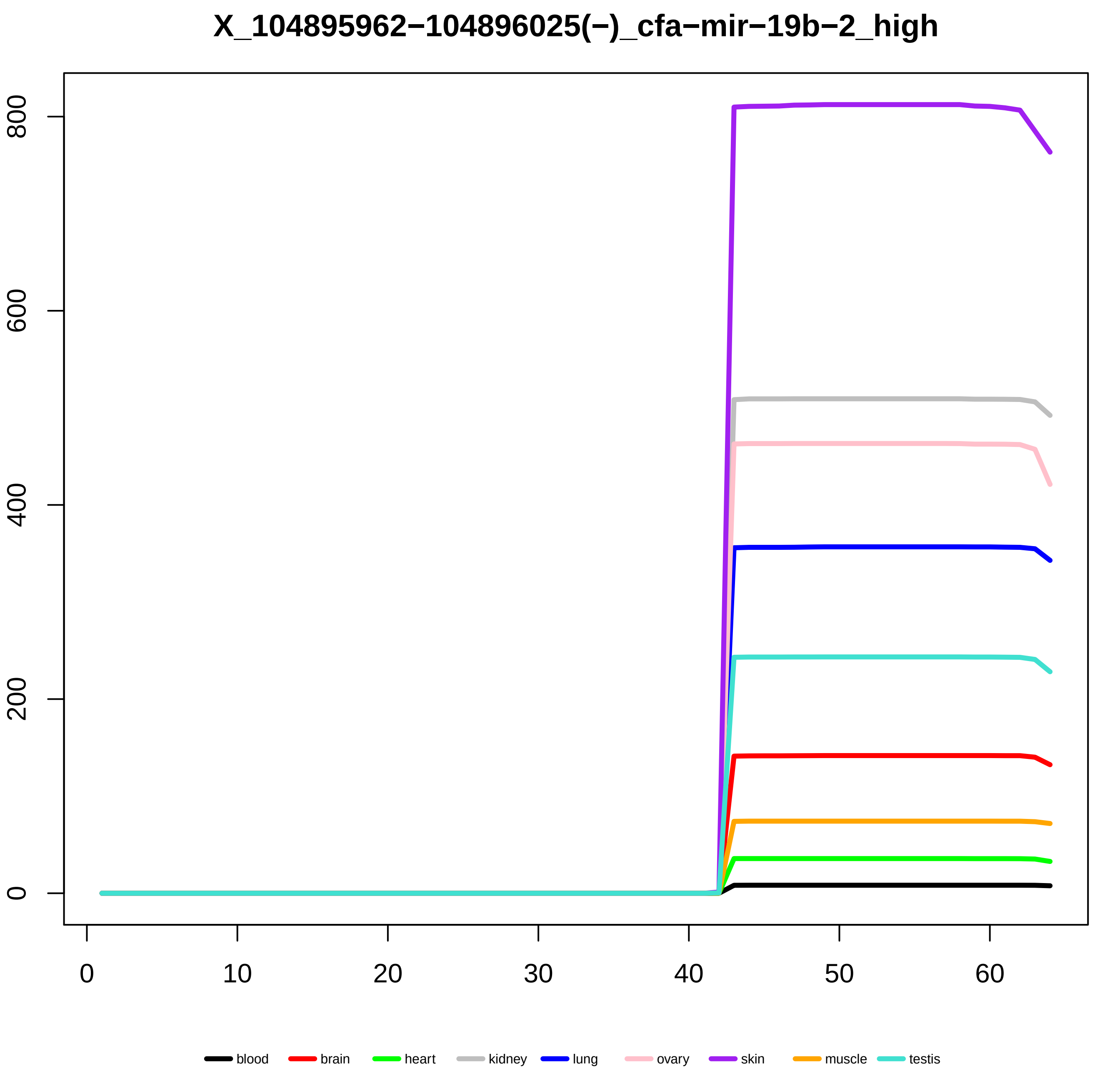

X_104895962−104896025(−)_cfa−mir−19b−2_high
0
200
400
600
800
0
10
20
30
40
50
60
blood
brain
heart
kidney
lung
ovary
skin
muscle
testis

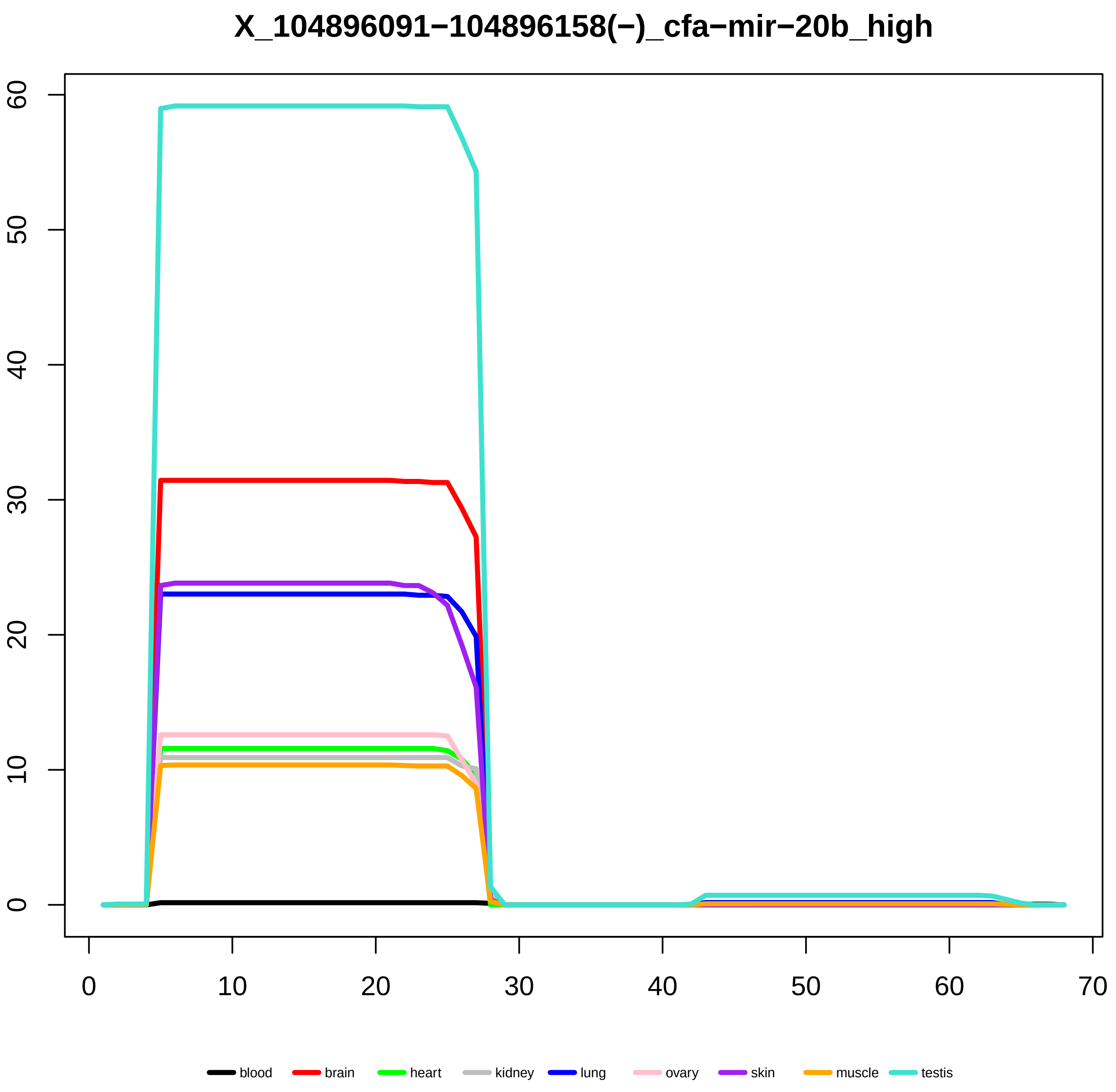
X_104896091−104896158(−)_cfa−mir−20b_high
0
10
20
30
40
50
60
0
10
20
30
40
50
60
70
blood
brain
heart
kidney
lung
ovary
skin
muscle
testis

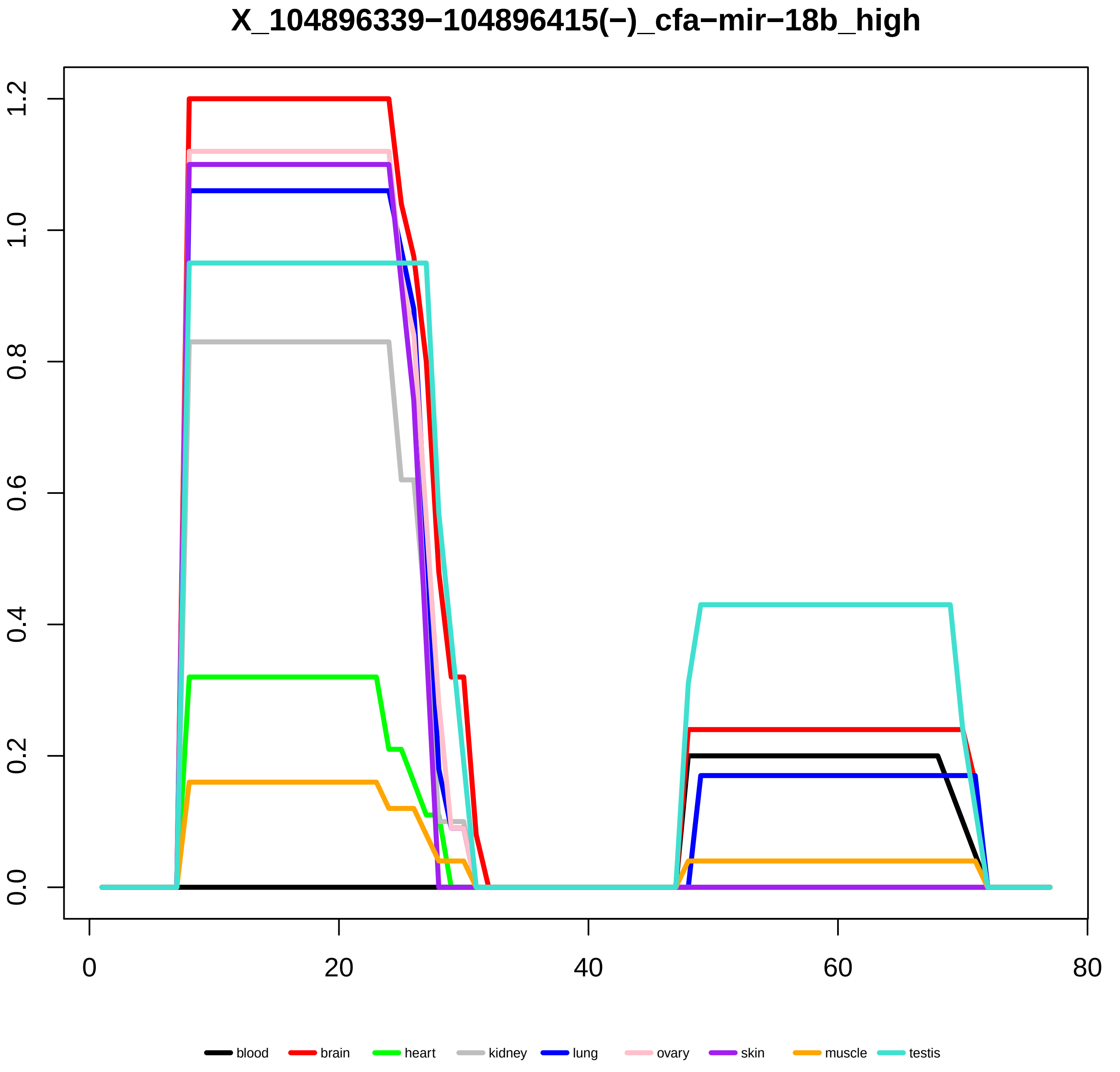

X_104896339−104896415(−)_cfa−mir−18b_high
0.0
0.2
0.4
0.6
0.8
1.0
1.2
0
20
40
60
80
blood
brain
heart
kidney
lung
ovary
skin
muscle
testis

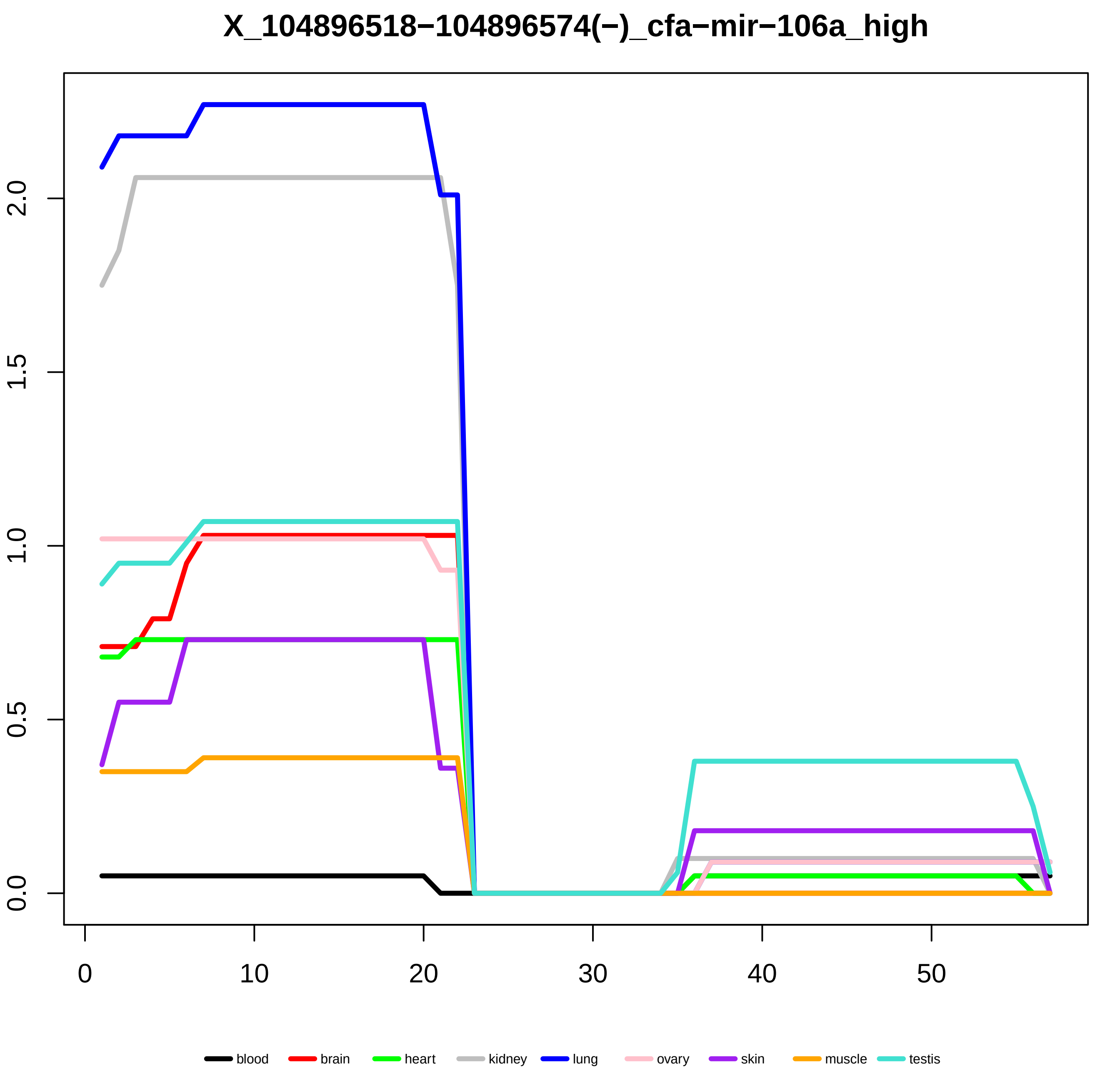
X_104896518−104896574(−)_cfa−mir−106a_high
0.0
0.5
1.0
1.5
2.0
0
10
20
30
40
50
blood
brain
heart
kidney
lung
ovary
skin
muscle
testis

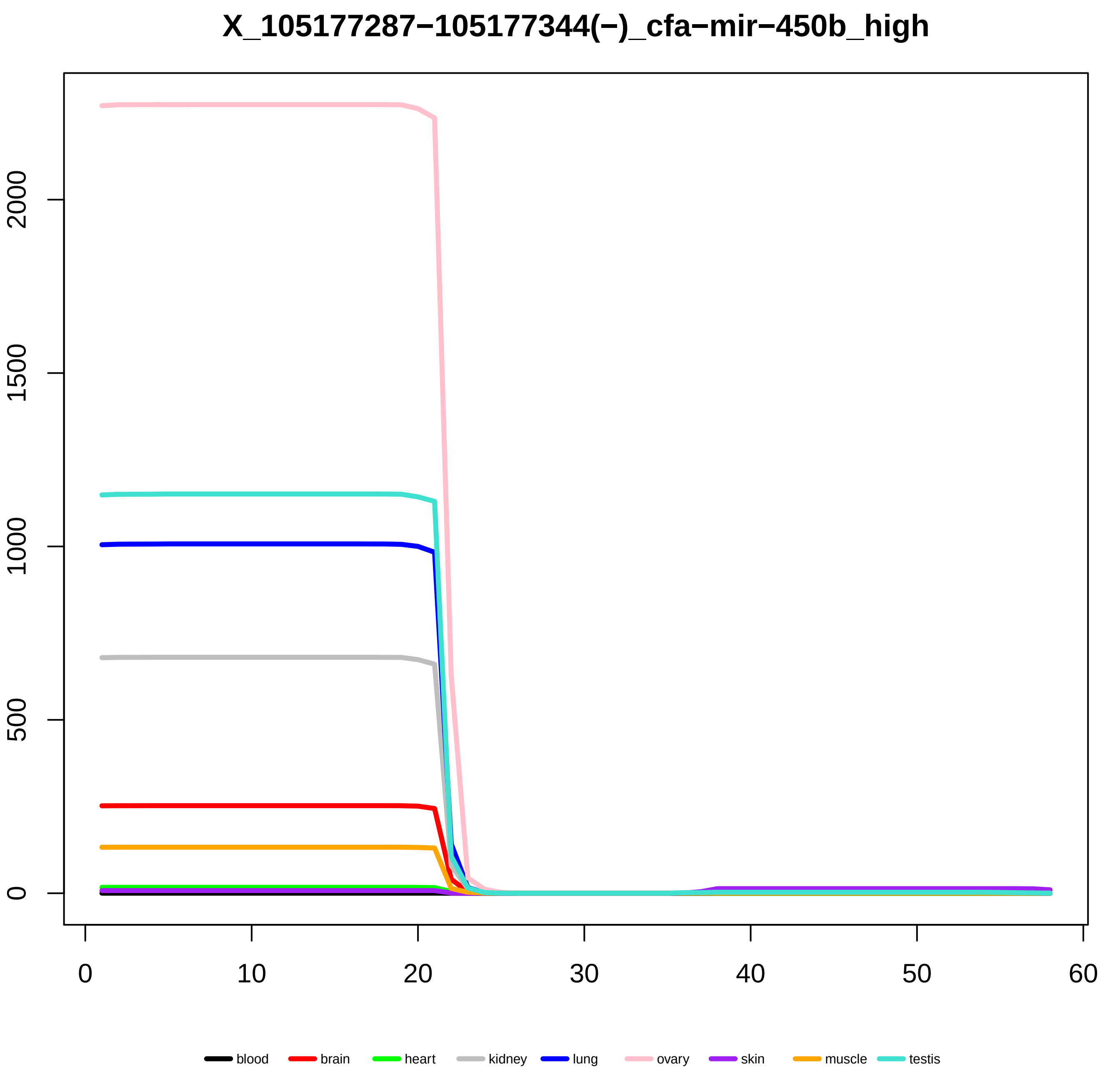

X_105177287−105177344(−)_cfa−mir−450b_high
0
500
1000
1500
2000
0
10
20
30
40
50
60
blood
brain
heart
kidney
lung
ovary
skin
muscle
testis

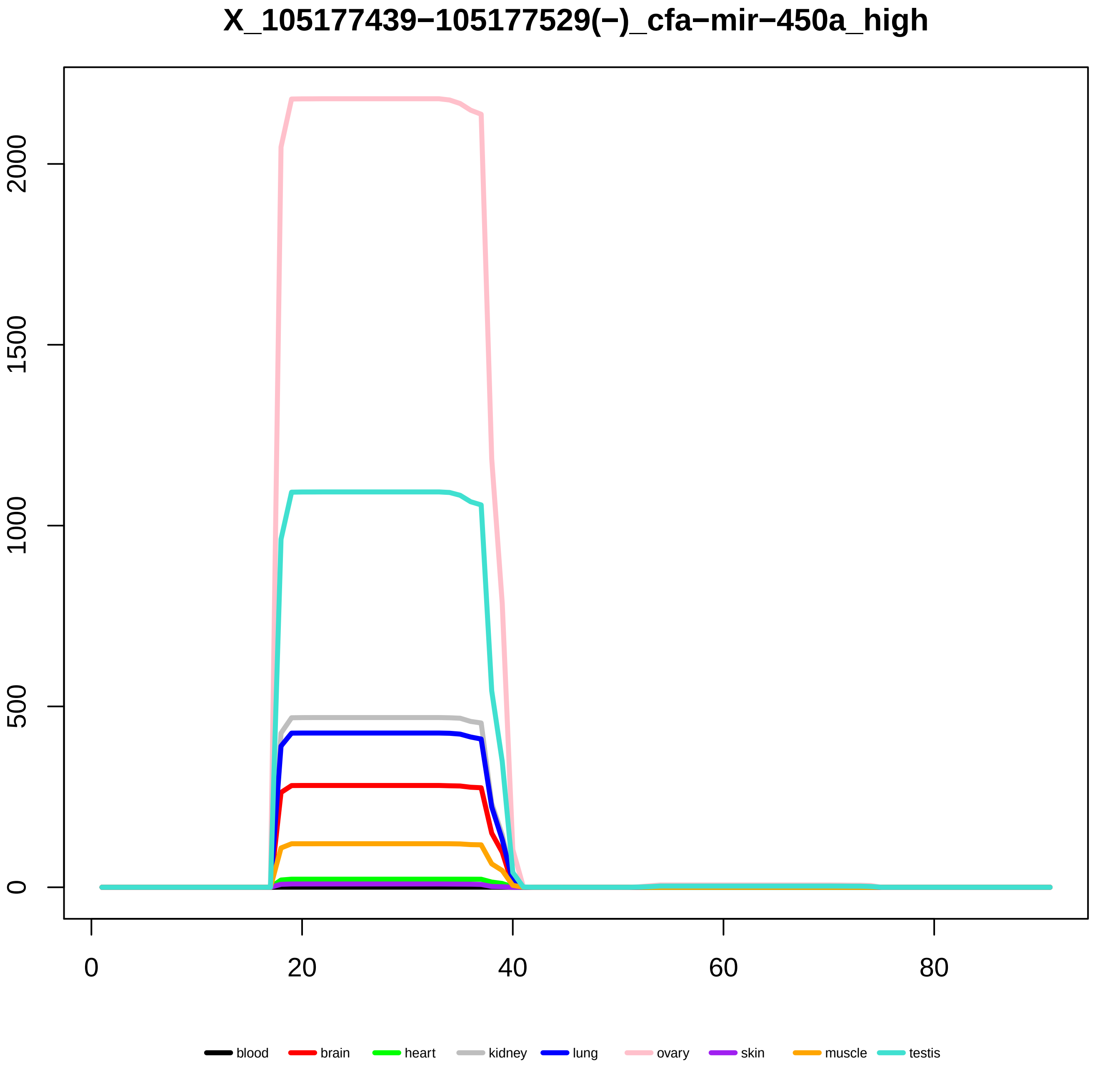

X_105177439−105177529(−)_cfa−mir−450a_high
0
500
1000
1500
2000
0
20
40
60
80
blood
brain
heart
kidney
lung
ovary
skin
muscle
testis

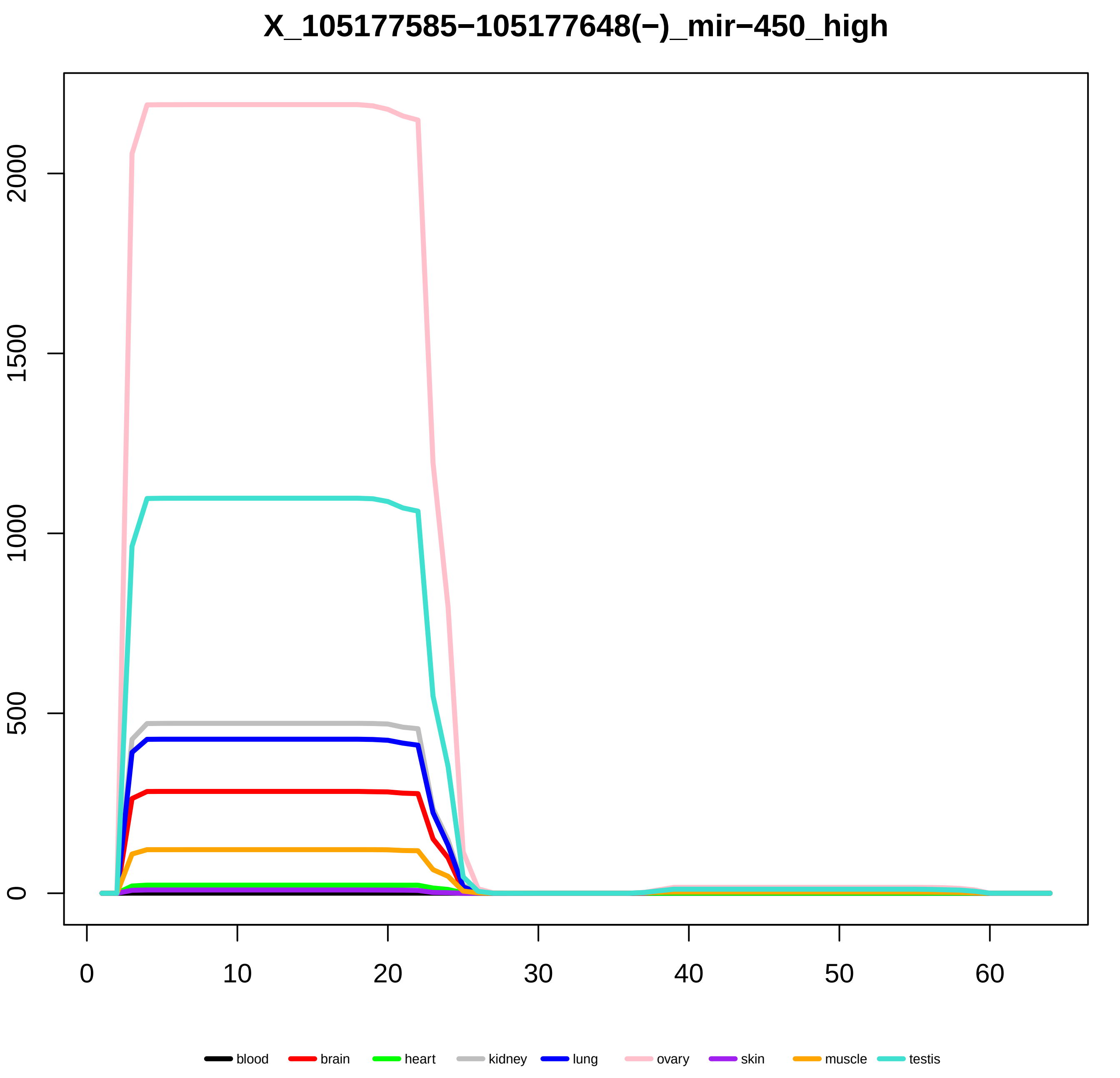
X_105177585−105177648(−)_mir−450_high
0
500
1000
1500
2000
0
10
20
30
40
50
60
blood
brain
heart
kidney
lung
ovary
skin
muscle
testis

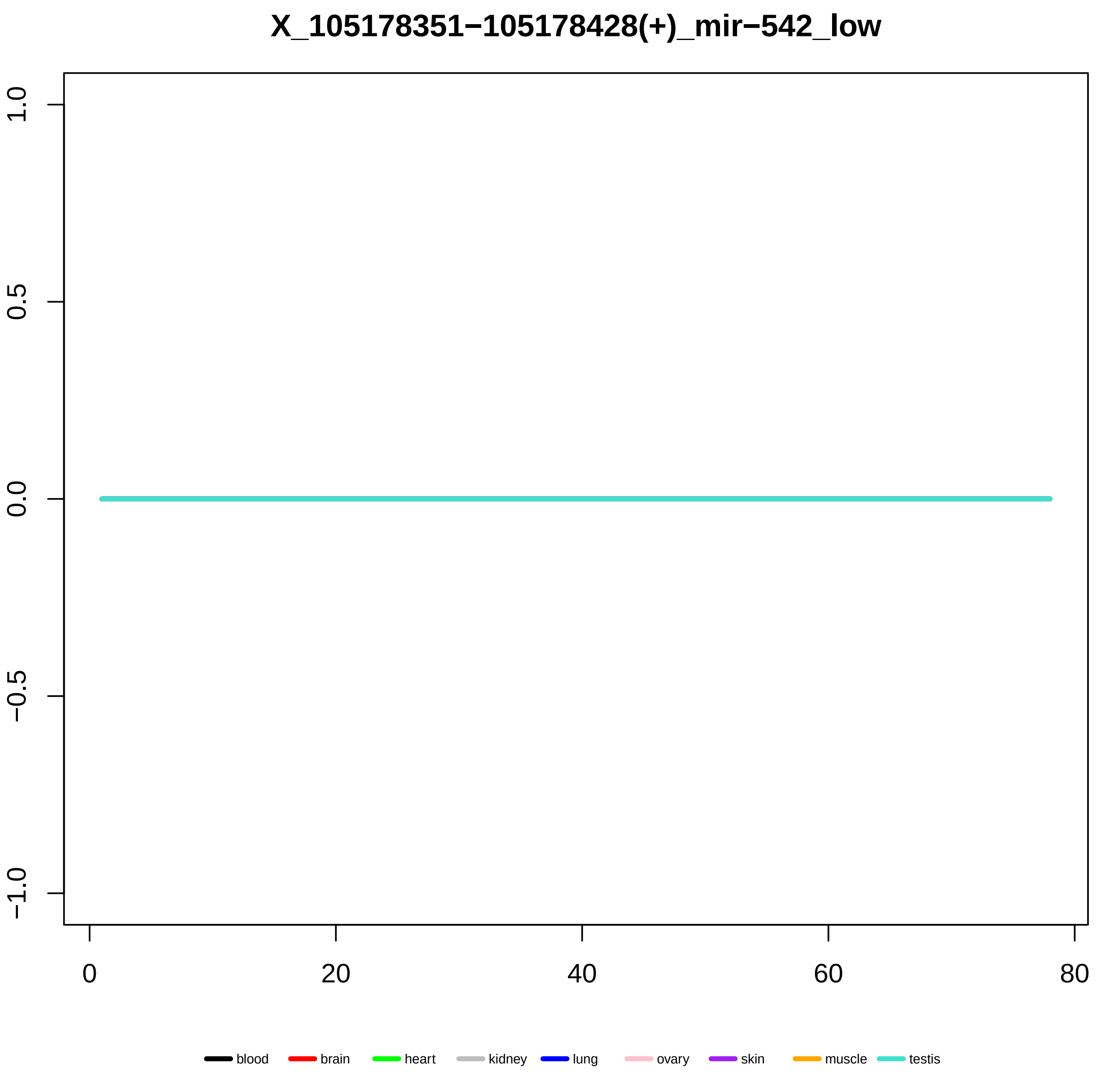
X_105178351−105178428(+)_mir−542_low
1.0
0.5
0.0
−0.5
−1.0
0
20
40
60
80
blood
brain
heart
kidney
lung
ovary
skin
muscle
testis

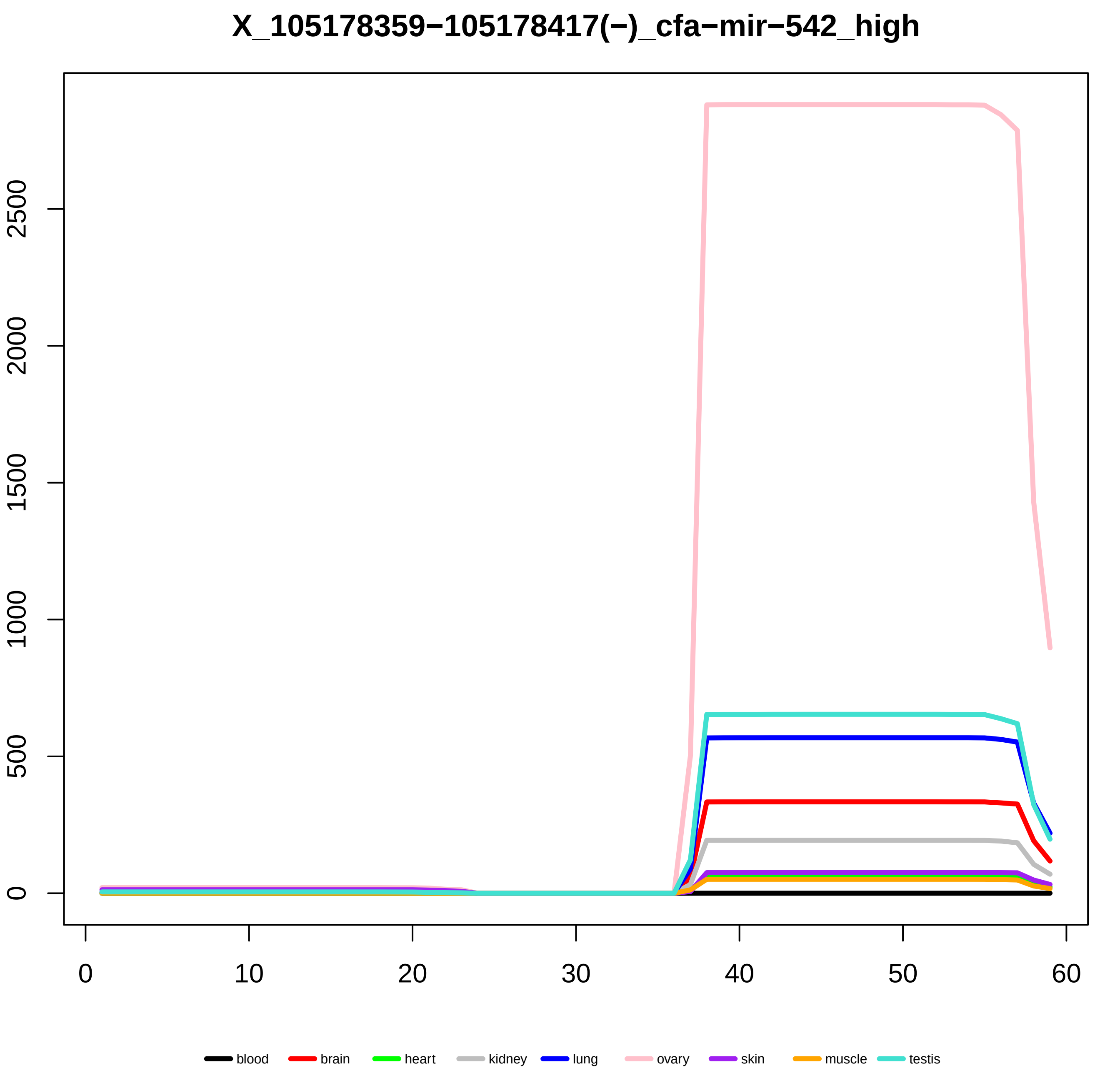
X_105178359−105178417(−)_cfa−mir−542_high
0
500
1000
1500
2000
2500
0
10
20
30
40
50
60
blood
brain
heart
kidney
lung
ovary
skin
muscle
testis

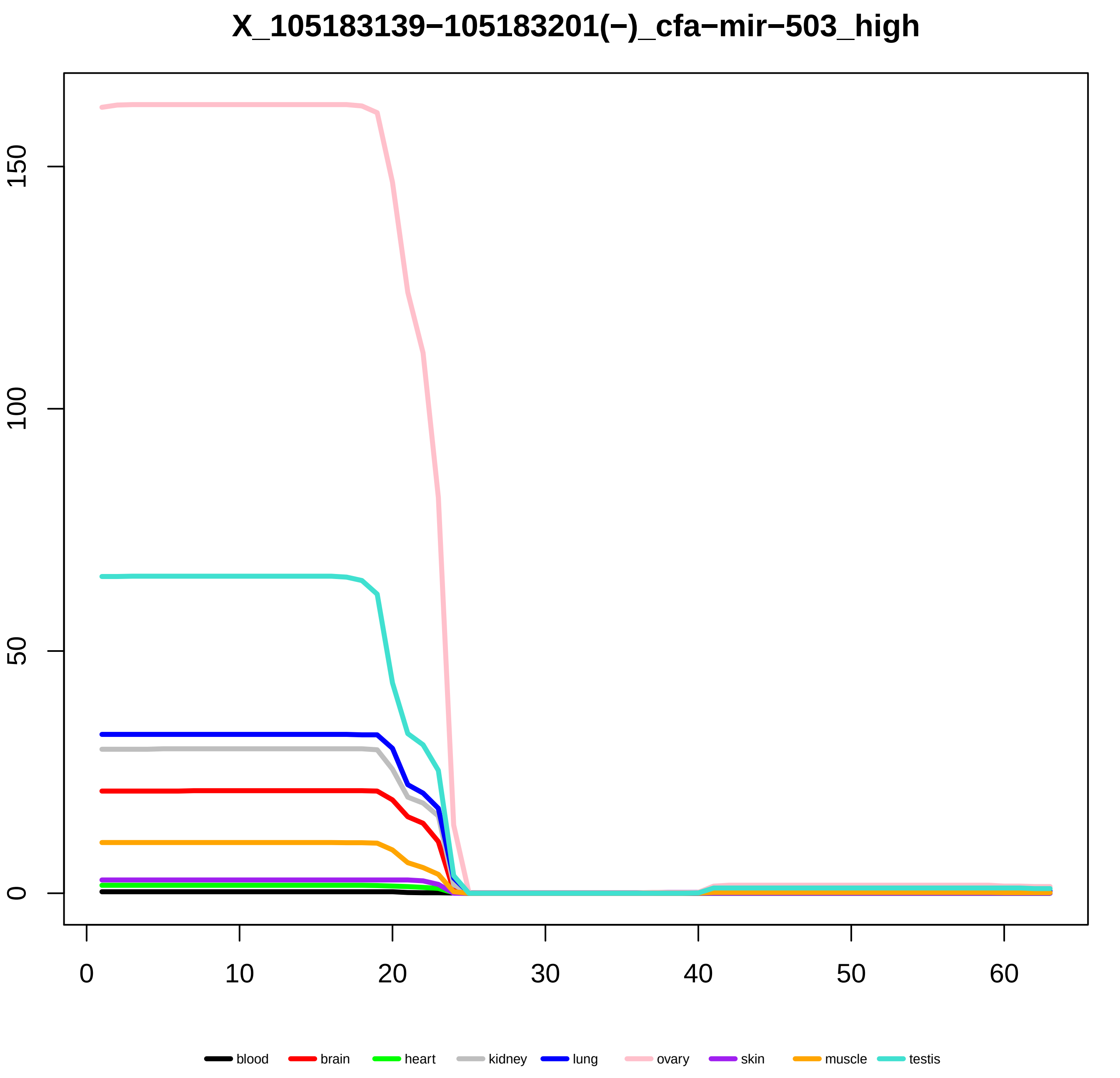
X_105183139−105183201(−)_cfa−mir−503_high
0
50
100
150
0
10
20
30
40
50
60
blood
brain
heart
kidney
lung
ovary
skin
muscle
testis

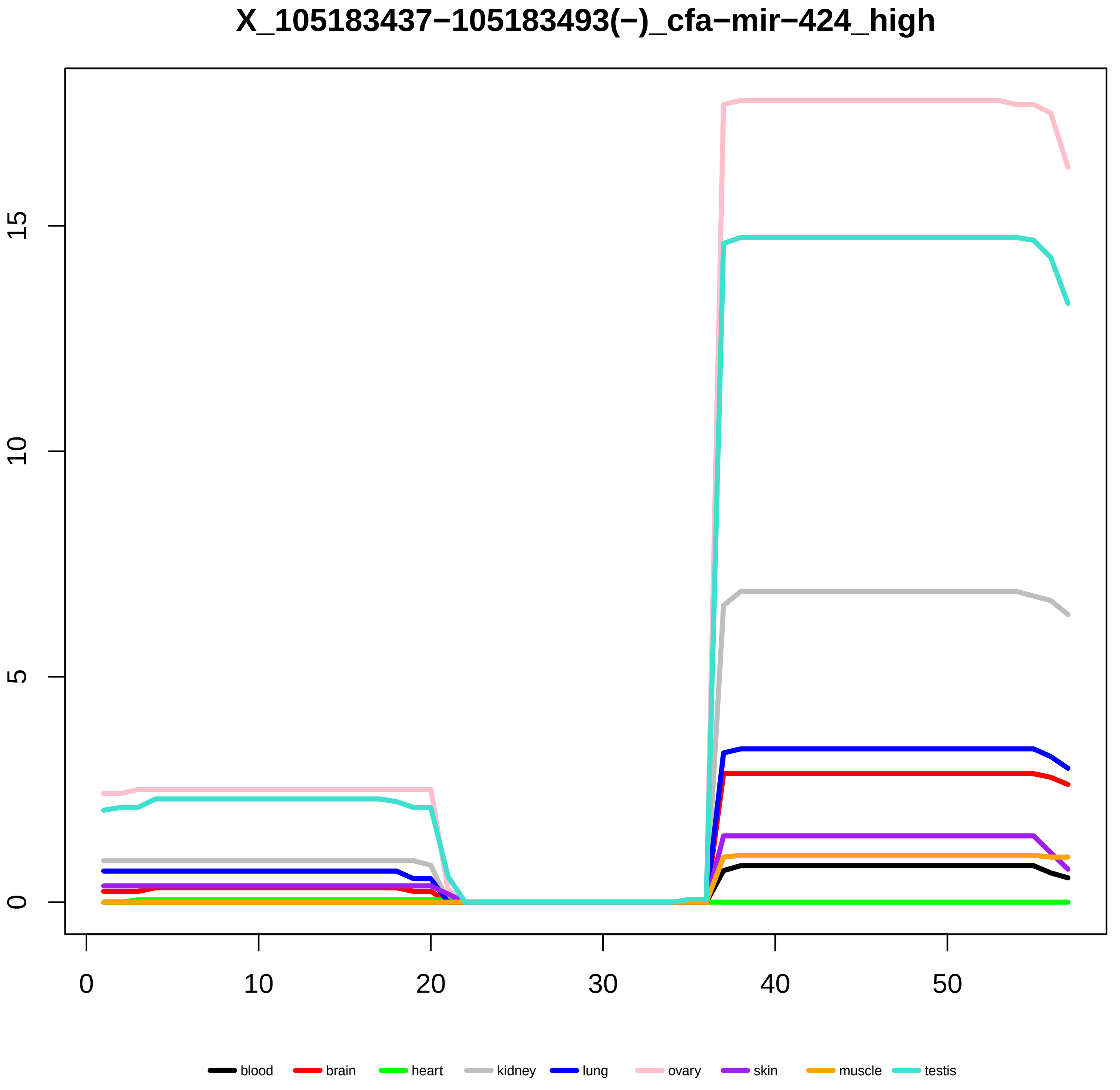

X_105183437−105183493(−)_cfa−mir−424_high
0
5
10
15
0
10
20
30
40
50
blood
brain
heart
kidney
lung
ovary
skin
muscle
testis

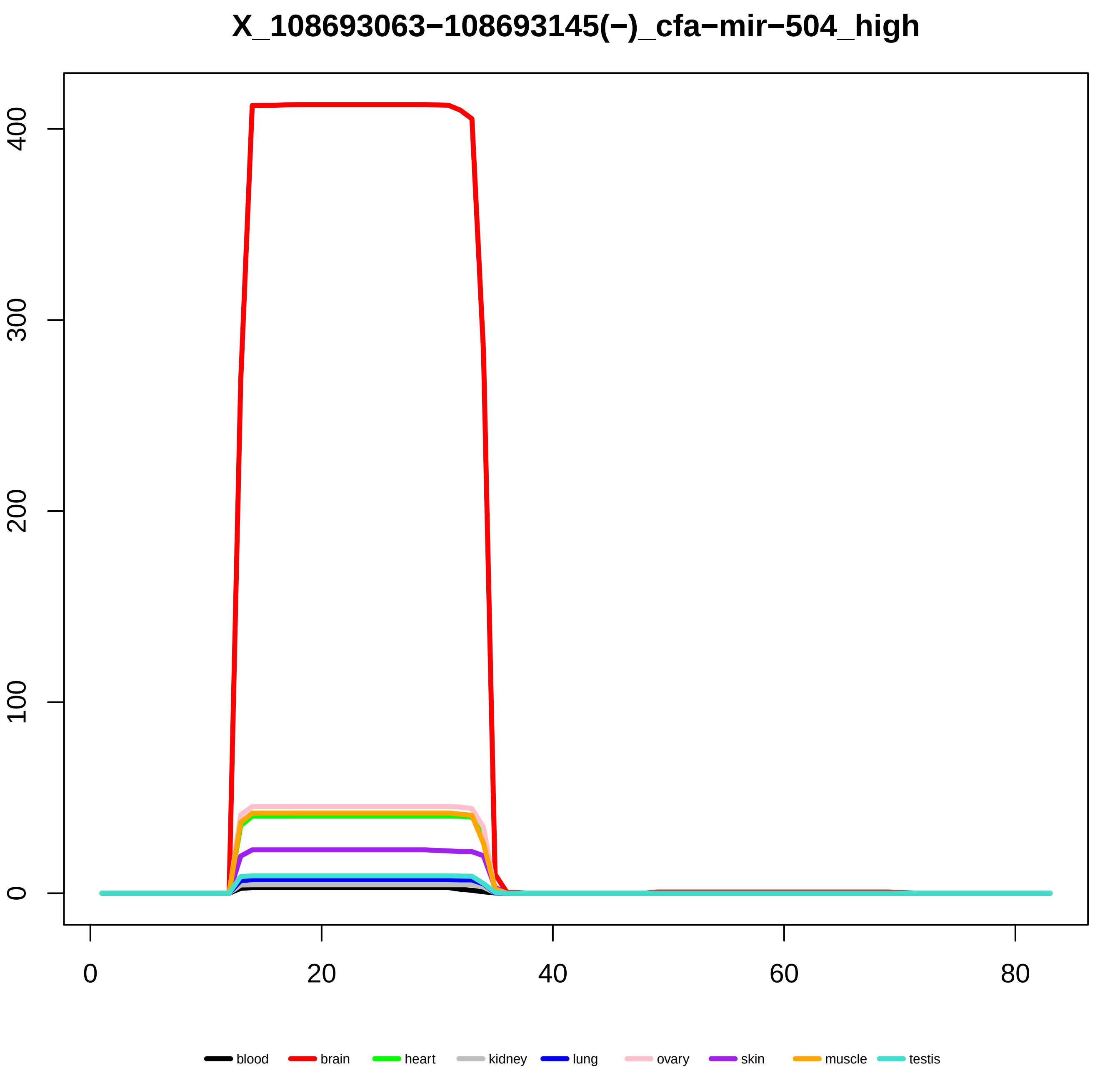
X_108693063−108693145(−)_cfa−mir−504_high
0
100
200
300
400
0
20
40
60
80
blood
brain
heart
kidney
lung
ovary
skin
muscle
testis

X_109866448−109866523(−)_cfa−mir−505_high

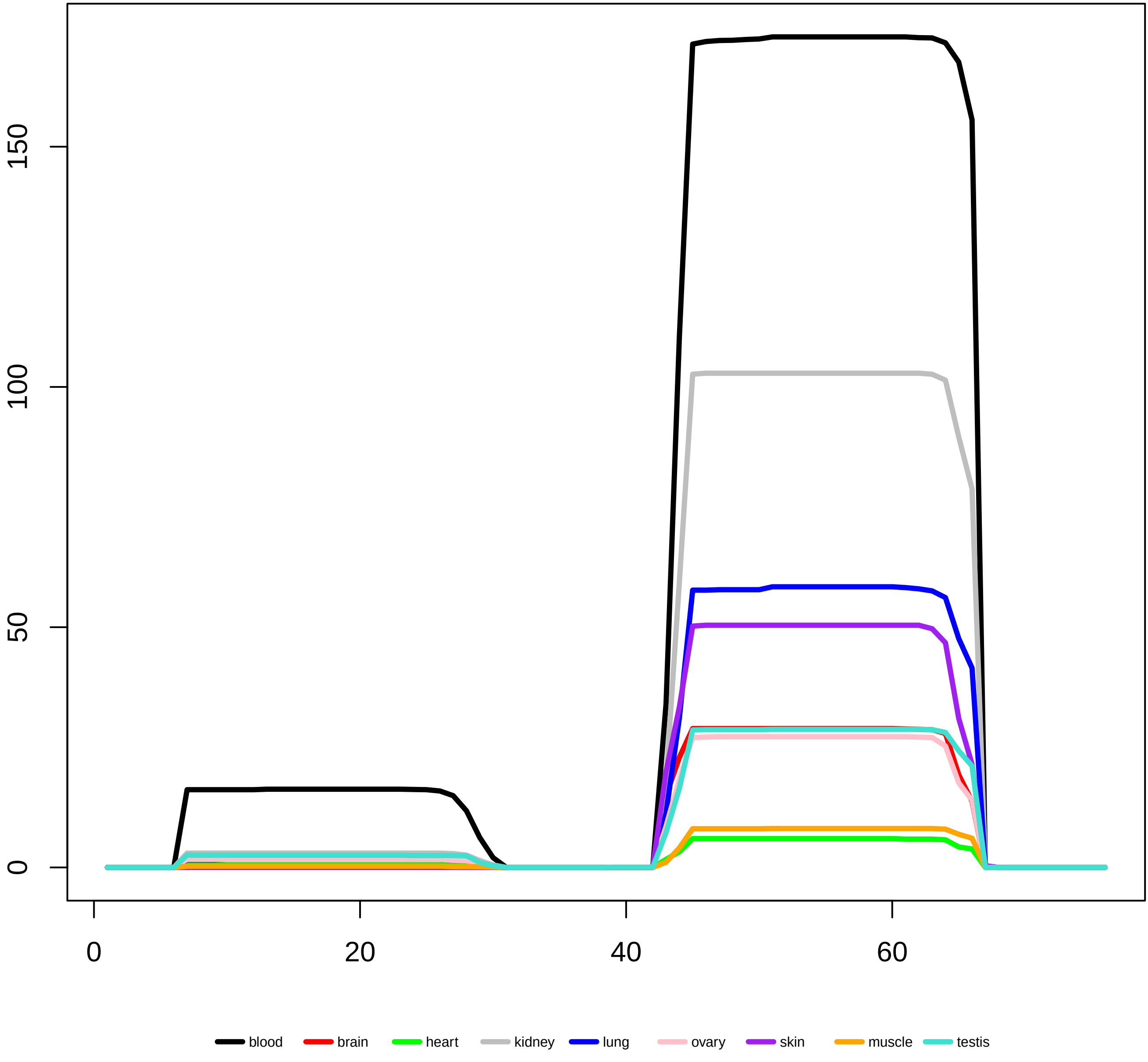

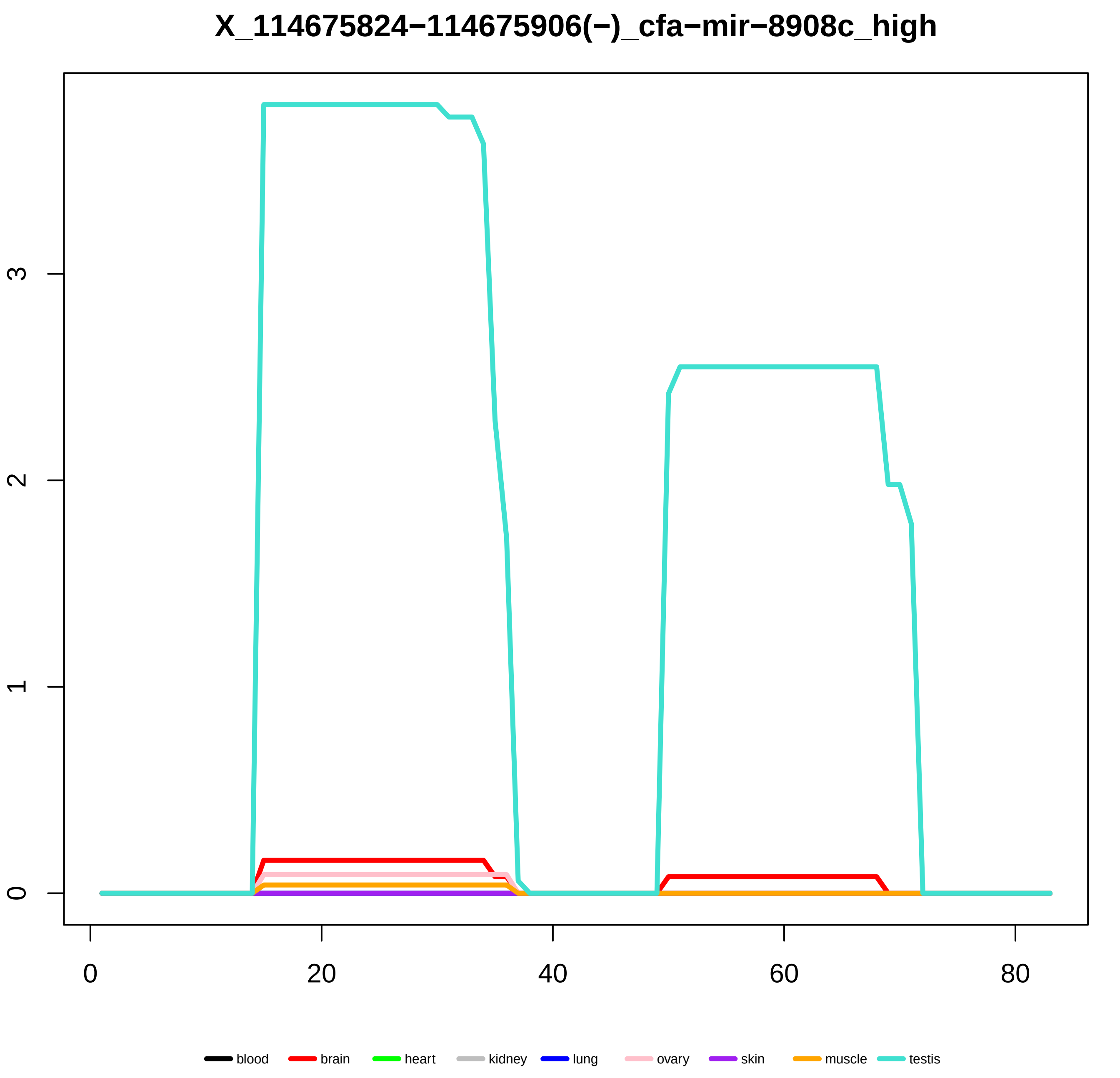

X_114675824−114675906(−)_cfa−mir−8908c_high
0
1
2
3
0
20
40
60
80
blood
brain
heart
kidney
lung
ovary
skin
muscle
testis

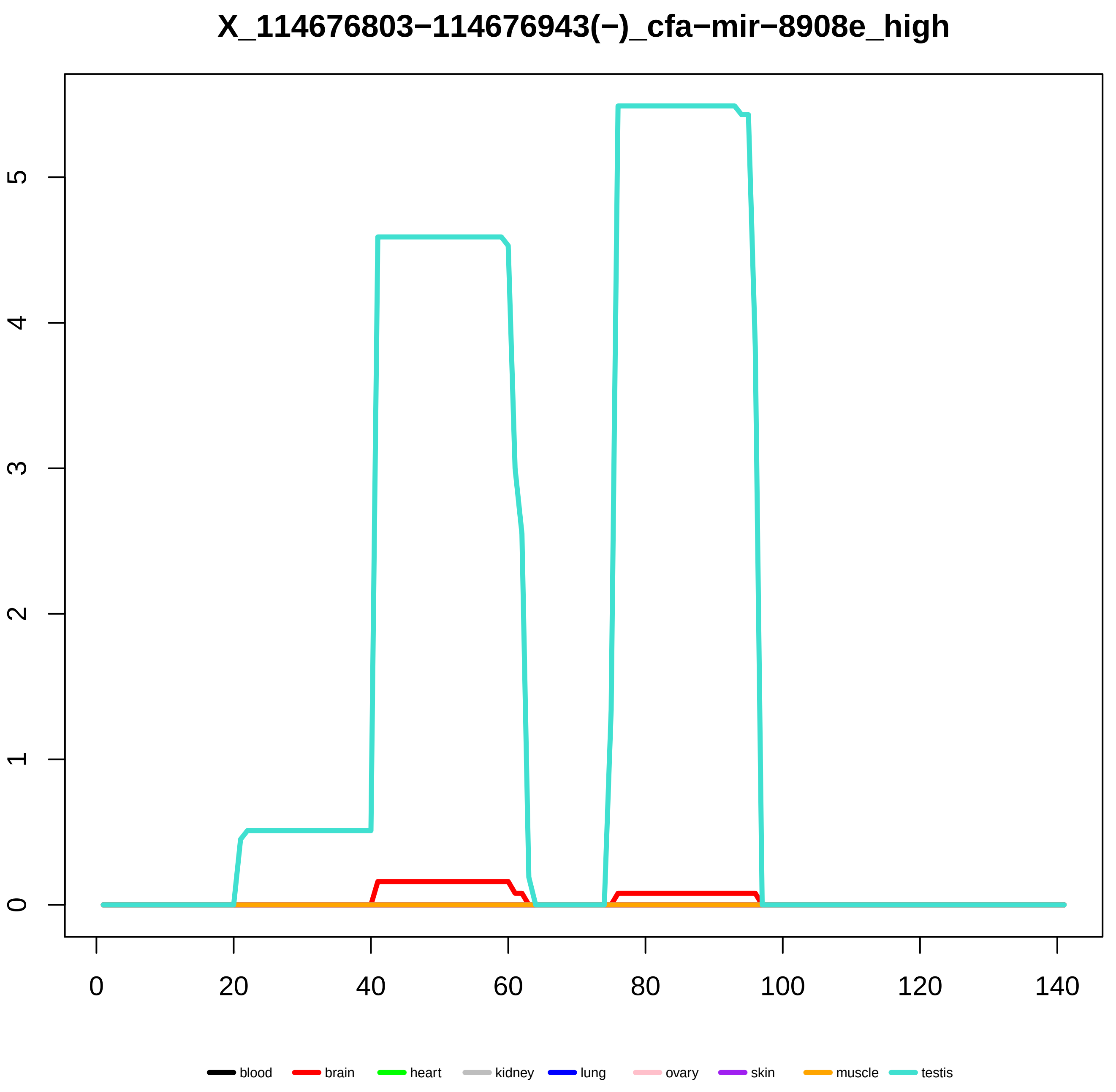

X_114676803−114676943(−)_cfa−mir−8908e_high
0
1
2
3
4
5
0
20
40
60
80
100
120
140
blood
brain
heart
kidney
lung
ovary
skin
muscle
testis

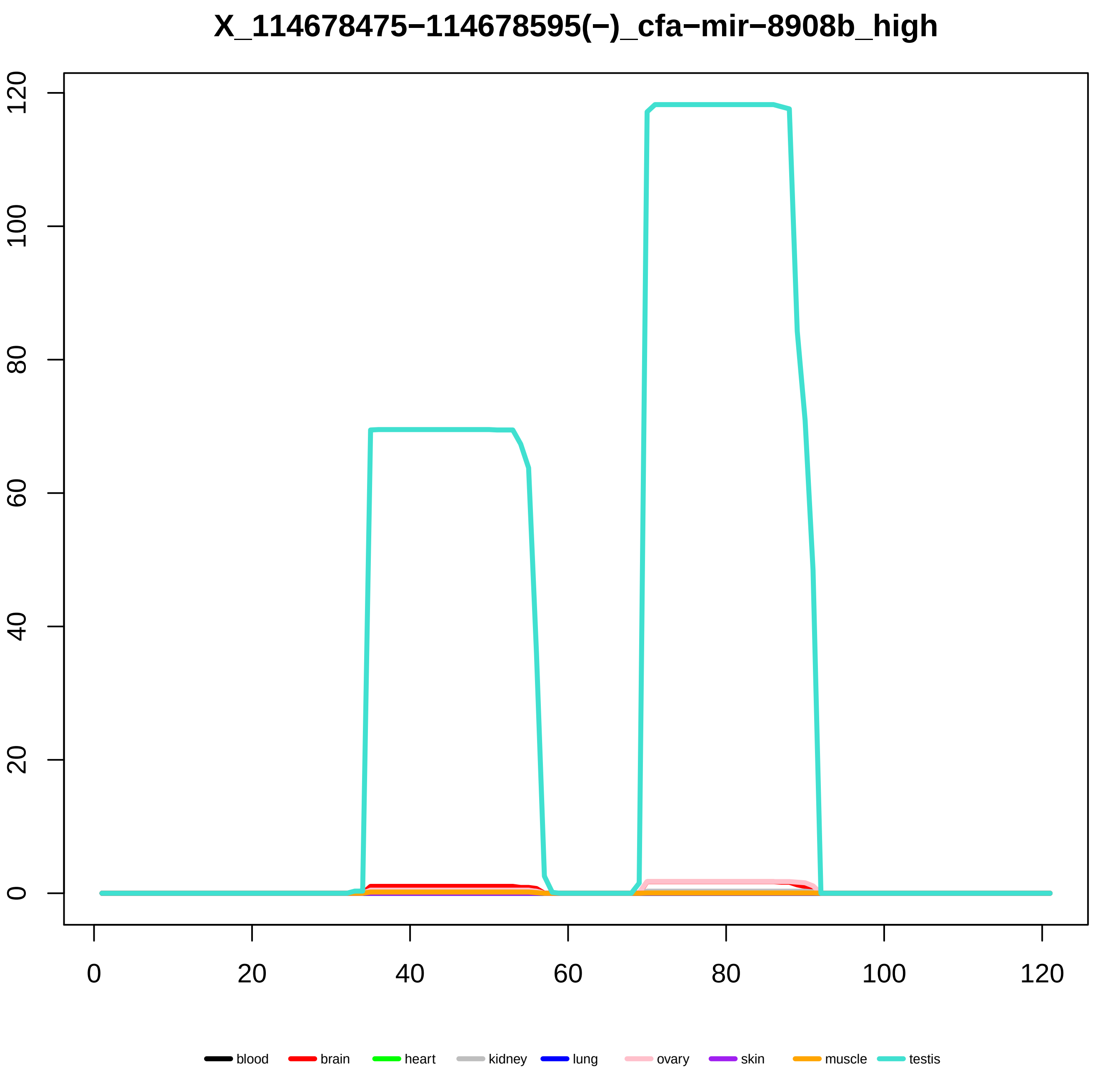
X_114678475−114678595(−)_cfa−mir−8908b_high
0
20
40
60
80
100
120
0
20
40
60
80
100
120
blood
brain
heart
kidney
lung
ovary
skin
muscle
testis

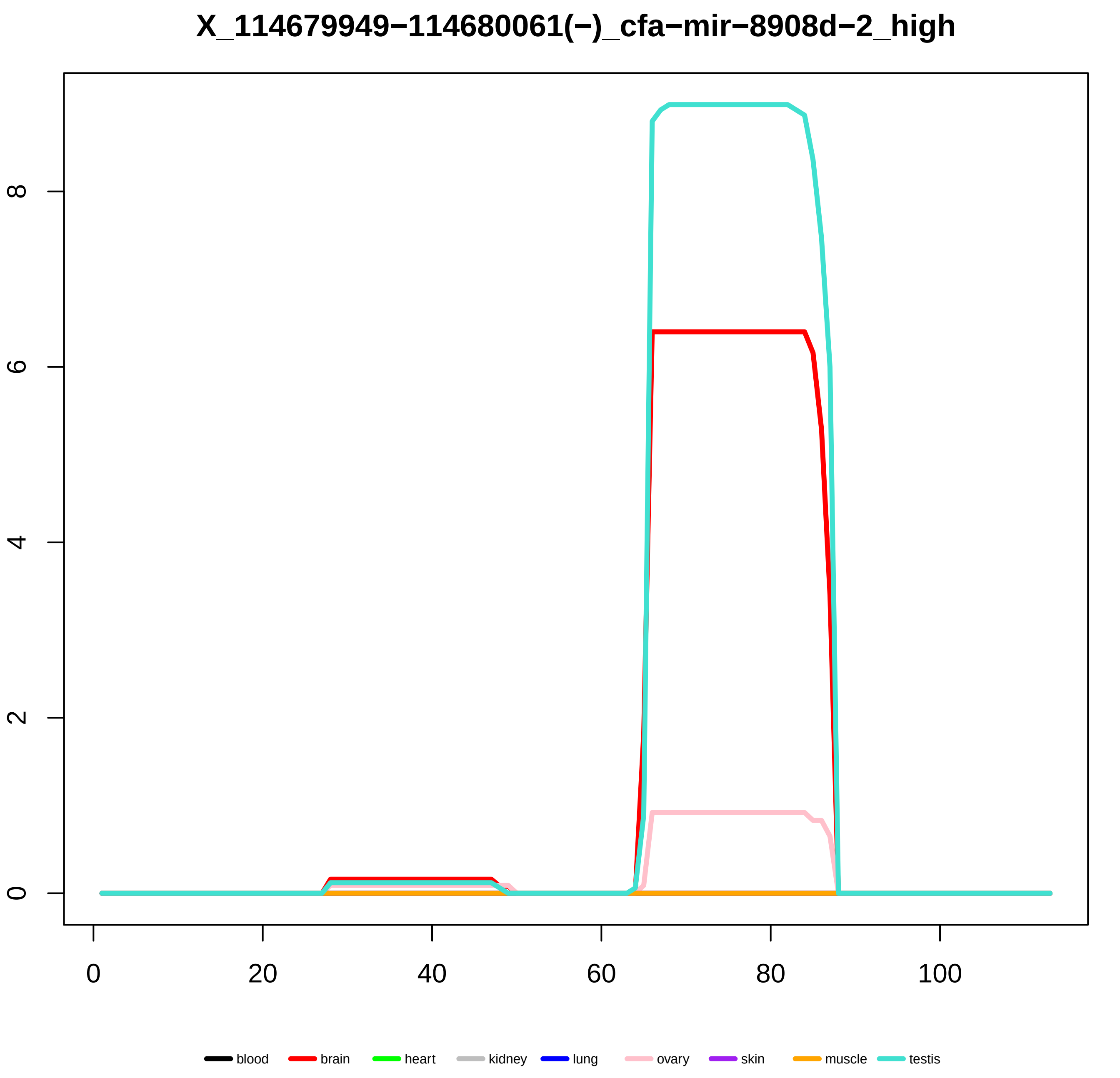

X_114679949−114680061(−)_cfa−mir−8908d−2_high
0
2
4
6
8
0
20
40
60
80
100
blood
brain
heart
kidney
lung
ovary
skin
muscle
testis

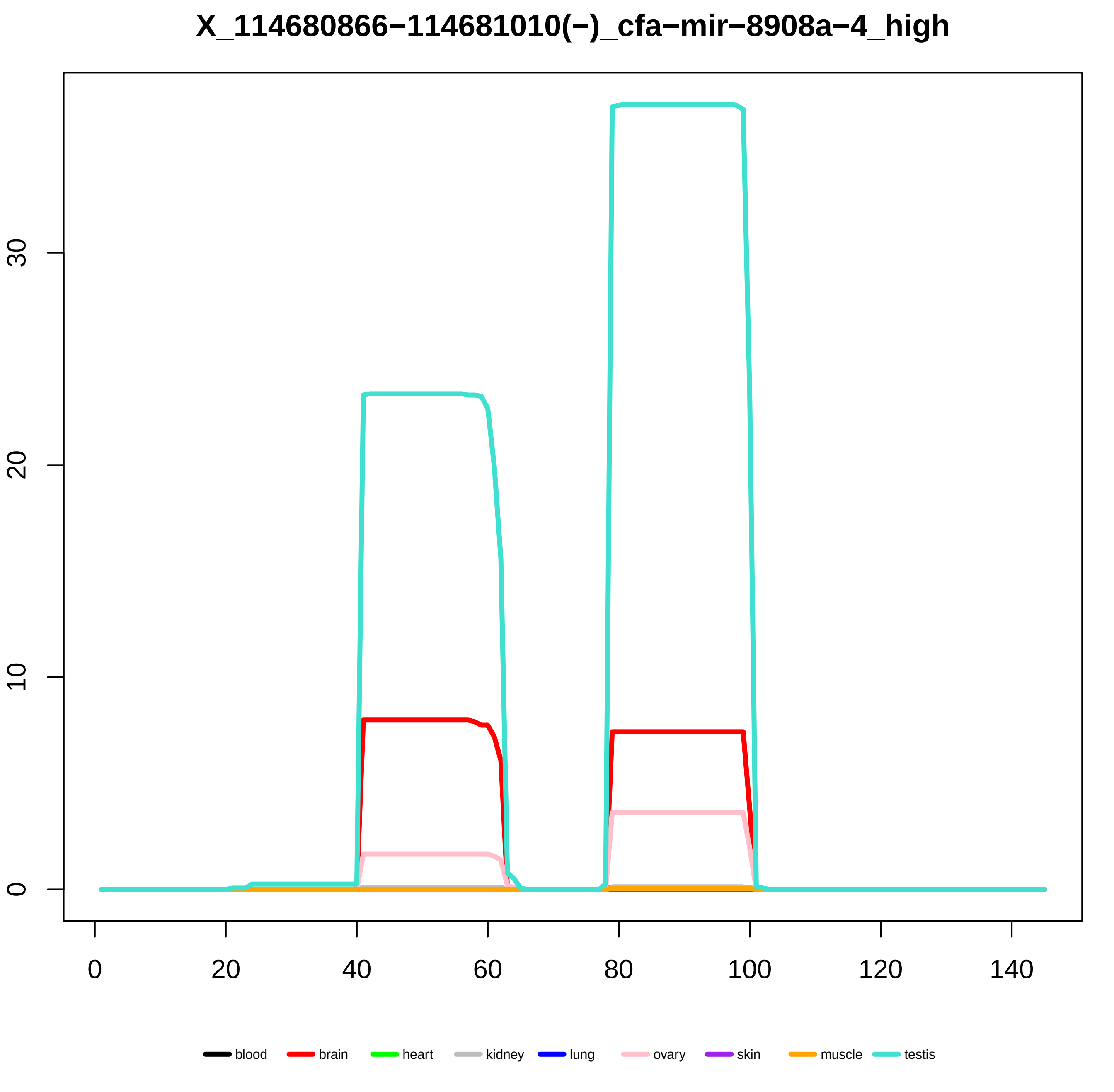
X_114680866−114681010(−)_cfa−mir−8908a−4_high
0
10
20
30
0
20
40
60
80
100
120
140
blood
brain
heart
kidney
lung
ovary
skin
muscle
testis

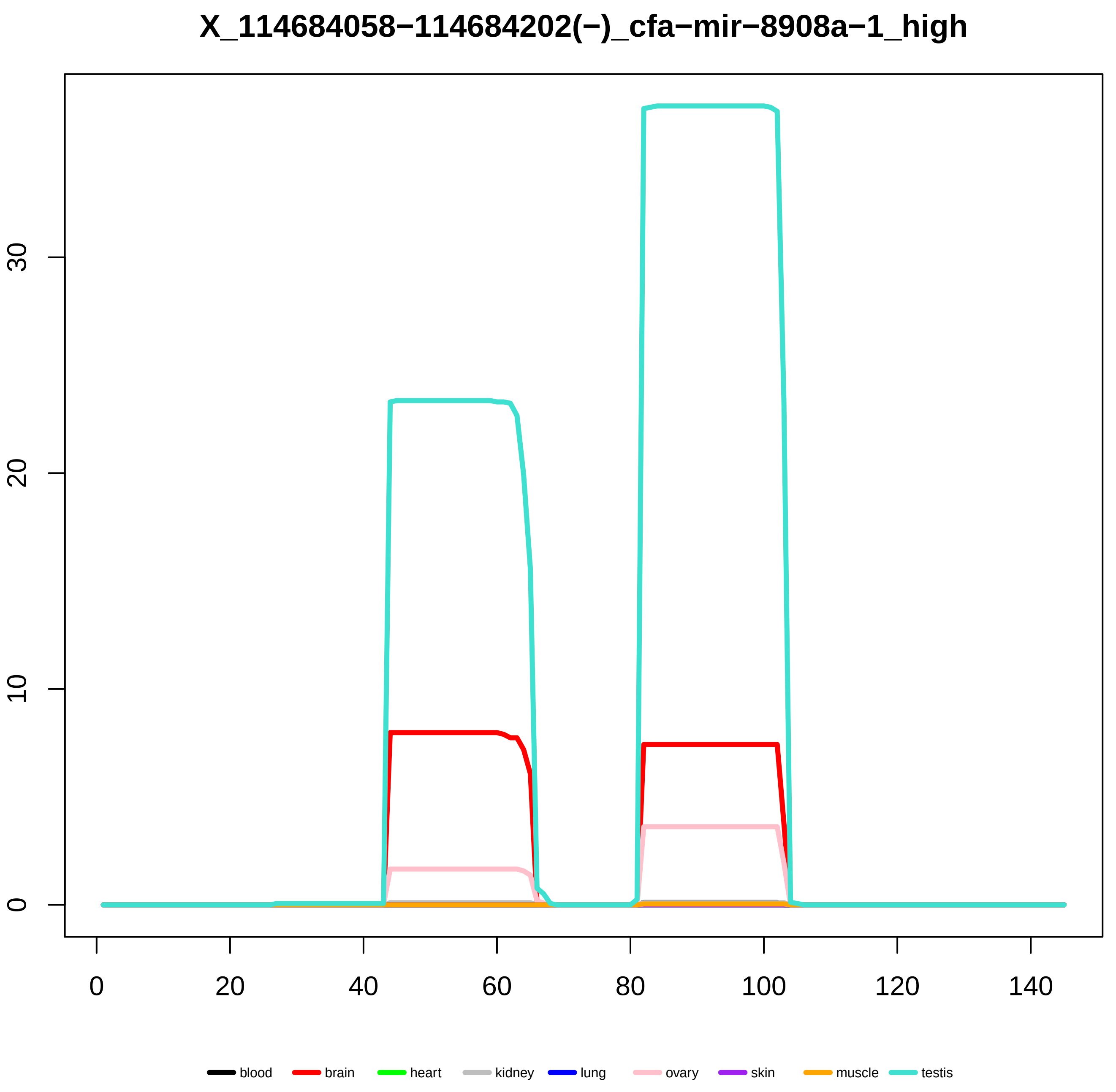
X_114684058−114684202(−)_cfa−mir−8908a−1_high
0
10
20
30
0
20
40
60
80
100
120
140
blood
brain
heart
kidney
lung
ovary
skin
muscle
testis

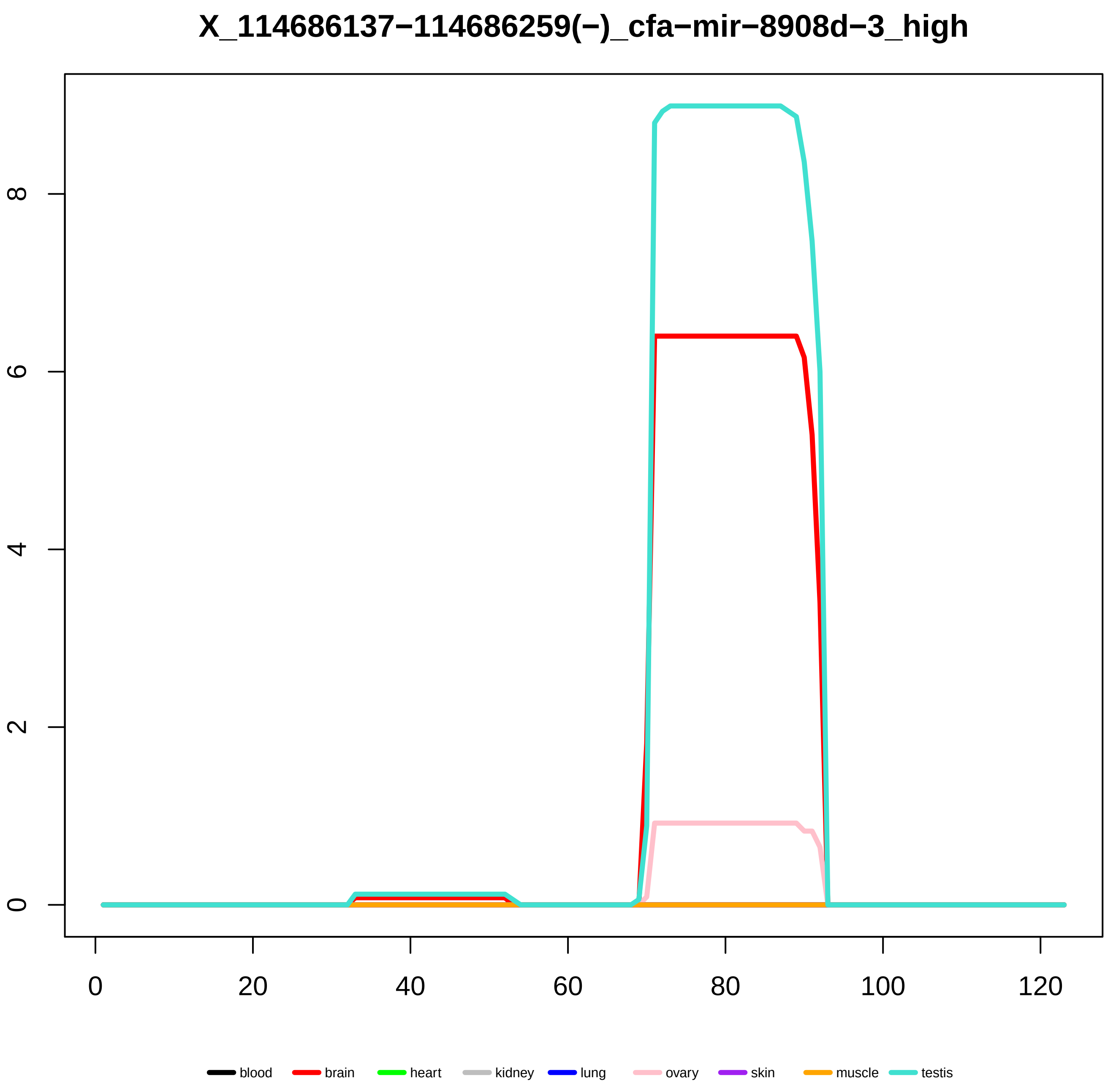

X_114686137−114686259(−)_cfa−mir−8908d−3_high
0
2
4
6
8
0
20
40
60
80
100
120
blood
brain
heart
kidney
lung
ovary
skin
muscle
testis

X_114686988−114687132(−)_cfa−mir−8908a−2_high

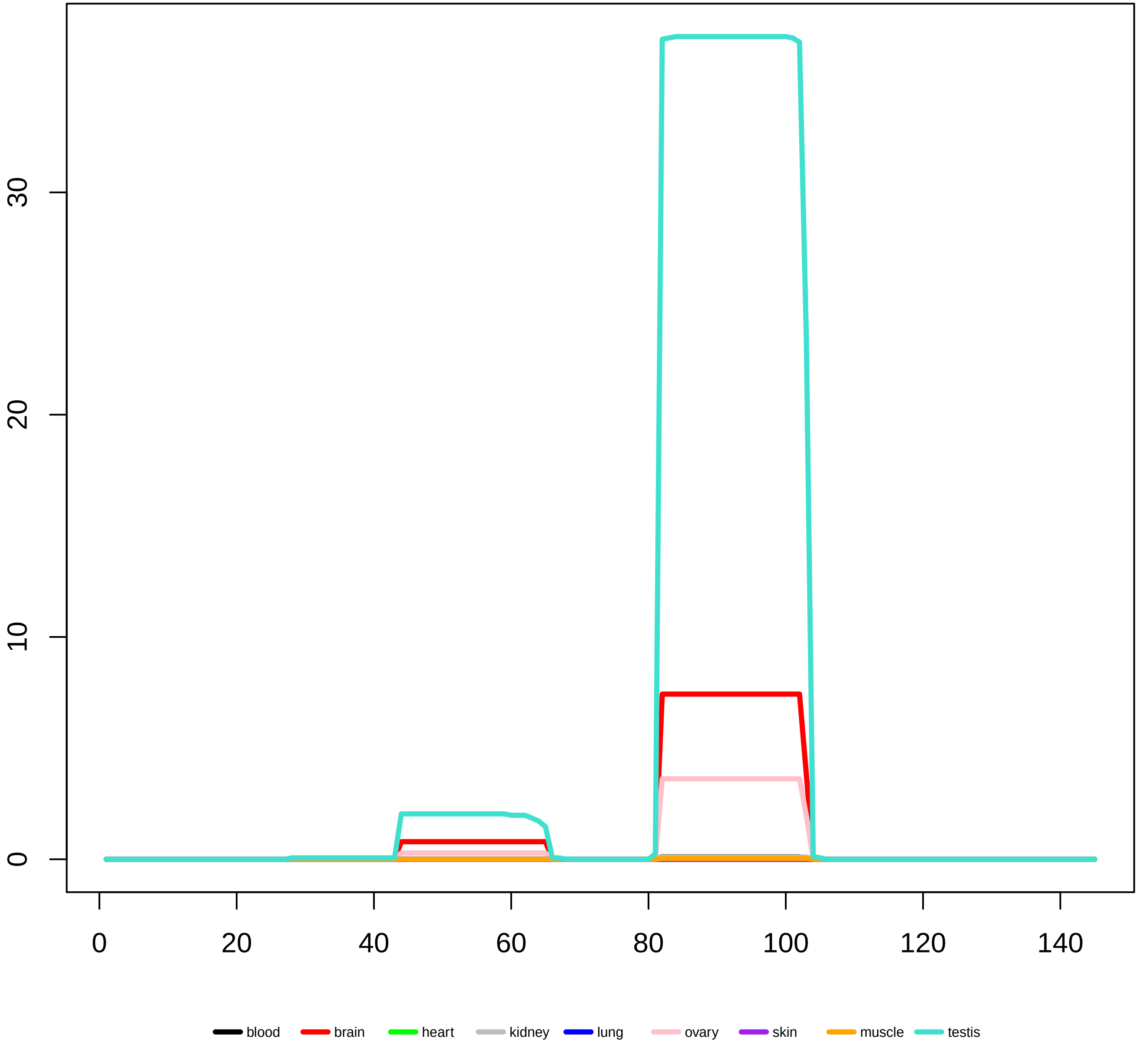

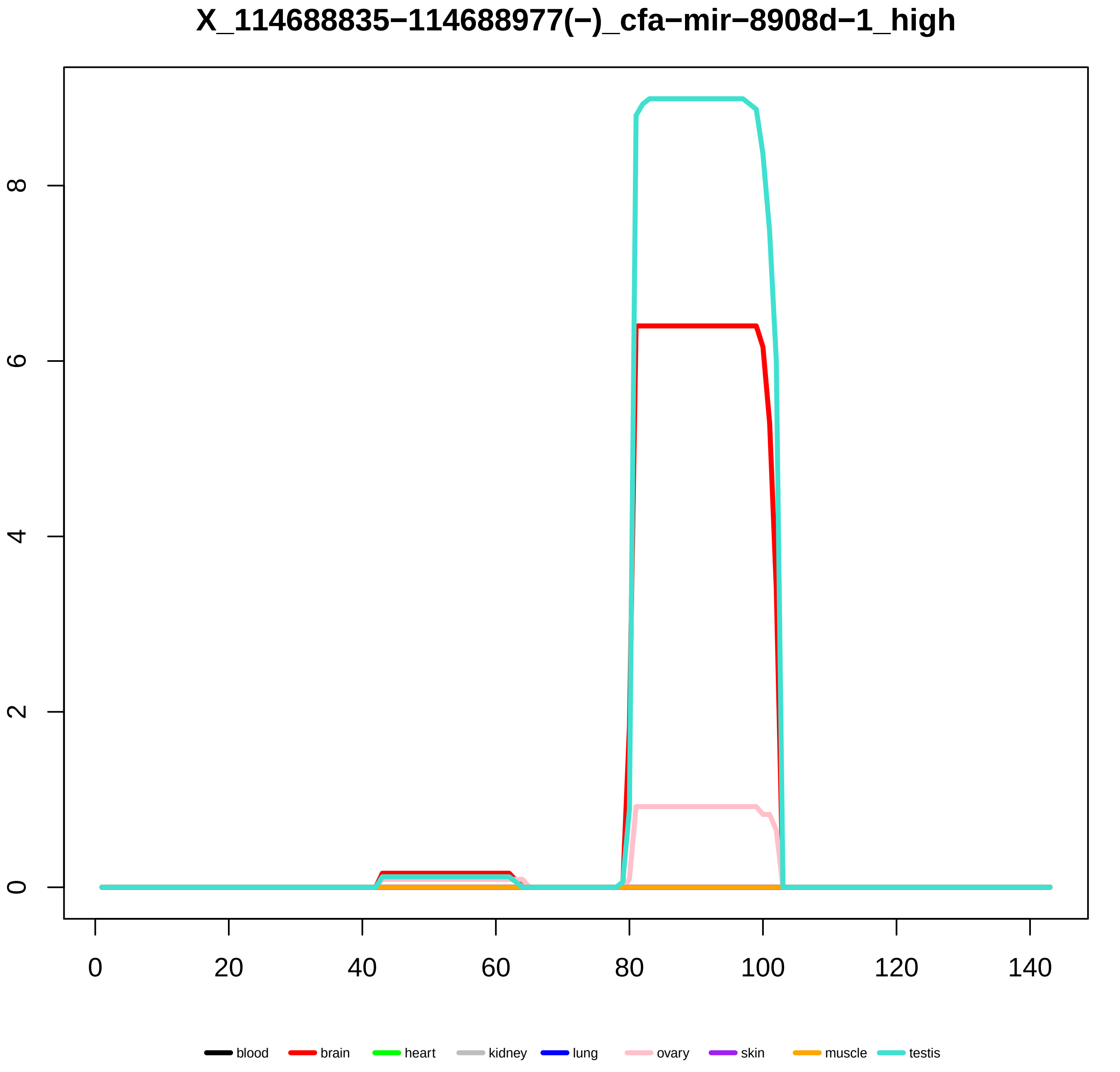

X_114688835−114688977(−)_cfa−mir−8908d−1_high
0
2
4
6
8
0
20
40
60
80
100
120
140
blood
brain
heart
kidney
lung
ovary
skin
muscle
testis

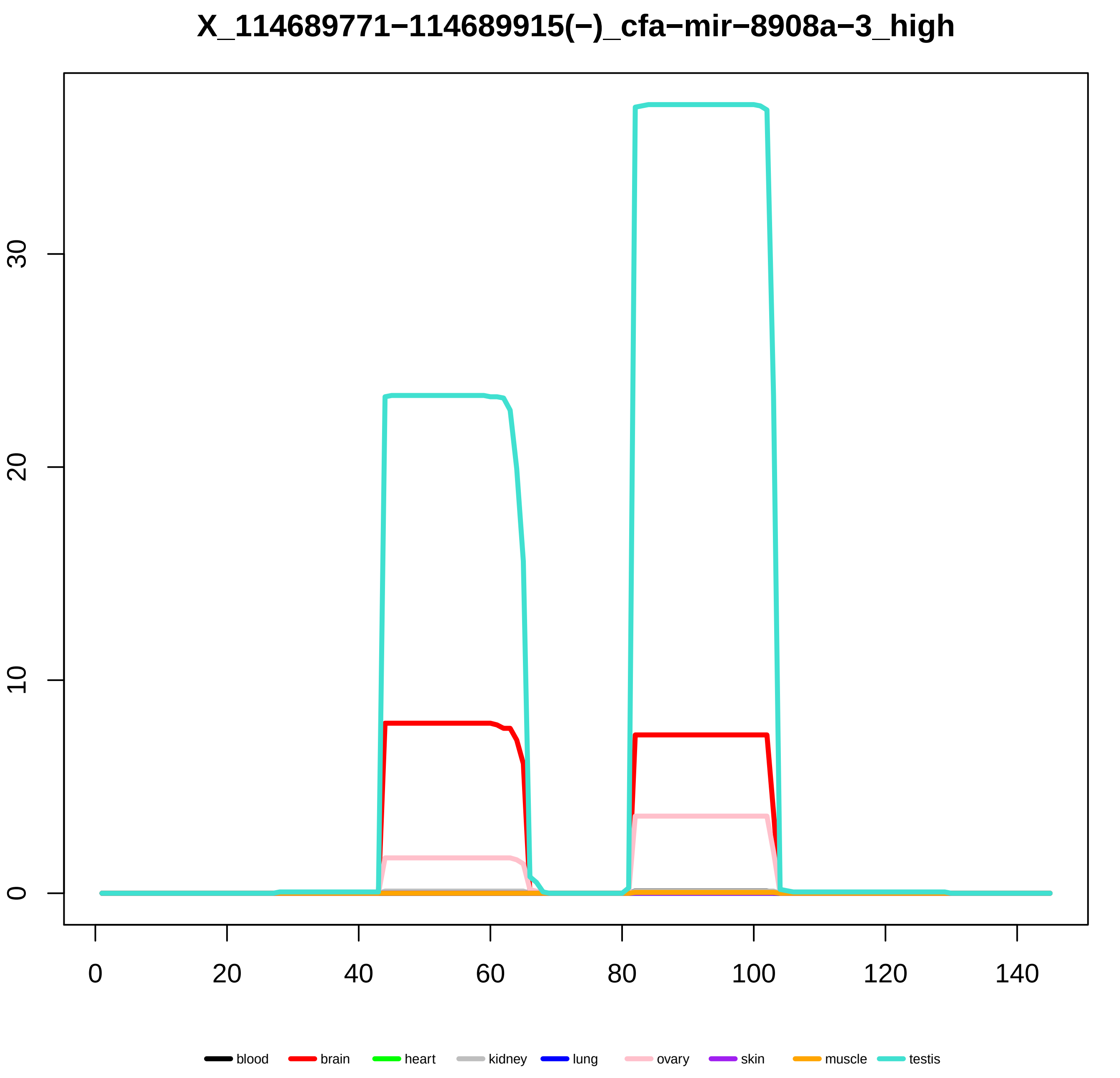

X_114689771−114689915(−)_cfa−mir−8908a−3_high
0
10
20
30
0
20
40
60
80
100
120
140
blood
brain
heart
kidney
lung
ovary
skin
muscle
testis

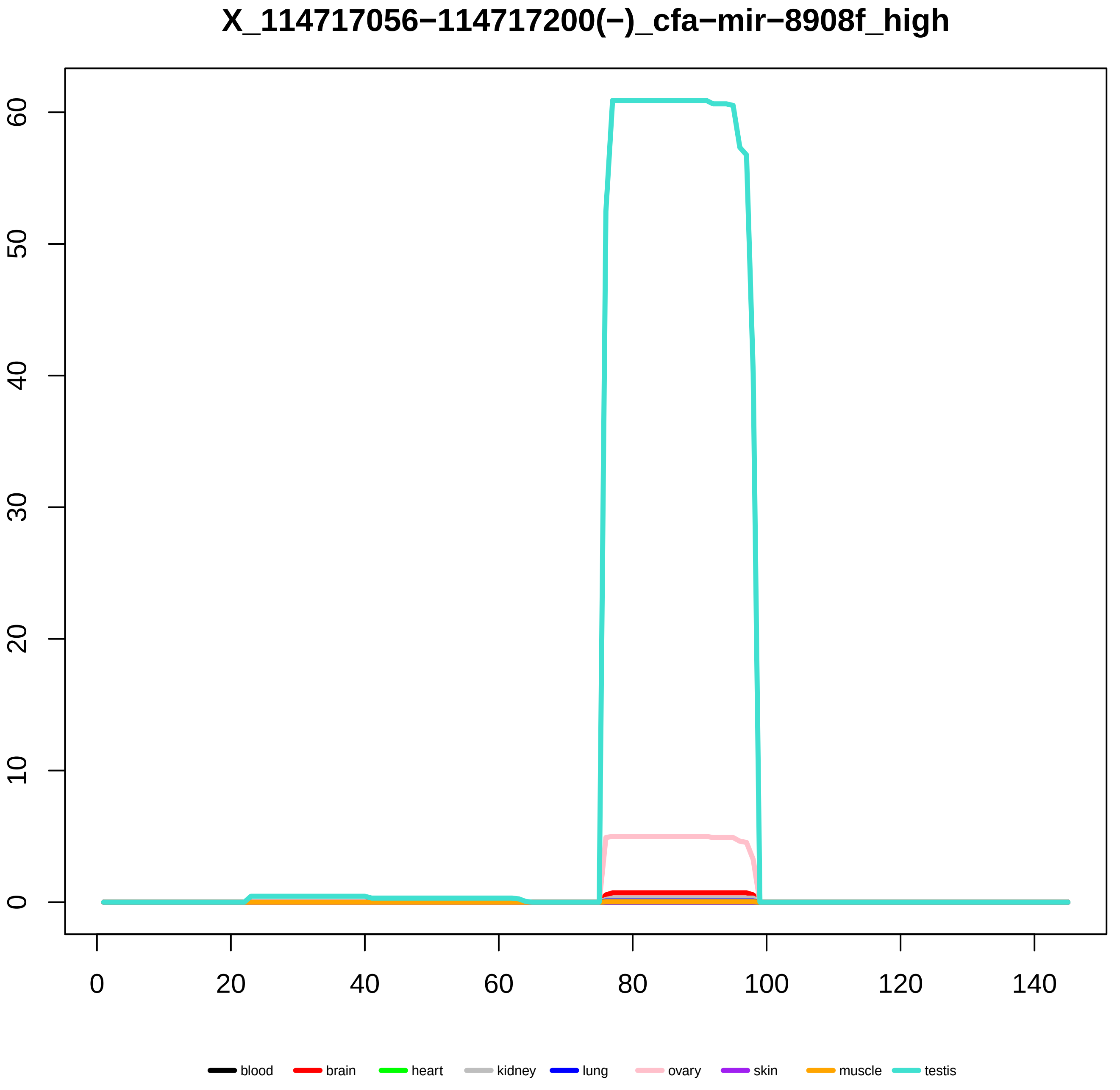
X_114717056−114717200(−)_cfa−mir−8908f_high
0
10
20
30
40
50
60
0
20
40
60
80
100
120
140
blood
brain
heart
kidney
lung
ovary
skin
muscle
testis

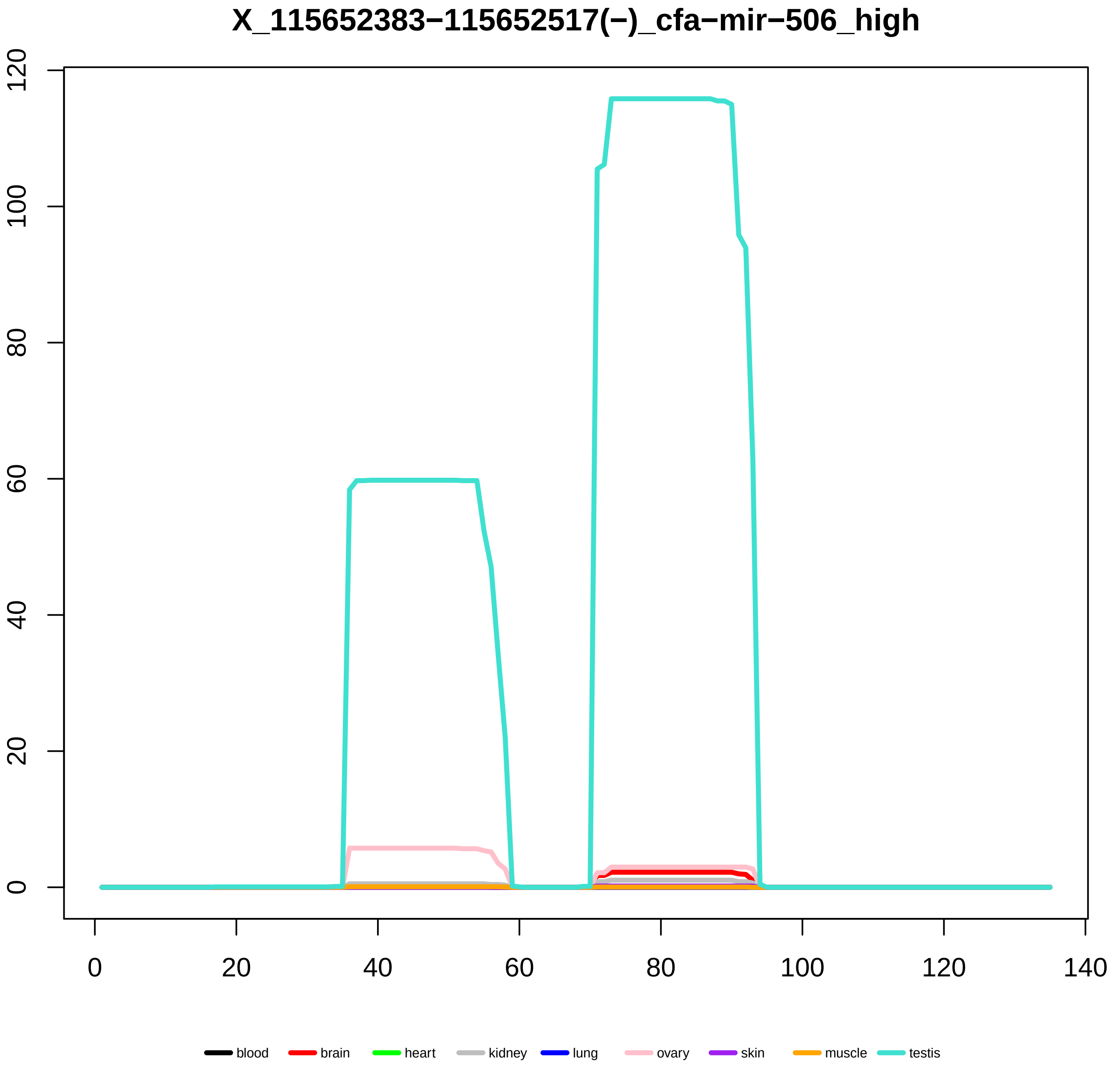
X_115652383−115652517(−)_cfa−mir−506_high
0
20
40
60
80
100
120
0
20
40
60
80
100
120
140
blood
brain
heart
kidney
lung
ovary
skin
muscle
testis

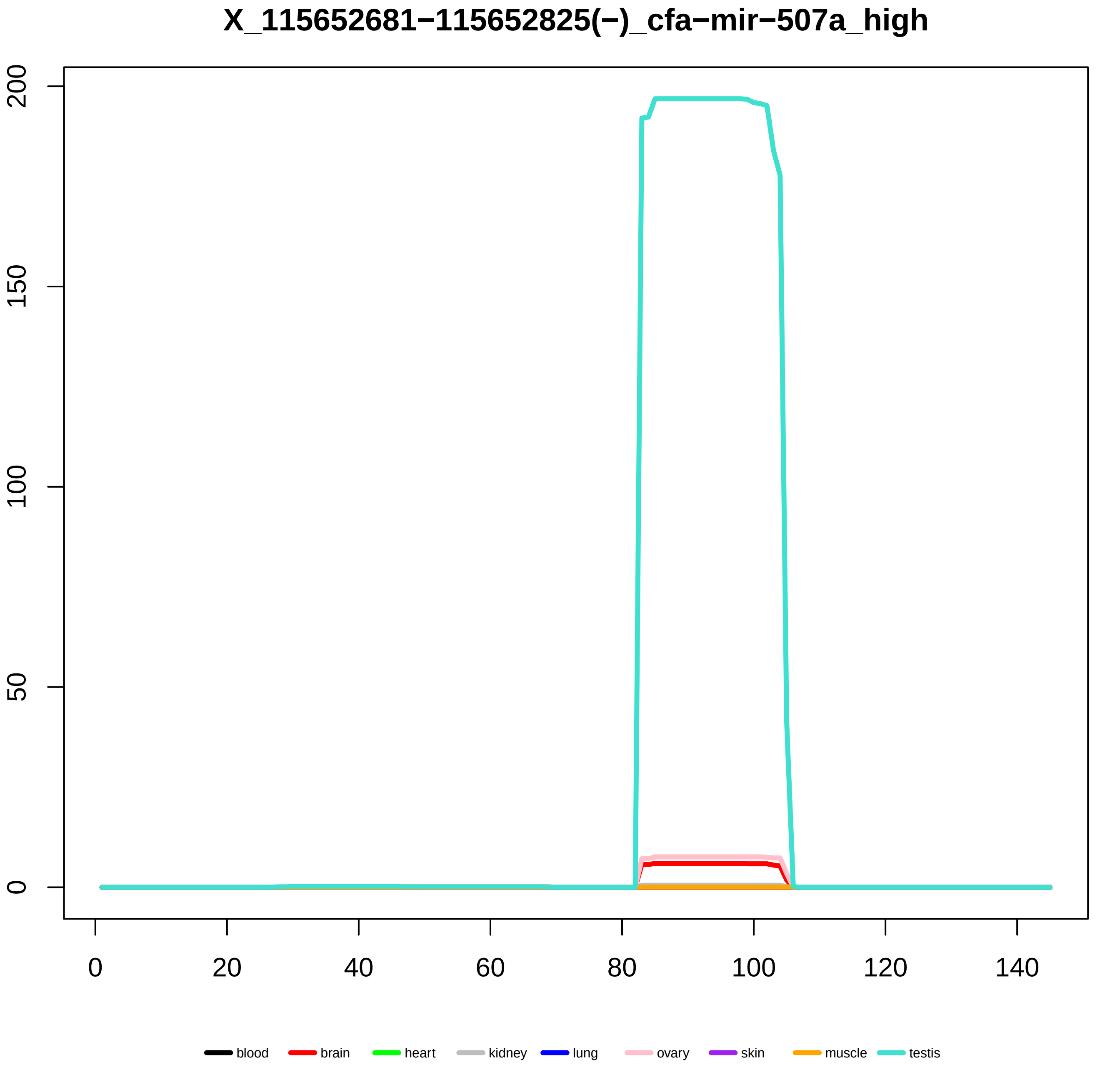

X_115652681−115652825(−)_cfa−mir−507a_high
200
150
100
50
0
0
20
40
60
80
100
120
140
blood
brain
heart
kidney
lung
ovary
skin
muscle
testis

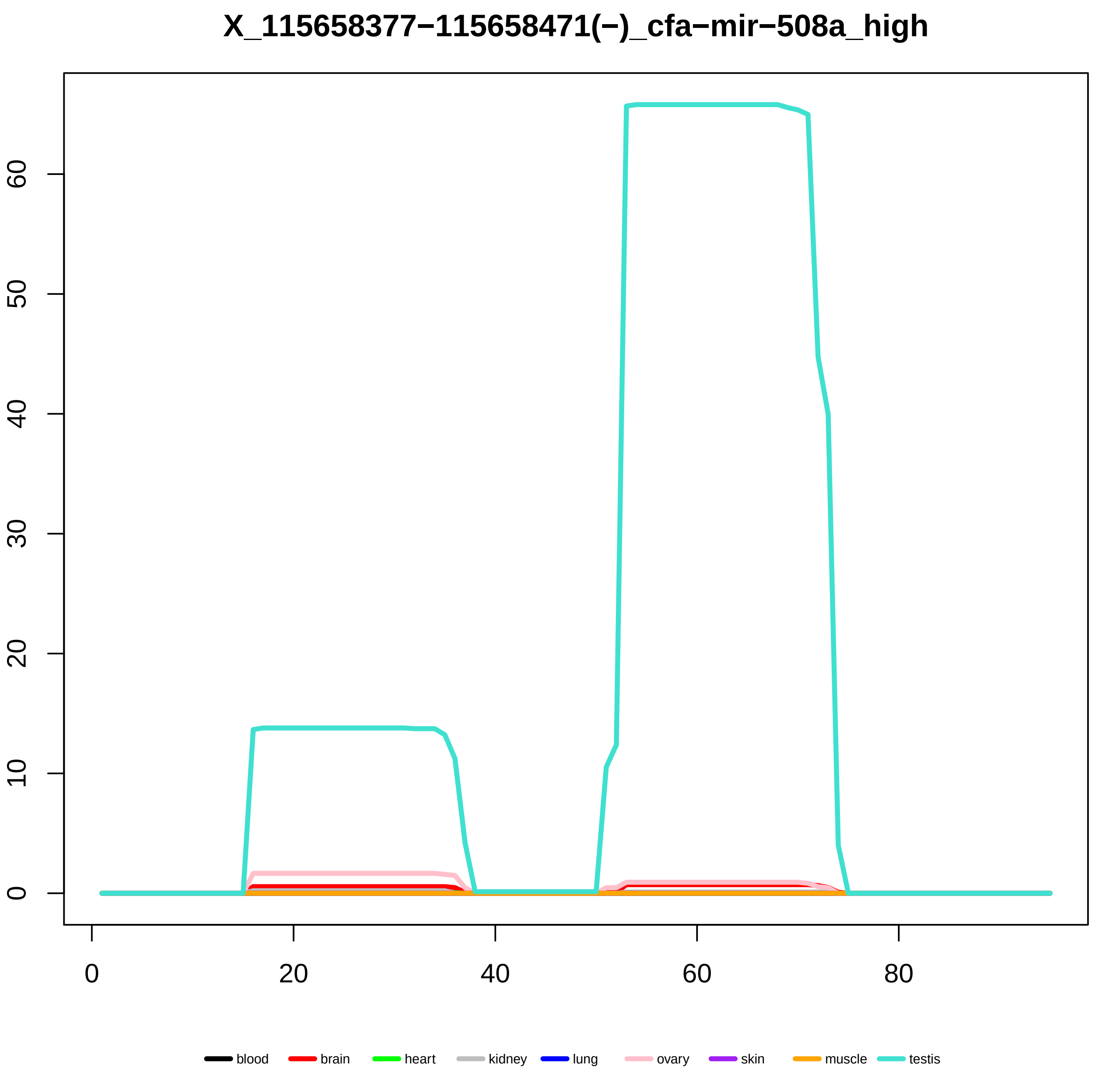

X_115658377−115658471(−)_cfa−mir−508a_high
0
10
20
30
40
50
60
0
20
40
60
80
blood
brain
heart
kidney
lung
ovary
skin
muscle
testis

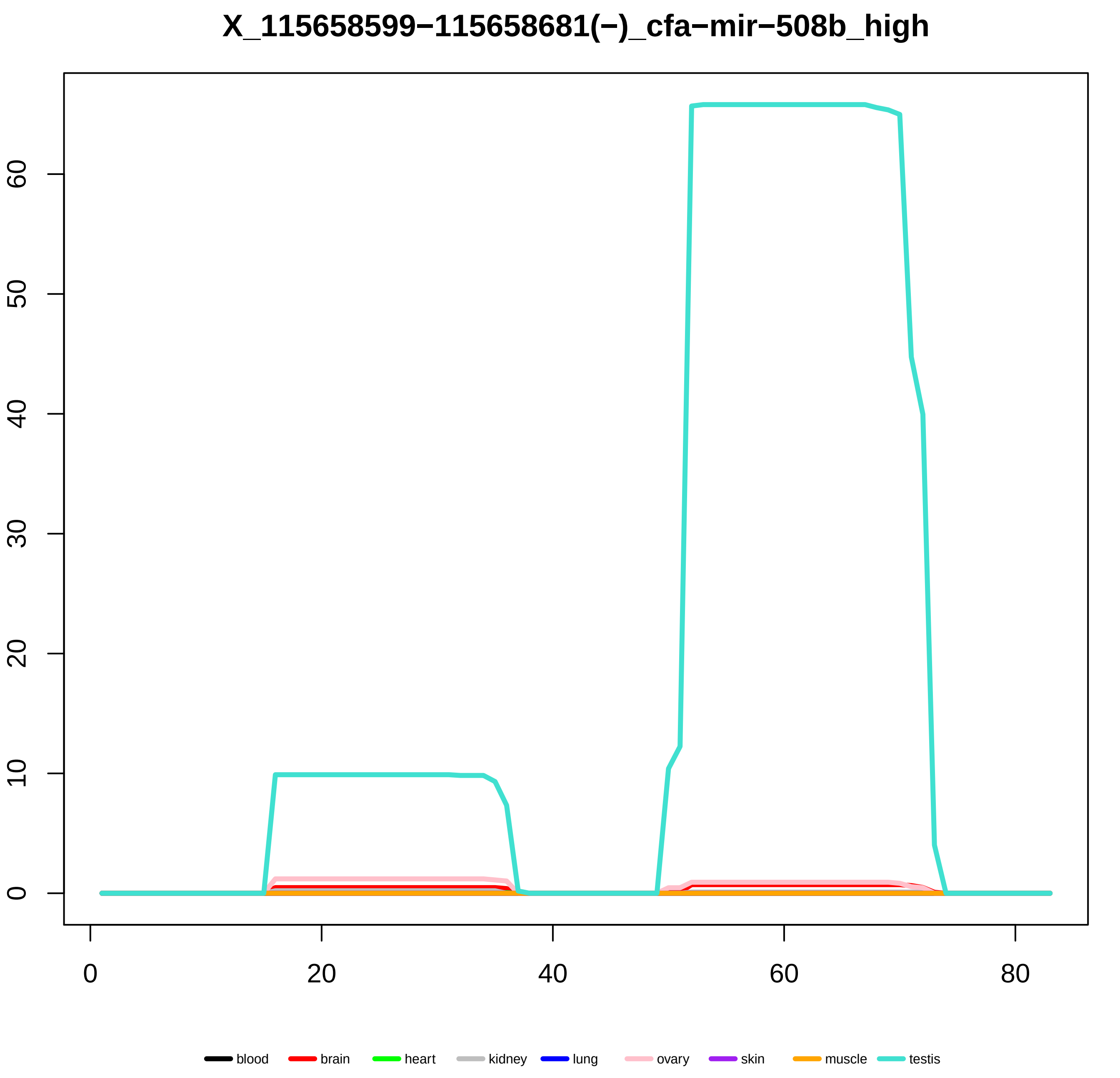

X_115658599−115658681(−)_cfa−mir−508b_high
0
10
20
30
40
50
60
0
20
40
60
80
blood
brain
heart
kidney
lung
ovary
skin
muscle
testis

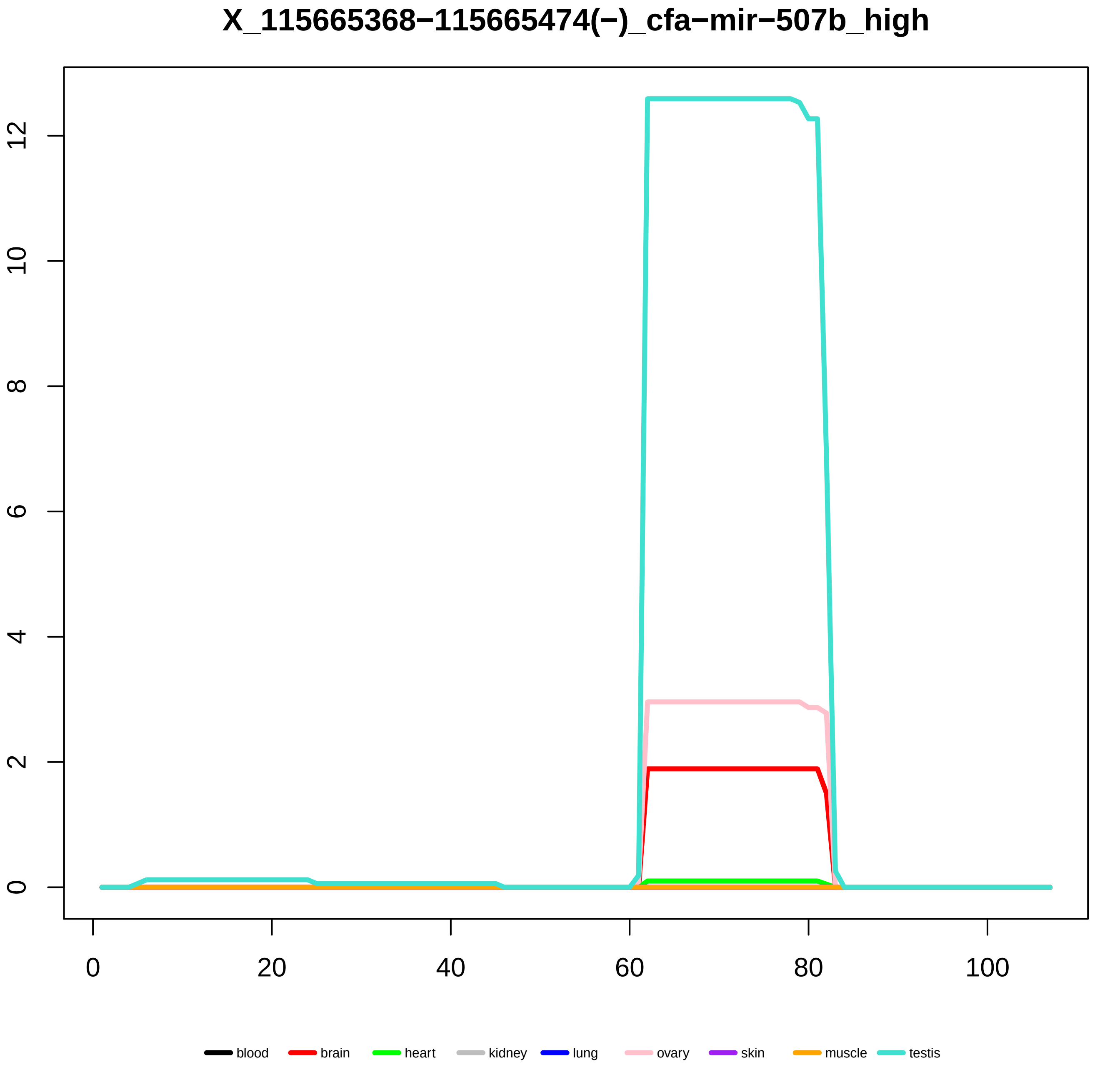

X_115665368−115665474(−)_cfa−mir−507b_high
0
2
4
6
8
10
12
0
20
40
60
80
100
blood
brain
heart
kidney
lung
ovary
skin
muscle
testis

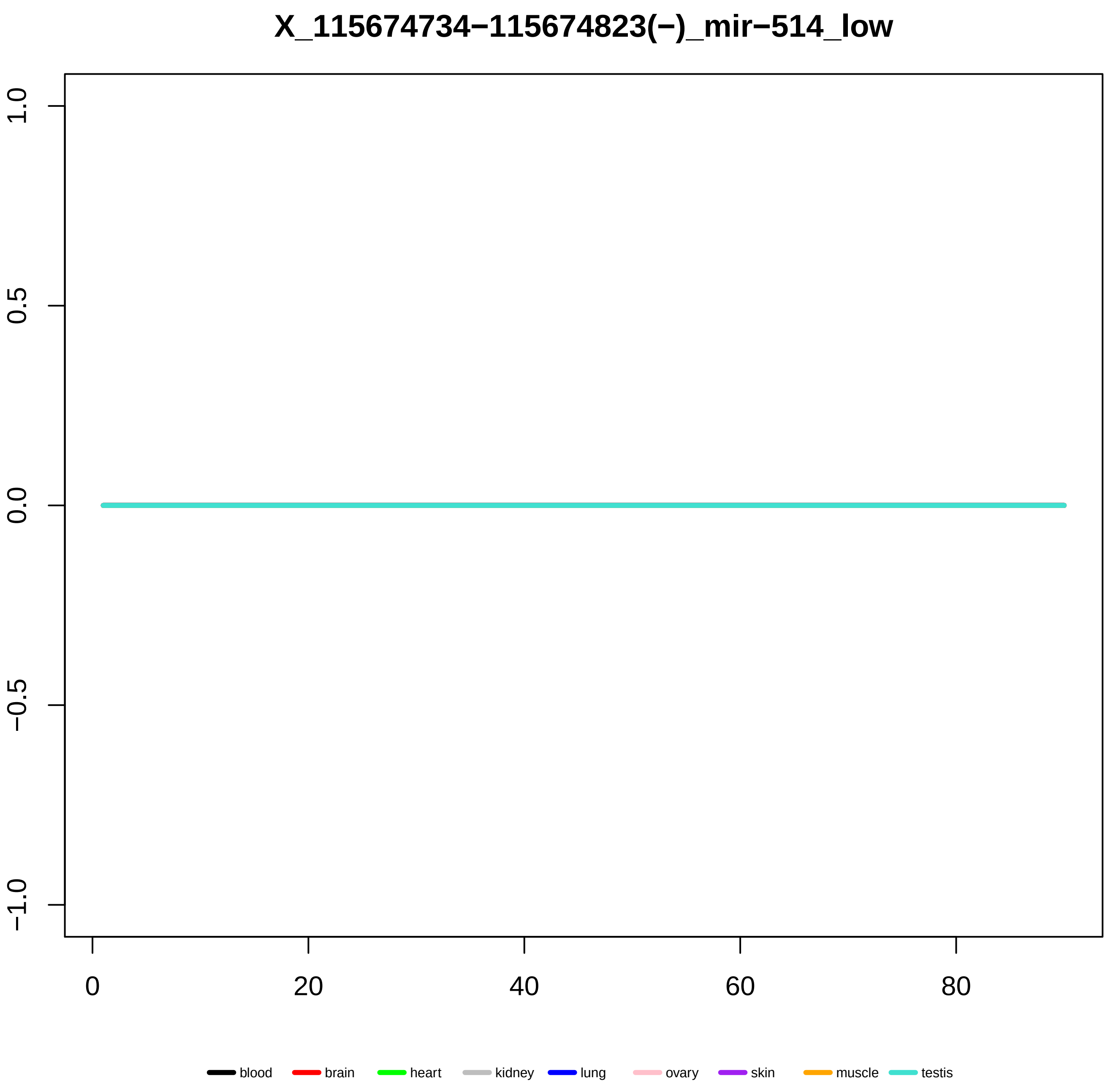
X_115674734−115674823(−)_mir−514_low
1.0
0.5
0.0
−0.5
−1.0
0
20
40
60
80
blood
brain
heart
kidney
lung
ovary
skin
muscle
testis

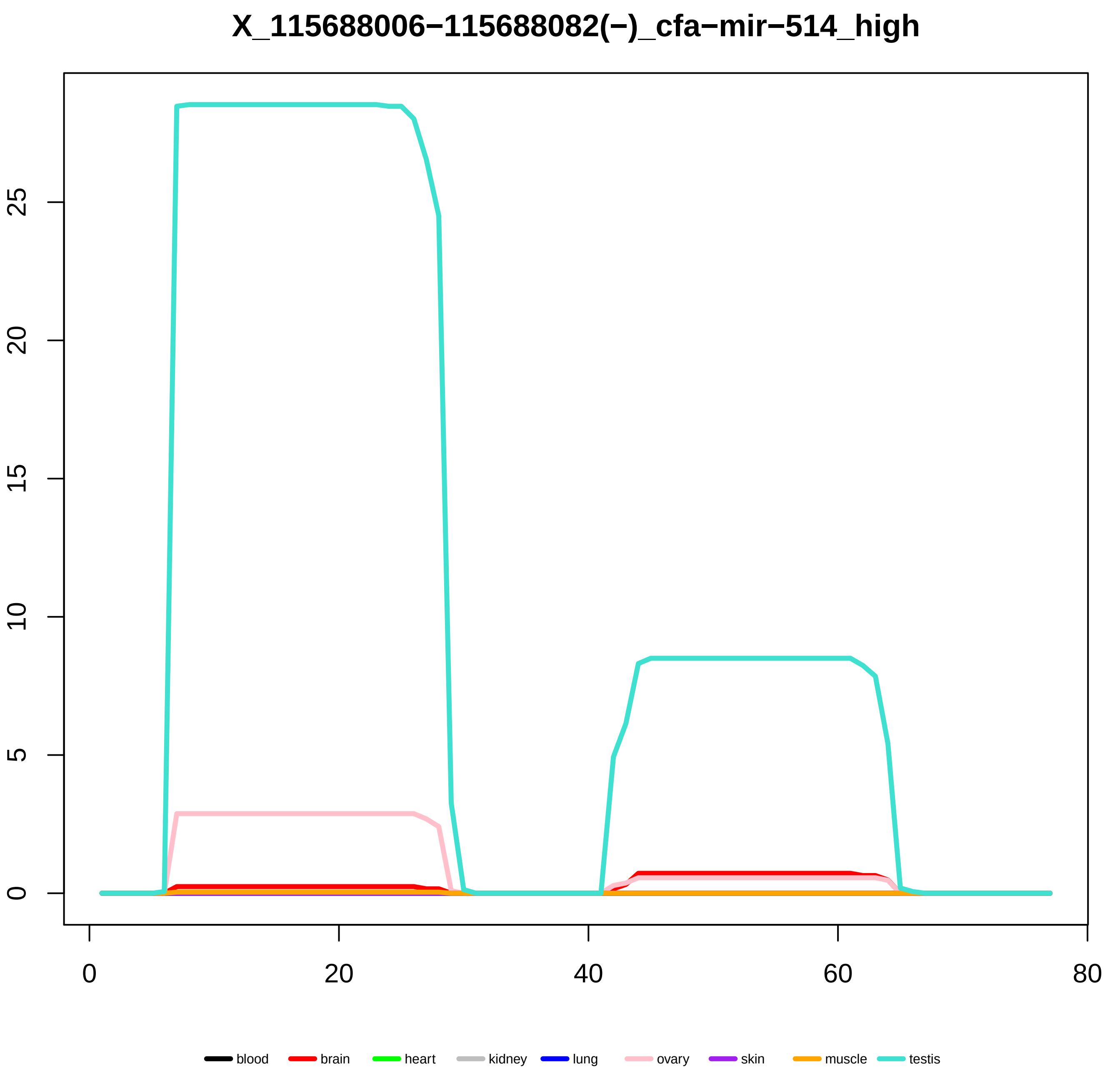
X_115688006−115688082(−)_cfa−mir−514_high
0
5
10
15
20
25
0
20
40
60
80
blood
brain
heart
kidney
lung
ovary
skin
muscle
testis

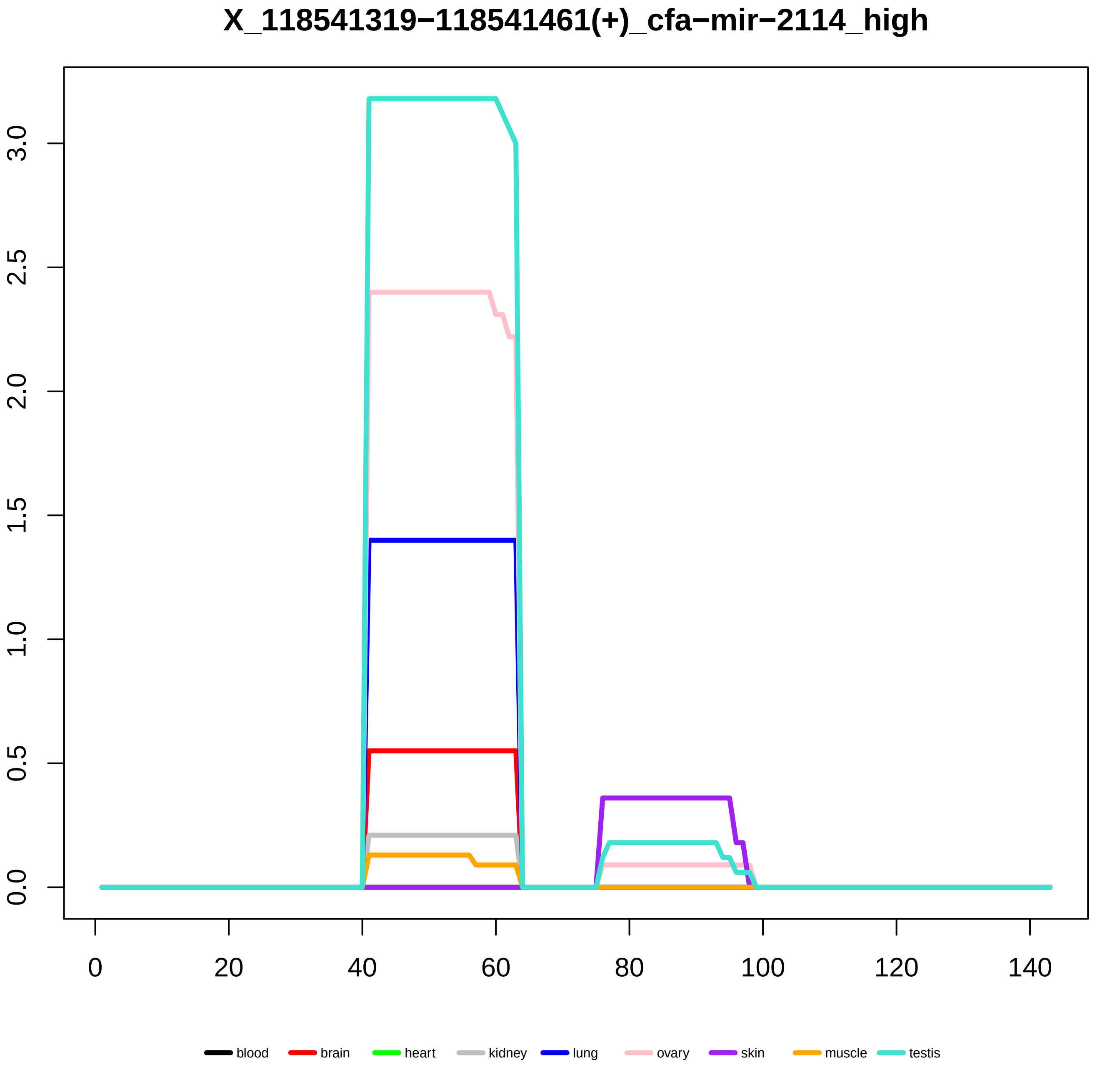

X_118541319−118541461(+)_cfa−mir−2114_high
0.0
0.5
1.0
1.5
2.0
2.5
3.0
0
20
40
60
80
100
120
140
blood
brain
heart
kidney
lung
ovary
skin
muscle
testis

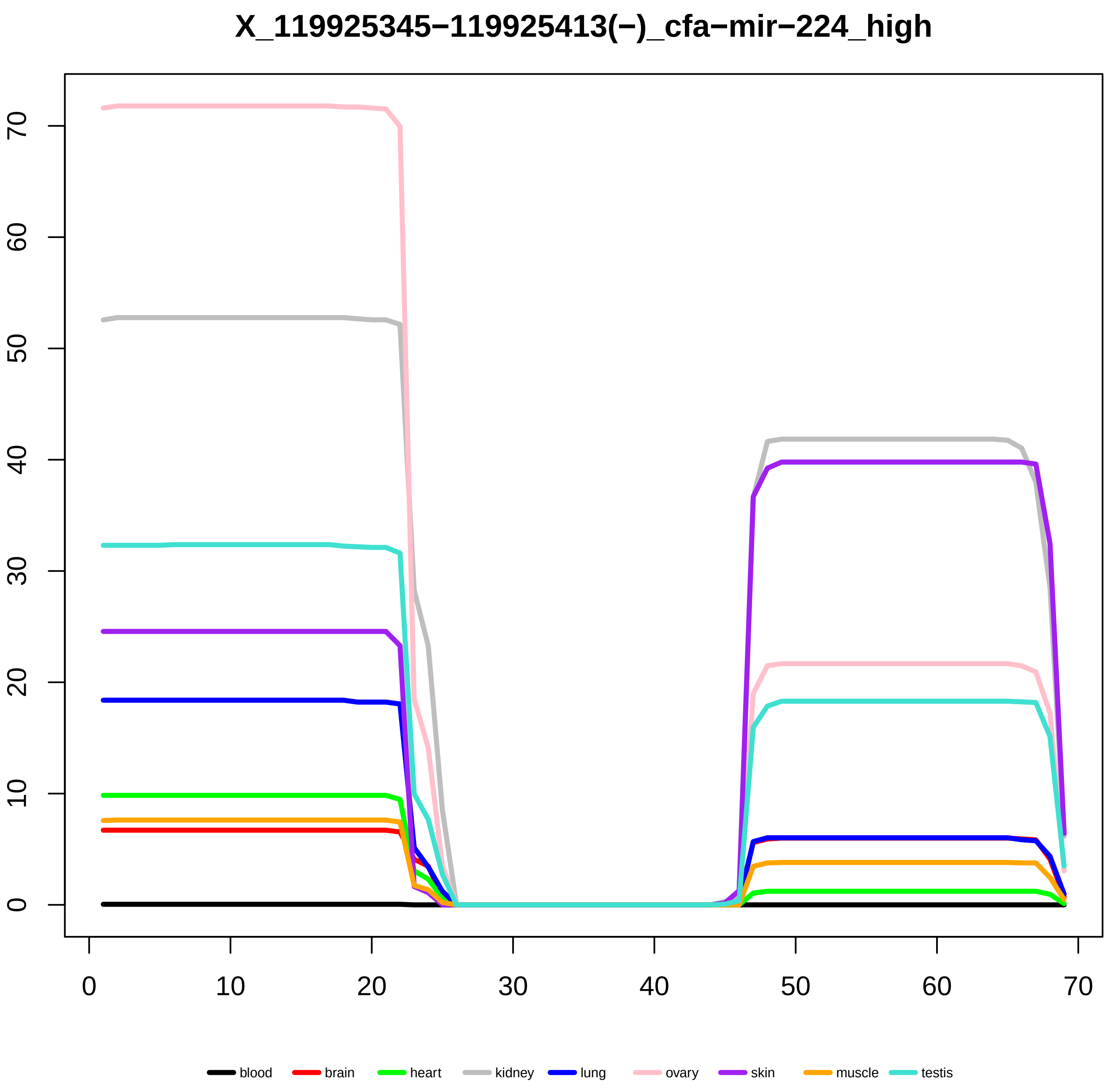
X_119925345−119925413(−)_cfa−mir−224_high
0
10
20
30
40
50
60
70
0
10
20
30
40
50
60
70
blood
brain
heart
kidney
lung
ovary
skin
muscle
testis

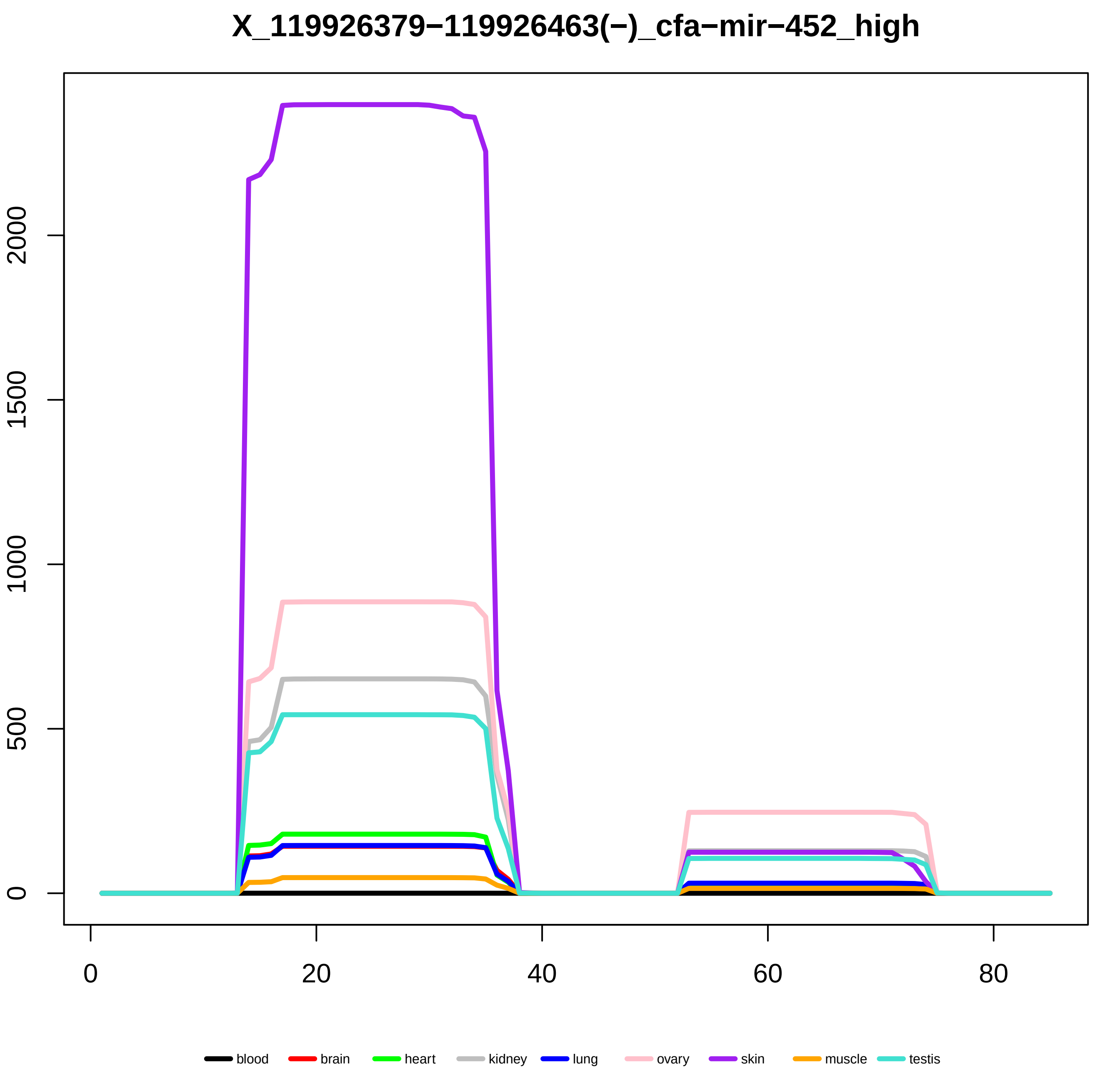

X_119926379−119926463(−)_cfa−mir−452_high
0
500
1000
1500
2000
0
20
40
60
80
blood
brain
heart
kidney
lung
ovary
skin
muscle
testis

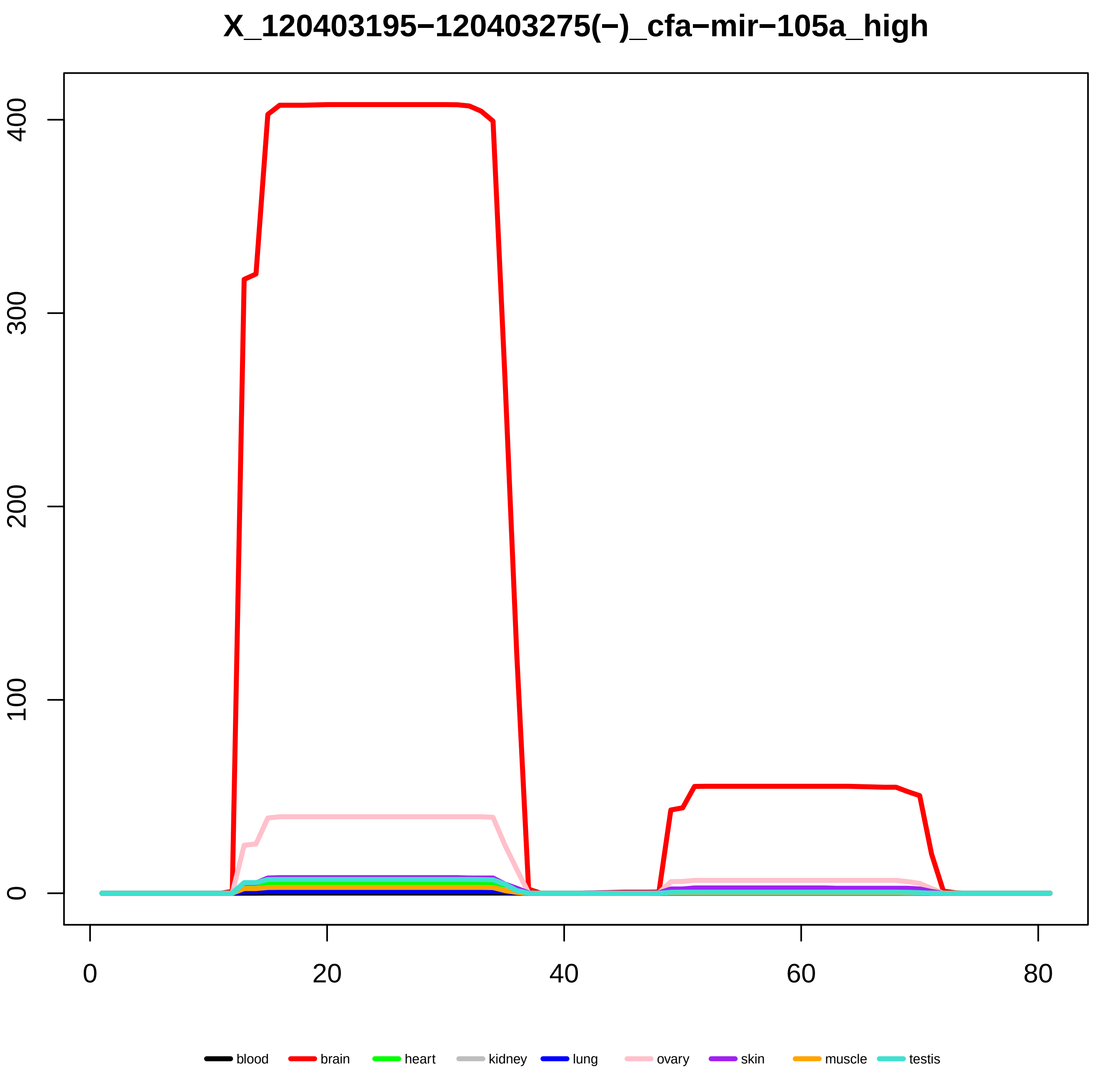
X_120403195−120403275(−)_cfa−mir−105a_high
0
100
200
300
400
0
20
40
60
80
blood
brain
heart
kidney
lung
ovary
skin
muscle
testis

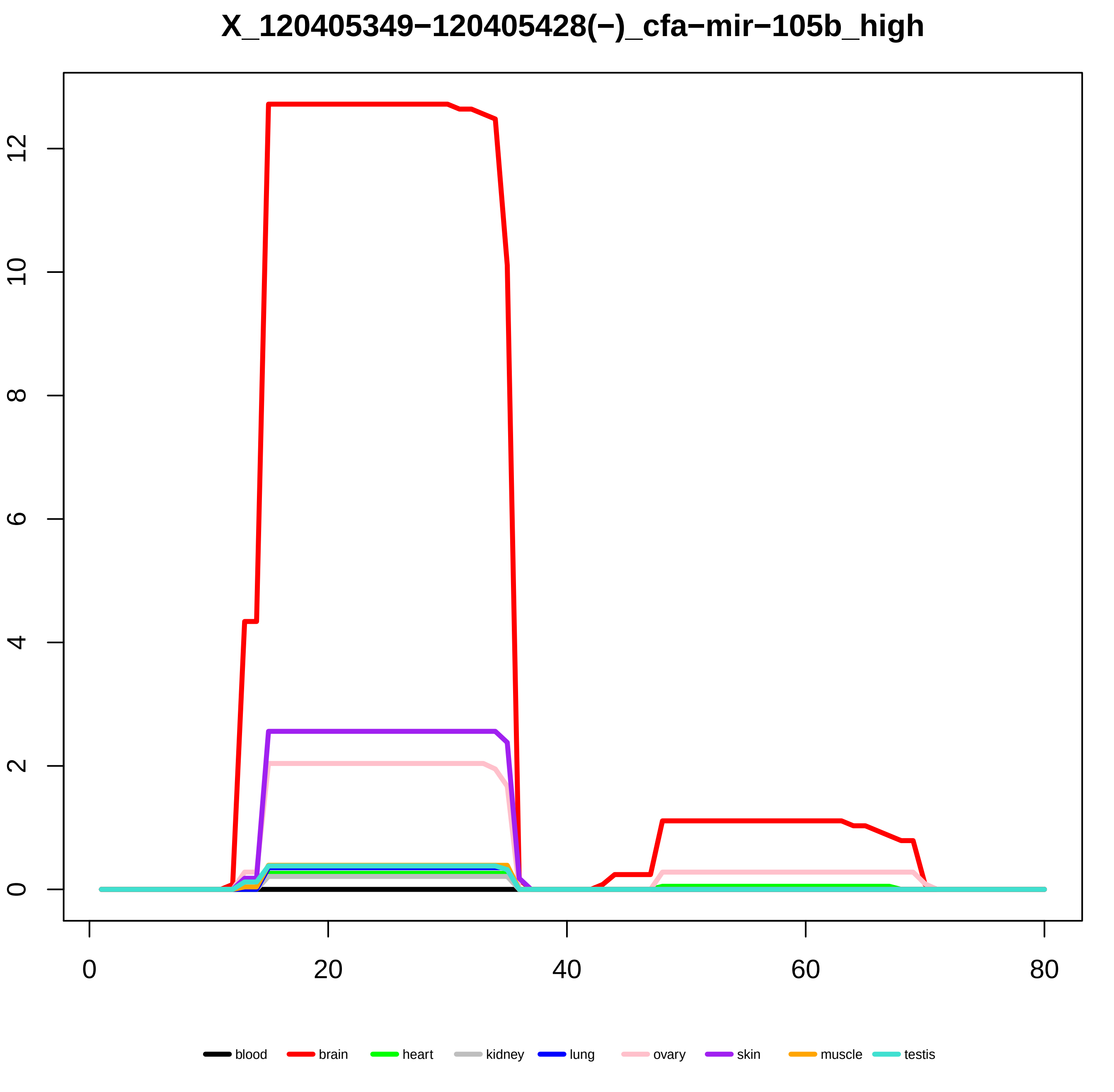
X_120405349−120405428(−)_cfa−mir−105b_high
0
2
4
6
8
10
12
0
20
40
60
80
blood
brain
heart
kidney
lung
ovary
skin
muscle
testis

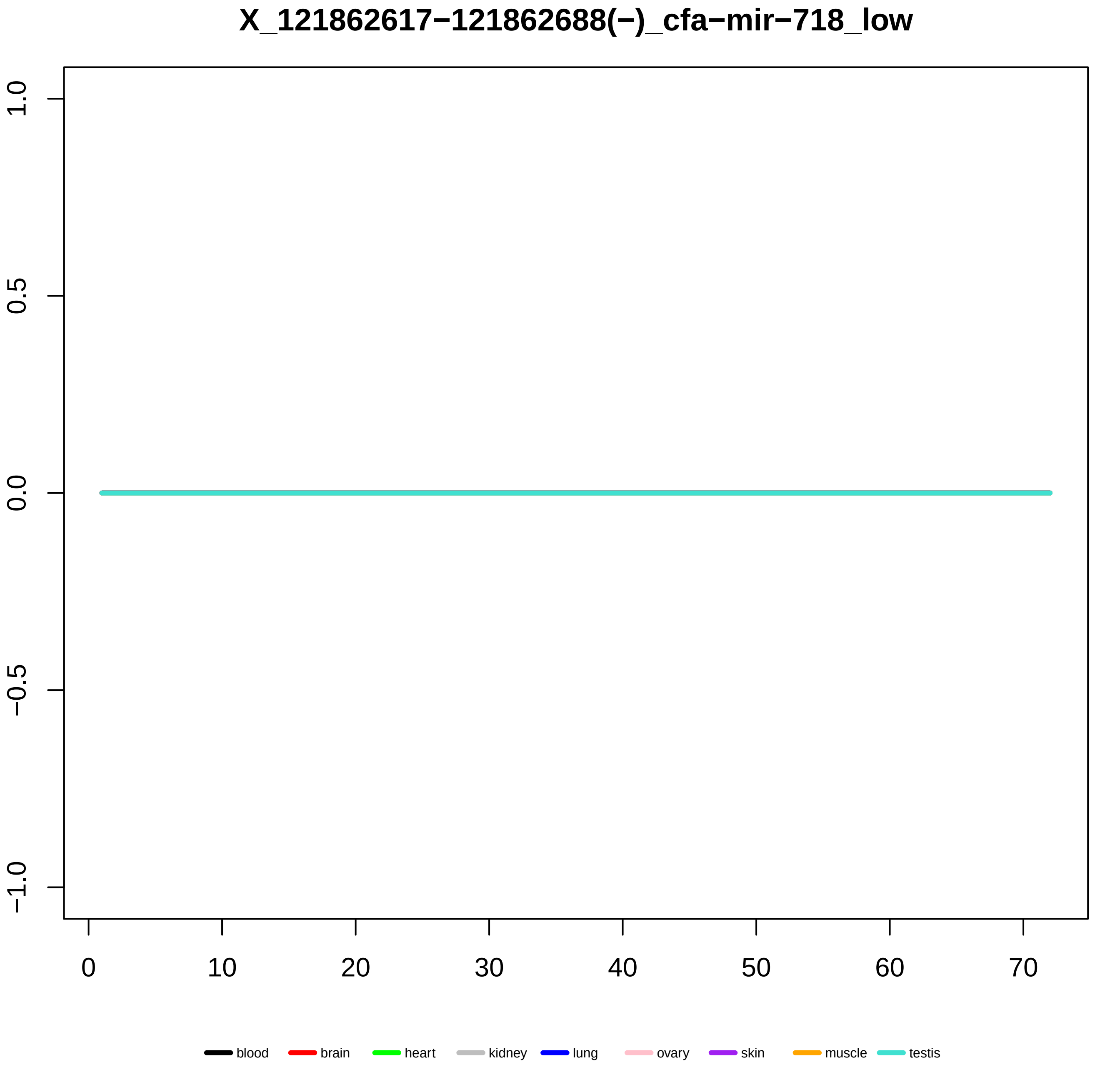
X_121862617−121862688(−)_cfa−mir−718_low
1.0
0.5
0.0
−0.5
−1.0
0
10
20
30
40
50
60
70
blood
brain
heart
kidney
lung
ovary
skin
muscle
testis

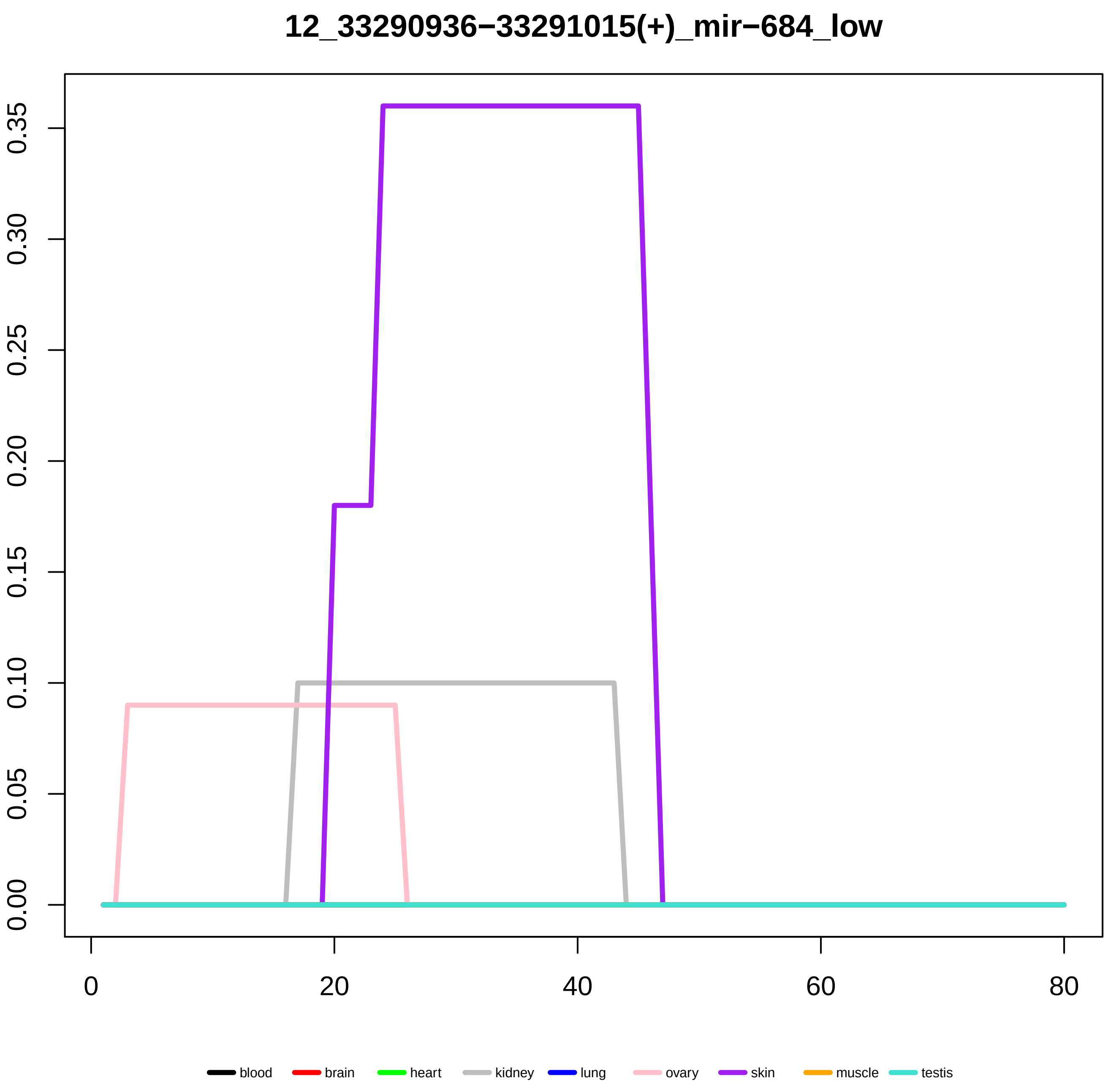
12_33290936−33291015(+)_mir−684_low
0.00
0.05
0.10
0.15
0.20
0.25
0.30
0.35
0
20
40
60
80
blood
brain
heart
kidney
lung
ovary
skin
muscle
testis

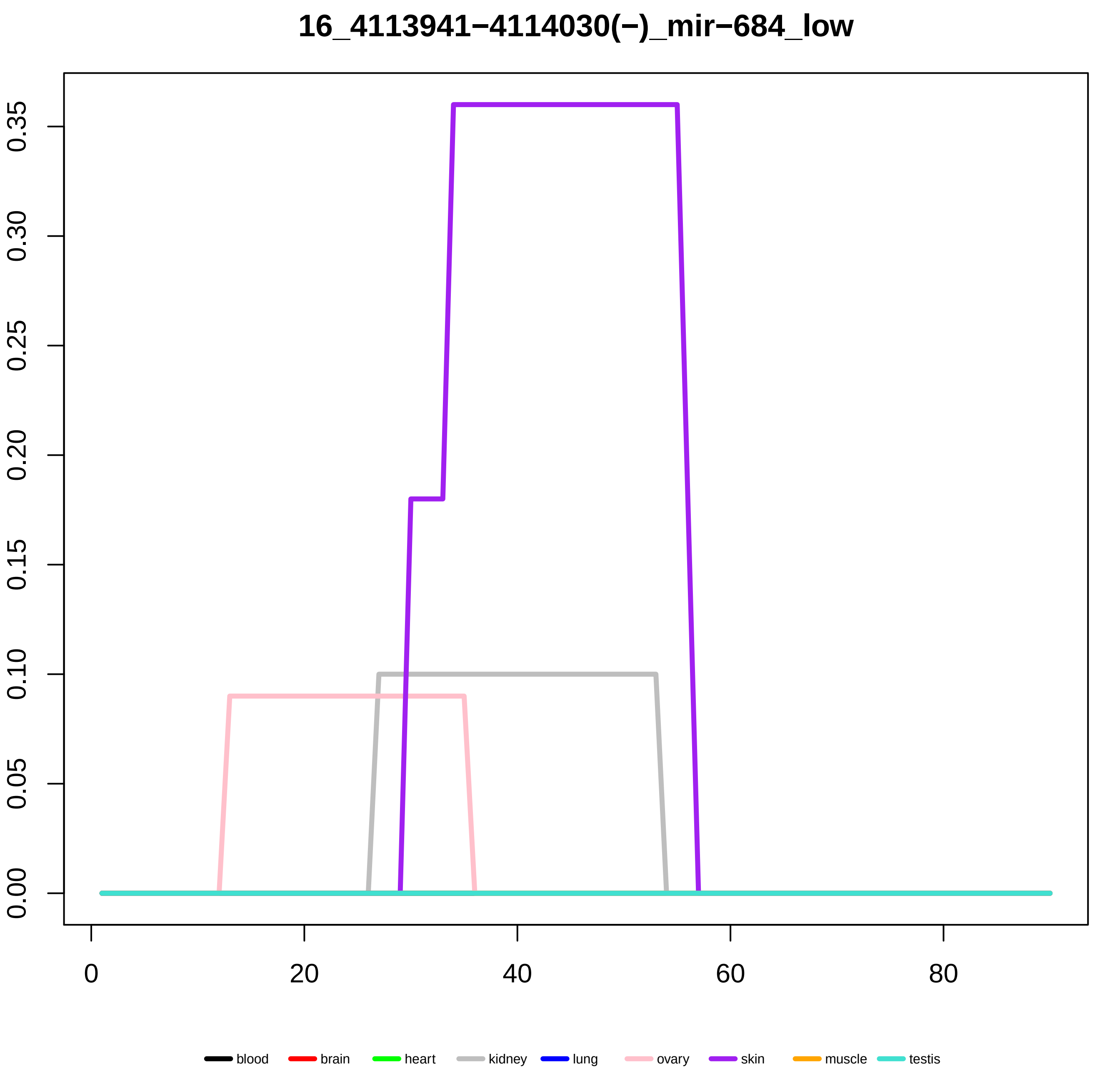

16_4113941−4114030(−)_mir−684_low
0.00
0.05
0.10
0.15
0.20
0.25
0.30
0.35
0
20
40
60
80
blood
brain
heart
kidney
lung
ovary
skin
muscle
testis

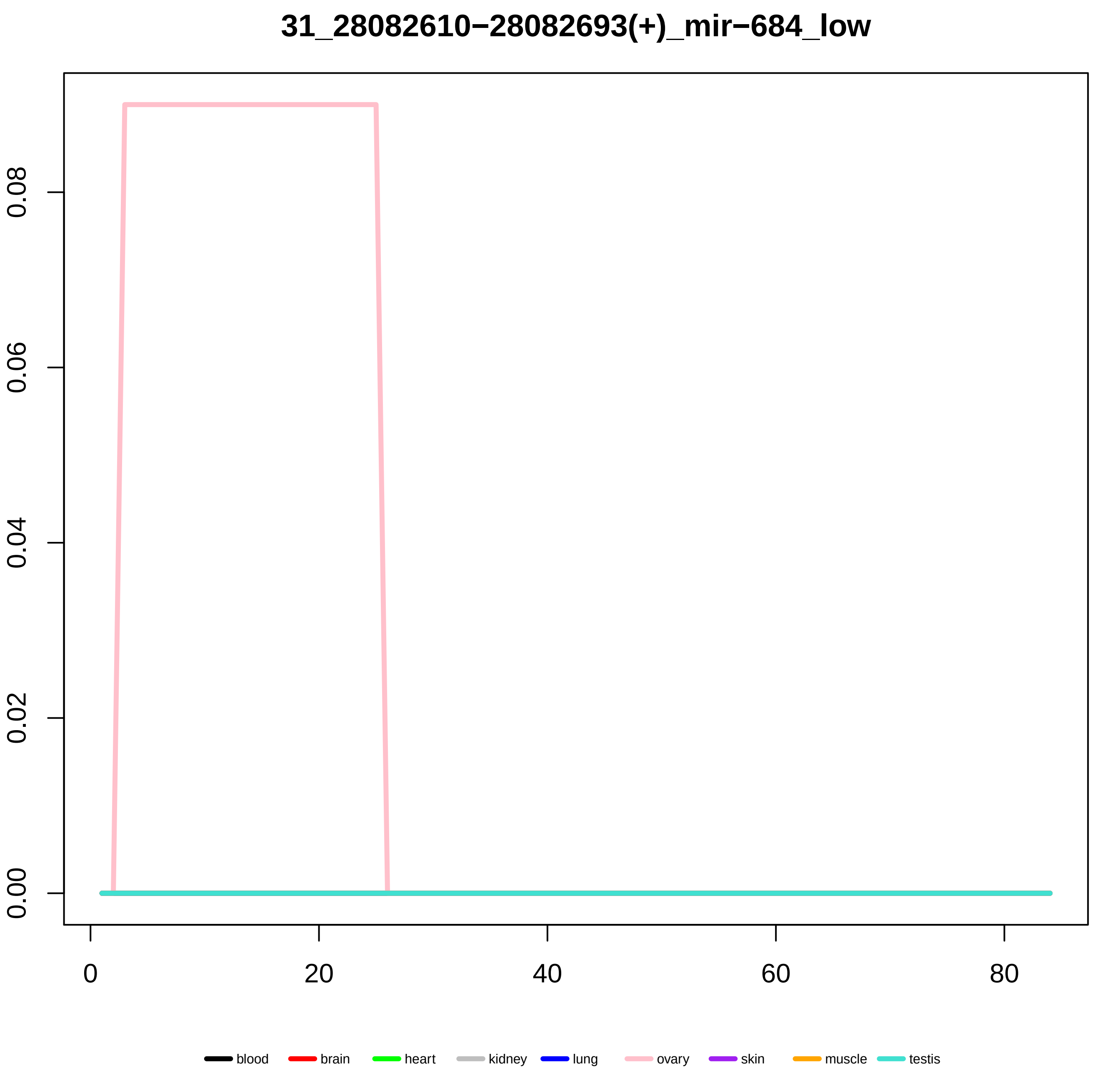
31_28082610−28082693(+)_mir−684_low
0.00
0.02
0.04
0.06
0.08
0
20
40
60
80
blood
brain
heart
kidney
lung
ovary
skin
muscle
testis

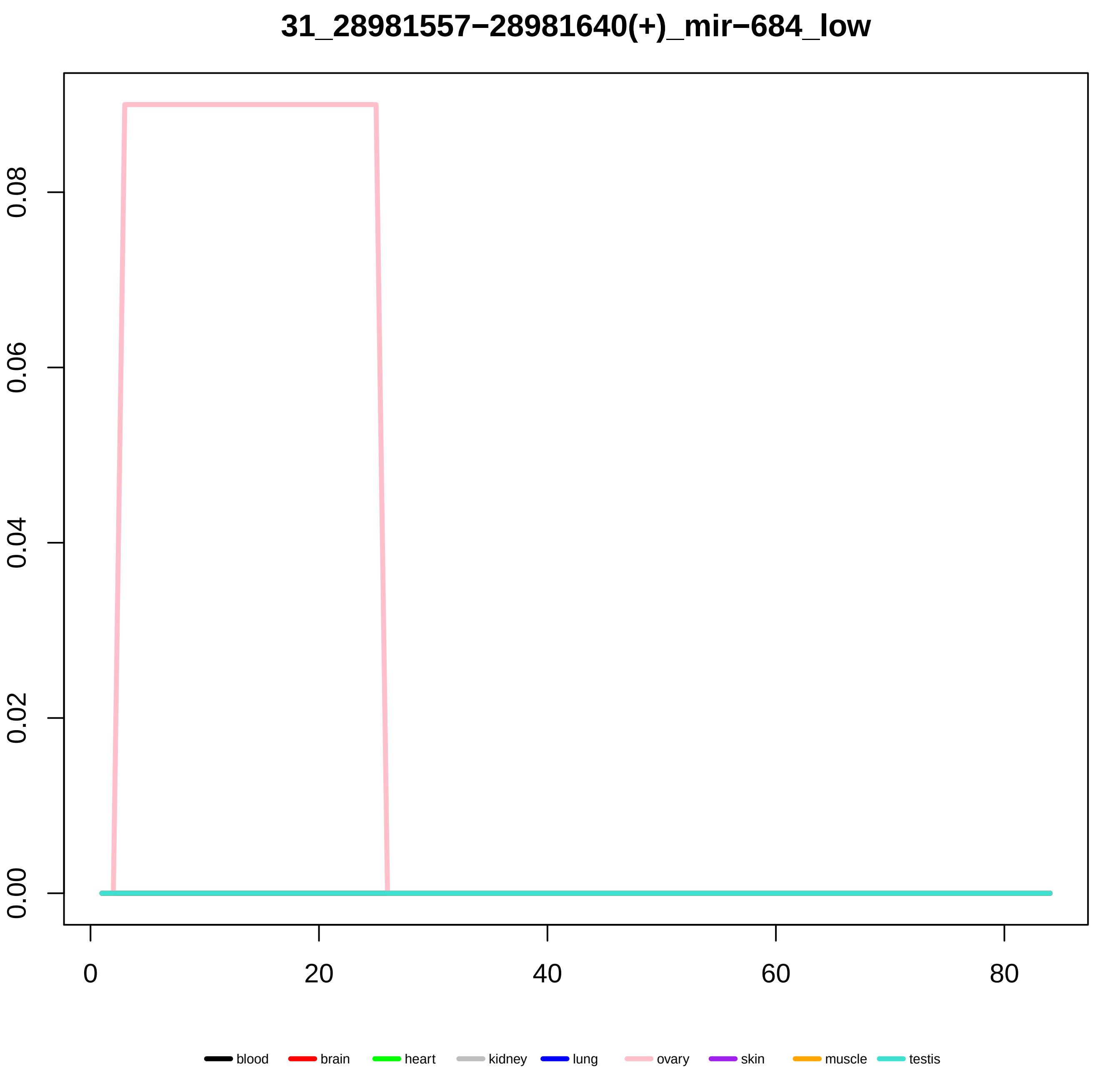
31_28981557−28981640(+)_mir−684_low
0.00
0.02
0.04
0.06
0.08
0
20
40
60
80
blood
brain
heart
kidney
lung
ovary
skin
muscle
testis

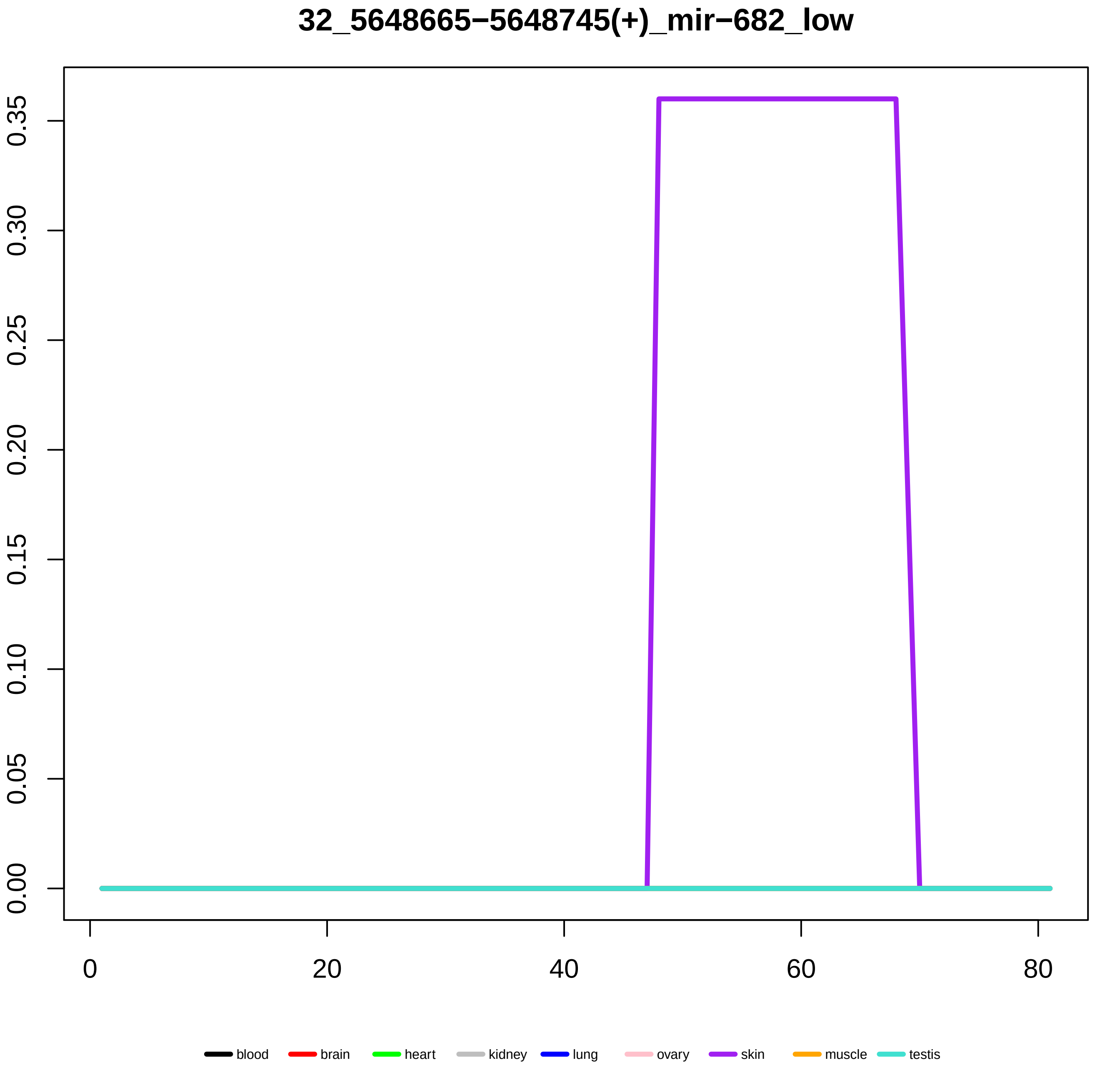
32_5648665−5648745(+)_mir−682_low
0.00
0.05
0.10
0.15
0.20
0.25
0.30
0.35
0
20
40
60
80
blood
brain
heart
kidney
lung
ovary
skin
muscle
testis

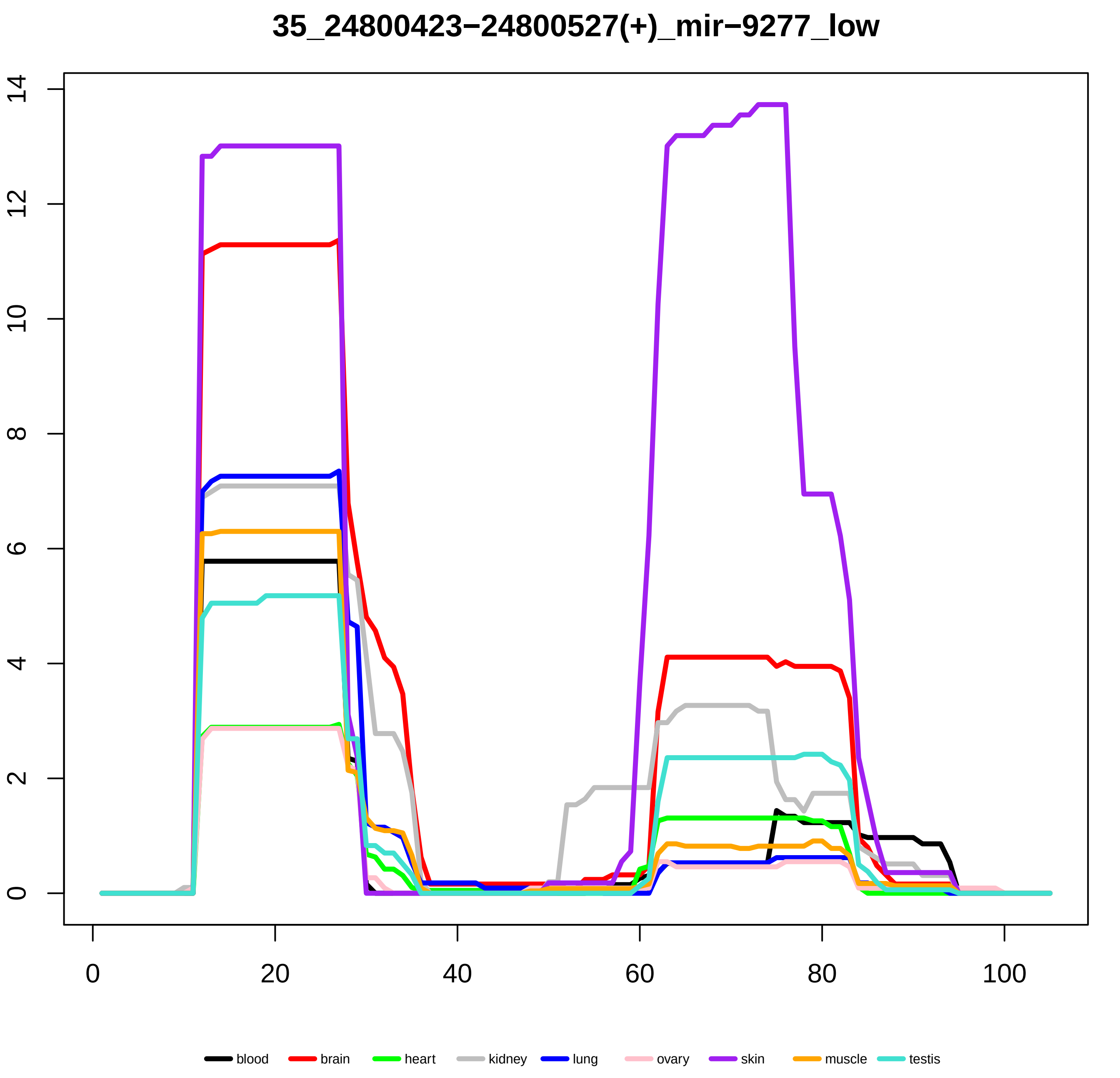

35_24800423−24800527(+)_mir−9277_low
0
2
4
6
8
10
12
14
0
20
40
60
80
100
blood
brain
heart
kidney
lung
ovary
skin
muscle
testis

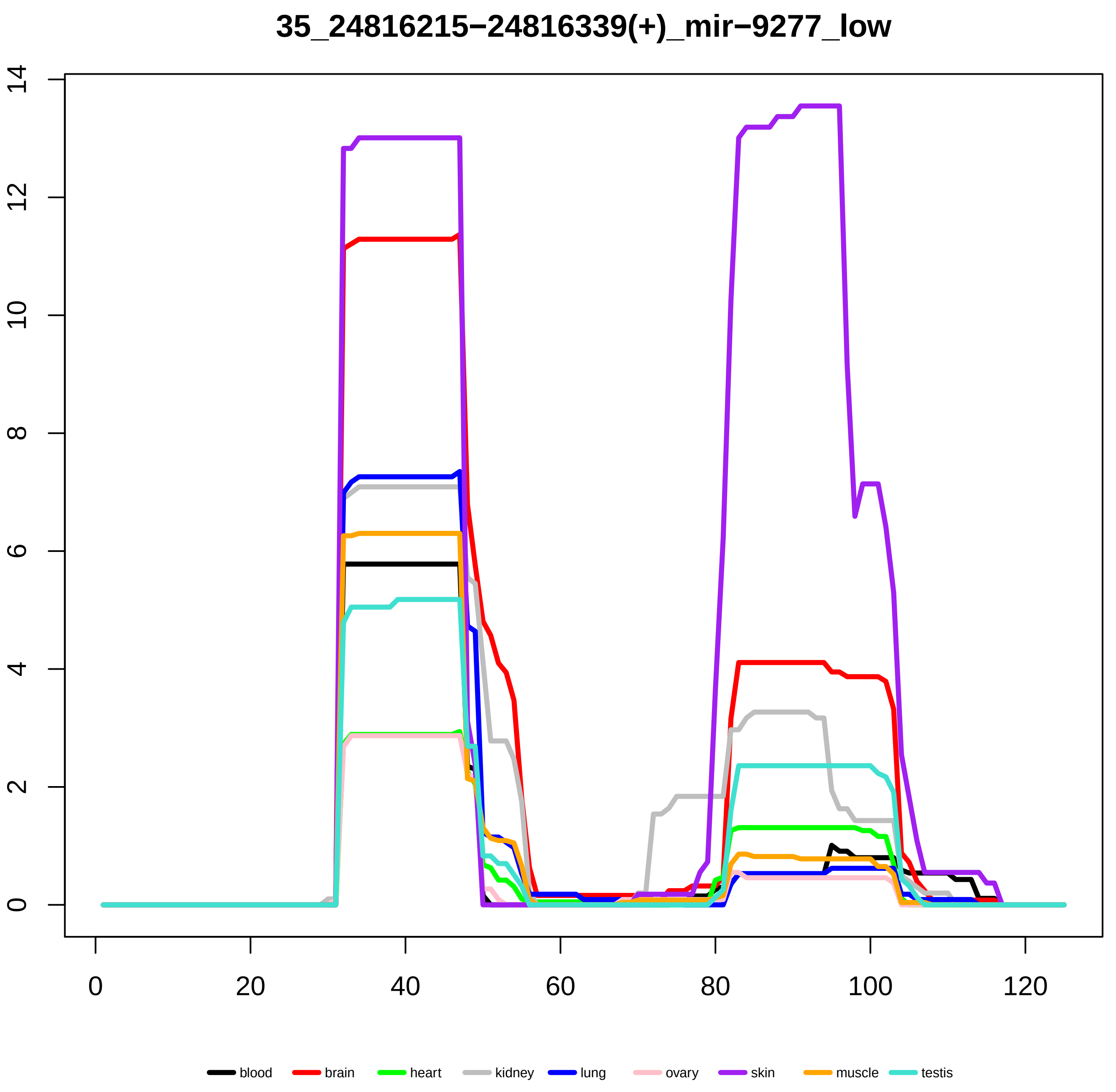

35_24816215−24816339(+)_mir−9277_low
0
2
4
6
8
10
12
14
0
20
40
60
80
100
120
blood
brain
heart
kidney
lung
ovary
skin
muscle
testis

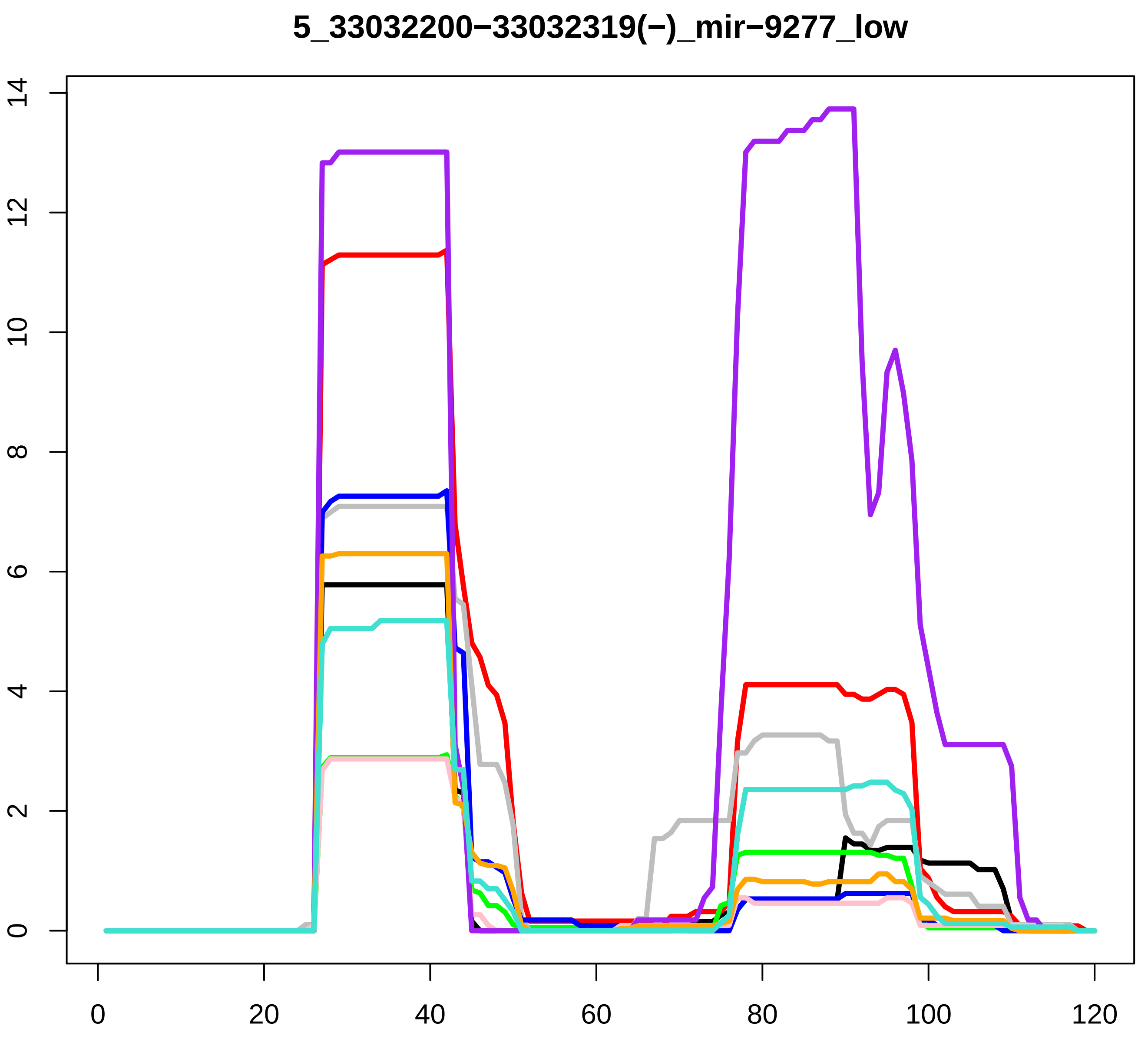
5_33032200−33032319(−)_mir−9277_low
0
2
4
6
8
10
12
14
0
20
40
60
80
100
120
