# Supplementary material for: An Improved microRNA Annotation of the Canine Genome
Source: PLoS One. 2016 Apr 27;11(4):e0153453. doi: 10.1371/journal.pone.0153453 (PMC4847789; doi:10.1371/journal.pone.0153453)
Supplement: S9 Fig — High counts for the first and tenth bin indicate the expected extreme values for the 3’/5’ proportions. (PDF) [file pone.0153453.s009.pdf]

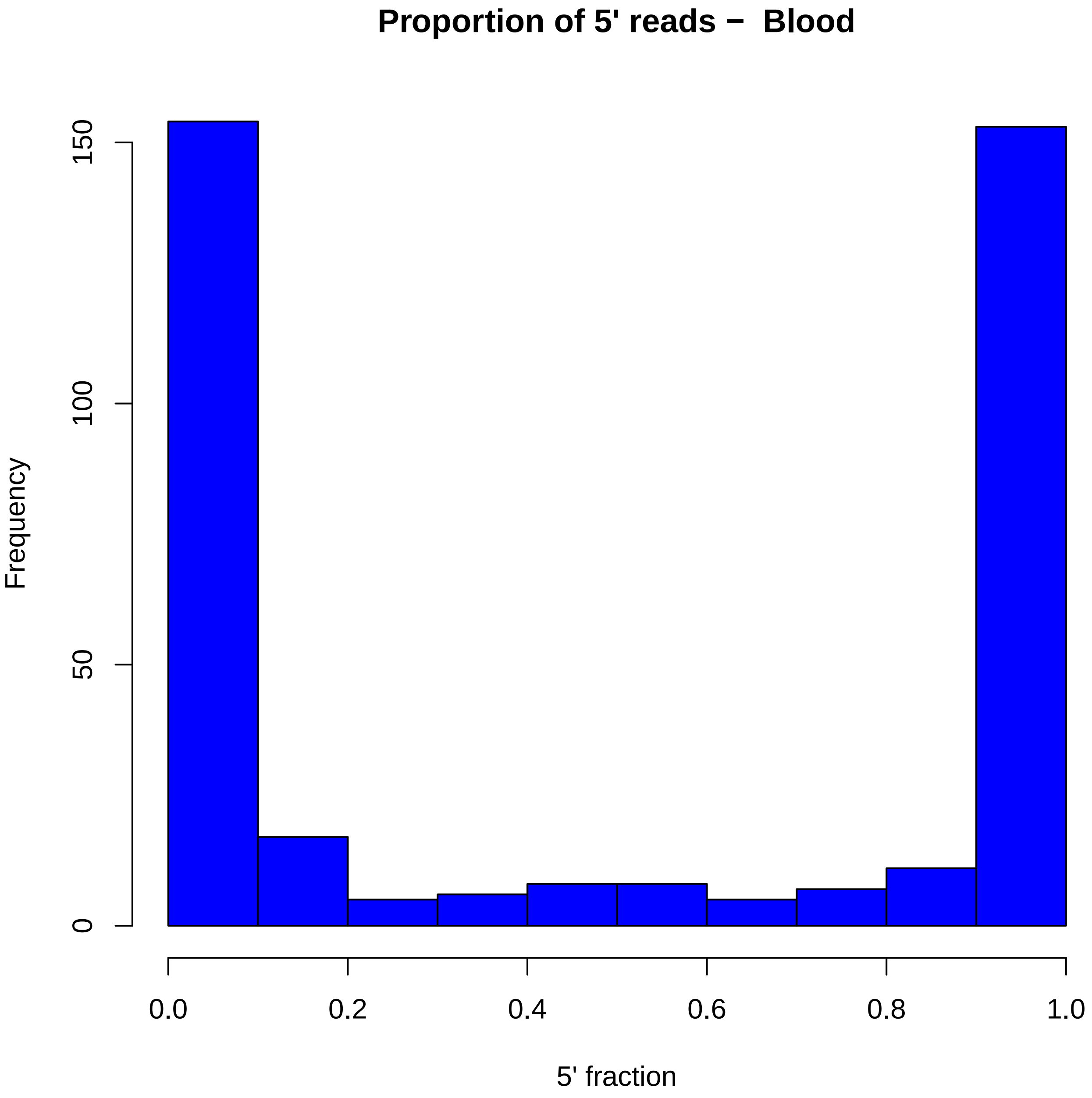

Proportion of 5' reads − Blood
Frequency
0
50
100
150
0.0
0.2
0.4
0.6
0.8
1.0
5' fraction

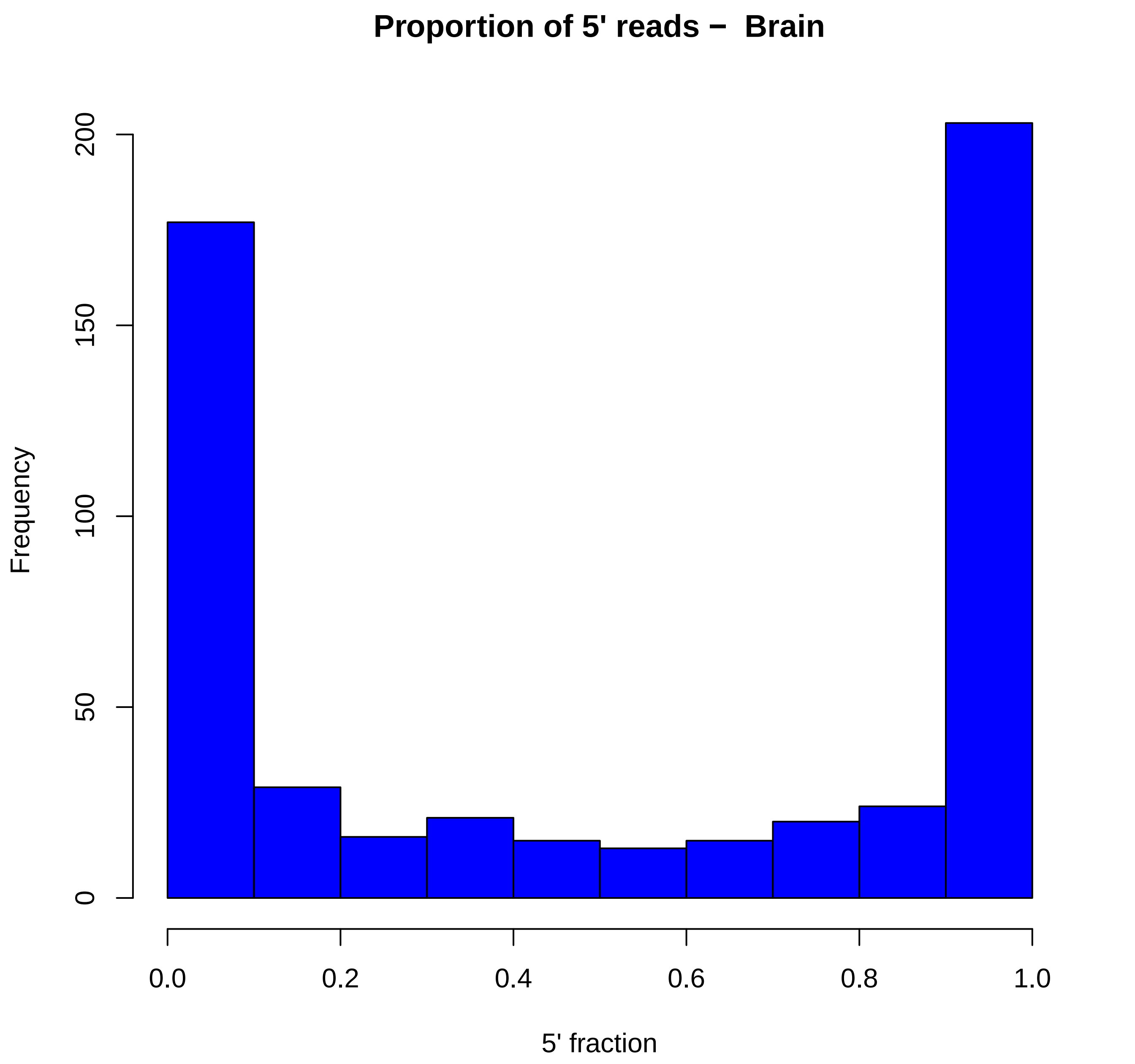

Proportion of 5' reads − Brain
Frequency
0
50
100
150
200
0.0
0.2
0.4
0.6
0.8
1.0
5' fraction

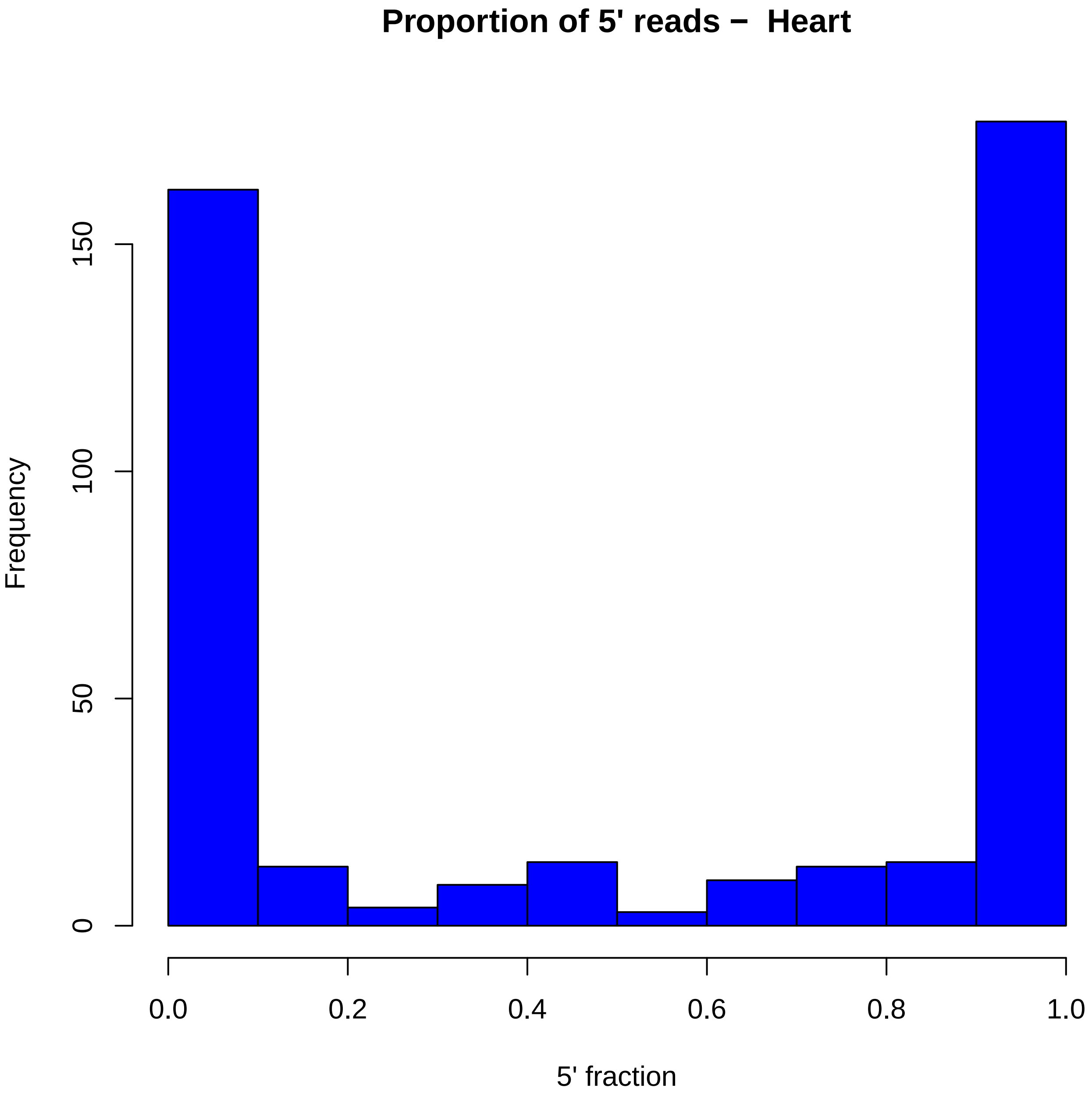

Proportion of 5' reads − Heart
Frequency
0
50
100
150
0.0
0.2
0.4
0.6
0.8
1.0
5' fraction

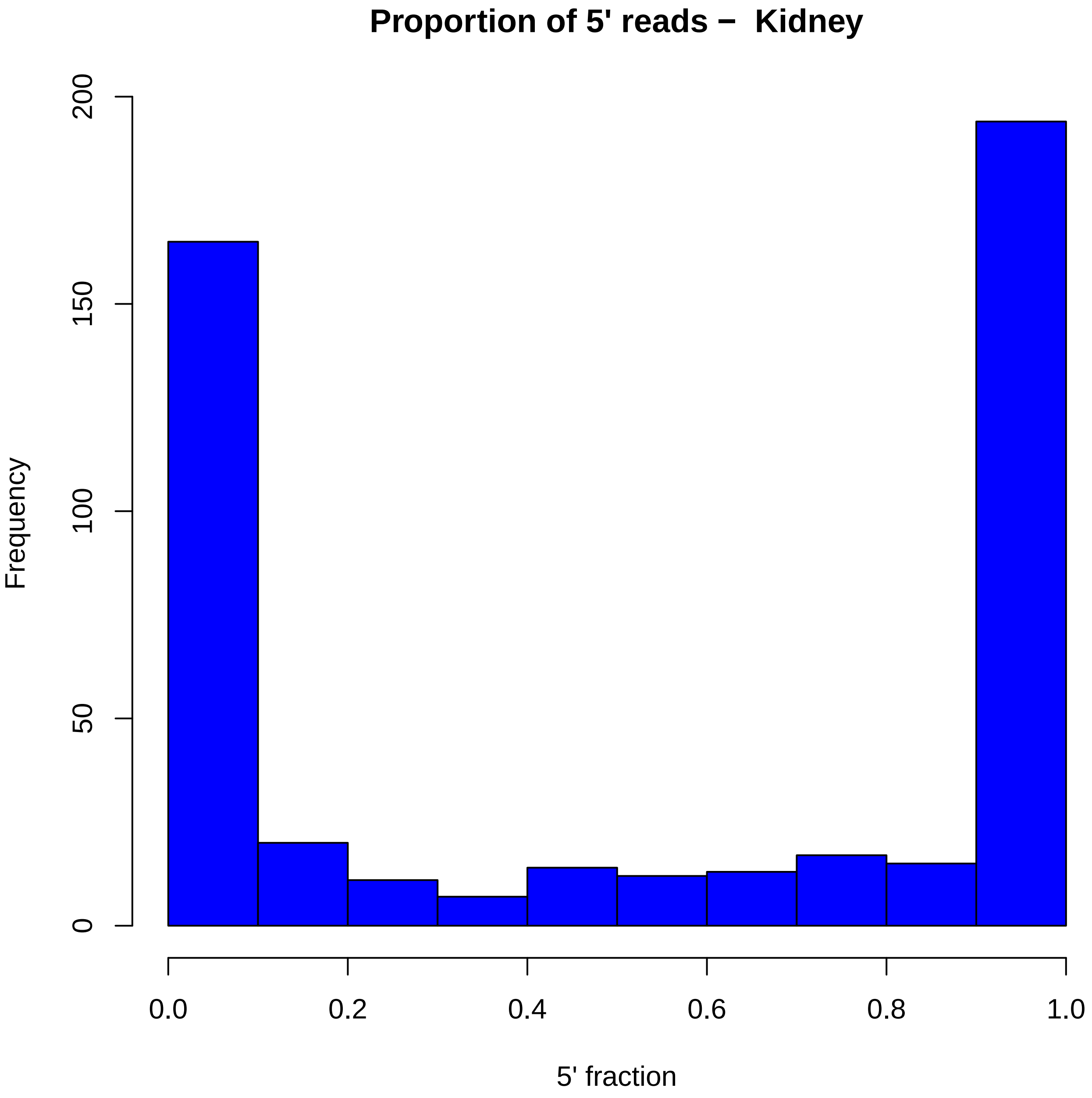
Proportion of 5' reads − Kidney
Frequency
0
50
100
150
200
0.0
0.2
0.4
0.6
0.8
1.0
5' fraction

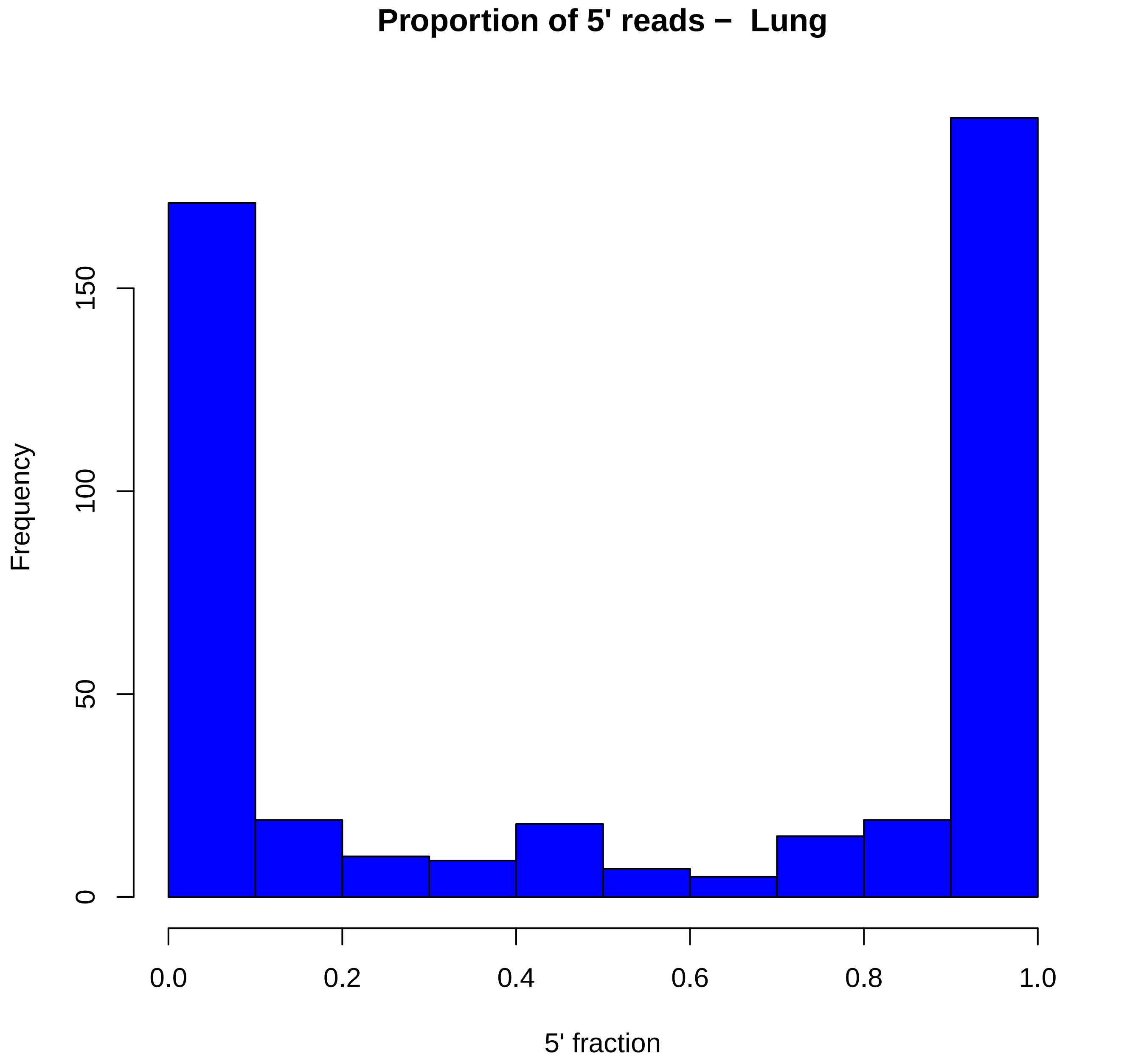
Proportion of 5' reads − Lung
Frequency
0
50
100
150
0.0
0.2
0.4
0.6
0.8
1.0
5' fraction

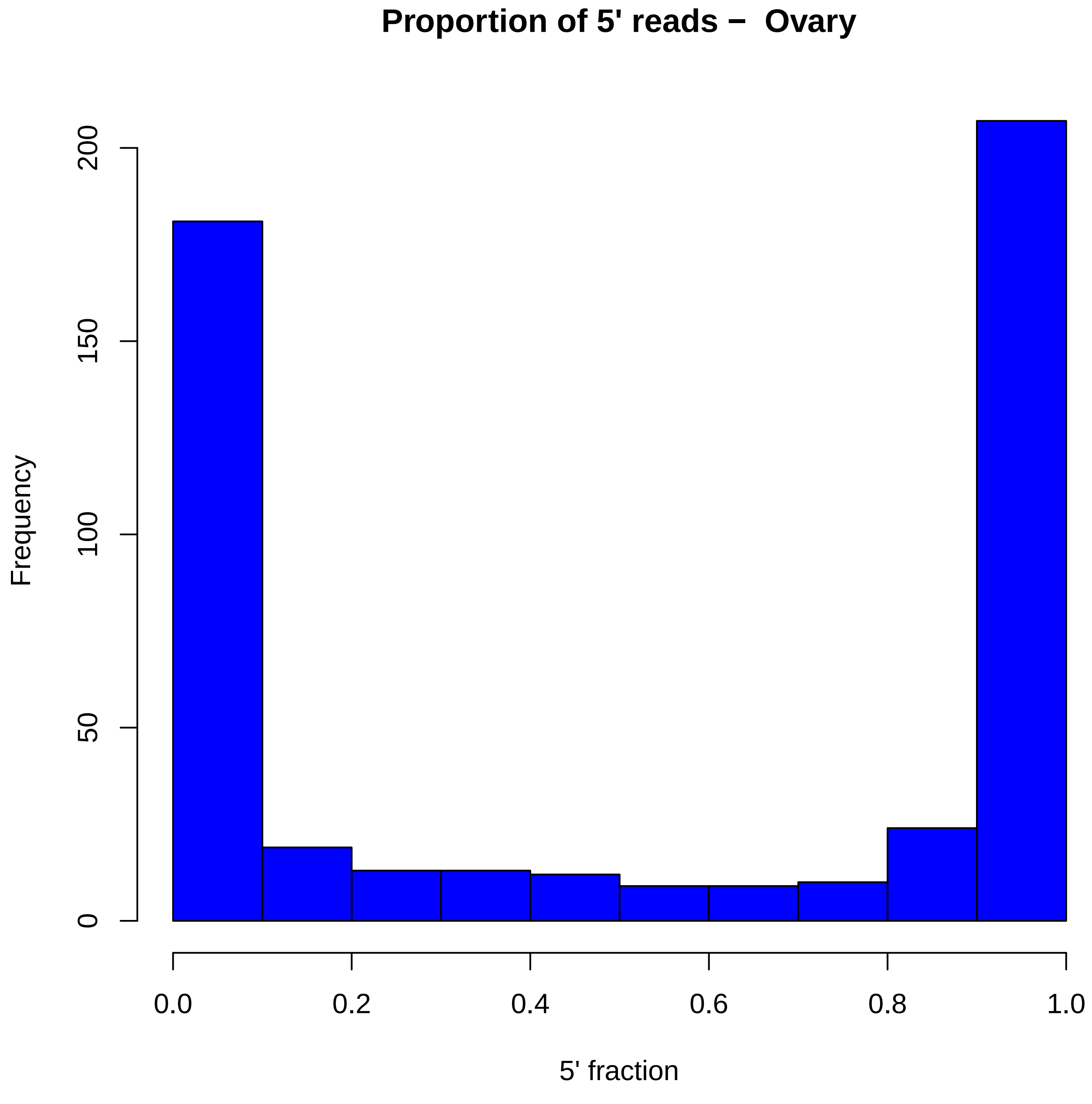
Proportion of 5' reads − Ovary
Frequency
0
50
100
150
200
0.0
0.2
0.4
0.6
0.8
1.0
5' fraction

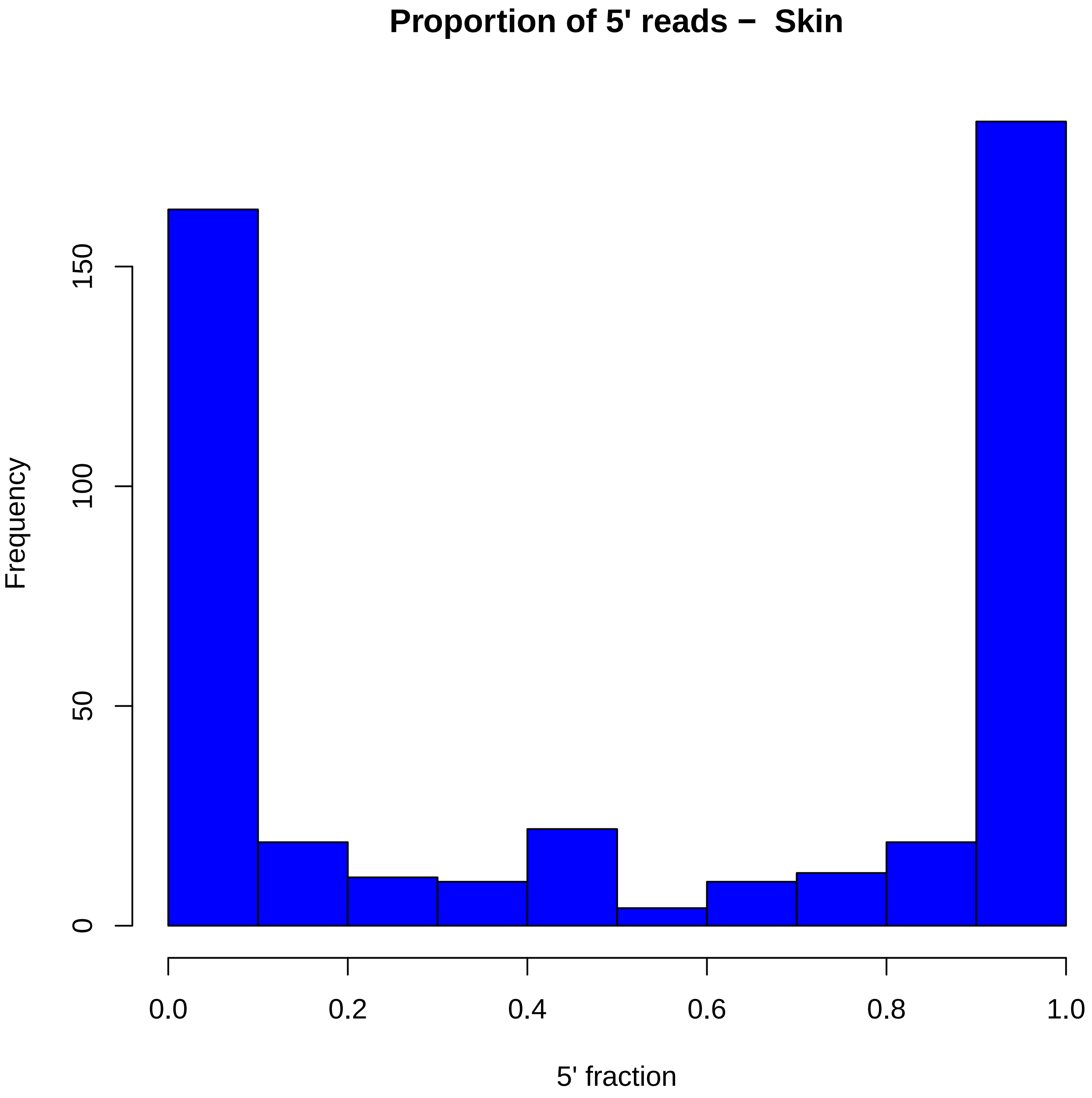

Proportion of 5' reads − Skin
Frequency
0
50
100
150
0.0
0.2
0.4
0.6
0.8
1.0
5' fraction

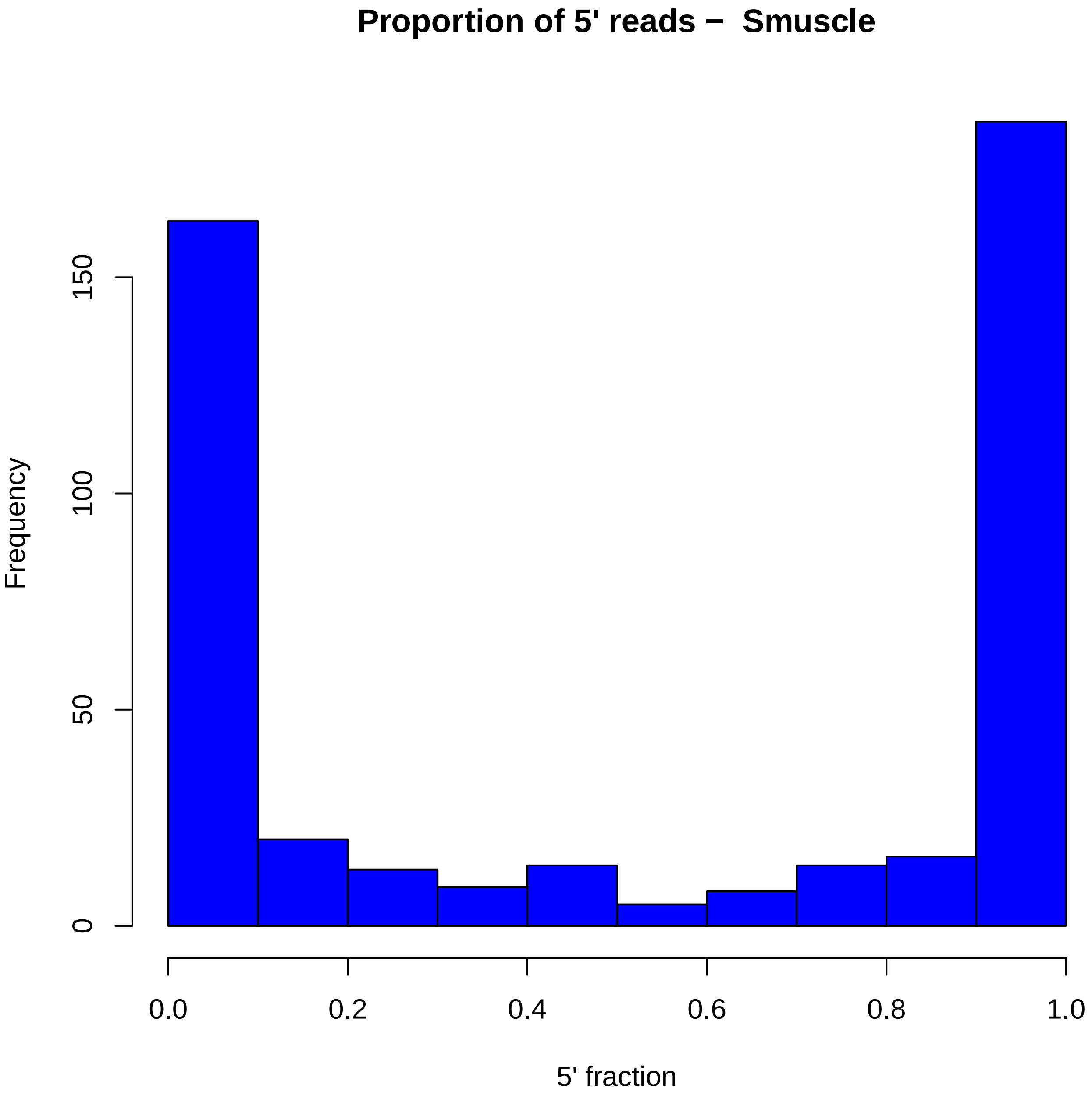

Proportion of 5' reads − Smuscle
Frequency
0
50
100
150
0.0
0.2
0.4
0.6
0.8
1.0
5' fraction

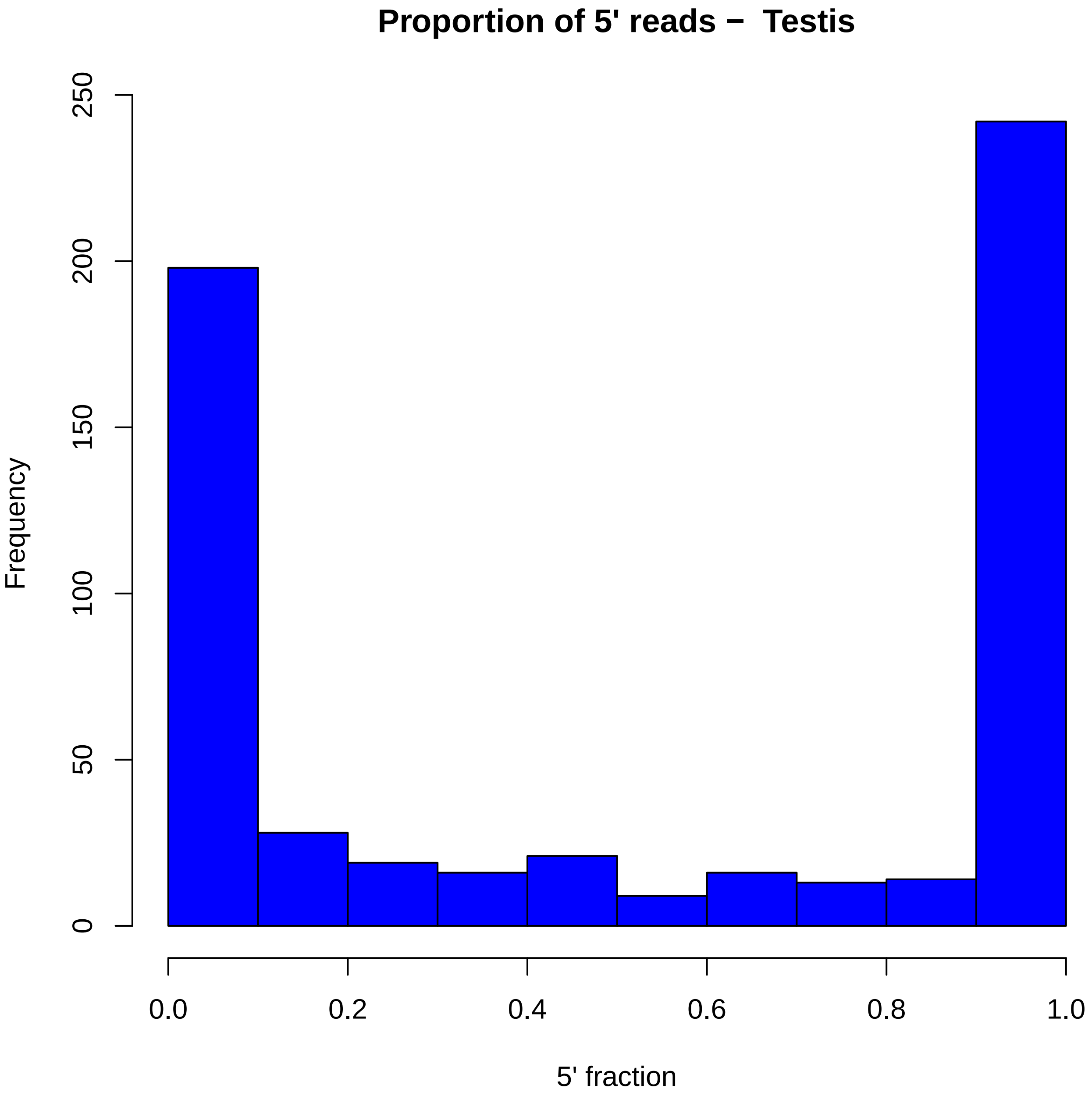
Proportion of 5' reads − Testis
Frequency
0
50
100
150
200
250
0.0
0.2
0.4
0.6
0.8
1.0
5' fraction
