# Supplementary material for: An Improved microRNA Annotation of the Canine Genome
Source: PLoS One. 2016 Apr 27;11(4):e0153453. doi: 10.1371/journal.pone.0153453 (PMC4847789; doi:10.1371/journal.pone.0153453)
Supplement: S10 Fig — Counts of reads at the 5’ end are plotted against the counts at the 3’ end. Different symbols indicate different tissues, as described by the legend. (PDF) [file pone.0153453.s010.pdf]

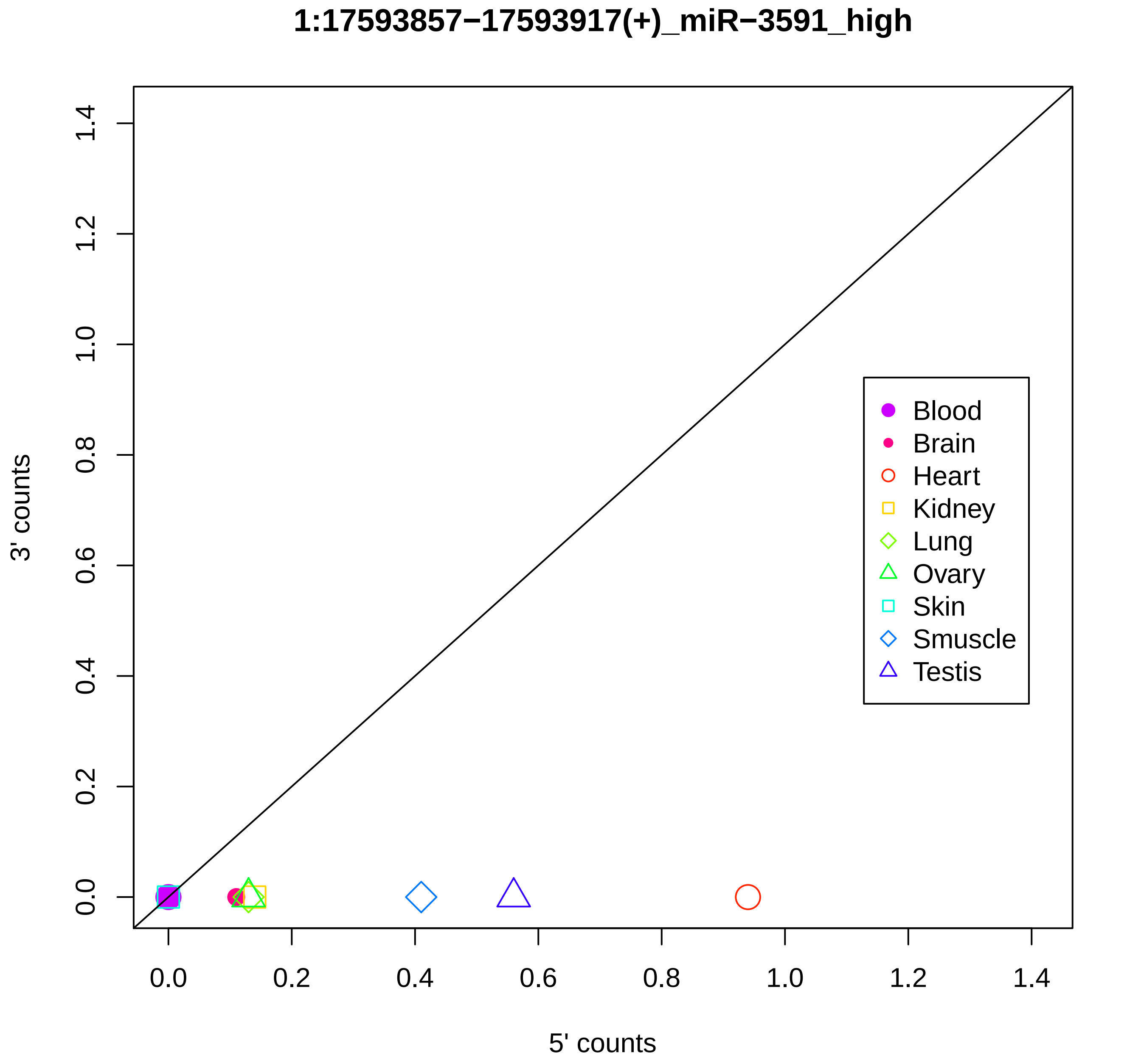
1:17593857−17593917(+)_miR−3591_high
3' counts
5' counts
0.0
0.2
0.4
0.6
0.8
1.0
1.2
1.4
Blood
Brain
Heart
Kidney
Lung
Ovary
Skin
Smuscle
Testis

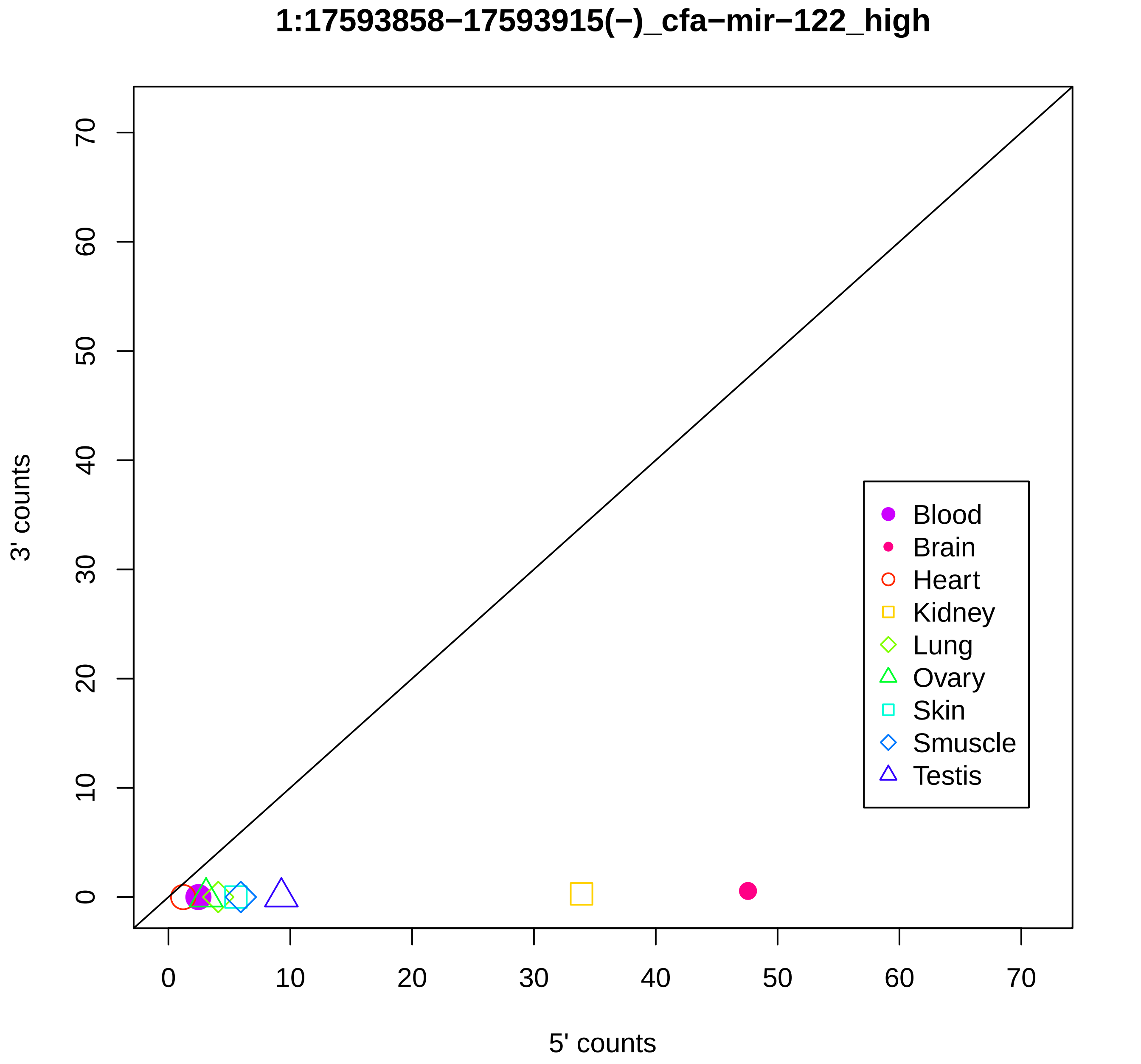

1:17593858−17593915(−)_cfa−mir−122_high
3' counts
5' counts
0
10
20
30
40
50
60
70
Blood
Brain
Heart
Kidney
Lung
Ovary
Skin
Smuscle
Testis

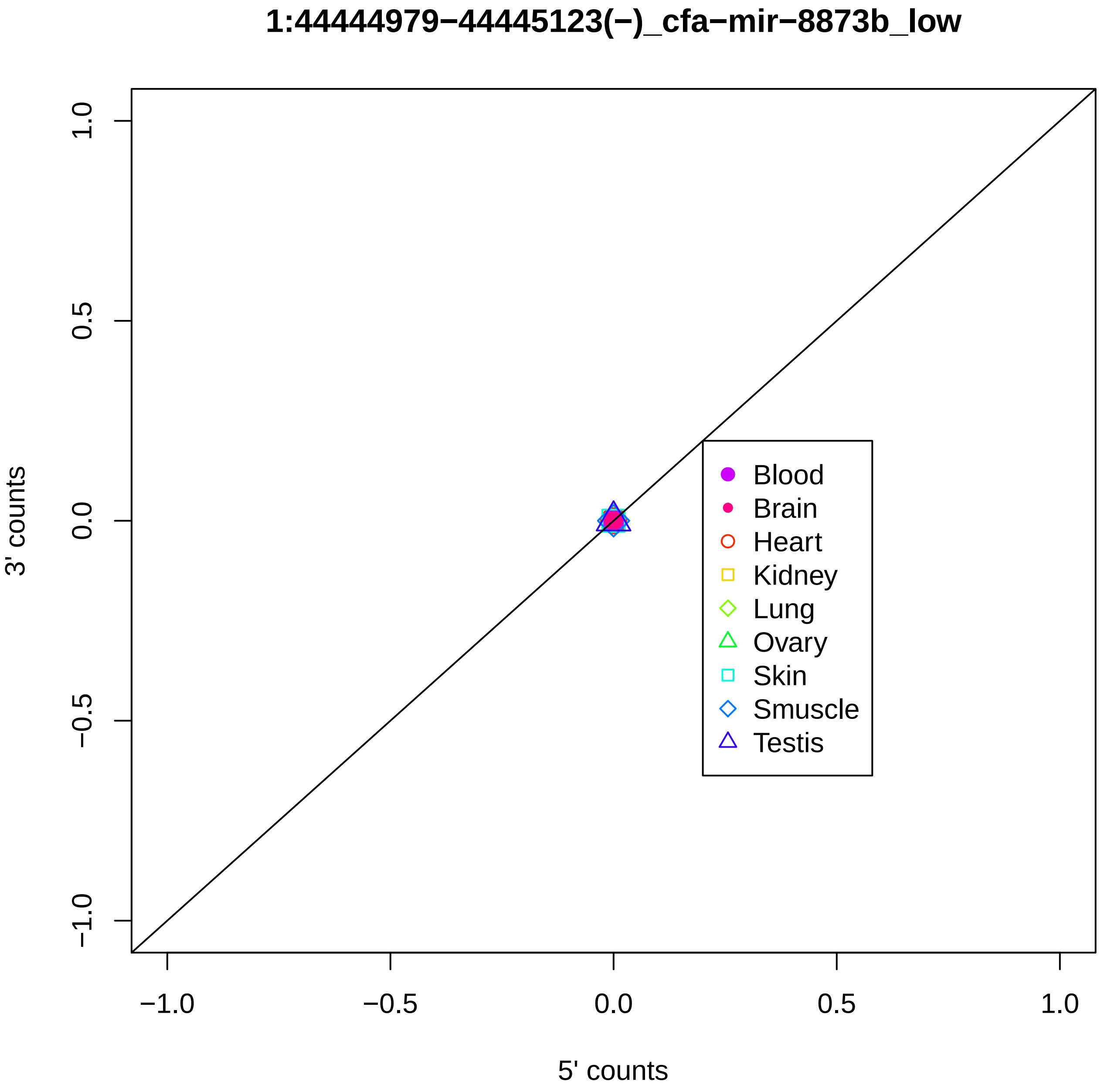

1:44444979−44445123(−)_cfa−mir−8873b_low
Blood
Brain
Heart
Kidney
Lung
Ovary
Skin
Smuscle
Testis
1.0
0.5
0.0
−0.5
−1.0
3' counts
−1.0
−0.5
0.0
0.5
1.0
5' counts

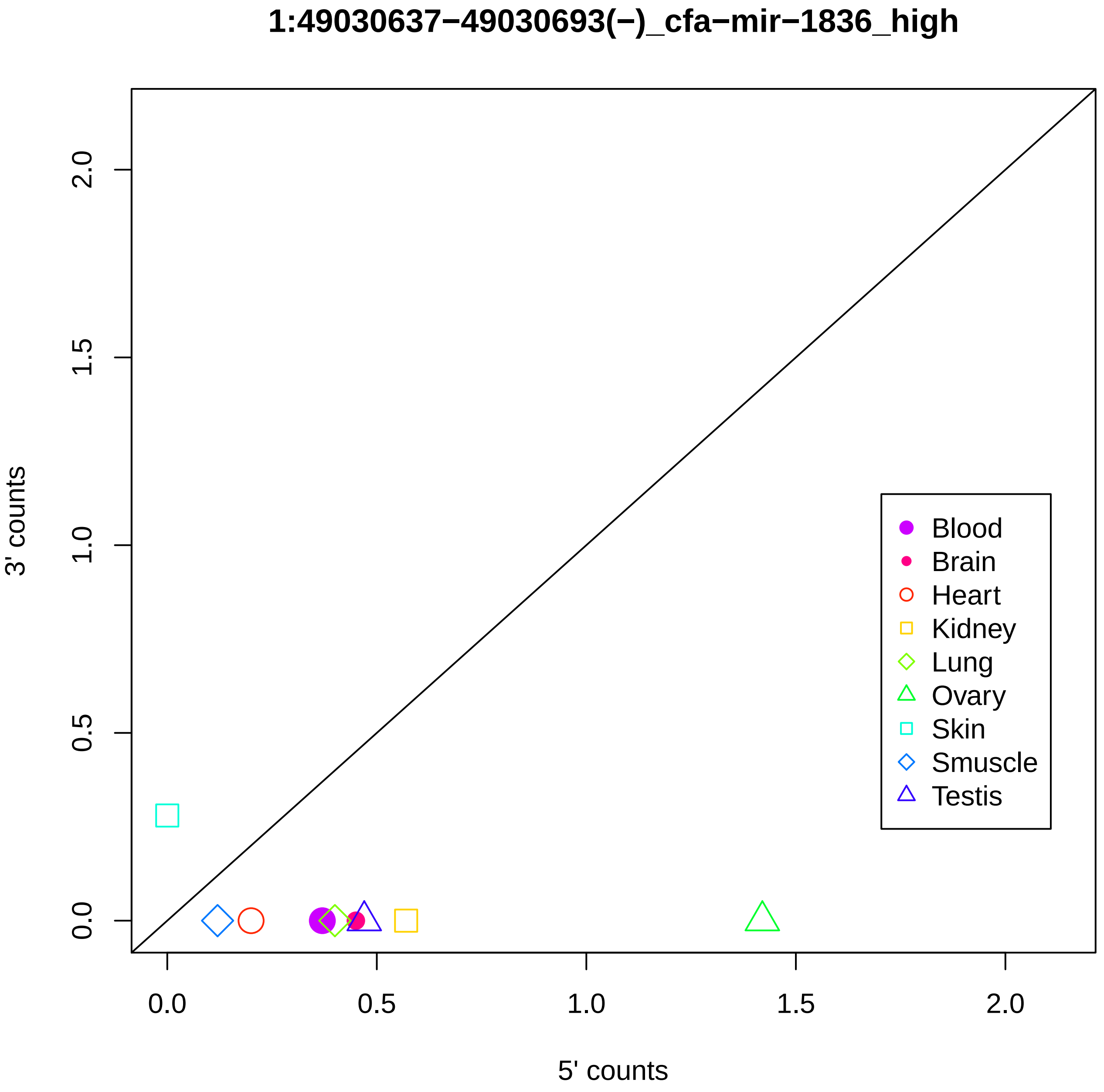
1:49030637−49030693(−)_cfa−mir−1836_high
3' counts
5' counts
0.0
0.5
1.0
1.5
2.0
0.0
0.5
1.0
1.5
2.0
Blood
Brain
Heart
Kidney
Lung
Ovary
Skin
Smuscle
Testis

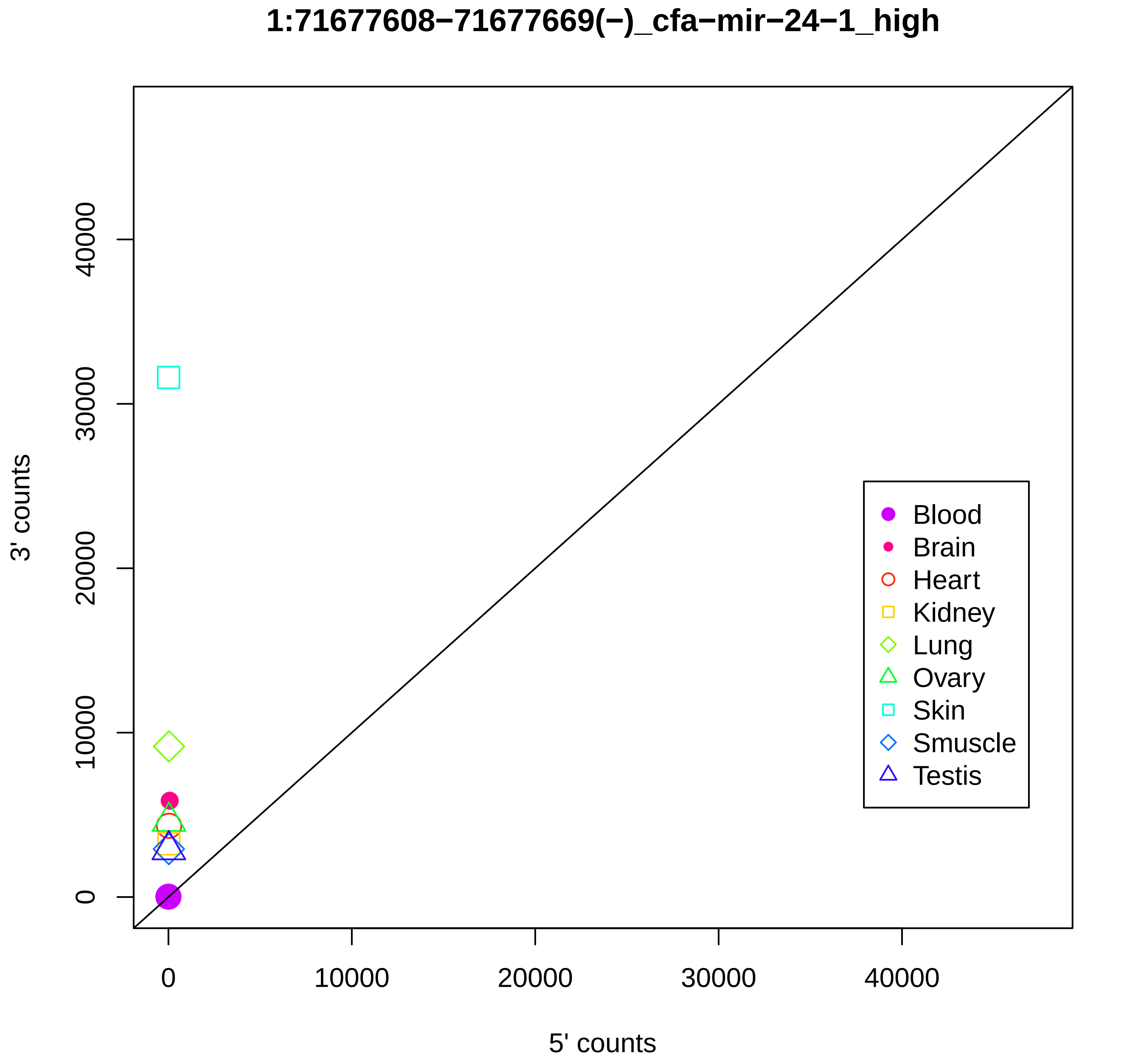

1:71677608−71677669(−)_cfa−mir−24−1_high
3' counts
5' counts
0
10000
20000
30000
40000
Blood
Brain
Heart
Kidney
Lung
Ovary
Skin
Smuscle
Testis

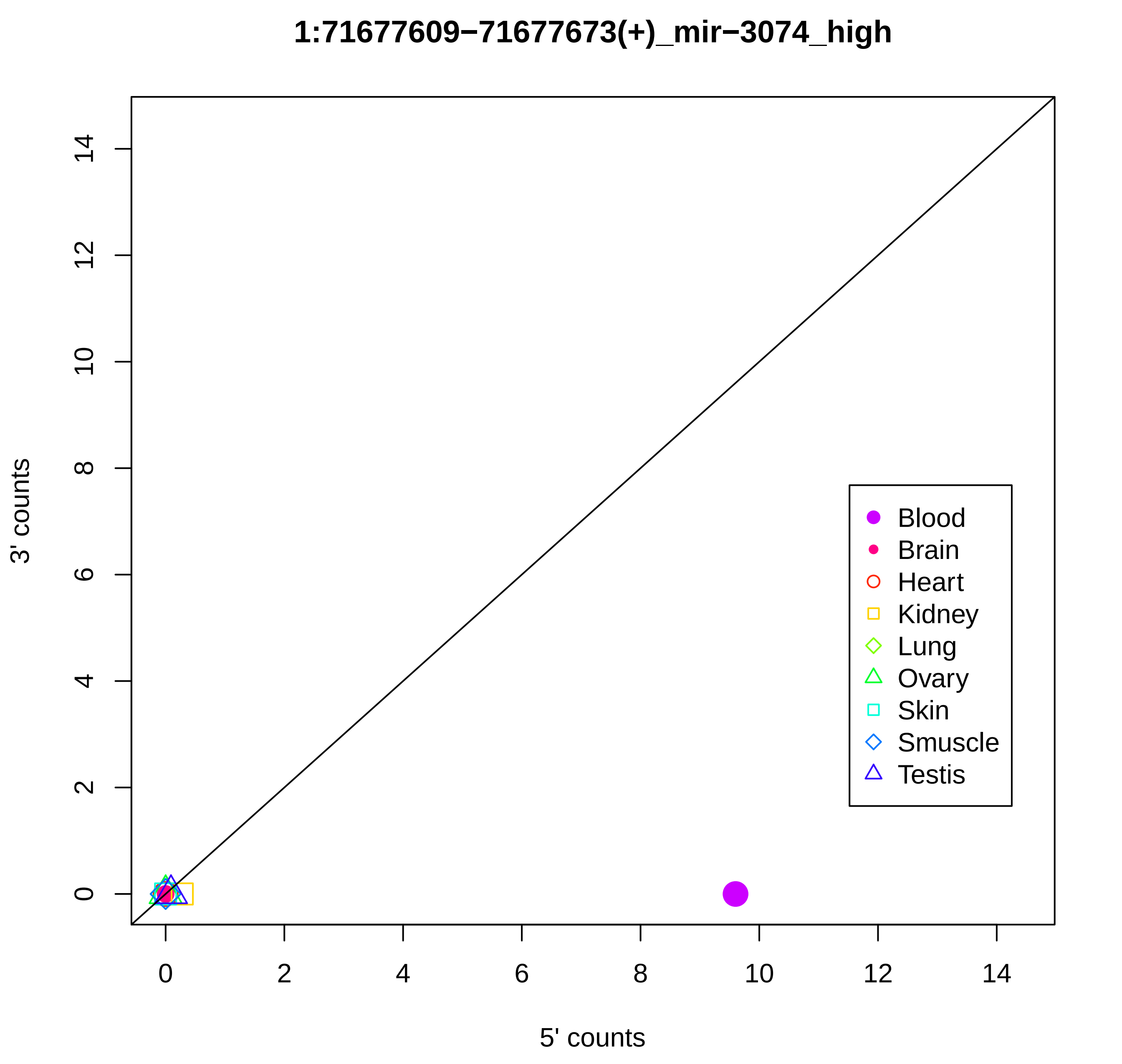

1:71677609−71677673(+)_mir−3074_high
3' counts
5' counts
0
2
4
6
8
10
12
14
Blood
Brain
Heart
Kidney
Lung
Ovary
Skin
Smuscle
Testis

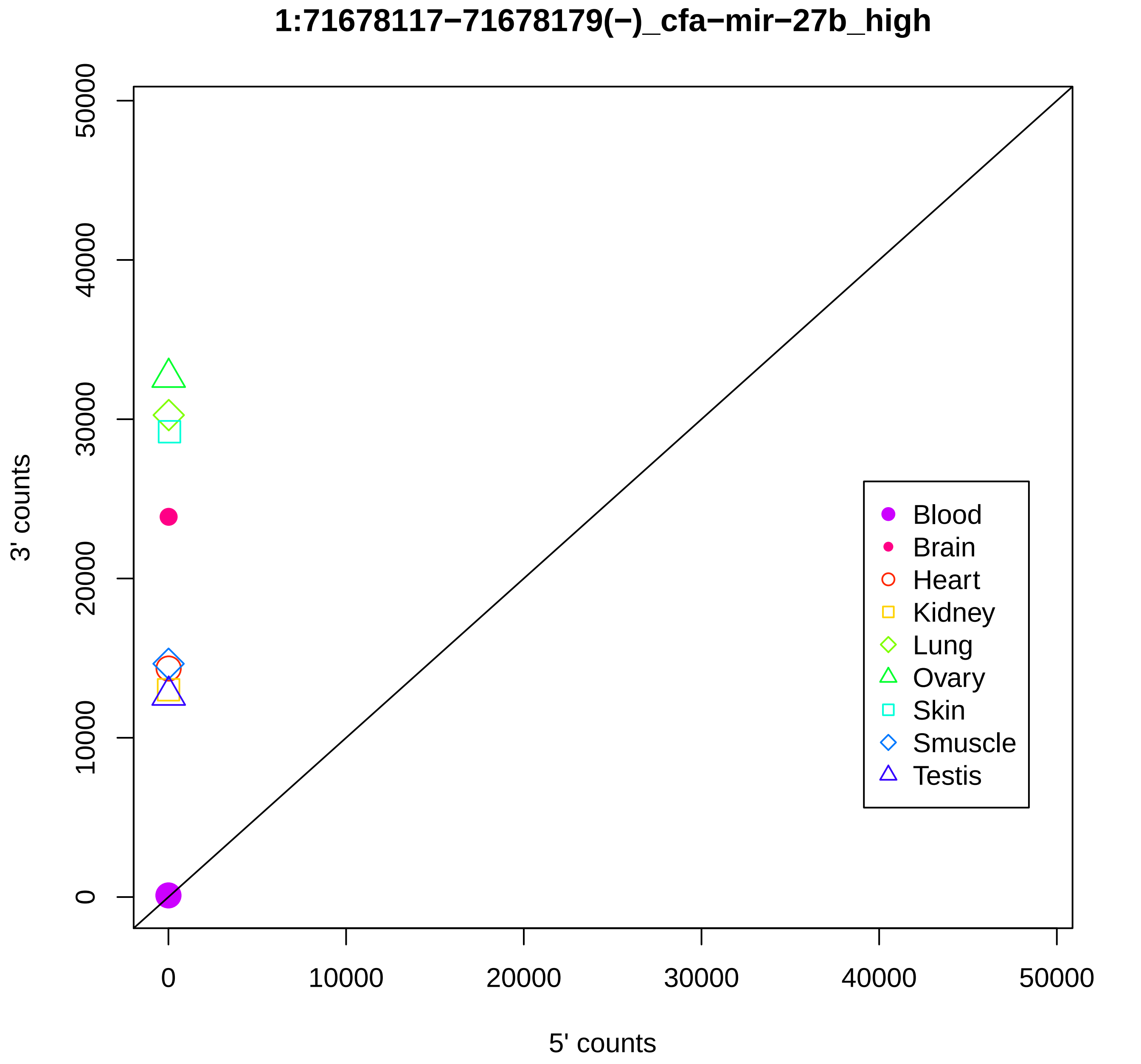
1:71678117−71678179(−)_cfa−mir−27b_high
3' counts
5' counts
0
10000
20000
30000
40000
50000
Blood
Brain
Heart
Kidney
Lung
Ovary
Skin
Smuscle
Testis

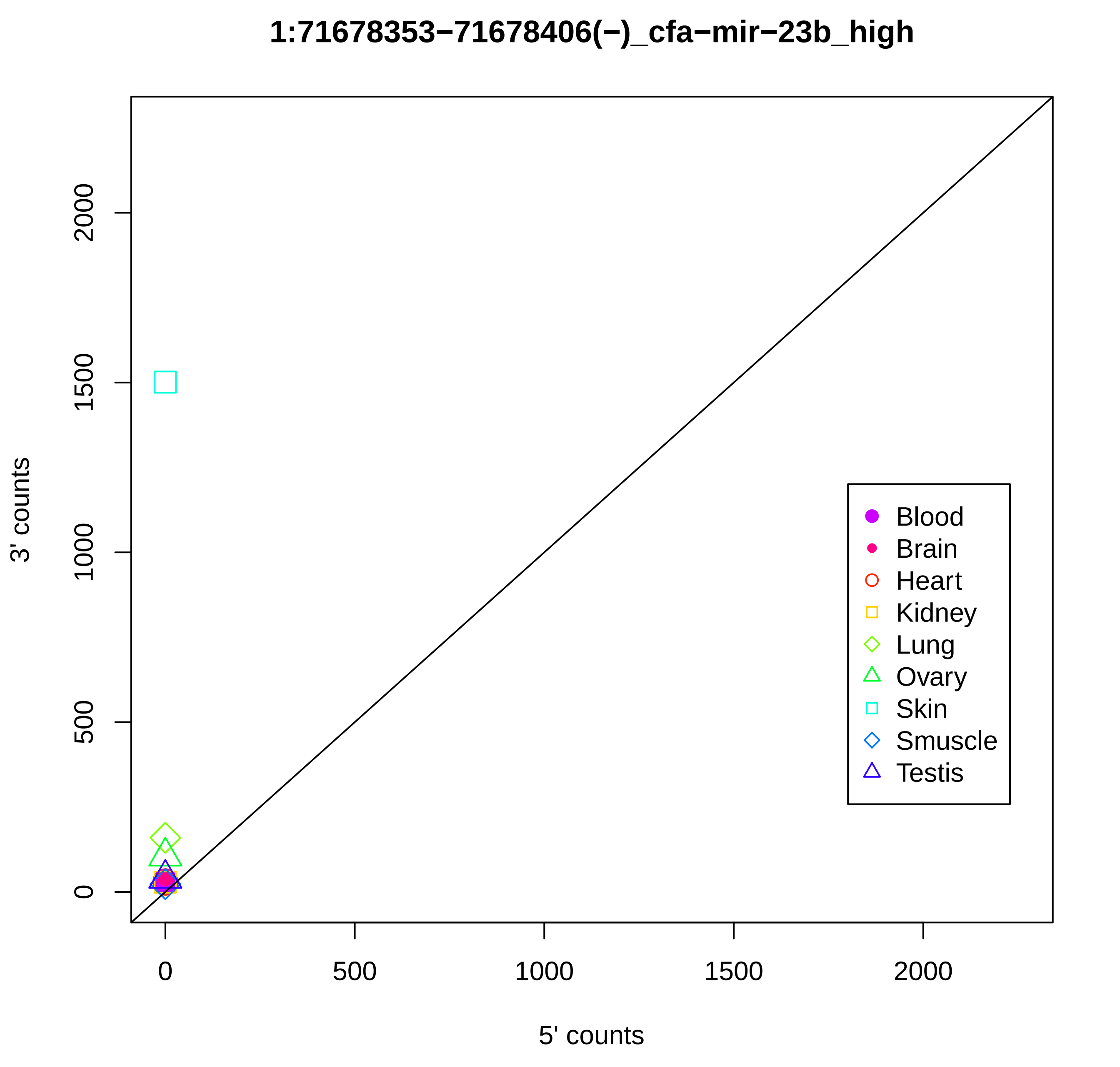
1:71678353−71678406(−)_cfa−mir−23b_high
3' counts
5' counts
0
500
1000
1500
2000
0
500
1000
1500
2000
Blood
Brain
Heart
Kidney
Lung
Ovary
Skin
Smuscle
Testis

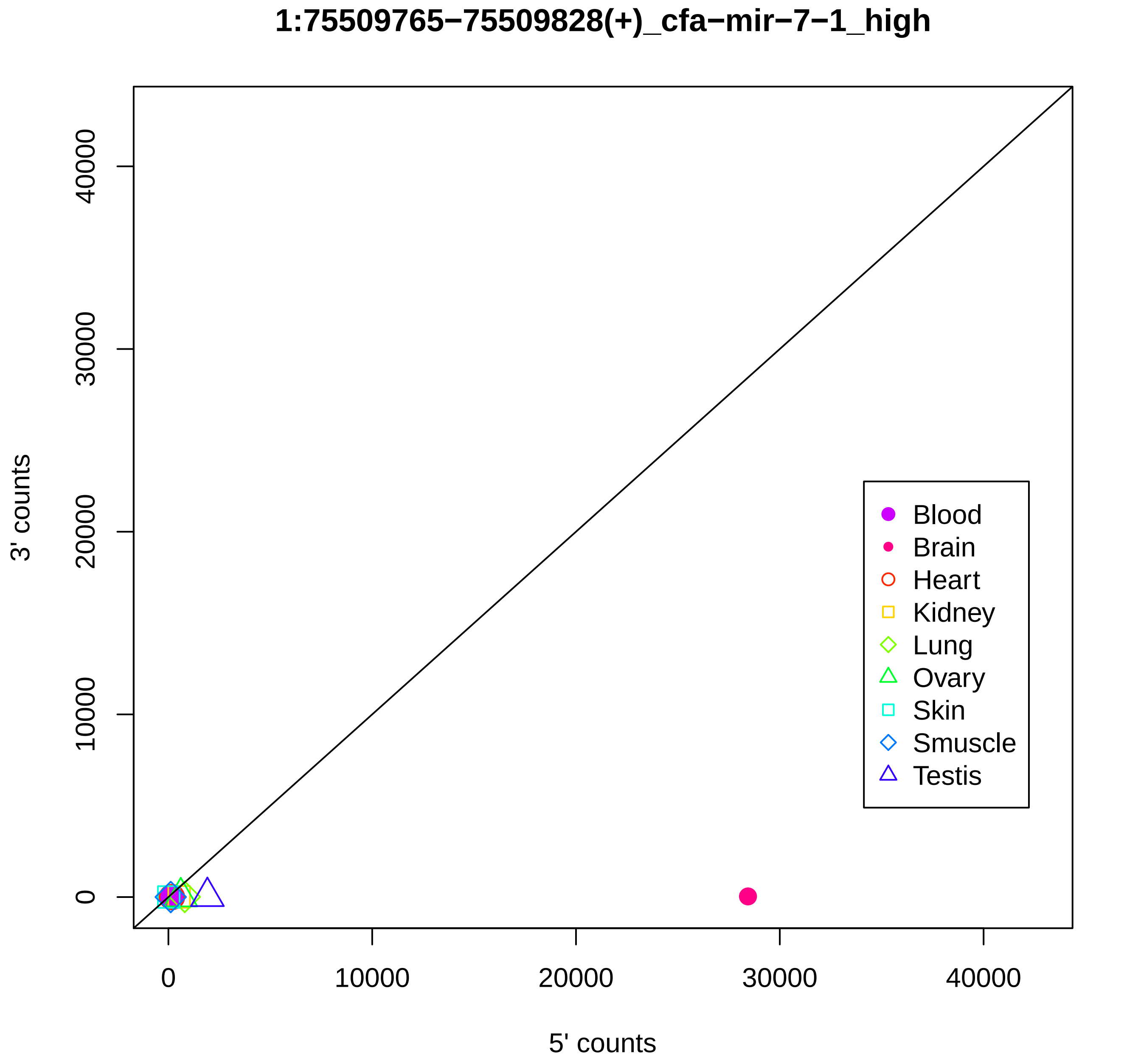
1:75509765−75509828(+)_cfa−mir−7−1_high
3' counts
0
10000
20000
30000
40000
5' counts
0
10000
20000
30000
40000
Blood
Brain
Heart
Kidney
Lung
Ovary
Skin
Smuscle
Testis

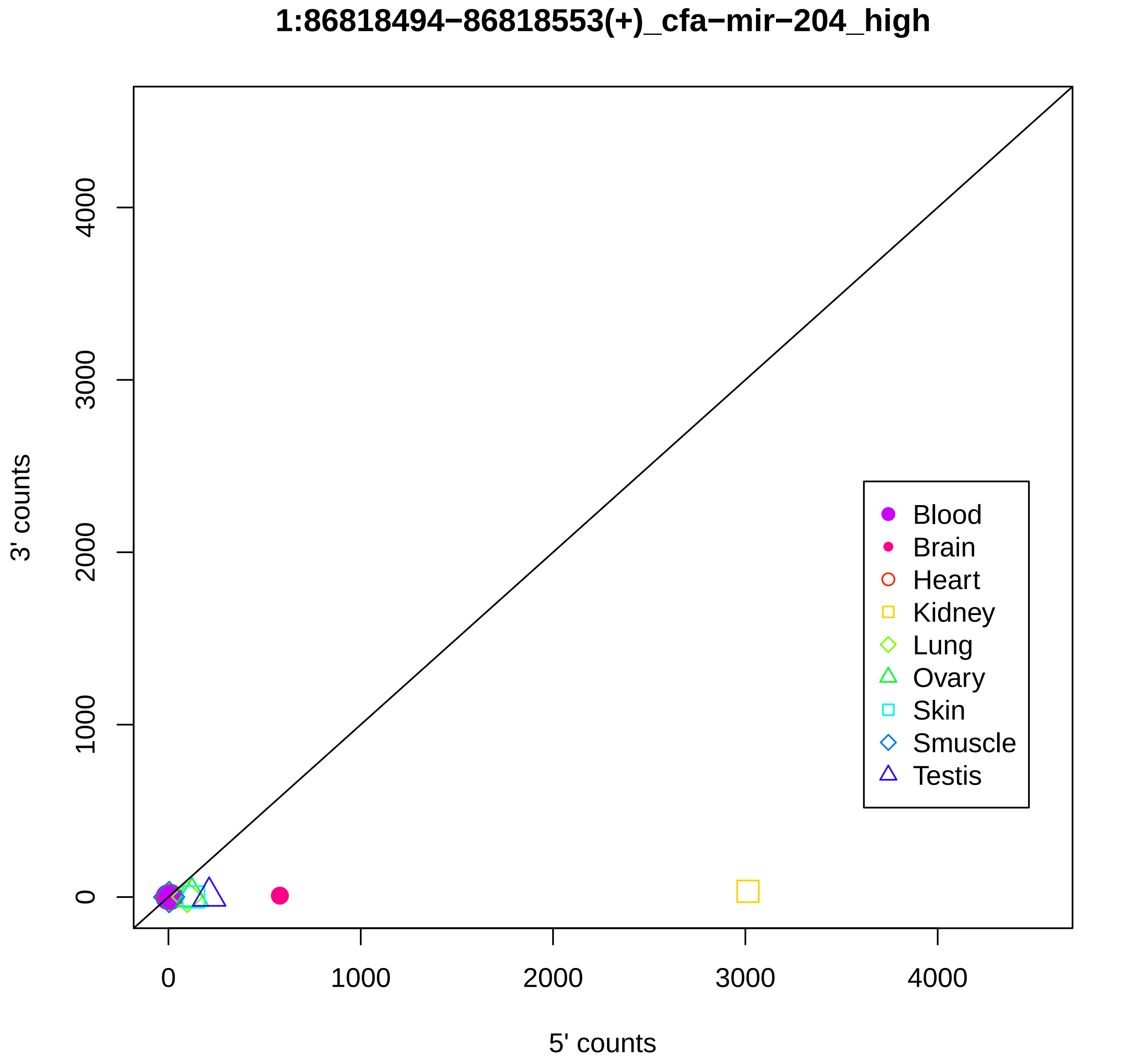
1:86818494−86818553(+)_cfa−mir−204_high
3' counts
5' counts
0
1000
2000
3000
4000
0
1000
2000
3000
4000
Blood
Brain
Heart
Kidney
Lung
Ovary
Skin
Smuscle
Testis

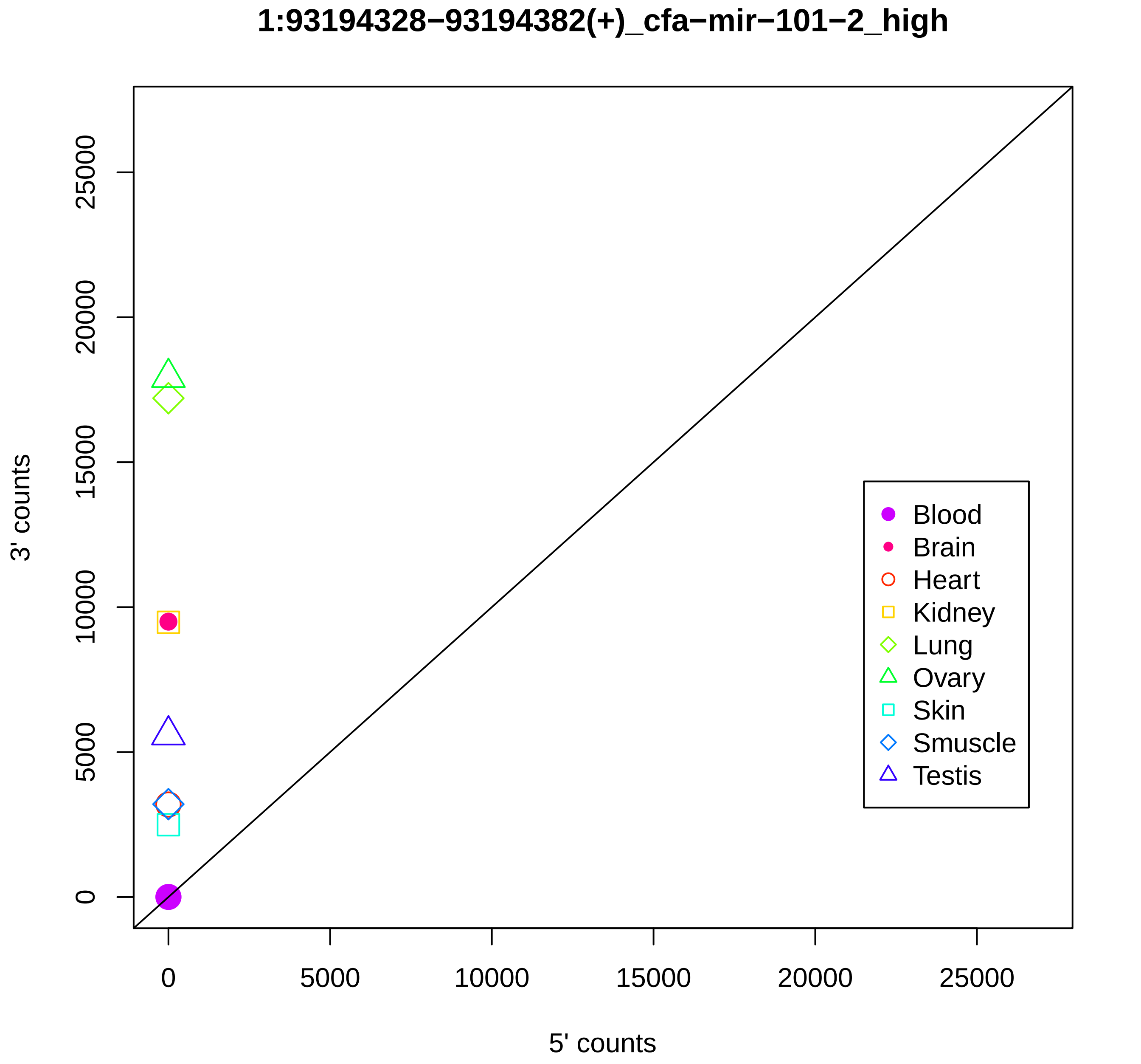

1:93194328−93194382(+)_cfa−mir−101−2_high
3' counts
5' counts
0
5000
10000
15000
20000
25000
Blood
Brain
Heart
Kidney
Lung
Ovary
Skin
Smuscle
Testis

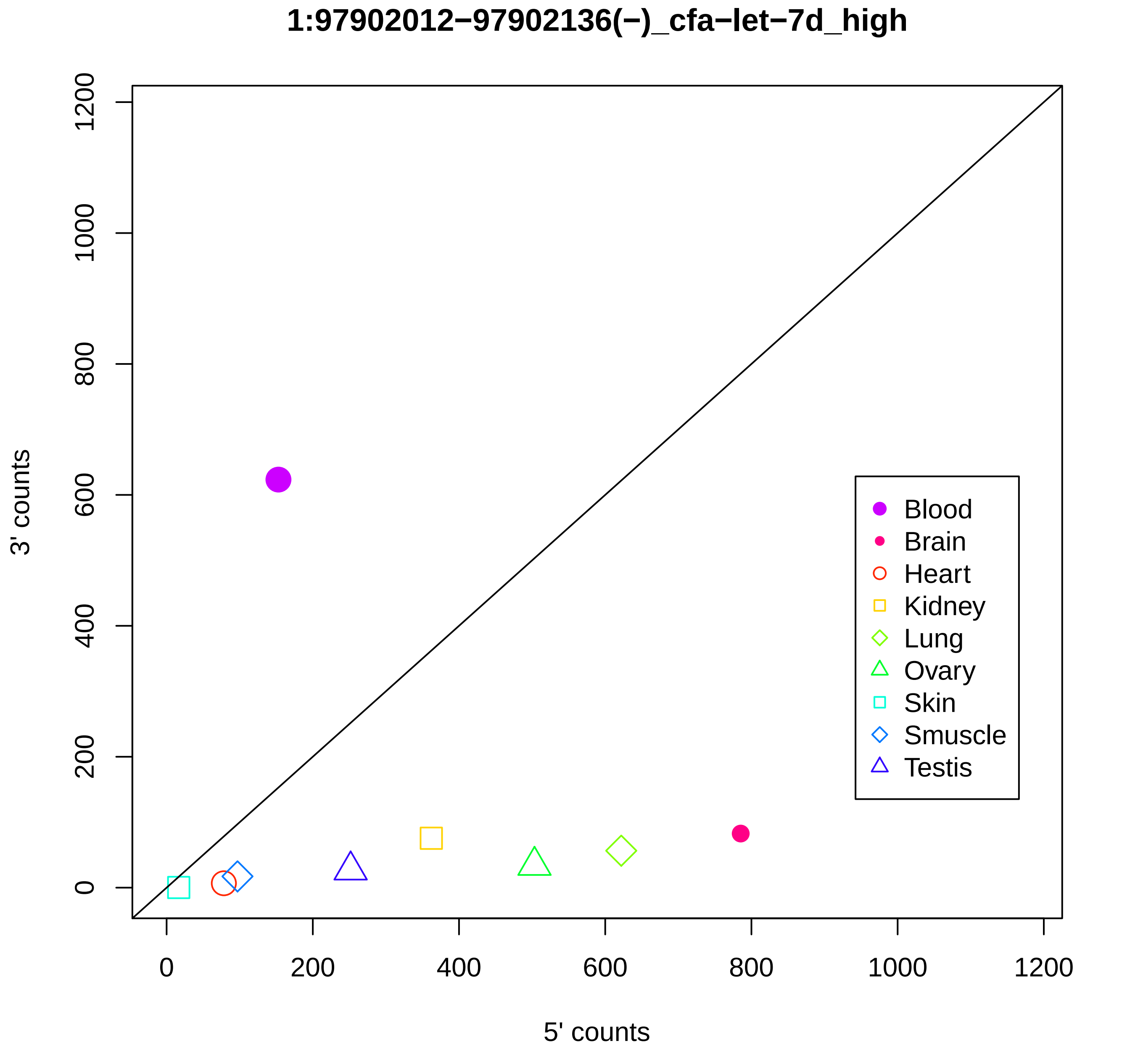

1:97902012−97902136(−)_cfa−let−7d_high
3' counts
5' counts
0
200
400
600
800
1000
1200
Blood
Brain
Heart
Kidney
Lung
Ovary
Skin
Smuscle
Testis

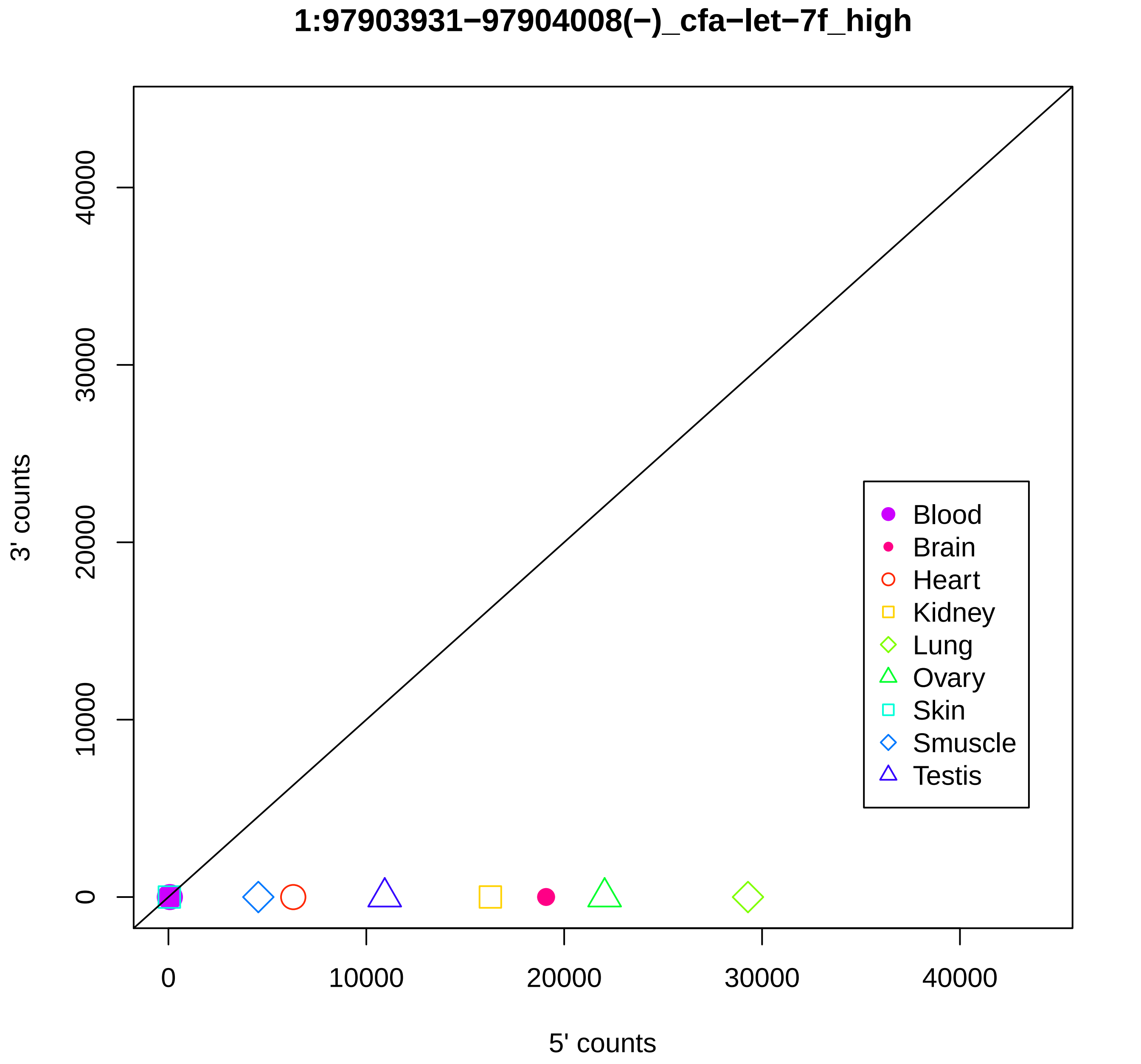

1:97903931−97904008(−)_cfa−let−7f_high
3' counts
5' counts
0
10000
20000
30000
40000
Blood
Brain
Heart
Kidney
Lung
Ovary
Skin
Smuscle
Testis

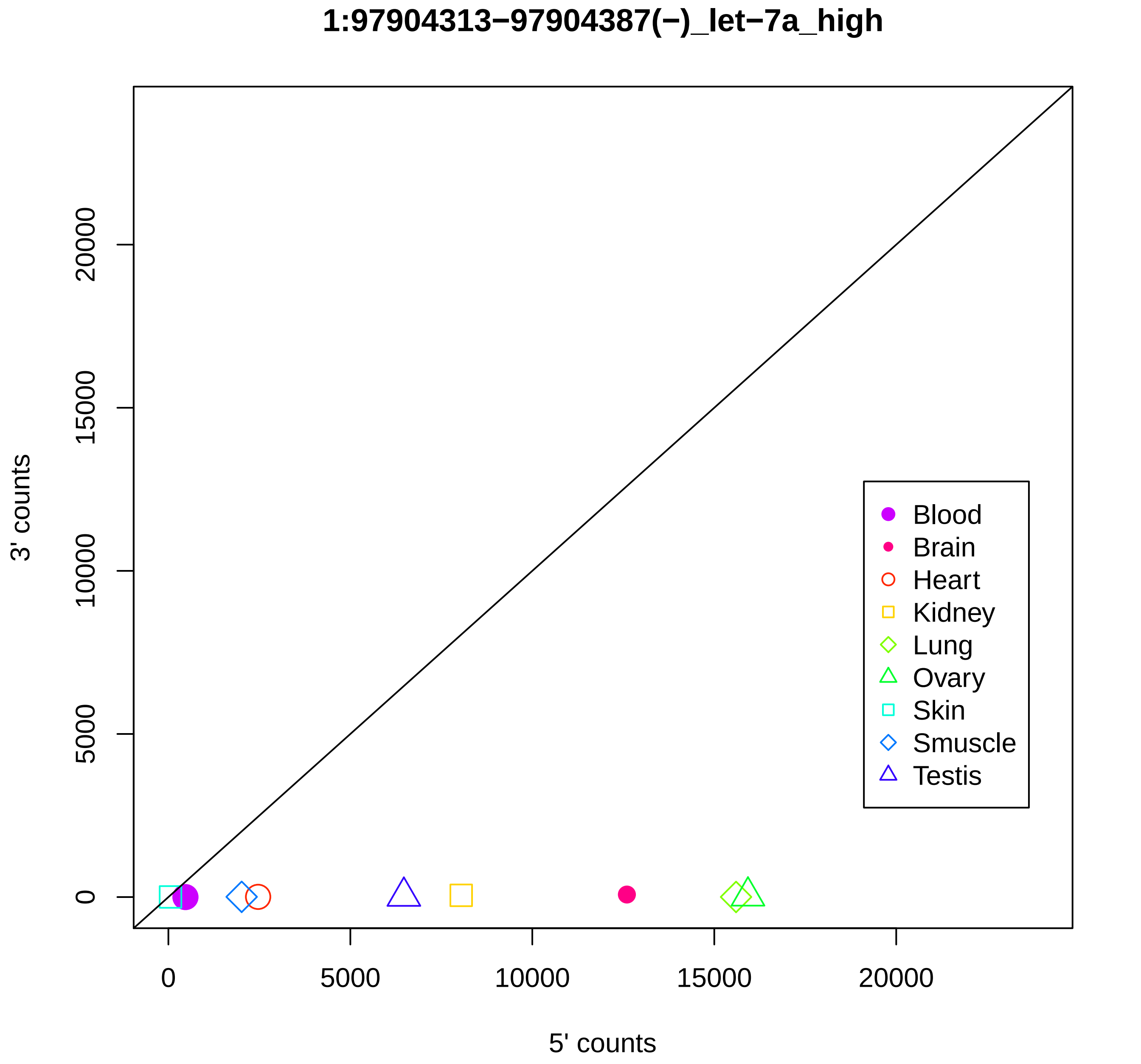

1:97904313−97904387(−)_let−7a_high
3' counts
0
5000
10000
15000
20000
5' counts
0
5000
10000
15000
20000
Blood
Brain
Heart
Kidney
Lung
Ovary
Skin
Smuscle
Testis

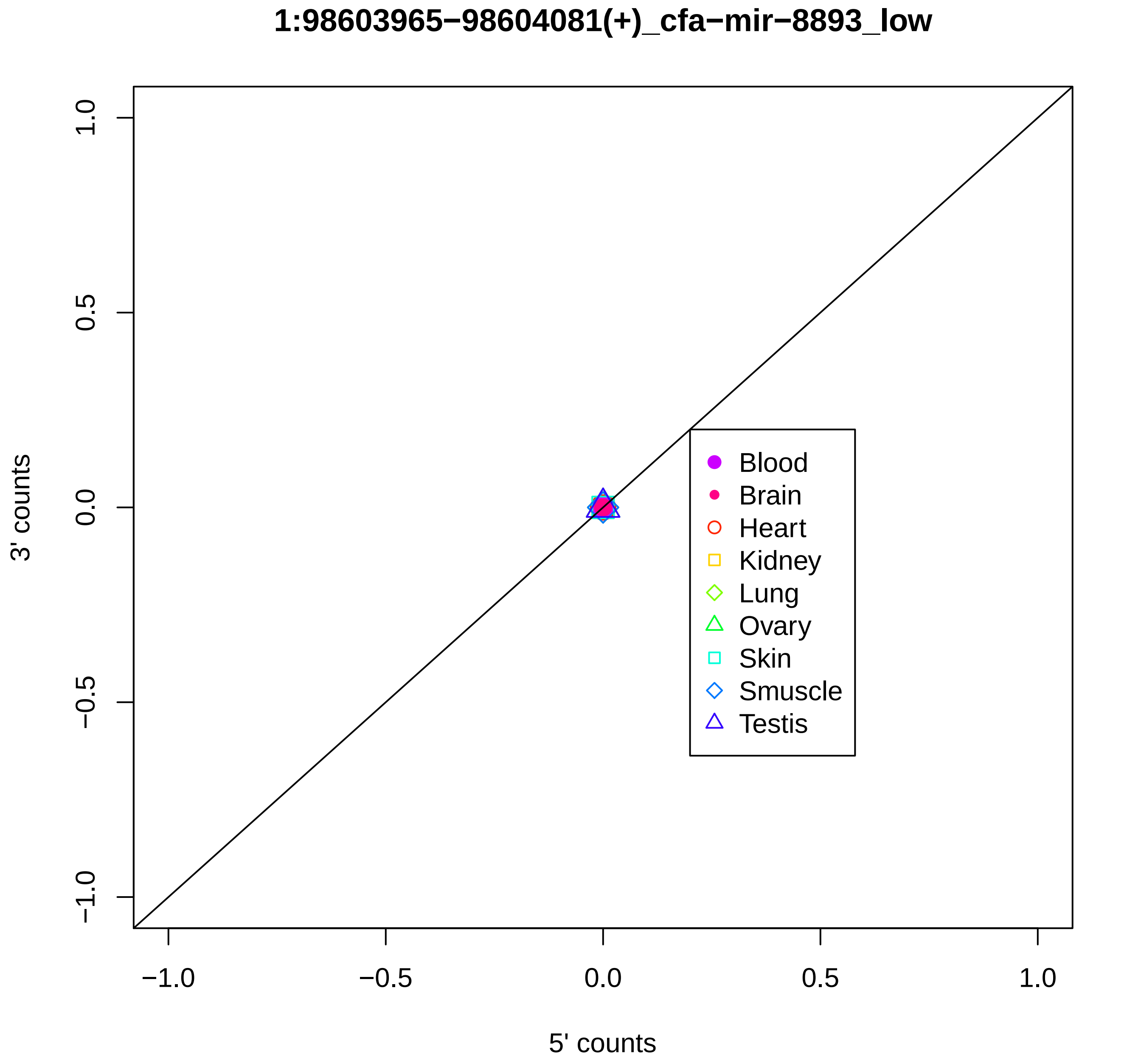
1:98603965−98604081(+)_cfa−mir−8893_low
1.0
0.5
0.0
−0.5
−1.0
3' counts
−1.0
−0.5
0.0
0.5
1.0
5' counts
Blood
Brain
Heart
Kidney
Lung
Ovary
Skin
Smuscle
Testis

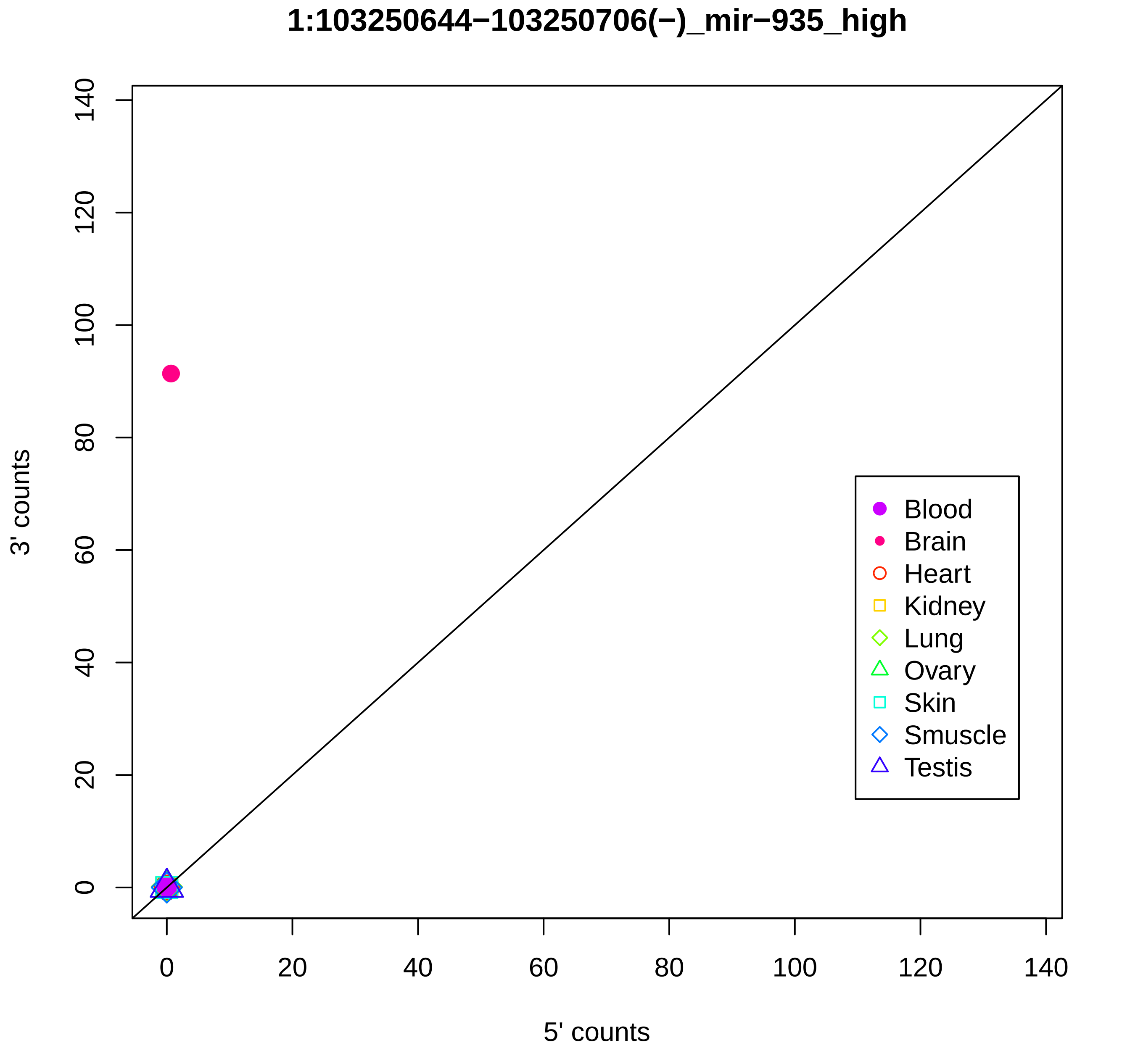
1:103250644−103250706(−)_mir−935_high
3' counts
5' counts
0
20
40
60
80
100
120
140
Blood
Brain
Heart
Kidney
Lung
Ovary
Skin
Smuscle
Testis

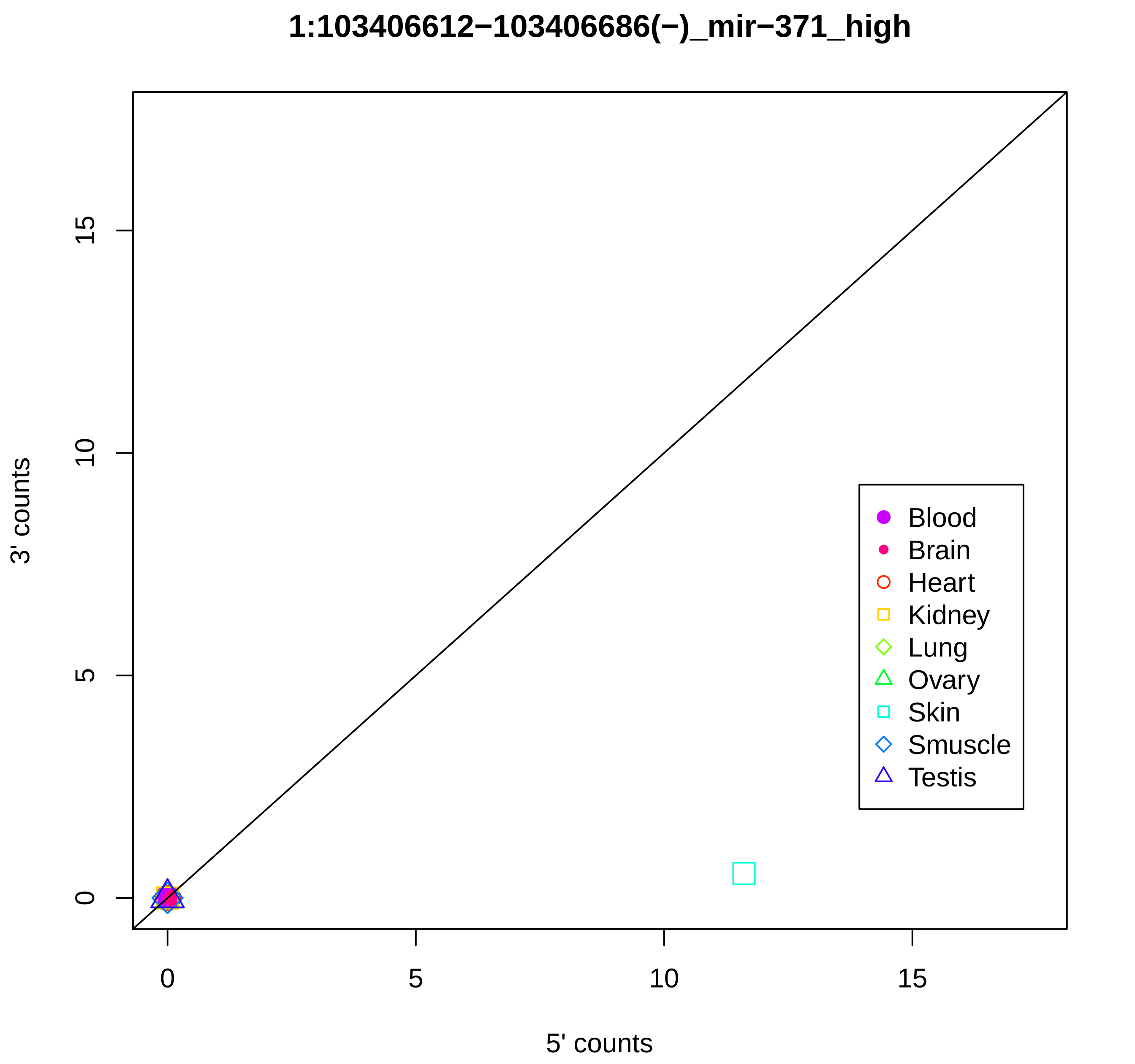

1:103406612−103406686(−)_mir−371_high
3' counts
5' counts
0
5
10
15
0
5
10
15
Blood
Brain
Heart
Kidney
Lung
Ovary
Skin
Smuscle
Testis

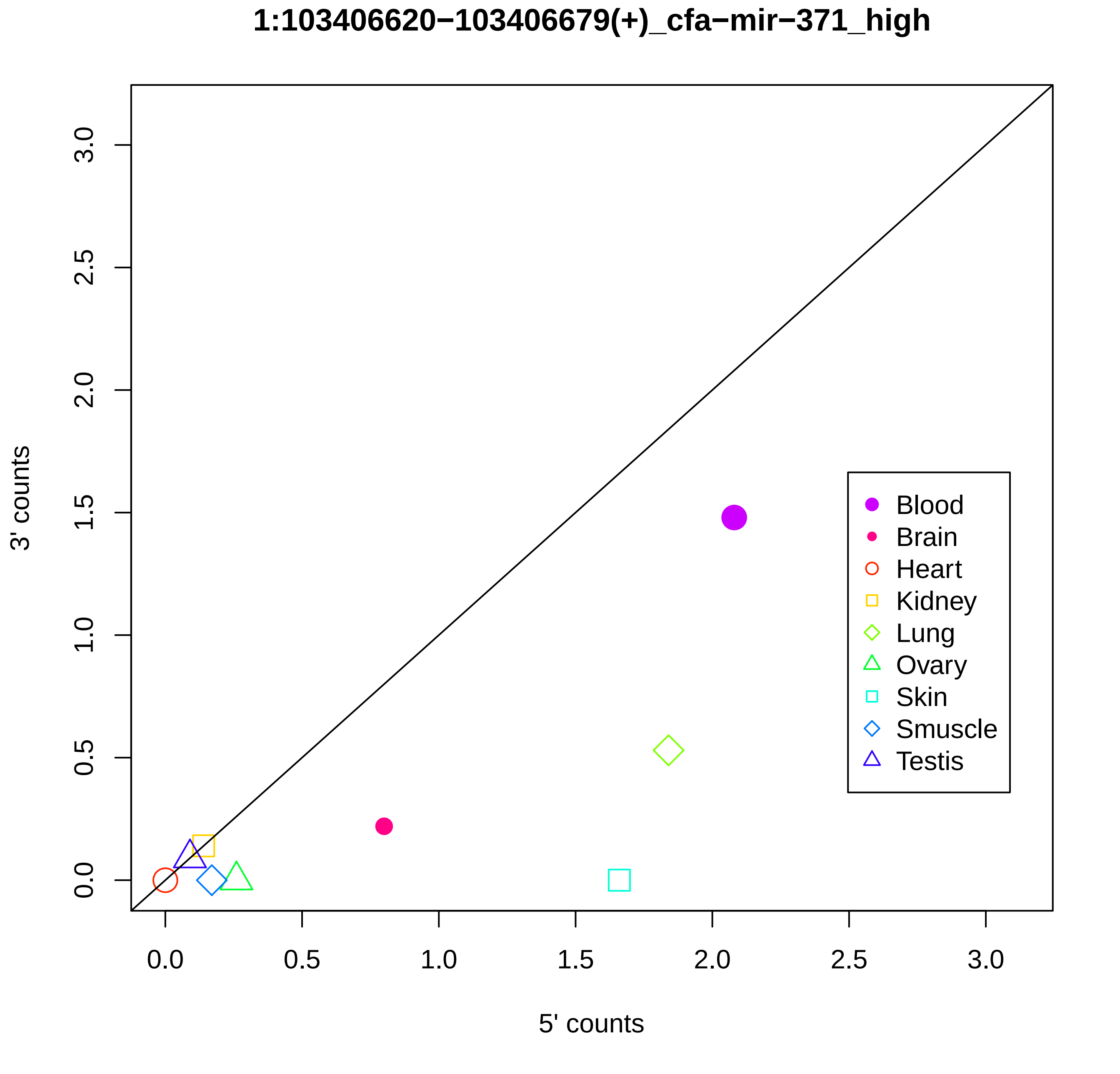
1:103406620−103406679(+)_cfa−mir−371_high
3' counts
5' counts
0.0
0.5
1.0
1.5
2.0
2.5
3.0
Blood
Brain
Heart
Kidney
Lung
Ovary
Skin
Smuscle
Testis

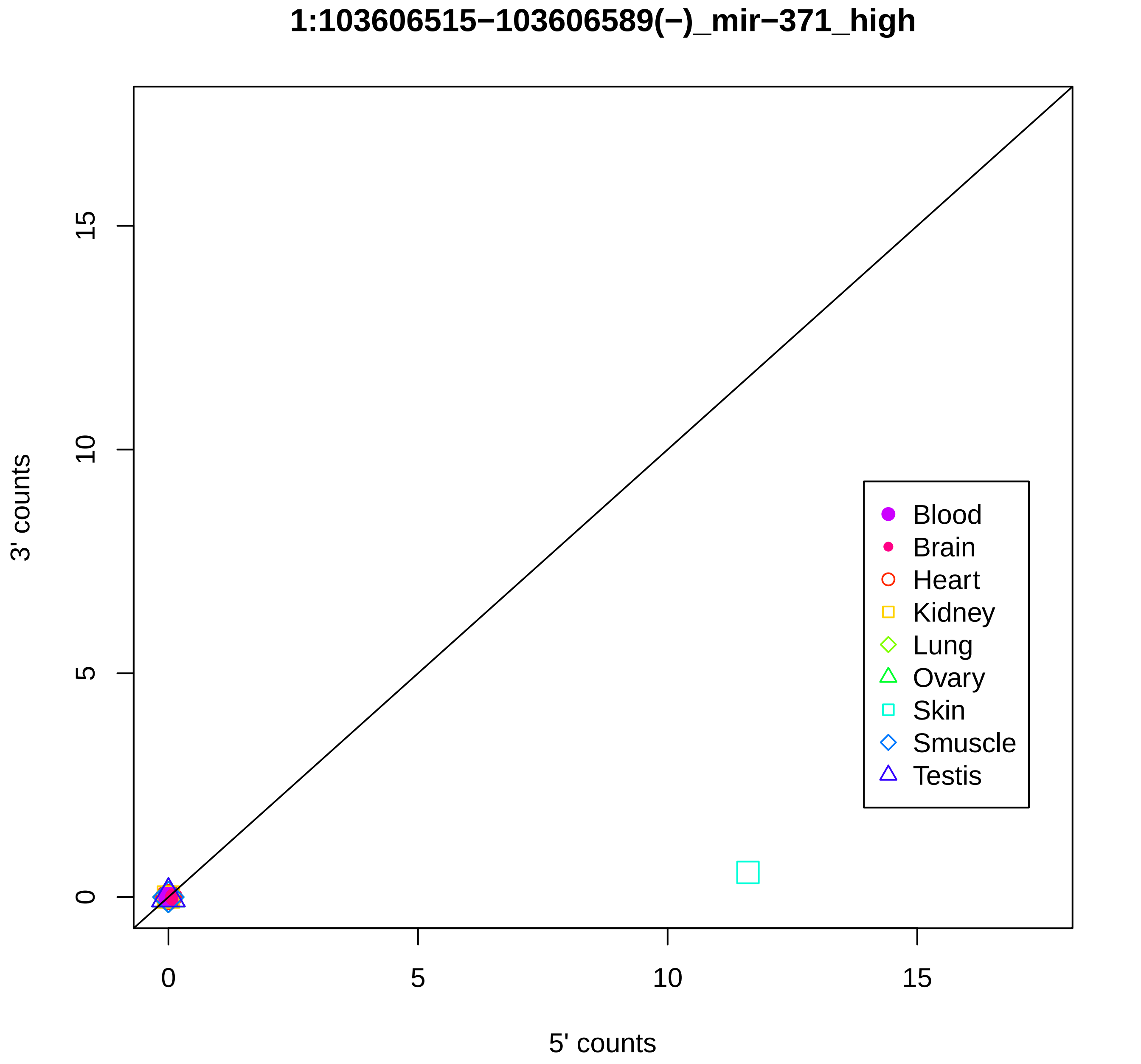

1:103606515−103606589(−)_mir−371_high
3' counts
5' counts
0
5
10
15
0
5
10
15
Blood
Brain
Heart
Kidney
Lung
Ovary
Skin
Smuscle
Testis

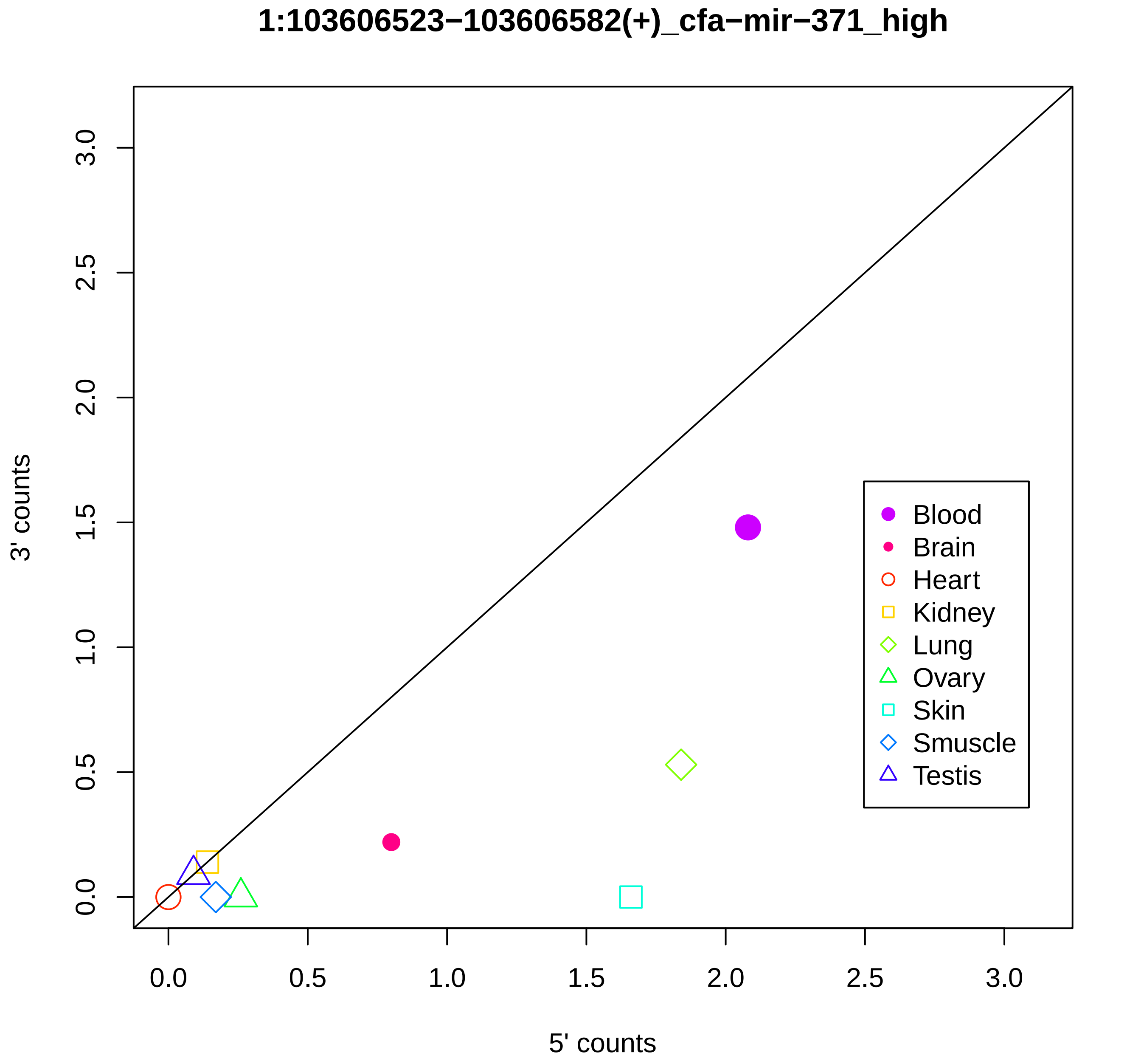

1:103606523−103606582(+)_cfa−mir−371_high
3' counts
5' counts
0.0
0.5
1.0
1.5
2.0
2.5
3.0
Blood
Brain
Heart
Kidney
Lung
Ovary
Skin
Smuscle
Testis

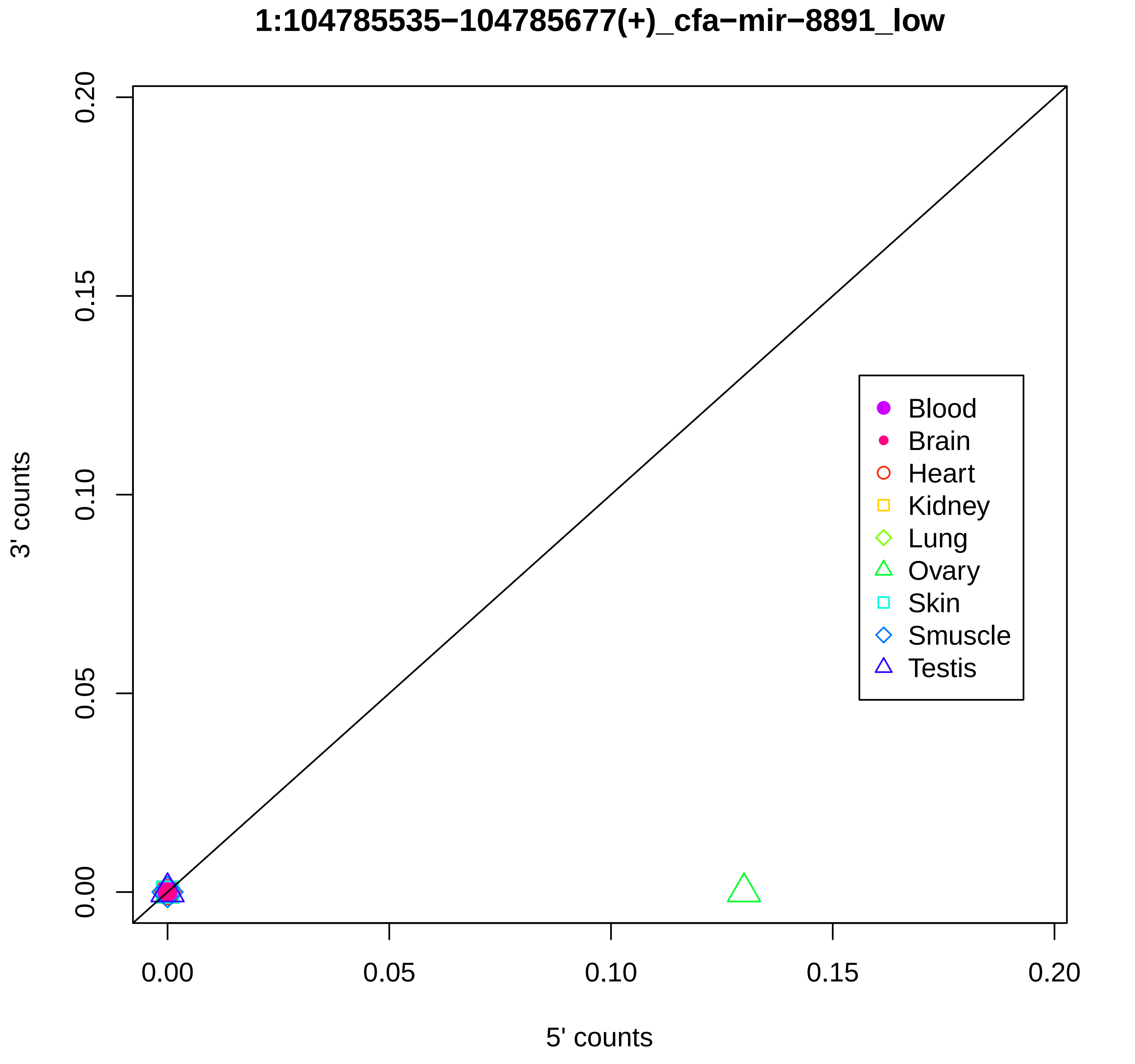

1:104785535−104785677(+)_cfa−mir−8891_low
3' counts
5' counts
0.00
0.05
0.10
0.15
0.20
0.00
0.05
0.10
0.15
0.20
Blood
Brain
Heart
Kidney
Lung
Ovary
Skin
Smuscle
Testis

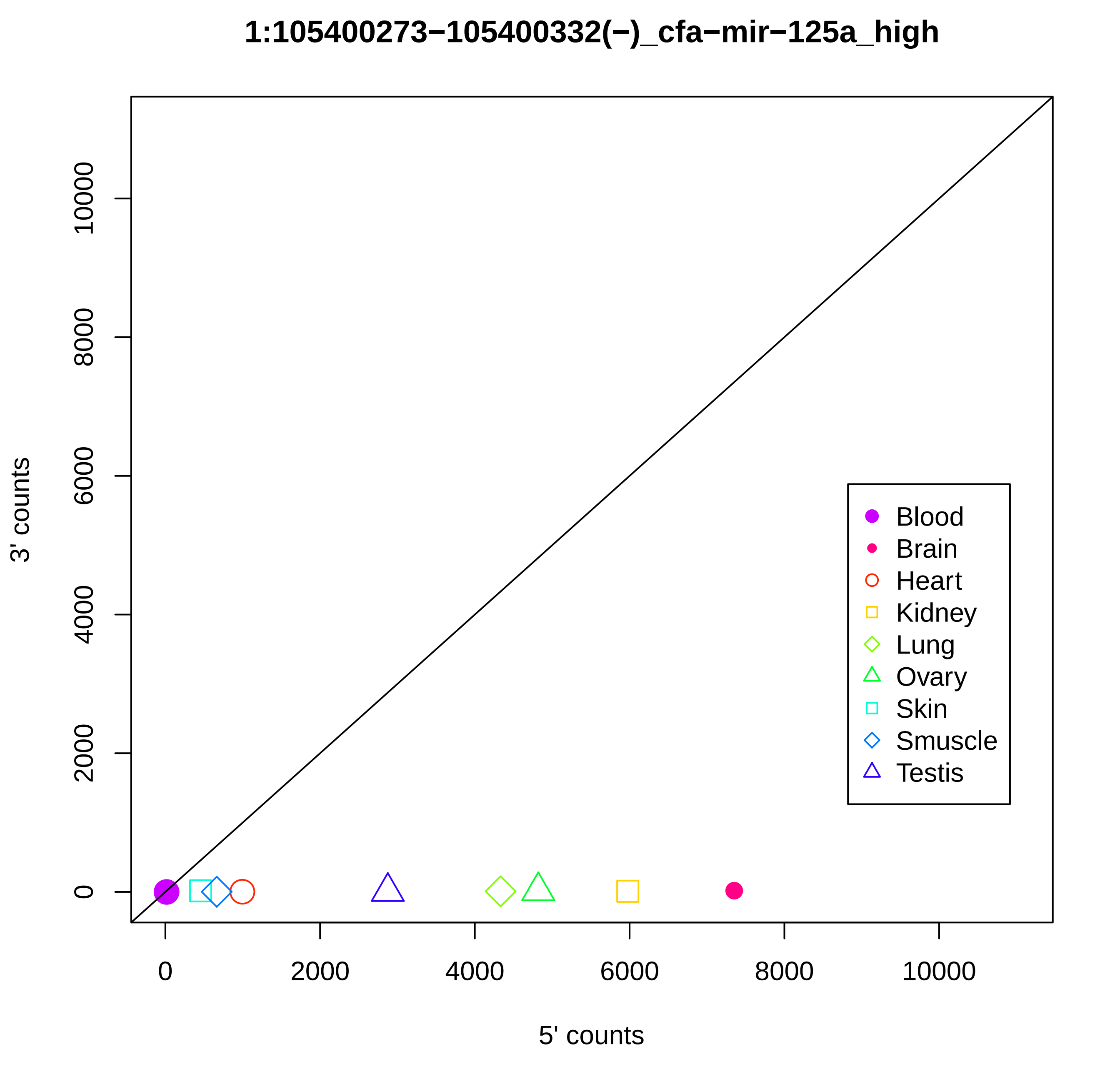

1:105400273−105400332(−)_cfa−mir−125a_high
3' counts
5' counts
0
2000
4000
6000
8000
10000
Blood
Brain
Heart
Kidney
Lung
Ovary
Skin
Smuscle
Testis

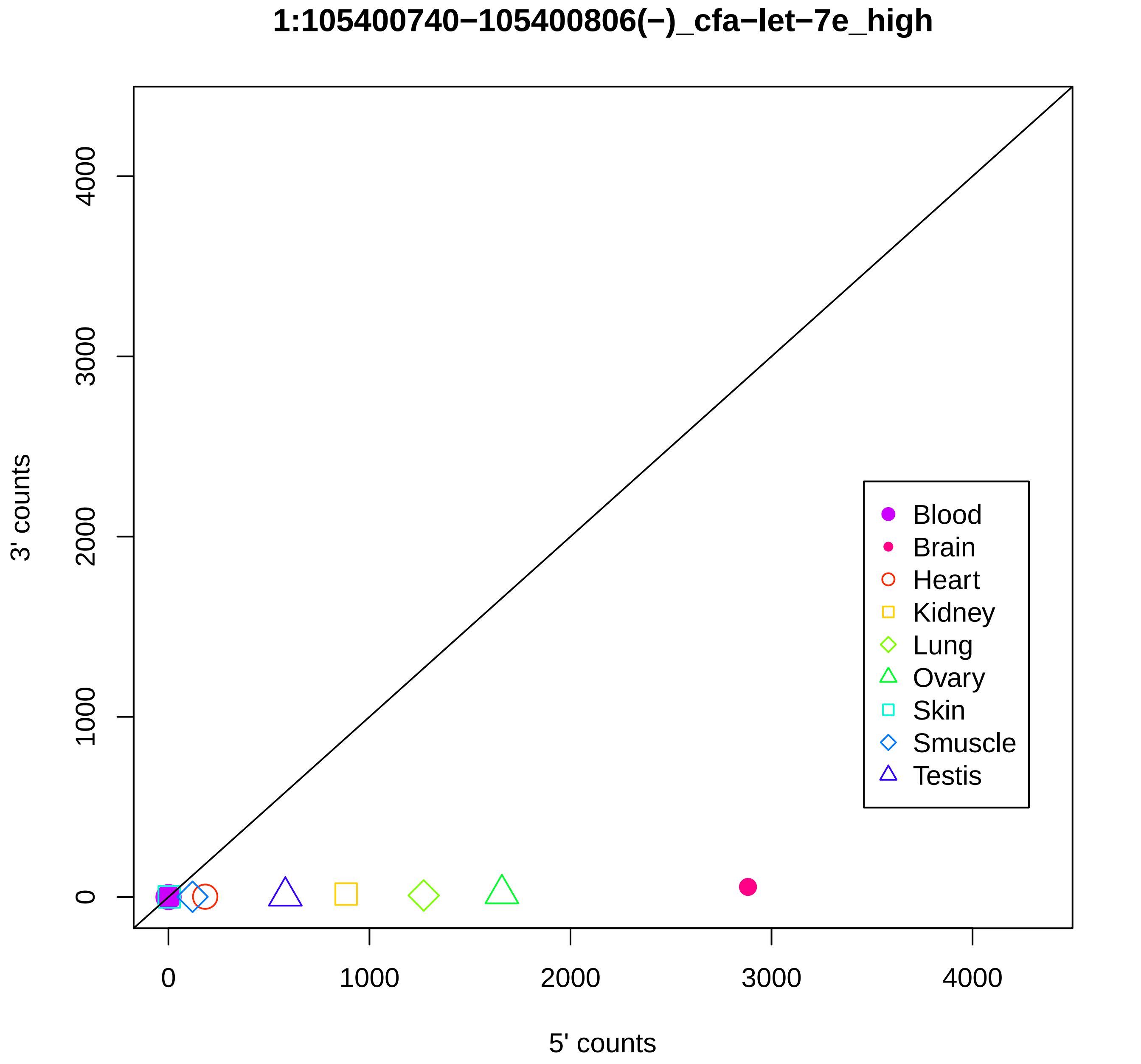

1:105400740−105400806(−)_cfa−let−7e_high
3' counts
5' counts
0
1000
2000
3000
4000
0
1000
2000
3000
4000
Blood
Brain
Heart
Kidney
Lung
Ovary
Skin
Smuscle
Testis

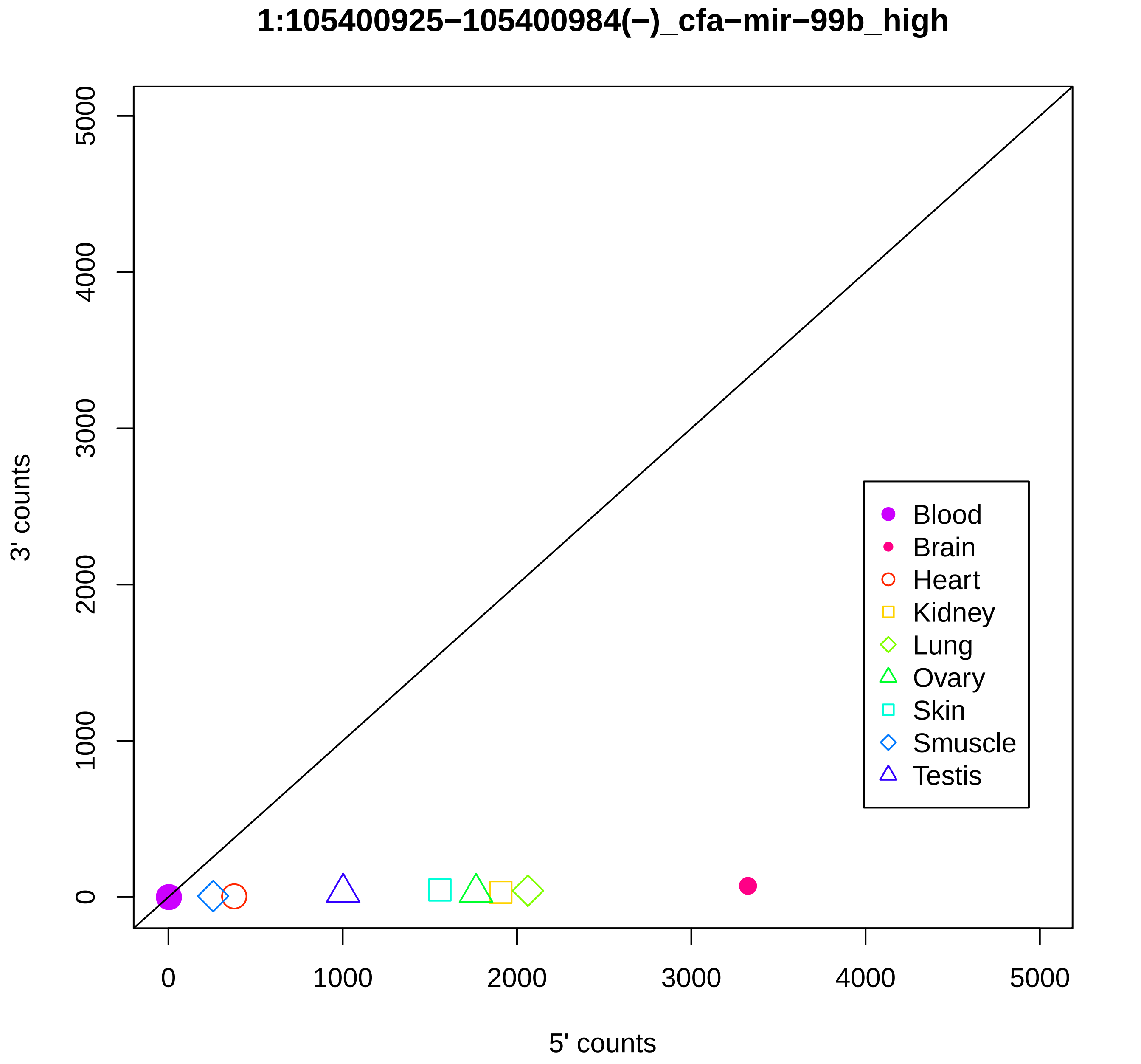

1:105400925−105400984(−)_cfa−mir−99b_high
3' counts
5' counts
0
1000
2000
3000
4000
5000
Blood
Brain
Heart
Kidney
Lung
Ovary
Skin
Smuscle
Testis

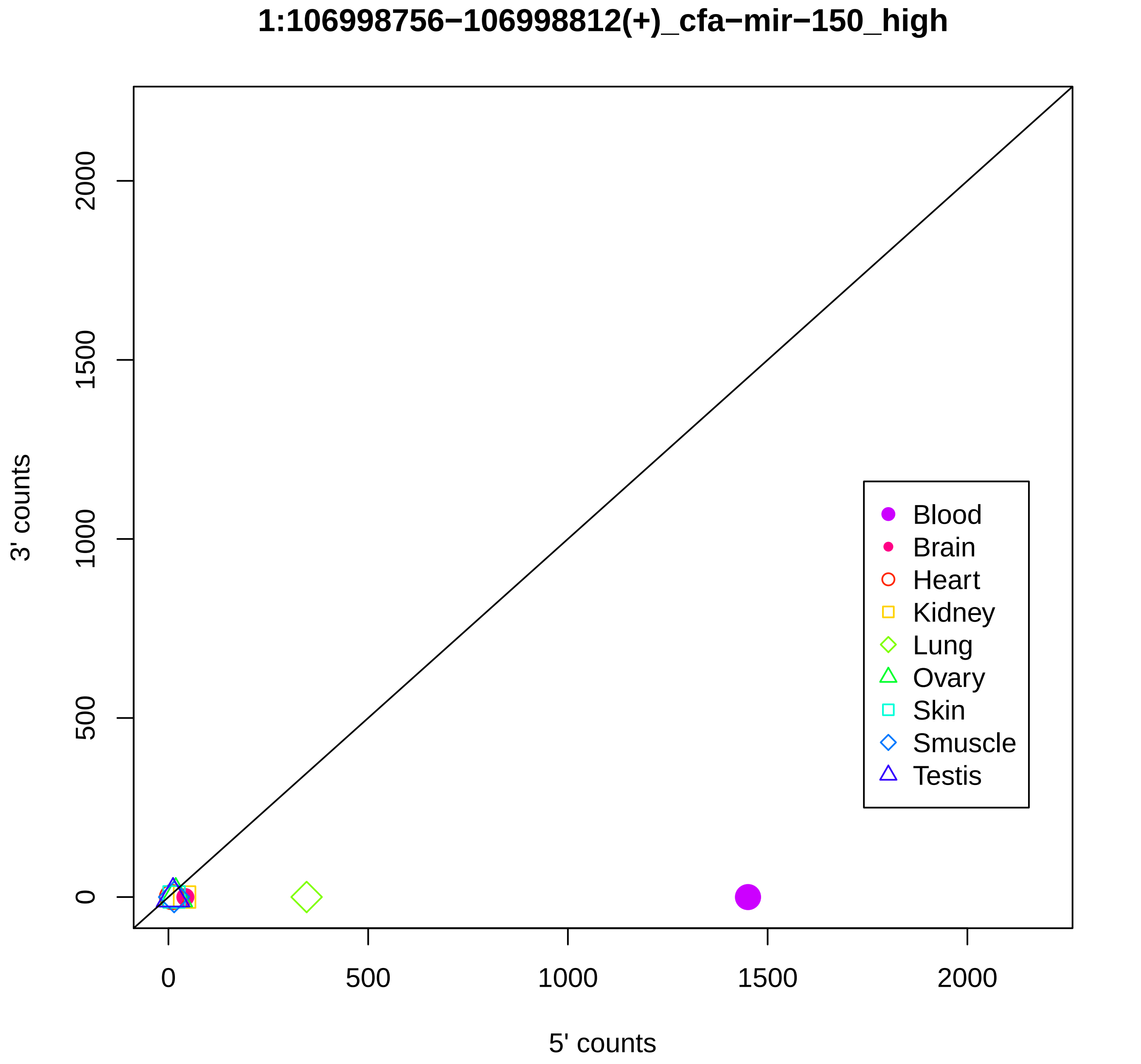

1:106998756−106998812(+)_cfa−mir−150_high
3' counts
5' counts
0
500
1000
1500
2000
Blood
Brain
Heart
Kidney
Lung
Ovary
Skin
Smuscle
Testis

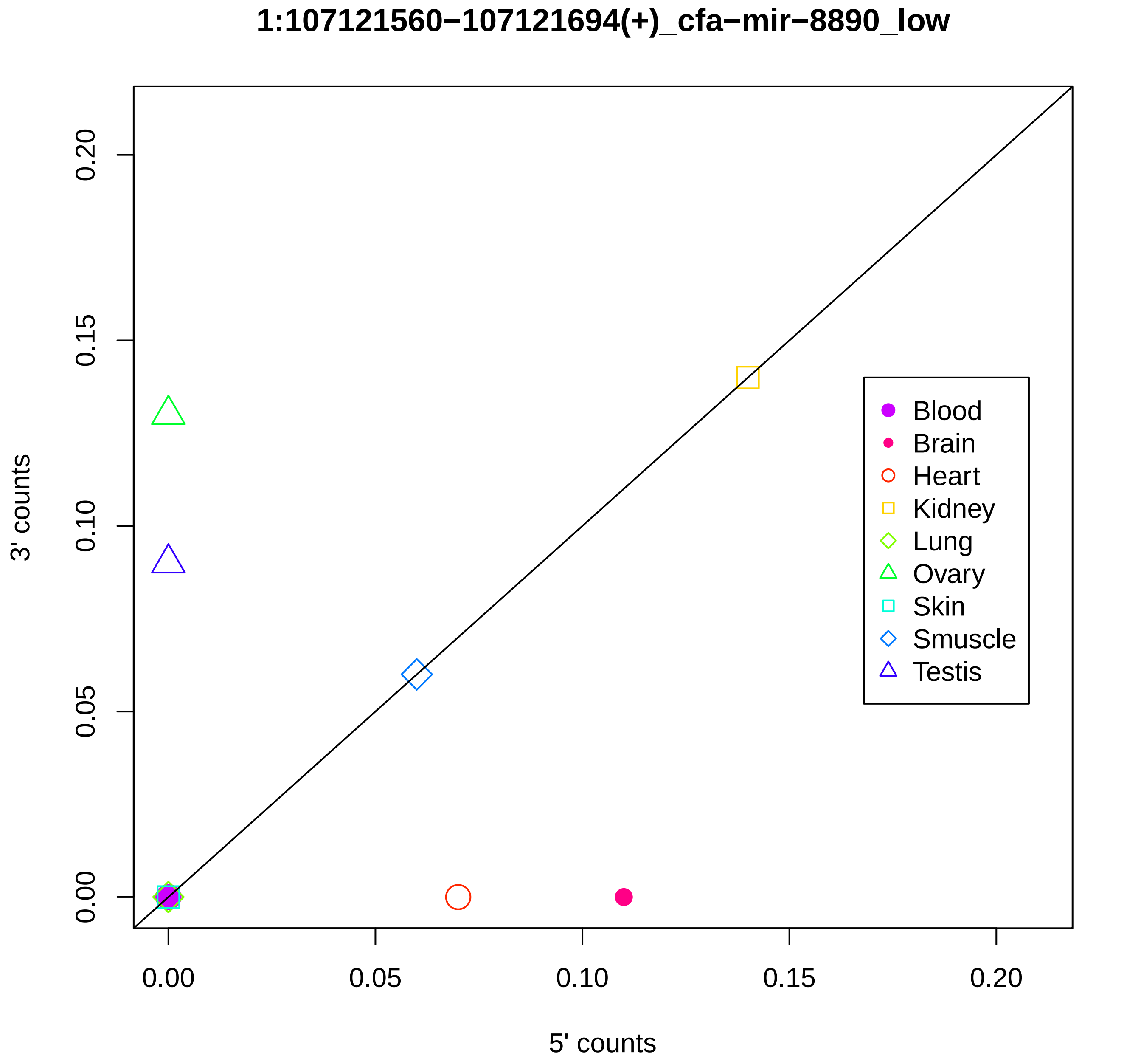
1:107121560−107121694(+)_cfa−mir−8890_low
3' counts
5' counts
0.00
0.05
0.10
0.15
0.20
Blood
Brain
Heart
Kidney
Lung
Ovary
Skin
Smuscle
Testis

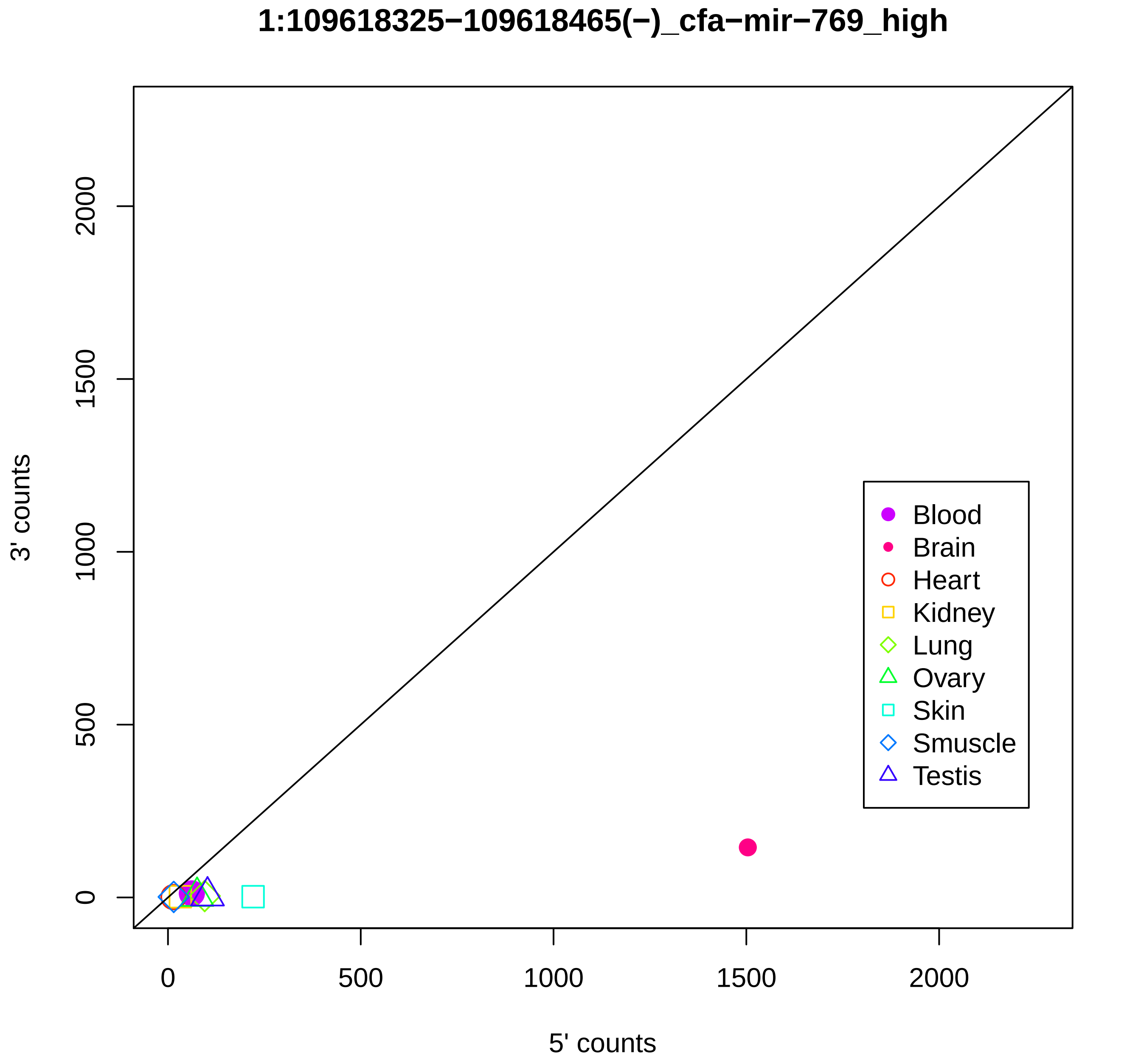

1:109618325−109618465(−)_cfa−mir−769_high
3' counts
5' counts
0
500
1000
1500
2000
0
500
1000
1500
2000
Blood
Brain
Heart
Kidney
Lung
Ovary
Skin
Smuscle
Testis

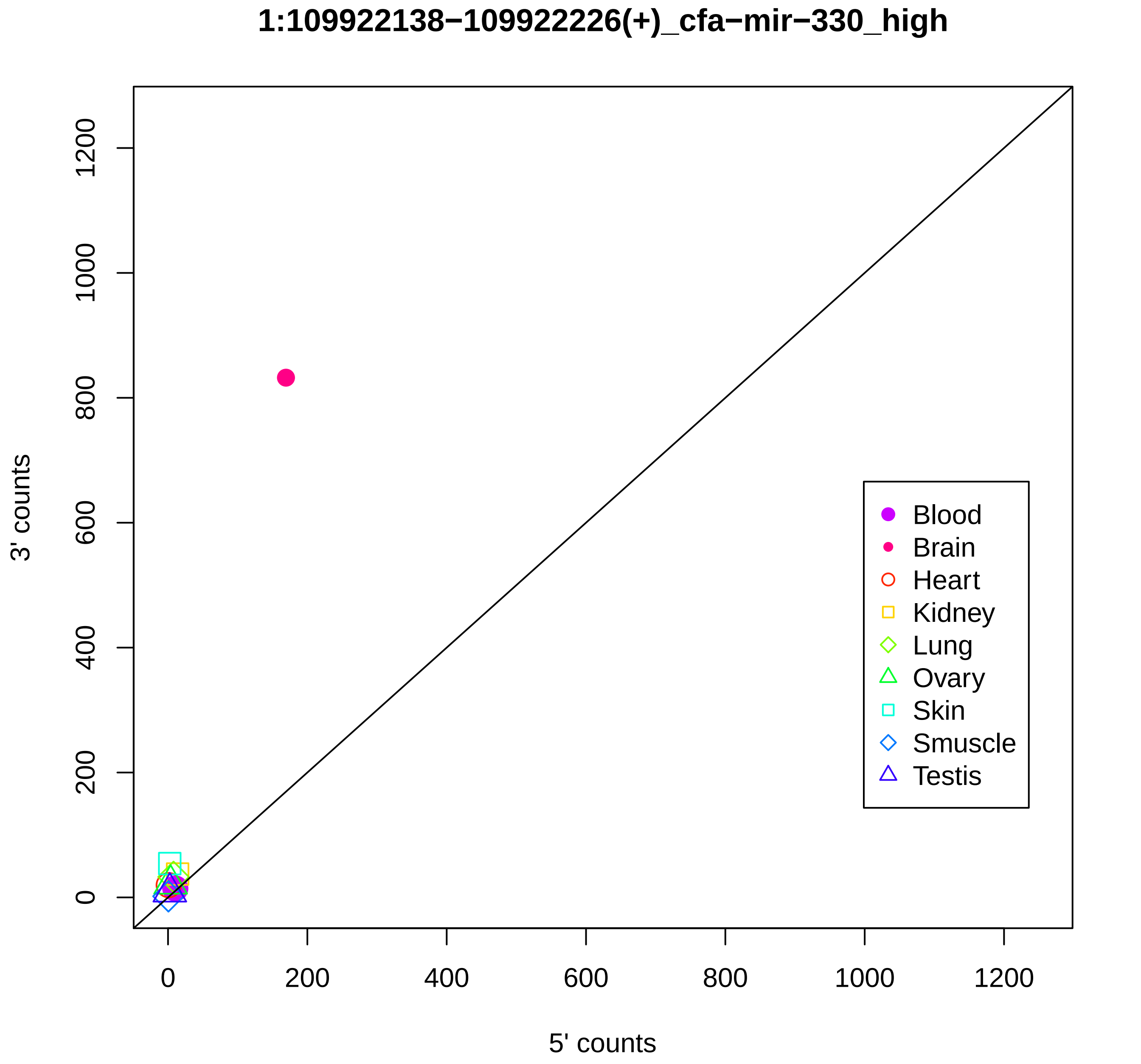

1:109922138−109922226(+)_cfa−mir−330_high
3' counts
5' counts
0
200
400
600
800
1000
1200
Blood
Brain
Heart
Kidney
Lung
Ovary
Skin
Smuscle
Testis

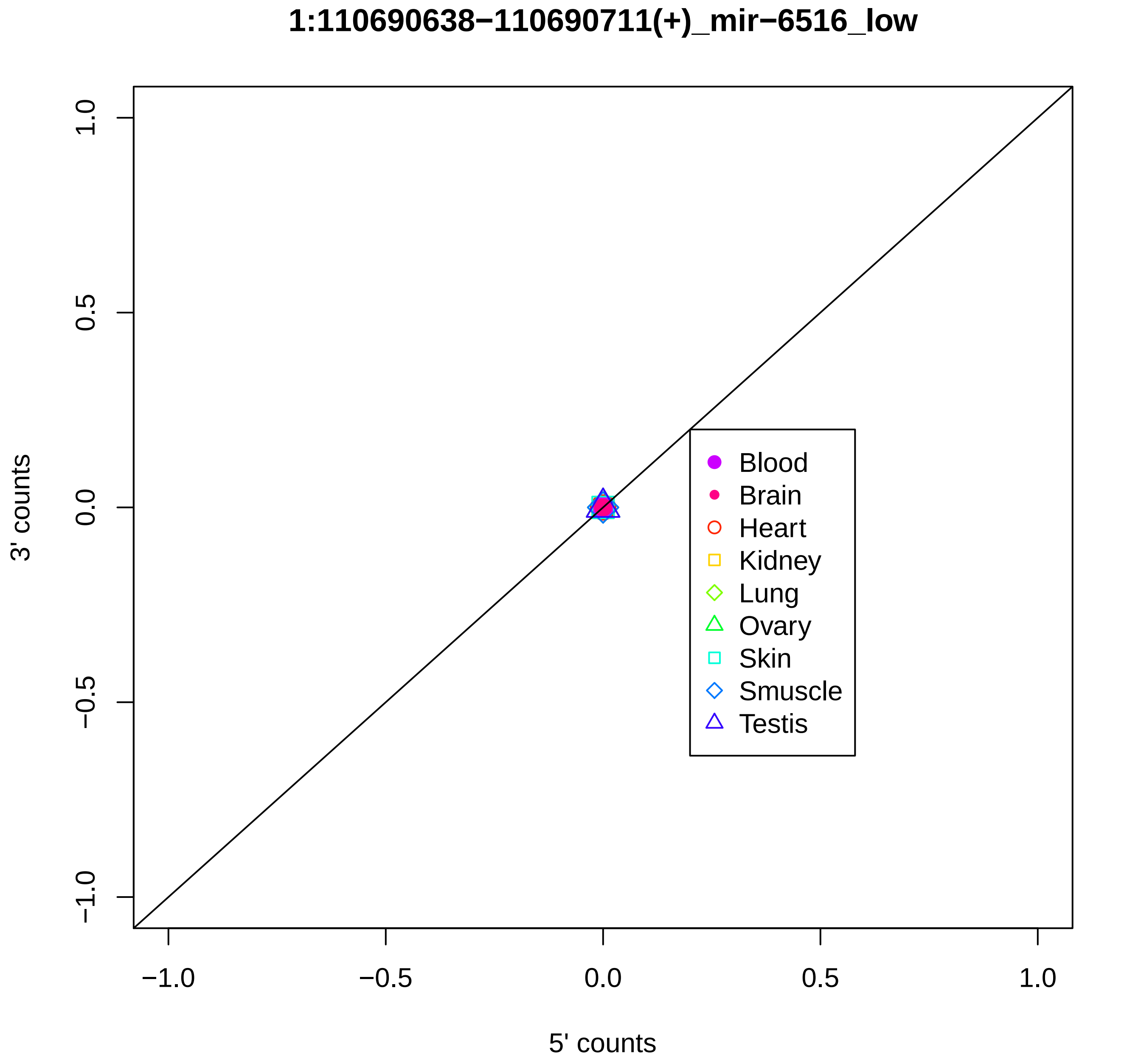
1:110690638−110690711(+)_mir−6516_low
3' counts
5' counts
1.0
0.5
0.0
−0.5
−1.0
Blood
Brain
Heart
Kidney
Lung
Ovary
Skin
Smuscle
Testis

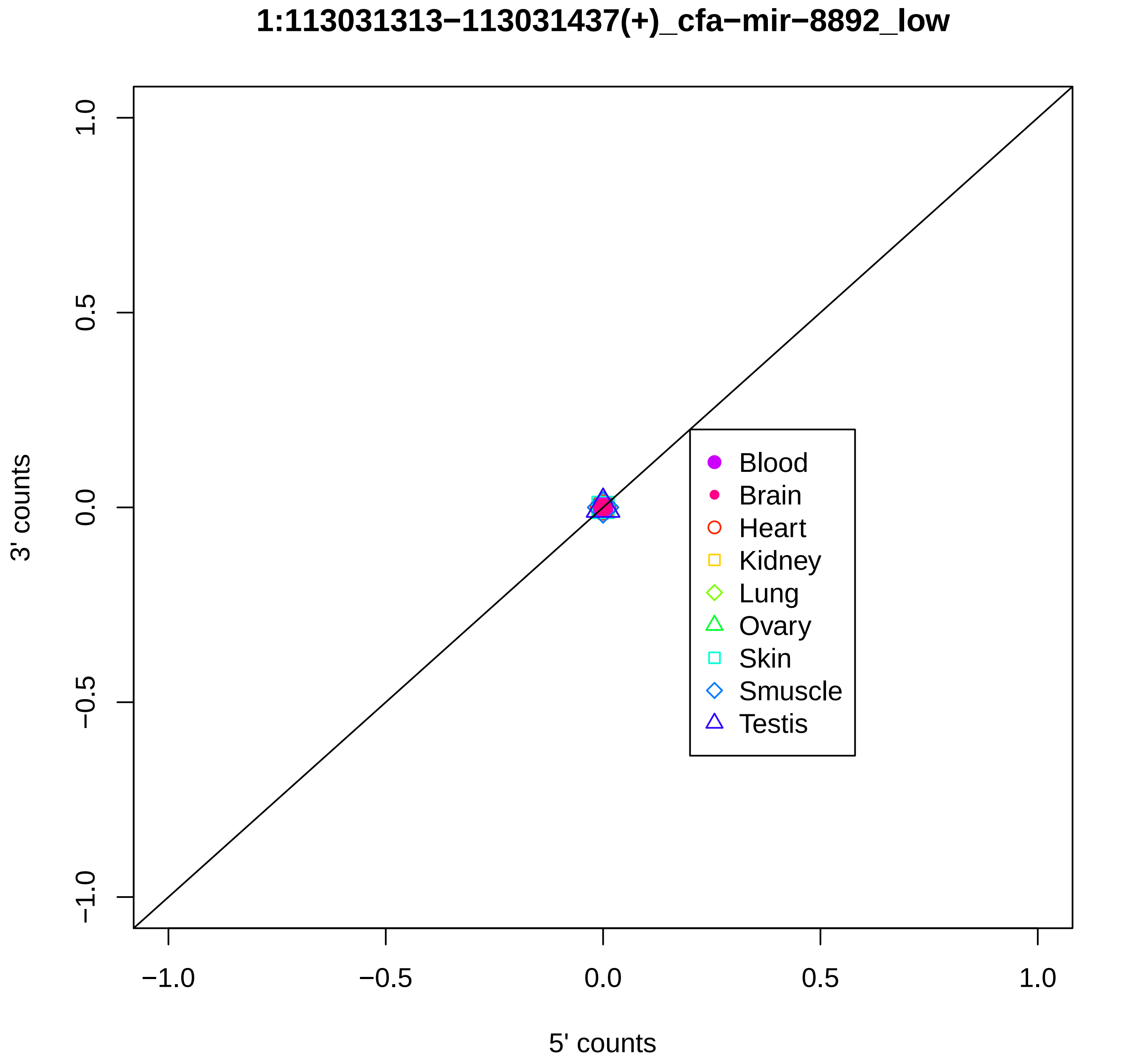

1:113031313−113031437(+)_cfa−mir−8892_low
3' counts
5' counts
−1.0
−0.5
0.0
0.5
1.0
Blood
Brain
Heart
Kidney
Lung
Ovary
Skin
Smuscle
Testis

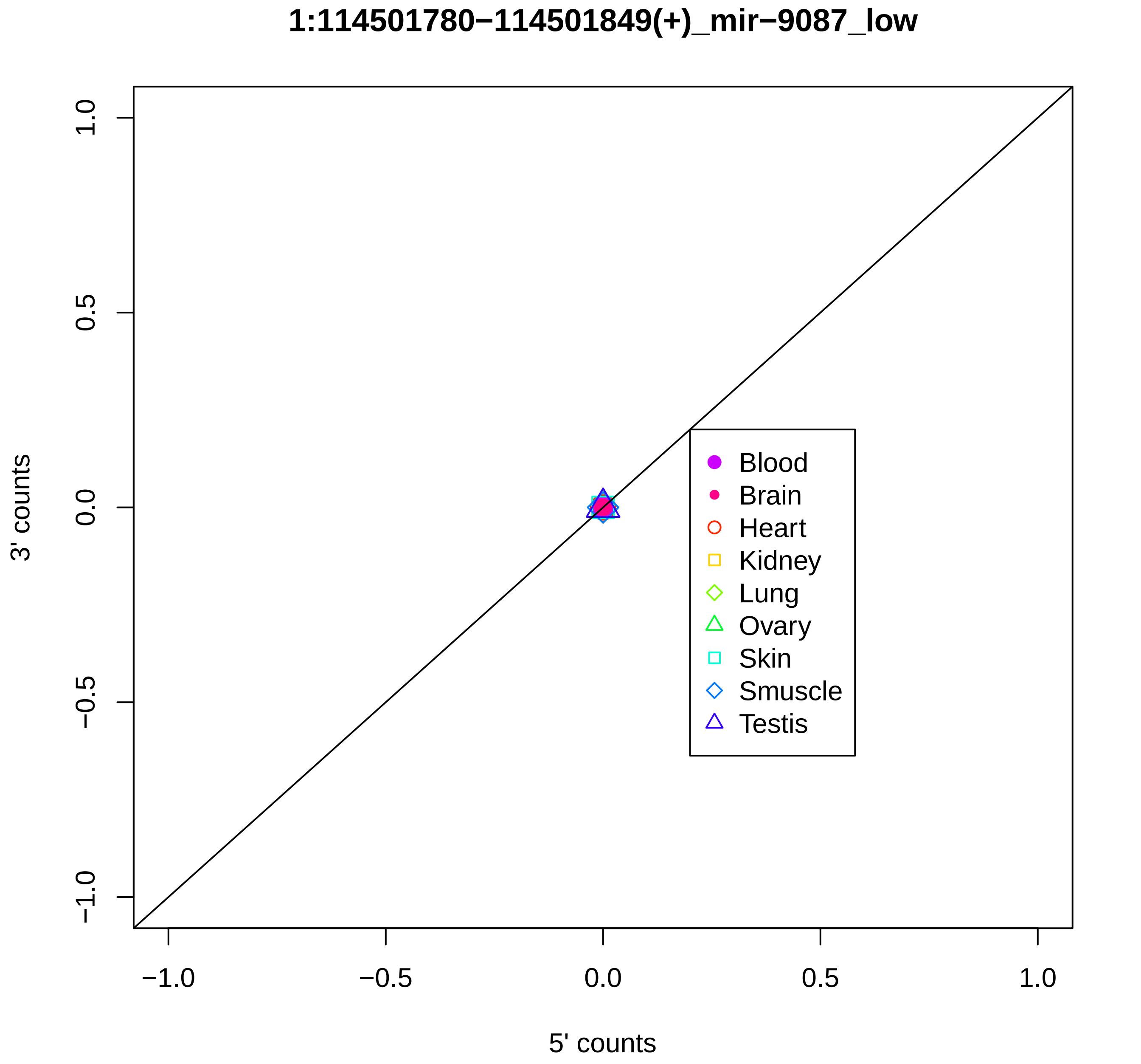
1:114501780−114501849(+)_mir−9087_low
3' counts
5' counts
1.0
0.5
0.0
−0.5
−1.0
Blood
Brain
Heart
Kidney
Lung
Ovary
Skin
Smuscle
Testis

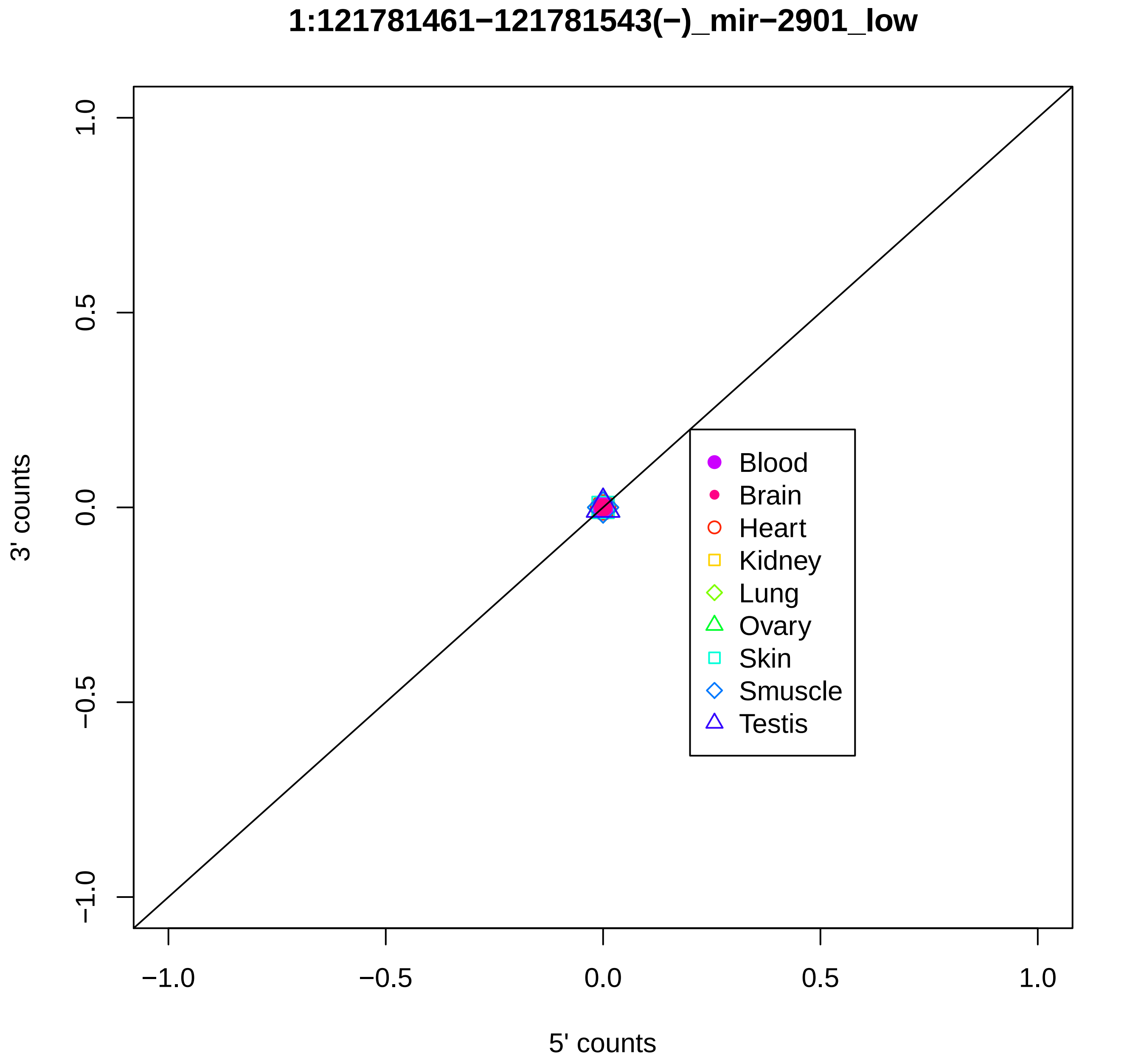

1:121781461−121781543(−)_mir−2901_low
3' counts
5' counts
−1.0
−0.5
0.0
0.5
1.0
Blood
Brain
Heart
Kidney
Lung
Ovary
Skin
Smuscle
Testis

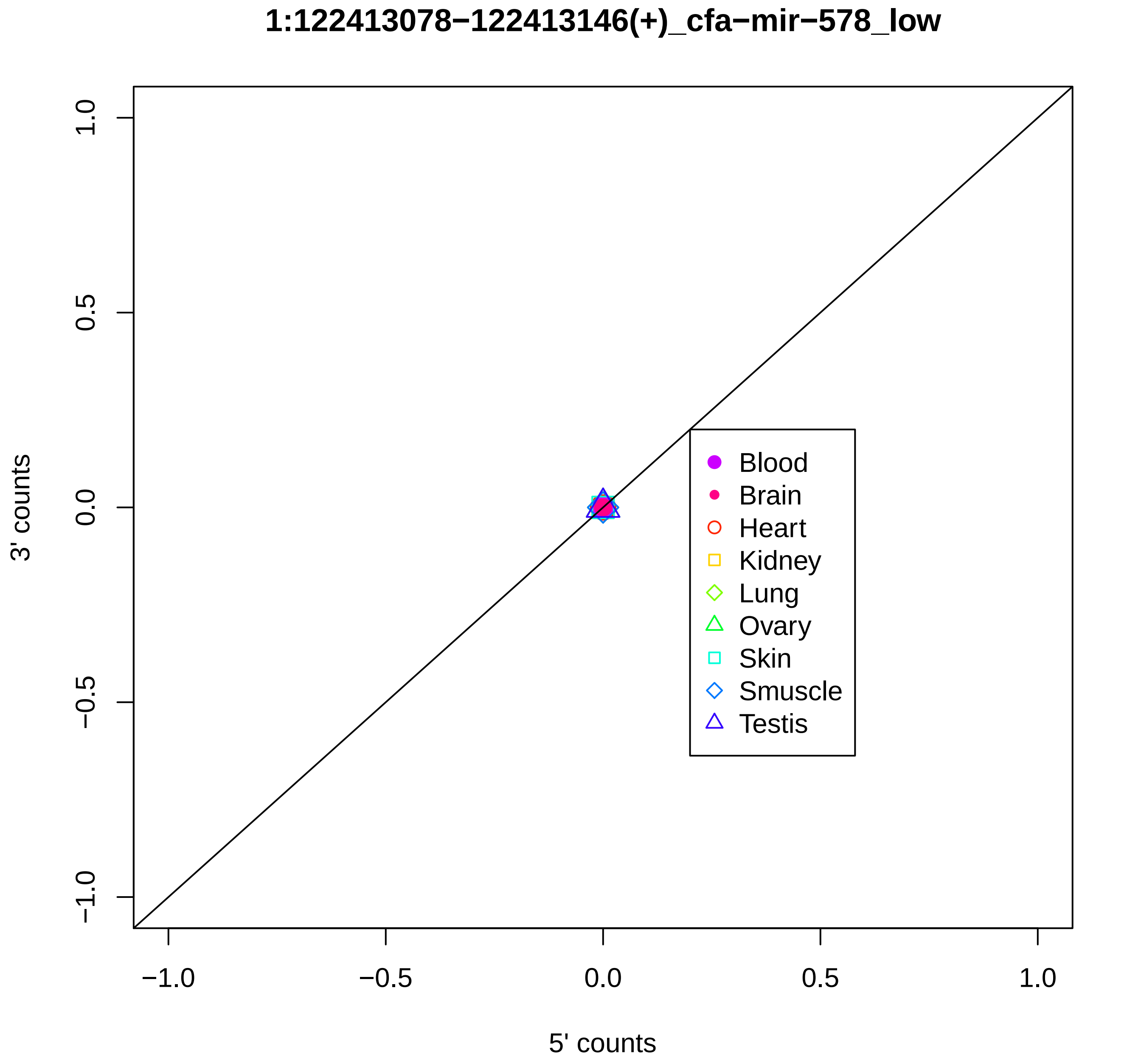

1:122413078−122413146(+)_cfa−mir−578_low
1.0
0.5
0.0
−0.5
−1.0
3' counts
−1.0
−0.5
0.0
0.5
1.0
5' counts
Blood
Brain
Heart
Kidney
Lung
Ovary
Skin
Smuscle
Testis

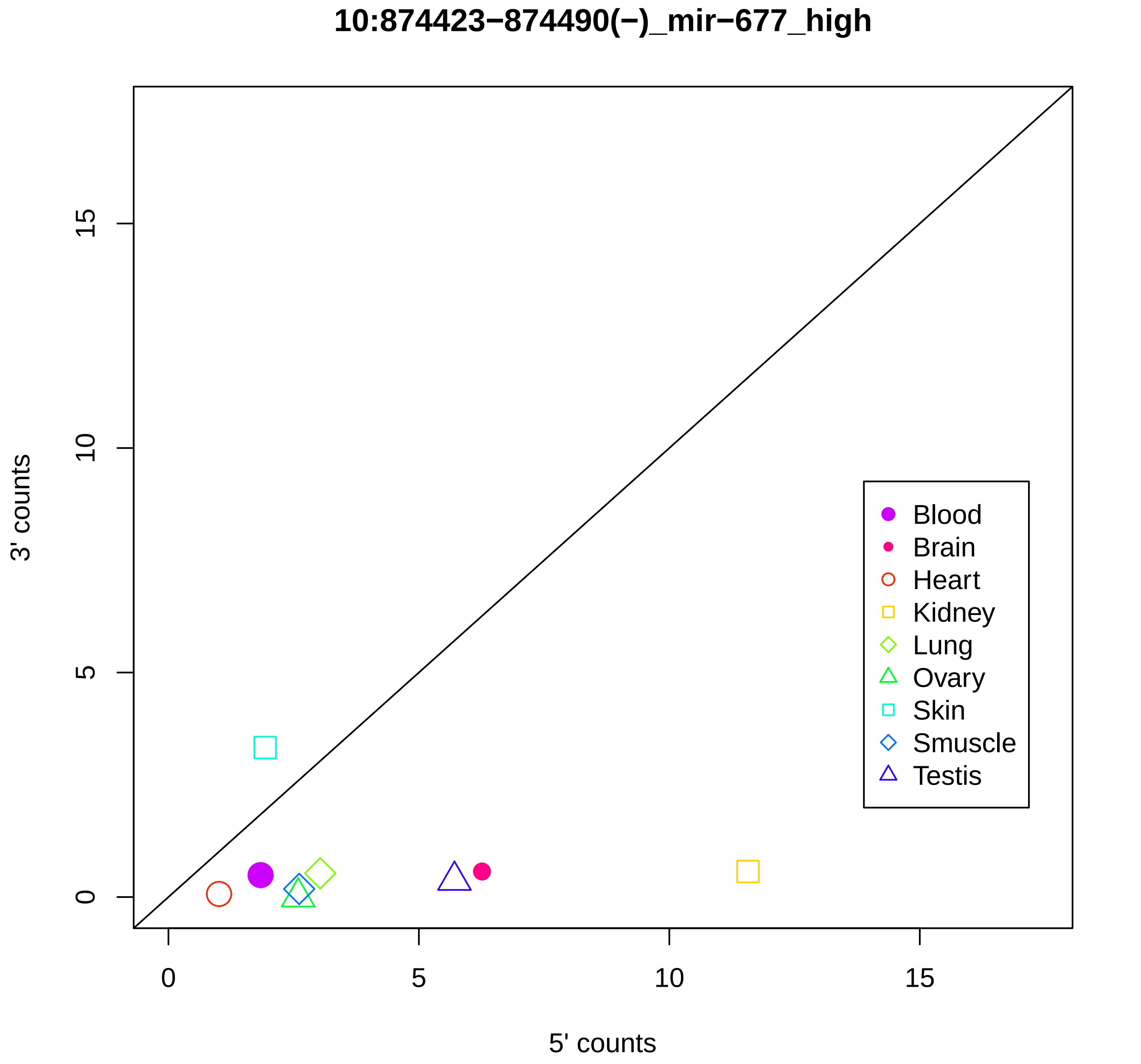

10:874423−874490(−)_mir−677_high
3' counts
5' counts
0
5
10
15
Blood
Brain
Heart
Kidney
Lung
Ovary
Skin
Smuscle
Testis

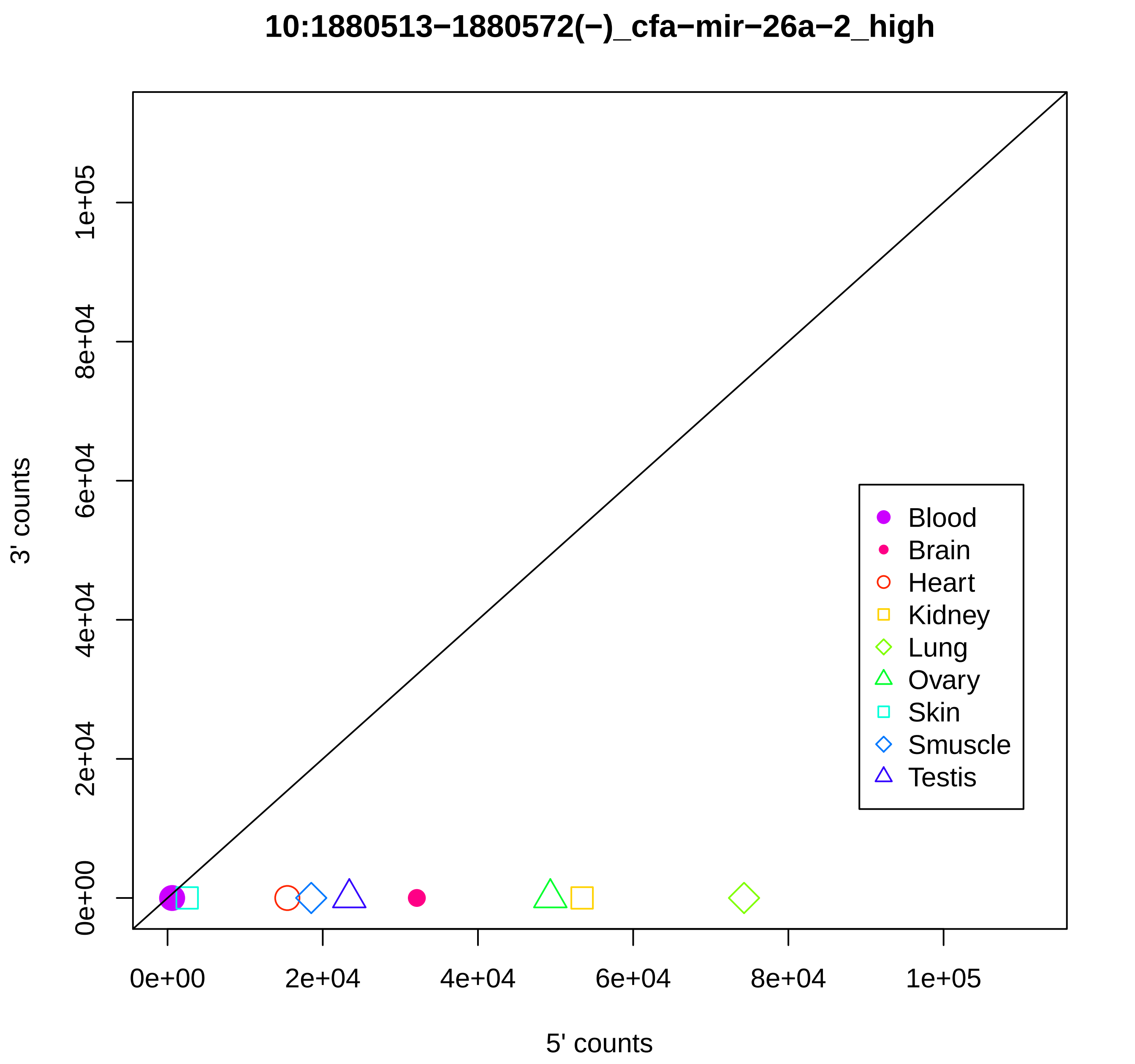

10:1880513−1880572(−)_cfa−mir−26a−2_high
3' counts
5' counts
0e+00
2e+04
4e+04
6e+04
8e+04
1e+05
Blood
Brain
Heart
Kidney
Lung
Ovary
Skin
Smuscle
Testis

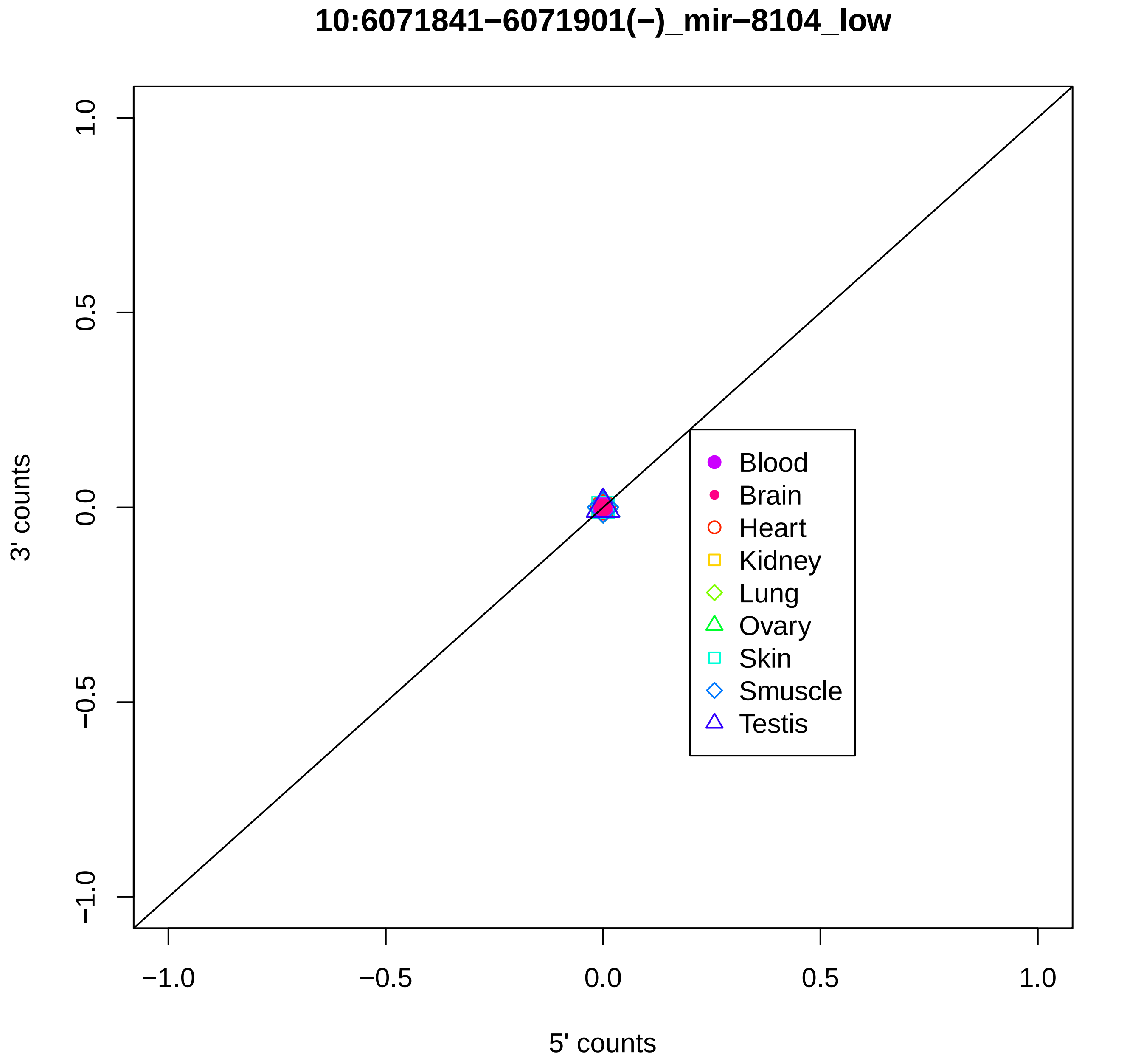

10:6071841−6071901(−)_mir−8104_low
3' counts
5' counts
1.0
0.5
0.0
−0.5
−1.0
Blood
Brain
Heart
Kidney
Lung
Ovary
Skin
Smuscle
Testis

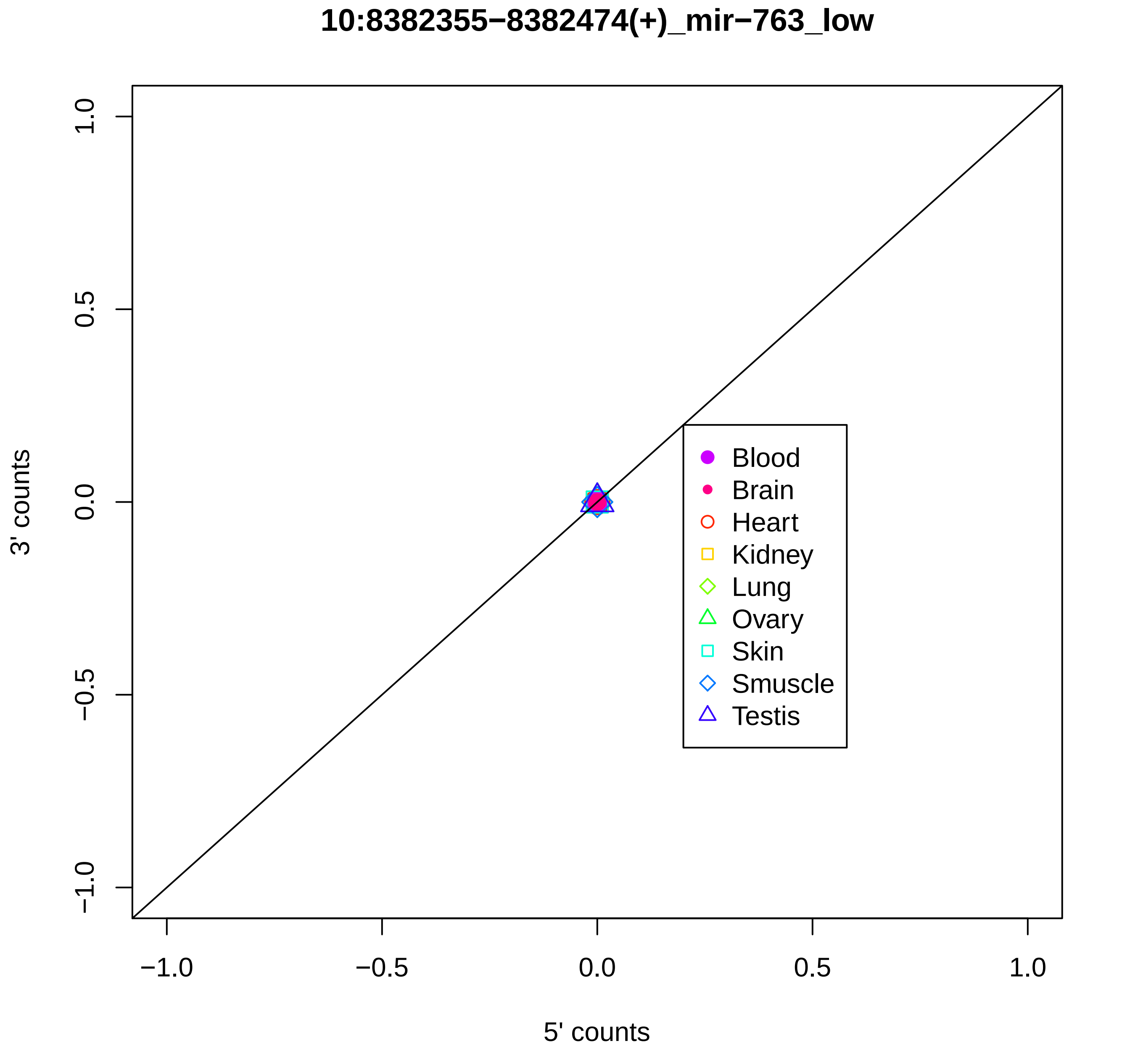

10:8382355−8382474(+)_mir−763_low
3' counts
5' counts
1.0
0.5
0.0
−0.5
−1.0
Blood
Brain
Heart
Kidney
Lung
Ovary
Skin
Smuscle
Testis

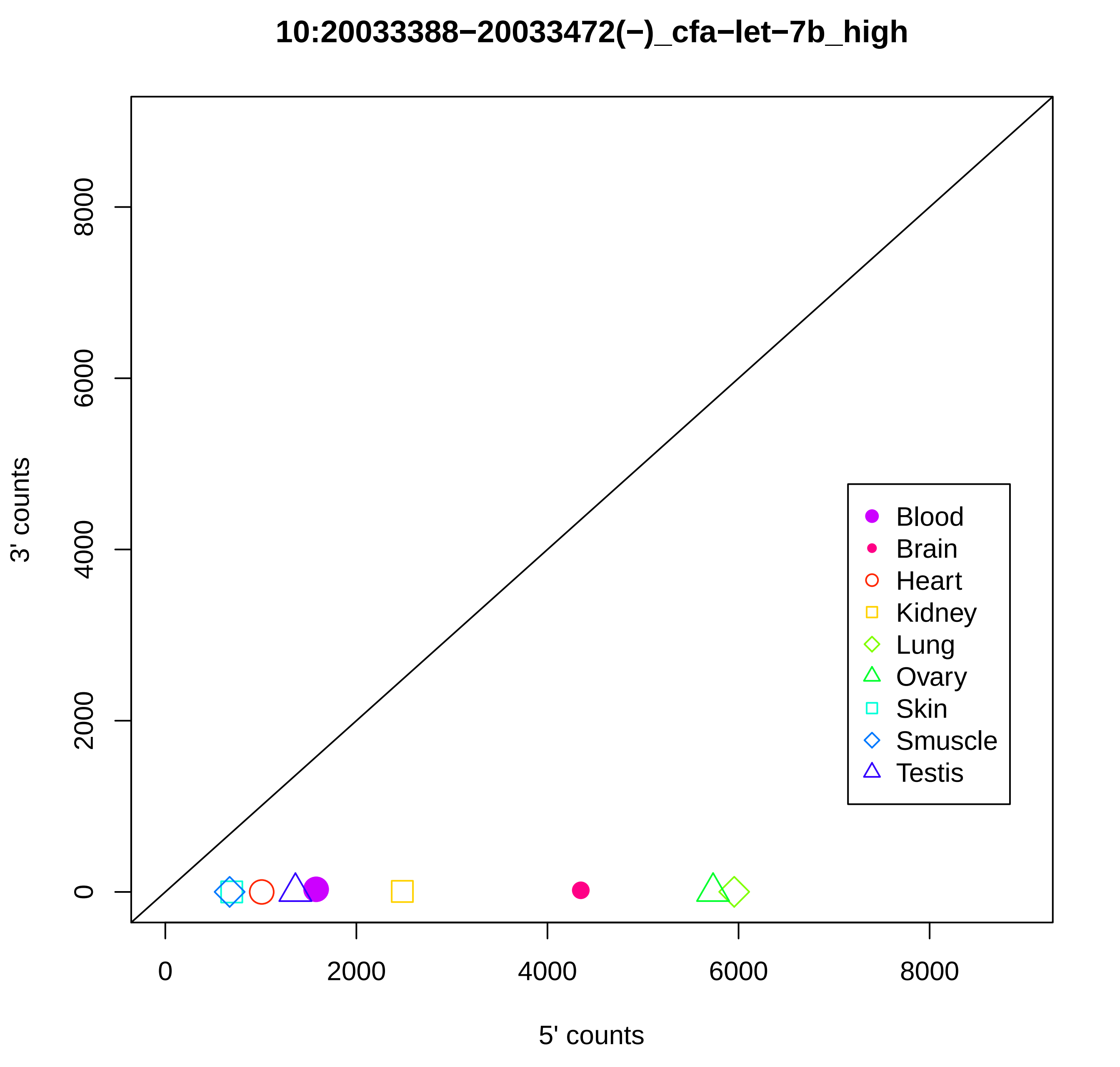
10:20033388−20033472(−)_cfa−let−7b_high
3' counts
5' counts
0
2000
4000
6000
8000
0
2000
4000
6000
8000
Blood
Brain
Heart
Kidney
Lung
Ovary
Skin
Smuscle
Testis

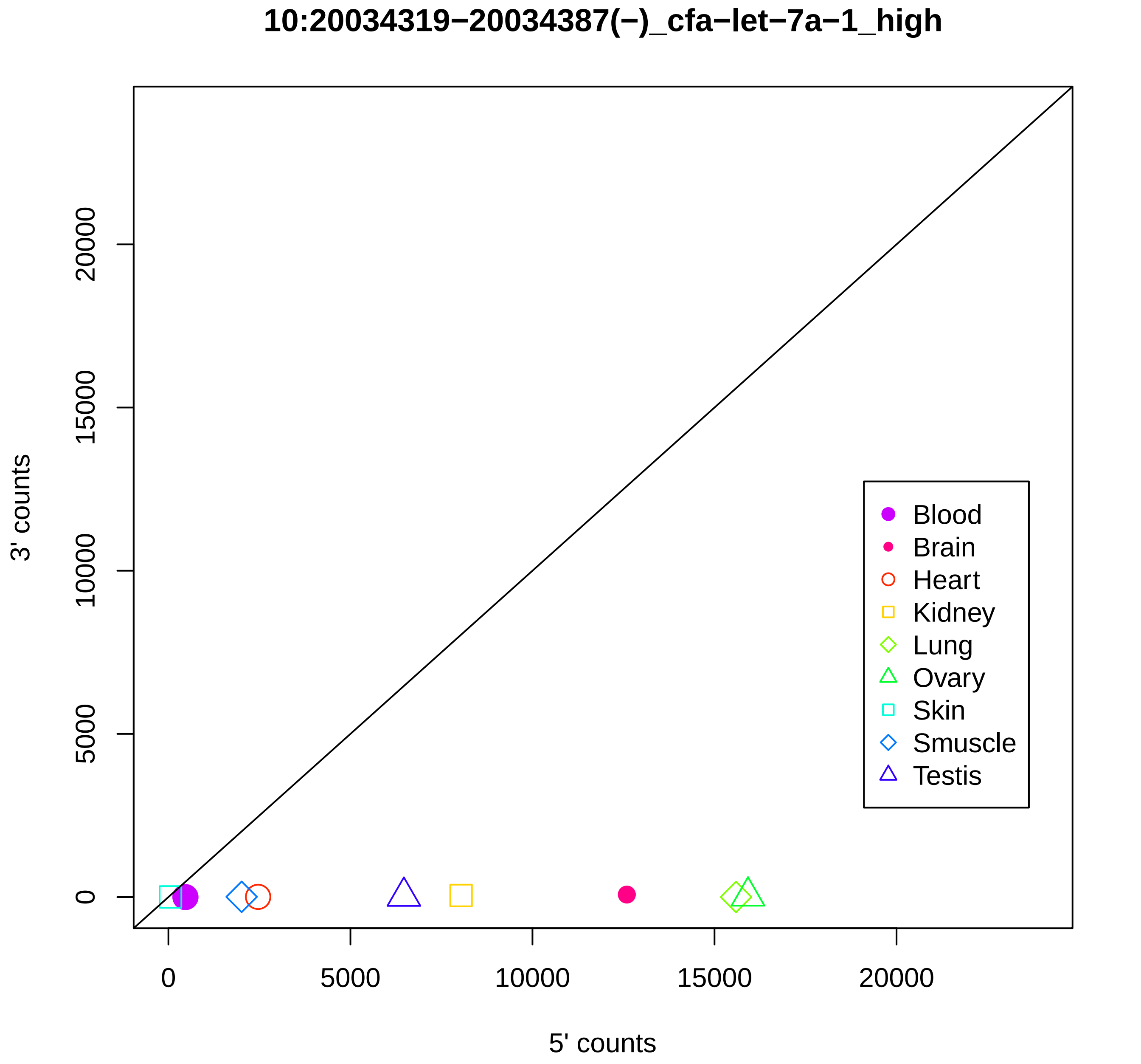
10:20034319−20034387(−)_cfa−let−7a−1_high
3' counts
0
5000
10000
15000
20000
5' counts
0
5000
10000
15000
20000
Blood
Brain
Heart
Kidney
Lung
Ovary
Skin
Smuscle
Testis

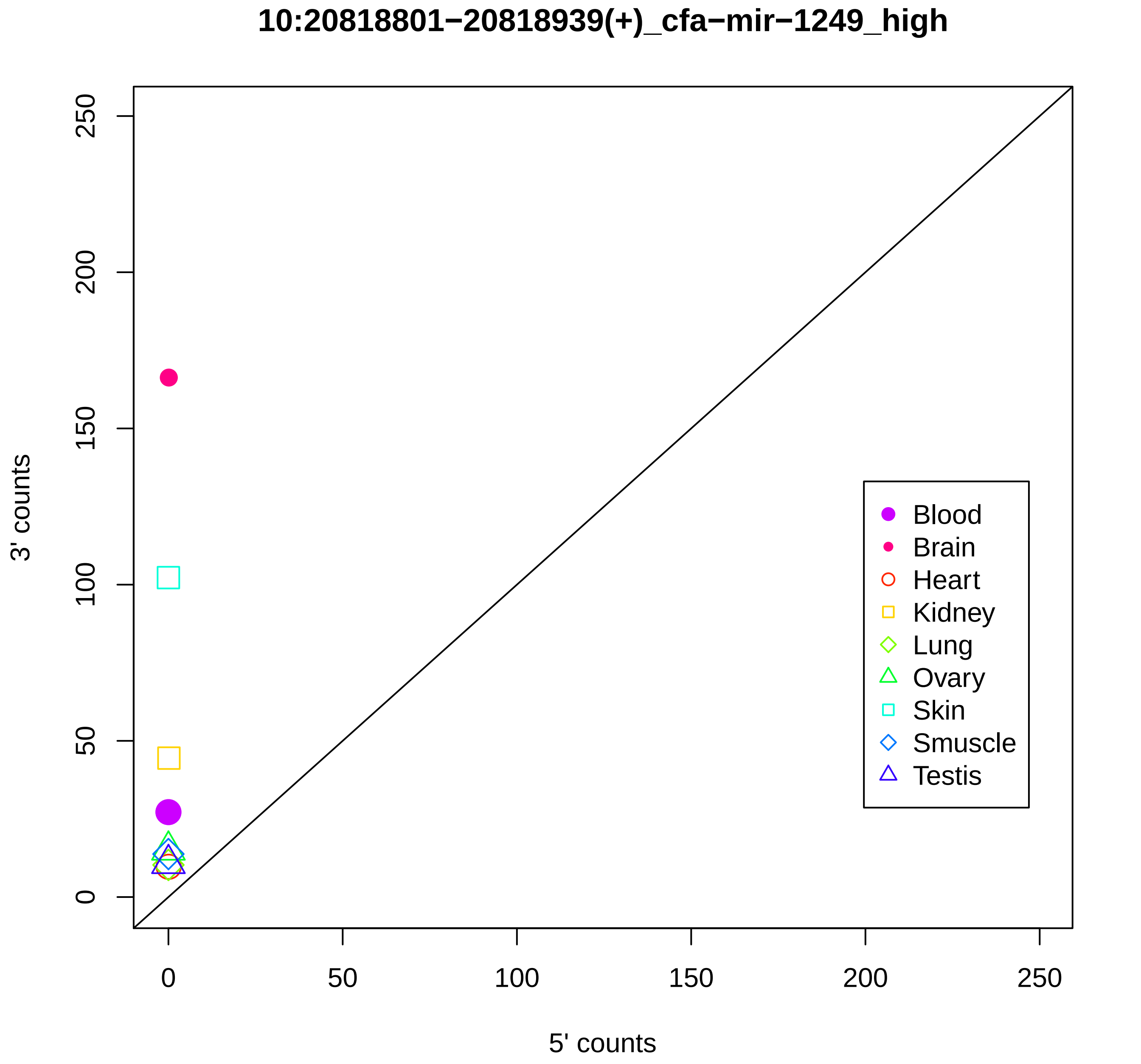
10:20818801−20818939(+)_cfa−mir−1249_high
3' counts
5' counts
0
50
100
150
200
250
Blood
Brain
Heart
Kidney
Lung
Ovary
Skin
Smuscle
Testis

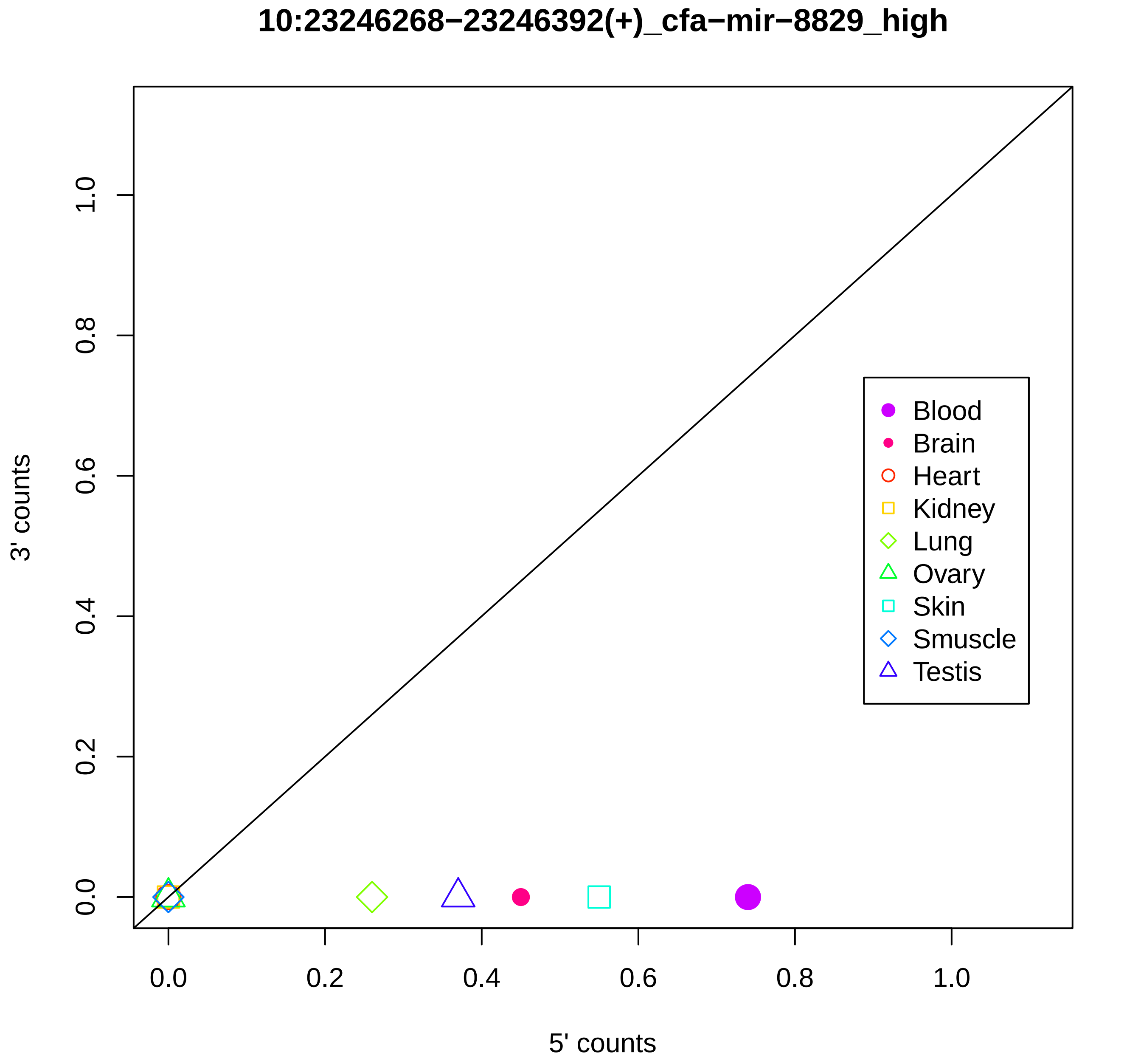

10:23246268−23246392(+)_cfa−mir−8829_high
3' counts
5' counts
0.0
0.2
0.4
0.6
0.8
1.0
Blood
Brain
Heart
Kidney
Lung
Ovary
Skin
Smuscle
Testis

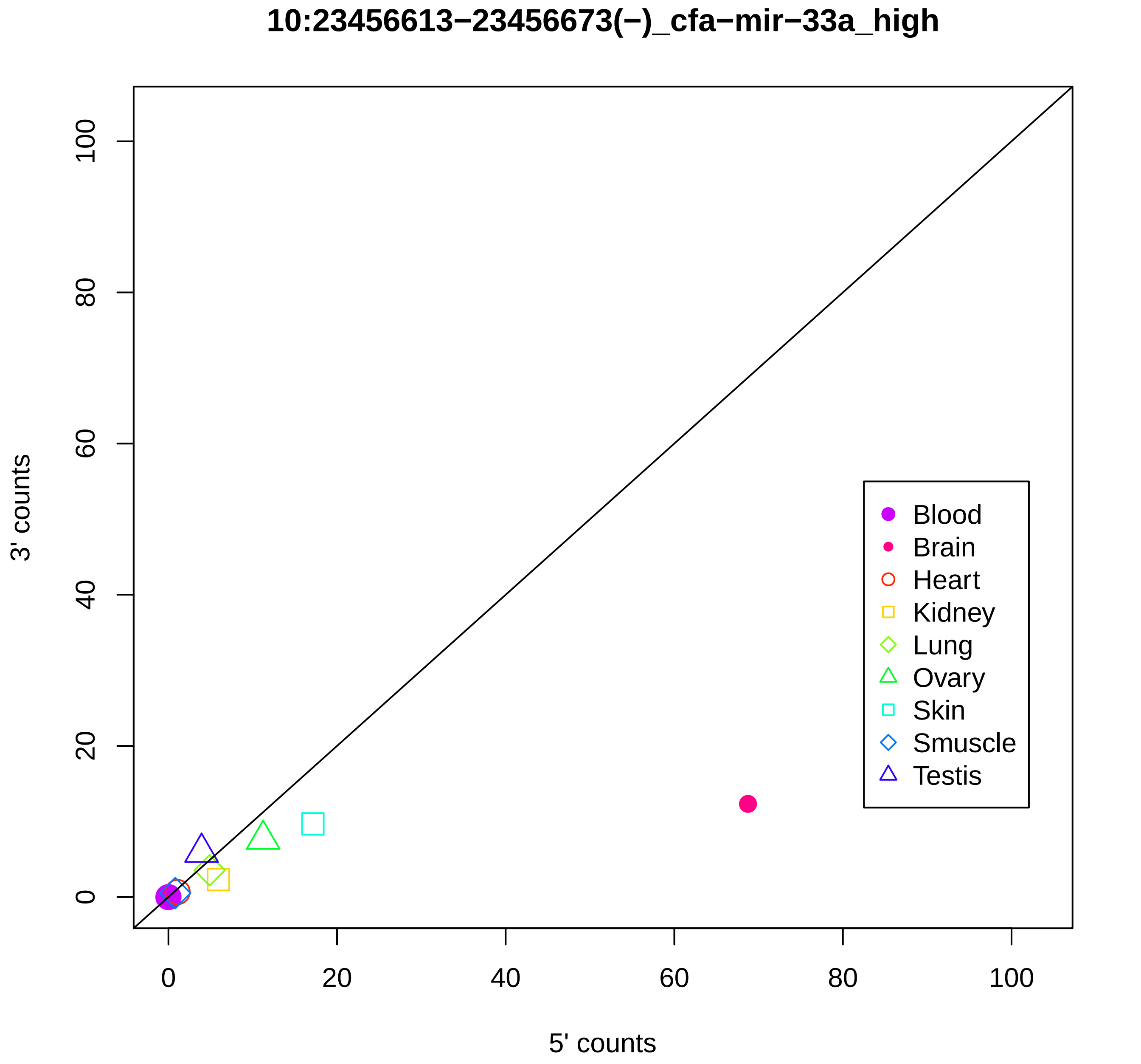

10:23456613−23456673(−)_cfa−mir−33a_high
3' counts
5' counts
0
20
40
60
80
100
Blood
Brain
Heart
Kidney
Lung
Ovary
Skin
Smuscle
Testis

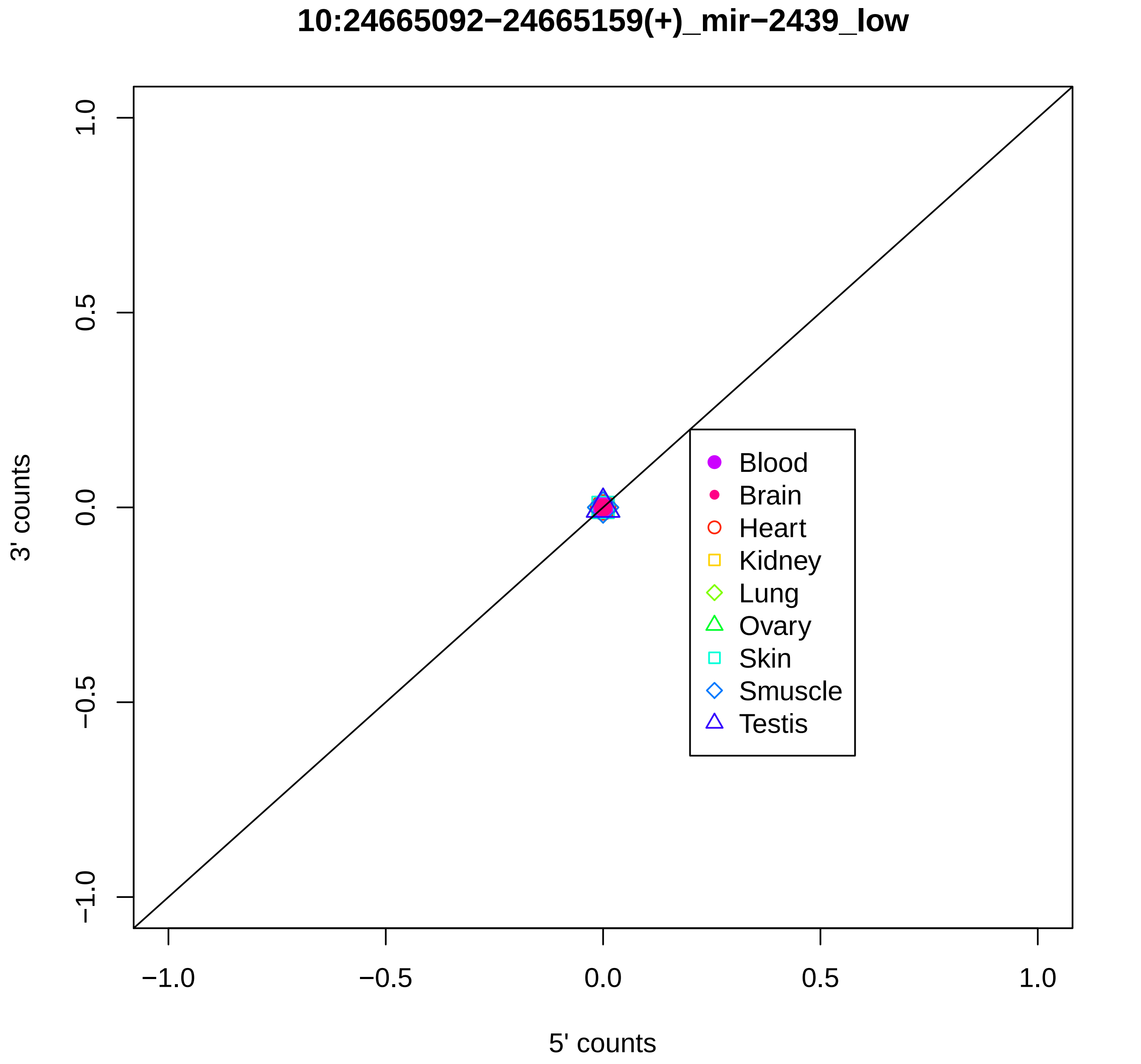
10:24665092−24665159(+)_mir−2439_low
1.0
0.5
0.0
−0.5
−1.0
3' counts
−1.0
−0.5
0.0
0.5
1.0
5' counts
Blood
Brain
Heart
Kidney
Lung
Ovary
Skin
Smuscle
Testis

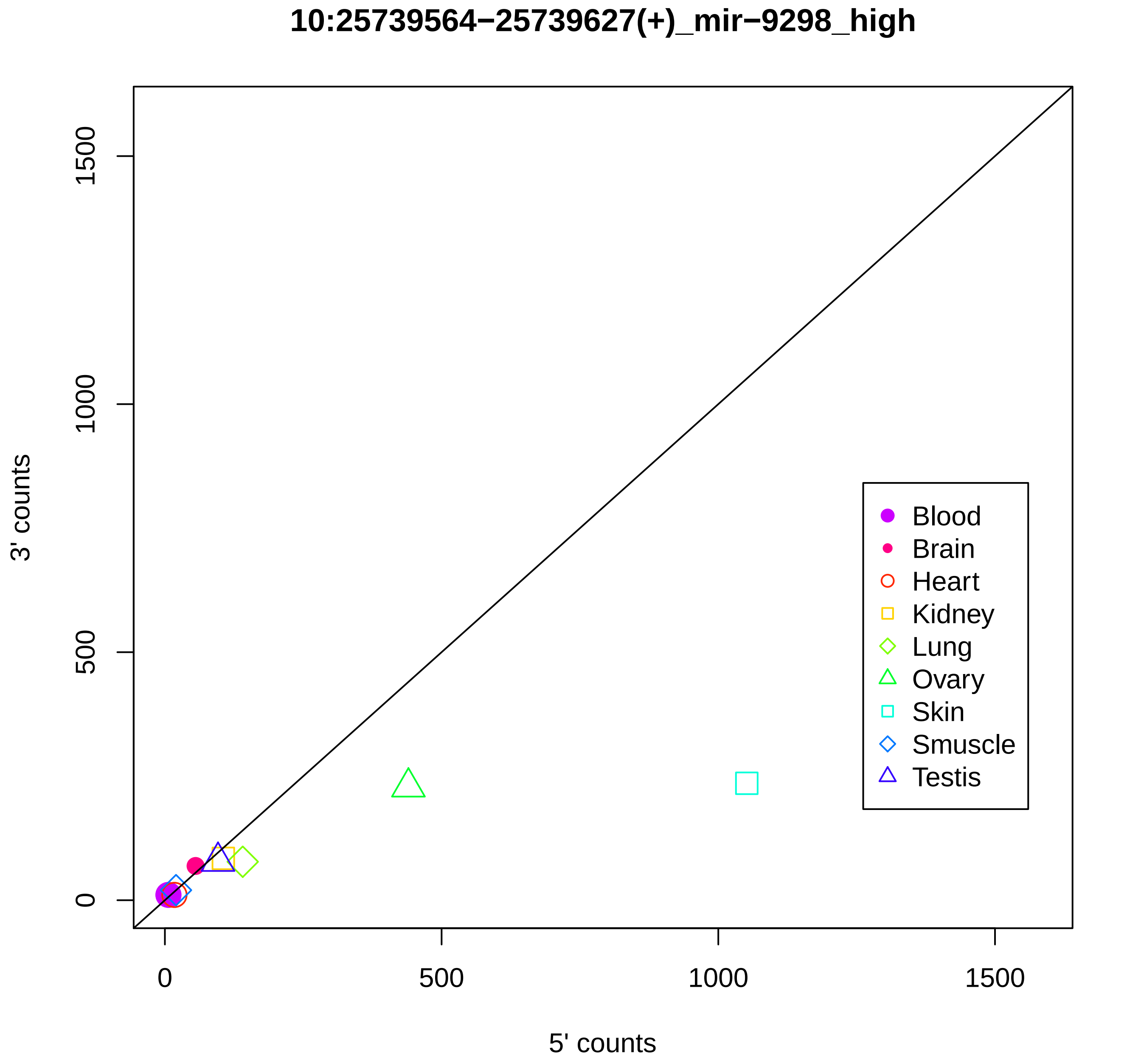

10:25739564−25739627(+)_mir−9298_high
3' counts
5' counts
0
500
1000
1500
0
500
1000
1500
Blood
Brain
Heart
Kidney
Lung
Ovary
Skin
Smuscle
Testis

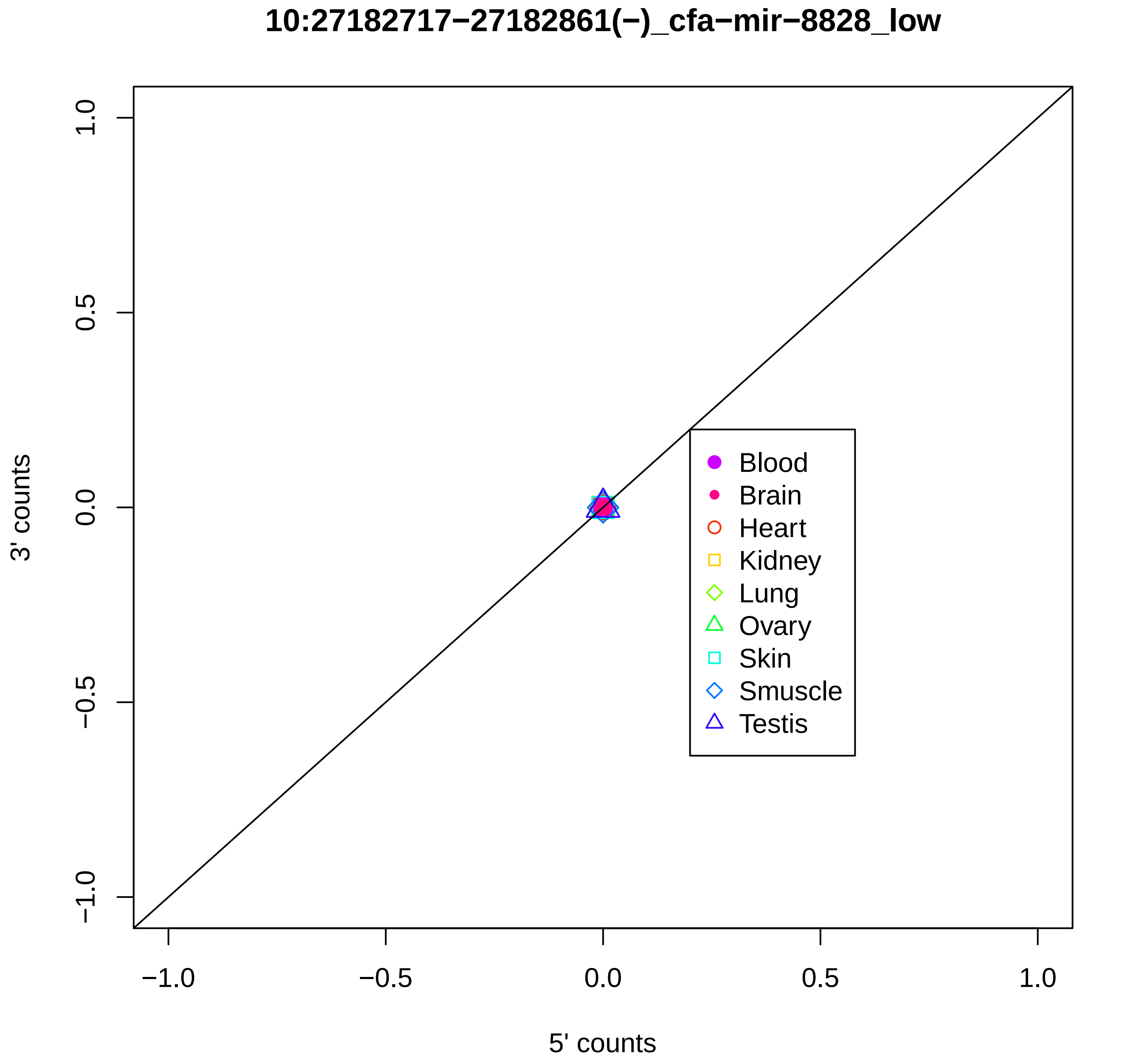
10:27182717−27182861(−)_cfa−mir−8828_low
3' counts
5' counts
−1.0
−0.5
0.0
0.5
1.0
Blood
Brain
Heart
Kidney
Lung
Ovary
Skin
Smuscle
Testis

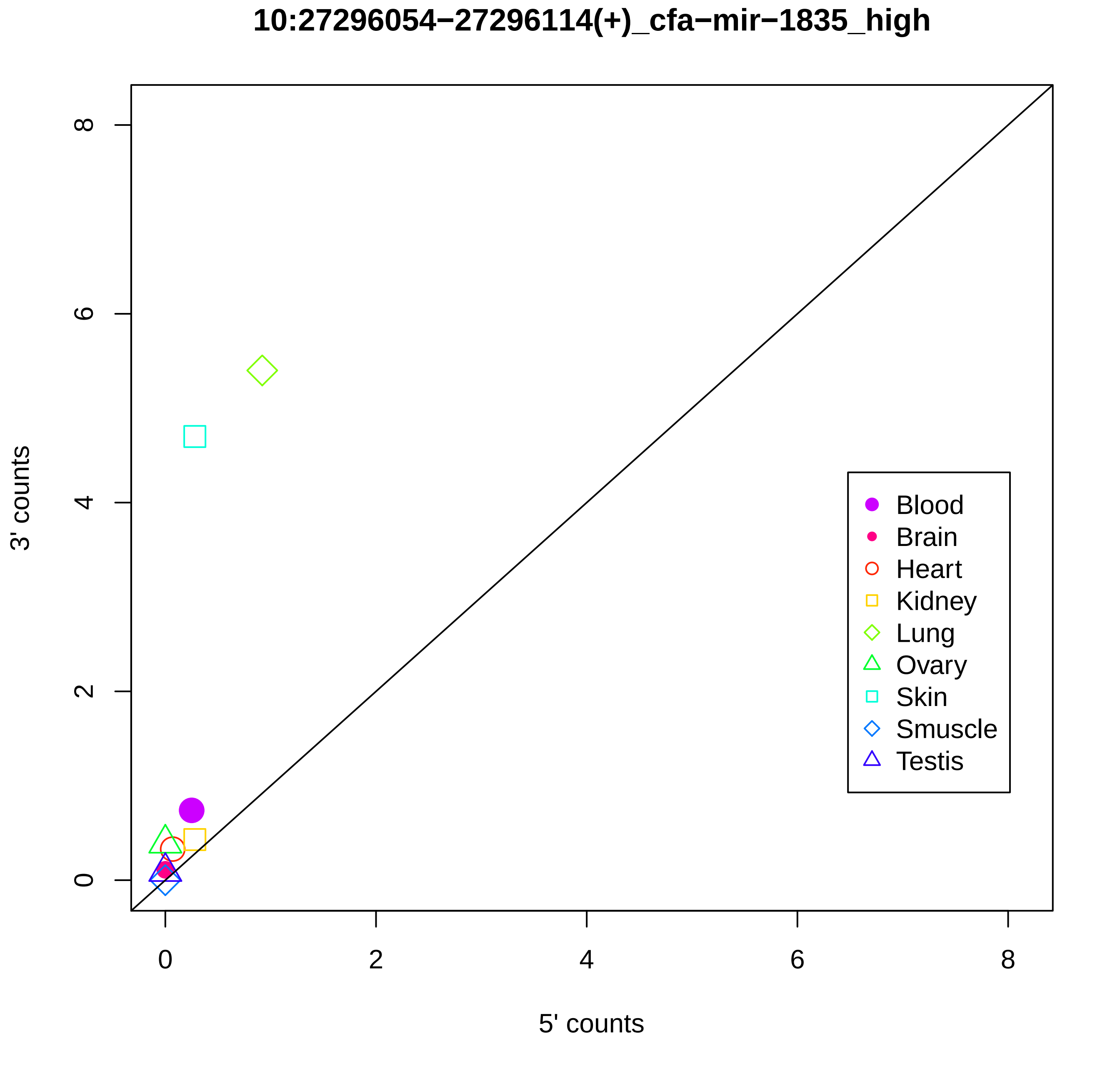
10:27296054−27296114(+)_cfa−mir−1835_high
3' counts
5' counts
0
2
4
6
8
0
2
4
6
8
Blood
Brain
Heart
Kidney
Lung
Ovary
Skin
Smuscle
Testis

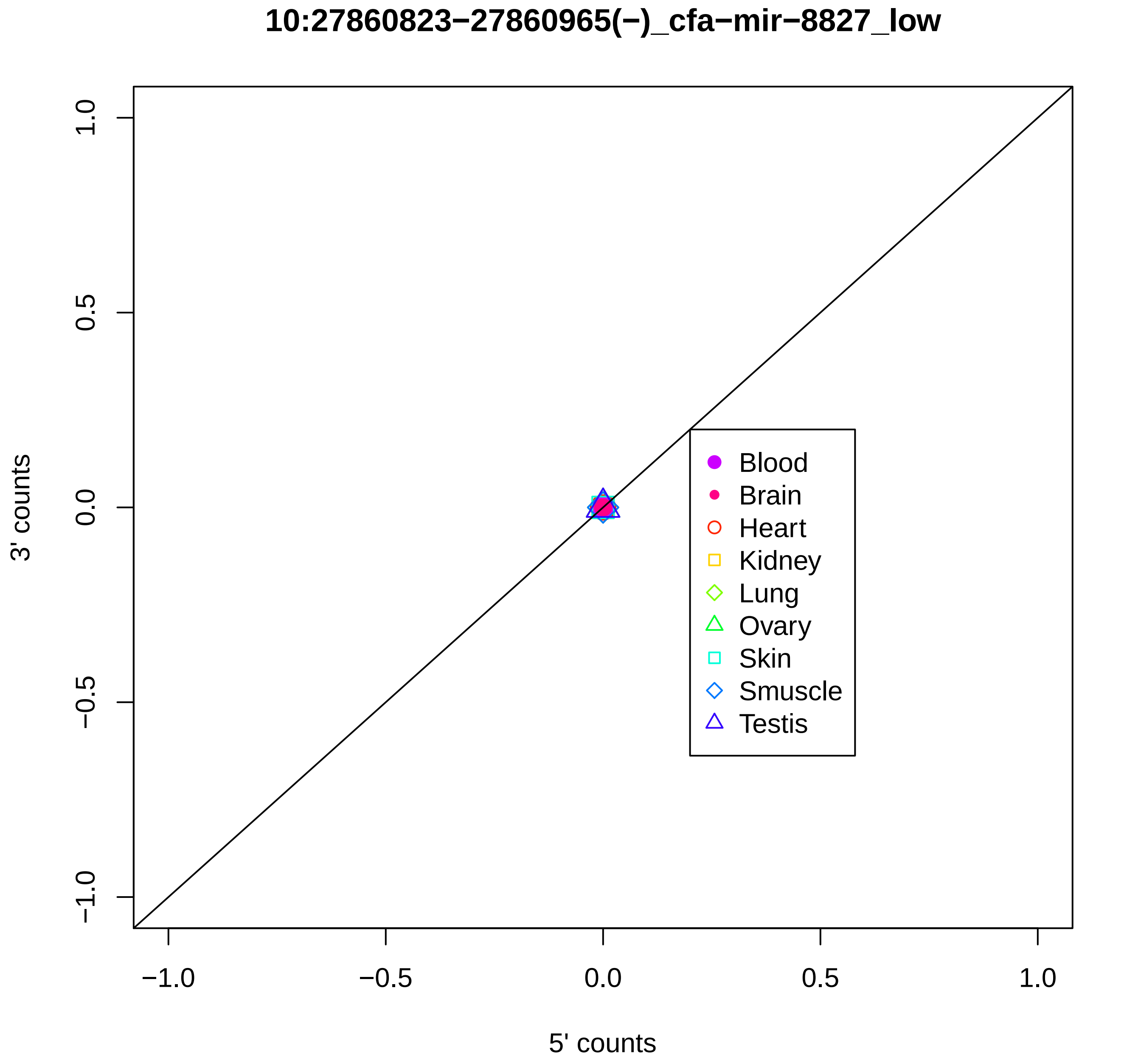

10:27860823−27860965(−)_cfa−mir−8827_low
3' counts
5' counts
−1.0
−0.5
0.0
0.5
1.0
Blood
Brain
Heart
Kidney
Lung
Ovary
Skin
Smuscle
Testis

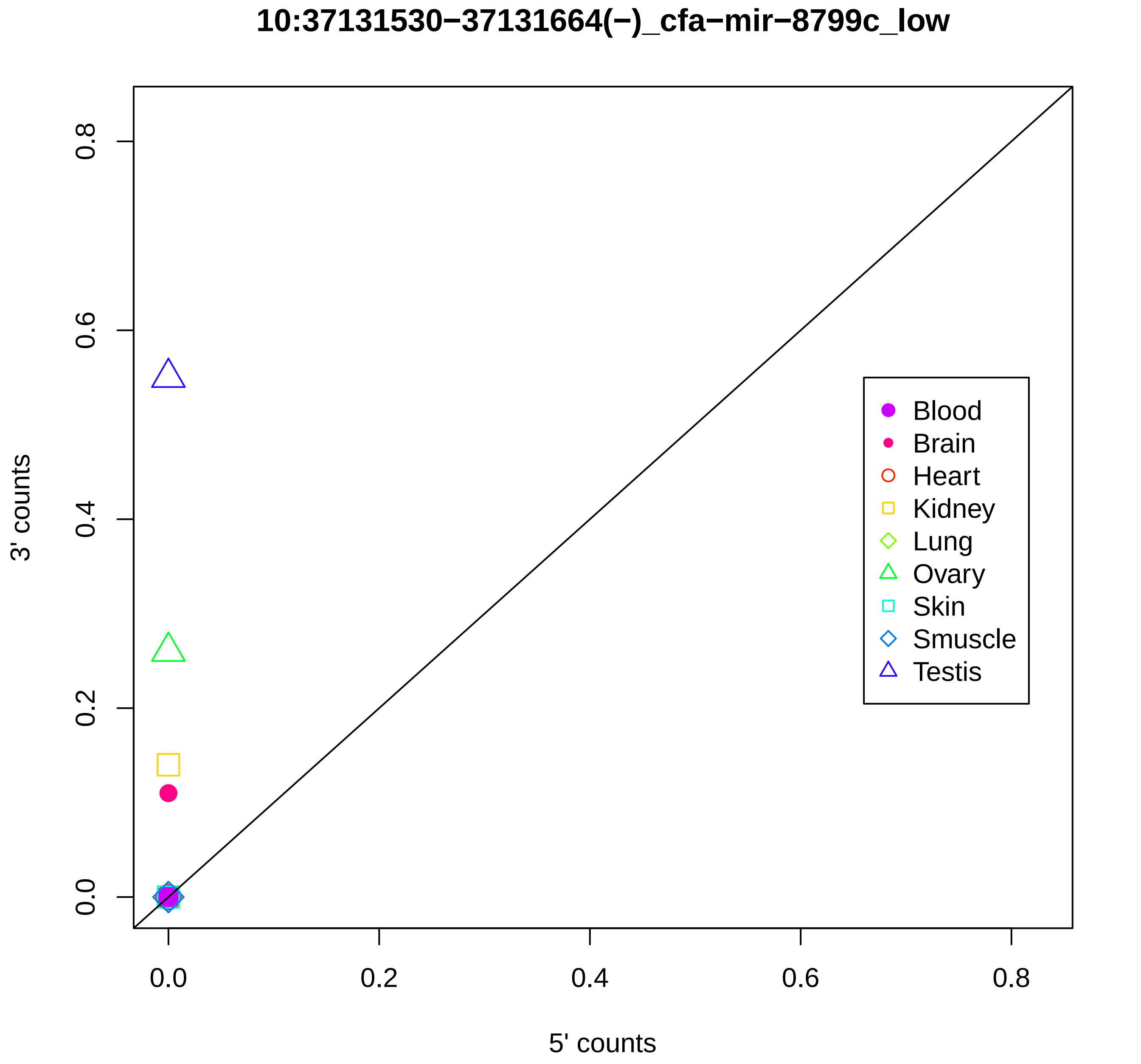
10:37131530−37131664(−)_cfa−mir−8799c_low
3' counts
5' counts
0.0
0.2
0.4
0.6
0.8
Blood
Brain
Heart
Kidney
Lung
Ovary
Skin
Smuscle
Testis

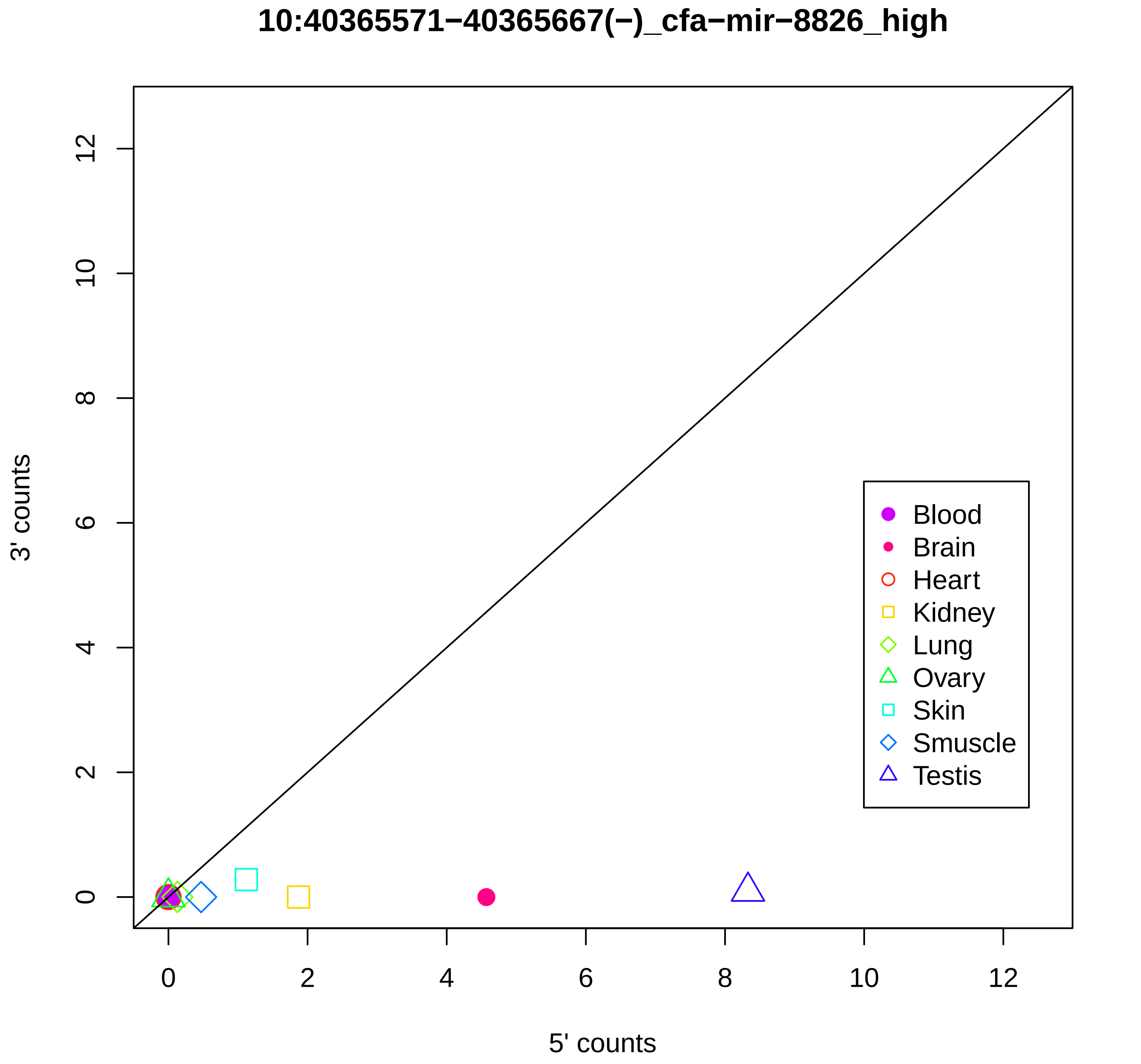

10:40365571−40365667(−)_cfa−mir−8826_high
3' counts
5' counts
0
2
4
6
8
10
12
Blood
Brain
Heart
Kidney
Lung
Ovary
Skin
Smuscle
Testis

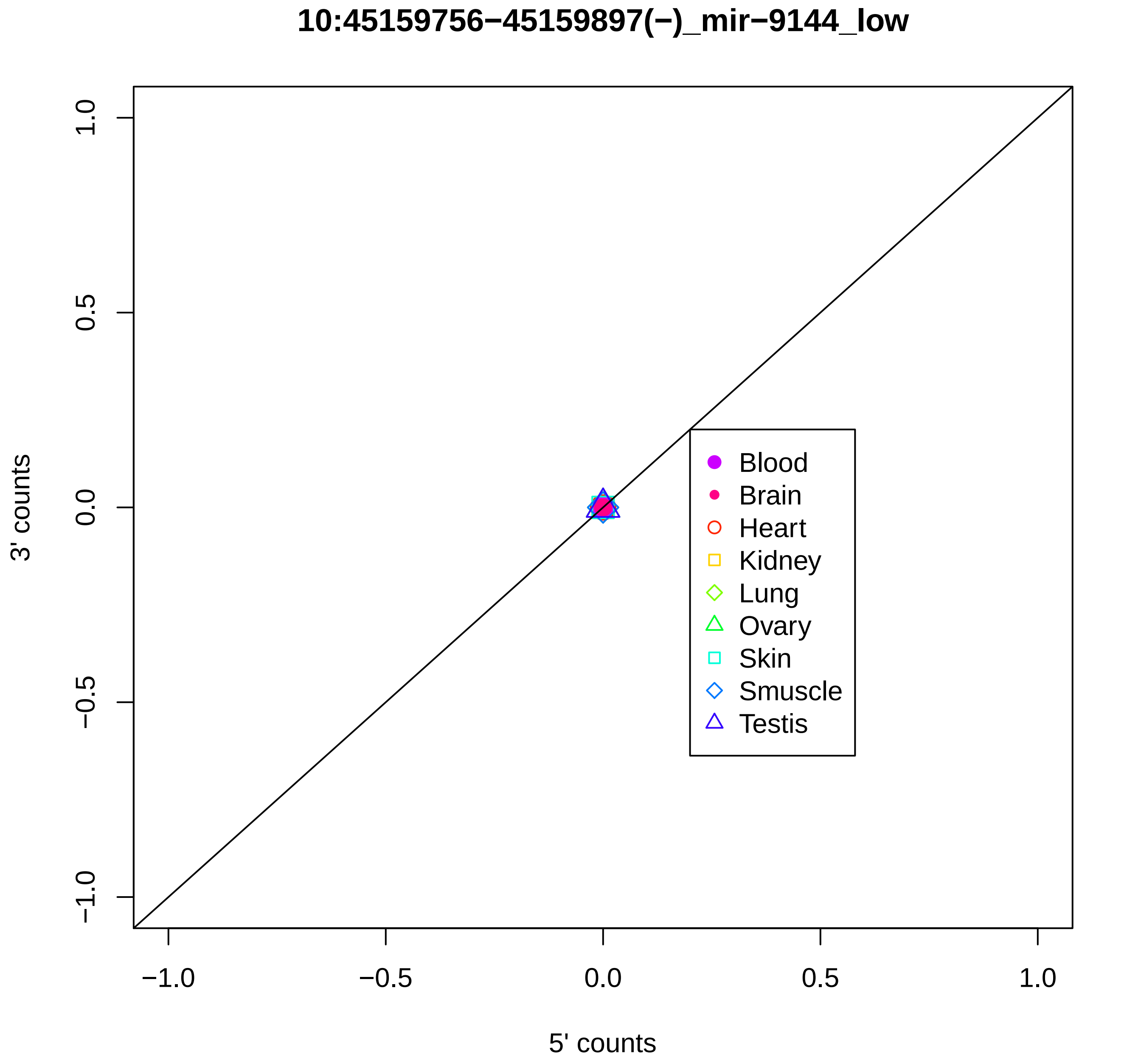
10:45159756−45159897(−)_mir−9144_low
1.0
0.5
0.0
−0.5
−1.0
3' counts
5' counts
Blood
Brain
Heart
Kidney
Lung
Ovary
Skin
Smuscle
Testis

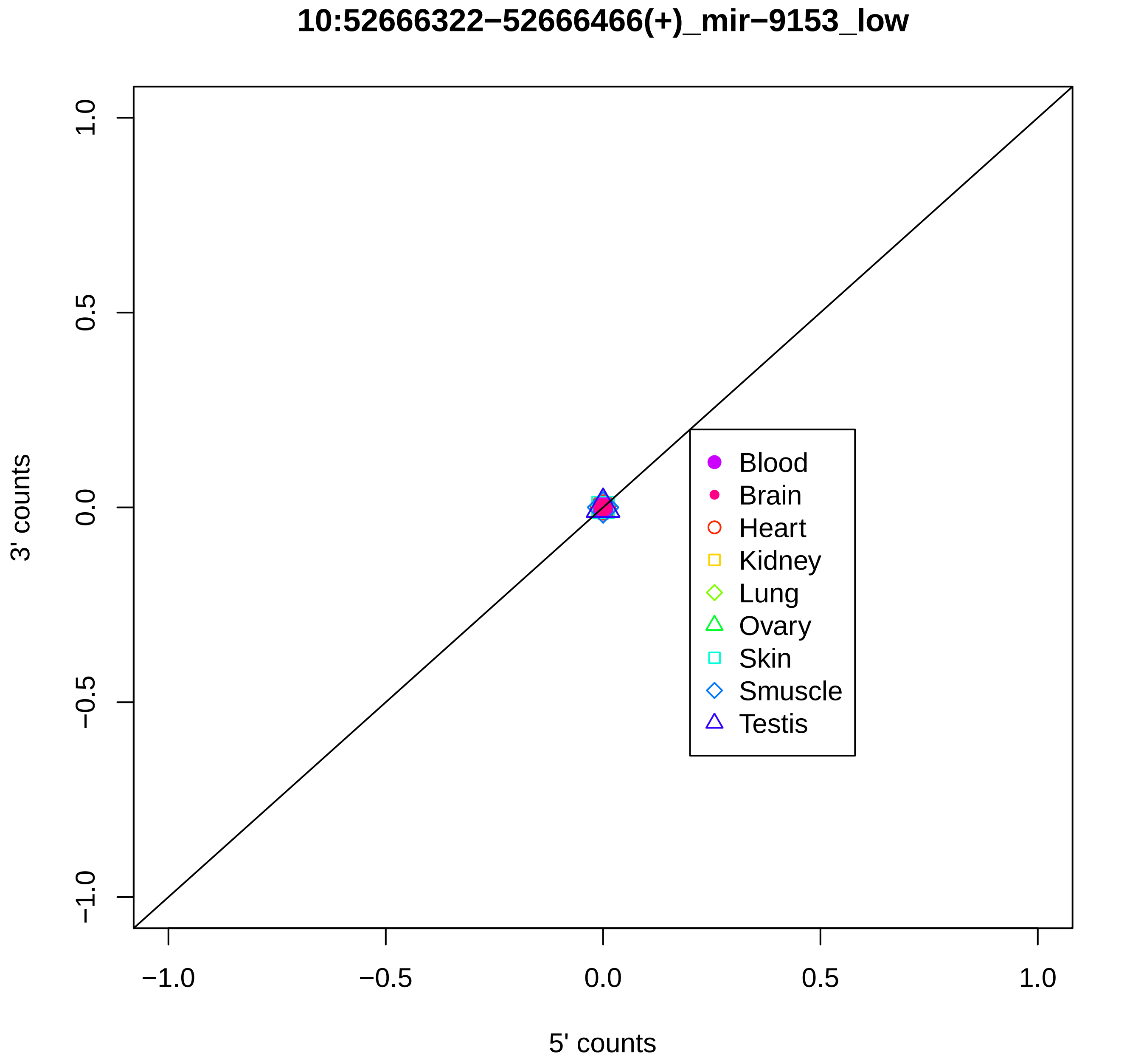

10:52666322−52666466(+)_mir−9153_low
1.0
0.5
0.0
−0.5
−1.0
3' counts
−1.0
−0.5
0.0
0.5
1.0
5' counts
Blood
Brain
Heart
Kidney
Lung
Ovary
Skin
Smuscle
Testis

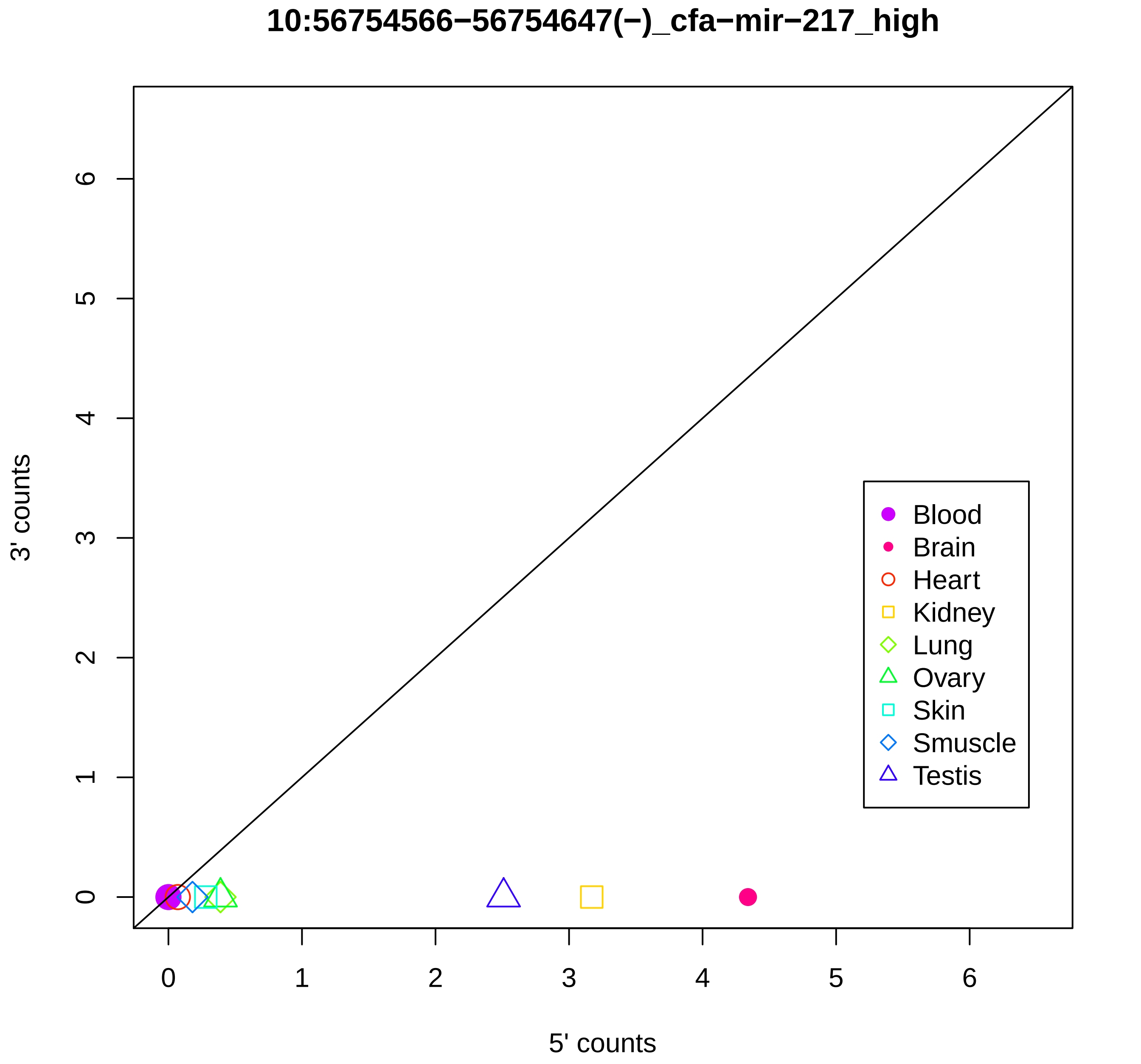
10:56754566−56754647(−)_cfa−mir−217_high
3' counts
5' counts
0
1
2
3
4
5
6
Blood
Brain
Heart
Kidney
Lung
Ovary
Skin
Smuscle
Testis

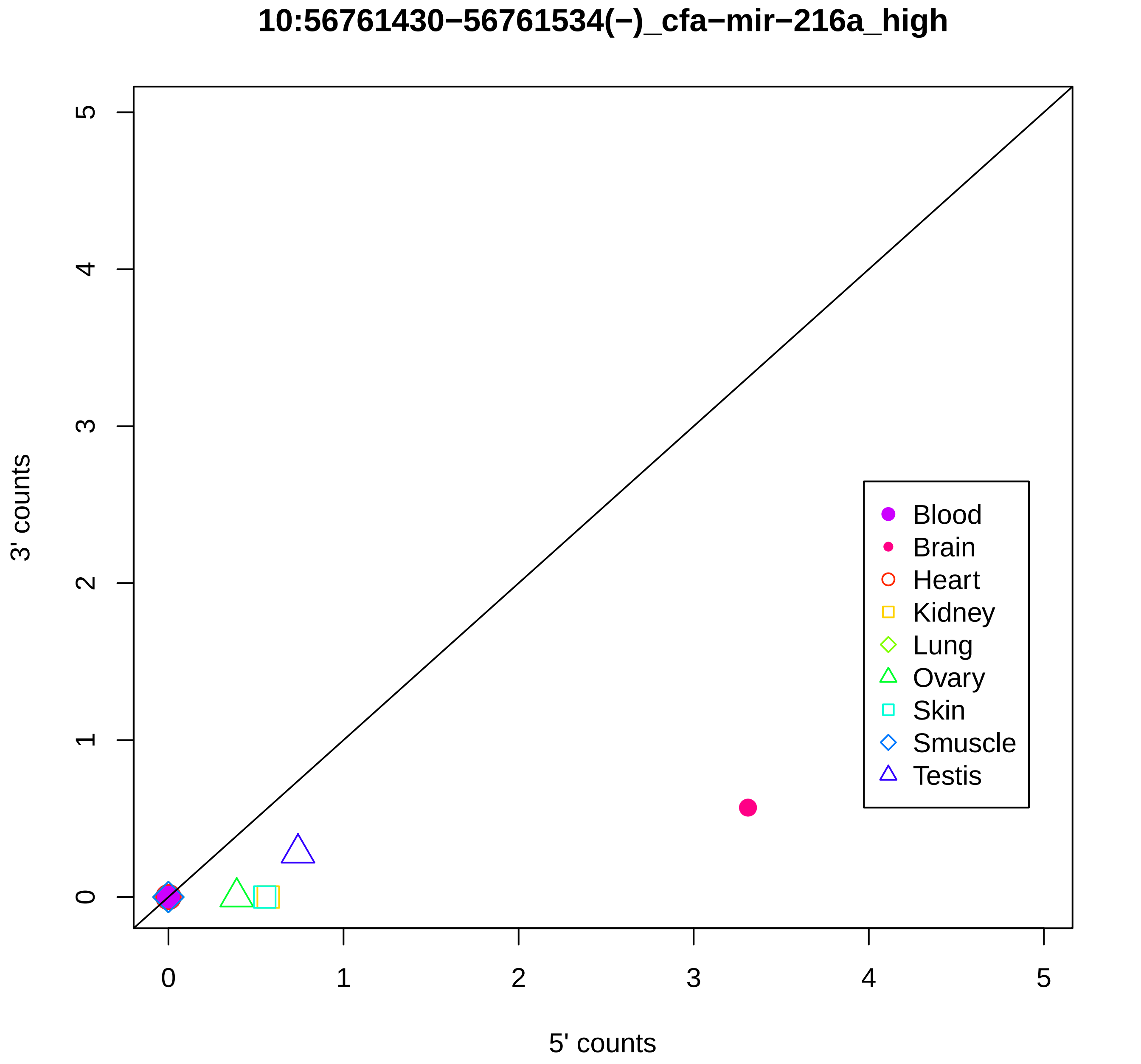

10:56761430−56761534(−)_cfa−mir−216a_high
3' counts
5' counts
0
1
2
3
4
5
0
1
2
3
4
5
Blood
Brain
Heart
Kidney
Lung
Ovary
Skin
Smuscle
Testis

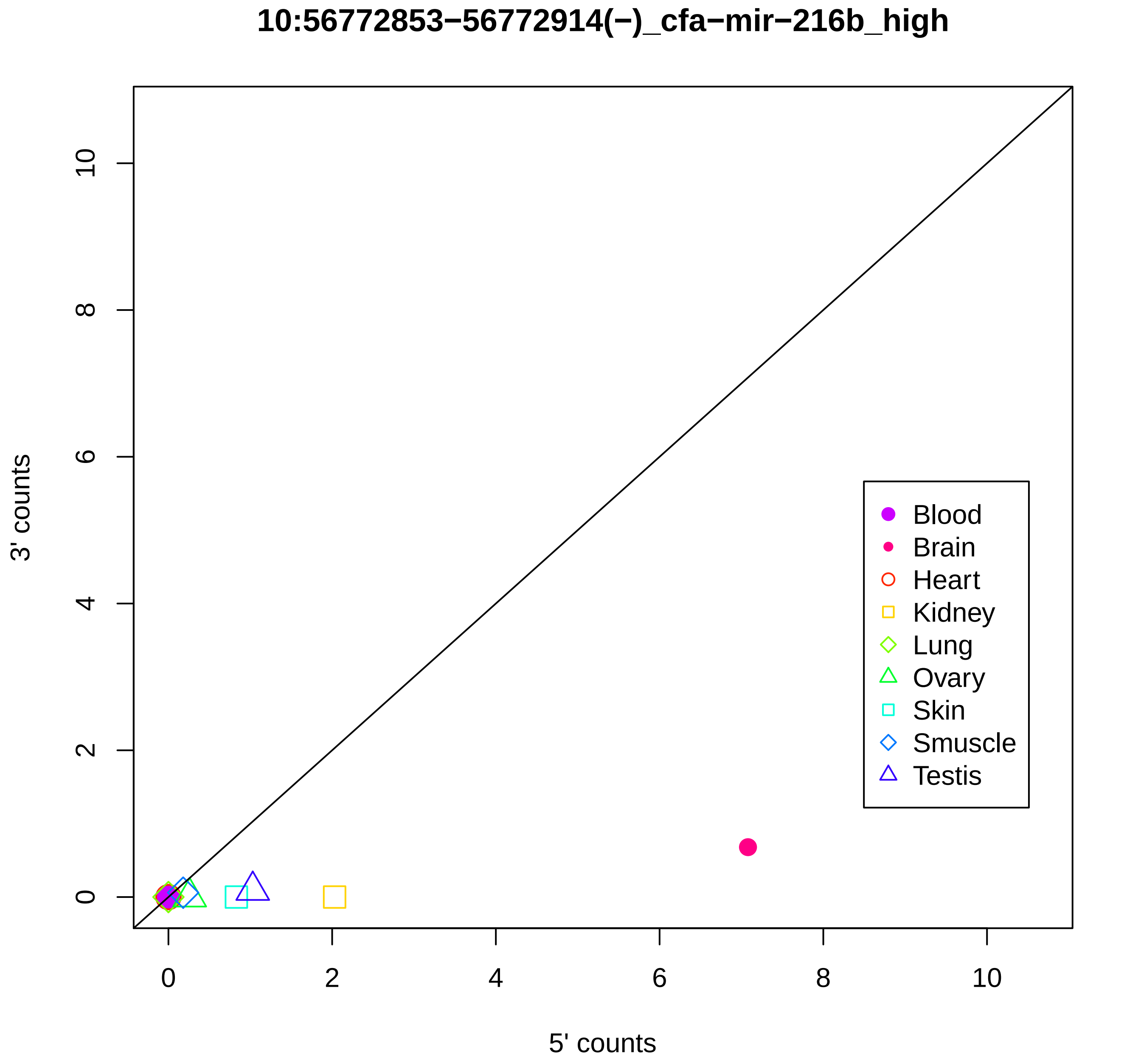
10:56772853−56772914(−)_cfa−mir−216b_high
3' counts
5' counts
0
2
4
6
8
10
0
2
4
6
8
10
Blood
Brain
Heart
Kidney
Lung
Ovary
Skin
Smuscle
Testis

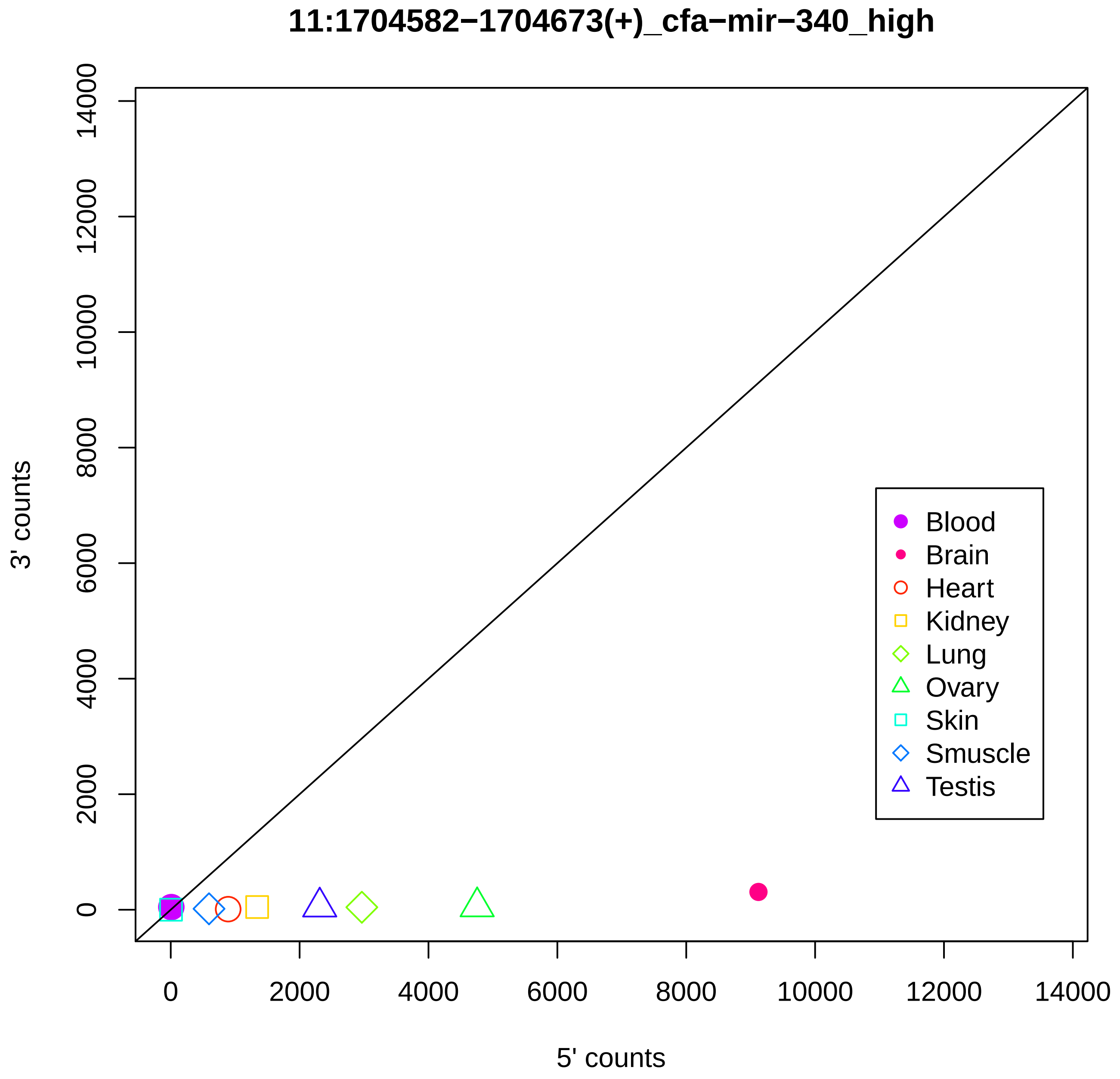

11:1704582−1704673(+)_cfa−mir−340_high
3' counts
5' counts
0
2000
4000
6000
8000
10000
12000
14000
Blood
Brain
Heart
Kidney
Lung
Ovary
Skin
Smuscle
Testis

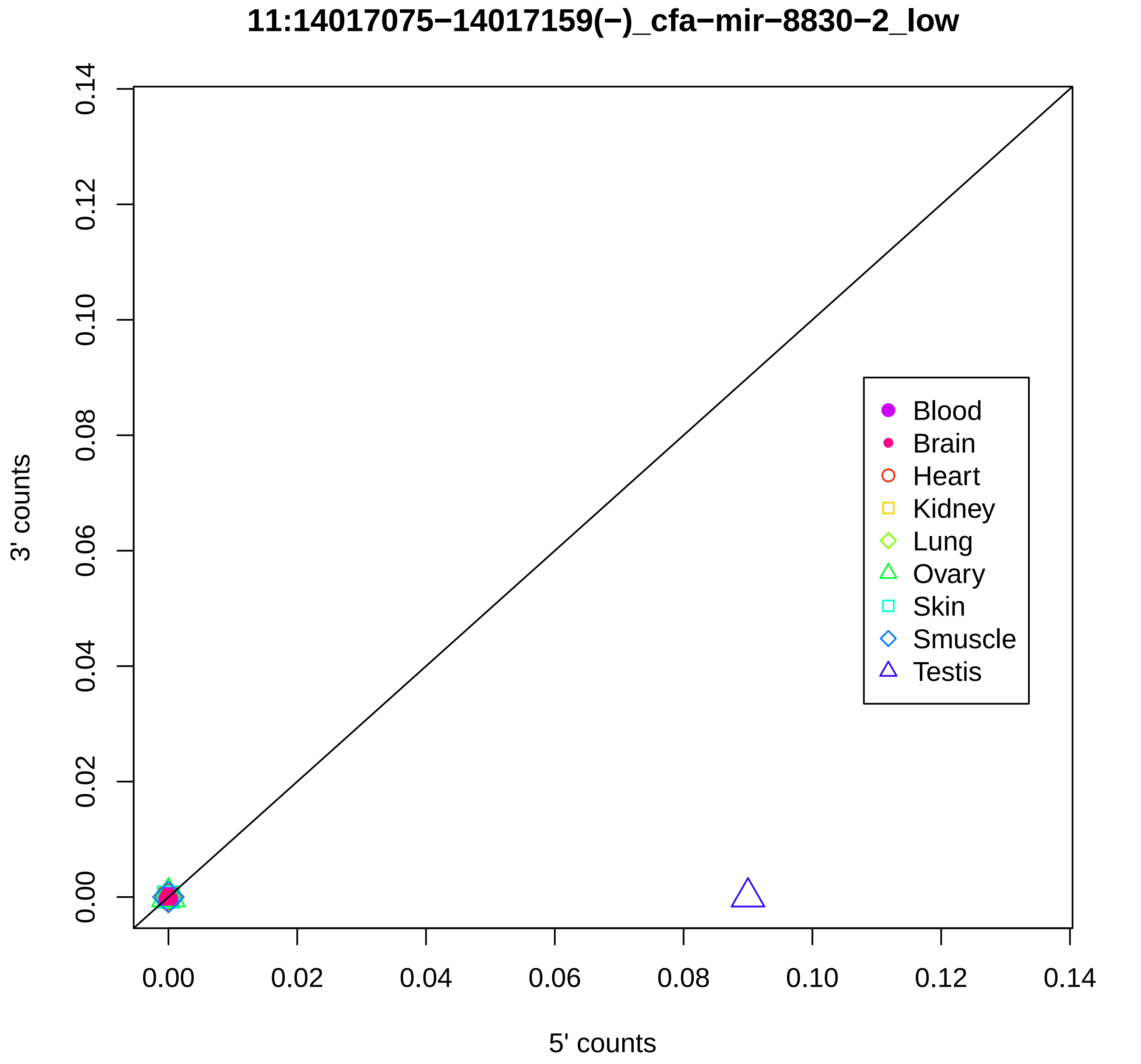

11:14017075−14017159(−)_cfa−mir−8830−2_low
3' counts
5' counts
0.00
0.02
0.04
0.06
0.08
0.10
0.12
0.14
Blood
Brain
Heart
Kidney
Lung
Ovary
Skin
Smuscle
Testis

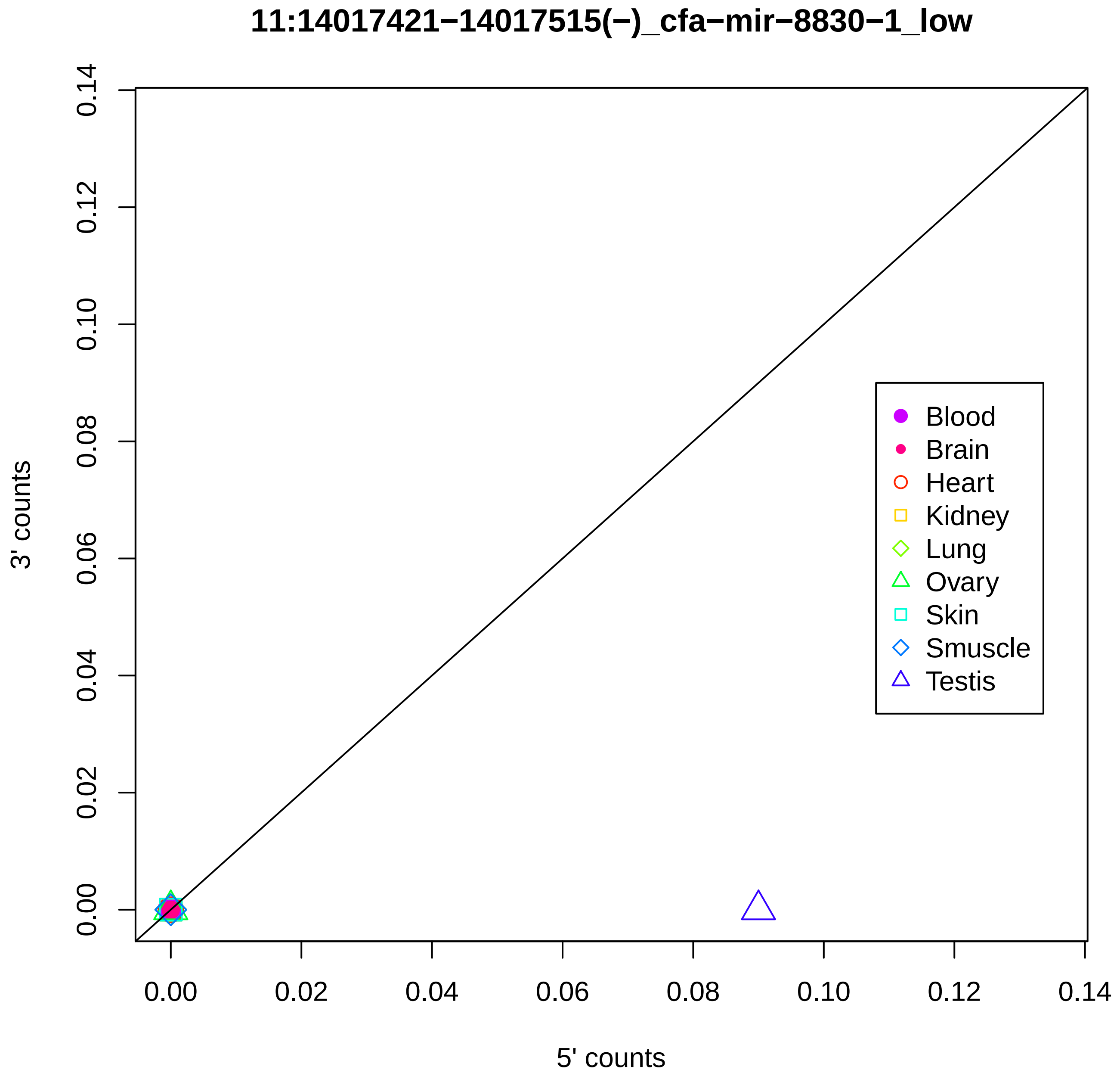
11:14017421−14017515(−)_cfa−mir−8830−1_low
3' counts
5' counts
0.00
0.02
0.04
0.06
0.08
0.10
0.12
0.14
Blood
Brain
Heart
Kidney
Lung
Ovary
Skin
Smuscle
Testis

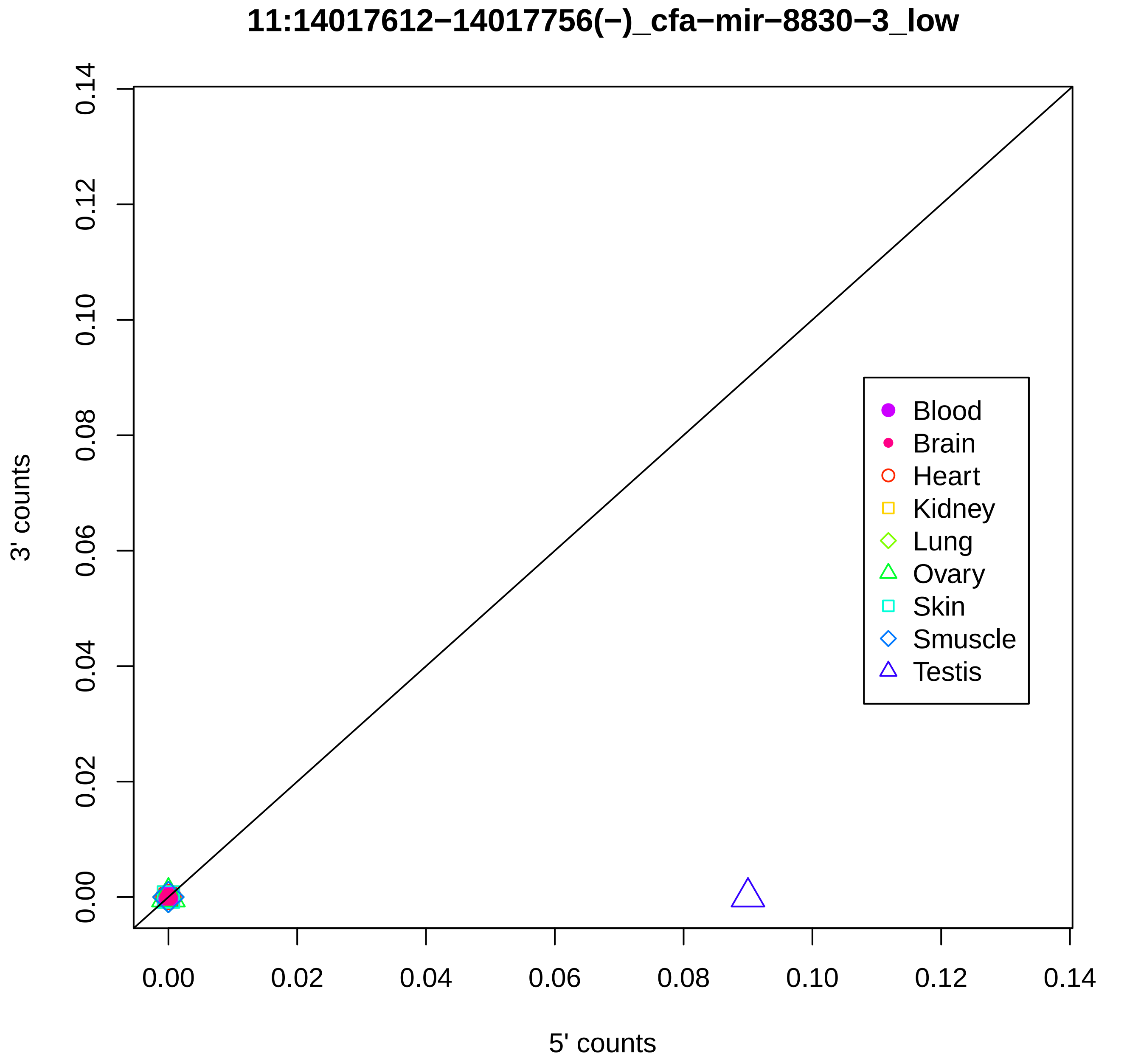

11:14017612−14017756(−)_cfa−mir−8830−3_low
3' counts
0.00
0.02
0.04
0.06
0.08
0.10
0.12
0.14
5' counts
0.00
0.02
0.04
0.06
0.08
0.10
0.12
0.14
Blood
Brain
Heart
Kidney
Lung
Ovary
Skin
Smuscle
Testis

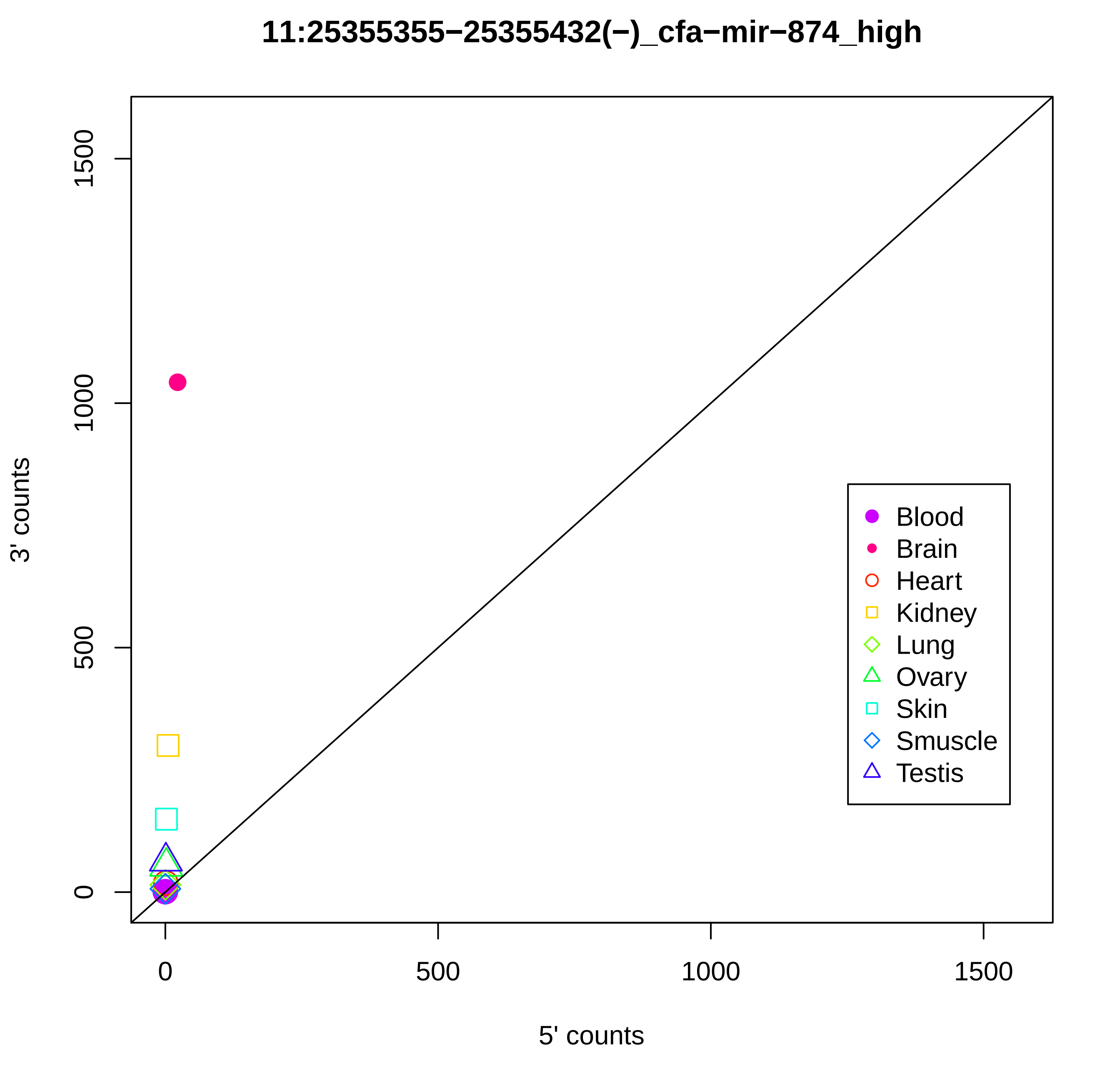
11:25355355−25355432(−)_cfa−mir−874_high
3' counts
5' counts
0
500
1000
1500
0
500
1000
1500
Blood
Brain
Heart
Kidney
Lung
Ovary
Skin
Smuscle
Testis

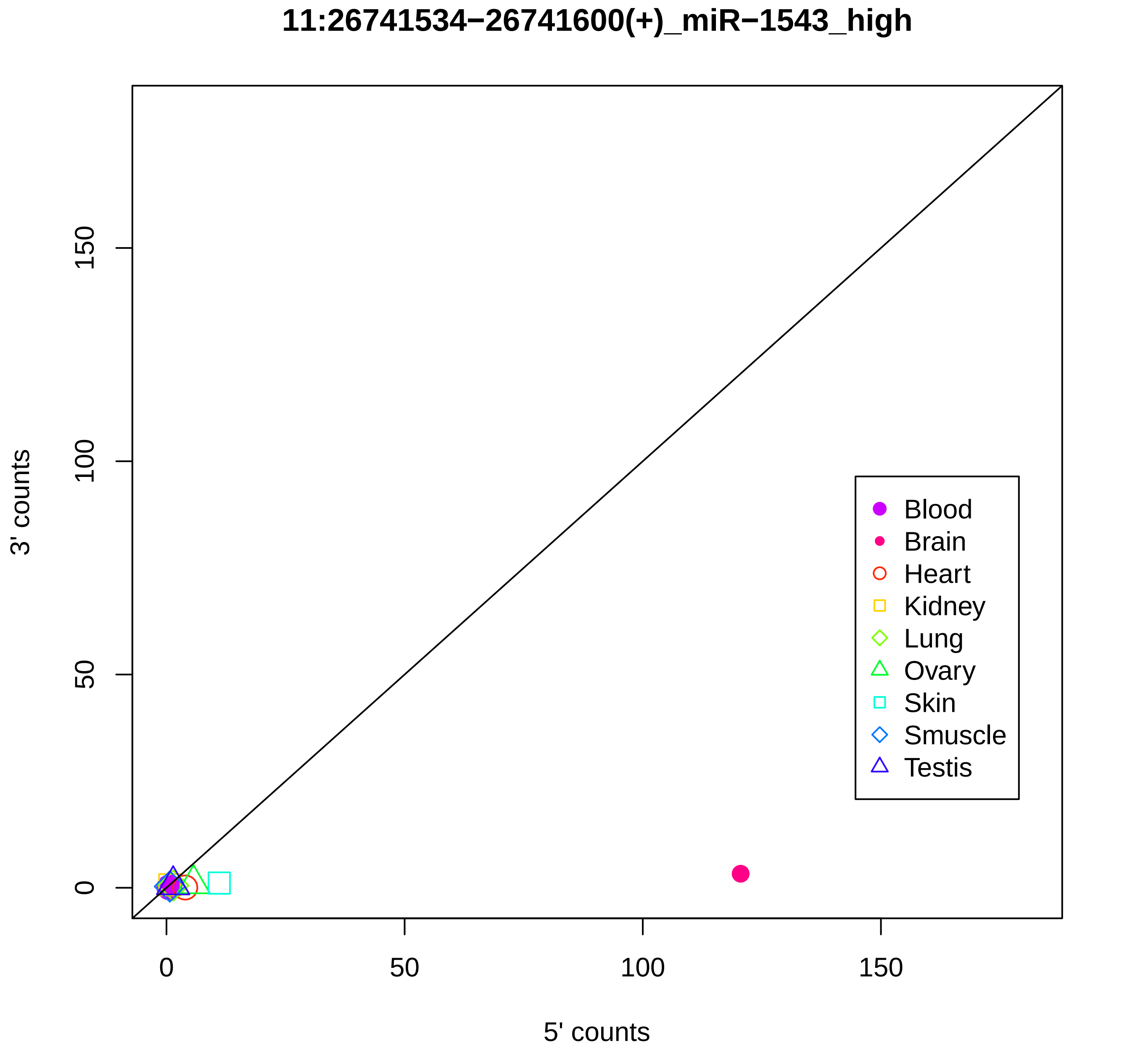

11:26741534−26741600(+)_miR−1543_high
3' counts
5' counts
0
50
100
150
0
50
100
150
Blood
Brain
Heart
Kidney
Lung
Ovary
Skin
Smuscle
Testis

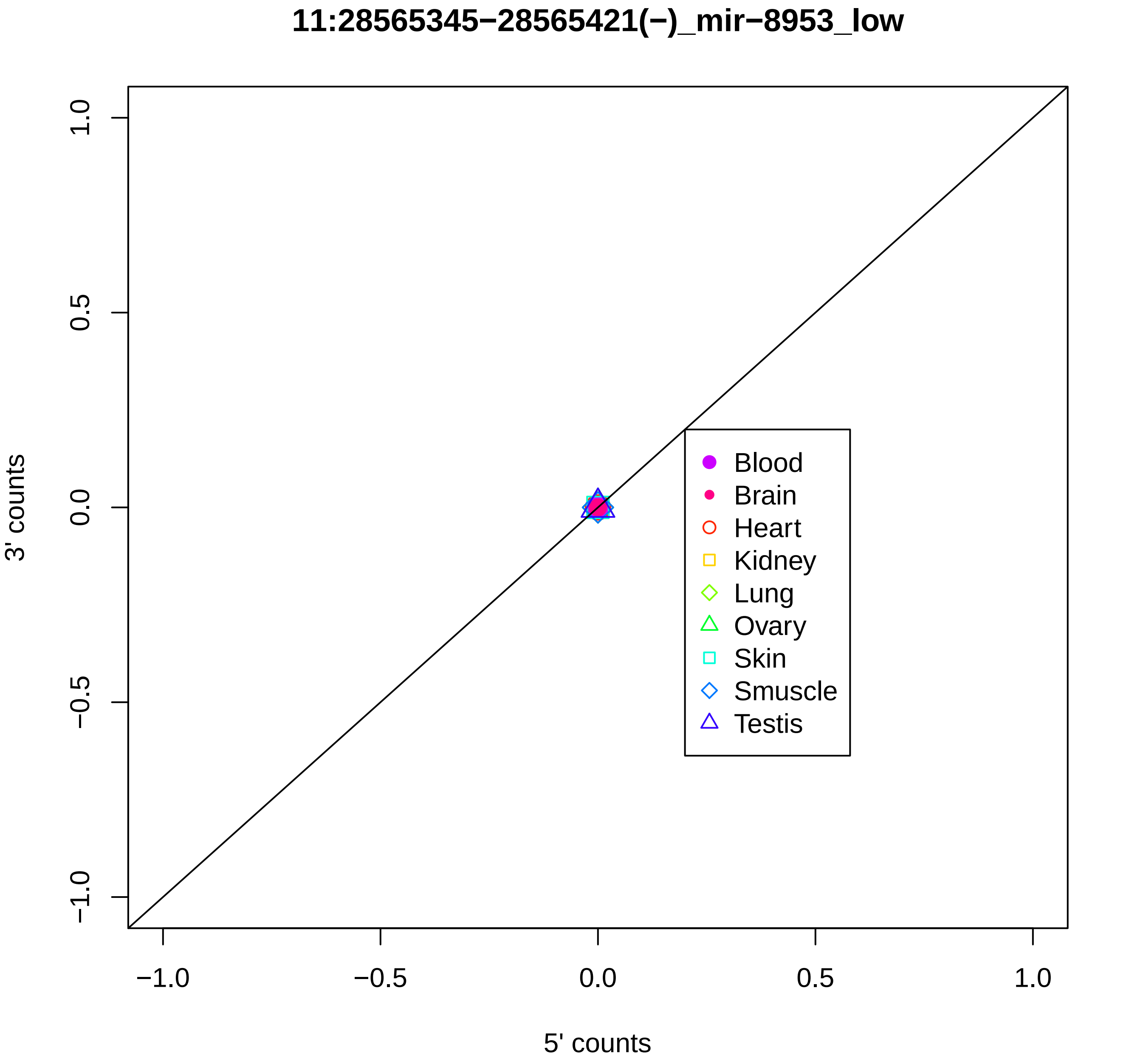

11:28565345−28565421(−)_mir−8953_low
3' counts
5' counts
−1.0
−0.5
0.0
0.5
1.0
Blood
Brain
Heart
Kidney
Lung
Ovary
Skin
Smuscle
Testis

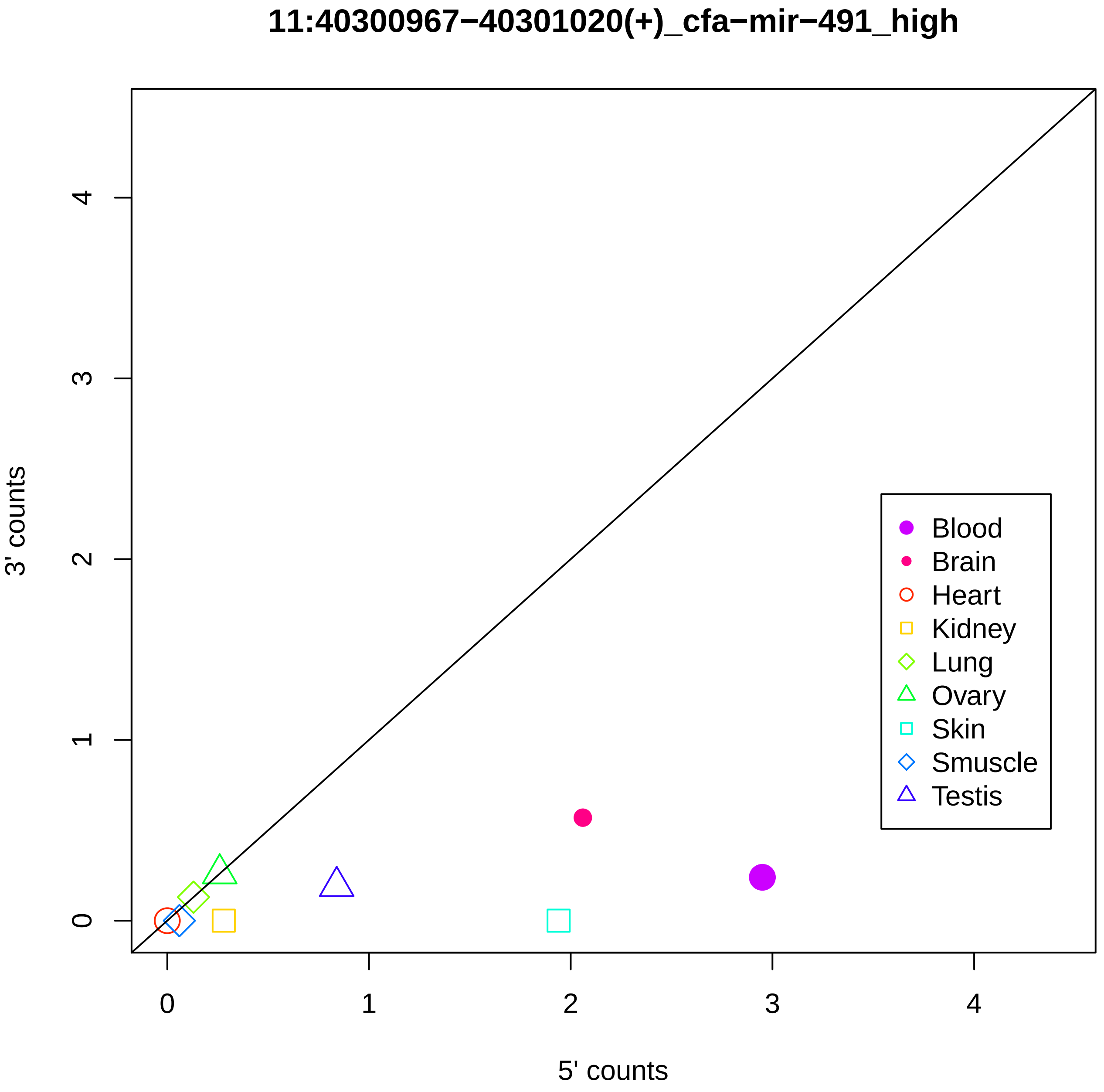

11:40300967−40301020(+)_cfa−mir−491_high
3' counts
5' counts
0
1
2
3
4
0
1
2
3
4
Blood
Brain
Heart
Kidney
Lung
Ovary
Skin
Smuscle
Testis

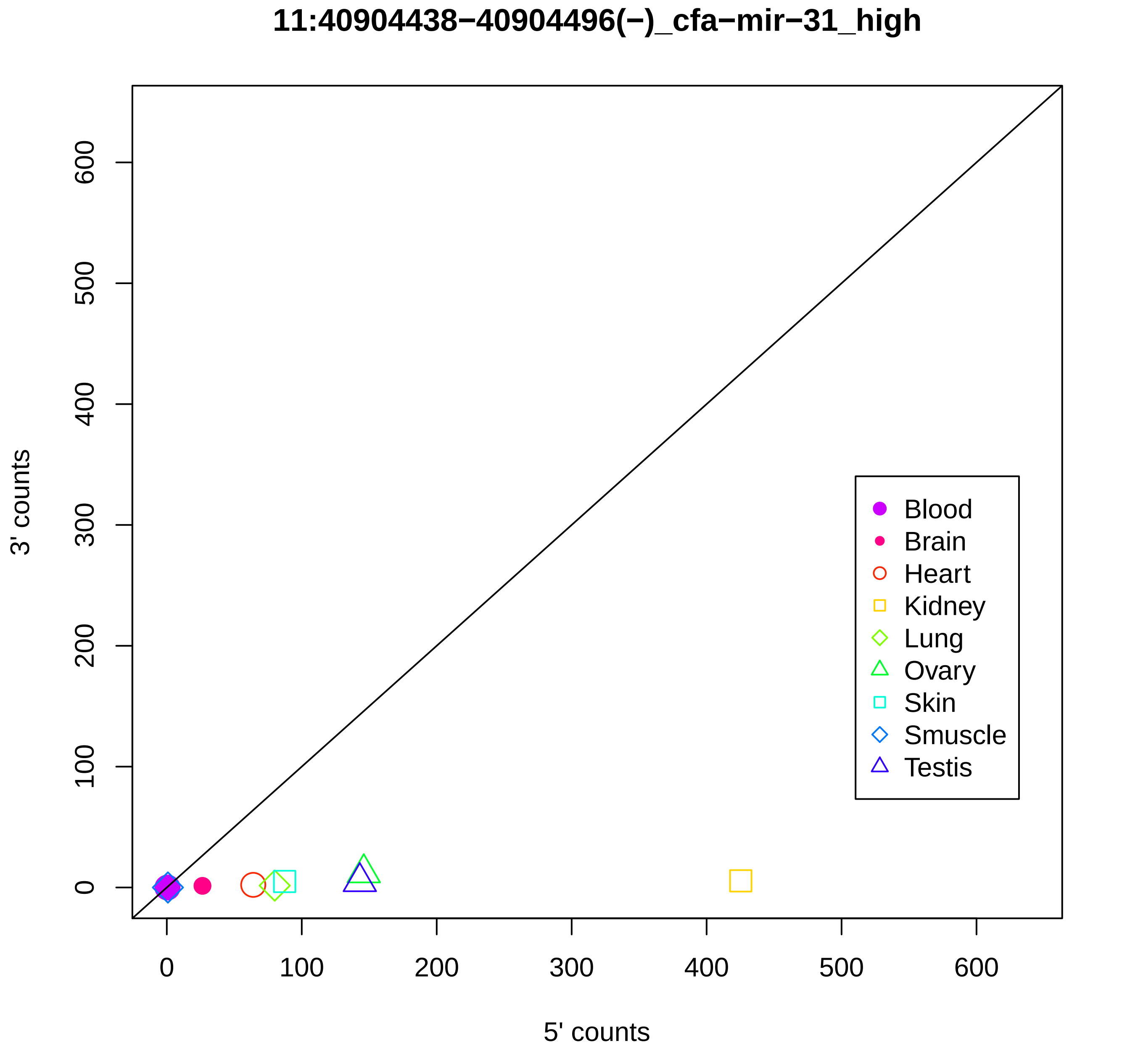

11:40904438−40904496(−)_cfa−mir−31_high
3' counts
5' counts
0
100
200
300
400
500
600
0
100
200
300
400
500
600
Blood
Brain
Heart
Kidney
Lung
Ovary
Skin
Smuscle
Testis

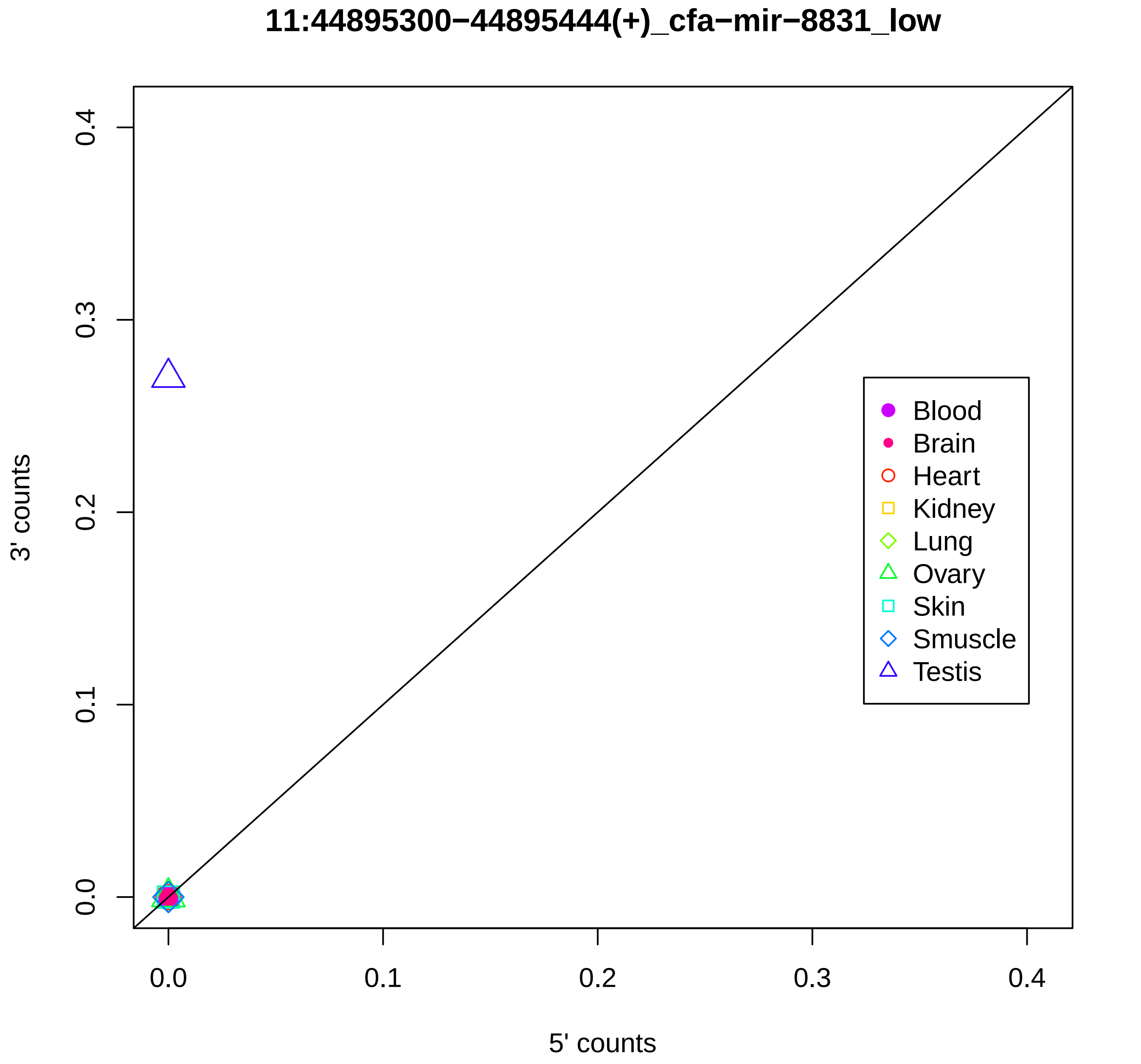

11:44895300−44895444(+)_cfa−mir−8831_low
3' counts
5' counts
0.0
0.1
0.2
0.3
0.4
Blood
Brain
Heart
Kidney
Lung
Ovary
Skin
Smuscle
Testis

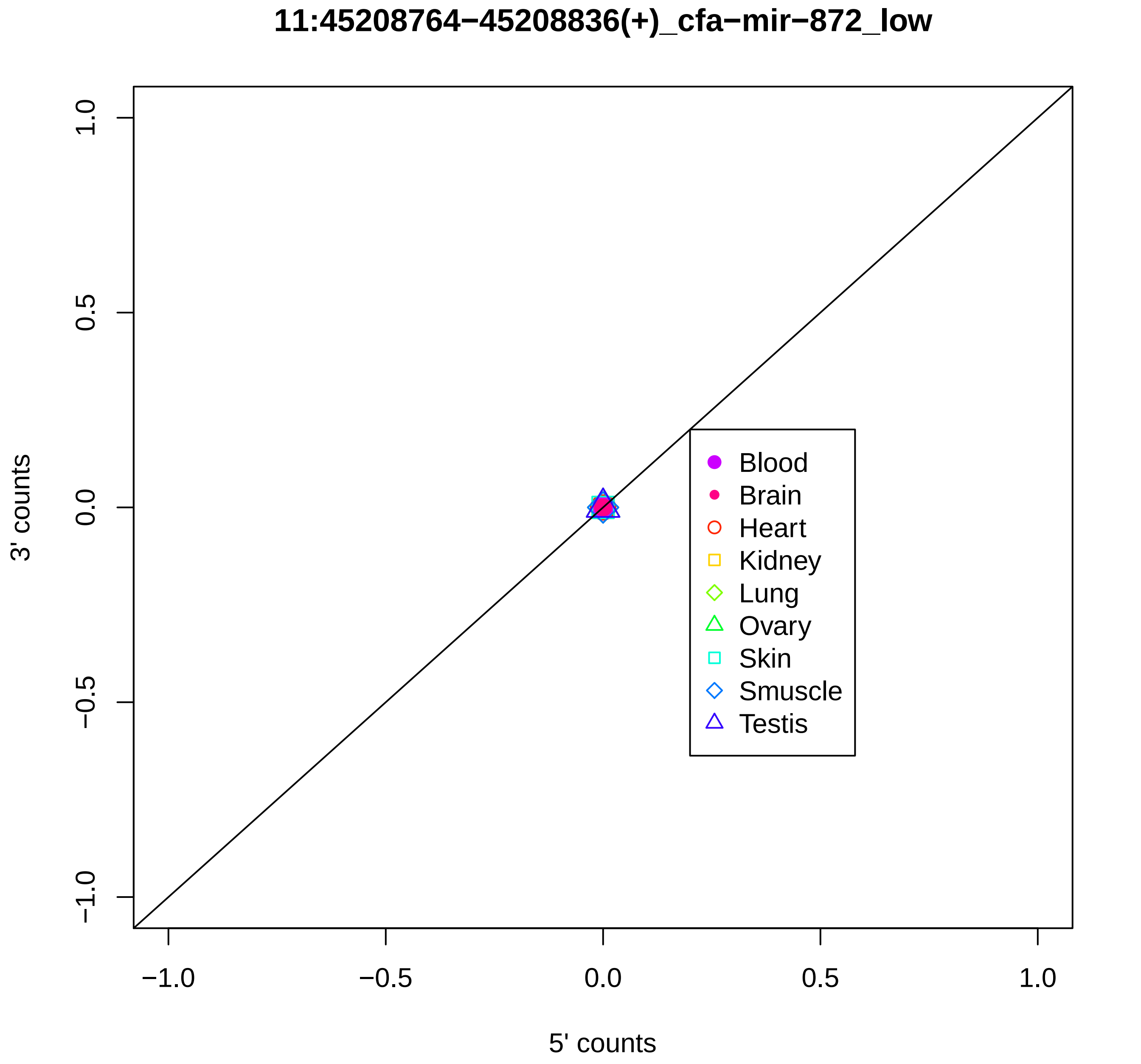
11:45208764−45208836(+)_cfa−mir−872_low
Blood
Brain
Heart
Kidney
Lung
Ovary
Skin
Smuscle
Testis
1.0
0.5
0.0
−0.5
−1.0
3' counts
−1.0
−0.5
0.0
0.5
1.0
5' counts

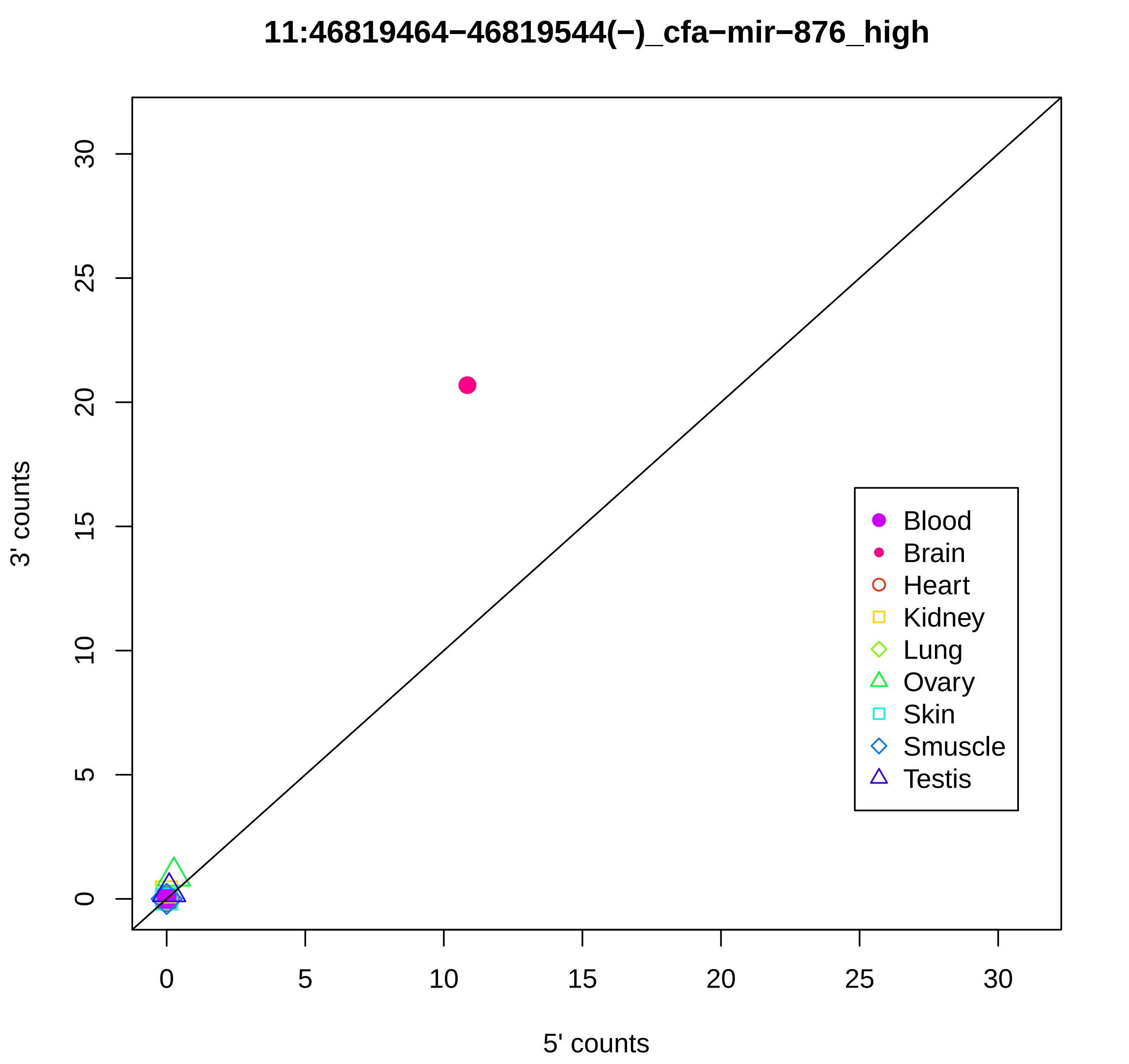

11:46819464−46819544(−)_cfa−mir−876_high
3' counts
5' counts
0
5
10
15
20
25
30
Blood
Brain
Heart
Kidney
Lung
Ovary
Skin
Smuscle
Testis

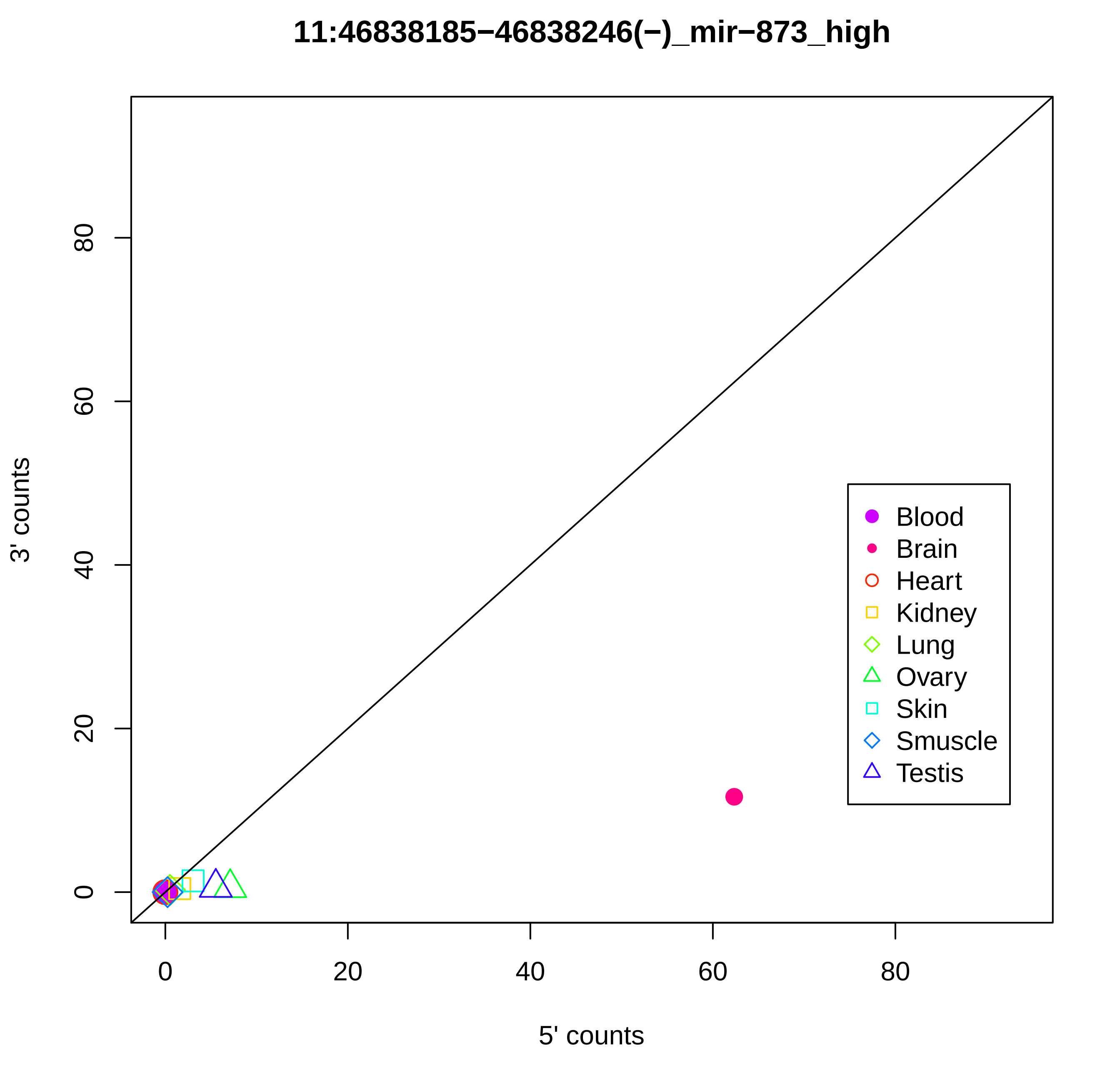

11:46838185−46838246(−)_mir−873_high
3' counts
5' counts
0
20
40
60
80
0
20
40
60
80
Blood
Brain
Heart
Kidney
Lung
Ovary
Skin
Smuscle
Testis

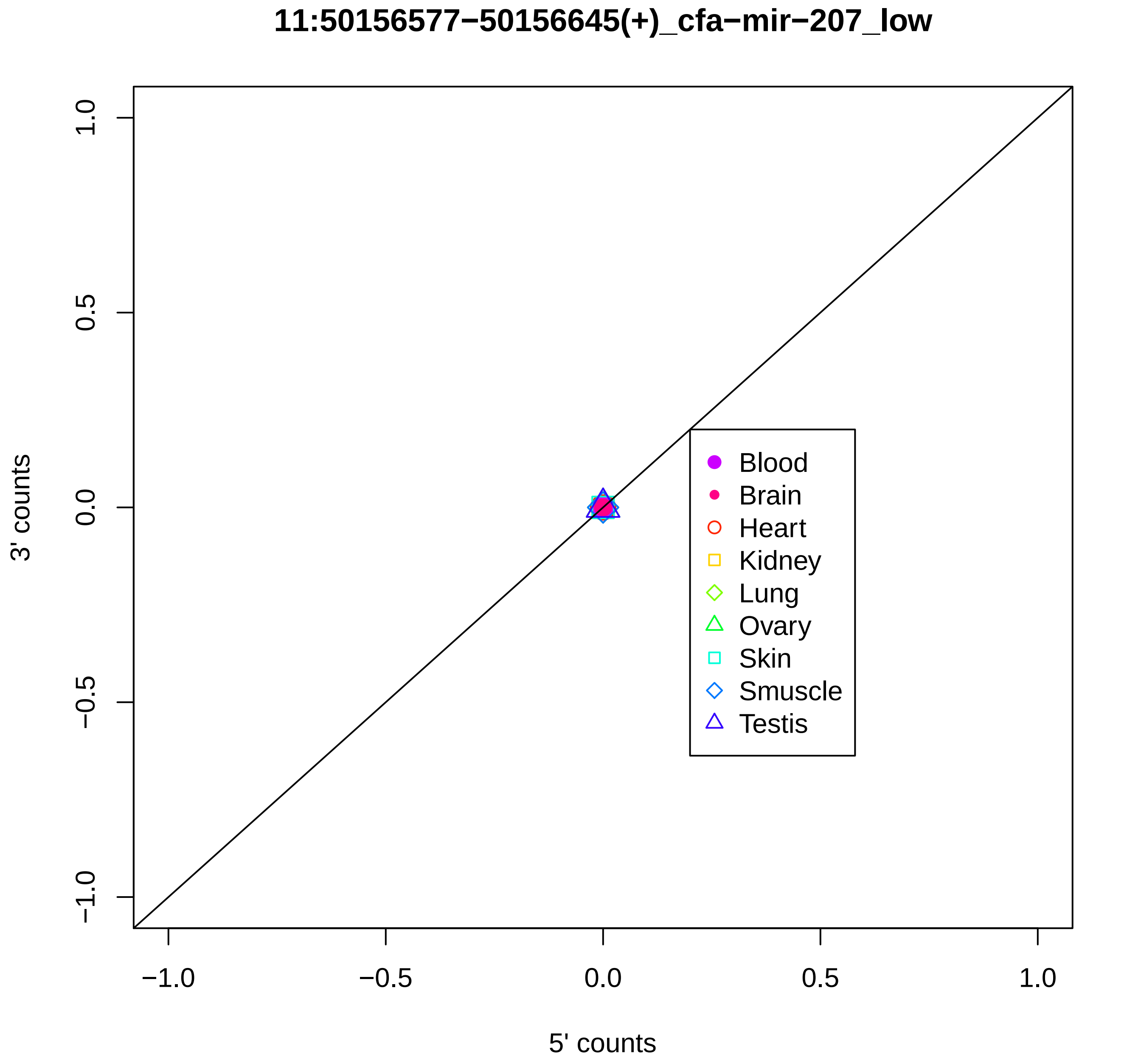

11:50156577−50156645(+)_cfa−mir−207_low
3' counts
5' counts
1.0
0.5
0.0
−0.5
−1.0
−1.0
−0.5
0.0
0.5
1.0
Blood
Brain
Heart
Kidney
Lung
Ovary
Skin
Smuscle
Testis

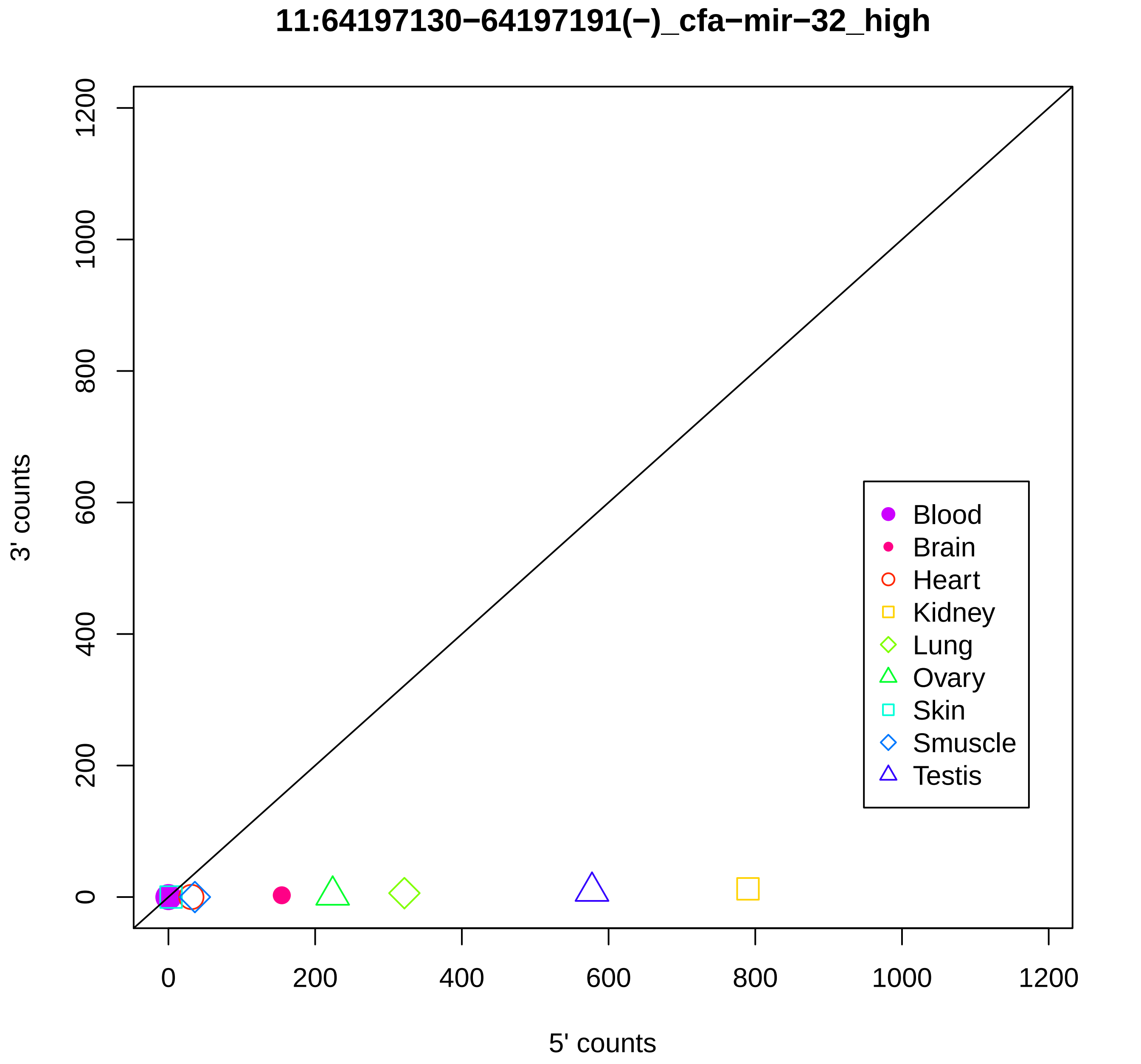

11:64197130−64197191(−)_cfa−mir−32_high
3' counts
5' counts
0
200
400
600
800
1000
1200
Blood
Brain
Heart
Kidney
Lung
Ovary
Skin
Smuscle
Testis

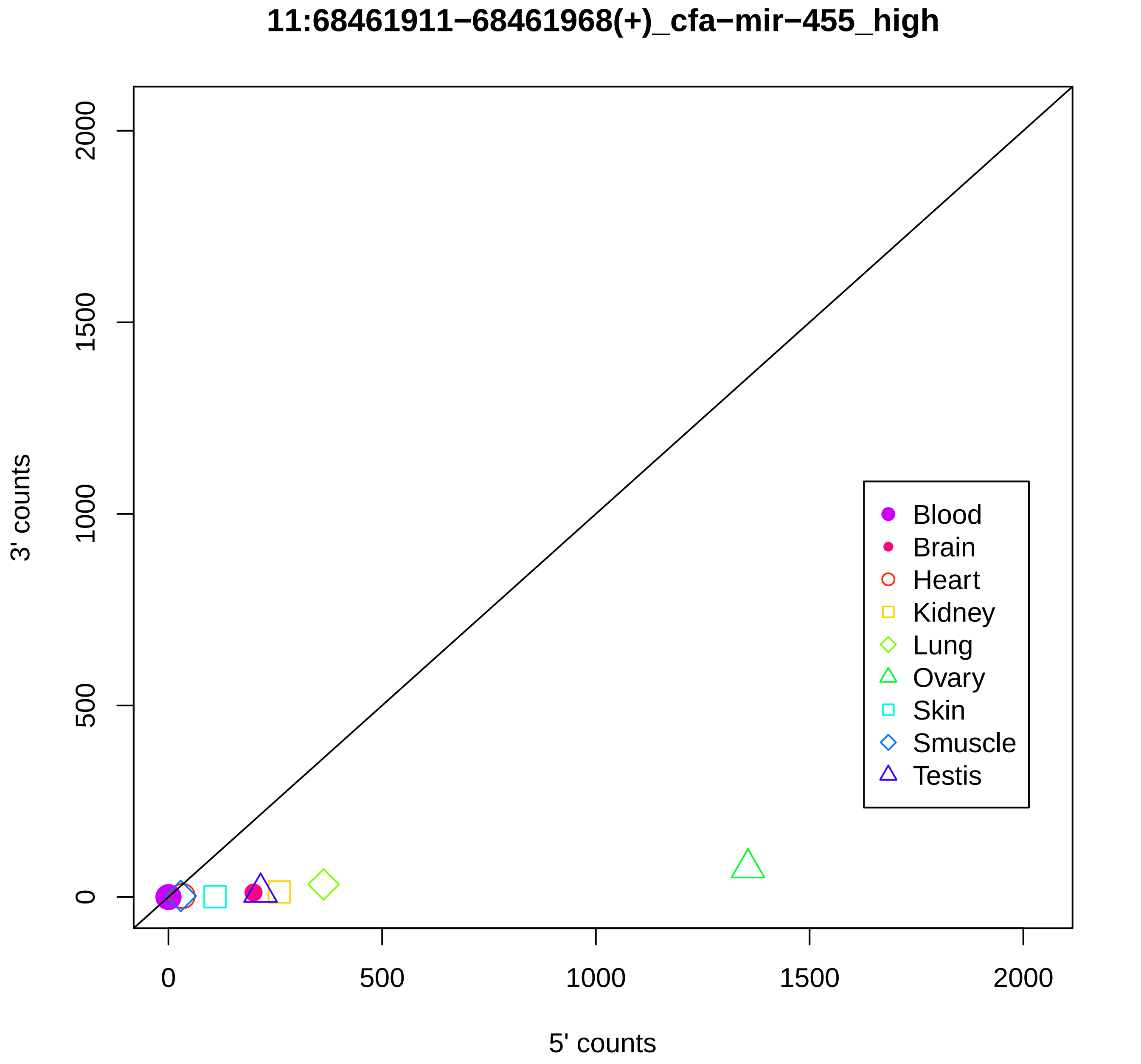

11:68461911−68461968(+)_cfa−mir−455_high
3' counts
5' counts
0
500
1000
1500
2000
0
500
1000
1500
2000
Blood
Brain
Heart
Kidney
Lung
Ovary
Skin
Smuscle
Testis

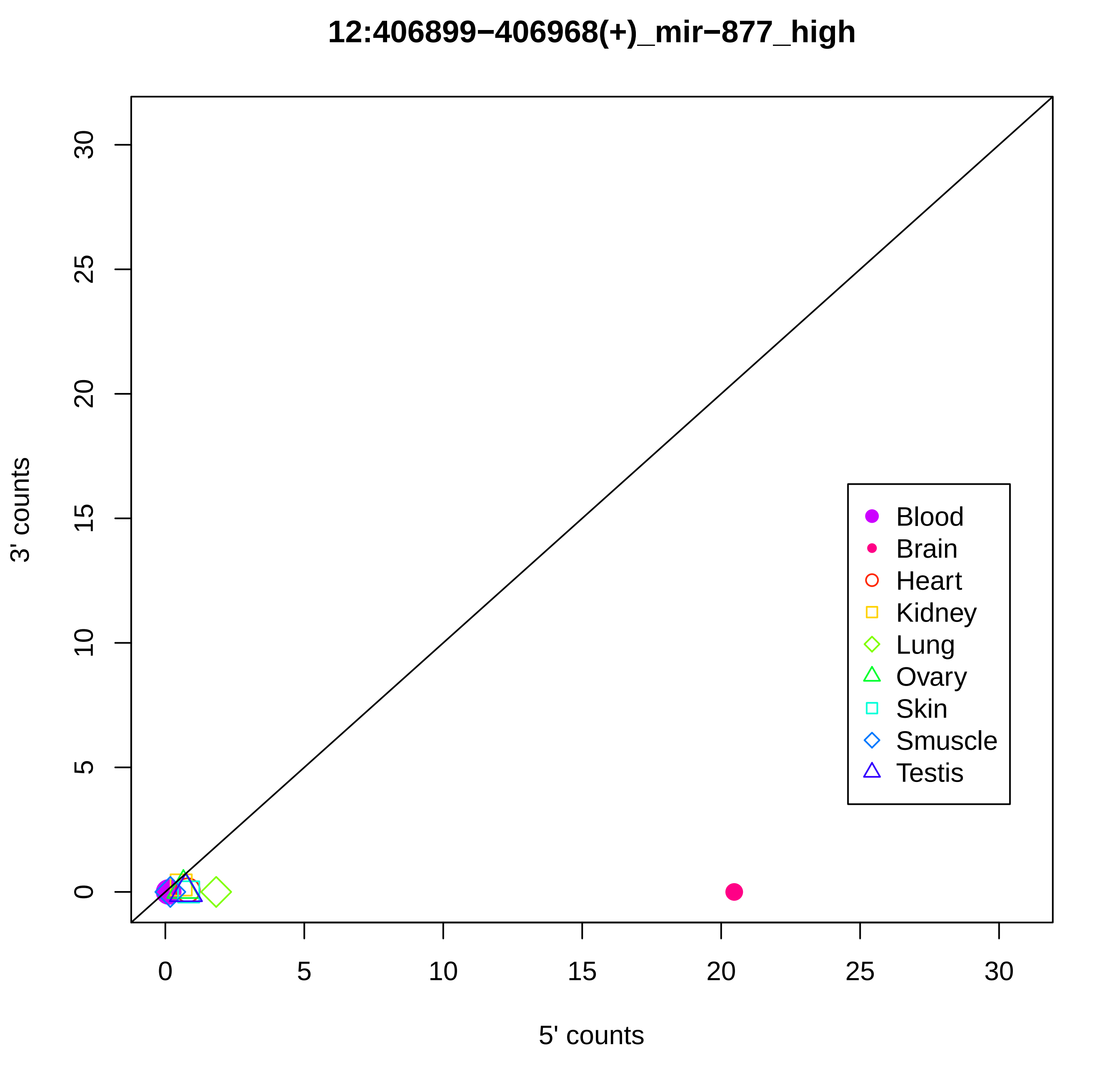

12:406899−406968(+)_mir−877_high
3' counts
5' counts
0
5
10
15
20
25
30
0
5
10
15
20
25
30
Blood
Brain
Heart
Kidney
Lung
Ovary
Skin
Smuscle
Testis

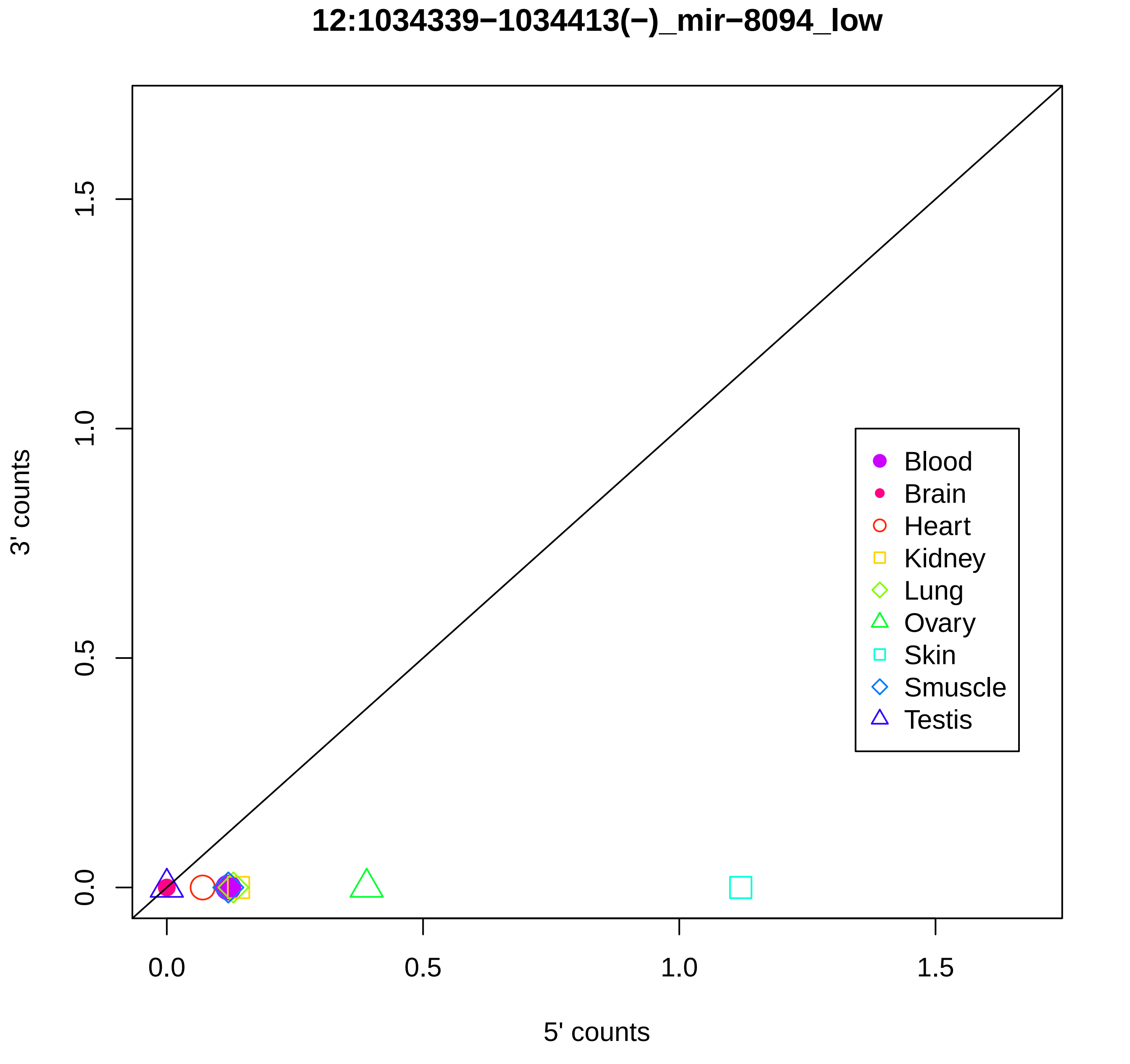
12:1034339−1034413(−)_mir−8094_low
3' counts
5' counts
0.0
0.5
1.0
1.5
0.0
0.5
1.0
1.5
Blood
Brain
Heart
Kidney
Lung
Ovary
Skin
Smuscle
Testis

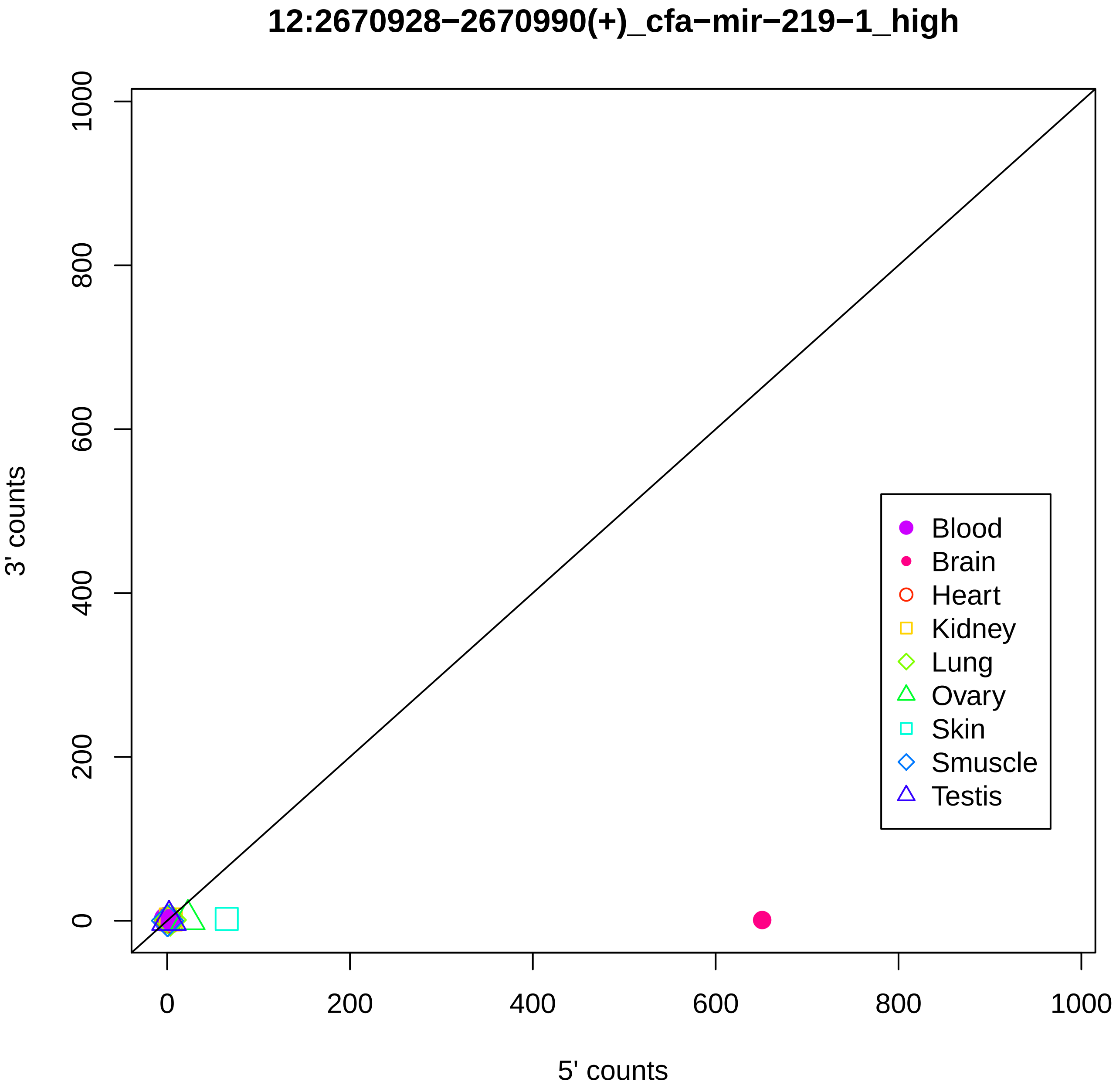

12:2670928−2670990(+)_cfa−mir−219−1_high
3' counts
5' counts
0
200
400
600
800
1000
Blood
Brain
Heart
Kidney
Lung
Ovary
Skin
Smuscle
Testis

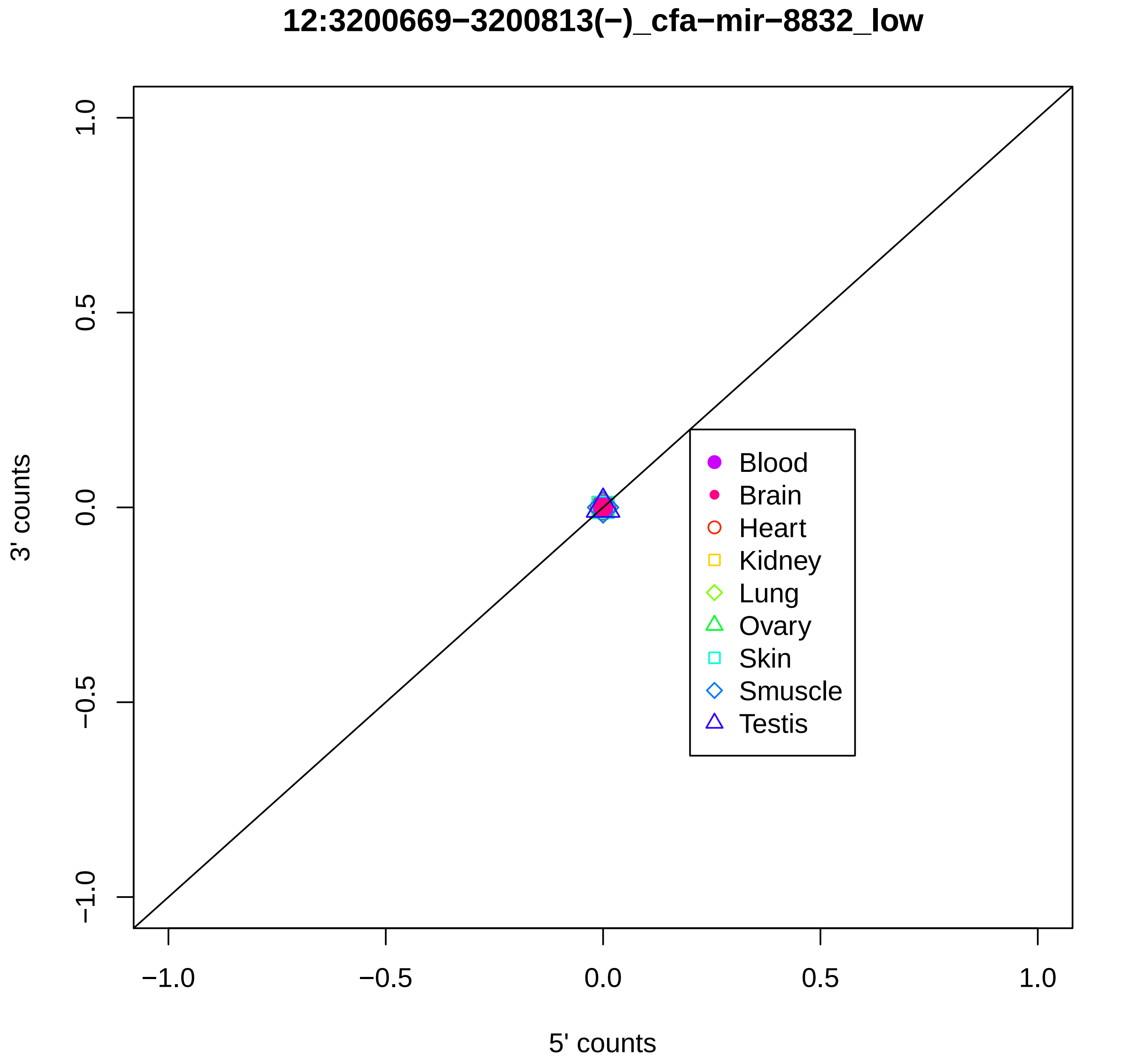
12:3200669−3200813(−)_cfa−mir−8832_low
3' counts
5' counts
1.0
0.5
0.0
−0.5
−1.0
Blood
Brain
Heart
Kidney
Lung
Ovary
Skin
Smuscle
Testis

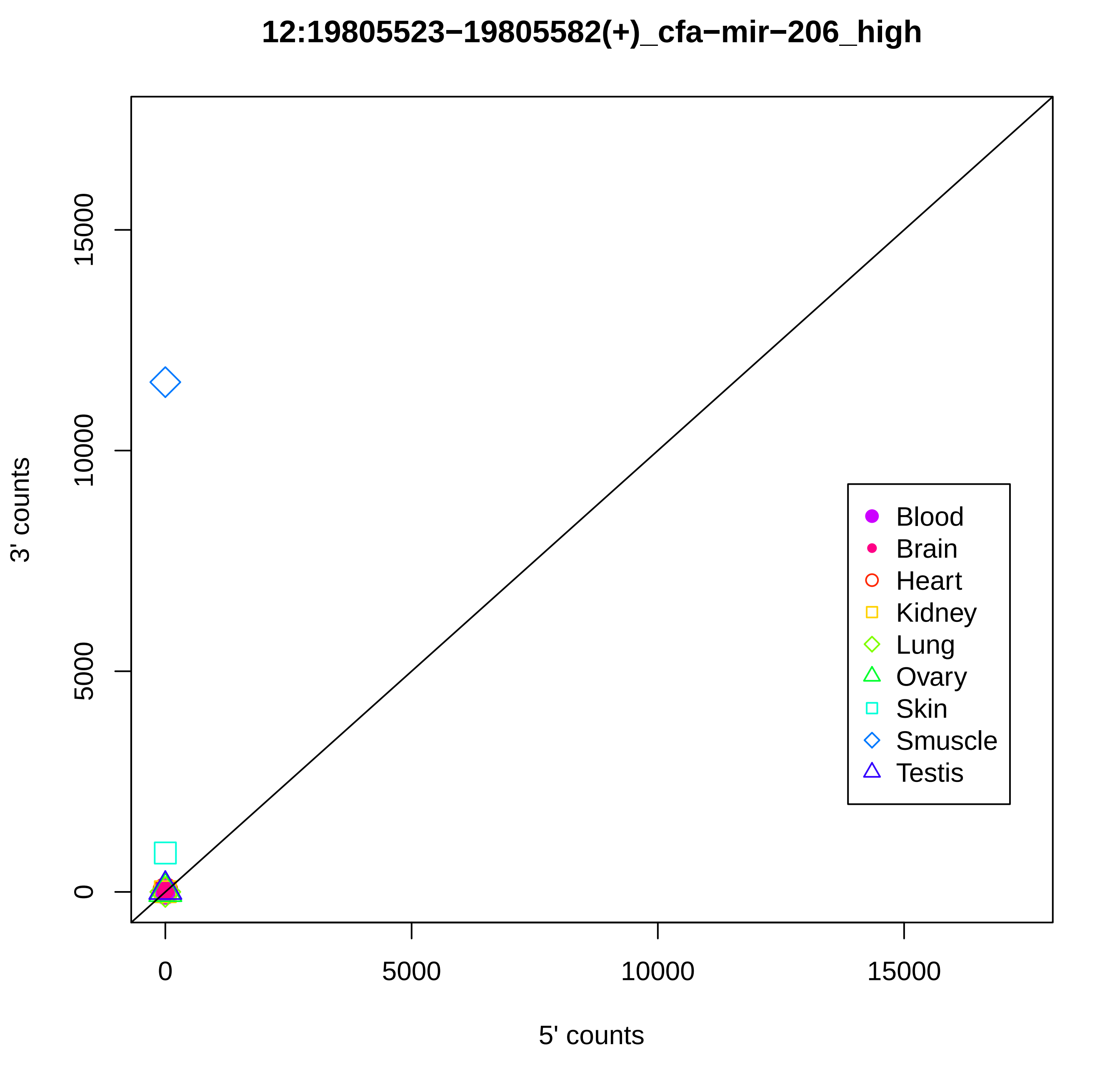
12:19805523−19805582(+)_cfa−mir−206_high
3' counts
5' counts
0
5000
10000
15000
0
5000
10000
15000
Blood
Brain
Heart
Kidney
Lung
Ovary
Skin
Smuscle
Testis

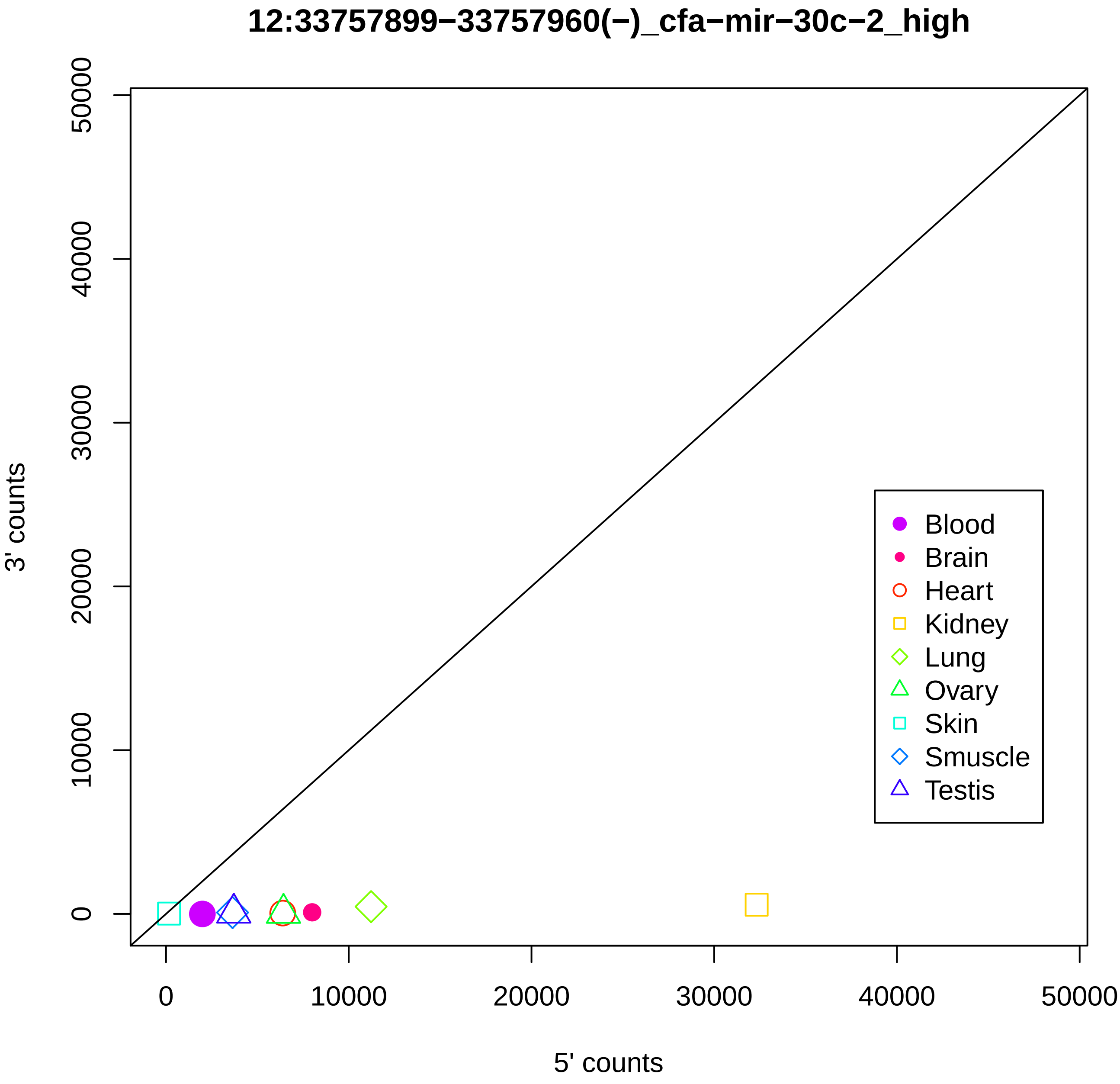
12:33757899−33757960(−)_cfa−mir−30c−2_high
3' counts
5' counts
0
10000
20000
30000
40000
50000
Blood
Brain
Heart
Kidney
Lung
Ovary
Skin
Smuscle
Testis

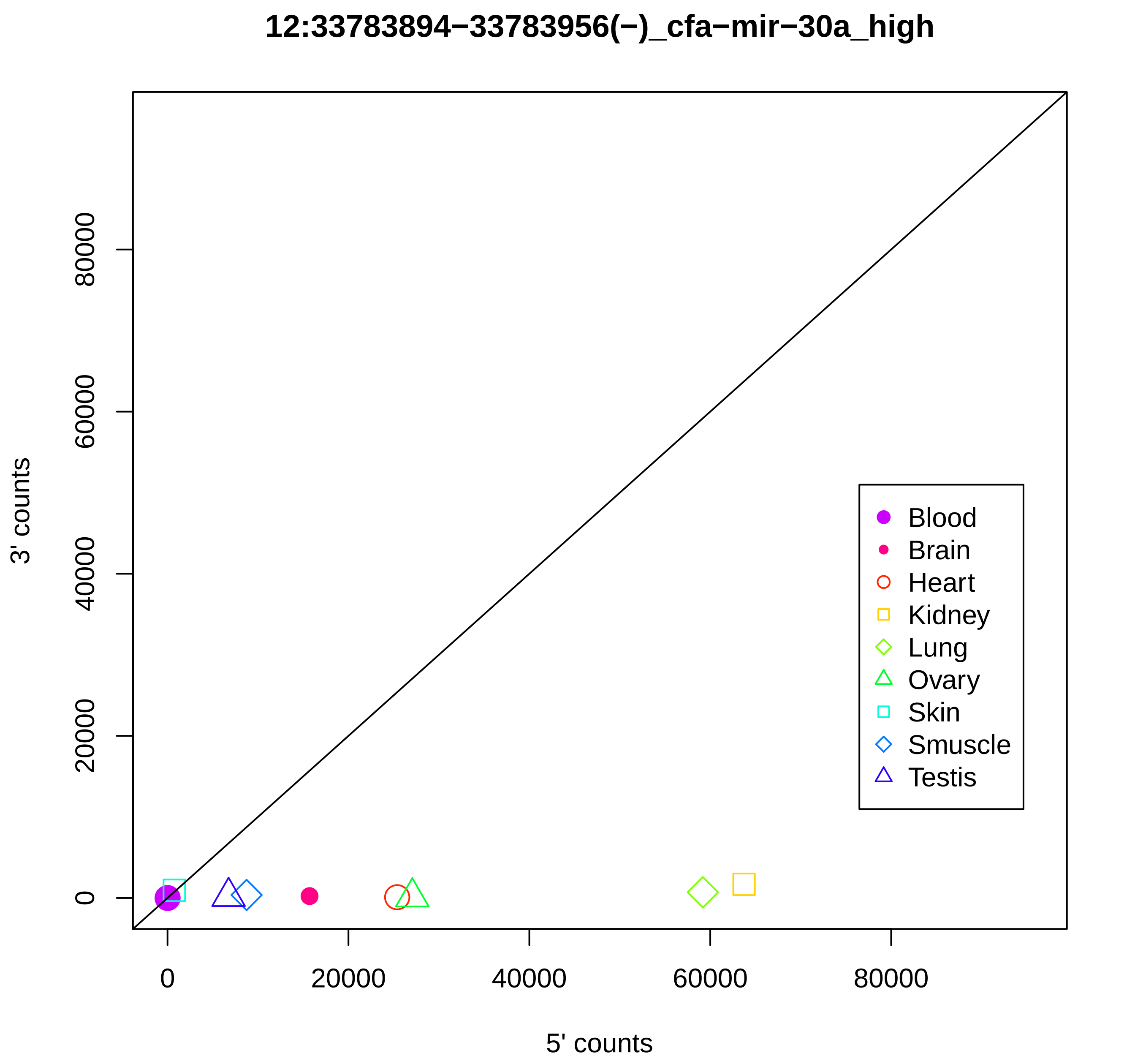
12:33783894−33783956(−)_cfa−mir−30a_high
3' counts
5' counts
0
20000
40000
60000
80000
Blood
Brain
Heart
Kidney
Lung
Ovary
Skin
Smuscle
Testis

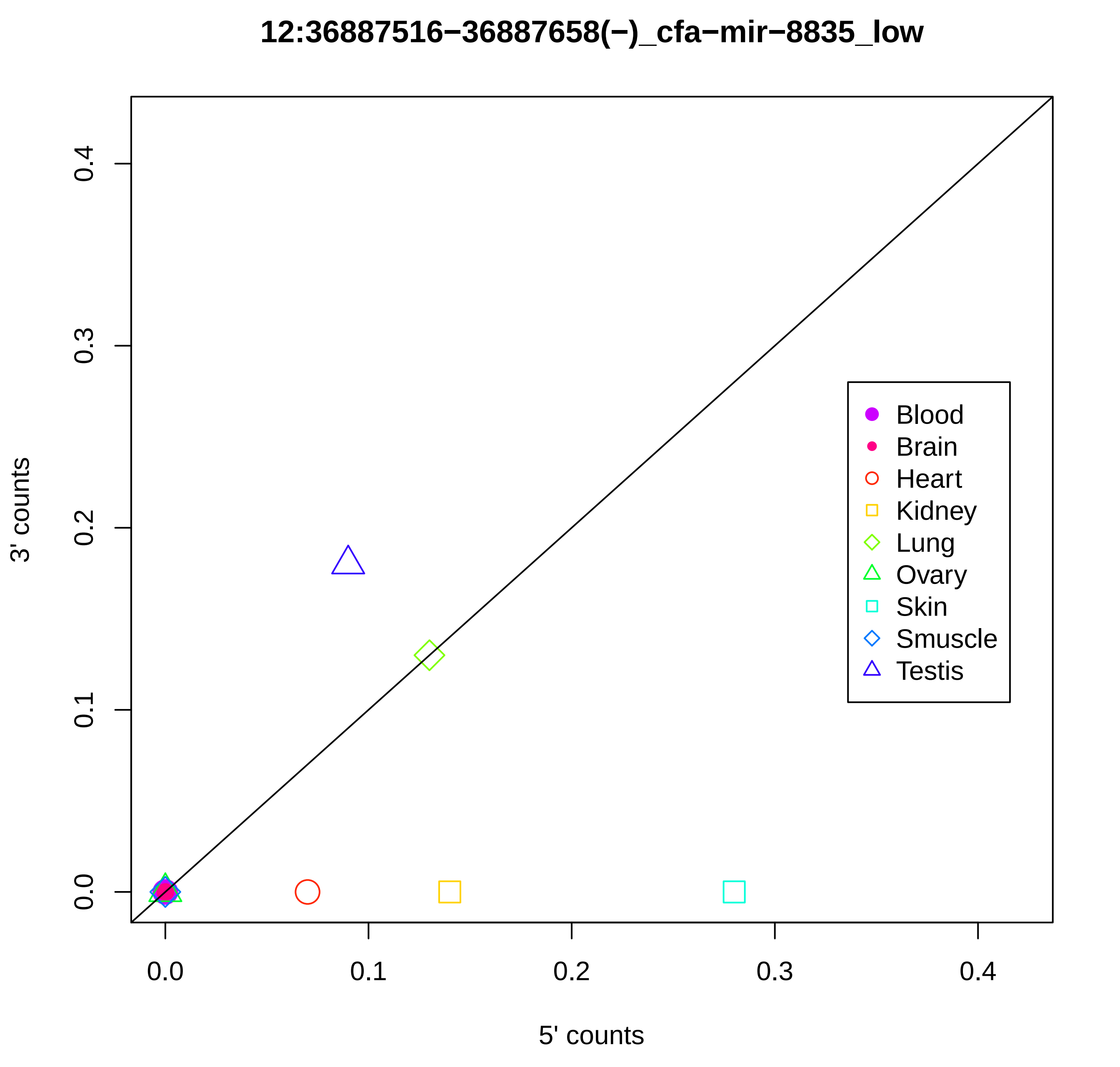

12:36887516−36887658(−)_cfa−mir−8835_low
3' counts
5' counts
0.0
0.1
0.2
0.3
0.4
Blood
Brain
Heart
Kidney
Lung
Ovary
Skin
Smuscle
Testis

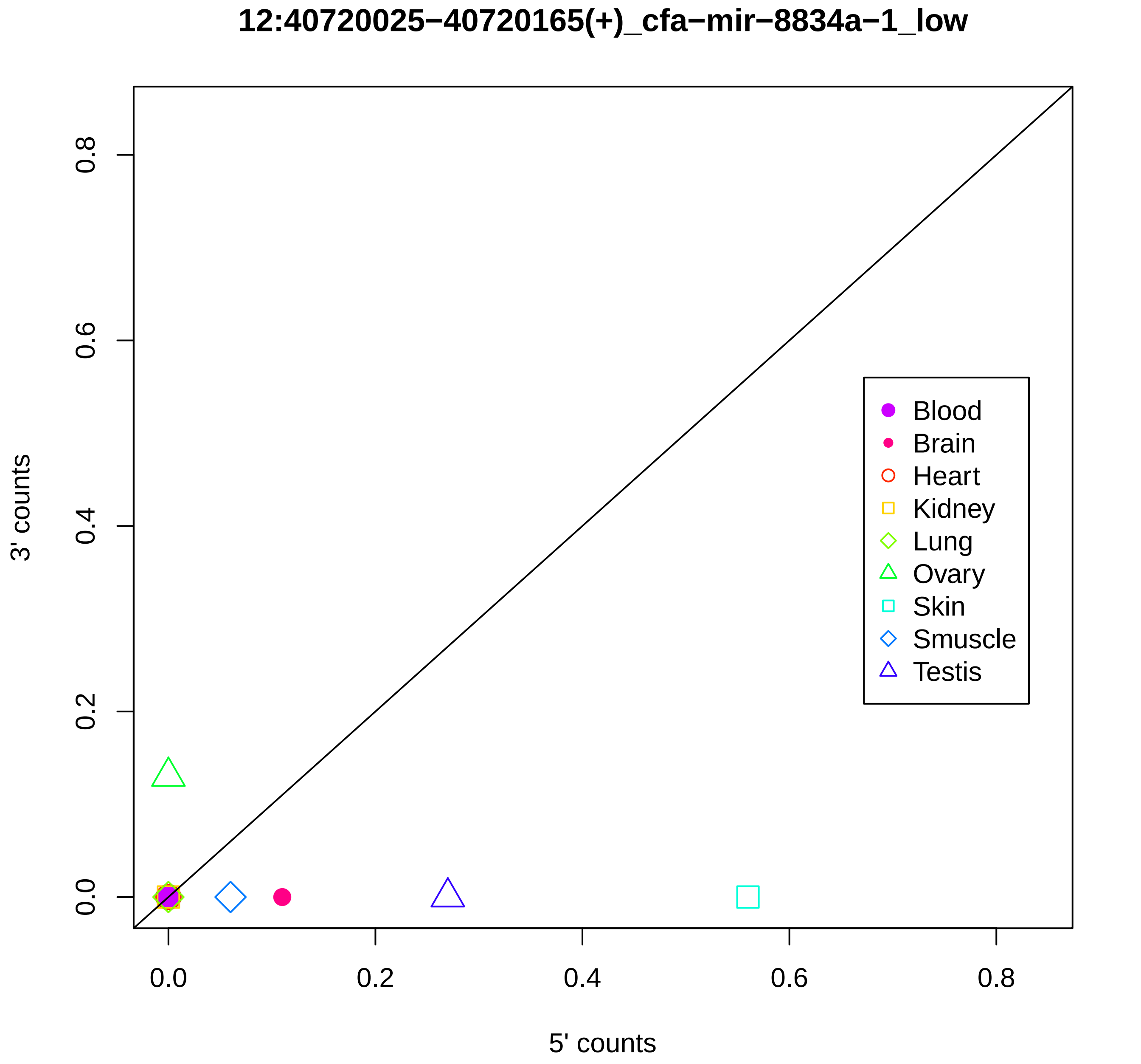
12:40720025−40720165(+)_cfa−mir−8834a−1_low
3' counts
5' counts
0.0
0.2
0.4
0.6
0.8
Blood
Brain
Heart
Kidney
Lung
Ovary
Skin
Smuscle
Testis

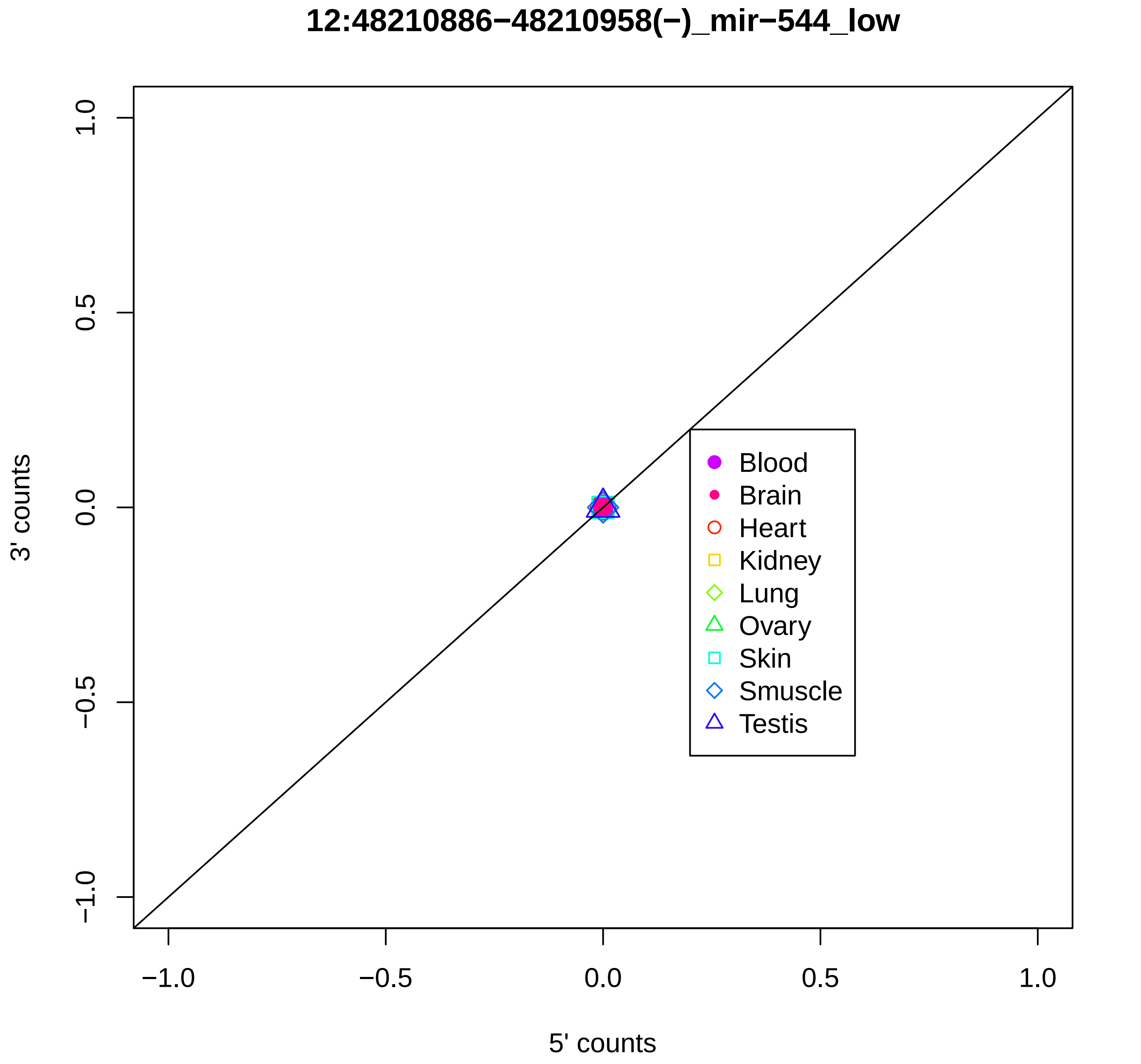

12:48210886−48210958(−)_mir−544_low
3' counts
5' counts
1.0
0.5
0.0
−0.5
−1.0
Blood
Brain
Heart
Kidney
Lung
Ovary
Skin
Smuscle
Testis

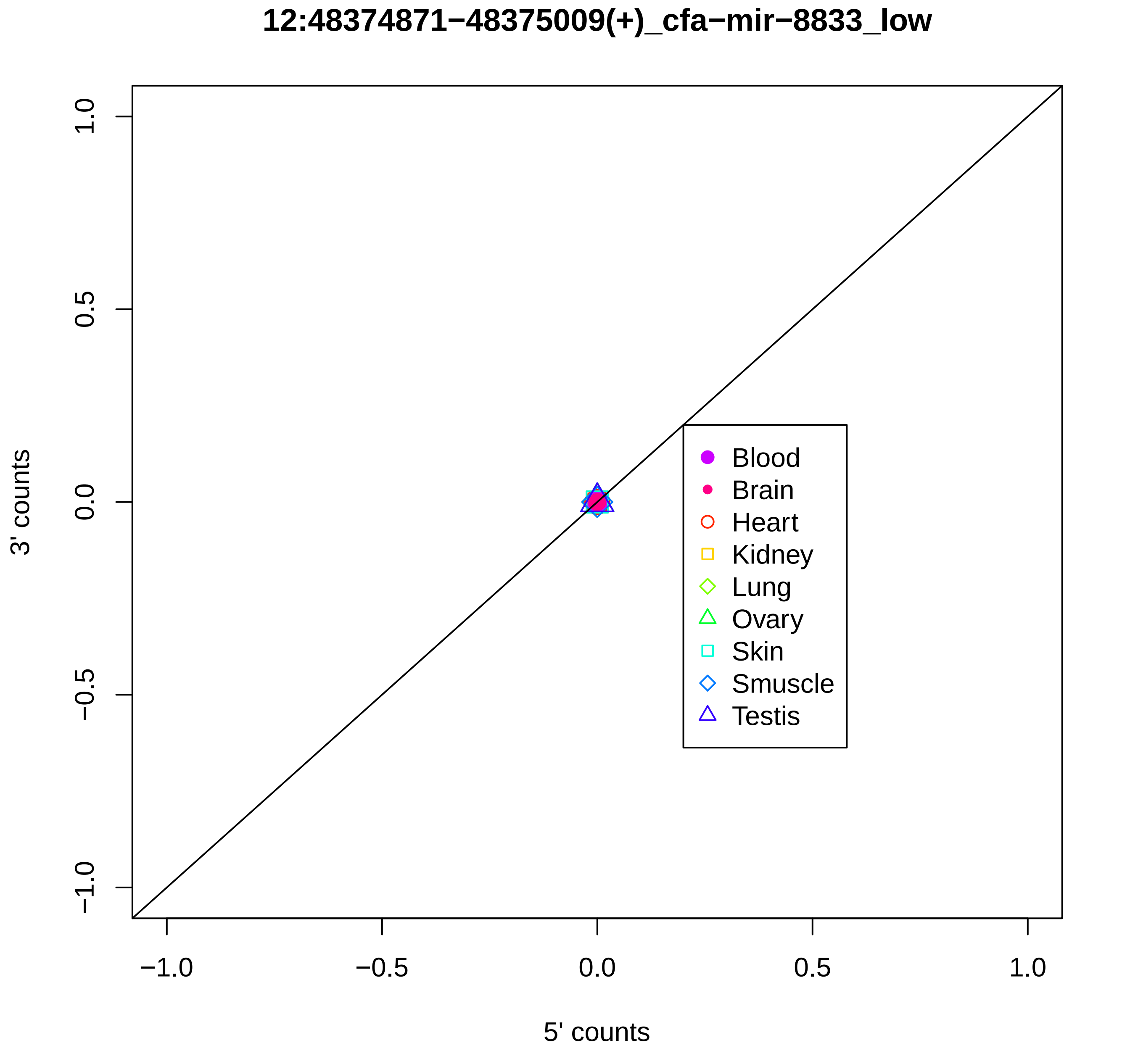

12:48374871−48375009(+)_cfa−mir−8833_low
Blood
Brain
Heart
Kidney
Lung
Ovary
Skin
Smuscle
Testis
1.0
0.5
0.0
−0.5
−1.0
3' counts
−1.0
−0.5
0.0
0.5
1.0
5' counts

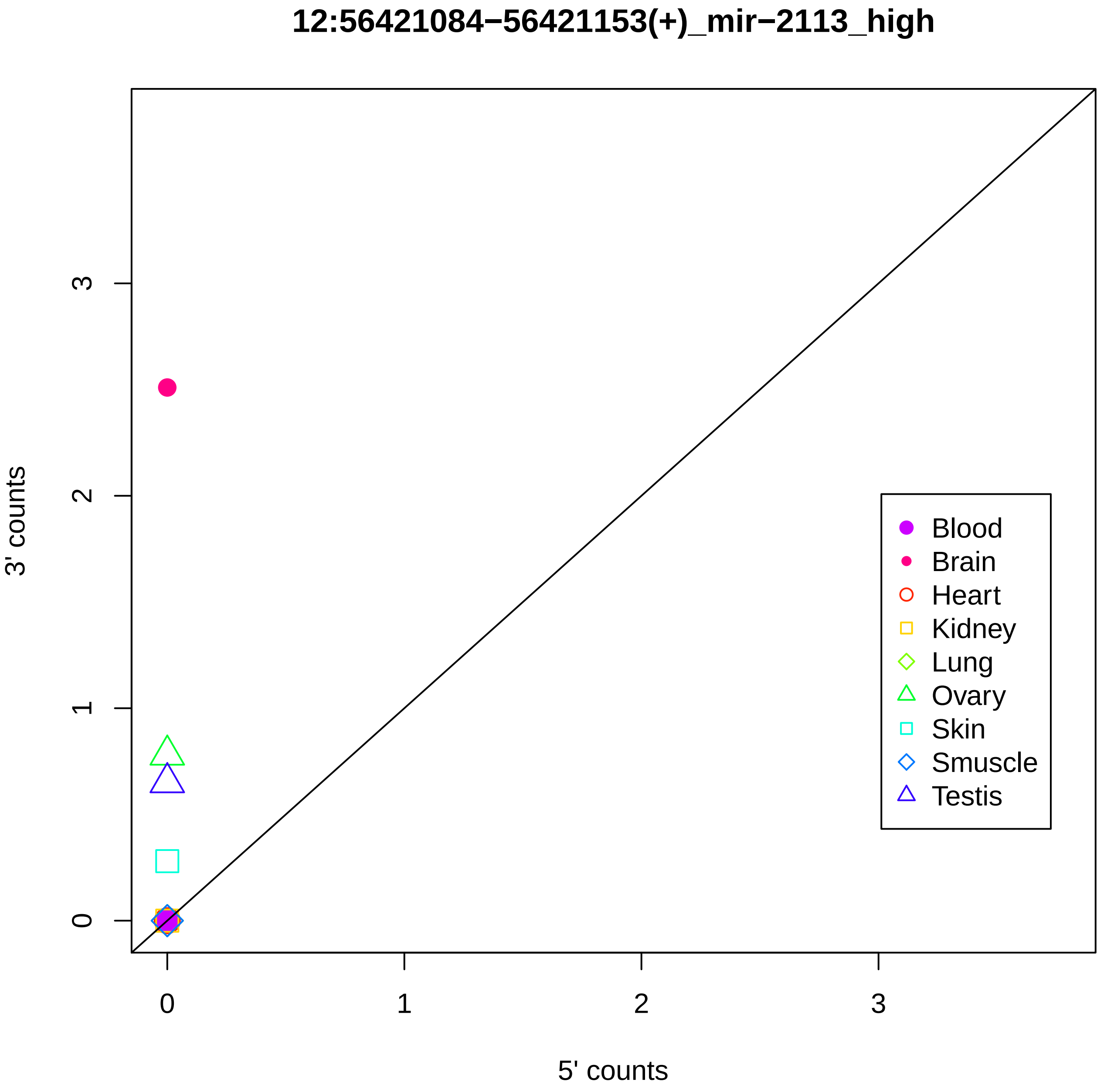
12:56421084−56421153(+)_mir−2113_high
3' counts
5' counts
0
1
2
3
0
1
2
3
Blood
Brain
Heart
Kidney
Lung
Ovary
Skin
Smuscle
Testis

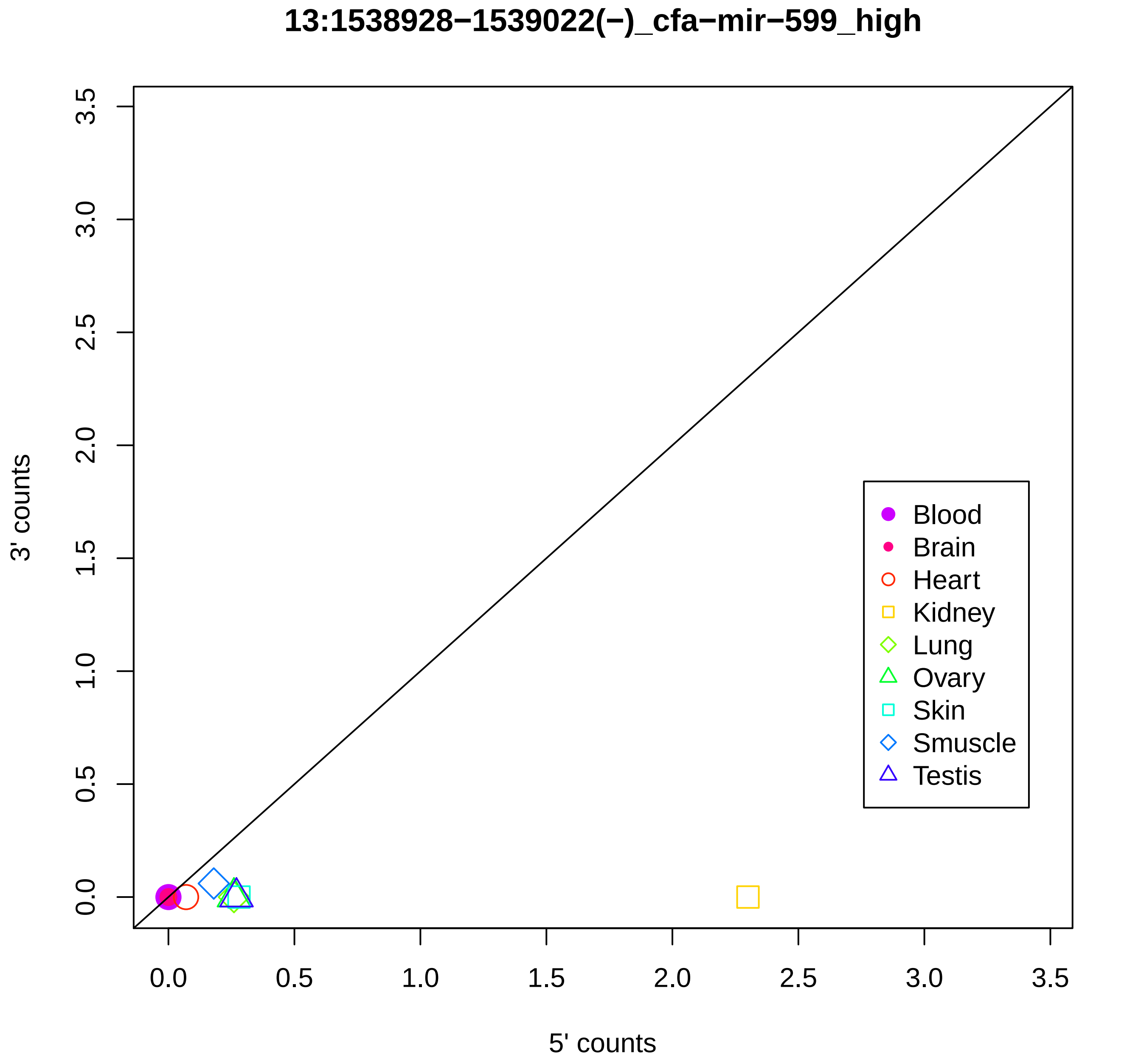
13:1538928−1539022(−)_cfa−mir−599_high
3' counts
5' counts
0.0
0.5
1.0
1.5
2.0
2.5
3.0
3.5
Blood
Brain
Heart
Kidney
Lung
Ovary
Skin
Smuscle
Testis

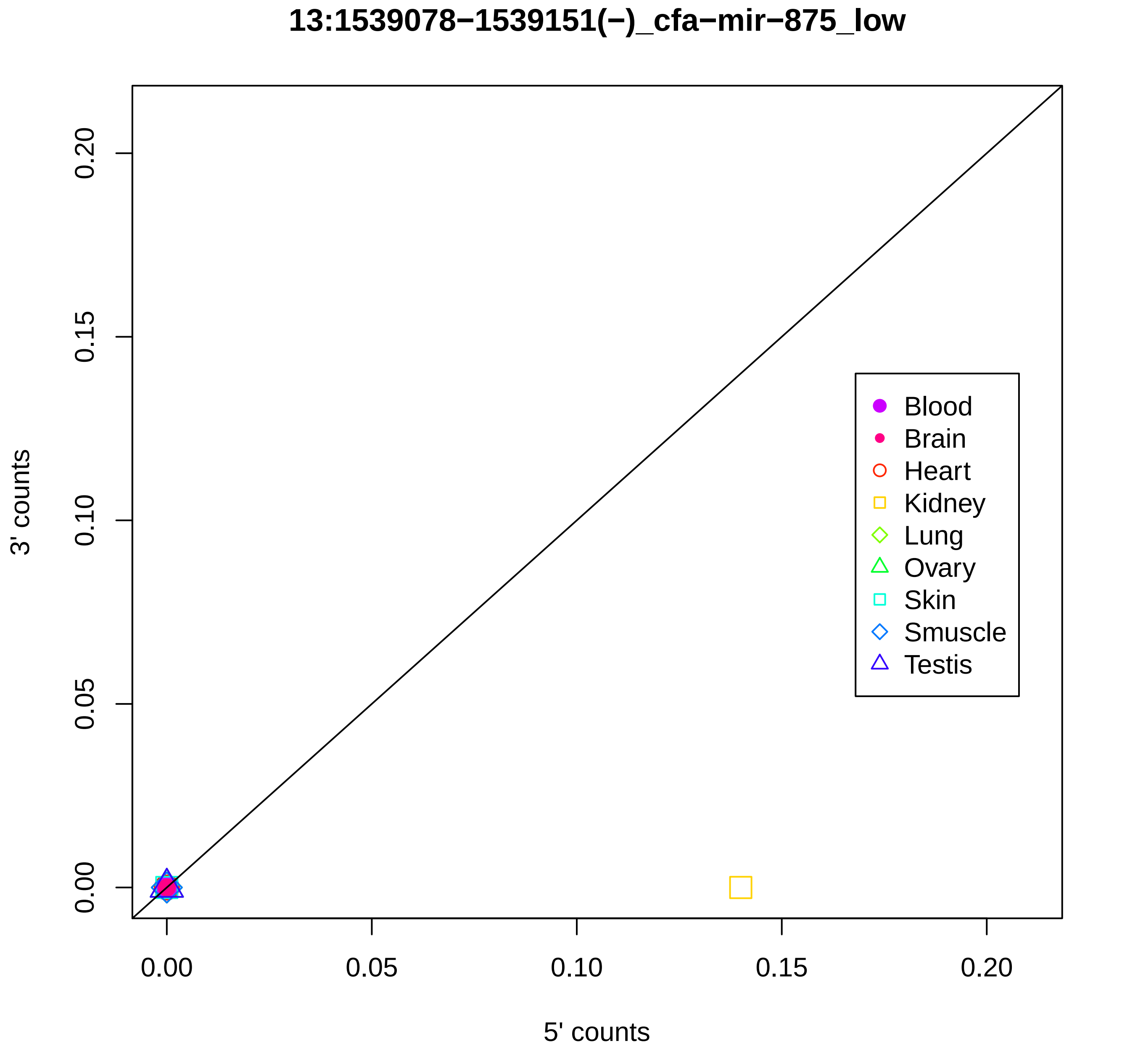
13:1539078−1539151(−)_cfa−mir−875_low
3' counts
5' counts
0.00
0.05
0.10
0.15
0.20
0.00
0.05
0.10
0.15
0.20
Blood
Brain
Heart
Kidney
Lung
Ovary
Skin
Smuscle
Testis

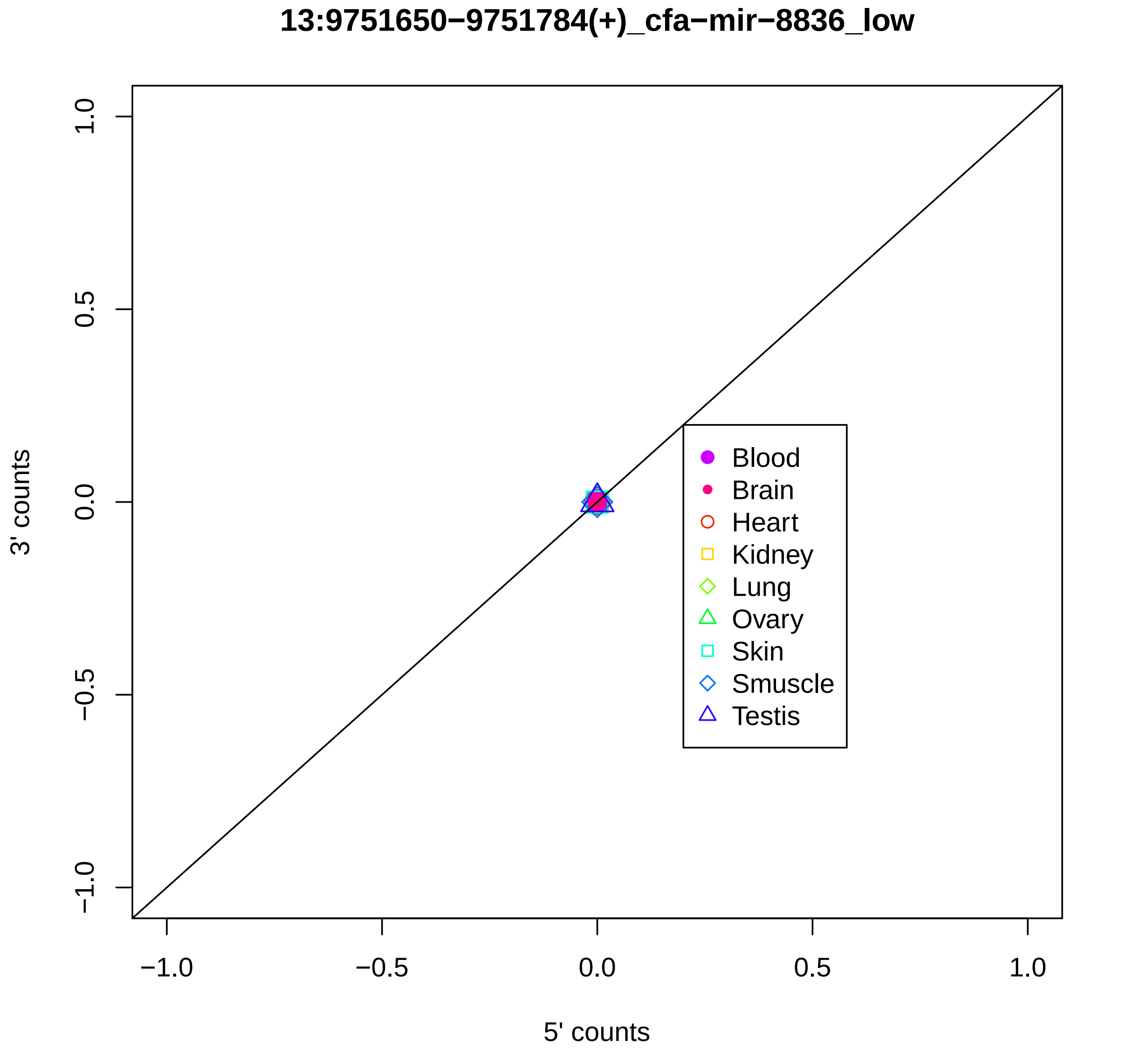
13:9751650−9751784(+)_cfa−mir−8836_low
3' counts
5' counts
1.0
0.5
0.0
−0.5
−1.0
−1.0
−0.5
0.0
0.5
1.0
Blood
Brain
Heart
Kidney
Lung
Ovary
Skin
Smuscle
Testis

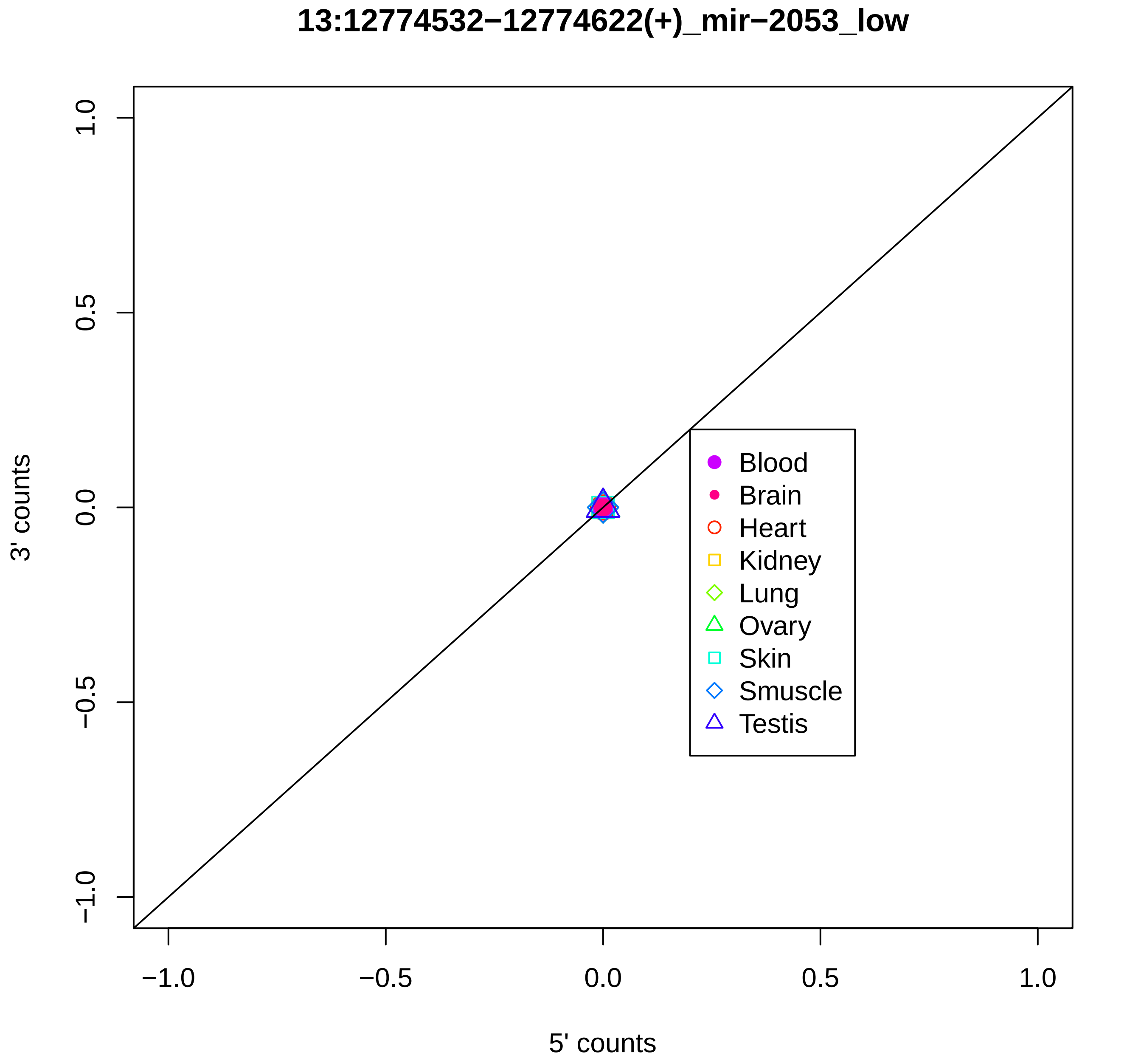

13:12774532−12774622(+)_mir−2053_low
3' counts
5' counts
1.0
0.5
0.0
−0.5
−1.0
−1.0
−0.5
0.0
0.5
1.0
Blood
Brain
Heart
Kidney
Lung
Ovary
Skin
Smuscle
Testis

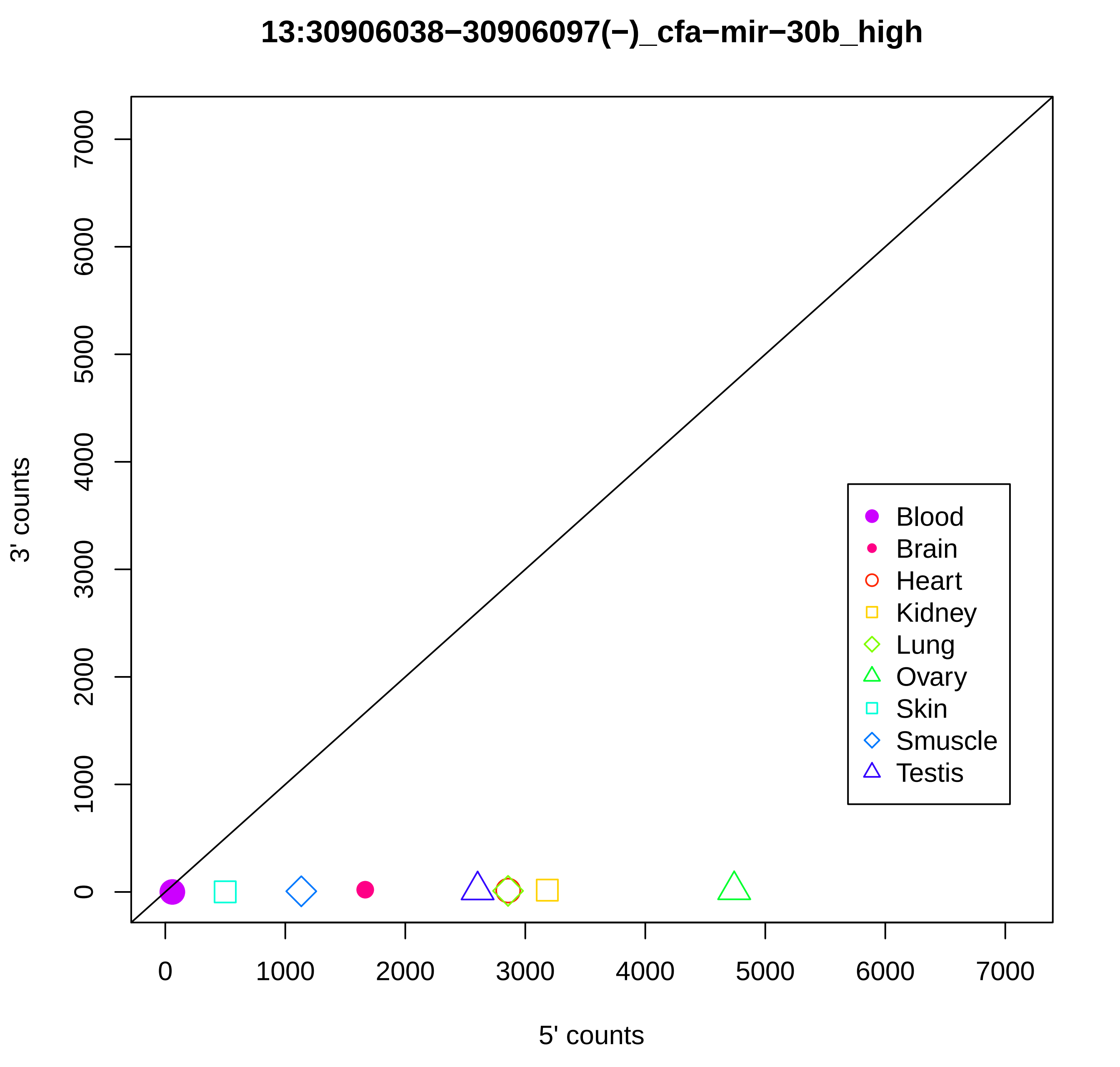
13:30906038−30906097(−)_cfa−mir−30b_high
3' counts
5' counts
0
1000
2000
3000
4000
5000
6000
7000
0
1000
2000
3000
4000
5000
6000
7000
Blood
Brain
Heart
Kidney
Lung
Ovary
Skin
Smuscle
Testis

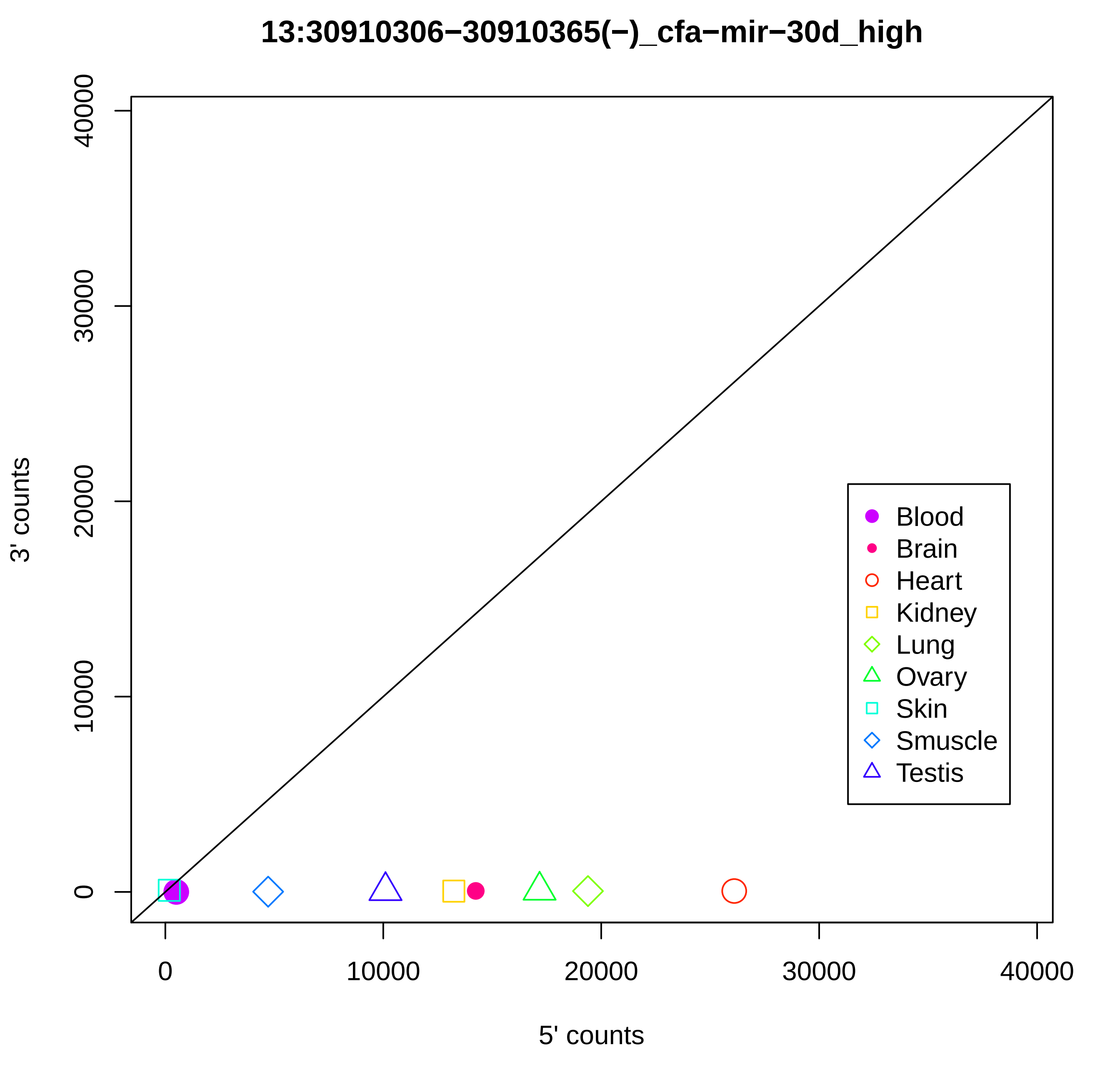

13:30910306−30910365(−)_cfa−mir−30d_high
3' counts
5' counts
0
10000
20000
30000
40000
0
10000
20000
30000
40000
Blood
Brain
Heart
Kidney
Lung
Ovary
Skin
Smuscle
Testis

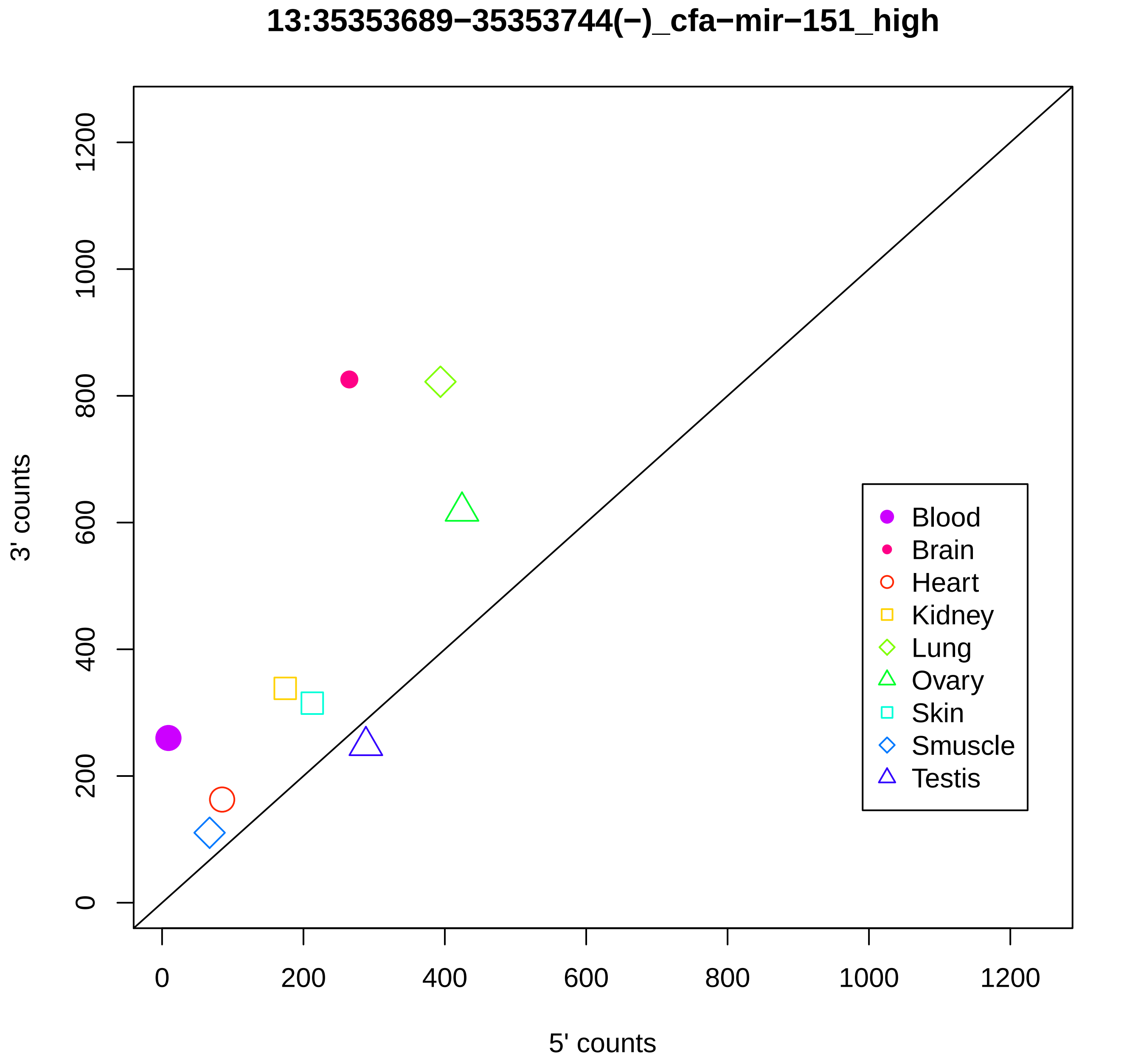

13:35353689−35353744(−)_cfa−mir−151_high
3' counts
5' counts
0
200
400
600
800
1000
1200
Blood
Brain
Heart
Kidney
Lung
Ovary
Skin
Smuscle
Testis

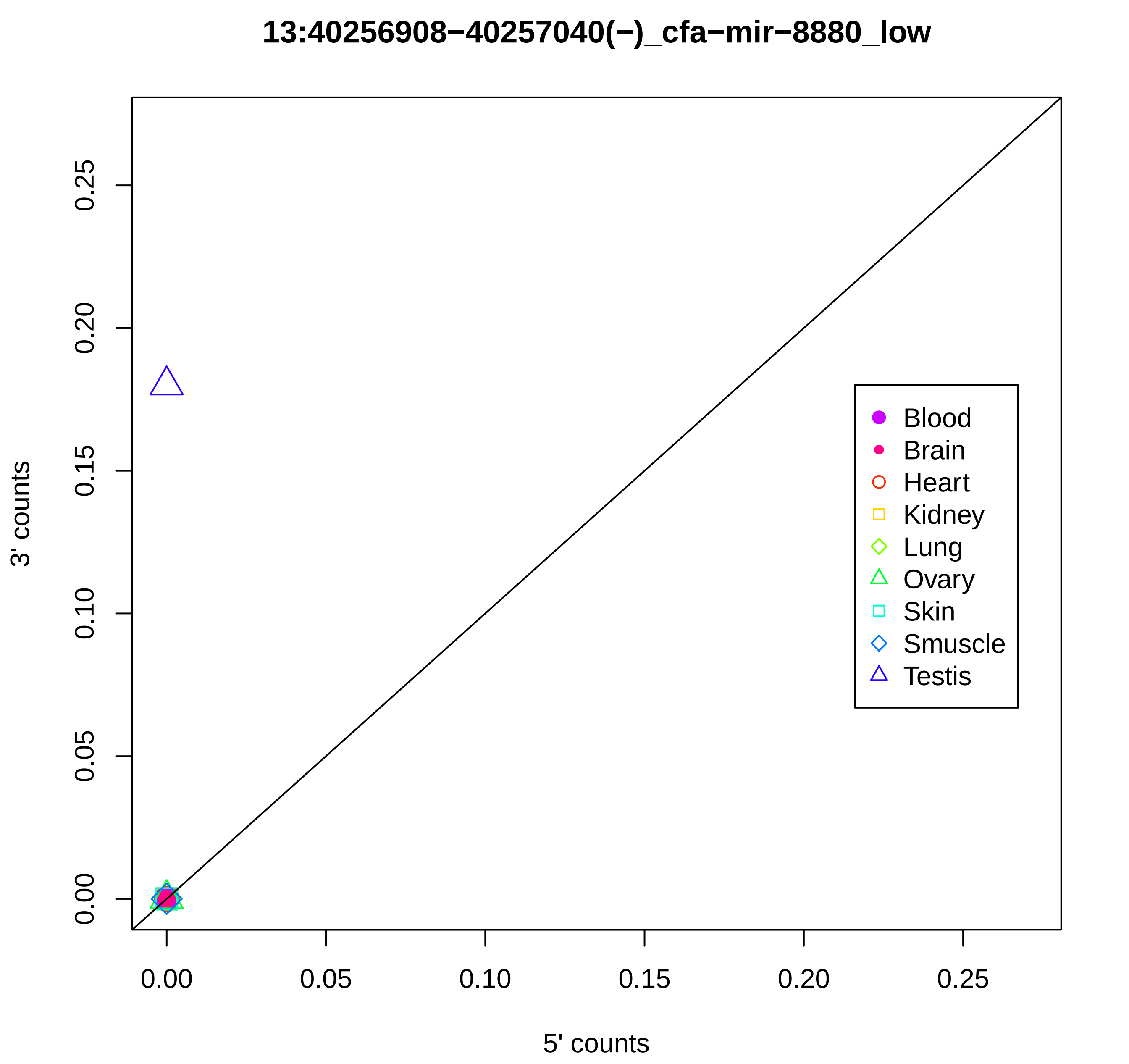
13:40256908−40257040(−)_cfa−mir−8880_low
3' counts
5' counts
0.00
0.05
0.10
0.15
0.20
0.25
Blood
Brain
Heart
Kidney
Lung
Ovary
Skin
Smuscle
Testis

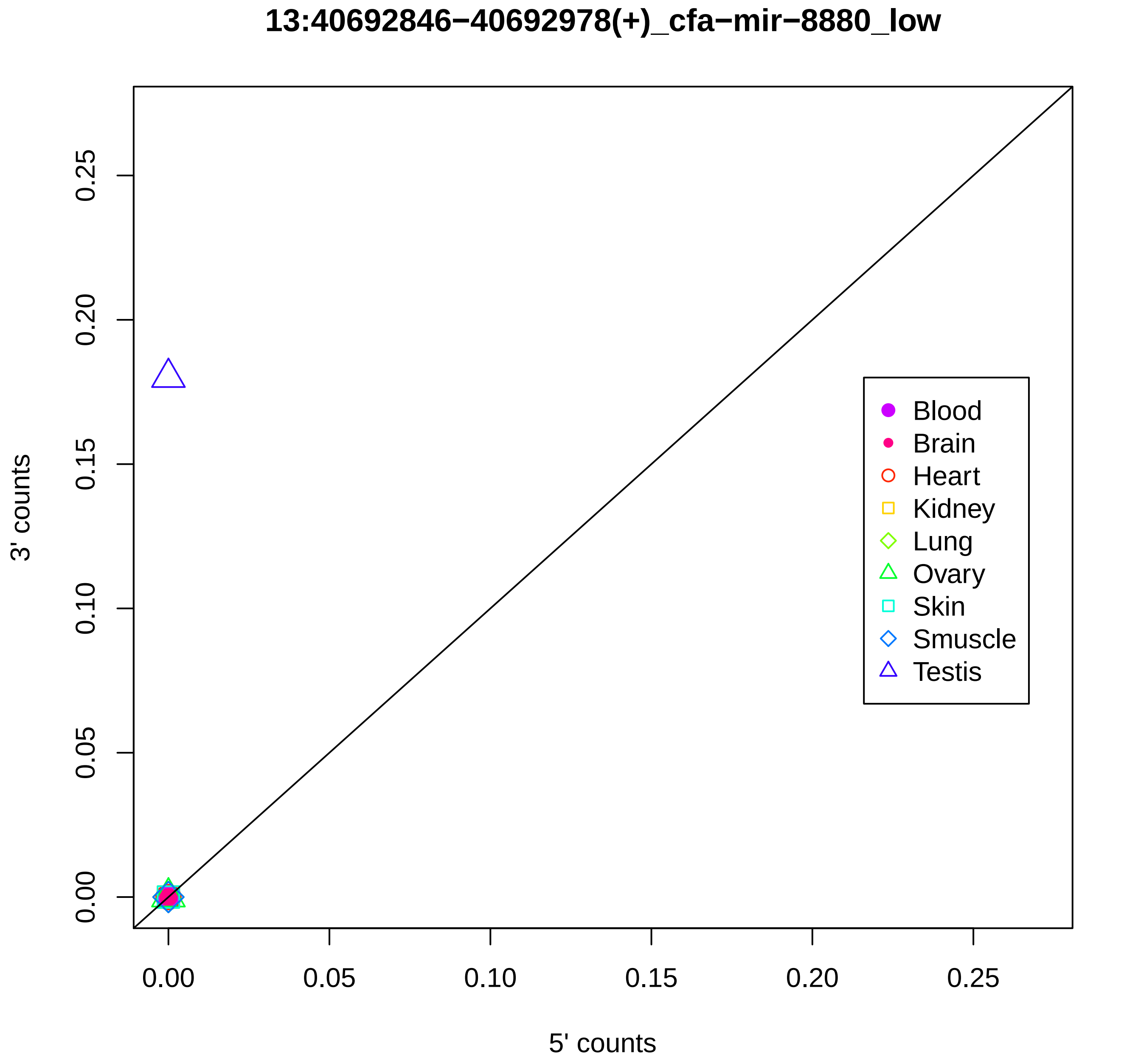

13:40692846−40692978(+)_cfa−mir−8880_low
3' counts
5' counts
0.00
0.05
0.10
0.15
0.20
0.25
Blood
Brain
Heart
Kidney
Lung
Ovary
Skin
Smuscle
Testis

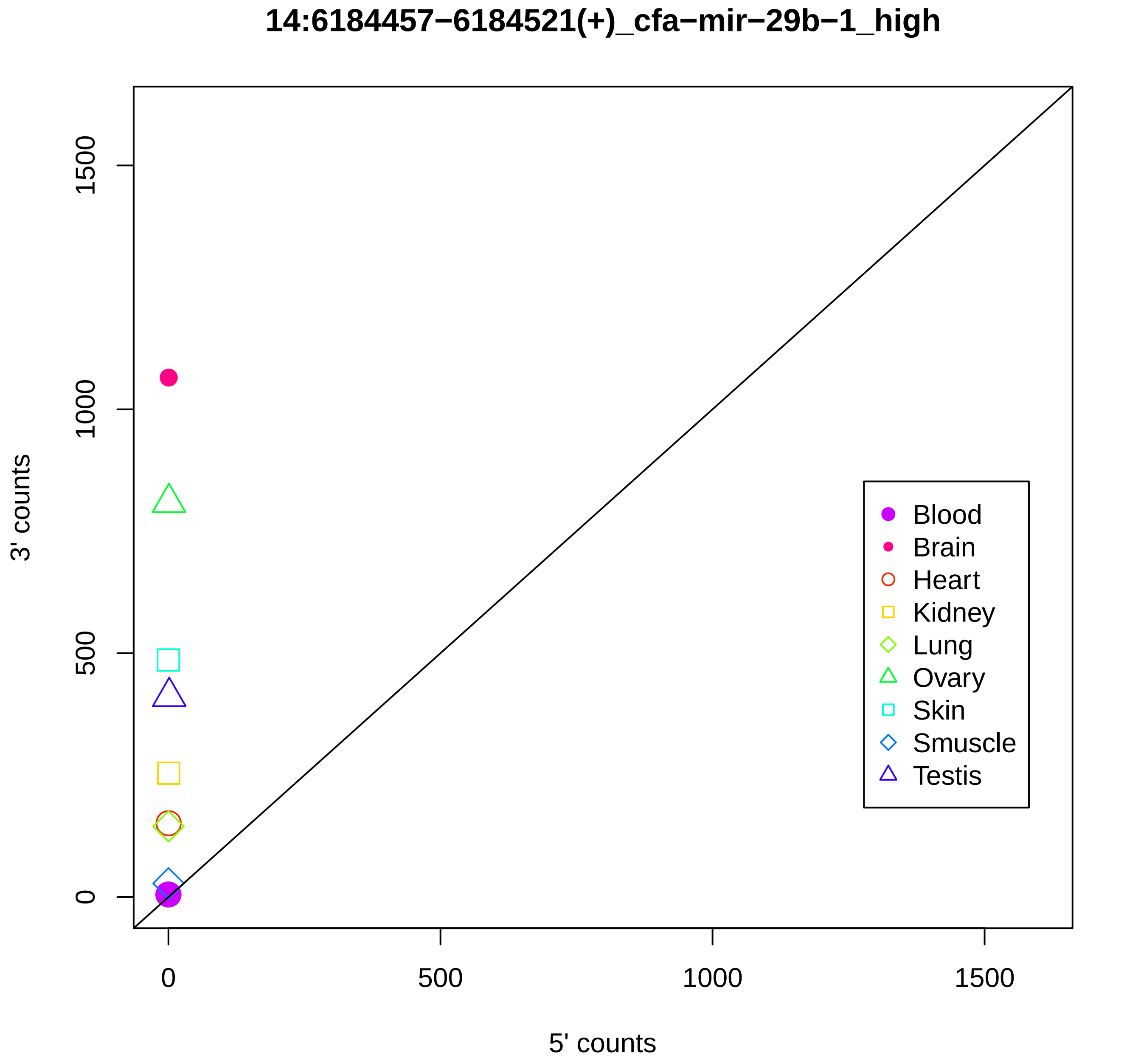
14:6184457−6184521(+)_cfa−mir−29b−1_high
3' counts
5' counts
0
500
1000
1500
0
500
1000
1500
Blood
Brain
Heart
Kidney
Lung
Ovary
Skin
Smuscle
Testis

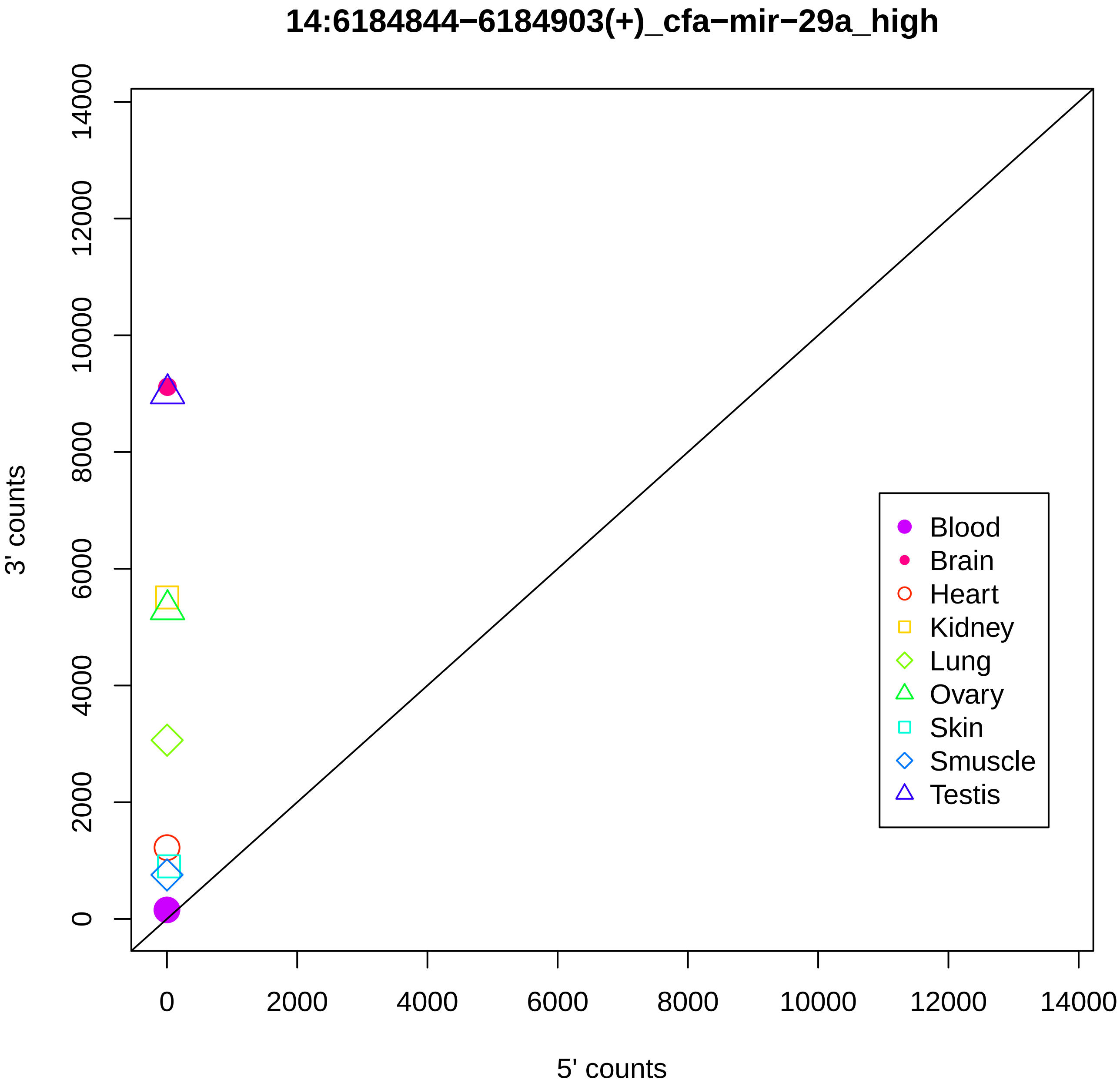
14:6184844−6184903(+)_cfa−mir−29a_high
3' counts
5' counts
0
2000
4000
6000
8000
10000
12000
14000
Blood
Brain
Heart
Kidney
Lung
Ovary
Skin
Smuscle
Testis

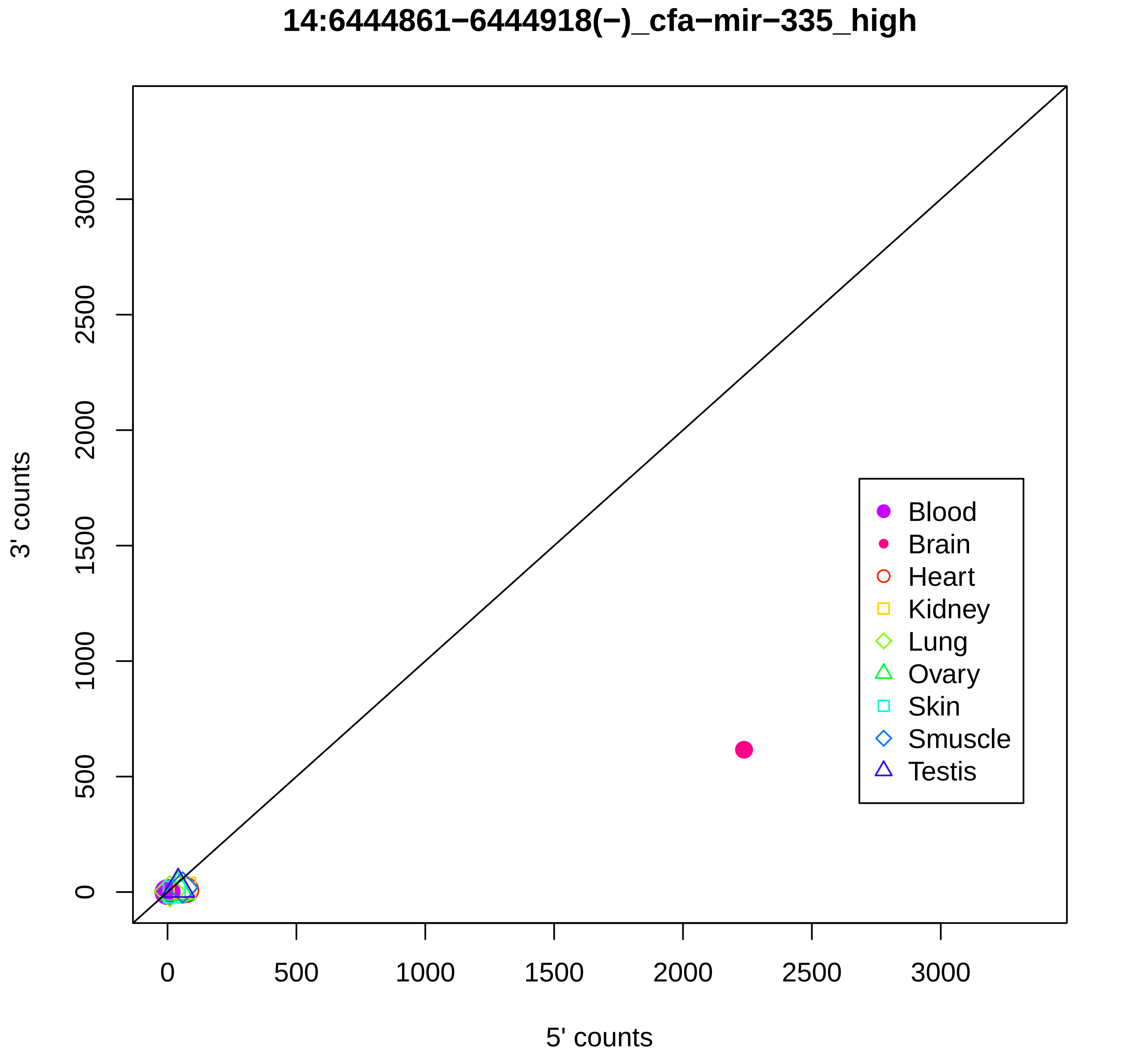
14:6444861−6444918(−)_cfa−mir−335_high
3' counts
5' counts
0
500
1000
1500
2000
2500
3000
Blood
Brain
Heart
Kidney
Lung
Ovary
Skin
Smuscle
Testis

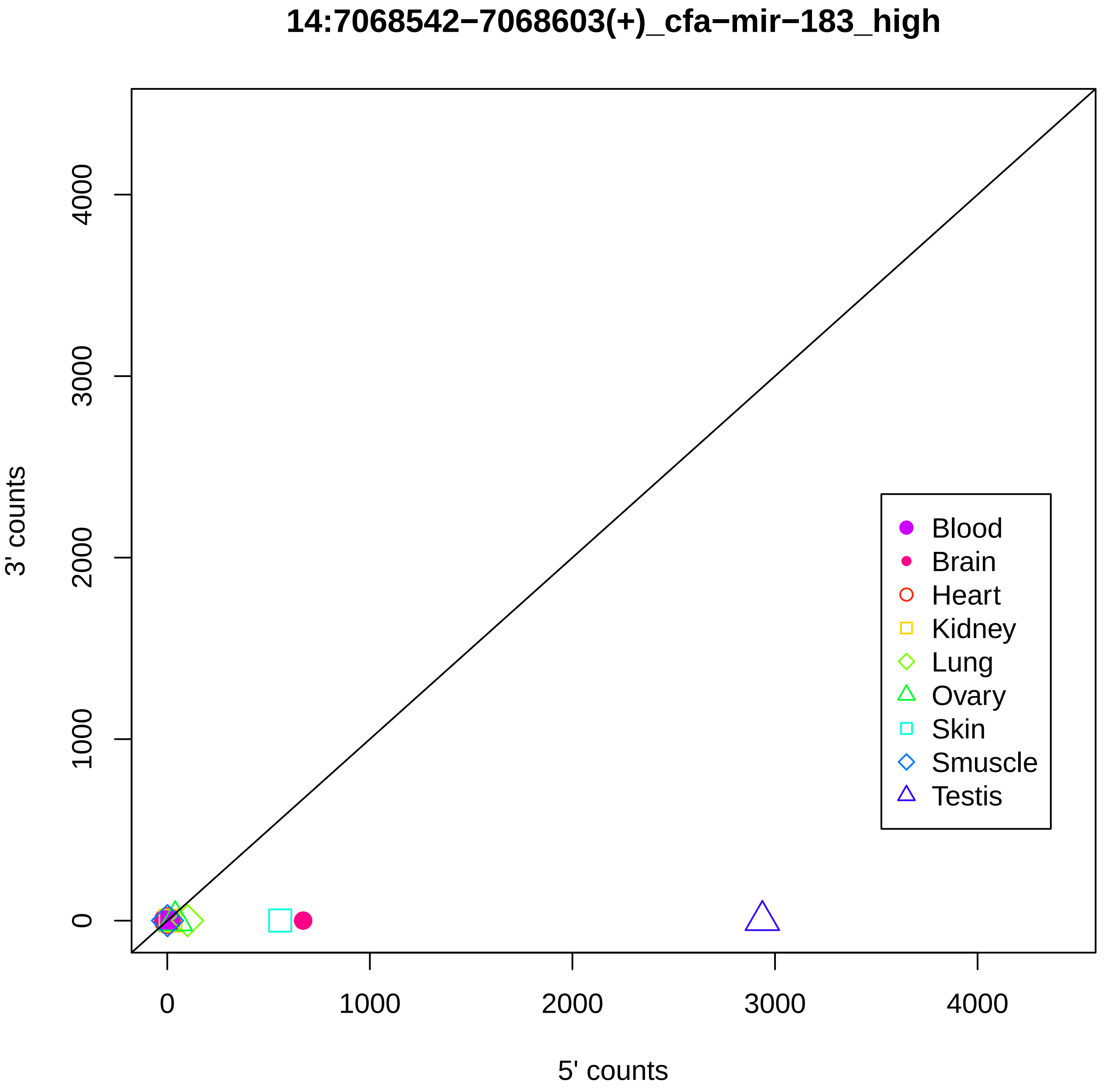
14:7068542−7068603(+)_cfa−mir−183_high
3' counts
5' counts
0
1000
2000
3000
4000
0
1000
2000
3000
4000
Blood
Brain
Heart
Kidney
Lung
Ovary
Skin
Smuscle
Testis

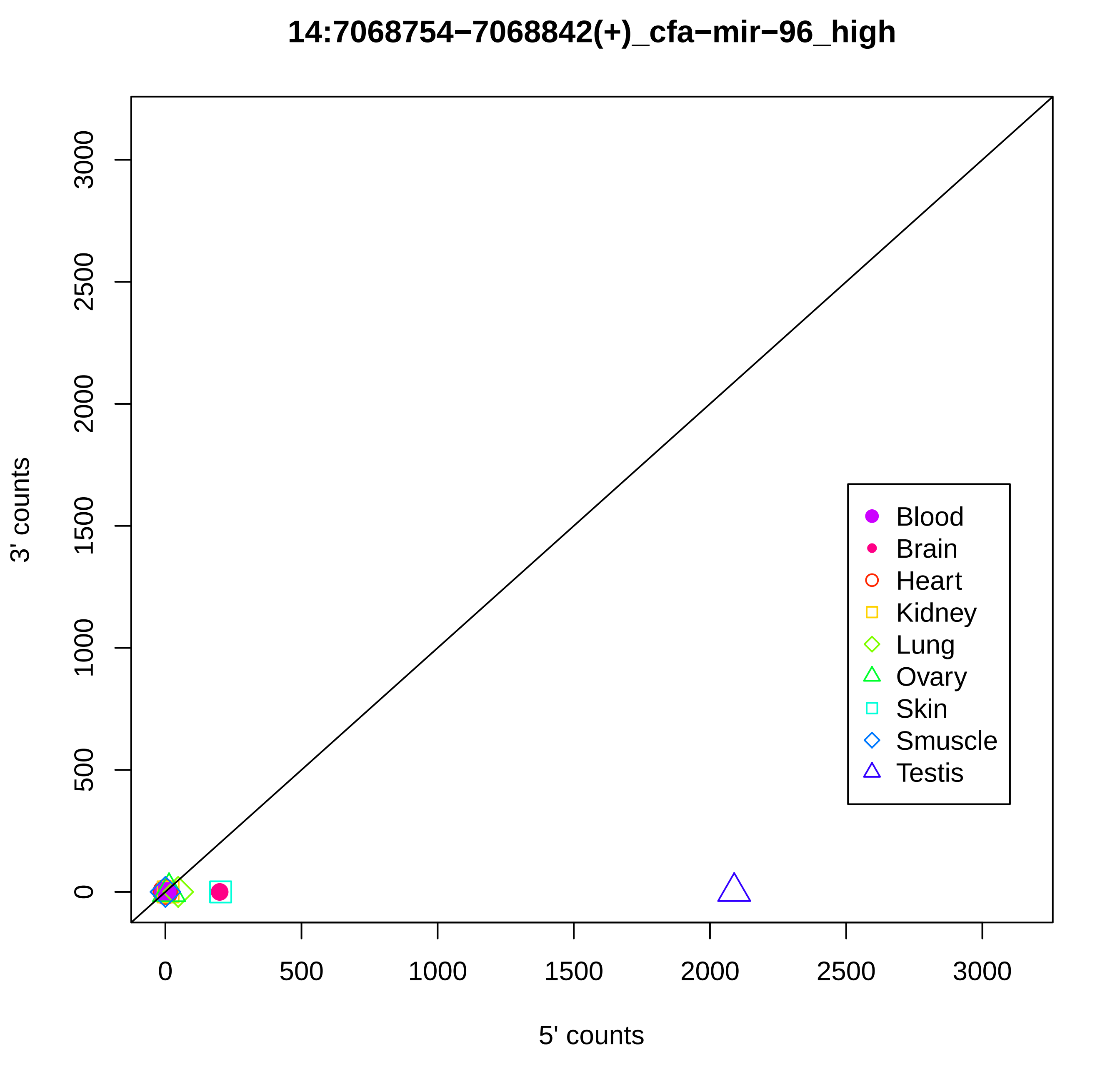
14:7068754−7068842(+)_cfa−mir−96_high
3' counts
5' counts
0
500
1000
1500
2000
2500
3000
0
500
1000
1500
2000
2500
3000
Blood
Brain
Heart
Kidney
Lung
Ovary
Skin
Smuscle
Testis

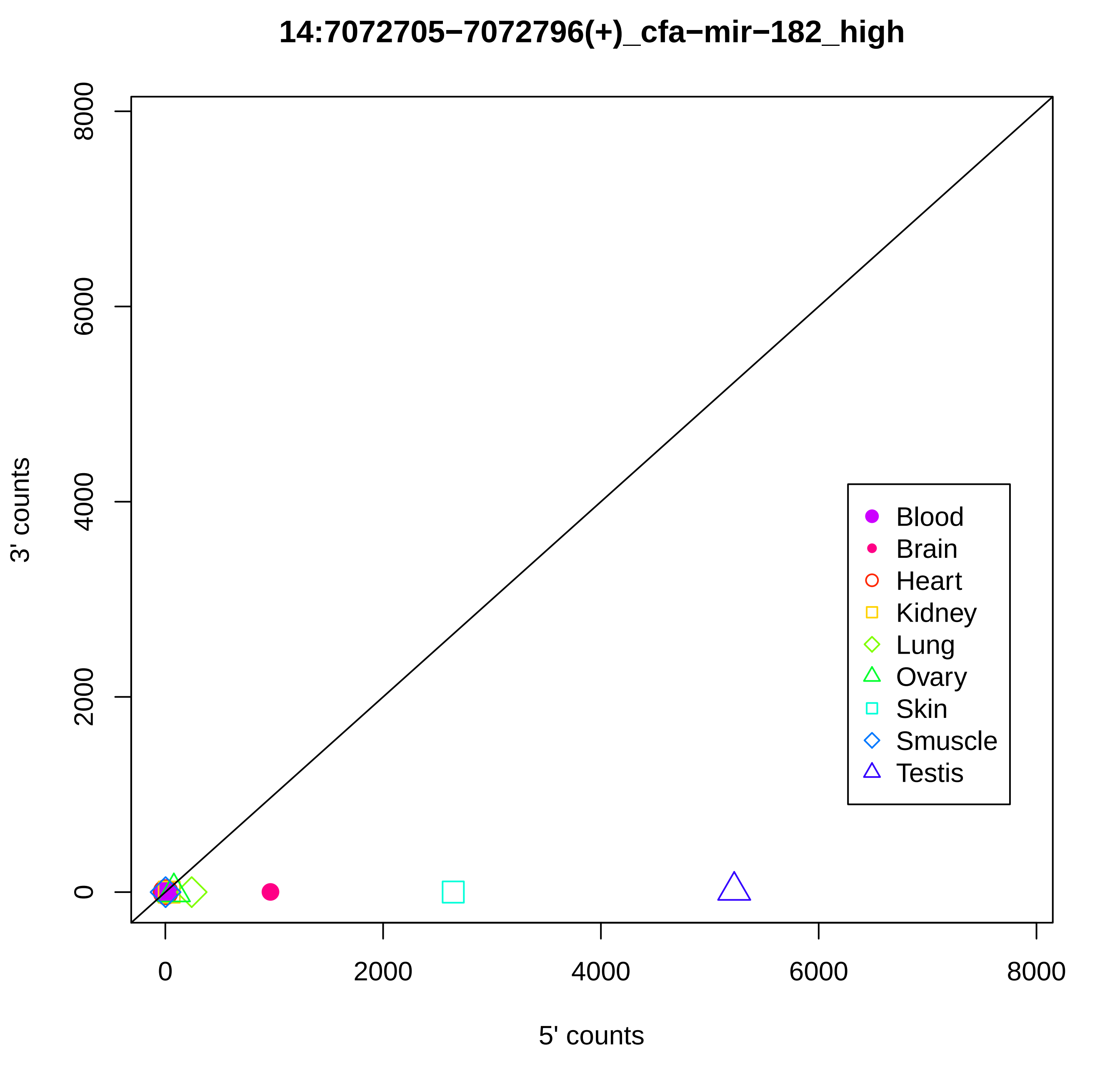

14:7072705−7072796(+)_cfa−mir−182_high
3' counts
5' counts
0
2000
4000
6000
8000
0
2000
4000
6000
8000
Blood
Brain
Heart
Kidney
Lung
Ovary
Skin
Smuscle
Testis

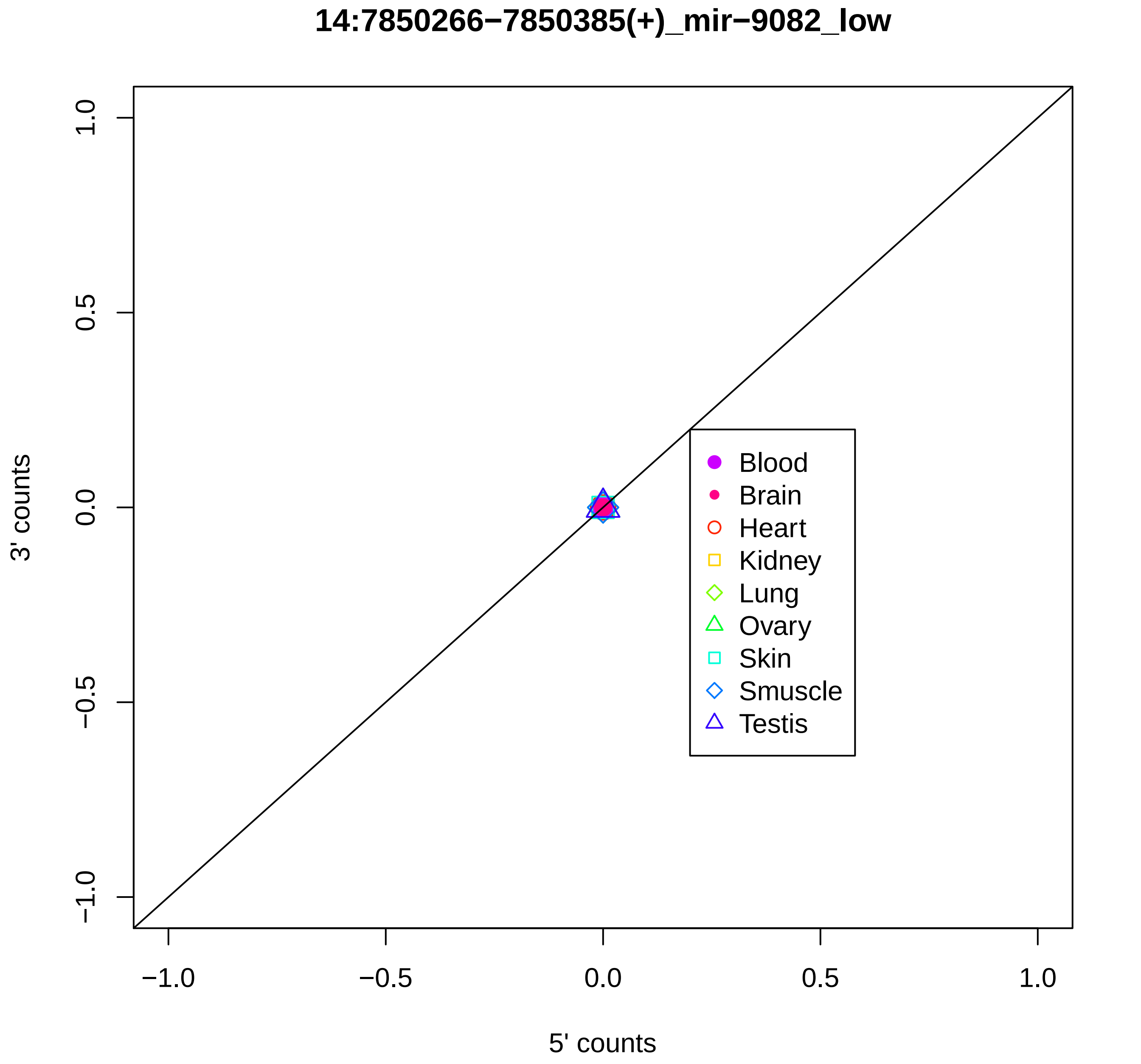

14:7850266−7850385(+)_mir−9082_low
3' counts
5' counts
−1.0
−0.5
0.0
0.5
1.0
Blood
Brain
Heart
Kidney
Lung
Ovary
Skin
Smuscle
Testis

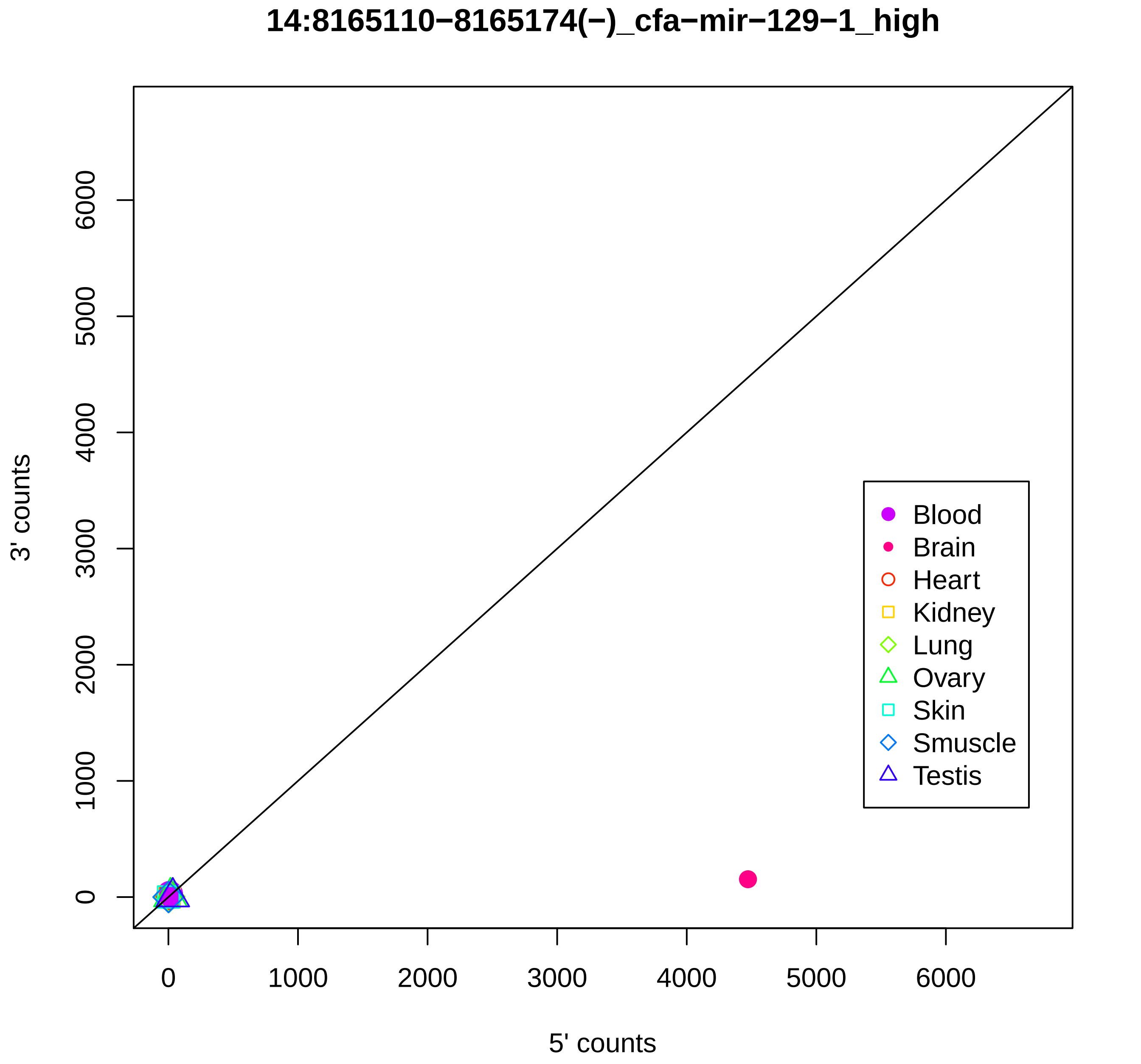

14:8165110−8165174(−)_cfa−mir−129−1_high
3' counts
5' counts
0
1000
2000
3000
4000
5000
6000
Blood
Brain
Heart
Kidney
Lung
Ovary
Skin
Smuscle
Testis

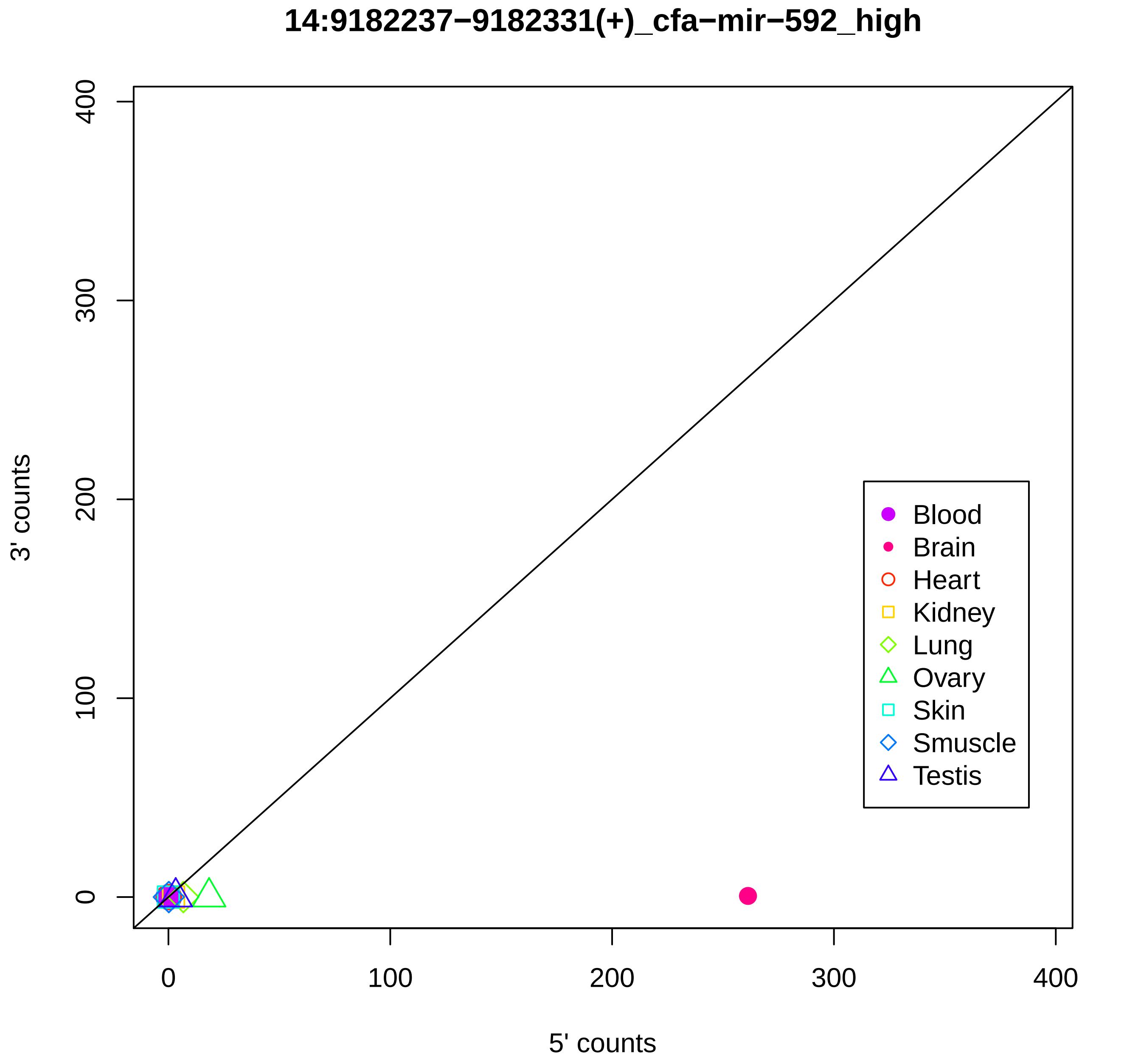

14:9182237−9182331(+)_cfa−mir−592_high
3' counts
5' counts
0
100
200
300
400
0
100
200
300
400
Blood
Brain
Heart
Kidney
Lung
Ovary
Skin
Smuscle
Testis

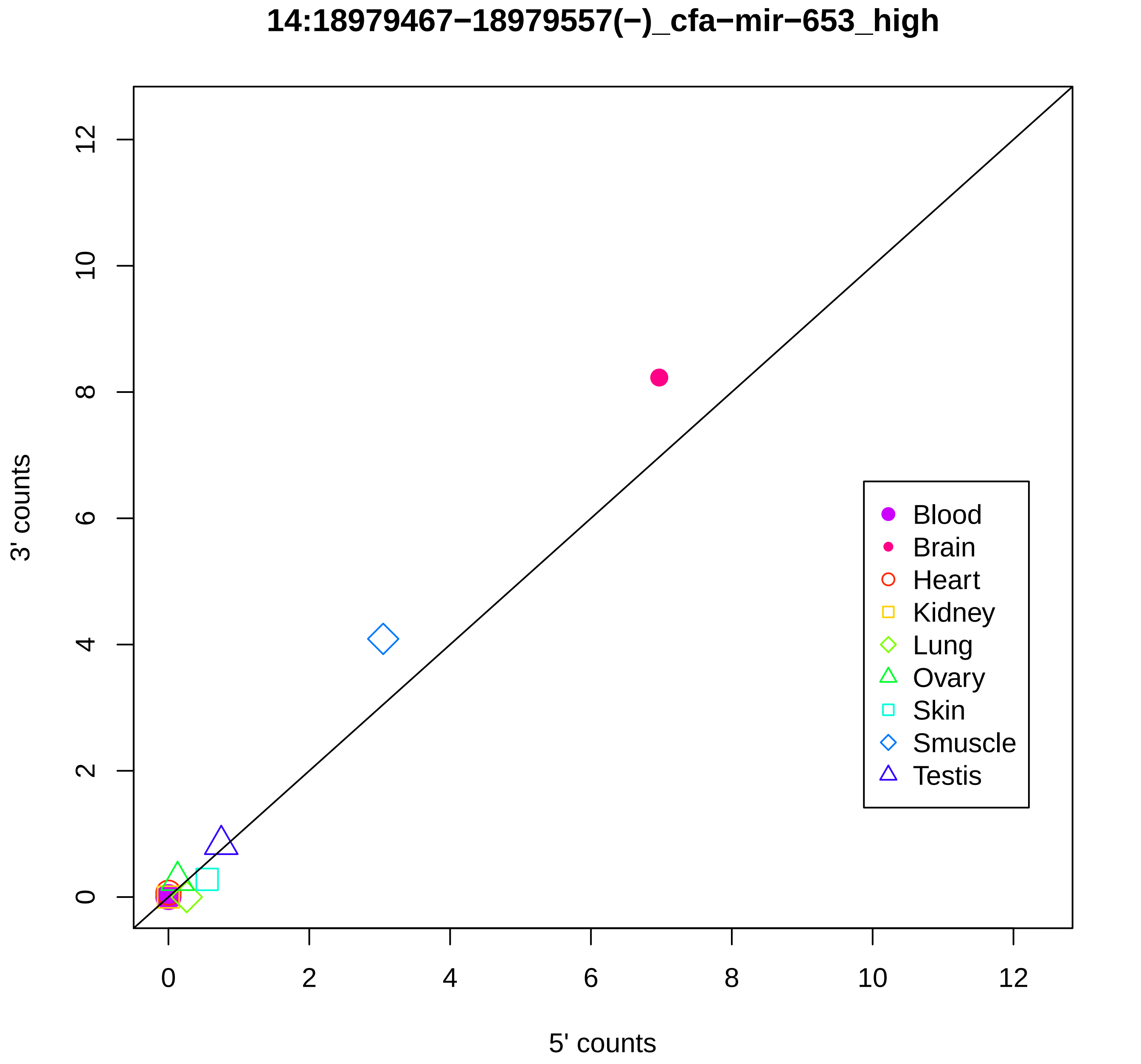

14:18979467−18979557(−)_cfa−mir−653_high
3' counts
5' counts
0
2
4
6
8
10
12
Blood
Brain
Heart
Kidney
Lung
Ovary
Skin
Smuscle
Testis

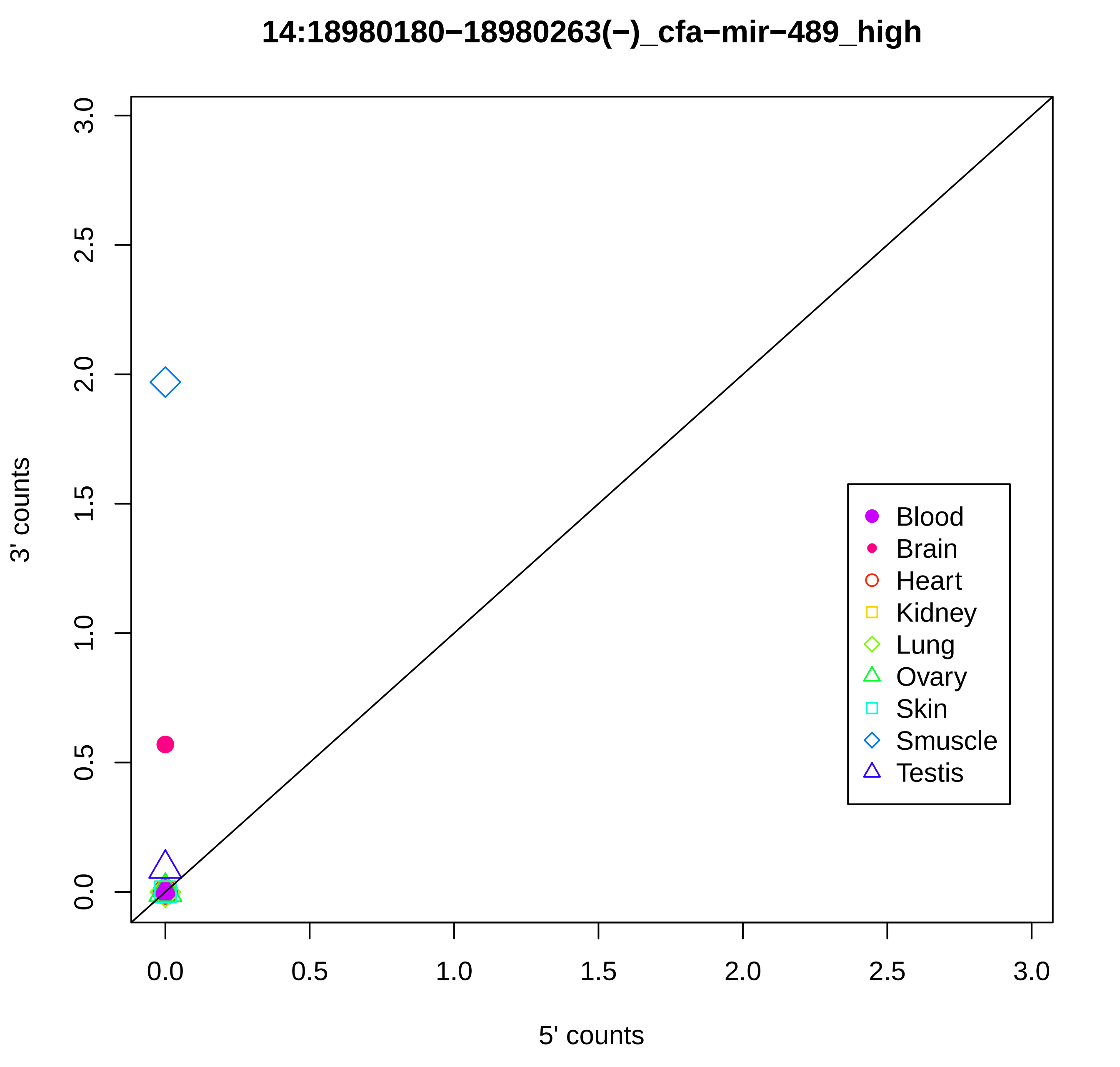
14:18980180−18980263(−)_cfa−mir−489_high
3' counts
5' counts
0.0
0.5
1.0
1.5
2.0
2.5
3.0
Blood
Brain
Heart
Kidney
Lung
Ovary
Skin
Smuscle
Testis

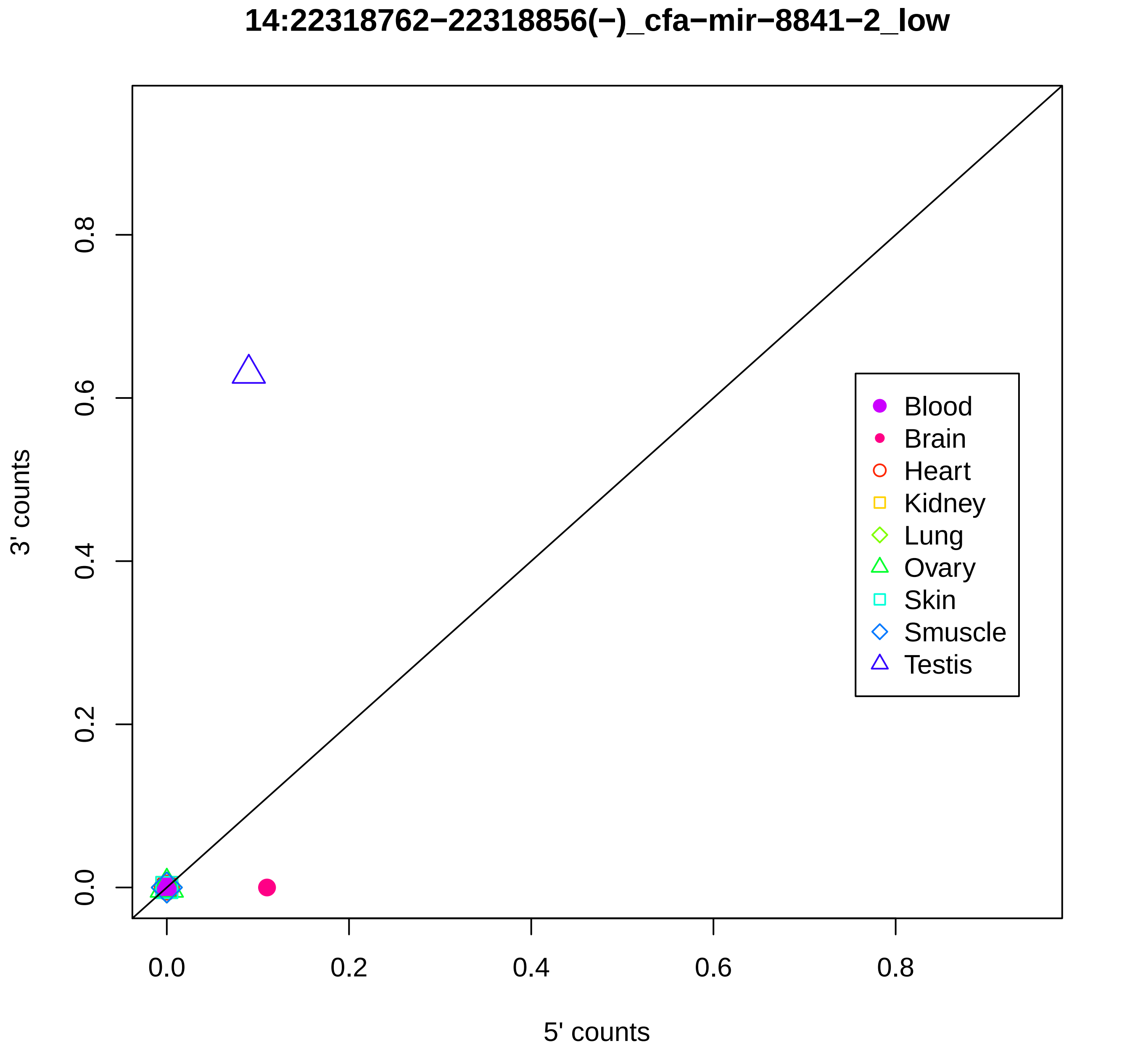
14:22318762−22318856(−)_cfa−mir−8841−2_low
3' counts
5' counts
0.0
0.2
0.4
0.6
0.8
Blood
Brain
Heart
Kidney
Lung
Ovary
Skin
Smuscle
Testis

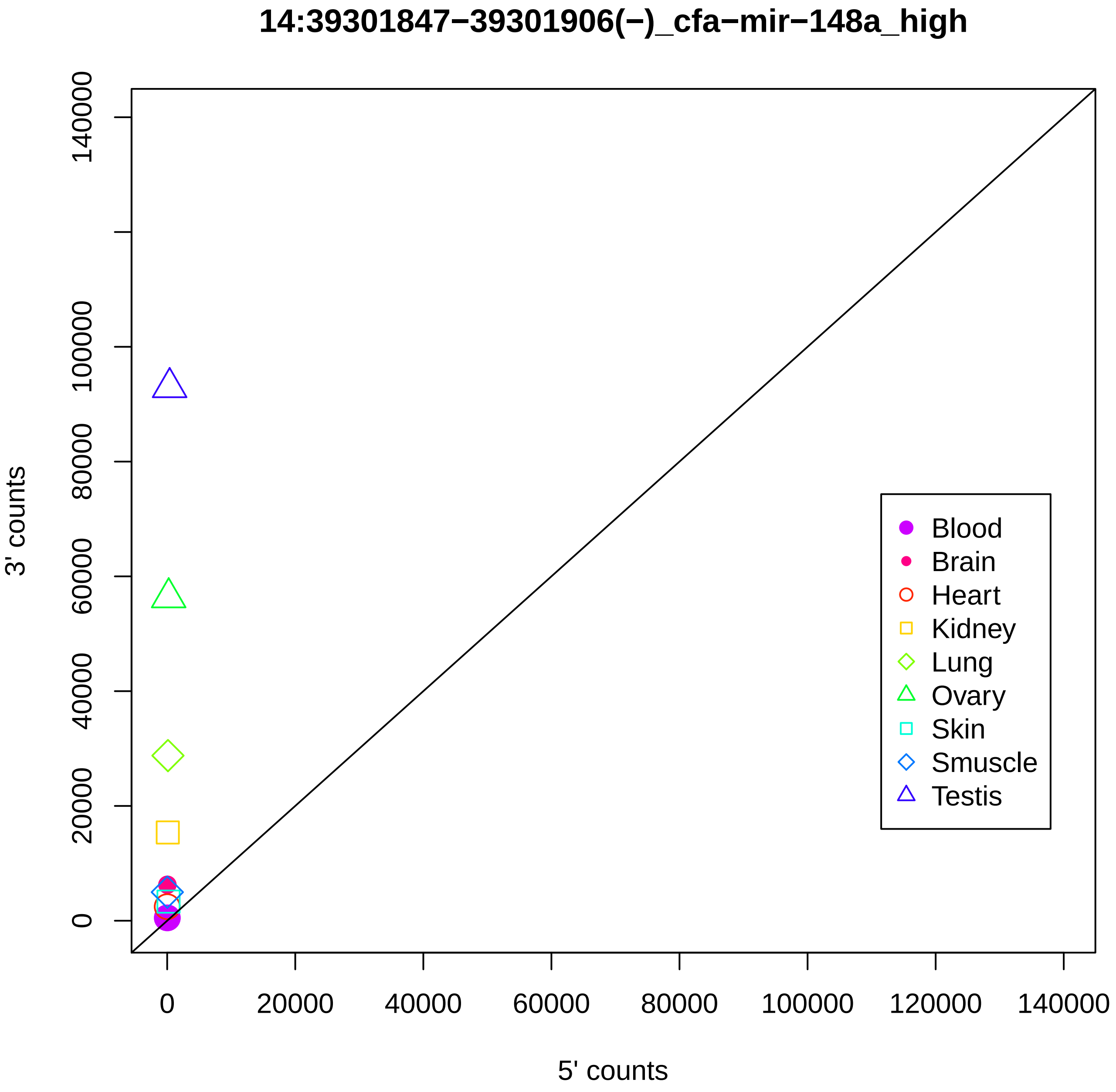

14:39301847−39301906(−)_cfa−mir−148a_high
3' counts
5' counts
0
20000
40000
60000
80000
100000
120000
140000
Blood
Brain
Heart
Kidney
Lung
Ovary
Skin
Smuscle
Testis

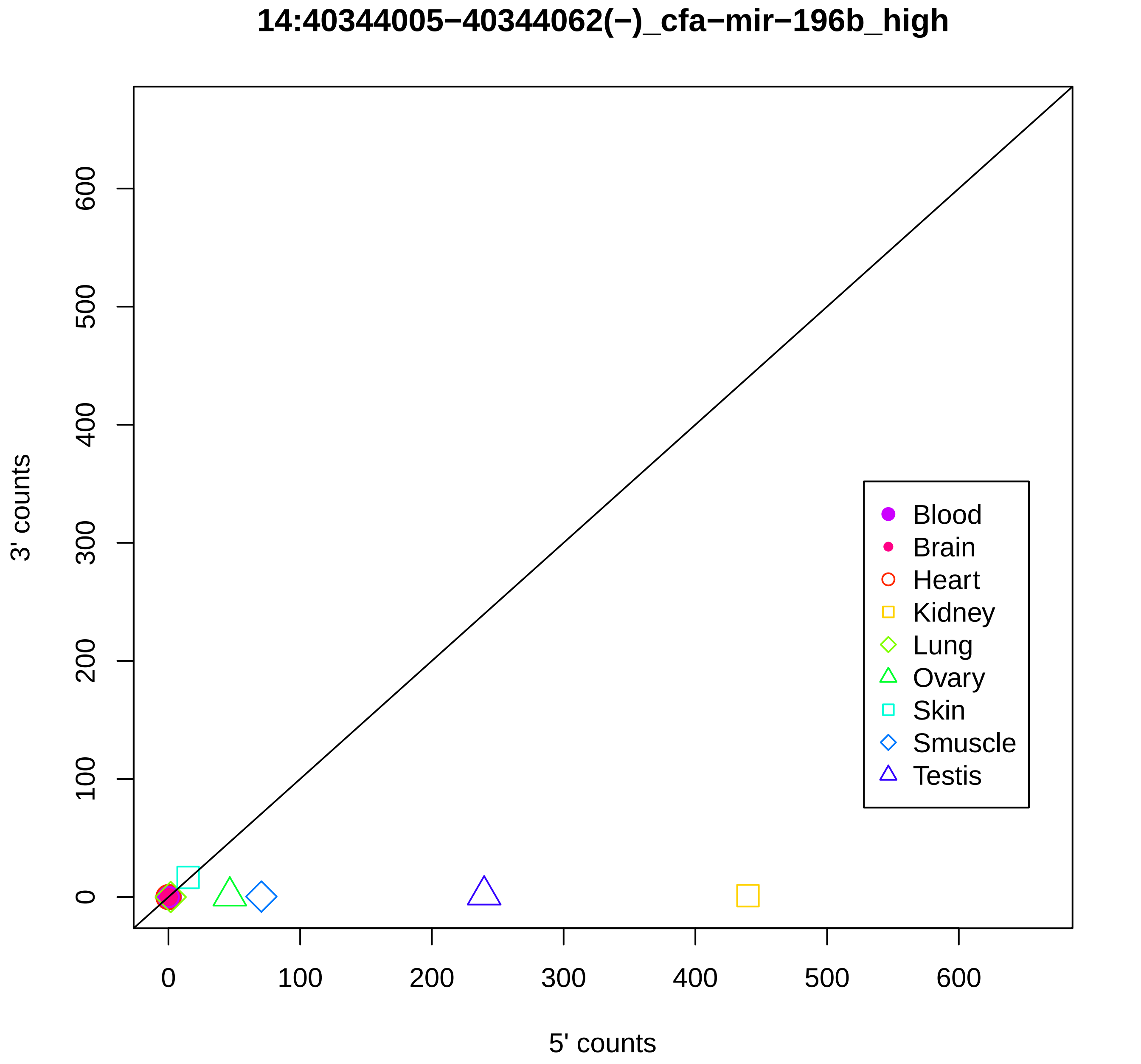

14:40344005−40344062(−)_cfa−mir−196b_high
3' counts
5' counts
0
100
200
300
400
500
600
Blood
Brain
Heart
Kidney
Lung
Ovary
Skin
Smuscle
Testis

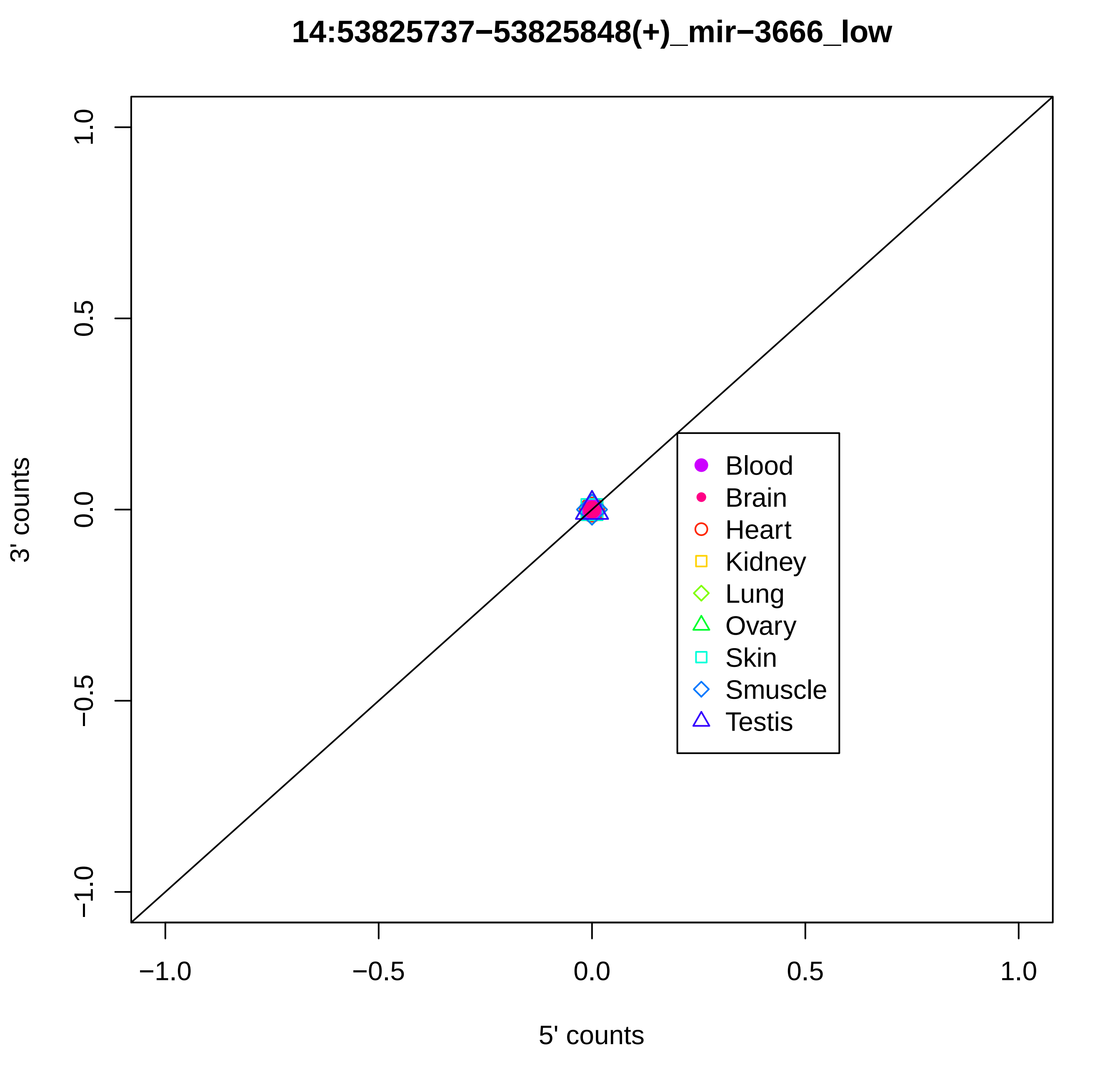
14:53825737−53825848(+)_mir−3666_low
3' counts
5' counts
1.0
0.5
0.0
−0.5
−1.0
−1.0
−0.5
0.0
0.5
1.0
Blood
Brain
Heart
Kidney
Lung
Ovary
Skin
Smuscle
Testis

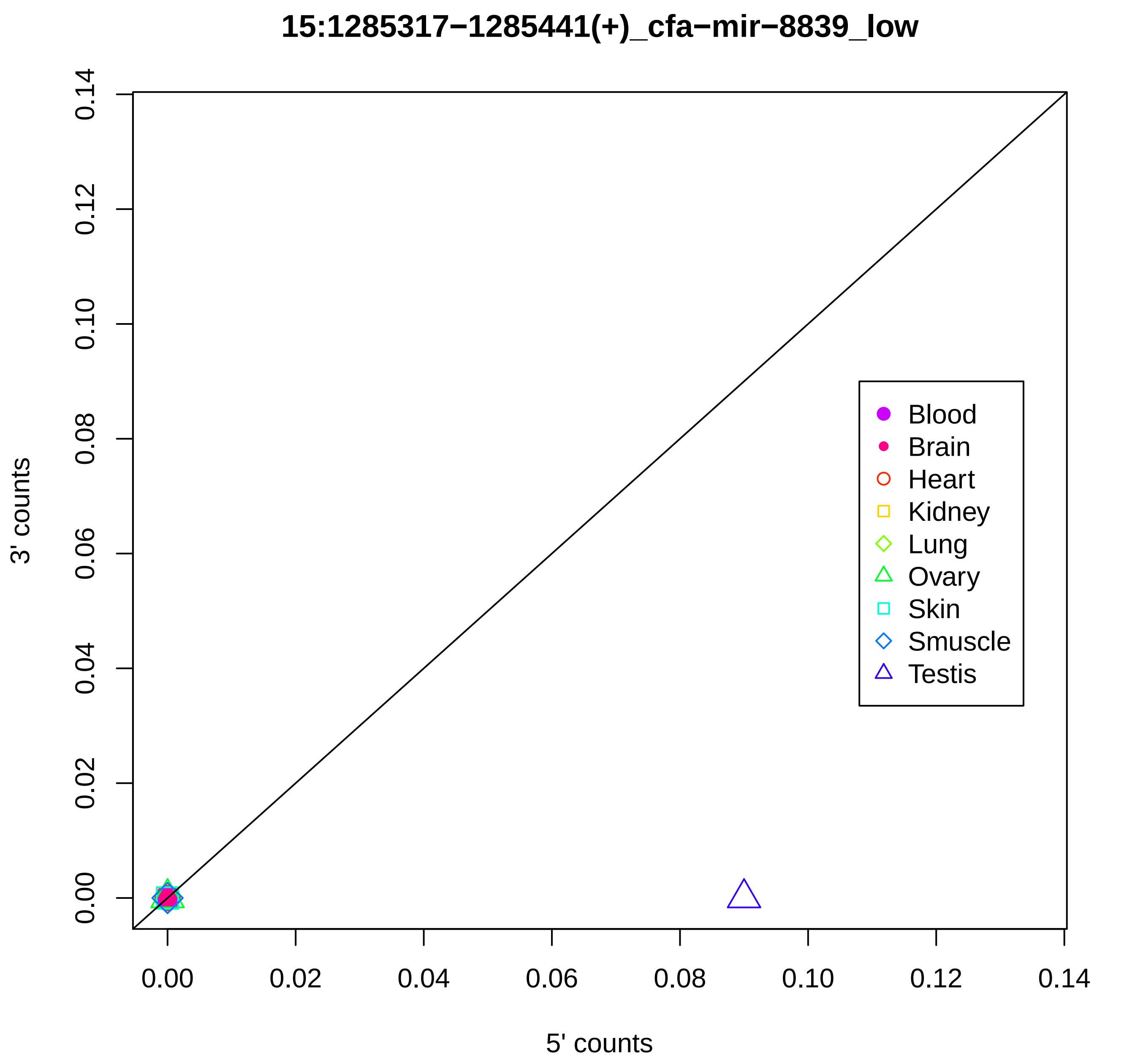
15:1285317−1285441(+)_cfa−mir−8839_low
3' counts
5' counts
0.00
0.02
0.04
0.06
0.08
0.10
0.12
0.14
Blood
Brain
Heart
Kidney
Lung
Ovary
Skin
Smuscle
Testis

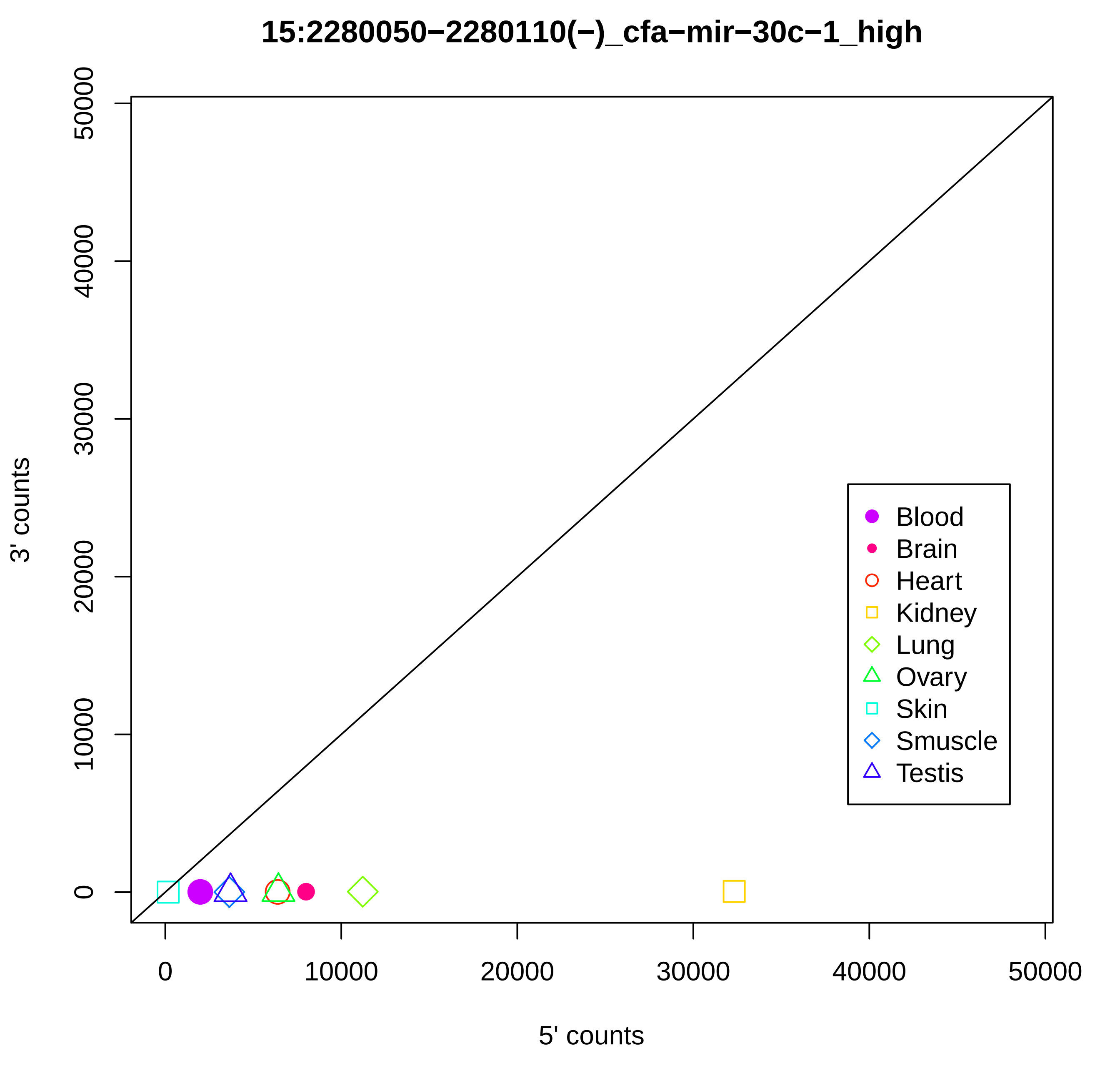
15:2280050−2280110(−)_cfa−mir−30c−1_high
3' counts
5' counts
0
10000
20000
30000
40000
50000
Blood
Brain
Heart
Kidney
Lung
Ovary
Skin
Smuscle
Testis

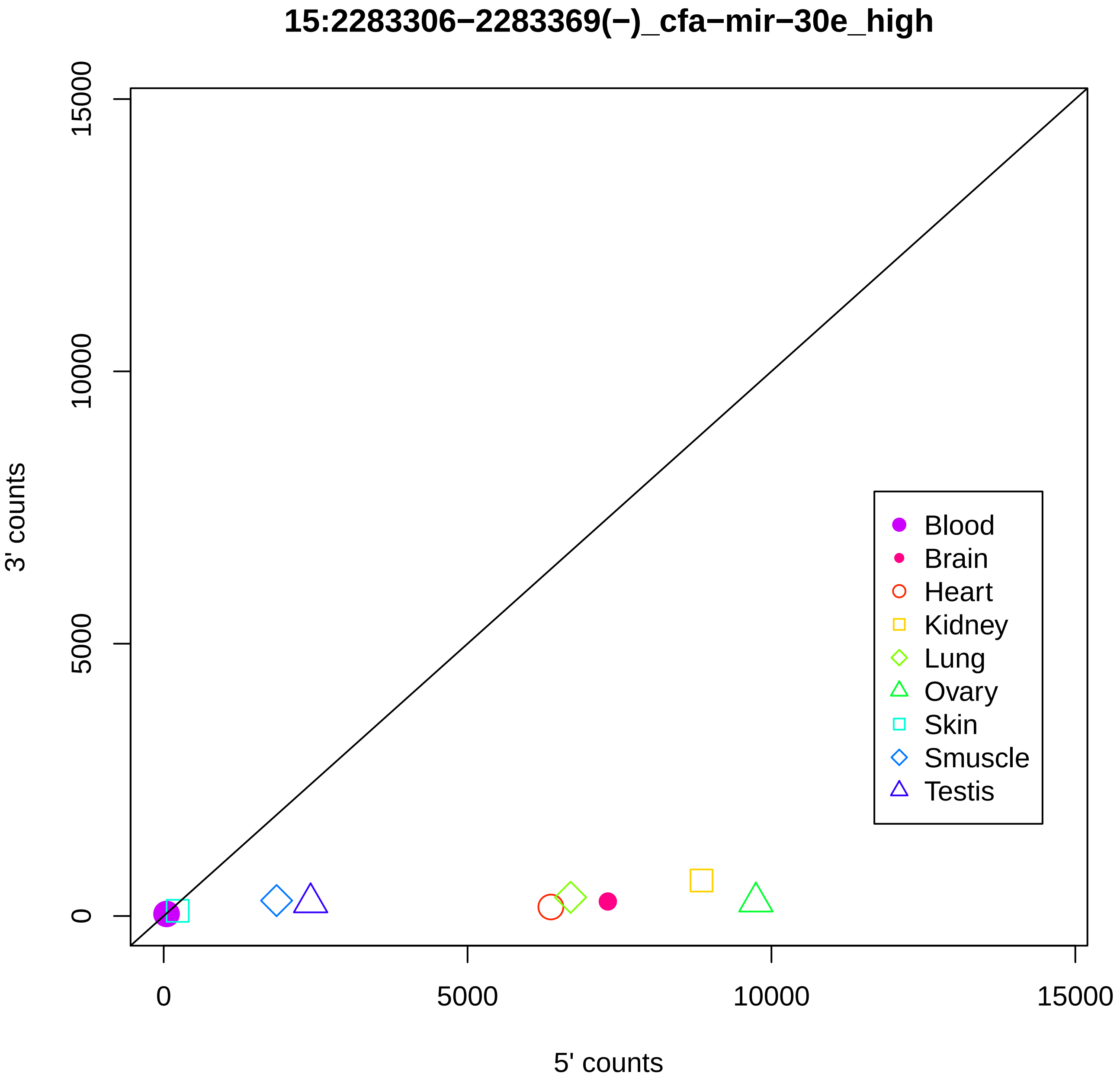
15:2283306−2283369(−)_cfa−mir−30e_high
3' counts
5' counts
0
5000
10000
15000
0
5000
10000
15000
Blood
Brain
Heart
Kidney
Lung
Ovary
Skin
Smuscle
Testis

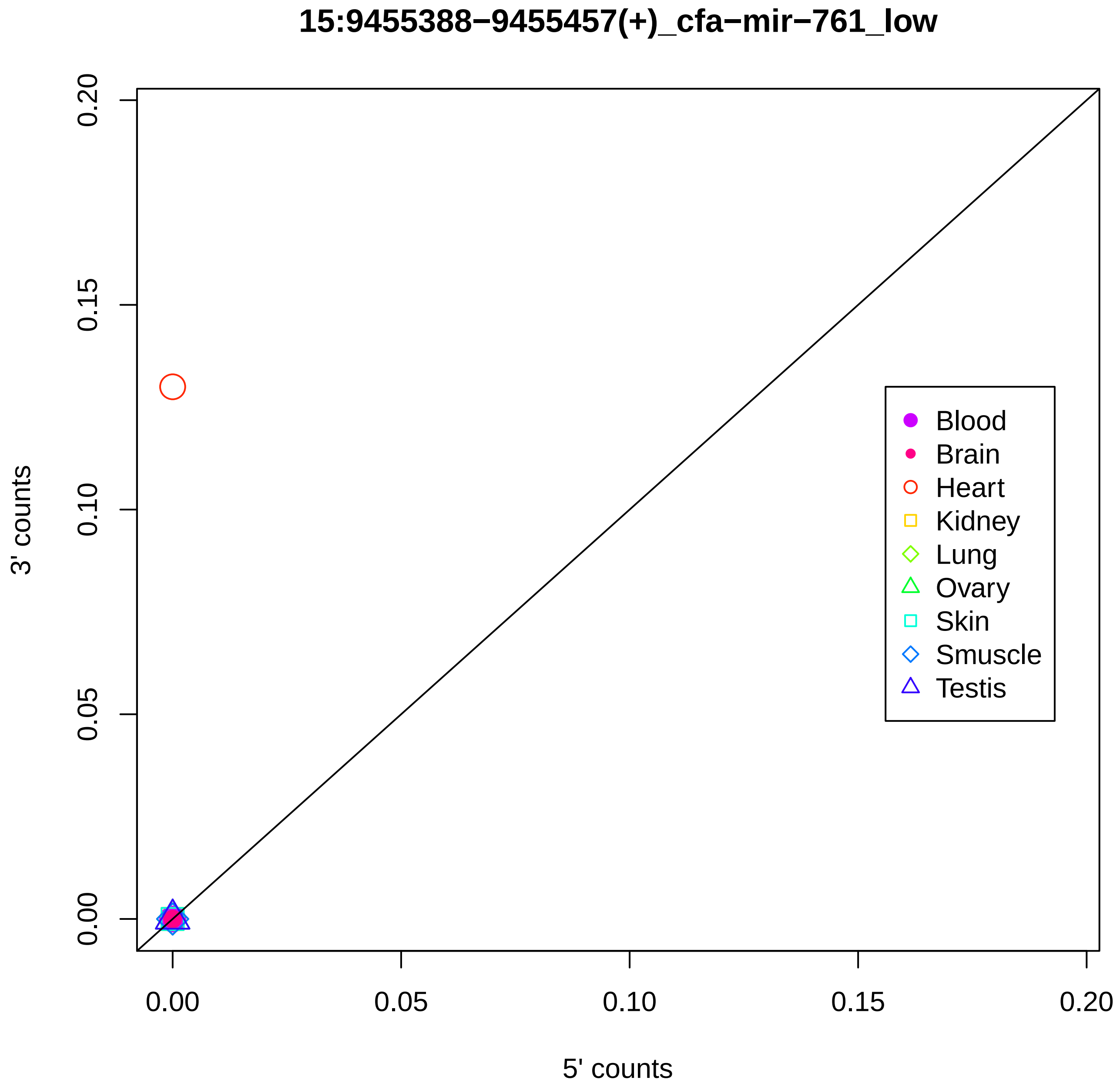

15:9455388−9455457(+)_cfa−mir−761_low
0.00
0.05
0.10
0.15
0.20
3' counts
5' counts
Blood
Brain
Heart
Kidney
Lung
Ovary
Skin
Smuscle
Testis

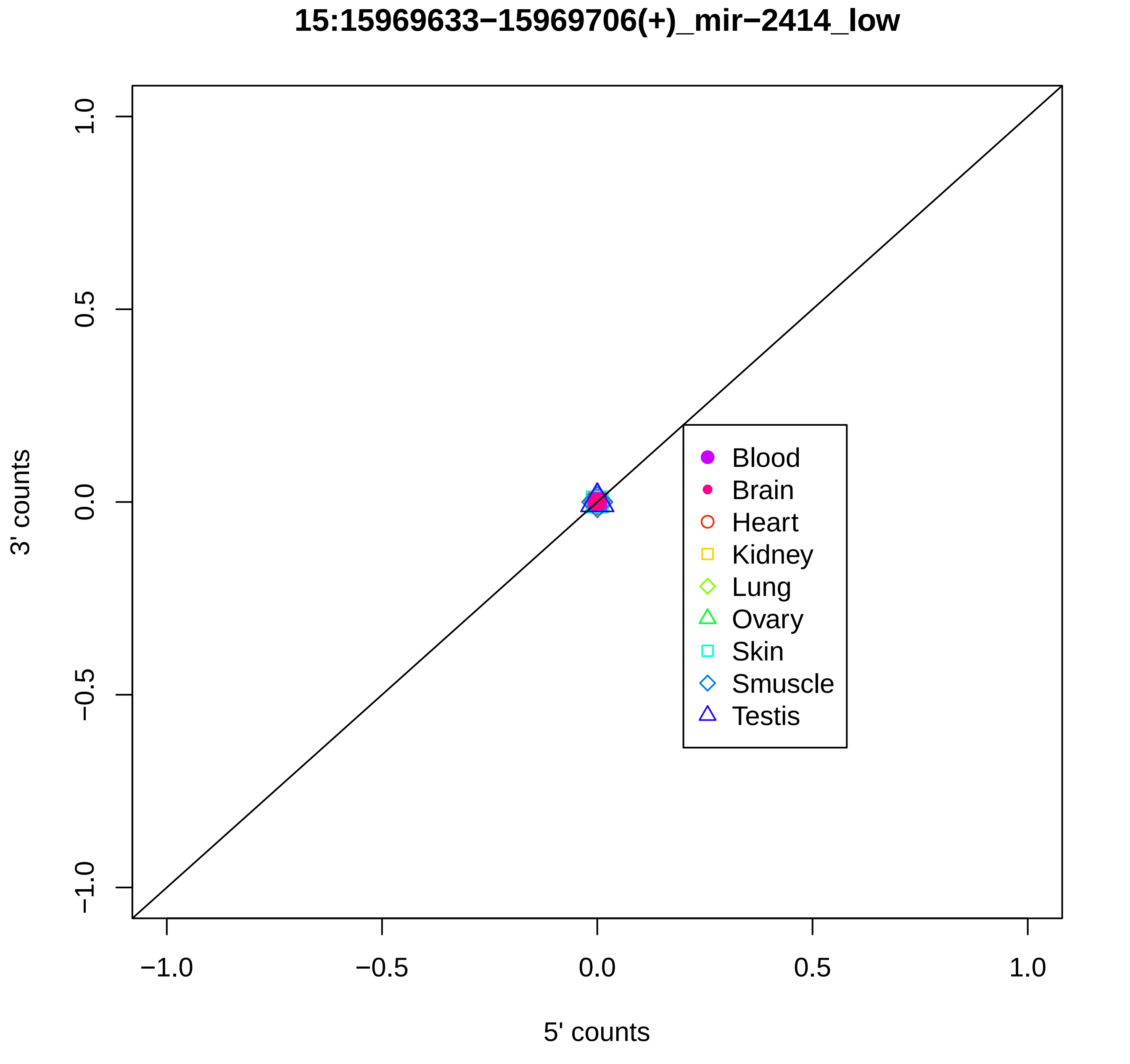
15:15969633−15969706(+)_mir−2414_low
3' counts
5' counts
1.0
0.5
0.0
−0.5
−1.0
−1.0
−0.5
0.0
0.5
1.0
Blood
Brain
Heart
Kidney
Lung
Ovary
Skin
Smuscle
Testis

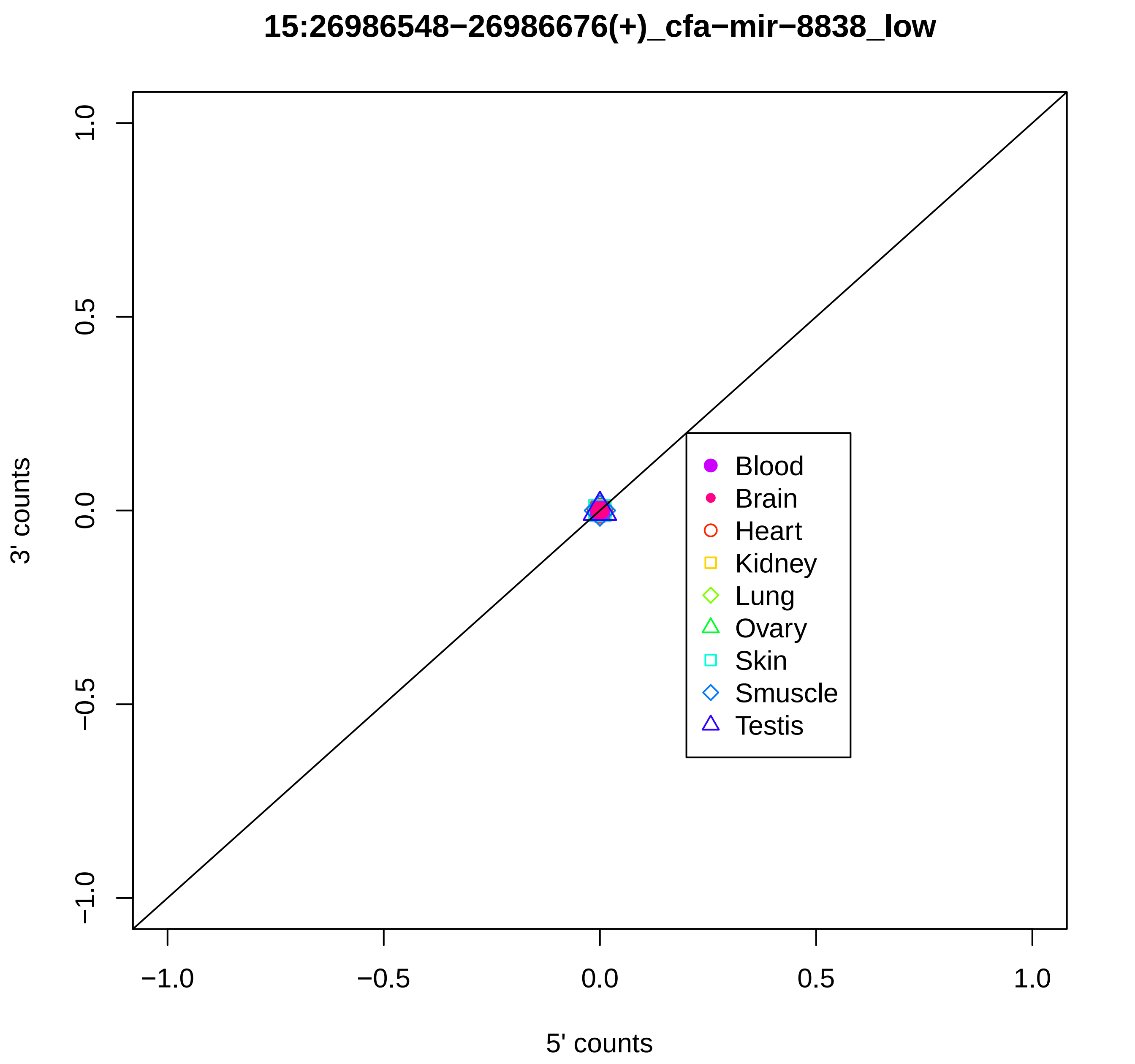

15:26986548−26986676(+)_cfa−mir−8838_low
3' counts
5' counts
−1.0
−0.5
0.0
0.5
1.0
Blood
Brain
Heart
Kidney
Lung
Ovary
Skin
Smuscle
Testis

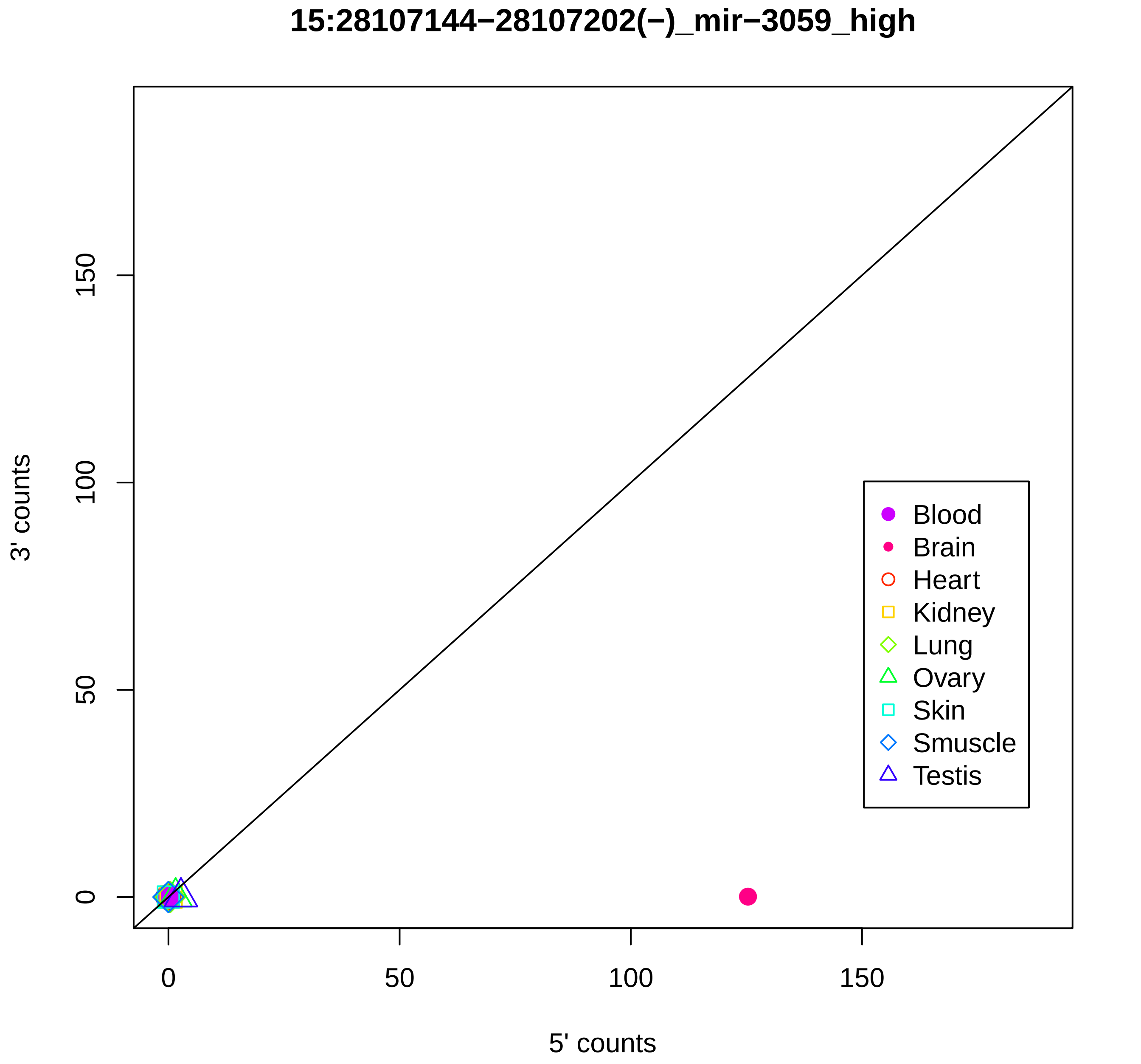

15:28107144−28107202(−)_mir−3059_high
3' counts
5' counts
0
50
100
150
Blood
Brain
Heart
Kidney
Lung
Ovary
Skin
Smuscle
Testis

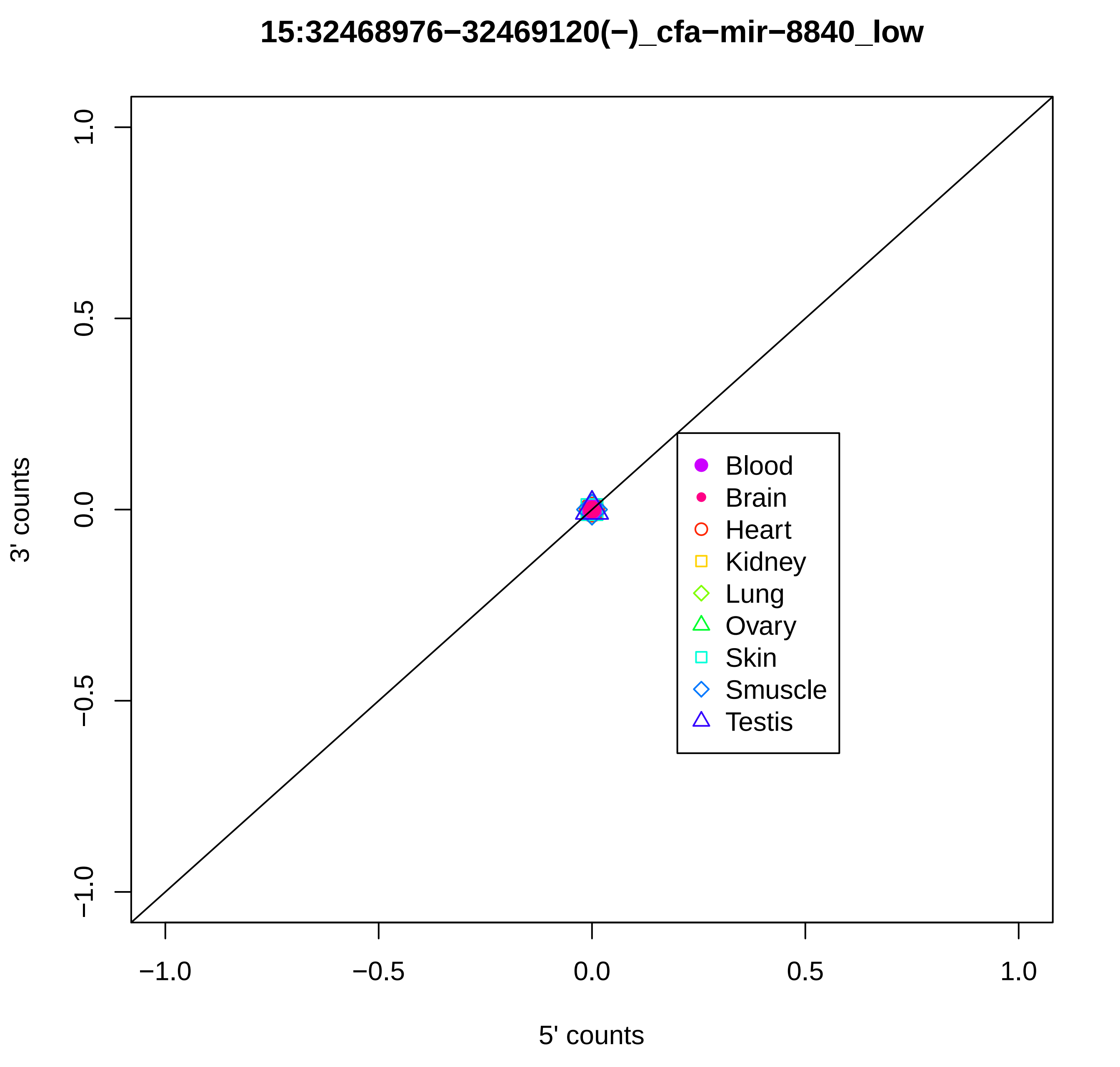
15:32468976−32469120(−)_cfa−mir−8840_low
1.0
0.5
0.0
−0.5
−1.0
3' counts
−1.0
−0.5
0.0
0.5
1.0
5' counts
Blood
Brain
Heart
Kidney
Lung
Ovary
Skin
Smuscle
Testis

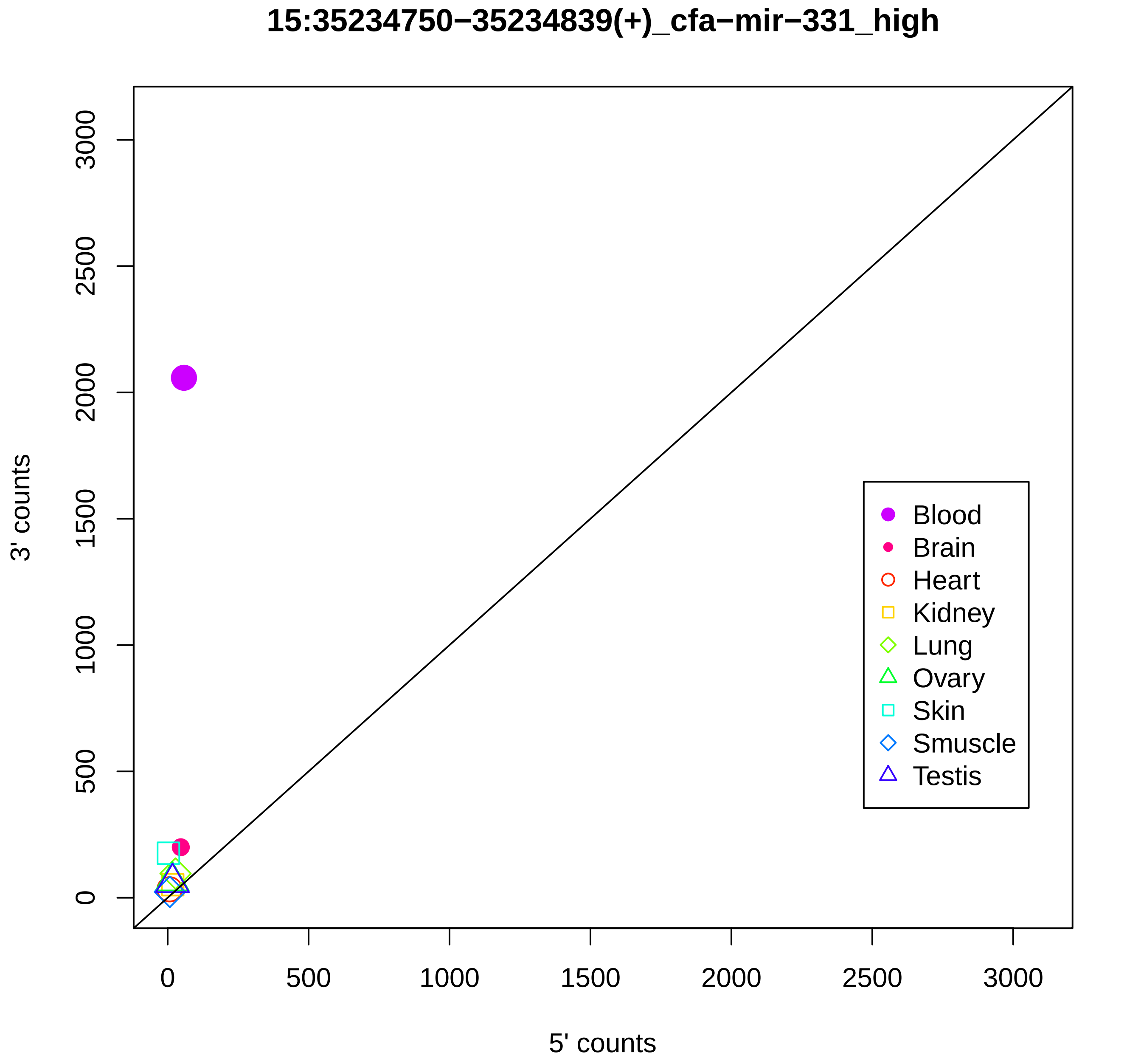

15:35234750−35234839(+)_cfa−mir−331_high
3' counts
5' counts
0
500
1000
1500
2000
2500
3000
Blood
Brain
Heart
Kidney
Lung
Ovary
Skin
Smuscle
Testis

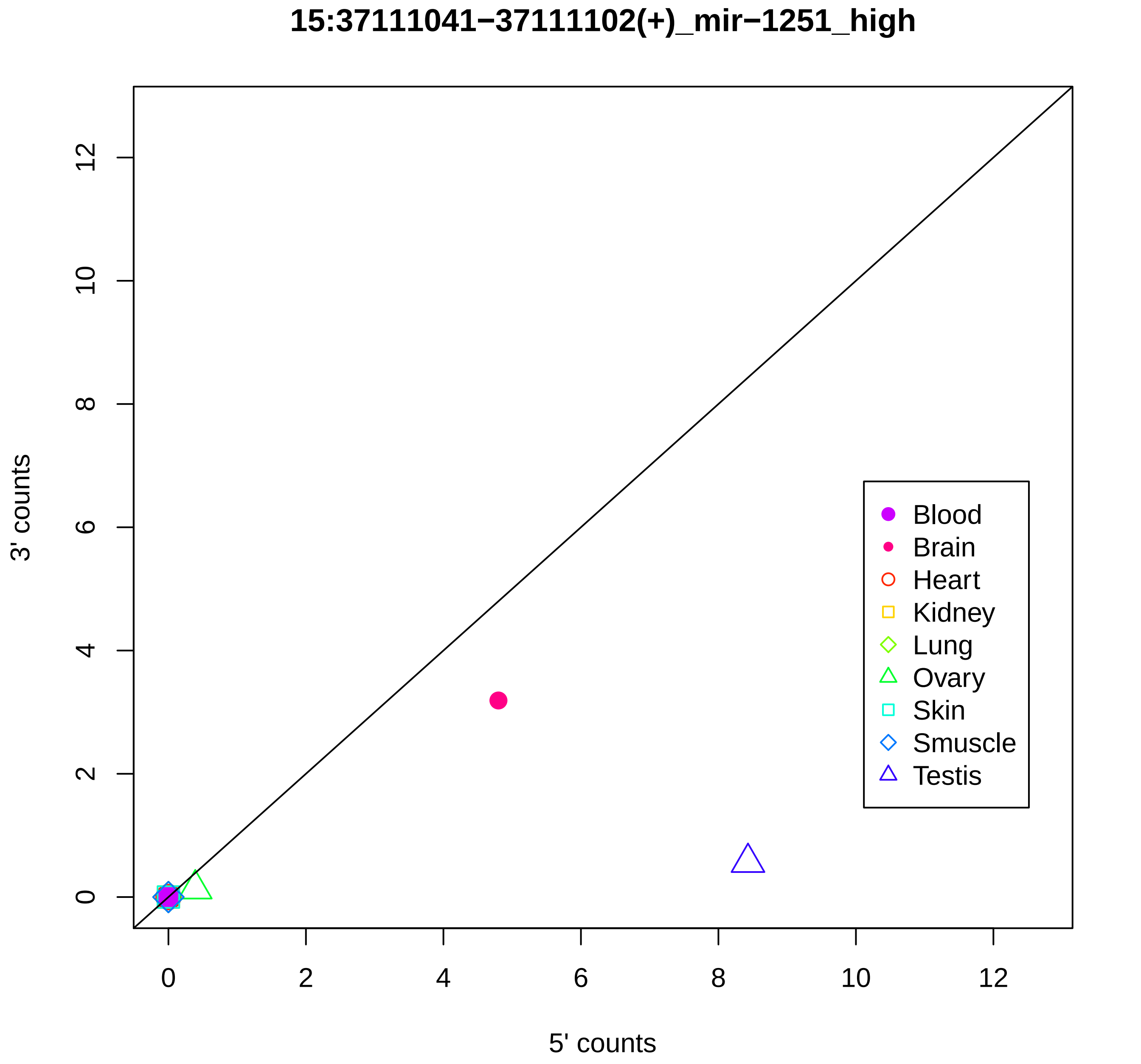

15:37111041−37111102(+)_mir−1251_high
3' counts
5' counts
0
2
4
6
8
10
12
Blood
Brain
Heart
Kidney
Lung
Ovary
Skin
Smuscle
Testis

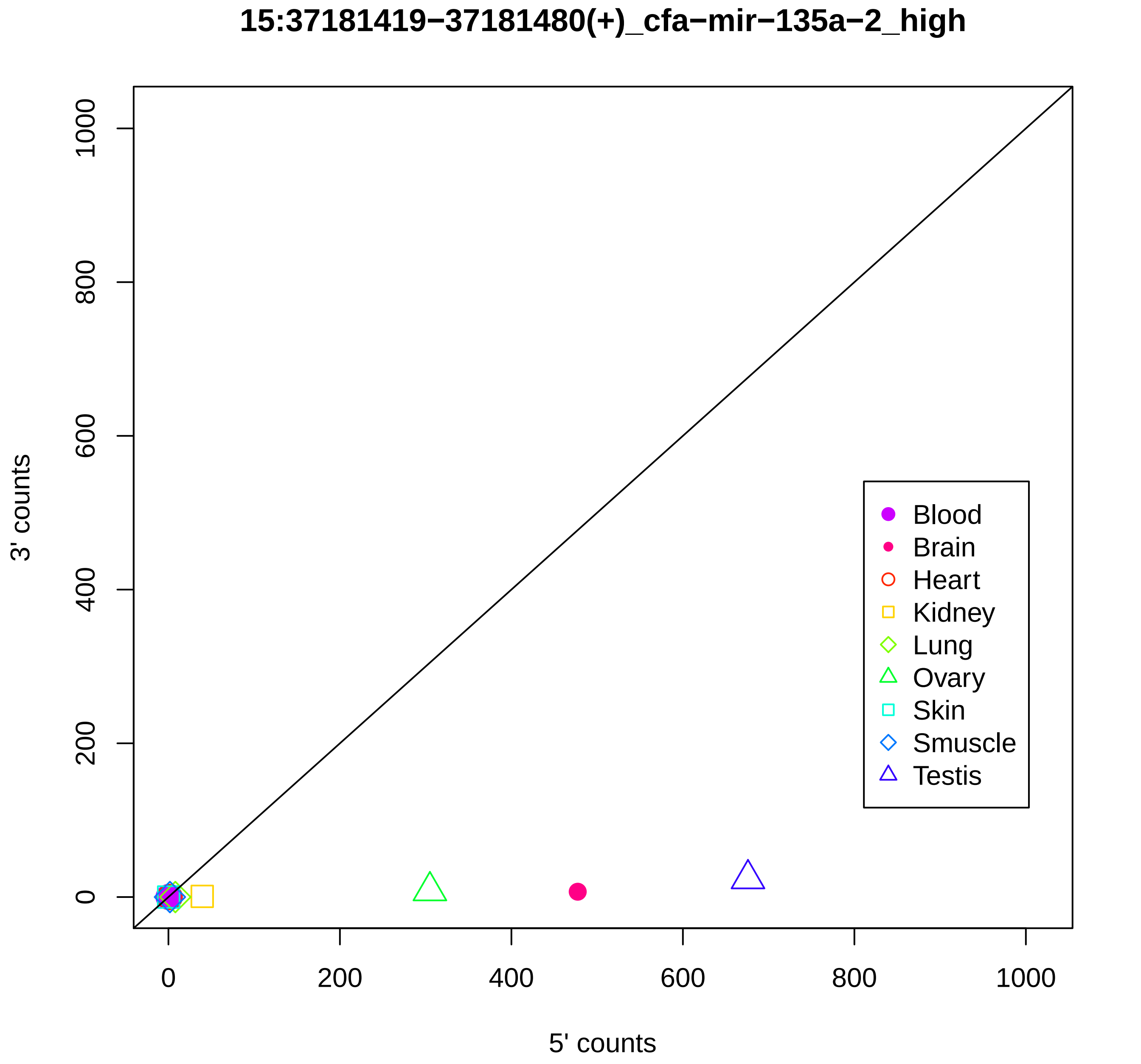
15:37181419−37181480(+)_cfa−mir−135a−2_high
3' counts
5' counts
0
200
400
600
800
1000
Blood
Brain
Heart
Kidney
Lung
Ovary
Skin
Smuscle
Testis

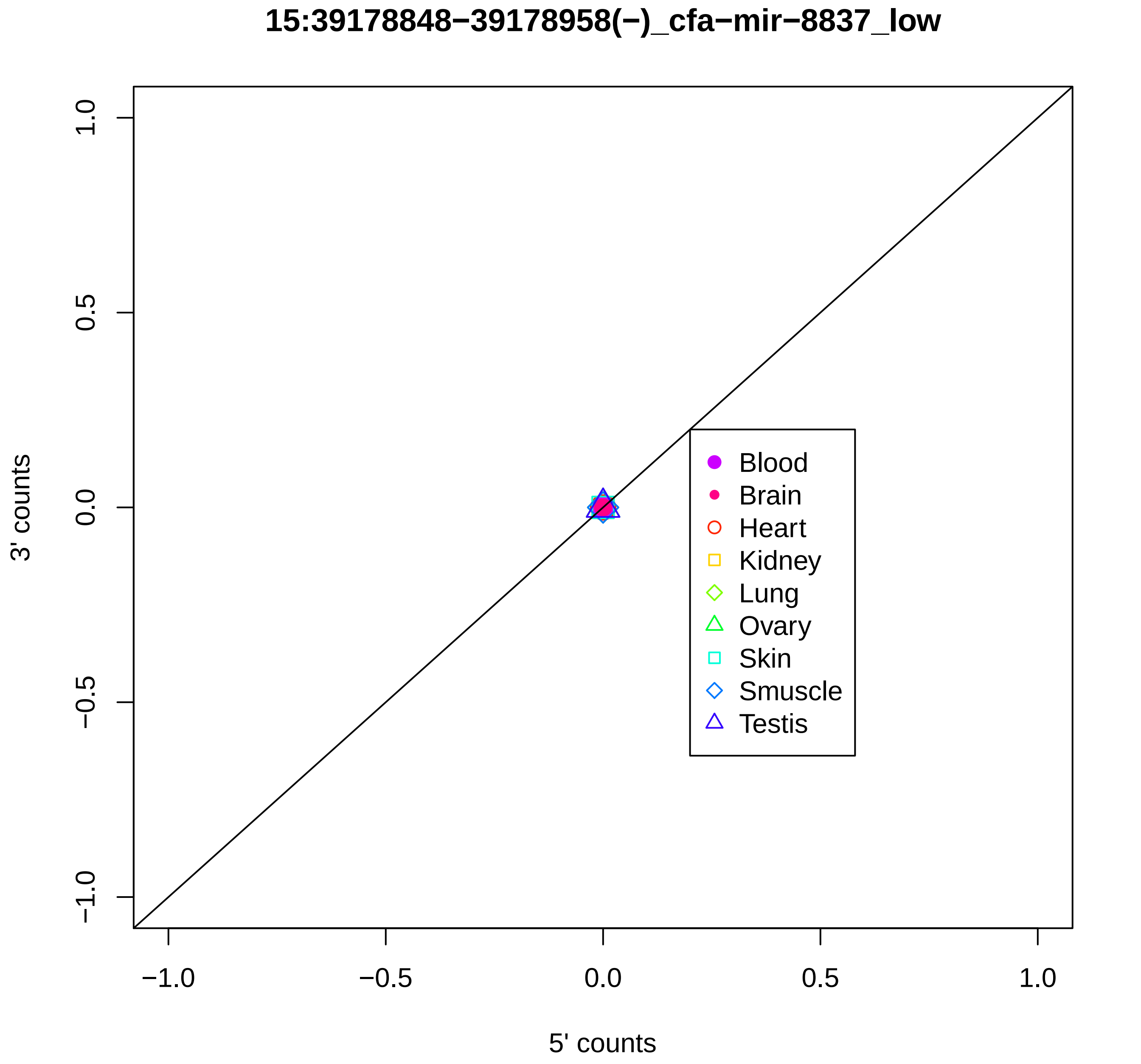

15:39178848−39178958(−)_cfa−mir−8837_low
3' counts
5' counts
1.0
0.5
0.0
−0.5
−1.0
Blood
Brain
Heart
Kidney
Lung
Ovary
Skin
Smuscle
Testis

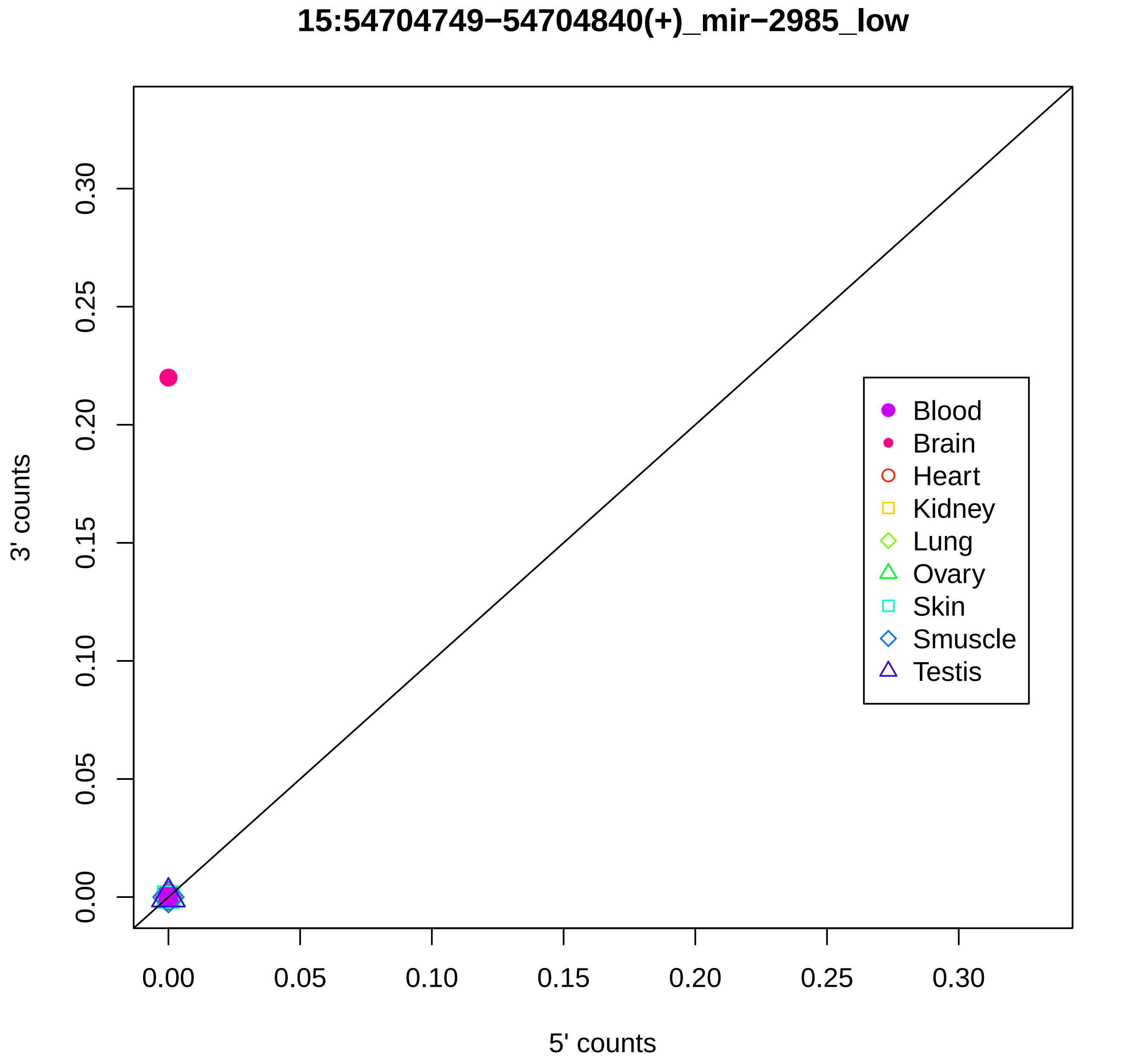

15:54704749−54704840(+)_mir−2985_low
3' counts
5' counts
0.00
0.05
0.10
0.15
0.20
0.25
0.30
Blood
Brain
Heart
Kidney
Lung
Ovary
Skin
Smuscle
Testis

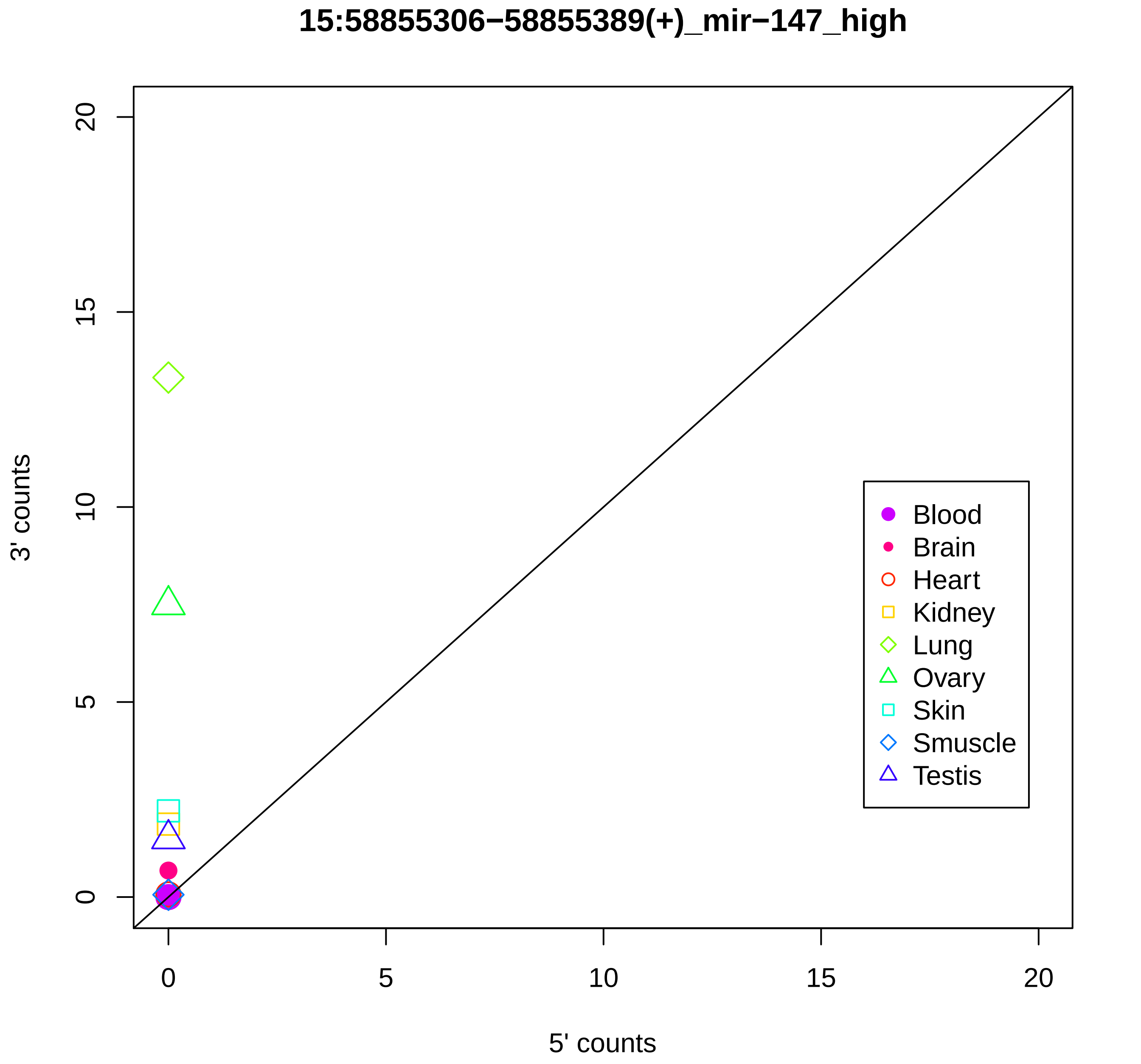
15:58855306−58855389(+)_mir−147_high
3' counts
5' counts
0
5
10
15
20
0
5
10
15
20
Blood
Brain
Heart
Kidney
Lung
Ovary
Skin
Smuscle
Testis

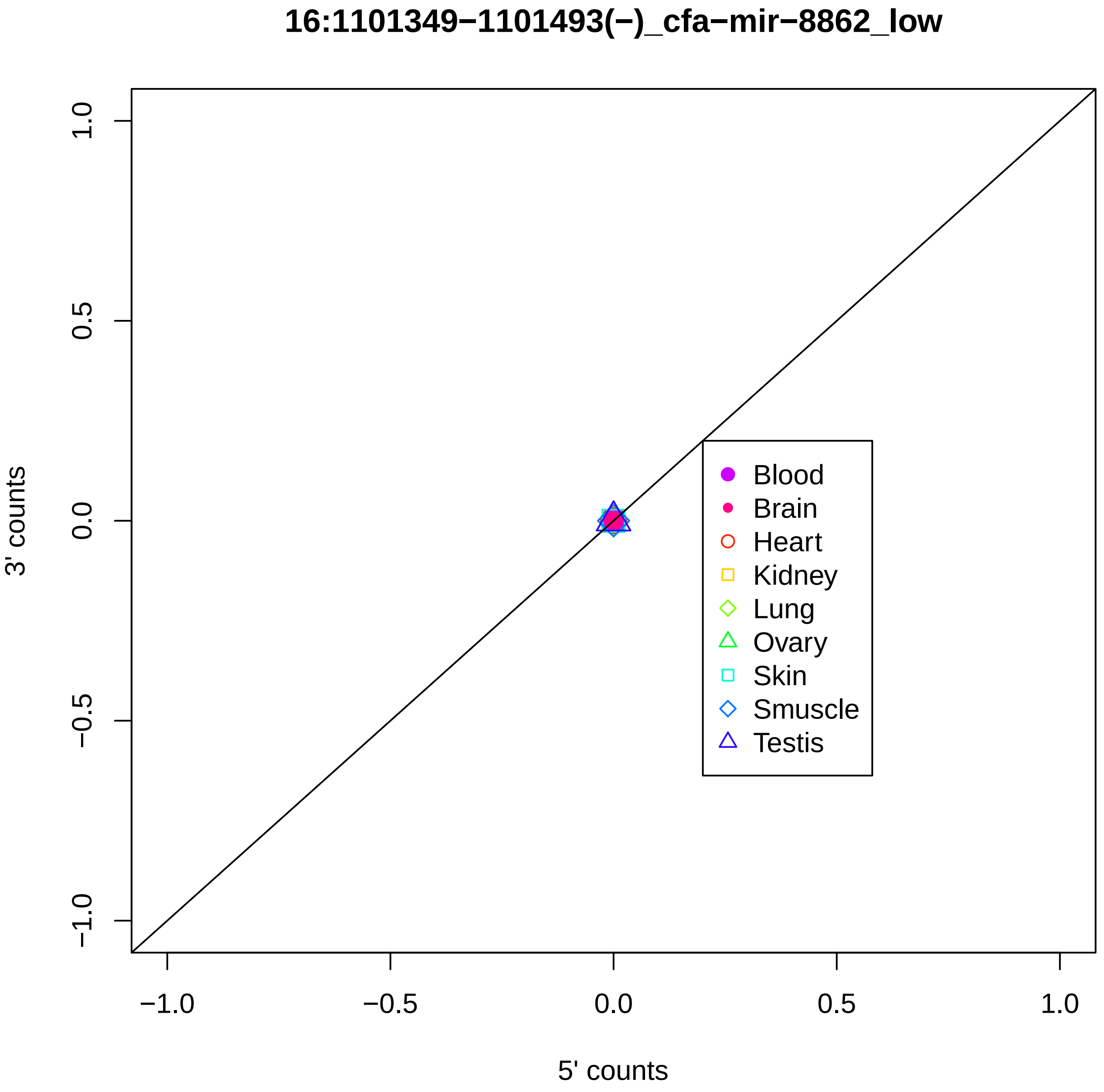
16:1101349−1101493(−)_cfa−mir−8862_low
3' counts
5' counts
−1.0
−0.5
0.0
0.5
1.0
Blood
Brain
Heart
Kidney
Lung
Ovary
Skin
Smuscle
Testis

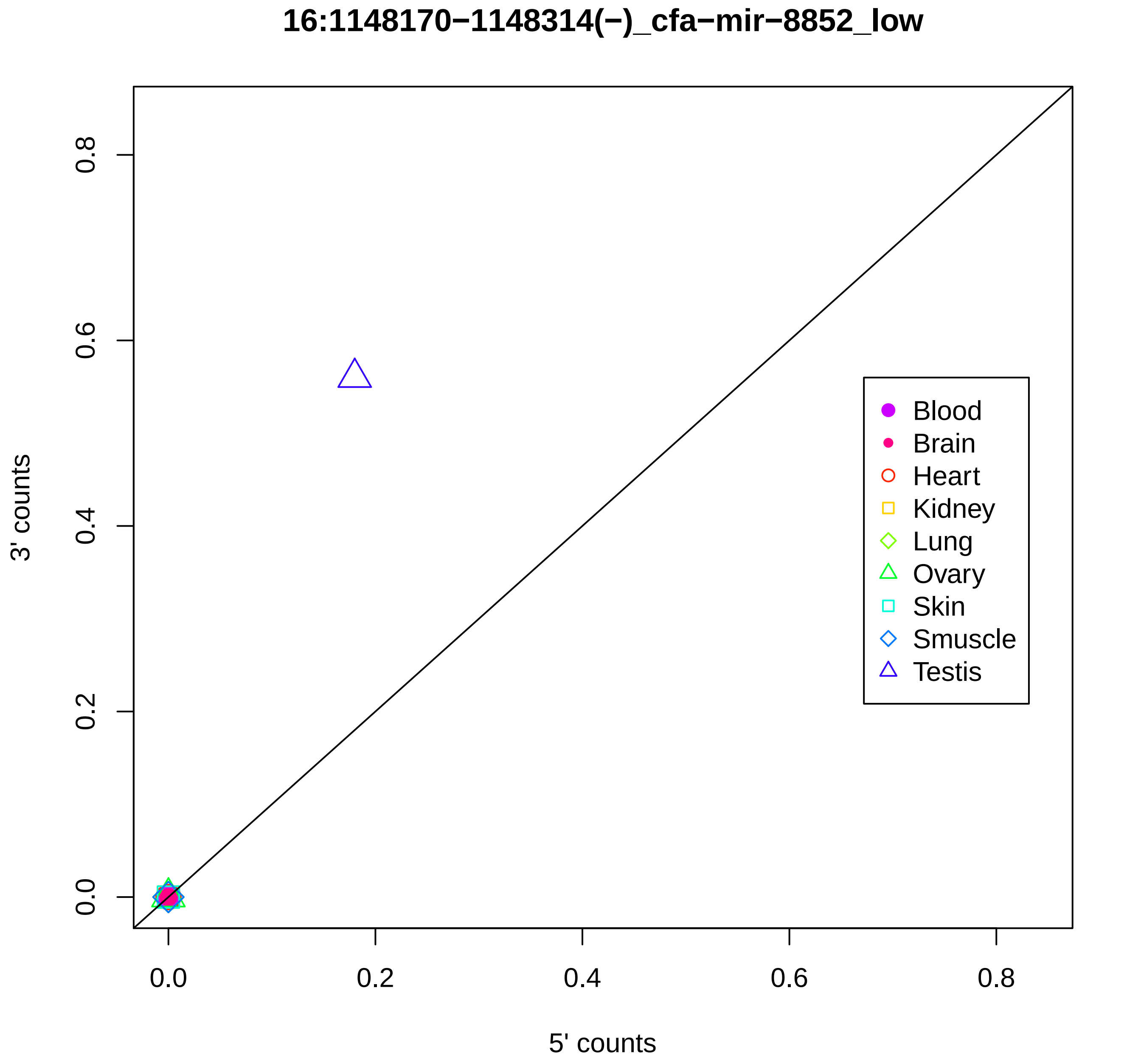

16:1148170−1148314(−)_cfa−mir−8852_low
3' counts
5' counts
0.0
0.2
0.4
0.6
0.8
Blood
Brain
Heart
Kidney
Lung
Ovary
Skin
Smuscle
Testis

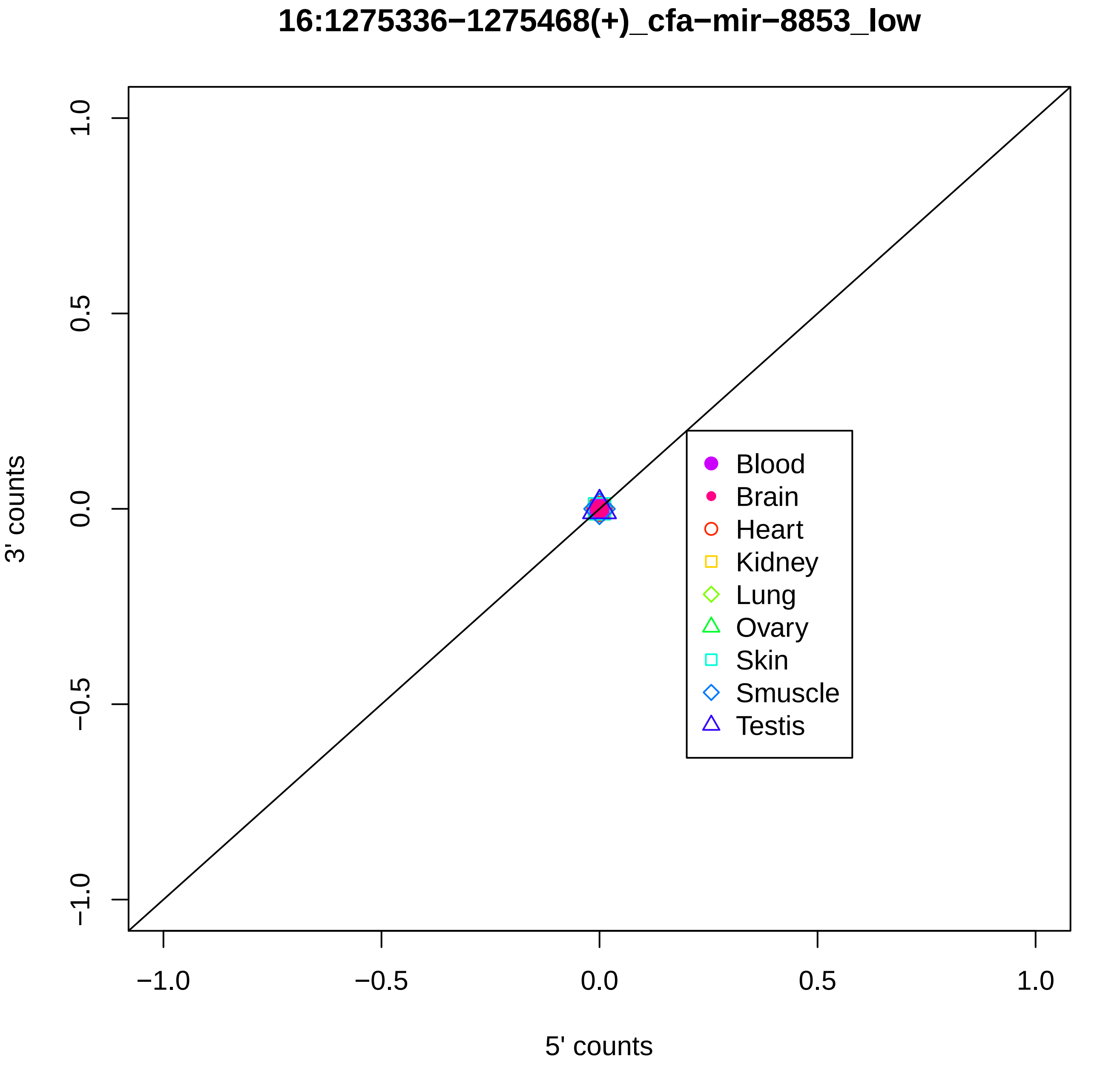

16:1275336−1275468(+)_cfa−mir−8853_low
3' counts
5' counts
1.0
0.5
0.0
−0.5
−1.0
−1.0
−0.5
0.0
0.5
1.0
Blood
Brain
Heart
Kidney
Lung
Ovary
Skin
Smuscle
Testis

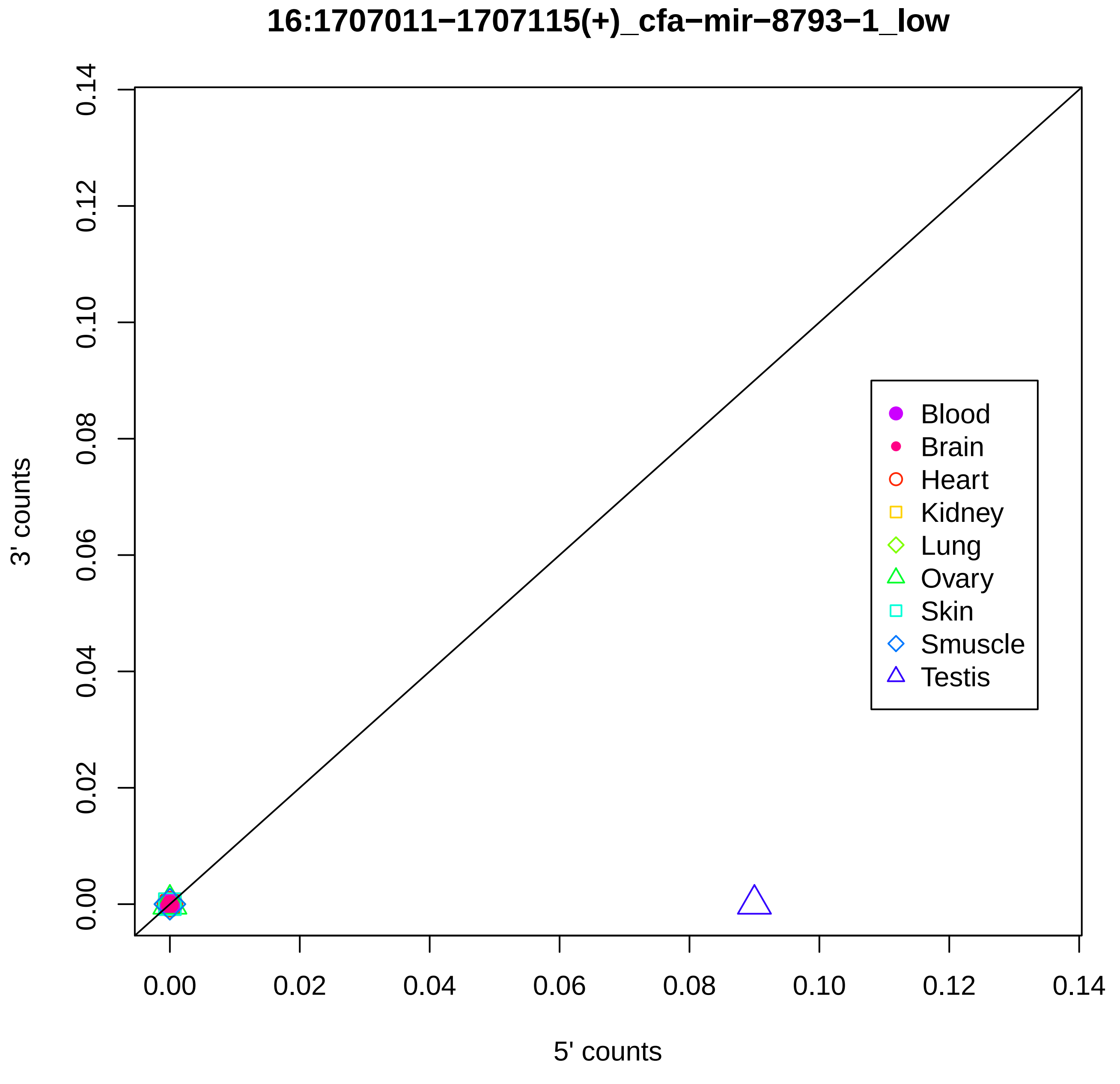

16:1707011−1707115(+)_cfa−mir−8793−1_low
3' counts
0.00
0.02
0.04
0.06
0.08
0.10
0.12
0.14
5' counts
Blood
Brain
Heart
Kidney
Lung
Ovary
Skin
Smuscle
Testis

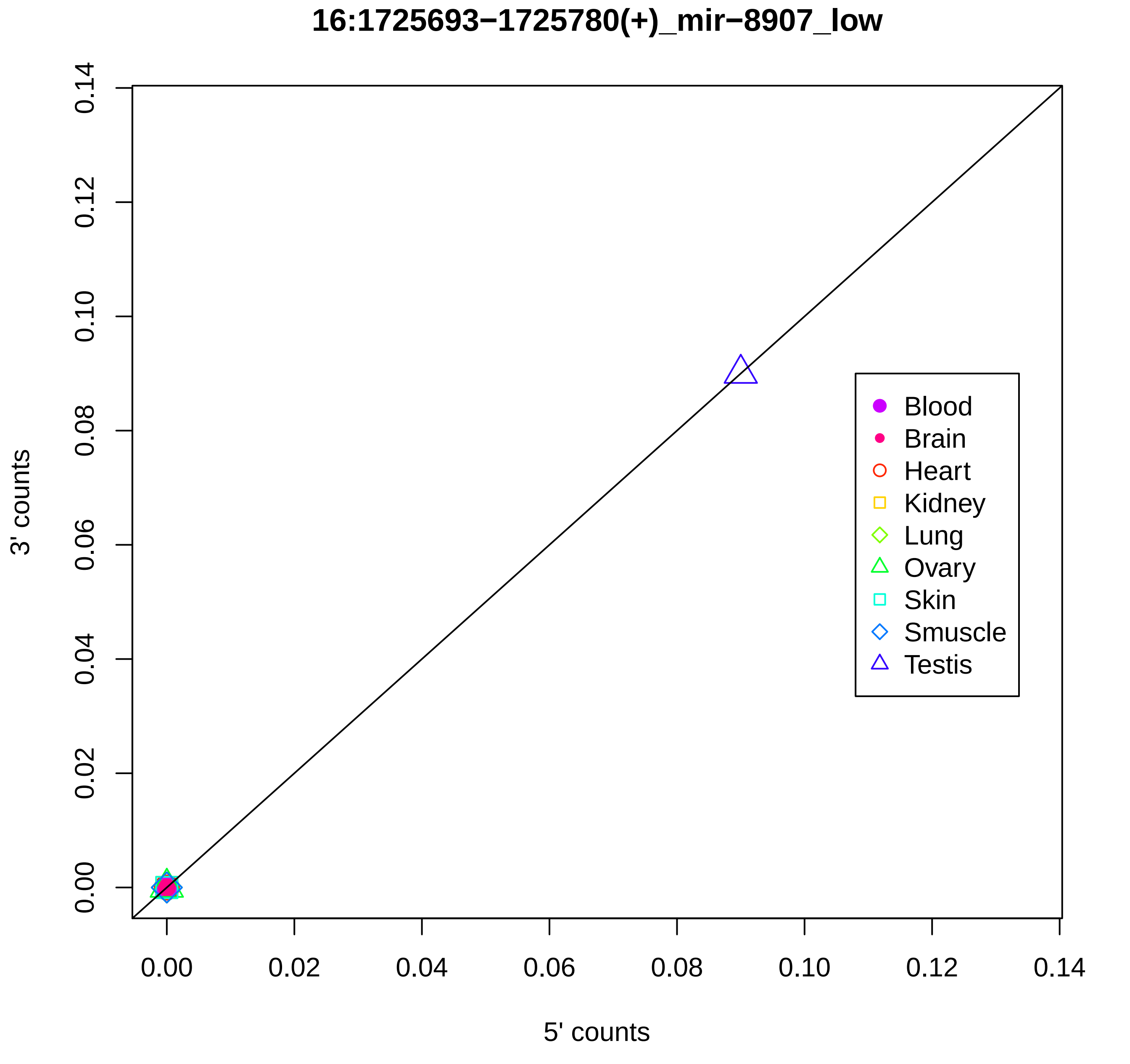

16:1725693−1725780(+)_mir−8907_low
3' counts
5' counts
0.00
0.02
0.04
0.06
0.08
0.10
0.12
0.14
Blood
Brain
Heart
Kidney
Lung
Ovary
Skin
Smuscle
Testis

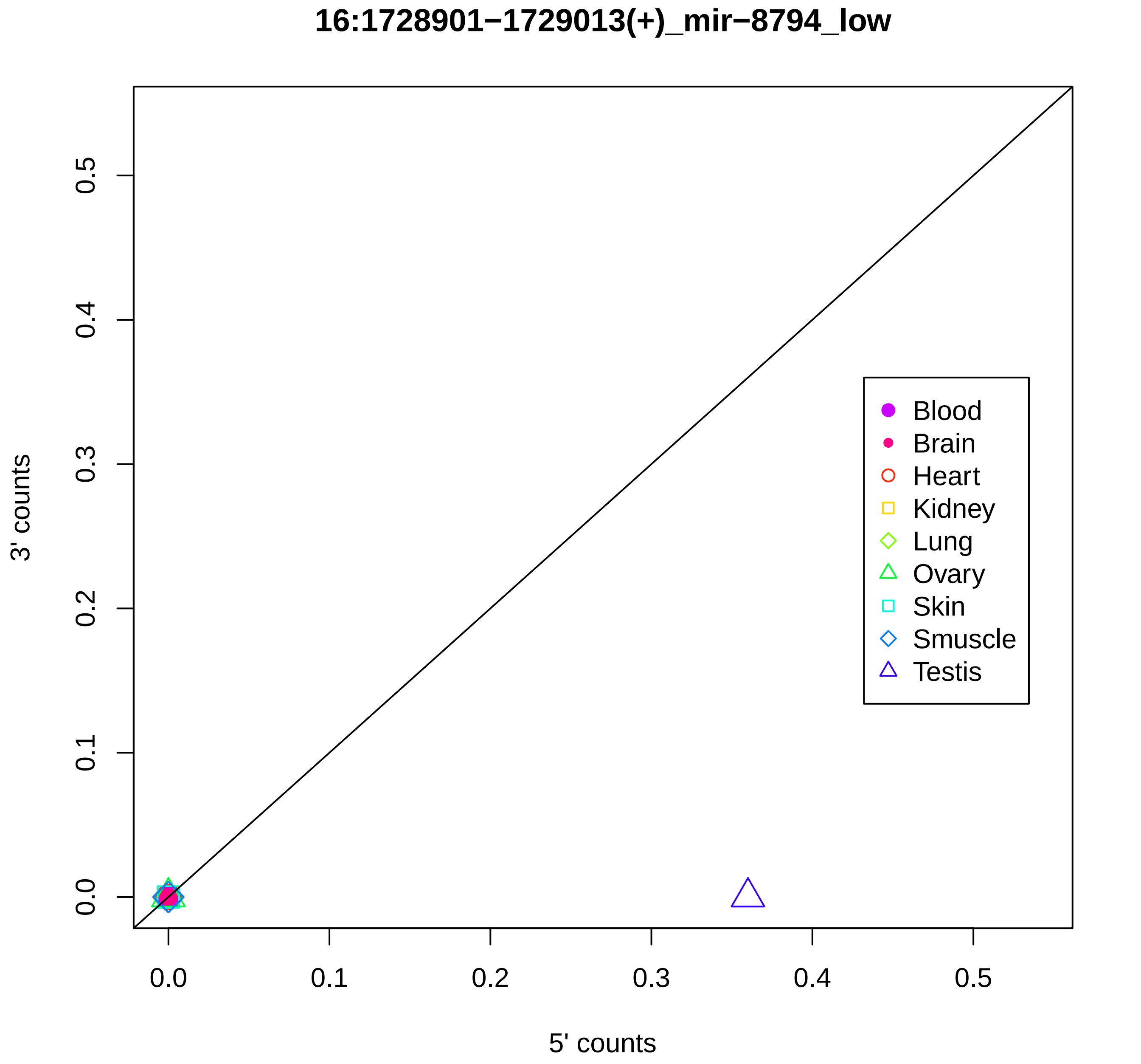

16:1728901−1729013(+)_mir−8794_low
3' counts
5' counts
0.0
0.1
0.2
0.3
0.4
0.5
Blood
Brain
Heart
Kidney
Lung
Ovary
Skin
Smuscle
Testis

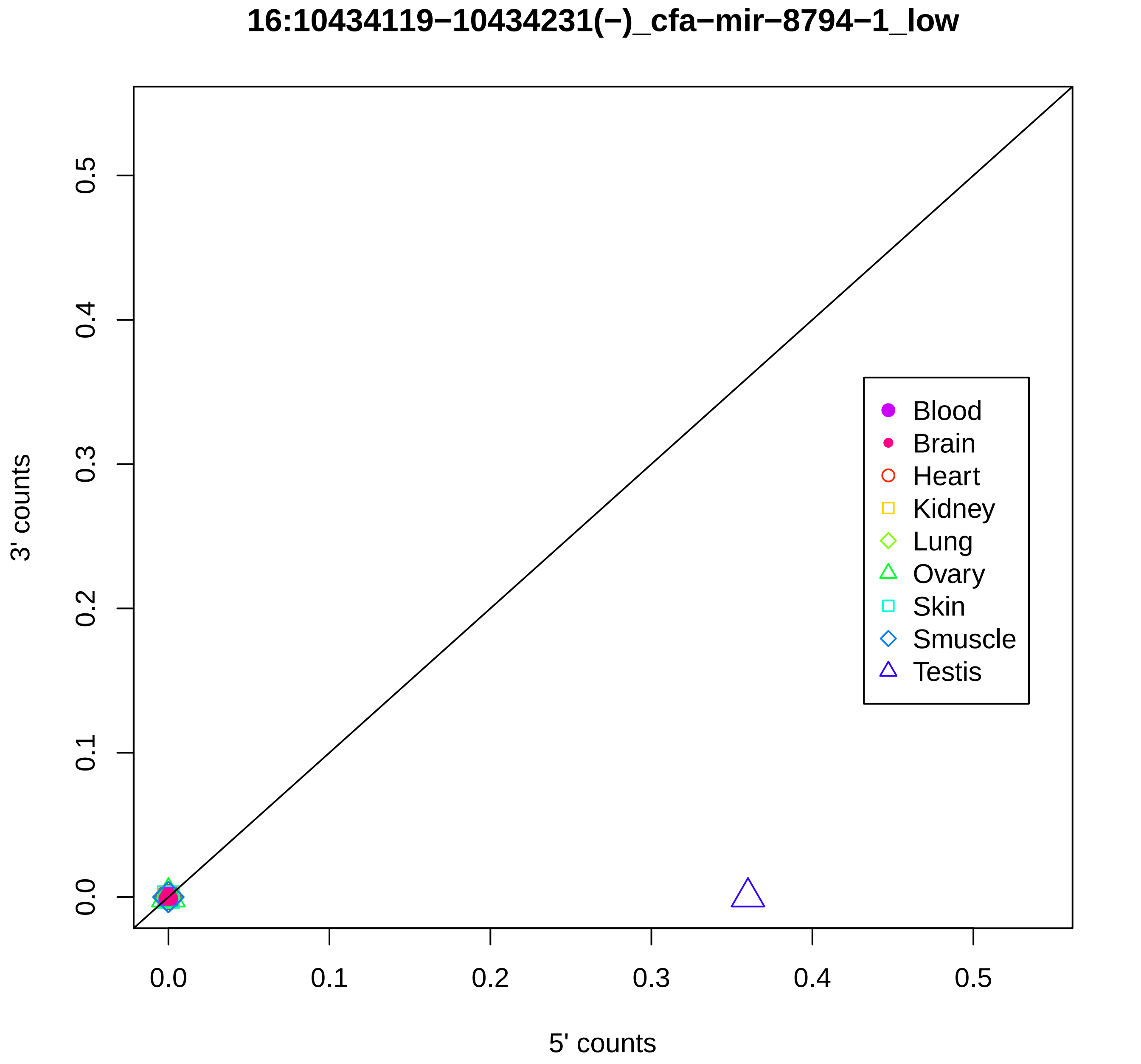

16:10434119−10434231(−)_cfa−mir−8794−1_low
3' counts
5' counts
0.0
0.1
0.2
0.3
0.4
0.5
Blood
Brain
Heart
Kidney
Lung
Ovary
Skin
Smuscle
Testis

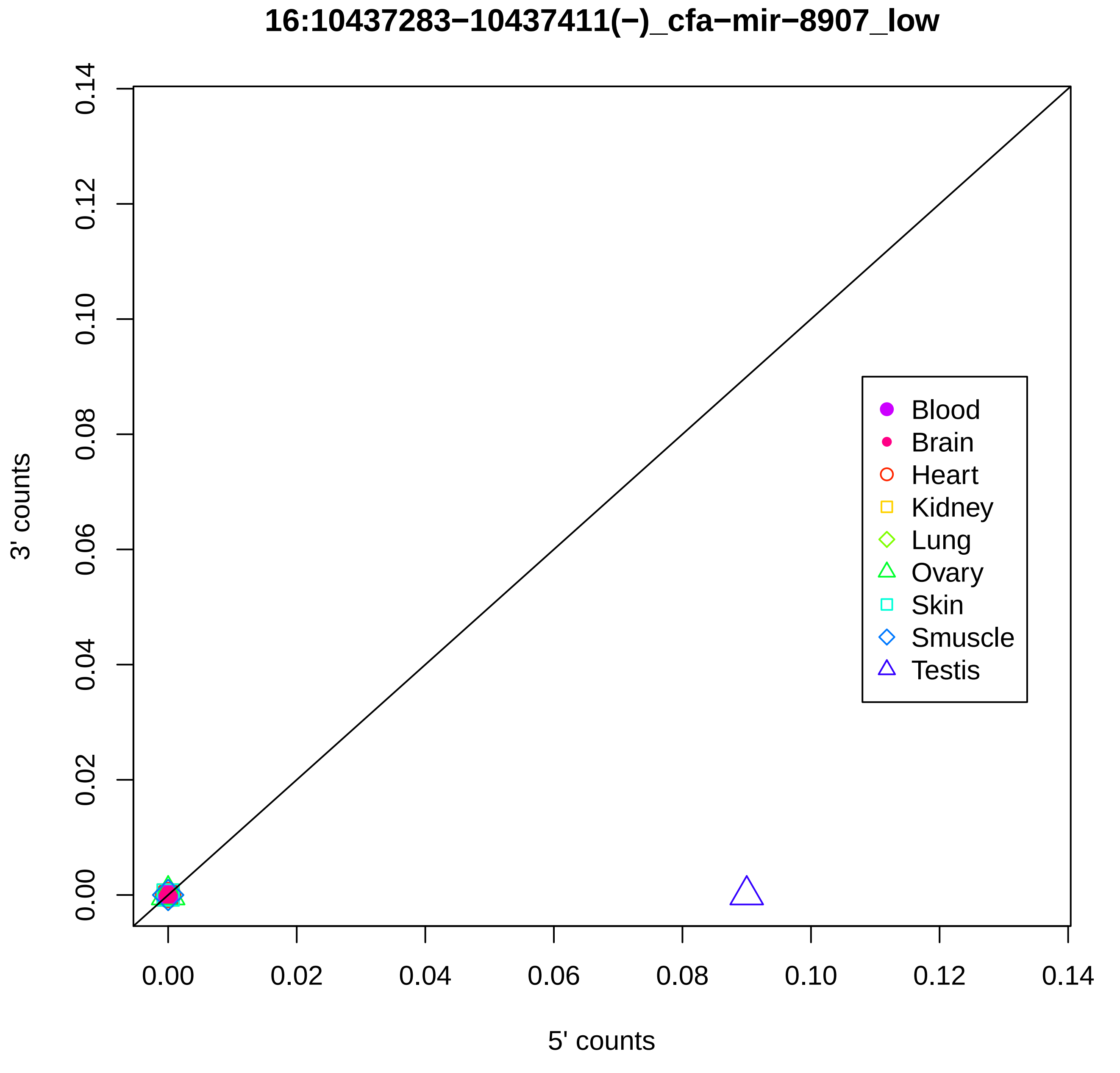

16:10437283−10437411(−)_cfa−mir−8907_low
3' counts
5' counts
0.00
0.02
0.04
0.06
0.08
0.10
0.12
0.14
Blood
Brain
Heart
Kidney
Lung
Ovary
Skin
Smuscle
Testis

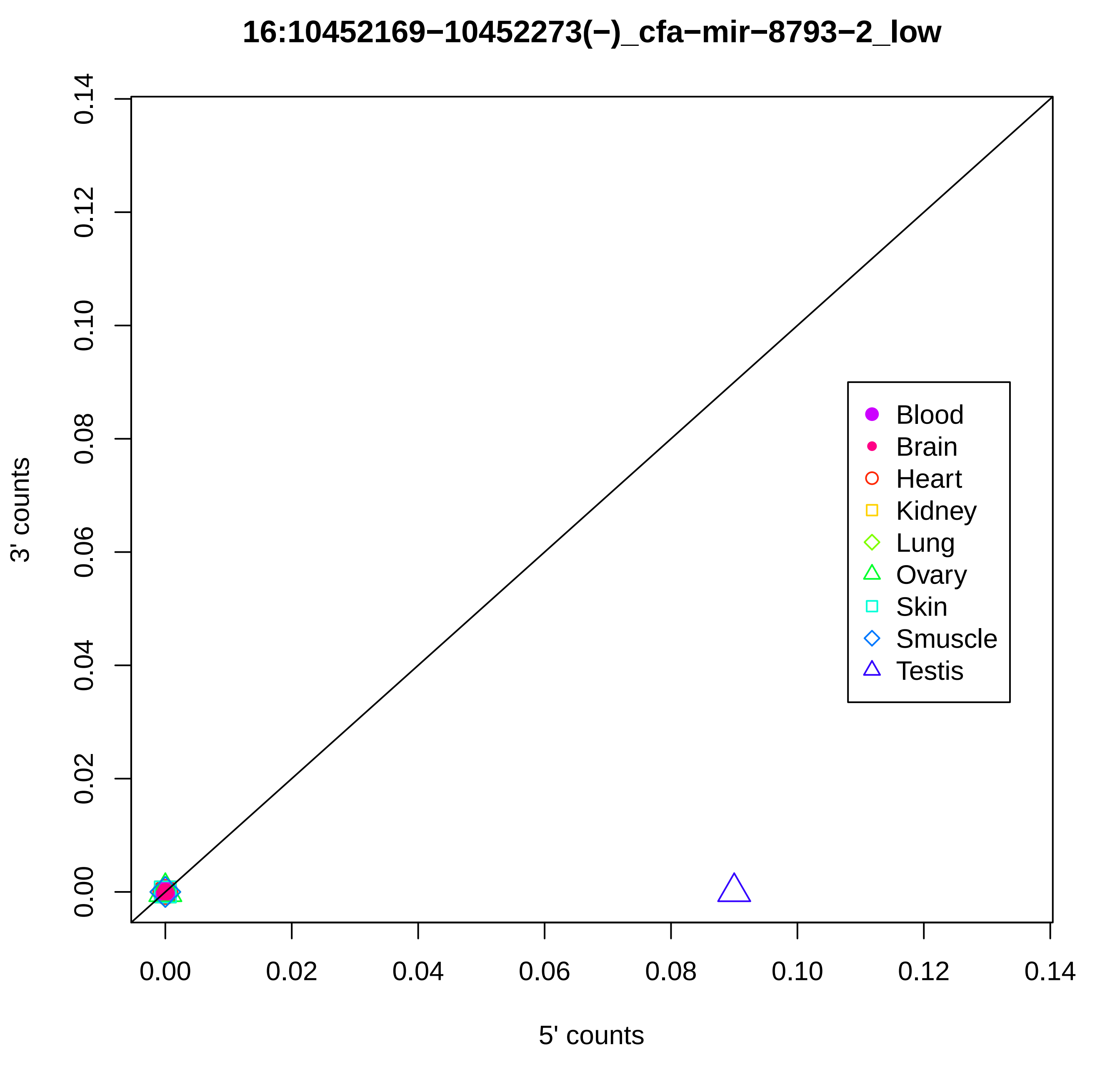
16:10452169−10452273(−)_cfa−mir−8793−2_low
3' counts
5' counts
0.00
0.02
0.04
0.06
0.08
0.10
0.12
0.14
Blood
Brain
Heart
Kidney
Lung
Ovary
Skin
Smuscle
Testis

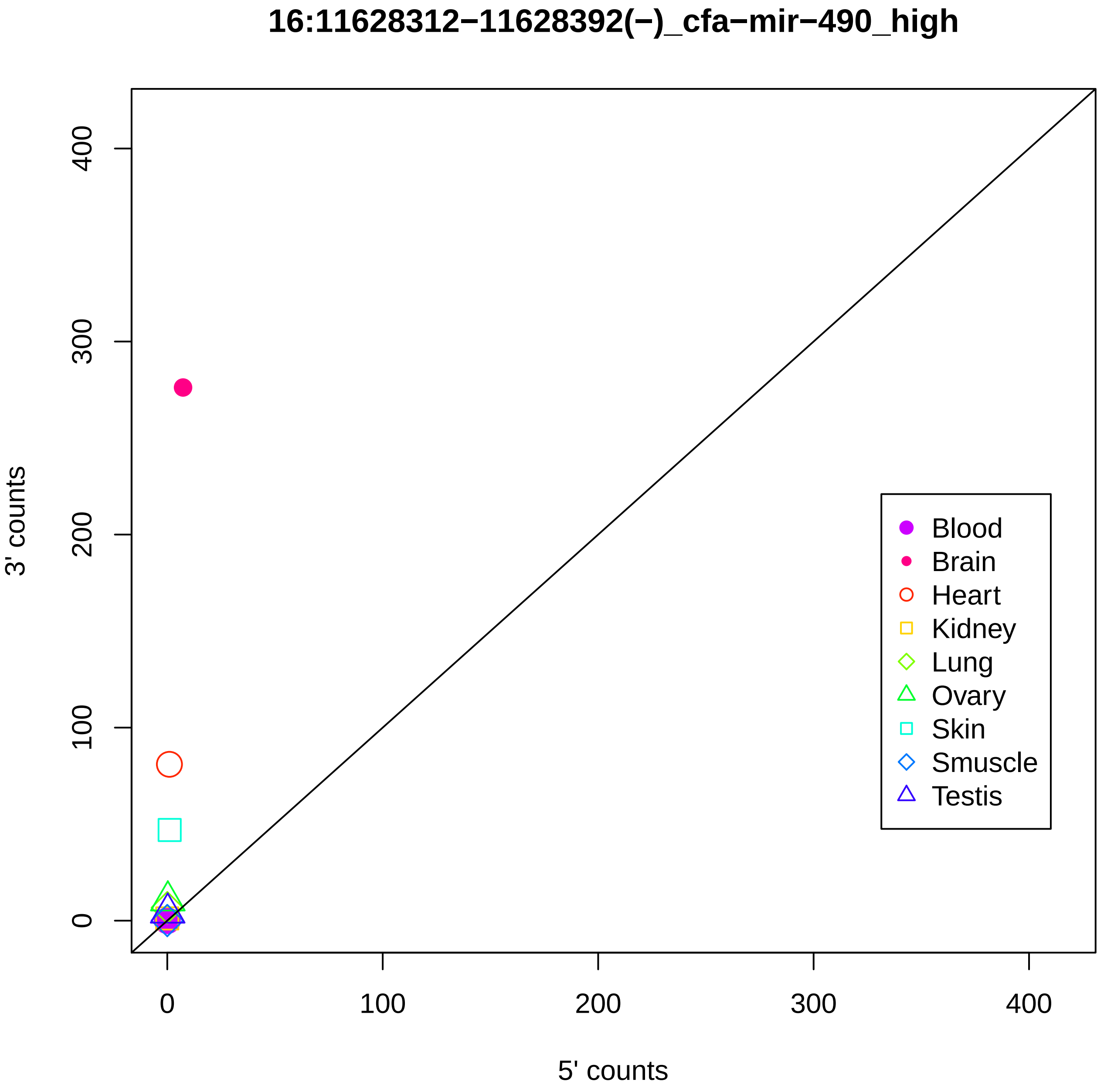

16:11628312−11628392(−)_cfa−mir−490_high
3' counts
5' counts
0
100
200
300
400
0
100
200
300
400
Blood
Brain
Heart
Kidney
Lung
Ovary
Skin
Smuscle
Testis

16:12928966−12929066(+)_cfa−mir−8847_low

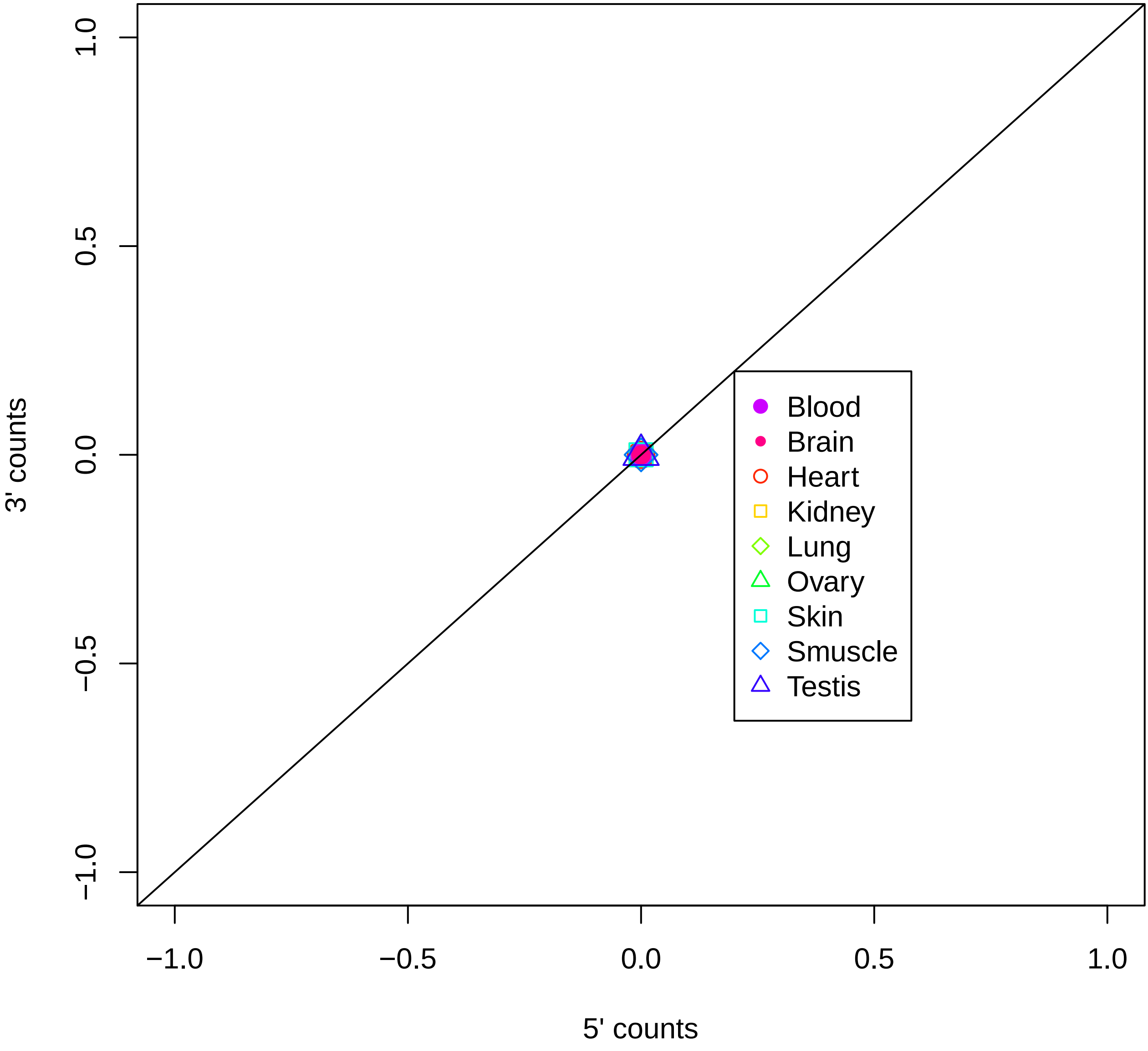

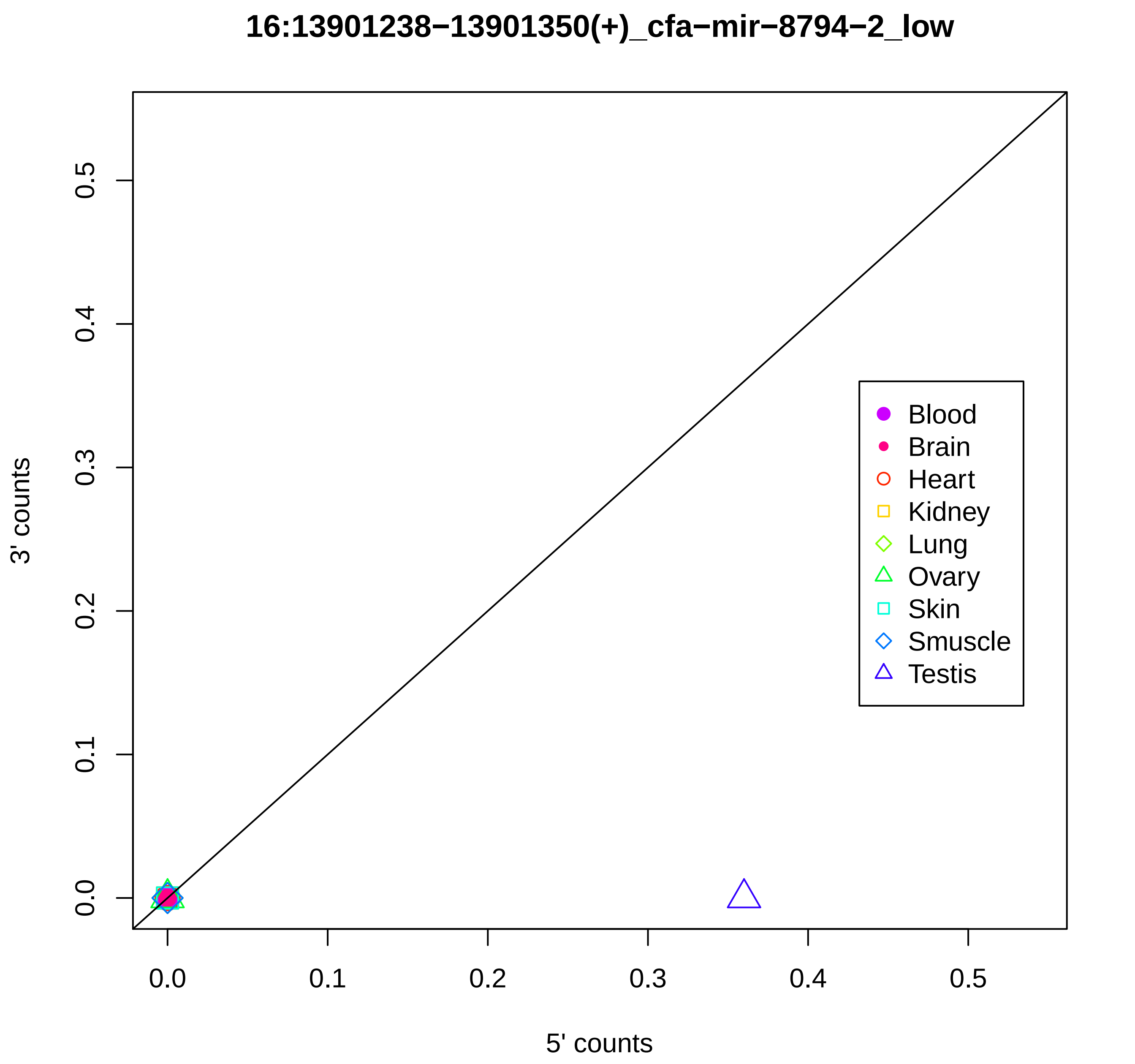
16:13901238−13901350(+)_cfa−mir−8794−2_low
3' counts
5' counts
0.0
0.1
0.2
0.3
0.4
0.5
Blood
Brain
Heart
Kidney
Lung
Ovary
Skin
Smuscle
Testis

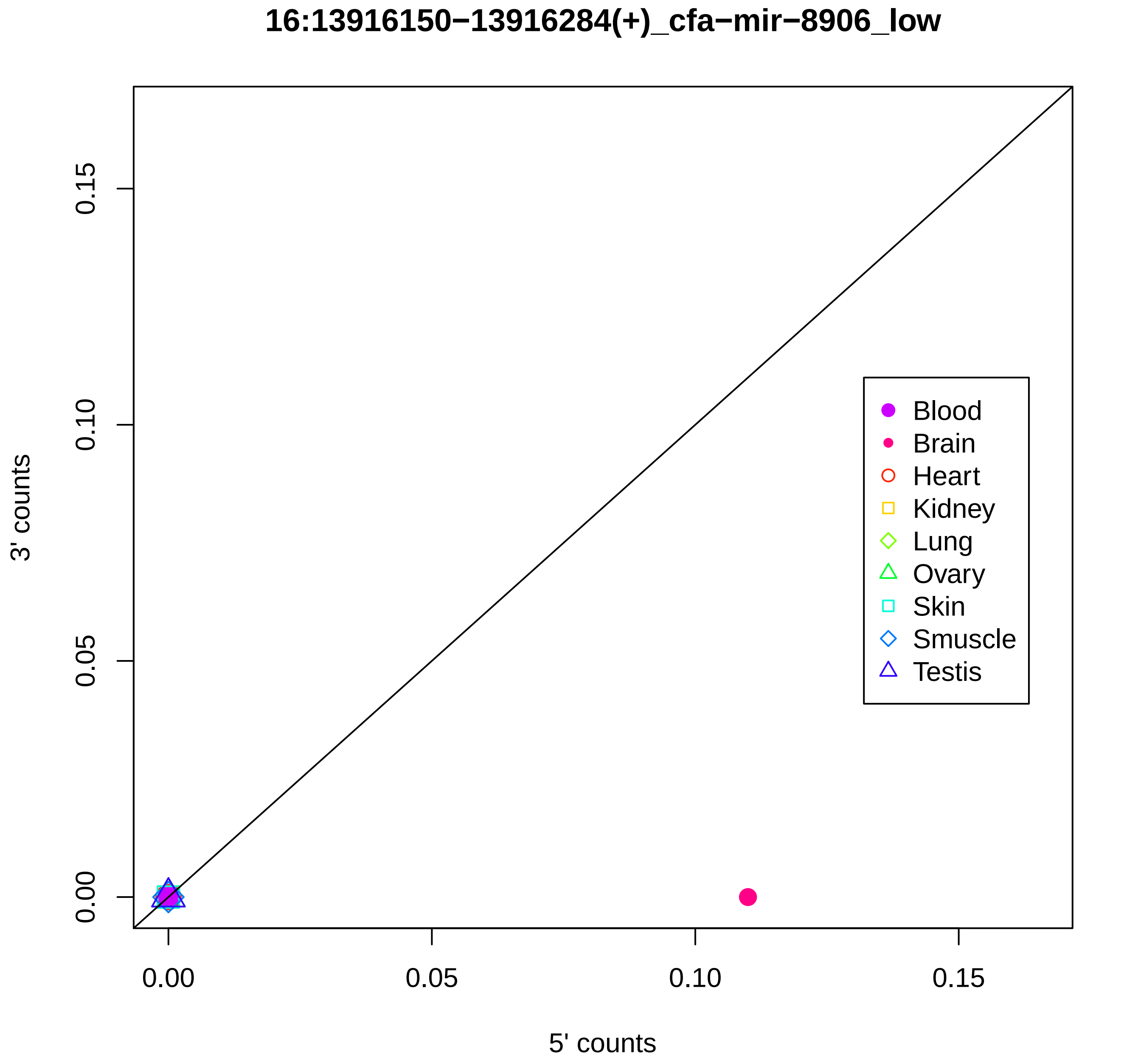
16:13916150−13916284(+)_cfa−mir−8906_low
3' counts
5' counts
0.00
0.05
0.10
0.15
Blood
Brain
Heart
Kidney
Lung
Ovary
Skin
Smuscle
Testis

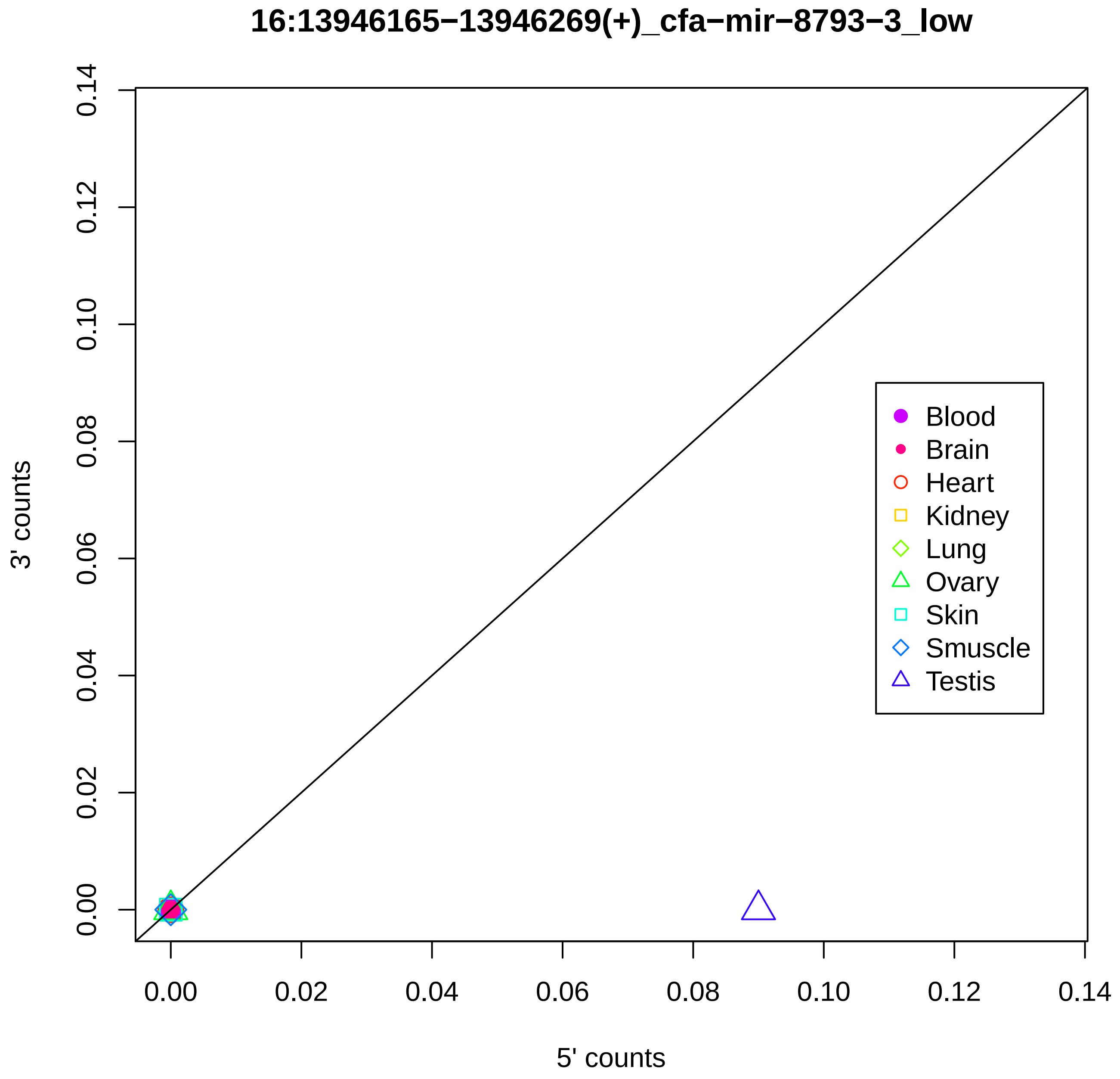

16:13946165−13946269(+)_cfa−mir−8793−3_low
3' counts
5' counts
0.00
0.02
0.04
0.06
0.08
0.10
0.12
0.14
Blood
Brain
Heart
Kidney
Lung
Ovary
Skin
Smuscle
Testis

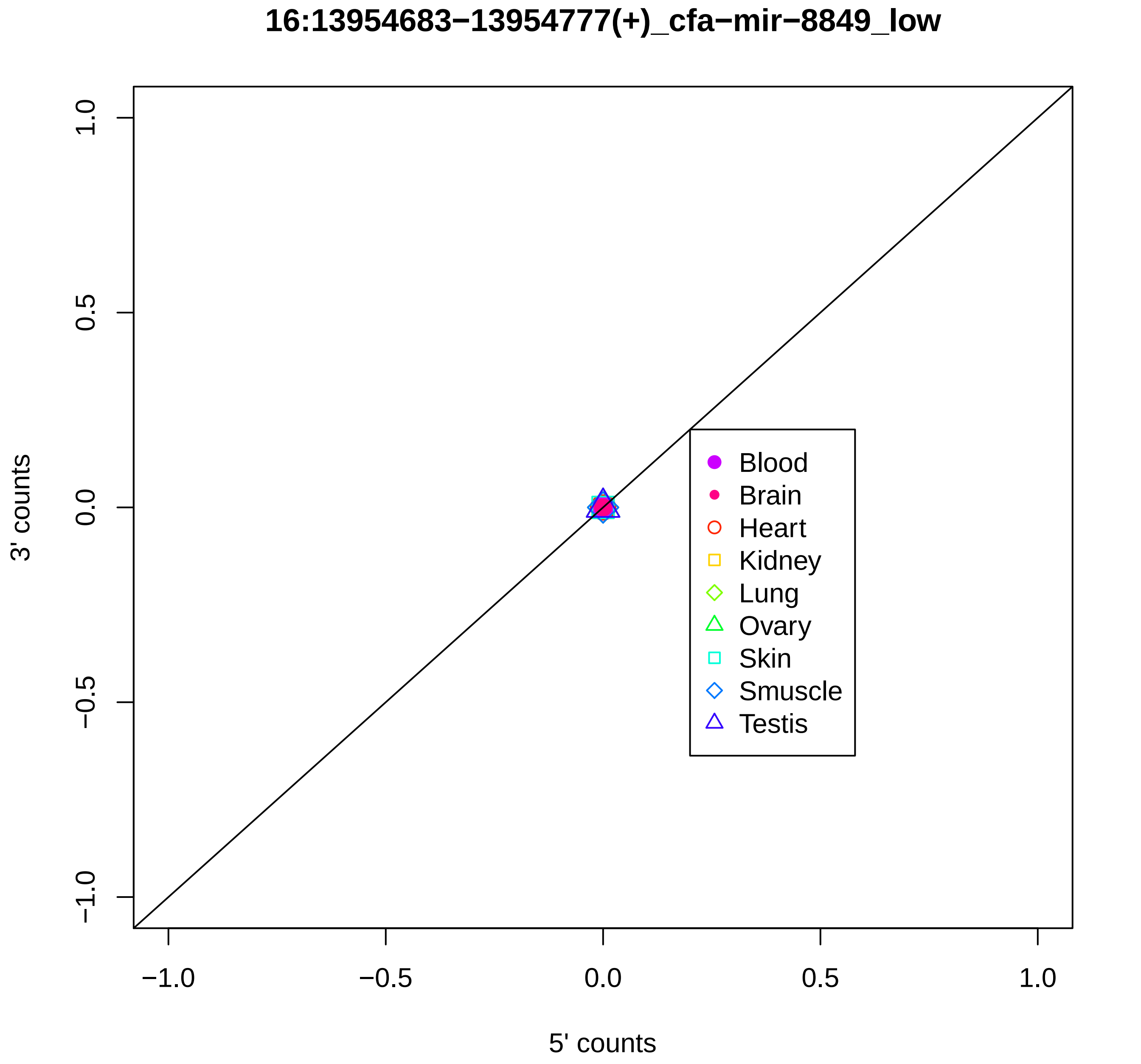
16:13954683−13954777(+)_cfa−mir−8849_low
3' counts
5' counts
1.0
0.5
0.0
−0.5
−1.0
Blood
Brain
Heart
Kidney
Lung
Ovary
Skin
Smuscle
Testis

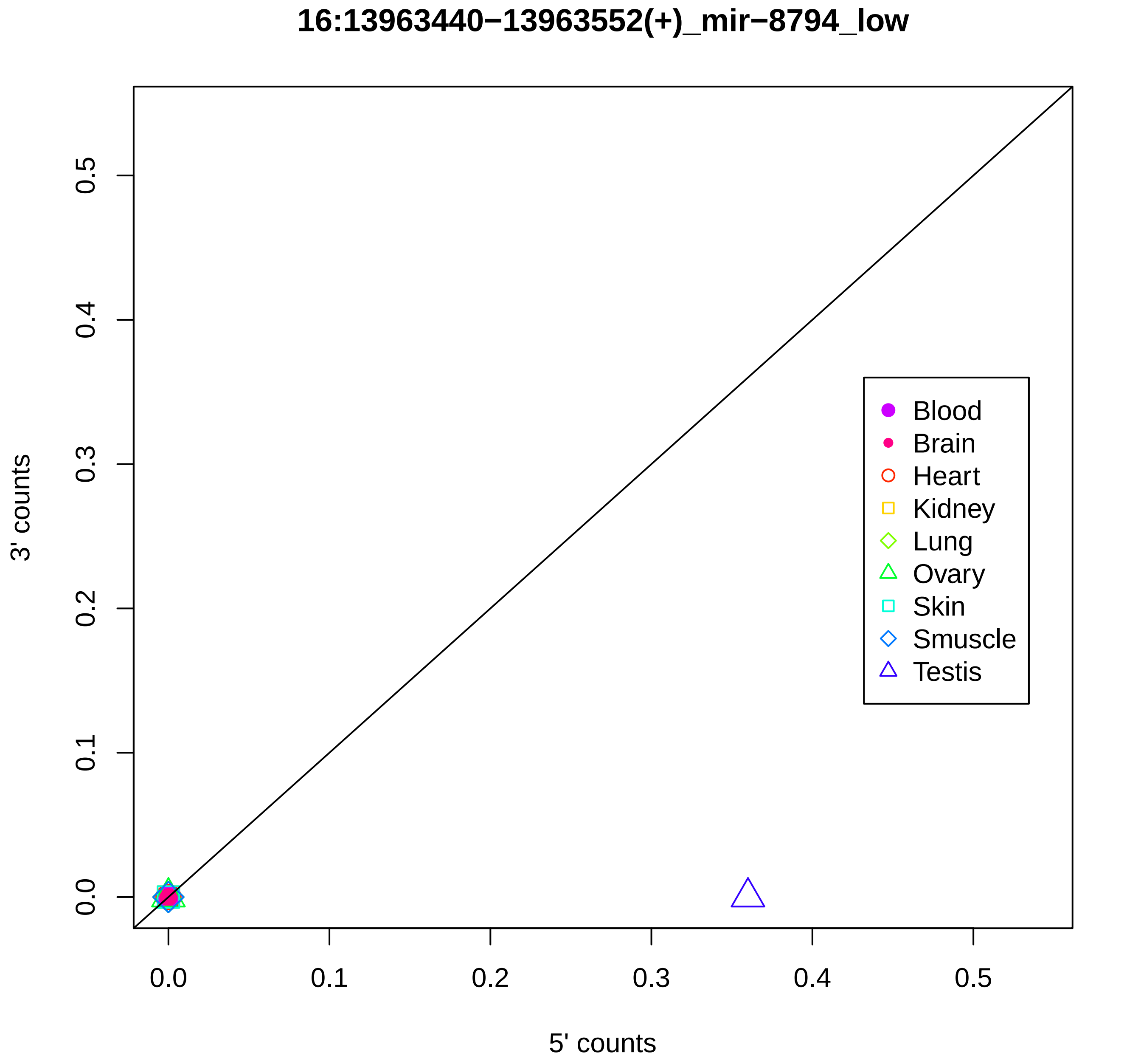
16:13963440−13963552(+)_mir−8794_low
3' counts
5' counts
0.0
0.1
0.2
0.3
0.4
0.5
Blood
Brain
Heart
Kidney
Lung
Ovary
Skin
Smuscle
Testis

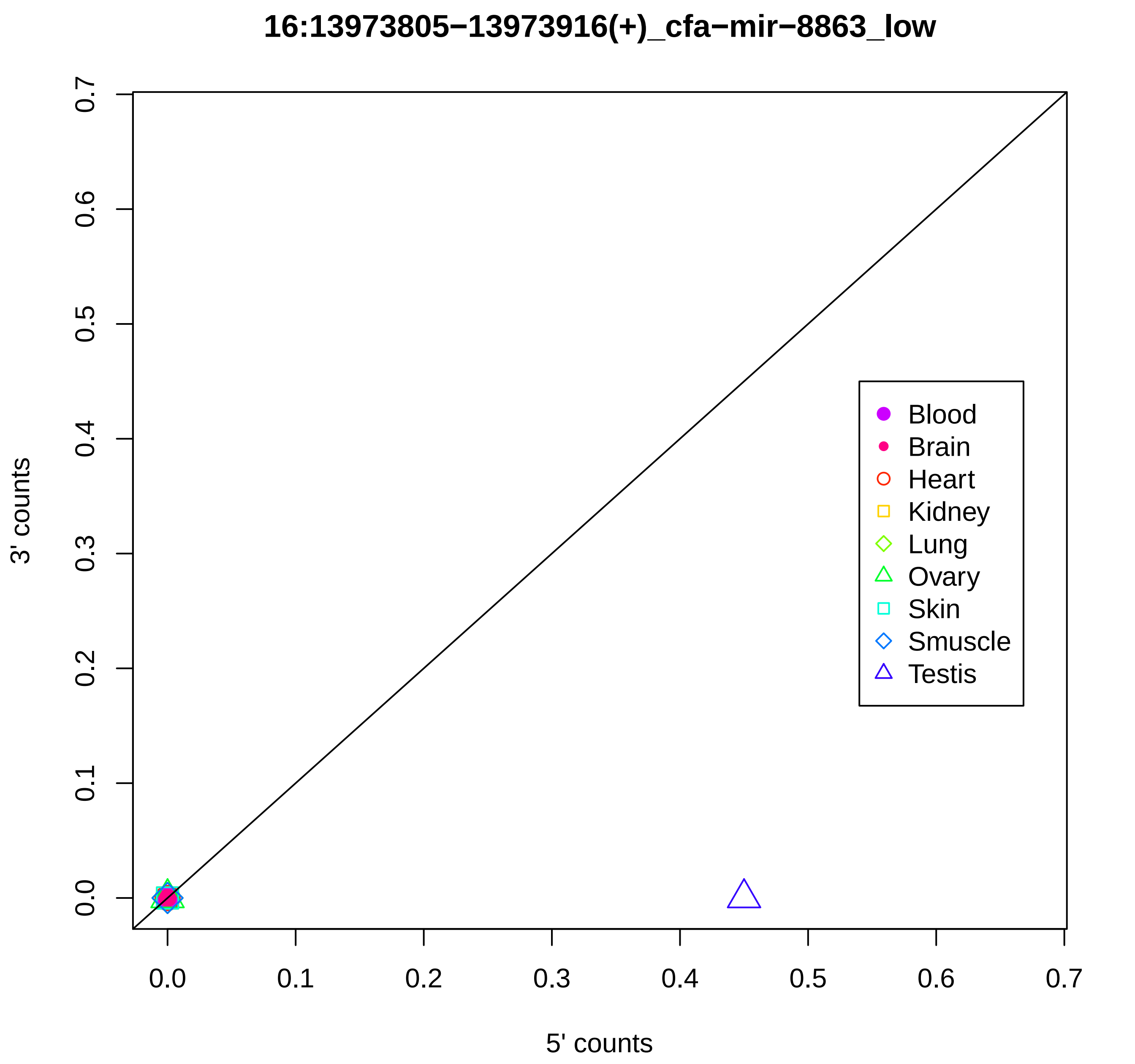

16:13973805−13973916(+)_cfa−mir−8863_low
3' counts
5' counts
0.0
0.1
0.2
0.3
0.4
0.5
0.6
0.7
Blood
Brain
Heart
Kidney
Lung
Ovary
Skin
Smuscle
Testis

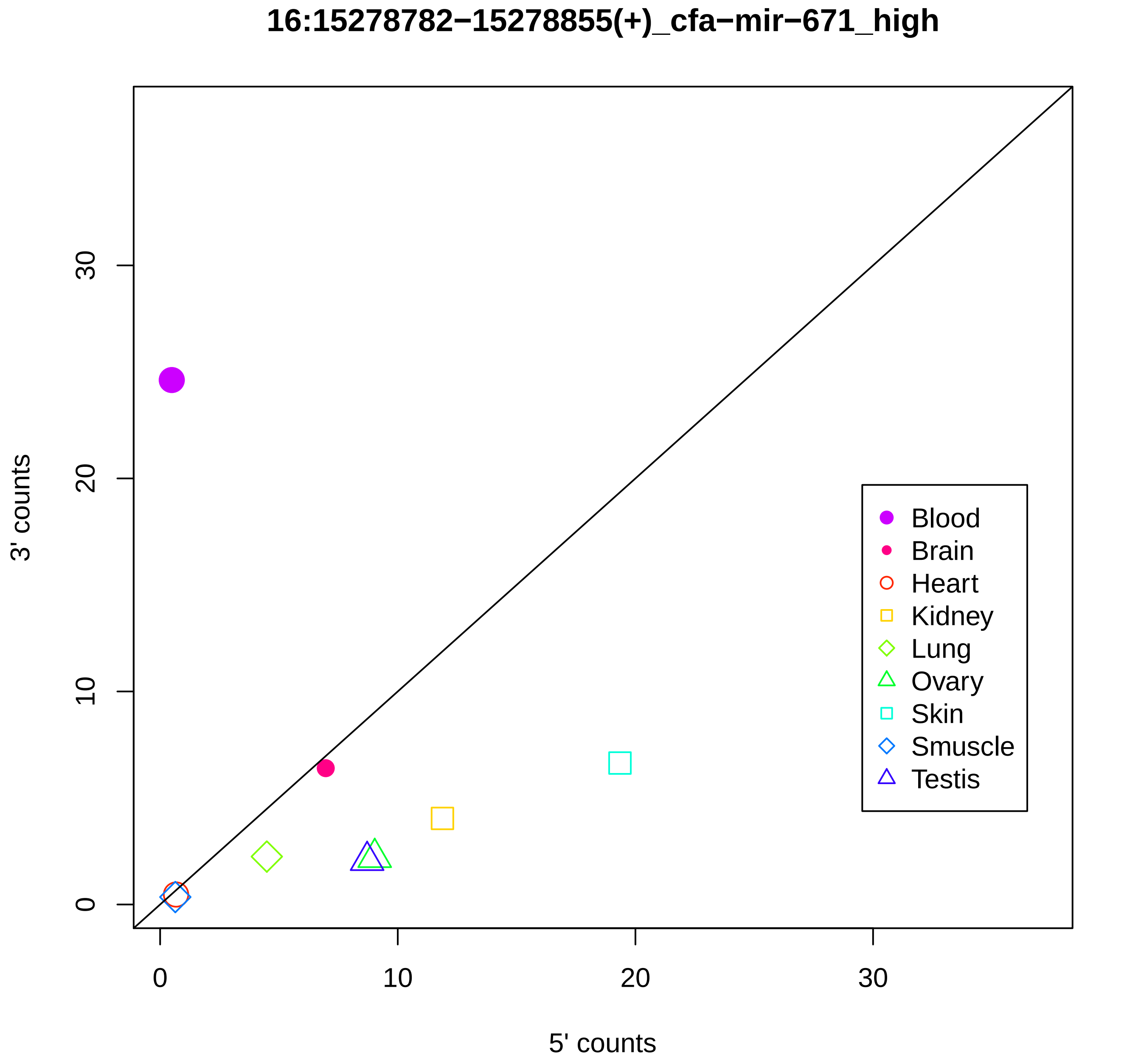
16:15278782−15278855(+)_cfa−mir−671_high
3' counts
5' counts
0
10
20
30
0
10
20
30
Blood
Brain
Heart
Kidney
Lung
Ovary
Skin
Smuscle
Testis

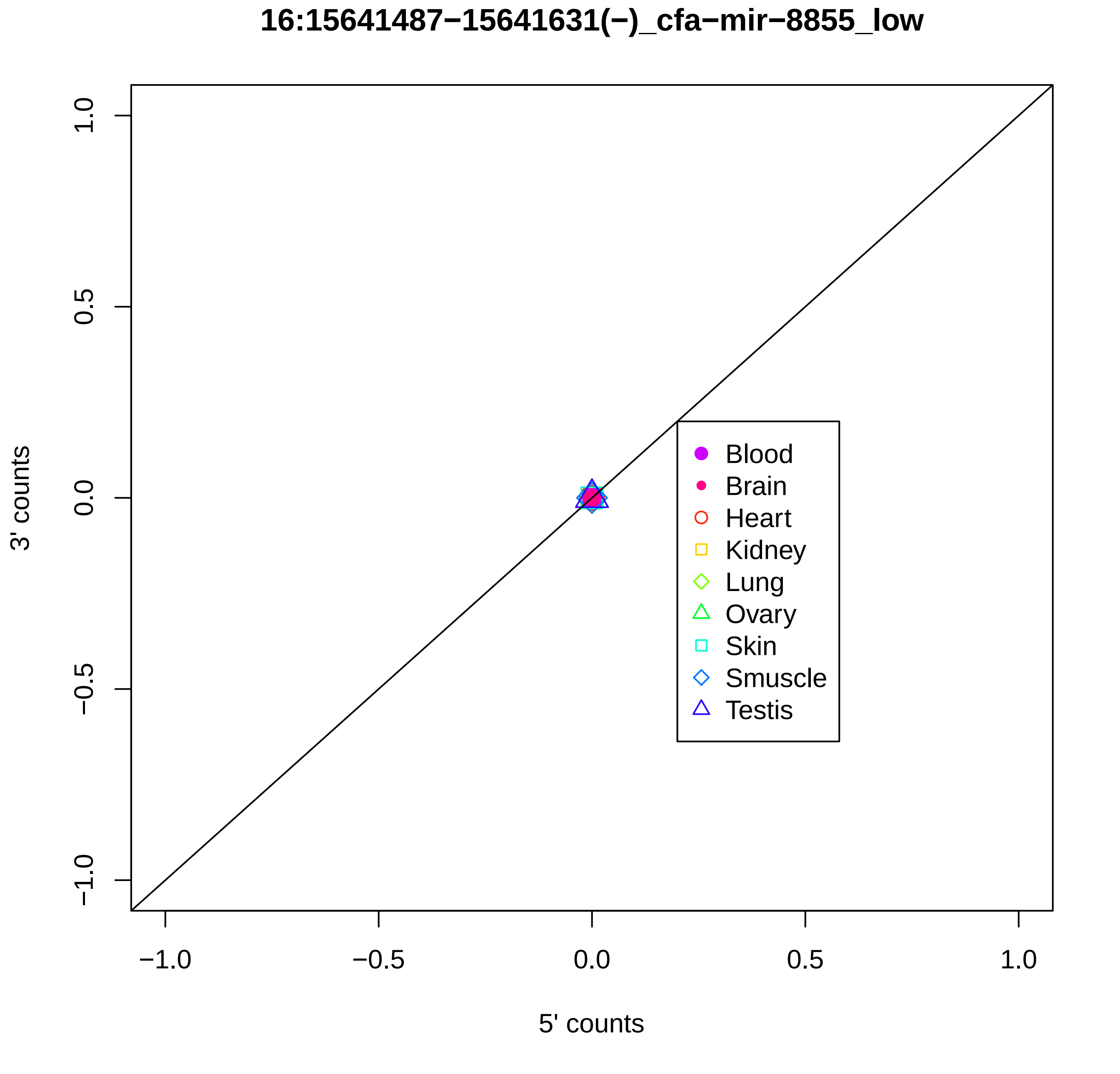

16:15641487−15641631(−)_cfa−mir−8855_low
3' counts
5' counts
1.0
0.5
0.0
−0.5
−1.0
Blood
Brain
Heart
Kidney
Lung
Ovary
Skin
Smuscle
Testis

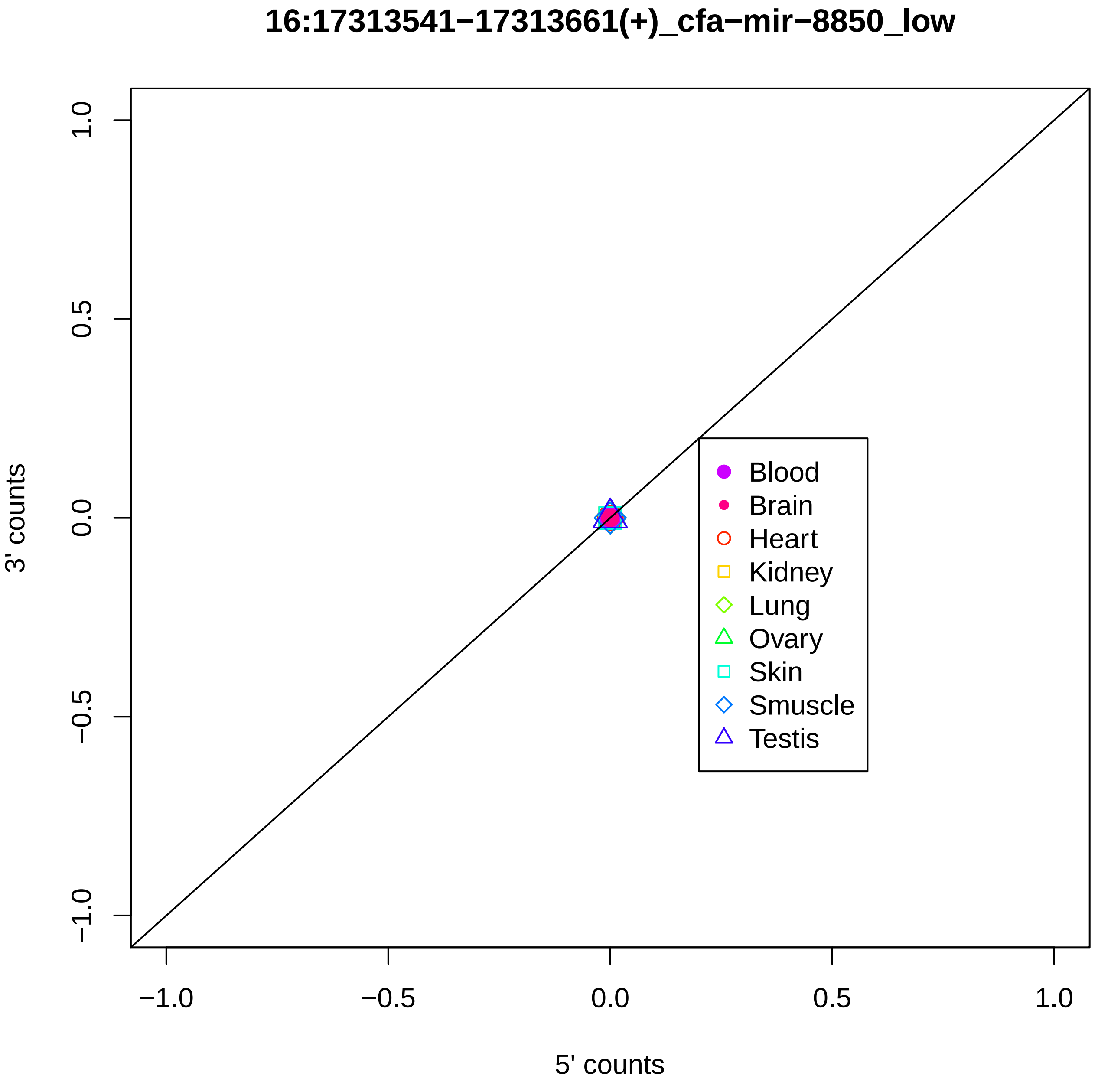

16:17313541−17313661(+)_cfa−mir−8850_low
1.0
0.5
0.0
−0.5
−1.0
3' counts
5' counts
Blood
Brain
Heart
Kidney
Lung
Ovary
Skin
Smuscle
Testis

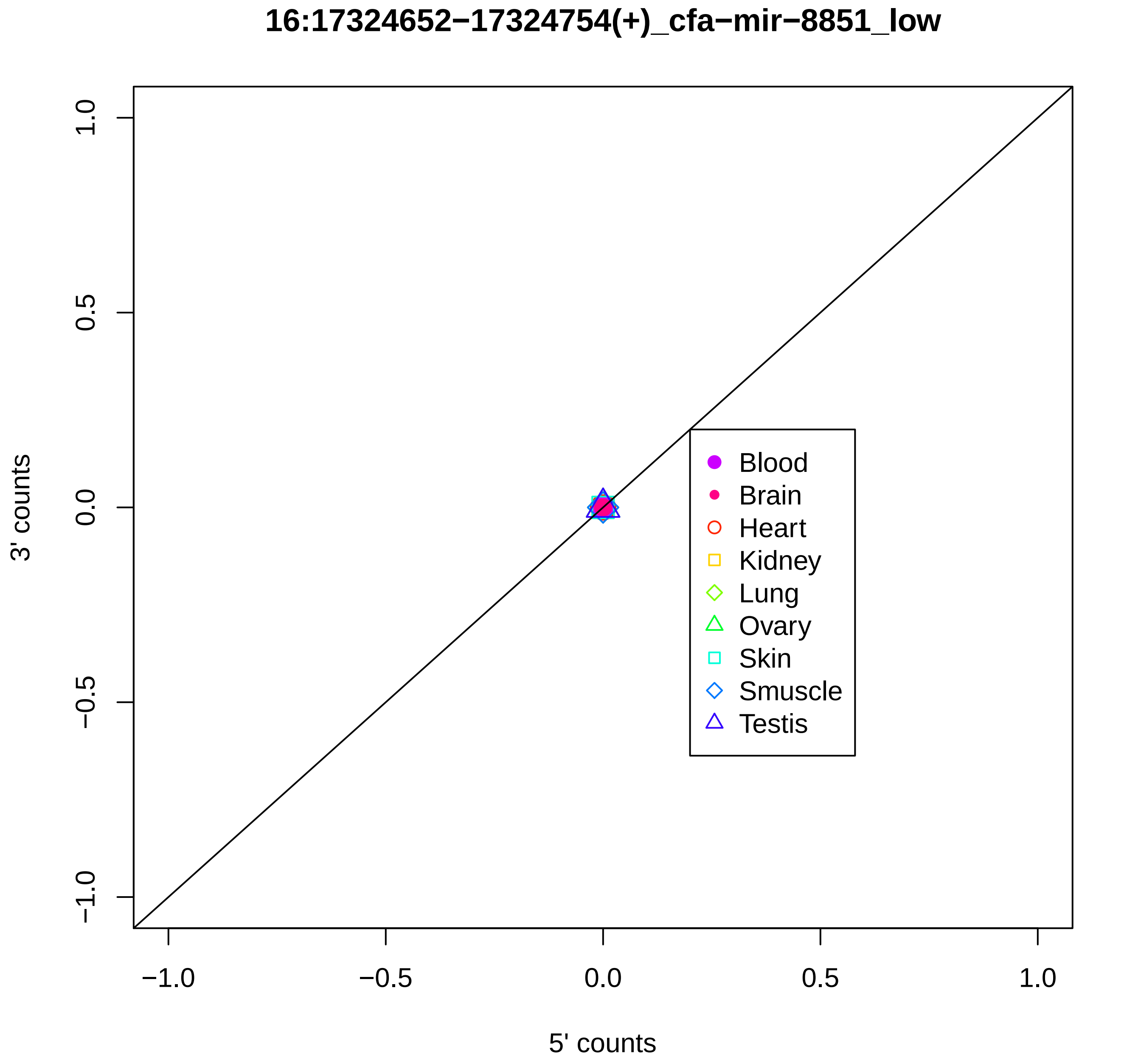

16:17324652−17324754(+)_cfa−mir−8851_low
3' counts
5' counts
1.0
0.5
0.0
−0.5
−1.0
Blood
Brain
Heart
Kidney
Lung
Ovary
Skin
Smuscle
Testis

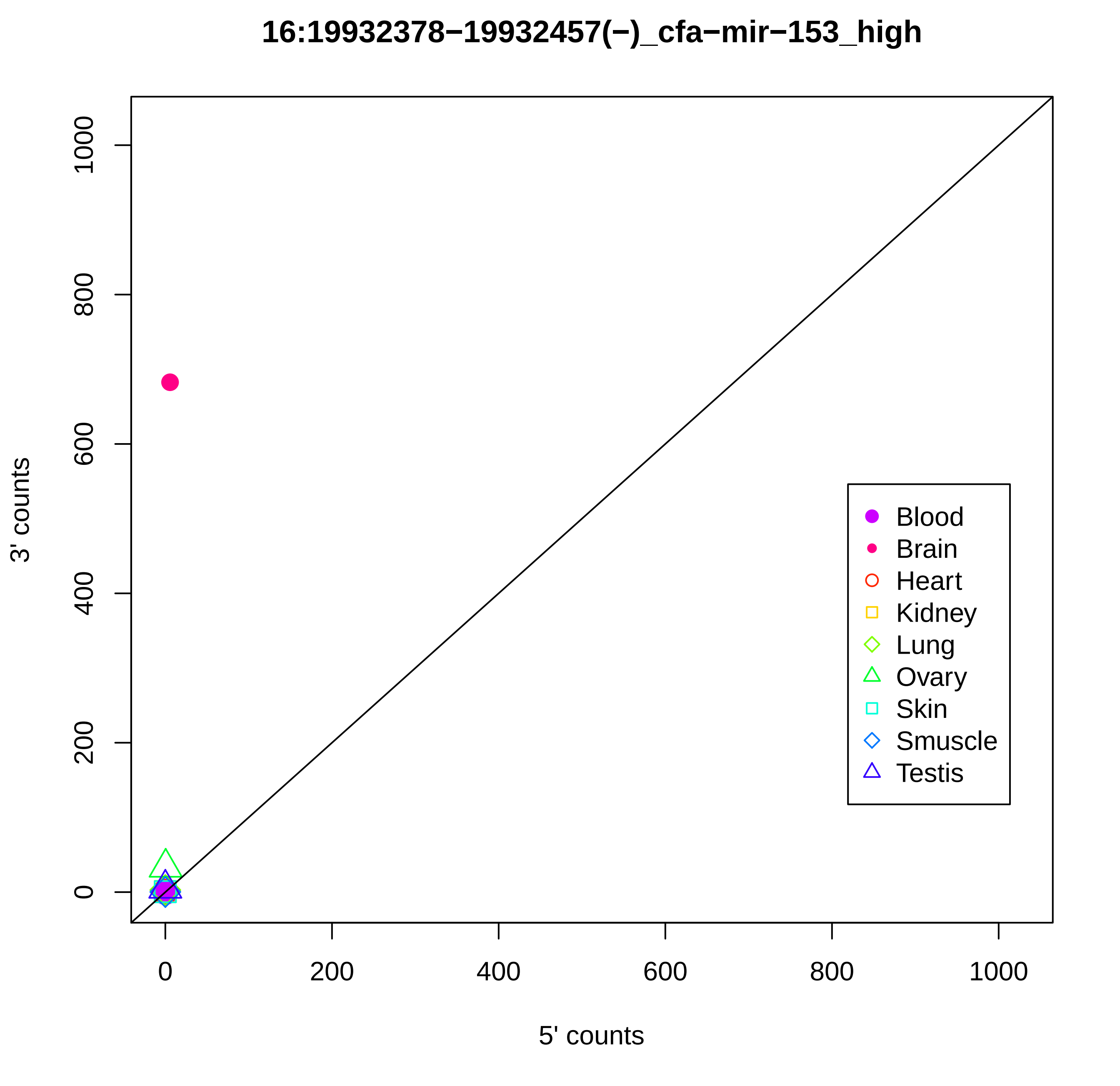
16:19932378−19932457(−)_cfa−mir−153_high
3' counts
5' counts
0
200
400
600
800
1000
0
200
400
600
800
1000
Blood
Brain
Heart
Kidney
Lung
Ovary
Skin
Smuscle
Testis

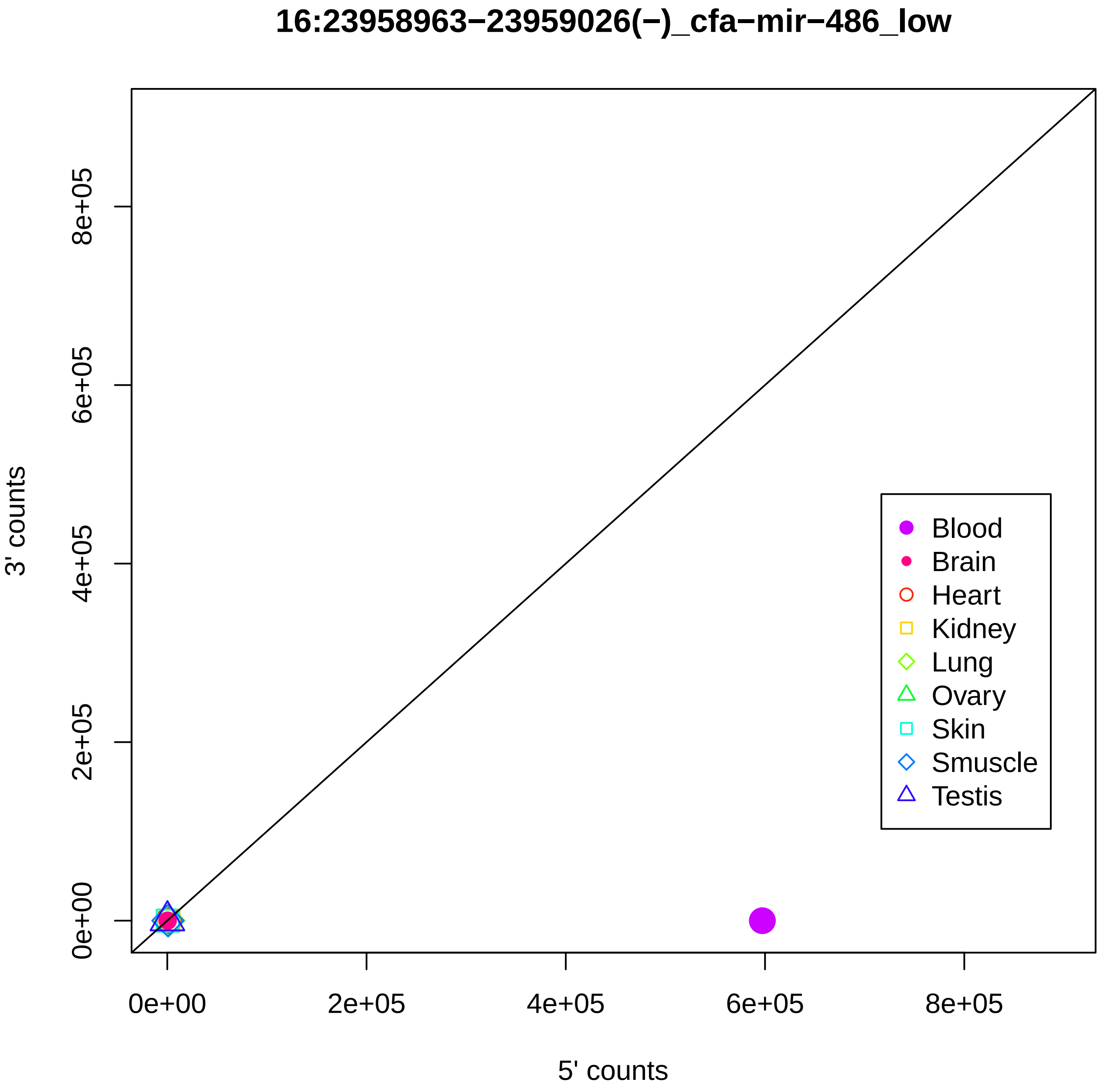

16:23958963−23959026(−)_cfa−mir−486_low
3' counts
5' counts
0e+00
2e+05
4e+05
6e+05
8e+05
Blood
Brain
Heart
Kidney
Lung
Ovary
Skin
Smuscle
Testis

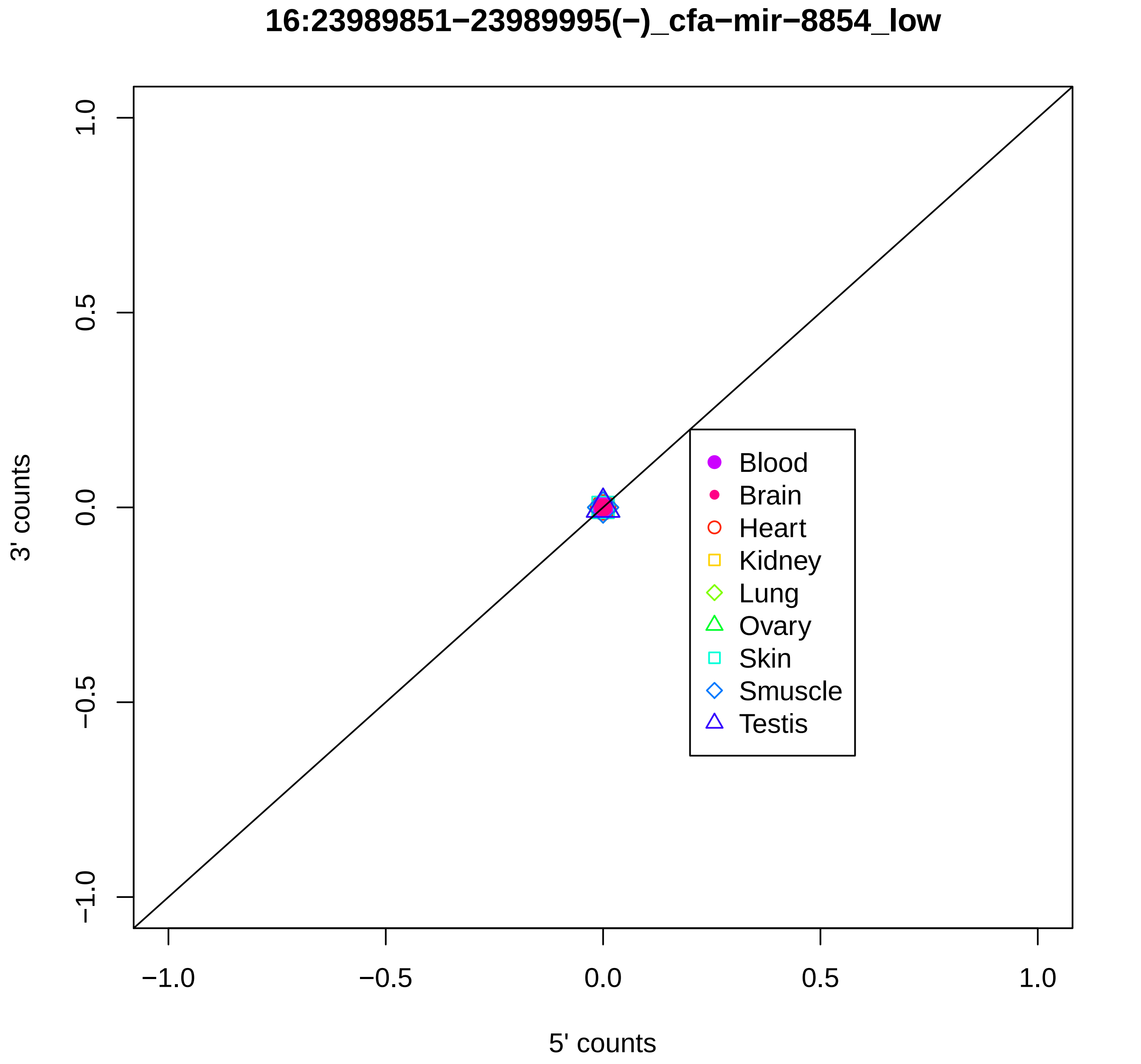
16:23989851−23989995(−)_cfa−mir−8854_low
3' counts
5' counts
−1.0
−0.5
0.0
0.5
1.0
−1.0
−0.5
0.0
0.5
1.0
Blood
Brain
Heart
Kidney
Lung
Ovary
Skin
Smuscle
Testis

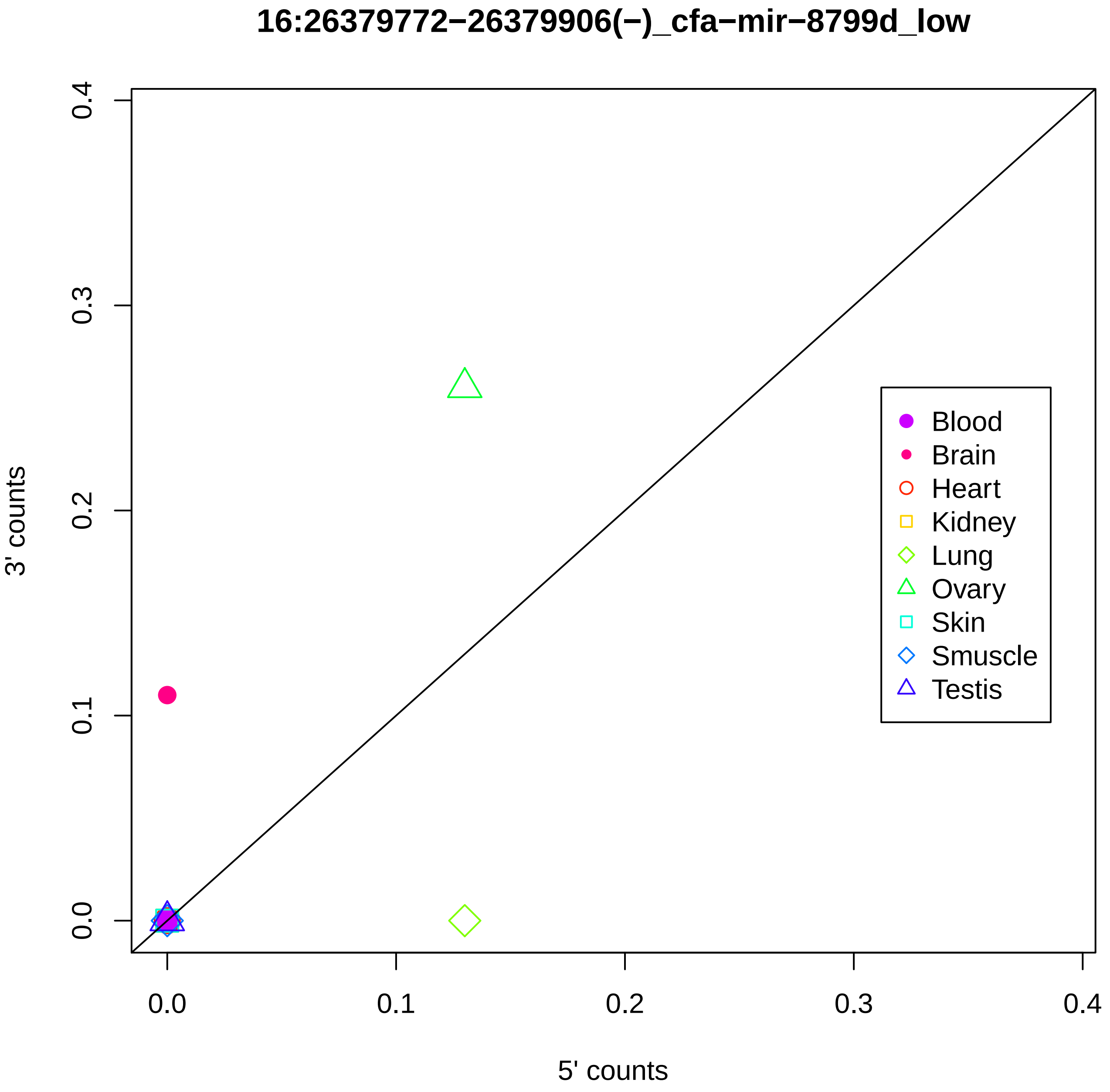

16:26379772−26379906(−)_cfa−mir−8799d_low
3' counts
5' counts
0.0
0.1
0.2
0.3
0.4
Blood
Brain
Heart
Kidney
Lung
Ovary
Skin
Smuscle
Testis

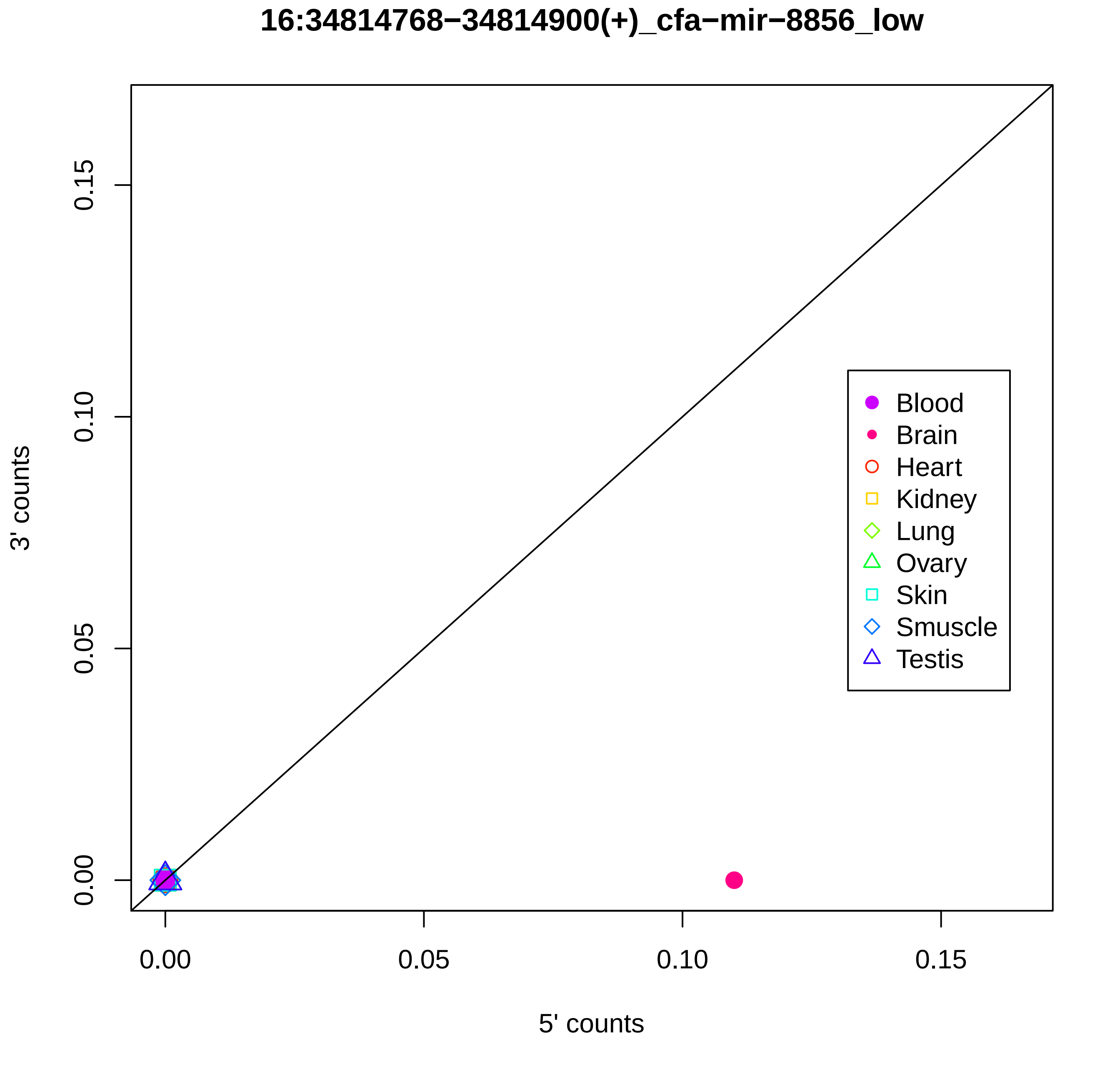
16:34814768−34814900(+)_cfa−mir−8856_low
3' counts
5' counts
0.00
0.05
0.10
0.15
0.00
0.05
0.10
0.15
Blood
Brain
Heart
Kidney
Lung
Ovary
Skin
Smuscle
Testis

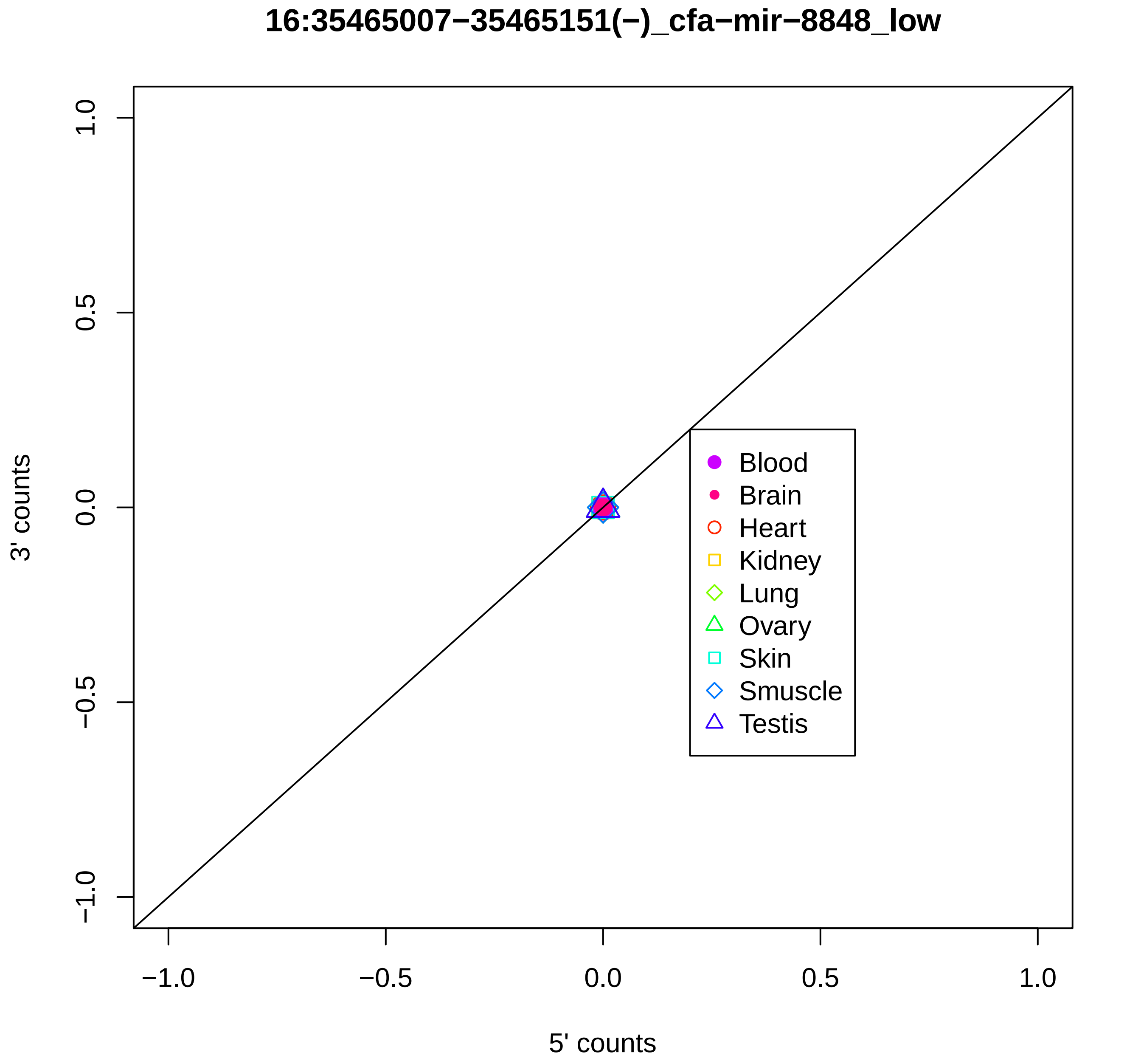

16:35465007−35465151(−)_cfa−mir−8848_low
3' counts
5' counts
−1.0
−0.5
0.0
0.5
1.0
Blood
Brain
Heart
Kidney
Lung
Ovary
Skin
Smuscle
Testis

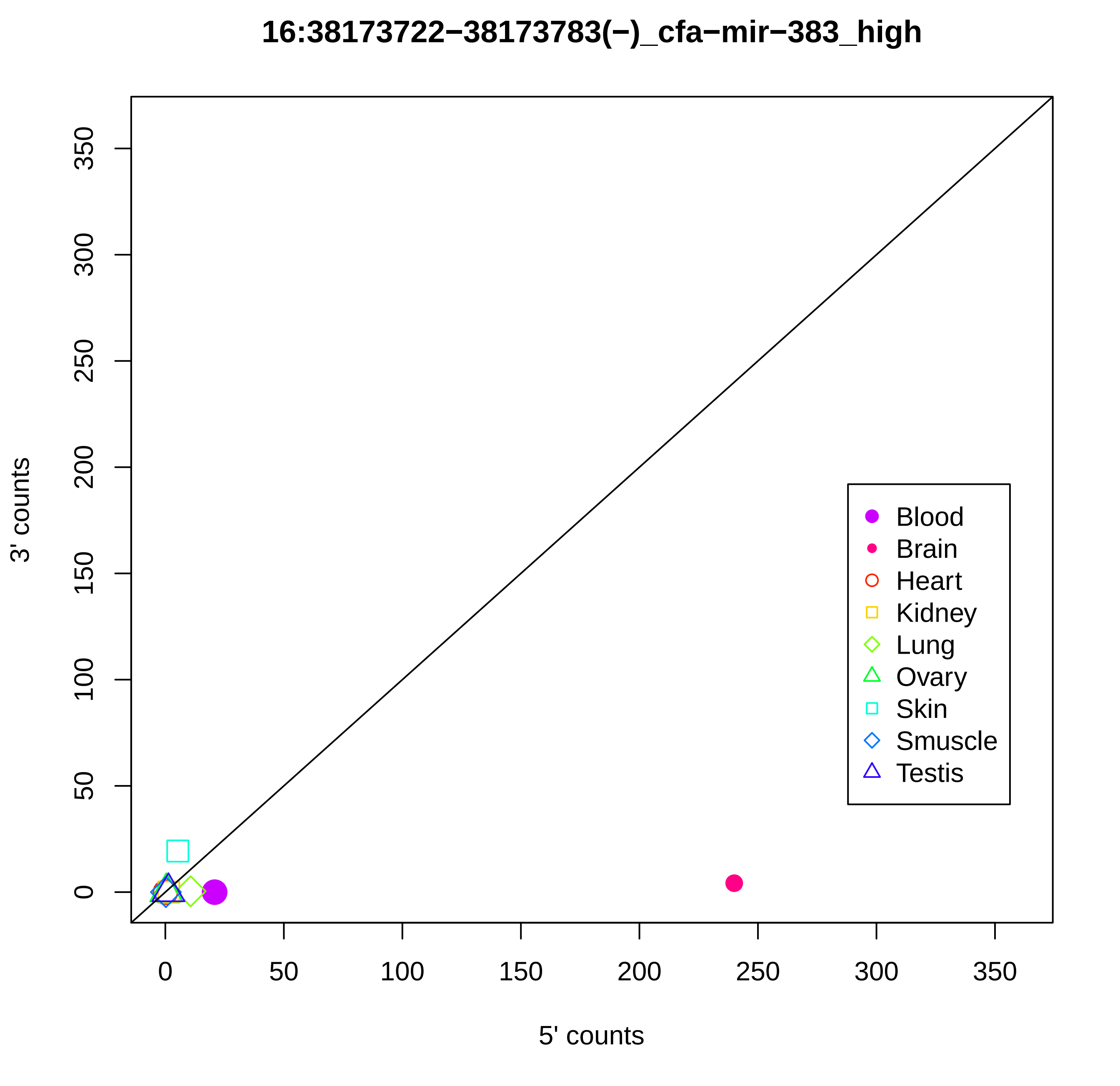

16:38173722−38173783(−)_cfa−mir−383_high
3' counts
5' counts
0
50
100
150
200
250
300
350
0
50
100
150
200
250
300
350
Blood
Brain
Heart
Kidney
Lung
Ovary
Skin
Smuscle
Testis

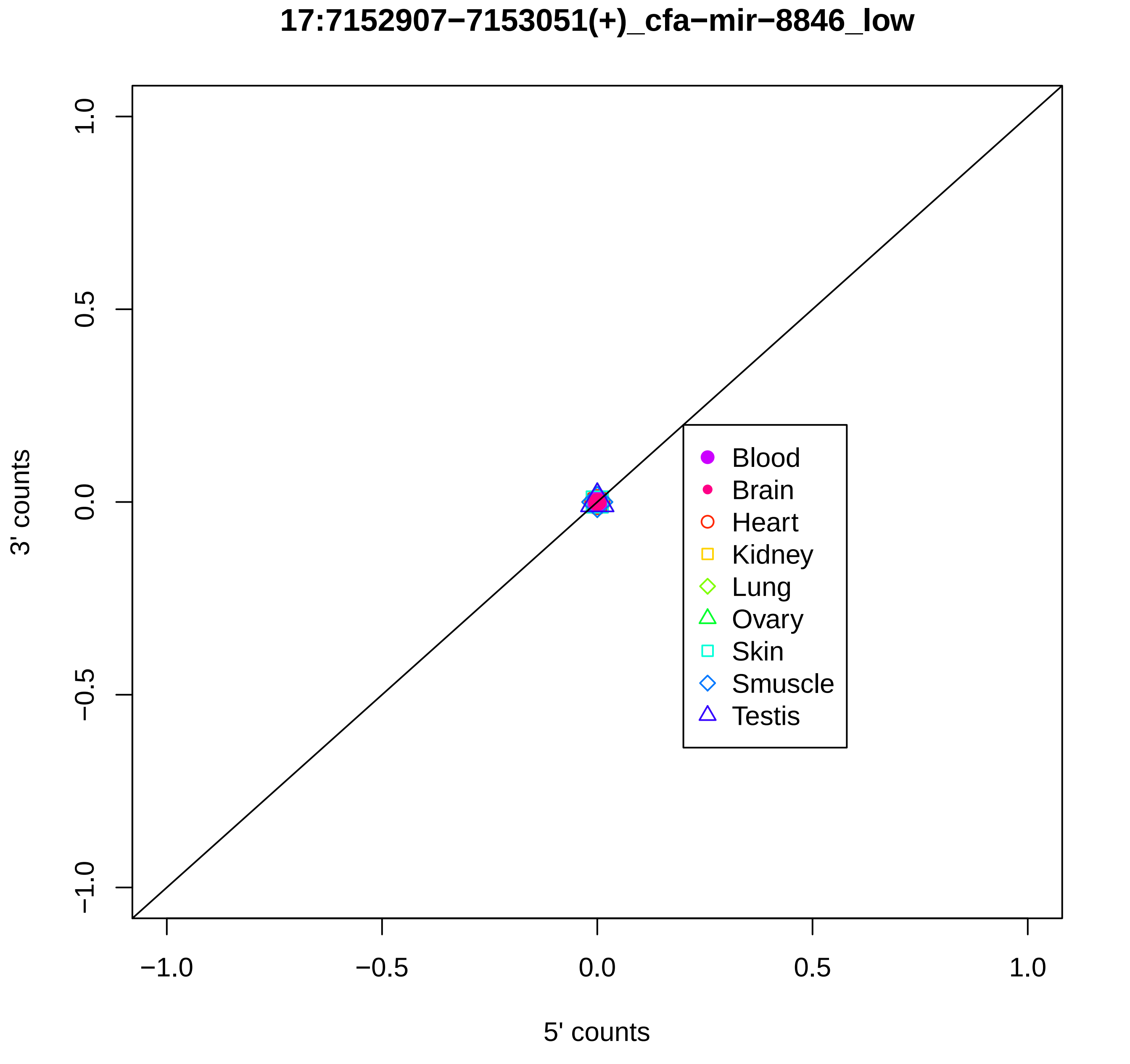

17:7152907−7153051(+)_cfa−mir−8846_low
1.0
0.5
0.0
−0.5
−1.0
3' counts
5' counts
Blood
Brain
Heart
Kidney
Lung
Ovary
Skin
Smuscle
Testis

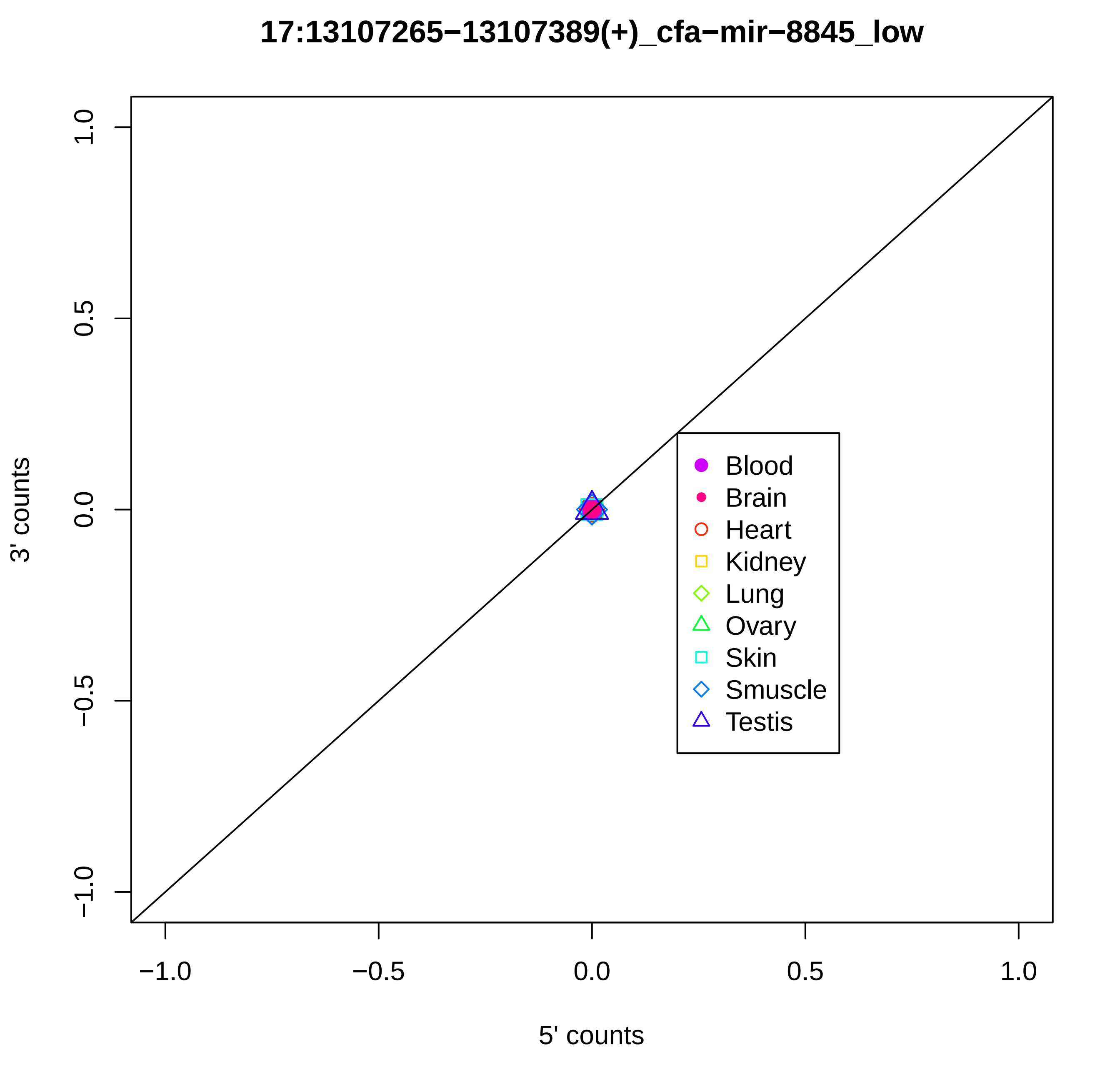

17:13107265−13107389(+)_cfa−mir−8845_low
Blood
Brain
Heart
Kidney
Lung
Ovary
Skin
Smuscle
Testis
1.0
0.5
0.0
−0.5
−1.0
3' counts
−1.0
−0.5
0.0
0.5
1.0
5' counts

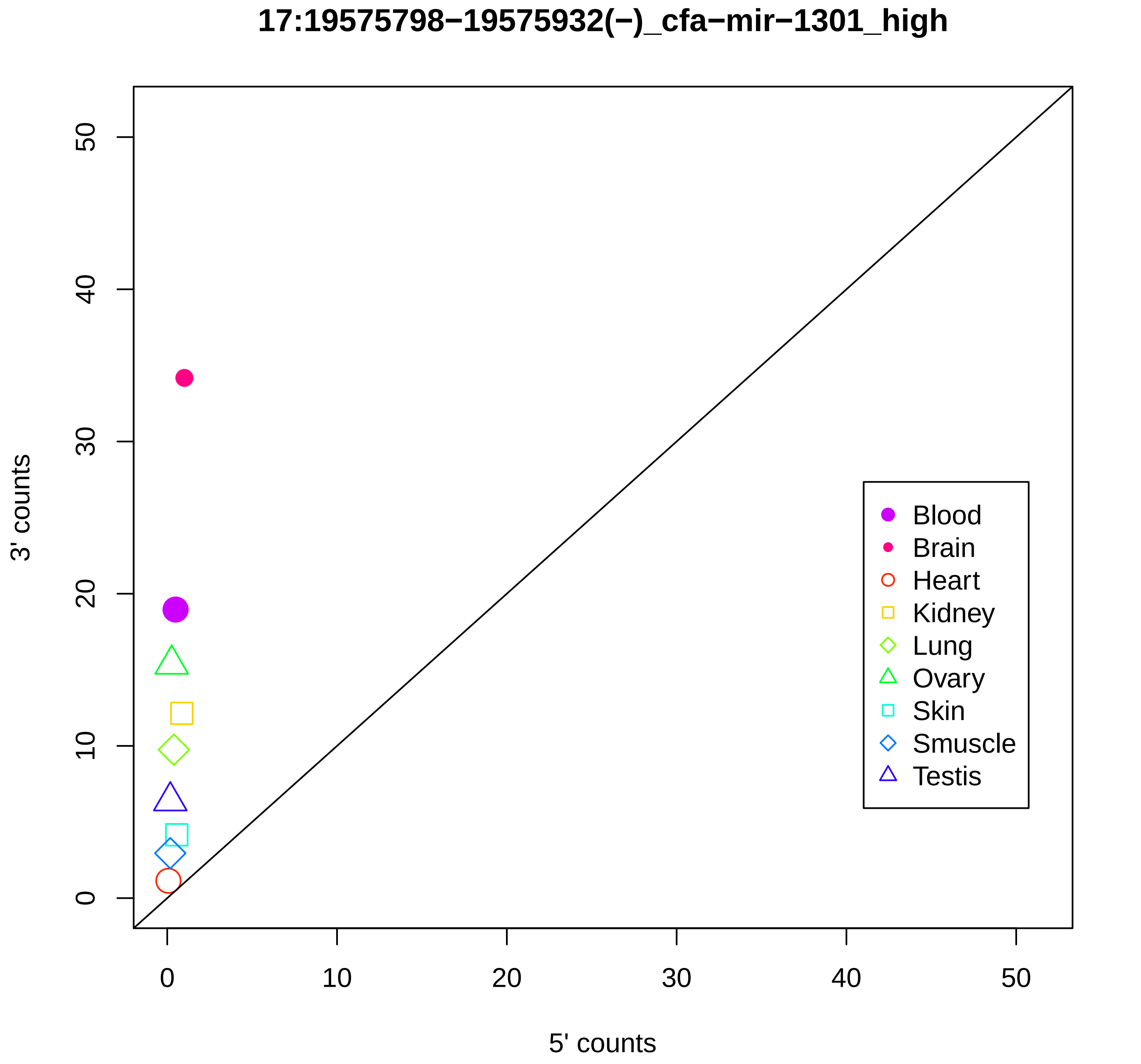

17:19575798−19575932(−)_cfa−mir−1301_high
3' counts
5' counts
0
10
20
30
40
50
Blood
Brain
Heart
Kidney
Lung
Ovary
Skin
Smuscle
Testis

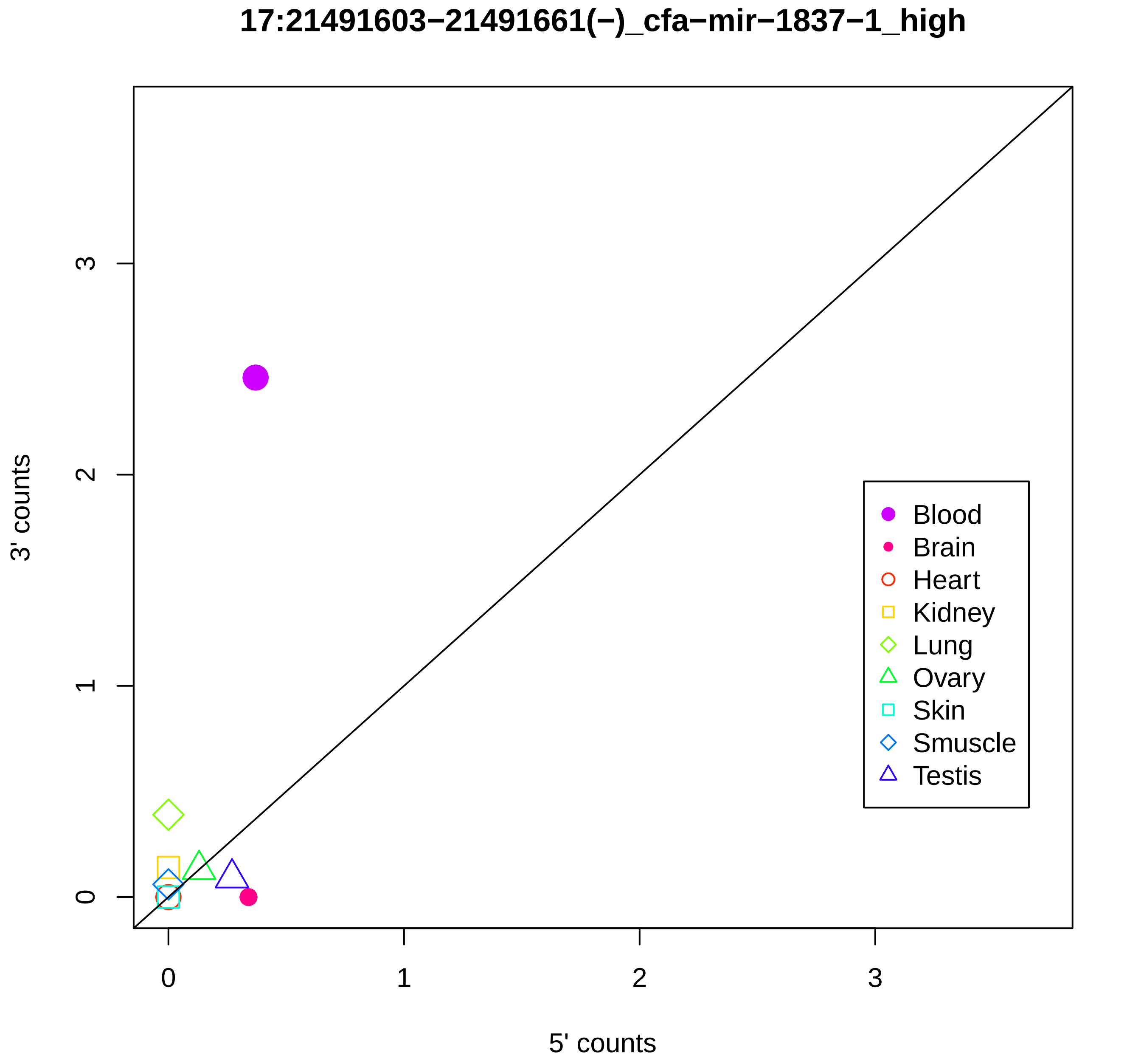
17:21491603−21491661(−)_cfa−mir−1837−1_high
3' counts
5' counts
0
1
2
3
0
1
2
3
Blood
Brain
Heart
Kidney
Lung
Ovary
Skin
Smuscle
Testis

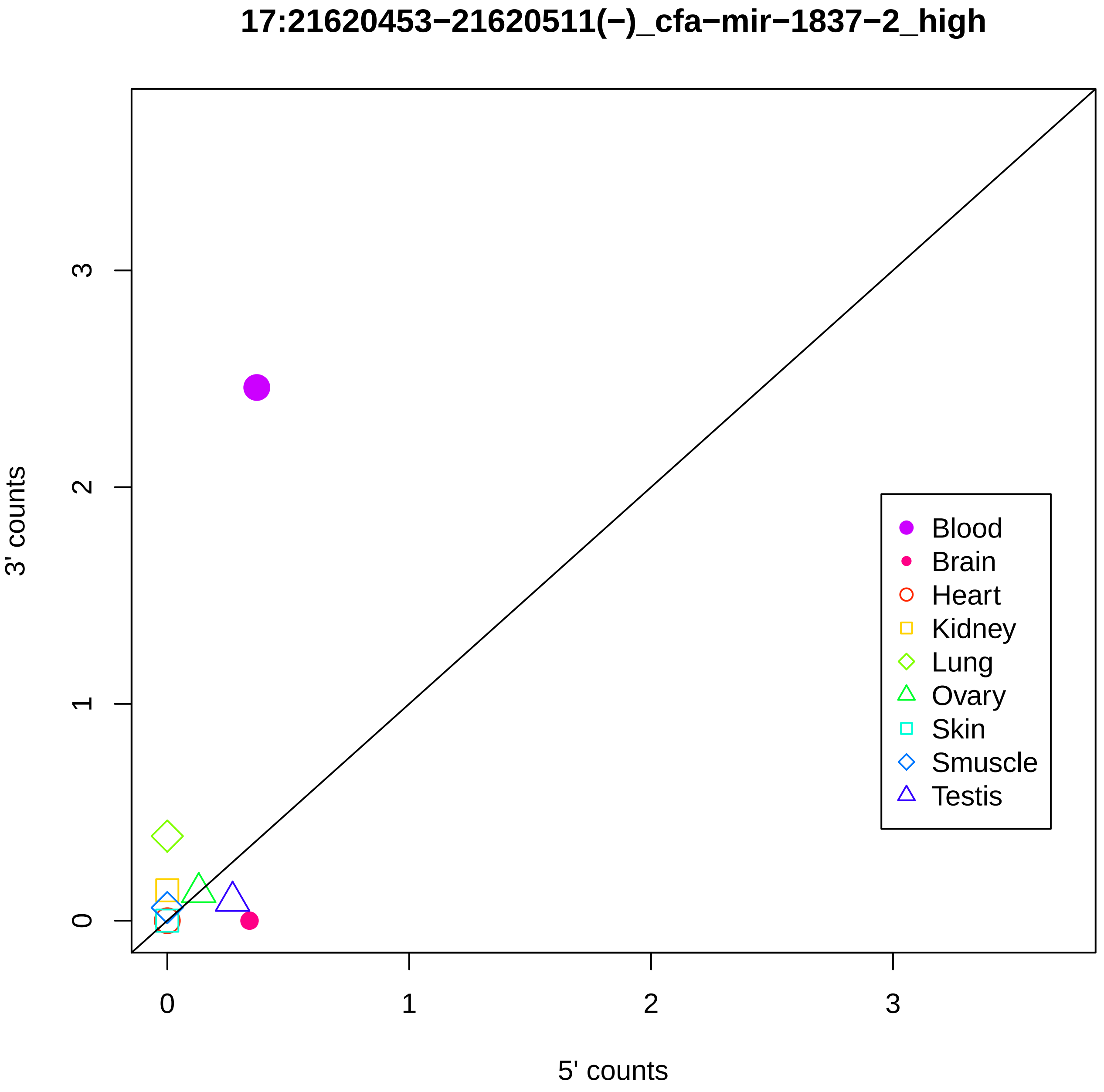

17:21620453−21620511(−)_cfa−mir−1837−2_high
3' counts
5' counts
0
1
2
3
0
1
2
3
Blood
Brain
Heart
Kidney
Lung
Ovary
Skin
Smuscle
Testis

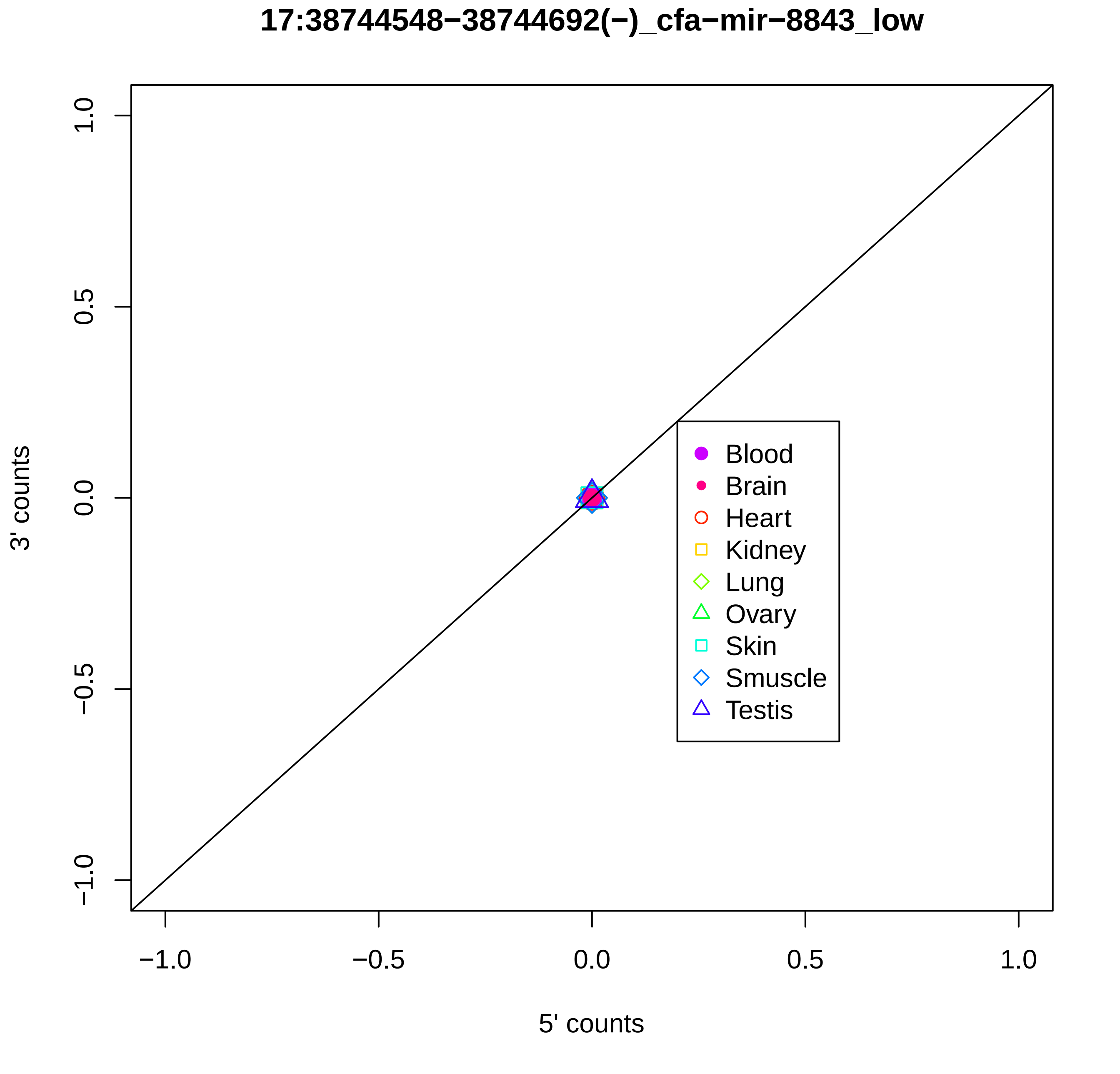

17:38744548−38744692(−)_cfa−mir−8843_low
Blood
Brain
Heart
Kidney
Lung
Ovary
Skin
Smuscle
Testis
3' counts
5' counts
−1.0
−0.5
0.0
0.5
1.0

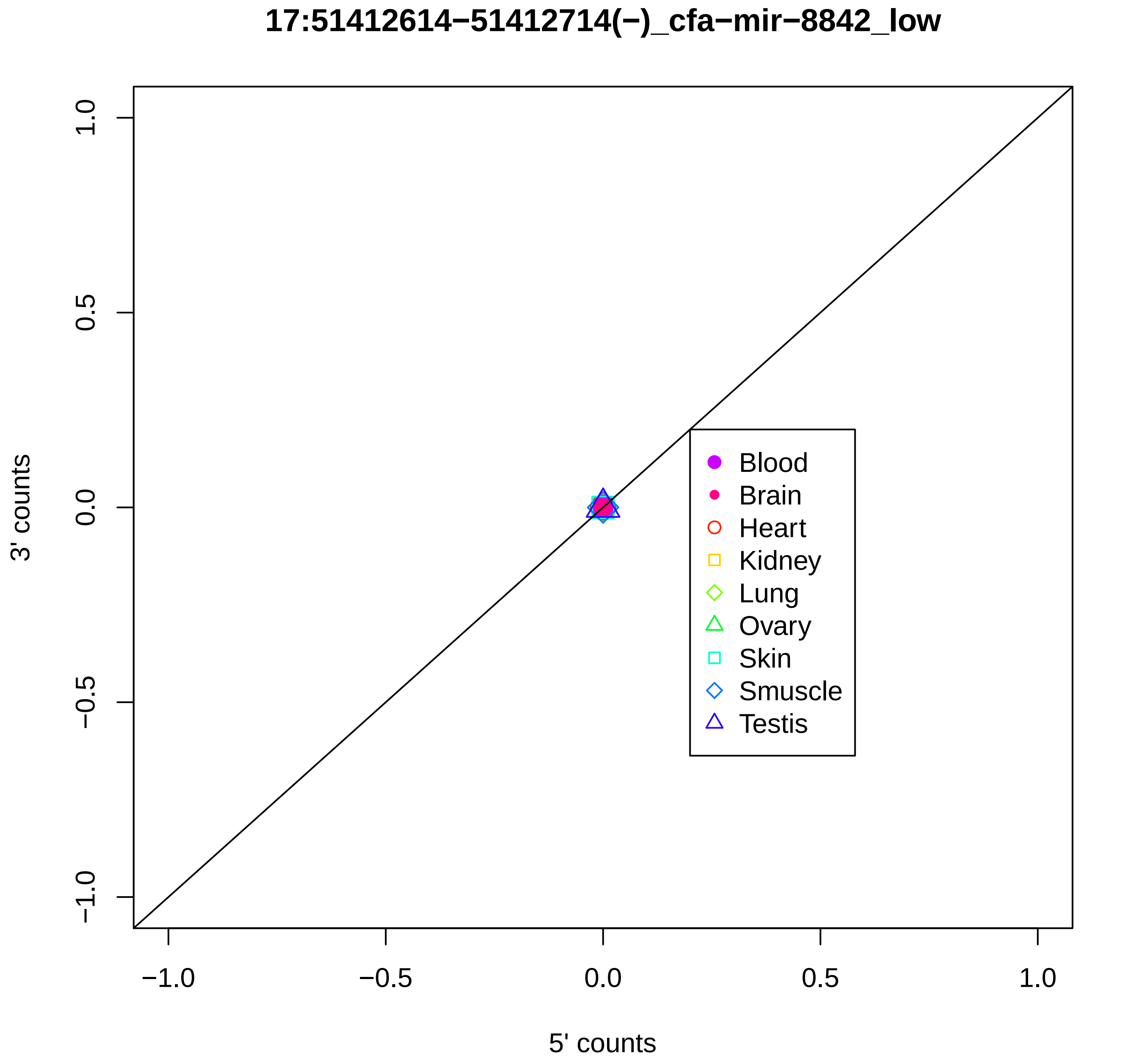

17:51412614−51412714(−)_cfa−mir−8842_low
3' counts
5' counts
1.0
0.5
0.0
−0.5
−1.0
Blood
Brain
Heart
Kidney
Lung
Ovary
Skin
Smuscle
Testis

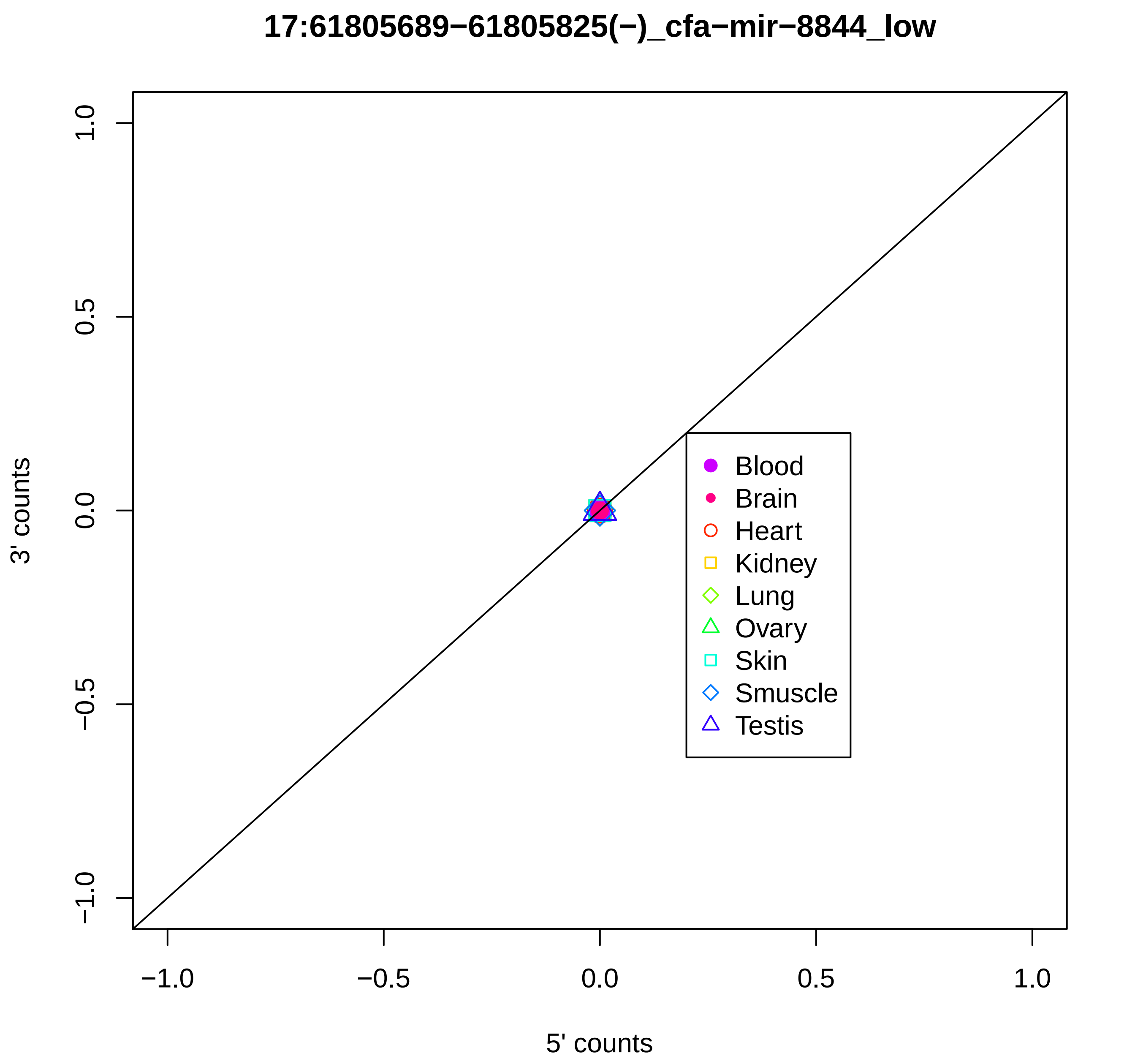
17:61805689−61805825(−)_cfa−mir−8844_low
Blood
Brain
Heart
Kidney
Lung
Ovary
Skin
Smuscle
Testis
3' counts
5' counts
−1.0
−0.5
0.0
0.5
1.0

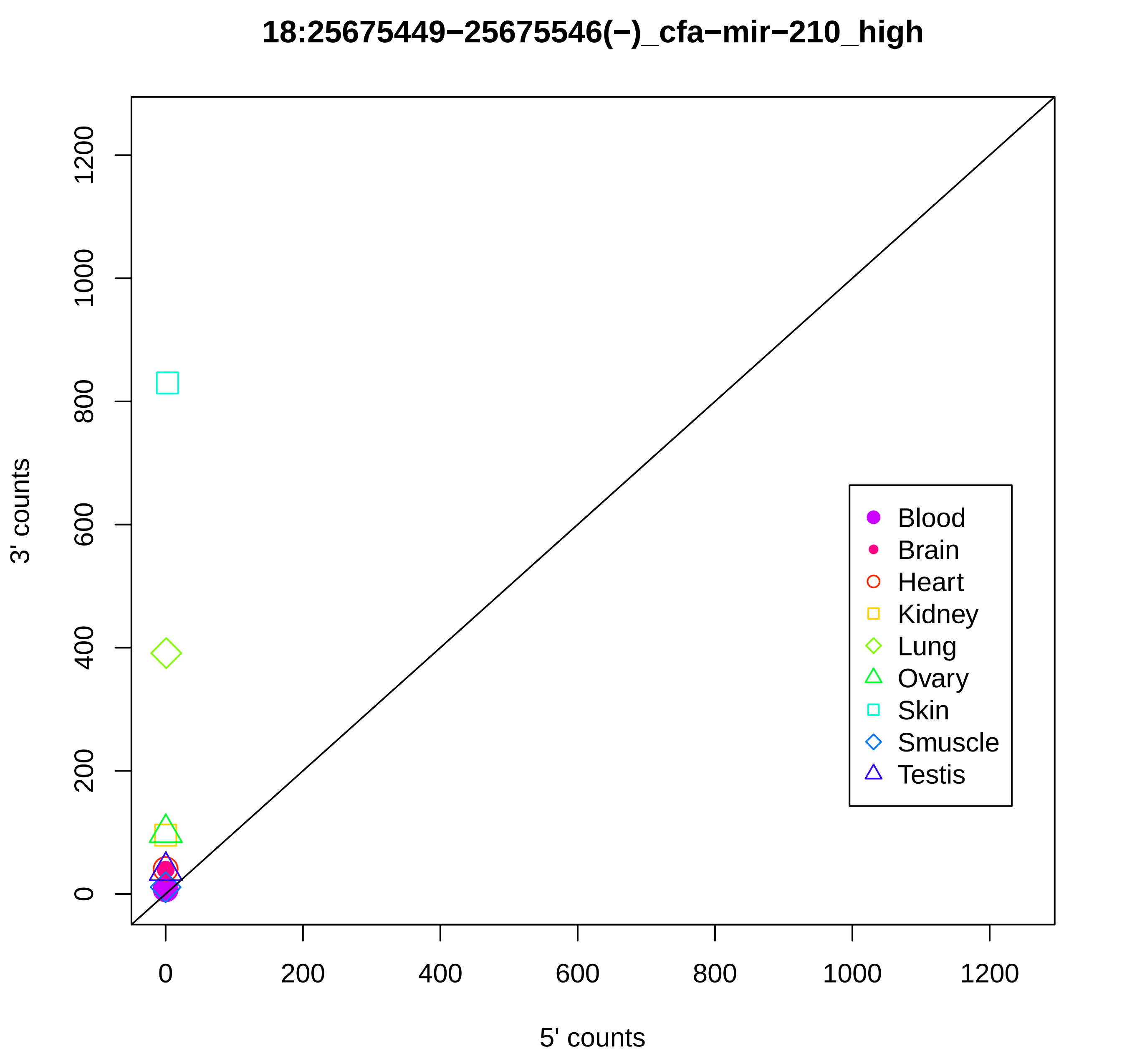

18:25675449−25675546(−)_cfa−mir−210_high
3' counts
5' counts
0
200
400
600
800
1000
1200
Blood
Brain
Heart
Kidney
Lung
Ovary
Skin
Smuscle
Testis

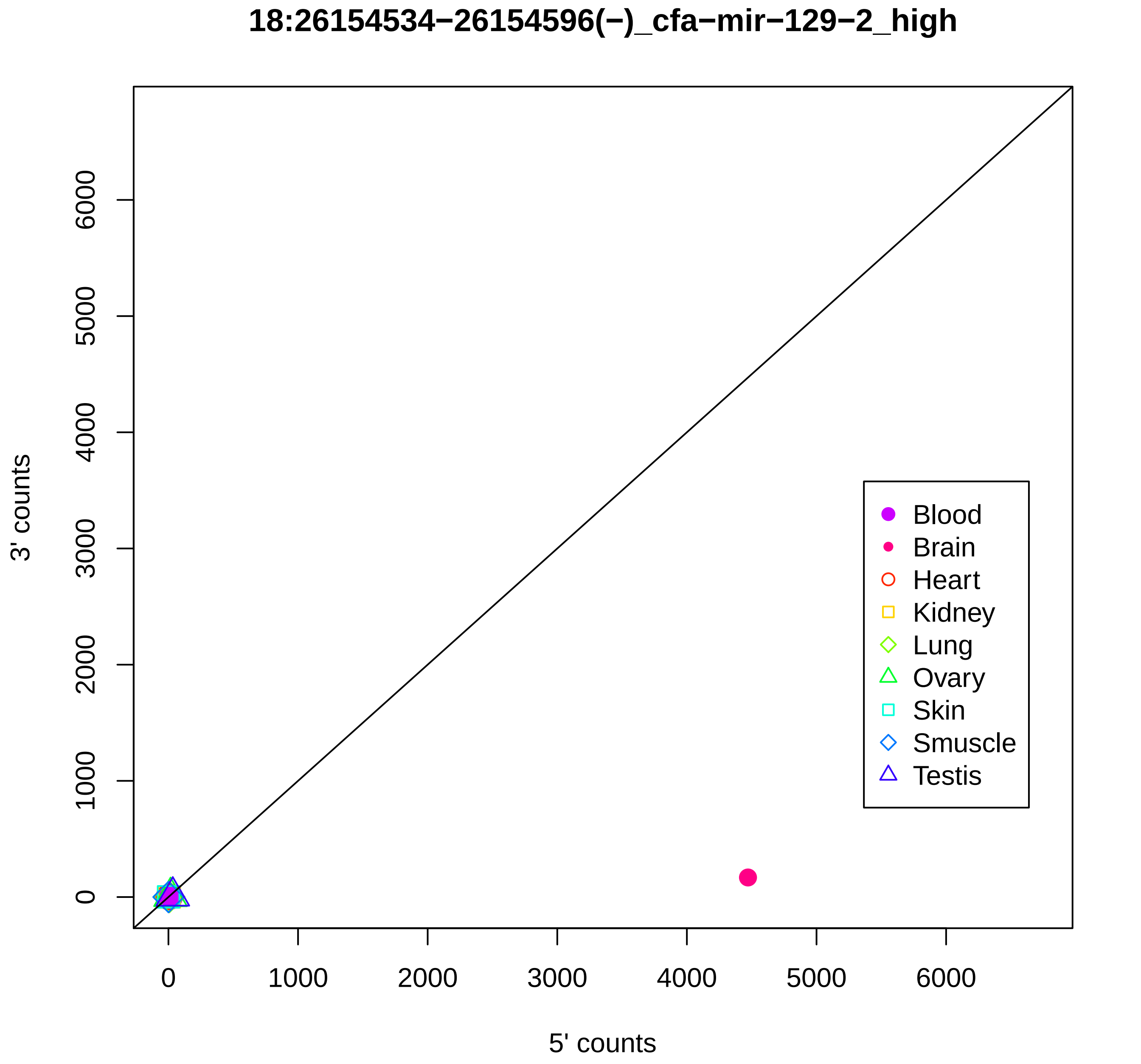

18:26154534−26154596(−)_cfa−mir−129−2_high
3' counts
0
1000
2000
3000
4000
5000
6000
5' counts
0
1000
2000
3000
4000
5000
6000
Blood
Brain
Heart
Kidney
Lung
Ovary
Skin
Smuscle
Testis

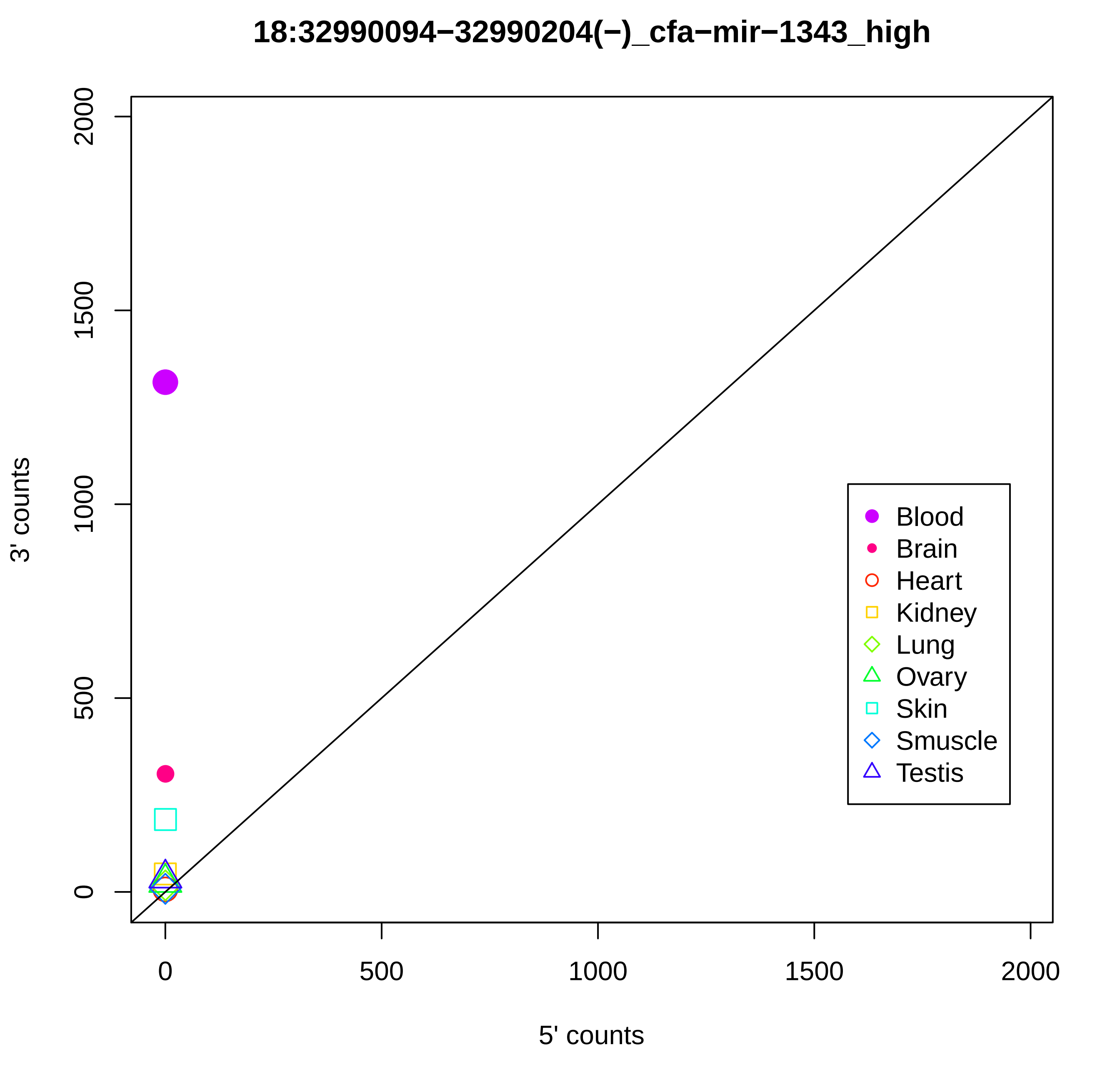

18:32990094−32990204(−)_cfa−mir−1343_high
3' counts
5' counts
0
500
1000
1500
2000
0
500
1000
1500
2000
Blood
Brain
Heart
Kidney
Lung
Ovary
Skin
Smuscle
Testis

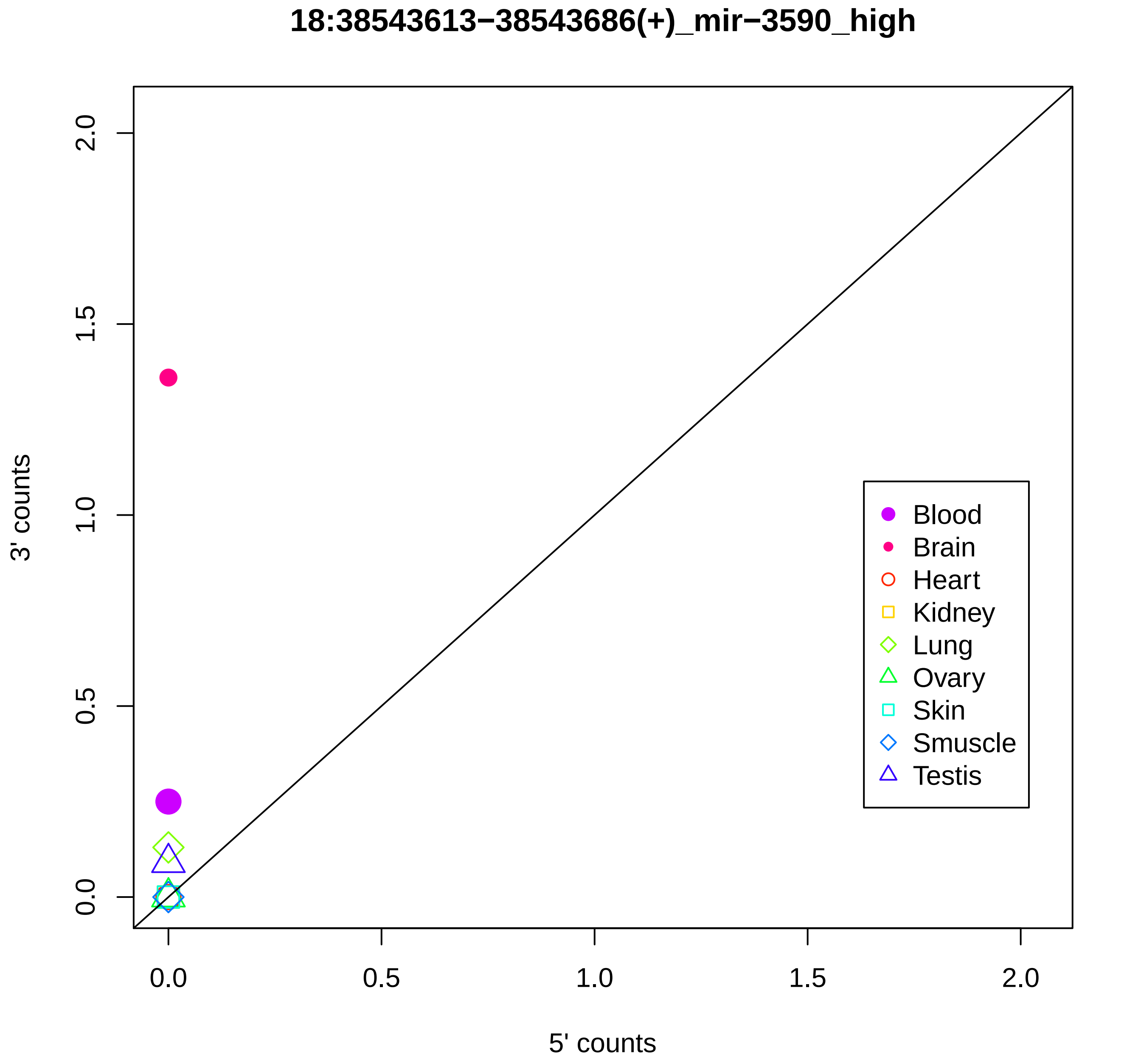

18:38543613−38543686(+)_mir−3590_high
3' counts
5' counts
0.0
0.5
1.0
1.5
2.0
Blood
Brain
Heart
Kidney
Lung
Ovary
Skin
Smuscle
Testis

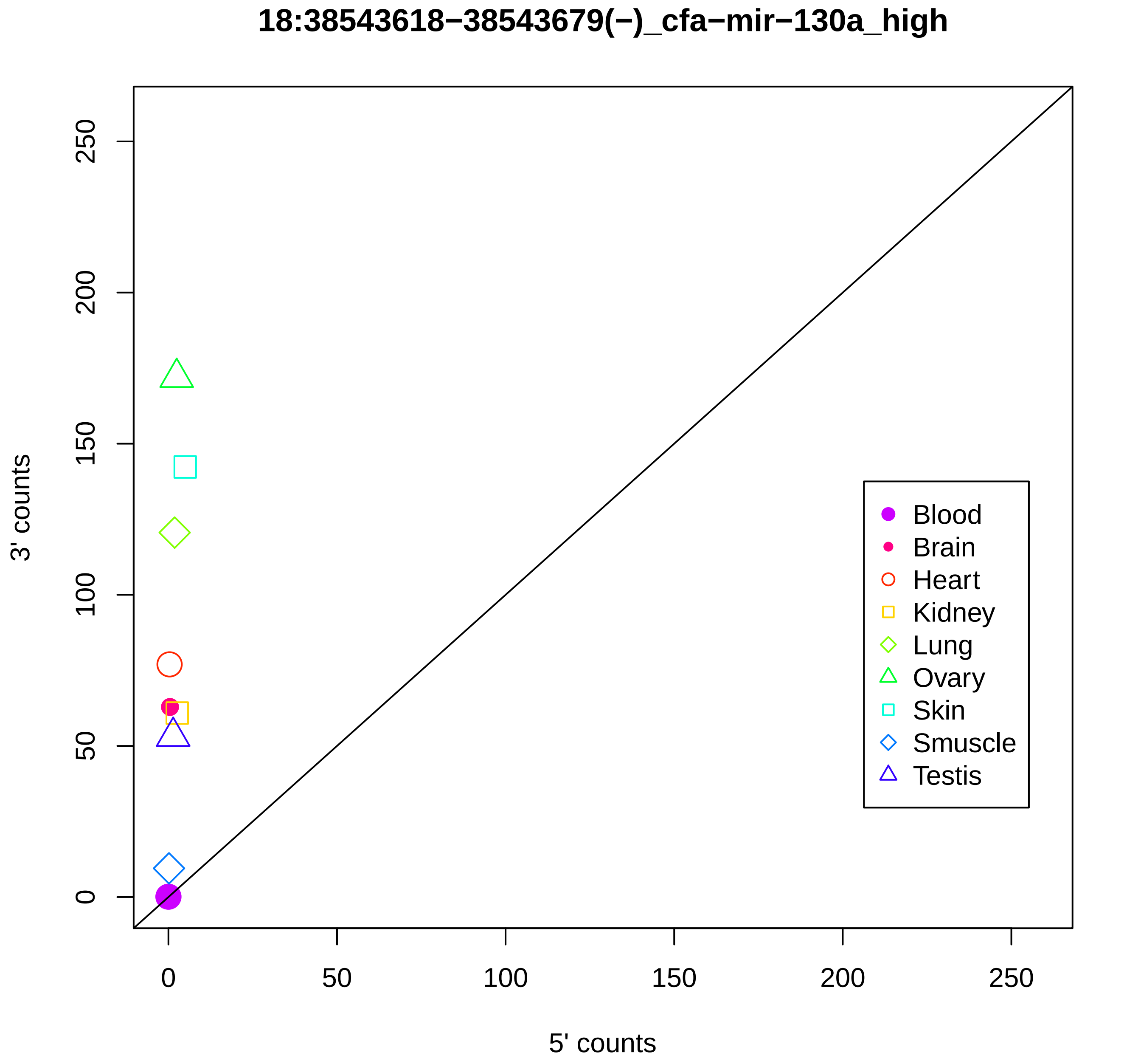

18:38543618−38543679(−)_cfa−mir−130a_high
3' counts
5' counts
0
50
100
150
200
250
Blood
Brain
Heart
Kidney
Lung
Ovary
Skin
Smuscle
Testis

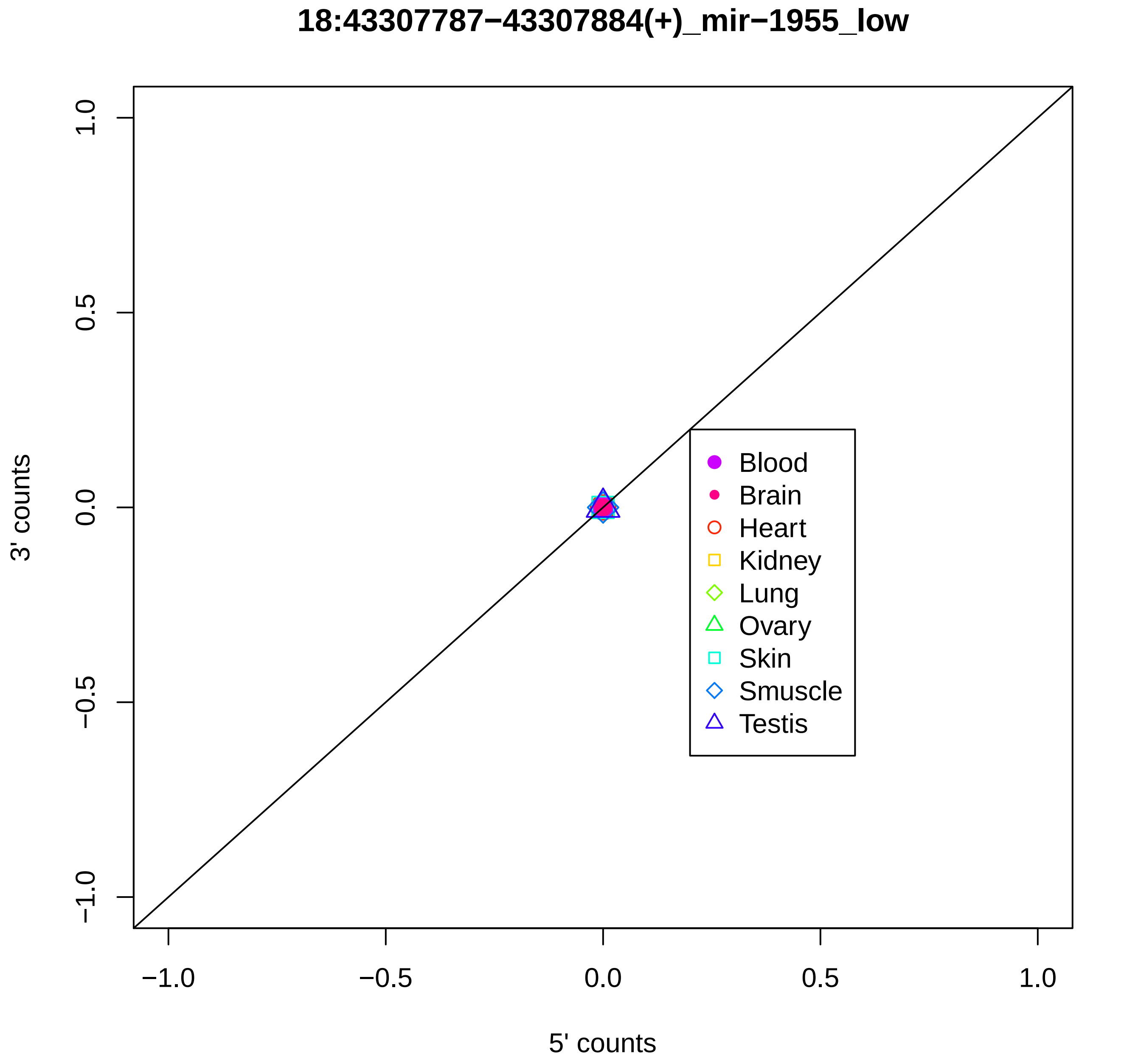
18:43307787−43307884(+)_mir−1955_low
3' counts
5' counts
1.0
0.5
0.0
−0.5
−1.0
Blood
Brain
Heart
Kidney
Lung
Ovary
Skin
Smuscle
Testis

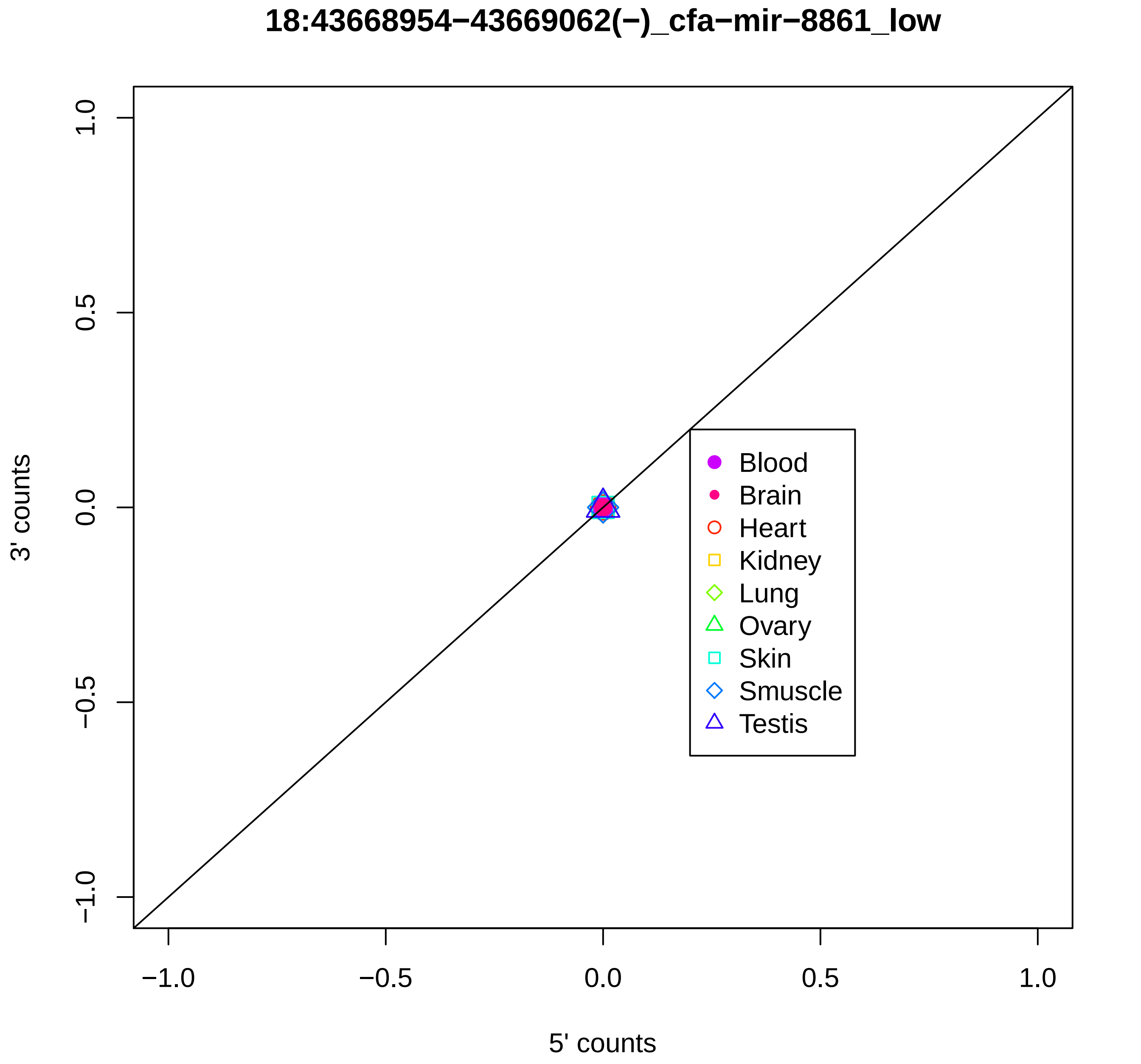
18:43668954−43669062(−)_cfa−mir−8861_low
3' counts
5' counts
1.0
0.5
0.0
−0.5
−1.0
Blood
Brain
Heart
Kidney
Lung
Ovary
Skin
Smuscle
Testis

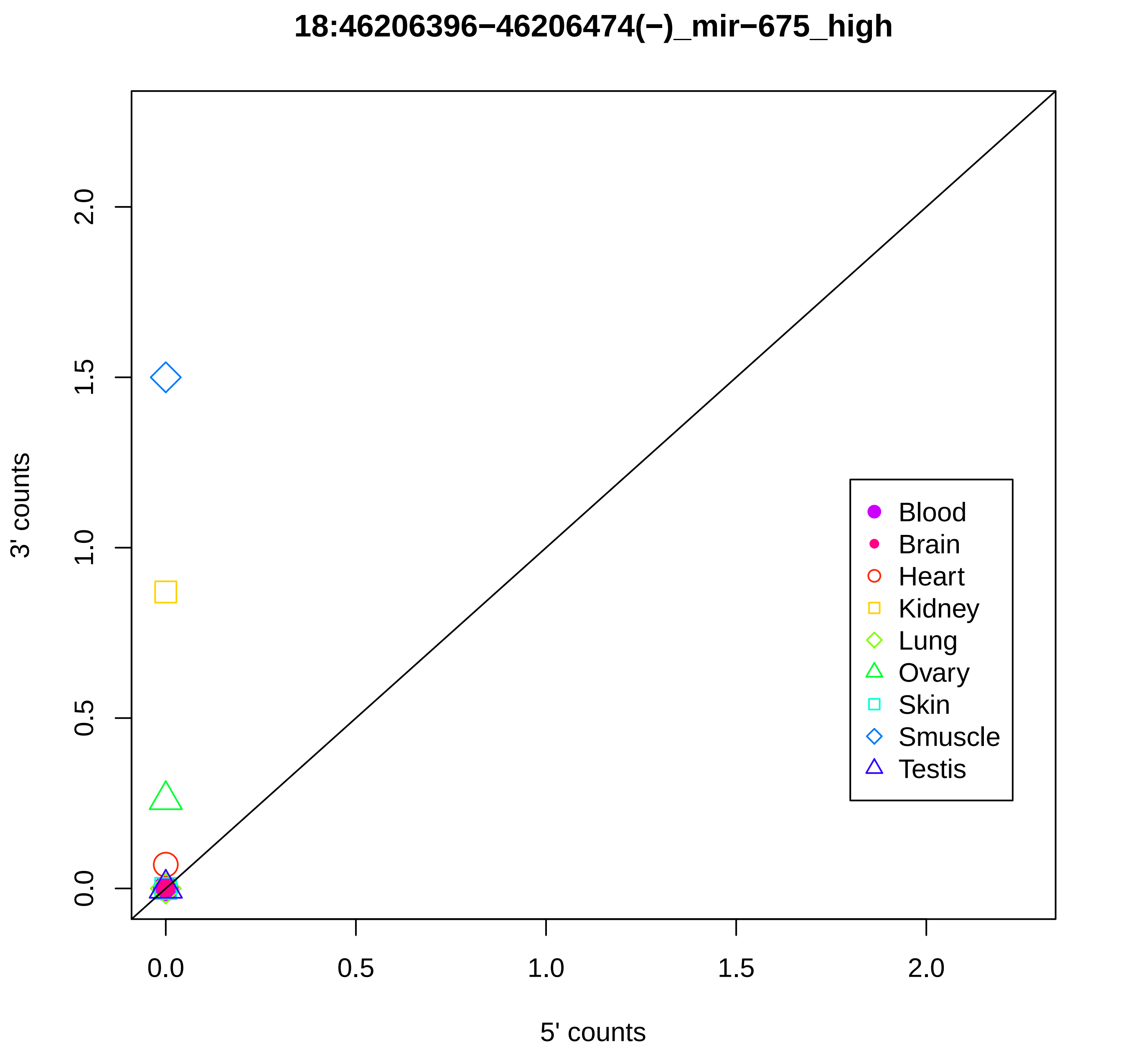

18:46206396−46206474(−)_mir−675_high
3' counts
5' counts
0.0
0.5
1.0
1.5
2.0
Blood
Brain
Heart
Kidney
Lung
Ovary
Skin
Smuscle
Testis

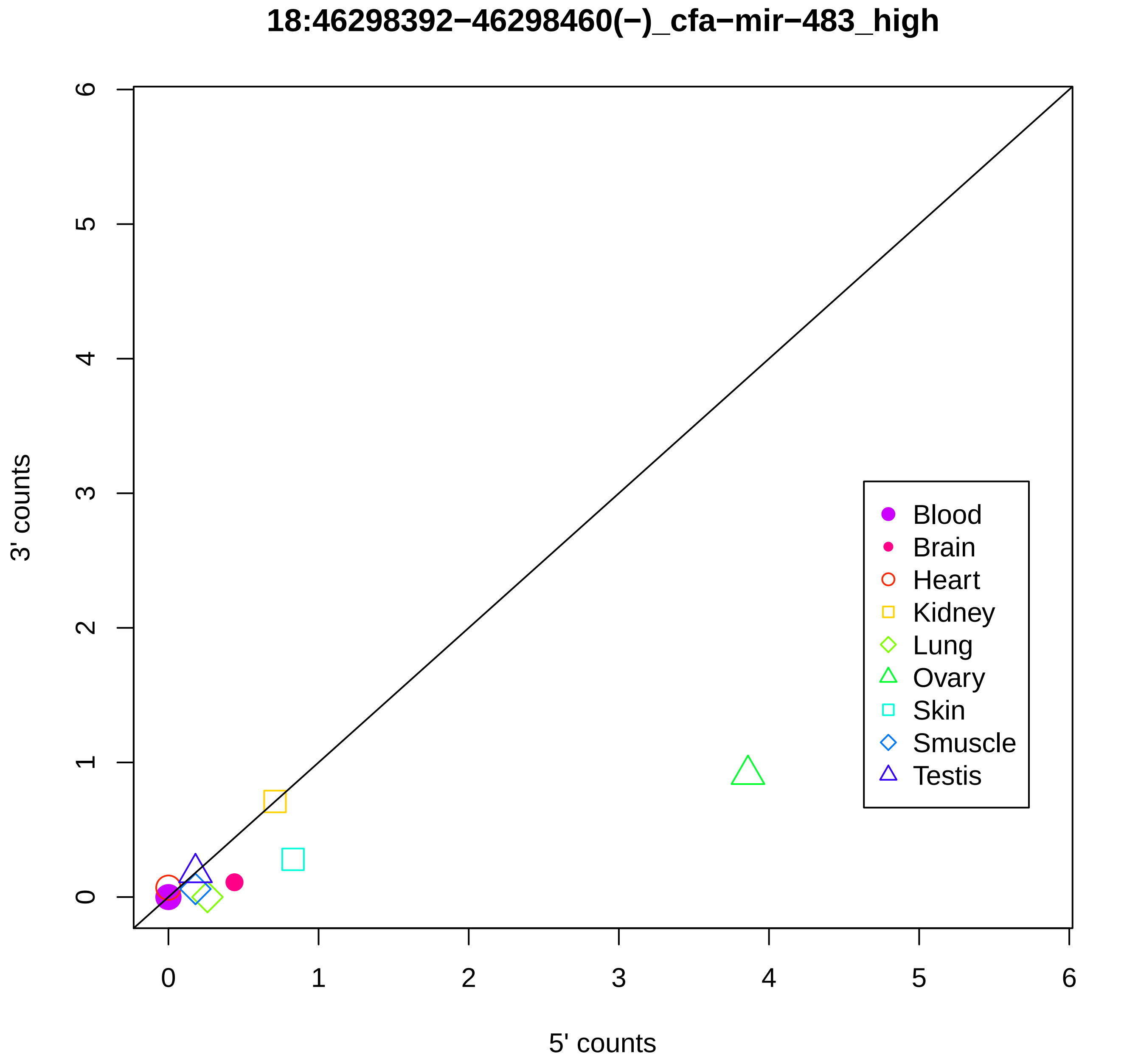

18:46298392−46298460(−)_cfa−mir−483_high
3' counts
5' counts
0
1
2
3
4
5
6
Blood
Brain
Heart
Kidney
Lung
Ovary
Skin
Smuscle
Testis

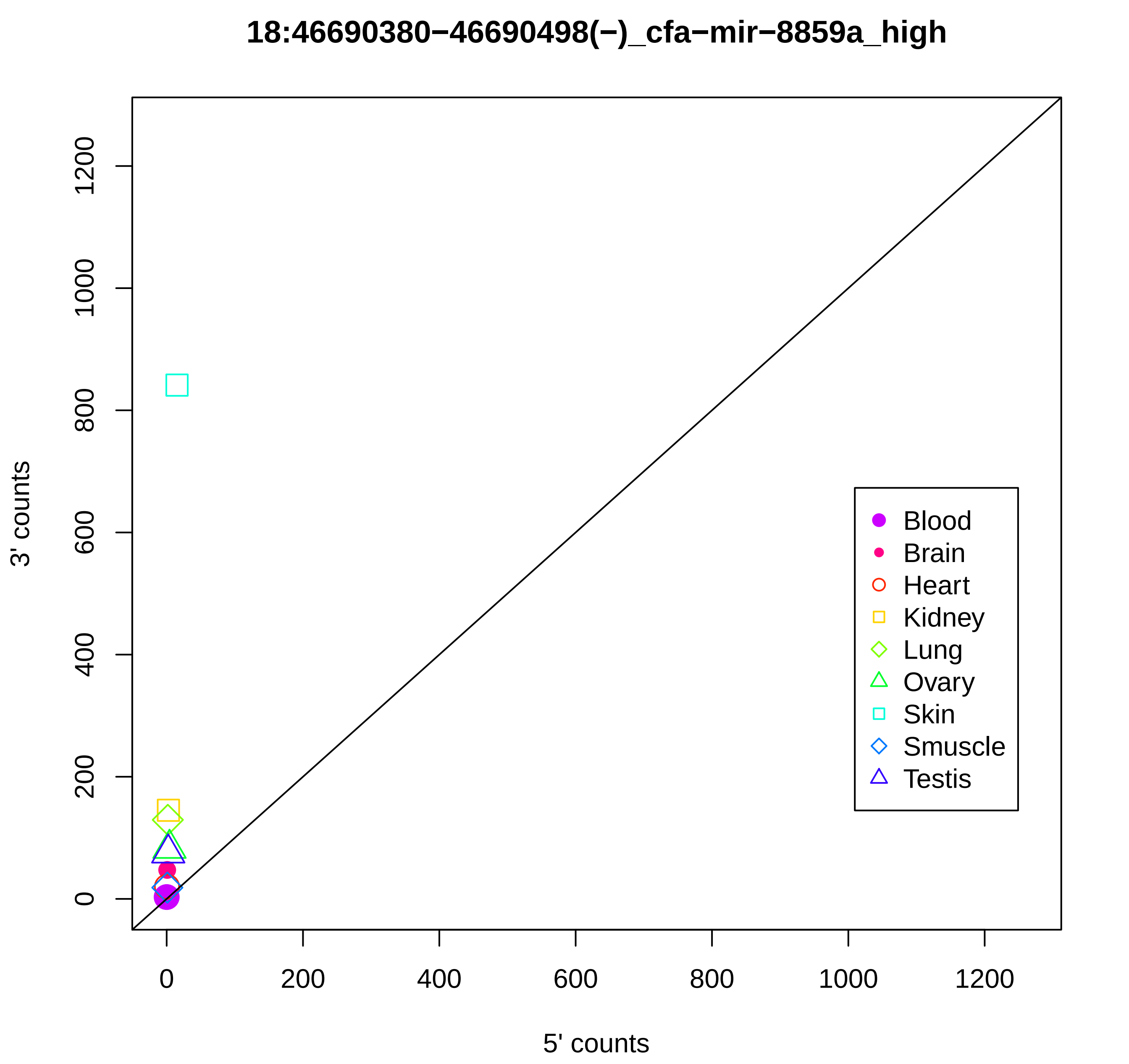
18:46690380−46690498(−)_cfa−mir−8859a_high
3' counts
5' counts
0
200
400
600
800
1000
1200
Blood
Brain
Heart
Kidney
Lung
Ovary
Skin
Smuscle
Testis

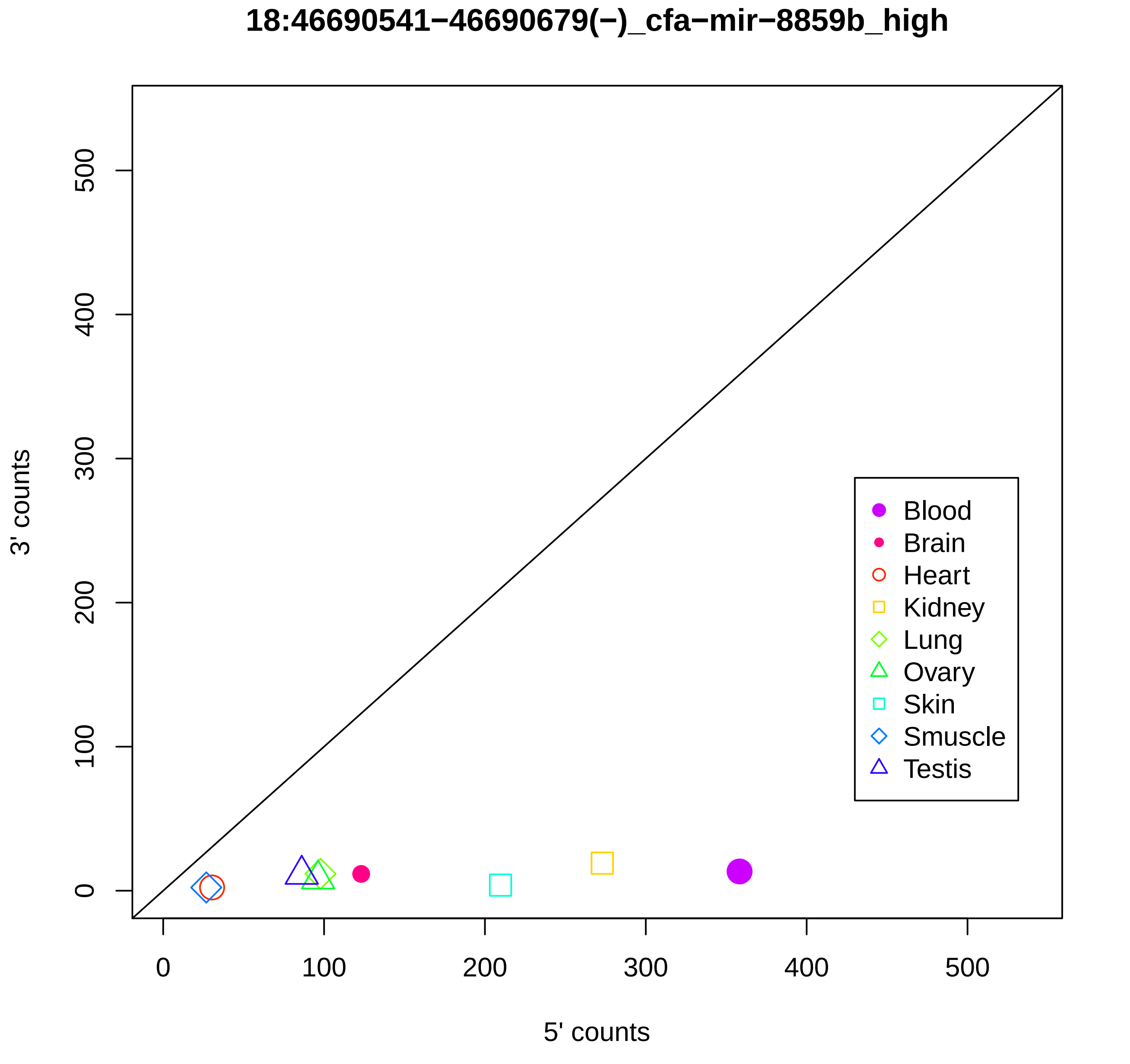

18:46690541−46690679(−)_cfa−mir−8859b_high
3' counts
5' counts
0
100
200
300
400
500
Blood
Brain
Heart
Kidney
Lung
Ovary
Skin
Smuscle
Testis

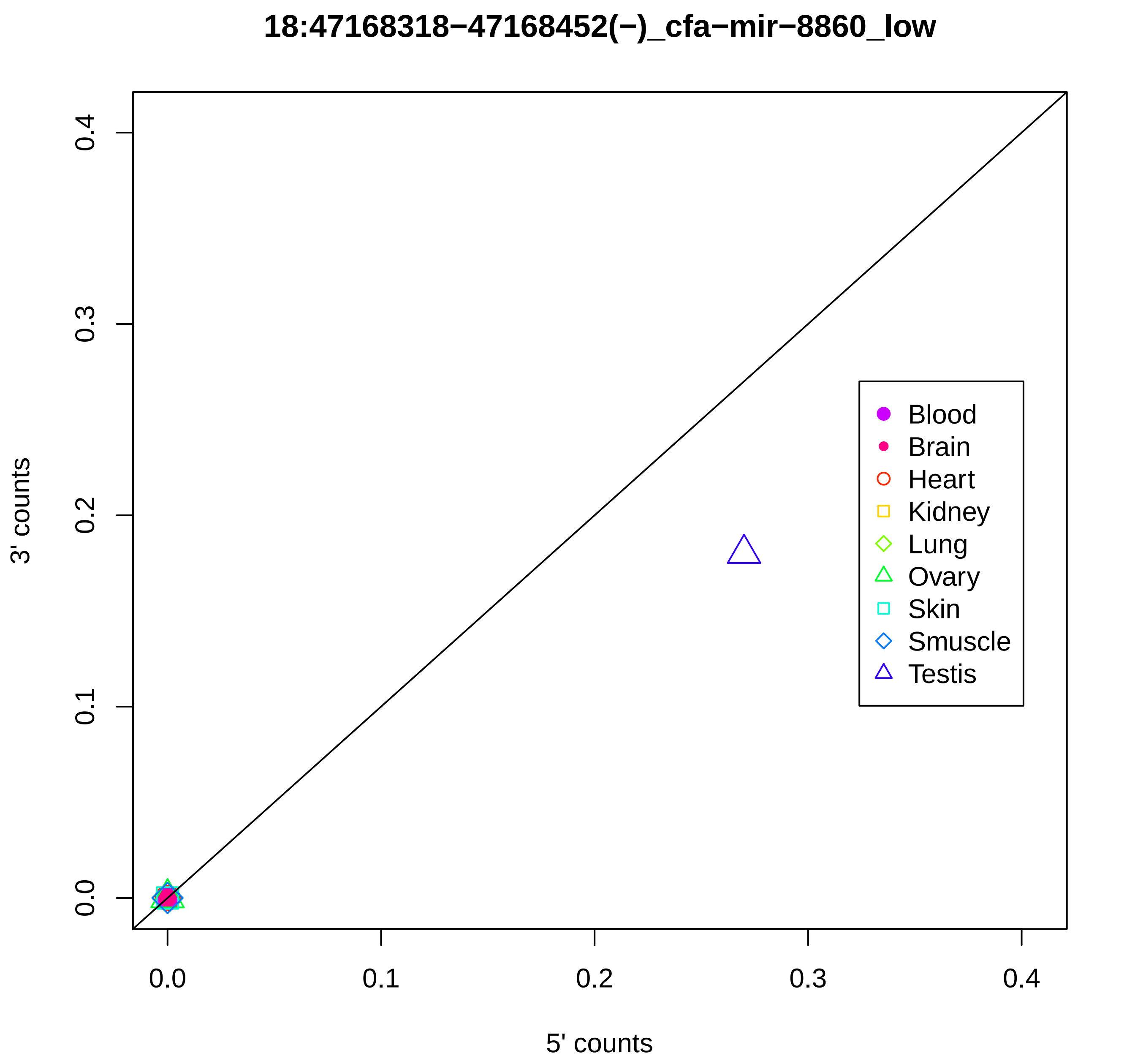

18:47168318−47168452(−)_cfa−mir−8860_low
3' counts
5' counts
0.0
0.1
0.2
0.3
0.4
Blood
Brain
Heart
Kidney
Lung
Ovary
Skin
Smuscle
Testis

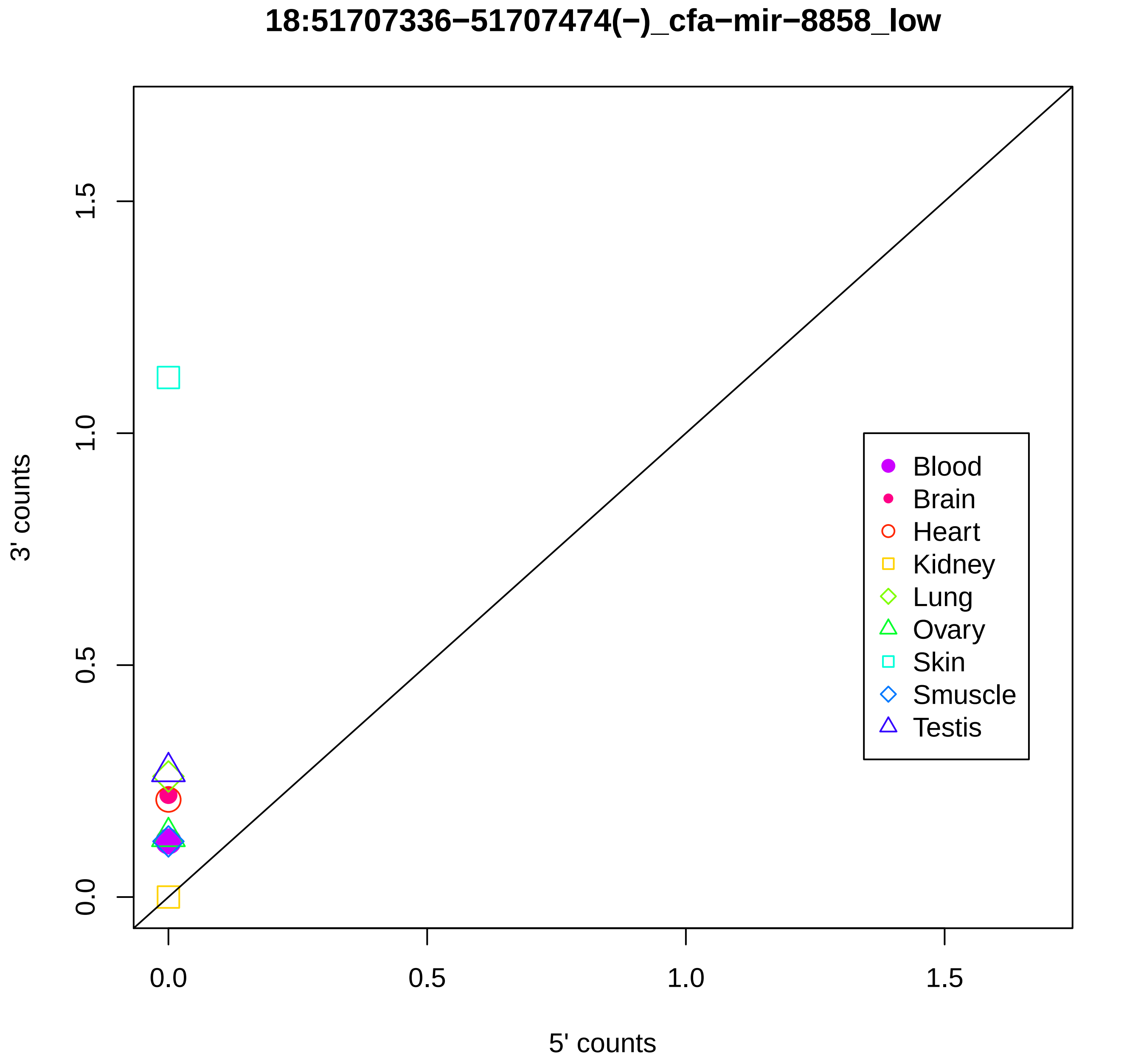

18:51707336−51707474(−)_cfa−mir−8858_low
3' counts
5' counts
0.0
0.5
1.0
1.5
0.0
0.5
1.0
1.5
Blood
Brain
Heart
Kidney
Lung
Ovary
Skin
Smuscle
Testis

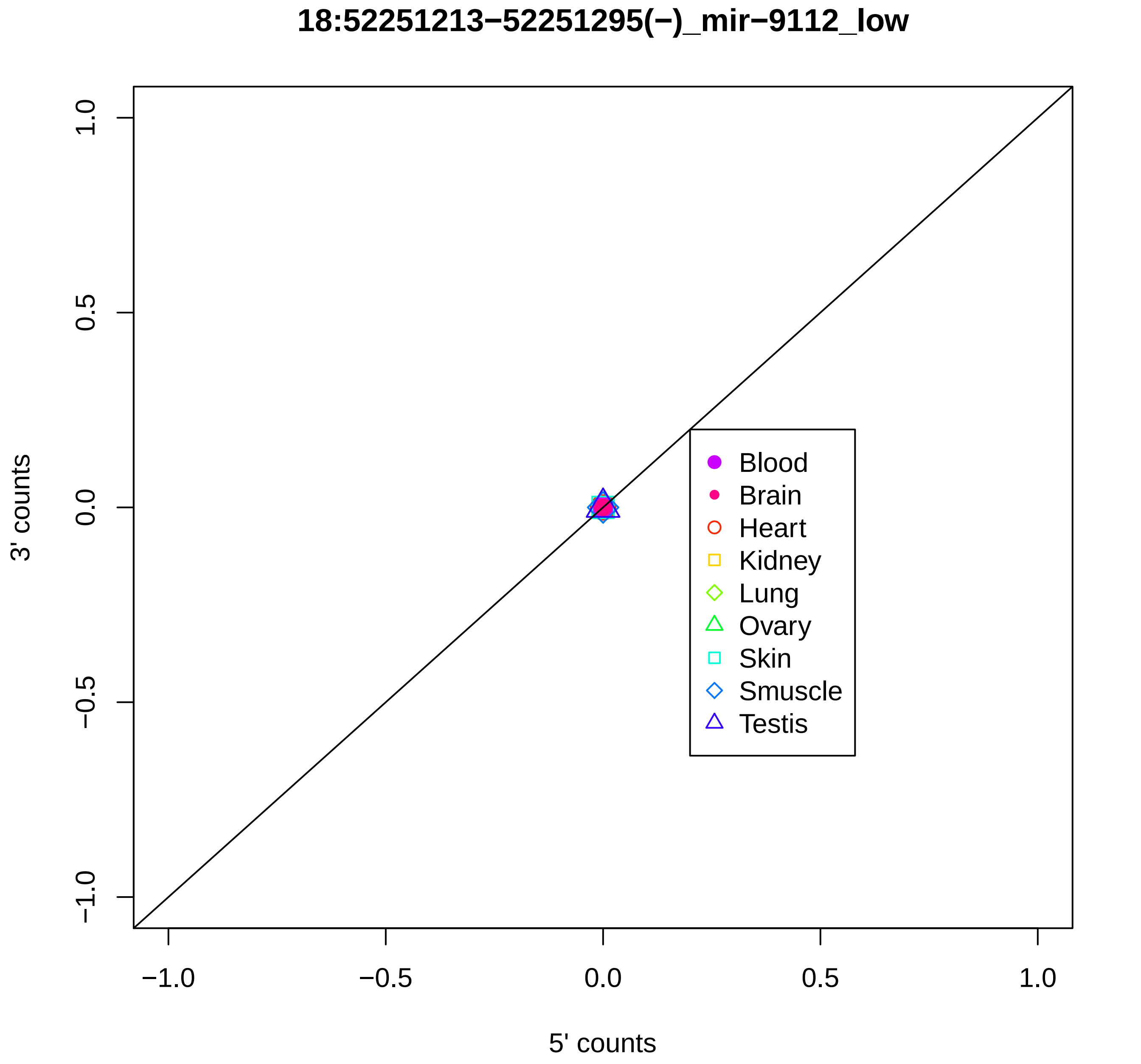

18:52251213−52251295(−)_mir−9112_low
3' counts
5' counts
1.0
0.5
0.0
−0.5
−1.0
Blood
Brain
Heart
Kidney
Lung
Ovary
Skin
Smuscle
Testis

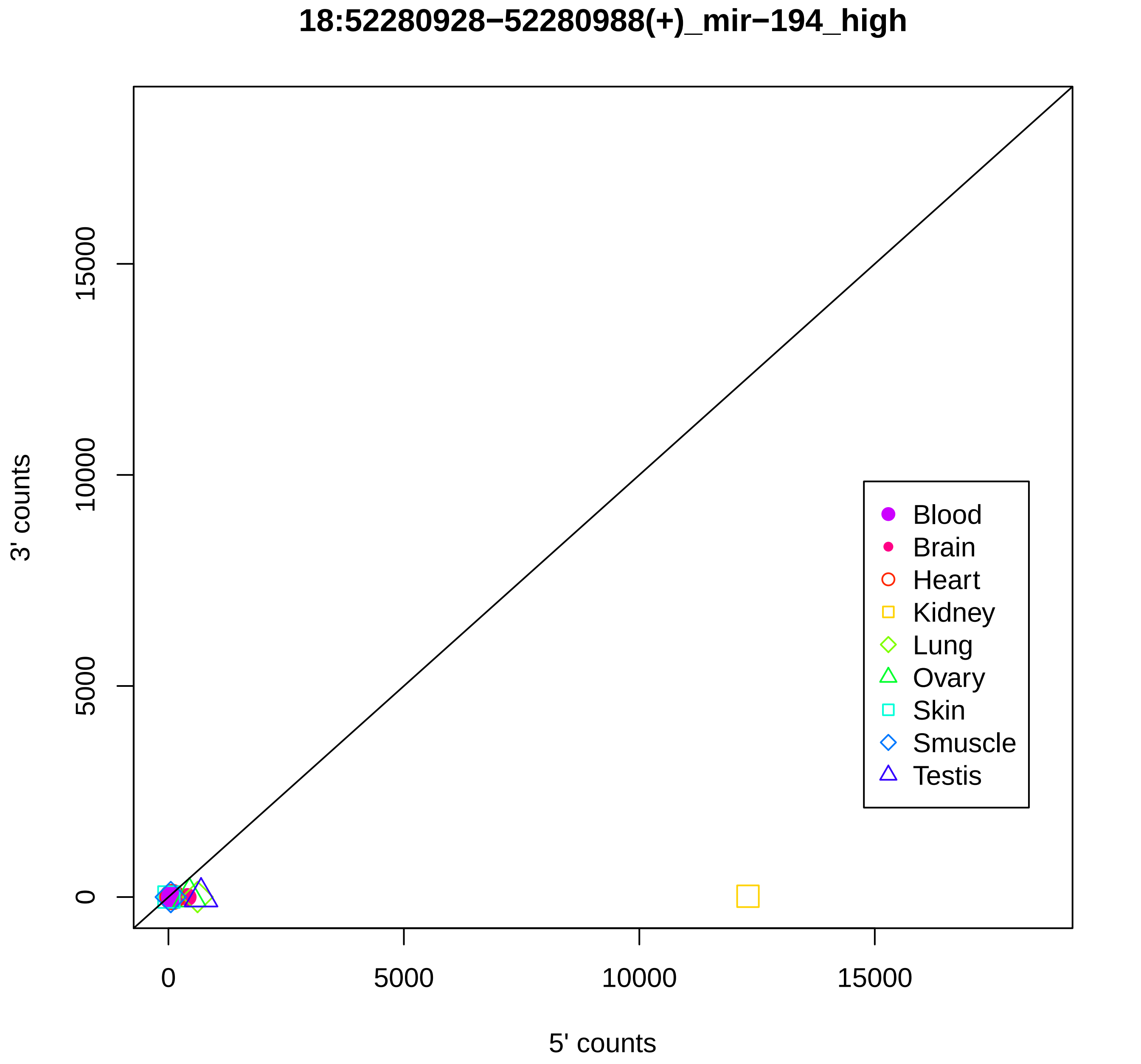

18:52280928−52280988(+)_mir−194_high
3' counts
15000
10000
5000
0
5' counts
0
5000
10000
15000
Blood
Brain
Heart
Kidney
Lung
Ovary
Skin
Smuscle
Testis

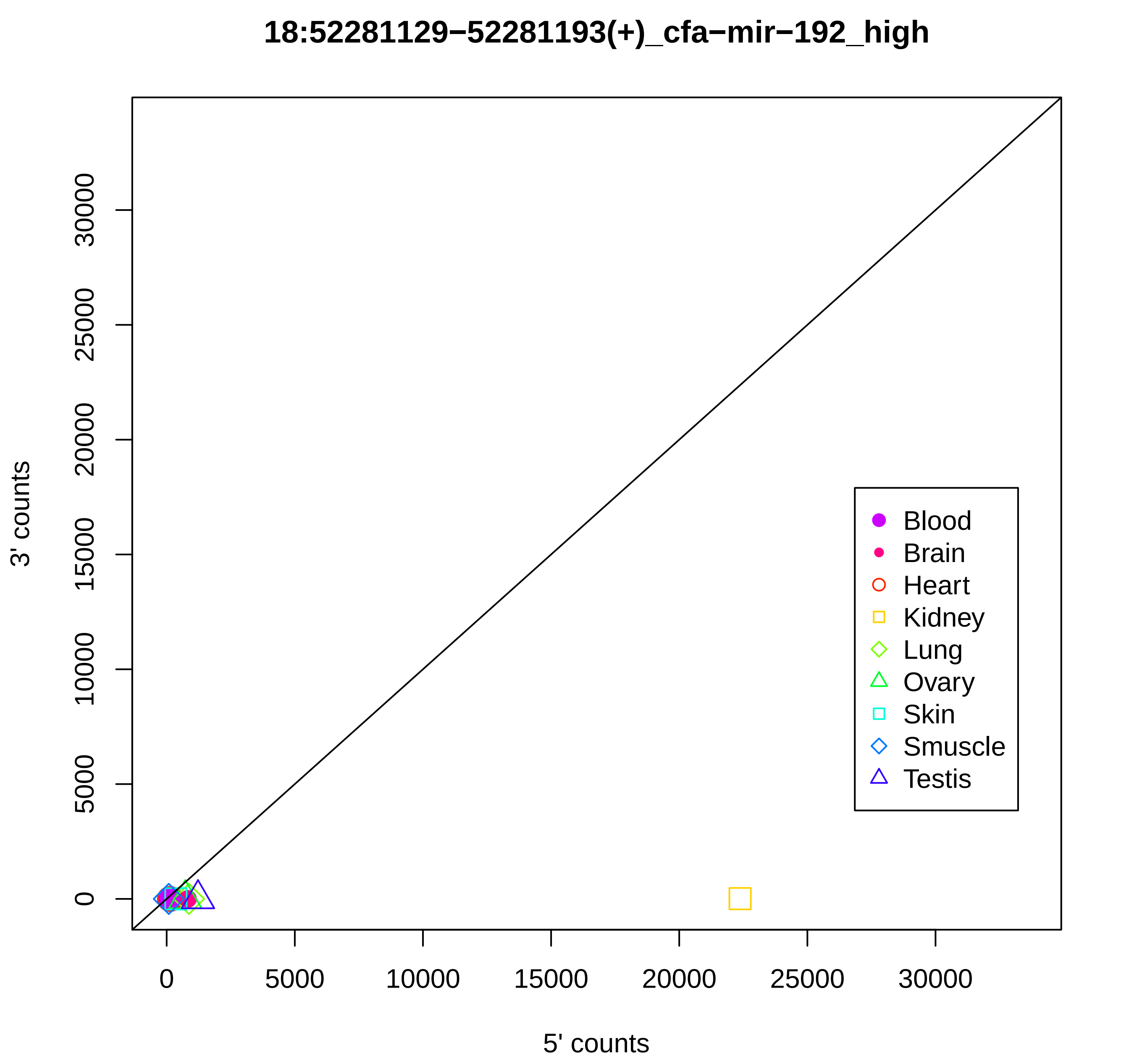

18:52281129−52281193(+)_cfa−mir−192_high
3' counts
5' counts
0
5000
10000
15000
20000
25000
30000
Blood
Brain
Heart
Kidney
Lung
Ovary
Skin
Smuscle
Testis

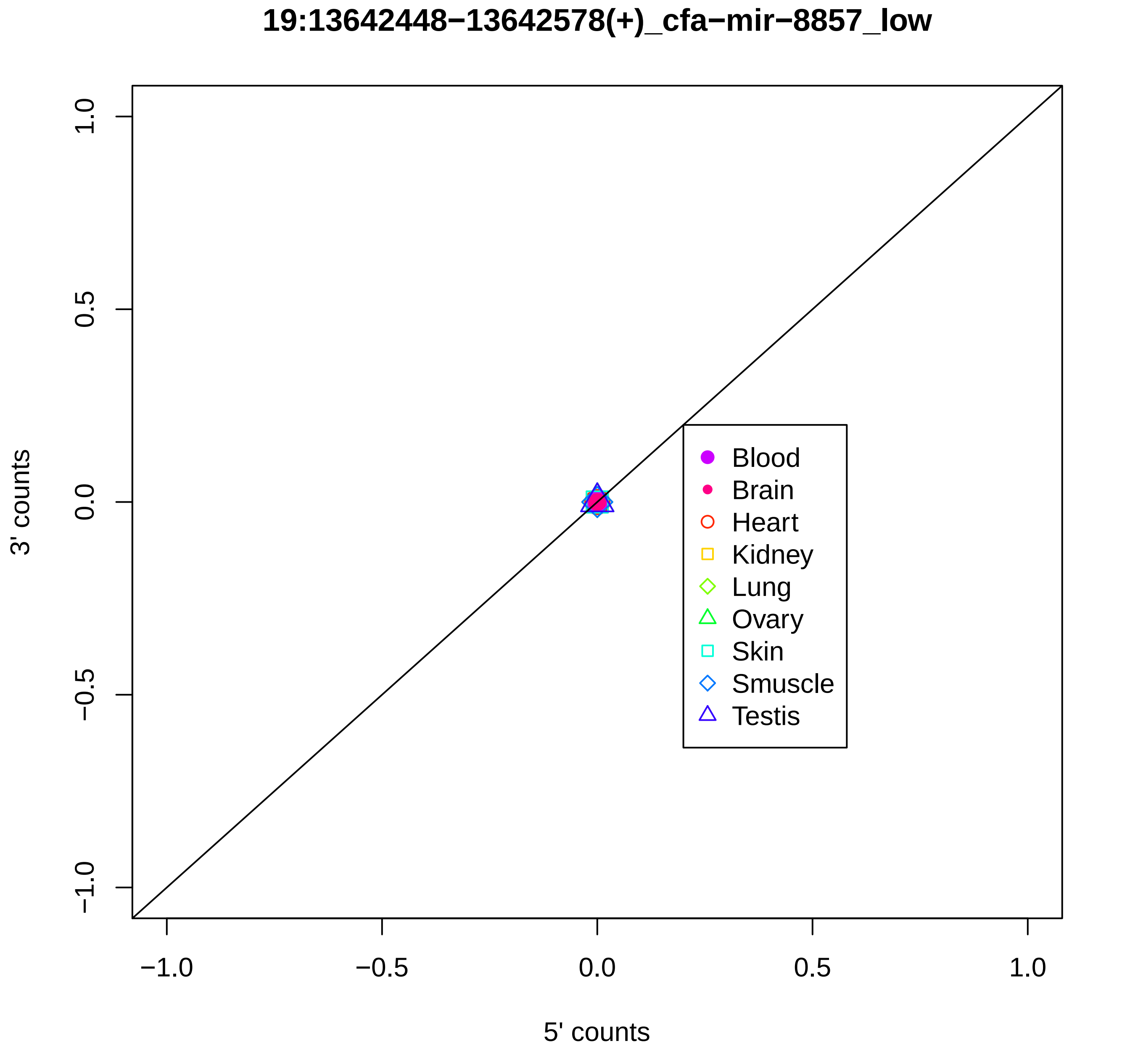

19:13642448−13642578(+)_cfa−mir−8857_low
3' counts
5' counts
1.0
0.5
0.0
−0.5
−1.0
Blood
Brain
Heart
Kidney
Lung
Ovary
Skin
Smuscle
Testis

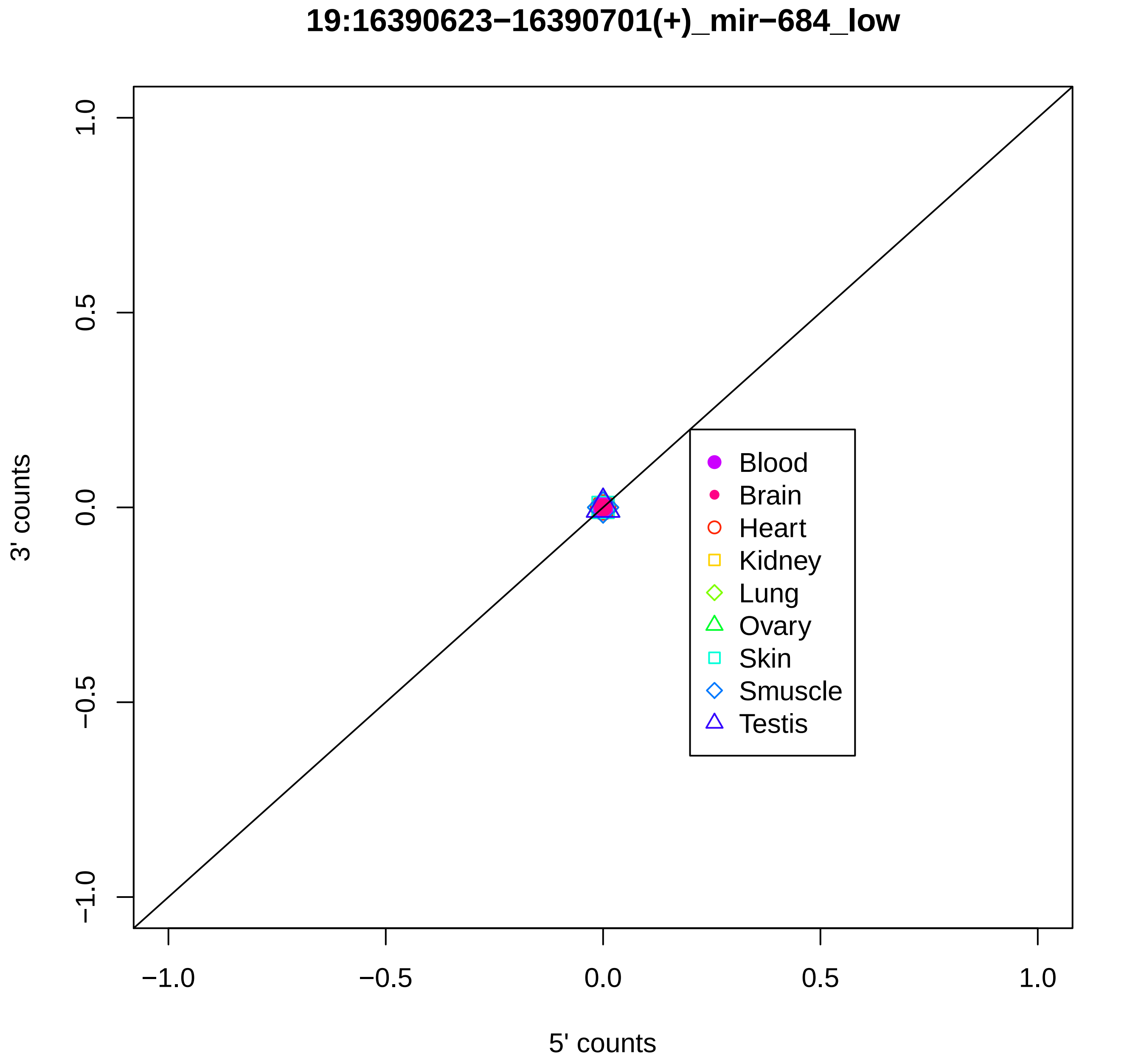

19:16390623−16390701(+)_mir−684_low
3' counts
5' counts
1.0
0.5
0.0
−0.5
−1.0
Blood
Brain
Heart
Kidney
Lung
Ovary
Skin
Smuscle
Testis

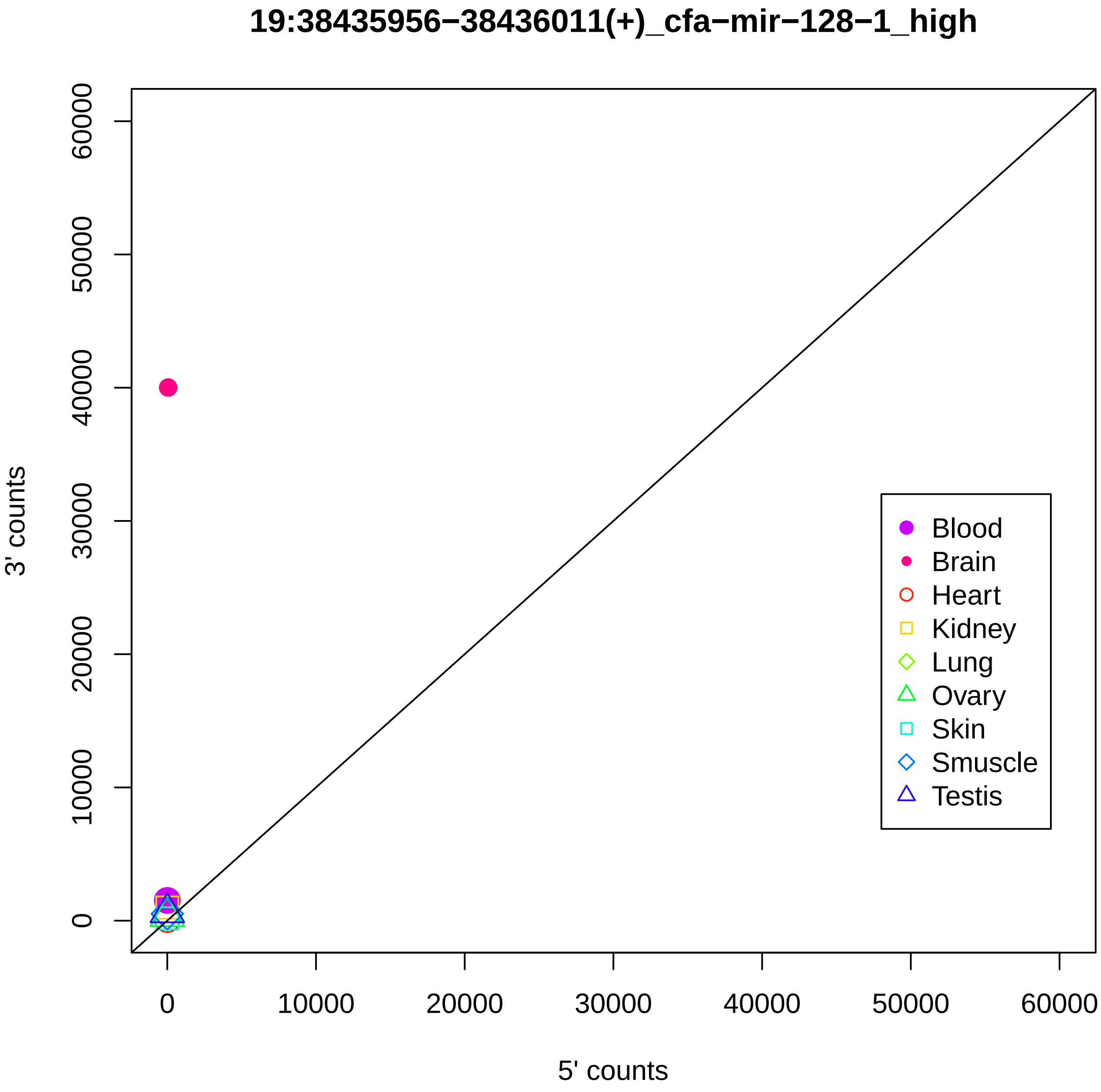
19:38435956−38436011(+)_cfa−mir−128−1_high
3' counts
5' counts
0
10000
20000
30000
40000
50000
60000
Blood
Brain
Heart
Kidney
Lung
Ovary
Skin
Smuscle
Testis

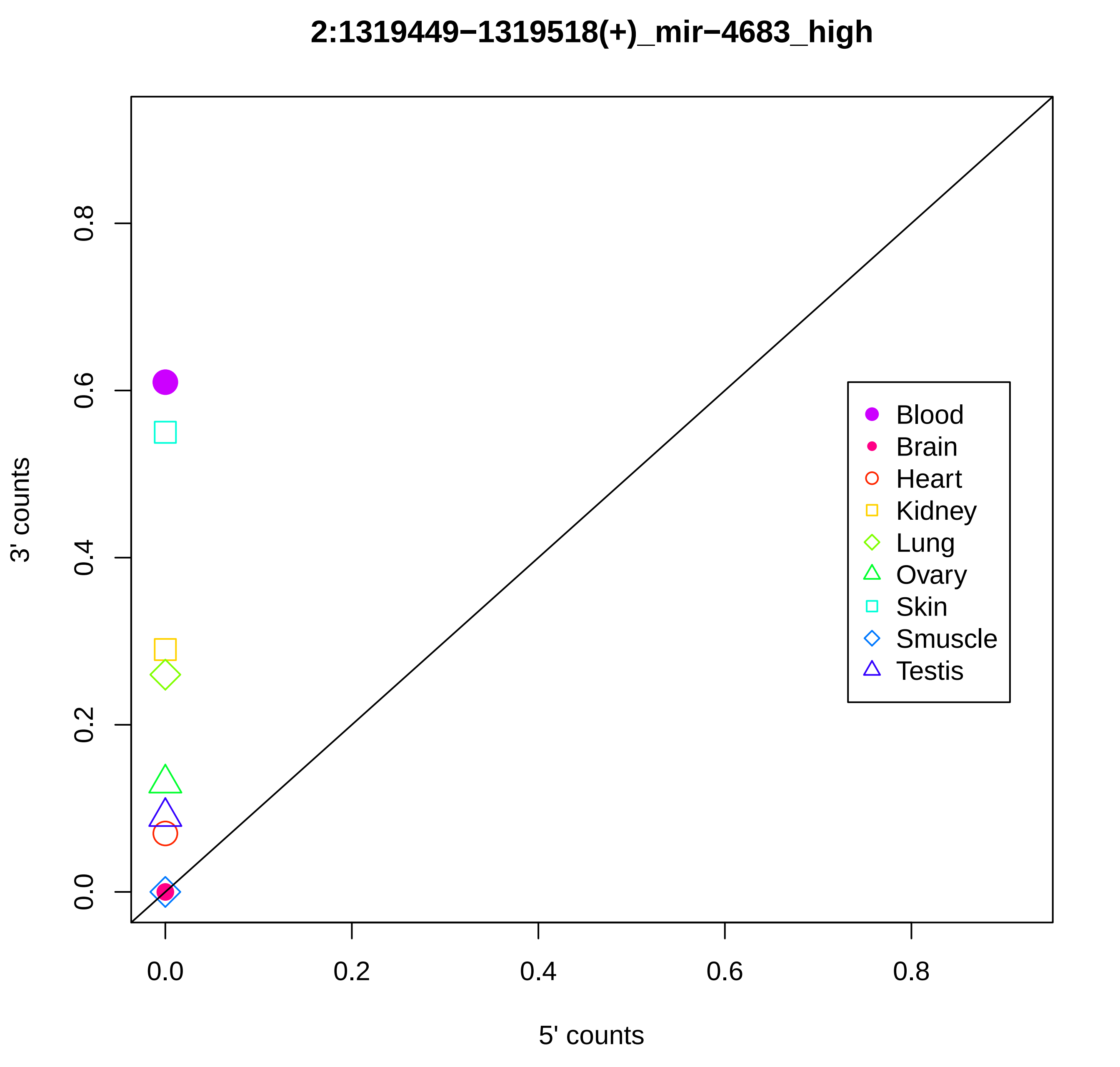
2:1319449−1319518(+)_mir−4683_high
3' counts
5' counts
0.0
0.2
0.4
0.6
0.8
Blood
Brain
Heart
Kidney
Lung
Ovary
Skin
Smuscle
Testis

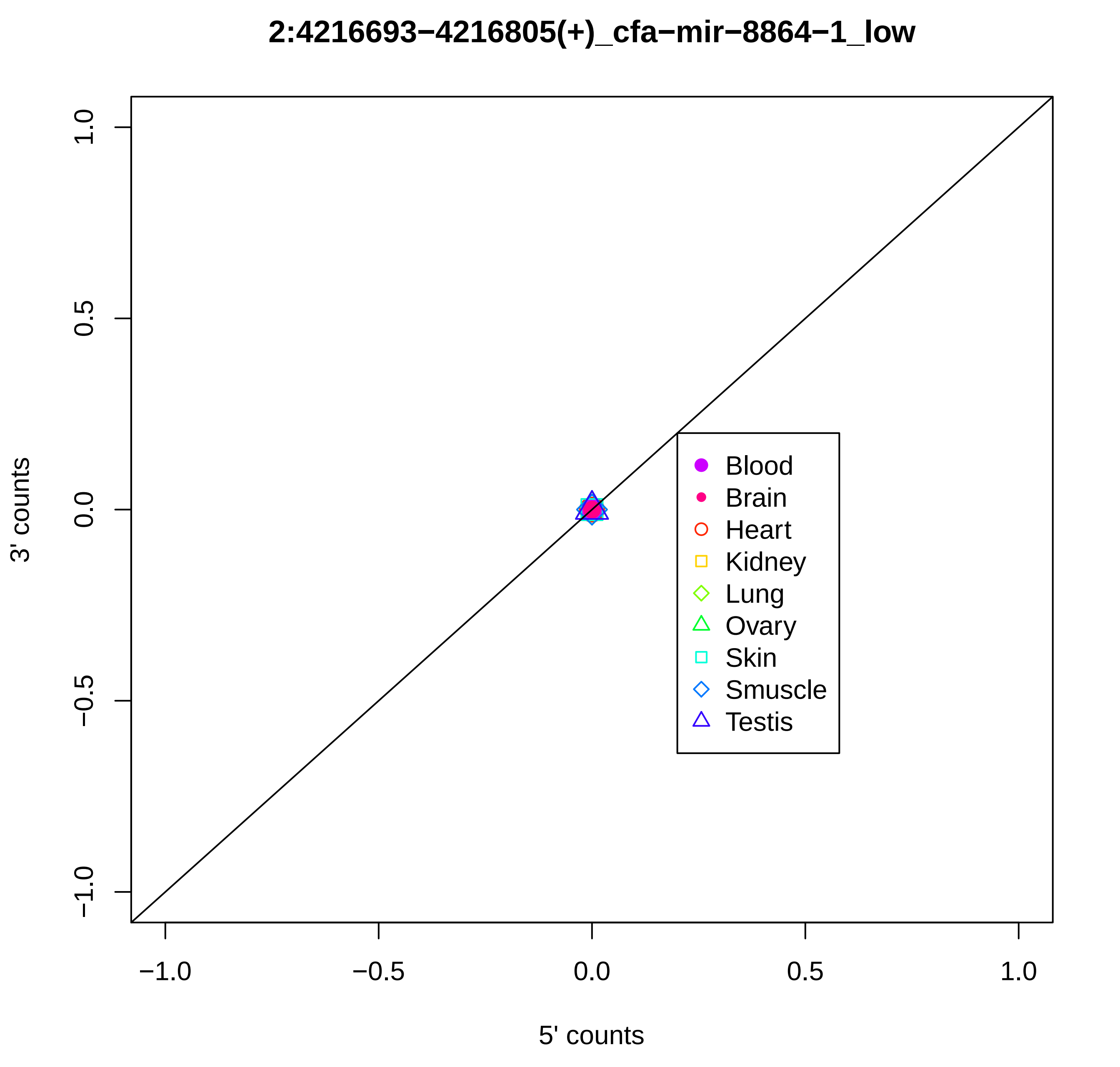
2:4216693−4216805(+)_cfa−mir−8864−1_low
3' counts
5' counts
1.0
0.5
0.0
−0.5
−1.0
−1.0
−0.5
0.0
0.5
1.0
Blood
Brain
Heart
Kidney
Lung
Ovary
Skin
Smuscle
Testis

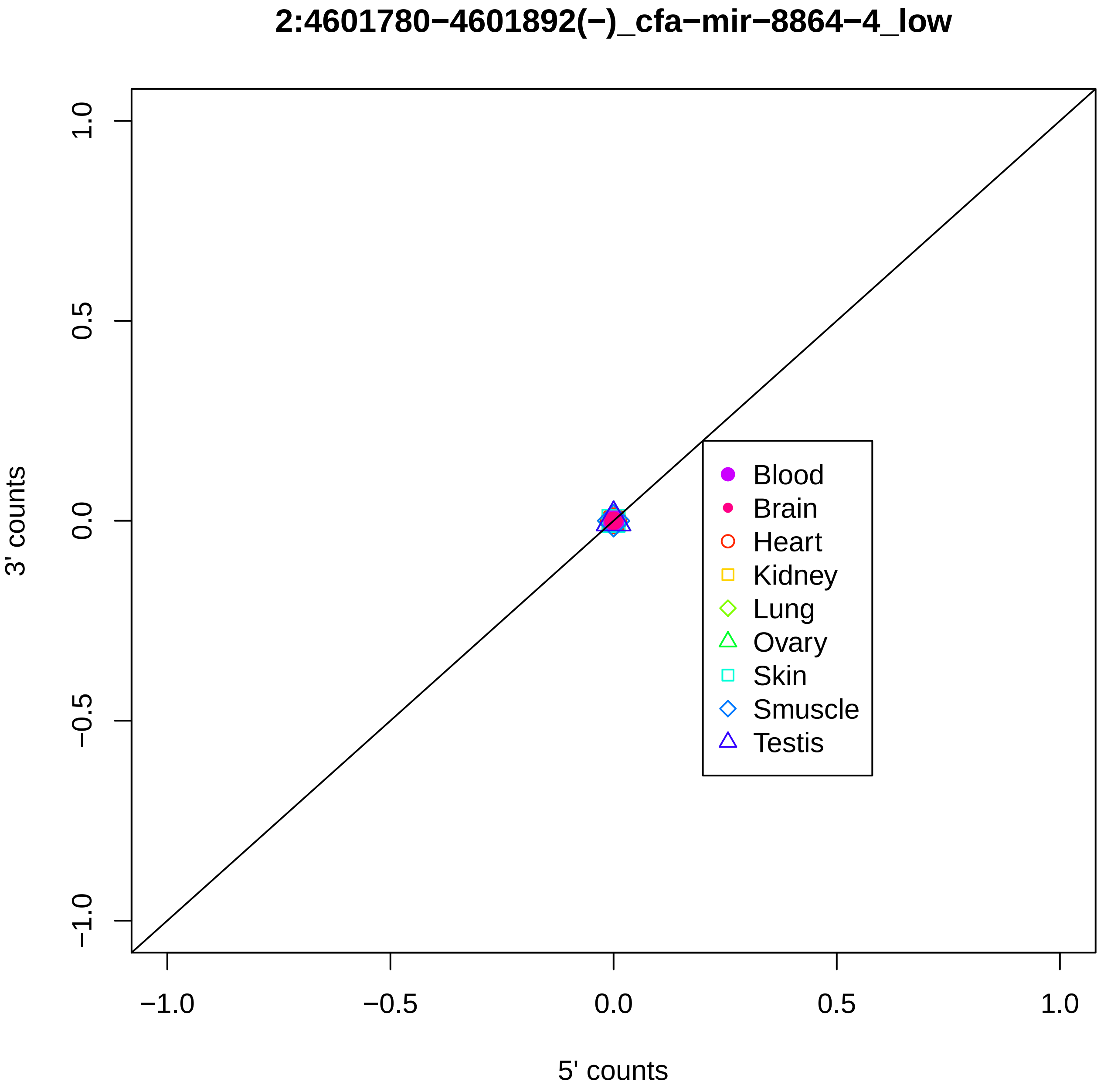
2:4601780−4601892(−)_cfa−mir−8864−4_low
3' counts
5' counts
1.0
0.5
0.0
−0.5
−1.0
Blood
Brain
Heart
Kidney
Lung
Ovary
Skin
Smuscle
Testis

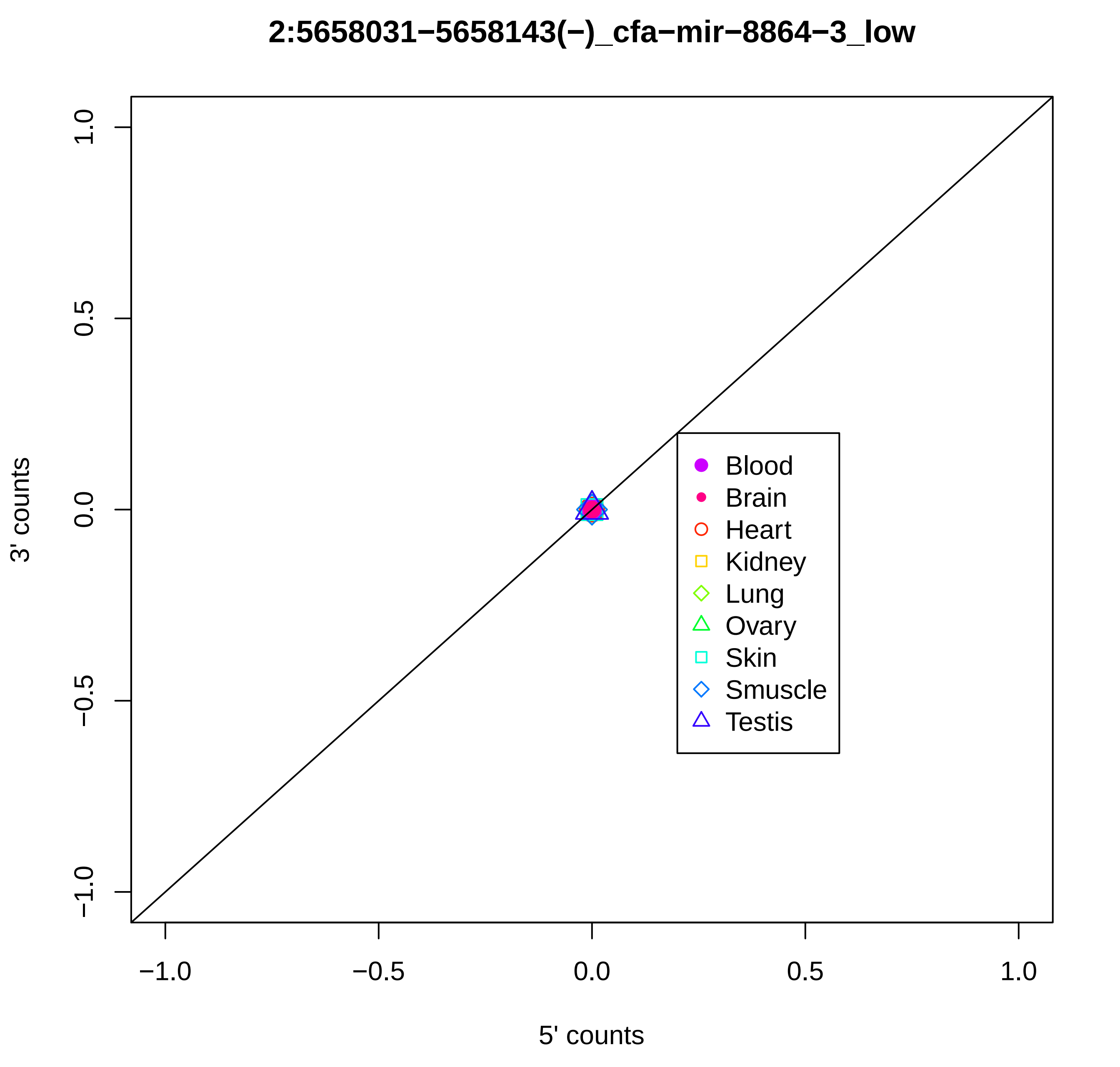
2:5658031−5658143(−)_cfa−mir−8864−3_low
3' counts
5' counts
1.0
0.5
0.0
−0.5
−1.0
Blood
Brain
Heart
Kidney
Lung
Ovary
Skin
Smuscle
Testis

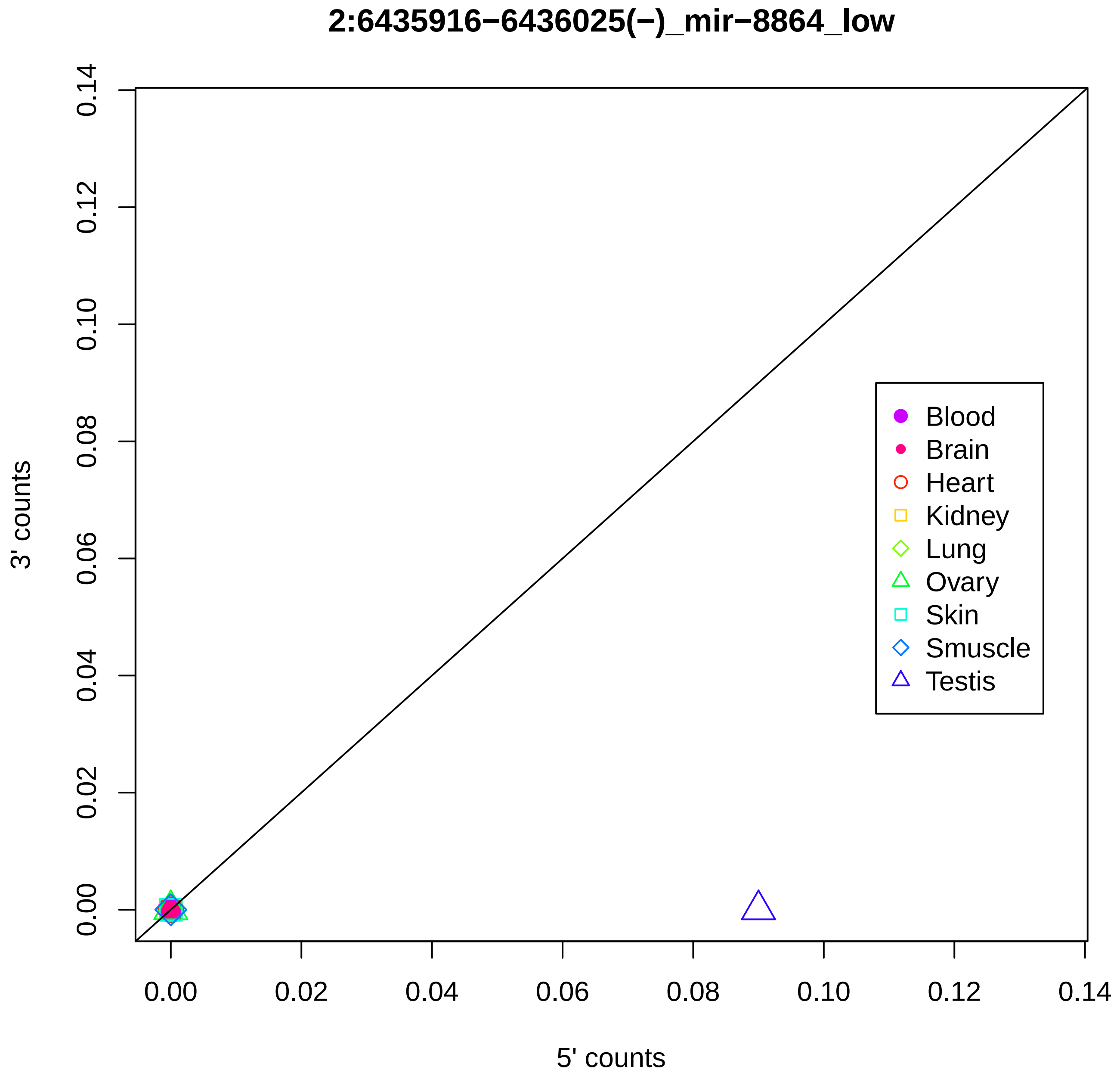

2:6435916−6436025(−)_mir−8864_low
3' counts
5' counts
0.00
0.02
0.04
0.06
0.08
0.10
0.12
0.14
Blood
Brain
Heart
Kidney
Lung
Ovary
Skin
Smuscle
Testis

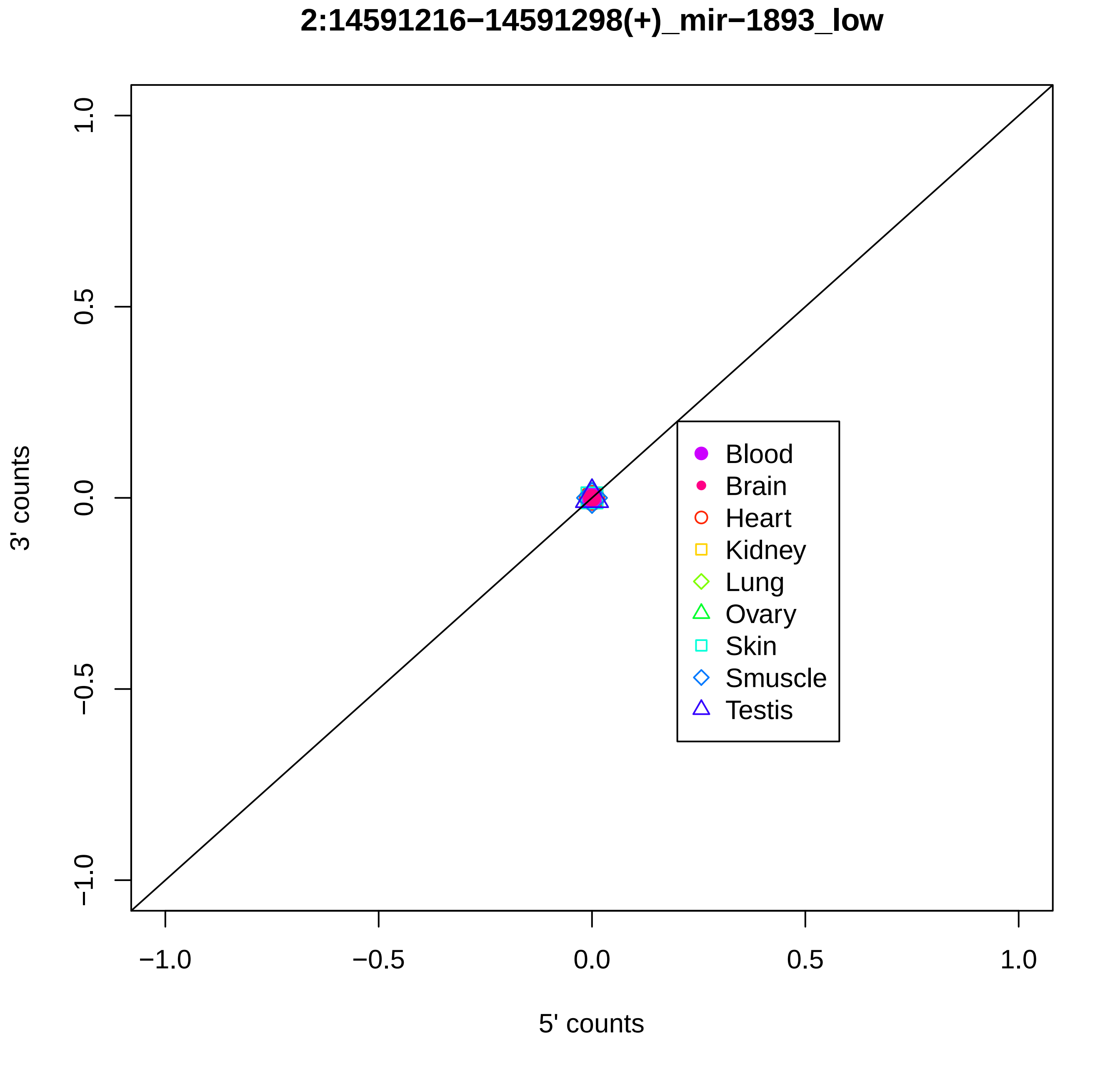

2:14591216−14591298(+)_mir−1893_low
3' counts
5' counts
−1.0
−0.5
0.0
0.5
1.0
Blood
Brain
Heart
Kidney
Lung
Ovary
Skin
Smuscle
Testis

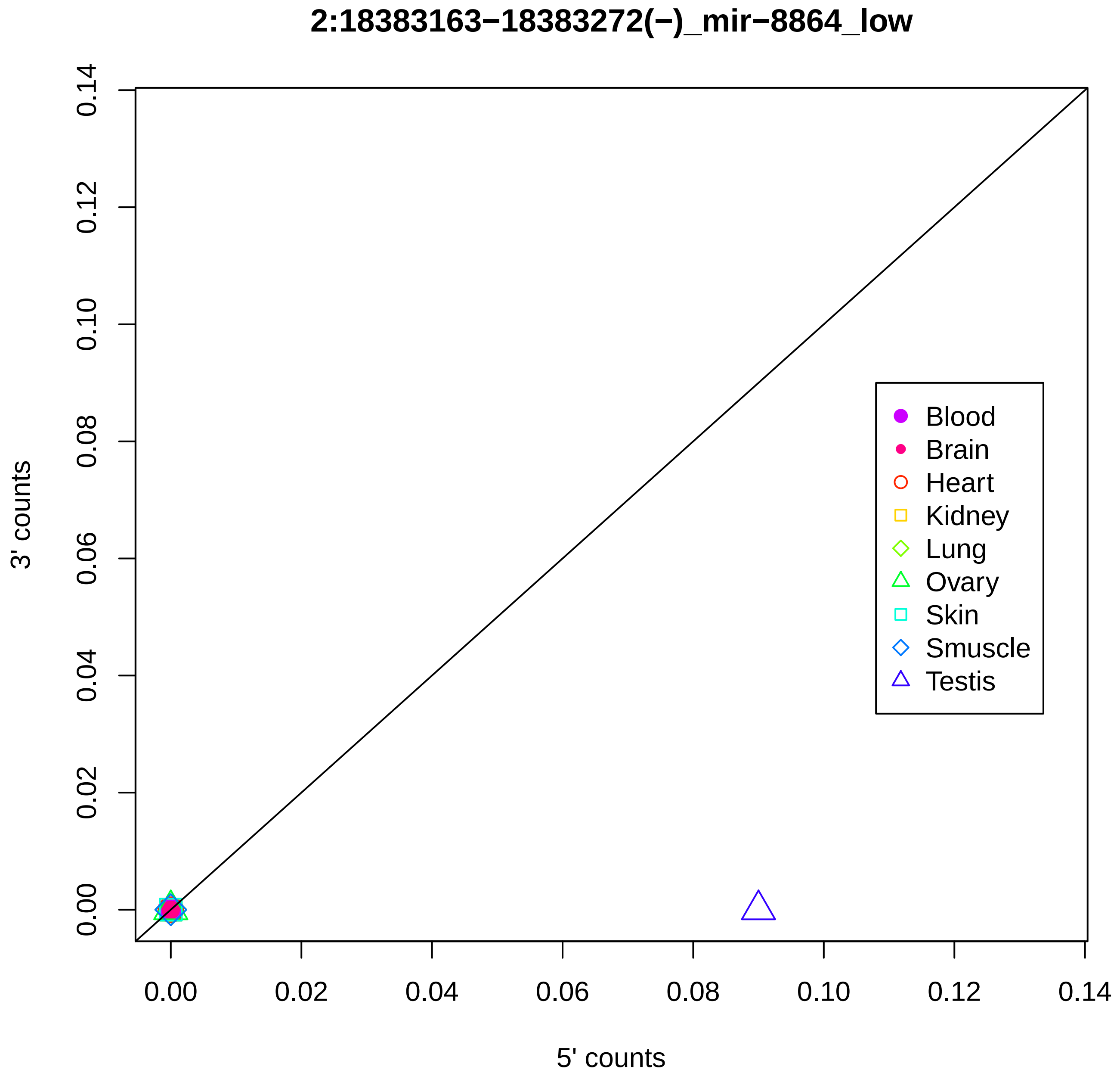
2:18383163−18383272(−)_mir−8864_low
3' counts
5' counts
0.00
0.02
0.04
0.06
0.08
0.10
0.12
0.14
Blood
Brain
Heart
Kidney
Lung
Ovary
Skin
Smuscle
Testis

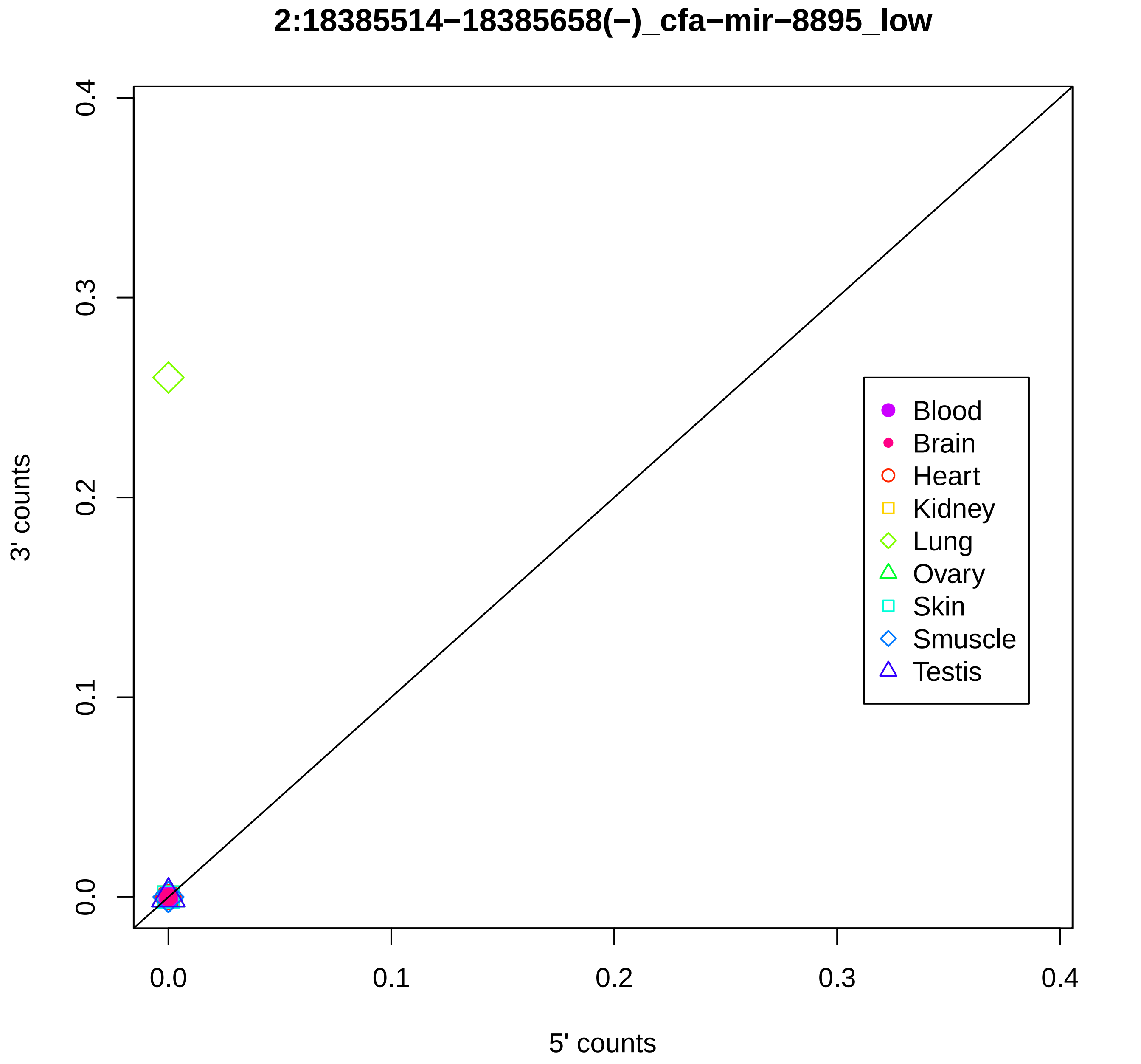
2:18385514−18385658(−)_cfa−mir−8895_low
3' counts
5' counts
0.0
0.1
0.2
0.3
0.4
Blood
Brain
Heart
Kidney
Lung
Ovary
Skin
Smuscle
Testis

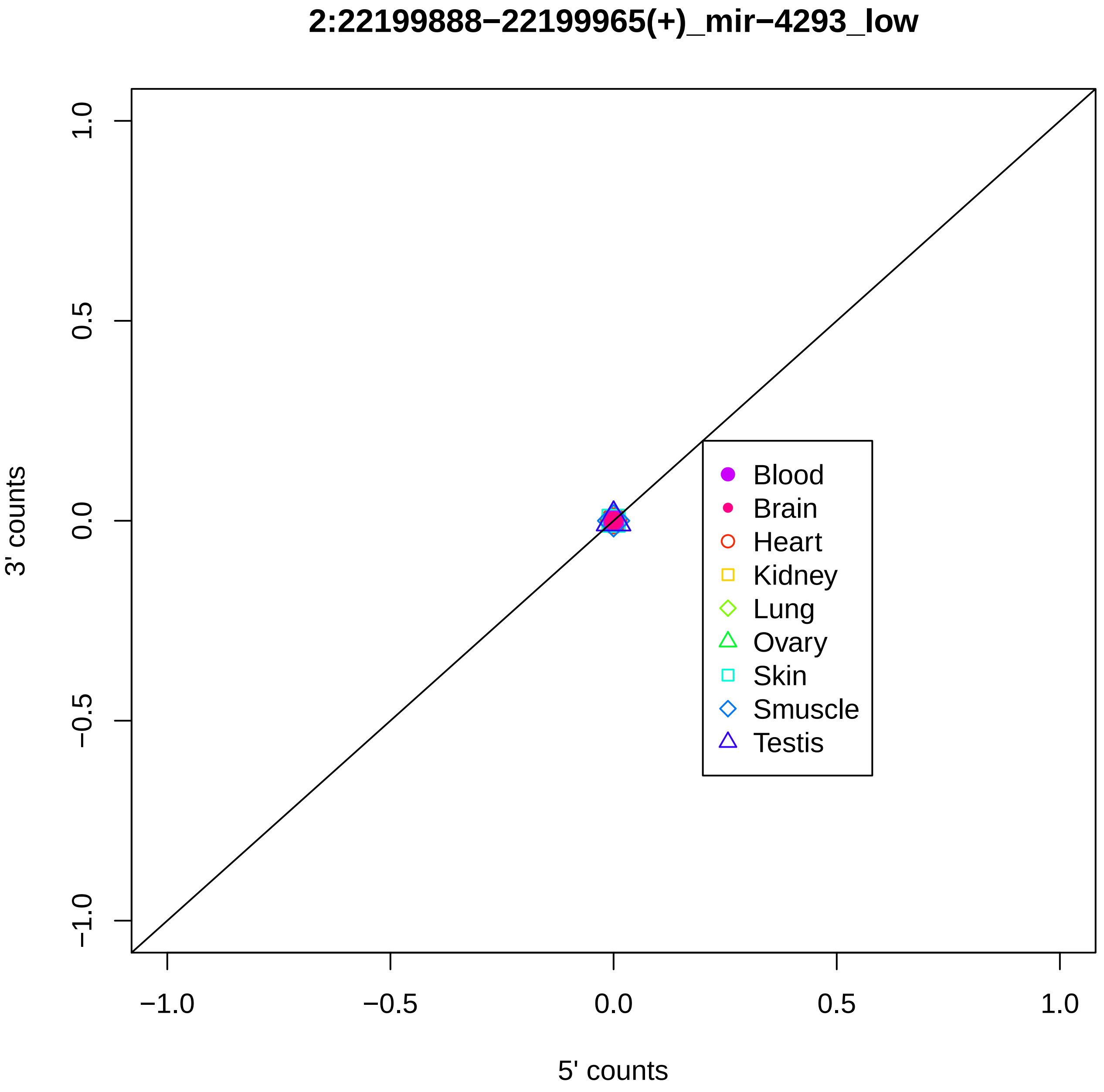

2:22199888−22199965(+)_mir−4293_low
3' counts
5' counts
1.0
0.5
0.0
−0.5
−1.0
Blood
Brain
Heart
Kidney
Lung
Ovary
Skin
Smuscle
Testis

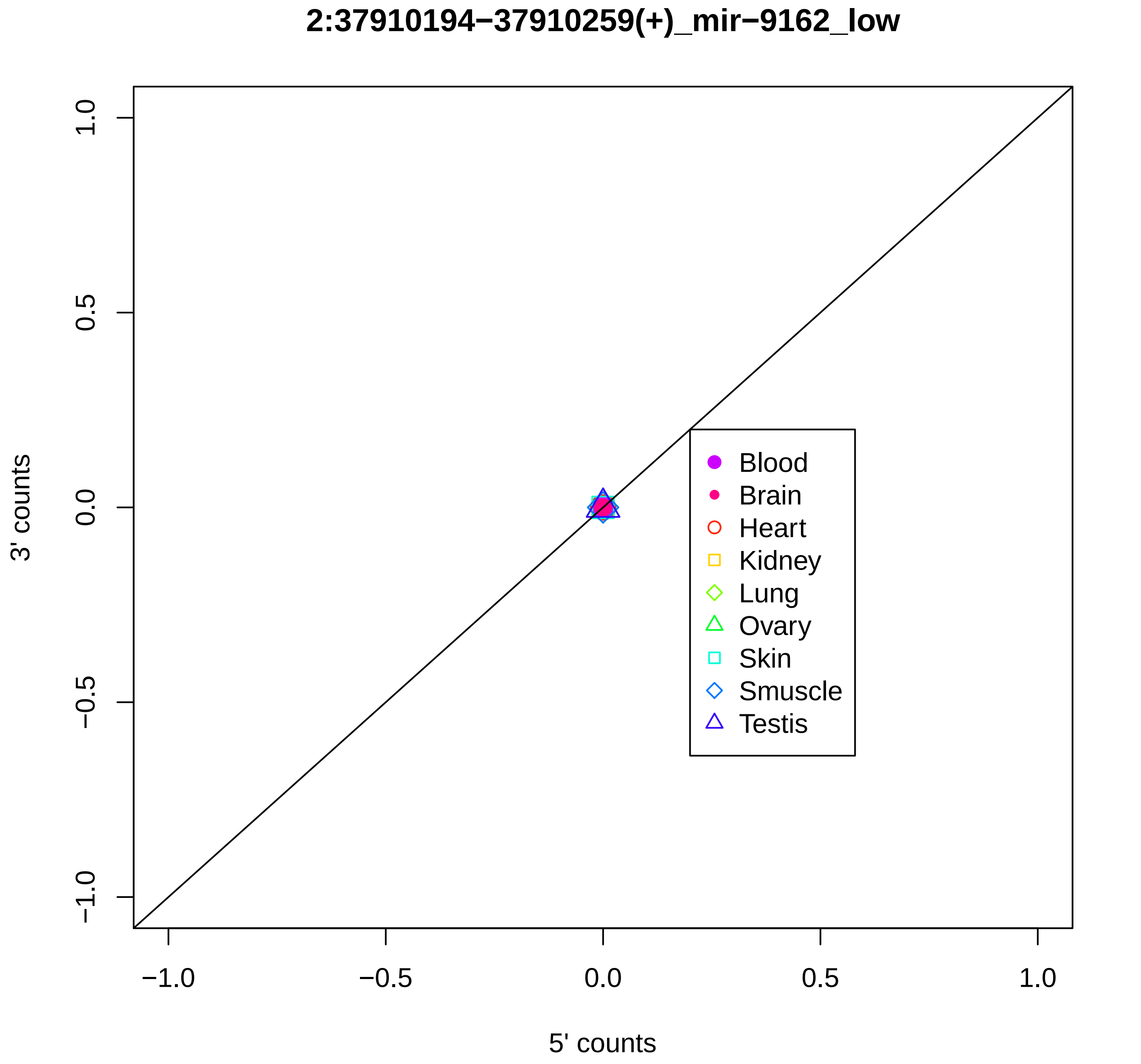

2:37910194−37910259(+)_mir−9162_low
3' counts
5' counts
1.0
0.5
0.0
−0.5
−1.0
Blood
Brain
Heart
Kidney
Lung
Ovary
Skin
Smuscle
Testis

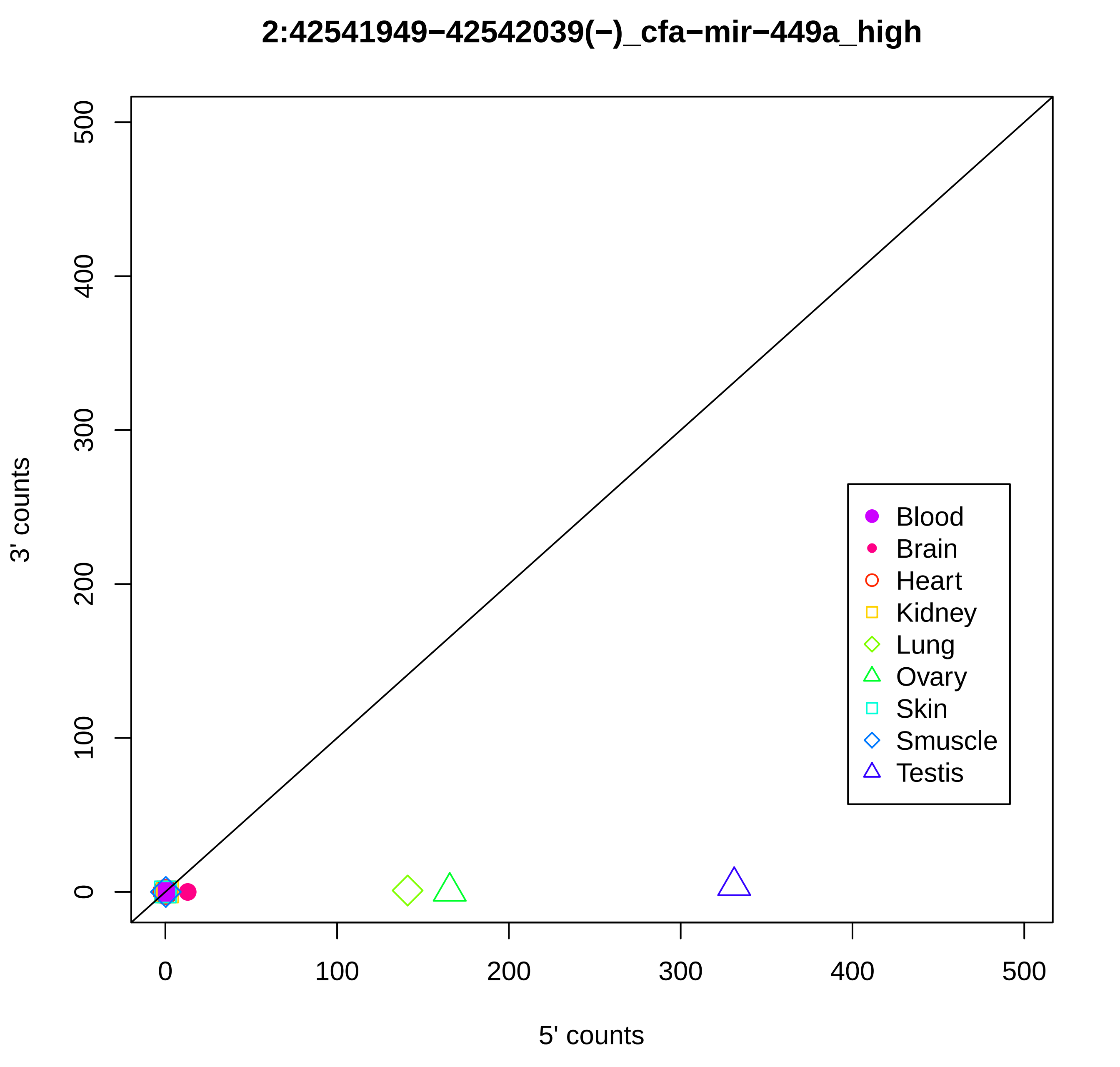
2:42541949−42542039(−)_cfa−mir−449a_high
3' counts
5' counts
0
100
200
300
400
500
0
100
200
300
400
500
Blood
Brain
Heart
Kidney
Lung
Ovary
Skin
Smuscle
Testis

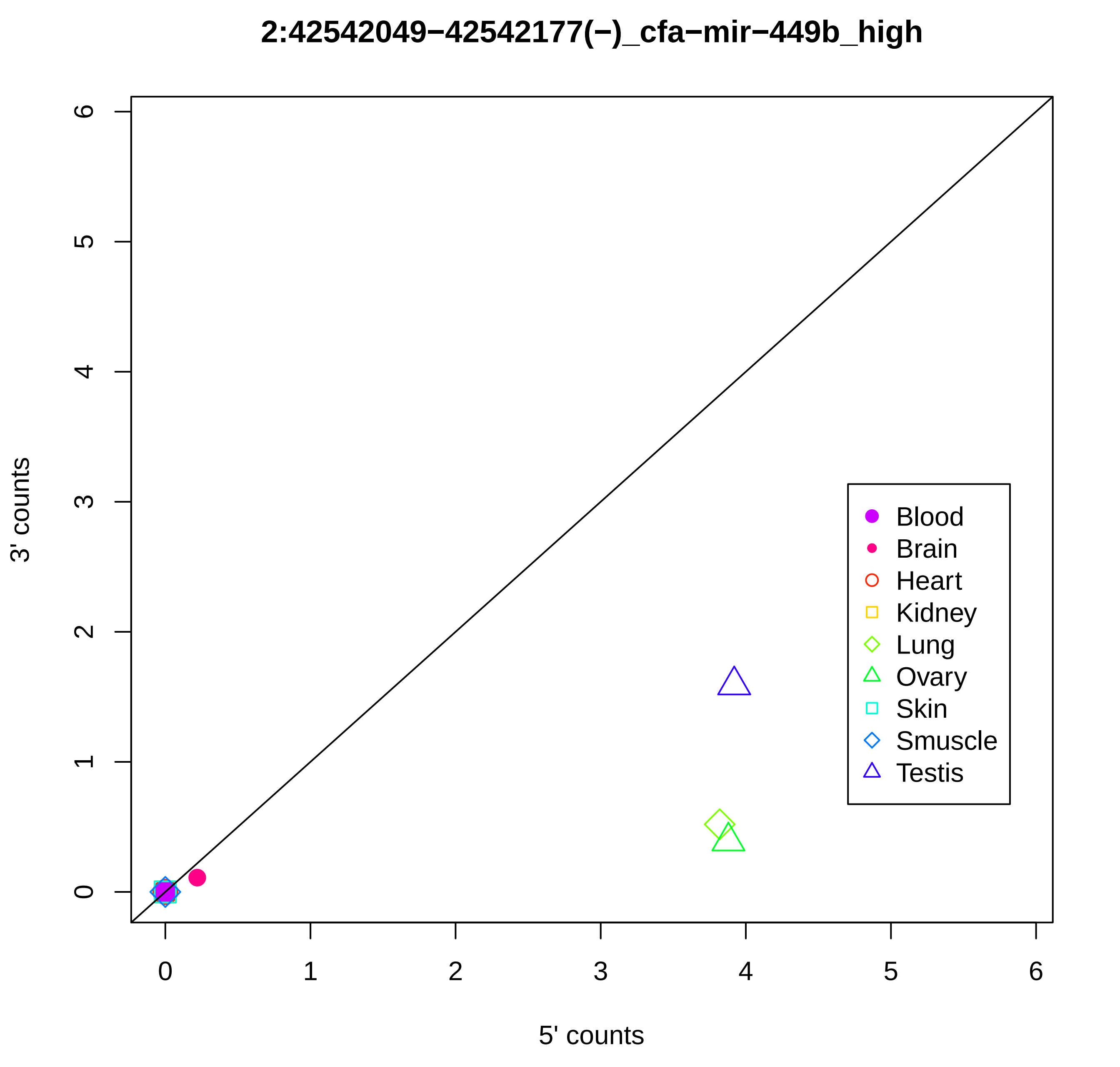
2:42542049−42542177(−)_cfa−mir−449b_high
3' counts
5' counts
0
1
2
3
4
5
6
Blood
Brain
Heart
Kidney
Lung
Ovary
Skin
Smuscle
Testis

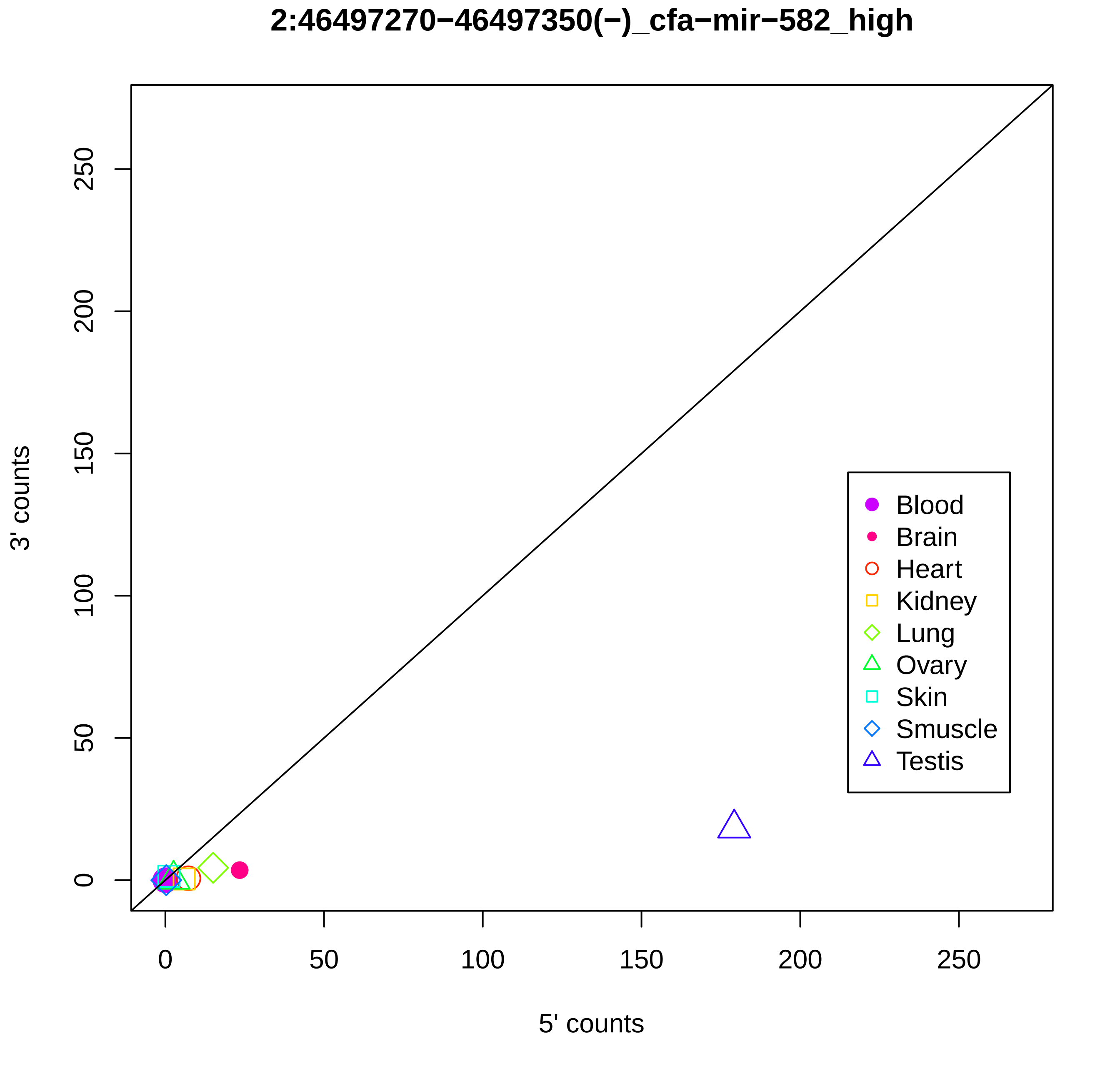

2:46497270−46497350(−)_cfa−mir−582_high
3' counts
5' counts
0
50
100
150
200
250
Blood
Brain
Heart
Kidney
Lung
Ovary
Skin
Smuscle
Testis

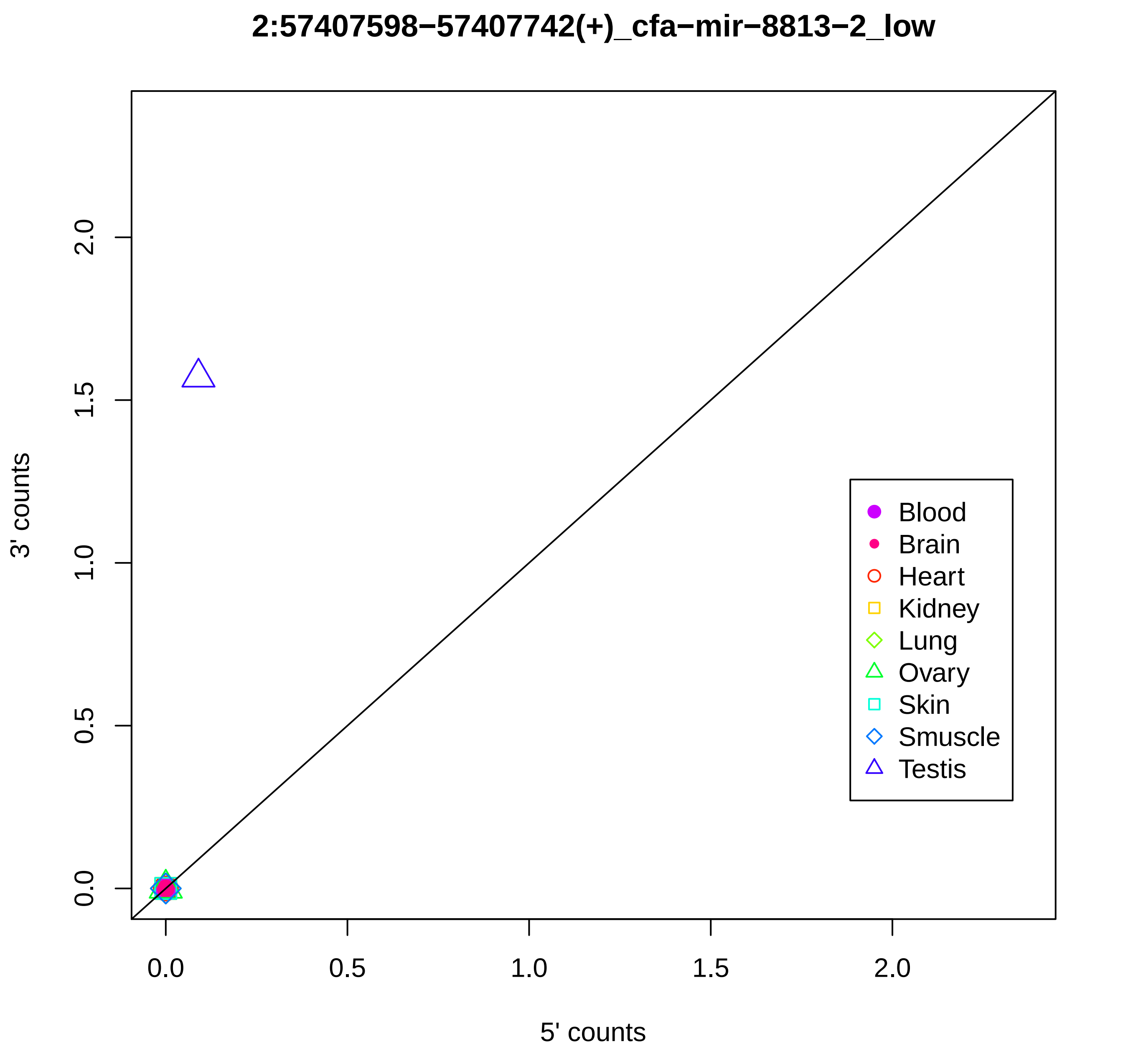

2:57407598−57407742(+)_cfa−mir−8813−2_low
3' counts
5' counts
0.0
0.5
1.0
1.5
2.0
0.0
0.5
1.0
1.5
2.0
Blood
Brain
Heart
Kidney
Lung
Ovary
Skin
Smuscle
Testis

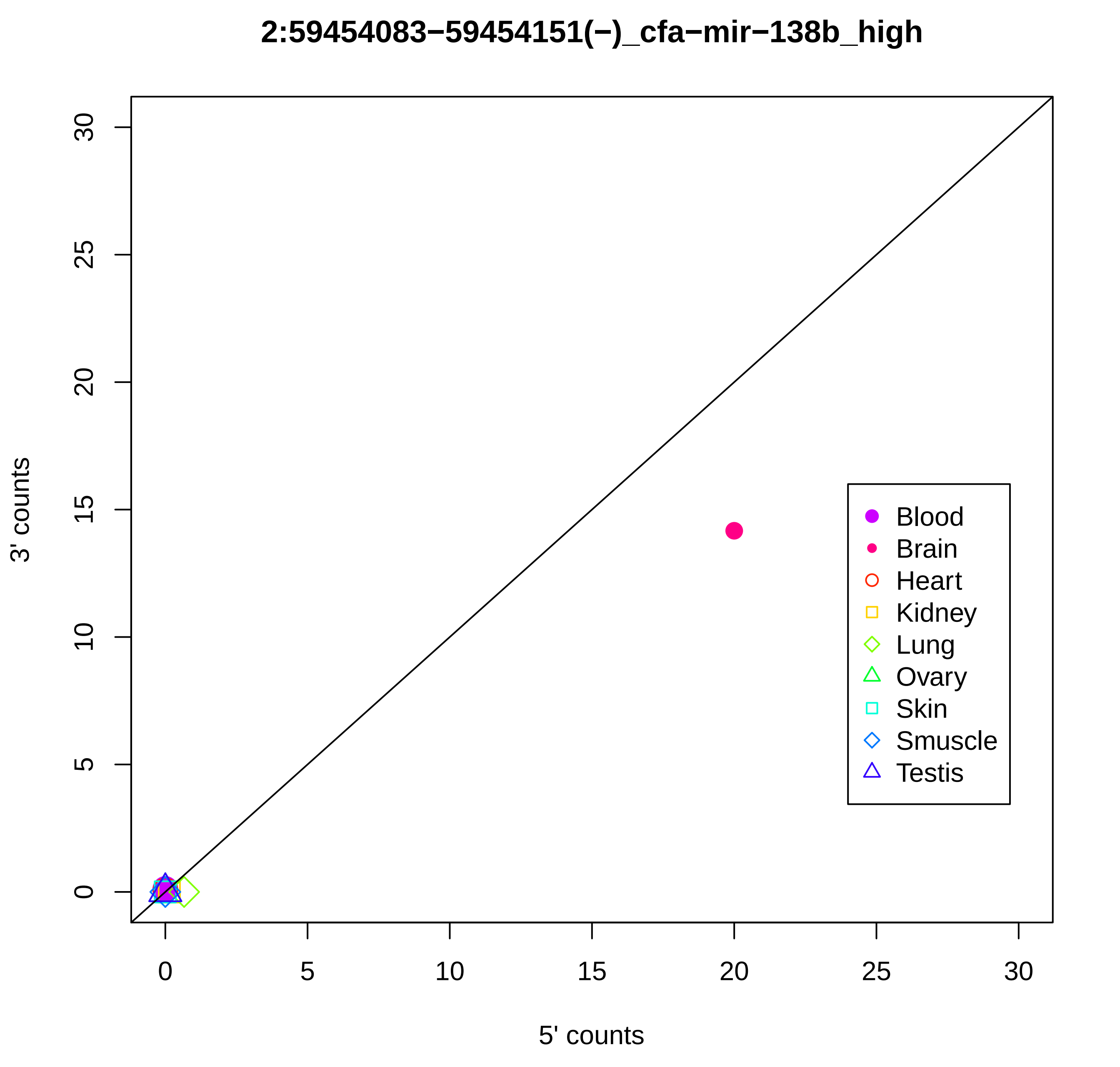

2:59454083−59454151(−)_cfa−mir−138b_high
3' counts
5' counts
0
5
10
15
20
25
30
Blood
Brain
Heart
Kidney
Lung
Ovary
Skin
Smuscle
Testis

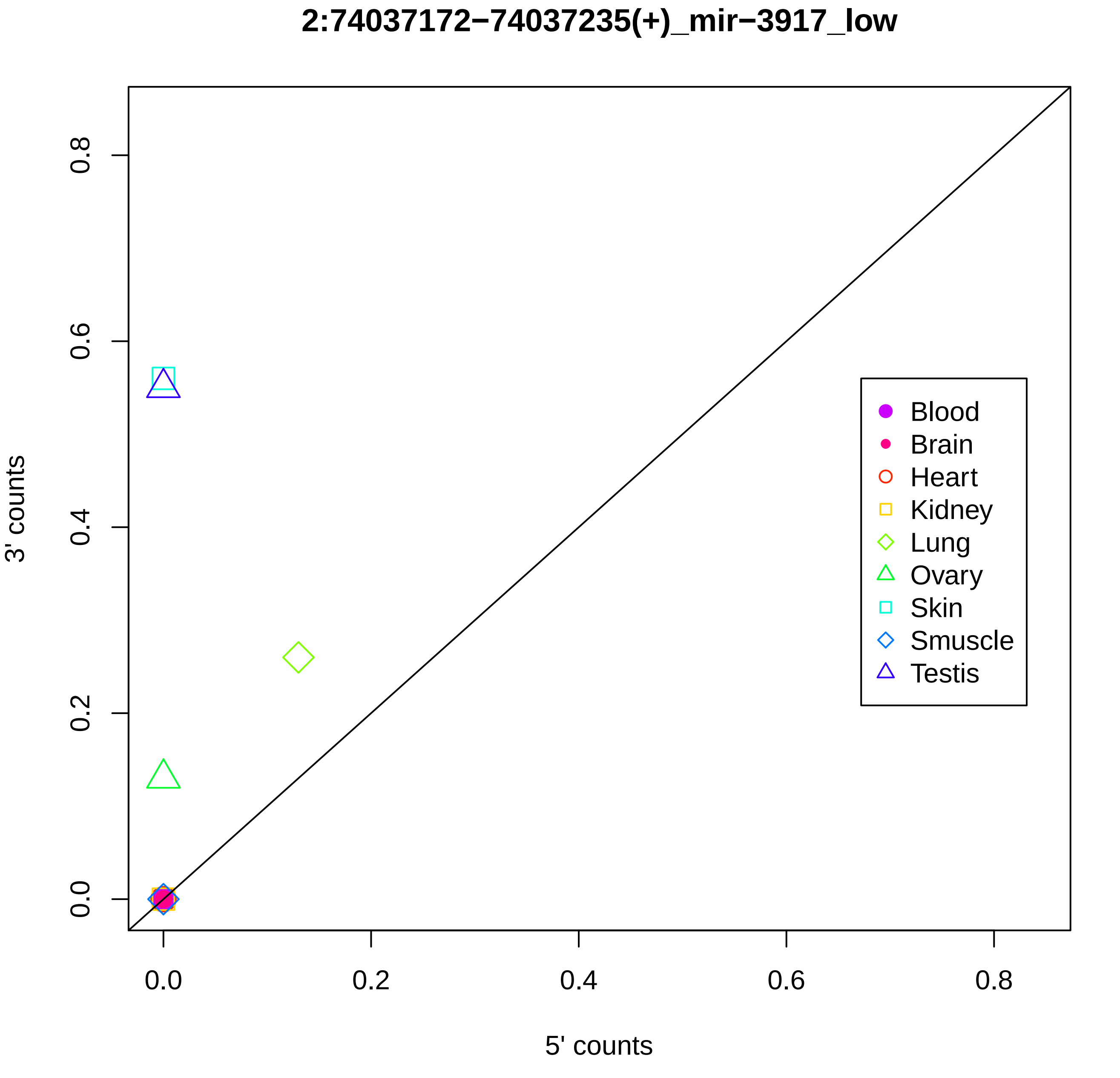

2:74037172−74037235(+)_mir−3917_low
3' counts
5' counts
0.0
0.2
0.4
0.6
0.8
Blood
Brain
Heart
Kidney
Lung
Ovary
Skin
Smuscle
Testis

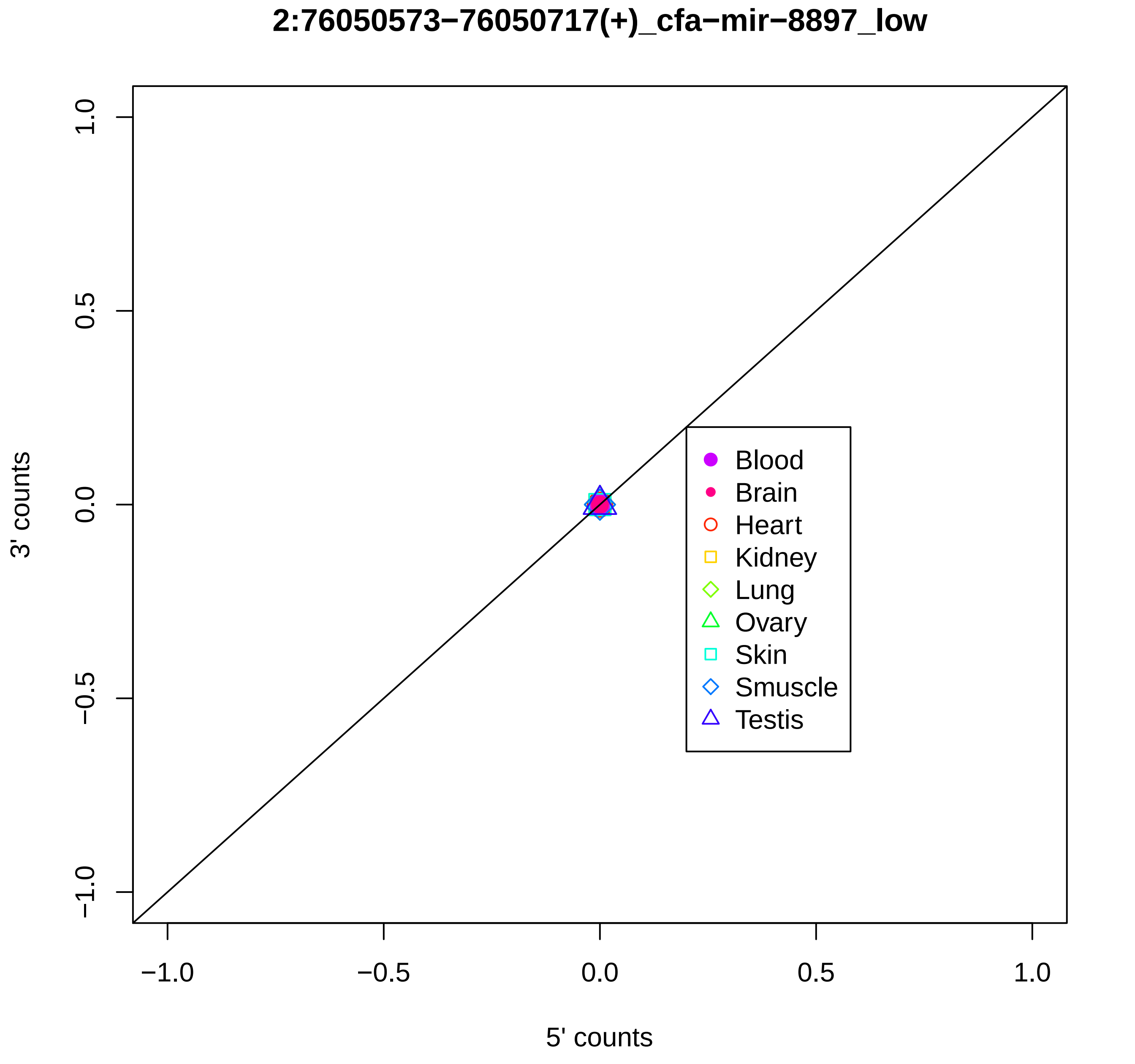
2:76050573−76050717(+)_cfa−mir−8897_low
3' counts
5' counts
1.0
0.5
0.0
−0.5
−1.0
Blood
Brain
Heart
Kidney
Lung
Ovary
Skin
Smuscle
Testis

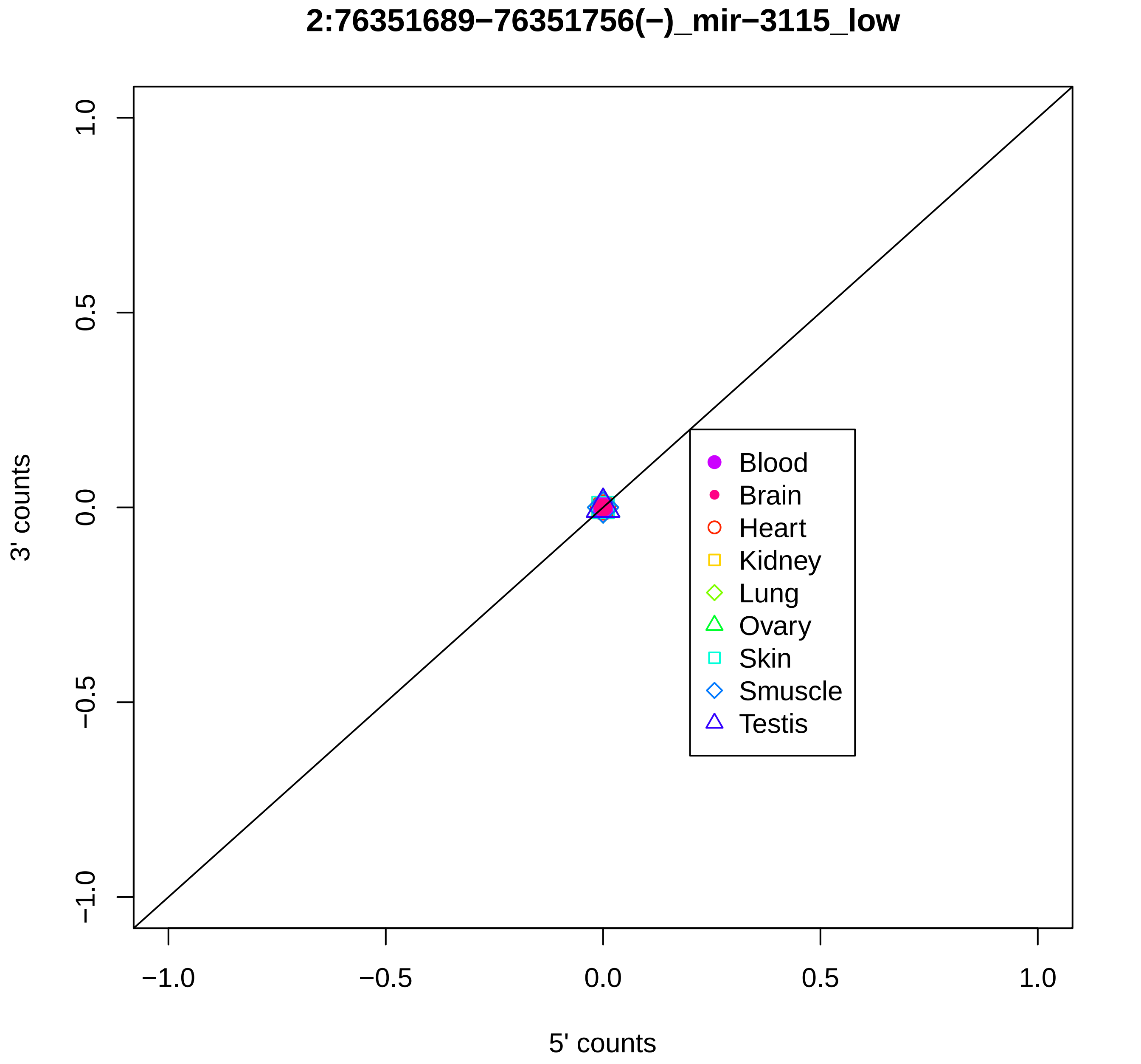

2:76351689−76351756(−)_mir−3115_low
3' counts
5' counts
1.0
0.5
0.0
−0.5
−1.0
Blood
Brain
Heart
Kidney
Lung
Ovary
Skin
Smuscle
Testis

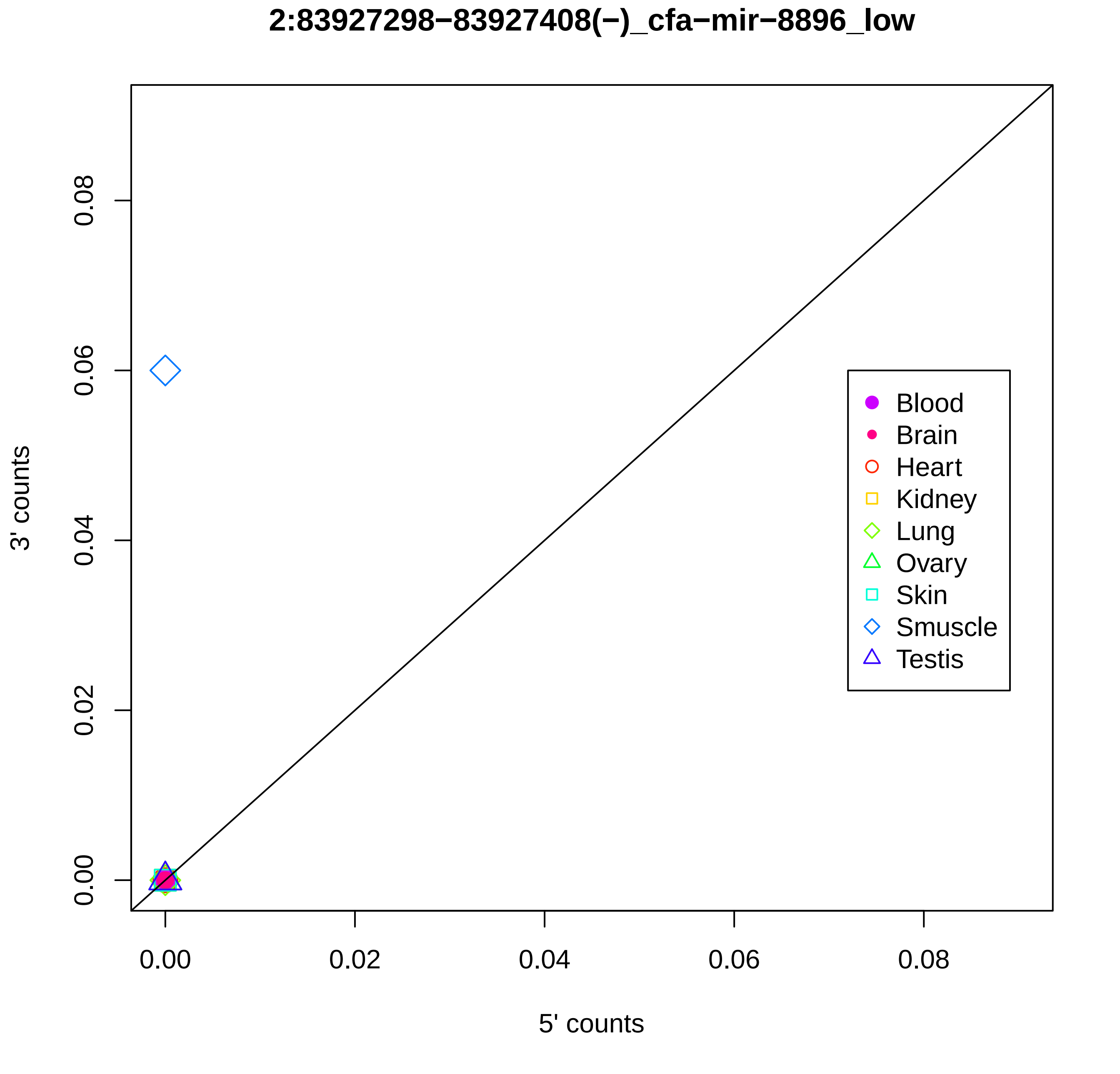

2:83927298−83927408(−)_cfa−mir−8896_low
3' counts
5' counts
0.00
0.02
0.04
0.06
0.08
Blood
Brain
Heart
Kidney
Lung
Ovary
Skin
Smuscle
Testis

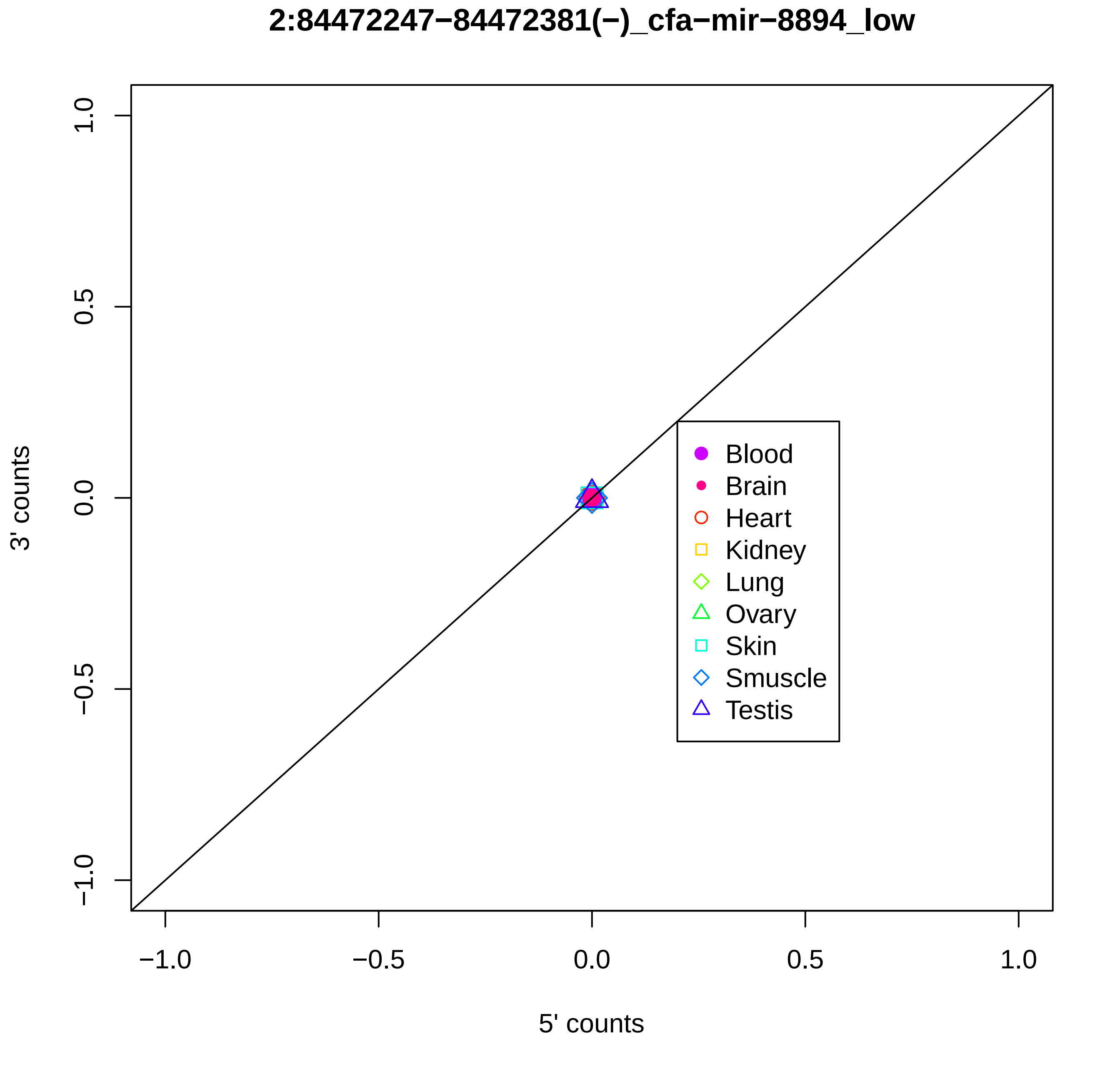

2:84472247−84472381(−)_cfa−mir−8894_low
1.0
0.5
0.0
−0.5
−1.0
3' counts
−1.0
−0.5
0.0
0.5
1.0
5' counts
Blood
Brain
Heart
Kidney
Lung
Ovary
Skin
Smuscle
Testis

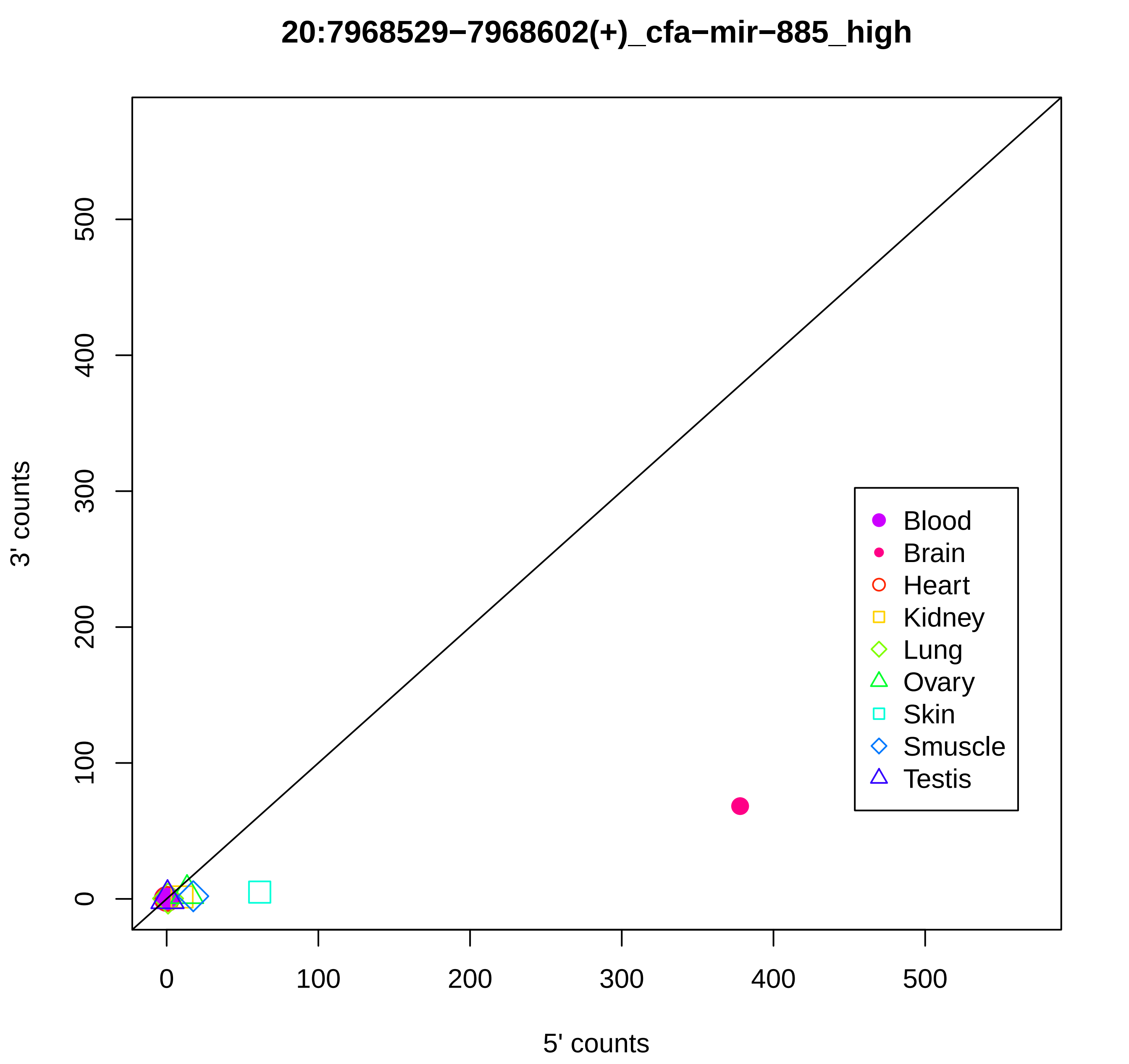
20:7968529−7968602(+)_cfa−mir−885_high
3' counts
0
100
200
300
400
500
5' counts
Blood
Brain
Heart
Kidney
Lung
Ovary
Skin
Smuscle
Testis

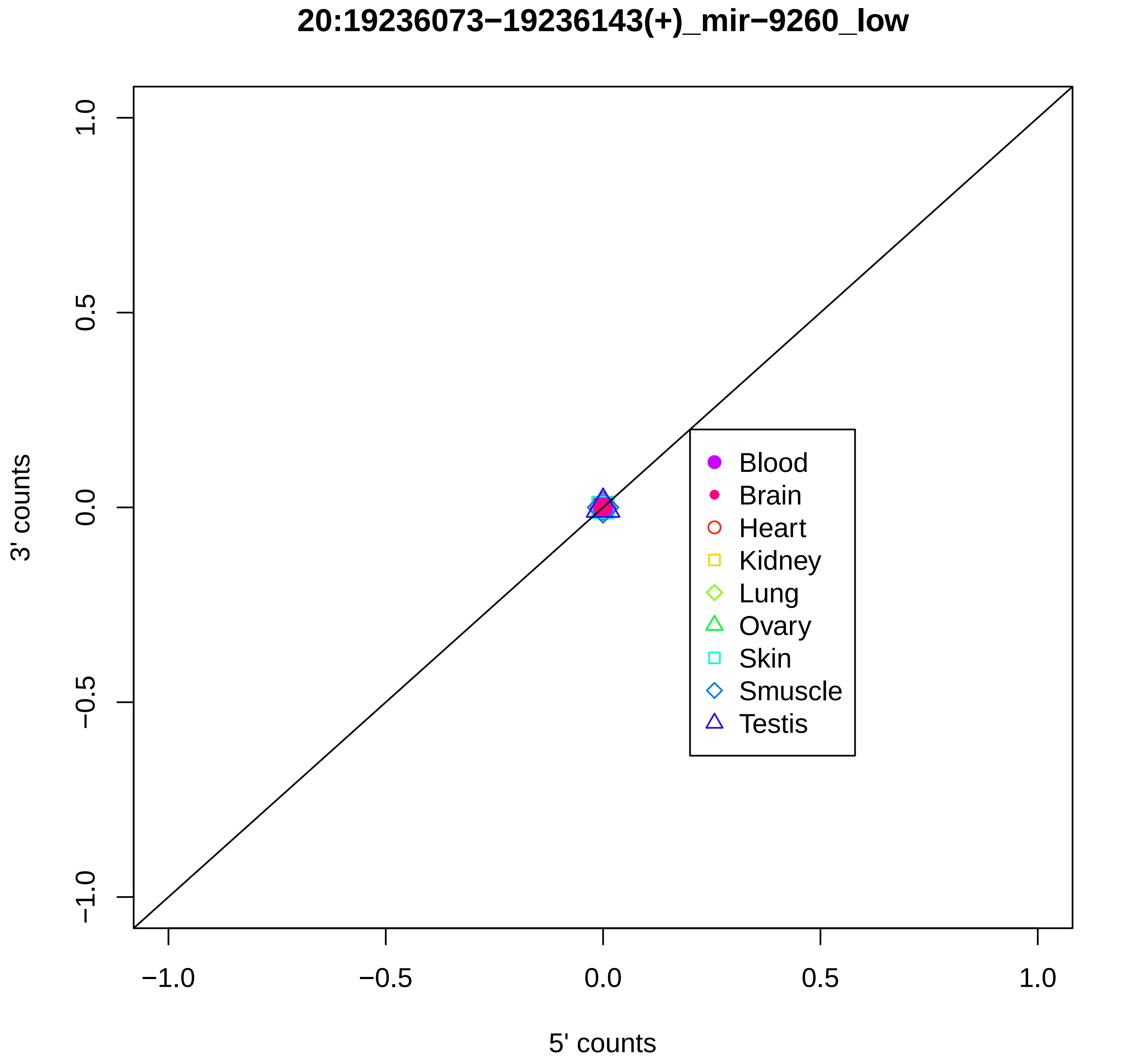

20:19236073−19236143(+)_mir−9260_low
1.0
0.5
0.0
−0.5
−1.0
3' counts
−1.0
−0.5
0.0
0.5
1.0
5' counts
Blood
Brain
Heart
Kidney
Lung
Ovary
Skin
Smuscle
Testis

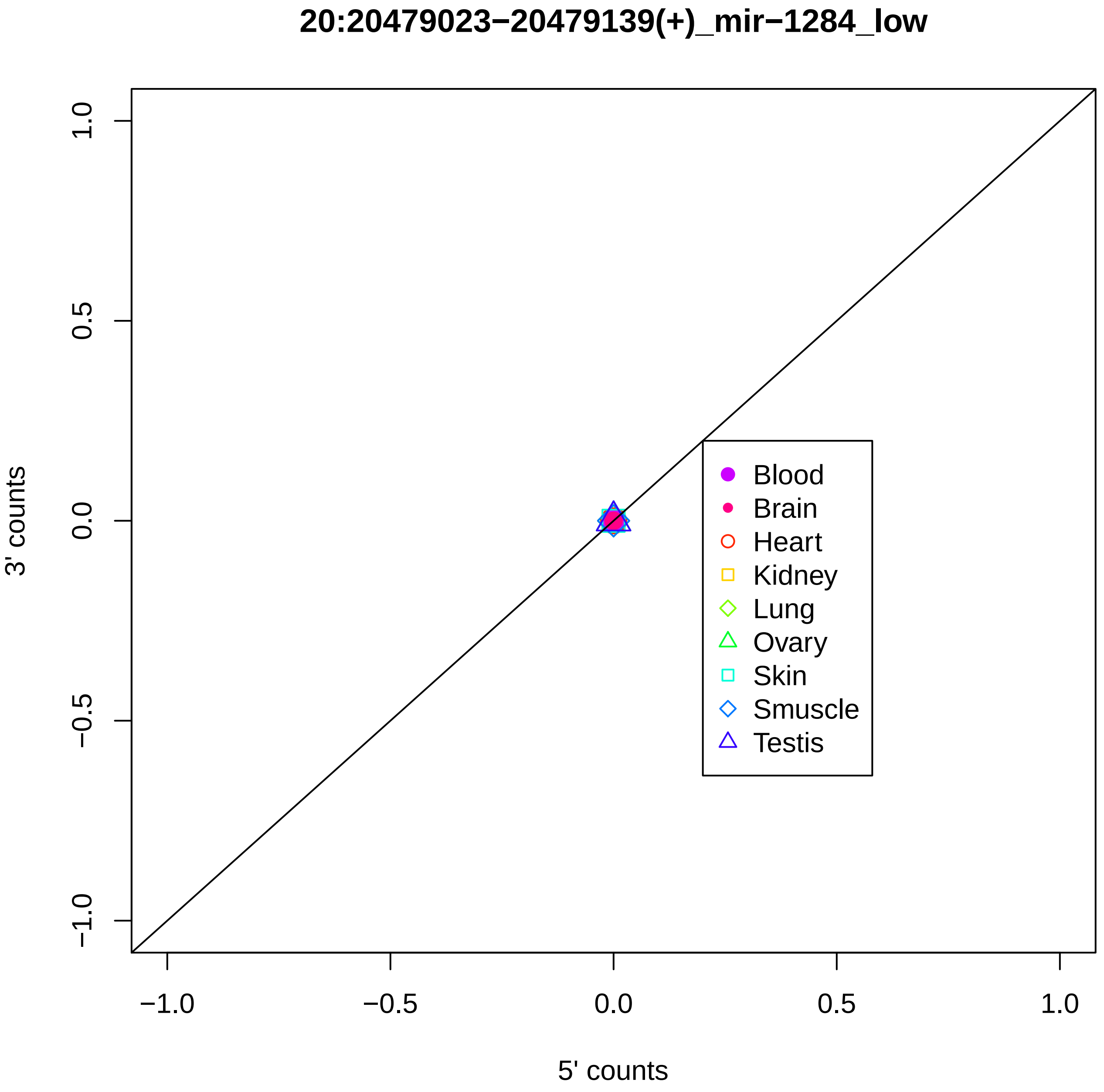

20:20479023−20479139(+)_mir−1284_low
3' counts
5' counts
1.0
0.5
0.0
−0.5
−1.0
−1.0
−0.5
0.0
0.5
1.0
Blood
Brain
Heart
Kidney
Lung
Ovary
Skin
Smuscle
Testis

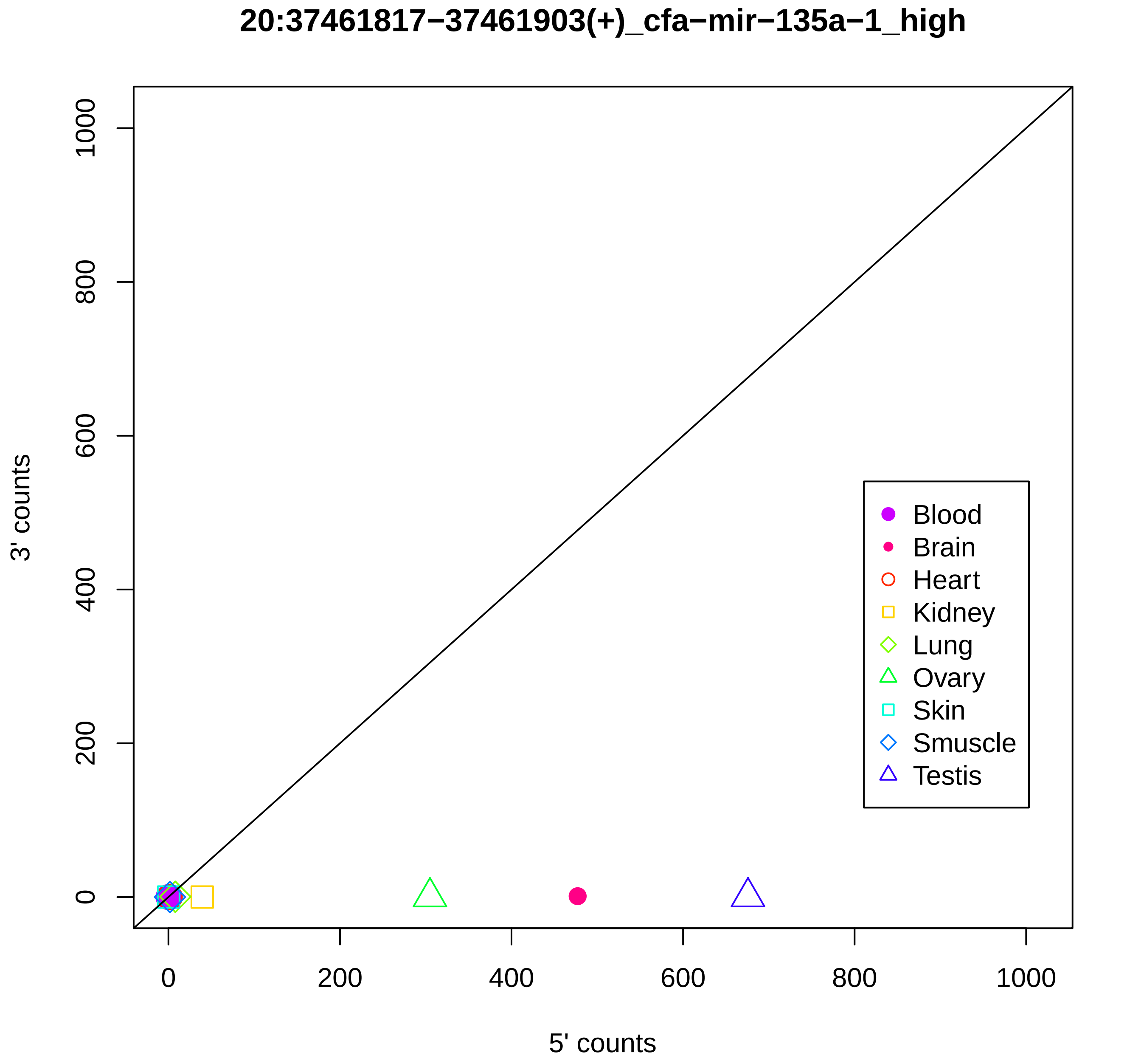

20:37461817−37461903(+)_cfa−mir−135a−1_high
3' counts
5' counts
0
200
400
600
800
1000
Blood
Brain
Heart
Kidney
Lung
Ovary
Skin
Smuscle
Testis

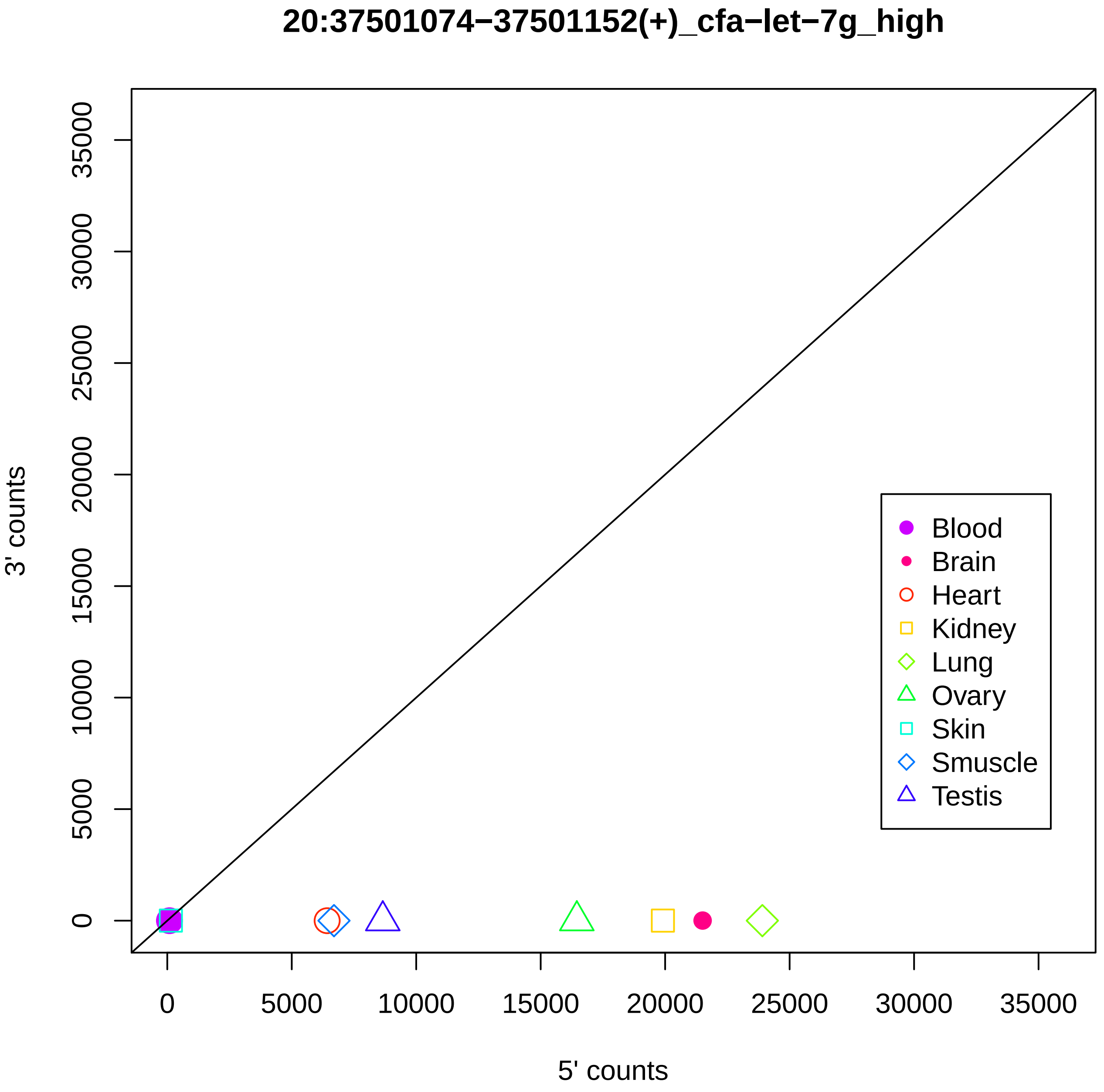

20:37501074−37501152(+)_cfa−let−7g_high
3' counts
5' counts
0
5000
10000
15000
20000
25000
30000
35000
Blood
Brain
Heart
Kidney
Lung
Ovary
Skin
Smuscle
Testis

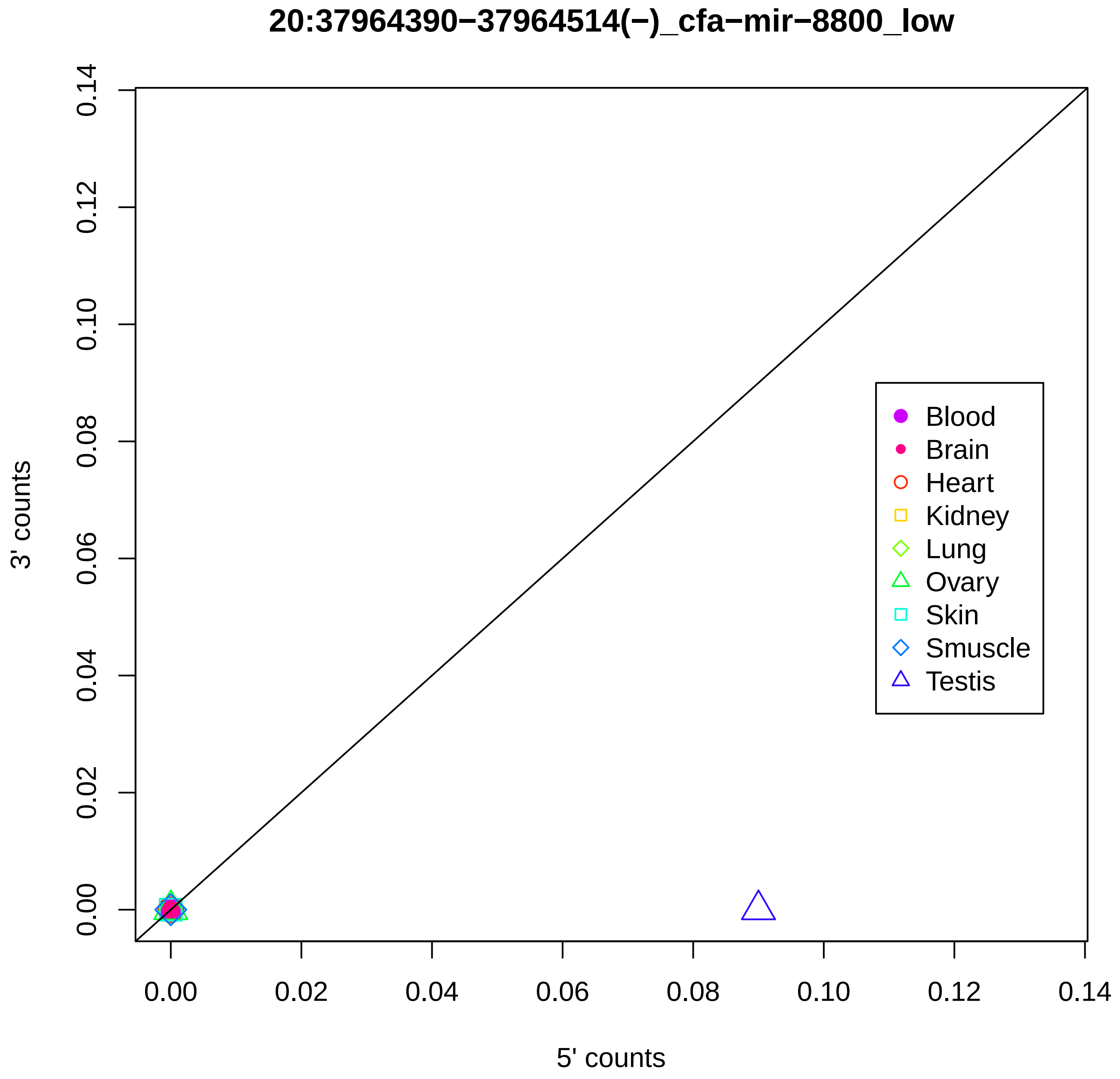

20:37964390−37964514(−)_cfa−mir−8800_low
3' counts
5' counts
0.00
0.02
0.04
0.06
0.08
0.10
0.12
0.14
Blood
Brain
Heart
Kidney
Lung
Ovary
Skin
Smuscle
Testis

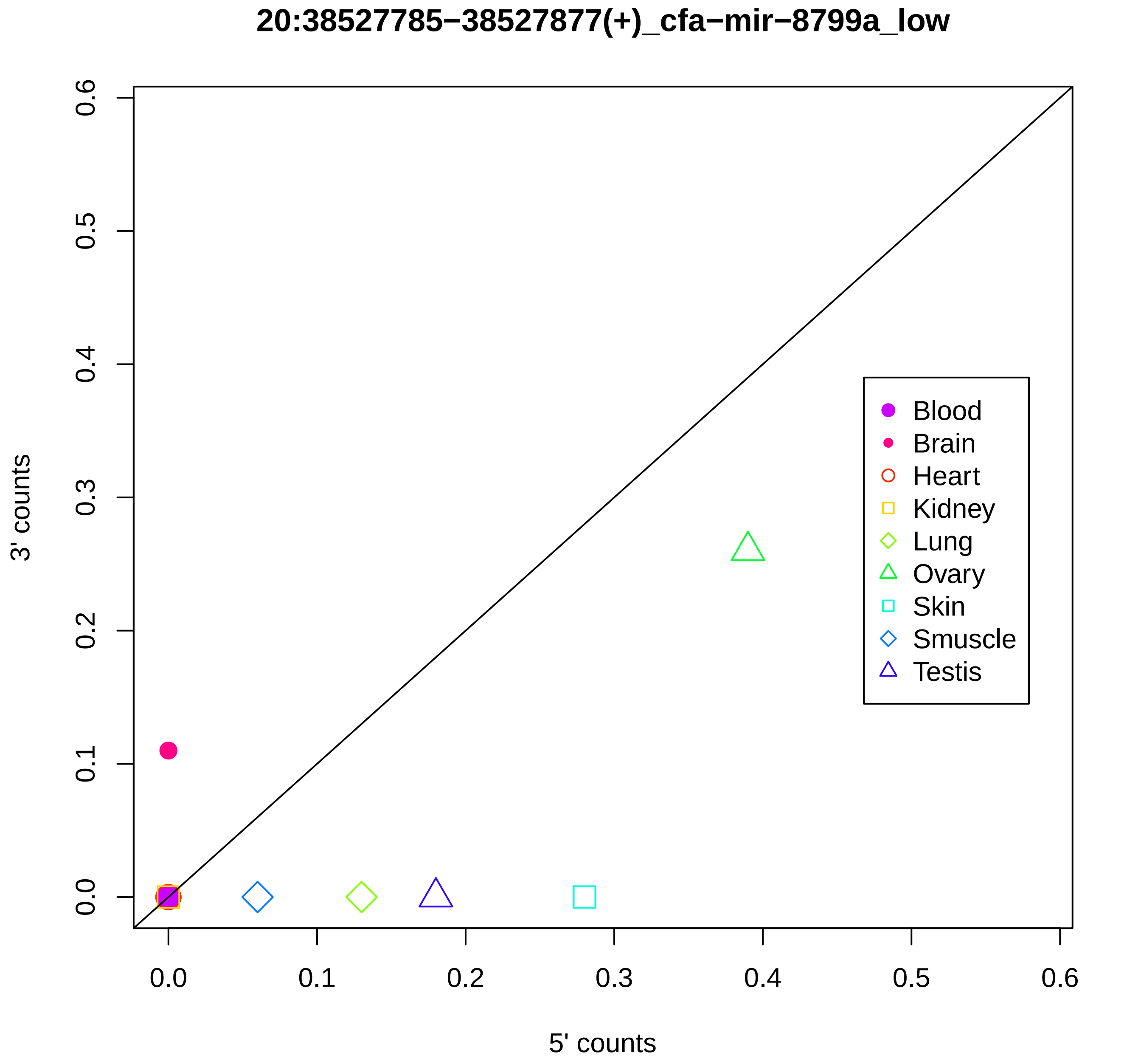

20:38527785−38527877(+)_cfa−mir−8799a_low
3' counts
5' counts
0.0
0.1
0.2
0.3
0.4
0.5
0.6
Blood
Brain
Heart
Kidney
Lung
Ovary
Skin
Smuscle
Testis

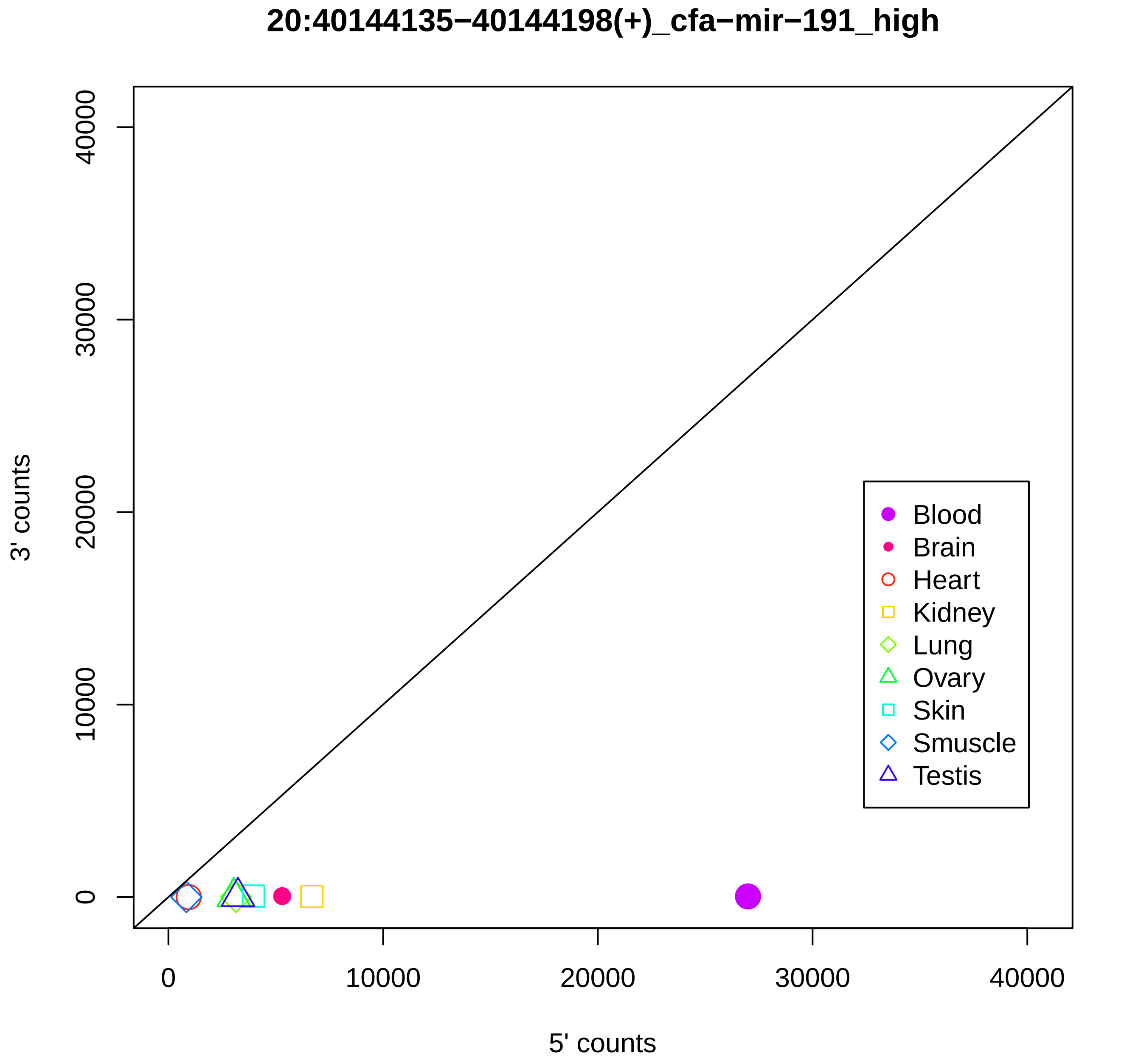
20:40144135−40144198(+)_cfa−mir−191_high
3' counts
5' counts
0
10000
20000
30000
40000
Blood
Brain
Heart
Kidney
Lung
Ovary
Skin
Smuscle
Testis

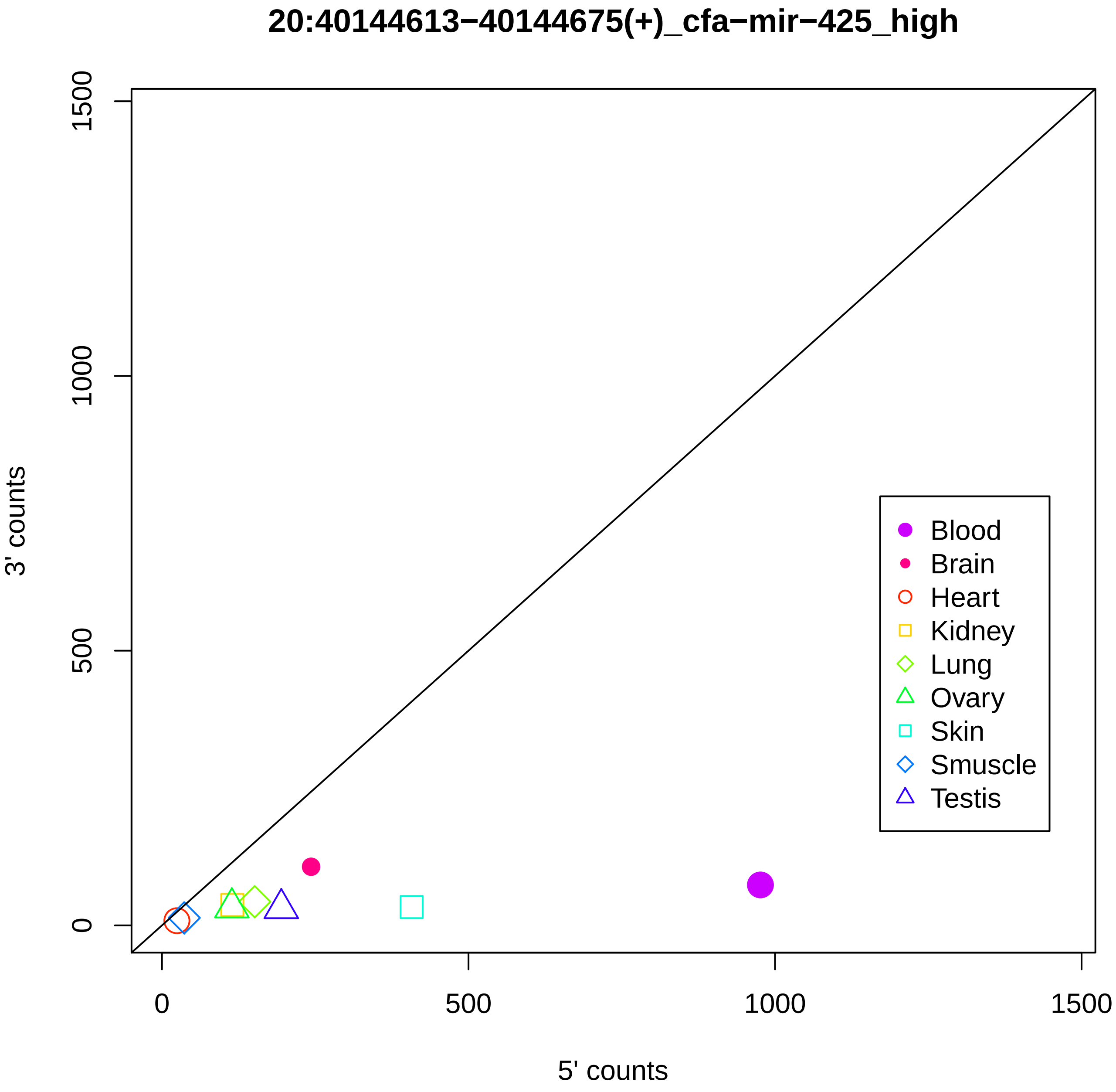
20:40144613−40144675(+)_cfa−mir−425_high
3' counts
5' counts
0
500
1000
1500
Blood
Brain
Heart
Kidney
Lung
Ovary
Skin
Smuscle
Testis

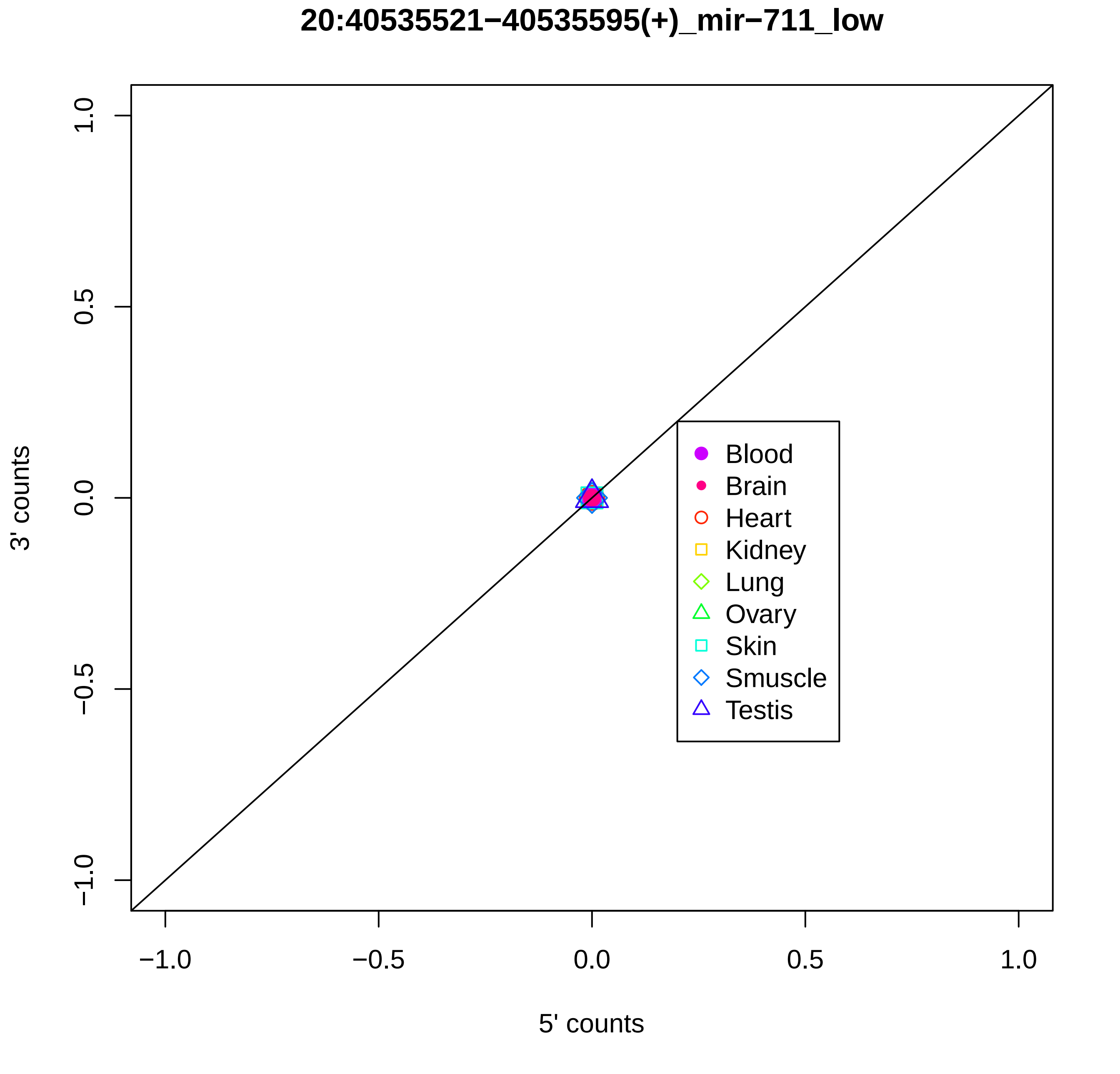

20:40535521−40535595(+)_mir−711_low
3' counts
5' counts
−1.0
−0.5
0.0
0.5
1.0
Blood
Brain
Heart
Kidney
Lung
Ovary
Skin
Smuscle
Testis

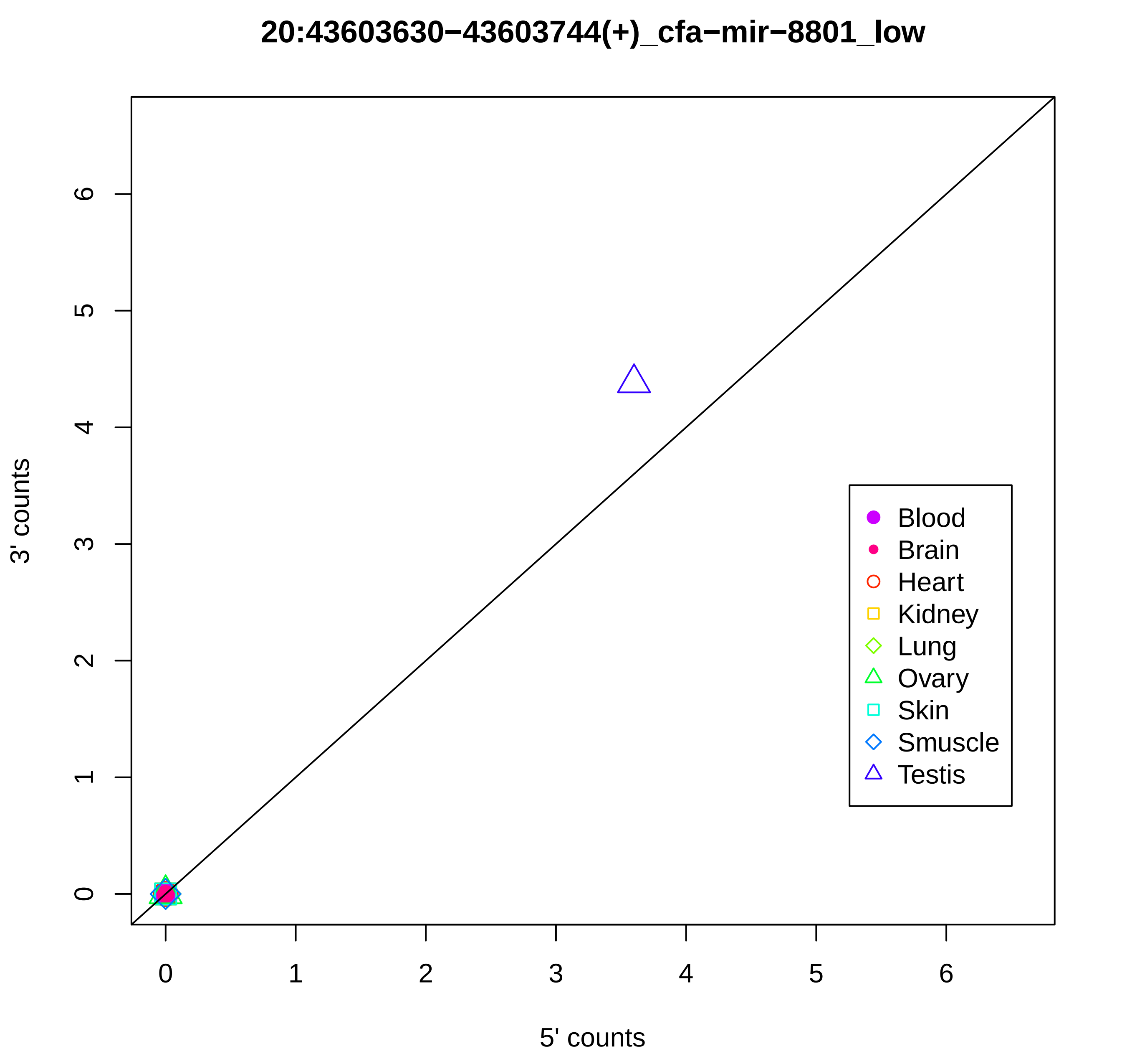

20:43603630−43603744(+)_cfa−mir−8801_low
3' counts
5' counts
0
1
2
3
4
5
6
Blood
Brain
Heart
Kidney
Lung
Ovary
Skin
Smuscle
Testis

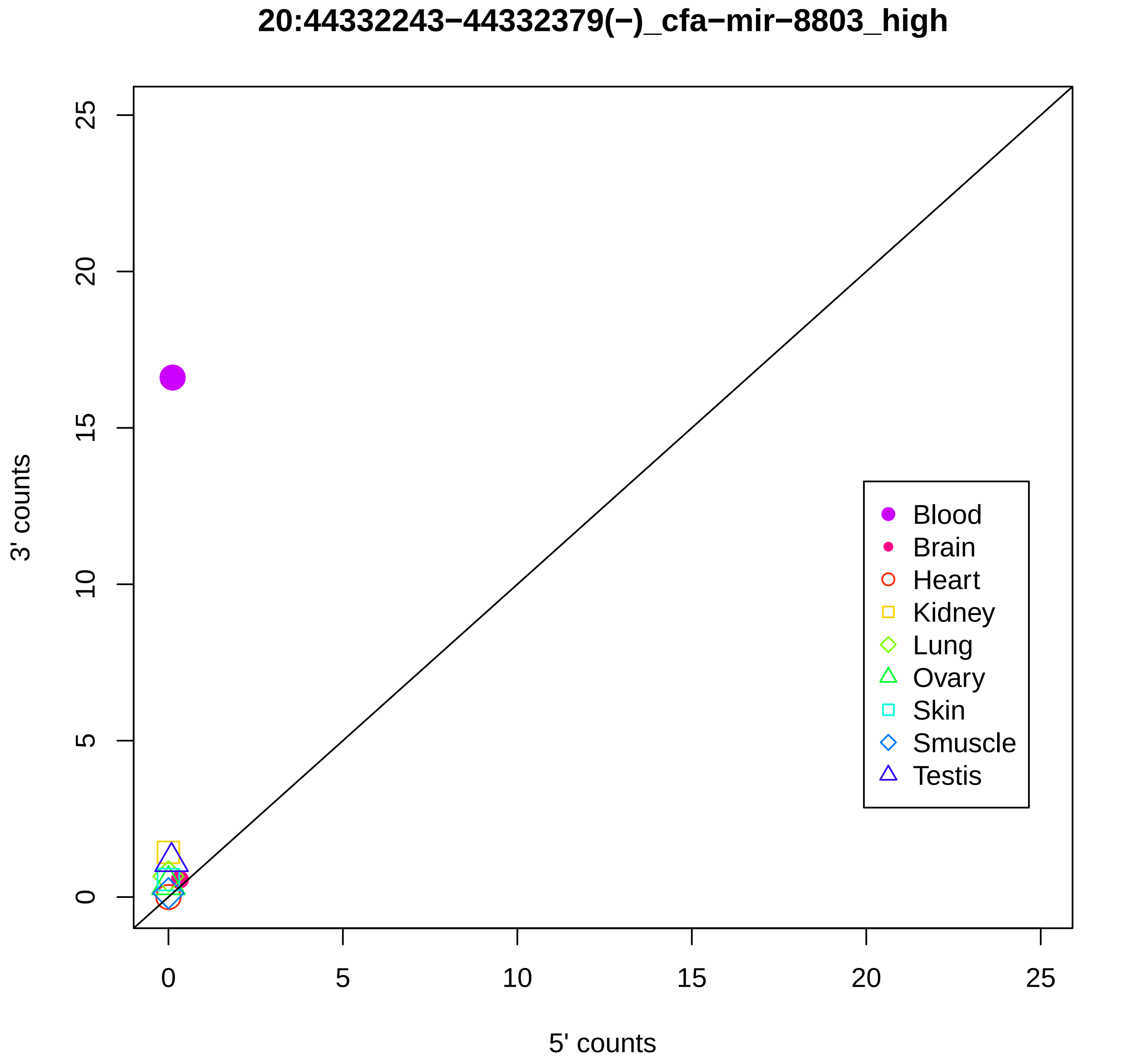
20:44332243−44332379(−)_cfa−mir−8803_high
3' counts
5' counts
0
5
10
15
20
25
Blood
Brain
Heart
Kidney
Lung
Ovary
Skin
Smuscle
Testis

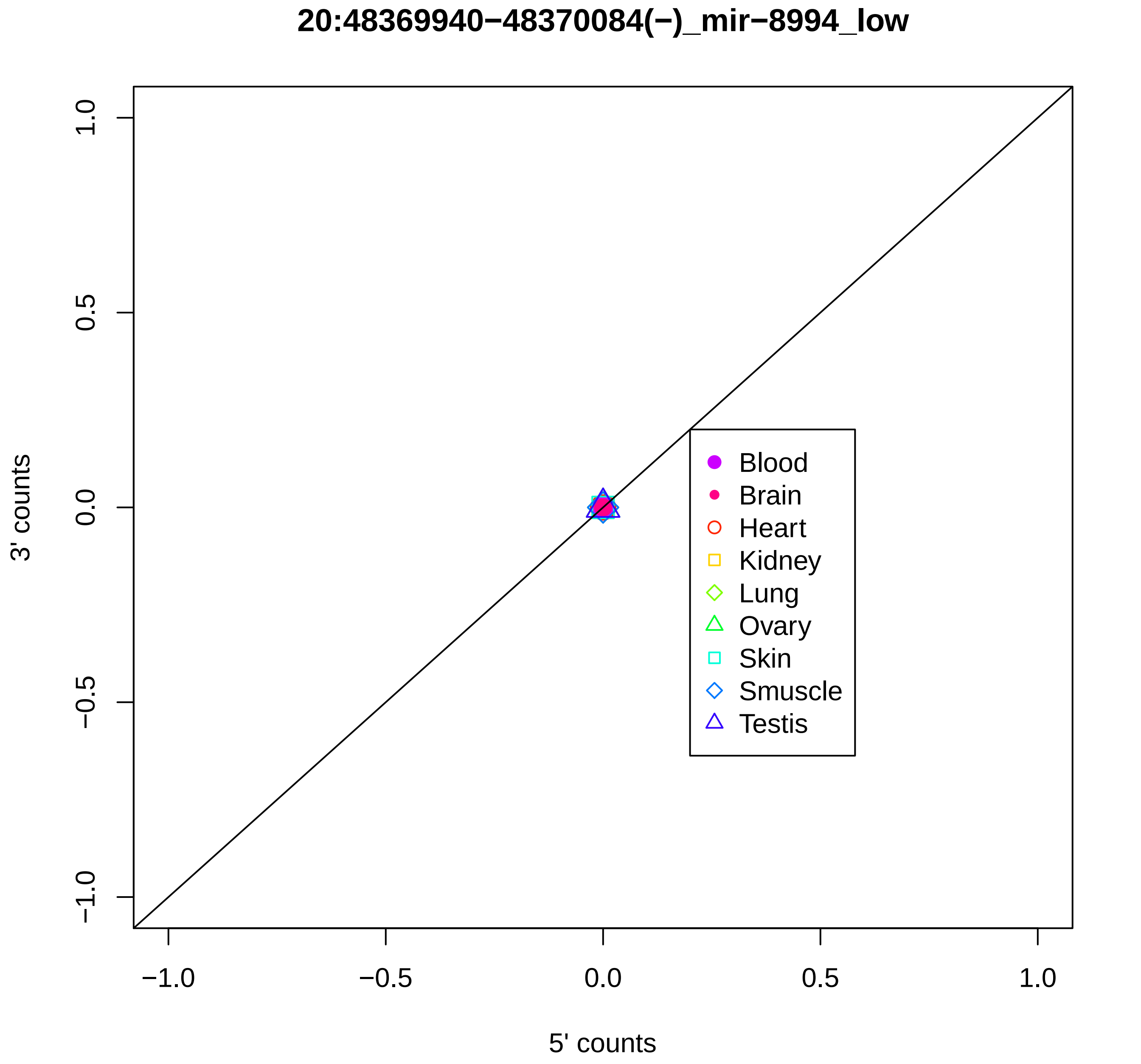

20:48369940−48370084(−)_mir−8994_low
3' counts
5' counts
1.0
0.5
0.0
−0.5
−1.0
Blood
Brain
Heart
Kidney
Lung
Ovary
Skin
Smuscle
Testis

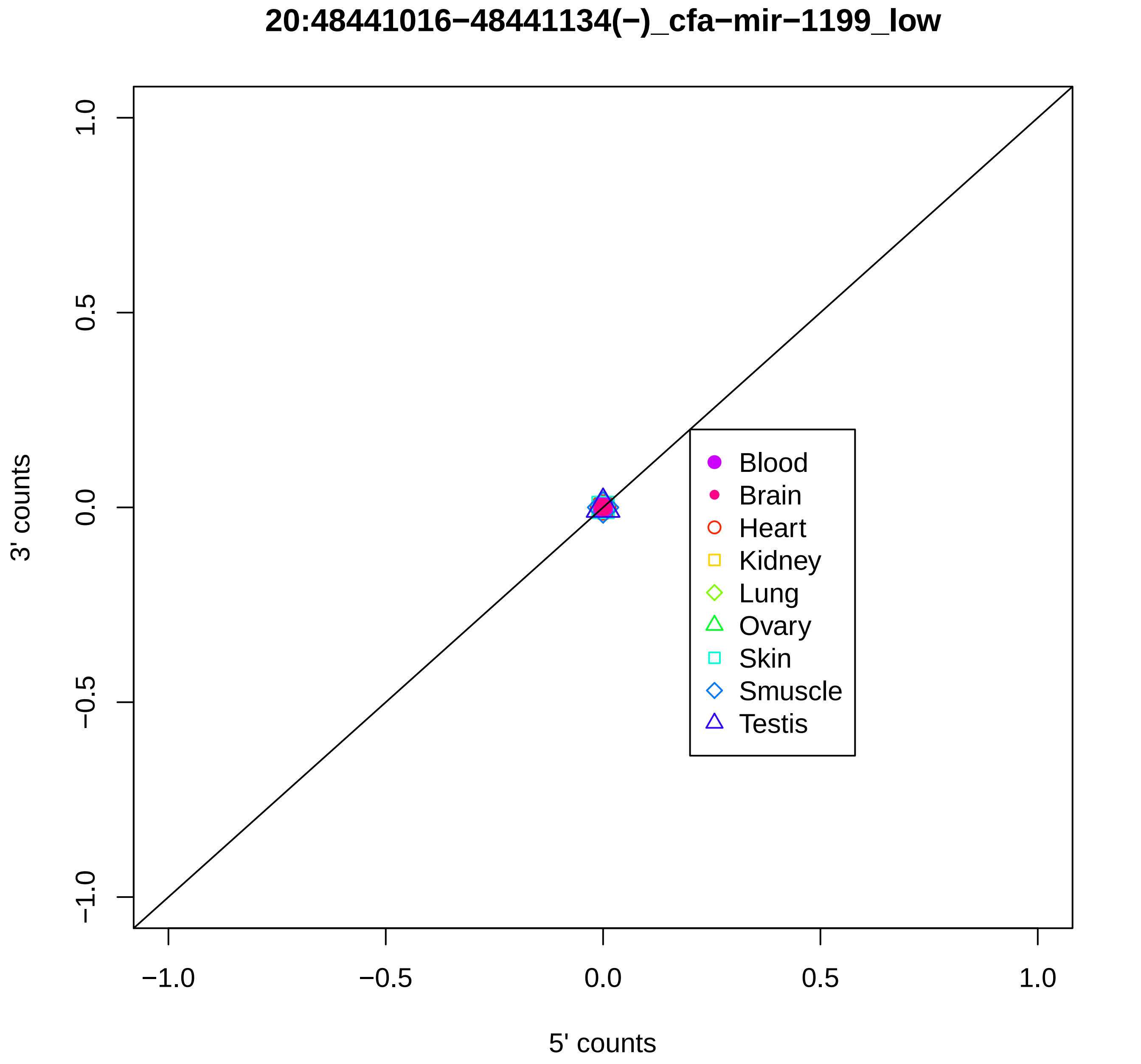
20:48441016−48441134(−)_cfa−mir−1199_low
3' counts
5' counts
−1.0
−0.5
0.0
0.5
1.0
Blood
Brain
Heart
Kidney
Lung
Ovary
Skin
Smuscle
Testis

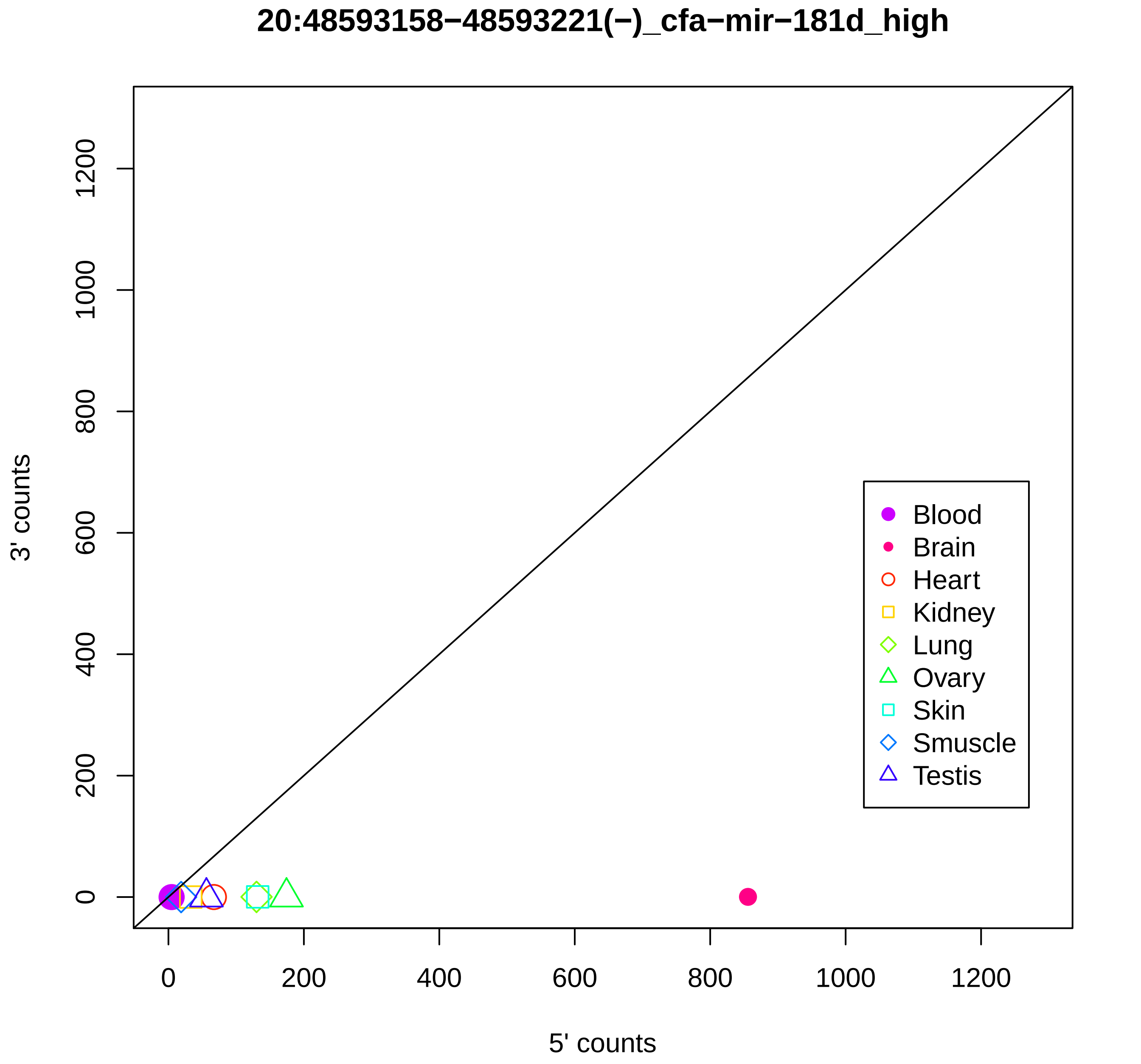

20:48593158−48593221(−)_cfa−mir−181d_high
3' counts
5' counts
0
200
400
600
800
1000
1200
Blood
Brain
Heart
Kidney
Lung
Ovary
Skin
Smuscle
Testis

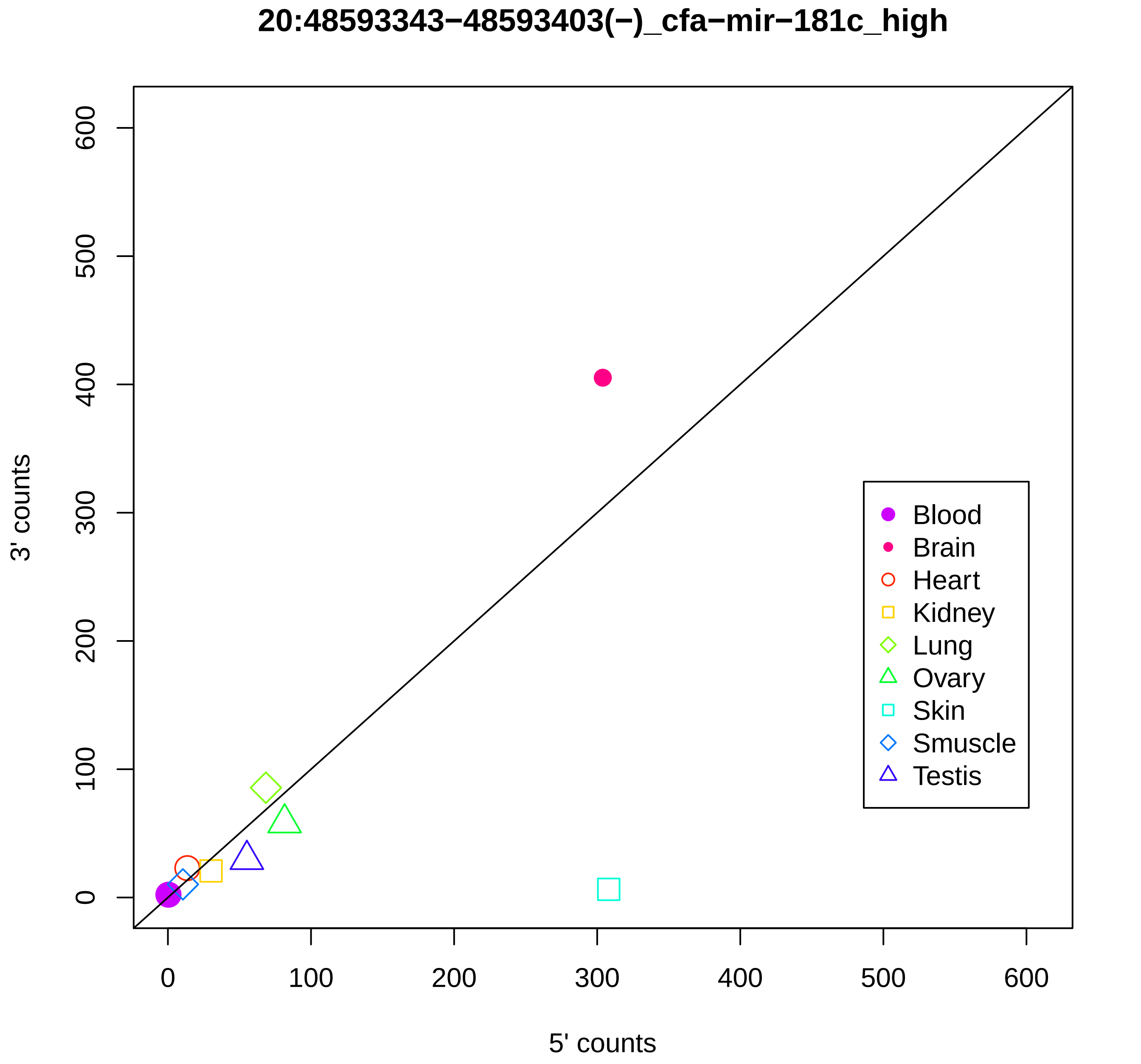

20:48593343−48593403(−)_cfa−mir−181c_high
3' counts
5' counts
0
100
200
300
400
500
600
Blood
Brain
Heart
Kidney
Lung
Ovary
Skin
Smuscle
Testis

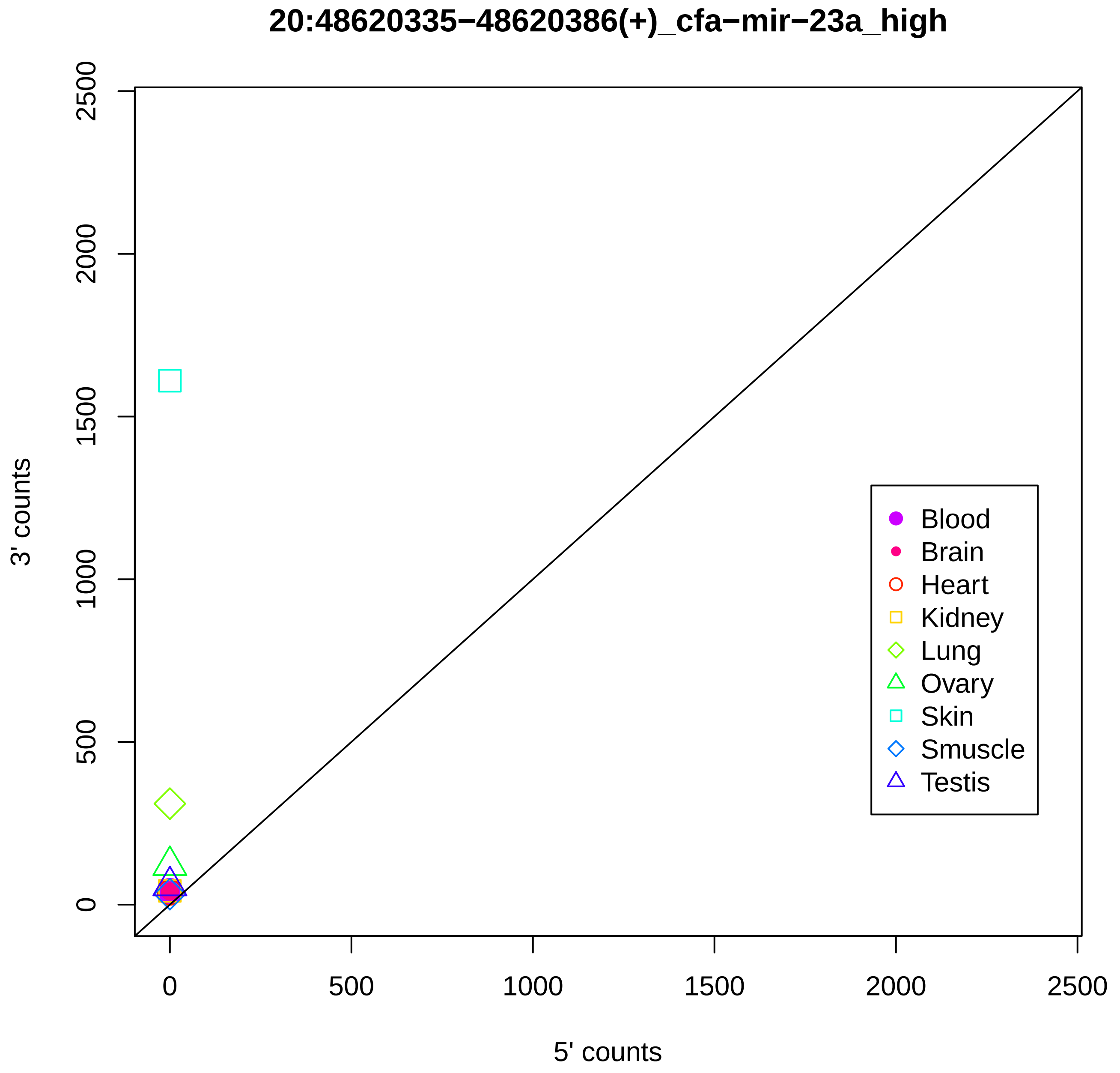

20:48620335−48620386(+)_cfa−mir−23a_high
3' counts
5' counts
0
500
1000
1500
2000
2500
Blood
Brain
Heart
Kidney
Lung
Ovary
Skin
Smuscle
Testis

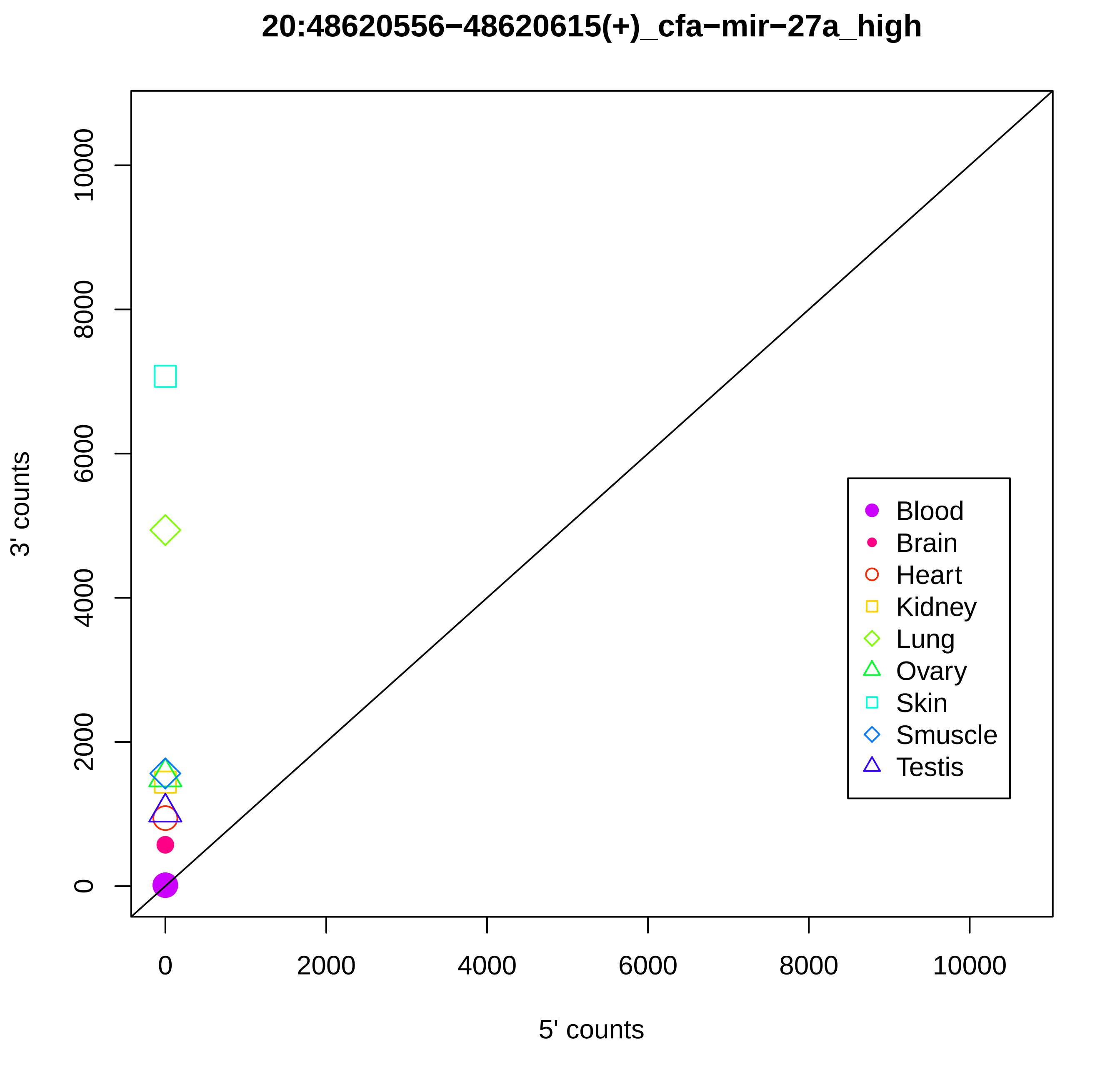

20:48620556−48620615(+)_cfa−mir−27a_high
3' counts
5' counts
0
2000
4000
6000
8000
10000
Blood
Brain
Heart
Kidney
Lung
Ovary
Skin
Smuscle
Testis

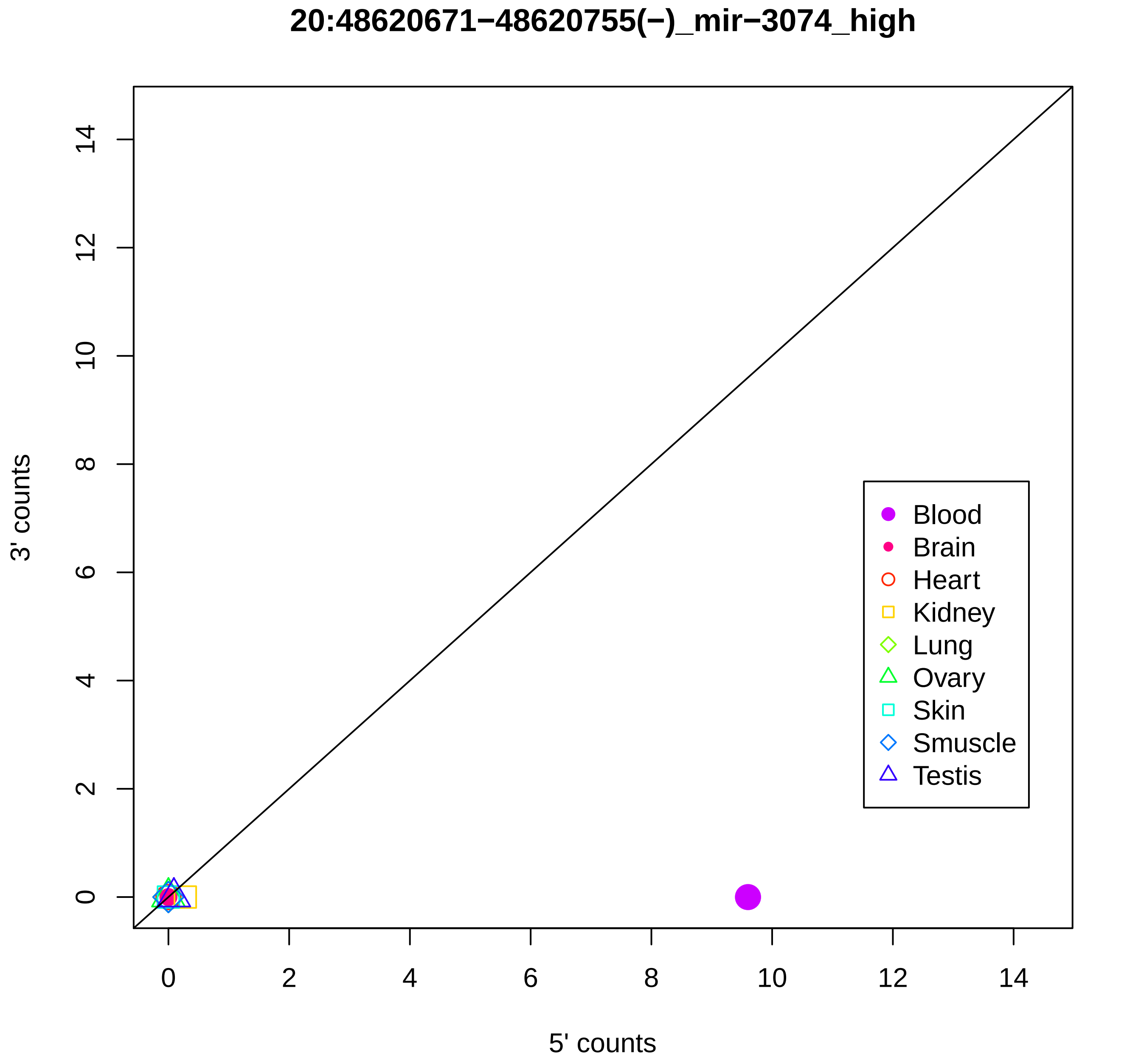
20:48620671−48620755(−)_mir−3074_high
3' counts
5' counts
0
2
4
6
8
10
12
14
Blood
Brain
Heart
Kidney
Lung
Ovary
Skin
Smuscle
Testis

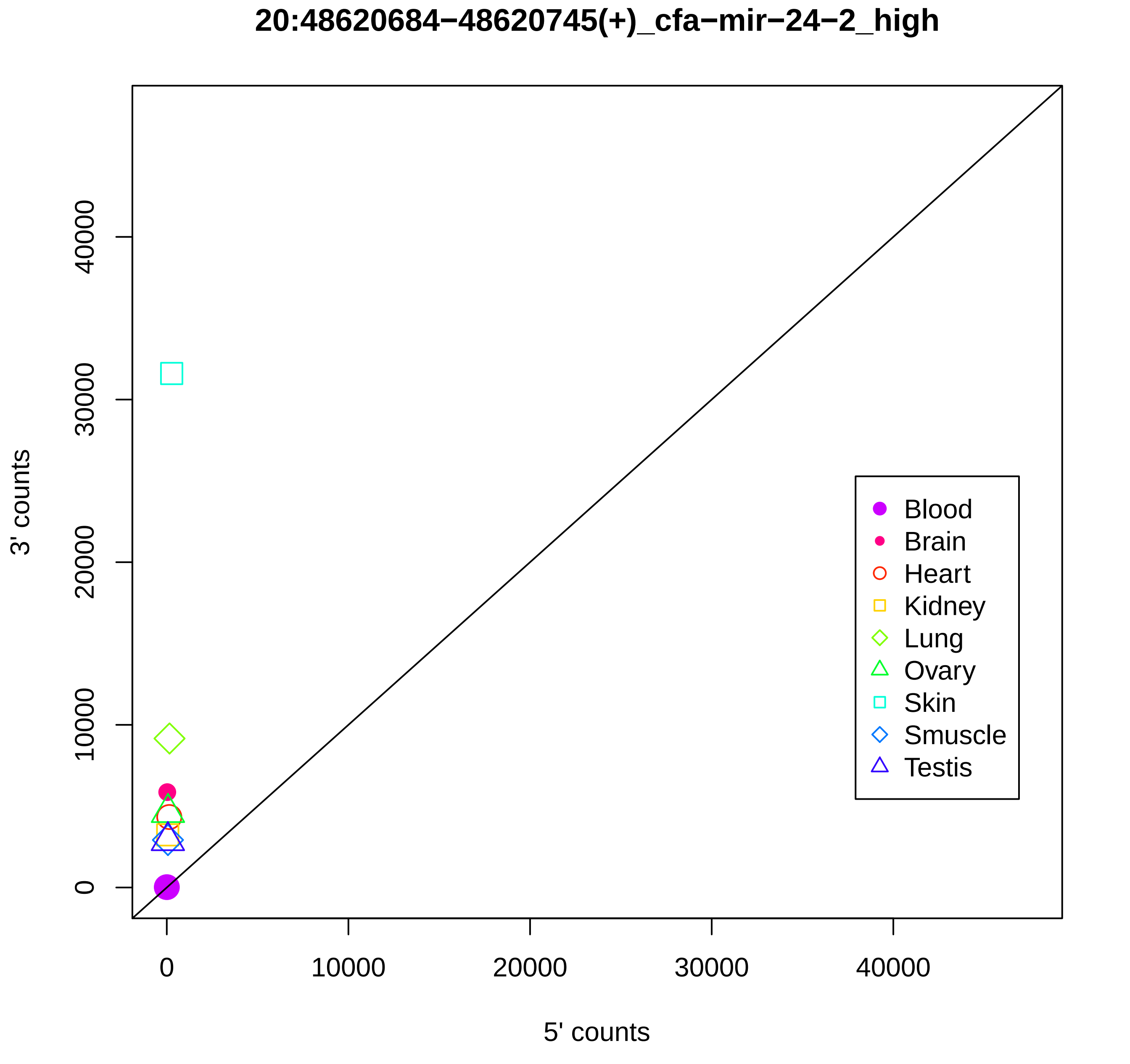

20:48620684−48620745(+)_cfa−mir−24−2_high
3' counts
5' counts
0
10000
20000
30000
40000
Blood
Brain
Heart
Kidney
Lung
Ovary
Skin
Smuscle
Testis

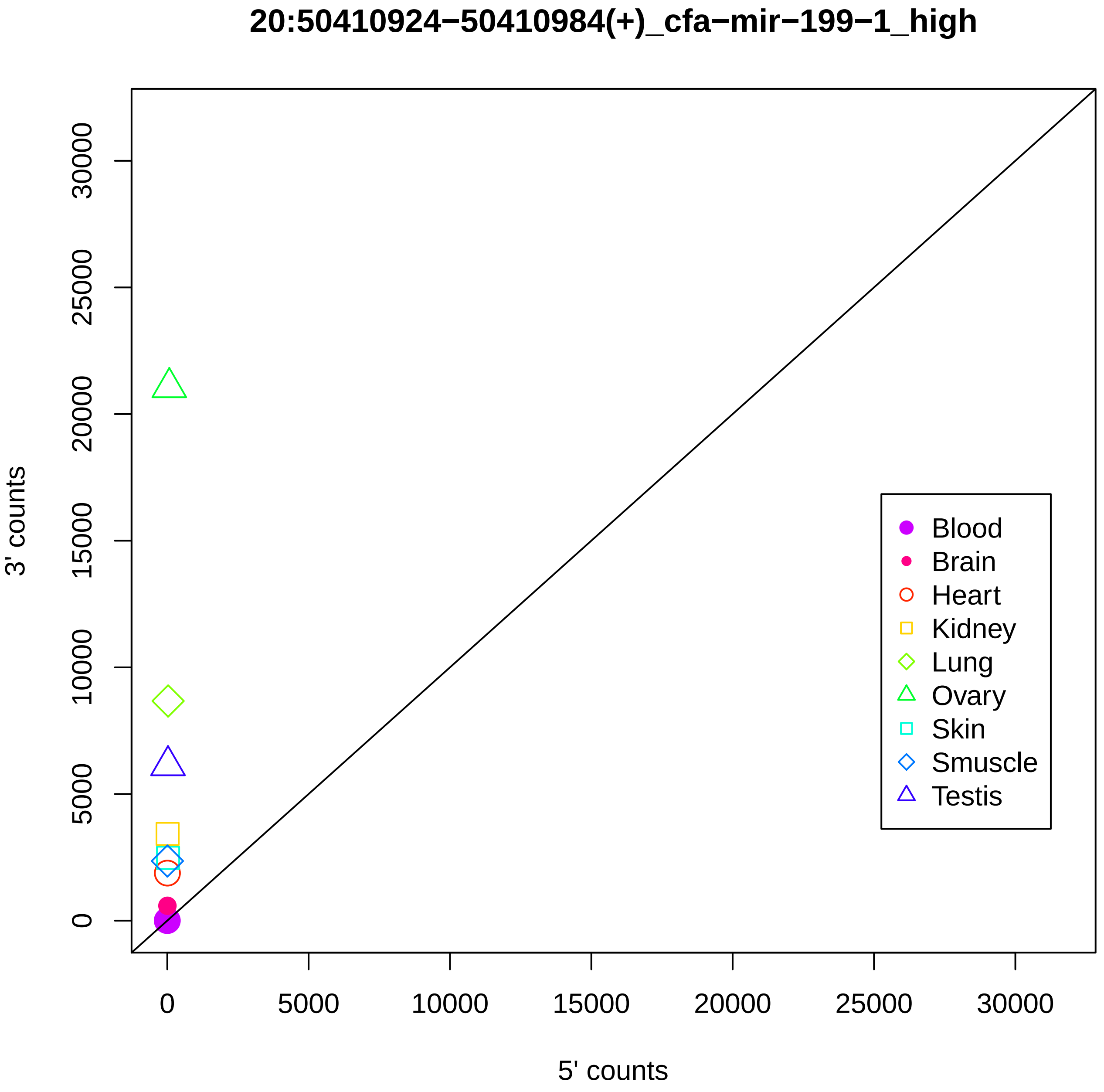

20:50410924−50410984(+)_cfa−mir−199−1_high
3' counts
5' counts
0
5000
10000
15000
20000
25000
30000
Blood
Brain
Heart
Kidney
Lung
Ovary
Skin
Smuscle
Testis

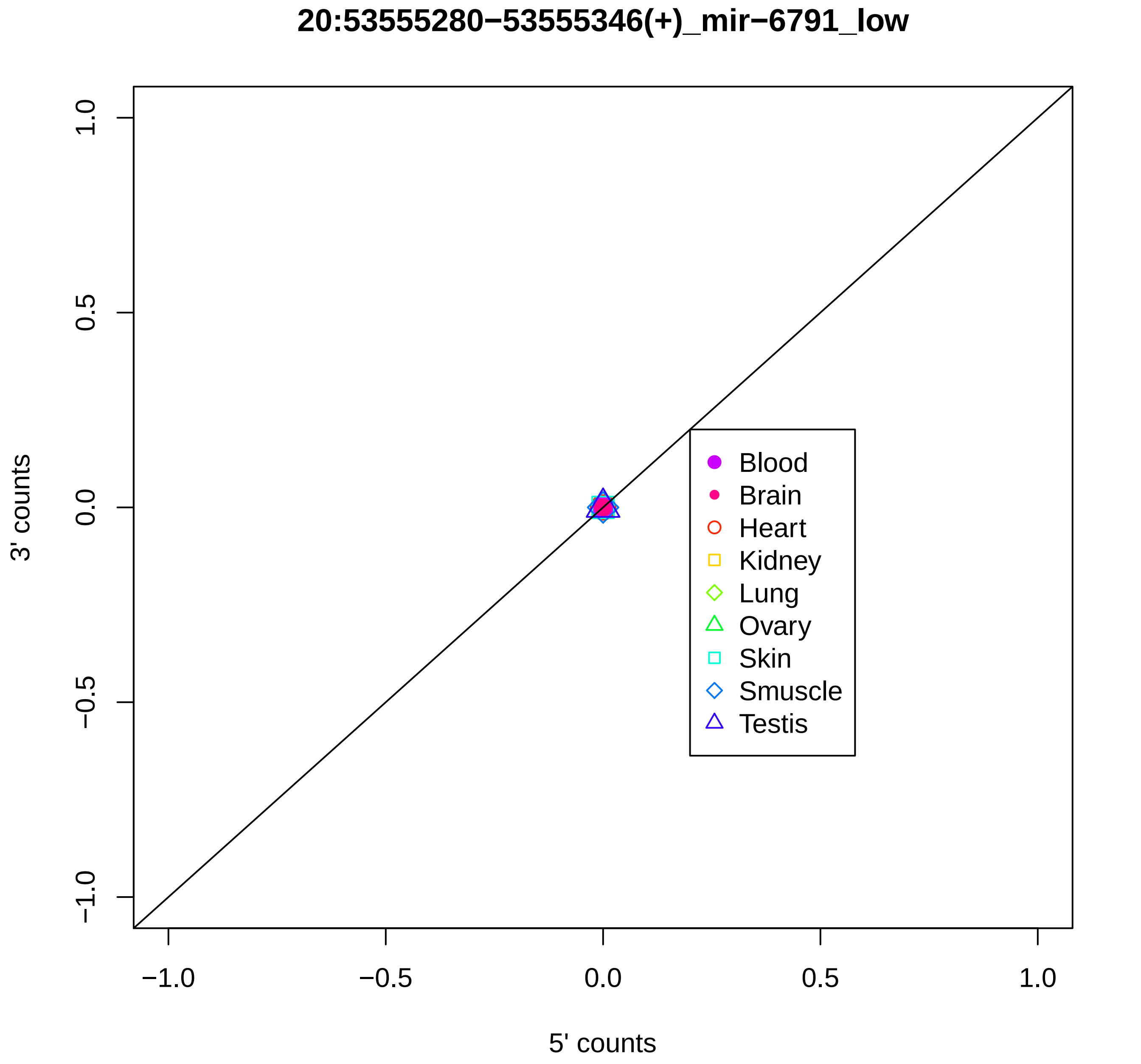

20:53555280−53555346(+)_mir−6791_low
3' counts
5' counts
1.0
0.5
0.0
−0.5
−1.0
Blood
Brain
Heart
Kidney
Lung
Ovary
Skin
Smuscle
Testis

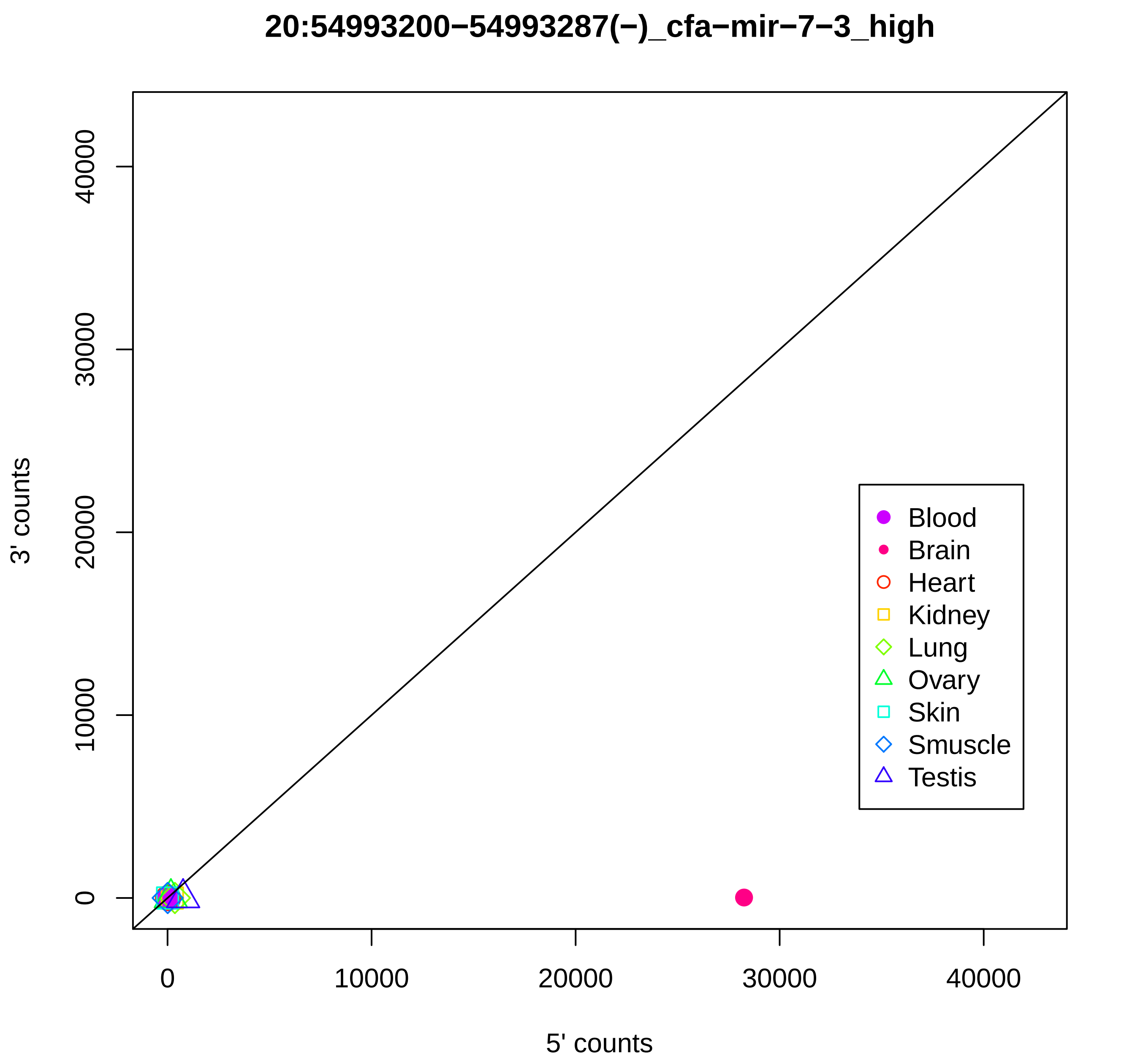
20:54993200−54993287(−)_cfa−mir−7−3_high
3' counts
0
10000
20000
30000
40000
5' counts
0
10000
20000
30000
40000
Blood
Brain
Heart
Kidney
Lung
Ovary
Skin
Smuscle
Testis

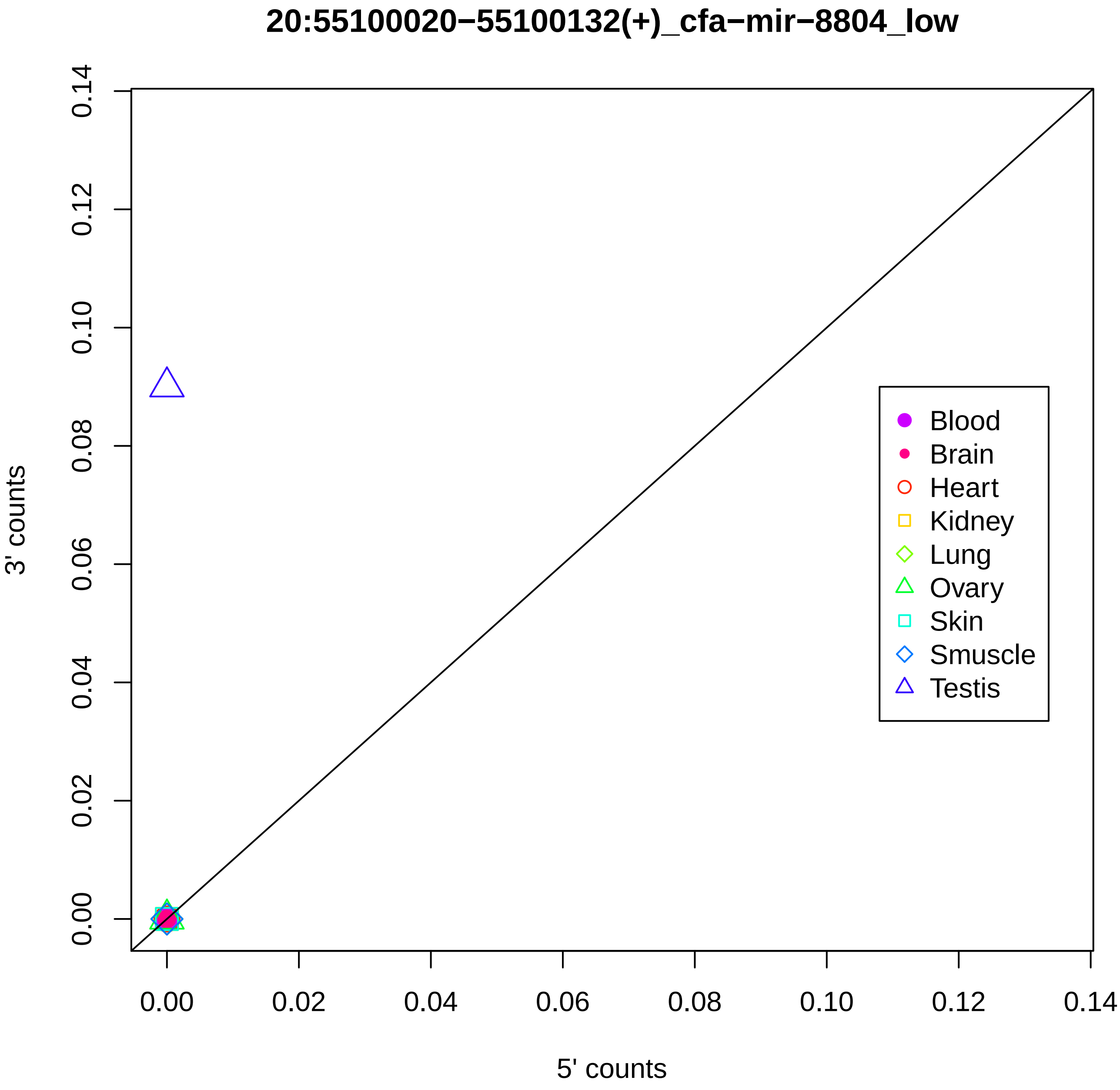

20:55100020−55100132(+)_cfa−mir−8804_low
3' counts
5' counts
0.00
0.02
0.04
0.06
0.08
0.10
0.12
0.14
Blood
Brain
Heart
Kidney
Lung
Ovary
Skin
Smuscle
Testis

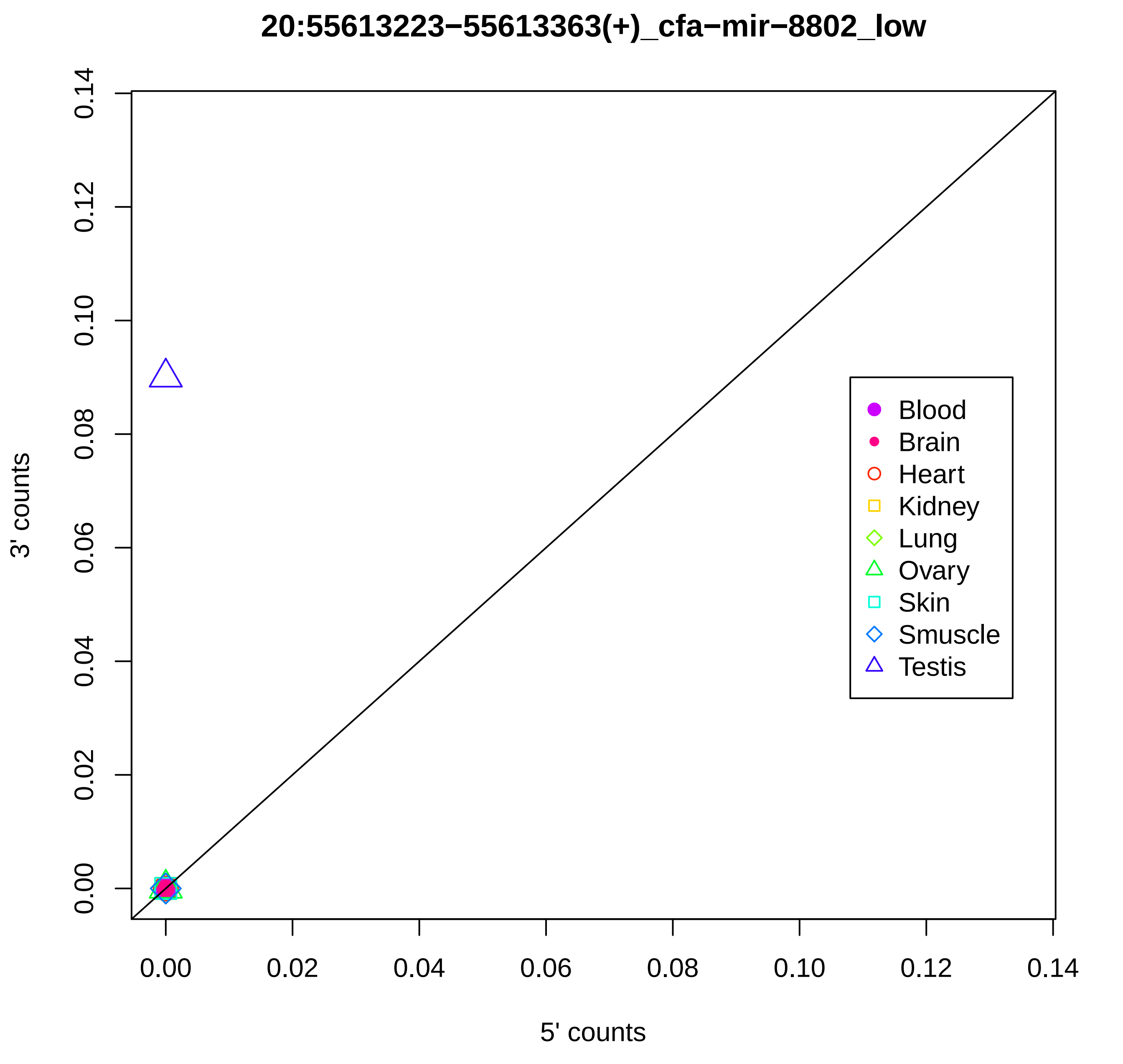

20:55613223−55613363(+)_cfa−mir−8802_low
3' counts
5' counts
0.00
0.02
0.04
0.06
0.08
0.10
0.12
0.14
Blood
Brain
Heart
Kidney
Lung
Ovary
Skin
Smuscle
Testis

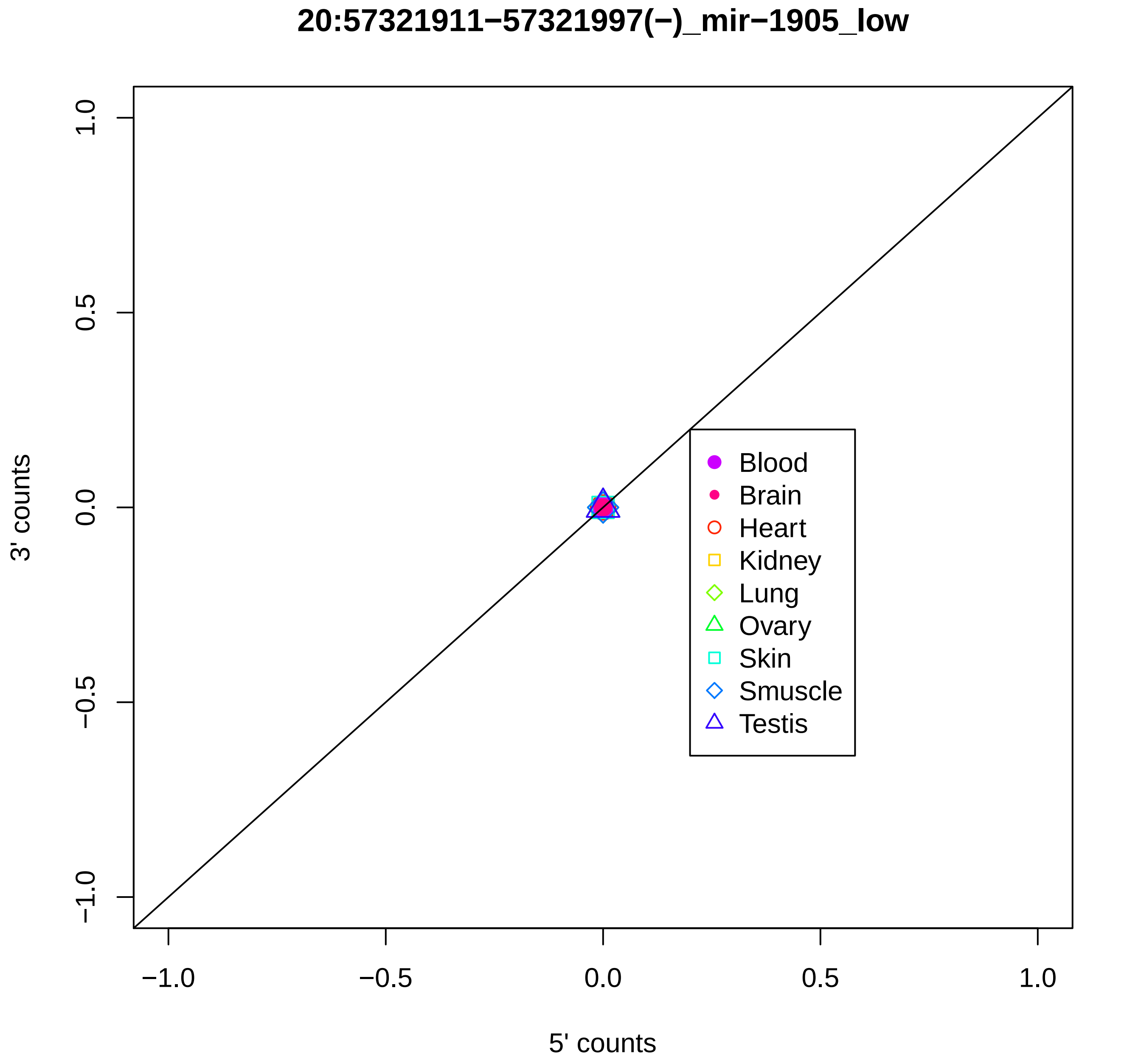
20:57321911−57321997(−)_mir−1905_low
Blood
Brain
Heart
Kidney
Lung
Ovary
Skin
Smuscle
Testis
1.0
0.5
0.0
−0.5
−1.0
3' counts
−1.0
−0.5
0.0
0.5
1.0
5' counts

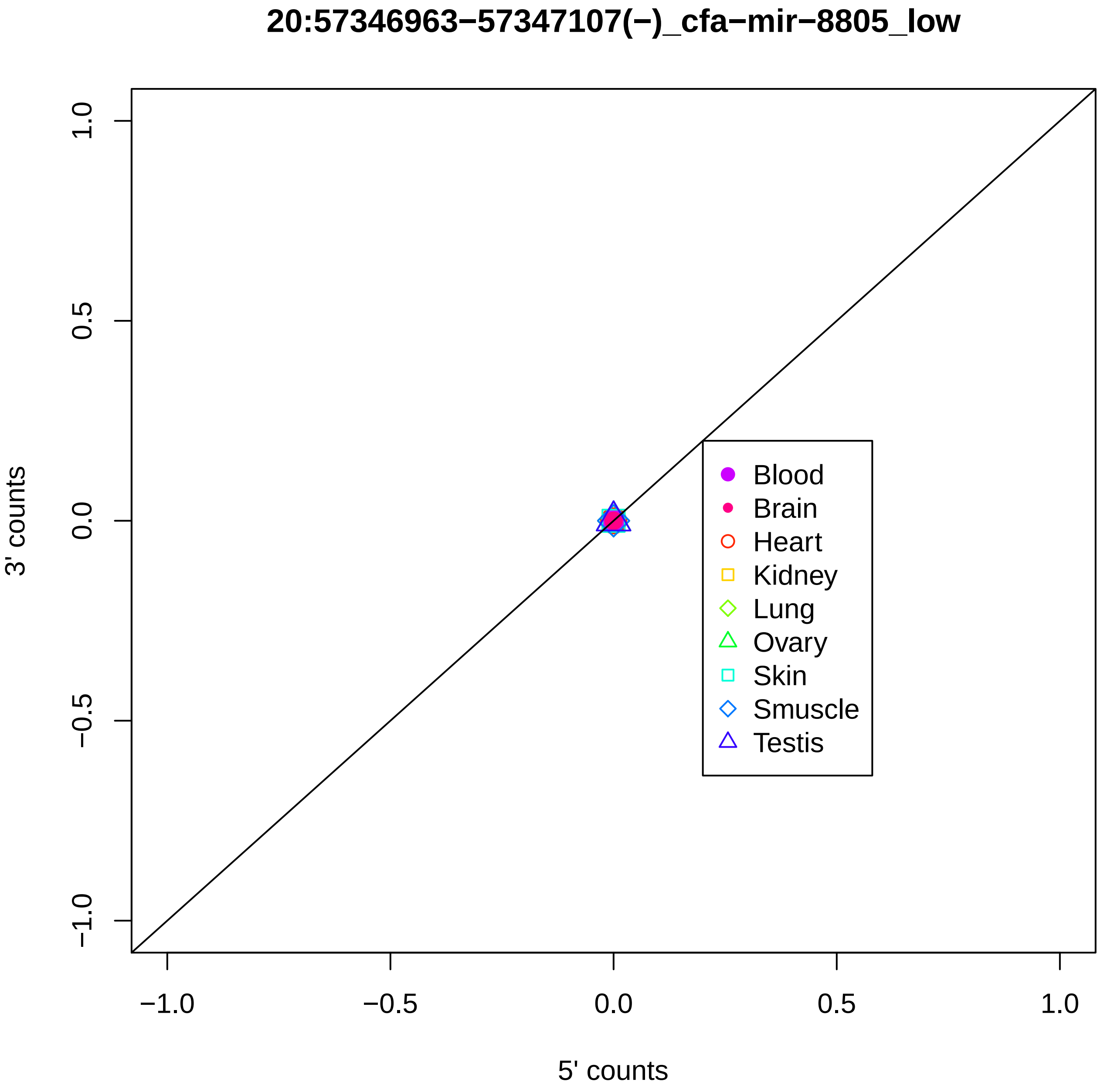
20:57346963−57347107(−)_cfa−mir−8805_low
3' counts
5' counts
1.0
0.5
0.0
−0.5
−1.0
Blood
Brain
Heart
Kidney
Lung
Ovary
Skin
Smuscle
Testis

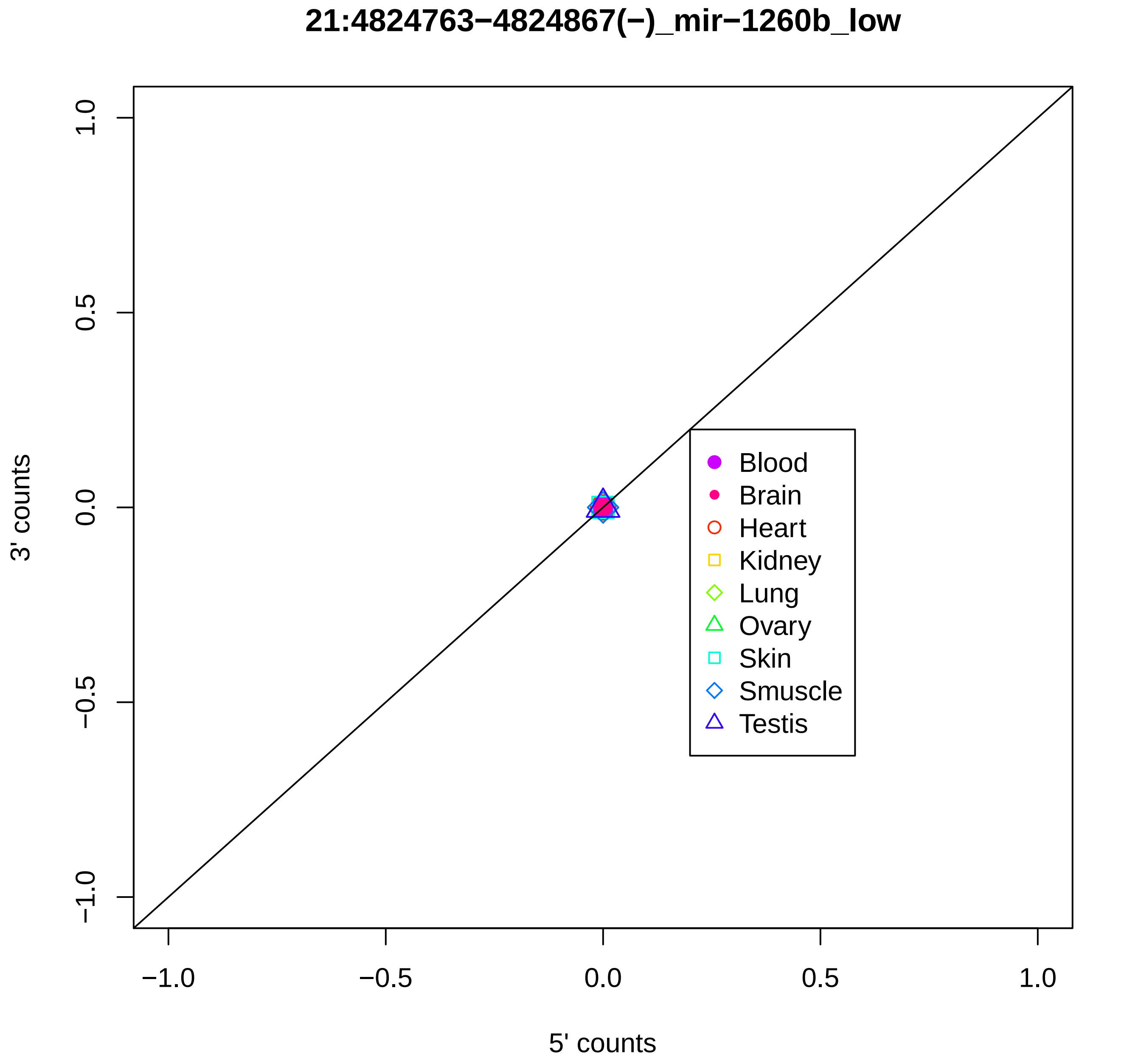

21:4824763−4824867(−)_mir−1260b_low
3' counts
5' counts
1.0
0.5
0.0
−0.5
−1.0
Blood
Brain
Heart
Kidney
Lung
Ovary
Skin
Smuscle
Testis

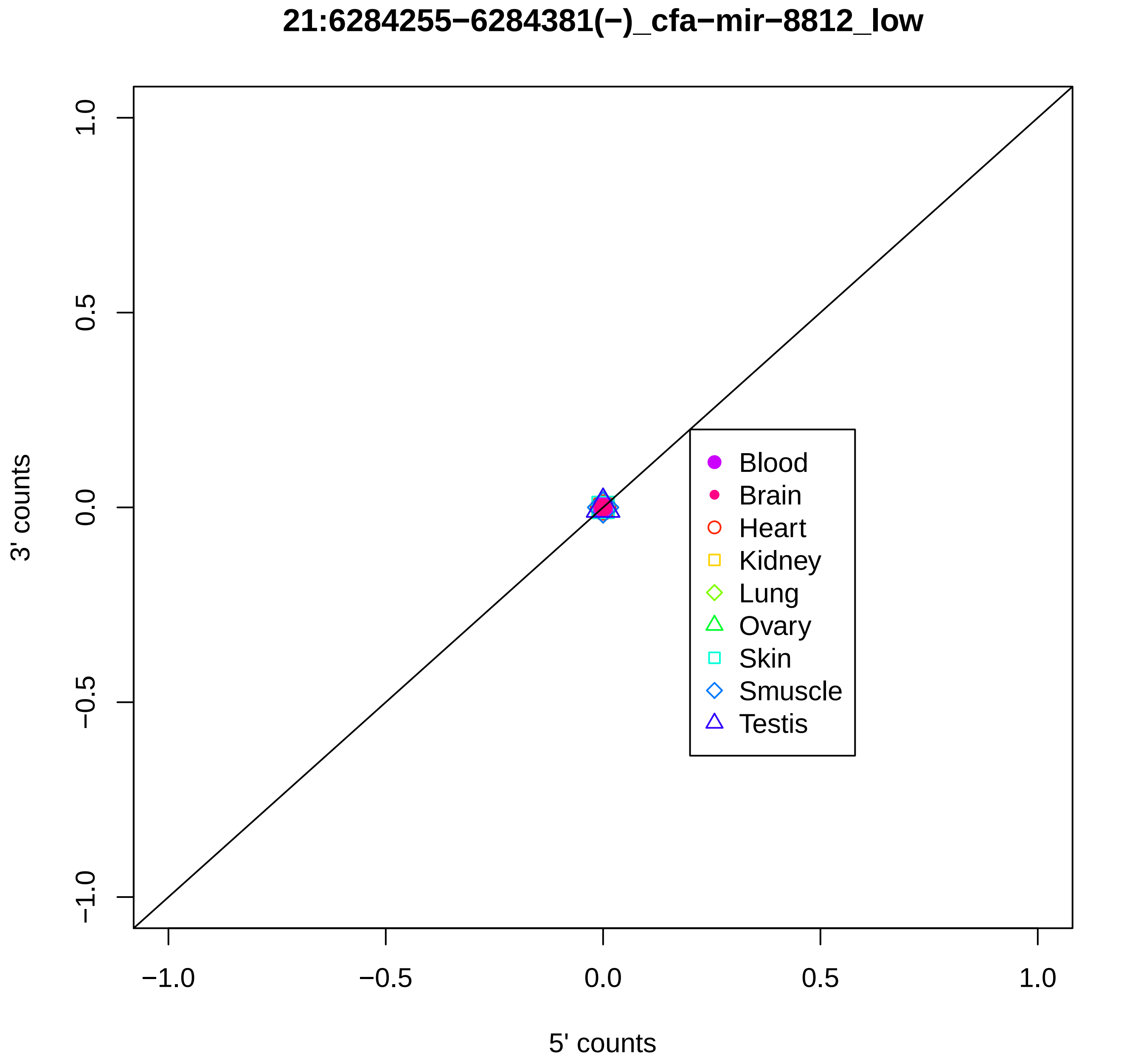

21:6284255−6284381(−)_cfa−mir−8812_low
3' counts
5' counts
−1.0
−0.5
0.0
0.5
1.0
Blood
Brain
Heart
Kidney
Lung
Ovary
Skin
Smuscle
Testis

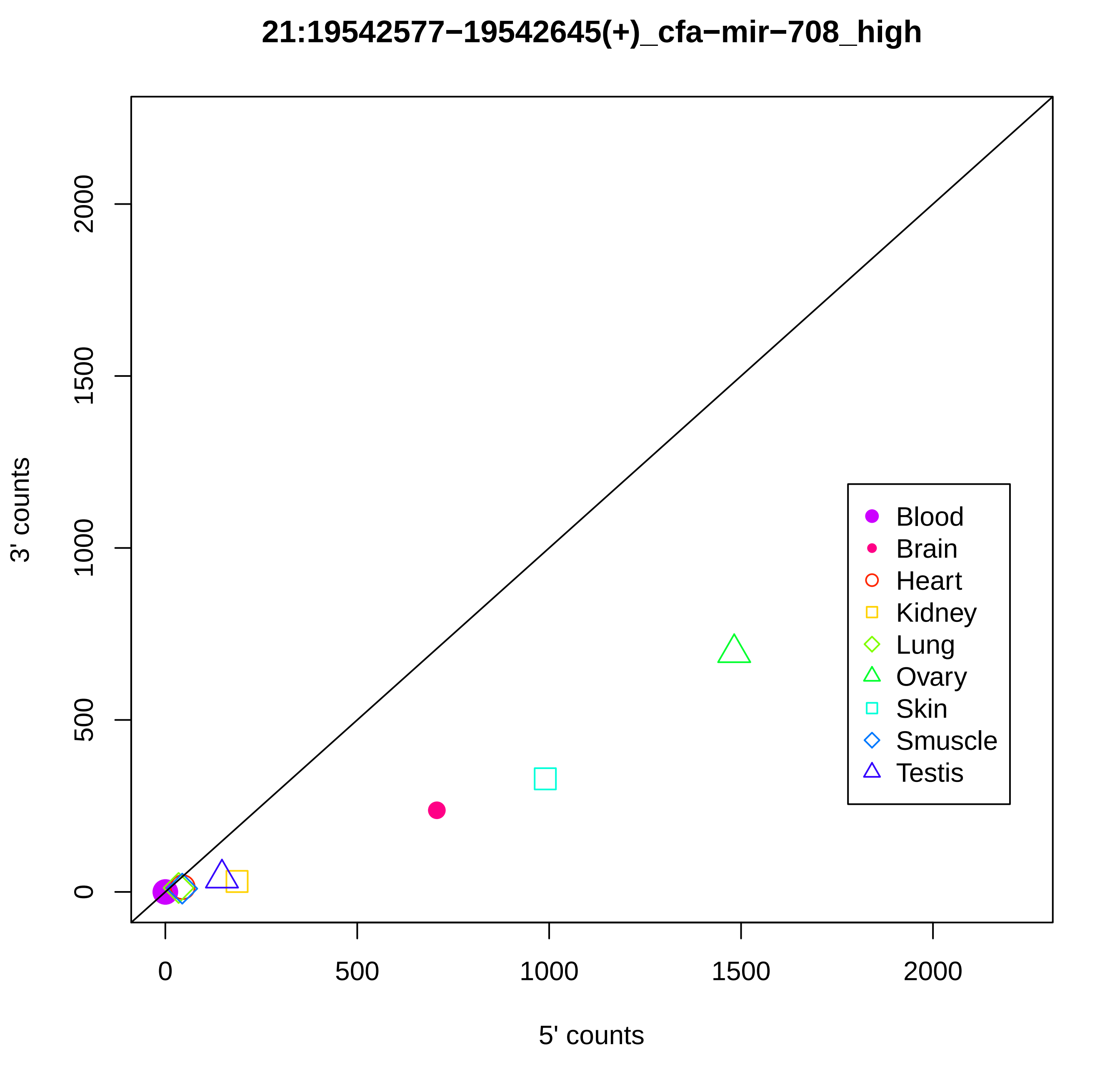

21:19542577−19542645(+)_cfa−mir−708_high
3' counts
5' counts
0
500
1000
1500
2000
0
500
1000
1500
2000
Blood
Brain
Heart
Kidney
Lung
Ovary
Skin
Smuscle
Testis

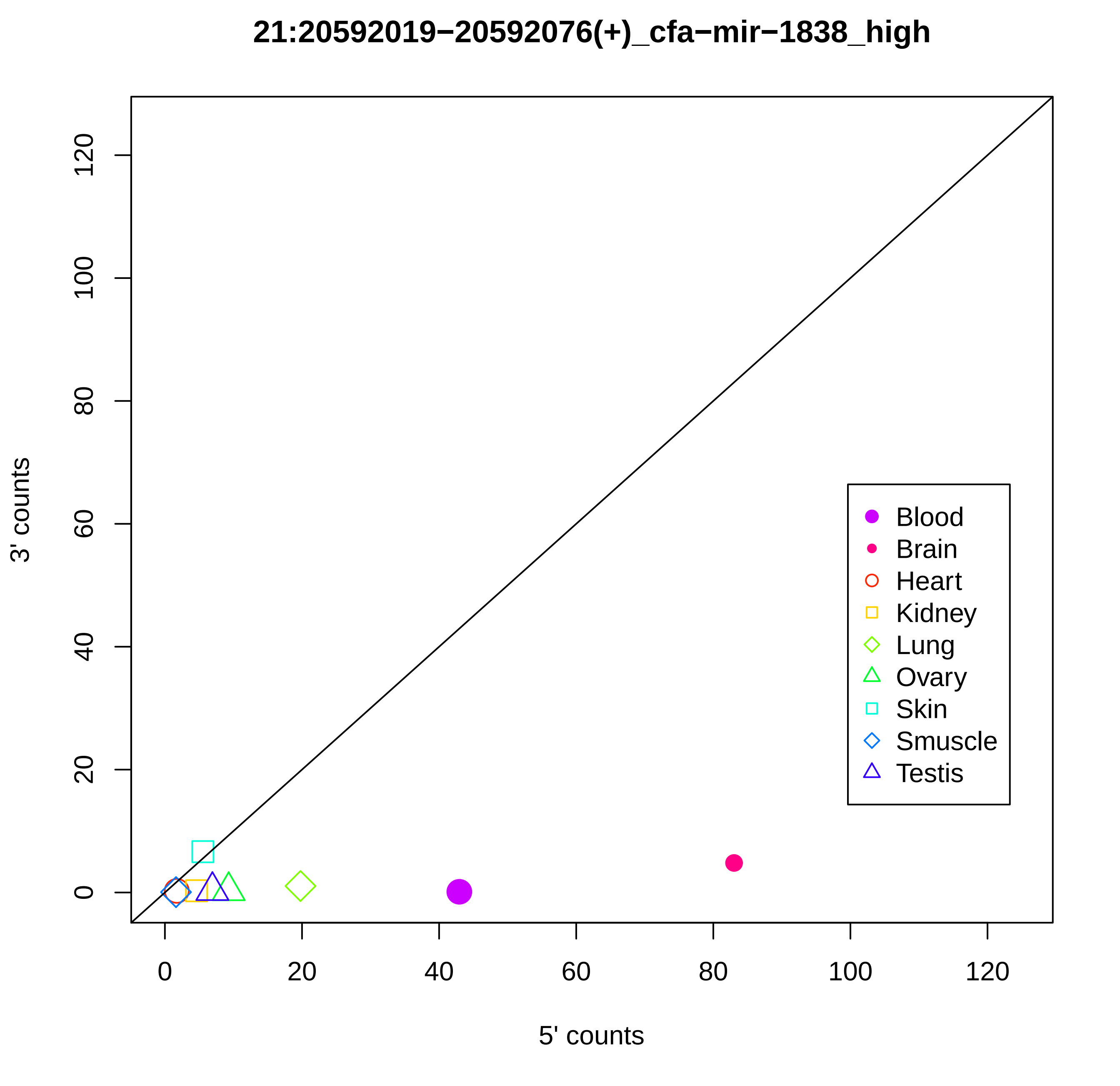

21:20592019−20592076(+)_cfa−mir−1838_high
3' counts
5' counts
0
20
40
60
80
100
120
Blood
Brain
Heart
Kidney
Lung
Ovary
Skin
Smuscle
Testis

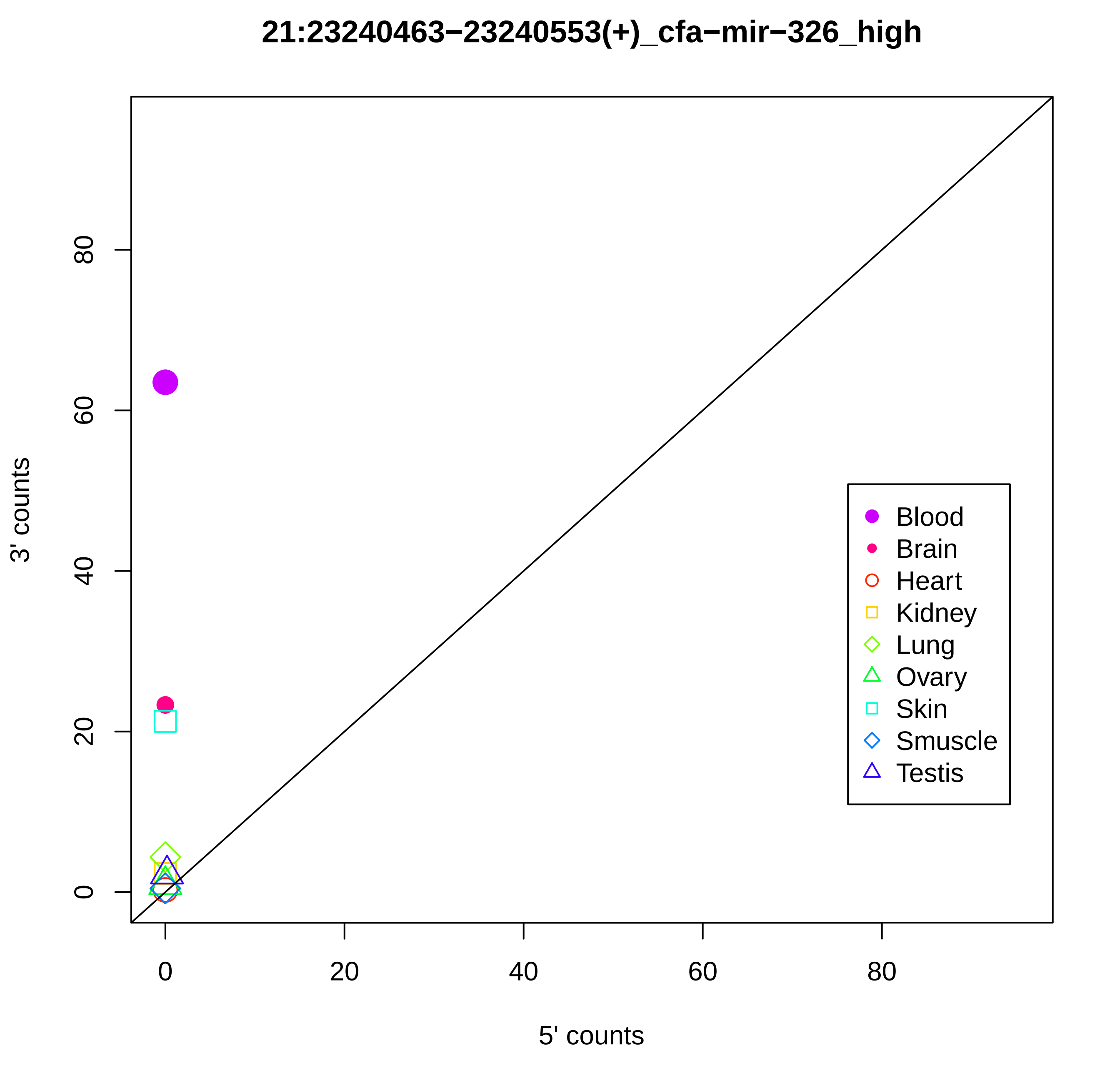

21:23240463−23240553(+)_cfa−mir−326_high
3' counts
5' counts
0
20
40
60
80
0
20
40
60
80
Blood
Brain
Heart
Kidney
Lung
Ovary
Skin
Smuscle
Testis

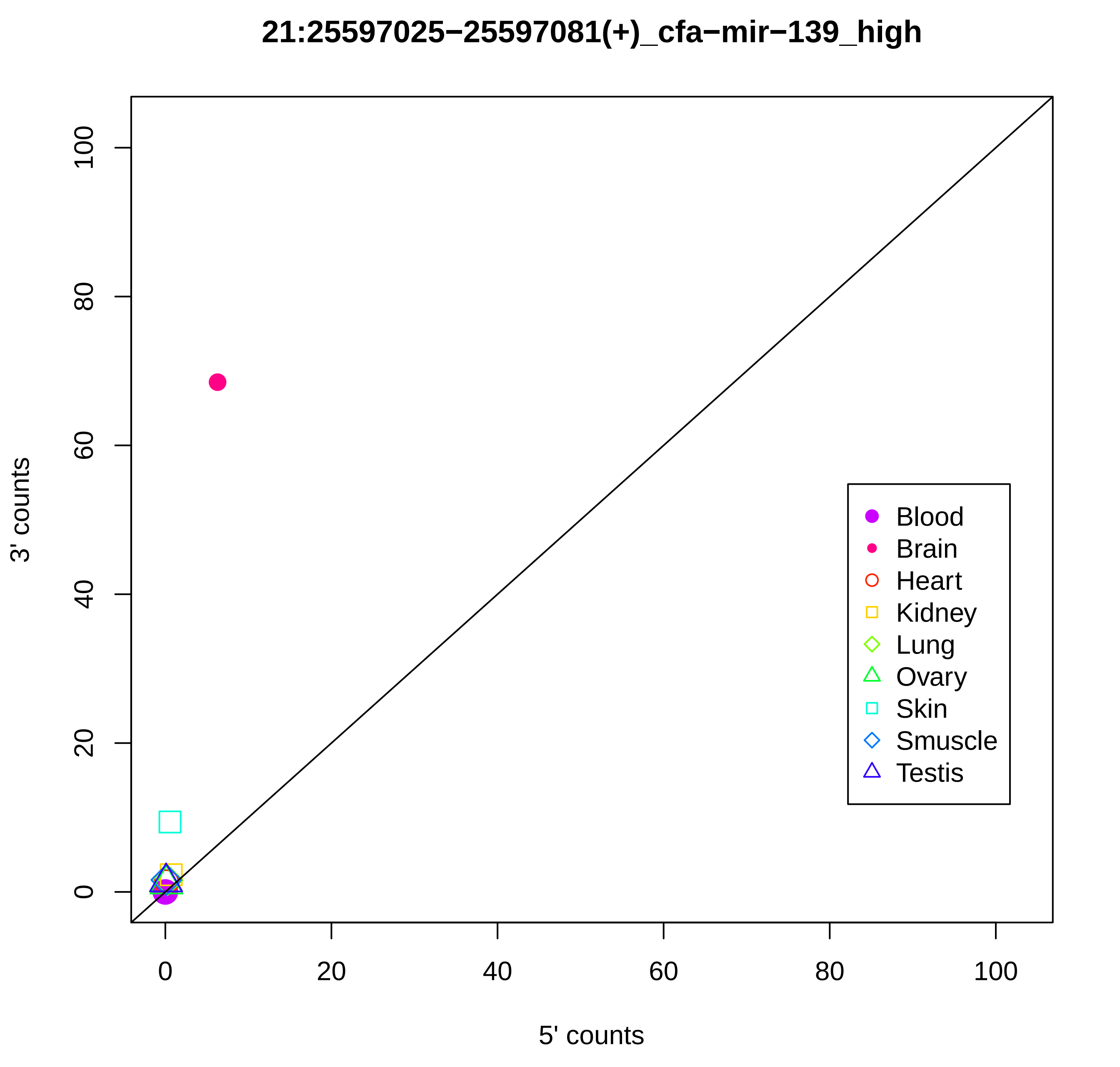

21:25597025−25597081(+)_cfa−mir−139_high
3' counts
5' counts
0
20
40
60
80
100
Blood
Brain
Heart
Kidney
Lung
Ovary
Skin
Smuscle
Testis

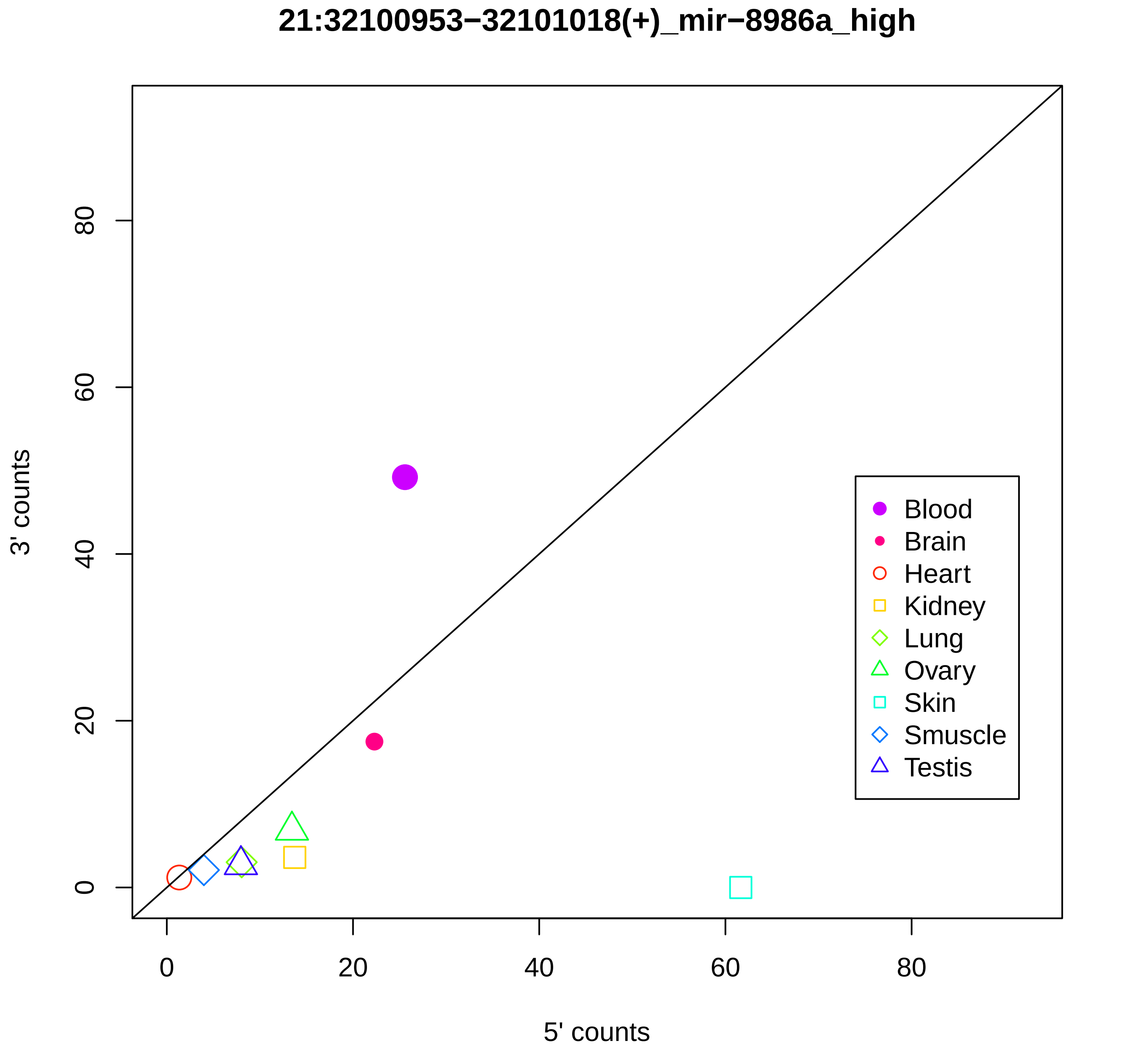

21:32100953−32101018(+)_mir−8986a_high
3' counts
5' counts
0
20
40
60
80
0
20
40
60
80
Blood
Brain
Heart
Kidney
Lung
Ovary
Skin
Smuscle
Testis

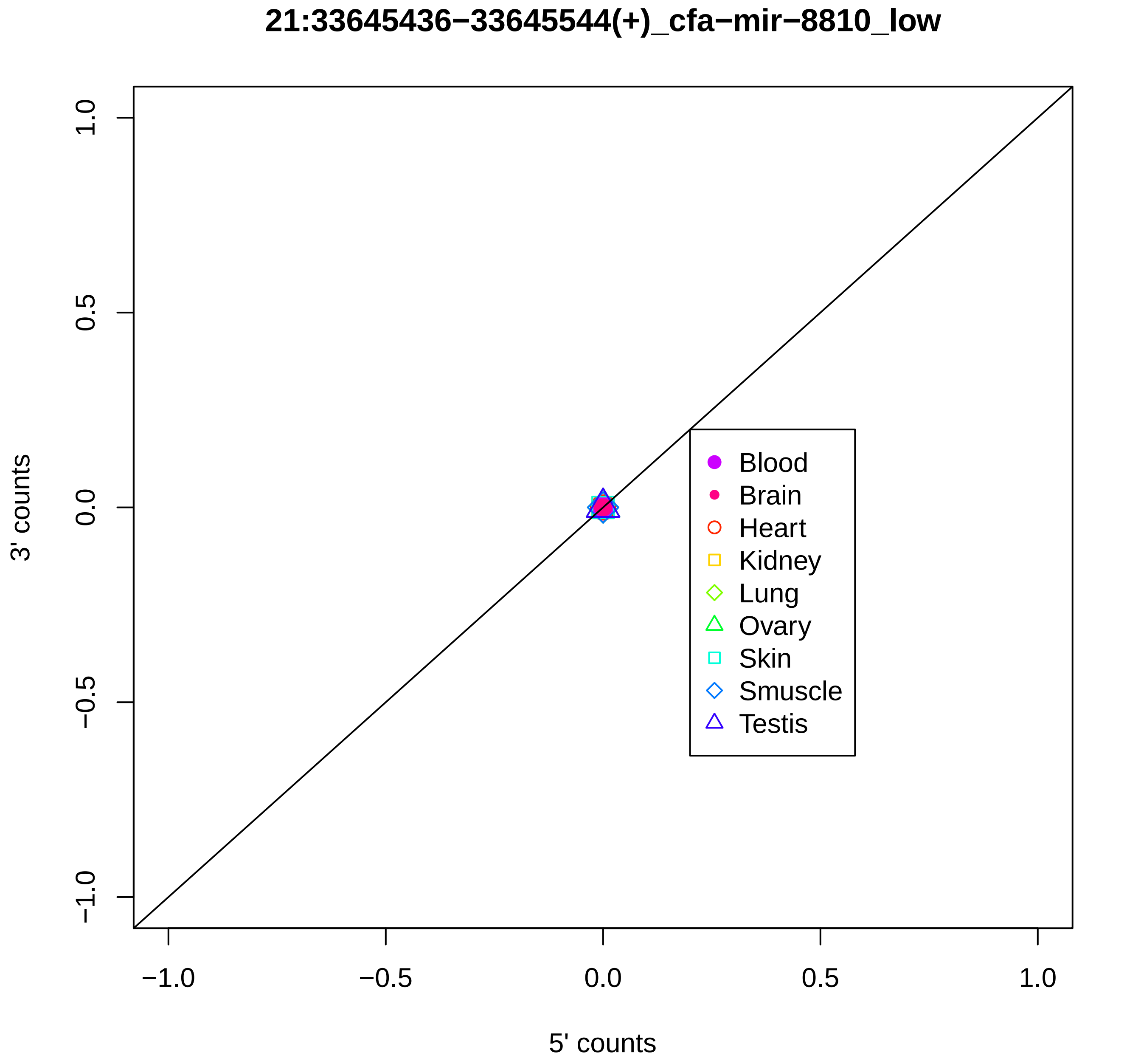

21:33645436−33645544(+)_cfa−mir−8810_low
1.0
0.5
0.0
−0.5
−1.0
3' counts
−1.0
−0.5
0.0
0.5
1.0
5' counts
Blood
Brain
Heart
Kidney
Lung
Ovary
Skin
Smuscle
Testis

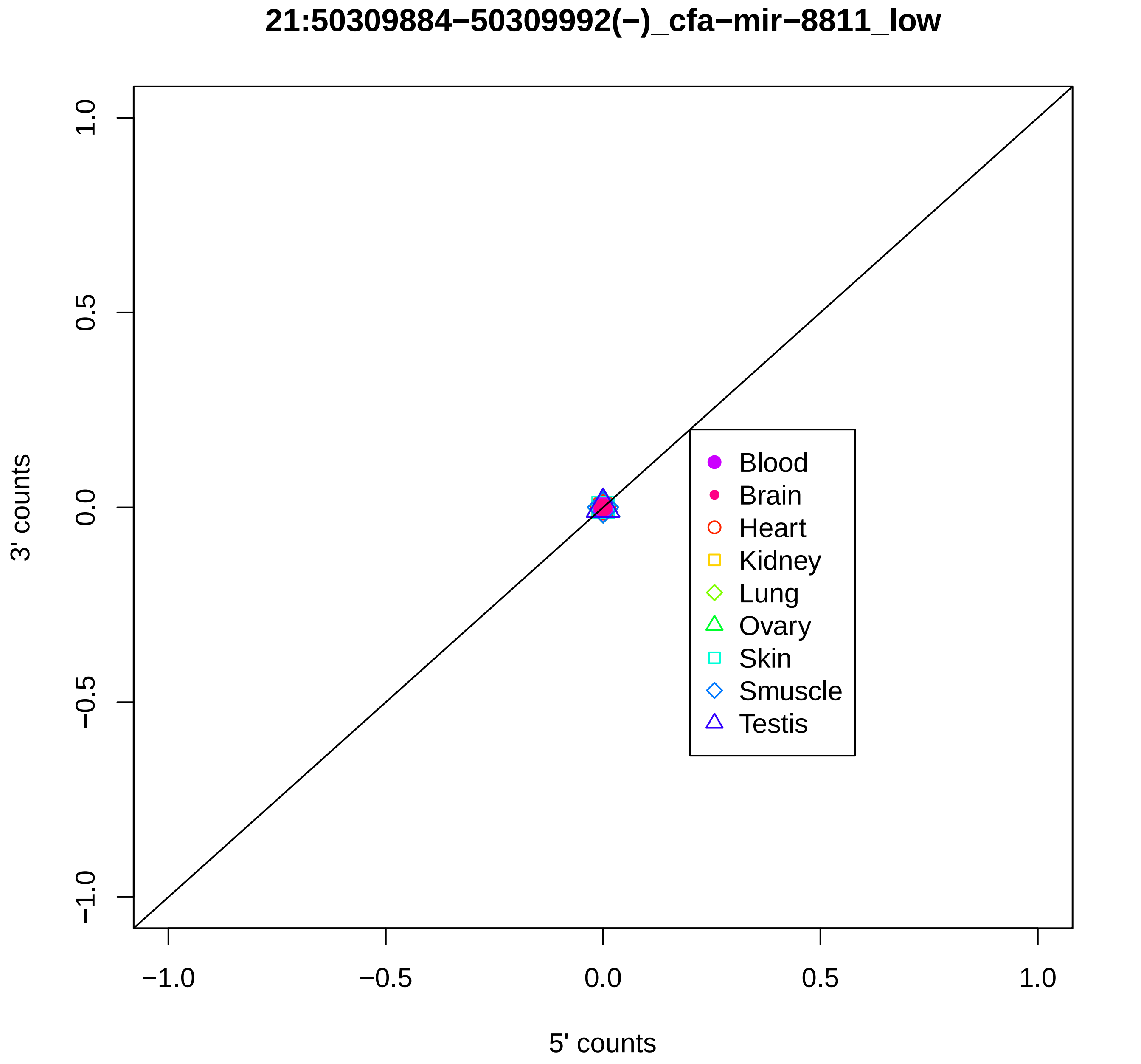
21:50309884−50309992(−)_cfa−mir−8811_low
3' counts
5' counts
−1.0
−0.5
0.0
0.5
1.0
Blood
Brain
Heart
Kidney
Lung
Ovary
Skin
Smuscle
Testis

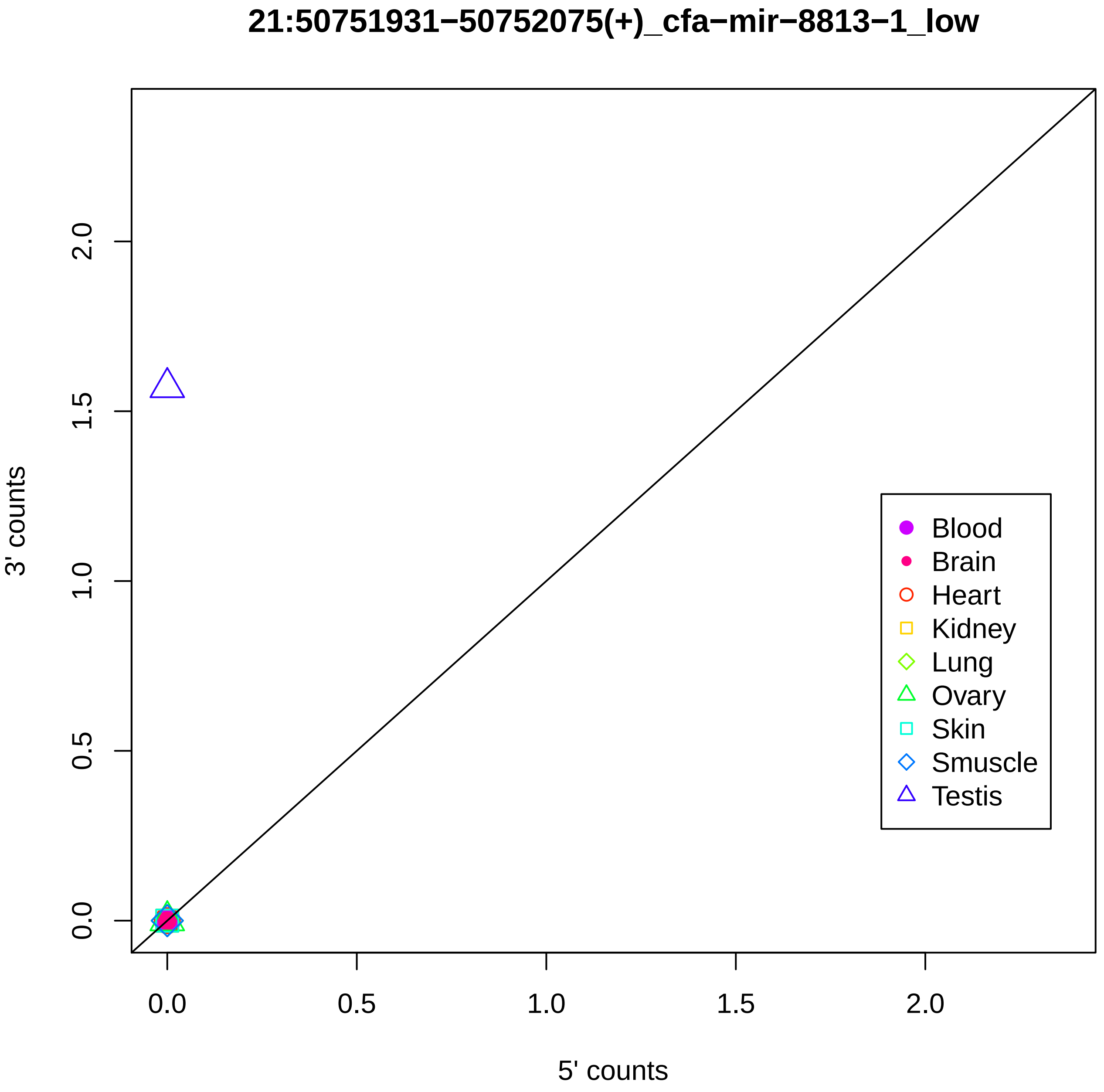
21:50751931−50752075(+)_cfa−mir−8813−1_low
3' counts
5' counts
0.0
0.5
1.0
1.5
2.0
Blood
Brain
Heart
Kidney
Lung
Ovary
Skin
Smuscle
Testis

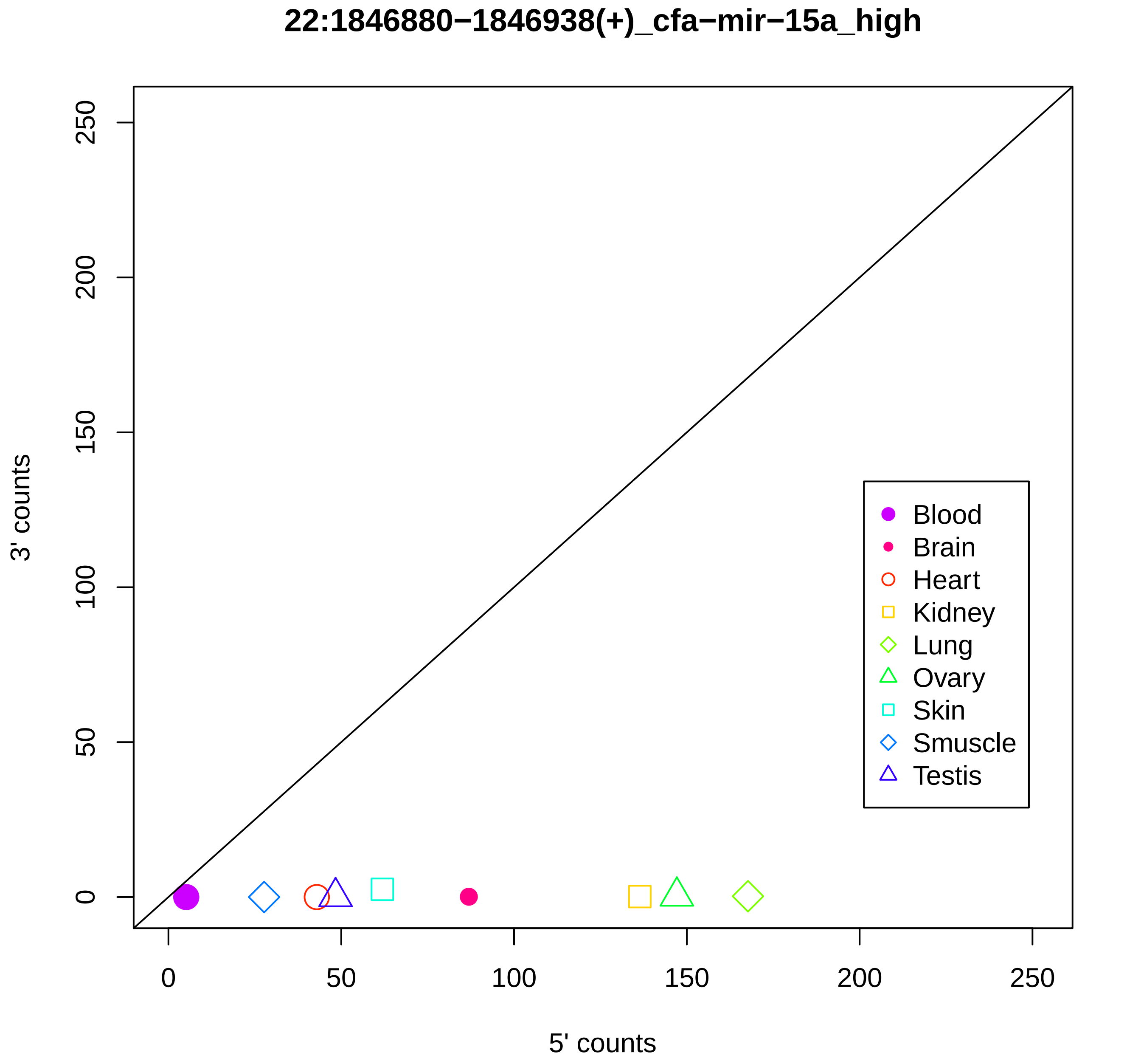

22:1846880−1846938(+)_cfa−mir−15a_high
3' counts
5' counts
0
50
100
150
200
250
Blood
Brain
Heart
Kidney
Lung
Ovary
Skin
Smuscle
Testis

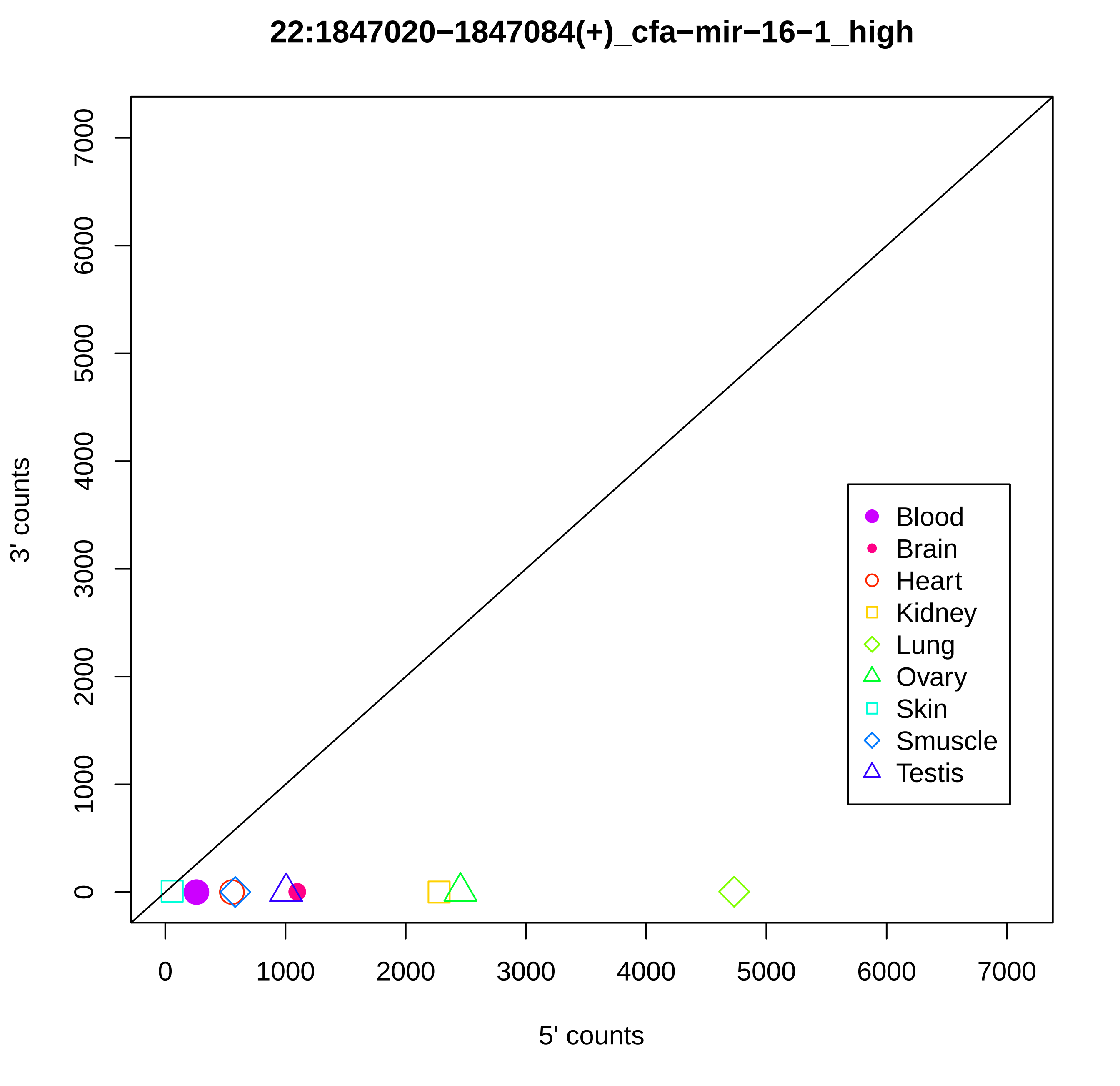

22:1847020−1847084(+)_cfa−mir−16−1_high
3' counts
5' counts
0
1000
2000
3000
4000
5000
6000
7000
Blood
Brain
Heart
Kidney
Lung
Ovary
Skin
Smuscle
Testis

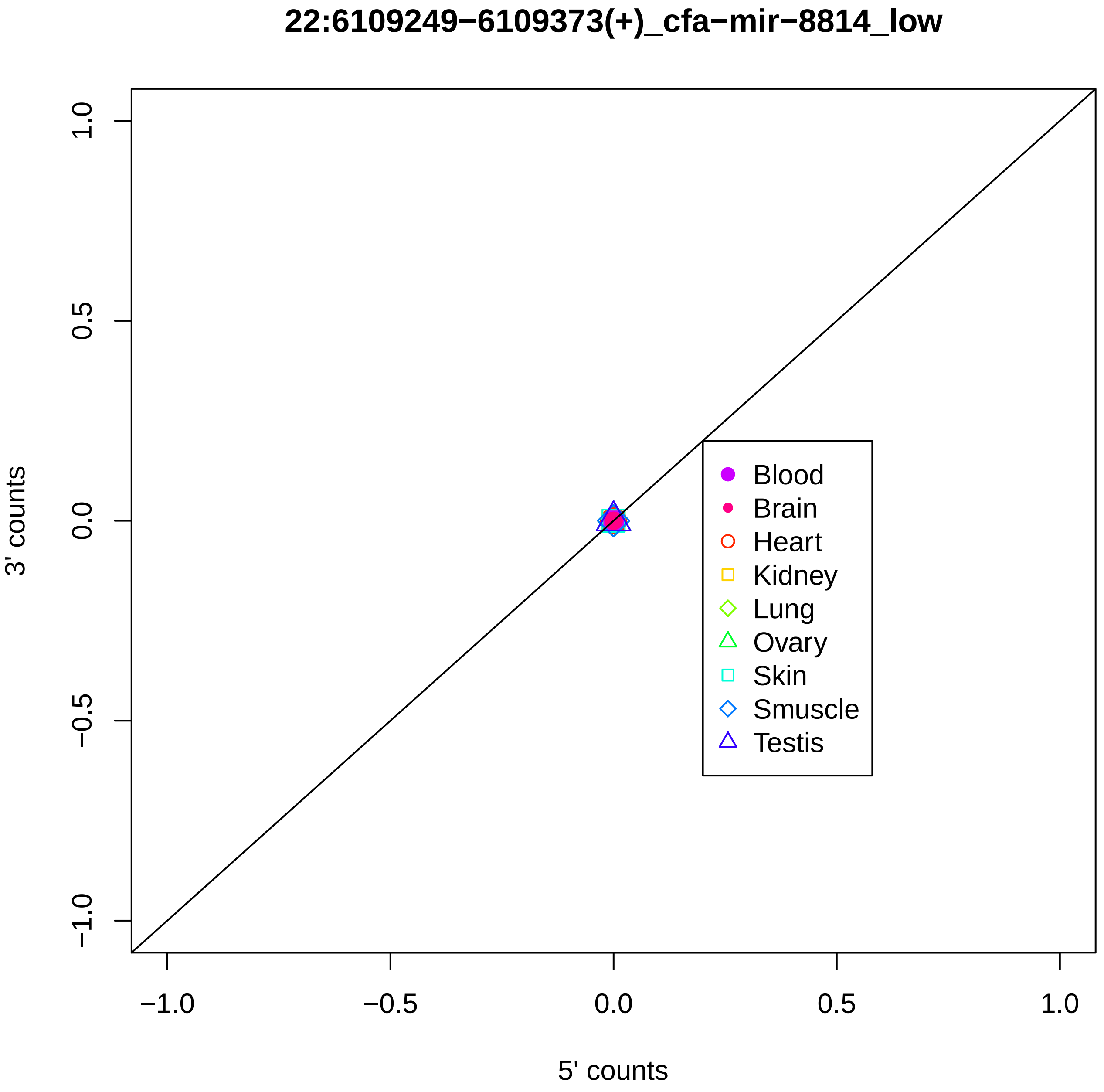
22:6109249−6109373(+)_cfa−mir−8814_low
3' counts
5' counts
−1.0
−0.5
0.0
0.5
1.0
Blood
Brain
Heart
Kidney
Lung
Ovary
Skin
Smuscle
Testis

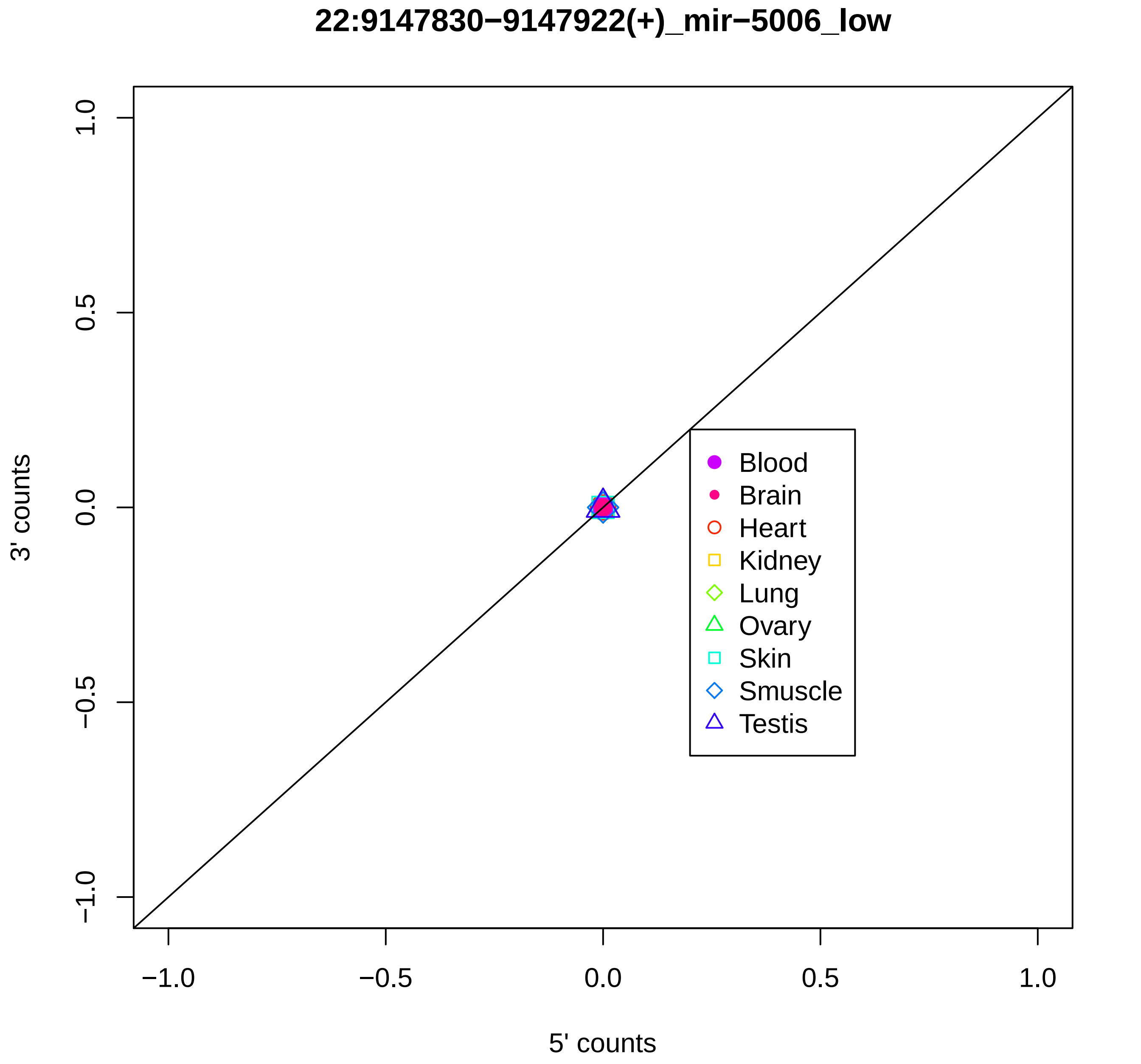

22:9147830−9147922(+)_mir−5006_low
3' counts
5' counts
1.0
0.5
0.0
−0.5
−1.0
Blood
Brain
Heart
Kidney
Lung
Ovary
Skin
Smuscle
Testis

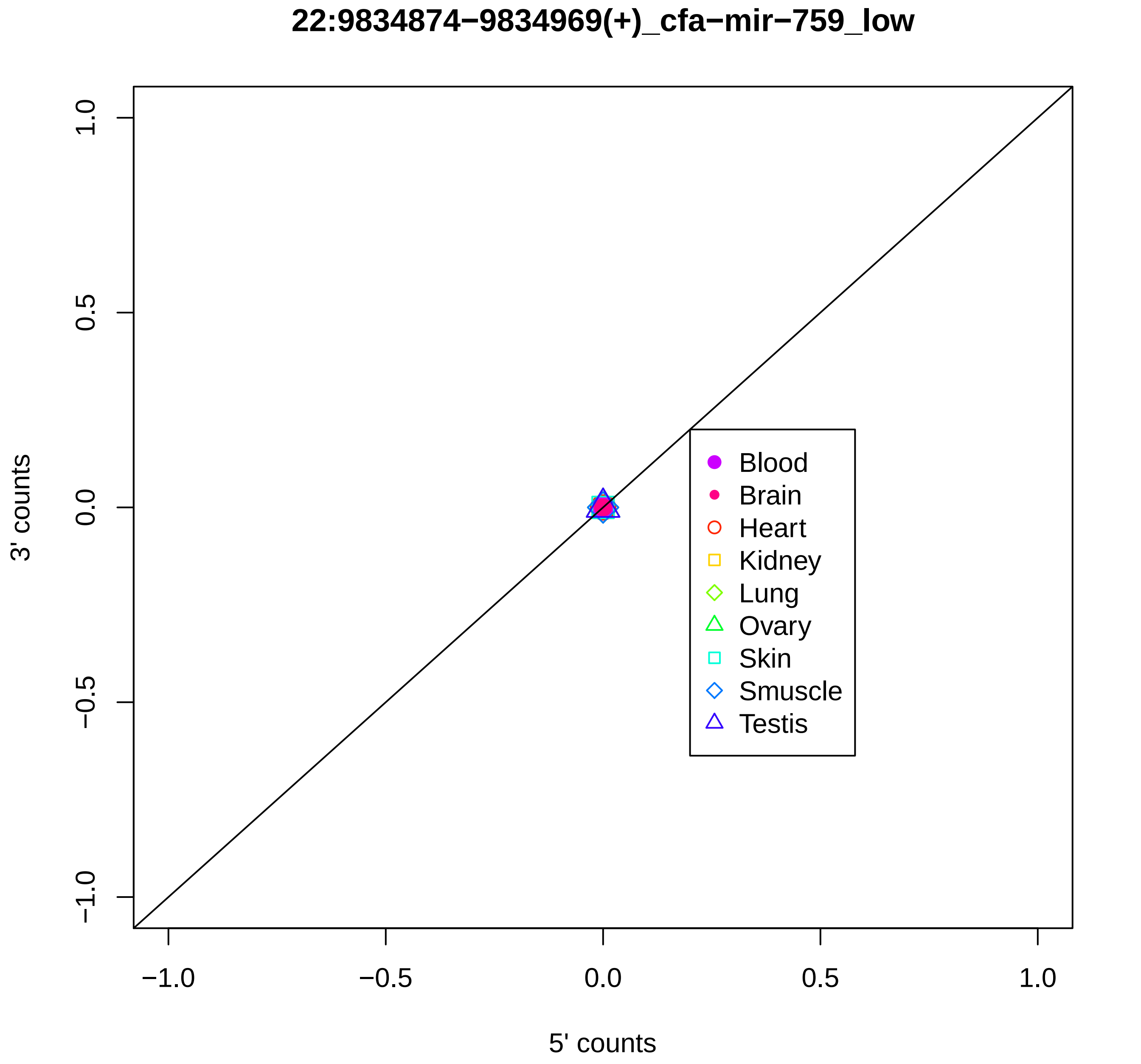
22:9834874−9834969(+)_cfa−mir−759_low
3' counts
5' counts
−1.0
−0.5
0.0
0.5
1.0
Blood
Brain
Heart
Kidney
Lung
Ovary
Skin
Smuscle
Testis

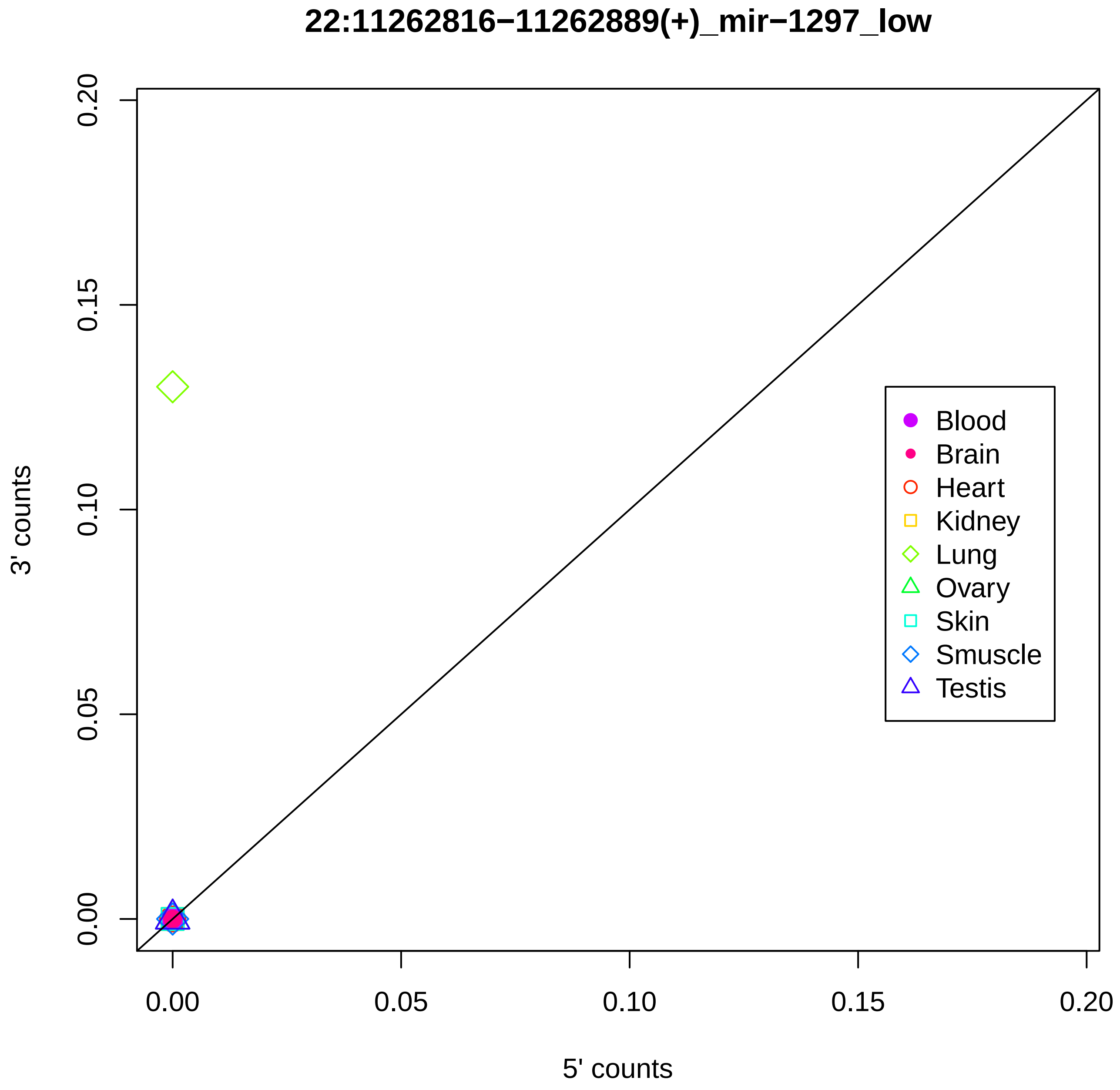
22:11262816−11262889(+)_mir−1297_low
3' counts
5' counts
0.00
0.05
0.10
0.15
0.20
Blood
Brain
Heart
Kidney
Lung
Ovary
Skin
Smuscle
Testis

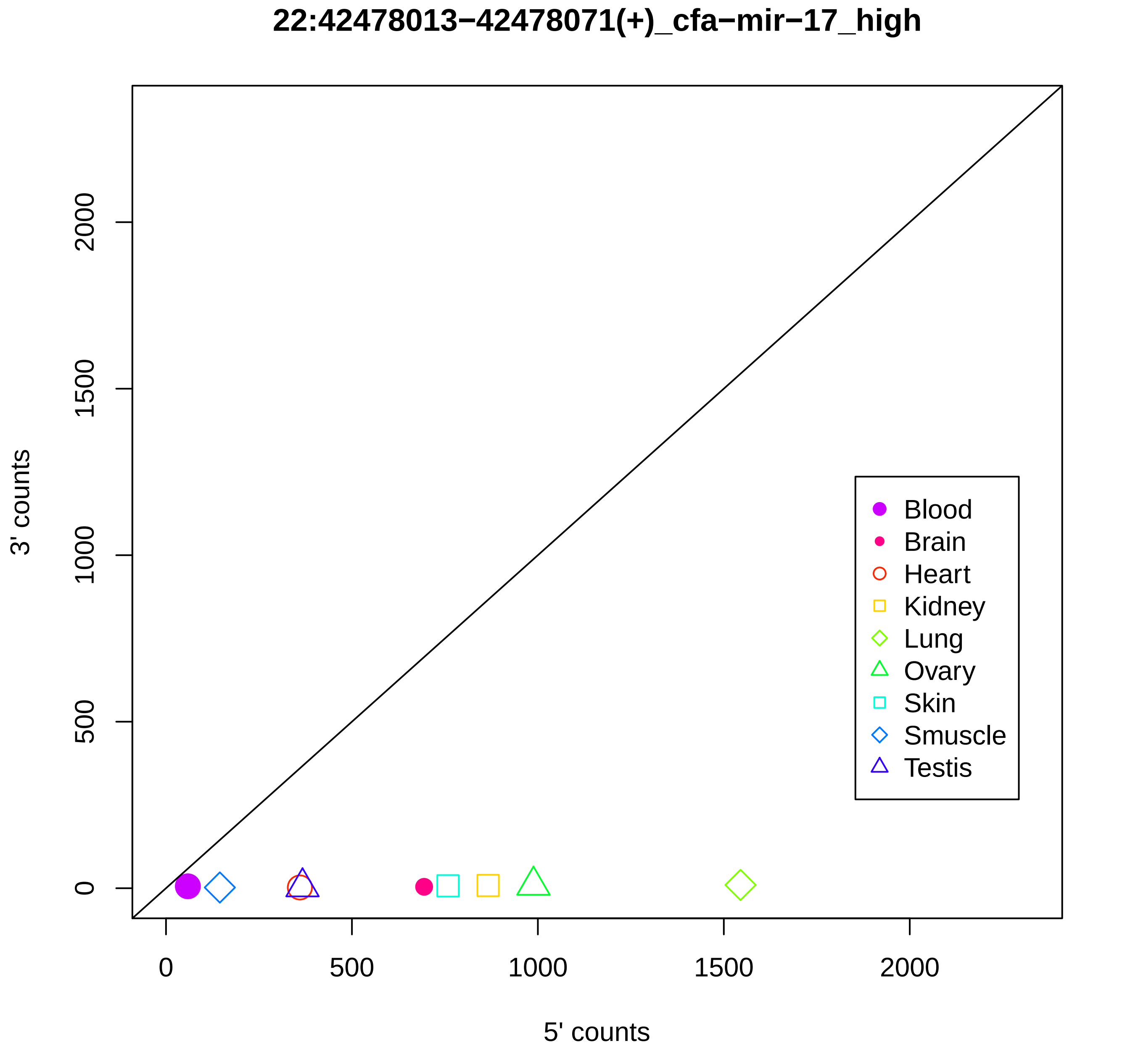

22:42478013−42478071(+)_cfa−mir−17_high
3' counts
0
500
1000
1500
2000
5' counts
0
500
1000
1500
2000
Blood
Brain
Heart
Kidney
Lung
Ovary
Skin
Smuscle
Testis

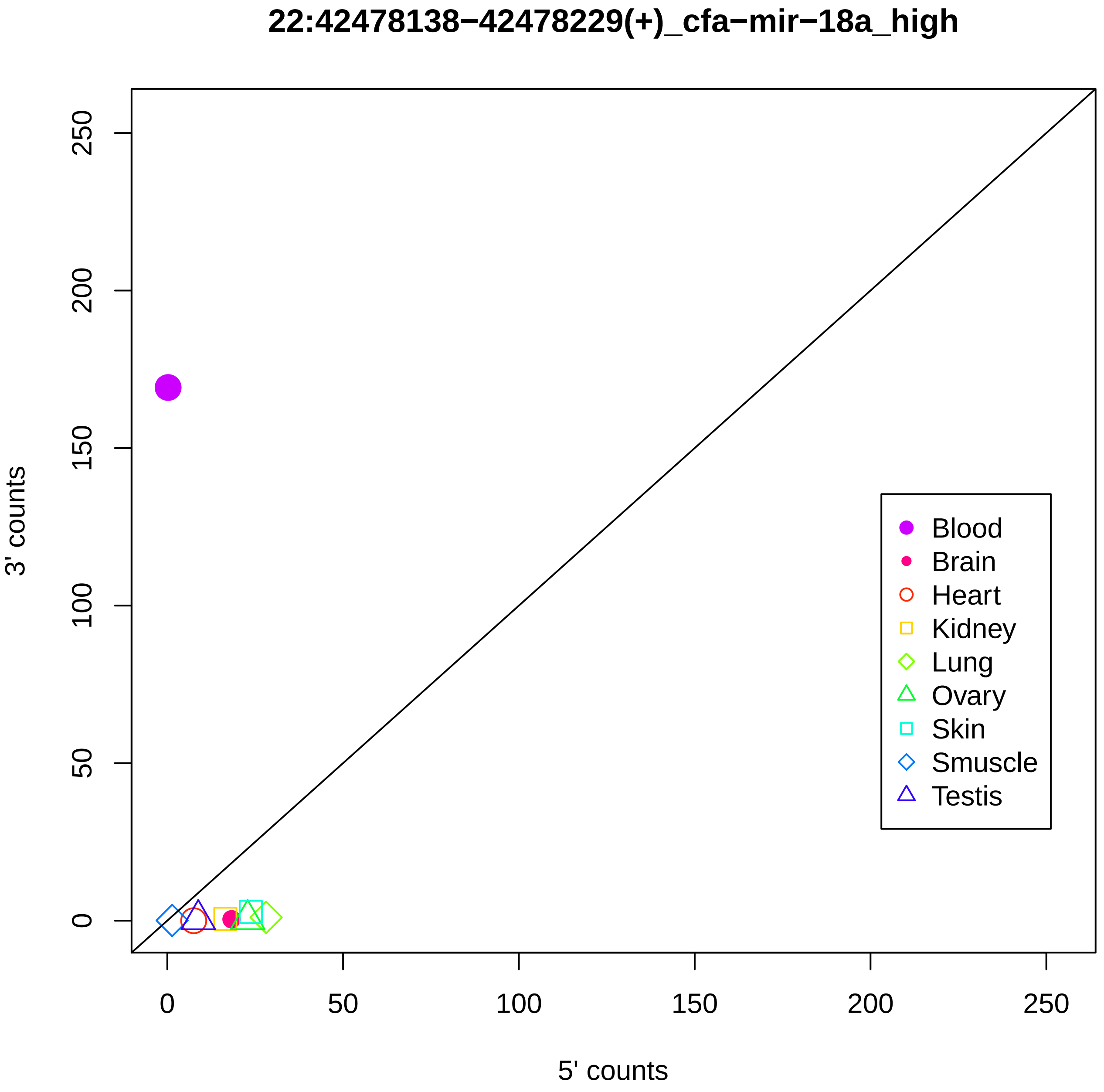
22:42478138−42478229(+)_cfa−mir−18a_high
3' counts
5' counts
0
50
100
150
200
250
Blood
Brain
Heart
Kidney
Lung
Ovary
Skin
Smuscle
Testis

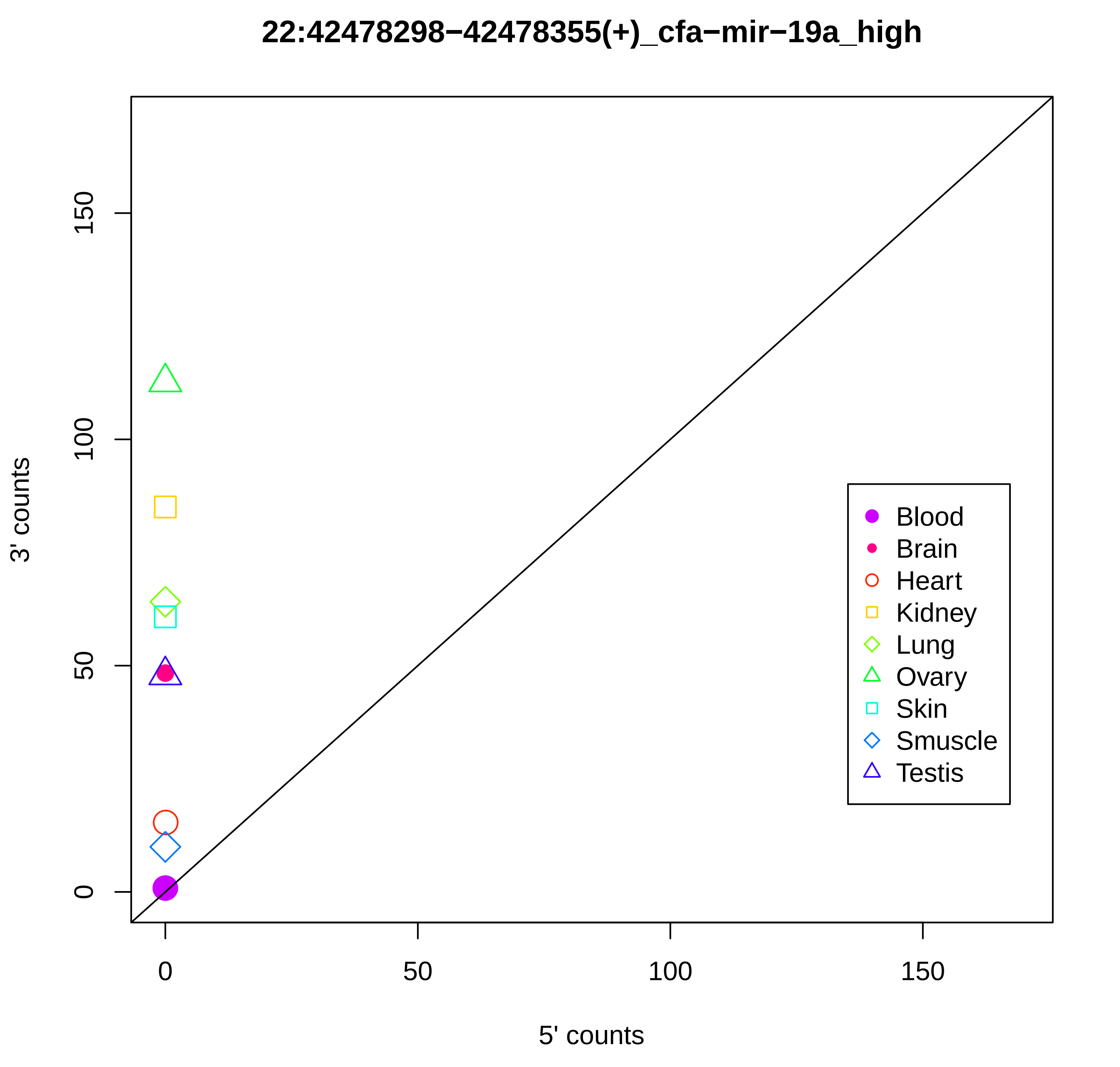

22:42478298−42478355(+)_cfa−mir−19a_high
3' counts
5' counts
0
50
100
150
0
50
100
150
Blood
Brain
Heart
Kidney
Lung
Ovary
Skin
Smuscle
Testis

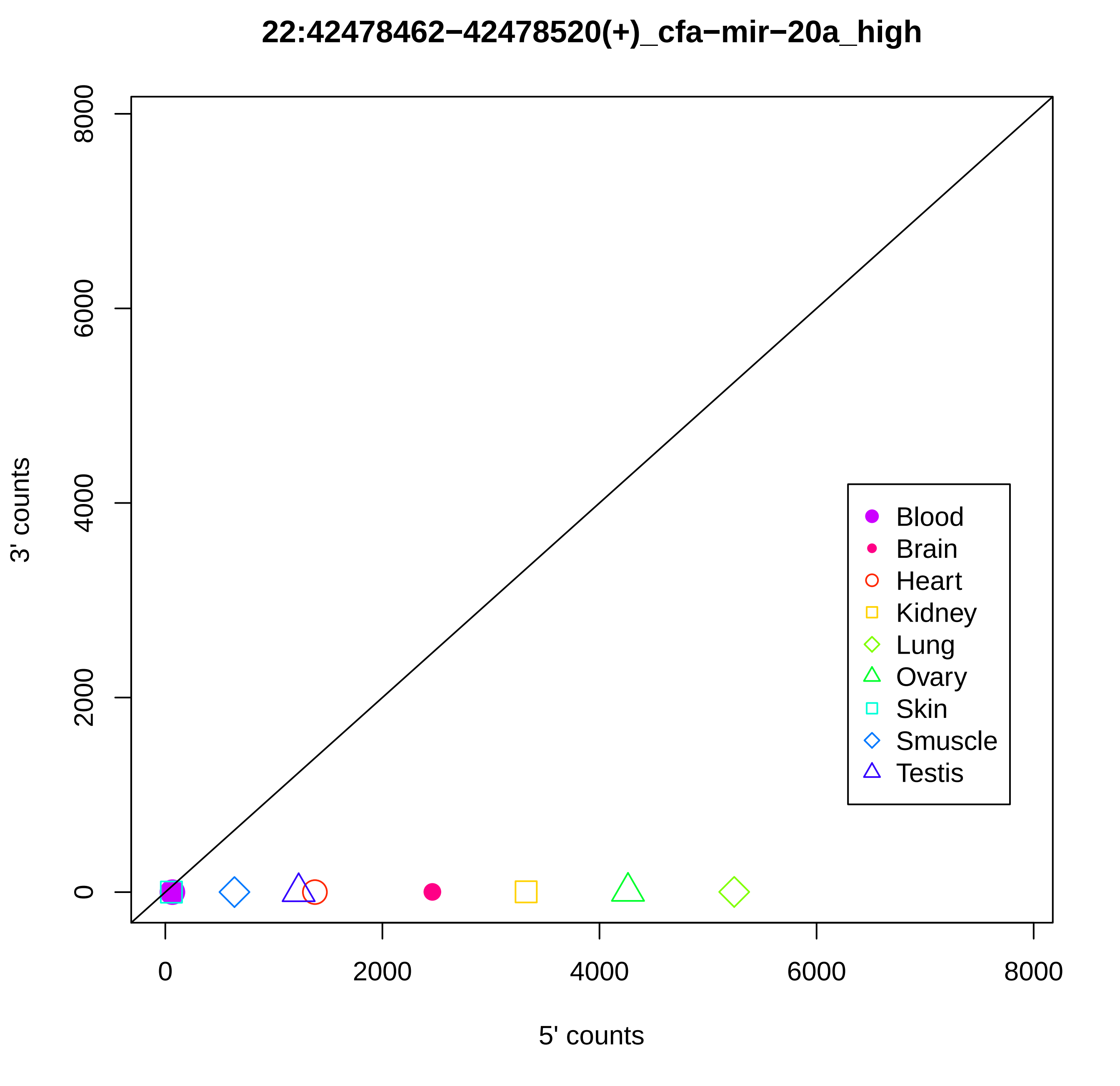

22:42478462−42478520(+)_cfa−mir−20a_high
3' counts
5' counts
0
2000
4000
6000
8000
0
2000
4000
6000
8000
Blood
Brain
Heart
Kidney
Lung
Ovary
Skin
Smuscle
Testis

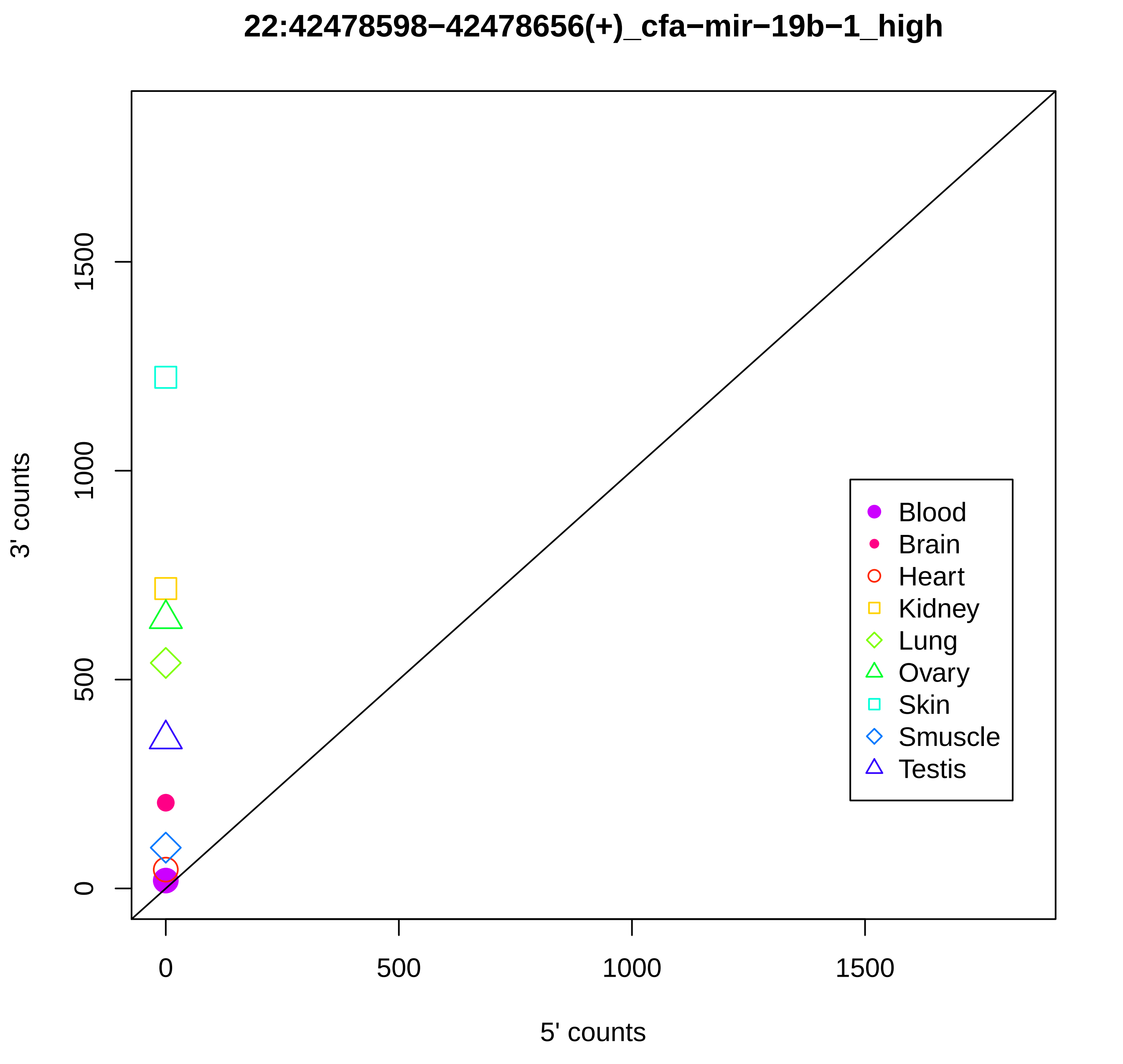

22:42478598−42478656(+)_cfa−mir−19b−1_high
3' counts
5' counts
0
500
1000
1500
0
500
1000
1500
Blood
Brain
Heart
Kidney
Lung
Ovary
Skin
Smuscle
Testis

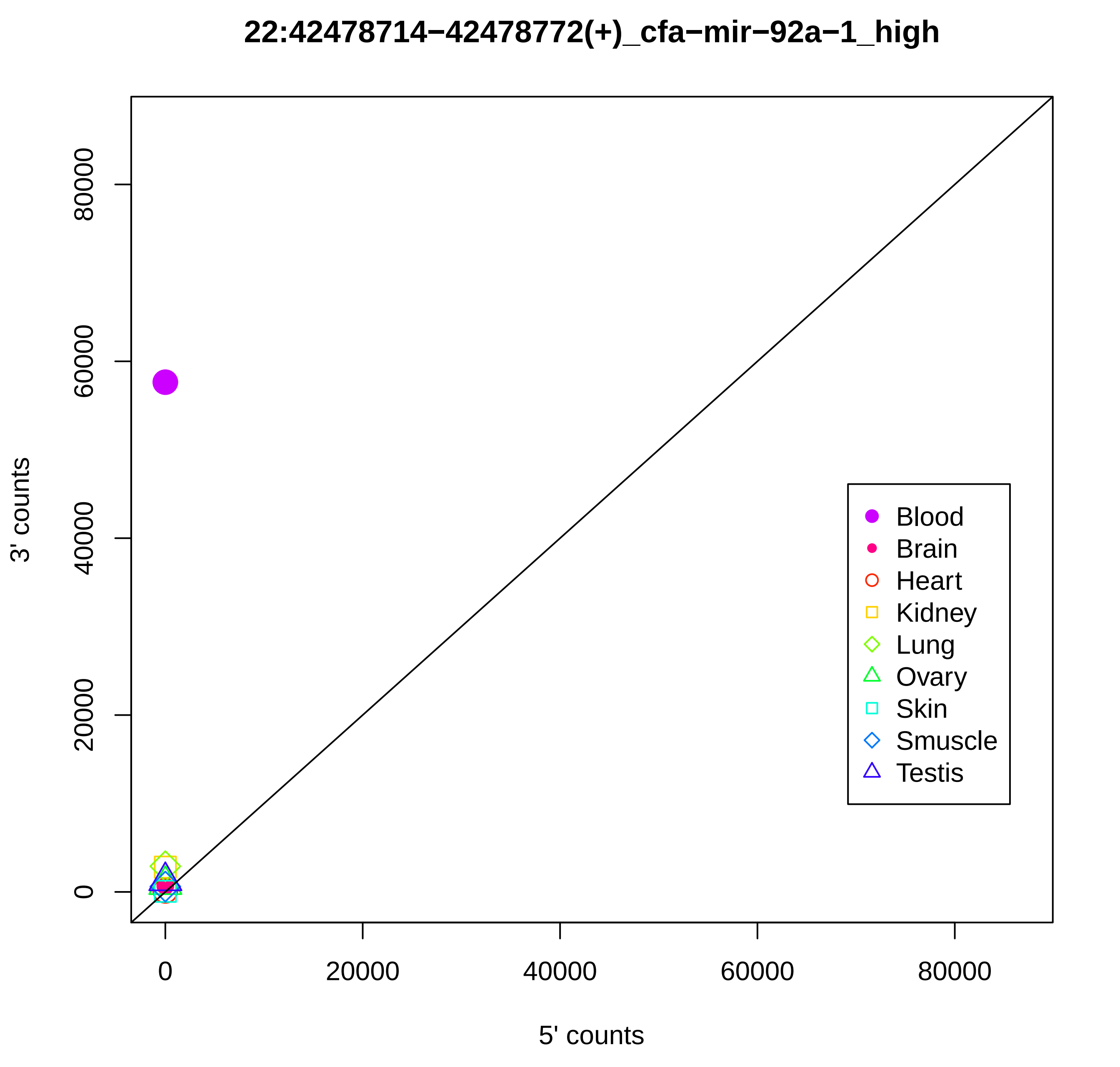

22:42478714−42478772(+)_cfa−mir−92a−1_high
3' counts
5' counts
0
20000
40000
60000
80000
Blood
Brain
Heart
Kidney
Lung
Ovary
Skin
Smuscle
Testis

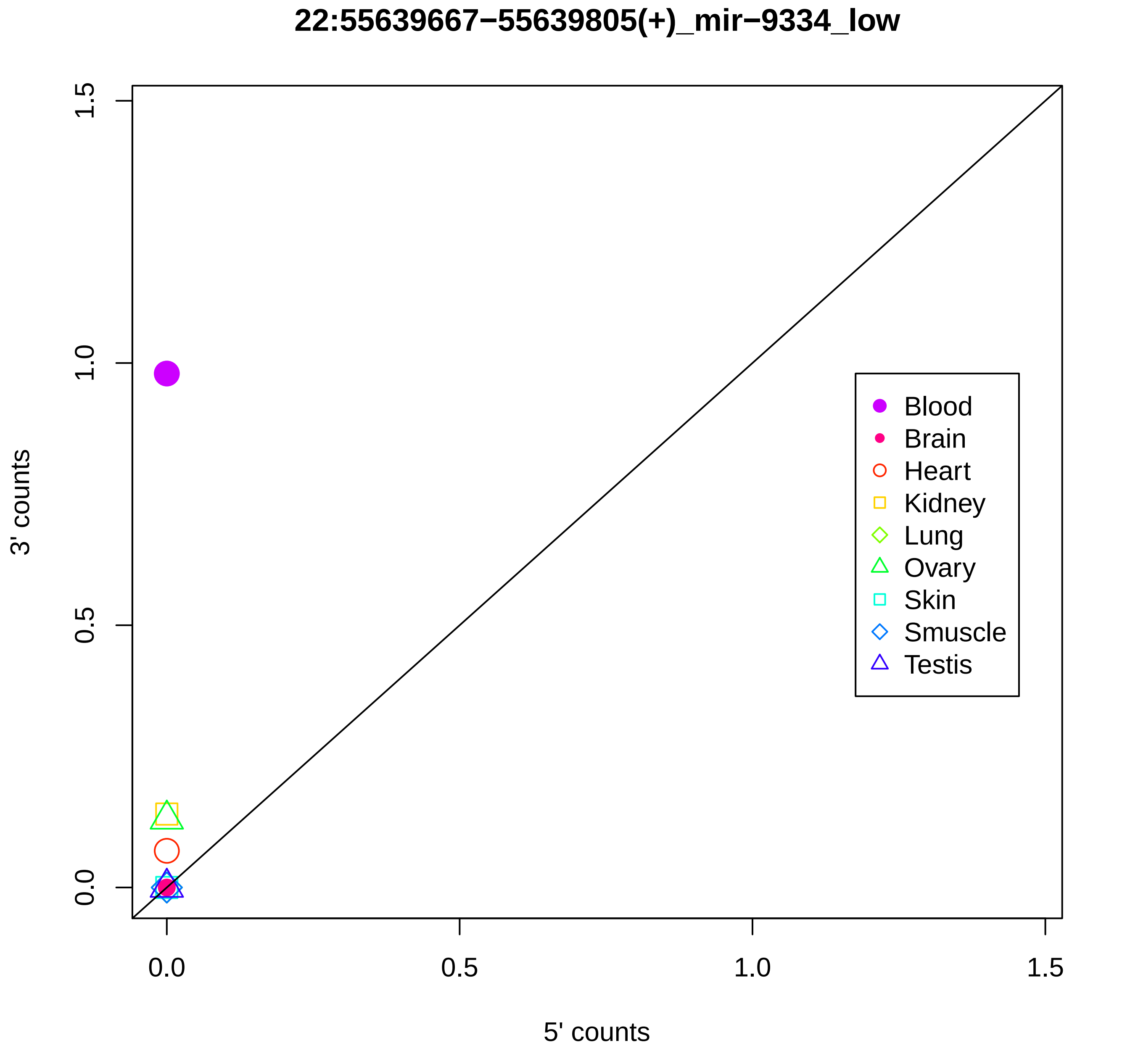

22:55639667−55639805(+)_mir−9334_low
3' counts
5' counts
0.0
0.5
1.0
1.5
0.0
0.5
1.0
1.5
Blood
Brain
Heart
Kidney
Lung
Ovary
Skin
Smuscle
Testis

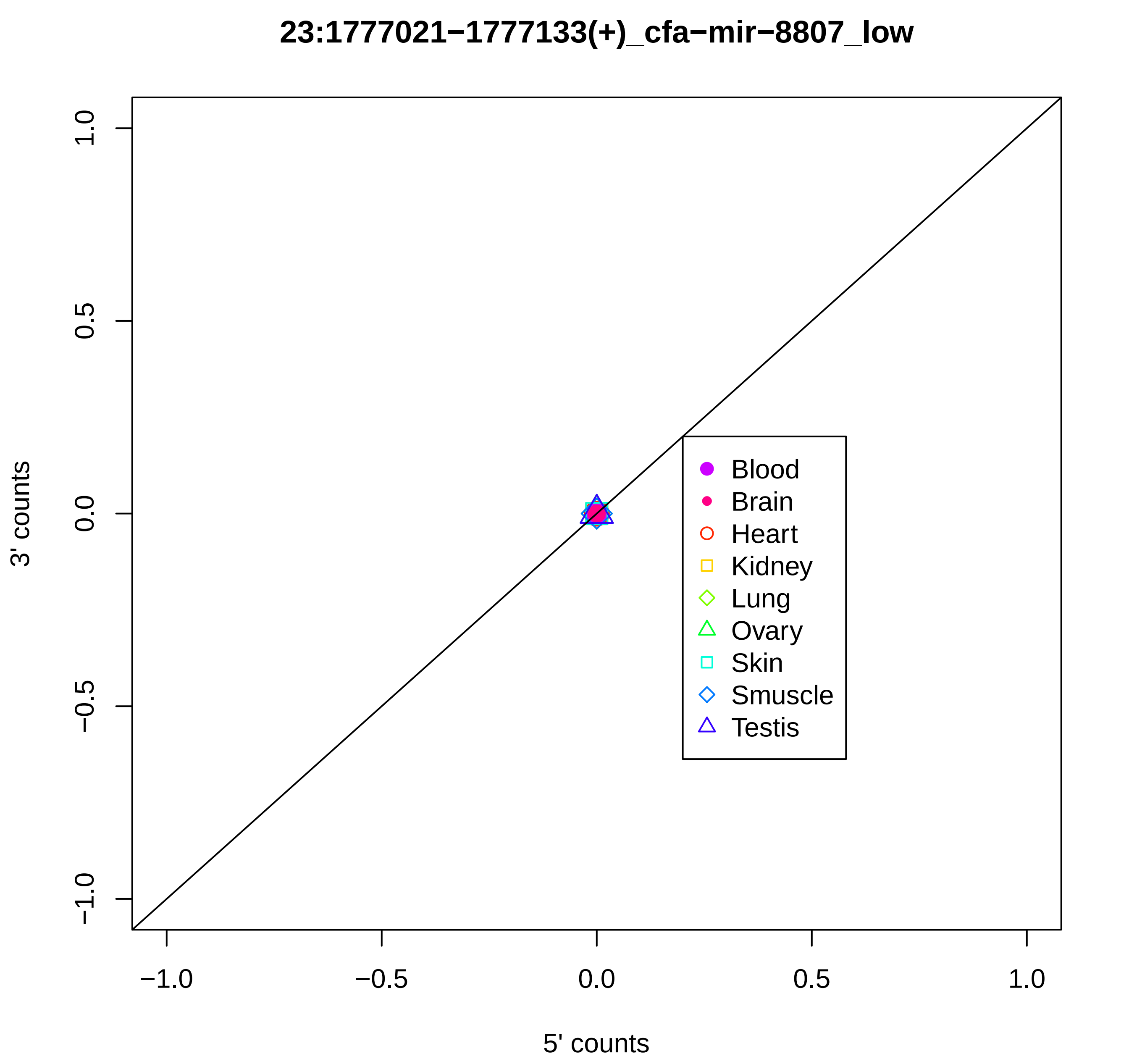

23:1777021−1777133(+)_cfa−mir−8807_low
1.0
0.5
0.0
−0.5
−1.0
3' counts
−1.0
−0.5
0.0
0.5
1.0
5' counts
Blood
Brain
Heart
Kidney
Lung
Ovary
Skin
Smuscle
Testis

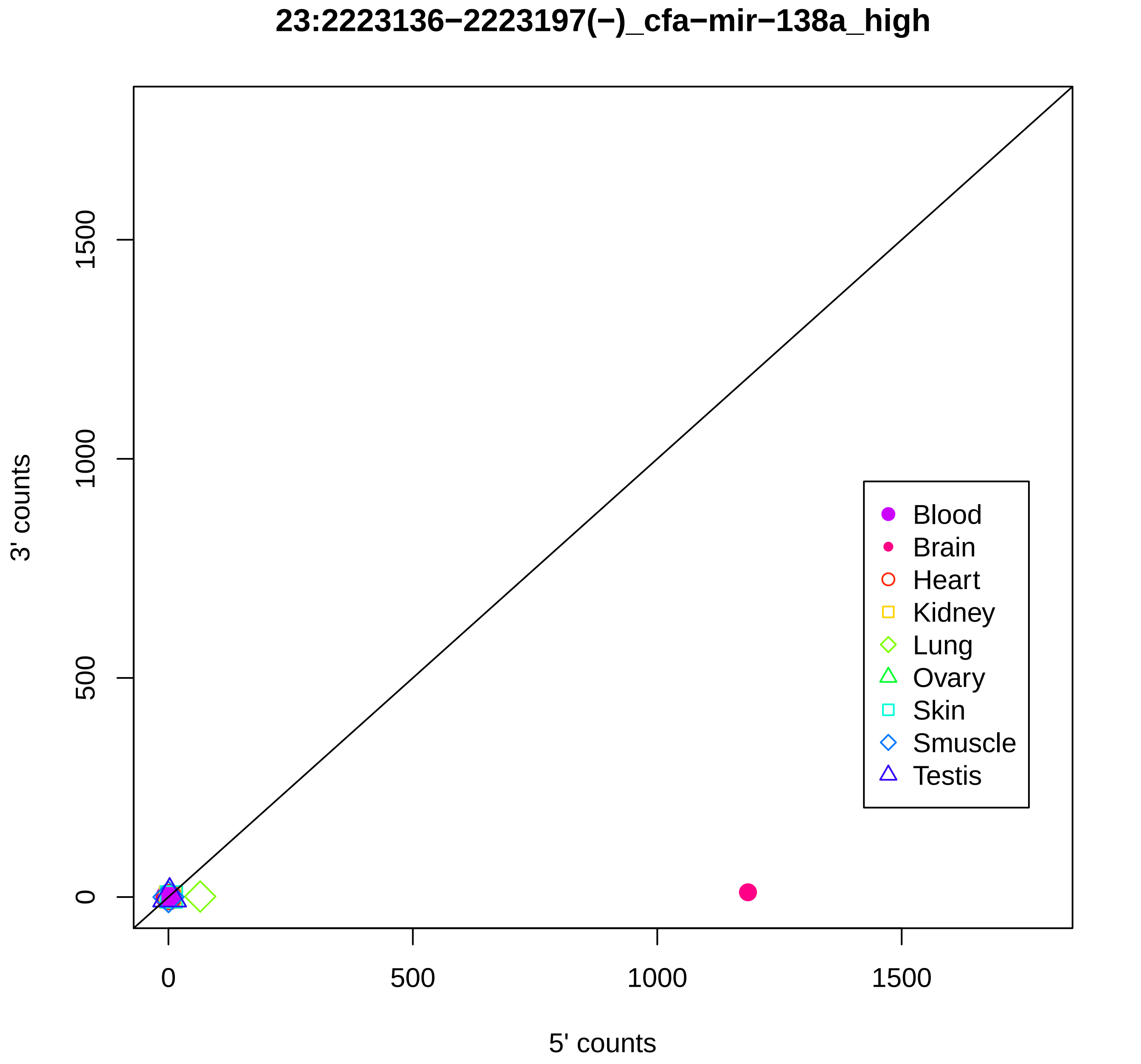
23:2223136−2223197(−)_cfa−mir−138a_high
3' counts
5' counts
0
500
1000
1500
0
500
1000
1500
Blood
Brain
Heart
Kidney
Lung
Ovary
Skin
Smuscle
Testis

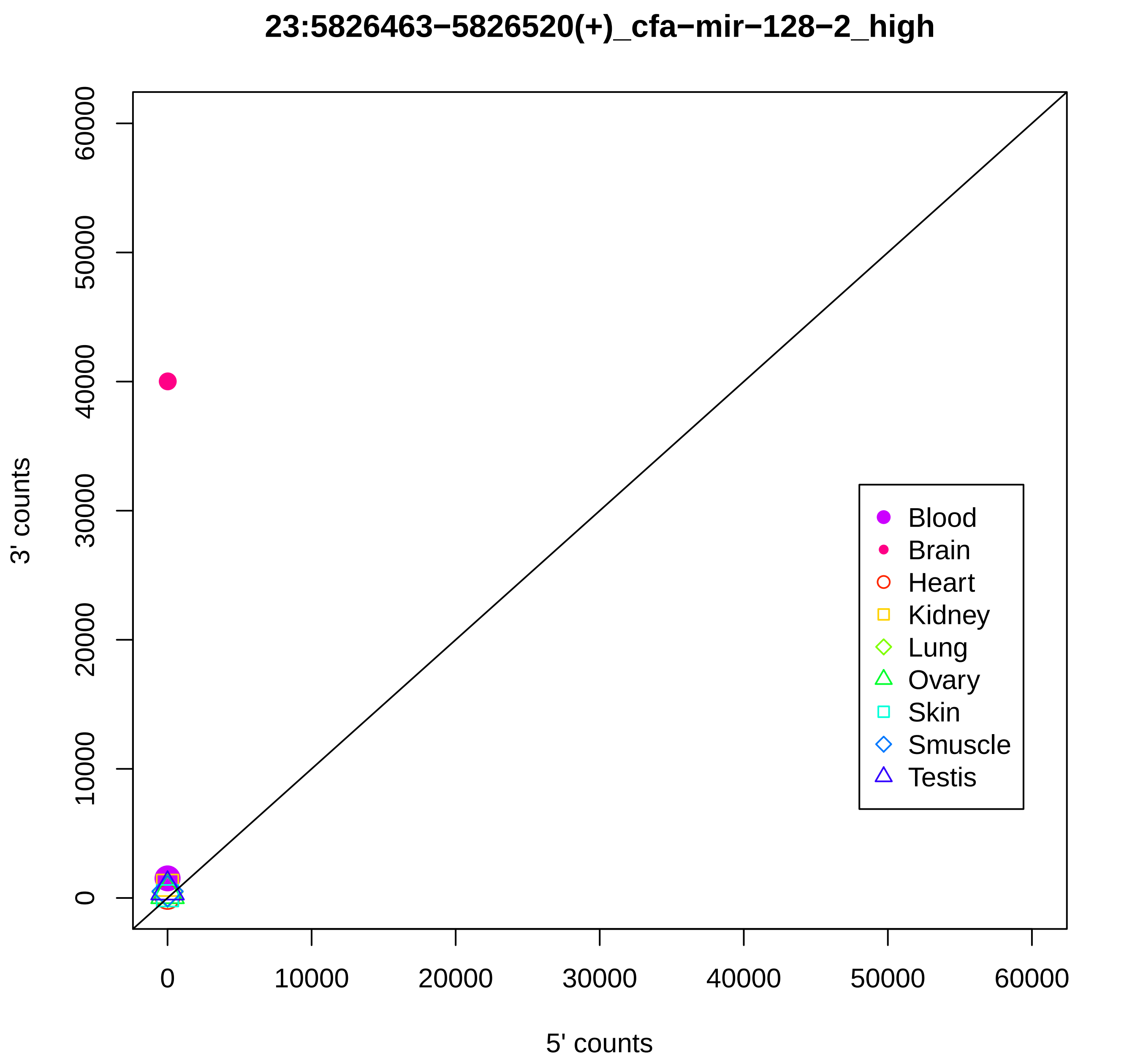

23:5826463−5826520(+)_cfa−mir−128−2_high
3' counts
5' counts
0
10000
20000
30000
40000
50000
60000
Blood
Brain
Heart
Kidney
Lung
Ovary
Skin
Smuscle
Testis

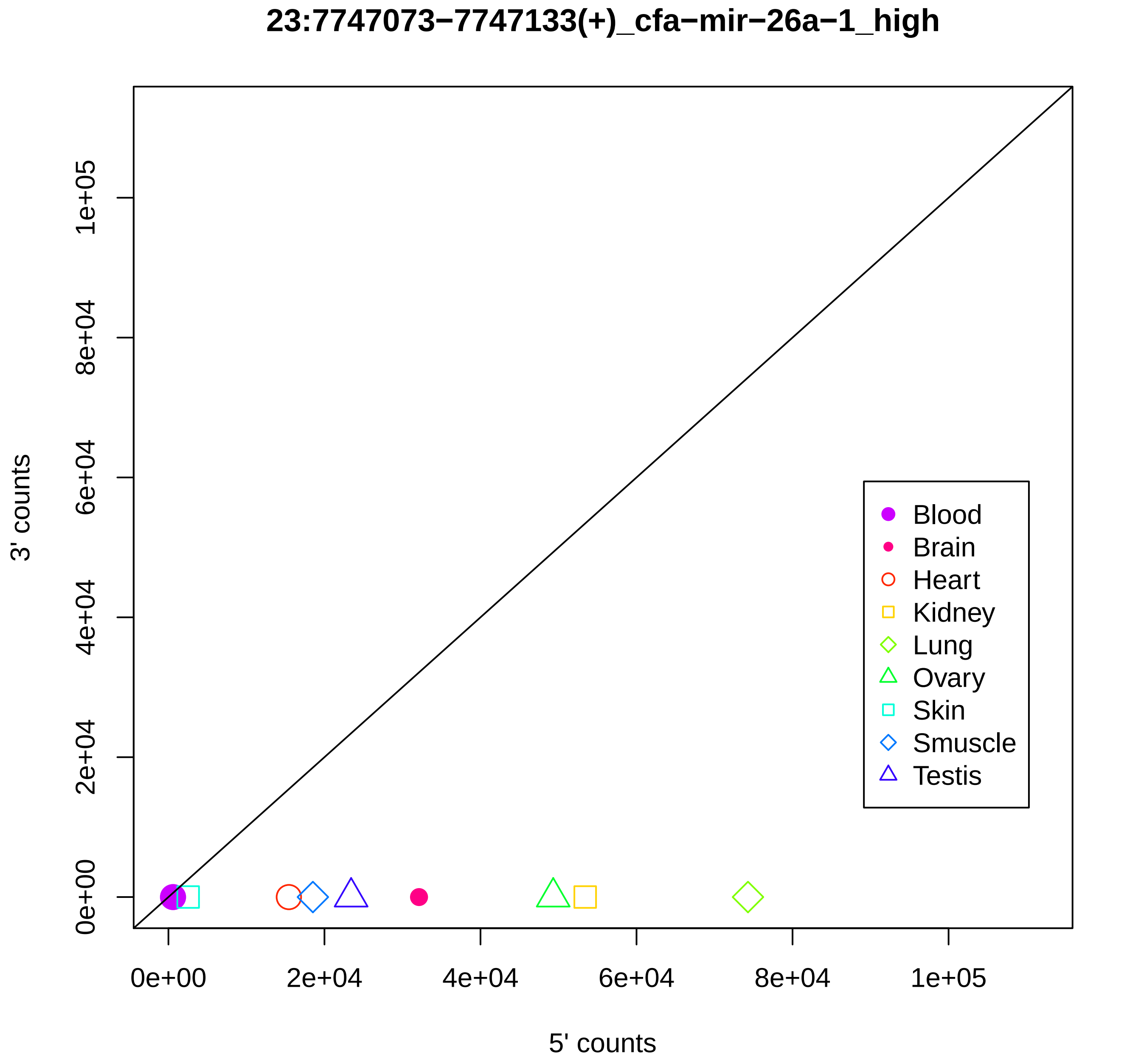

23:7747073−7747133(+)_cfa−mir−26a−1_high
3' counts
5' counts
0e+00
2e+04
4e+04
6e+04
8e+04
1e+05
Blood
Brain
Heart
Kidney
Lung
Ovary
Skin
Smuscle
Testis

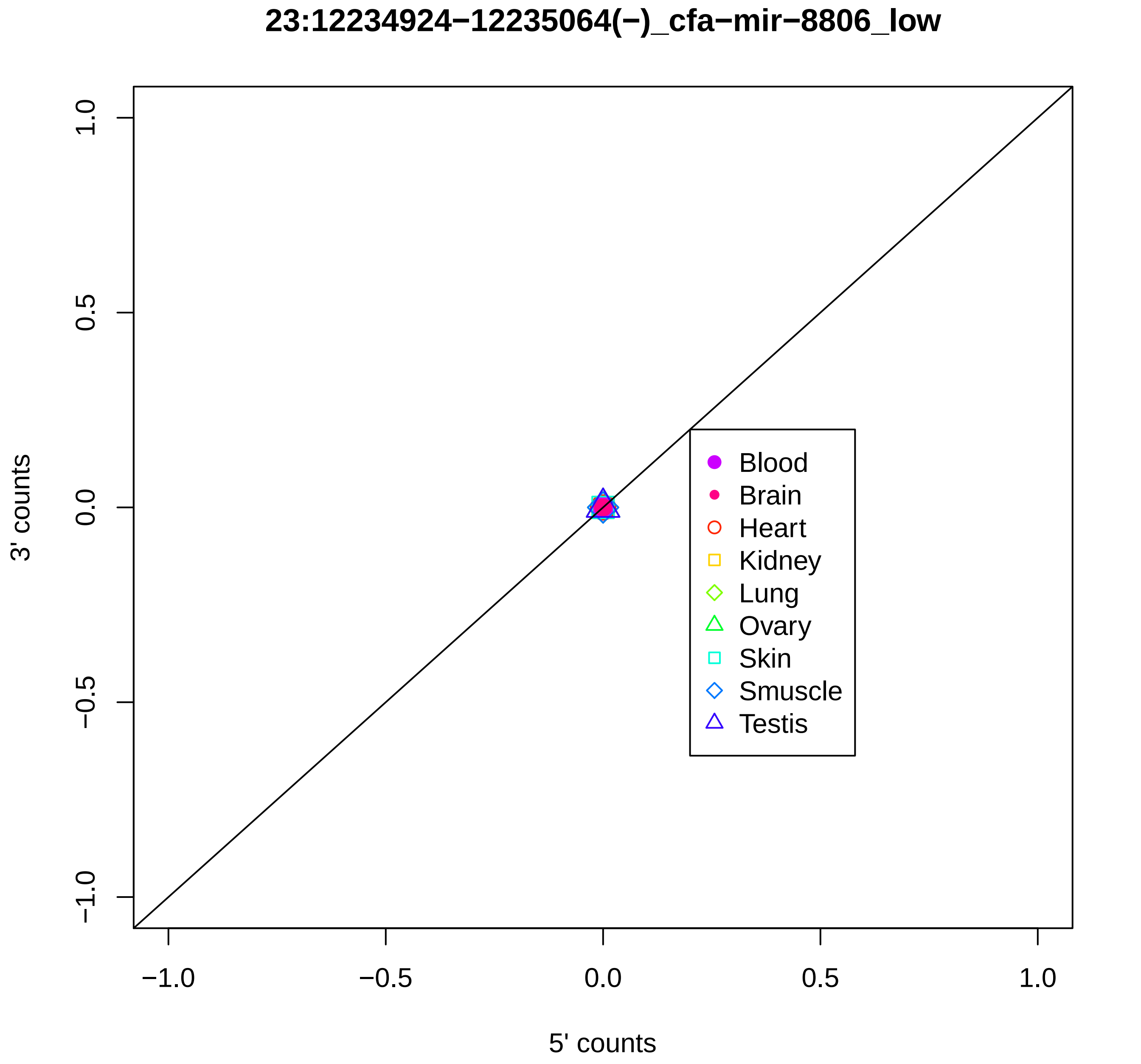

23:12234924−12235064(−)_cfa−mir−8806_low
1.0
0.5
0.0
−0.5
−1.0
3' counts
−1.0
−0.5
0.0
0.5
1.0
5' counts
Blood
Brain
Heart
Kidney
Lung
Ovary
Skin
Smuscle
Testis

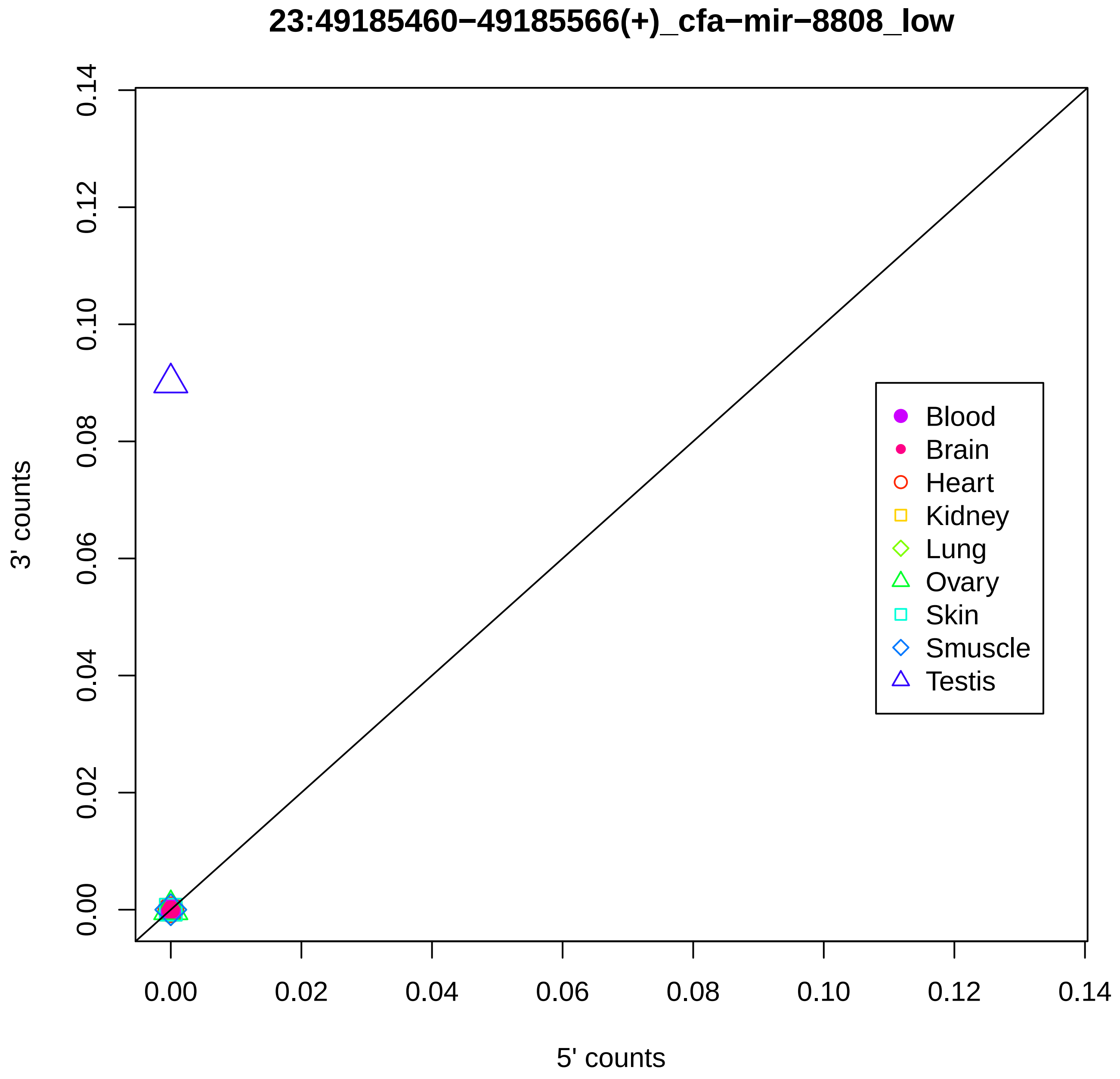
23:49185460−49185566(+)_cfa−mir−8808_low
3' counts
5' counts
0.00
0.02
0.04
0.06
0.08
0.10
0.12
0.14
Blood
Brain
Heart
Kidney
Lung
Ovary
Skin
Smuscle
Testis

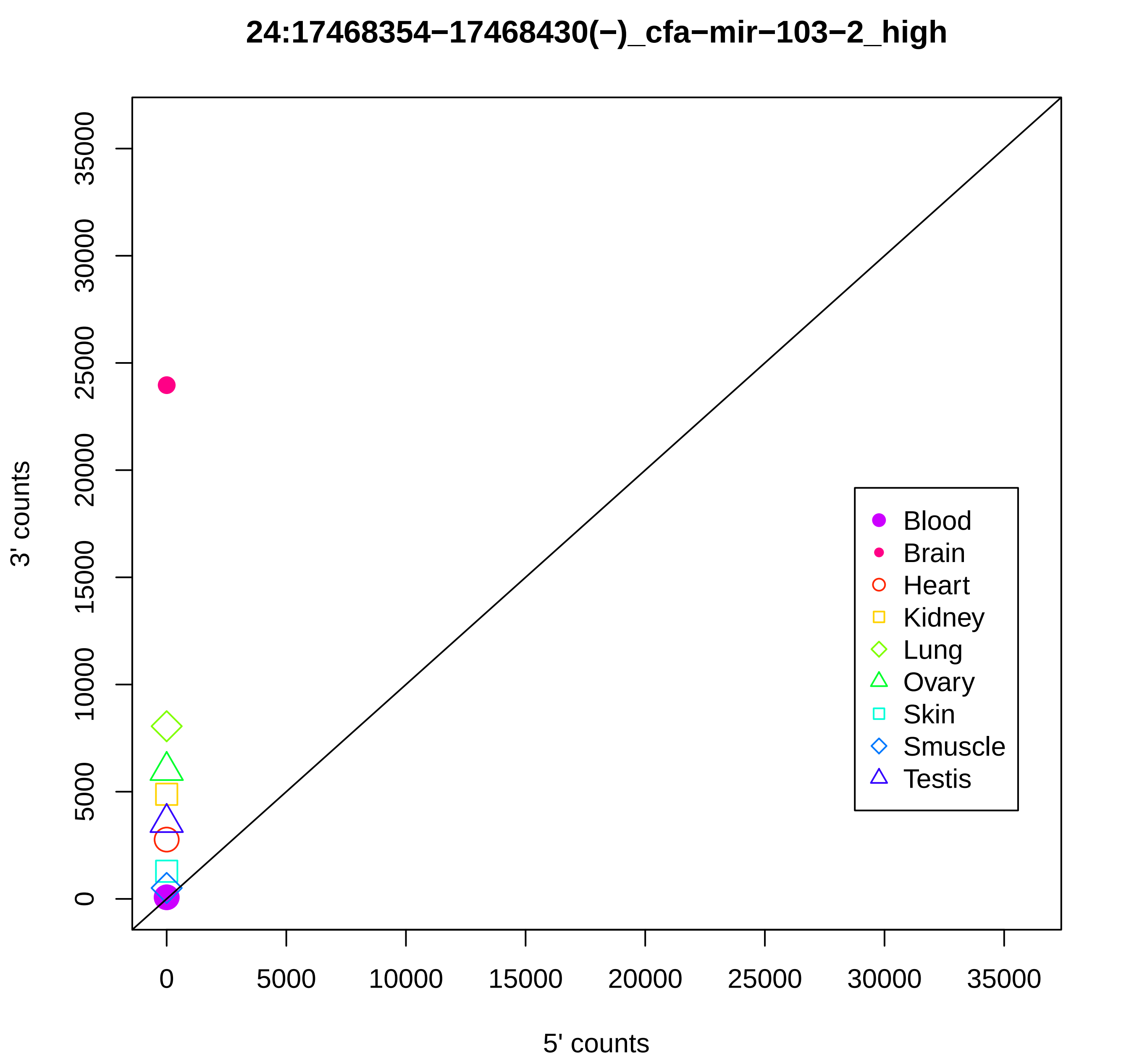

24:17468354−17468430(−)_cfa−mir−103−2_high
3' counts
5' counts
0
5000
10000
15000
20000
25000
30000
35000
Blood
Brain
Heart
Kidney
Lung
Ovary
Skin
Smuscle
Testis

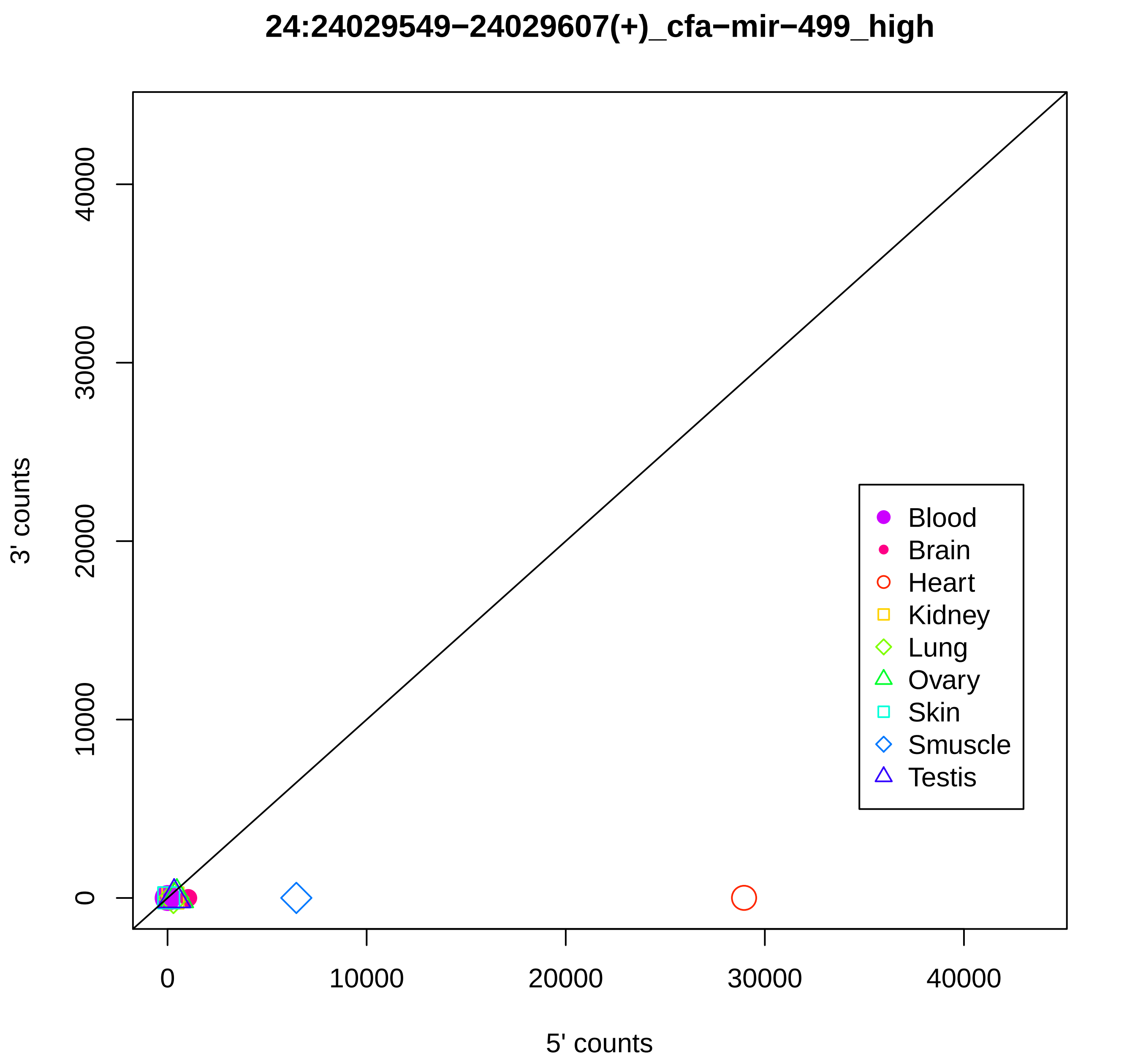
24:24029549−24029607(+)_cfa−mir−499_high
3' counts
5' counts
0
10000
20000
30000
40000
Blood
Brain
Heart
Kidney
Lung
Ovary
Skin
Smuscle
Testis

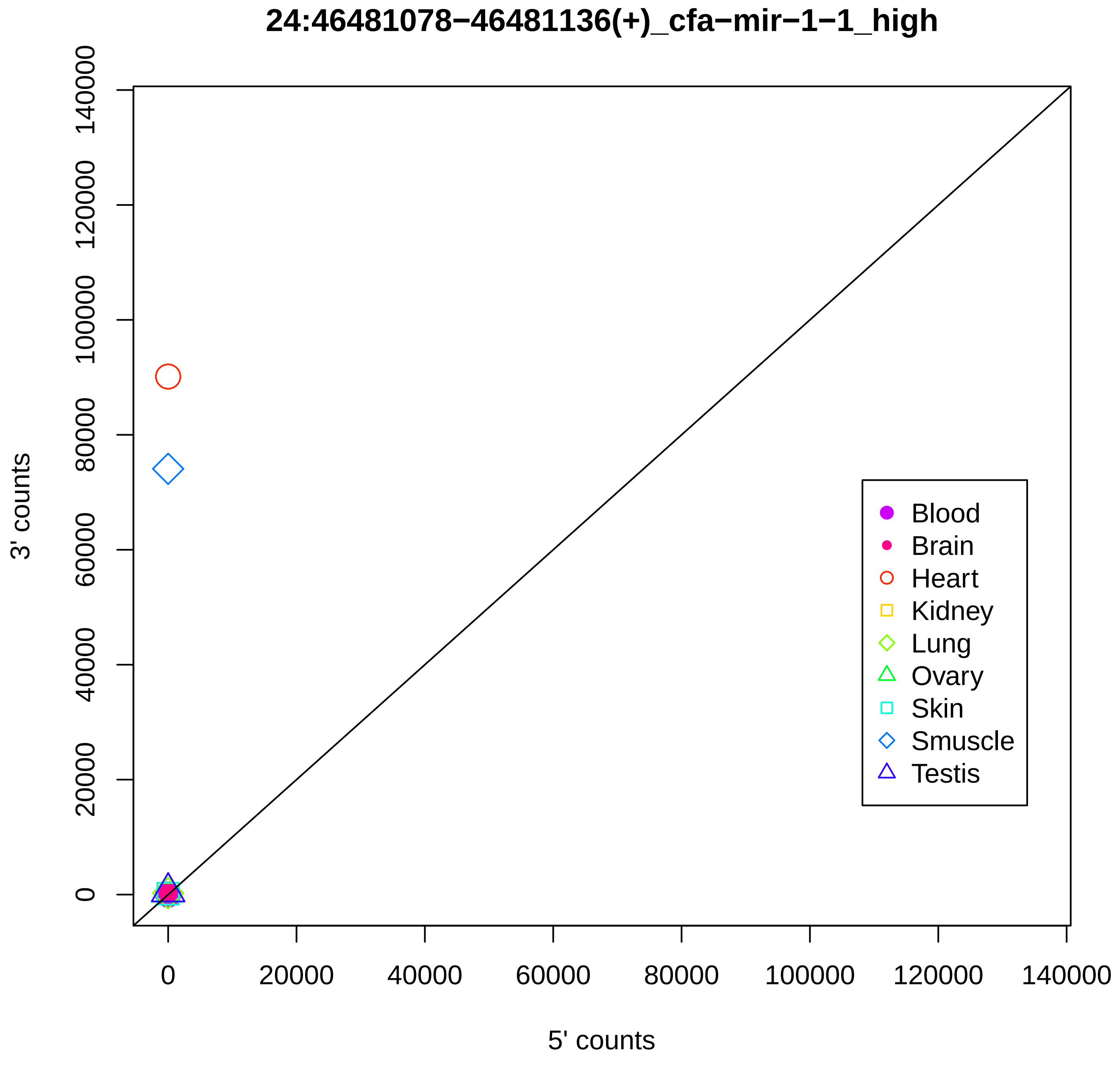
24:46481078−46481136(+)_cfa−mir−1−1_high
3' counts
5' counts
0
20000
40000
60000
80000
100000
120000
140000
Blood
Brain
Heart
Kidney
Lung
Ovary
Skin
Smuscle
Testis

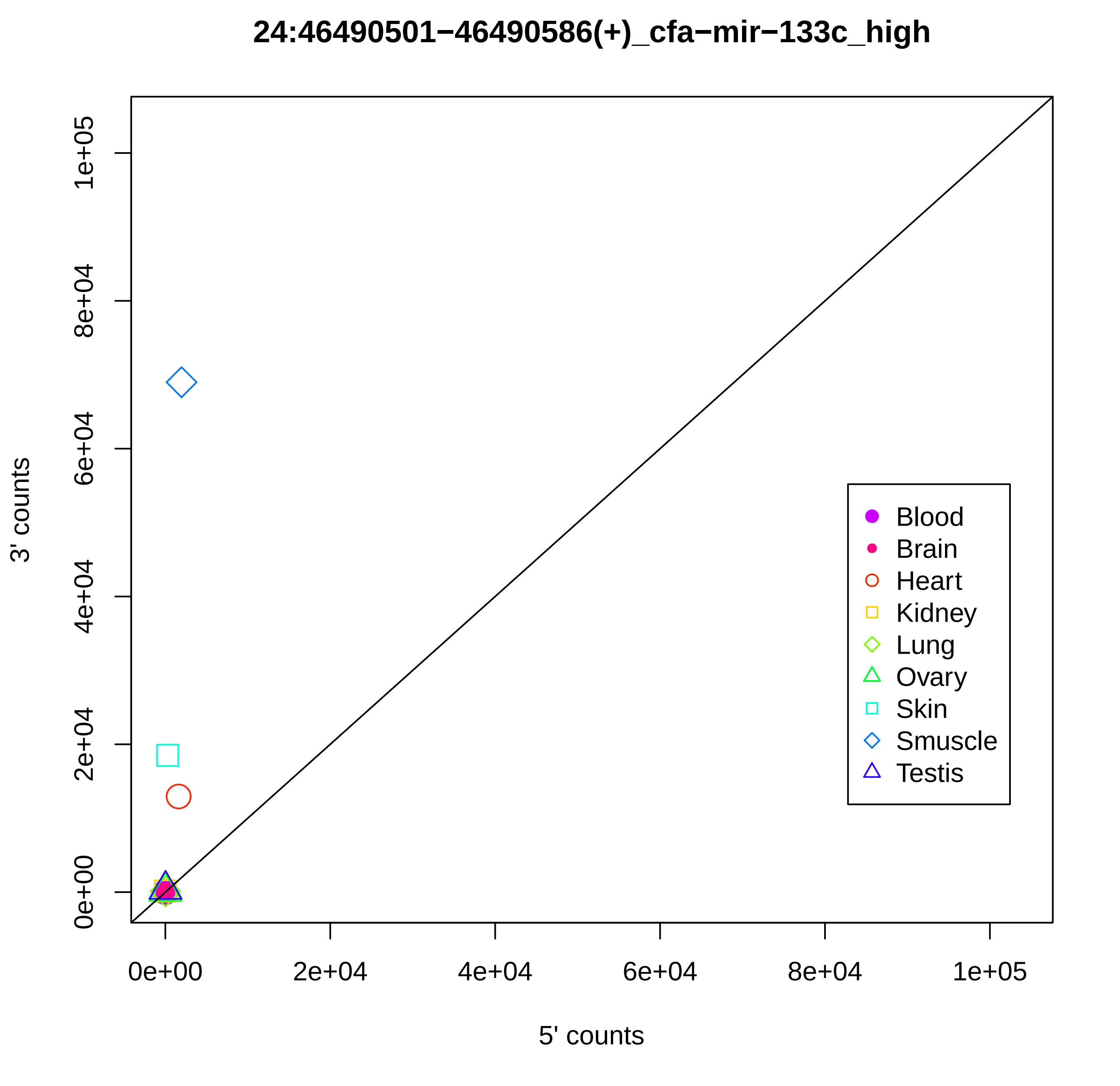

24:46490501−46490586(+)_cfa−mir−133c_high
3' counts
5' counts
0e+00
2e+04
4e+04
6e+04
8e+04
1e+05
Blood
Brain
Heart
Kidney
Lung
Ovary
Skin
Smuscle
Testis

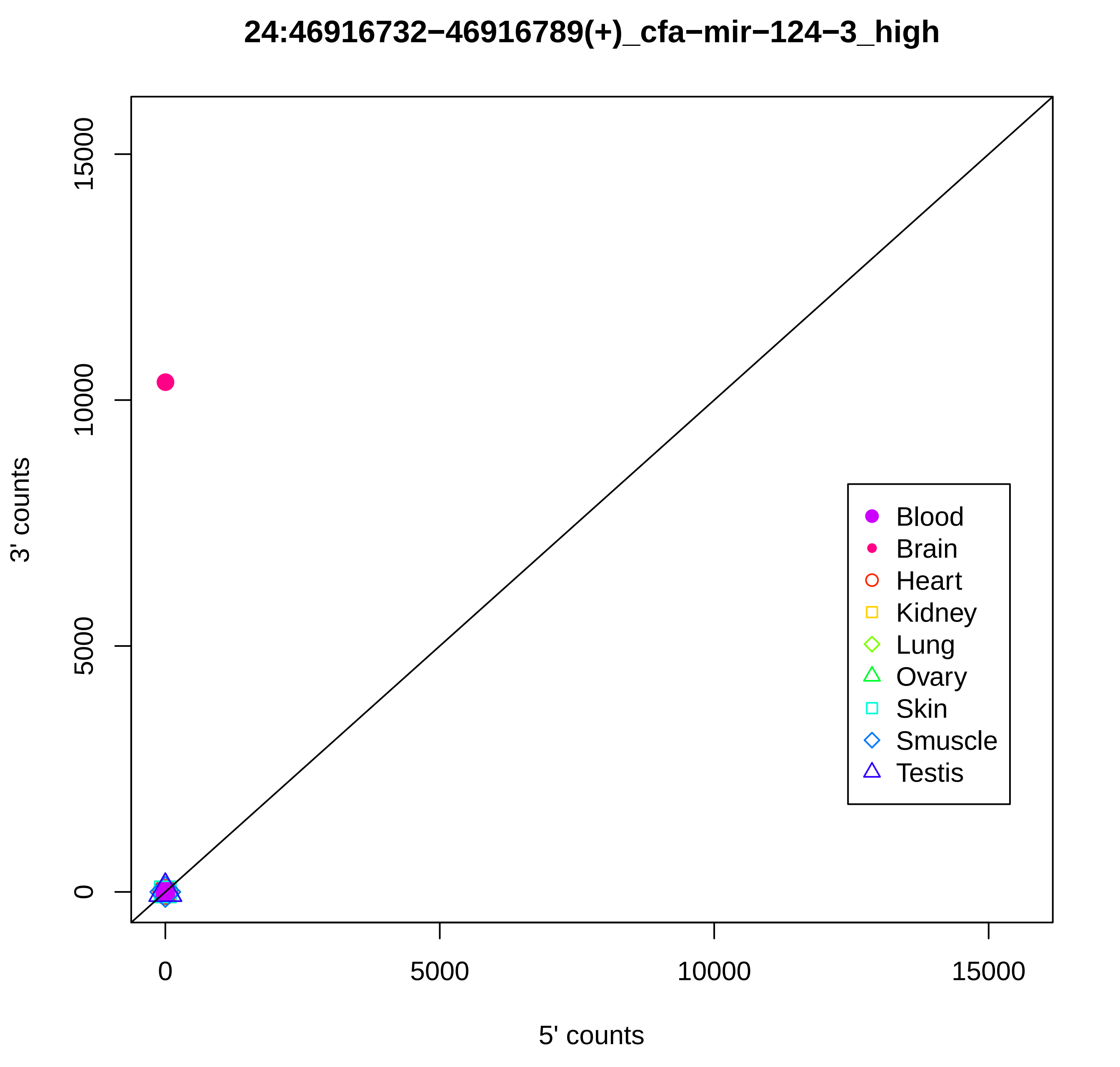

24:46916732−46916789(+)_cfa−mir−124−3_high
3' counts
5' counts
0
5000
10000
15000
Blood
Brain
Heart
Kidney
Lung
Ovary
Skin
Smuscle
Testis

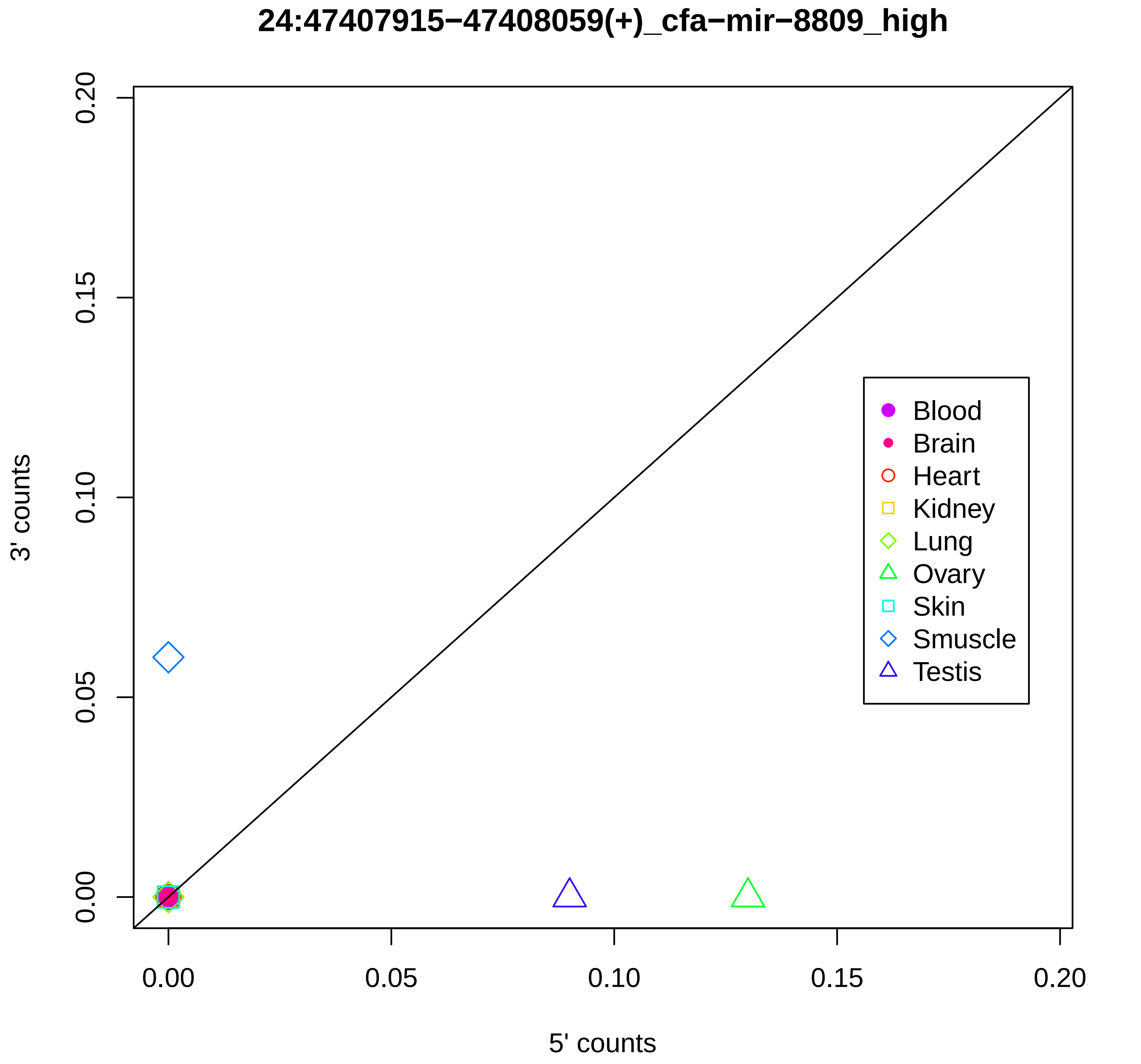

24:47407915−47408059(+)_cfa−mir−8809_high
0.00
0.05
0.10
0.15
0.20
3' counts
5' counts
Blood
Brain
Heart
Kidney
Lung
Ovary
Skin
Smuscle
Testis

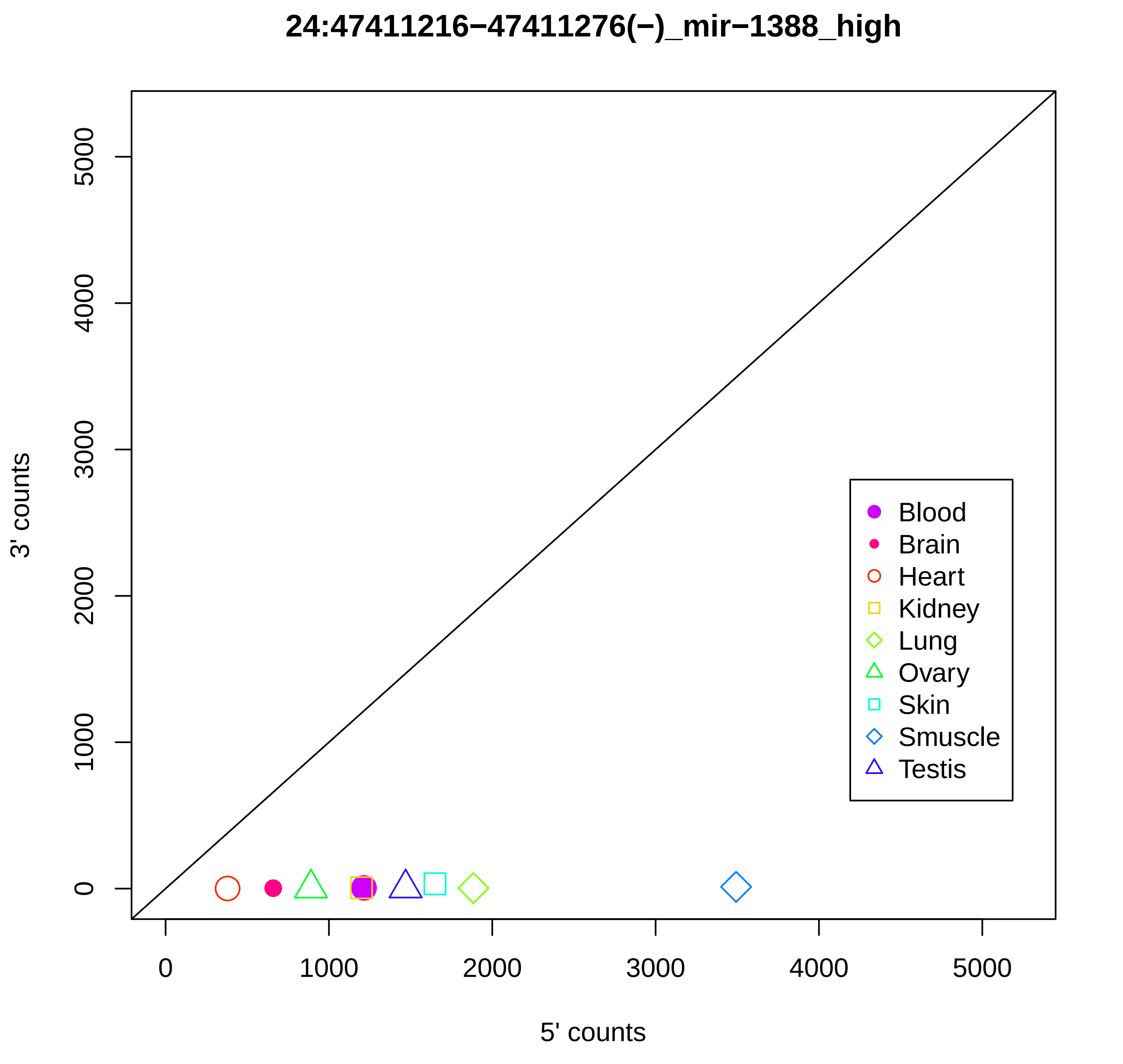
24:47411216−47411276(−)_mir−1388_high
3' counts
5' counts
0
1000
2000
3000
4000
5000
0
1000
2000
3000
4000
5000
Blood
Brain
Heart
Kidney
Lung
Ovary
Skin
Smuscle
Testis

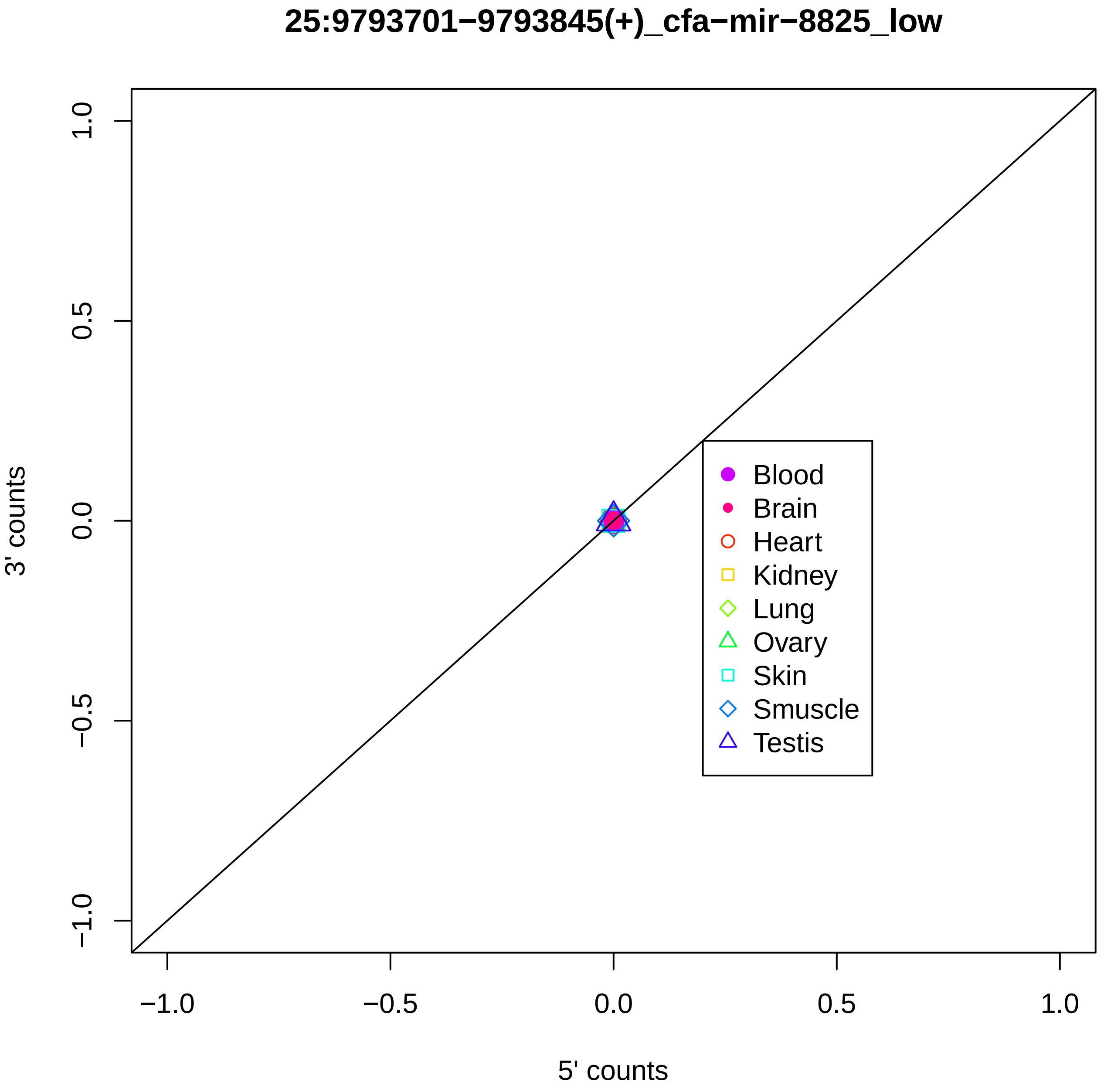

25:9793701−9793845(+)_cfa−mir−8825_low
3' counts
5' counts
−1.0
−0.5
0.0
0.5
1.0
Blood
Brain
Heart
Kidney
Lung
Ovary
Skin
Smuscle
Testis

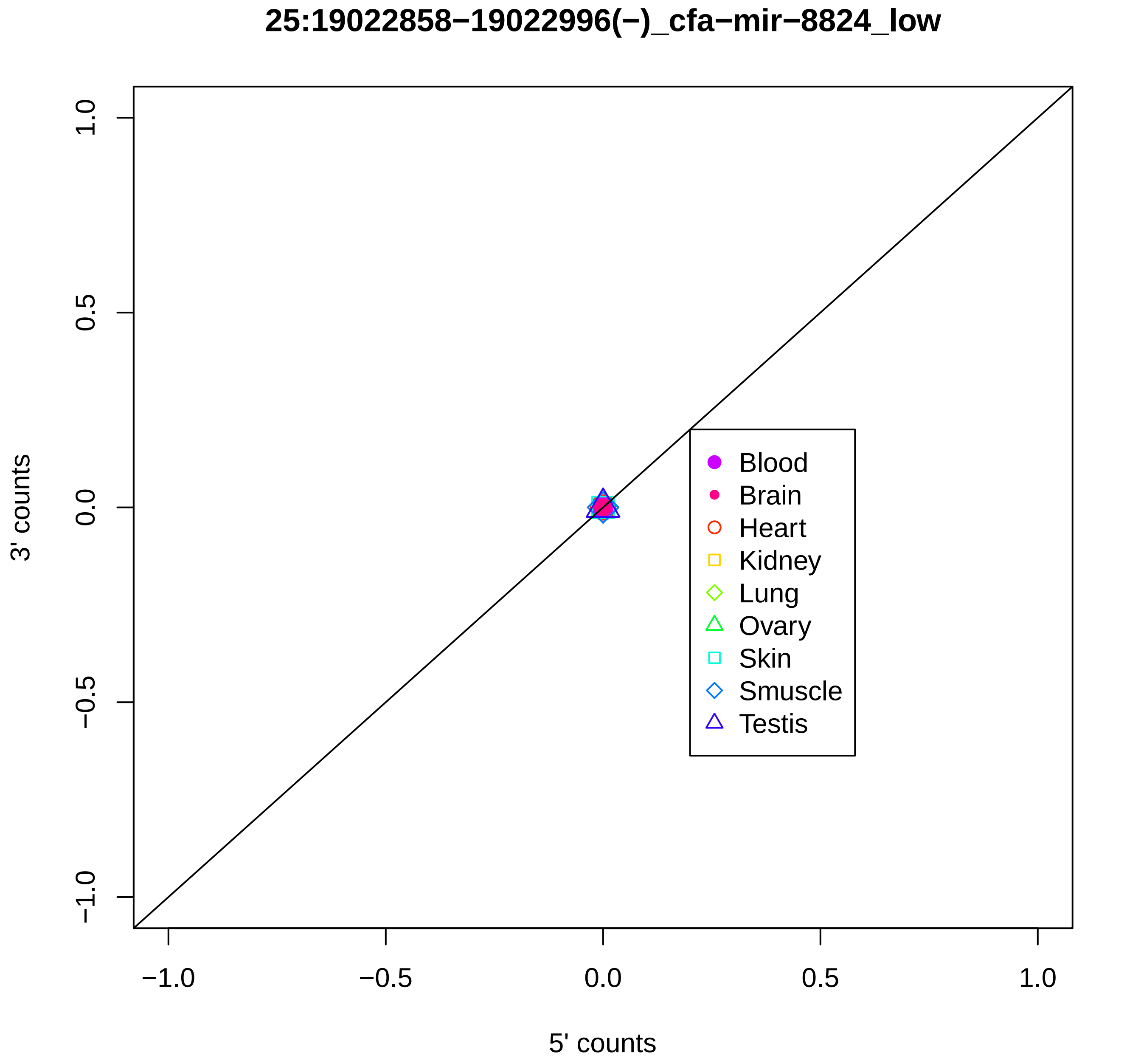

25:19022858−19022996(−)_cfa−mir−8824_low
3' counts
5' counts
1.0
0.5
0.0
−0.5
−1.0
Blood
Brain
Heart
Kidney
Lung
Ovary
Skin
Smuscle
Testis

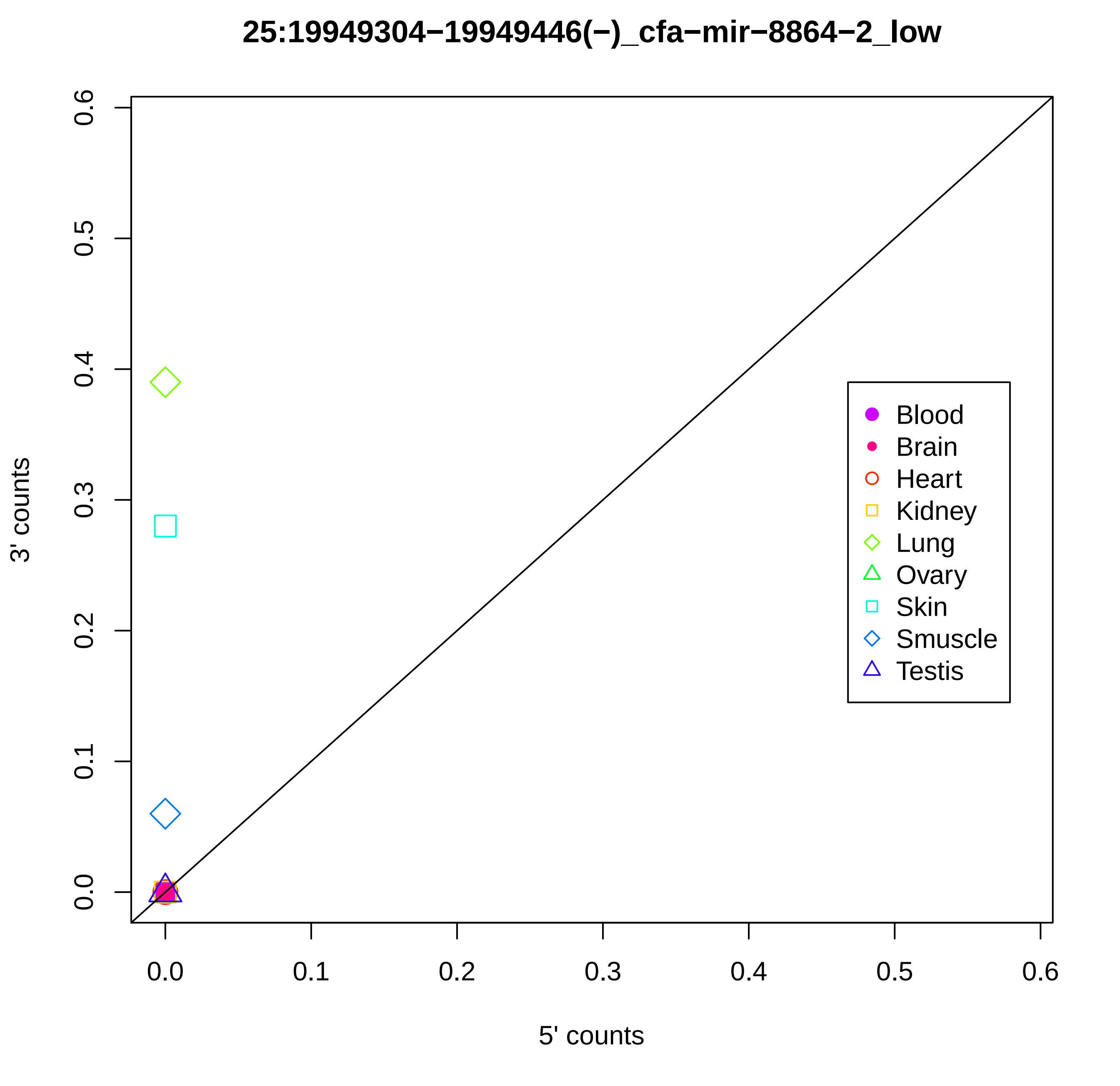

25:19949304−19949446(−)_cfa−mir−8864−2_low
3' counts
5' counts
0.0
0.1
0.2
0.3
0.4
0.5
0.6
Blood
Brain
Heart
Kidney
Lung
Ovary
Skin
Smuscle
Testis

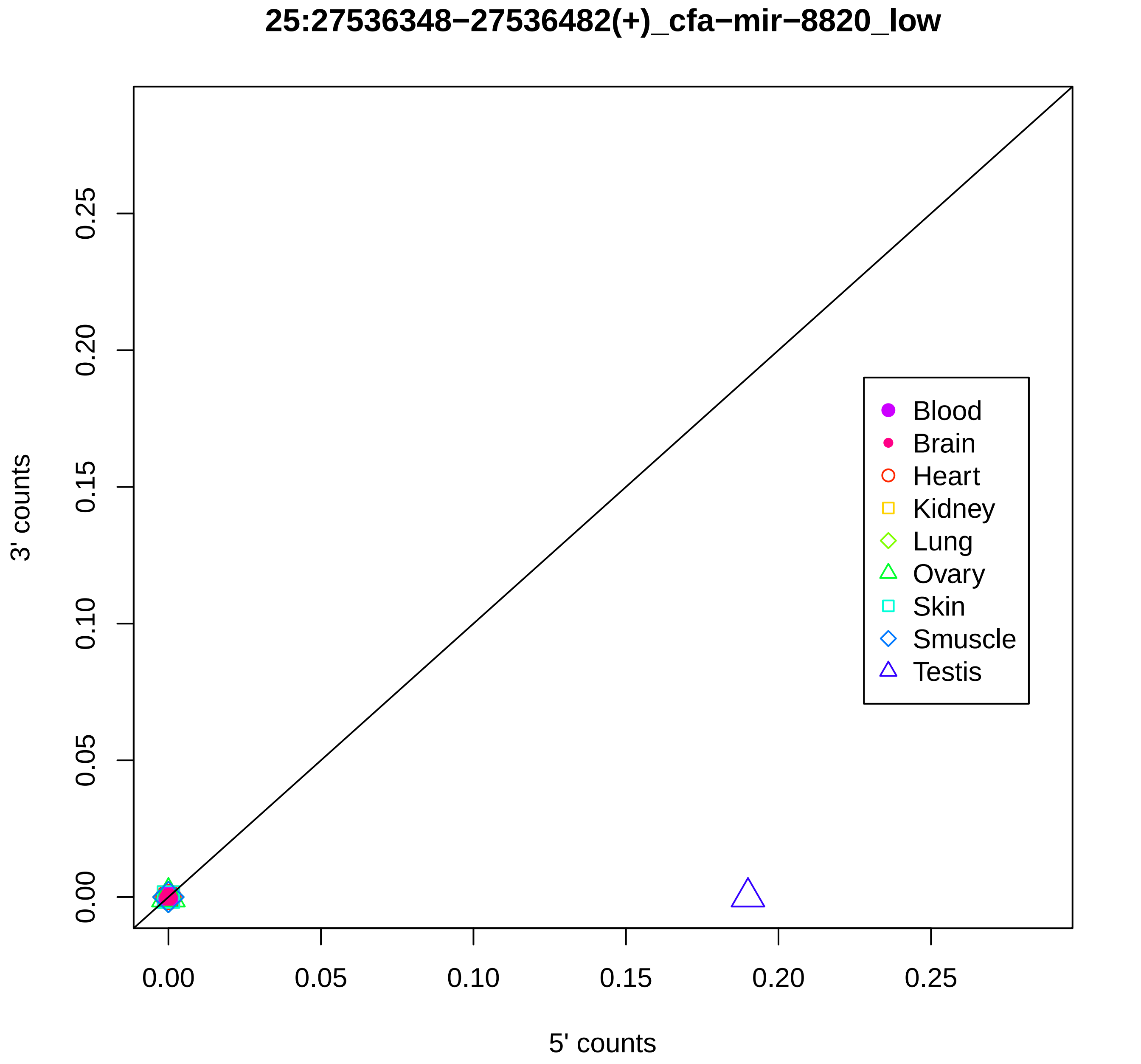

25:27536348−27536482(+)_cfa−mir−8820_low
3' counts
5' counts
0.00
0.05
0.10
0.15
0.20
0.25
Blood
Brain
Heart
Kidney
Lung
Ovary
Skin
Smuscle
Testis

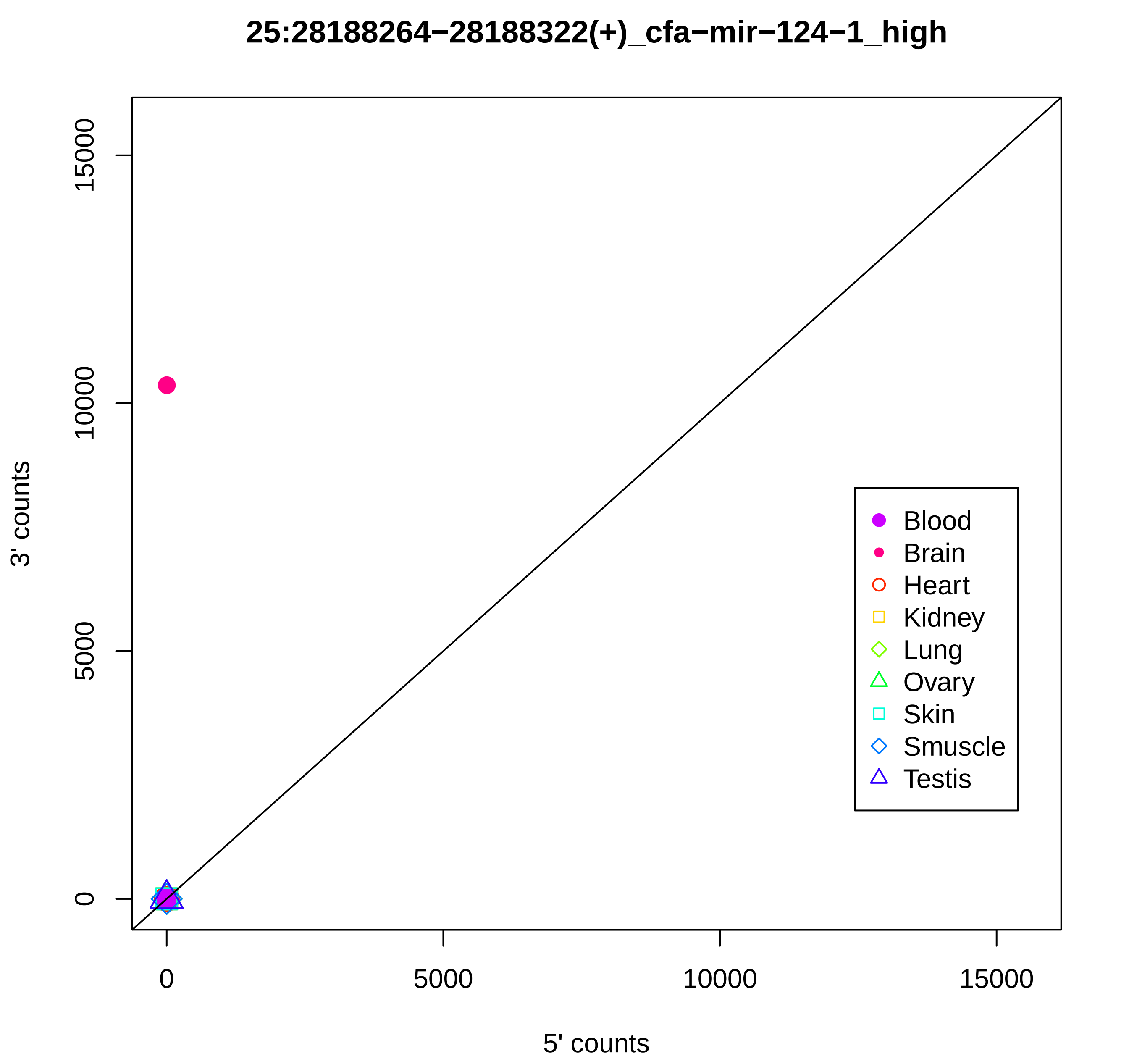

25:28188264−28188322(+)_cfa−mir−124−1_high
3' counts
5' counts
0
5000
10000
15000
0
5000
10000
15000
Blood
Brain
Heart
Kidney
Lung
Ovary
Skin
Smuscle
Testis

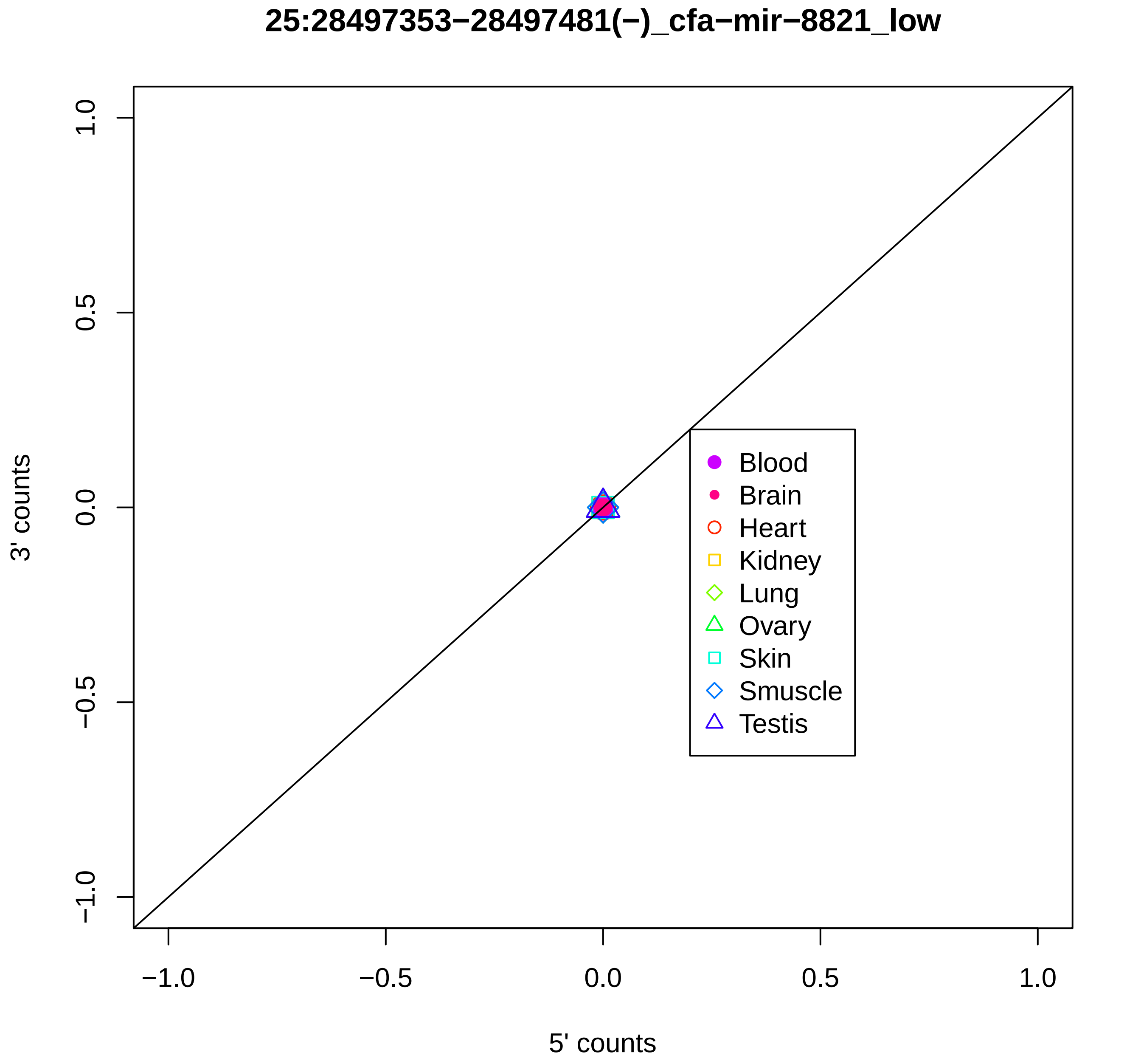

25:28497353−28497481(−)_cfa−mir−8821_low
3' counts
5' counts
−1.0
−0.5
0.0
0.5
1.0
Blood
Brain
Heart
Kidney
Lung
Ovary
Skin
Smuscle
Testis

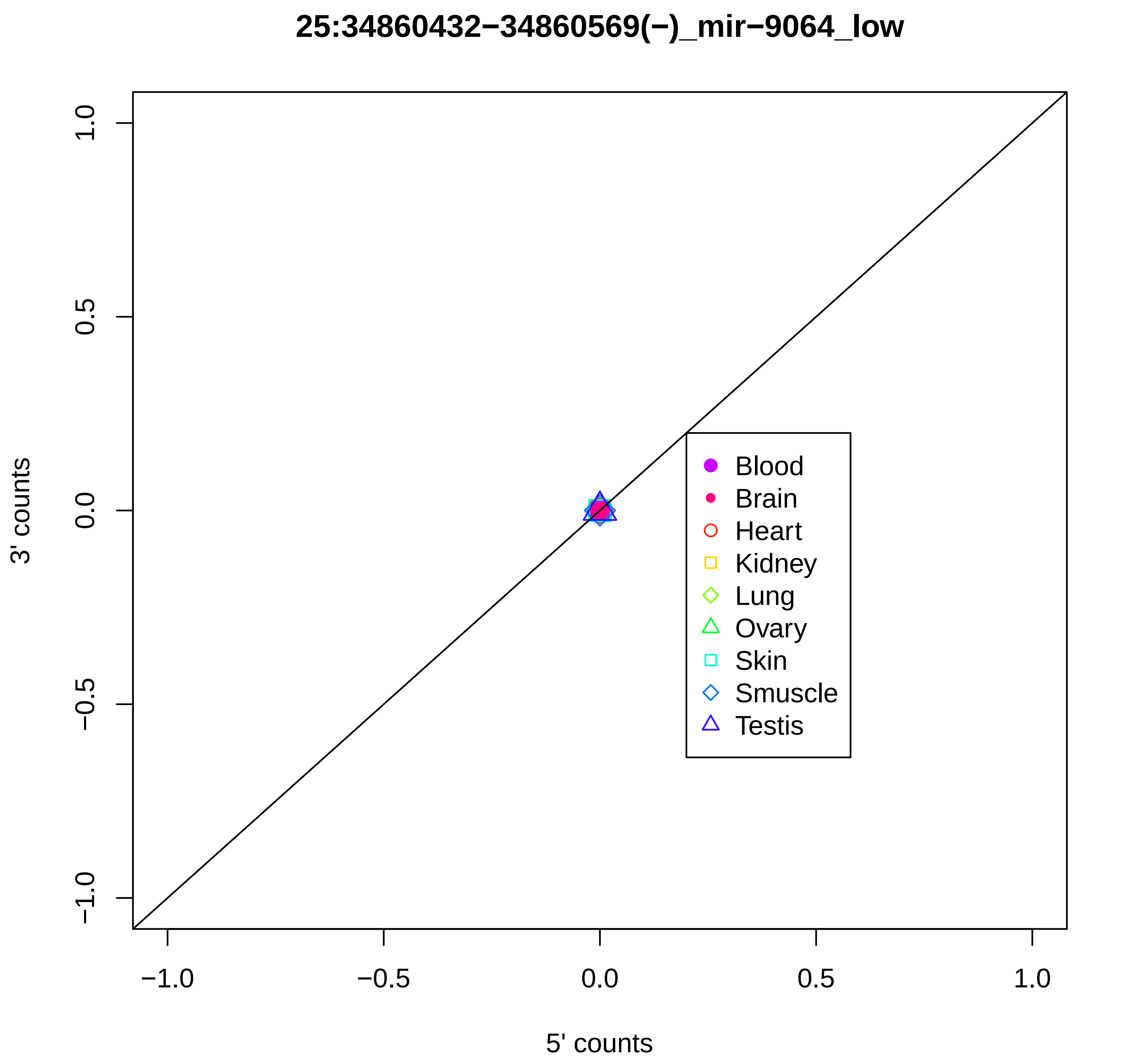

25:34860432−34860569(−)_mir−9064_low
3' counts
5' counts
1.0
0.5
0.0
−0.5
−1.0
Blood
Brain
Heart
Kidney
Lung
Ovary
Skin
Smuscle
Testis

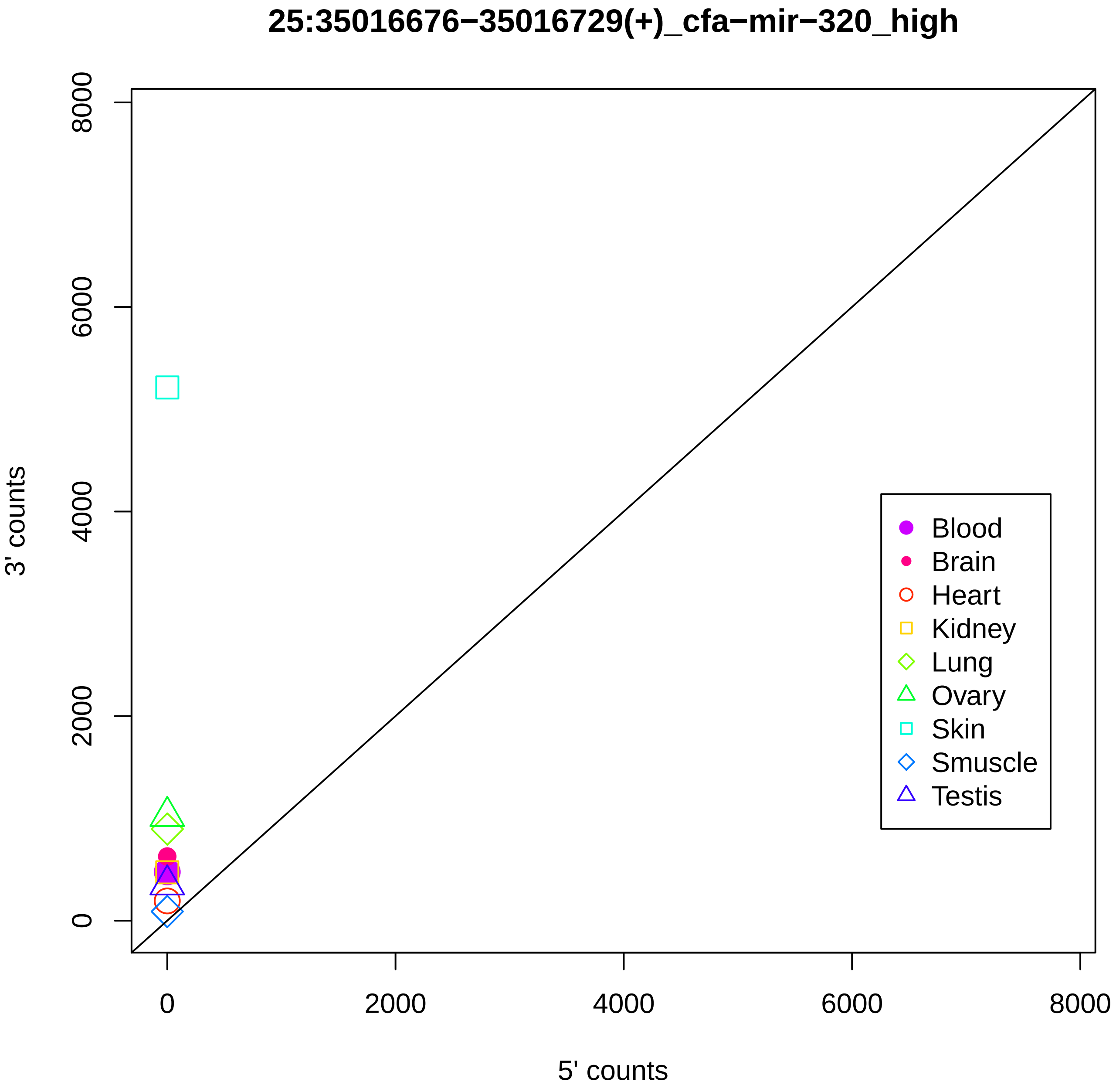

25:35016676−35016729(+)_cfa−mir−320_high
3' counts
5' counts
0
2000
4000
6000
8000
0
2000
4000
6000
8000
Blood
Brain
Heart
Kidney
Lung
Ovary
Skin
Smuscle
Testis

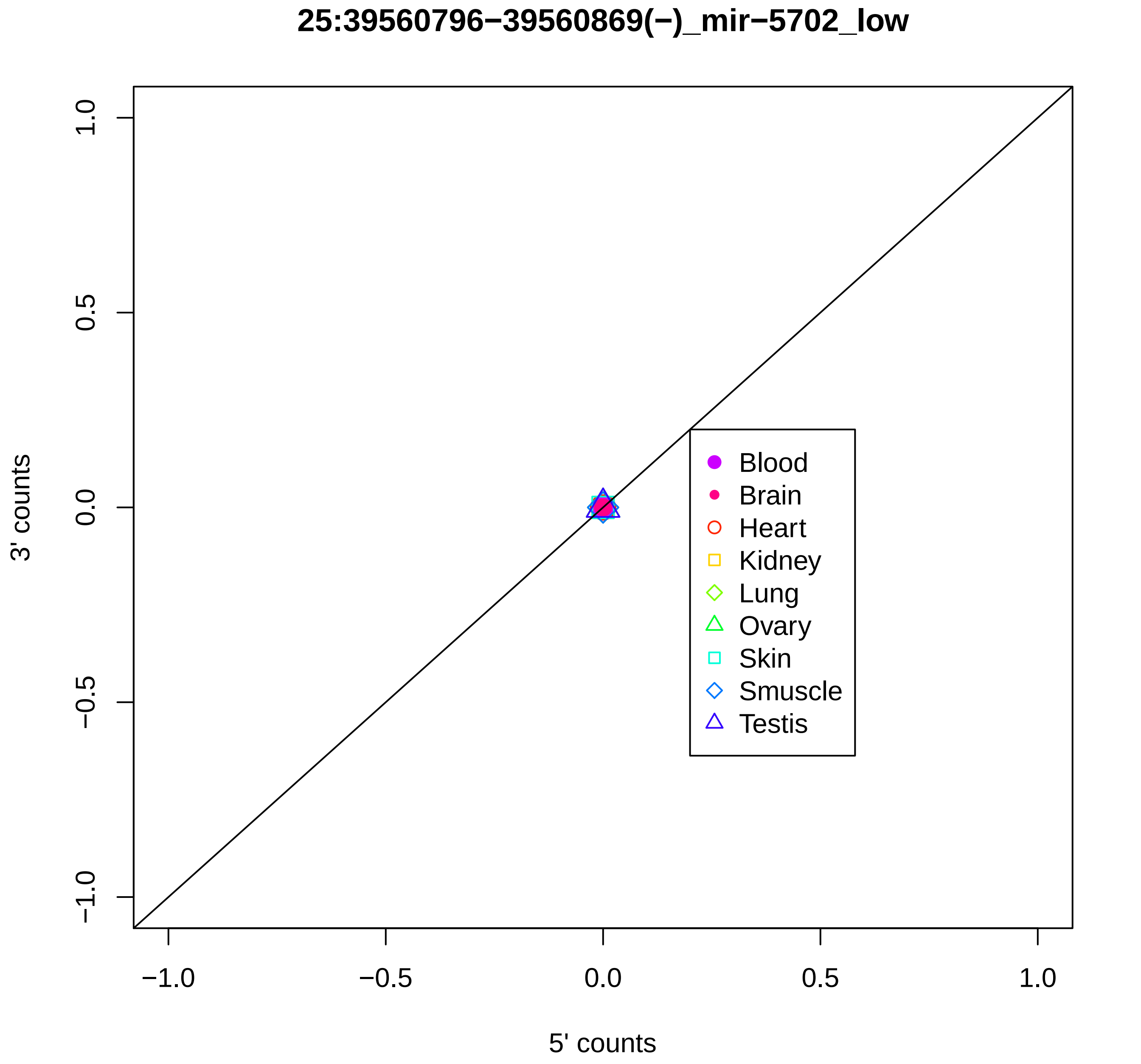
25:39560796−39560869(−)_mir−5702_low
3' counts
5' counts
−1.0
−0.5
0.0
0.5
1.0
Blood
Brain
Heart
Kidney
Lung
Ovary
Skin
Smuscle
Testis

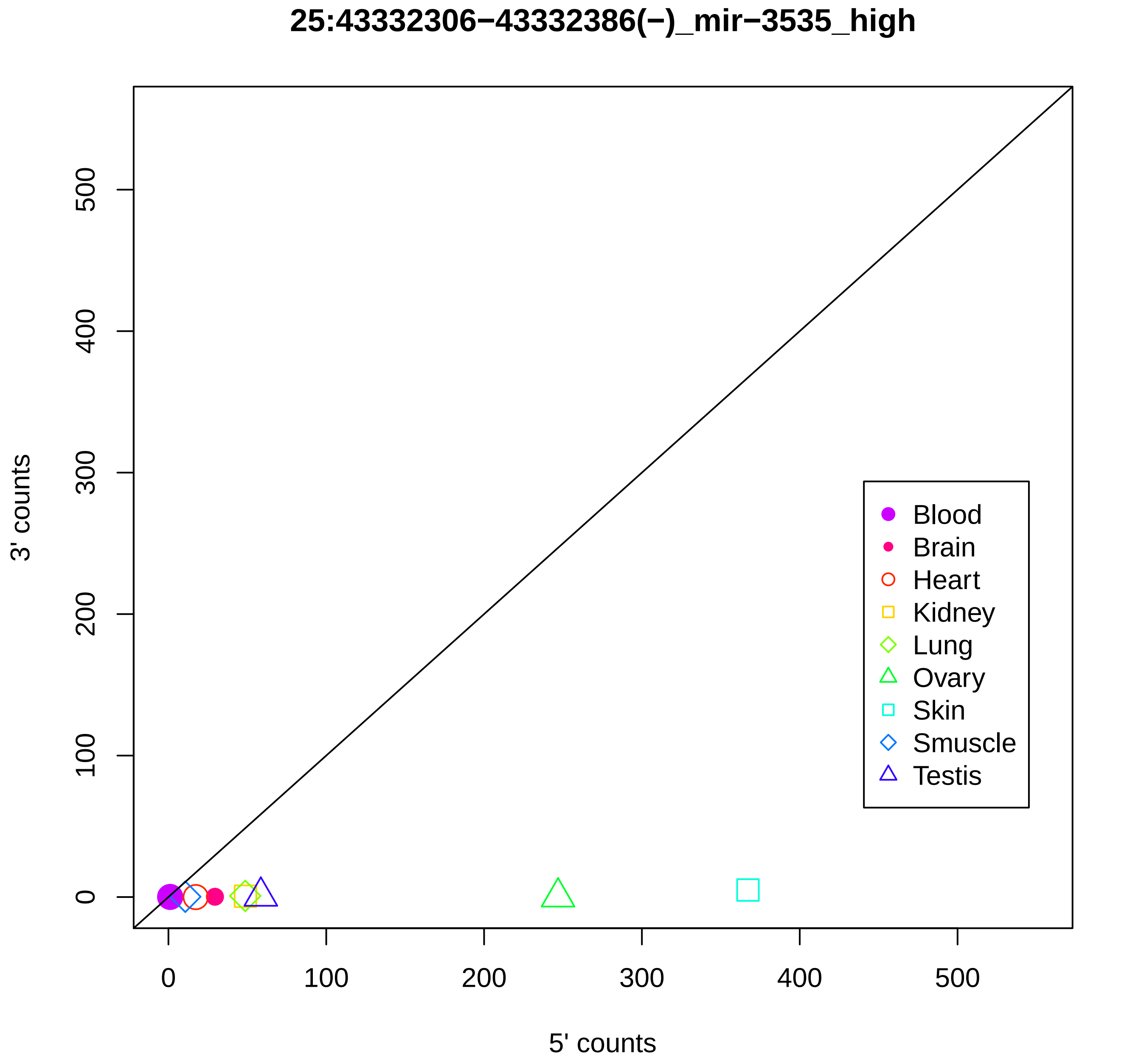
25:43332306−43332386(−)_mir−3535_high
3' counts
5' counts
0
100
200
300
400
500
0
100
200
300
400
500
Blood
Brain
Heart
Kidney
Lung
Ovary
Skin
Smuscle
Testis

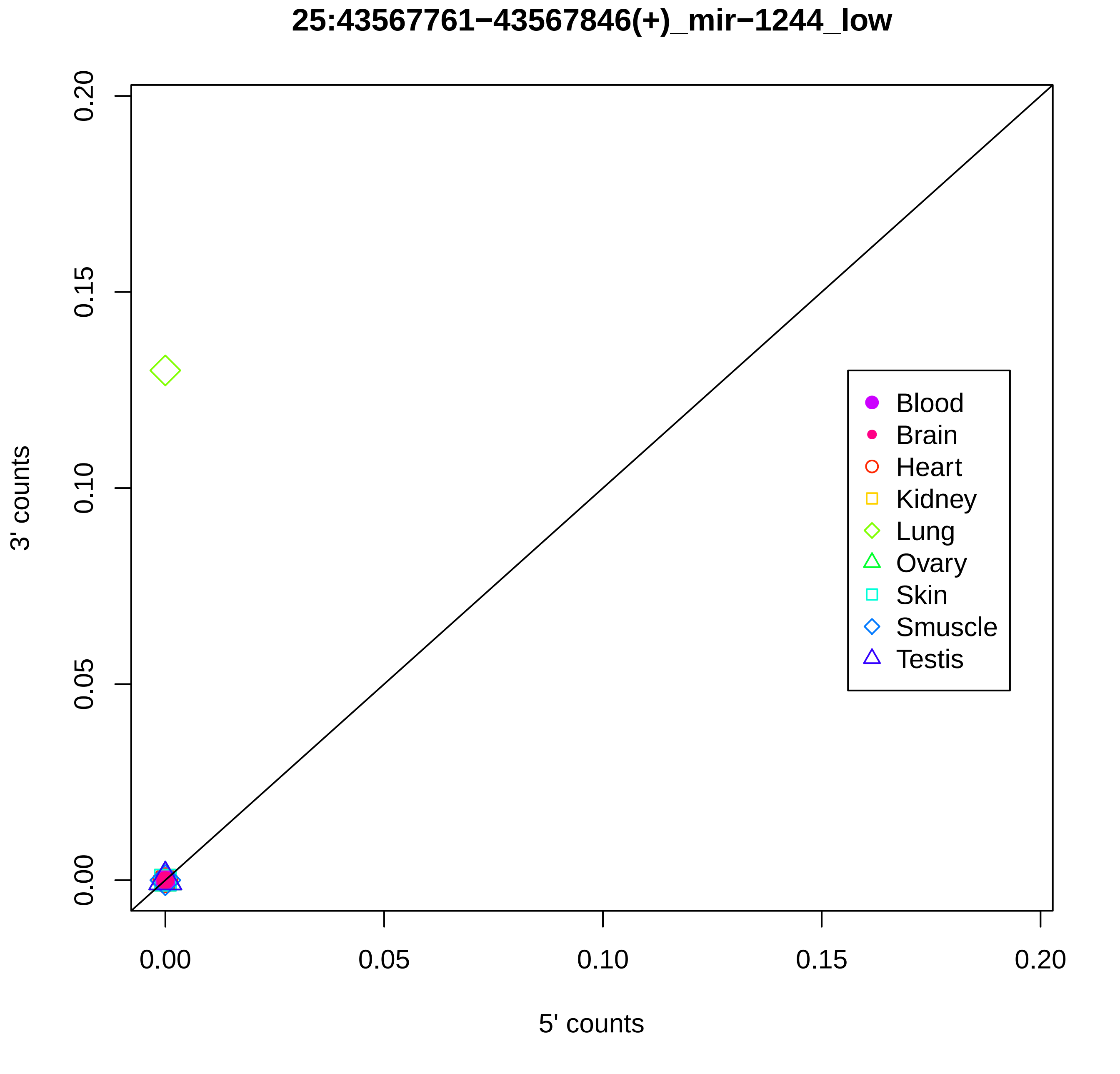

25:43567761−43567846(+)_mir−1244_low
3' counts
5' counts
0.00
0.05
0.10
0.15
0.20
Blood
Brain
Heart
Kidney
Lung
Ovary
Skin
Smuscle
Testis

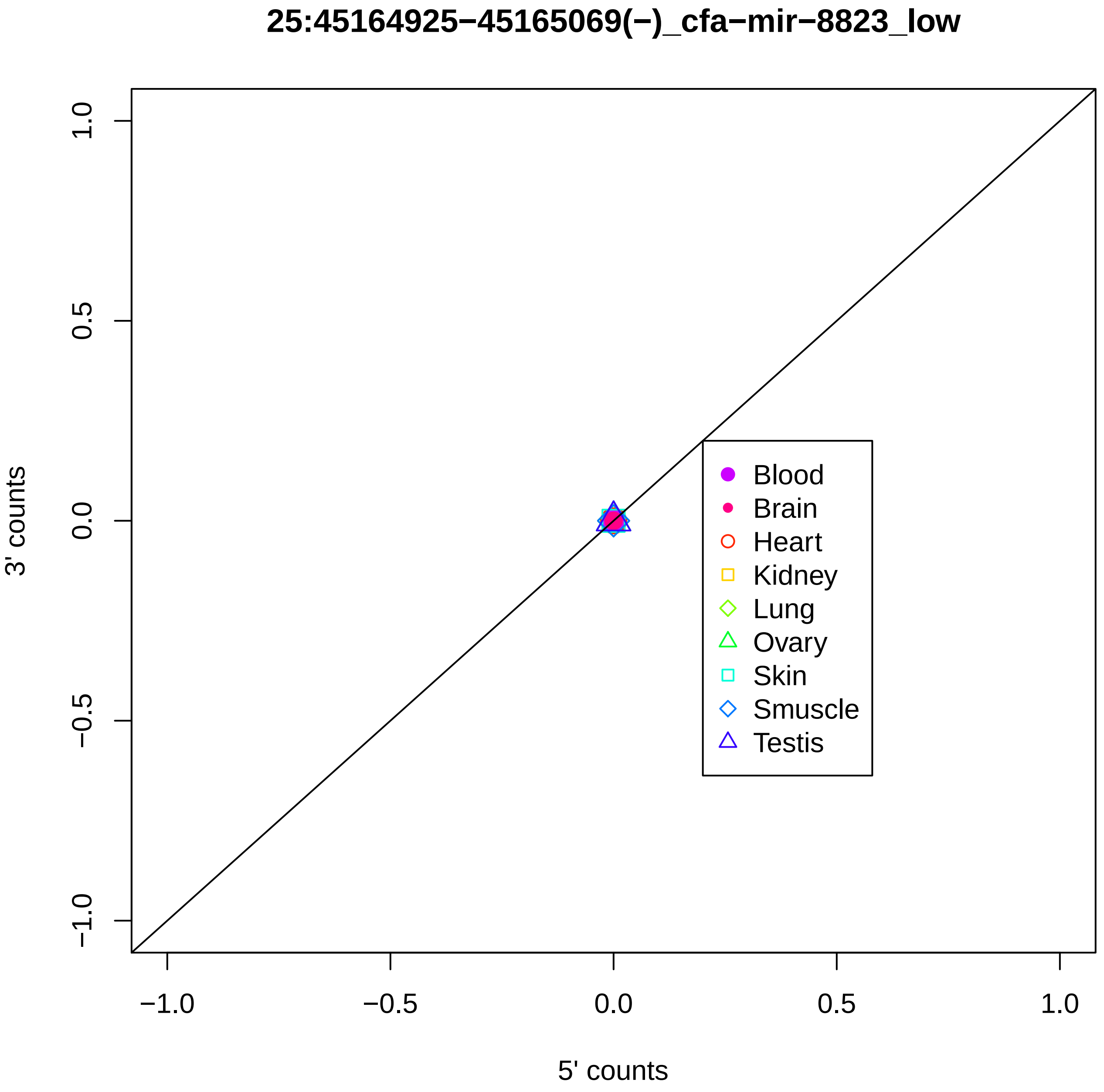

25:45164925−45165069(−)_cfa−mir−8823_low
Blood
Brain
Heart
Kidney
Lung
Ovary
Skin
Smuscle
Testis
1.0
0.5
0.0
−0.5
−1.0
3' counts
−1.0
−0.5
0.0
0.5
1.0
5' counts

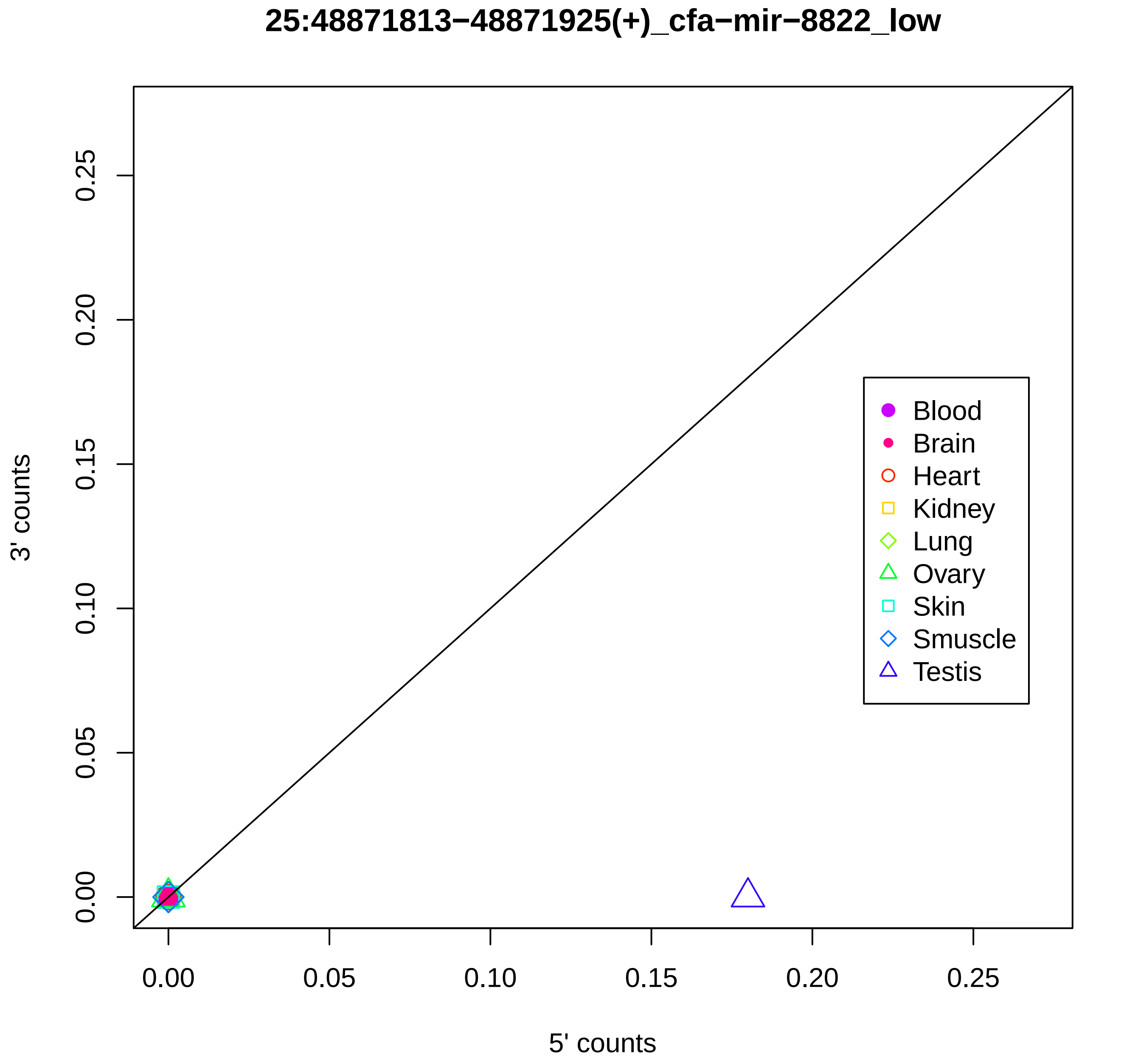
25:48871813−48871925(+)_cfa−mir−8822_low
3' counts
5' counts
0.00
0.05
0.10
0.15
0.20
0.25
Blood
Brain
Heart
Kidney
Lung
Ovary
Skin
Smuscle
Testis

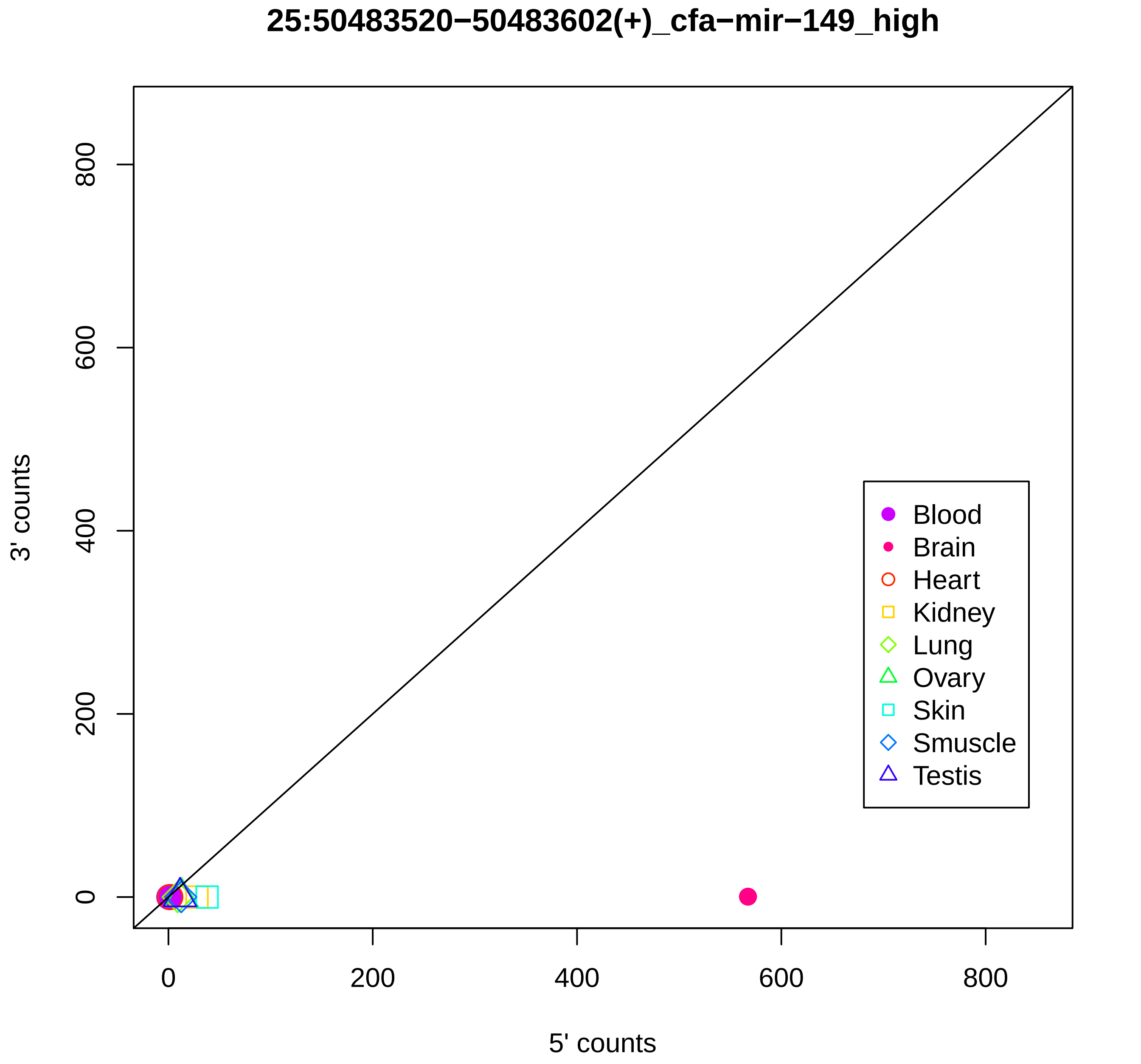

25:50483520−50483602(+)_cfa−mir−149_high
3' counts
5' counts
0
200
400
600
800
0
200
400
600
800
Blood
Brain
Heart
Kidney
Lung
Ovary
Skin
Smuscle
Testis

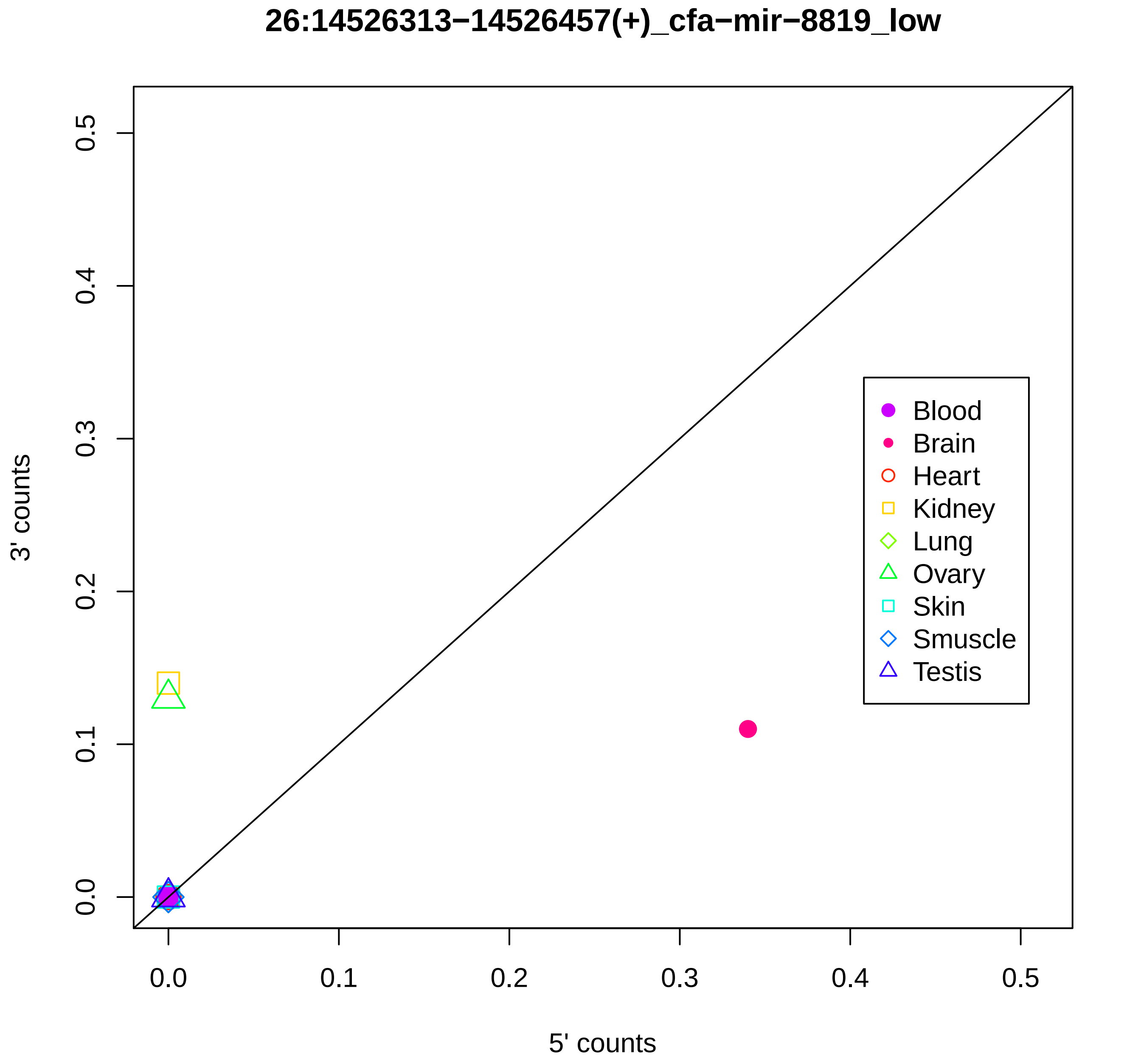

26:14526313−14526457(+)_cfa−mir−8819_low
3' counts
5' counts
0.0
0.1
0.2
0.3
0.4
0.5
Blood
Brain
Heart
Kidney
Lung
Ovary
Skin
Smuscle
Testis

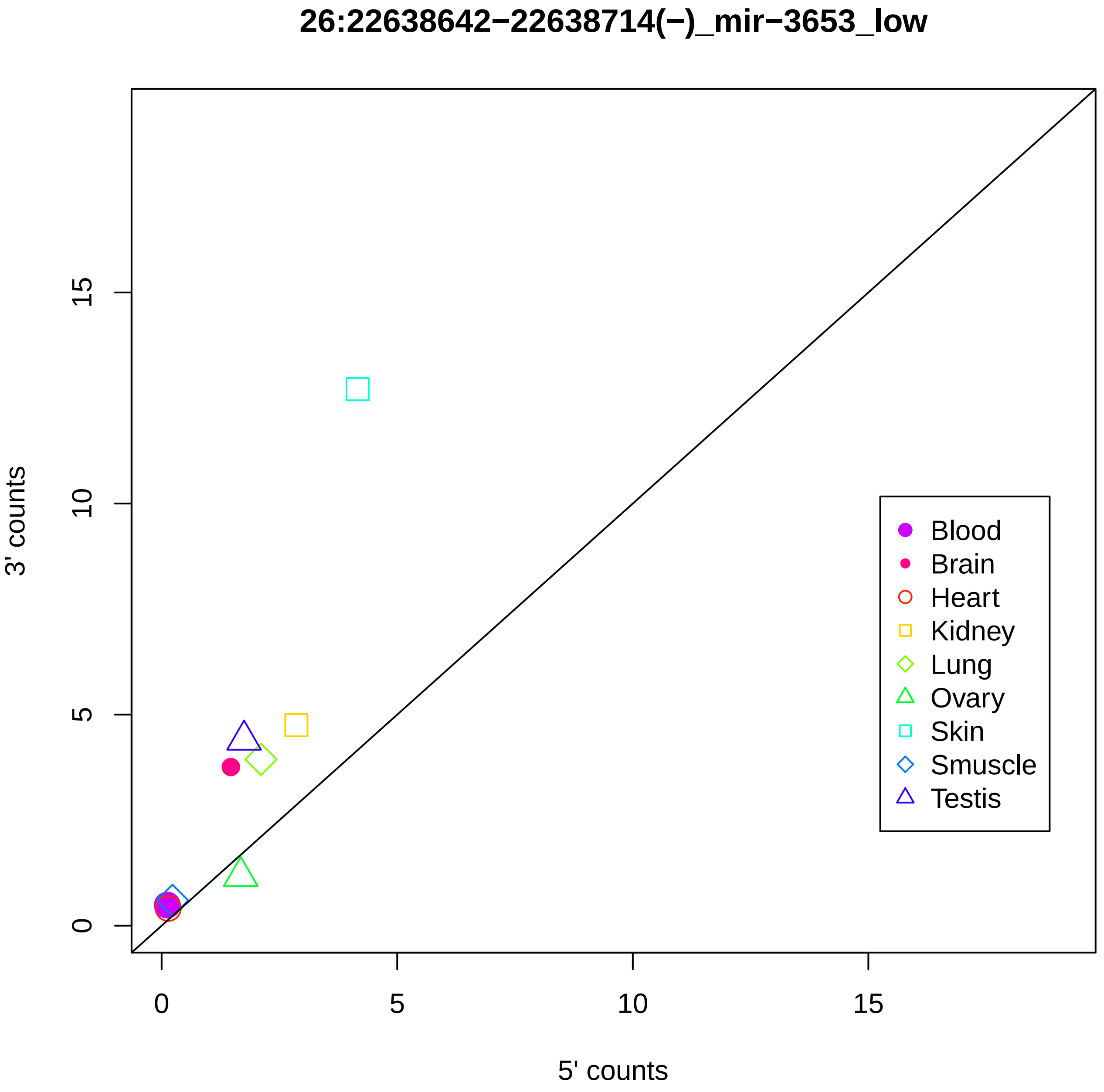

26:22638642−22638714(−)_mir−3653_low
3' counts
5' counts
0
5
10
15
0
5
10
15
Blood
Brain
Heart
Kidney
Lung
Ovary
Skin
Smuscle
Testis

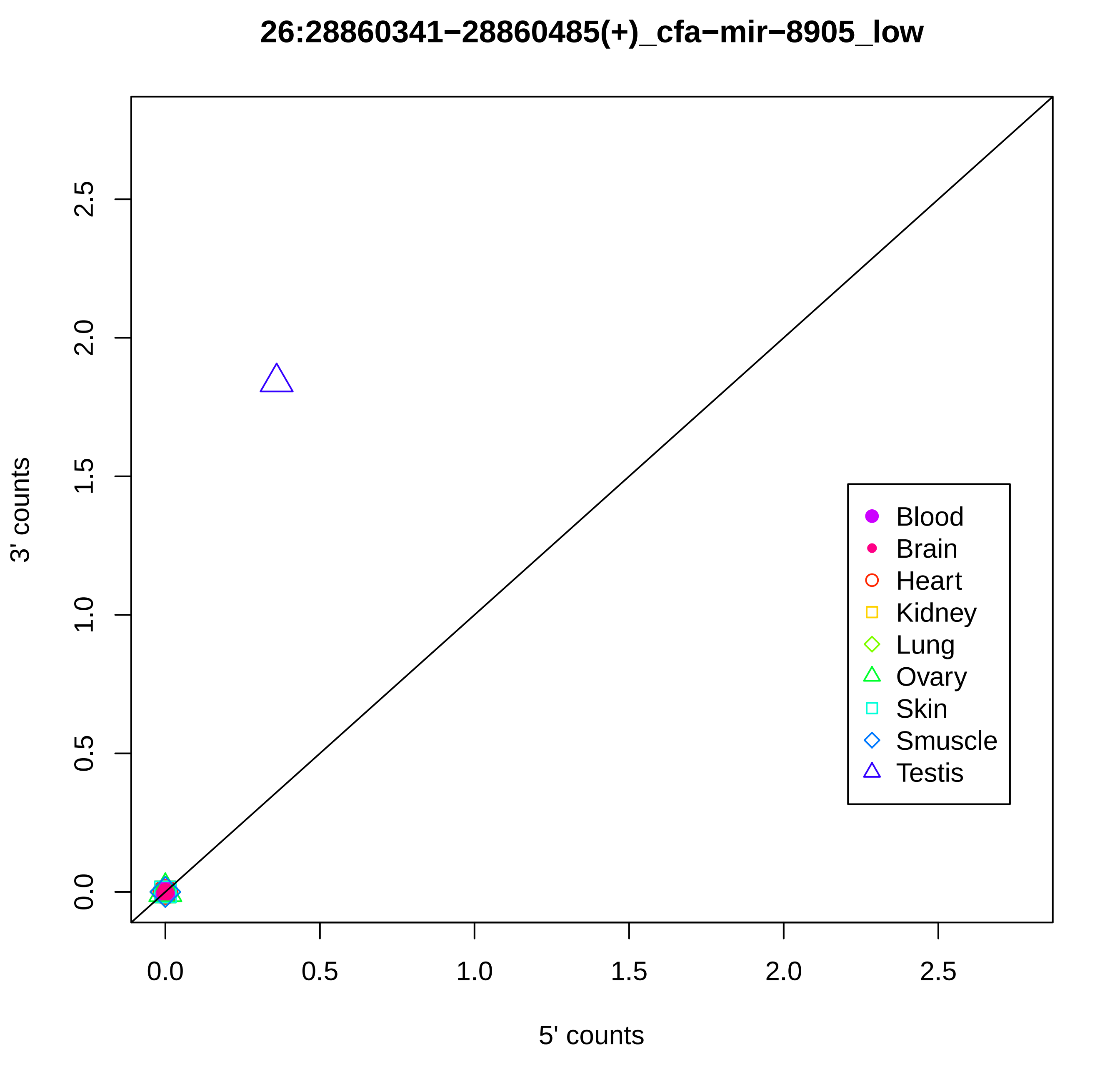
26:28860341−28860485(+)_cfa−mir−8905_low
3' counts
5' counts
0.0
0.5
1.0
1.5
2.0
2.5
Blood
Brain
Heart
Kidney
Lung
Ovary
Skin
Smuscle
Testis

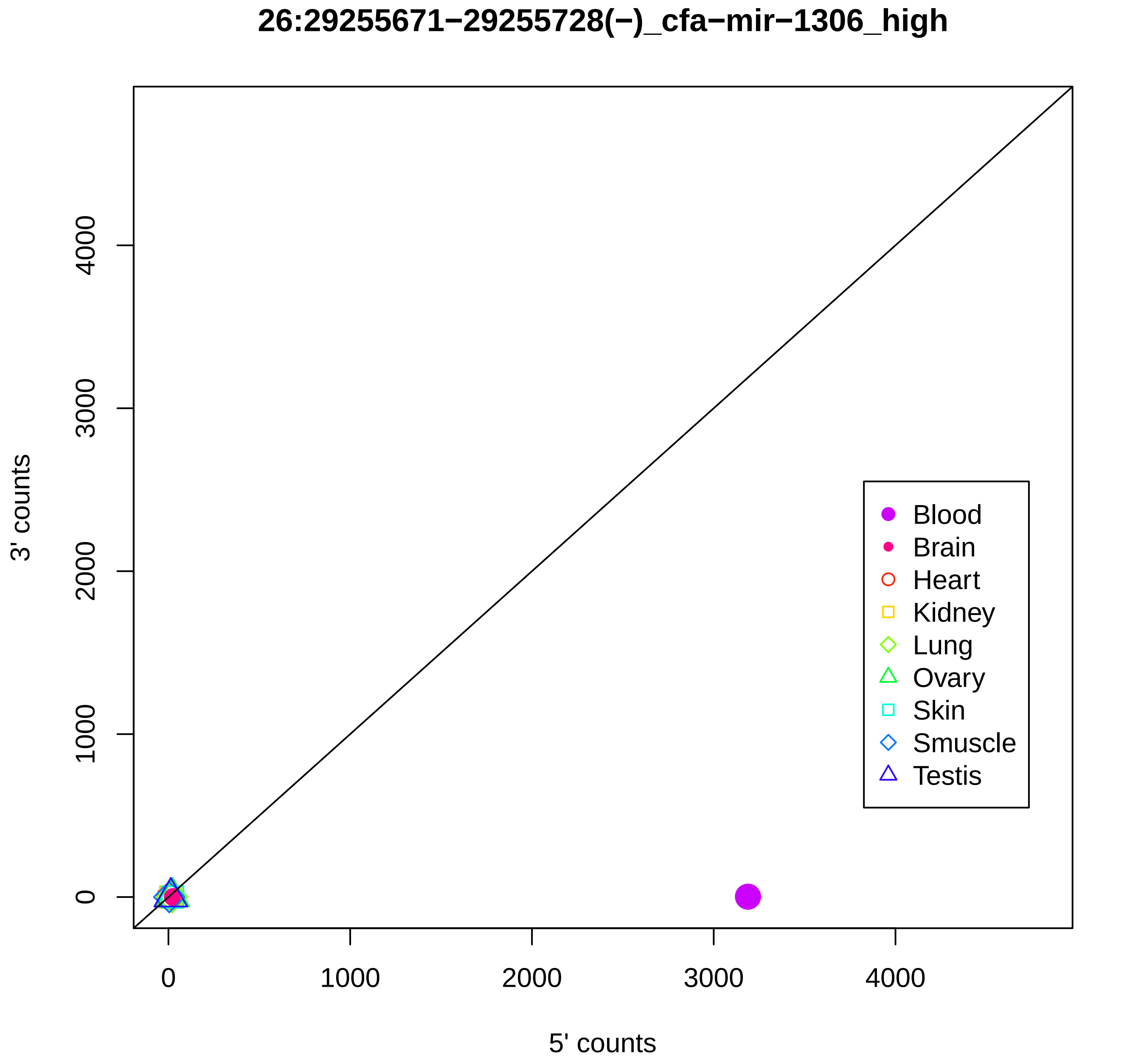
26:29255671−29255728(−)_cfa−mir−1306_high
3' counts
5' counts
0
1000
2000
3000
4000
0
1000
2000
3000
4000
Blood
Brain
Heart
Kidney
Lung
Ovary
Skin
Smuscle
Testis

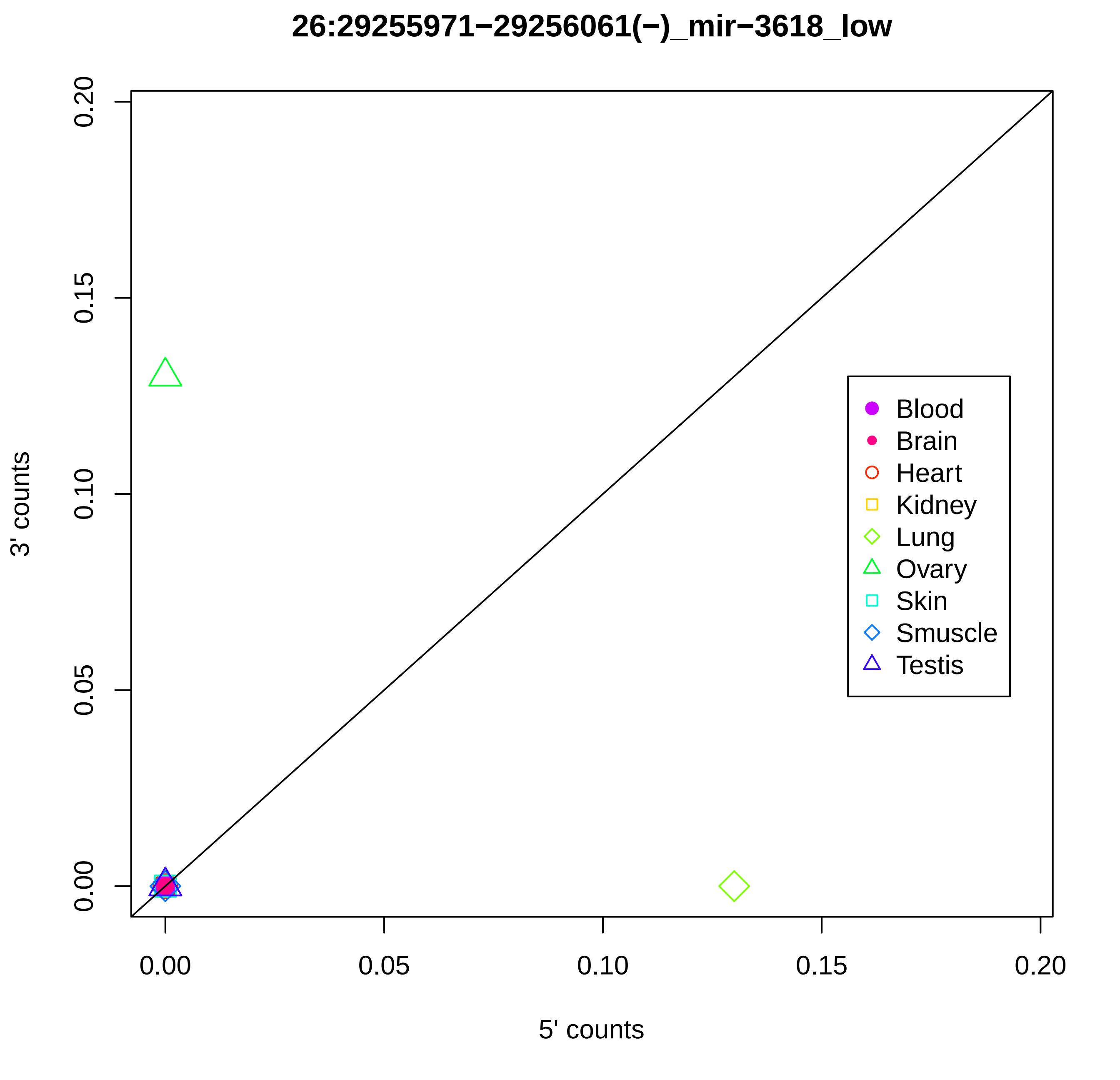
26:29255971−29256061(−)_mir−3618_low
3' counts
5' counts
0.00
0.05
0.10
0.15
0.20
0.00
0.05
0.10
0.15
0.20
Blood
Brain
Heart
Kidney
Lung
Ovary
Skin
Smuscle
Testis

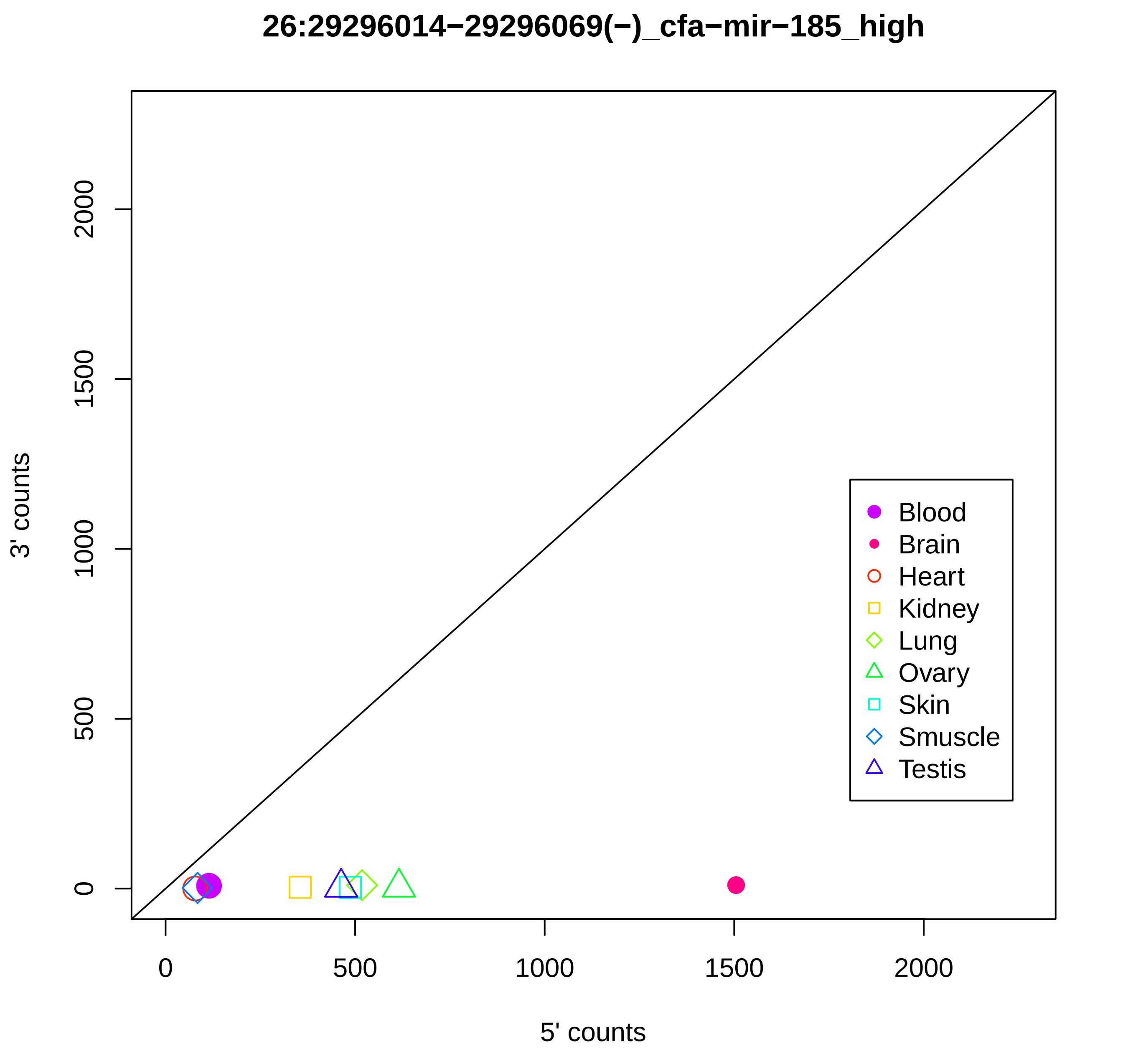

26:29296014−29296069(−)_cfa−mir−185_high
3' counts
5' counts
0
500
1000
1500
2000
Blood
Brain
Heart
Kidney
Lung
Ovary
Skin
Smuscle
Testis

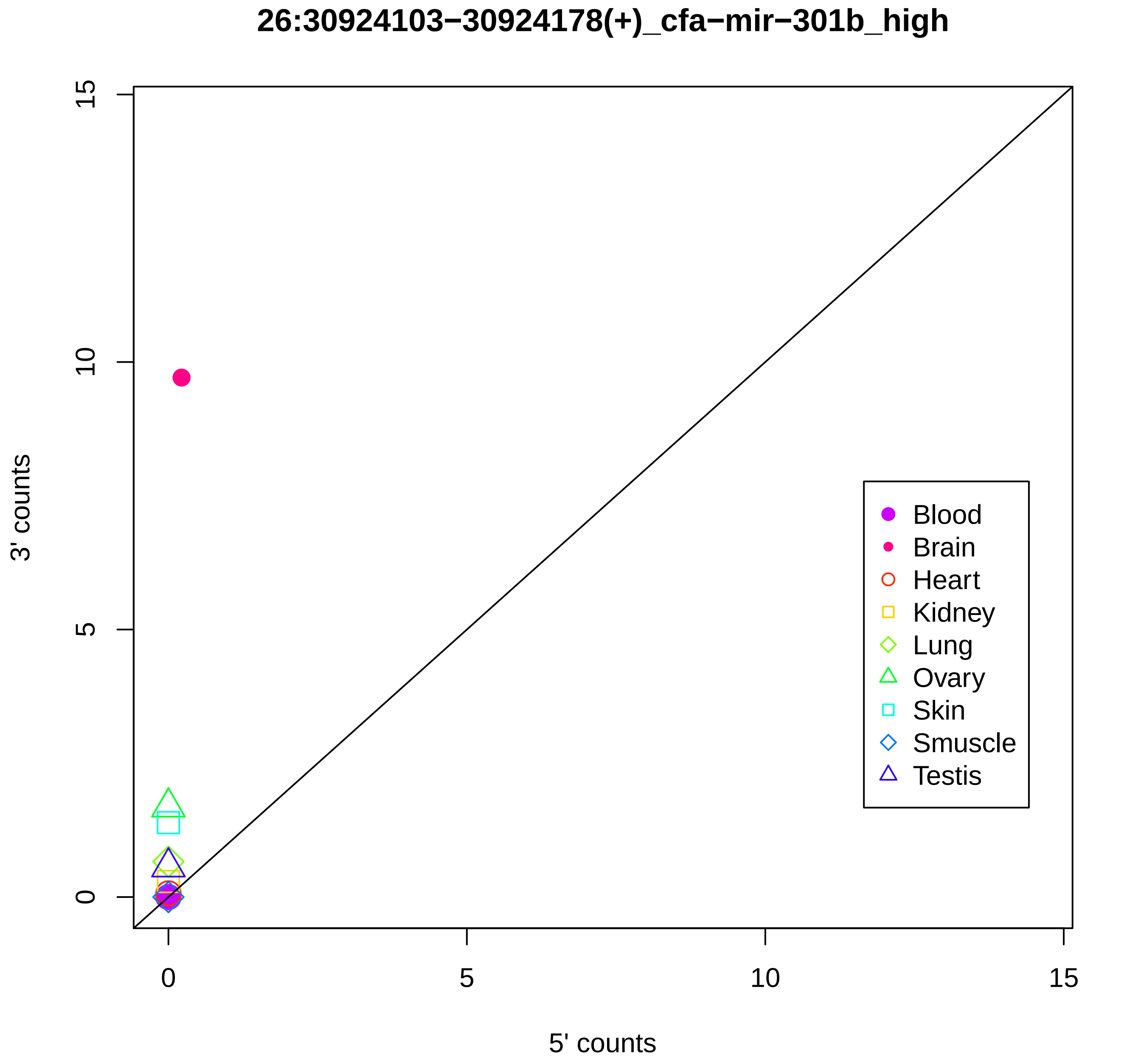
26:30924103−30924178(+)_cfa−mir−301b_high
3' counts
5' counts
0
5
10
15
Blood
Brain
Heart
Kidney
Lung
Ovary
Skin
Smuscle
Testis

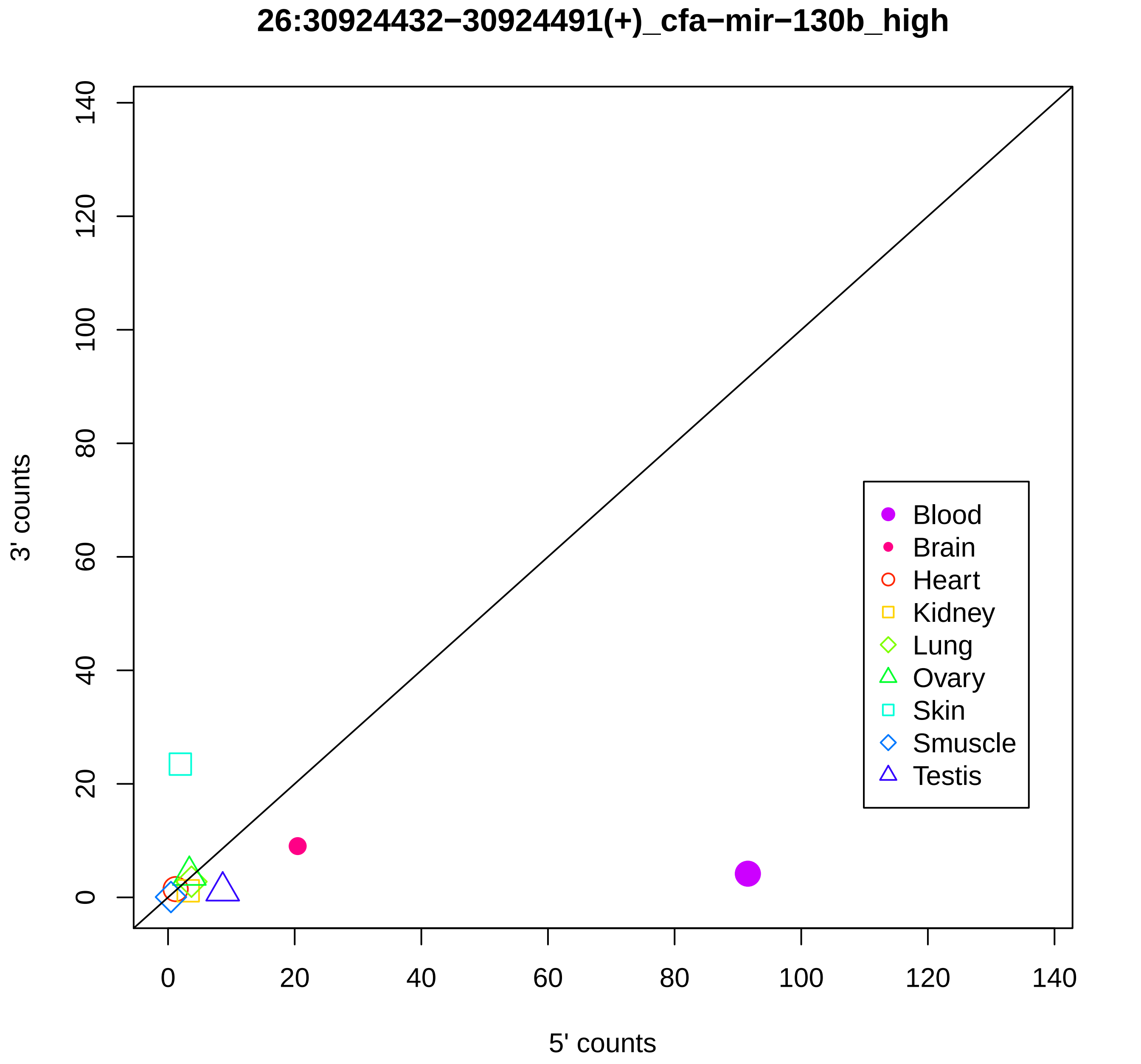
26:30924432−30924491(+)_cfa−mir−130b_high
3' counts
5' counts
0
20
40
60
80
100
120
140
Blood
Brain
Heart
Kidney
Lung
Ovary
Skin
Smuscle
Testis

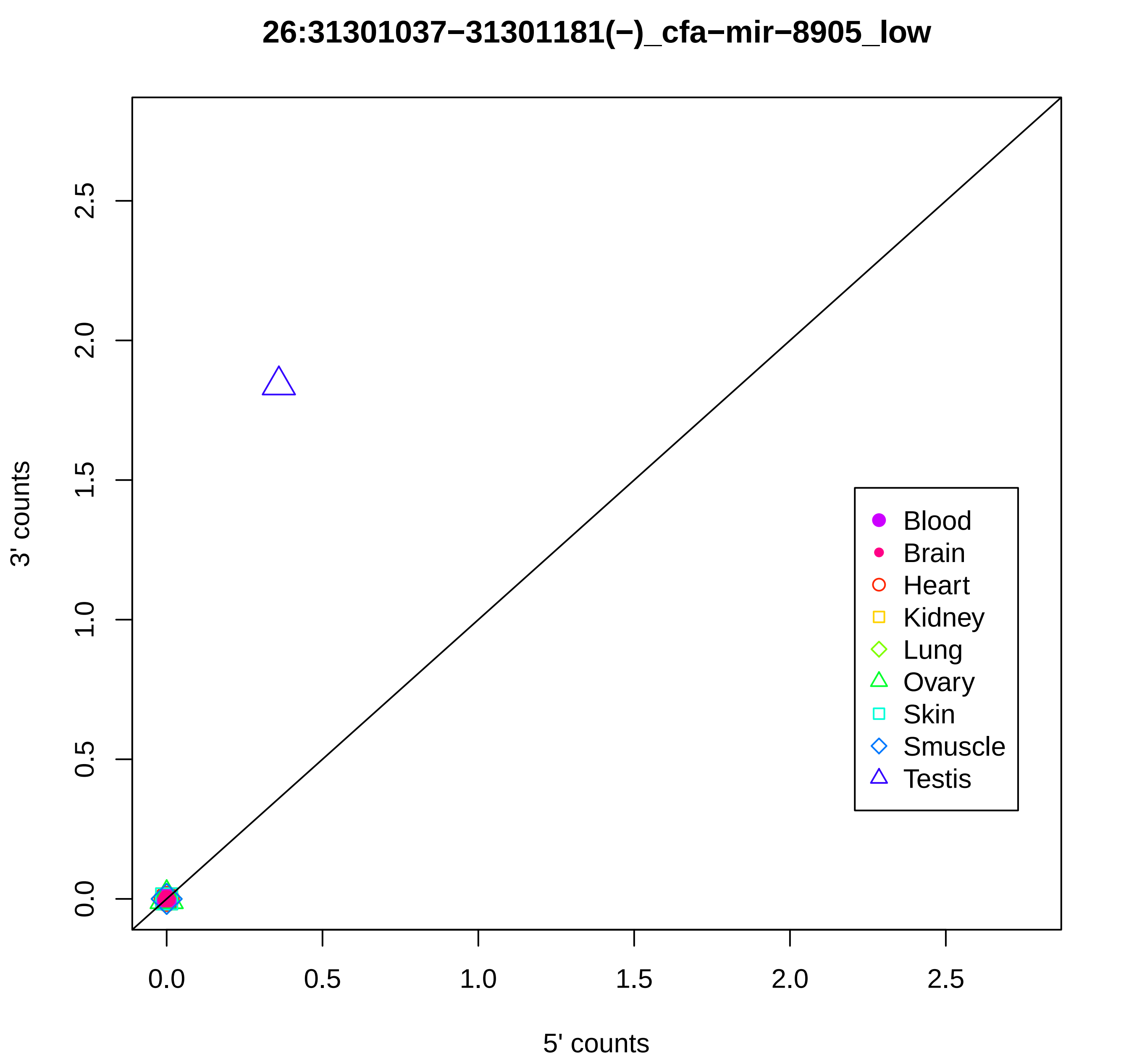
26:31301037−31301181(−)_cfa−mir−8905_low
3' counts
5' counts
0.0
0.5
1.0
1.5
2.0
2.5
Blood
Brain
Heart
Kidney
Lung
Ovary
Skin
Smuscle
Testis

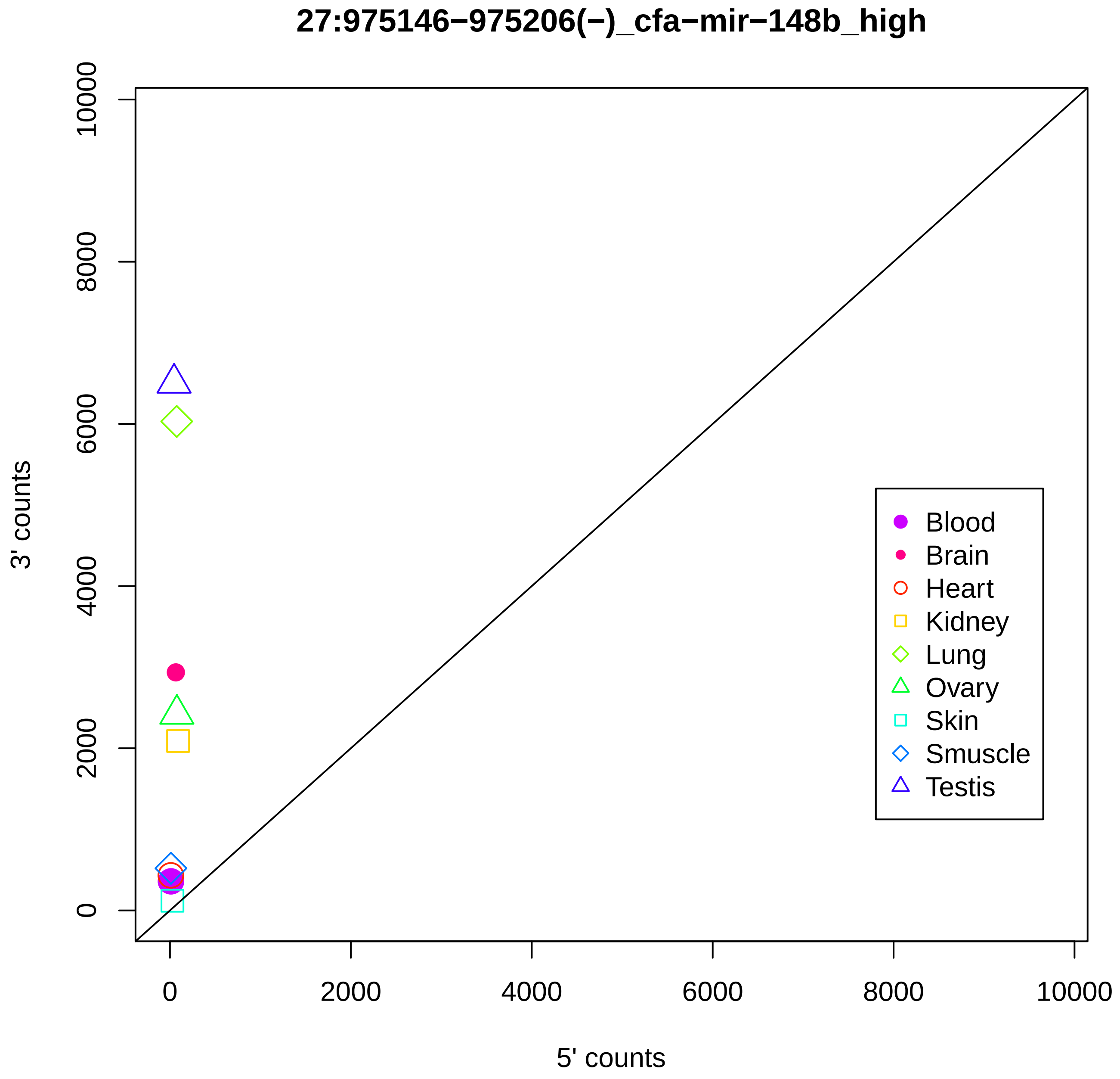
27:975146−975206(−)_cfa−mir−148b_high
3' counts
5' counts
0
2000
4000
6000
8000
10000
Blood
Brain
Heart
Kidney
Lung
Ovary
Skin
Smuscle
Testis

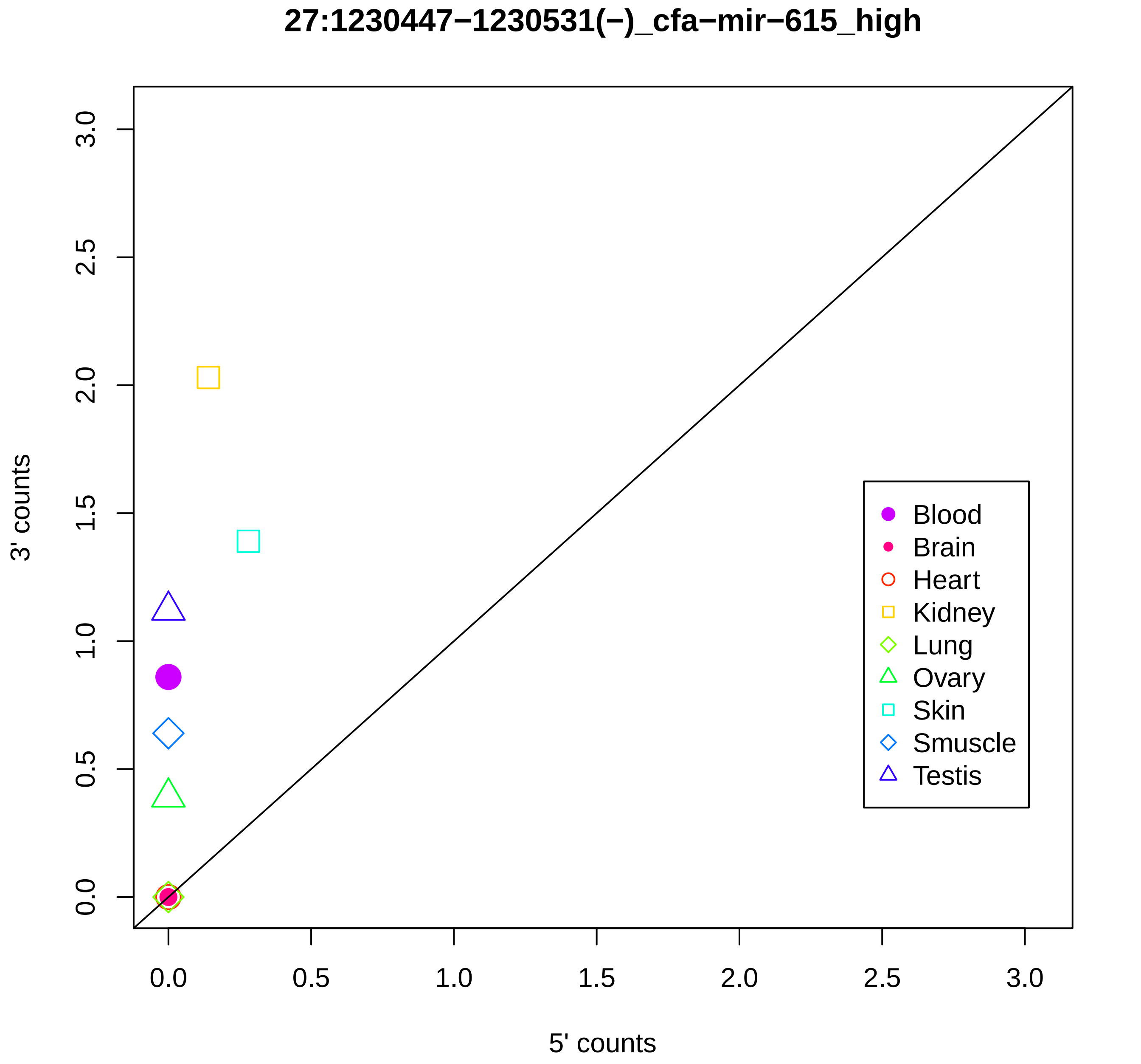
27:1230447−1230531(−)_cfa−mir−615_high
3' counts
5' counts
0.0
0.5
1.0
1.5
2.0
2.5
3.0
Blood
Brain
Heart
Kidney
Lung
Ovary
Skin
Smuscle
Testis

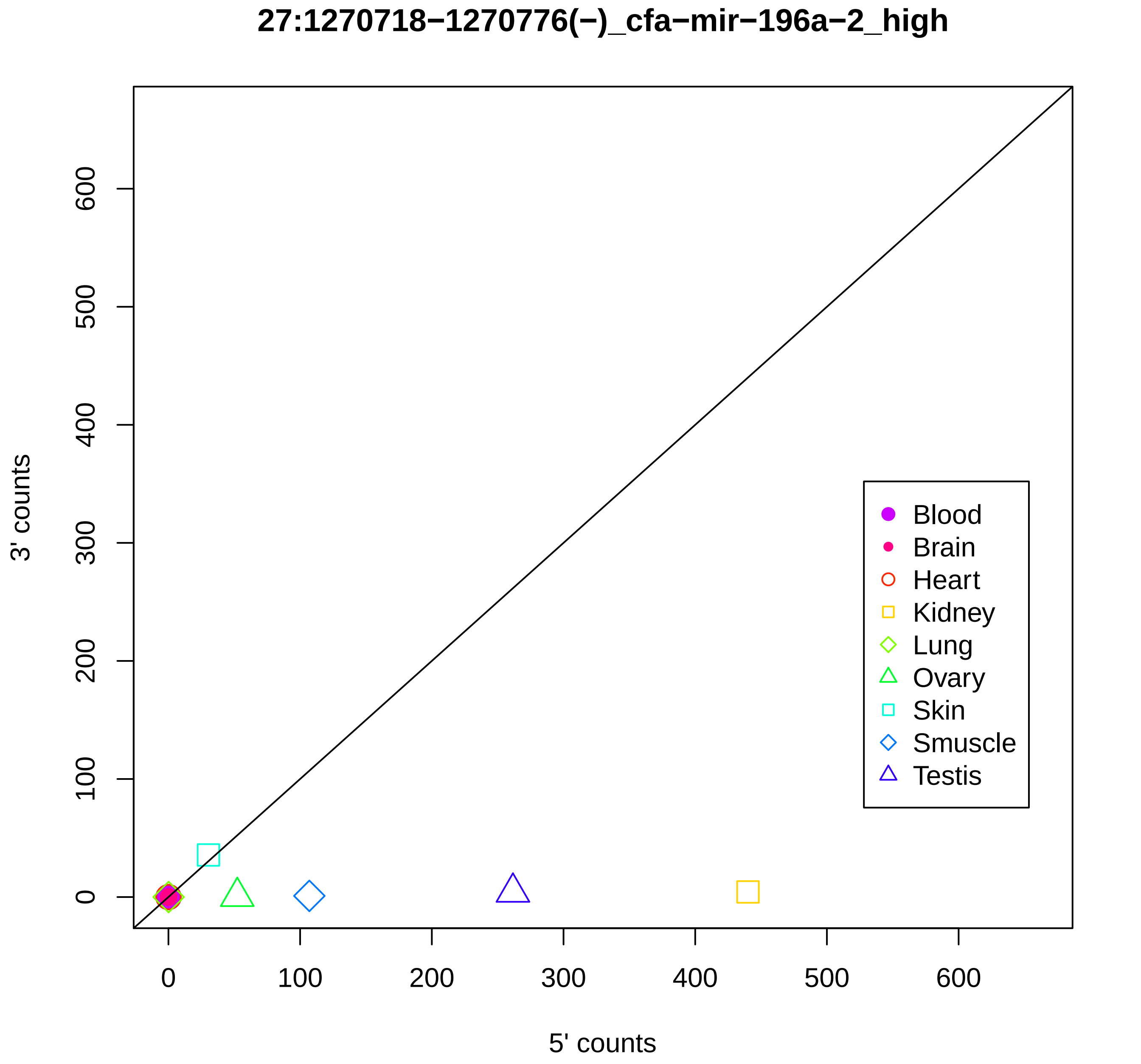
27:1270718−1270776(−)_cfa−mir−196a−2_high
3' counts
5' counts
0
100
200
300
400
500
600
Blood
Brain
Heart
Kidney
Lung
Ovary
Skin
Smuscle
Testis

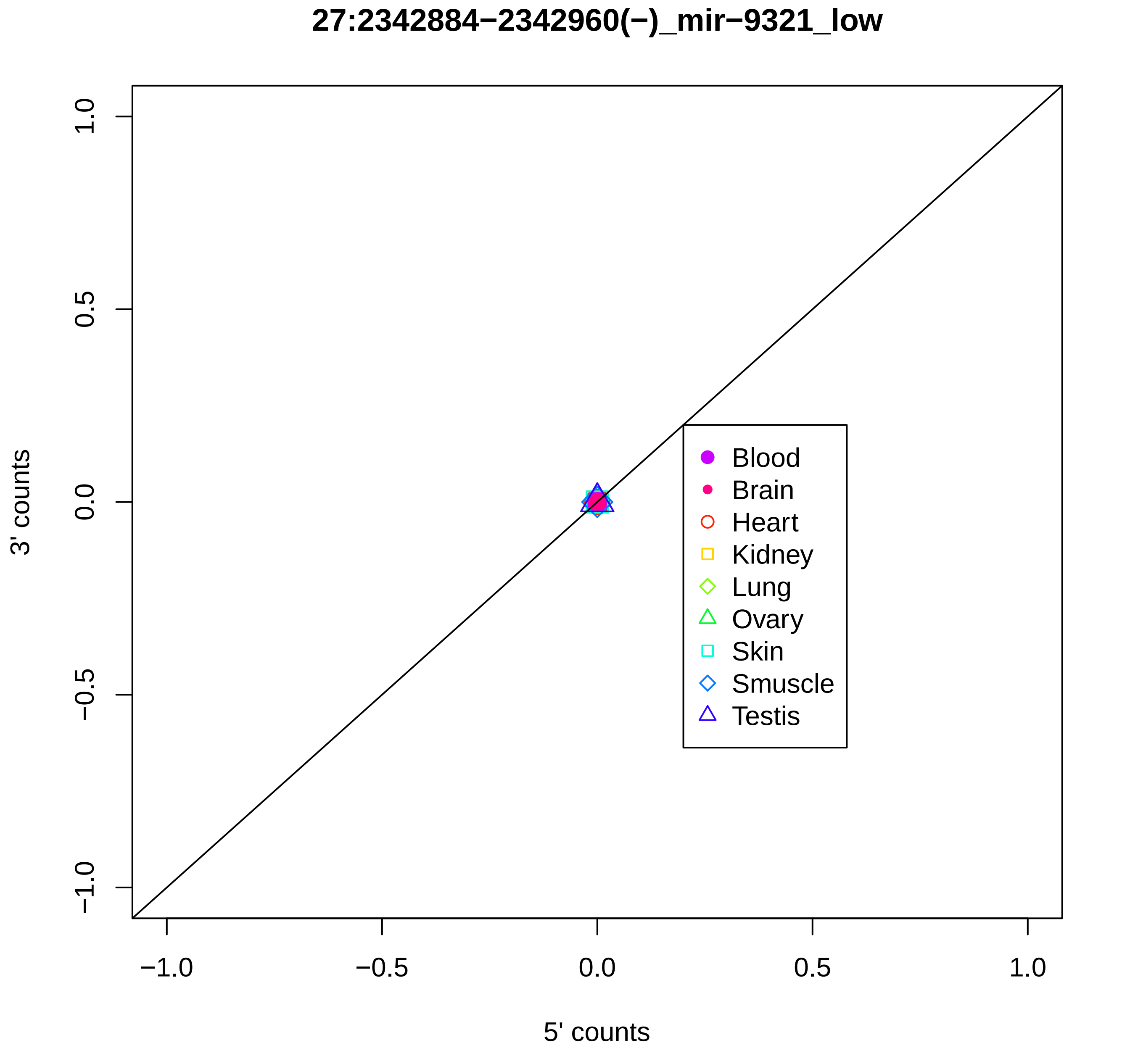

27:2342884−2342960(−)_mir−9321_low
3' counts
5' counts
1.0
0.5
0.0
−0.5
−1.0
Blood
Brain
Heart
Kidney
Lung
Ovary
Skin
Smuscle
Testis

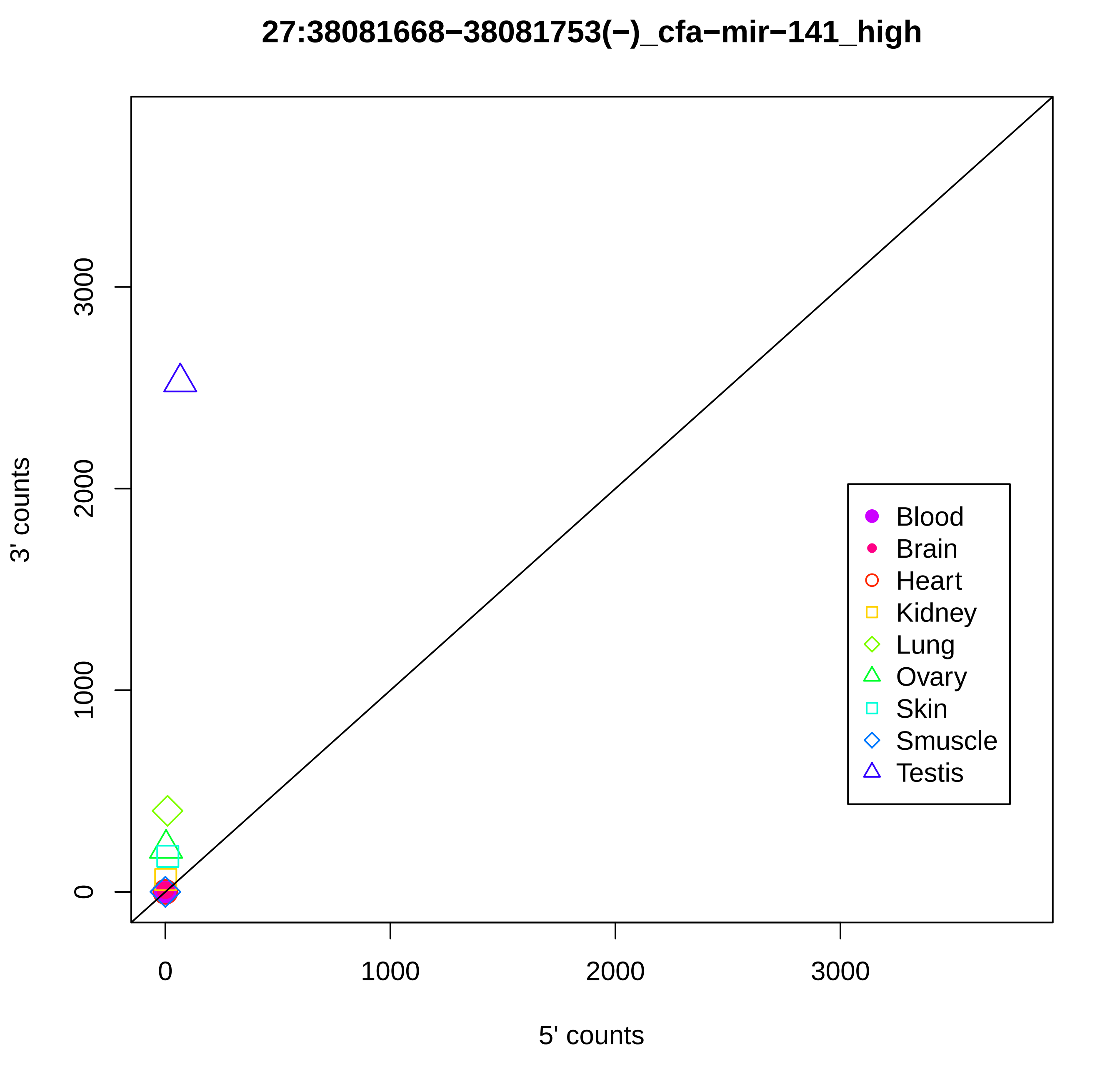

27:38081668−38081753(−)_cfa−mir−141_high
3' counts
0
1000
2000
3000
5' counts
0
1000
2000
3000
Blood
Brain
Heart
Kidney
Lung
Ovary
Skin
Smuscle
Testis

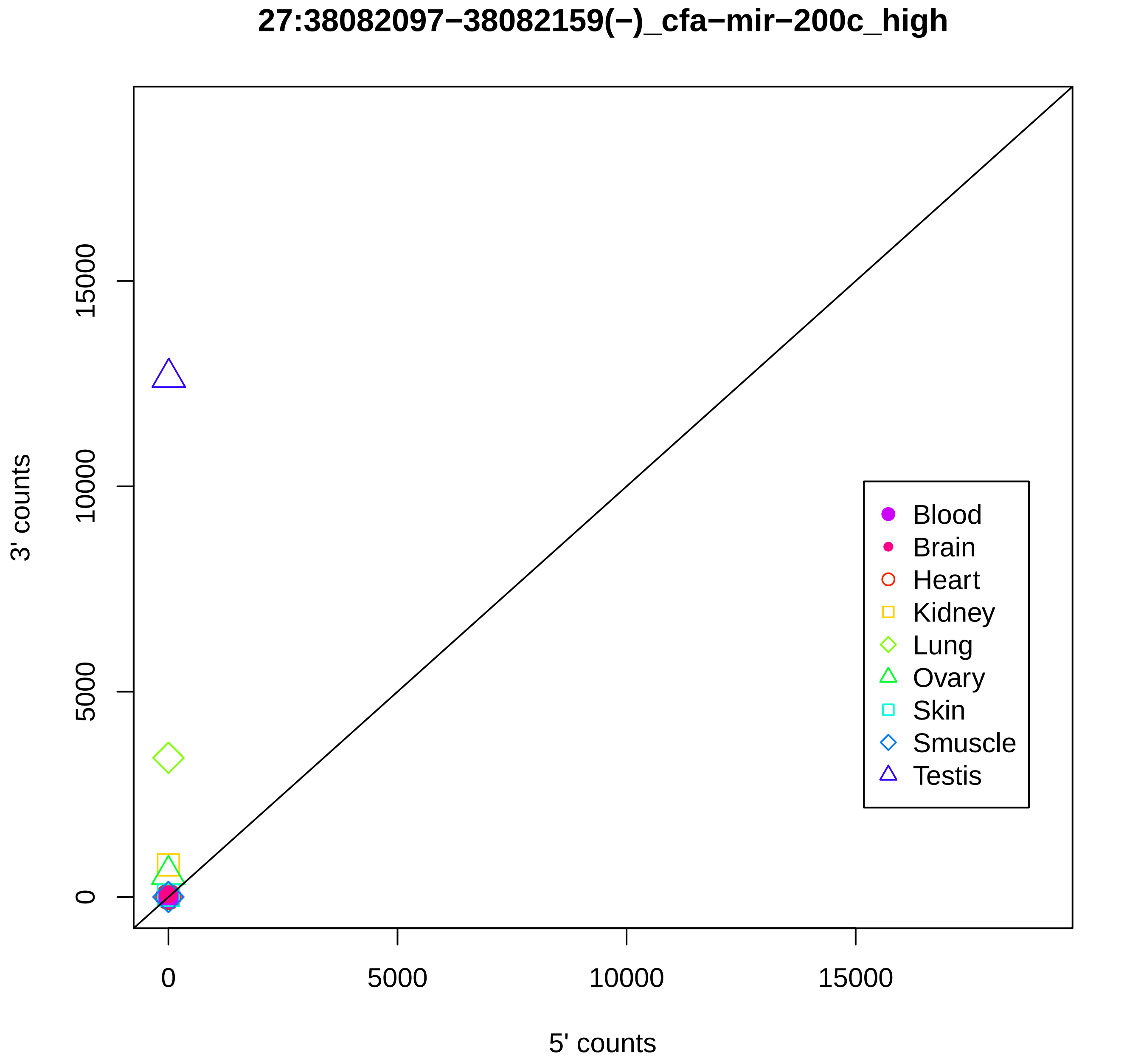

27:38082097−38082159(−)_cfa−mir−200c_high
3' counts
5' counts
0
5000
10000
15000
0
5000
10000
15000
Blood
Brain
Heart
Kidney
Lung
Ovary
Skin
Smuscle
Testis

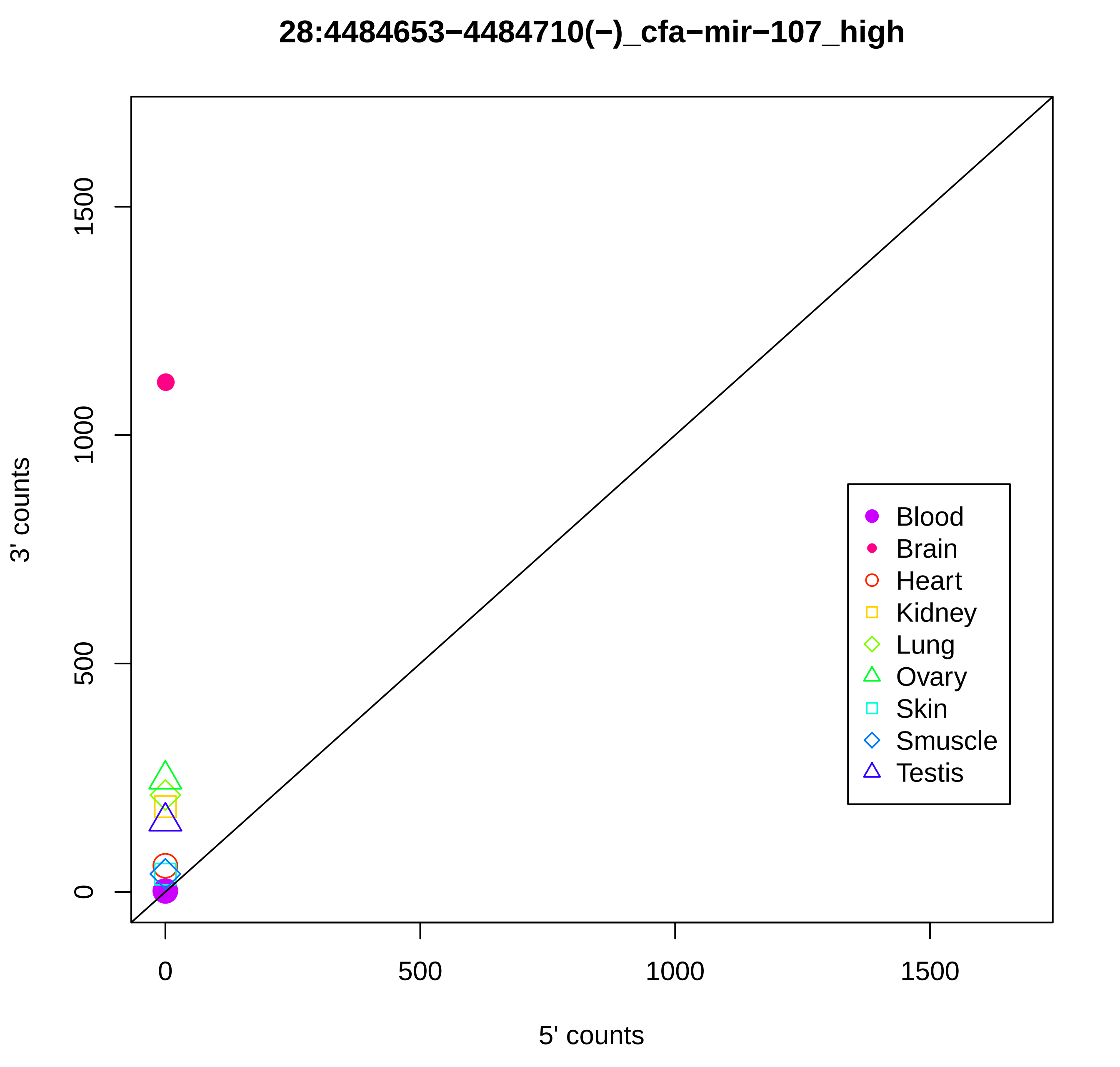

28:4484653−4484710(−)_cfa−mir−107_high
3' counts
5' counts
0
500
1000
1500
0
500
1000
1500
Blood
Brain
Heart
Kidney
Lung
Ovary
Skin
Smuscle
Testis

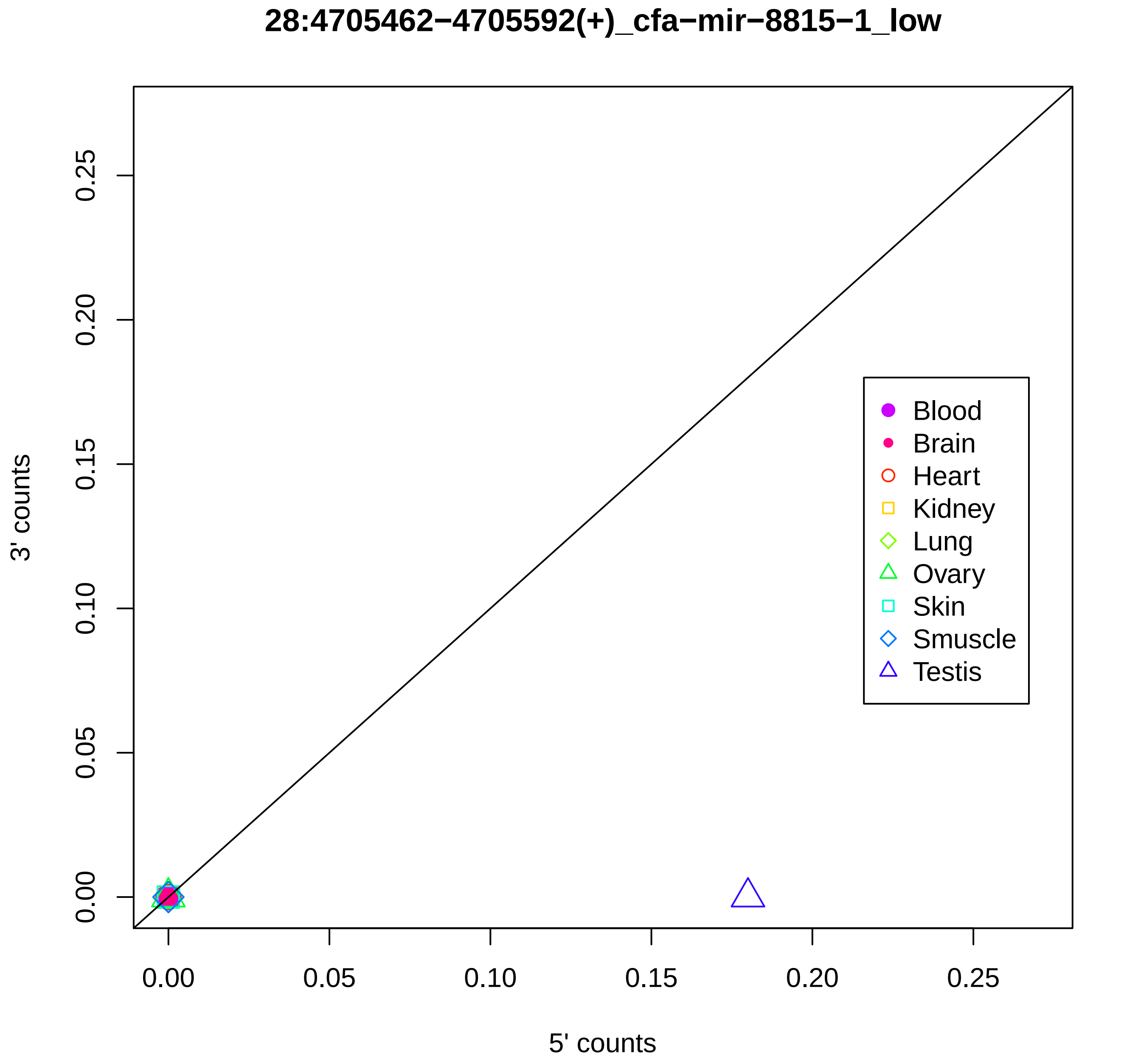

28:4705462−4705592(+)_cfa−mir−8815−1_low
3' counts
5' counts
0.00
0.05
0.10
0.15
0.20
0.25
Blood
Brain
Heart
Kidney
Lung
Ovary
Skin
Smuscle
Testis

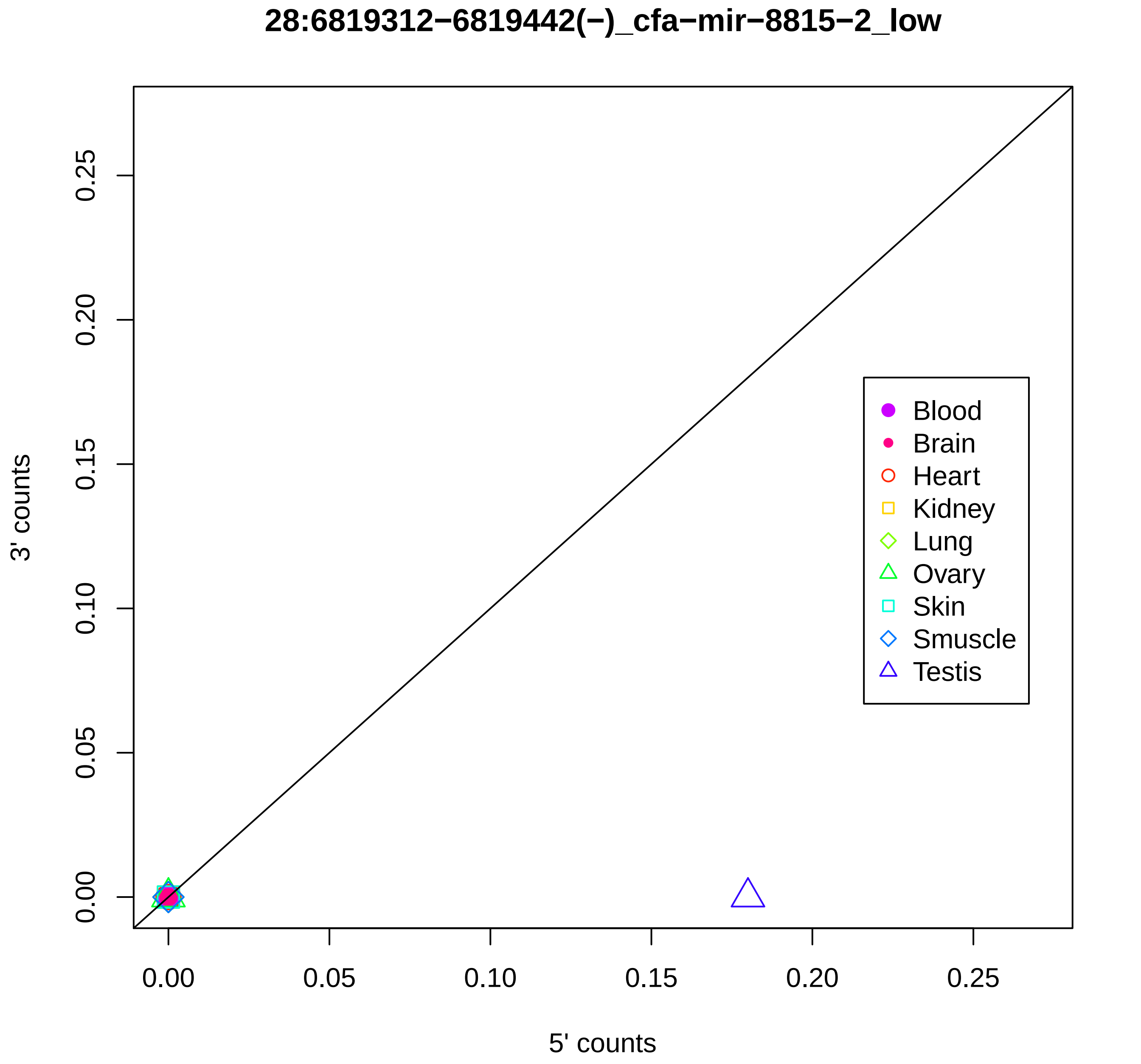

28:6819312−6819442(−)_cfa−mir−8815−2_low
3' counts
5' counts
0.00
0.05
0.10
0.15
0.20
0.25
Blood
Brain
Heart
Kidney
Lung
Ovary
Skin
Smuscle
Testis

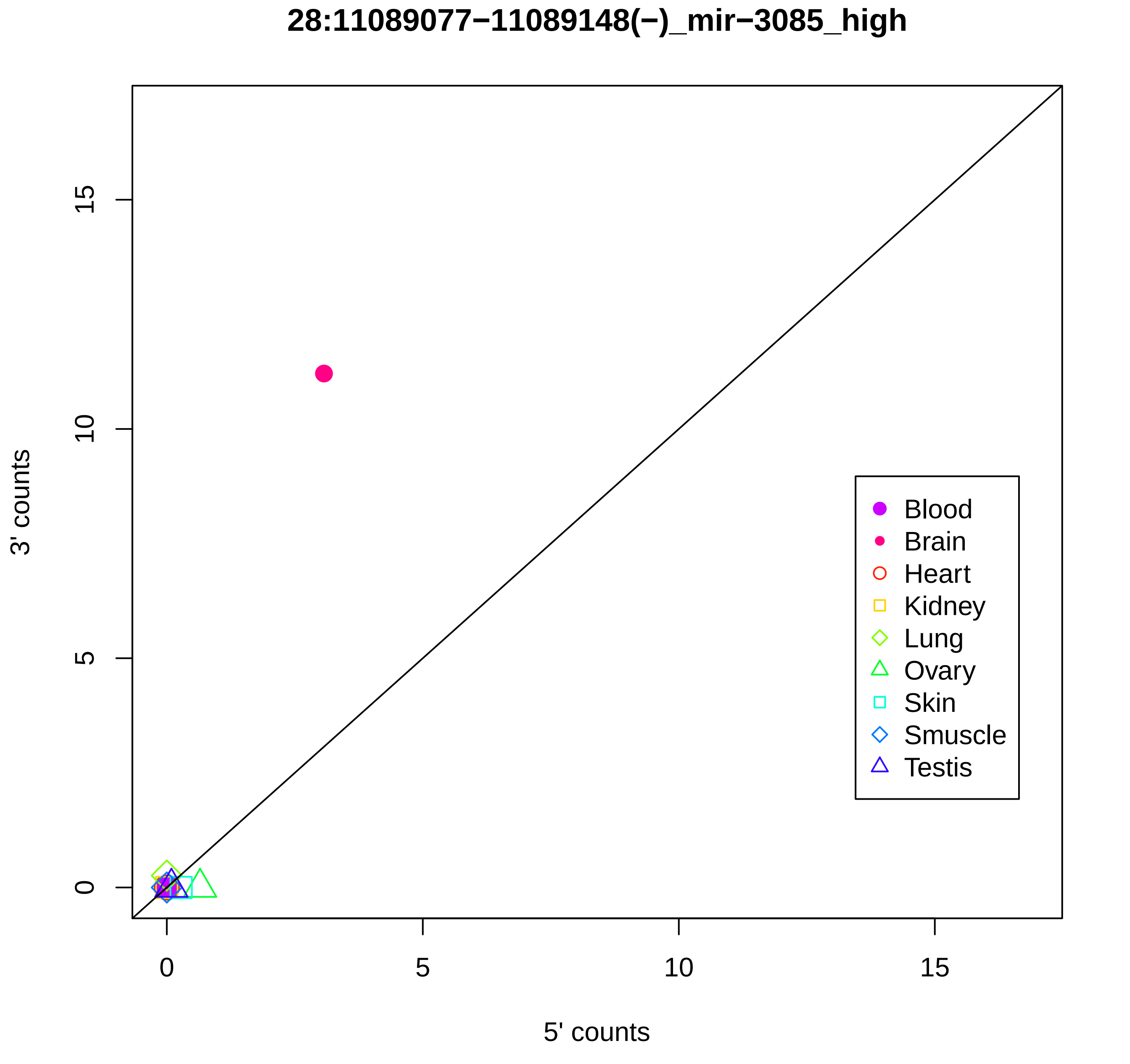
28:11089077−11089148(−)_mir−3085_high
3' counts
5' counts
0
5
10
15
0
5
10
15
Blood
Brain
Heart
Kidney
Lung
Ovary
Skin
Smuscle
Testis

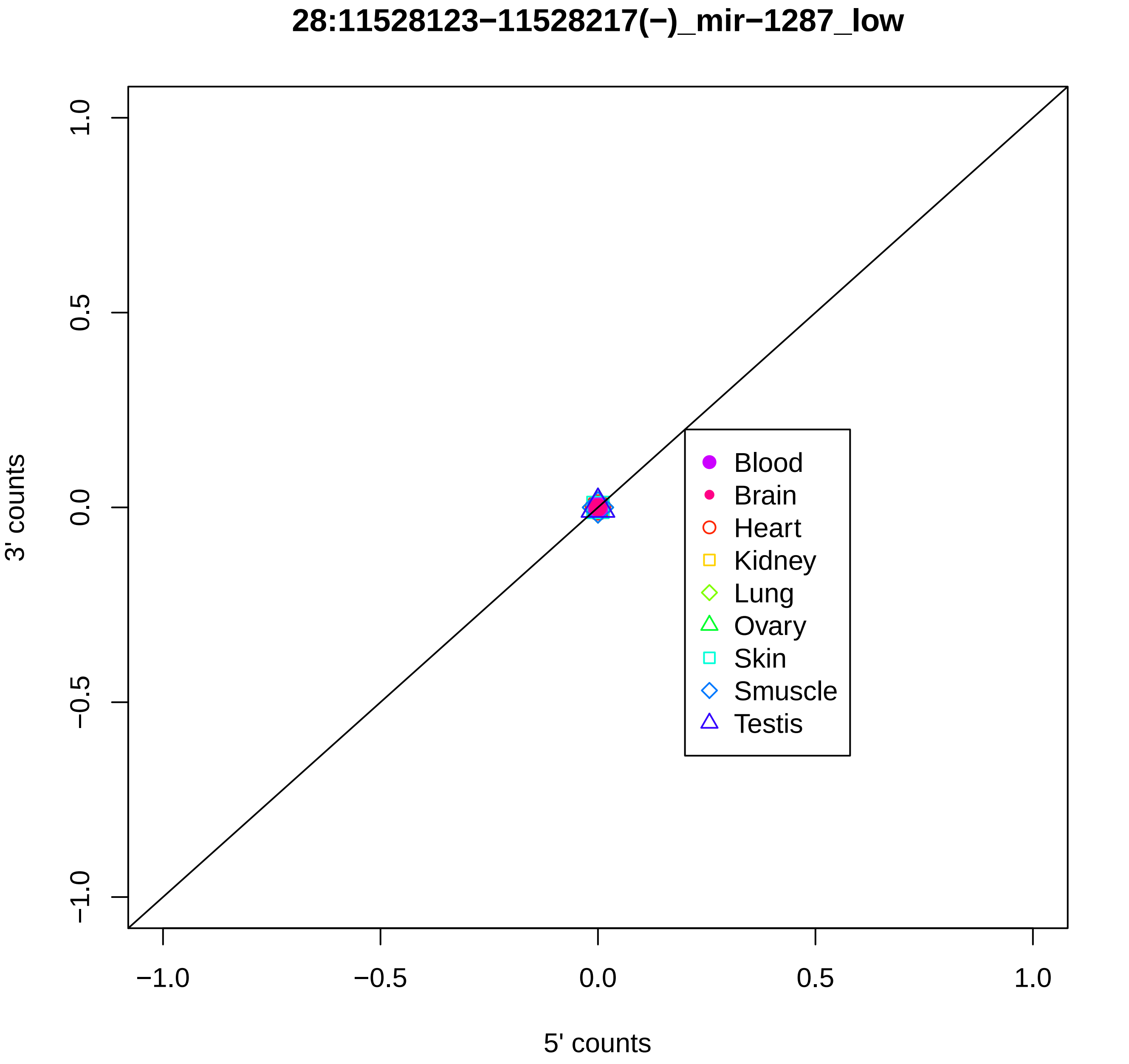
28:11528123−11528217(−)_mir−1287_low
3' counts
5' counts
−1.0
−0.5
0.0
0.5
1.0
Blood
Brain
Heart
Kidney
Lung
Ovary
Skin
Smuscle
Testis

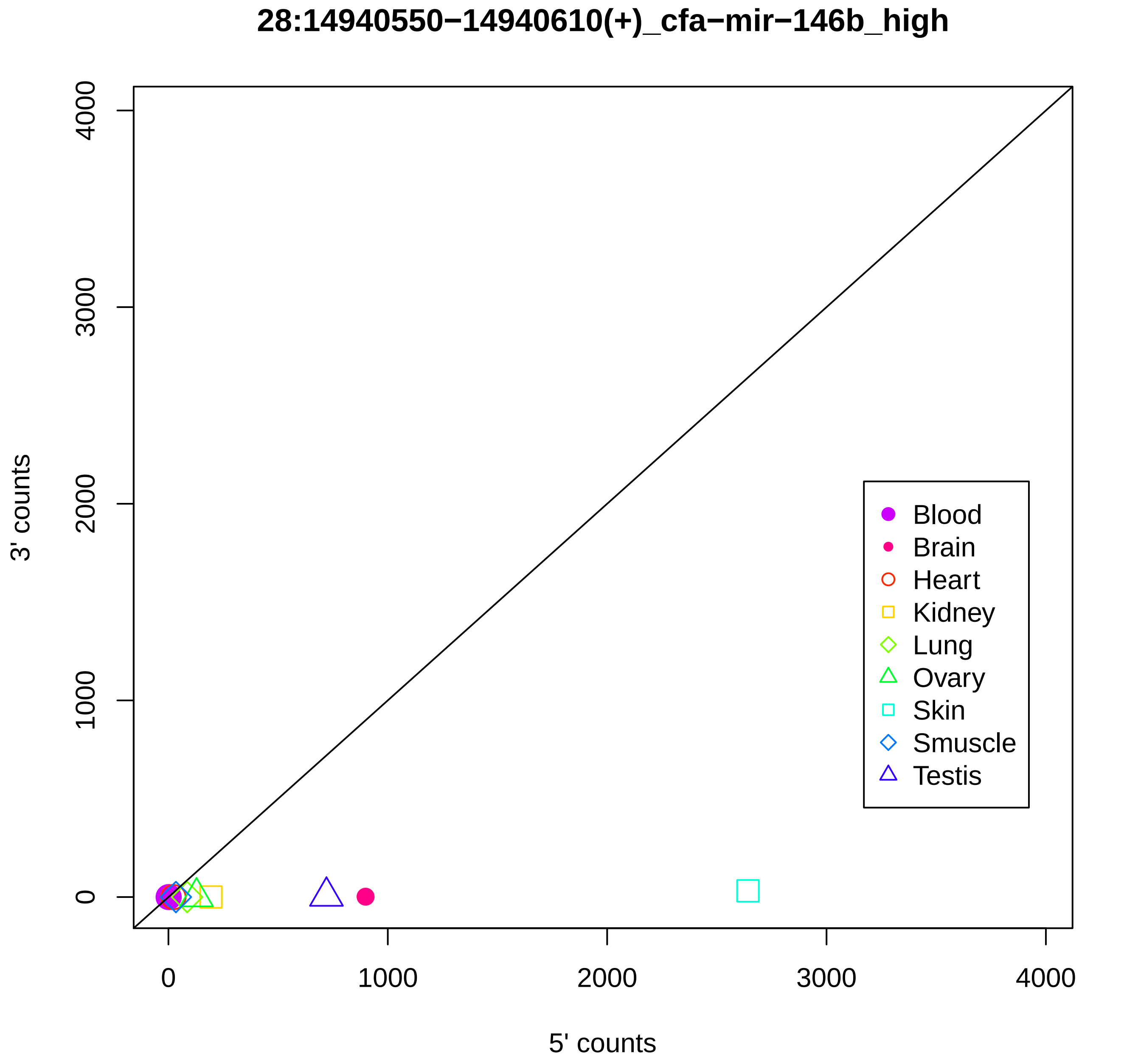
28:14940550−14940610(+)_cfa−mir−146b_high
3' counts
5' counts
0
1000
2000
3000
4000
0
1000
2000
3000
4000
Blood
Brain
Heart
Kidney
Lung
Ovary
Skin
Smuscle
Testis

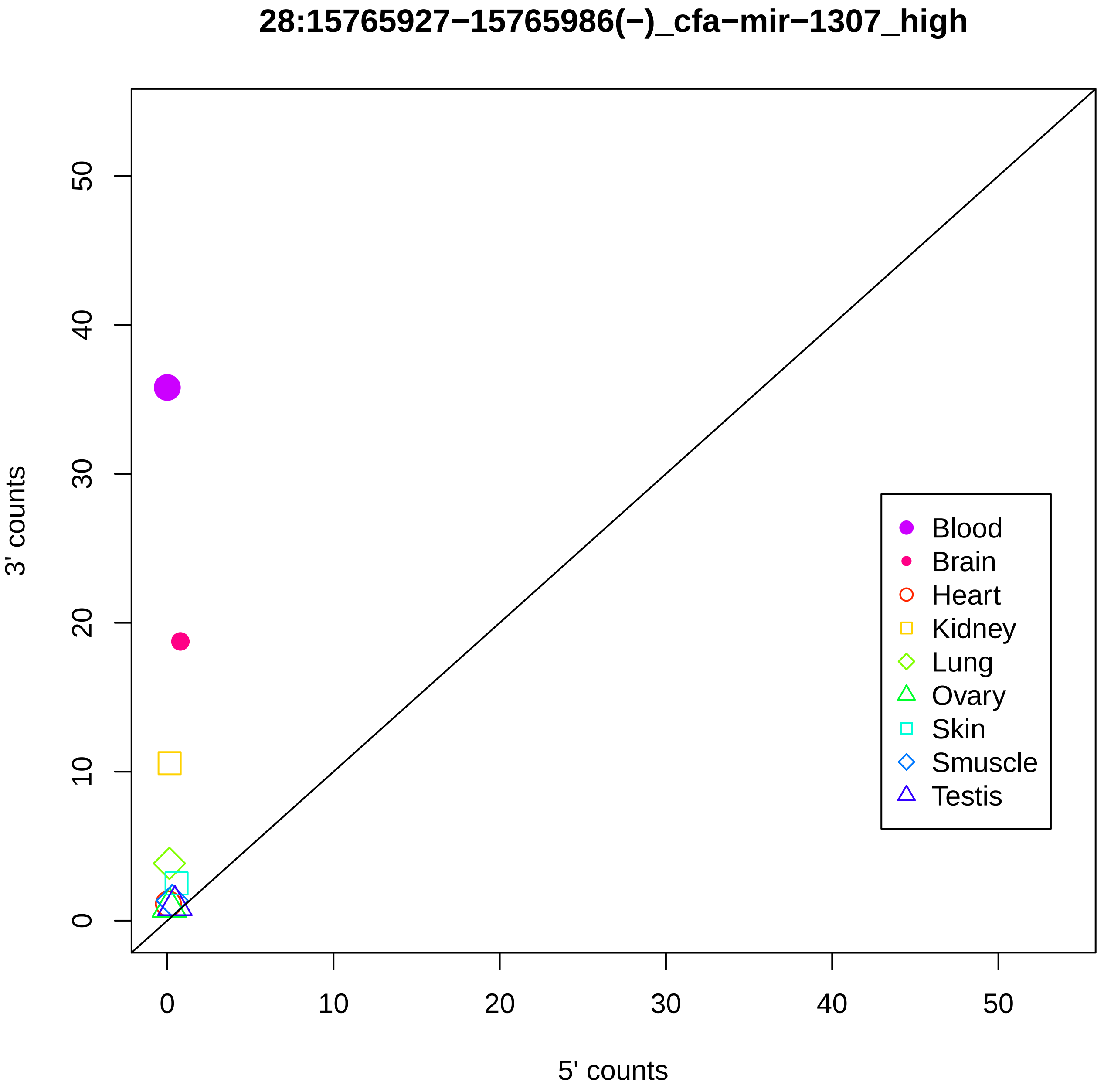

28:15765927−15765986(−)_cfa−mir−1307_high
3' counts
5' counts
0
10
20
30
40
50
0
10
20
30
40
50
Blood
Brain
Heart
Kidney
Lung
Ovary
Skin
Smuscle
Testis

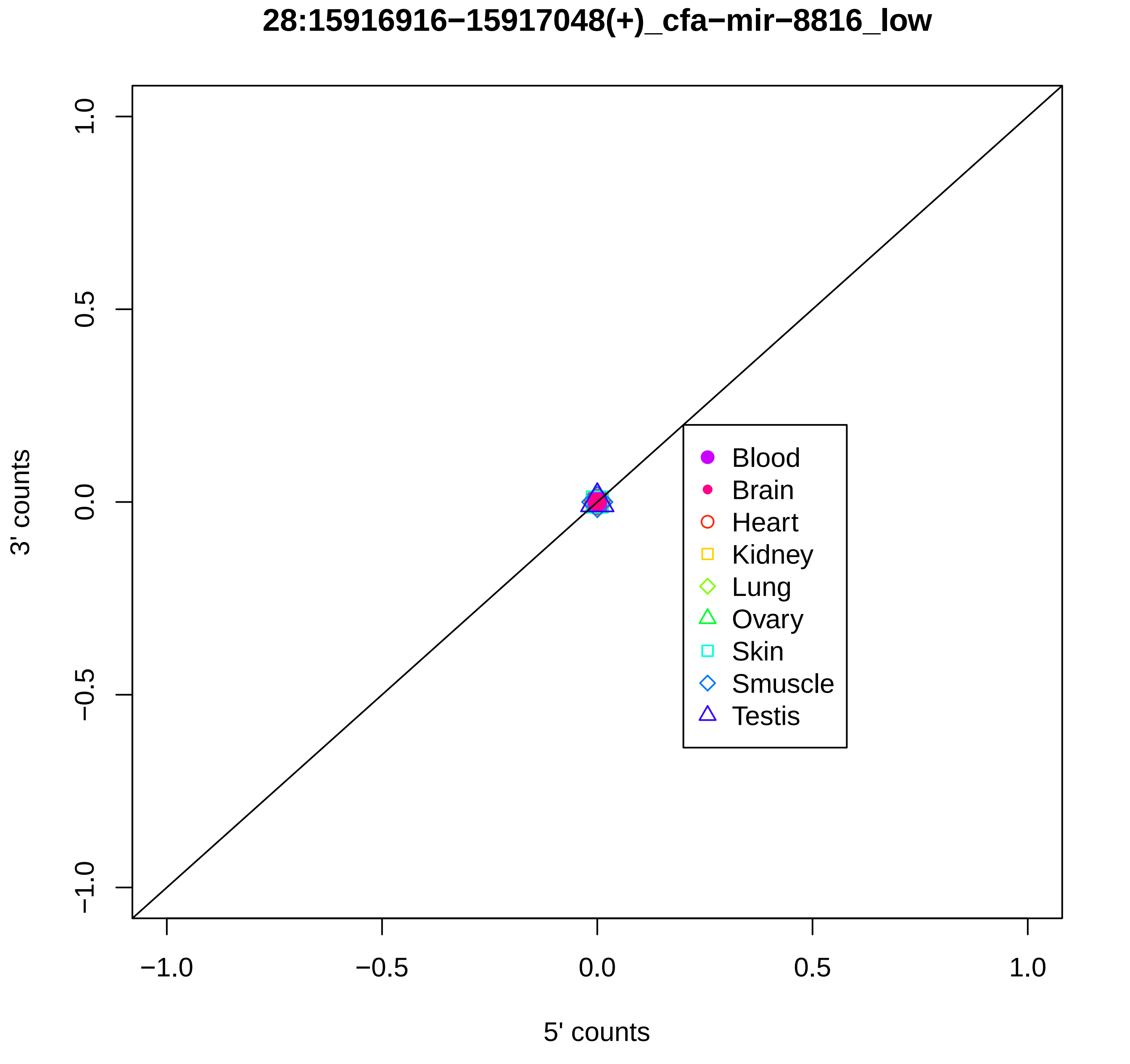
28:15916916−15917048(+)_cfa−mir−8816_low
3' counts
5' counts
1.0
0.5
0.0
−0.5
−1.0
−1.0
−0.5
0.0
0.5
1.0
Blood
Brain
Heart
Kidney
Lung
Ovary
Skin
Smuscle
Testis

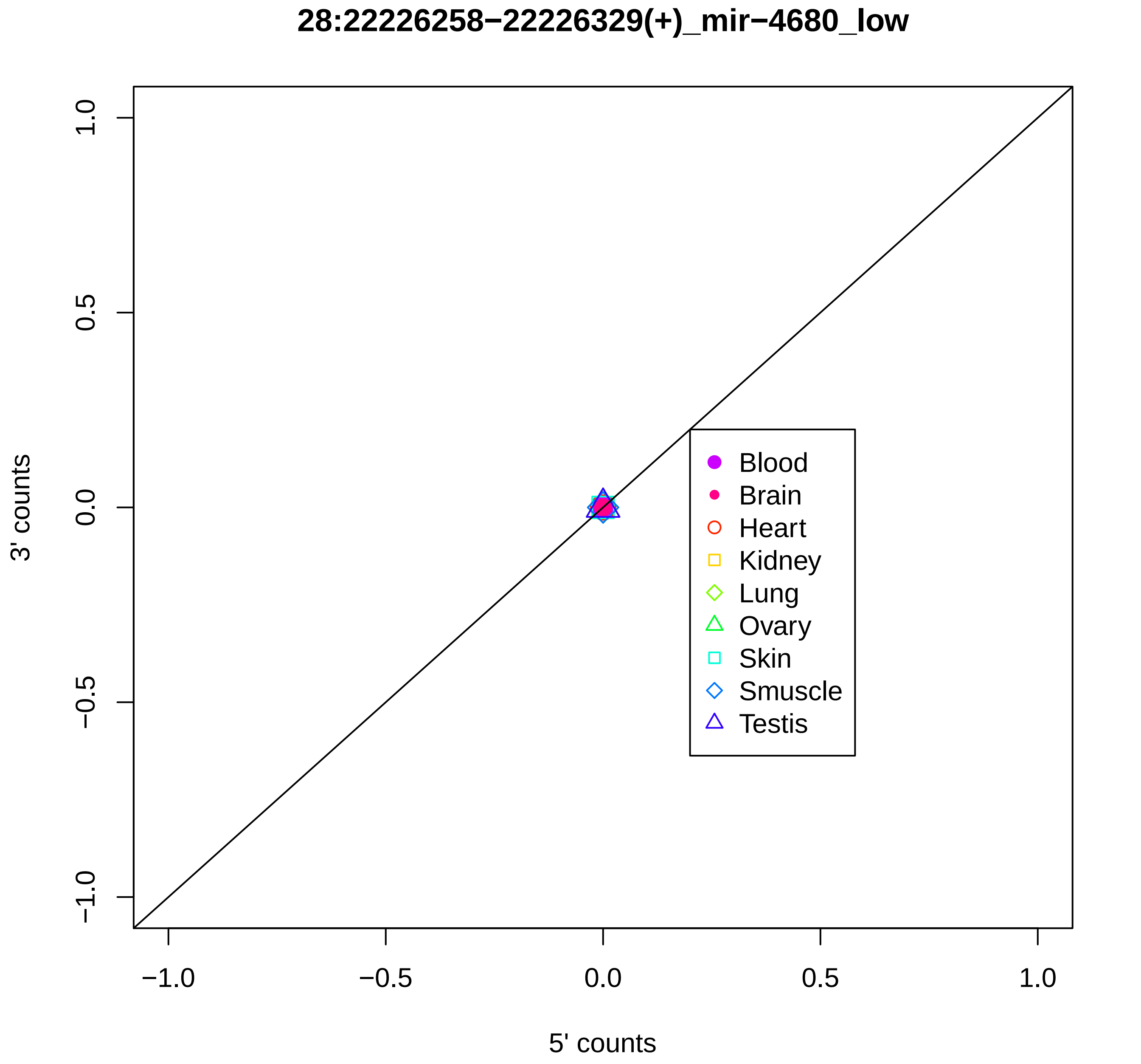

28:22226258−22226329(+)_mir−4680_low
3' counts
5' counts
1.0
0.5
0.0
−0.5
−1.0
Blood
Brain
Heart
Kidney
Lung
Ovary
Skin
Smuscle
Testis

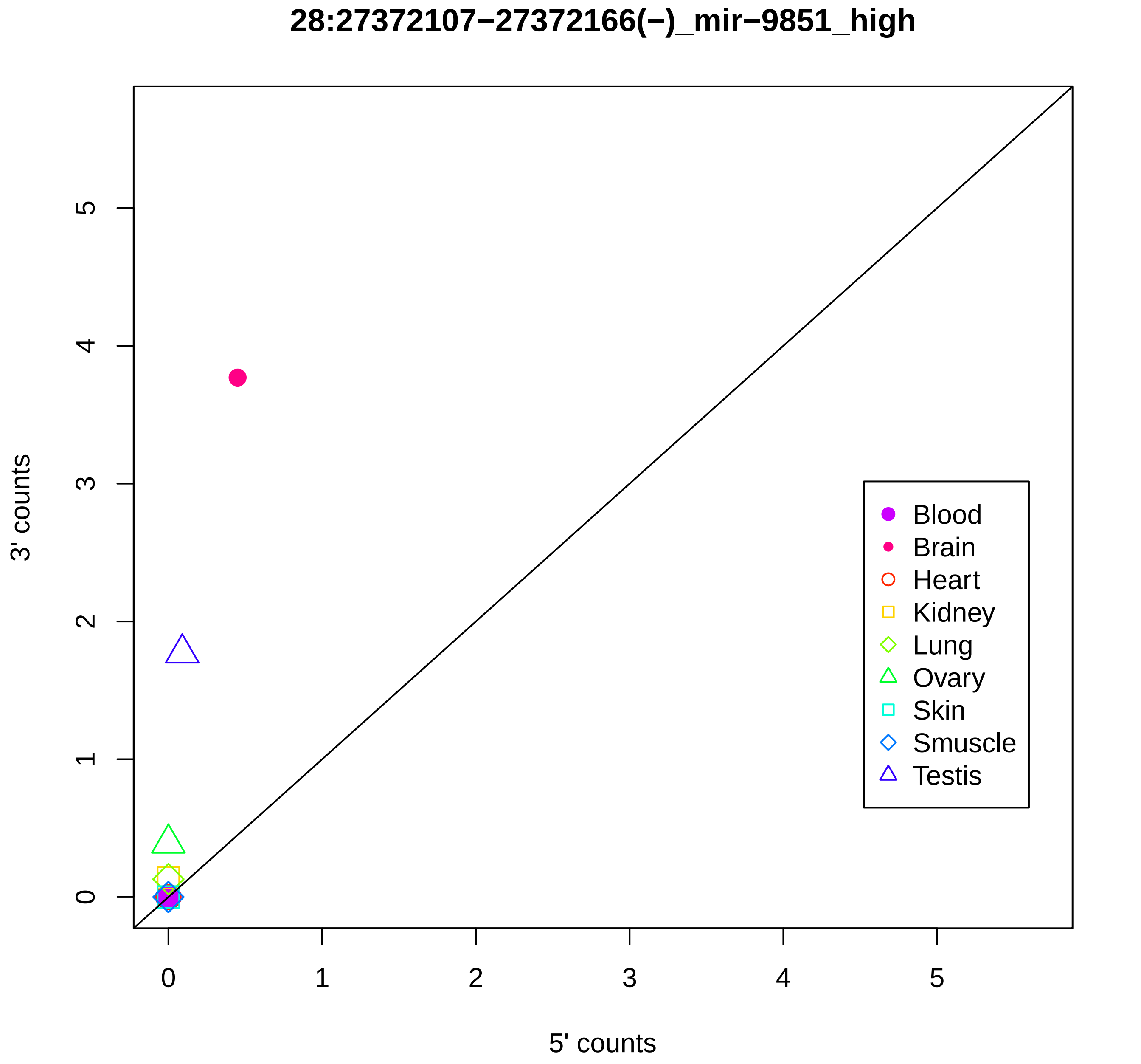

28:27372107−27372166(−)_mir−9851_high
3' counts
5' counts
0
1
2
3
4
5
Blood
Brain
Heart
Kidney
Lung
Ovary
Skin
Smuscle
Testis

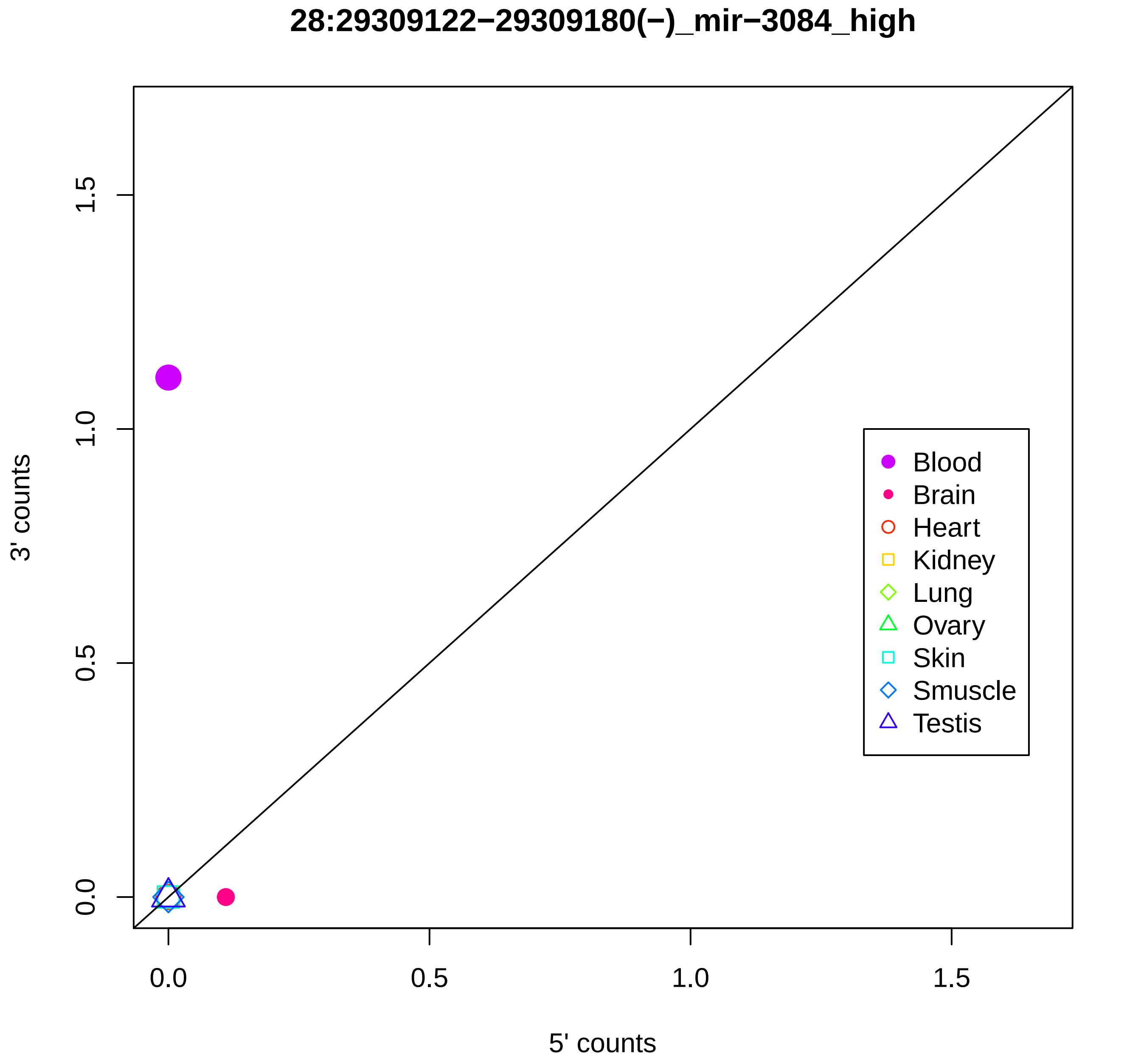

28:29309122−29309180(−)_mir−3084_high
3' counts
5' counts
0.0
0.5
1.0
1.5
Blood
Brain
Heart
Kidney
Lung
Ovary
Skin
Smuscle
Testis

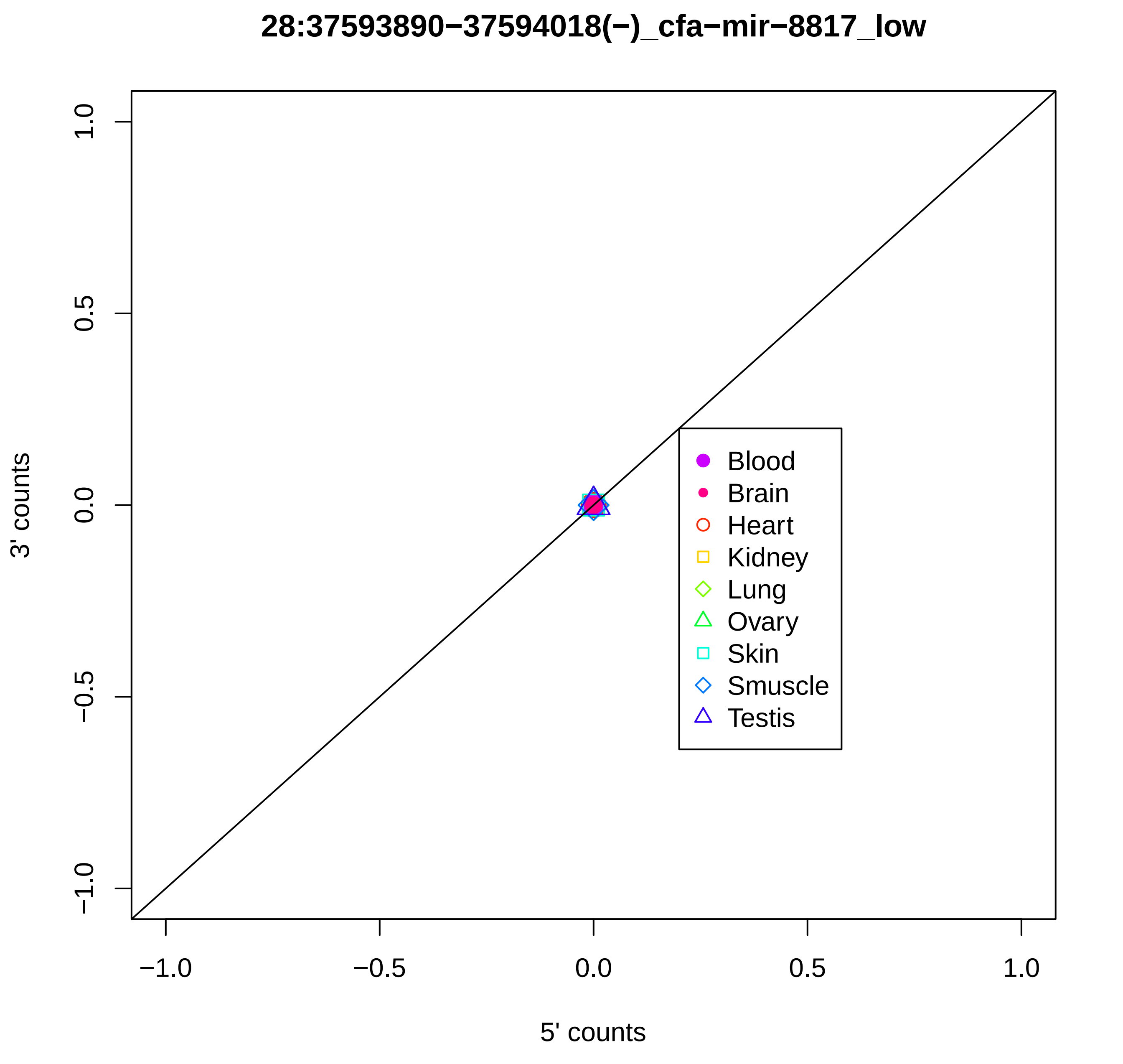
28:37593890−37594018(−)_cfa−mir−8817_low
3' counts
5' counts
1.0
0.5
0.0
−0.5
−1.0
−1.0
−0.5
0.0
0.5
1.0
Blood
Brain
Heart
Kidney
Lung
Ovary
Skin
Smuscle
Testis

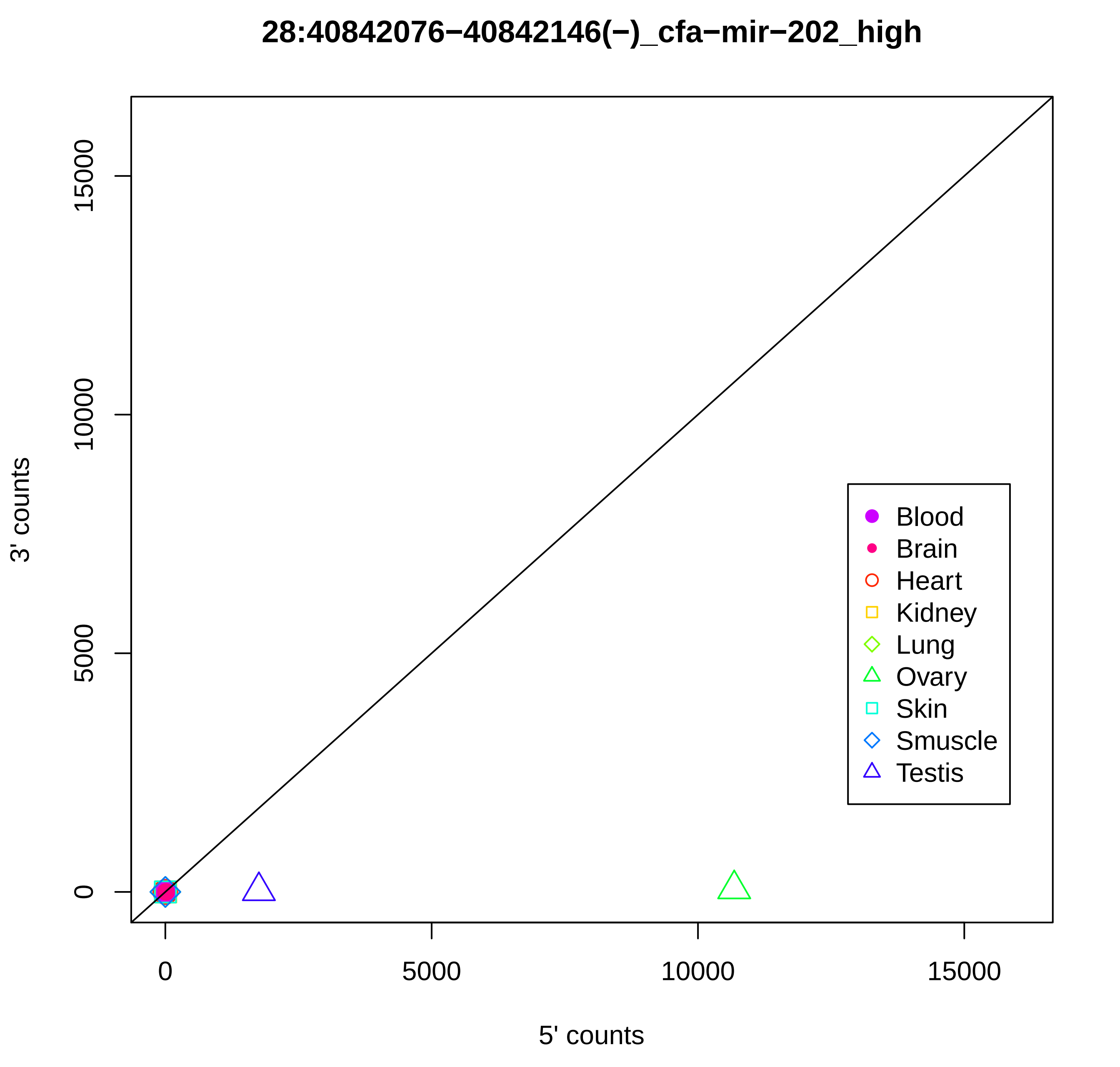

28:40842076−40842146(−)_cfa−mir−202_high
3' counts
0
5000
10000
15000
5' counts
0
5000
10000
15000
Blood
Brain
Heart
Kidney
Lung
Ovary
Skin
Smuscle
Testis

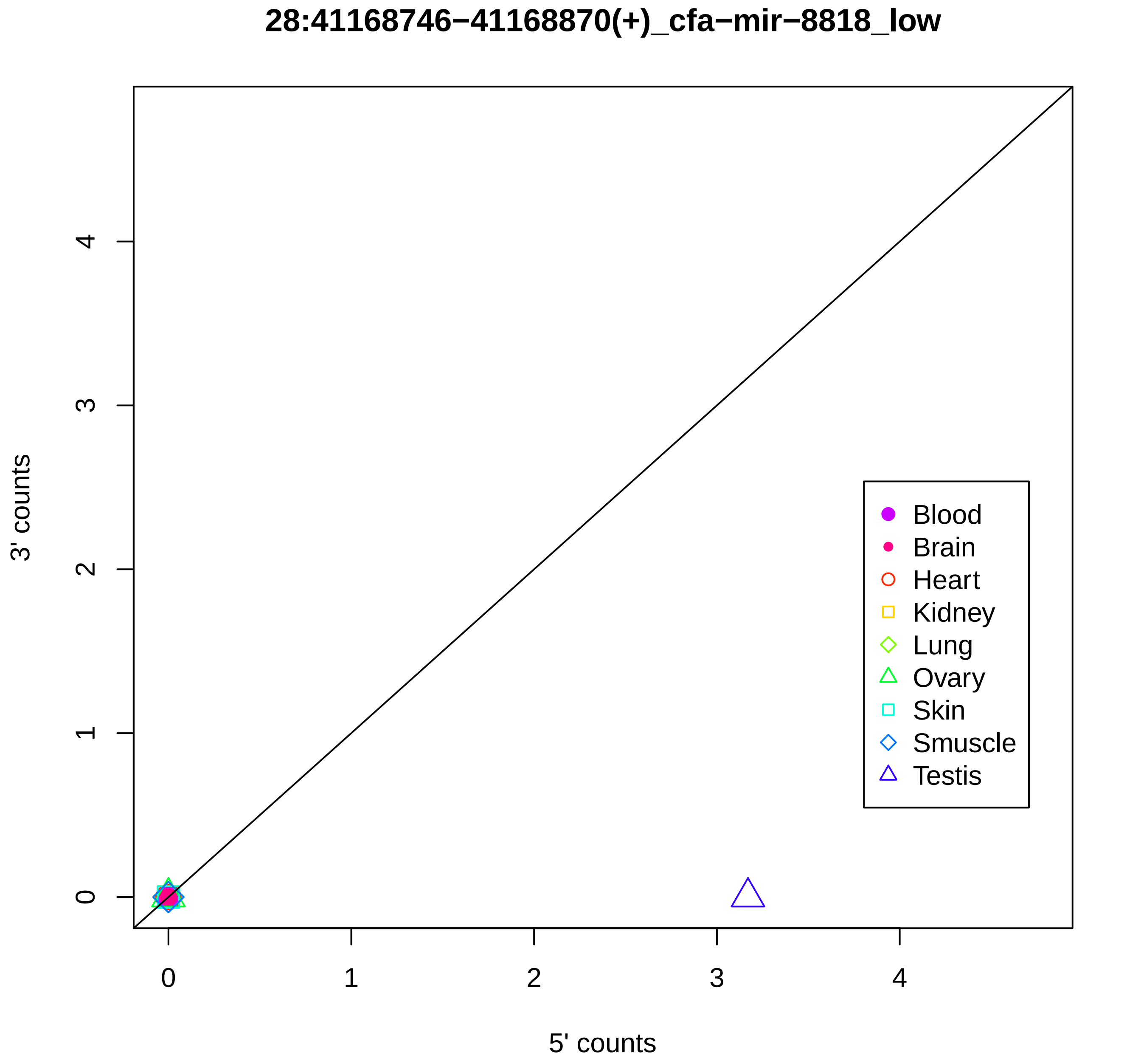
28:41168746−41168870(+)_cfa−mir−8818_low
3' counts
5' counts
0
1
2
3
4
0
1
2
3
4
Blood
Brain
Heart
Kidney
Lung
Ovary
Skin
Smuscle
Testis

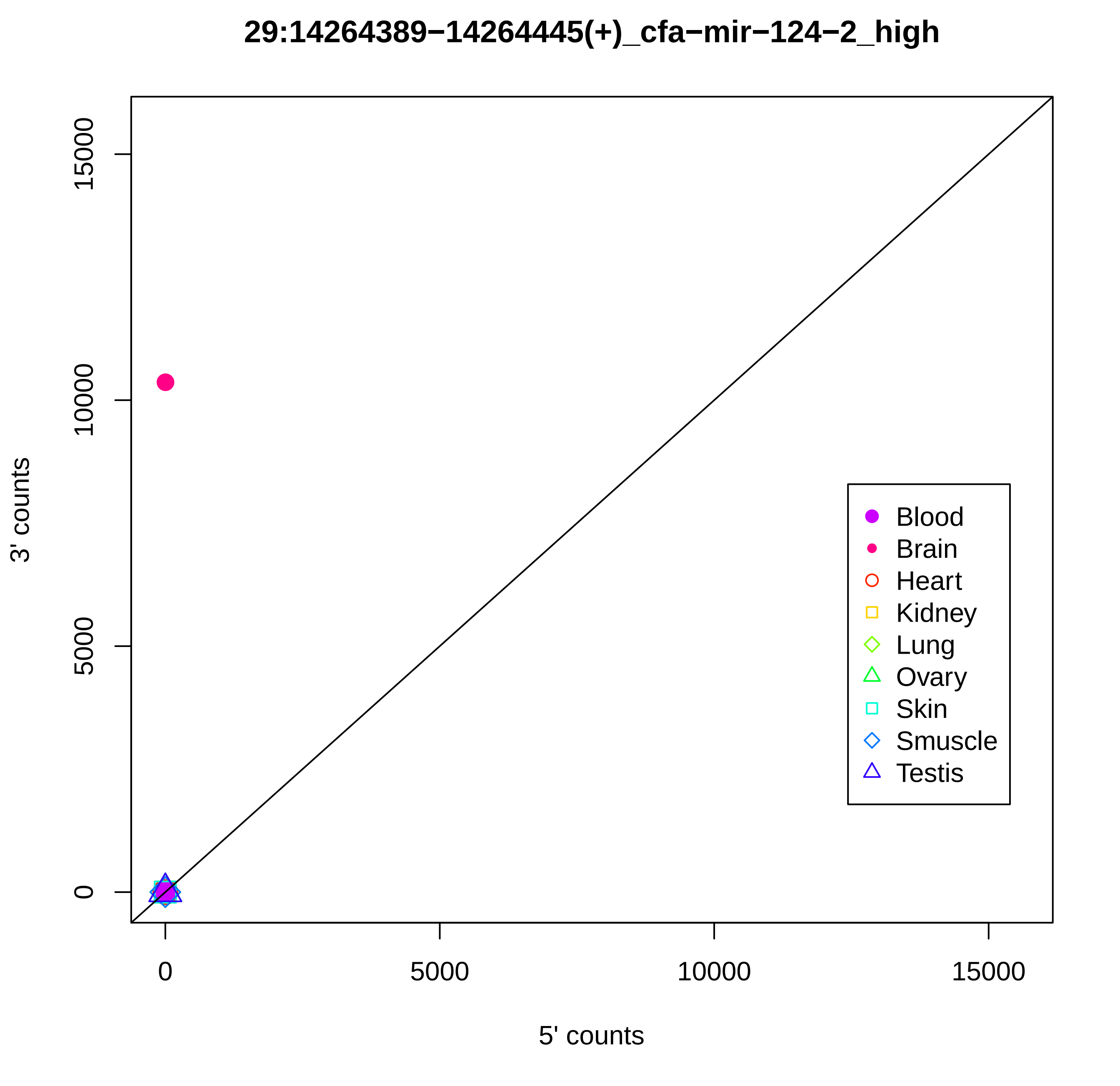

29:14264389−14264445(+)_cfa−mir−124−2_high
15000
10000
5000
0
3' counts
0
5000
10000
15000
5' counts
Blood
Brain
Heart
Kidney
Lung
Ovary
Skin
Smuscle
Testis

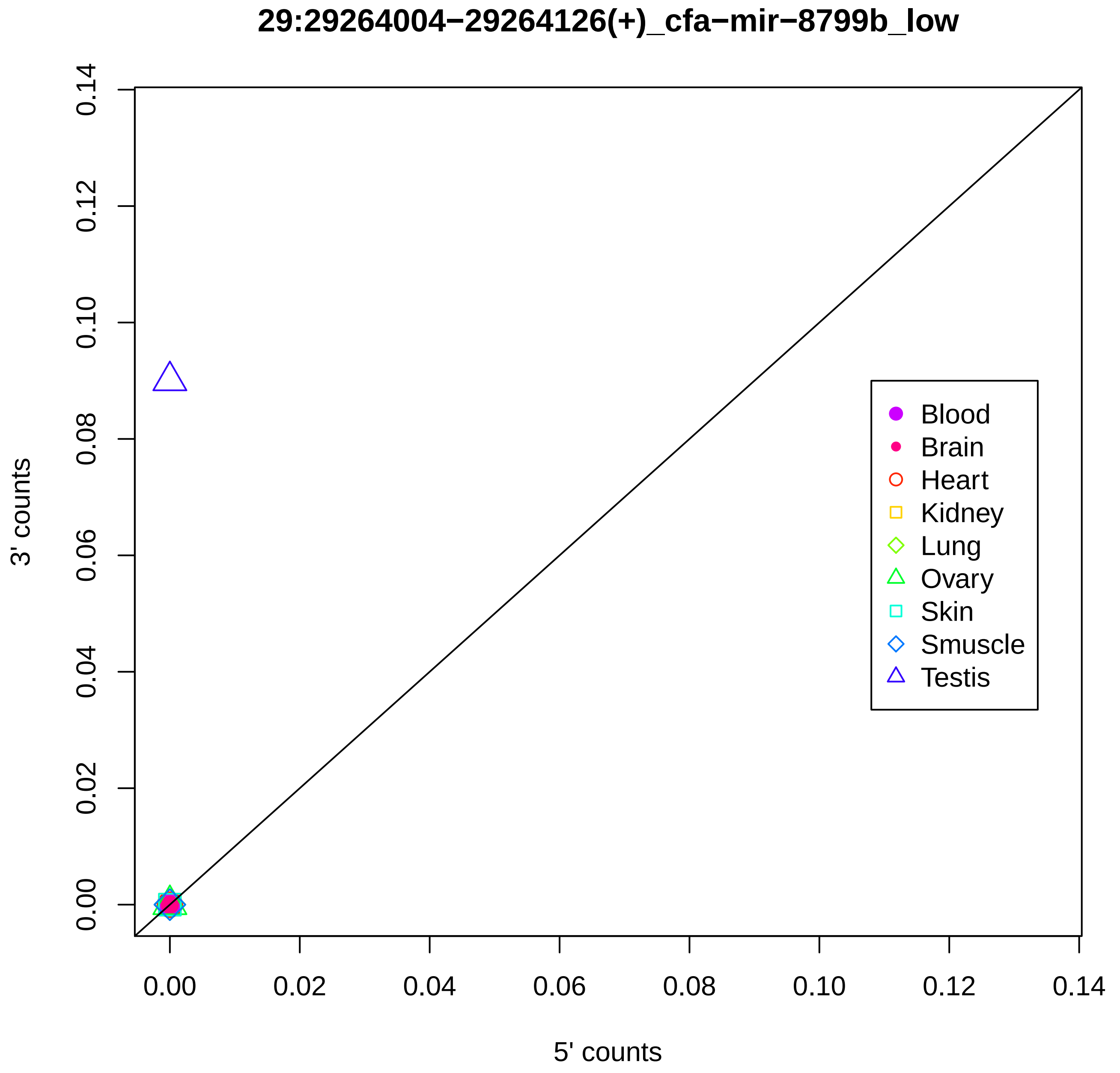

29:29264004−29264126(+)_cfa−mir−8799b_low
3' counts
5' counts
0.00
0.02
0.04
0.06
0.08
0.10
0.12
0.14
Blood
Brain
Heart
Kidney
Lung
Ovary
Skin
Smuscle
Testis

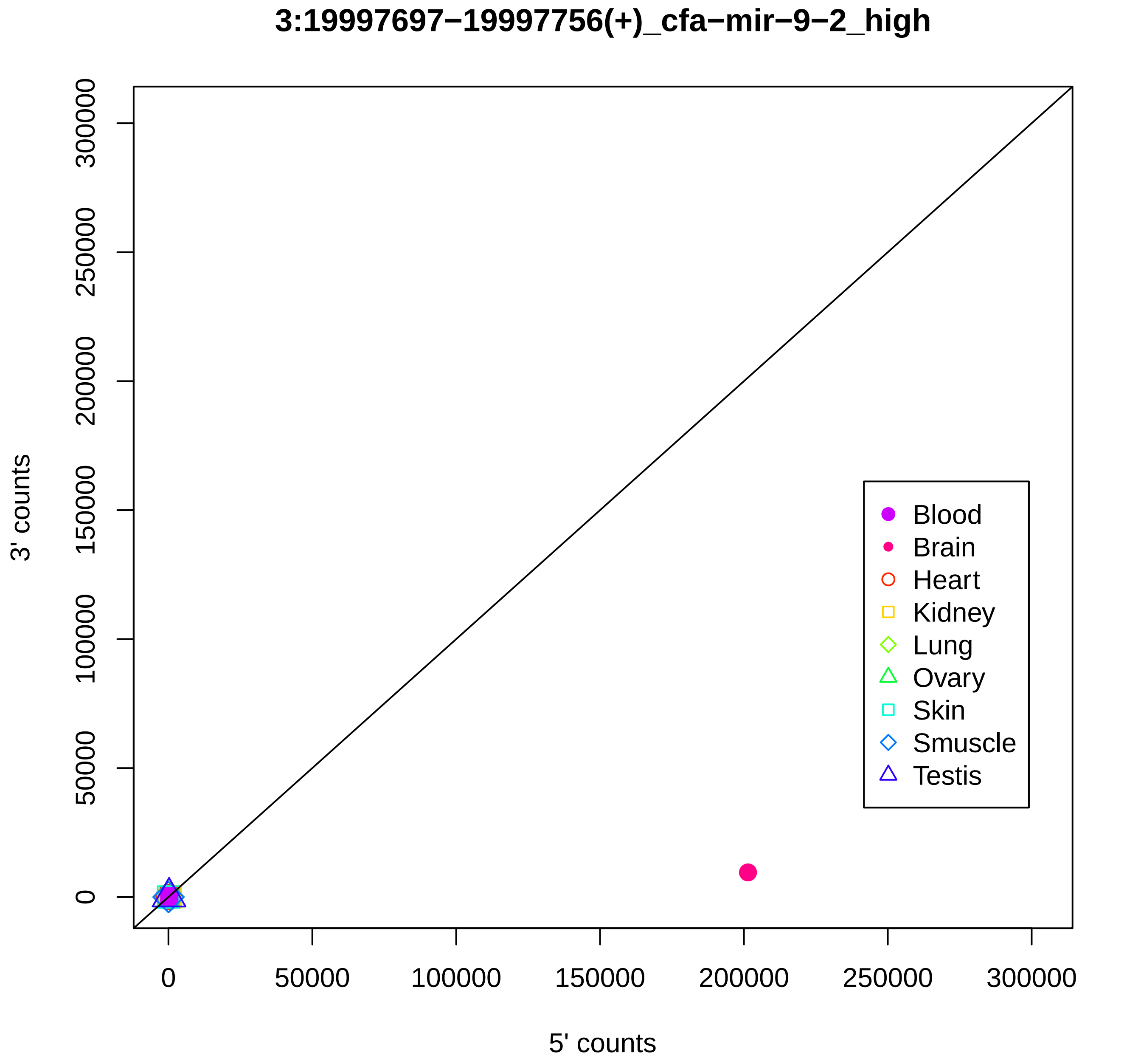
3:19997697−19997756(+)_cfa−mir−9−2_high
3' counts
5' counts
0
50000
100000
150000
200000
250000
300000
Blood
Brain
Heart
Kidney
Lung
Ovary
Skin
Smuscle
Testis

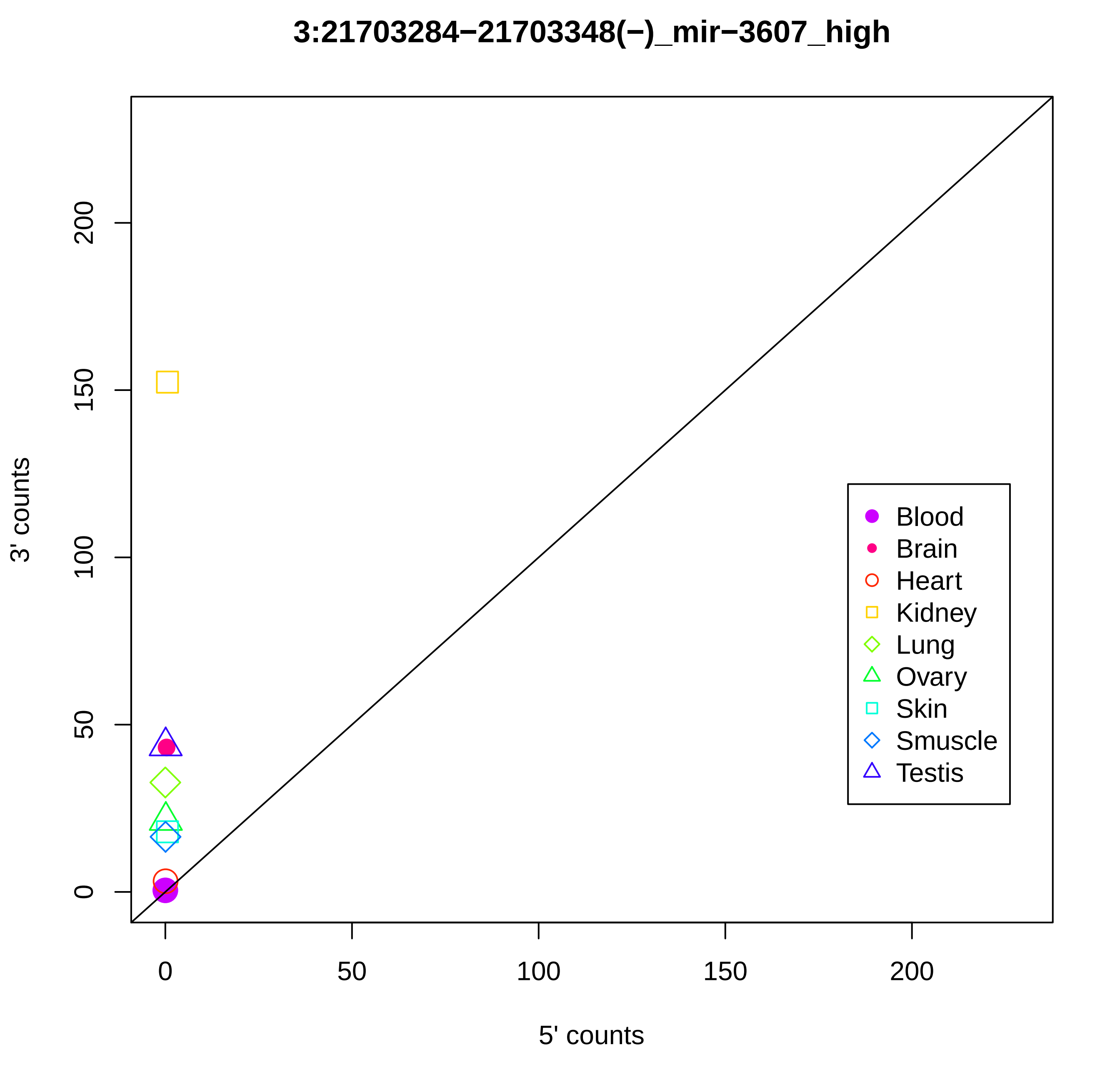

3:21703284−21703348(−)_mir−3607_high
3' counts
5' counts
0
50
100
150
200
0
50
100
150
200
Blood
Brain
Heart
Kidney
Lung
Ovary
Skin
Smuscle
Testis

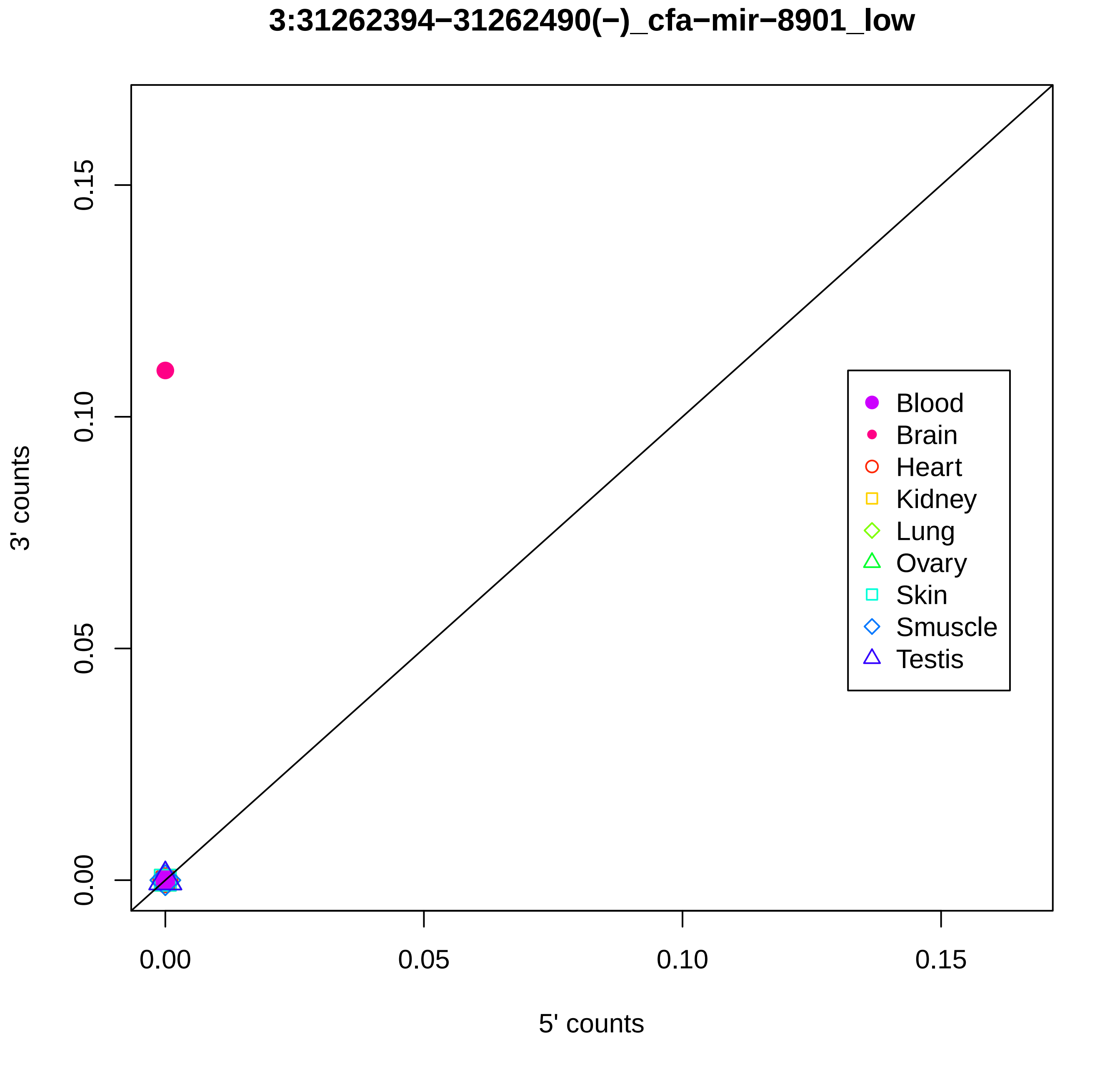

3:31262394−31262490(−)_cfa−mir−8901_low
3' counts
5' counts
0.00
0.05
0.10
0.15
0.00
0.05
0.10
0.15
Blood
Brain
Heart
Kidney
Lung
Ovary
Skin
Smuscle
Testis

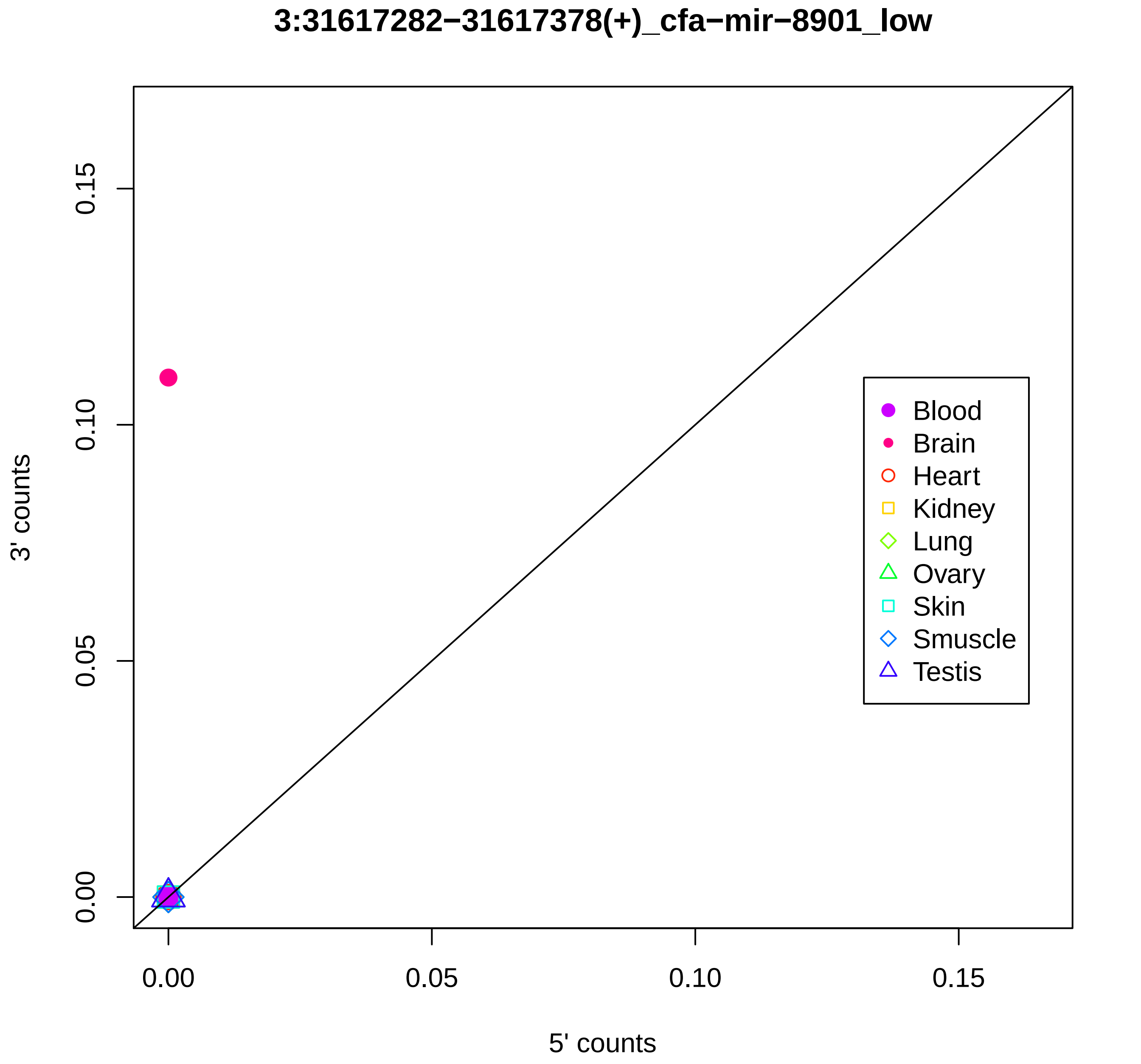
3:31617282−31617378(+)_cfa−mir−8901_low
3' counts
5' counts
0.00
0.05
0.10
0.15
0.00
0.05
0.10
0.15
Blood
Brain
Heart
Kidney
Lung
Ovary
Skin
Smuscle
Testis

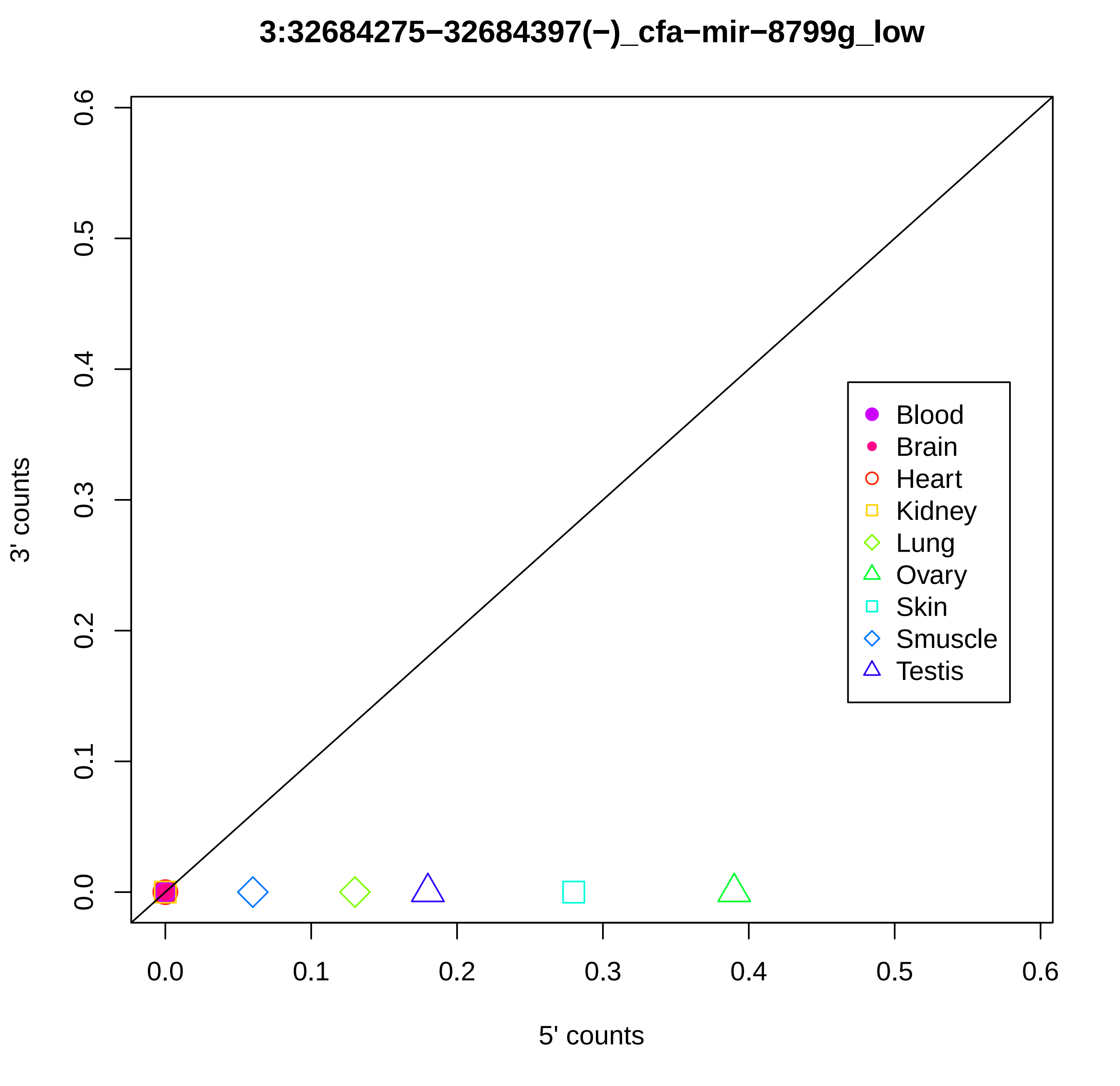

3:32684275−32684397(−)_cfa−mir−8799g_low
3' counts
5' counts
0.0
0.1
0.2
0.3
0.4
0.5
0.6
Blood
Brain
Heart
Kidney
Lung
Ovary
Skin
Smuscle
Testis

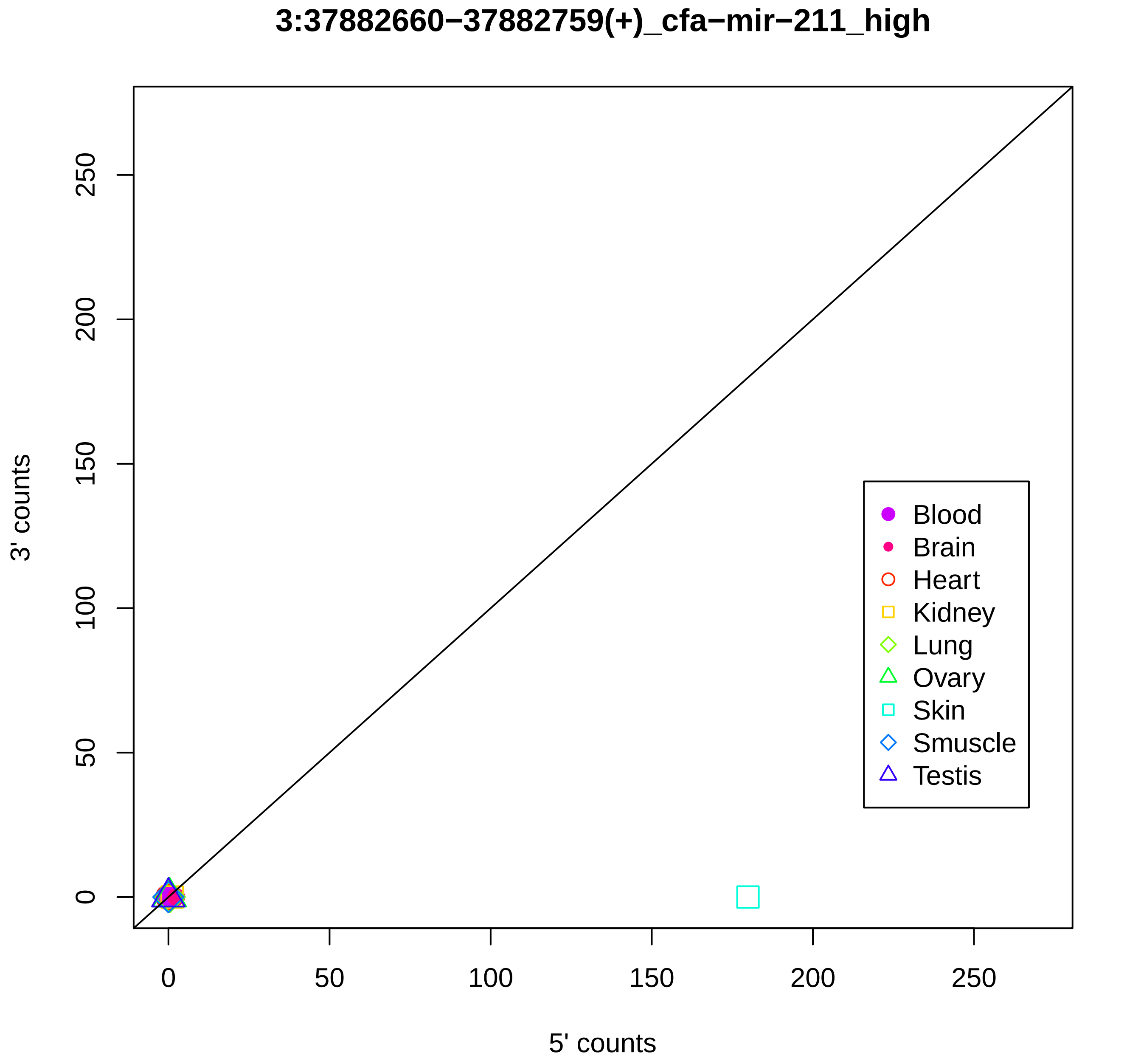

3:37882660−37882759(+)_cfa−mir−211_high
3' counts
5' counts
0
50
100
150
200
250
0
50
100
150
200
250
Blood
Brain
Heart
Kidney
Lung
Ovary
Skin
Smuscle
Testis

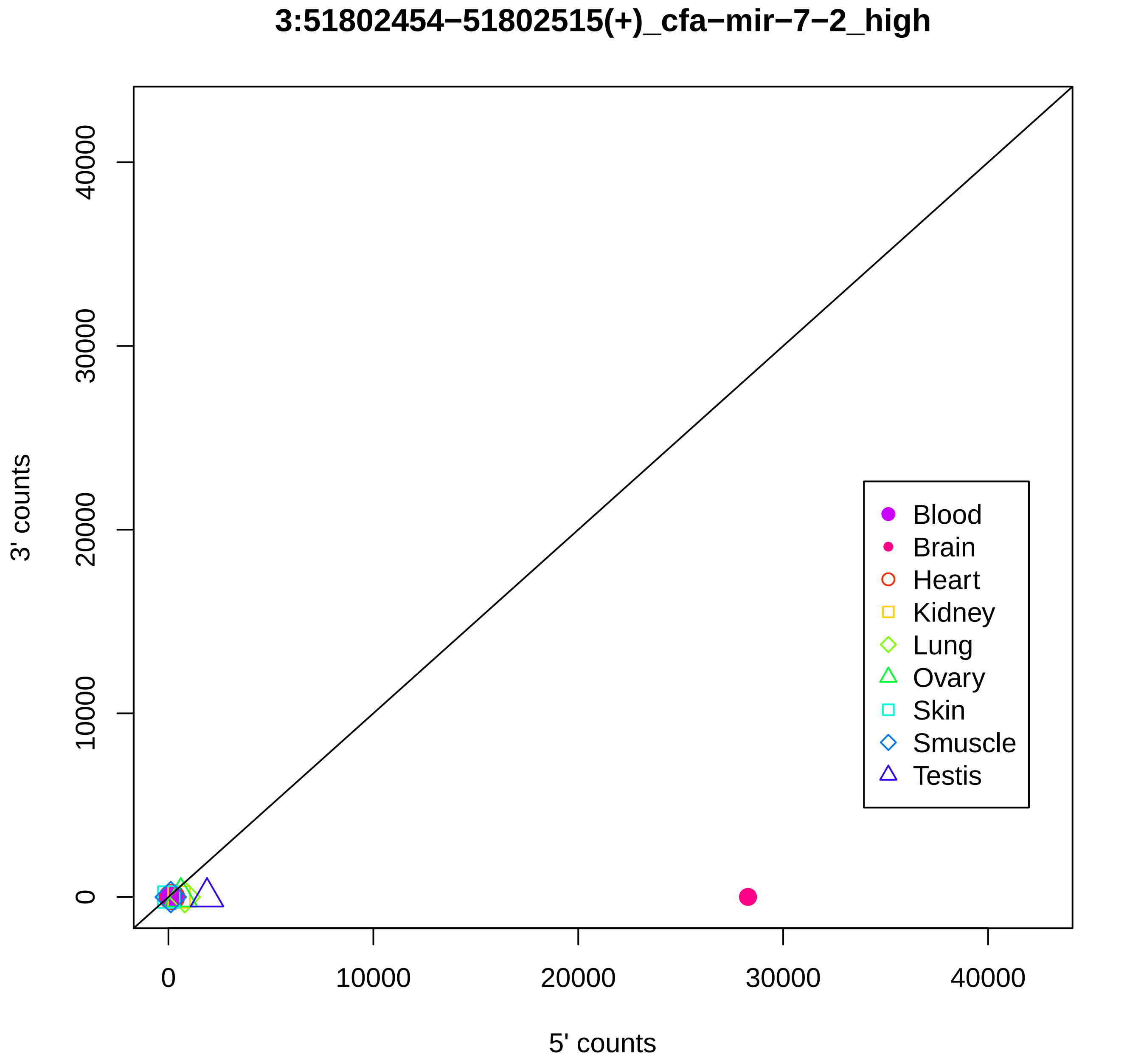

3:51802454−51802515(+)_cfa−mir−7−2_high
3' counts
5' counts
0
10000
20000
30000
40000
Blood
Brain
Heart
Kidney
Lung
Ovary
Skin
Smuscle
Testis

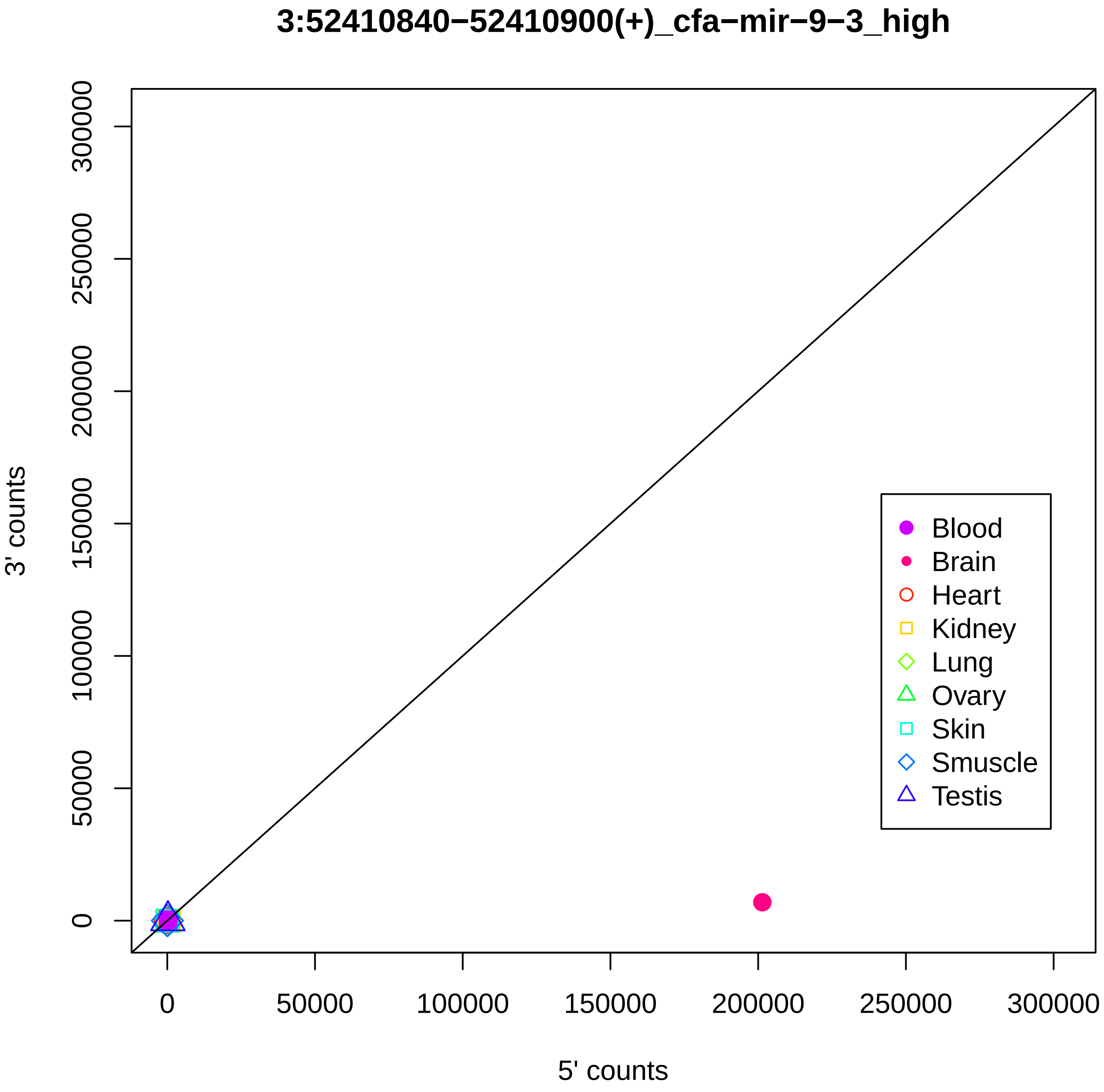
3:52410840−52410900(+)_cfa−mir−9−3_high
3' counts
5' counts
0
50000
100000
150000
200000
250000
300000
Blood
Brain
Heart
Kidney
Lung
Ovary
Skin
Smuscle
Testis

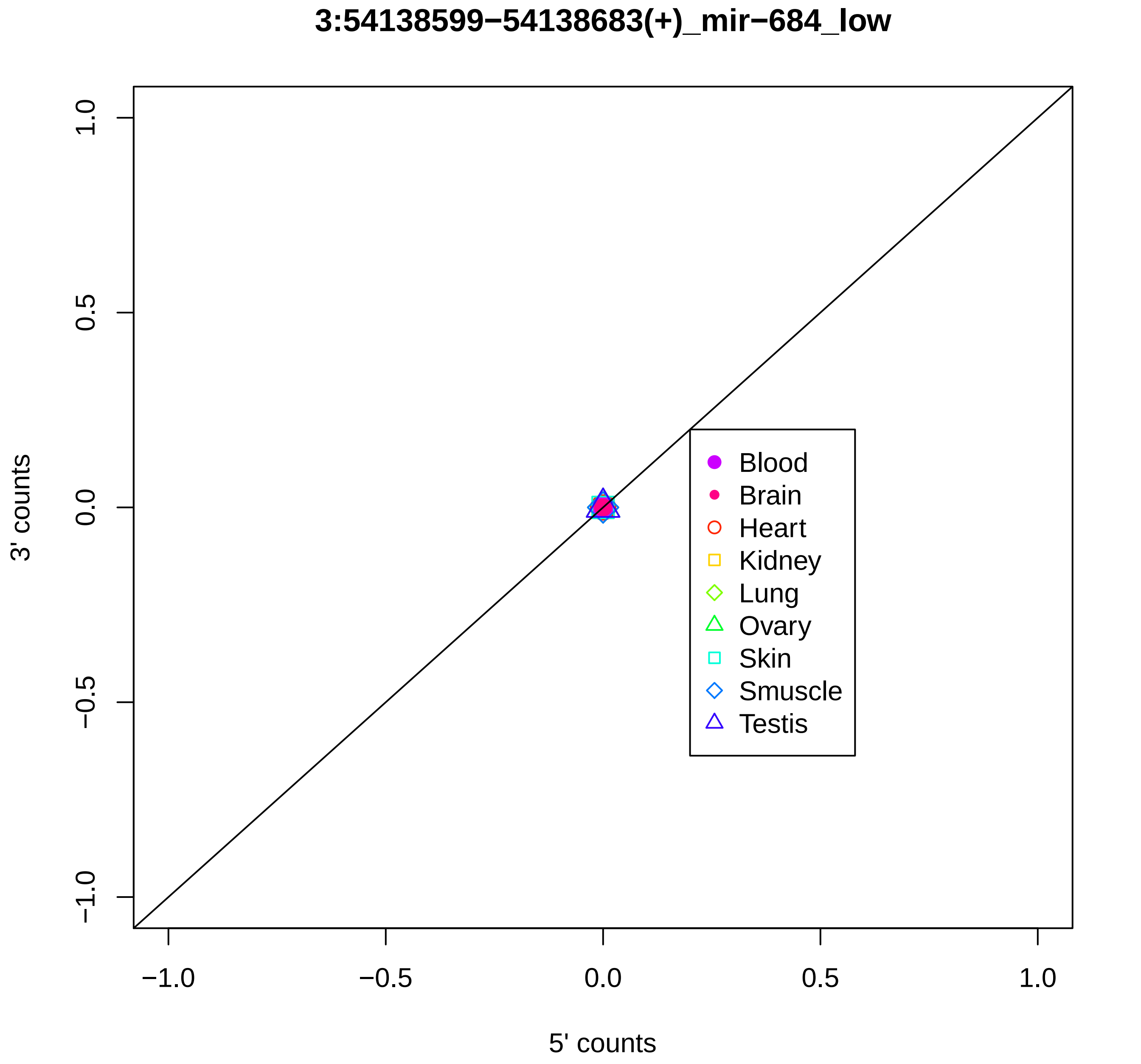

3:54138599−54138683(+)_mir−684_low
3' counts
5' counts
1.0
0.5
0.0
−0.5
−1.0
Blood
Brain
Heart
Kidney
Lung
Ovary
Skin
Smuscle
Testis

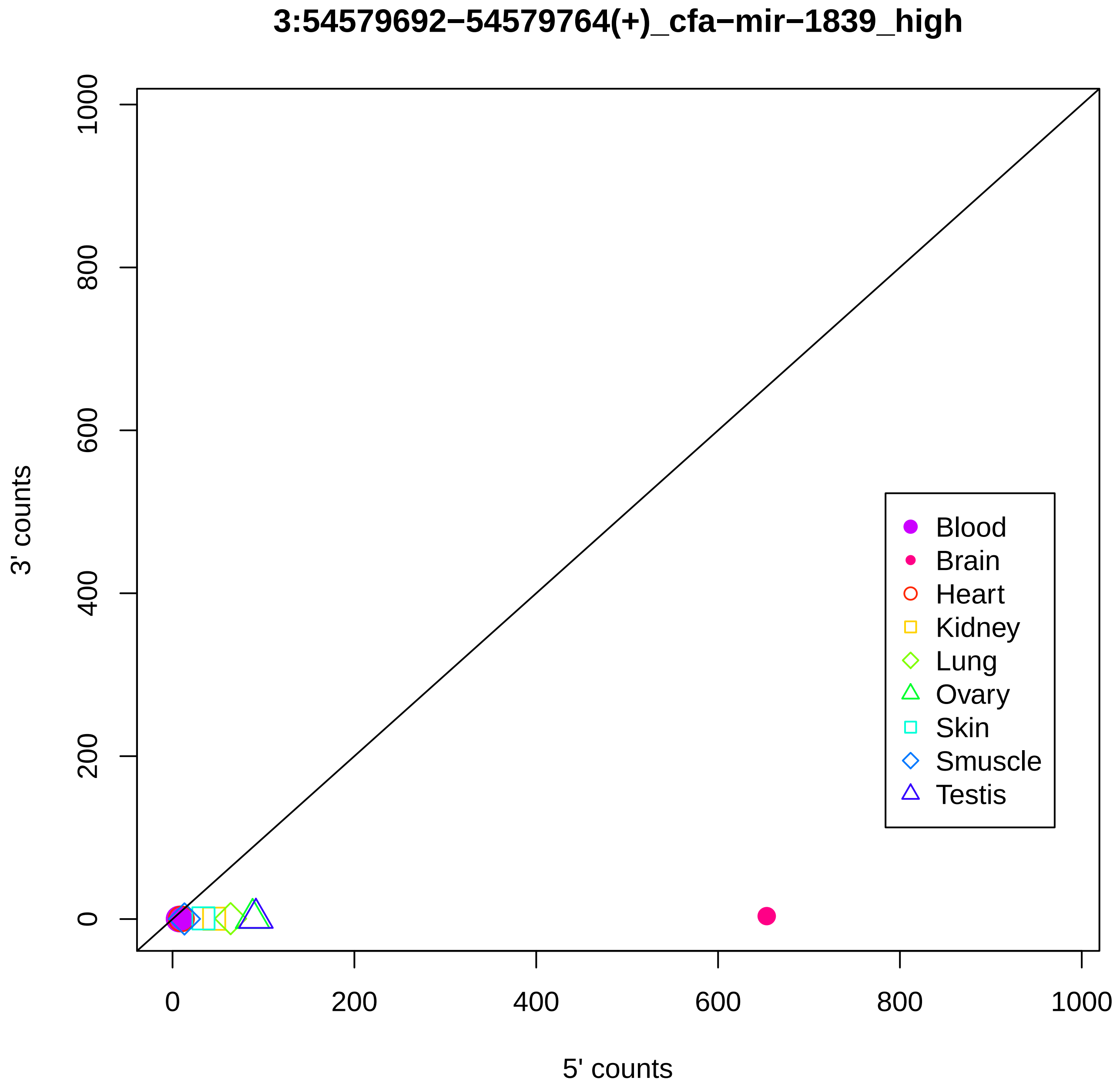
3:54579692−54579764(+)_cfa−mir−1839_high
3' counts
5' counts
0
200
400
600
800
1000
0
200
400
600
800
1000
Blood
Brain
Heart
Kidney
Lung
Ovary
Skin
Smuscle
Testis

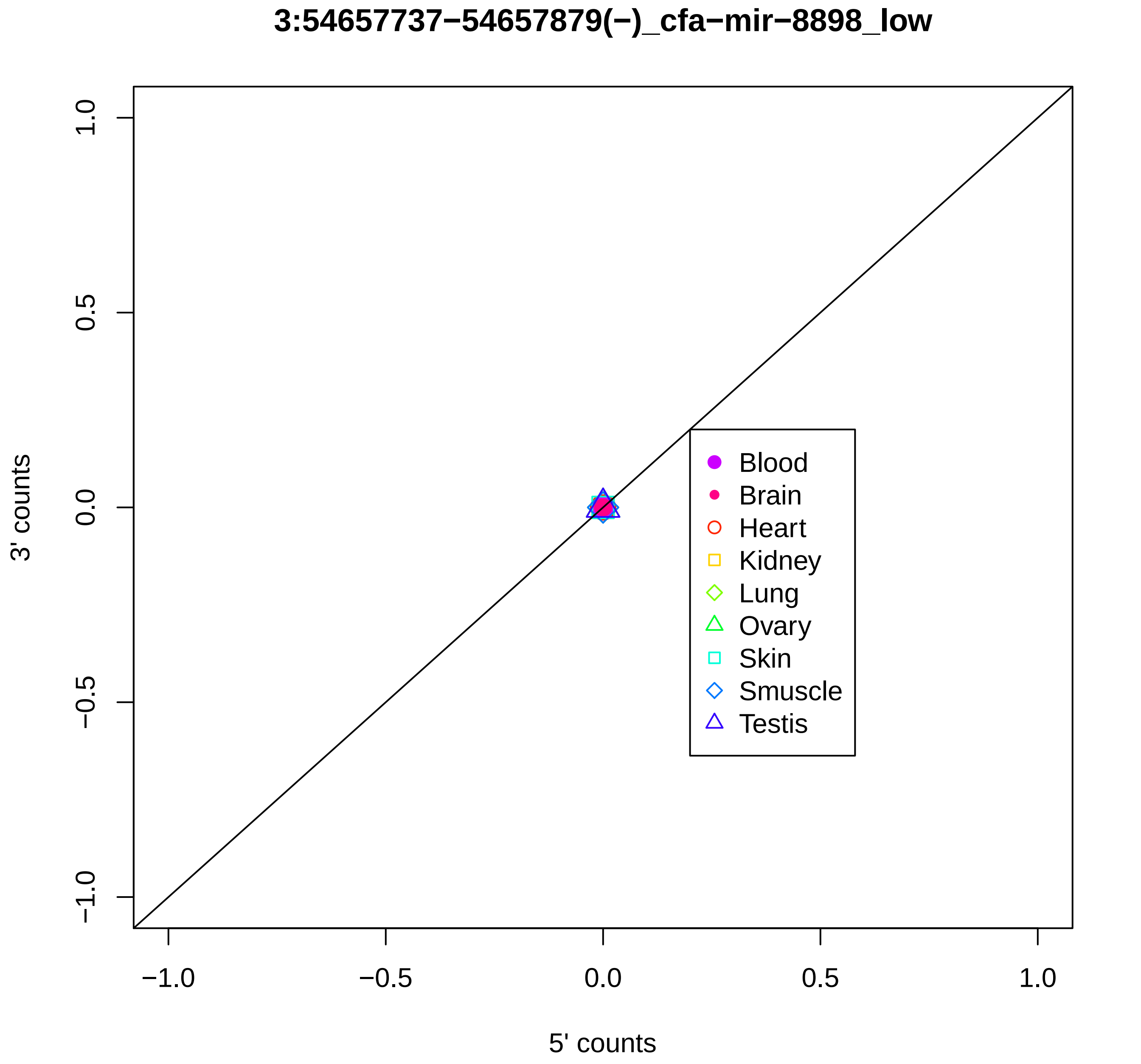

3:54657737−54657879(−)_cfa−mir−8898_low
3' counts
5' counts
1.0
0.5
0.0
−0.5
−1.0
Blood
Brain
Heart
Kidney
Lung
Ovary
Skin
Smuscle
Testis

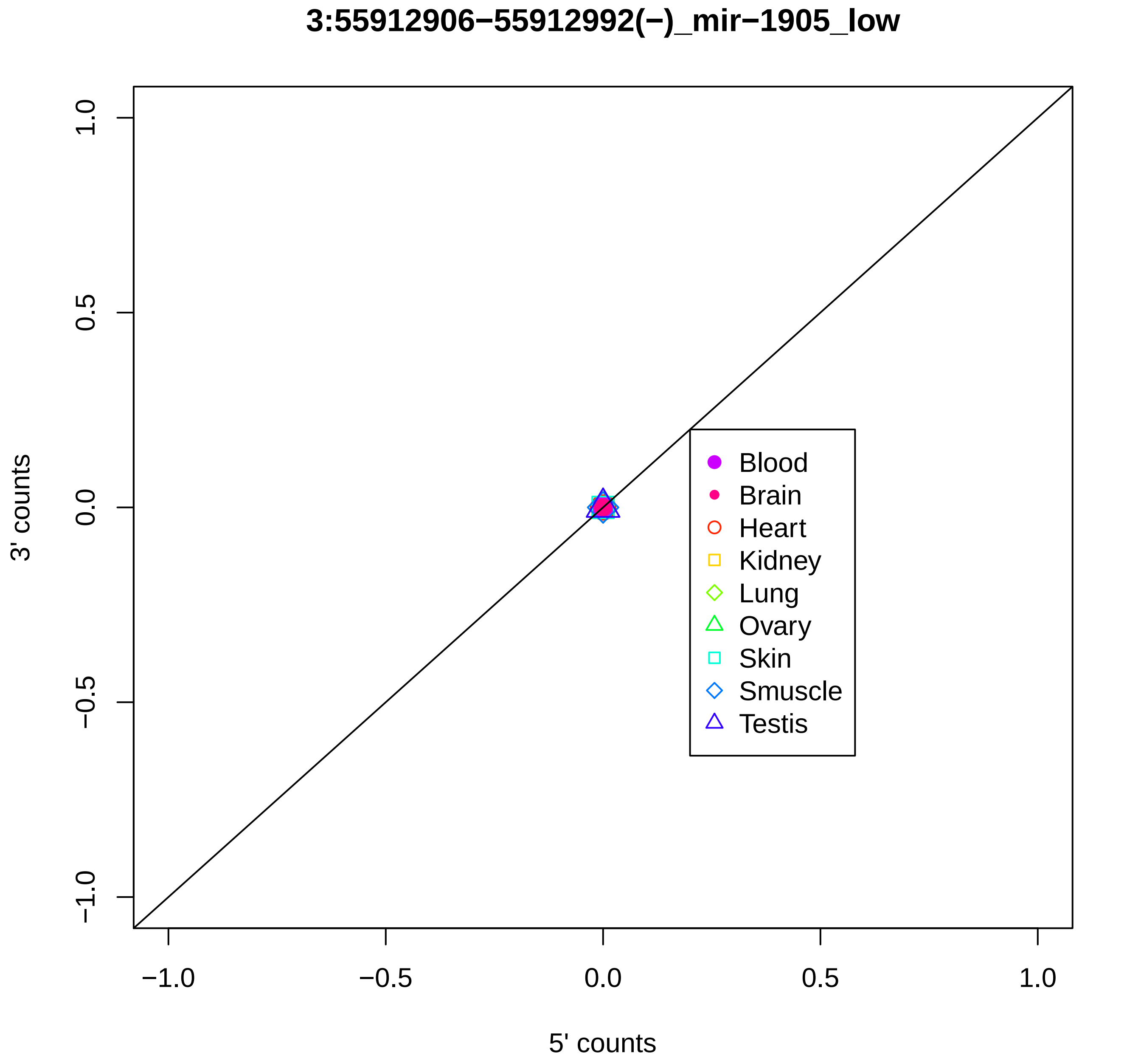
3:55912906−55912992(−)_mir−1905_low
3' counts
5' counts
1.0
0.5
0.0
−0.5
−1.0
−1.0
−0.5
0.0
0.5
1.0
Blood
Brain
Heart
Kidney
Lung
Ovary
Skin
Smuscle
Testis

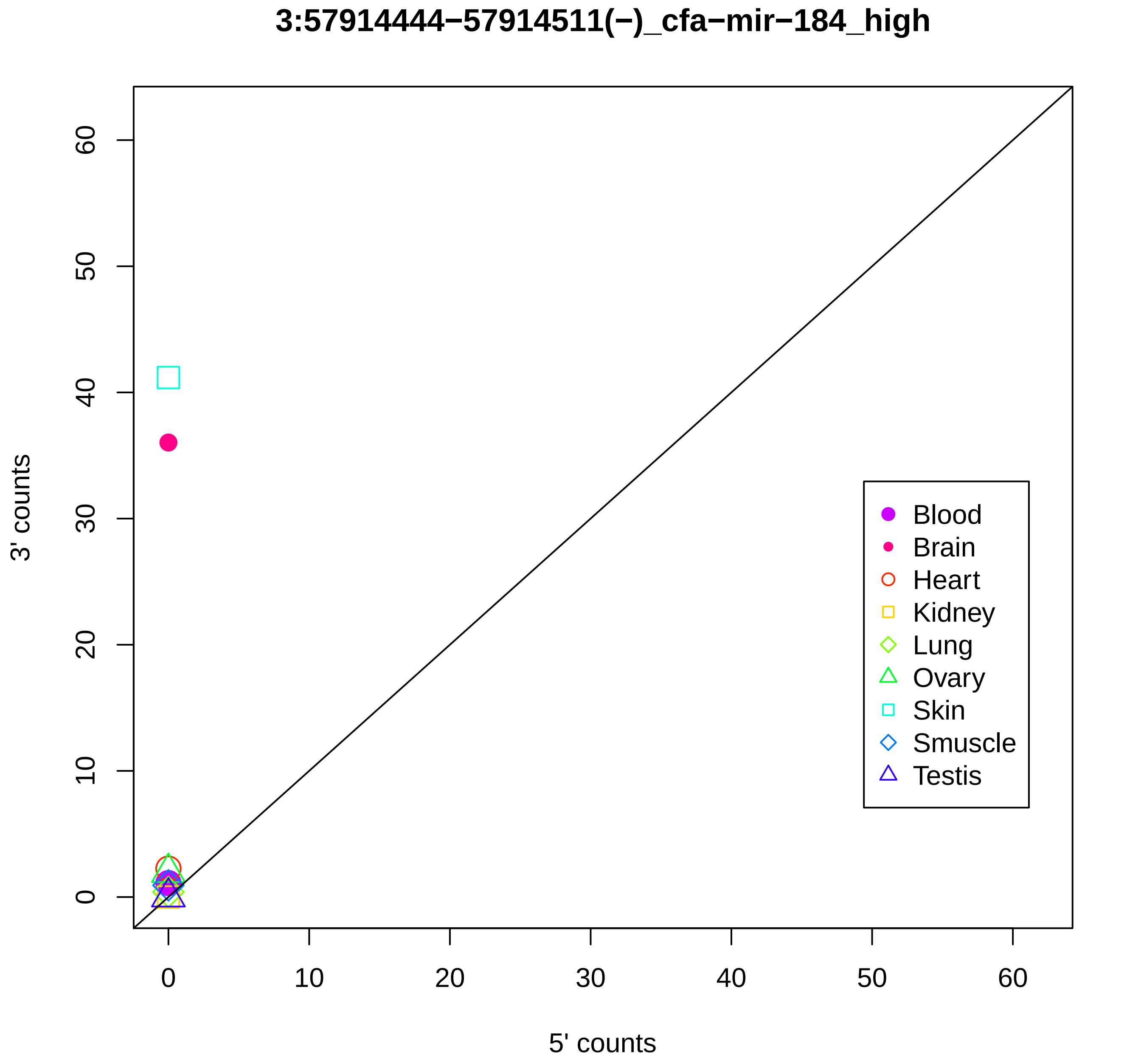

3:57914444−57914511(−)_cfa−mir−184_high
3' counts
5' counts
0
10
20
30
40
50
60
Blood
Brain
Heart
Kidney
Lung
Ovary
Skin
Smuscle
Testis

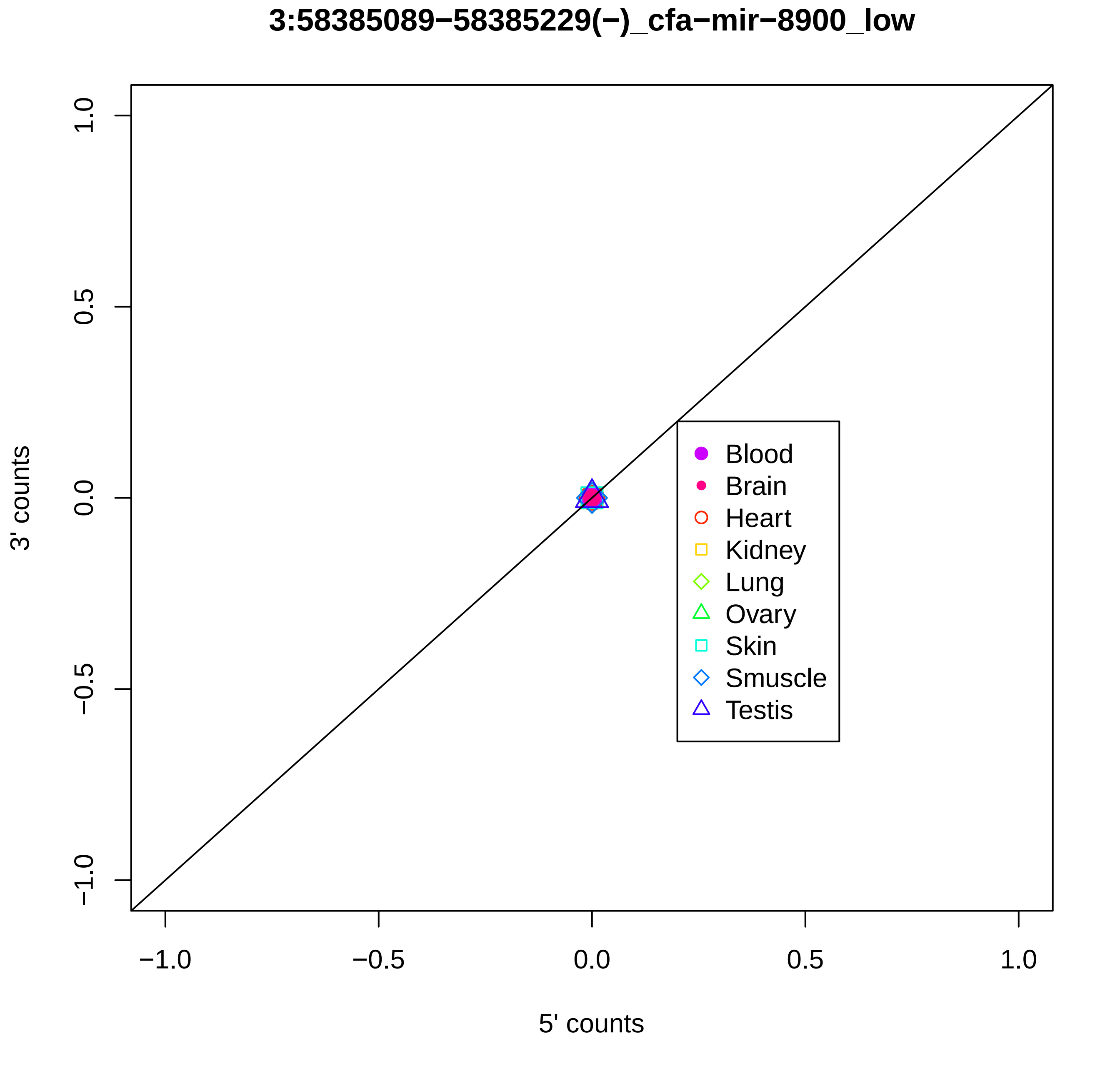

3:58385089−58385229(−)_cfa−mir−8900_low
3' counts
5' counts
1.0
0.5
0.0
−0.5
−1.0
Blood
Brain
Heart
Kidney
Lung
Ovary
Skin
Smuscle
Testis

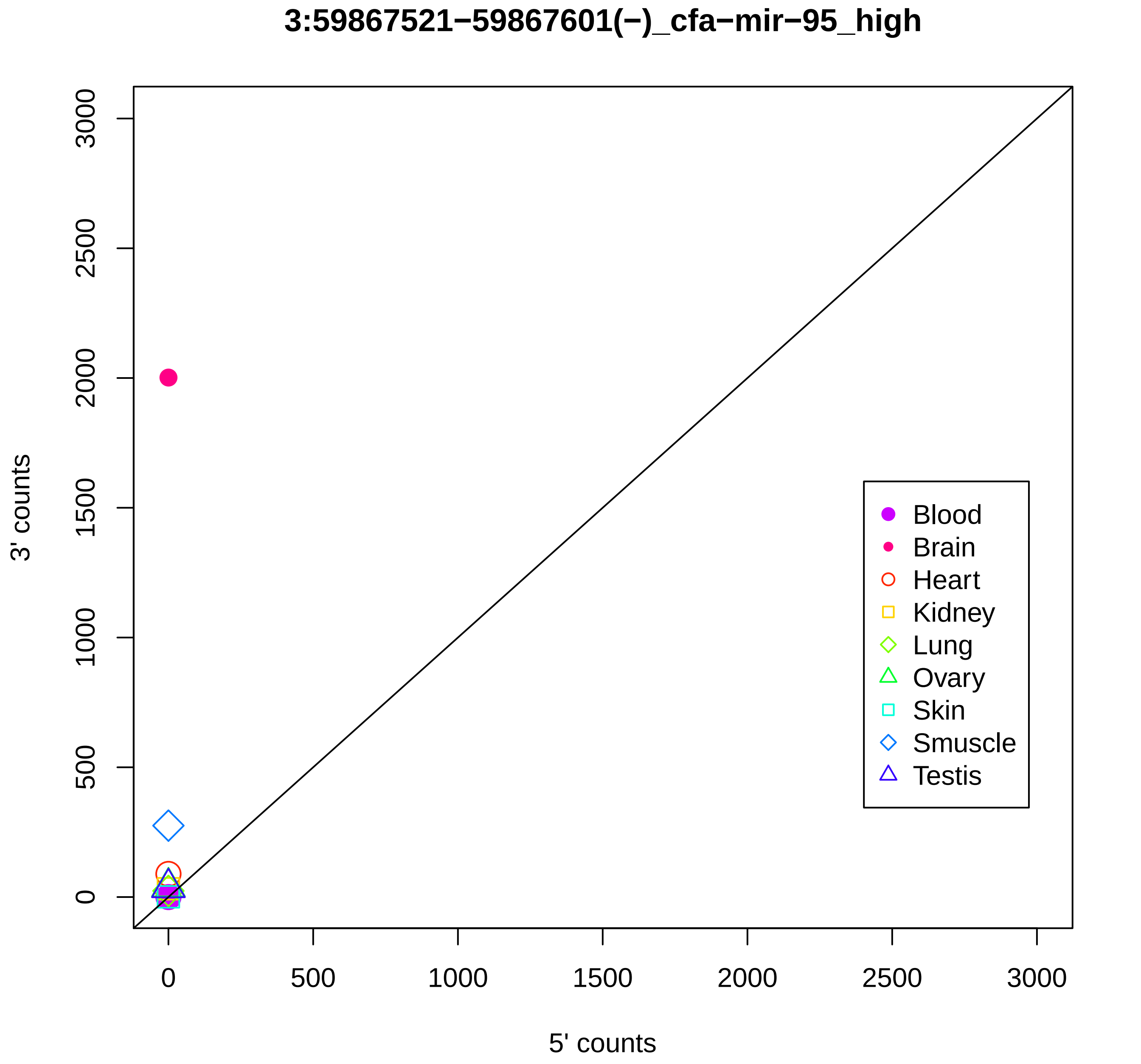

3:59867521−59867601(−)_cfa−mir−95_high
3' counts
5' counts
0
500
1000
1500
2000
2500
3000
Blood
Brain
Heart
Kidney
Lung
Ovary
Skin
Smuscle
Testis

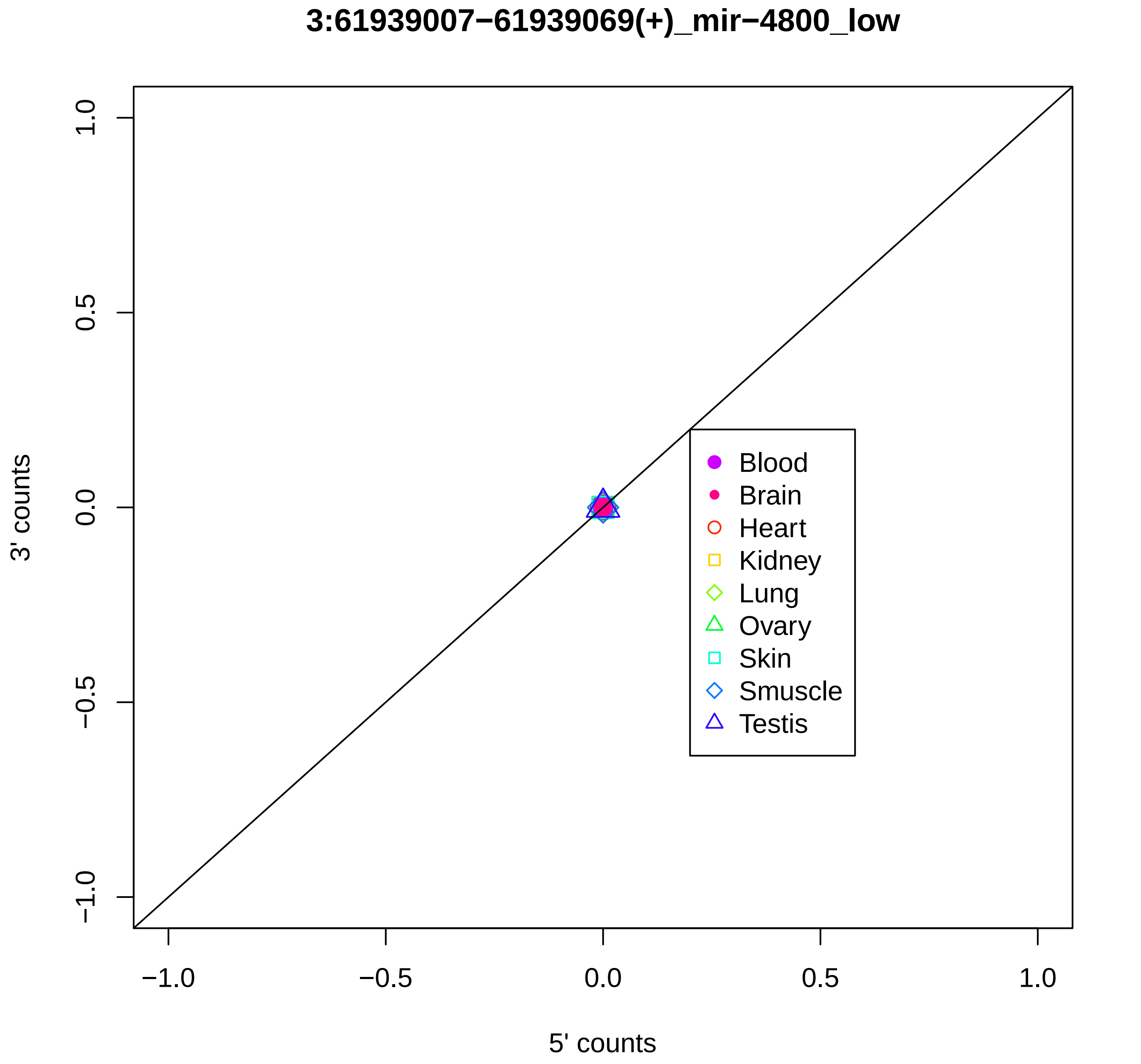
3:61939007−61939069(+)_mir−4800_low
3' counts
5' counts
−1.0
−0.5
0.0
0.5
1.0
Blood
Brain
Heart
Kidney
Lung
Ovary
Skin
Smuscle
Testis

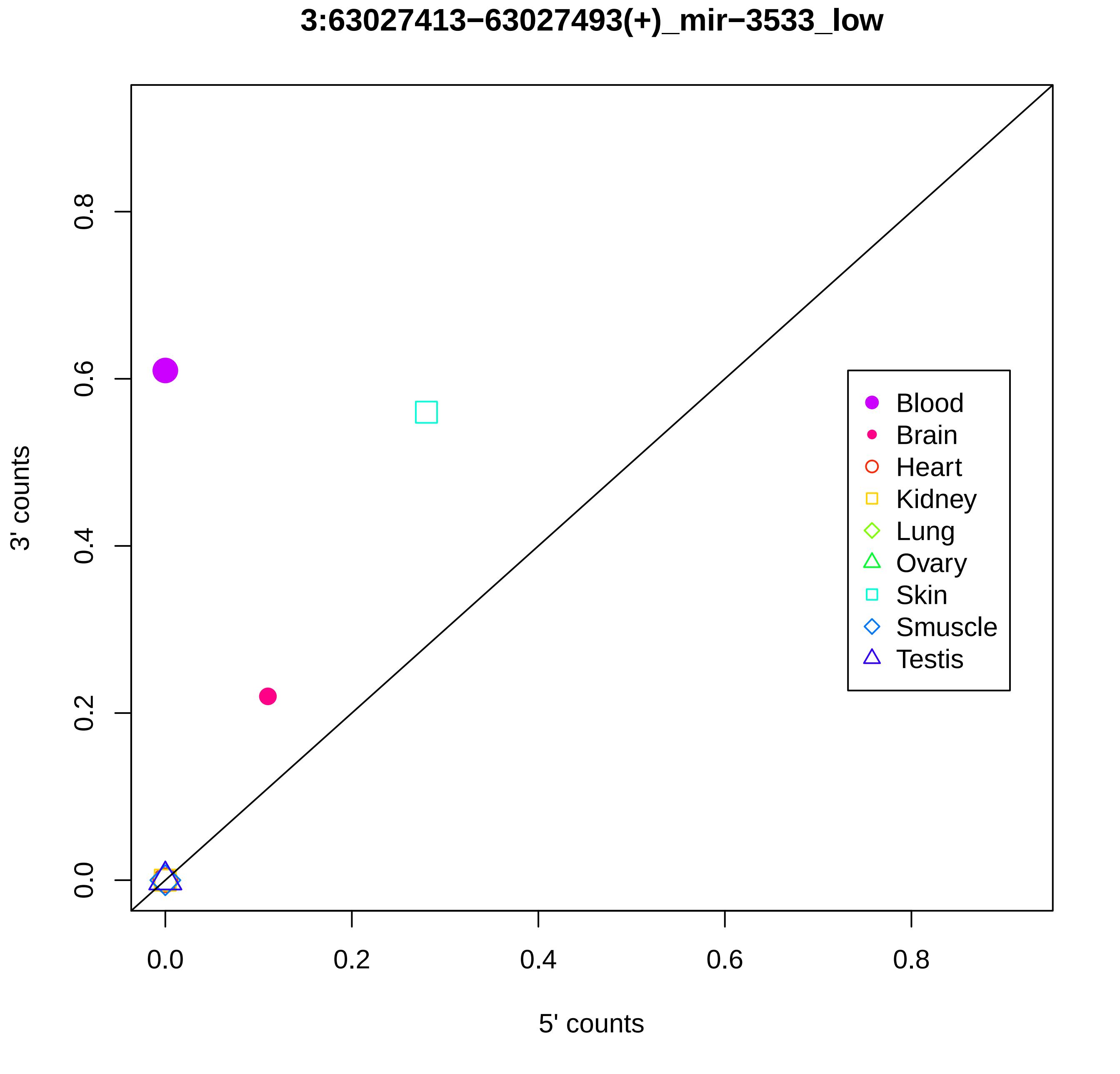

3:63027413−63027493(+)_mir−3533_low
3' counts
5' counts
0.0
0.2
0.4
0.6
0.8
Blood
Brain
Heart
Kidney
Lung
Ovary
Skin
Smuscle
Testis

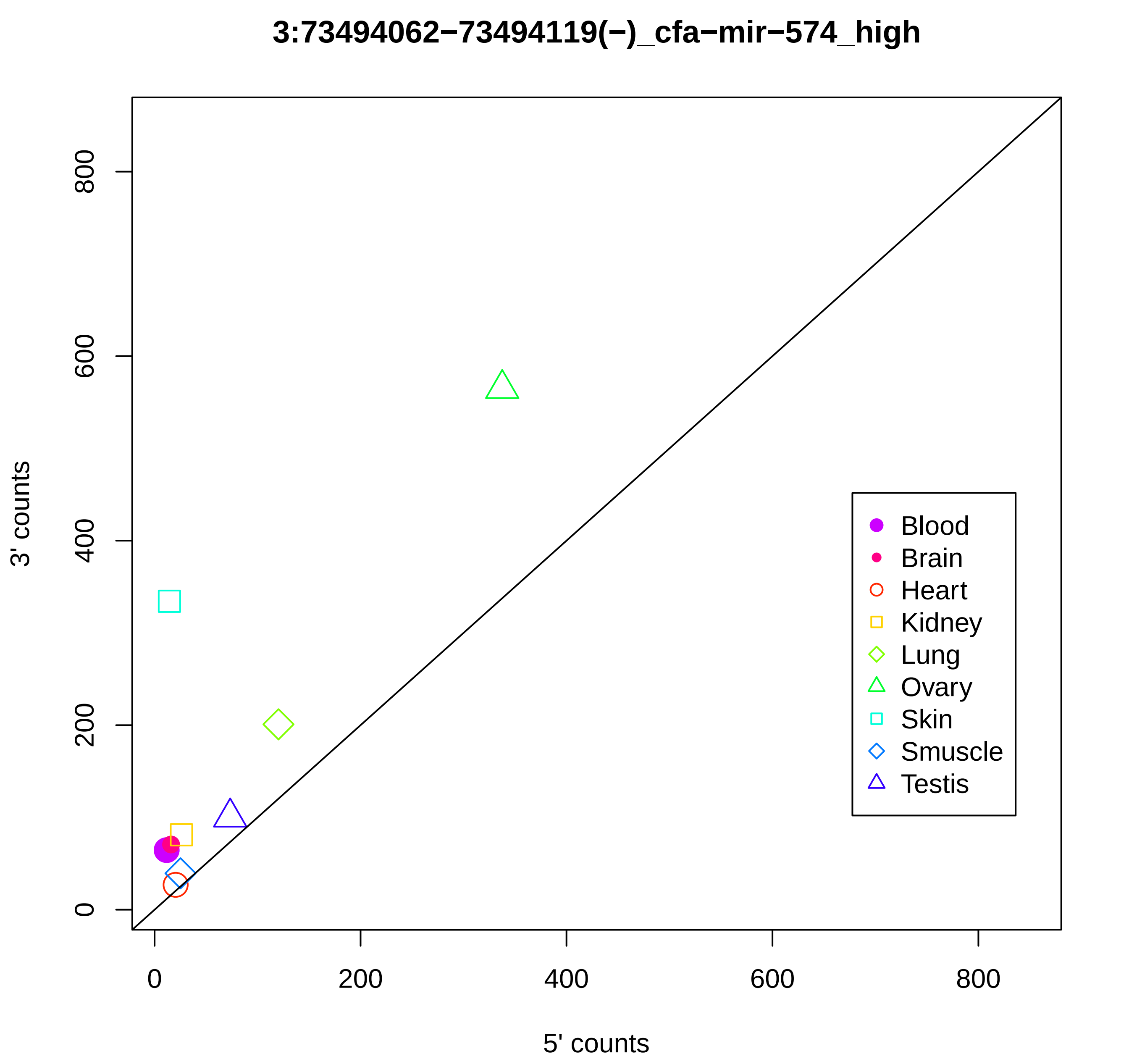

3:73494062−73494119(−)_cfa−mir−574_high
3' counts
5' counts
0
200
400
600
800
0
200
400
600
800
Blood
Brain
Heart
Kidney
Lung
Ovary
Skin
Smuscle
Testis

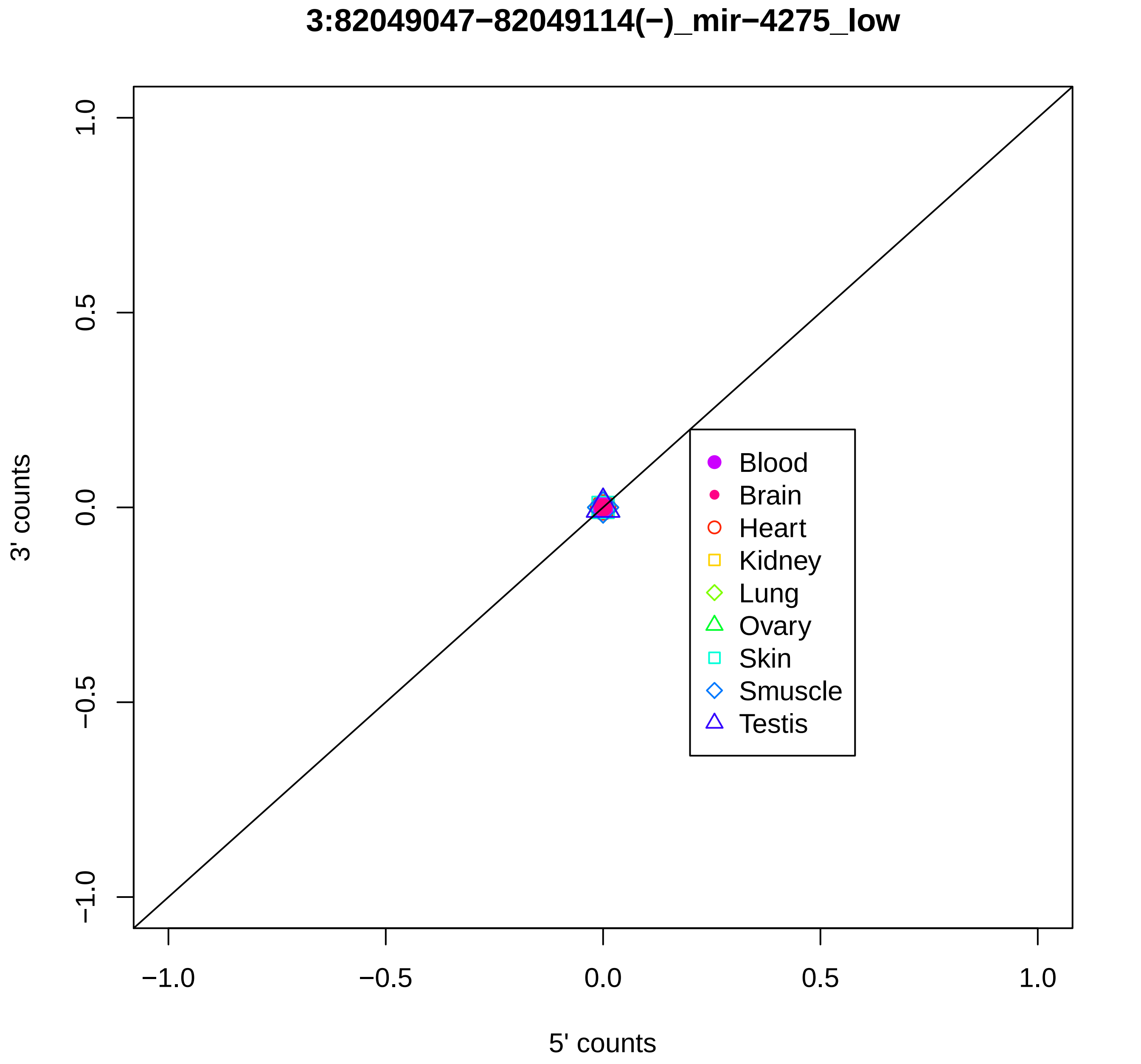

3:82049047−82049114(−)_mir−4275_low
3' counts
5' counts
1.0
0.5
0.0
−0.5
−1.0
Blood
Brain
Heart
Kidney
Lung
Ovary
Skin
Smuscle
Testis

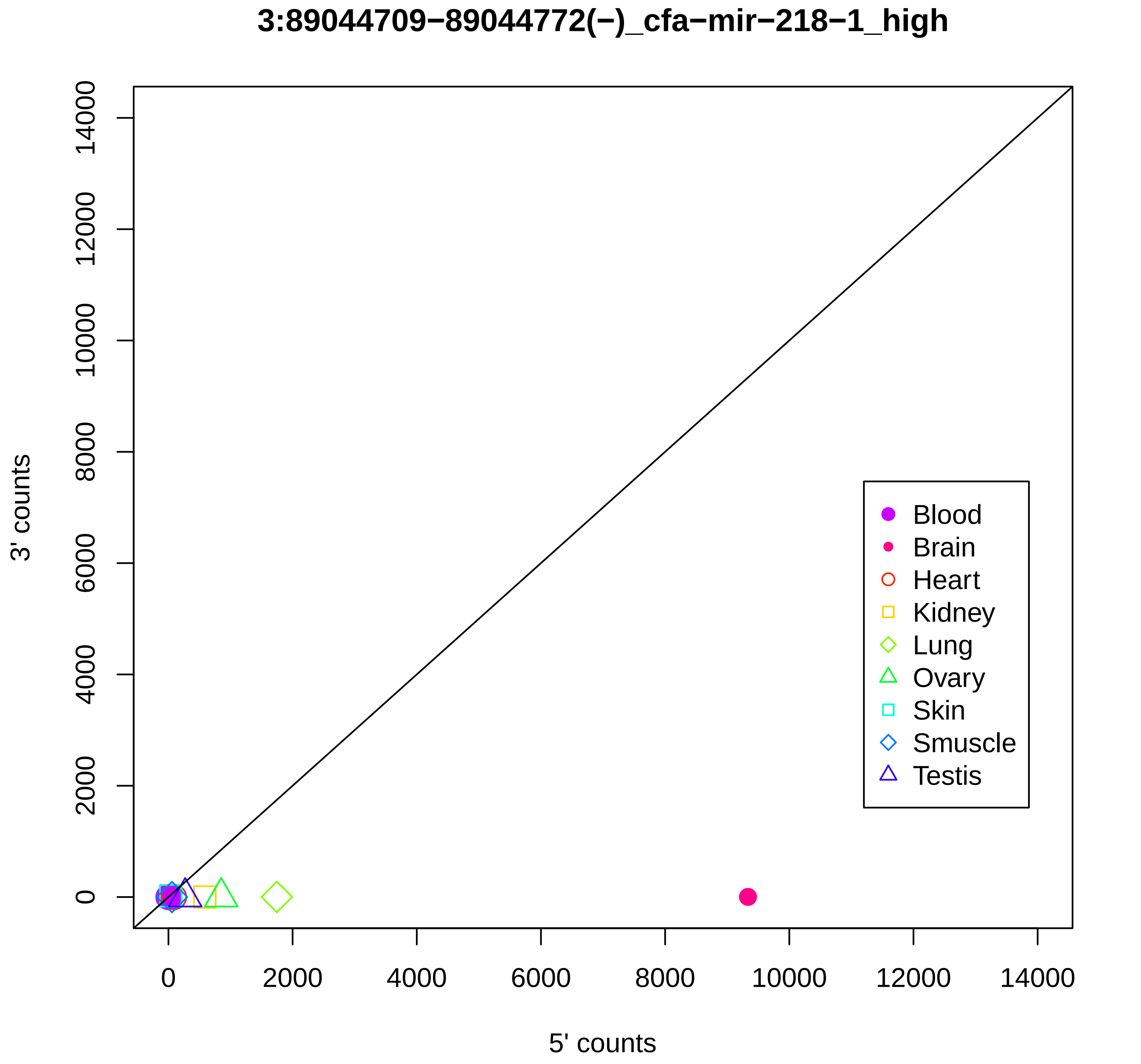
3:89044709−89044772(−)_cfa−mir−218−1_high
3' counts
5' counts
0
2000
4000
6000
8000
10000
12000
14000
Blood
Brain
Heart
Kidney
Lung
Ovary
Skin
Smuscle
Testis

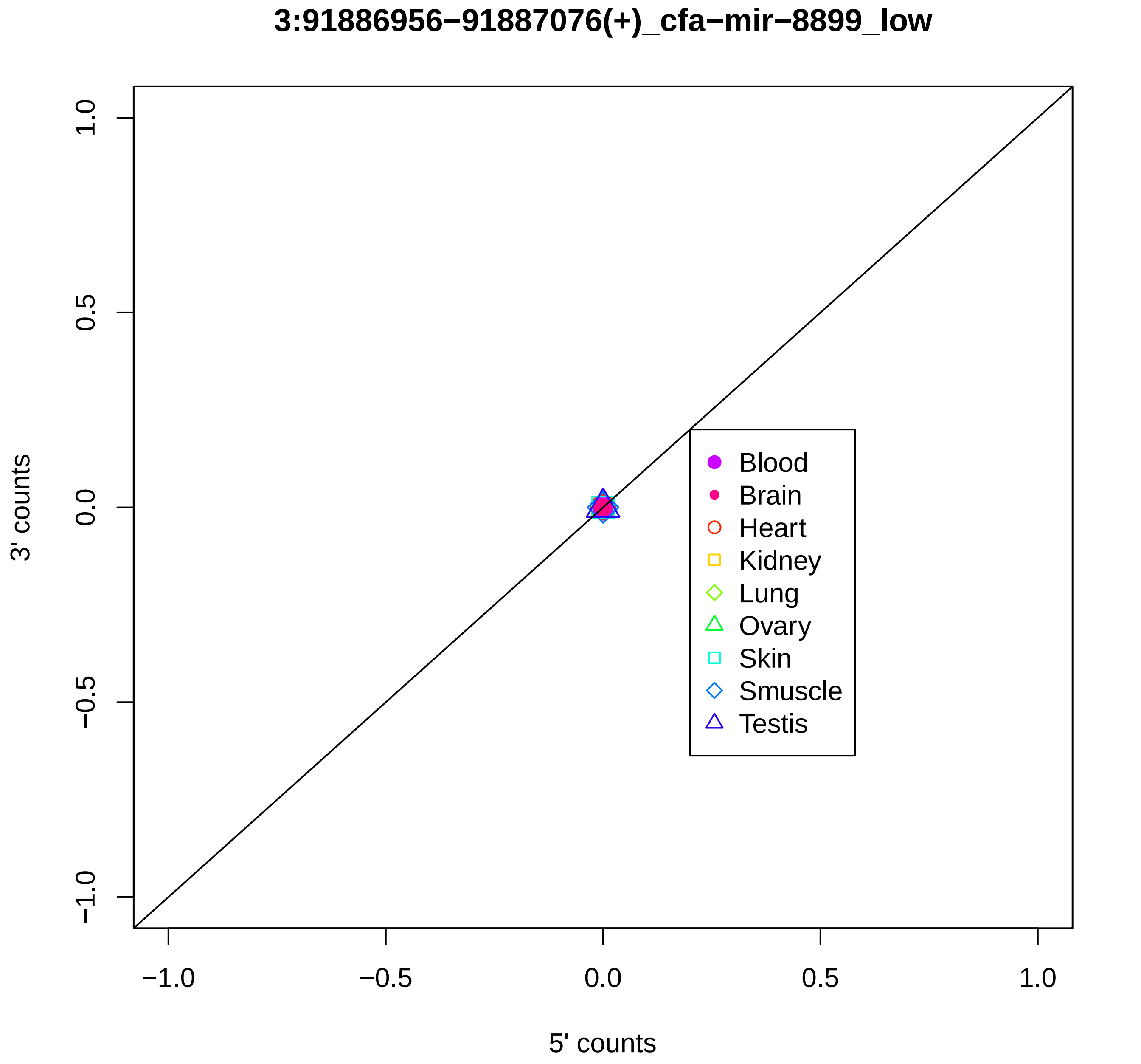

3:91886956−91887076(+)_cfa−mir−8899_low
3' counts
5' counts
1.0
0.5
0.0
−0.5
−1.0
Blood
Brain
Heart
Kidney
Lung
Ovary
Skin
Smuscle
Testis

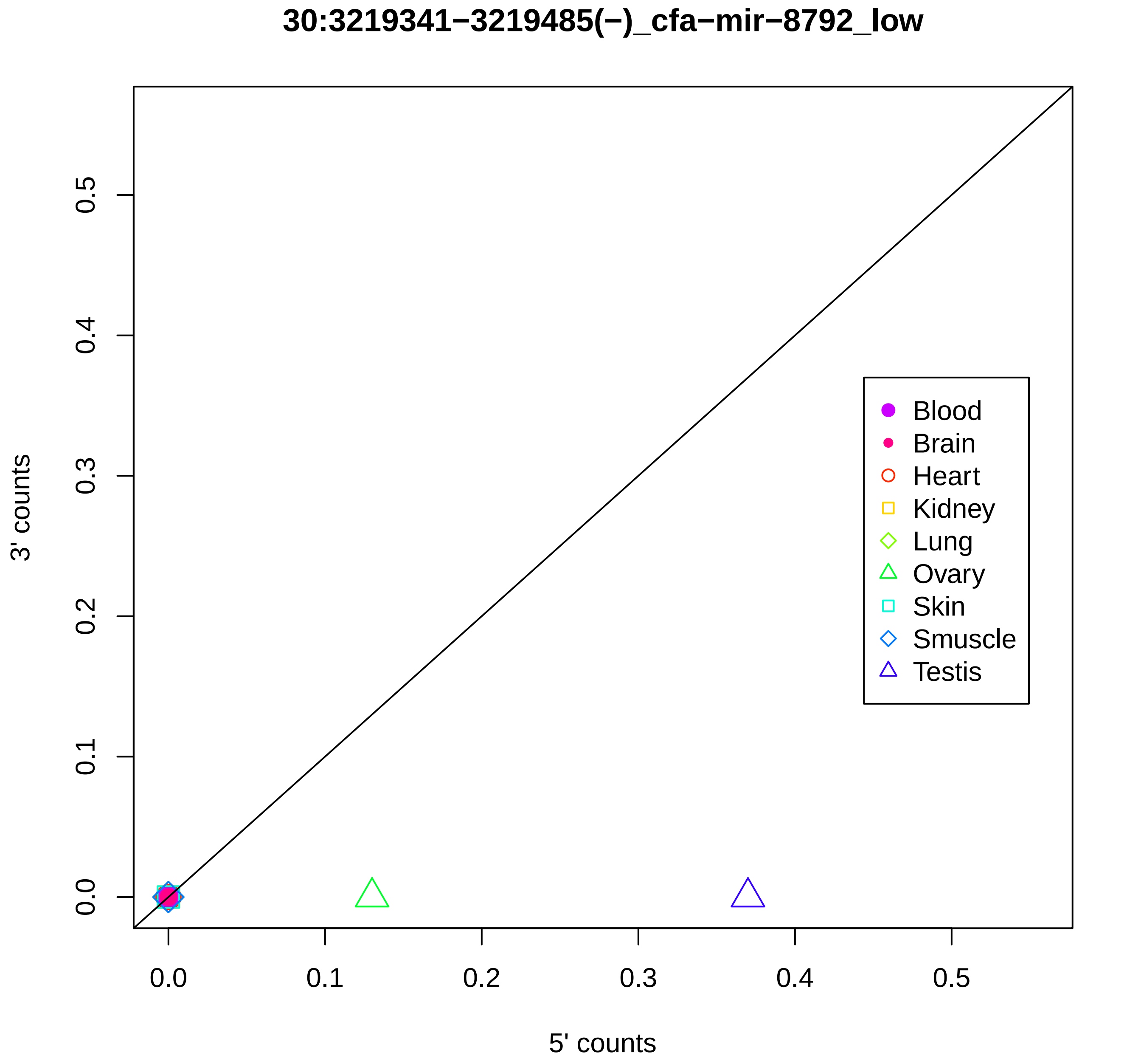

30:3219341−3219485(−)_cfa−mir−8792_low
3' counts
5' counts
0.0
0.1
0.2
0.3
0.4
0.5
Blood
Brain
Heart
Kidney
Lung
Ovary
Skin
Smuscle
Testis

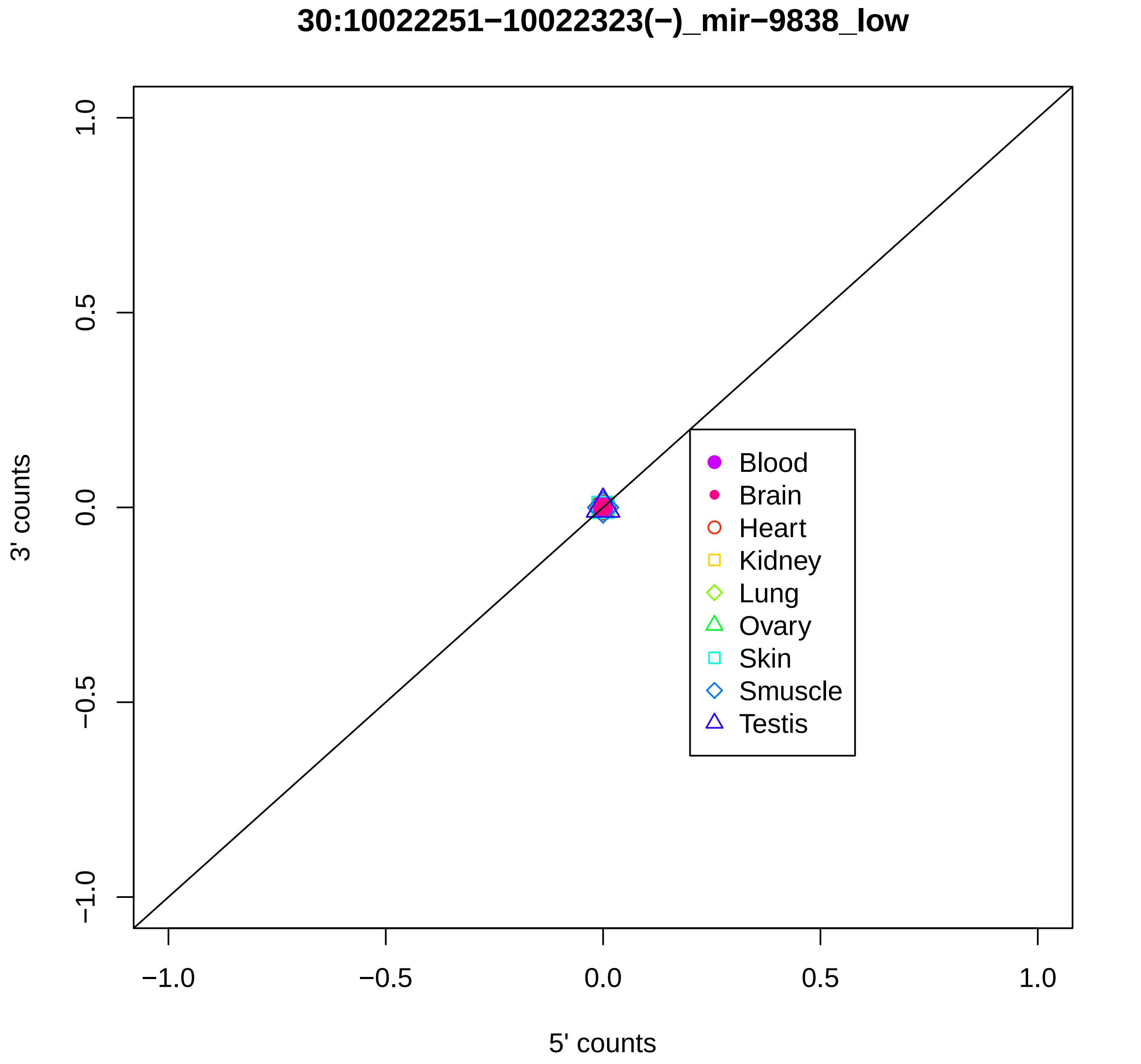
30:10022251−10022323(−)_mir−9838_low
1.0
0.5
0.0
−0.5
−1.0
3' counts
−1.0
−0.5
0.0
0.5
1.0
5' counts
Blood
Brain
Heart
Kidney
Lung
Ovary
Skin
Smuscle
Testis

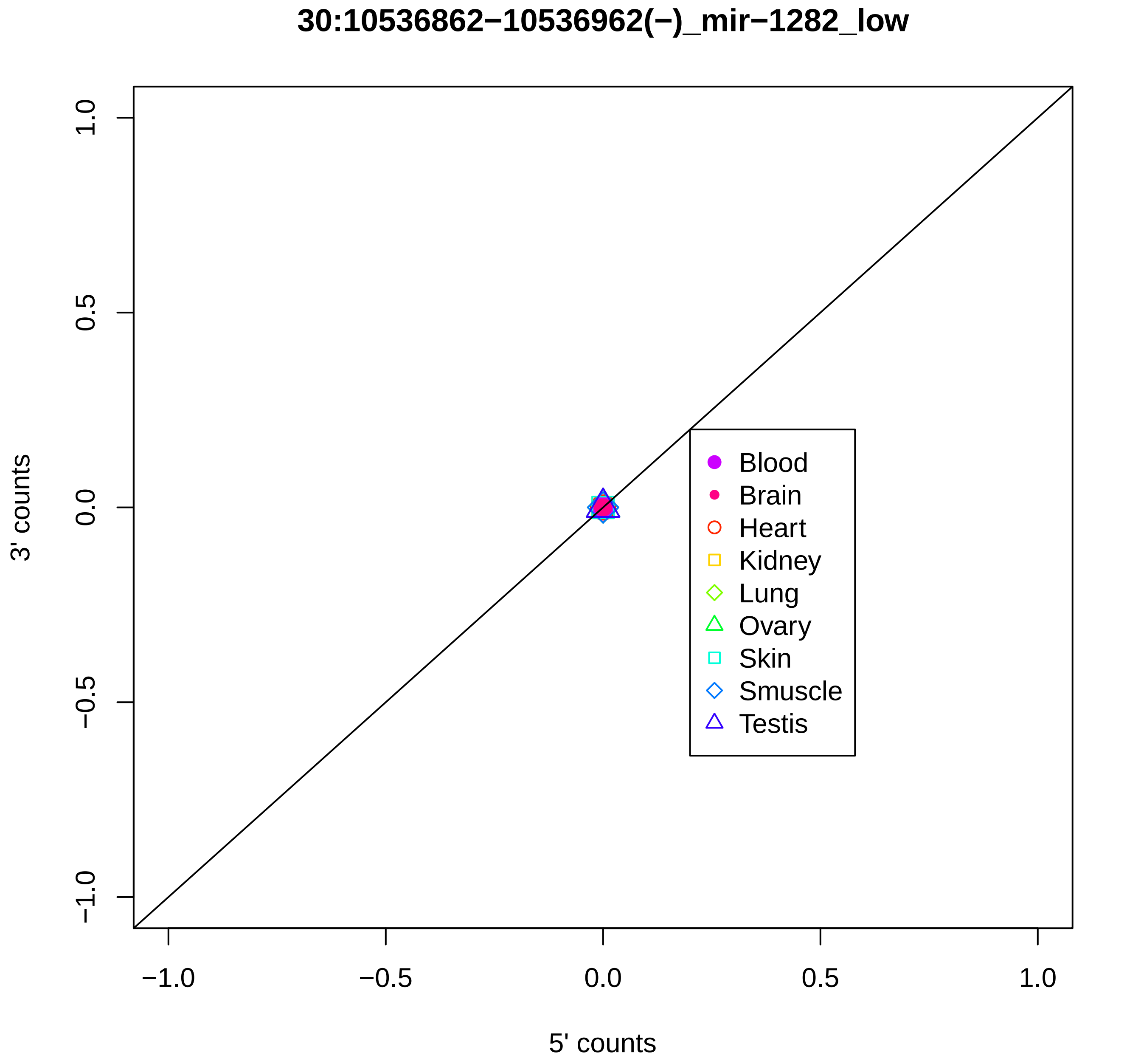

30:10536862−10536962(−)_mir−1282_low
3' counts
5' counts
1.0
0.5
0.0
−0.5
−1.0
Blood
Brain
Heart
Kidney
Lung
Ovary
Skin
Smuscle
Testis

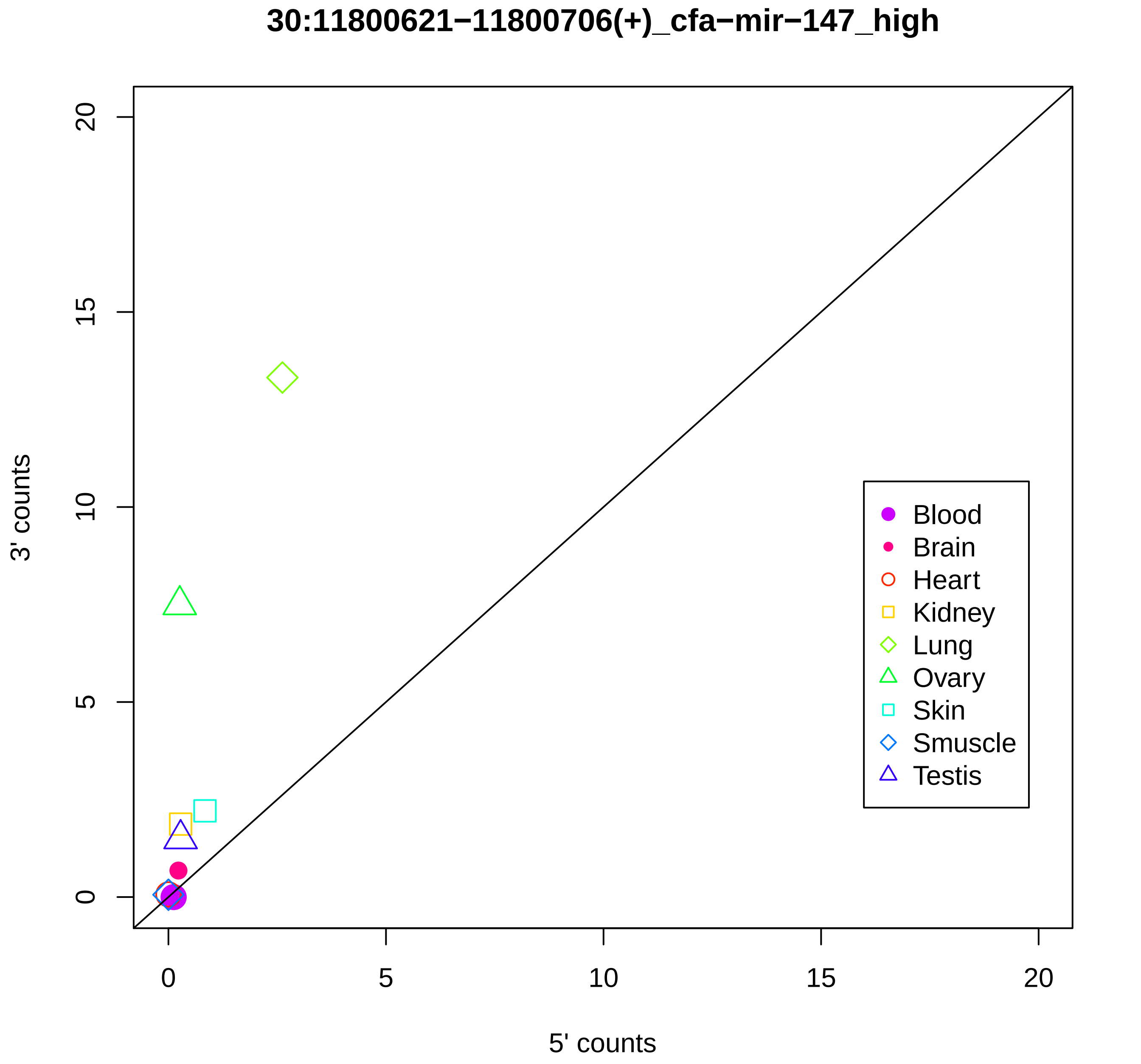

30:11800621−11800706(+)_cfa−mir−147_high
3' counts
5' counts
0
5
10
15
20
0
5
10
15
20
Blood
Brain
Heart
Kidney
Lung
Ovary
Skin
Smuscle
Testis

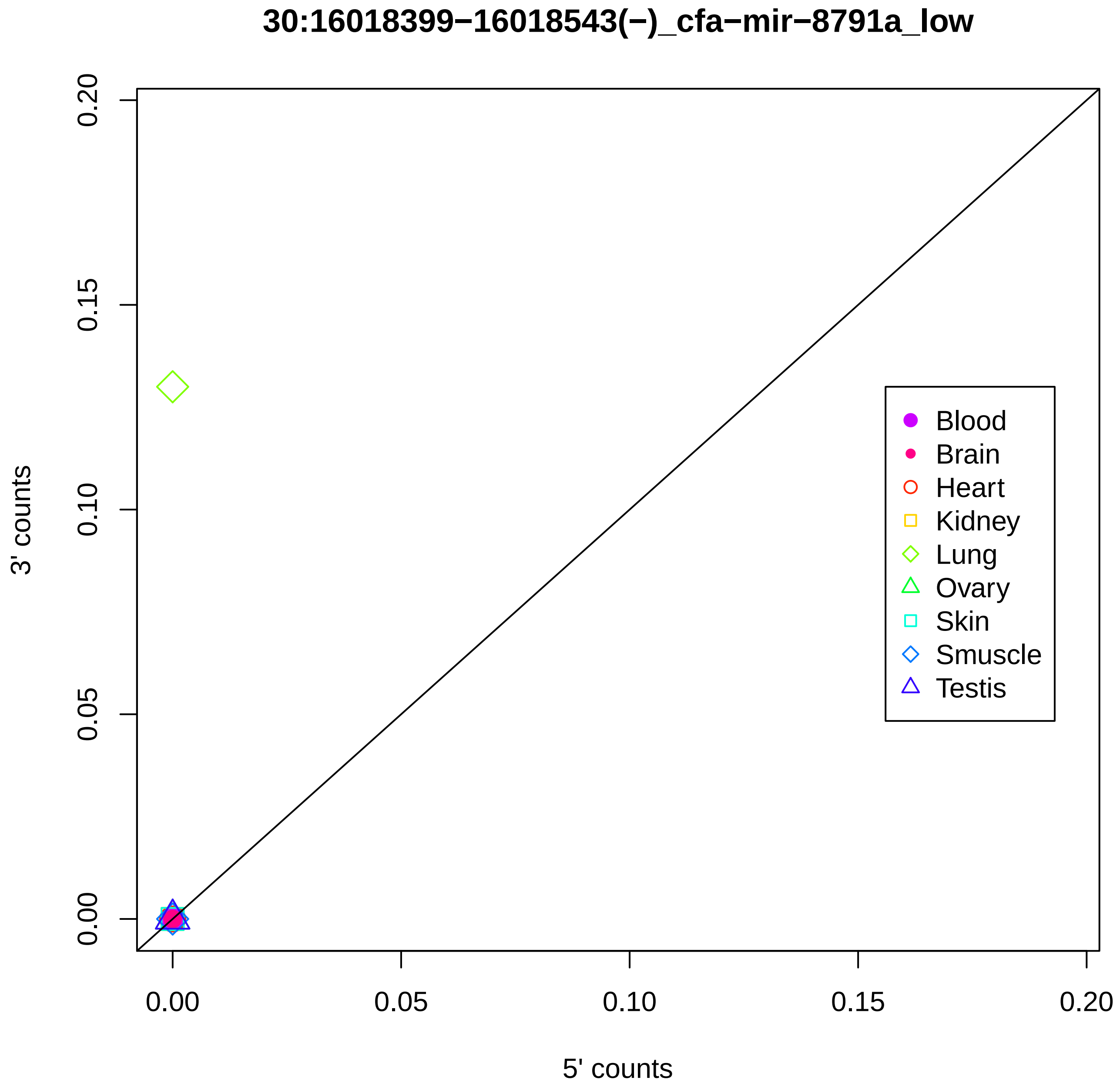

30:16018399−16018543(−)_cfa−mir−8791a_low
3' counts
5' counts
0.00
0.05
0.10
0.15
0.20
Blood
Brain
Heart
Kidney
Lung
Ovary
Skin
Smuscle
Testis

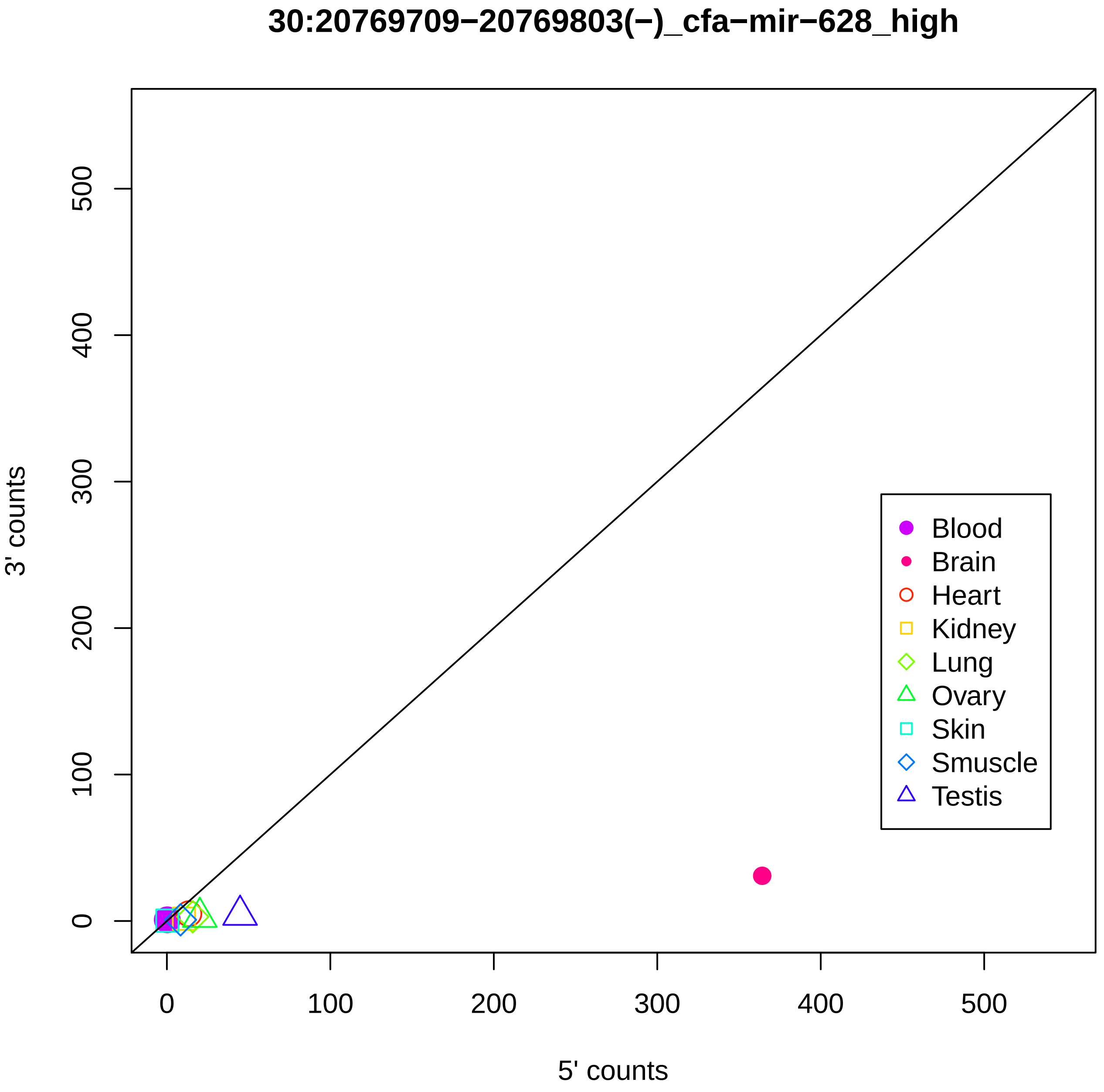

30:20769709−20769803(−)_cfa−mir−628_high
3' counts
5' counts
0
100
200
300
400
500
Blood
Brain
Heart
Kidney
Lung
Ovary
Skin
Smuscle
Testis

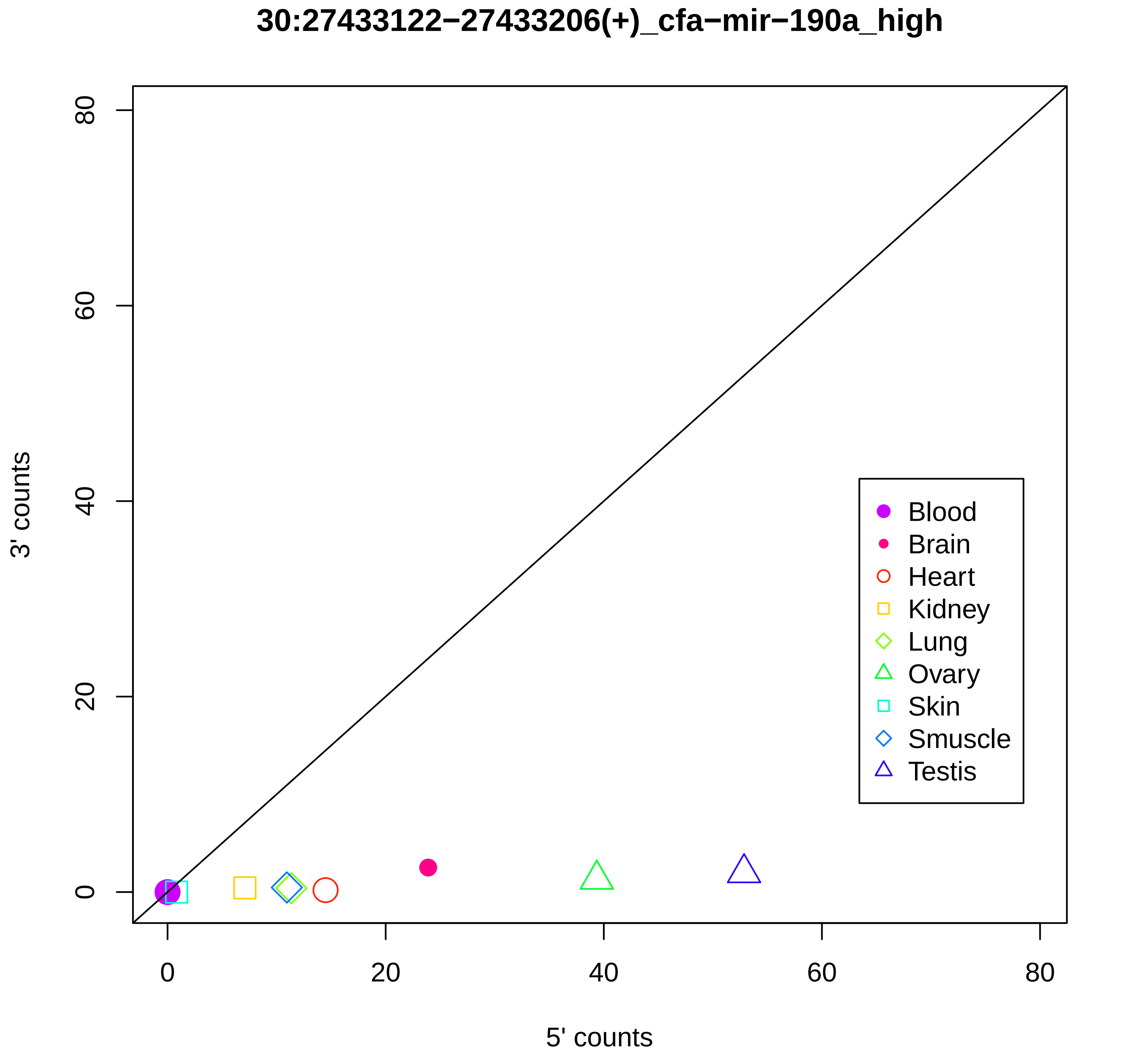

30:27433122−27433206(+)_cfa−mir−190a_high
3' counts
5' counts
0
20
40
60
80
0
20
40
60
80
Blood
Brain
Heart
Kidney
Lung
Ovary
Skin
Smuscle
Testis

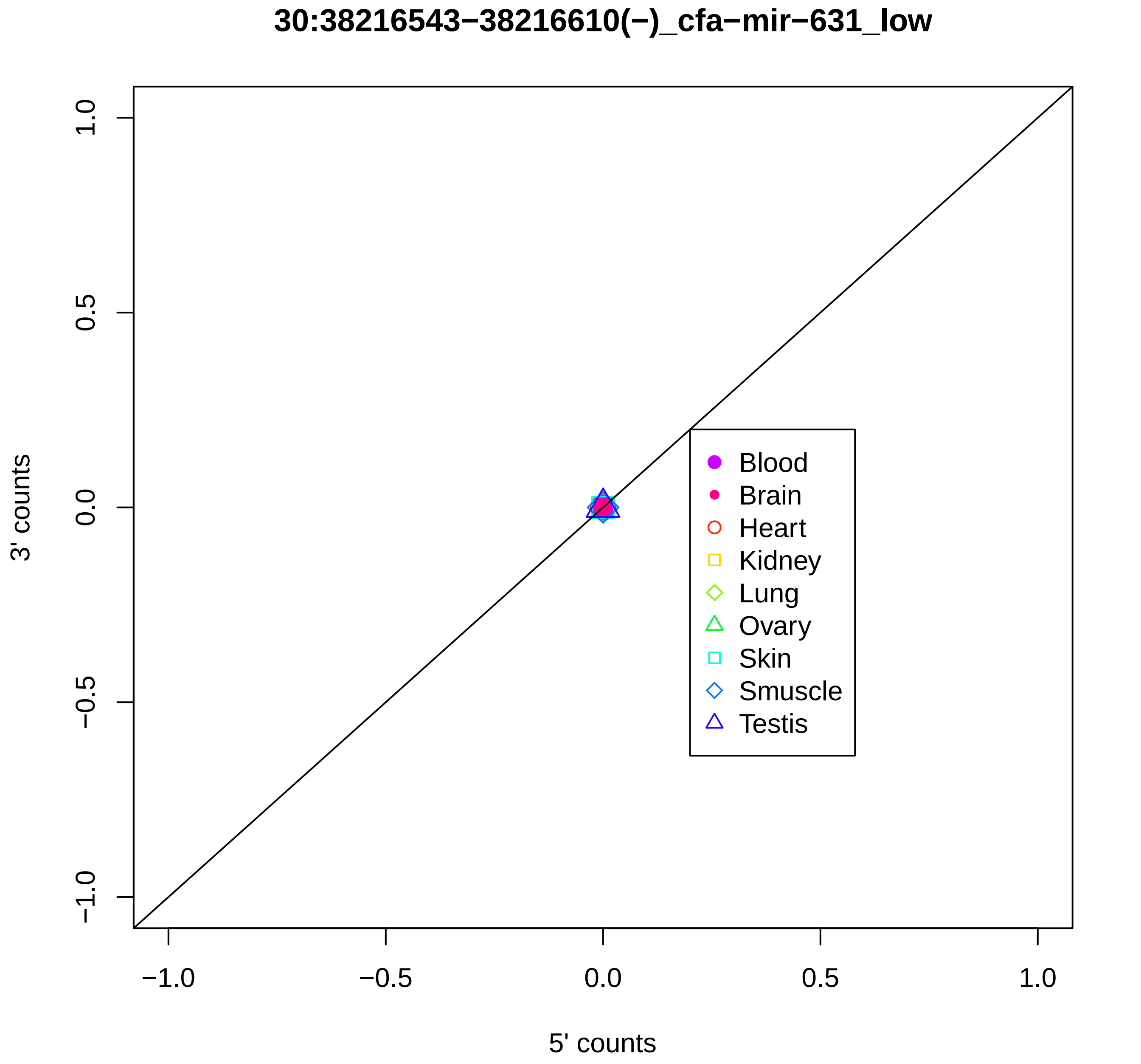
30:38216543−38216610(−)_cfa−mir−631_low
3' counts
5' counts
−1.0
−0.5
0.0
0.5
1.0
Blood
Brain
Heart
Kidney
Lung
Ovary
Skin
Smuscle
Testis

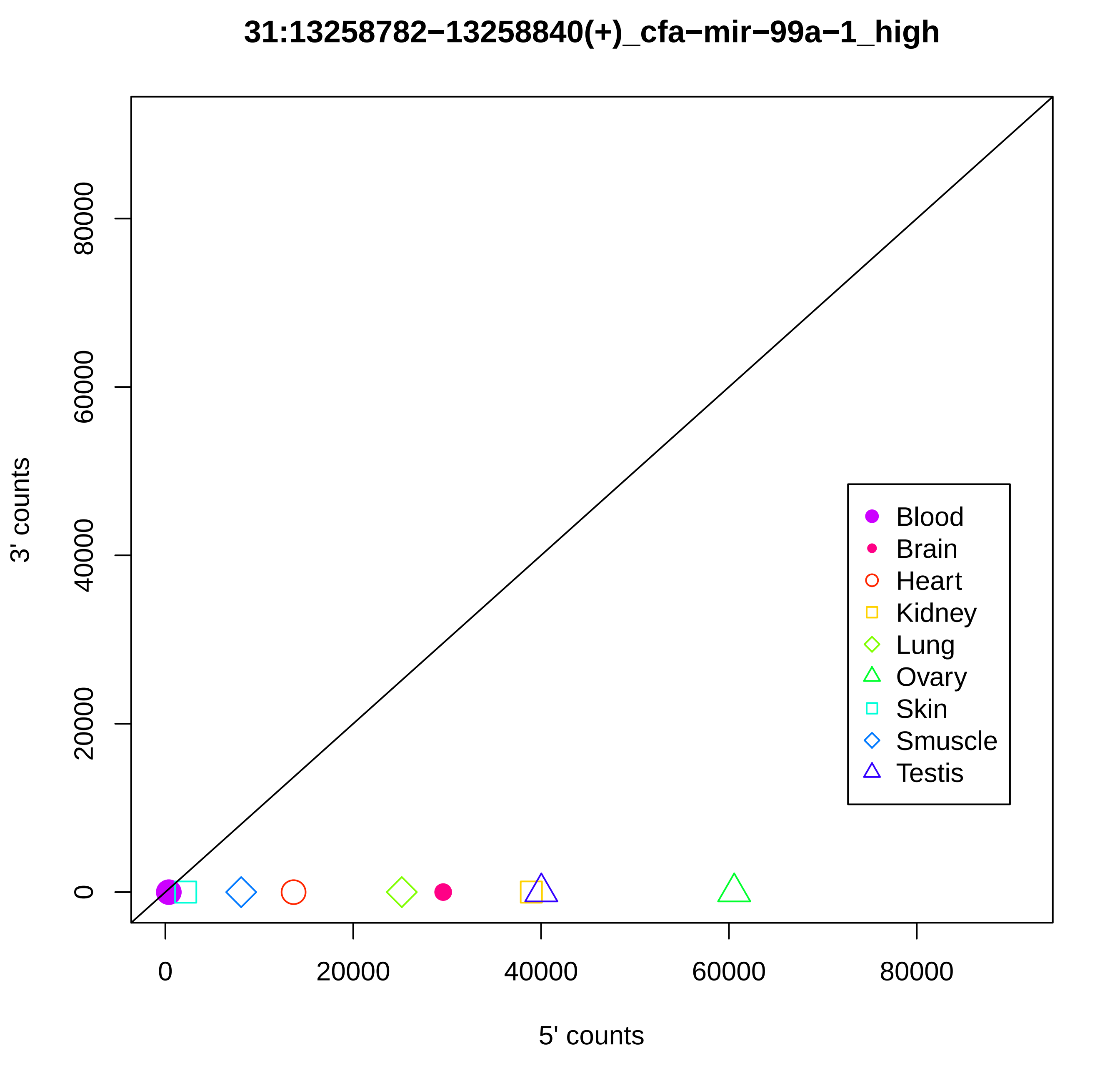
31:13258782−13258840(+)_cfa−mir−99a−1_high
3' counts
5' counts
0
20000
40000
60000
80000
Blood
Brain
Heart
Kidney
Lung
Ovary
Skin
Smuscle
Testis

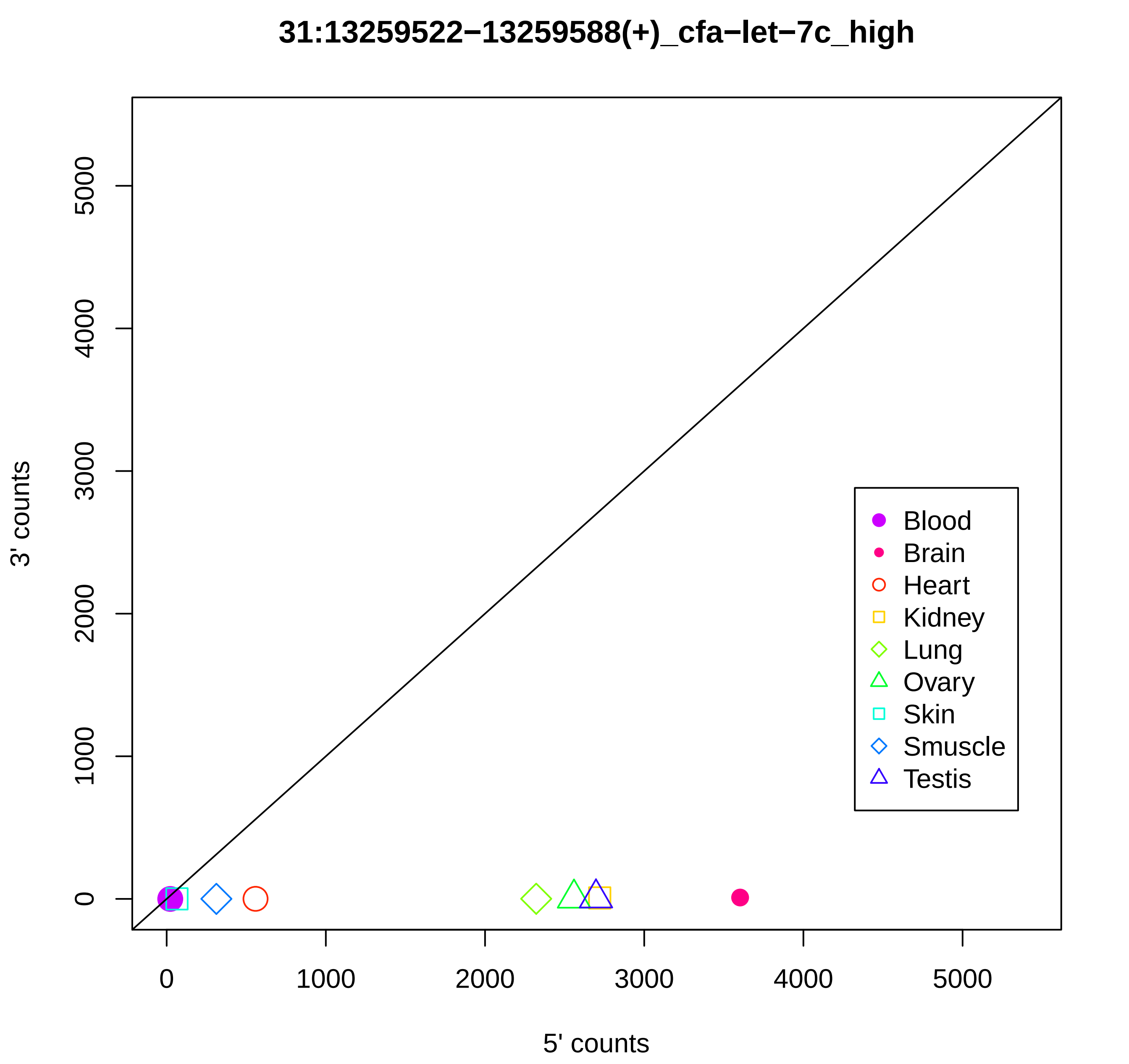
31:13259522−13259588(+)_cfa−let−7c_high
3' counts
5' counts
0
1000
2000
3000
4000
5000
0
1000
2000
3000
4000
5000
Blood
Brain
Heart
Kidney
Lung
Ovary
Skin
Smuscle
Testis

31:13304980−13305040(+)_cfa−mir−125b−2_high

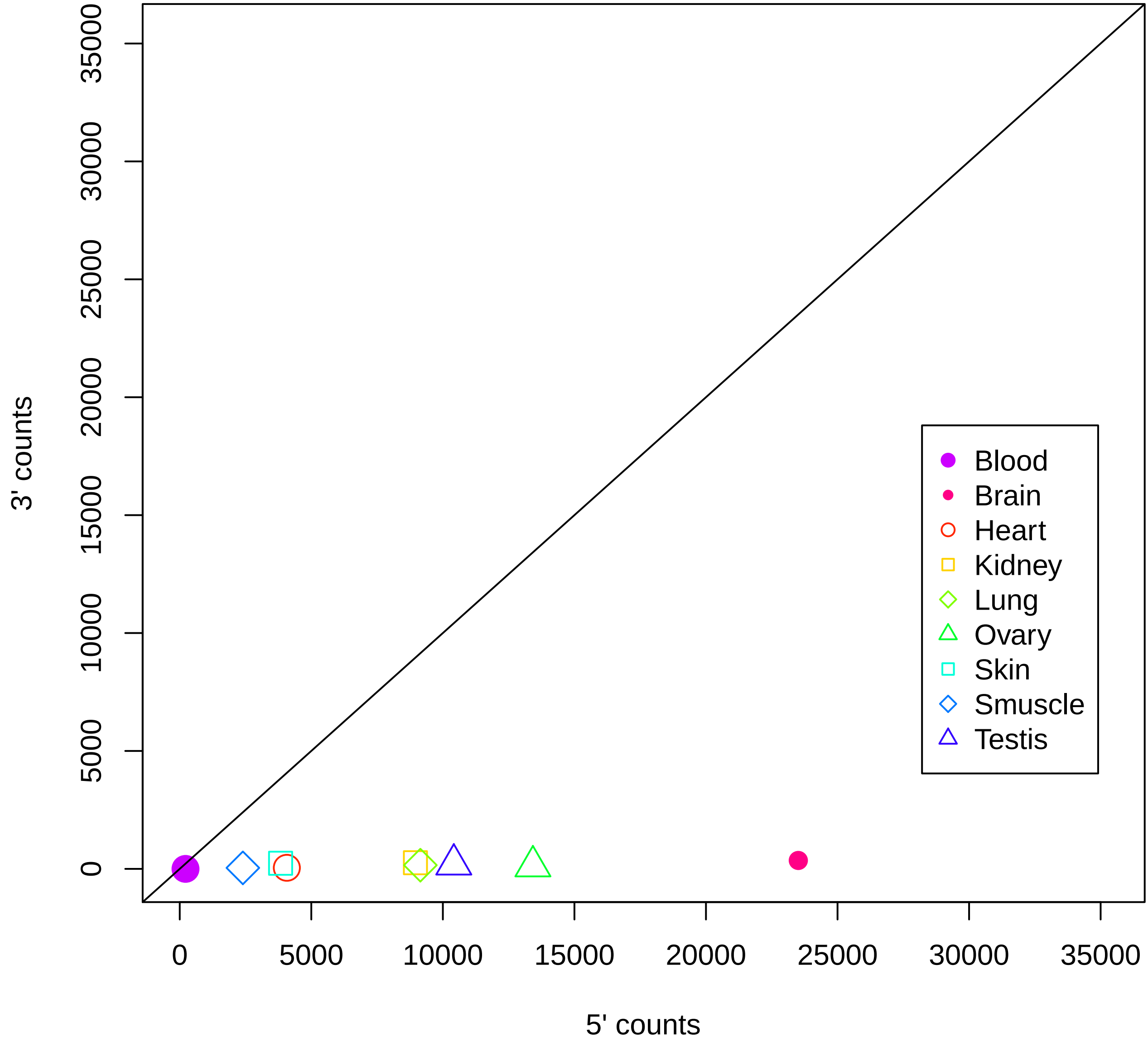

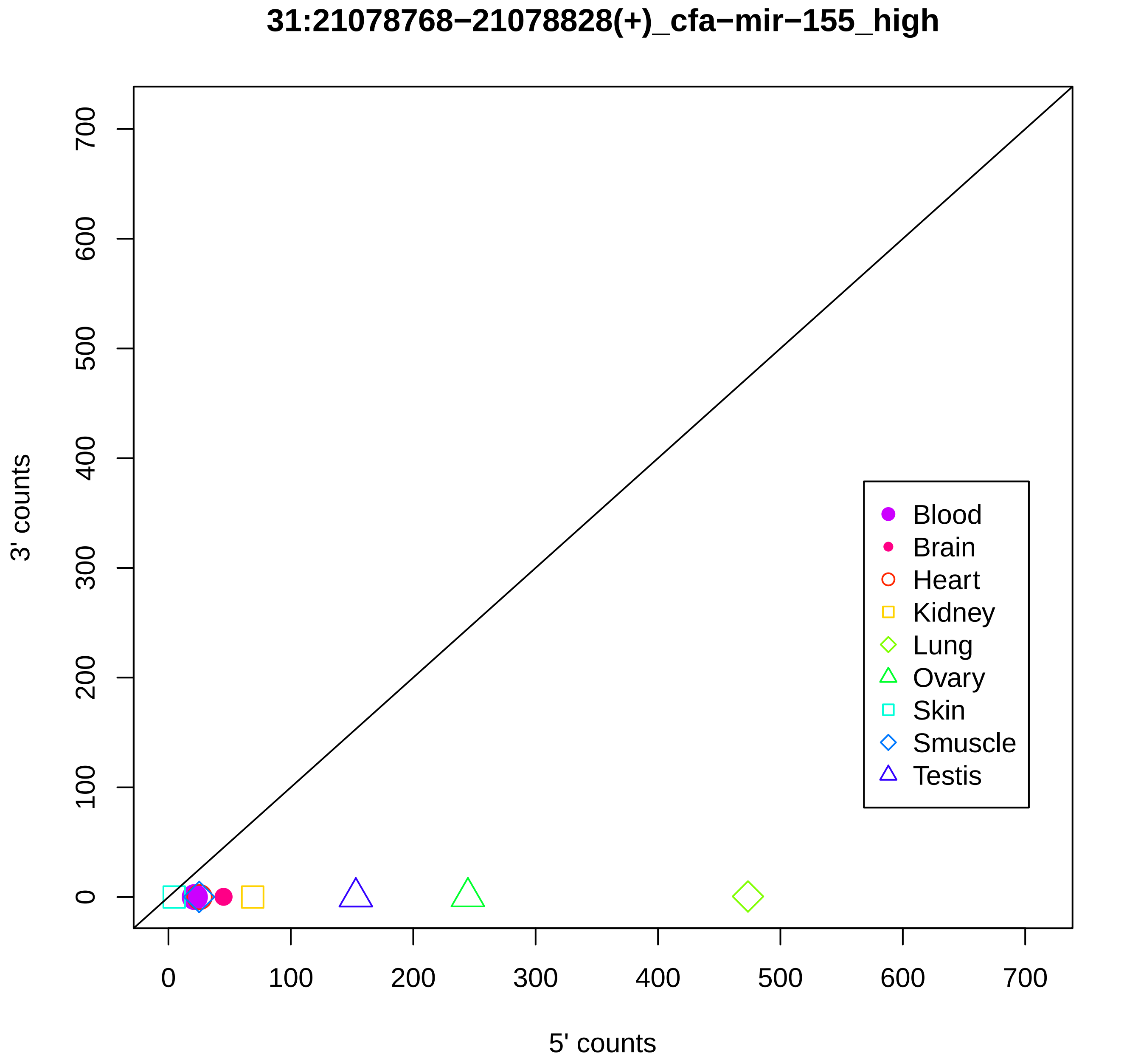

31:21078768−21078828(+)_cfa−mir−155_high
3' counts
5' counts
0
100
200
300
400
500
600
700
Blood
Brain
Heart
Kidney
Lung
Ovary
Skin
Smuscle
Testis

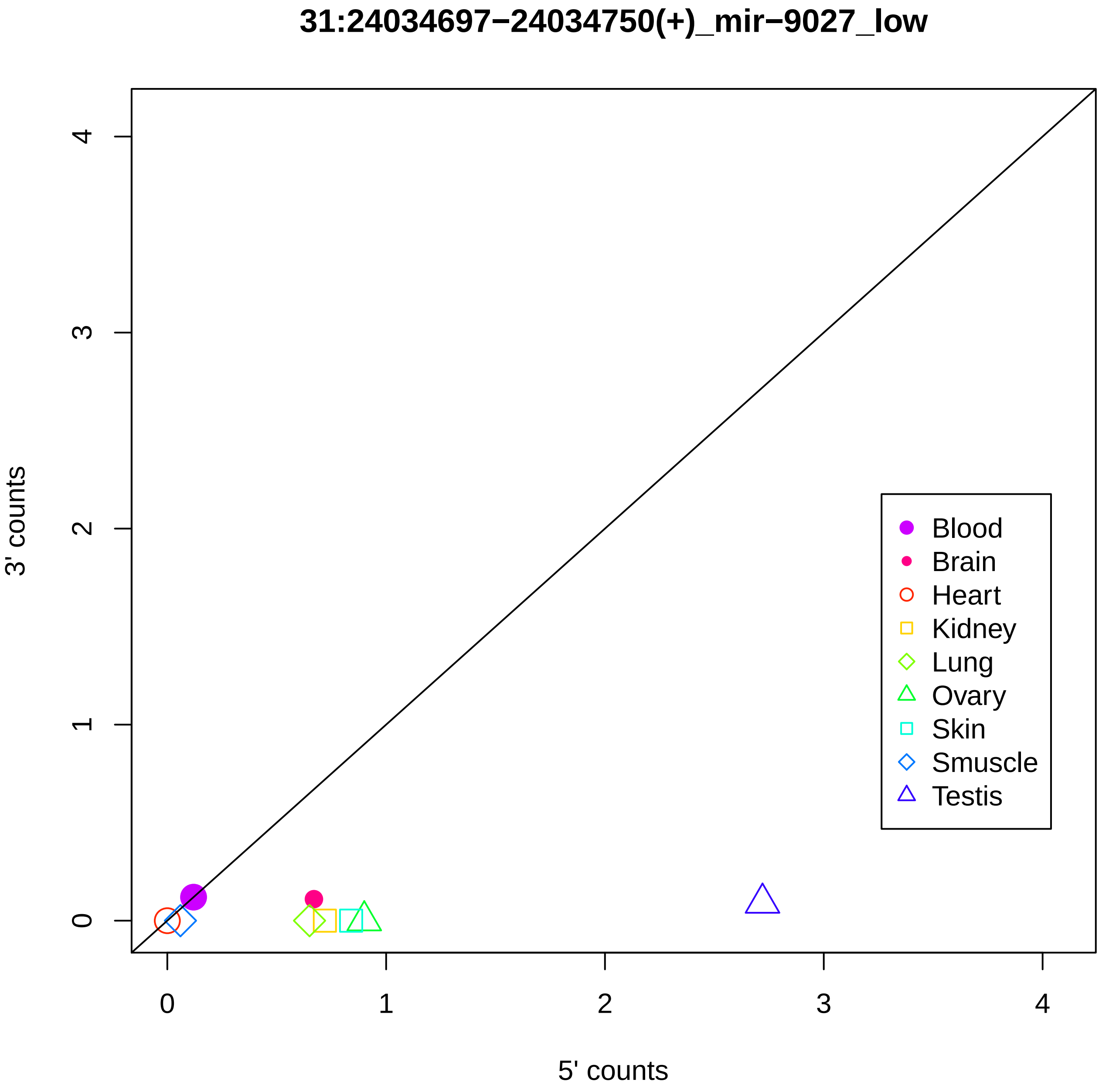
31:24034697−24034750(+)_mir−9027_low
3' counts
5' counts
0
1
2
3
4
Blood
Brain
Heart
Kidney
Lung
Ovary
Skin
Smuscle
Testis

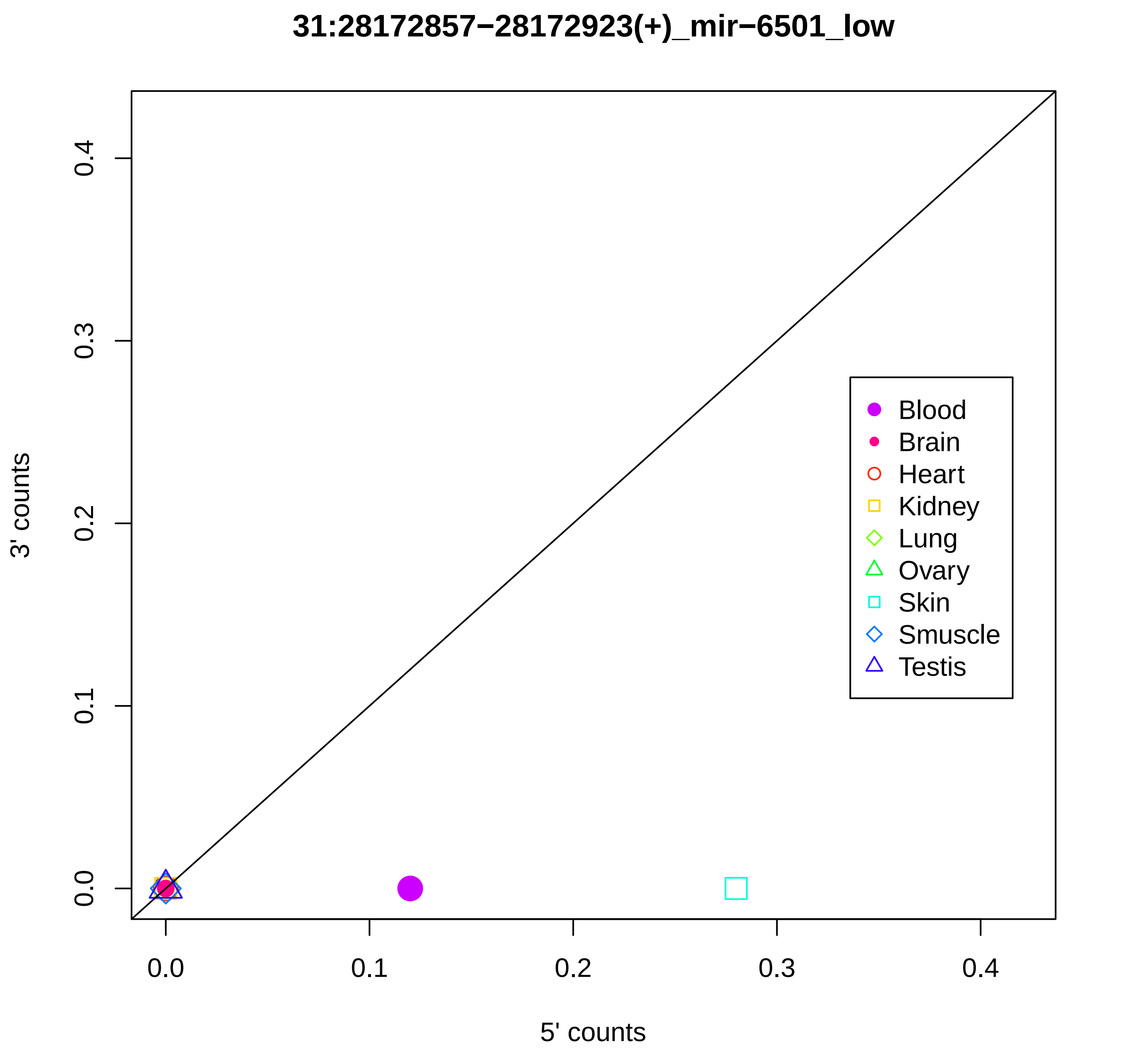
31:28172857−28172923(+)_mir−6501_low
3' counts
5' counts
0.0
0.1
0.2
0.3
0.4
Blood
Brain
Heart
Kidney
Lung
Ovary
Skin
Smuscle
Testis

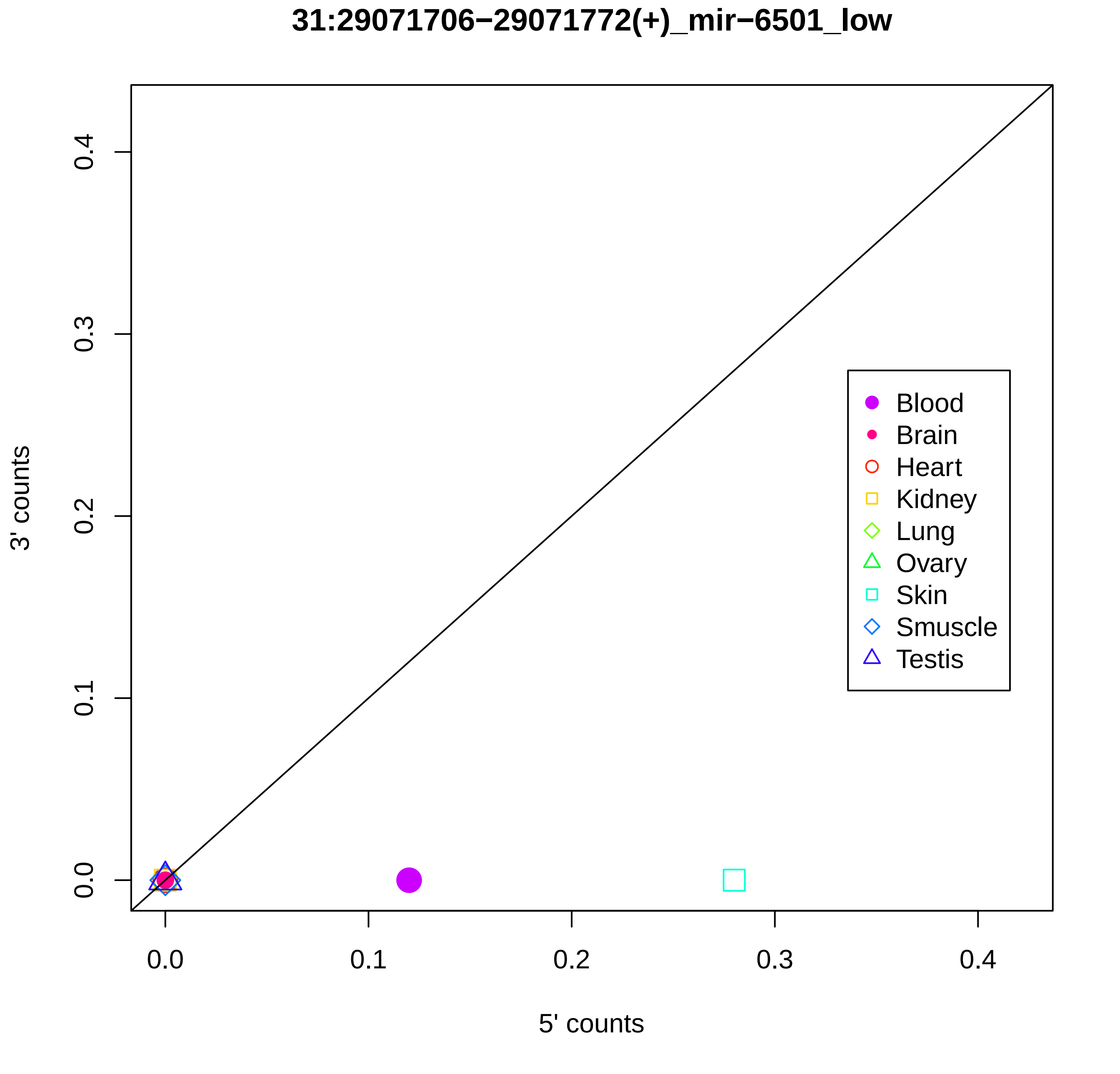

31:29071706−29071772(+)_mir−6501_low
3' counts
5' counts
0.0
0.1
0.2
0.3
0.4
0.0
0.1
0.2
0.3
0.4
Blood
Brain
Heart
Kidney
Lung
Ovary
Skin
Smuscle
Testis

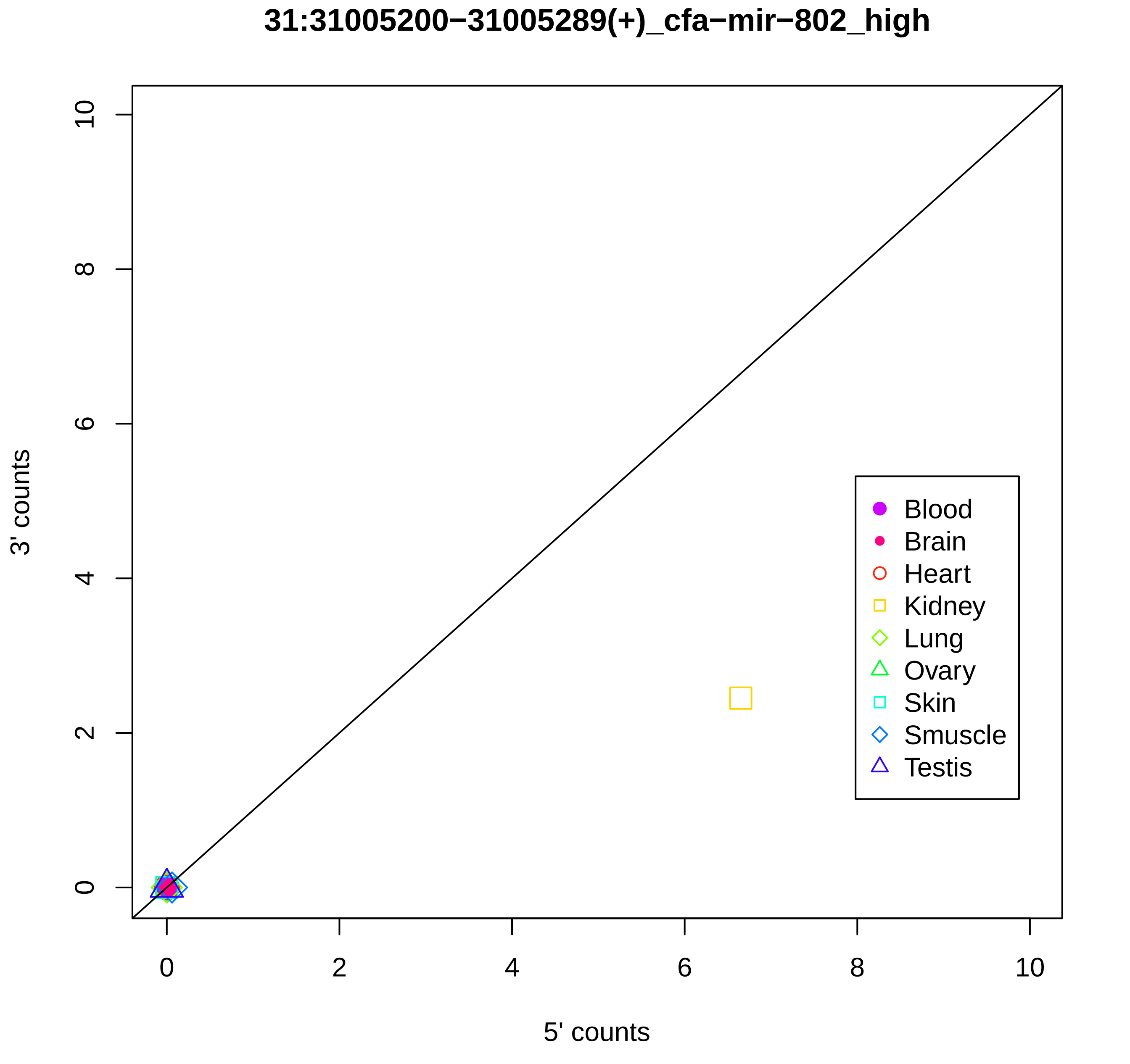
31:31005200−31005289(+)_cfa−mir−802_high
3' counts
5' counts
0
2
4
6
8
10
0
2
4
6
8
10
Blood
Brain
Heart
Kidney
Lung
Ovary
Skin
Smuscle
Testis

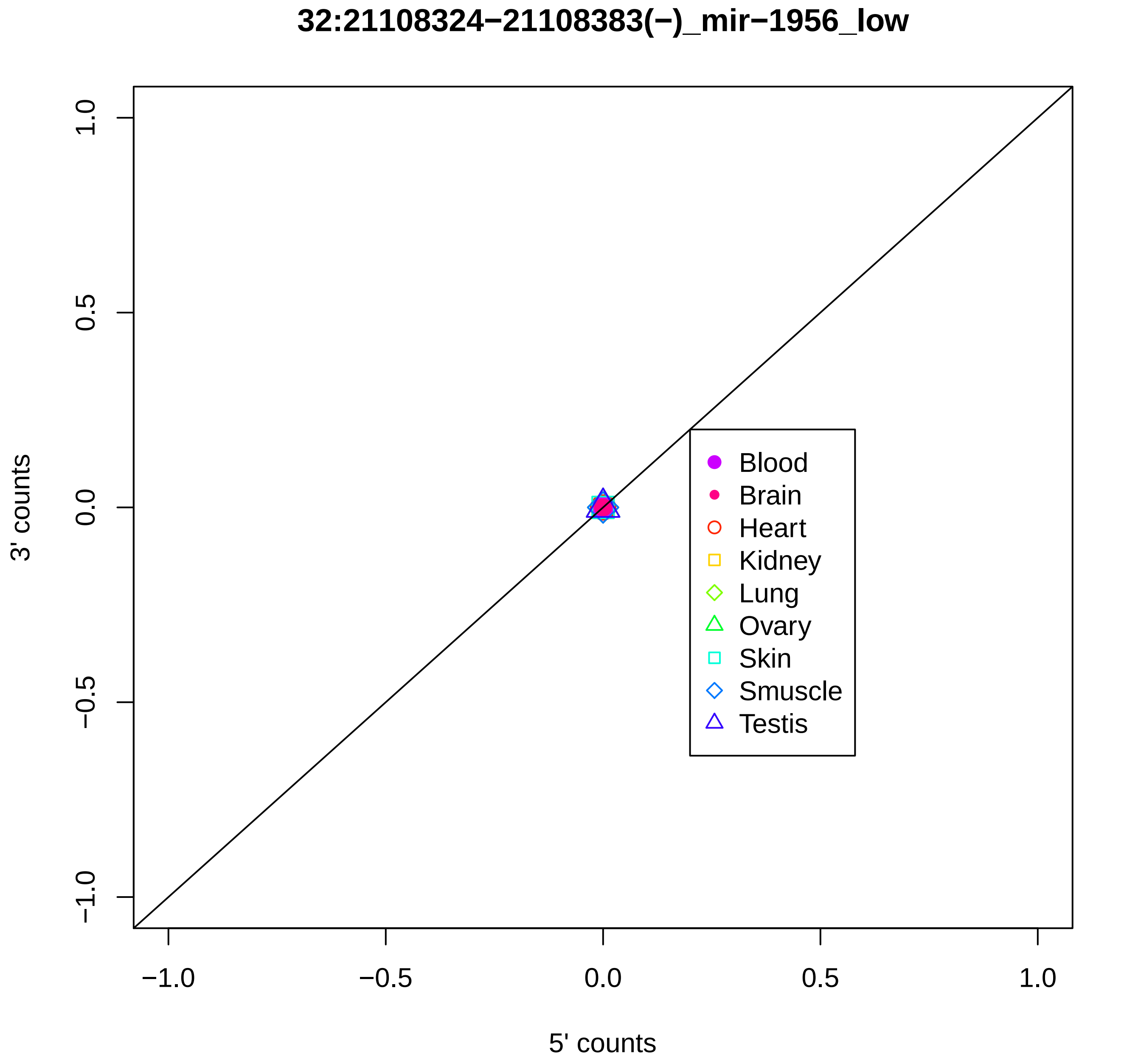
32:21108324−21108383(−)_mir−1956_low
3' counts
5' counts
1.0
0.5
0.0
−0.5
−1.0
Blood
Brain
Heart
Kidney
Lung
Ovary
Skin
Smuscle
Testis

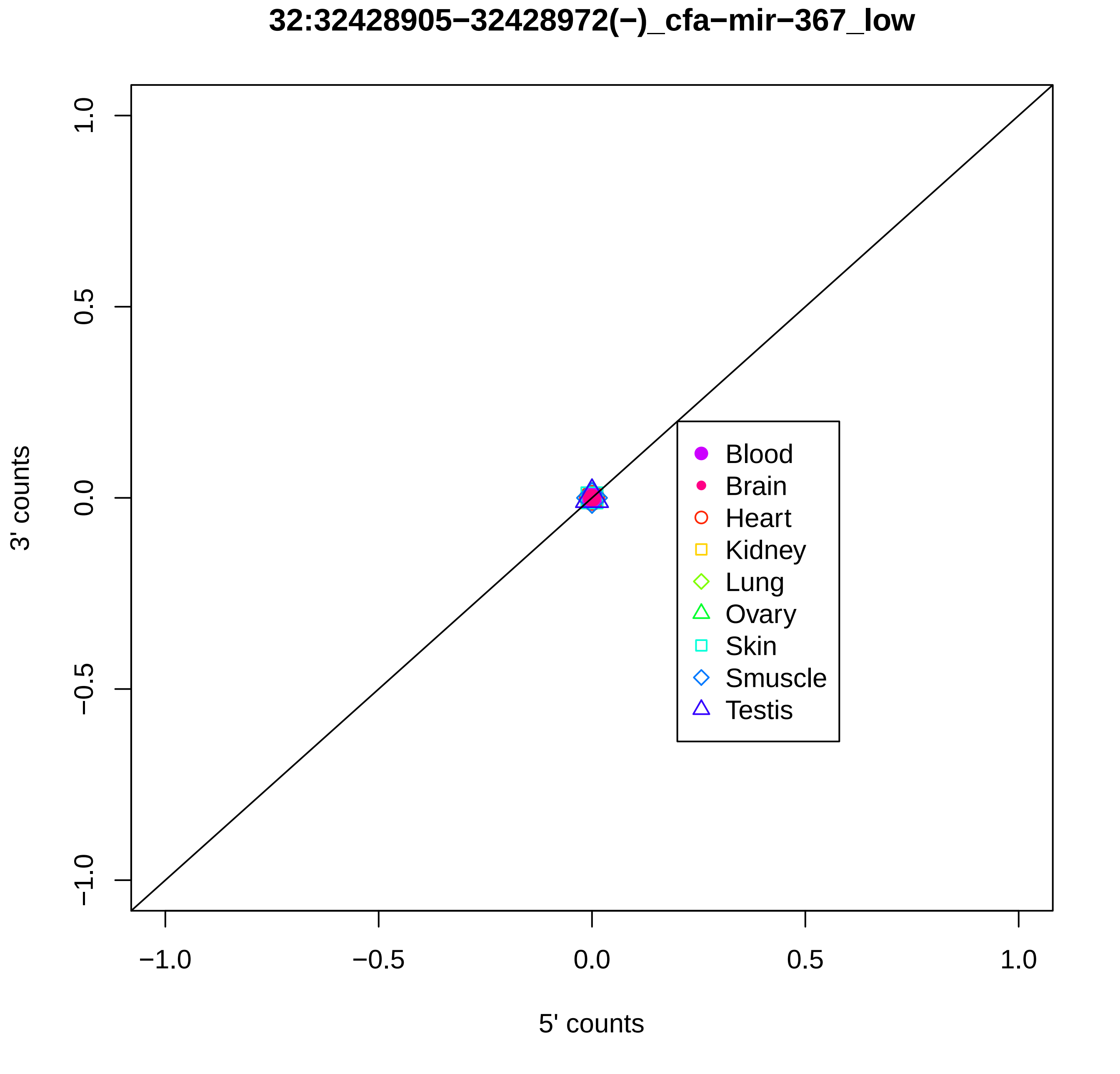
32:32428905−32428972(−)_cfa−mir−367_low
3' counts
5' counts
−1.0
−0.5
0.0
0.5
1.0
Blood
Brain
Heart
Kidney
Lung
Ovary
Skin
Smuscle
Testis

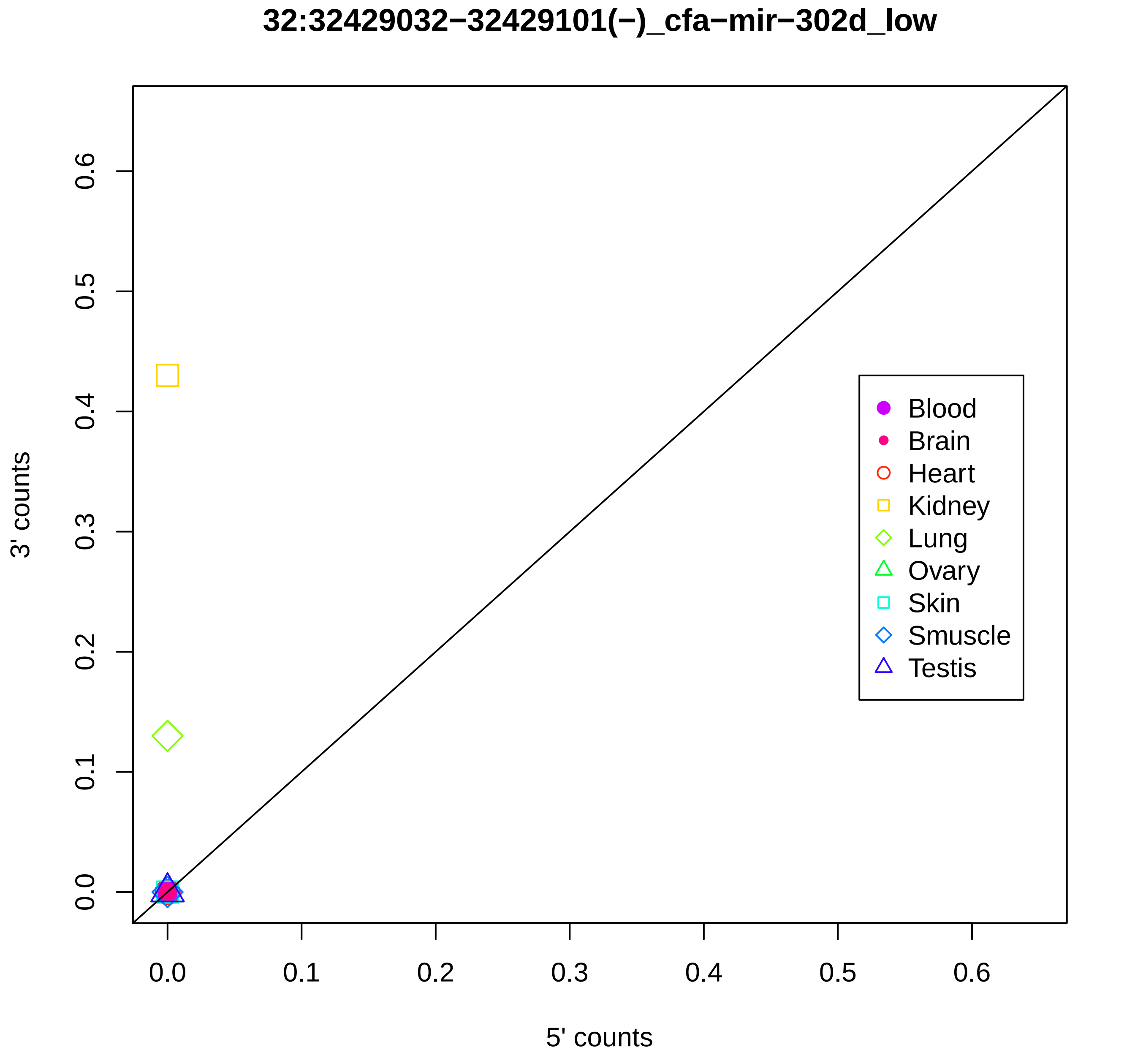
32:32429032−32429101(−)_cfa−mir−302d_low
3' counts
5' counts
0.0
0.1
0.2
0.3
0.4
0.5
0.6
Blood
Brain
Heart
Kidney
Lung
Ovary
Skin
Smuscle
Testis

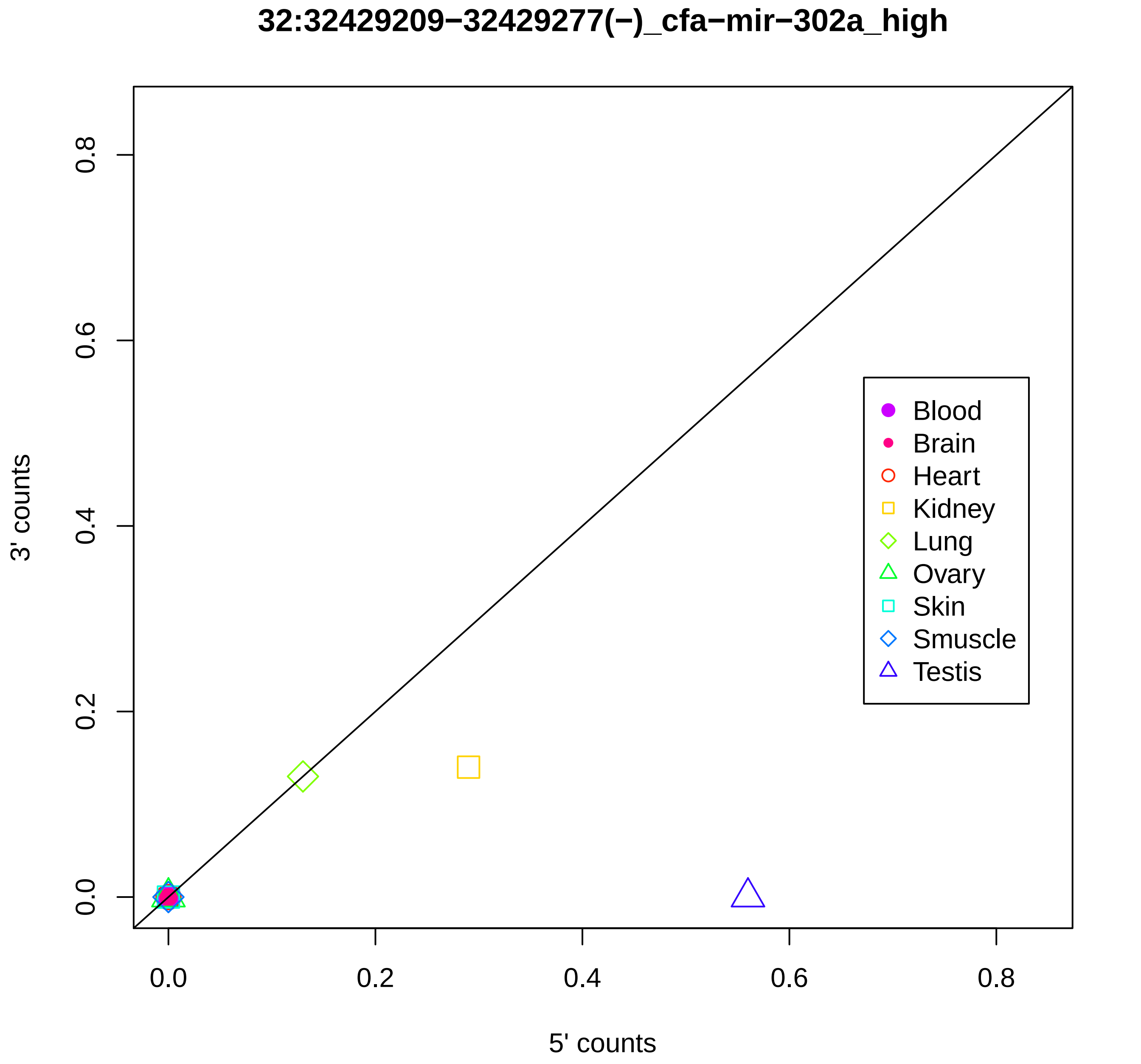
32:32429209−32429277(−)_cfa−mir−302a_high
3' counts
5' counts
0.0
0.2
0.4
0.6
0.8
0.0
0.2
0.4
0.6
0.8
Blood
Brain
Heart
Kidney
Lung
Ovary
Skin
Smuscle
Testis

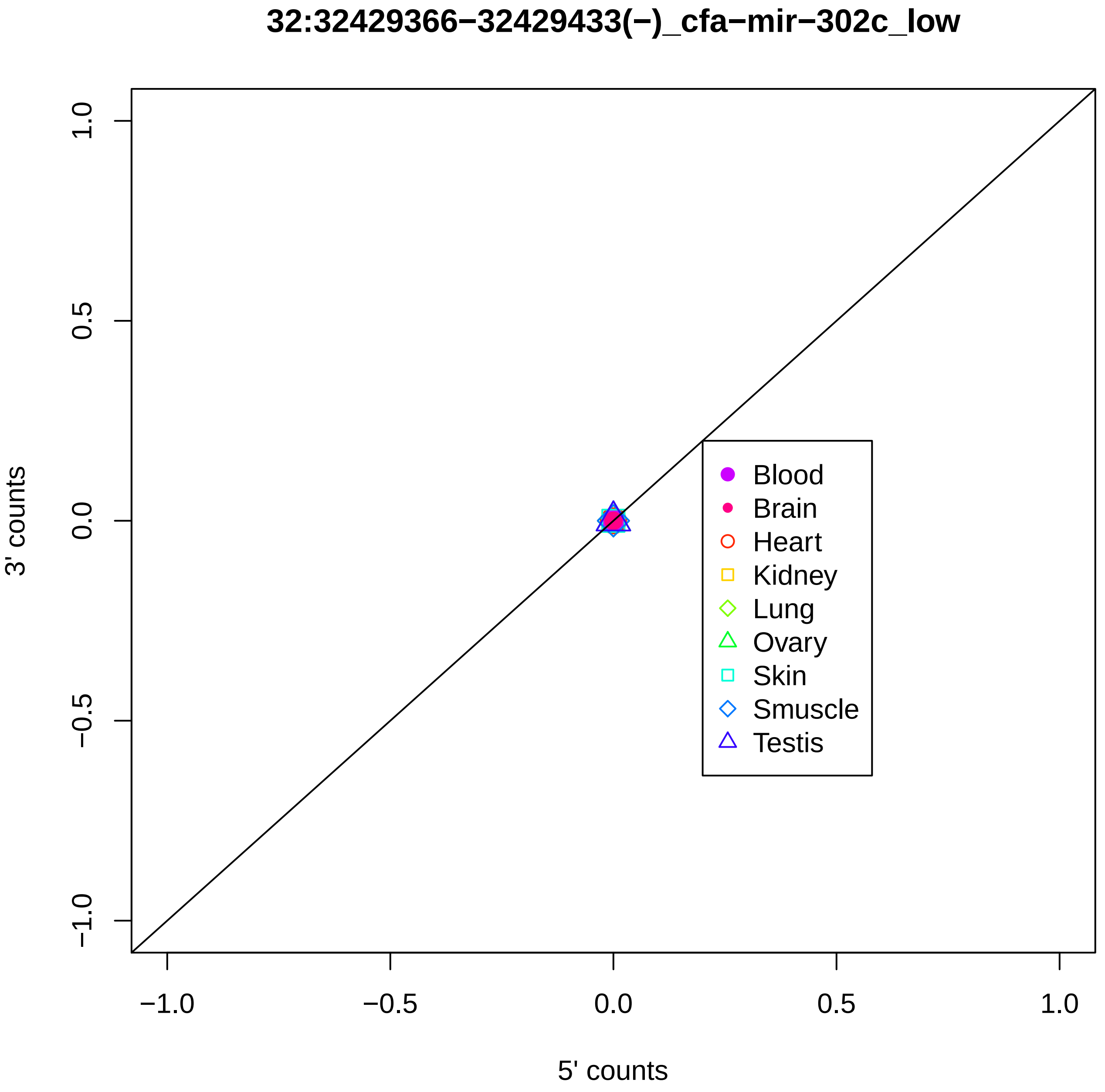

32:32429366−32429433(−)_cfa−mir−302c_low
3' counts
5' counts
1.0
0.5
0.0
−0.5
−1.0
Blood
Brain
Heart
Kidney
Lung
Ovary
Skin
Smuscle
Testis

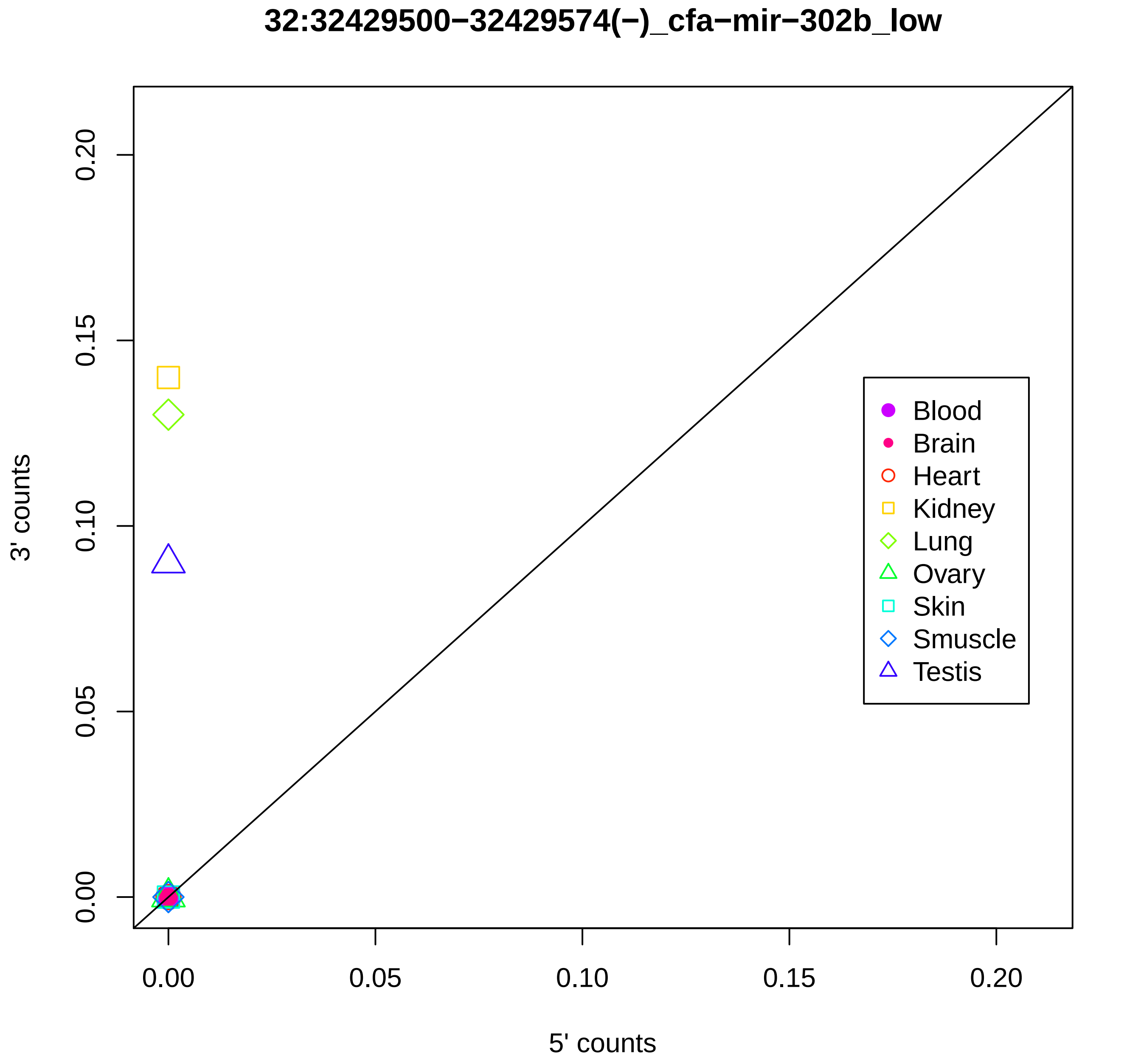
32:32429500−32429574(−)_cfa−mir−302b_low
3' counts
5' counts
0.00
0.05
0.10
0.15
0.20
Blood
Brain
Heart
Kidney
Lung
Ovary
Skin
Smuscle
Testis

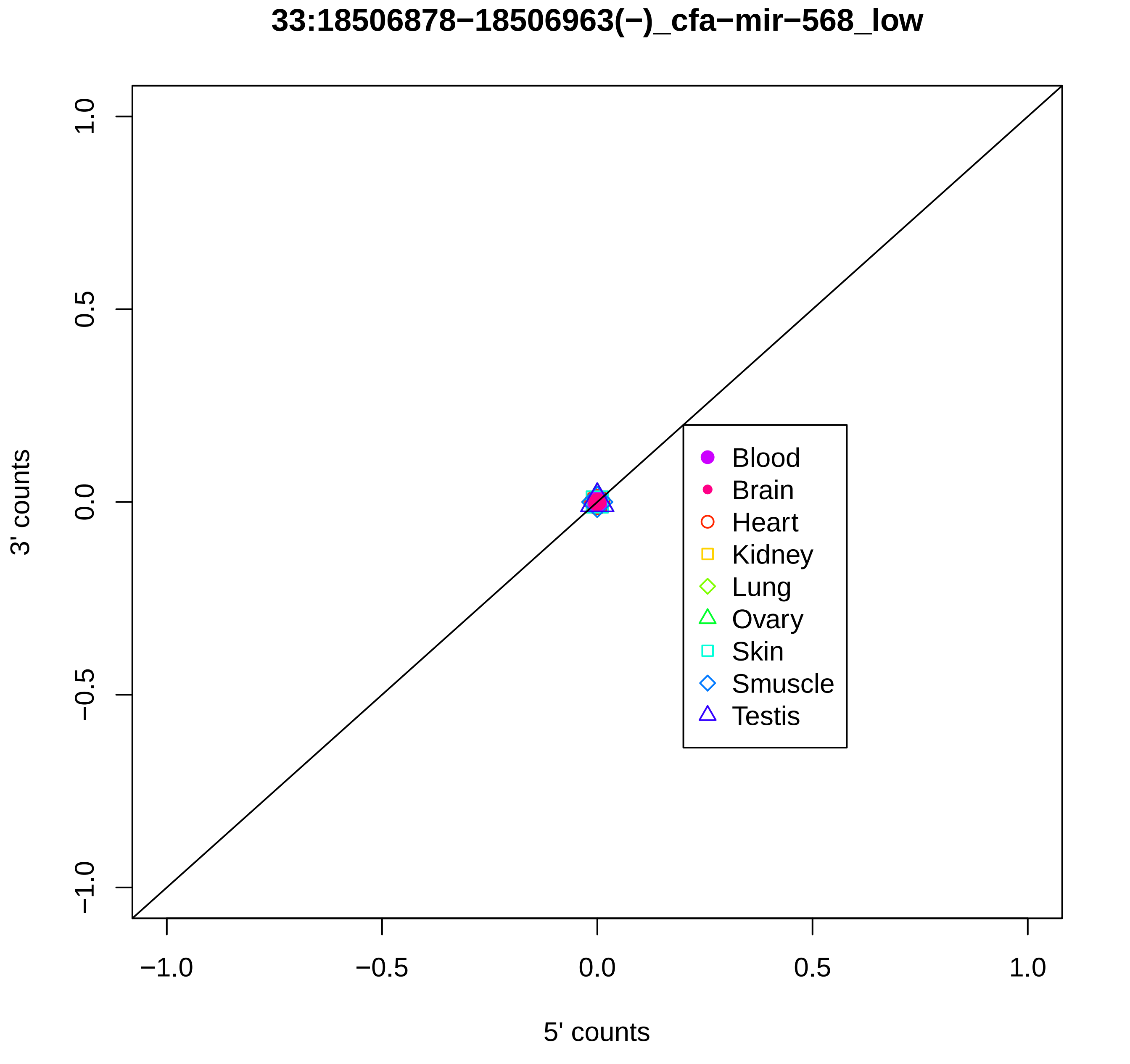

33:18506878−18506963(−)_cfa−mir−568_low
3' counts
5' counts
1.0
0.5
0.0
−0.5
−1.0
−1.0
−0.5
0.0
0.5
1.0
Blood
Brain
Heart
Kidney
Lung
Ovary
Skin
Smuscle
Testis

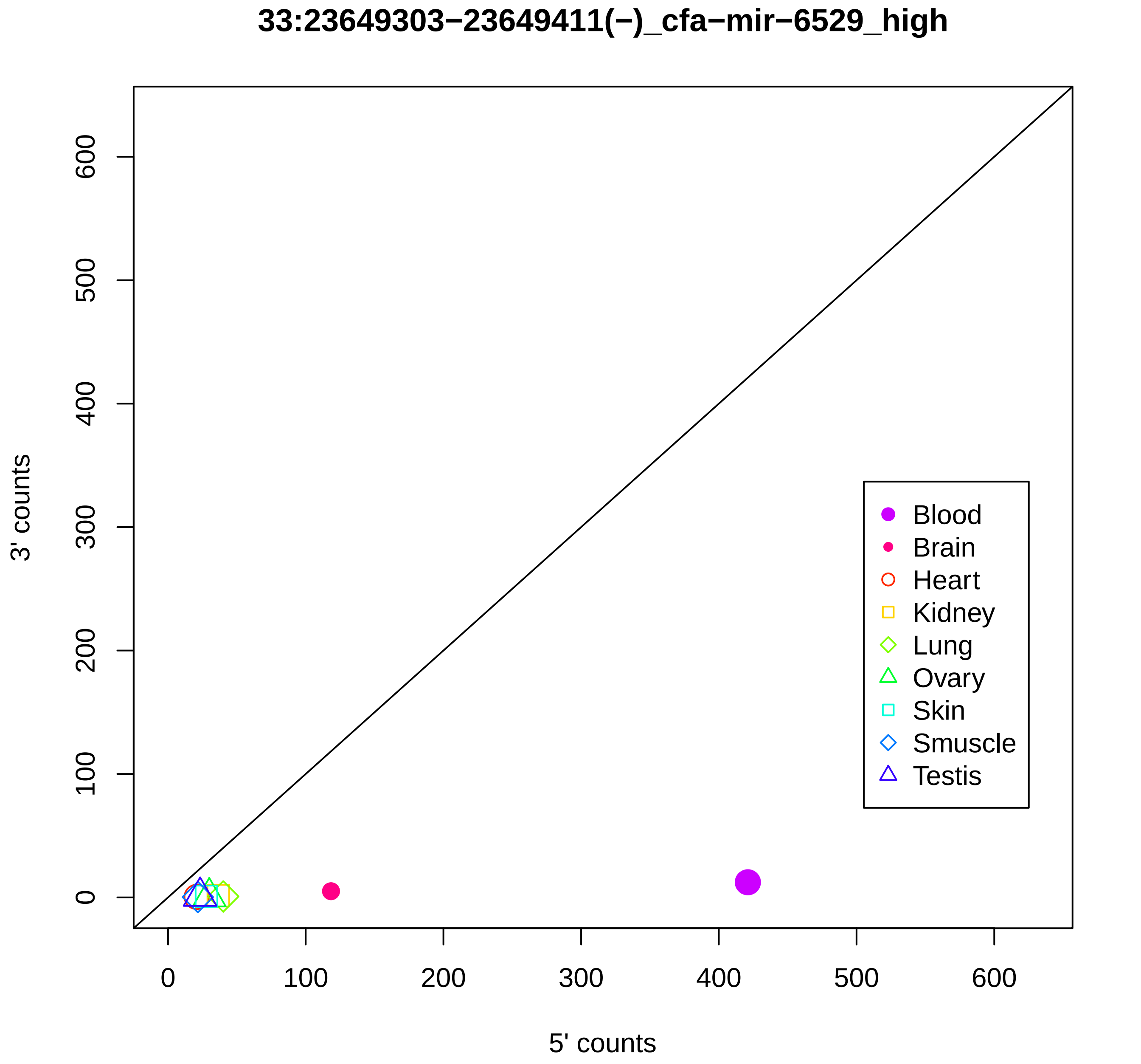

33:23649303−23649411(−)_cfa−mir−6529_high
3' counts
5' counts
0
100
200
300
400
500
600
0
100
200
300
400
500
600
Blood
Brain
Heart
Kidney
Lung
Ovary
Skin
Smuscle
Testis

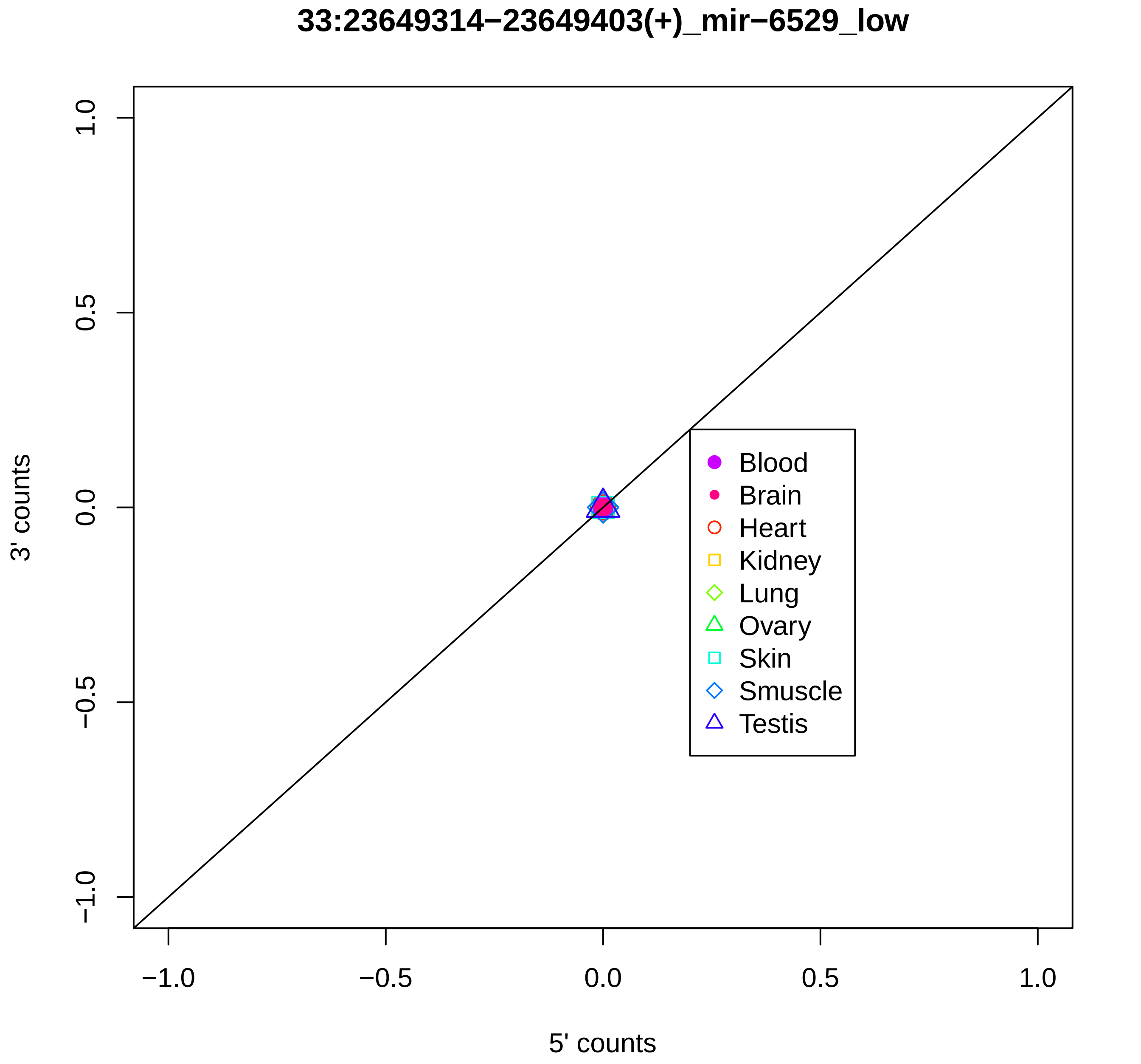
33:23649314−23649403(+)_mir−6529_low
3' counts
5' counts
1.0
0.5
0.0
−0.5
−1.0
−1.0
−0.5
0.0
0.5
1.0
Blood
Brain
Heart
Kidney
Lung
Ovary
Skin
Smuscle
Testis

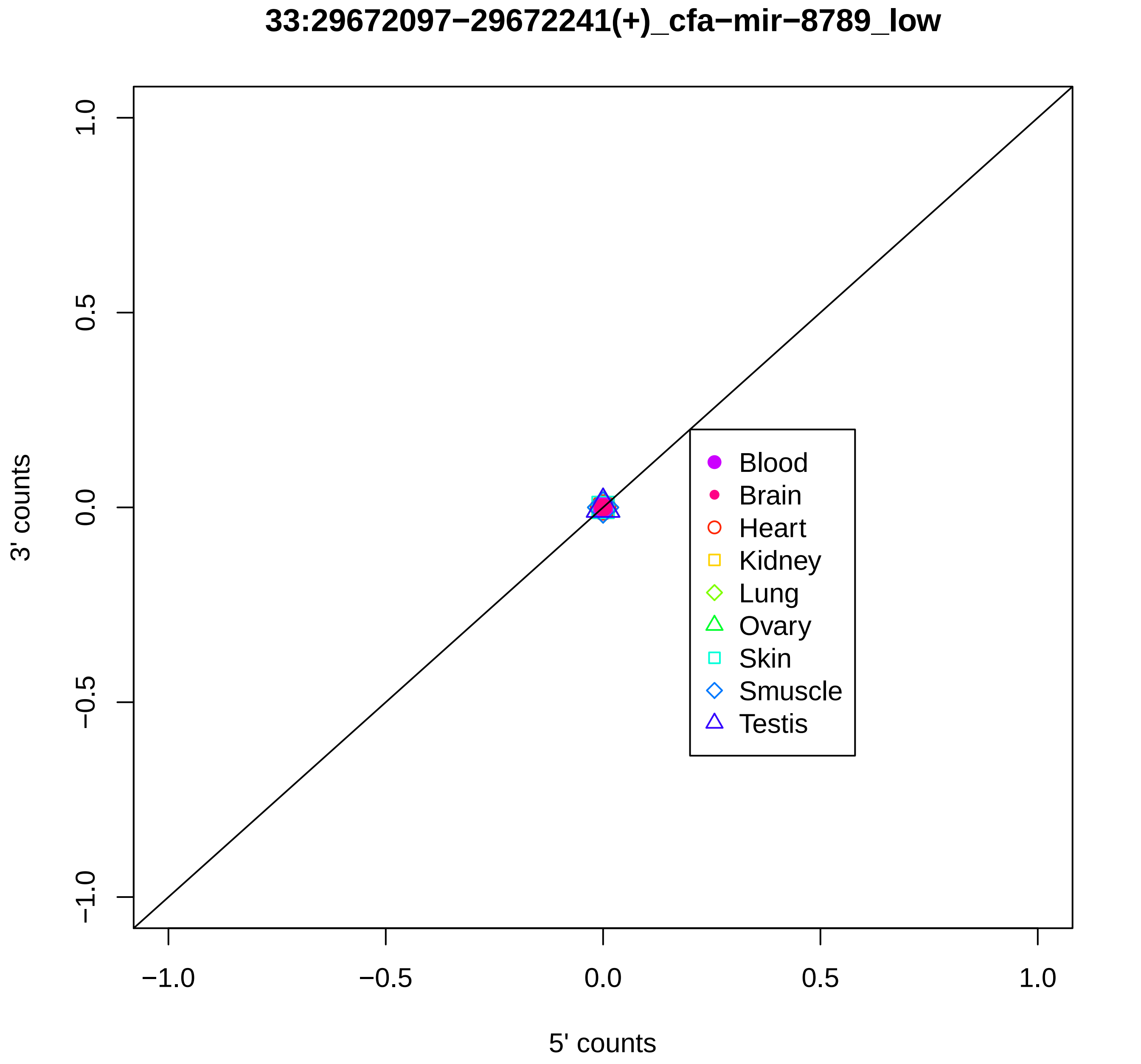
33:29672097−29672241(+)_cfa−mir−8789_low
3' counts
5' counts
1.0
0.5
0.0
−0.5
−1.0
Blood
Brain
Heart
Kidney
Lung
Ovary
Skin
Smuscle
Testis

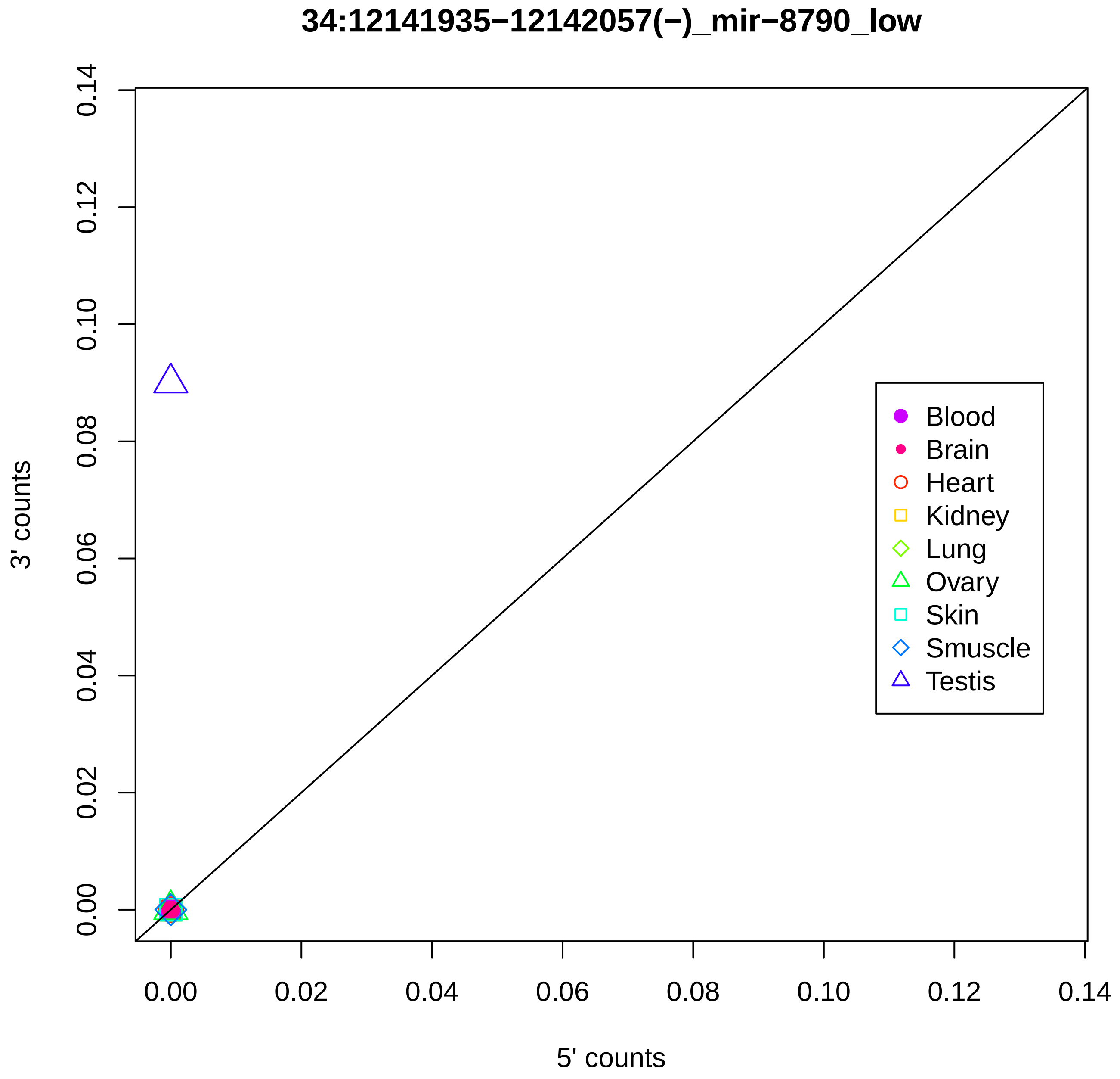

34:12141935−12142057(−)_mir−8790_low
3' counts
5' counts
0.00
0.02
0.04
0.06
0.08
0.10
0.12
0.14
Blood
Brain
Heart
Kidney
Lung
Ovary
Skin
Smuscle
Testis

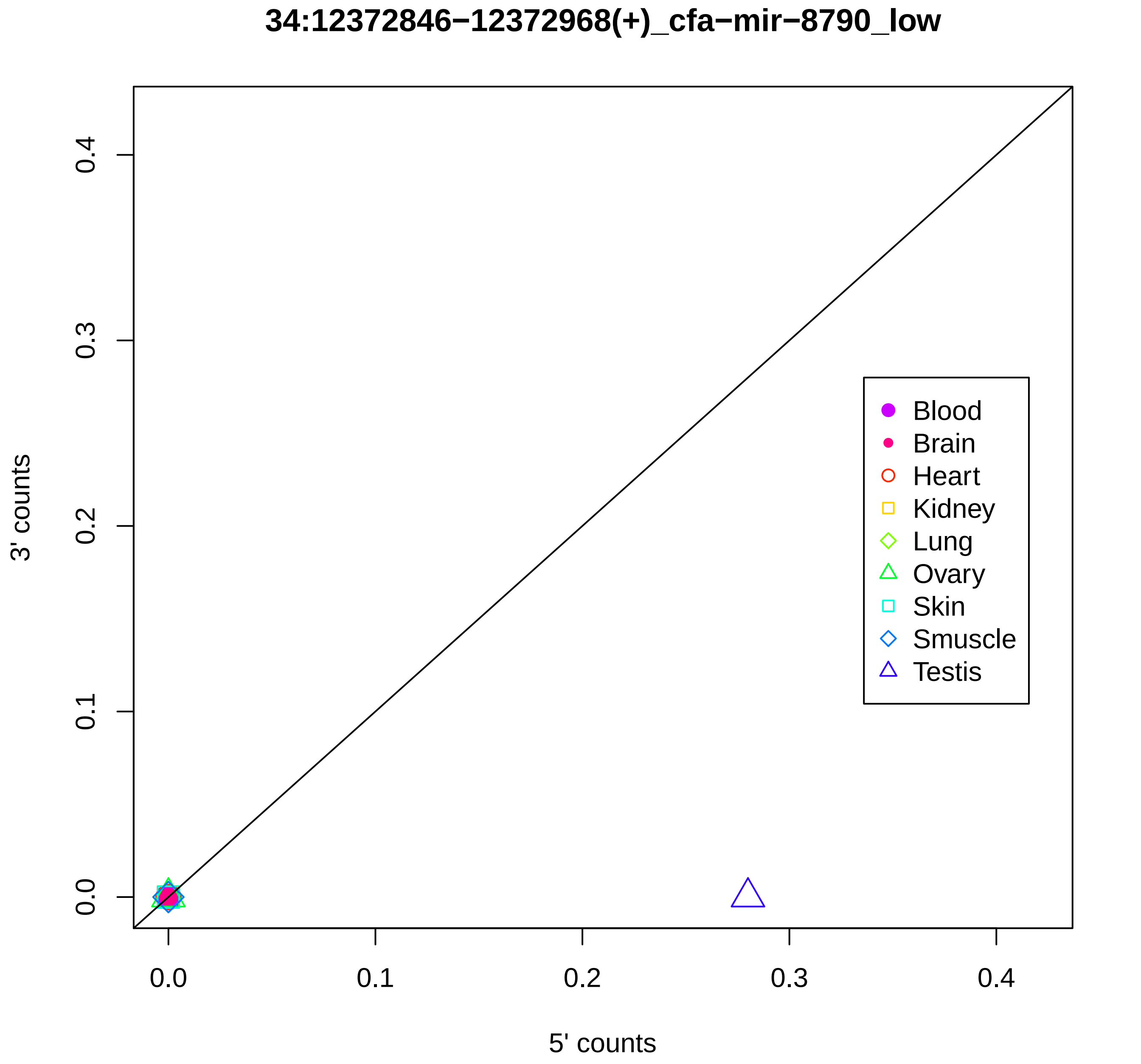

34:12372846−12372968(+)_cfa−mir−8790_low
3' counts
5' counts
0.0
0.1
0.2
0.3
0.4
Blood
Brain
Heart
Kidney
Lung
Ovary
Skin
Smuscle
Testis

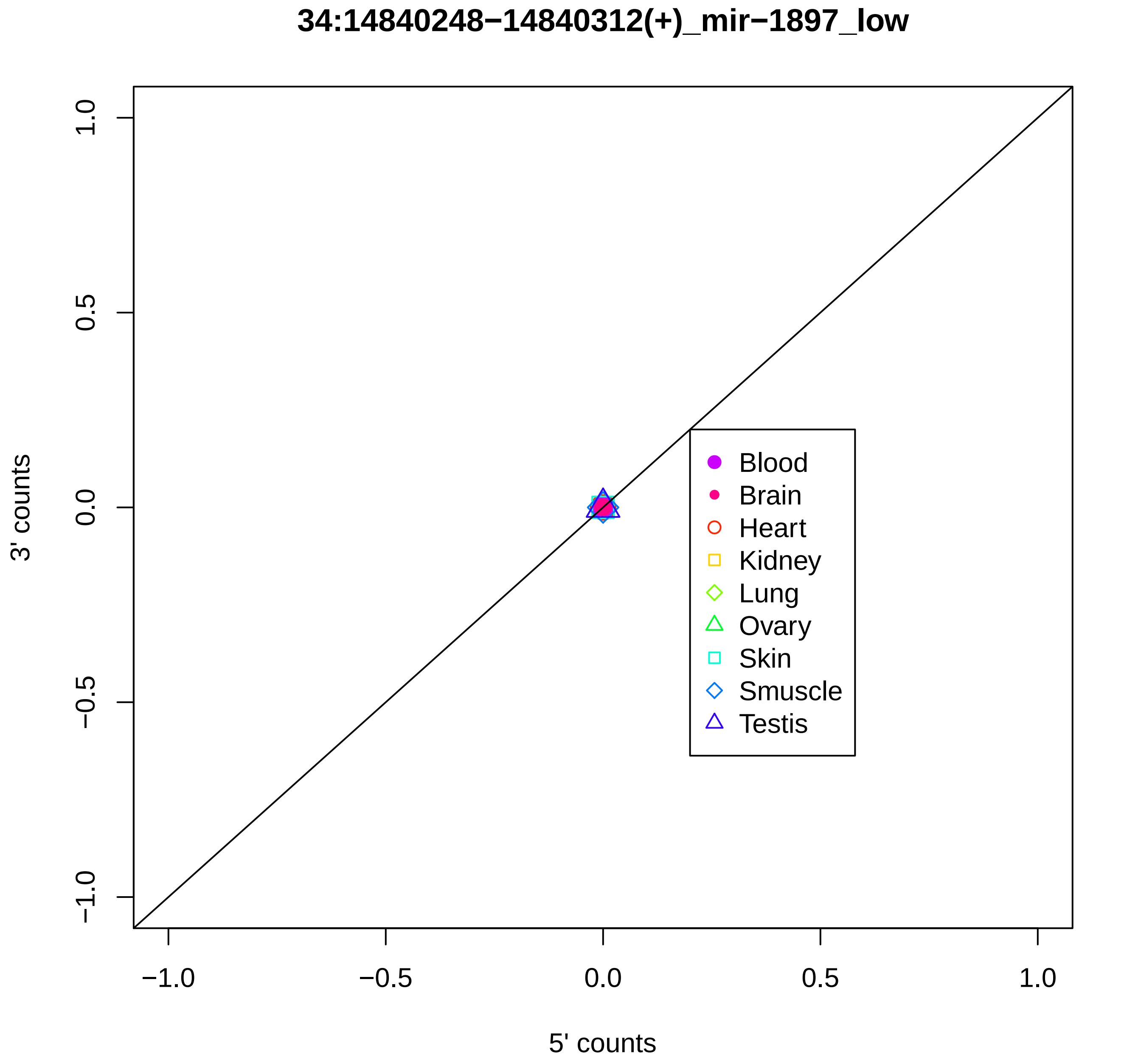

34:14840248−14840312(+)_mir−1897_low
1.0
0.5
0.0
−0.5
−1.0
3' counts
−1.0
−0.5
0.0
0.5
1.0
5' counts
Blood
Brain
Heart
Kidney
Lung
Ovary
Skin
Smuscle
Testis

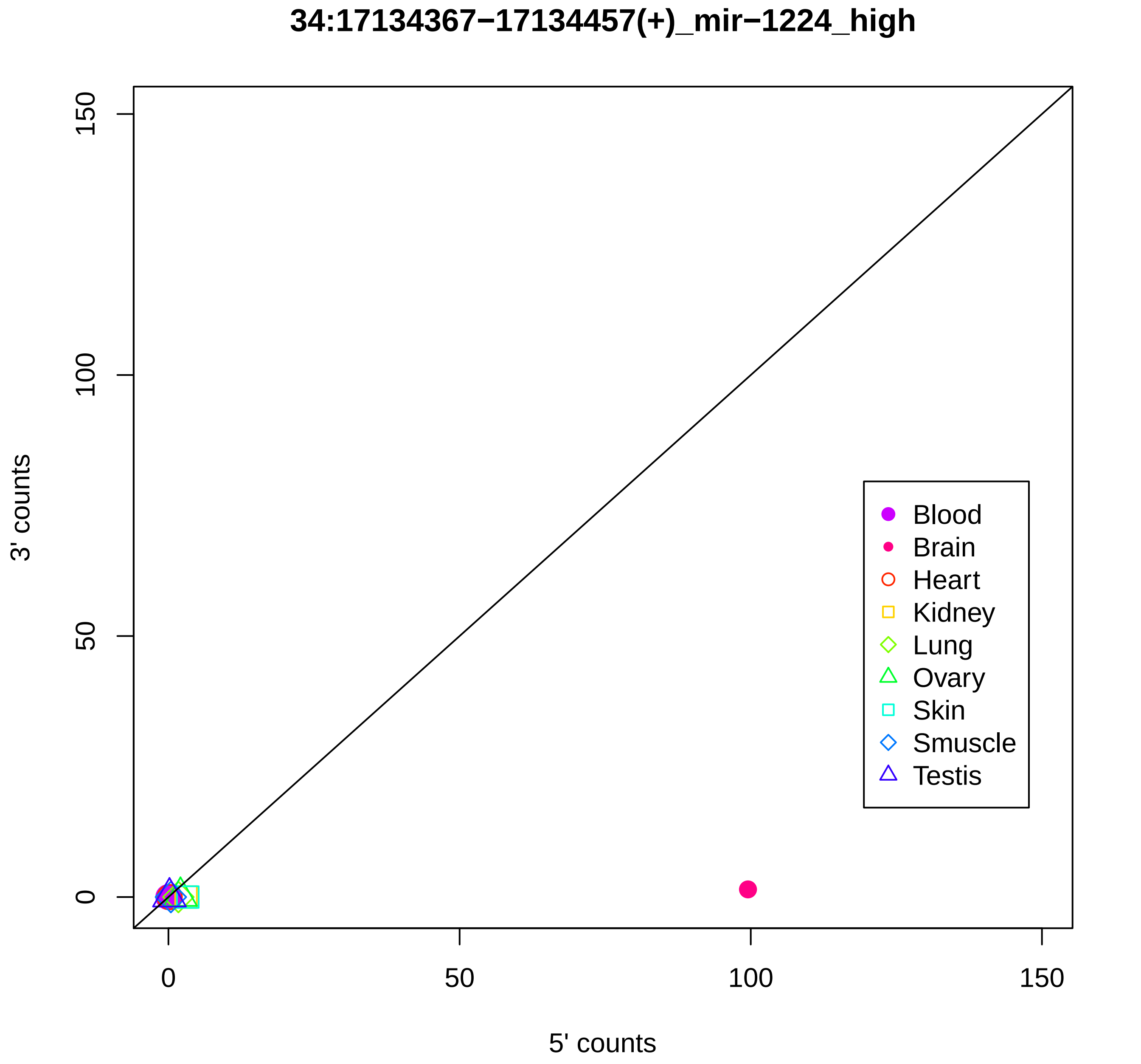

34:17134367−17134457(+)_mir−1224_high
3' counts
5' counts
0
50
100
150
0
50
100
150
Blood
Brain
Heart
Kidney
Lung
Ovary
Skin
Smuscle
Testis

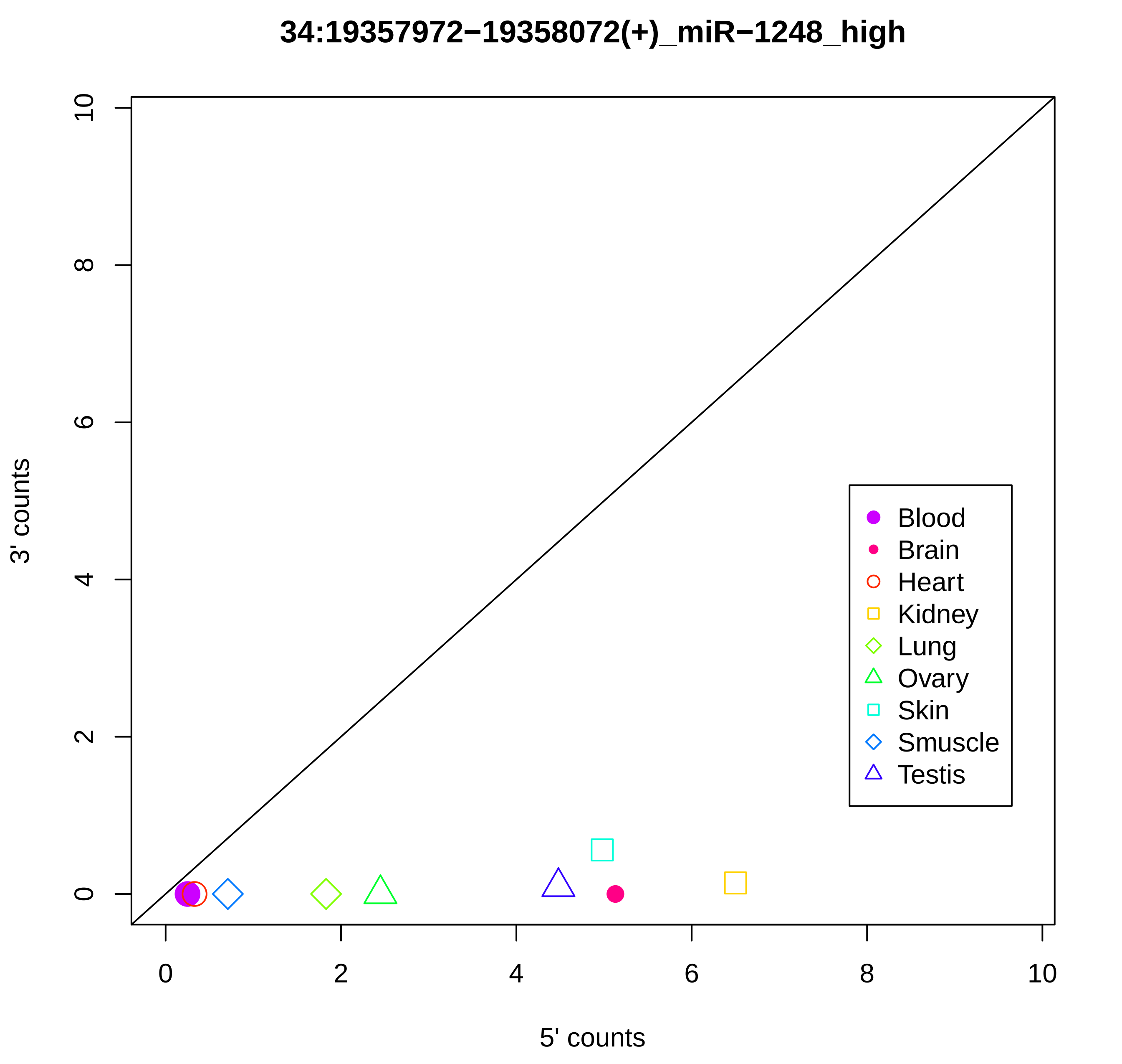

34:19357972−19358072(+)_miR−1248_high
3' counts
5' counts
0
2
4
6
8
10
0
2
4
6
8
10
Blood
Brain
Heart
Kidney
Lung
Ovary
Skin
Smuscle
Testis

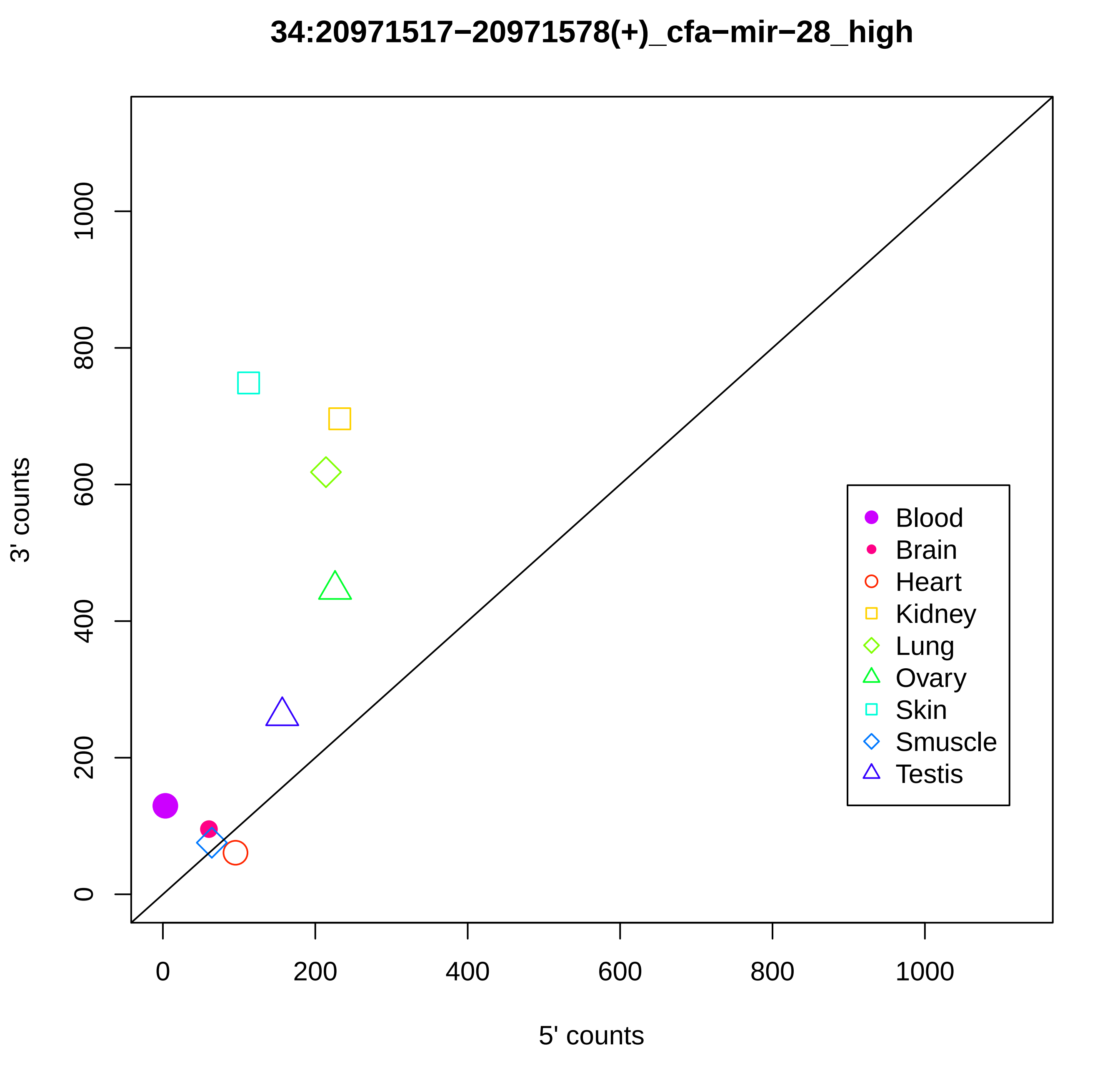
34:20971517−20971578(+)_cfa−mir−28_high
3' counts
5' counts
0
200
400
600
800
1000
0
200
400
600
800
1000
Blood
Brain
Heart
Kidney
Lung
Ovary
Skin
Smuscle
Testis

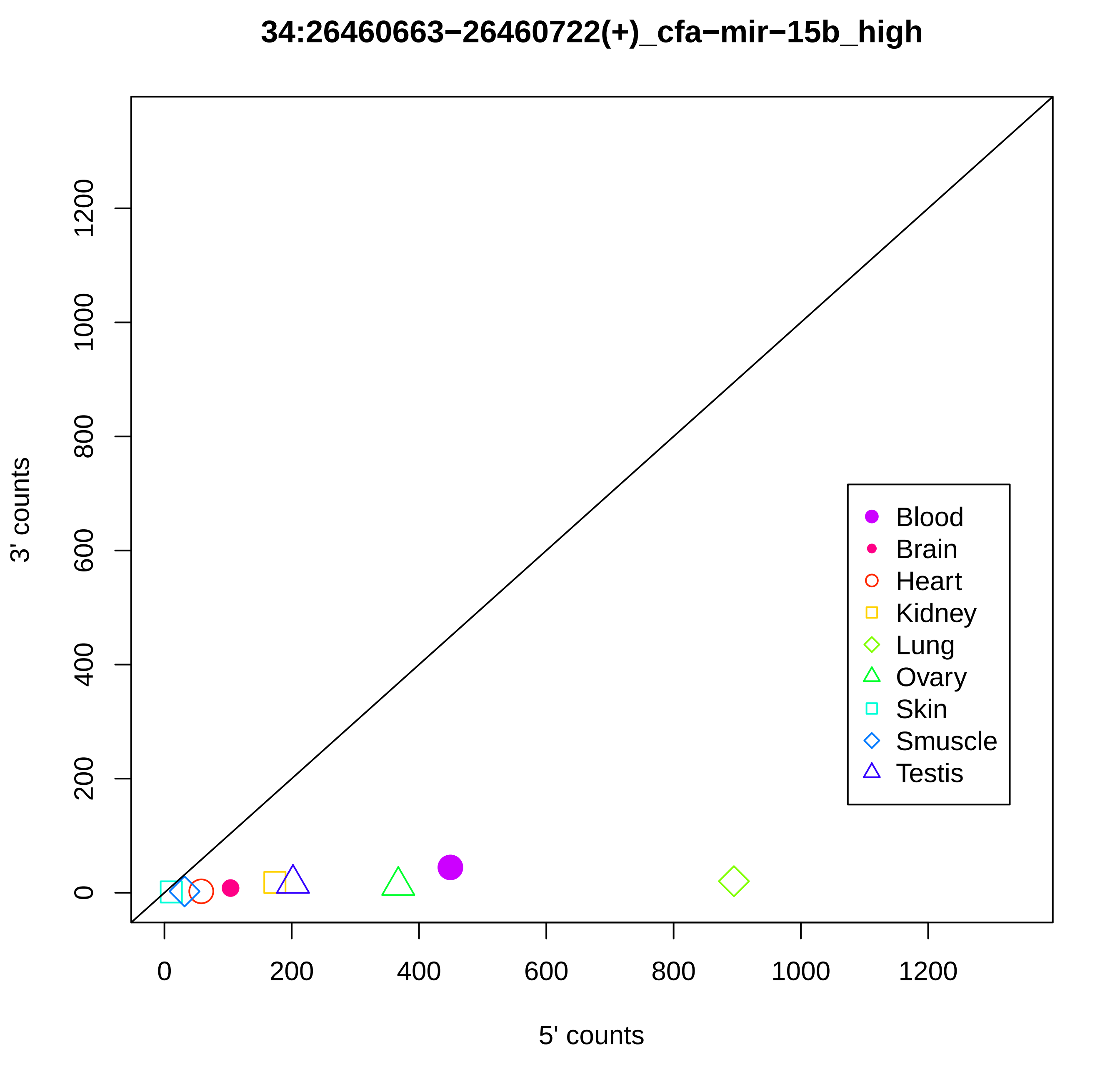

34:26460663−26460722(+)_cfa−mir−15b_high
3' counts
5' counts
0
200
400
600
800
1000
1200
Blood
Brain
Heart
Kidney
Lung
Ovary
Skin
Smuscle
Testis

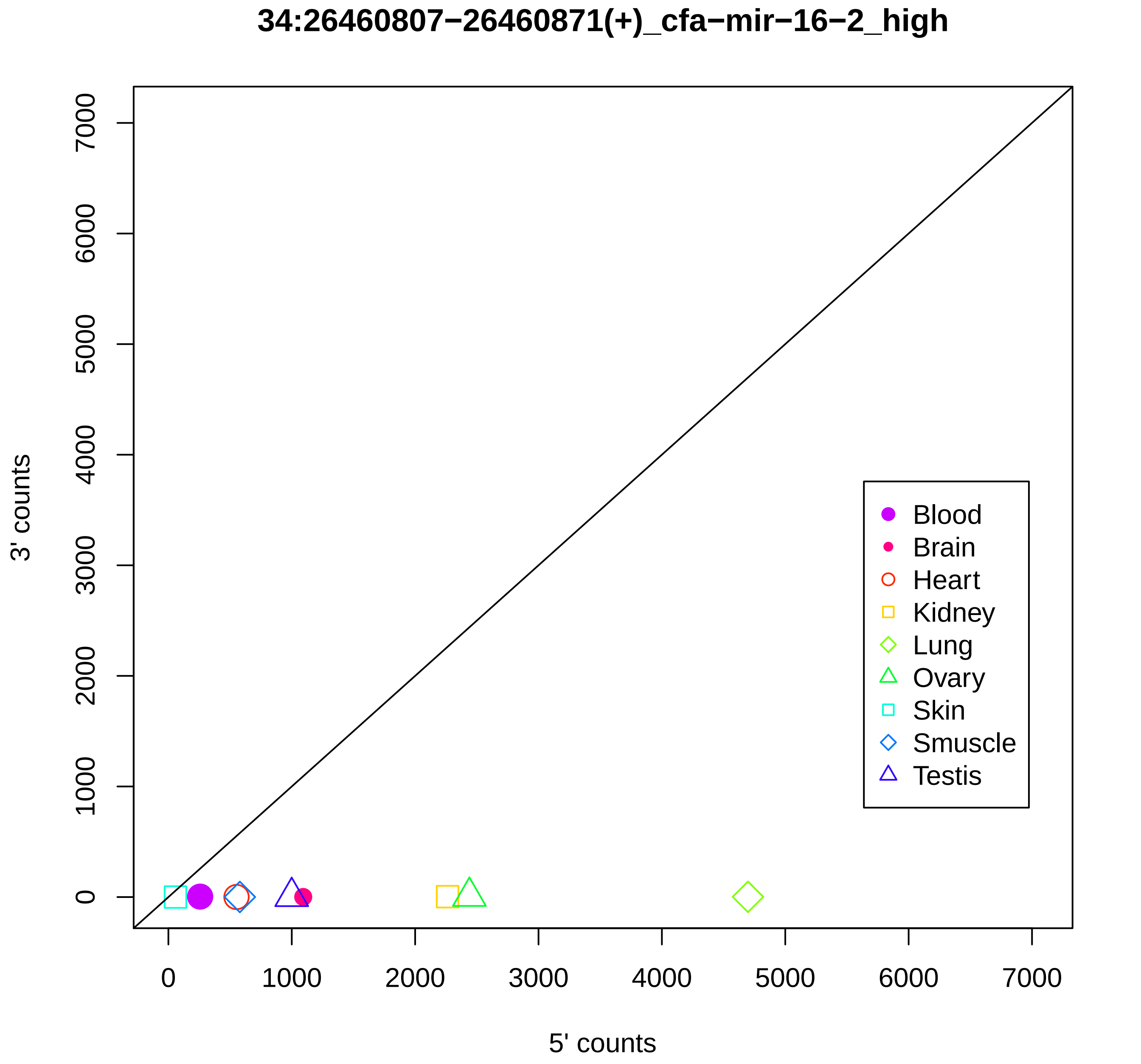

34:26460807−26460871(+)_cfa−mir−16−2_high
3' counts
5' counts
0
1000
2000
3000
4000
5000
6000
7000
Blood
Brain
Heart
Kidney
Lung
Ovary
Skin
Smuscle
Testis

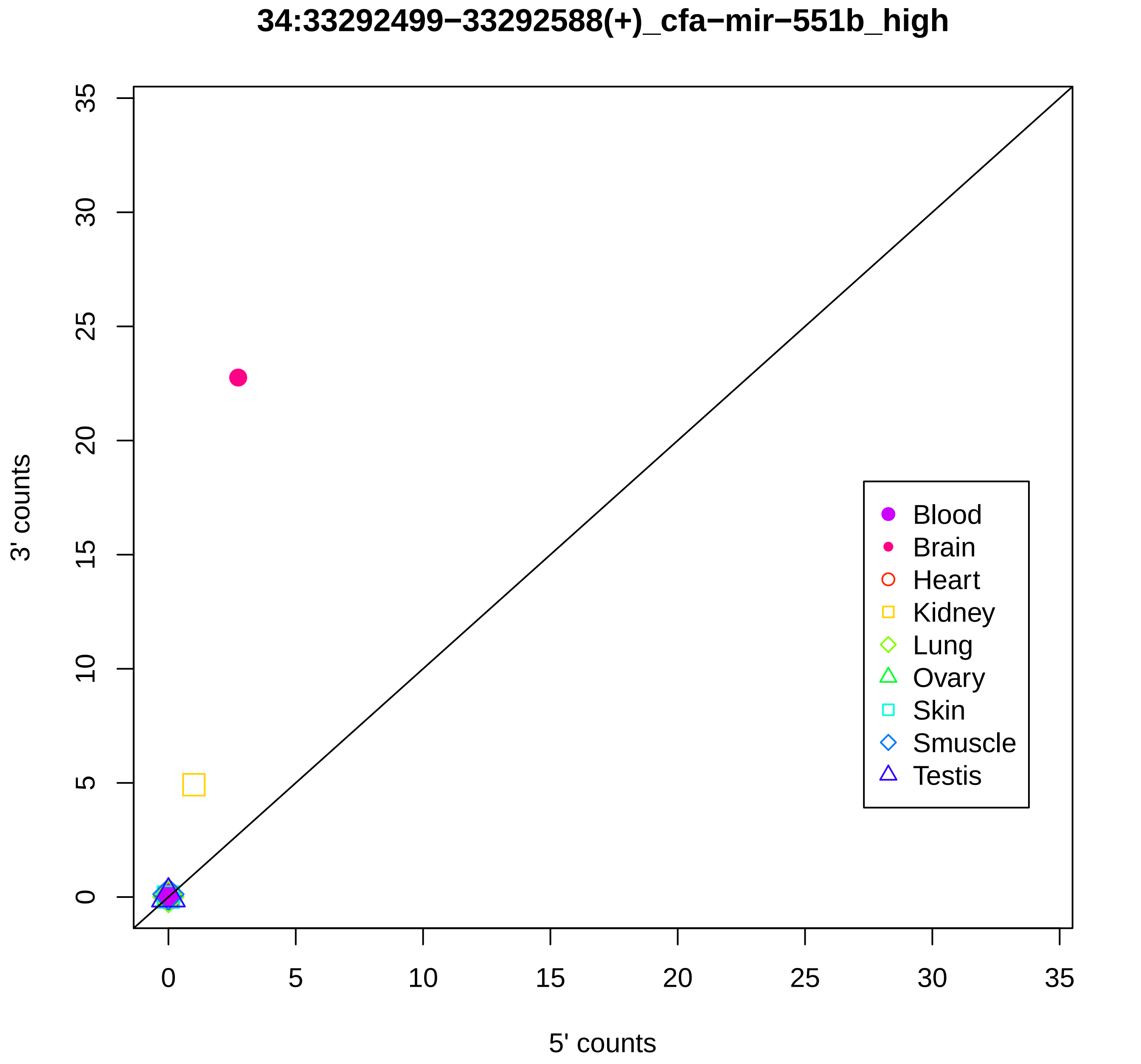
34:33292499−33292588(+)_cfa−mir−551b_high
3' counts
5' counts
0
5
10
15
20
25
30
35
Blood
Brain
Heart
Kidney
Lung
Ovary
Skin
Smuscle
Testis

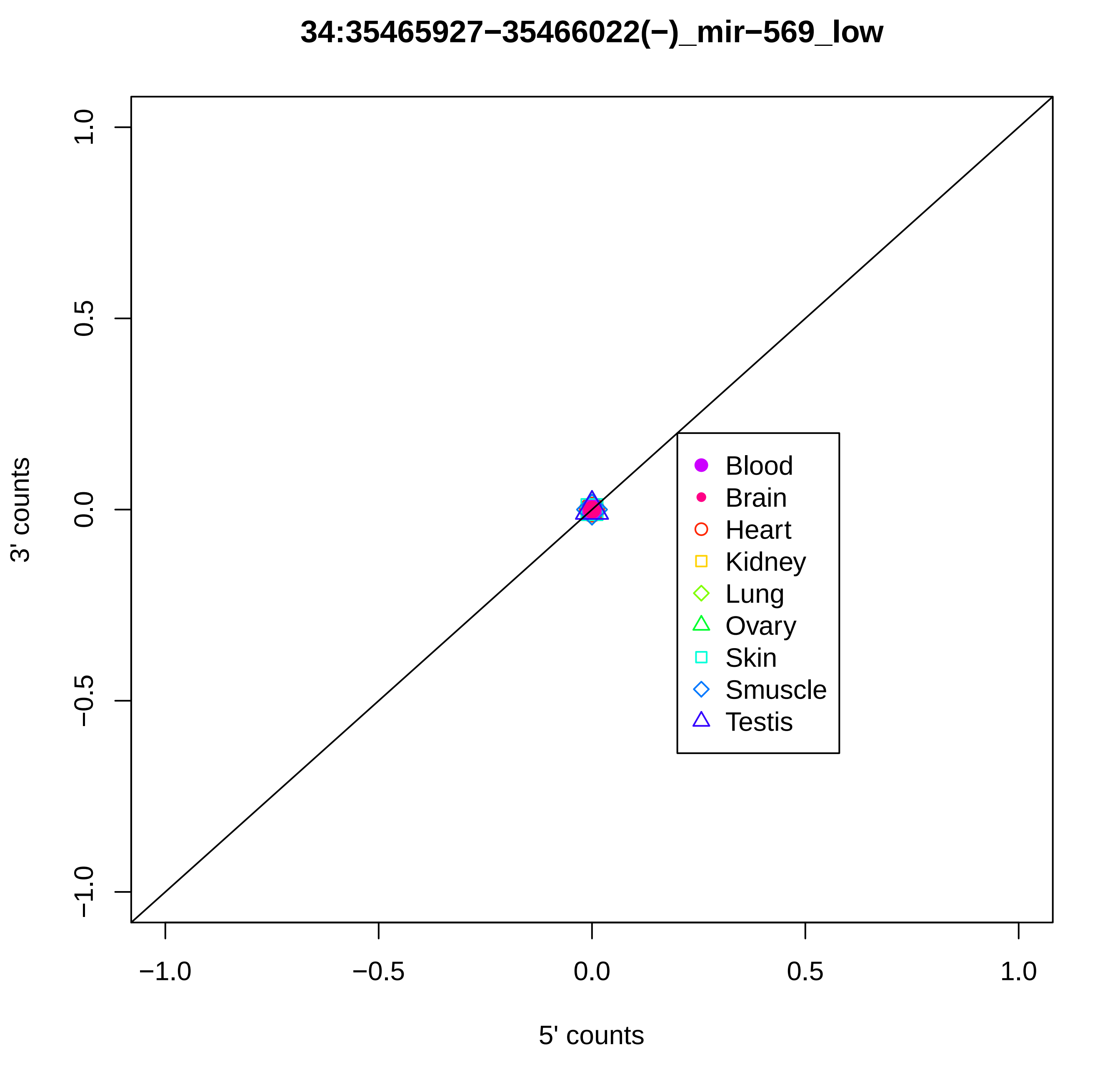
34:35465927−35466022(−)_mir−569_low
3' counts
5' counts
1.0
0.5
0.0
−0.5
−1.0
−1.0
−0.5
0.0
0.5
1.0
Blood
Brain
Heart
Kidney
Lung
Ovary
Skin
Smuscle
Testis

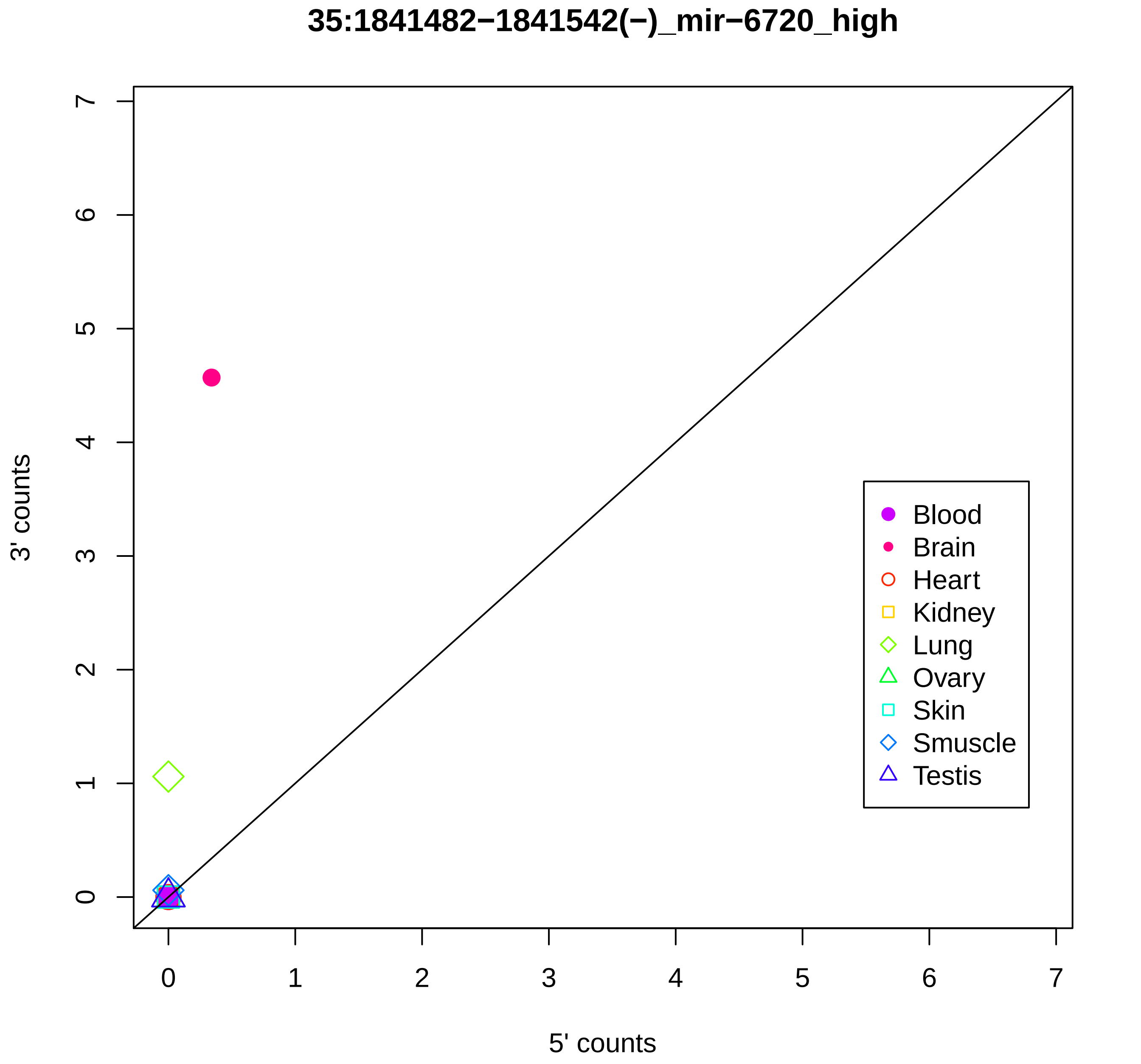

35:1841482−1841542(−)_mir−6720_high
3' counts
5' counts
0
1
2
3
4
5
6
7
Blood
Brain
Heart
Kidney
Lung
Ovary
Skin
Smuscle
Testis

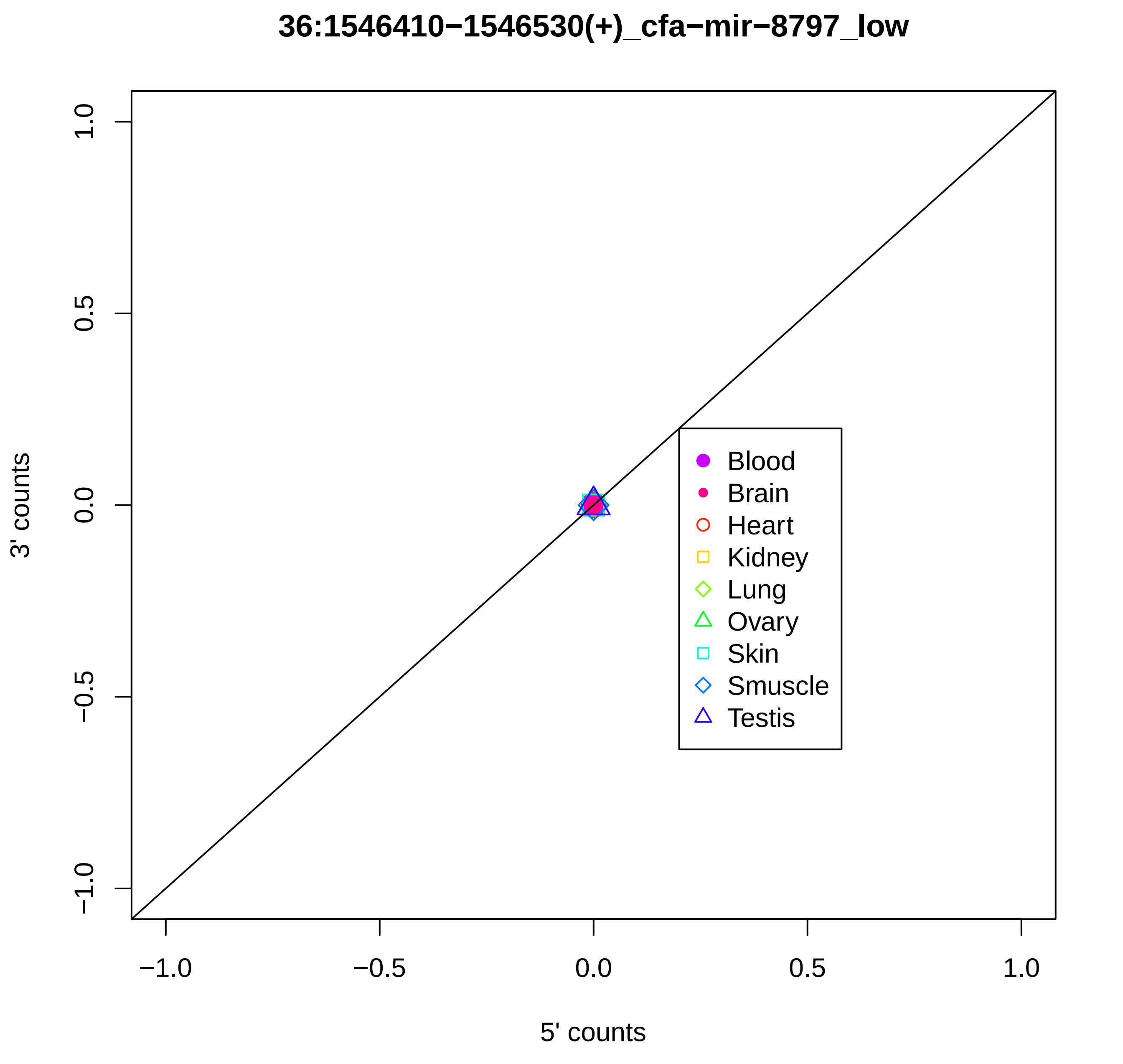

36:1546410−1546530(+)_cfa−mir−8797_low
3' counts
5' counts
−1.0
−0.5
0.0
0.5
1.0
Blood
Brain
Heart
Kidney
Lung
Ovary
Skin
Smuscle
Testis

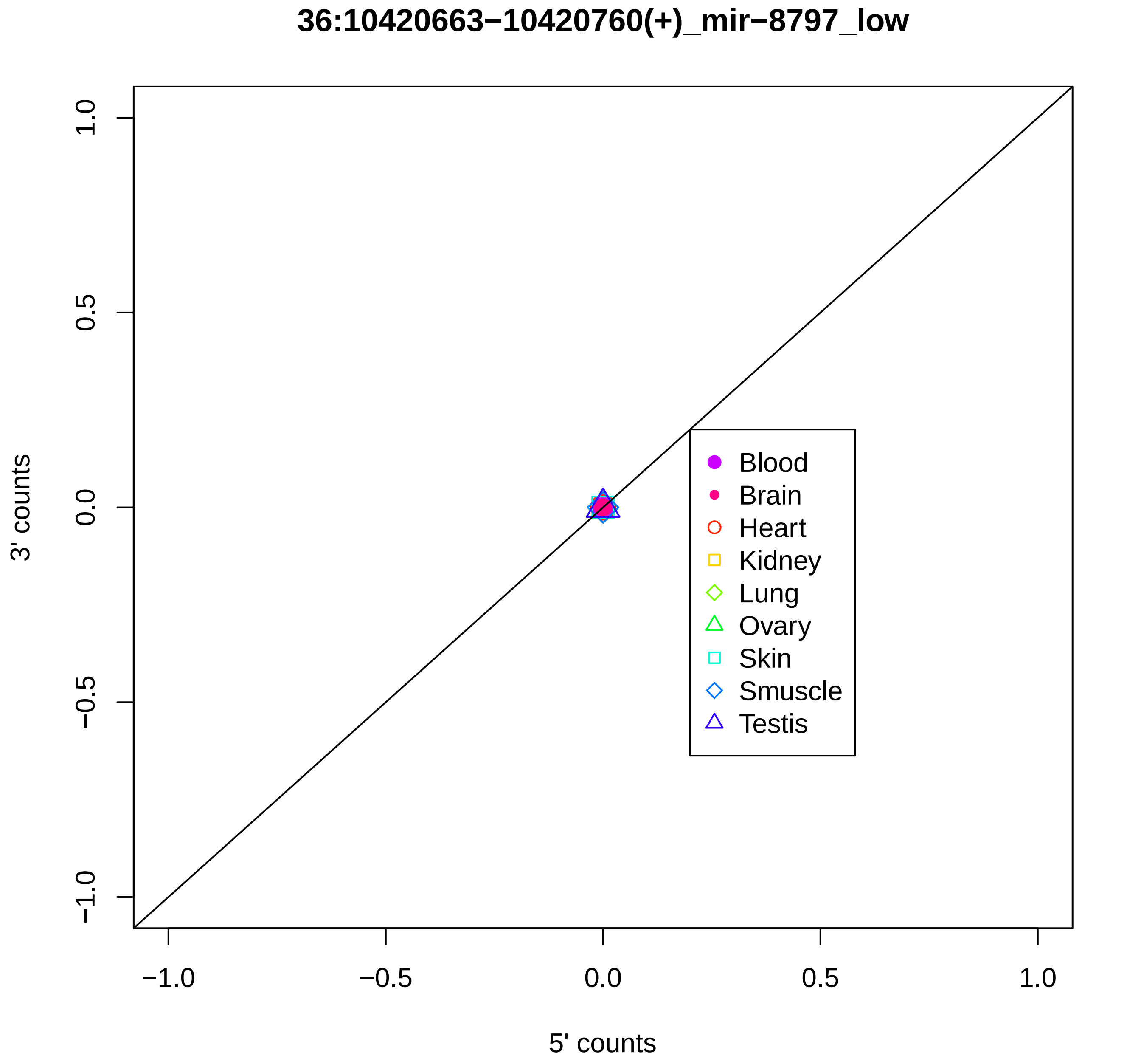

36:10420663−10420760(+)_mir−8797_low
3' counts
5' counts
−1.0
−0.5
0.0
0.5
1.0
Blood
Brain
Heart
Kidney
Lung
Ovary
Skin
Smuscle
Testis

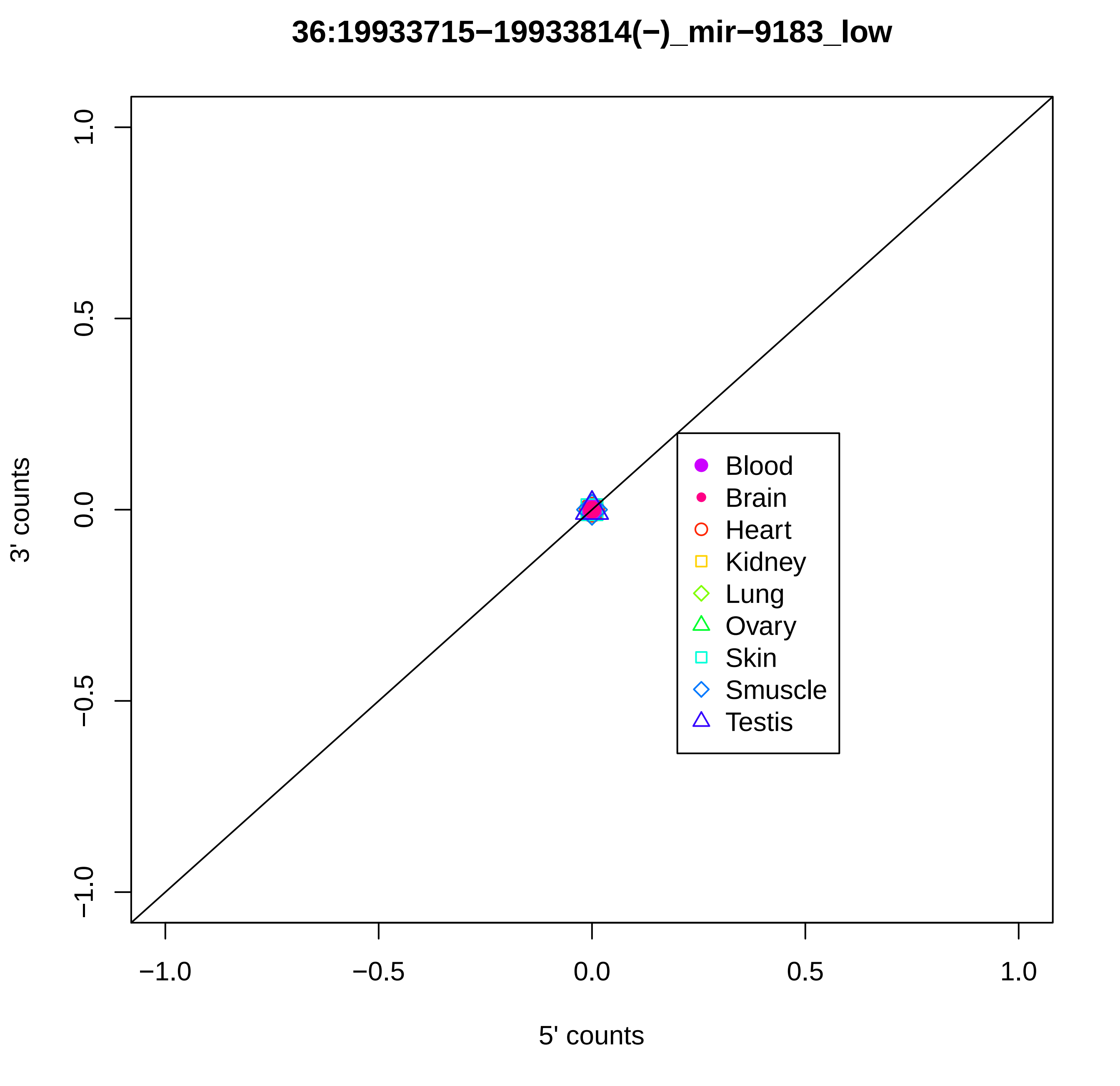

36:19933715−19933814(−)_mir−9183_low
Blood
Brain
Heart
Kidney
Lung
Ovary
Skin
Smuscle
Testis
3' counts
5' counts
−1.0
−0.5
0.0
0.5
1.0

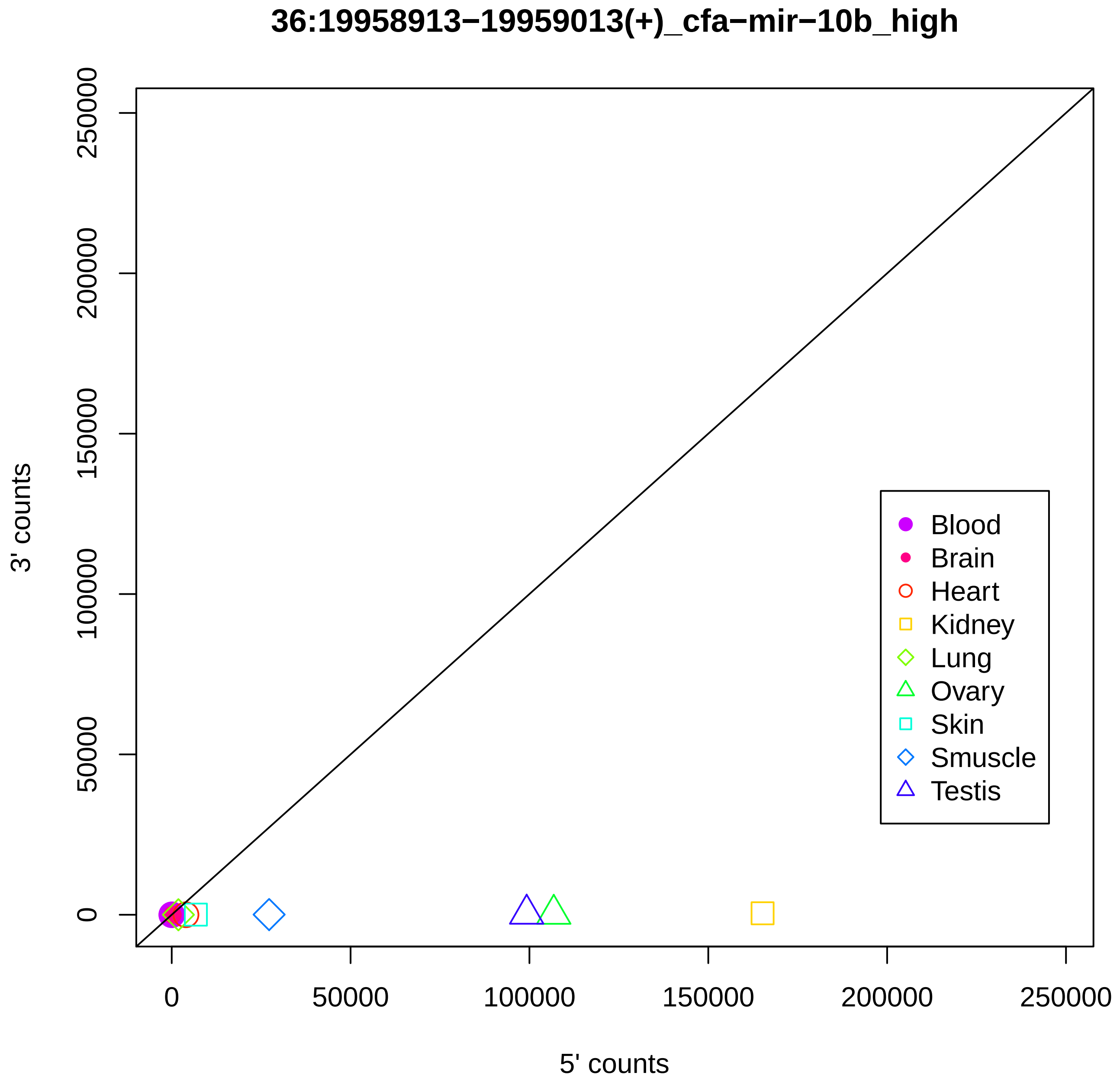

36:19958913−19959013(+)_cfa−mir−10b_high
3' counts
0
50000
100000
150000
200000
250000
5' counts
0
50000
100000
150000
200000
250000
Blood
Brain
Heart
Kidney
Lung
Ovary
Skin
Smuscle
Testis

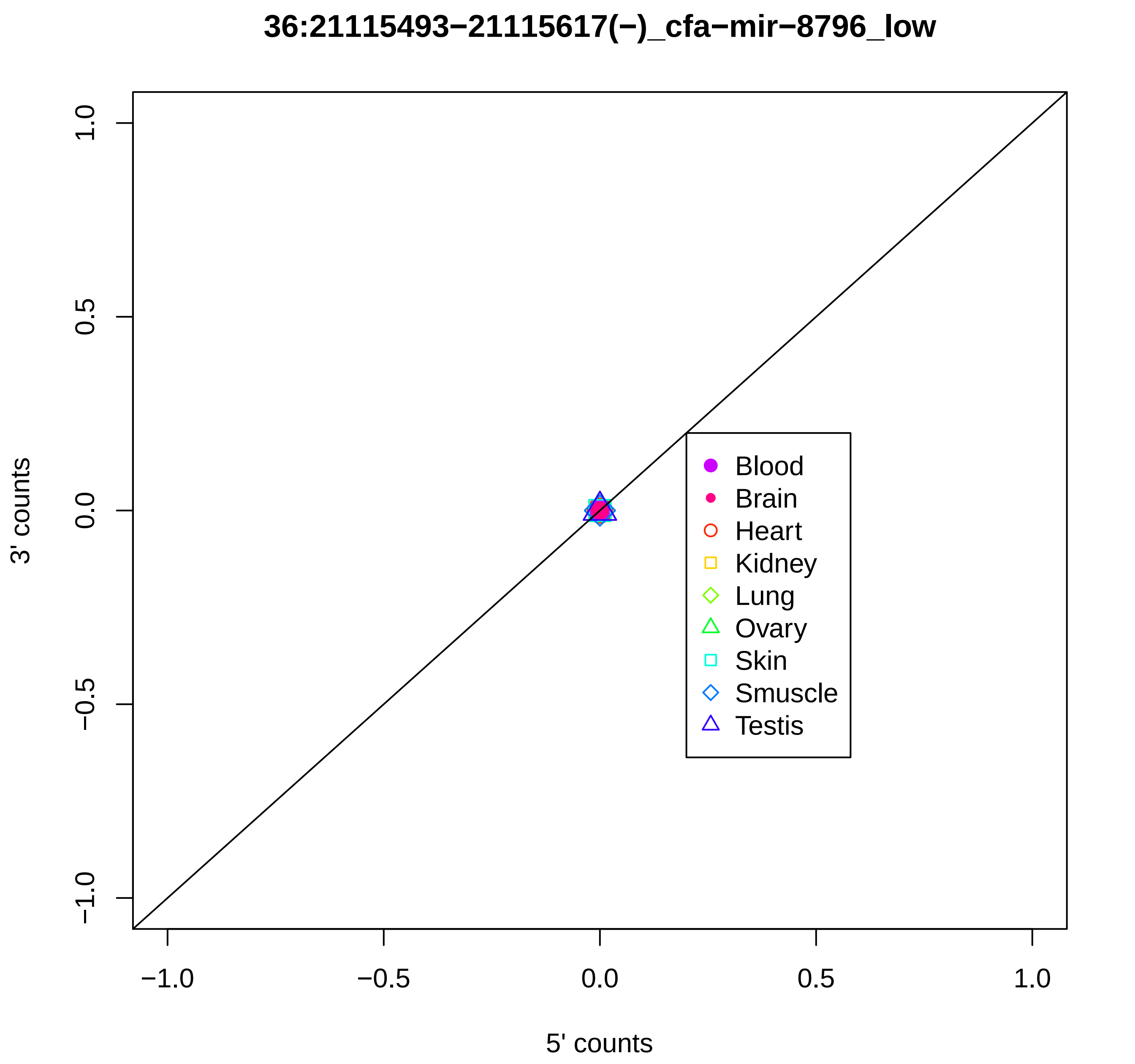

36:21115493−21115617(−)_cfa−mir−8796_low
Blood
Brain
Heart
Kidney
Lung
Ovary
Skin
Smuscle
Testis
3' counts
5' counts
−1.0
−0.5
0.0
0.5
1.0

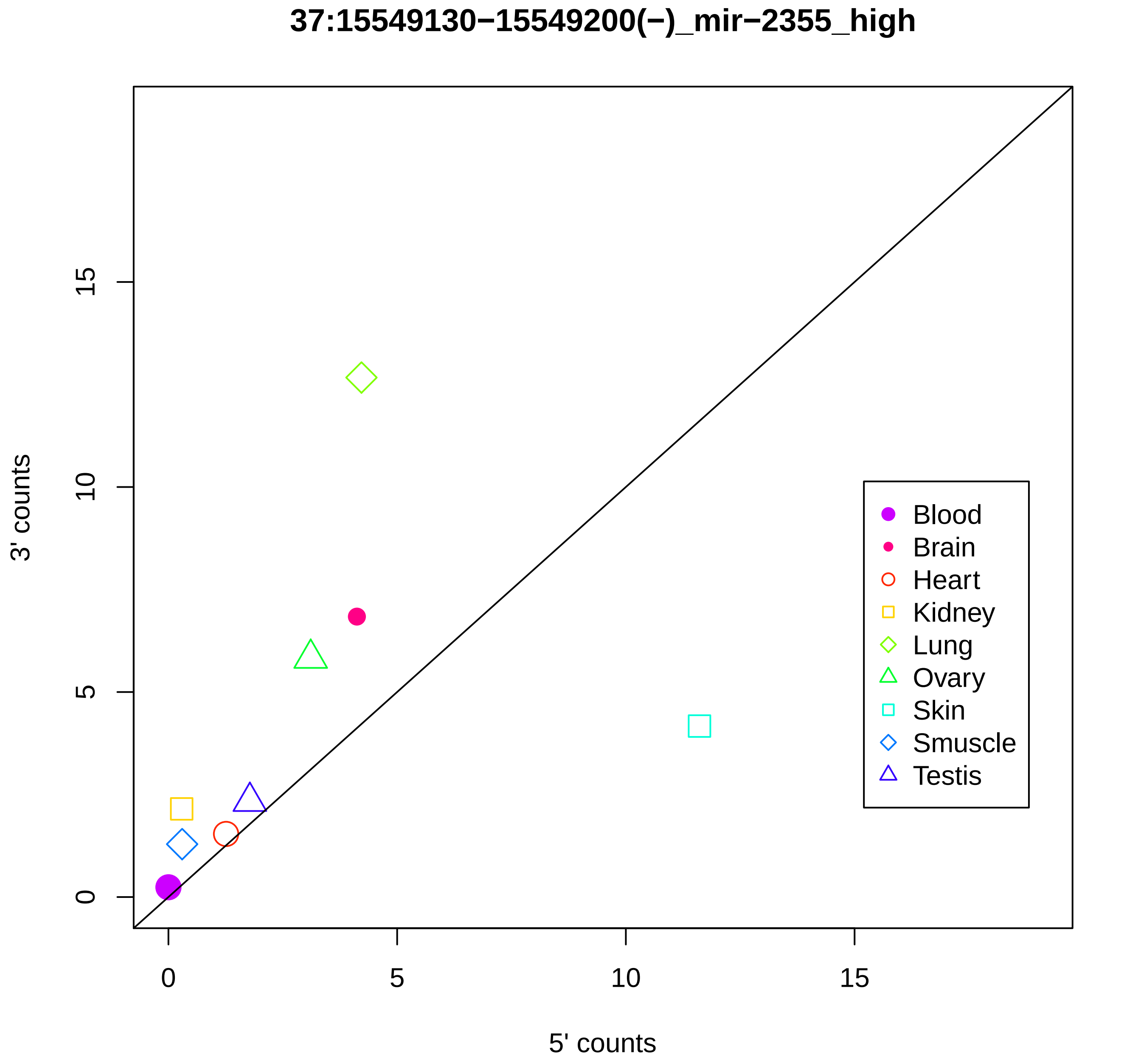
37:15549130−15549200(−)_mir−2355_high
0
5
10
15
5' counts
3' counts
Blood
Brain
Heart
Kidney
Lung
Ovary
Skin
Smuscle
Testis

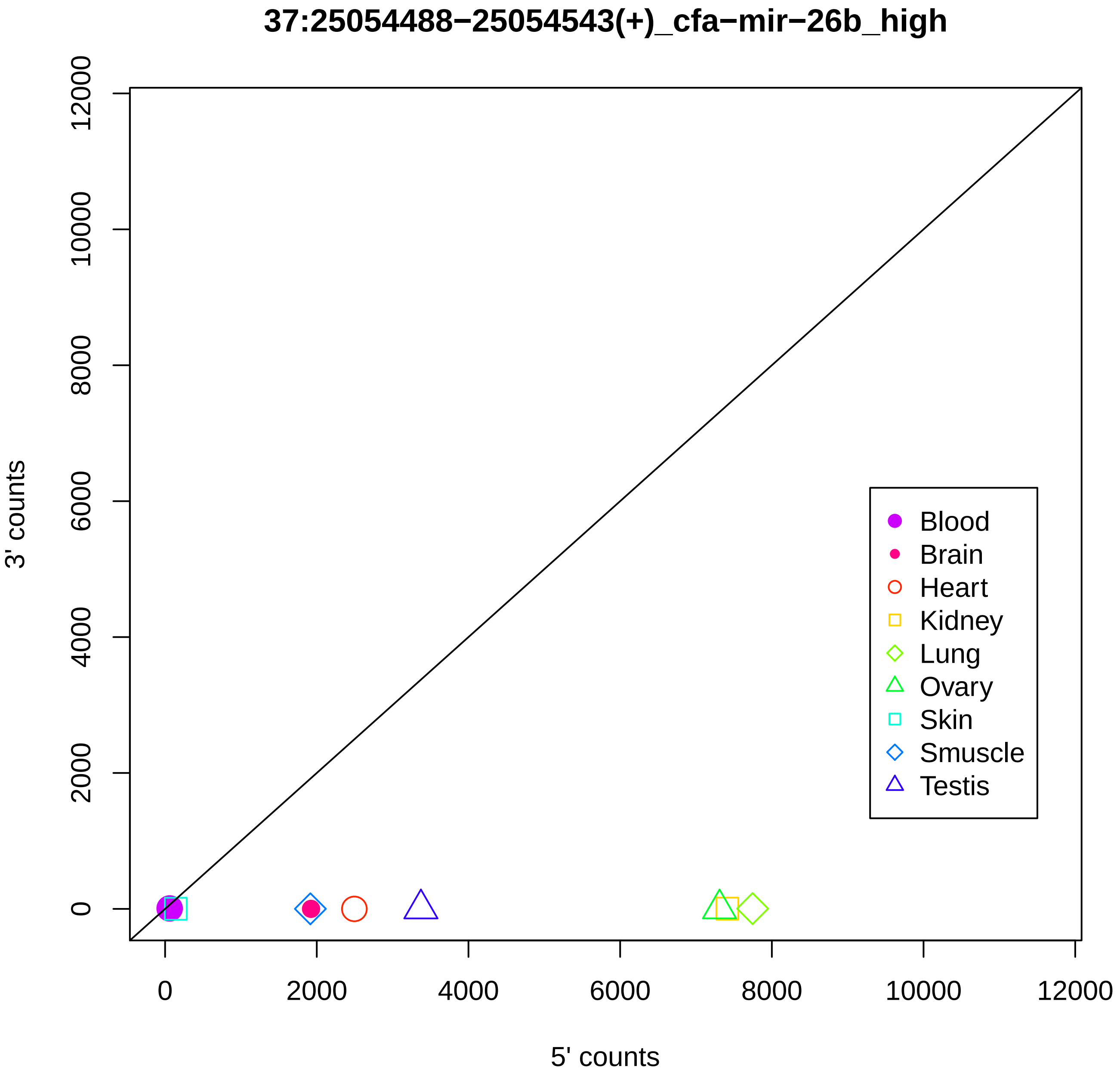

37:25054488−25054543(+)_cfa−mir−26b_high
3' counts
5' counts
0
2000
4000
6000
8000
10000
12000
Blood
Brain
Heart
Kidney
Lung
Ovary
Skin
Smuscle
Testis

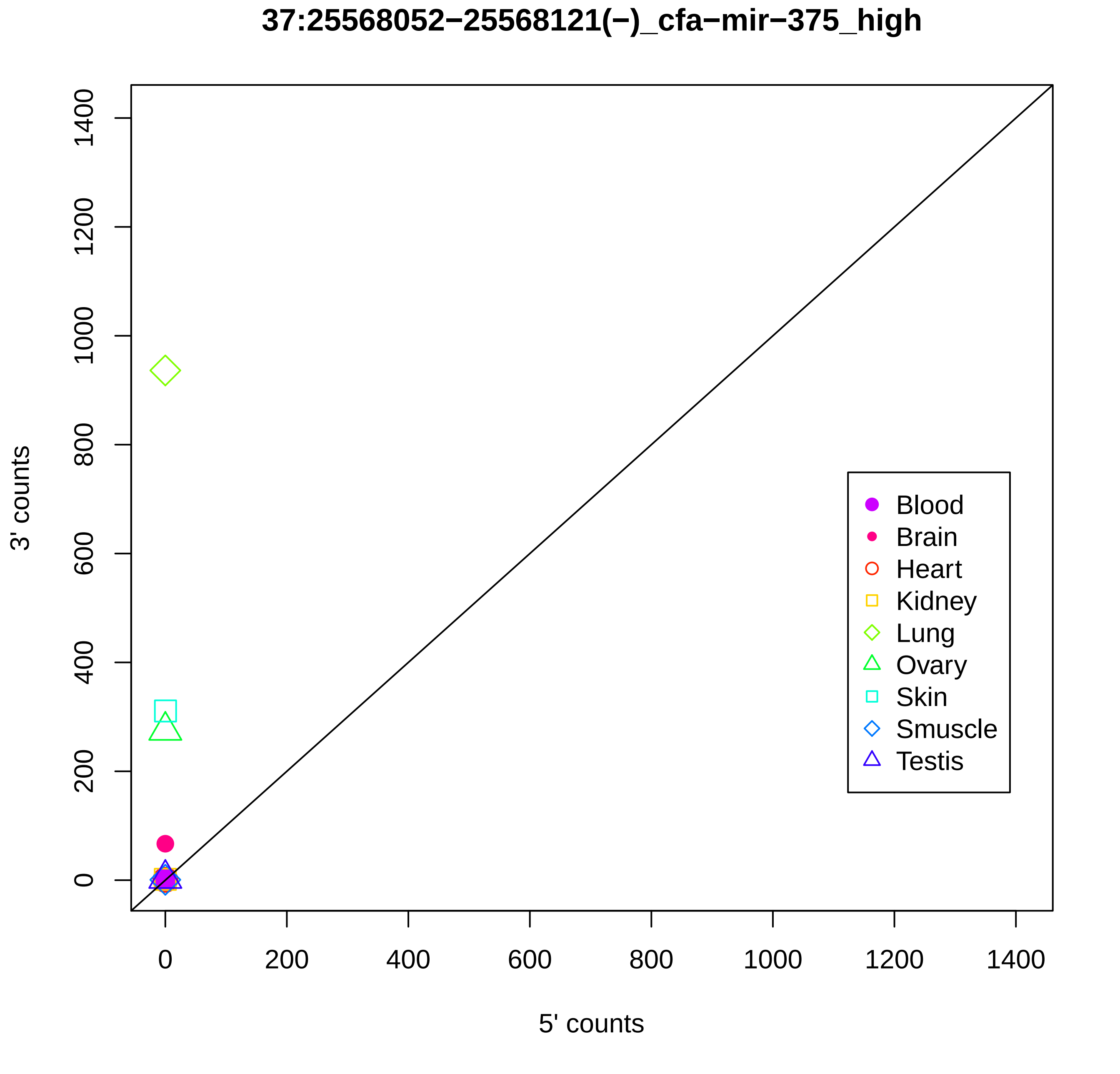
37:25568052−25568121(−)_cfa−mir−375_high
3' counts
5' counts
0
200
400
600
800
1000
1200
1400
Blood
Brain
Heart
Kidney
Lung
Ovary
Skin
Smuscle
Testis

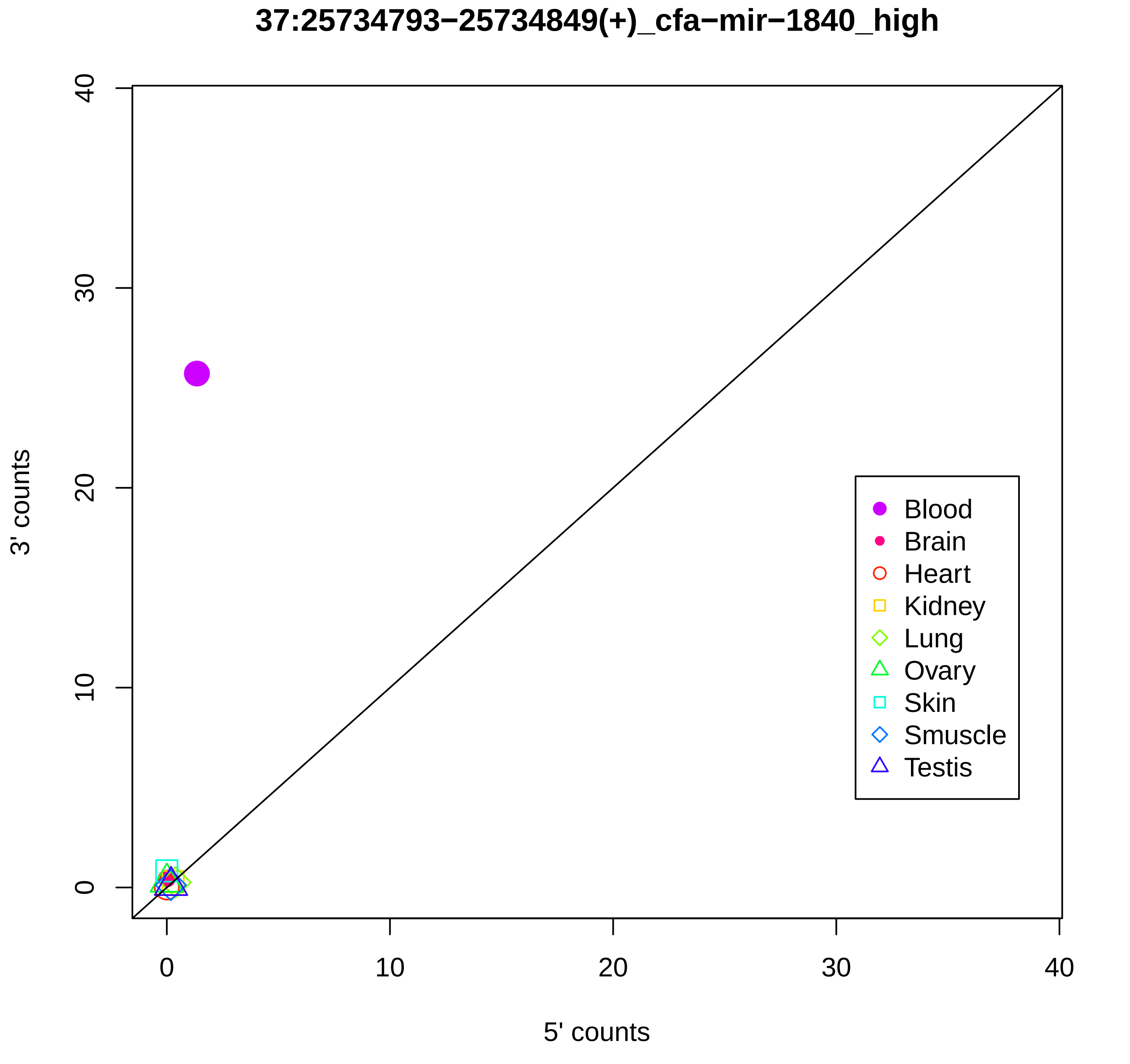
37:25734793−25734849(+)_cfa−mir−1840_high
3' counts
5' counts
0
10
20
30
40
Blood
Brain
Heart
Kidney
Lung
Ovary
Skin
Smuscle
Testis

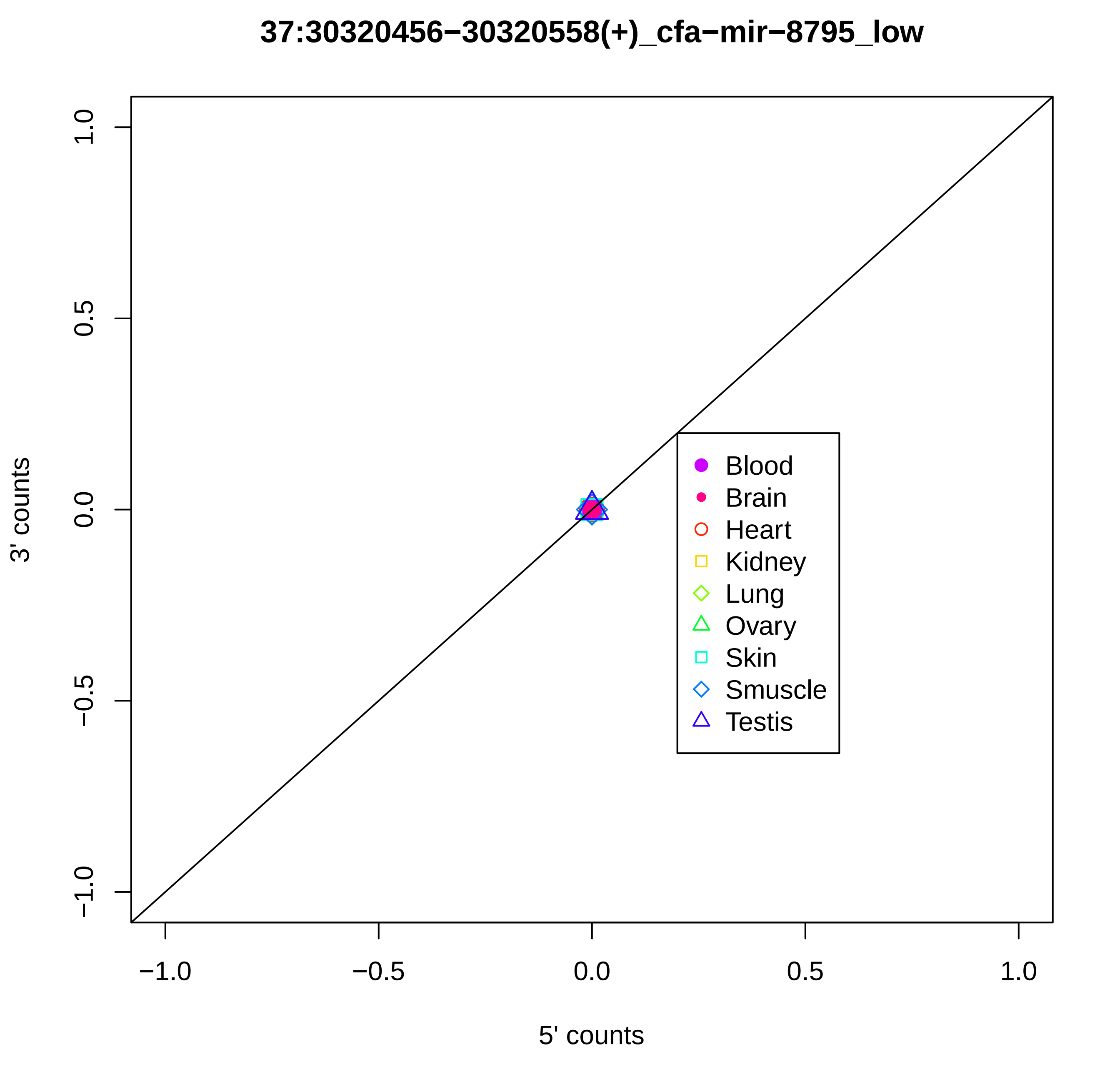

37:30320456−30320558(+)_cfa−mir−8795_low
3' counts
5' counts
1.0
0.5
0.0
−0.5
−1.0
Blood
Brain
Heart
Kidney
Lung
Ovary
Skin
Smuscle
Testis

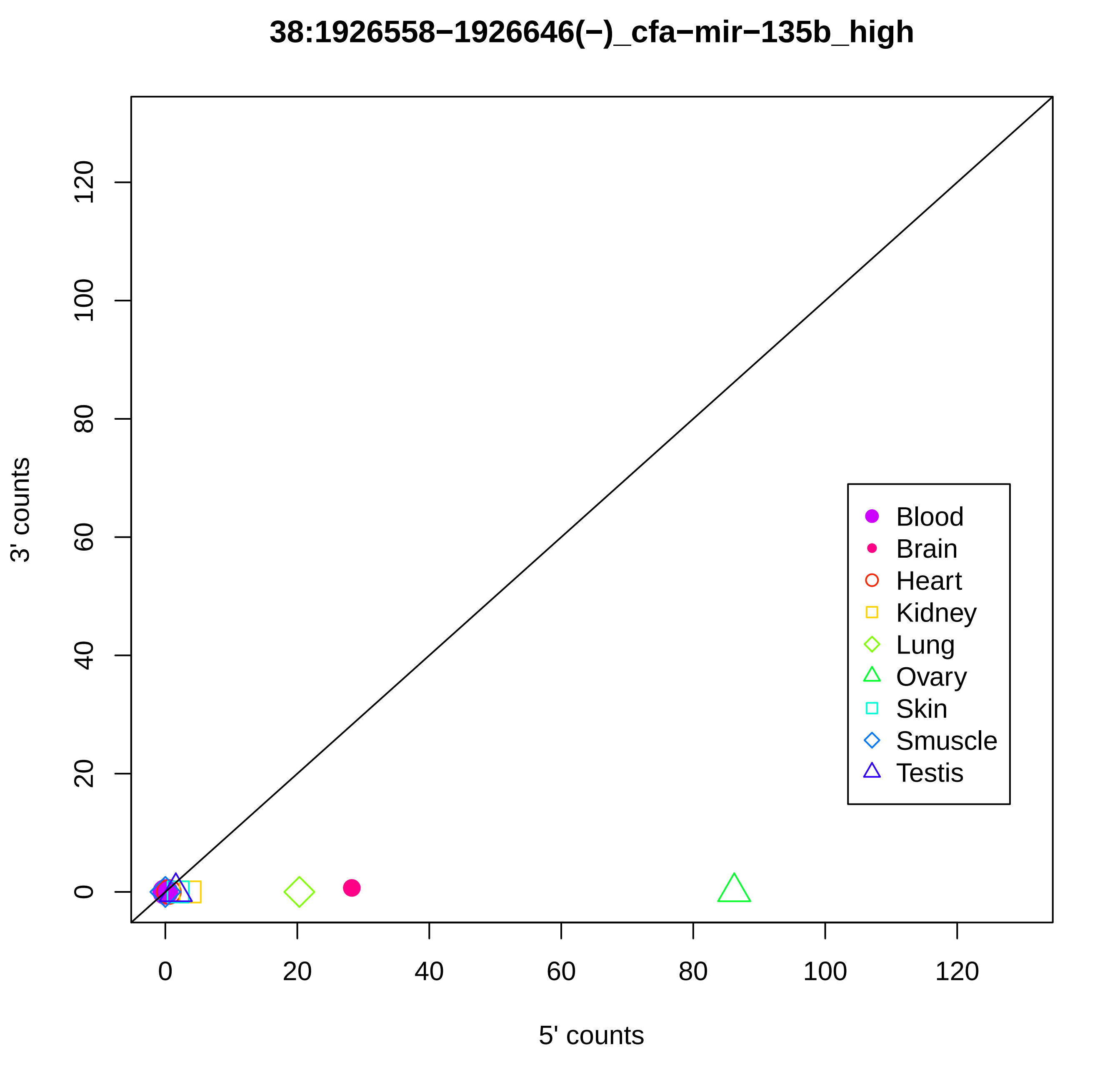

38:1926558−1926646(−)_cfa−mir−135b_high
3' counts
5' counts
0
20
40
60
80
100
120
Blood
Brain
Heart
Kidney
Lung
Ovary
Skin
Smuscle
Testis

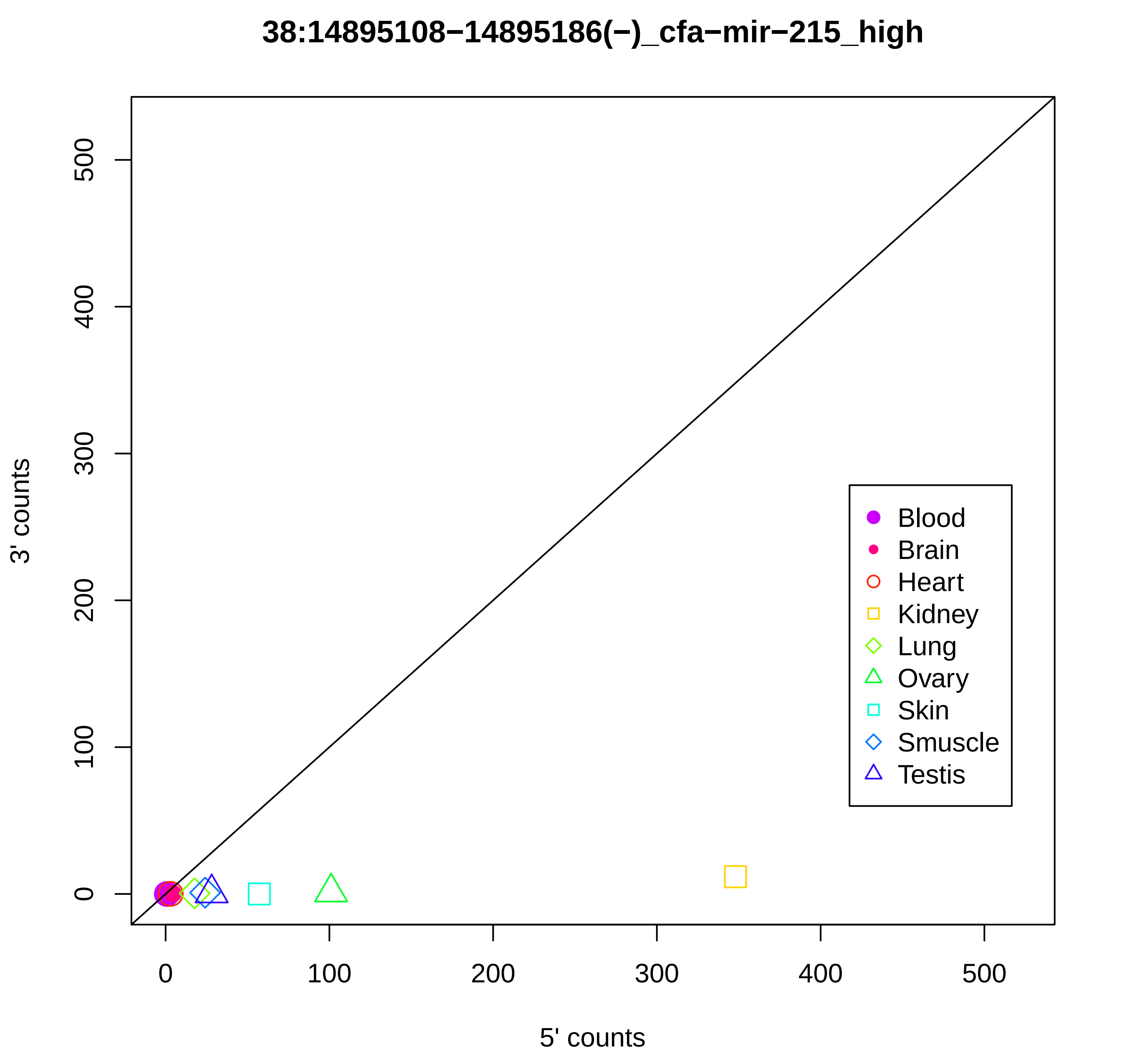

38:14895108−14895186(−)_cfa−mir−215_high
3' counts
5' counts
0
100
200
300
400
500
0
100
200
300
400
500
Blood
Brain
Heart
Kidney
Lung
Ovary
Skin
Smuscle
Testis

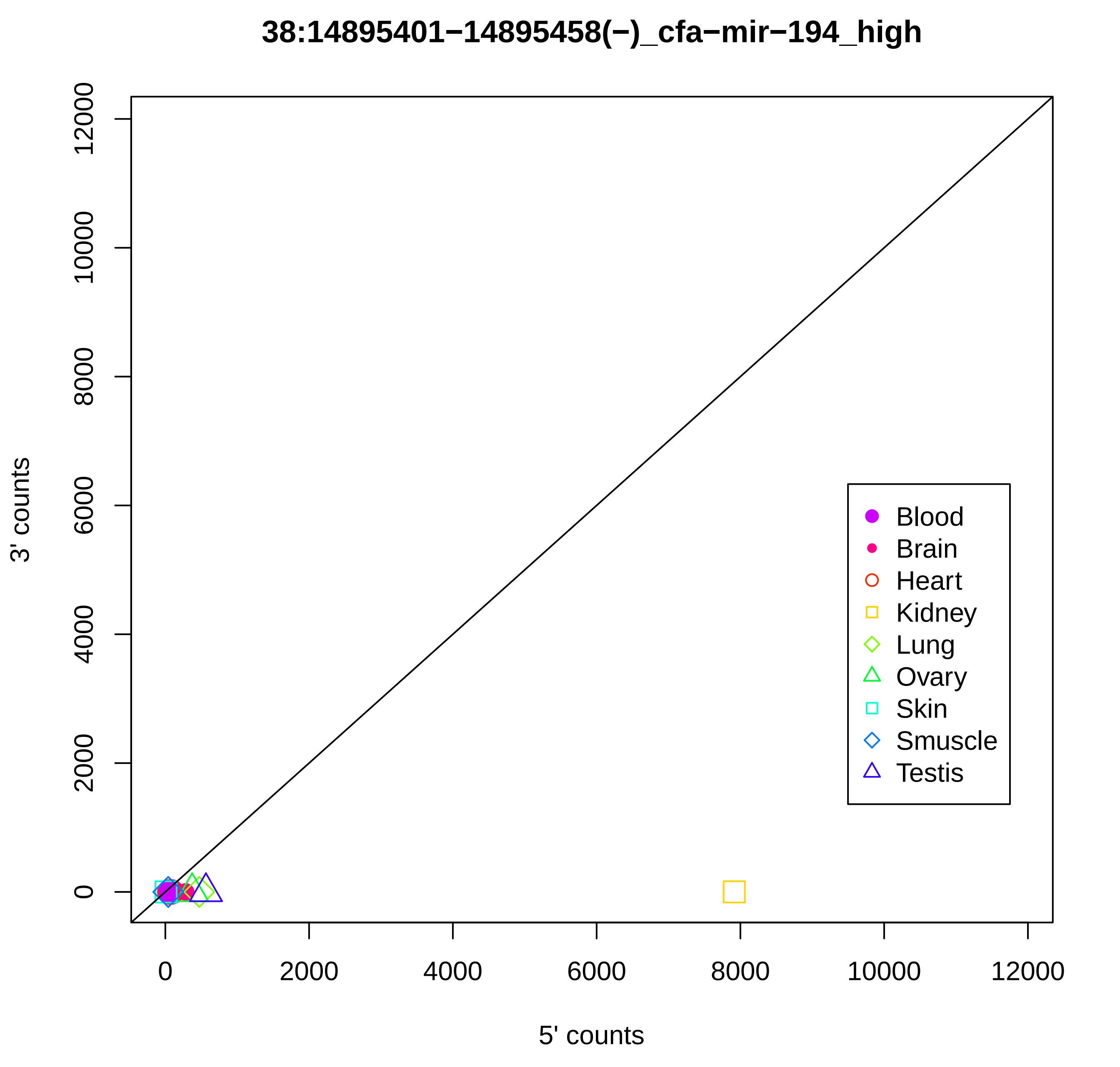

38:14895401−14895458(−)_cfa−mir−194_high
3' counts
5' counts
0
2000
4000
6000
8000
10000
12000
Blood
Brain
Heart
Kidney
Lung
Ovary
Skin
Smuscle
Testis

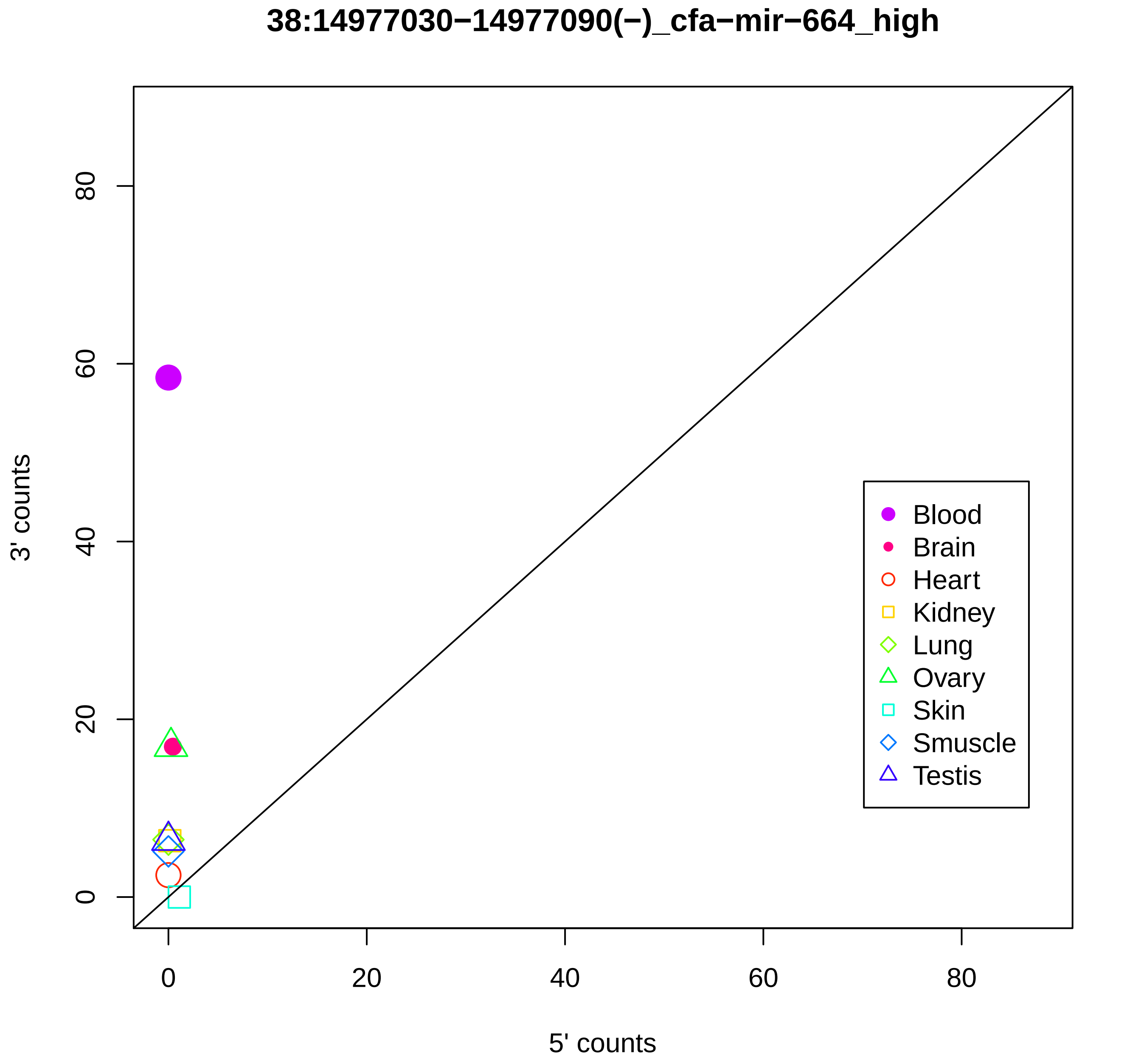
38:14977030−14977090(−)_cfa−mir−664_high
3' counts
5' counts
0
20
40
60
80
0
20
40
60
80
Blood
Brain
Heart
Kidney
Lung
Ovary
Skin
Smuscle
Testis

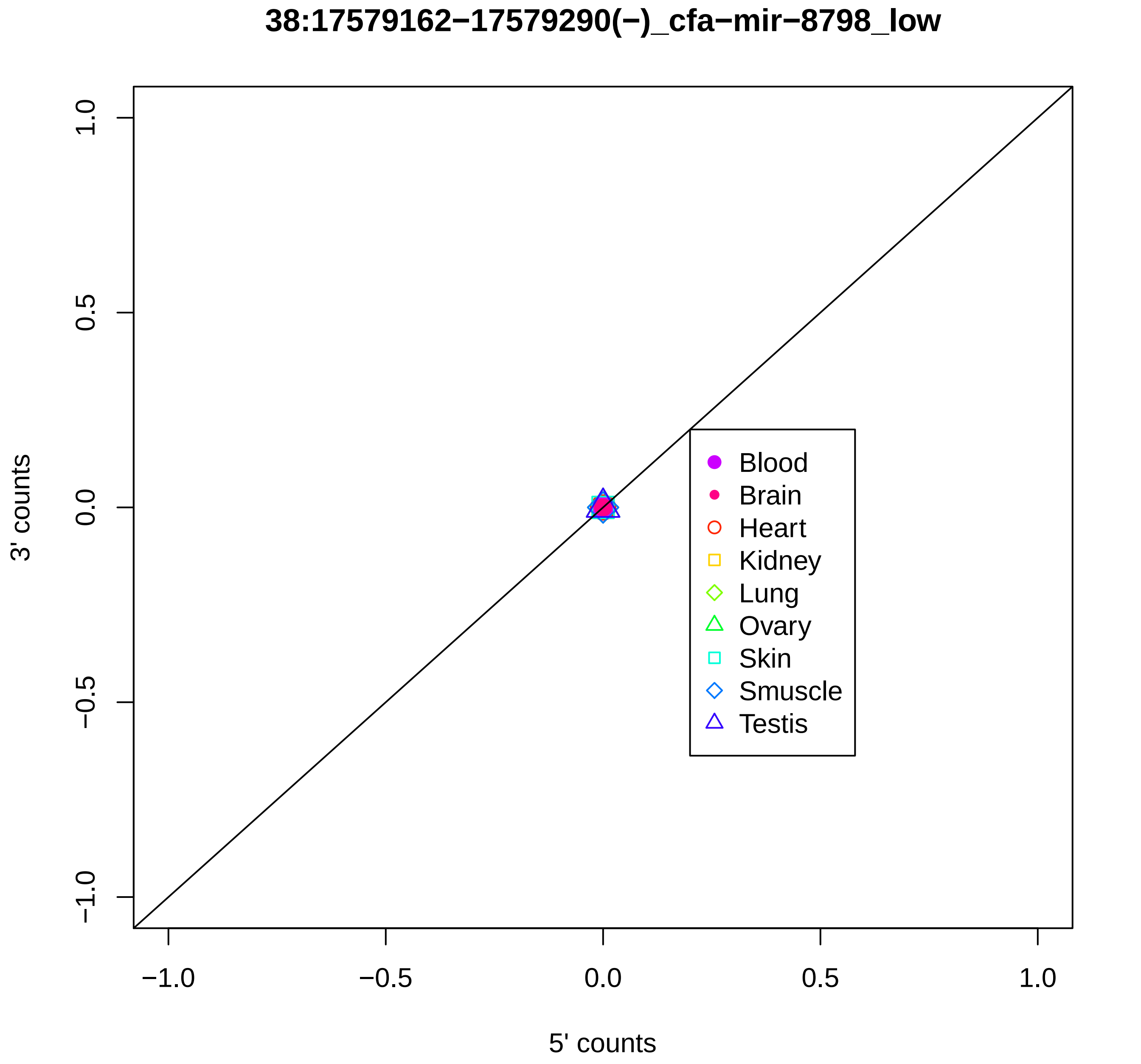

38:17579162−17579290(−)_cfa−mir−8798_low
3' counts
5' counts
−1.0
−0.5
0.0
0.5
1.0
Blood
Brain
Heart
Kidney
Lung
Ovary
Skin
Smuscle
Testis

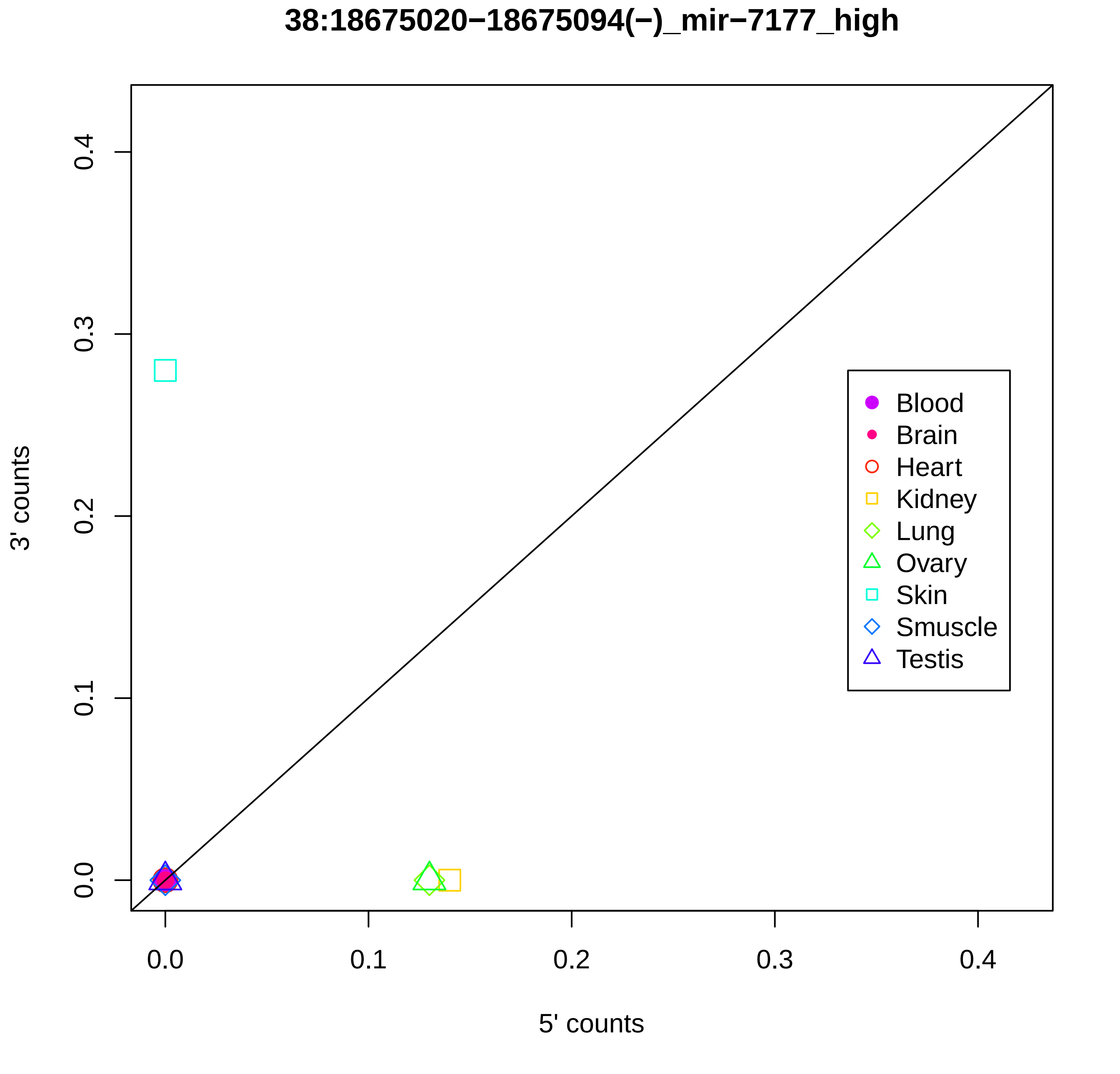

38:18675020−18675094(−)_mir−7177_high
3' counts
5' counts
0.0
0.1
0.2
0.3
0.4
Blood
Brain
Heart
Kidney
Lung
Ovary
Skin
Smuscle
Testis

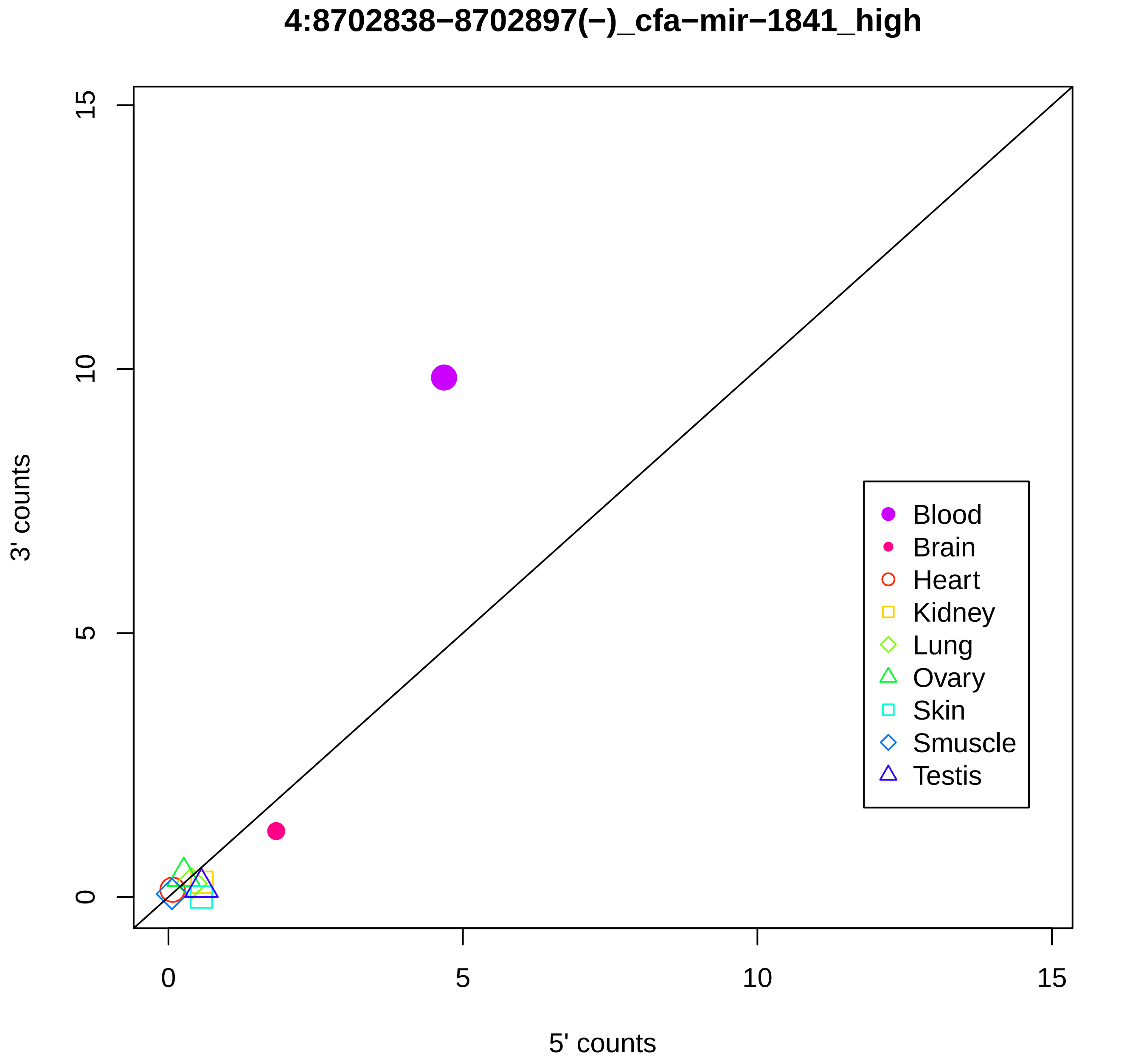

4:8702838−8702897(−)_cfa−mir−1841_high
3' counts
5' counts
0
5
10
15
0
5
10
15
Blood
Brain
Heart
Kidney
Lung
Ovary
Skin
Smuscle
Testis

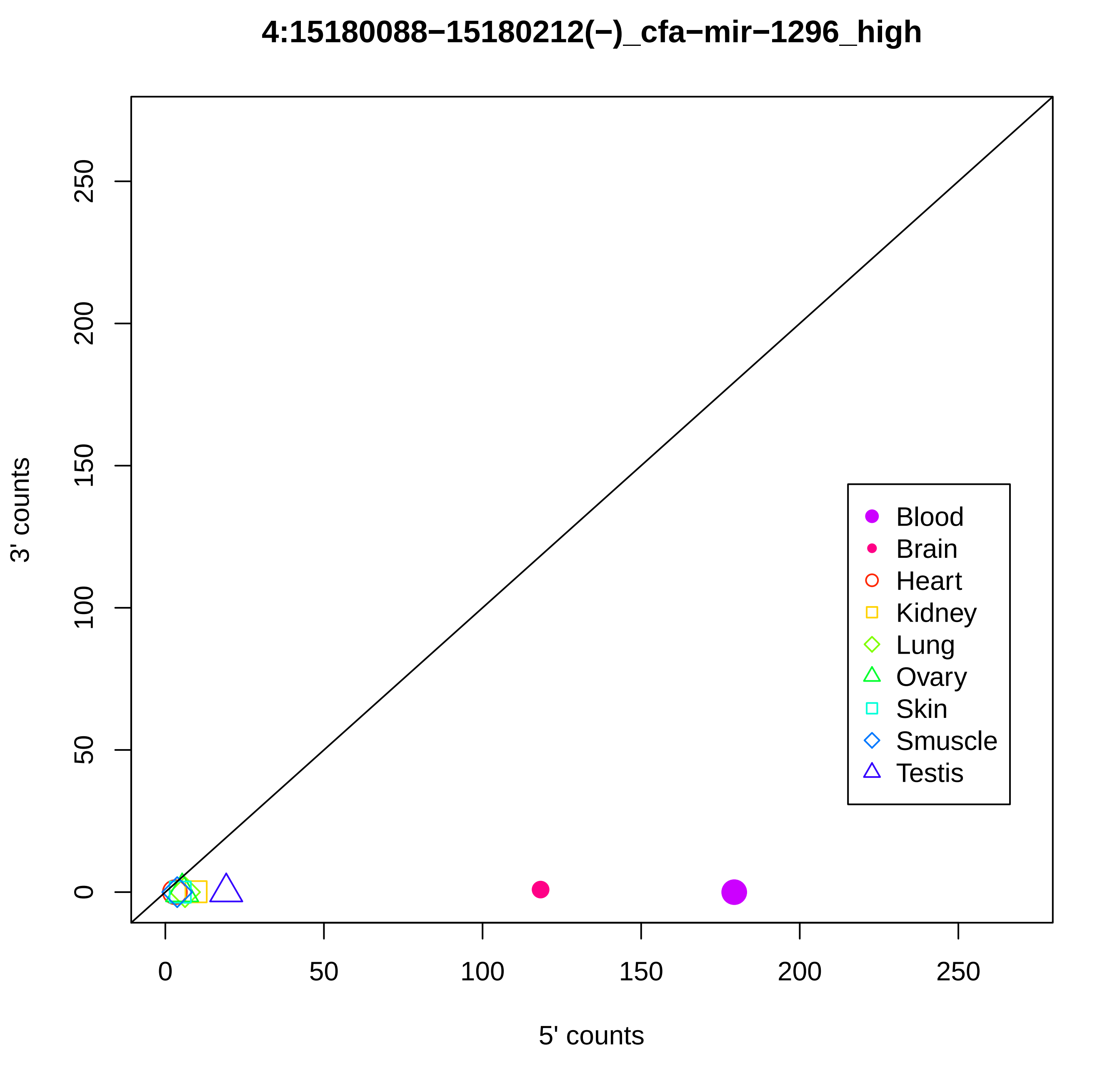

4:15180088−15180212(−)_cfa−mir−1296_high
3' counts
5' counts
0
50
100
150
200
250
Blood
Brain
Heart
Kidney
Lung
Ovary
Skin
Smuscle
Testis

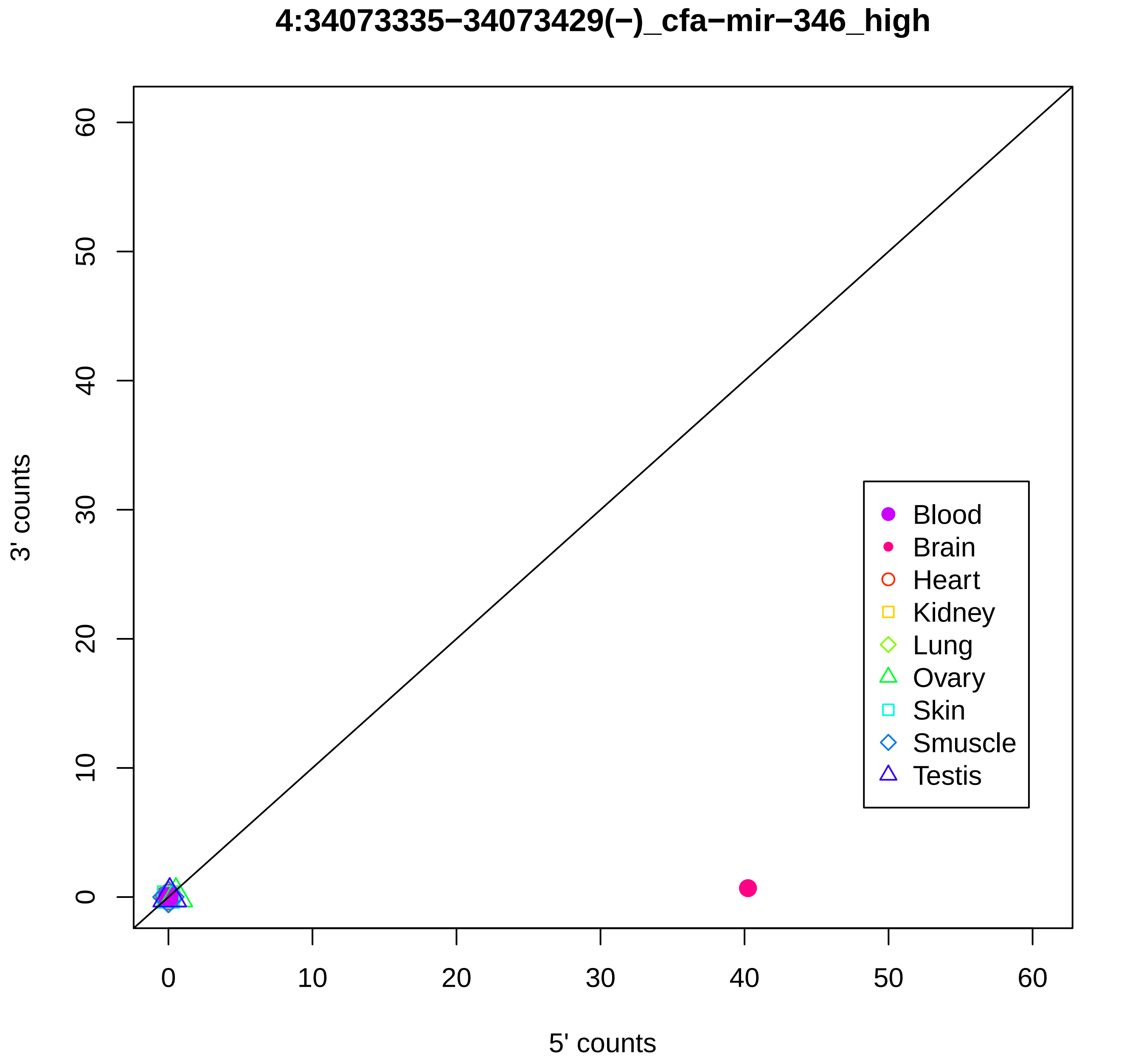

4:34073335−34073429(−)_cfa−mir−346_high
3' counts
5' counts
0
10
20
30
40
50
60
Blood
Brain
Heart
Kidney
Lung
Ovary
Skin
Smuscle
Testis

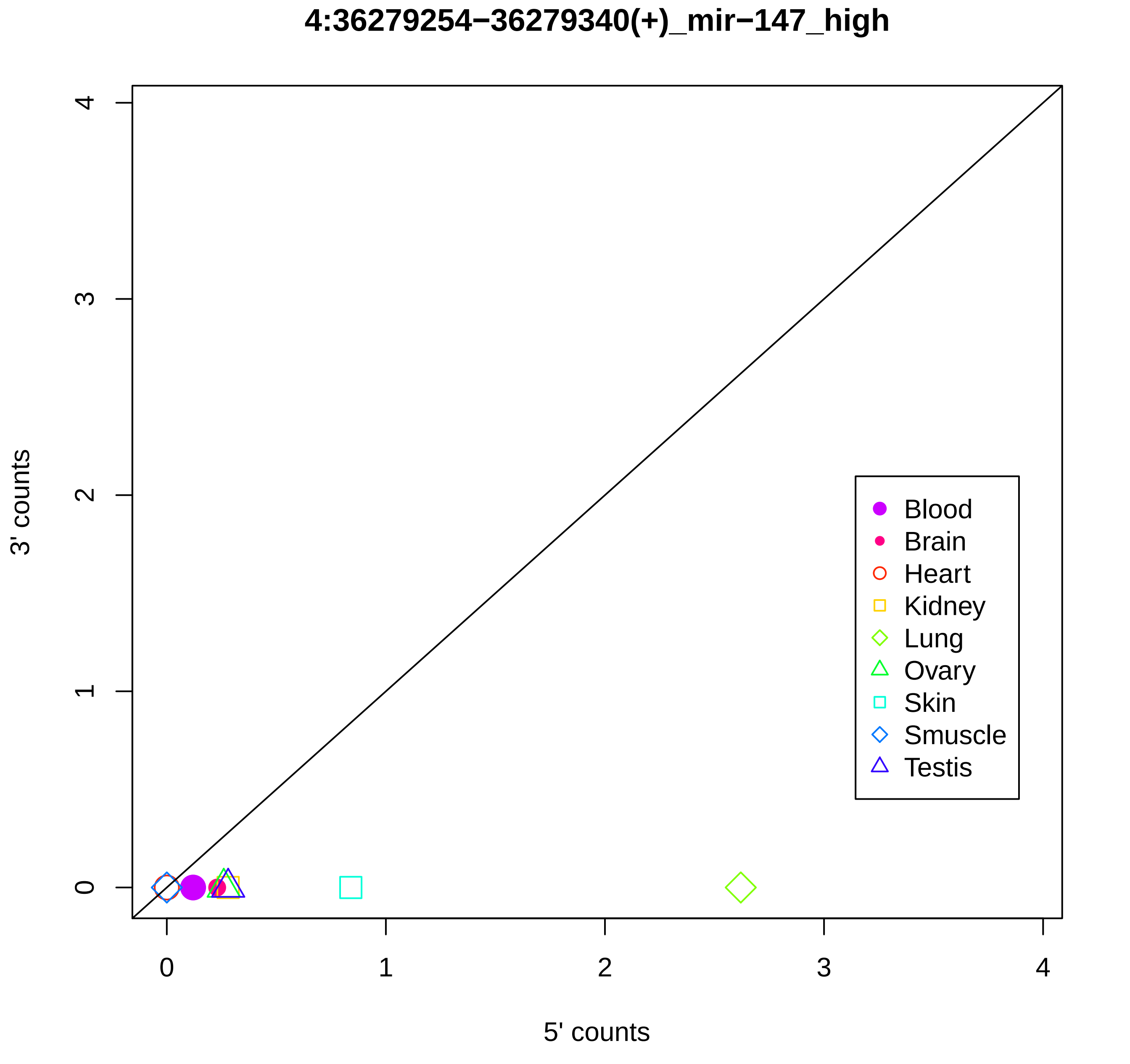
4:36279254−36279340(+)_mir−147_high
3' counts
5' counts
0
1
2
3
4
Blood
Brain
Heart
Kidney
Lung
Ovary
Skin
Smuscle
Testis

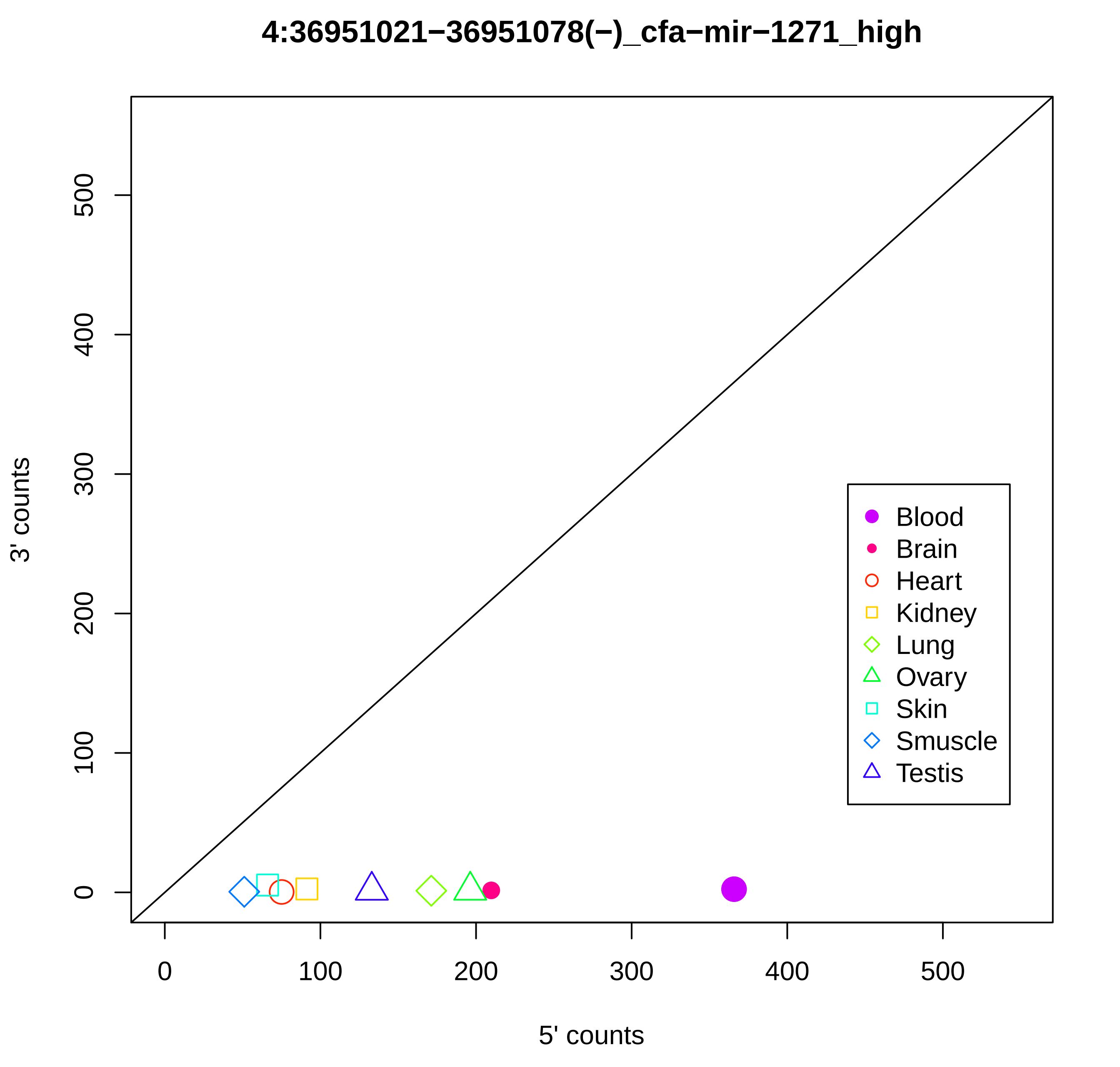
4:36951021−36951078(−)_cfa−mir−1271_high
3' counts
5' counts
0
100
200
300
400
500
0
100
200
300
400
500
Blood
Brain
Heart
Kidney
Lung
Ovary
Skin
Smuscle
Testis

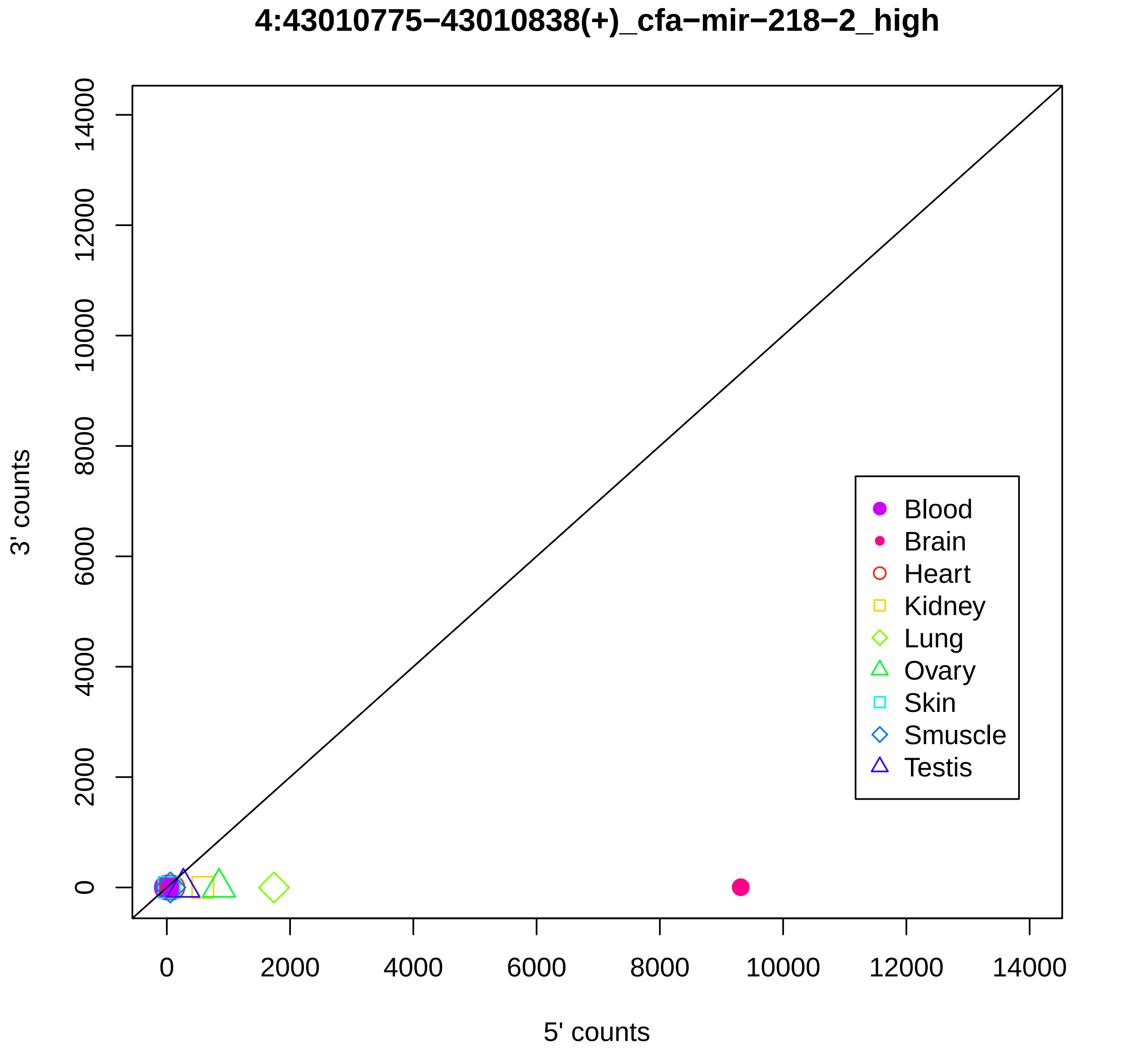
4:43010775−43010838(+)_cfa−mir−218−2_high
3' counts
5' counts
0
2000
4000
6000
8000
10000
12000
14000
Blood
Brain
Heart
Kidney
Lung
Ovary
Skin
Smuscle
Testis

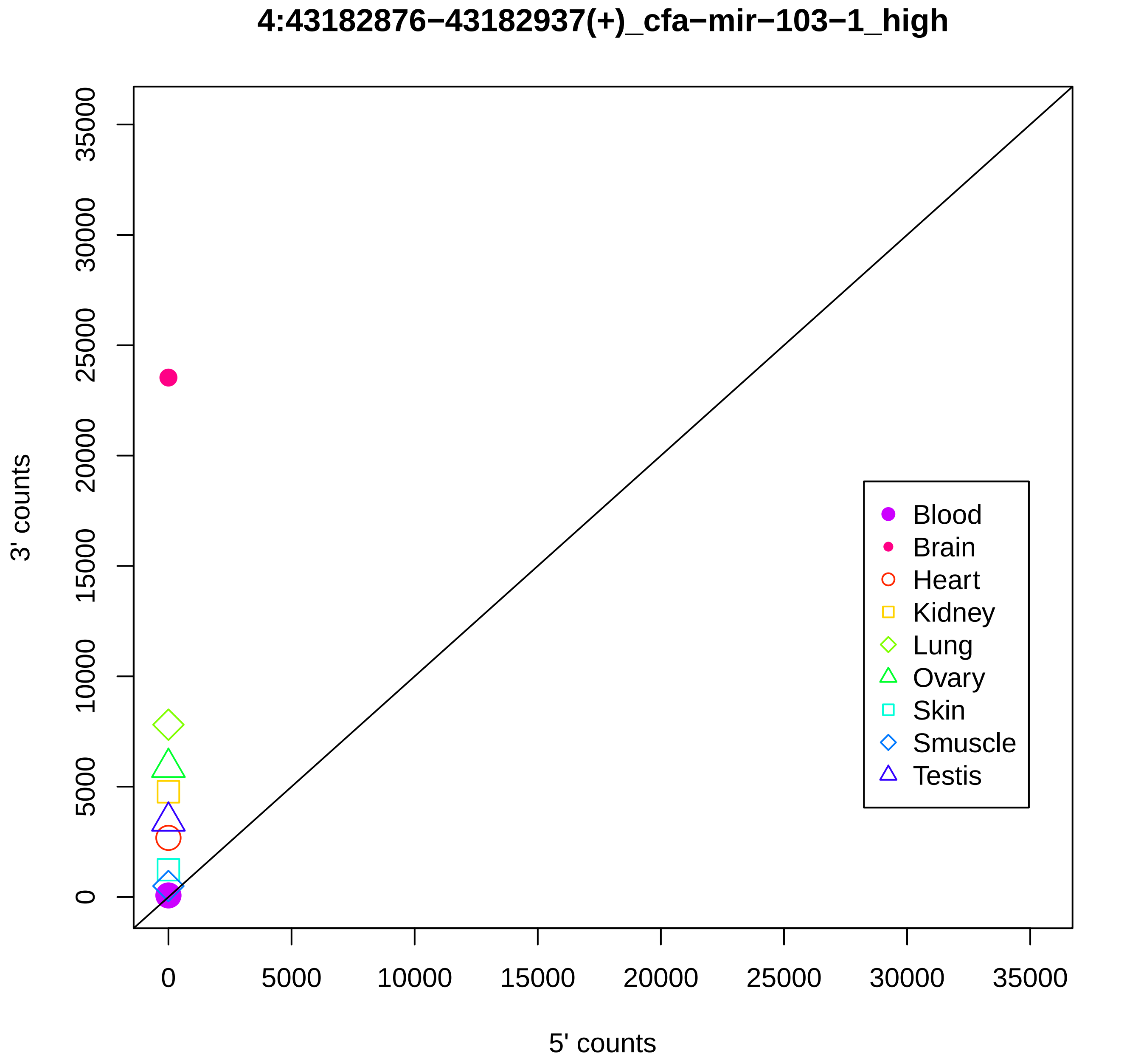
4:43182876−43182937(+)_cfa−mir−103−1_high
3' counts
5' counts
0
5000
10000
15000
20000
25000
30000
35000
Blood
Brain
Heart
Kidney
Lung
Ovary
Skin
Smuscle
Testis

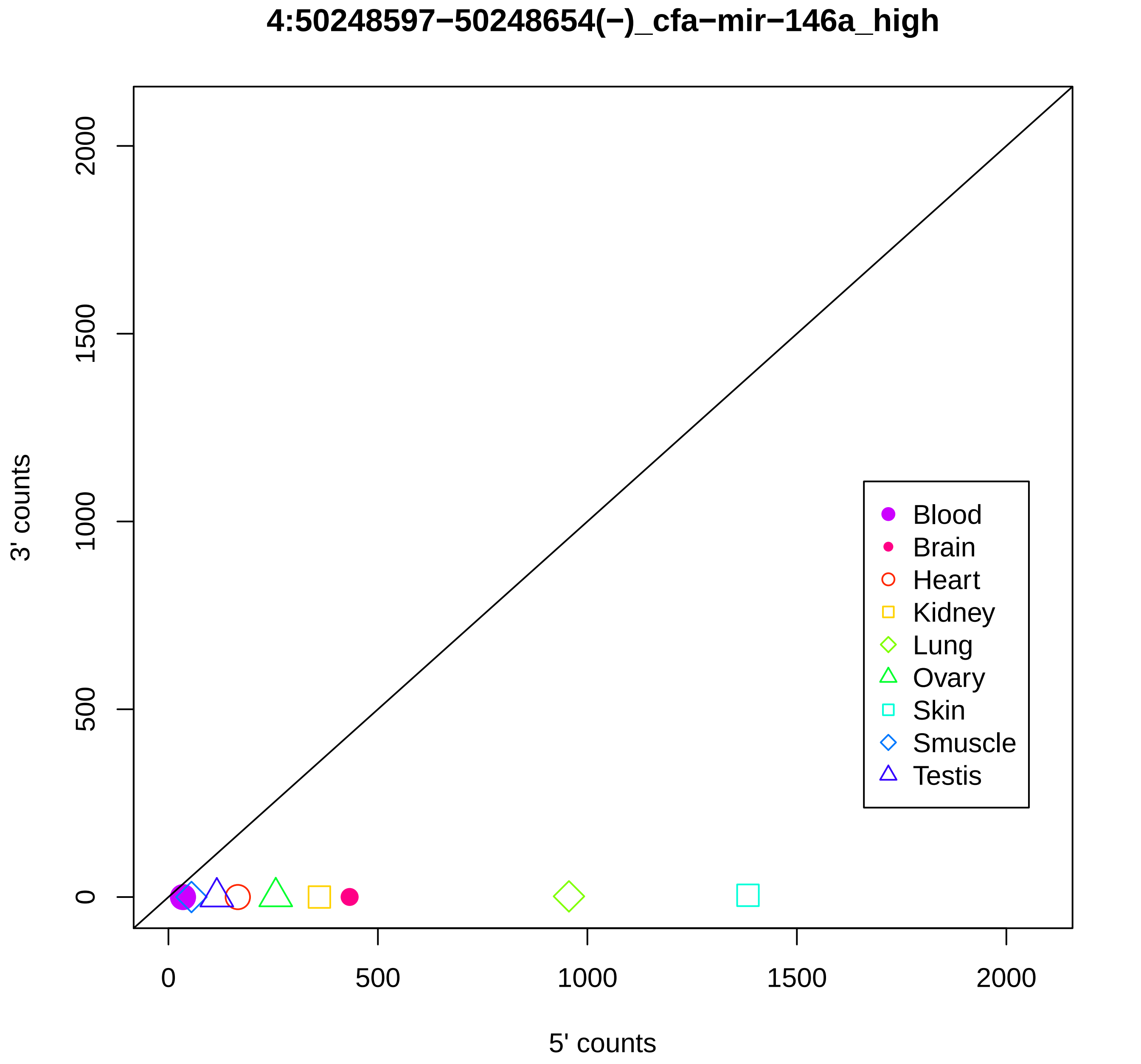
4:50248597−50248654(−)_cfa−mir−146a_high
3' counts
5' counts
0
500
1000
1500
2000
Blood
Brain
Heart
Kidney
Lung
Ovary
Skin
Smuscle
Testis

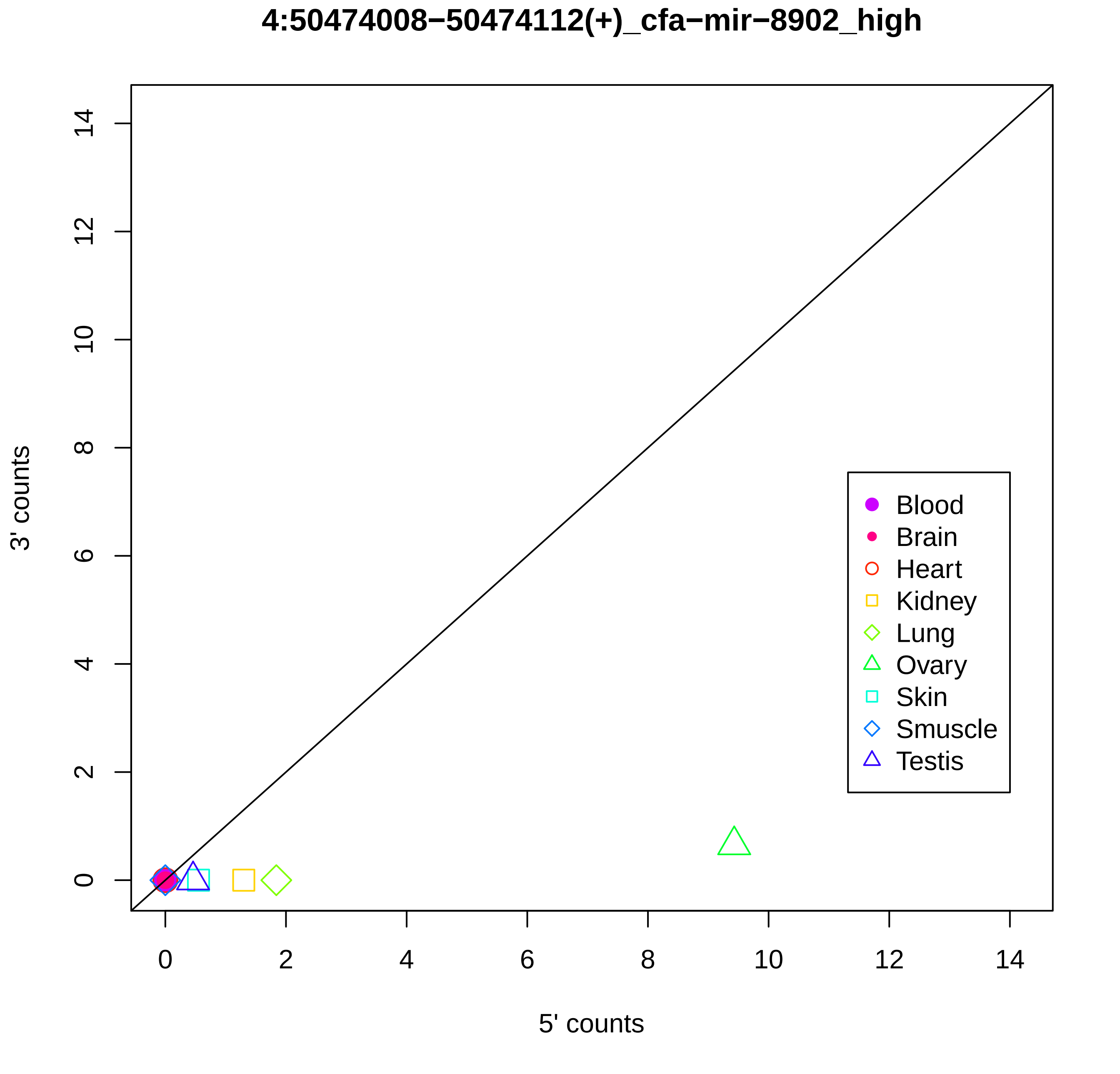

4:50474008−50474112(+)_cfa−mir−8902_high
3' counts
5' counts
0
2
4
6
8
10
12
14
Blood
Brain
Heart
Kidney
Lung
Ovary
Skin
Smuscle
Testis

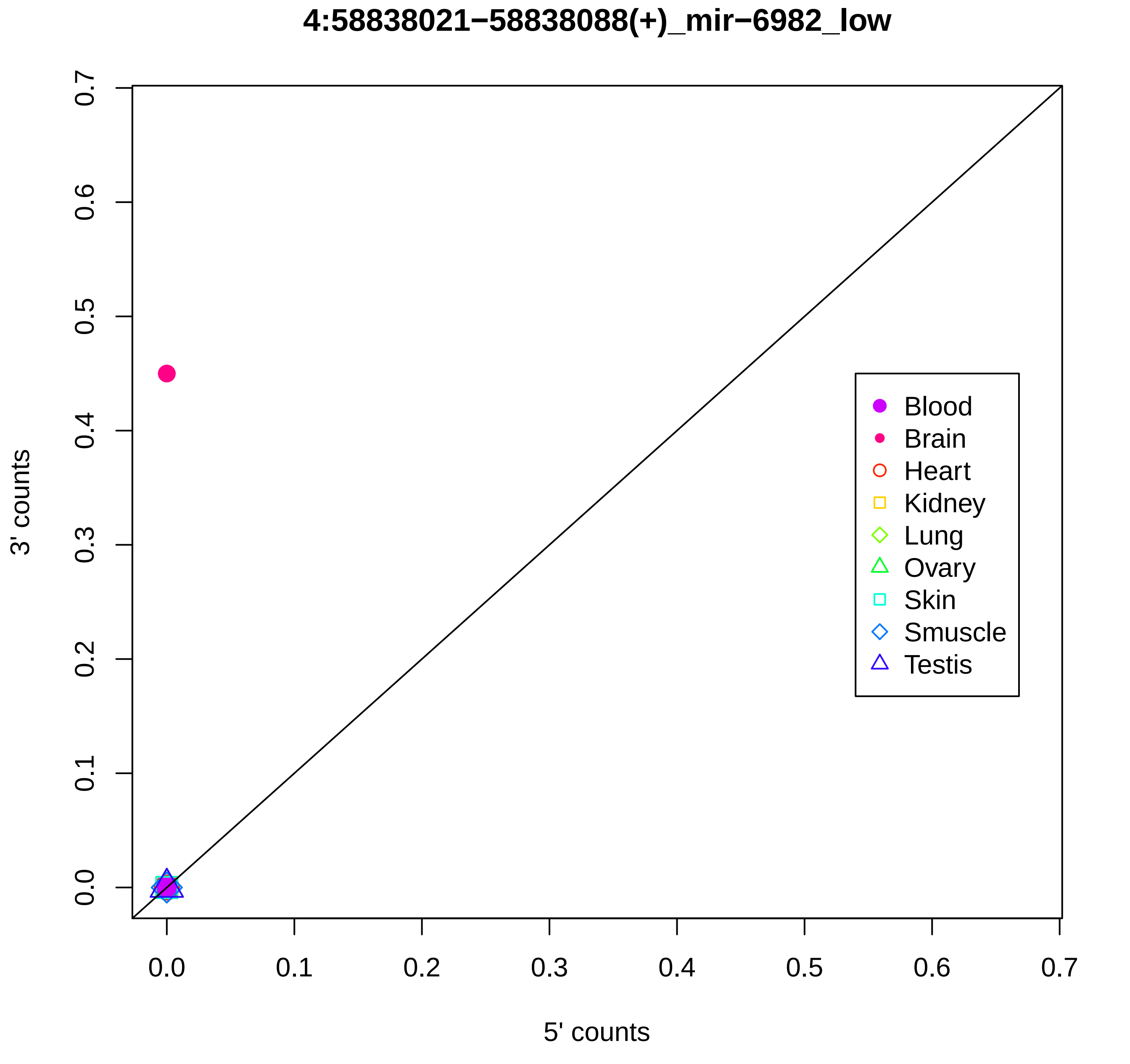

4:58838021−58838088(+)_mir−6982_low
3' counts
5' counts
0.0
0.1
0.2
0.3
0.4
0.5
0.6
0.7
Blood
Brain
Heart
Kidney
Lung
Ovary
Skin
Smuscle
Testis

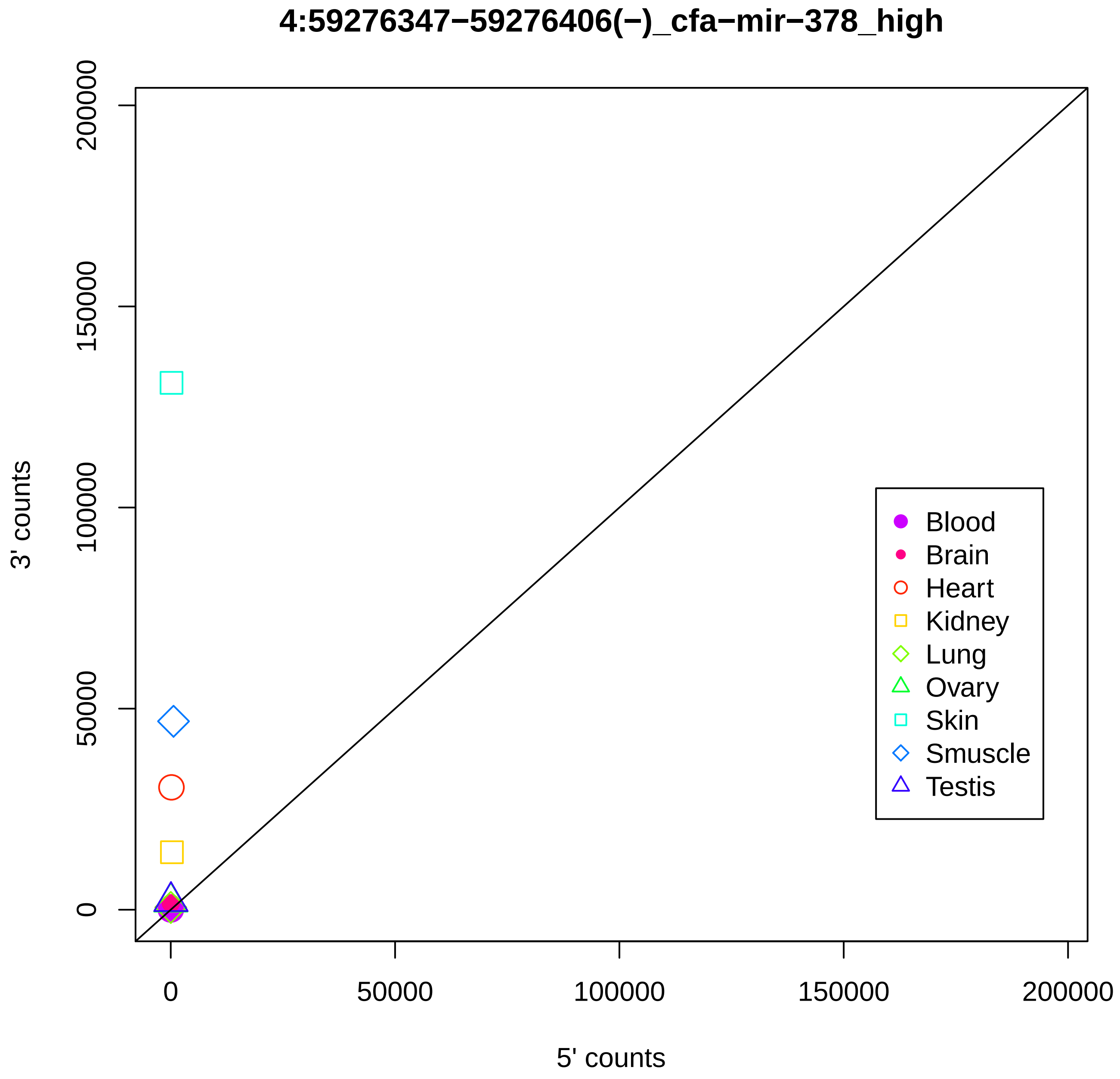

4:59276347−59276406(−)_cfa−mir−378_high
3' counts
5' counts
0
50000
100000
150000
200000
Blood
Brain
Heart
Kidney
Lung
Ovary
Skin
Smuscle
Testis

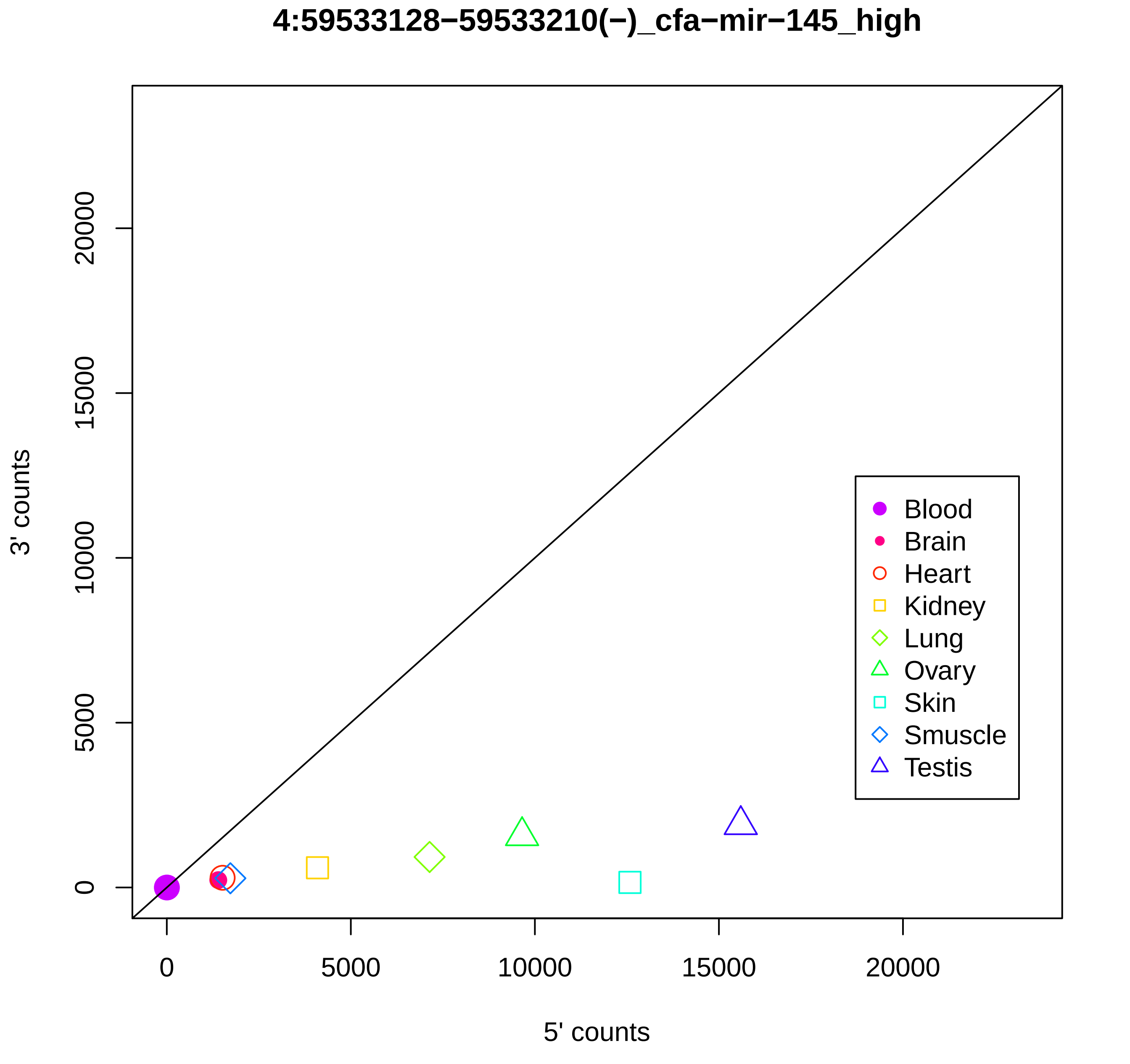
4:59533128−59533210(−)_cfa−mir−145_high
3' counts
5' counts
0
5000
10000
15000
20000
Blood
Brain
Heart
Kidney
Lung
Ovary
Skin
Smuscle
Testis

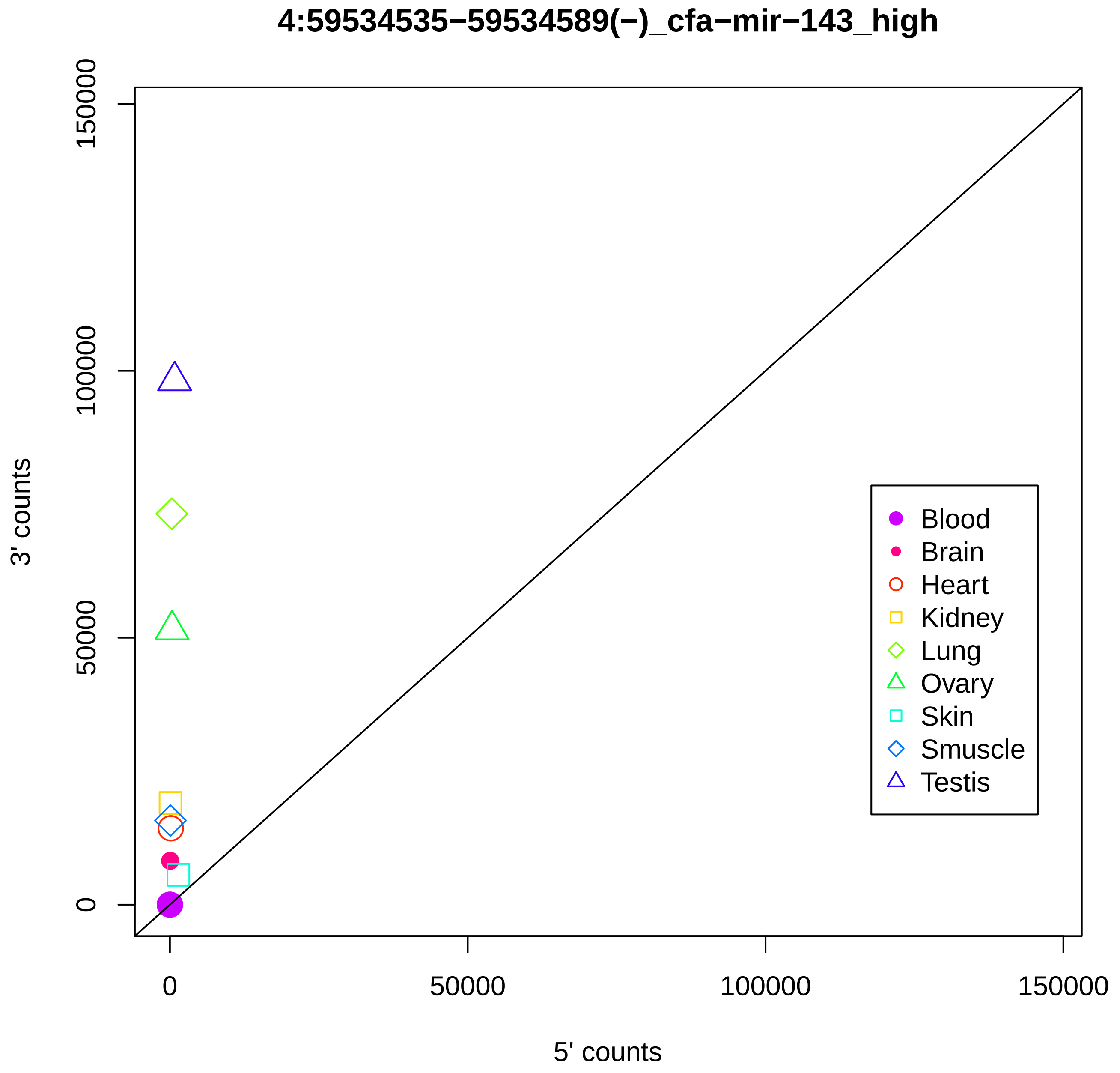

4:59534535−59534589(−)_cfa−mir−143_high
3' counts
5' counts
0
50000
100000
150000
Blood
Brain
Heart
Kidney
Lung
Ovary
Skin
Smuscle
Testis

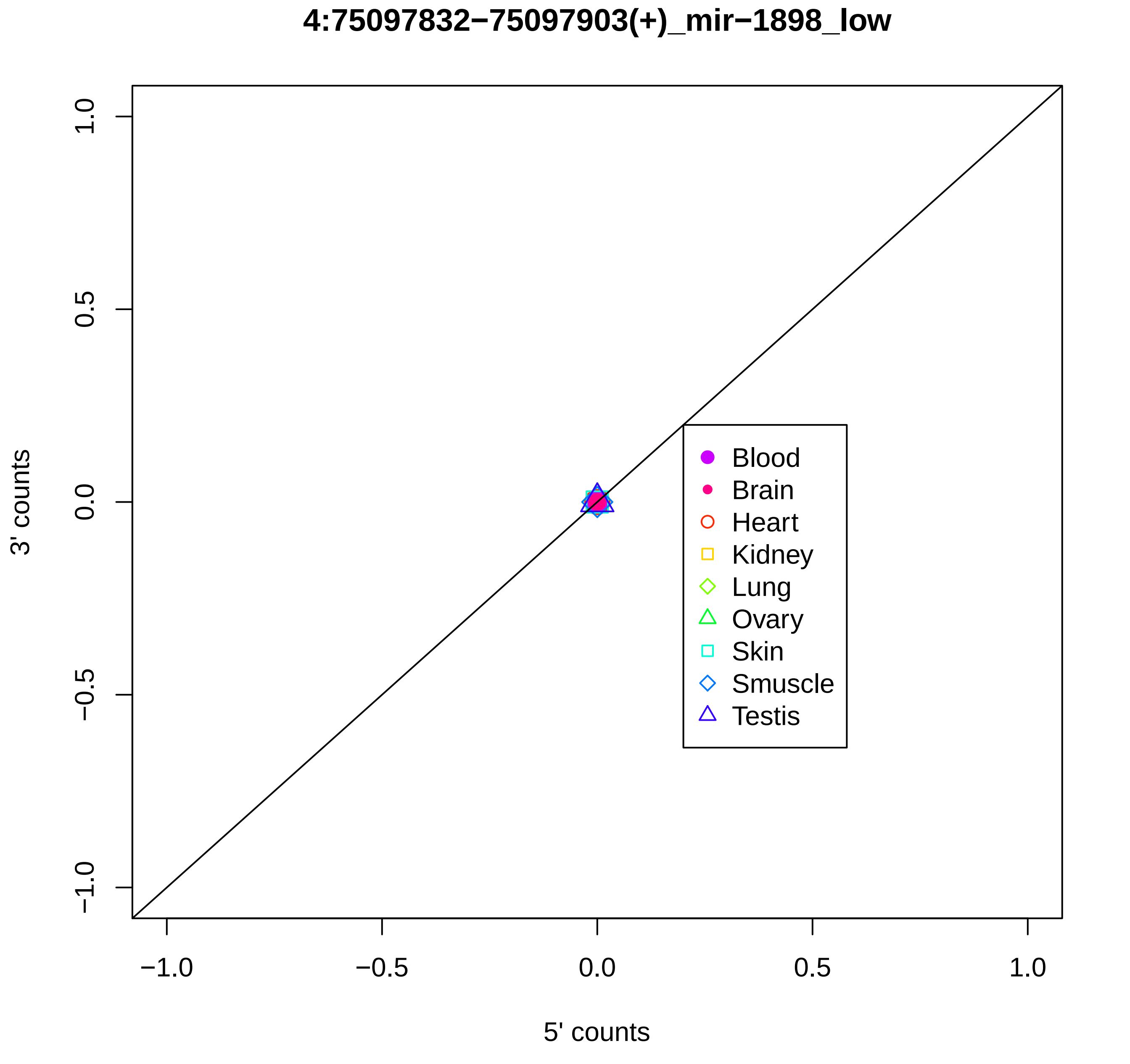
4:75097832−75097903(+)_mir−1898_low
3' counts
5' counts
1.0
0.5
0.0
−0.5
−1.0
−1.0
−0.5
0.0
0.5
1.0
Blood
Brain
Heart
Kidney
Lung
Ovary
Skin
Smuscle
Testis

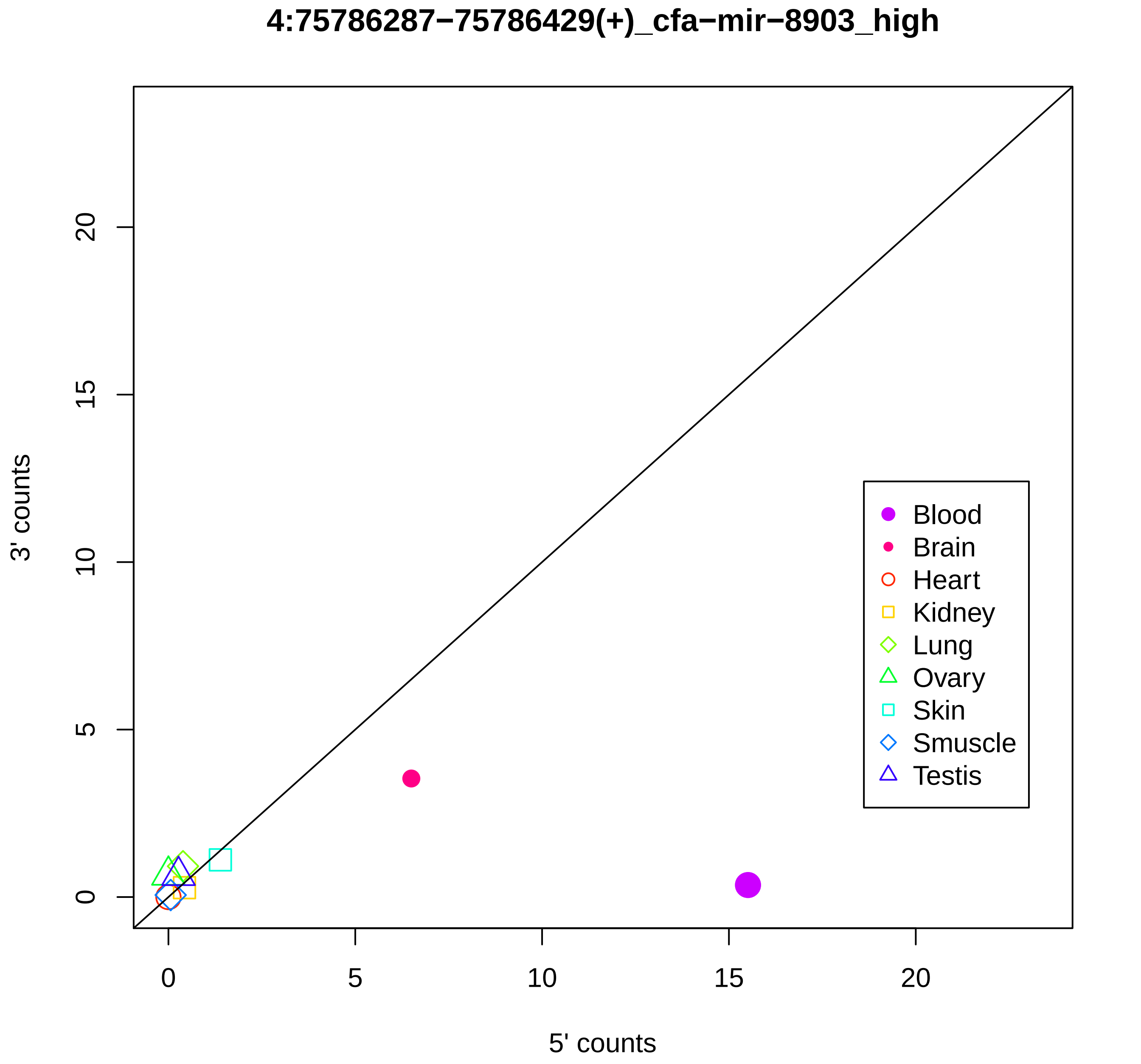

4:75786287−75786429(+)_cfa−mir−8903_high
3' counts
5' counts
0
5
10
15
20
0
5
10
15
20
Blood
Brain
Heart
Kidney
Lung
Ovary
Skin
Smuscle
Testis

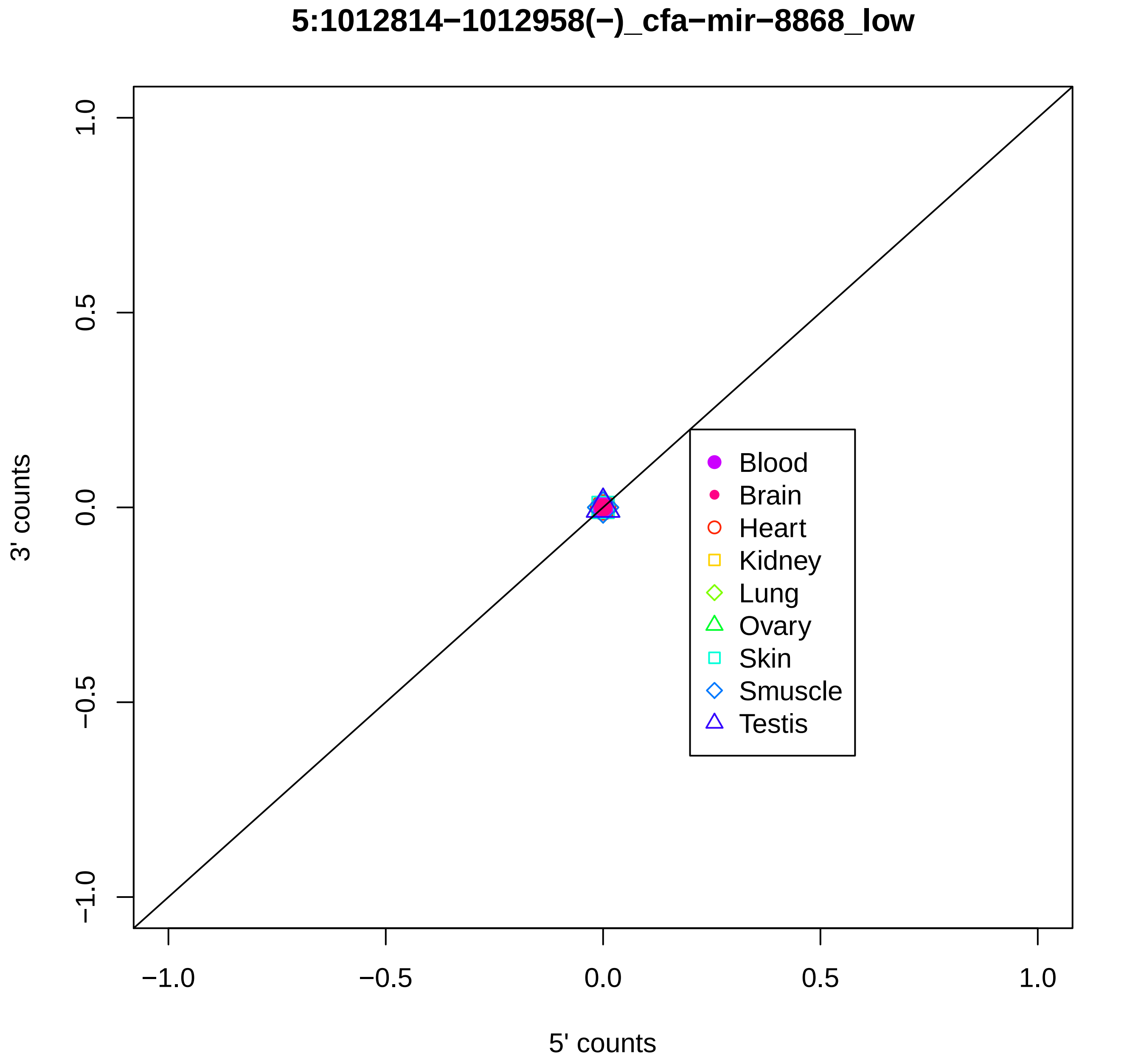
5:1012814−1012958(−)_cfa−mir−8868_low
3' counts
5' counts
1.0
0.5
0.0
−0.5
−1.0
−1.0
−0.5
0.0
0.5
1.0
Blood
Brain
Heart
Kidney
Lung
Ovary
Skin
Smuscle
Testis

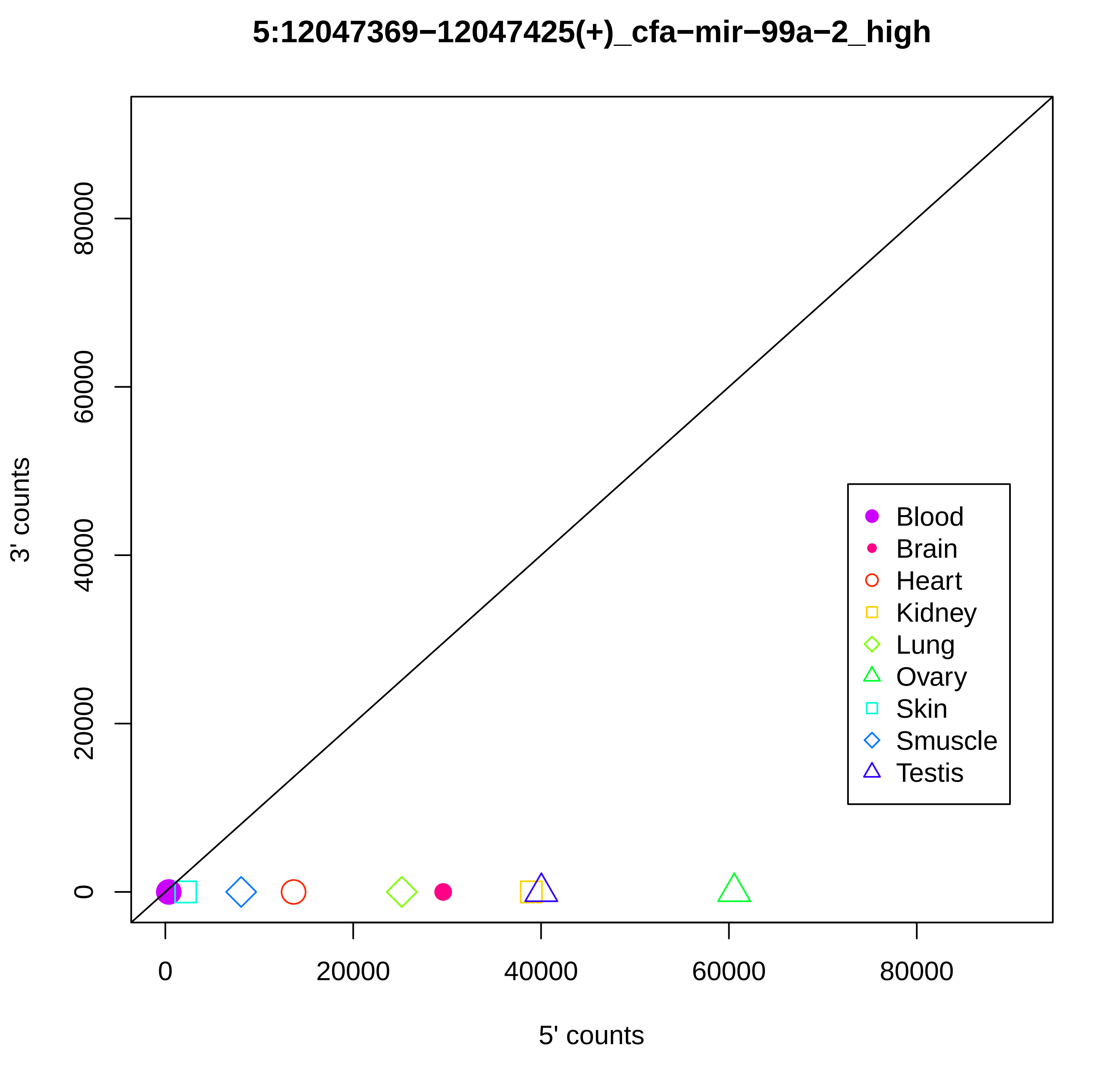

5:12047369−12047425(+)_cfa−mir−99a−2_high
3' counts
5' counts
0
20000
40000
60000
80000
Blood
Brain
Heart
Kidney
Lung
Ovary
Skin
Smuscle
Testis

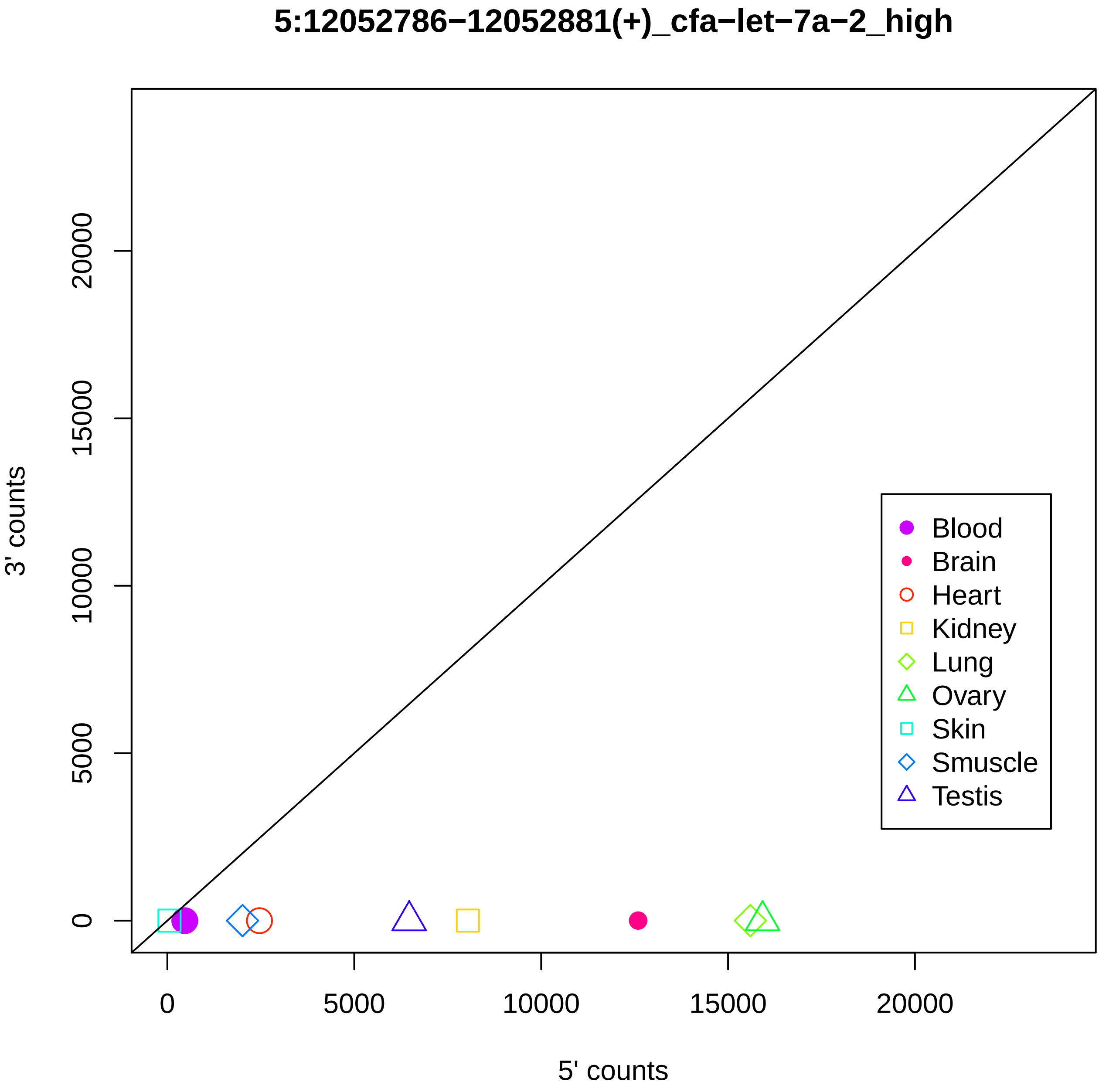

5:12052786−12052881(+)_cfa−let−7a−2_high
3' counts
20000
15000
10000
5000
0
0
5000
10000
15000
20000
5' counts
Blood
Brain
Heart
Kidney
Lung
Ovary
Skin
Smuscle
Testis

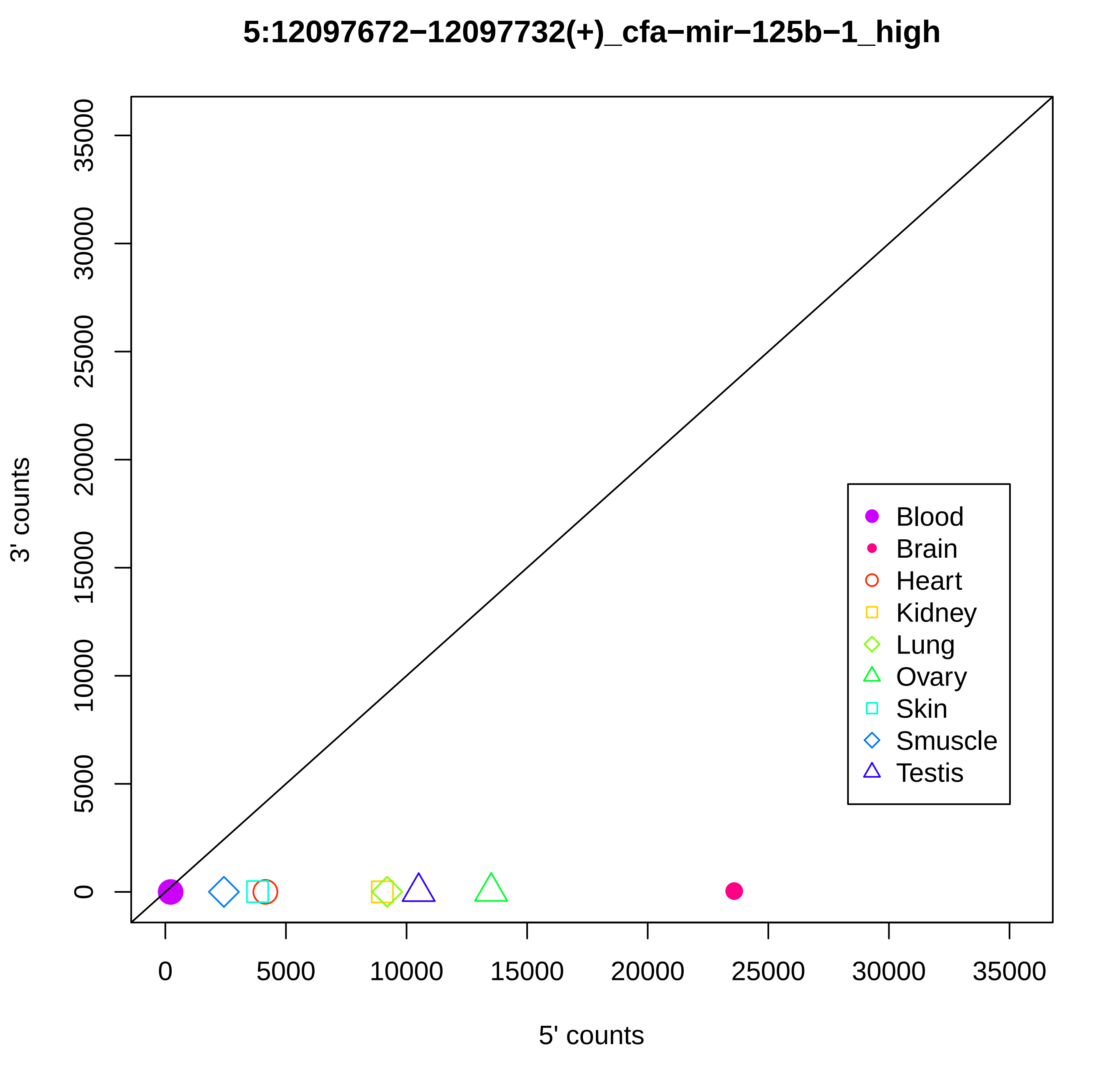

5:12097672−12097732(+)_cfa−mir−125b−1_high
3' counts
5' counts
0
5000
10000
15000
20000
25000
30000
35000
Blood
Brain
Heart
Kidney
Lung
Ovary
Skin
Smuscle
Testis

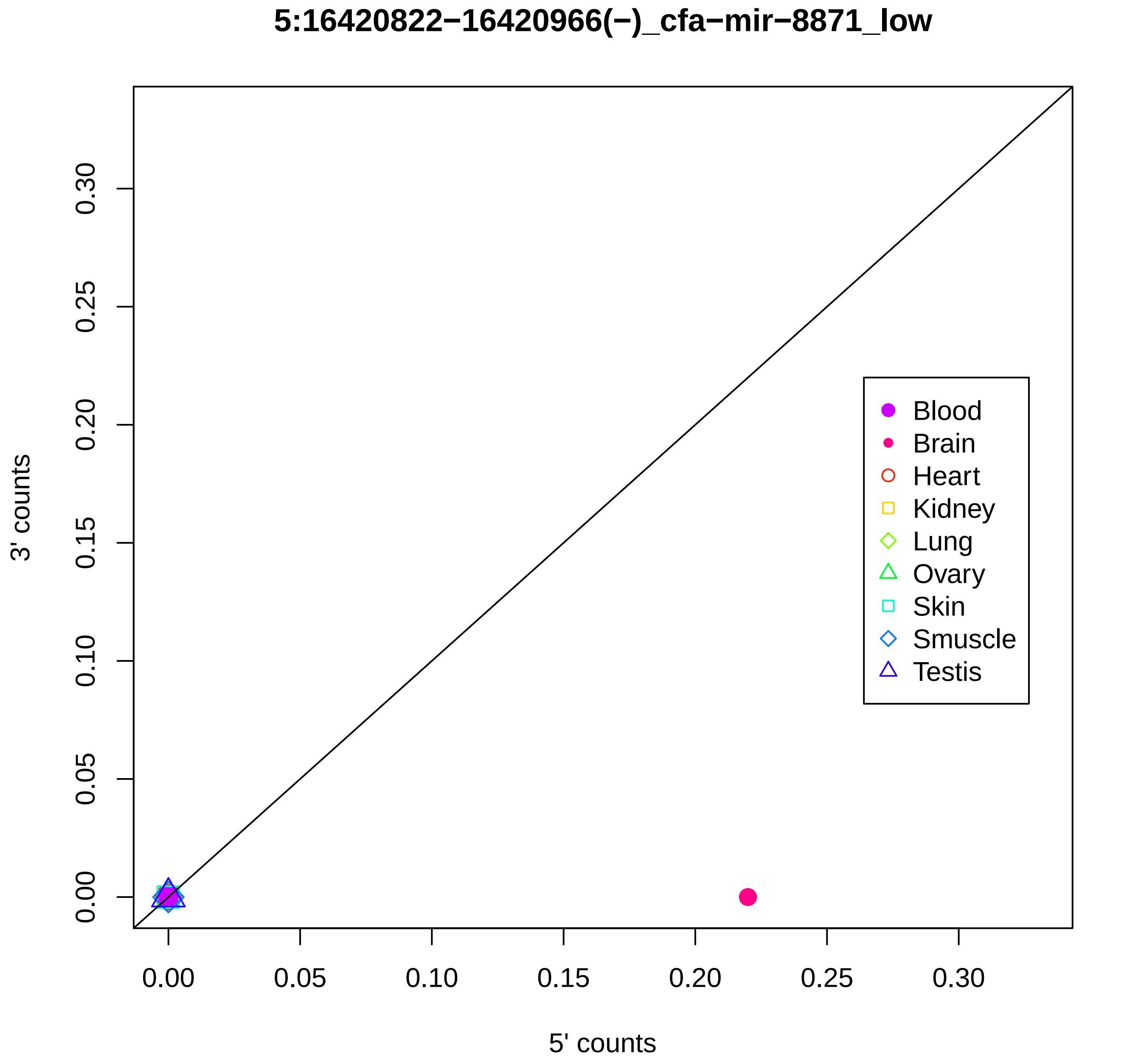
5:16420822−16420966(−)_cfa−mir−8871_low
3' counts
5' counts
0.00
0.05
0.10
0.15
0.20
0.25
0.30
Blood
Brain
Heart
Kidney
Lung
Ovary
Skin
Smuscle
Testis

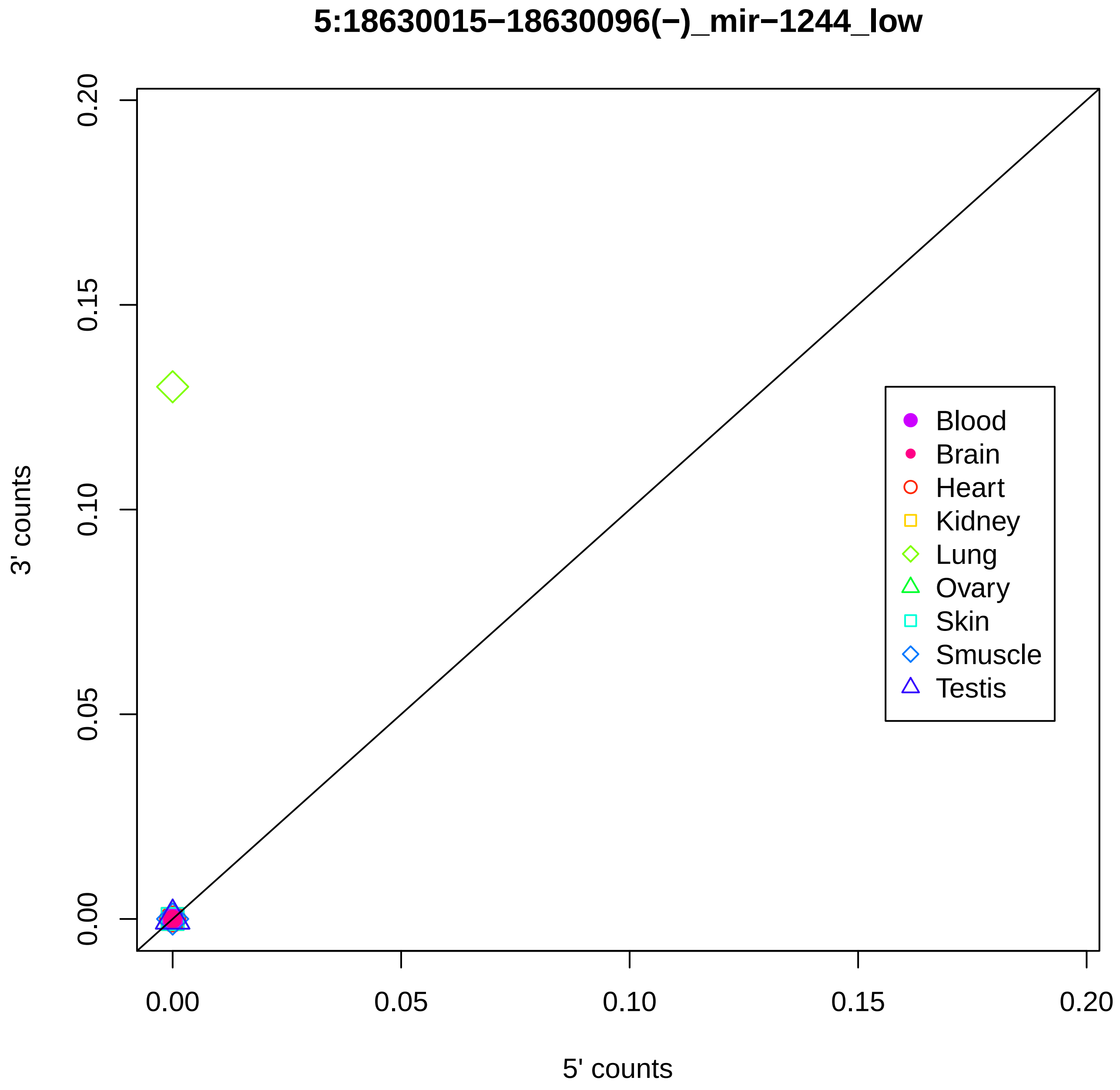

5:18630015−18630096(−)_mir−1244_low
3' counts
5' counts
0.00
0.05
0.10
0.15
0.20
Blood
Brain
Heart
Kidney
Lung
Ovary
Skin
Smuscle
Testis

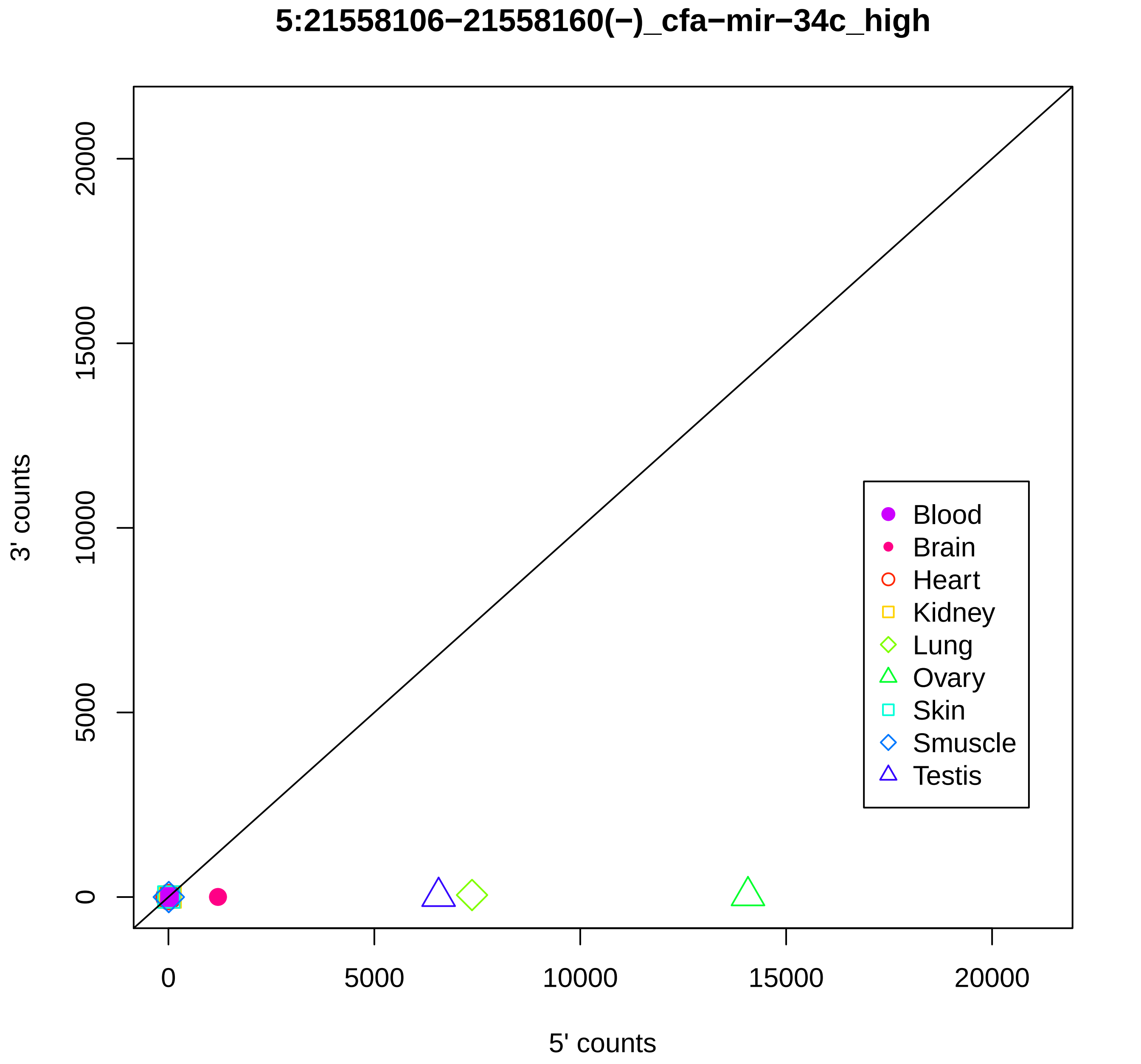
5:21558106−21558160(−)_cfa−mir−34c_high
3' counts
0
5000
10000
15000
20000
5' counts
0
5000
10000
15000
20000
Blood
Brain
Heart
Kidney
Lung
Ovary
Skin
Smuscle
Testis

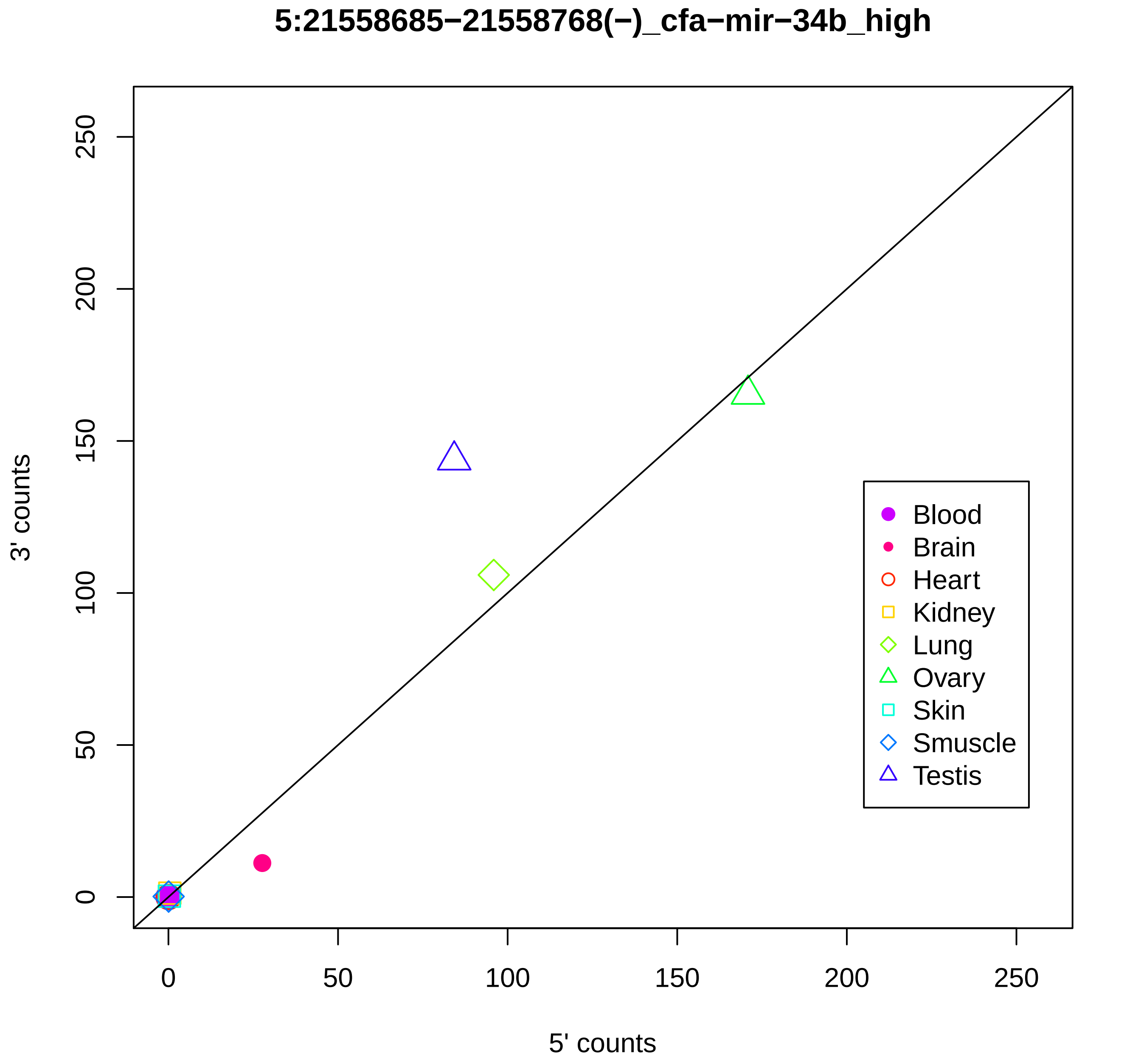
5:21558685−21558768(−)_cfa−mir−34b_high
3' counts
5' counts
0
50
100
150
200
250
Blood
Brain
Heart
Kidney
Lung
Ovary
Skin
Smuscle
Testis

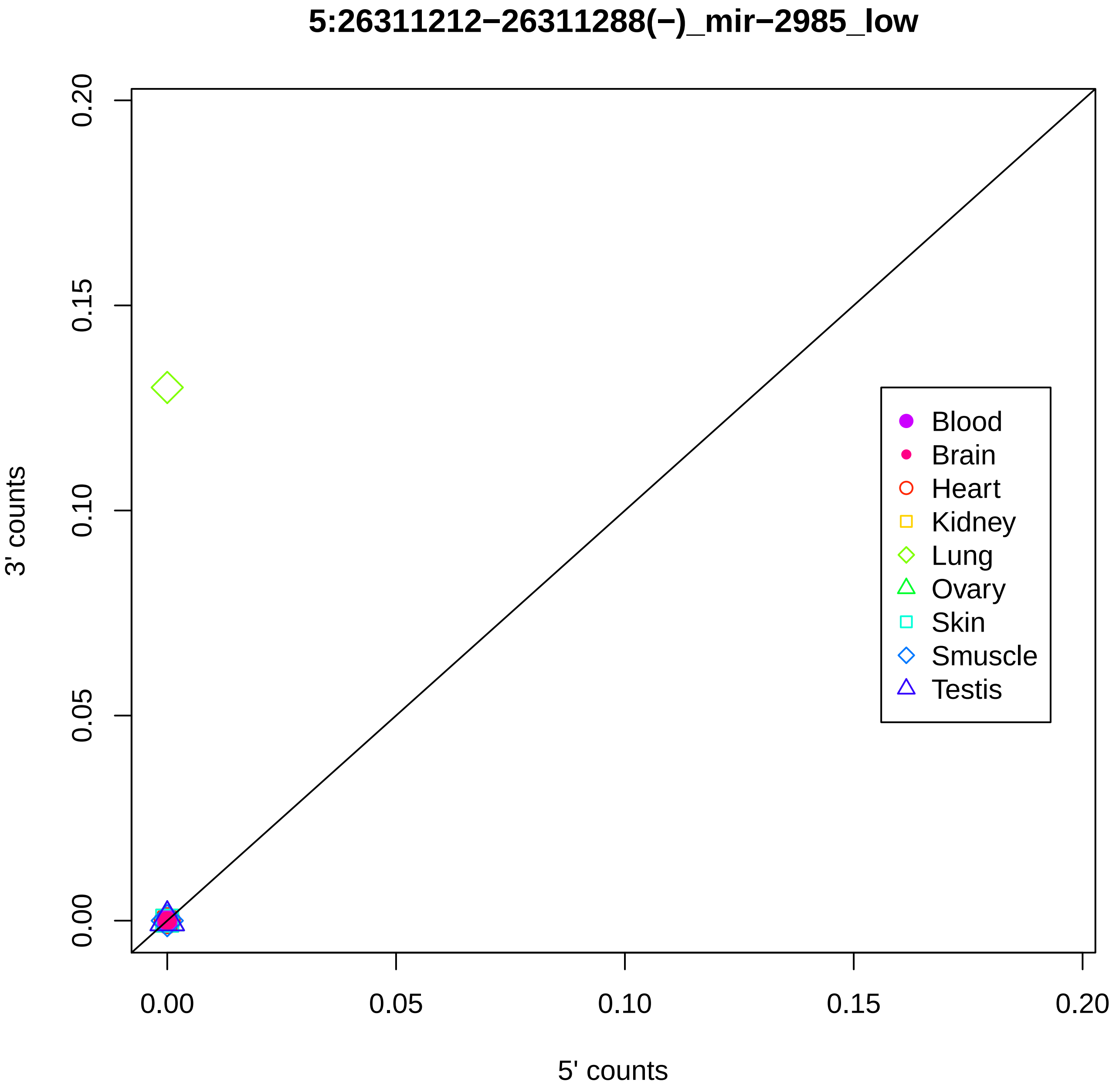
5:26311212−26311288(−)_mir−2985_low
3' counts
5' counts
0.00
0.05
0.10
0.15
0.20
Blood
Brain
Heart
Kidney
Lung
Ovary
Skin
Smuscle
Testis

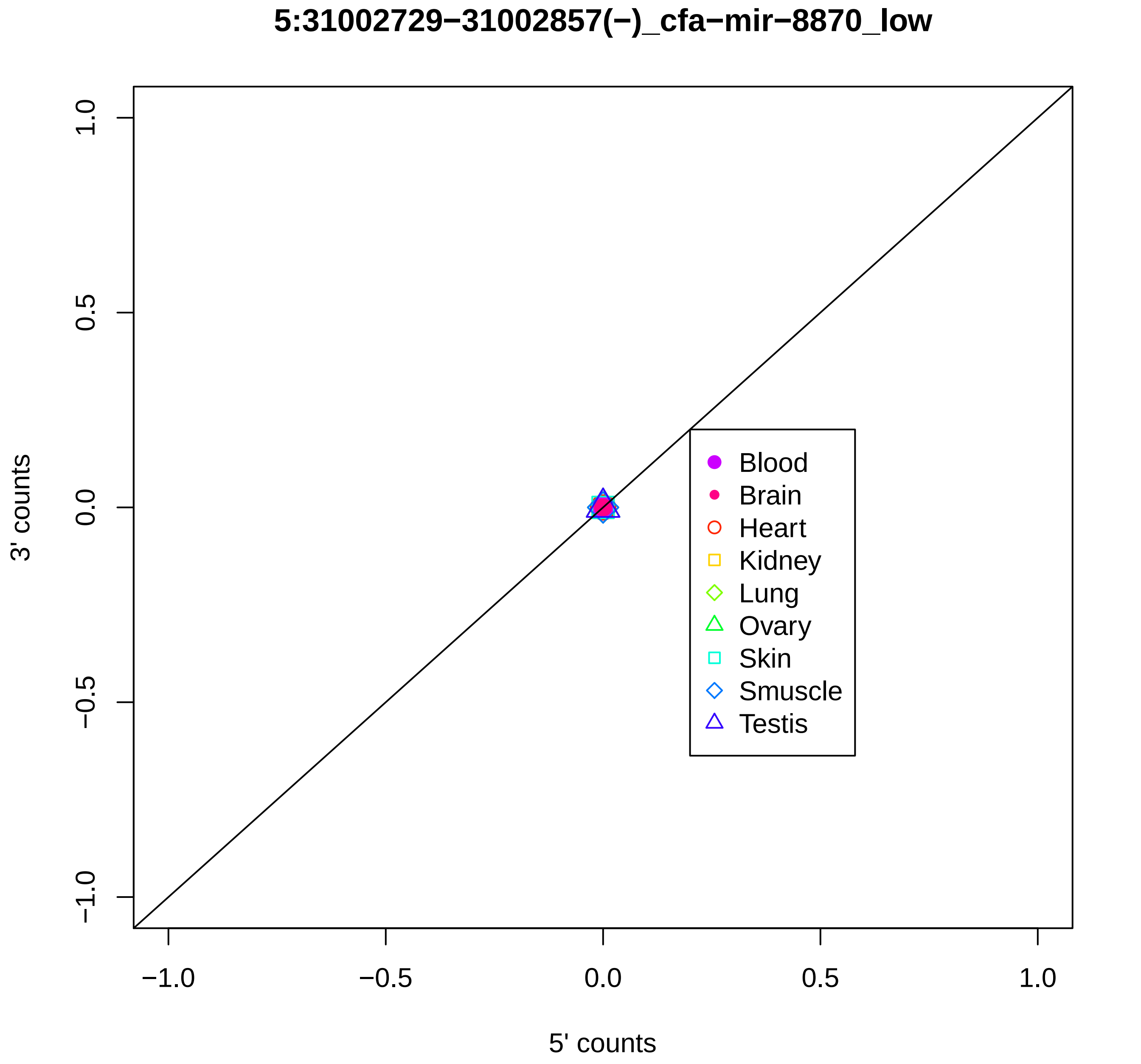

5:31002729−31002857(−)_cfa−mir−8870_low
3' counts
5' counts
1.0
0.5
0.0
−0.5
−1.0
Blood
Brain
Heart
Kidney
Lung
Ovary
Skin
Smuscle
Testis

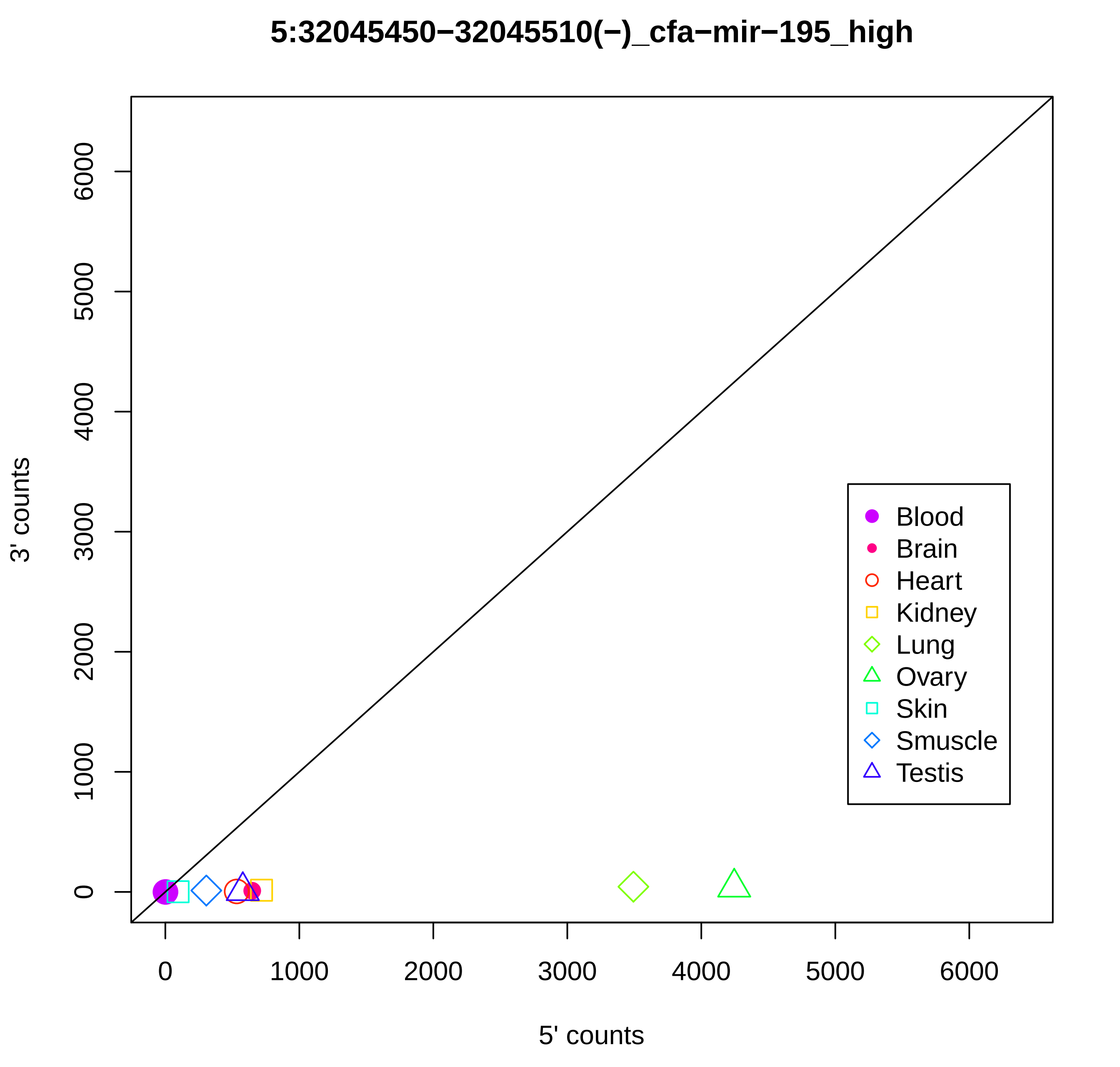

5:32045450−32045510(−)_cfa−mir−195_high
3' counts
5' counts
0
1000
2000
3000
4000
5000
6000
Blood
Brain
Heart
Kidney
Lung
Ovary
Skin
Smuscle
Testis

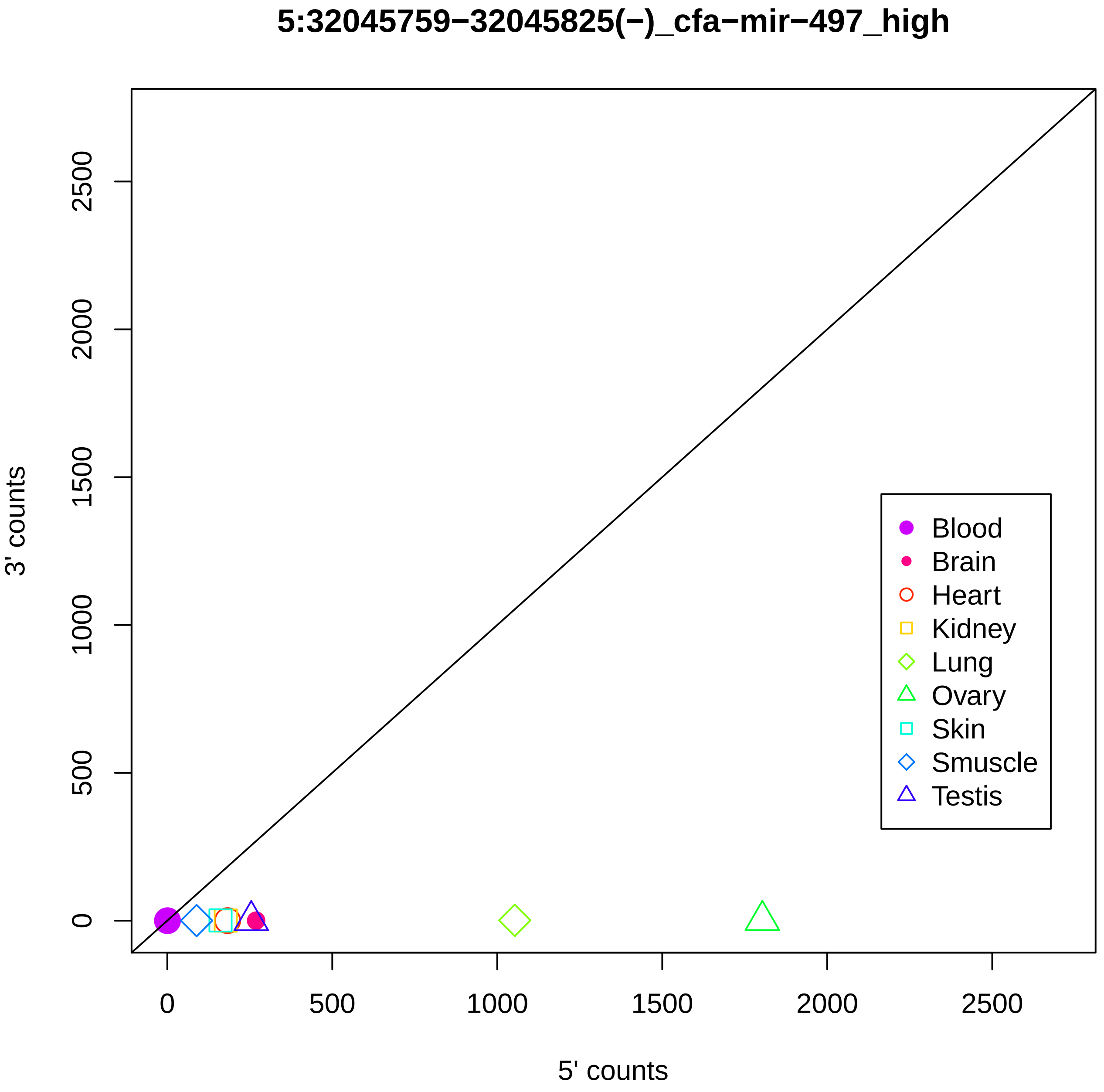
5:32045759−32045825(−)_cfa−mir−497_high
3' counts
5' counts
0
500
1000
1500
2000
2500
Blood
Brain
Heart
Kidney
Lung
Ovary
Skin
Smuscle
Testis

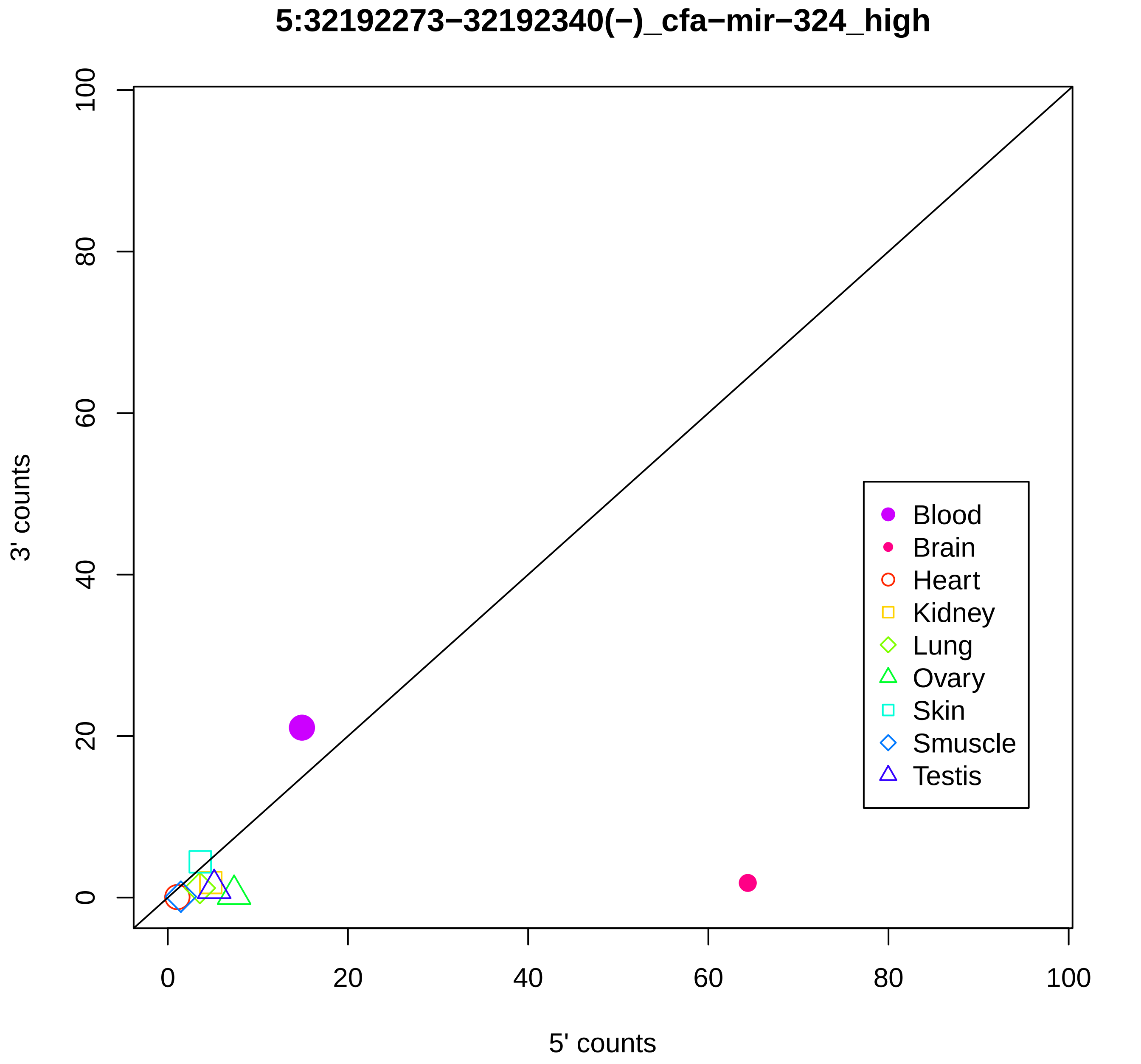

5:32192273−32192340(−)_cfa−mir−324_high
3' counts
5' counts
0
20
40
60
80
100
Blood
Brain
Heart
Kidney
Lung
Ovary
Skin
Smuscle
Testis

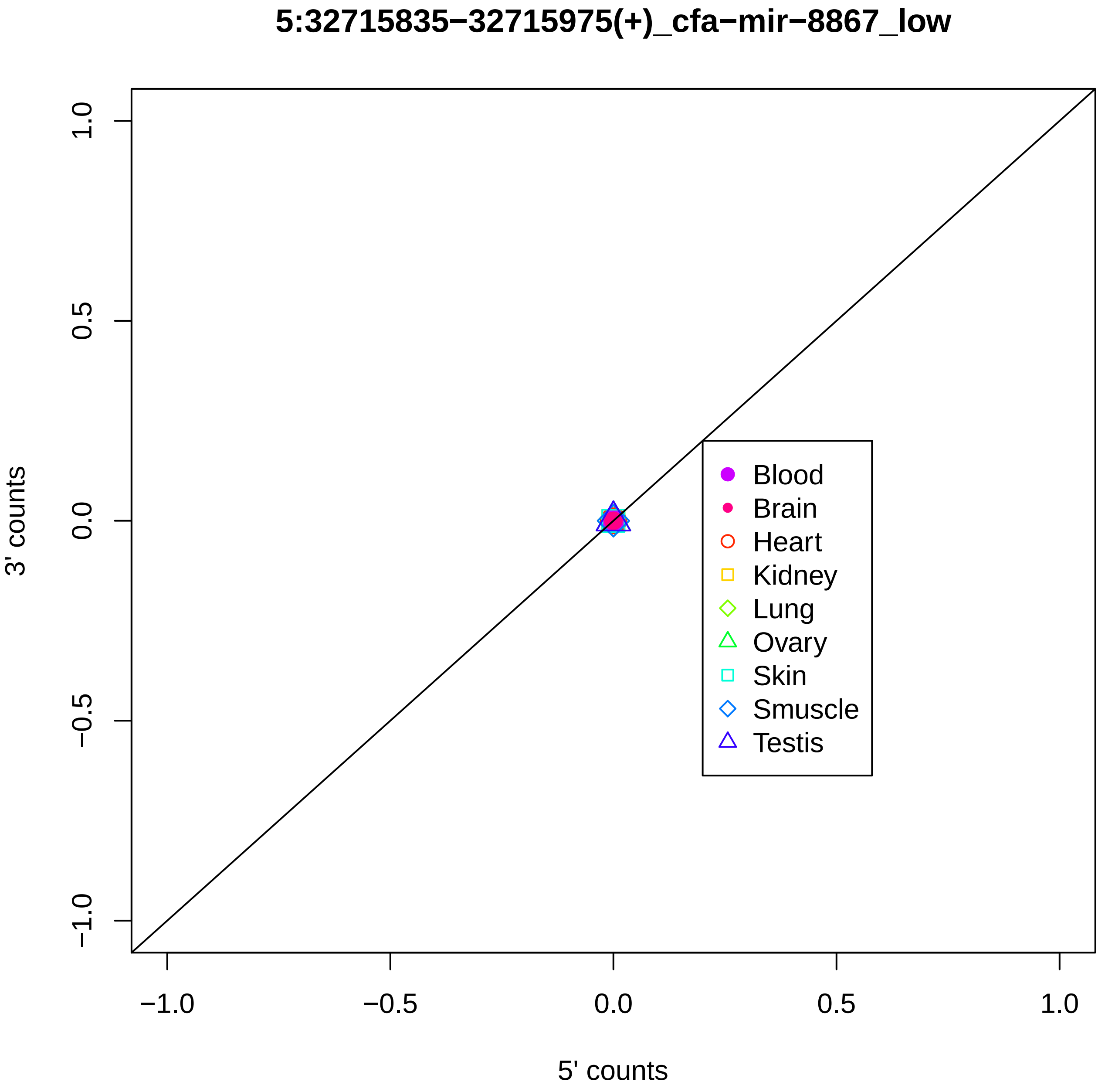

5:32715835−32715975(+)_cfa−mir−8867_low
3' counts
5' counts
−1.0
−0.5
0.0
0.5
1.0
Blood
Brain
Heart
Kidney
Lung
Ovary
Skin
Smuscle
Testis

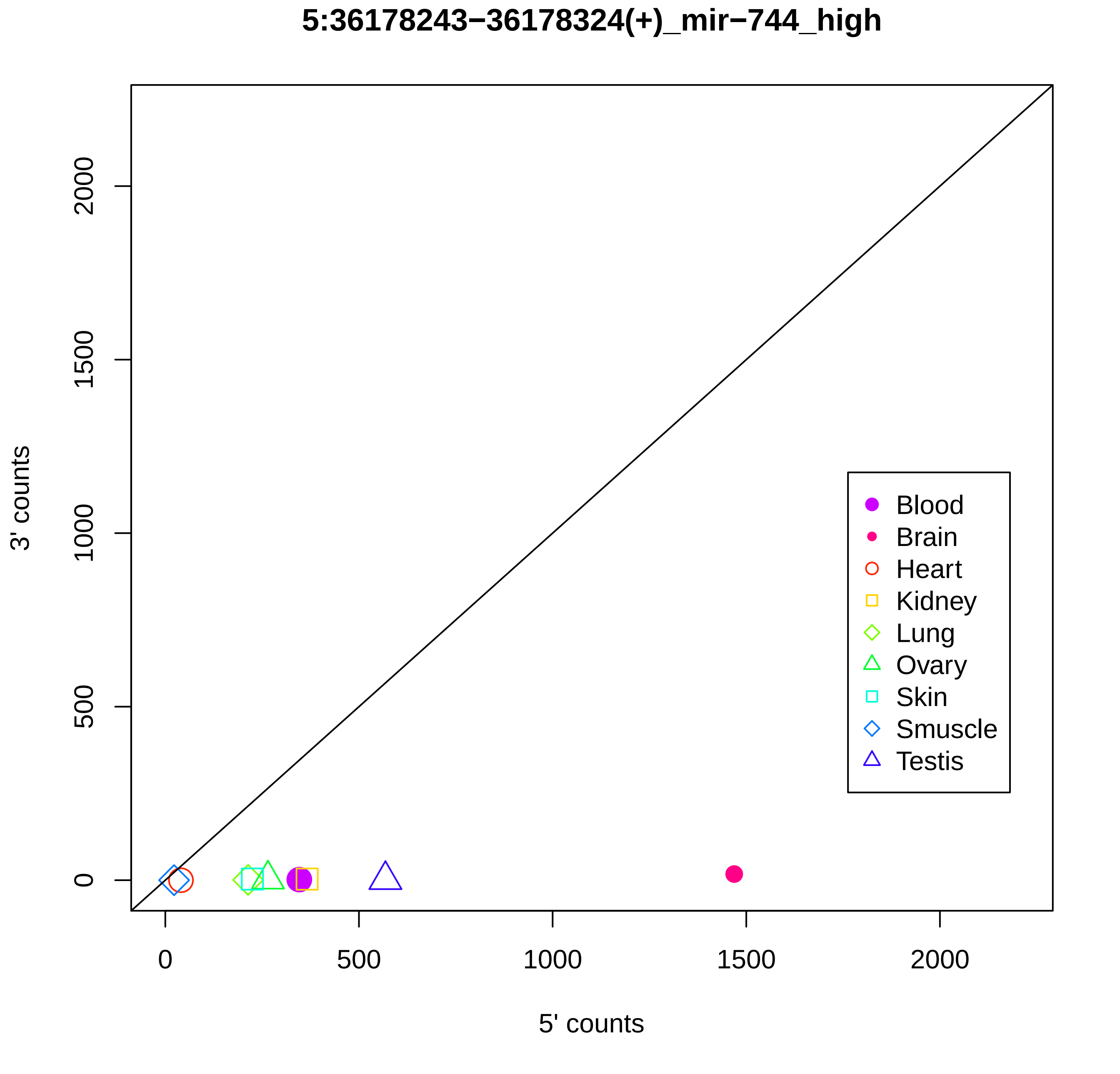

5:36178243−36178324(+)_mir−744_high
3' counts
5' counts
0
500
1000
1500
2000
Blood
Brain
Heart
Kidney
Lung
Ovary
Skin
Smuscle
Testis

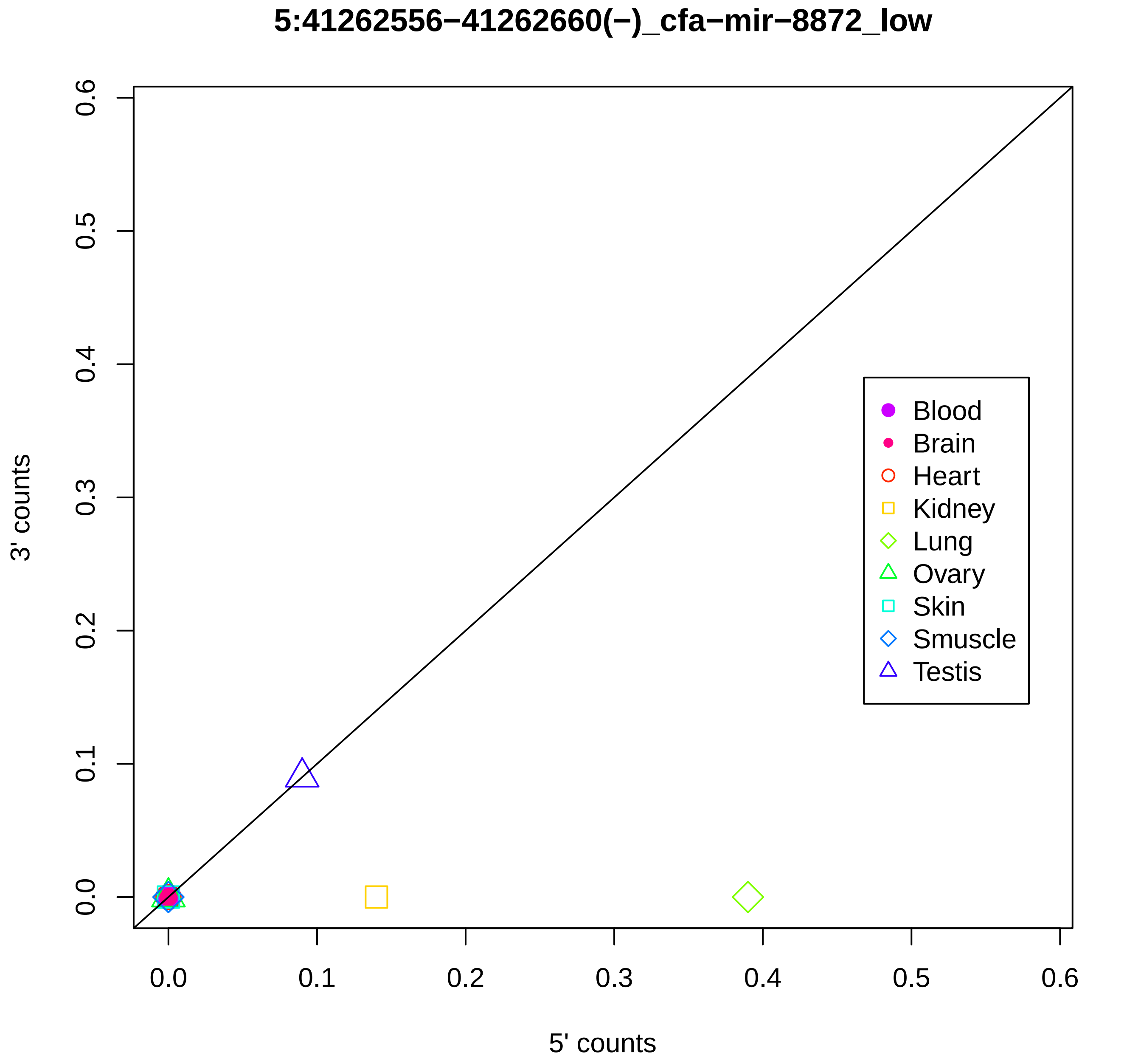
5:41262556−41262660(−)_cfa−mir−8872_low
3' counts
5' counts
0.0
0.1
0.2
0.3
0.4
0.5
0.6
Blood
Brain
Heart
Kidney
Lung
Ovary
Skin
Smuscle
Testis

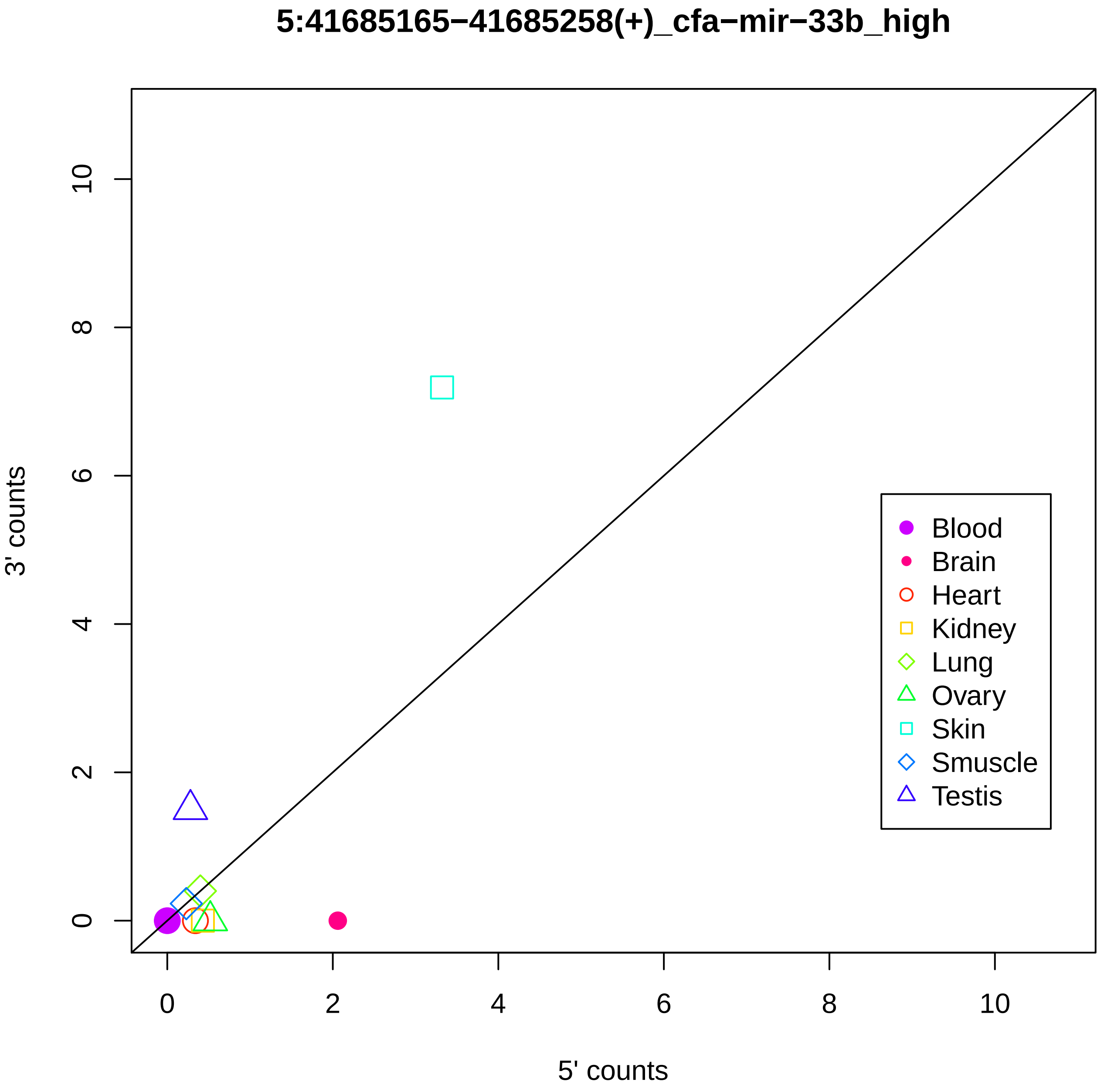
5:41685165−41685258(+)_cfa−mir−33b_high
3' counts
5' counts
0
2
4
6
8
10
0
2
4
6
8
10
Blood
Brain
Heart
Kidney
Lung
Ovary
Skin
Smuscle
Testis

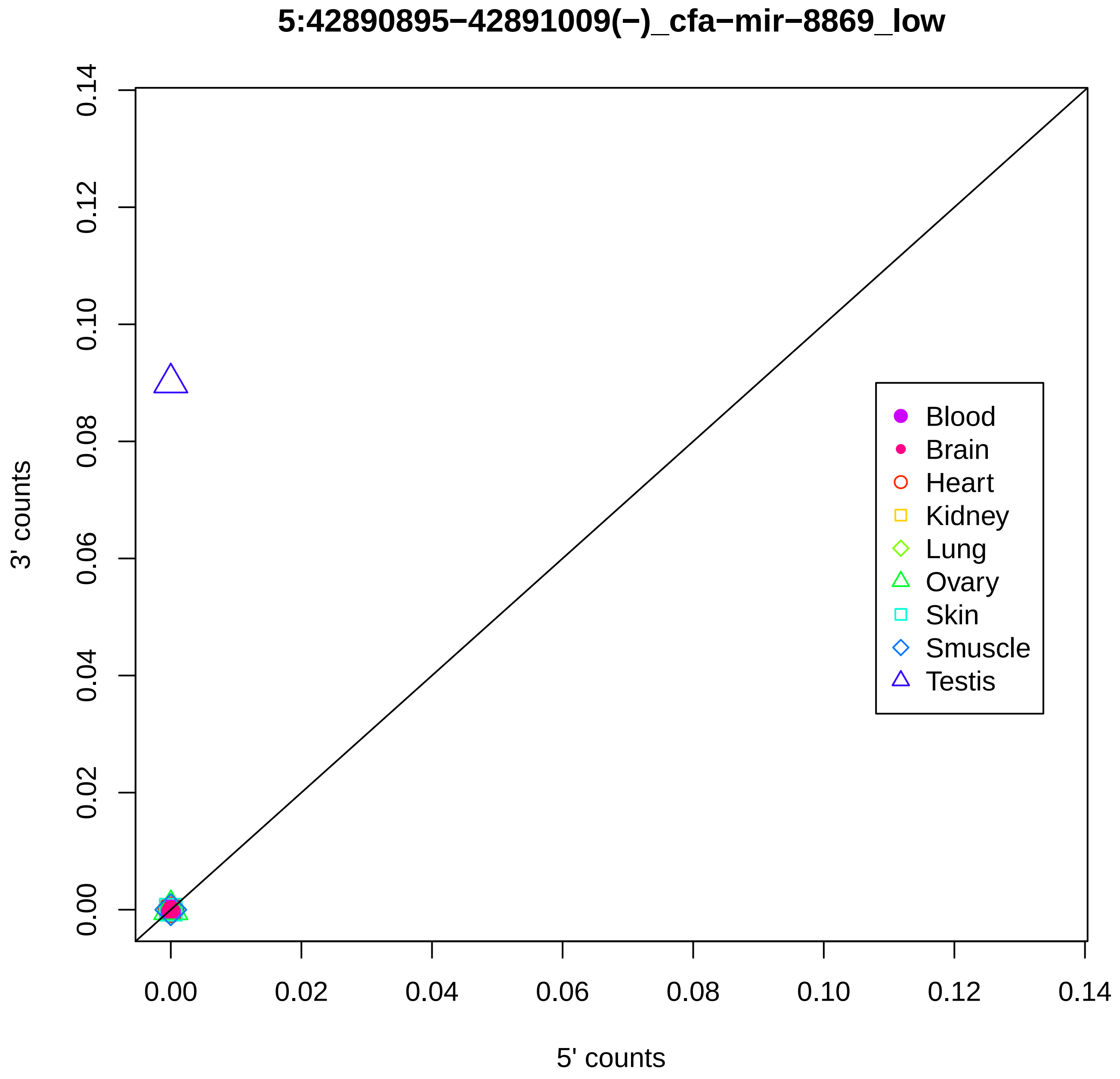
5:42890895−42891009(−)_cfa−mir−8869_low
3' counts
5' counts
0.00
0.02
0.04
0.06
0.08
0.10
0.12
0.14
Blood
Brain
Heart
Kidney
Lung
Ovary
Skin
Smuscle
Testis

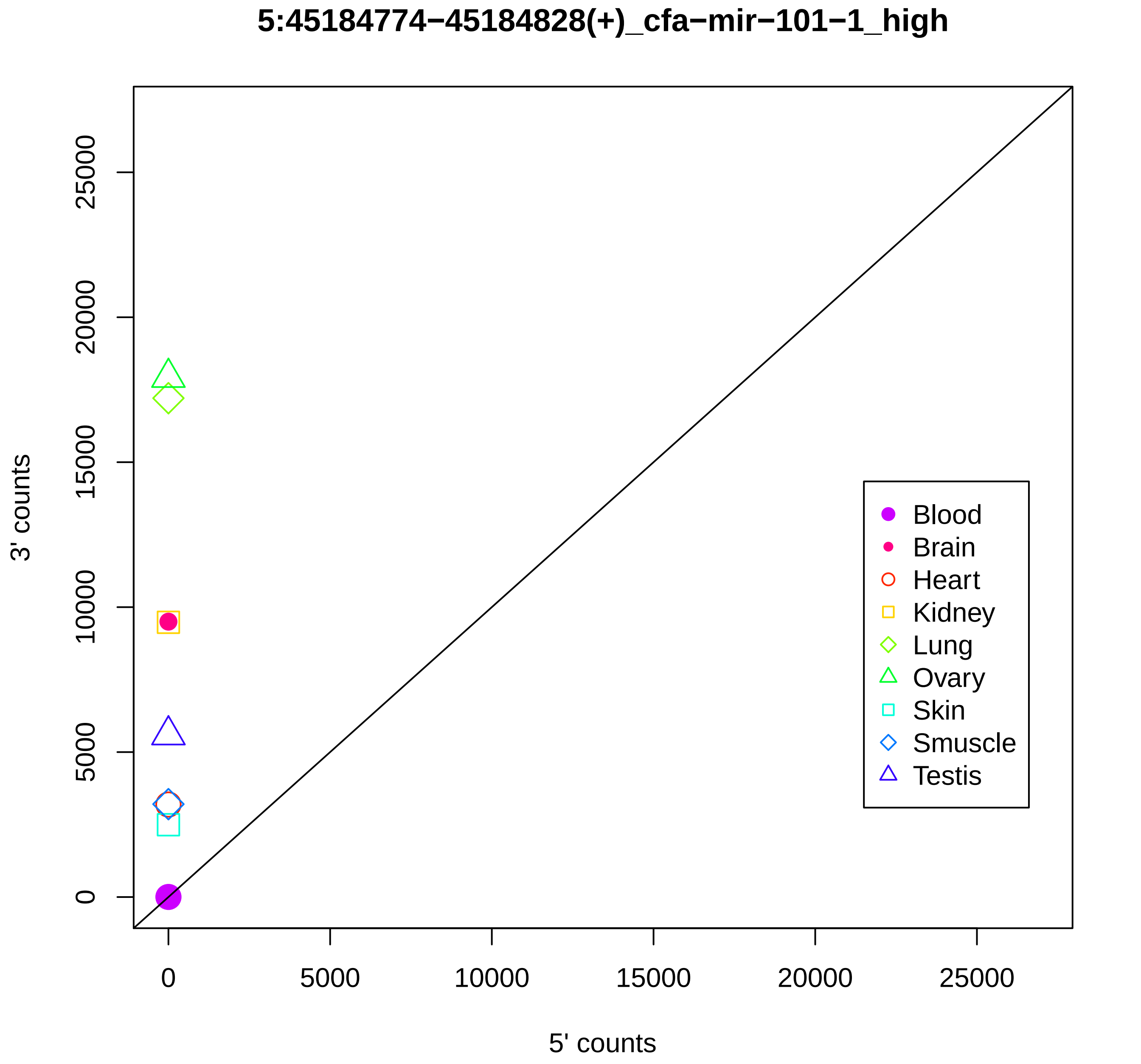

5:45184774−45184828(+)_cfa−mir−101−1_high
3' counts
5' counts
0
5000
10000
15000
20000
25000
Blood
Brain
Heart
Kidney
Lung
Ovary
Skin
Smuscle
Testis

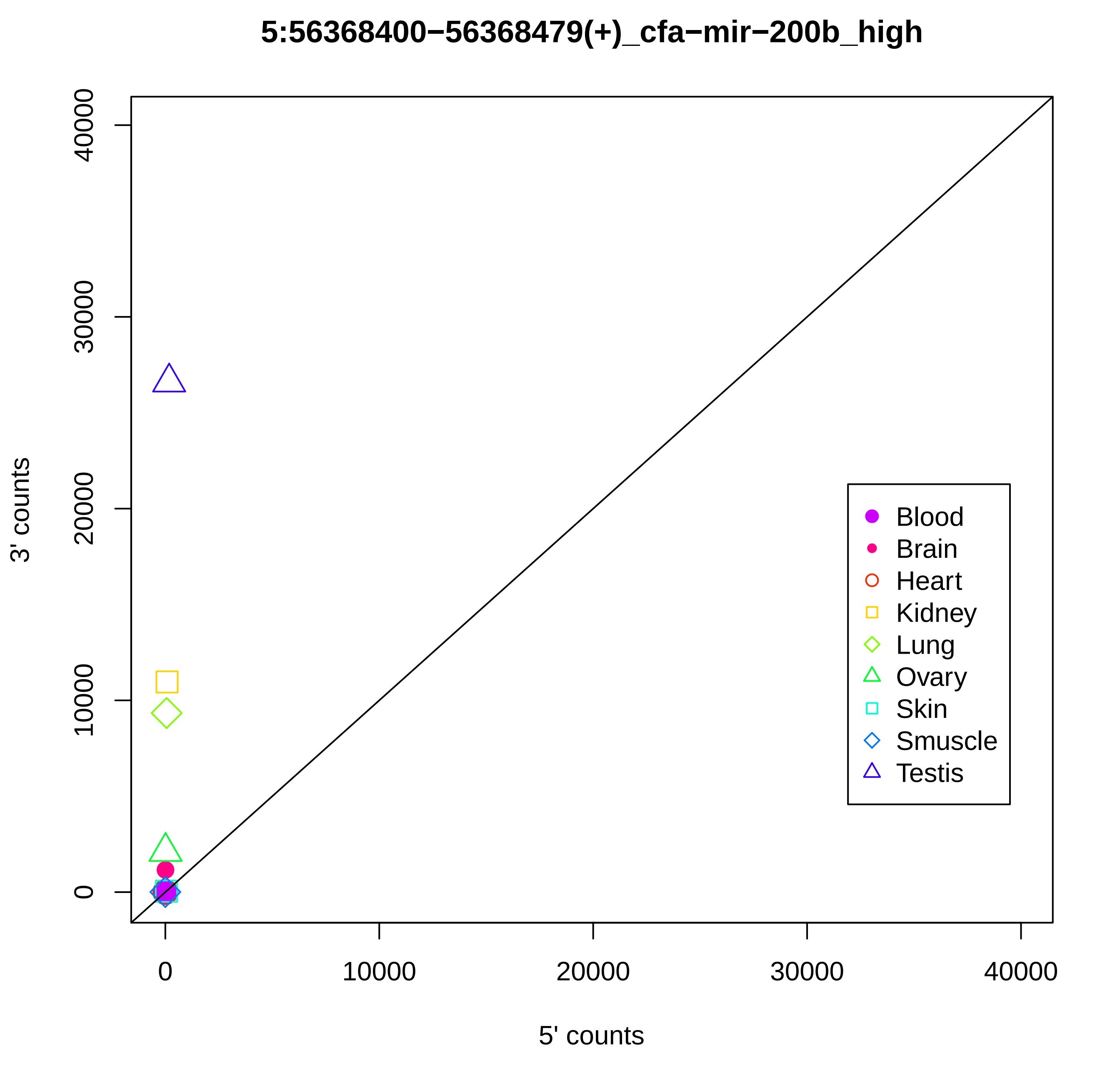

5:56368400−56368479(+)_cfa−mir−200b_high
3' counts
5' counts
0
10000
20000
30000
40000
Blood
Brain
Heart
Kidney
Lung
Ovary
Skin
Smuscle
Testis

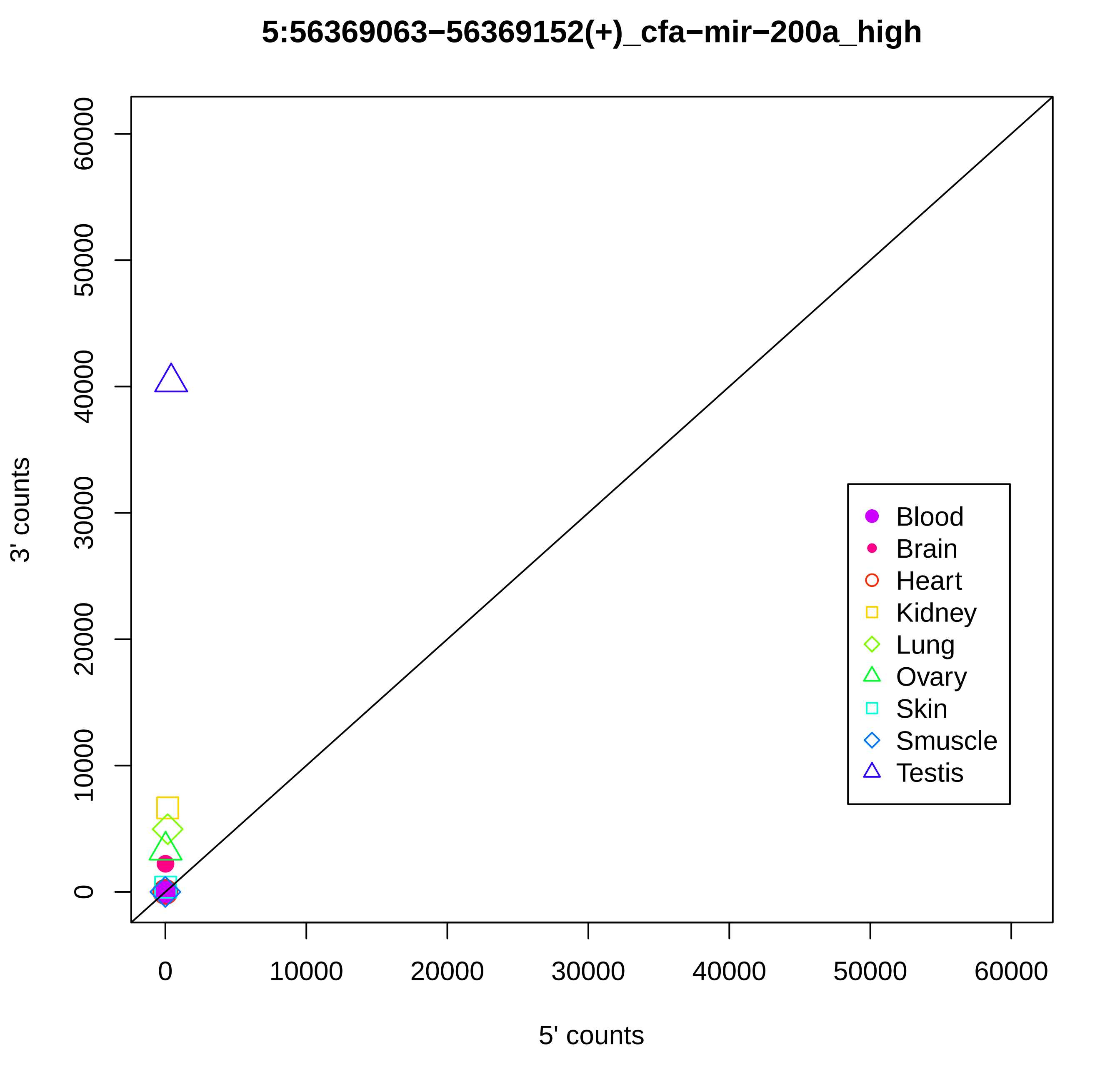

5:56369063−56369152(+)_cfa−mir−200a_high
3' counts
5' counts
0
10000
20000
30000
40000
50000
60000
Blood
Brain
Heart
Kidney
Lung
Ovary
Skin
Smuscle
Testis

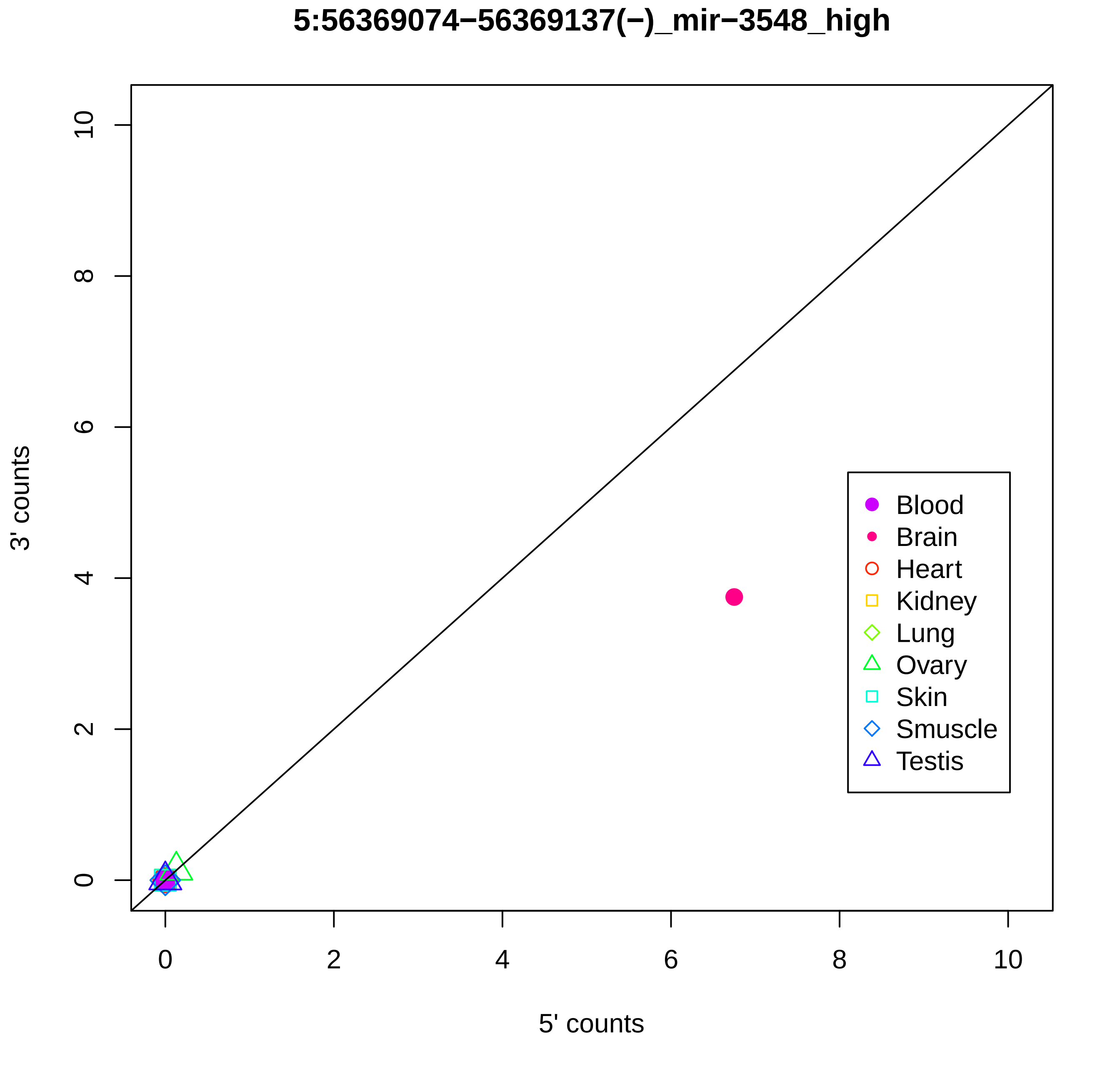
5:56369074−56369137(−)_mir−3548_high
3' counts
5' counts
0
2
4
6
8
10
0
2
4
6
8
10
Blood
Brain
Heart
Kidney
Lung
Ovary
Skin
Smuscle
Testis

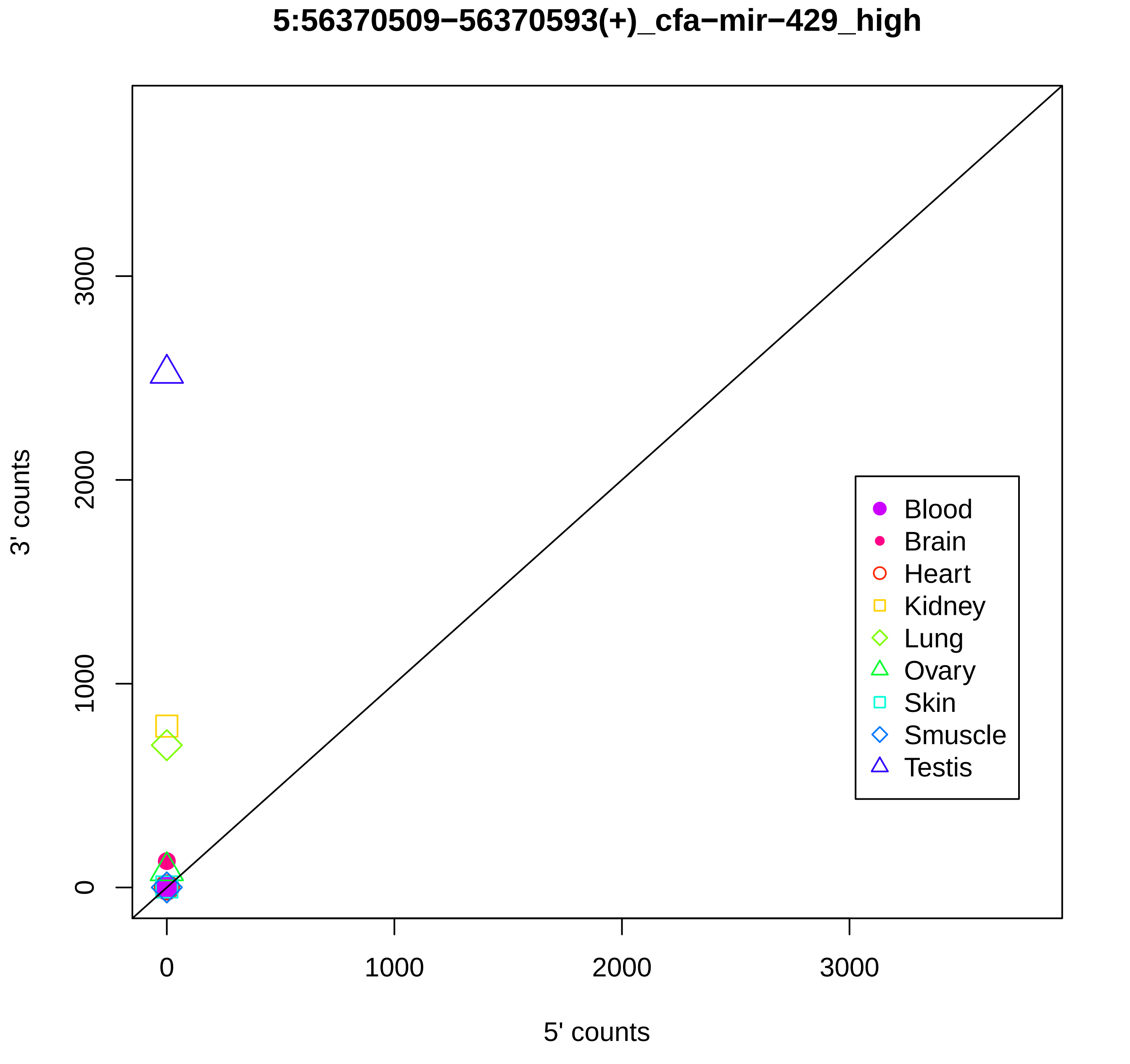

5:56370509−56370593(+)_cfa−mir−429_high
3' counts
5' counts
0
1000
2000
3000
0
1000
2000
3000
Blood
Brain
Heart
Kidney
Lung
Ovary
Skin
Smuscle
Testis

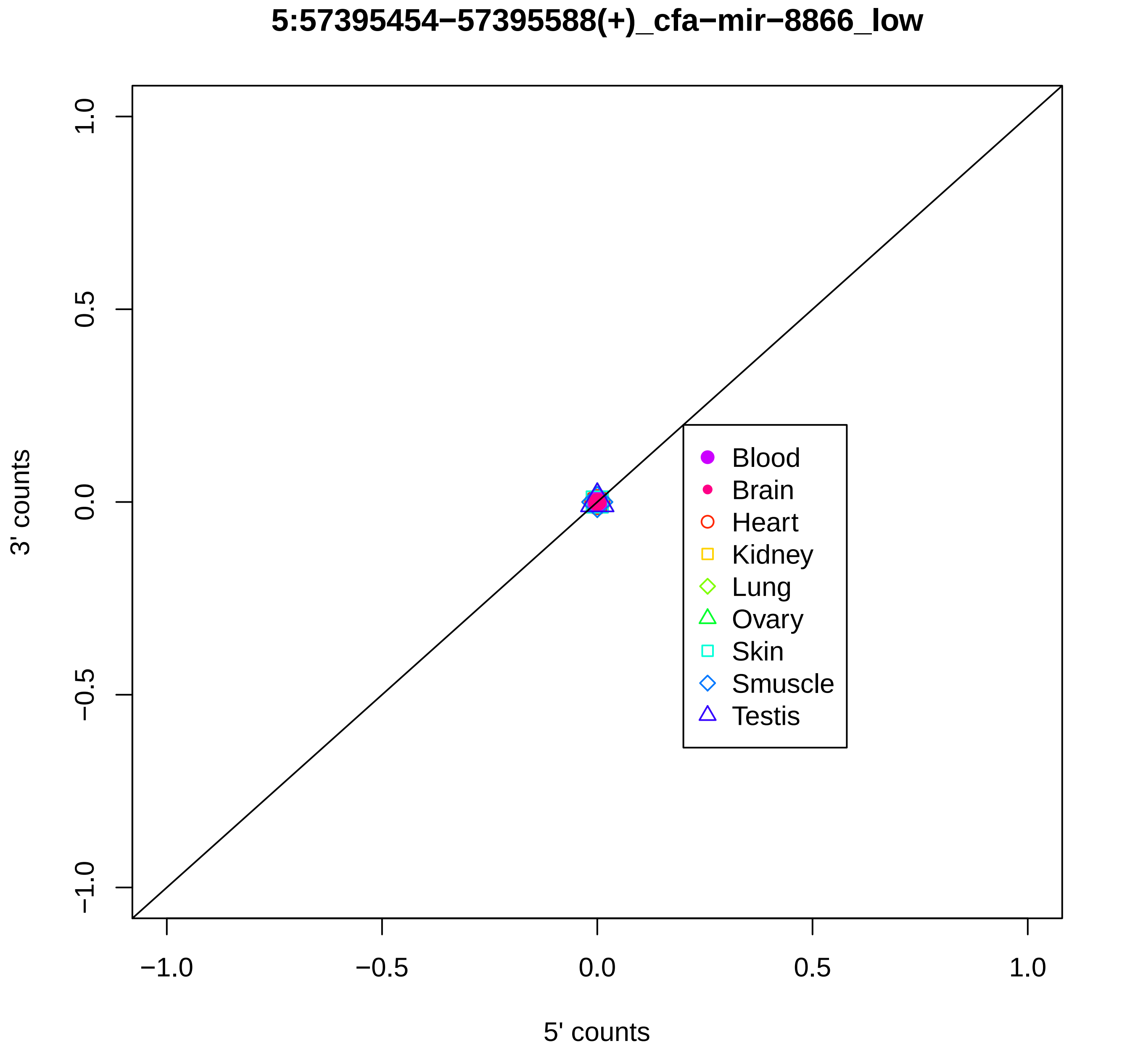

5:57395454−57395588(+)_cfa−mir−8866_low
Blood
Brain
Heart
Kidney
Lung
Ovary
Skin
Smuscle
Testis
−1.0
−0.5
0.0
0.5
1.0
5' counts
3' counts

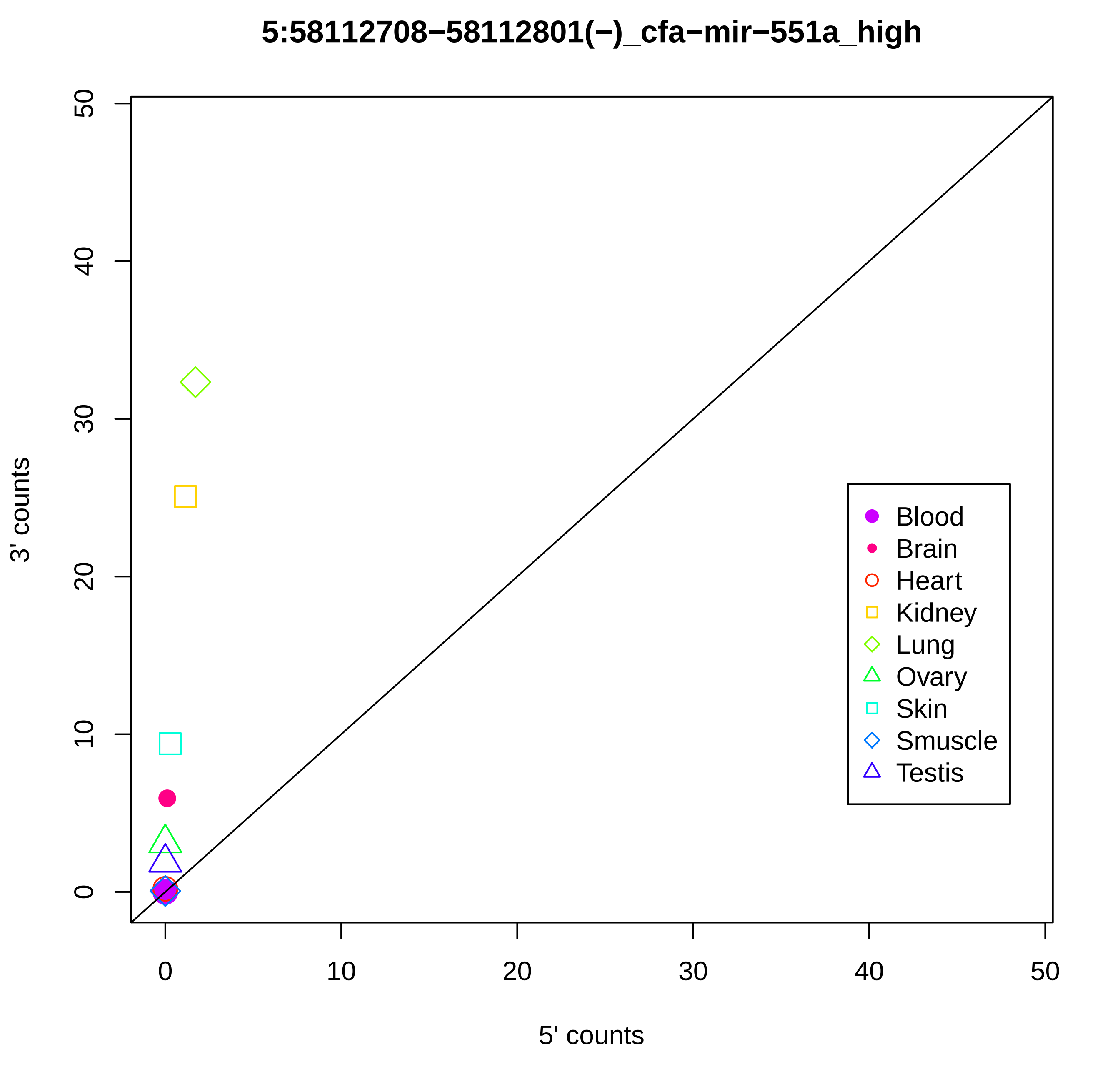
5:58112708−58112801(−)_cfa−mir−551a_high
3' counts
5' counts
0
10
20
30
40
50
0
10
20
30
40
50
Blood
Brain
Heart
Kidney
Lung
Ovary
Skin
Smuscle
Testis

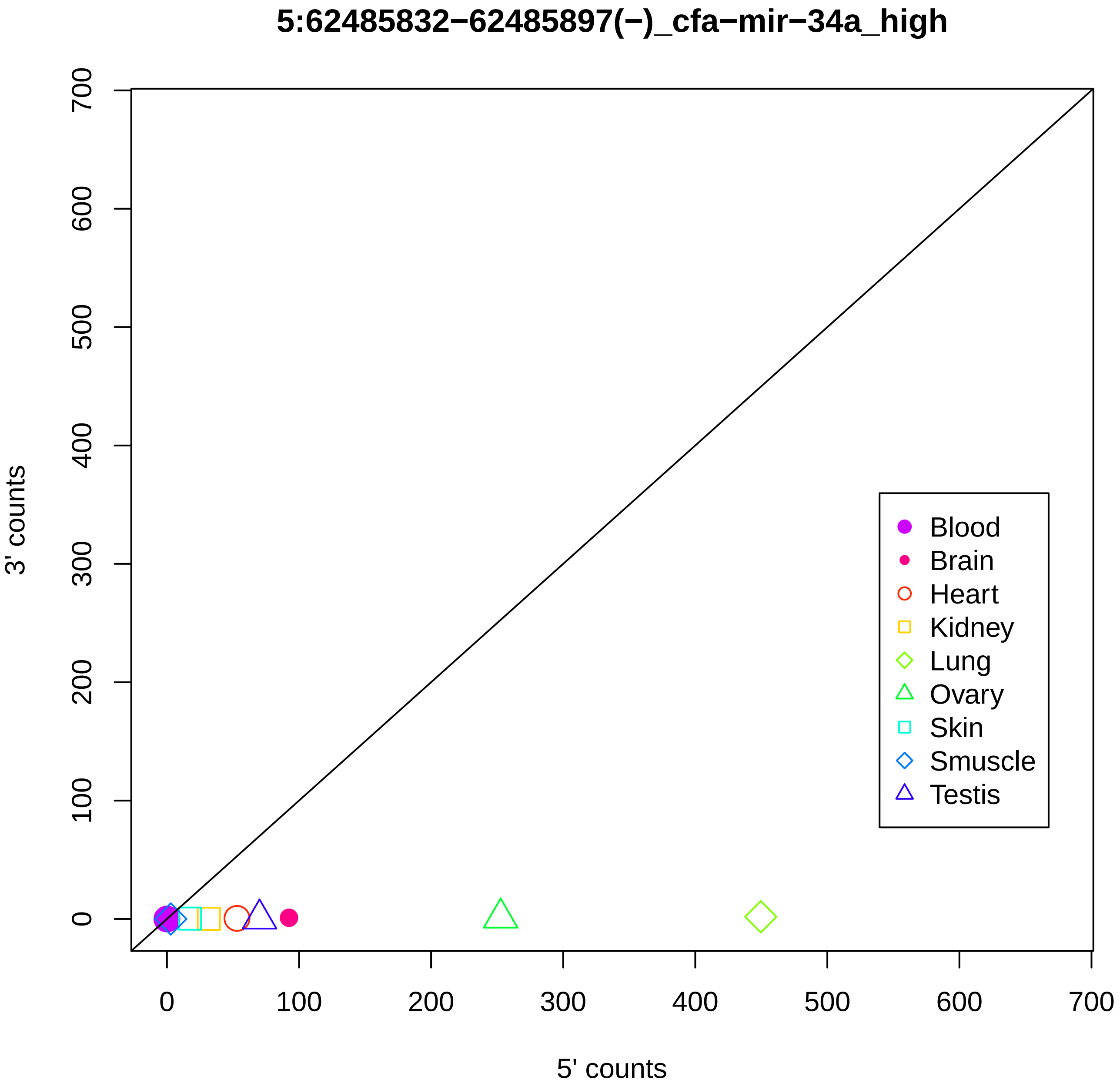
5:62485832−62485897(−)_cfa−mir−34a_high
3' counts
5' counts
0
100
200
300
400
500
600
700
Blood
Brain
Heart
Kidney
Lung
Ovary
Skin
Smuscle
Testis

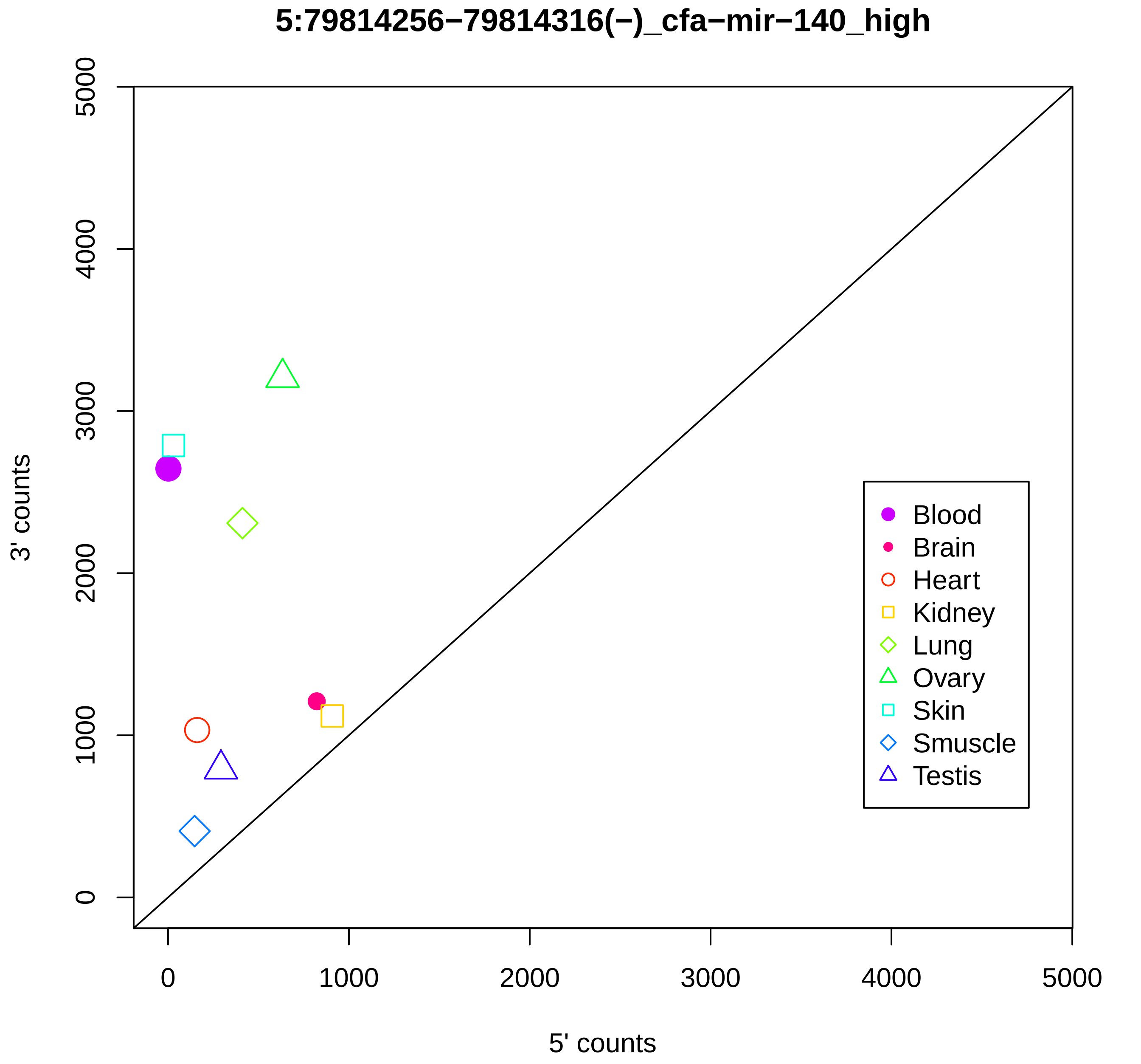

5:79814256−79814316(−)_cfa−mir−140_high
3' counts
5' counts
0
1000
2000
3000
4000
5000
Blood
Brain
Heart
Kidney
Lung
Ovary
Skin
Smuscle
Testis

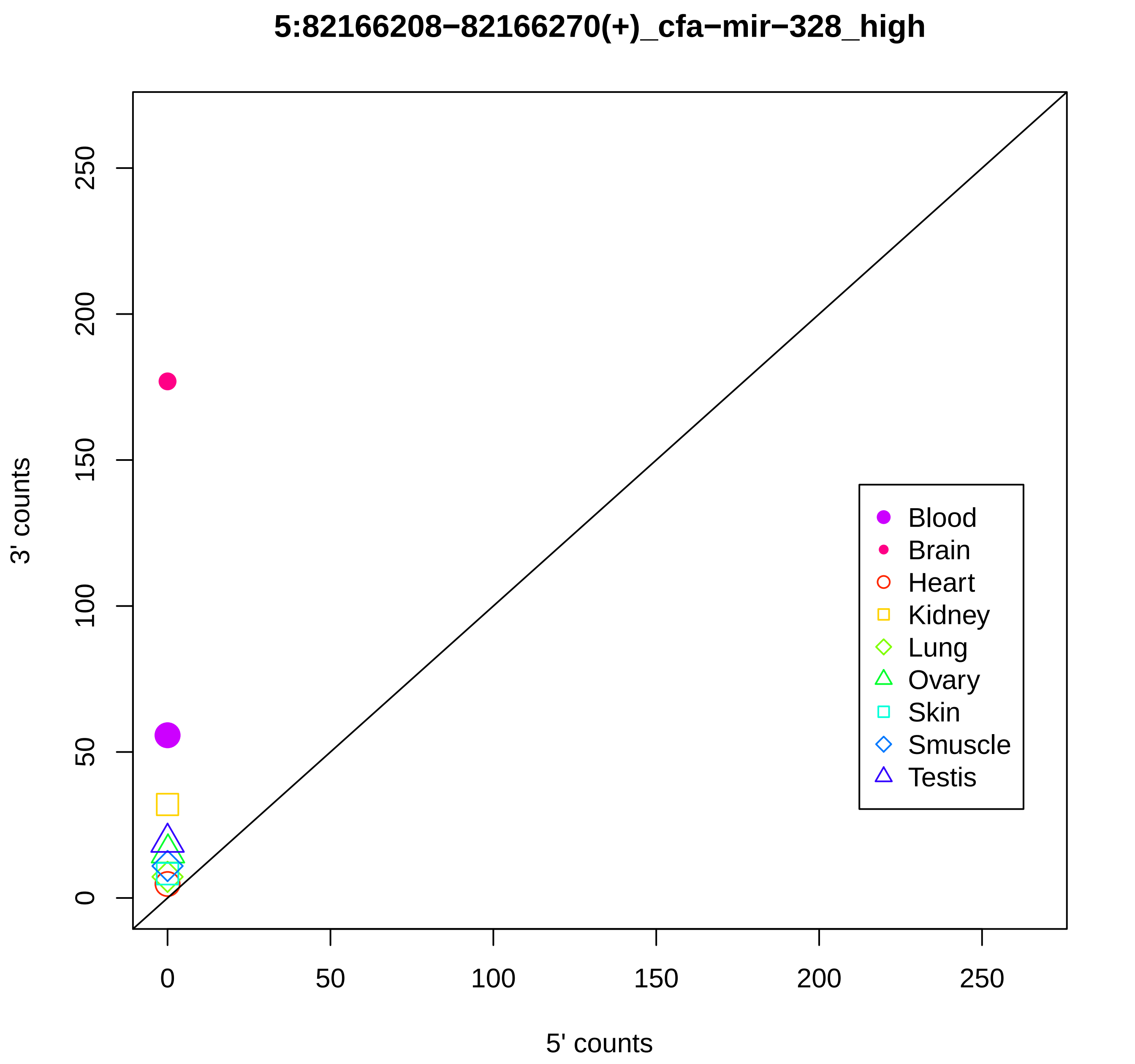

5:82166208−82166270(+)_cfa−mir−328_high
3' counts
5' counts
0
50
100
150
200
250
0
50
100
150
200
250
Blood
Brain
Heart
Kidney
Lung
Ovary
Skin
Smuscle
Testis

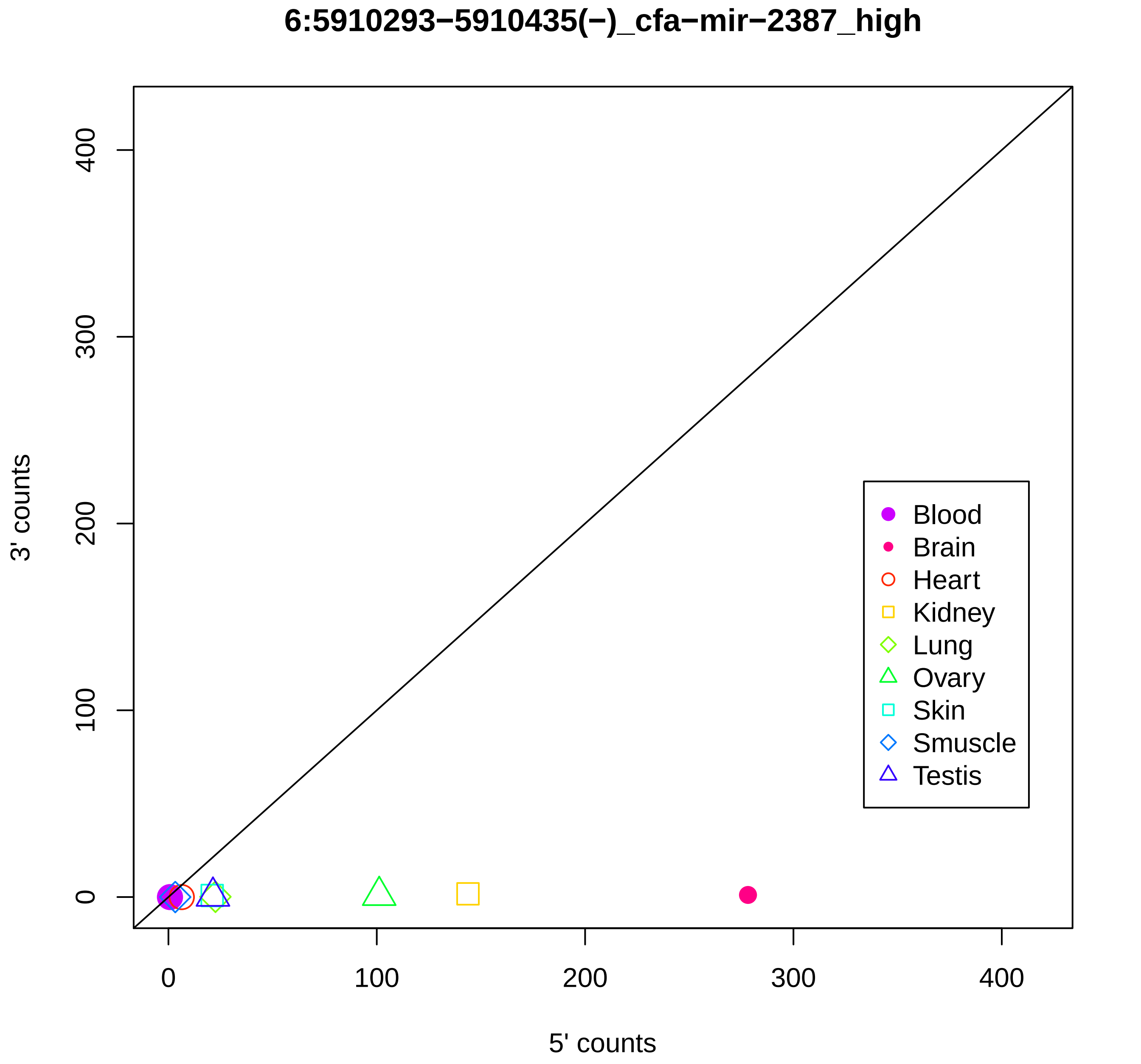
6:5910293−5910435(−)_cfa−mir−2387_high
3' counts
5' counts
0
100
200
300
400
0
100
200
300
400
Blood
Brain
Heart
Kidney
Lung
Ovary
Skin
Smuscle
Testis

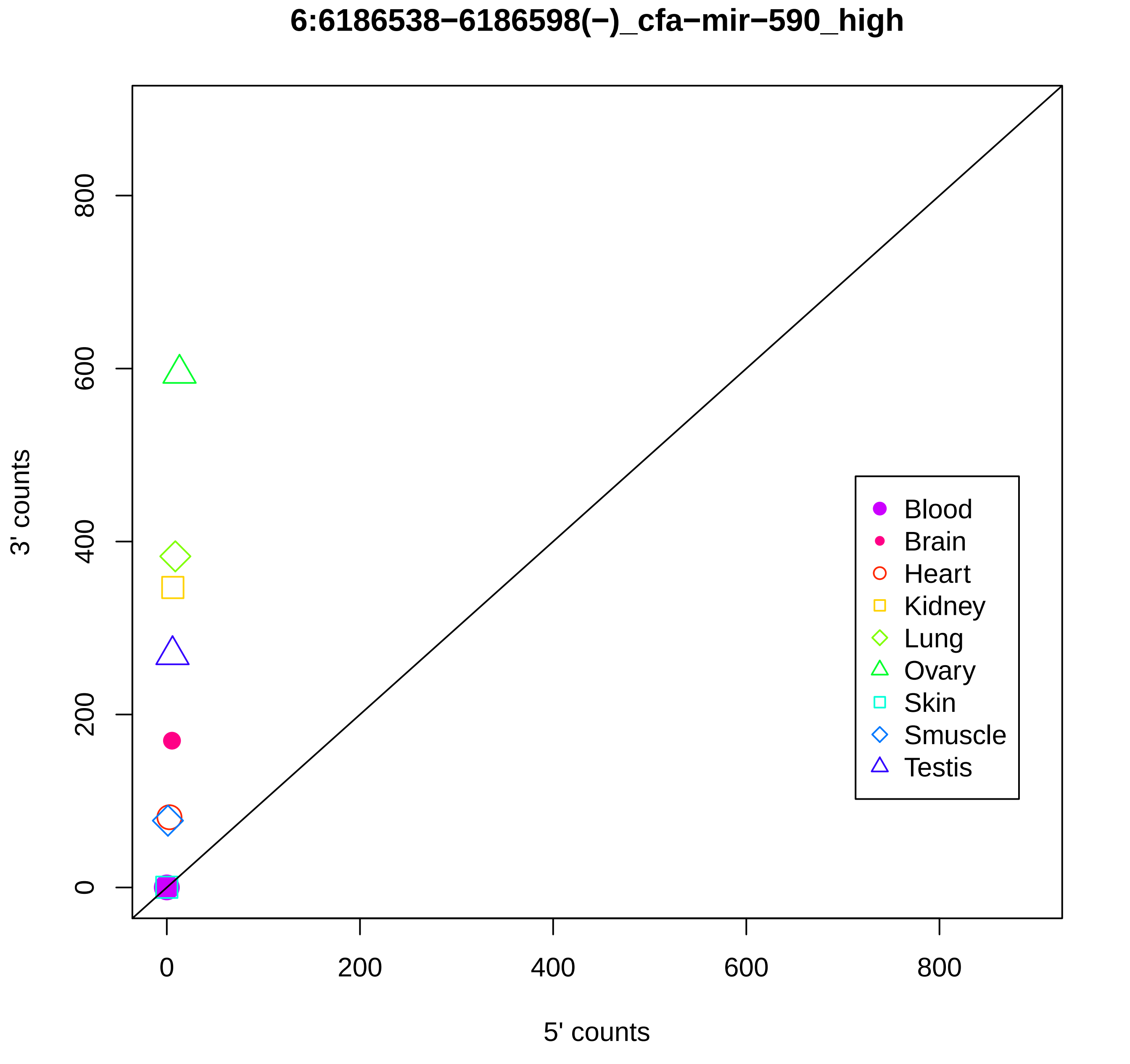
6:6186538−6186598(−)_cfa−mir−590_high
3' counts
5' counts
0
200
400
600
800
0
200
400
600
800
Blood
Brain
Heart
Kidney
Lung
Ovary
Skin
Smuscle
Testis

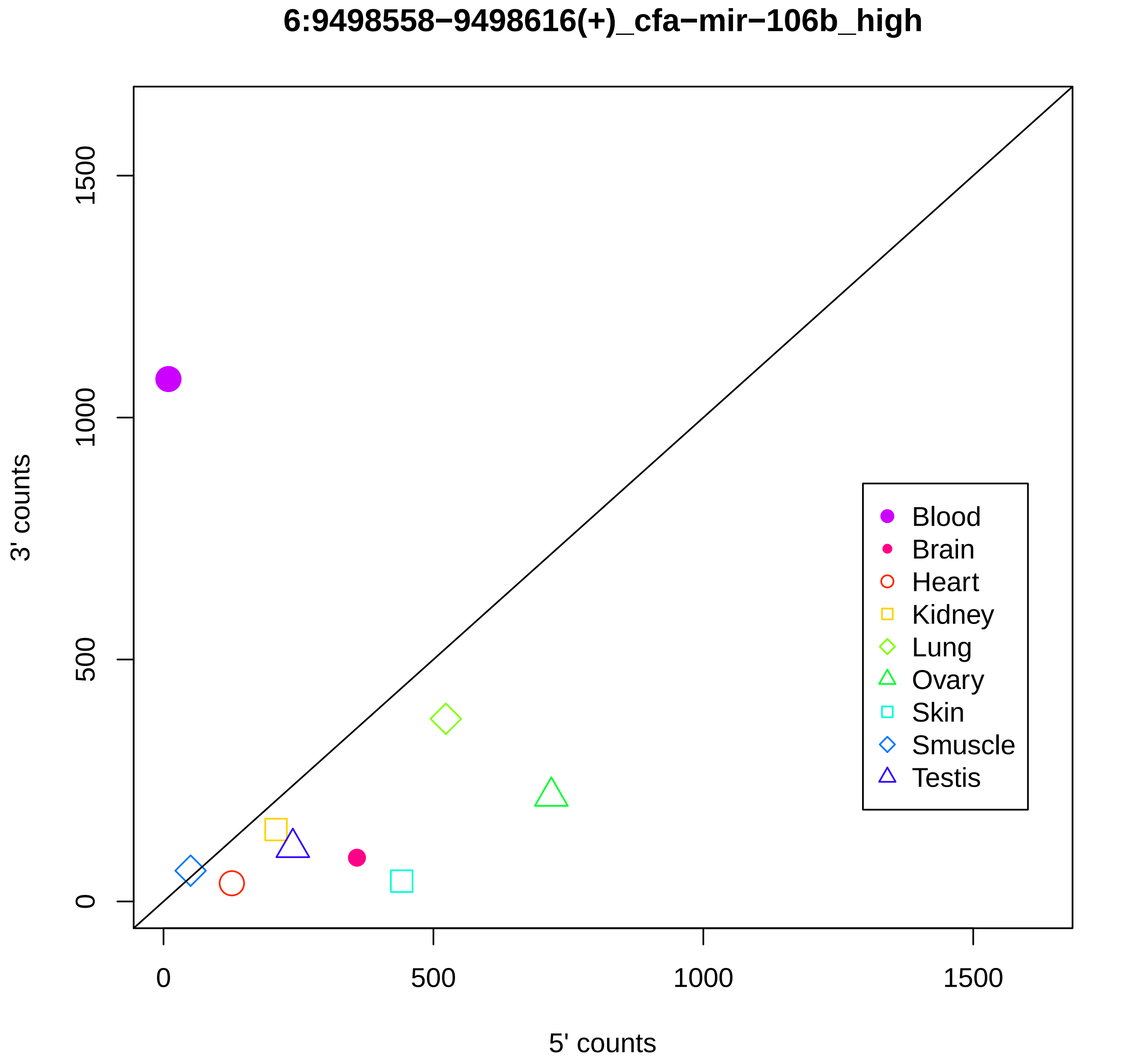
6:9498558−9498616(+)_cfa−mir−106b_high
3' counts
5' counts
0
500
1000
1500
Blood
Brain
Heart
Kidney
Lung
Ovary
Skin
Smuscle
Testis

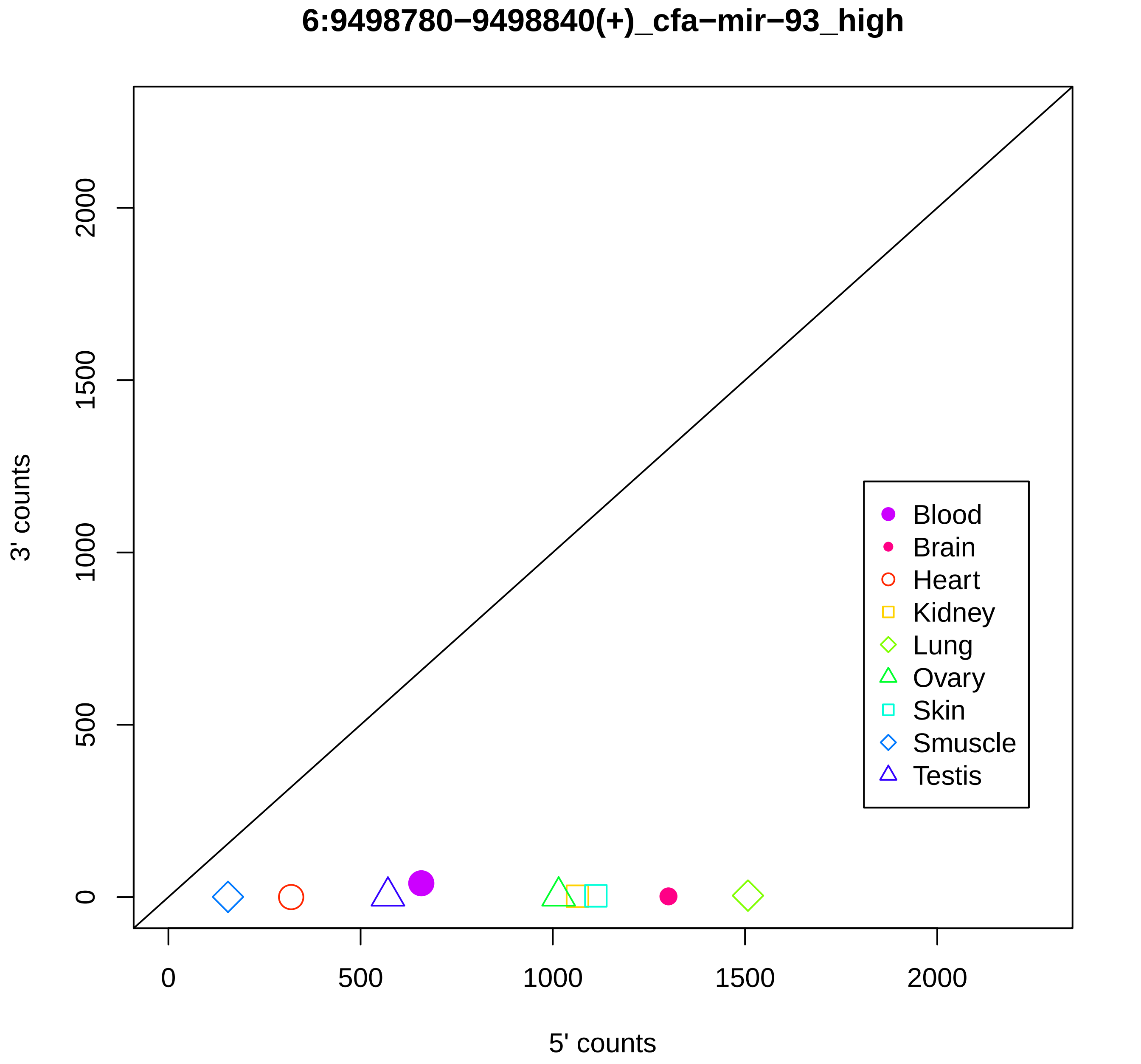

6:9498780−9498840(+)_cfa−mir−93_high
3' counts
5' counts
0
500
1000
1500
2000
Blood
Brain
Heart
Kidney
Lung
Ovary
Skin
Smuscle
Testis

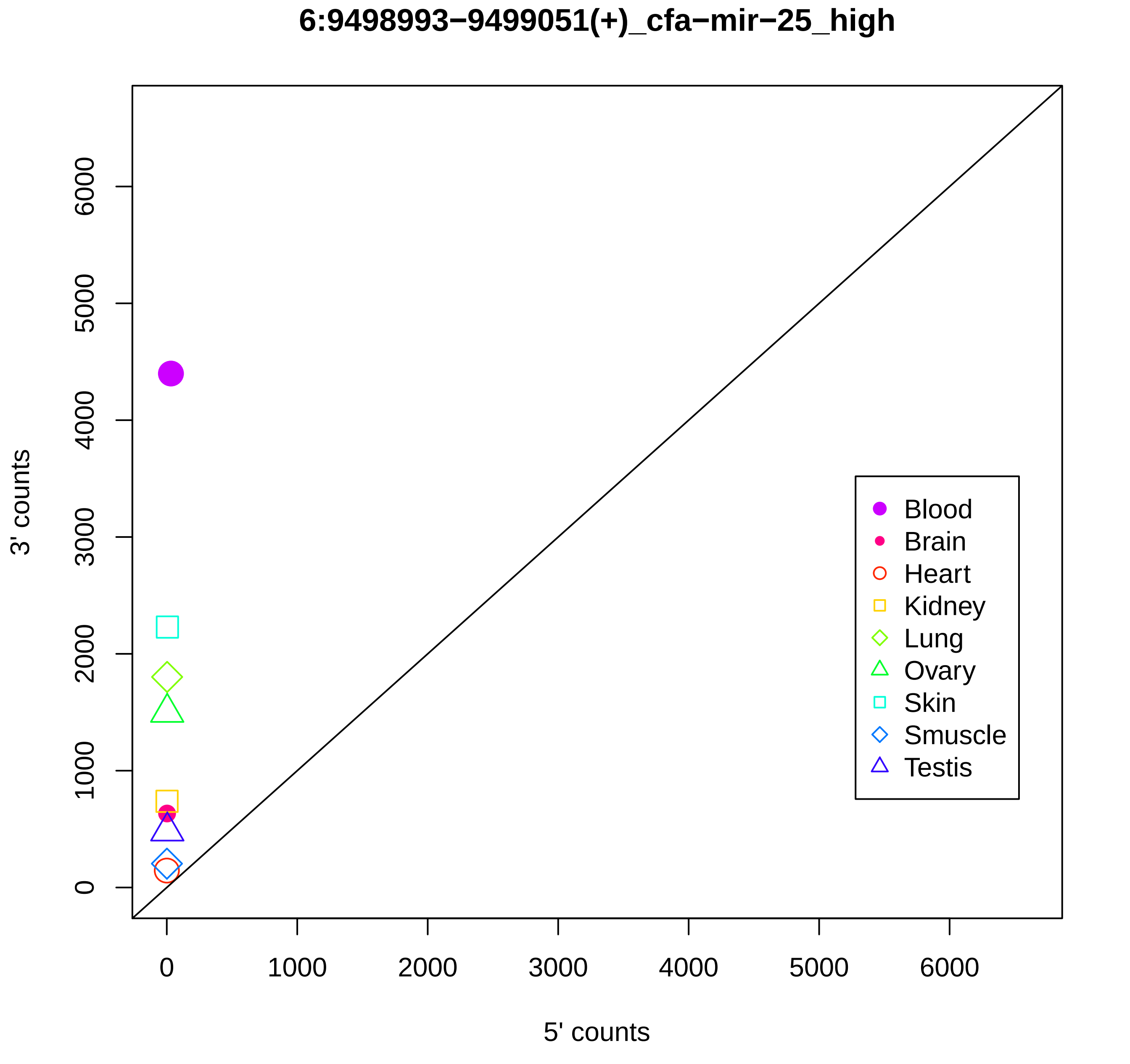

6:9498993−9499051(+)_cfa−mir−25_high
3' counts
5' counts
0
1000
2000
3000
4000
5000
6000
0
1000
2000
3000
4000
5000
6000
Blood
Brain
Heart
Kidney
Lung
Ovary
Skin
Smuscle
Testis

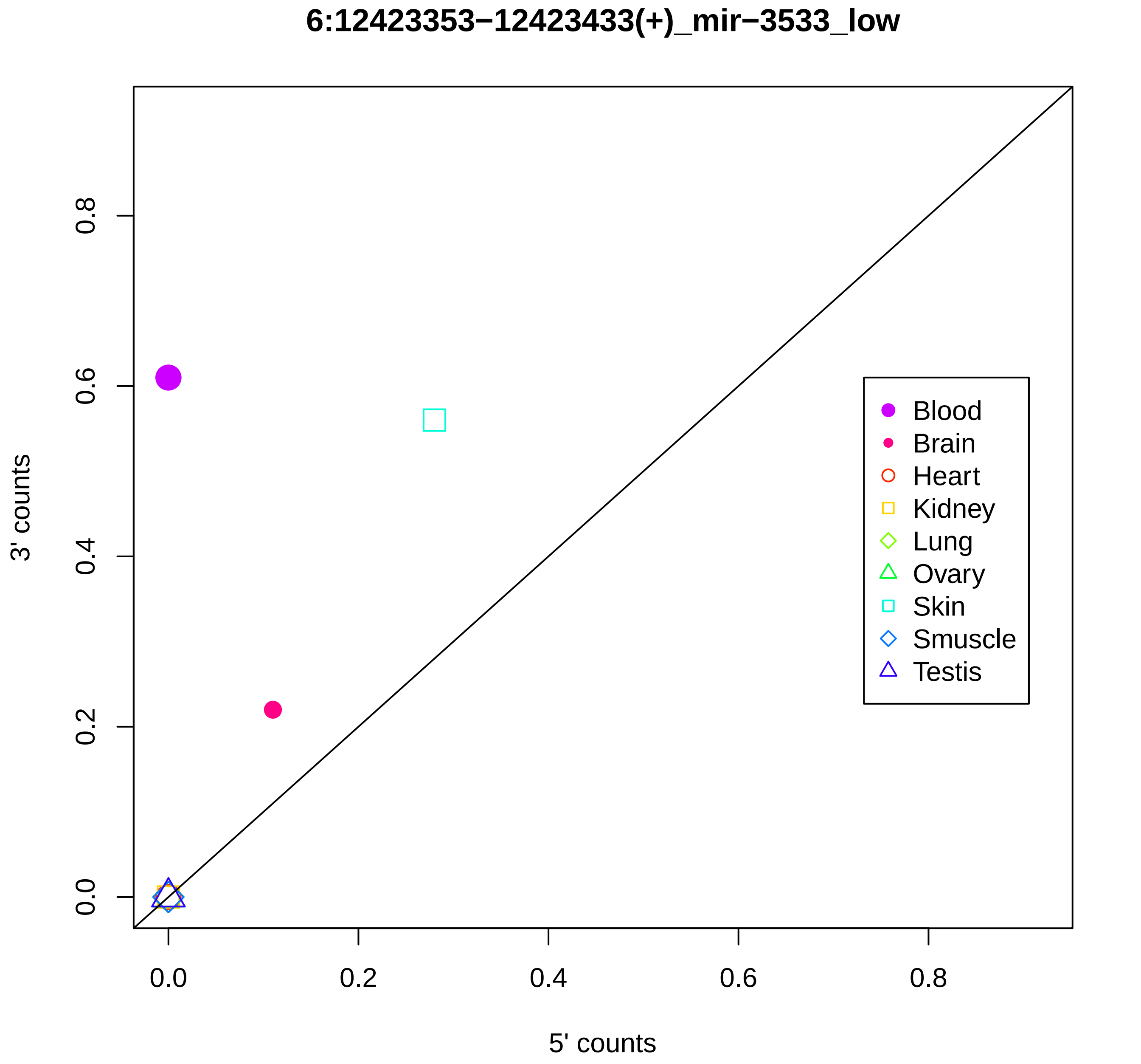

6:12423353−12423433(+)_mir−3533_low
Blood
Brain
Heart
Kidney
Lung
Ovary
Skin
Smuscle
Testis
3' counts
5' counts
0.0
0.2
0.4
0.6
0.8

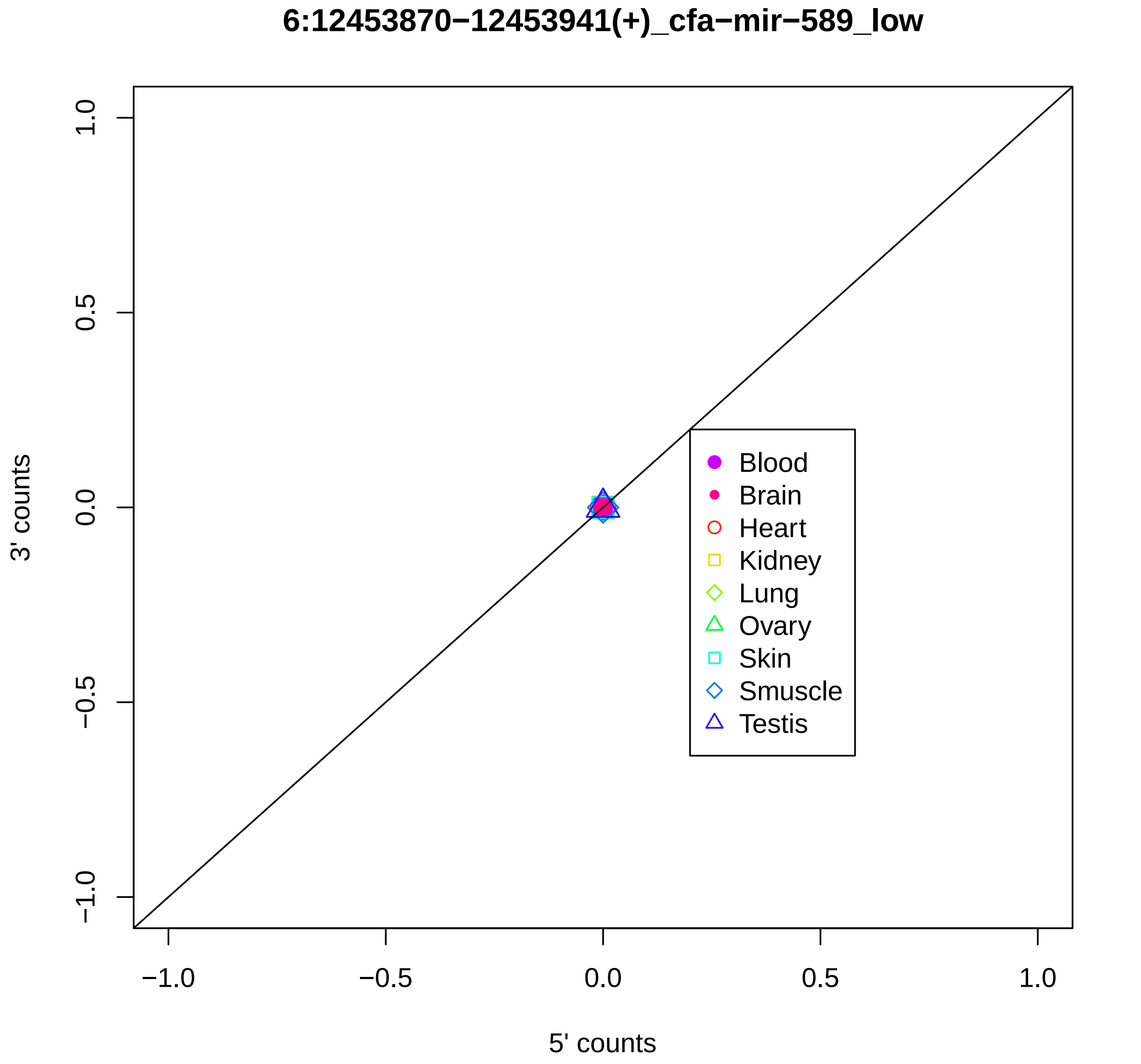

6:12453870−12453941(+)_cfa−mir−589_low
Blood
Brain
Heart
Kidney
Lung
Ovary
Skin
Smuscle
Testis
1.0
0.5
0.0
−0.5
−1.0
3' counts
−1.0
−0.5
0.0
0.5
1.0
5' counts

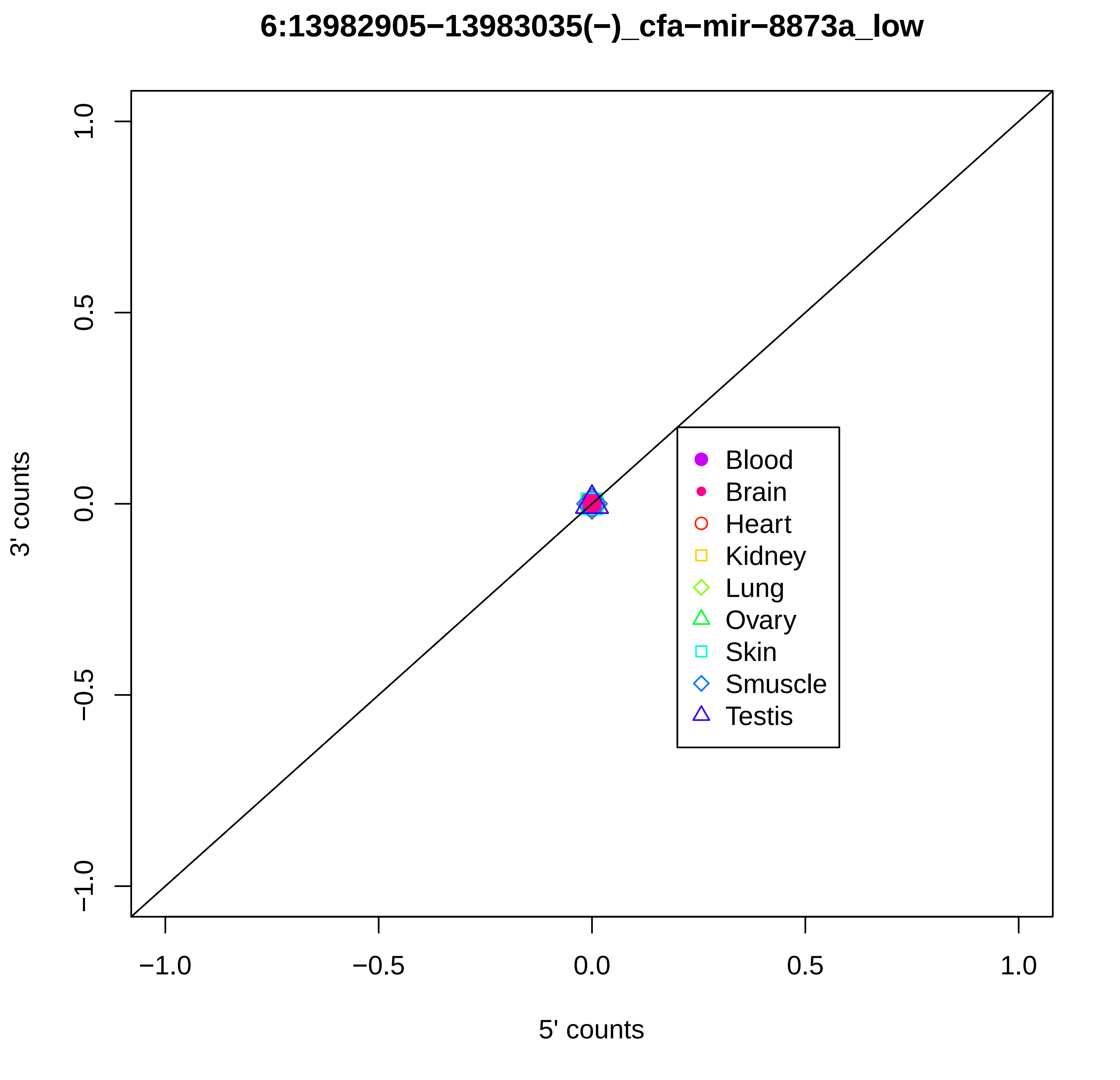
6:13982905−13983035(−)_cfa−mir−8873a_low
Blood
Brain
Heart
Kidney
Lung
Ovary
Skin
Smuscle
Testis
1.0
0.5
0.0
−0.5
−1.0
3' counts
−1.0
−0.5
0.0
0.5
1.0
5' counts

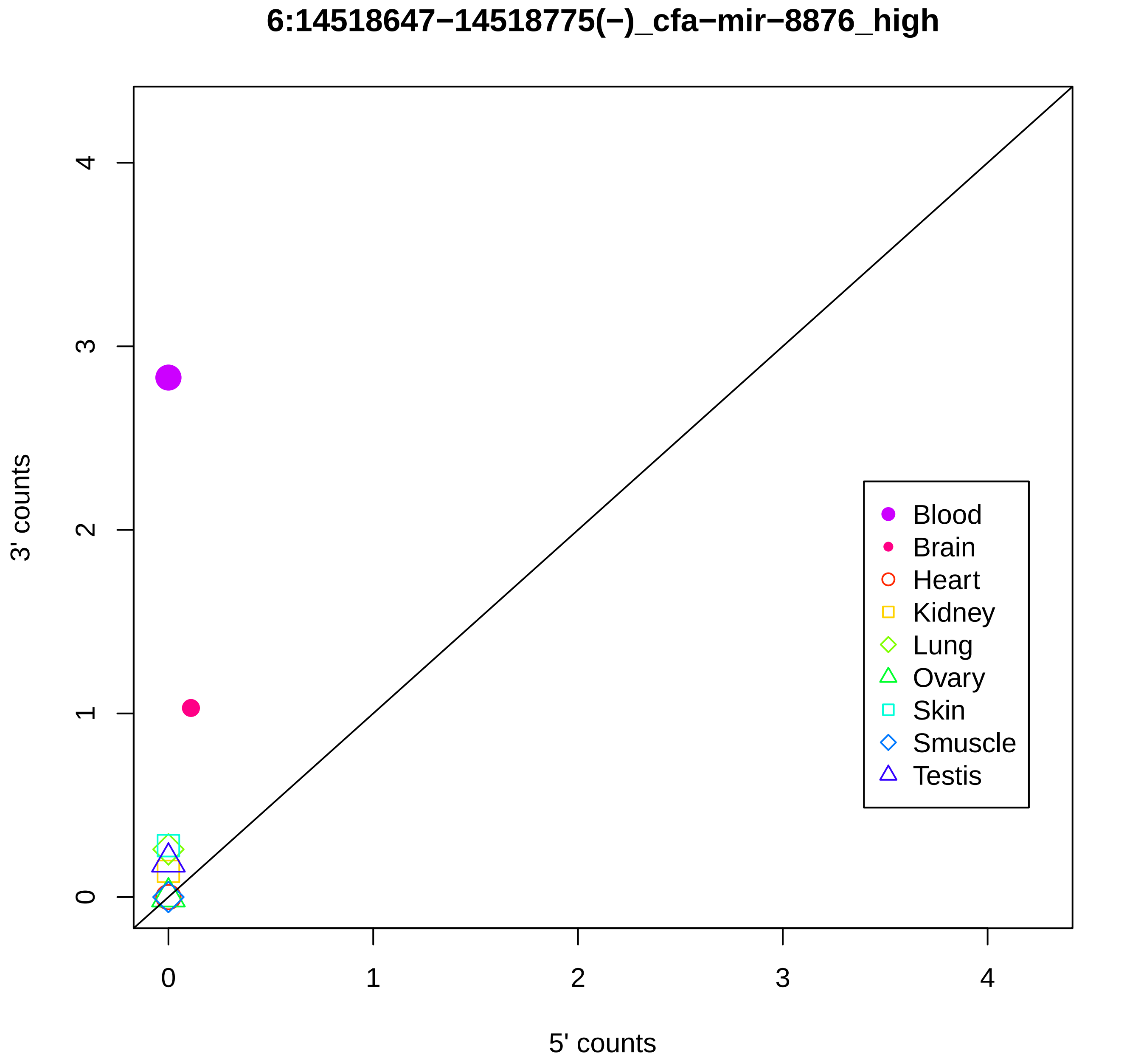

6:14518647−14518775(−)_cfa−mir−8876_high
3' counts
5' counts
0
1
2
3
4
0
1
2
3
4
Blood
Brain
Heart
Kidney
Lung
Ovary
Skin
Smuscle
Testis

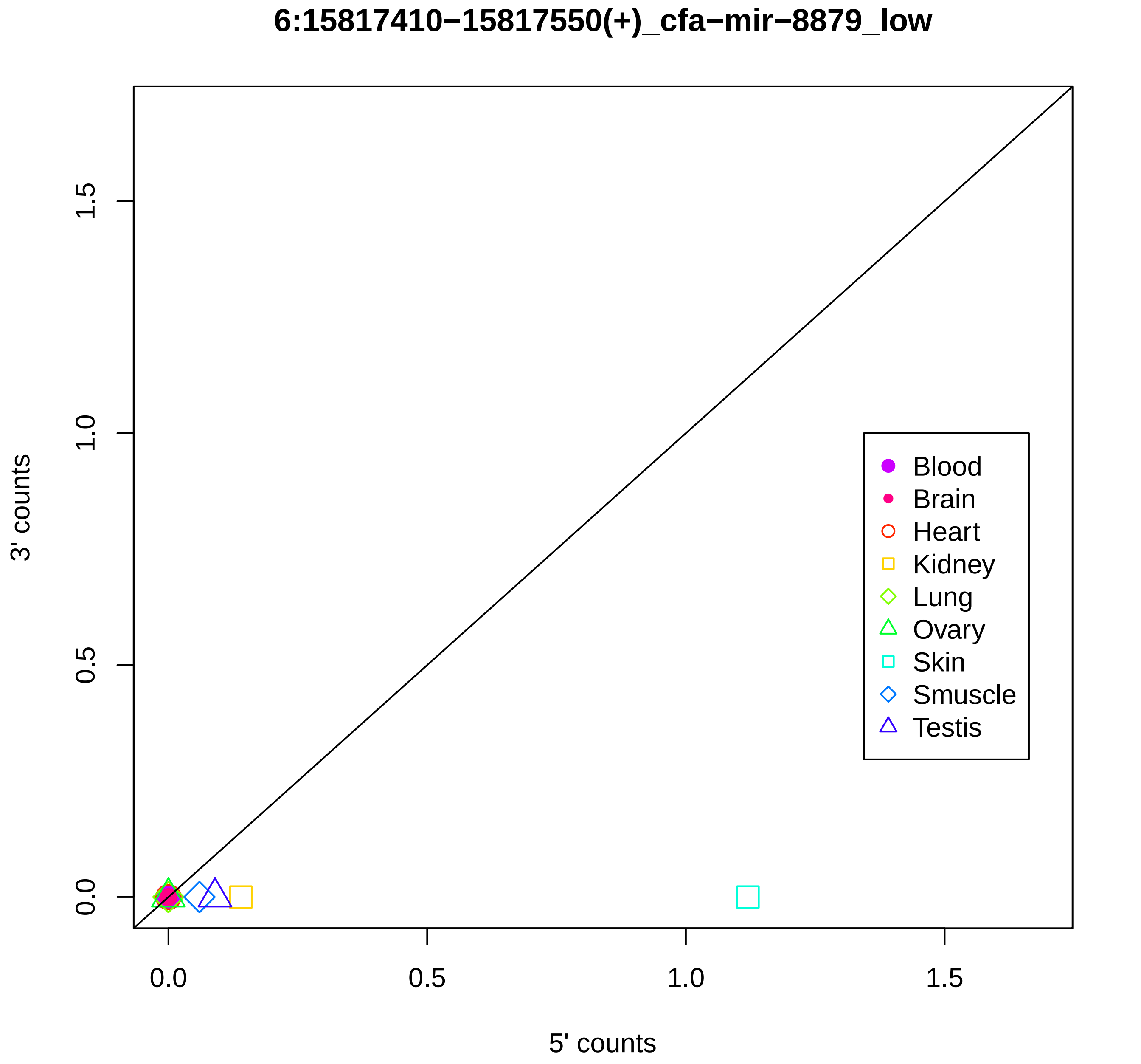

6:15817410−15817550(+)_cfa−mir−8879_low
3' counts
5' counts
0.0
0.5
1.0
1.5
0.0
0.5
1.0
1.5
Blood
Brain
Heart
Kidney
Lung
Ovary
Skin
Smuscle
Testis

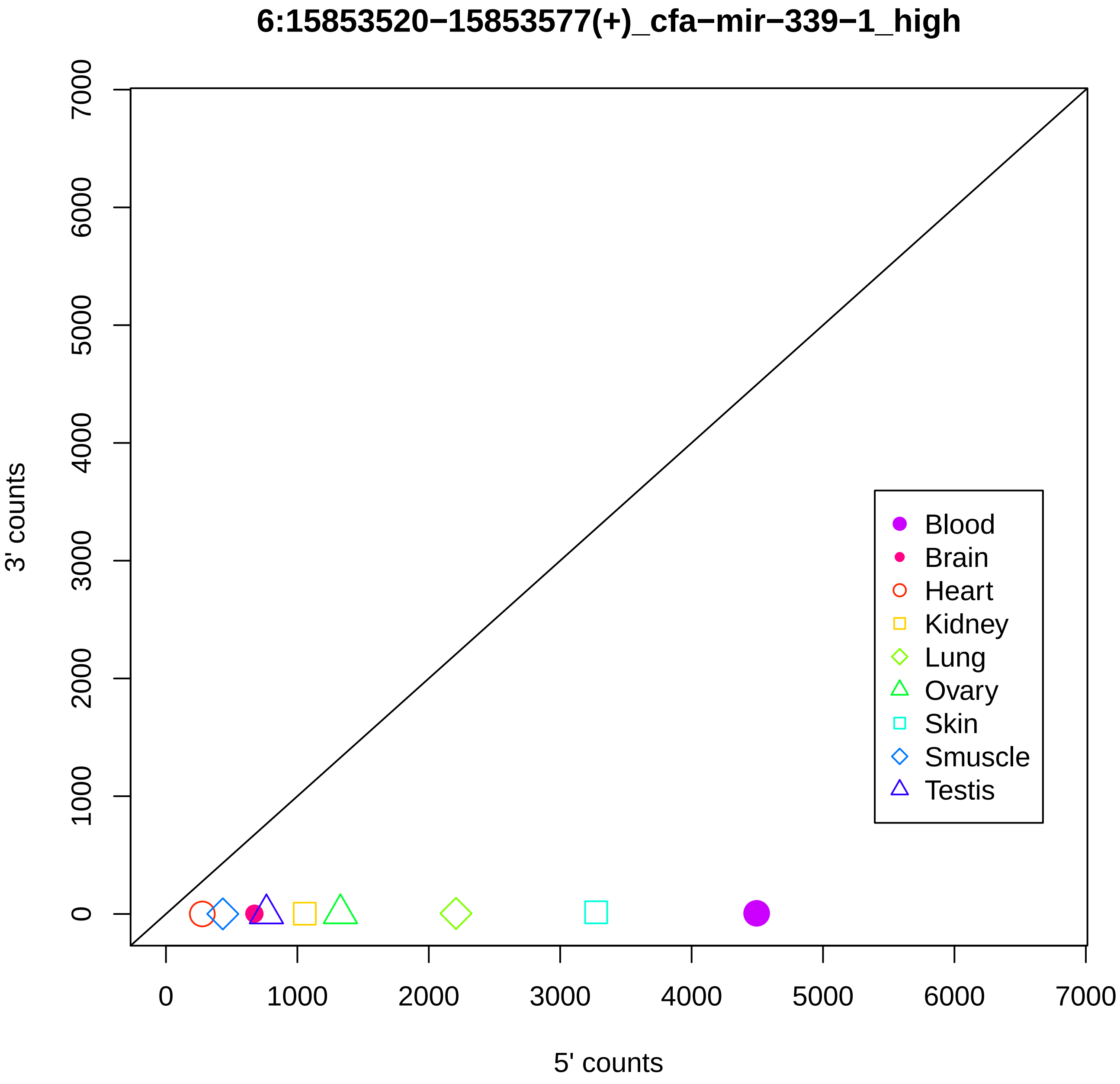

6:15853520−15853577(+)_cfa−mir−339−1_high
3' counts
5' counts
0
1000
2000
3000
4000
5000
6000
7000
Blood
Brain
Heart
Kidney
Lung
Ovary
Skin
Smuscle
Testis

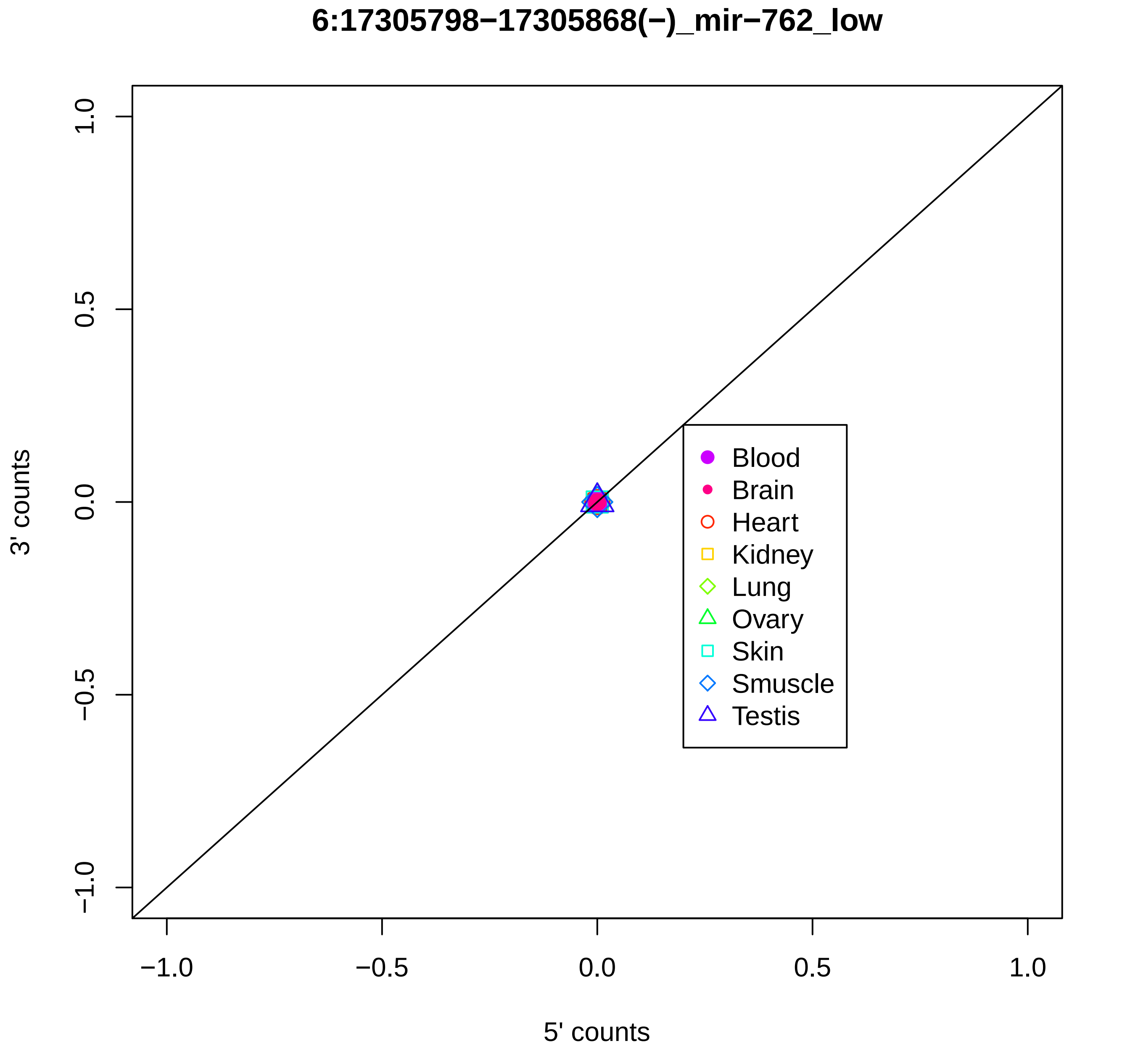

6:17305798−17305868(−)_mir−762_low
3' counts
5' counts
1.0
0.5
0.0
−0.5
−1.0
−1.0
−0.5
0.0
0.5
1.0
Blood
Brain
Heart
Kidney
Lung
Ovary
Skin
Smuscle
Testis

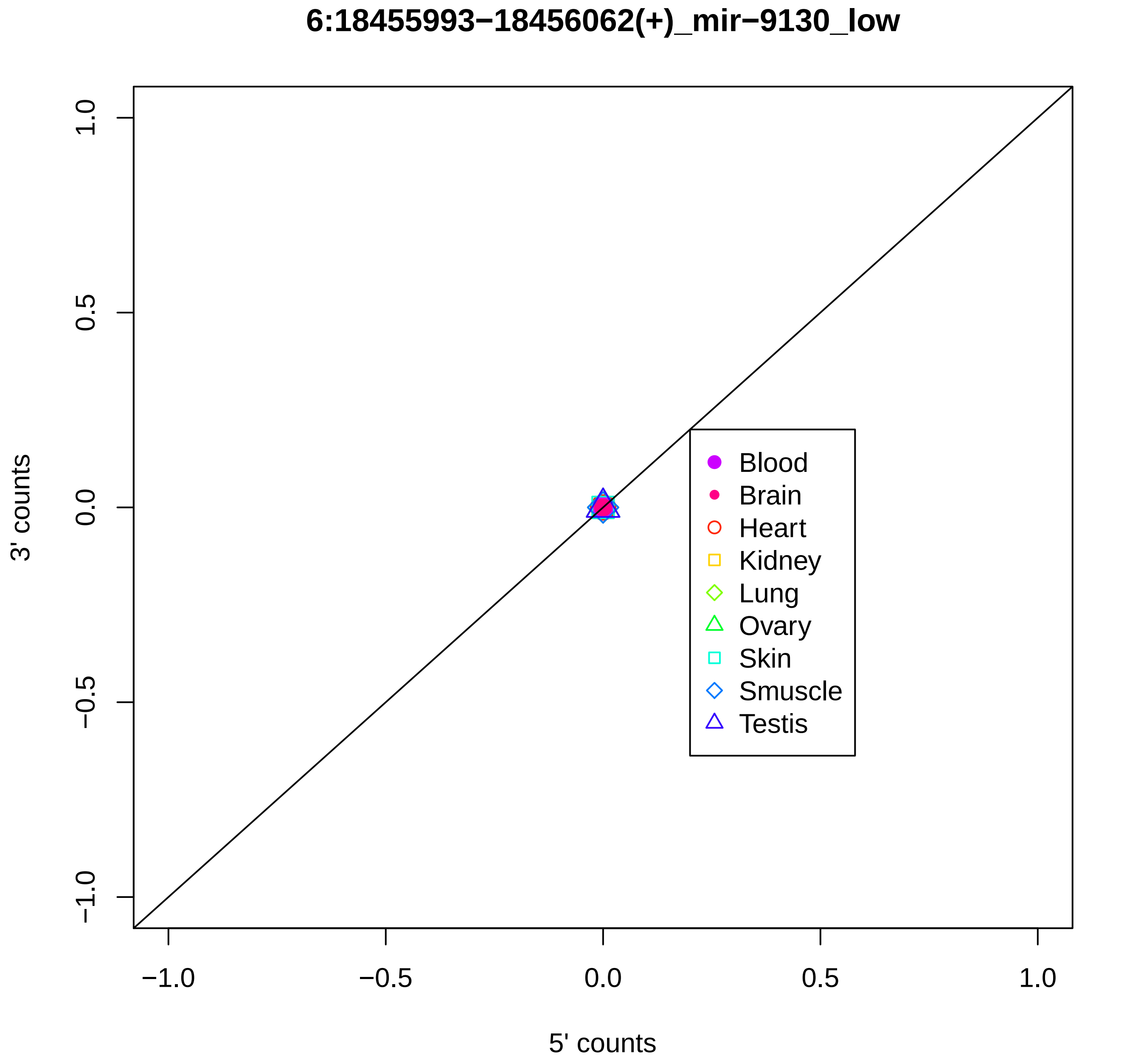

6:18455993−18456062(+)_mir−9130_low
3' counts
5' counts
−1.0
−0.5
0.0
0.5
1.0
Blood
Brain
Heart
Kidney
Lung
Ovary
Skin
Smuscle
Testis

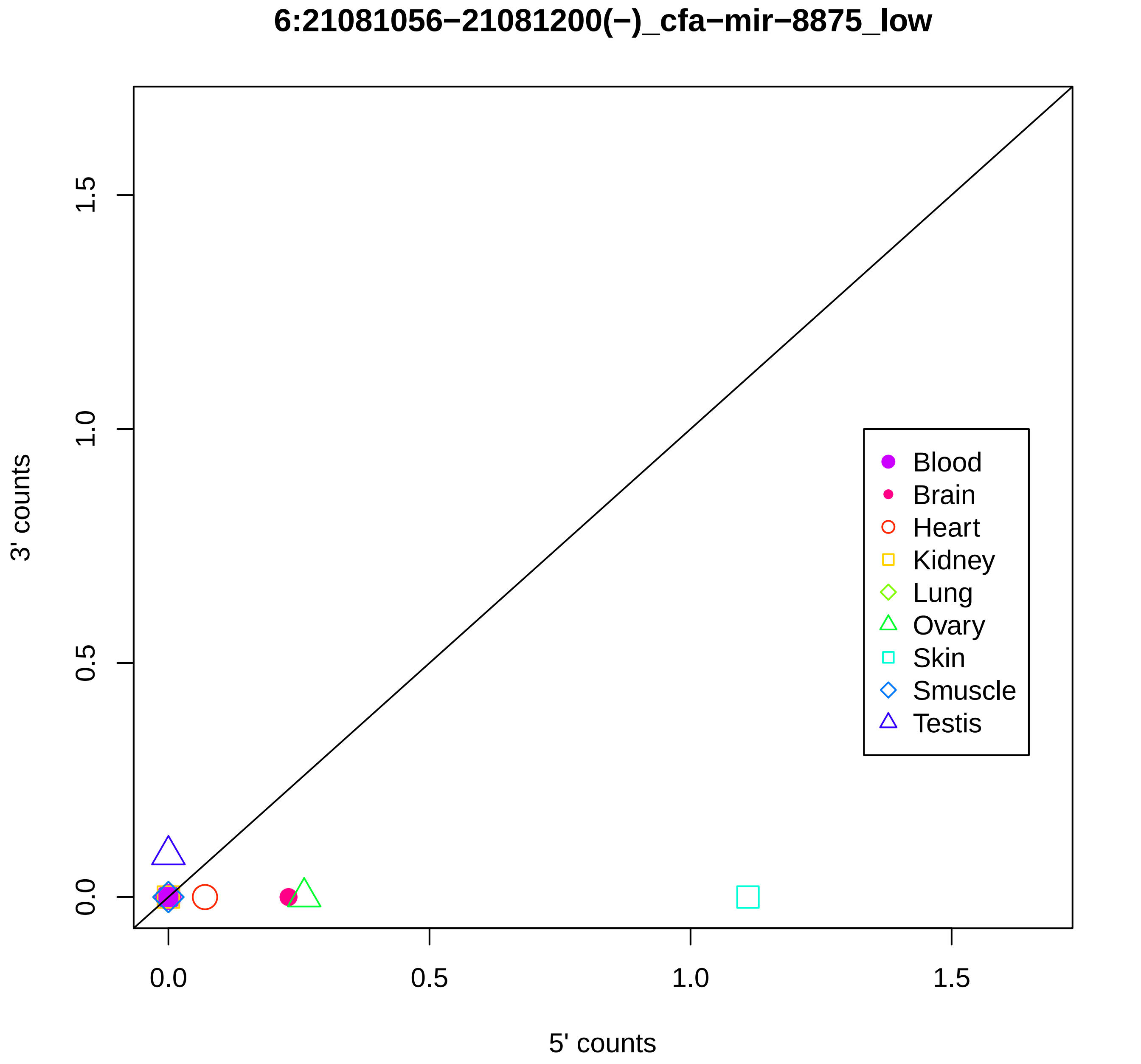

6:21081056−21081200(−)_cfa−mir−8875_low
3' counts
5' counts
0.0
0.5
1.0
1.5
Blood
Brain
Heart
Kidney
Lung
Ovary
Skin
Smuscle
Testis

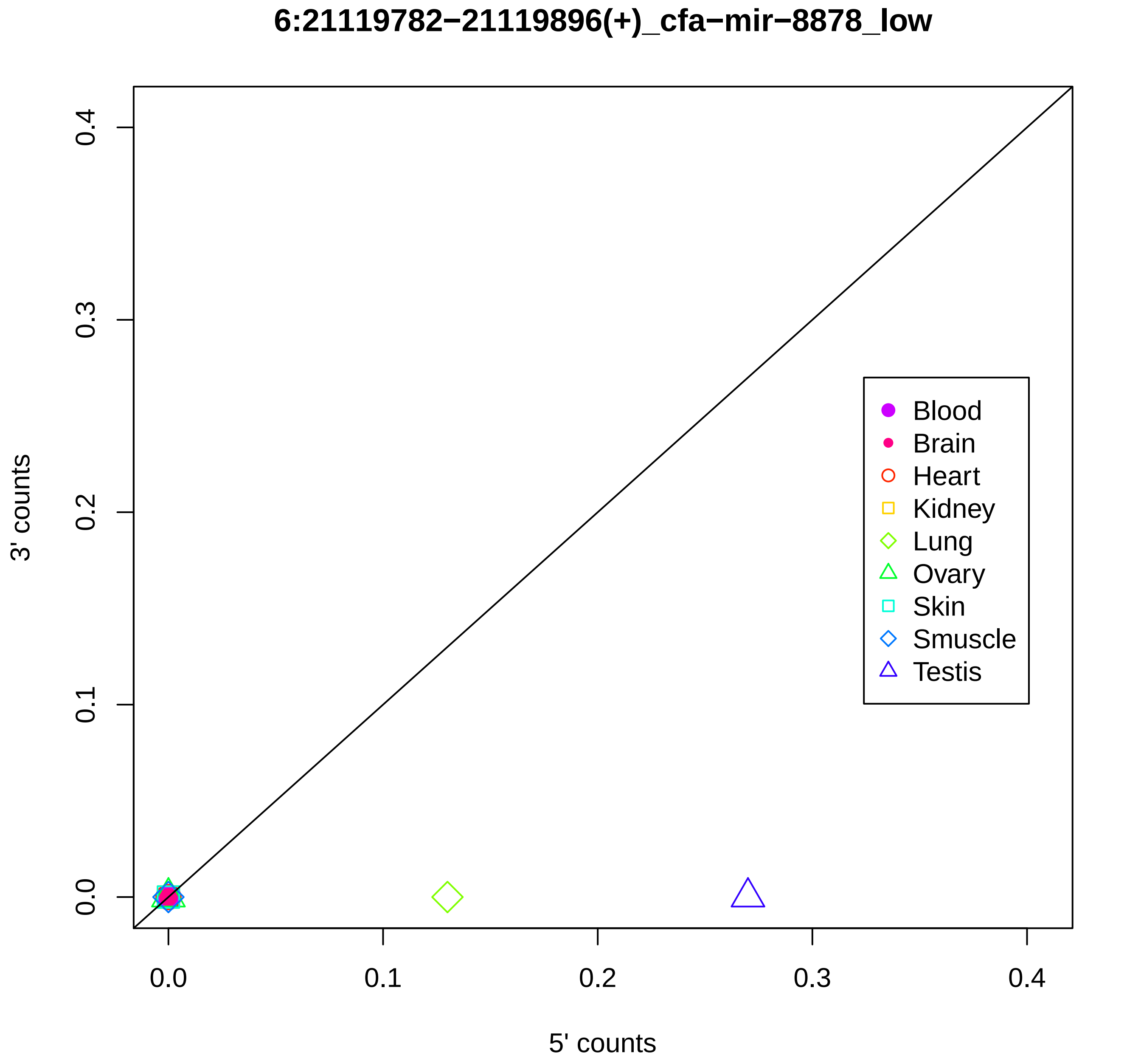

6:21119782−21119896(+)_cfa−mir−8878_low
3' counts
5' counts
0.0
0.1
0.2
0.3
0.4
Blood
Brain
Heart
Kidney
Lung
Ovary
Skin
Smuscle
Testis

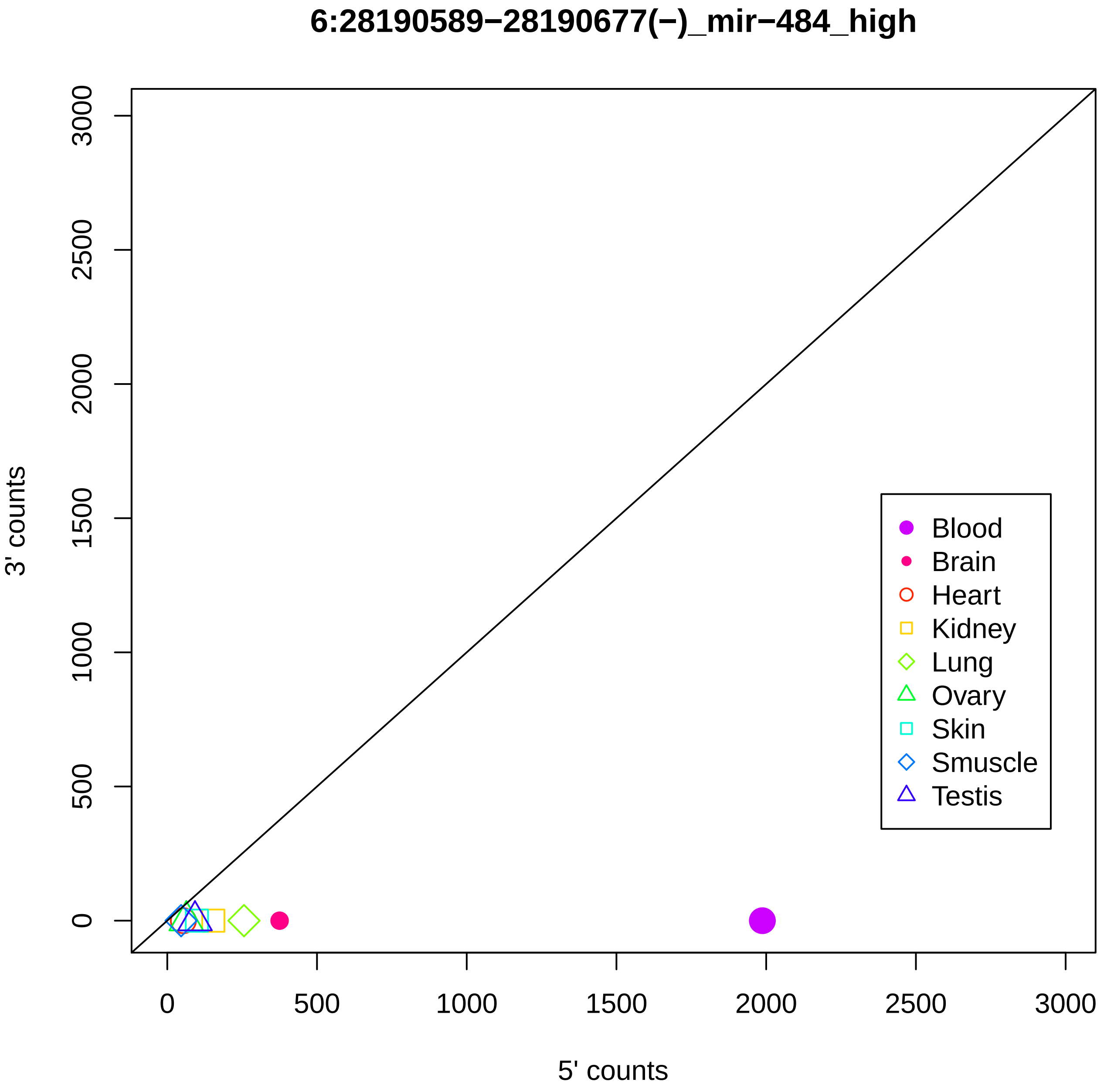
6:28190589−28190677(−)_mir−484_high
3' counts
5' counts
0
500
1000
1500
2000
2500
3000
Blood
Brain
Heart
Kidney
Lung
Ovary
Skin
Smuscle
Testis

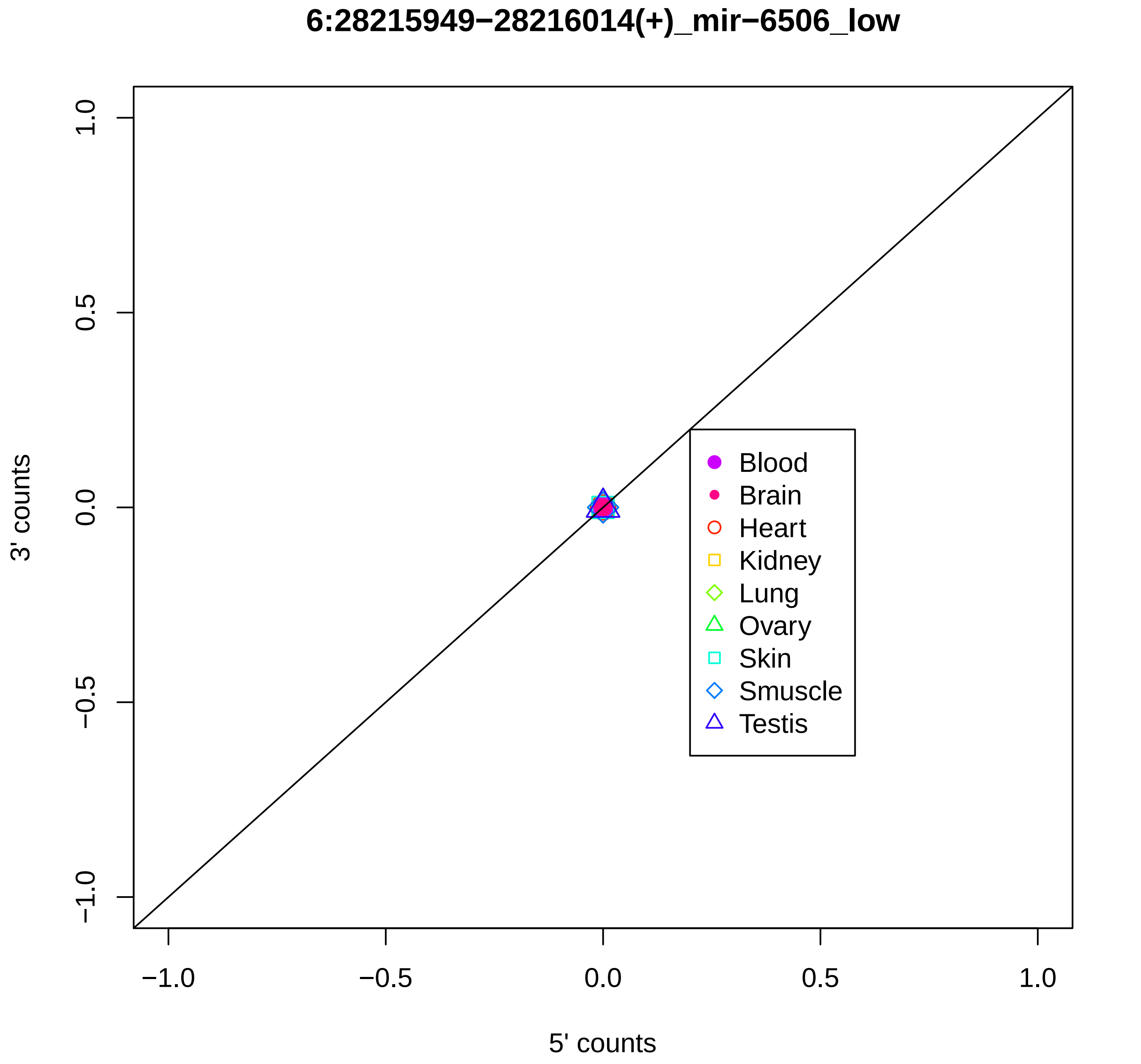

6:28215949−28216014(+)_mir−6506_low
3' counts
5' counts
1.0
0.5
0.0
−0.5
−1.0
Blood
Brain
Heart
Kidney
Lung
Ovary
Skin
Smuscle
Testis

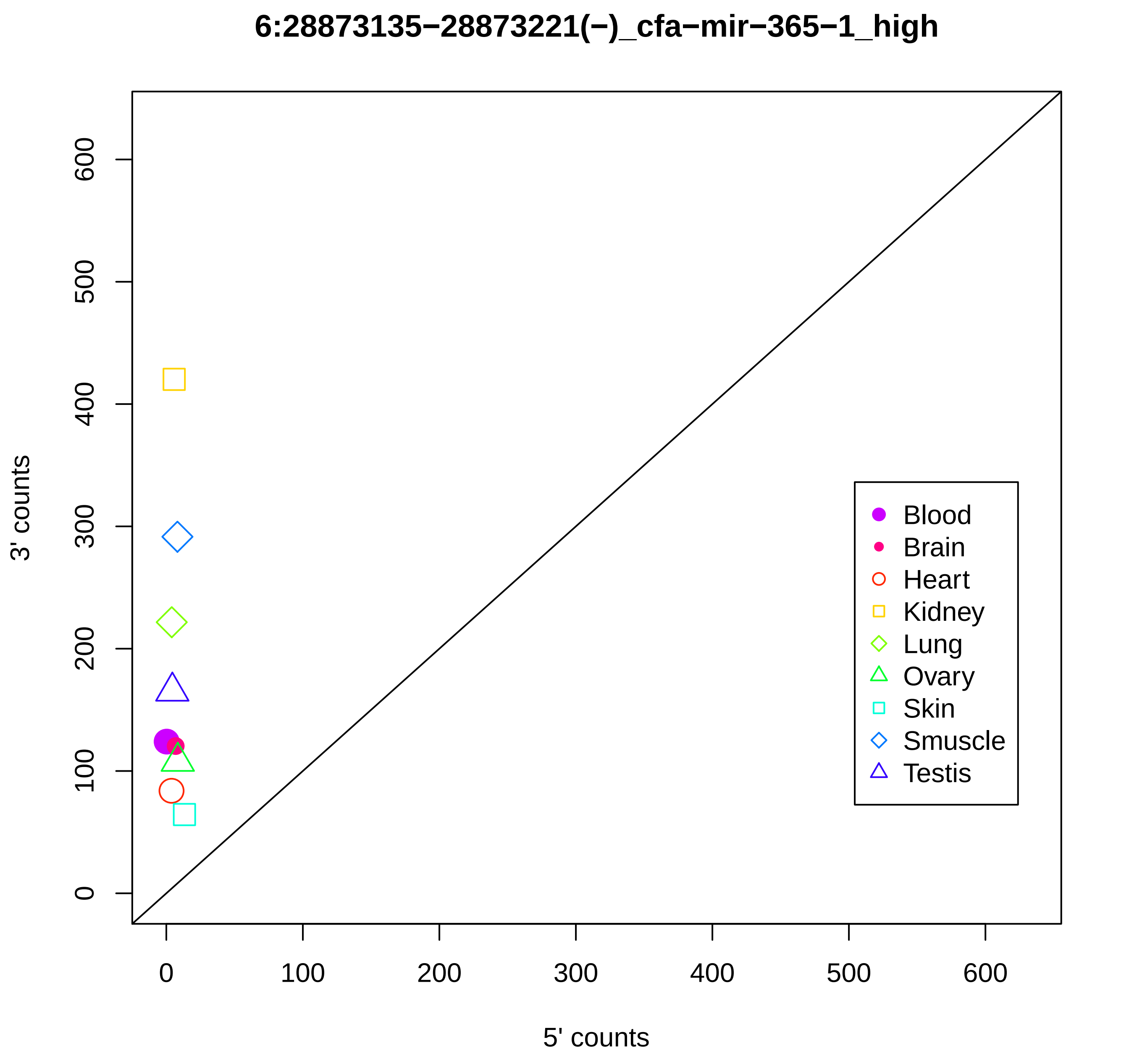

6:28873135−28873221(−)_cfa−mir−365−1_high
3' counts
0
100
200
300
400
500
600
5' counts
0
100
200
300
400
500
600
Blood
Brain
Heart
Kidney
Lung
Ovary
Skin
Smuscle
Testis

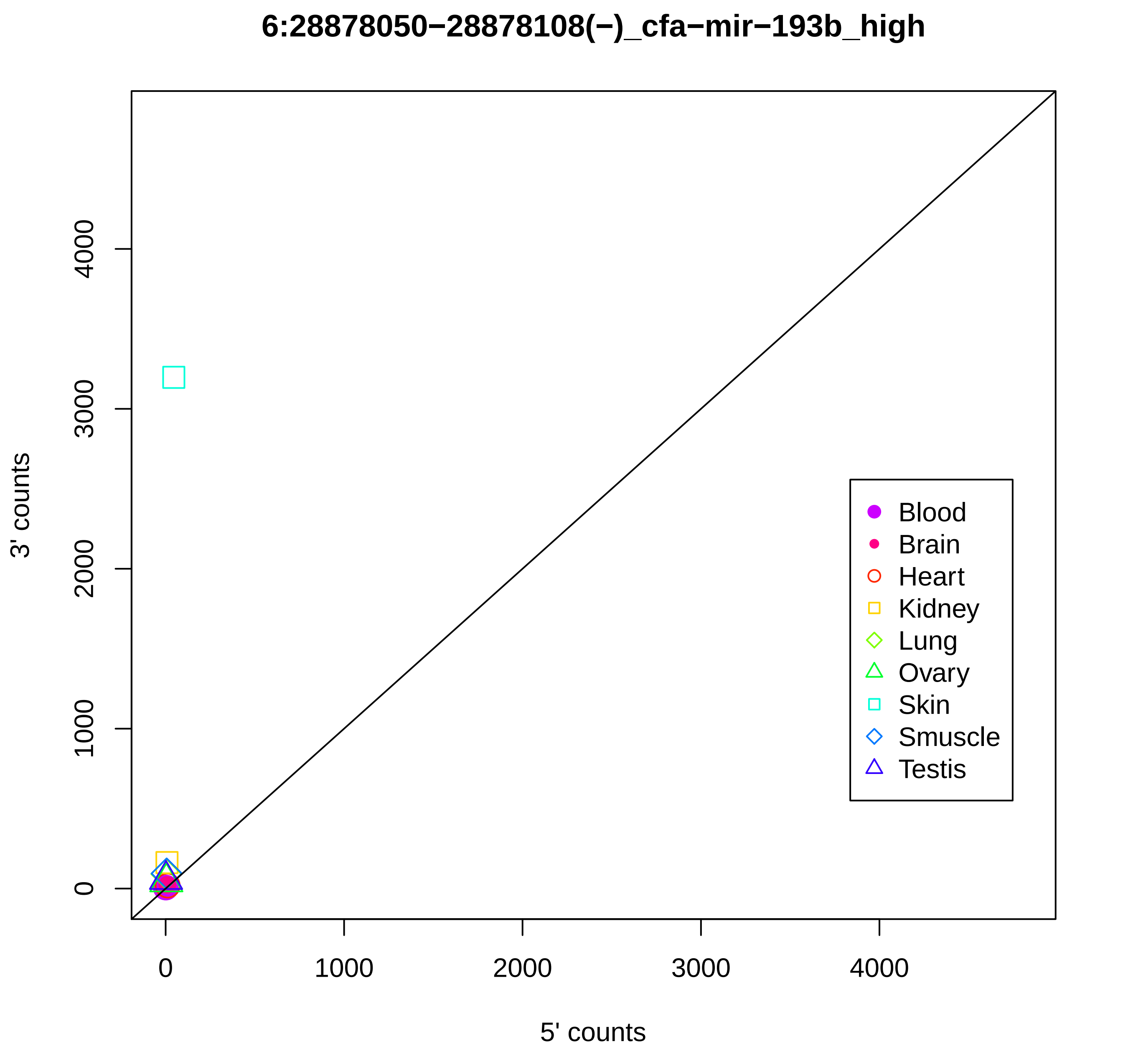

6:28878050−28878108(−)_cfa−mir−193b_high
3' counts
5' counts
0
1000
2000
3000
4000
0
1000
2000
3000
4000
Blood
Brain
Heart
Kidney
Lung
Ovary
Skin
Smuscle
Testis

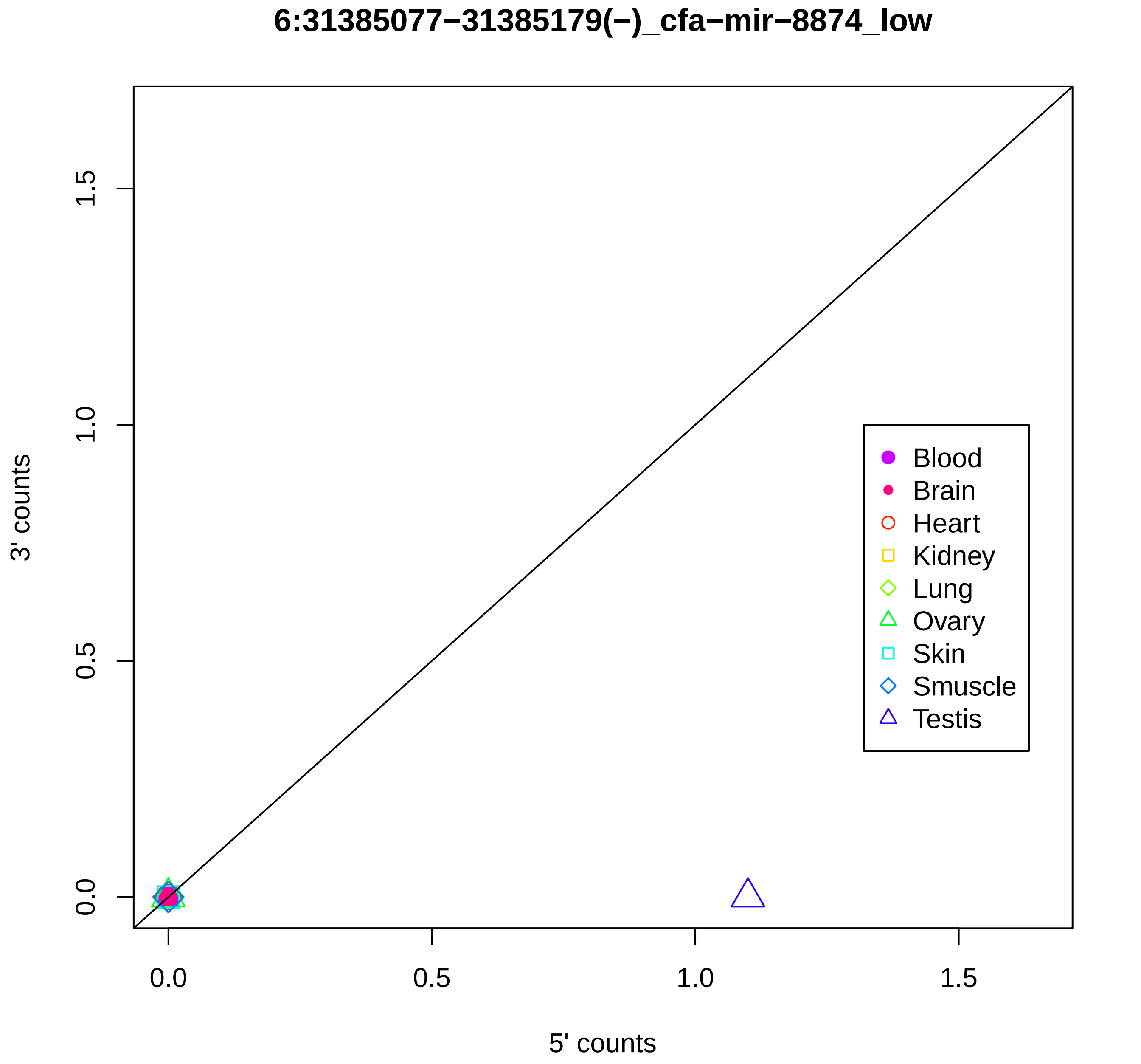

6:31385077−31385179(−)_cfa−mir−8874_low
3' counts
5' counts
0.0
0.5
1.0
1.5
Blood
Brain
Heart
Kidney
Lung
Ovary
Skin
Smuscle
Testis

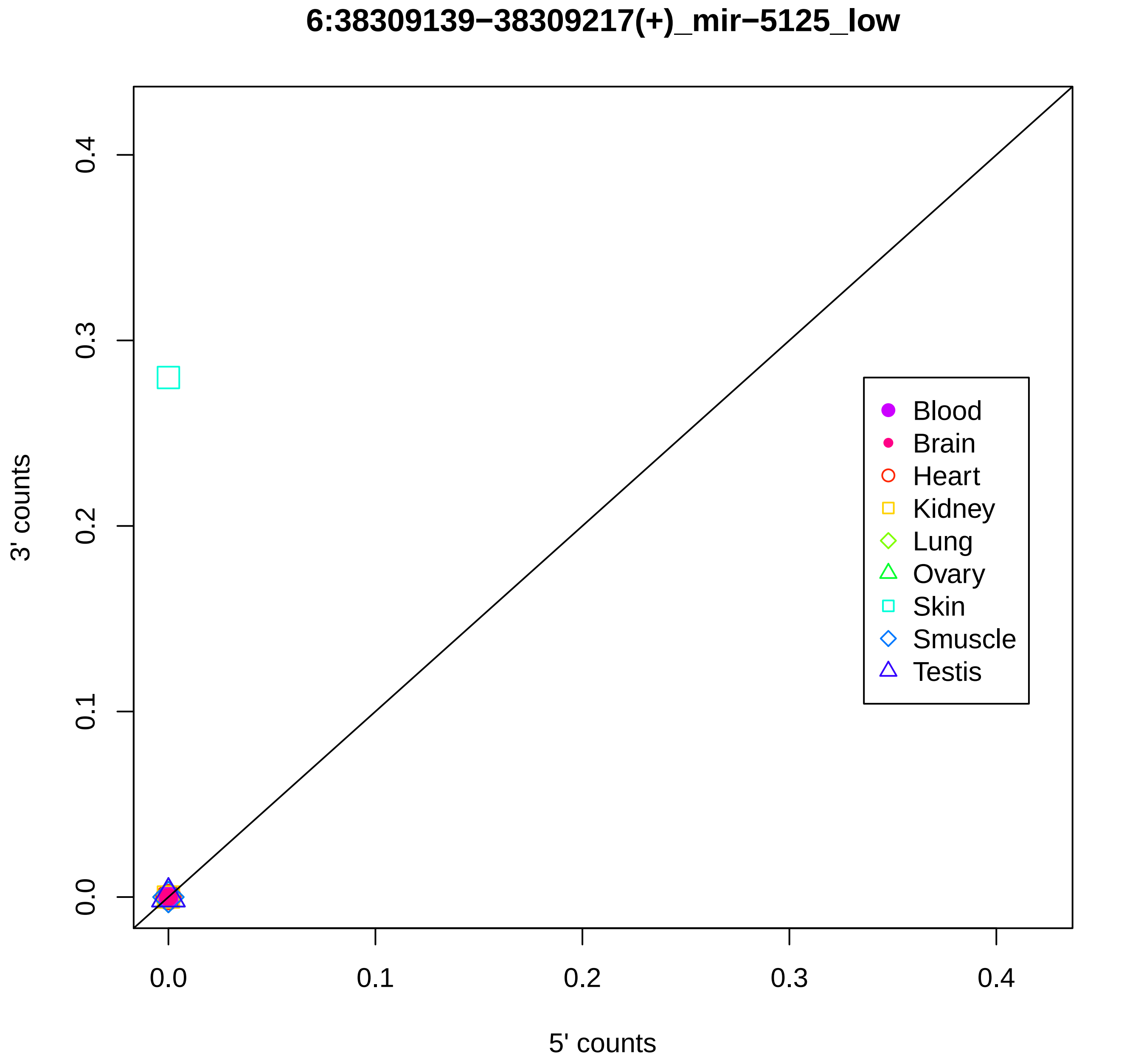

6:38309139−38309217(+)_mir−5125_low
3' counts
5' counts
0.0
0.1
0.2
0.3
0.4
Blood
Brain
Heart
Kidney
Lung
Ovary
Skin
Smuscle
Testis

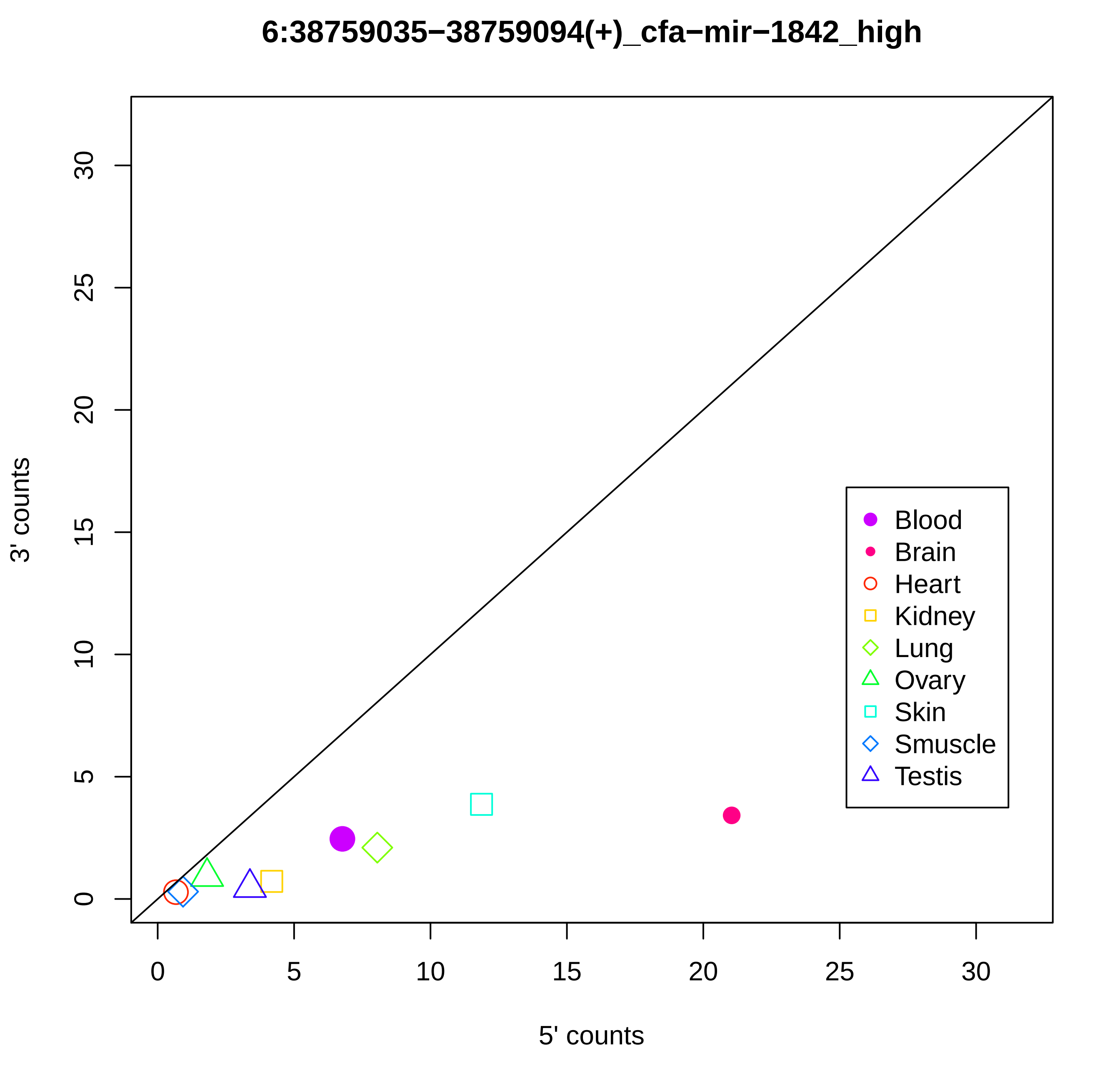

6:38759035−38759094(+)_cfa−mir−1842_high
3' counts
5' counts
0
5
10
15
20
25
30
Blood
Brain
Heart
Kidney
Lung
Ovary
Skin
Smuscle
Testis

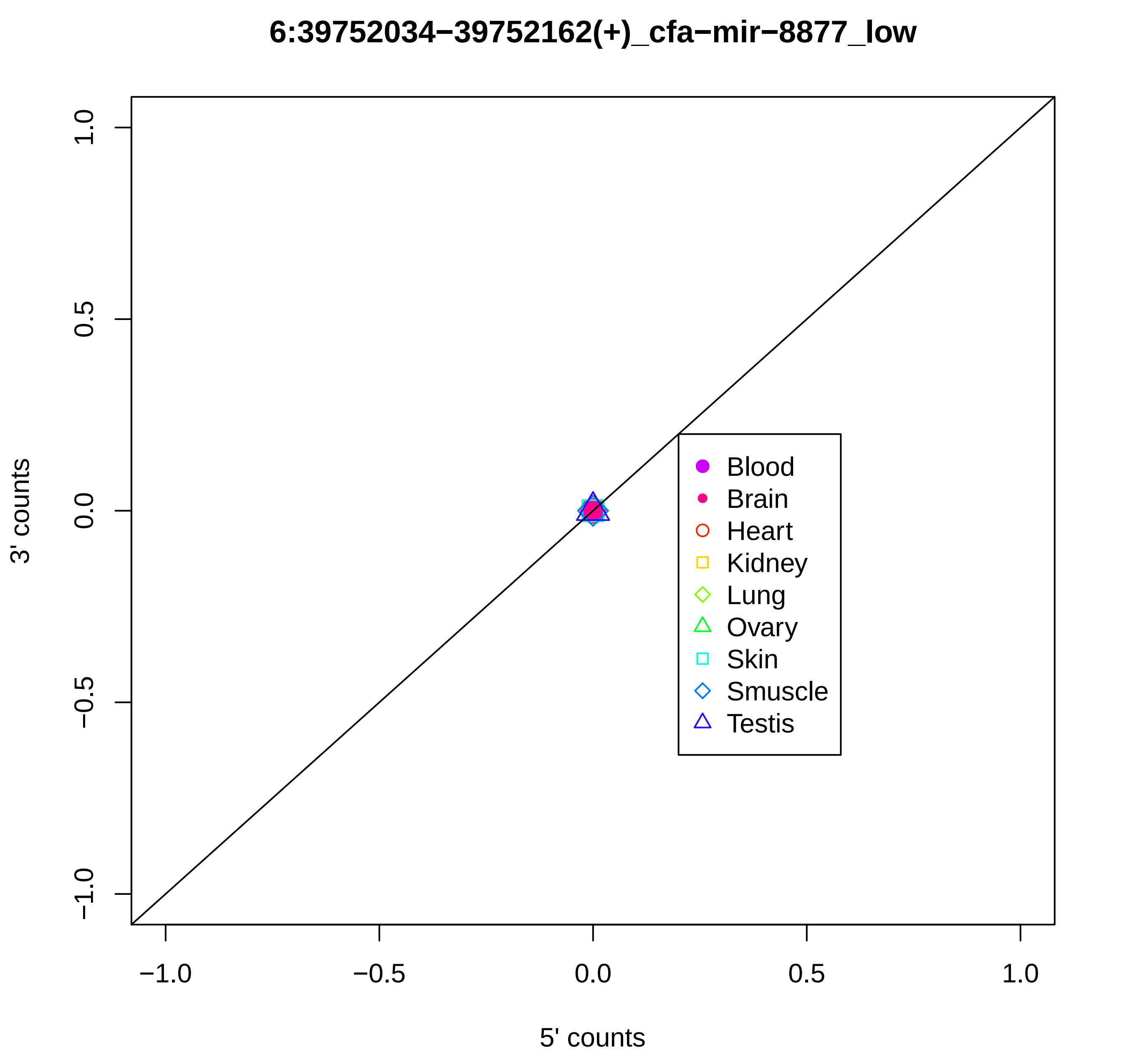

6:39752034−39752162(+)_cfa−mir−8877_low
1.0
0.5
0.0
−0.5
−1.0
3' counts
Blood
Brain
Heart
Kidney
Lung
Ovary
Skin
Smuscle
Testis
−1.0
−0.5
0.0
0.5
1.0
5' counts

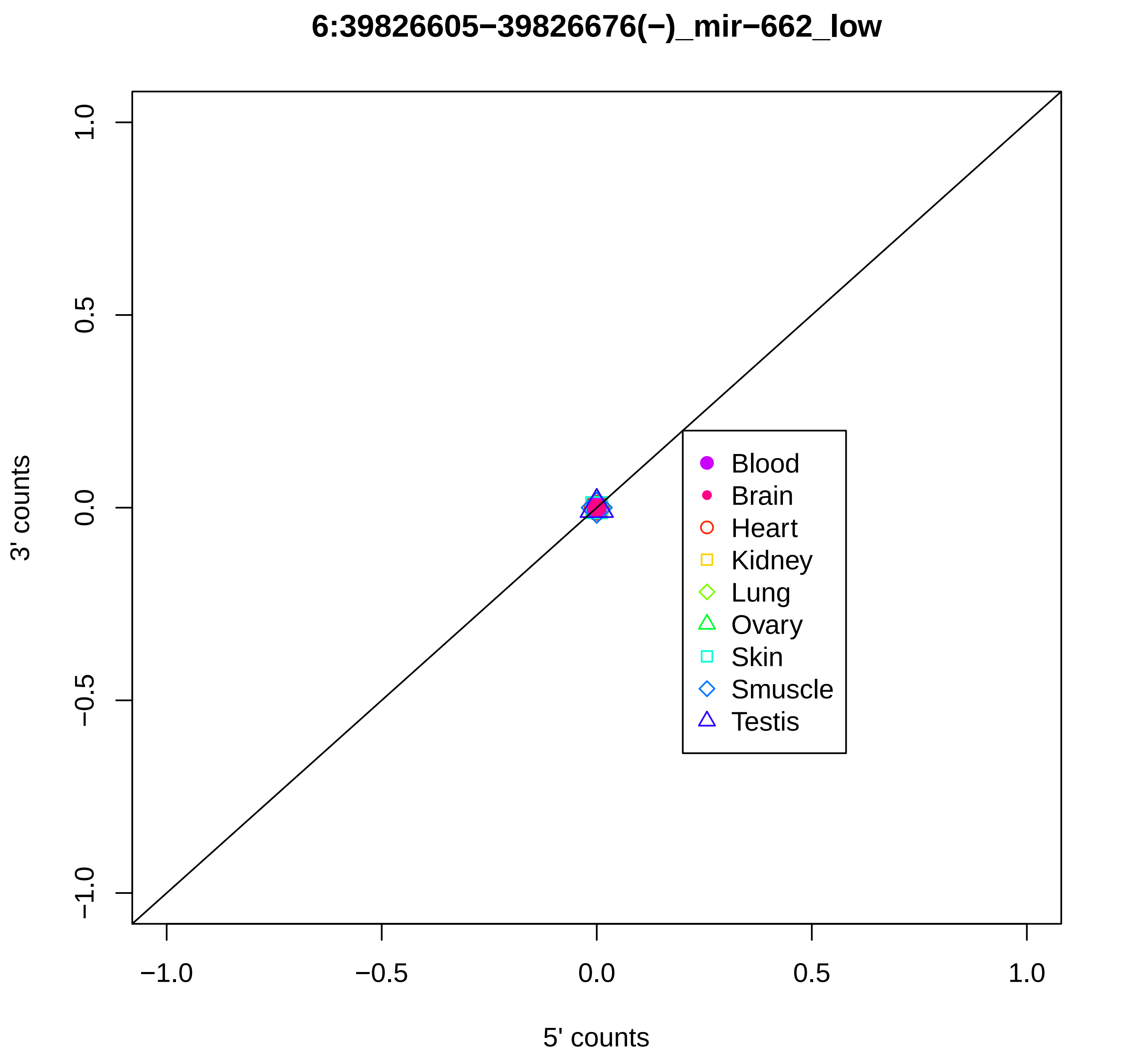
6:39826605−39826676(−)_mir−662_low
3' counts
5' counts
−1.0
−0.5
0.0
0.5
1.0
Blood
Brain
Heart
Kidney
Lung
Ovary
Skin
Smuscle
Testis

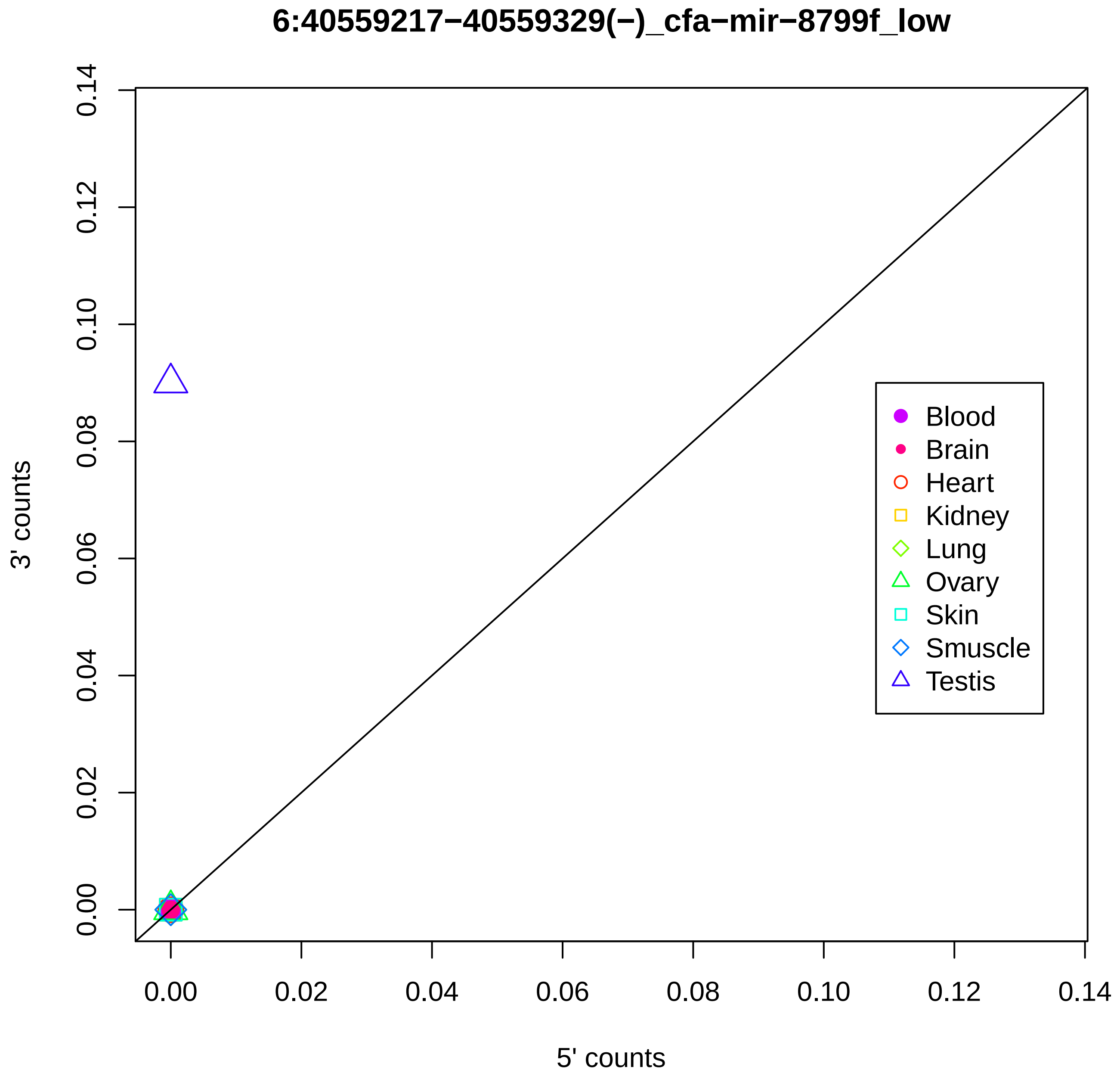

6:40559217−40559329(−)_cfa−mir−8799f_low
3' counts
5' counts
0.00
0.02
0.04
0.06
0.08
0.10
0.12
0.14
Blood
Brain
Heart
Kidney
Lung
Ovary
Skin
Smuscle
Testis

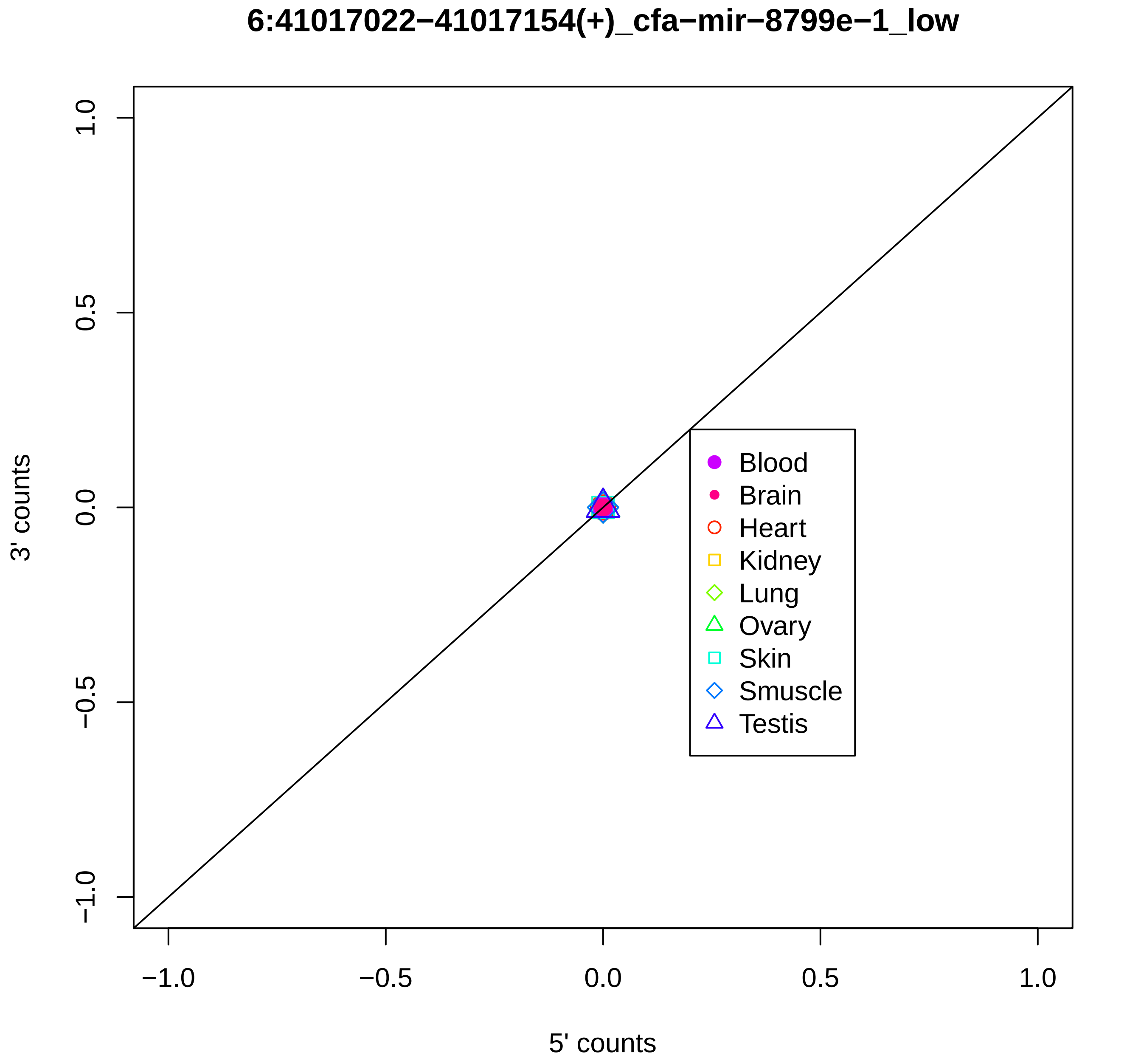

6:41017022−41017154(+)_cfa−mir−8799e−1_low
3' counts
5' counts
1.0
0.5
0.0
−0.5
−1.0
Blood
Brain
Heart
Kidney
Lung
Ovary
Skin
Smuscle
Testis

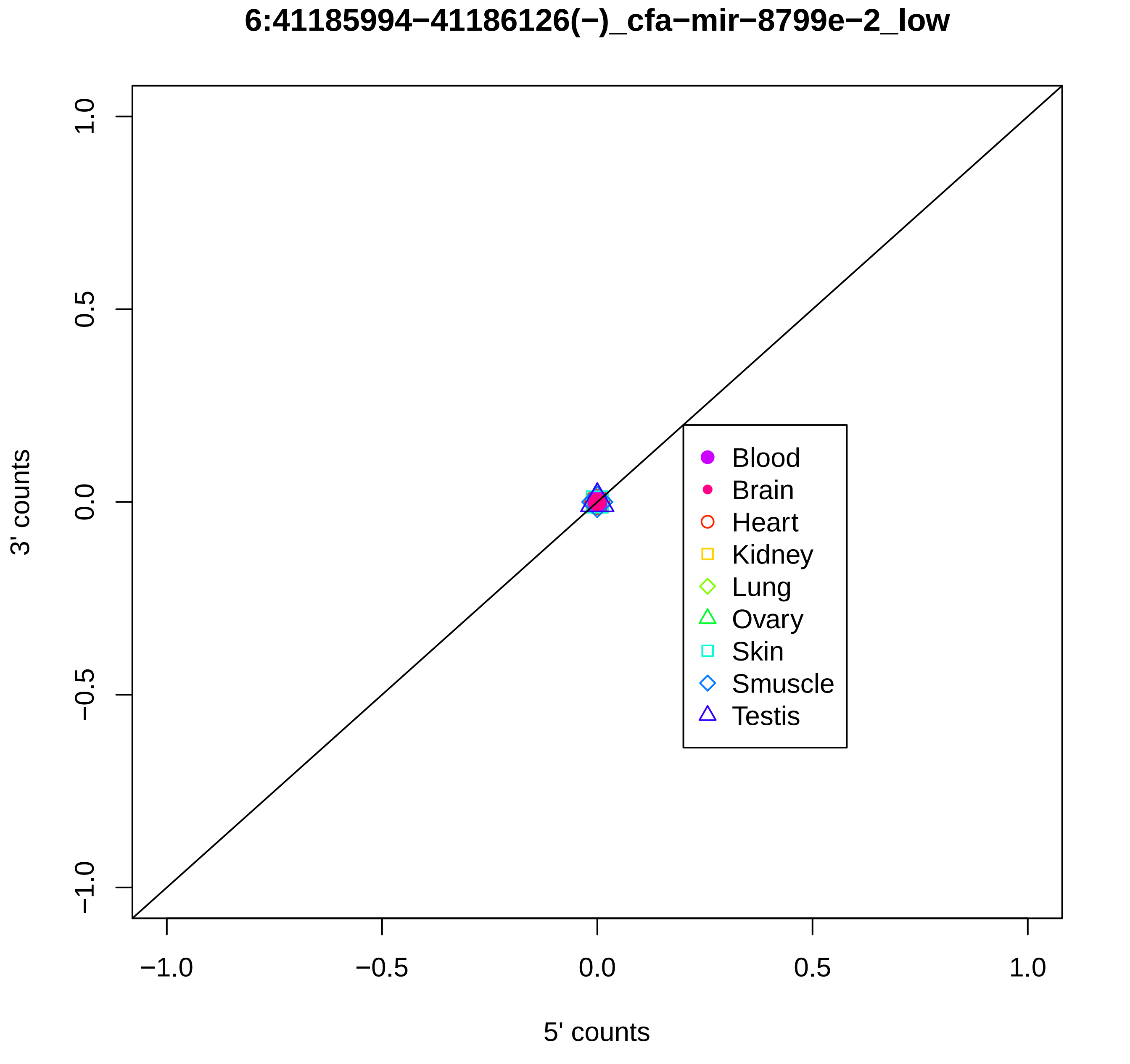

6:41185994−41186126(−)_cfa−mir−8799e−2_low
3' counts
5' counts
−1.0
−0.5
0.0
0.5
1.0
Blood
Brain
Heart
Kidney
Lung
Ovary
Skin
Smuscle
Testis

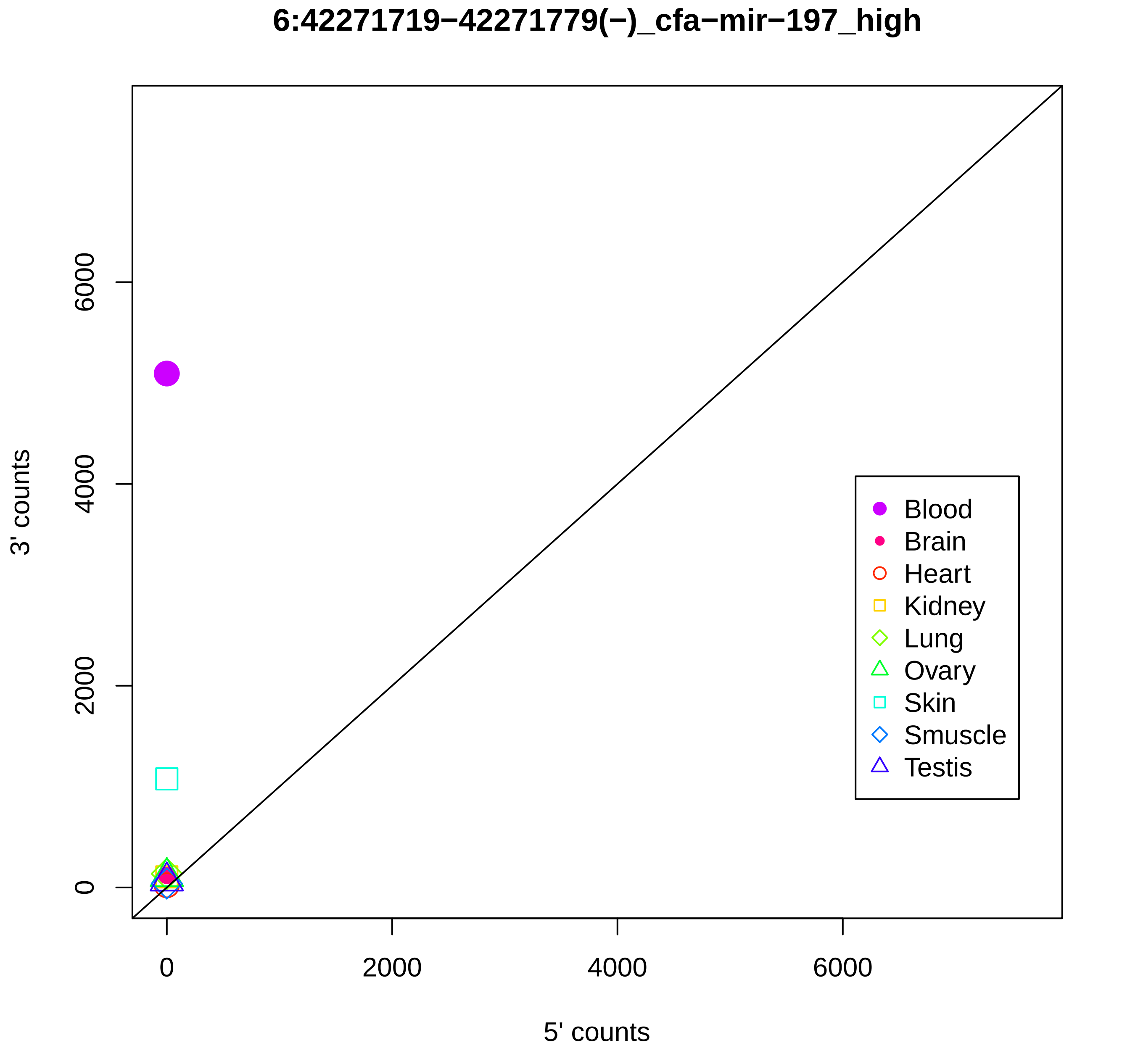

6:42271719−42271779(−)_cfa−mir−197_high
3' counts
5' counts
0
2000
4000
6000
0
2000
4000
6000
Blood
Brain
Heart
Kidney
Lung
Ovary
Skin
Smuscle
Testis

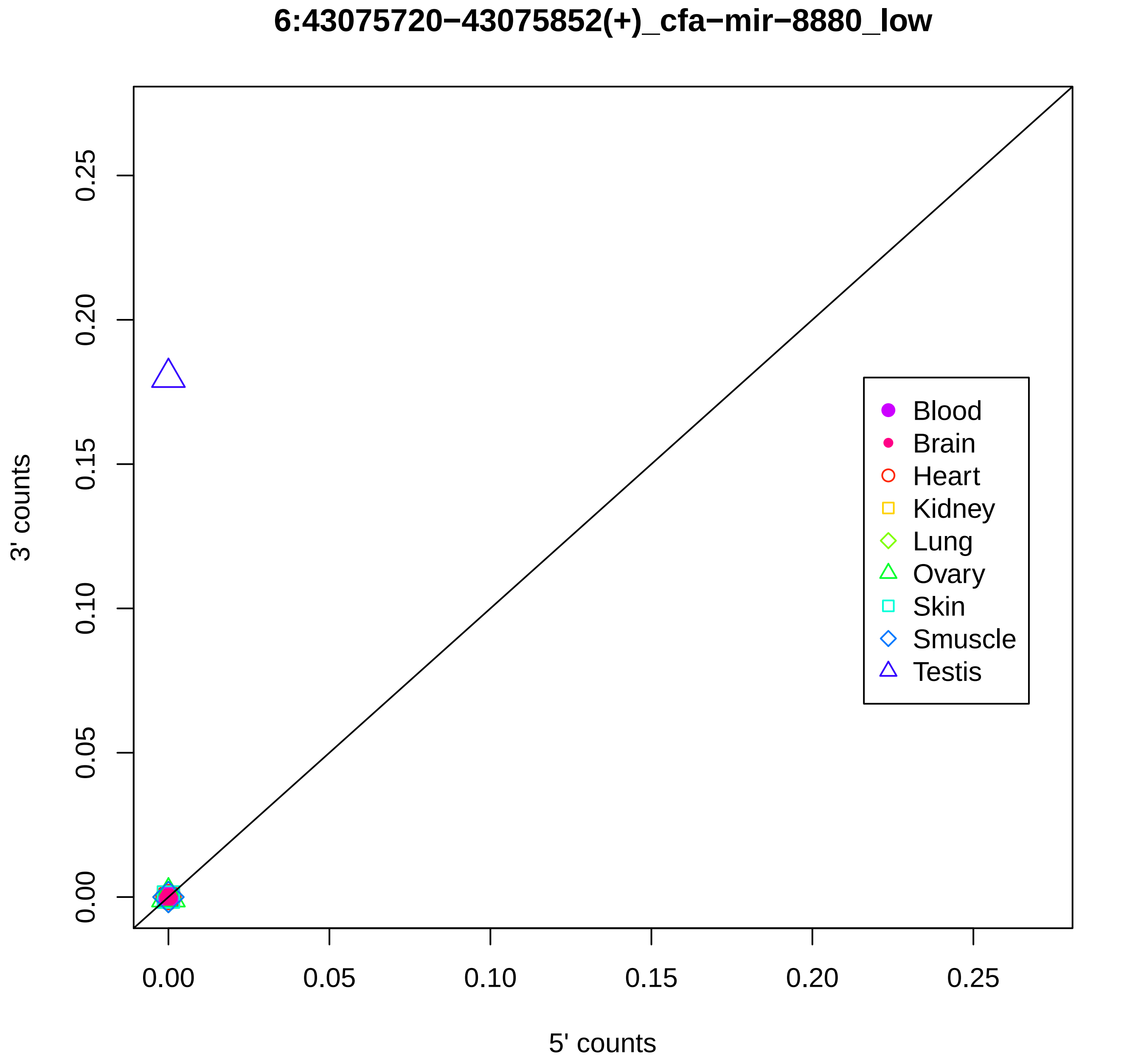

6:43075720−43075852(+)_cfa−mir−8880_low
3' counts
5' counts
0.00
0.05
0.10
0.15
0.20
0.25
Blood
Brain
Heart
Kidney
Lung
Ovary
Skin
Smuscle
Testis

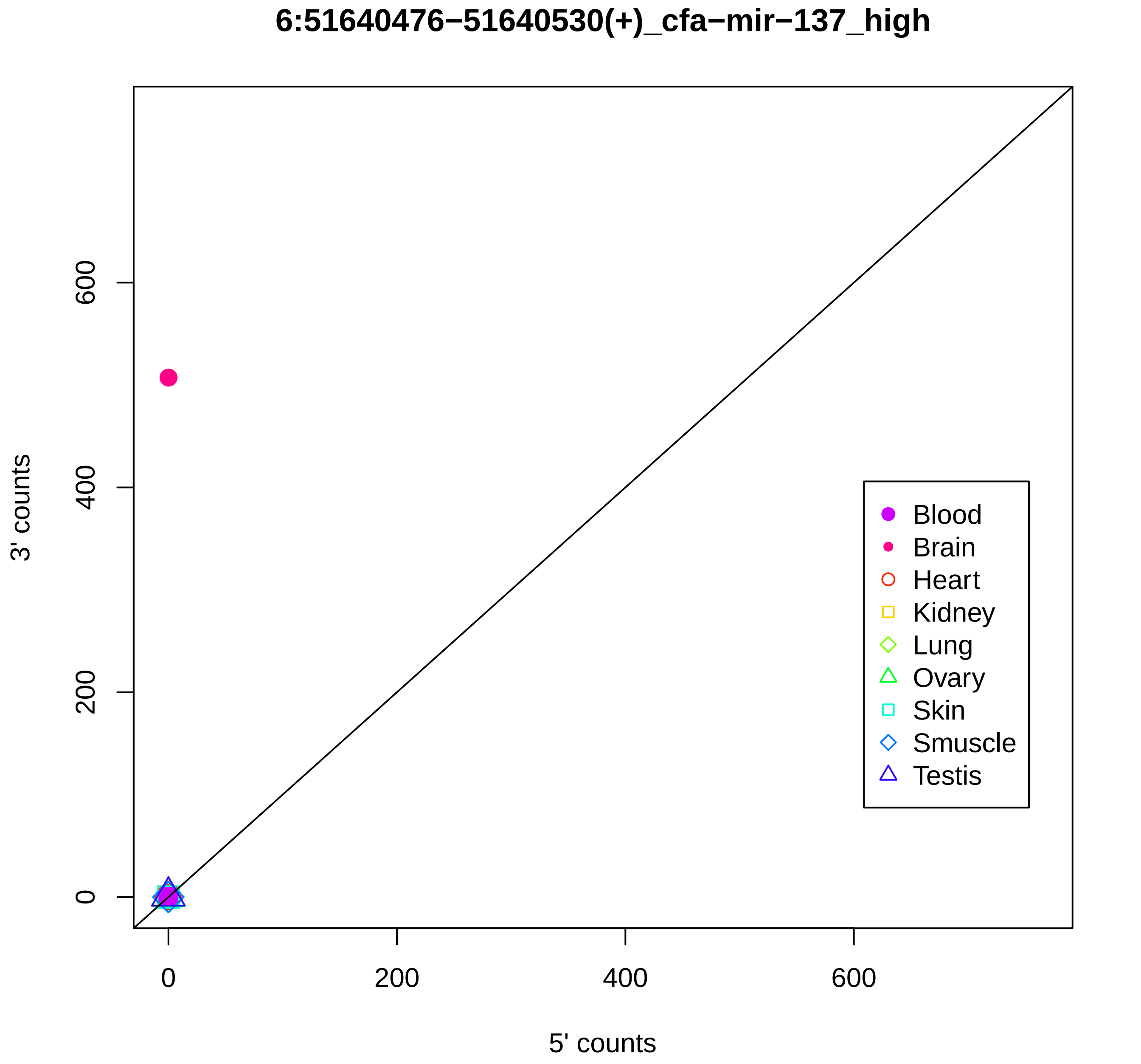
6:51640476−51640530(+)_cfa−mir−137_high
3' counts
5' counts
0
200
400
600
Blood
Brain
Heart
Kidney
Lung
Ovary
Skin
Smuscle
Testis

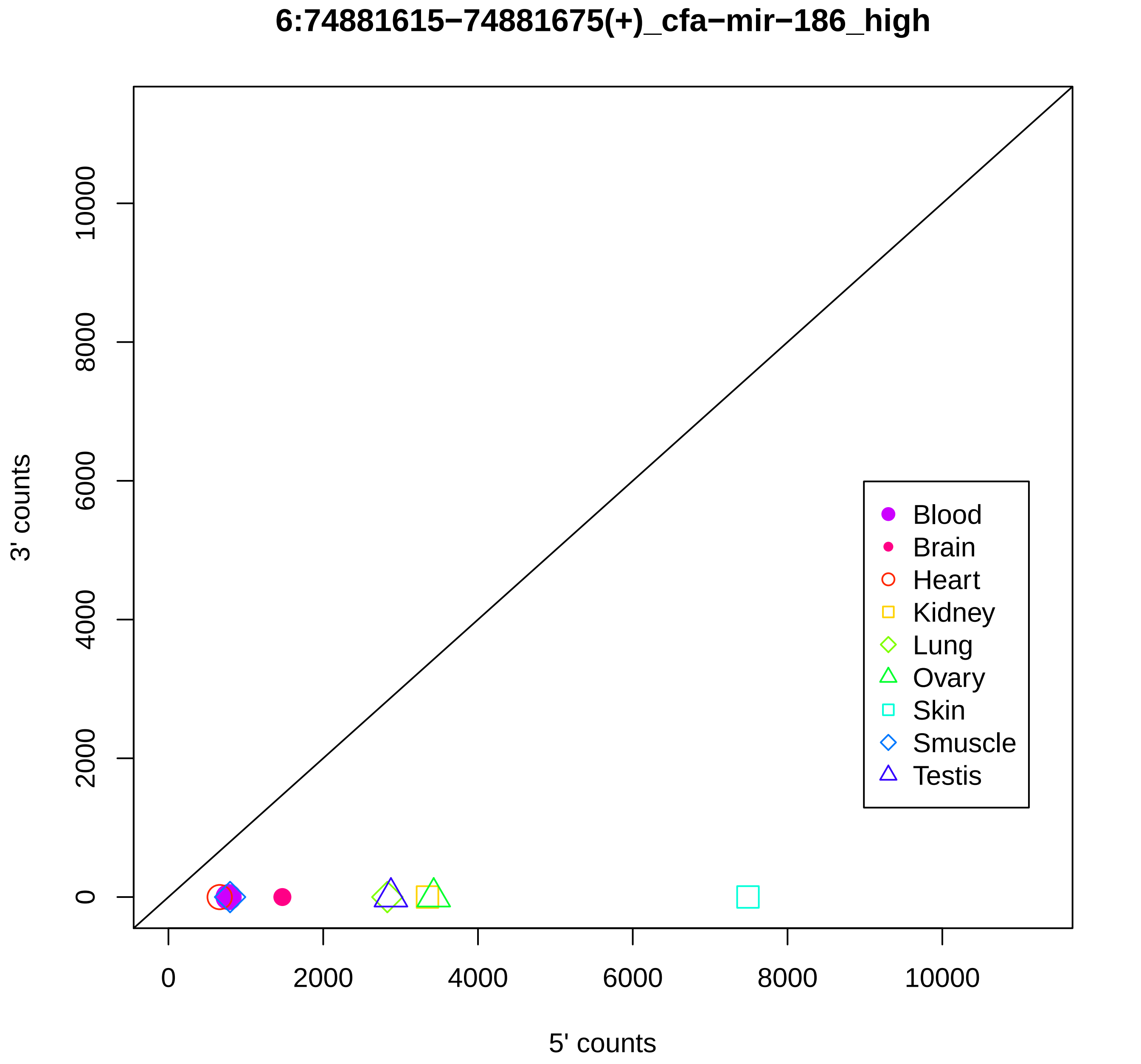

6:74881615−74881675(+)_cfa−mir−186_high
3' counts
5' counts
0
2000
4000
6000
8000
10000
Blood
Brain
Heart
Kidney
Lung
Ovary
Skin
Smuscle
Testis

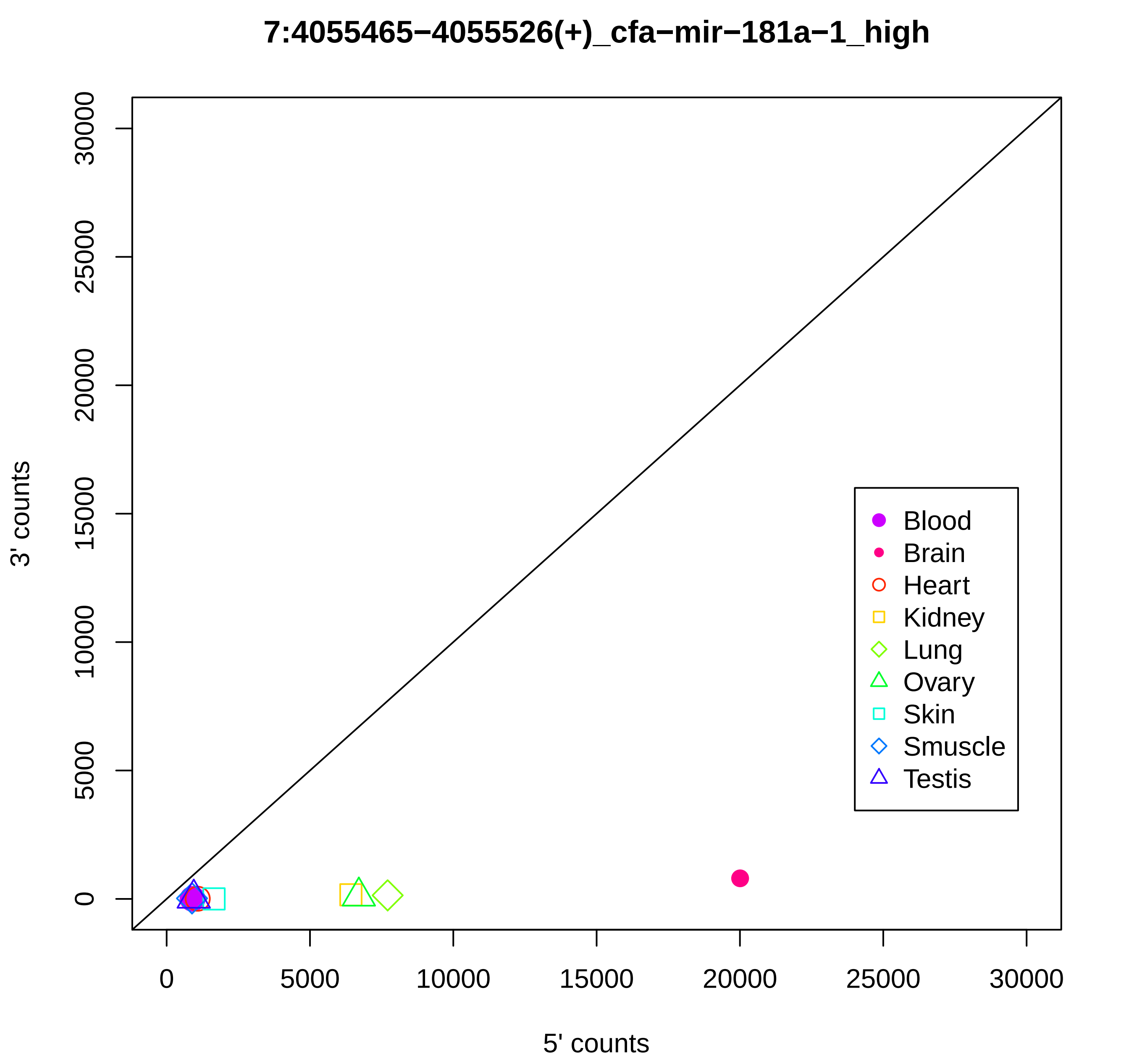

7:4055465−4055526(+)_cfa−mir−181a−1_high
3' counts
5' counts
0
5000
10000
15000
20000
25000
30000
Blood
Brain
Heart
Kidney
Lung
Ovary
Skin
Smuscle
Testis

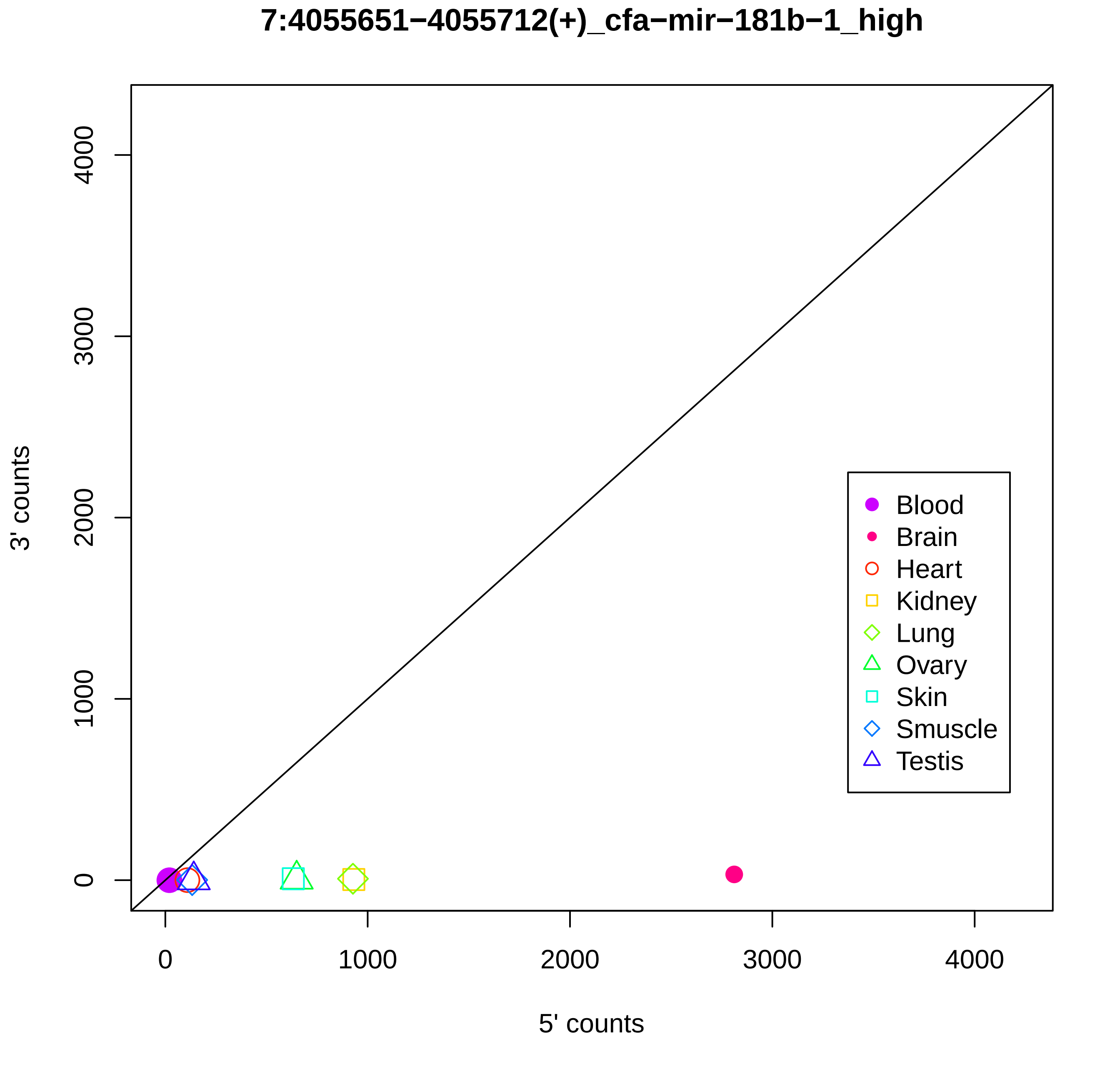

7:4055651−4055712(+)_cfa−mir−181b−1_high
3' counts
5' counts
0
1000
2000
3000
4000
0
1000
2000
3000
4000
Blood
Brain
Heart
Kidney
Lung
Ovary
Skin
Smuscle
Testis

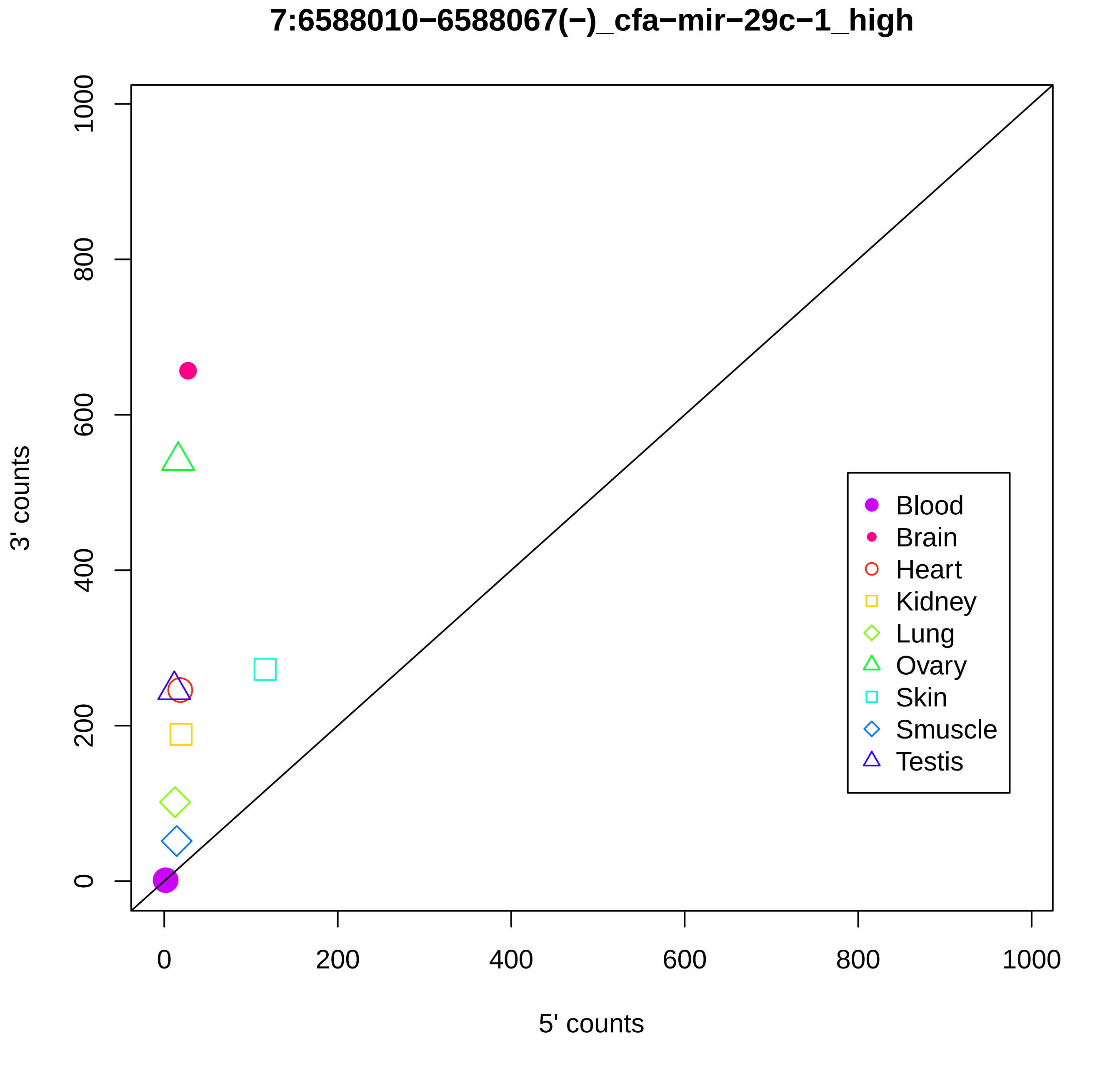

7:6588010−6588067(−)_cfa−mir−29c−1_high
3' counts
5' counts
0
200
400
600
800
1000
Blood
Brain
Heart
Kidney
Lung
Ovary
Skin
Smuscle
Testis

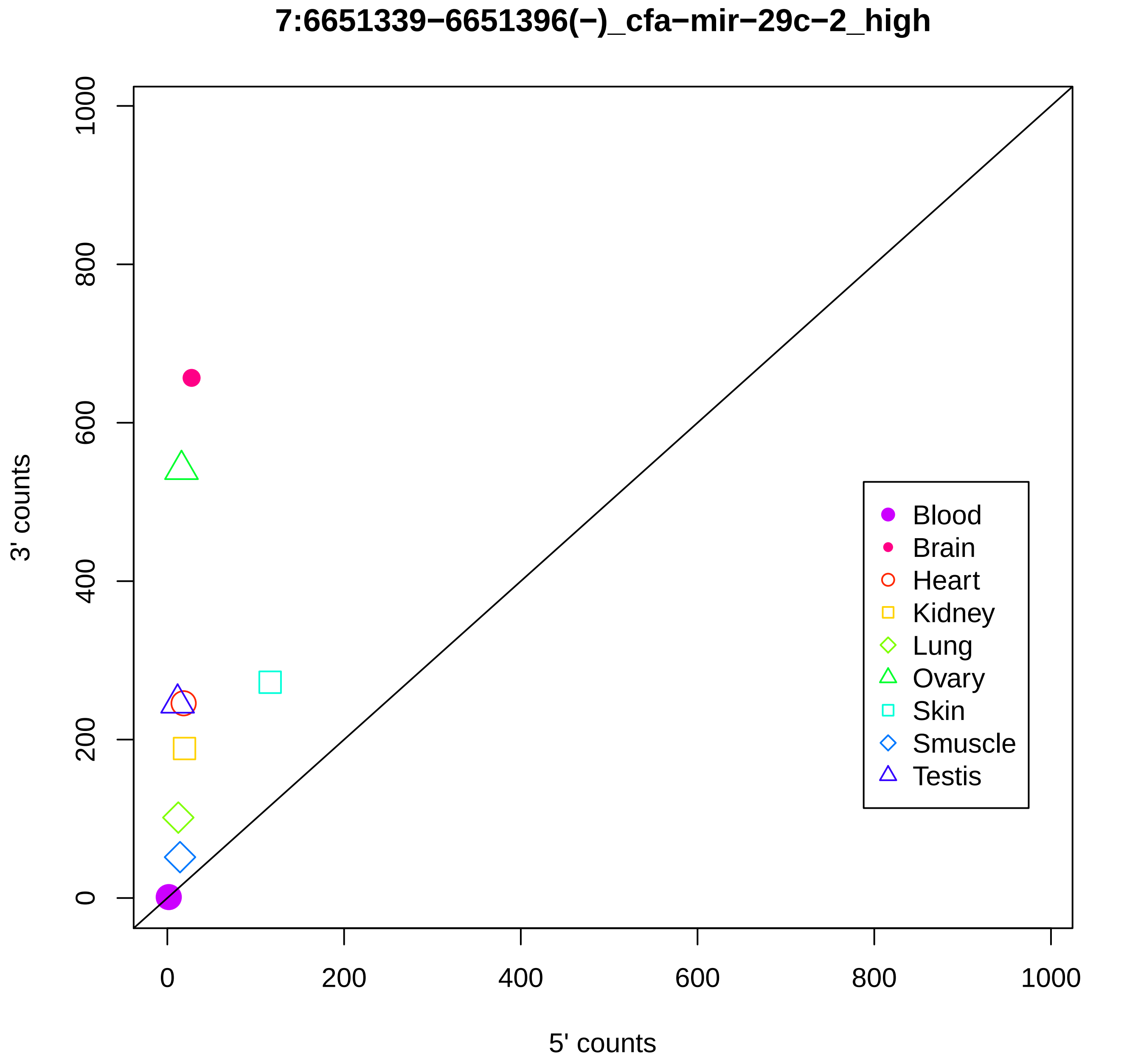
7:6651339−6651396(−)_cfa−mir−29c−2_high
3' counts
5' counts
0
200
400
600
800
1000
0
200
400
600
800
1000
Blood
Brain
Heart
Kidney
Lung
Ovary
Skin
Smuscle
Testis

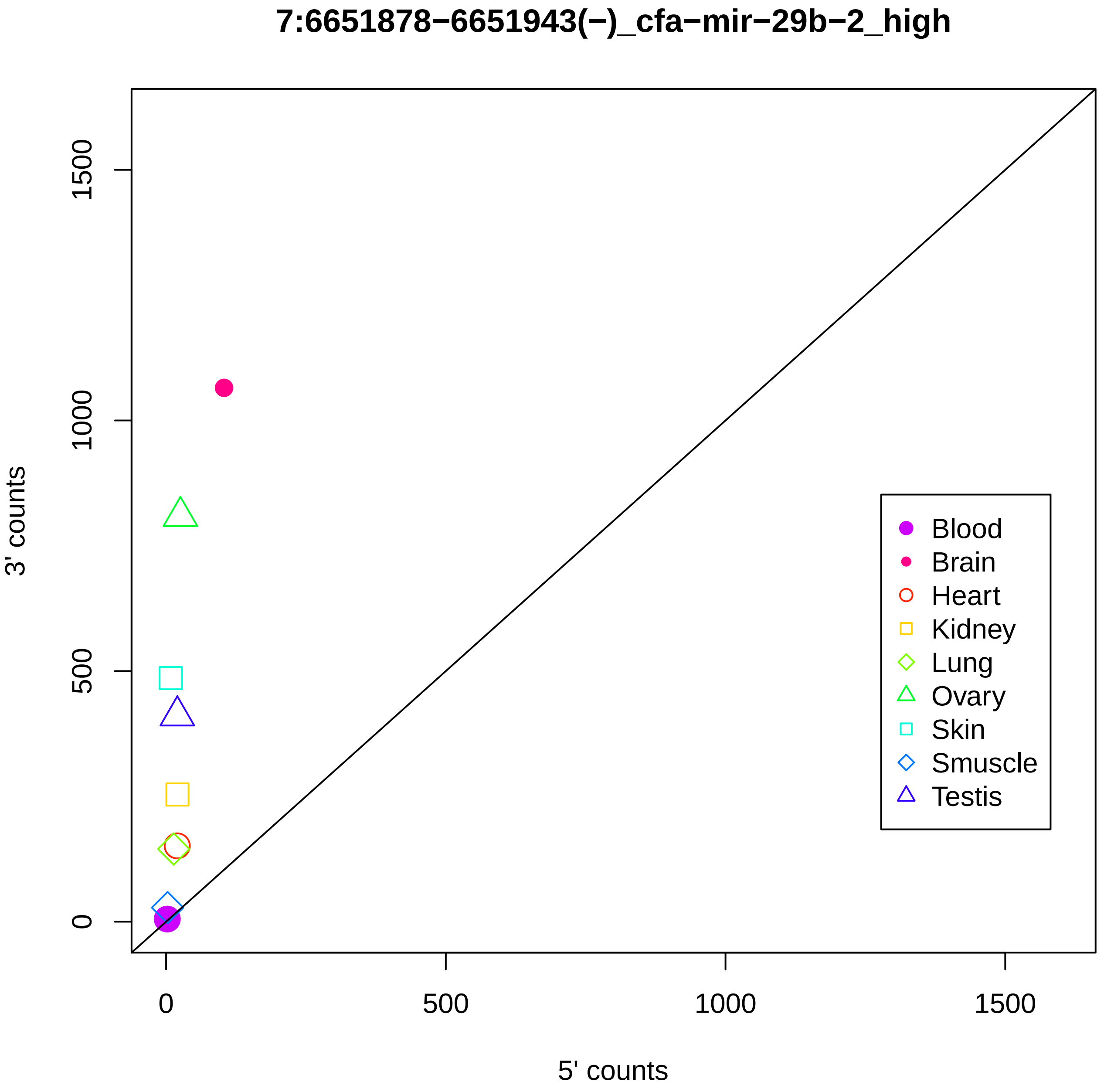
7:6651878−6651943(−)_cfa−mir−29b−2_high
3' counts
5' counts
0
500
1000
1500
0
500
1000
1500
Blood
Brain
Heart
Kidney
Lung
Ovary
Skin
Smuscle
Testis

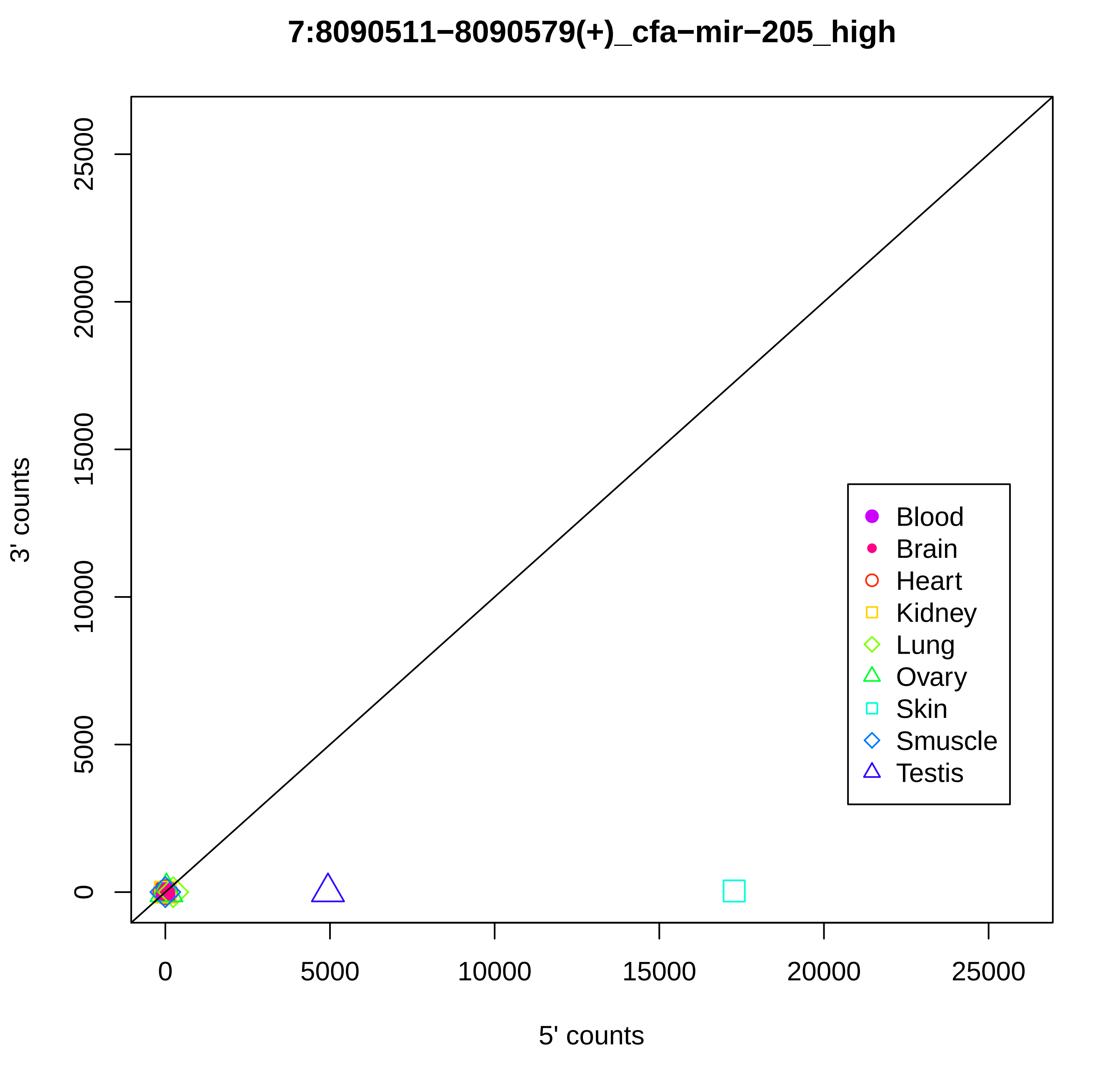
7:8090511−8090579(+)_cfa−mir−205_high
3' counts
5' counts
0
5000
10000
15000
20000
25000
Blood
Brain
Heart
Kidney
Lung
Ovary
Skin
Smuscle
Testis

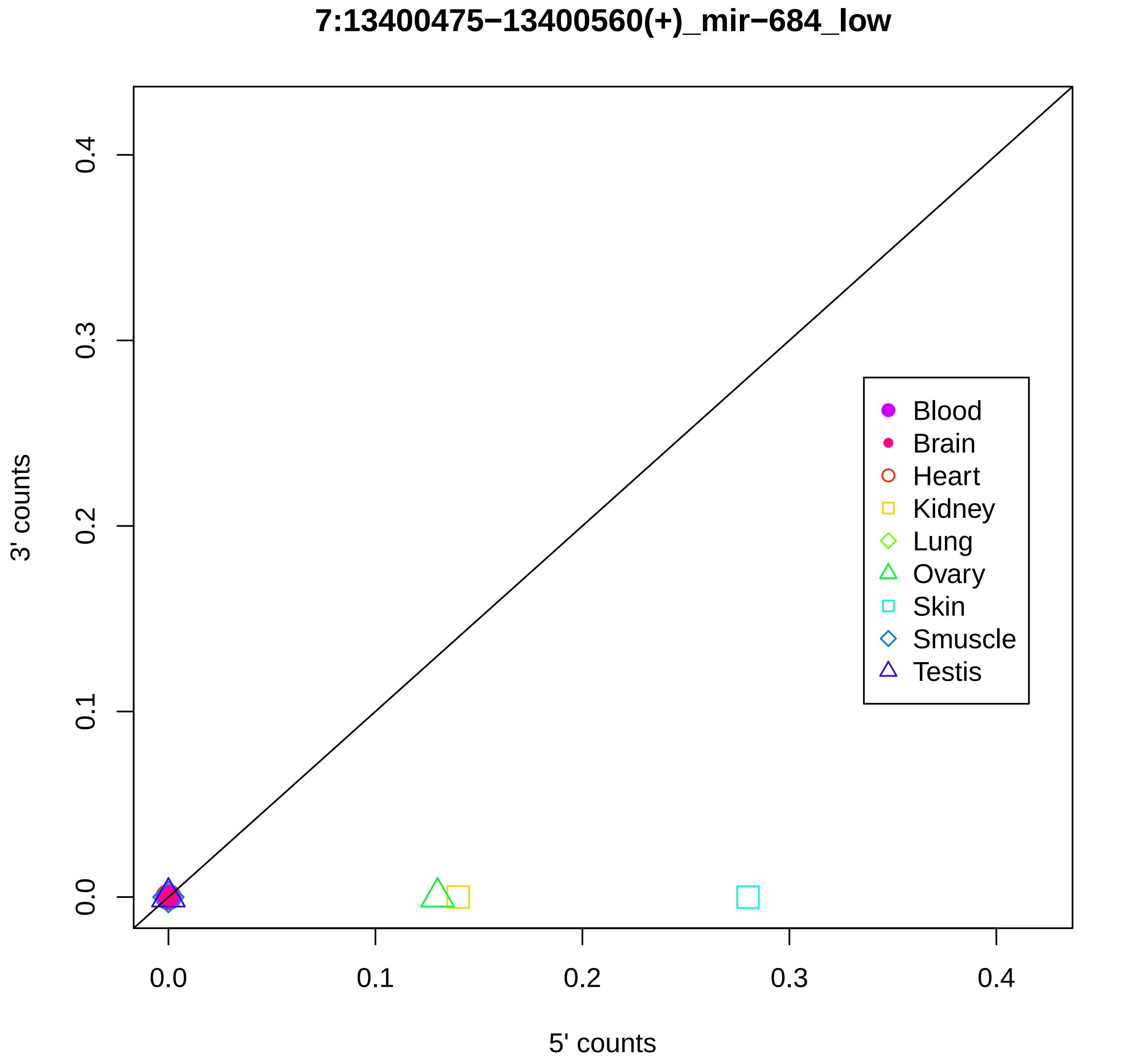
7:13400475−13400560(+)_mir−684_low
3' counts
5' counts
0.0
0.1
0.2
0.3
0.4
Blood
Brain
Heart
Kidney
Lung
Ovary
Skin
Smuscle
Testis

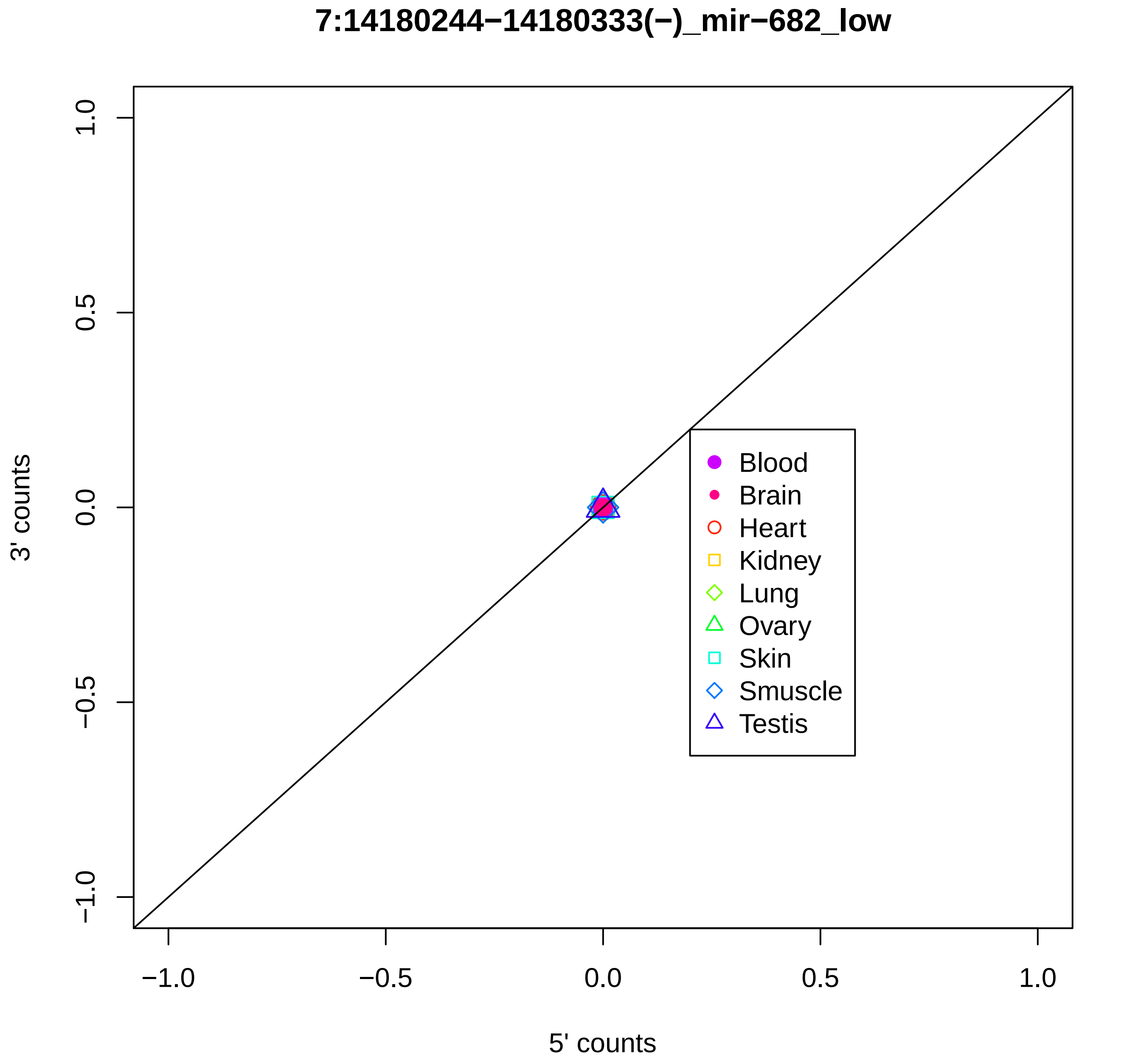
7:14180244−14180333(−)_mir−682_low
3' counts
5' counts
−1.0
−0.5
0.0
0.5
1.0
Blood
Brain
Heart
Kidney
Lung
Ovary
Skin
Smuscle
Testis

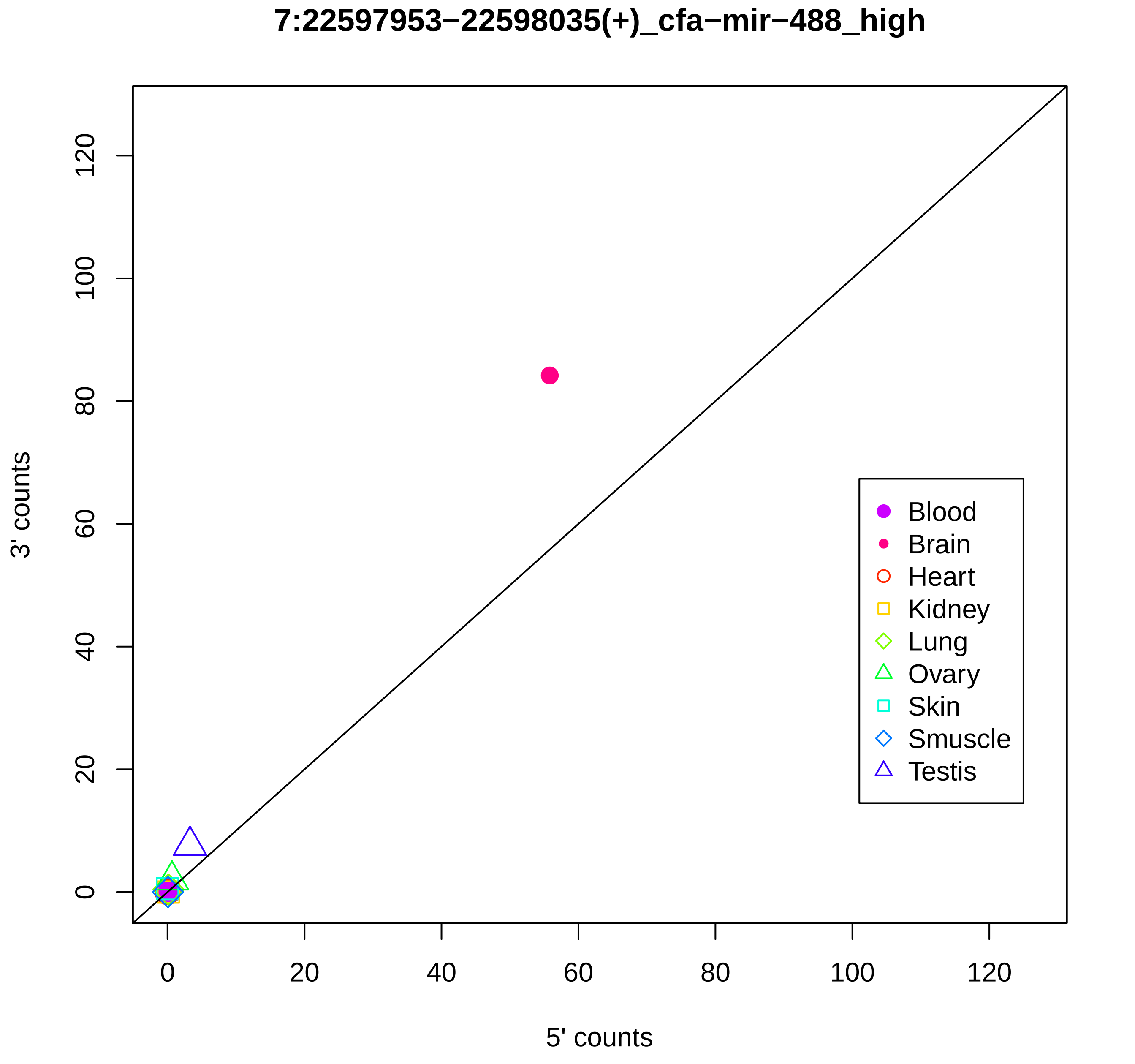
7:22597953−22598035(+)_cfa−mir−488_high
3' counts
5' counts
0
20
40
60
80
100
120
Blood
Brain
Heart
Kidney
Lung
Ovary
Skin
Smuscle
Testis

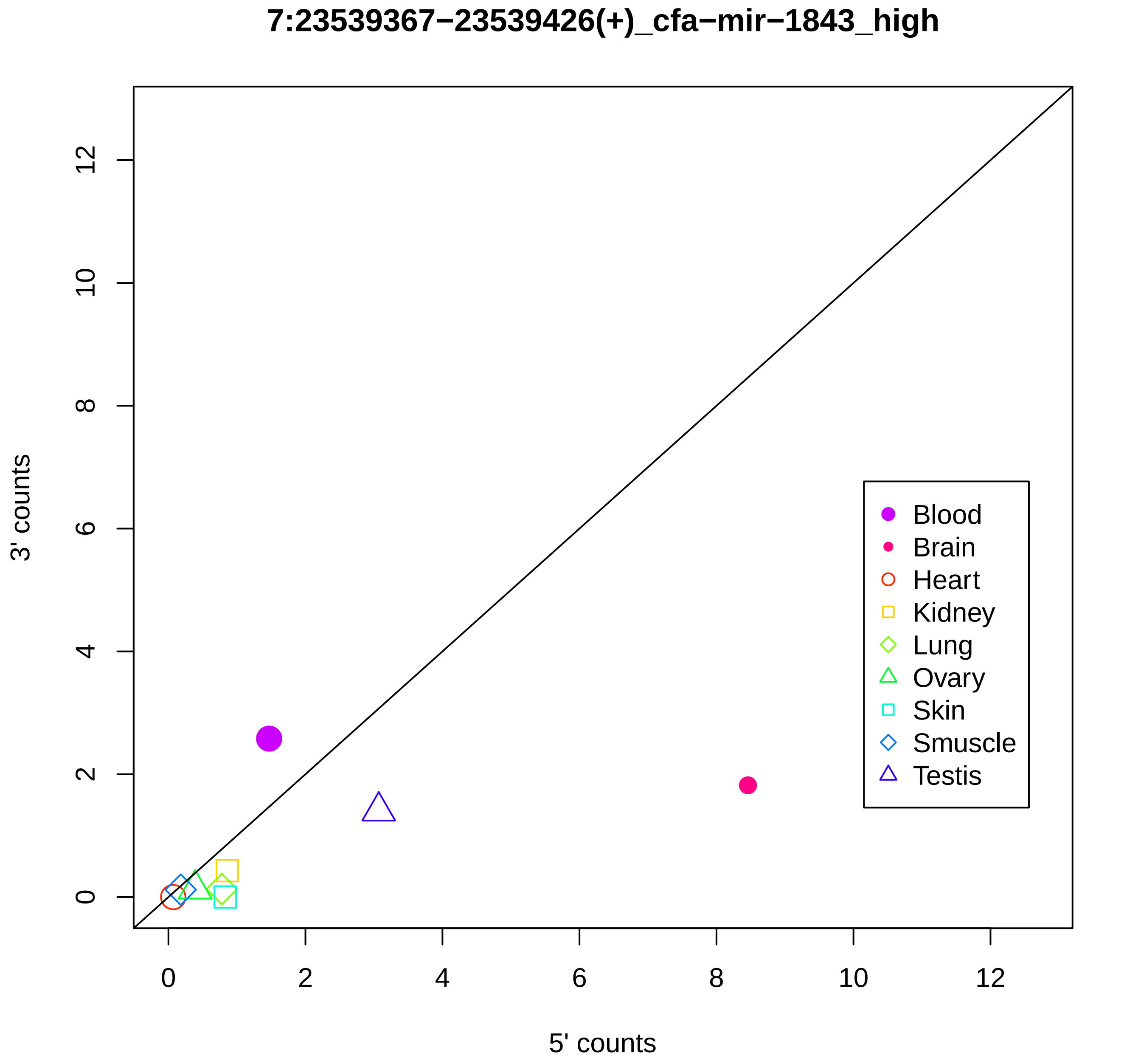
7:23539367−23539426(+)_cfa−mir−1843_high
3' counts
5' counts
0
2
4
6
8
10
12
Blood
Brain
Heart
Kidney
Lung
Ovary
Skin
Smuscle
Testis

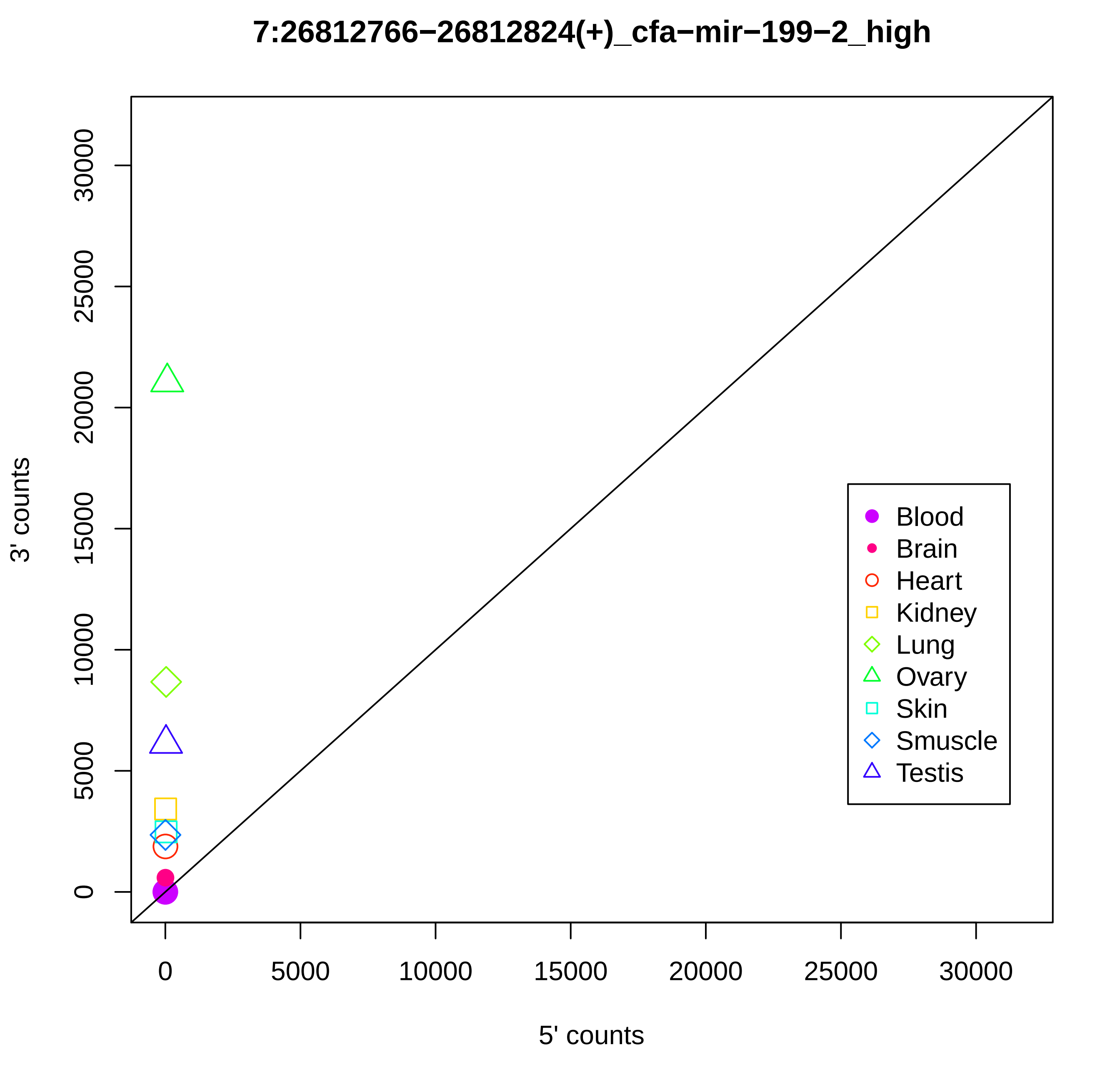

7:26812766−26812824(+)_cfa−mir−199−2_high
3' counts
5' counts
0
5000
10000
15000
20000
25000
30000
Blood
Brain
Heart
Kidney
Lung
Ovary
Skin
Smuscle
Testis

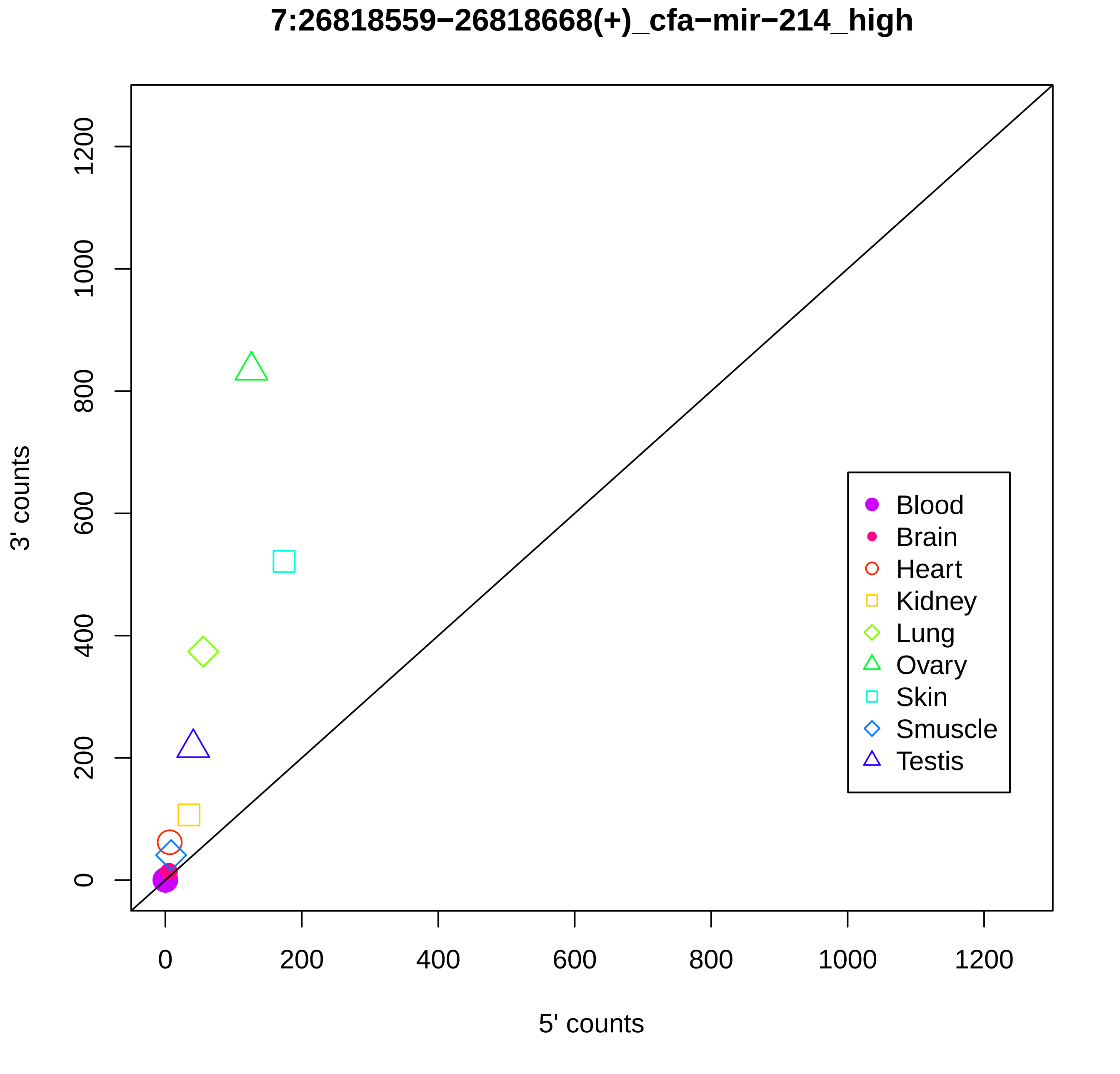

7:26818559−26818668(+)_cfa−mir−214_high
3' counts
5' counts
0
200
400
600
800
1000
1200
Blood
Brain
Heart
Kidney
Lung
Ovary
Skin
Smuscle
Testis

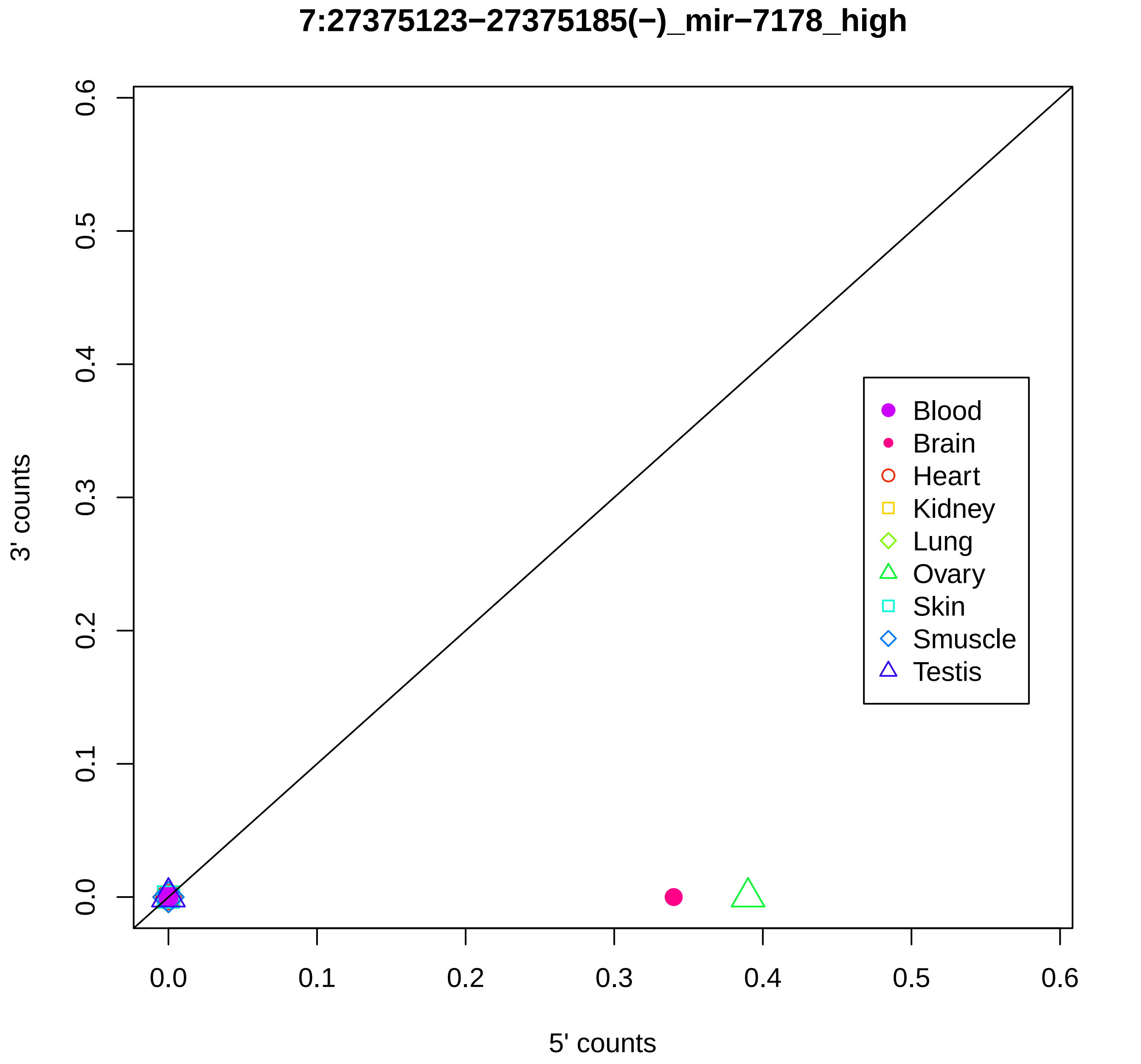

7:27375123−27375185(−)_mir−7178_high
3' counts
5' counts
0.0
0.1
0.2
0.3
0.4
0.5
0.6
Blood
Brain
Heart
Kidney
Lung
Ovary
Skin
Smuscle
Testis

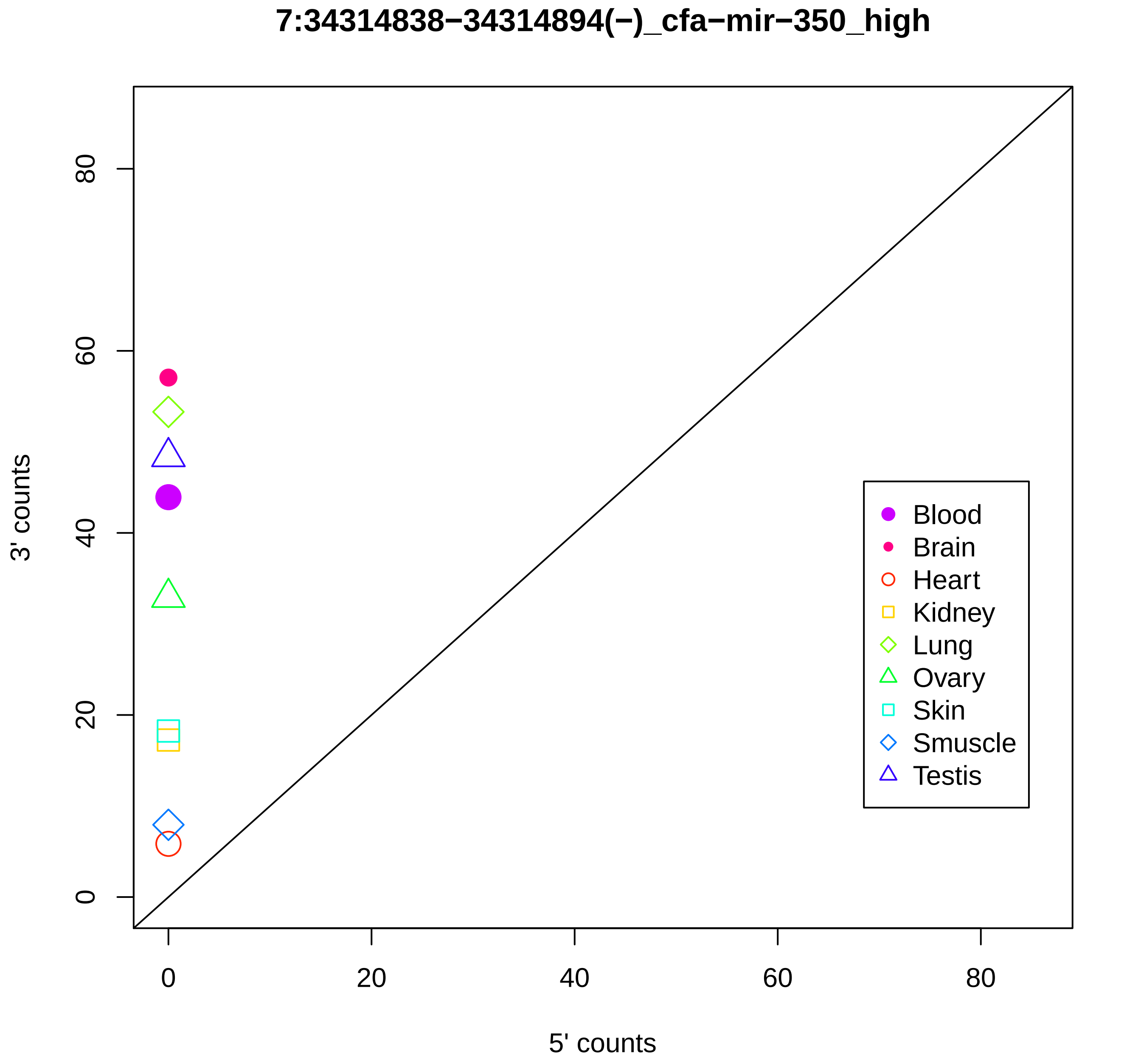

7:34314838−34314894(−)_cfa−mir−350_high
3' counts
5' counts
0
20
40
60
80
0
20
40
60
80
Blood
Brain
Heart
Kidney
Lung
Ovary
Skin
Smuscle
Testis

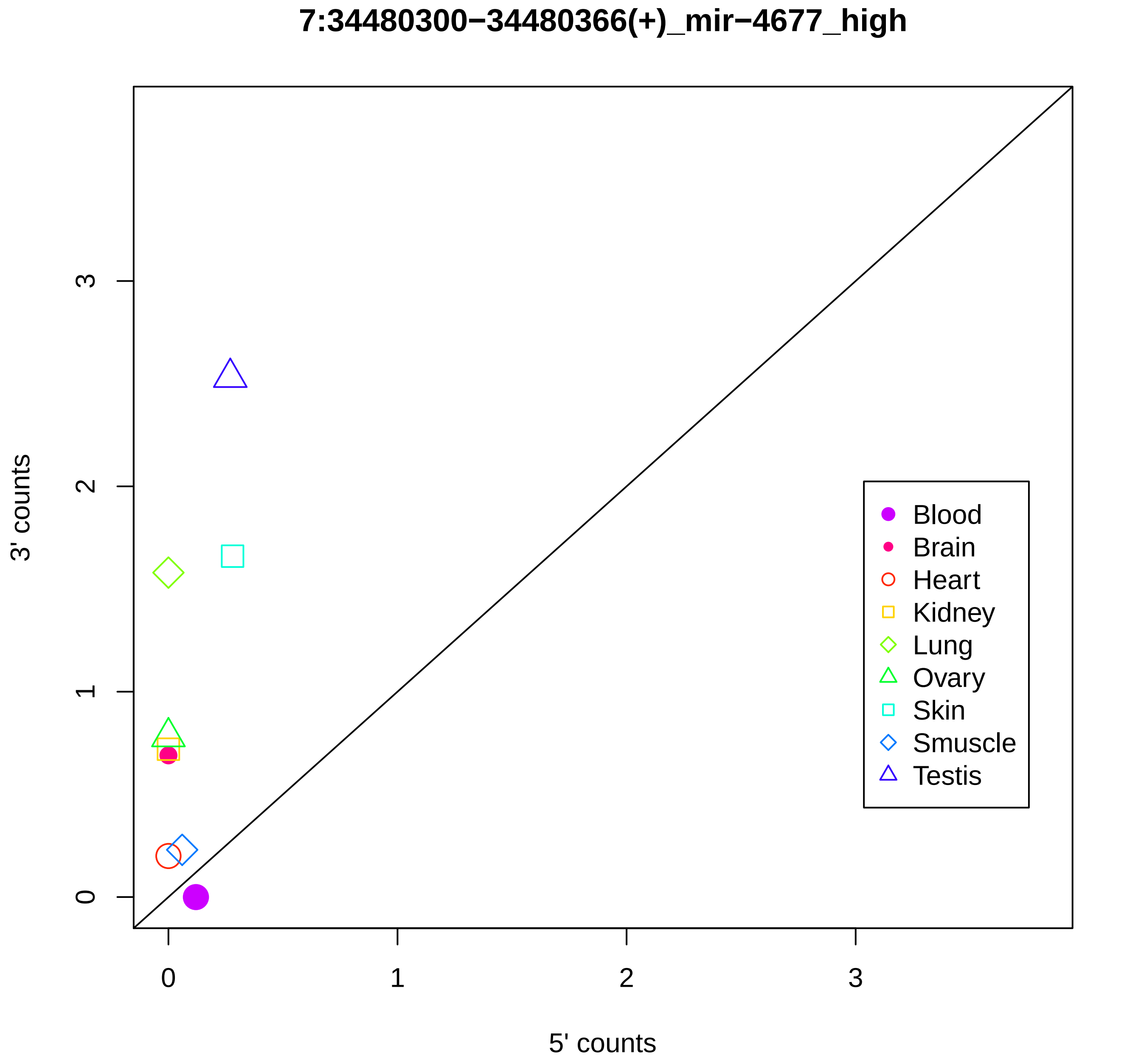

7:34480300−34480366(+)_mir−4677_high
3' counts
5' counts
0
1
2
3
0
1
2
3
Blood
Brain
Heart
Kidney
Lung
Ovary
Skin
Smuscle
Testis

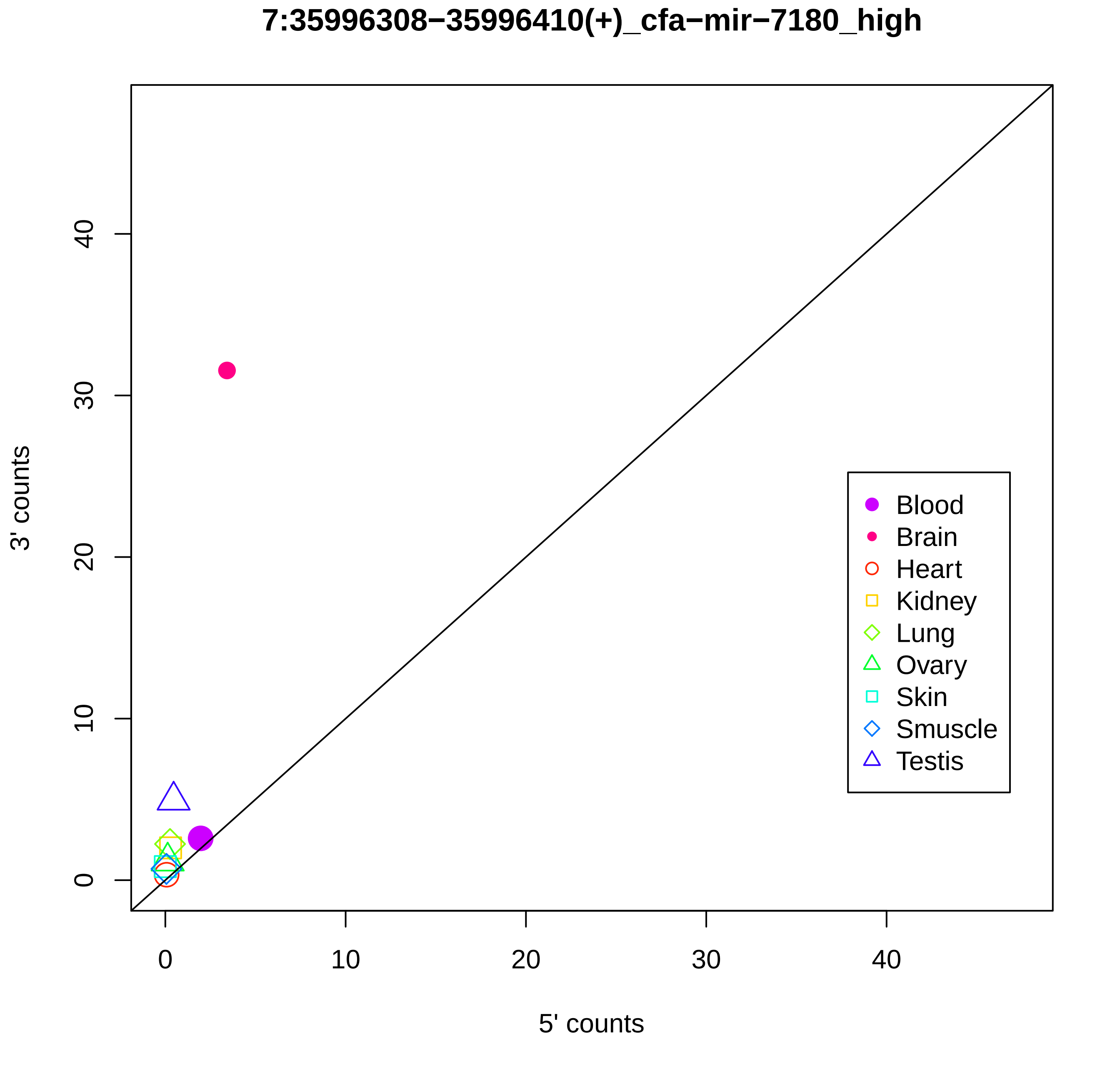

7:35996308−35996410(+)_cfa−mir−7180_high
3' counts
5' counts
0
10
20
30
40
Blood
Brain
Heart
Kidney
Lung
Ovary
Skin
Smuscle
Testis

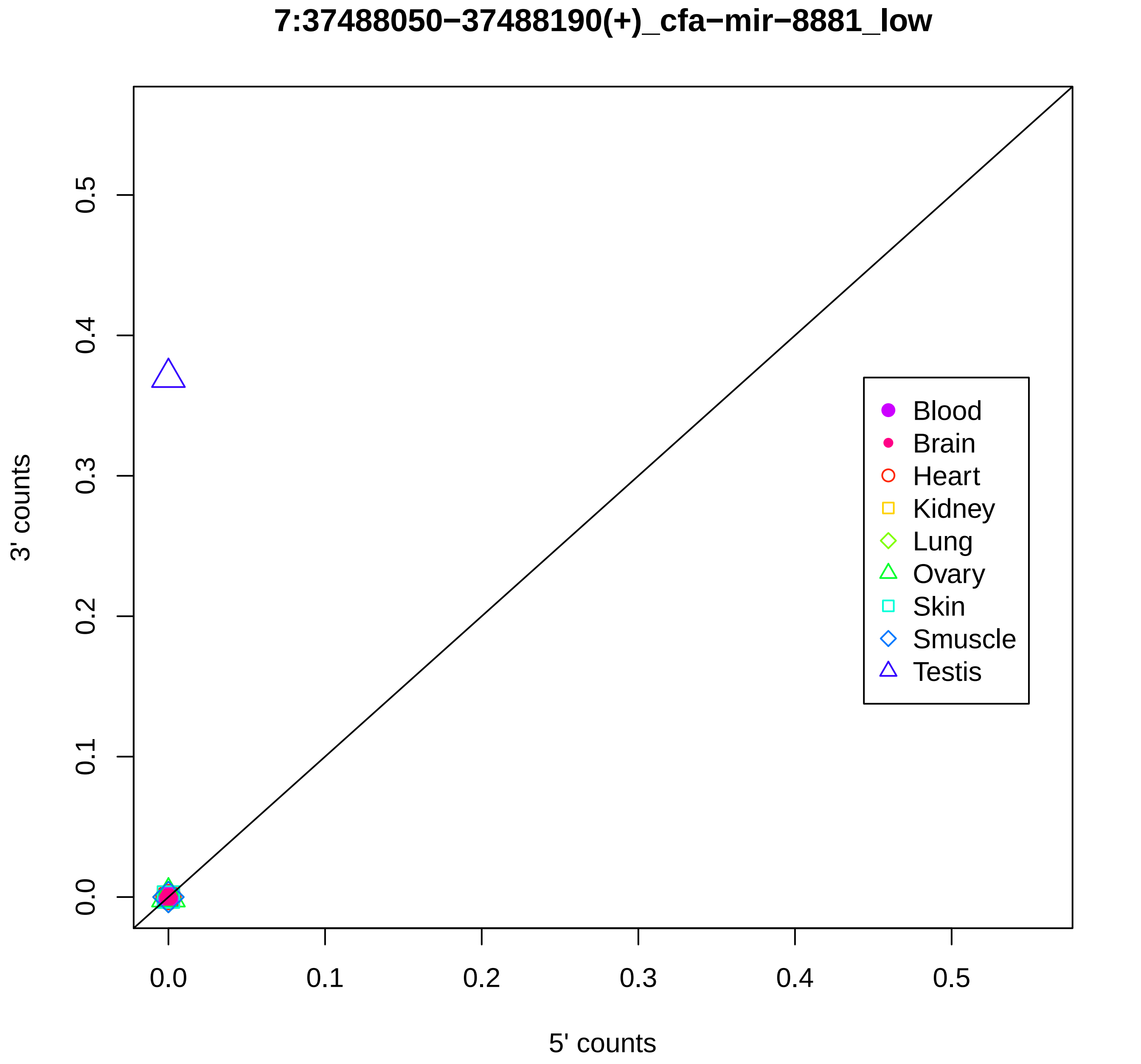

7:37488050−37488190(+)_cfa−mir−8881_low
3' counts
5' counts
0.0
0.1
0.2
0.3
0.4
0.5
Blood
Brain
Heart
Kidney
Lung
Ovary
Skin
Smuscle
Testis

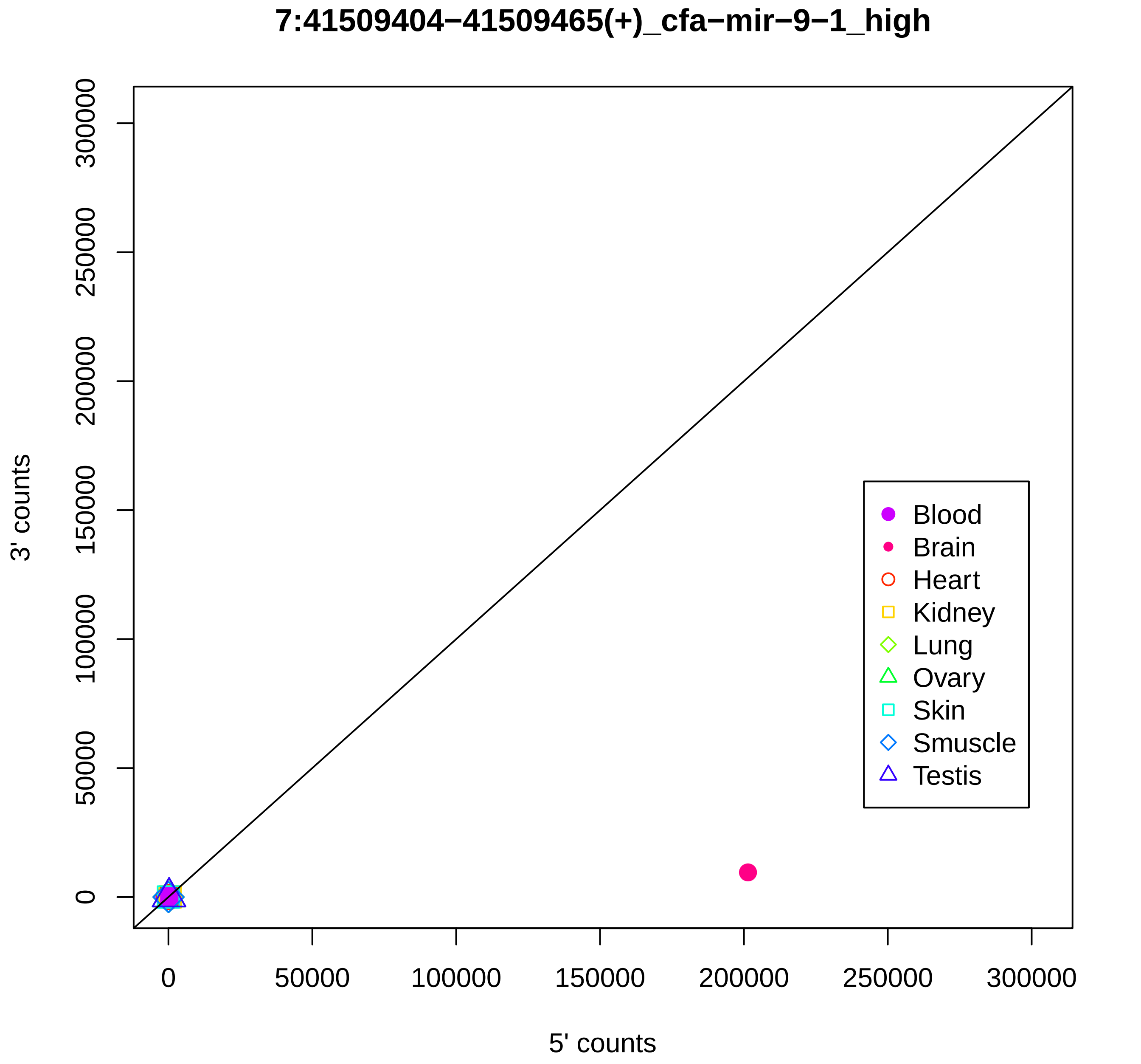
7:41509404−41509465(+)_cfa−mir−9−1_high
3' counts
5' counts
0
50000
100000
150000
200000
250000
300000
Blood
Brain
Heart
Kidney
Lung
Ovary
Skin
Smuscle
Testis

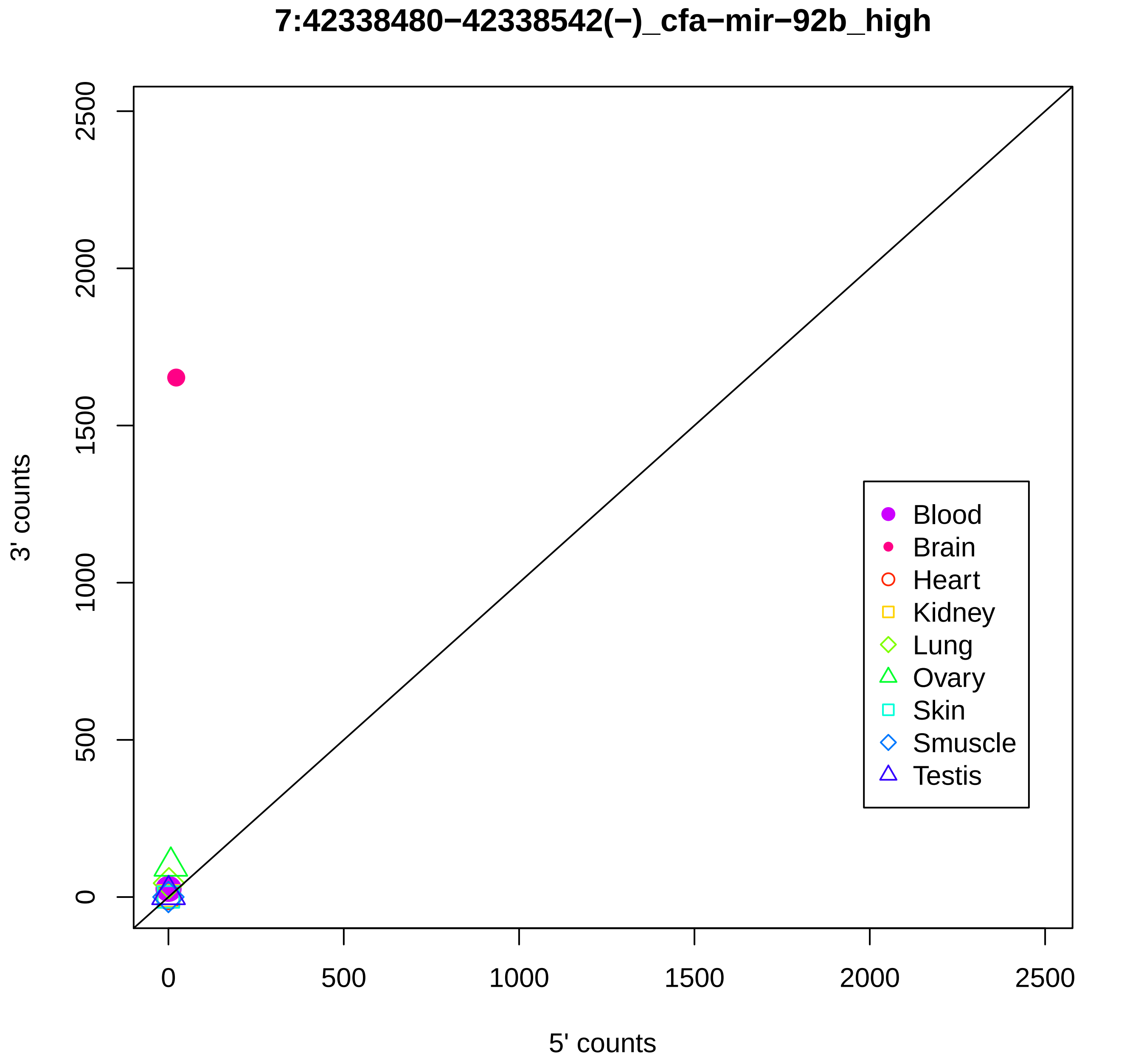

7:42338480−42338542(−)_cfa−mir−92b_high
3' counts
5' counts
0
500
1000
1500
2000
2500
Blood
Brain
Heart
Kidney
Lung
Ovary
Skin
Smuscle
Testis

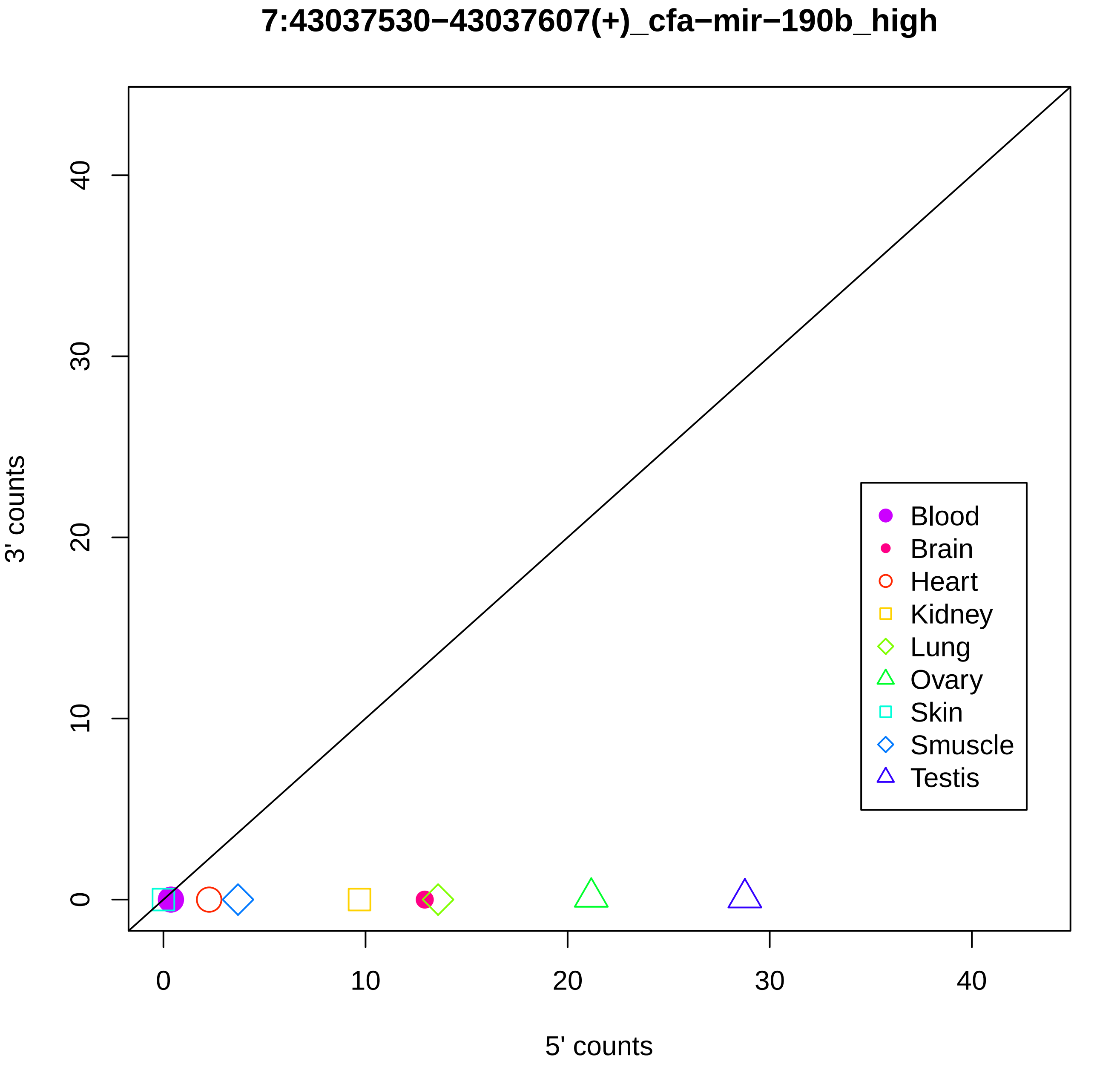

7:43037530−43037607(+)_cfa−mir−190b_high
3' counts
5' counts
0
10
20
30
40
Blood
Brain
Heart
Kidney
Lung
Ovary
Skin
Smuscle
Testis

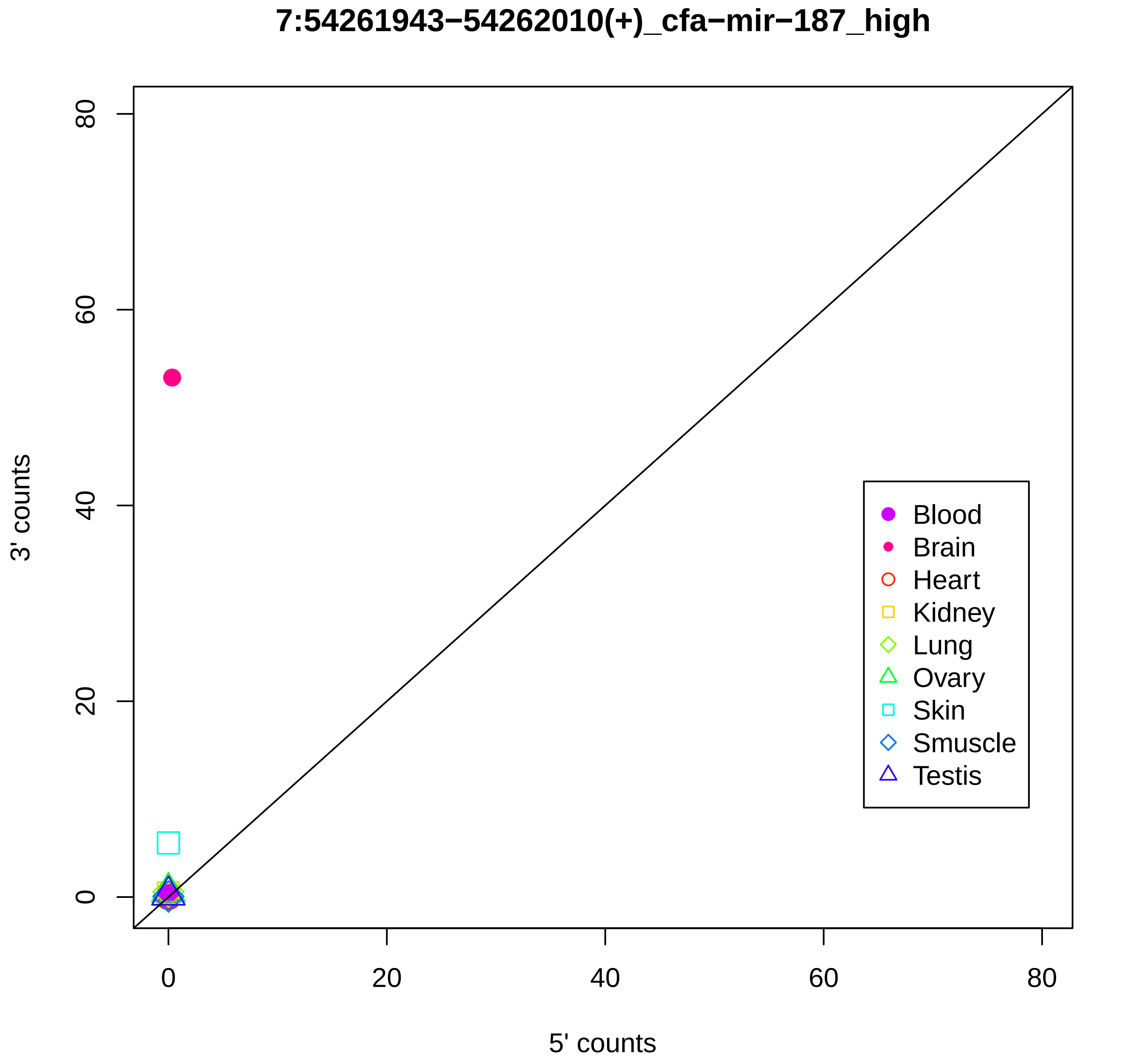

7:54261943−54262010(+)_cfa−mir−187_high
3' counts
5' counts
0
20
40
60
80
0
20
40
60
80
Blood
Brain
Heart
Kidney
Lung
Ovary
Skin
Smuscle
Testis

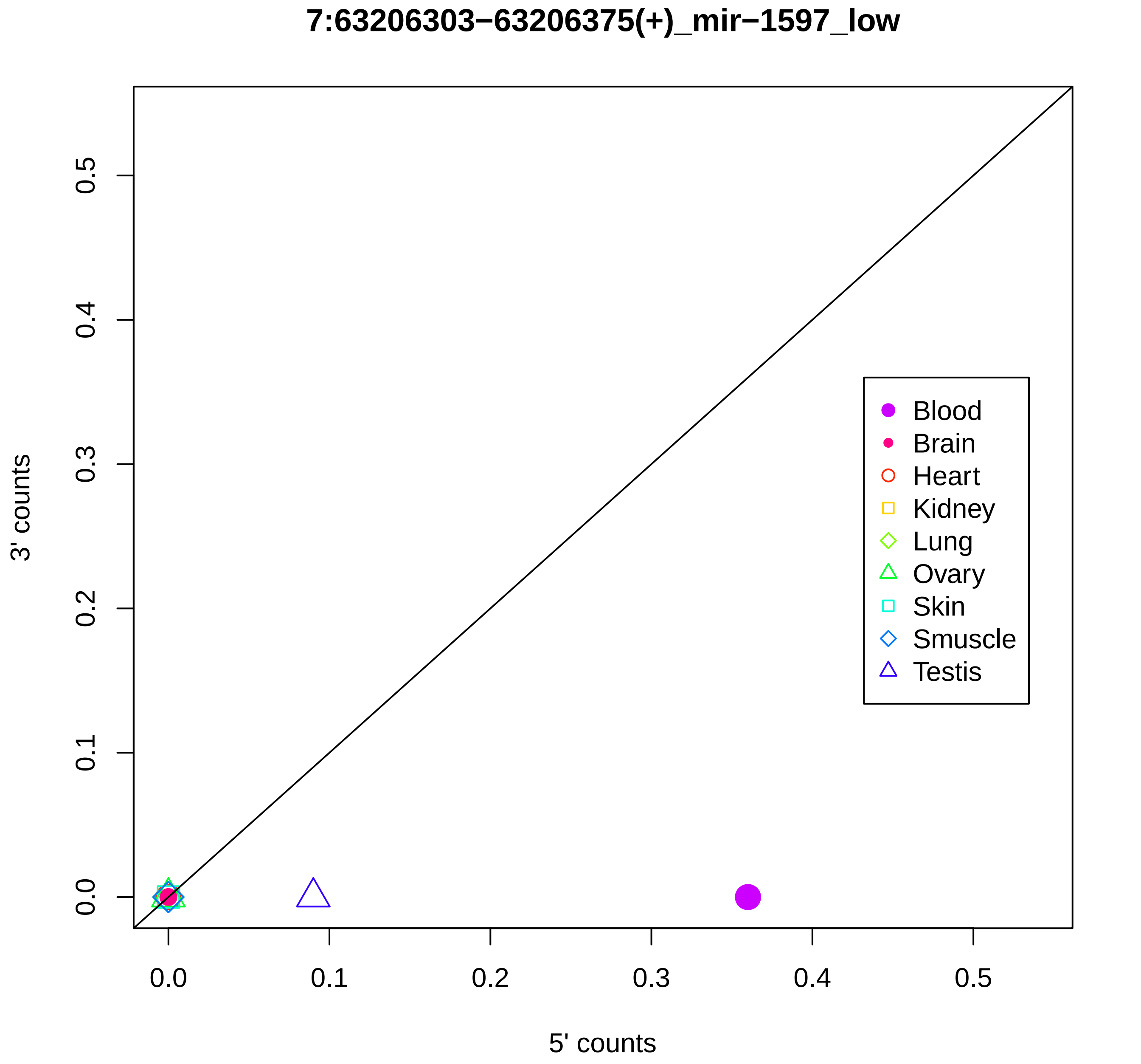
7:63206303−63206375(+)_mir−1597_low
3' counts
5' counts
0.0
0.1
0.2
0.3
0.4
0.5
Blood
Brain
Heart
Kidney
Lung
Ovary
Skin
Smuscle
Testis

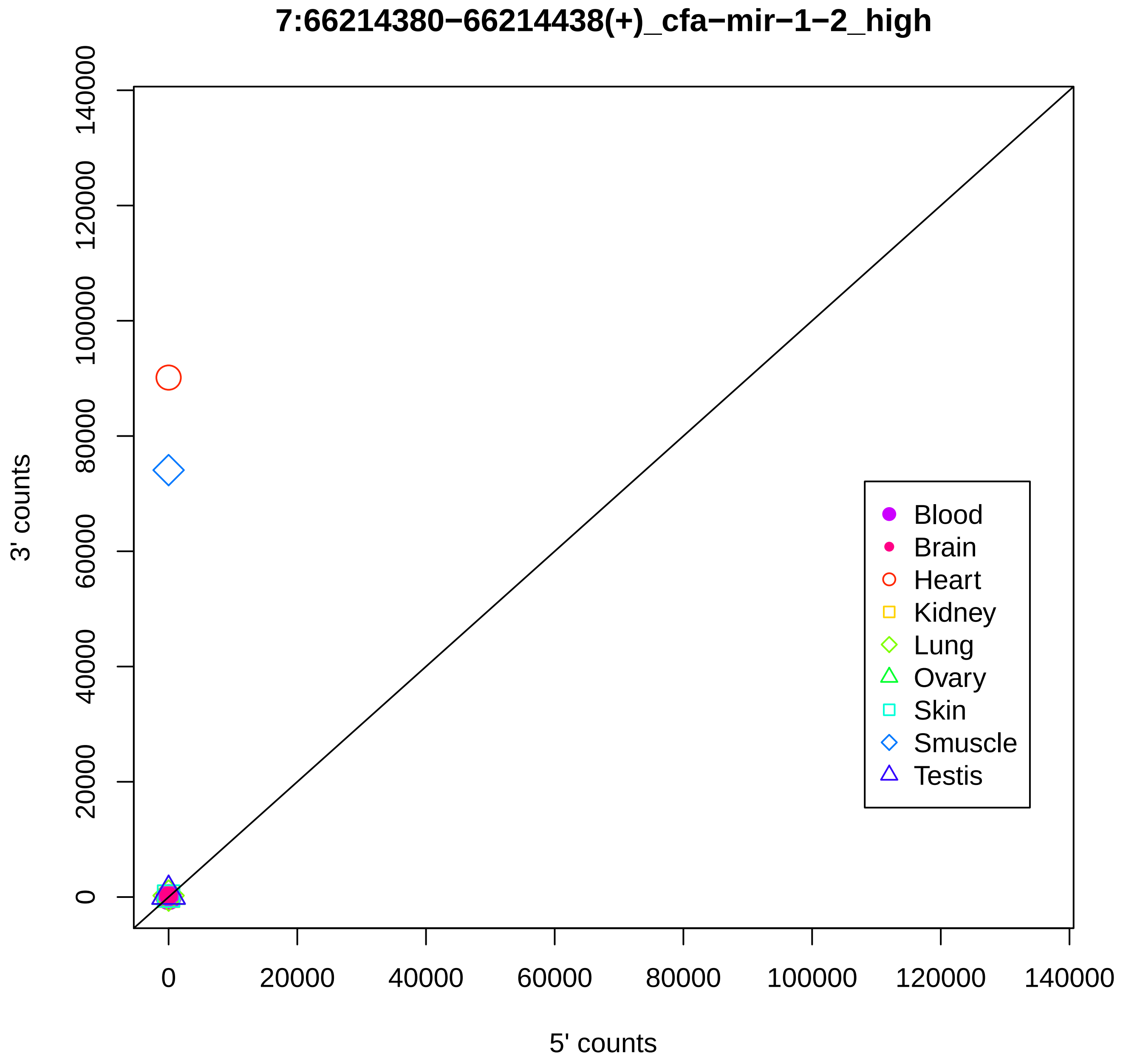
7:66214380−66214438(+)_cfa−mir−1−2_high
3' counts
5' counts
0
20000
40000
60000
80000
100000
120000
140000
Blood
Brain
Heart
Kidney
Lung
Ovary
Skin
Smuscle
Testis

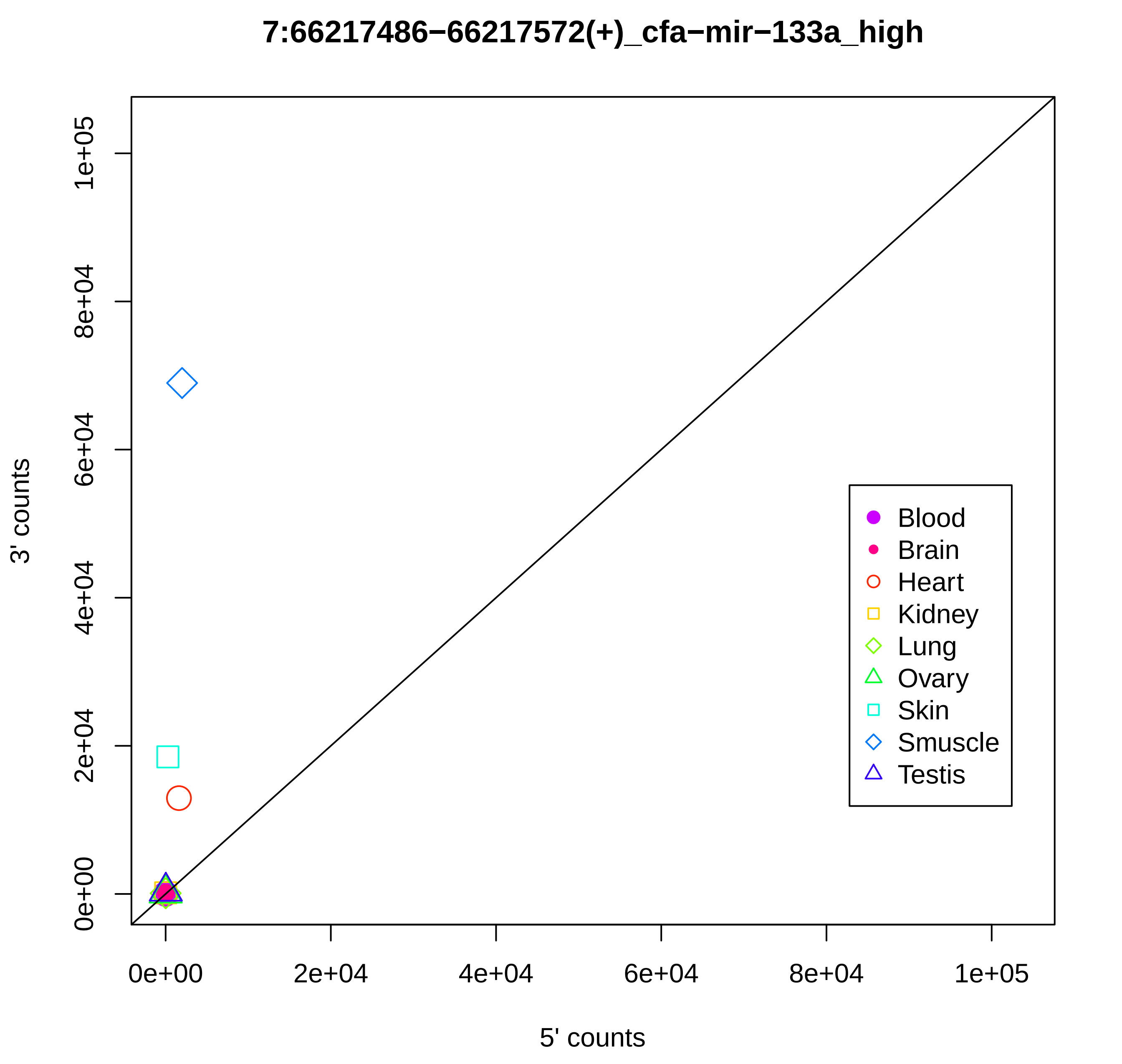

7:66217486−66217572(+)_cfa−mir−133a_high
3' counts
5' counts
0e+00
2e+04
4e+04
6e+04
8e+04
1e+05
Blood
Brain
Heart
Kidney
Lung
Ovary
Skin
Smuscle
Testis

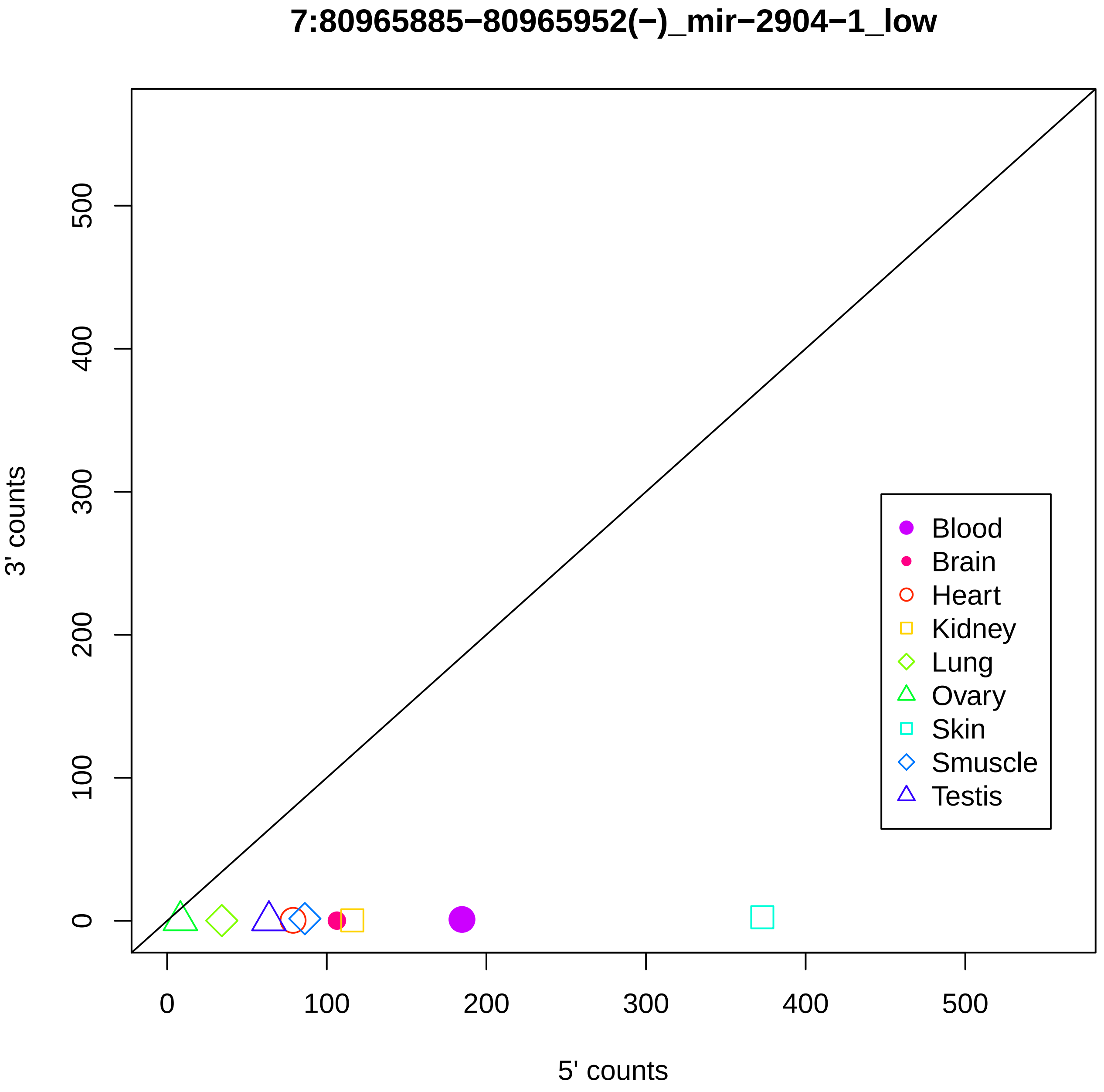
7:80965885−80965952(−)_mir−2904−1_low
3' counts
5' counts
0
100
200
300
400
500
Blood
Brain
Heart
Kidney
Lung
Ovary
Skin
Smuscle
Testis

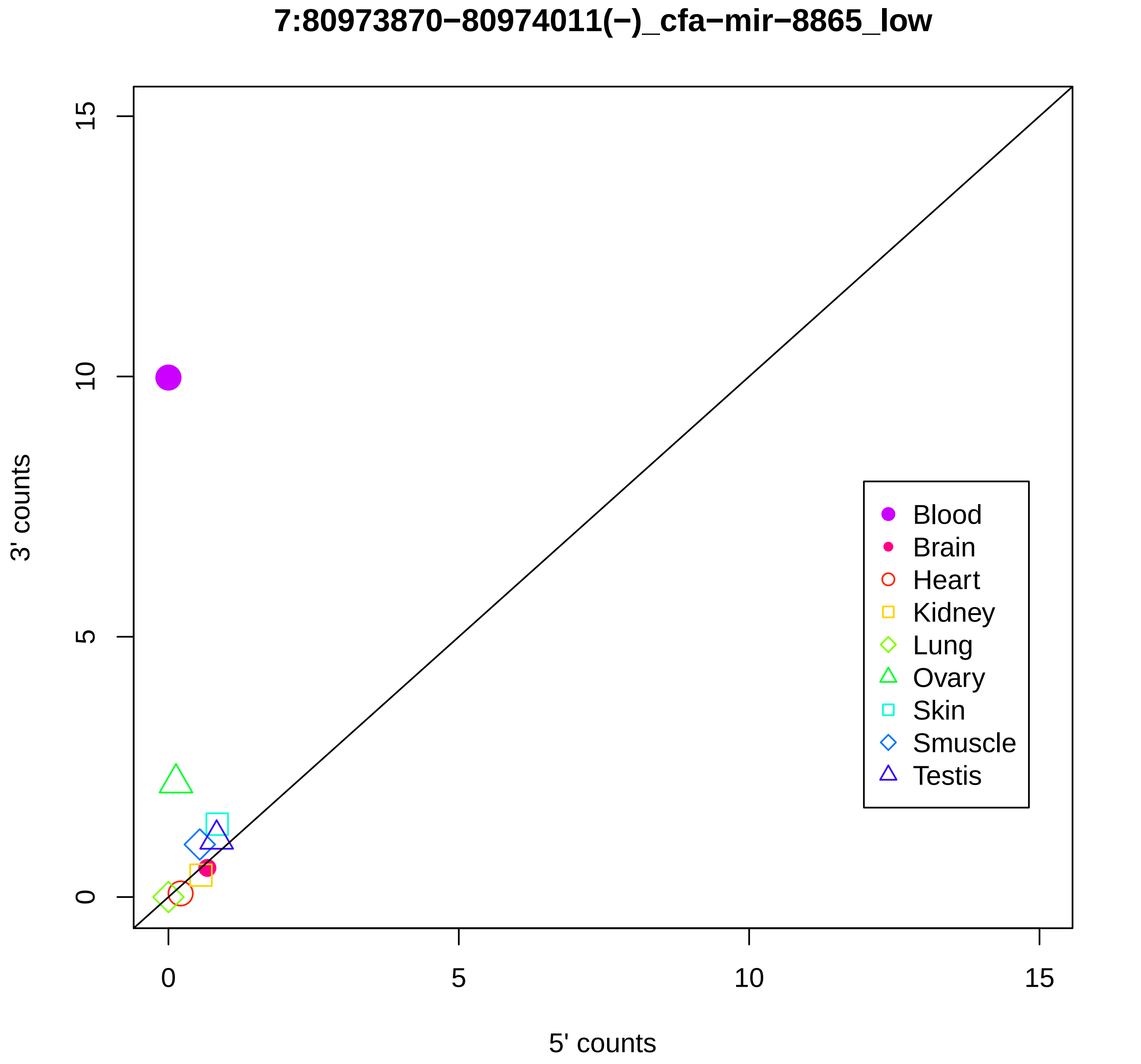
7:80973870−80974011(−)_cfa−mir−8865_low
3' counts
5' counts
0
5
10
15
Blood
Brain
Heart
Kidney
Lung
Ovary
Skin
Smuscle
Testis

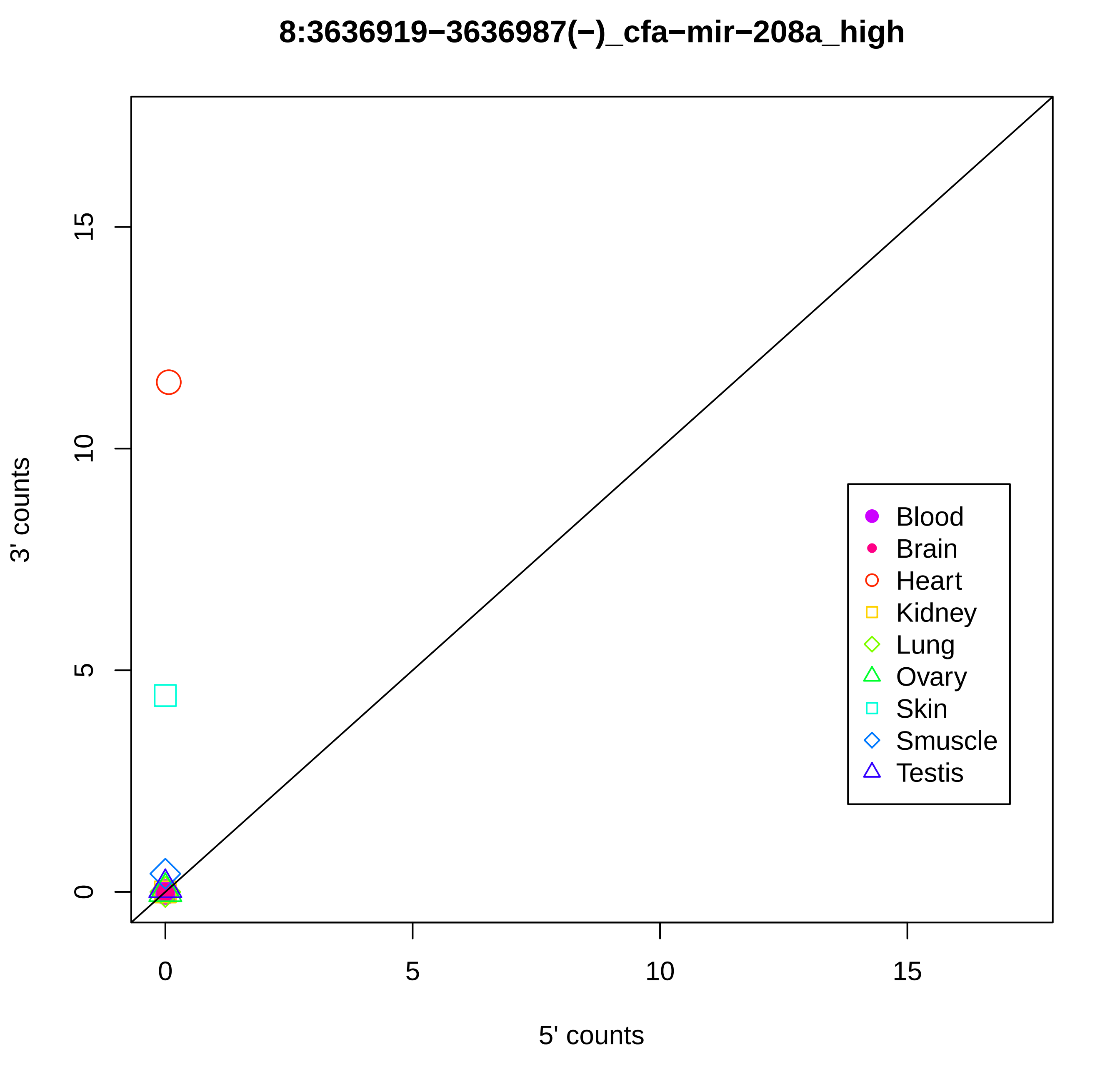

8:3636919−3636987(−)_cfa−mir−208a_high
3' counts
5' counts
0
5
10
15
0
5
10
15
Blood
Brain
Heart
Kidney
Lung
Ovary
Skin
Smuscle
Testis

8:3665163−3665236(−)_cfa−mir−208b_high

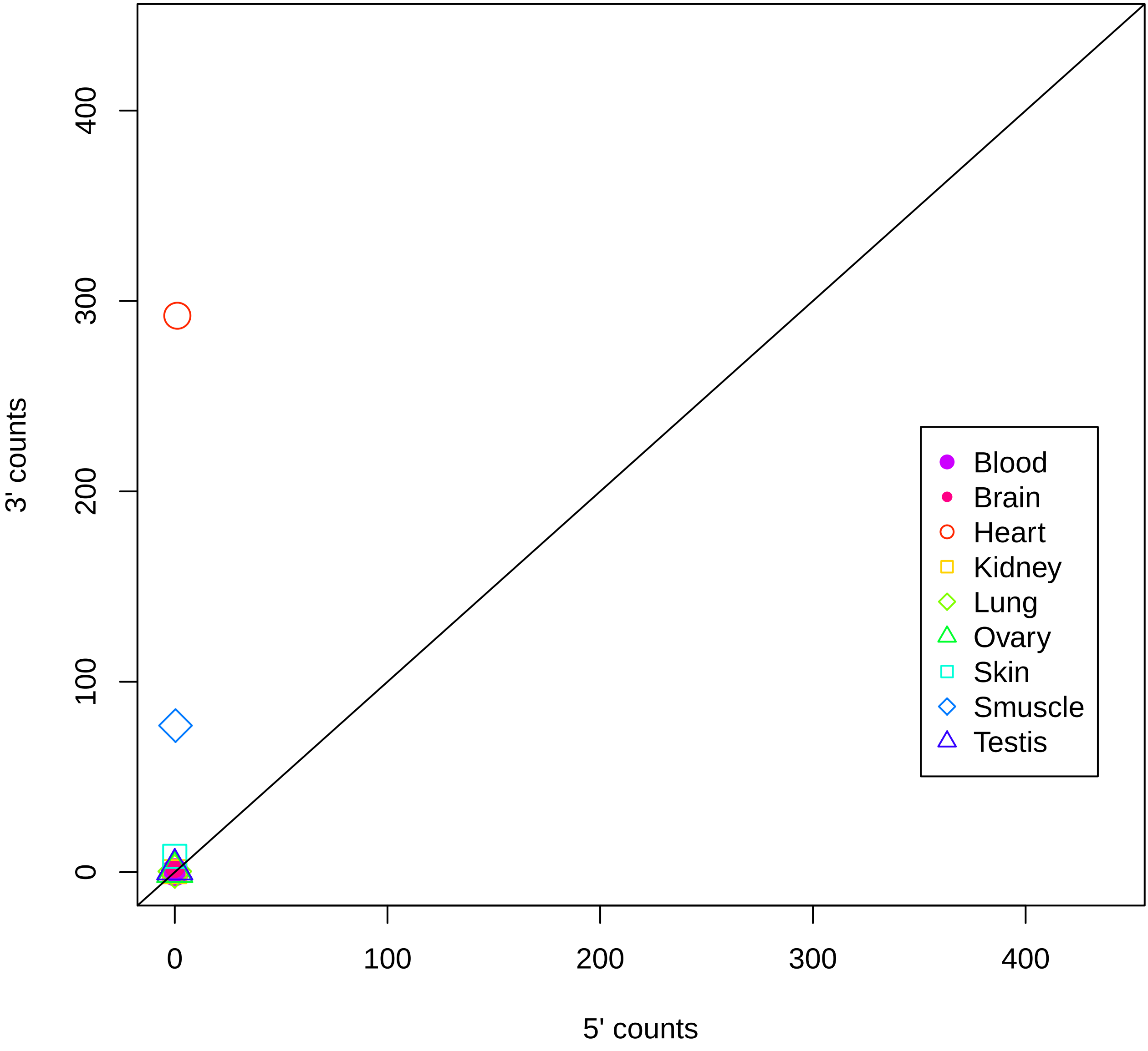

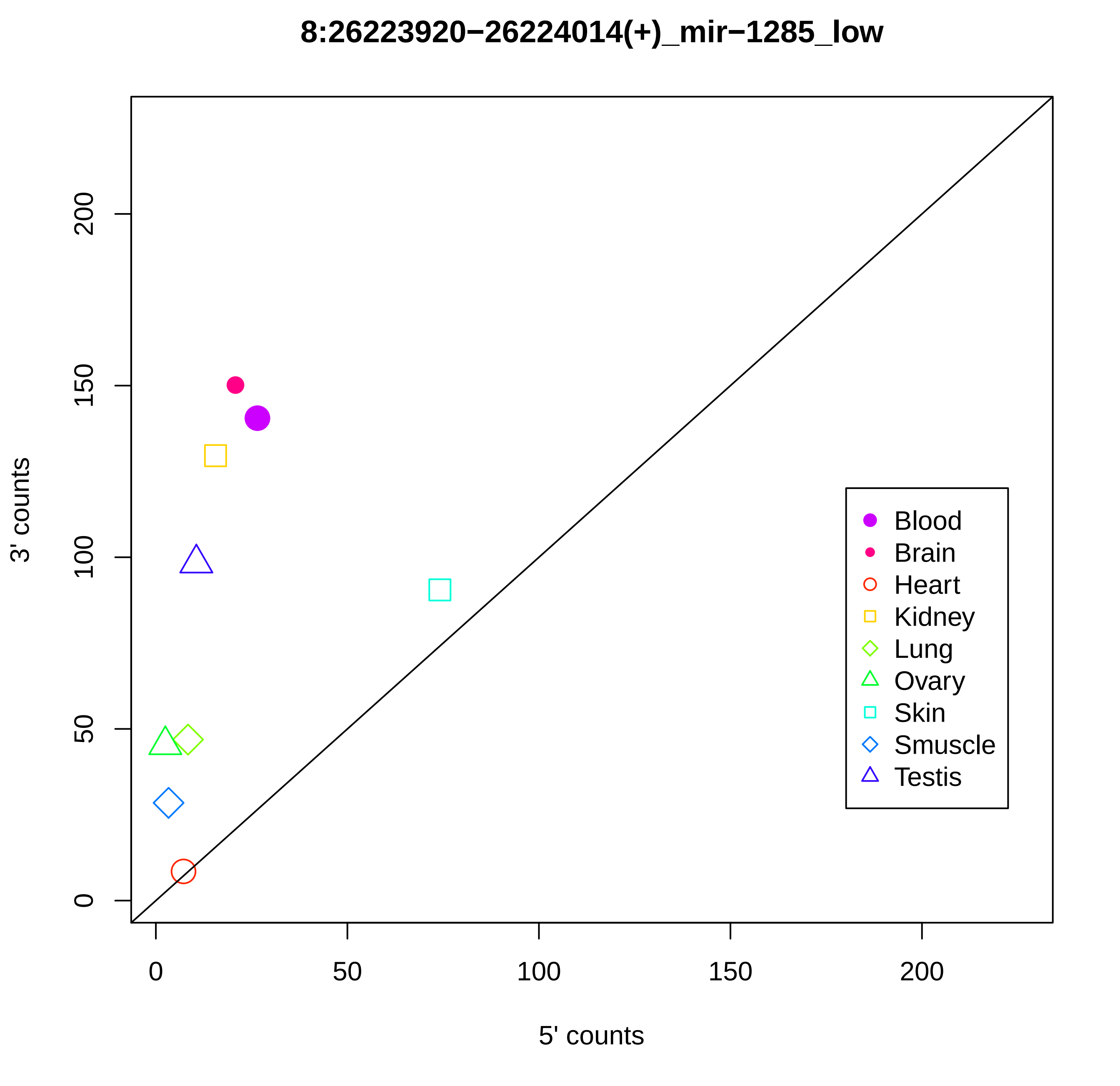
8:26223920−26224014(+)_mir−1285_low
3' counts
5' counts
0
50
100
150
200
Blood
Brain
Heart
Kidney
Lung
Ovary
Skin
Smuscle
Testis

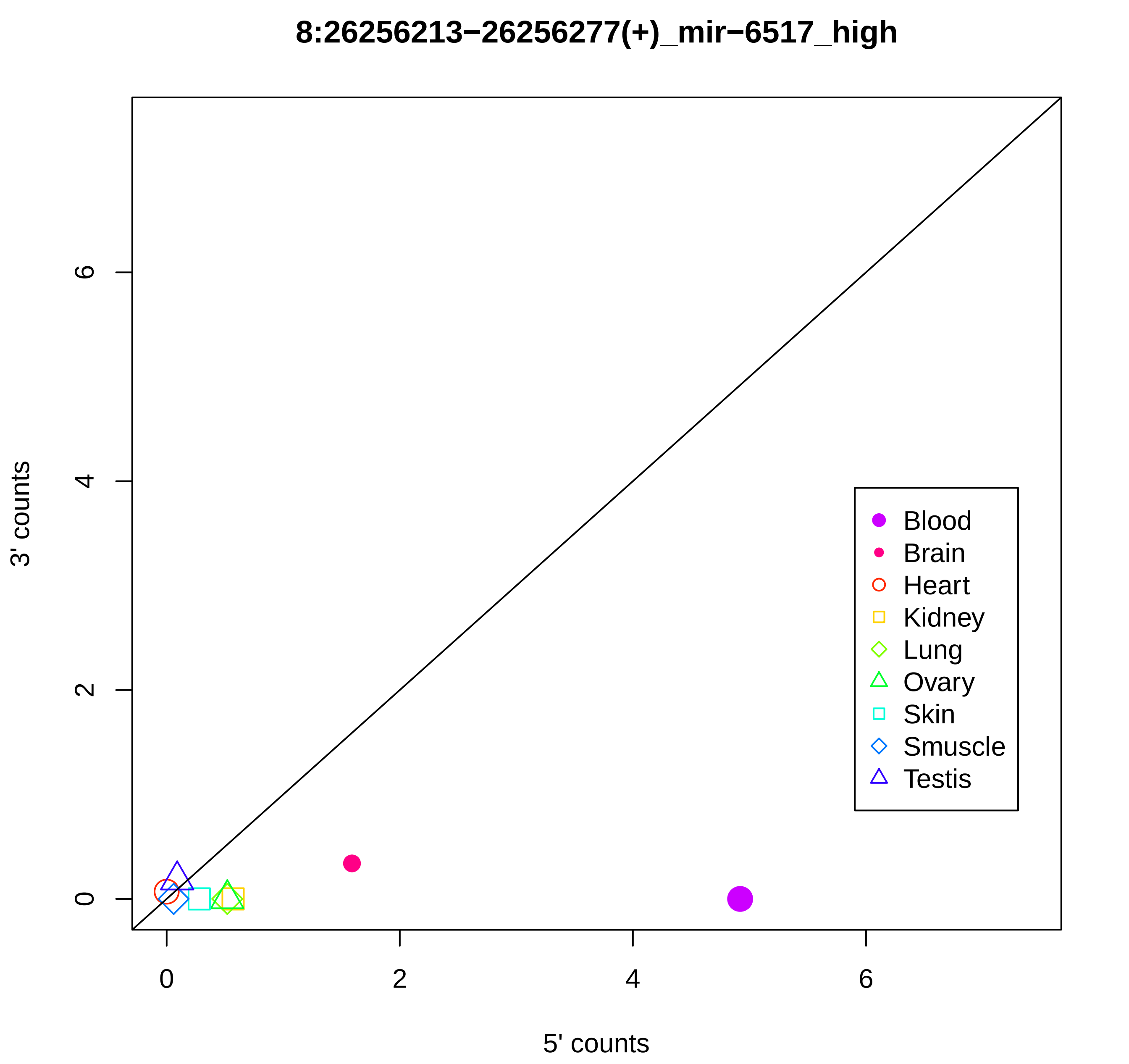
8:26256213−26256277(+)_mir−6517_high
3' counts
5' counts
0
2
4
6
0
2
4
6
Blood
Brain
Heart
Kidney
Lung
Ovary
Skin
Smuscle
Testis

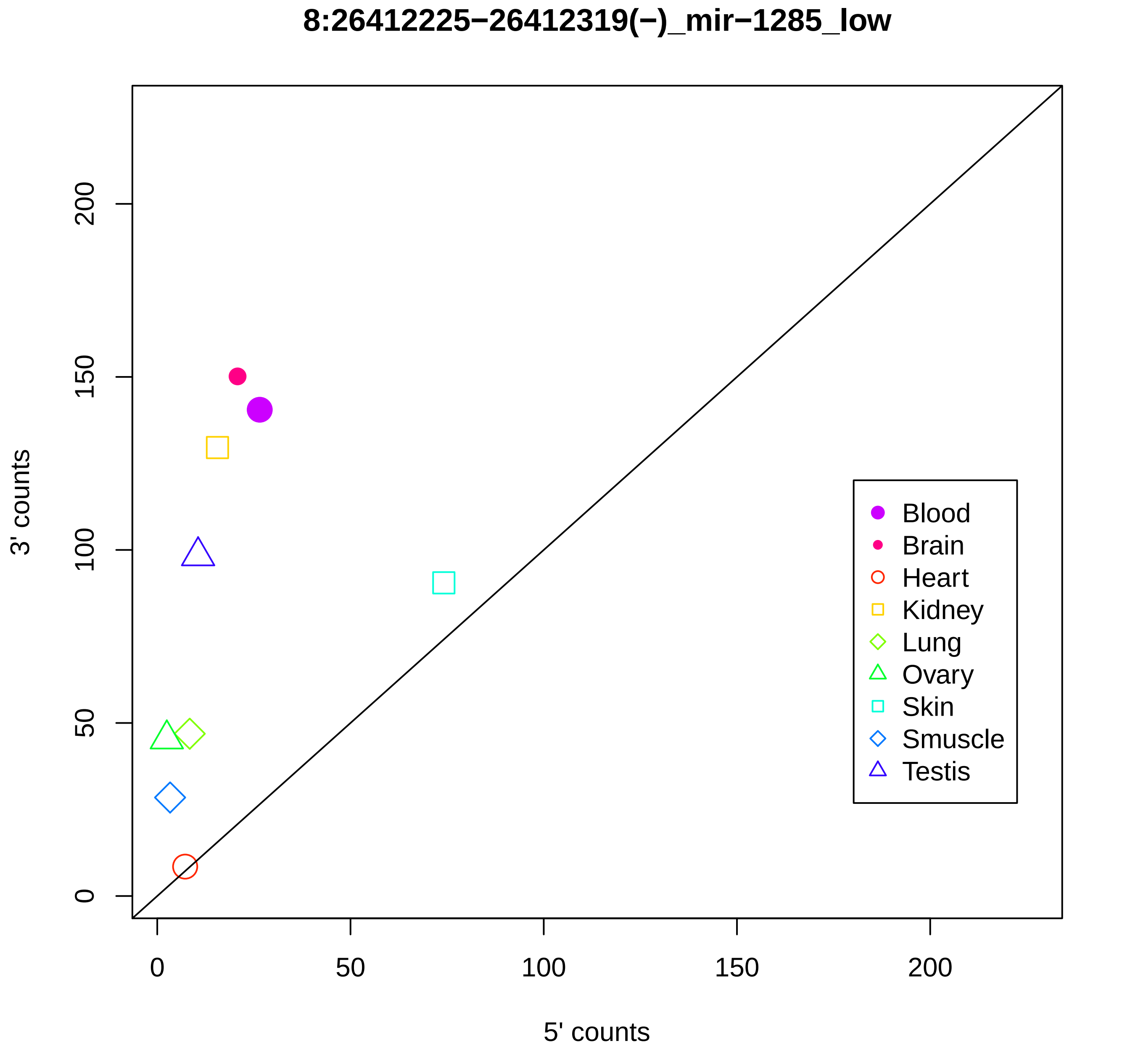

8:26412225−26412319(−)_mir−1285_low
Blood
Brain
Heart
Kidney
Lung
Ovary
Skin
Smuscle
Testis
0
50
100
150
200
3' counts
5' counts

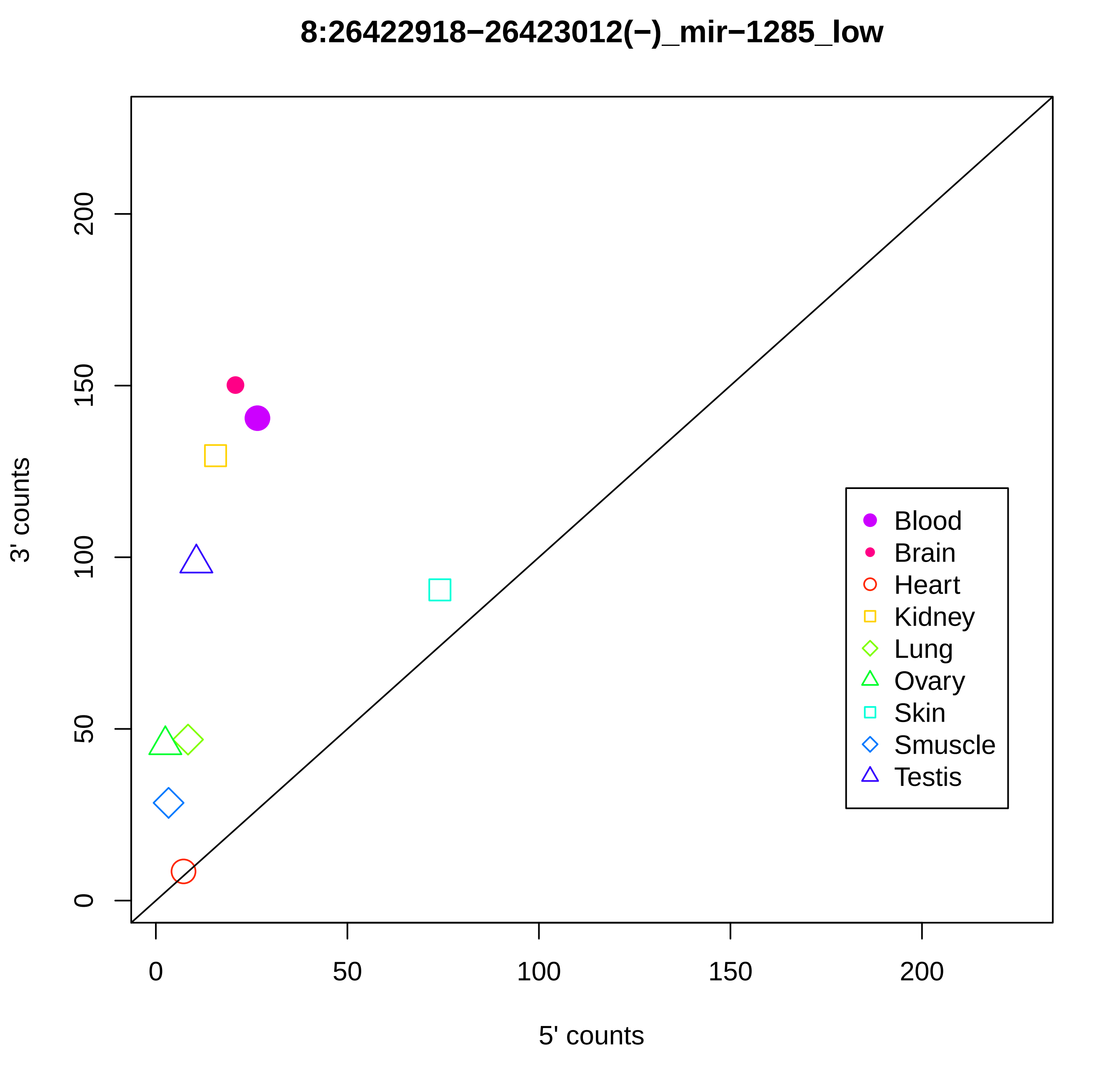

8:26422918−26423012(−)_mir−1285_low
3' counts
0
50
100
150
200
5' counts
0
50
100
150
200
Blood
Brain
Heart
Kidney
Lung
Ovary
Skin
Smuscle
Testis

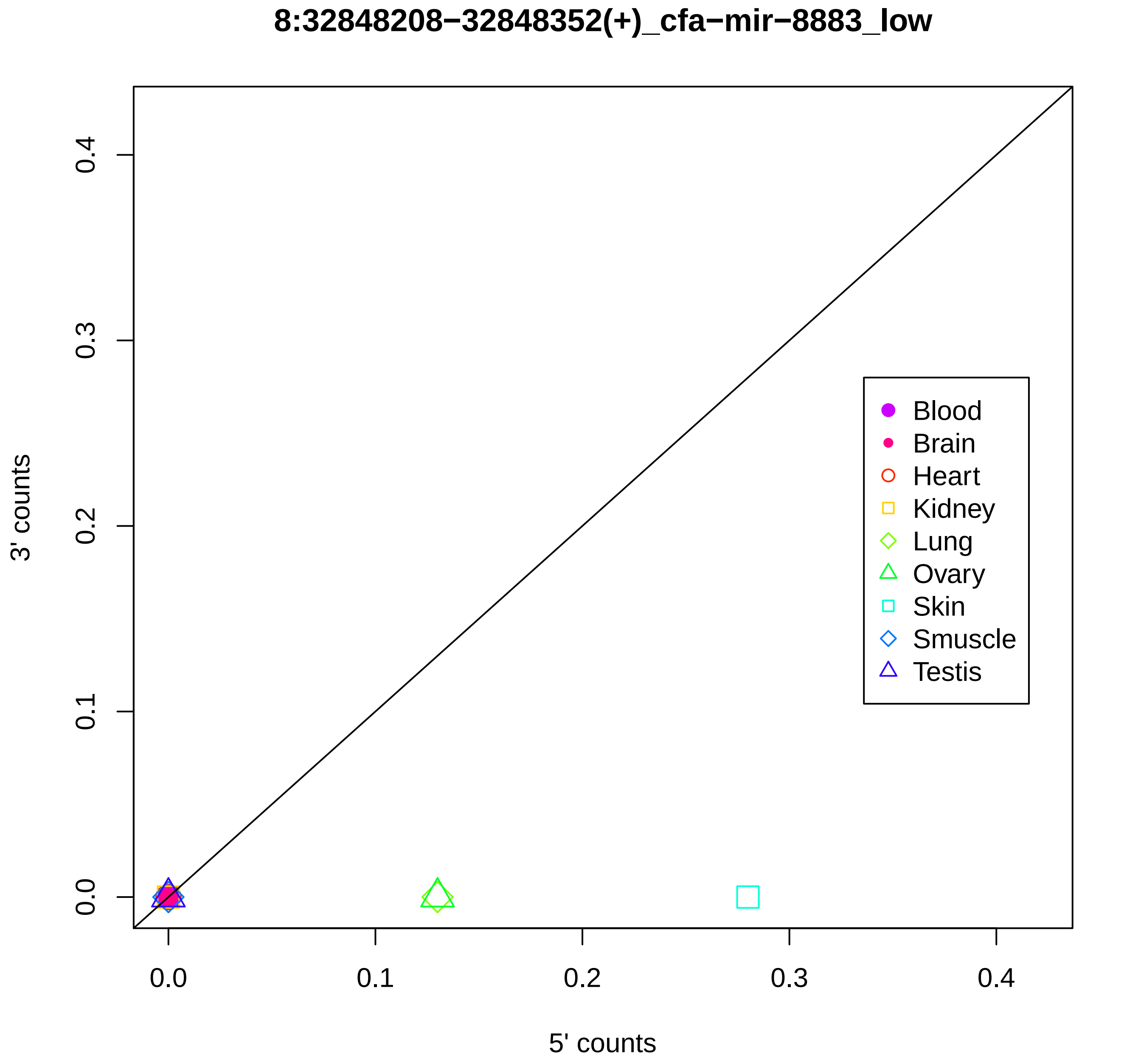
8:32848208−32848352(+)_cfa−mir−8883_low
3' counts
5' counts
0.0
0.1
0.2
0.3
0.4
Blood
Brain
Heart
Kidney
Lung
Ovary
Skin
Smuscle
Testis

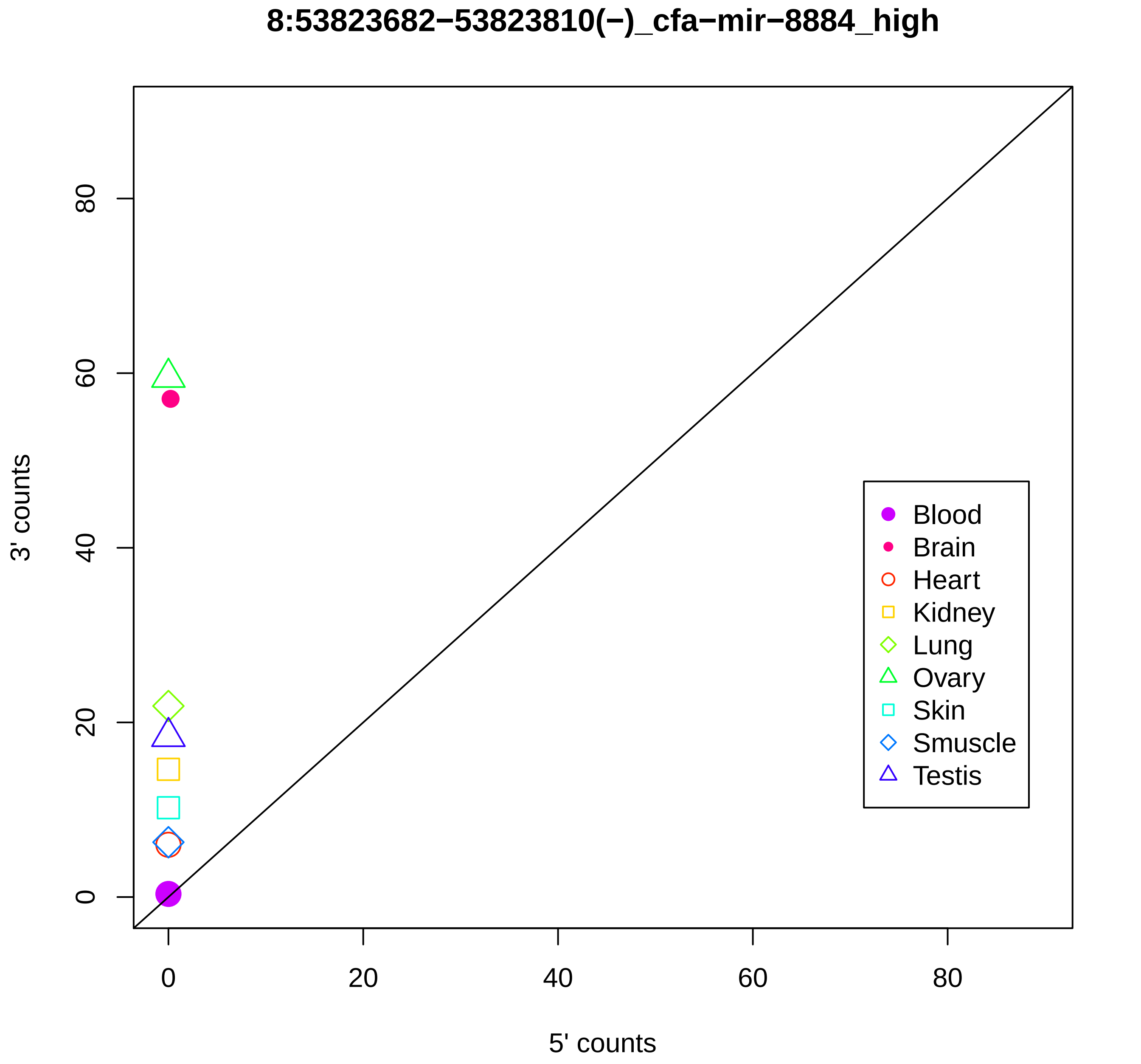

8:53823682−53823810(−)_cfa−mir−8884_high
3' counts
5' counts
0
20
40
60
80
0
20
40
60
80
Blood
Brain
Heart
Kidney
Lung
Ovary
Skin
Smuscle
Testis

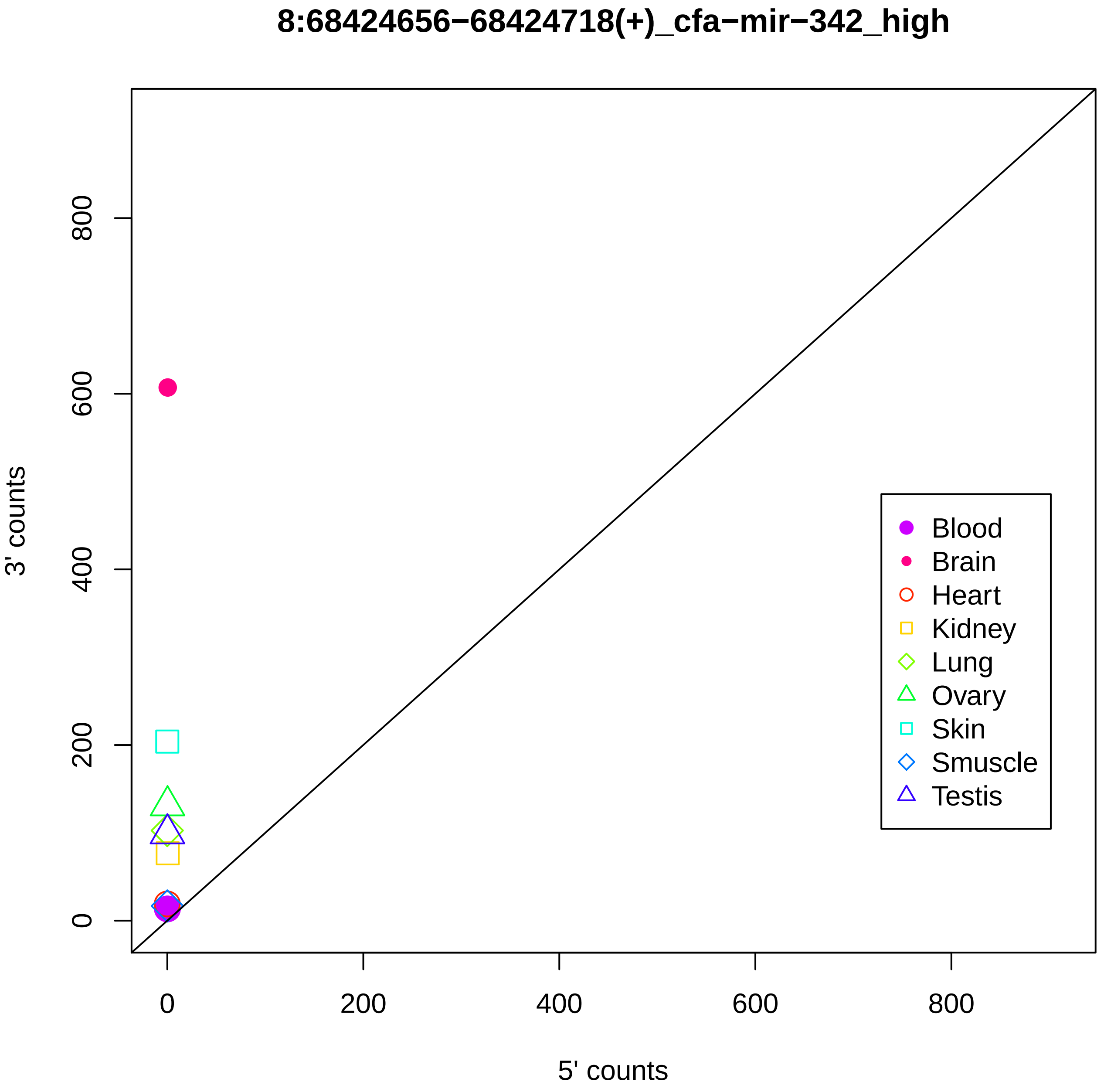

8:68424656−68424718(+)_cfa−mir−342_high
3' counts
5' counts
0
200
400
600
800
0
200
400
600
800
Blood
Brain
Heart
Kidney
Lung
Ovary
Skin
Smuscle
Testis

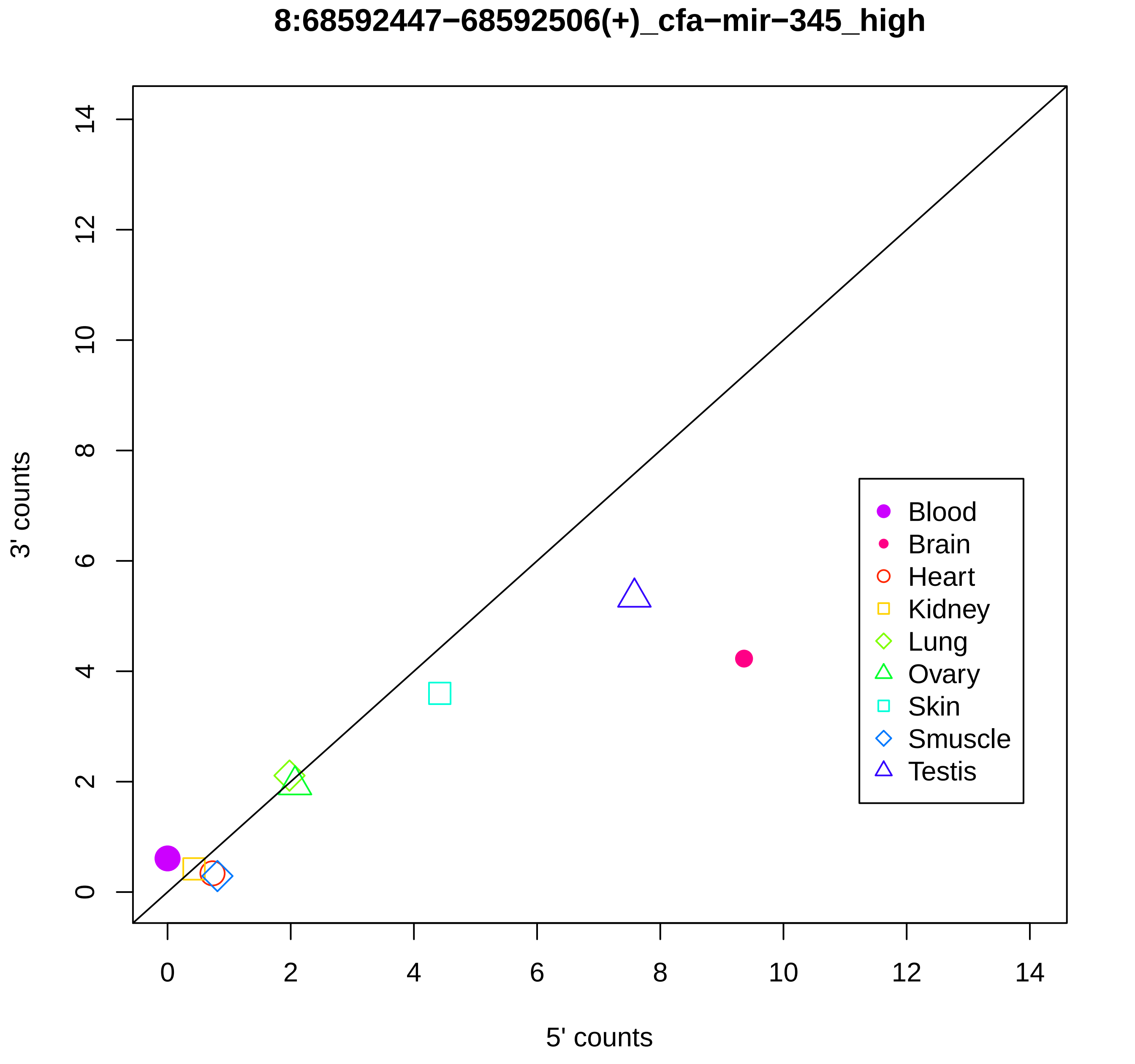

8:68592447−68592506(+)_cfa−mir−345_high
3' counts
5' counts
0
2
4
6
8
10
12
14
Blood
Brain
Heart
Kidney
Lung
Ovary
Skin
Smuscle
Testis

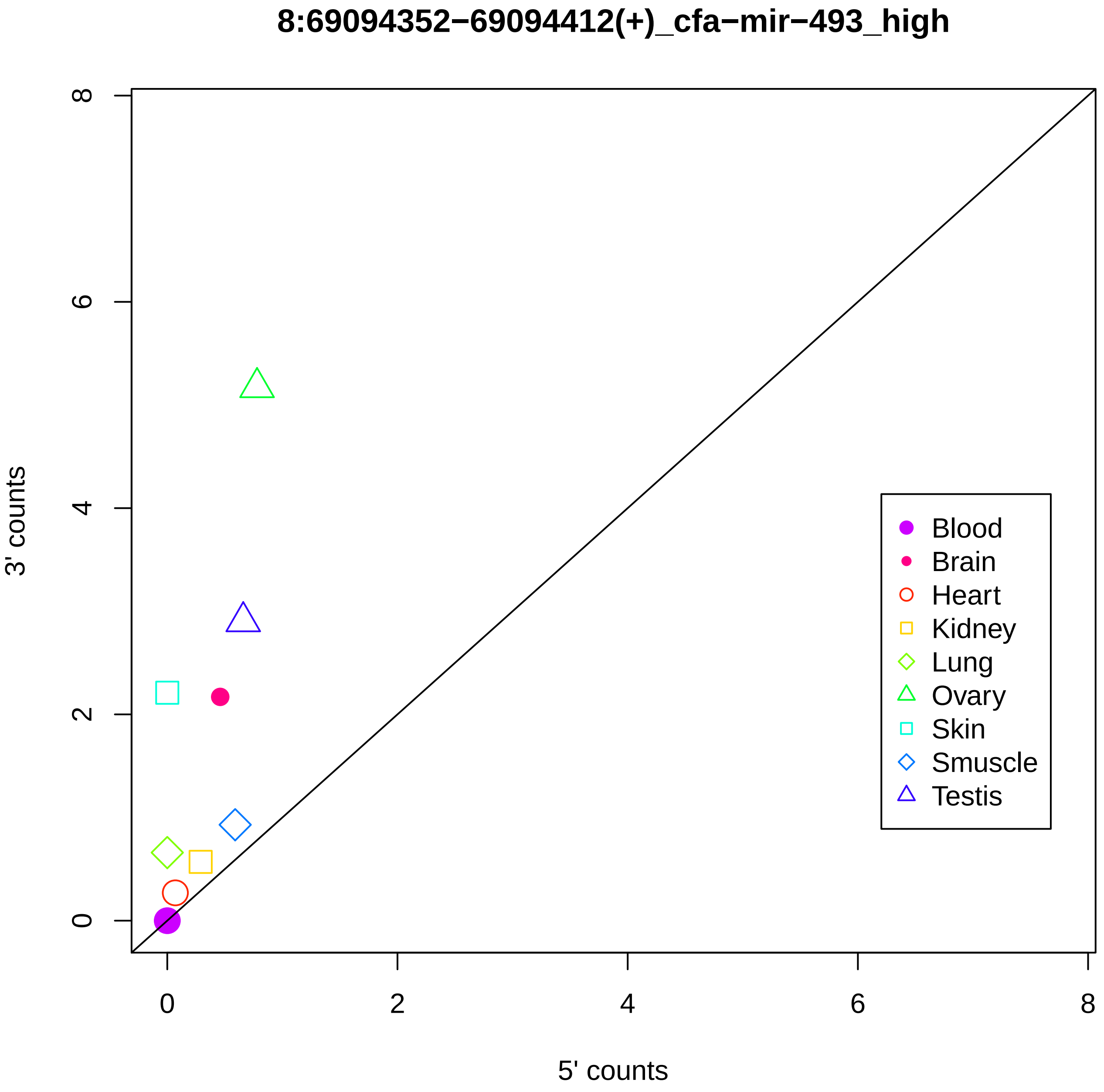

8:69094352−69094412(+)_cfa−mir−493_high
3' counts
5' counts
0
2
4
6
8
Blood
Brain
Heart
Kidney
Lung
Ovary
Skin
Smuscle
Testis

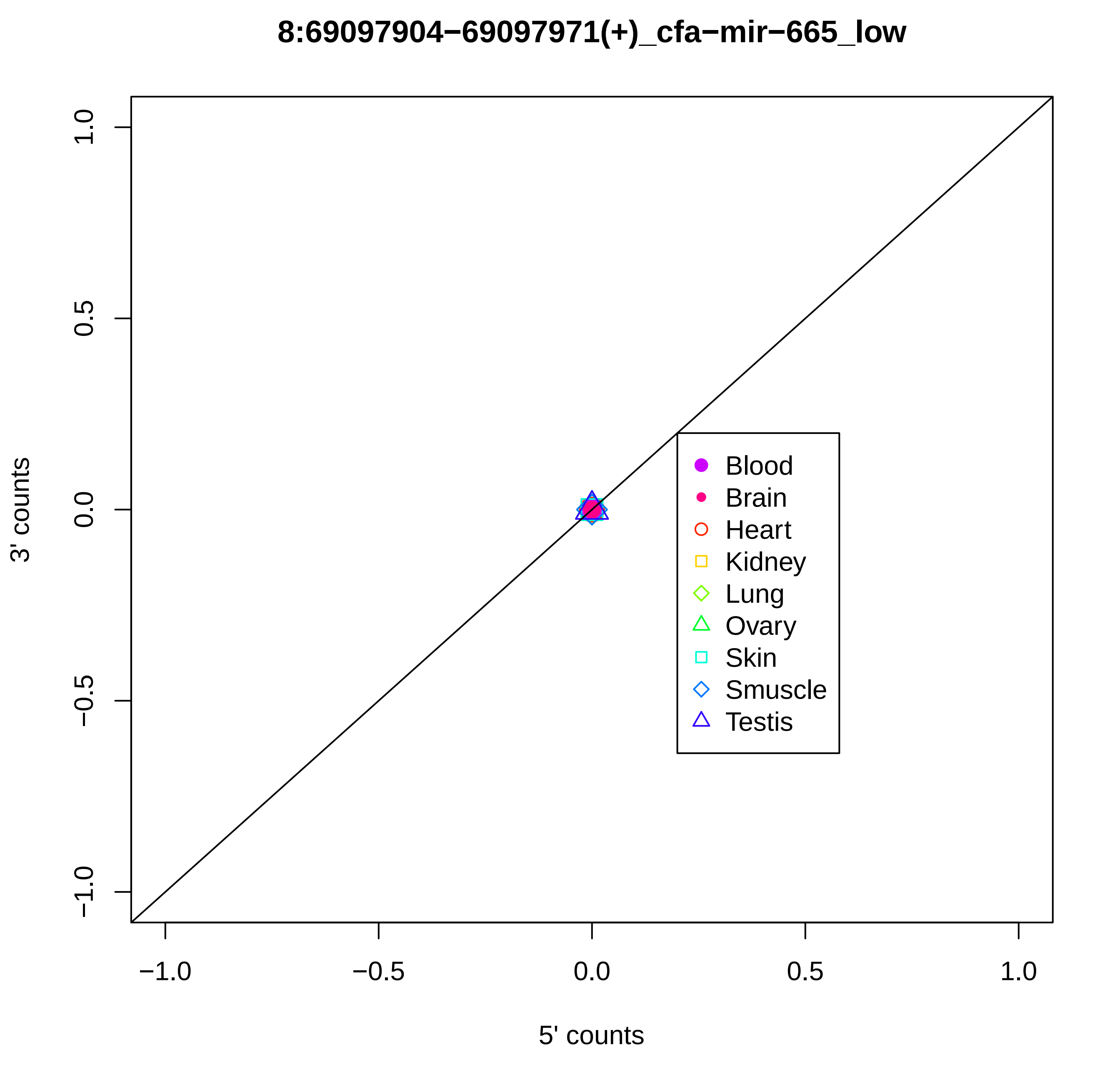

8:69097904−69097971(+)_cfa−mir−665_low
Blood
Brain
Heart
Kidney
Lung
Ovary
Skin
Smuscle
Testis
3' counts
5' counts
−1.0
−0.5
0.0
0.5
1.0

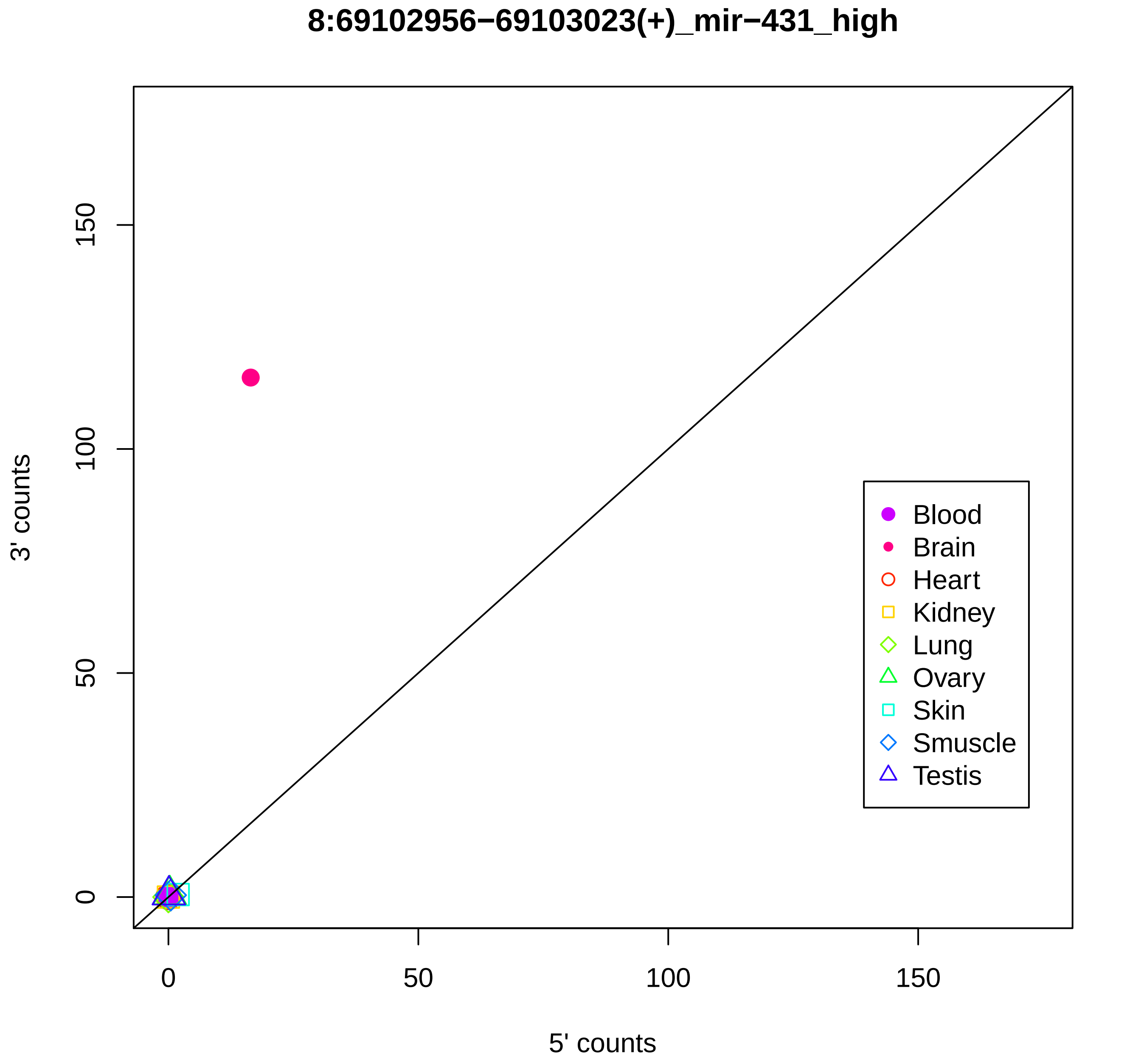

8:69102956−69103023(+)_mir−431_high
3' counts
5' counts
0
50
100
150
0
50
100
150
Blood
Brain
Heart
Kidney
Lung
Ovary
Skin
Smuscle
Testis

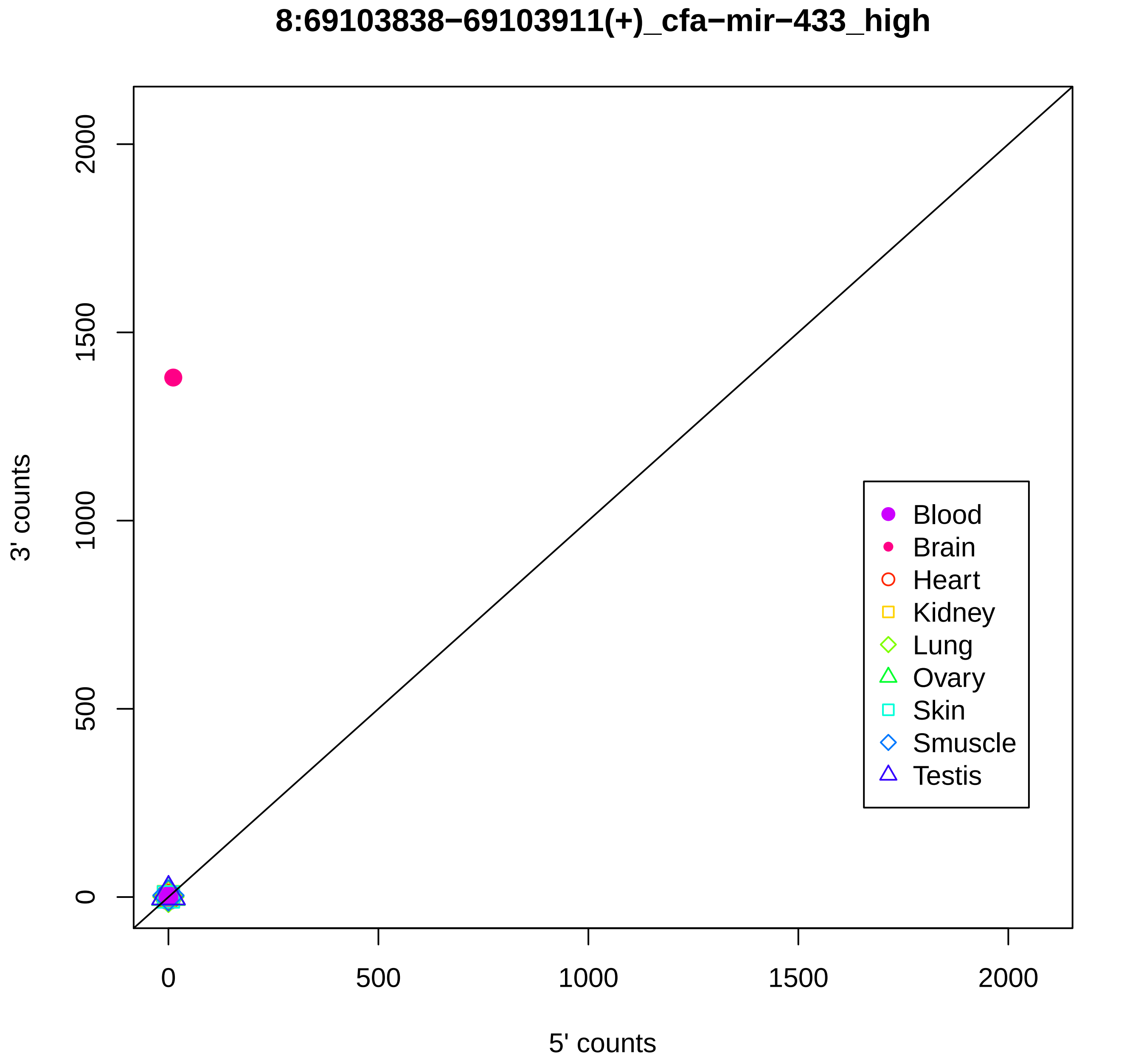
8:69103838−69103911(+)_cfa−mir−433_high
3' counts
5' counts
0
500
1000
1500
2000
Blood
Brain
Heart
Kidney
Lung
Ovary
Skin
Smuscle
Testis

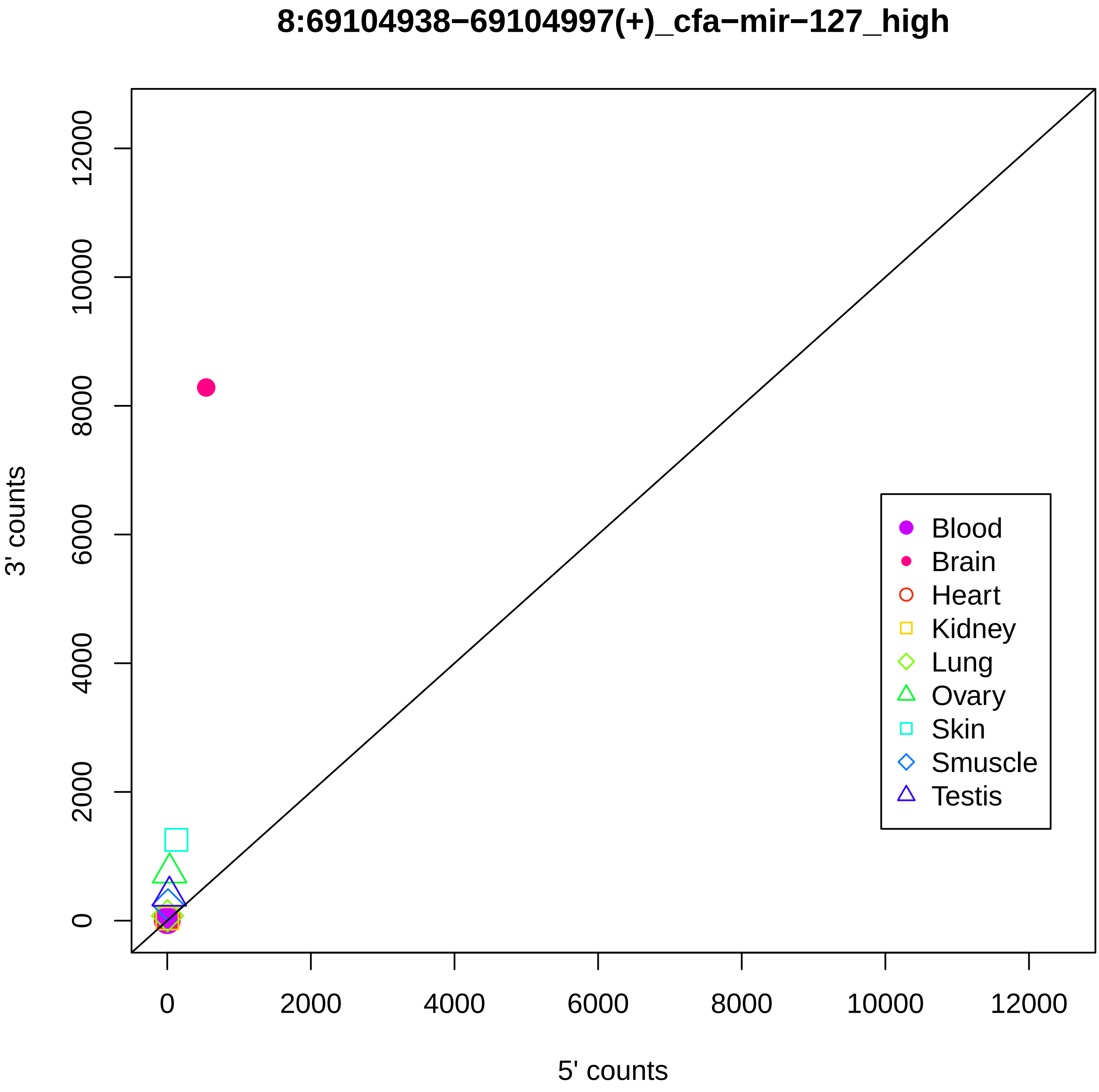
8:69104938−69104997(+)_cfa−mir−127_high
3' counts
5' counts
0
2000
4000
6000
8000
10000
12000
Blood
Brain
Heart
Kidney
Lung
Ovary
Skin
Smuscle
Testis

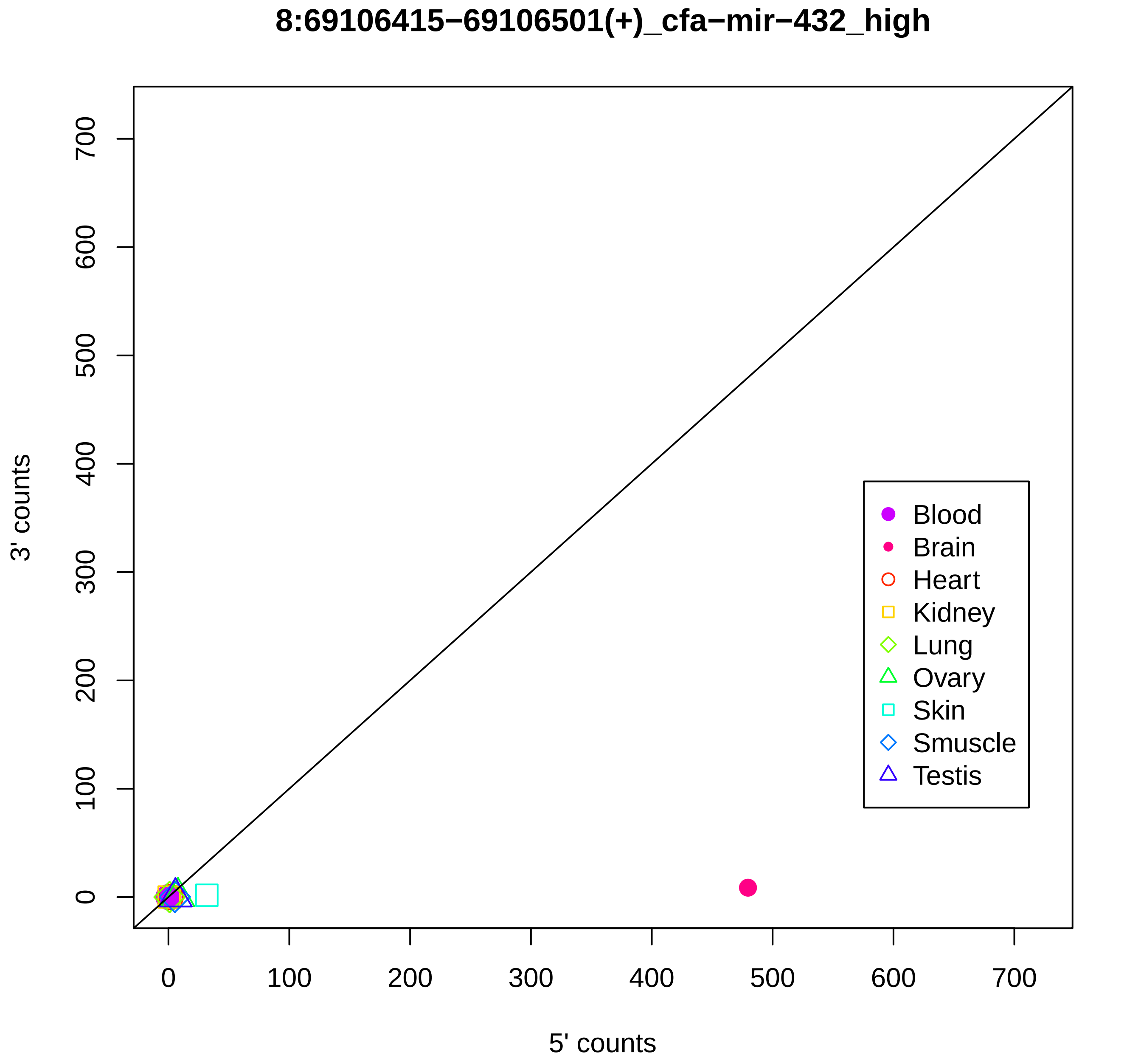

8:69106415−69106501(+)_cfa−mir−432_high
3' counts
5' counts
0
100
200
300
400
500
600
700
Blood
Brain
Heart
Kidney
Lung
Ovary
Skin
Smuscle
Testis

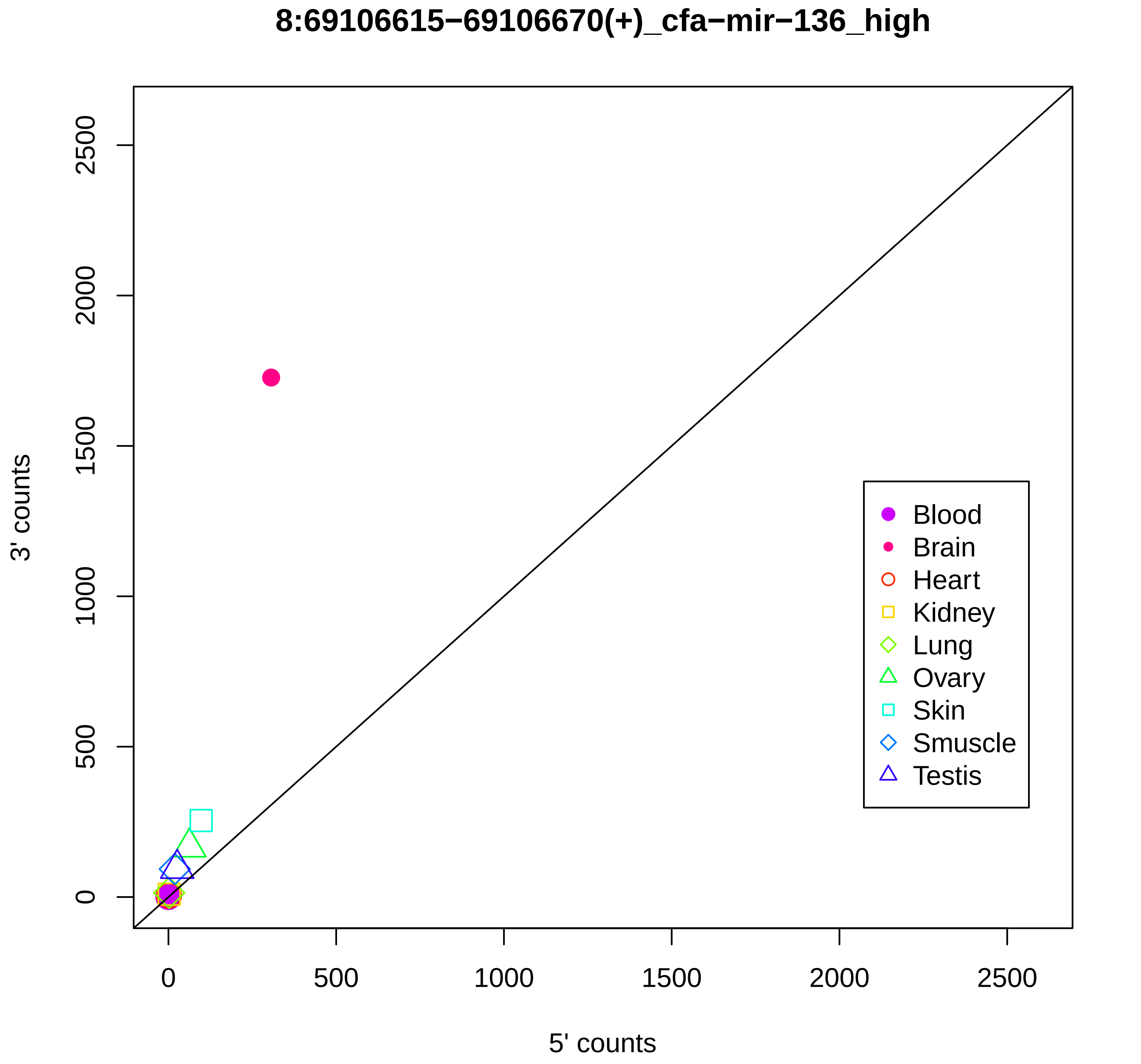
8:69106615−69106670(+)_cfa−mir−136_high
3' counts
5' counts
0
500
1000
1500
2000
2500
Blood
Brain
Heart
Kidney
Lung
Ovary
Skin
Smuscle
Testis

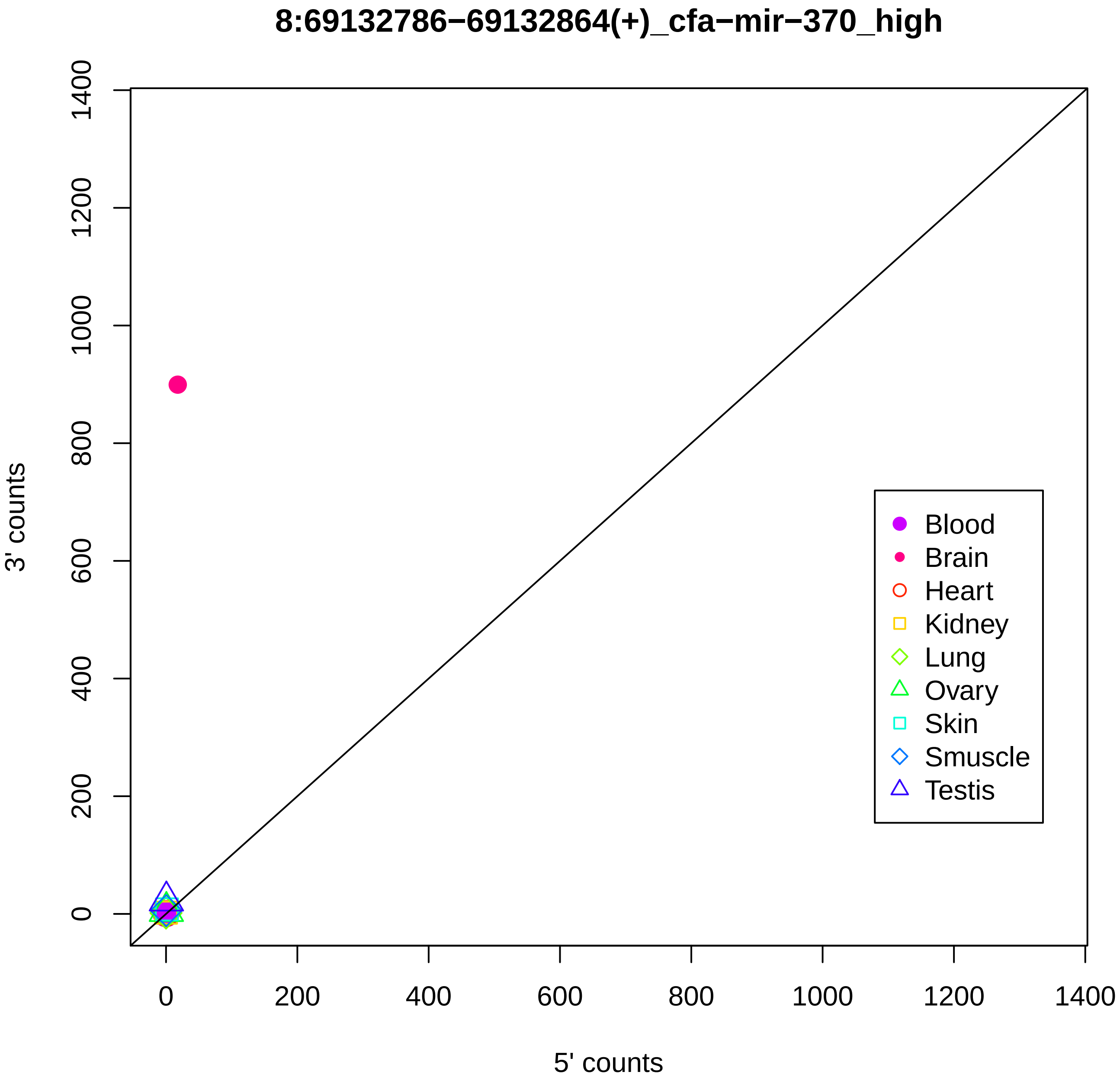
8:69132786−69132864(+)_cfa−mir−370_high
3' counts
5' counts
0
200
400
600
800
1000
1200
1400
Blood
Brain
Heart
Kidney
Lung
Ovary
Skin
Smuscle
Testis

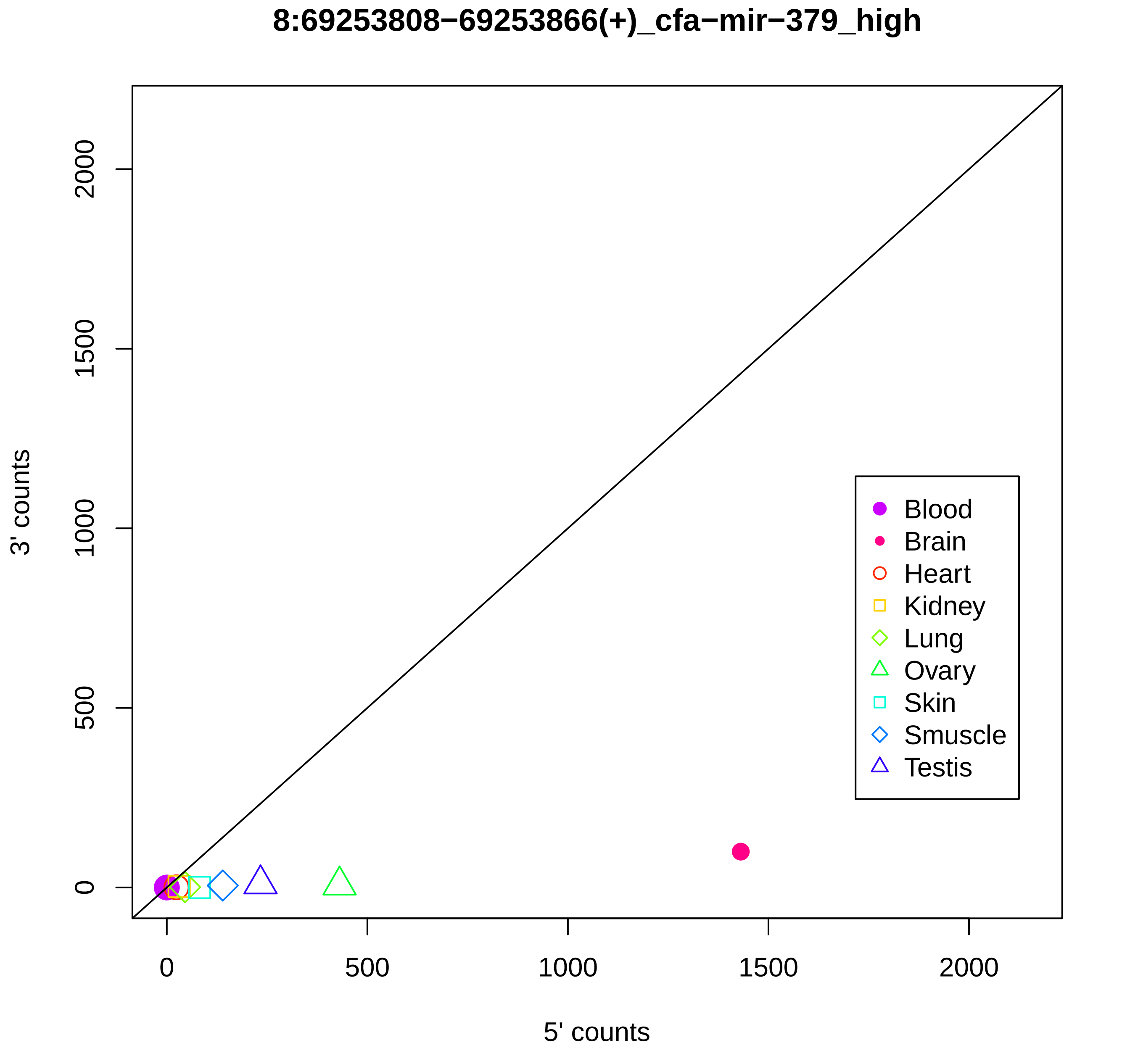
8:69253808−69253866(+)_cfa−mir−379_high
3' counts
5' counts
0
500
1000
1500
2000
0
500
1000
1500
2000
Blood
Brain
Heart
Kidney
Lung
Ovary
Skin
Smuscle
Testis

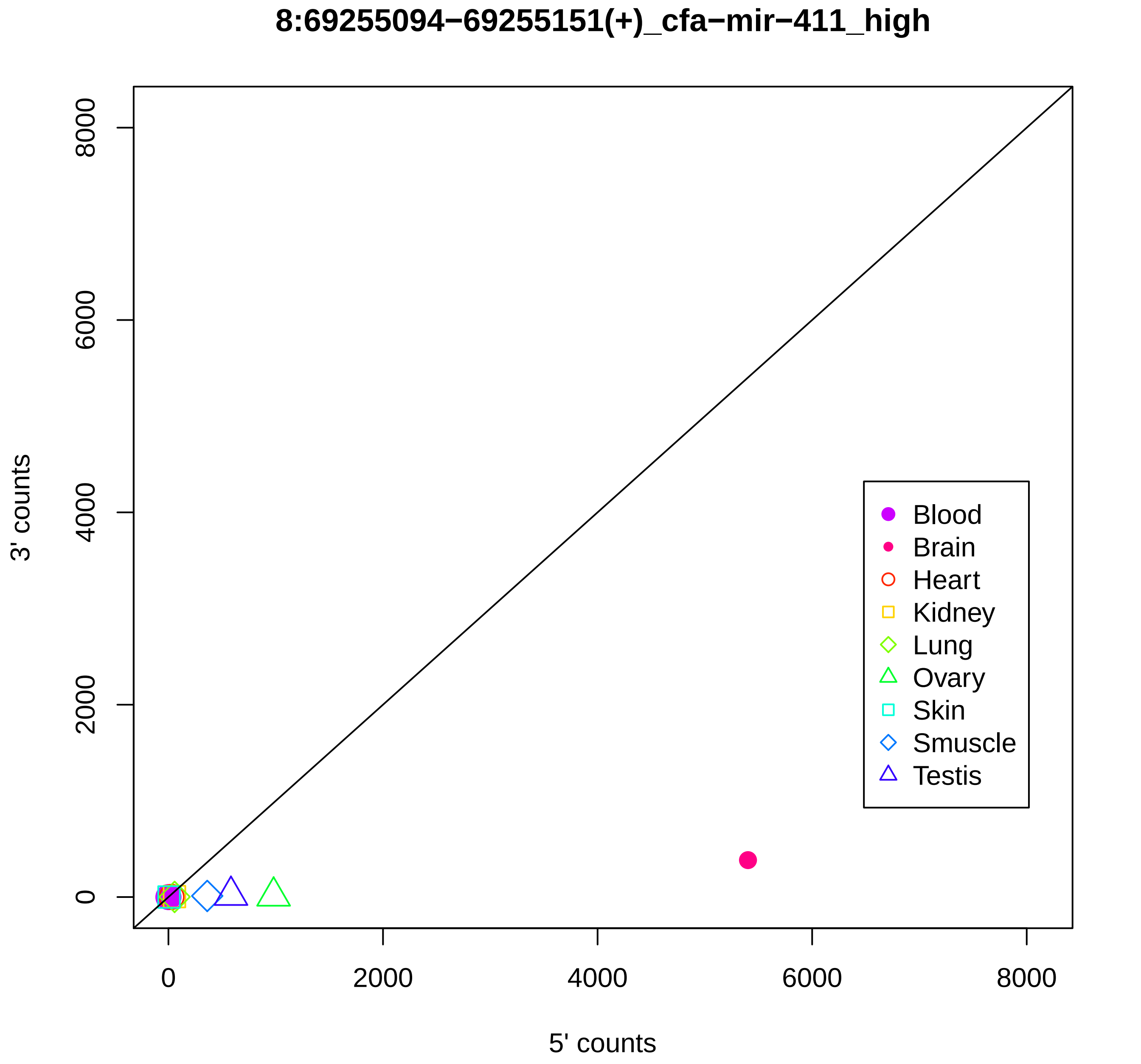

8:69255094−69255151(+)_cfa−mir−411_high
3' counts
5' counts
0
2000
4000
6000
8000
0
2000
4000
6000
8000
Blood
Brain
Heart
Kidney
Lung
Ovary
Skin
Smuscle
Testis

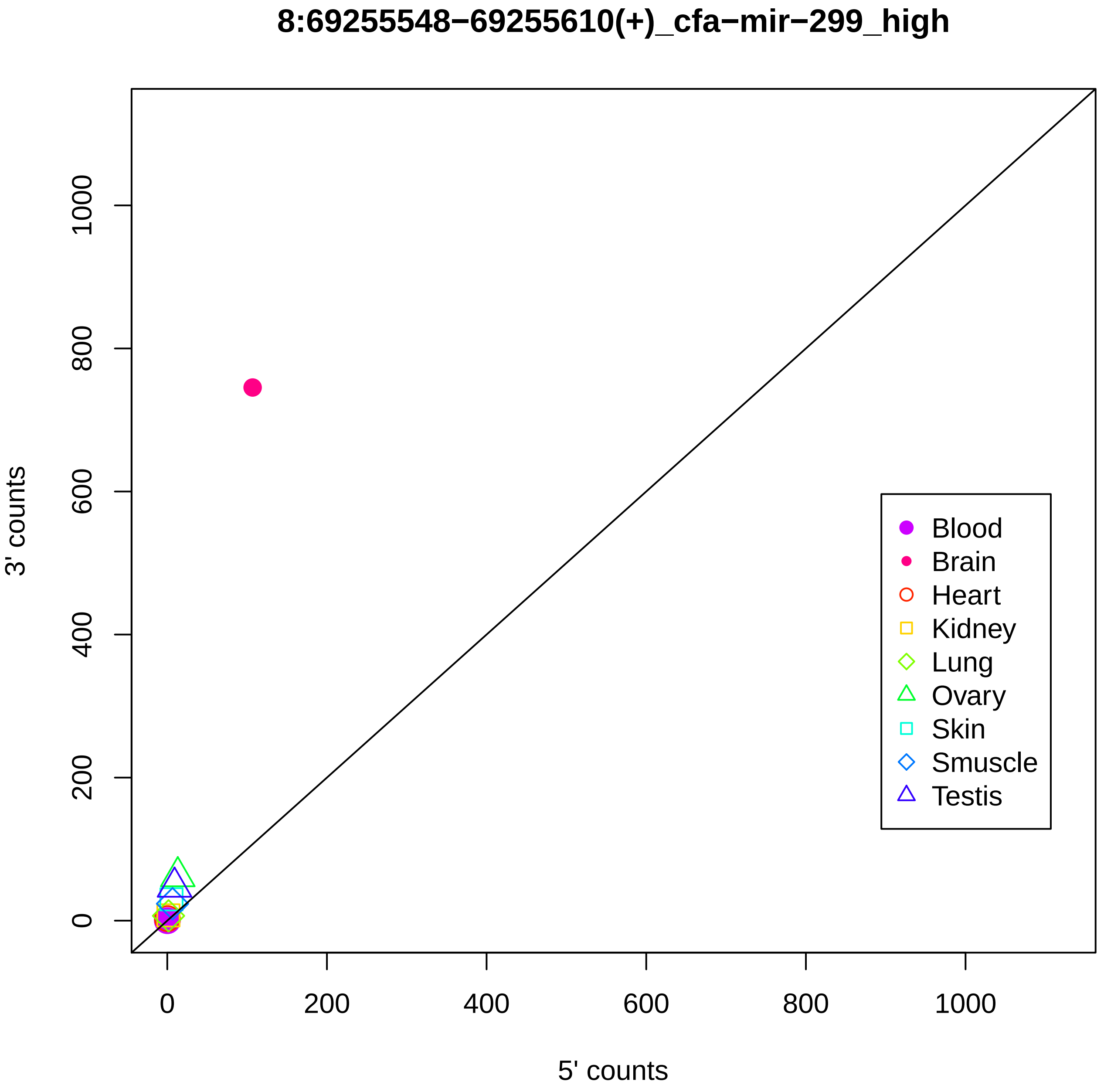
8:69255548−69255610(+)_cfa−mir−299_high
3' counts
5' counts
0
200
400
600
800
1000
0
200
400
600
800
1000
Blood
Brain
Heart
Kidney
Lung
Ovary
Skin
Smuscle
Testis

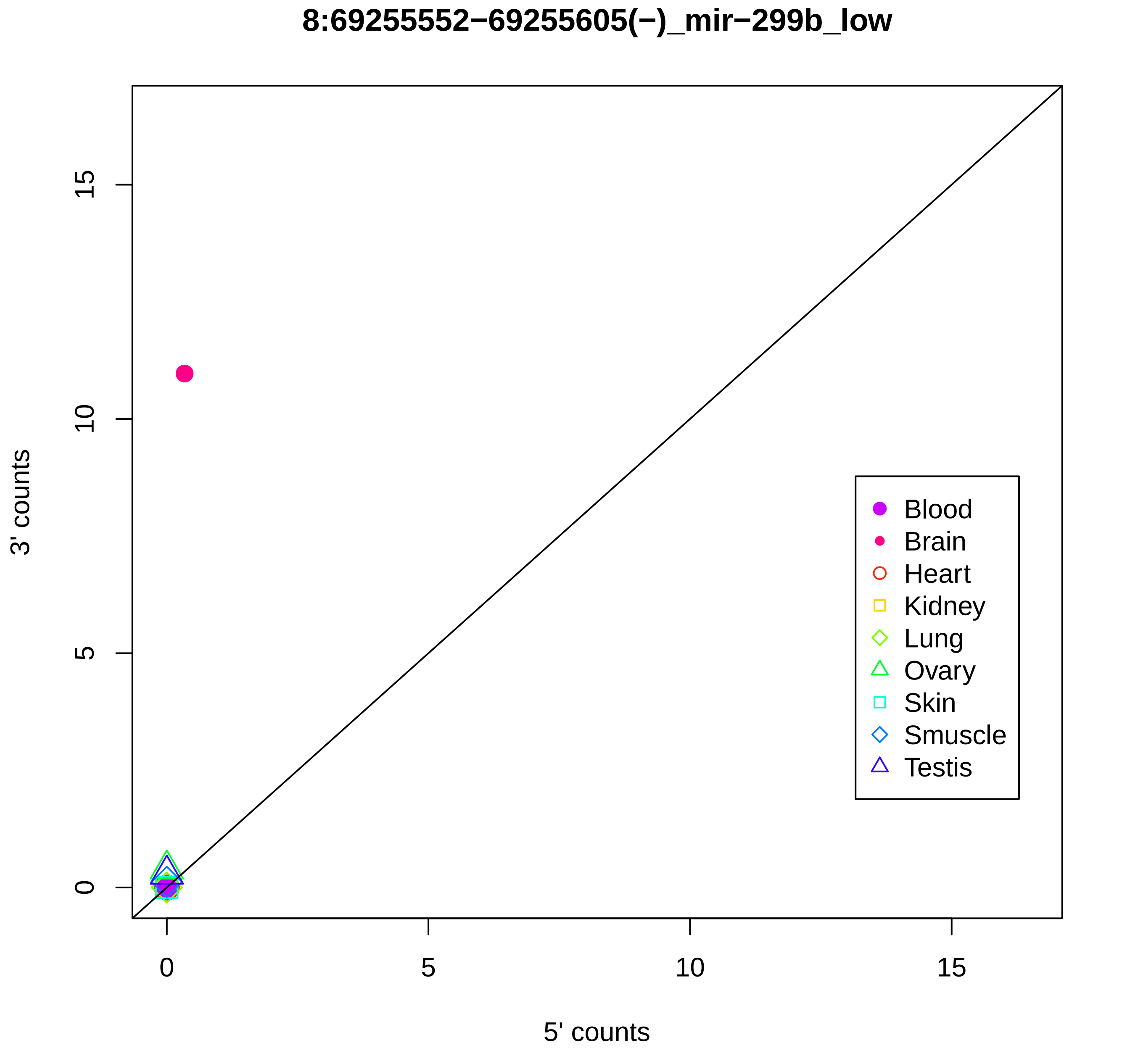

8:69255552−69255605(−)_mir−299b_low
3' counts
5' counts
0
5
10
15
0
5
10
15
Blood
Brain
Heart
Kidney
Lung
Ovary
Skin
Smuscle
Testis

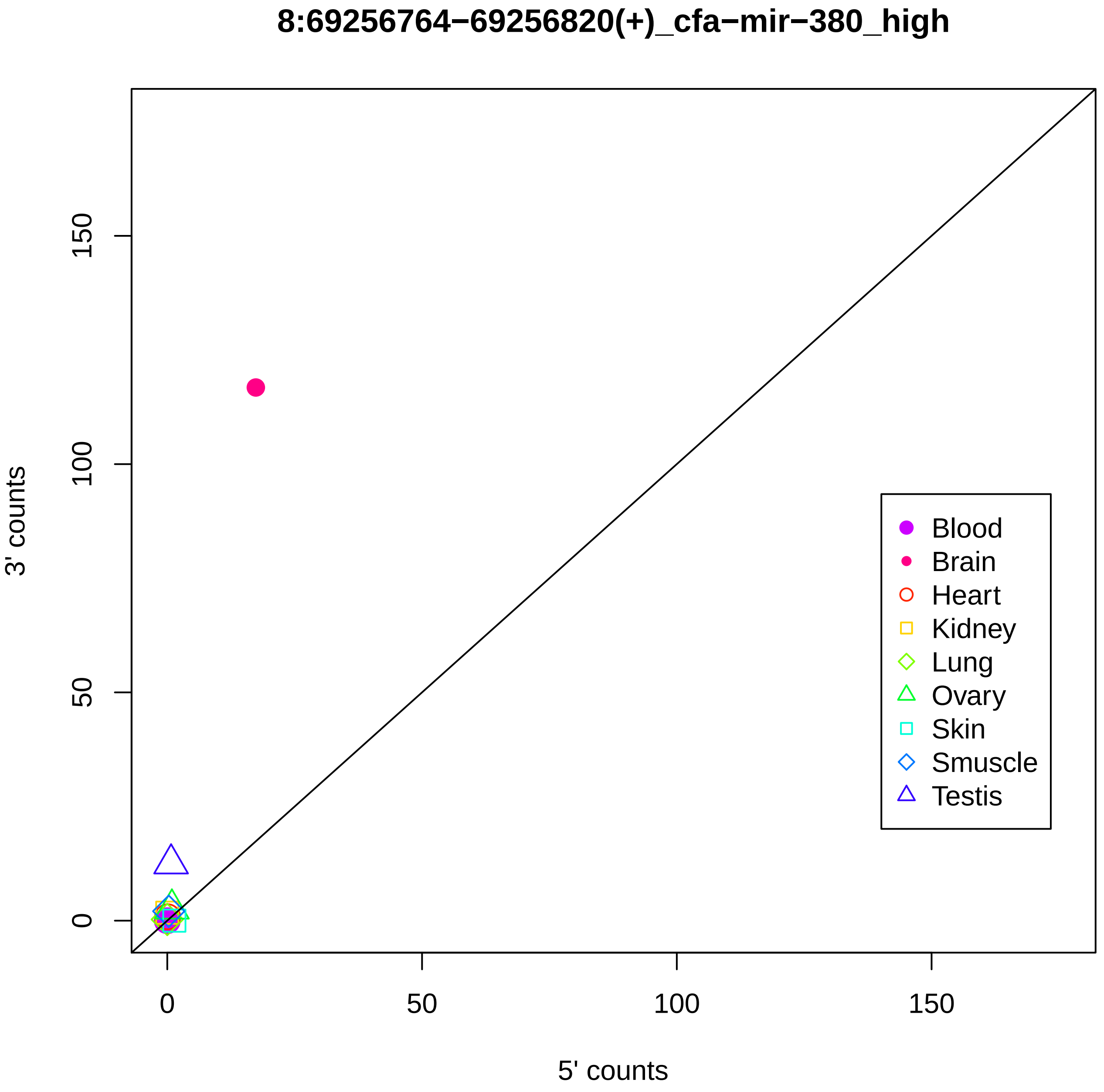

8:69256764−69256820(+)_cfa−mir−380_high
3' counts
5' counts
0
50
100
150
0
50
100
150
Blood
Brain
Heart
Kidney
Lung
Ovary
Skin
Smuscle
Testis

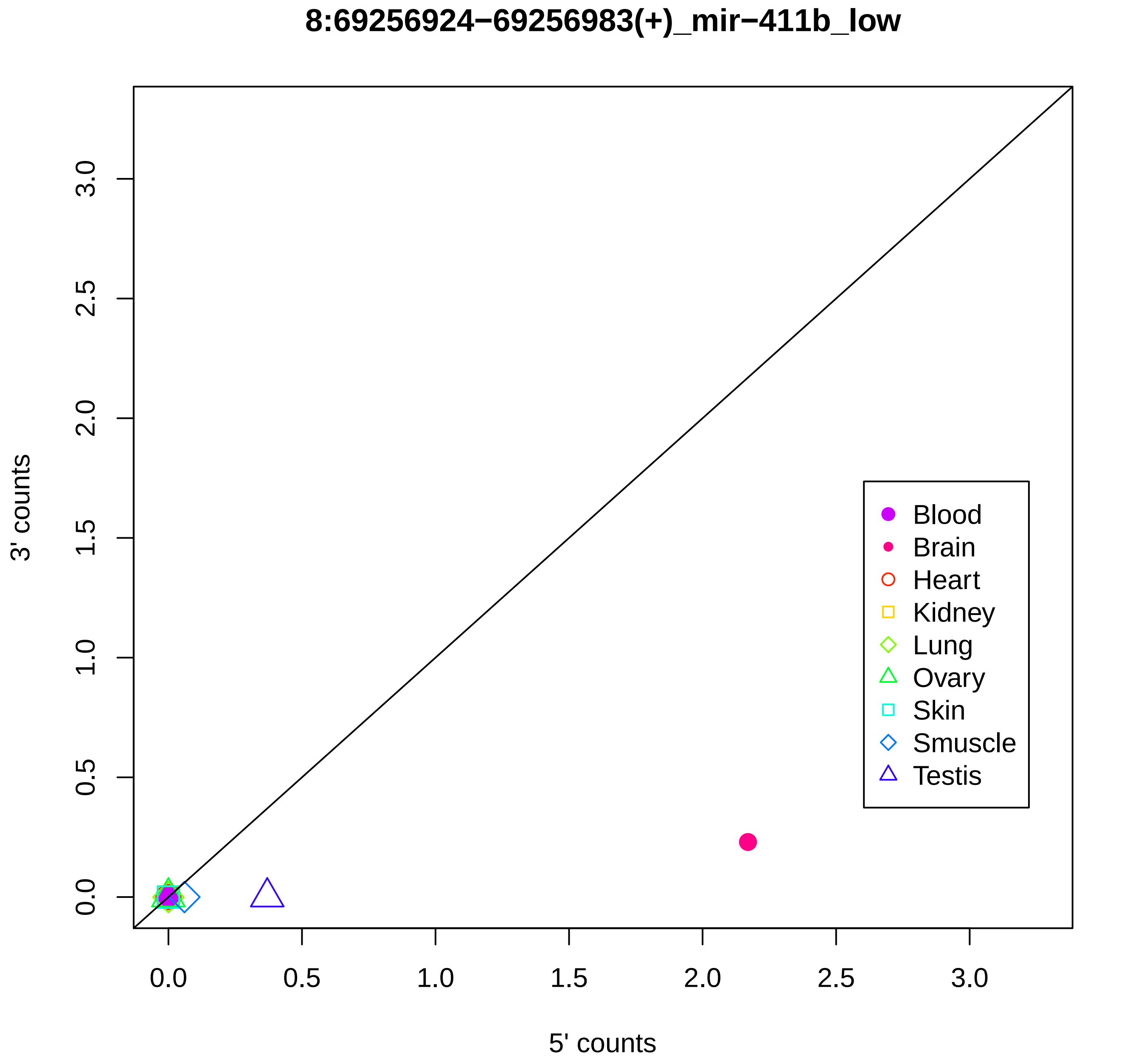

8:69256924−69256983(+)_mir−411b_low
3' counts
5' counts
0.0
0.5
1.0
1.5
2.0
2.5
3.0
Blood
Brain
Heart
Kidney
Lung
Ovary
Skin
Smuscle
Testis

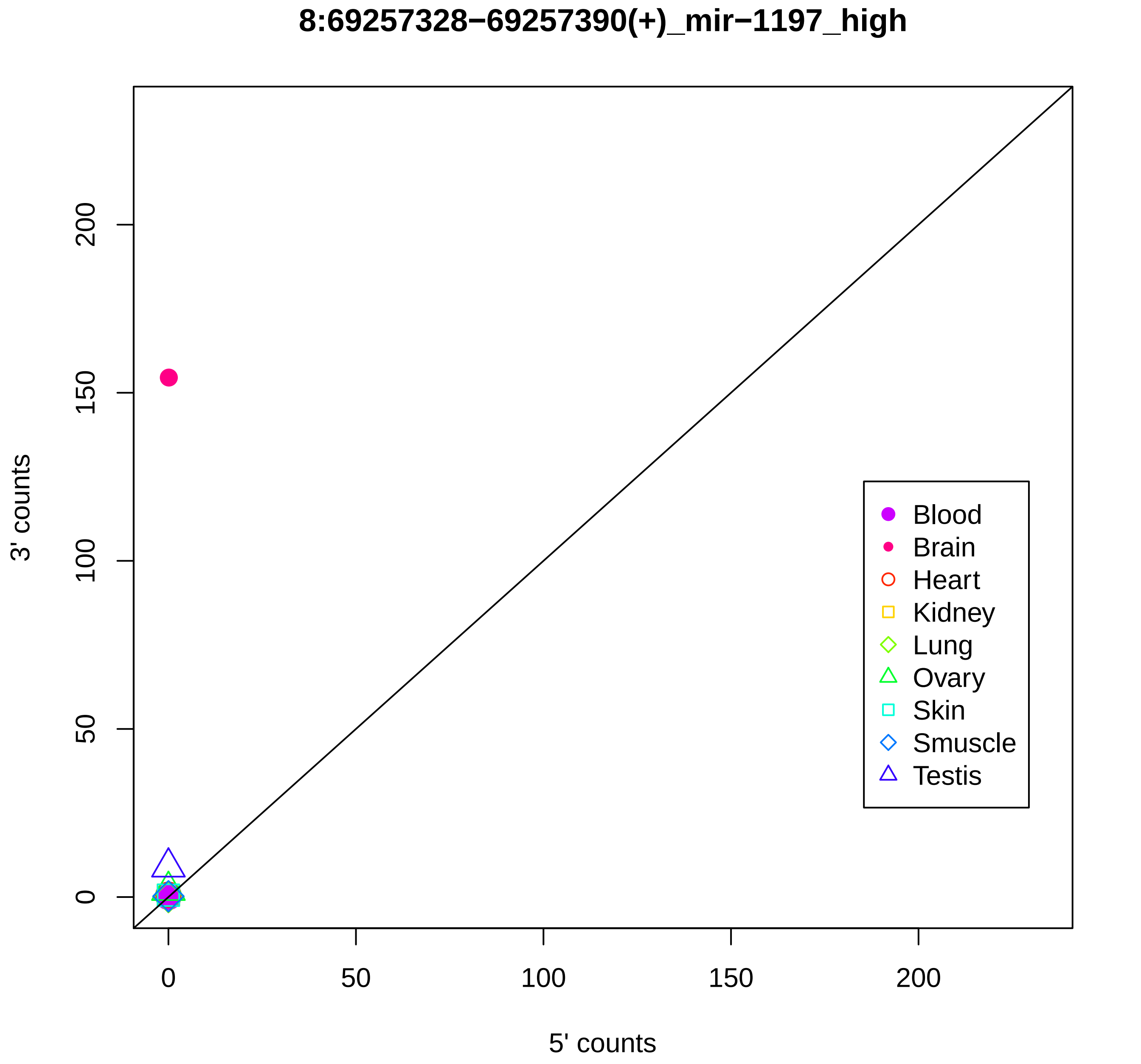
8:69257328−69257390(+)_mir−1197_high
3' counts
5' counts
0
50
100
150
200
0
50
100
150
200
Blood
Brain
Heart
Kidney
Lung
Ovary
Skin
Smuscle
Testis

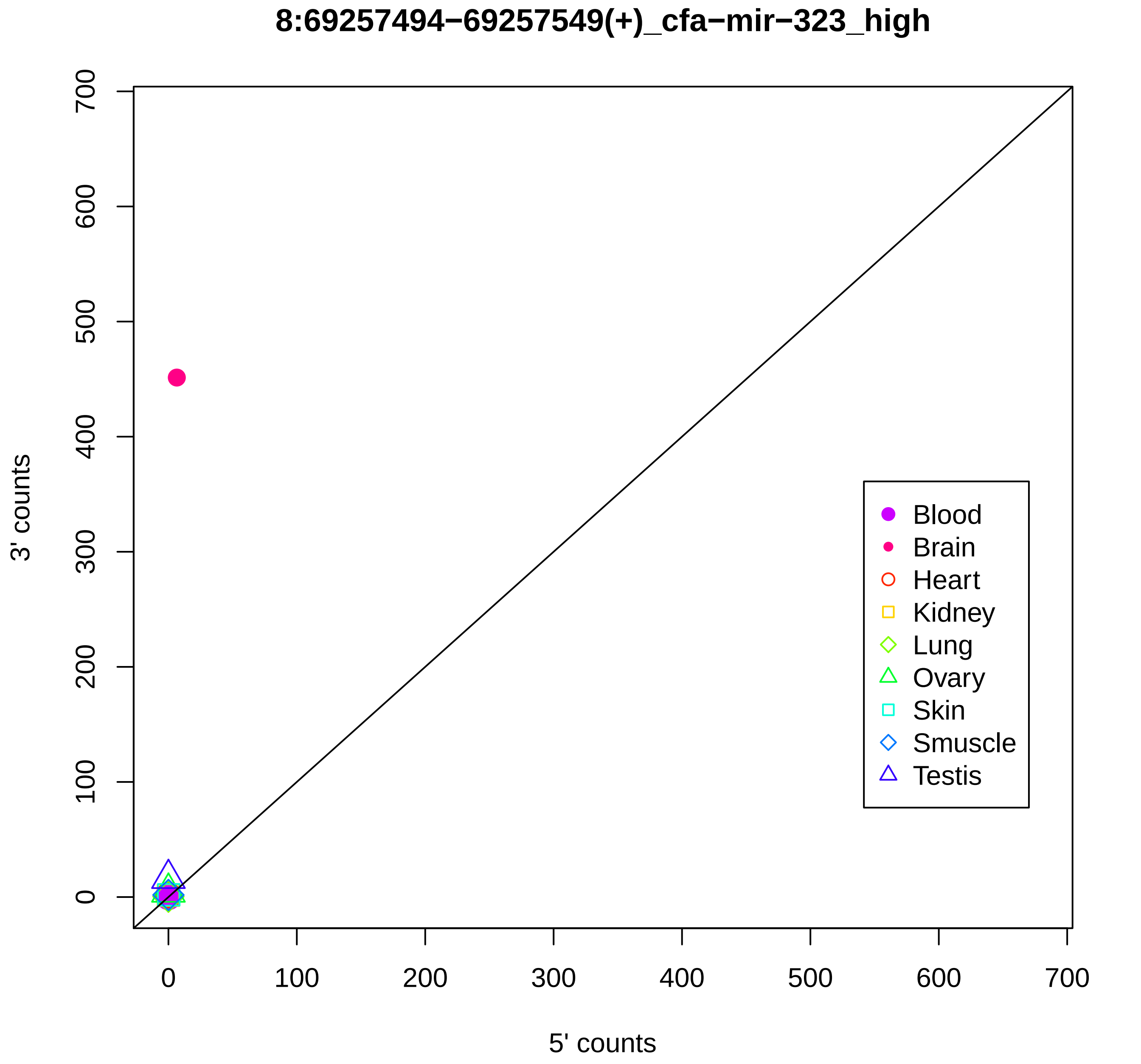

8:69257494−69257549(+)_cfa−mir−323_high
3' counts
5' counts
0
100
200
300
400
500
600
700
0
100
200
300
400
500
600
700
Blood
Brain
Heart
Kidney
Lung
Ovary
Skin
Smuscle
Testis

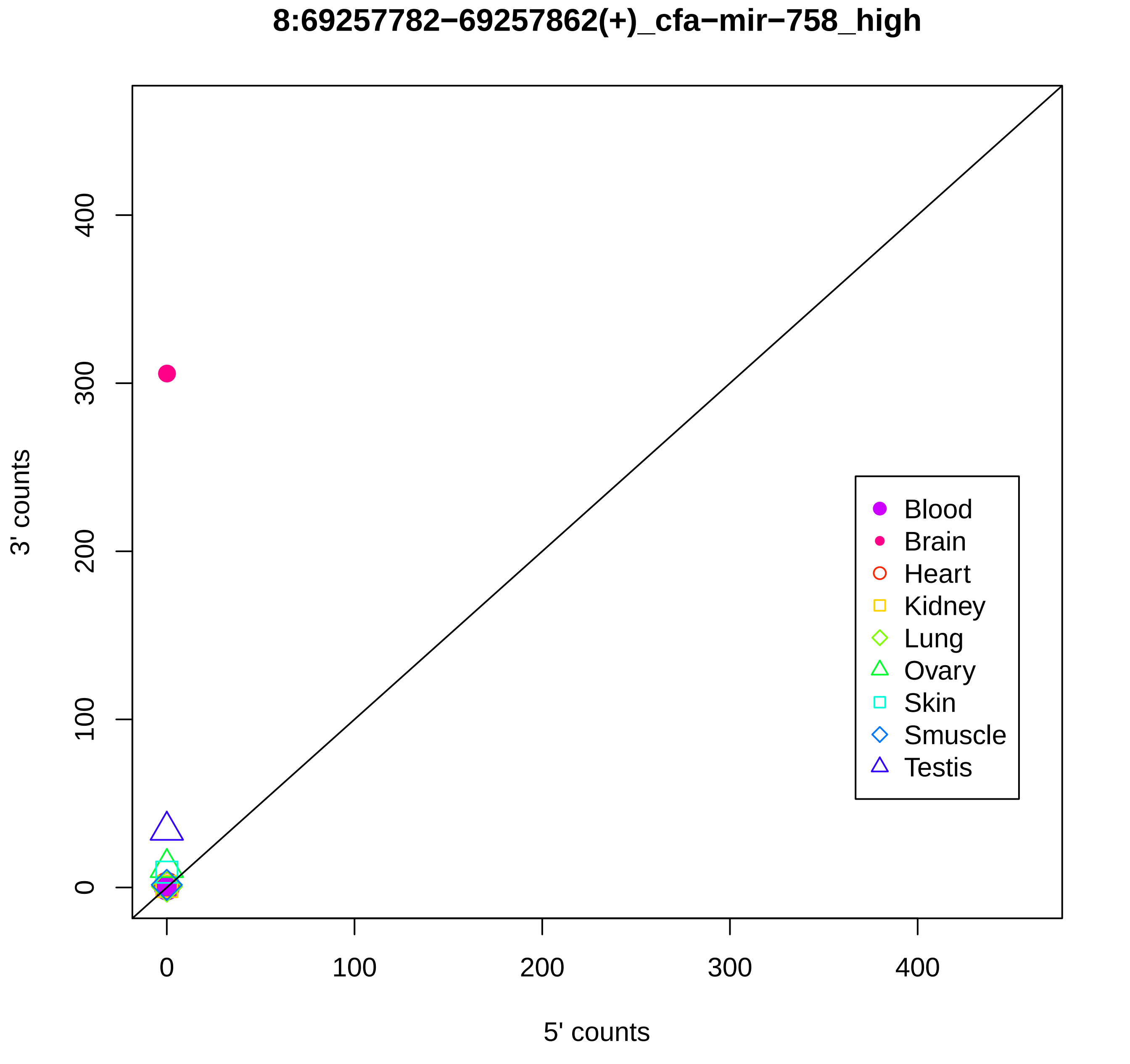

8:69257782−69257862(+)_cfa−mir−758_high
3' counts
5' counts
0
100
200
300
400
0
100
200
300
400
Blood
Brain
Heart
Kidney
Lung
Ovary
Skin
Smuscle
Testis

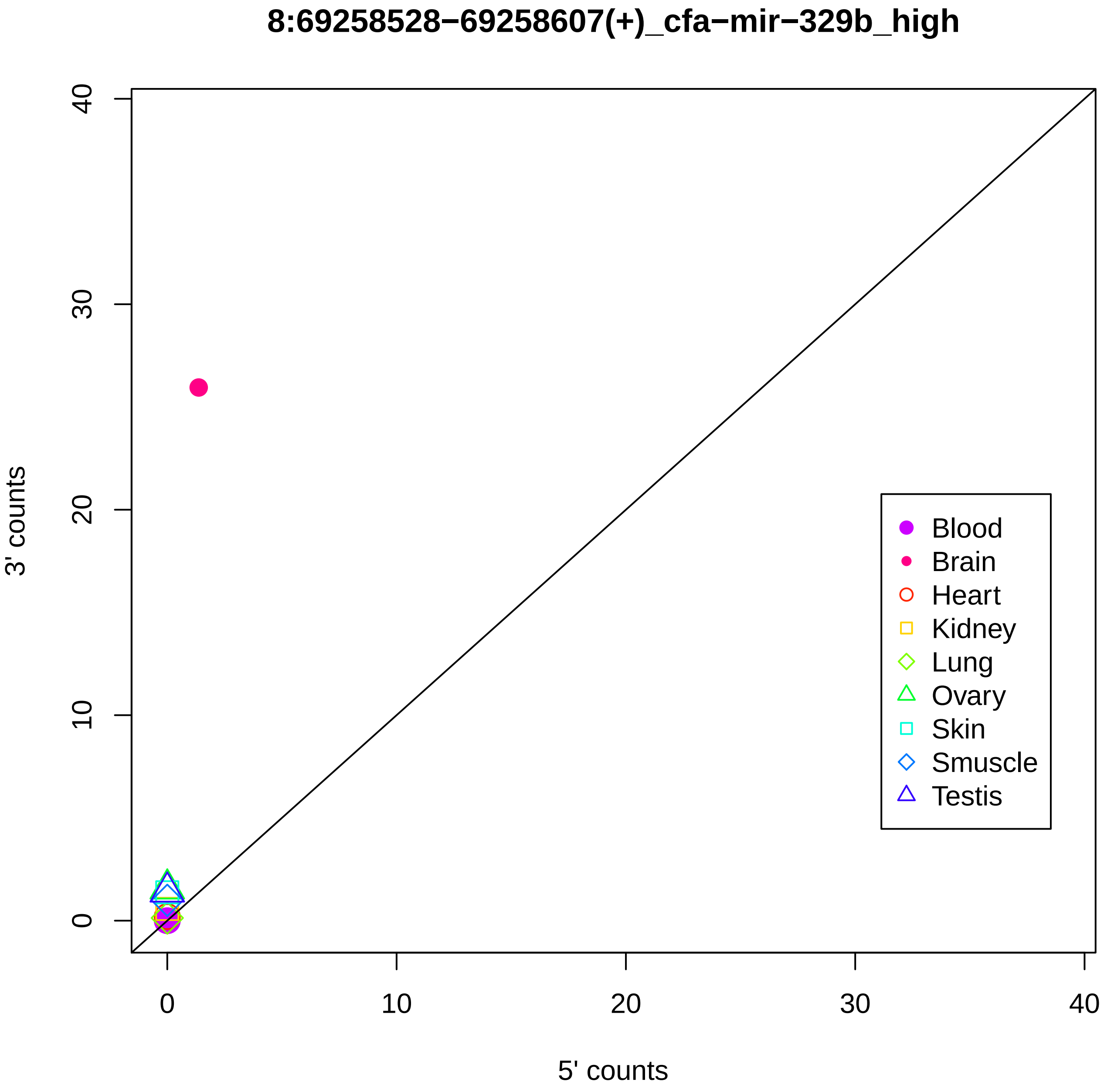

8:69258528−69258607(+)_cfa−mir−329b_high
3' counts
5' counts
0
10
20
30
40
0
10
20
30
40
Blood
Brain
Heart
Kidney
Lung
Ovary
Skin
Smuscle
Testis

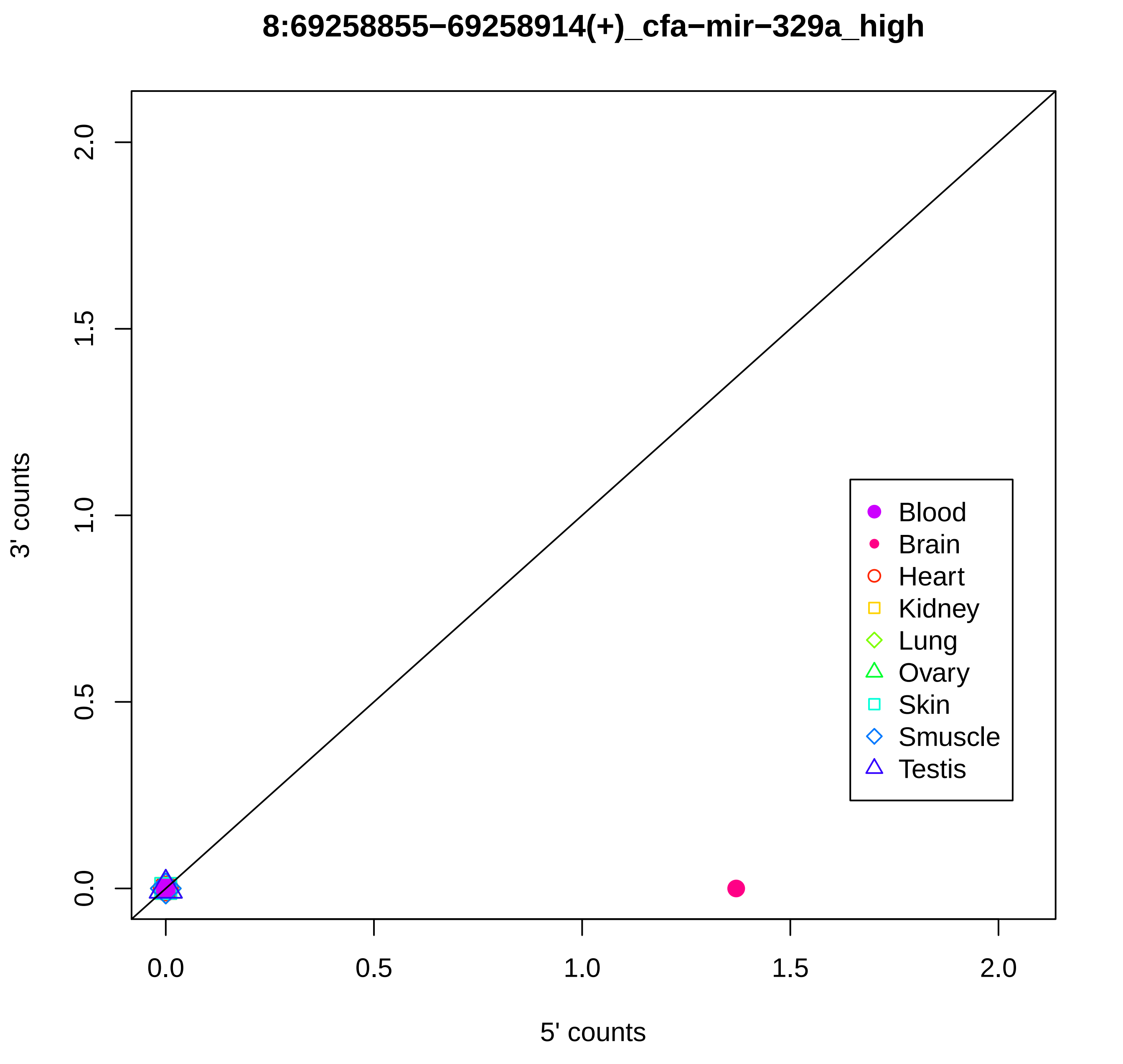
8:69258855−69258914(+)_cfa−mir−329a_high
3' counts
5' counts
0.0
0.5
1.0
1.5
2.0
0.0
0.5
1.0
1.5
2.0
Blood
Brain
Heart
Kidney
Lung
Ovary
Skin
Smuscle
Testis

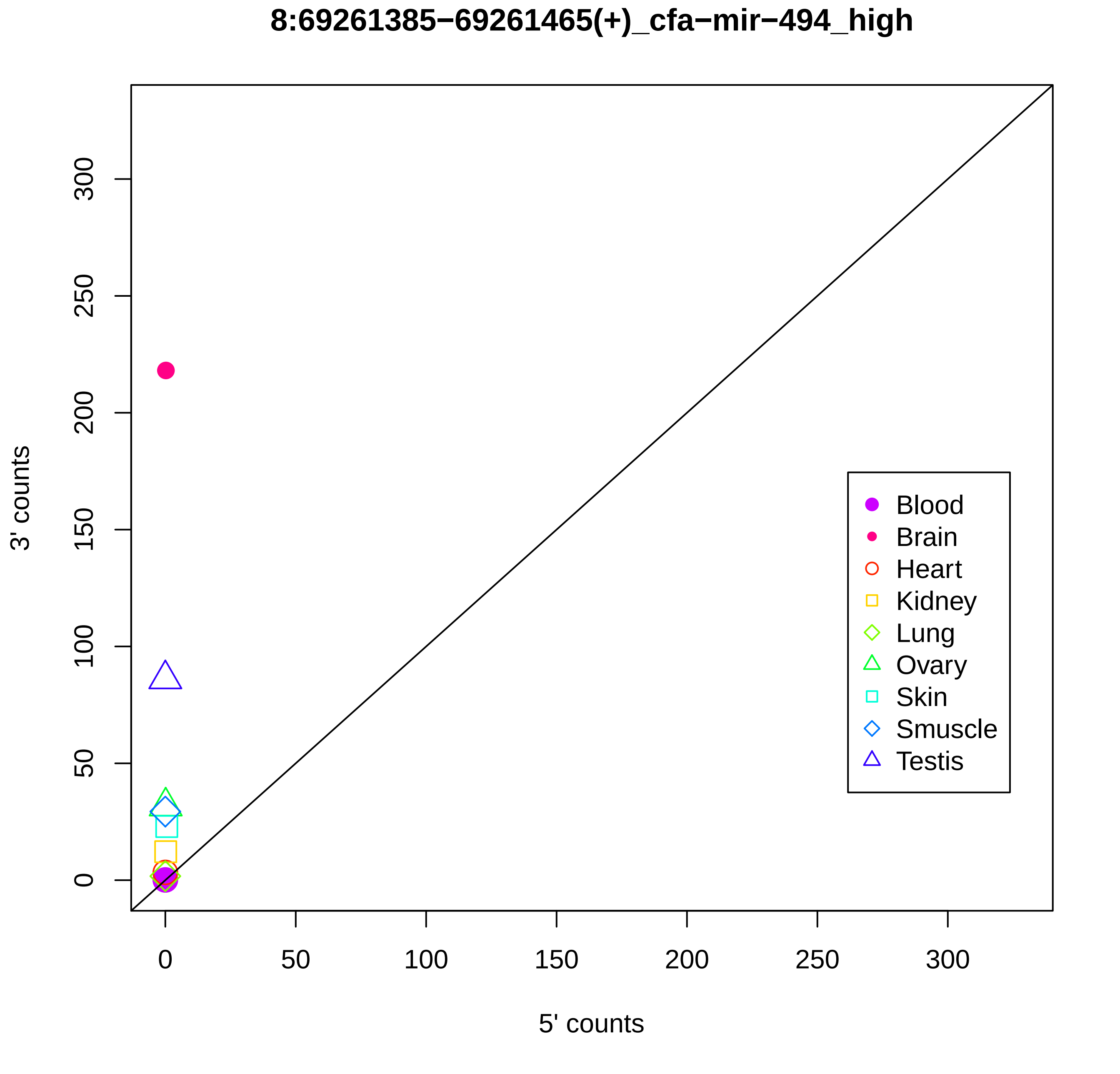

8:69261385−69261465(+)_cfa−mir−494_high
3' counts
5' counts
0
50
100
150
200
250
300
Blood
Brain
Heart
Kidney
Lung
Ovary
Skin
Smuscle
Testis

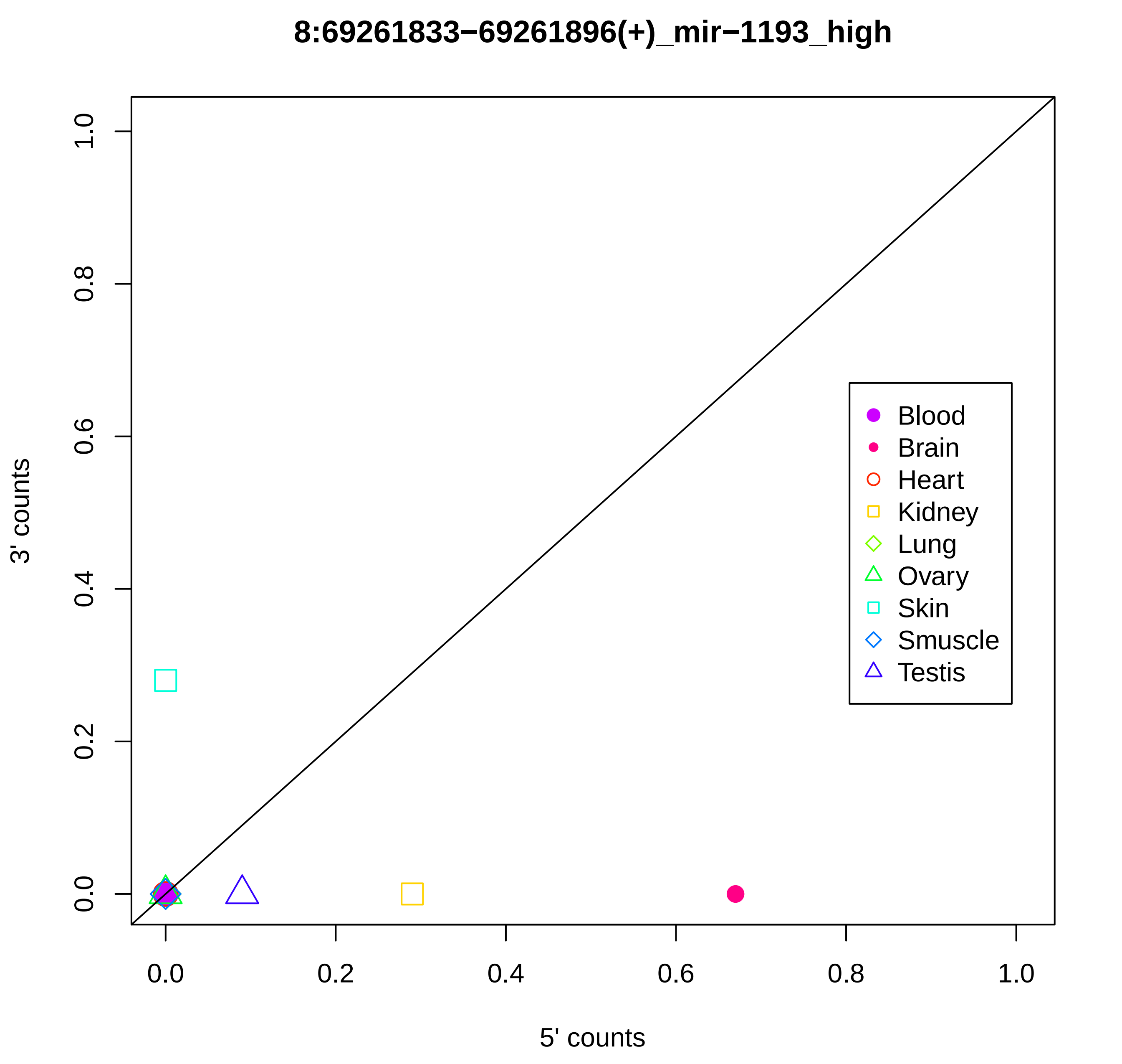

8:69261833−69261896(+)_mir−1193_high
3' counts
5' counts
0.0
0.2
0.4
0.6
0.8
1.0
Blood
Brain
Heart
Kidney
Lung
Ovary
Skin
Smuscle
Testis

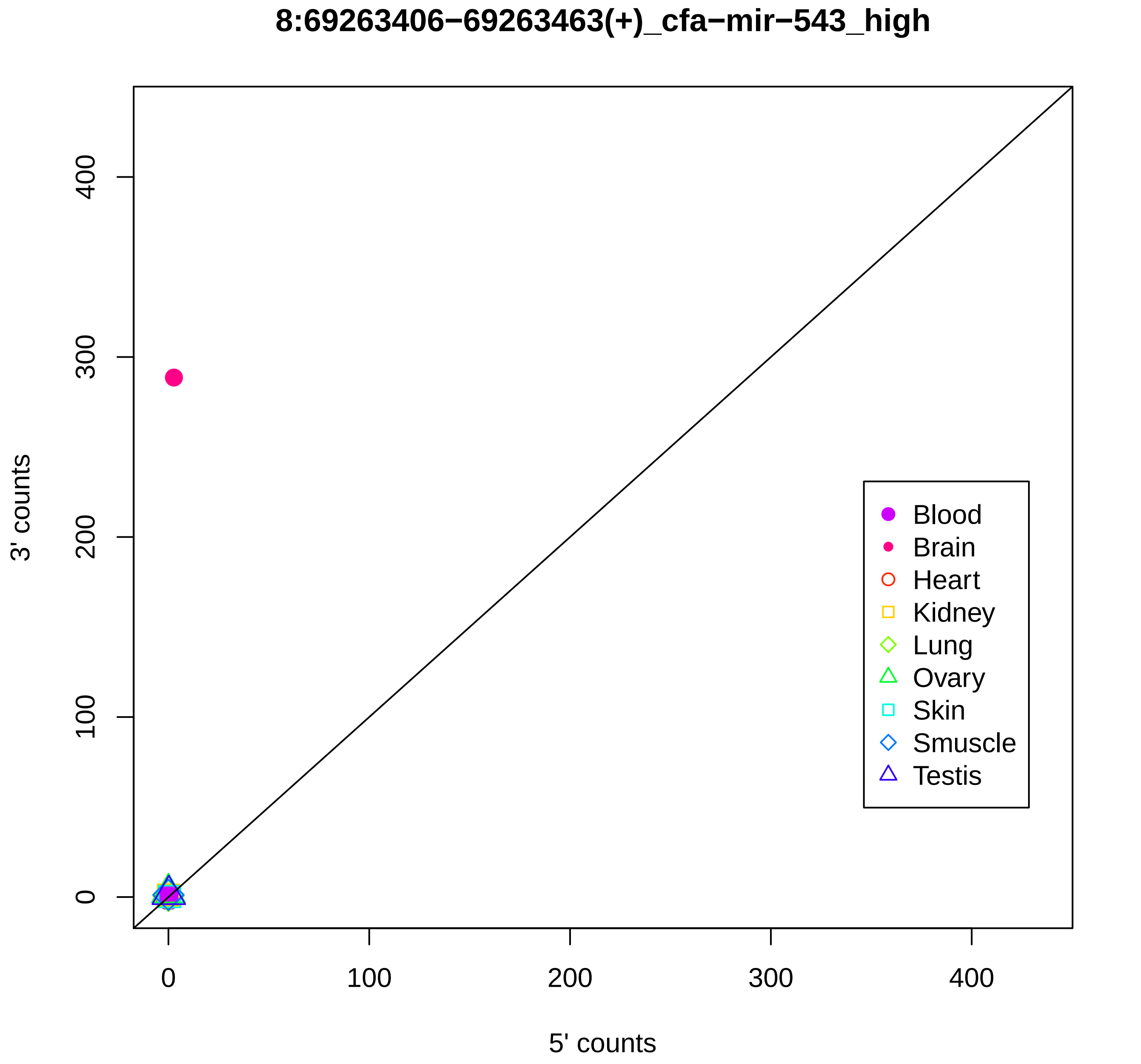

8:69263406−69263463(+)_cfa−mir−543_high
3' counts
5' counts
0
100
200
300
400
0
100
200
300
400
Blood
Brain
Heart
Kidney
Lung
Ovary
Skin
Smuscle
Testis

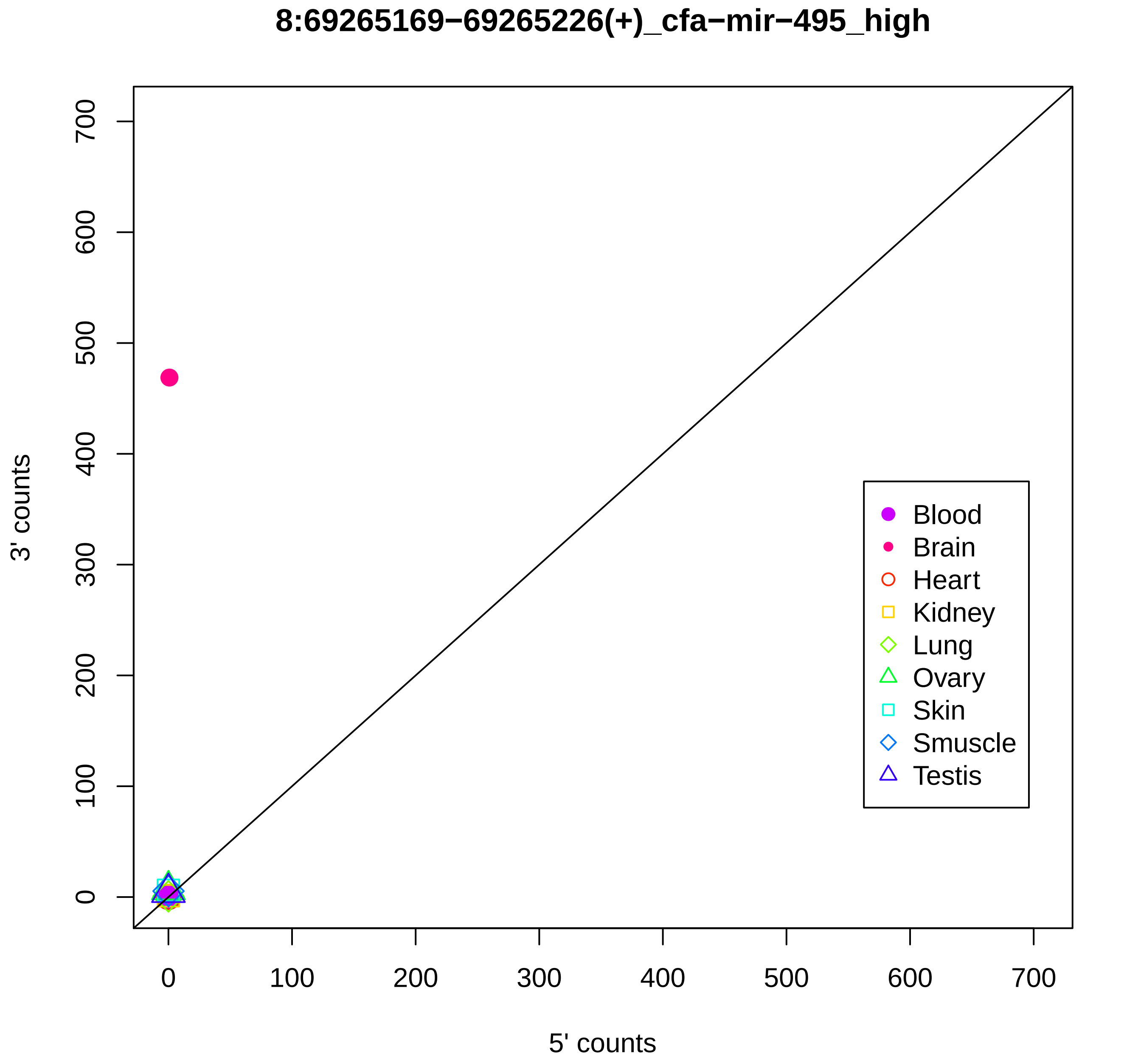

8:69265169−69265226(+)_cfa−mir−495_high
3' counts
5' counts
0
100
200
300
400
500
600
700
Blood
Brain
Heart
Kidney
Lung
Ovary
Skin
Smuscle
Testis

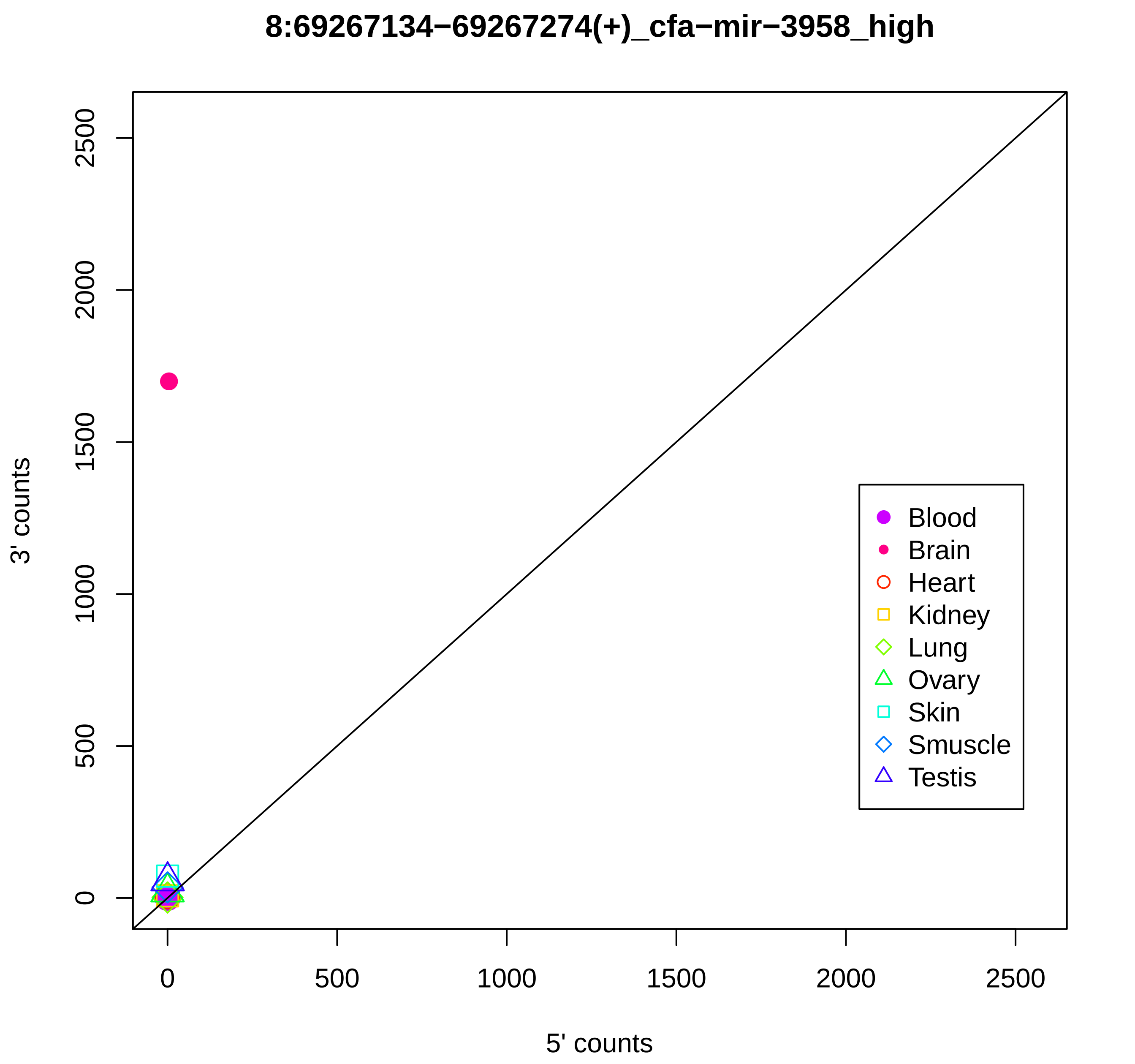

8:69267134−69267274(+)_cfa−mir−3958_high
3' counts
5' counts
0
500
1000
1500
2000
2500
Blood
Brain
Heart
Kidney
Lung
Ovary
Skin
Smuscle
Testis

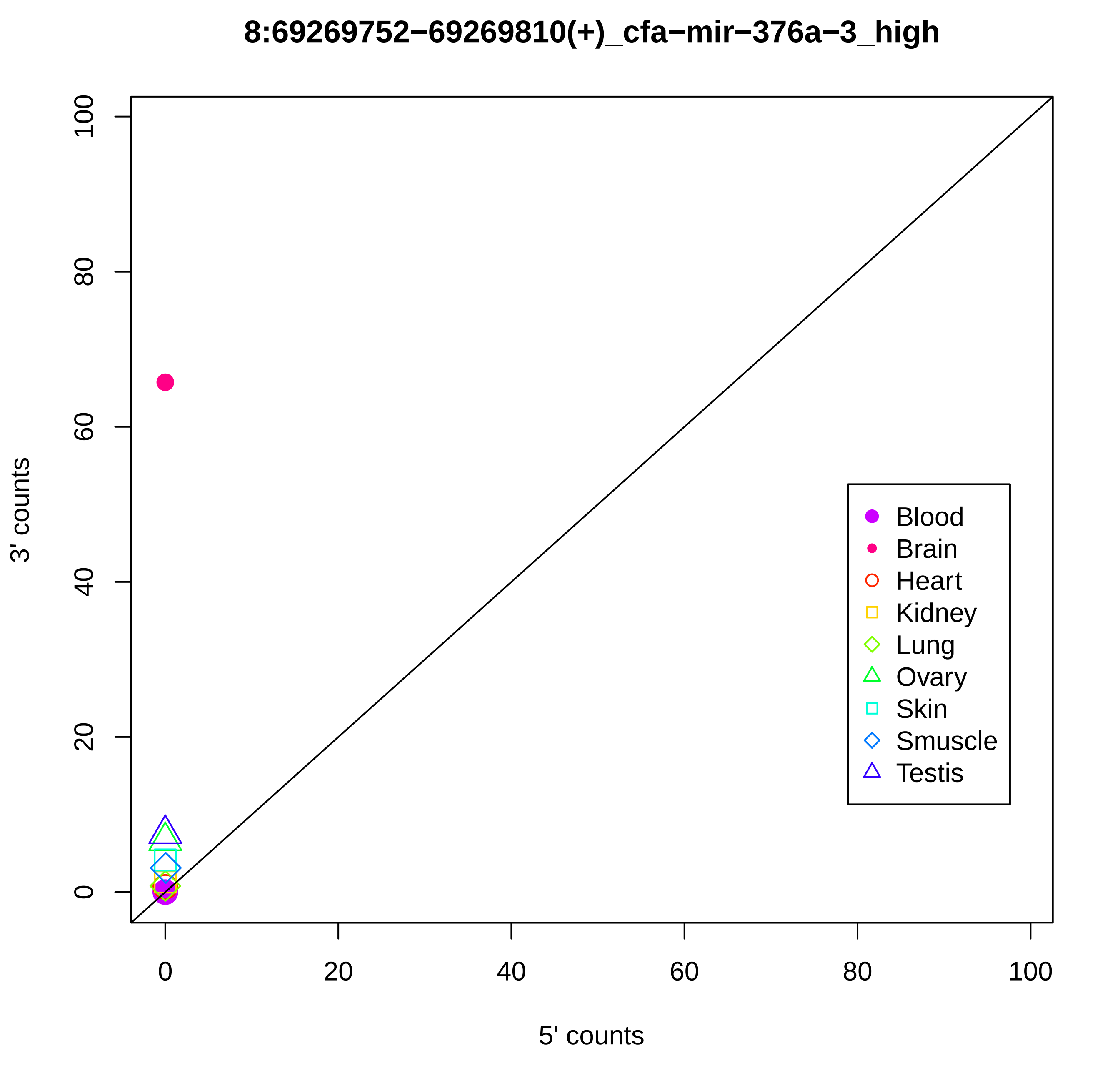

8:69269752−69269810(+)_cfa−mir−376a−3_high
3' counts
5' counts
0
20
40
60
80
100
0
20
40
60
80
100
Blood
Brain
Heart
Kidney
Lung
Ovary
Skin
Smuscle
Testis

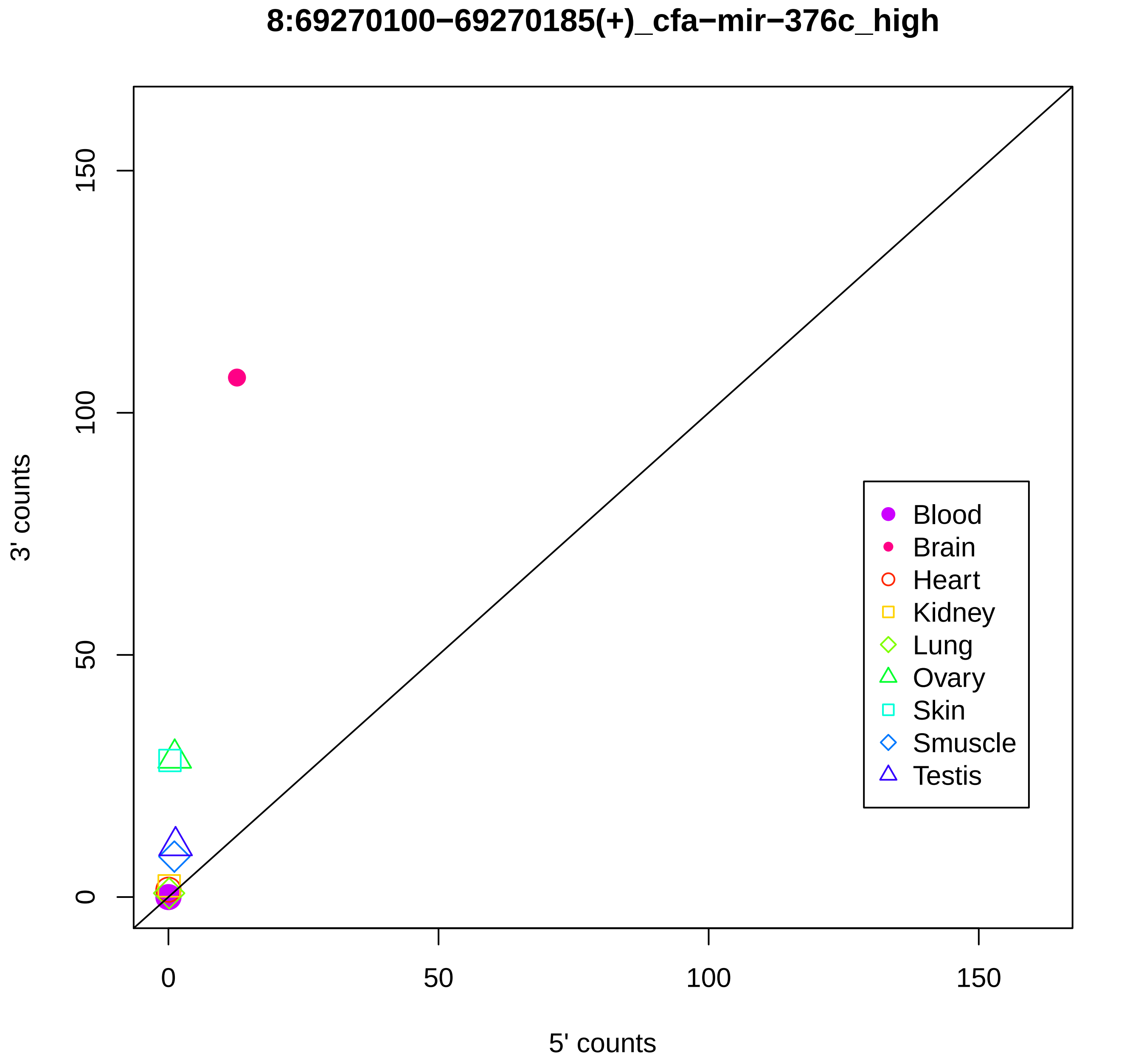

8:69270100−69270185(+)_cfa−mir−376c_high
3' counts
5' counts
0
50
100
150
0
50
100
150
Blood
Brain
Heart
Kidney
Lung
Ovary
Skin
Smuscle
Testis

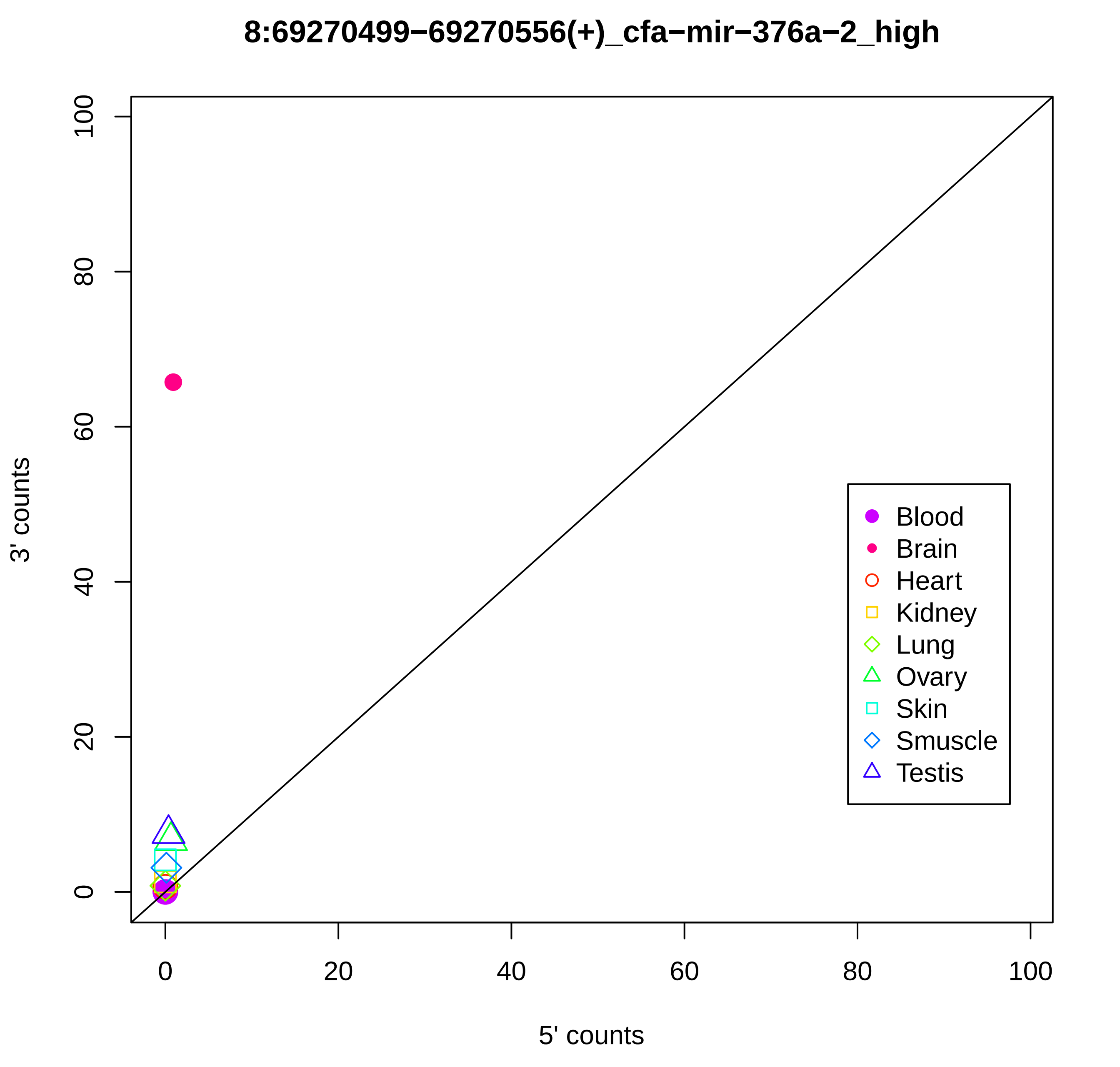
8:69270499−69270556(+)_cfa−mir−376a−2_high
3' counts
5' counts
0
20
40
60
80
100
Blood
Brain
Heart
Kidney
Lung
Ovary
Skin
Smuscle
Testis

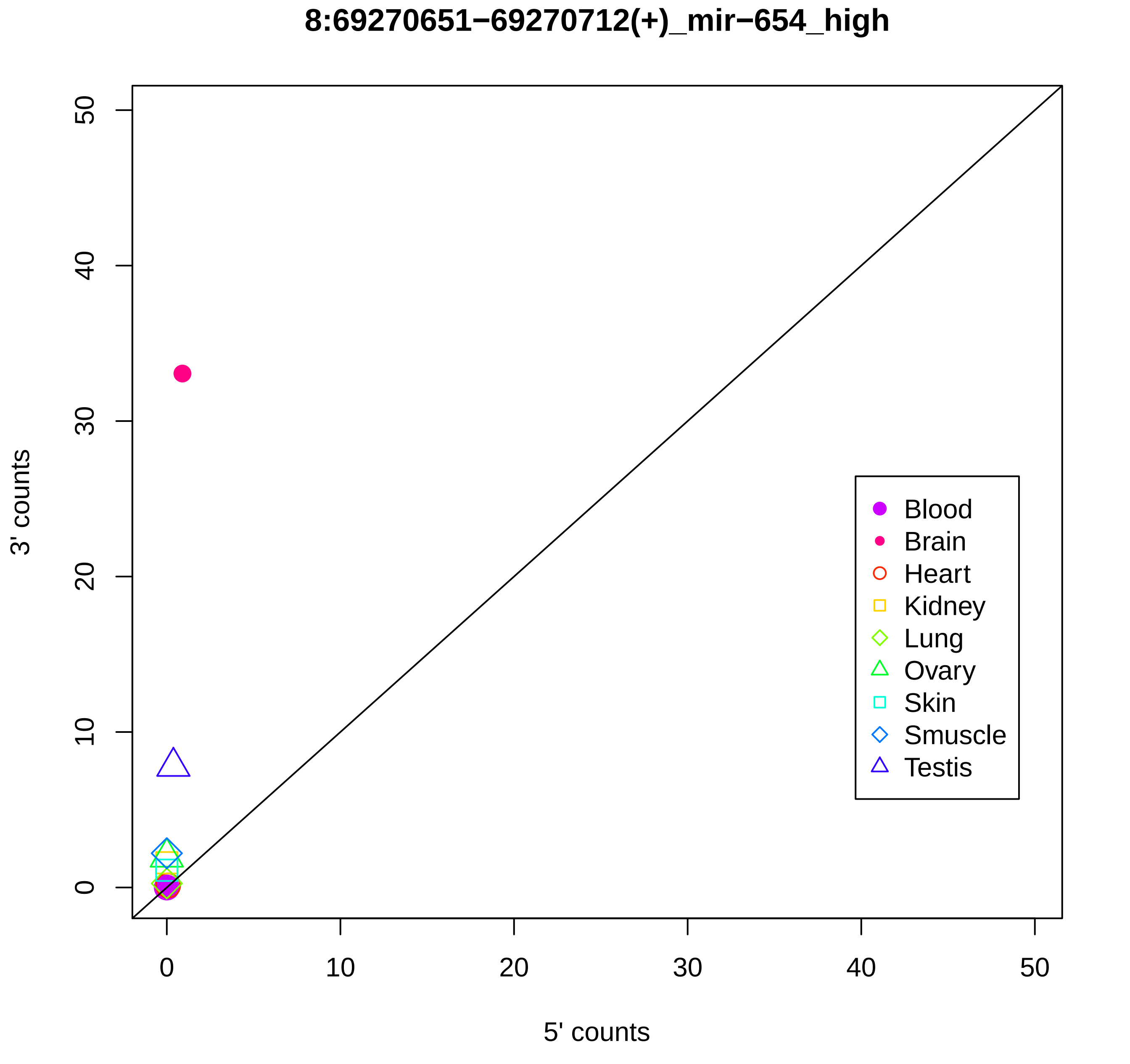

8:69270651−69270712(+)_mir−654_high
3' counts
5' counts
0
10
20
30
40
50
0
10
20
30
40
50
Blood
Brain
Heart
Kidney
Lung
Ovary
Skin
Smuscle
Testis

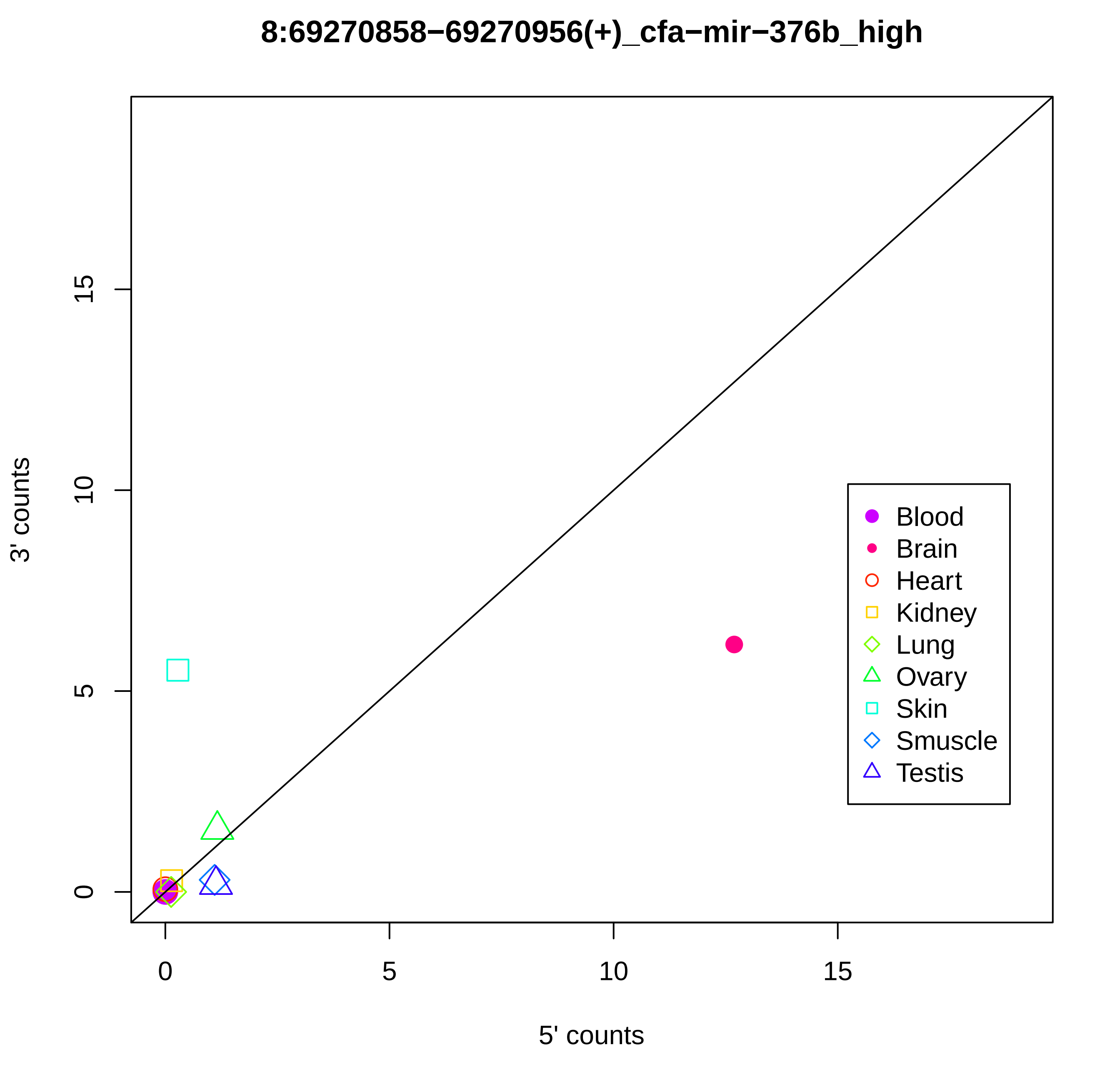

8:69270858−69270956(+)_cfa−mir−376b_high
3' counts
5' counts
0
5
10
15
Blood
Brain
Heart
Kidney
Lung
Ovary
Skin
Smuscle
Testis

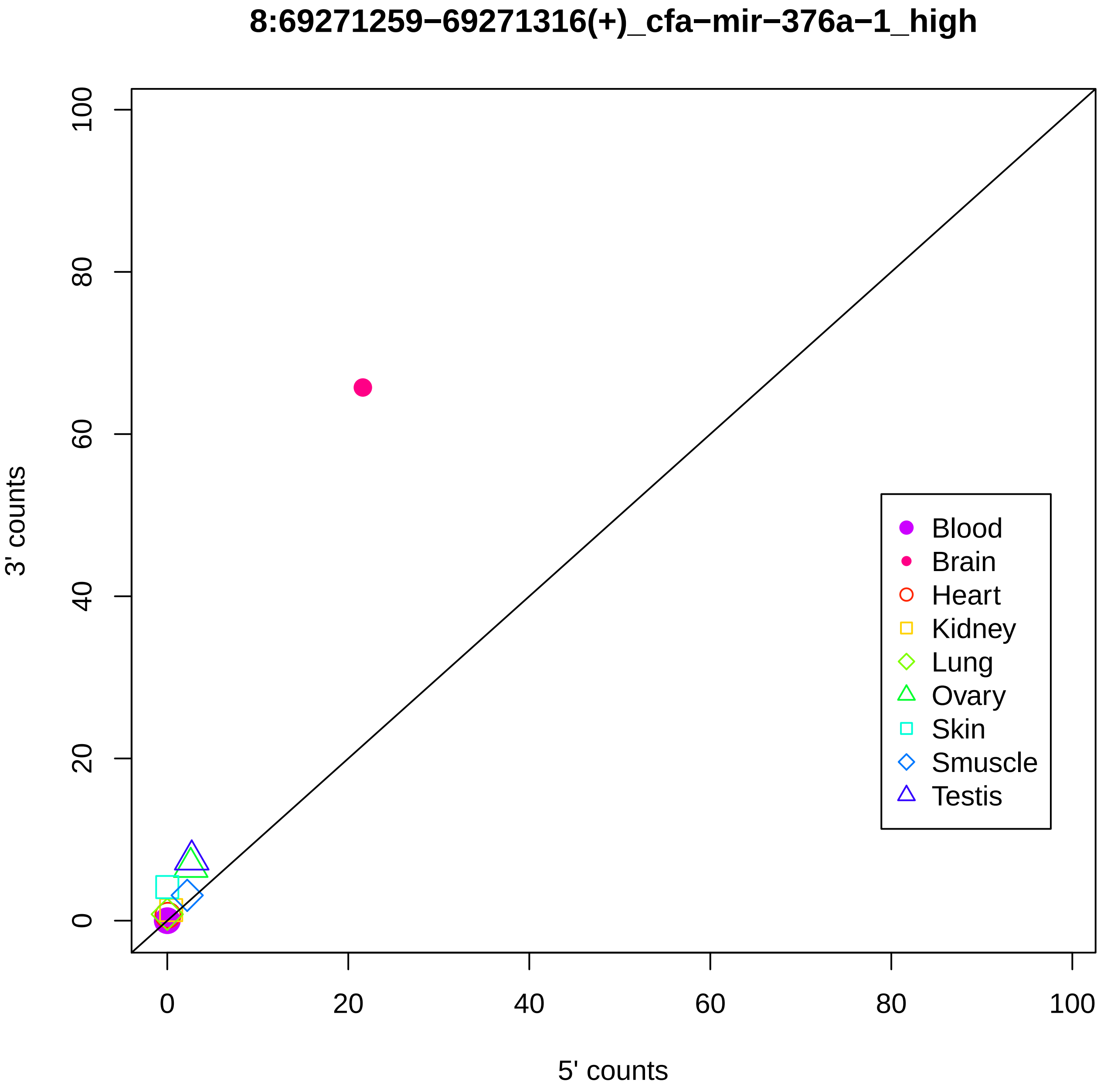

8:69271259−69271316(+)_cfa−mir−376a−1_high
3' counts
5' counts
0
20
40
60
80
100
Blood
Brain
Heart
Kidney
Lung
Ovary
Skin
Smuscle
Testis

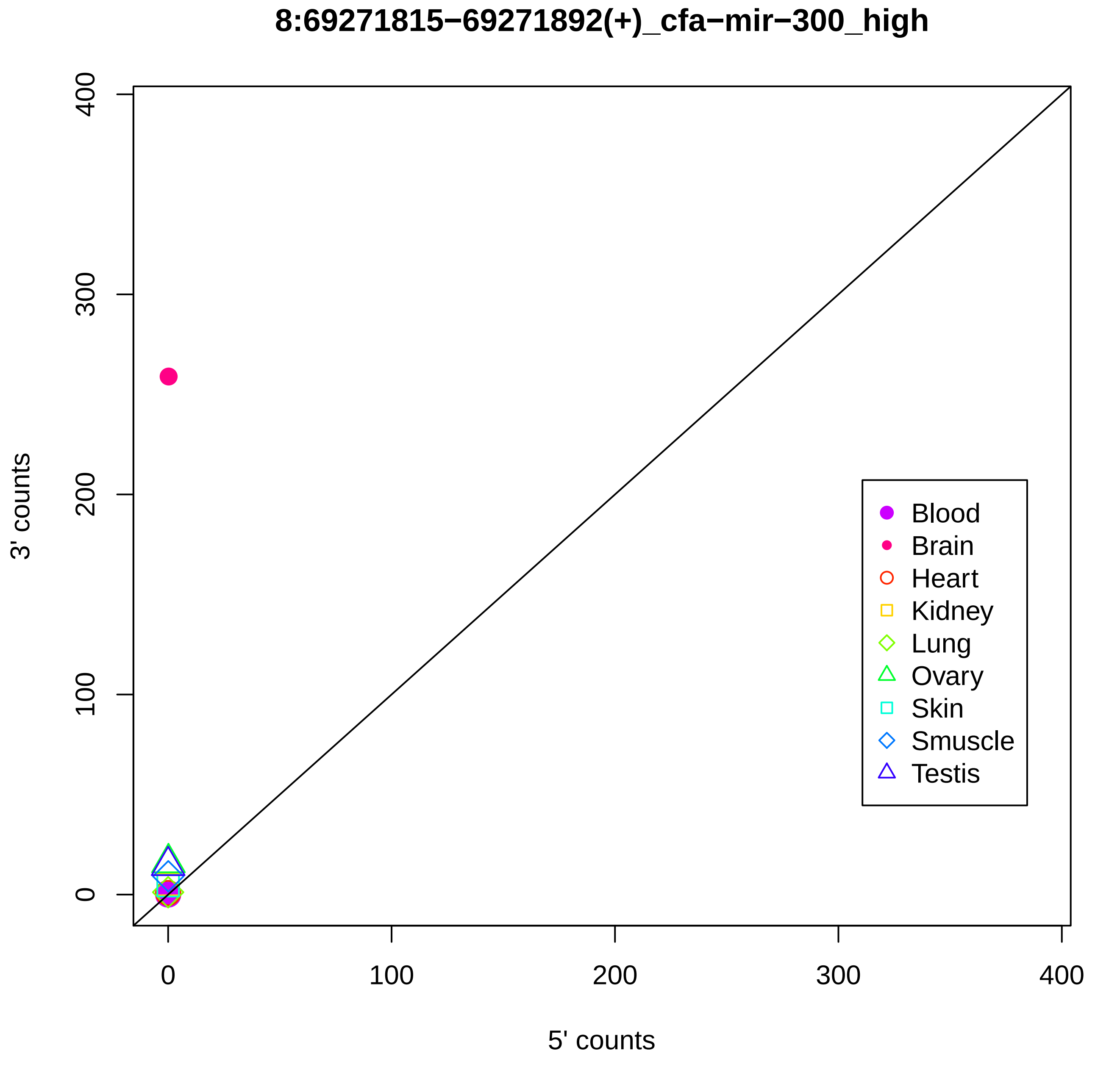

8:69271815−69271892(+)_cfa−mir−300_high
3' counts
5' counts
0
100
200
300
400
0
100
200
300
400
Blood
Brain
Heart
Kidney
Lung
Ovary
Skin
Smuscle
Testis

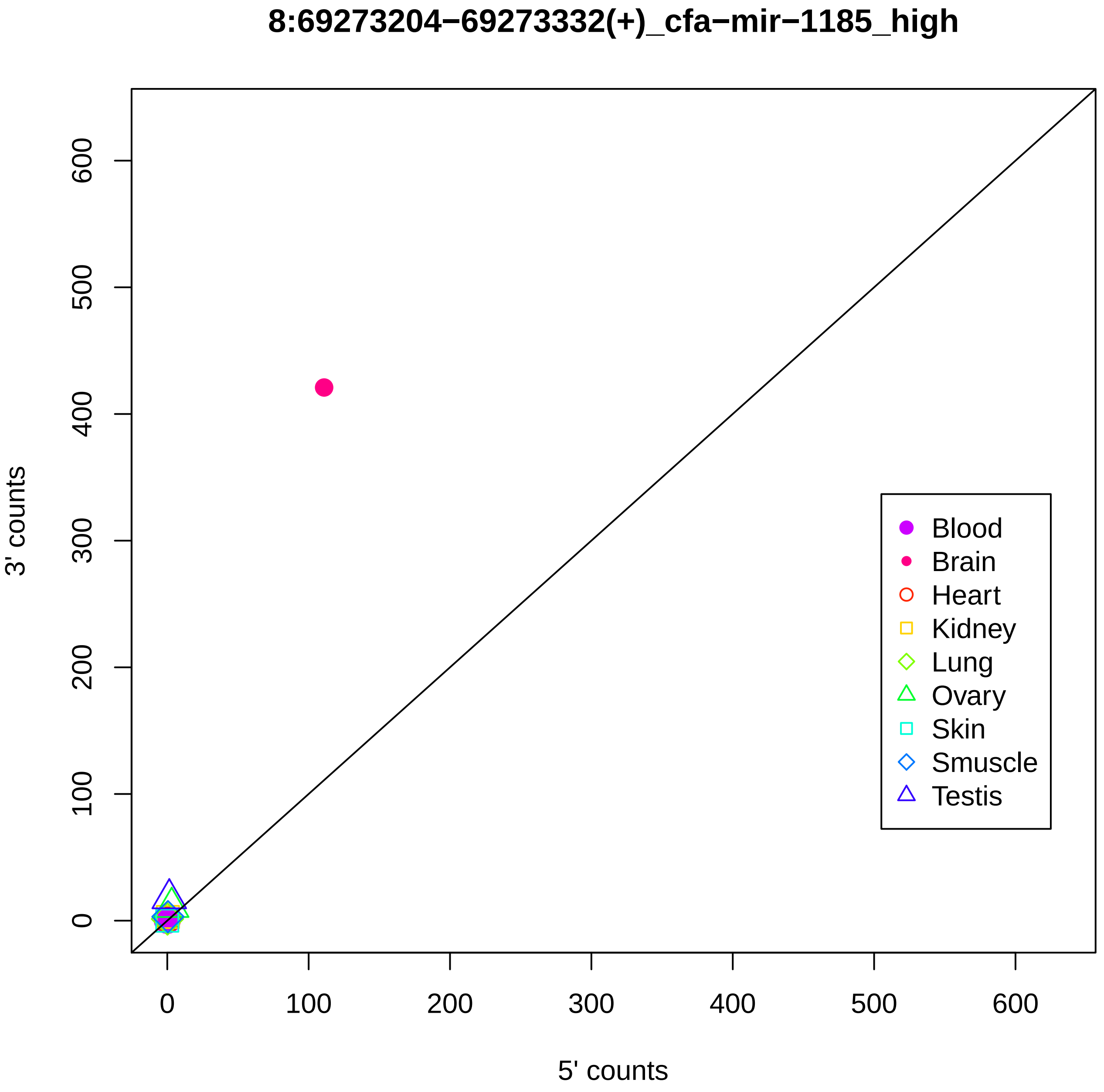
8:69273204−69273332(+)_cfa−mir−1185_high
3' counts
5' counts
0
100
200
300
400
500
600
0
100
200
300
400
500
600
Blood
Brain
Heart
Kidney
Lung
Ovary
Skin
Smuscle
Testis

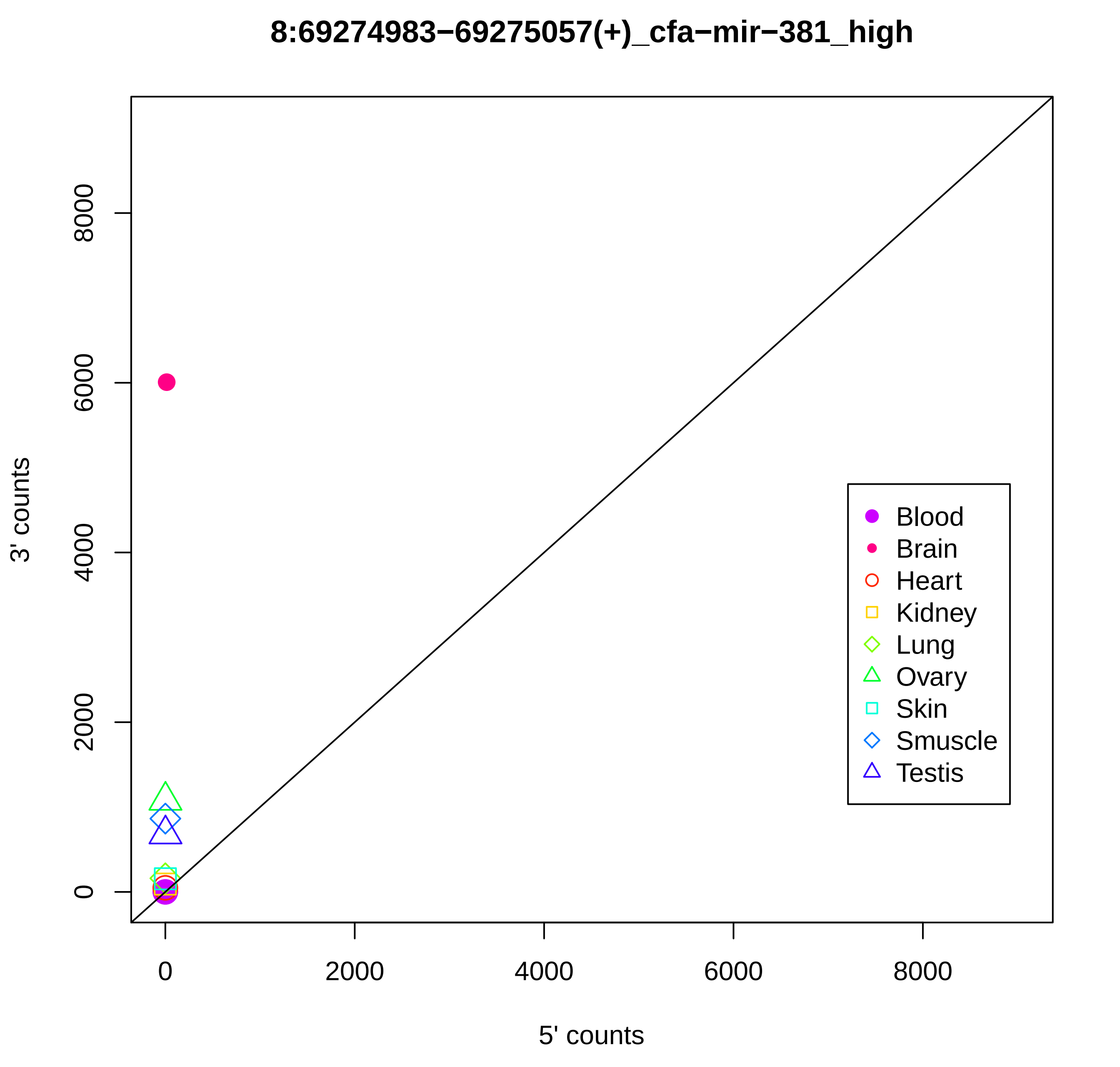
8:69274983−69275057(+)_cfa−mir−381_high
3' counts
5' counts
0
2000
4000
6000
8000
0
2000
4000
6000
8000
Blood
Brain
Heart
Kidney
Lung
Ovary
Skin
Smuscle
Testis

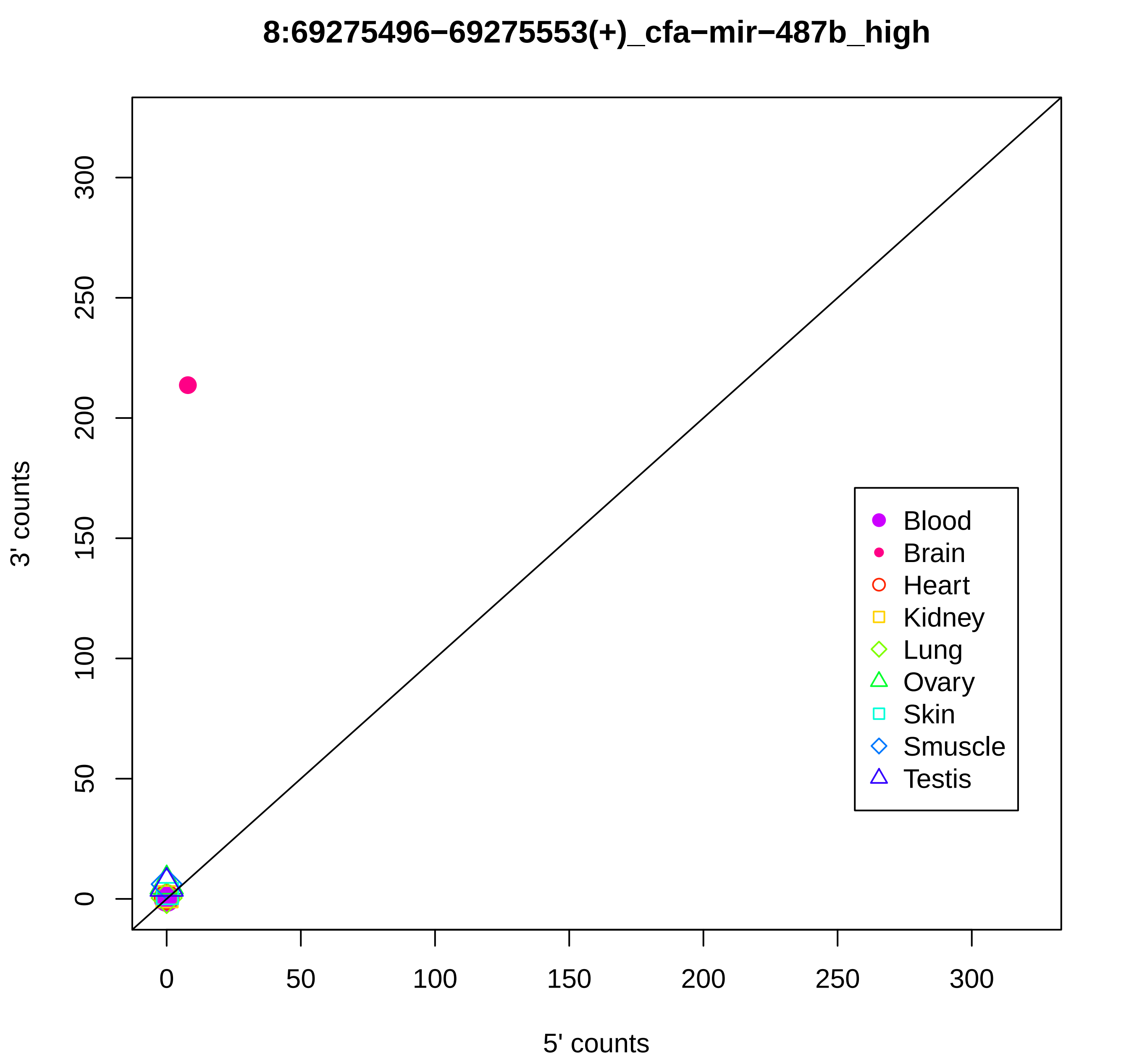

8:69275496−69275553(+)_cfa−mir−487b_high
3' counts
5' counts
0
50
100
150
200
250
300
Blood
Brain
Heart
Kidney
Lung
Ovary
Skin
Smuscle
Testis

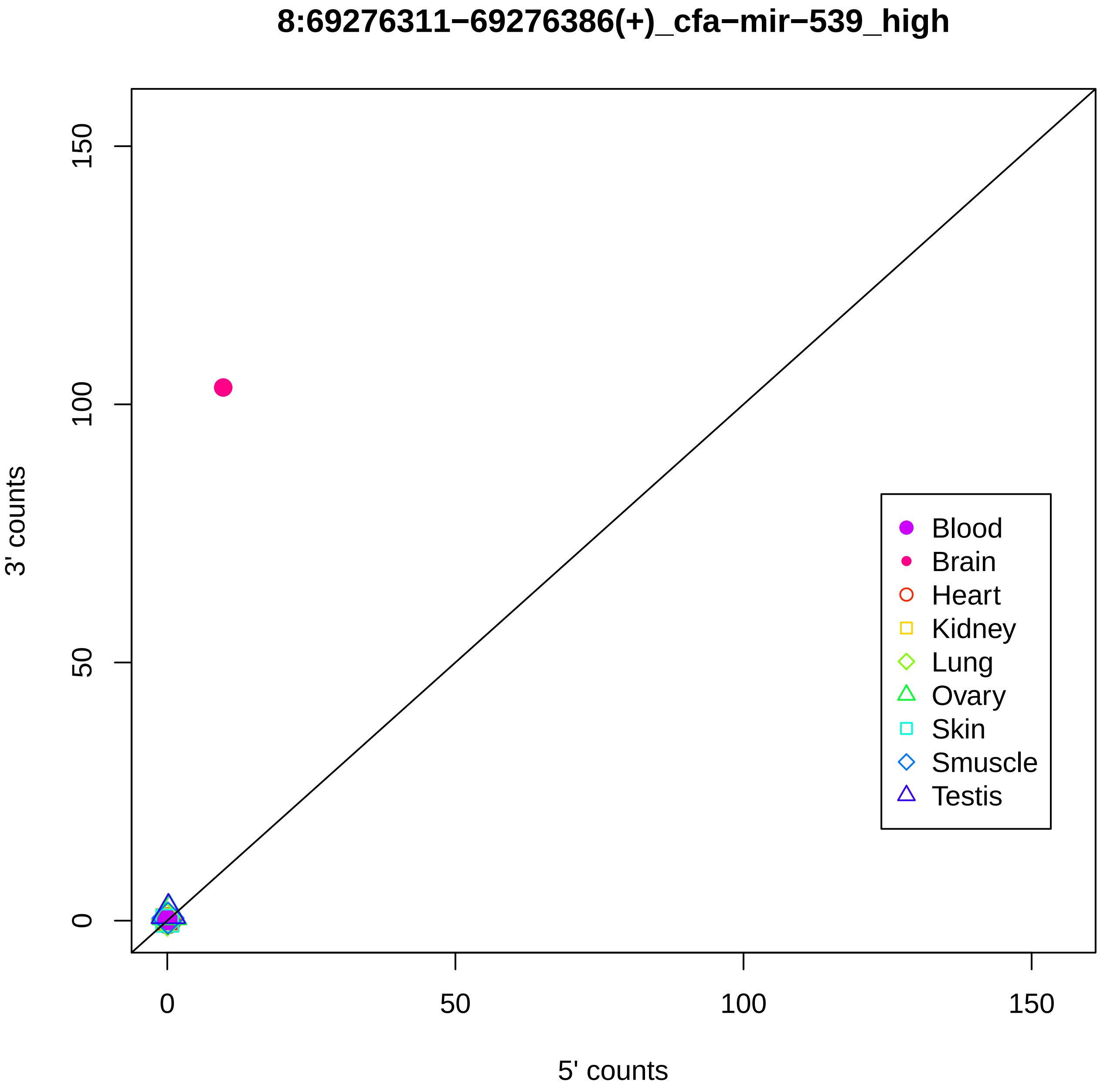
8:69276311−69276386(+)_cfa−mir−539_high
3' counts
5' counts
0
50
100
150
0
50
100
150
Blood
Brain
Heart
Kidney
Lung
Ovary
Skin
Smuscle
Testis

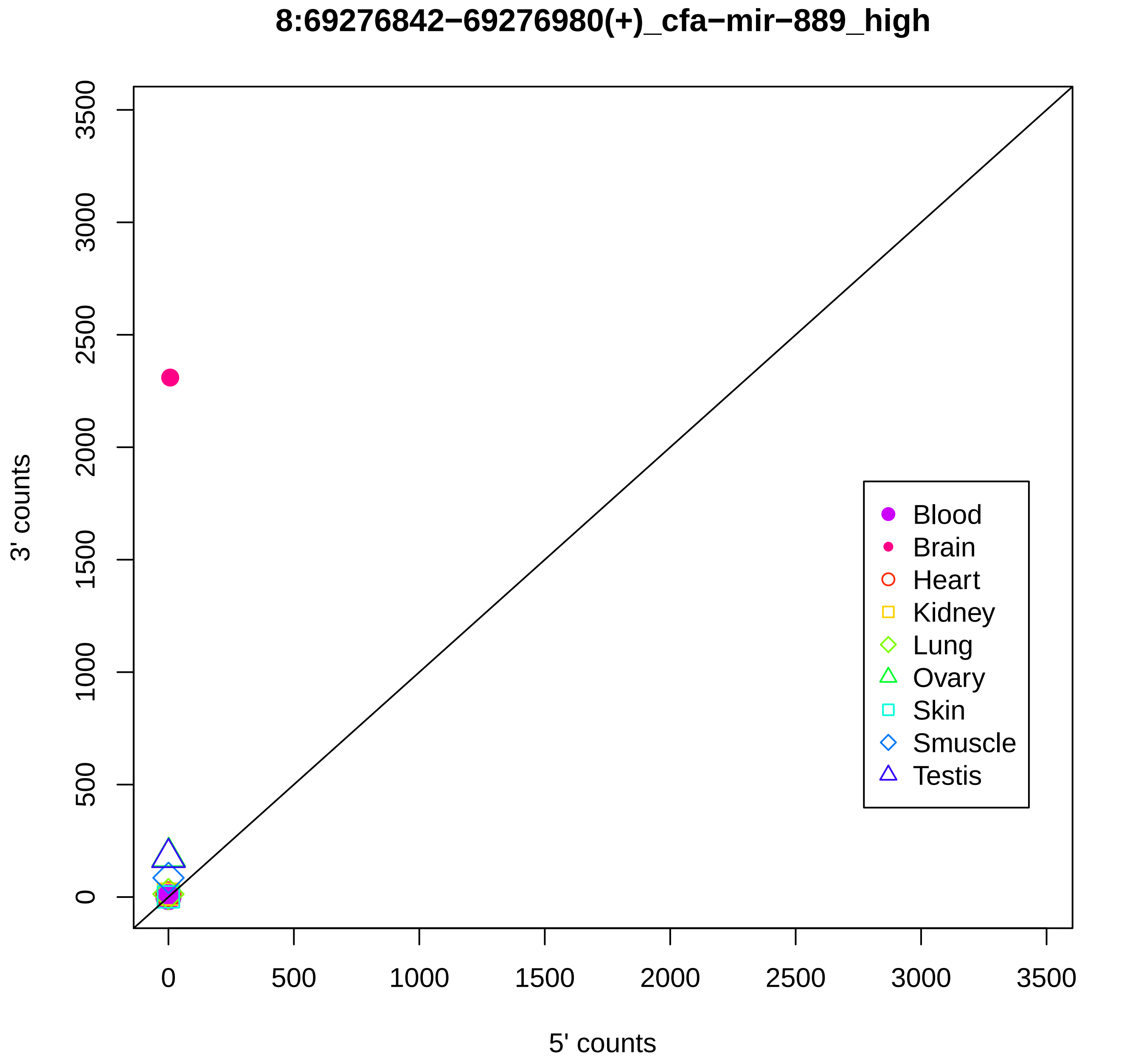
8:69276842−69276980(+)_cfa−mir−889_high
3' counts
5' counts
0
500
1000
1500
2000
2500
3000
3500
Blood
Brain
Heart
Kidney
Lung
Ovary
Skin
Smuscle
Testis

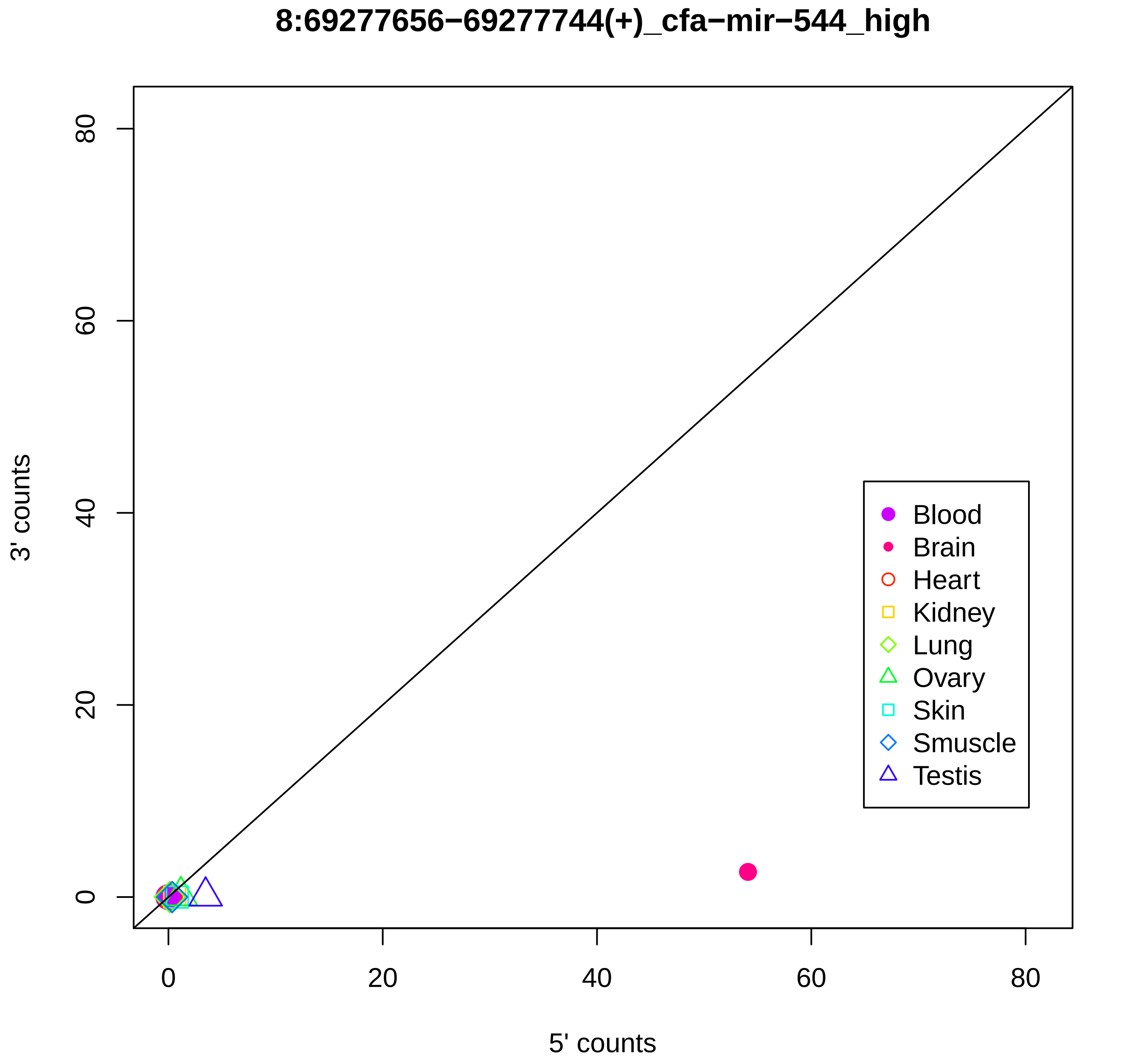

8:69277656−69277744(+)_cfa−mir−544_high
3' counts
5' counts
0
20
40
60
80
0
20
40
60
80
Blood
Brain
Heart
Kidney
Lung
Ovary
Skin
Smuscle
Testis

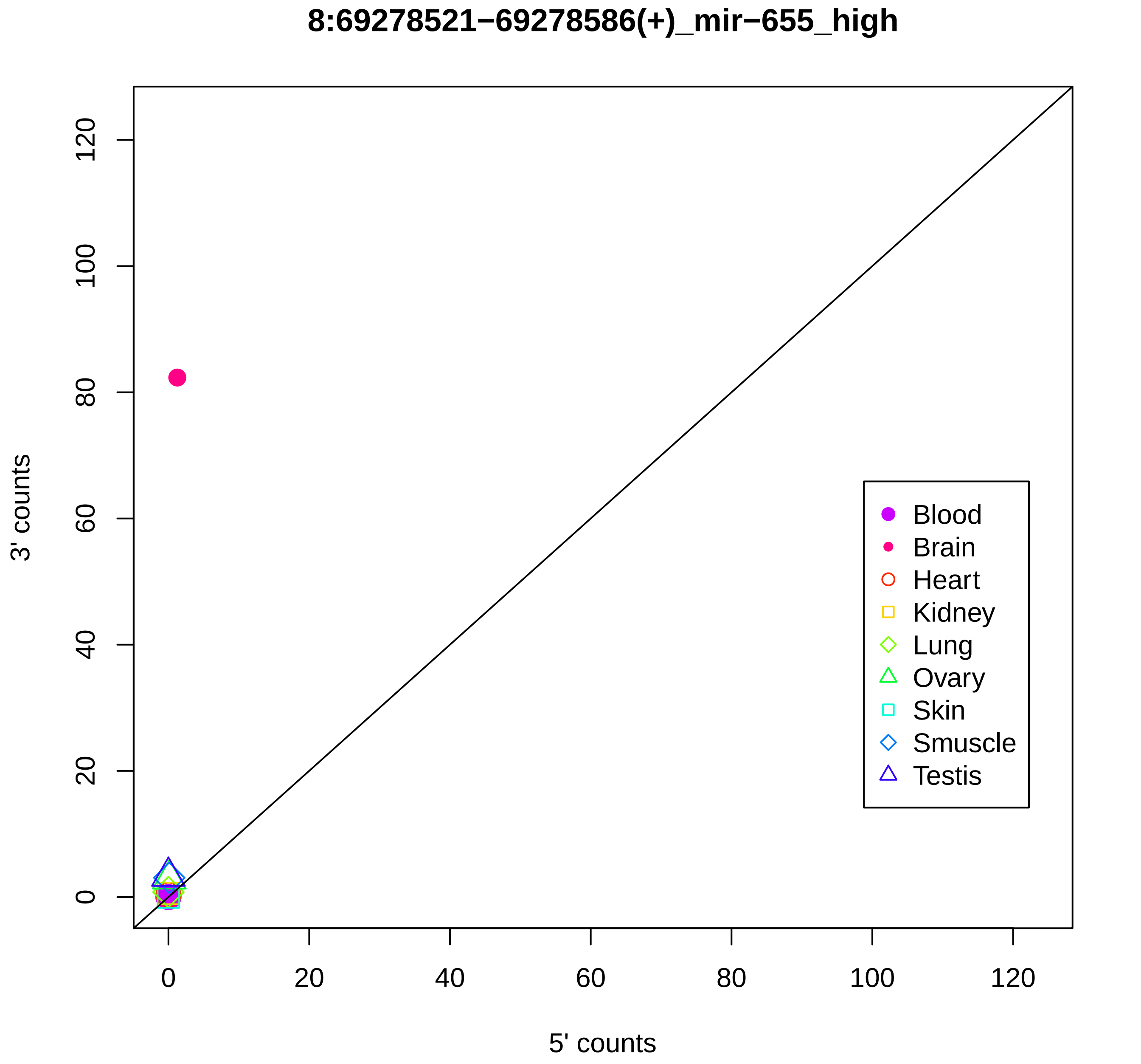
8:69278521−69278586(+)_mir−655_high
3' counts
5' counts
0
20
40
60
80
100
120
Blood
Brain
Heart
Kidney
Lung
Ovary
Skin
Smuscle
Testis

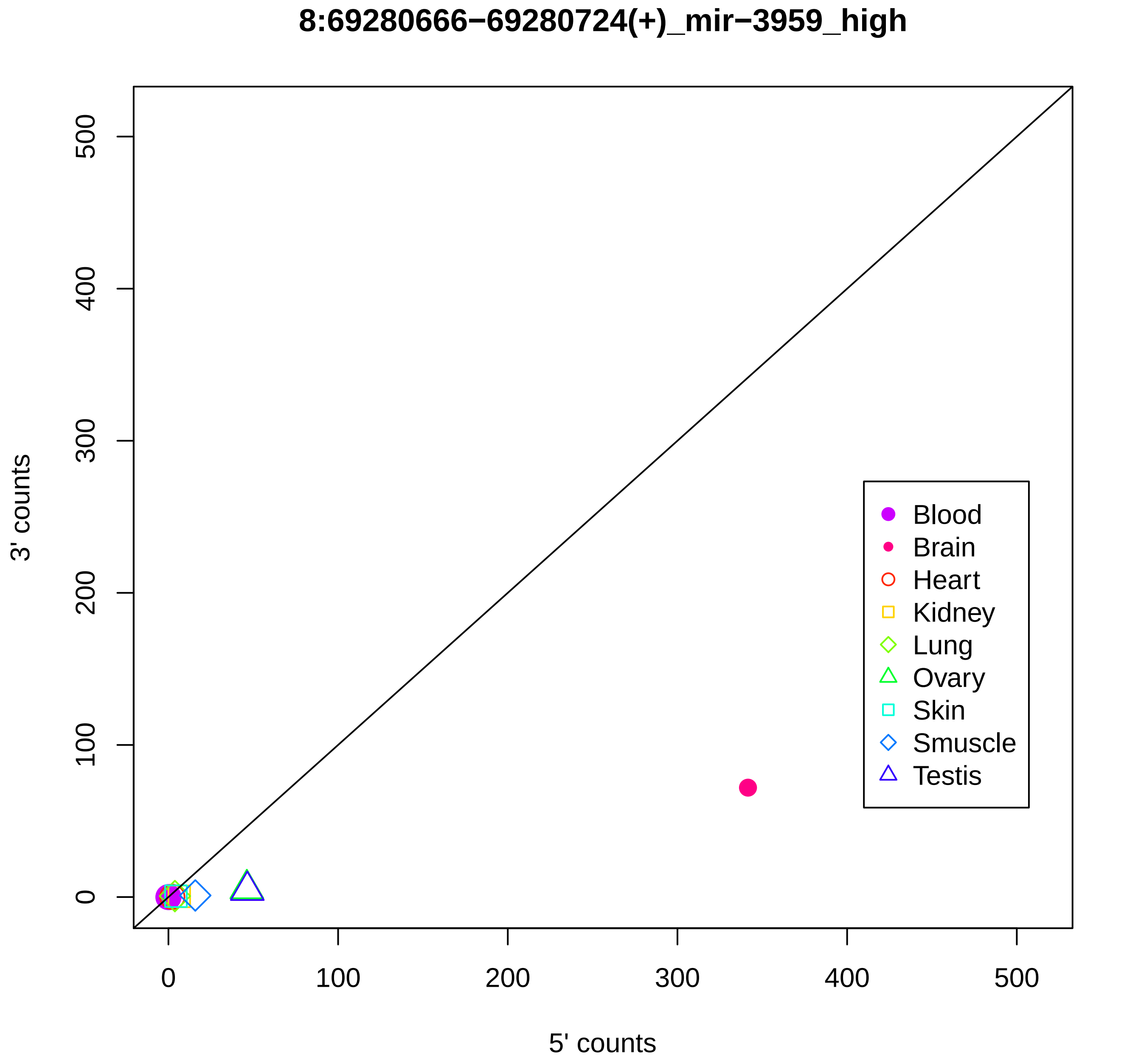

8:69280666−69280724(+)_mir−3959_high
3' counts
5' counts
0
100
200
300
400
500
Blood
Brain
Heart
Kidney
Lung
Ovary
Skin
Smuscle
Testis

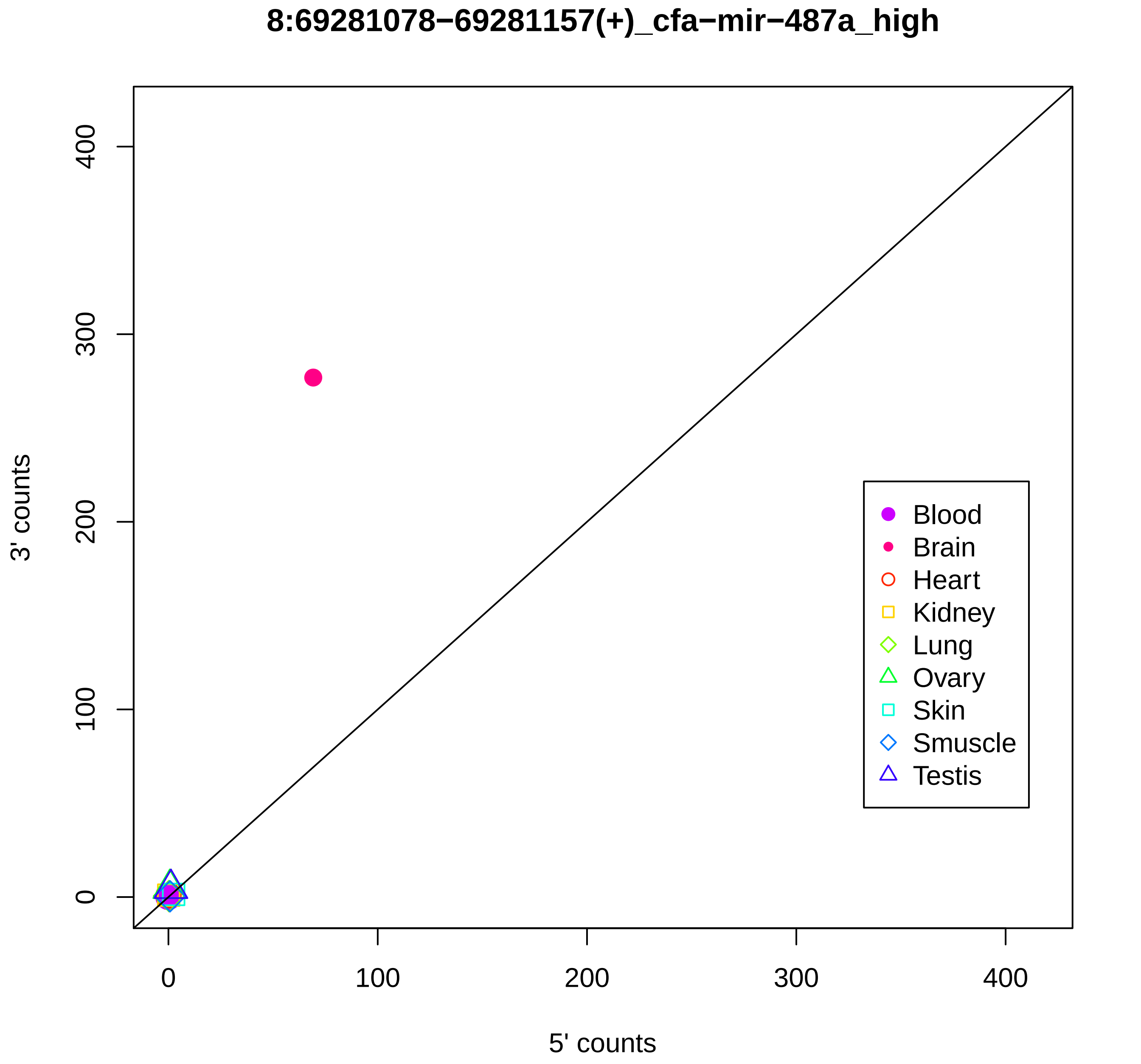

8:69281078−69281157(+)_cfa−mir−487a_high
3' counts
5' counts
0
100
200
300
400
0
100
200
300
400
Blood
Brain
Heart
Kidney
Lung
Ovary
Skin
Smuscle
Testis

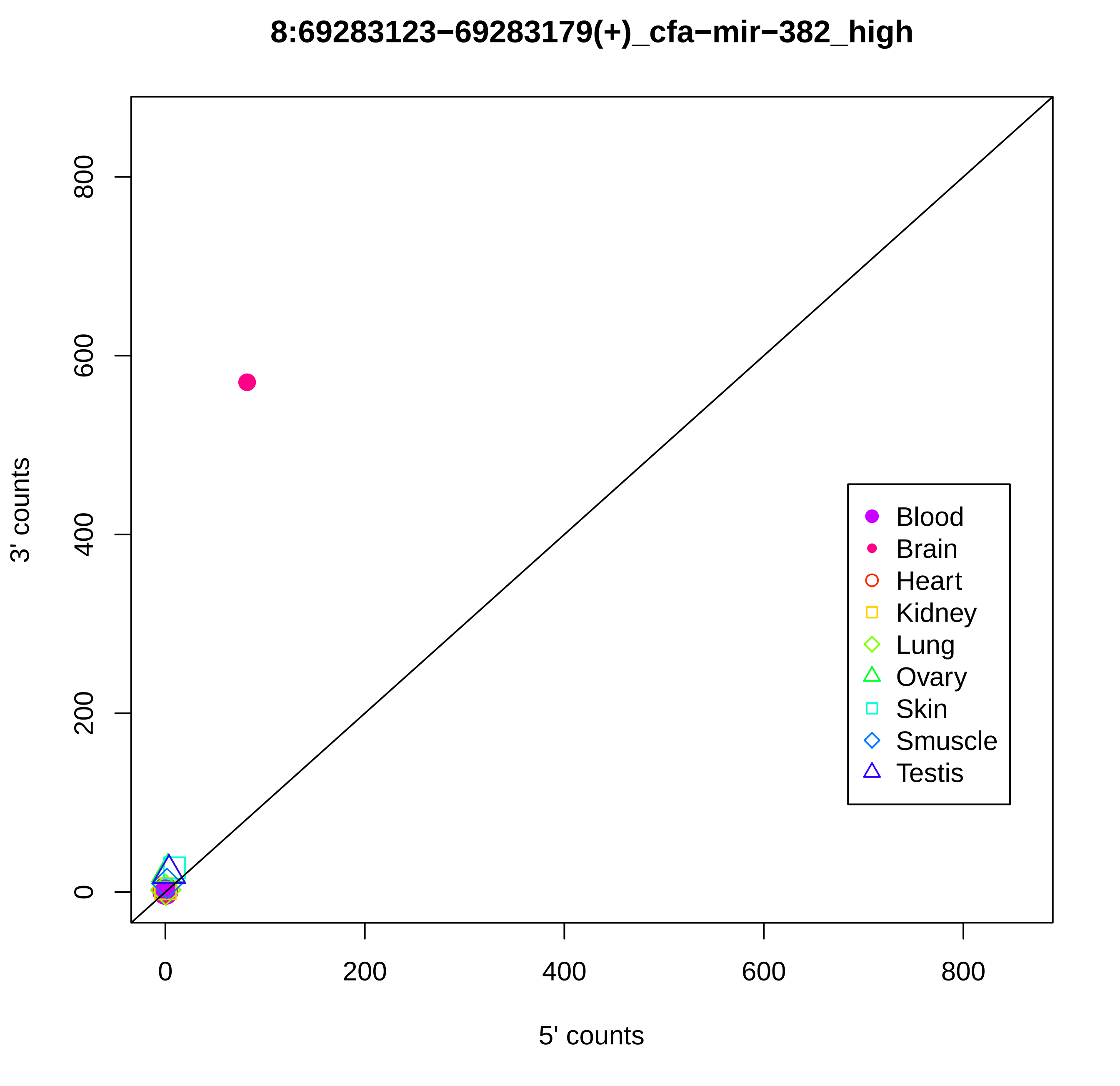
8:69283123−69283179(+)_cfa−mir−382_high
3' counts
5' counts
0
200
400
600
800
0
200
400
600
800
Blood
Brain
Heart
Kidney
Lung
Ovary
Skin
Smuscle
Testis

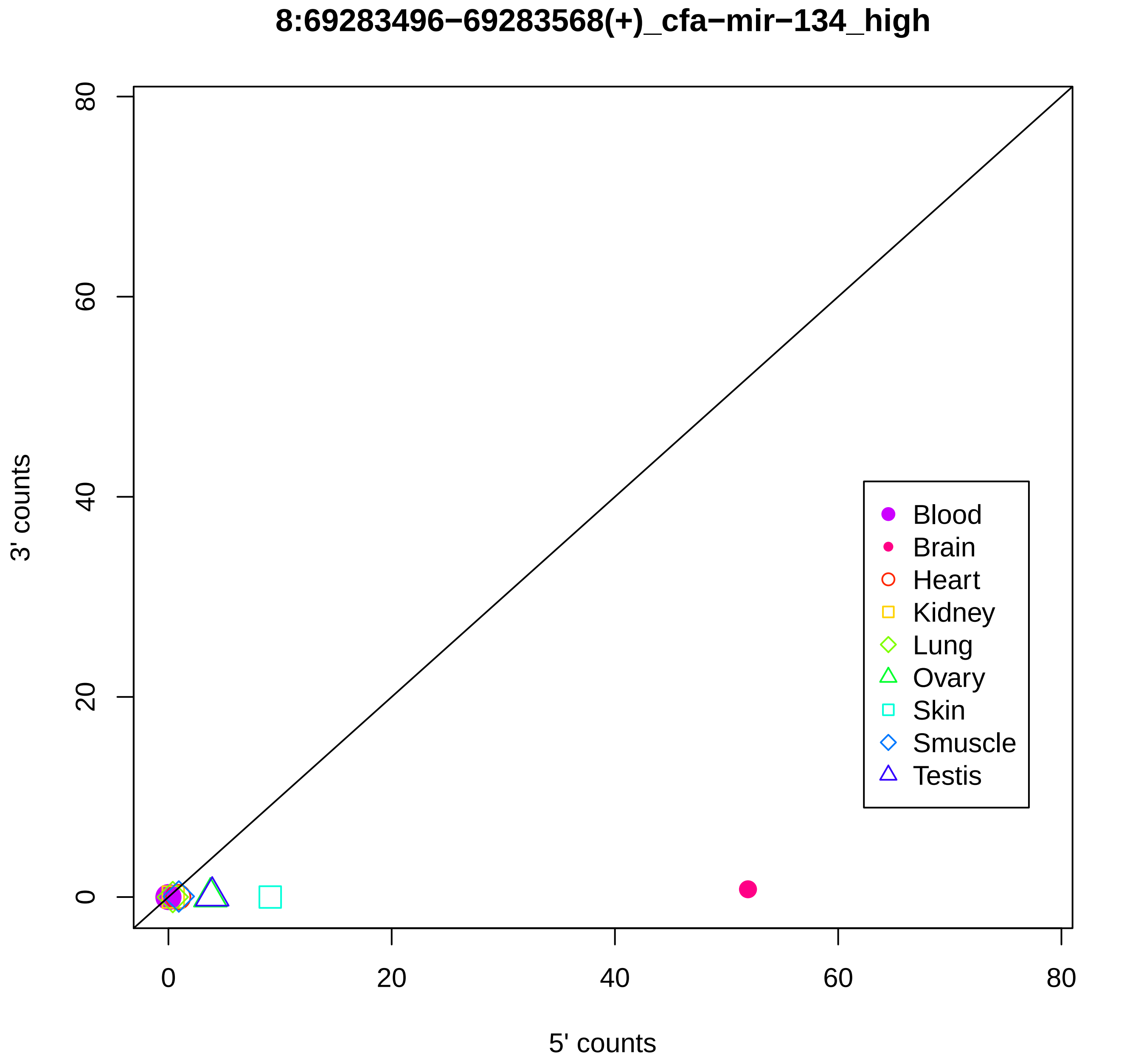
8:69283496−69283568(+)_cfa−mir−134_high
3' counts
5' counts
0
20
40
60
80
0
20
40
60
80
Blood
Brain
Heart
Kidney
Lung
Ovary
Skin
Smuscle
Testis

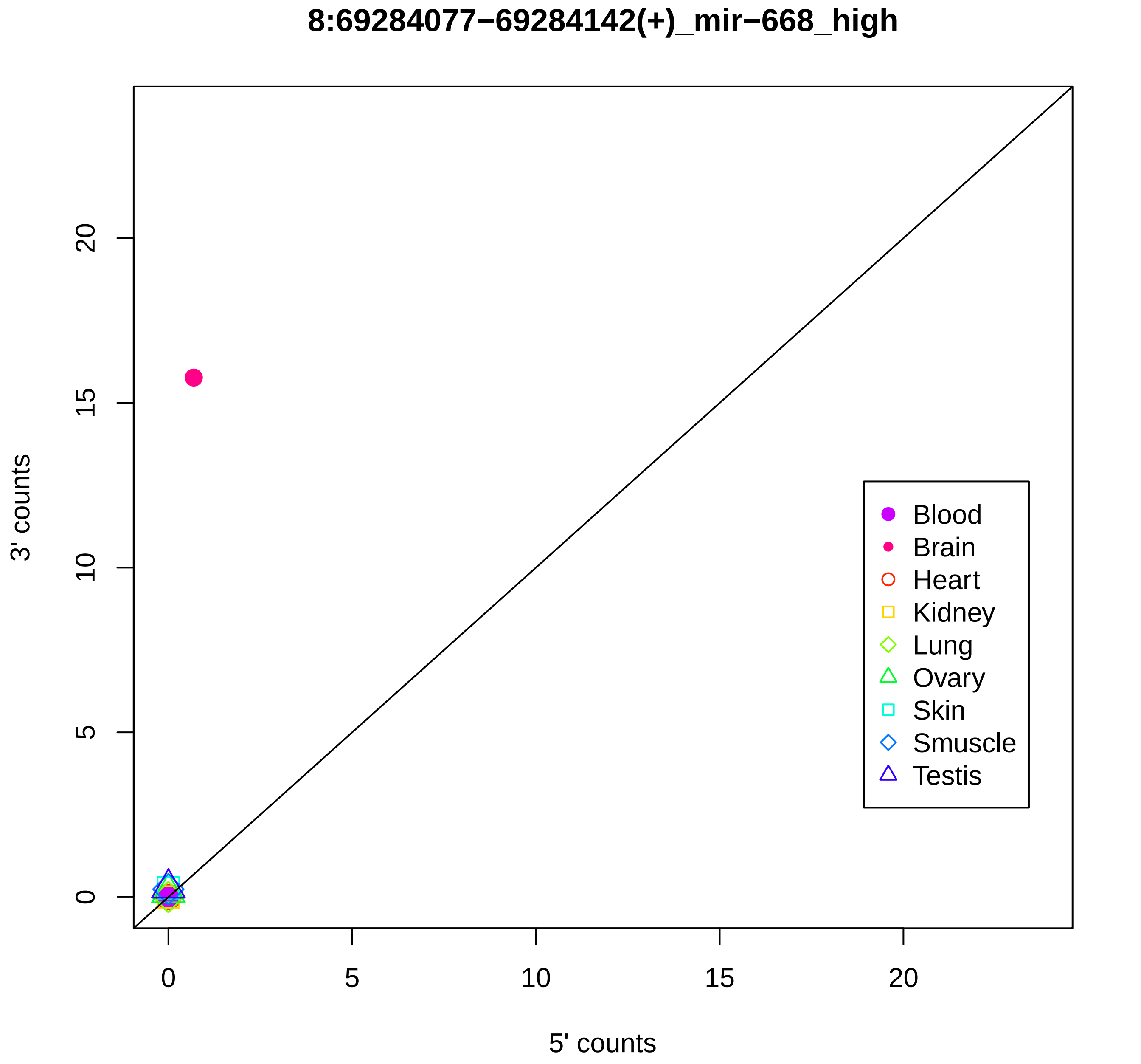
8:69284077−69284142(+)_mir−668_high
3' counts
5' counts
0
5
10
15
20
Blood
Brain
Heart
Kidney
Lung
Ovary
Skin
Smuscle
Testis

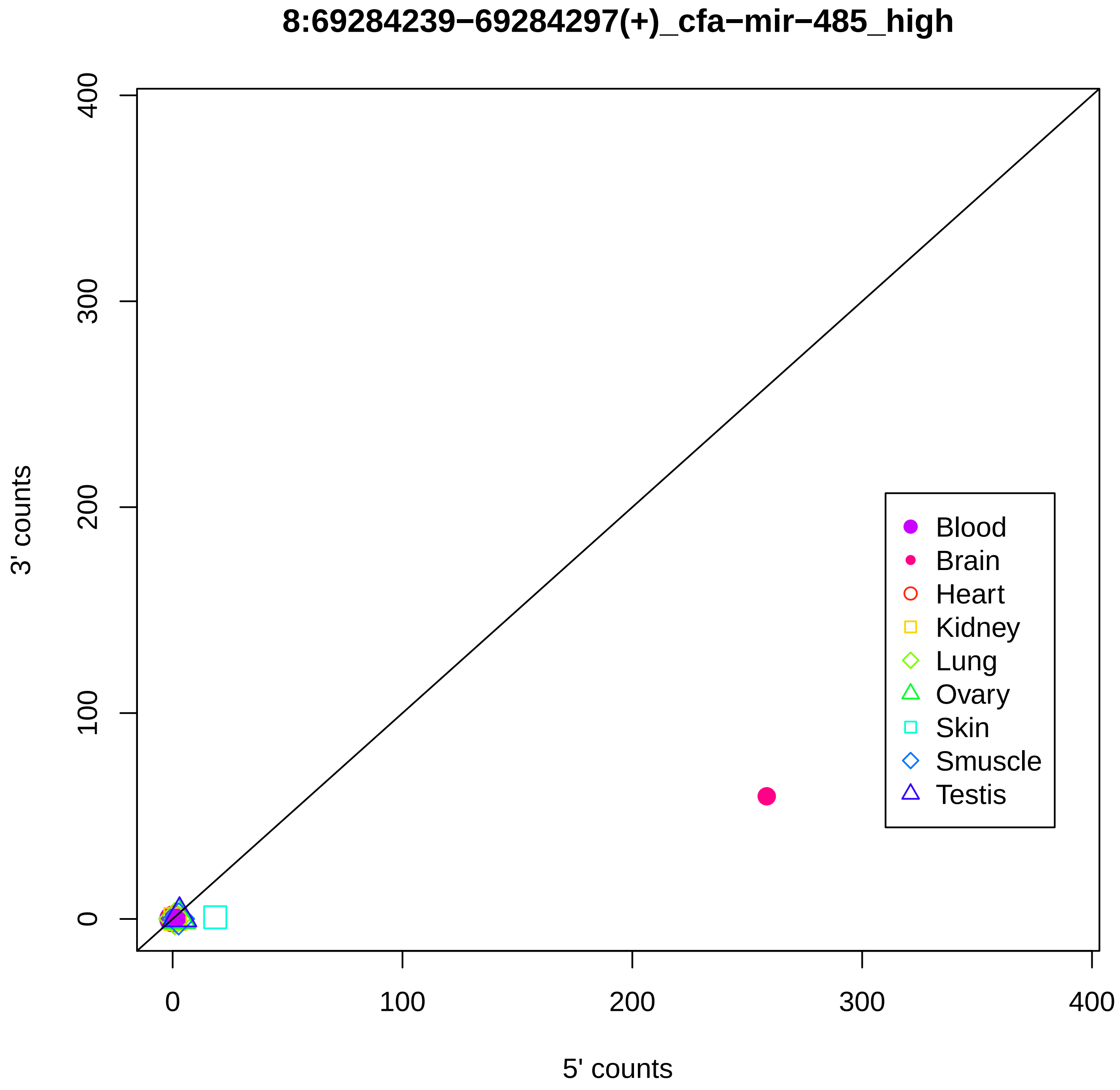

8:69284239−69284297(+)_cfa−mir−485_high
3' counts
5' counts
0
100
200
300
400
0
100
200
300
400
Blood
Brain
Heart
Kidney
Lung
Ovary
Skin
Smuscle
Testis

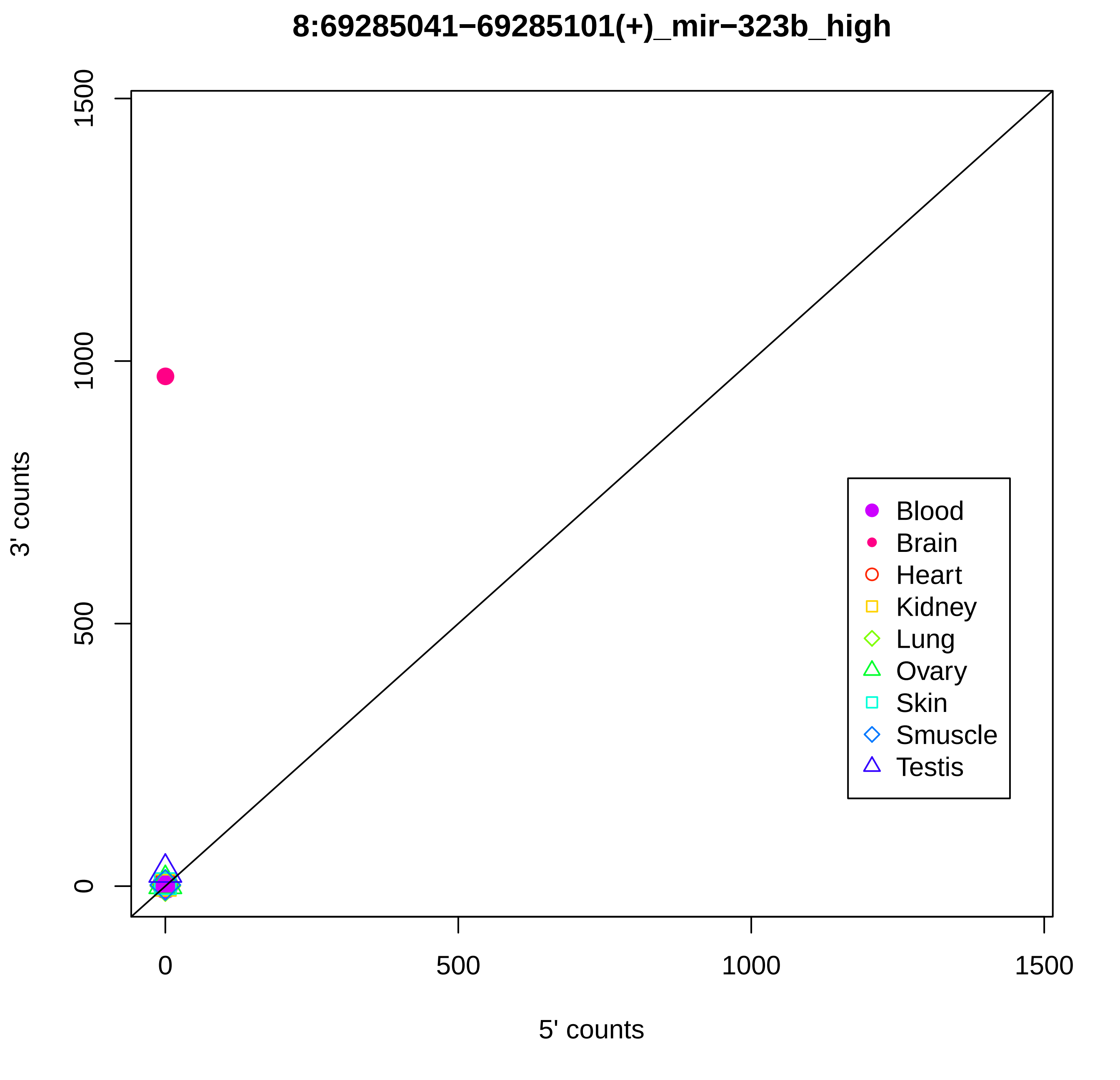

8:69285041−69285101(+)_mir−323b_high
3' counts
5' counts
0
500
1000
1500
0
500
1000
1500
Blood
Brain
Heart
Kidney
Lung
Ovary
Skin
Smuscle
Testis

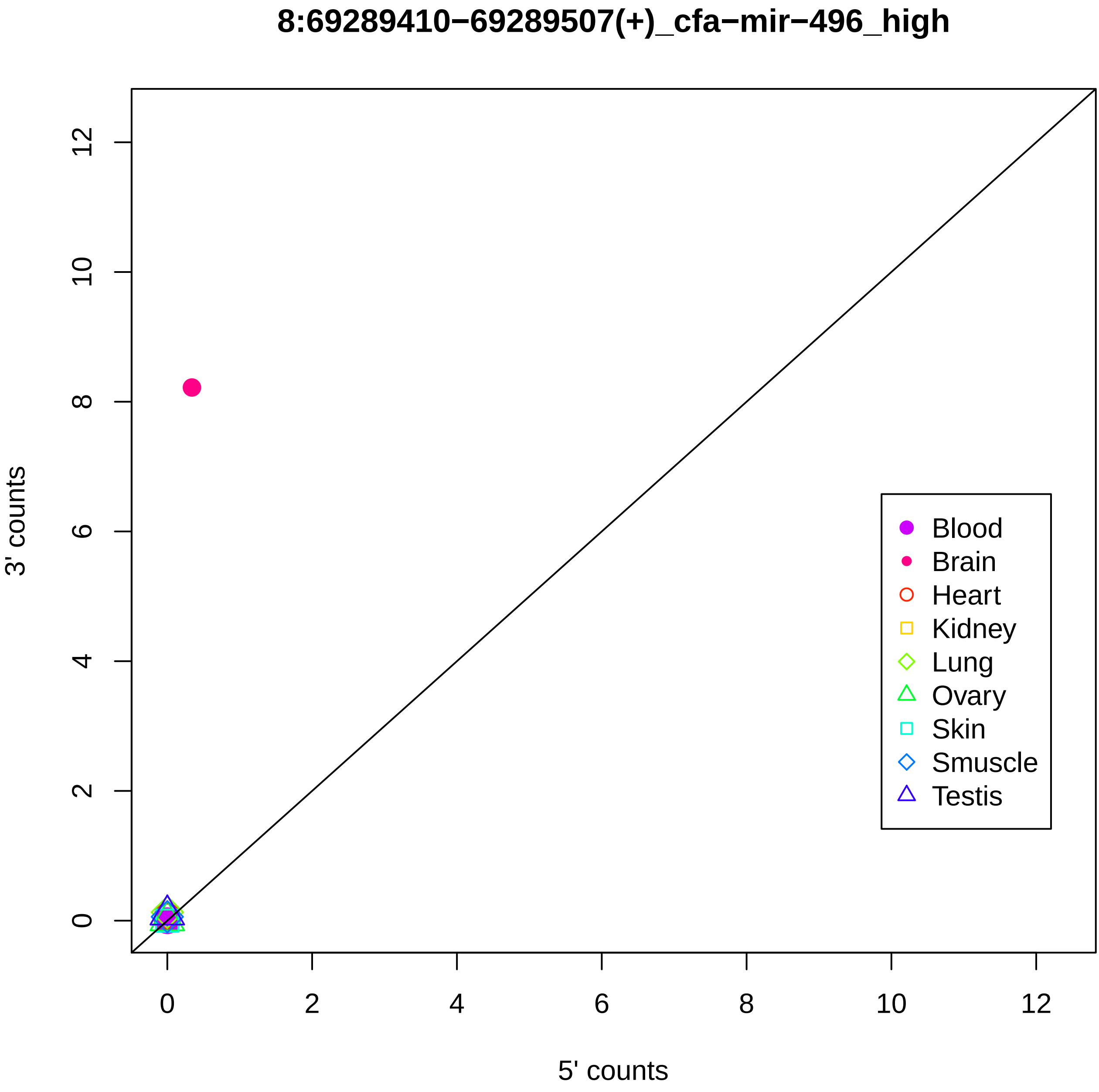
8:69289410−69289507(+)_cfa−mir−496_high
3' counts
5' counts
0
2
4
6
8
10
12
Blood
Brain
Heart
Kidney
Lung
Ovary
Skin
Smuscle
Testis

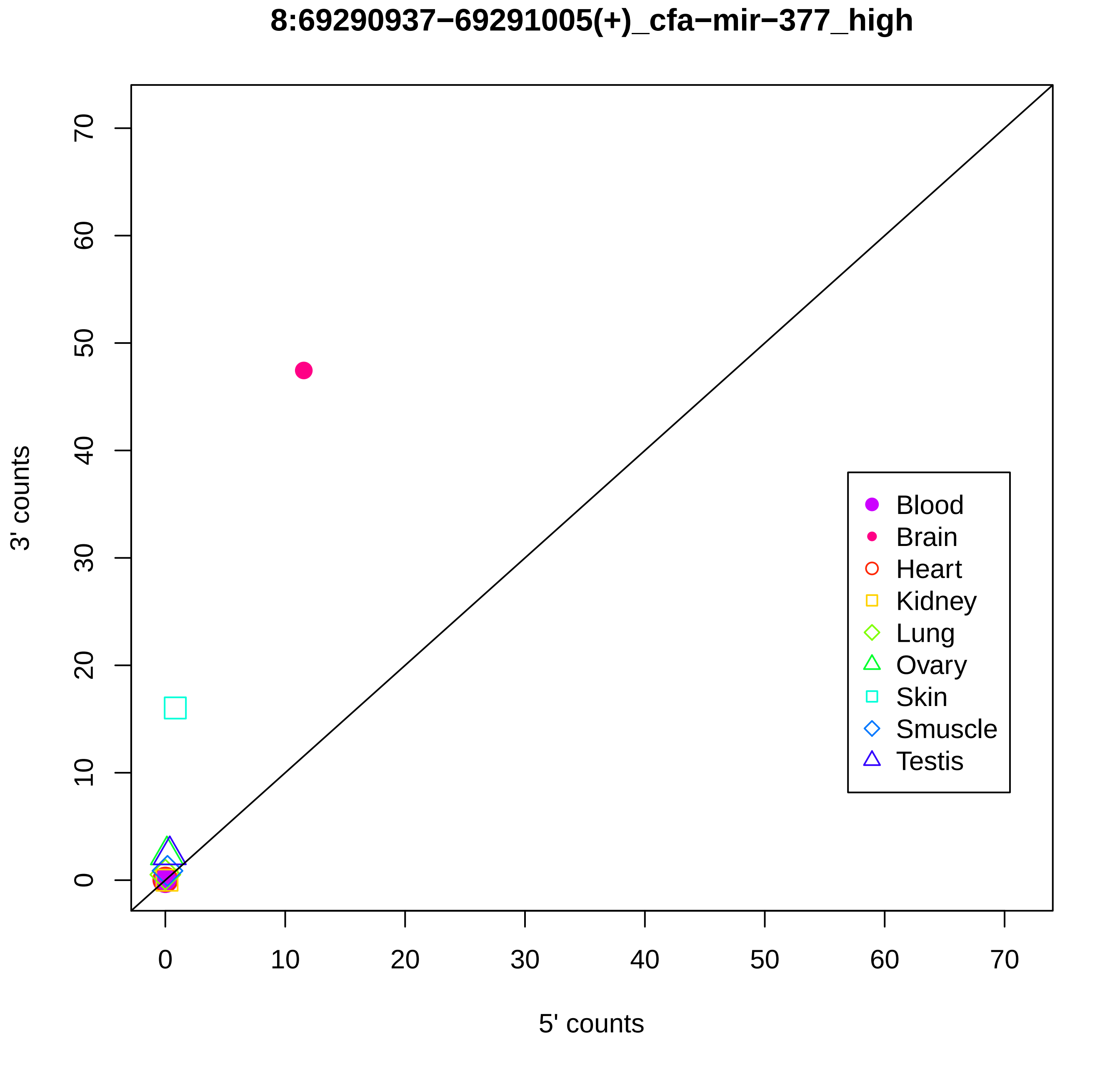

8:69290937−69291005(+)_cfa−mir−377_high
3' counts
5' counts
0
10
20
30
40
50
60
70
Blood
Brain
Heart
Kidney
Lung
Ovary
Skin
Smuscle
Testis

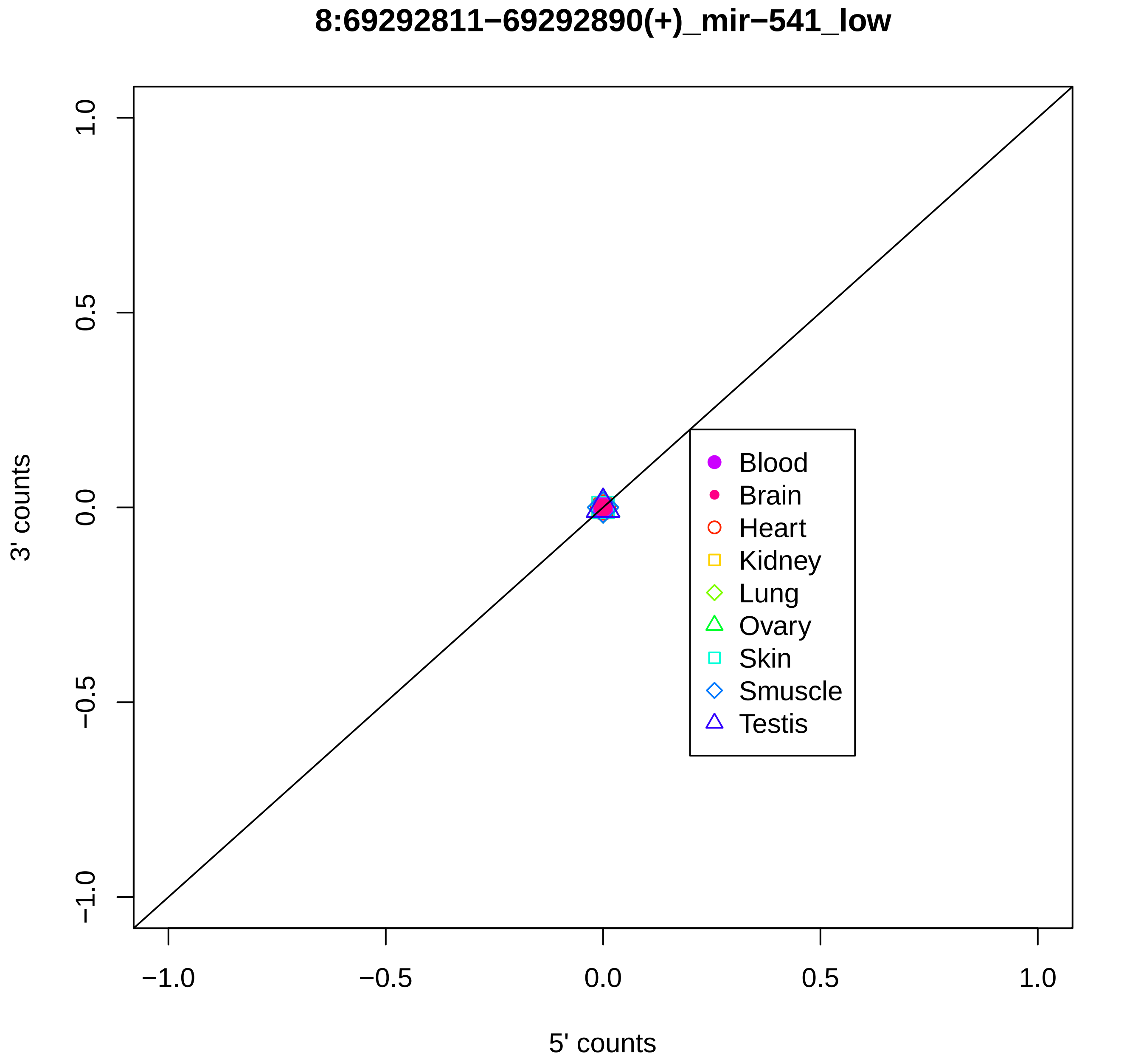

8:69292811−69292890(+)_mir−541_low
3' counts
5' counts
1.0
0.5
0.0
−0.5
−1.0
−1.0
−0.5
0.0
0.5
1.0
Blood
Brain
Heart
Kidney
Lung
Ovary
Skin
Smuscle
Testis

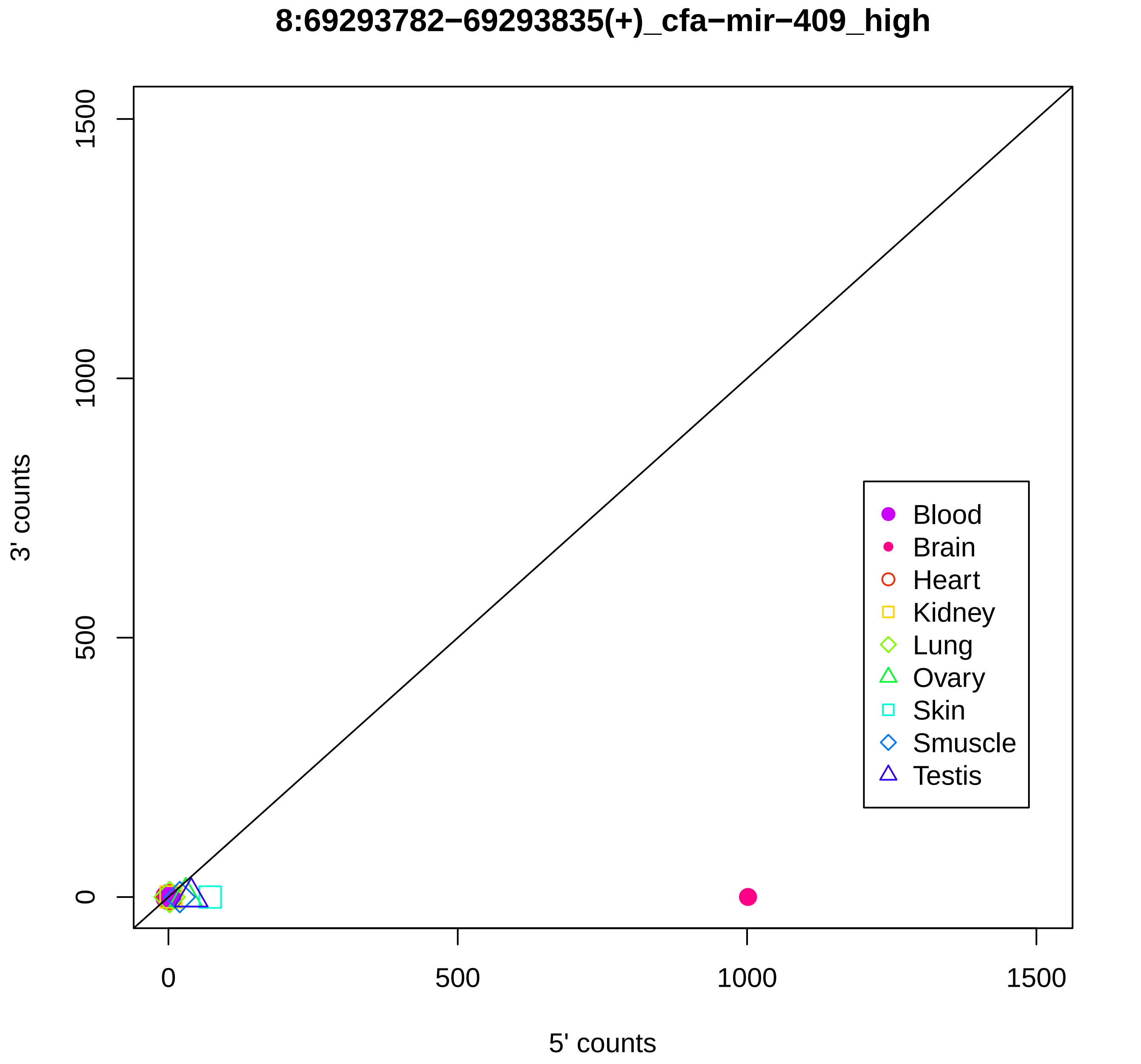

8:69293782−69293835(+)_cfa−mir−409_high
3' counts
5' counts
0
500
1000
1500
0
500
1000
1500
Blood
Brain
Heart
Kidney
Lung
Ovary
Skin
Smuscle
Testis

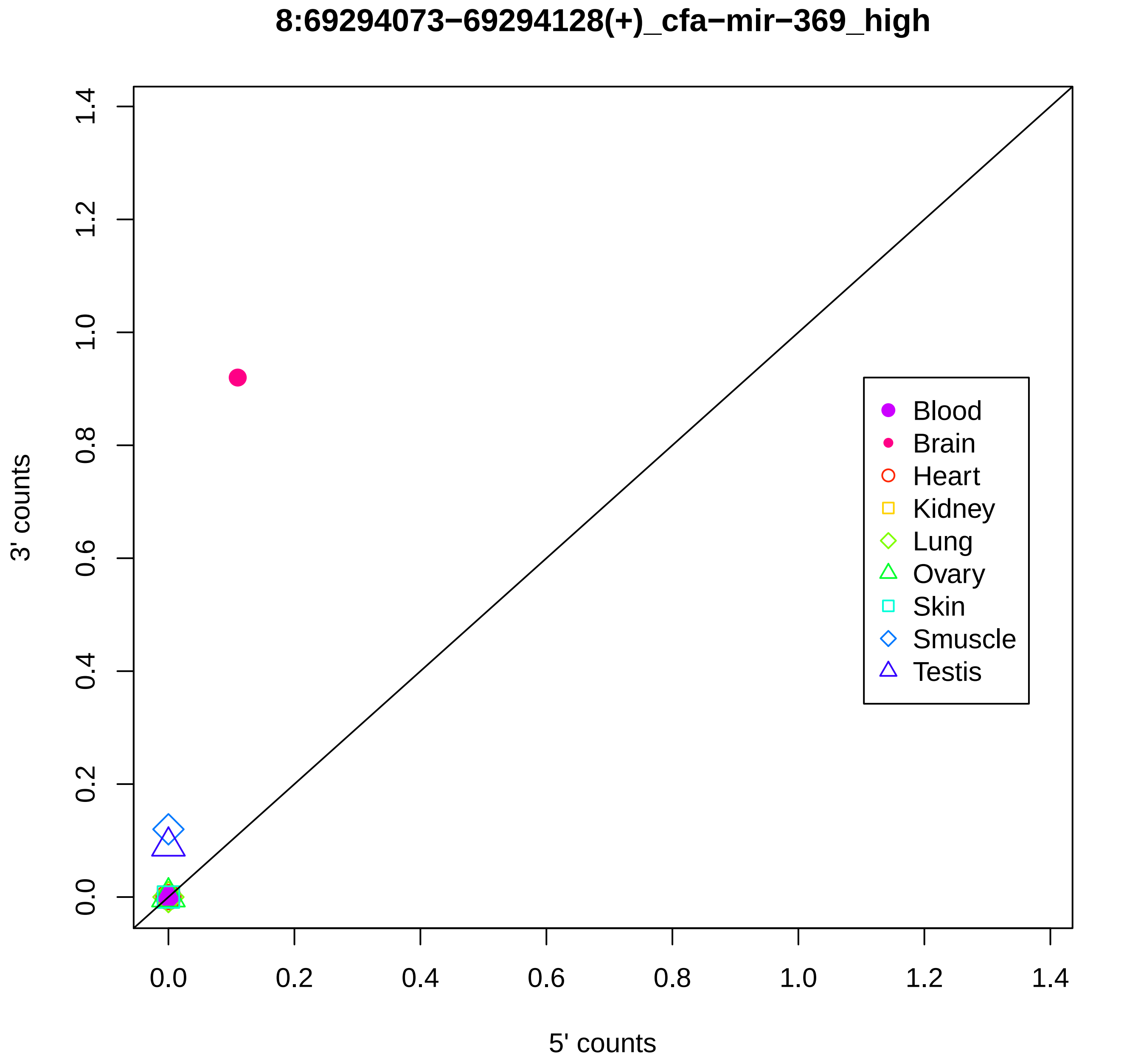

8:69294073−69294128(+)_cfa−mir−369_high
3' counts
5' counts
0.0
0.2
0.4
0.6
0.8
1.0
1.2
1.4
Blood
Brain
Heart
Kidney
Lung
Ovary
Skin
Smuscle
Testis

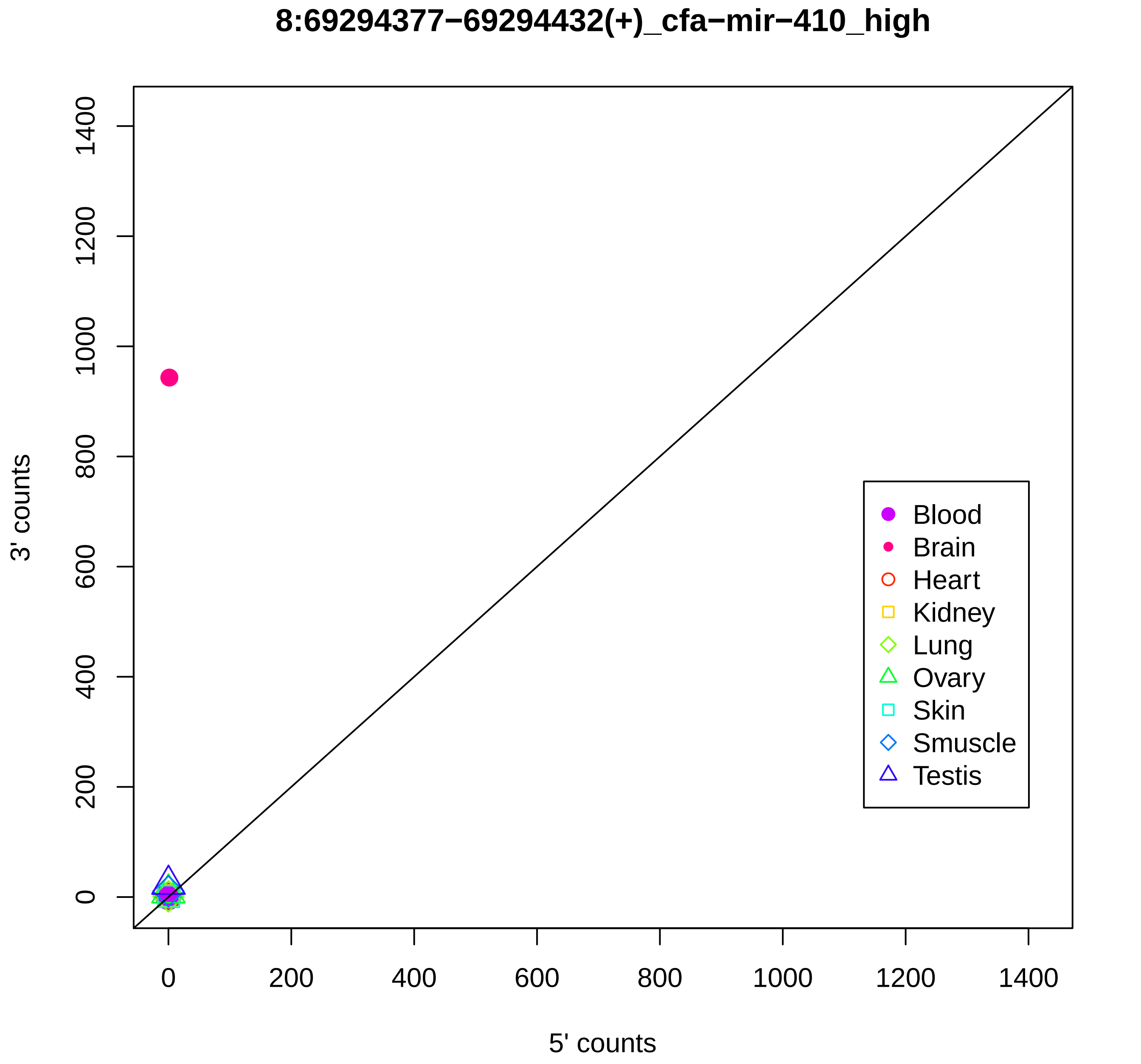

8:69294377−69294432(+)_cfa−mir−410_high
3' counts
5' counts
0
200
400
600
800
1000
1200
1400
0
200
400
600
800
1000
1200
1400
Blood
Brain
Heart
Kidney
Lung
Ovary
Skin
Smuscle
Testis

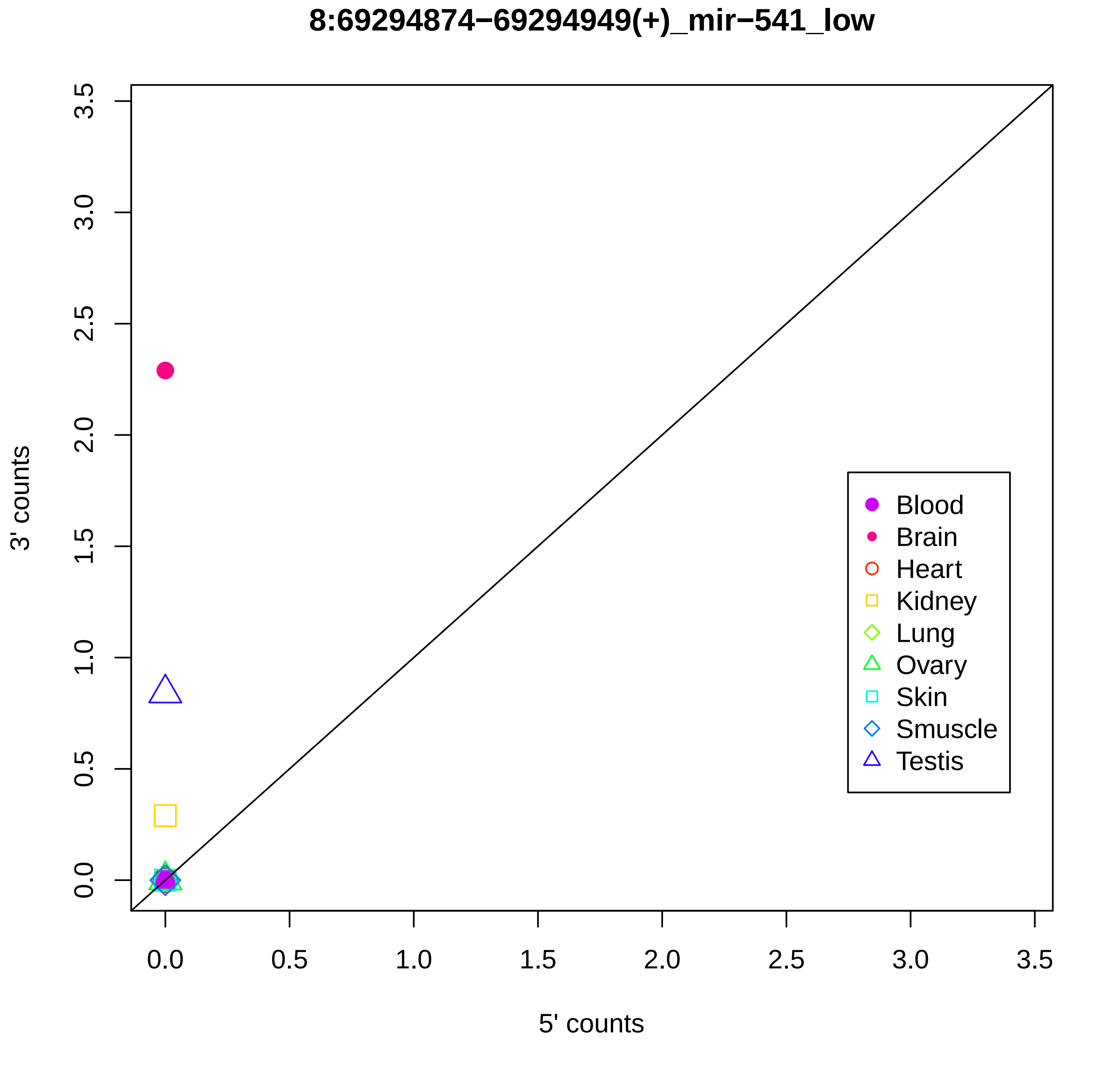

8:69294874−69294949(+)_mir−541_low
3' counts
5' counts
0.0
0.5
1.0
1.5
2.0
2.5
3.0
3.5
Blood
Brain
Heart
Kidney
Lung
Ovary
Skin
Smuscle
Testis

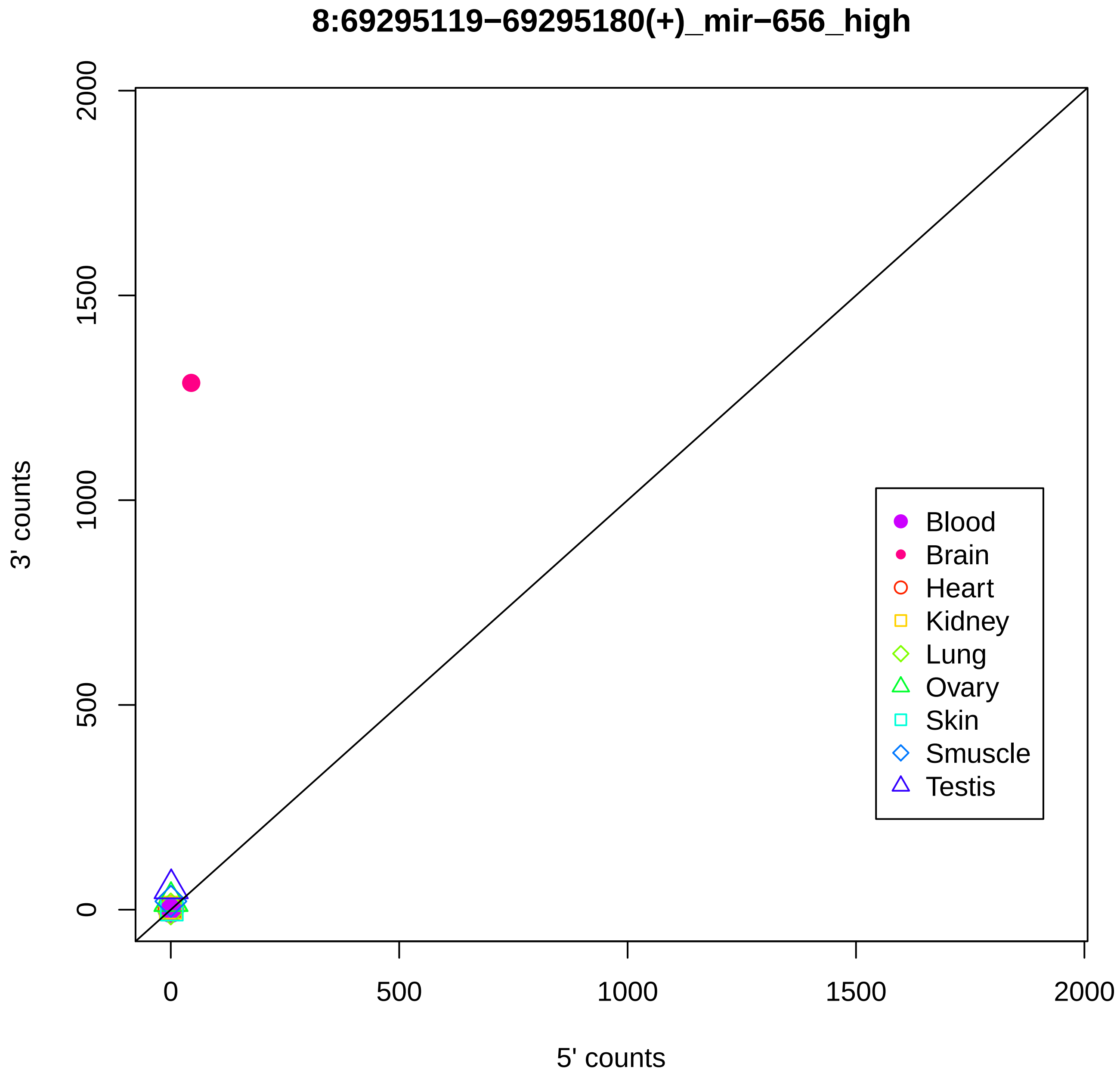

8:69295119−69295180(+)_mir−656_high
3' counts
5' counts
0
500
1000
1500
2000
Blood
Brain
Heart
Kidney
Lung
Ovary
Skin
Smuscle
Testis

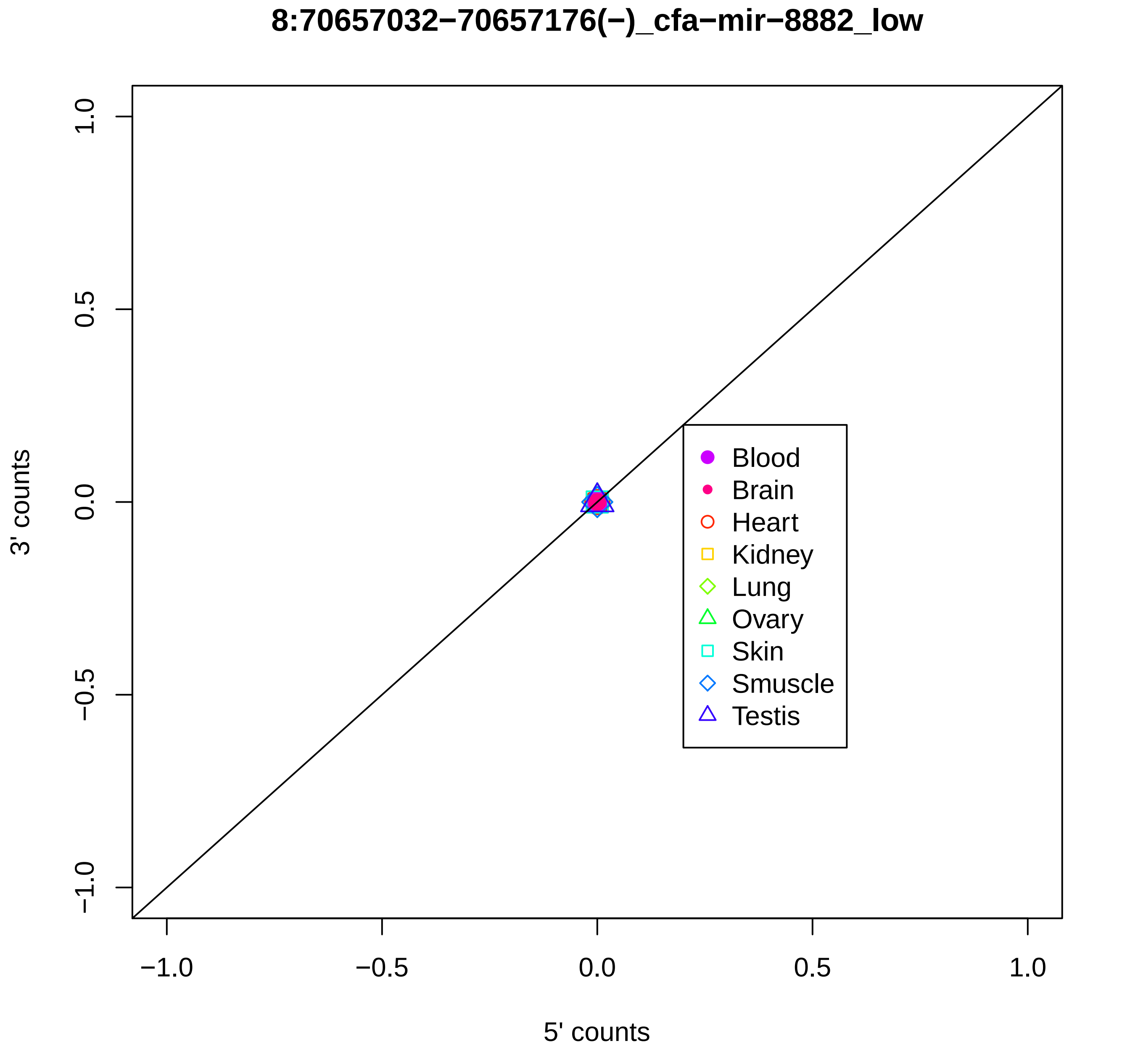

8:70657032−70657176(−)_cfa−mir−8882_low
1.0
0.5
0.0
−0.5
−1.0
3' counts
−1.0
−0.5
0.0
0.5
1.0
5' counts
Blood
Brain
Heart
Kidney
Lung
Ovary
Skin
Smuscle
Testis

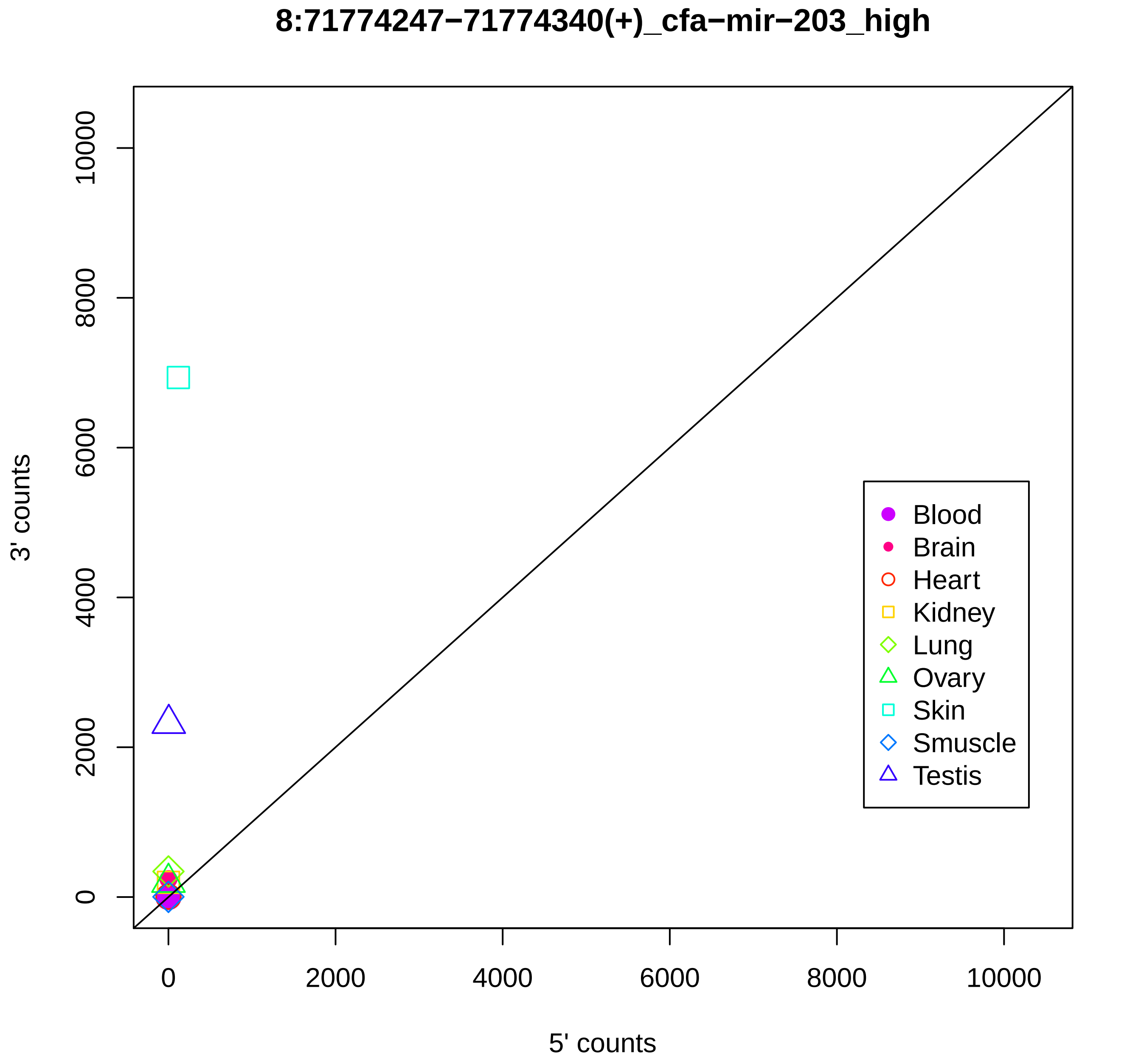
8:71774247−71774340(+)_cfa−mir−203_high
3' counts
5' counts
0
2000
4000
6000
8000
10000
Blood
Brain
Heart
Kidney
Lung
Ovary
Skin
Smuscle
Testis

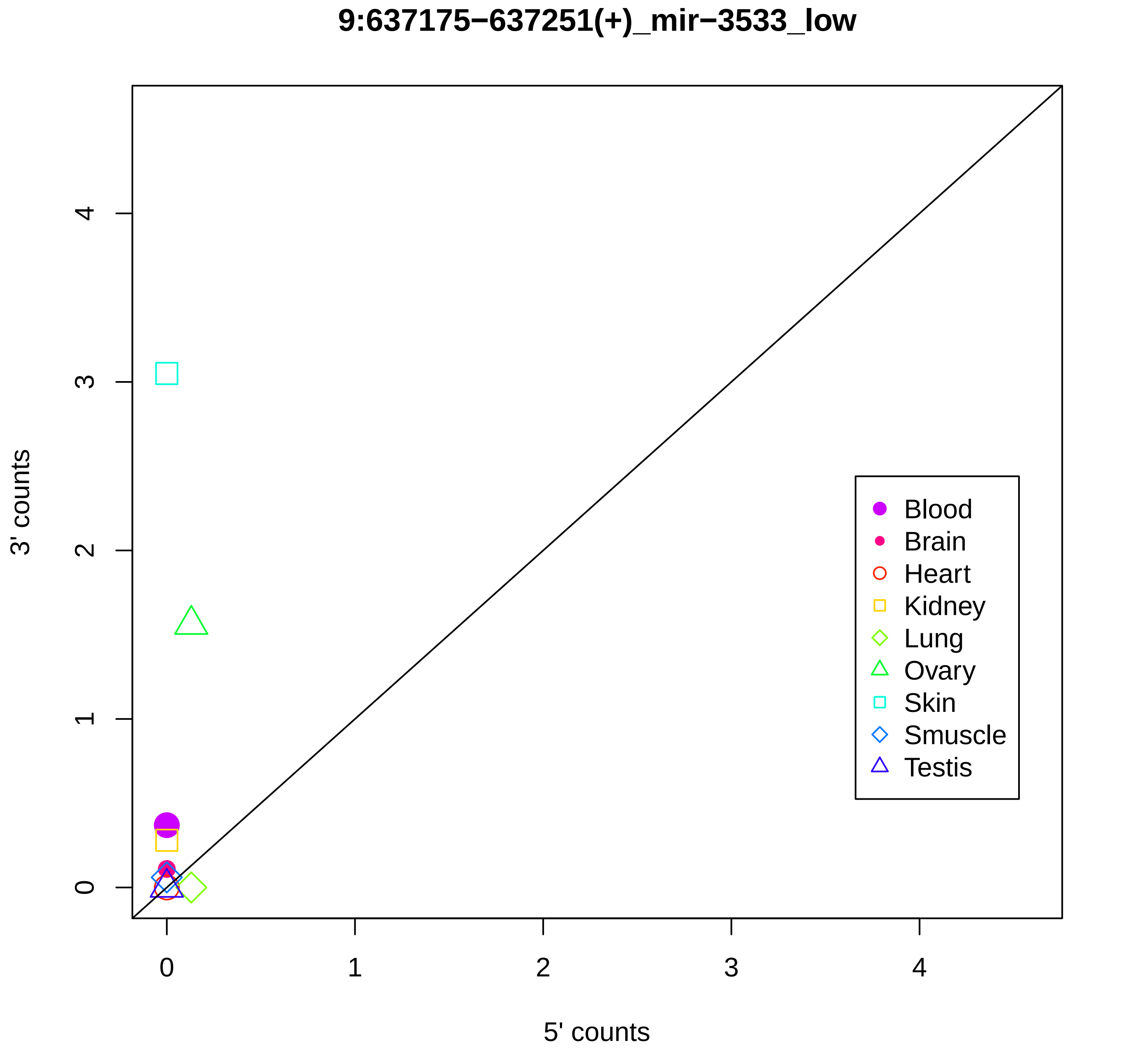

9:637175−637251(+)_mir−3533_low
3' counts
5' counts
0
1
2
3
4
0
1
2
3
4
Blood
Brain
Heart
Kidney
Lung
Ovary
Skin
Smuscle
Testis

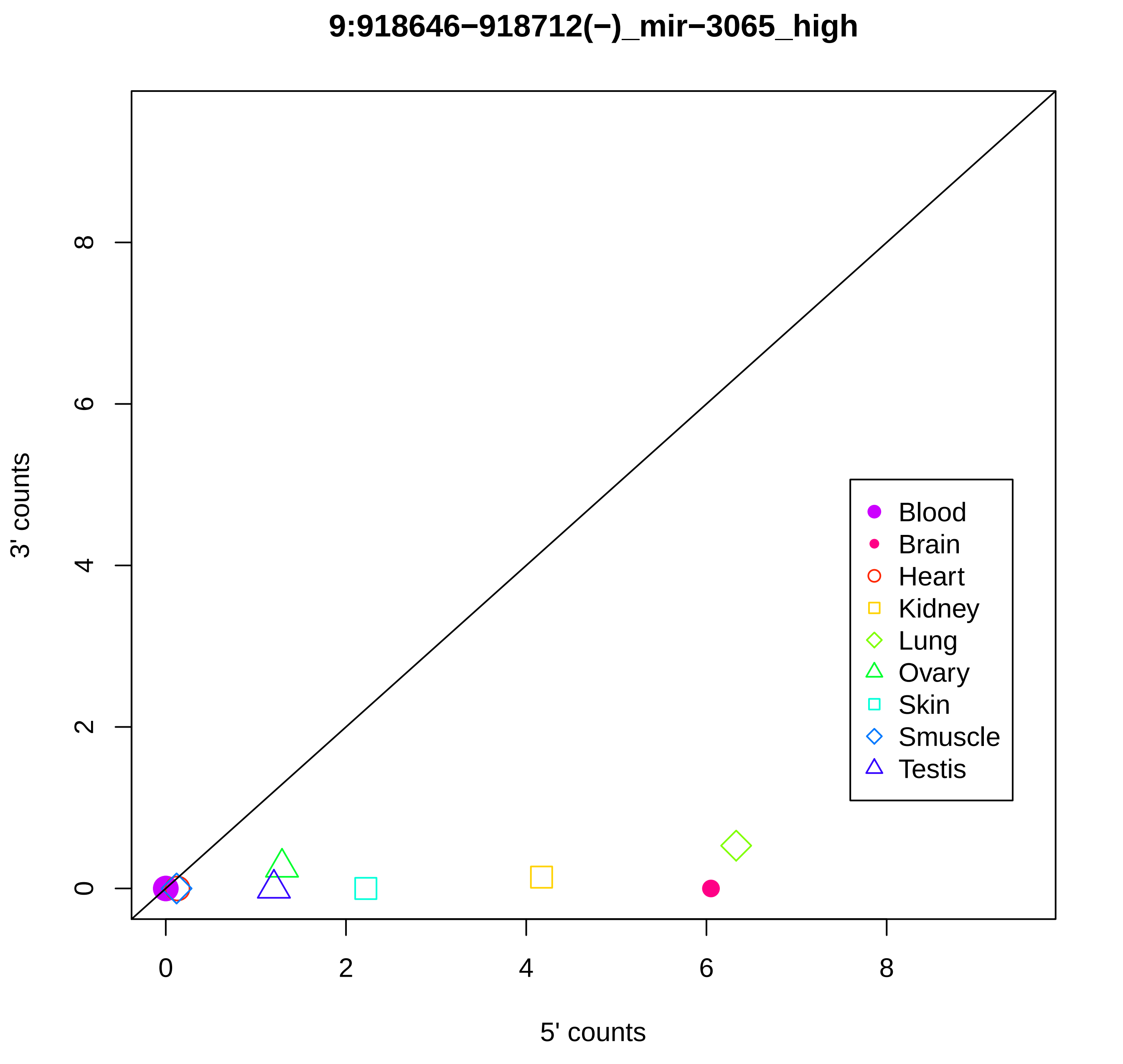

9:918646−918712(−)_mir−3065_high
3' counts
5' counts
0
2
4
6
8
0
2
4
6
8
Blood
Brain
Heart
Kidney
Lung
Ovary
Skin
Smuscle
Testis

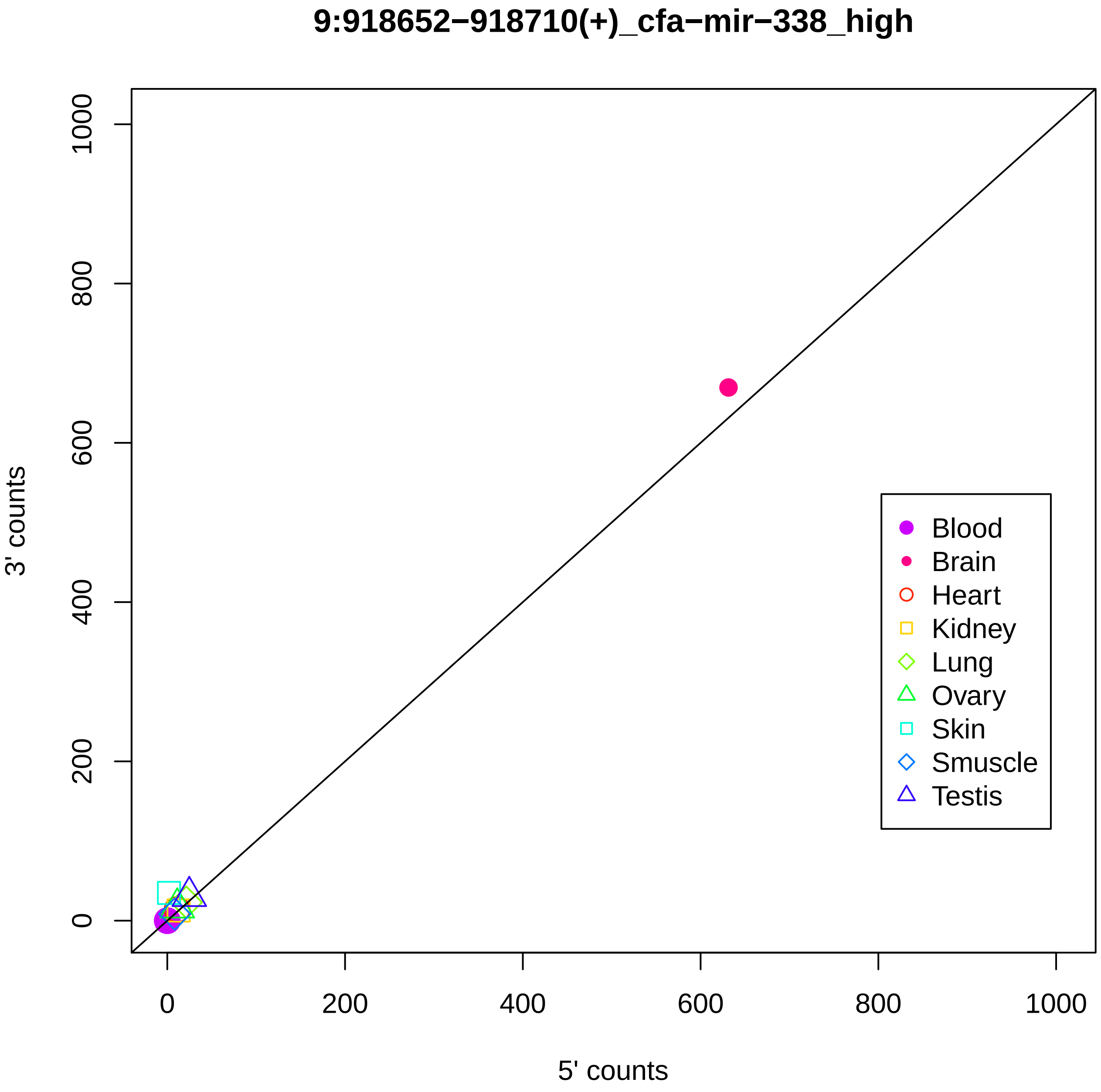
9:918652−918710(+)_cfa−mir−338_high
3' counts
5' counts
0
200
400
600
800
1000
0
200
400
600
800
1000
Blood
Brain
Heart
Kidney
Lung
Ovary
Skin
Smuscle
Testis

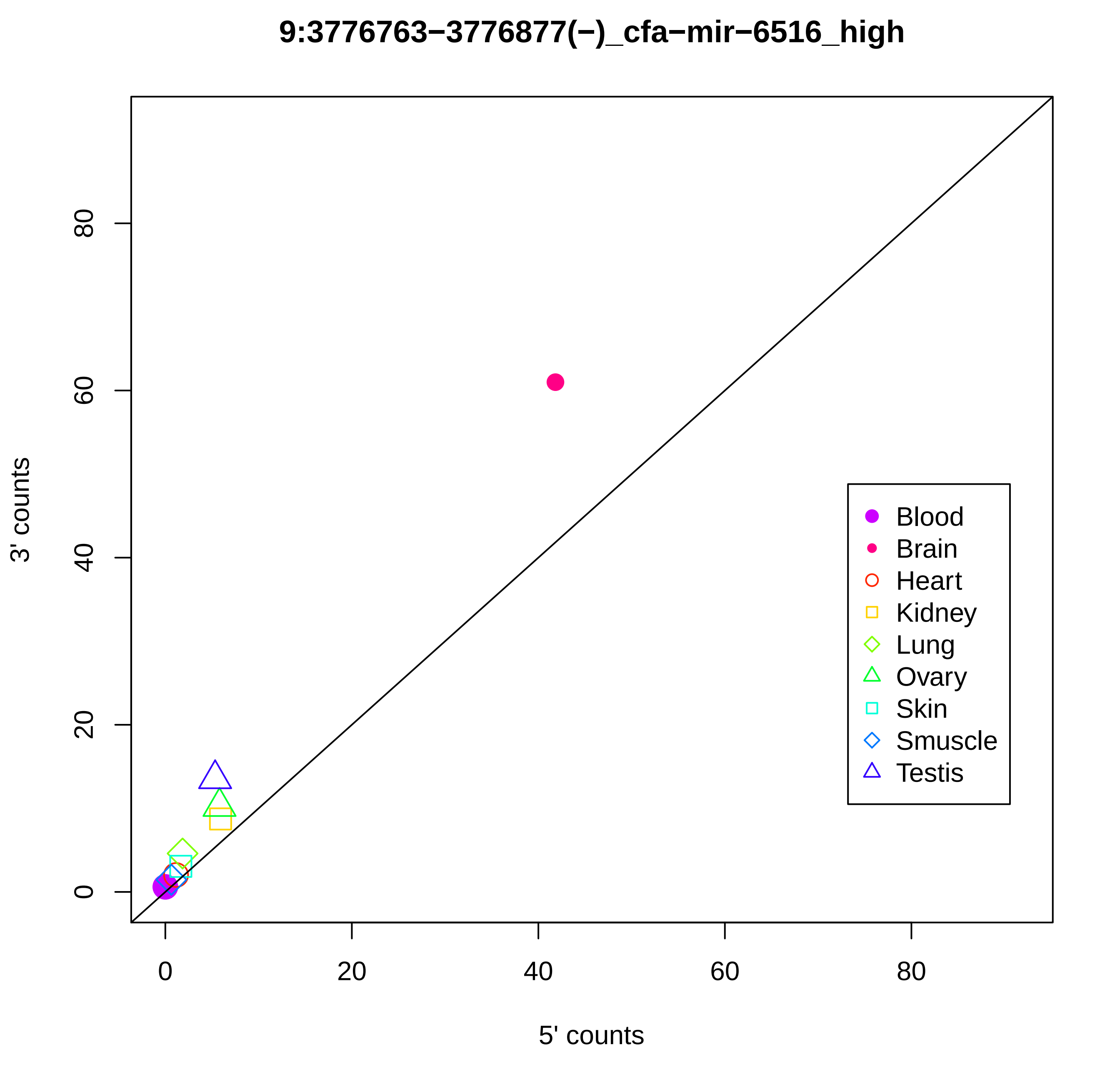
9:3776763−3776877(−)_cfa−mir−6516_high
3' counts
5' counts
0
20
40
60
80
0
20
40
60
80
Blood
Brain
Heart
Kidney
Lung
Ovary
Skin
Smuscle
Testis

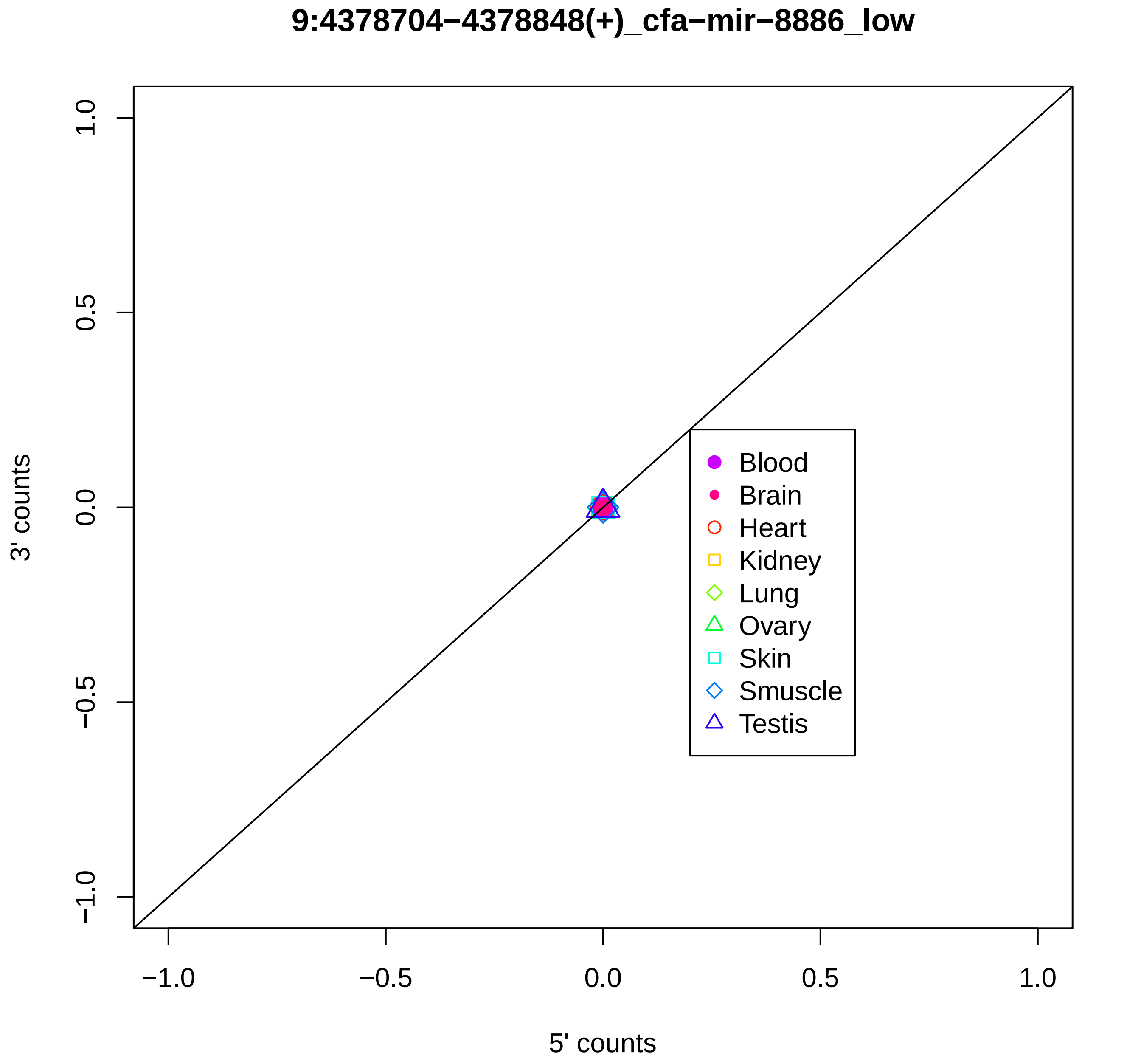
9:4378704−4378848(+)_cfa−mir−8886_low
3' counts
5' counts
−1.0
−0.5
0.0
0.5
1.0
Blood
Brain
Heart
Kidney
Lung
Ovary
Skin
Smuscle
Testis

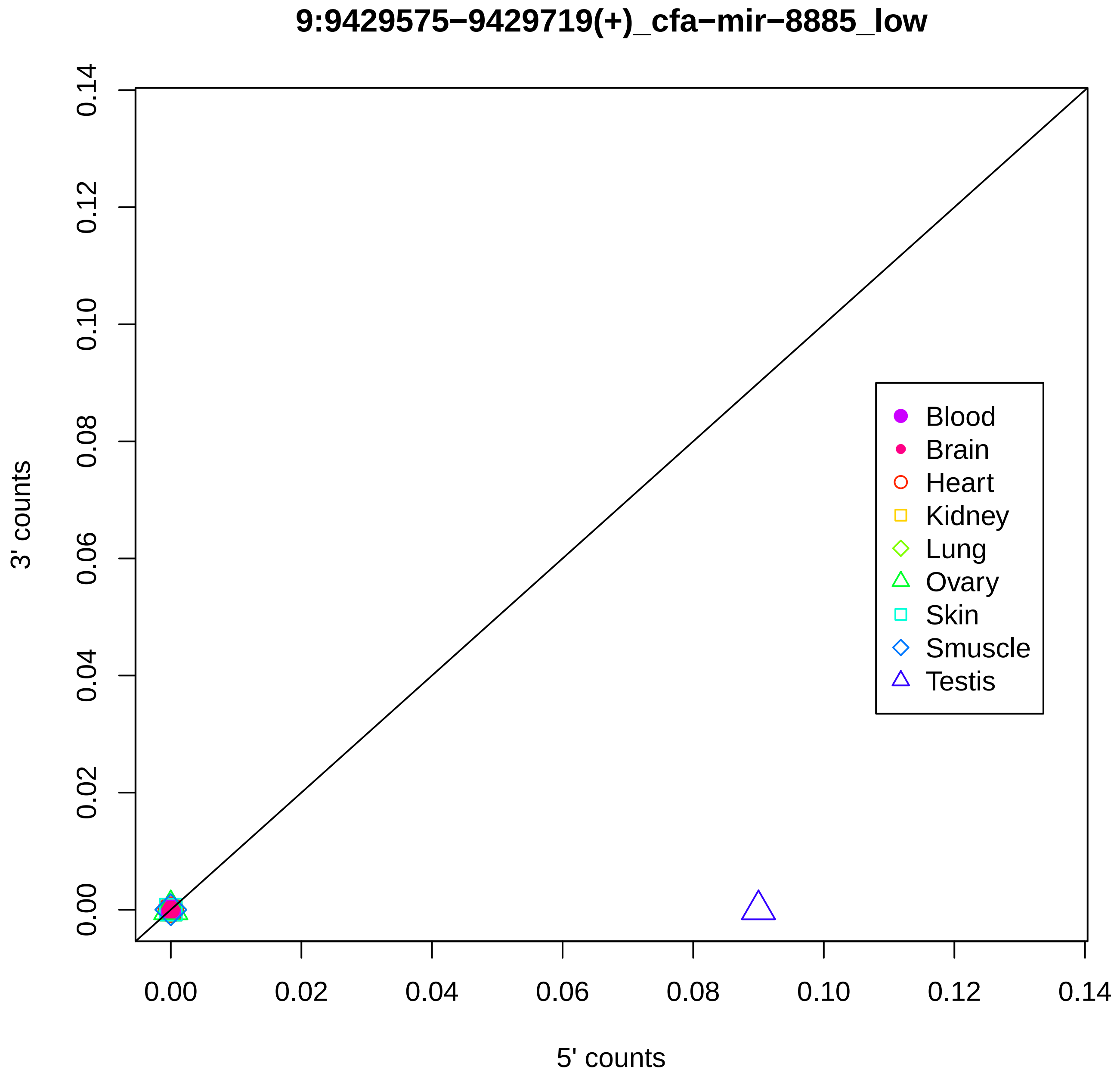

9:9429575−9429719(+)_cfa−mir−8885_low
3' counts
5' counts
0.00
0.02
0.04
0.06
0.08
0.10
0.12
0.14
Blood
Brain
Heart
Kidney
Lung
Ovary
Skin
Smuscle
Testis

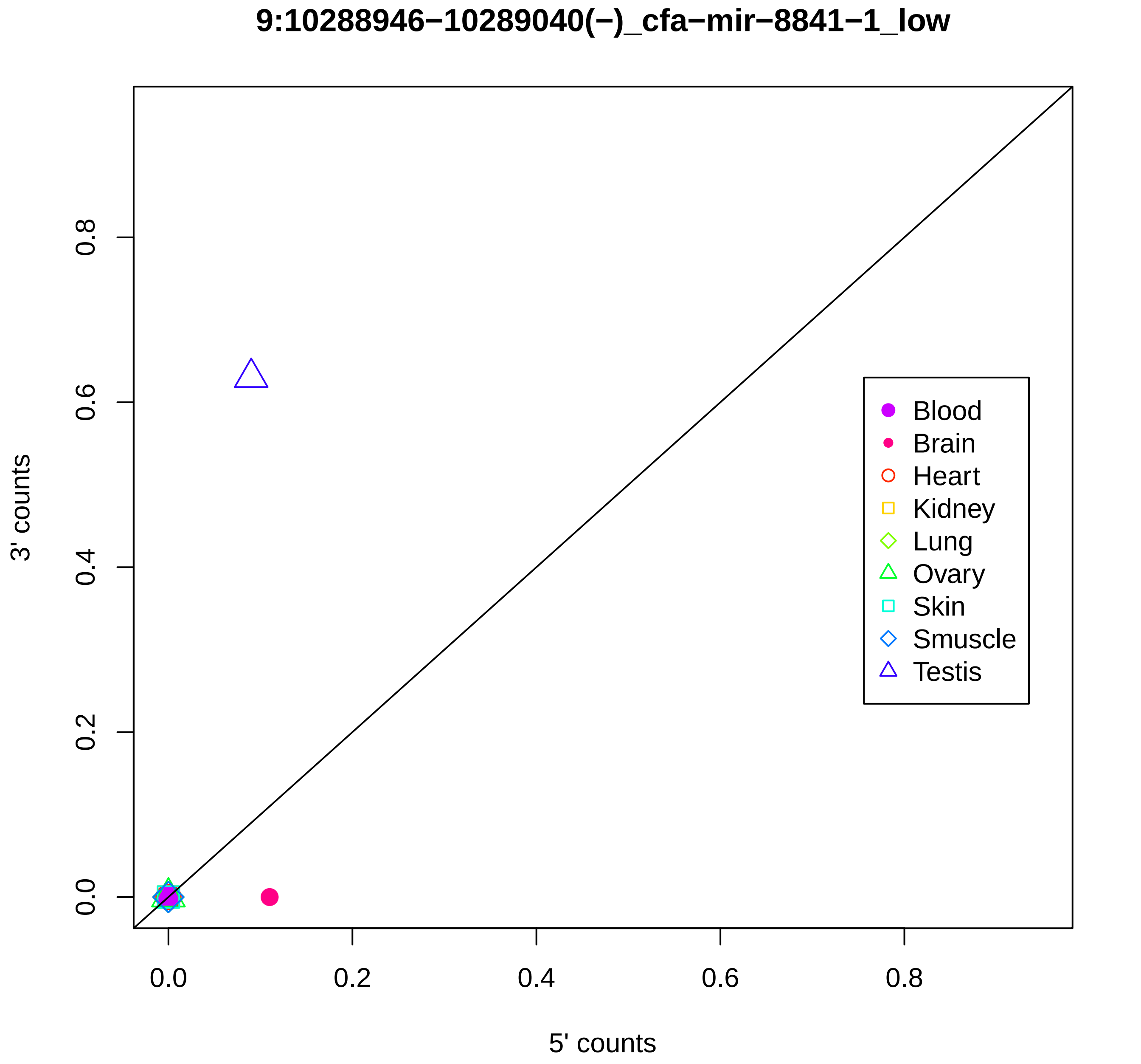

9:10288946−10289040(−)_cfa−mir−8841−1_low
3' counts
5' counts
0.0
0.2
0.4
0.6
0.8
Blood
Brain
Heart
Kidney
Lung
Ovary
Skin
Smuscle
Testis

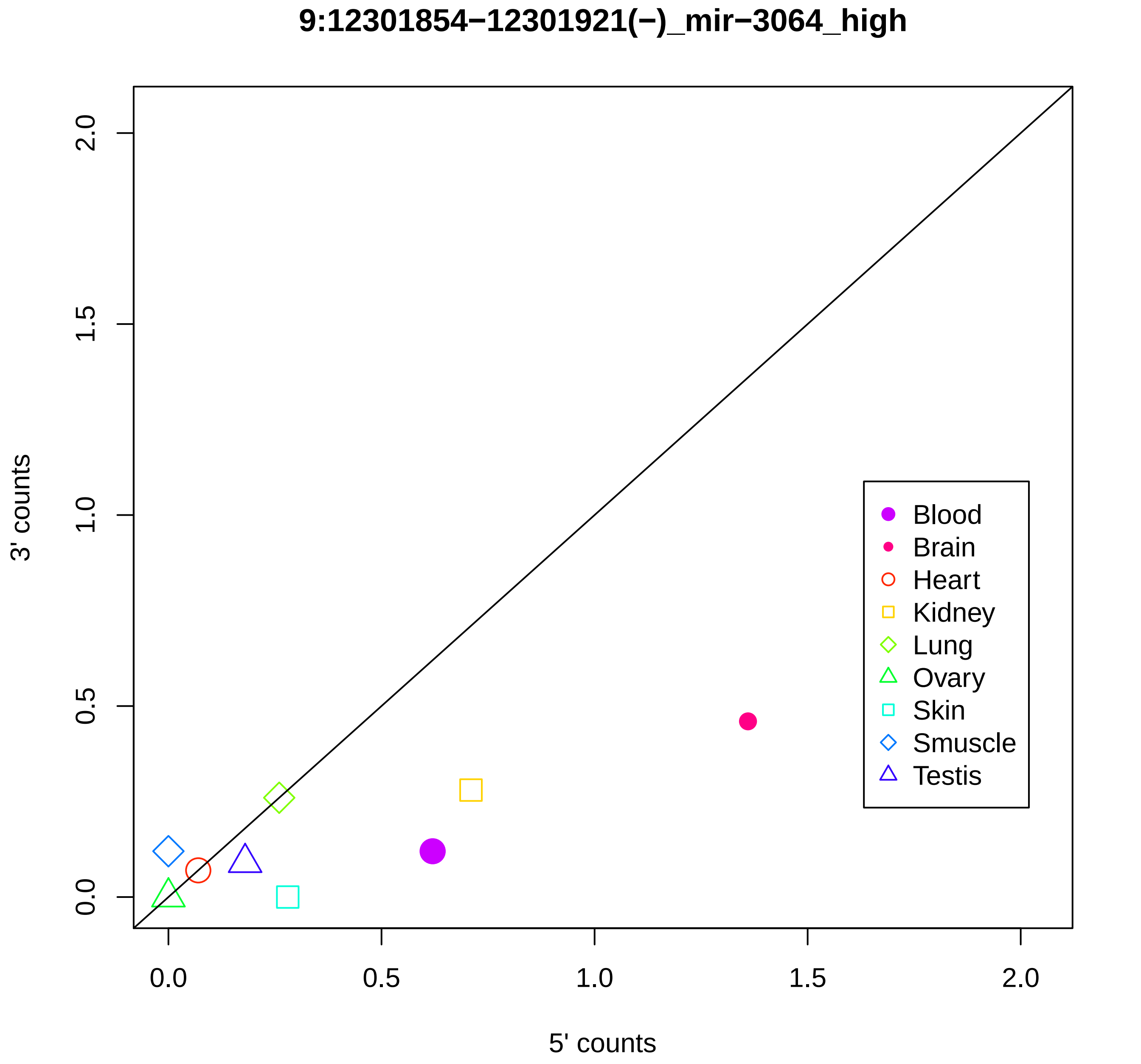
9:12301854−12301921(−)_mir−3064_high
3' counts
5' counts
0.0
0.5
1.0
1.5
2.0
0.0
0.5
1.0
1.5
2.0
Blood
Brain
Heart
Kidney
Lung
Ovary
Skin
Smuscle
Testis

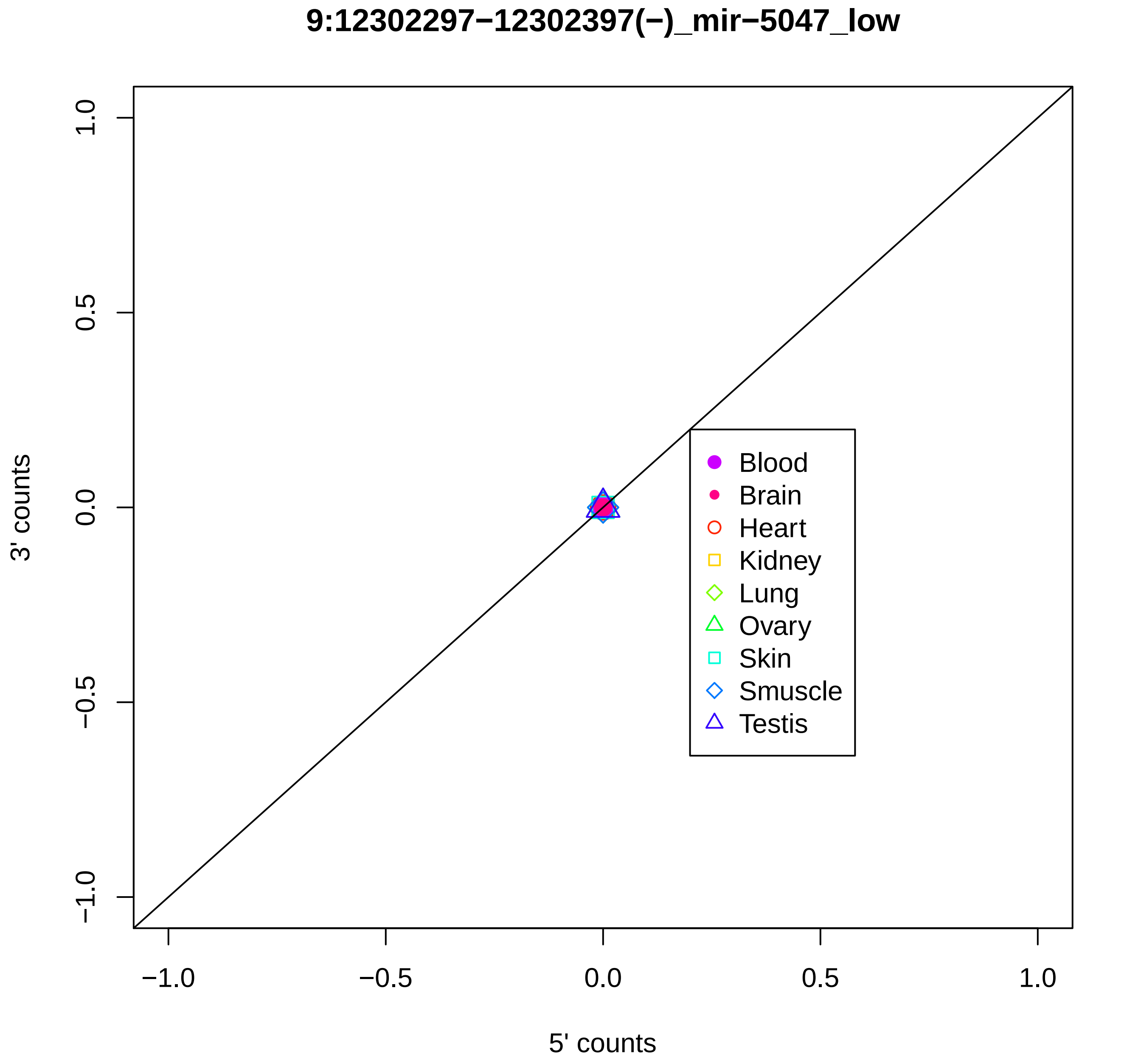

9:12302297−12302397(−)_mir−5047_low
3' counts
5' counts
1.0
0.5
0.0
−0.5
−1.0
Blood
Brain
Heart
Kidney
Lung
Ovary
Skin
Smuscle
Testis

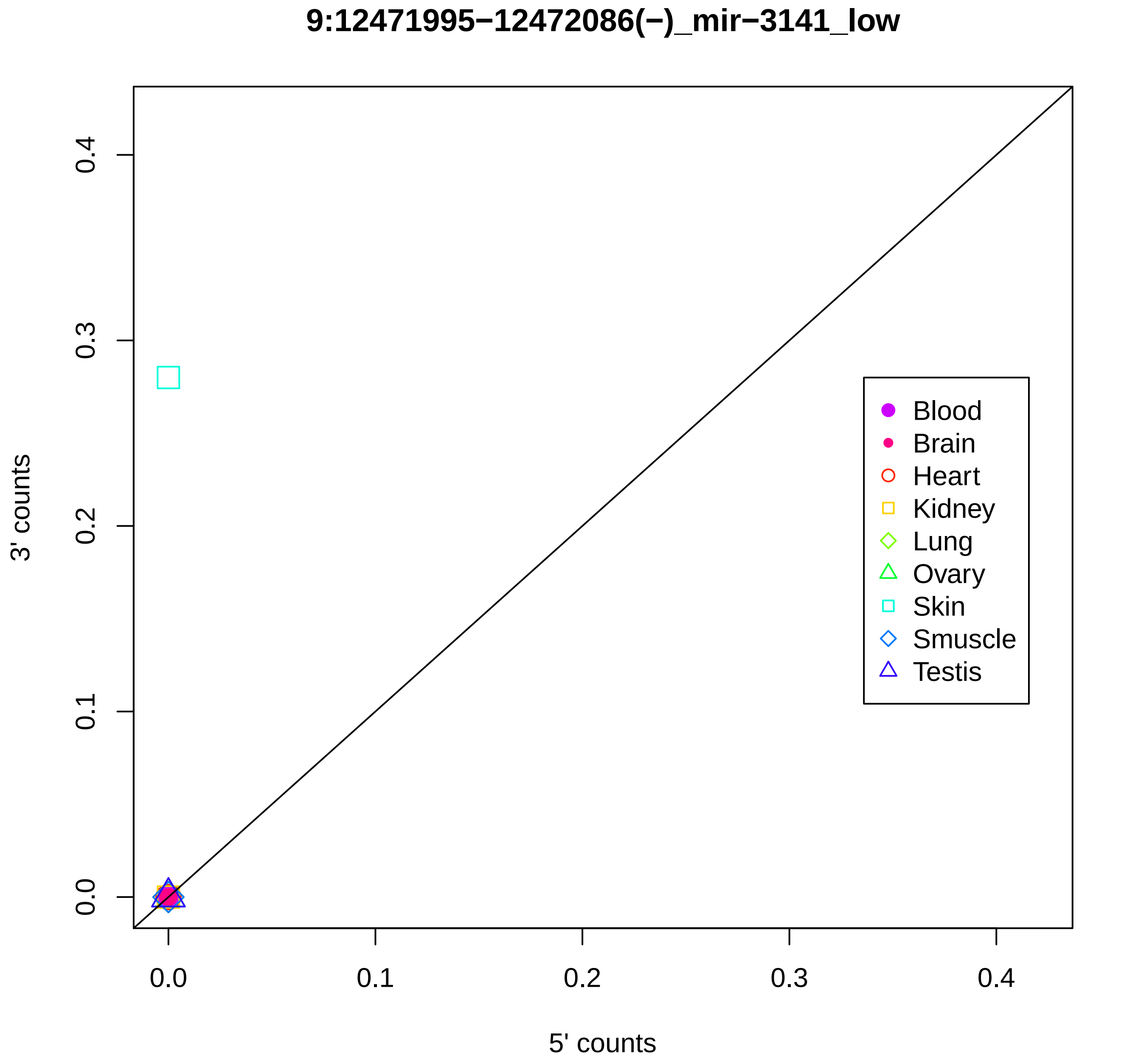

9:12471995−12472086(−)_mir−3141_low
3' counts
5' counts
0.0
0.1
0.2
0.3
0.4
Blood
Brain
Heart
Kidney
Lung
Ovary
Skin
Smuscle
Testis

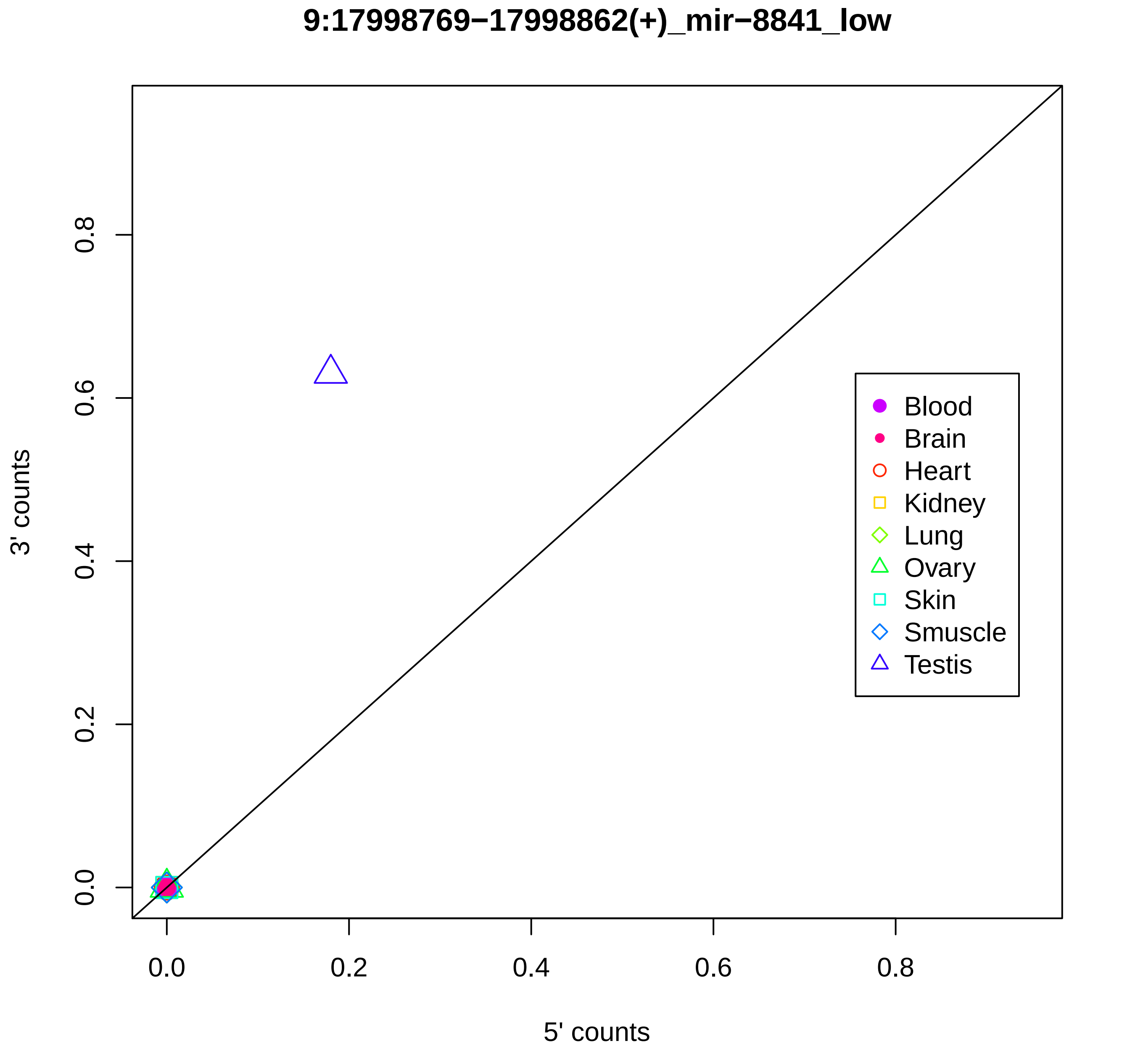

9:17998769−17998862(+)_mir−8841_low
3' counts
5' counts
0.0
0.2
0.4
0.6
0.8
Blood
Brain
Heart
Kidney
Lung
Ovary
Skin
Smuscle
Testis

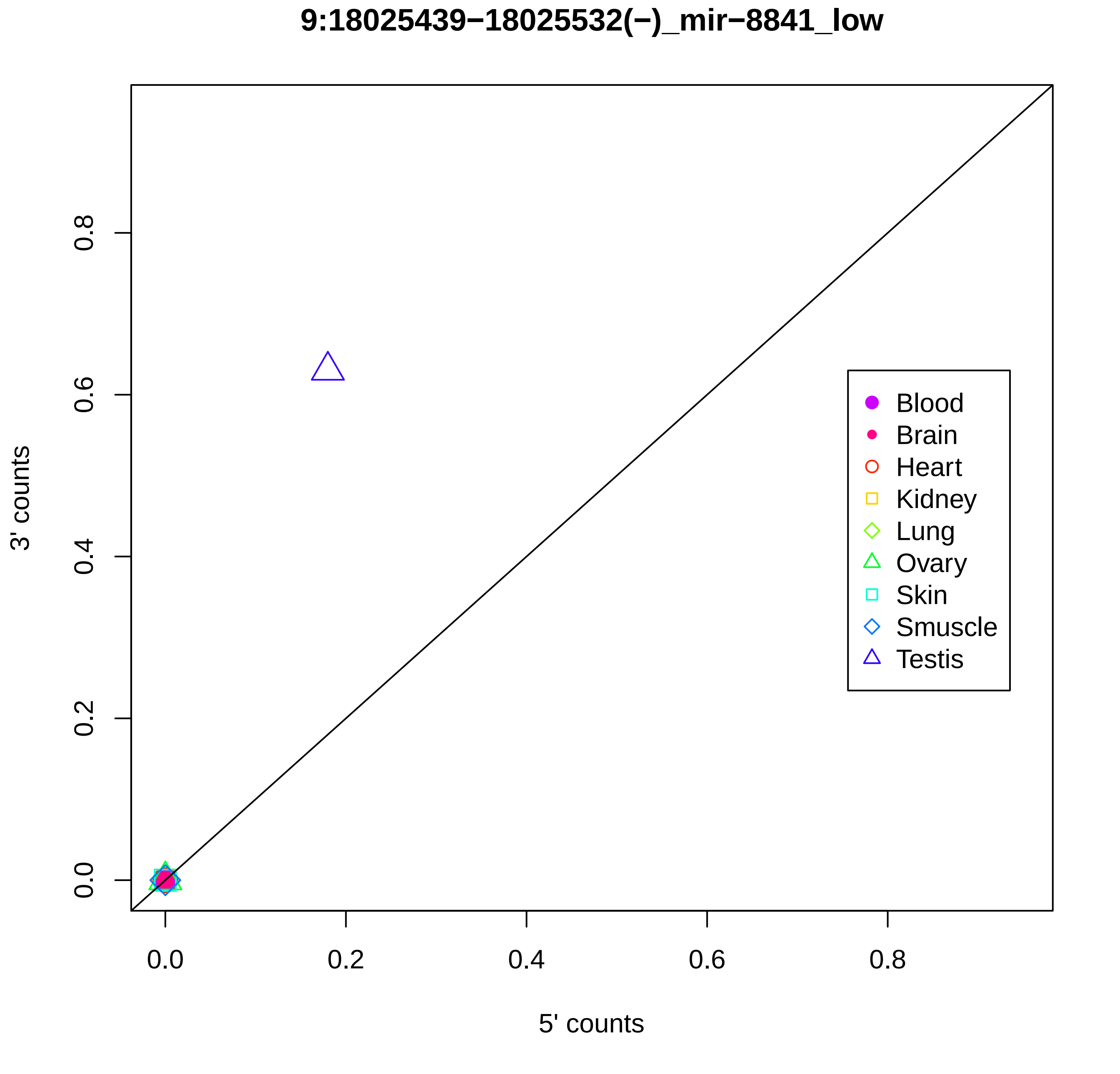
9:18025439−18025532(−)_mir−8841_low
3' counts
5' counts
0.0
0.2
0.4
0.6
0.8
0.0
0.2
0.4
0.6
0.8
Blood
Brain
Heart
Kidney
Lung
Ovary
Skin
Smuscle
Testis

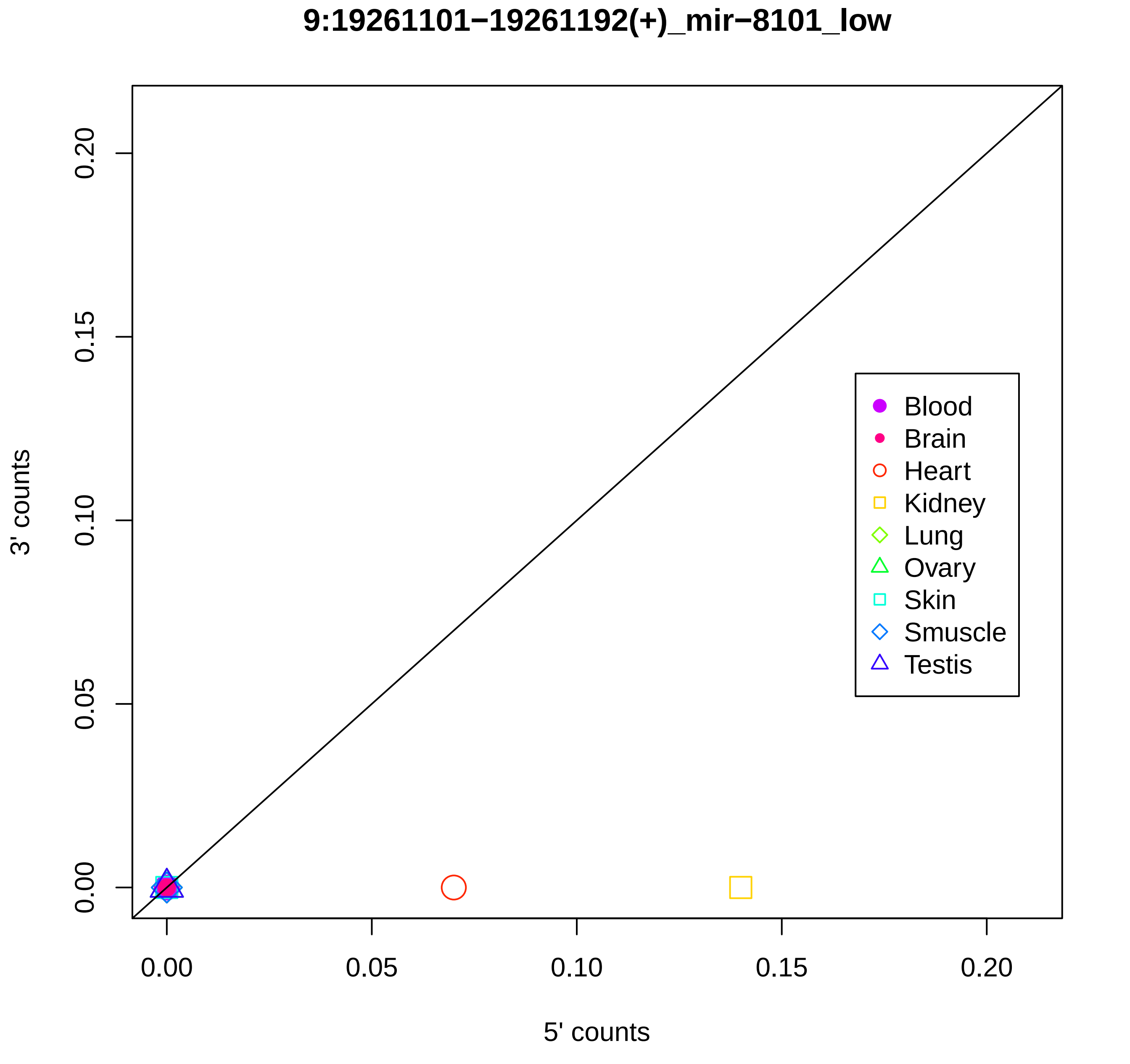

9:19261101−19261192(+)_mir−8101_low
3' counts
5' counts
0.00
0.05
0.10
0.15
0.20
0.00
0.05
0.10
0.15
0.20
Blood
Brain
Heart
Kidney
Lung
Ovary
Skin
Smuscle
Testis

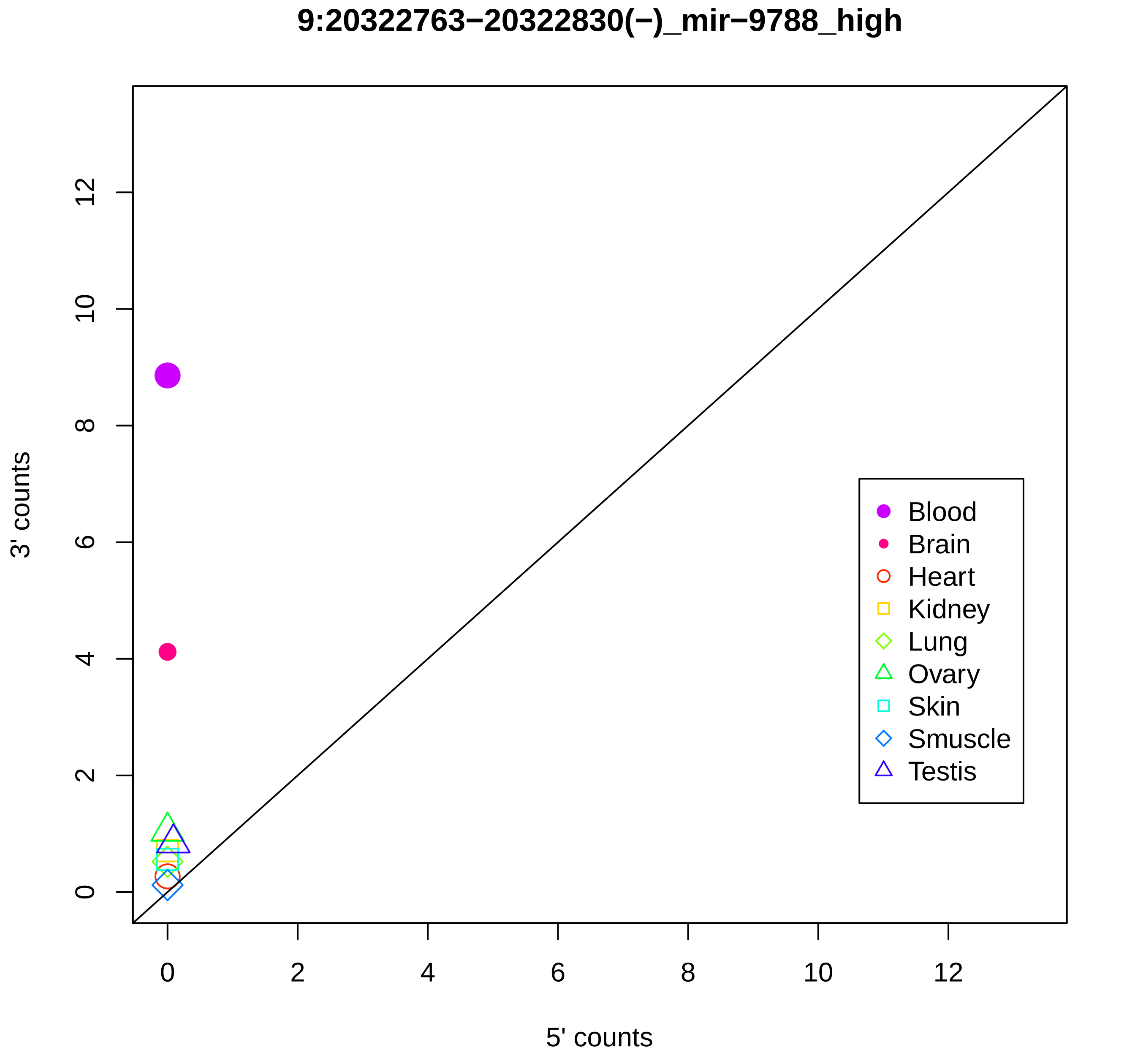

9:20322763−20322830(−)_mir−9788_high
3' counts
5' counts
0
2
4
6
8
10
12
Blood
Brain
Heart
Kidney
Lung
Ovary
Skin
Smuscle
Testis

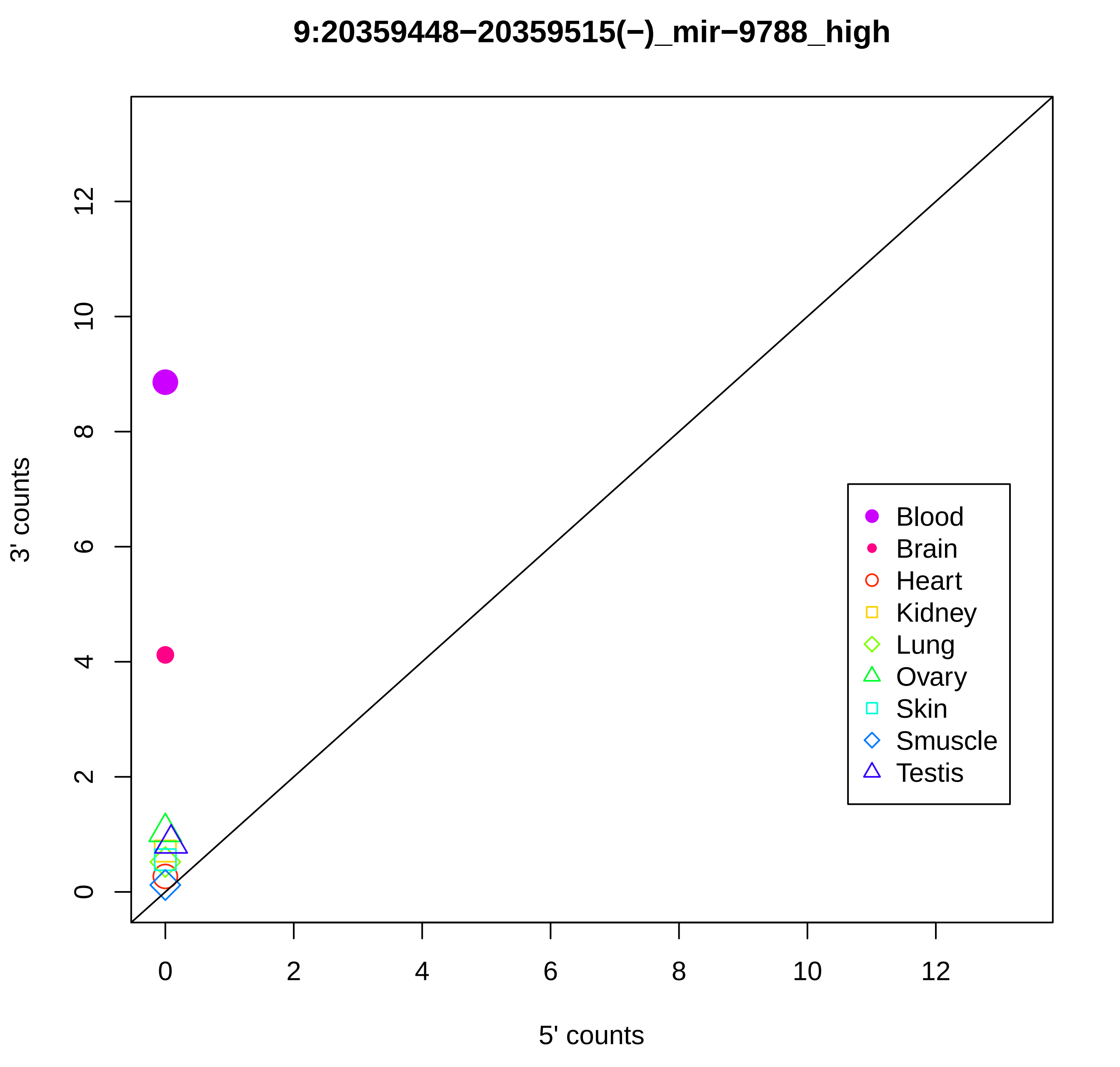

9:20359448−20359515(−)_mir−9788_high
3' counts
5' counts
0
2
4
6
8
10
12
Blood
Brain
Heart
Kidney
Lung
Ovary
Skin
Smuscle
Testis

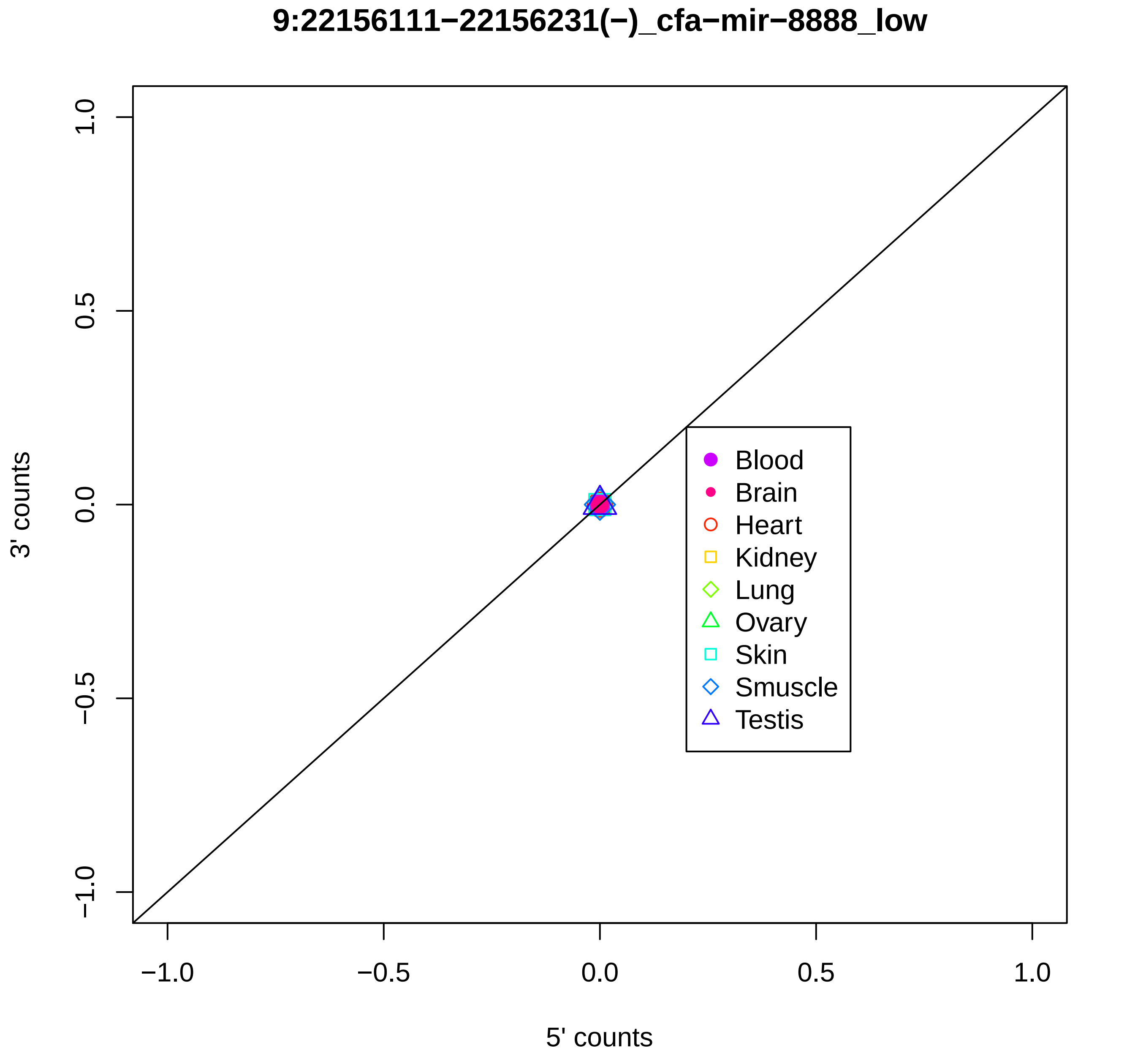

9:22156111−22156231(−)_cfa−mir−8888_low
1.0
0.5
0.0
−0.5
−1.0
3' counts
−1.0
−0.5
0.0
0.5
1.0
5' counts
Blood
Brain
Heart
Kidney
Lung
Ovary
Skin
Smuscle
Testis

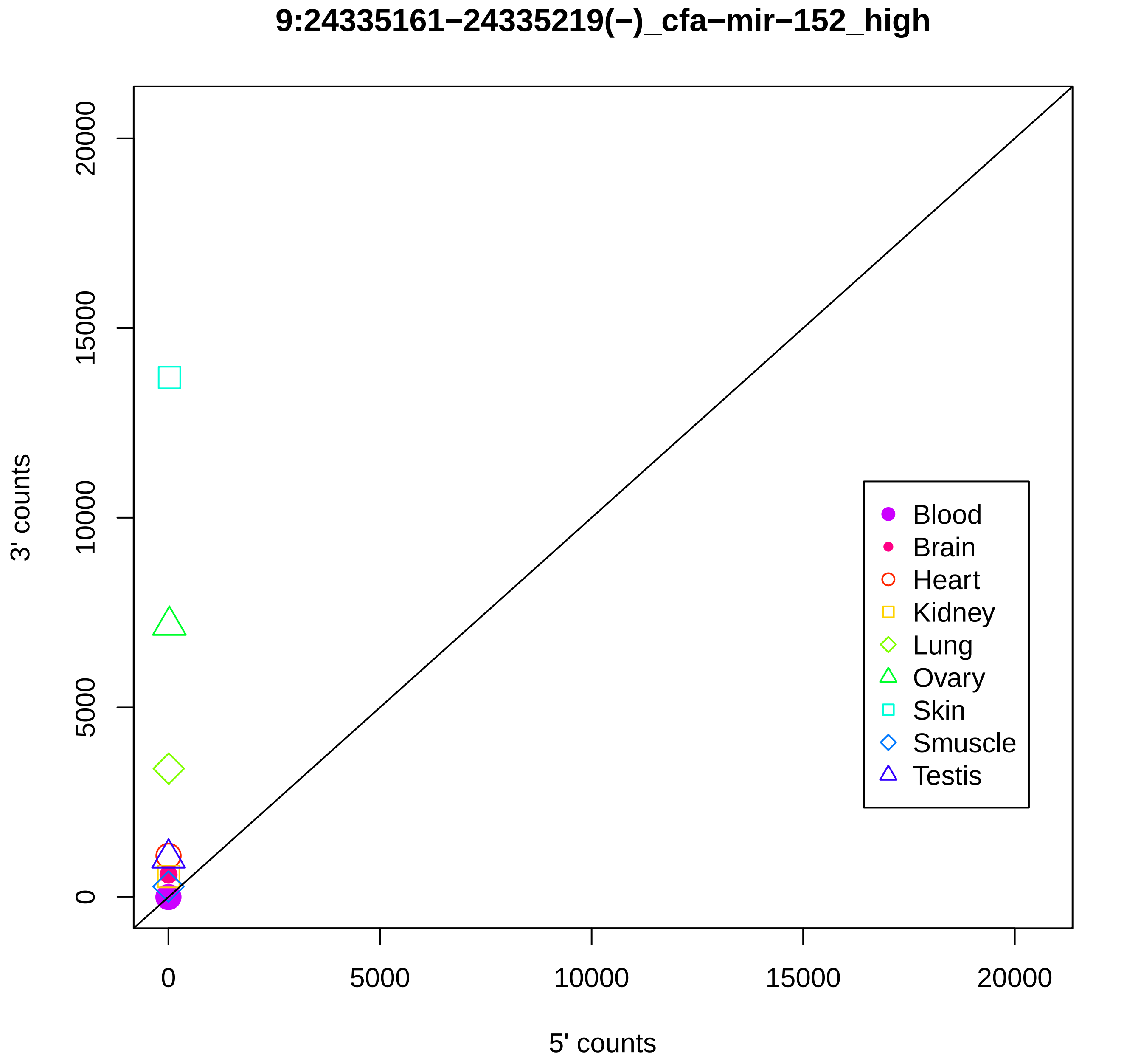

9:24335161−24335219(−)_cfa−mir−152_high
3' counts
5' counts
0
5000
10000
15000
20000
Blood
Brain
Heart
Kidney
Lung
Ovary
Skin
Smuscle
Testis

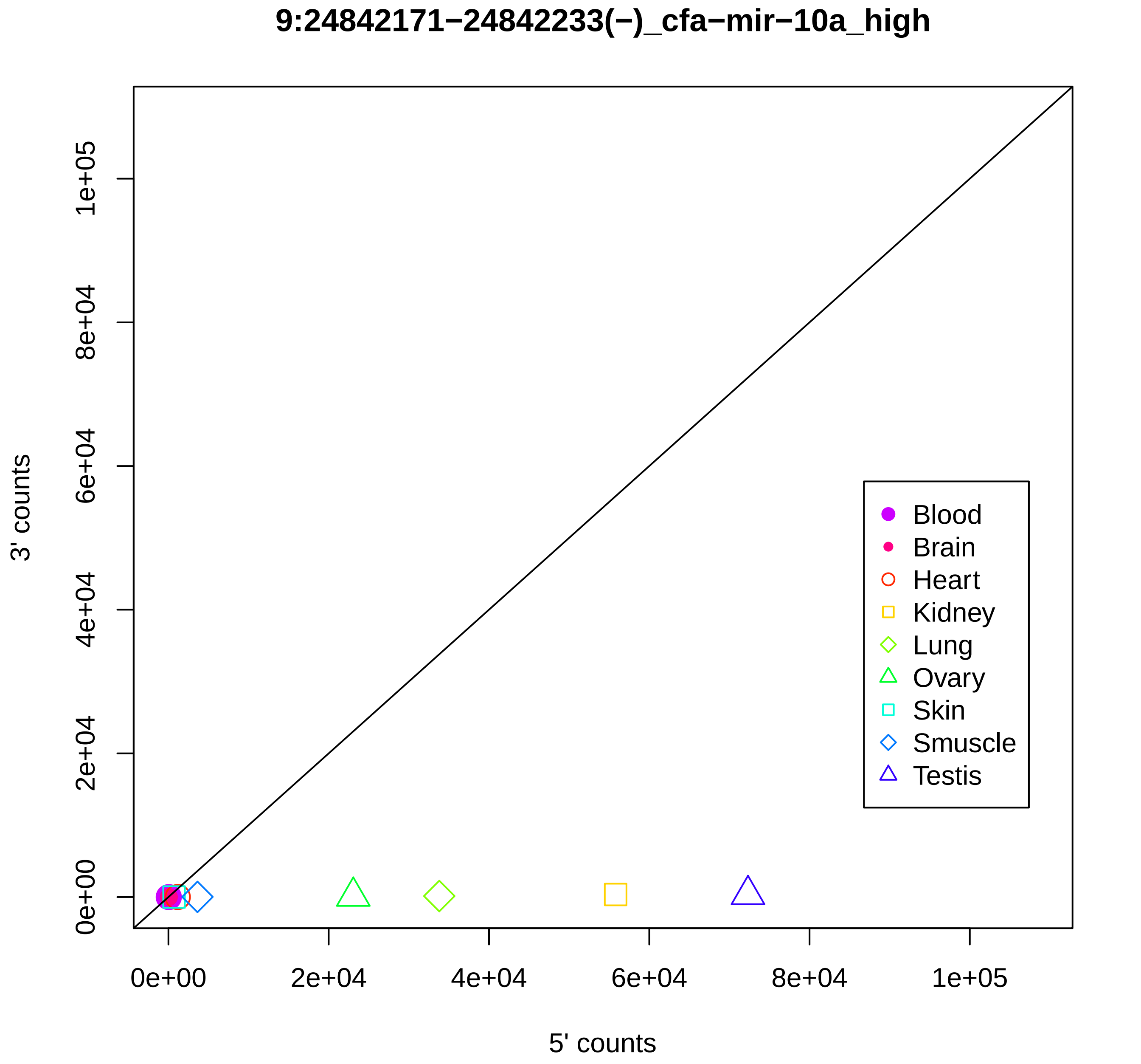
9:24842171−24842233(−)_cfa−mir−10a_high
3' counts
5' counts
0e+00
2e+04
4e+04
6e+04
8e+04
1e+05
Blood
Brain
Heart
Kidney
Lung
Ovary
Skin
Smuscle
Testis

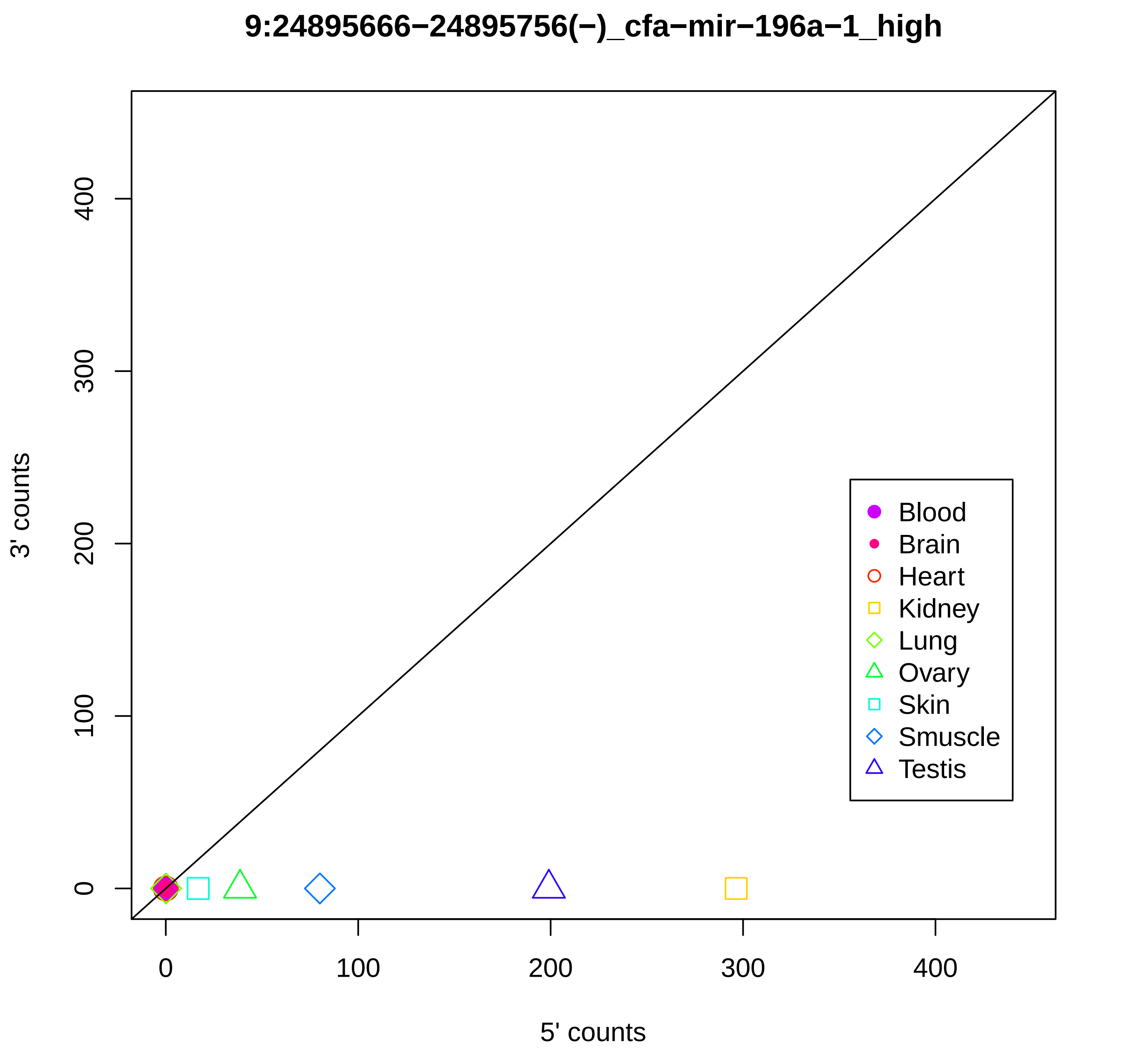
9:24895666−24895756(−)_cfa−mir−196a−1_high
3' counts
5' counts
0
100
200
300
400
0
100
200
300
400
Blood
Brain
Heart
Kidney
Lung
Ovary
Skin
Smuscle
Testis

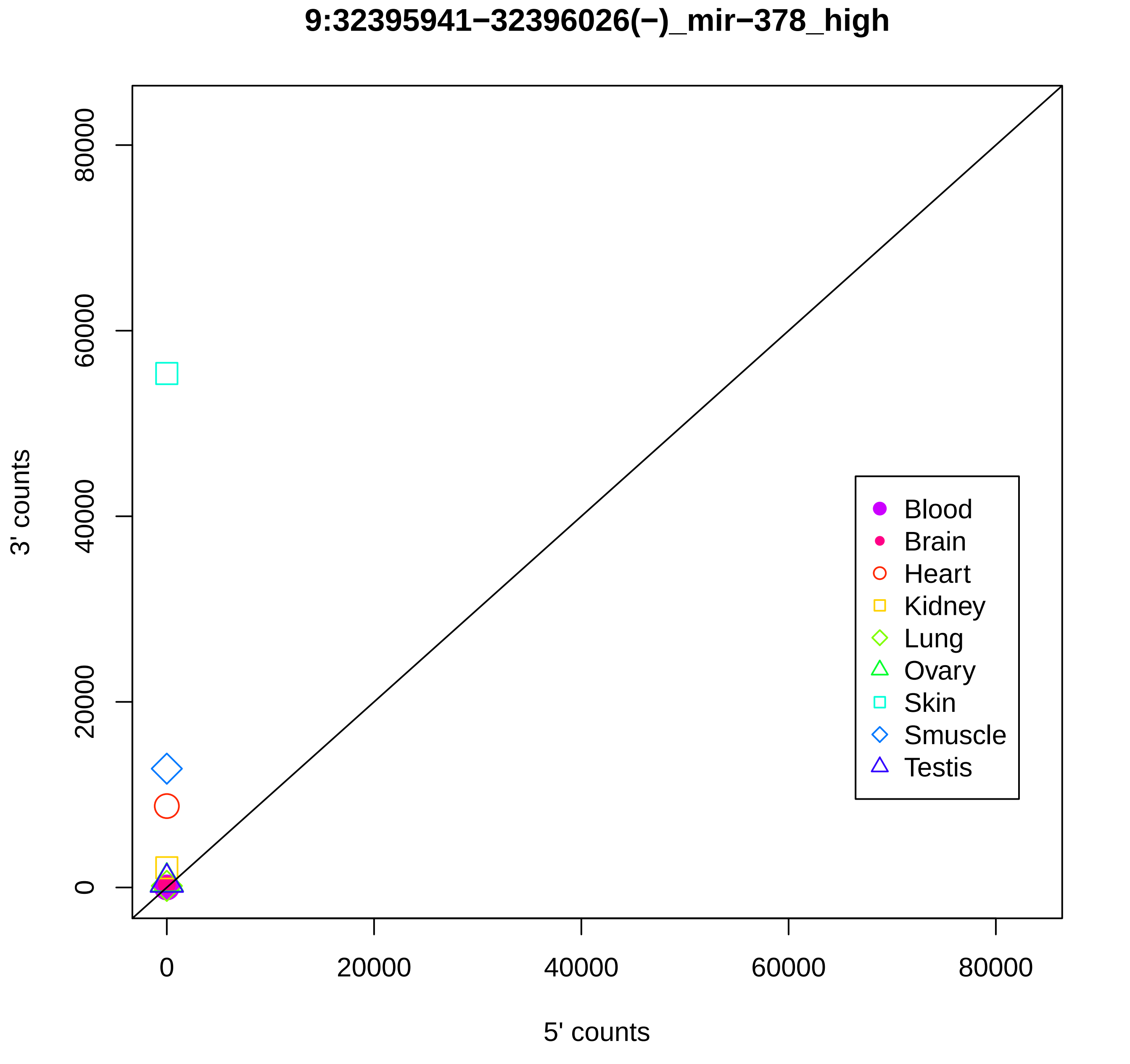

9:32395941−32396026(−)_mir−378_high
3' counts
5' counts
0
20000
40000
60000
80000
0
20000
40000
60000
80000
Blood
Brain
Heart
Kidney
Lung
Ovary
Skin
Smuscle
Testis

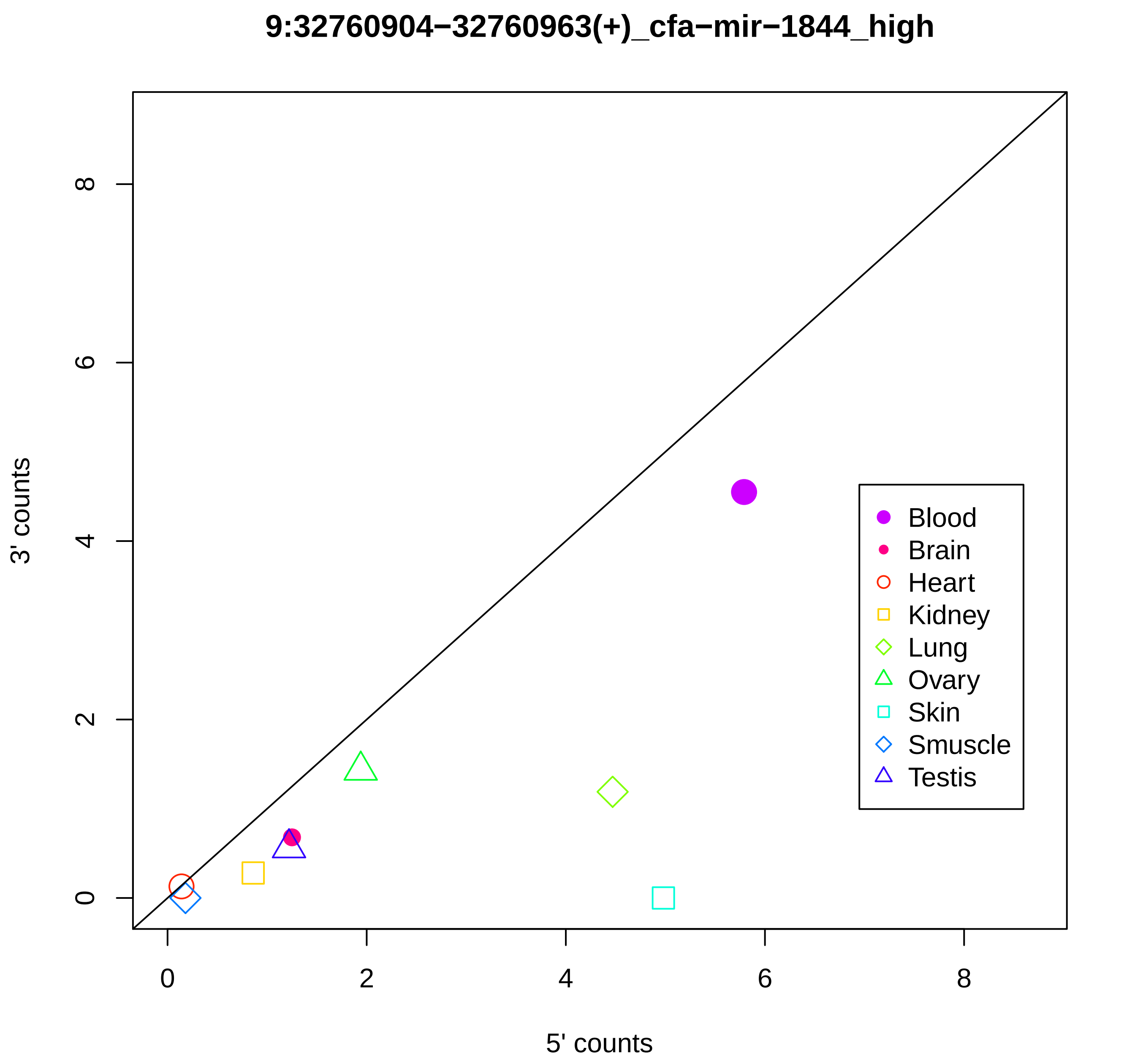

9:32760904−32760963(+)_cfa−mir−1844_high
3' counts
5' counts
0
2
4
6
8
0
2
4
6
8
Blood
Brain
Heart
Kidney
Lung
Ovary
Skin
Smuscle
Testis

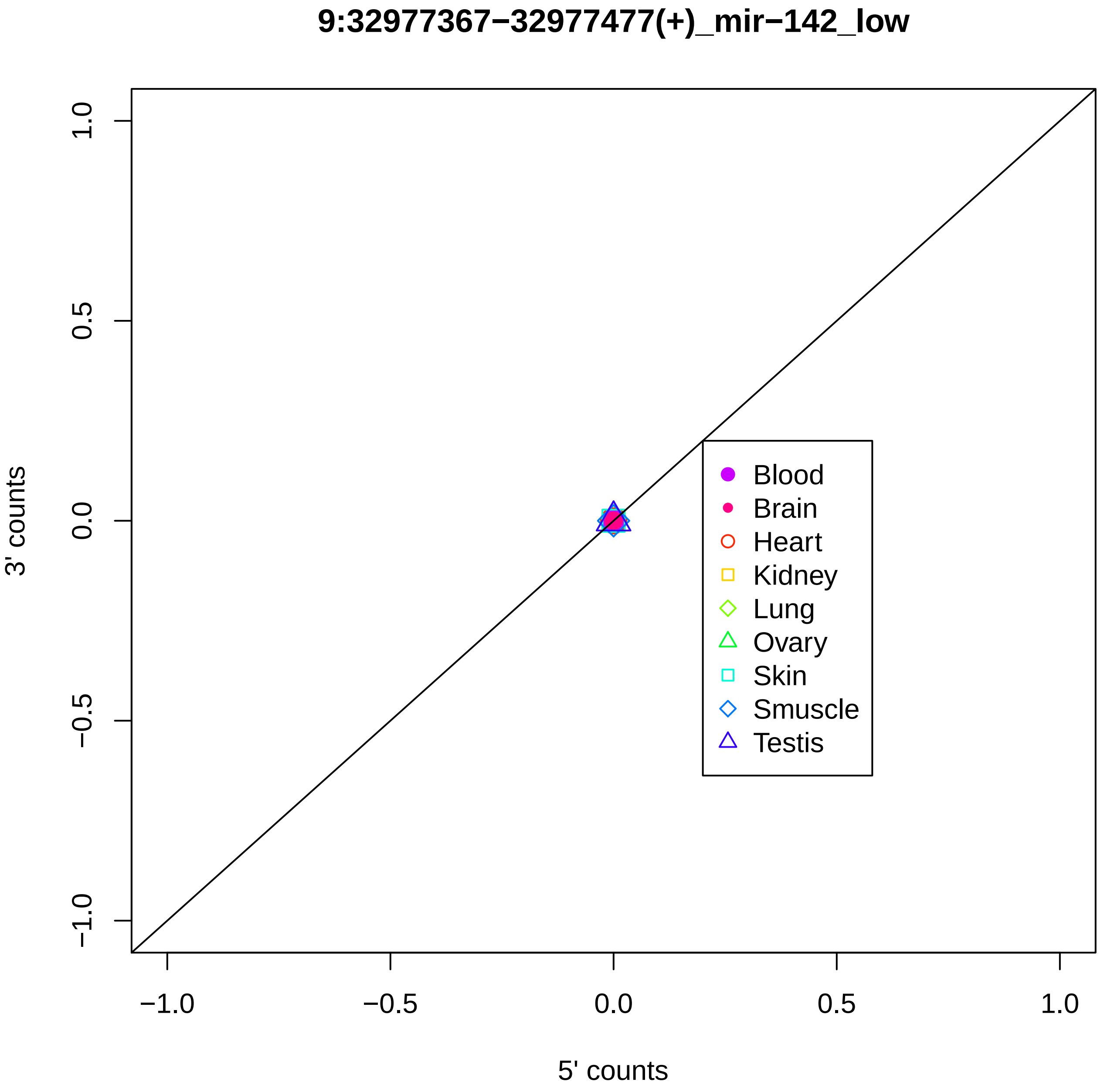

9:32977367−32977477(+)_mir−142_low
3' counts
1.0
0.5
0.0
−0.5
−1.0
5' counts
−1.0
−0.5
0.0
0.5
1.0
Blood
Brain
Heart
Kidney
Lung
Ovary
Skin
Smuscle
Testis

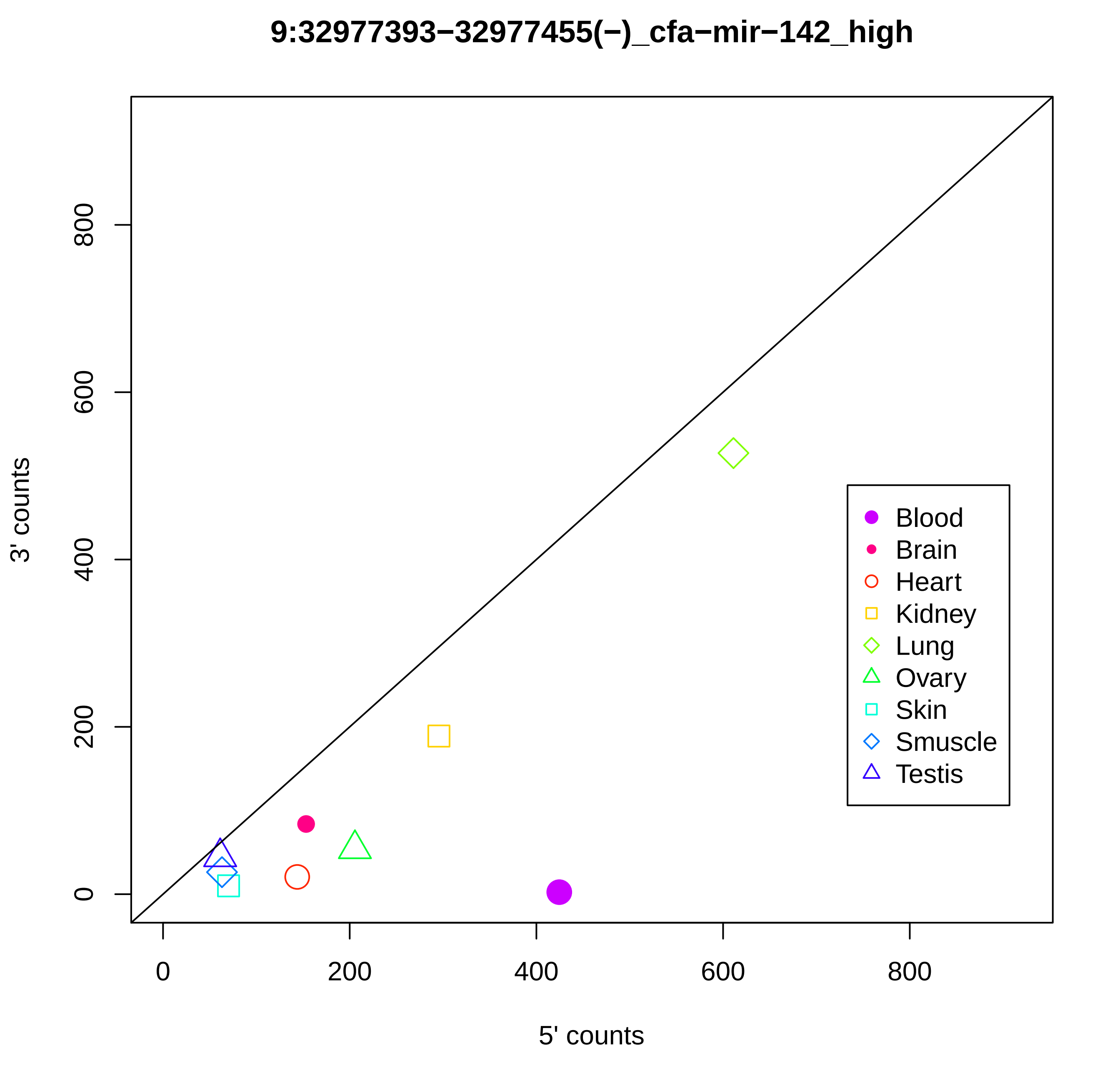

9:32977393−32977455(−)_cfa−mir−142_high
3' counts
5' counts
0
200
400
600
800
Blood
Brain
Heart
Kidney
Lung
Ovary
Skin
Smuscle
Testis

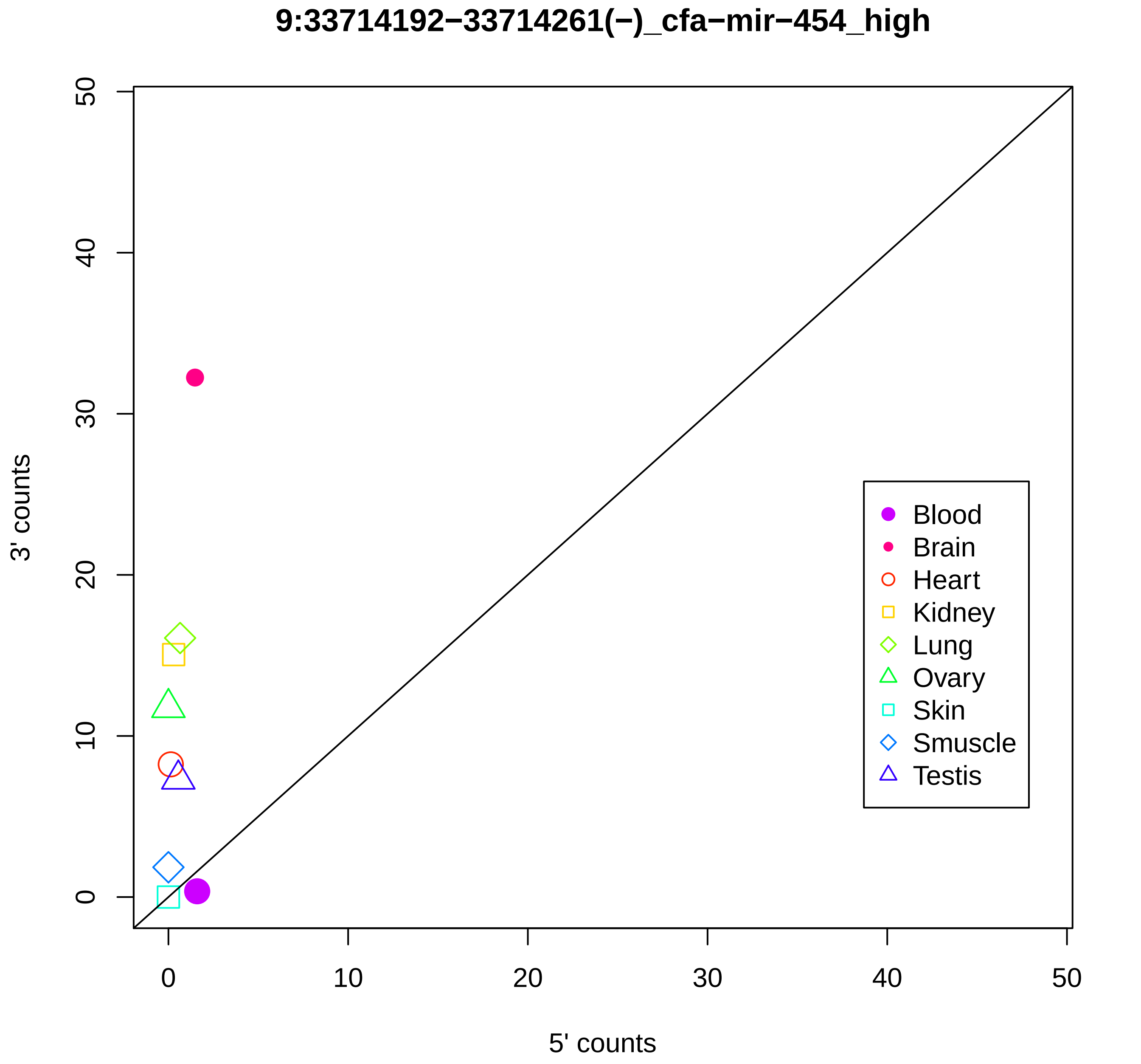

9:33714192−33714261(−)_cfa−mir−454_high
3' counts
5' counts
0
10
20
30
40
50
0
10
20
30
40
50
Blood
Brain
Heart
Kidney
Lung
Ovary
Skin
Smuscle
Testis

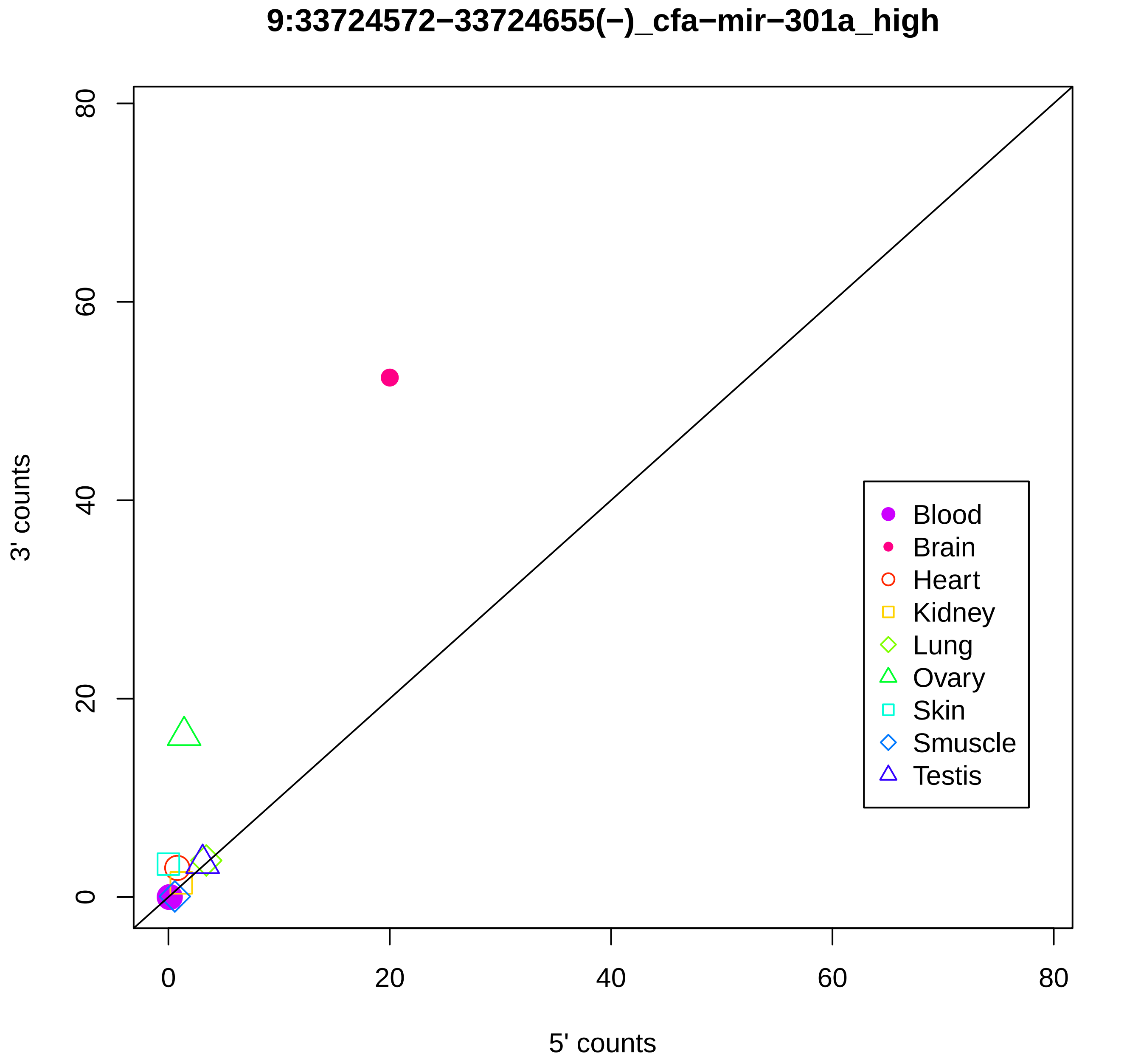
9:33724572−33724655(−)_cfa−mir−301a_high
3' counts
5' counts
0
20
40
60
80
0
20
40
60
80
Blood
Brain
Heart
Kidney
Lung
Ovary
Skin
Smuscle
Testis

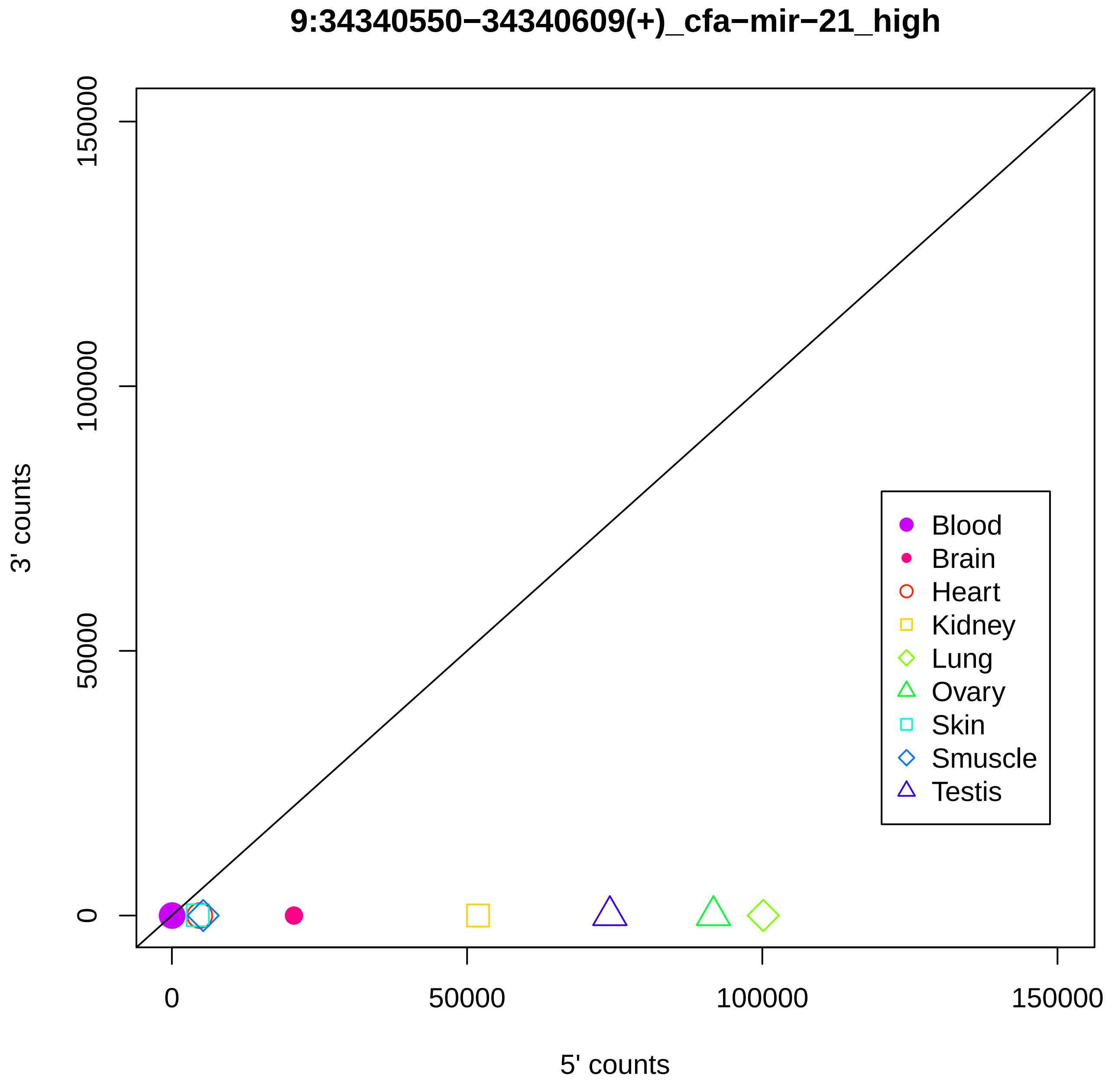

9:34340550−34340609(+)_cfa−mir−21_high
3' counts
5' counts
0
50000
100000
150000
0
50000
100000
150000
Blood
Brain
Heart
Kidney
Lung
Ovary
Skin
Smuscle
Testis

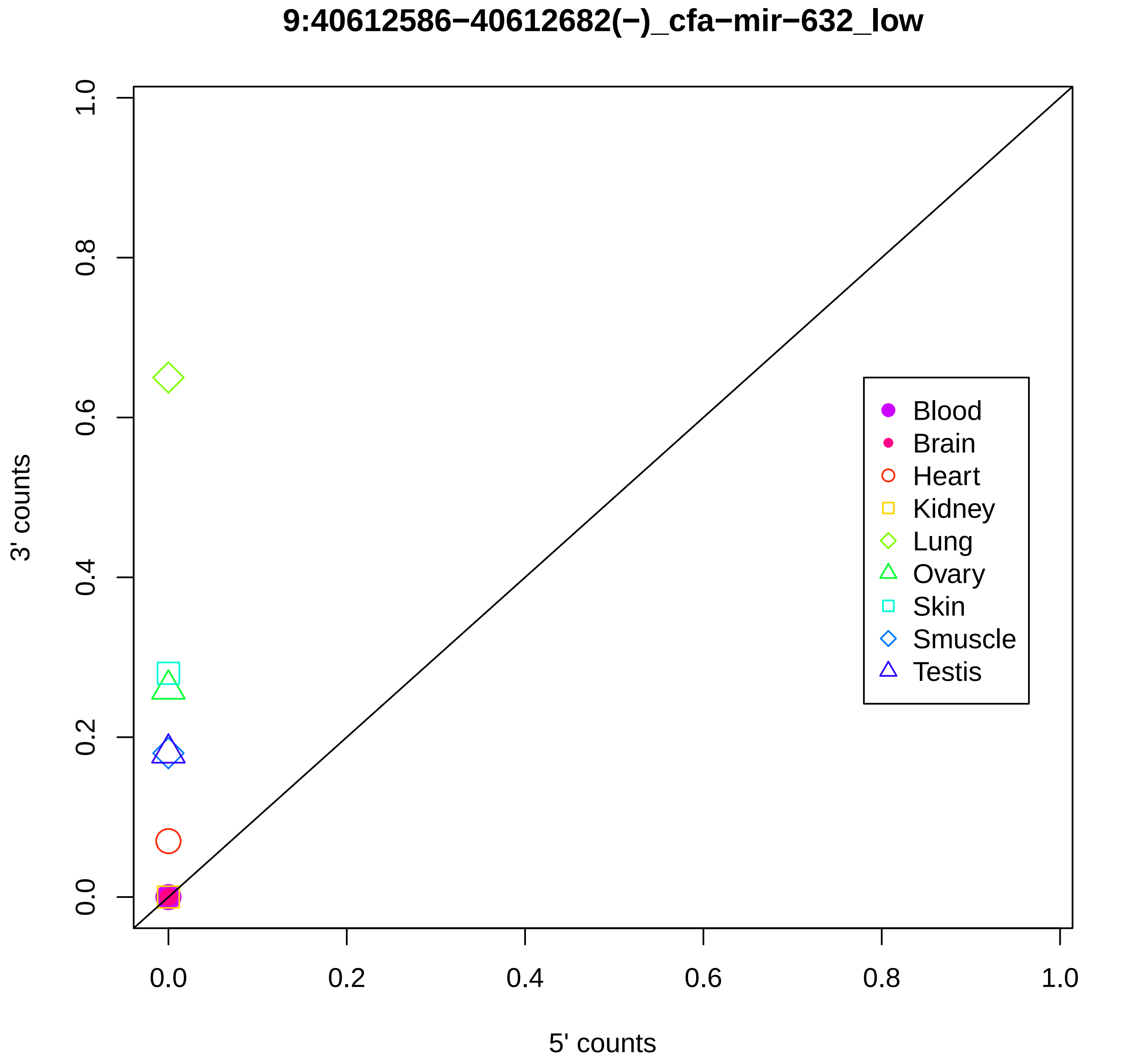

9:40612586−40612682(−)_cfa−mir−632_low
3' counts
5' counts
0.0
0.2
0.4
0.6
0.8
1.0
Blood
Brain
Heart
Kidney
Lung
Ovary
Skin
Smuscle
Testis

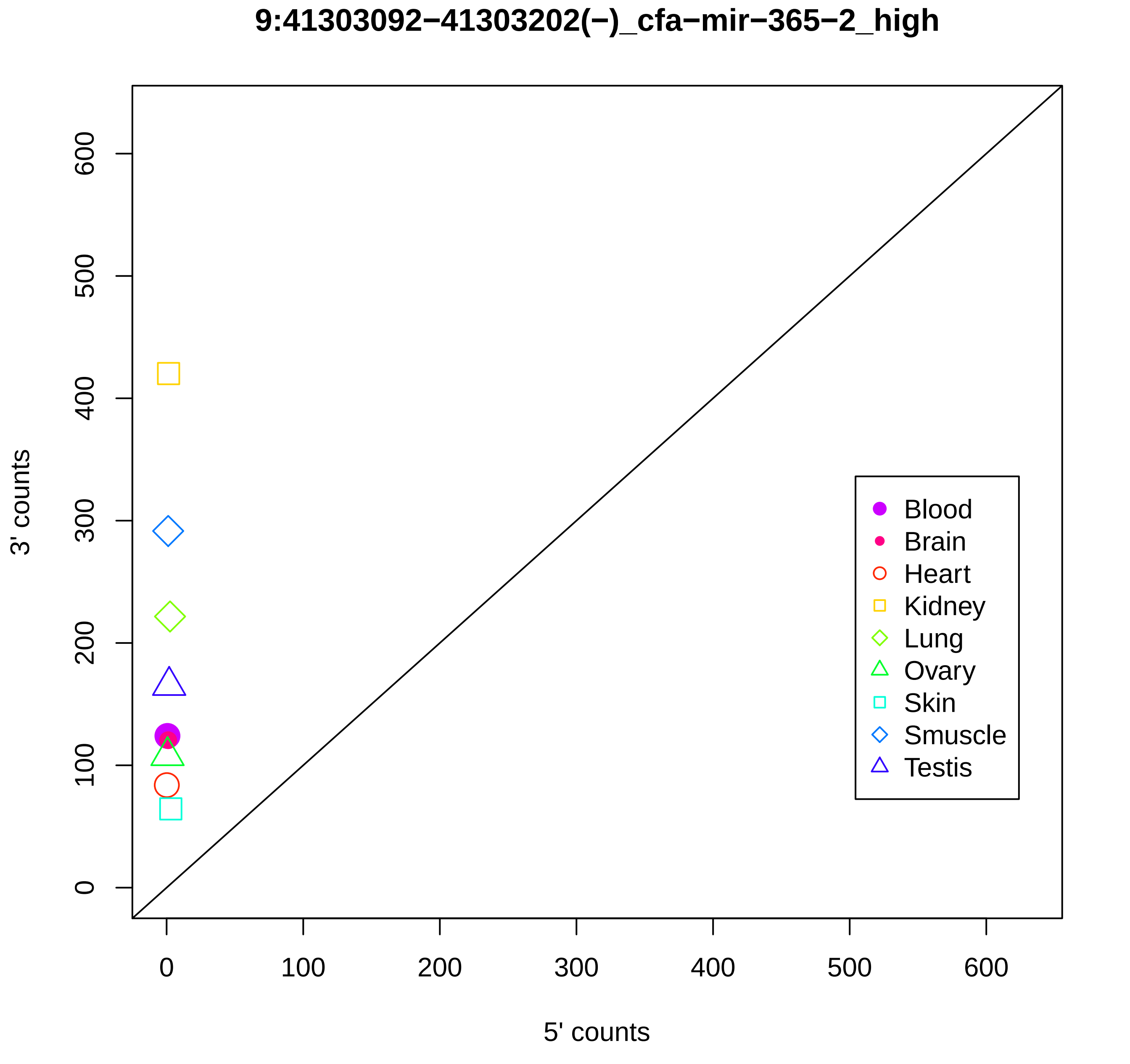

9:41303092−41303202(−)_cfa−mir−365−2_high
3' counts
5' counts
0
100
200
300
400
500
600
Blood
Brain
Heart
Kidney
Lung
Ovary
Skin
Smuscle
Testis

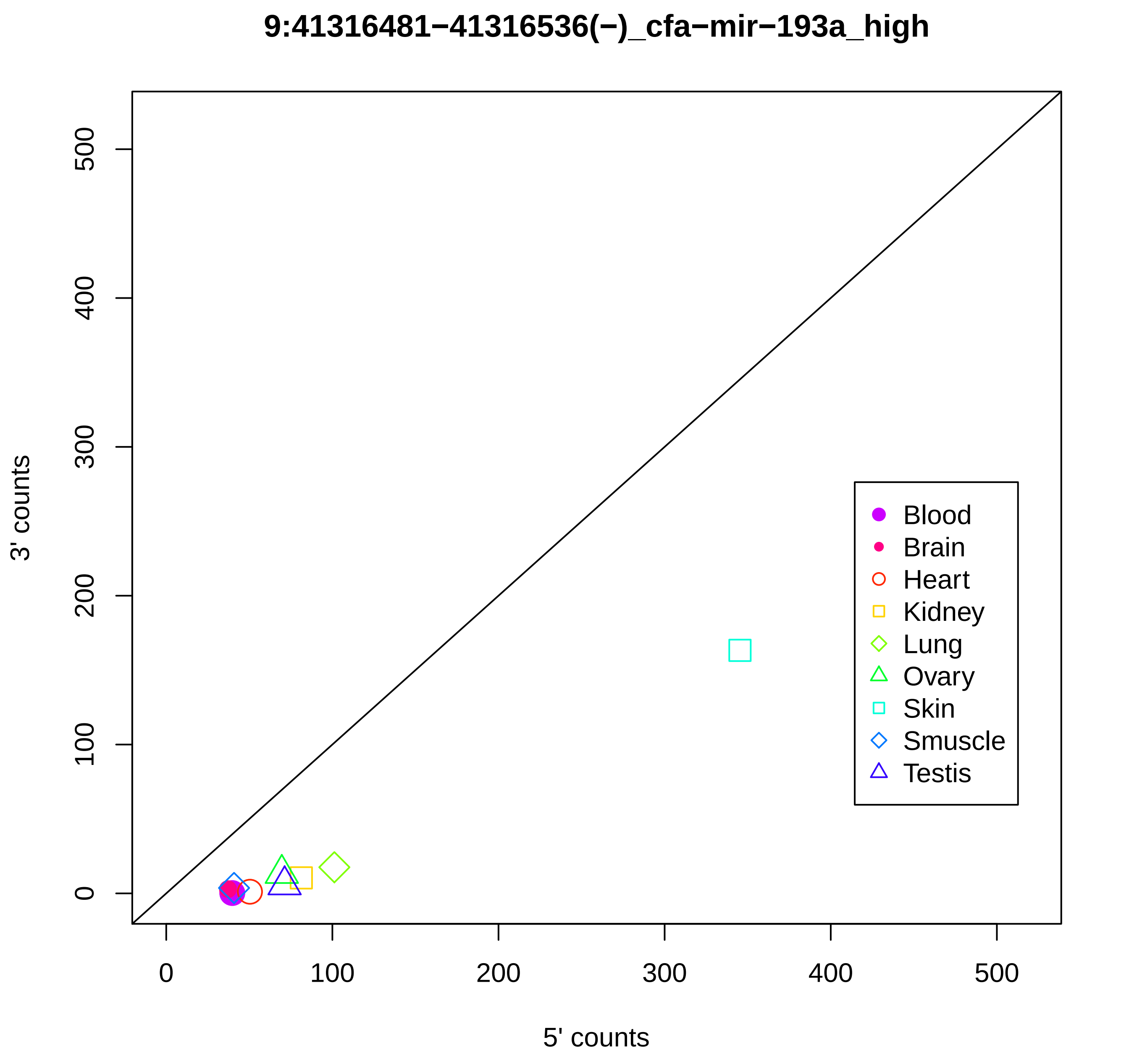

9:41316481−41316536(−)_cfa−mir−193a_high
3' counts
5' counts
0
100
200
300
400
500
0
100
200
300
400
500
Blood
Brain
Heart
Kidney
Lung
Ovary
Skin
Smuscle
Testis

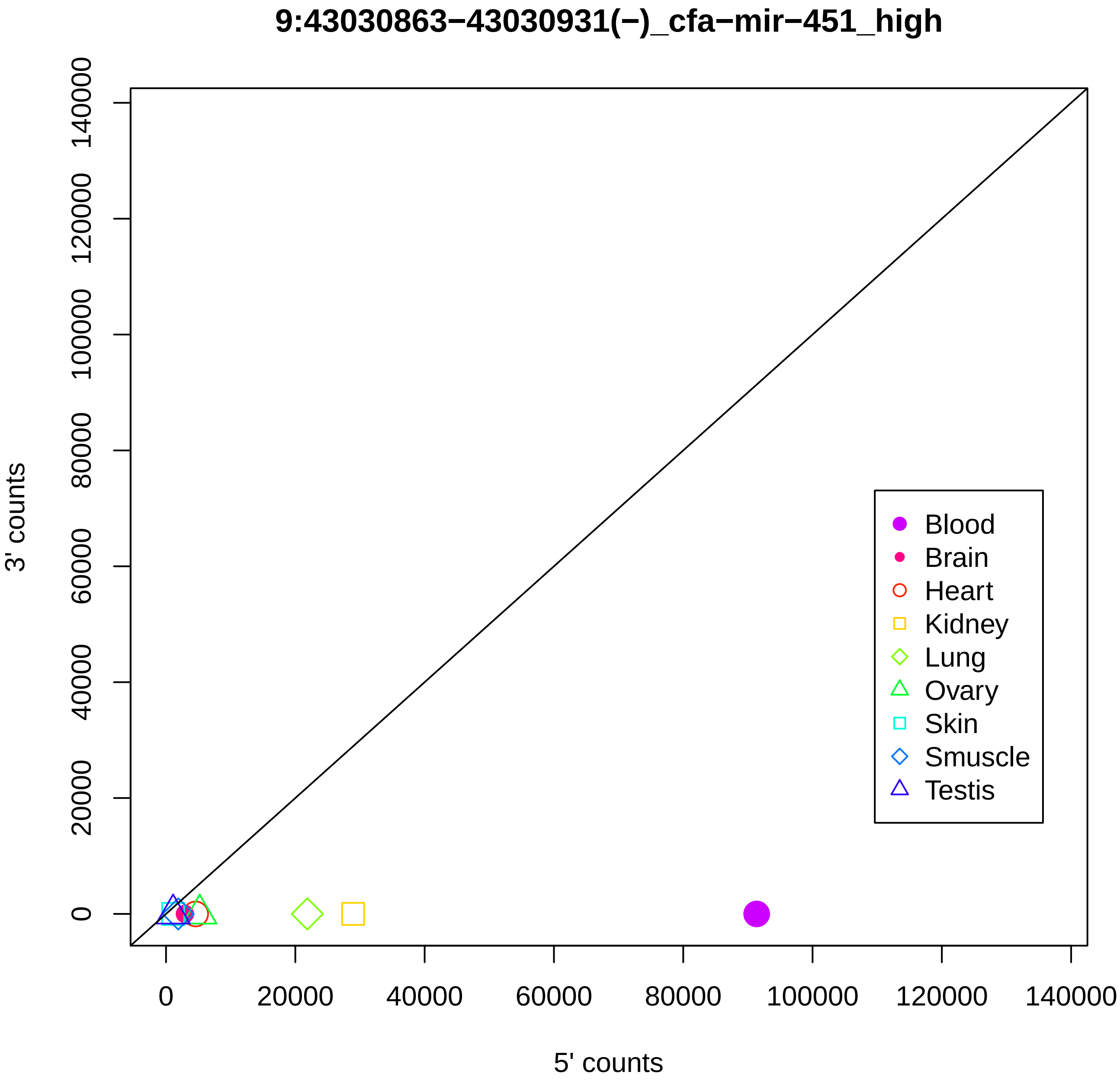
9:43030863−43030931(−)_cfa−mir−451_high
3' counts
5' counts
0
20000
40000
60000
80000
100000
120000
140000
Blood
Brain
Heart
Kidney
Lung
Ovary
Skin
Smuscle
Testis

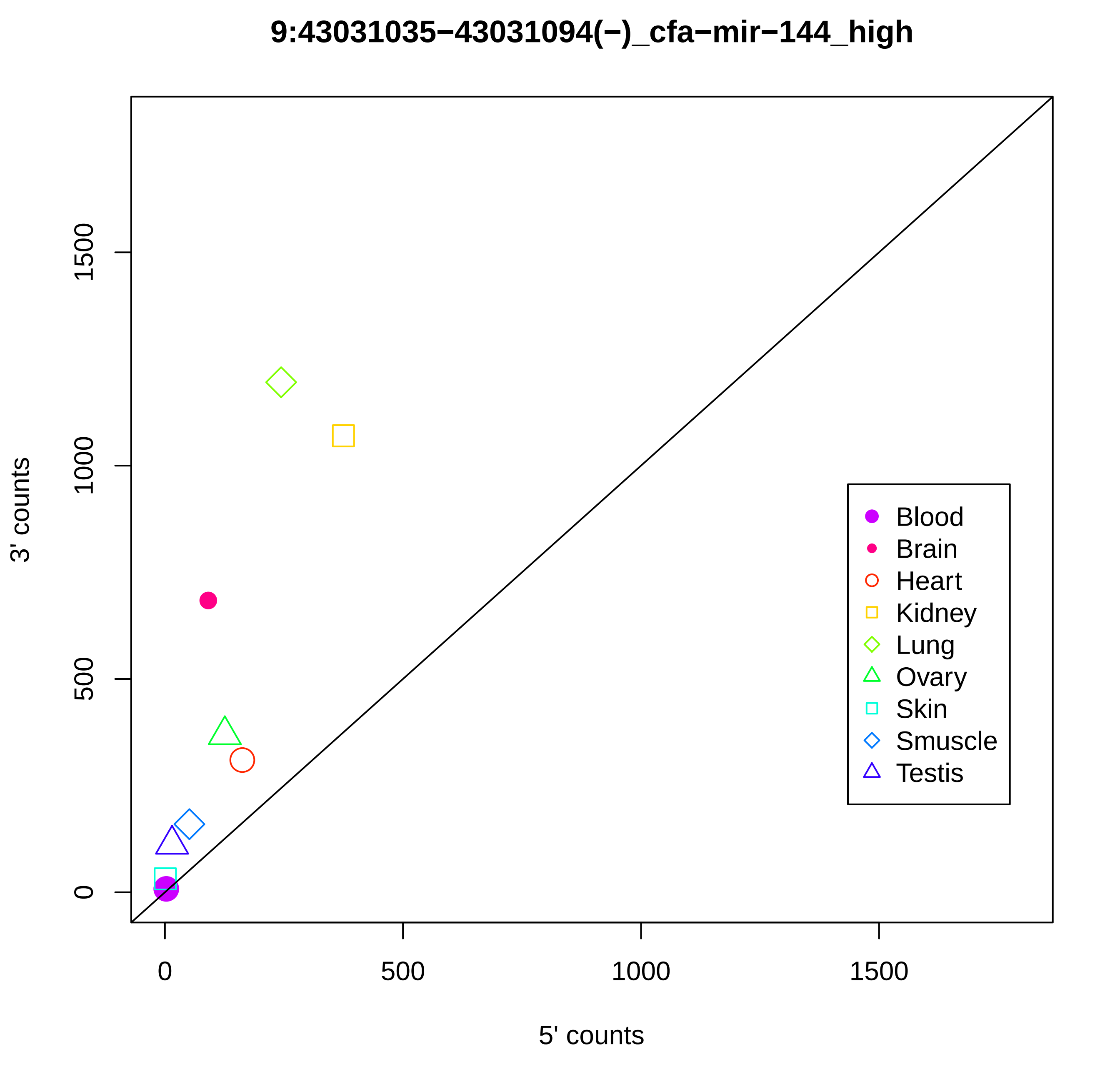

9:43031035−43031094(−)_cfa−mir−144_high
3' counts
5' counts
0
500
1000
1500
0
500
1000
1500
Blood
Brain
Heart
Kidney
Lung
Ovary
Skin
Smuscle
Testis

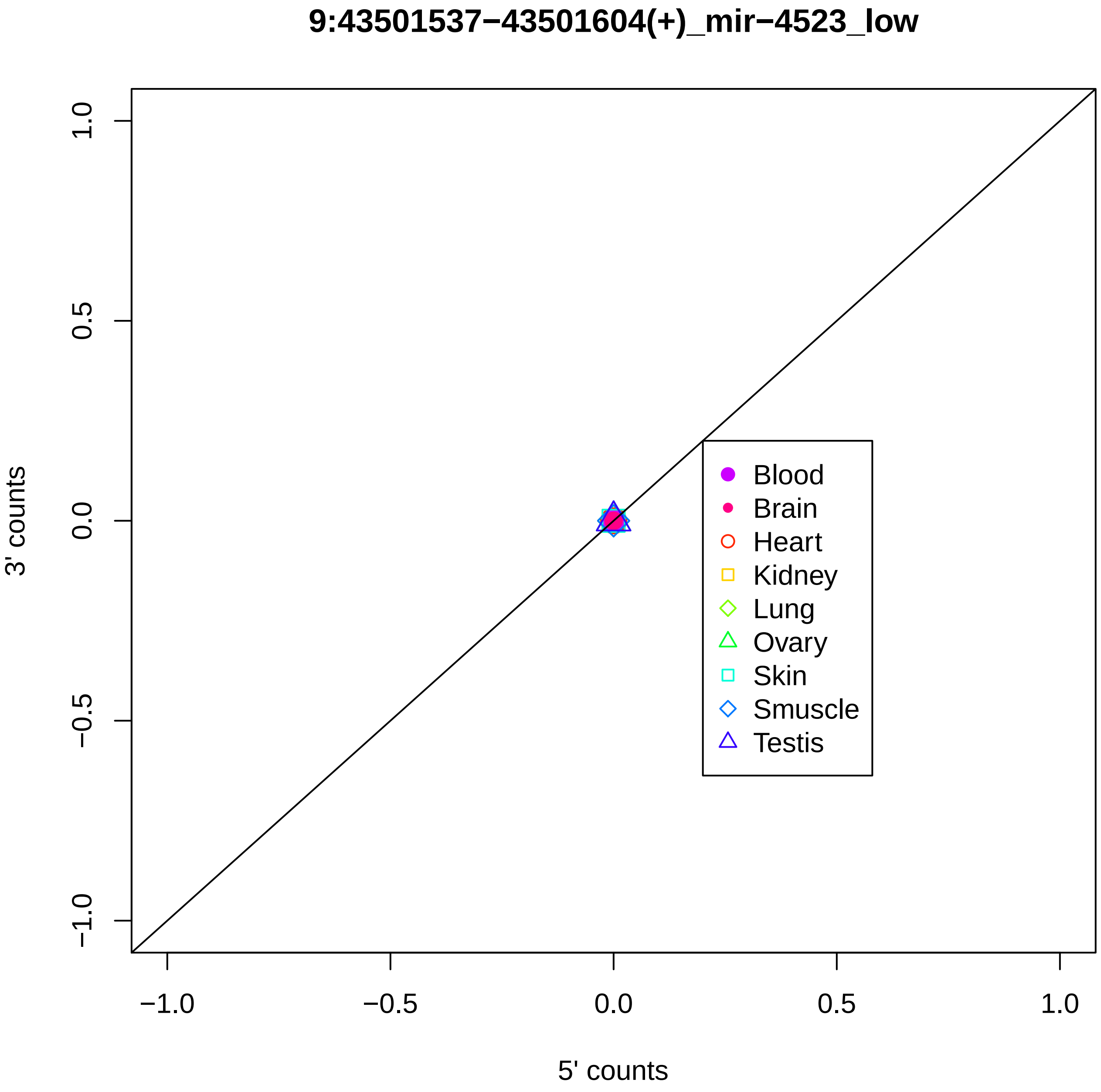
9:43501537−43501604(+)_mir−4523_low
1.0
0.5
0.0
−0.5
−1.0
3' counts
−1.0
−0.5
0.0
0.5
1.0
5' counts
Blood
Brain
Heart
Kidney
Lung
Ovary
Skin
Smuscle
Testis

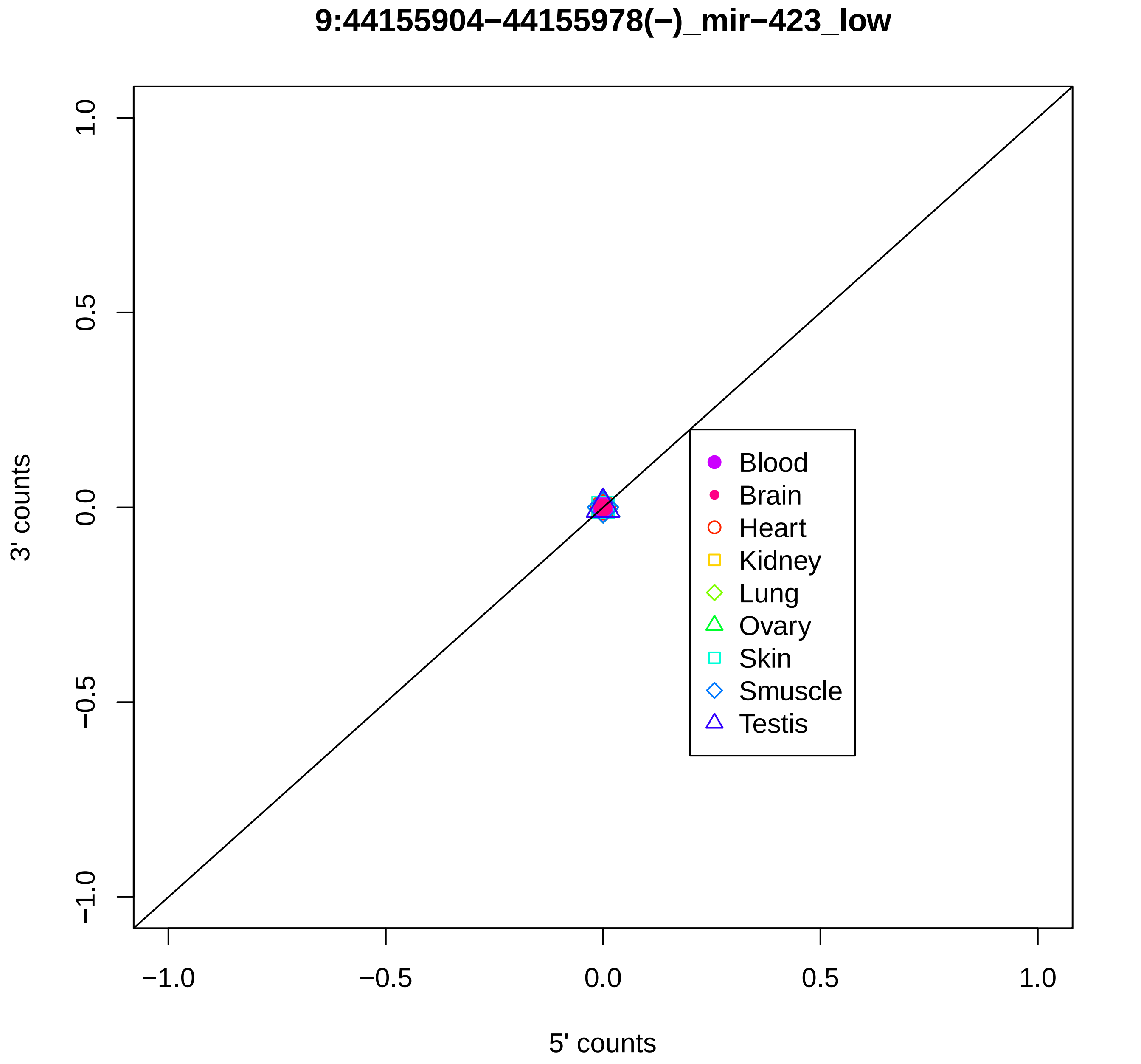

9:44155904−44155978(−)_mir−423_low
3' counts
5' counts
−1.0
−0.5
0.0
0.5
1.0
Blood
Brain
Heart
Kidney
Lung
Ovary
Skin
Smuscle
Testis

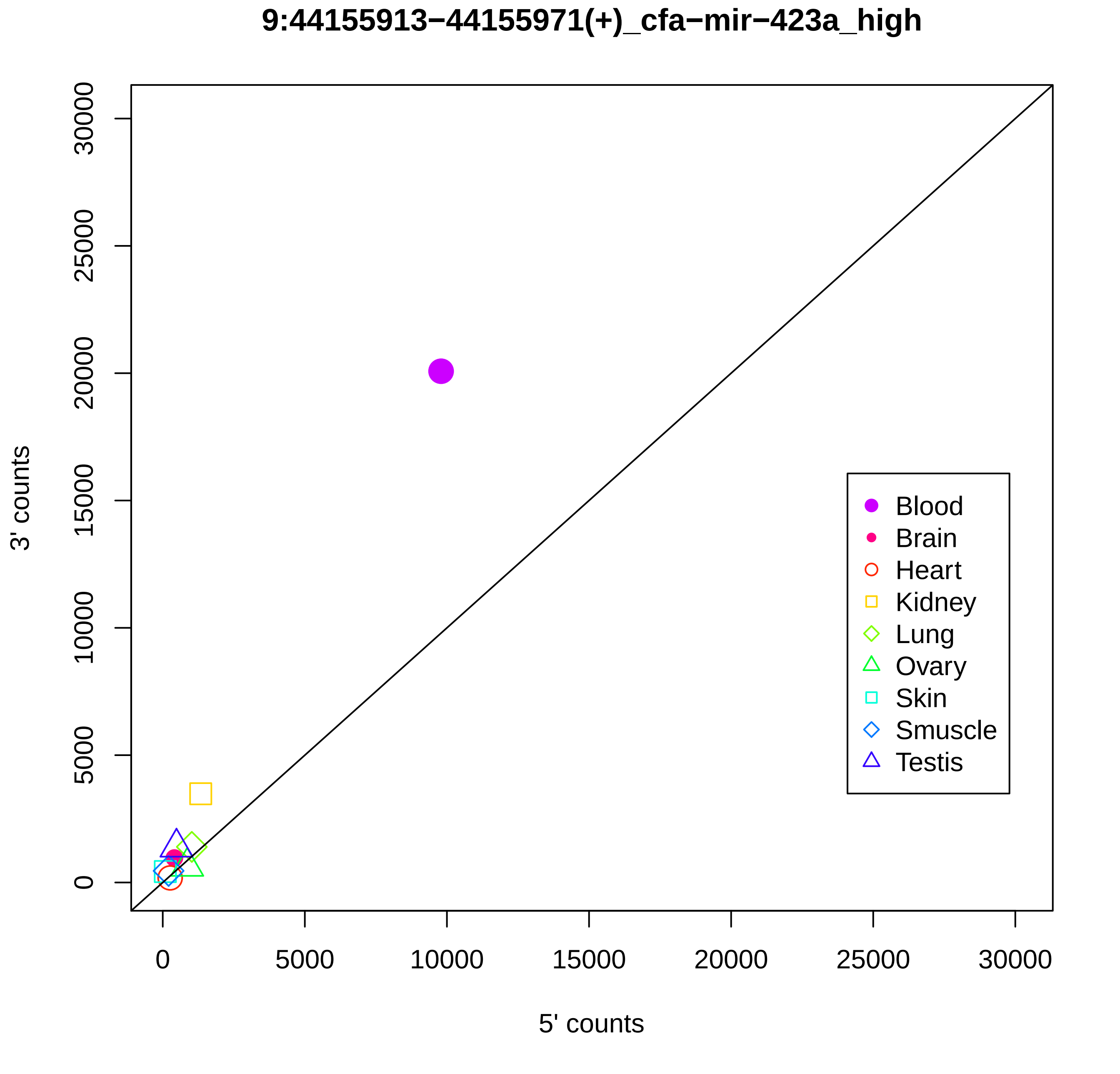

9:44155913−44155971(+)_cfa−mir−423a_high
3' counts
5' counts
0
5000
10000
15000
20000
25000
30000
Blood
Brain
Heart
Kidney
Lung
Ovary
Skin
Smuscle
Testis

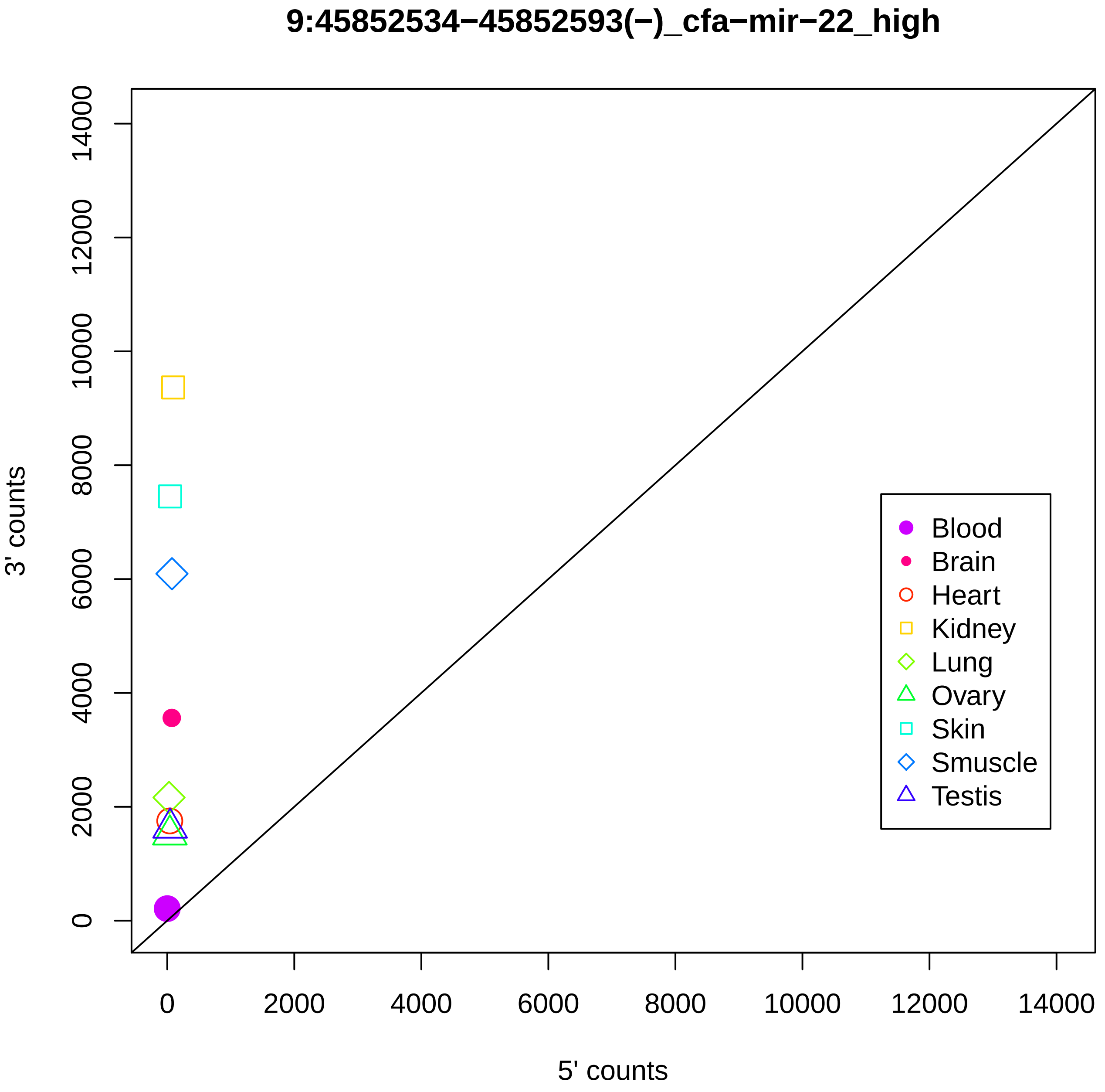
9:45852534−45852593(−)_cfa−mir−22_high
3' counts
0
2000
4000
6000
8000
10000
12000
14000
5' counts
Blood
Brain
Heart
Kidney
Lung
Ovary
Skin
Smuscle
Testis

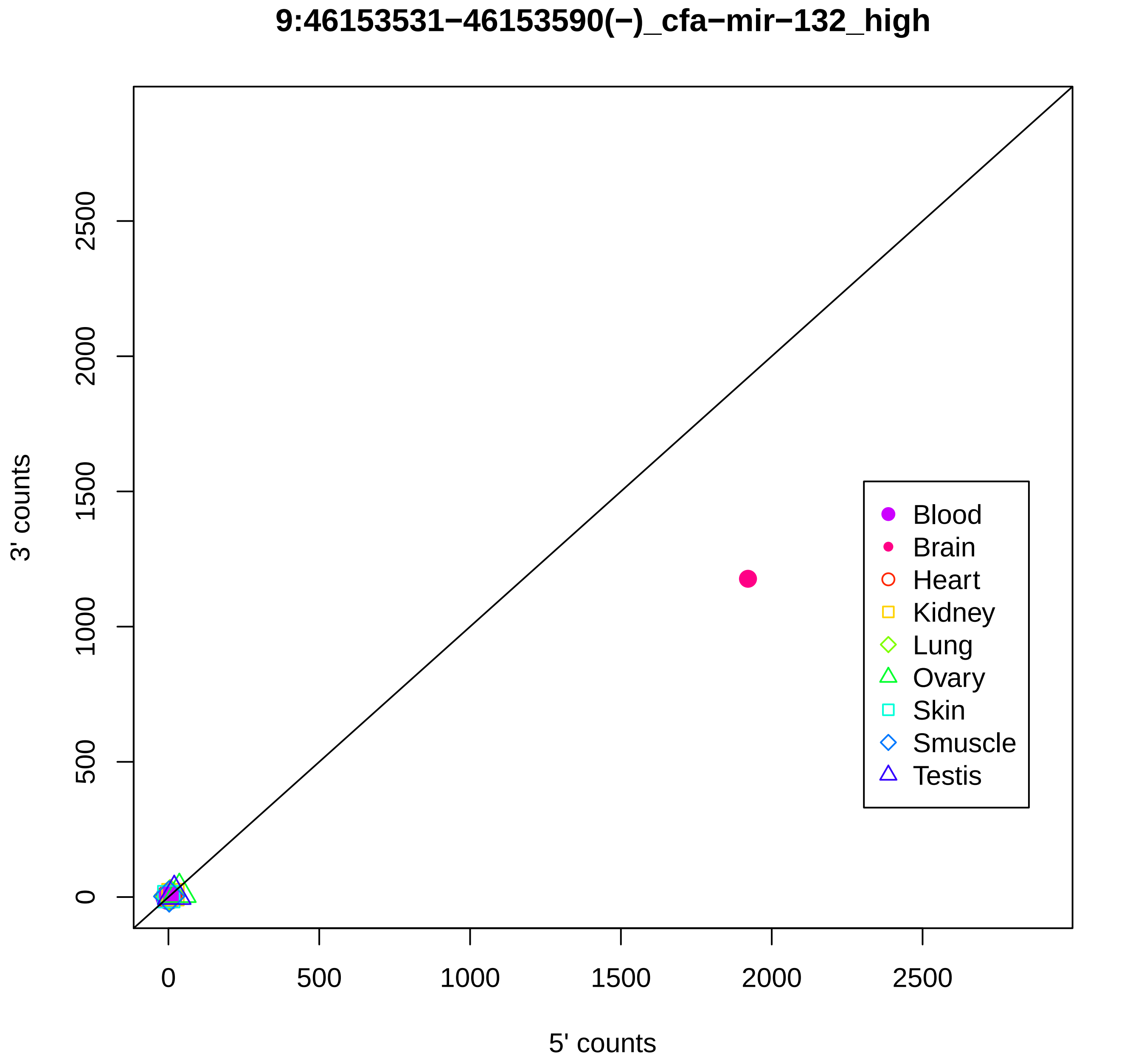

9:46153531−46153590(−)_cfa−mir−132_high
3' counts
5' counts
0
500
1000
1500
2000
2500
Blood
Brain
Heart
Kidney
Lung
Ovary
Skin
Smuscle
Testis

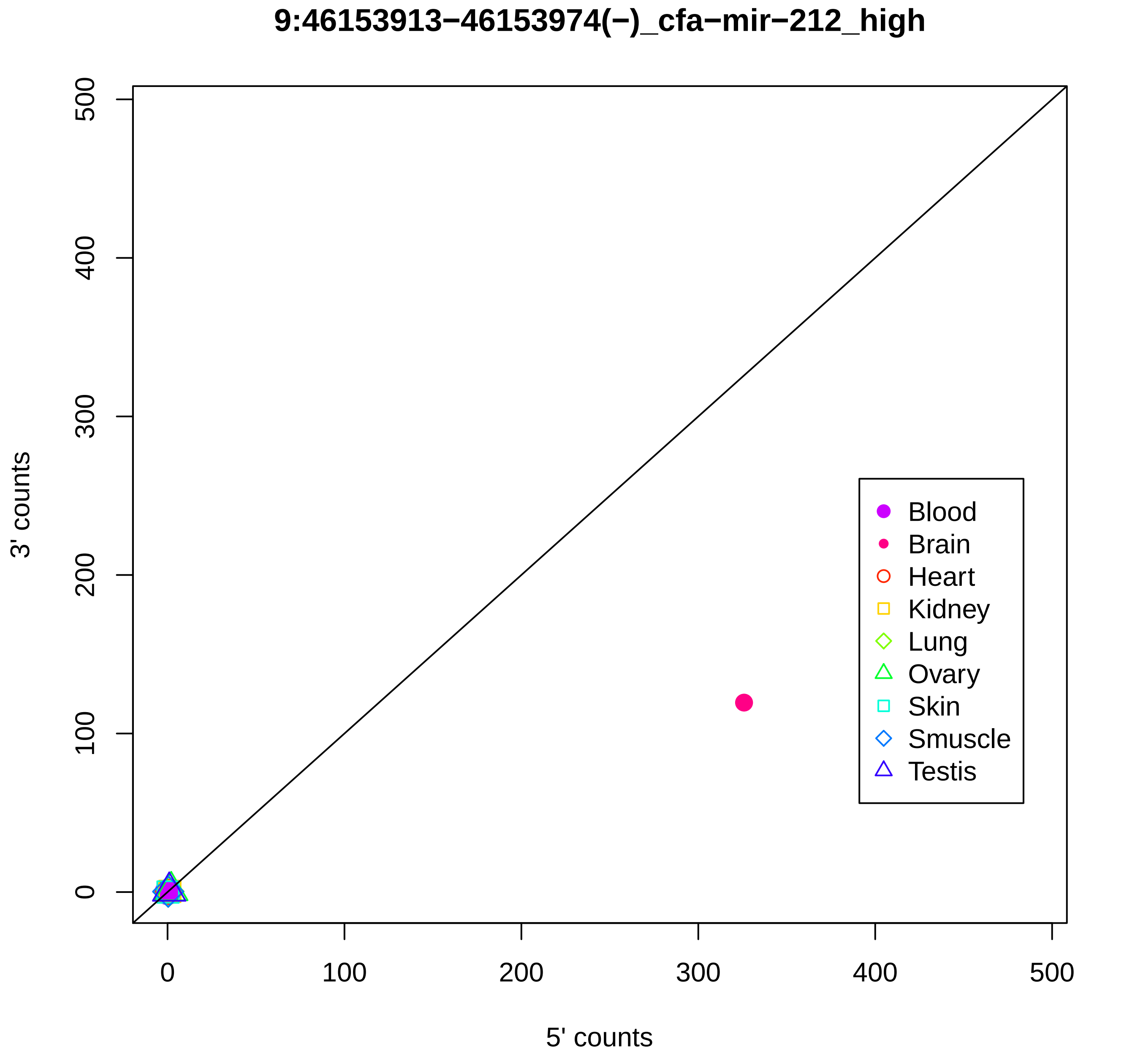

9:46153913−46153974(−)_cfa−mir−212_high
3' counts
5' counts
0
100
200
300
400
500
0
100
200
300
400
500
Blood
Brain
Heart
Kidney
Lung
Ovary
Skin
Smuscle
Testis

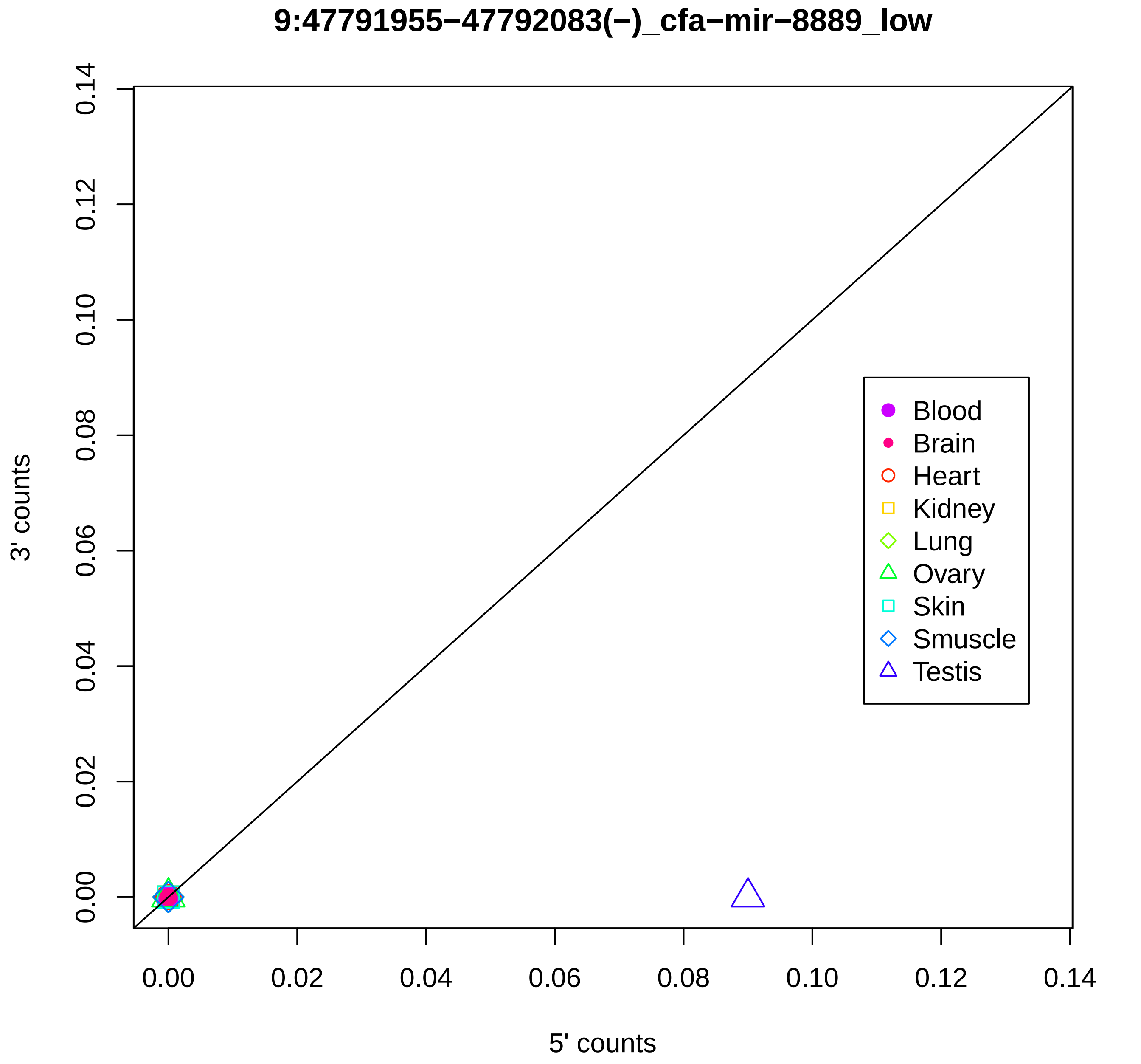

9:47791955−47792083(−)_cfa−mir−8889_low
3' counts
5' counts
0.00
0.02
0.04
0.06
0.08
0.10
0.12
0.14
Blood
Brain
Heart
Kidney
Lung
Ovary
Skin
Smuscle
Testis

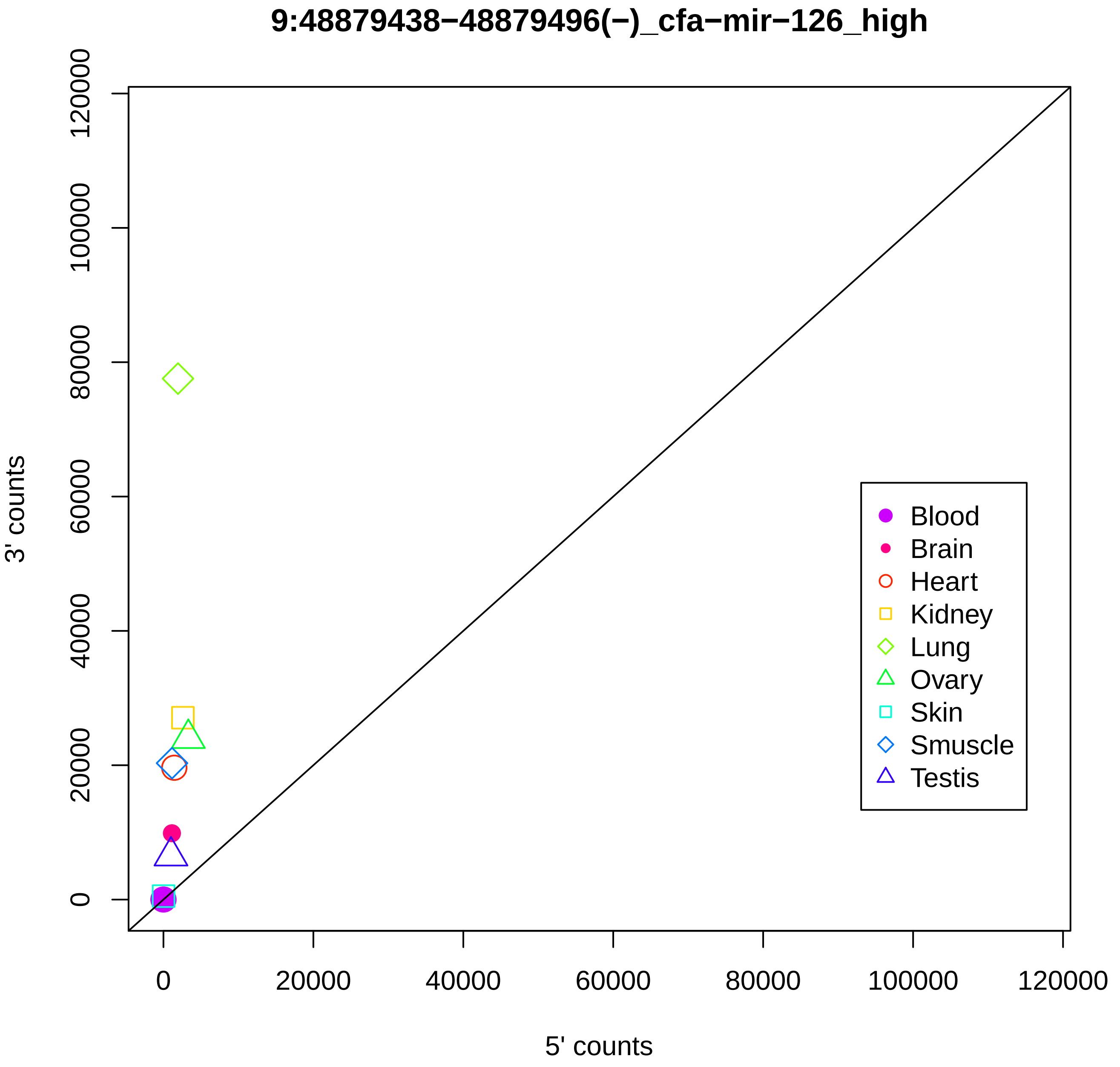

9:48879438−48879496(−)_cfa−mir−126_high
3' counts
5' counts
0
20000
40000
60000
80000
100000
120000
Blood
Brain
Heart
Kidney
Lung
Ovary
Skin
Smuscle
Testis

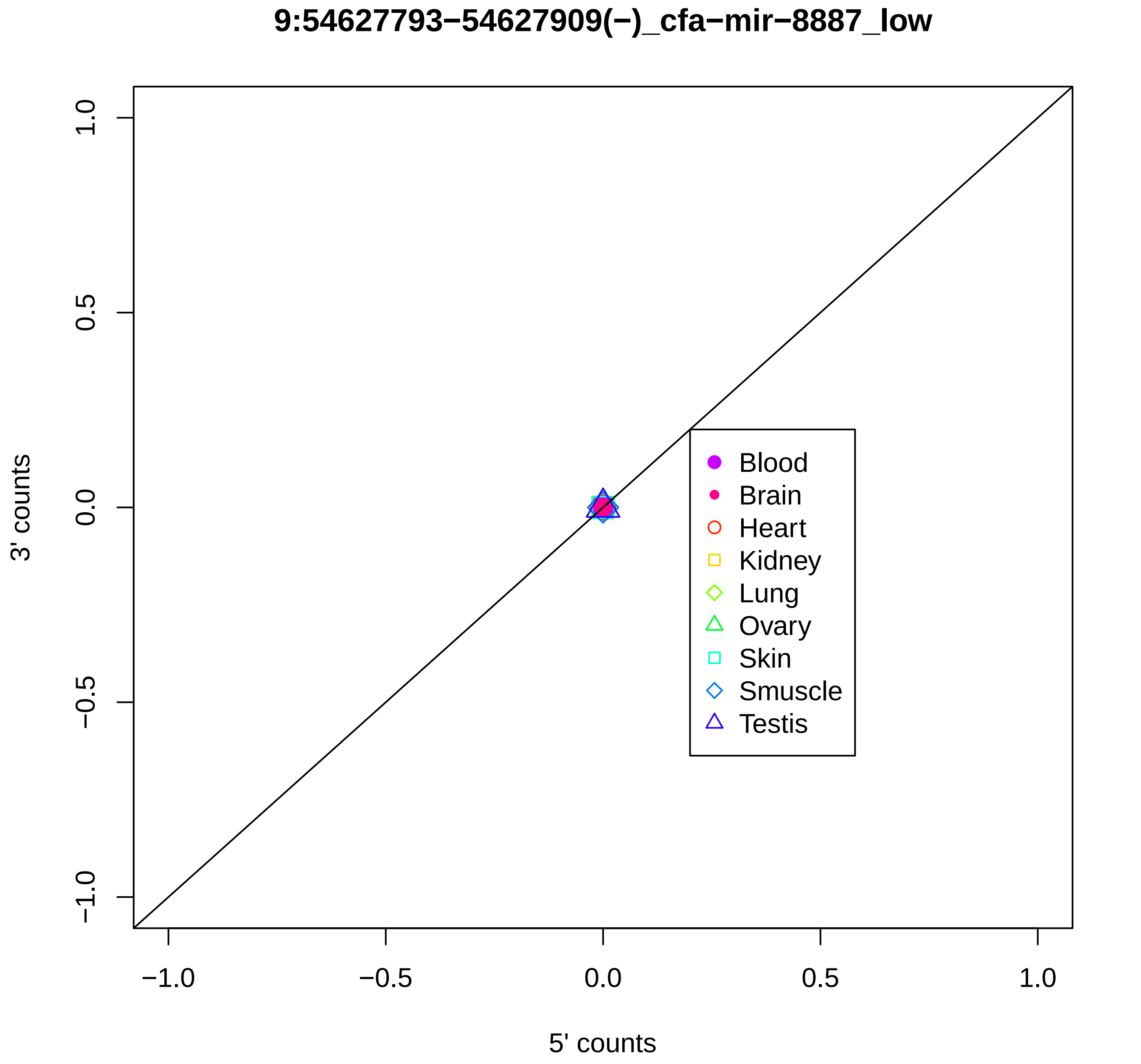

9:54627793−54627909(−)_cfa−mir−8887_low
3' counts
5' counts
−1.0
−0.5
0.0
0.5
1.0
Blood
Brain
Heart
Kidney
Lung
Ovary
Skin
Smuscle
Testis

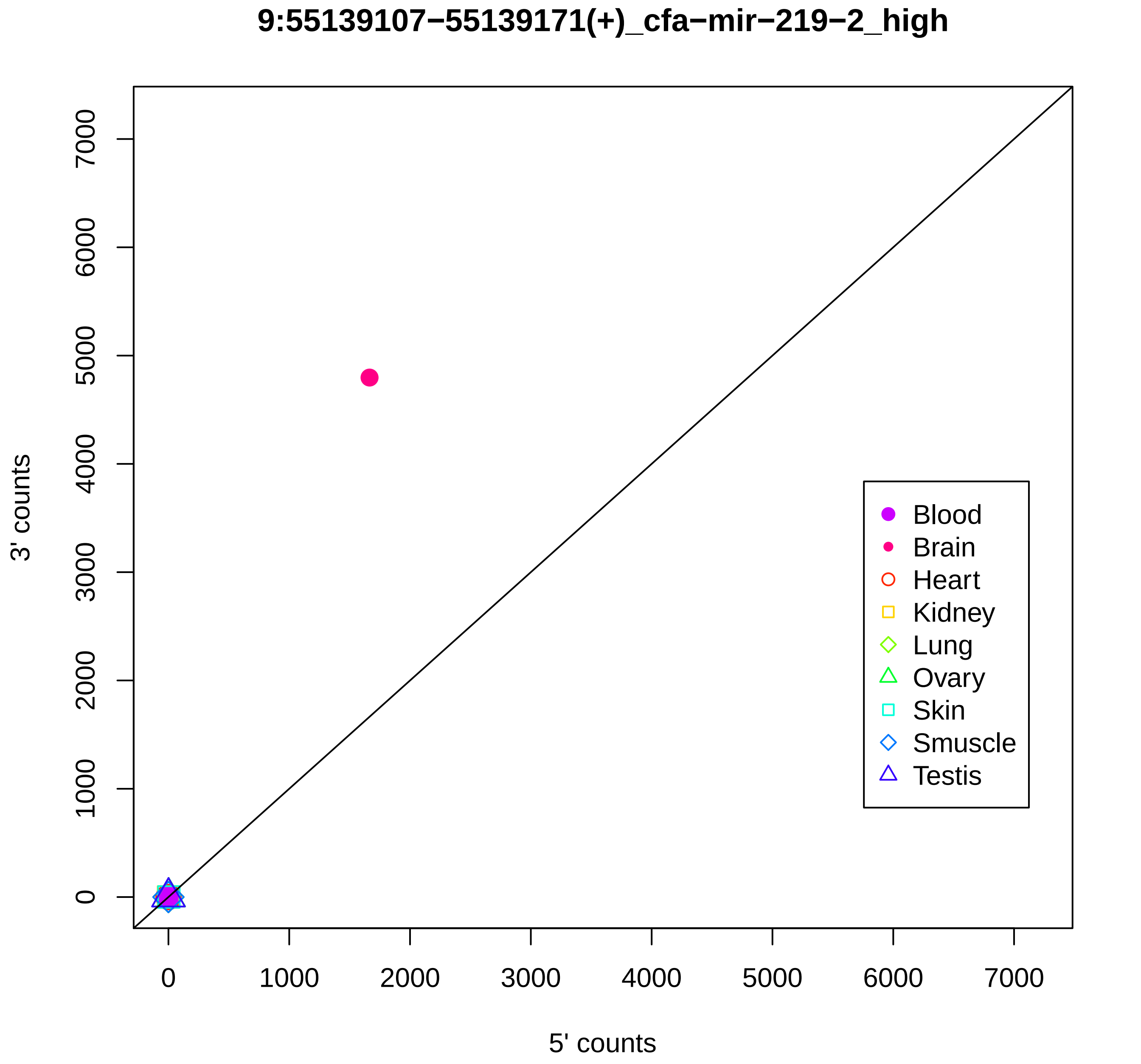

9:55139107−55139171(+)_cfa−mir−219−2_high
3' counts
5' counts
0
1000
2000
3000
4000
5000
6000
7000
Blood
Brain
Heart
Kidney
Lung
Ovary
Skin
Smuscle
Testis

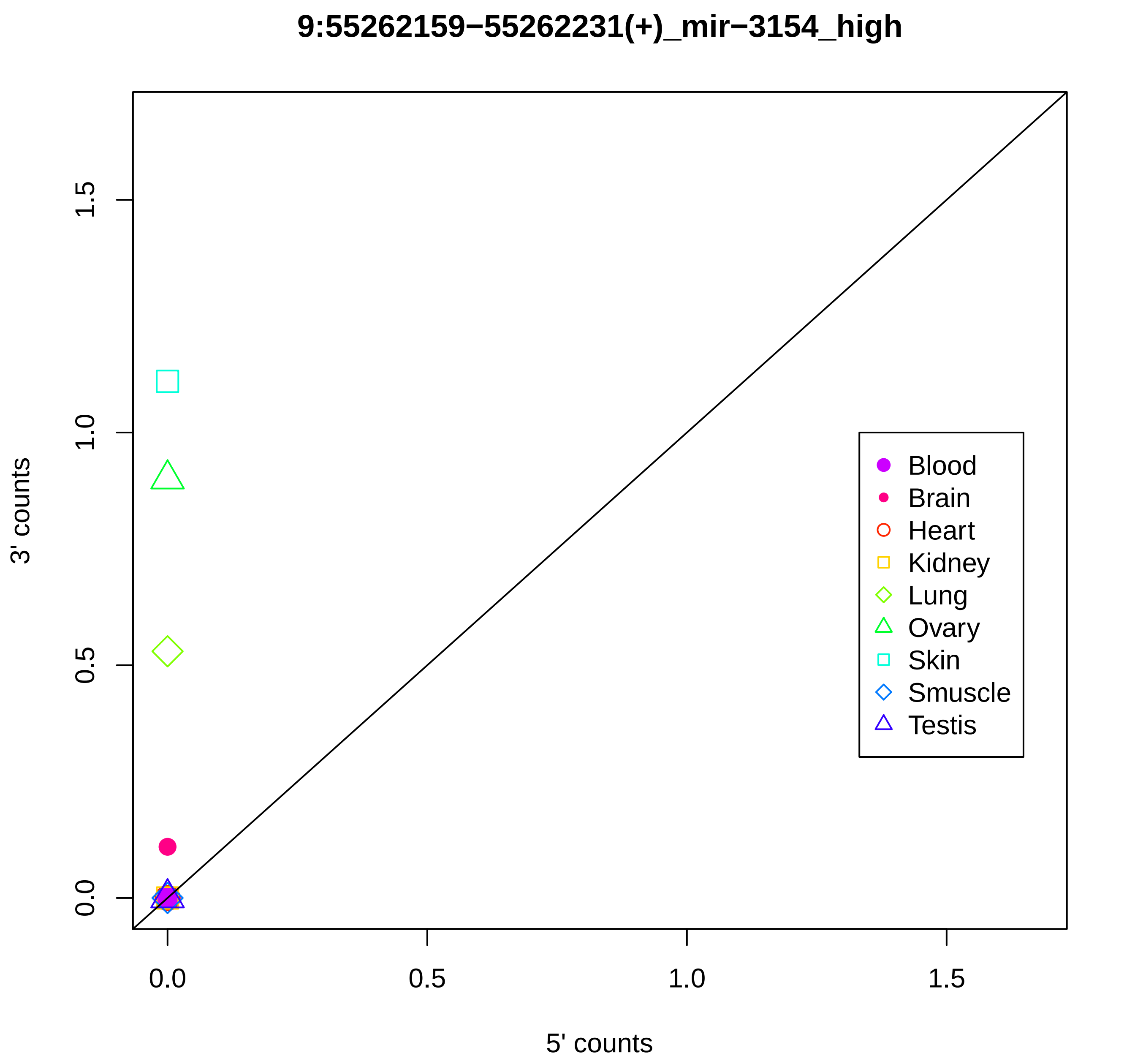
9:55262159−55262231(+)_mir−3154_high
3' counts
5' counts
0.0
0.5
1.0
1.5
Blood
Brain
Heart
Kidney
Lung
Ovary
Skin
Smuscle
Testis

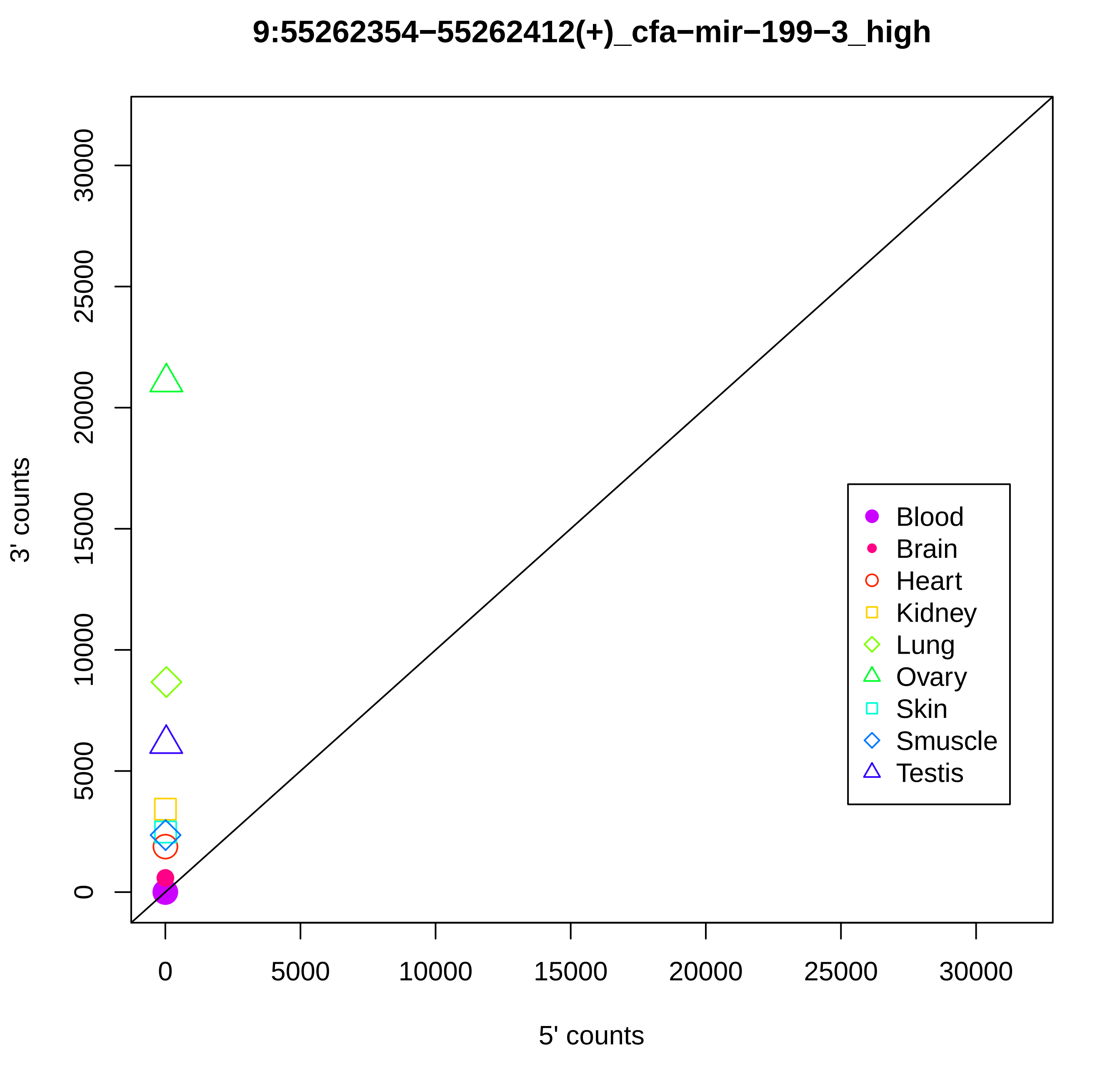

9:55262354−55262412(+)_cfa−mir−199−3_high
3' counts
5' counts
0
5000
10000
15000
20000
25000
30000
Blood
Brain
Heart
Kidney
Lung
Ovary
Skin
Smuscle
Testis

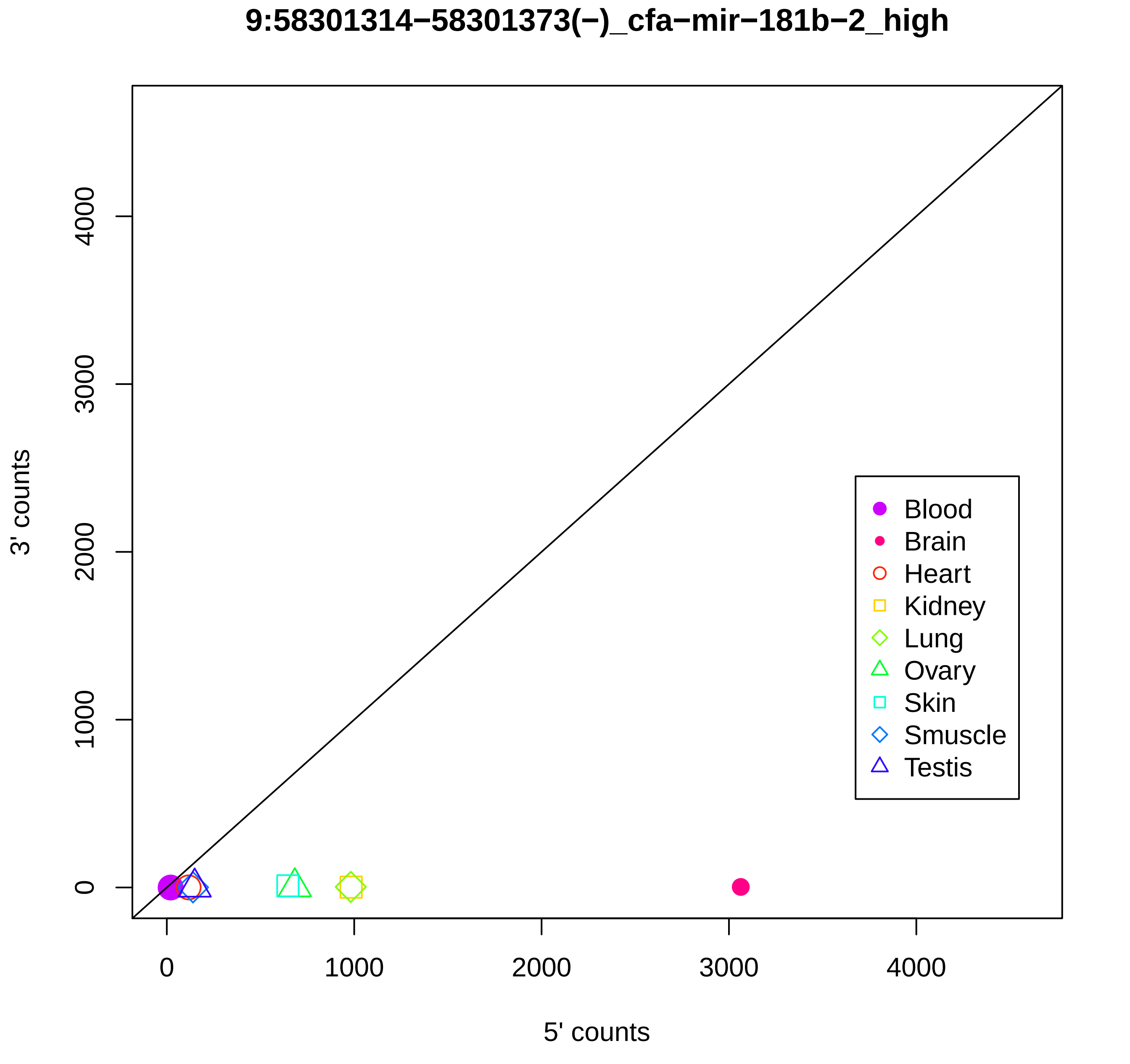

9:58301314−58301373(−)_cfa−mir−181b−2_high
3' counts
5' counts
0
1000
2000
3000
4000
Blood
Brain
Heart
Kidney
Lung
Ovary
Skin
Smuscle
Testis

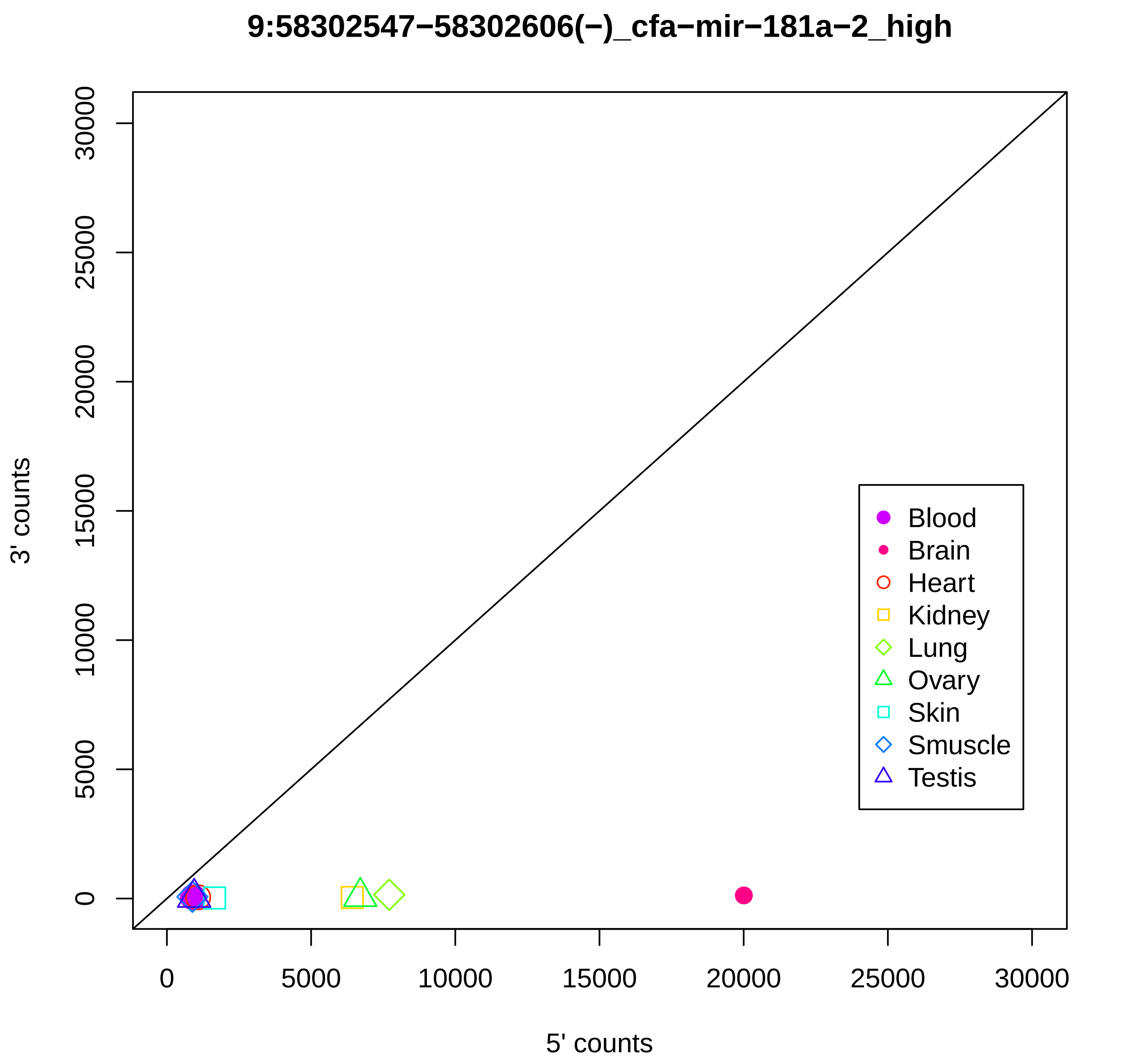

9:58302547−58302606(−)_cfa−mir−181a−2_high
3' counts
5' counts
0
5000
10000
15000
20000
25000
30000
Blood
Brain
Heart
Kidney
Lung
Ovary
Skin
Smuscle
Testis

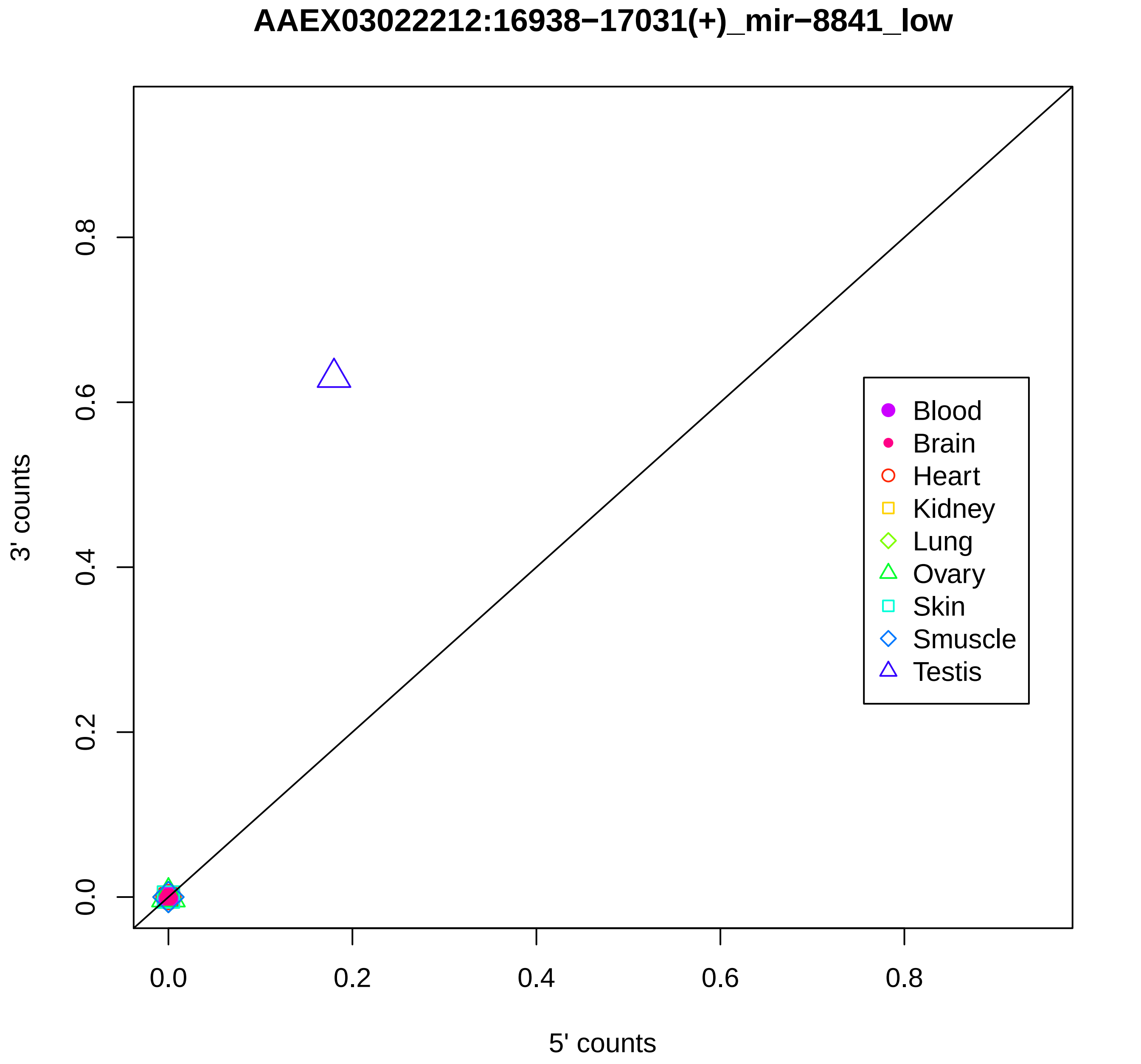

AAEX03022212:16938−17031(+)_mir−8841_low
3' counts
5' counts
0.0
0.2
0.4
0.6
0.8
Blood
Brain
Heart
Kidney
Lung
Ovary
Skin
Smuscle
Testis

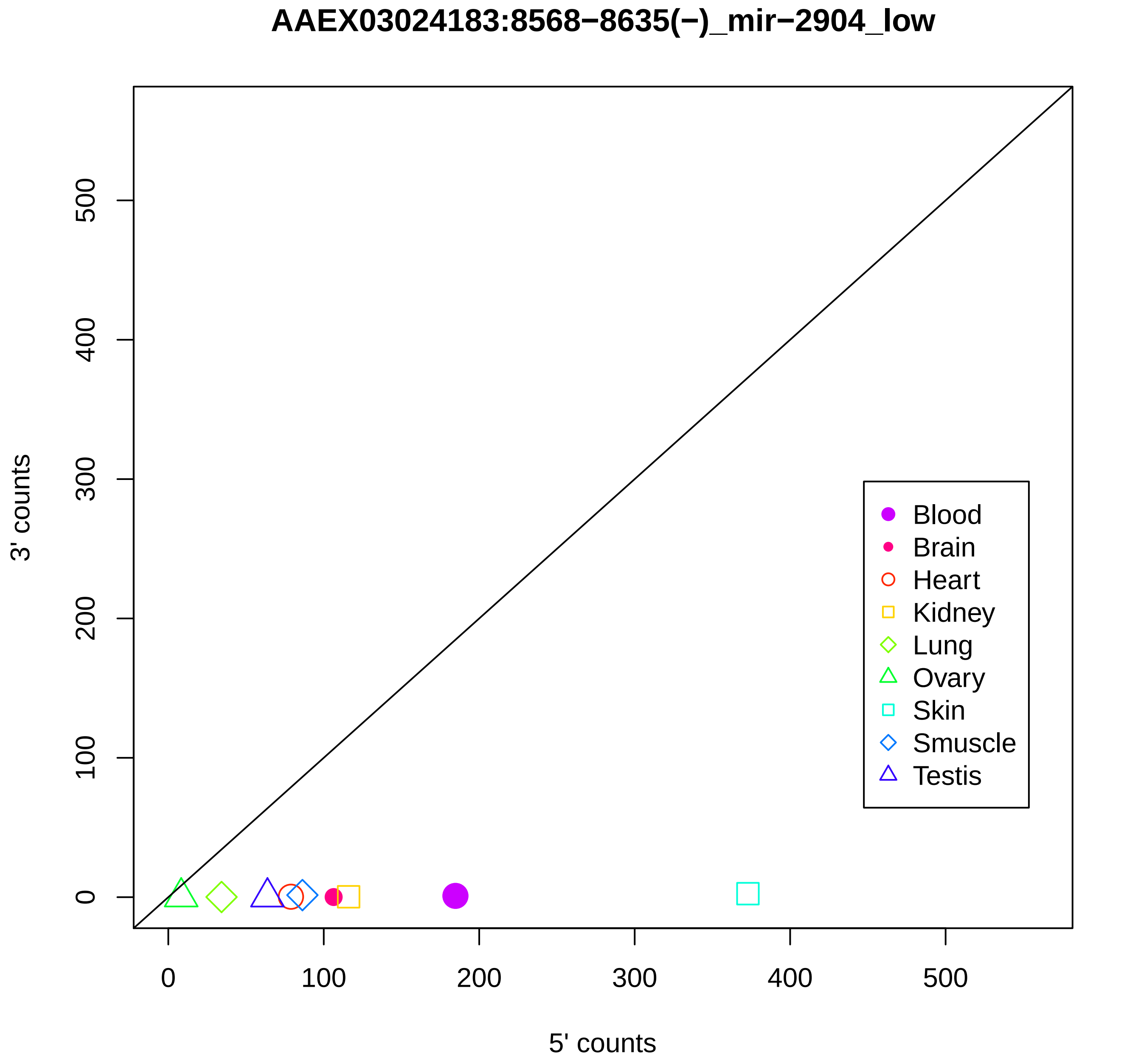

AAEX03024183:8568−8635(−)_mir−2904_low
3' counts
5' counts
0
100
200
300
400
500
0
100
200
300
400
500
Blood
Brain
Heart
Kidney
Lung
Ovary
Skin
Smuscle
Testis

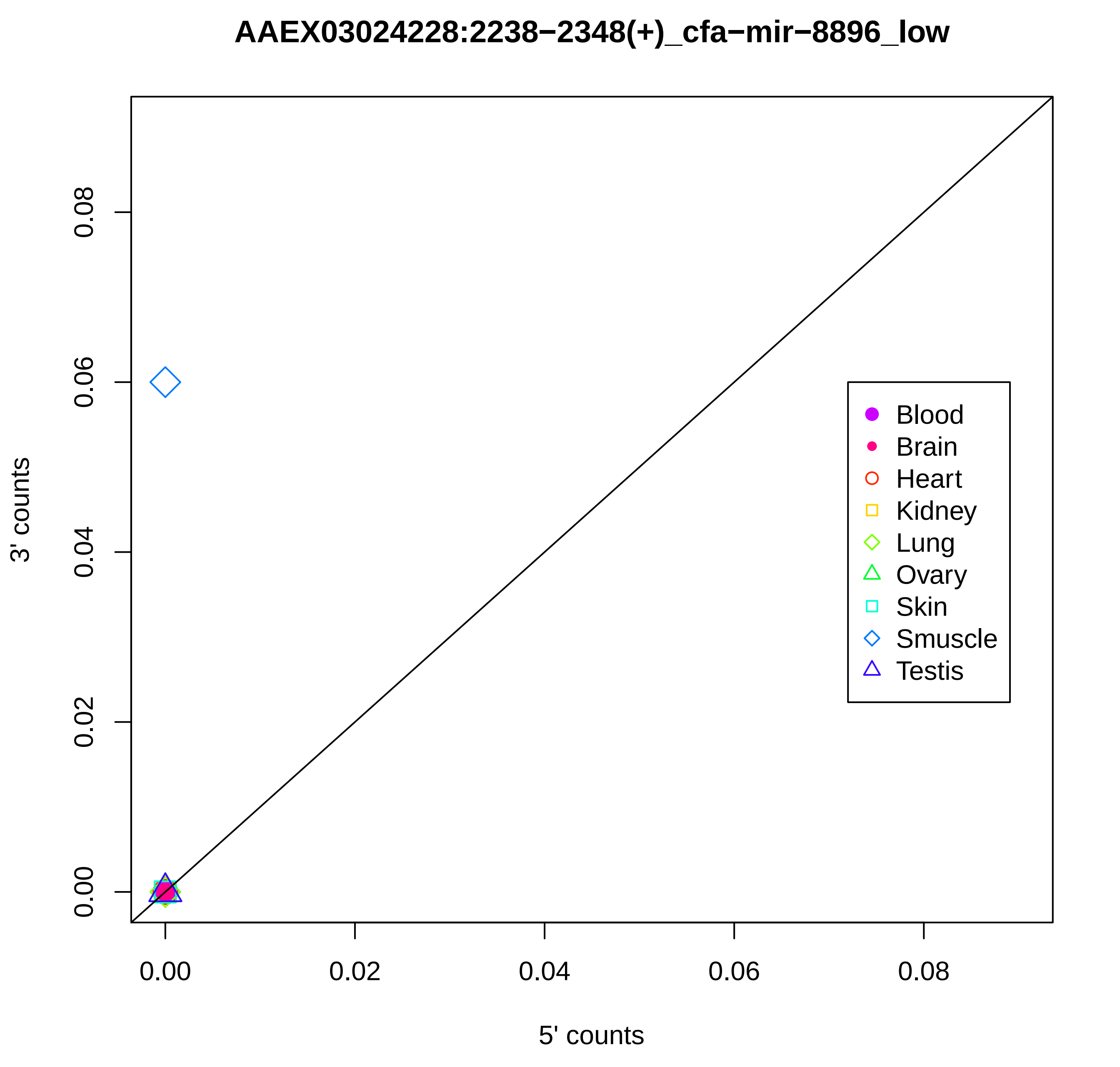

AAEX03024228:2238−2348(+)_cfa−mir−8896_low
3' counts
5' counts
0.00
0.02
0.04
0.06
0.08
0.00
0.02
0.04
0.06
0.08
Blood
Brain
Heart
Kidney
Lung
Ovary
Skin
Smuscle
Testis

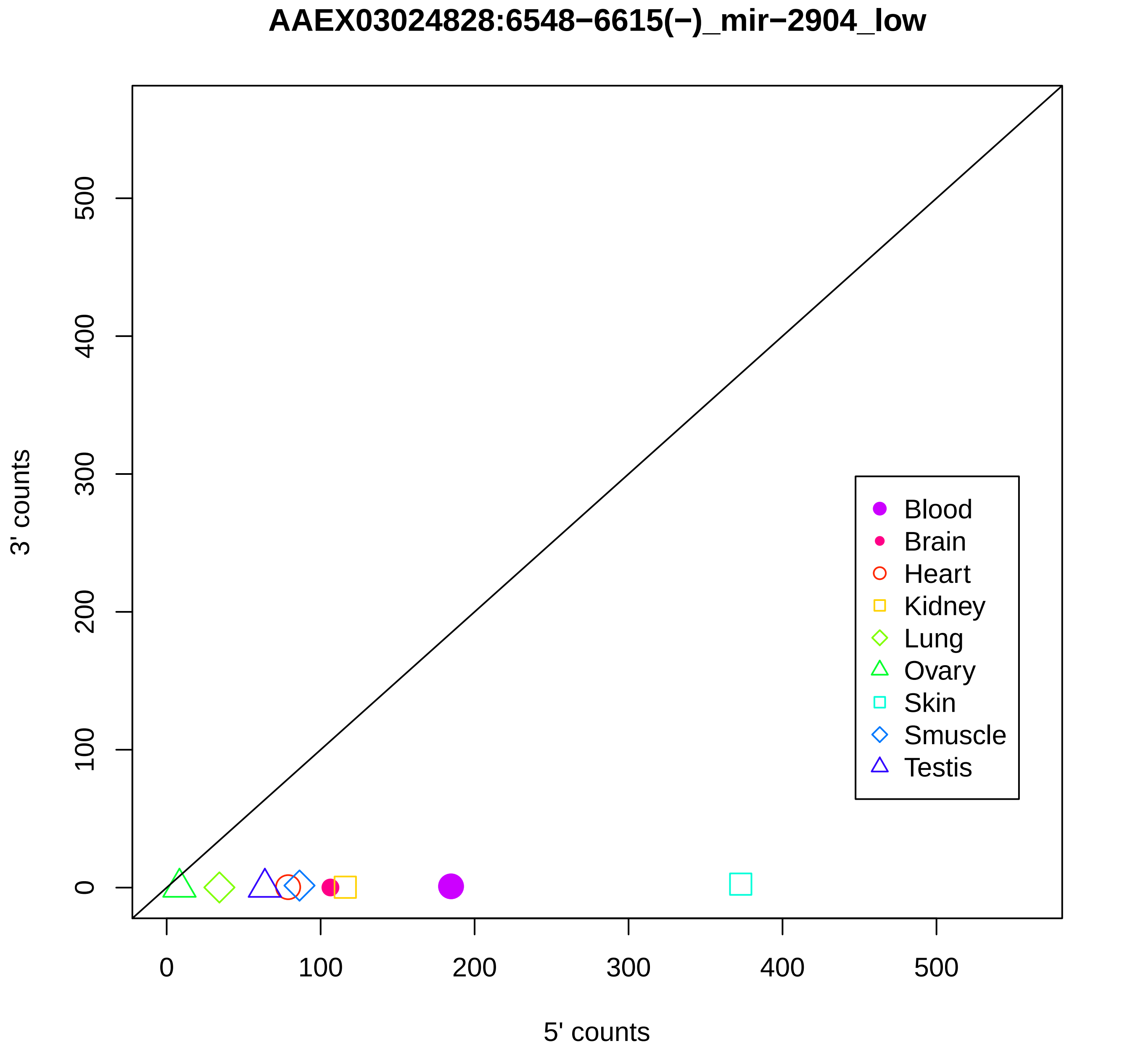

AAEX03024828:6548−6615(−)_mir−2904_low
3' counts
5' counts
0
100
200
300
400
500
Blood
Brain
Heart
Kidney
Lung
Ovary
Skin
Smuscle
Testis

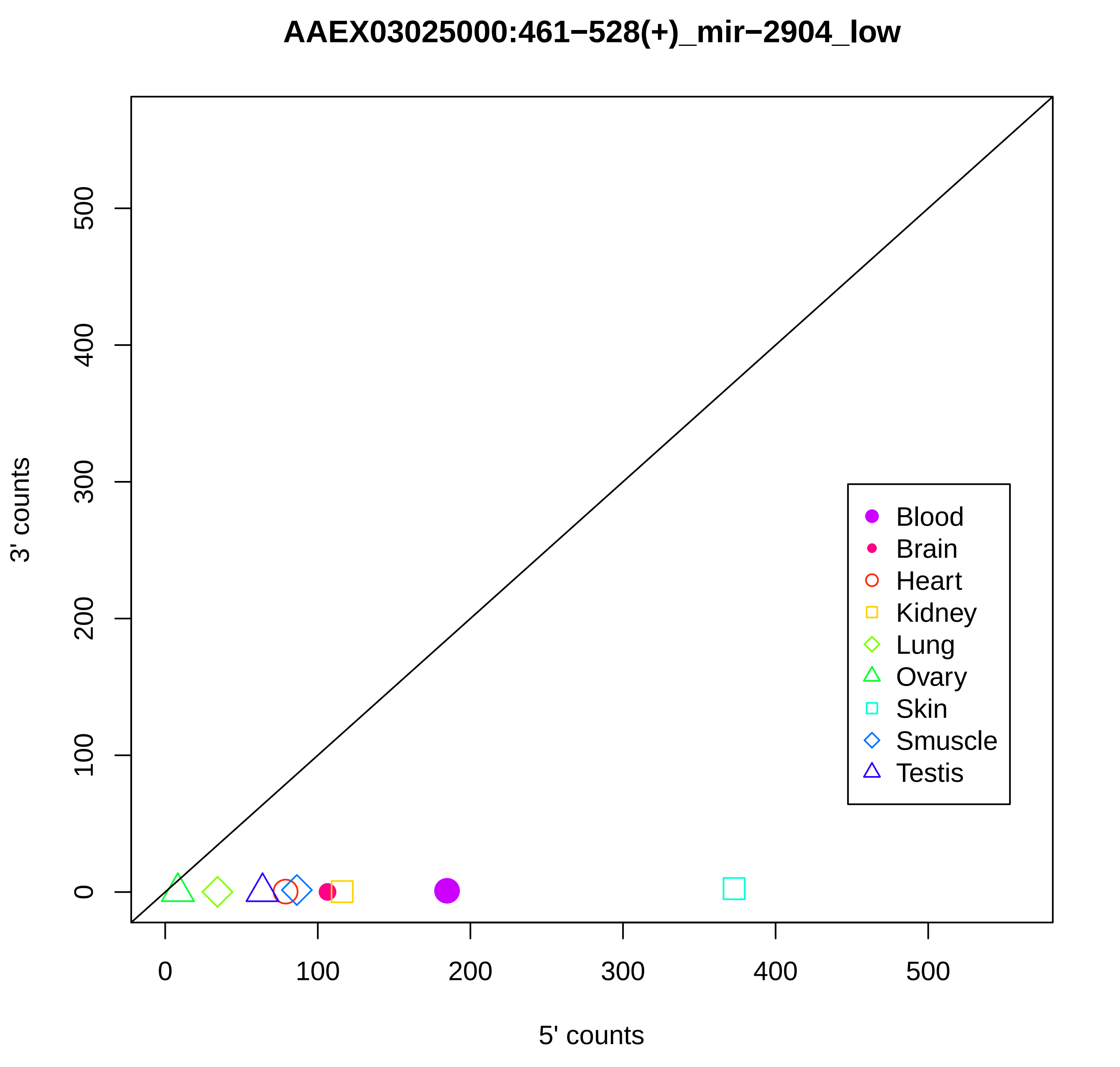
AAEX03025000:461−528(+)_mir−2904_low
3' counts
5' counts
0
100
200
300
400
500
0
100
200
300
400
500
Blood
Brain
Heart
Kidney
Lung
Ovary
Skin
Smuscle
Testis

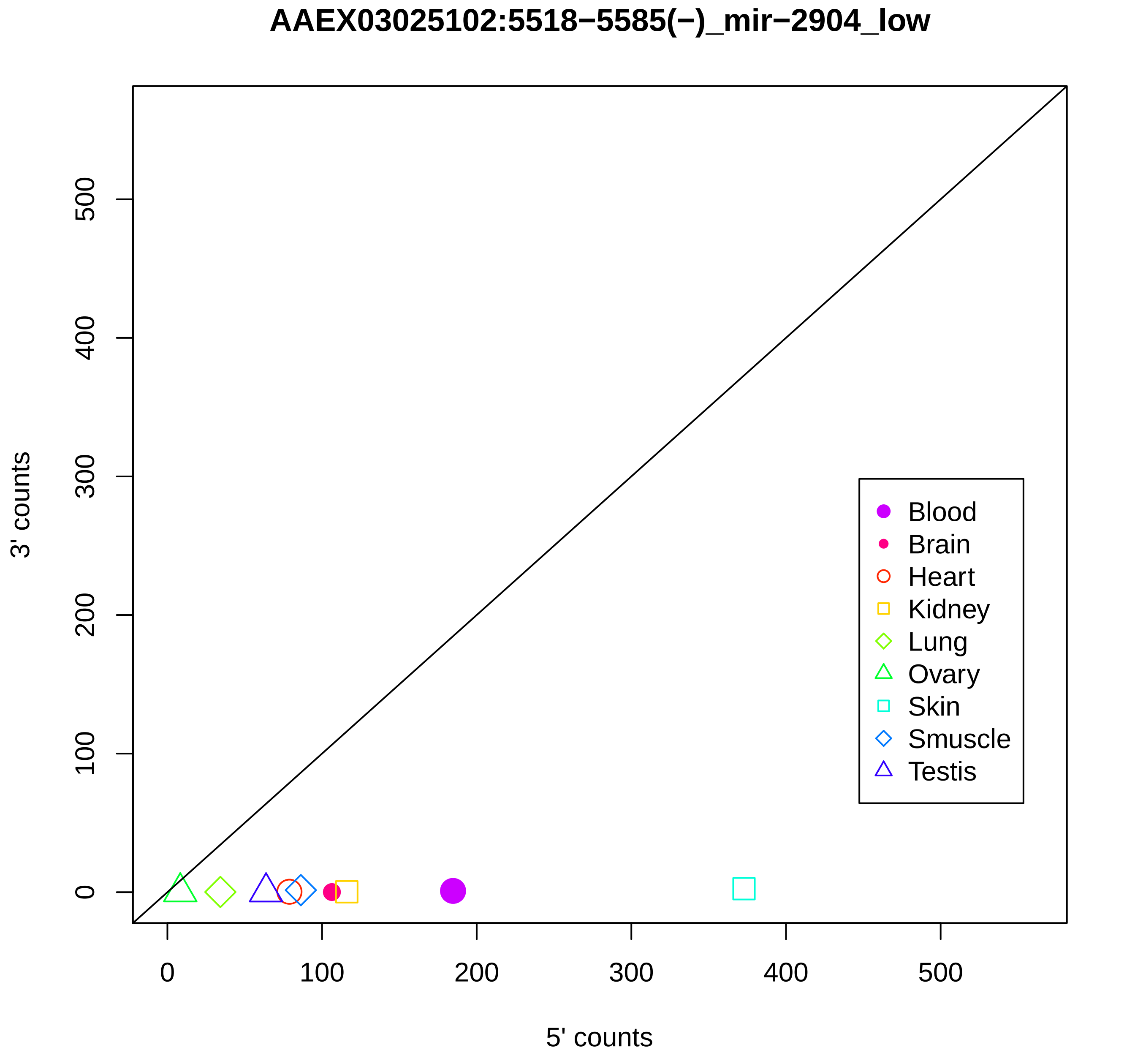
AAEX03025102:5518−5585(−)_mir−2904_low
3' counts
5' counts
0
100
200
300
400
500
Blood
Brain
Heart
Kidney
Lung
Ovary
Skin
Smuscle
Testis

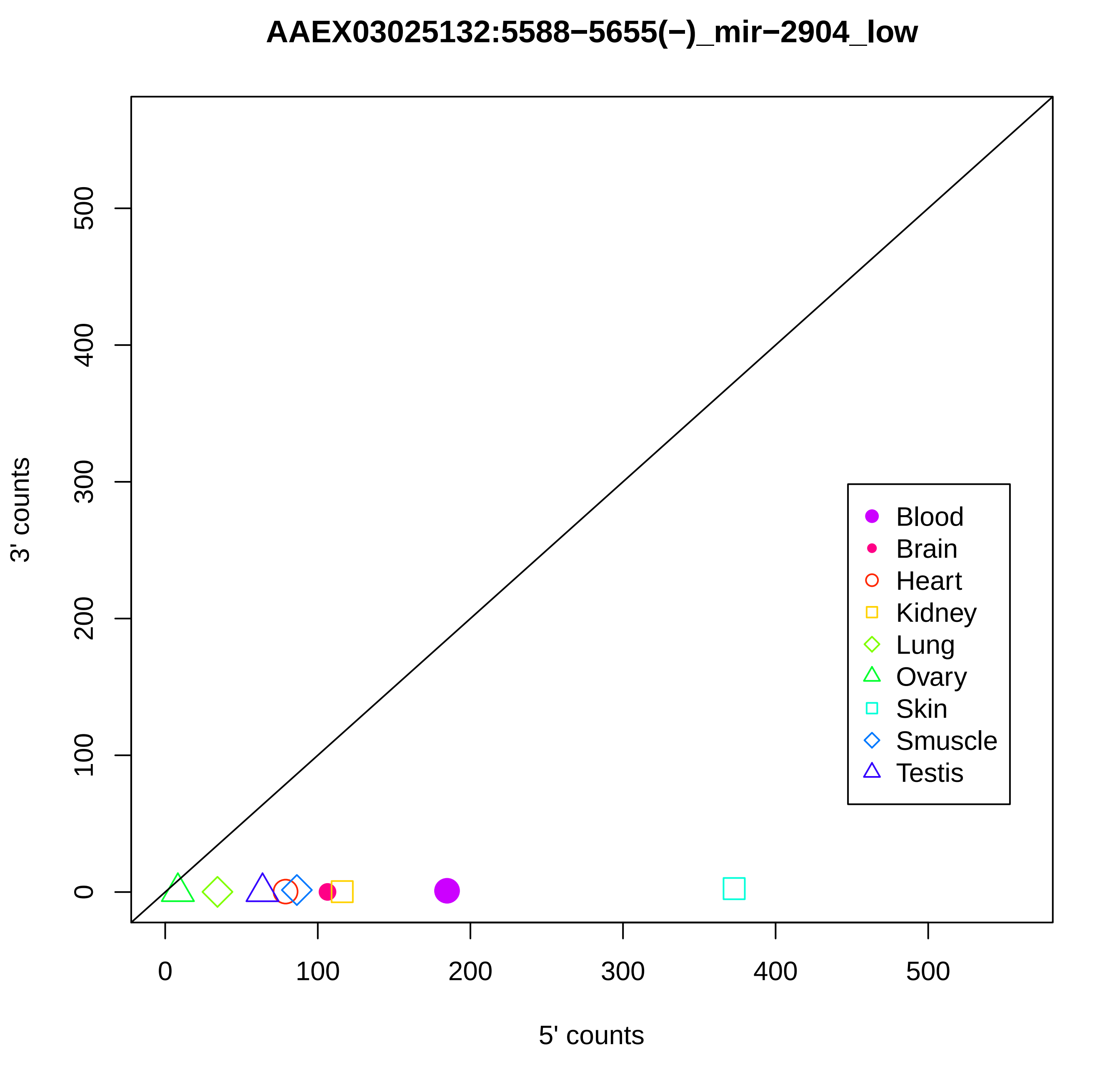

AAEX03025132:5588−5655(−)_mir−2904_low
3' counts
5' counts
0
100
200
300
400
500
Blood
Brain
Heart
Kidney
Lung
Ovary
Skin
Smuscle
Testis

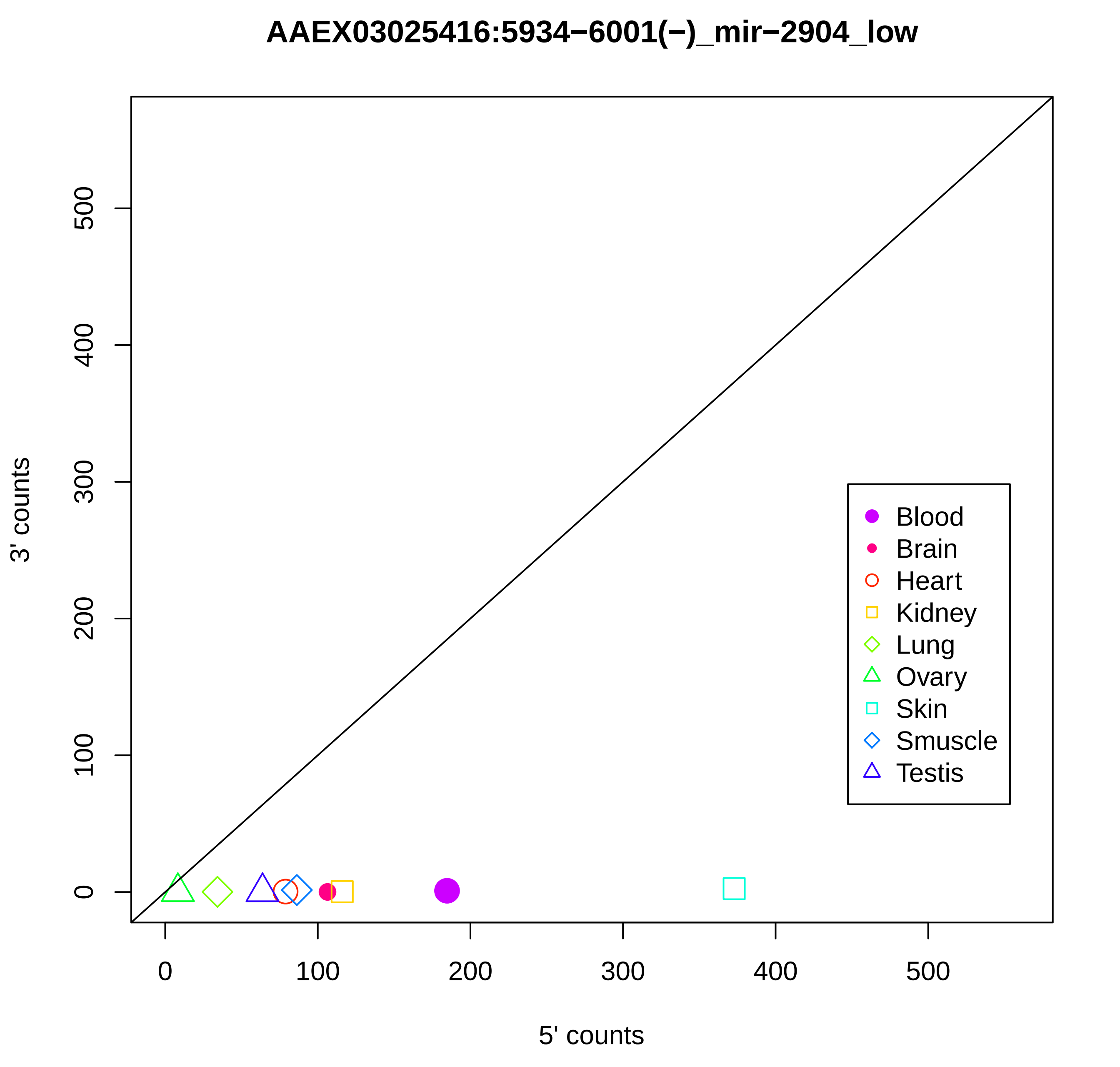

AAEX03025416:5934−6001(−)_mir−2904_low
3' counts
5' counts
0
100
200
300
400
500
Blood
Brain
Heart
Kidney
Lung
Ovary
Skin
Smuscle
Testis

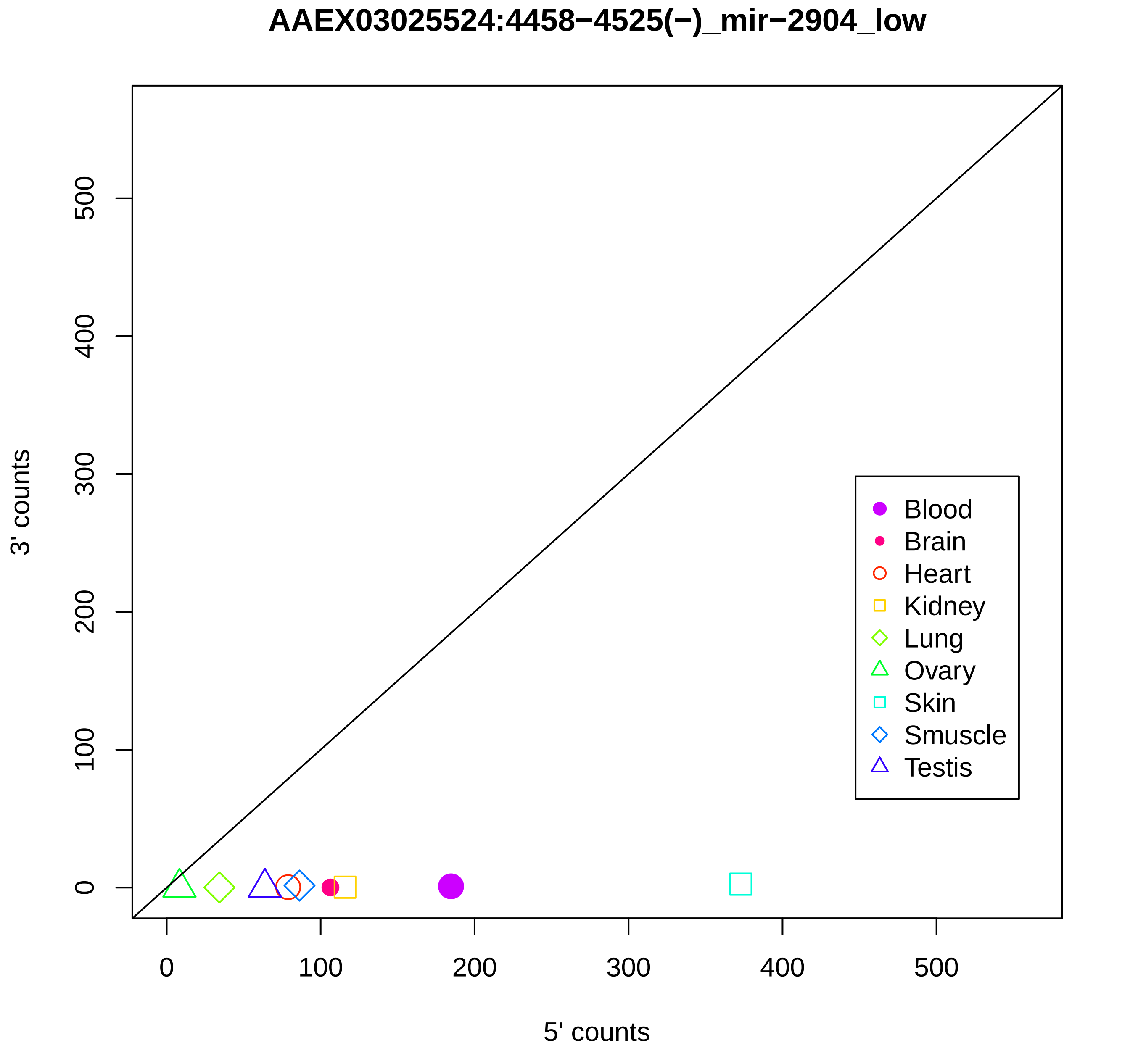

AAEX03025524:4458−4525(−)_mir−2904_low
3' counts
5' counts
0
100
200
300
400
500
0
100
200
300
400
500
Blood
Brain
Heart
Kidney
Lung
Ovary
Skin
Smuscle
Testis

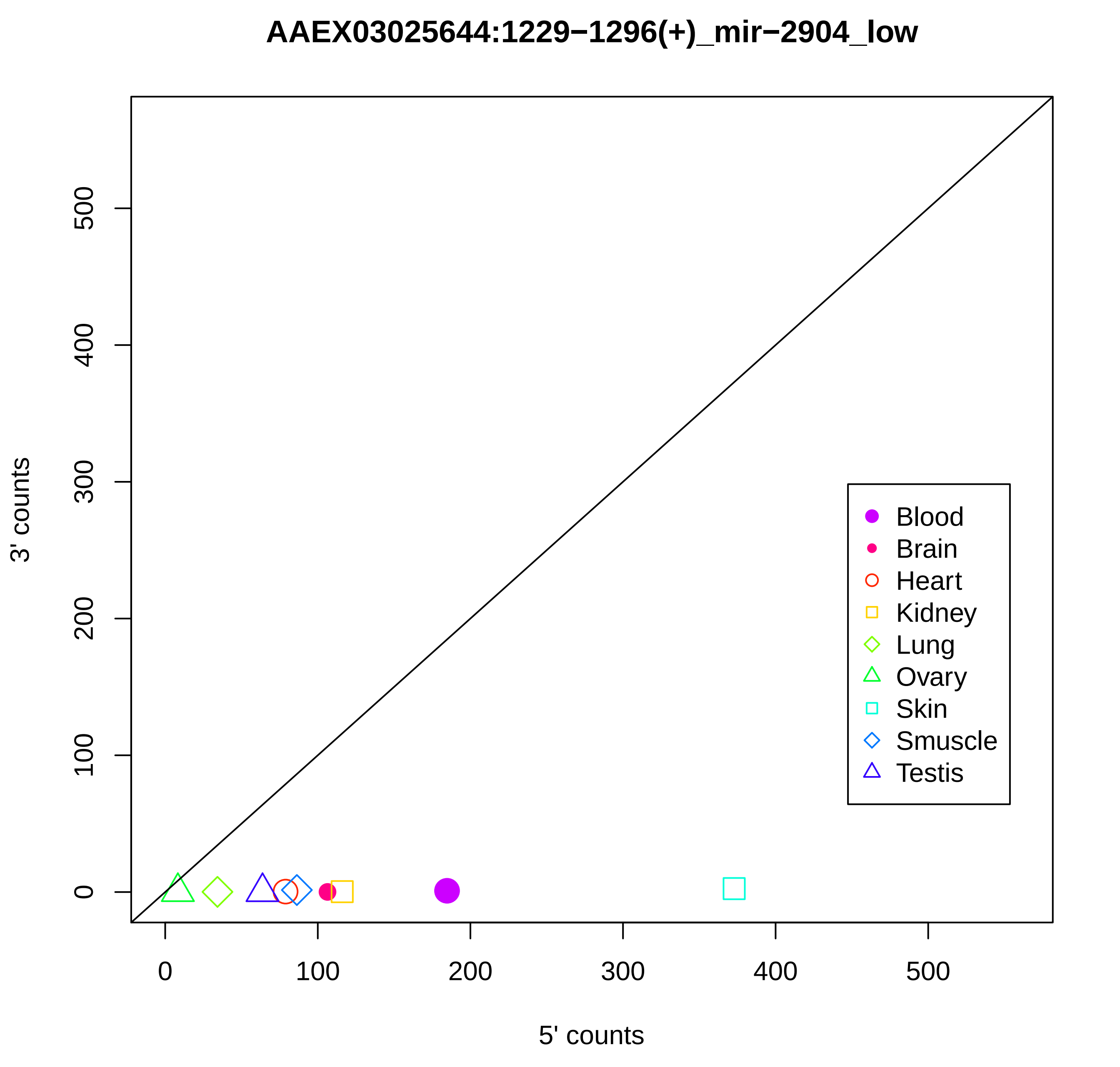

AAEX03025644:1229−1296(+)_mir−2904_low
3' counts
5' counts
0
100
200
300
400
500
Blood
Brain
Heart
Kidney
Lung
Ovary
Skin
Smuscle
Testis

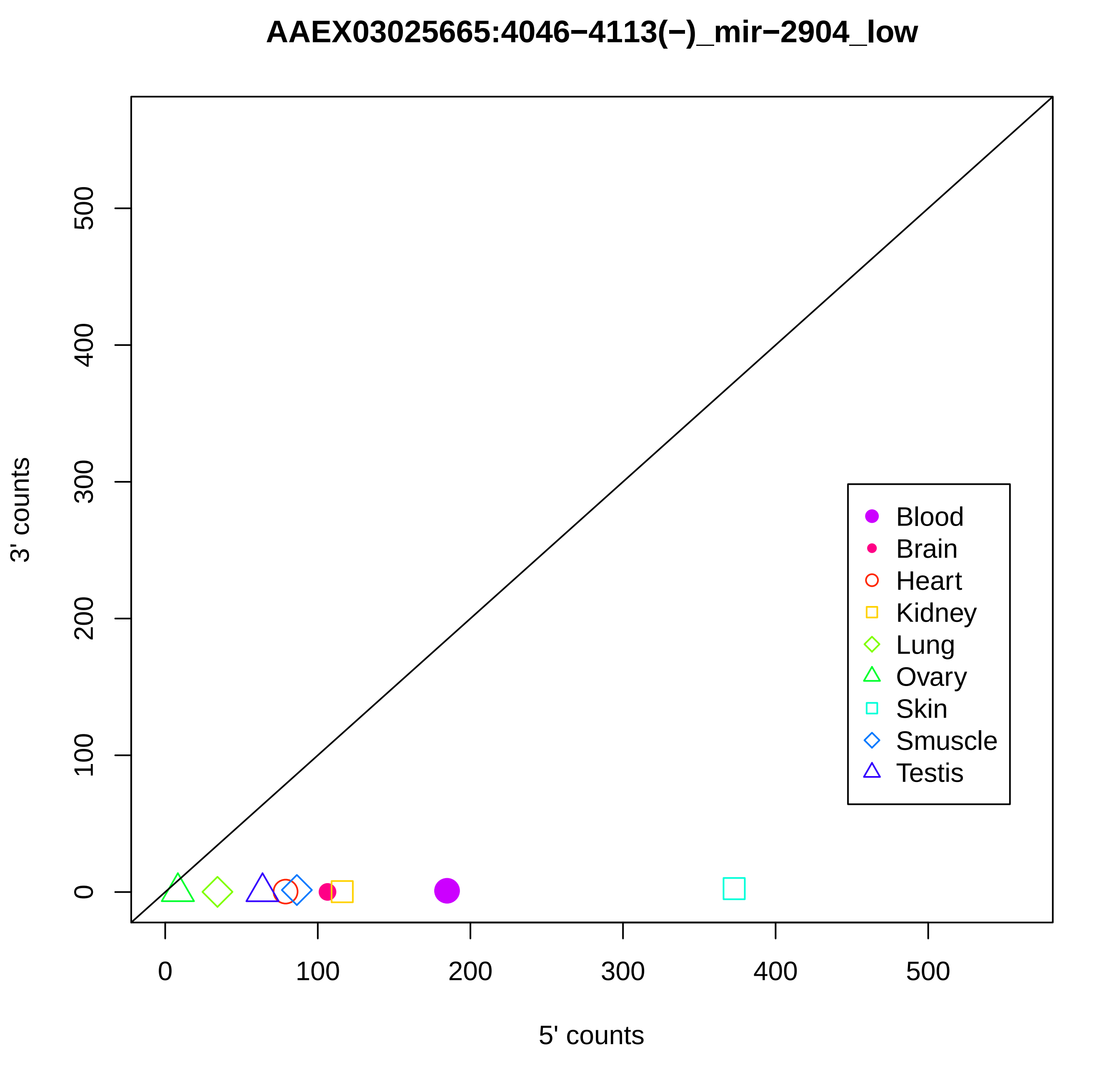
AAEX03025665:4046−4113(−)_mir−2904_low
3' counts
5' counts
0
100
200
300
400
500
0
100
200
300
400
500
Blood
Brain
Heart
Kidney
Lung
Ovary
Skin
Smuscle
Testis

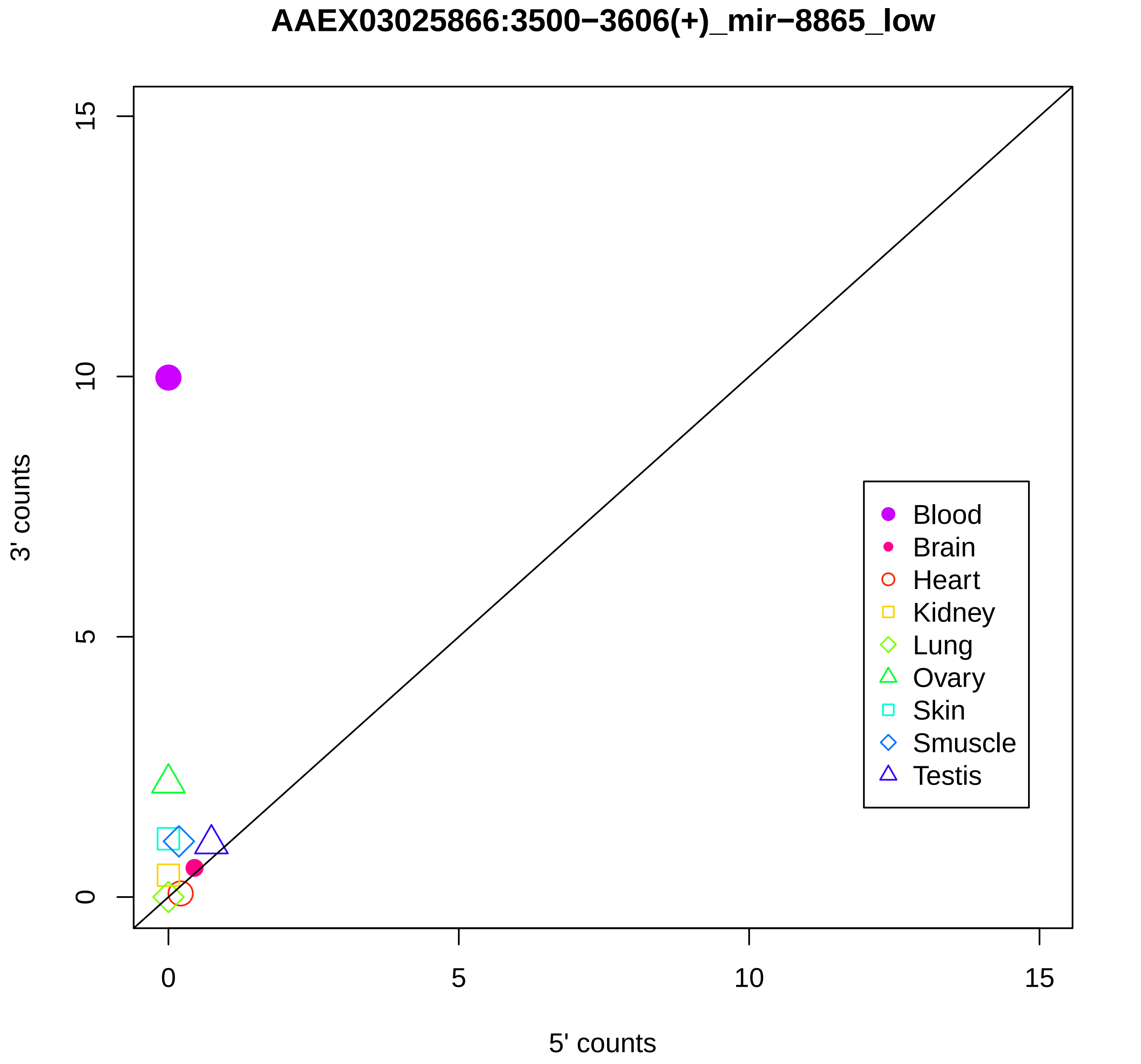

AAEX03025866:3500−3606(+)_mir−8865_low
3' counts
5' counts
0
5
10
15
0
5
10
15
Blood
Brain
Heart
Kidney
Lung
Ovary
Skin
Smuscle
Testis

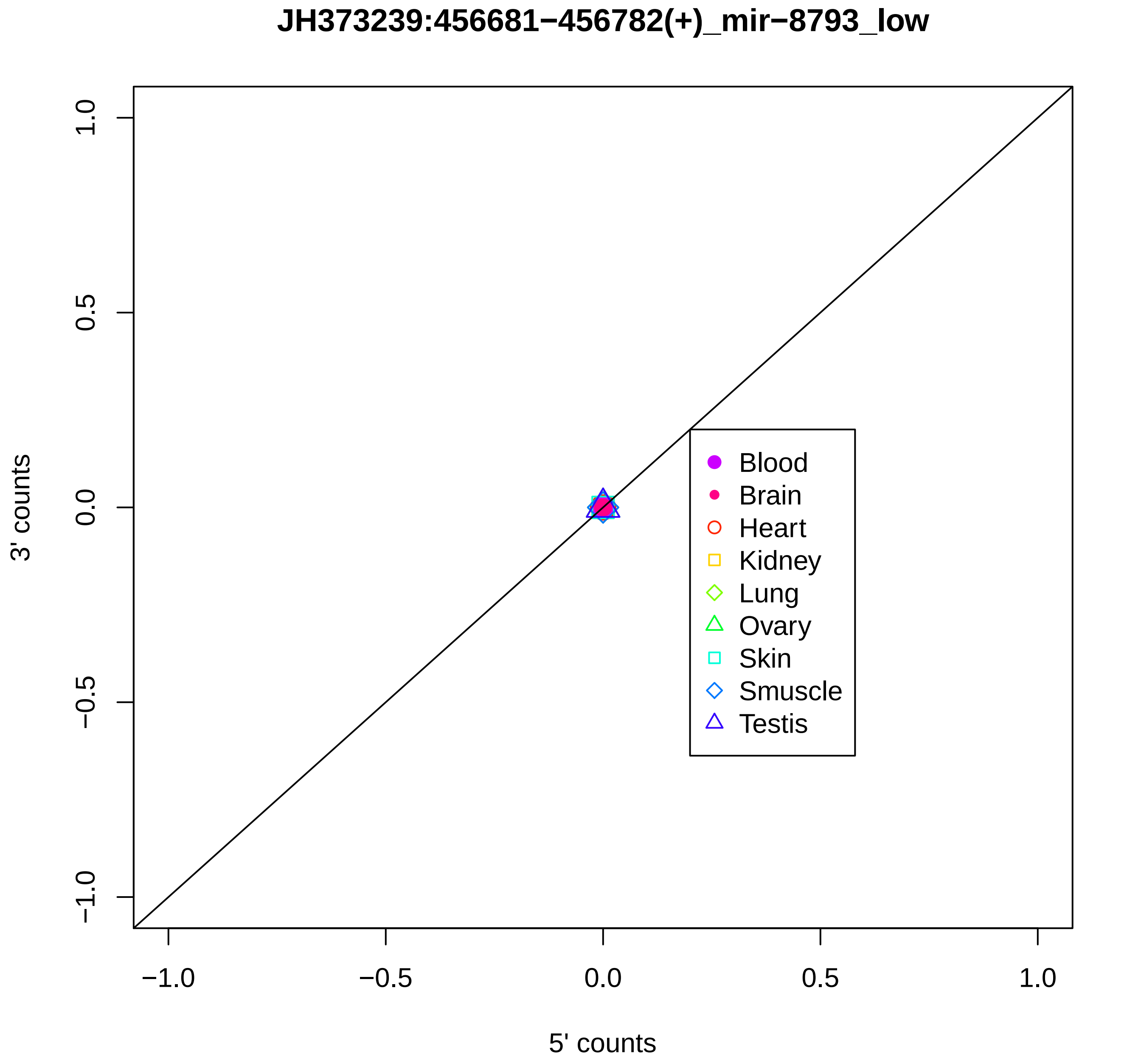

JH373239:456681−456782(+)_mir−8793_low
3' counts
5' counts
1.0
0.5
0.0
−0.5
−1.0
Blood
Brain
Heart
Kidney
Lung
Ovary
Skin
Smuscle
Testis

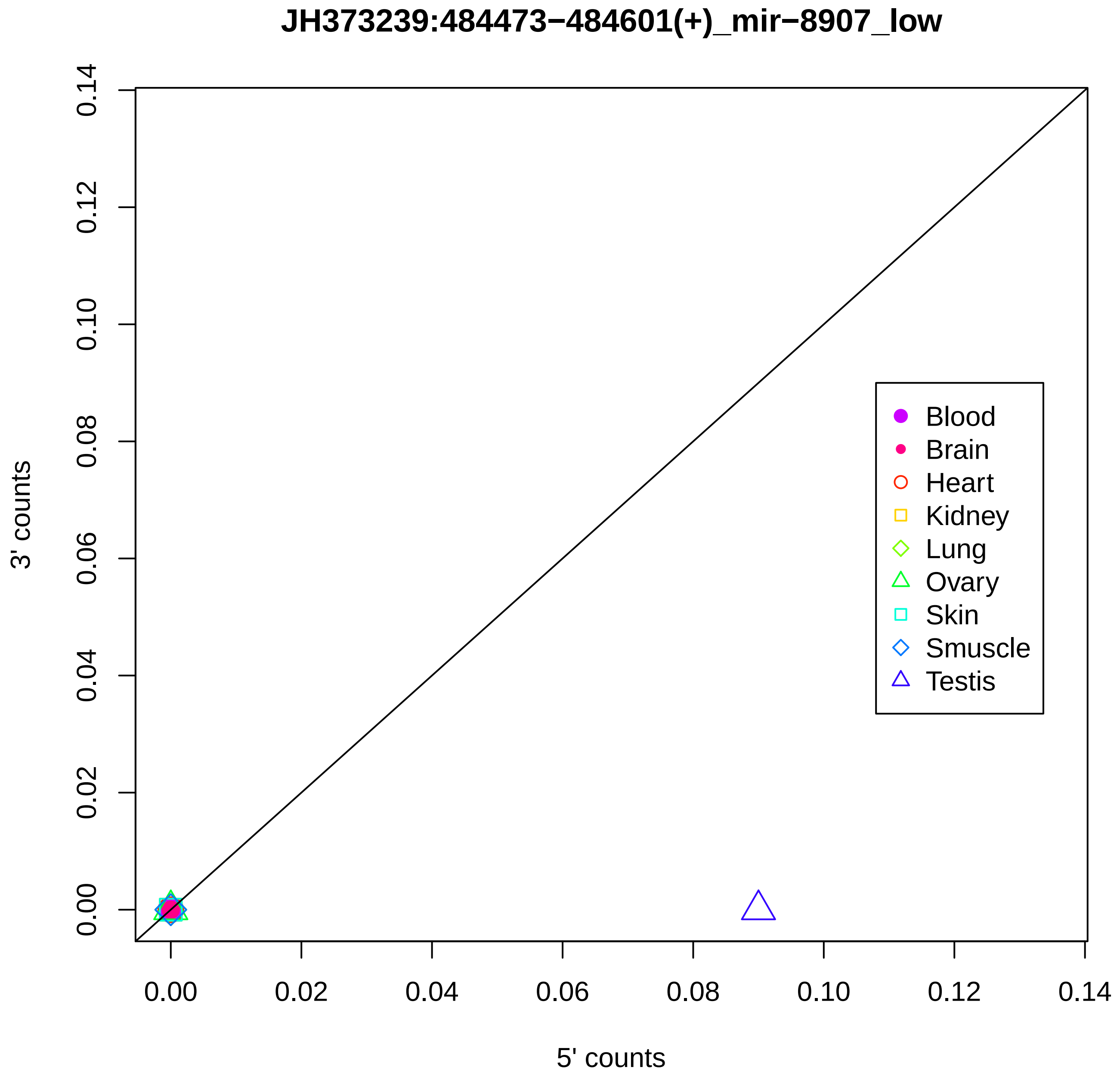

JH373239:484473−484601(+)_mir−8907_low
3' counts
5' counts
0.00
0.02
0.04
0.06
0.08
0.10
0.12
0.14
Blood
Brain
Heart
Kidney
Lung
Ovary
Skin
Smuscle
Testis

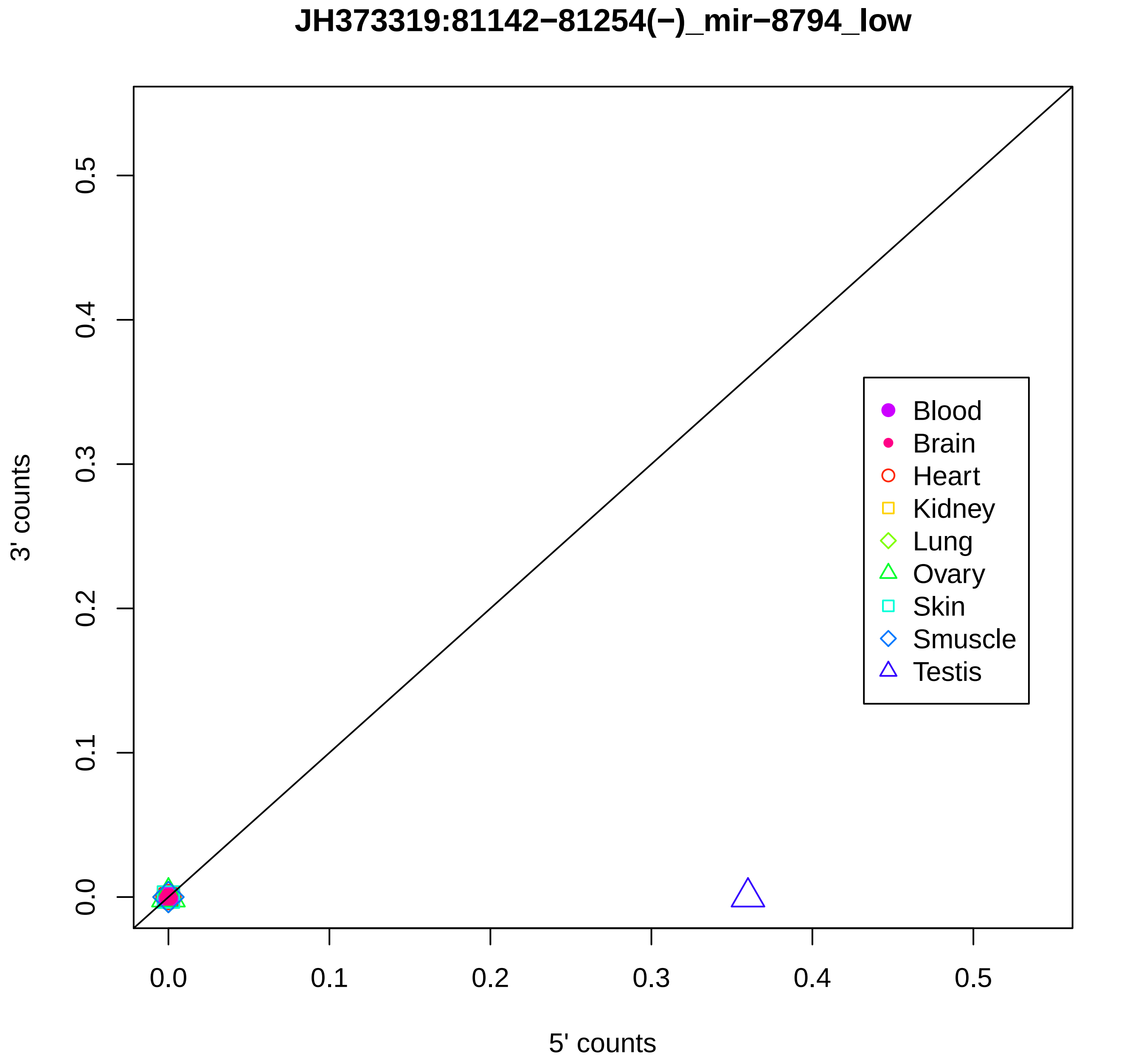

JH373319:81142−81254(−)_mir−8794_low
3' counts
5' counts
0.0
0.1
0.2
0.3
0.4
0.5
Blood
Brain
Heart
Kidney
Lung
Ovary
Skin
Smuscle
Testis

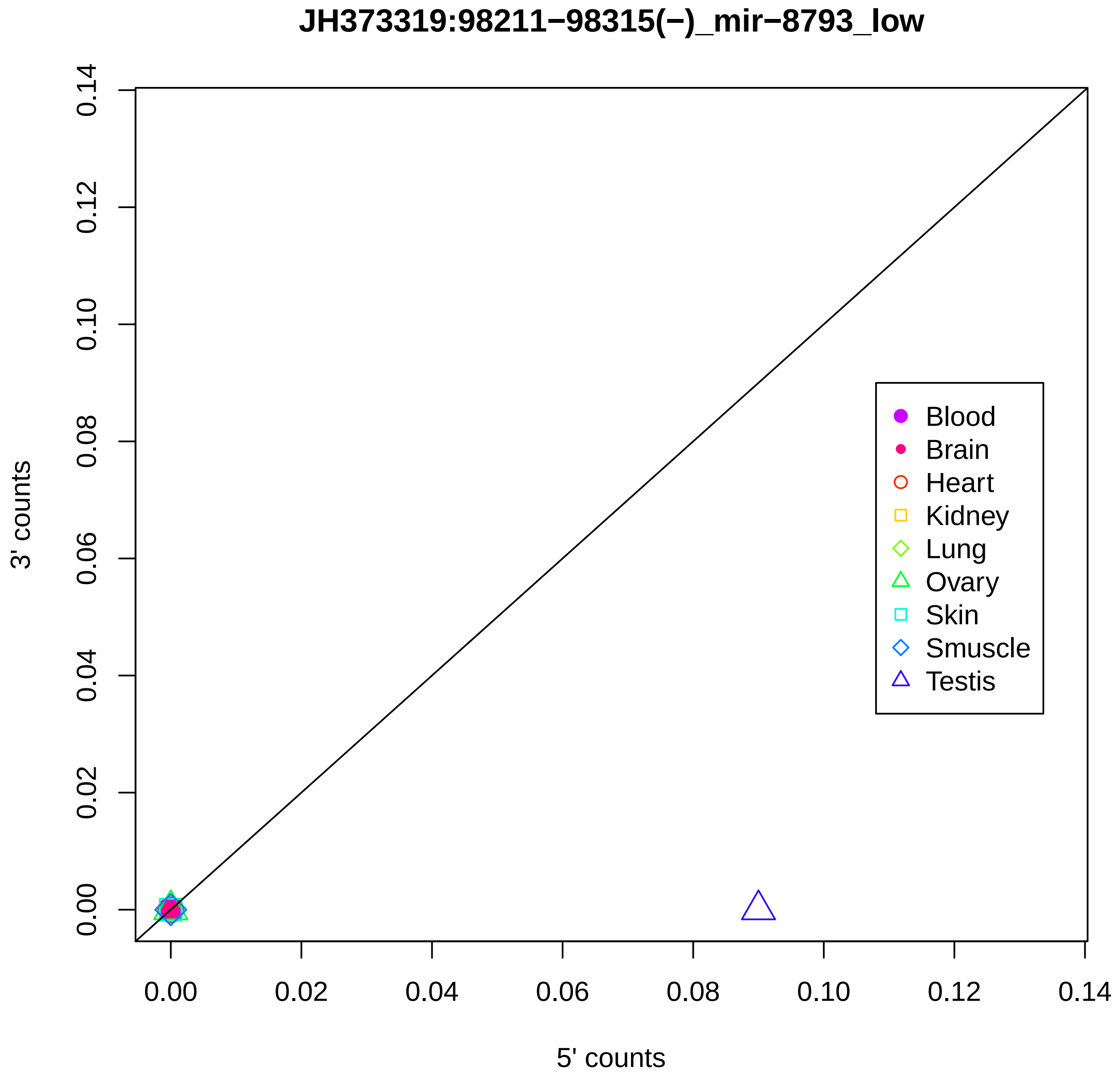
JH373319:98211−98315(−)_mir−8793_low
3' counts
5' counts
0.00
0.02
0.04
0.06
0.08
0.10
0.12
0.14
Blood
Brain
Heart
Kidney
Lung
Ovary
Skin
Smuscle
Testis

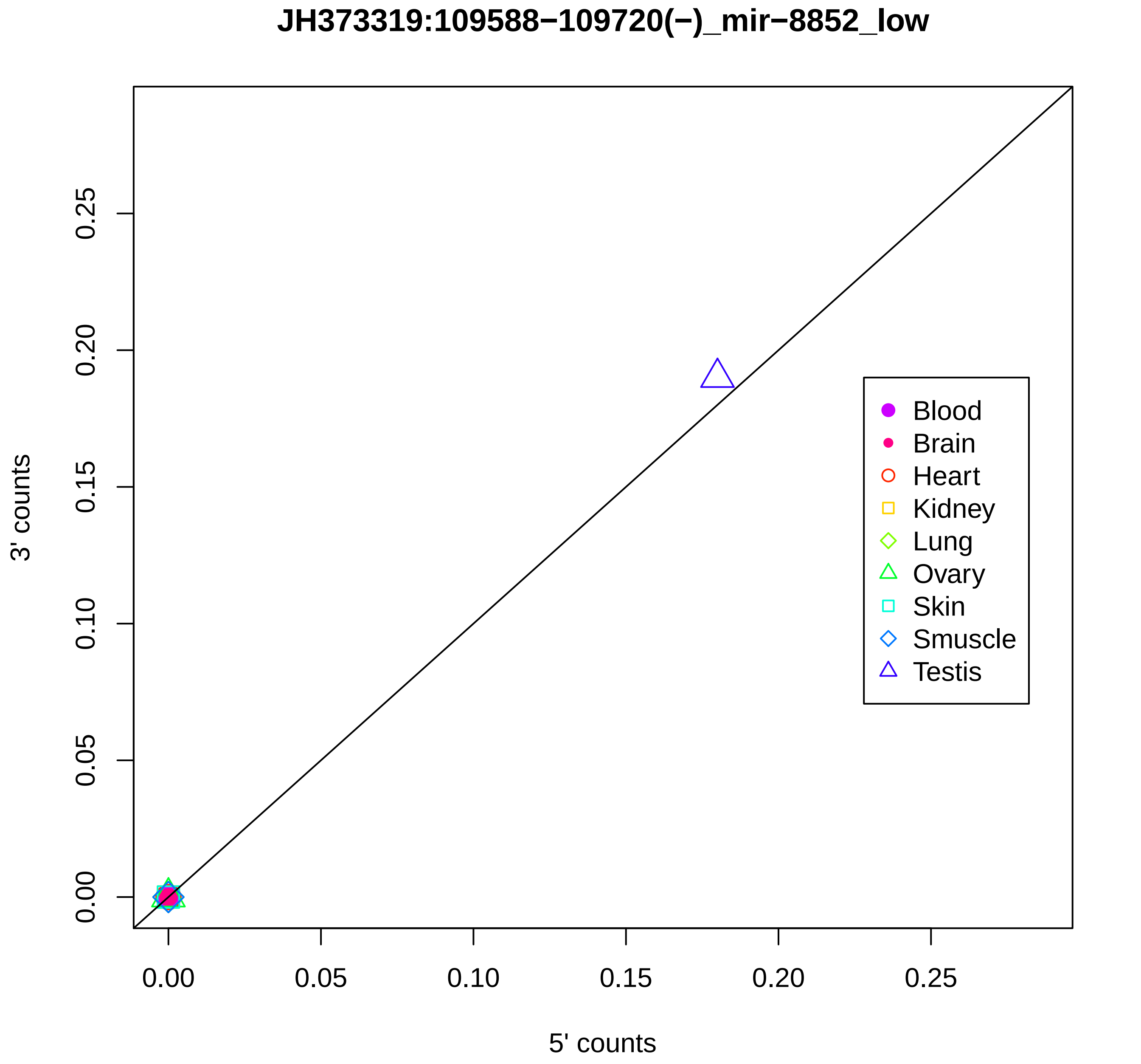

JH373319:109588−109720(−)_mir−8852_low
3' counts
5' counts
0.00
0.05
0.10
0.15
0.20
0.25
Blood
Brain
Heart
Kidney
Lung
Ovary
Skin
Smuscle
Testis

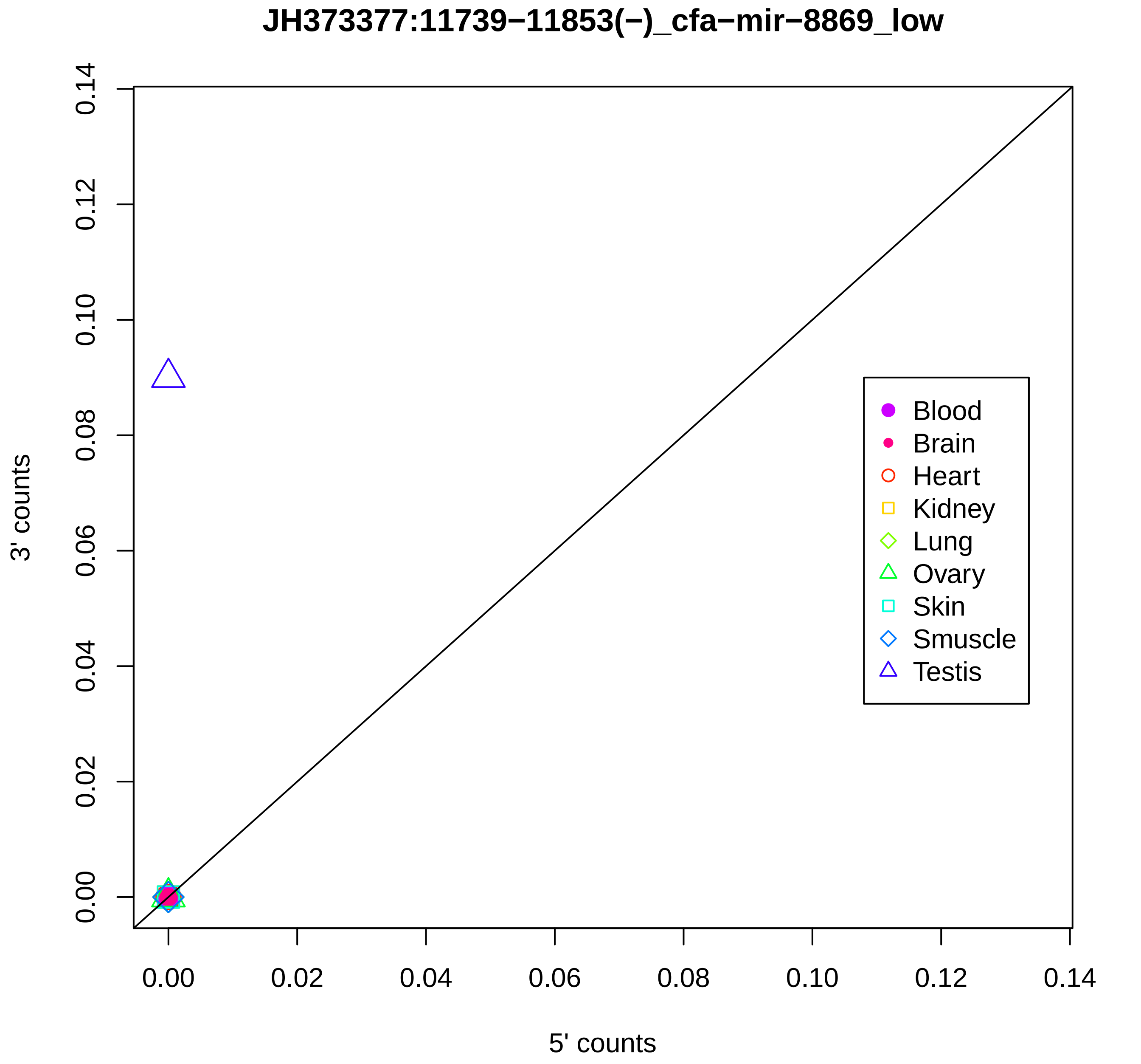

JH373377:11739−11853(−)_cfa−mir−8869_low
3' counts
5' counts
0.00
0.02
0.04
0.06
0.08
0.10
0.12
0.14
Blood
Brain
Heart
Kidney
Lung
Ovary
Skin
Smuscle
Testis

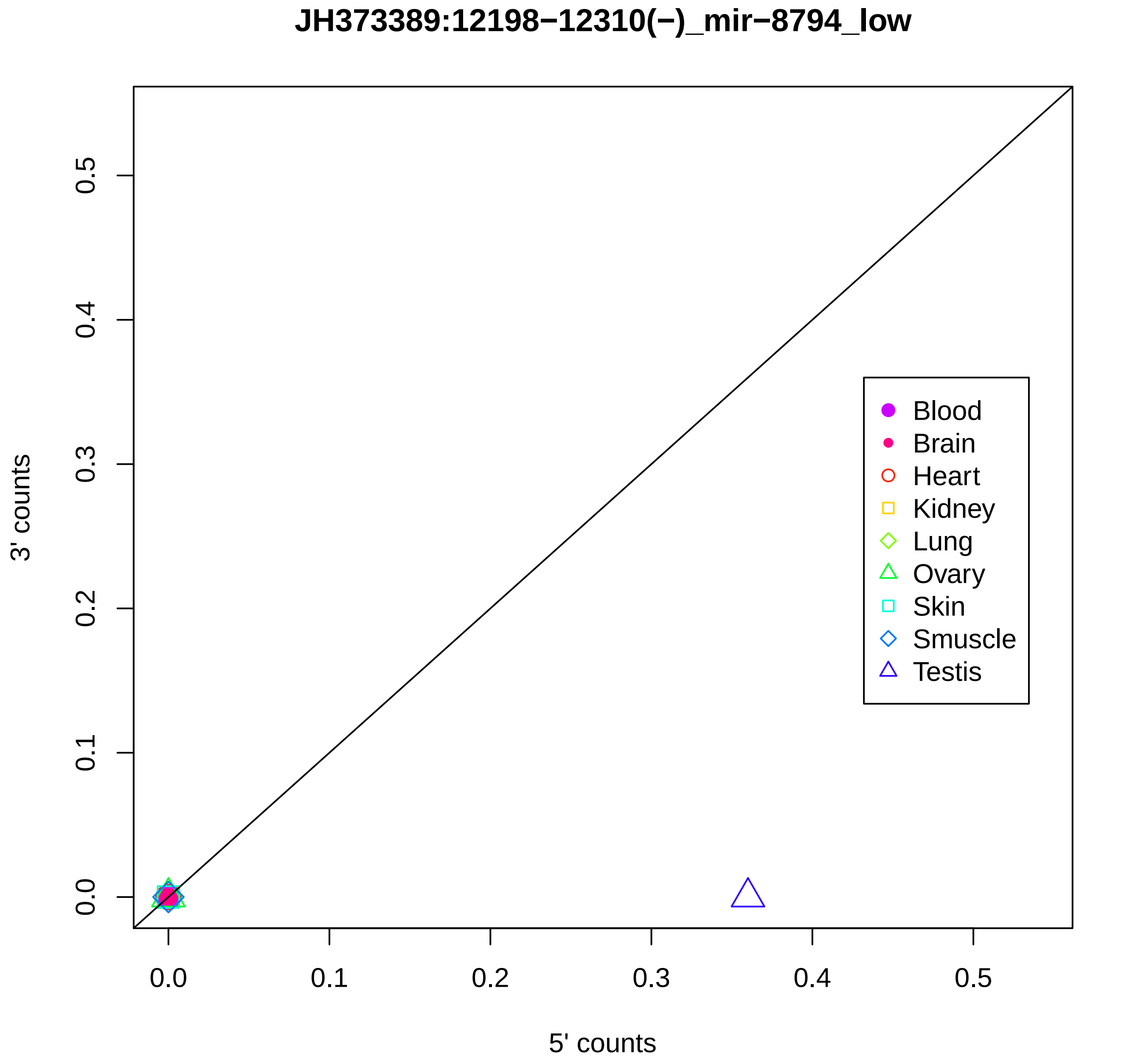

JH373389:12198−12310(−)_mir−8794_low
3' counts
5' counts
0.0
0.1
0.2
0.3
0.4
0.5
Blood
Brain
Heart
Kidney
Lung
Ovary
Skin
Smuscle
Testis

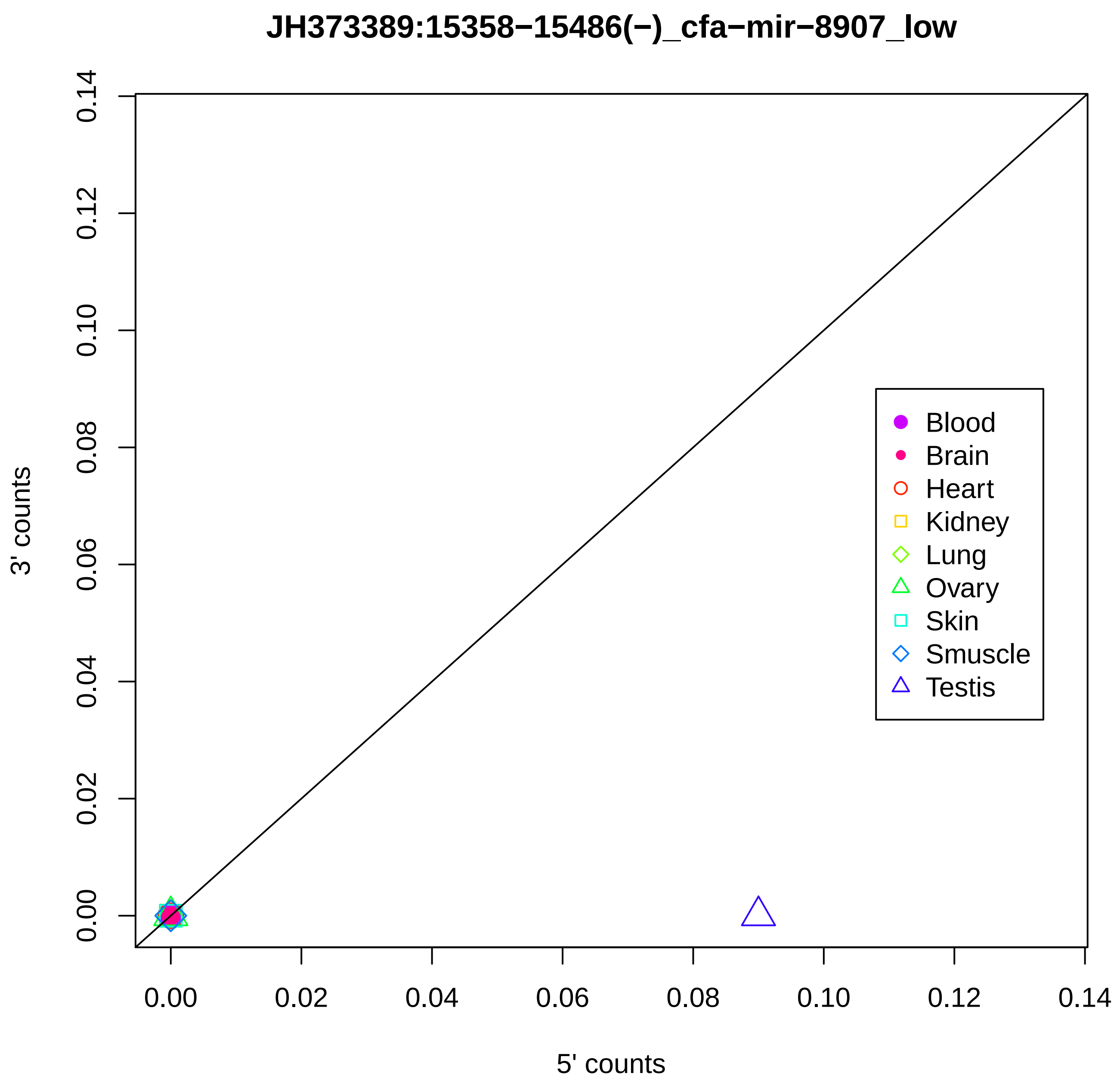

JH373389:15358−15486(−)_cfa−mir−8907_low
3' counts
5' counts
0.00
0.02
0.04
0.06
0.08
0.10
0.12
0.14
Blood
Brain
Heart
Kidney
Lung
Ovary
Skin
Smuscle
Testis

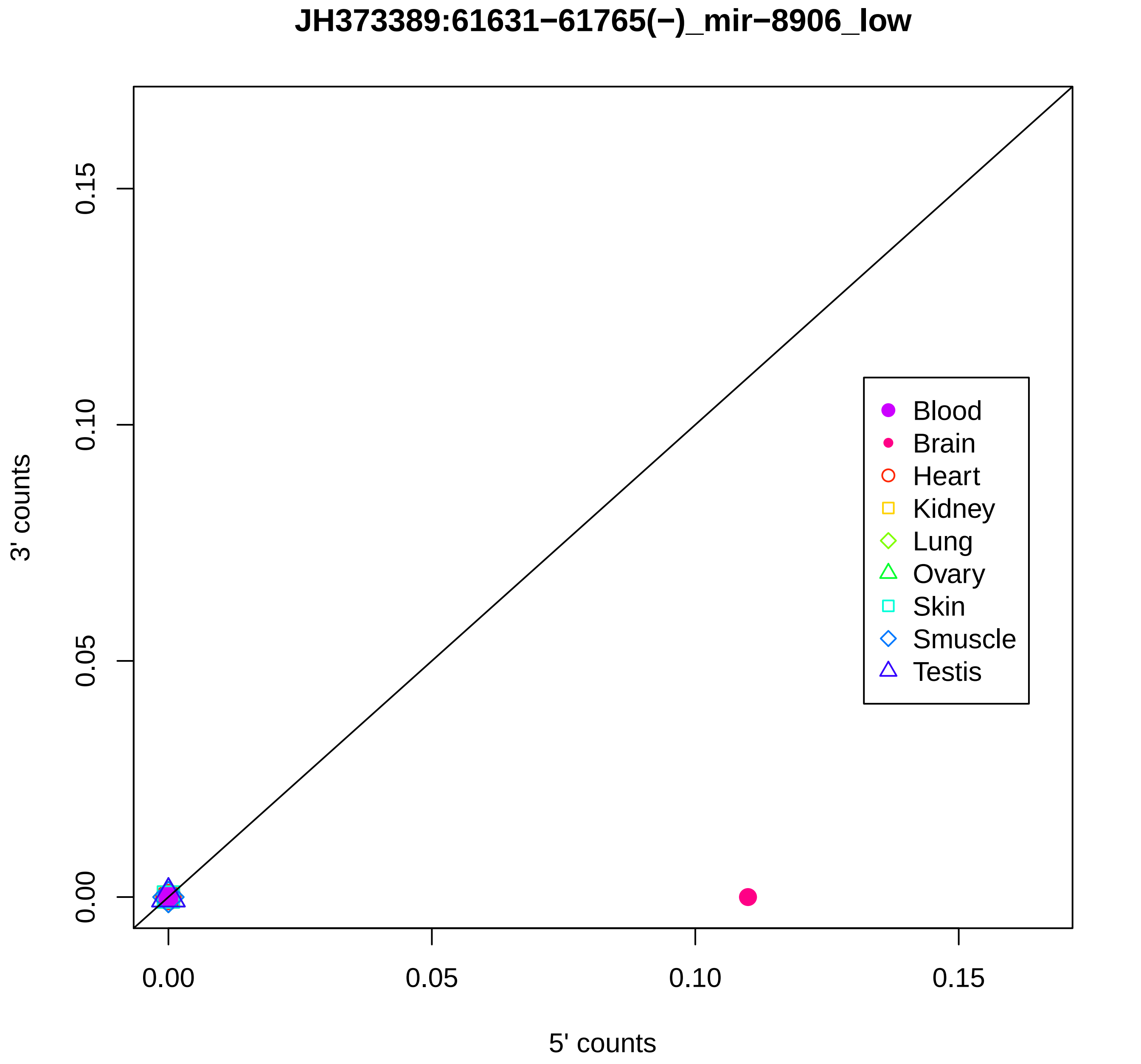

JH373389:61631−61765(−)_mir−8906_low
3' counts
5' counts
0.00
0.05
0.10
0.15
Blood
Brain
Heart
Kidney
Lung
Ovary
Skin
Smuscle
Testis

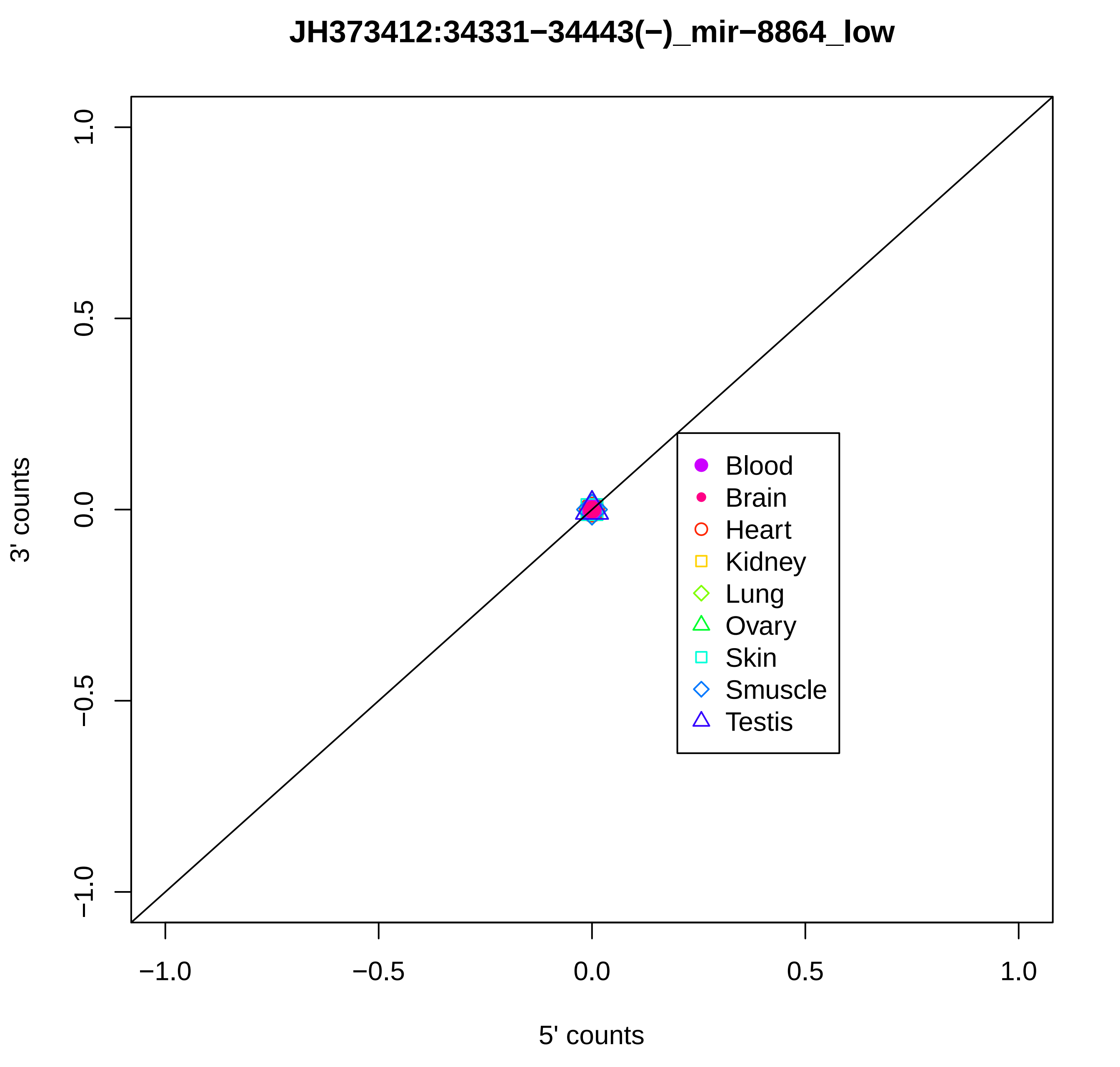

JH373412:34331−34443(−)_mir−8864_low
3' counts
5' counts
−1.0
−0.5
0.0
0.5
1.0
Blood
Brain
Heart
Kidney
Lung
Ovary
Skin
Smuscle
Testis

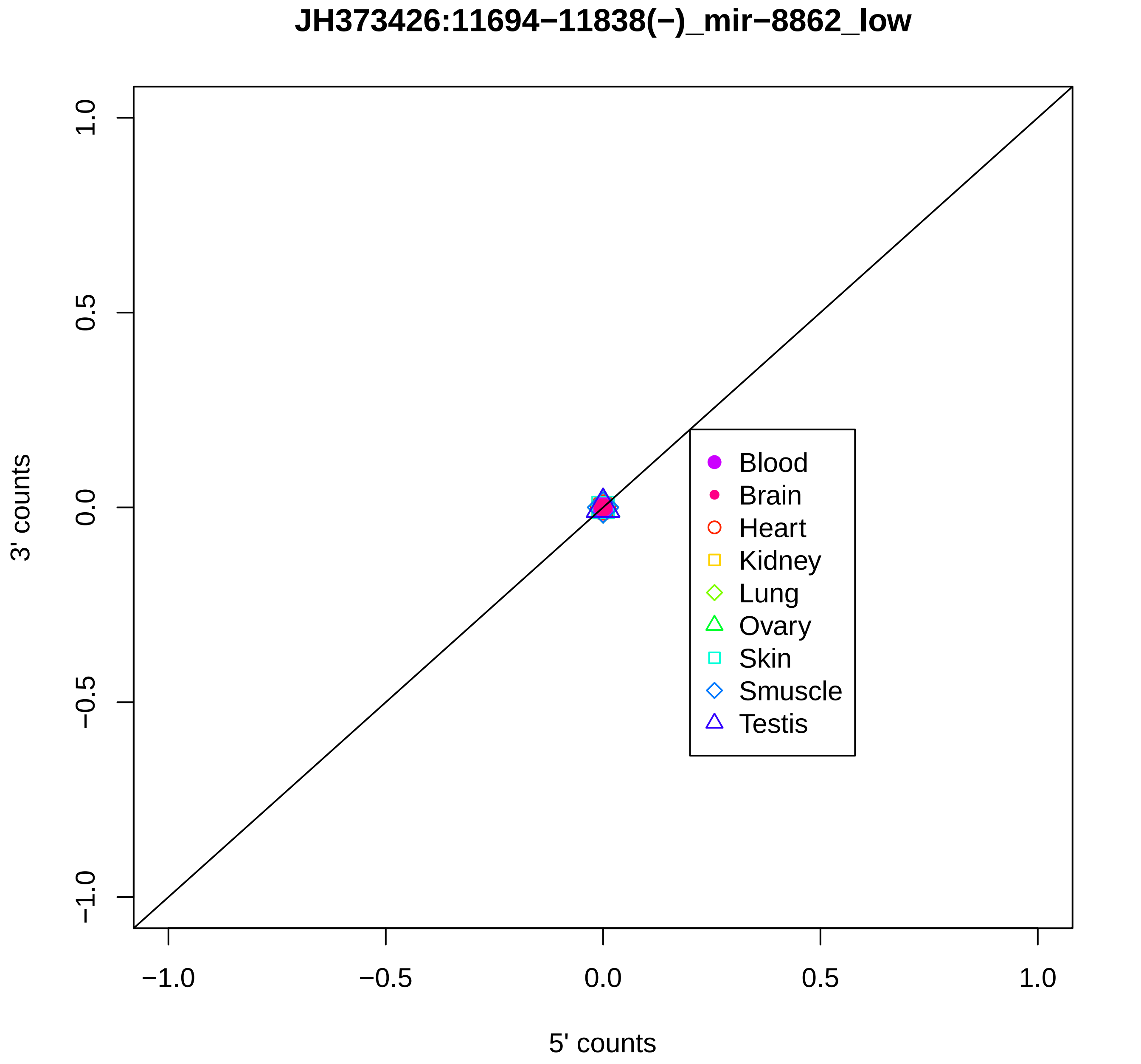
JH373426:11694−11838(−)_mir−8862_low
3' counts
5' counts
1.0
0.5
0.0
−0.5
−1.0
−1.0
−0.5
0.0
0.5
1.0
Blood
Brain
Heart
Kidney
Lung
Ovary
Skin
Smuscle
Testis

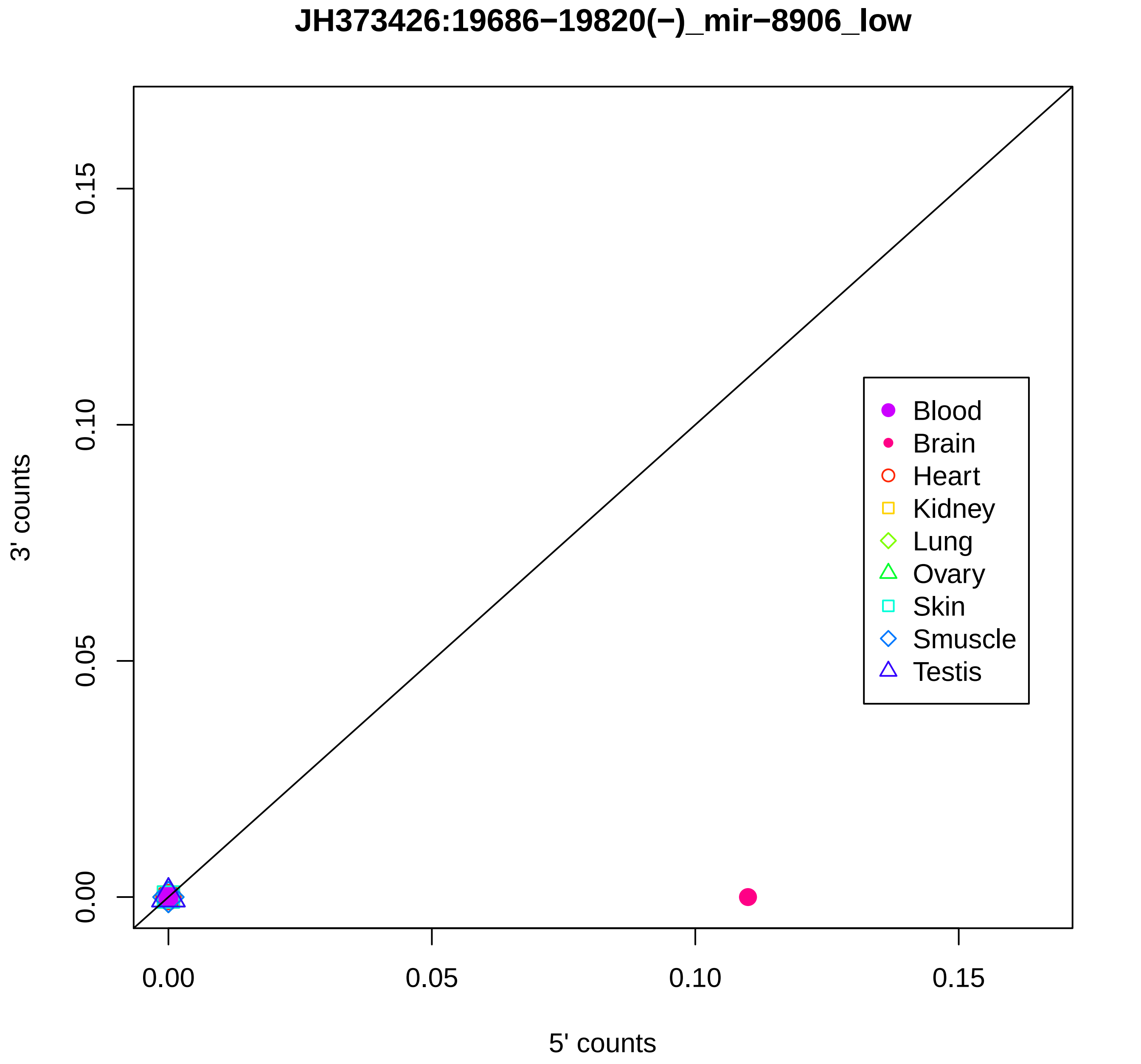
JH373426:19686−19820(−)_mir−8906_low
3' counts
5' counts
0.00
0.05
0.10
0.15
Blood
Brain
Heart
Kidney
Lung
Ovary
Skin
Smuscle
Testis

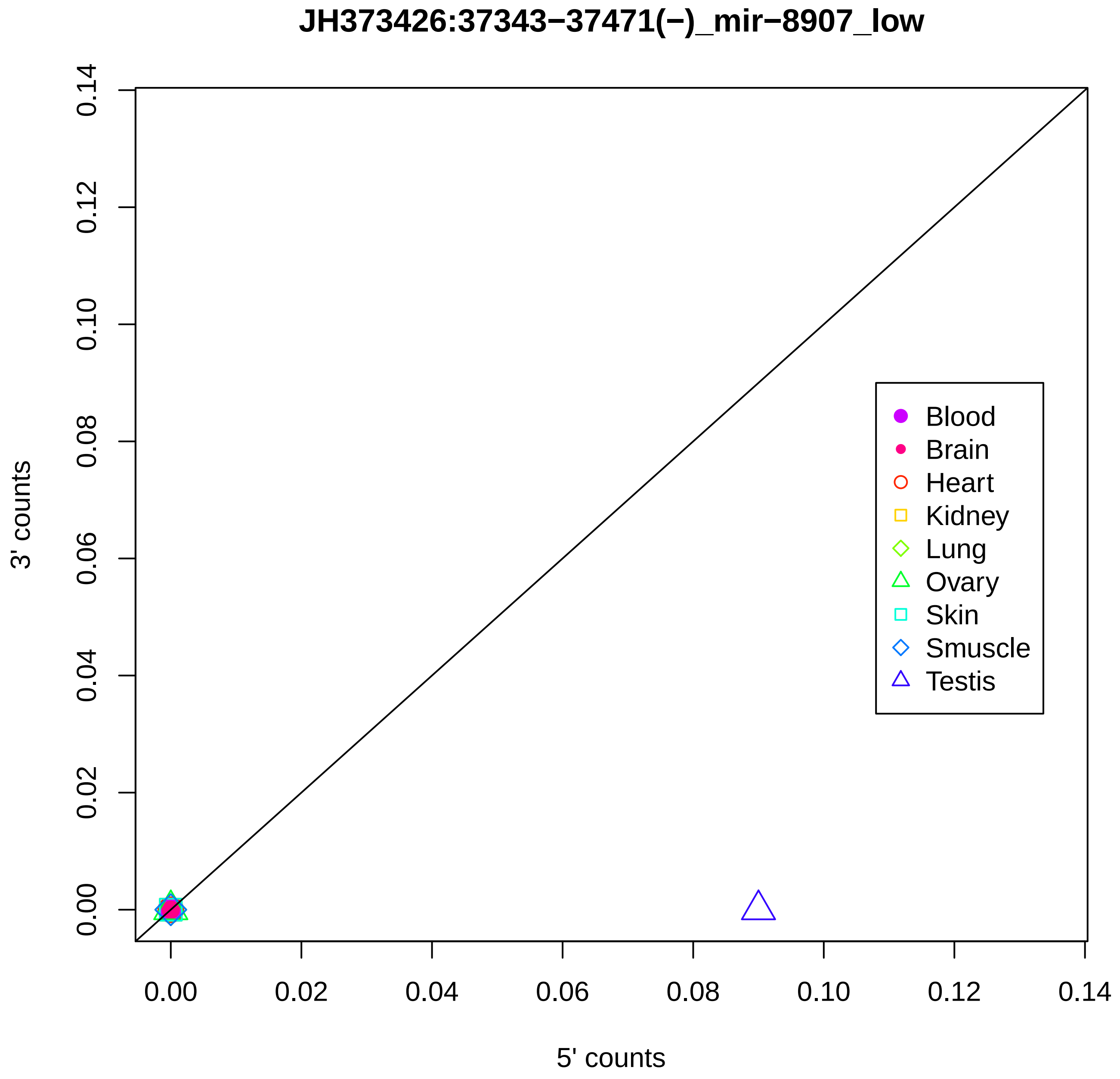

JH373426:37343−37471(−)_mir−8907_low
3' counts
5' counts
0.00
0.02
0.04
0.06
0.08
0.10
0.12
0.14
Blood
Brain
Heart
Kidney
Lung
Ovary
Skin
Smuscle
Testis

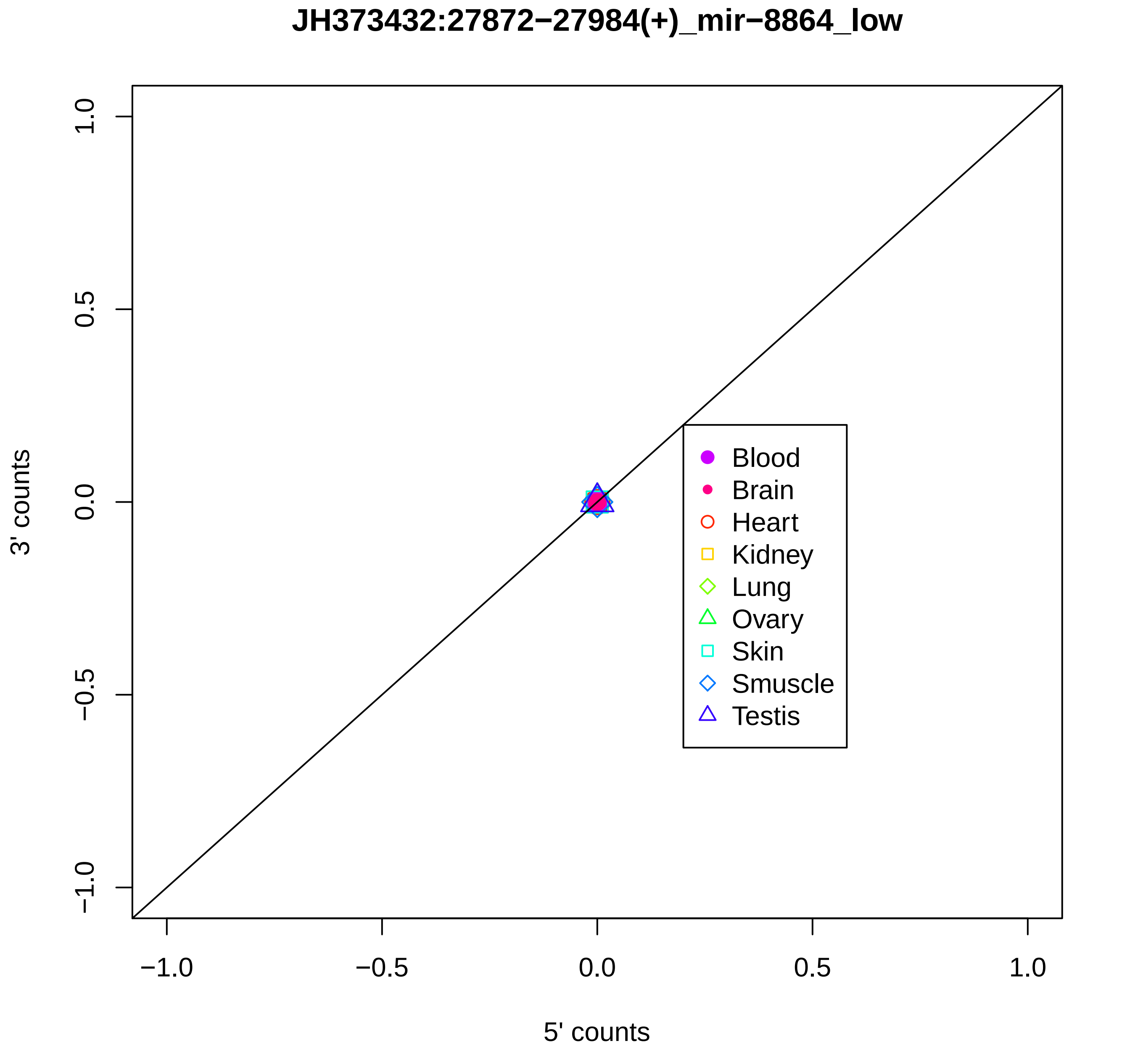

JH373432:27872−27984(+)_mir−8864_low
3' counts
5' counts
1.0
0.5
0.0
−0.5
−1.0
−1.0
−0.5
0.0
0.5
1.0
Blood
Brain
Heart
Kidney
Lung
Ovary
Skin
Smuscle
Testis

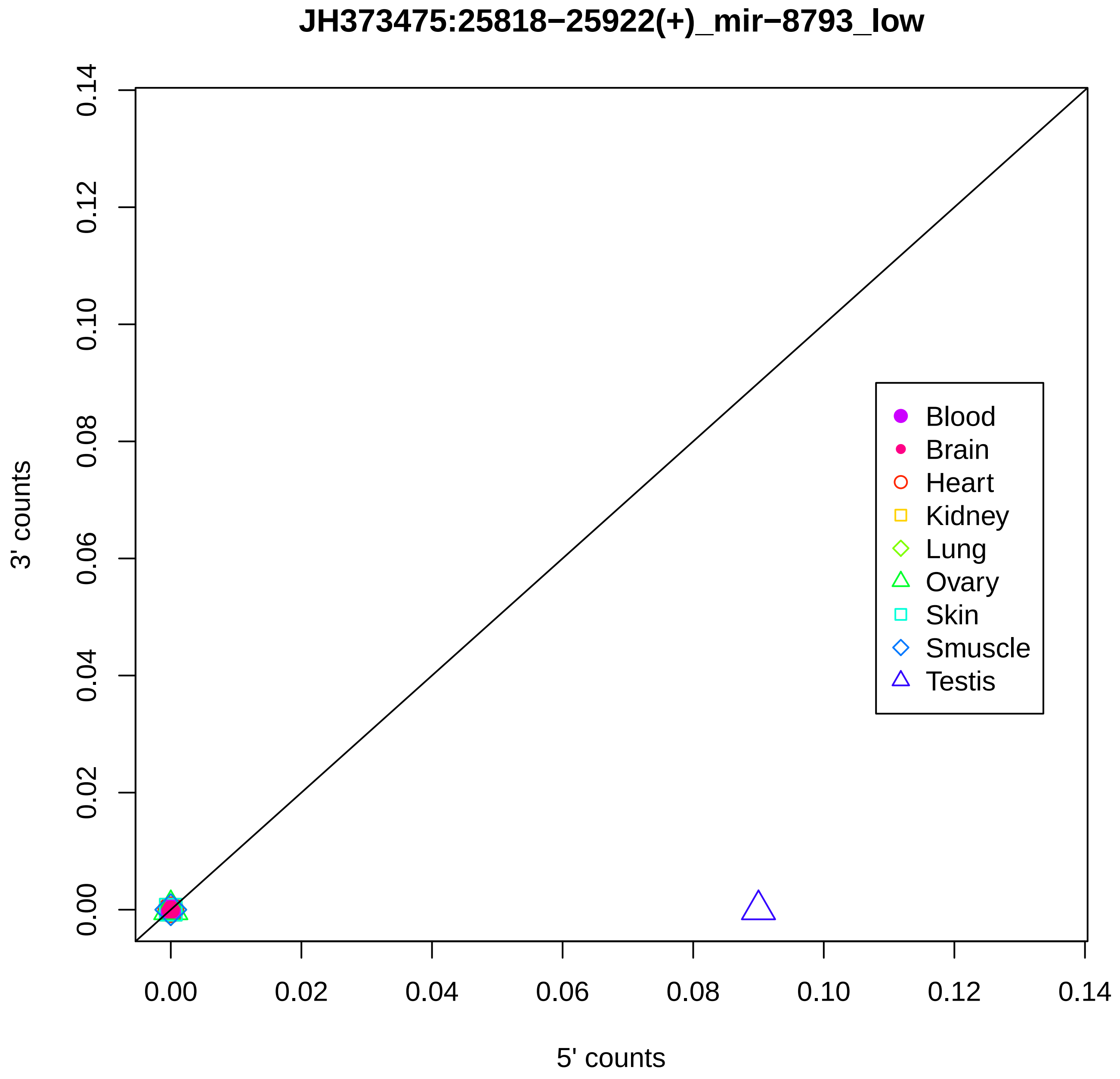
JH373475:25818−25922(+)_mir−8793_low
3' counts
5' counts
0.00
0.02
0.04
0.06
0.08
0.10
0.12
0.14
Blood
Brain
Heart
Kidney
Lung
Ovary
Skin
Smuscle
Testis

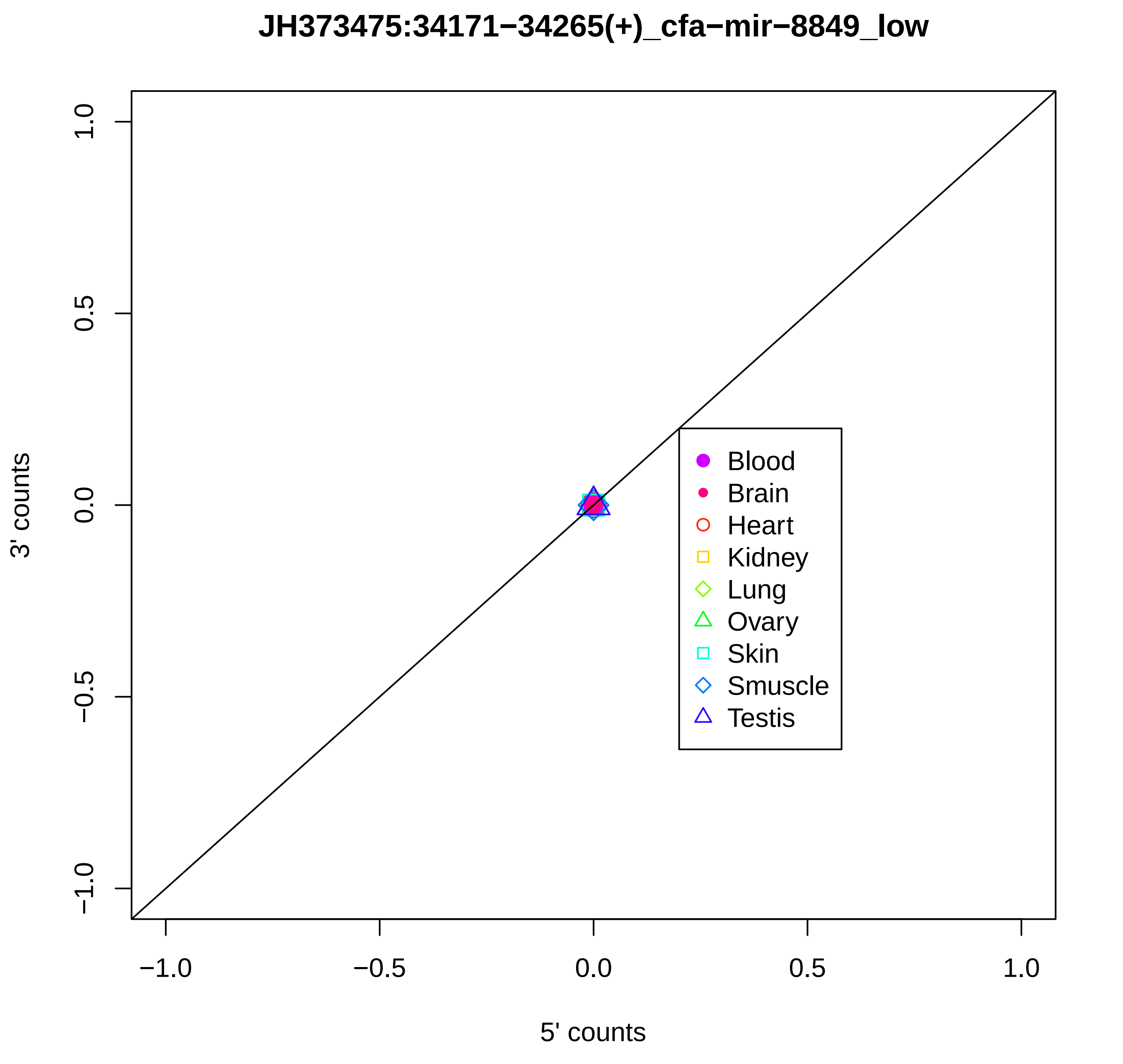
JH373475:34171−34265(+)_cfa−mir−8849_low
3' counts
5' counts
1.0
0.5
0.0
−0.5
−1.0
Blood
Brain
Heart
Kidney
Lung
Ovary
Skin
Smuscle
Testis

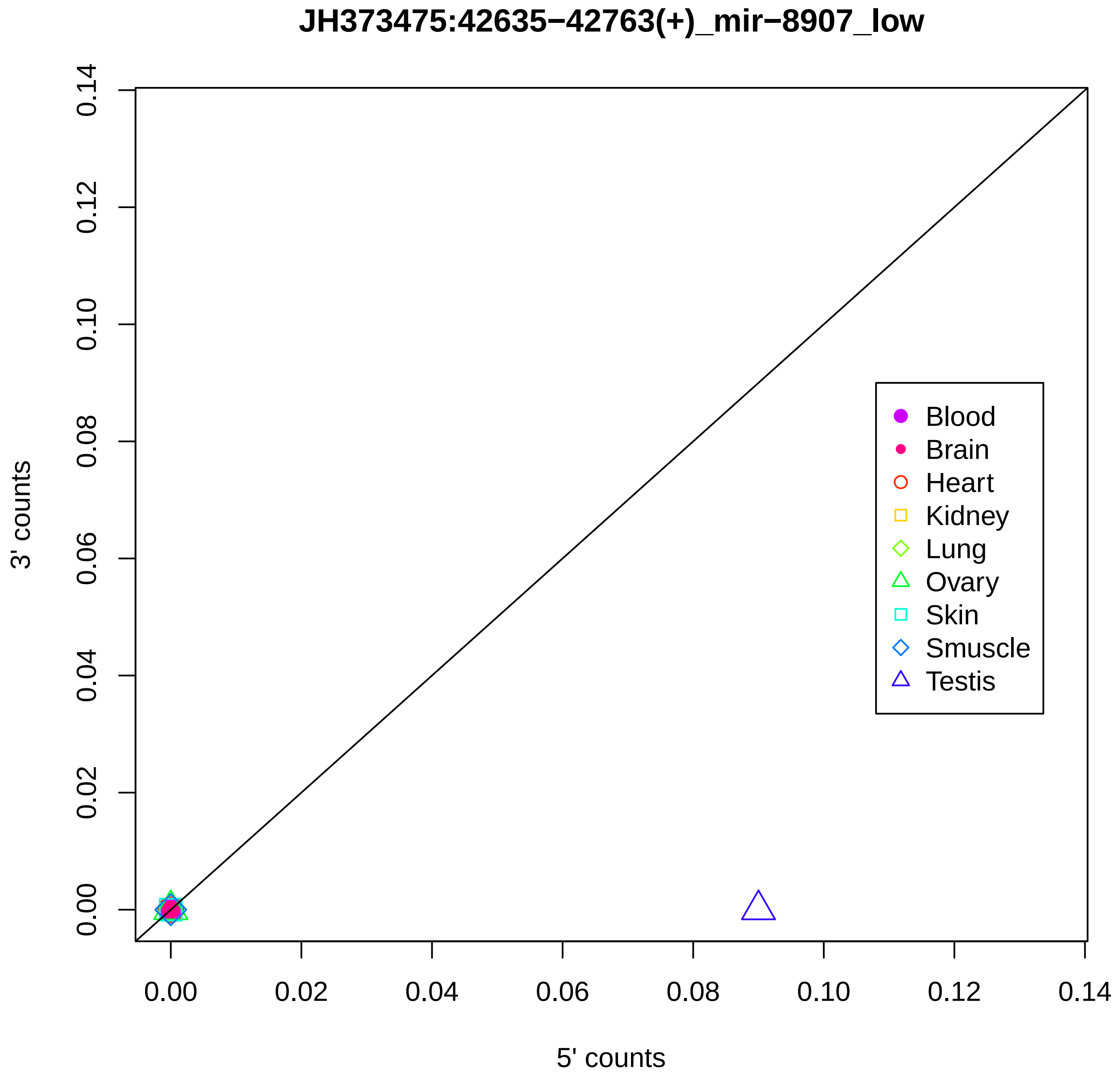

JH373475:42635−42763(+)_mir−8907_low
3' counts
5' counts
0.00
0.02
0.04
0.06
0.08
0.10
0.12
0.14
Blood
Brain
Heart
Kidney
Lung
Ovary
Skin
Smuscle
Testis

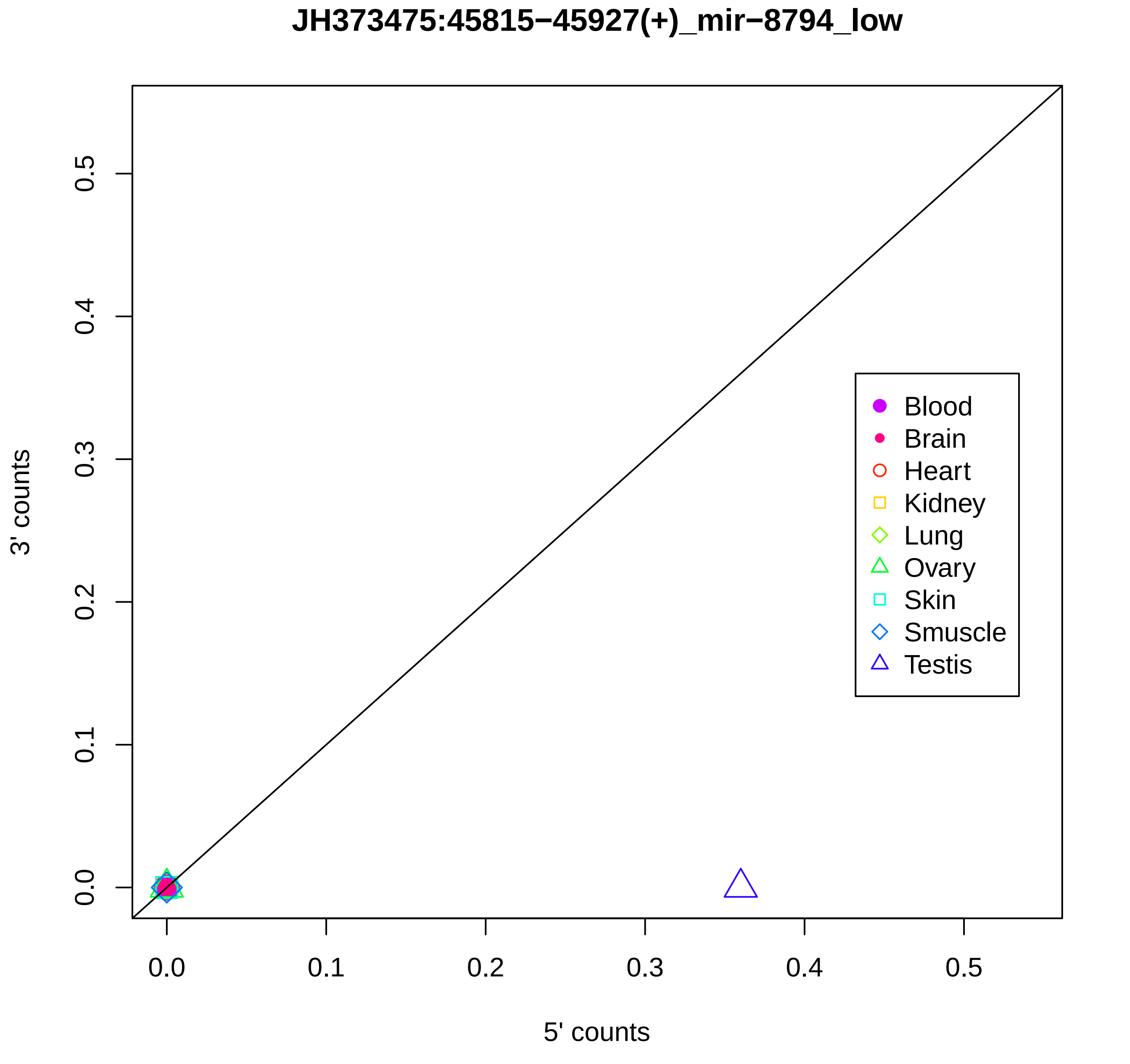
JH373475:45815−45927(+)_mir−8794_low
3' counts
5' counts
0.0
0.1
0.2
0.3
0.4
0.5
Blood
Brain
Heart
Kidney
Lung
Ovary
Skin
Smuscle
Testis

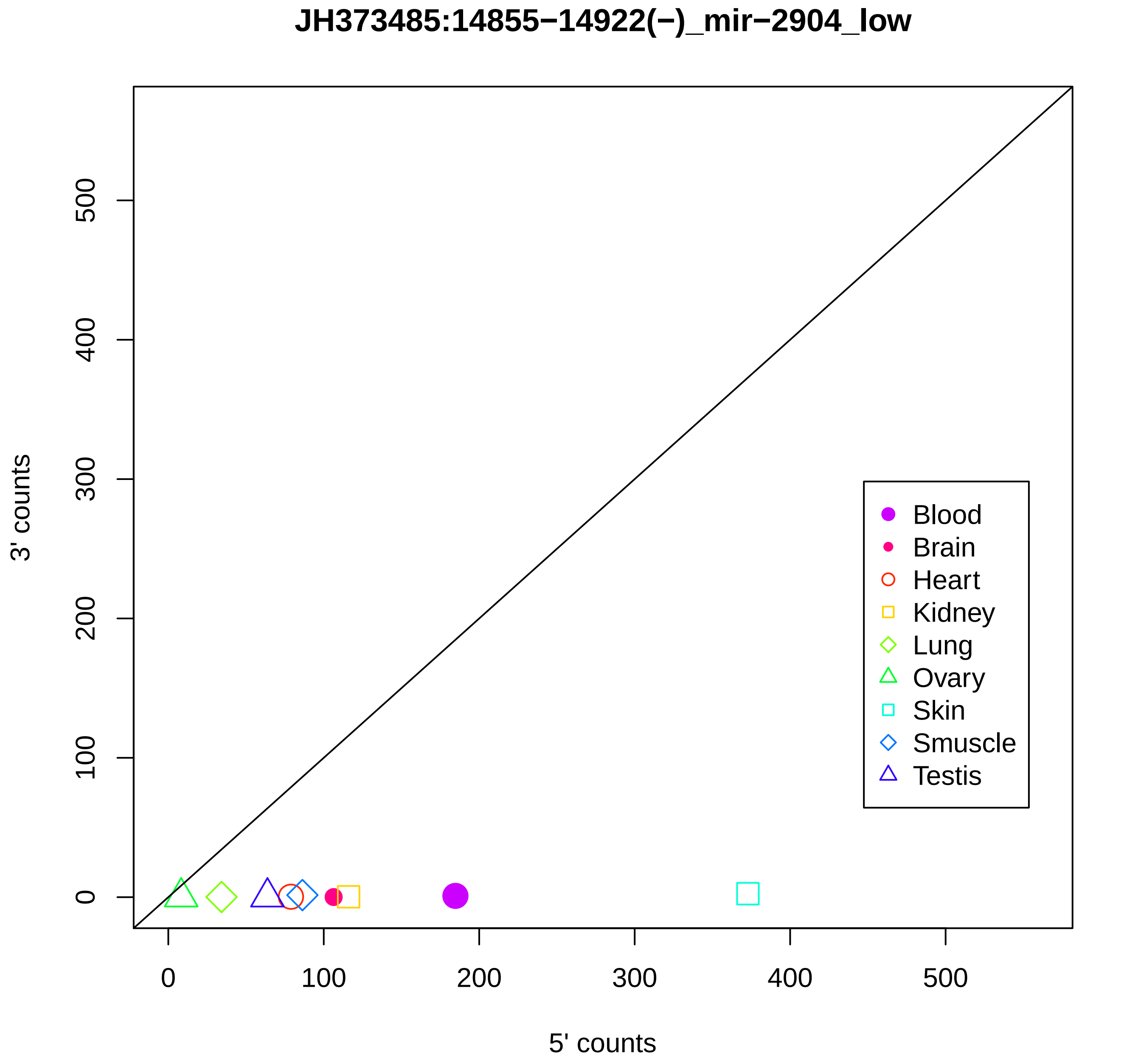

JH373485:14855−14922(−)_mir−2904_low
3' counts
5' counts
0
100
200
300
400
500
Blood
Brain
Heart
Kidney
Lung
Ovary
Skin
Smuscle
Testis

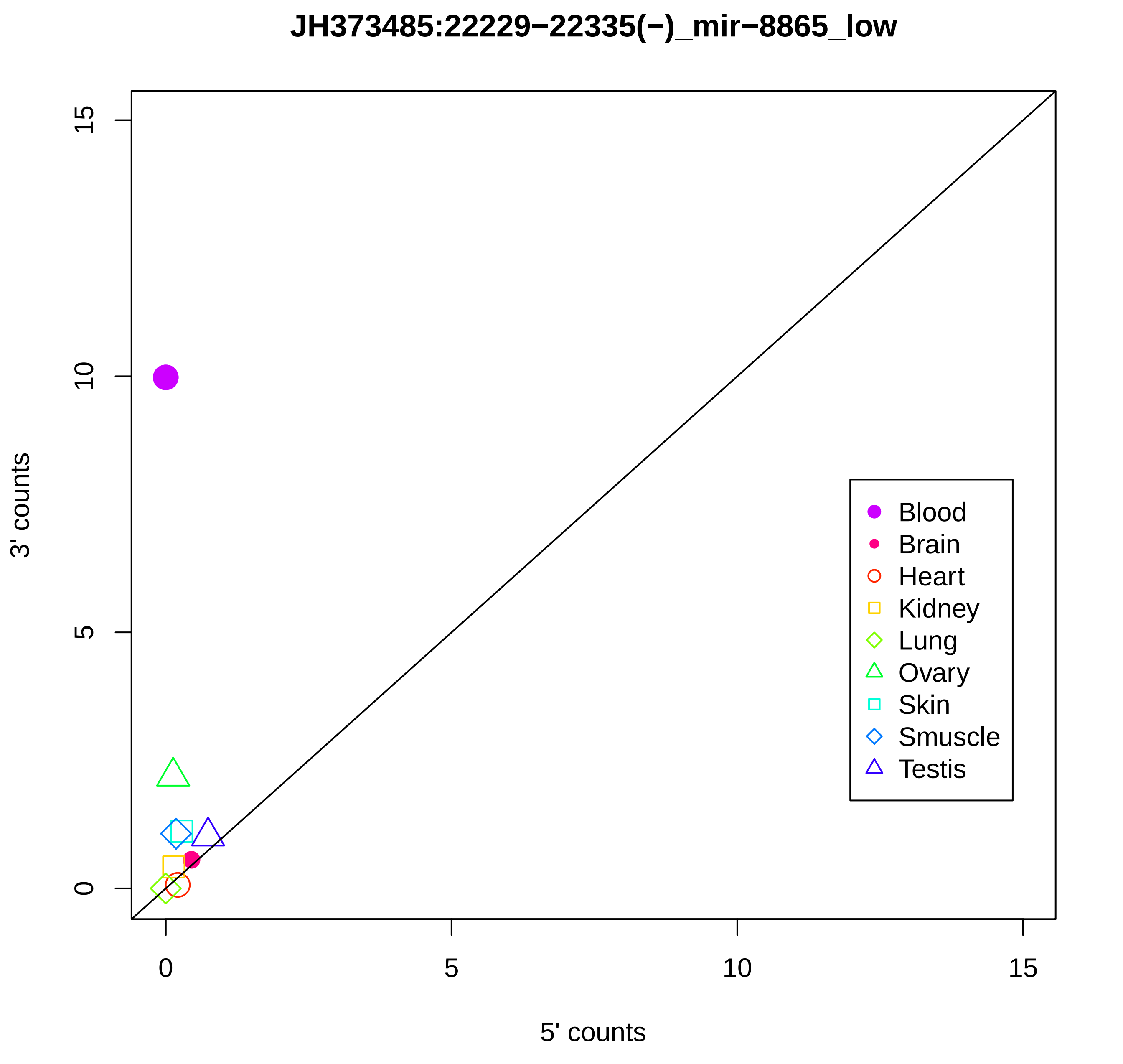
JH373485:22229−22335(−)_mir−8865_low
3' counts
5' counts
0
5
10
15
Blood
Brain
Heart
Kidney
Lung
Ovary
Skin
Smuscle
Testis

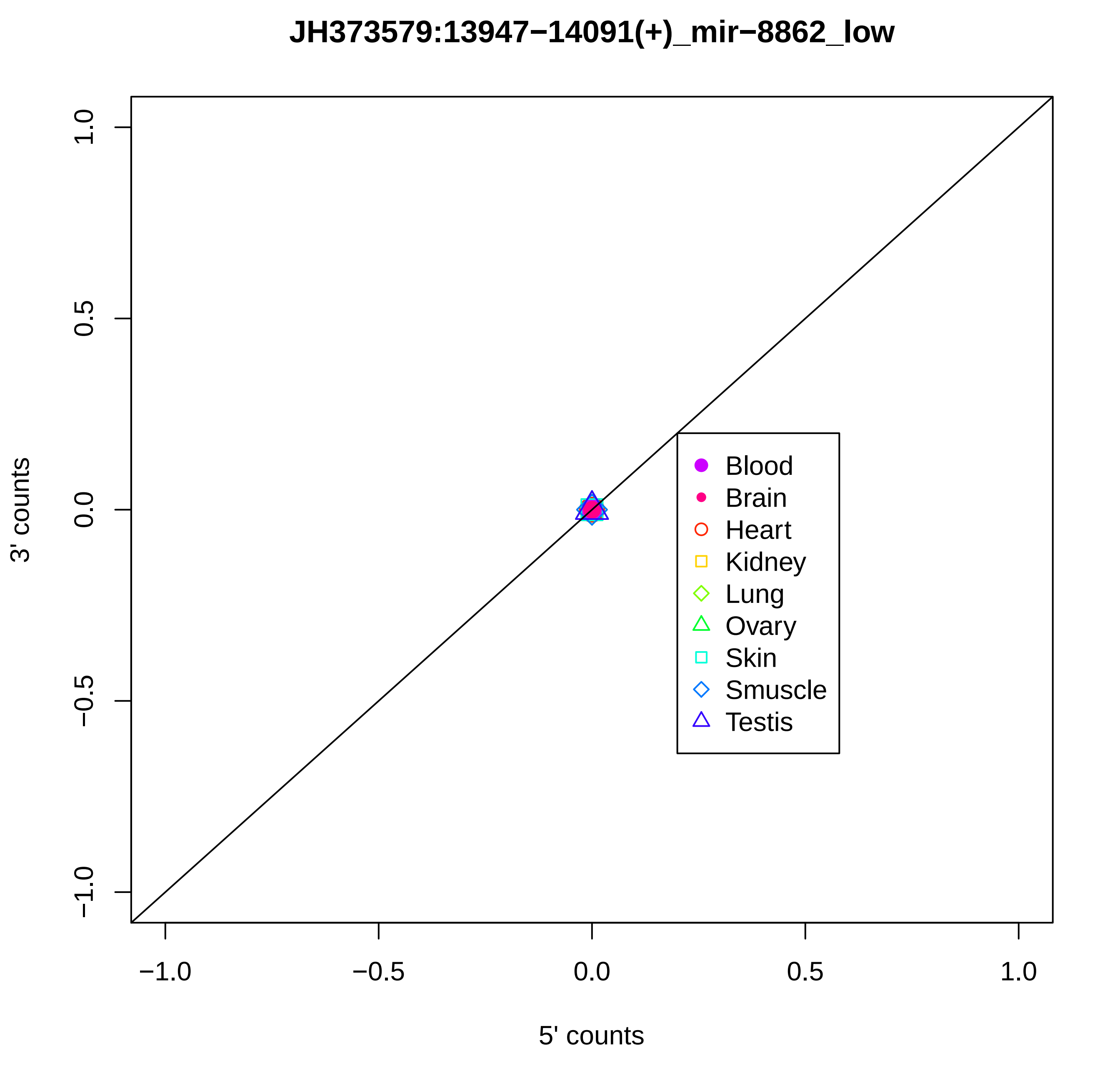

JH373579:13947−14091(+)_mir−8862_low
1.0
0.5
0.0
−0.5
−1.0
3' counts
−1.0
−0.5
0.0
0.5
1.0
5' counts
Blood
Brain
Heart
Kidney
Lung
Ovary
Skin
Smuscle
Testis

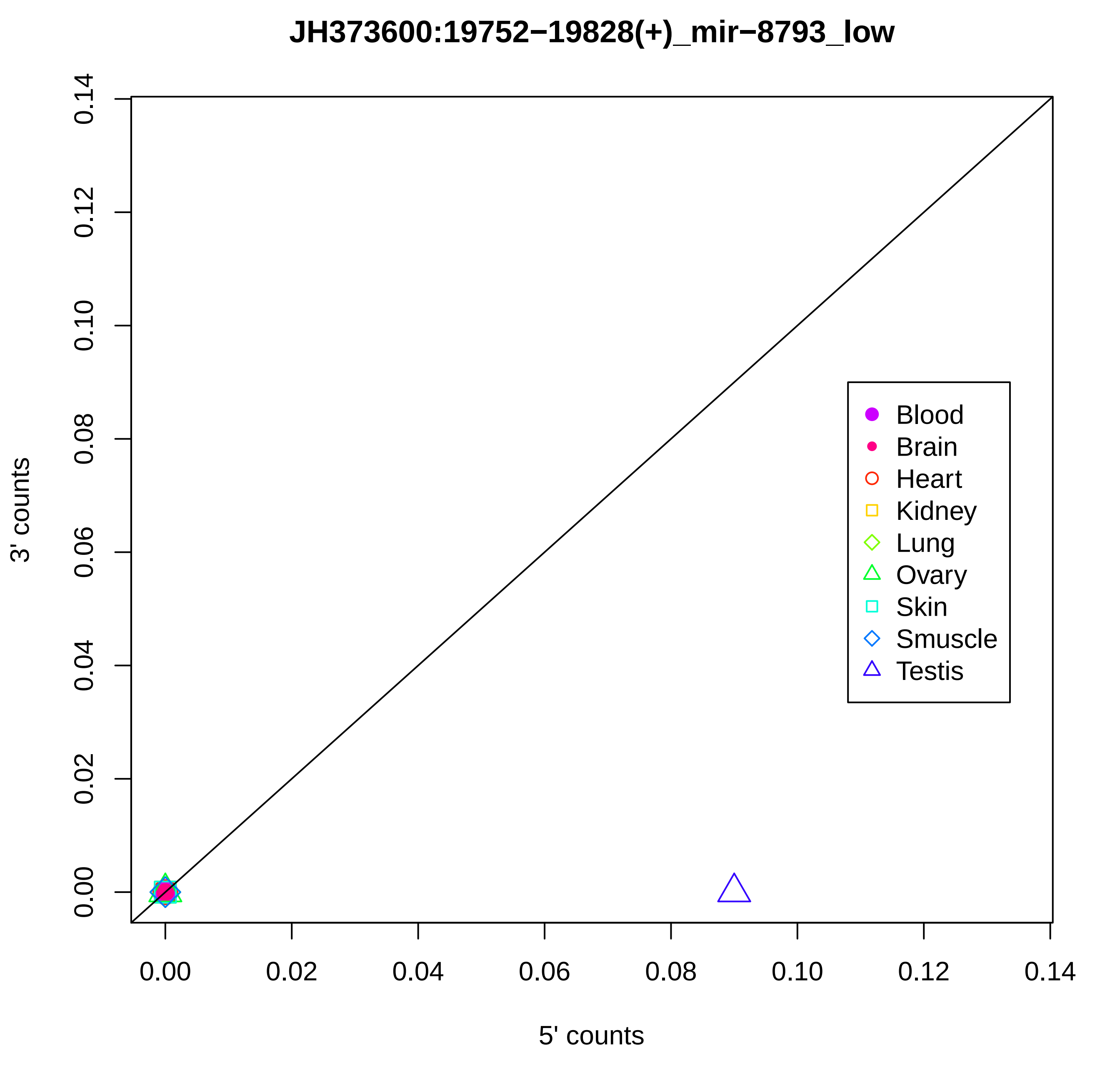
JH373600:19752−19828(+)_mir−8793_low
3' counts
0.00
0.02
0.04
0.06
0.08
0.10
0.12
0.14
5' counts
Blood
Brain
Heart
Kidney
Lung
Ovary
Skin
Smuscle
Testis

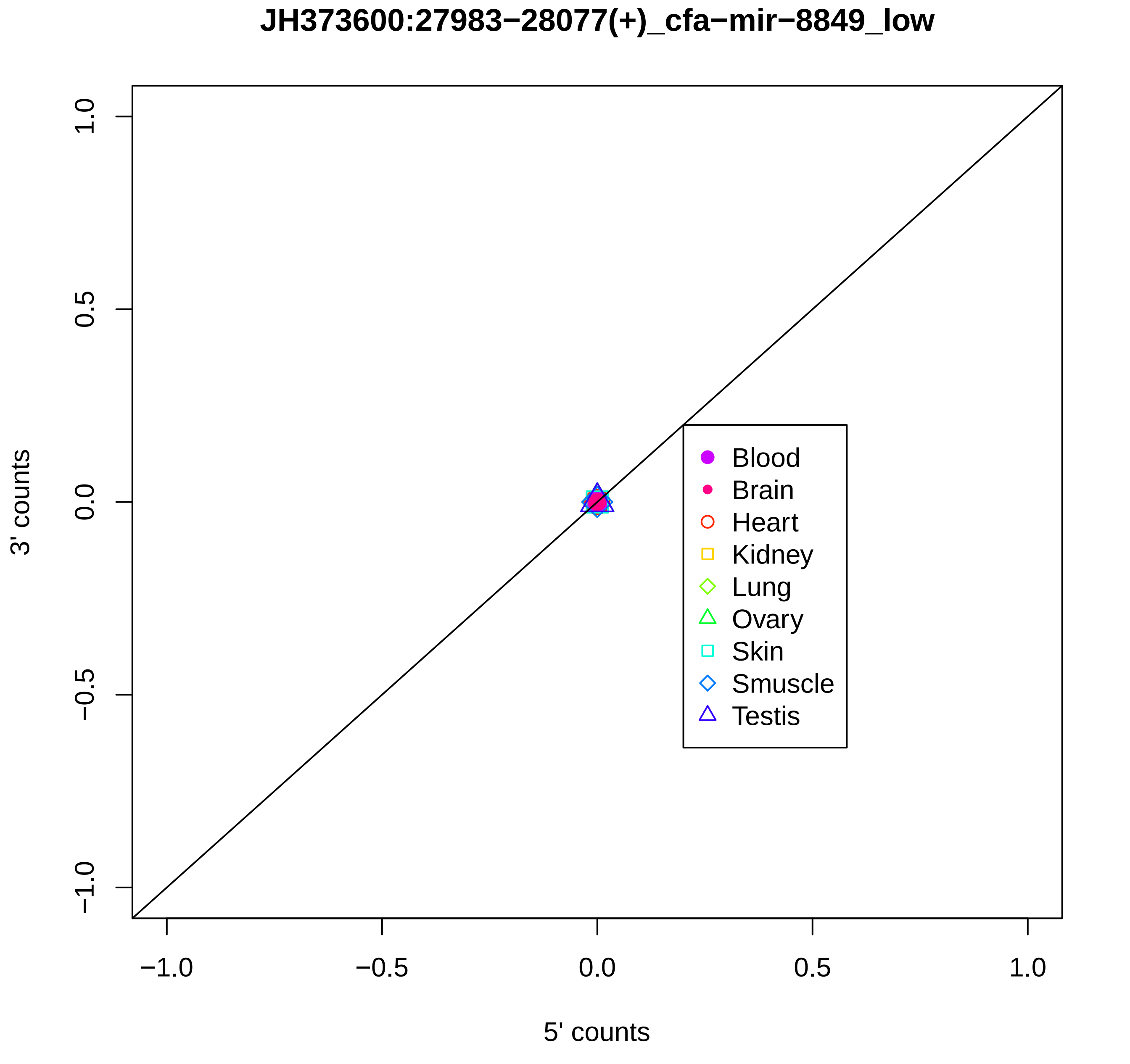

JH373600:27983−28077(+)_cfa−mir−8849_low
3' counts
5' counts
1.0
0.5
0.0
−0.5
−1.0
Blood
Brain
Heart
Kidney
Lung
Ovary
Skin
Smuscle
Testis

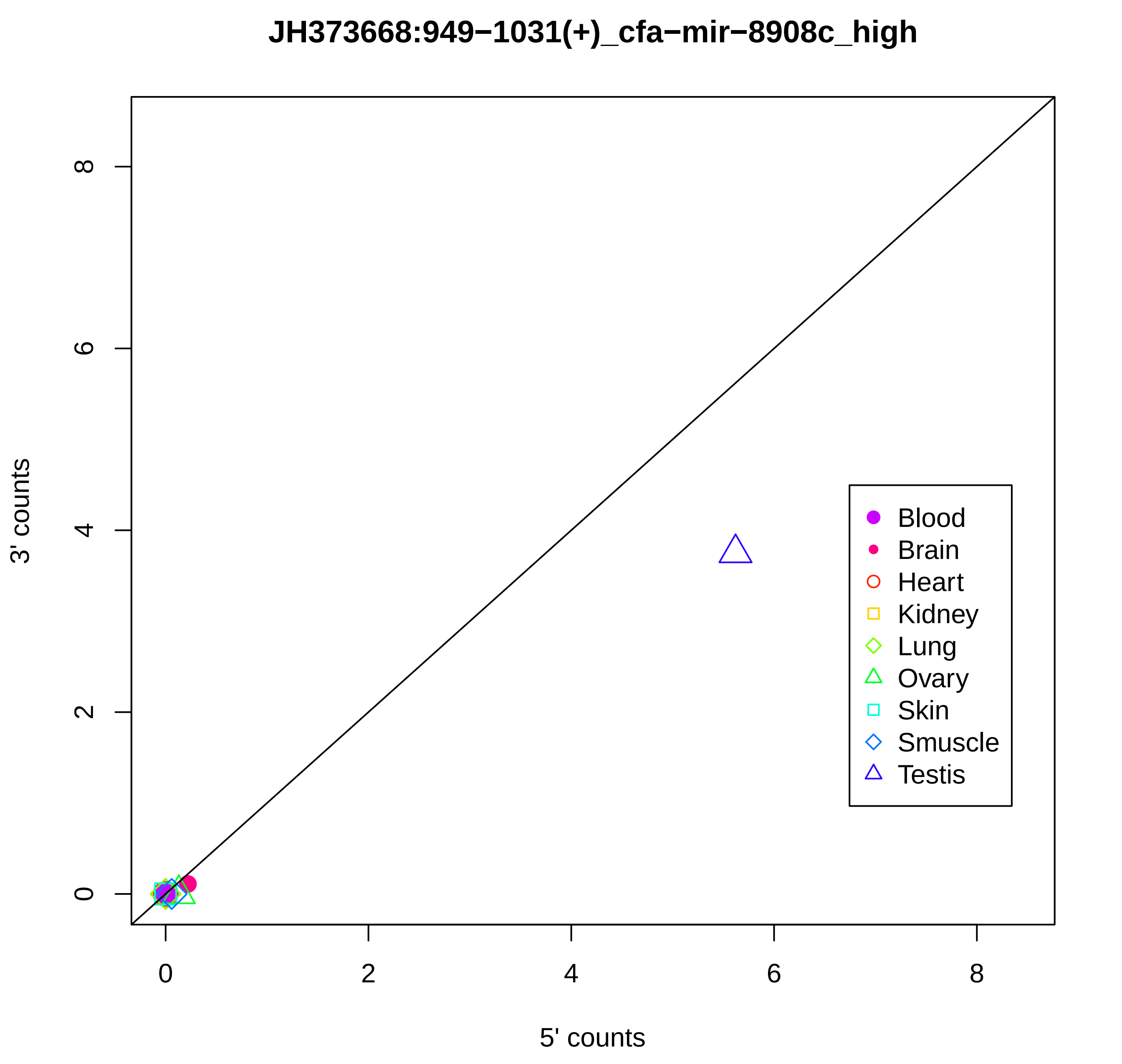

JH373668:949−1031(+)_cfa−mir−8908c_high
3' counts
5' counts
0
2
4
6
8
0
2
4
6
8
Blood
Brain
Heart
Kidney
Lung
Ovary
Skin
Smuscle
Testis

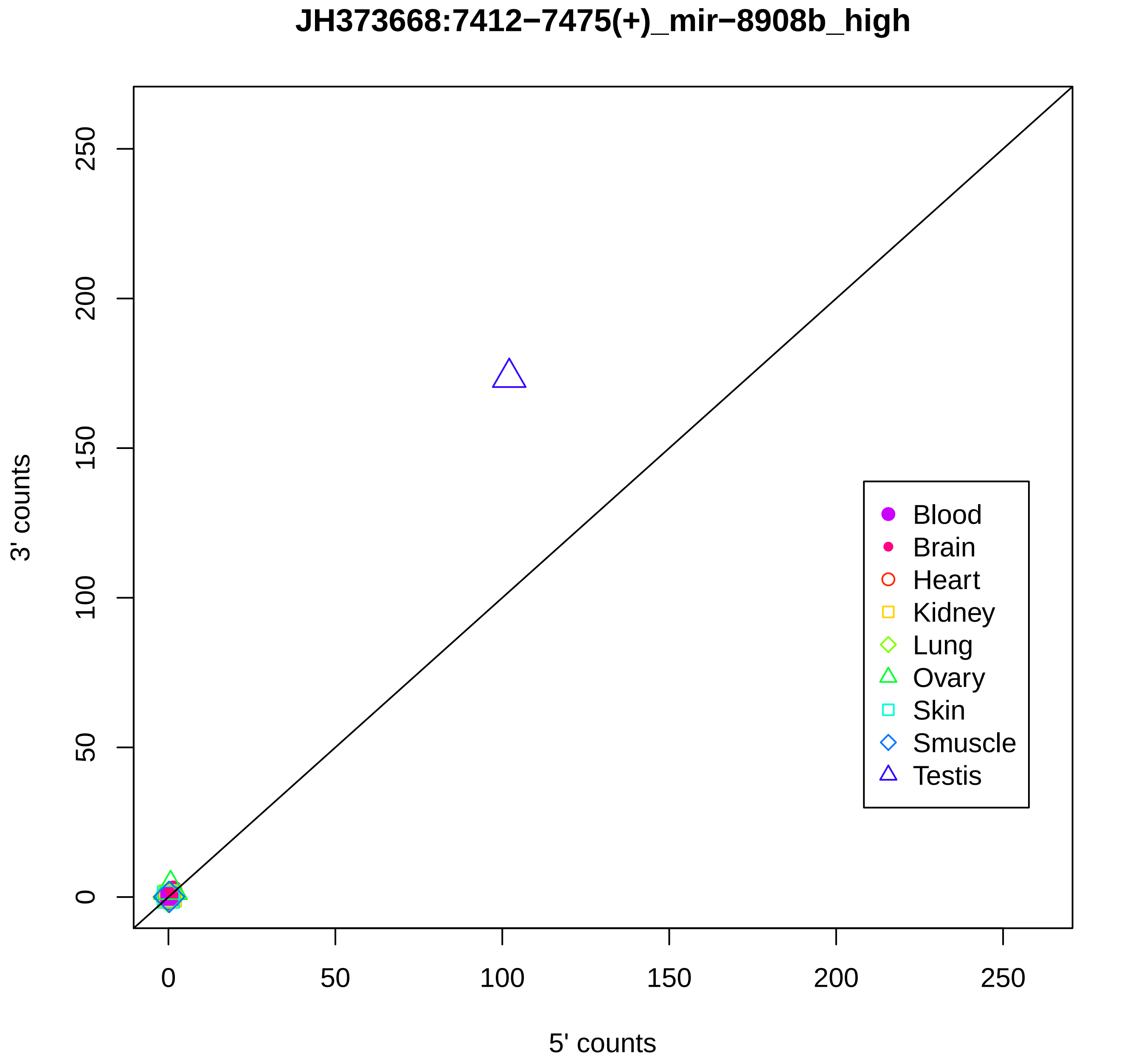
JH373668:7412−7475(+)_mir−8908b_high
3' counts
5' counts
0
50
100
150
200
250
Blood
Brain
Heart
Kidney
Lung
Ovary
Skin
Smuscle
Testis

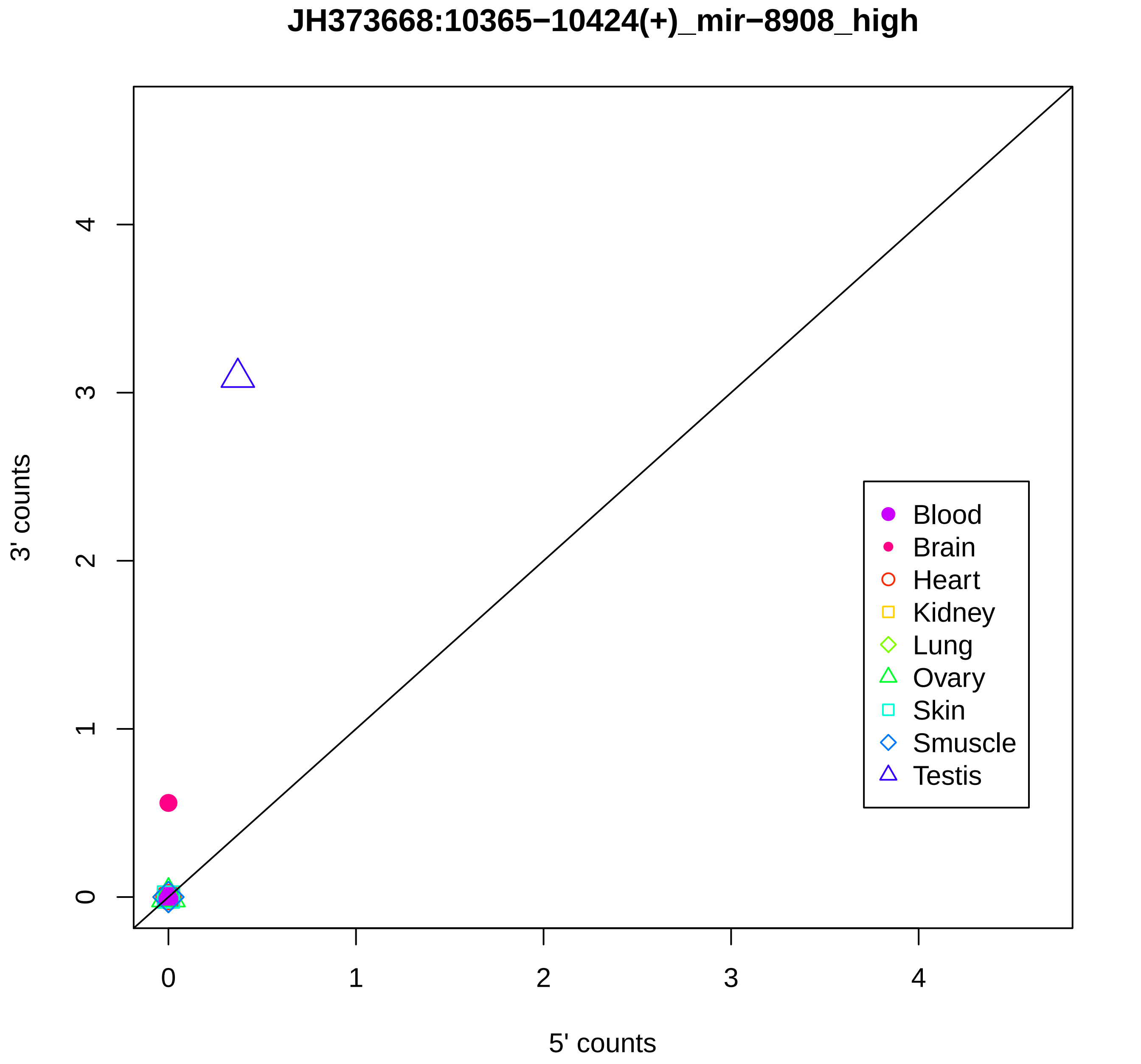

JH373668:10365−10424(+)_mir−8908_high
3' counts
5' counts
0
1
2
3
4
Blood
Brain
Heart
Kidney
Lung
Ovary
Skin
Smuscle
Testis

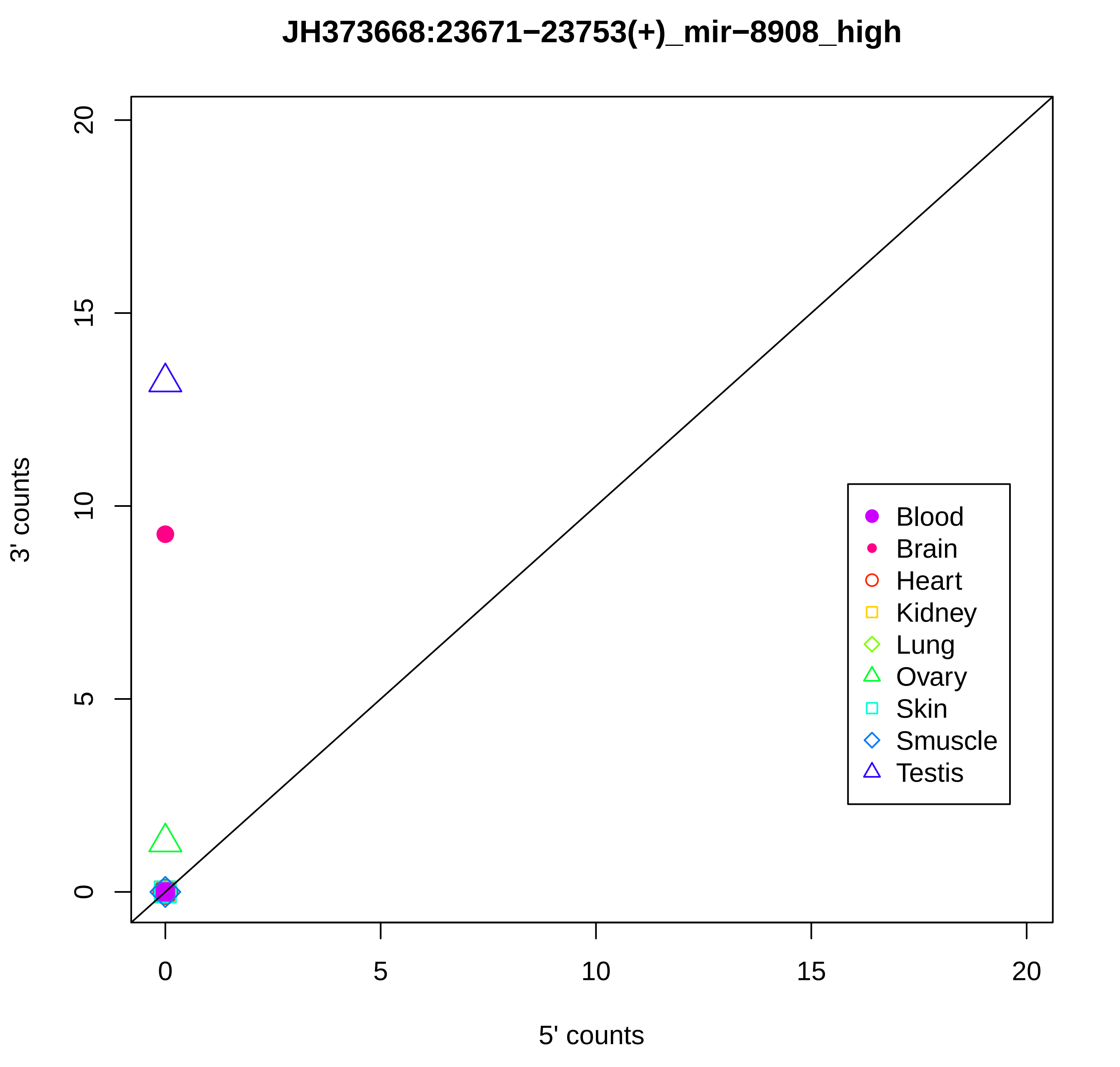

JH373668:23671−23753(+)_mir−8908_high
3' counts
5' counts
0
5
10
15
20
0
5
10
15
20
Blood
Brain
Heart
Kidney
Lung
Ovary
Skin
Smuscle
Testis

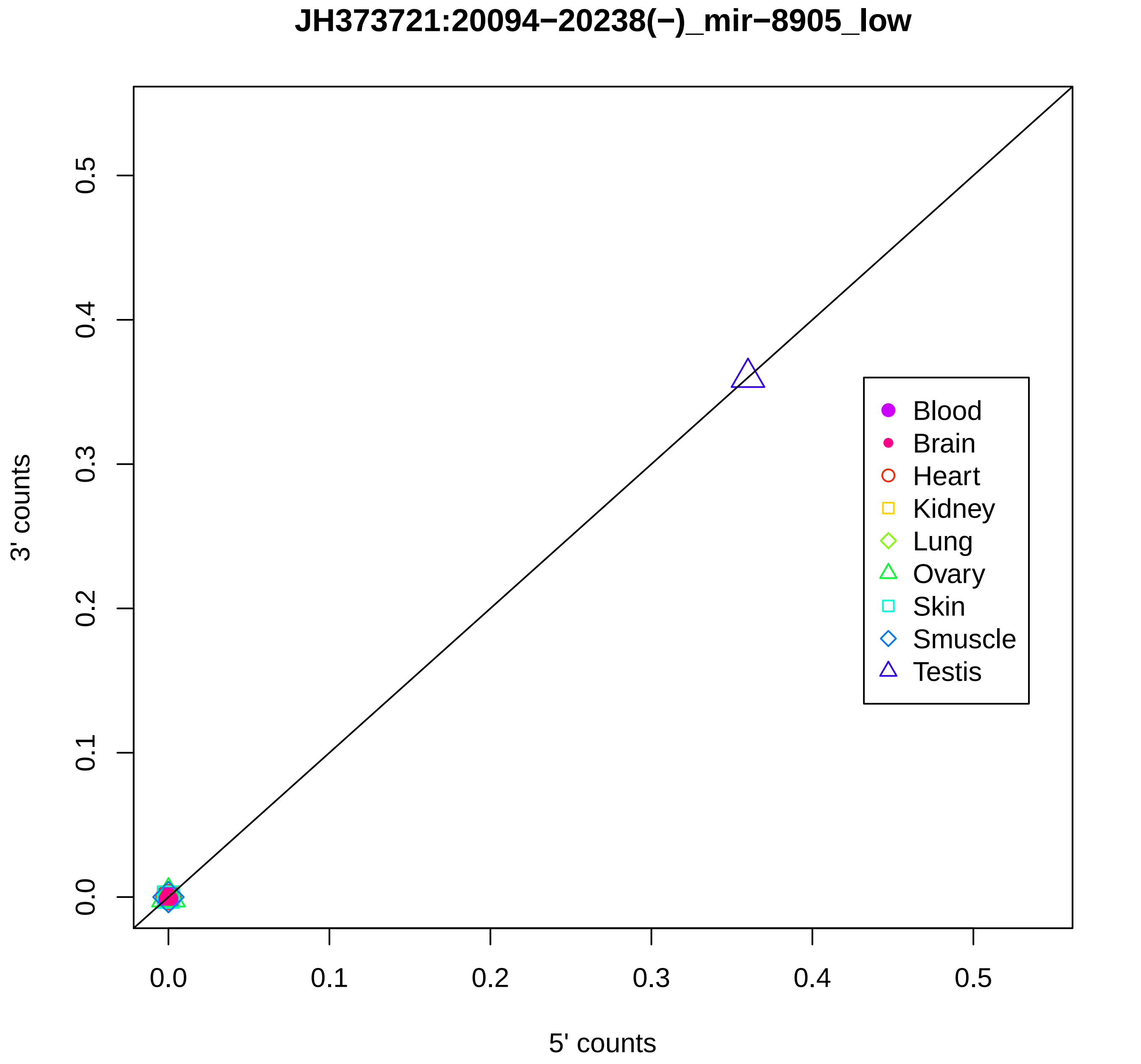

JH373721:20094−20238(−)_mir−8905_low
3' counts
5' counts
0.0
0.1
0.2
0.3
0.4
0.5
Blood
Brain
Heart
Kidney
Lung
Ovary
Skin
Smuscle
Testis

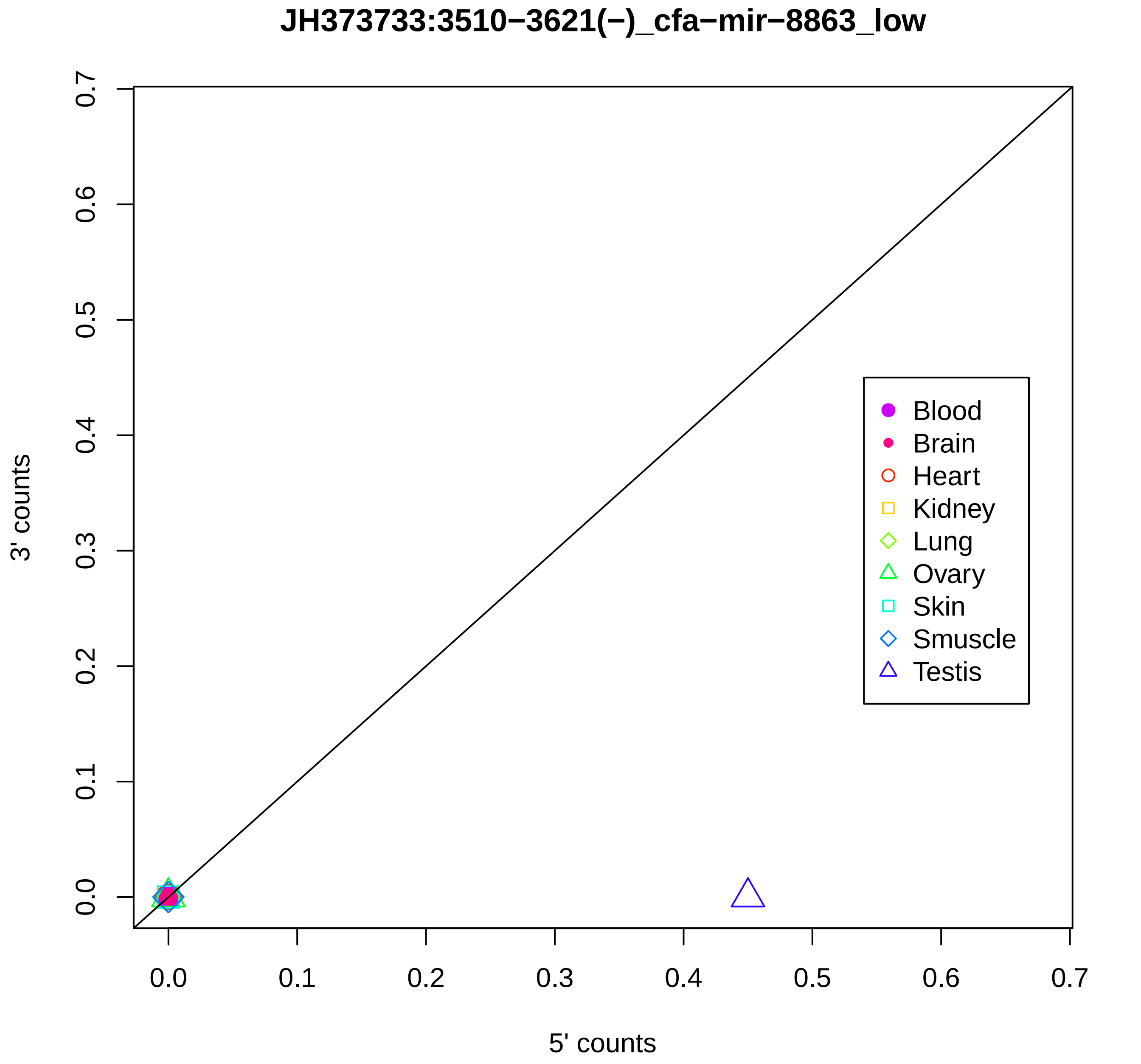

JH373733:3510−3621(−)_cfa−mir−8863_low
0.0
0.1
0.2
0.3
0.4
0.5
0.6
0.7
5' counts
3' counts
Blood
Brain
Heart
Kidney
Lung
Ovary
Skin
Smuscle
Testis

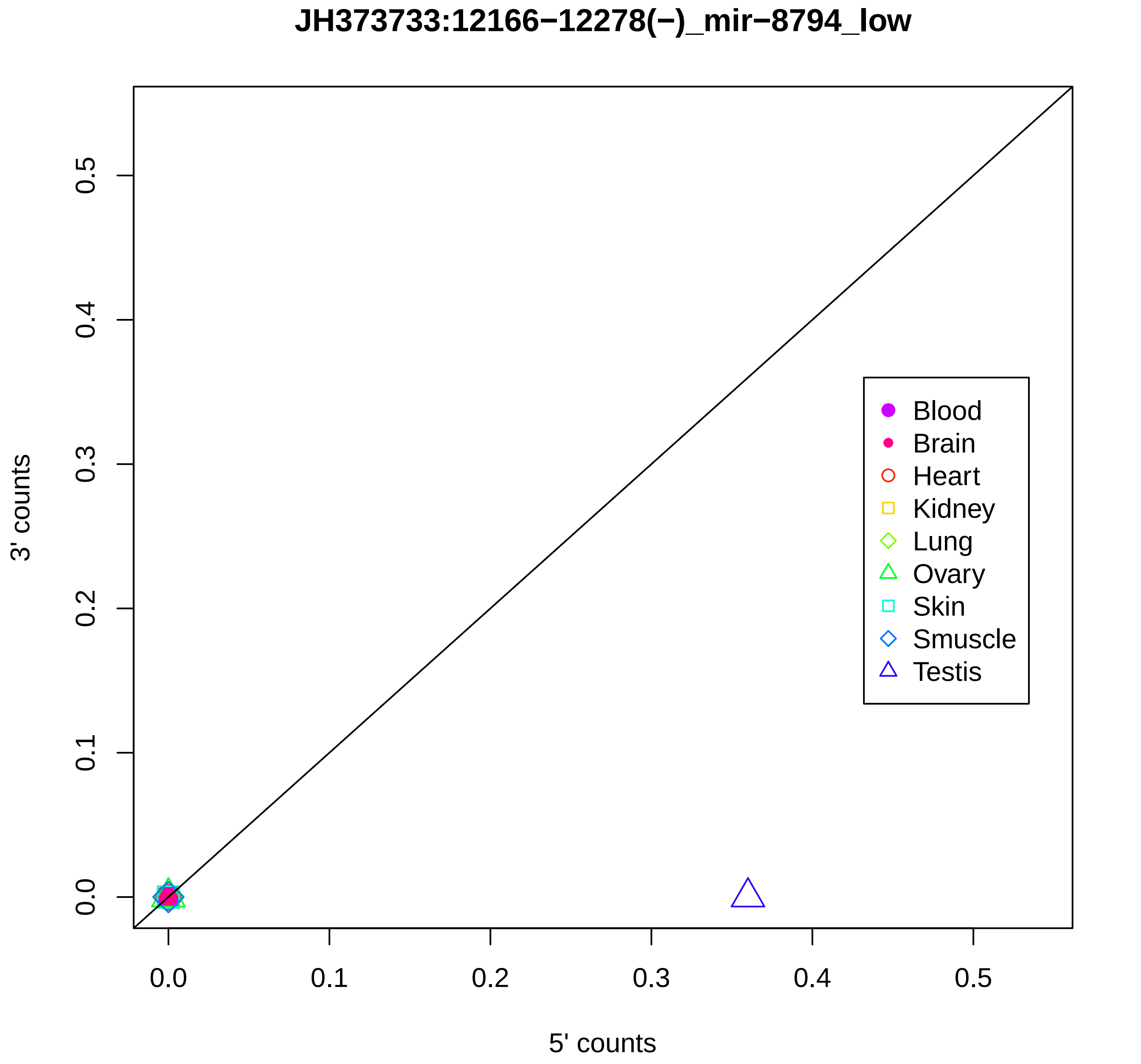

JH373733:12166−12278(−)_mir−8794_low
3' counts
5' counts
0.0
0.1
0.2
0.3
0.4
0.5
Blood
Brain
Heart
Kidney
Lung
Ovary
Skin
Smuscle
Testis

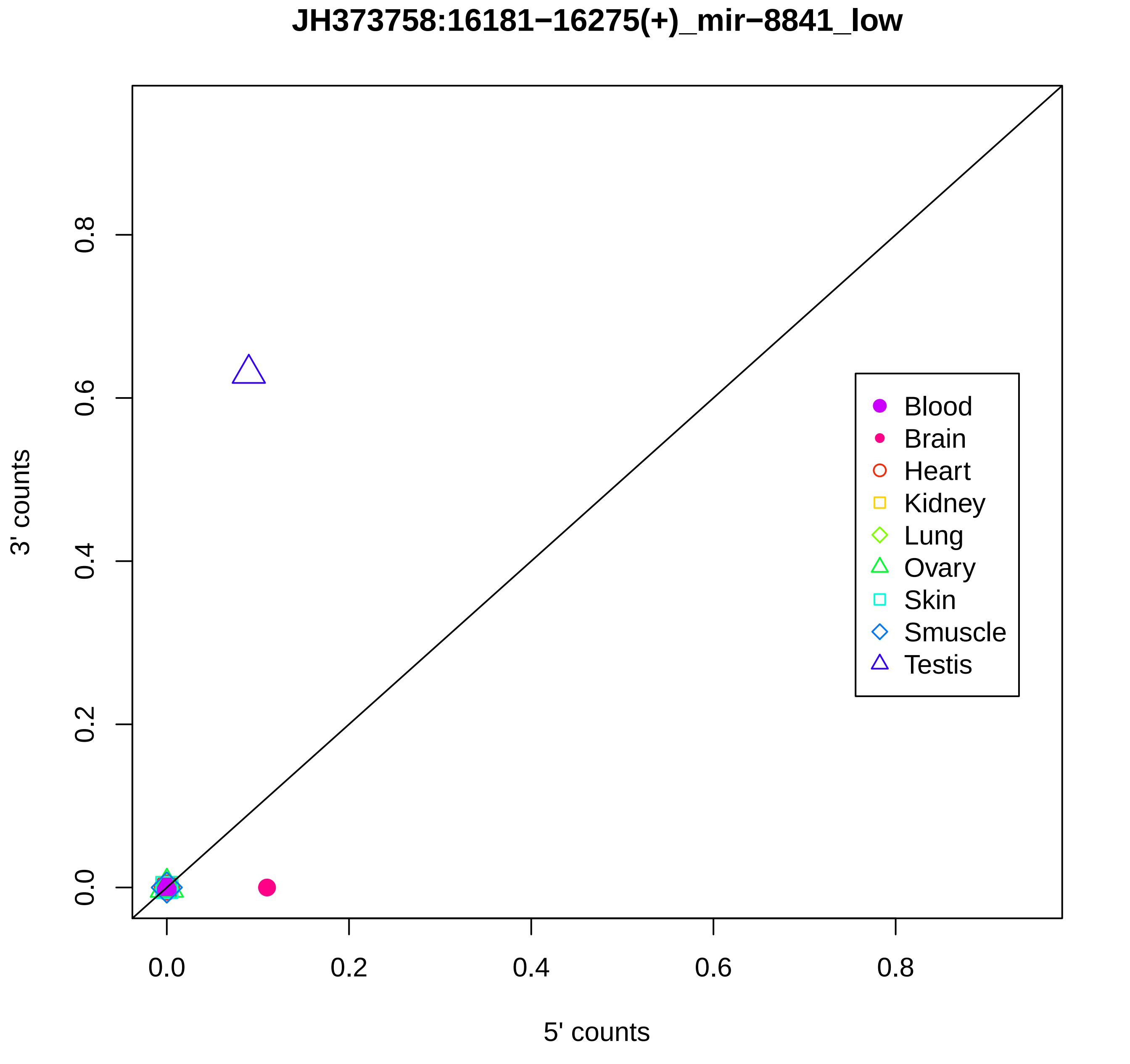

JH373758:16181−16275(+)_mir−8841_low
3' counts
5' counts
0.0
0.2
0.4
0.6
0.8
Blood
Brain
Heart
Kidney
Lung
Ovary
Skin
Smuscle
Testis

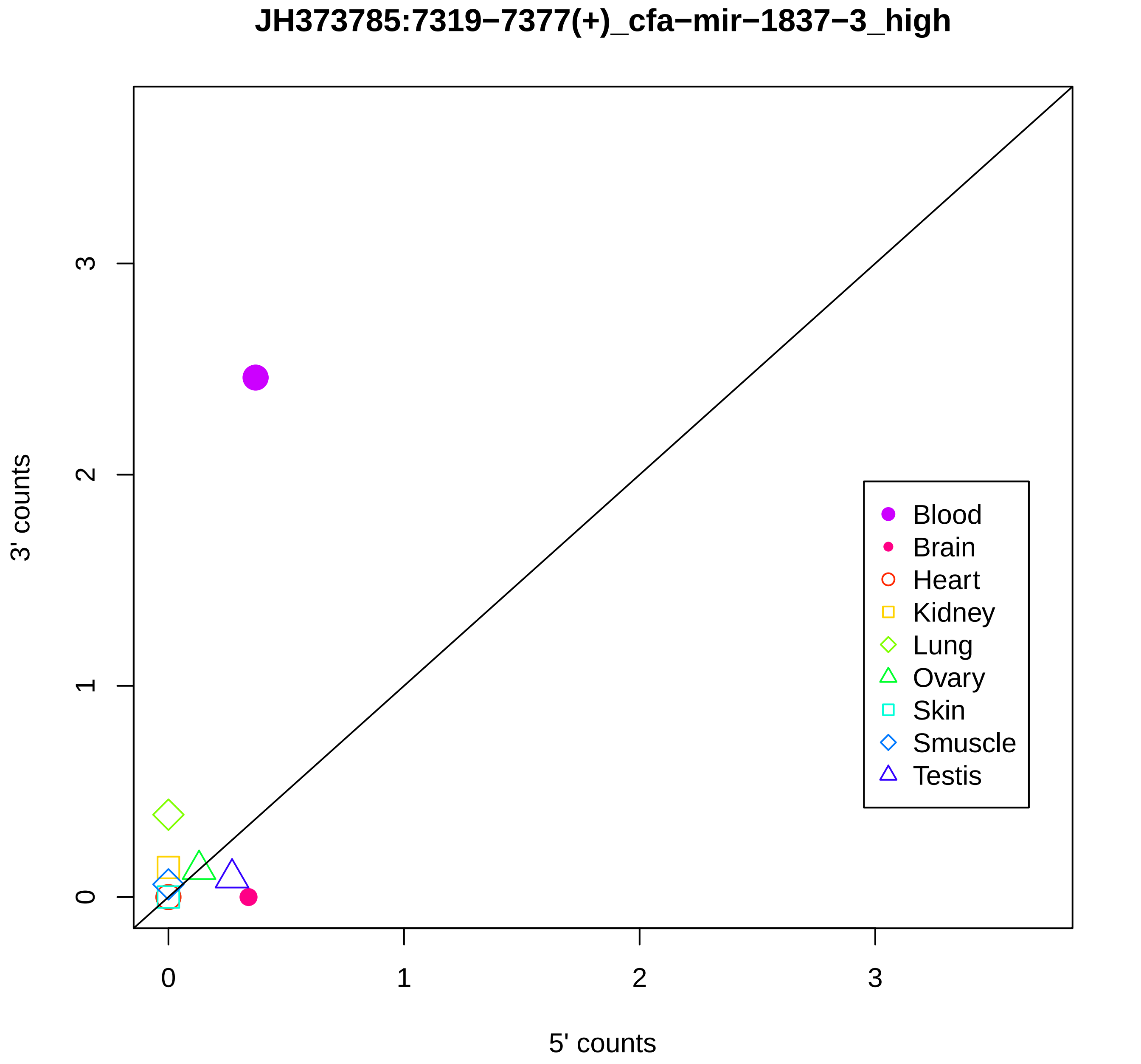
JH373785:7319−7377(+)_cfa−mir−1837−3_high
3' counts
5' counts
0
1
2
3
0
1
2
3
Blood
Brain
Heart
Kidney
Lung
Ovary
Skin
Smuscle
Testis

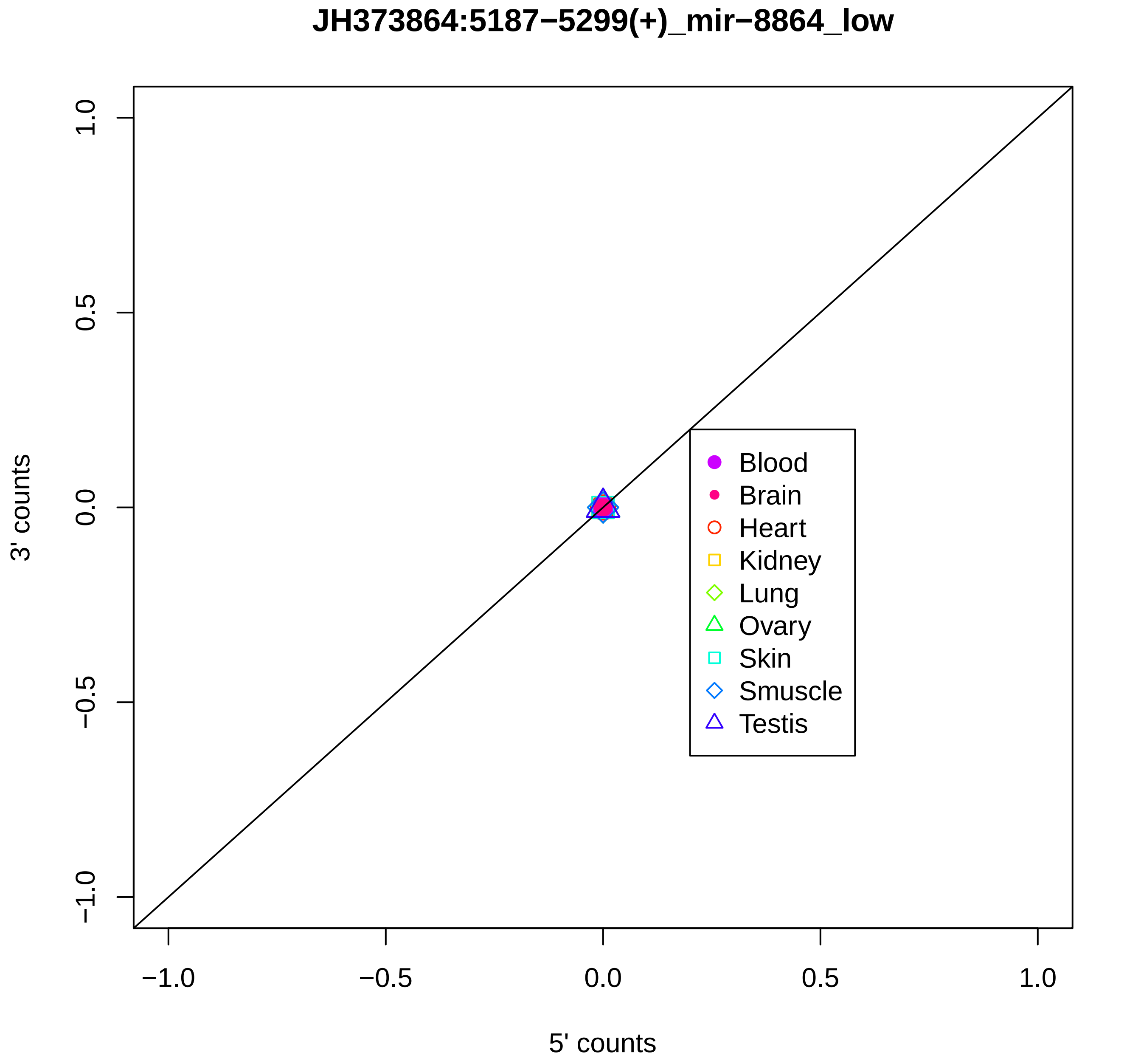

JH373864:5187−5299(+)_mir−8864_low
1.0
0.5
0.0
−0.5
−1.0
3' counts
−1.0
−0.5
0.0
0.5
1.0
5' counts
Blood
Brain
Heart
Kidney
Lung
Ovary
Skin
Smuscle
Testis

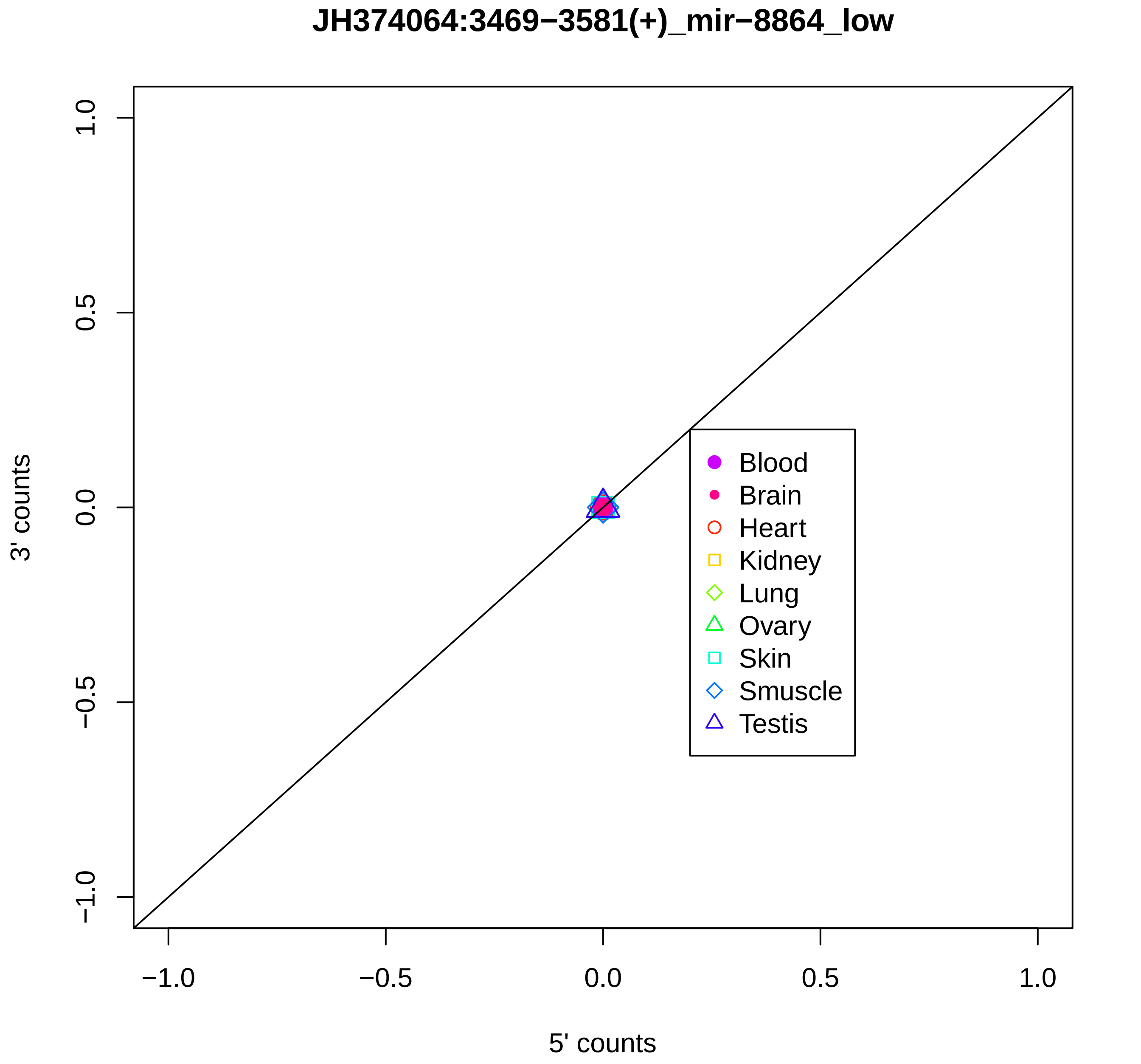

JH374064:3469−3581(+)_mir−8864_low
3' counts
5' counts
−1.0
−0.5
0.0
0.5
1.0
Blood
Brain
Heart
Kidney
Lung
Ovary
Skin
Smuscle
Testis

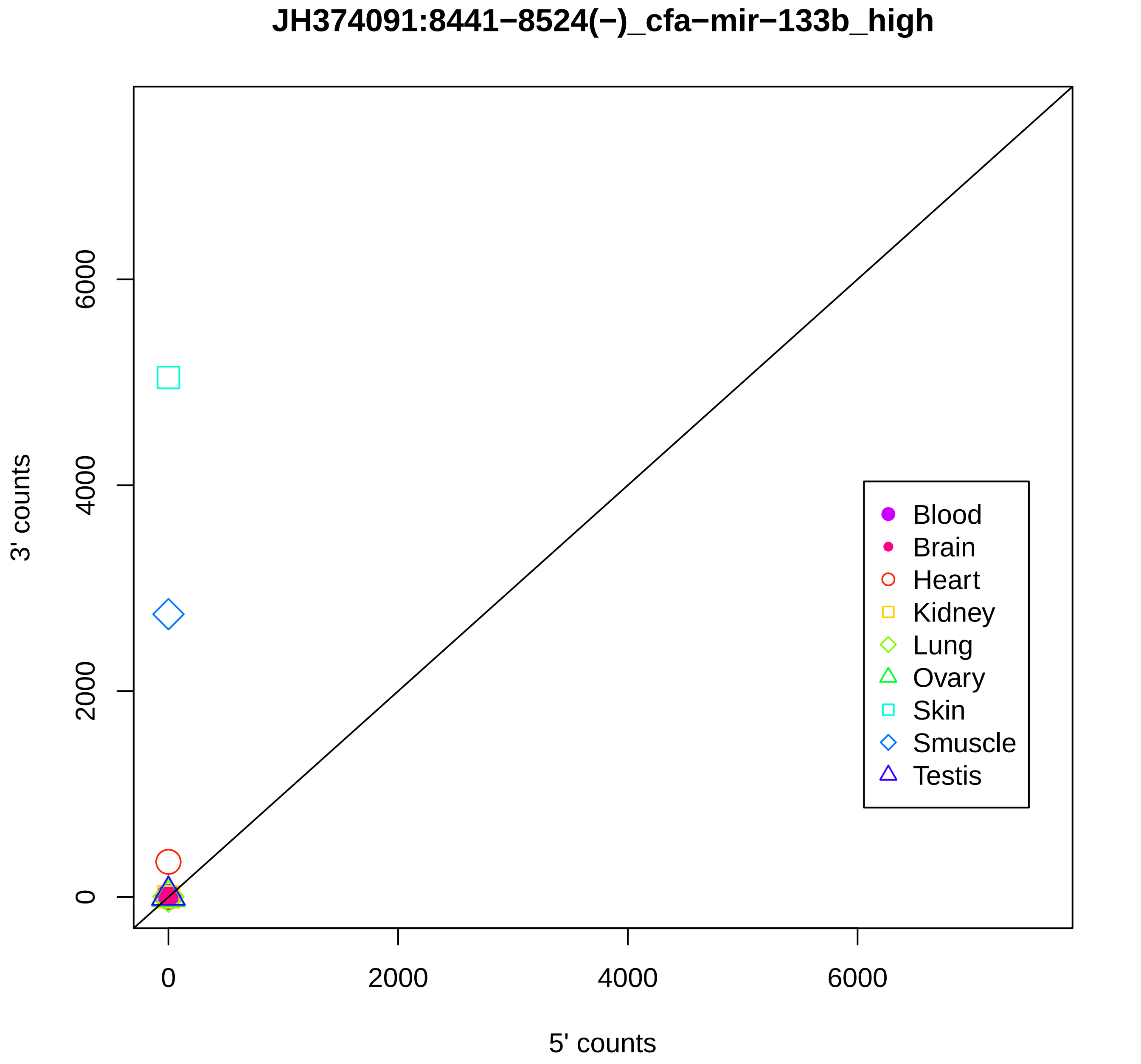

JH374091:8441−8524(−)_cfa−mir−133b_high
3' counts
5' counts
0
2000
4000
6000
0
2000
4000
6000
Blood
Brain
Heart
Kidney
Lung
Ovary
Skin
Smuscle
Testis

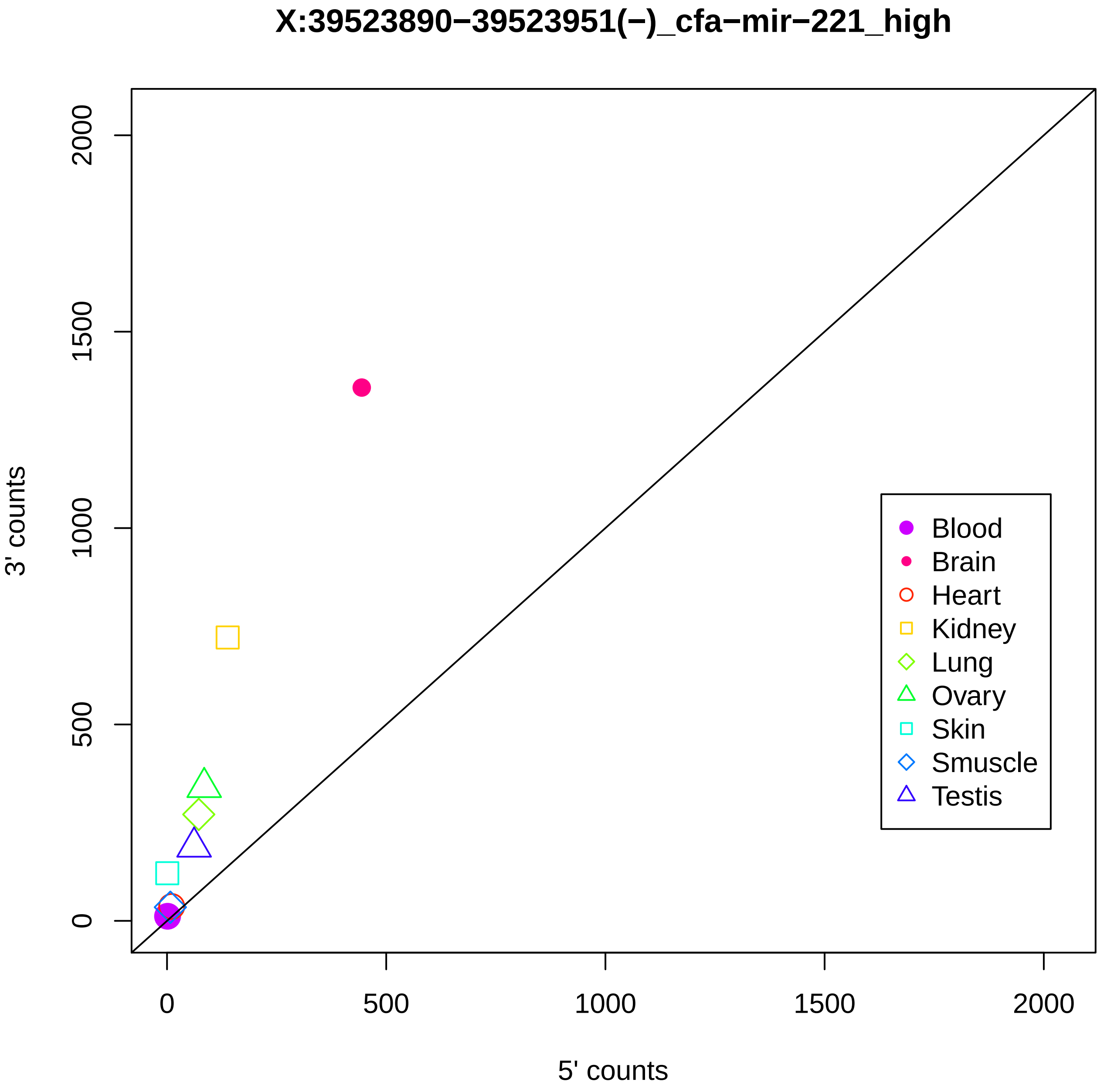

X:39523890−39523951(−)_cfa−mir−221_high
3' counts
5' counts
0
500
1000
1500
2000
Blood
Brain
Heart
Kidney
Lung
Ovary
Skin
Smuscle
Testis

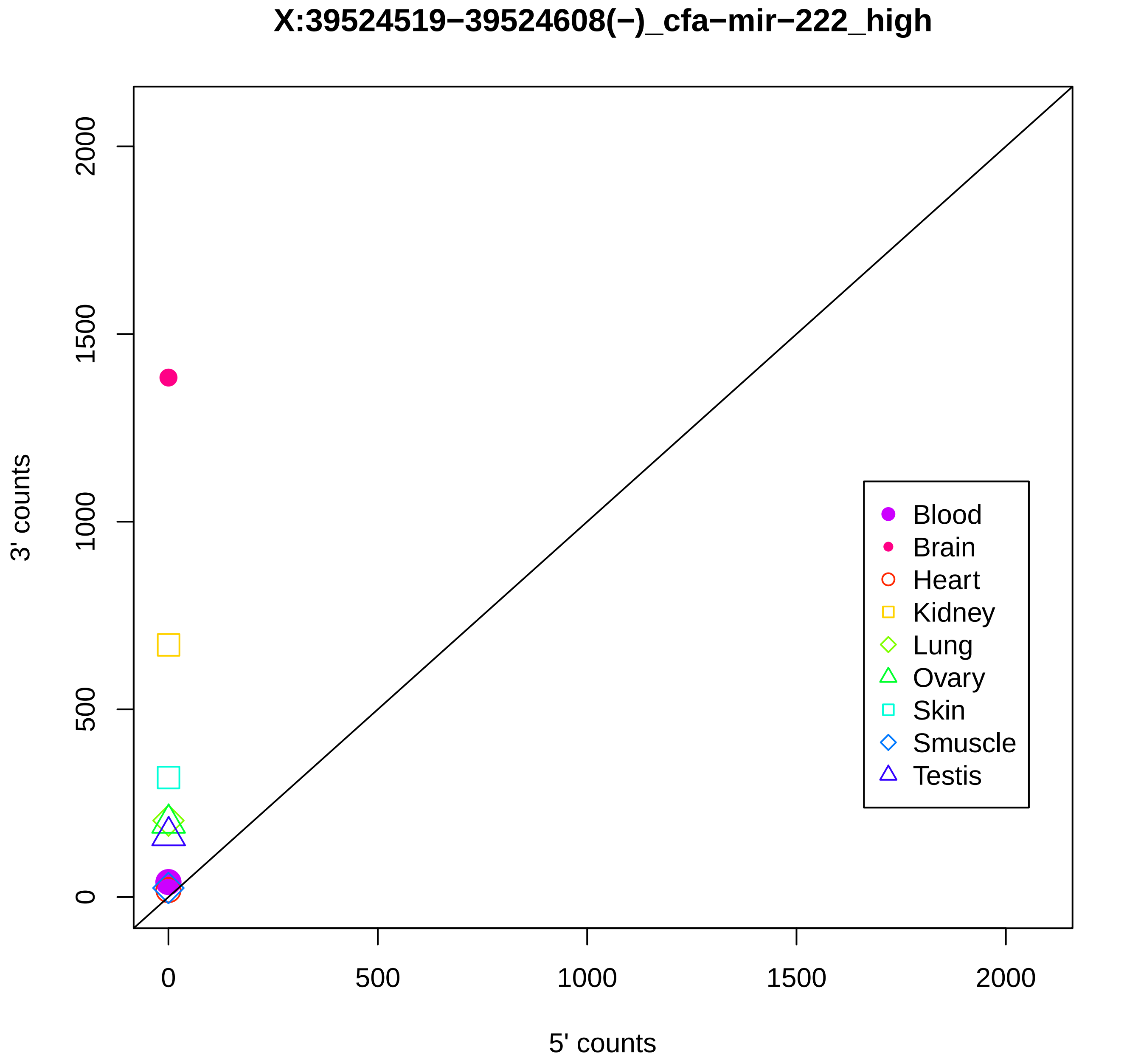

X:39524519−39524608(−)_cfa−mir−222_high
3' counts
5' counts
0
500
1000
1500
2000
Blood
Brain
Heart
Kidney
Lung
Ovary
Skin
Smuscle
Testis

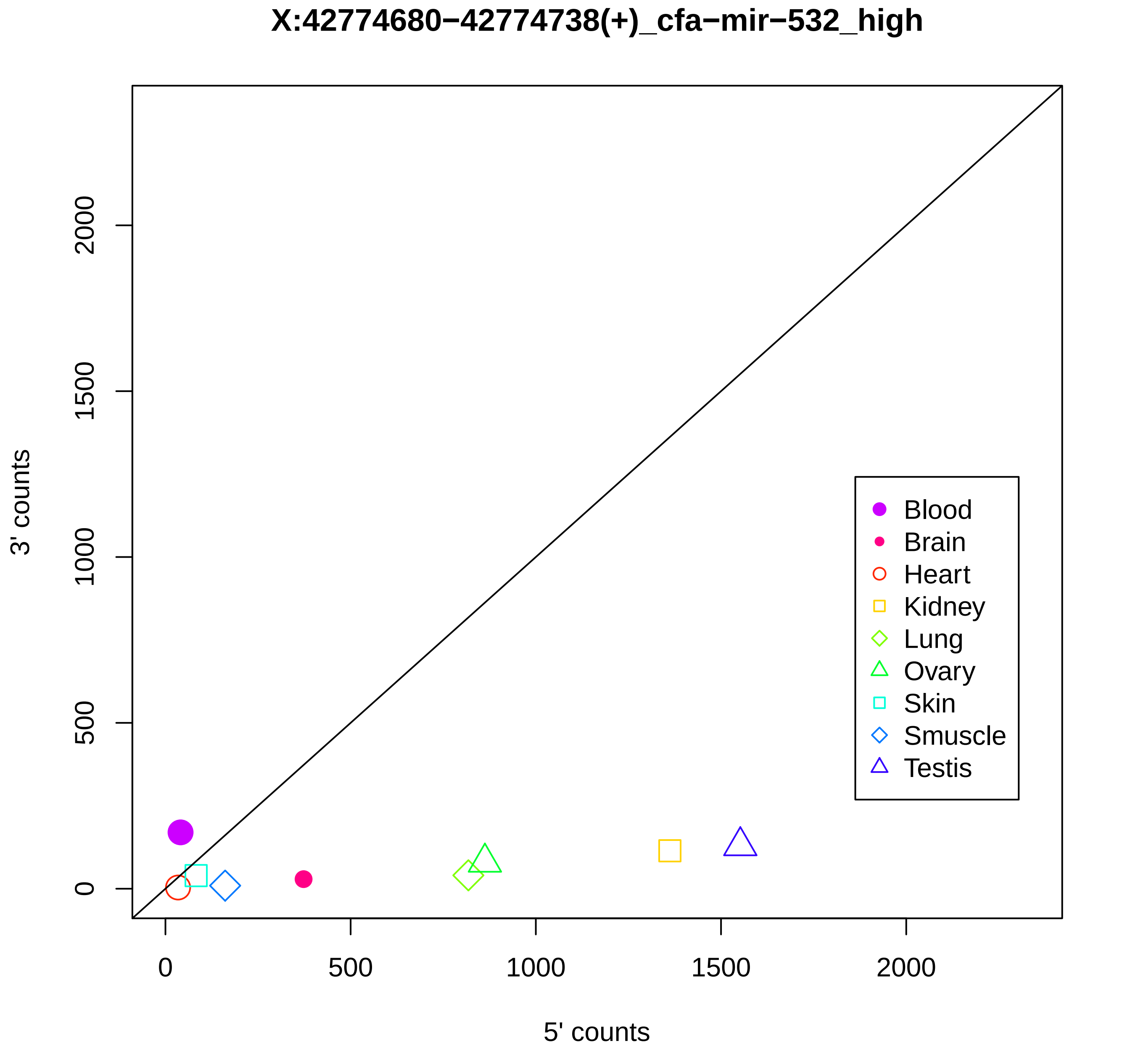

X:42774680−42774738(+)_cfa−mir−532_high
3' counts
5' counts
0
500
1000
1500
2000
Blood
Brain
Heart
Kidney
Lung
Ovary
Skin
Smuscle
Testis

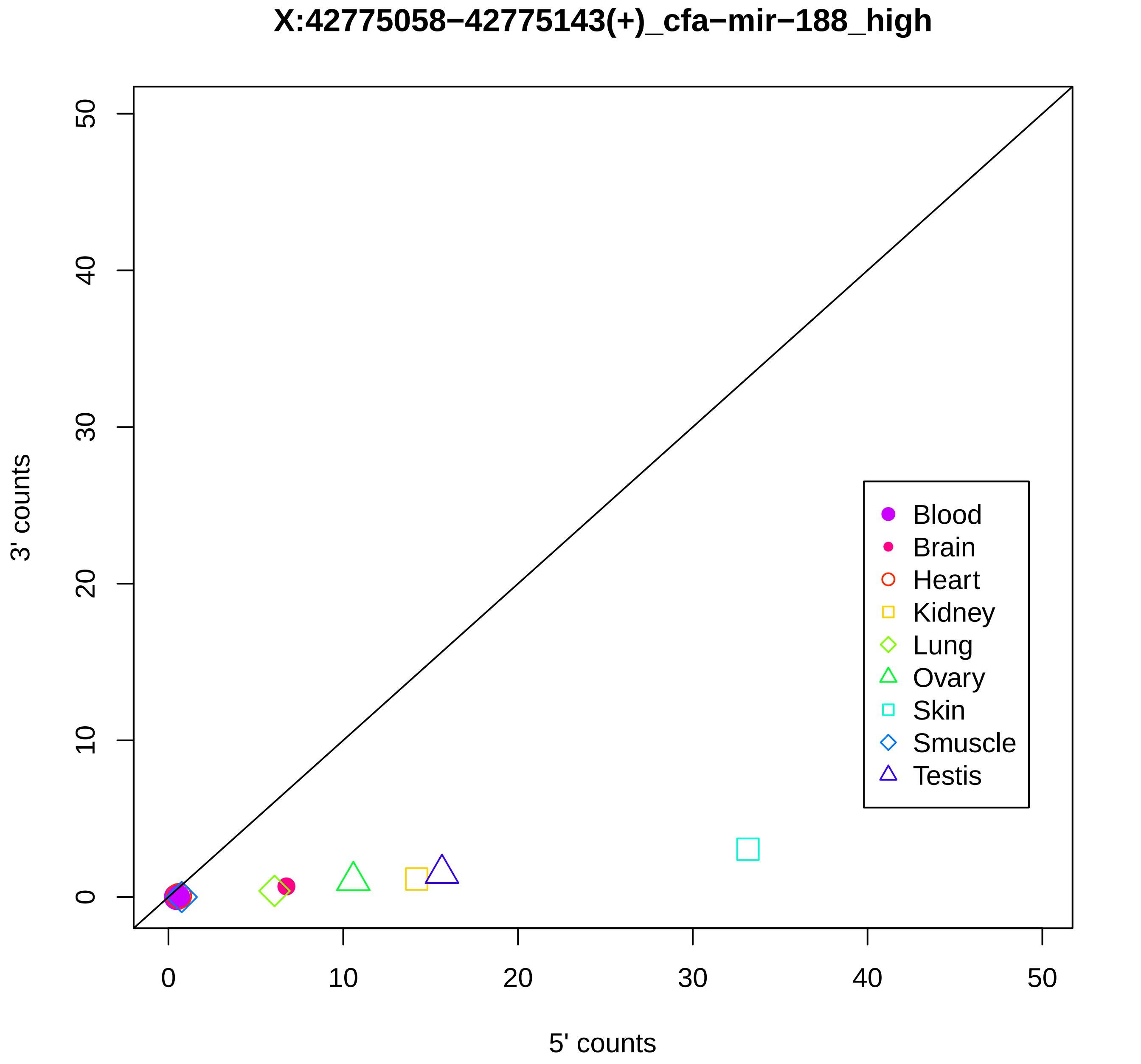
X:42775058−42775143(+)_cfa−mir−188_high
3' counts
5' counts
0
10
20
30
40
50
0
10
20
30
40
50
Blood
Brain
Heart
Kidney
Lung
Ovary
Skin
Smuscle
Testis

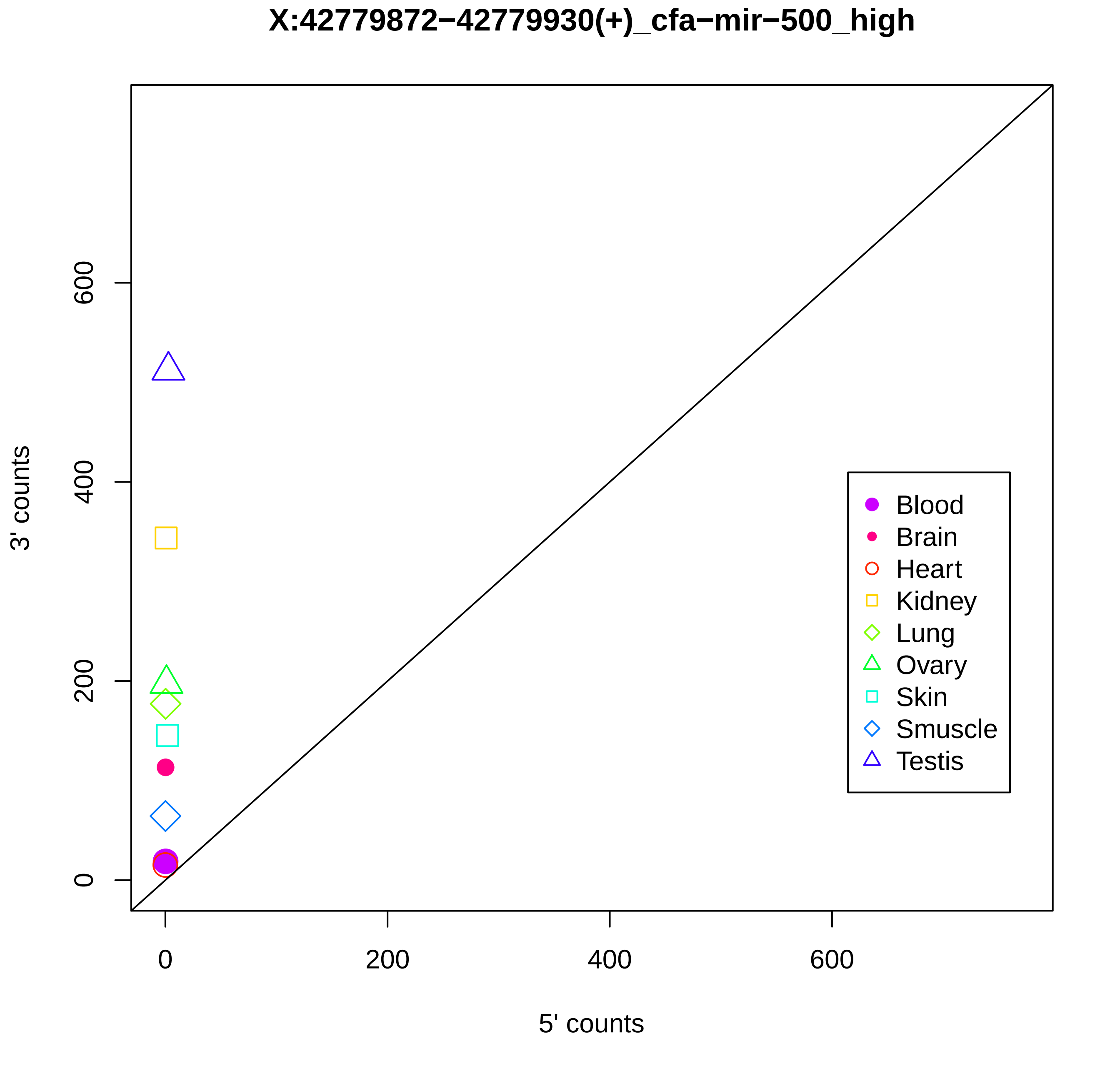

X:42779872−42779930(+)_cfa−mir−500_high
3' counts
0
200
400
600
5' counts
0
200
400
600
Blood
Brain
Heart
Kidney
Lung
Ovary
Skin
Smuscle
Testis

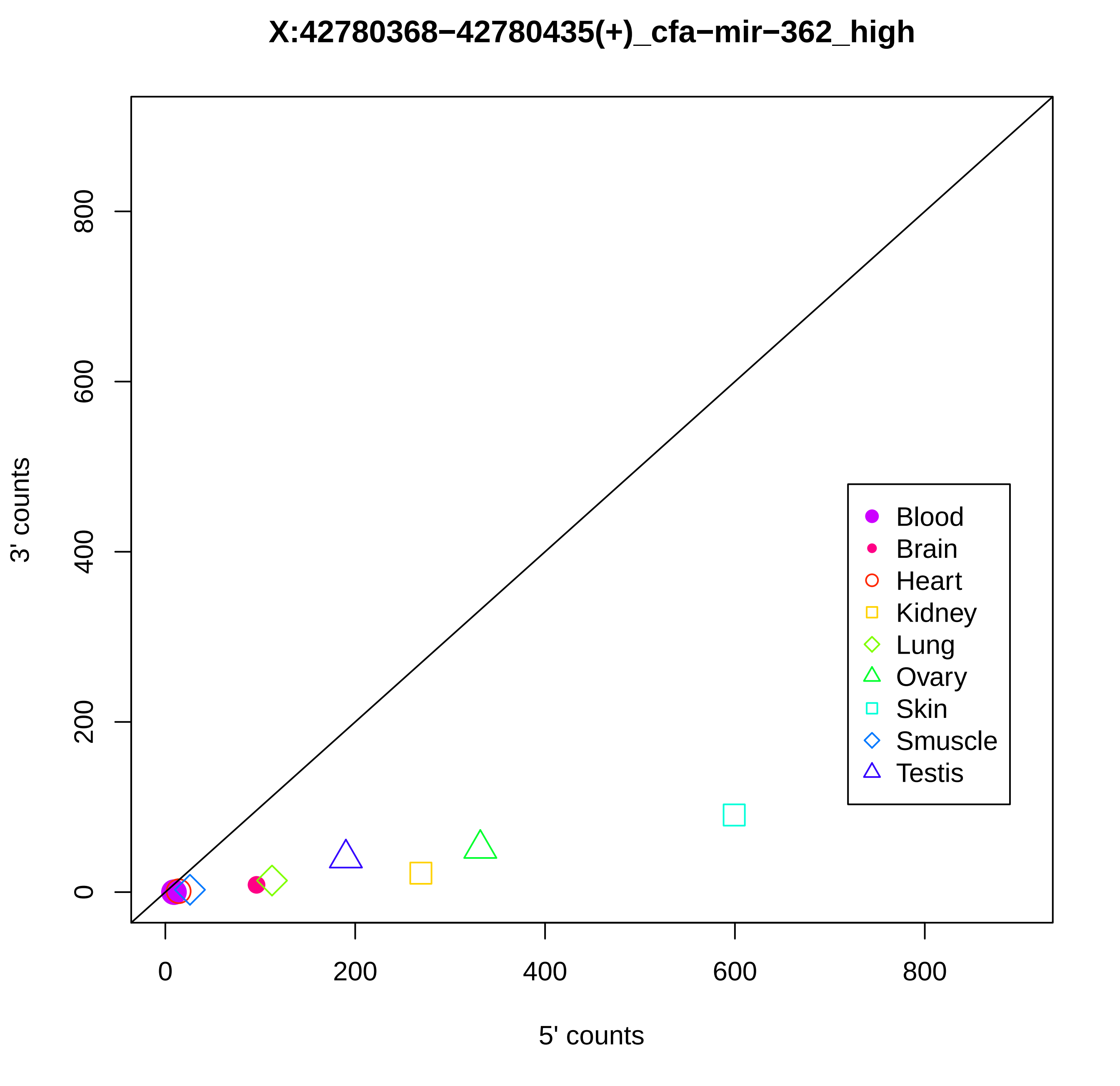
X:42780368−42780435(+)_cfa−mir−362_high
3' counts
5' counts
0
200
400
600
800
0
200
400
600
800
Blood
Brain
Heart
Kidney
Lung
Ovary
Skin
Smuscle
Testis

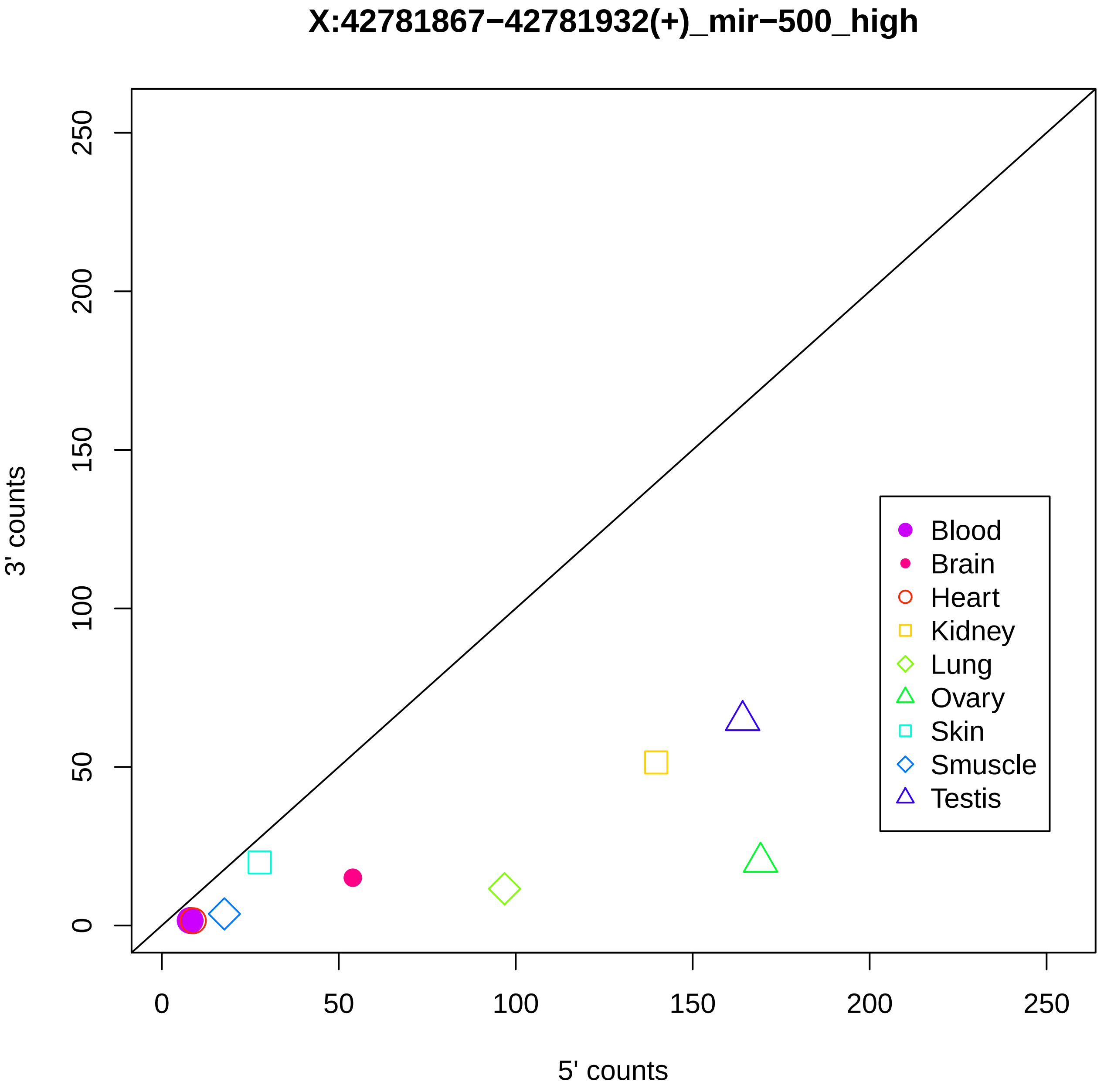
X:42781867−42781932(+)_mir−500_high
3' counts
5' counts
0
50
100
150
200
250
Blood
Brain
Heart
Kidney
Lung
Ovary
Skin
Smuscle
Testis

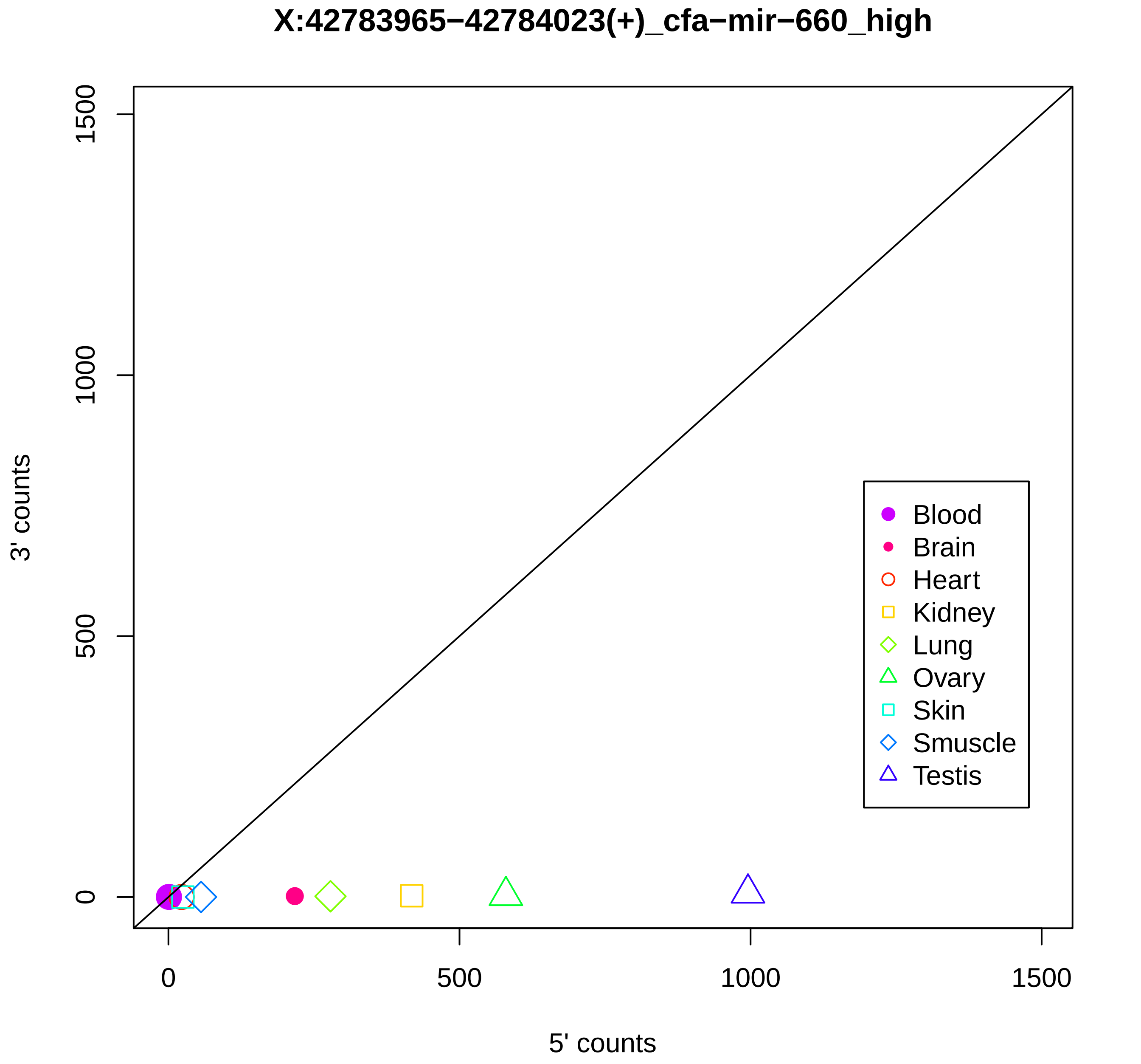

X:42783965−42784023(+)_cfa−mir−660_high
3' counts
5' counts
0
500
1000
1500
0
500
1000
1500
Blood
Brain
Heart
Kidney
Lung
Ovary
Skin
Smuscle
Testis

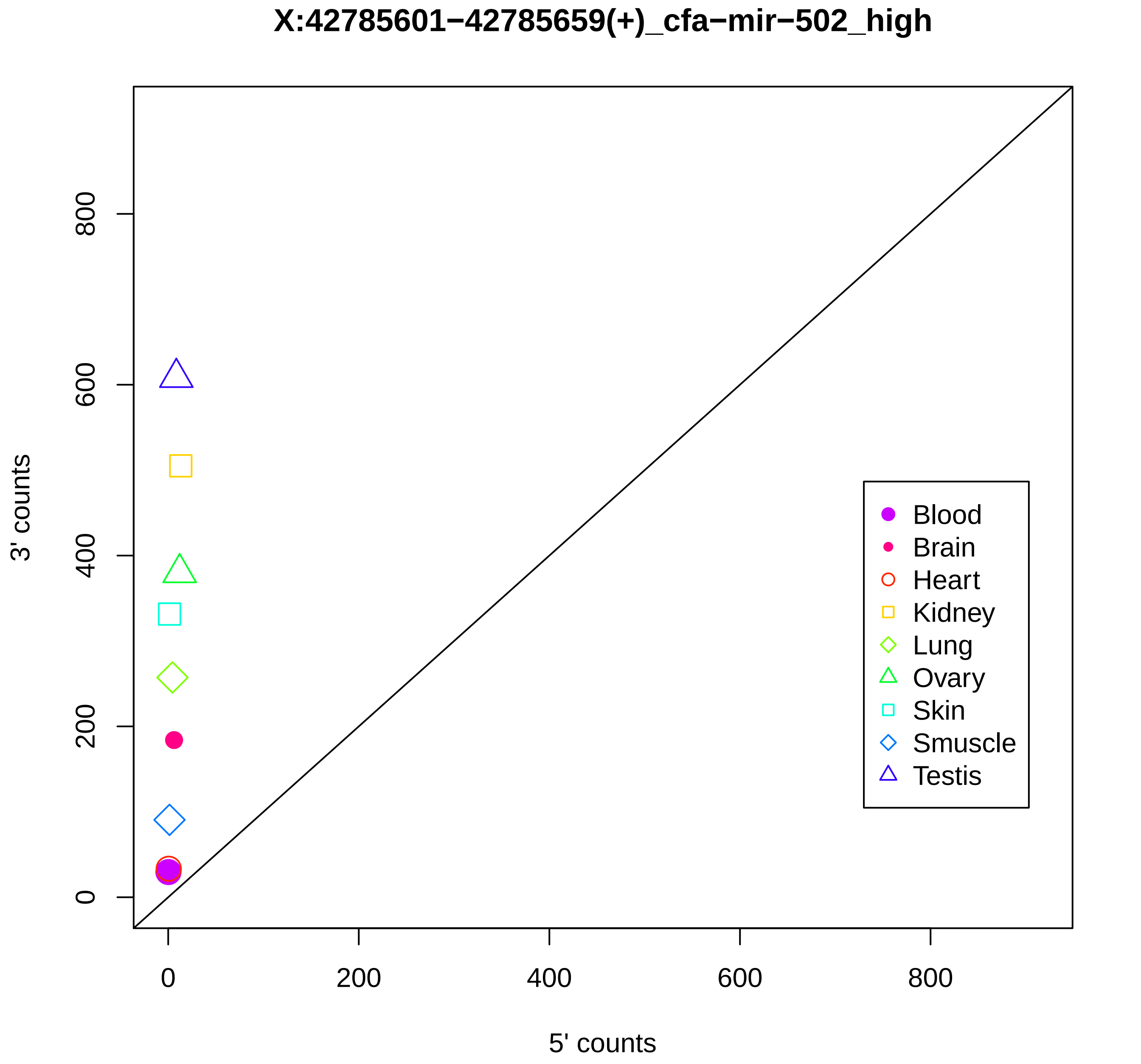

X:42785601−42785659(+)_cfa−mir−502_high
3' counts
5' counts
0
200
400
600
800
0
200
400
600
800
Blood
Brain
Heart
Kidney
Lung
Ovary
Skin
Smuscle
Testis

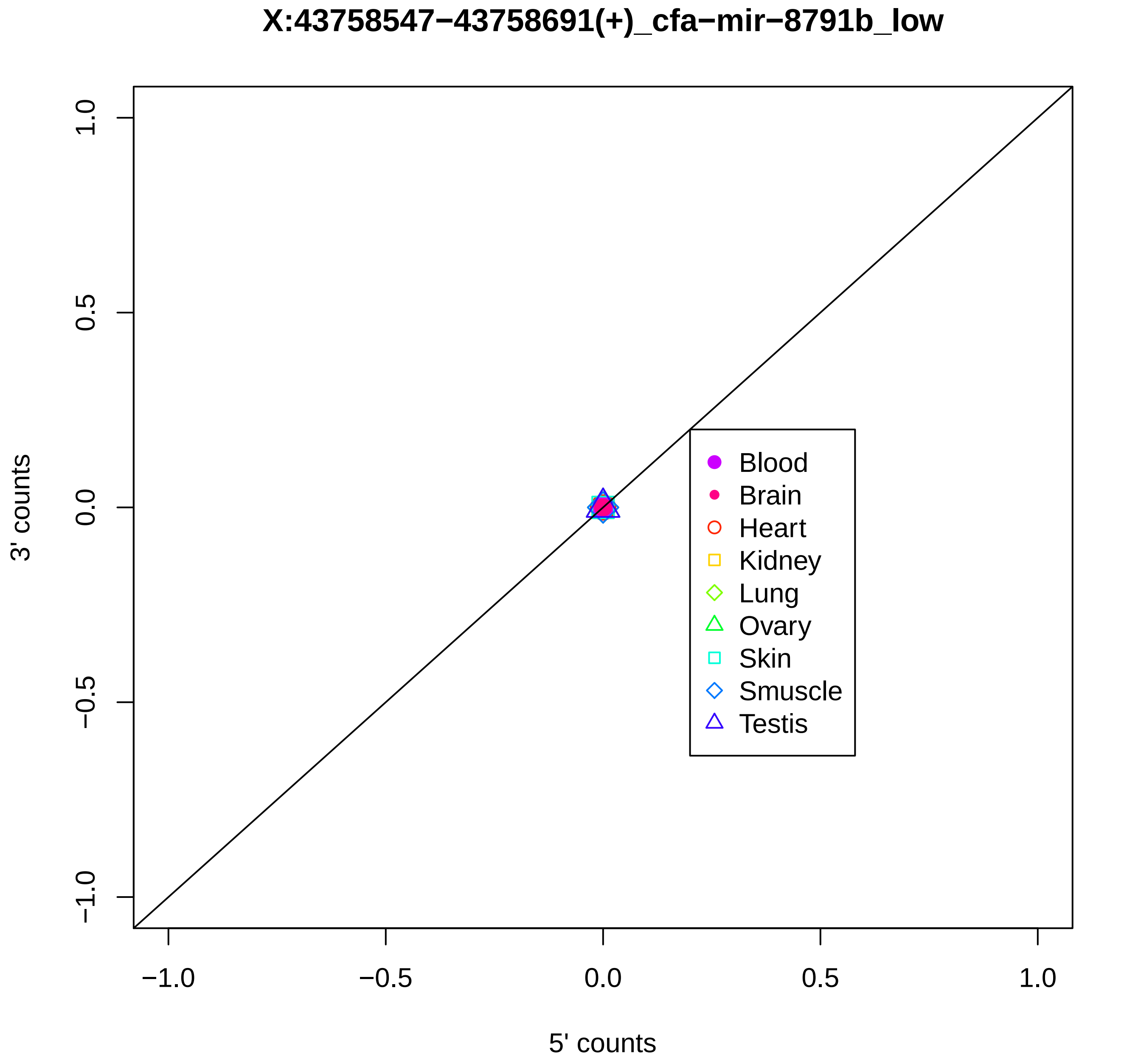

X:43758547−43758691(+)_cfa−mir−8791b_low
3' counts
5' counts
1.0
0.5
0.0
−0.5
−1.0
Blood
Brain
Heart
Kidney
Lung
Ovary
Skin
Smuscle
Testis

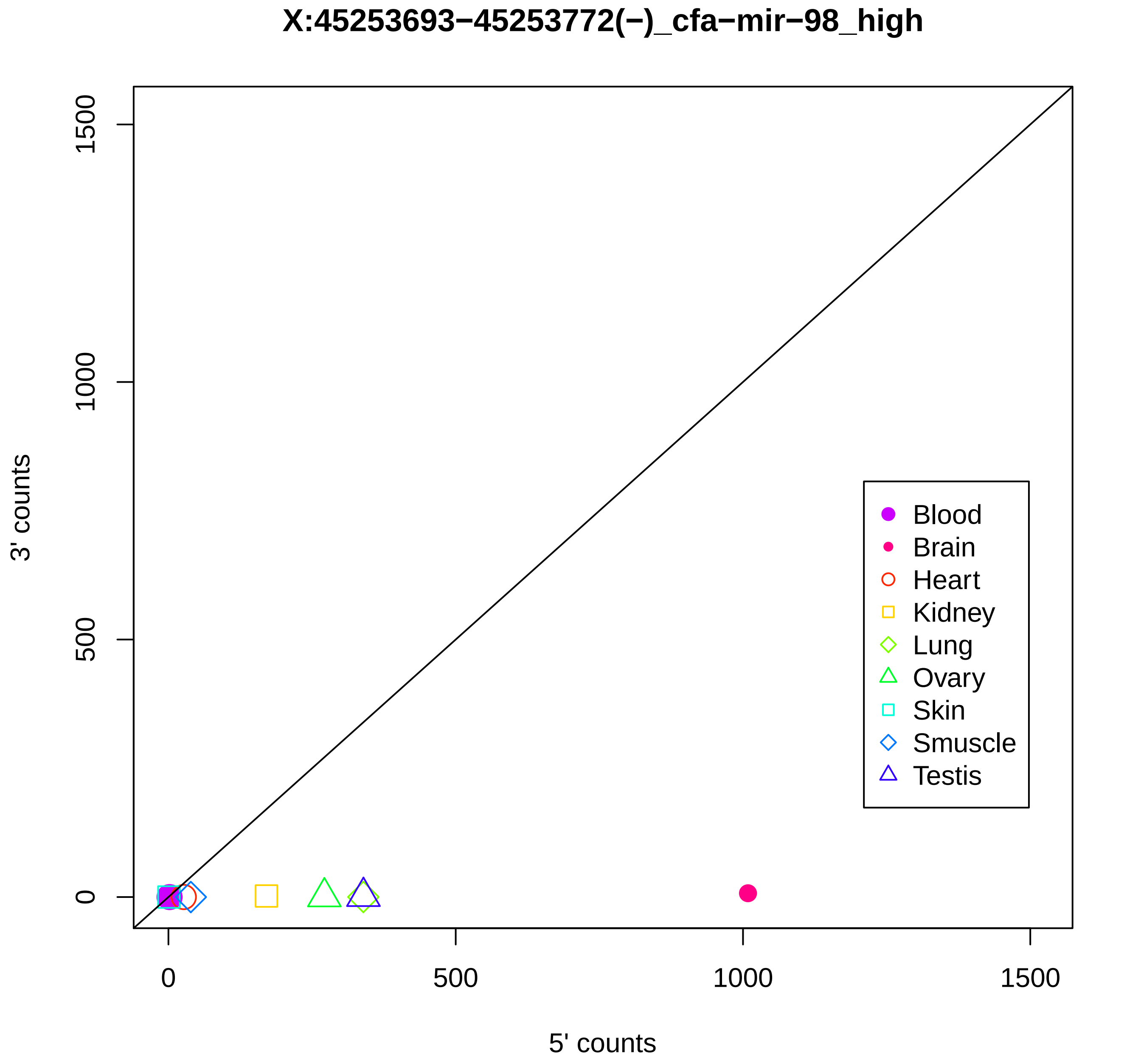
X:45253693−45253772(−)_cfa−mir−98_high
3' counts
5' counts
0
500
1000
1500
0
500
1000
1500
Blood
Brain
Heart
Kidney
Lung
Ovary
Skin
Smuscle
Testis

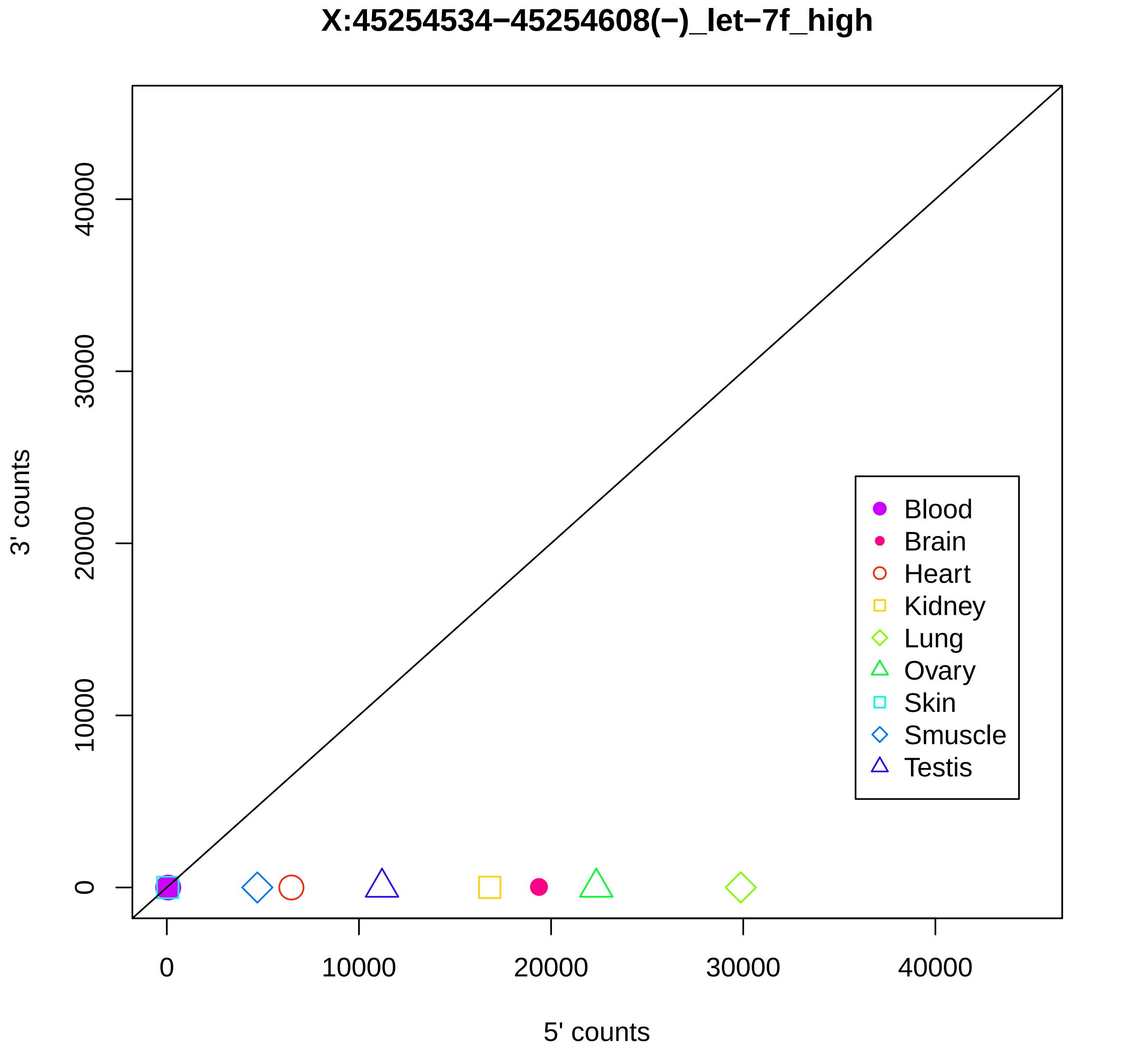
X:45254534−45254608(−)_let−7f_high
3' counts
5' counts
0
10000
20000
30000
40000
Blood
Brain
Heart
Kidney
Lung
Ovary
Skin
Smuscle
Testis

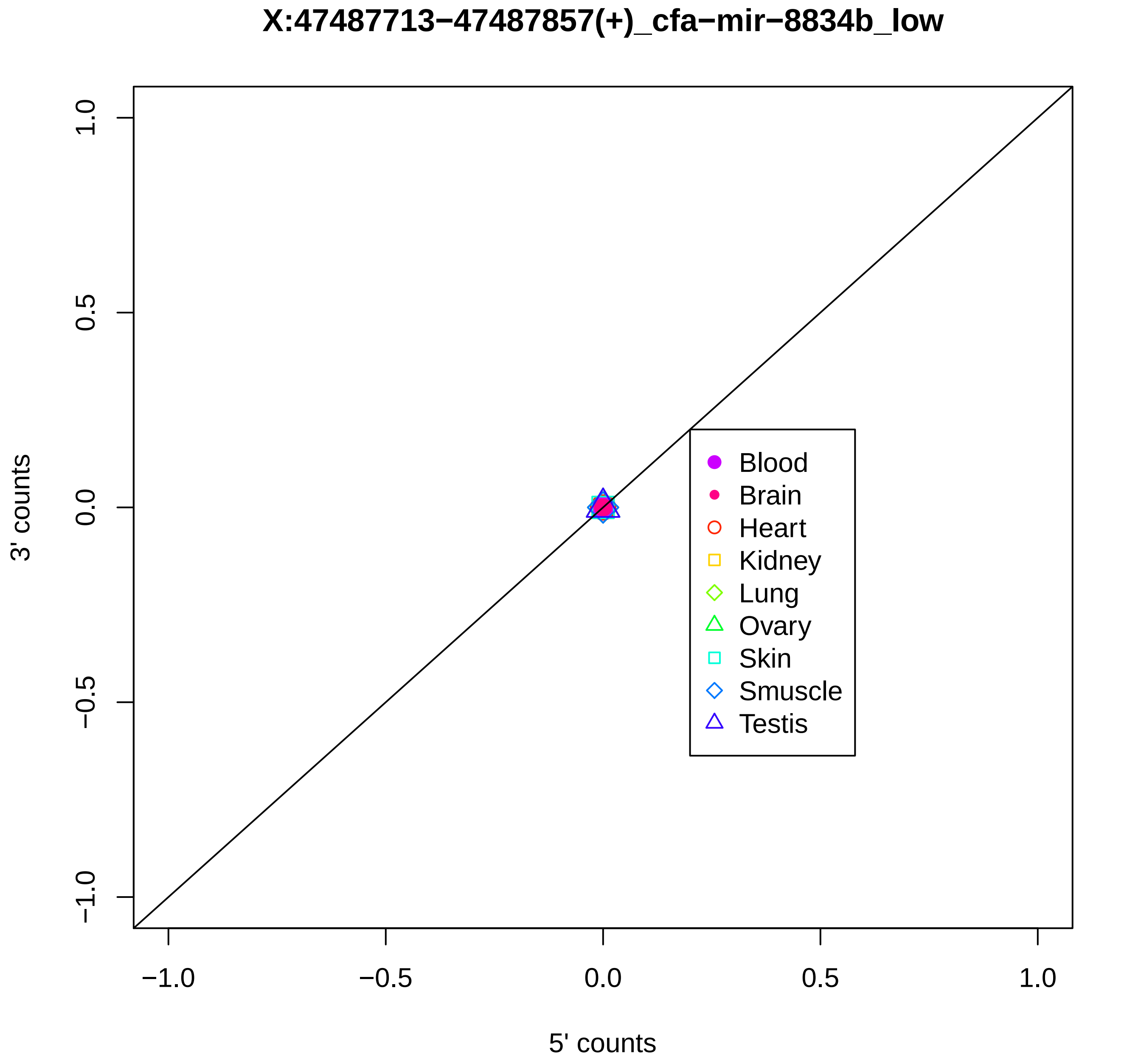

X:47487713−47487857(+)_cfa−mir−8834b_low
3' counts
5' counts
1.0
0.5
0.0
−0.5
−1.0
Blood
Brain
Heart
Kidney
Lung
Ovary
Skin
Smuscle
Testis

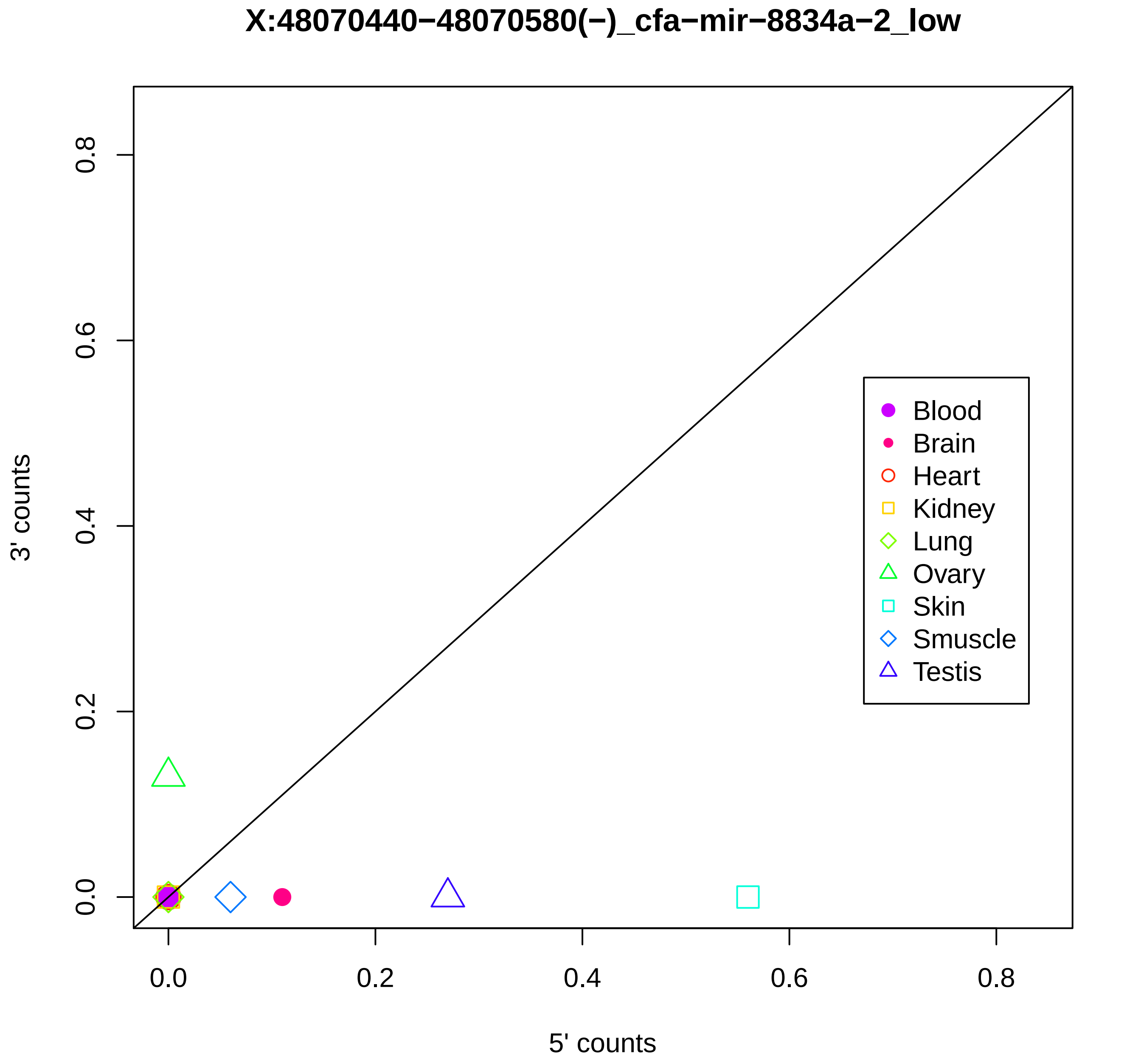
X:48070440−48070580(−)_cfa−mir−8834a−2_low
3' counts
5' counts
0.0
0.2
0.4
0.6
0.8
Blood
Brain
Heart
Kidney
Lung
Ovary
Skin
Smuscle
Testis

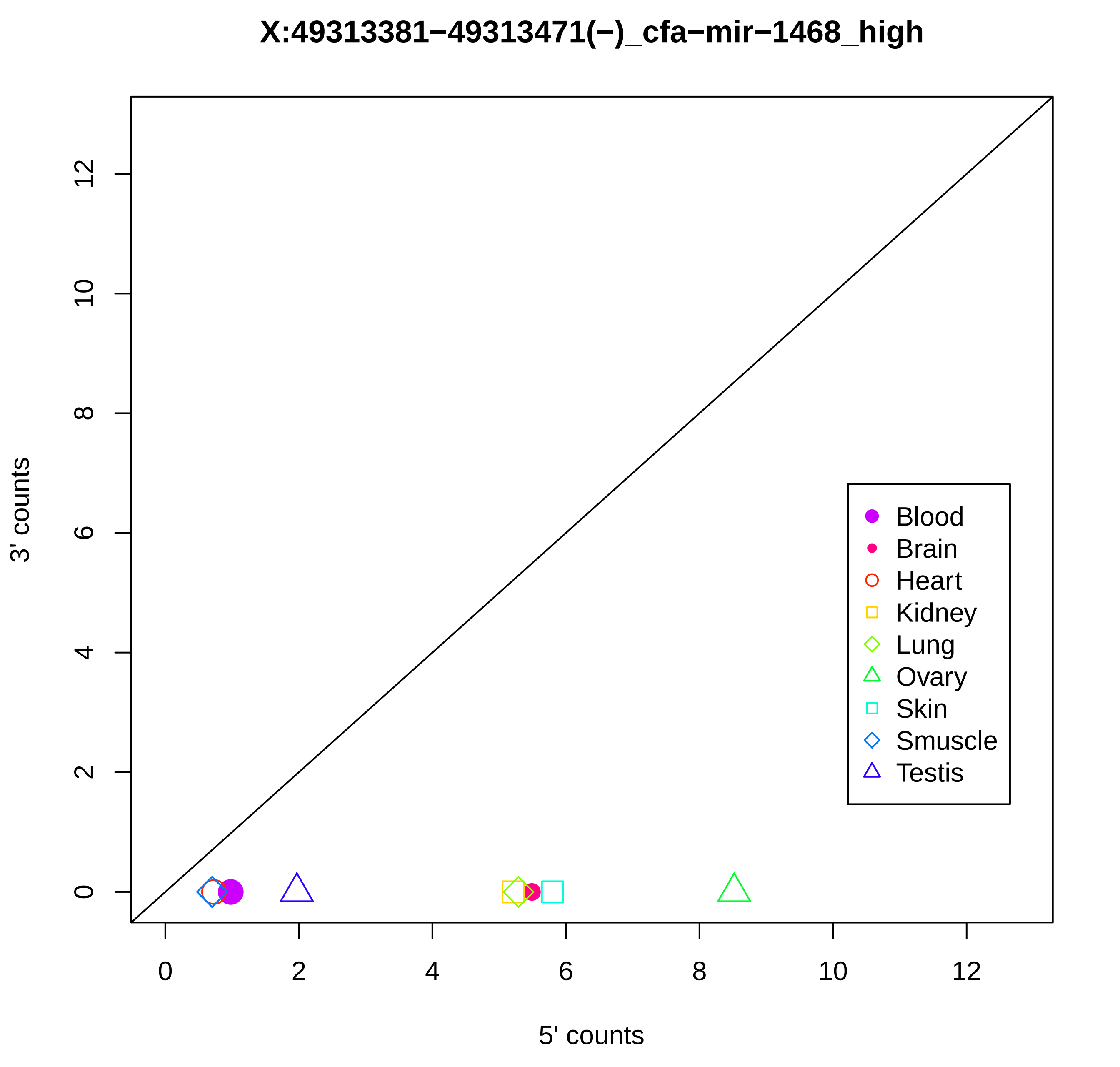

X:49313381−49313471(−)_cfa−mir−1468_high
3' counts
5' counts
0
2
4
6
8
10
12
Blood
Brain
Heart
Kidney
Lung
Ovary
Skin
Smuscle
Testis

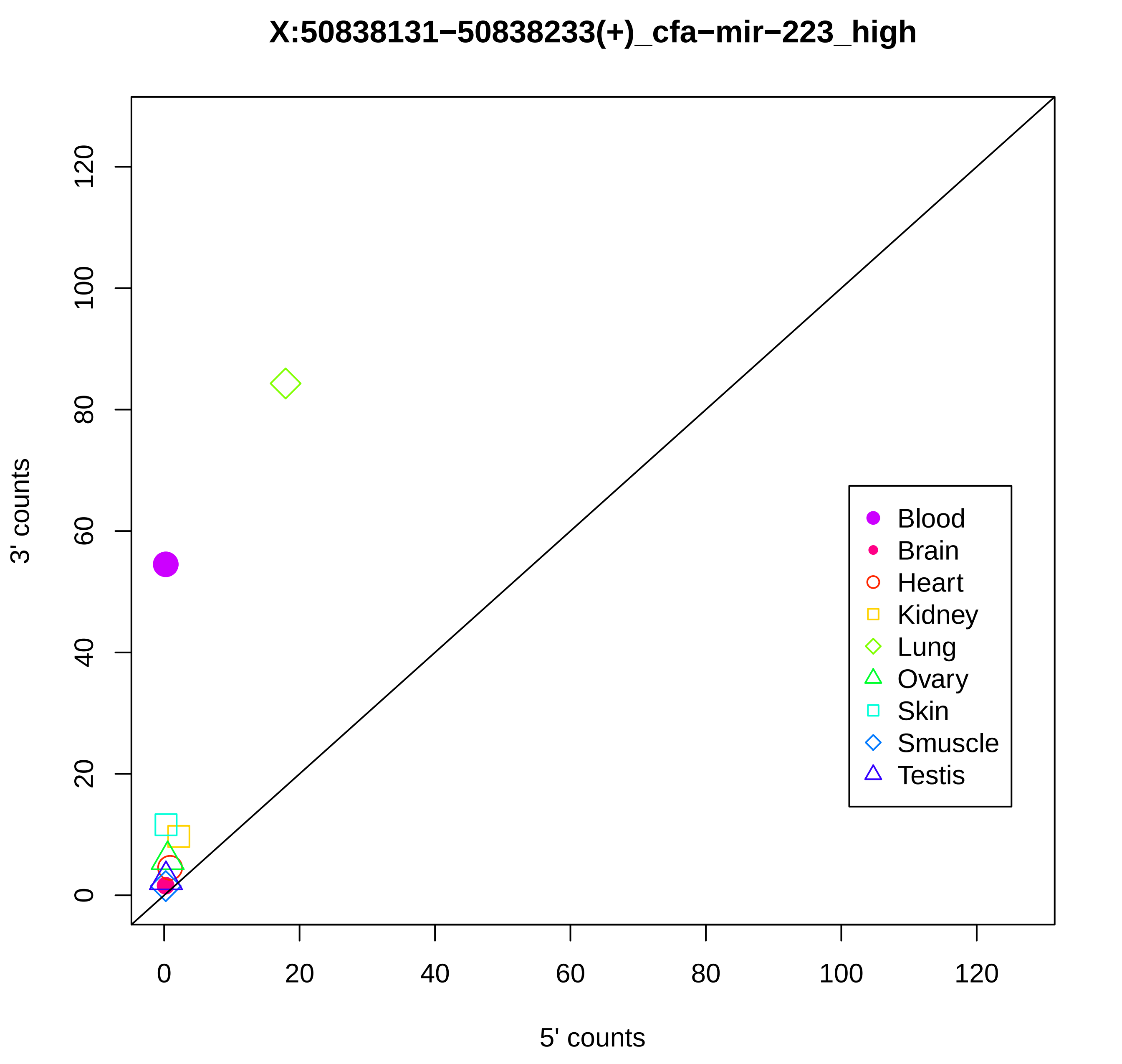
X:50838131−50838233(+)_cfa−mir−223_high
3' counts
5' counts
0
20
40
60
80
100
120
Blood
Brain
Heart
Kidney
Lung
Ovary
Skin
Smuscle
Testis

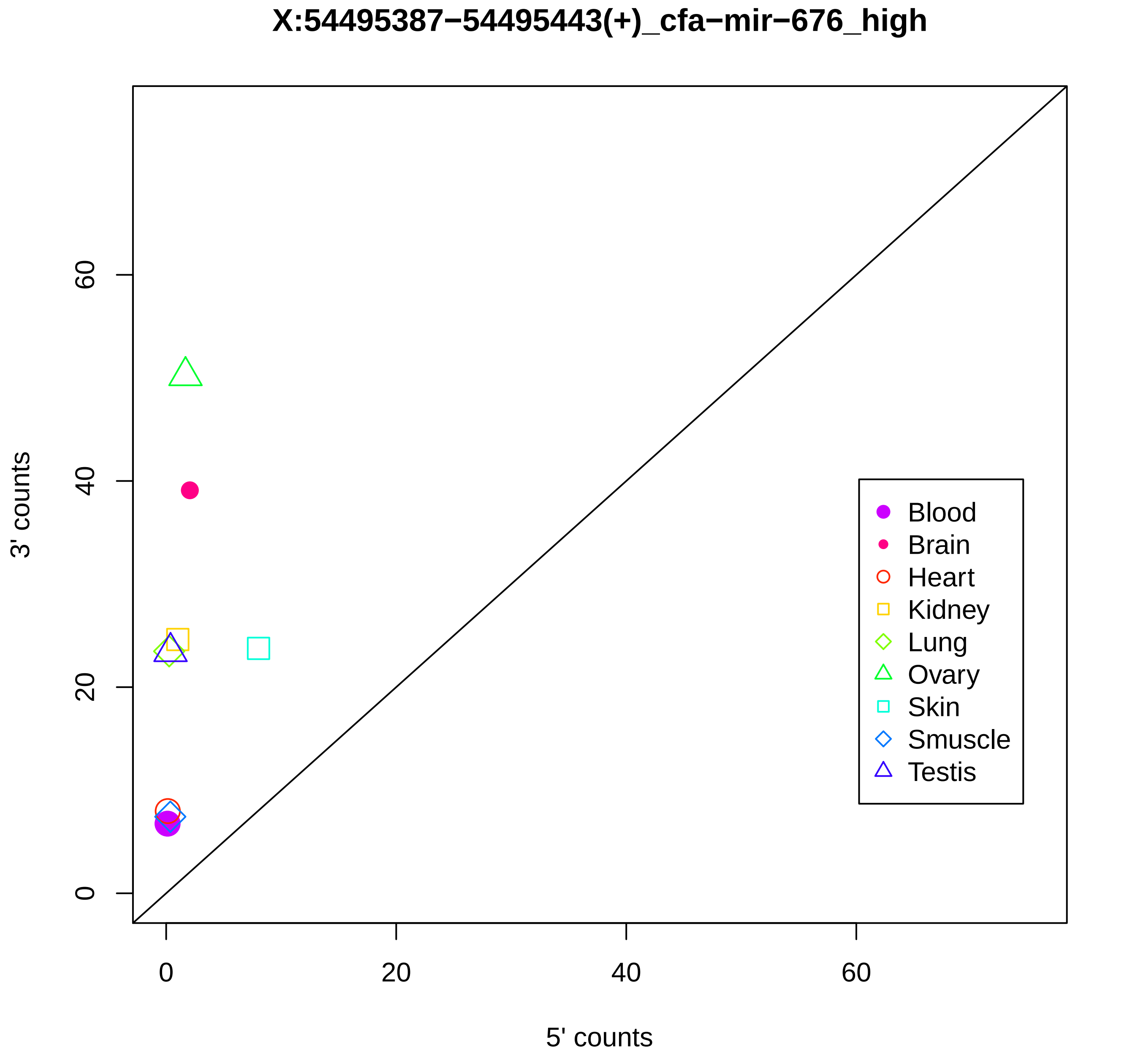

X:54495387−54495443(+)_cfa−mir−676_high
3' counts
5' counts
0
20
40
60
0
20
40
60
Blood
Brain
Heart
Kidney
Lung
Ovary
Skin
Smuscle
Testis

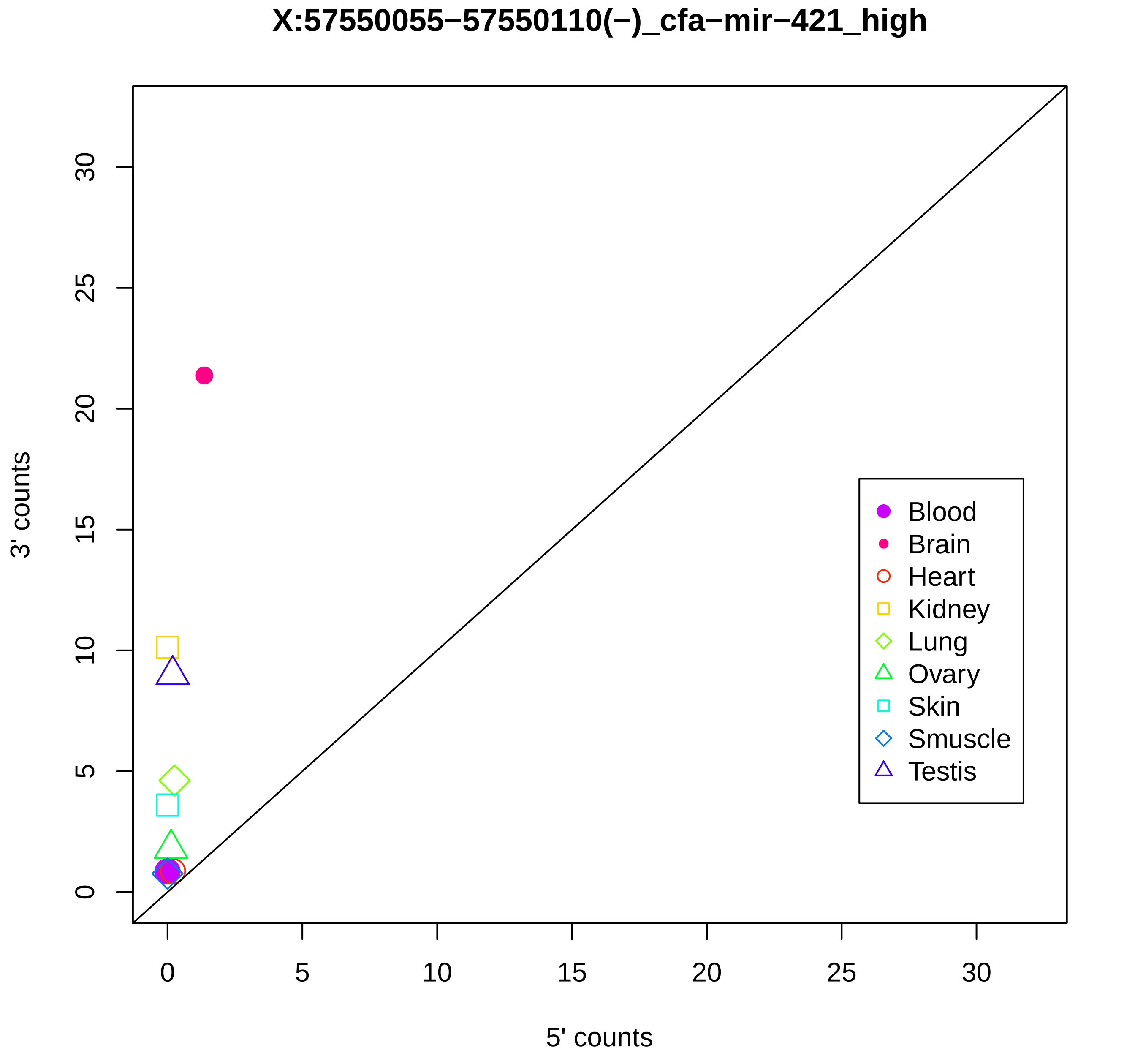
X:57550055−57550110(−)_cfa−mir−421_high
3' counts
5' counts
0
5
10
15
20
25
30
Blood
Brain
Heart
Kidney
Lung
Ovary
Skin
Smuscle
Testis

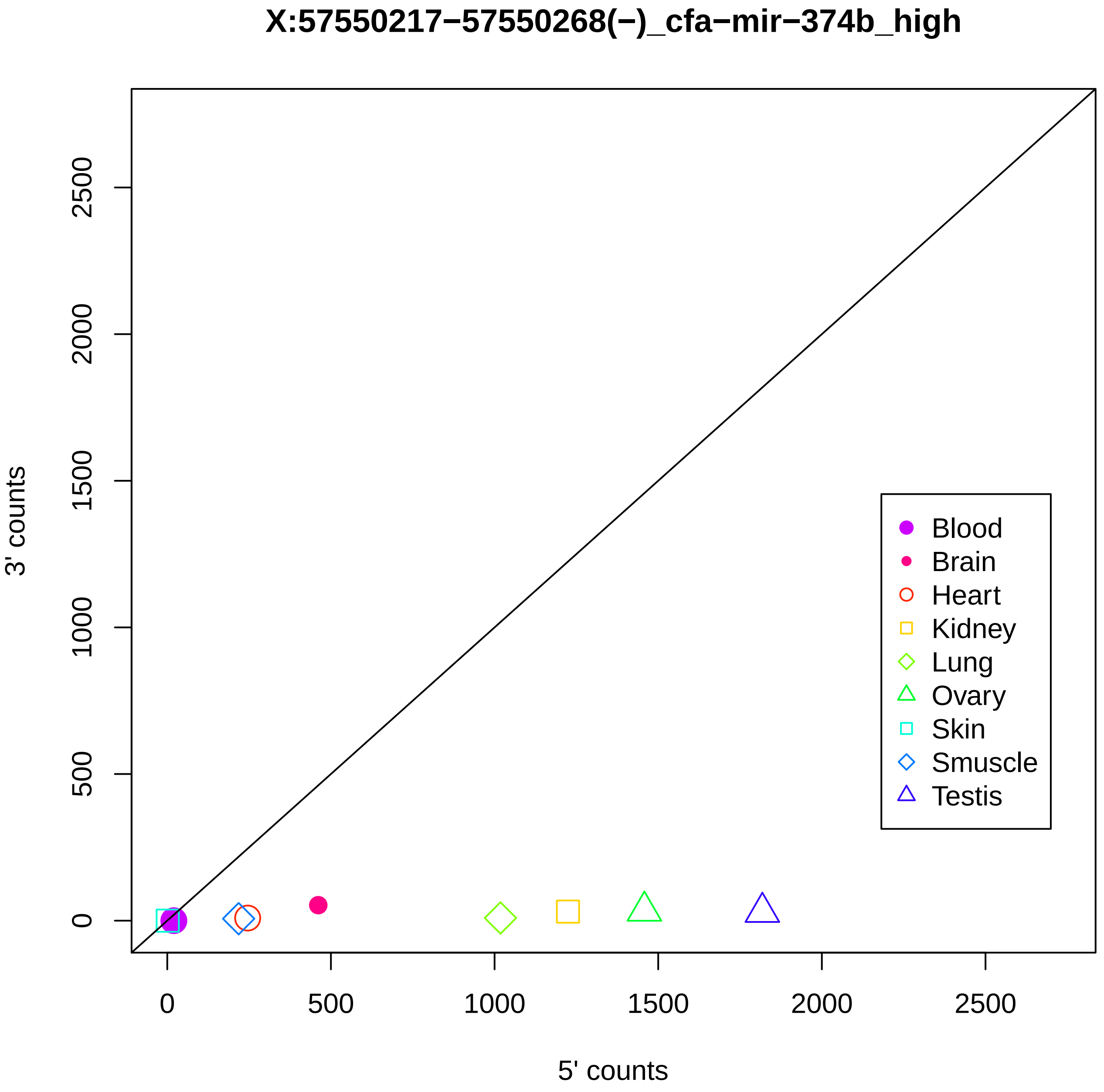
X:57550217−57550268(−)_cfa−mir−374b_high
3' counts
5' counts
0
500
1000
1500
2000
2500
Blood
Brain
Heart
Kidney
Lung
Ovary
Skin
Smuscle
Testis

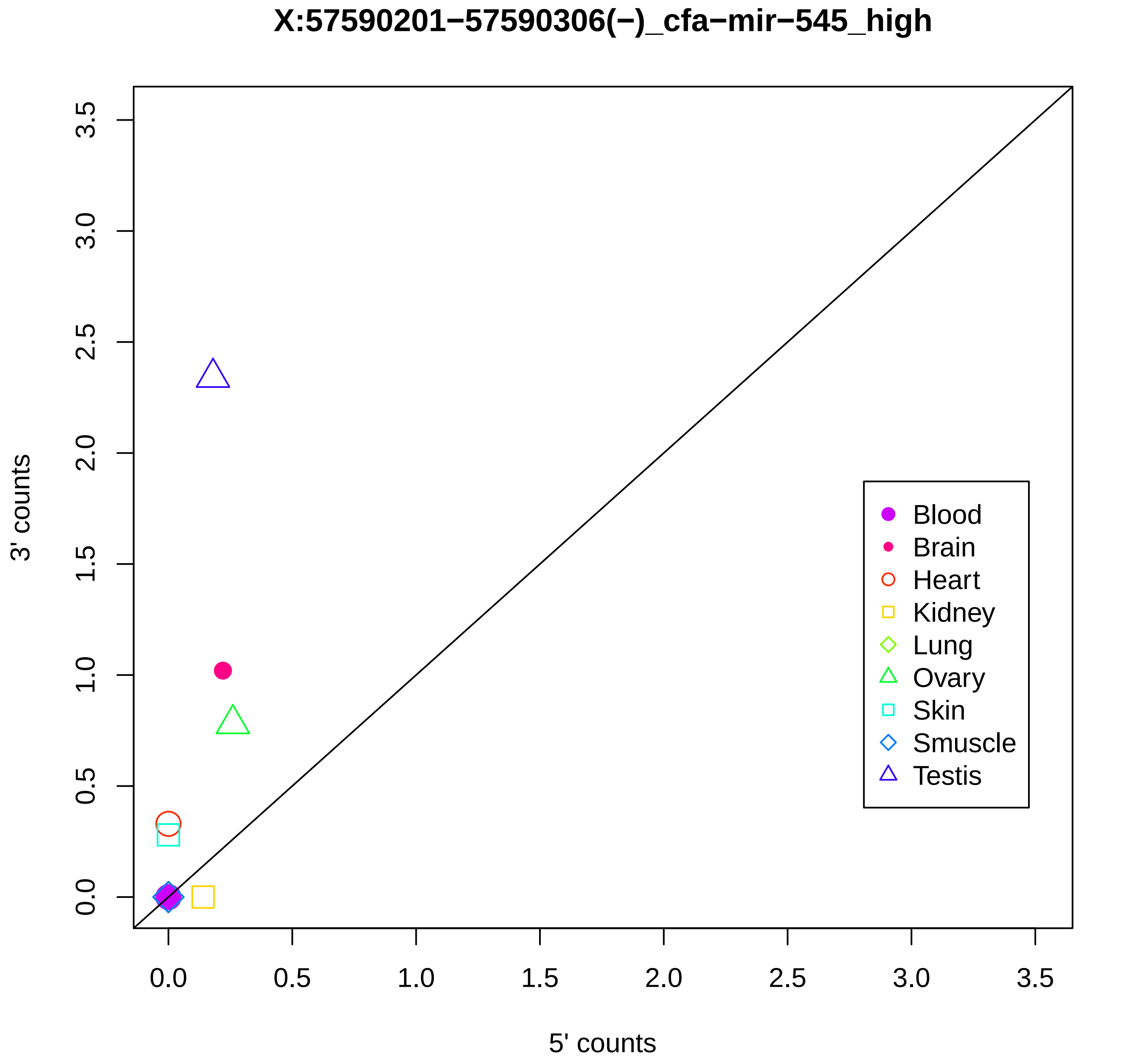

X:57590201−57590306(−)_cfa−mir−545_high
3' counts
5' counts
0.0
0.5
1.0
1.5
2.0
2.5
3.0
3.5
Blood
Brain
Heart
Kidney
Lung
Ovary
Skin
Smuscle
Testis

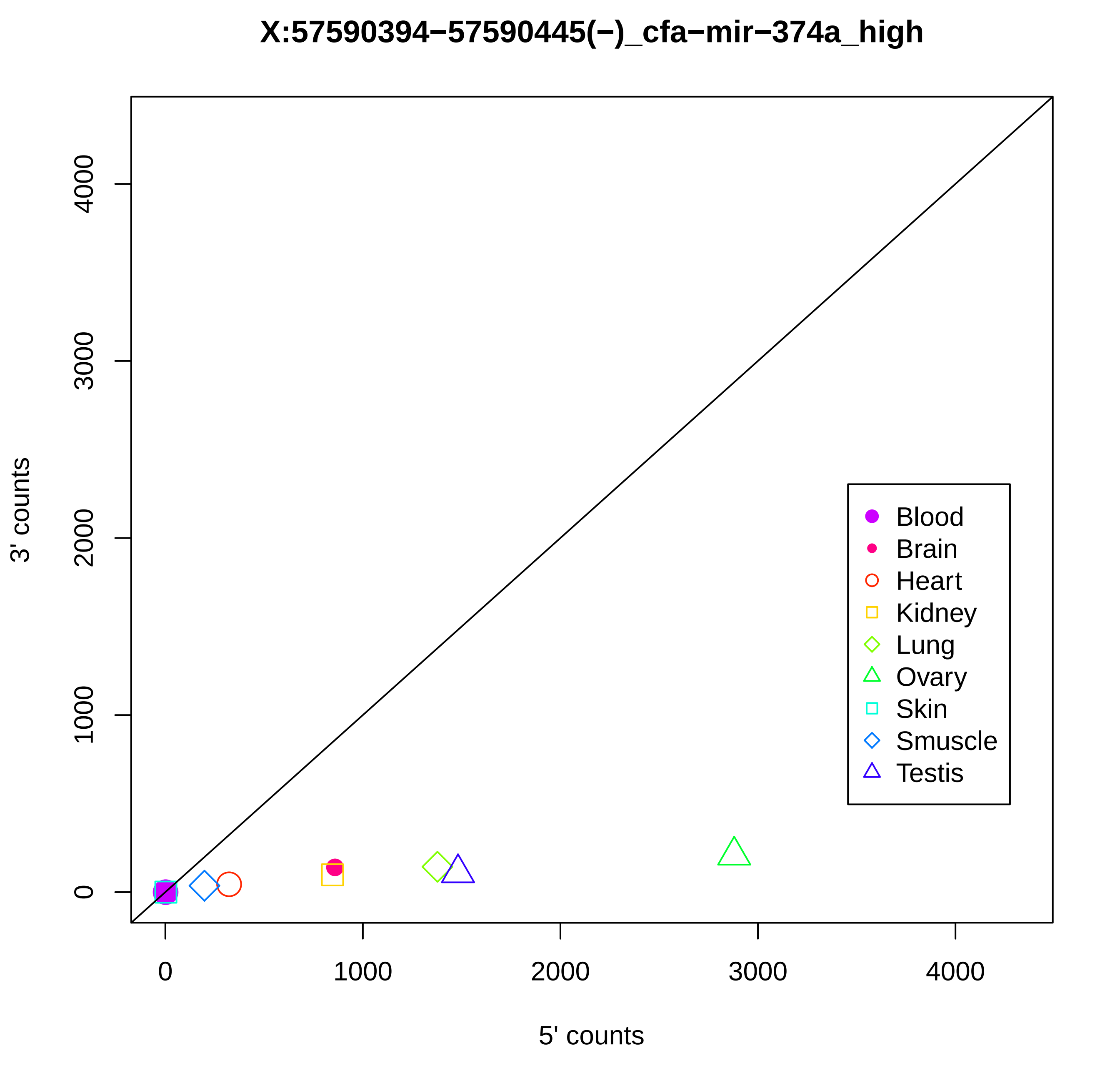

X:57590394−57590445(−)_cfa−mir−374a_high
3' counts
5' counts
0
1000
2000
3000
4000
0
1000
2000
3000
4000
Blood
Brain
Heart
Kidney
Lung
Ovary
Skin
Smuscle
Testis

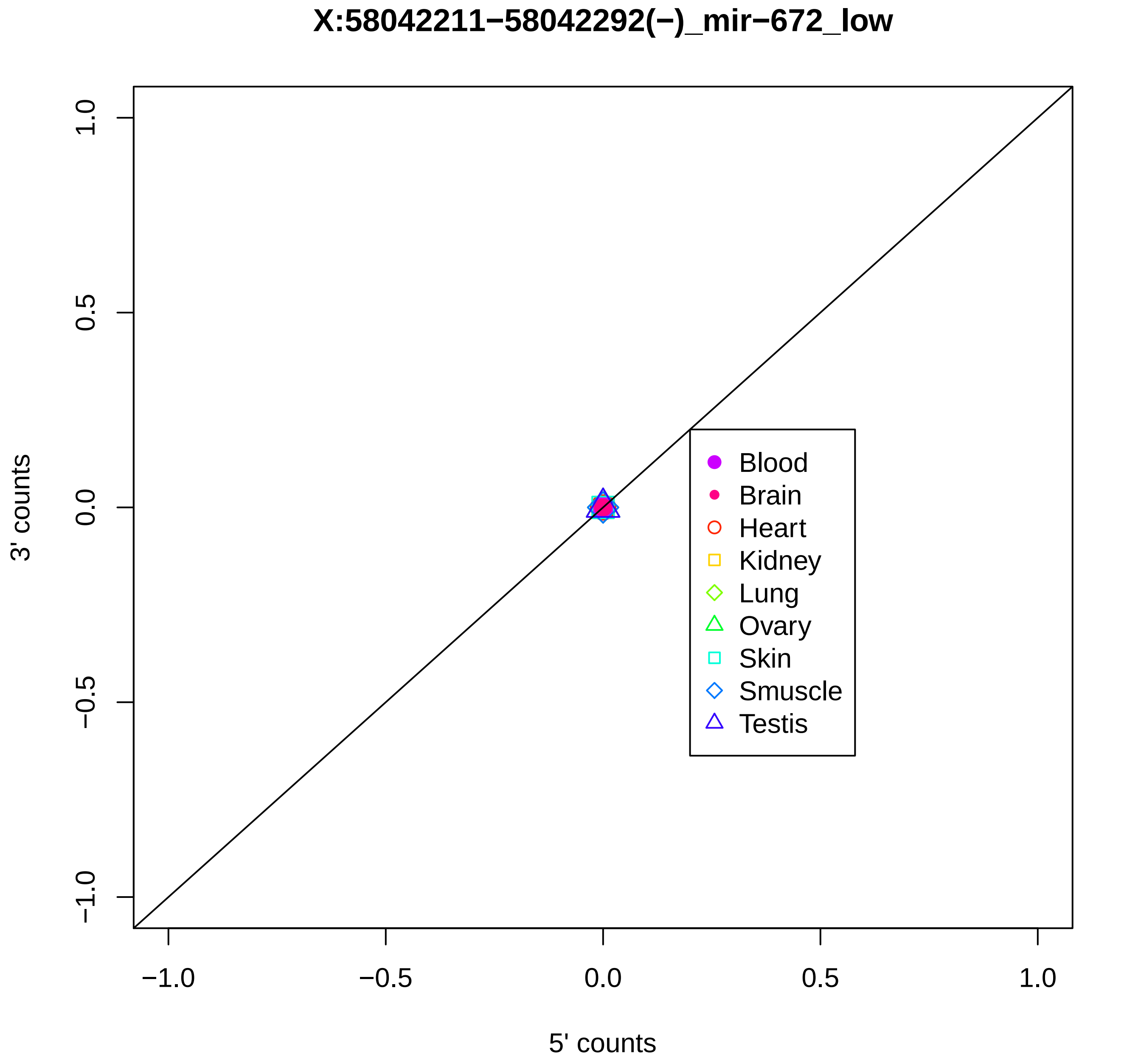
X:58042211−58042292(−)_mir−672_low
1.0
0.5
0.0
−0.5
−1.0
3' counts
−1.0
−0.5
0.0
0.5
1.0
5' counts
Blood
Brain
Heart
Kidney
Lung
Ovary
Skin
Smuscle
Testis

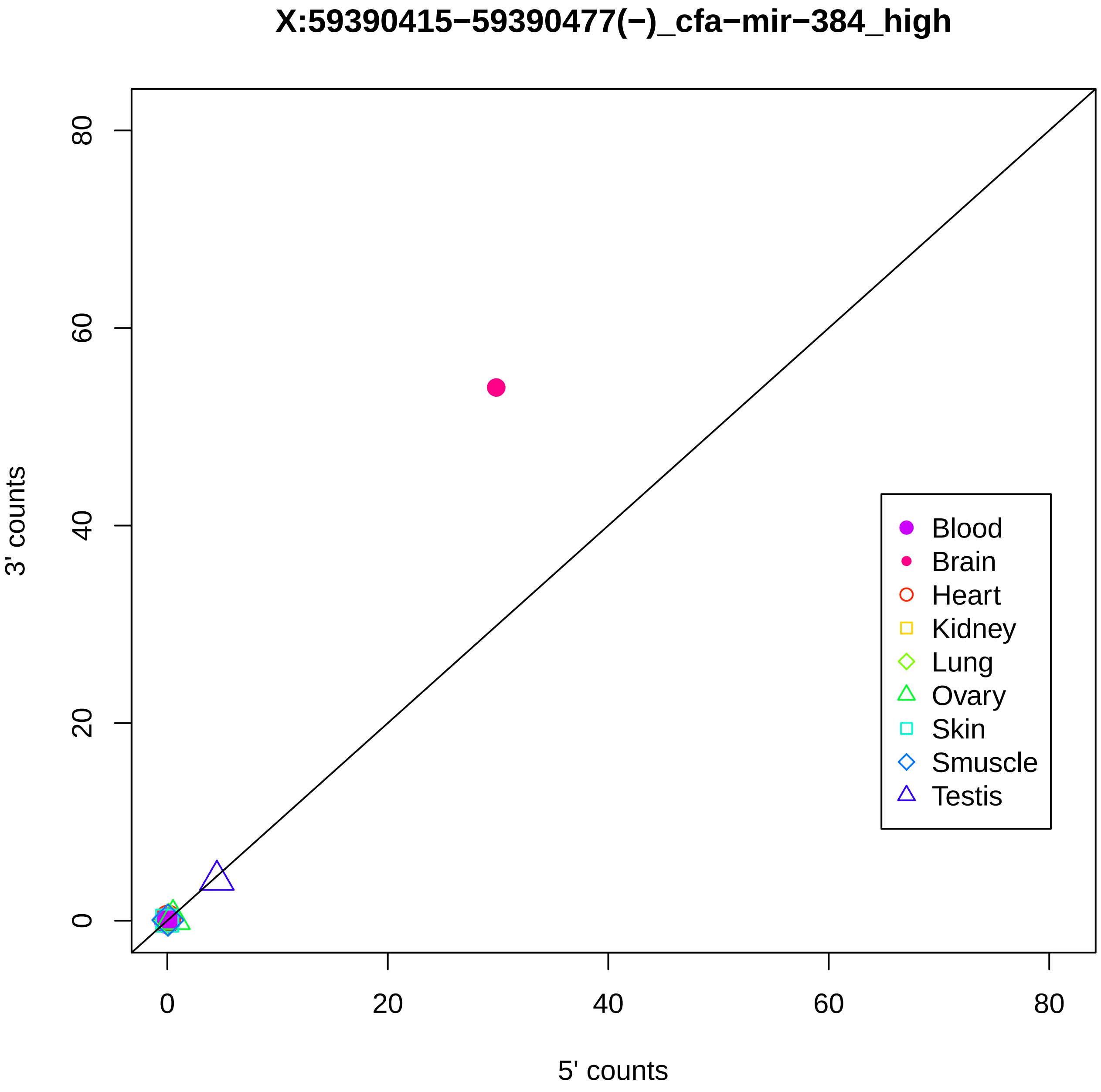

X:59390415−59390477(−)_cfa−mir−384_high
3' counts
5' counts
0
20
40
60
80
0
20
40
60
80
Blood
Brain
Heart
Kidney
Lung
Ovary
Skin
Smuscle
Testis

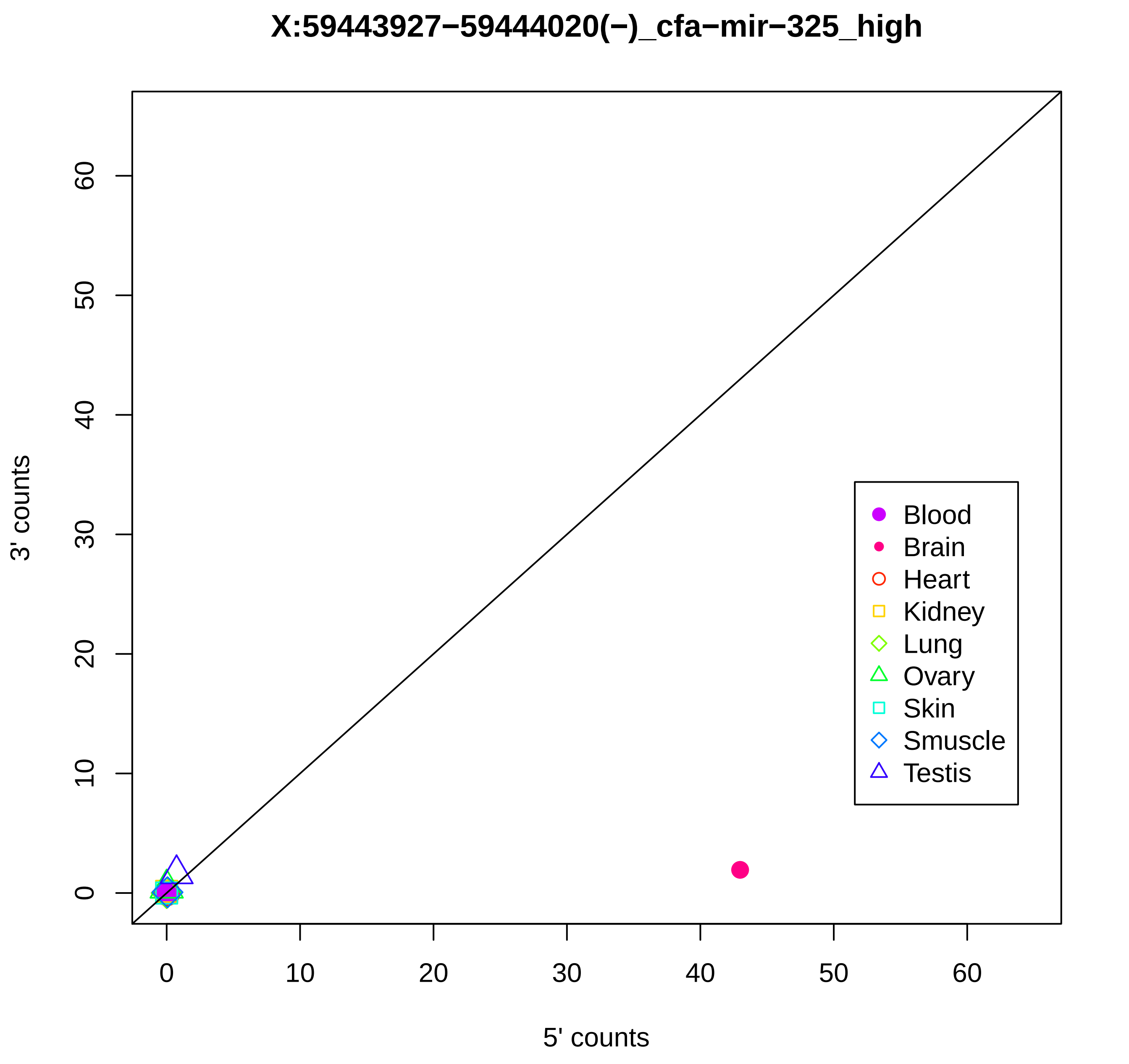

X:59443927−59444020(−)_cfa−mir−325_high
3' counts
5' counts
0
10
20
30
40
50
60
Blood
Brain
Heart
Kidney
Lung
Ovary
Skin
Smuscle
Testis

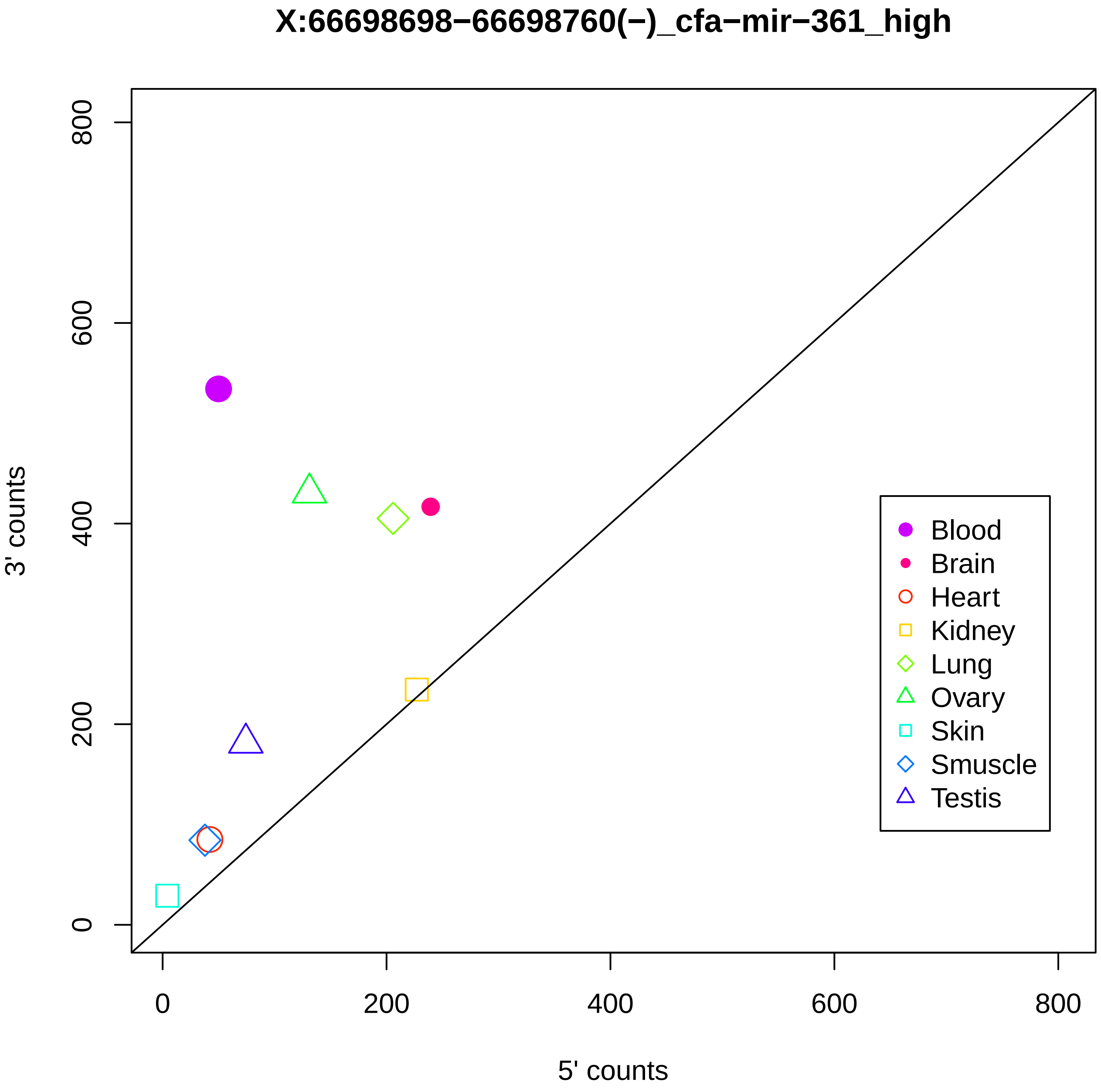

X:66698698−66698760(−)_cfa−mir−361_high
3' counts
5' counts
0
200
400
600
800
0
200
400
600
800
Blood
Brain
Heart
Kidney
Lung
Ovary
Skin
Smuscle
Testis

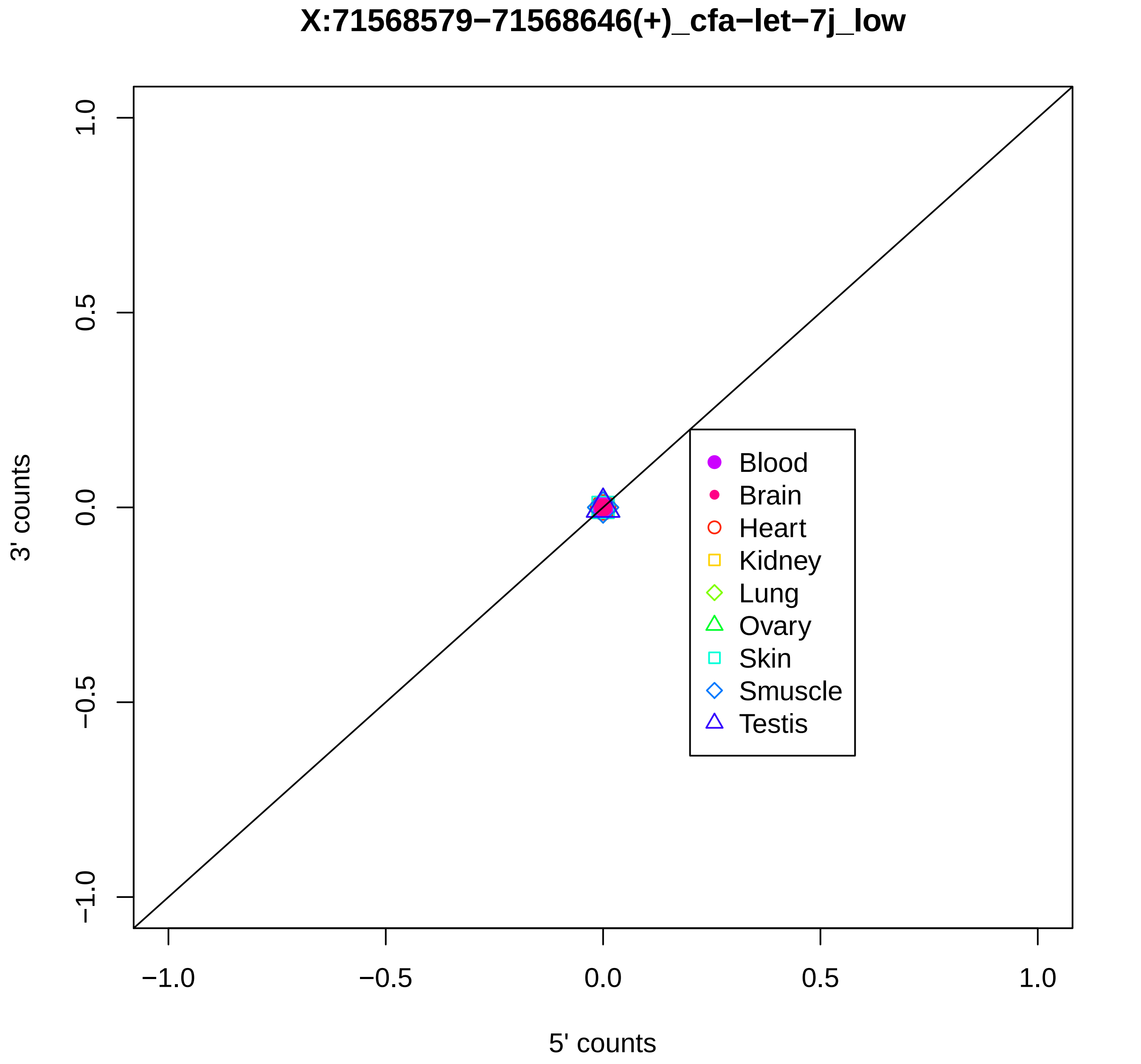

X:71568579−71568646(+)_cfa−let−7j_low
Blood
Brain
Heart
Kidney
Lung
Ovary
Skin
Smuscle
Testis
3' counts
5' counts
−1.0
−0.5
0.0
0.5
1.0

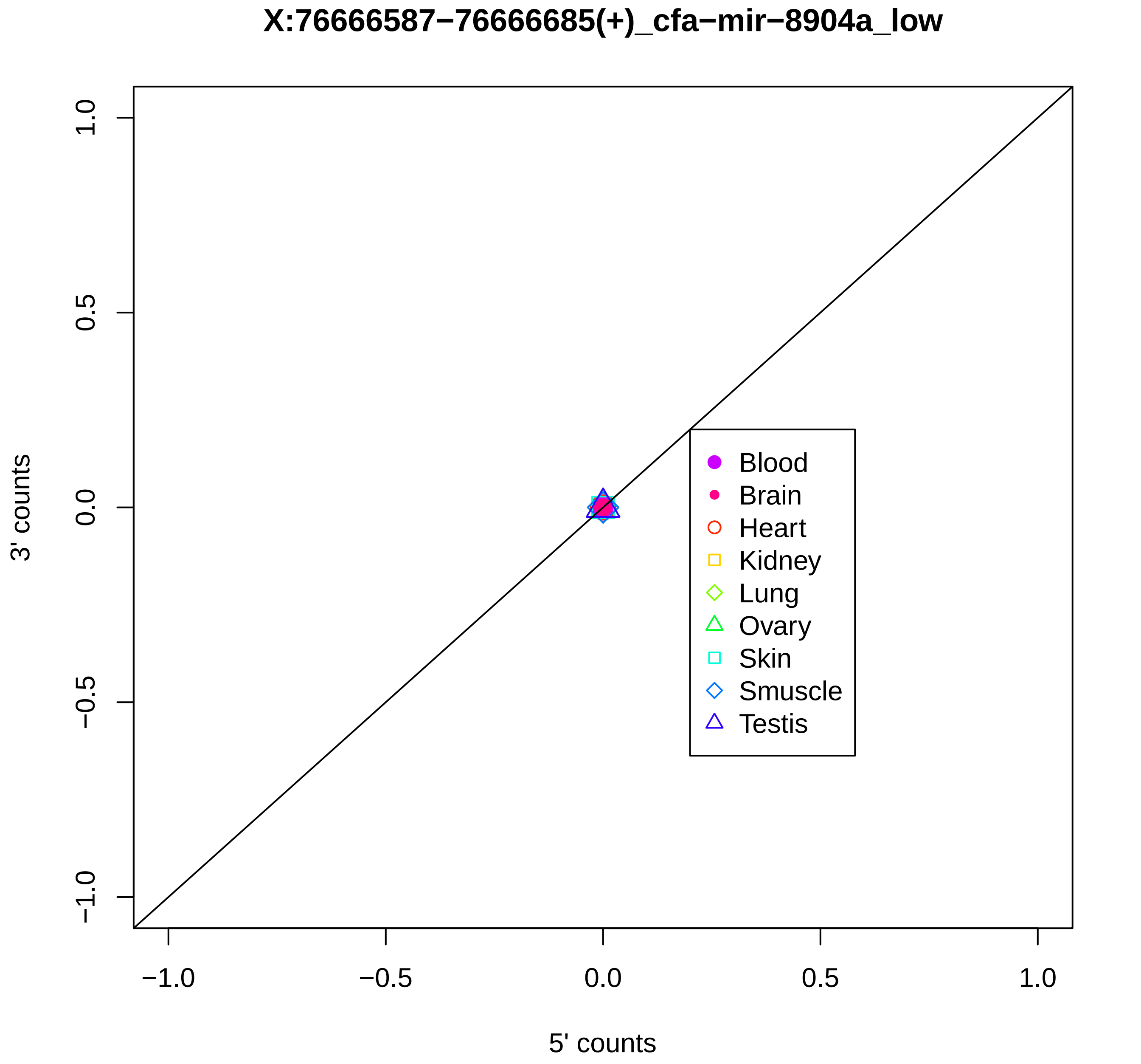
X:76666587−76666685(+)_cfa−mir−8904a_low
3' counts
5' counts
−1.0
−0.5
0.0
0.5
1.0
Blood
Brain
Heart
Kidney
Lung
Ovary
Skin
Smuscle
Testis

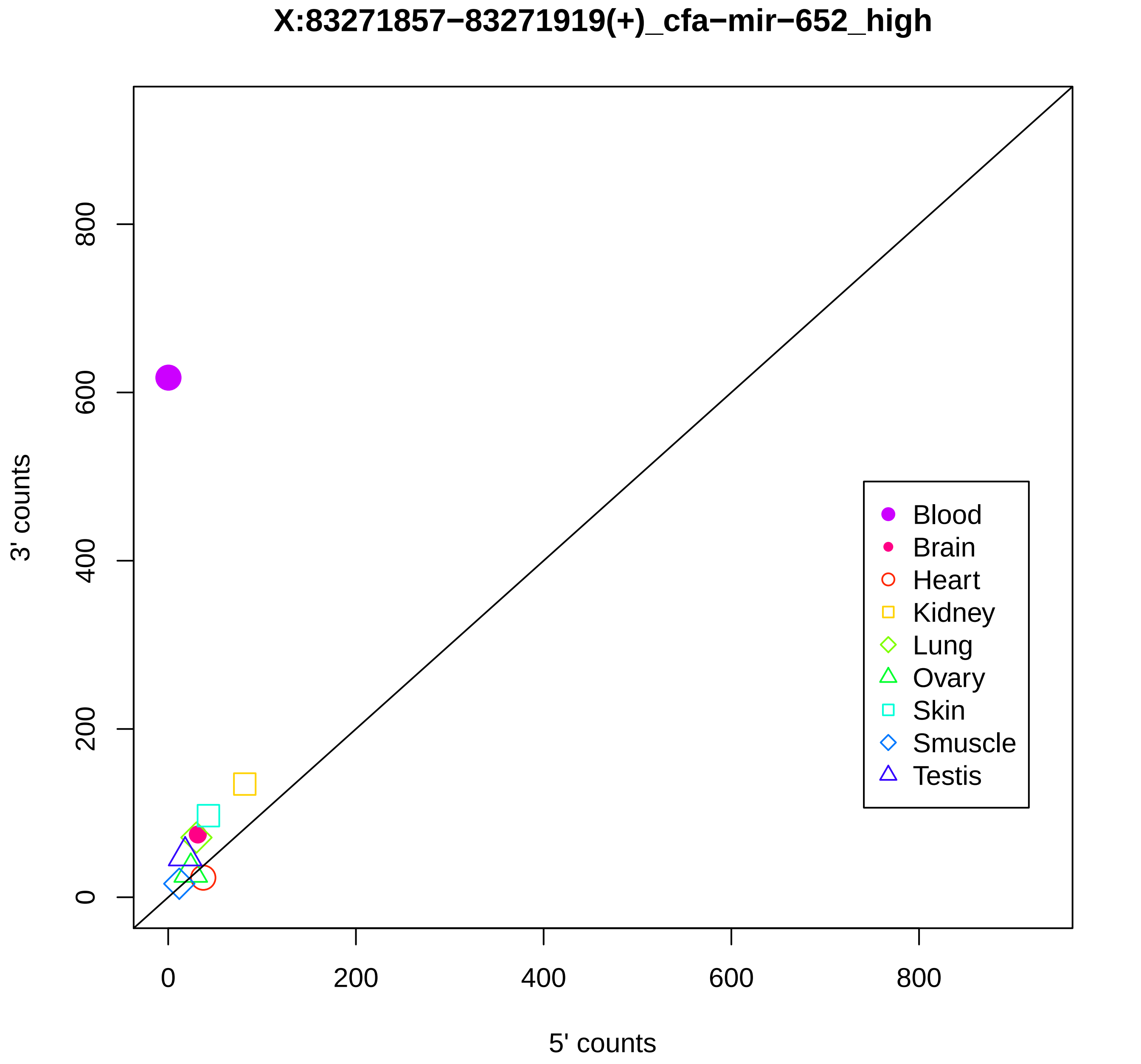
X:83271857−83271919(+)_cfa−mir−652_high
3' counts
5' counts
0
200
400
600
800
0
200
400
600
800
Blood
Brain
Heart
Kidney
Lung
Ovary
Skin
Smuscle
Testis

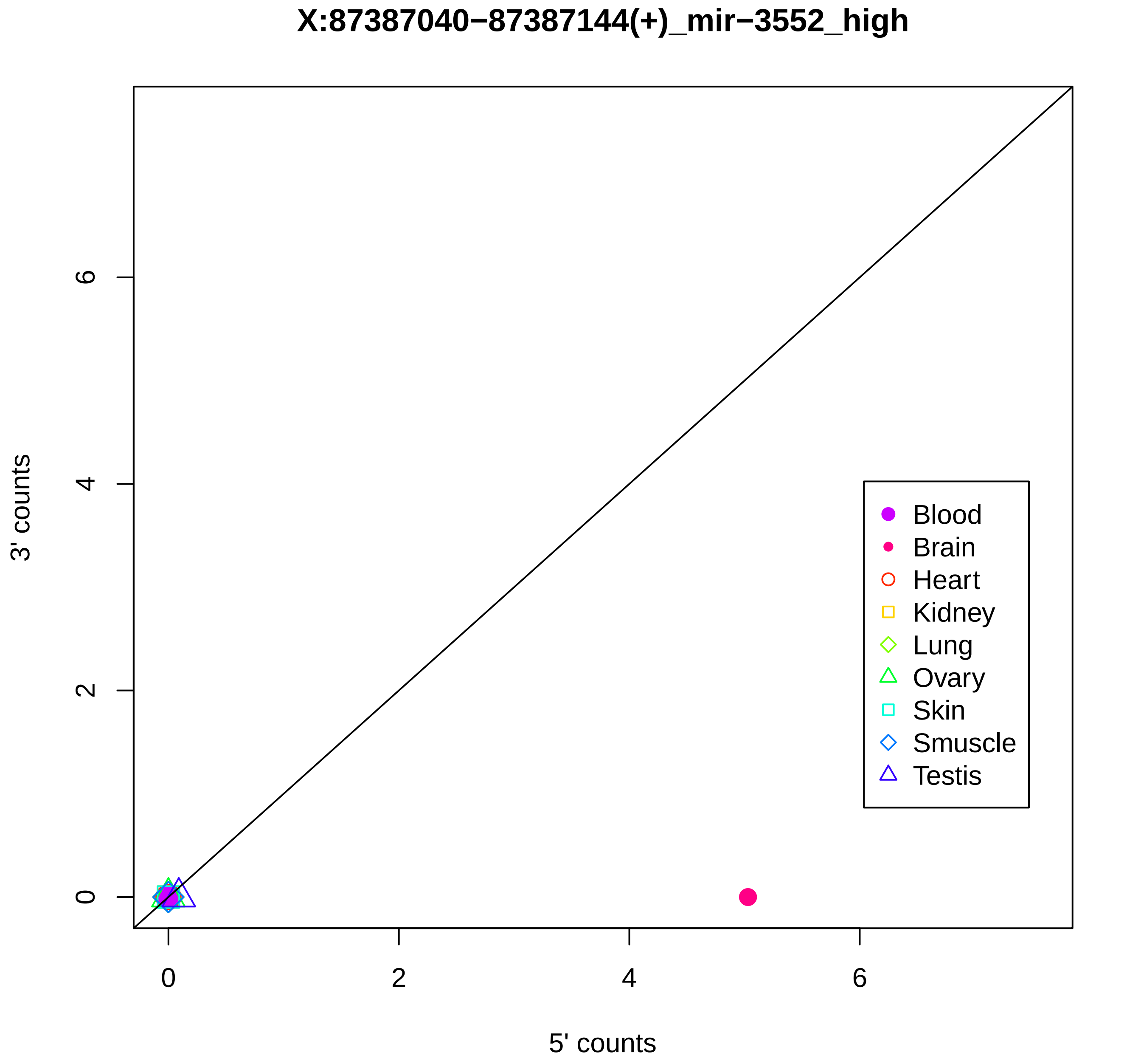

X:87387040−87387144(+)_mir−3552_high
3' counts
5' counts
0
2
4
6
0
2
4
6
Blood
Brain
Heart
Kidney
Lung
Ovary
Skin
Smuscle
Testis

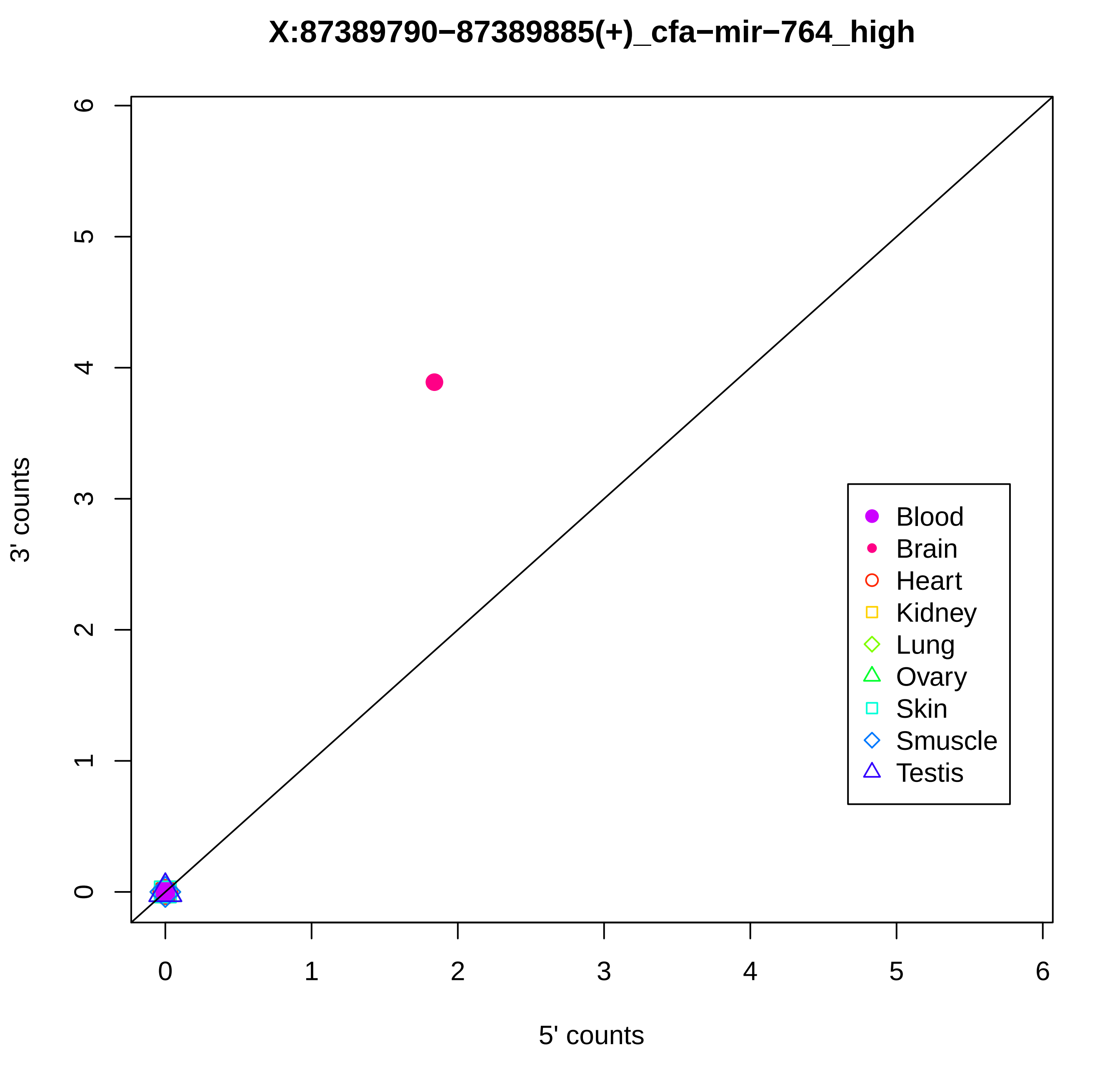

X:87389790−87389885(+)_cfa−mir−764_high
3' counts
5' counts
0
1
2
3
4
5
6
Blood
Brain
Heart
Kidney
Lung
Ovary
Skin
Smuscle
Testis

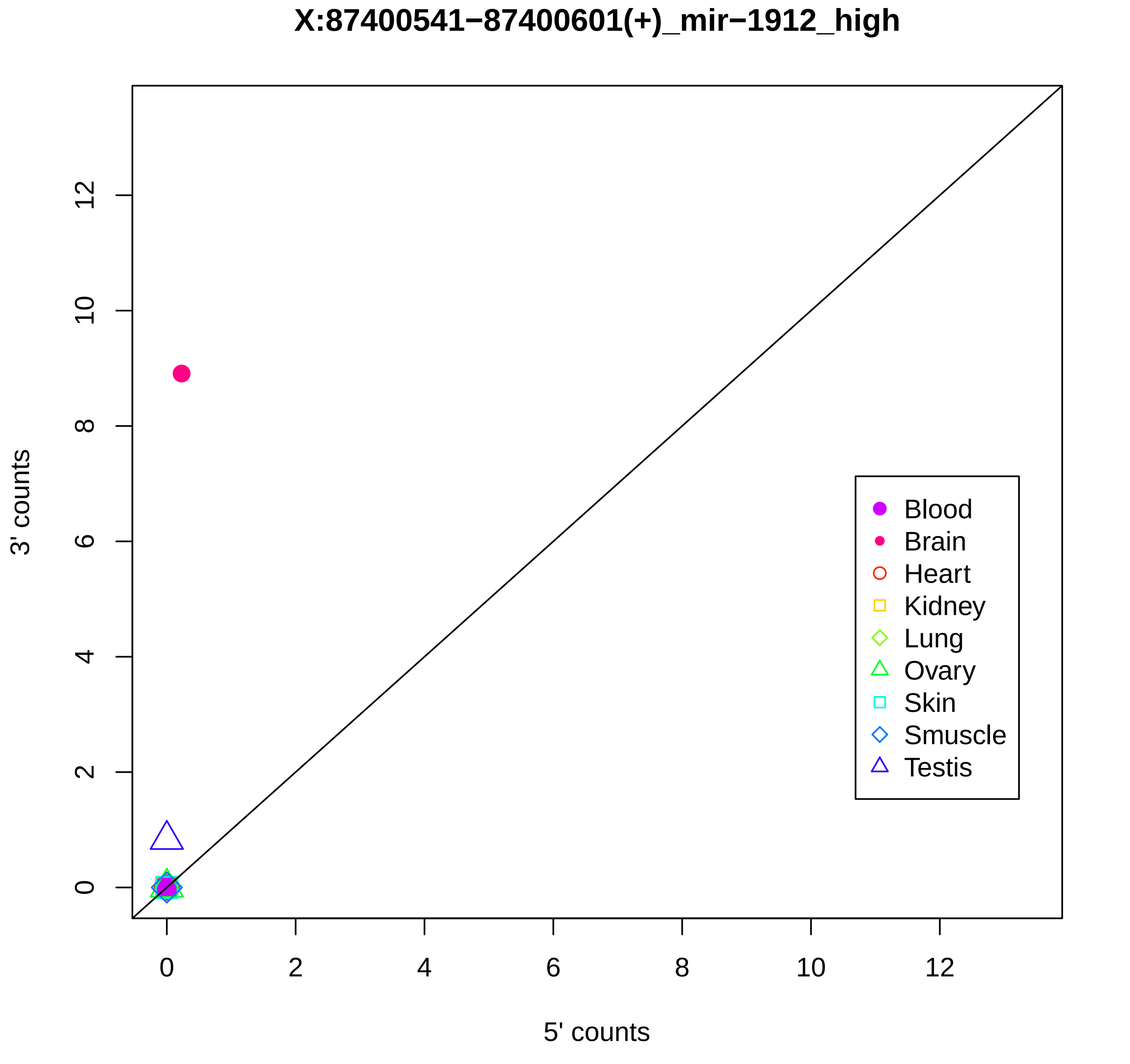
X:87400541−87400601(+)_mir−1912_high
3' counts
5' counts
0
2
4
6
8
10
12
Blood
Brain
Heart
Kidney
Lung
Ovary
Skin
Smuscle
Testis

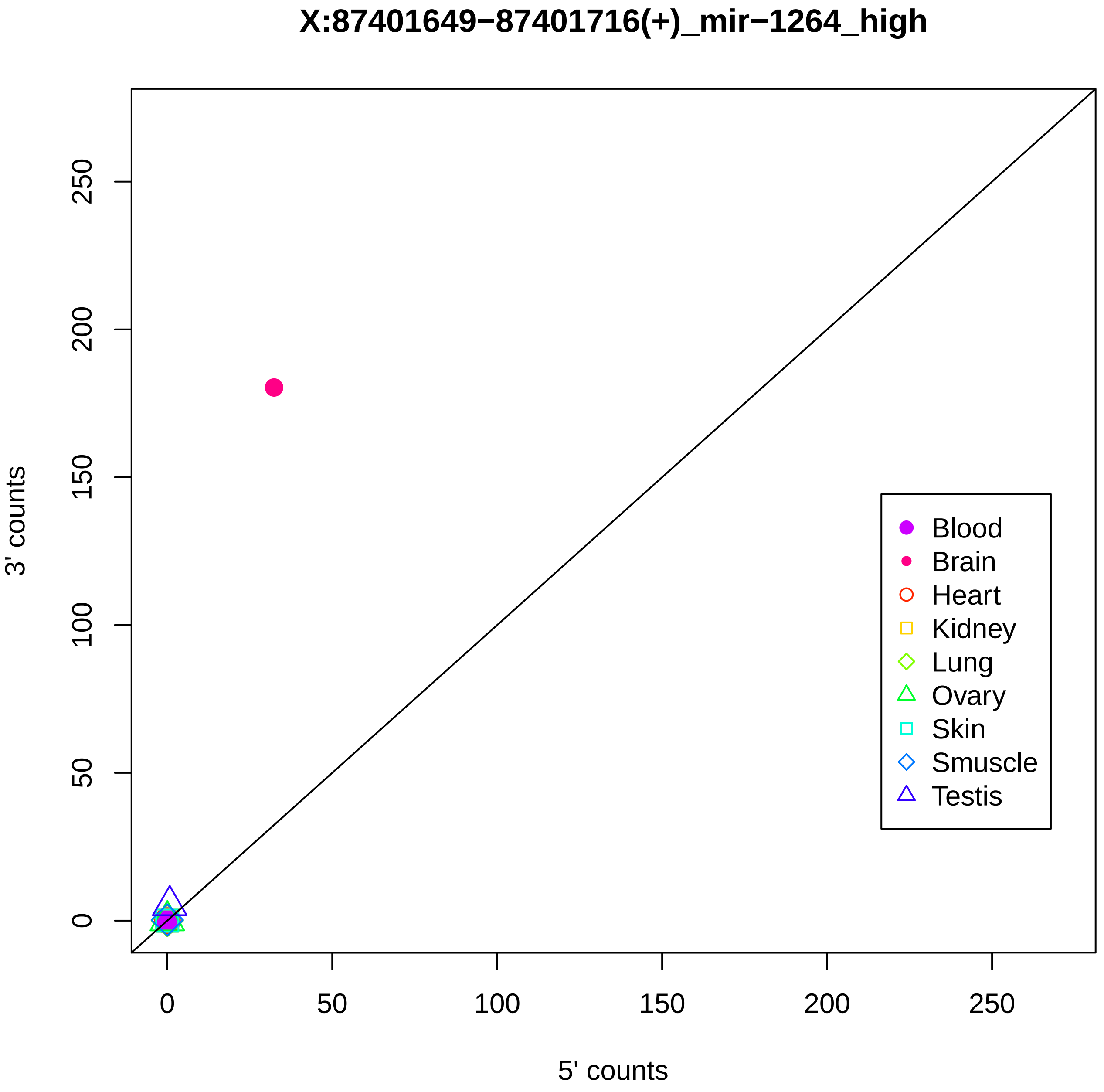

X:87401649−87401716(+)_mir−1264_high
3' counts
5' counts
0
50
100
150
200
250
Blood
Brain
Heart
Kidney
Lung
Ovary
Skin
Smuscle
Testis

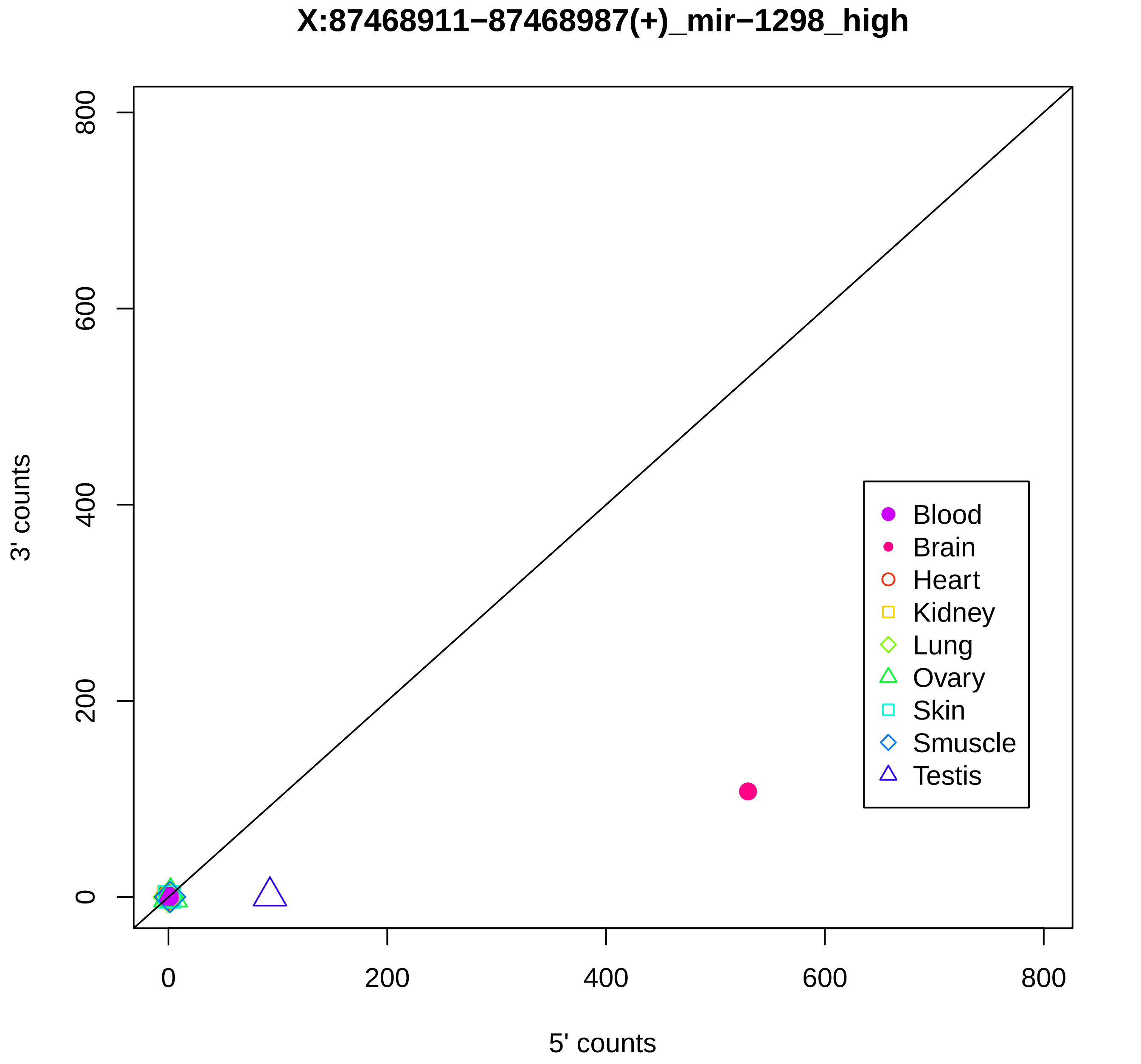
X:87468911−87468987(+)_mir−1298_high
3' counts
5' counts
0
200
400
600
800
Blood
Brain
Heart
Kidney
Lung
Ovary
Skin
Smuscle
Testis

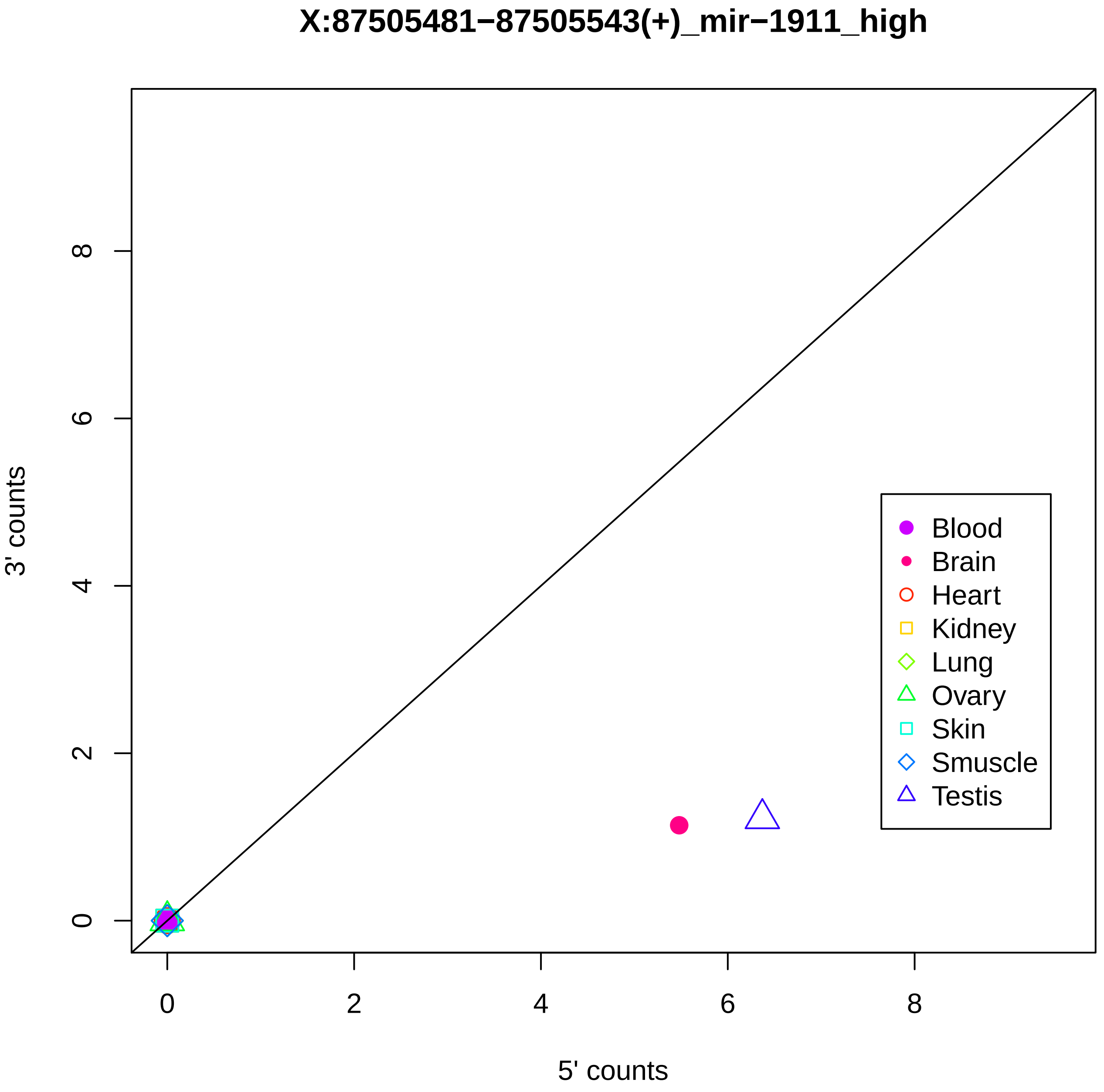
X:87505481−87505543(+)_mir−1911_high
3' counts
5' counts
0
2
4
6
8
0
2
4
6
8
Blood
Brain
Heart
Kidney
Lung
Ovary
Skin
Smuscle
Testis

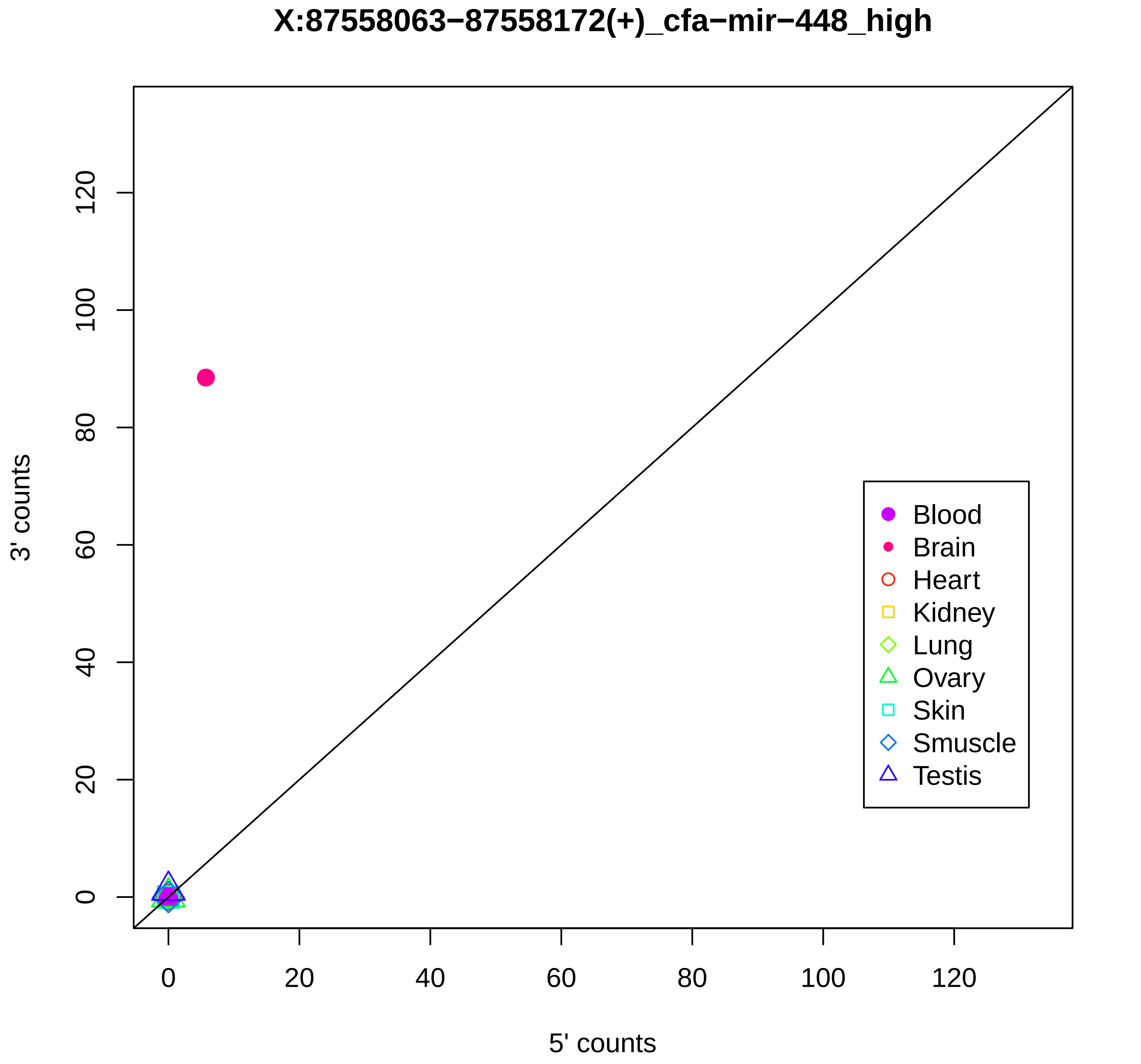

X:87558063−87558172(+)_cfa−mir−448_high
3' counts
5' counts
0
20
40
60
80
100
120
Blood
Brain
Heart
Kidney
Lung
Ovary
Skin
Smuscle
Testis

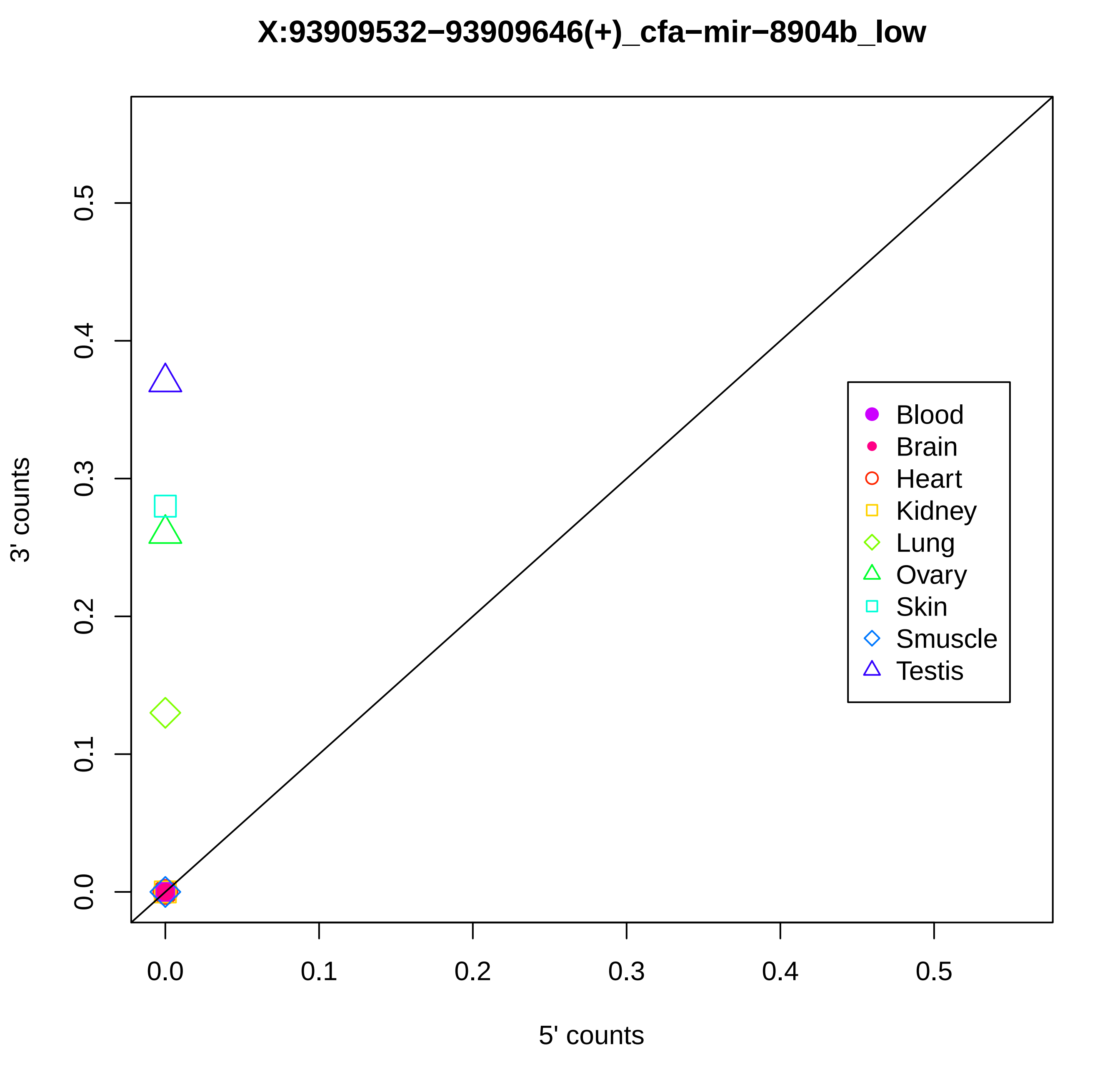

X:93909532−93909646(+)_cfa−mir−8904b_low
3' counts
5' counts
0.0
0.1
0.2
0.3
0.4
0.5
Blood
Brain
Heart
Kidney
Lung
Ovary
Skin
Smuscle
Testis

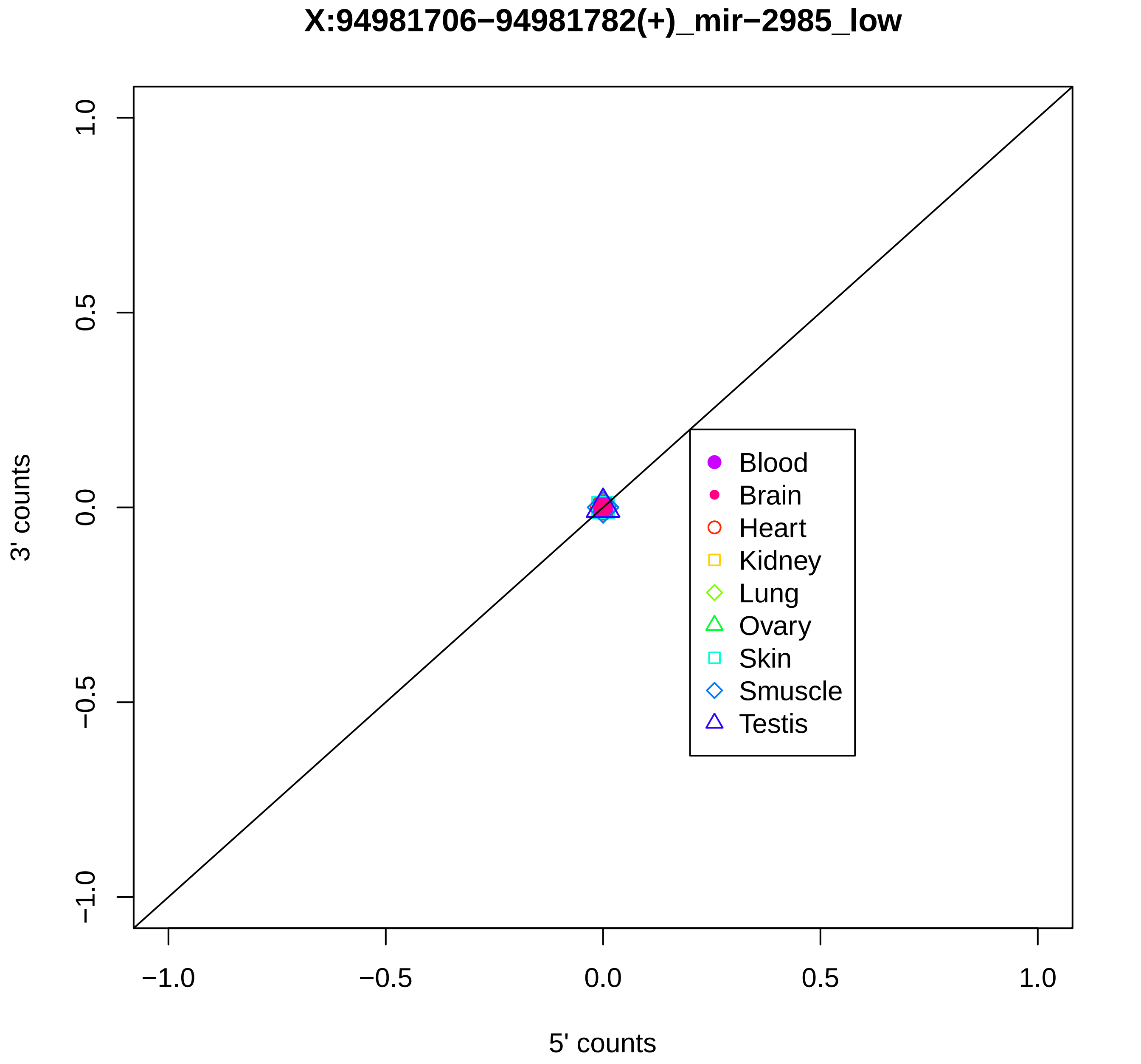

X:94981706−94981782(+)_mir−2985_low
3' counts
5' counts
1.0
0.5
0.0
−0.5
−1.0
Blood
Brain
Heart
Kidney
Lung
Ovary
Skin
Smuscle
Testis

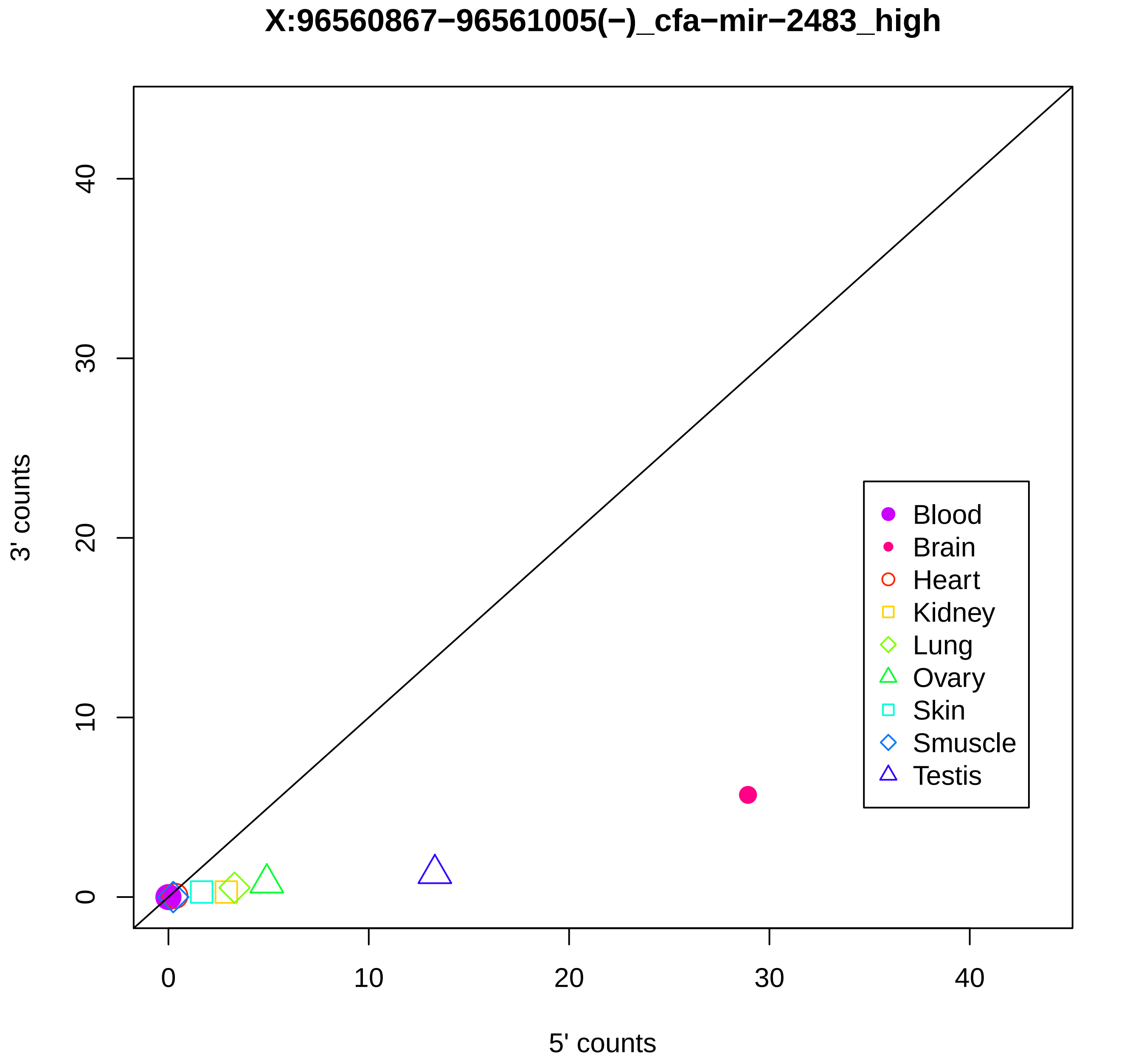

X:96560867−96561005(−)_cfa−mir−2483_high
3' counts
5' counts
0
10
20
30
40
0
10
20
30
40
Blood
Brain
Heart
Kidney
Lung
Ovary
Skin
Smuscle
Testis

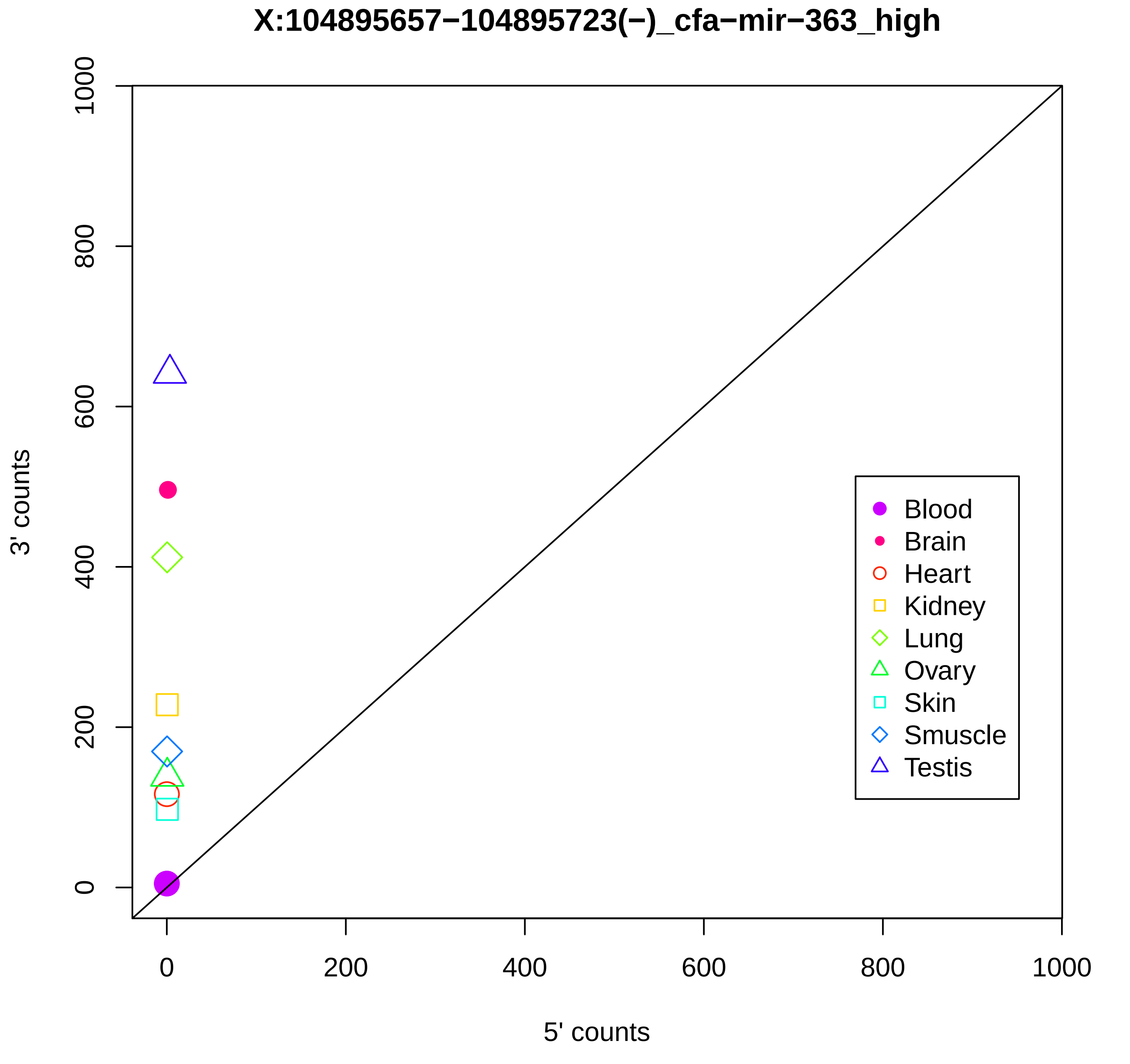
X:104895657−104895723(−)_cfa−mir−363_high
3' counts
5' counts
0
200
400
600
800
1000
Blood
Brain
Heart
Kidney
Lung
Ovary
Skin
Smuscle
Testis

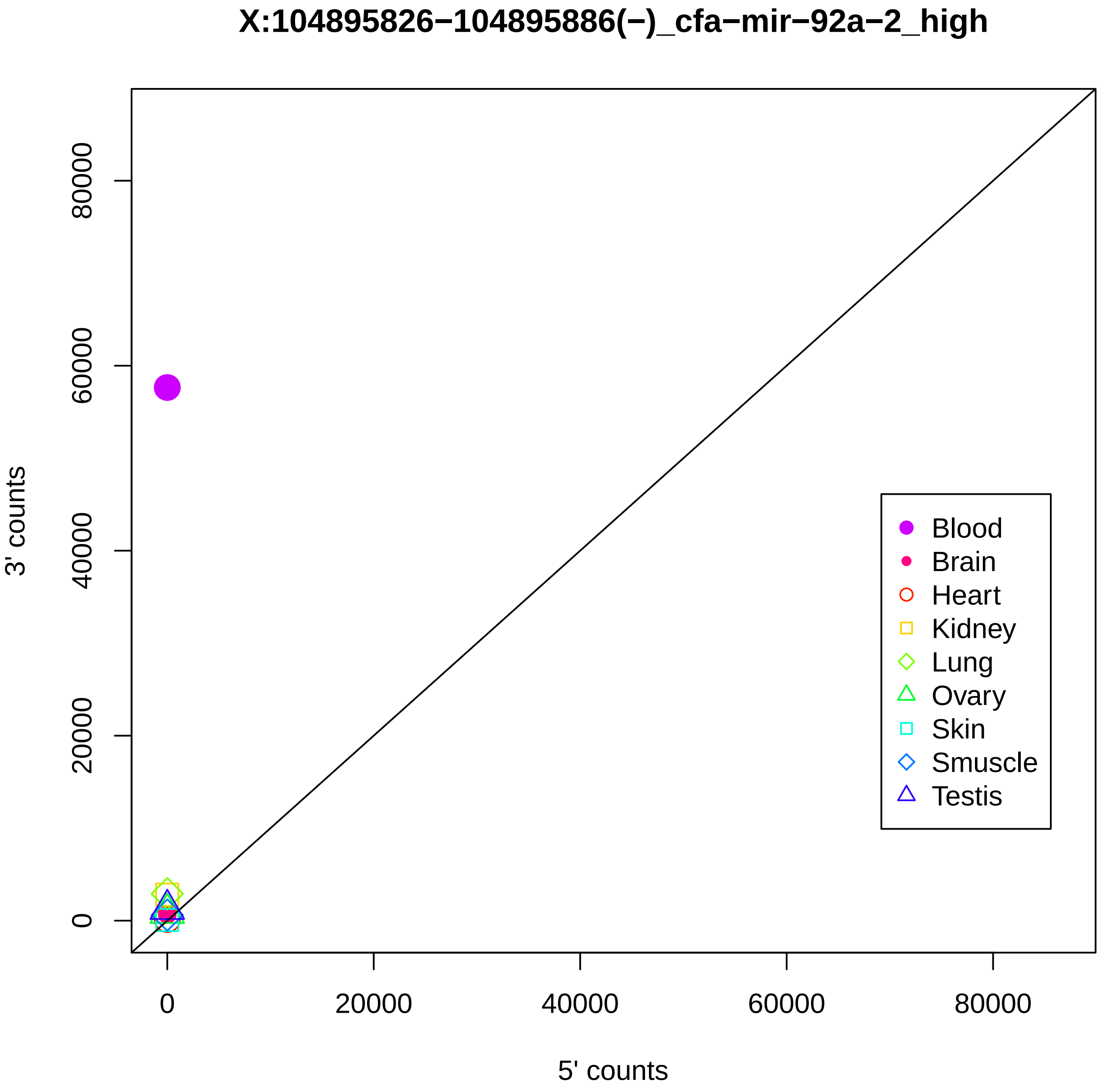

X:104895826−104895886(−)_cfa−mir−92a−2_high
3' counts
0
20000
40000
60000
80000
5' counts
0
20000
40000
60000
80000
Blood
Brain
Heart
Kidney
Lung
Ovary
Skin
Smuscle
Testis

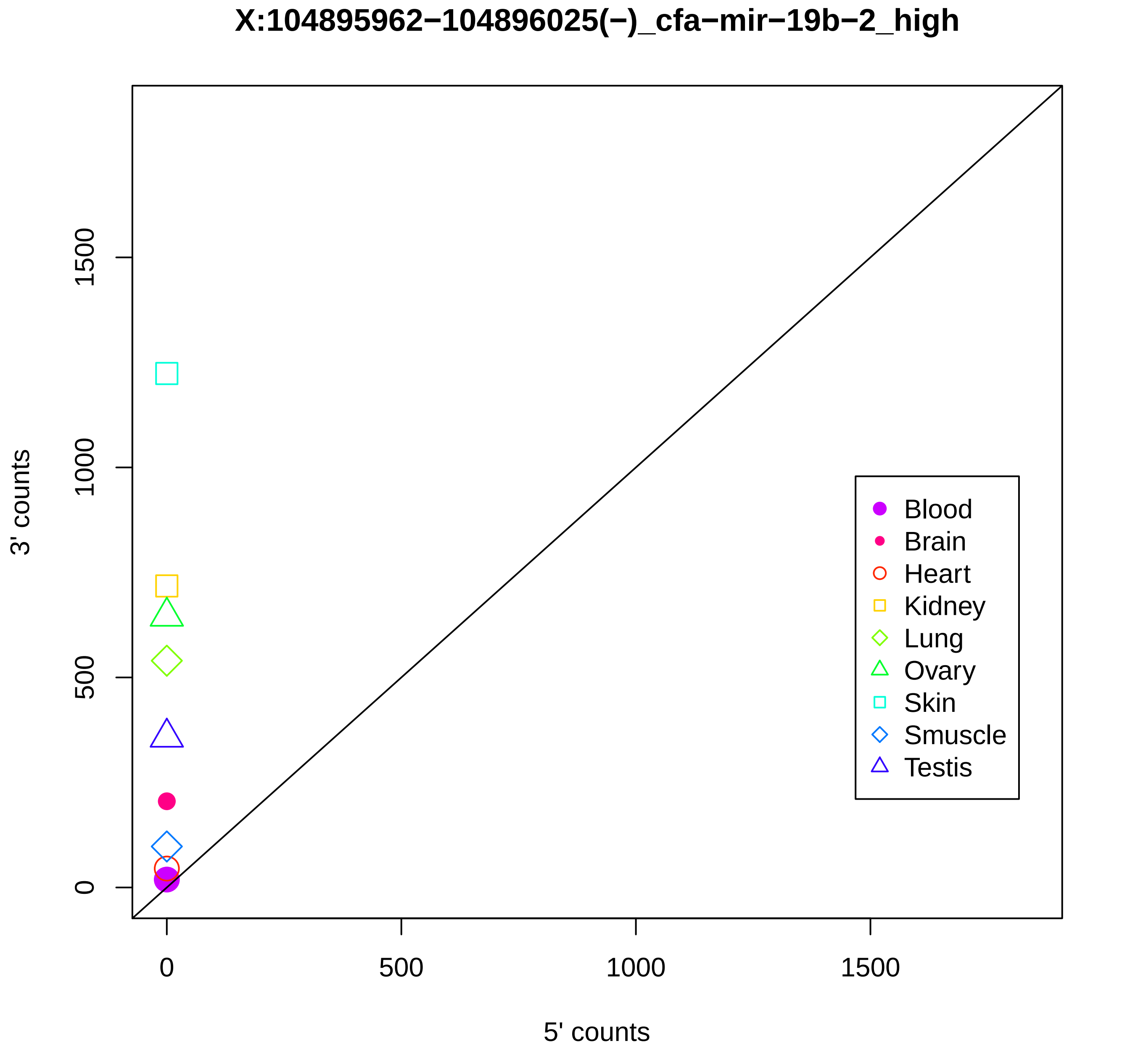

X:104895962−104896025(−)_cfa−mir−19b−2_high
3' counts
5' counts
0
500
1000
1500
0
500
1000
1500
Blood
Brain
Heart
Kidney
Lung
Ovary
Skin
Smuscle
Testis

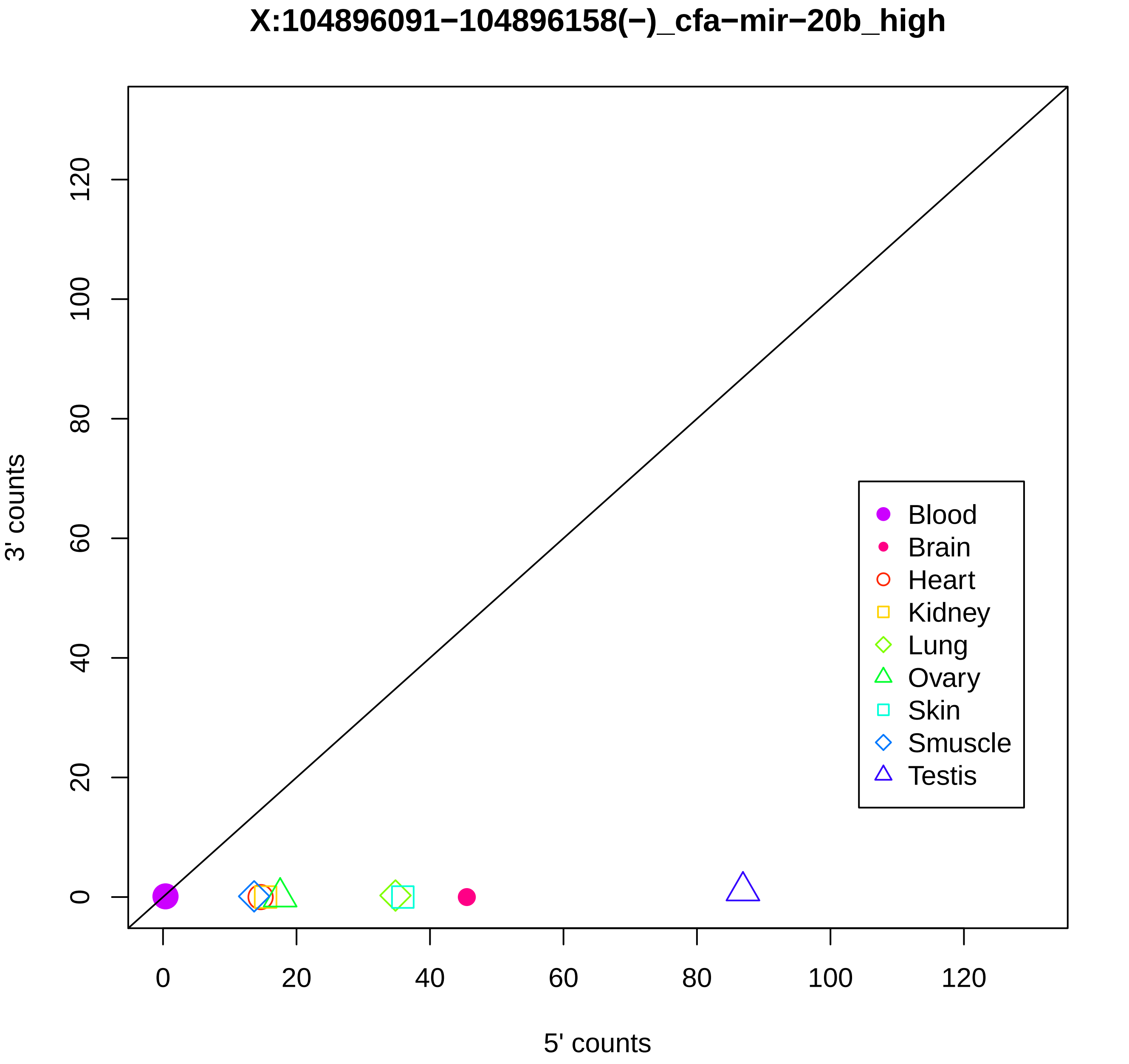

X:104896091−104896158(−)_cfa−mir−20b_high
3' counts
5' counts
0
20
40
60
80
100
120
Blood
Brain
Heart
Kidney
Lung
Ovary
Skin
Smuscle
Testis

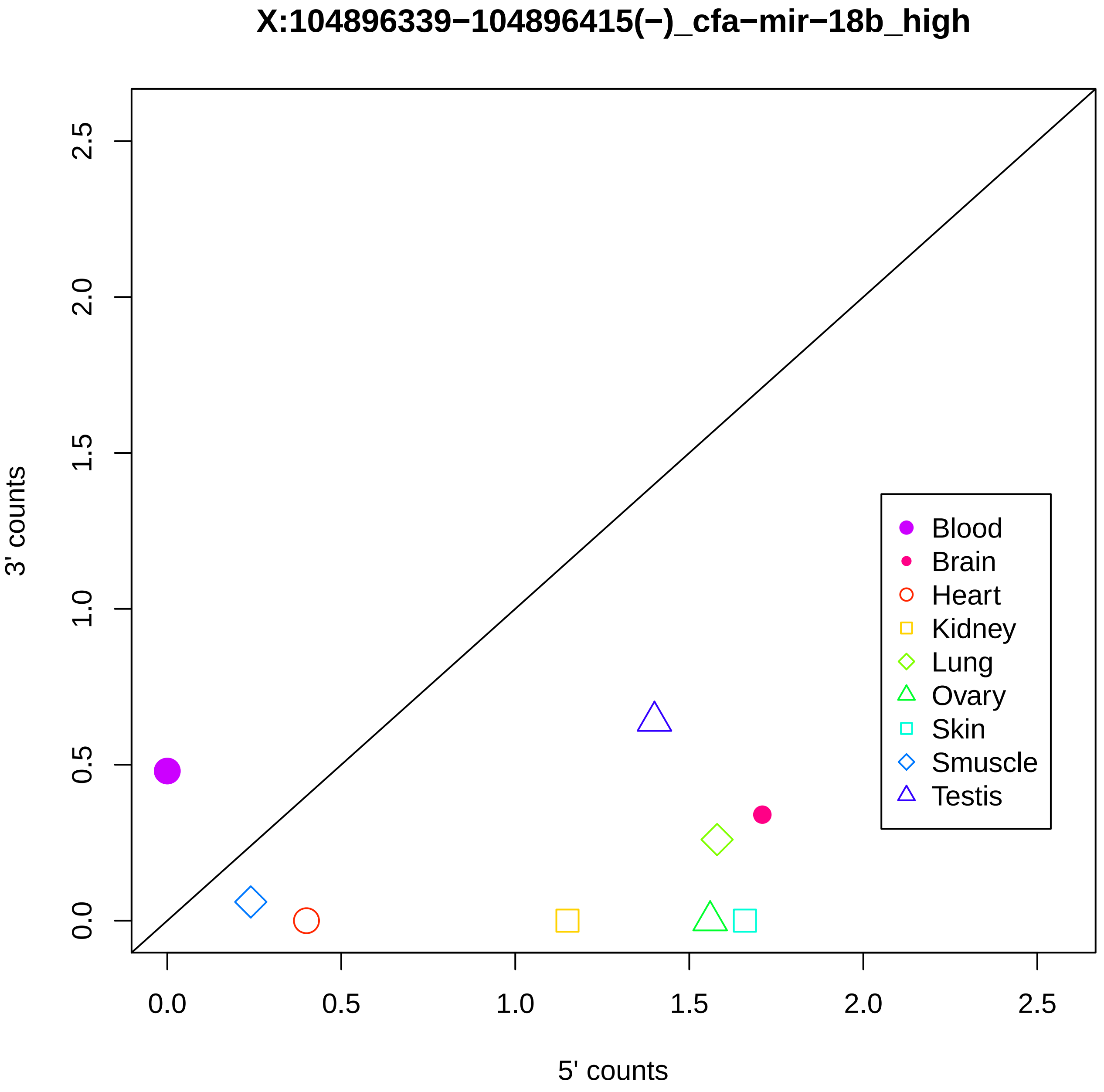

X:104896339−104896415(−)_cfa−mir−18b_high
3' counts
5' counts
0.0
0.5
1.0
1.5
2.0
2.5
Blood
Brain
Heart
Kidney
Lung
Ovary
Skin
Smuscle
Testis

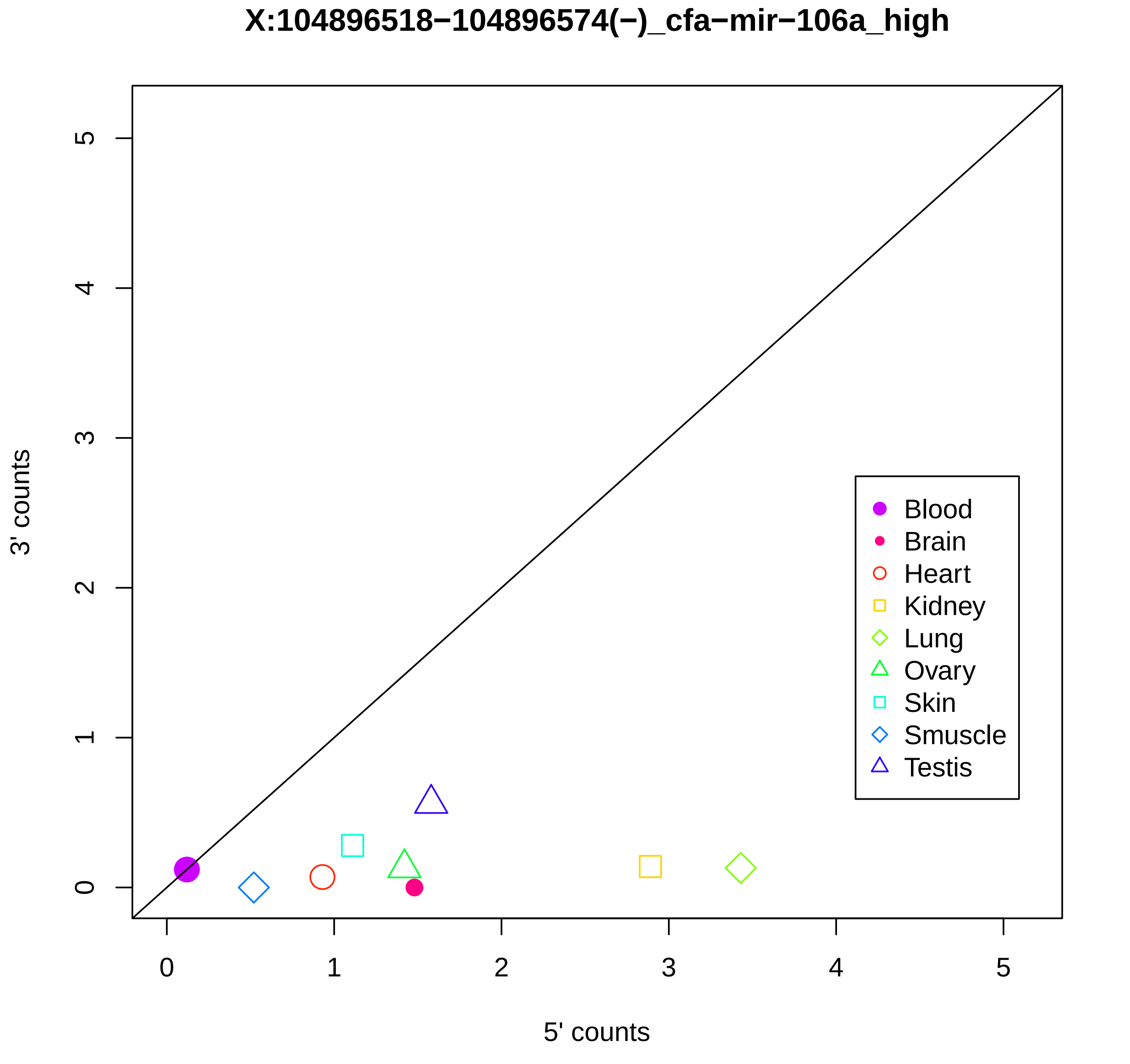

X:104896518−104896574(−)_cfa−mir−106a_high
3' counts
5' counts
0
1
2
3
4
5
Blood
Brain
Heart
Kidney
Lung
Ovary
Skin
Smuscle
Testis

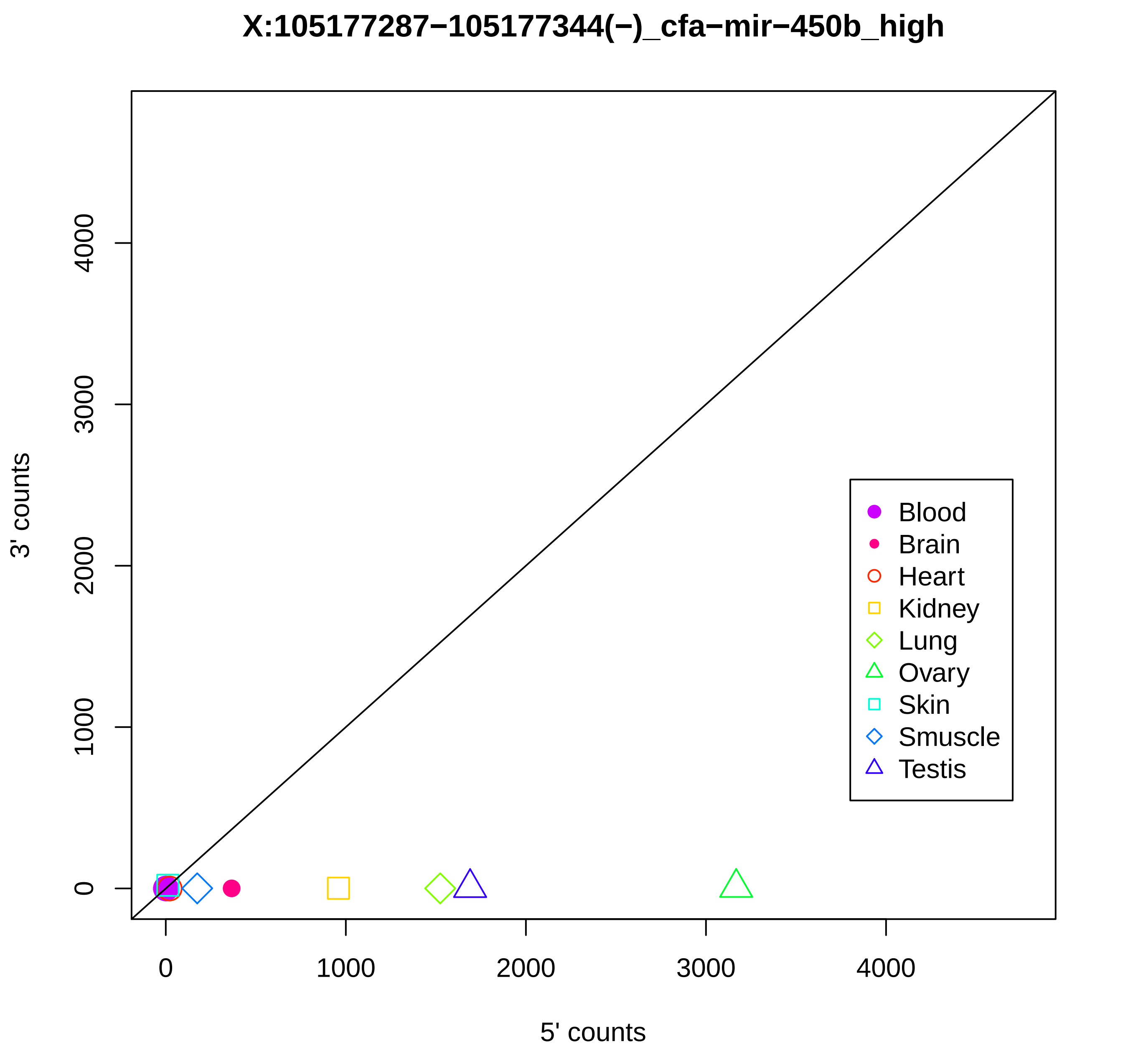

X:105177287−105177344(−)_cfa−mir−450b_high
3' counts
5' counts
0
1000
2000
3000
4000
0
1000
2000
3000
4000
Blood
Brain
Heart
Kidney
Lung
Ovary
Skin
Smuscle
Testis

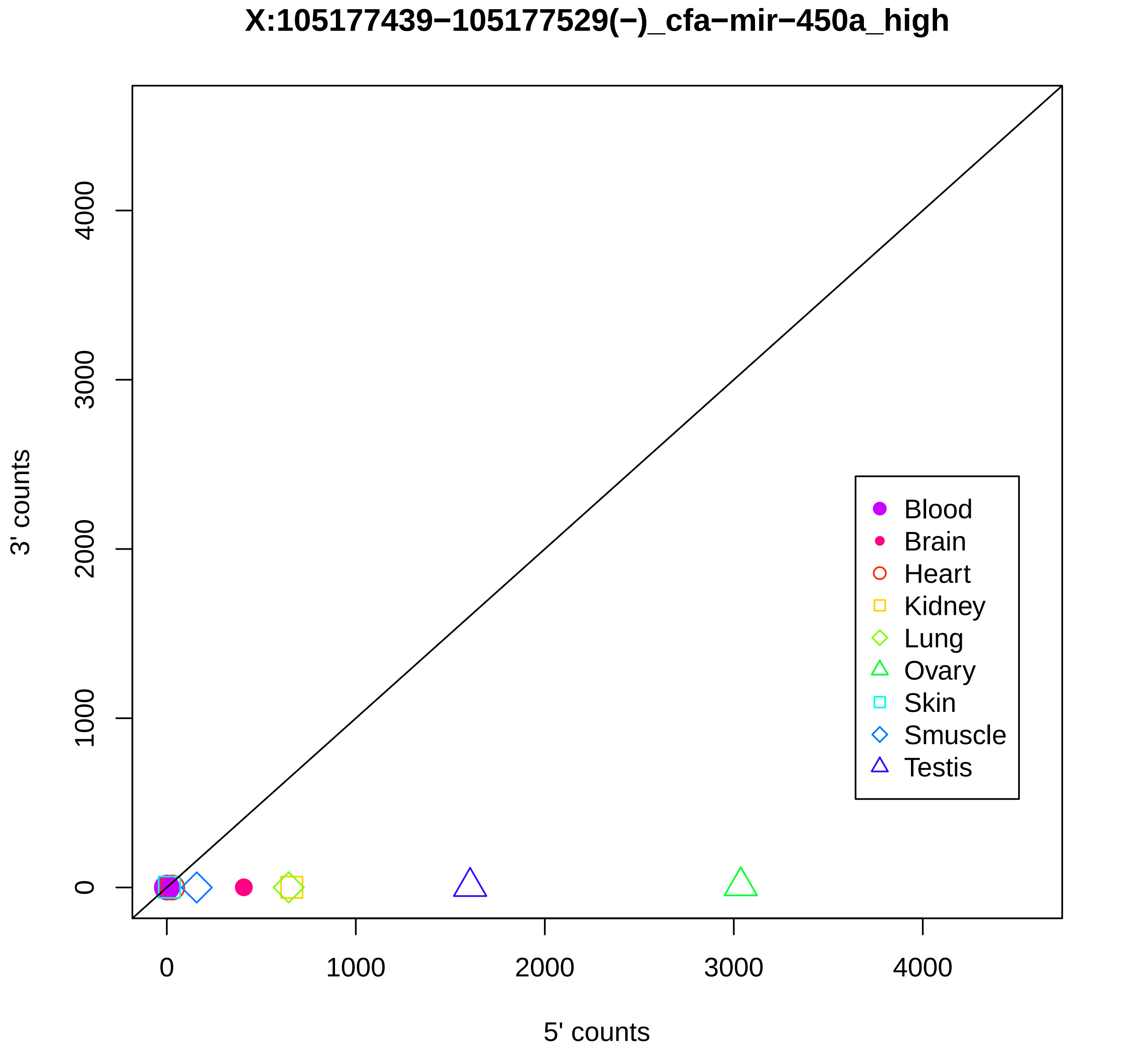

X:105177439−105177529(−)_cfa−mir−450a_high
3' counts
5' counts
0
1000
2000
3000
4000
0
1000
2000
3000
4000
Blood
Brain
Heart
Kidney
Lung
Ovary
Skin
Smuscle
Testis

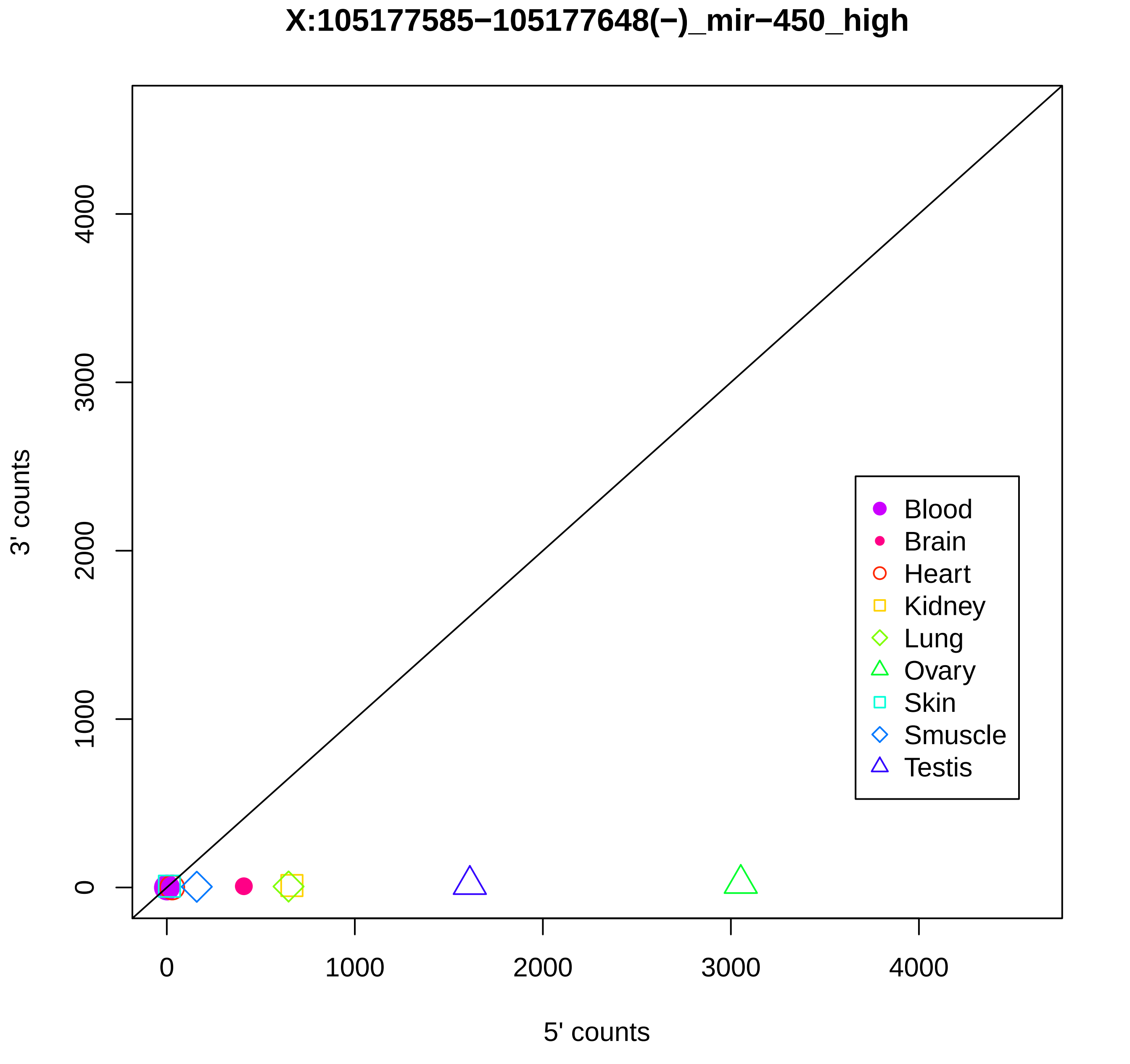
X:105177585−105177648(−)_mir−450_high
3' counts
5' counts
0
1000
2000
3000
4000
0
1000
2000
3000
4000
Blood
Brain
Heart
Kidney
Lung
Ovary
Skin
Smuscle
Testis

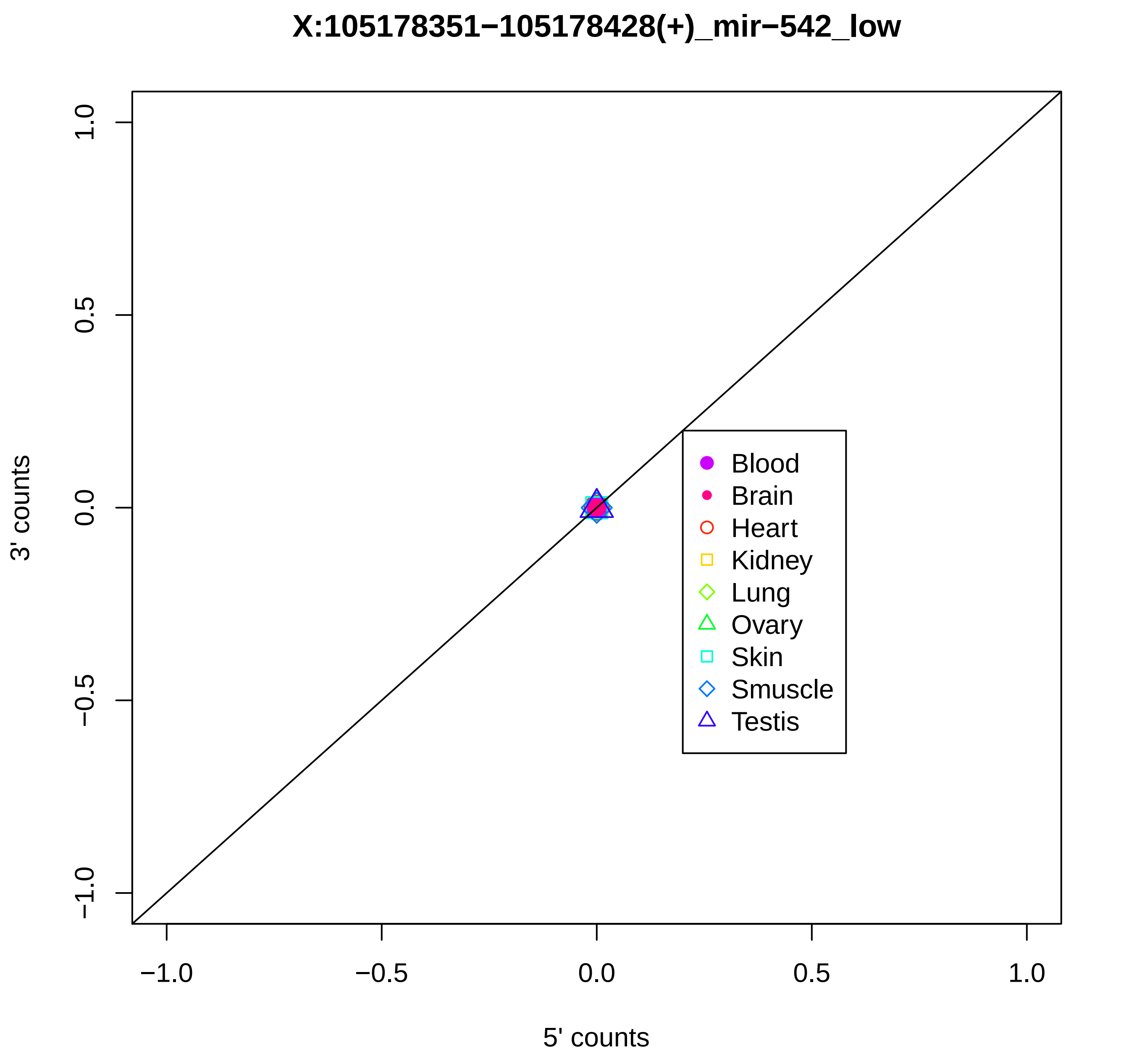

X:105178351−105178428(+)_mir−542_low
3' counts
5' counts
1.0
0.5
0.0
−0.5
−1.0
Blood
Brain
Heart
Kidney
Lung
Ovary
Skin
Smuscle
Testis

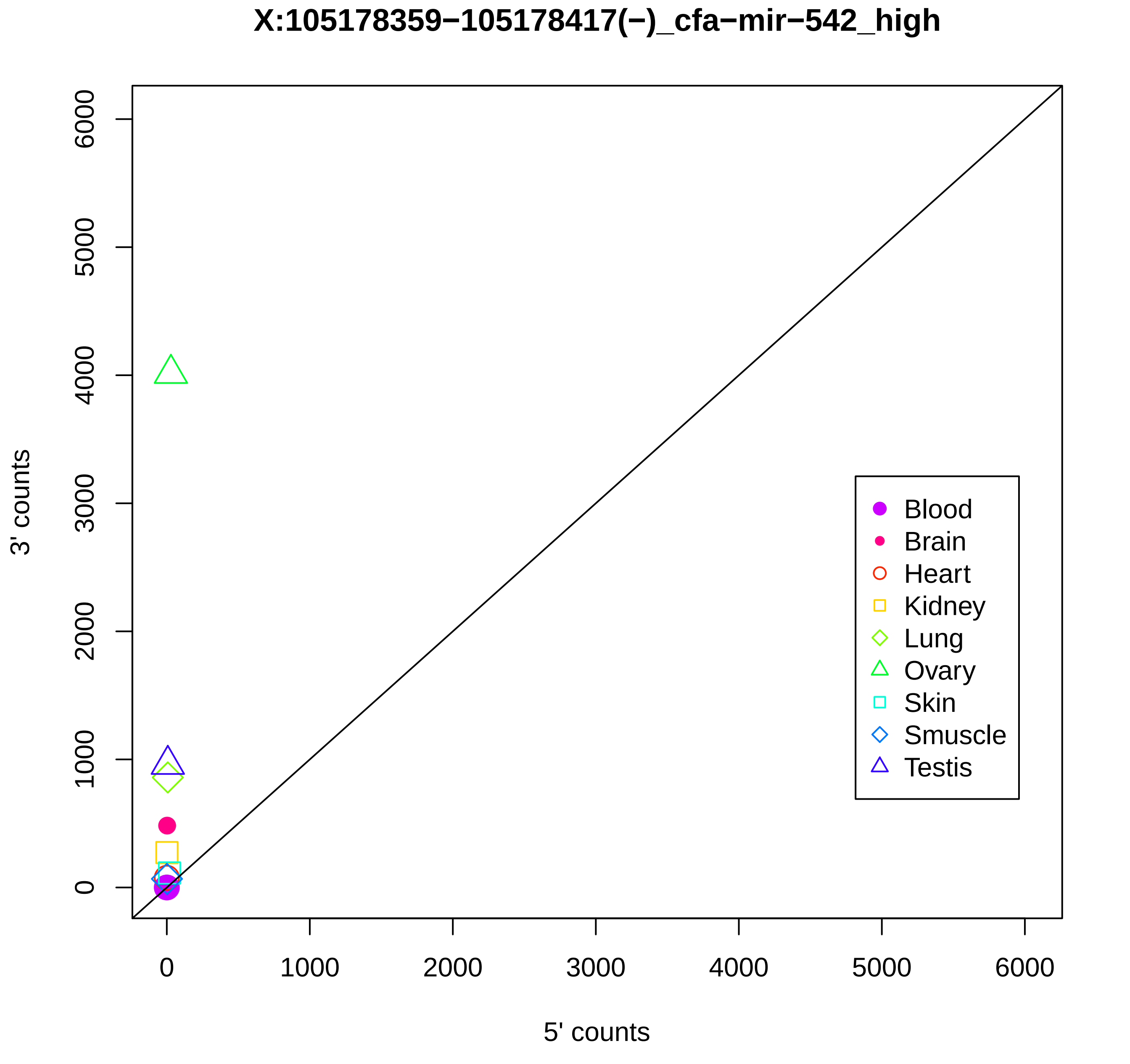

X:105178359−105178417(−)_cfa−mir−542_high
3' counts
5' counts
0
1000
2000
3000
4000
5000
6000
Blood
Brain
Heart
Kidney
Lung
Ovary
Skin
Smuscle
Testis

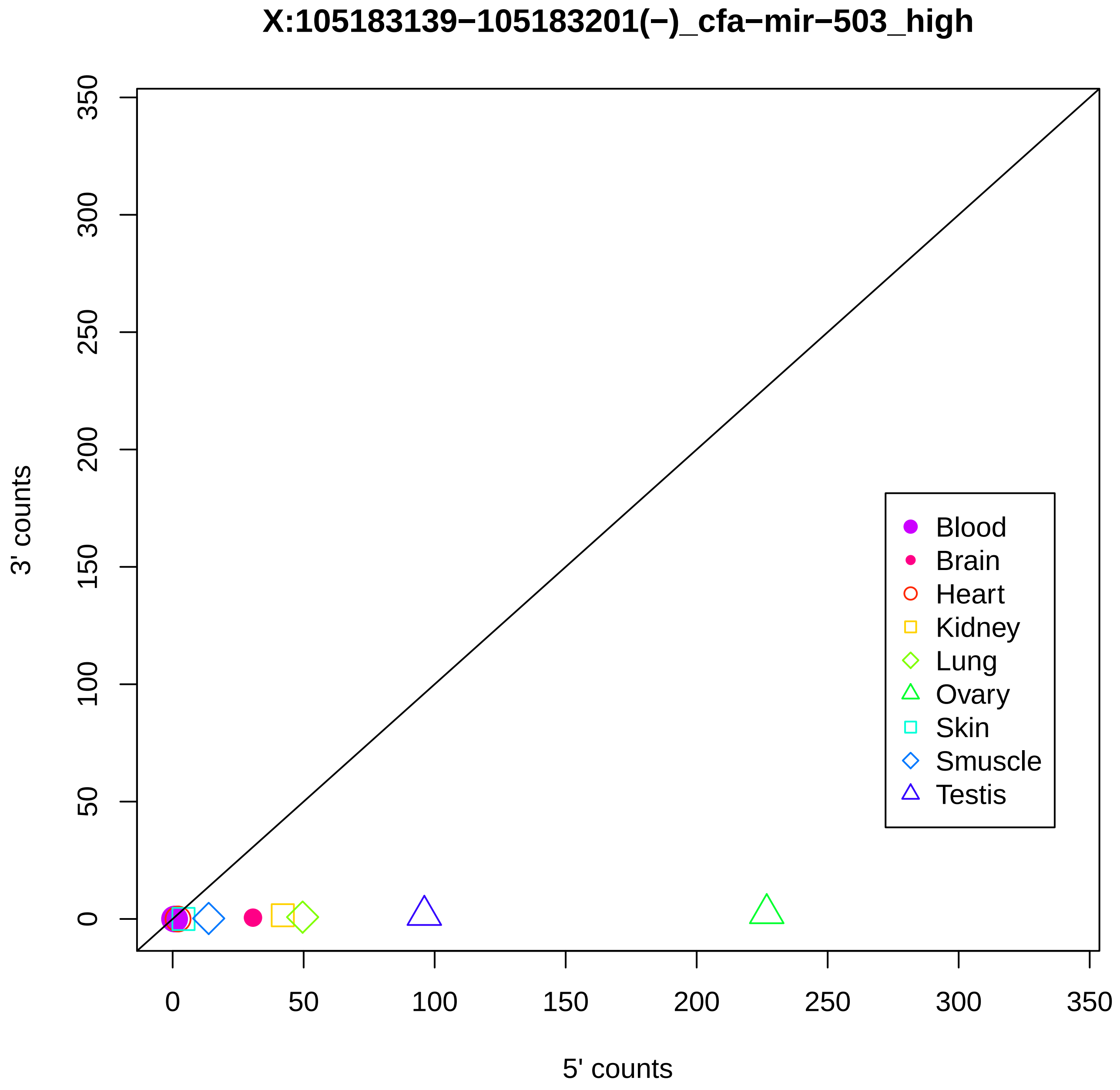

X:105183139−105183201(−)_cfa−mir−503_high
3' counts
5' counts
0
50
100
150
200
250
300
350
Blood
Brain
Heart
Kidney
Lung
Ovary
Skin
Smuscle
Testis

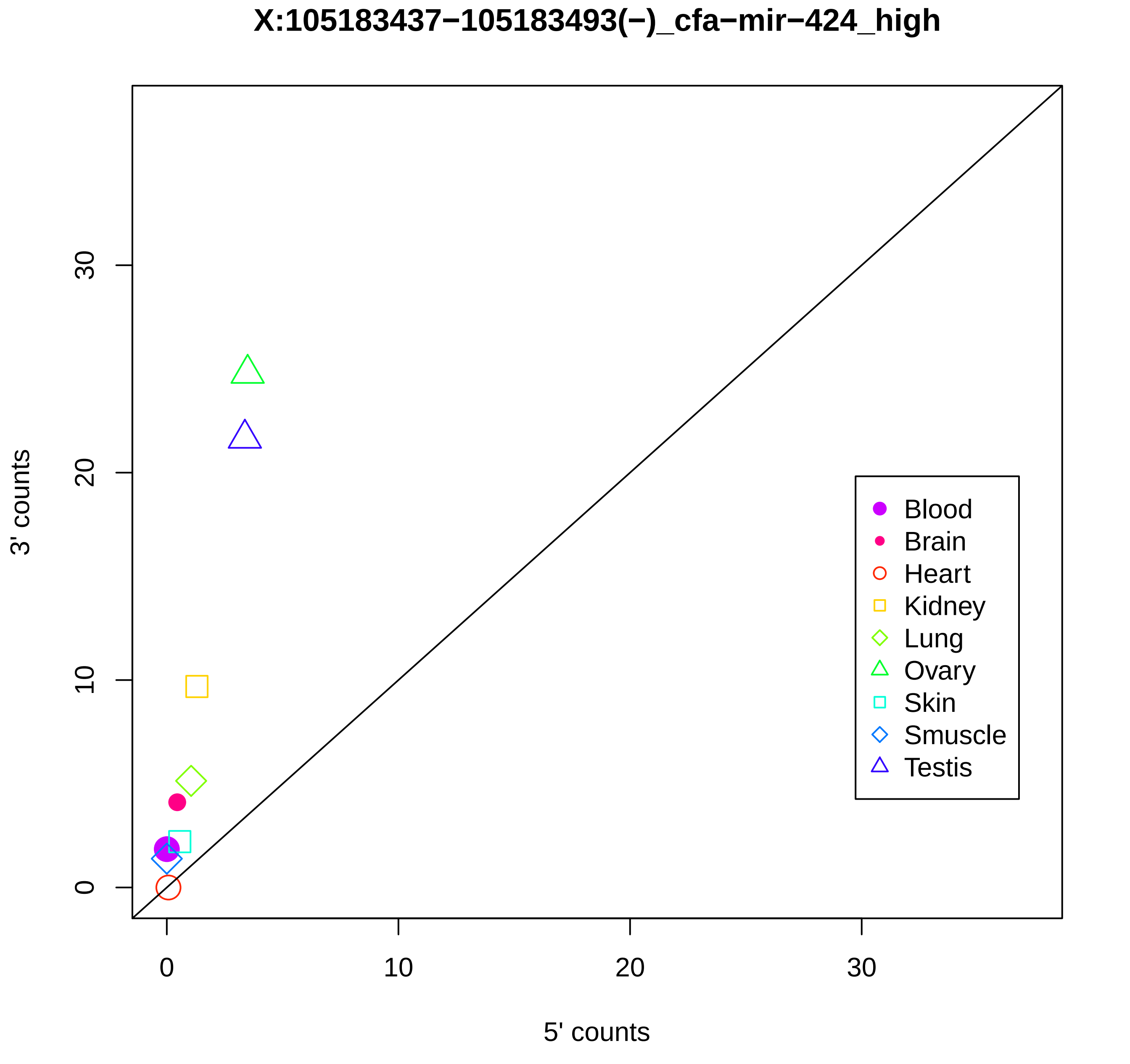
X:105183437−105183493(−)_cfa−mir−424_high
3' counts
0
10
20
30
5' counts
0
10
20
30
Blood
Brain
Heart
Kidney
Lung
Ovary
Skin
Smuscle
Testis

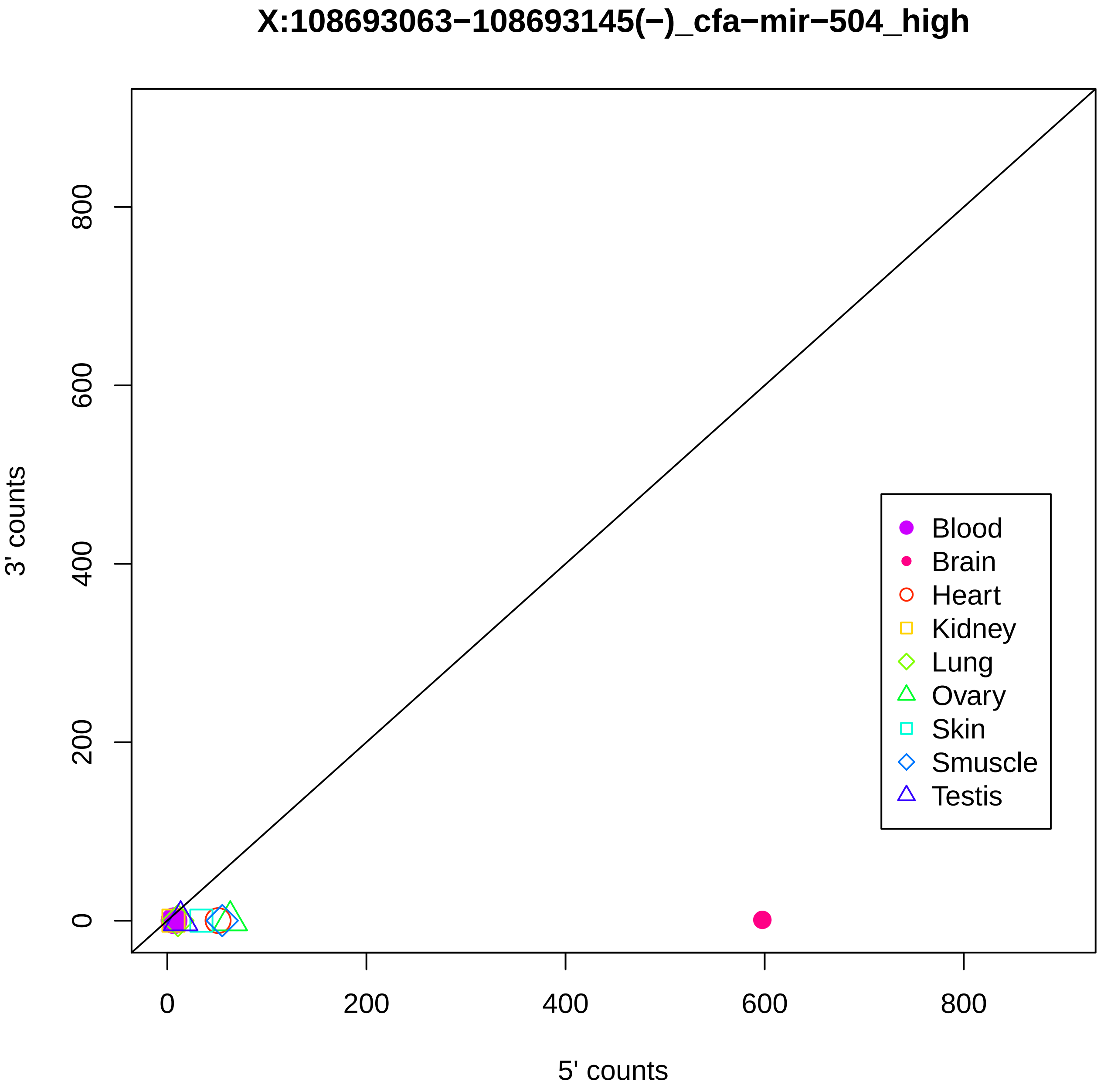

X:108693063−108693145(−)_cfa−mir−504_high
0
200
400
600
800
3' counts
5' counts
Blood
Brain
Heart
Kidney
Lung
Ovary
Skin
Smuscle
Testis

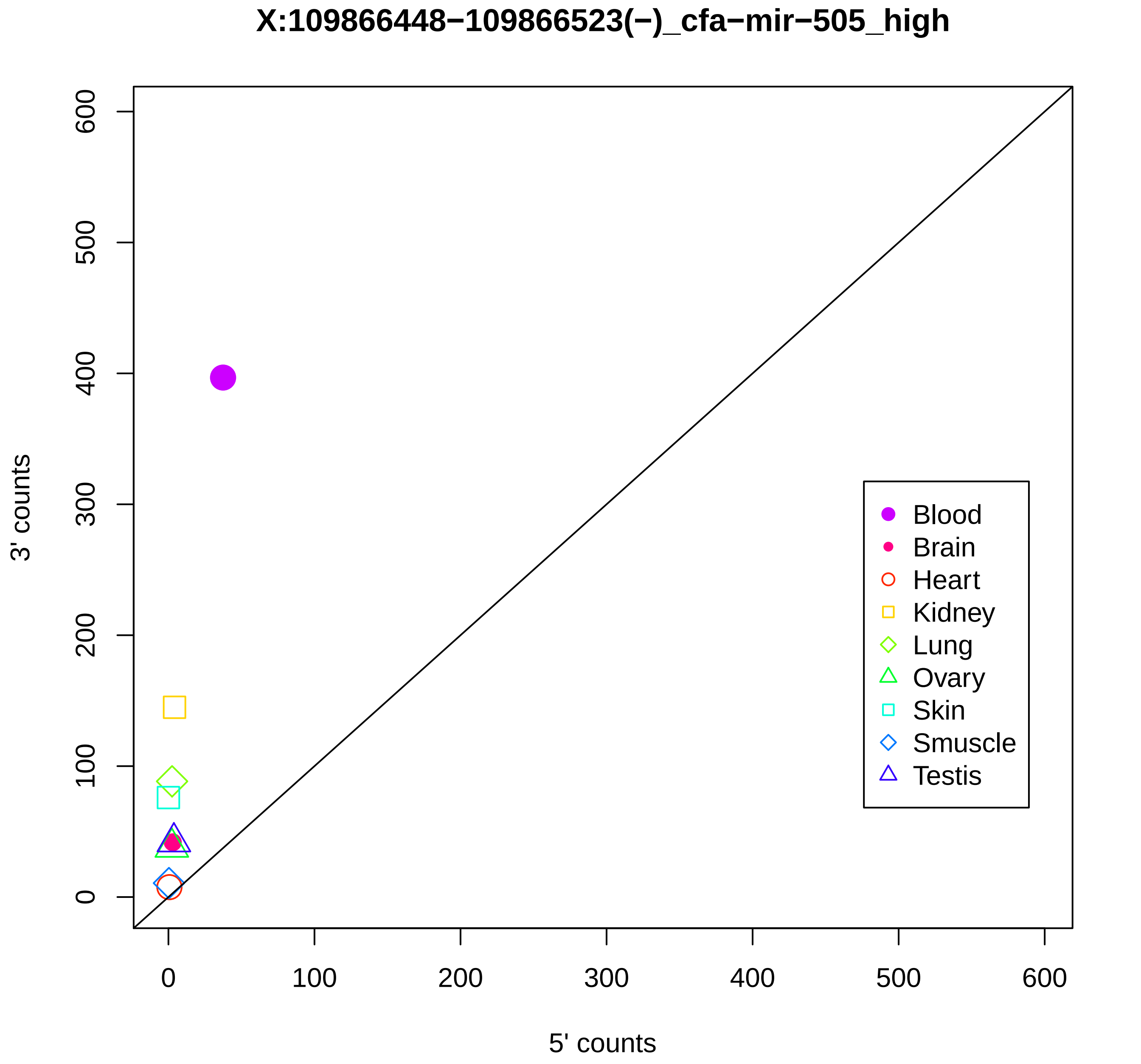
X:109866448−109866523(−)_cfa−mir−505_high
3' counts
5' counts
0
100
200
300
400
500
600
Blood
Brain
Heart
Kidney
Lung
Ovary
Skin
Smuscle
Testis

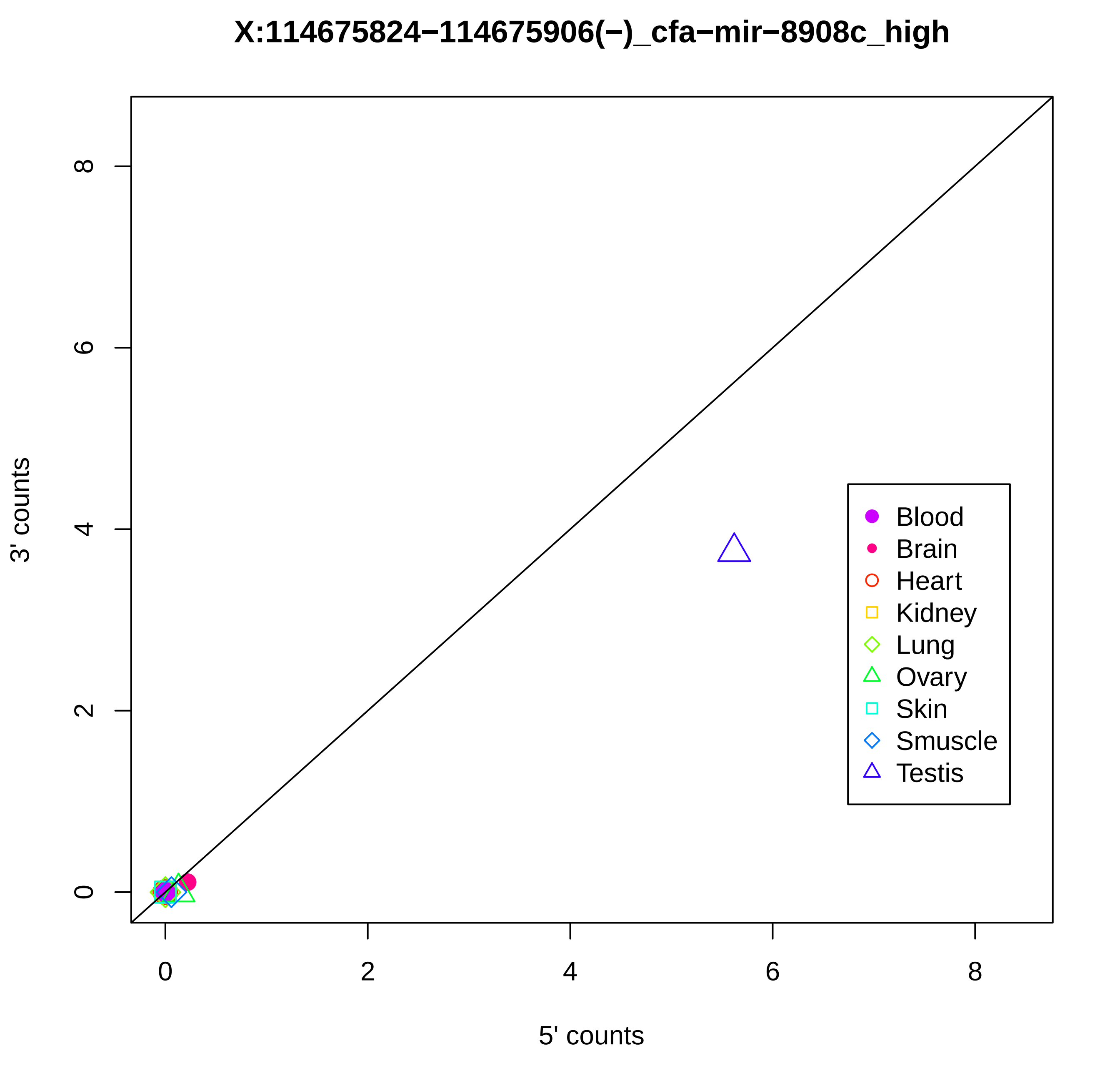
X:114675824−114675906(−)_cfa−mir−8908c_high
3' counts
5' counts
0
2
4
6
8
Blood
Brain
Heart
Kidney
Lung
Ovary
Skin
Smuscle
Testis

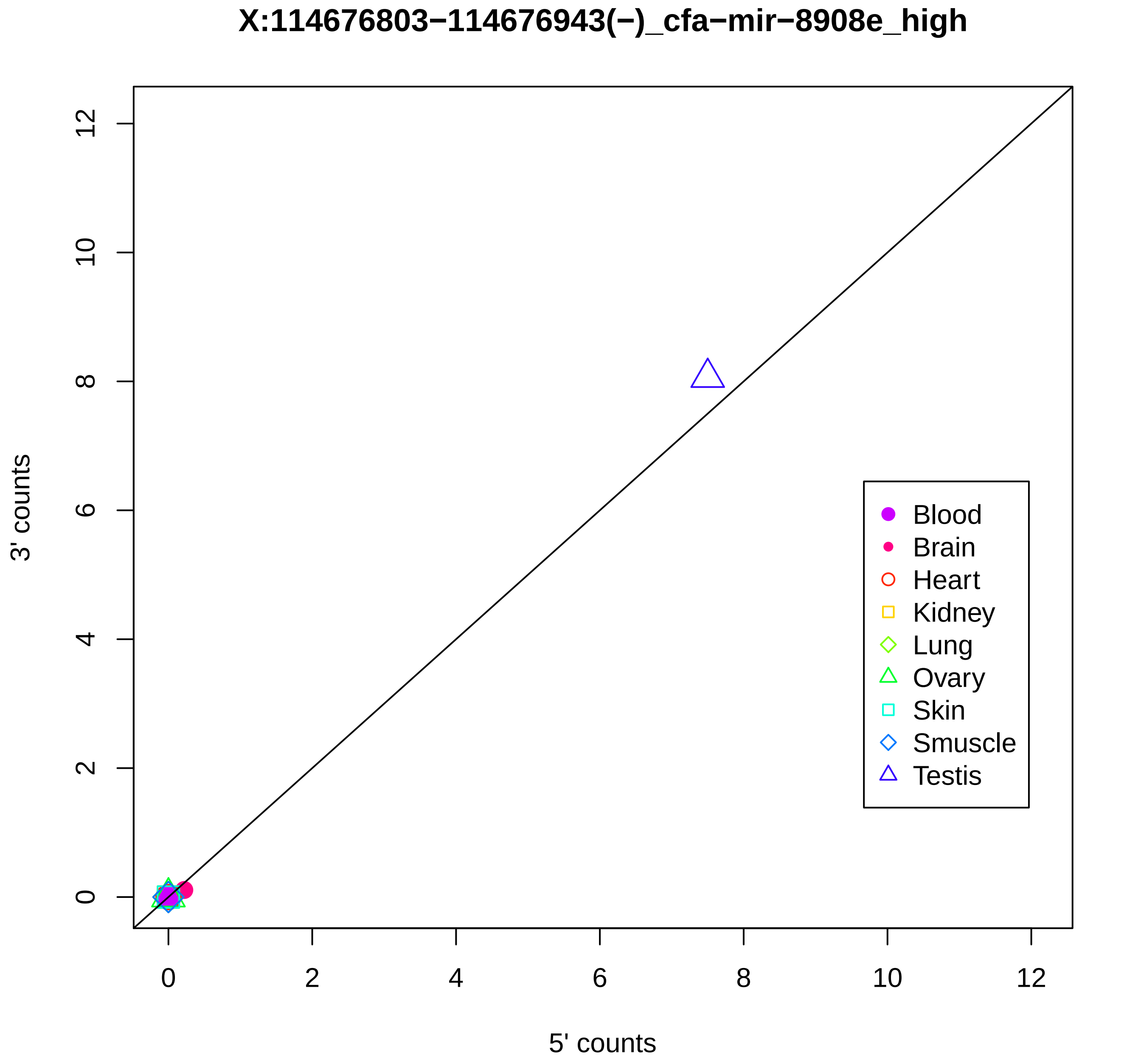
X:114676803−114676943(−)_cfa−mir−8908e_high
3' counts
5' counts
0
2
4
6
8
10
12
0
2
4
6
8
10
12
Blood
Brain
Heart
Kidney
Lung
Ovary
Skin
Smuscle
Testis

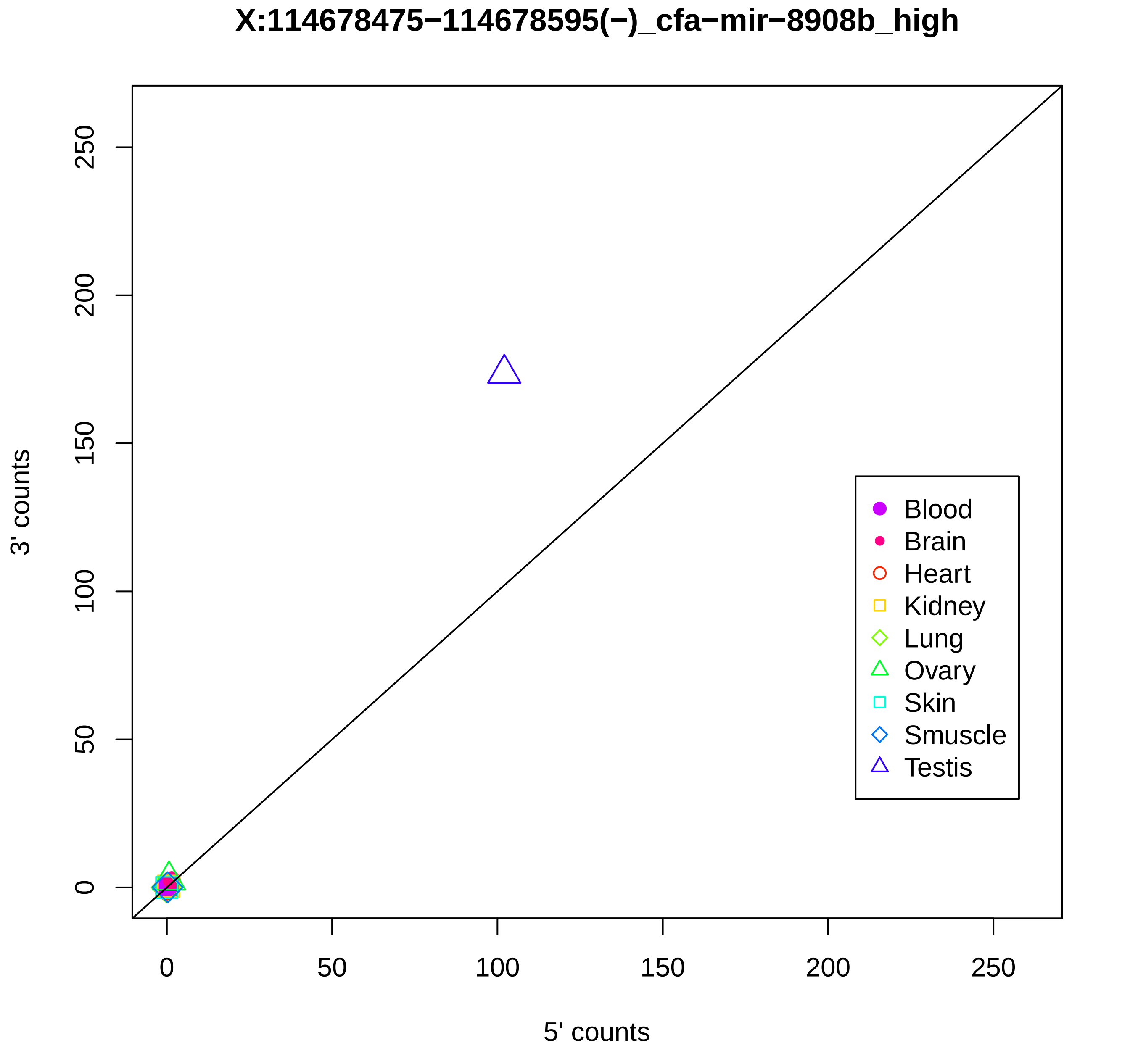

X:114678475−114678595(−)_cfa−mir−8908b_high
3' counts
5' counts
0
50
100
150
200
250
0
50
100
150
200
250
Blood
Brain
Heart
Kidney
Lung
Ovary
Skin
Smuscle
Testis

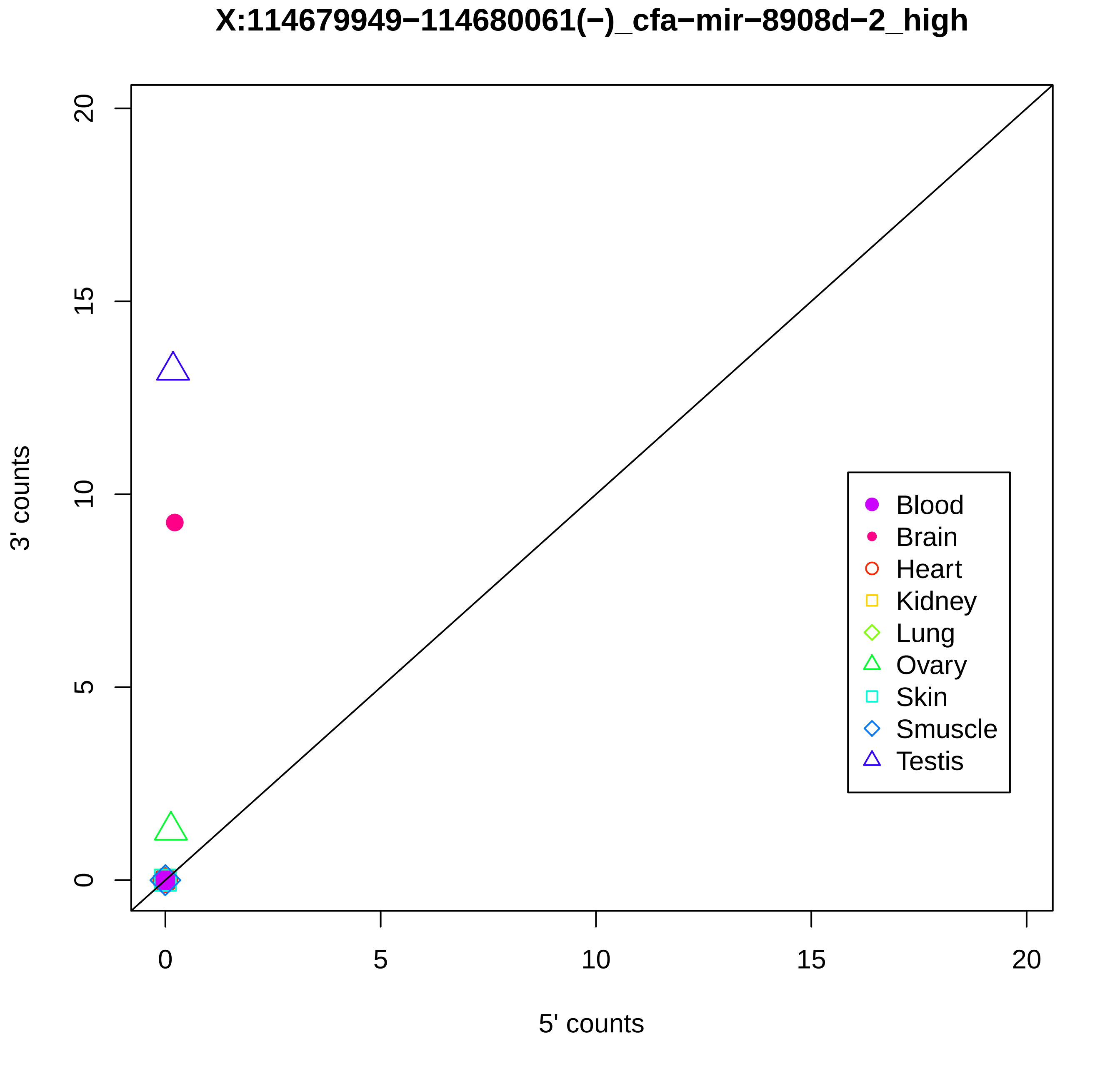
X:114679949−114680061(−)_cfa−mir−8908d−2_high
3' counts
5' counts
0
5
10
15
20
0
5
10
15
20
Blood
Brain
Heart
Kidney
Lung
Ovary
Skin
Smuscle
Testis

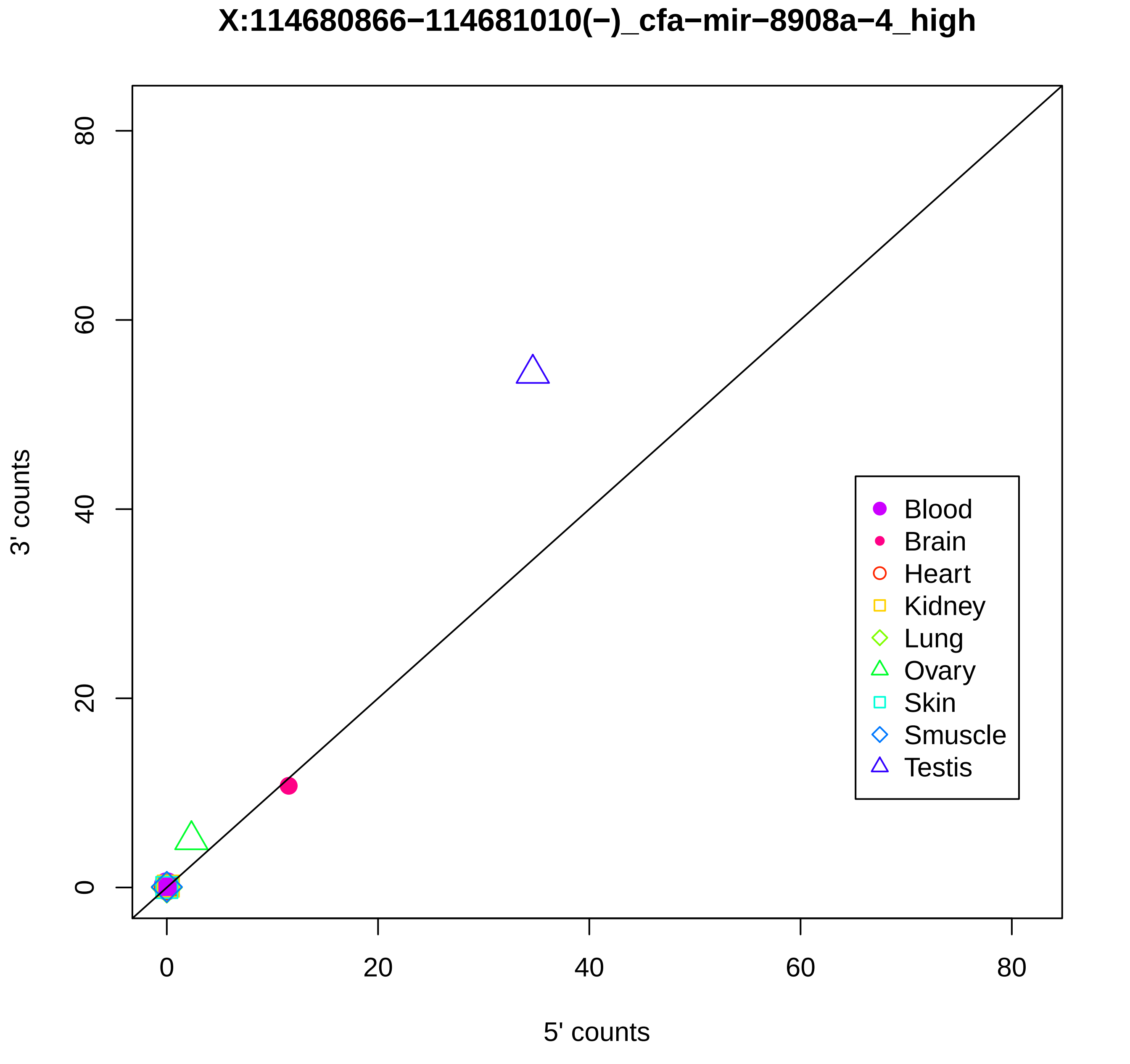
X:114680866−114681010(−)_cfa−mir−8908a−4_high
3' counts
5' counts
0
20
40
60
80
0
20
40
60
80
Blood
Brain
Heart
Kidney
Lung
Ovary
Skin
Smuscle
Testis

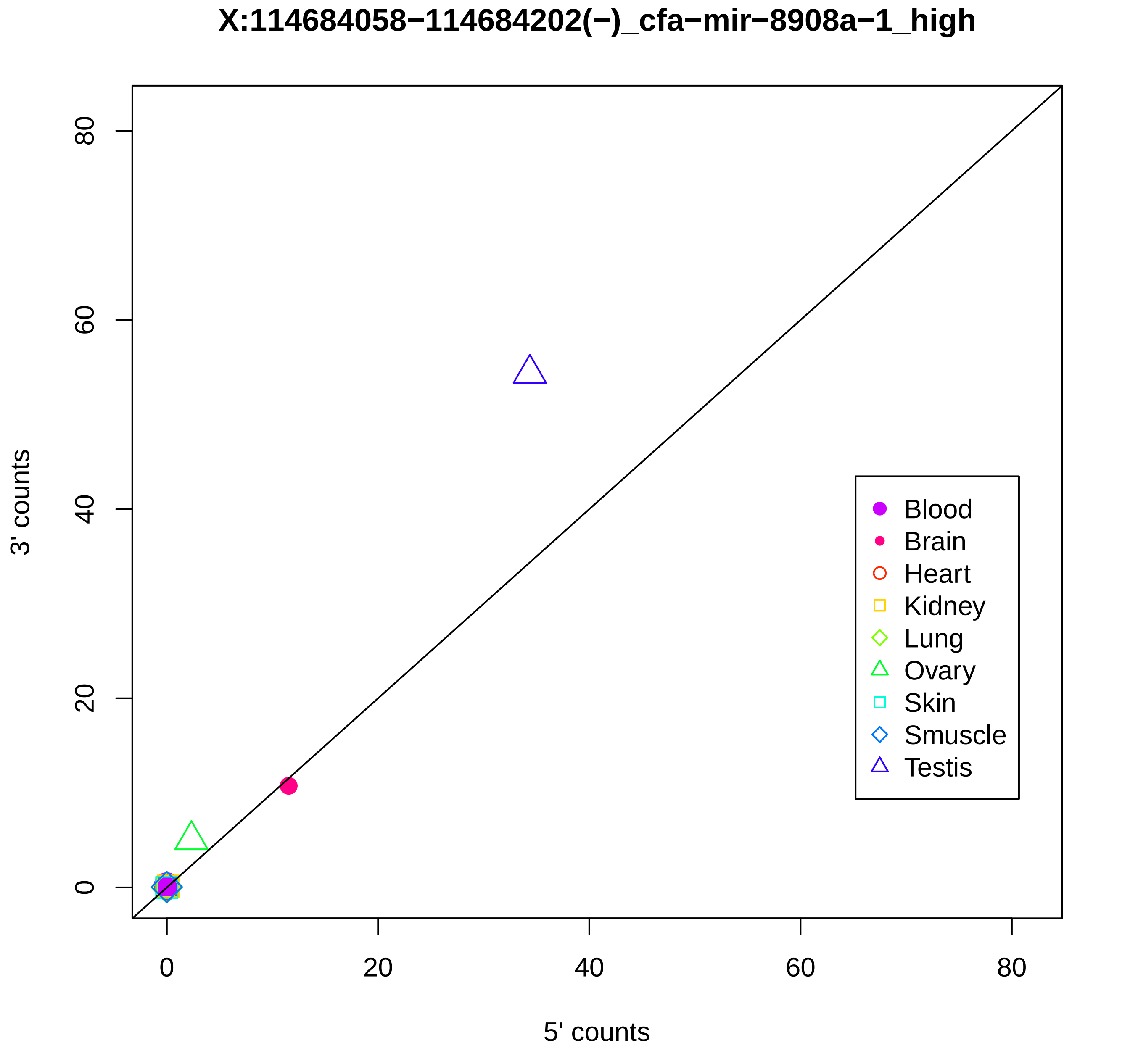

X:114684058−114684202(−)_cfa−mir−8908a−1_high
3' counts
5' counts
0
20
40
60
80
0
20
40
60
80
Blood
Brain
Heart
Kidney
Lung
Ovary
Skin
Smuscle
Testis

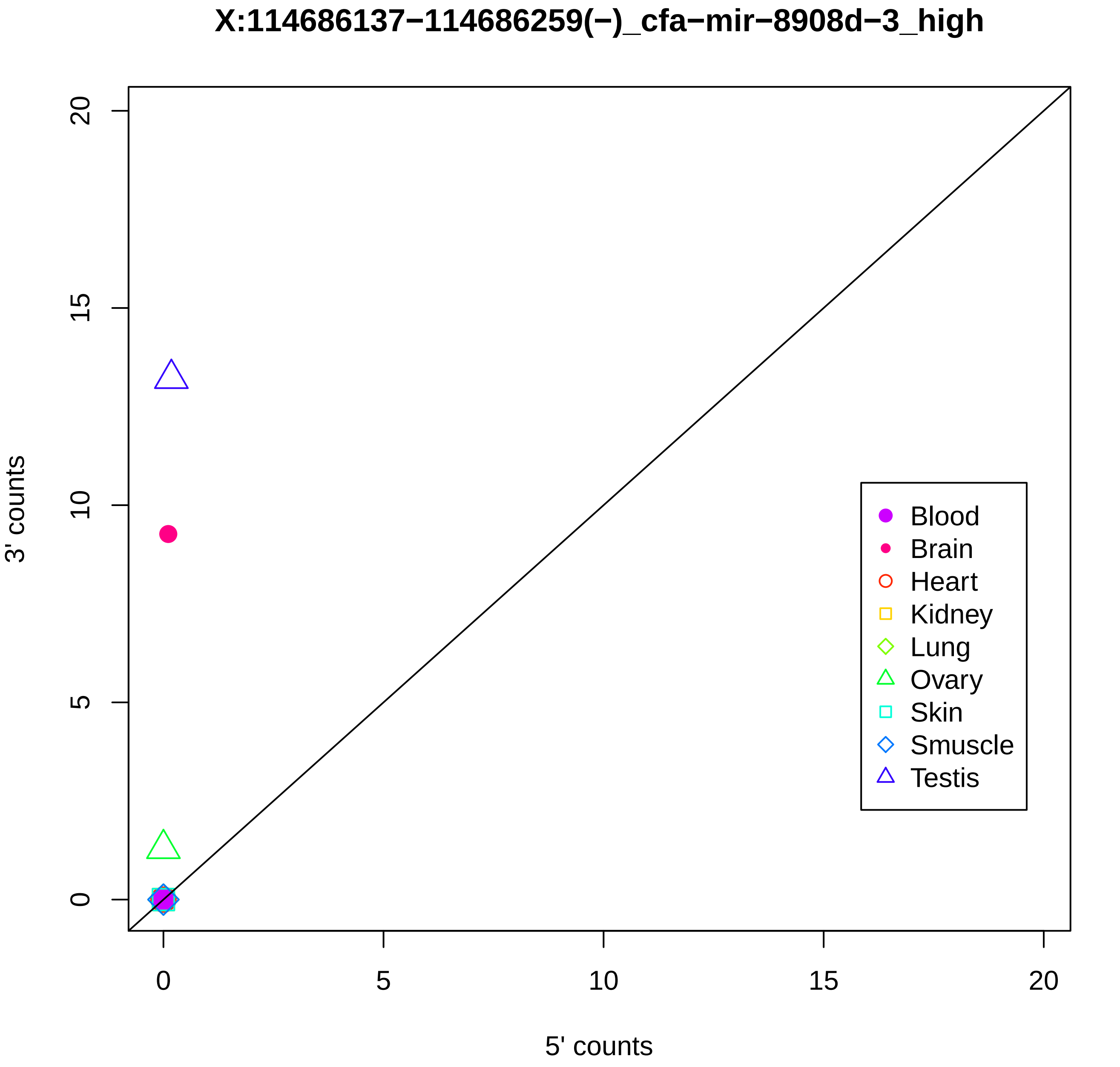

X:114686137−114686259(−)_cfa−mir−8908d−3_high
3' counts
5' counts
0
5
10
15
20
0
5
10
15
20
Blood
Brain
Heart
Kidney
Lung
Ovary
Skin
Smuscle
Testis

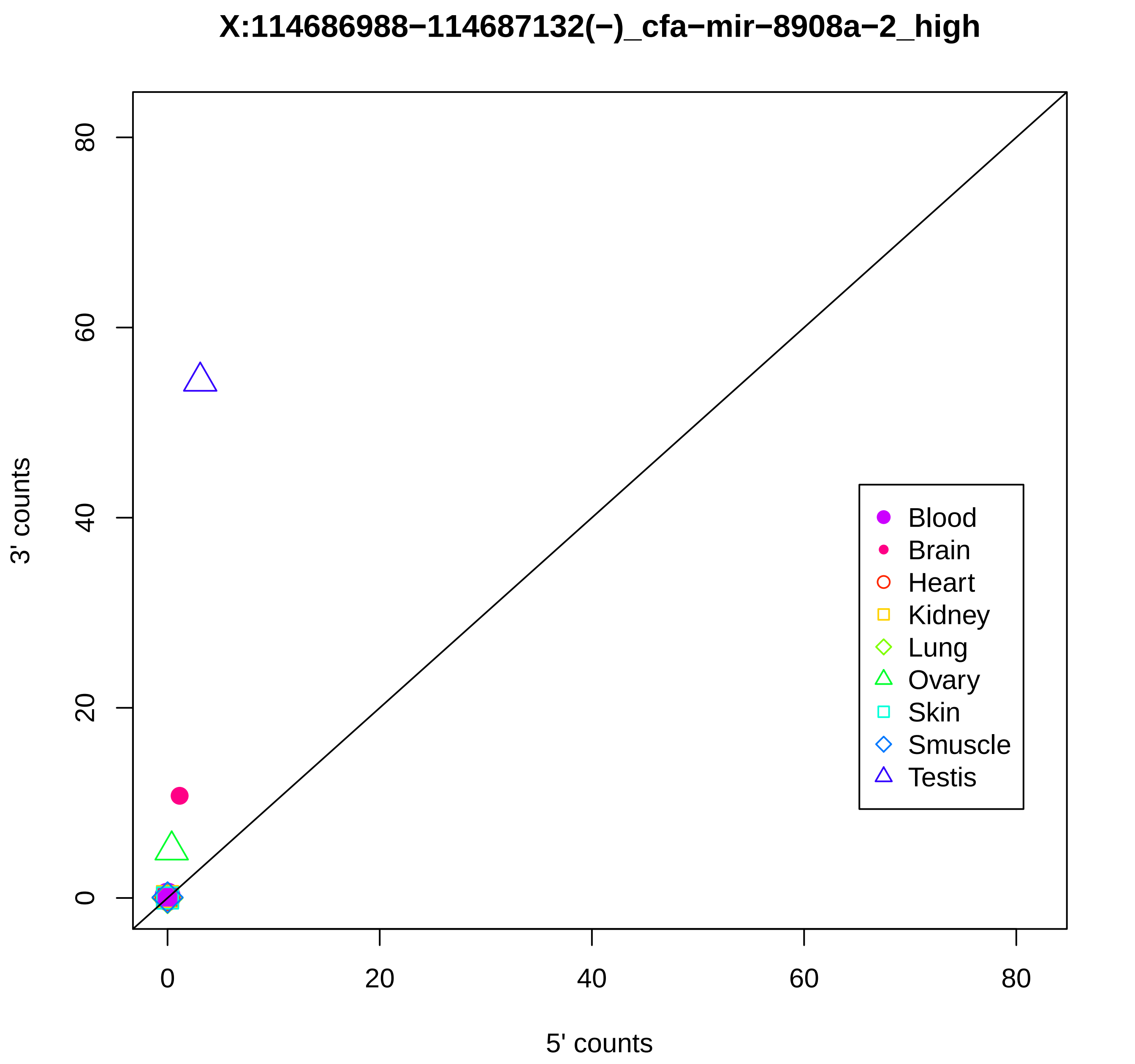

X:114686988−114687132(−)_cfa−mir−8908a−2_high
3' counts
5' counts
0
20
40
60
80
0
20
40
60
80
Blood
Brain
Heart
Kidney
Lung
Ovary
Skin
Smuscle
Testis

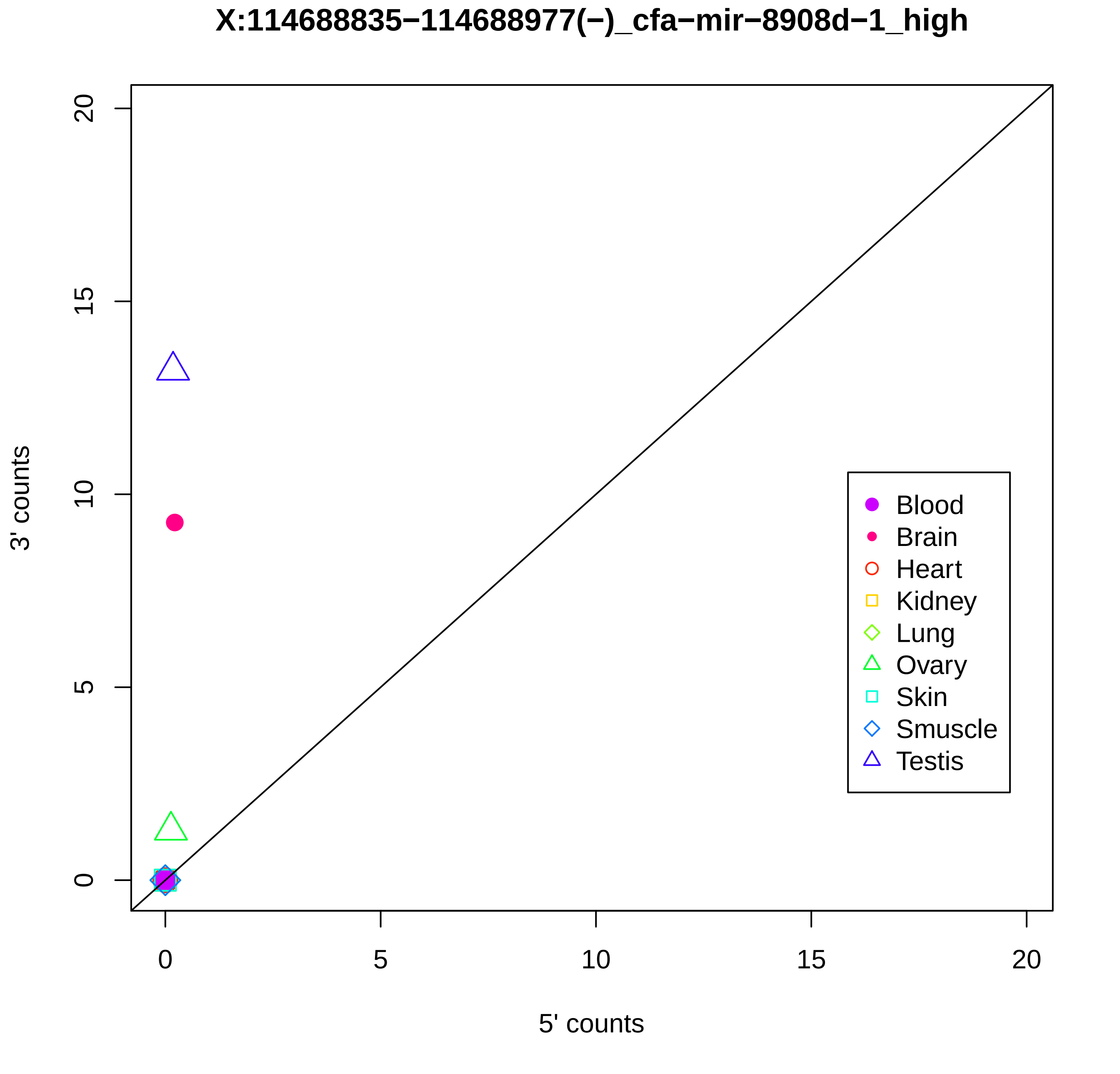

X:114688835−114688977(−)_cfa−mir−8908d−1_high
3' counts
5' counts
0
5
10
15
20
0
5
10
15
20
Blood
Brain
Heart
Kidney
Lung
Ovary
Skin
Smuscle
Testis

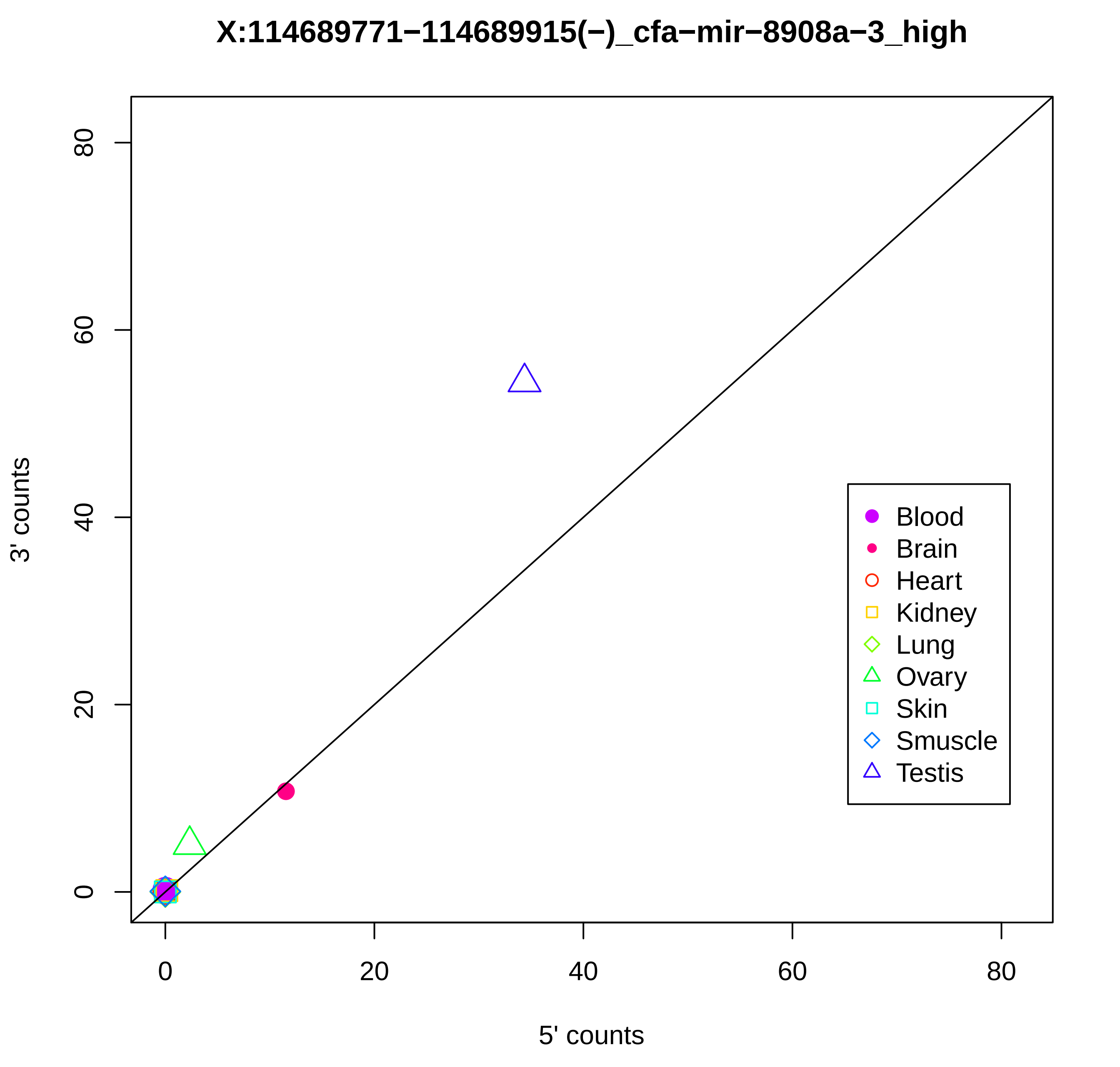

X:114689771−114689915(−)_cfa−mir−8908a−3_high
3' counts
5' counts
0
20
40
60
80
0
20
40
60
80
Blood
Brain
Heart
Kidney
Lung
Ovary
Skin
Smuscle
Testis

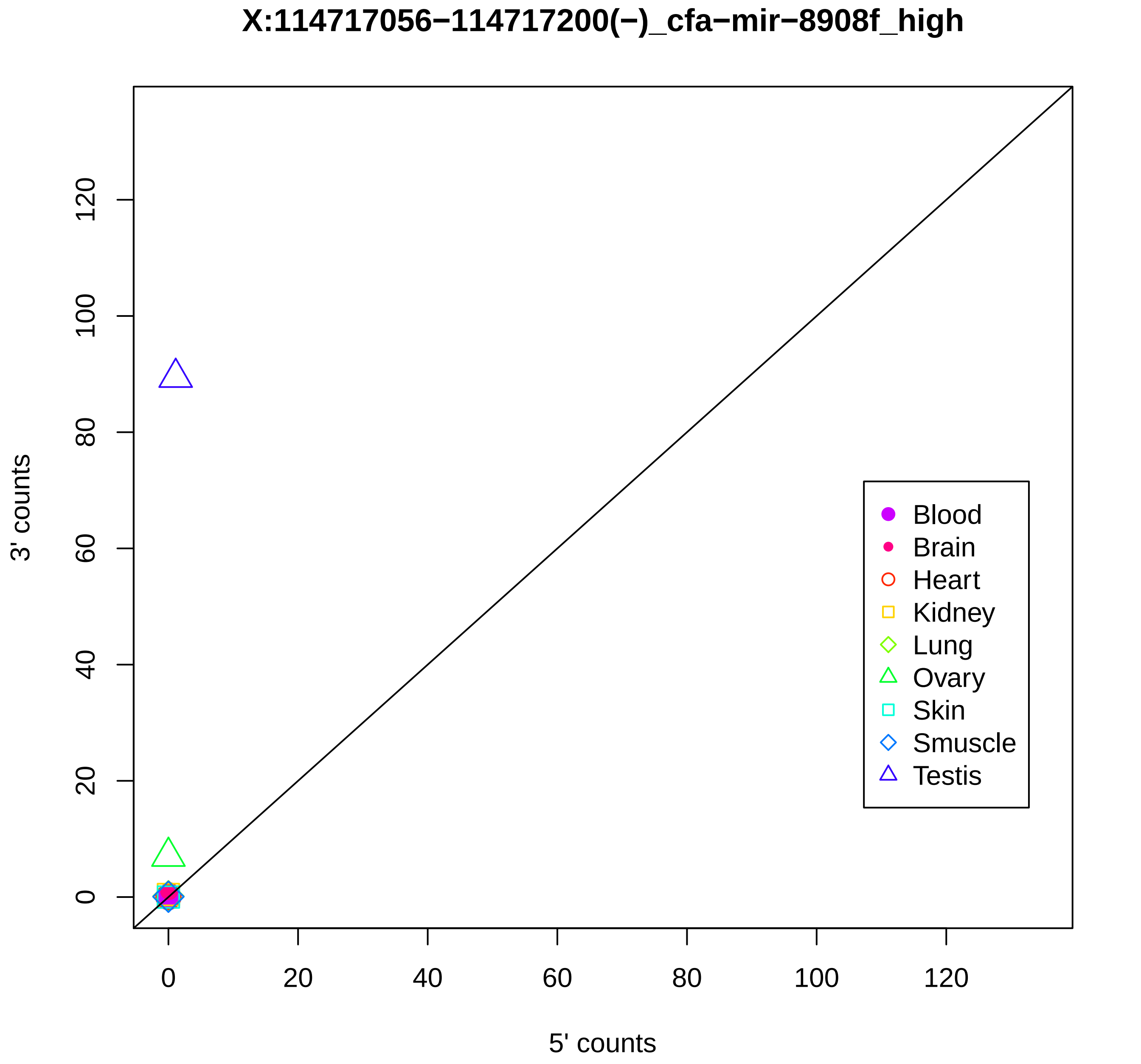
X:114717056−114717200(−)_cfa−mir−8908f_high
3' counts
5' counts
0
20
40
60
80
100
120
Blood
Brain
Heart
Kidney
Lung
Ovary
Skin
Smuscle
Testis

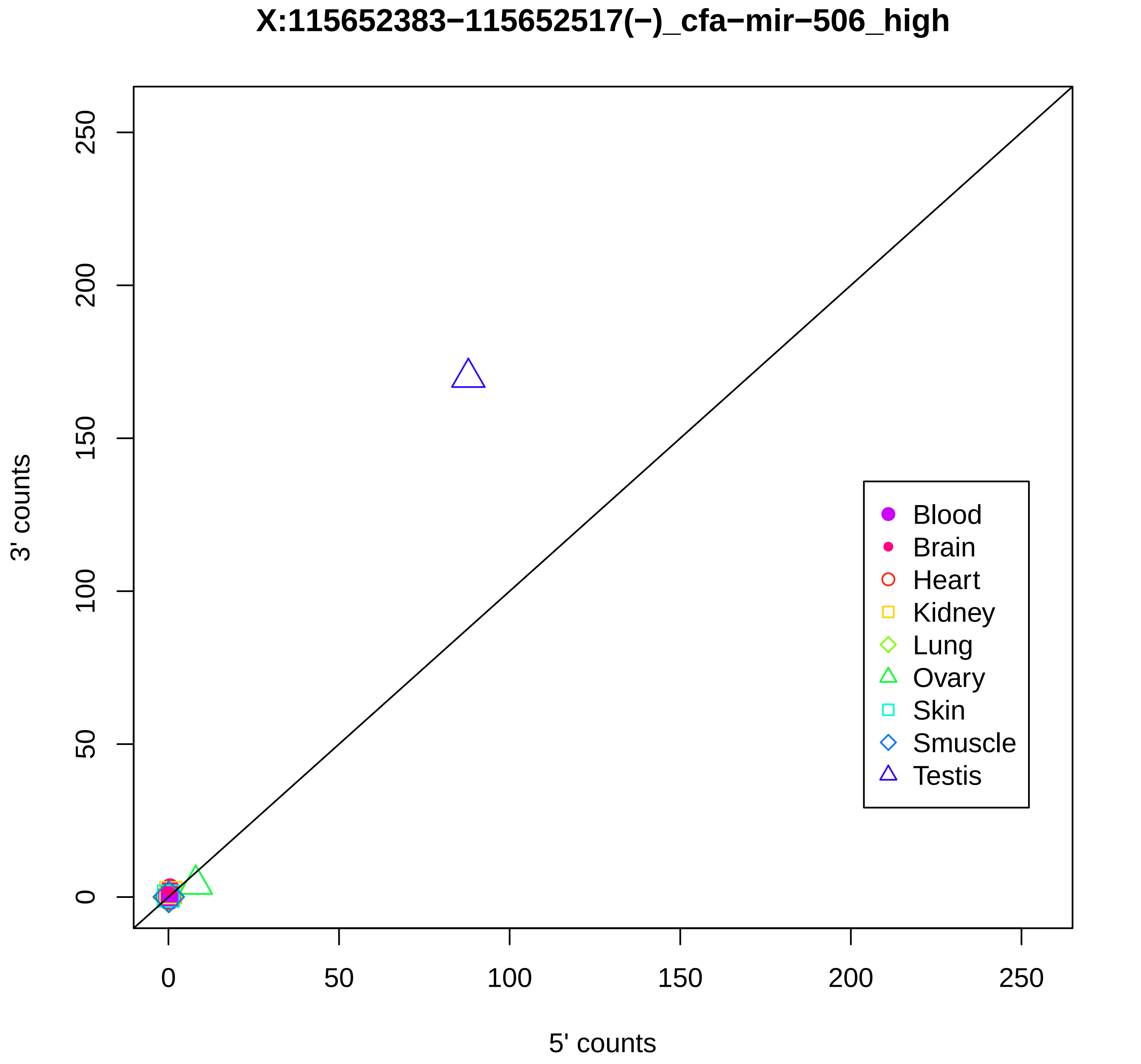

X:115652383−115652517(−)_cfa−mir−506_high
3' counts
5' counts
0
50
100
150
200
250
0
50
100
150
200
250
Blood
Brain
Heart
Kidney
Lung
Ovary
Skin
Smuscle
Testis

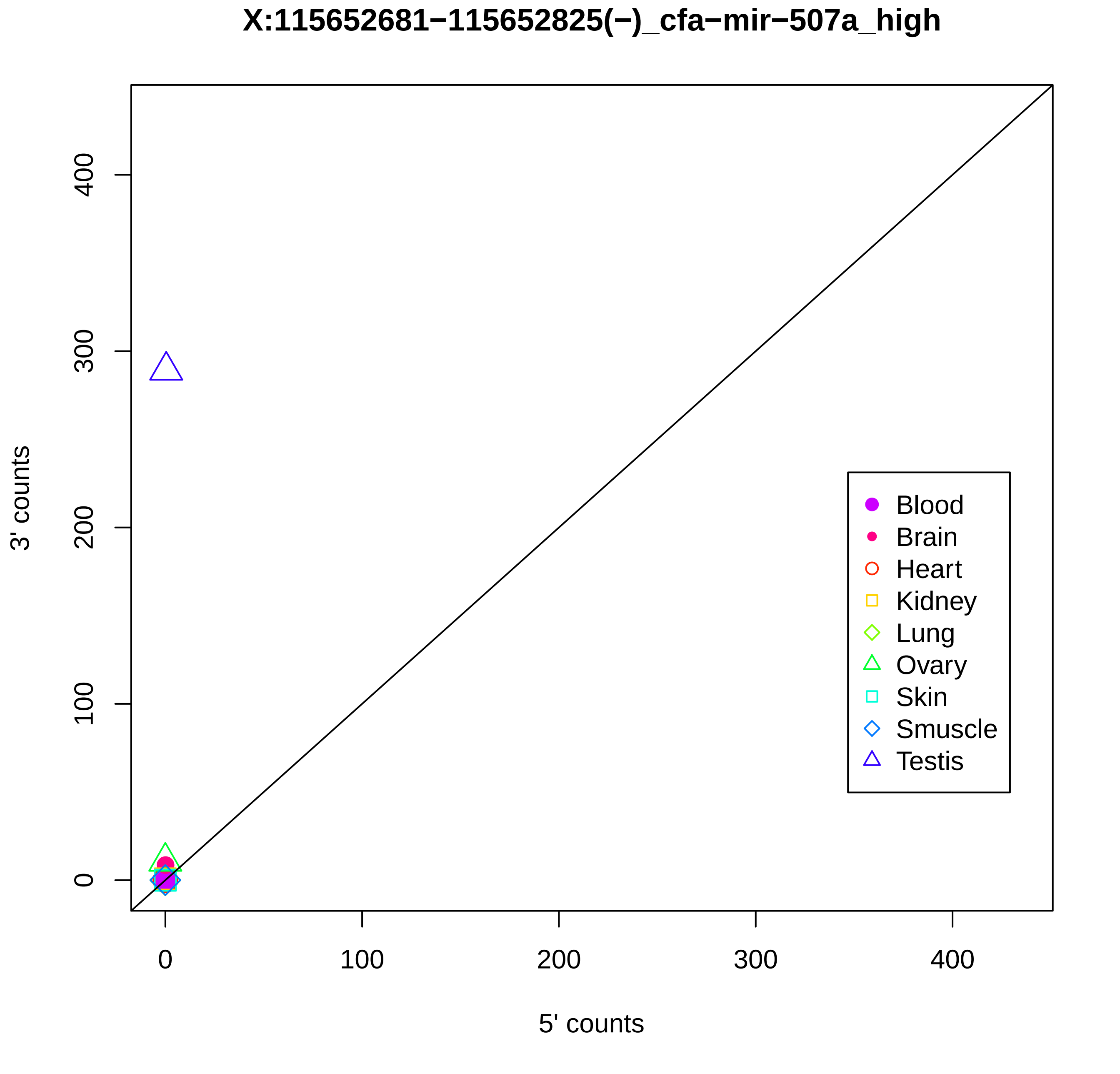
X:115652681−115652825(−)_cfa−mir−507a_high
3' counts
5' counts
0
100
200
300
400
0
100
200
300
400
Blood
Brain
Heart
Kidney
Lung
Ovary
Skin
Smuscle
Testis

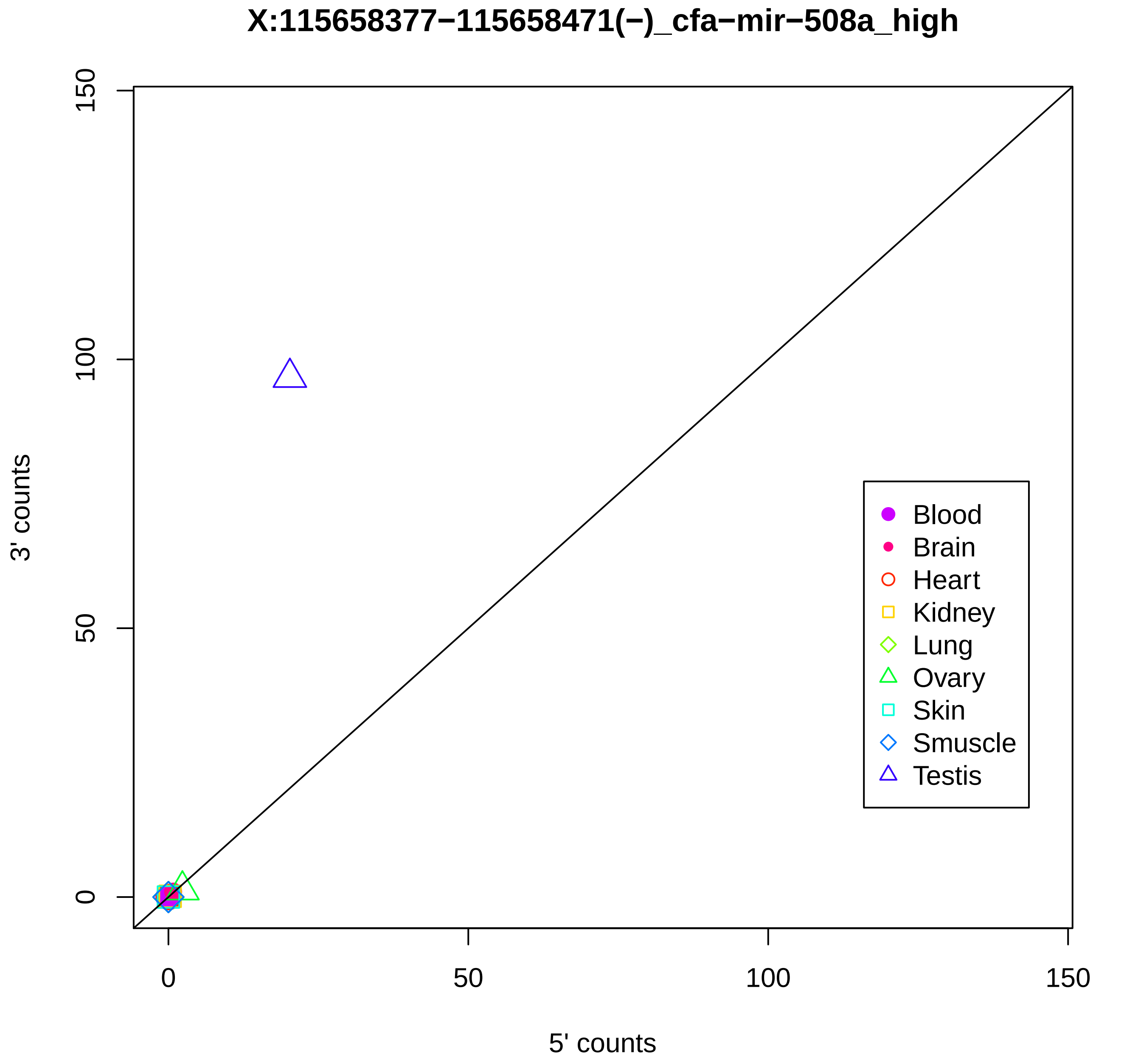
X:115658377−115658471(−)_cfa−mir−508a_high
3' counts
5' counts
0
50
100
150
0
50
100
150
Blood
Brain
Heart
Kidney
Lung
Ovary
Skin
Smuscle
Testis

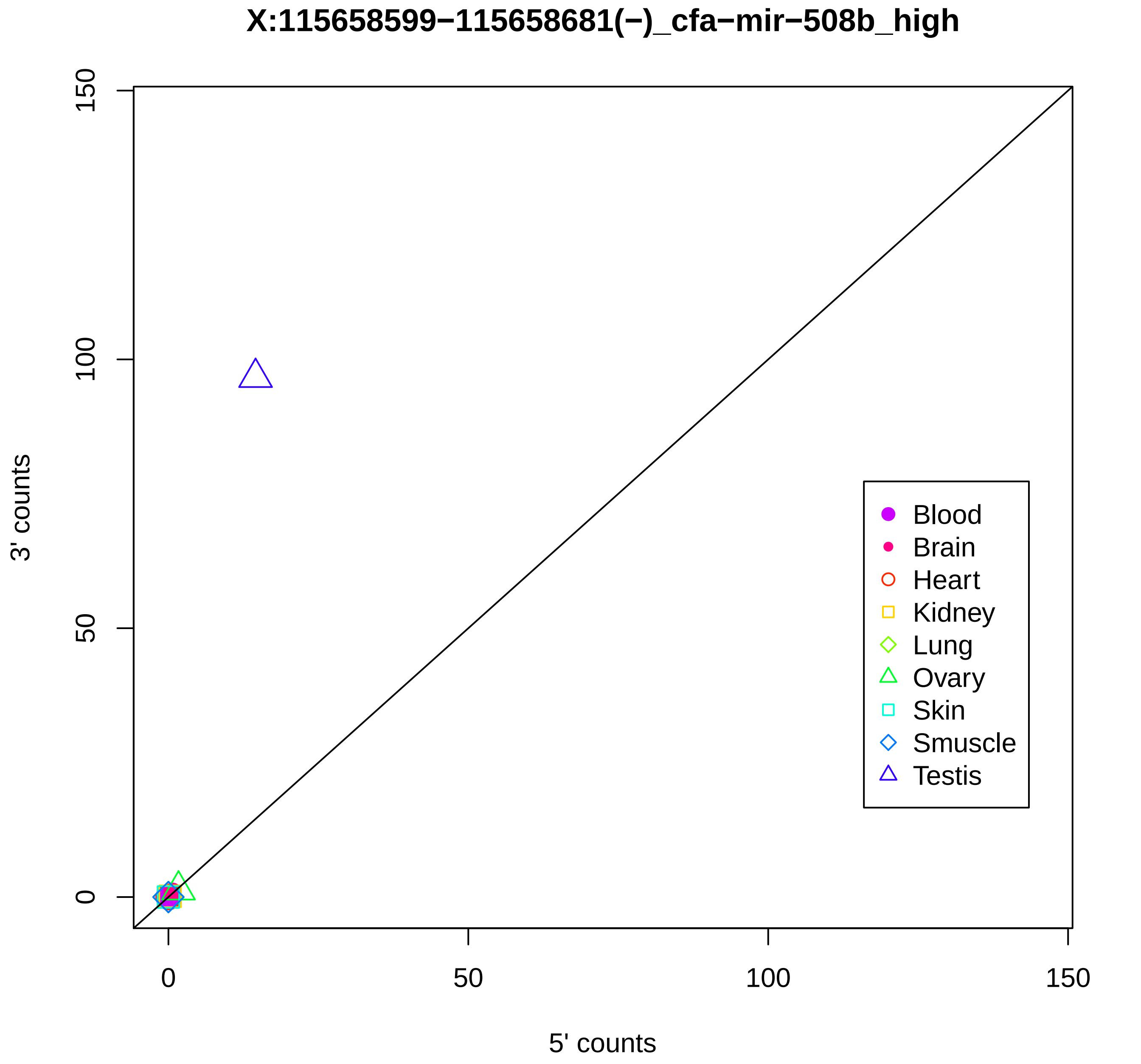
X:115658599−115658681(−)_cfa−mir−508b_high
150
100
50
0
3' counts
0
50
100
150
5' counts
Blood
Brain
Heart
Kidney
Lung
Ovary
Skin
Smuscle
Testis

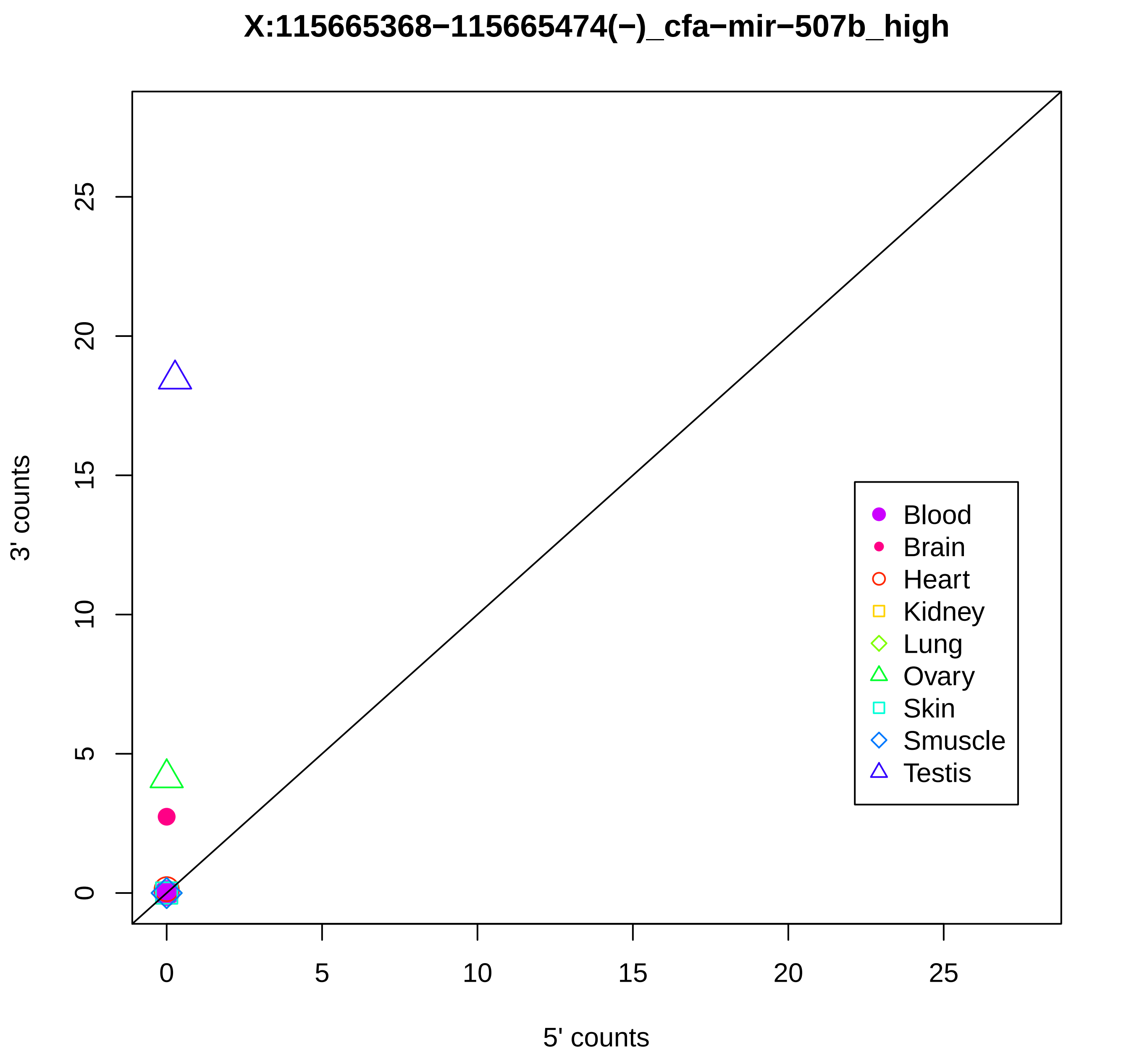

X:115665368−115665474(−)_cfa−mir−507b_high
3' counts
5' counts
0
5
10
15
20
25
Blood
Brain
Heart
Kidney
Lung
Ovary
Skin
Smuscle
Testis

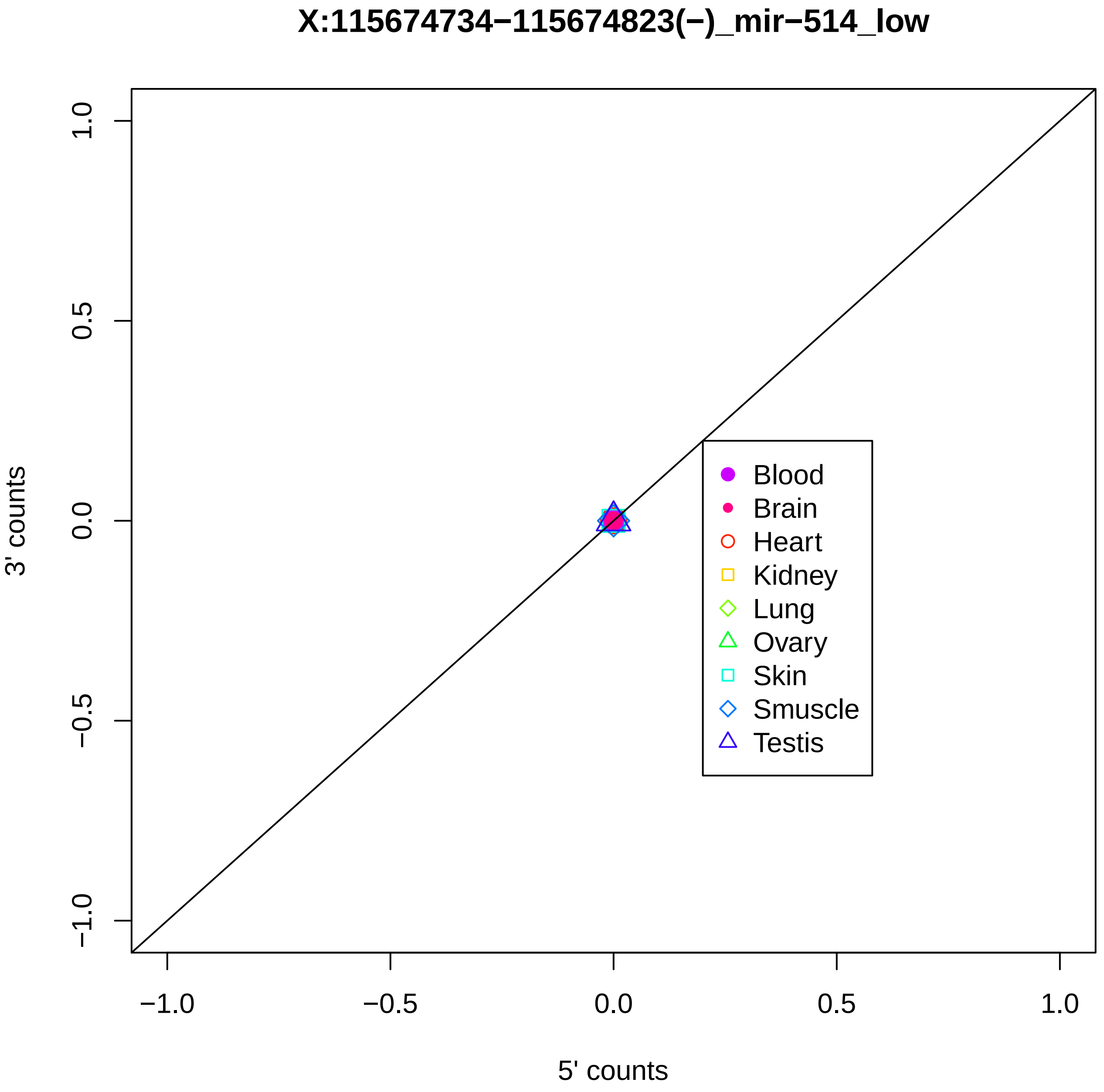

X:115674734−115674823(−)_mir−514_low
1.0
0.5
0.0
−0.5
−1.0
3' counts
−1.0
−0.5
0.0
0.5
1.0
5' counts
Blood
Brain
Heart
Kidney
Lung
Ovary
Skin
Smuscle
Testis

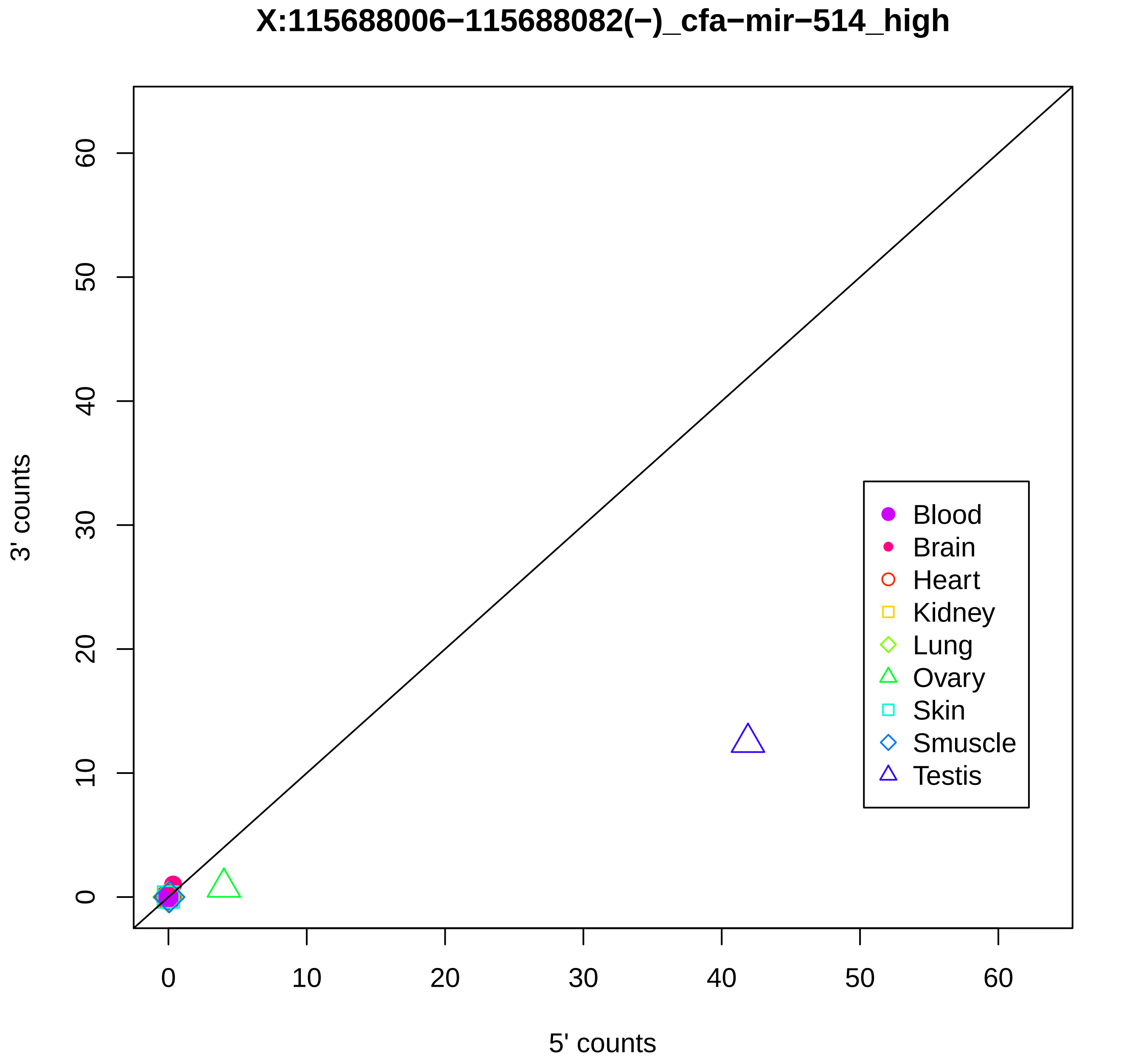

X:115688006−115688082(−)_cfa−mir−514_high
3' counts
5' counts
0
10
20
30
40
50
60
Blood
Brain
Heart
Kidney
Lung
Ovary
Skin
Smuscle
Testis

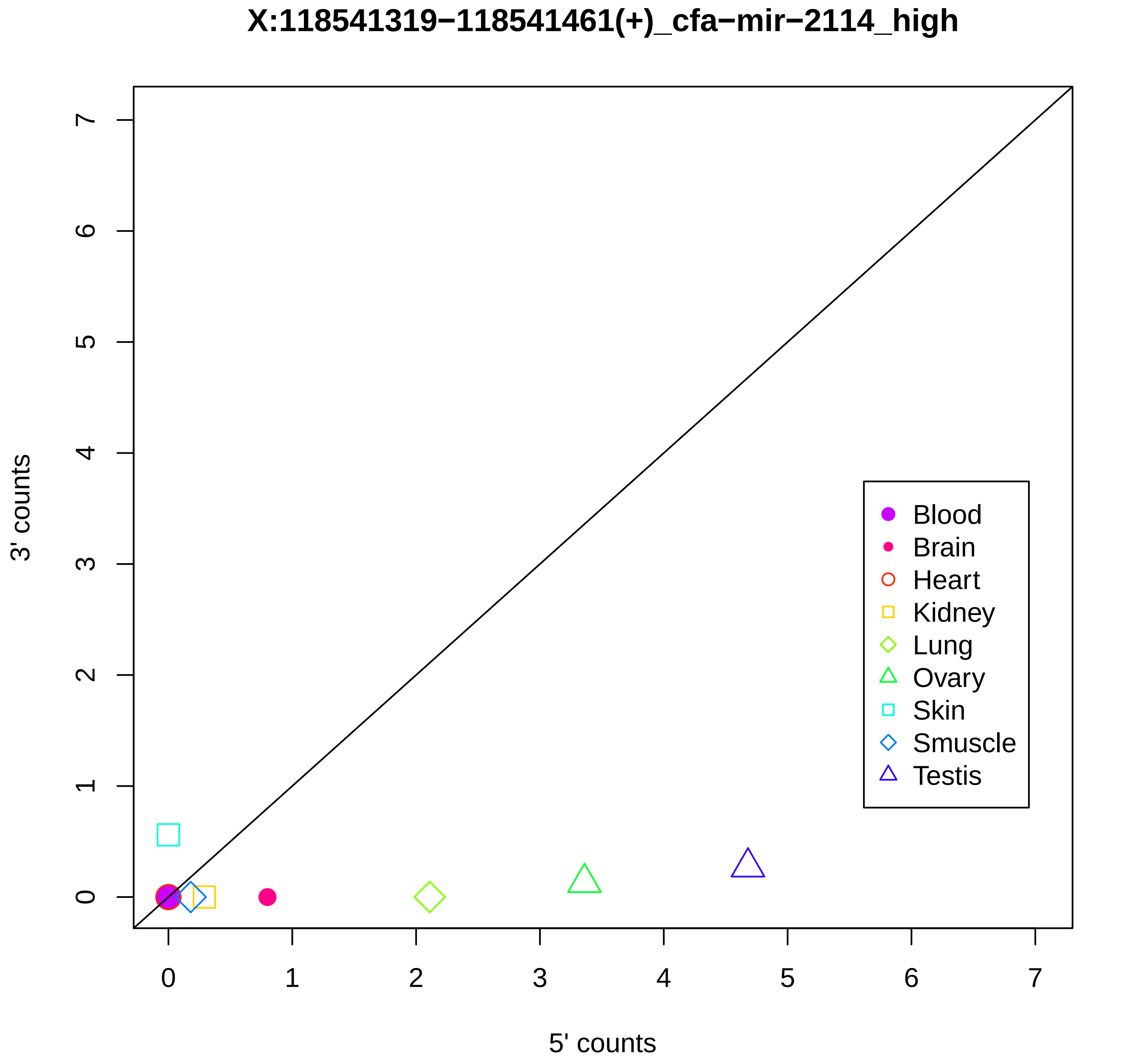

X:118541319−118541461(+)_cfa−mir−2114_high
3' counts
5' counts
0
1
2
3
4
5
6
7
0
1
2
3
4
5
6
7
Blood
Brain
Heart
Kidney
Lung
Ovary
Skin
Smuscle
Testis

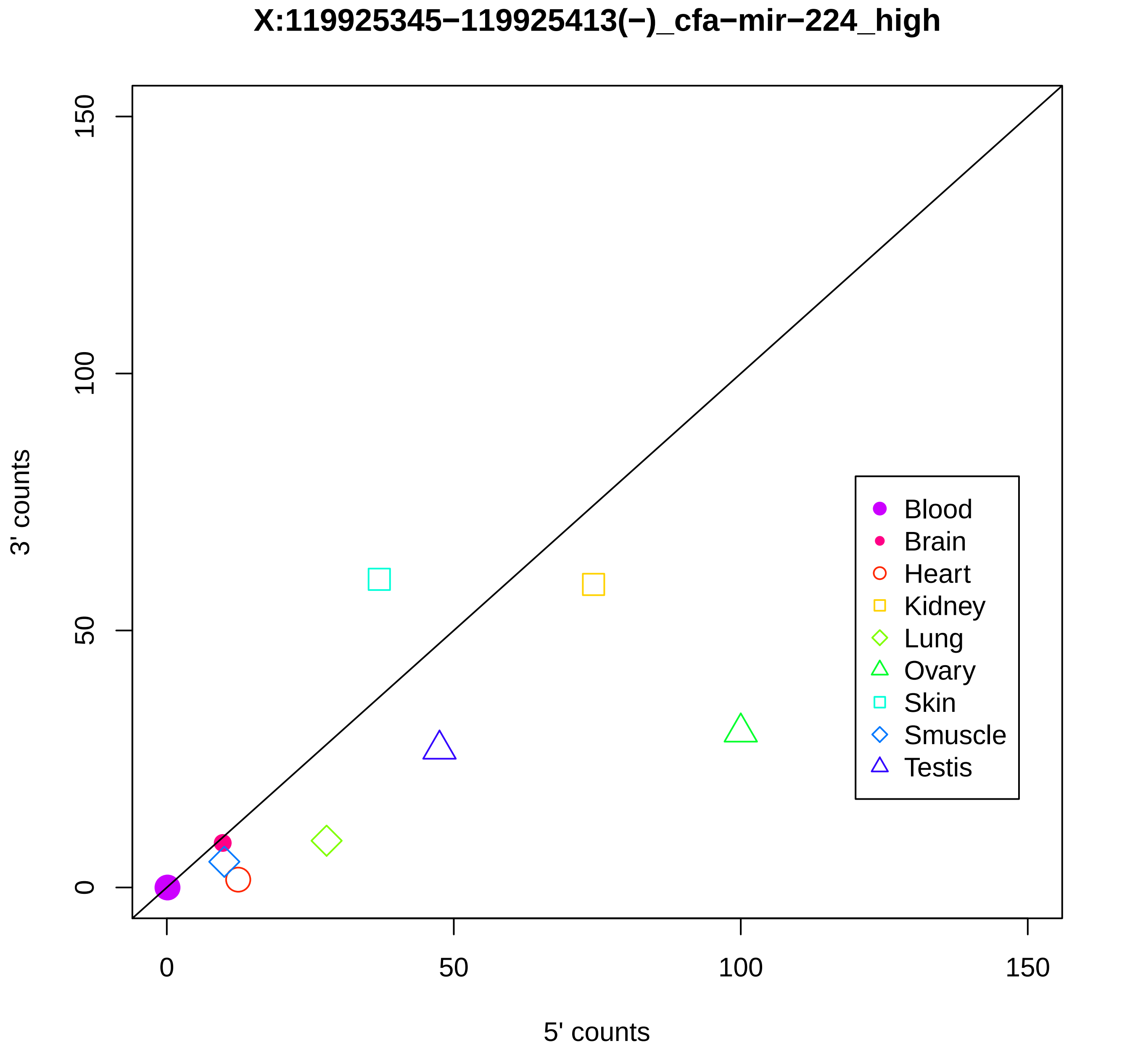

X:119925345−119925413(−)_cfa−mir−224_high
3' counts
5' counts
0
50
100
150
0
50
100
150
Blood
Brain
Heart
Kidney
Lung
Ovary
Skin
Smuscle
Testis

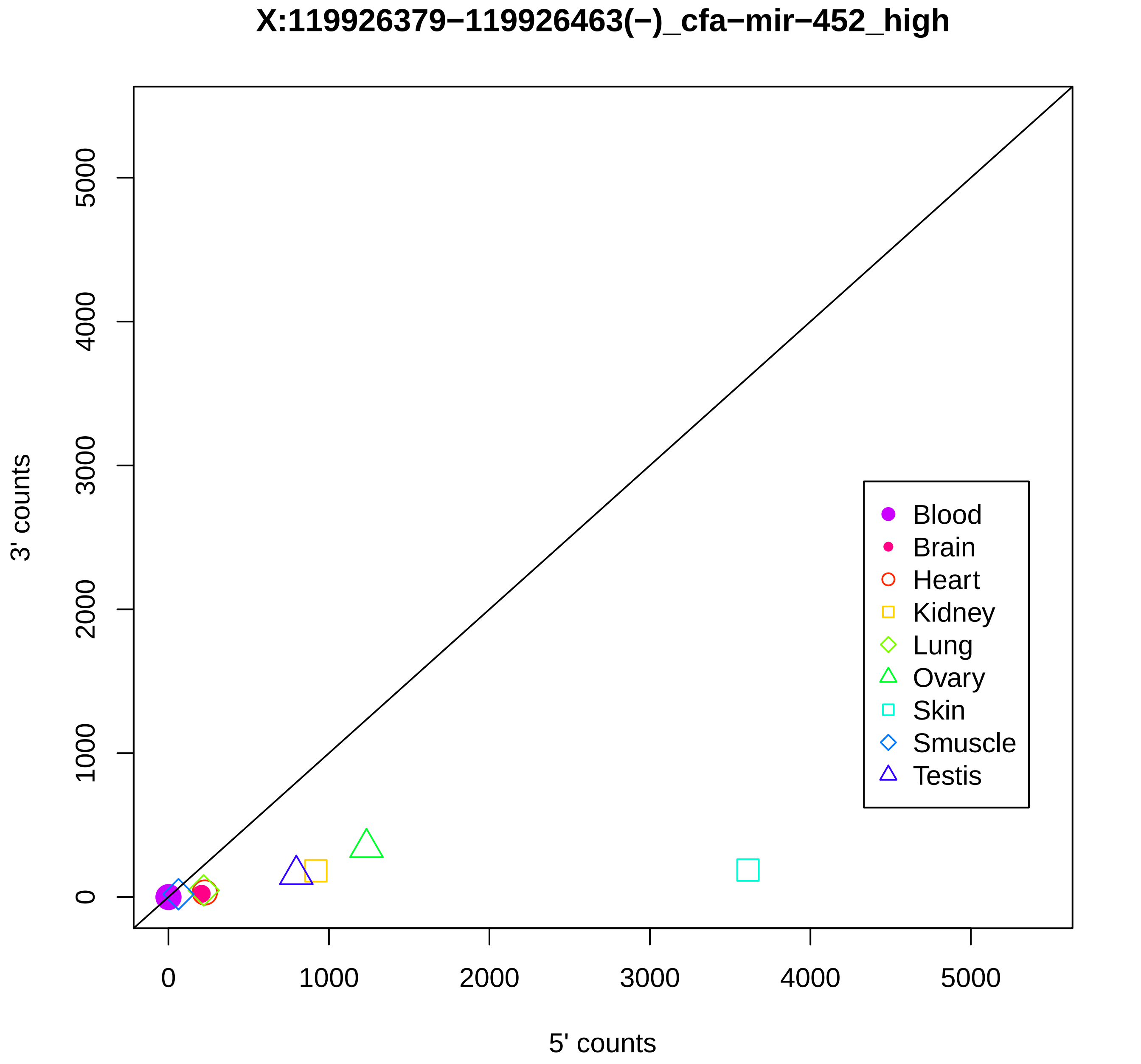

X:119926379−119926463(−)_cfa−mir−452_high
3' counts
5' counts
0
1000
2000
3000
4000
5000
Blood
Brain
Heart
Kidney
Lung
Ovary
Skin
Smuscle
Testis

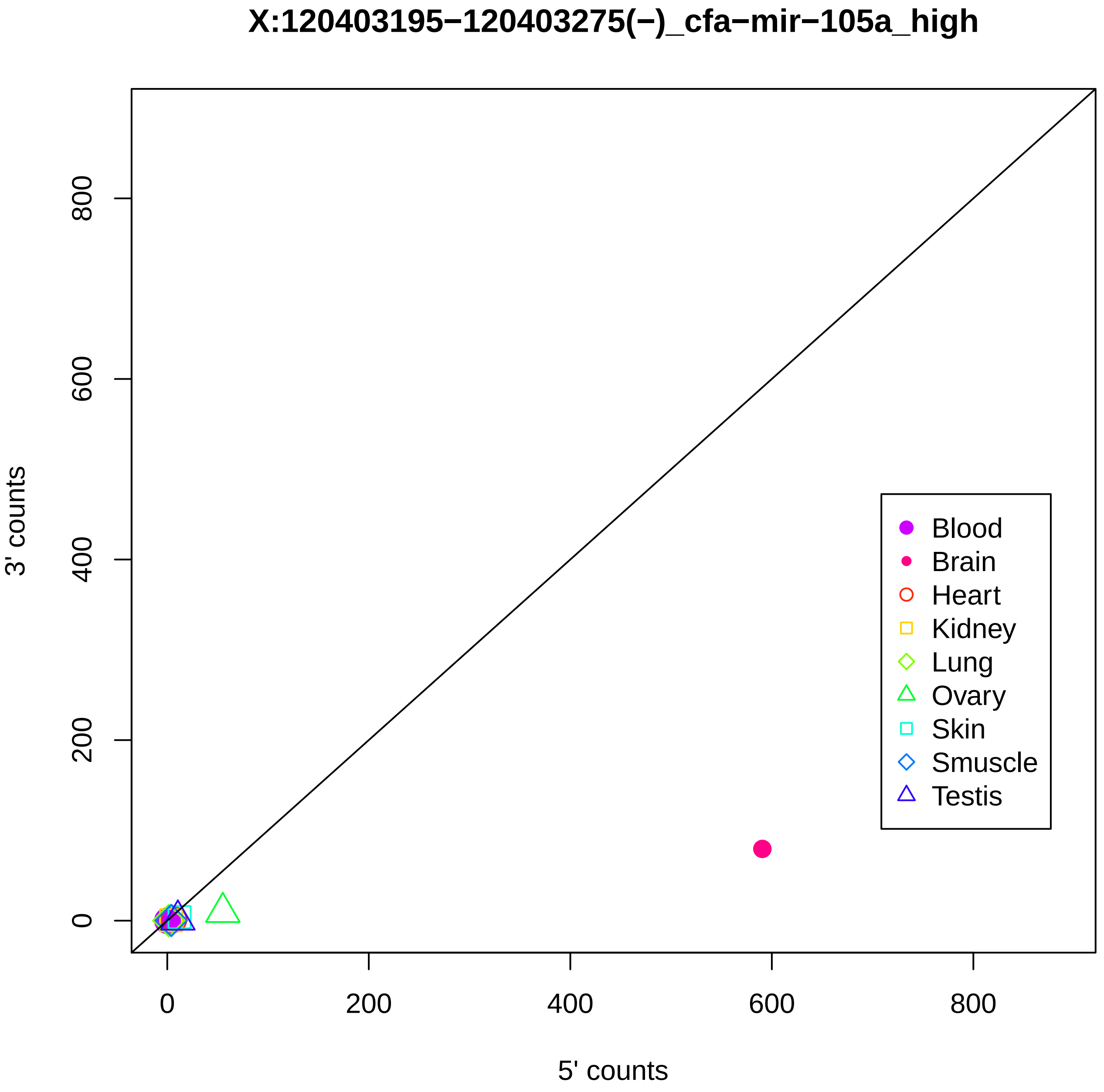
X:120403195−120403275(−)_cfa−mir−105a_high
3' counts
5' counts
0
200
400
600
800
Blood
Brain
Heart
Kidney
Lung
Ovary
Skin
Smuscle
Testis

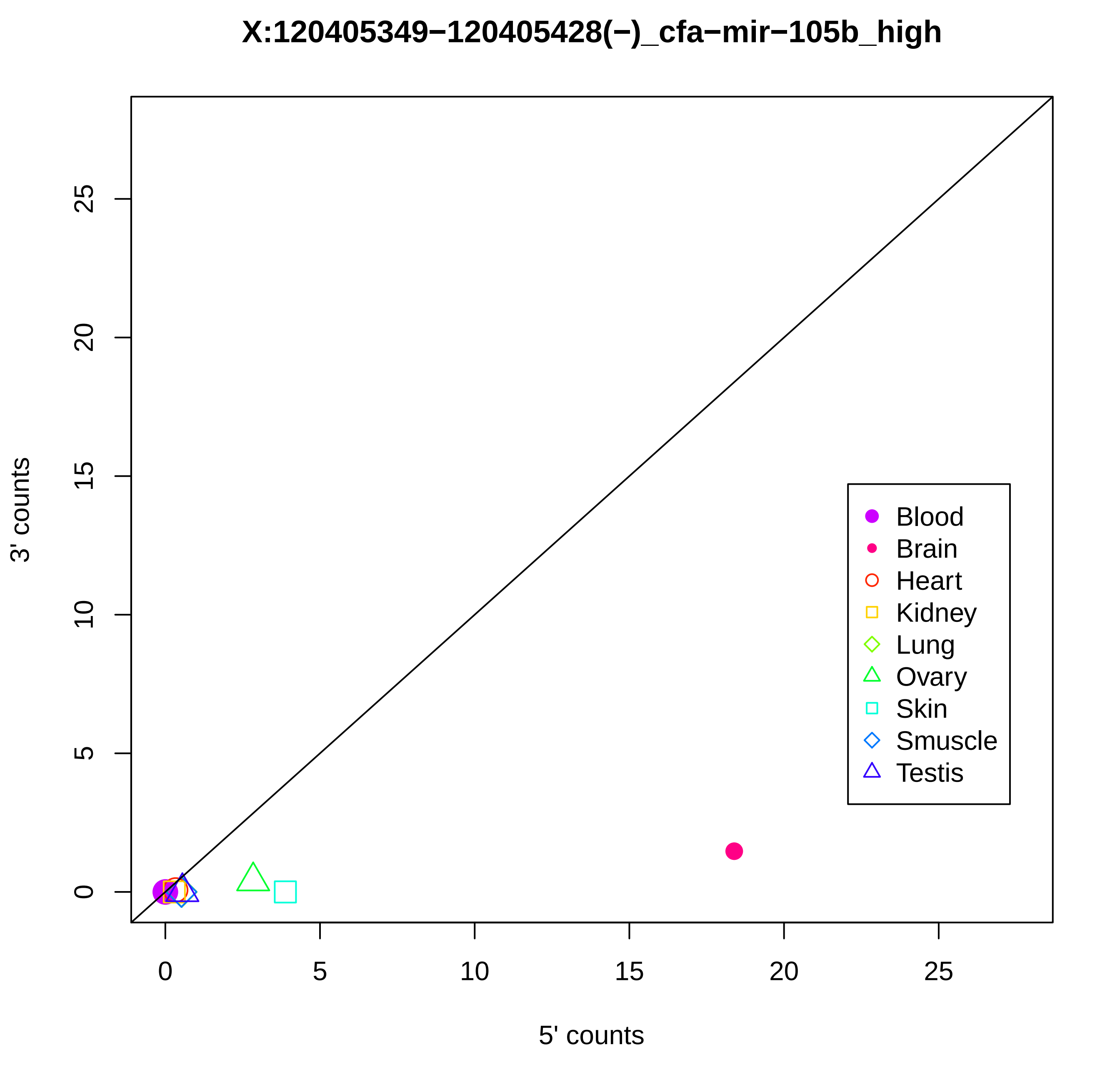

X:120405349−120405428(−)_cfa−mir−105b_high
3' counts
5' counts
0
5
10
15
20
25
0
5
10
15
20
25
Blood
Brain
Heart
Kidney
Lung
Ovary
Skin
Smuscle
Testis

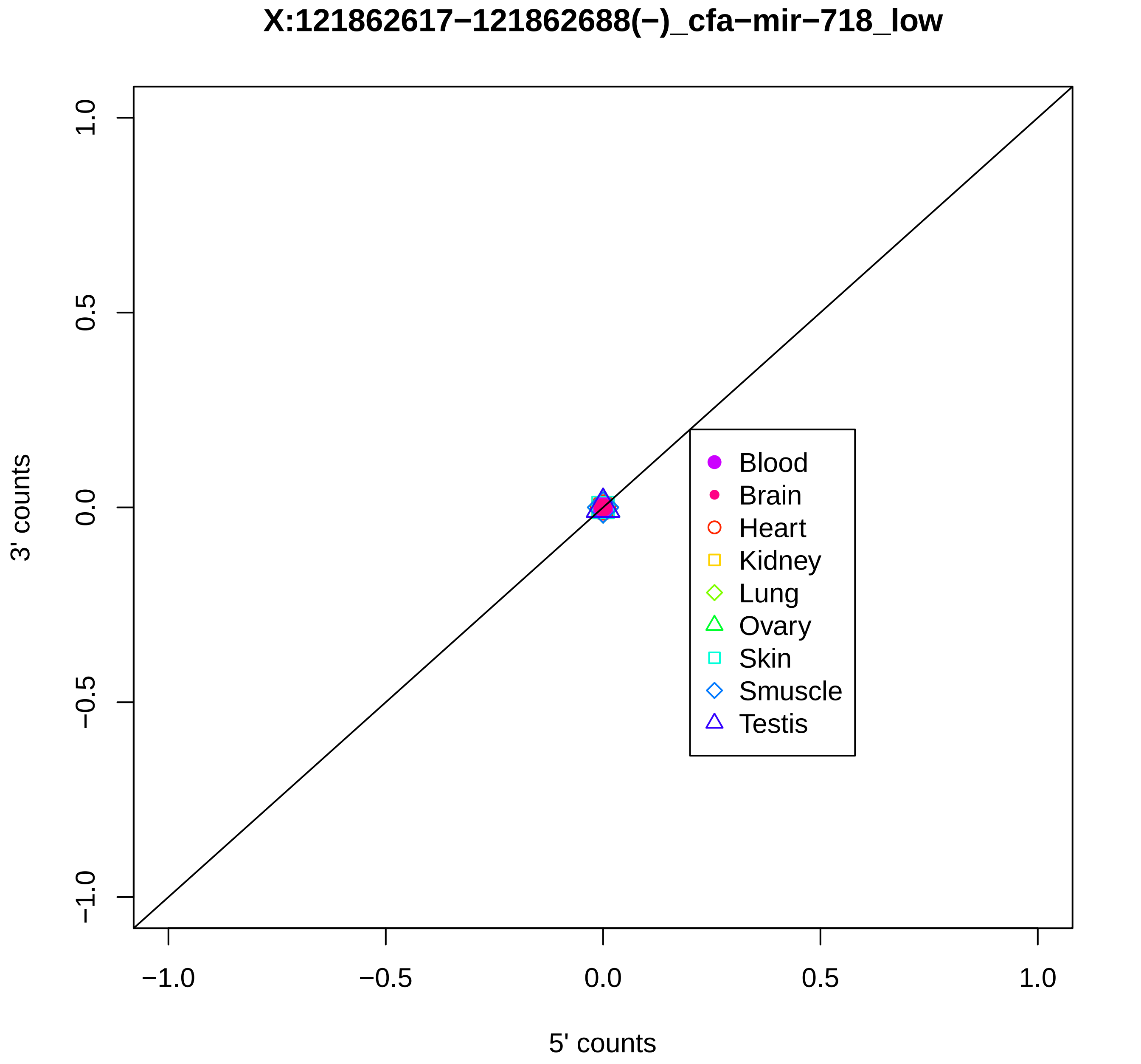

X:121862617−121862688(−)_cfa−mir−718_low
3' counts
5' counts
−1.0
−0.5
0.0
0.5
1.0
Blood
Brain
Heart
Kidney
Lung
Ovary
Skin
Smuscle
Testis

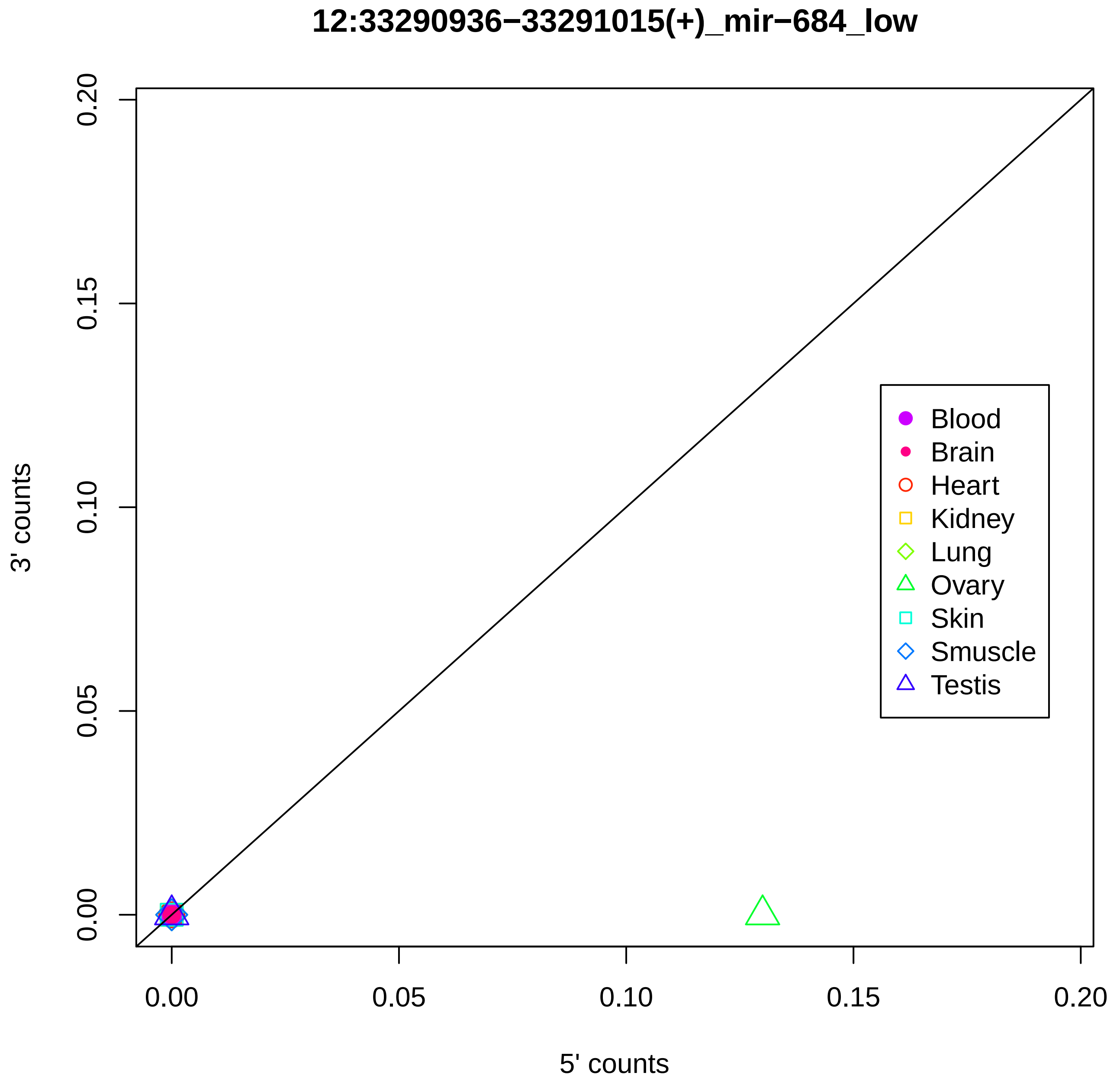
12:33290936−33291015(+)_mir−684_low
3' counts
5' counts
0.00
0.05
0.10
0.15
0.20
Blood
Brain
Heart
Kidney
Lung
Ovary
Skin
Smuscle
Testis

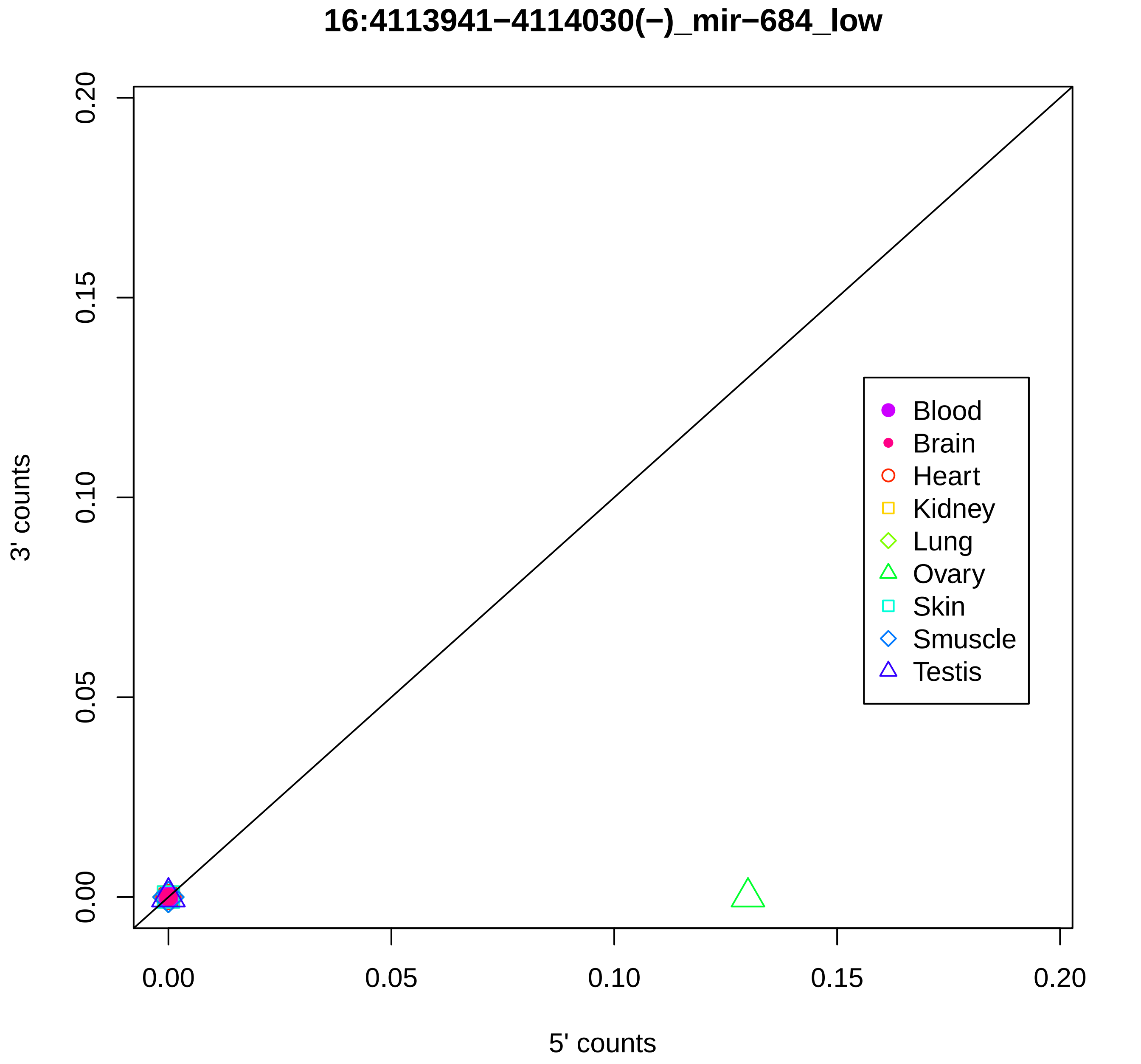
16:4113941−4114030(−)_mir−684_low
3' counts
5' counts
0.00
0.05
0.10
0.15
0.20
0.00
0.05
0.10
0.15
0.20
Blood
Brain
Heart
Kidney
Lung
Ovary
Skin
Smuscle
Testis

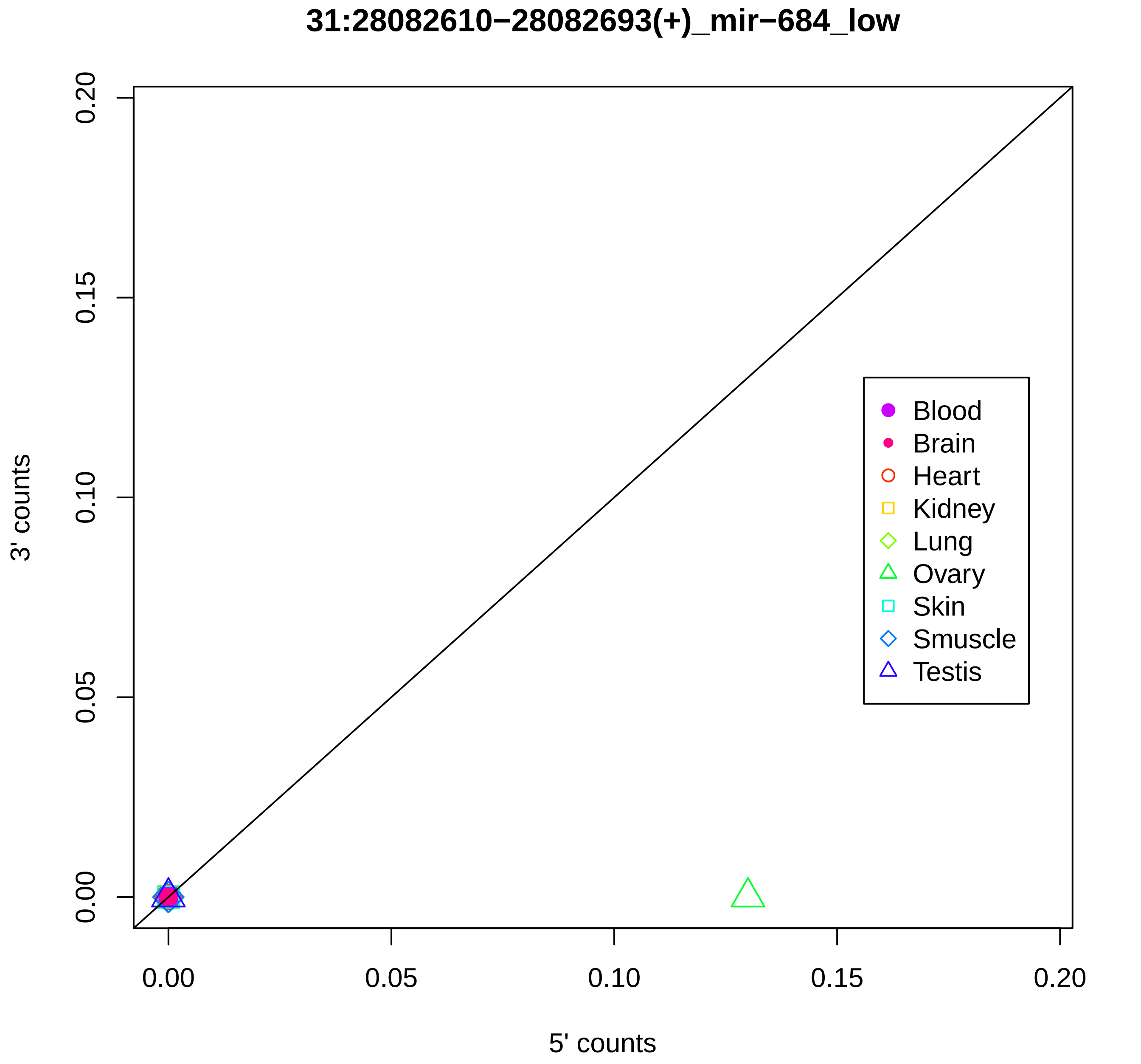

31:28082610−28082693(+)_mir−684_low
3' counts
5' counts
0.00
0.05
0.10
0.15
0.20
Blood
Brain
Heart
Kidney
Lung
Ovary
Skin
Smuscle
Testis

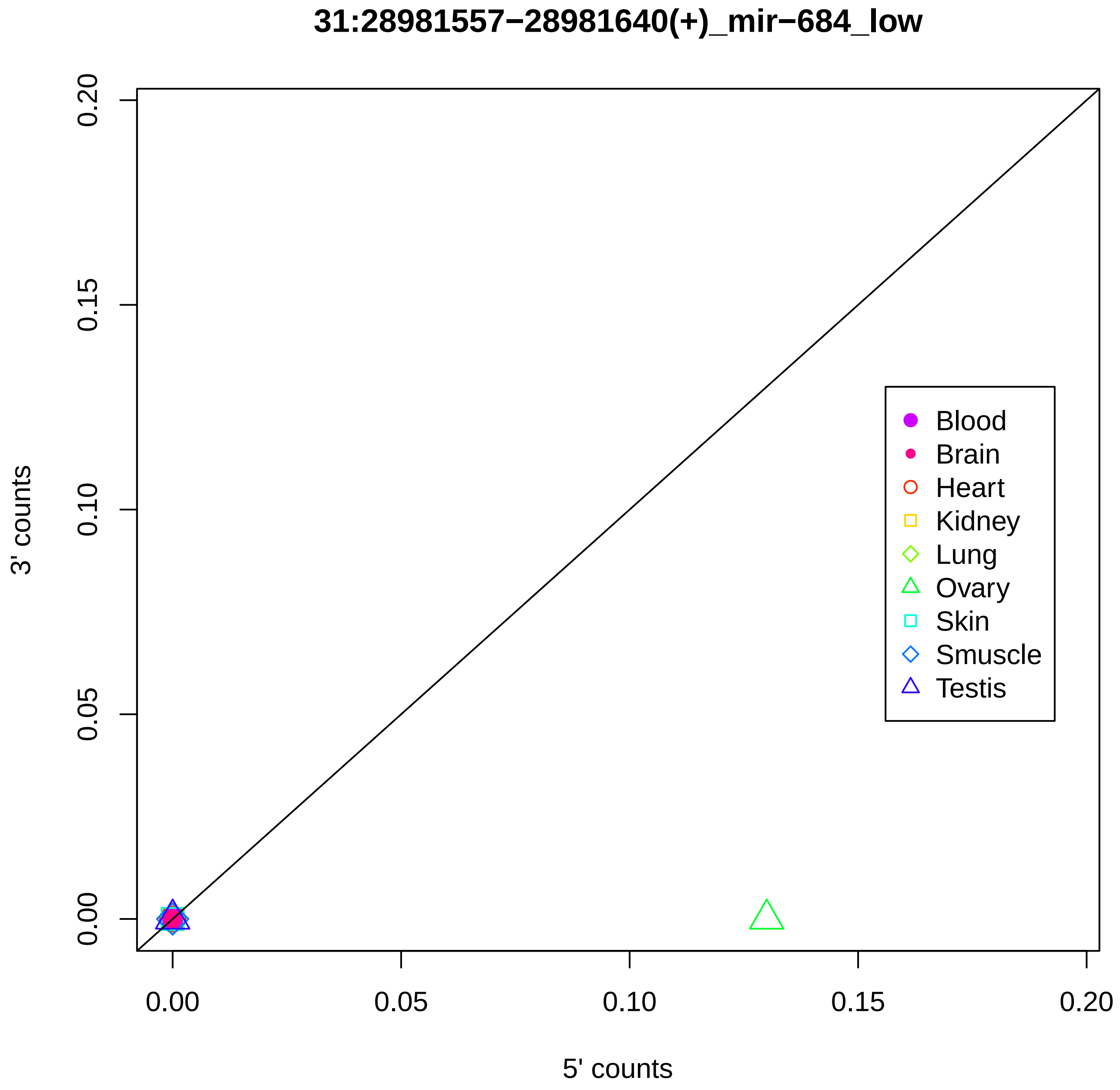

31:28981557−28981640(+)_mir−684_low
3' counts
5' counts
0.00
0.05
0.10
0.15
0.20
Blood
Brain
Heart
Kidney
Lung
Ovary
Skin
Smuscle
Testis

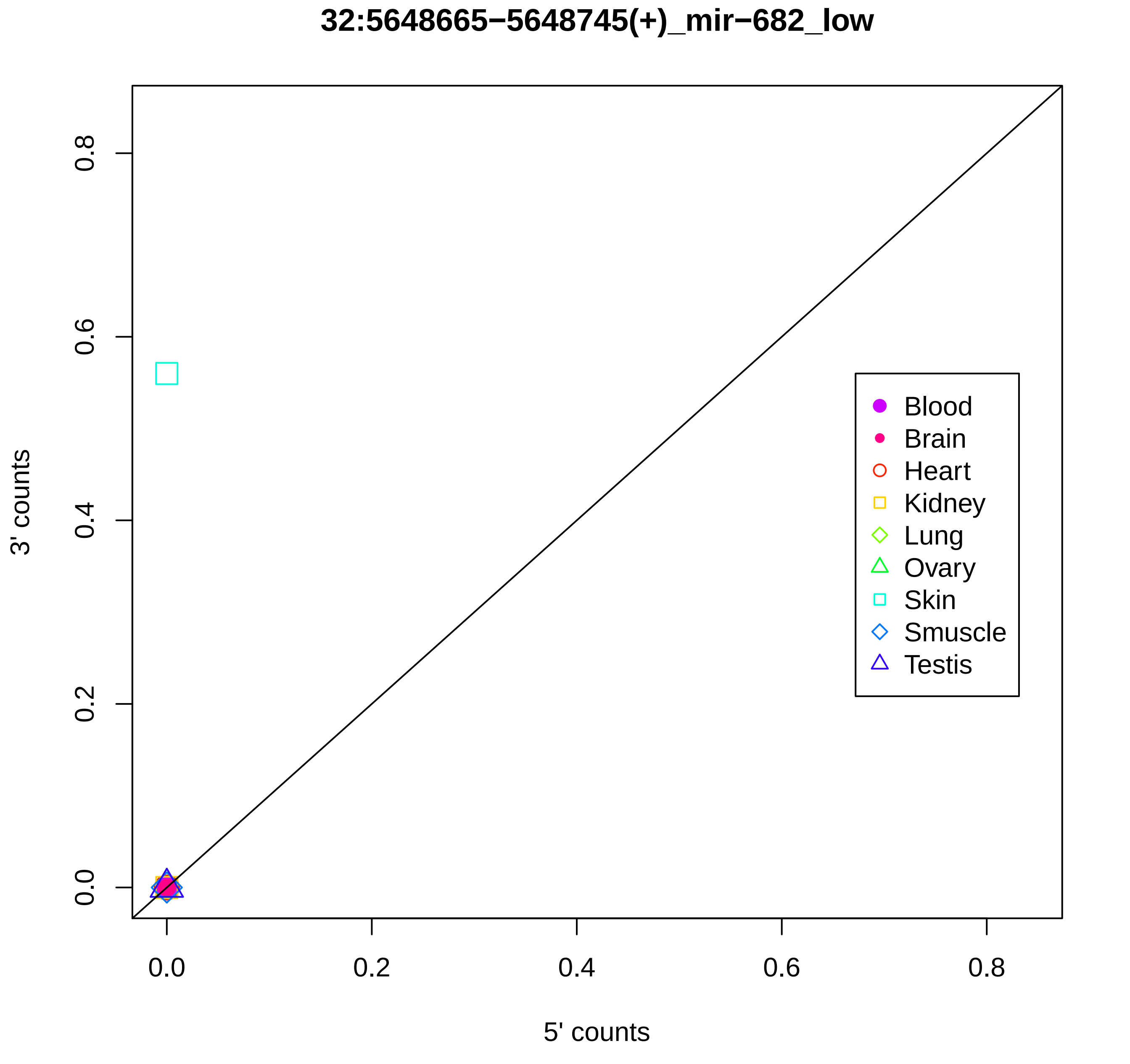

32:5648665−5648745(+)_mir−682_low
3' counts
5' counts
0.0
0.2
0.4
0.6
0.8
Blood
Brain
Heart
Kidney
Lung
Ovary
Skin
Smuscle
Testis

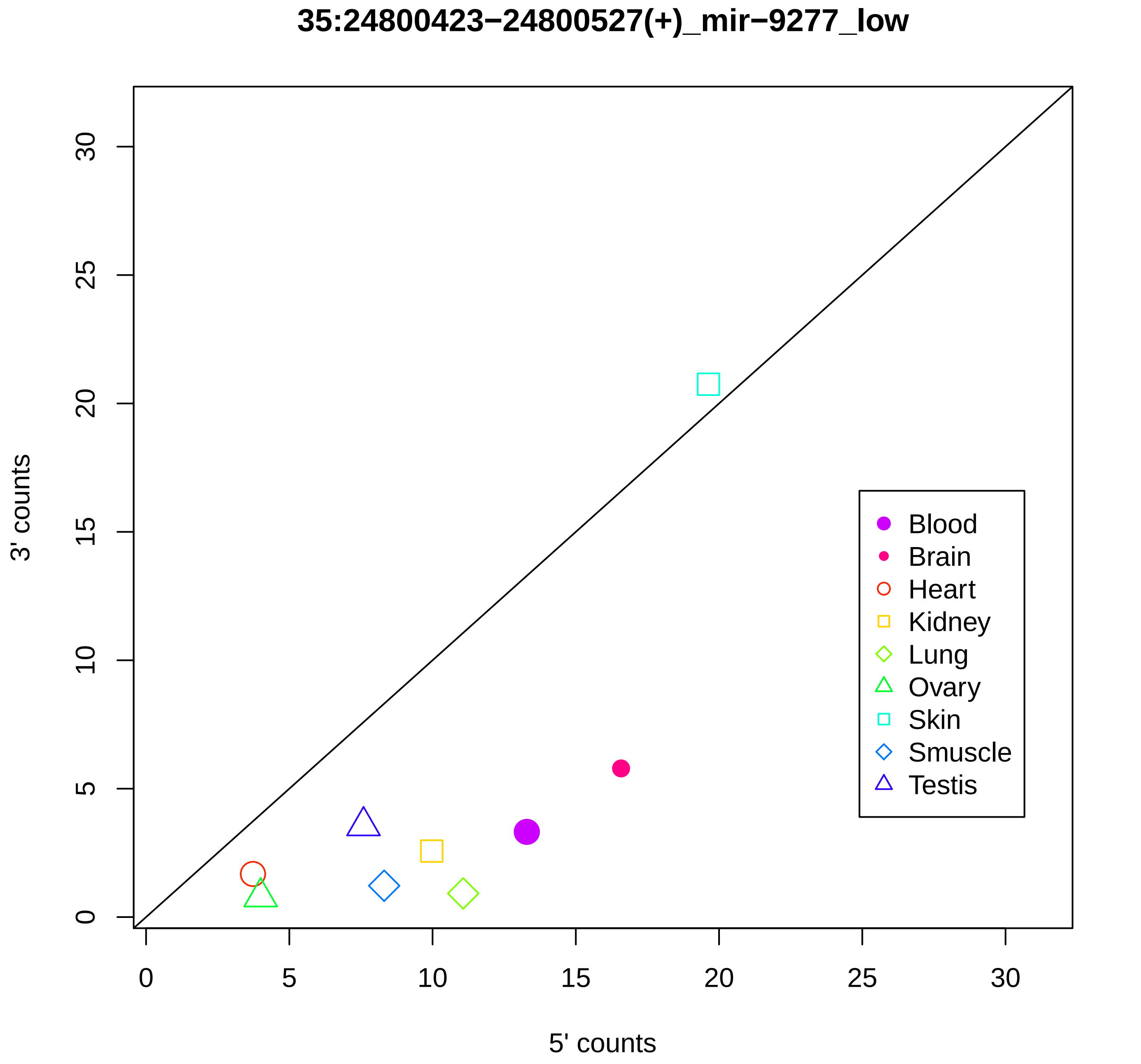
35:24800423−24800527(+)_mir−9277_low
3' counts
5' counts
0
5
10
15
20
25
30
Blood
Brain
Heart
Kidney
Lung
Ovary
Skin
Smuscle
Testis

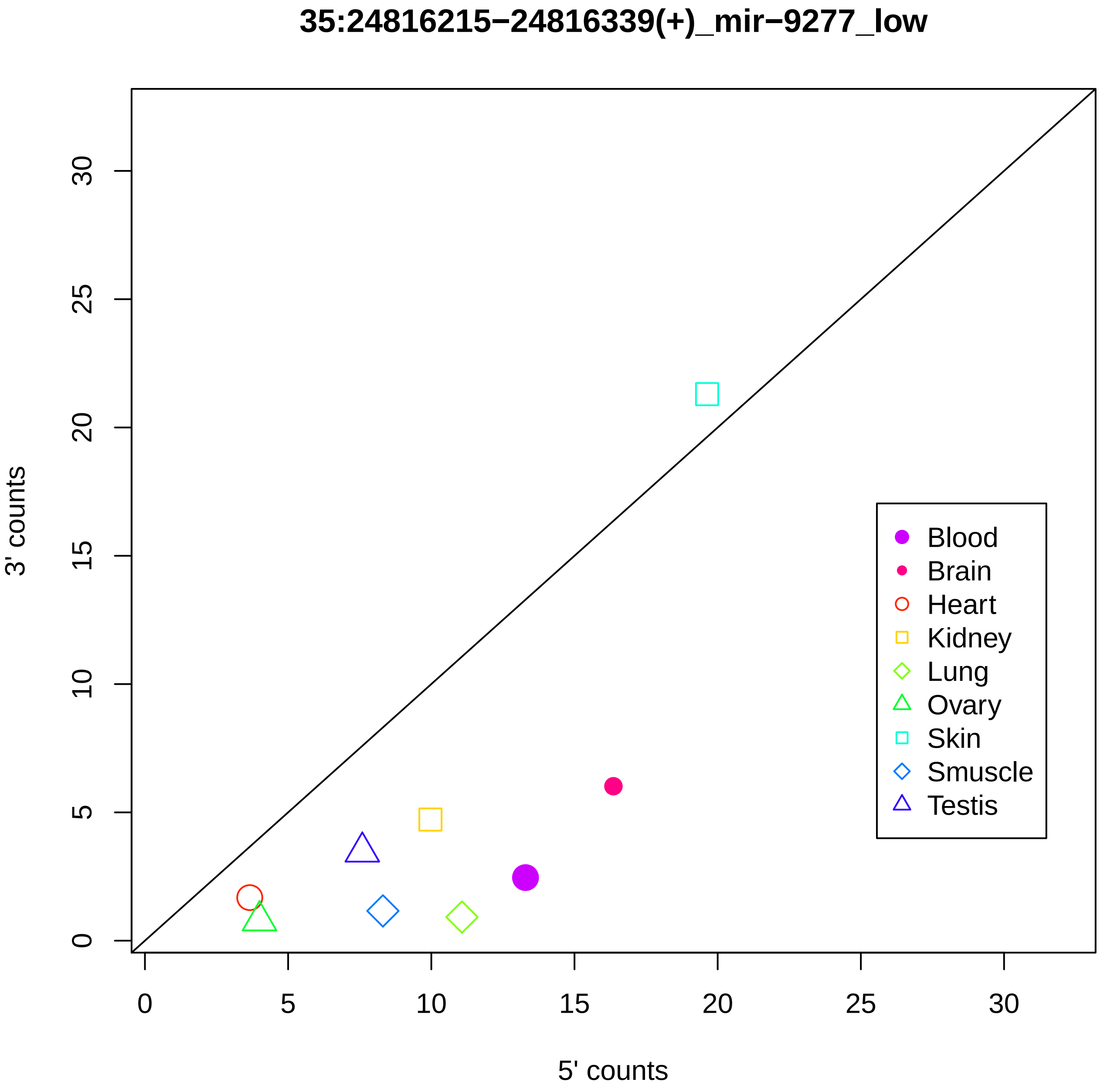
35:24816215−24816339(+)_mir−9277_low
3' counts
5' counts
0
5
10
15
20
25
30
Blood
Brain
Heart
Kidney
Lung
Ovary
Skin
Smuscle
Testis

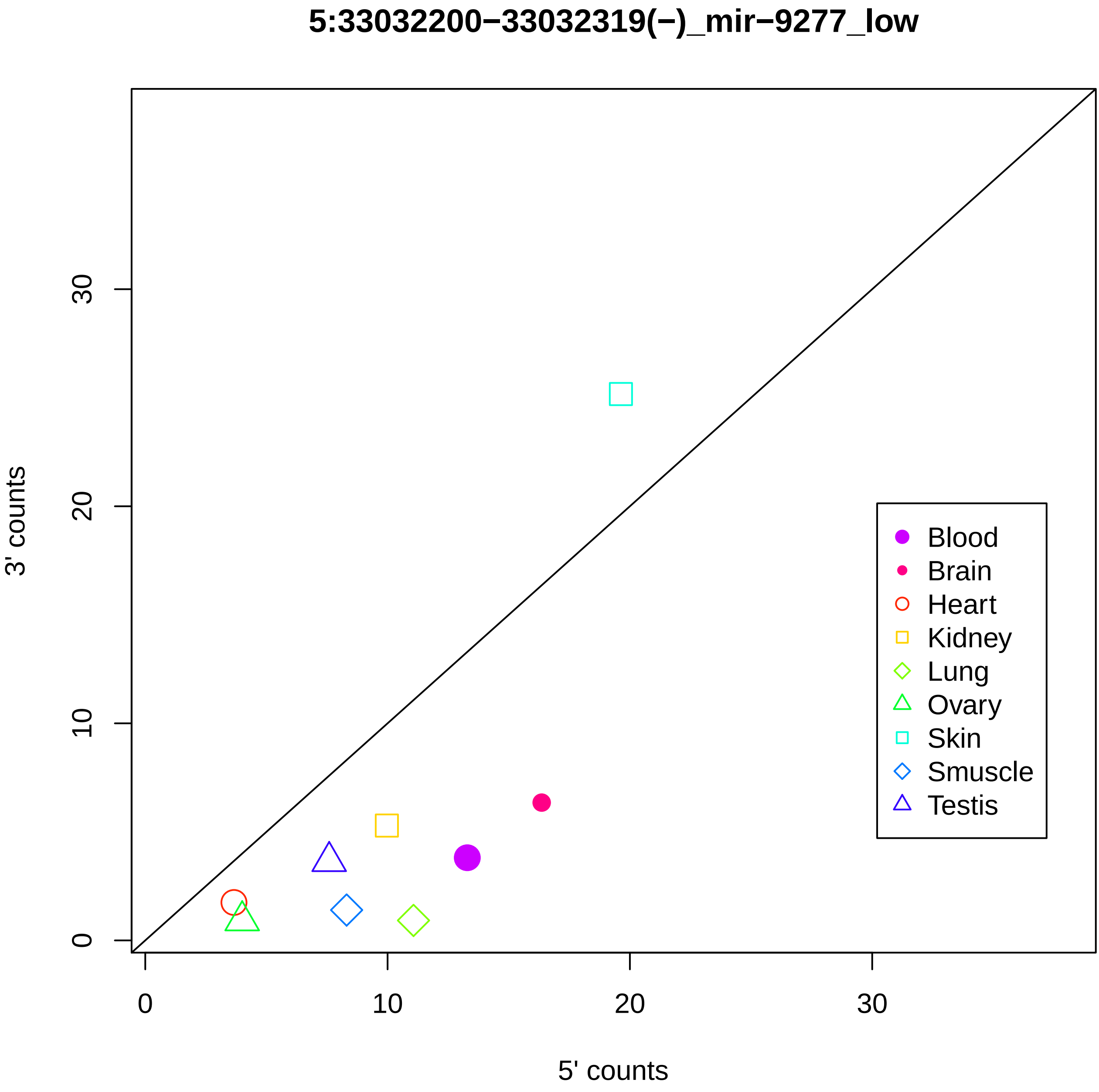
5:33032200−33032319(−)_mir−9277_low
3' counts
5' counts
0
10
20
30
Blood
Brain
Heart
Kidney
Lung
Ovary
Skin
Smuscle
Testis

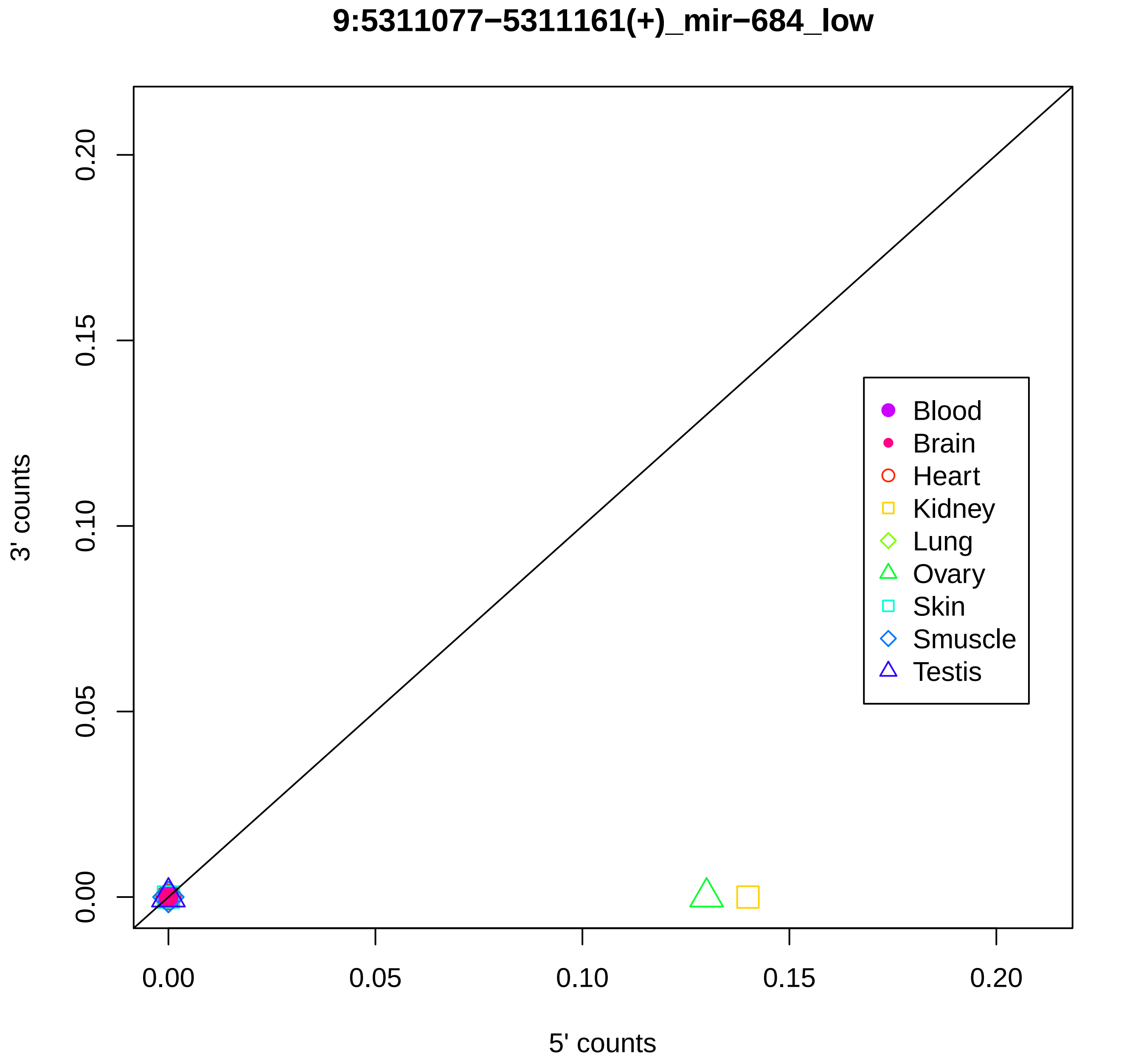

9:5311077−5311161(+)_mir−684_low
0.00
0.05
0.10
0.15
0.20
0.00
0.05
0.10
0.15
0.20
5' counts
3' counts
Blood
Brain
Heart
Kidney
Lung
Ovary
Skin
Smuscle
Testis

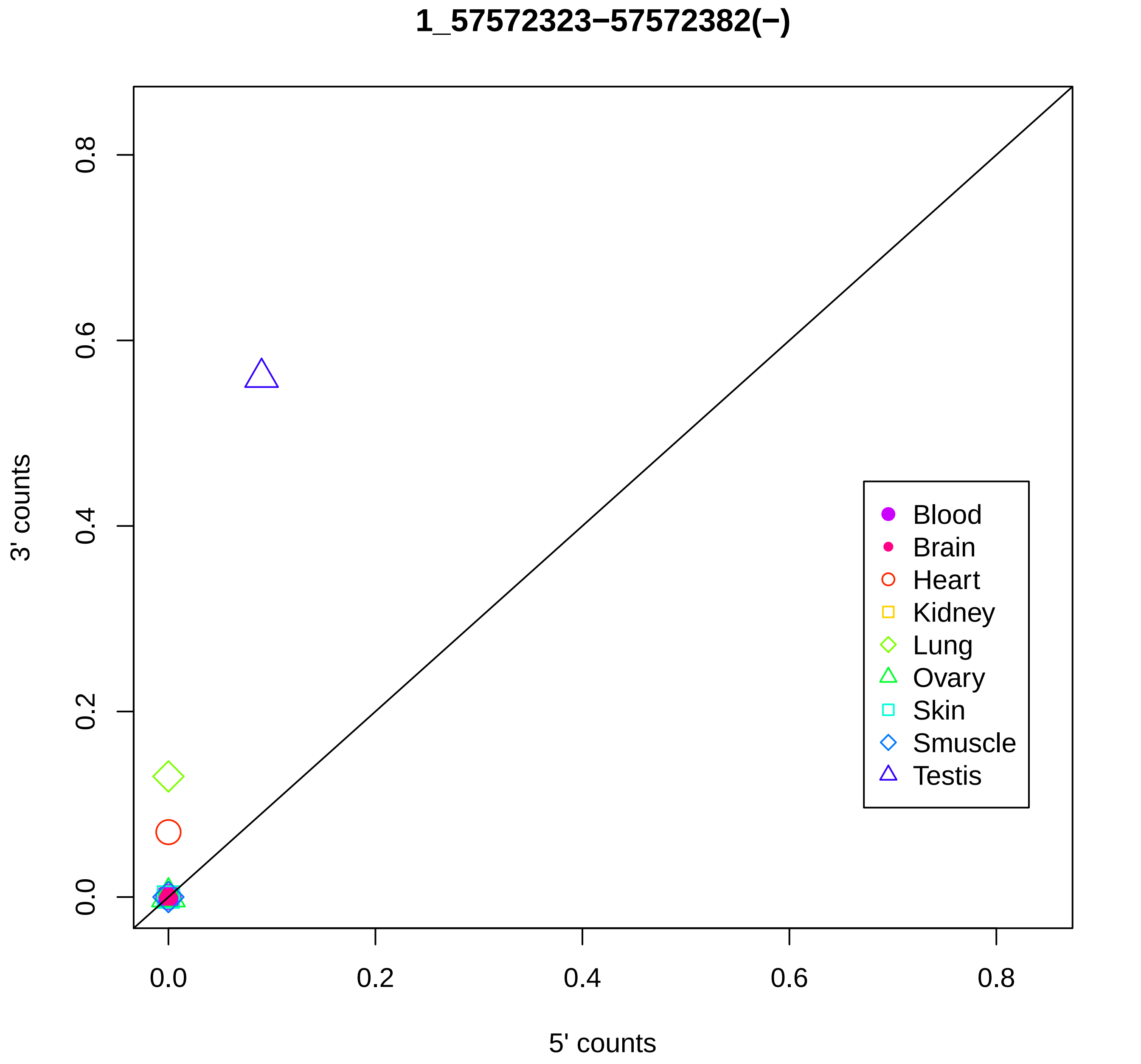

1_57572323−57572382(−)
3' counts
5' counts
0.0
0.2
0.4
0.6
0.8
Blood
Brain
Heart
Kidney
Lung
Ovary
Skin
Smuscle
Testis

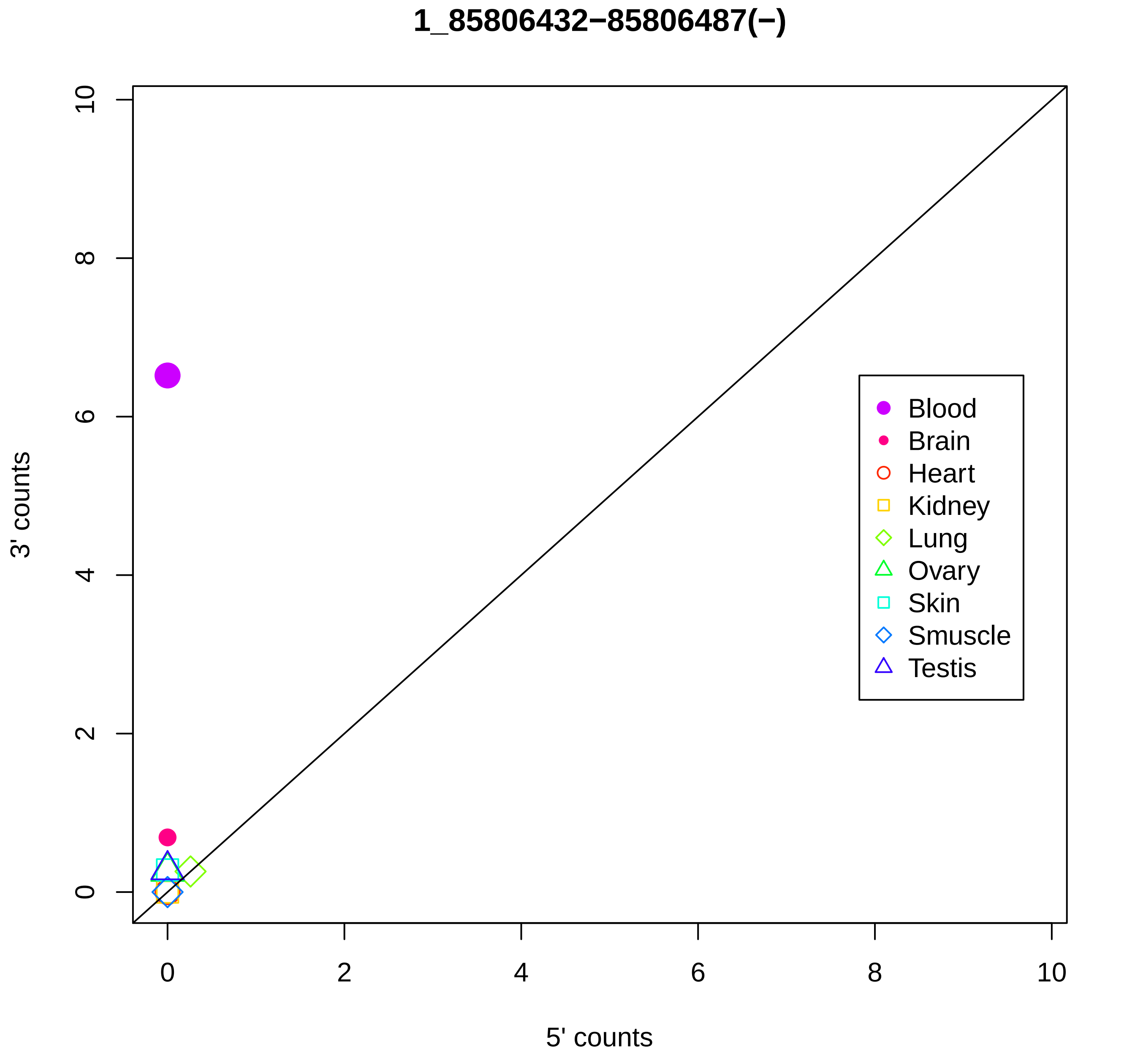

1_85806432−85806487(−)
3' counts
5' counts
0
2
4
6
8
10
Blood
Brain
Heart
Kidney
Lung
Ovary
Skin
Smuscle
Testis

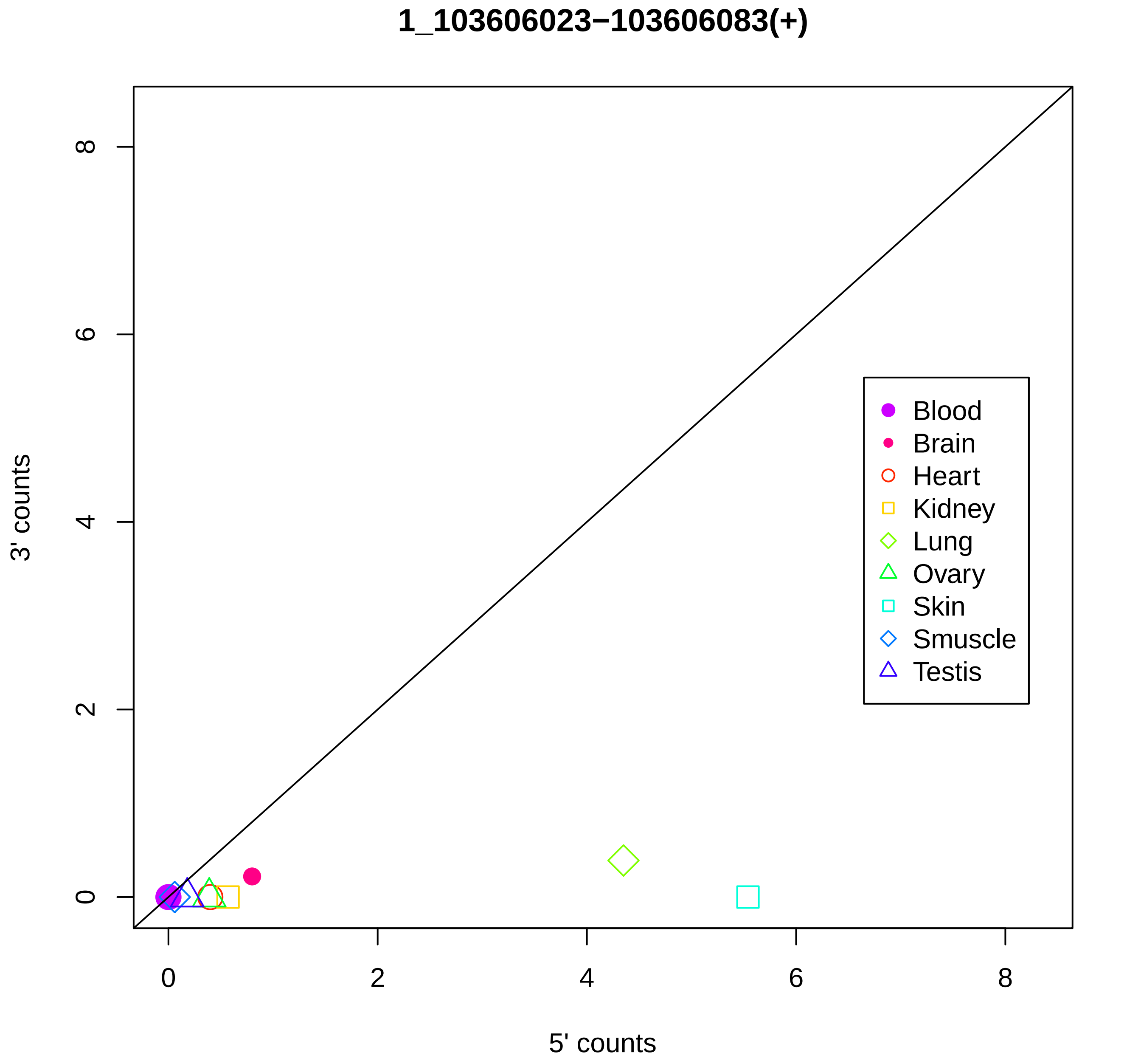

1_103606023−103606083(+)
3' counts
5' counts
0
2
4
6
8
Blood
Brain
Heart
Kidney
Lung
Ovary
Skin
Smuscle
Testis

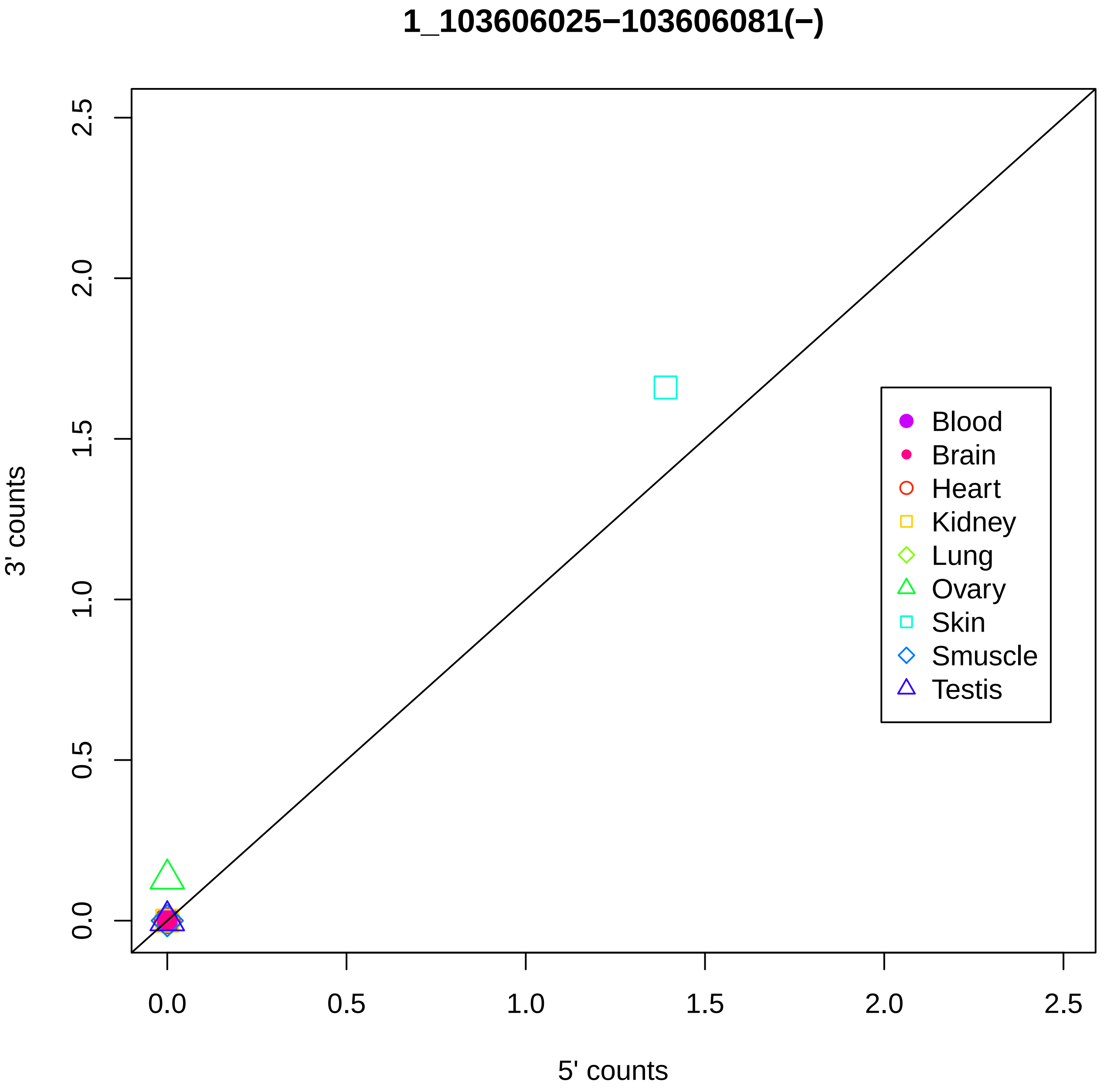
1_103606025−103606081(−)
3' counts
5' counts
0.0
0.5
1.0
1.5
2.0
2.5
Blood
Brain
Heart
Kidney
Lung
Ovary
Skin
Smuscle
Testis

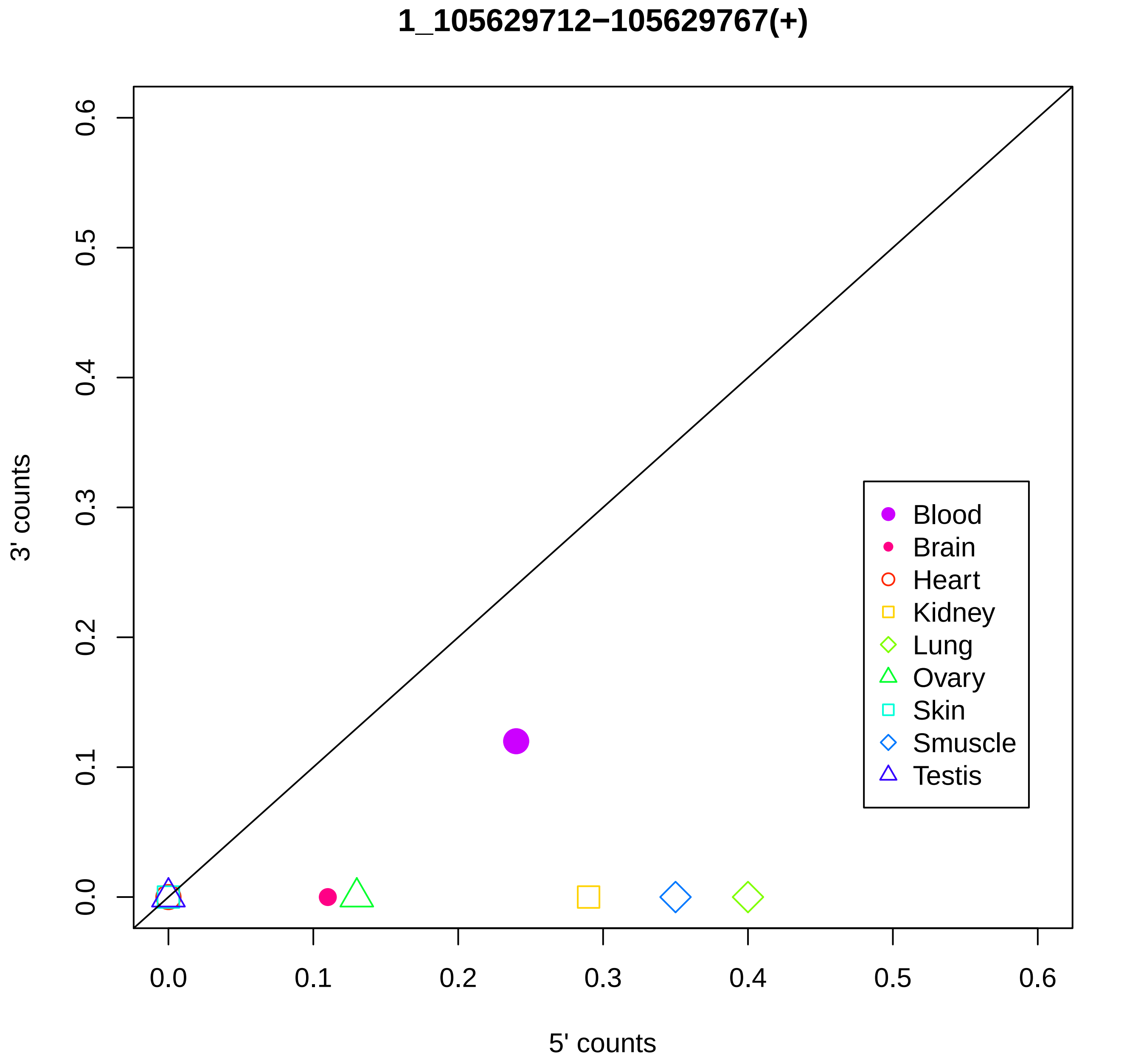
1_105629712−105629767(+)
3' counts
5' counts
0.0
0.1
0.2
0.3
0.4
0.5
0.6
Blood
Brain
Heart
Kidney
Lung
Ovary
Skin
Smuscle
Testis

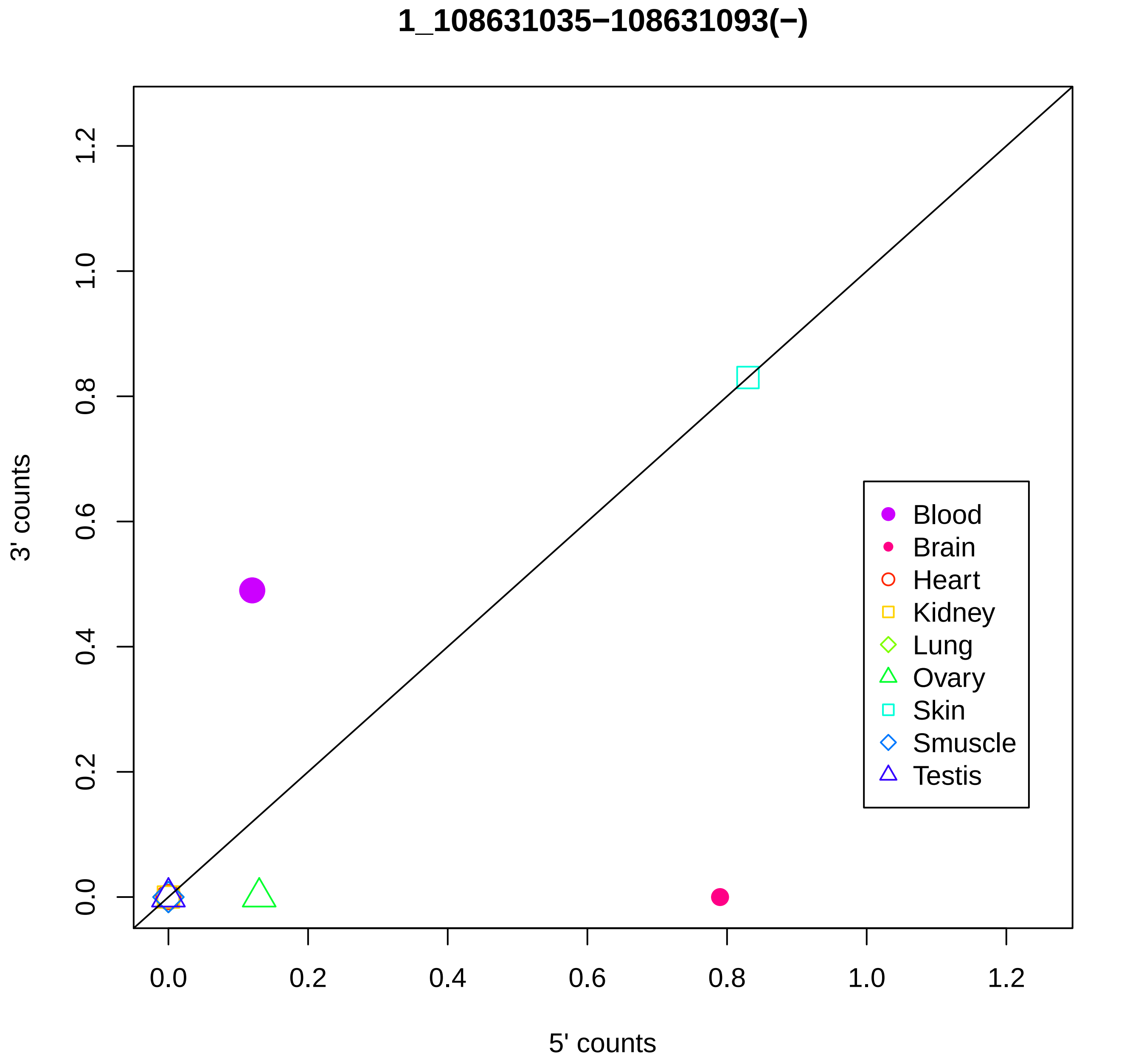

1_108631035−108631093(−)
3' counts
5' counts
0.0
0.2
0.4
0.6
0.8
1.0
1.2
Blood
Brain
Heart
Kidney
Lung
Ovary
Skin
Smuscle
Testis

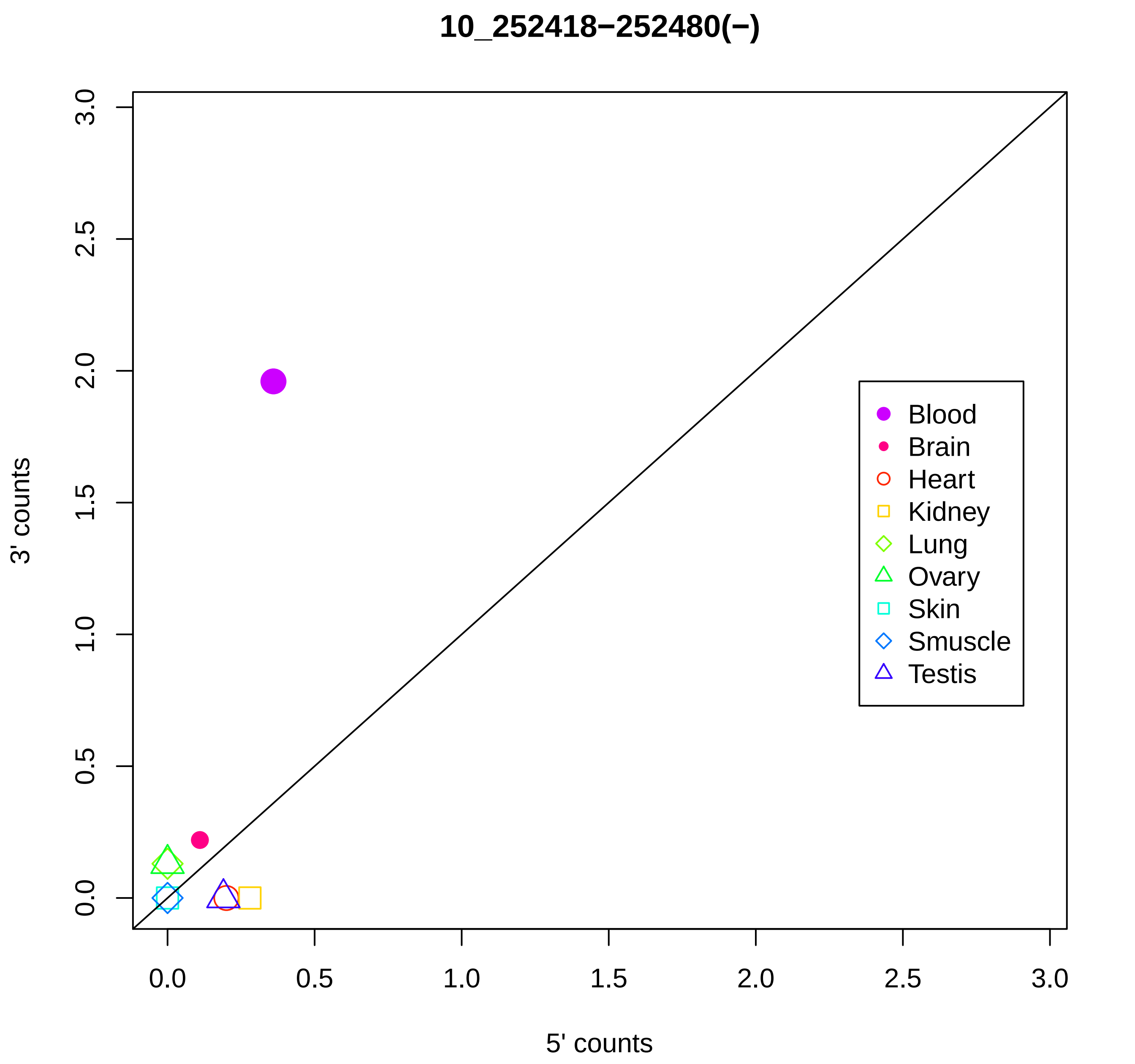

10_252418−252480(−)
3' counts
5' counts
0.0
0.5
1.0
1.5
2.0
2.5
3.0
Blood
Brain
Heart
Kidney
Lung
Ovary
Skin
Smuscle
Testis

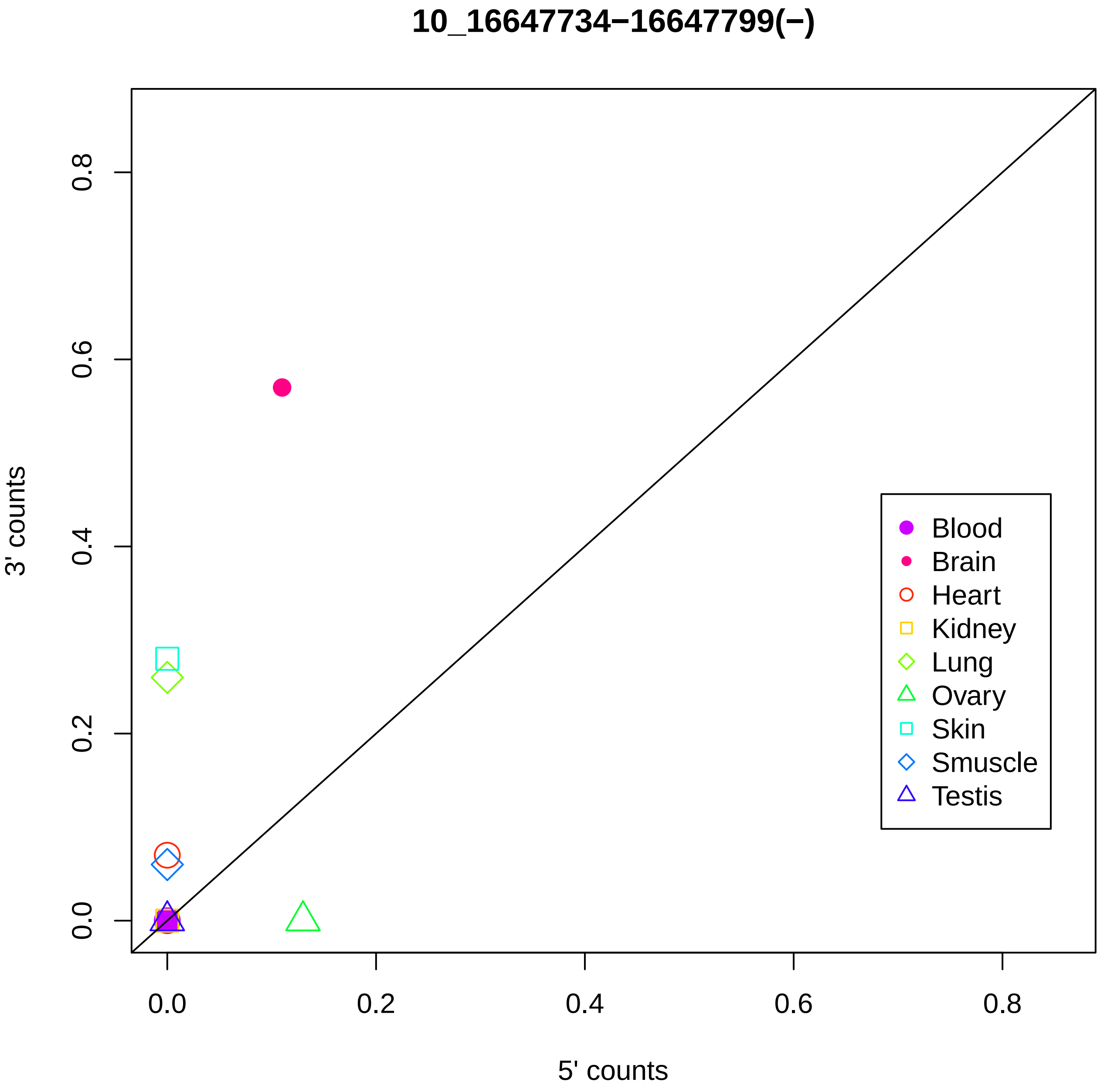

10_16647734−16647799(−)
3' counts
5' counts
0.0
0.2
0.4
0.6
0.8
0.0
0.2
0.4
0.6
0.8
Blood
Brain
Heart
Kidney
Lung
Ovary
Skin
Smuscle
Testis

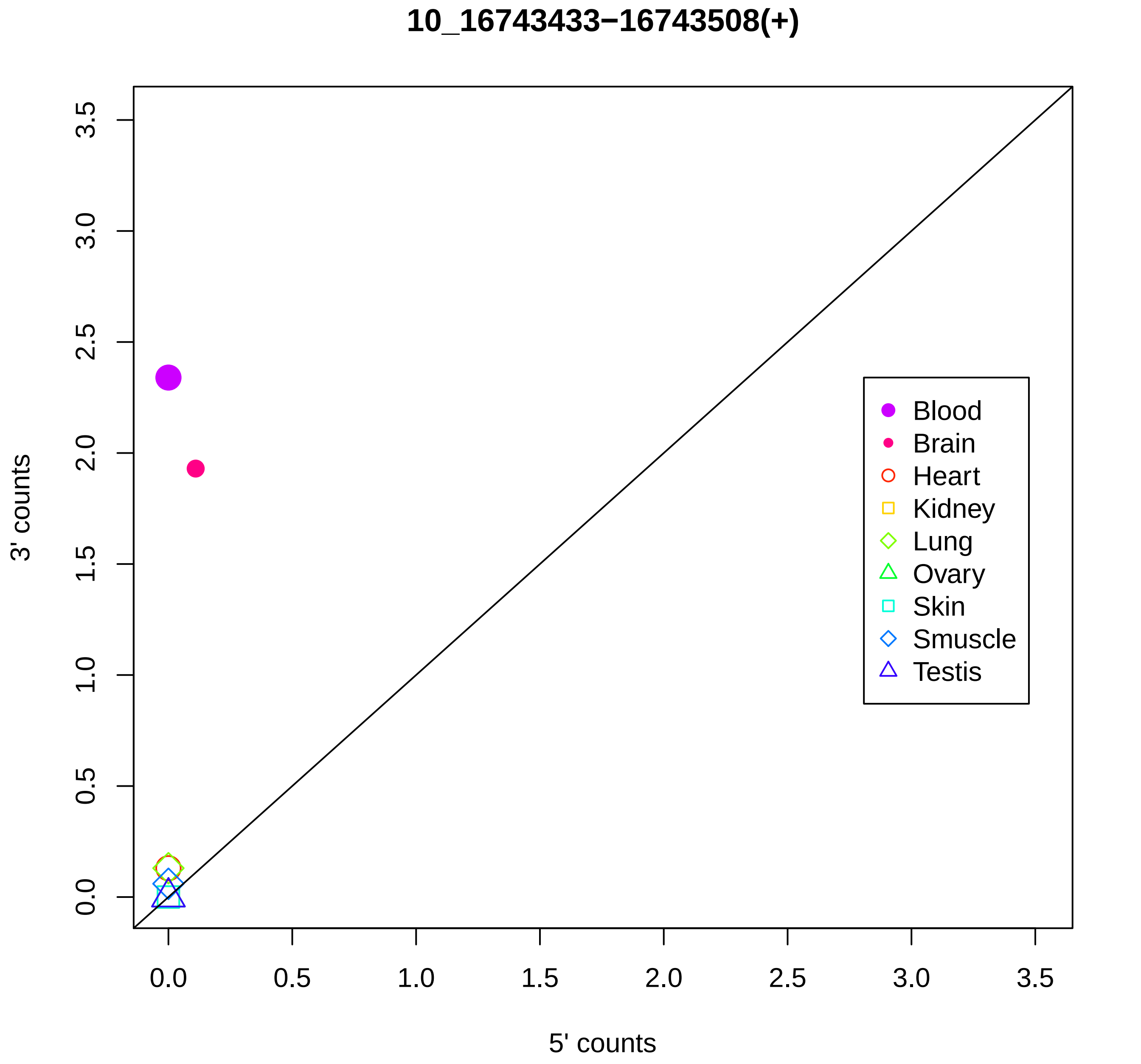
10_16743433−16743508(+)
3' counts
5' counts
0.0
0.5
1.0
1.5
2.0
2.5
3.0
3.5
Blood
Brain
Heart
Kidney
Lung
Ovary
Skin
Smuscle
Testis

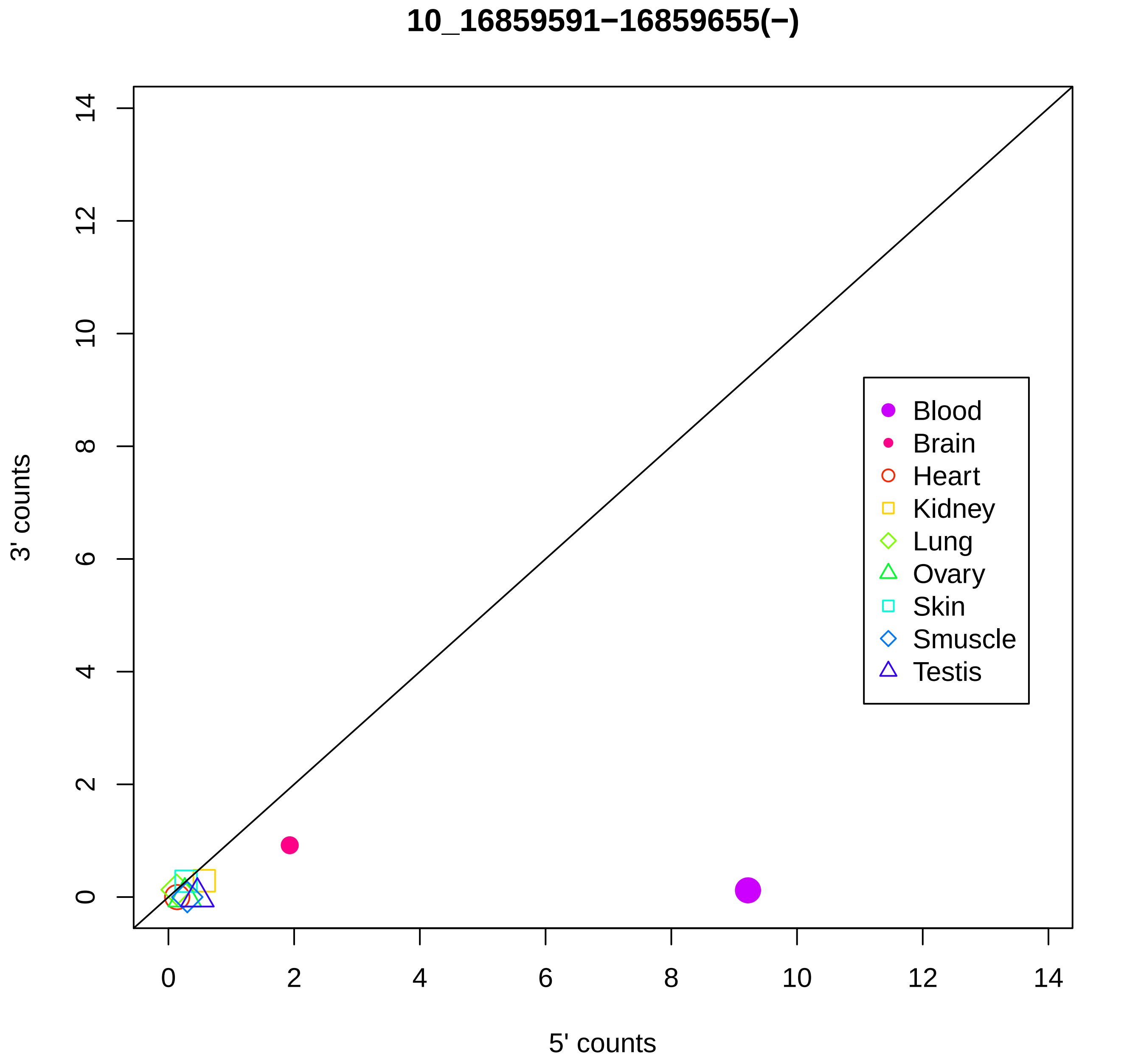
10_16859591−16859655(−)
3' counts
5' counts
0
2
4
6
8
10
12
14
Blood
Brain
Heart
Kidney
Lung
Ovary
Skin
Smuscle
Testis

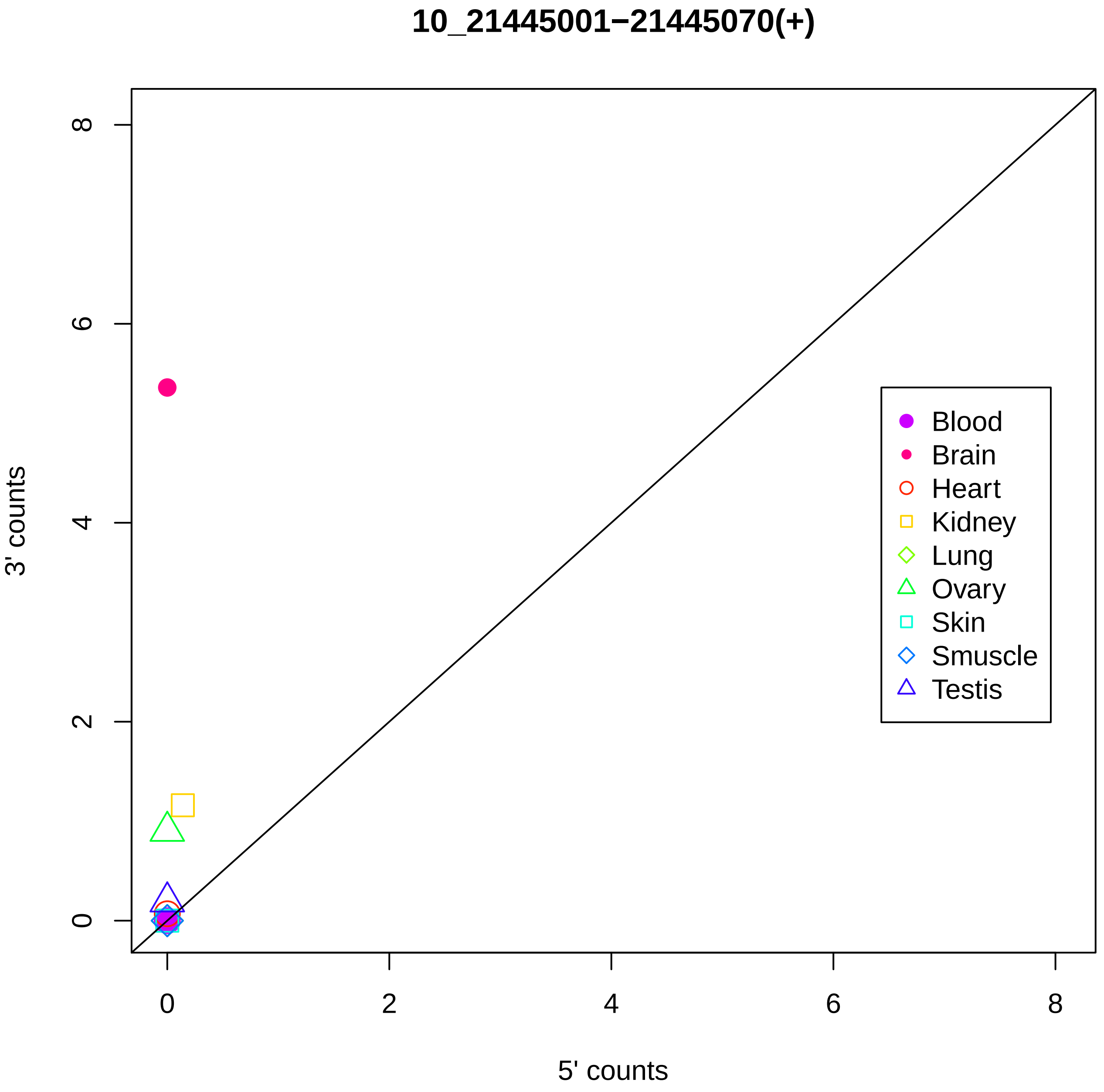

10_21445001−21445070(+)
3' counts
5' counts
0
2
4
6
8
Blood
Brain
Heart
Kidney
Lung
Ovary
Skin
Smuscle
Testis

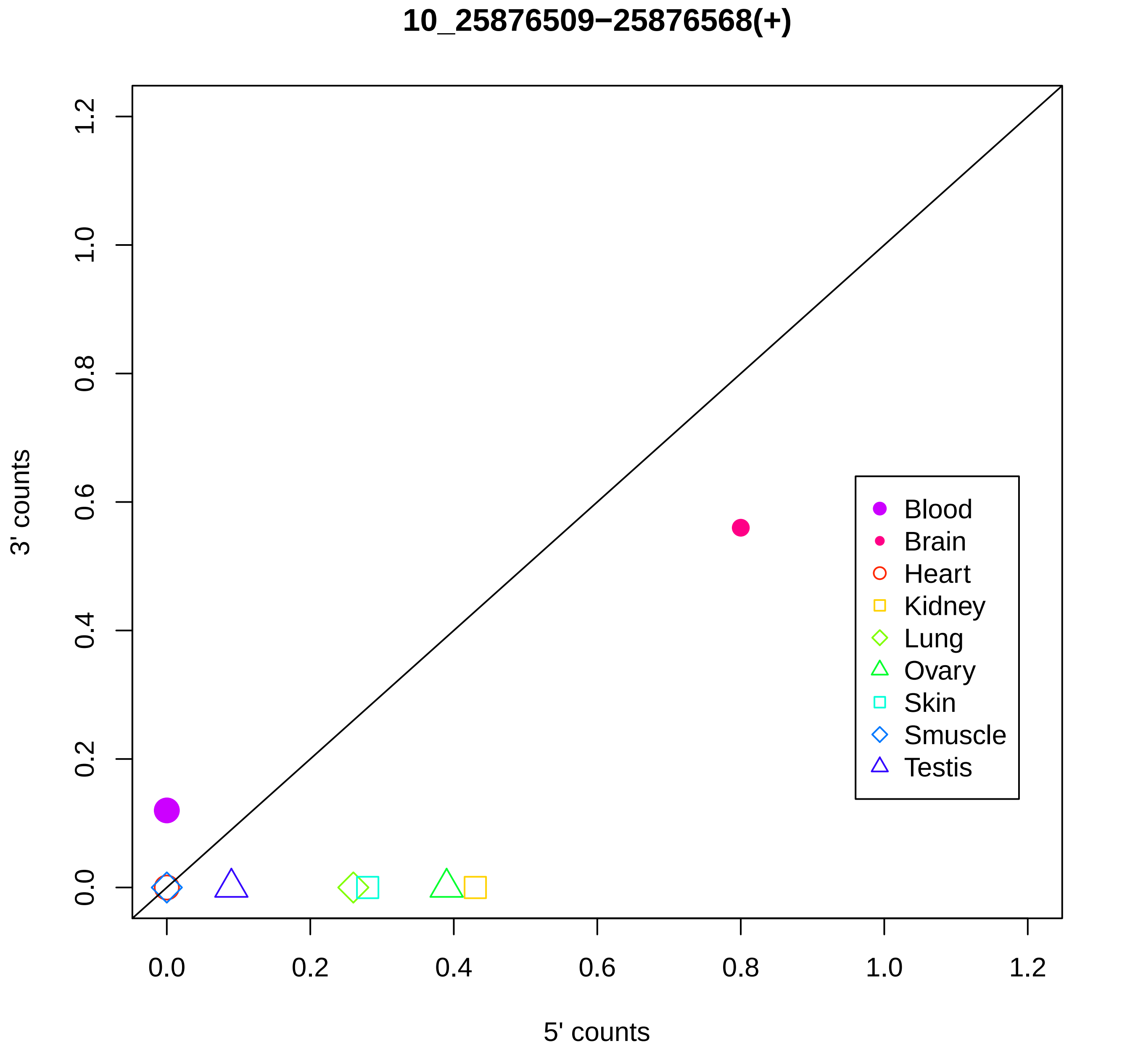

10_25876509−25876568(+)
3' counts
5' counts
0.0
0.2
0.4
0.6
0.8
1.0
1.2
Blood
Brain
Heart
Kidney
Lung
Ovary
Skin
Smuscle
Testis

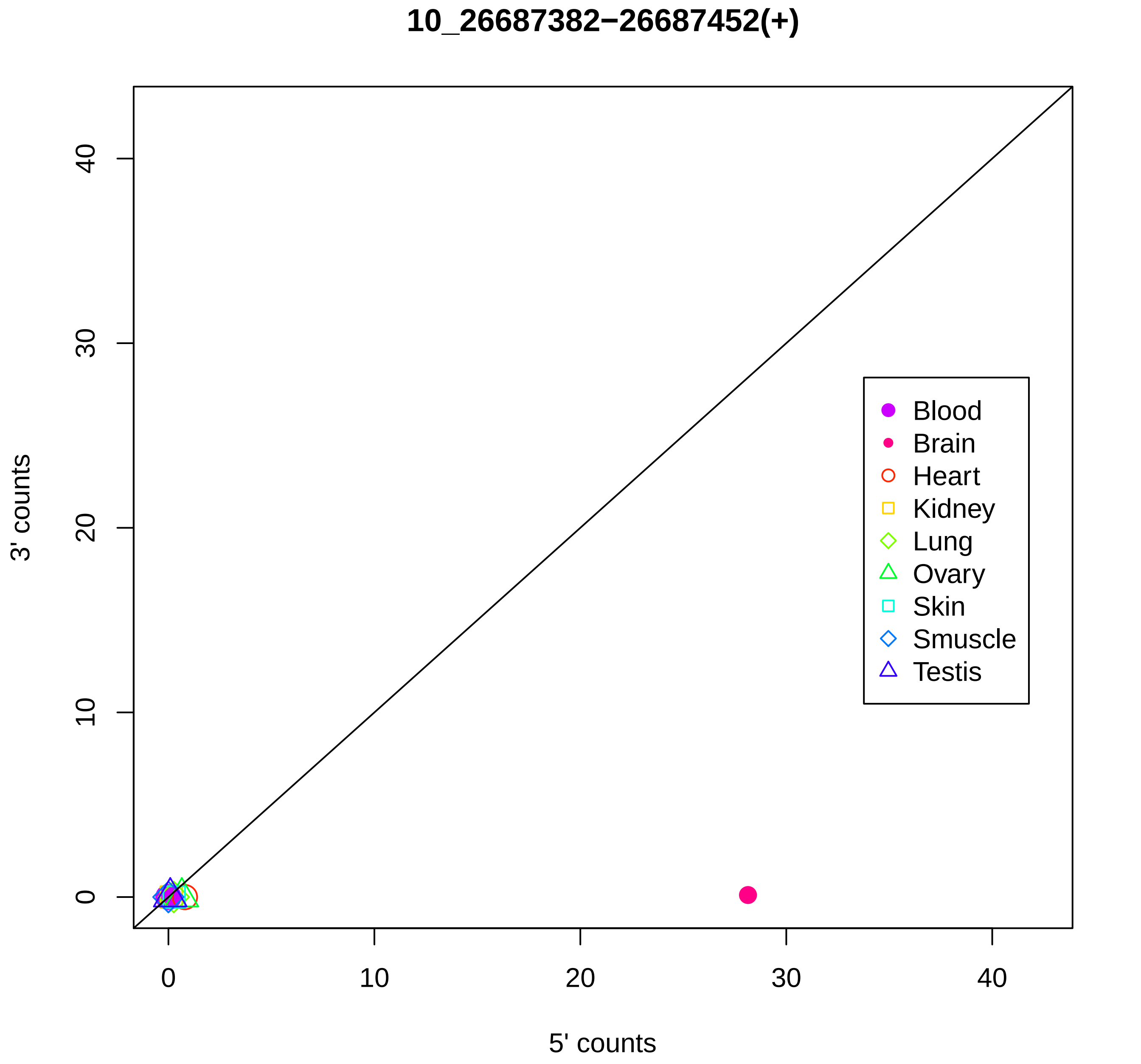

10_26687382−26687452(+)
3' counts
5' counts
0
10
20
30
40
0
10
20
30
40
Blood
Brain
Heart
Kidney
Lung
Ovary
Skin
Smuscle
Testis

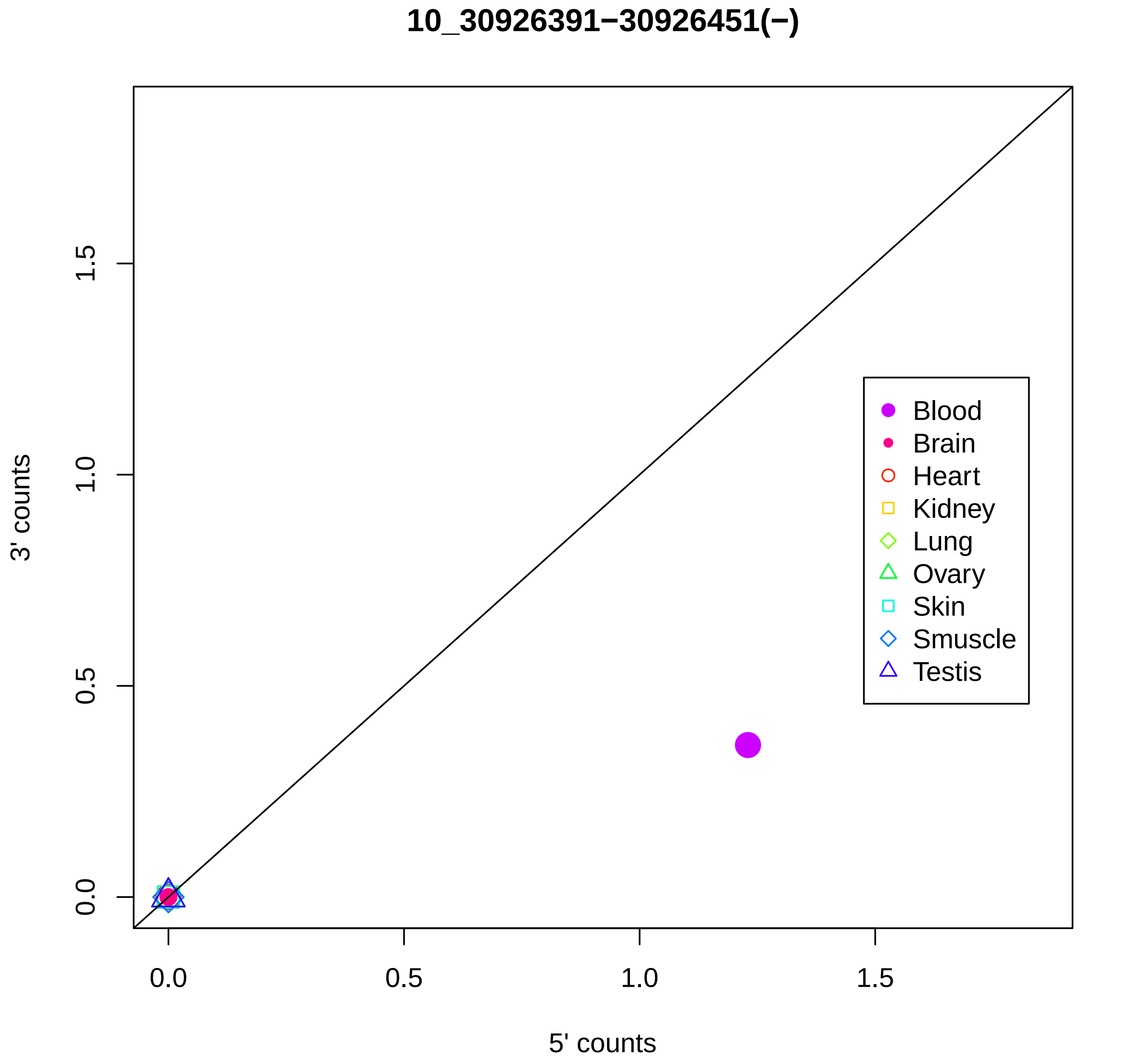

10_30926391−30926451(−)
3' counts
5' counts
0.0
0.5
1.0
1.5
Blood
Brain
Heart
Kidney
Lung
Ovary
Skin
Smuscle
Testis

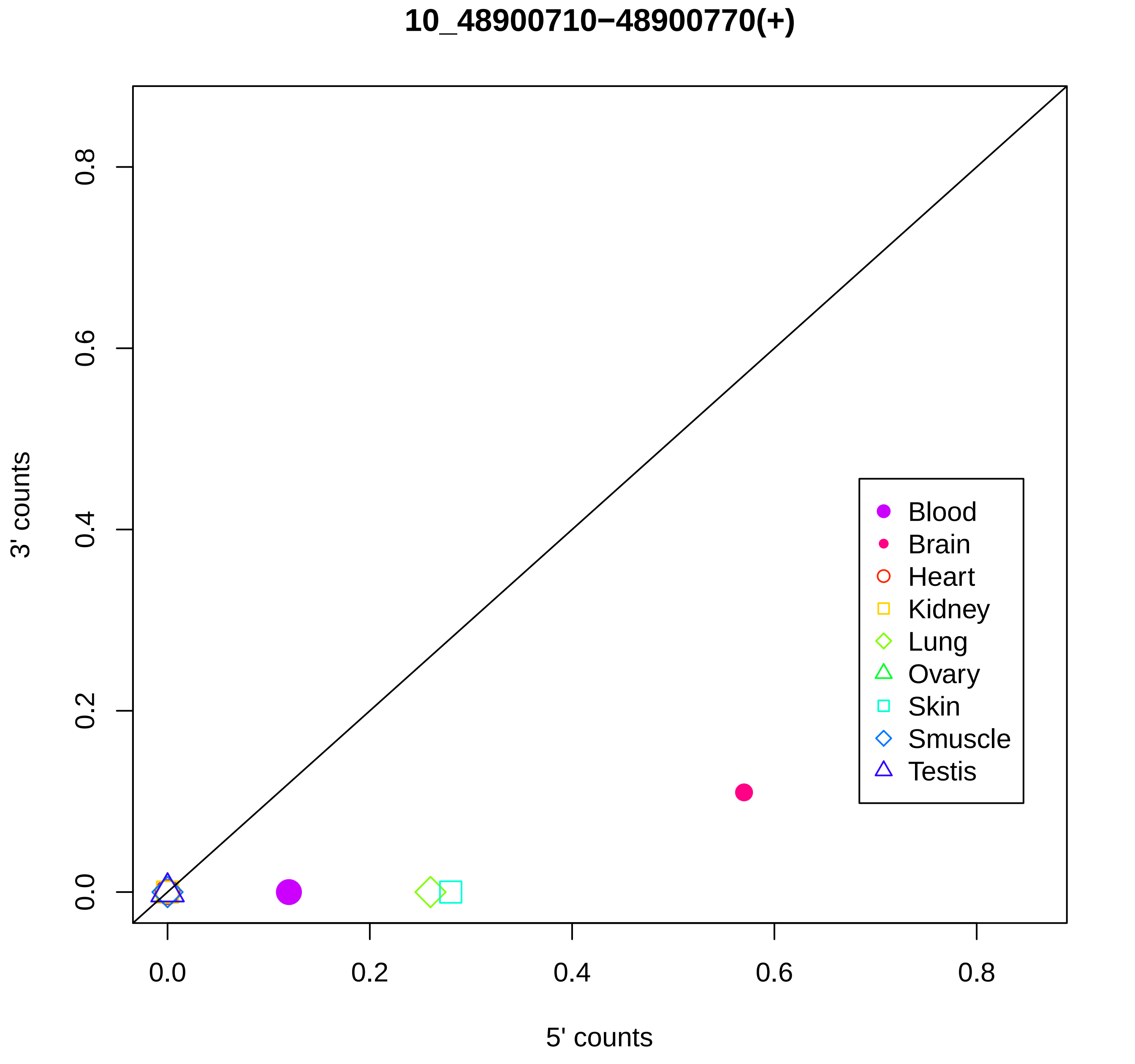
10_48900710−48900770(+)
0.0
0.2
0.4
0.6
0.8
3' counts
5' counts
Blood
Brain
Heart
Kidney
Lung
Ovary
Skin
Smuscle
Testis

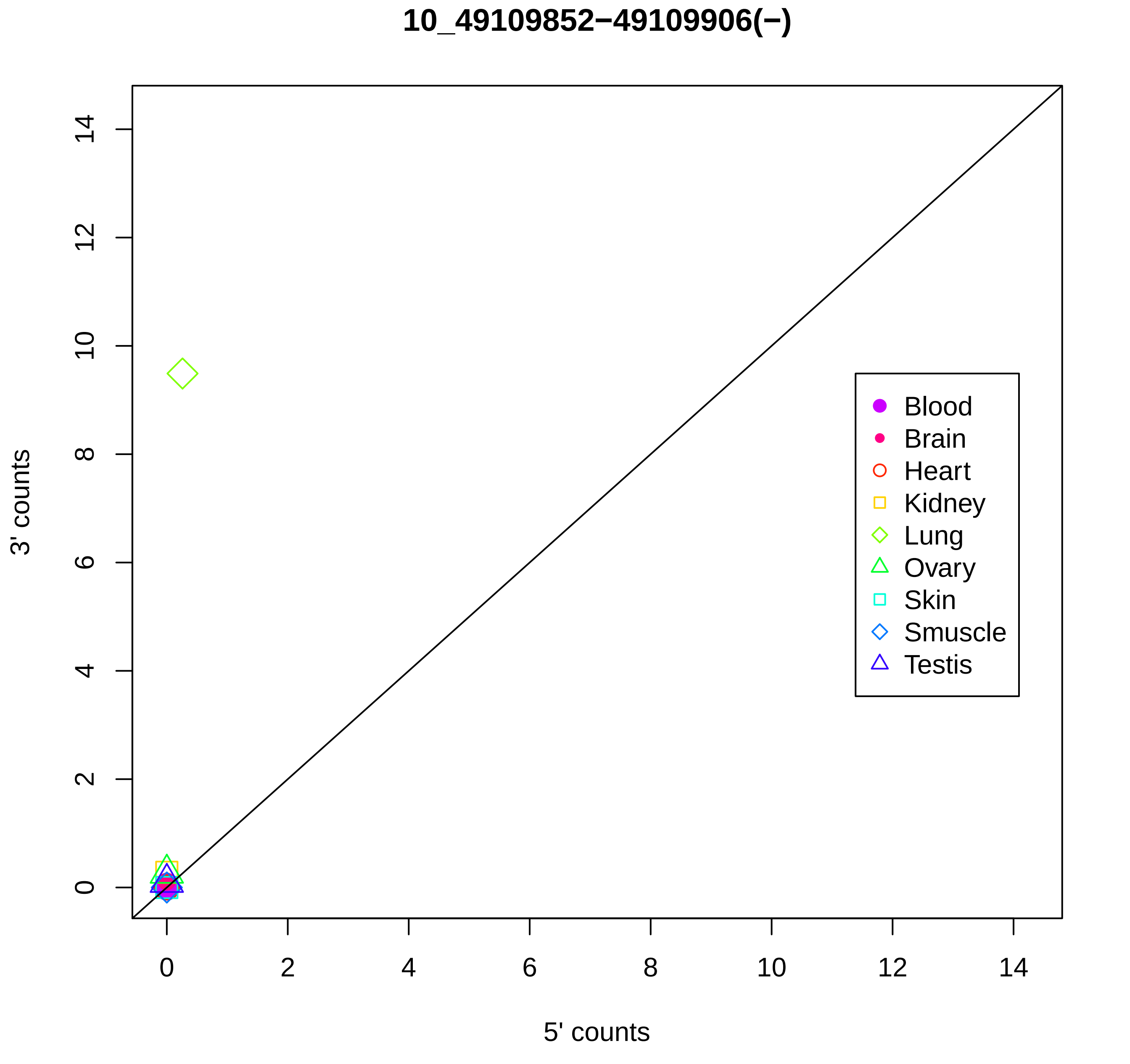

10_49109852−49109906(−)
3' counts
5' counts
0
2
4
6
8
10
12
14
Blood
Brain
Heart
Kidney
Lung
Ovary
Skin
Smuscle
Testis

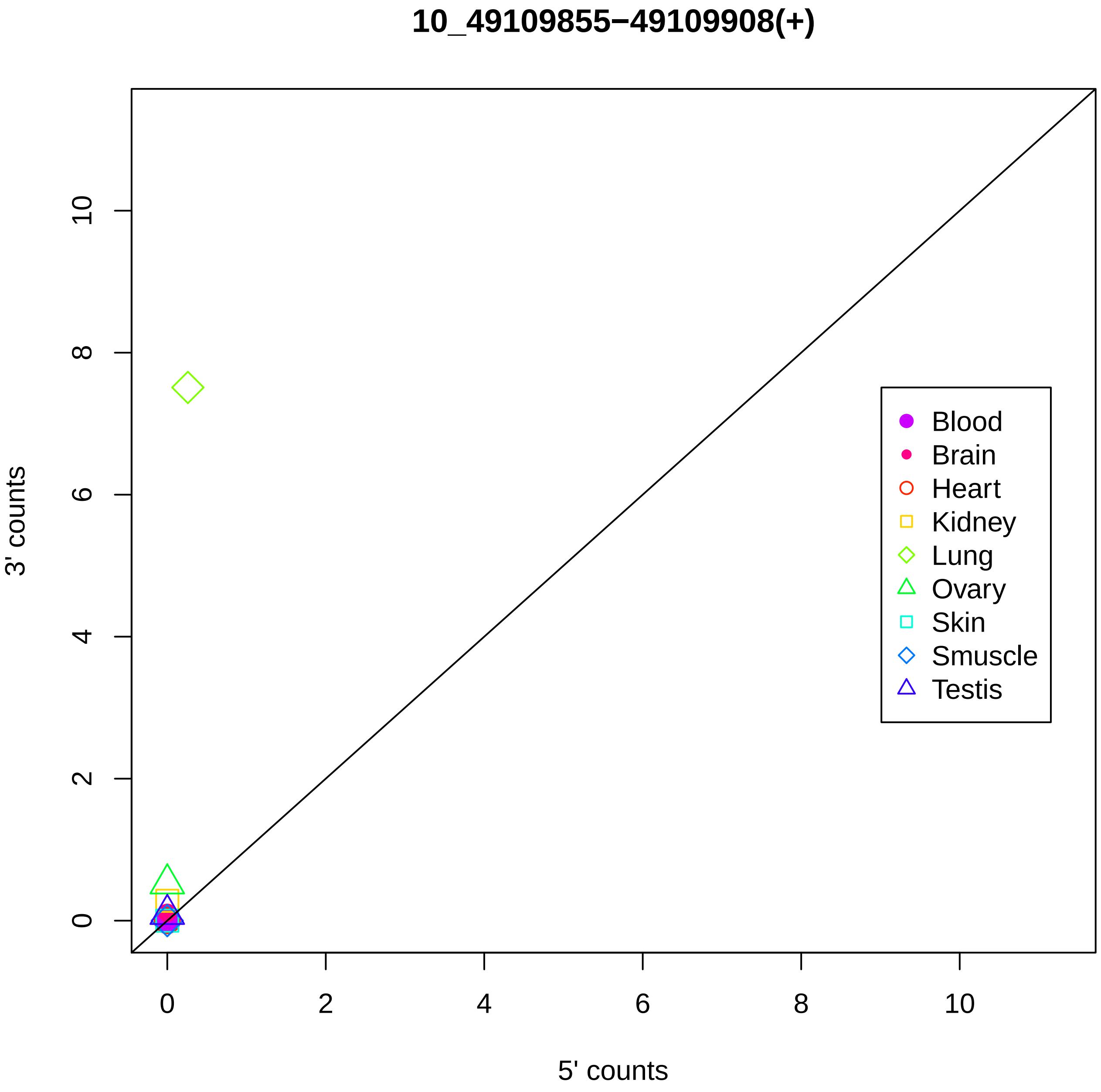
10_49109855−49109908(+)
3' counts
5' counts
0
2
4
6
8
10
Blood
Brain
Heart
Kidney
Lung
Ovary
Skin
Smuscle
Testis

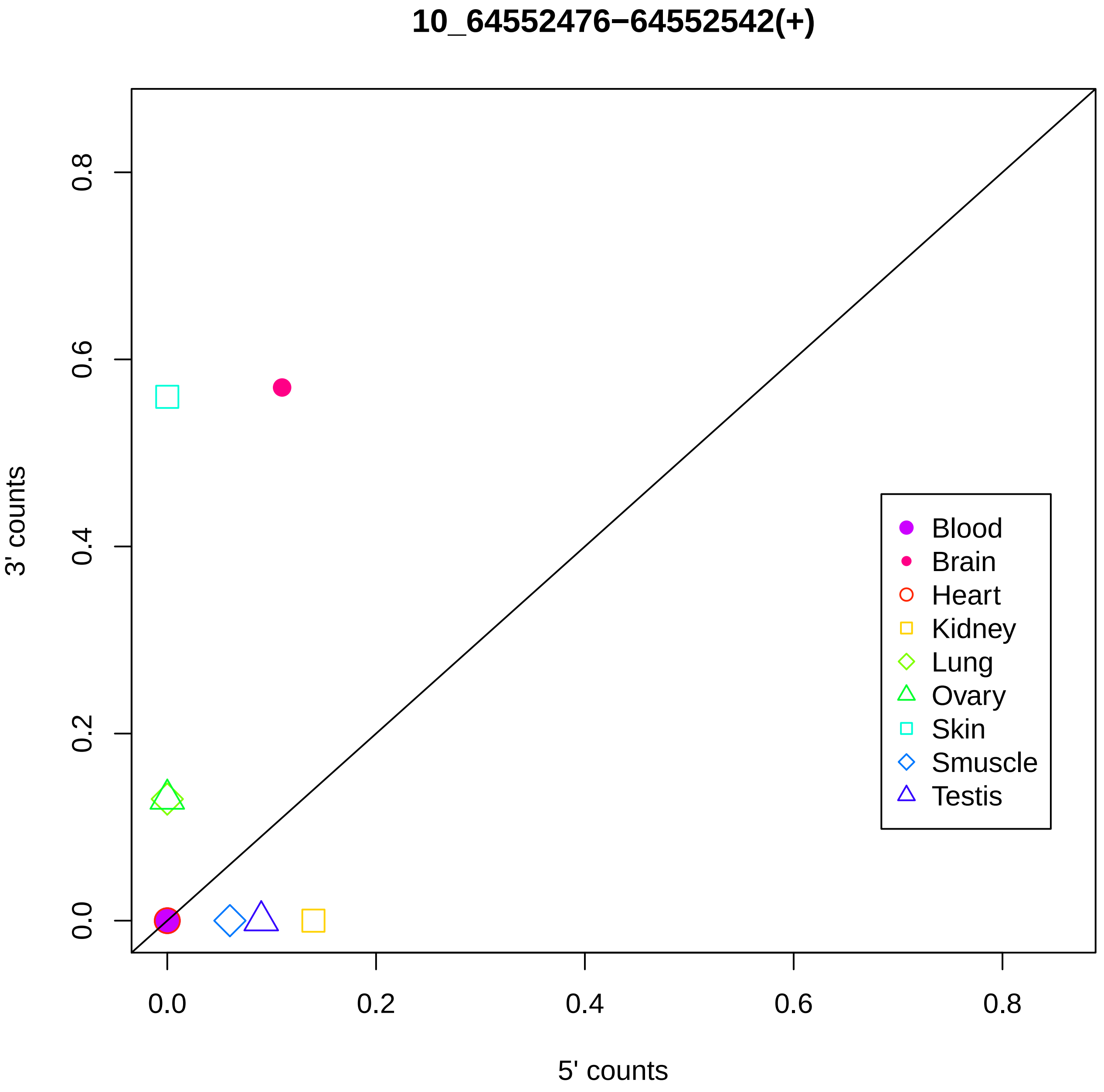

10_64552476−64552542(+)
3' counts
5' counts
0.0
0.2
0.4
0.6
0.8
Blood
Brain
Heart
Kidney
Lung
Ovary
Skin
Smuscle
Testis

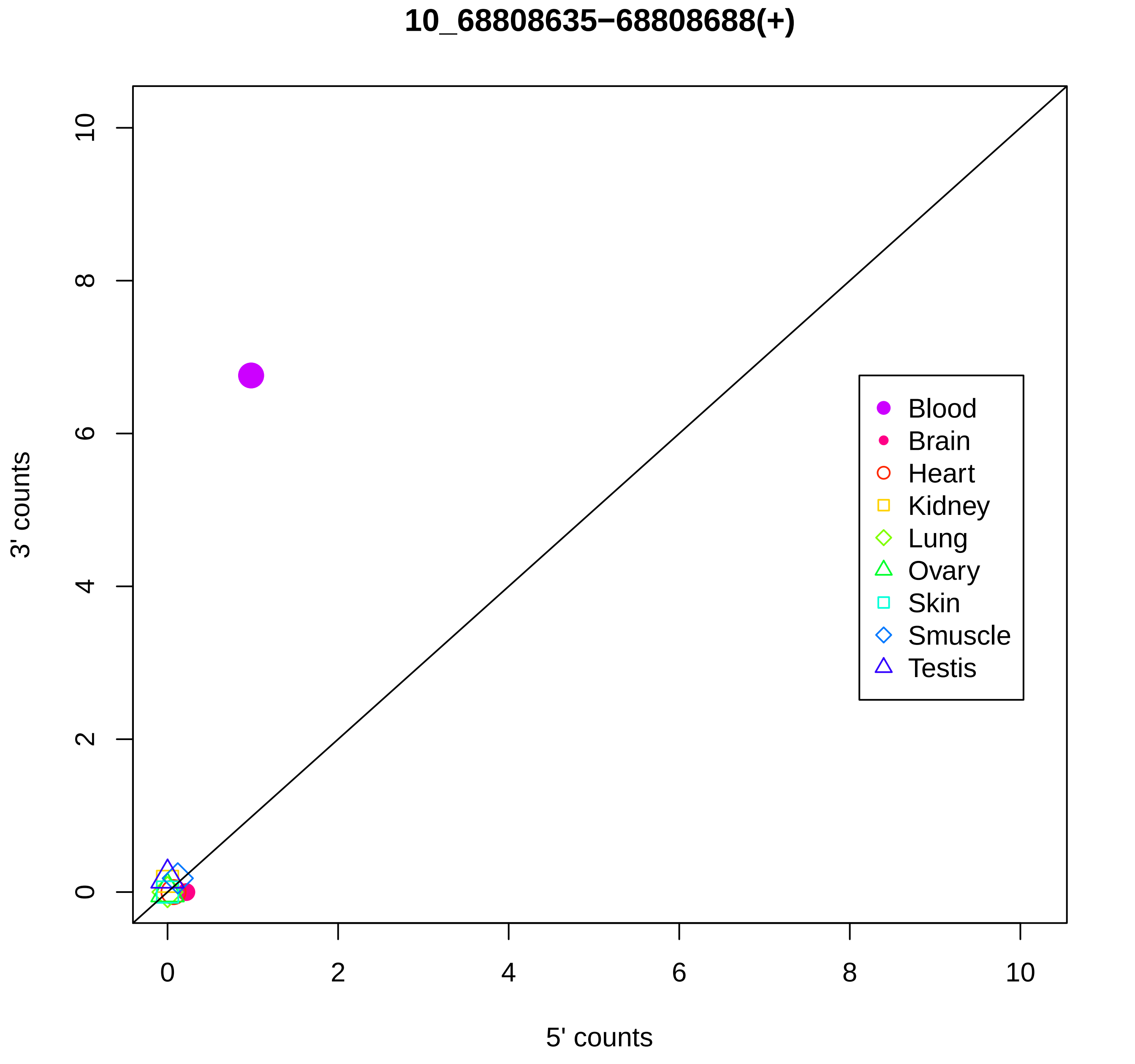

10_68808635−68808688(+)
3' counts
5' counts
0
2
4
6
8
10
Blood
Brain
Heart
Kidney
Lung
Ovary
Skin
Smuscle
Testis

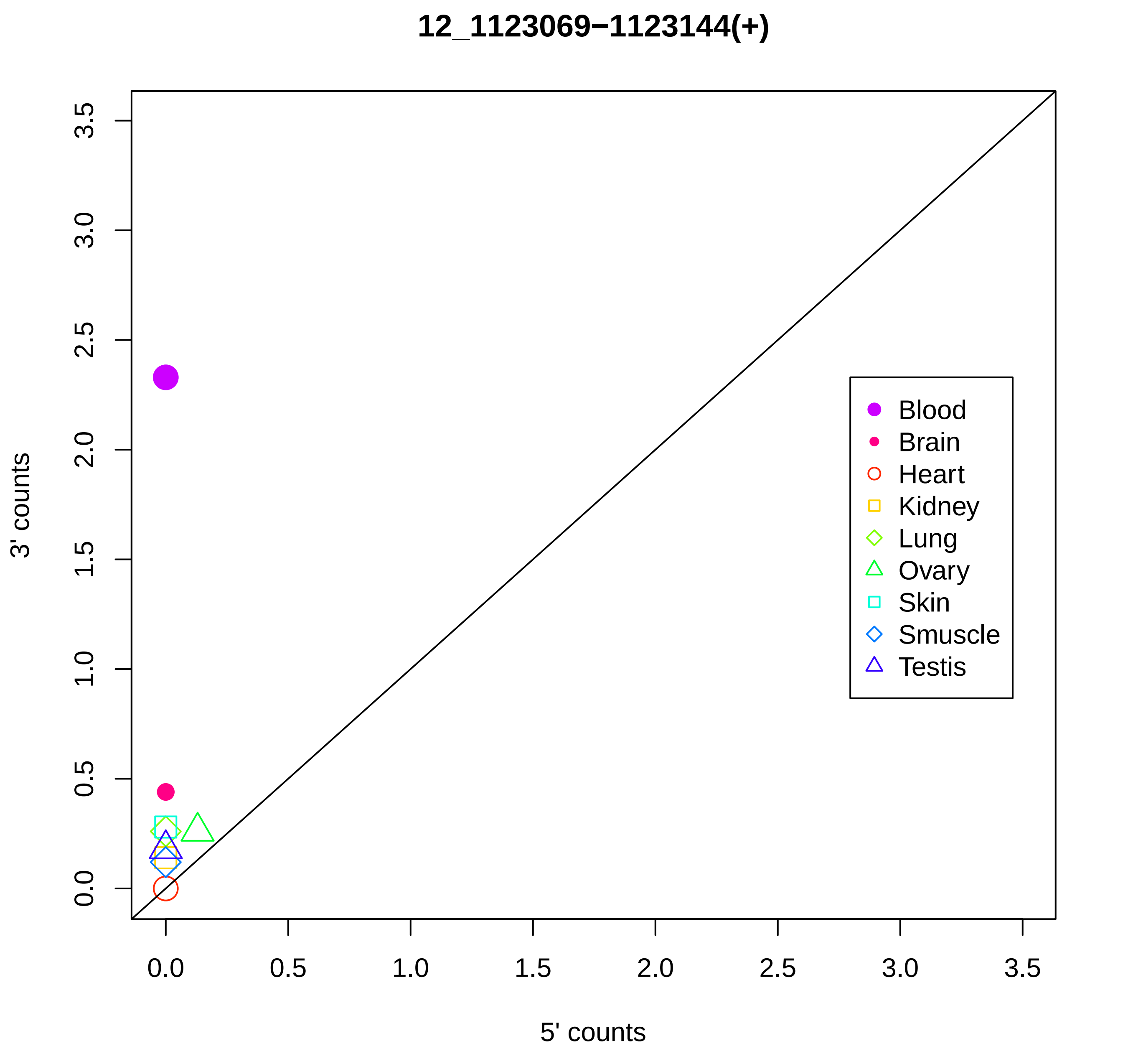

12_1123069−1123144(+)
3' counts
5' counts
0.0
0.5
1.0
1.5
2.0
2.5
3.0
3.5
Blood
Brain
Heart
Kidney
Lung
Ovary
Skin
Smuscle
Testis

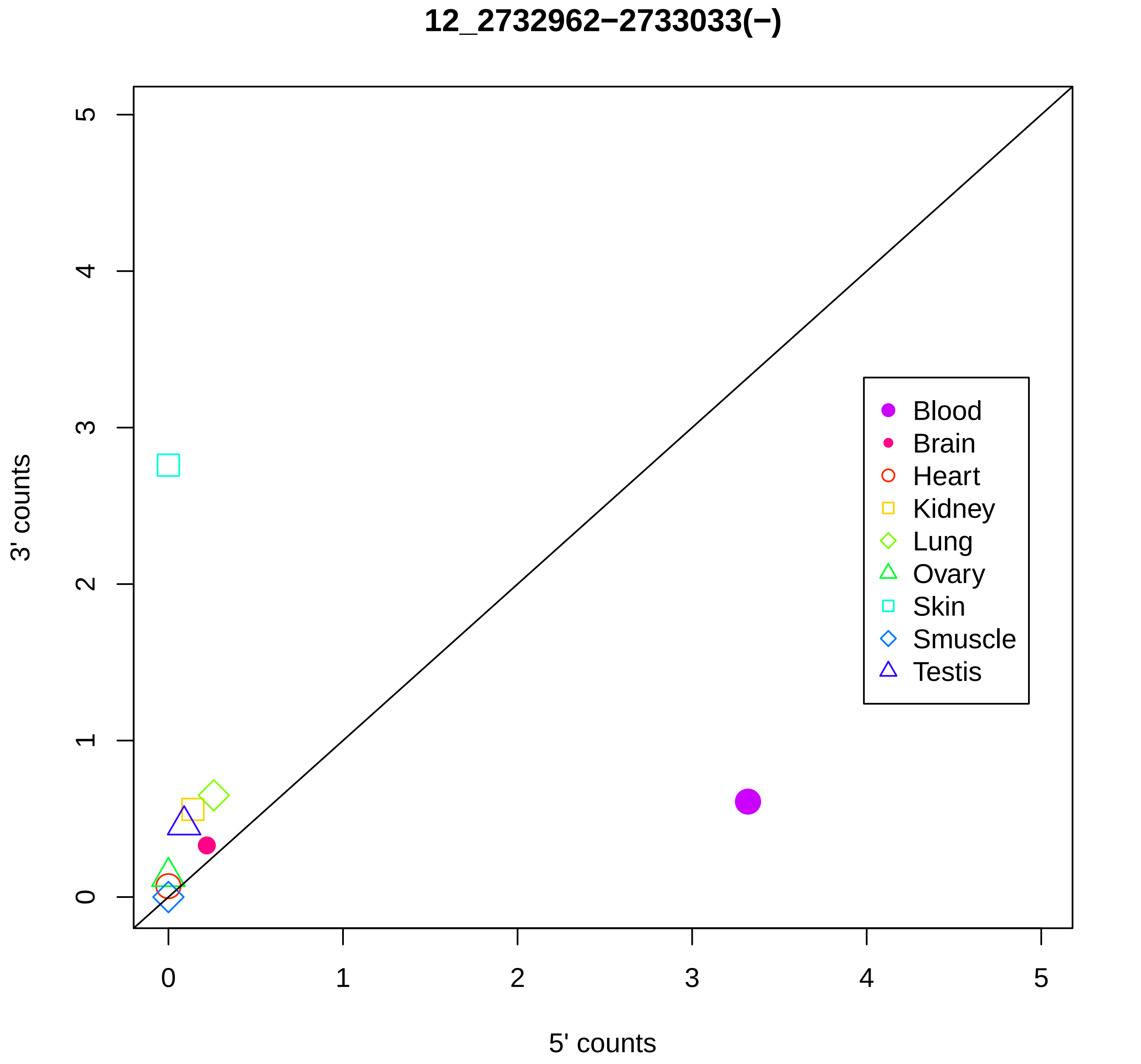

12_2732962−2733033(−)
3' counts
5' counts
0
1
2
3
4
5
Blood
Brain
Heart
Kidney
Lung
Ovary
Skin
Smuscle
Testis

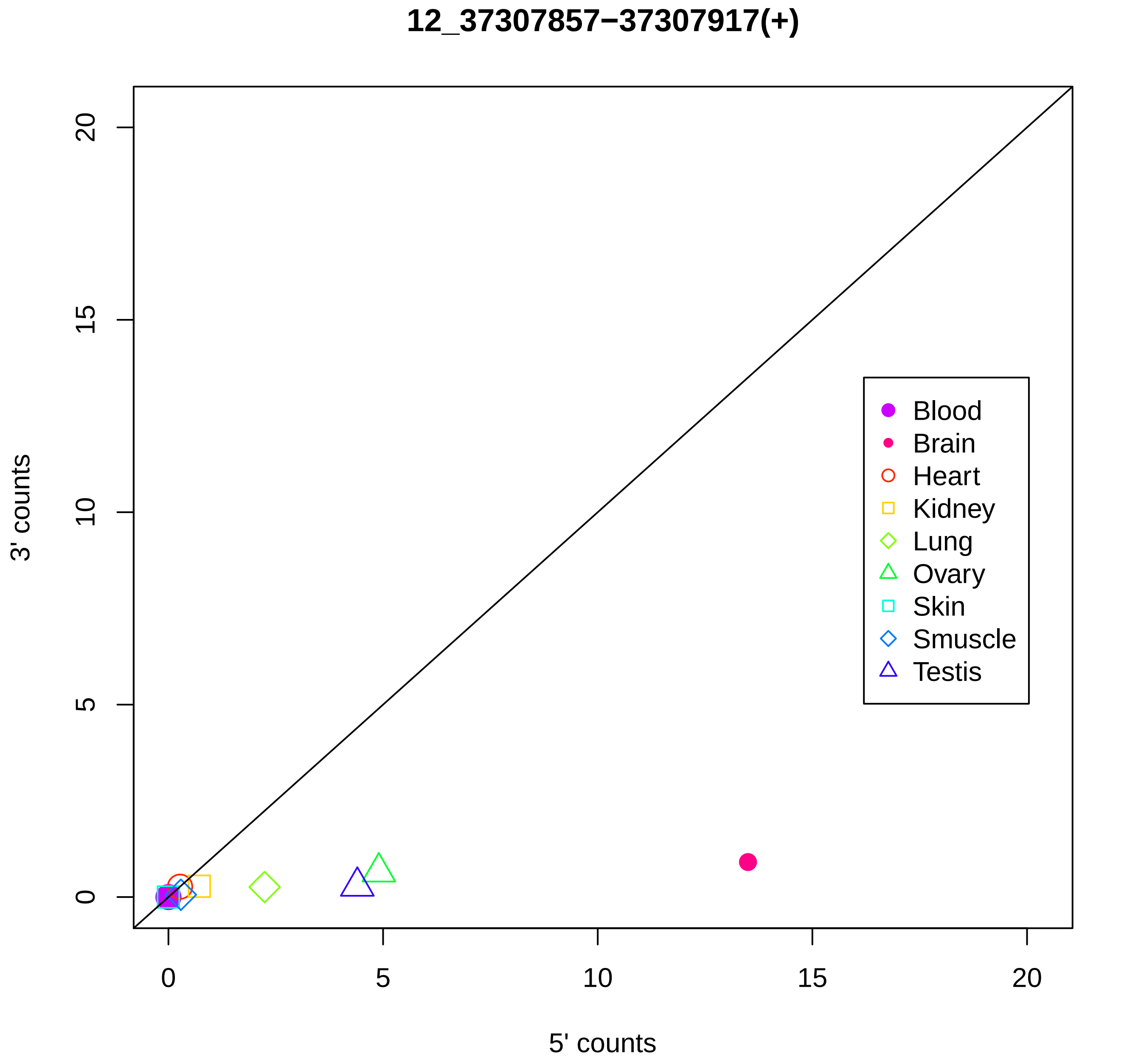

12_37307857−37307917(+)
3' counts
5' counts
0
5
10
15
20
0
5
10
15
20
Blood
Brain
Heart
Kidney
Lung
Ovary
Skin
Smuscle
Testis

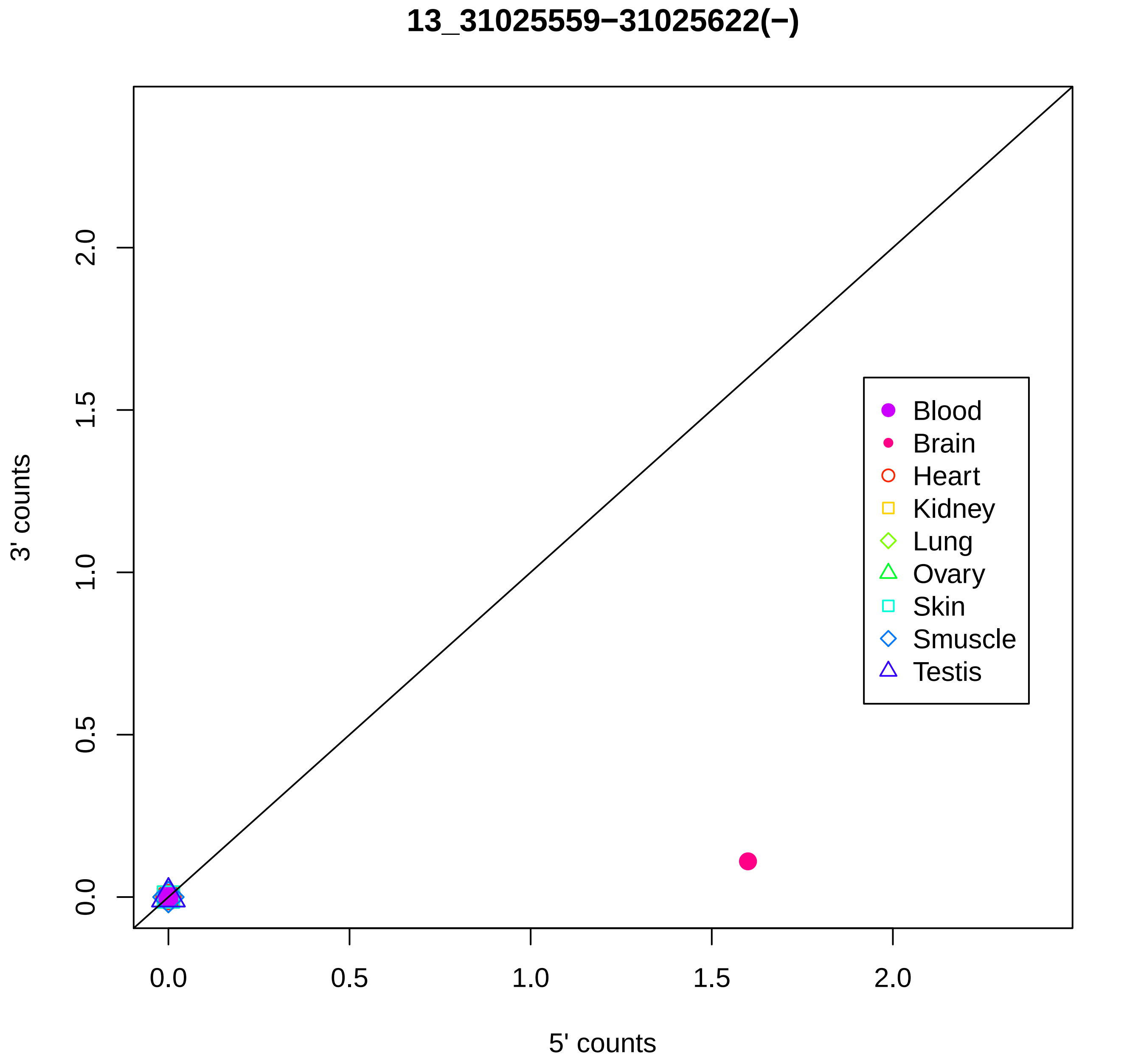

13_31025559−31025622(−)
3' counts
5' counts
0.0
0.5
1.0
1.5
2.0
Blood
Brain
Heart
Kidney
Lung
Ovary
Skin
Smuscle
Testis

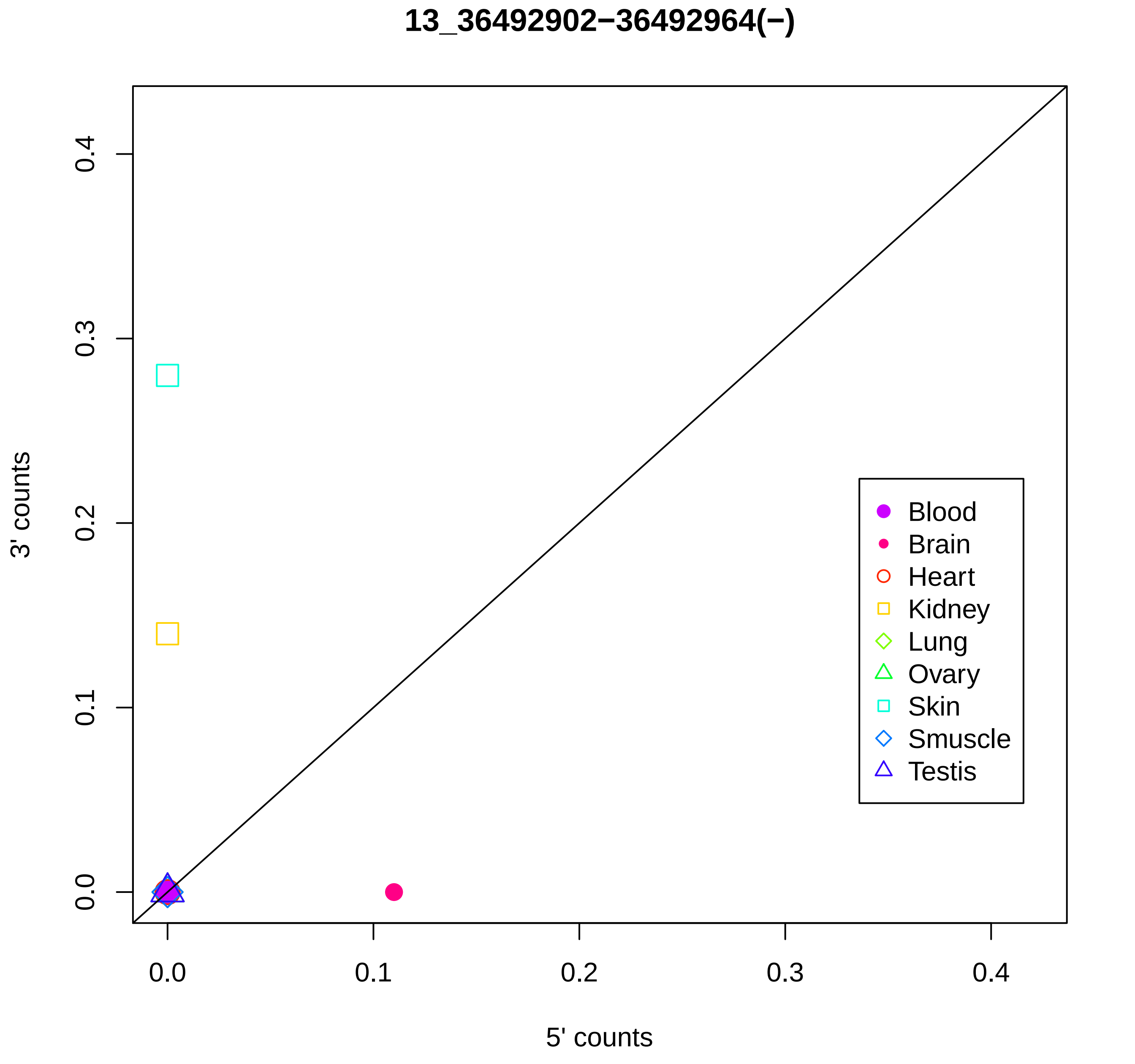

13_36492902−36492964(−)
3' counts
5' counts
0.0
0.1
0.2
0.3
0.4
Blood
Brain
Heart
Kidney
Lung
Ovary
Skin
Smuscle
Testis

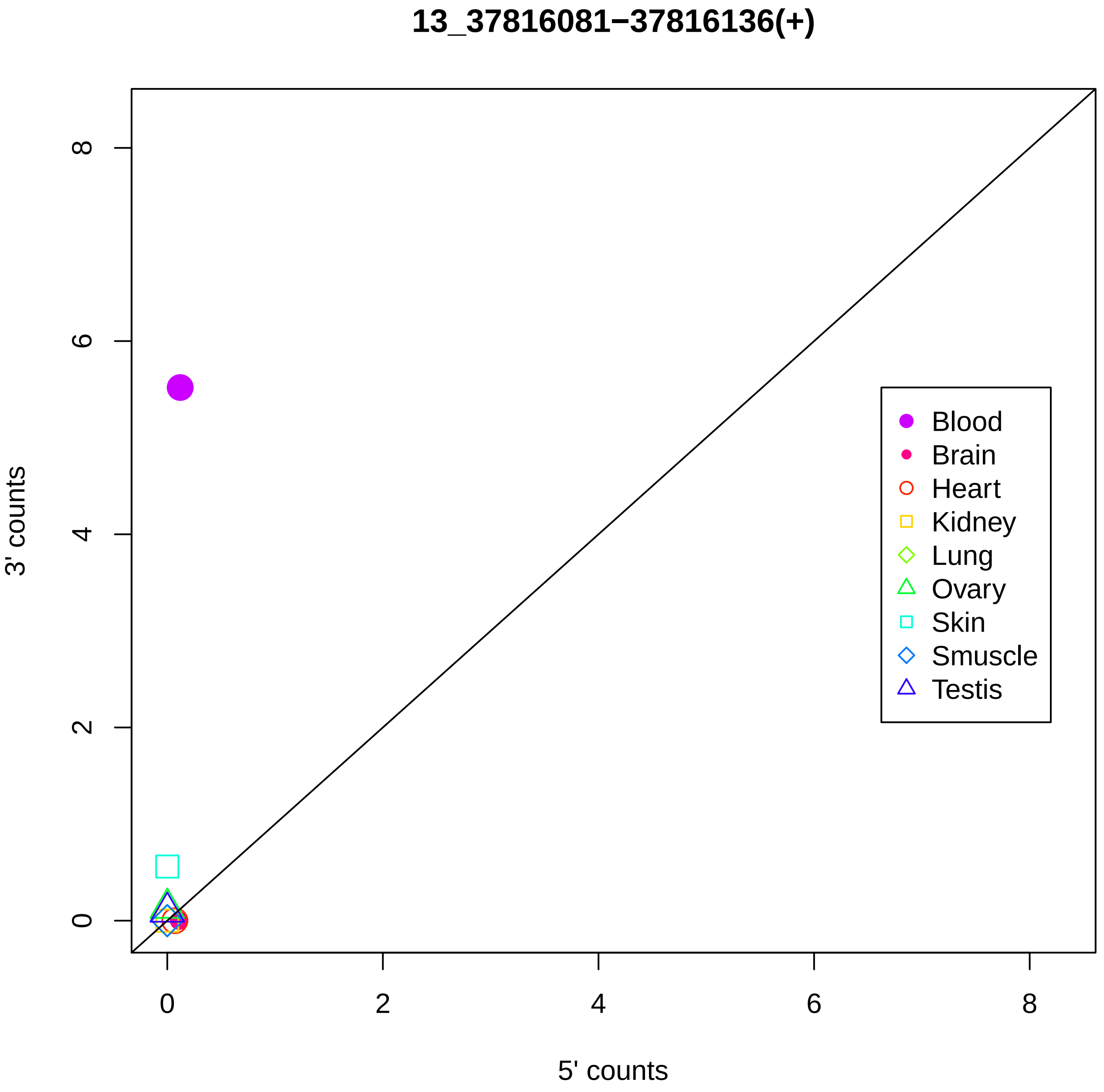
13_37816081−37816136(+)
Blood
Brain
Heart
Kidney
Lung
Ovary
Skin
Smuscle
Testis
3' counts
5' counts
0
2
4
6
8

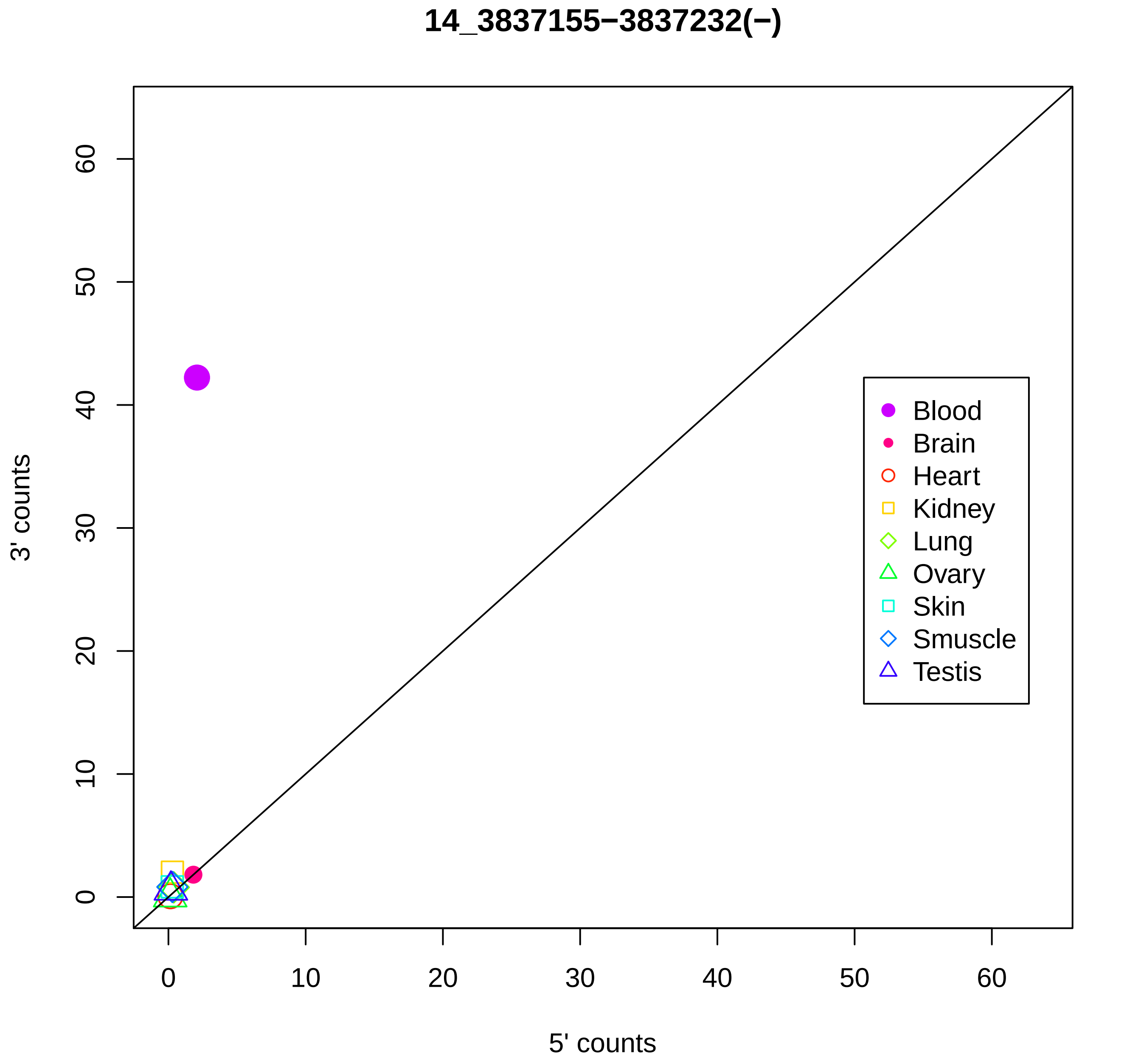

14_3837155−3837232(−)
3' counts
5' counts
0
10
20
30
40
50
60
Blood
Brain
Heart
Kidney
Lung
Ovary
Skin
Smuscle
Testis

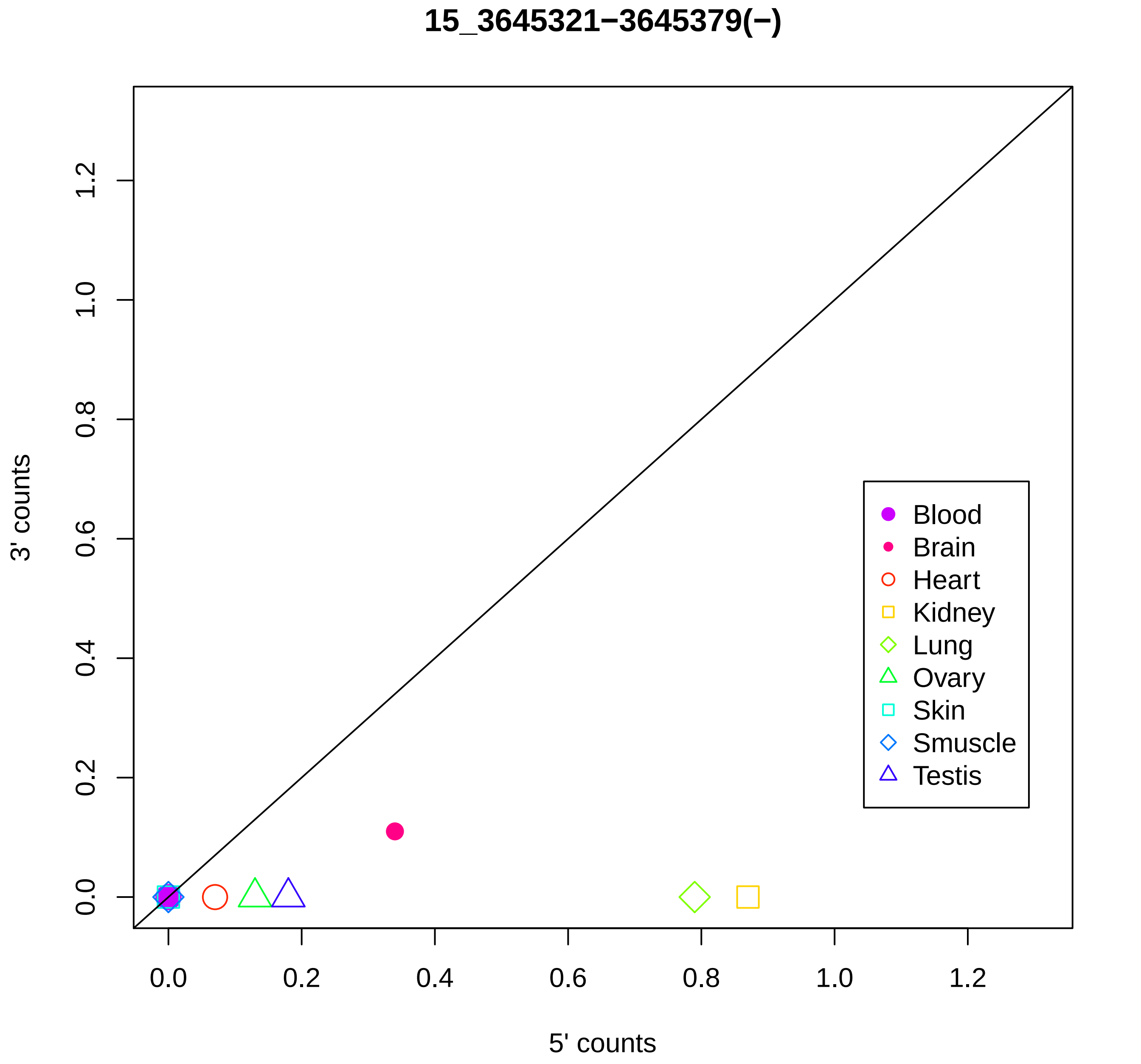

15_3645321−3645379(−)
Blood
Brain
Heart
Kidney
Lung
Ovary
Skin
Smuscle
Testis
0.0
0.2
0.4
0.6
0.8
1.0
1.2
5' counts
3' counts

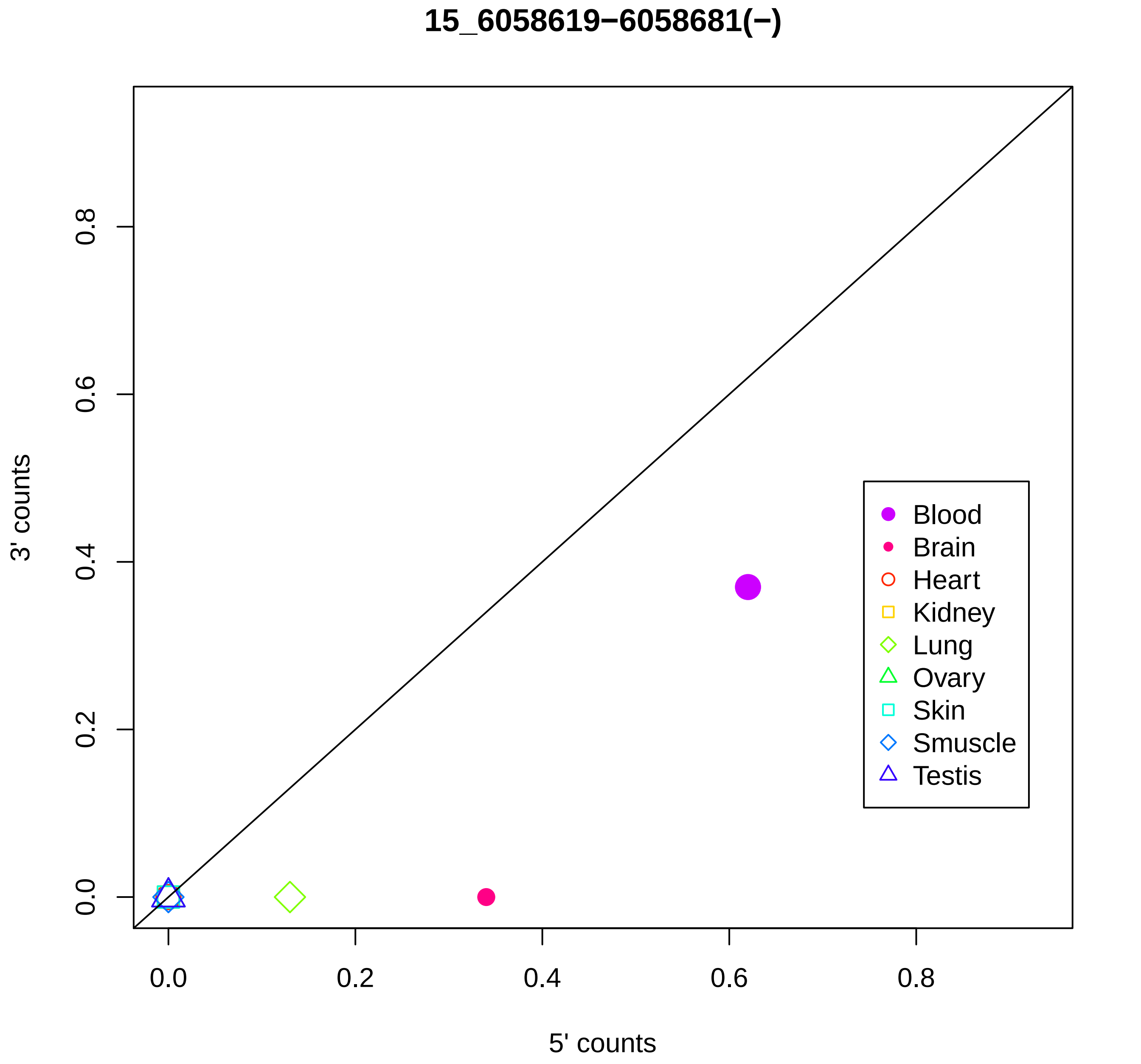

15_6058619−6058681(−)
3' counts
5' counts
0.0
0.2
0.4
0.6
0.8
Blood
Brain
Heart
Kidney
Lung
Ovary
Skin
Smuscle
Testis

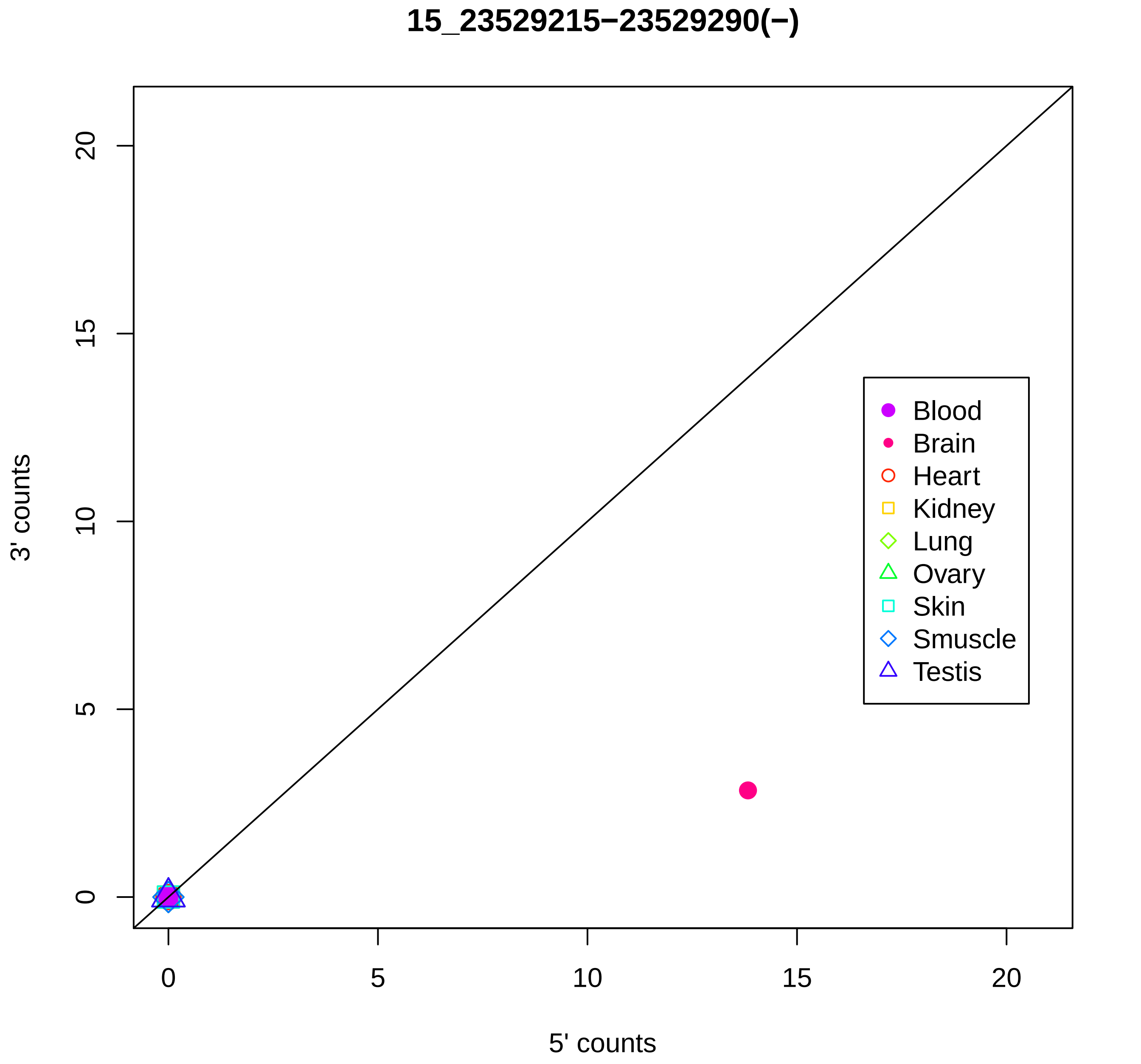

15_23529215−23529290(−)
3' counts
5' counts
0
5
10
15
20
Blood
Brain
Heart
Kidney
Lung
Ovary
Skin
Smuscle
Testis

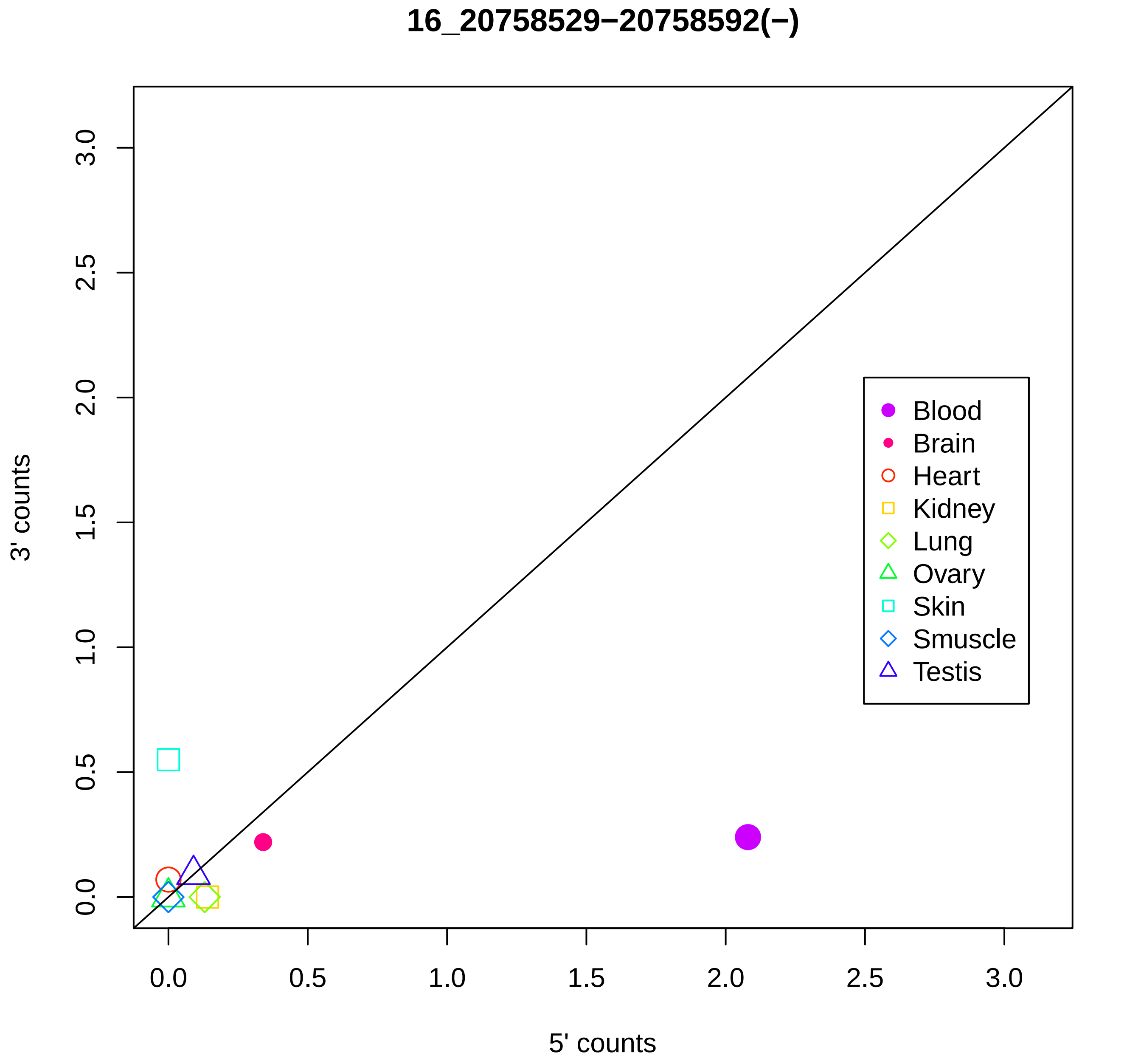
16_20758529−20758592(−)
3' counts
5' counts
0.0
0.5
1.0
1.5
2.0
2.5
3.0
Blood
Brain
Heart
Kidney
Lung
Ovary
Skin
Smuscle
Testis

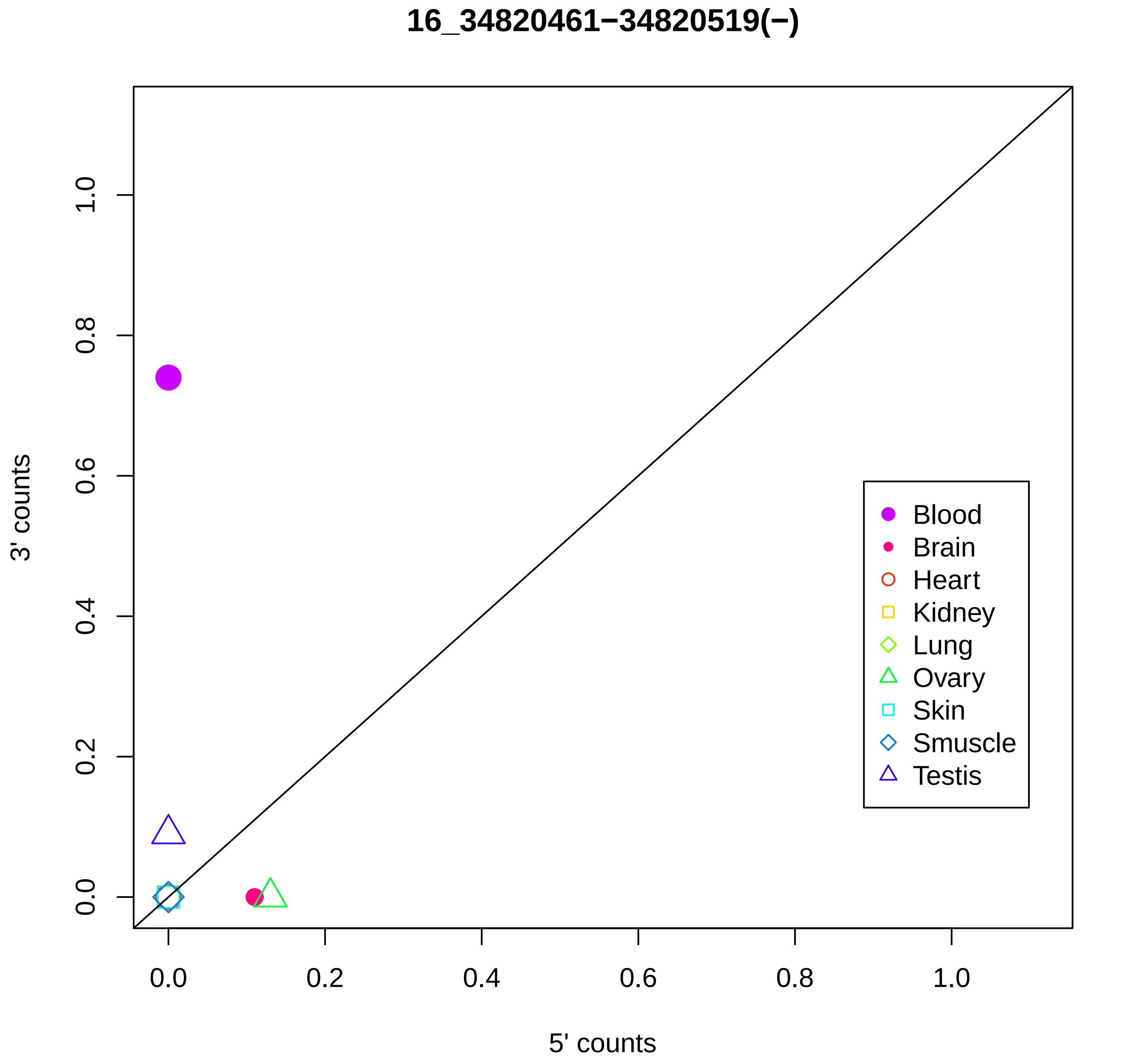
16_34820461−34820519(−)
3' counts
5' counts
0.0
0.2
0.4
0.6
0.8
1.0
Blood
Brain
Heart
Kidney
Lung
Ovary
Skin
Smuscle
Testis

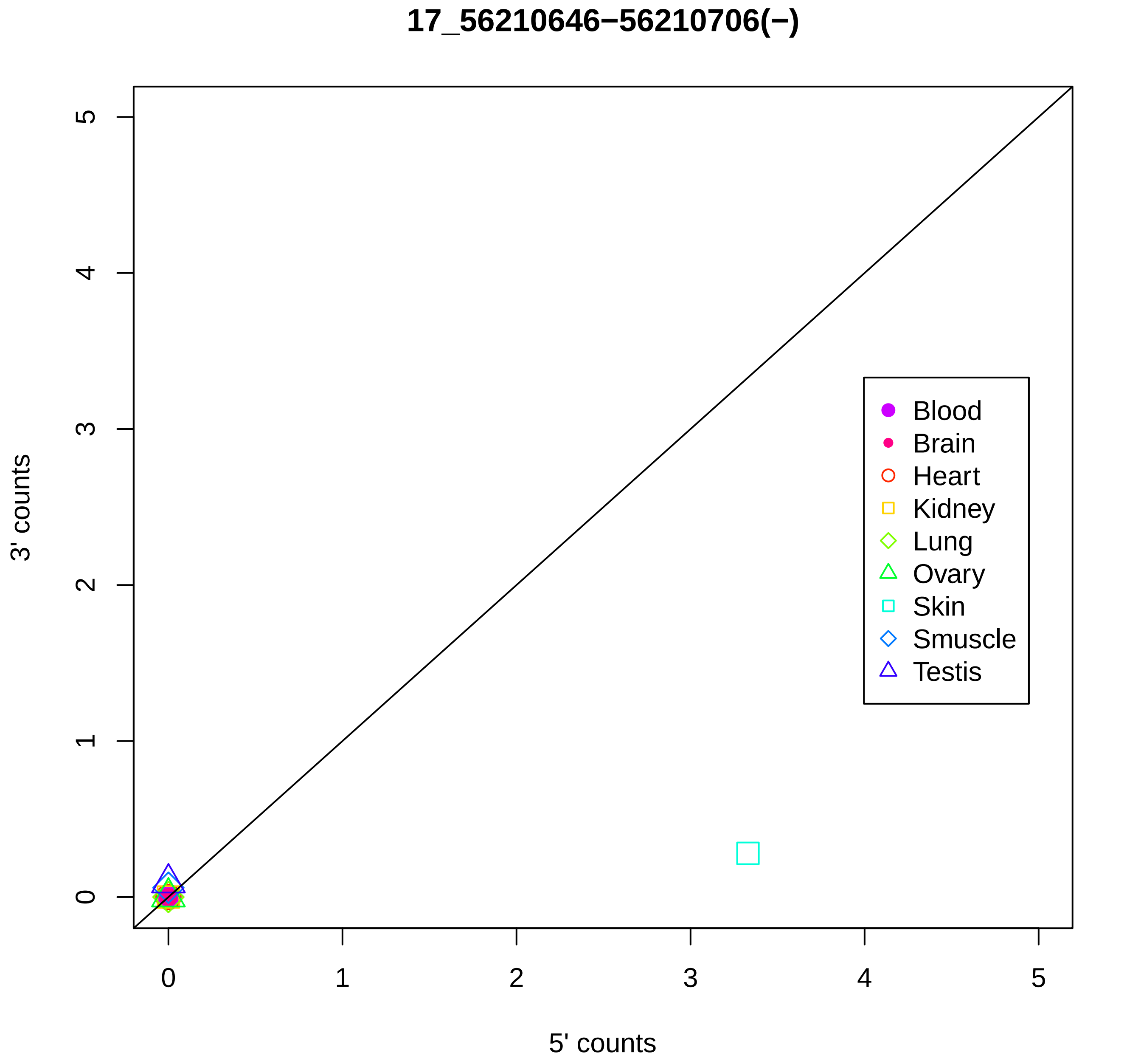

17_56210646−56210706(−)
3' counts
5' counts
0
1
2
3
4
5
Blood
Brain
Heart
Kidney
Lung
Ovary
Skin
Smuscle
Testis

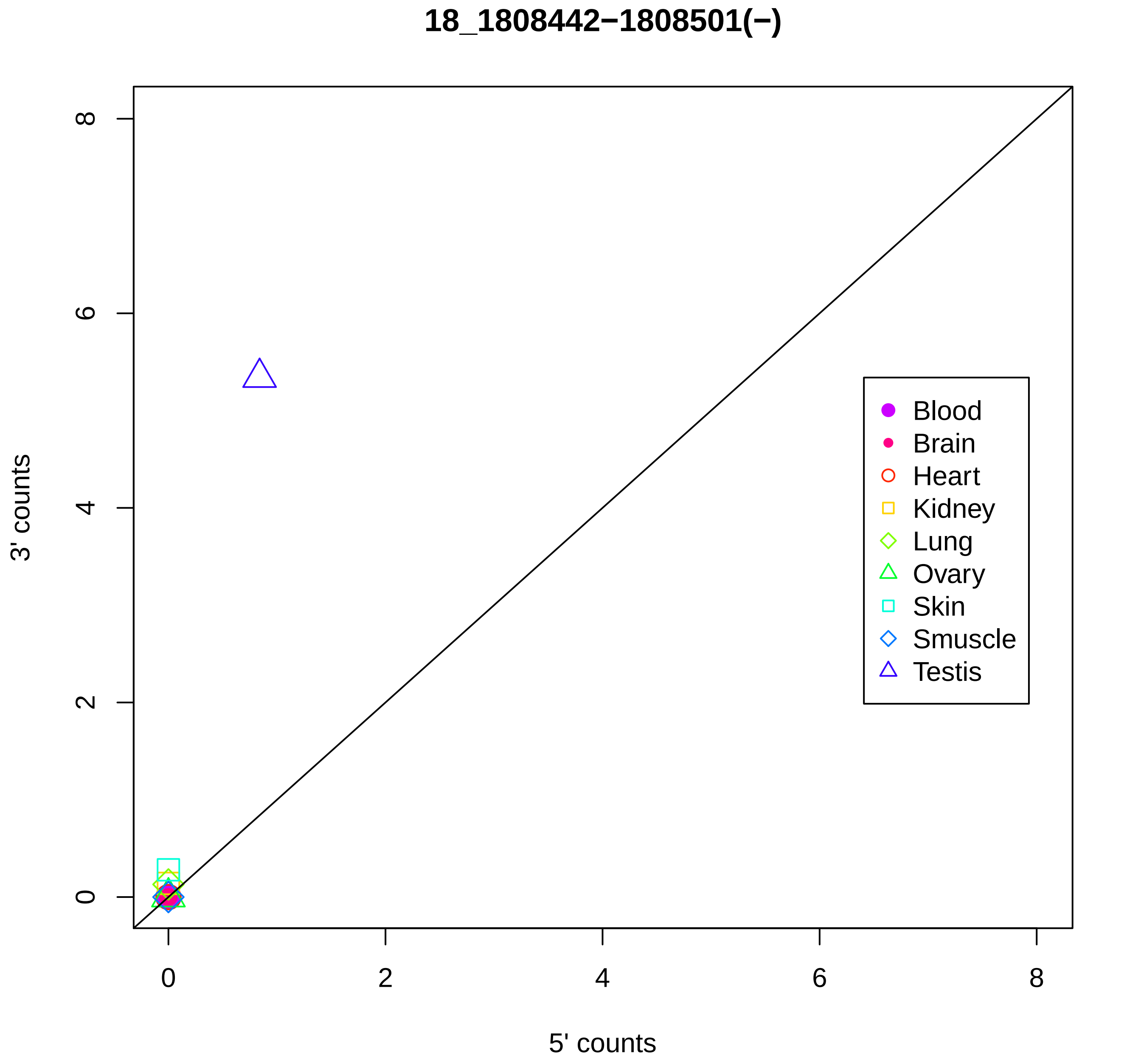
18_1808442−1808501(−)
3' counts
5' counts
0
2
4
6
8
0
2
4
6
8
Blood
Brain
Heart
Kidney
Lung
Ovary
Skin
Smuscle
Testis

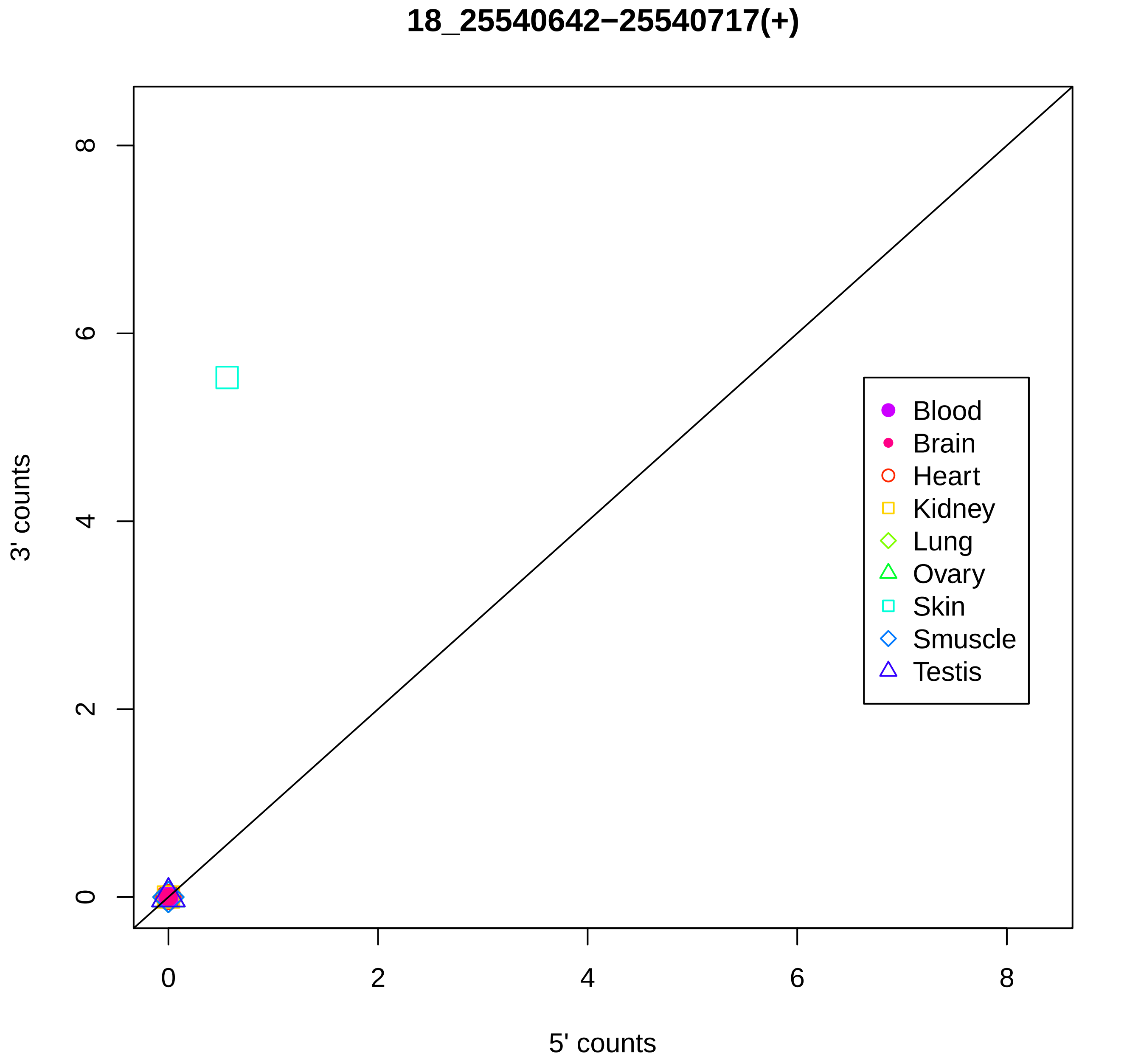

18_25540642−25540717(+)
3' counts
5' counts
0
2
4
6
8
0
2
4
6
8
Blood
Brain
Heart
Kidney
Lung
Ovary
Skin
Smuscle
Testis

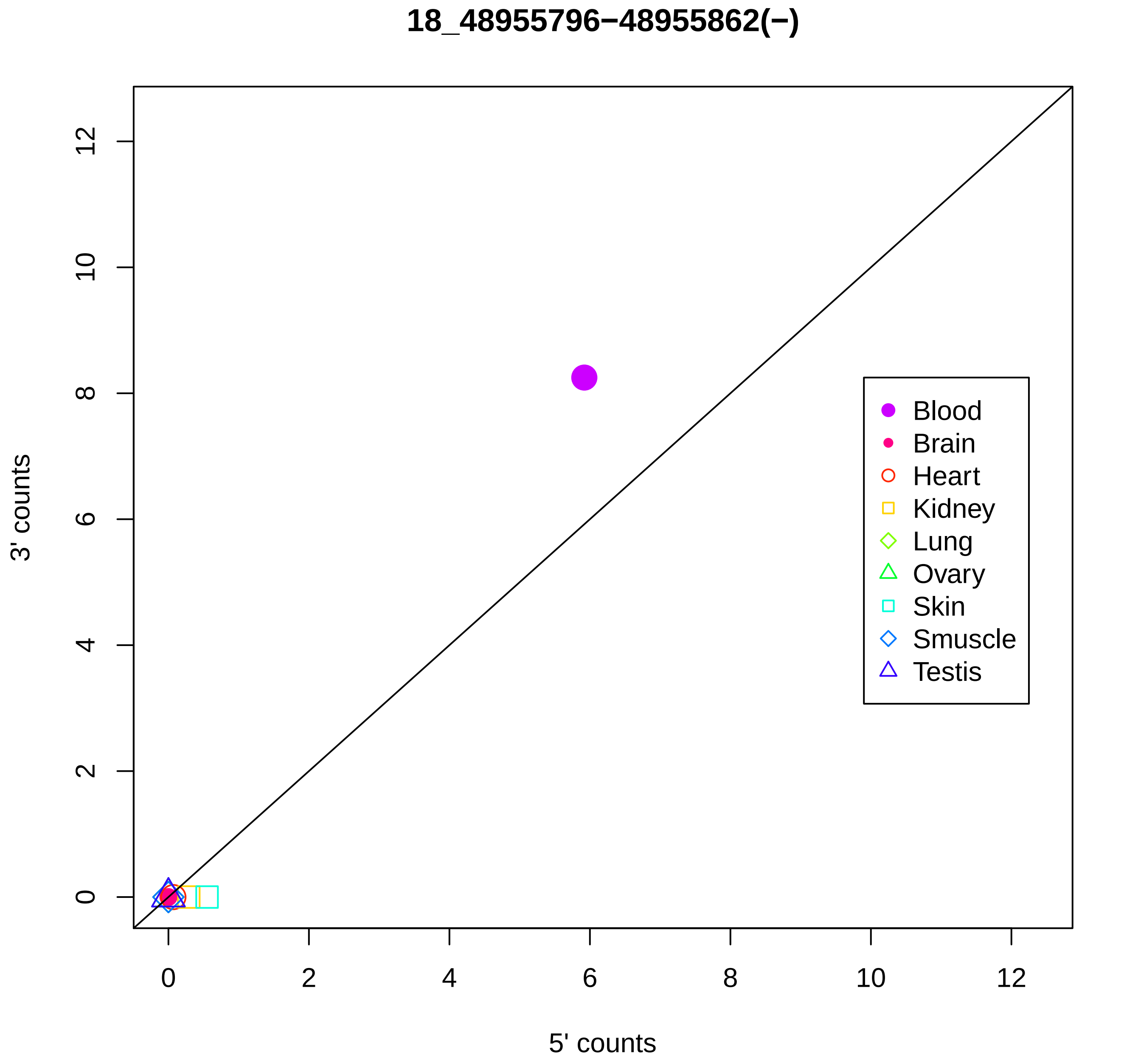
18_48955796−48955862(−)
3' counts
5' counts
0
2
4
6
8
10
12
Blood
Brain
Heart
Kidney
Lung
Ovary
Skin
Smuscle
Testis

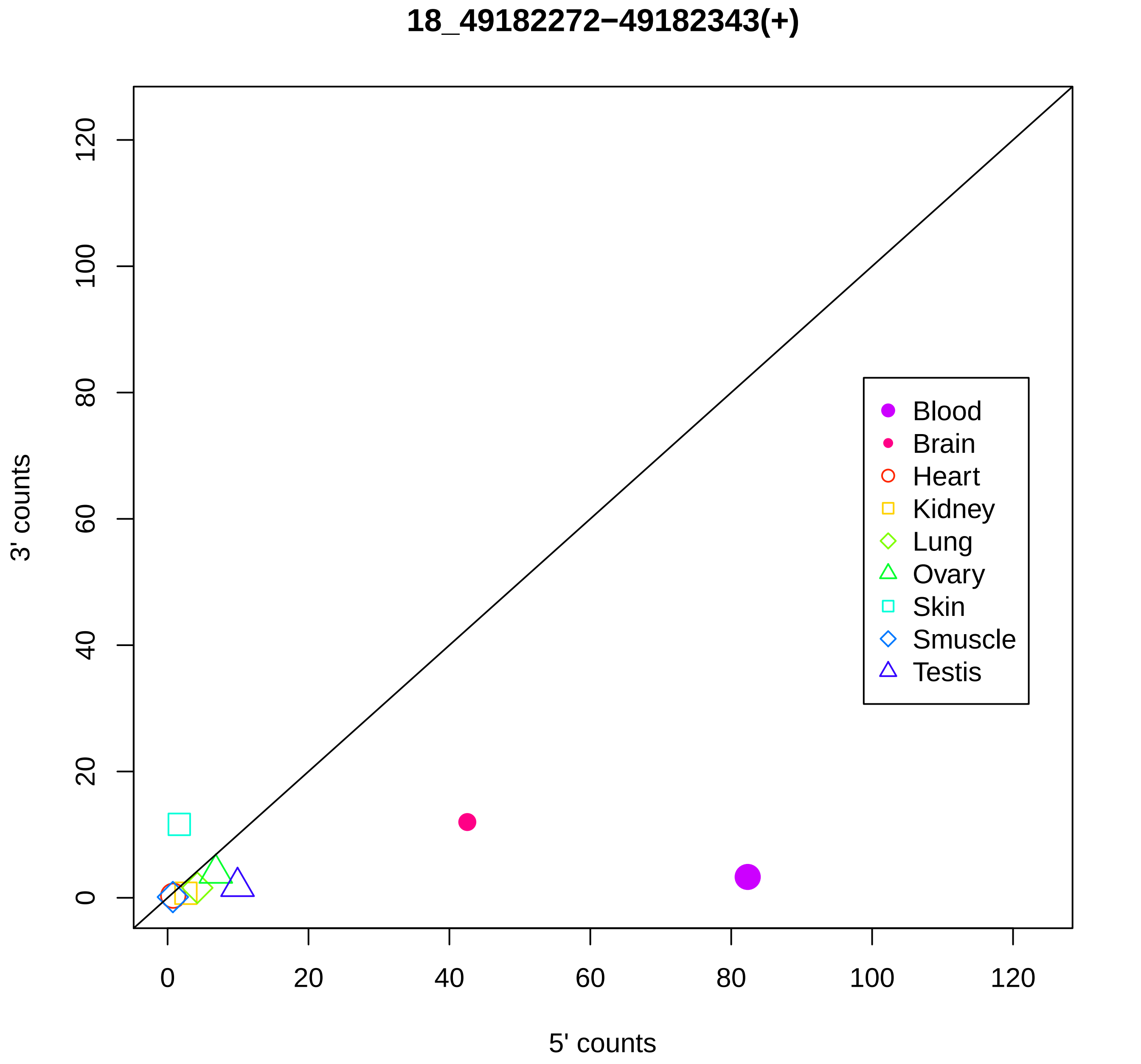

18_49182272−49182343(+)
3' counts
5' counts
0
20
40
60
80
100
120
Blood
Brain
Heart
Kidney
Lung
Ovary
Skin
Smuscle
Testis

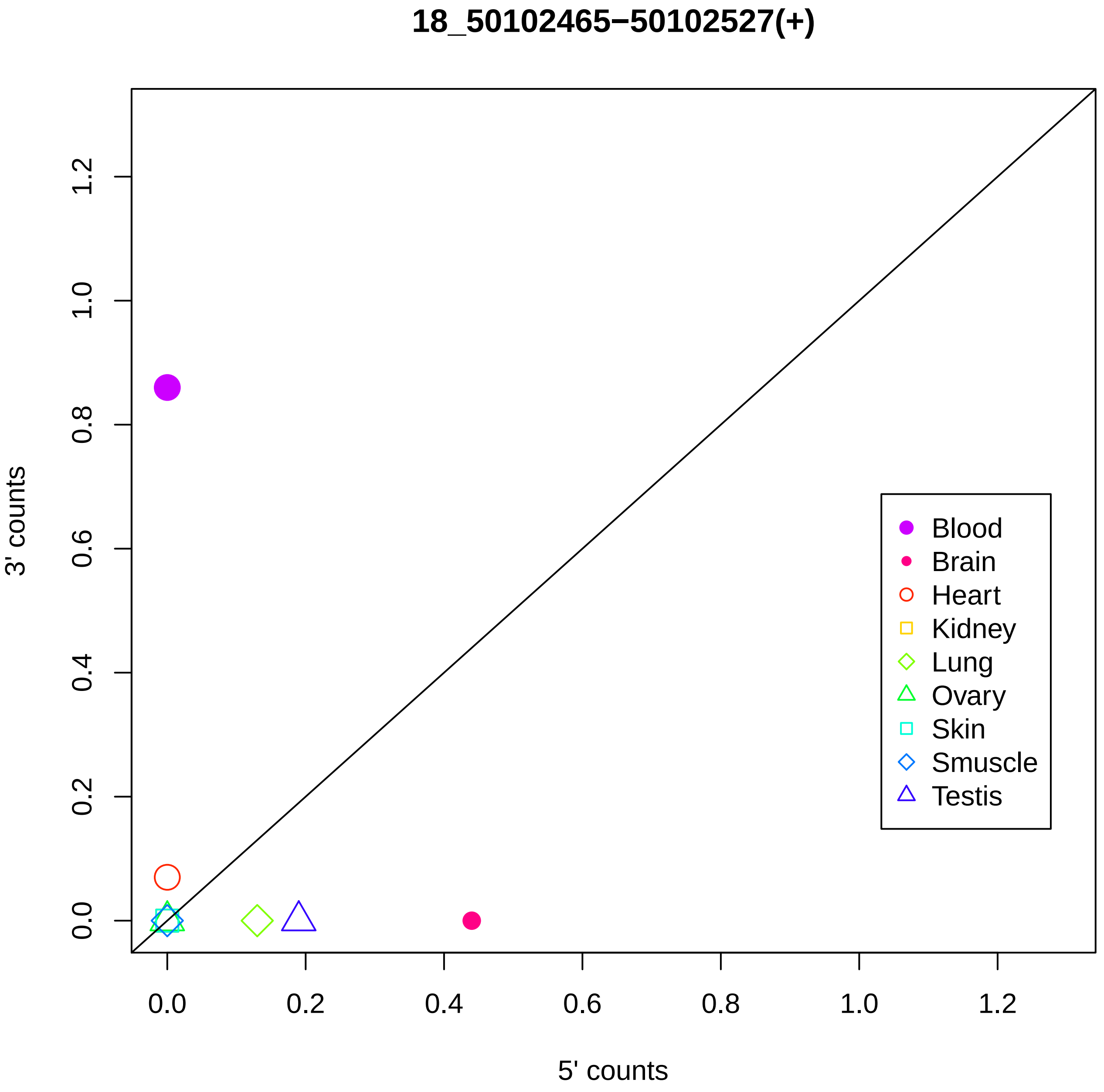

18_50102465−50102527(+)
3' counts
5' counts
0.0
0.2
0.4
0.6
0.8
1.0
1.2
Blood
Brain
Heart
Kidney
Lung
Ovary
Skin
Smuscle
Testis

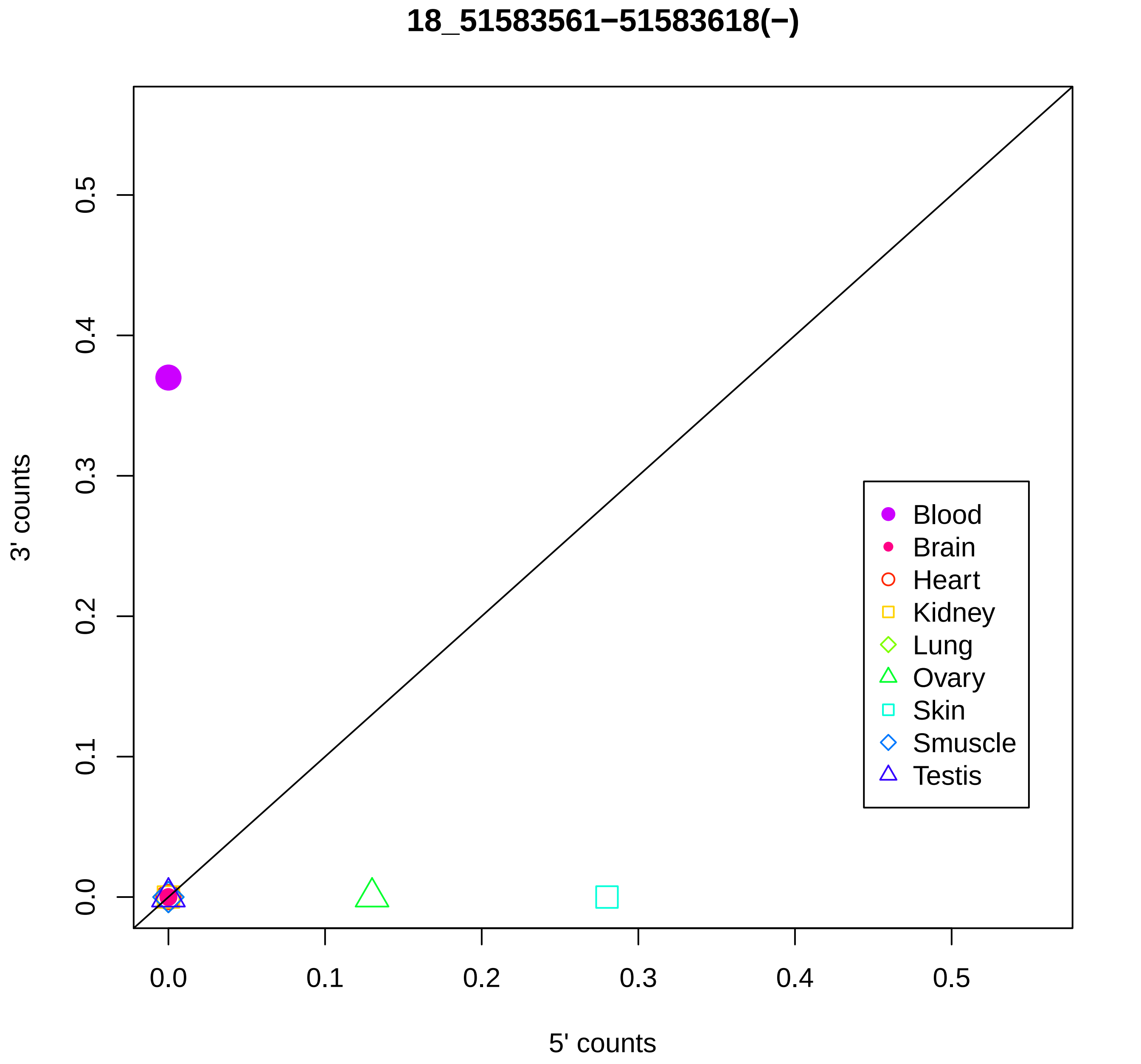
18_51583561−51583618(−)
3' counts
0.0
0.1
0.2
0.3
0.4
0.5
5' counts
0.0
0.1
0.2
0.3
0.4
0.5
Blood
Brain
Heart
Kidney
Lung
Ovary
Skin
Smuscle
Testis

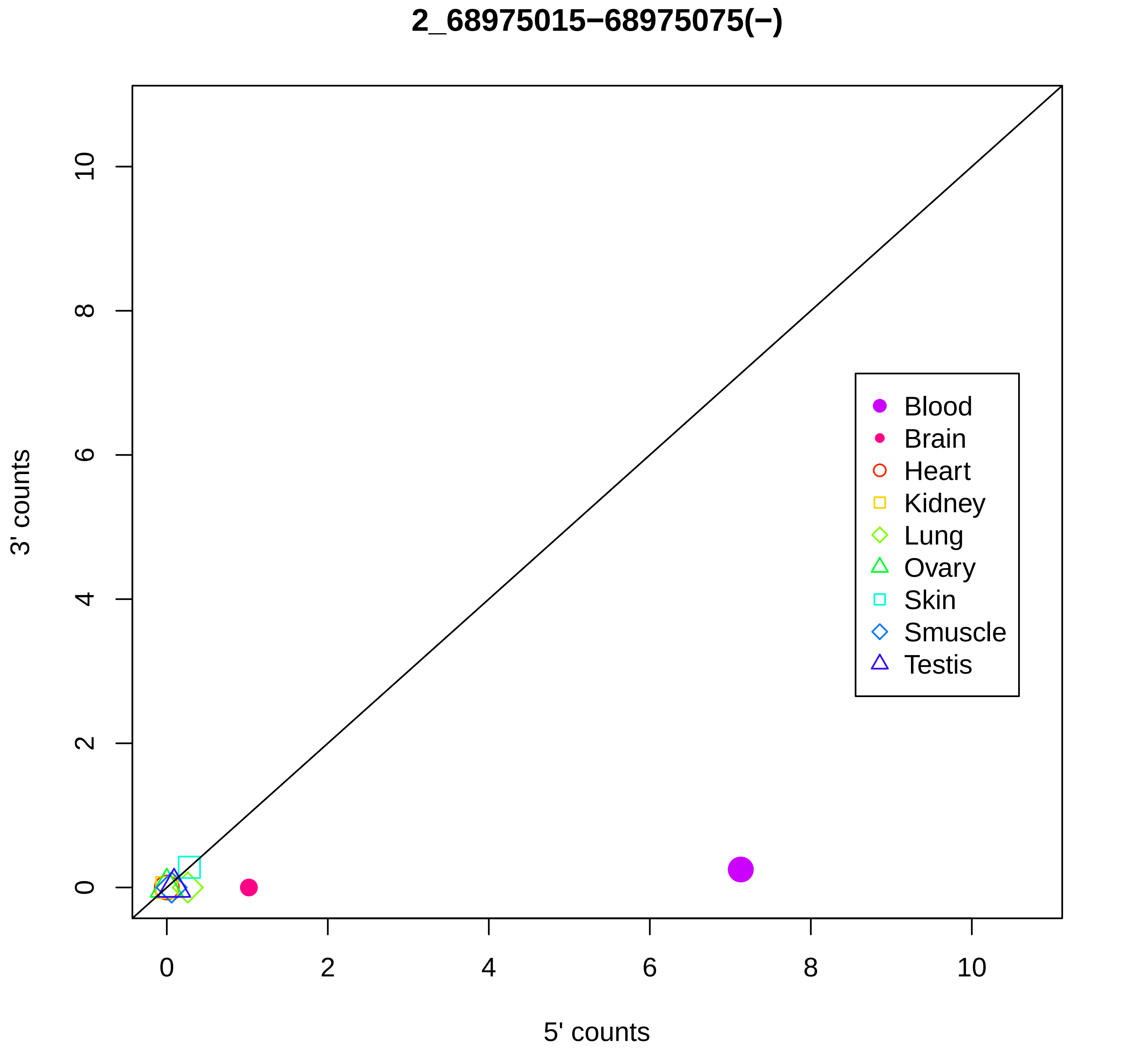

2_68975015−68975075(−)
3' counts
5' counts
0
2
4
6
8
10
Blood
Brain
Heart
Kidney
Lung
Ovary
Skin
Smuscle
Testis

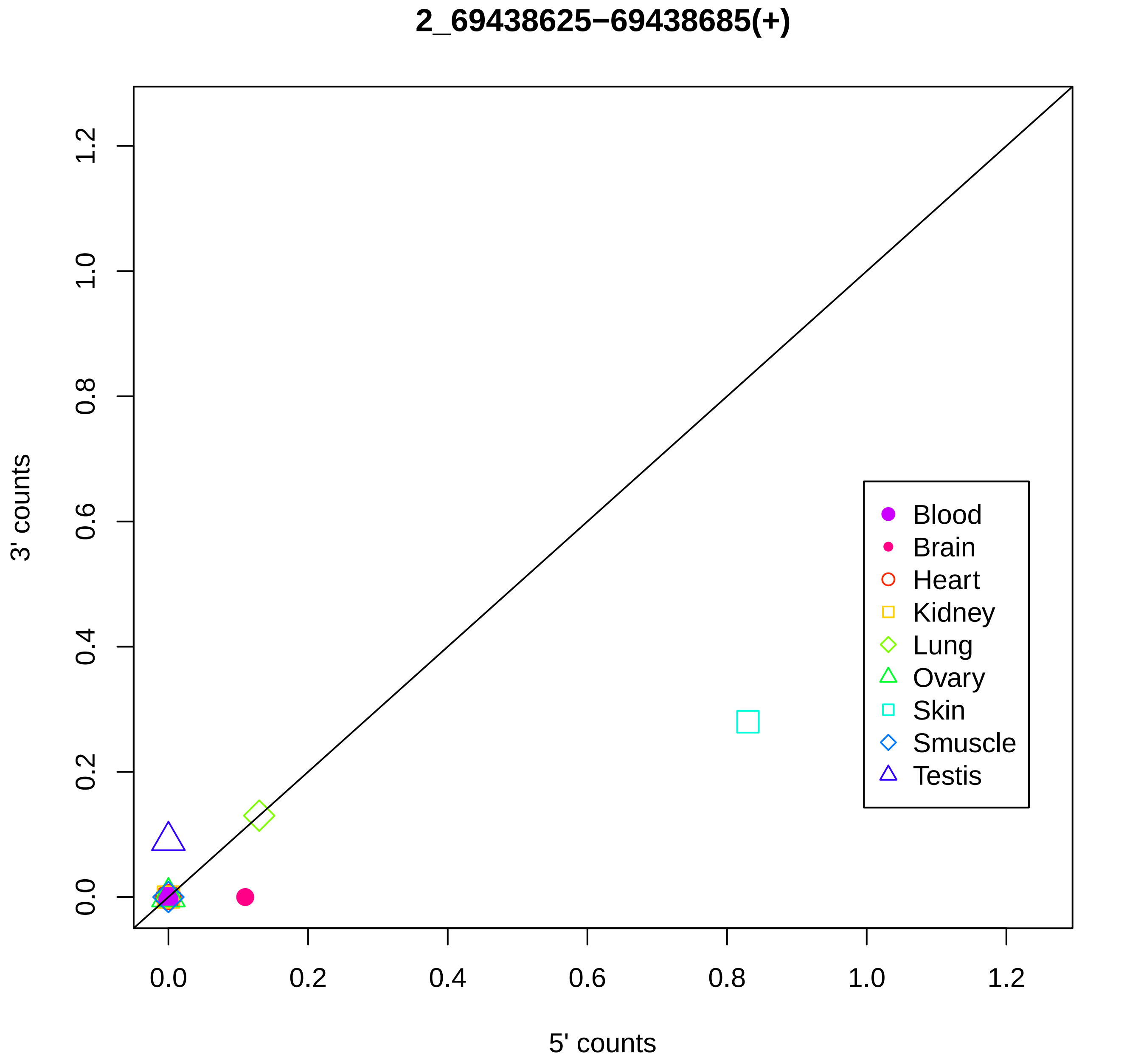
2_69438625−69438685(+)
3' counts
5' counts
0.0
0.2
0.4
0.6
0.8
1.0
1.2
Blood
Brain
Heart
Kidney
Lung
Ovary
Skin
Smuscle
Testis

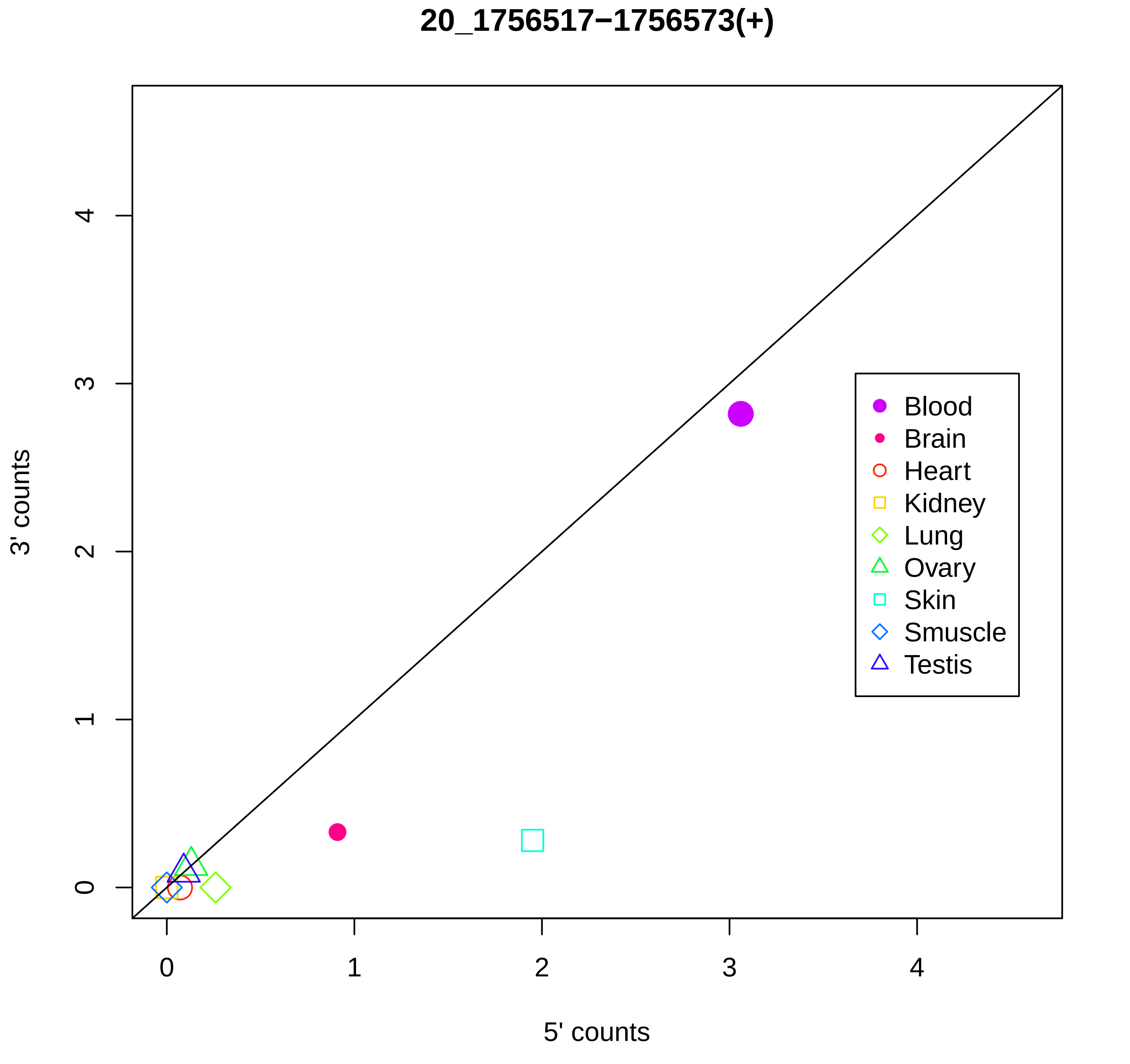

20_1756517−1756573(+)
3' counts
5' counts
0
1
2
3
4
0
1
2
3
4
Blood
Brain
Heart
Kidney
Lung
Ovary
Skin
Smuscle
Testis

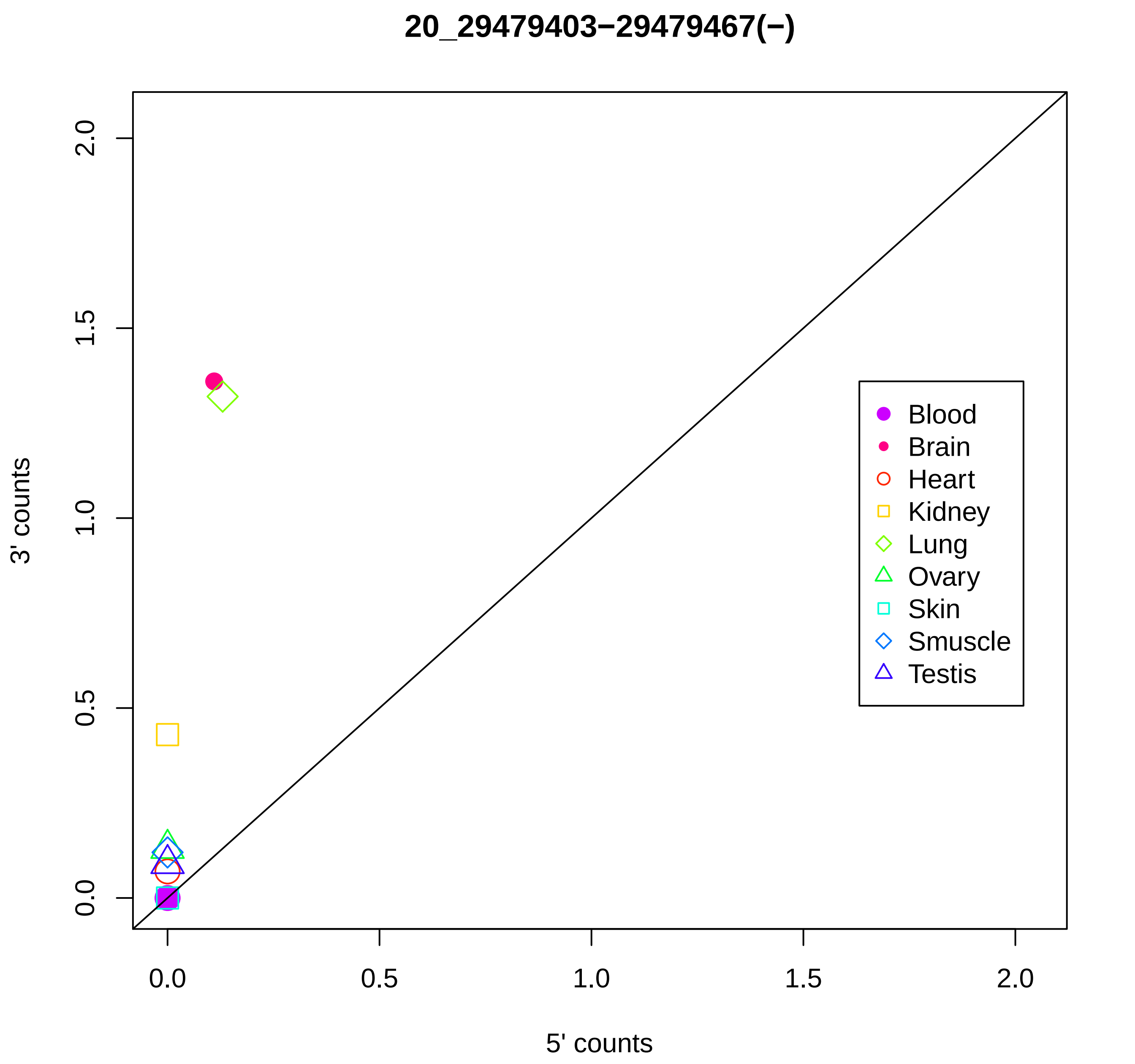

20_29479403−29479467(−)
3' counts
5' counts
0.0
0.5
1.0
1.5
2.0
Blood
Brain
Heart
Kidney
Lung
Ovary
Skin
Smuscle
Testis

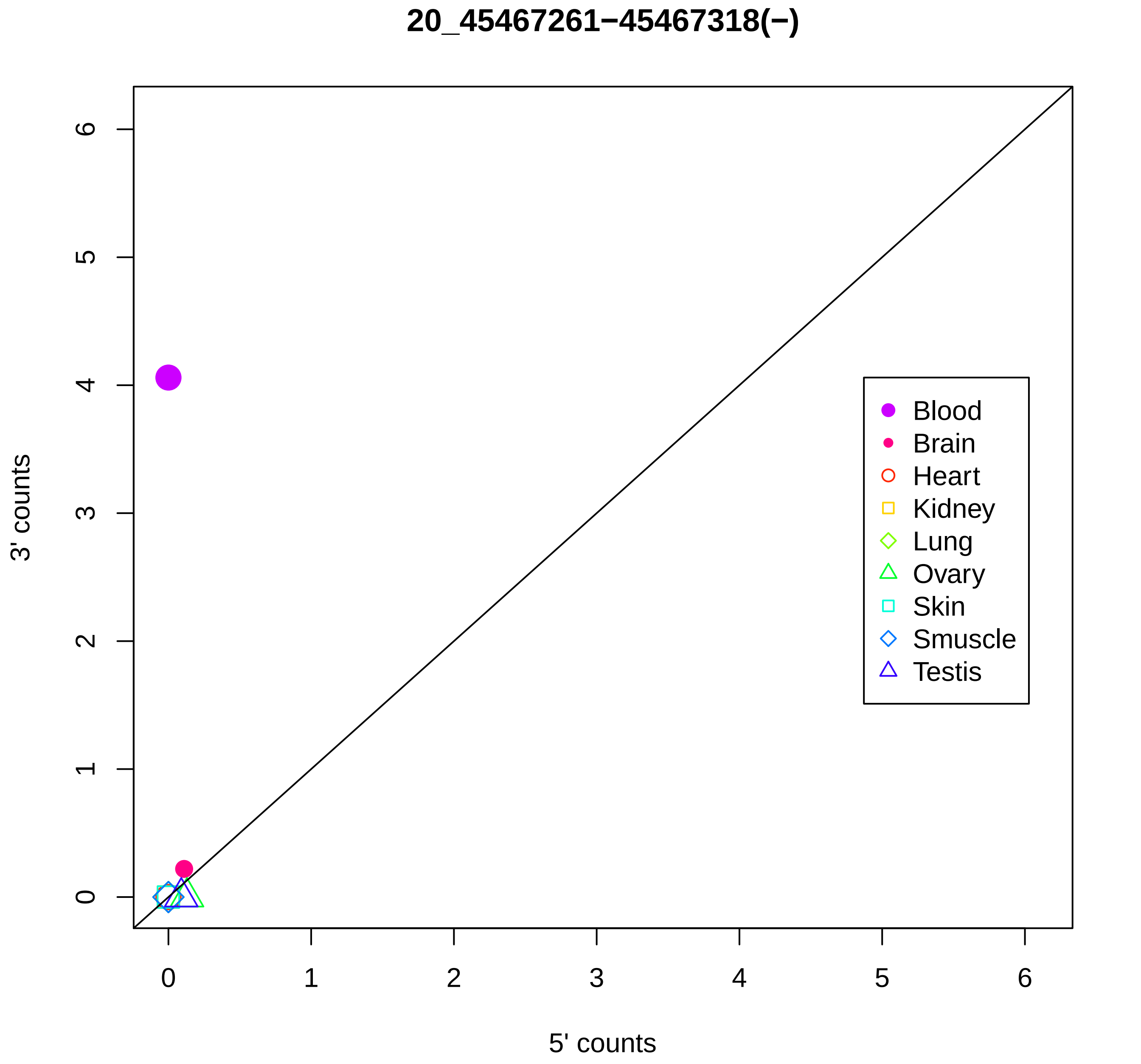

20_45467261−45467318(−)
3' counts
5' counts
0
1
2
3
4
5
6
Blood
Brain
Heart
Kidney
Lung
Ovary
Skin
Smuscle
Testis

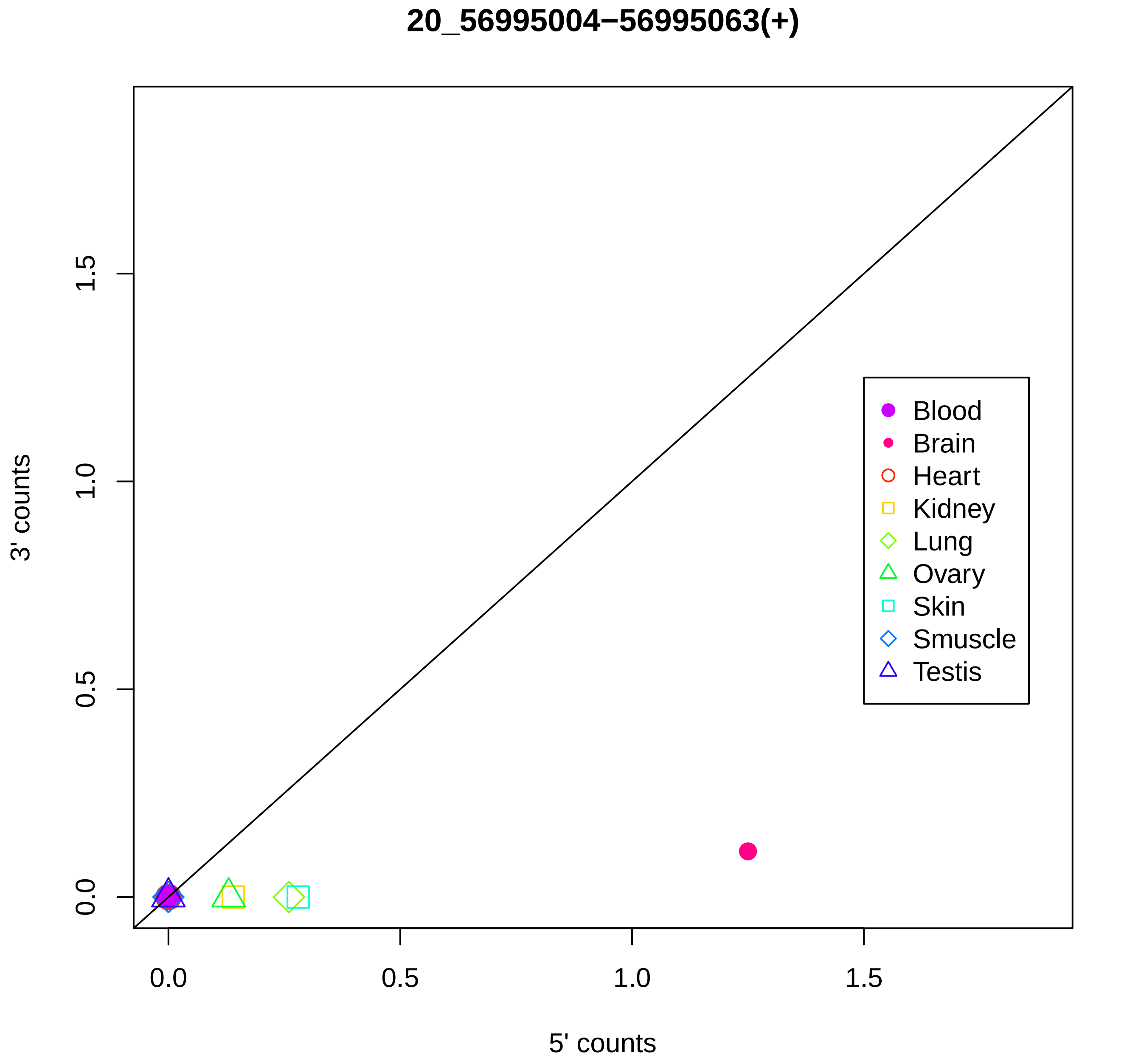

20_56995004−56995063(+)
3' counts
5' counts
0.0
0.5
1.0
1.5
0.0
0.5
1.0
1.5
Blood
Brain
Heart
Kidney
Lung
Ovary
Skin
Smuscle
Testis

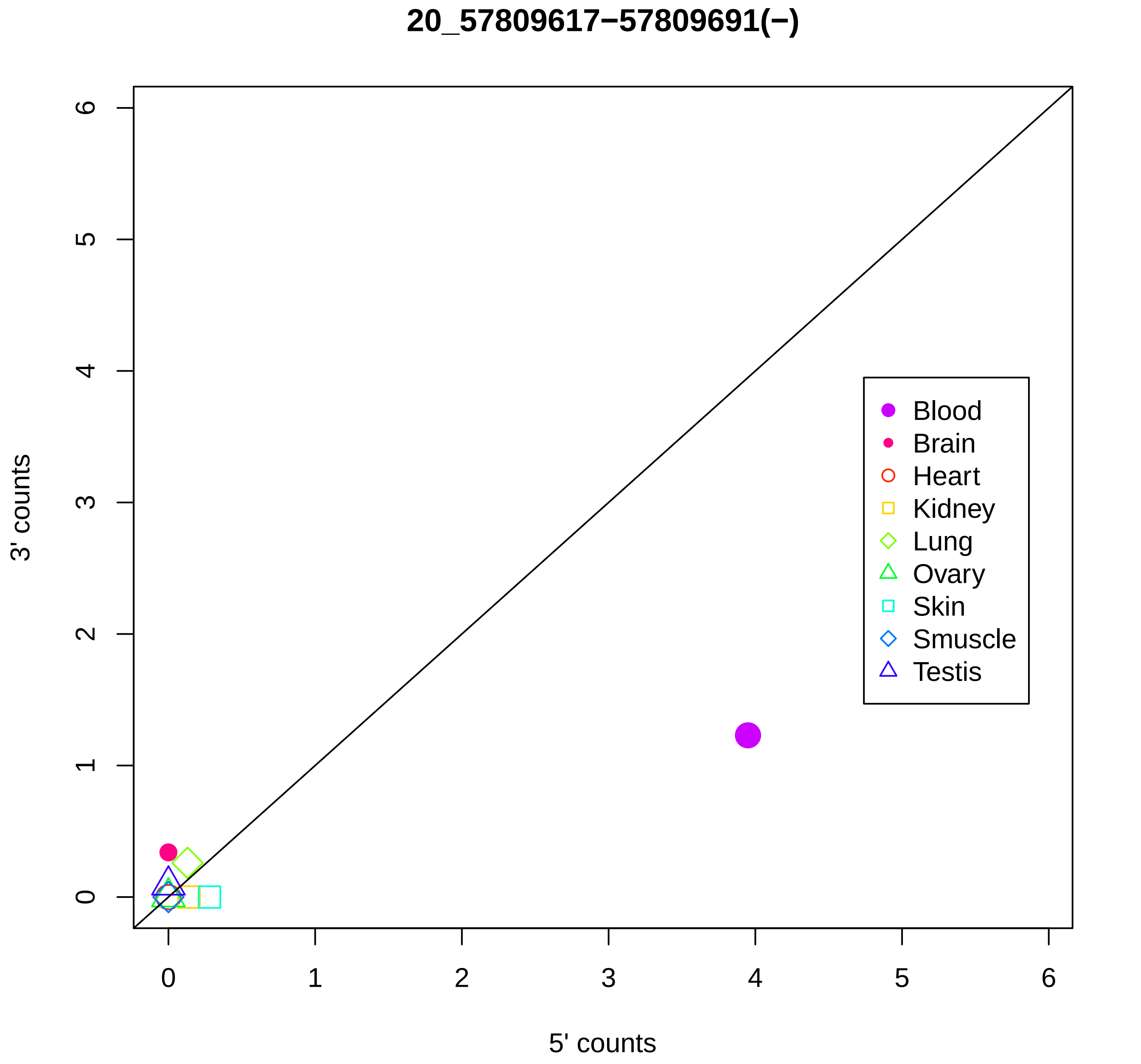
20_57809617−57809691(−)
3' counts
5' counts
0
1
2
3
4
5
6
Blood
Brain
Heart
Kidney
Lung
Ovary
Skin
Smuscle
Testis

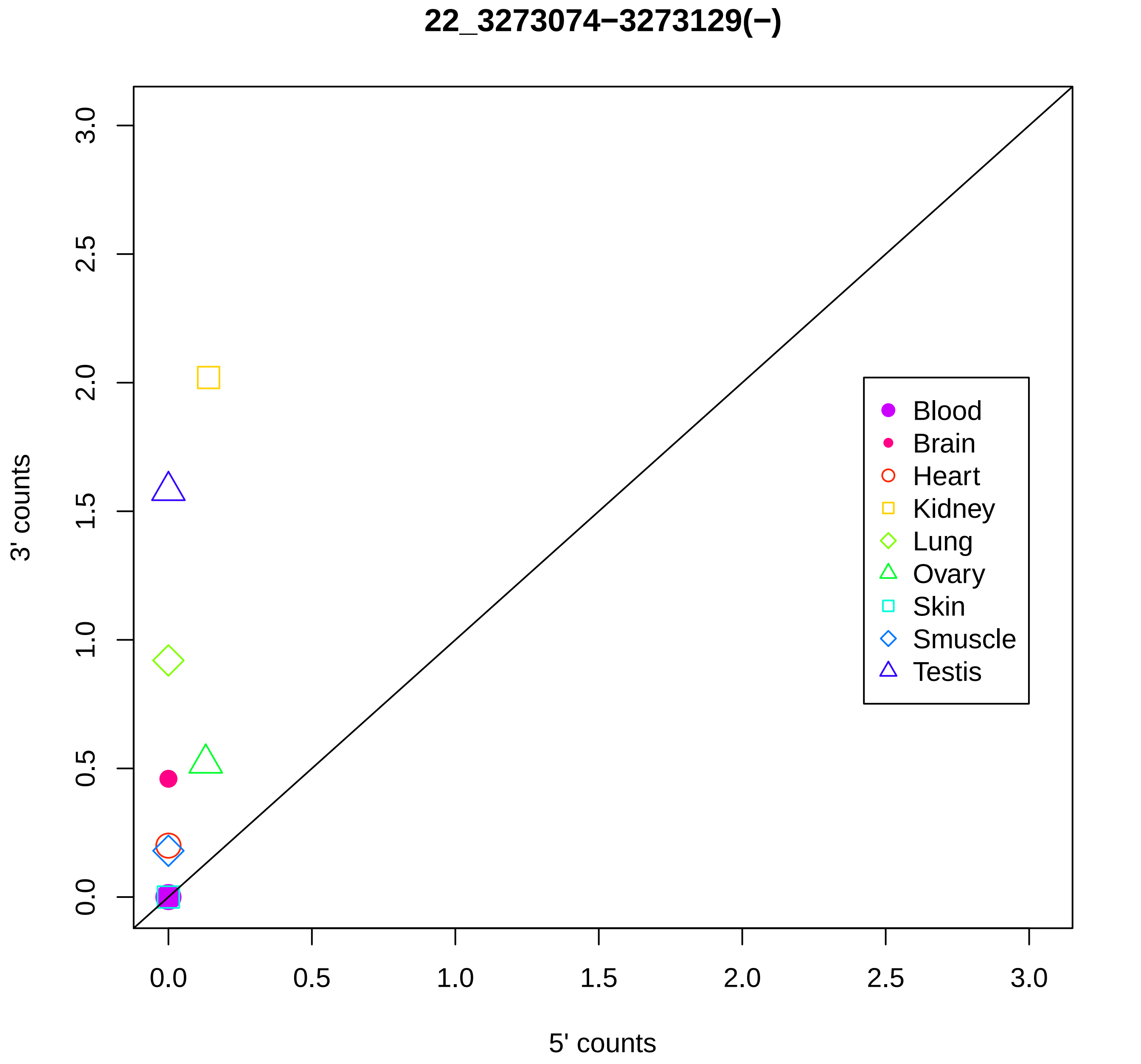
22_3273074−3273129(−)
3' counts
5' counts
0.0
0.5
1.0
1.5
2.0
2.5
3.0
Blood
Brain
Heart
Kidney
Lung
Ovary
Skin
Smuscle
Testis

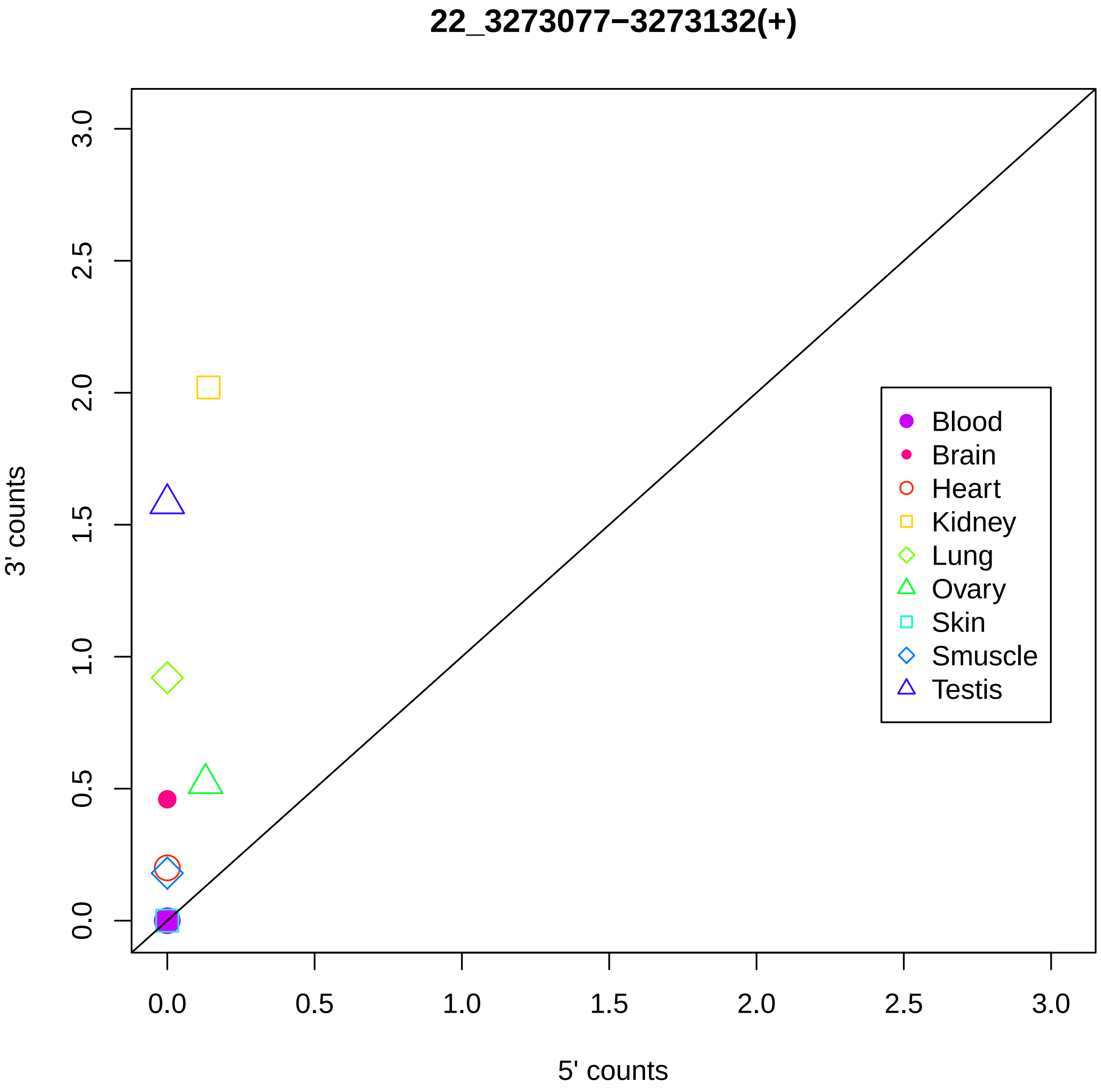

22_3273077−3273132(+)
3' counts
5' counts
0.0
0.5
1.0
1.5
2.0
2.5
3.0
Blood
Brain
Heart
Kidney
Lung
Ovary
Skin
Smuscle
Testis

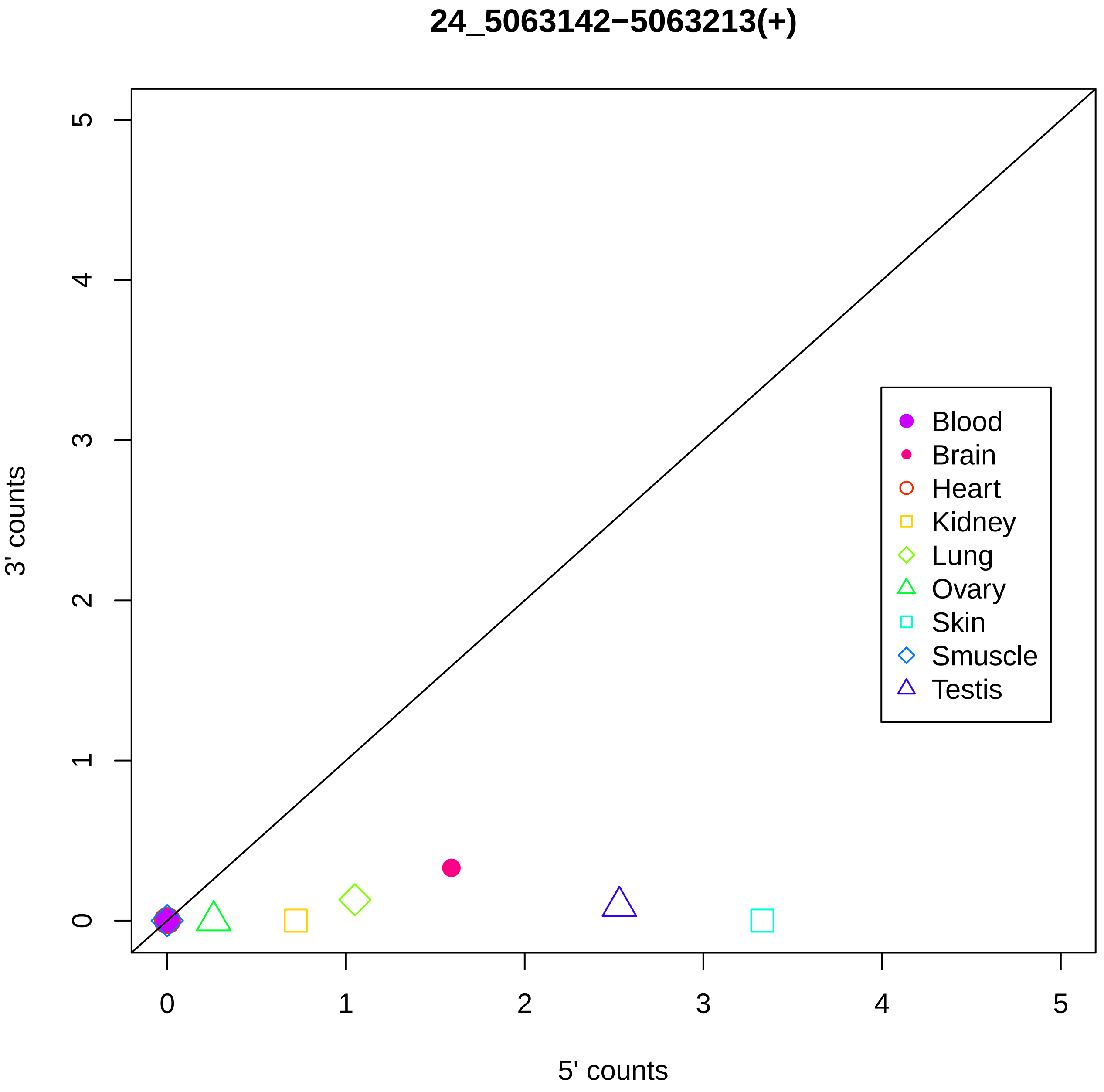

24_5063142−5063213(+)
3' counts
5' counts
0
1
2
3
4
5
Blood
Brain
Heart
Kidney
Lung
Ovary
Skin
Smuscle
Testis

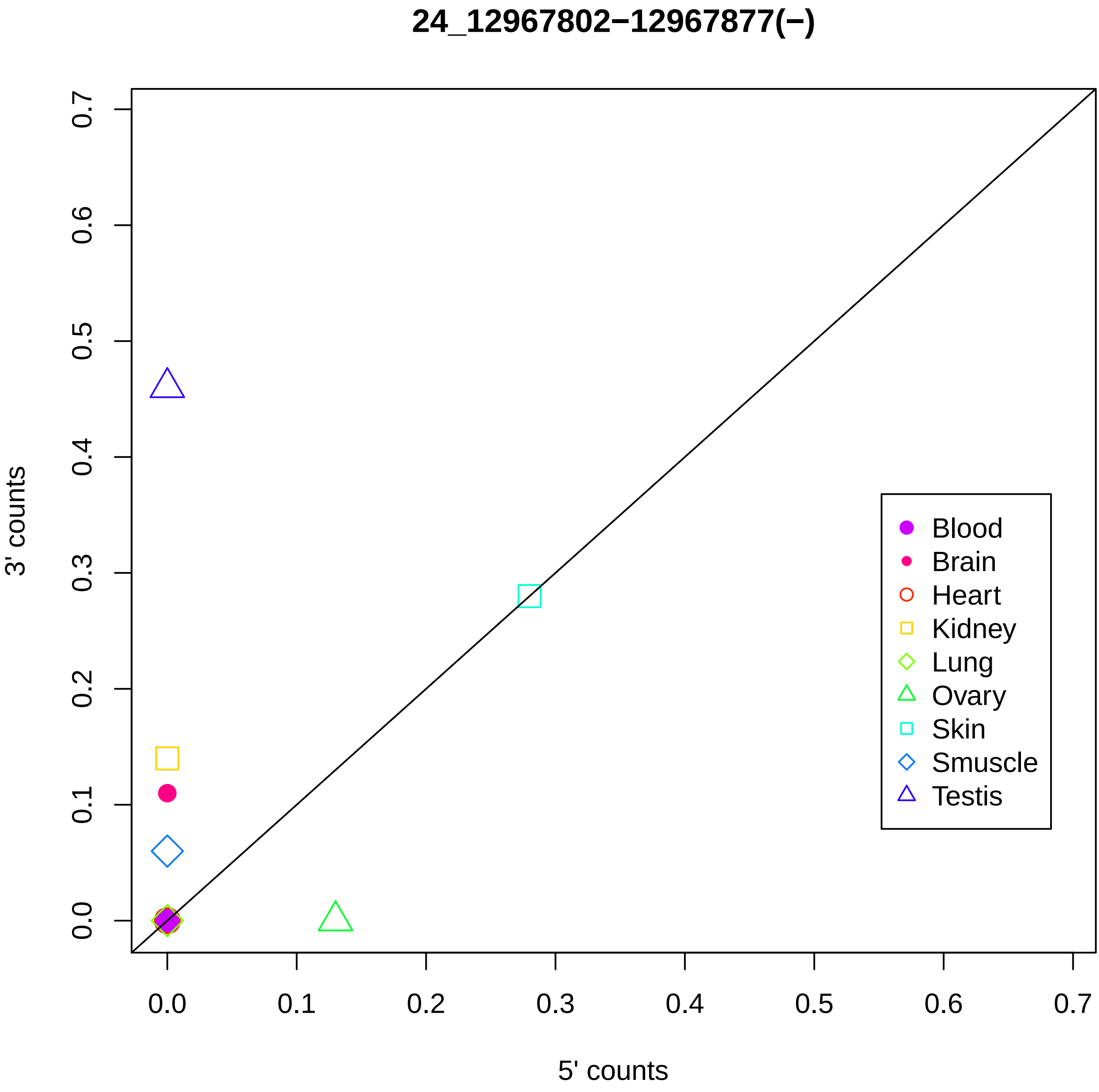
24_12967802−12967877(−)
3' counts
5' counts
0.0
0.1
0.2
0.3
0.4
0.5
0.6
0.7
Blood
Brain
Heart
Kidney
Lung
Ovary
Skin
Smuscle
Testis

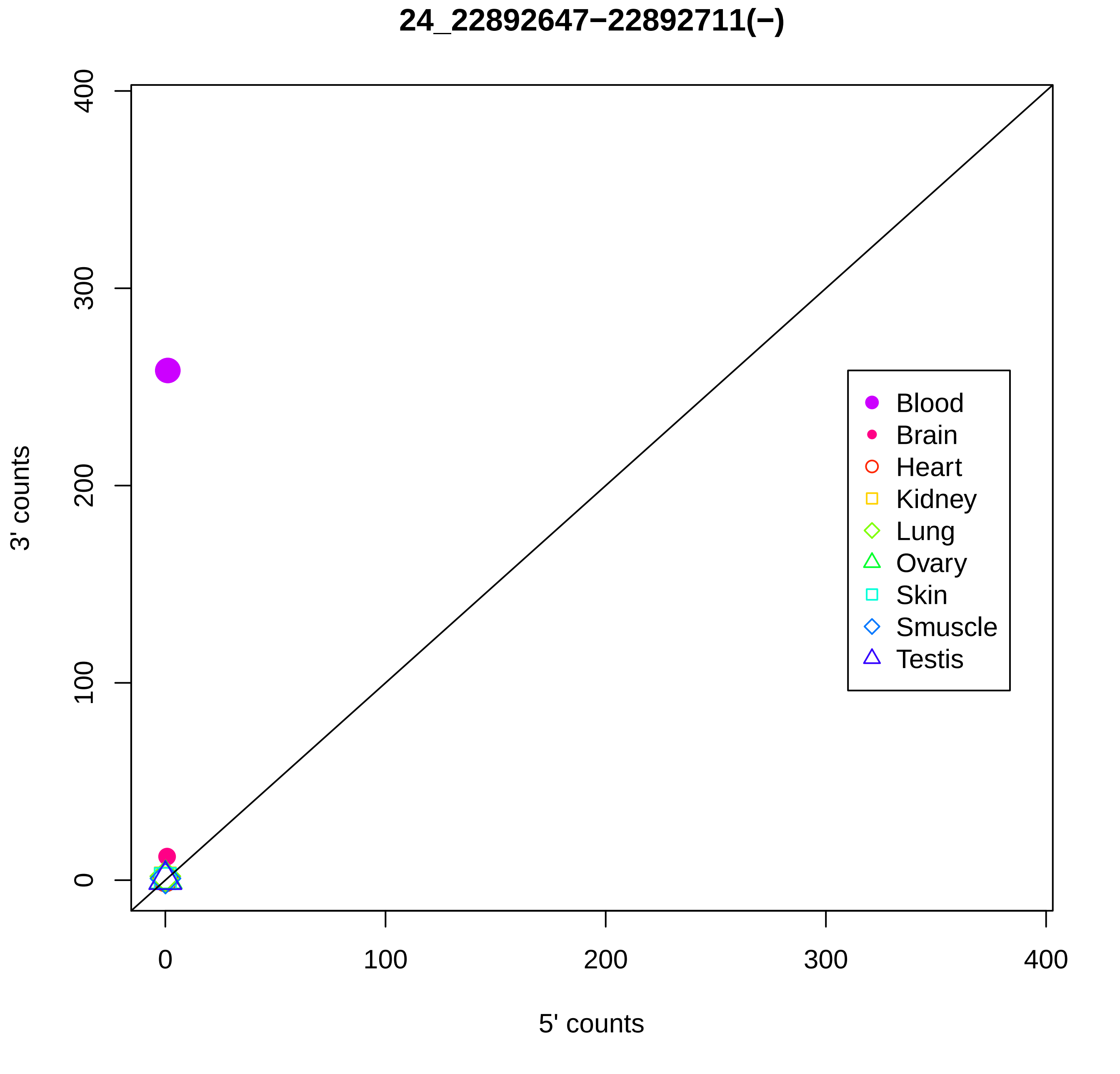

24_22892647−22892711(−)
3' counts
5' counts
0
100
200
300
400
0
100
200
300
400
Blood
Brain
Heart
Kidney
Lung
Ovary
Skin
Smuscle
Testis

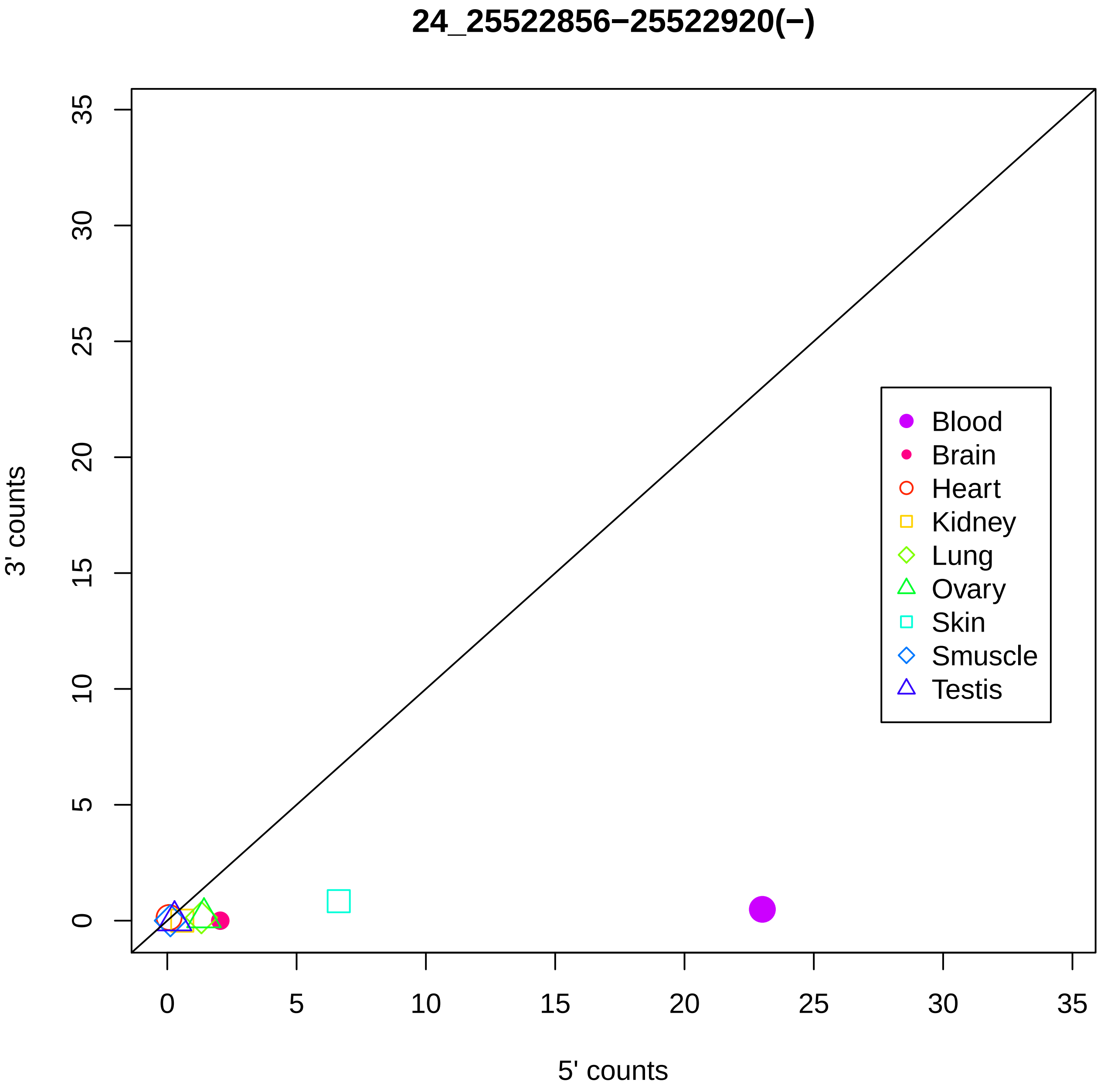

24_25522856−25522920(−)
3' counts
5' counts
0
5
10
15
20
25
30
35
Blood
Brain
Heart
Kidney
Lung
Ovary
Skin
Smuscle
Testis

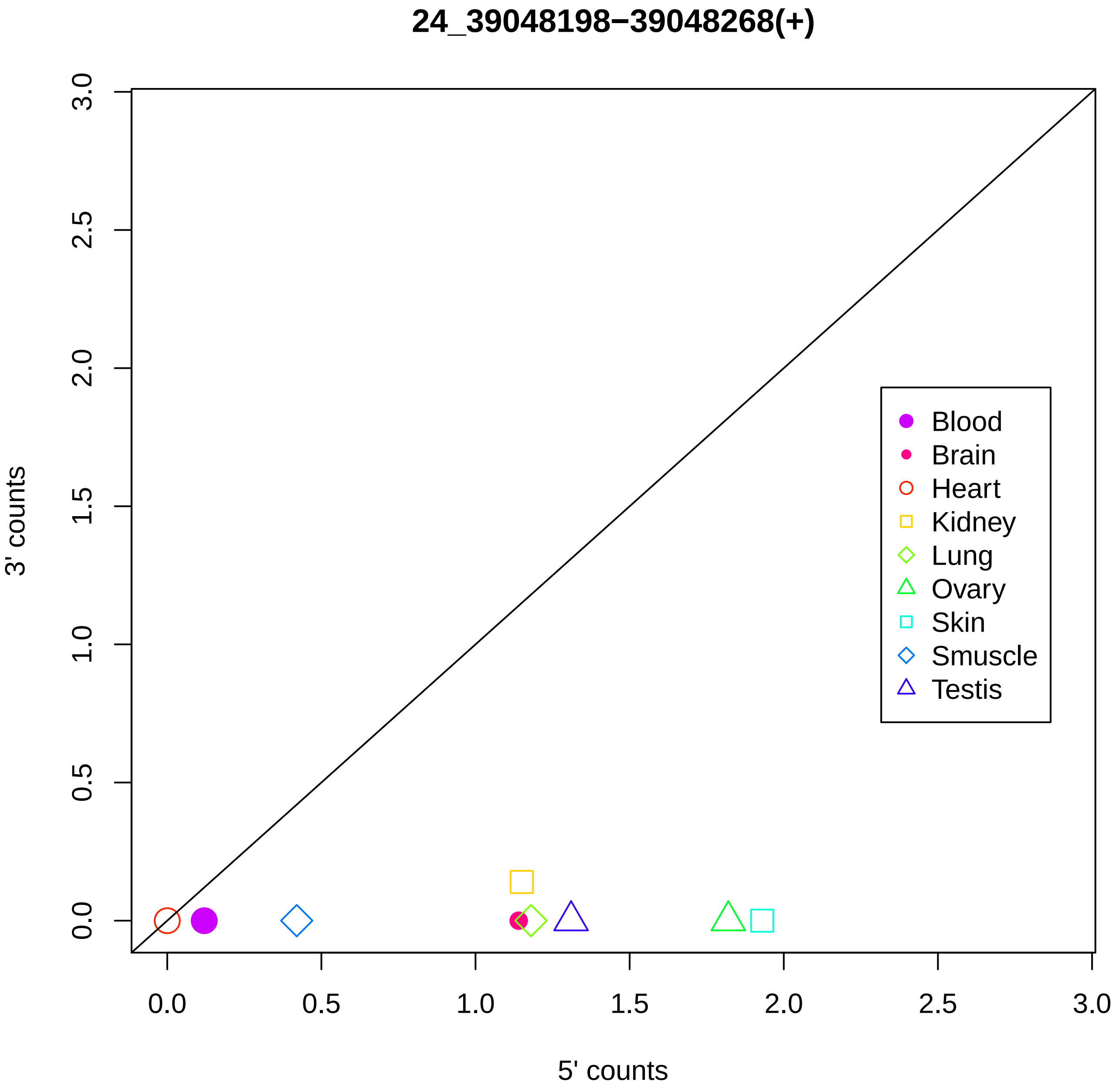

24_39048198−39048268(+)
3' counts
5' counts
0.0
0.5
1.0
1.5
2.0
2.5
3.0
Blood
Brain
Heart
Kidney
Lung
Ovary
Skin
Smuscle
Testis

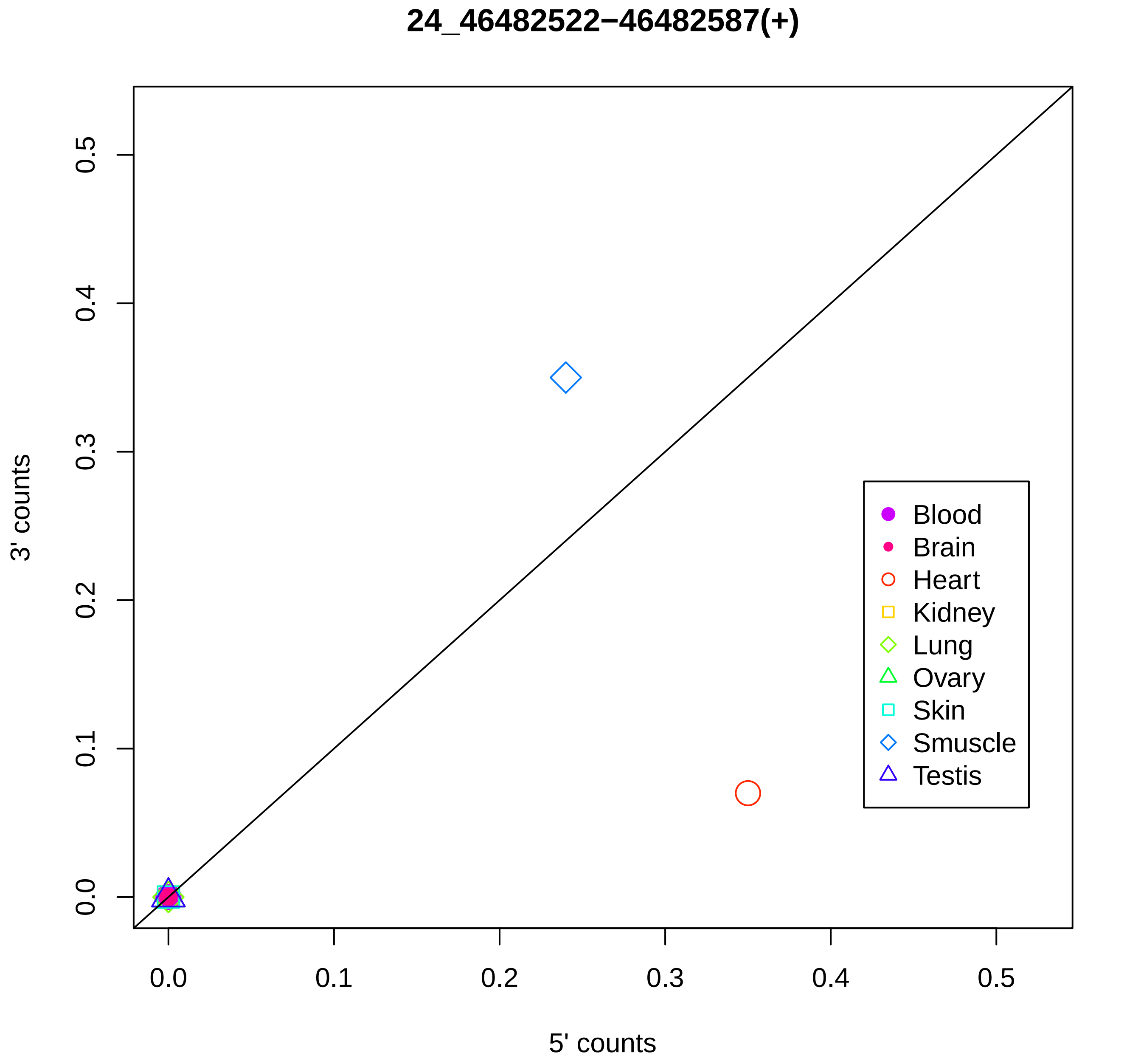

24_46482522−46482587(+)
3' counts
5' counts
0.0
0.1
0.2
0.3
0.4
0.5
Blood
Brain
Heart
Kidney
Lung
Ovary
Skin
Smuscle
Testis

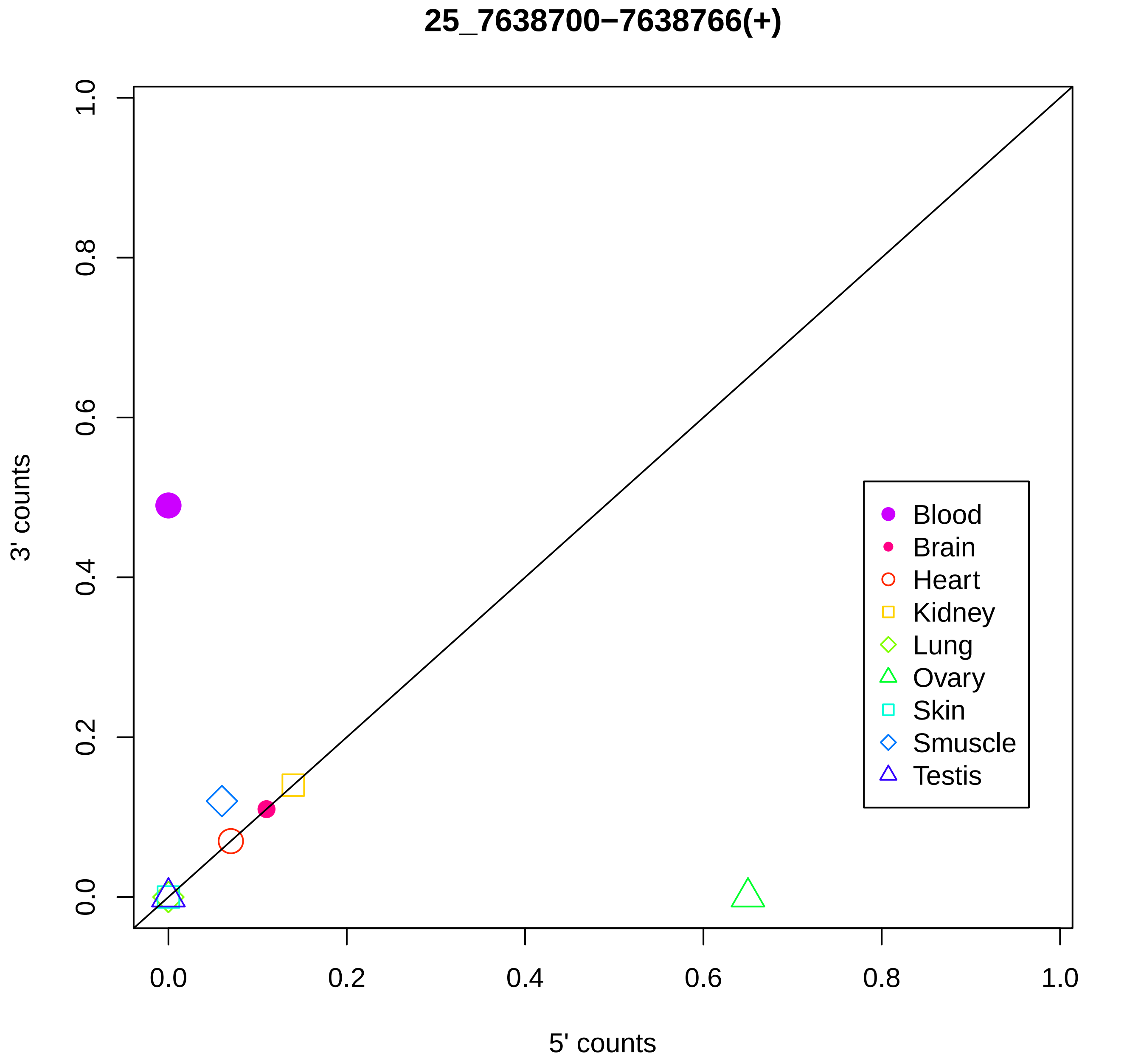

25_7638700−7638766(+)
3' counts
5' counts
0.0
0.2
0.4
0.6
0.8
1.0
Blood
Brain
Heart
Kidney
Lung
Ovary
Skin
Smuscle
Testis

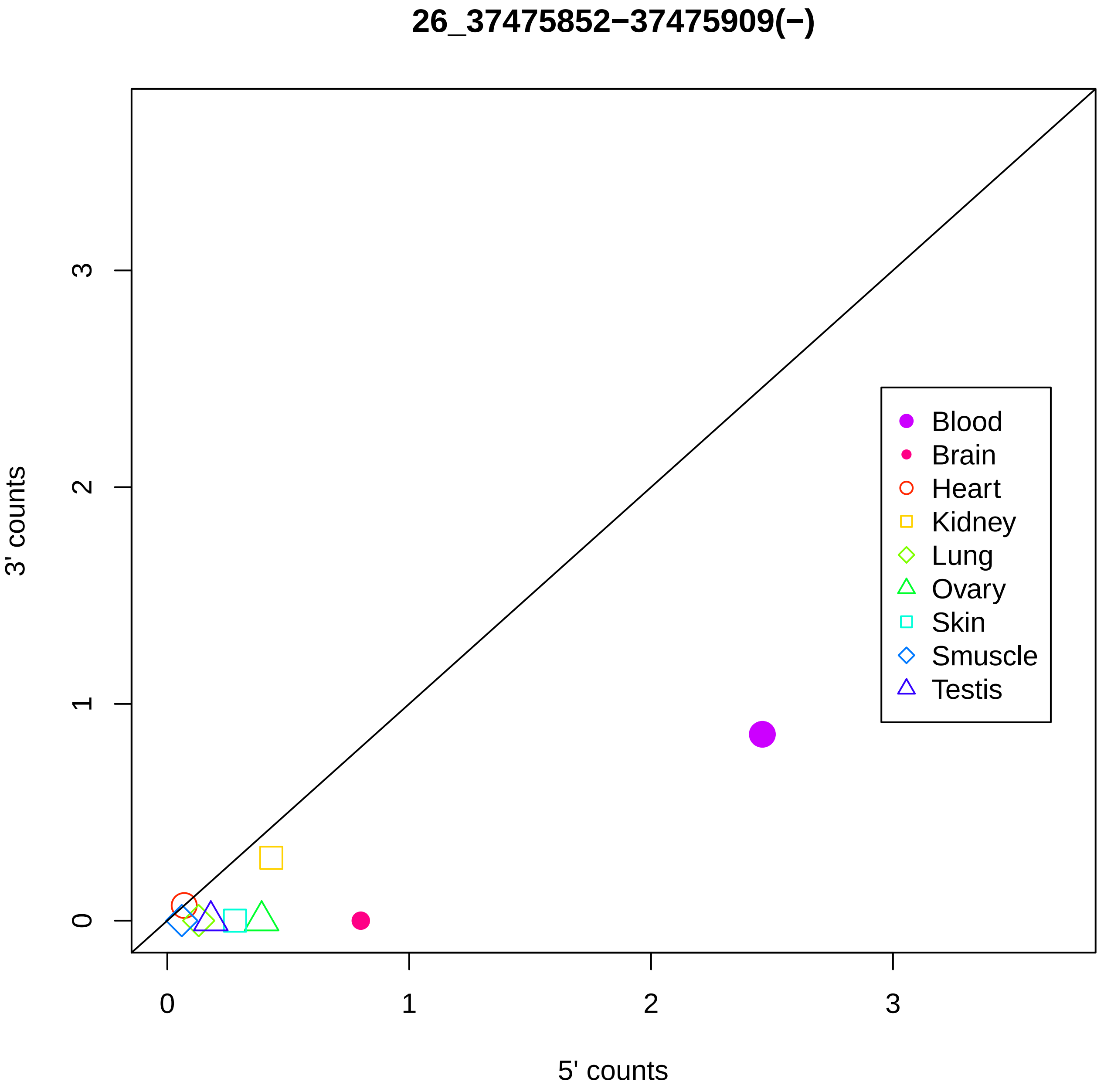
26_37475852−37475909(−)
3' counts
5' counts
0
1
2
3
0
1
2
3
Blood
Brain
Heart
Kidney
Lung
Ovary
Skin
Smuscle
Testis

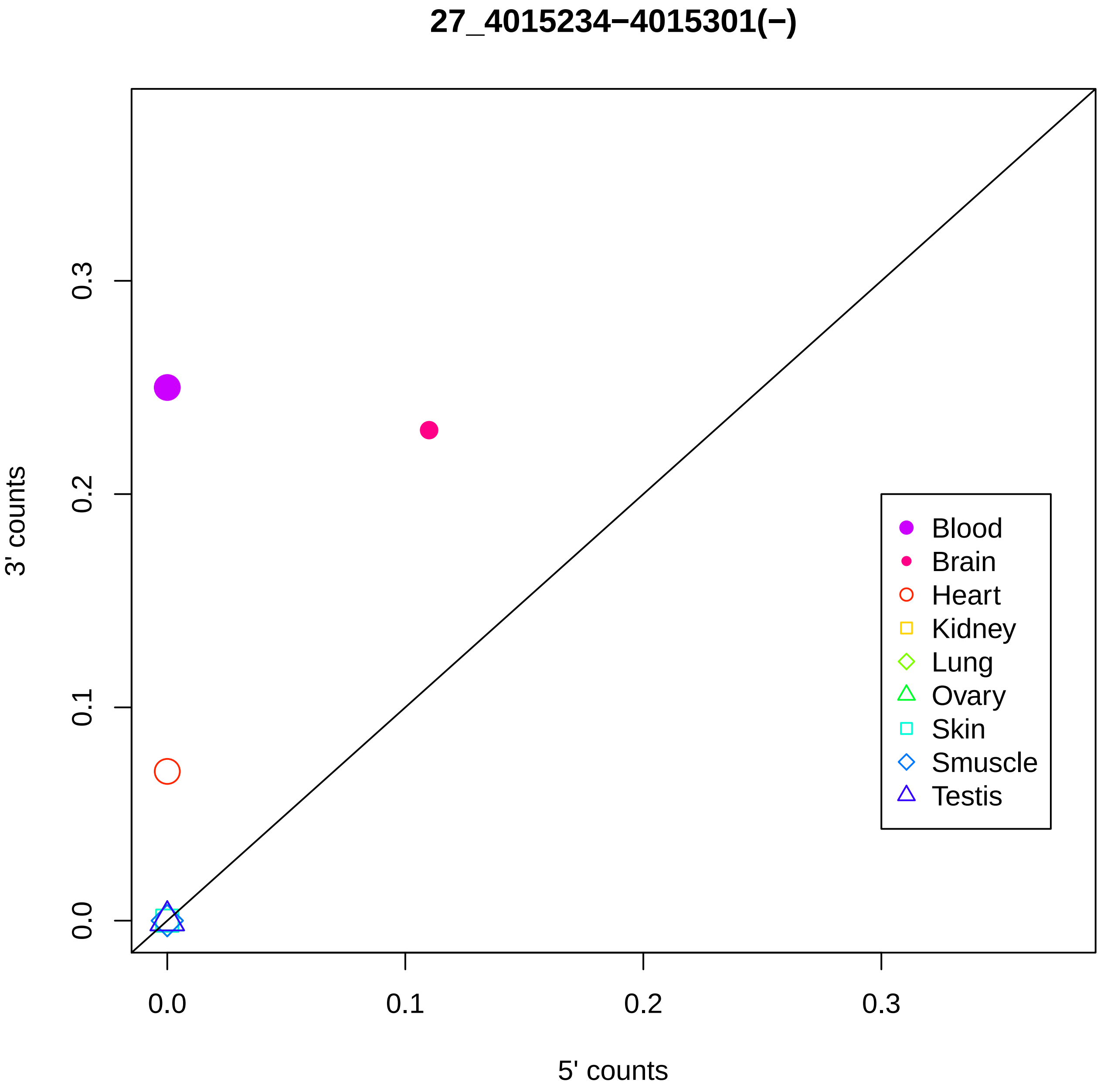

27_4015234−4015301(−)
3' counts
5' counts
0.0
0.1
0.2
0.3
0.0
0.1
0.2
0.3
Blood
Brain
Heart
Kidney
Lung
Ovary
Skin
Smuscle
Testis

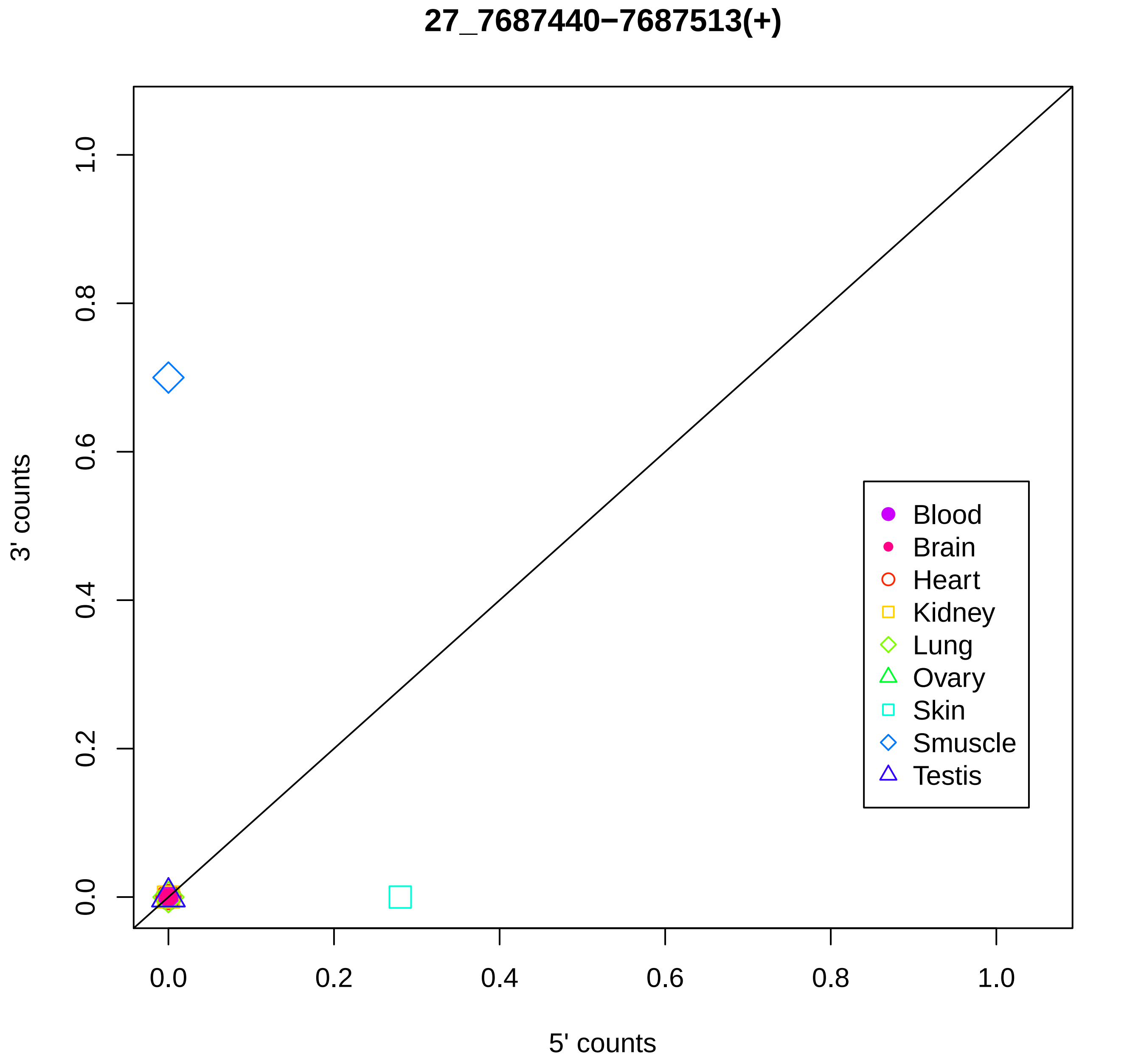
27_7687440−7687513(+)
1.0
0.8
0.6
0.4
0.2
0.0
3' counts
0.0
0.2
0.4
0.6
0.8
1.0
5' counts
Blood
Brain
Heart
Kidney
Lung
Ovary
Skin
Smuscle
Testis

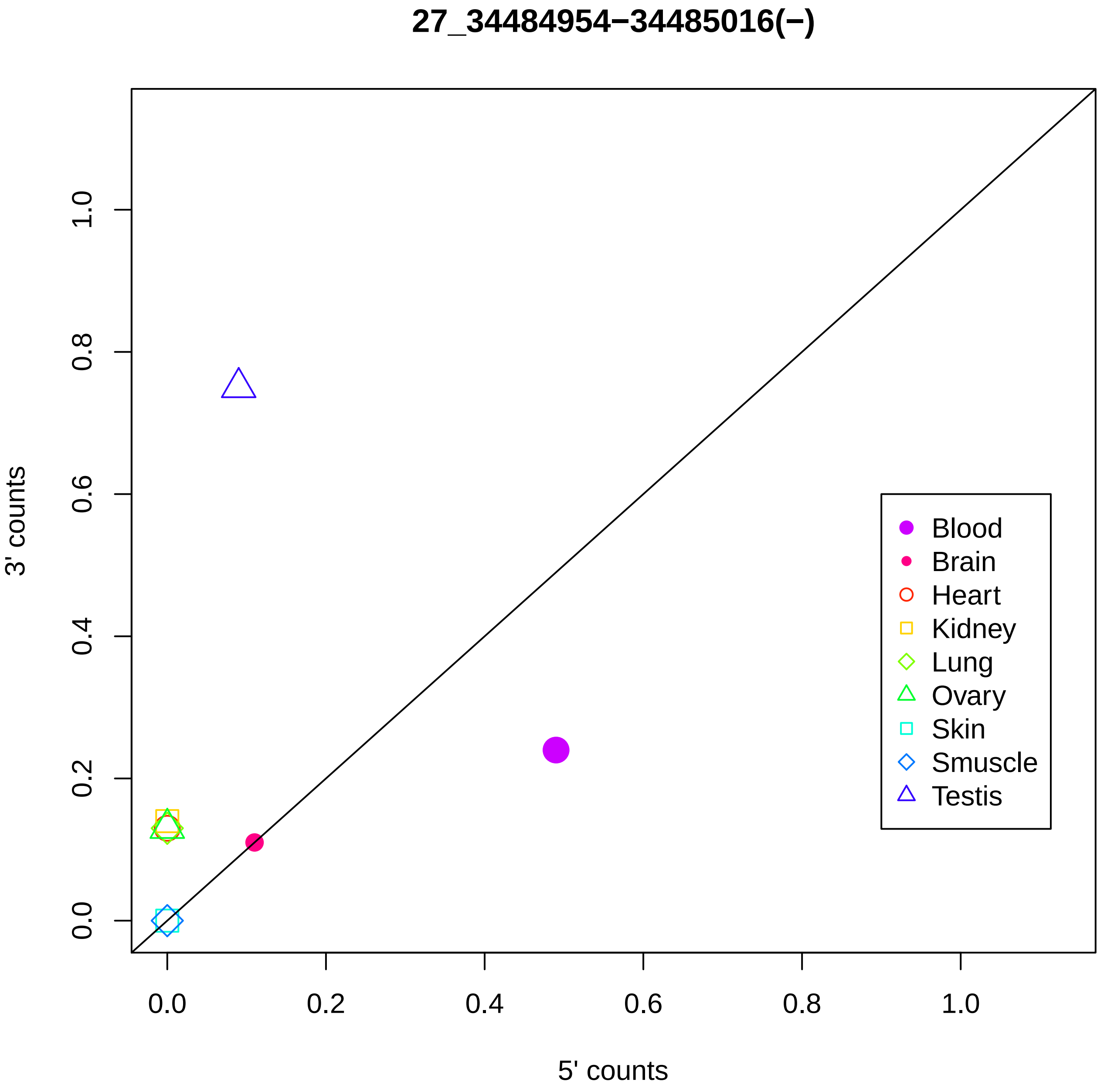
27_34484954−34485016(−)
3' counts
5' counts
0.0
0.2
0.4
0.6
0.8
1.0
Blood
Brain
Heart
Kidney
Lung
Ovary
Skin
Smuscle
Testis

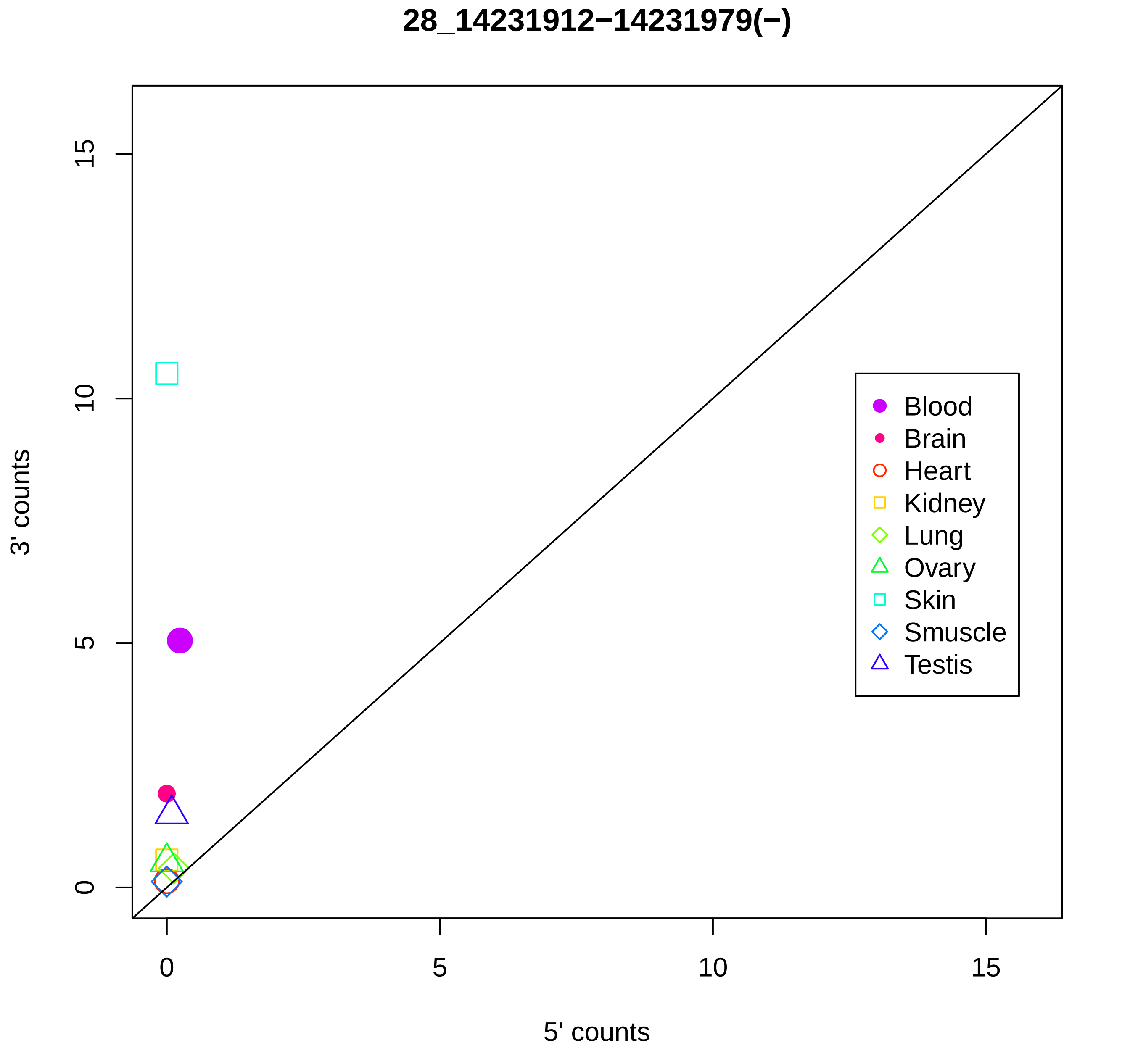
28_14231912−14231979(−)
3' counts
5' counts
0
5
10
15
0
5
10
15
Blood
Brain
Heart
Kidney
Lung
Ovary
Skin
Smuscle
Testis

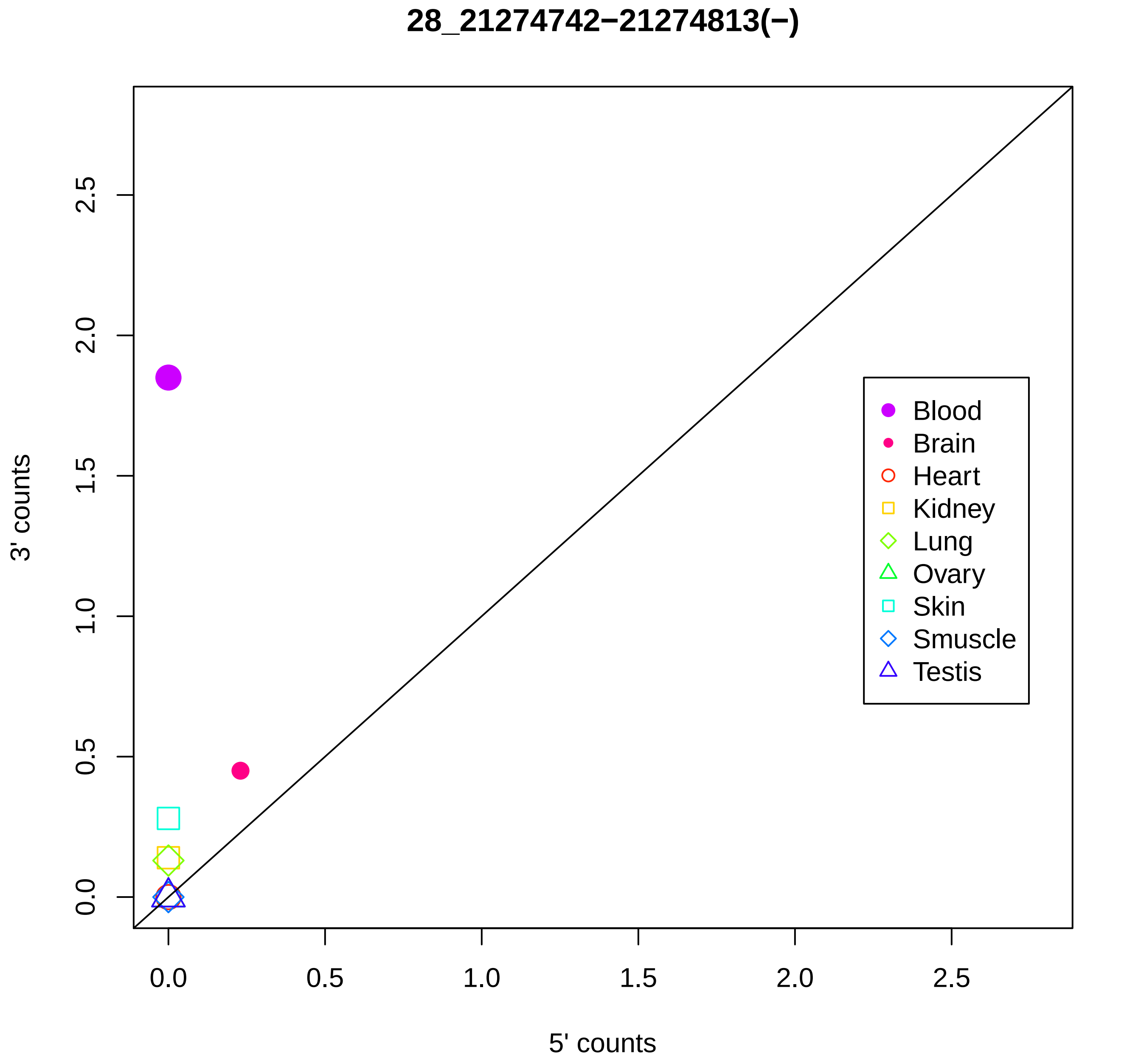

28_21274742−21274813(−)
3' counts
5' counts
0.0
0.5
1.0
1.5
2.0
2.5
Blood
Brain
Heart
Kidney
Lung
Ovary
Skin
Smuscle
Testis

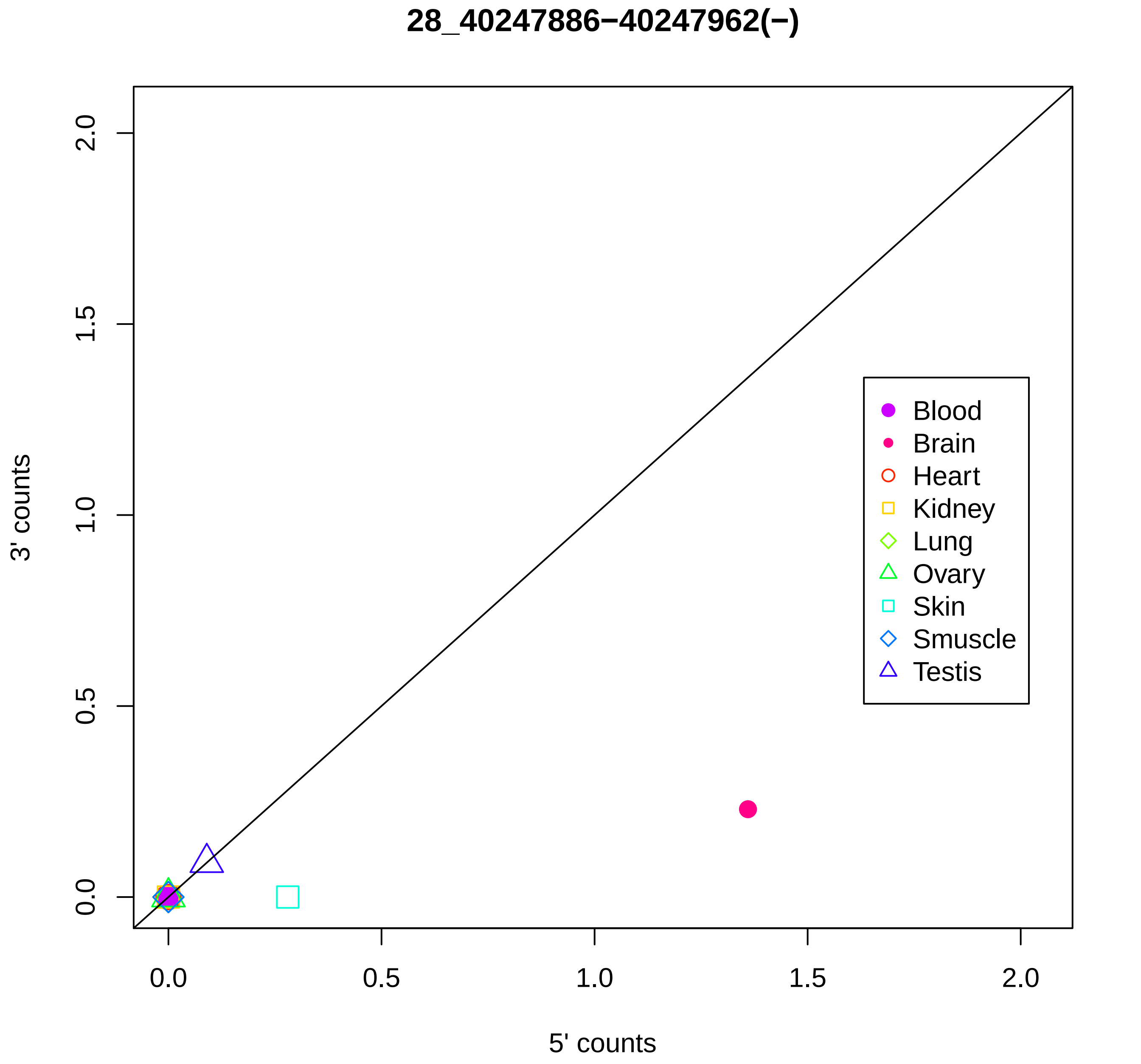
28_40247886−40247962(−)
3' counts
5' counts
0.0
0.5
1.0
1.5
2.0
Blood
Brain
Heart
Kidney
Lung
Ovary
Skin
Smuscle
Testis

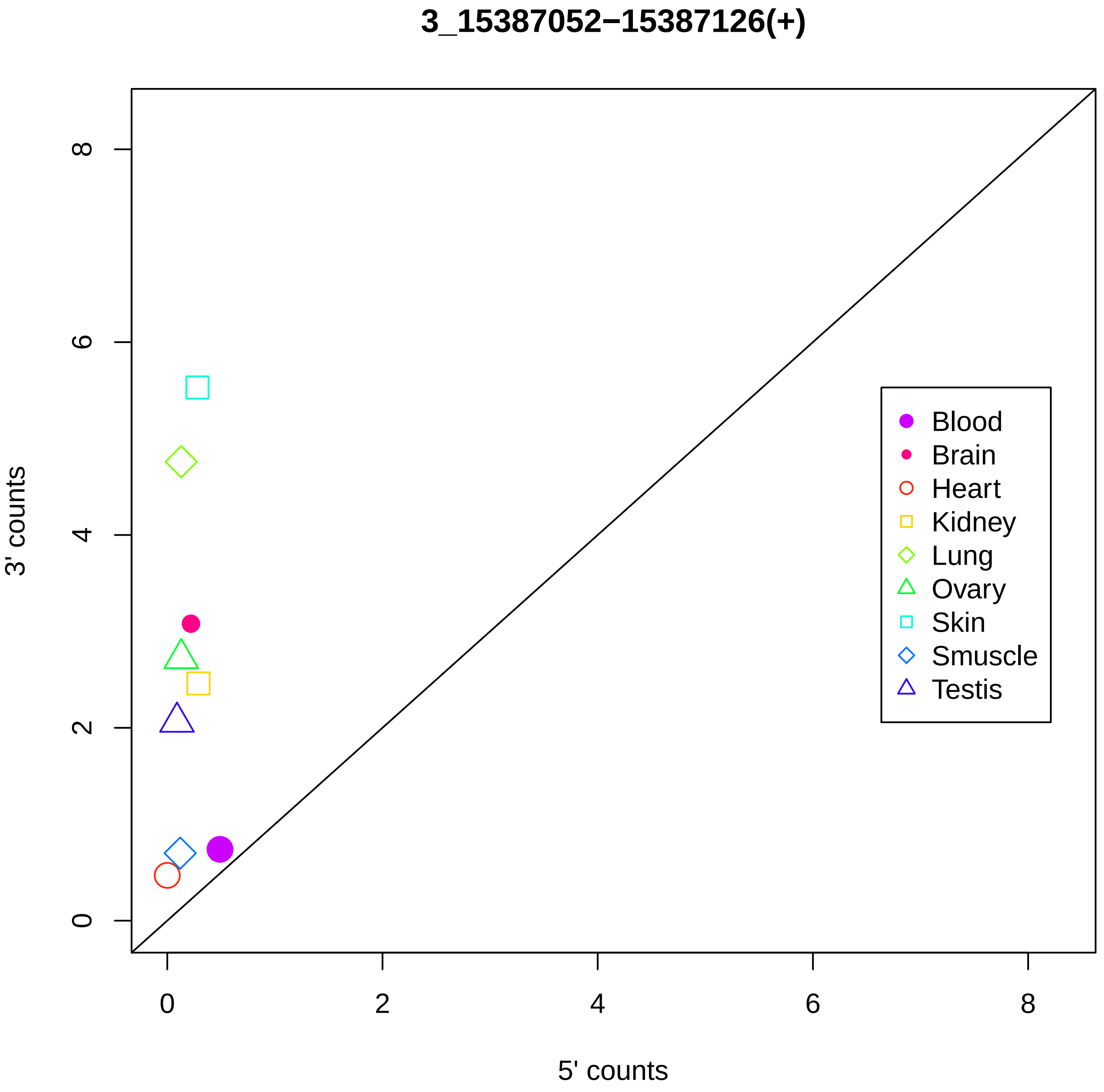
3_15387052−15387126(+)
Blood
Brain
Heart
Kidney
Lung
Ovary
Skin
Smuscle
Testis
3' counts
5' counts
0
2
4
6
8
0
2
4
6
8

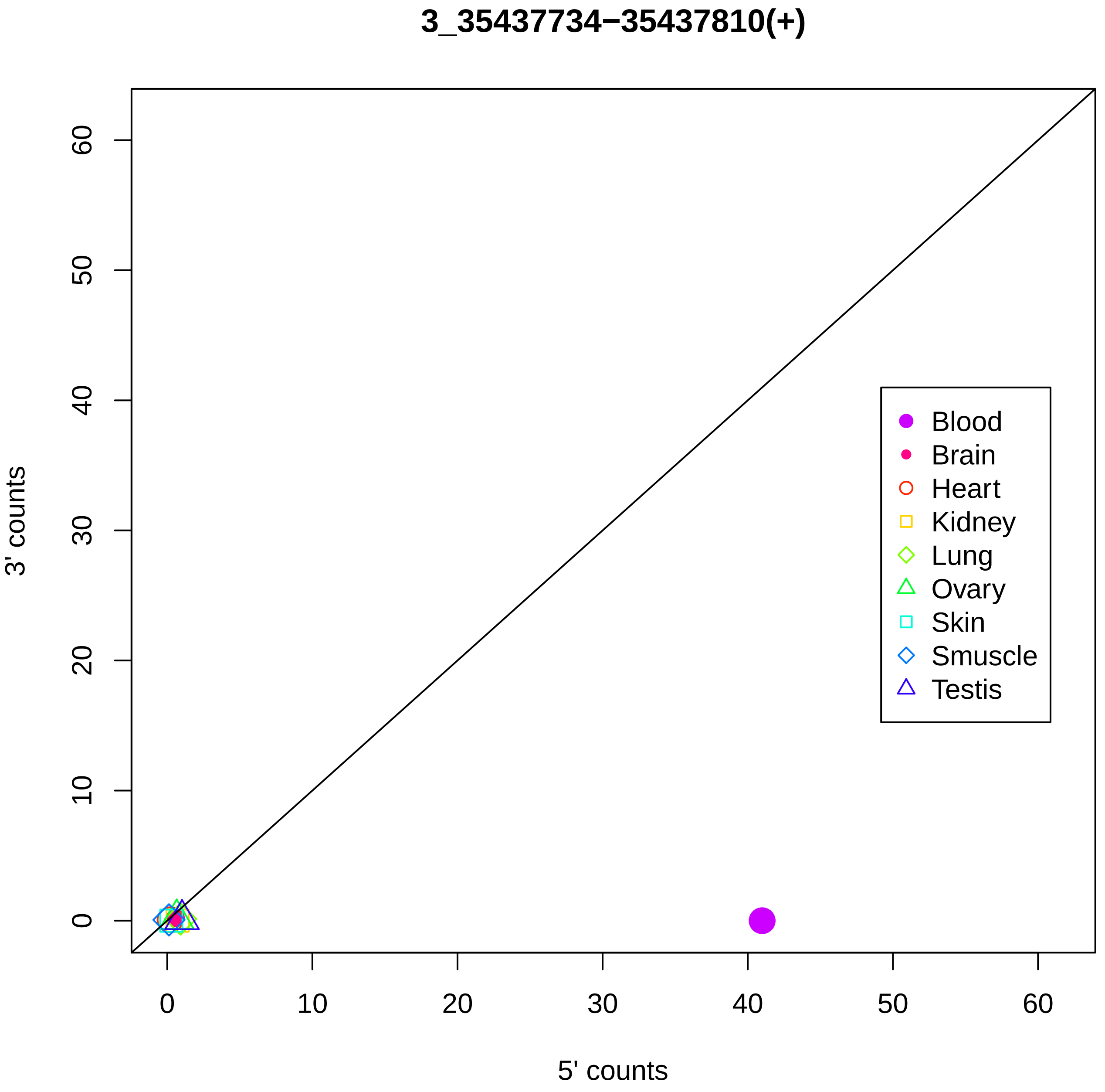

3_35437734−35437810(+)
3' counts
5' counts
0
10
20
30
40
50
60
Blood
Brain
Heart
Kidney
Lung
Ovary
Skin
Smuscle
Testis

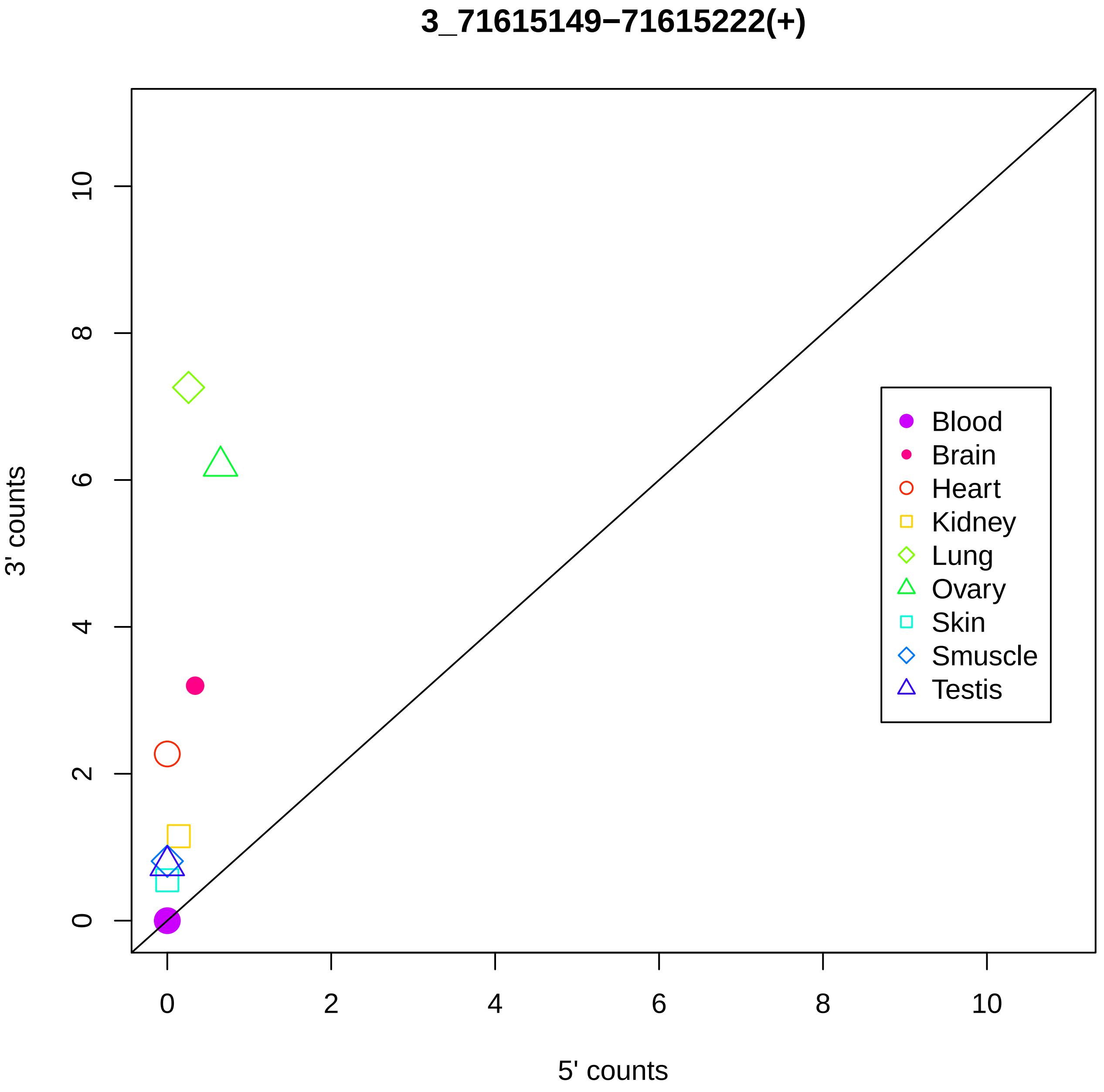

3_71615149−71615222(+)
3' counts
5' counts
0
2
4
6
8
10
Blood
Brain
Heart
Kidney
Lung
Ovary
Skin
Smuscle
Testis

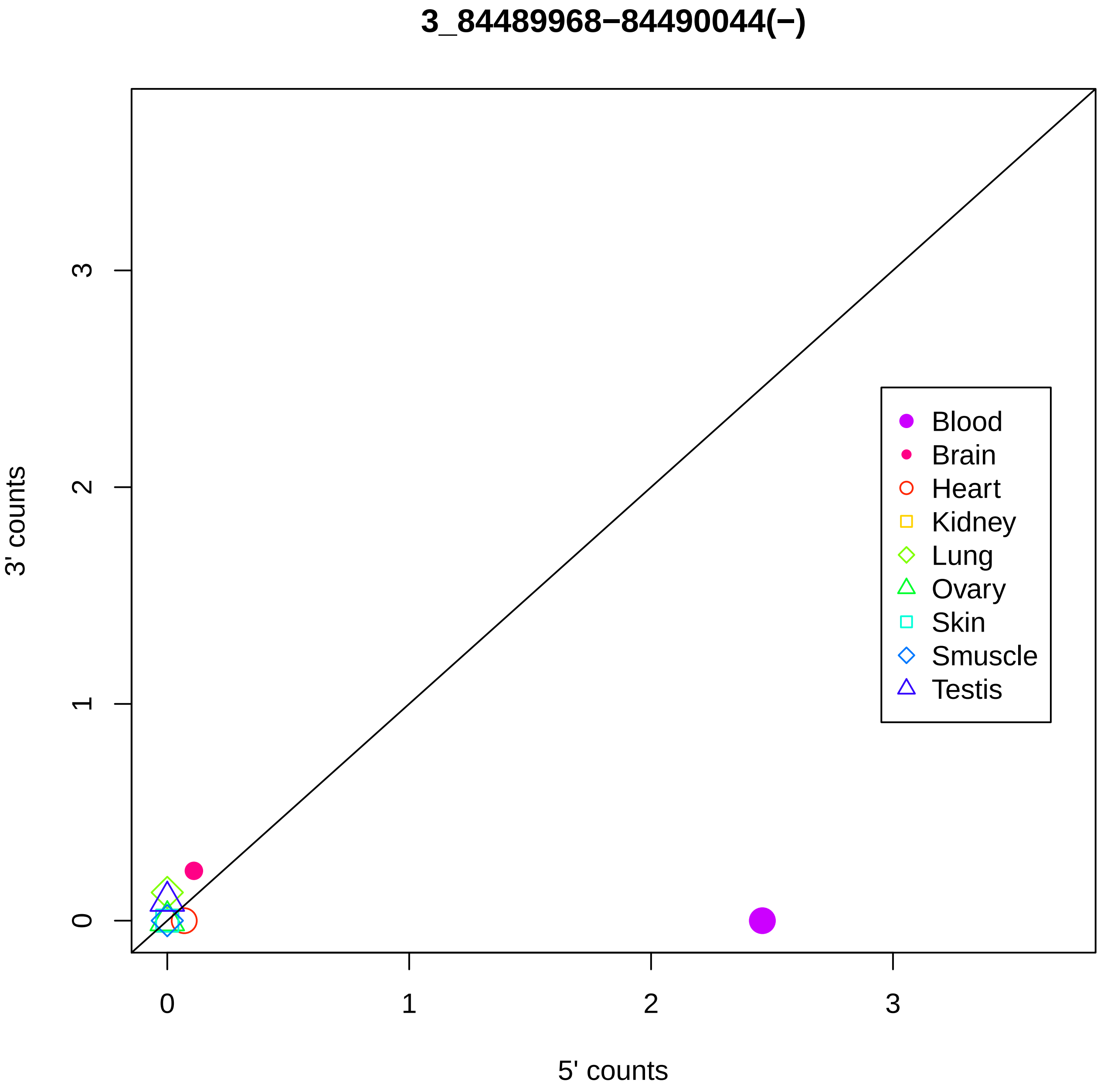

3_84489968−84490044(−)
3' counts
5' counts
0
1
2
3
0
1
2
3
Blood
Brain
Heart
Kidney
Lung
Ovary
Skin
Smuscle
Testis

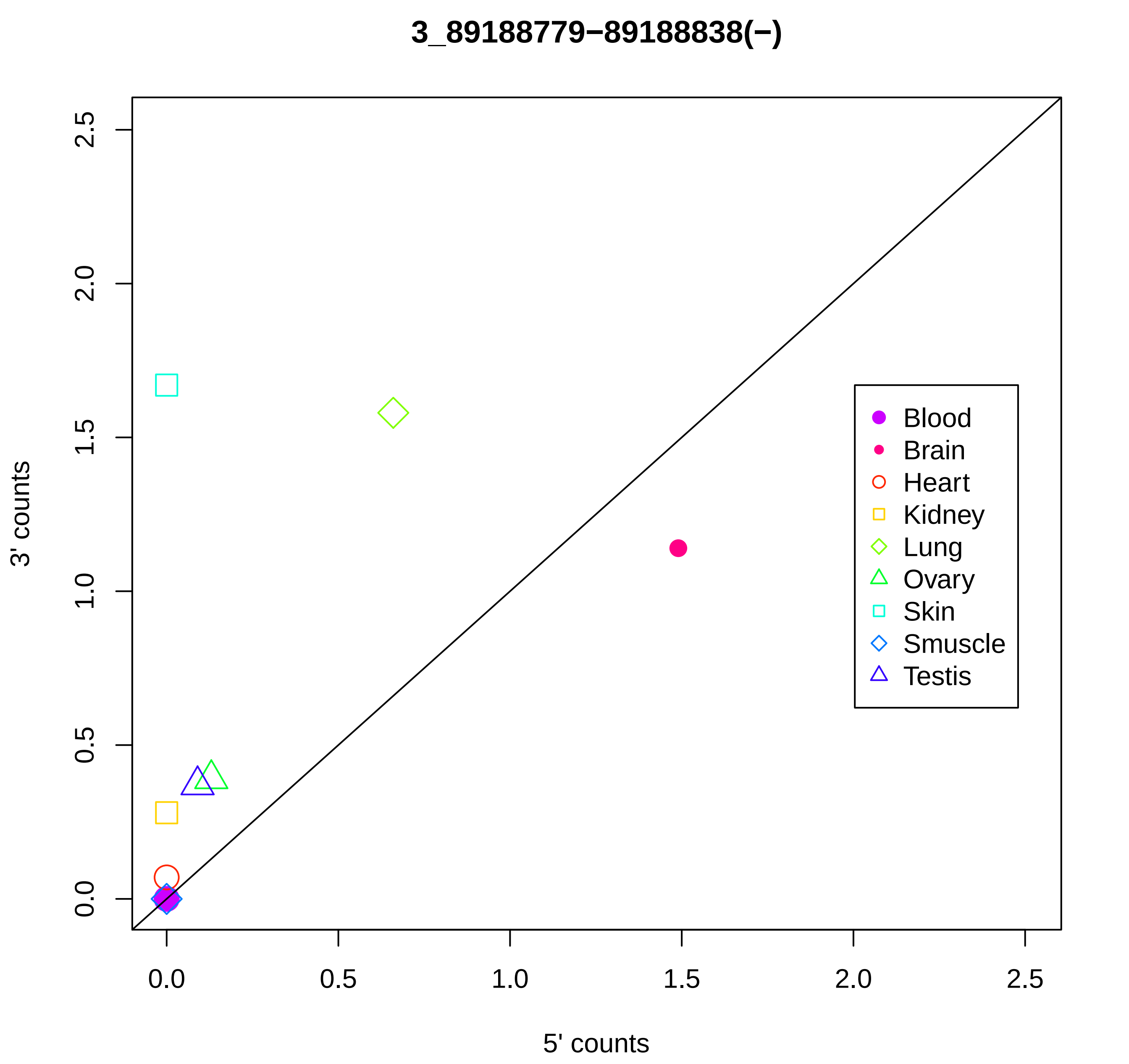

3_89188779−89188838(−)
3' counts
5' counts
0.0
0.5
1.0
1.5
2.0
2.5
Blood
Brain
Heart
Kidney
Lung
Ovary
Skin
Smuscle
Testis

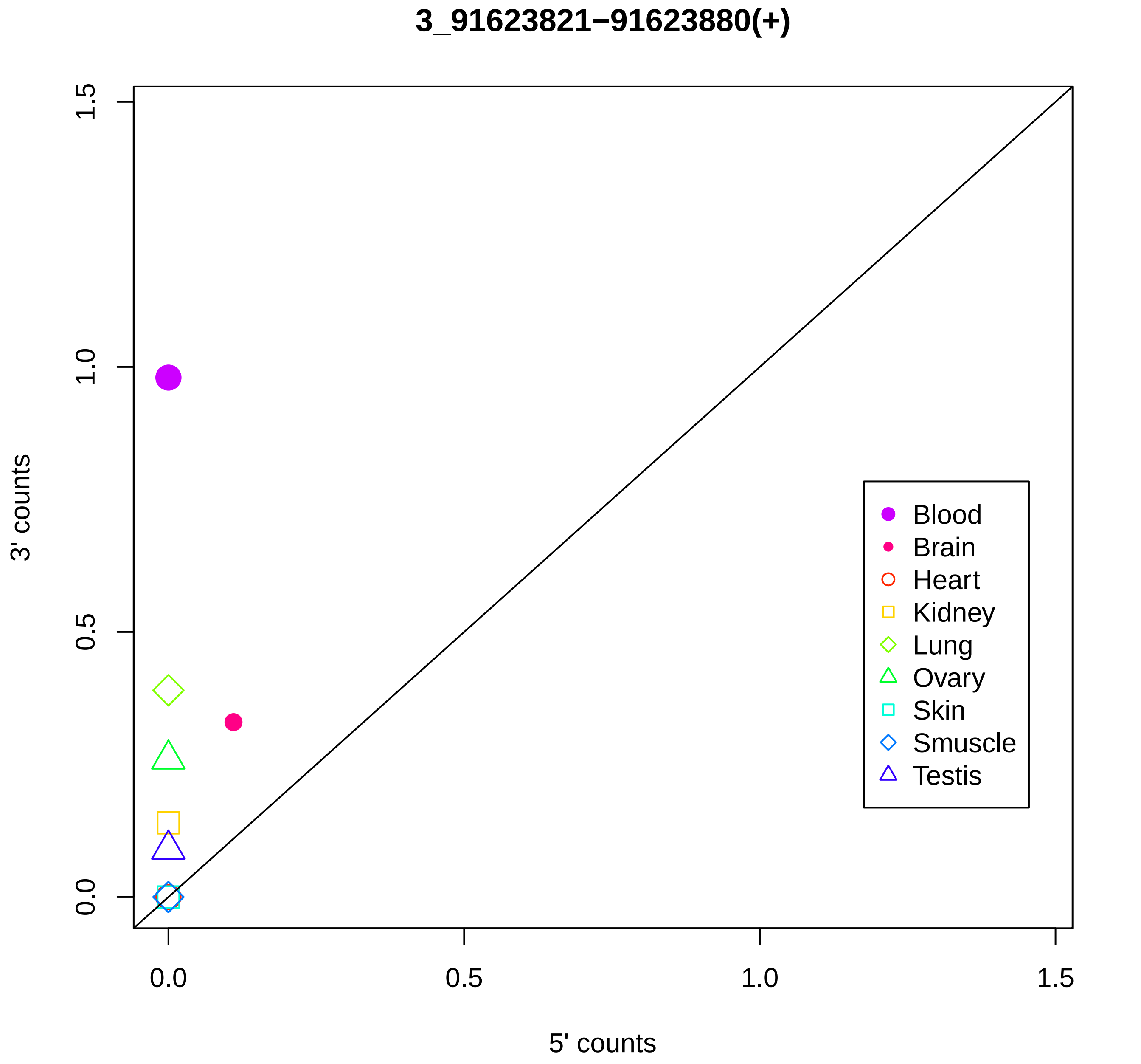

3_91623821−91623880(+)
3' counts
5' counts
0.0
0.5
1.0
1.5
0.0
0.5
1.0
1.5
Blood
Brain
Heart
Kidney
Lung
Ovary
Skin
Smuscle
Testis

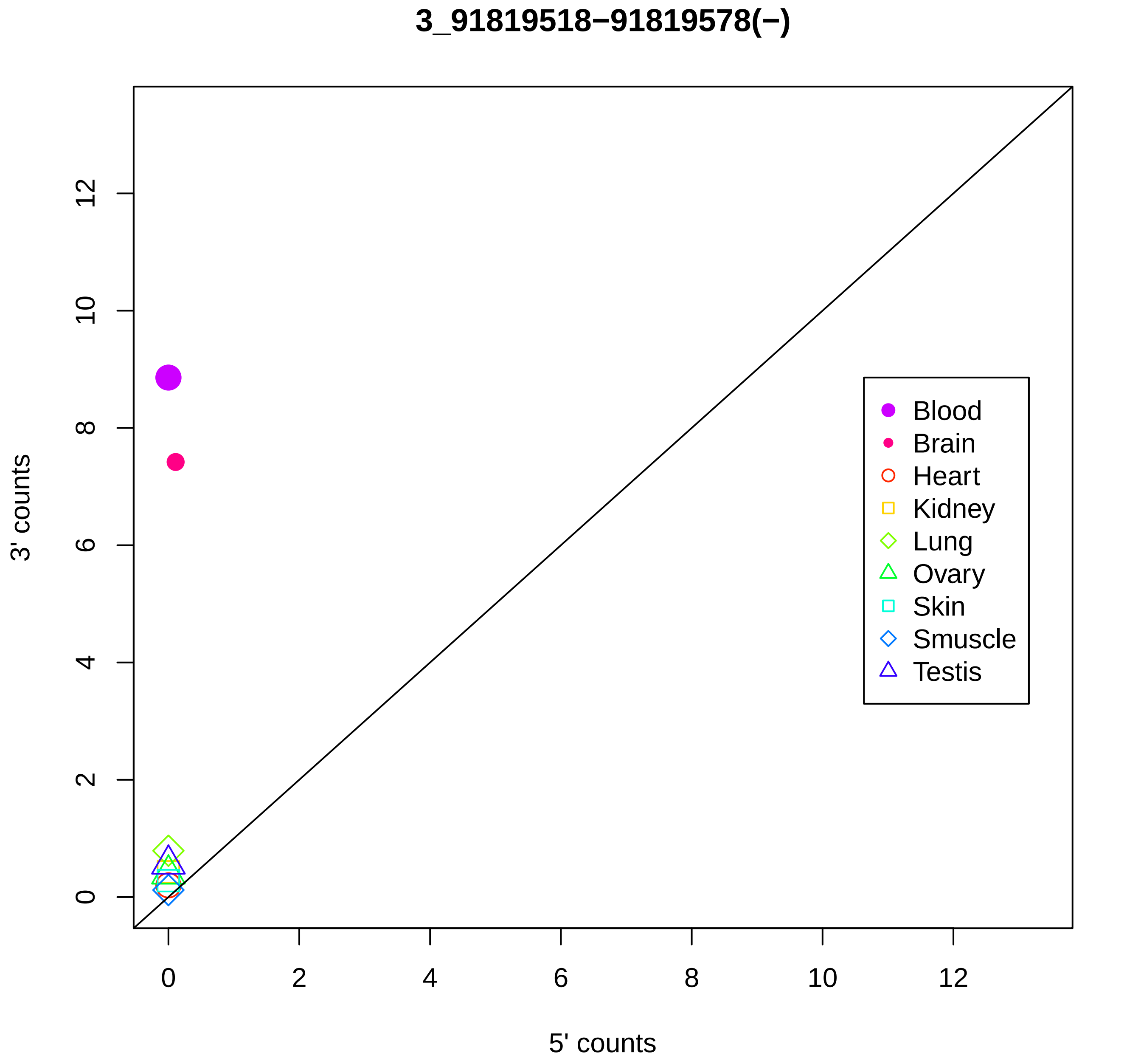

3_91819518−91819578(−)
Blood
Brain
Heart
Kidney
Lung
Ovary
Skin
Smuscle
Testis
0
2
4
6
8
10
12
5' counts
3' counts

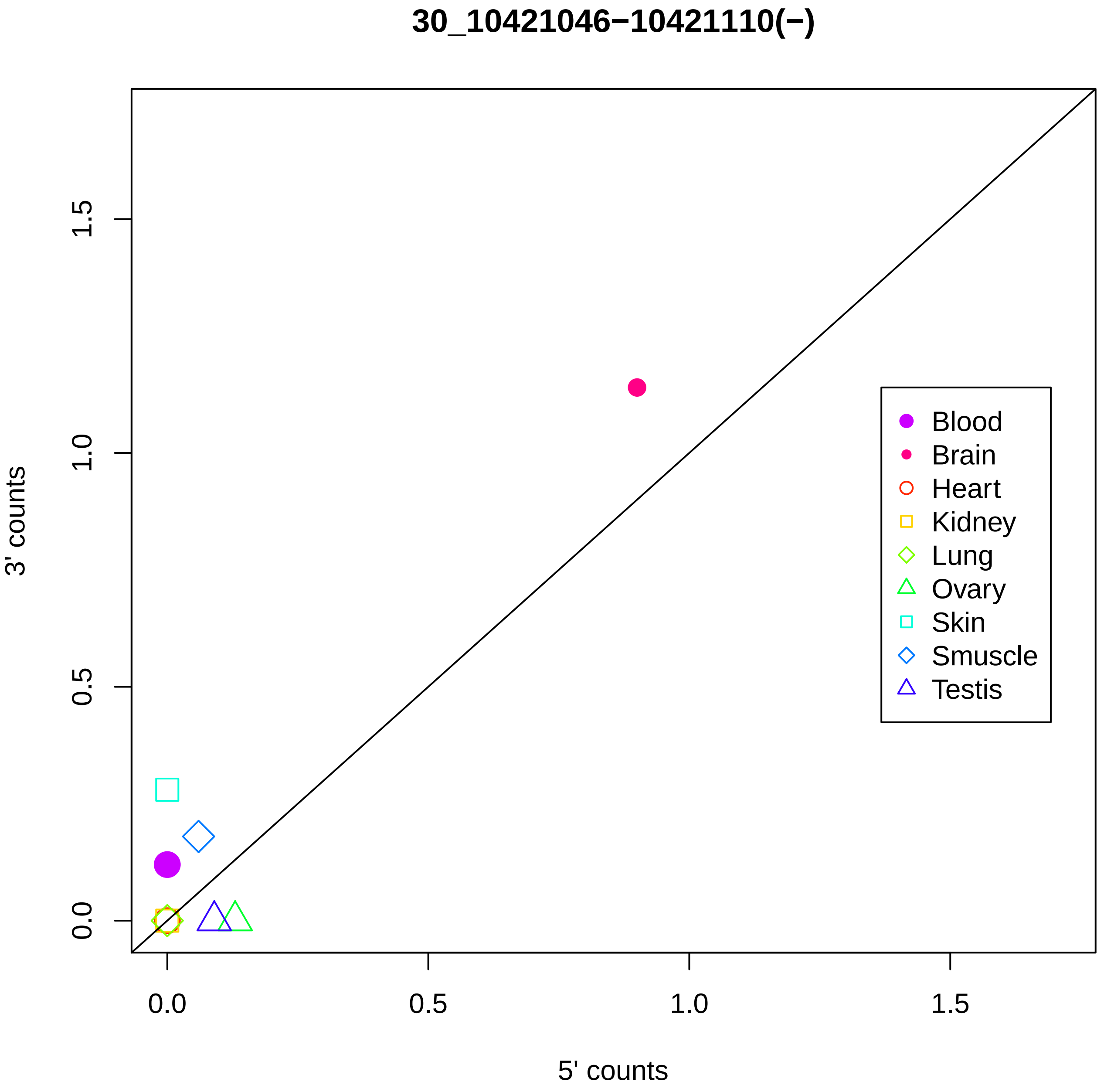

30_10421046−10421110(−)
Blood
Brain
Heart
Kidney
Lung
Ovary
Skin
Smuscle
Testis
0.0
0.5
1.0
1.5
5' counts
3' counts

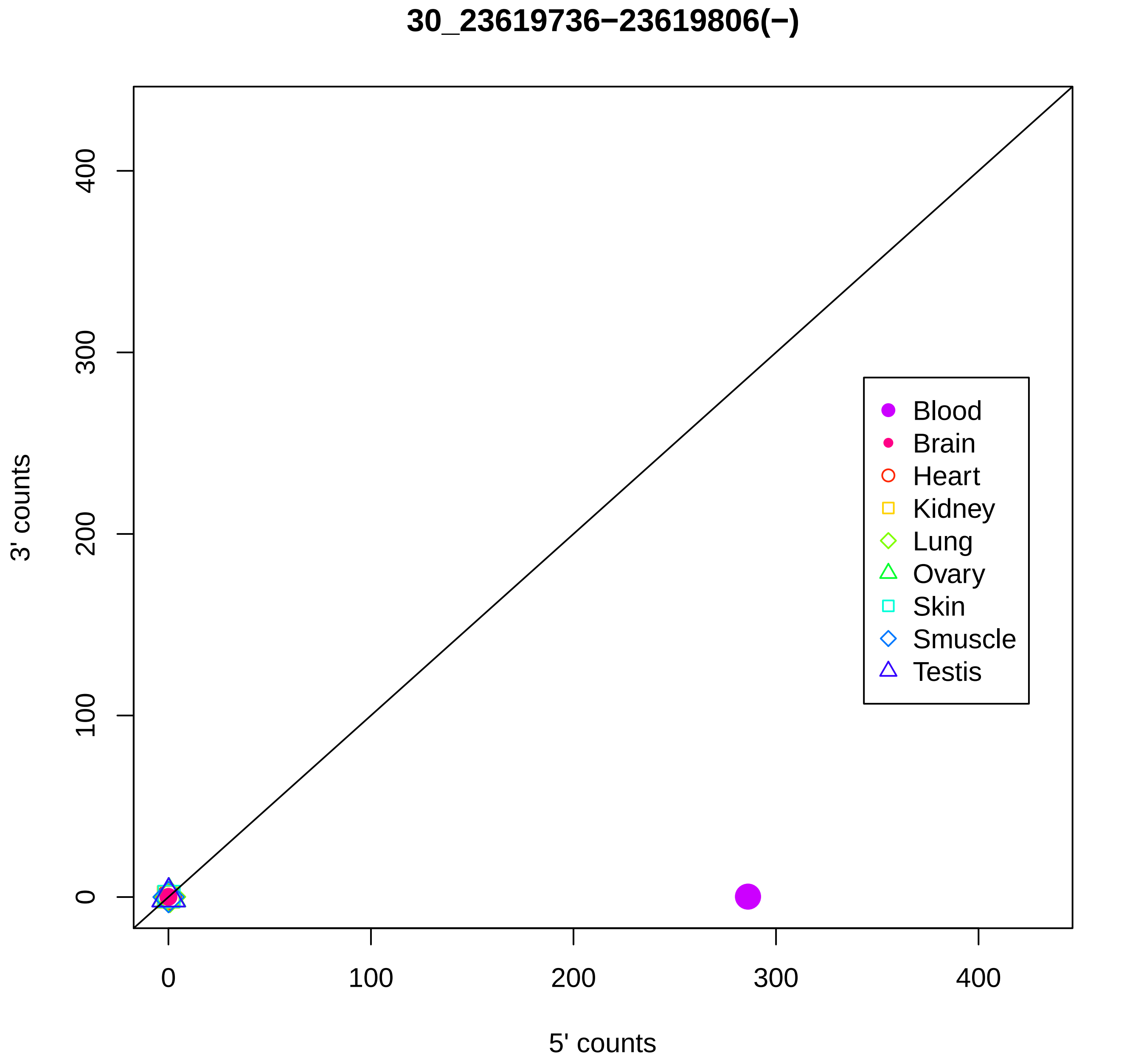
30_23619736−23619806(−)
3' counts
5' counts
0
100
200
300
400
0
100
200
300
400
Blood
Brain
Heart
Kidney
Lung
Ovary
Skin
Smuscle
Testis

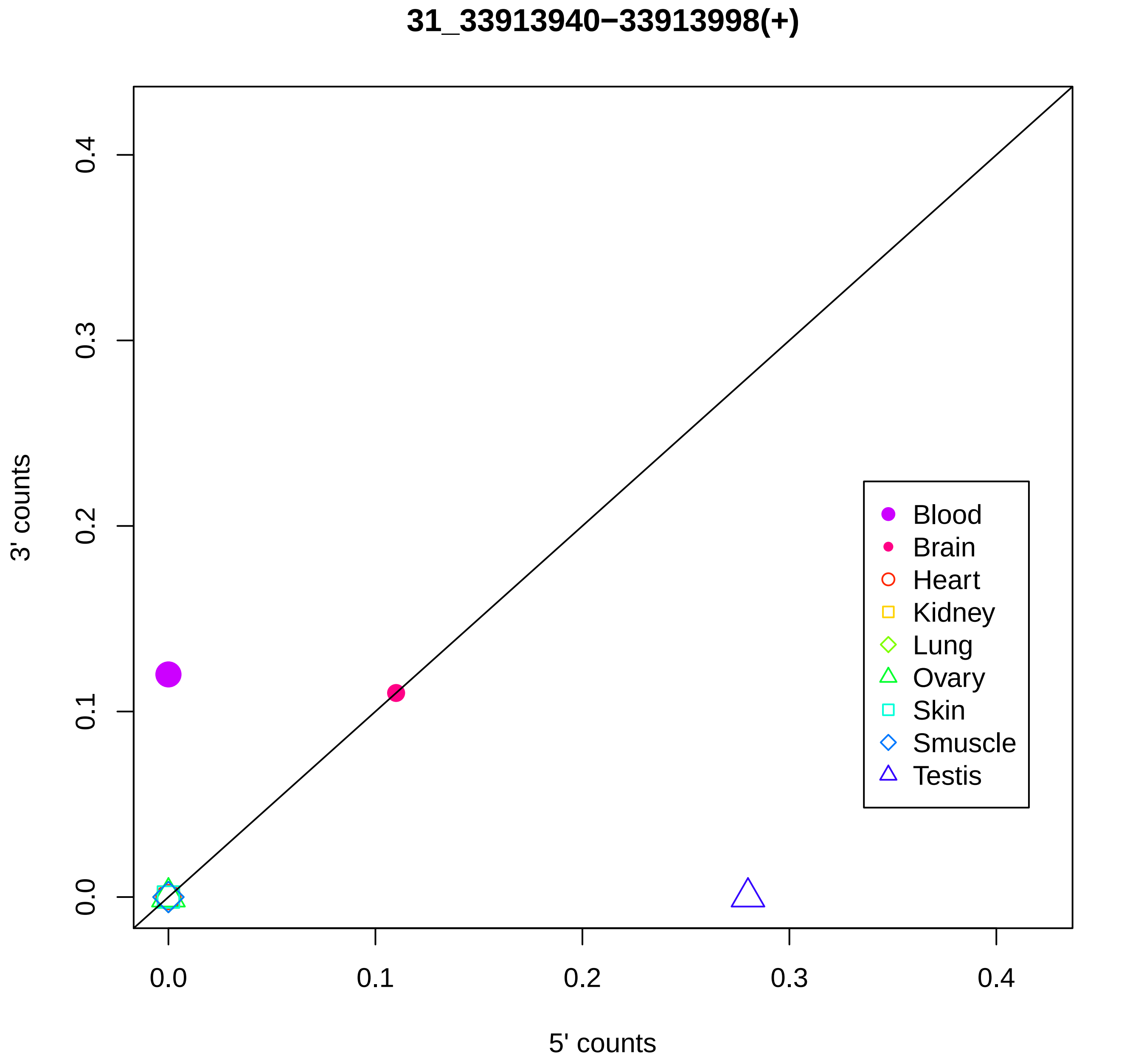
31_33913940−33913998(+)
3' counts
5' counts
0.0
0.1
0.2
0.3
0.4
Blood
Brain
Heart
Kidney
Lung
Ovary
Skin
Smuscle
Testis

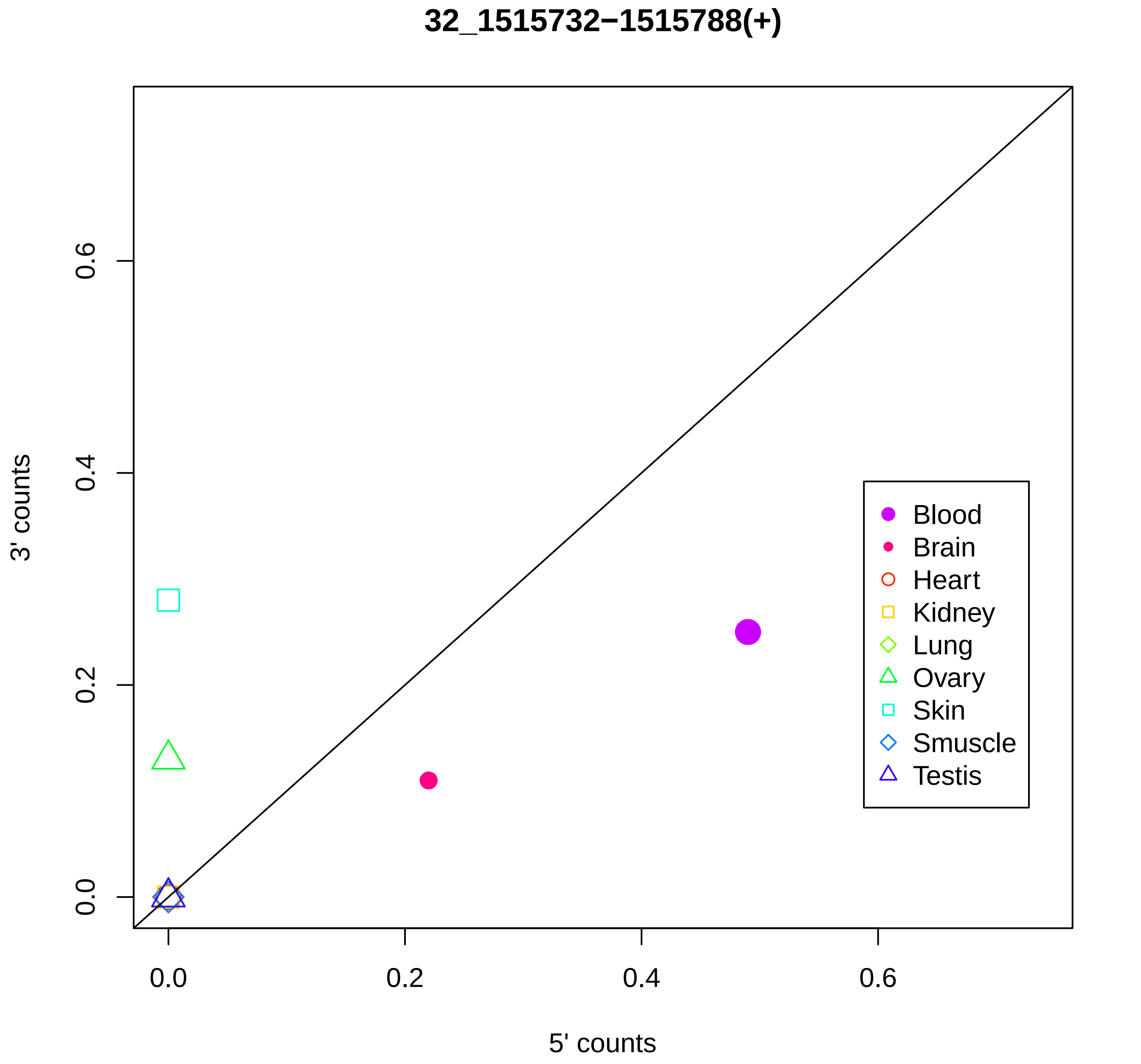
32_1515732−1515788(+)
3' counts
5' counts
0.0
0.2
0.4
0.6
Blood
Brain
Heart
Kidney
Lung
Ovary
Skin
Smuscle
Testis

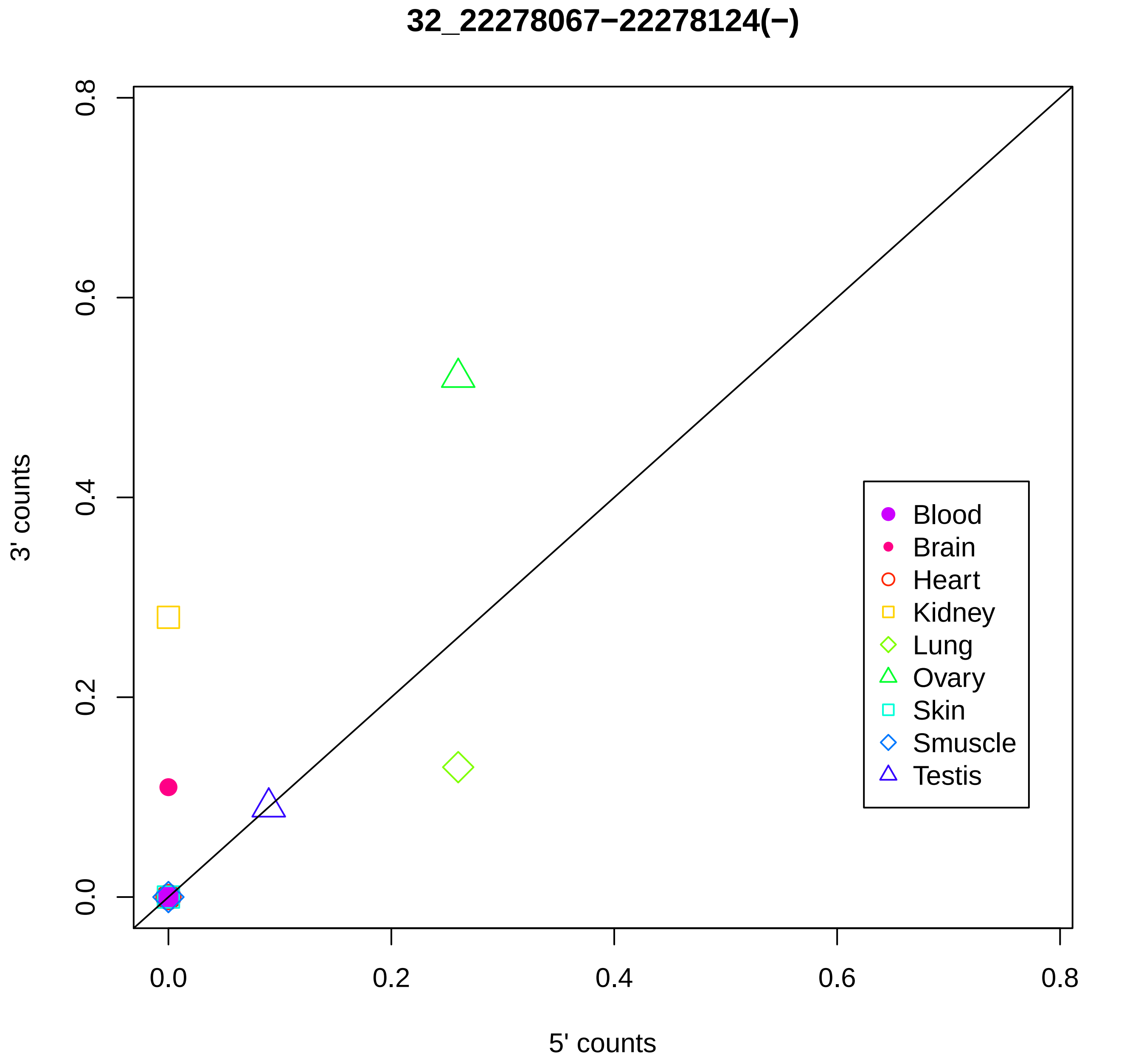
32_22278067−22278124(−)
3' counts
5' counts
0.0
0.2
0.4
0.6
0.8
Blood
Brain
Heart
Kidney
Lung
Ovary
Skin
Smuscle
Testis

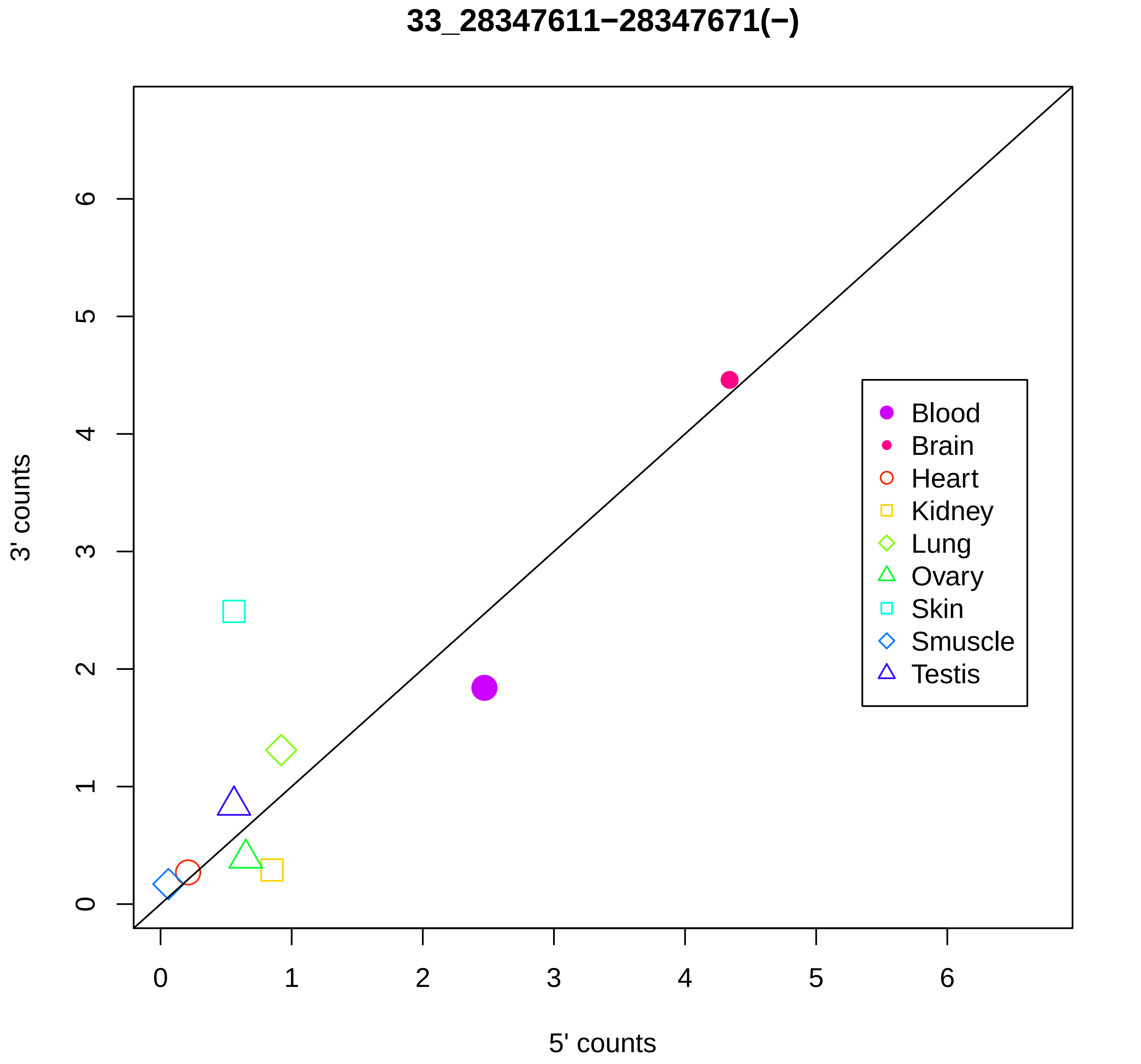

33_28347611−28347671(−)
3' counts
5' counts
0
1
2
3
4
5
6
Blood
Brain
Heart
Kidney
Lung
Ovary
Skin
Smuscle
Testis

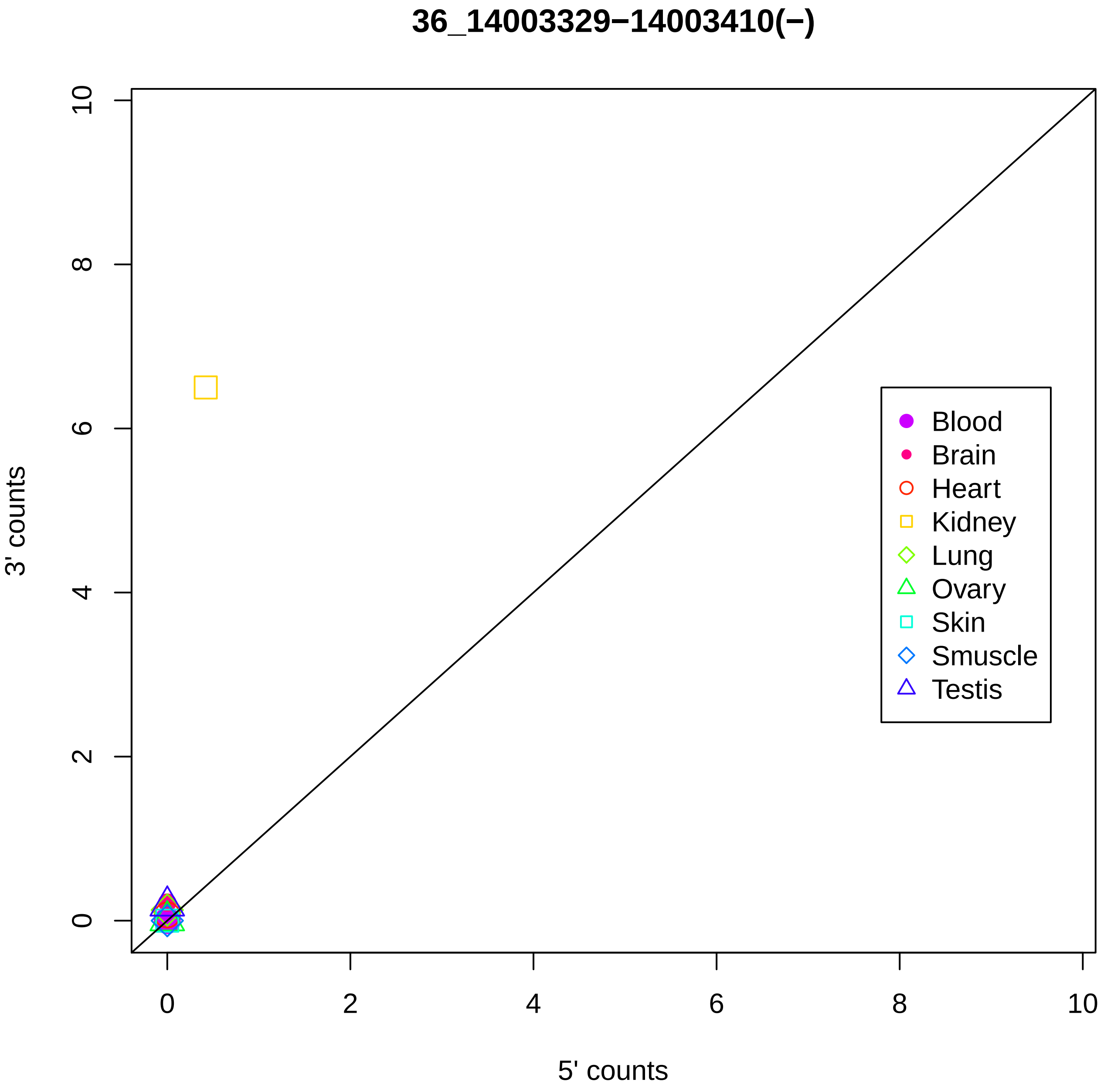

36_14003329−14003410(−)
3' counts
5' counts
0
2
4
6
8
10
Blood
Brain
Heart
Kidney
Lung
Ovary
Skin
Smuscle
Testis

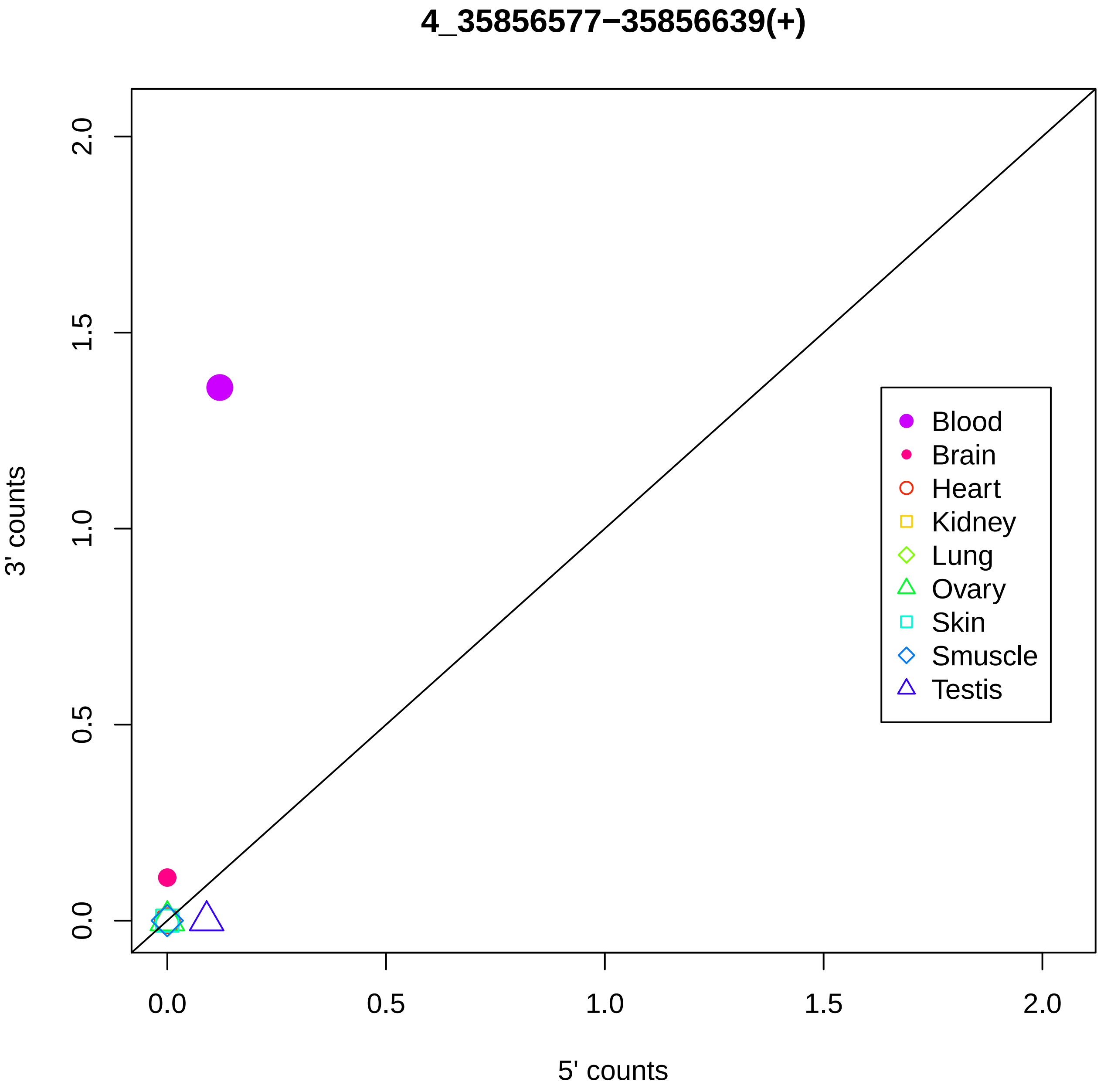
4_35856577−35856639(+)
3' counts
5' counts
0.0
0.5
1.0
1.5
2.0
Blood
Brain
Heart
Kidney
Lung
Ovary
Skin
Smuscle
Testis

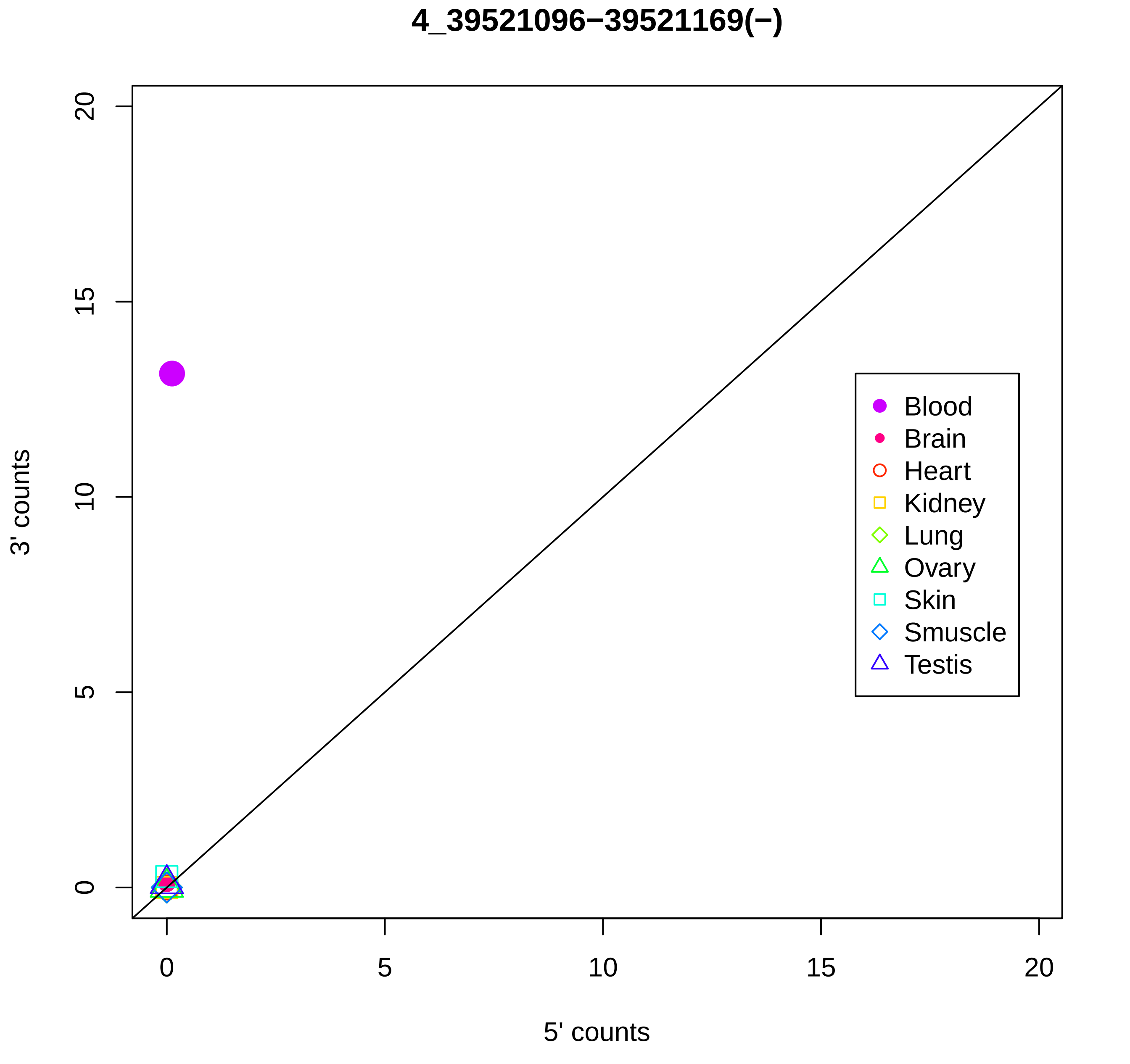

4_39521096−39521169(−)
3' counts
5' counts
0
5
10
15
20
0
5
10
15
20
Blood
Brain
Heart
Kidney
Lung
Ovary
Skin
Smuscle
Testis

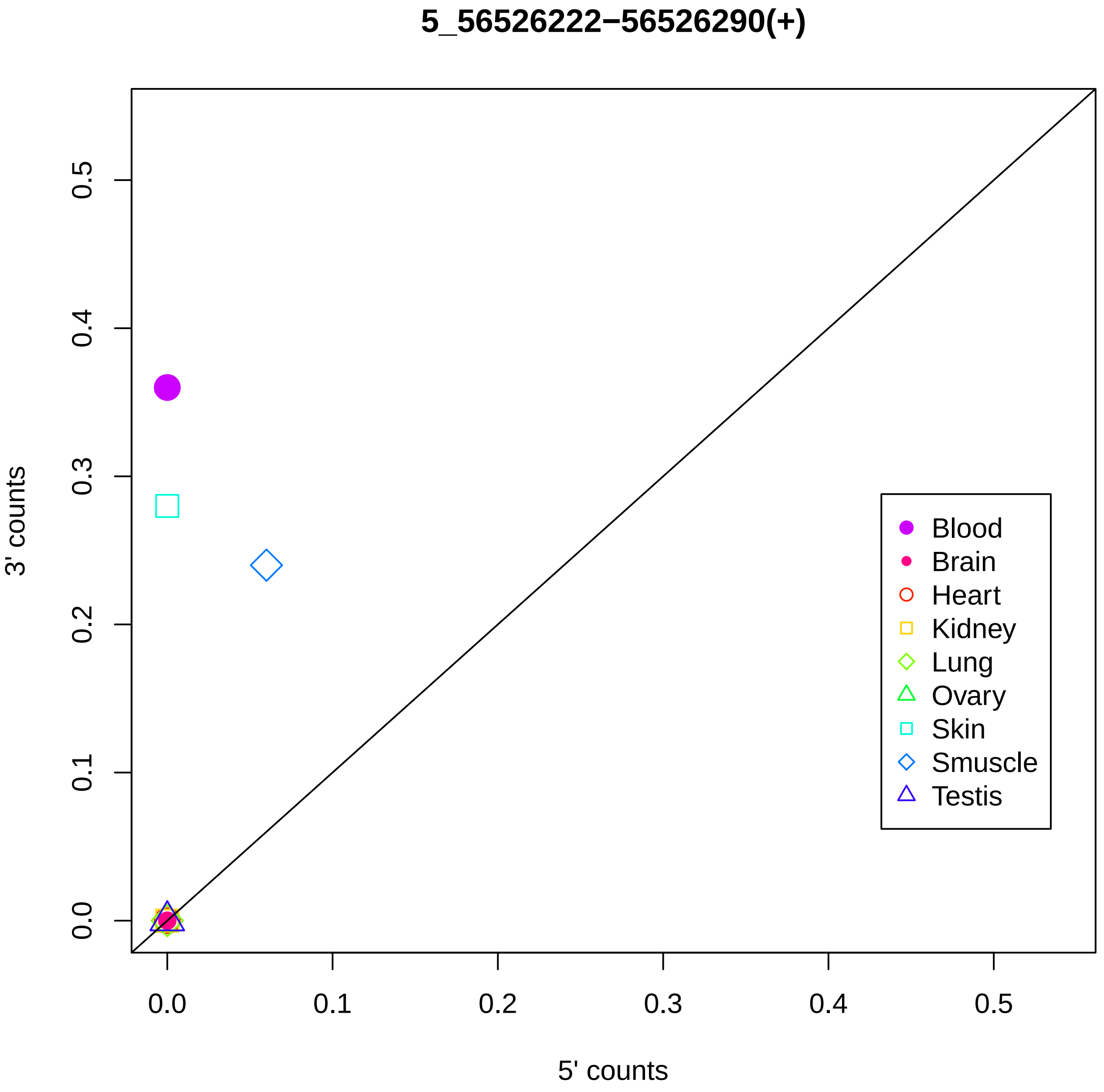

5_56526222−56526290(+)
3' counts
5' counts
0.0
0.1
0.2
0.3
0.4
0.5
Blood
Brain
Heart
Kidney
Lung
Ovary
Skin
Smuscle
Testis

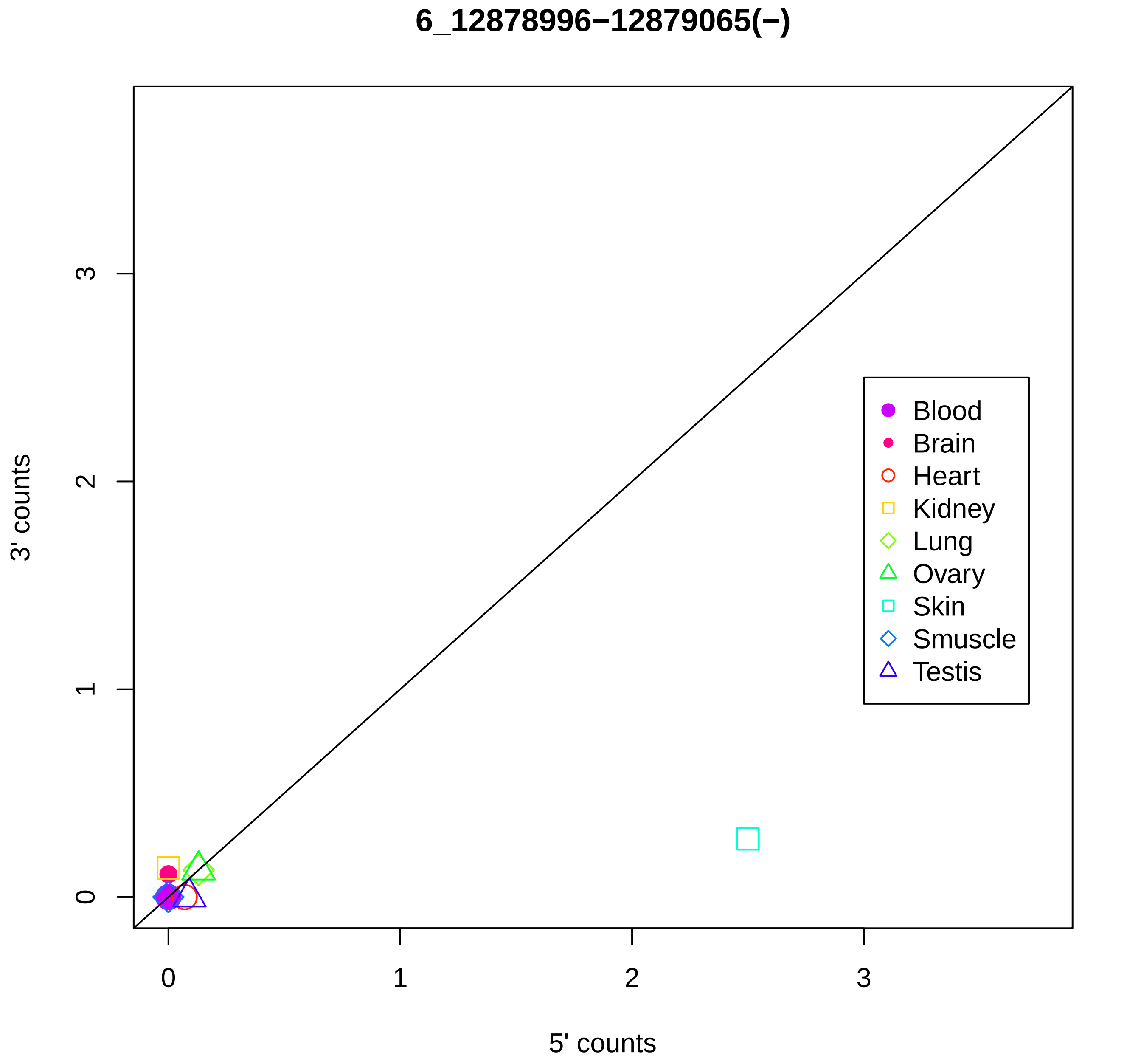

6_12878996−12879065(−)
3' counts
5' counts
0
1
2
3
0
1
2
3
Blood
Brain
Heart
Kidney
Lung
Ovary
Skin
Smuscle
Testis

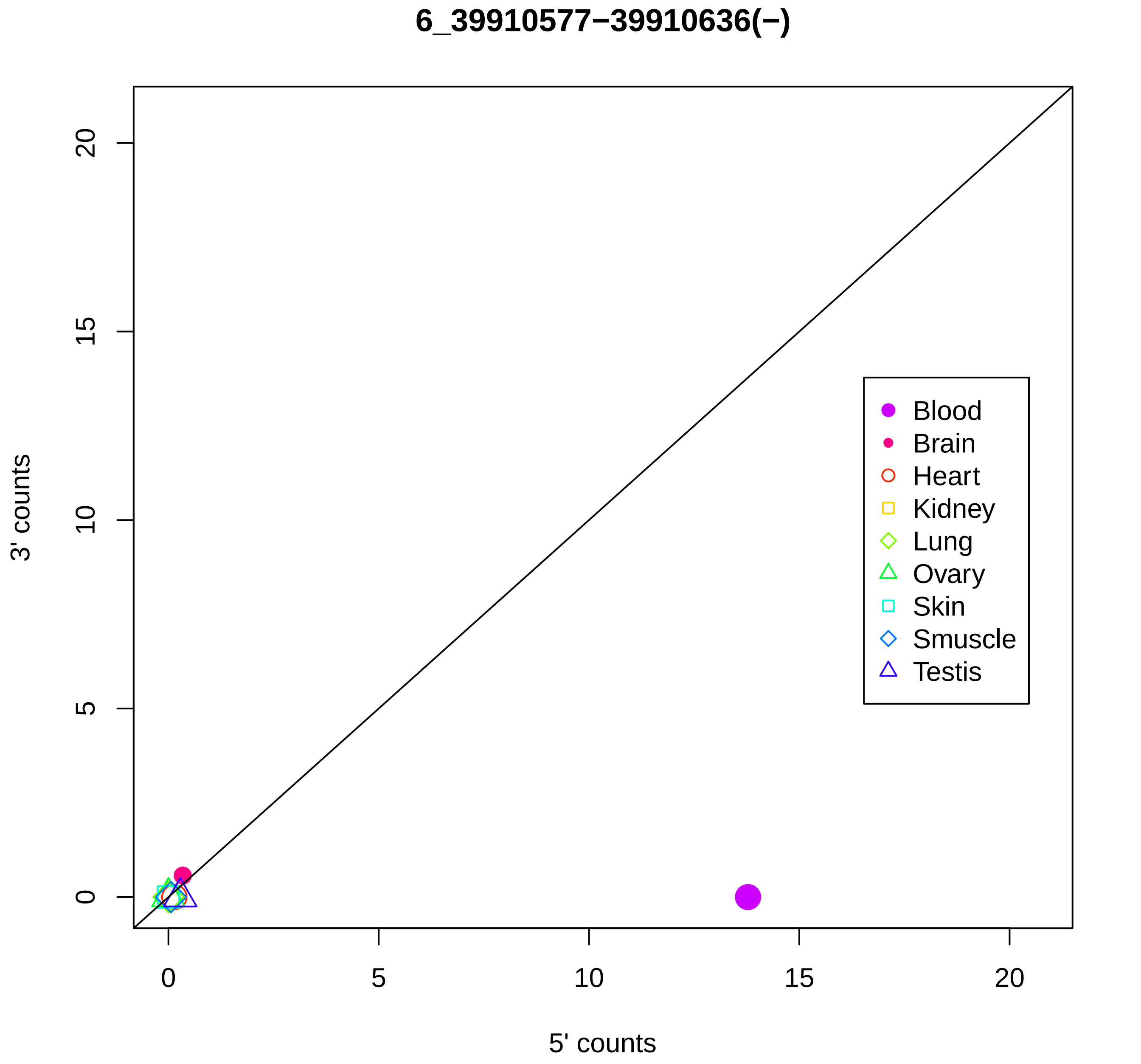

6_39910577−39910636(−)
3' counts
5' counts
0
5
10
15
20
Blood
Brain
Heart
Kidney
Lung
Ovary
Skin
Smuscle
Testis

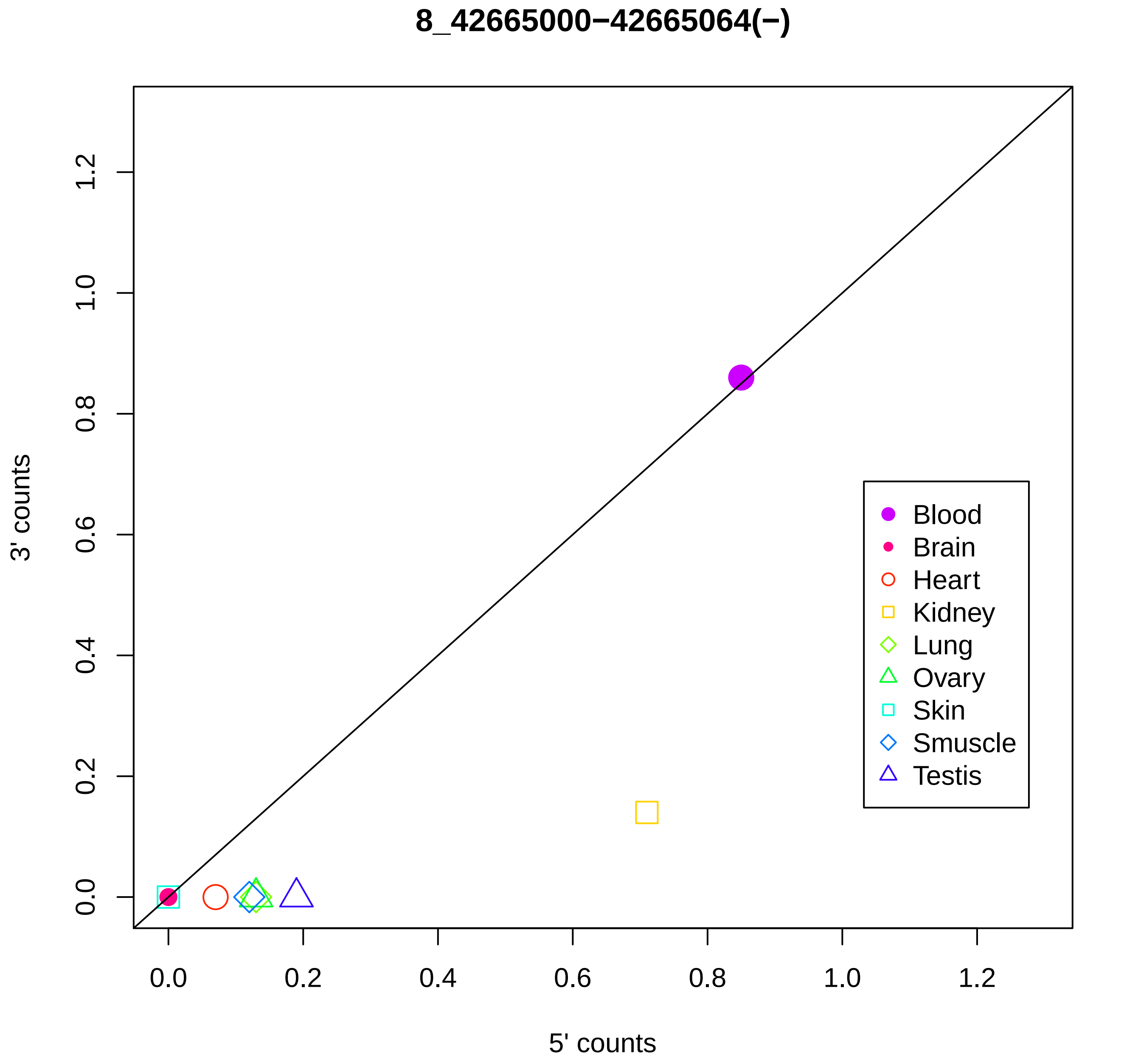

8_42665000−42665064(−)
3' counts
5' counts
0.0
0.2
0.4
0.6
0.8
1.0
1.2
Blood
Brain
Heart
Kidney
Lung
Ovary
Skin
Smuscle
Testis

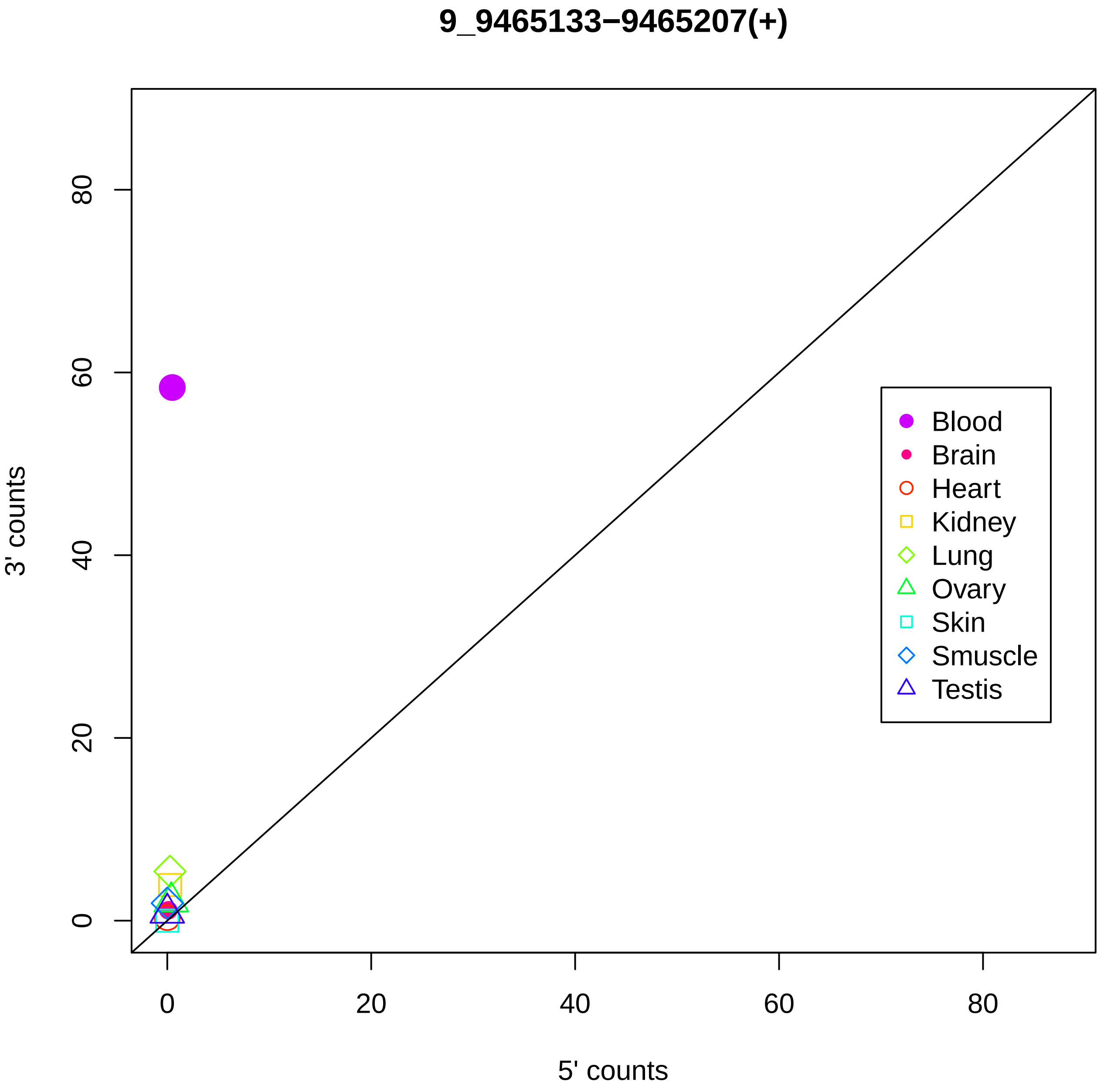

9_9465133−9465207(+)
80
60
40
20
0
3' counts
0
20
40
60
80
5' counts
Blood
Brain
Heart
Kidney
Lung
Ovary
Skin
Smuscle
Testis

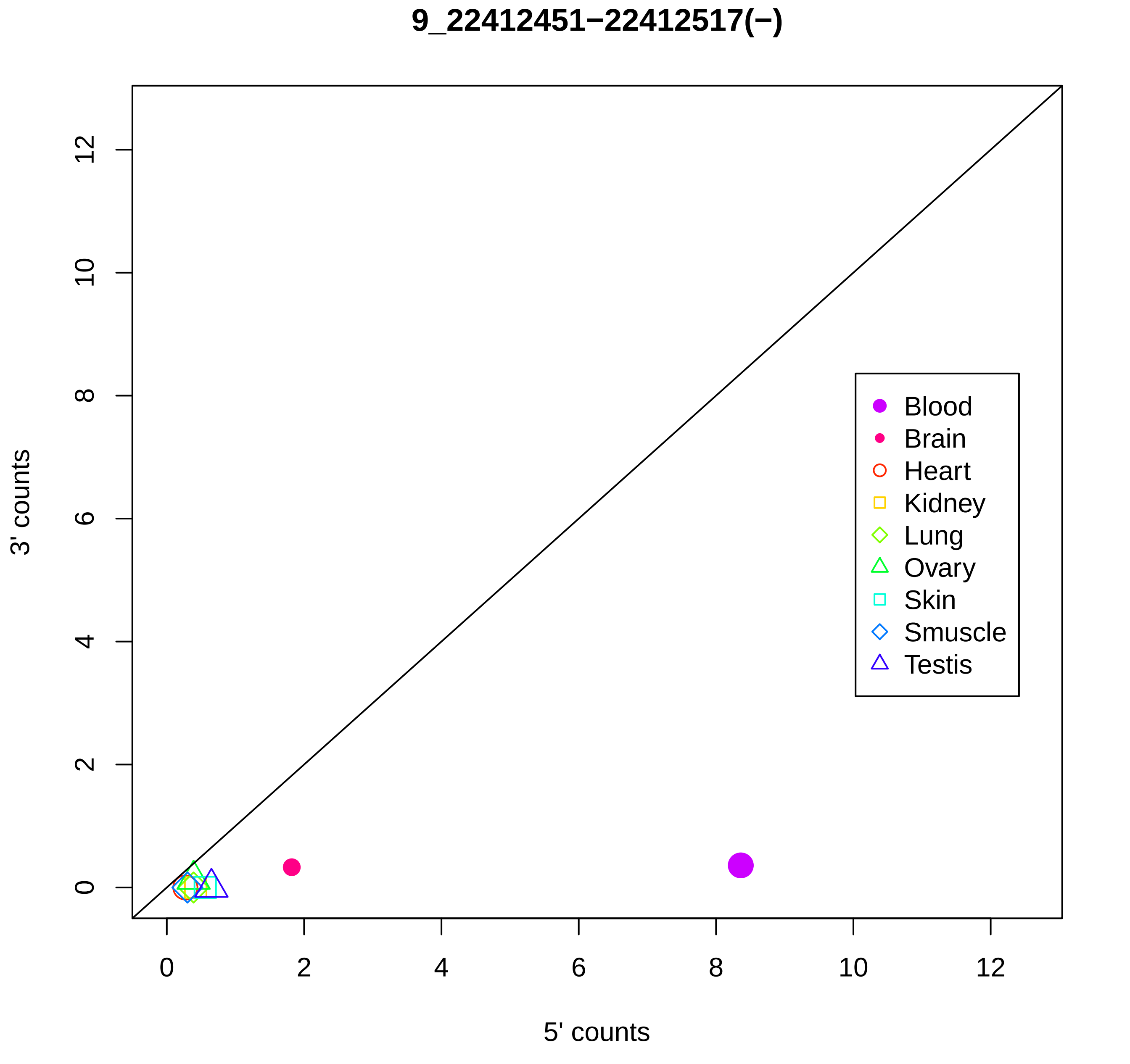

9_22412451−22412517(−)
3' counts
5' counts
0
2
4
6
8
10
12
Blood
Brain
Heart
Kidney
Lung
Ovary
Skin
Smuscle
Testis

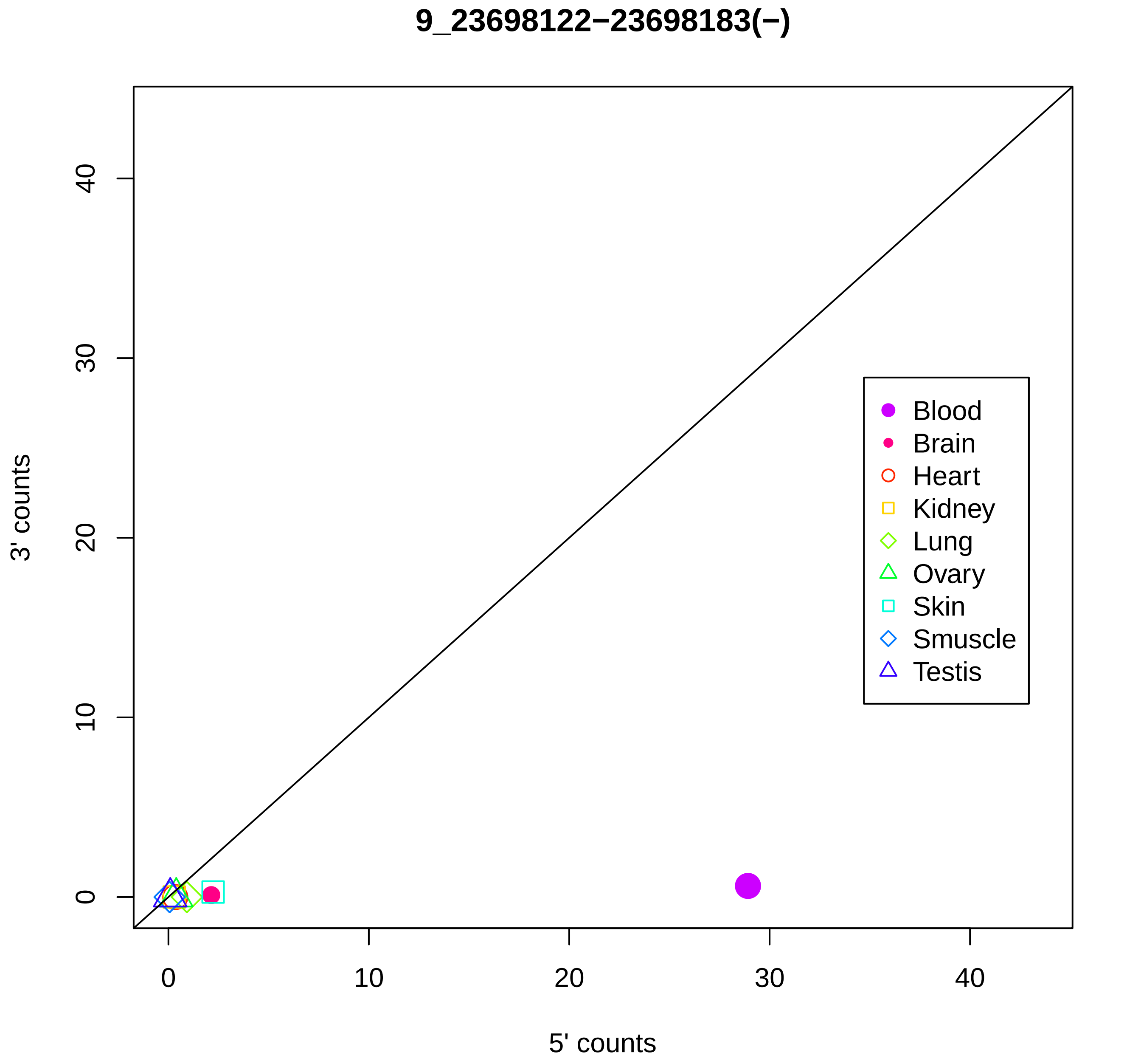

9_23698122−23698183(−)
3' counts
5' counts
0
10
20
30
40
0
10
20
30
40
Blood
Brain
Heart
Kidney
Lung
Ovary
Skin
Smuscle
Testis

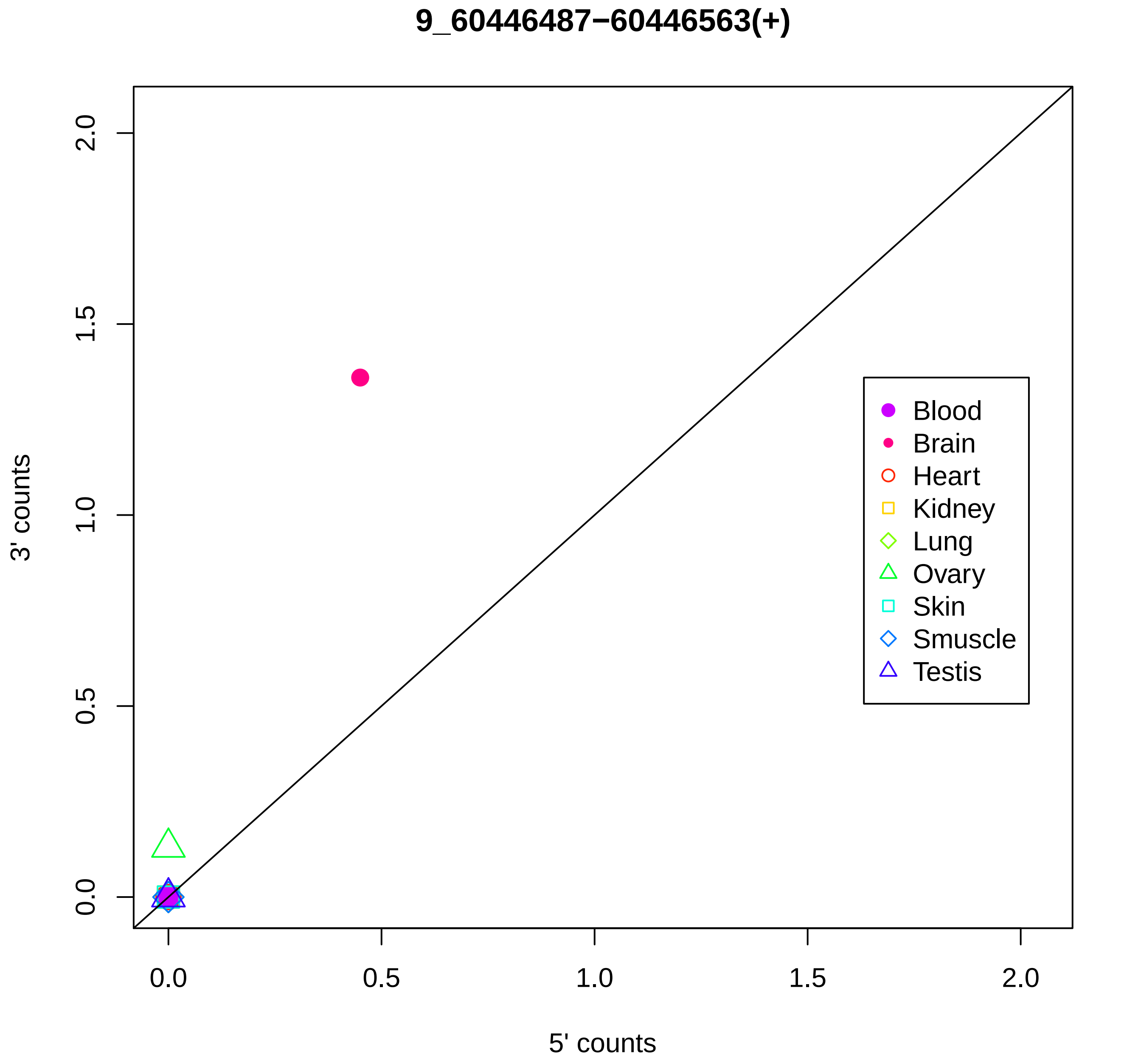

9_60446487−60446563(+)
3' counts
5' counts
0.0
0.5
1.0
1.5
2.0
Blood
Brain
Heart
Kidney
Lung
Ovary
Skin
Smuscle
Testis

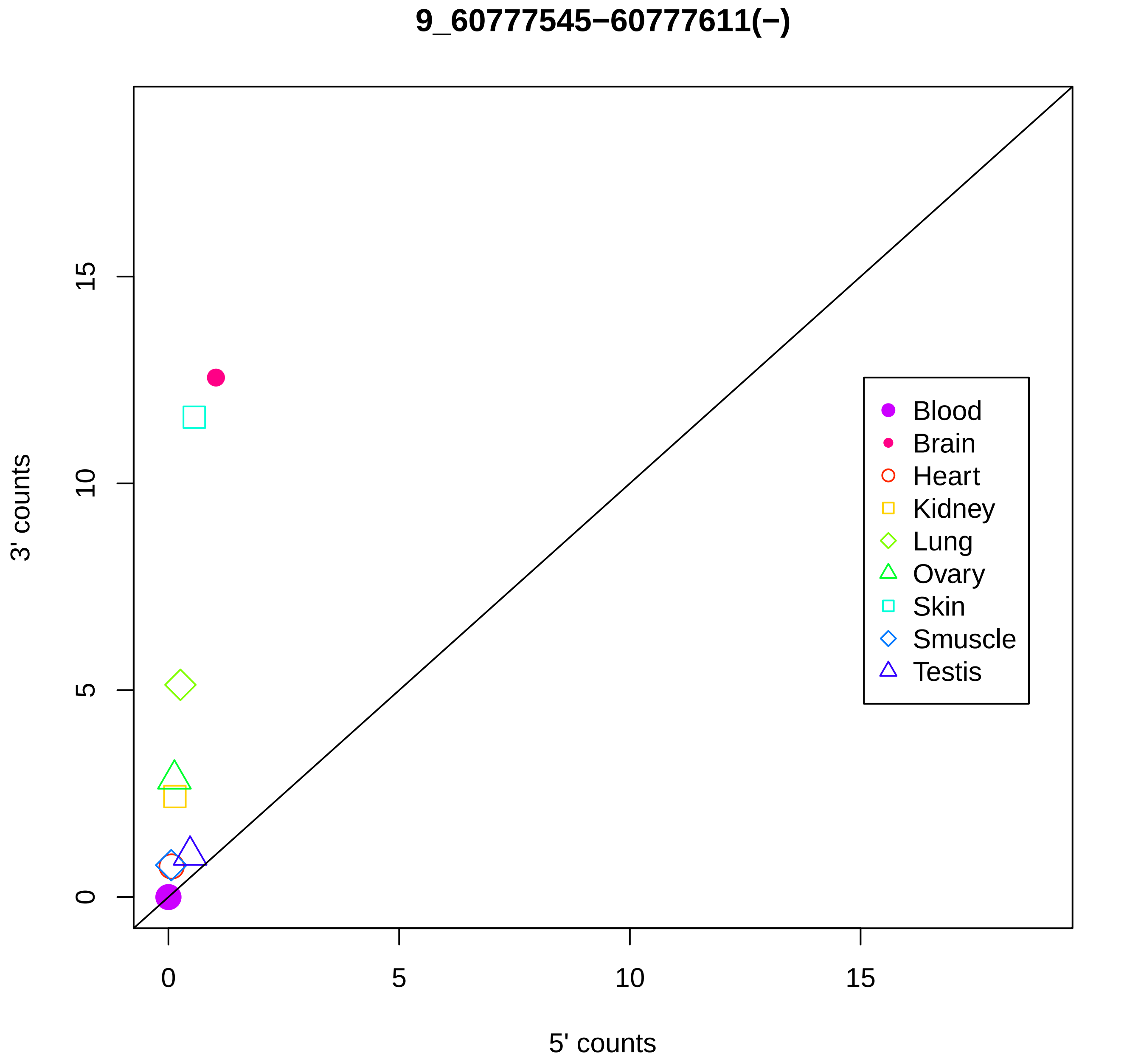

9_60777545−60777611(−)
3' counts
5' counts
0
5
10
15
0
5
10
15
Blood
Brain
Heart
Kidney
Lung
Ovary
Skin
Smuscle
Testis

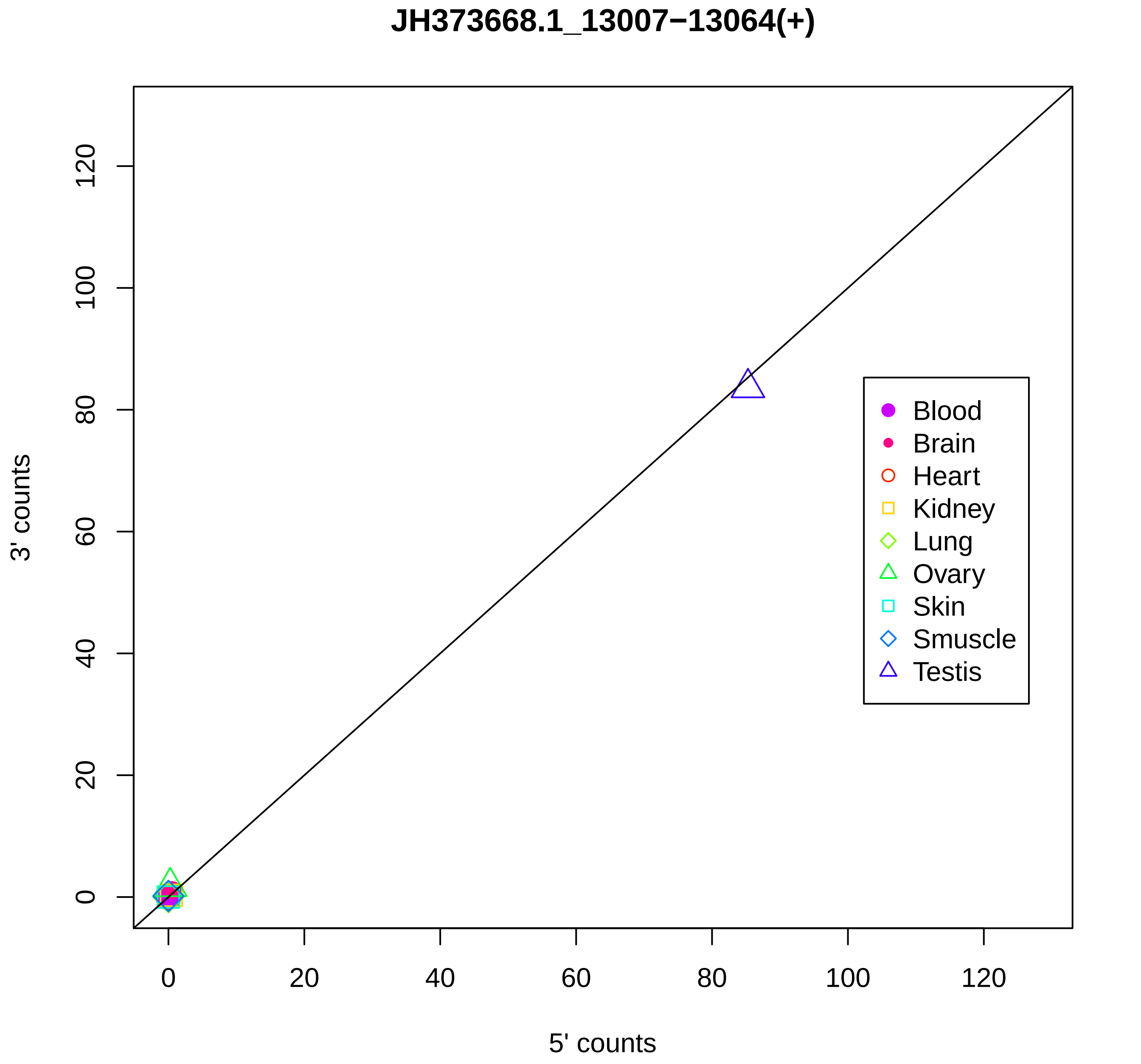

JH373668.1_13007−13064(+)
3' counts
5' counts
0
20
40
60
80
100
120
0
20
40
60
80
100
120
Blood
Brain
Heart
Kidney
Lung
Ovary
Skin
Smuscle
Testis

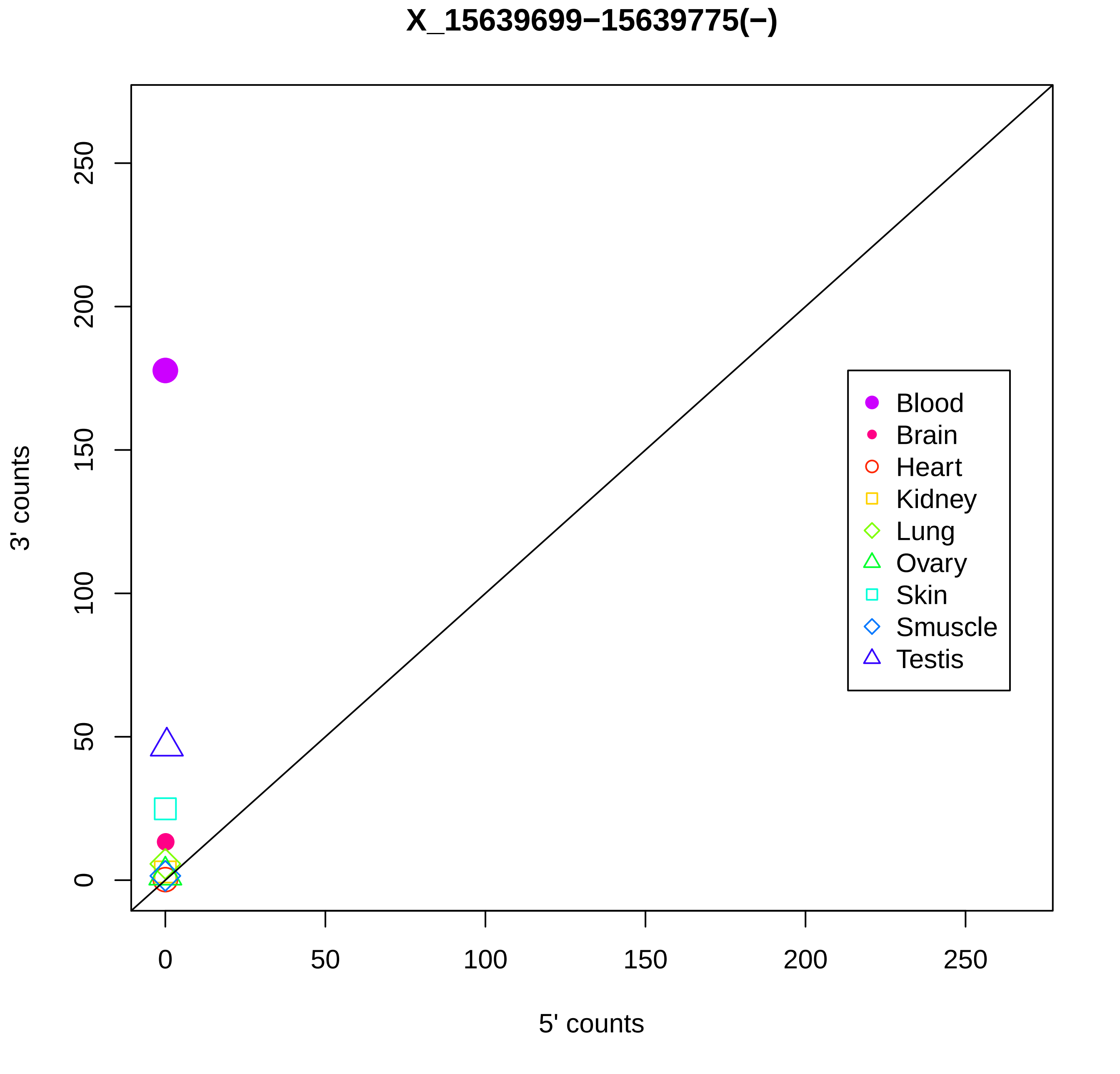
X_15639699−15639775(−)
3' counts
5' counts
0
50
100
150
200
250
Blood
Brain
Heart
Kidney
Lung
Ovary
Skin
Smuscle
Testis

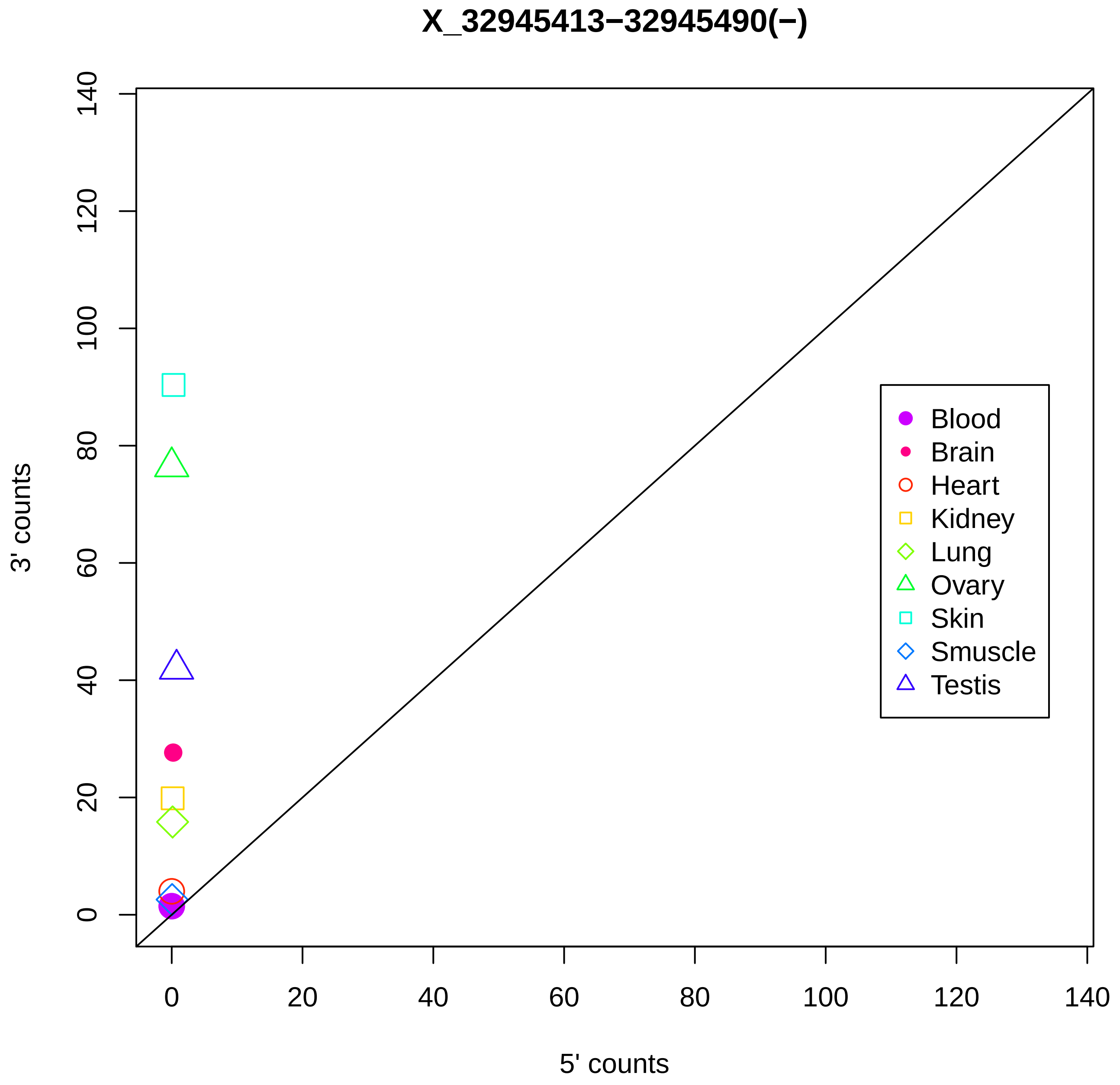

X_32945413−32945490(−)
Blood
Brain
Heart
Kidney
Lung
Ovary
Skin
Smuscle
Testis
3' counts
5' counts
0
20
40
60
80
100
120
140

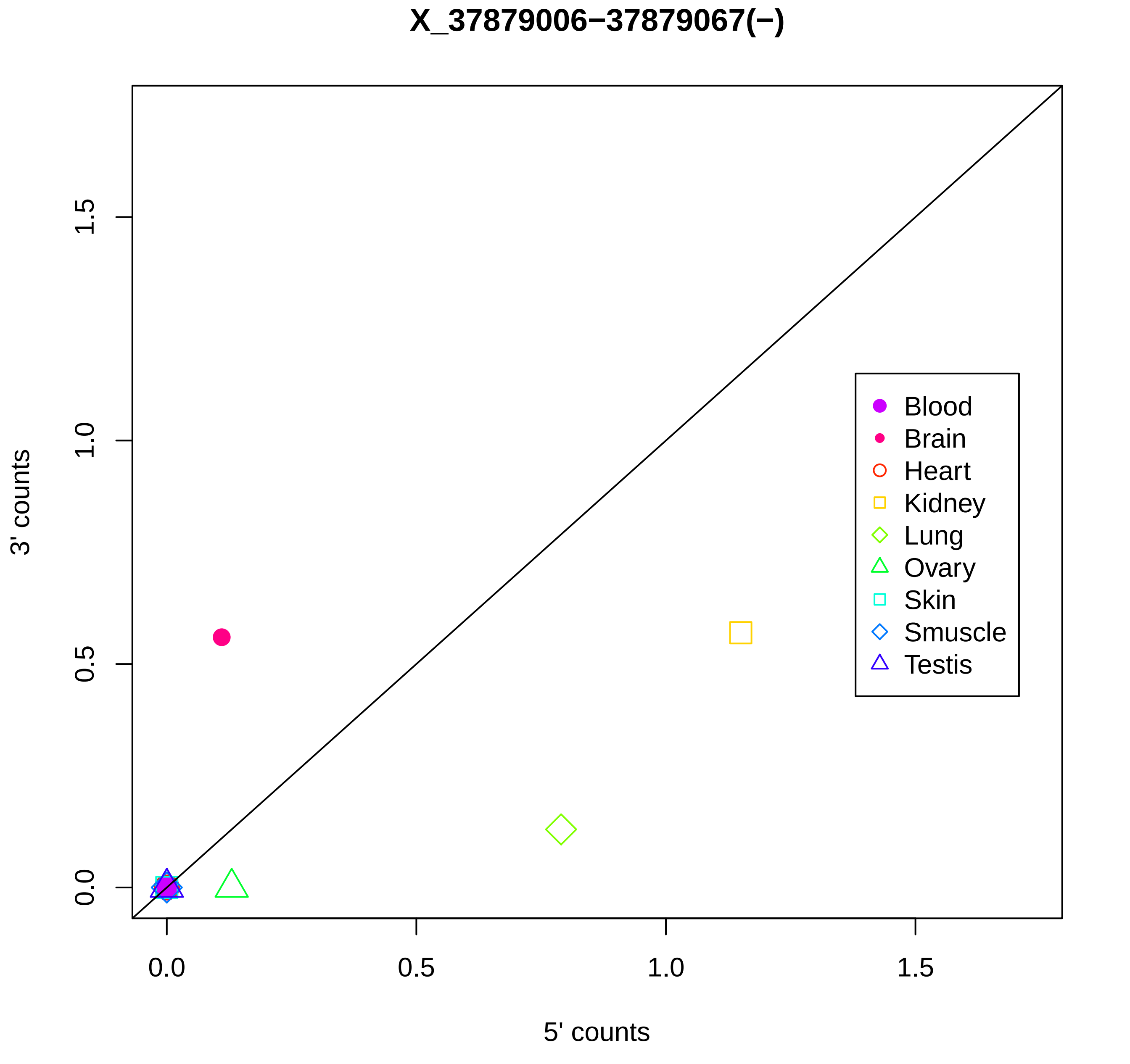
X_37879006−37879067(−)
3' counts
5' counts
0.0
0.5
1.0
1.5
0.0
0.5
1.0
1.5
Blood
Brain
Heart
Kidney
Lung
Ovary
Skin
Smuscle
Testis

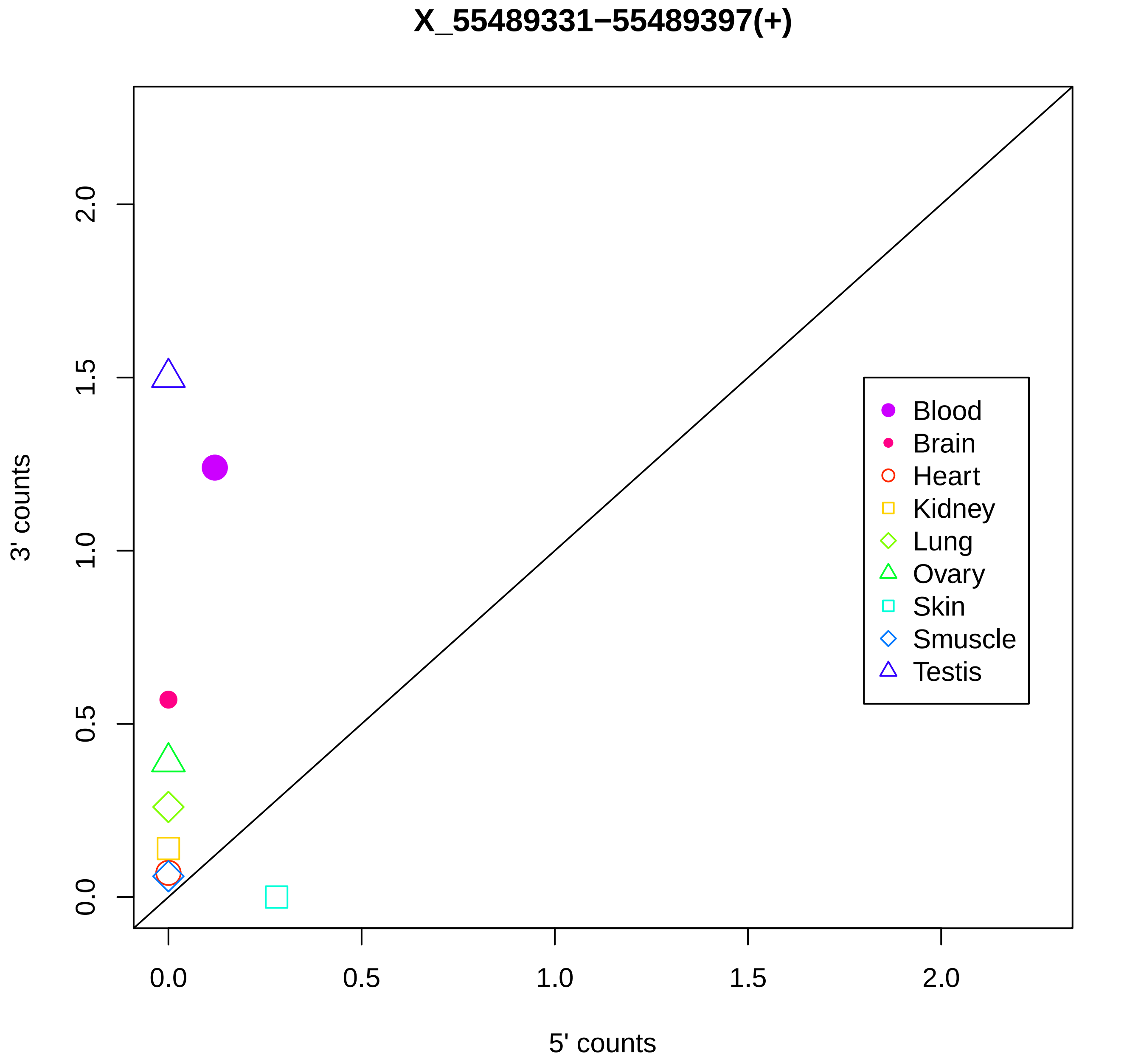
X_55489331−55489397(+)
3' counts
5' counts
0.0
0.5
1.0
1.5
2.0
Blood
Brain
Heart
Kidney
Lung
Ovary
Skin
Smuscle
Testis
