# Supplementary material for: An Improved microRNA Annotation of the Canine Genome
Source: PLoS One. 2016 Apr 27;11(4):e0153453. doi: 10.1371/journal.pone.0153453 (PMC4847789; doi:10.1371/journal.pone.0153453)
Supplement: S6 Fig — (PDF) [file pone.0153453.s006.pdf]

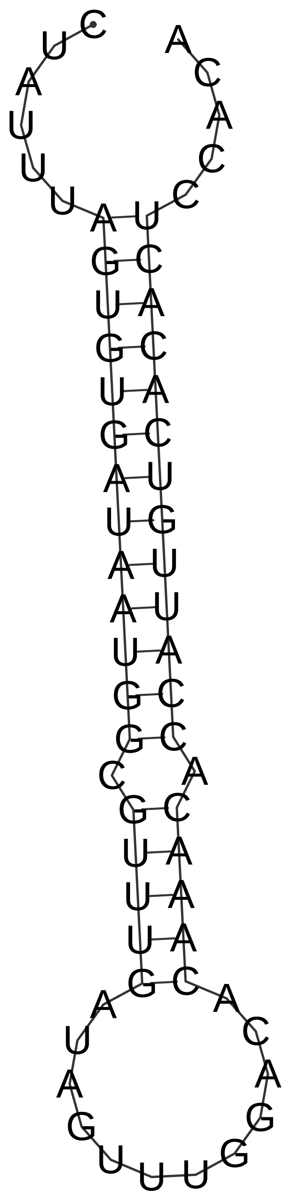

1_17593857-1759391_miR-3591_high

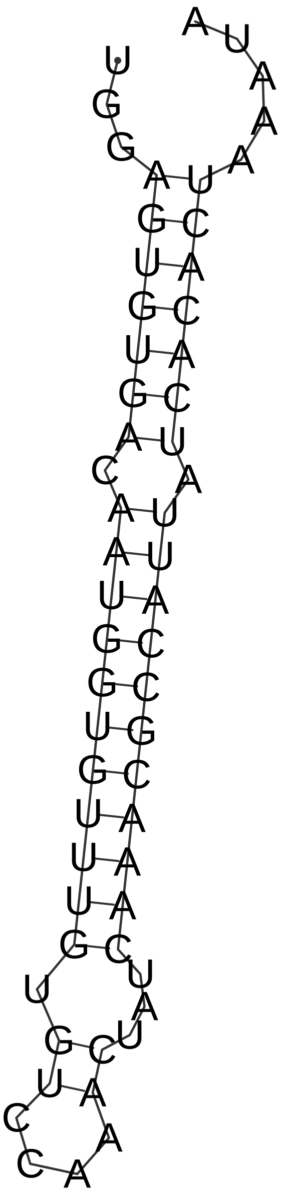

1_17593858-1759391_cfa-mir-122_high

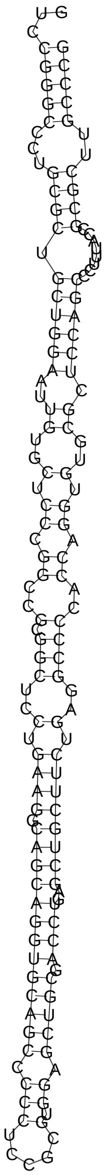

1_44444979-4444512_cfa-mir-8873b_low

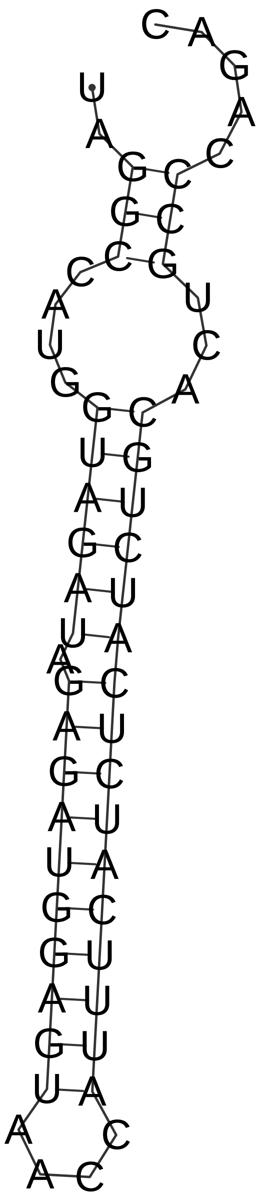

1_49030637-4903069_cfa-mir-1836_high

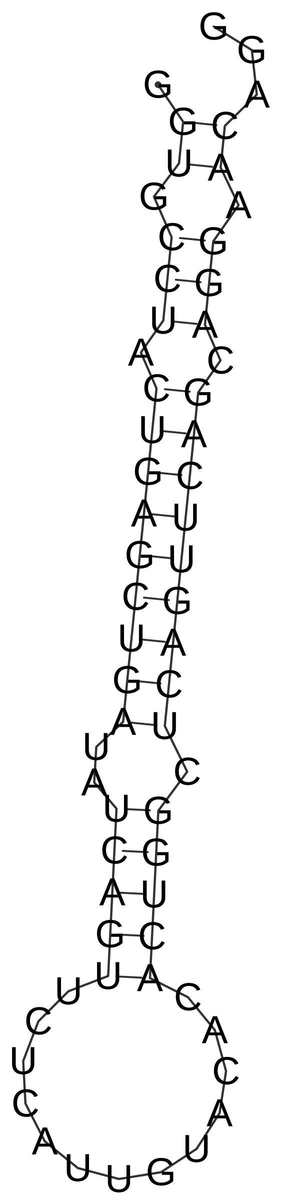

1_71677608-7167766_cfa-mir-24-1_high

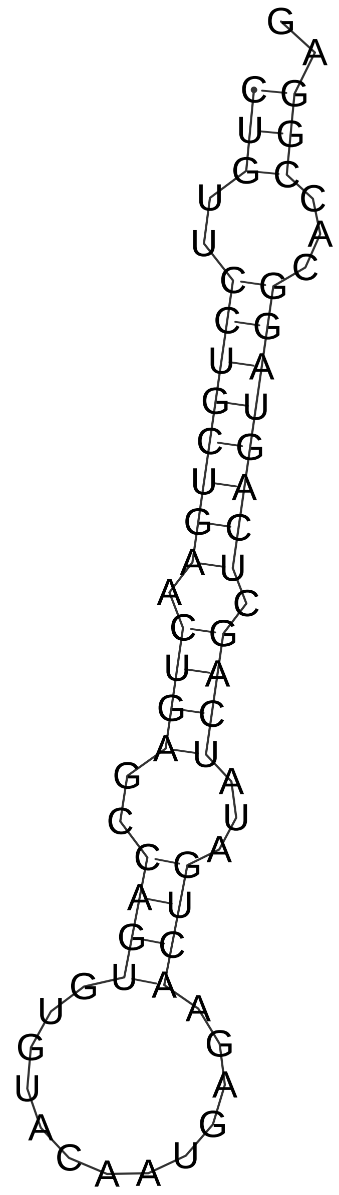

1_71677609-7167767_mir-3074_high

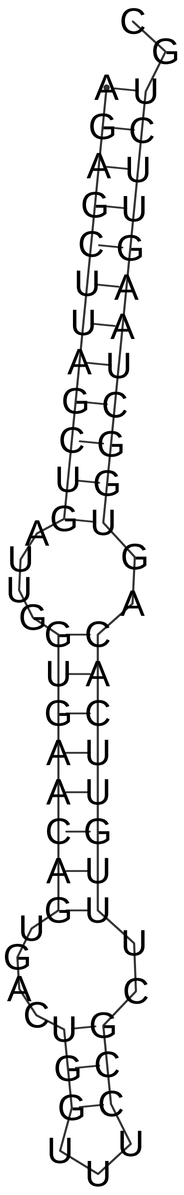

1_71678117-7167817_cfa-mir-27b_high

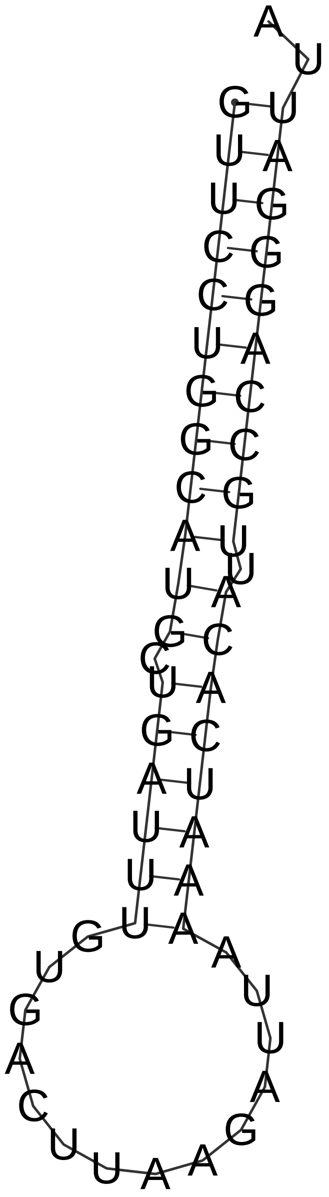

1_71678353-7167840_cfa-mir-23b_high

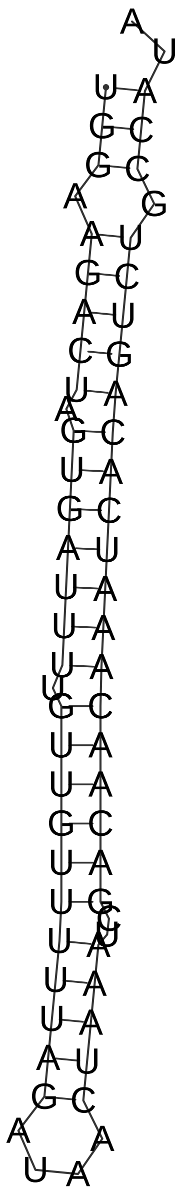

1_75509765-7550982_cfa-mir-7-1_high

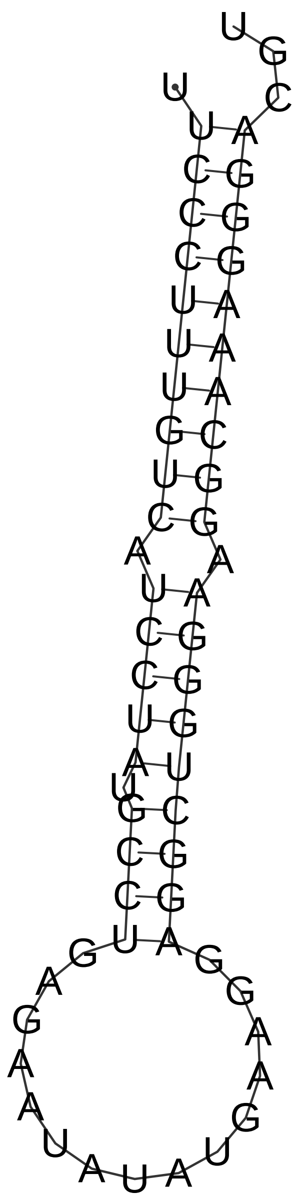

1_86818494-8681855_cfa-mir-204_high

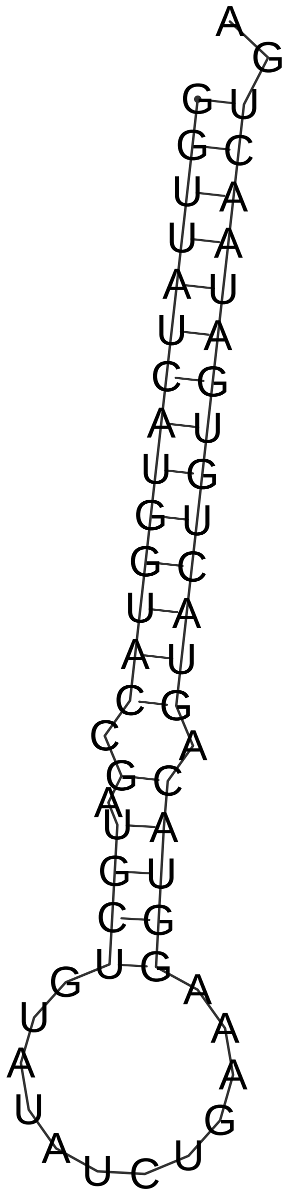

1_93194328-9319438_cfa-mir-101-2_high

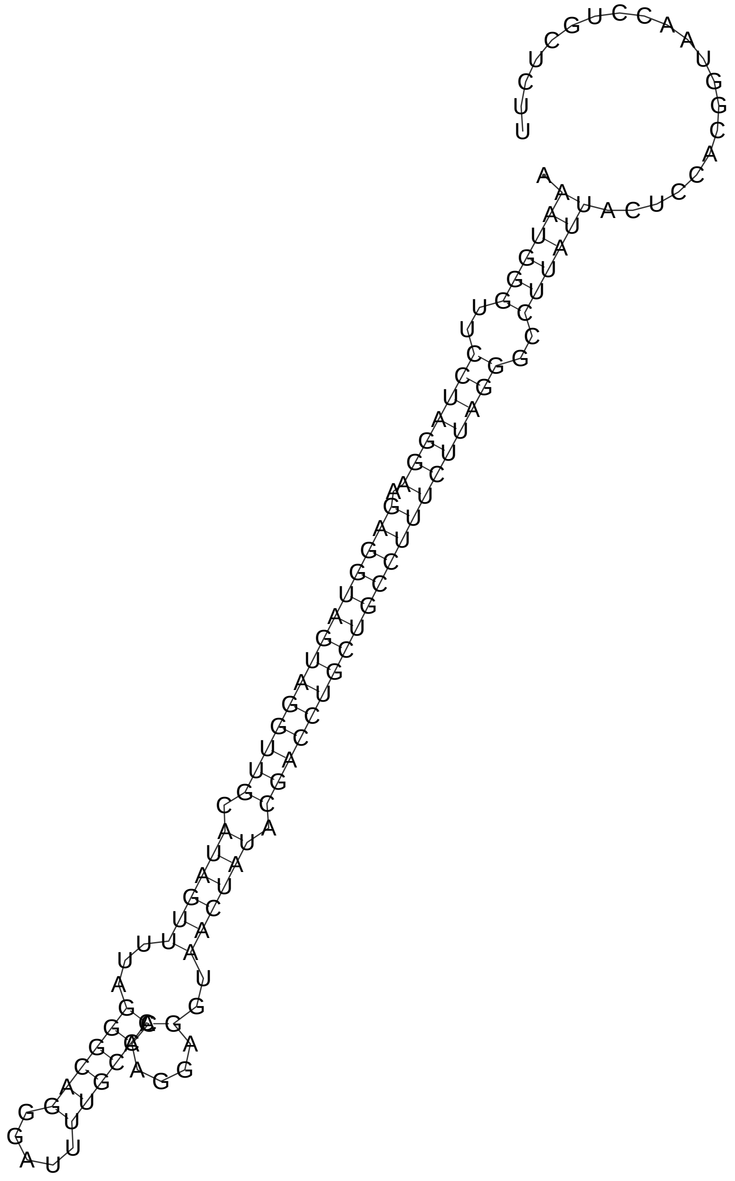

1_97902012-9790213_cfa-let-7d_high

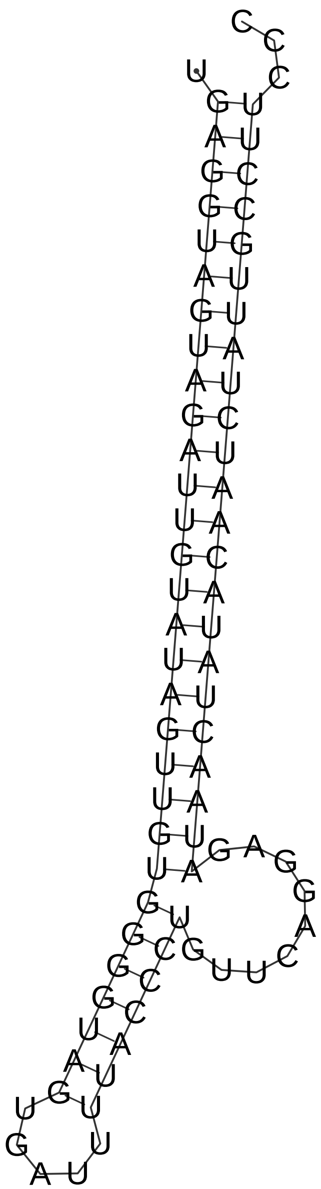

1_97903931-9790400_cfa-let-7f_high

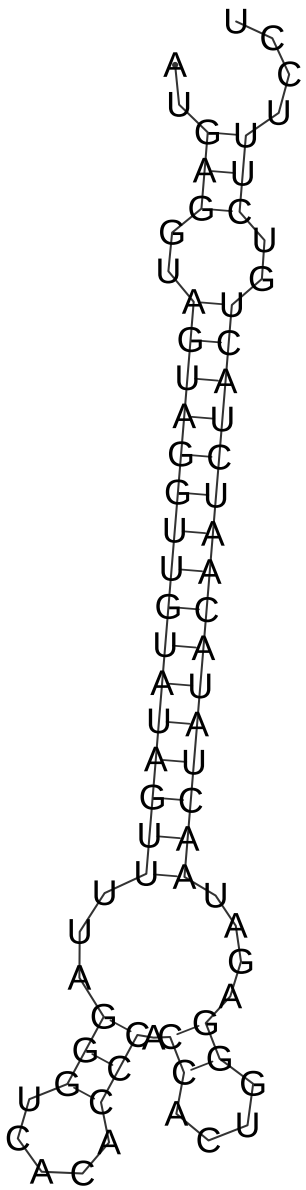

1_97904313-9790438_let-7a_high

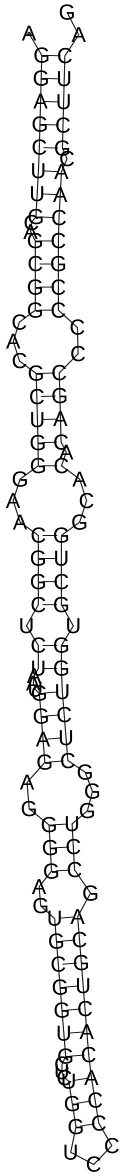

1_98603965-9860408_cfa-mir-8893_low

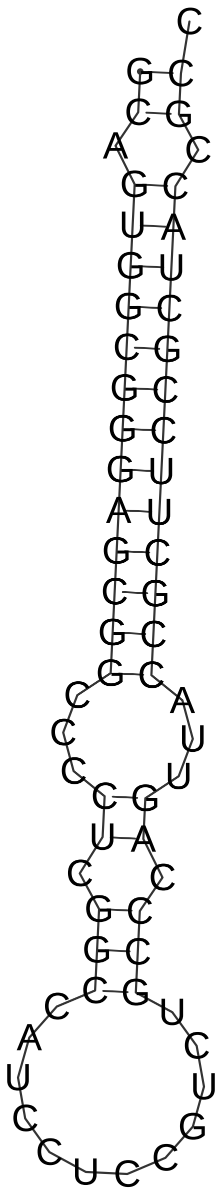

1_103250644-10325070_mir-935_high

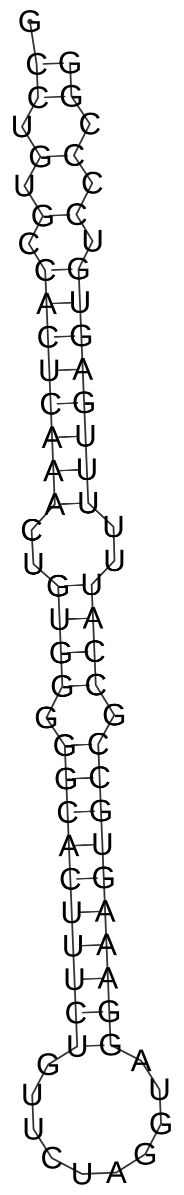

1_103406612-10340668_mir-371_high

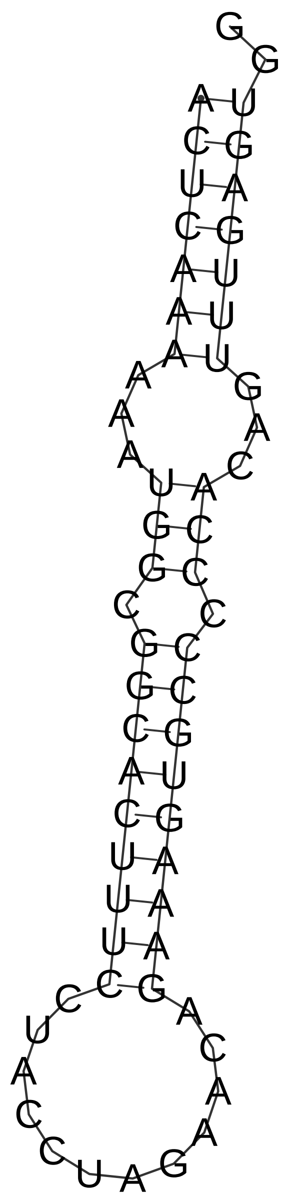

1_103406620-10340667_cfa-mir-371_high

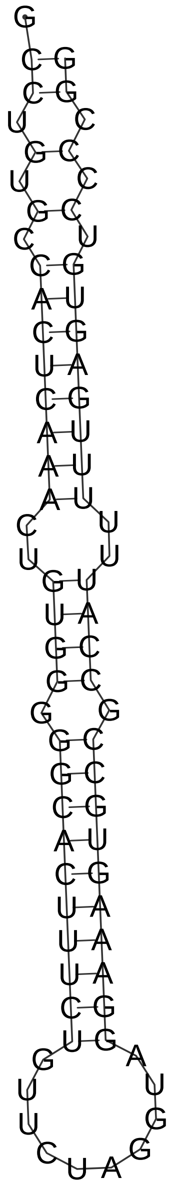

1_103606515-10360658_mir-371_high

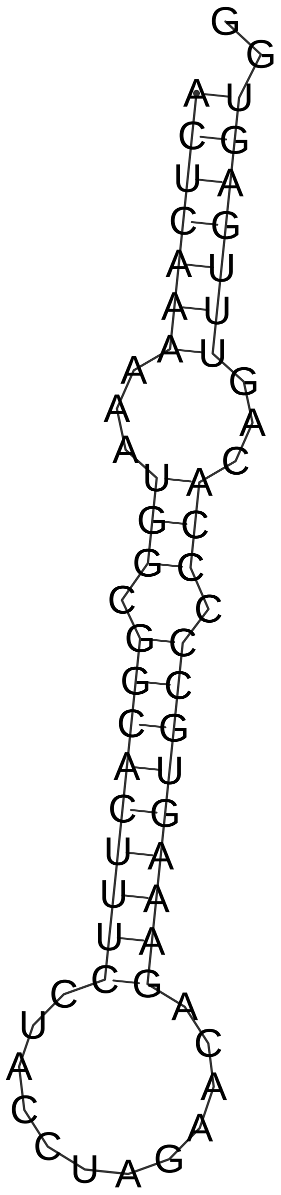

1_103606523-10360658_cfa-mir-371_high

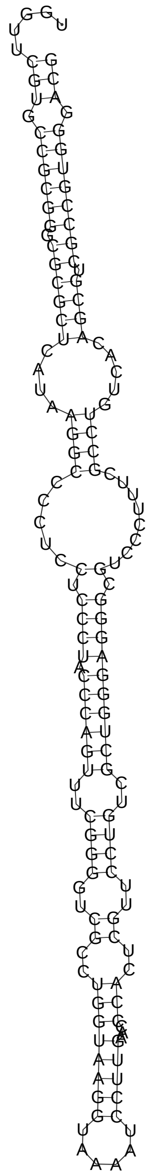

1_104785535-10478567_cfa-mir-8891_low

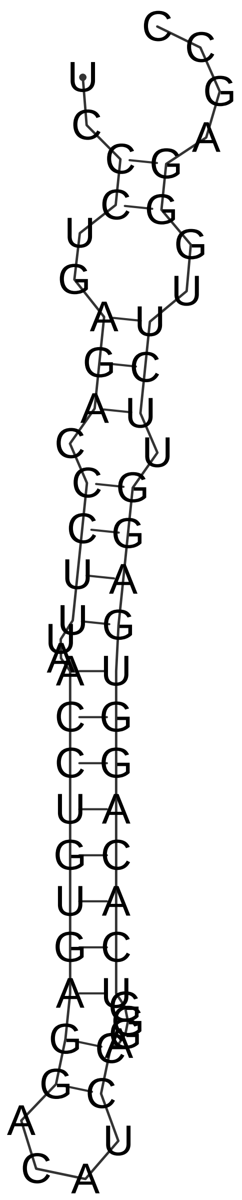

1_105400273-10540033_cfa-mir-125a_high

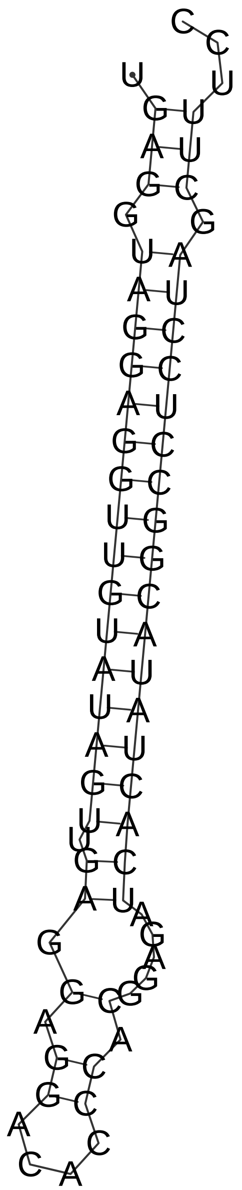

1_105400740-10540080_cfa-let-7e_high

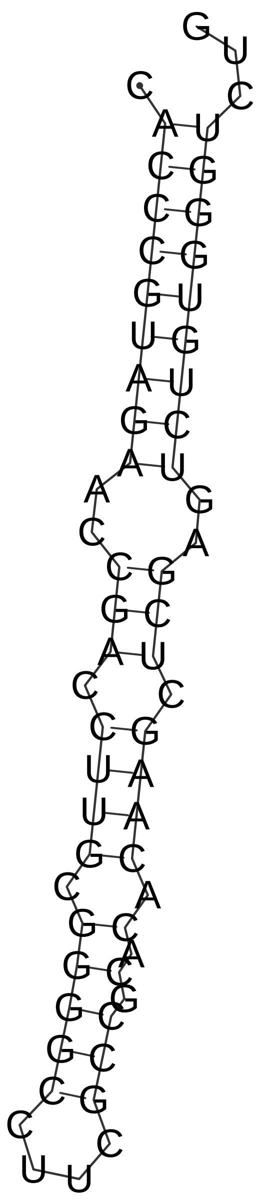

1_105400925-10540098_cfa-mir-99b_high

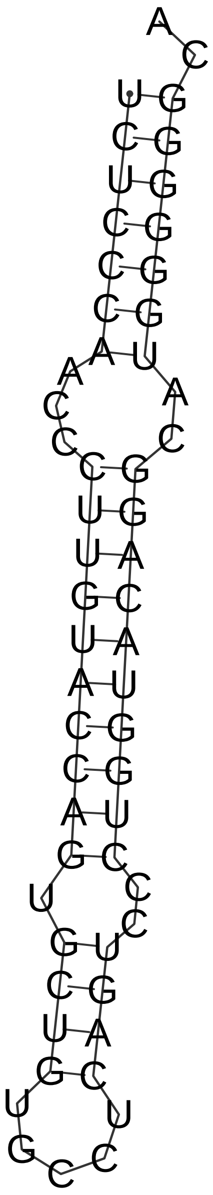

1_106998756-10699881_cfa-mir-150_high

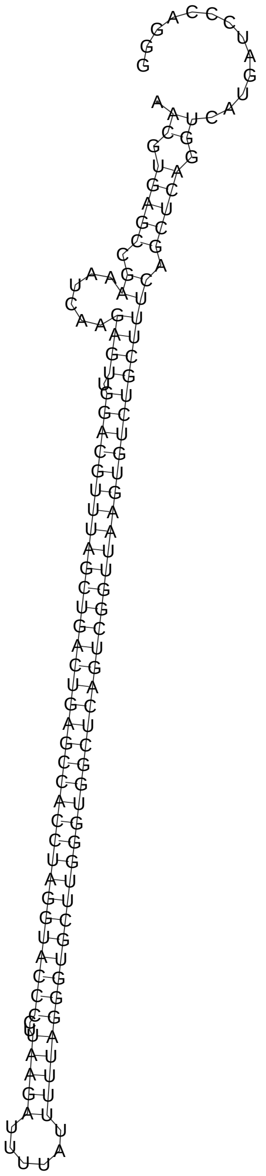

1_107121560-10712169_cfa-mir-8890_low

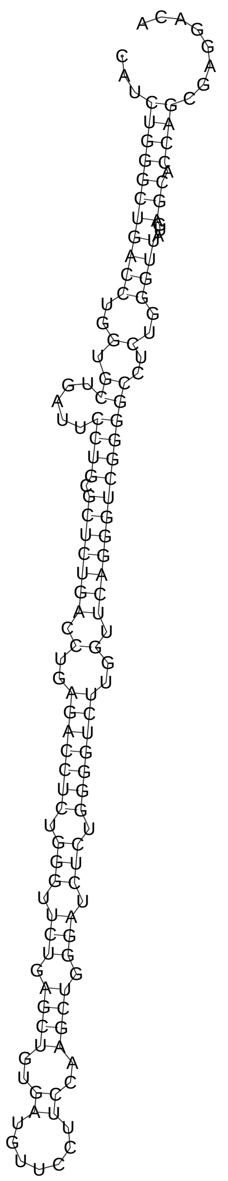

1_109618325-10961846_cfa-mir-769_high

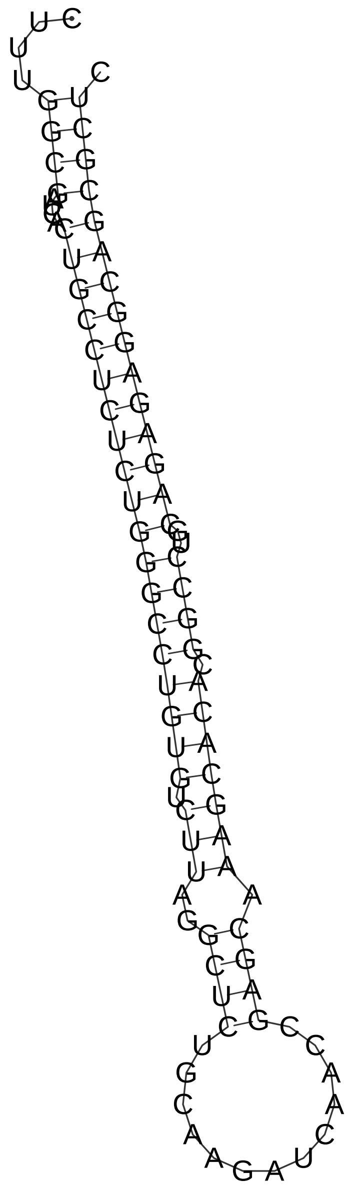

1_109922138-10992222_cfa-mir-330_high

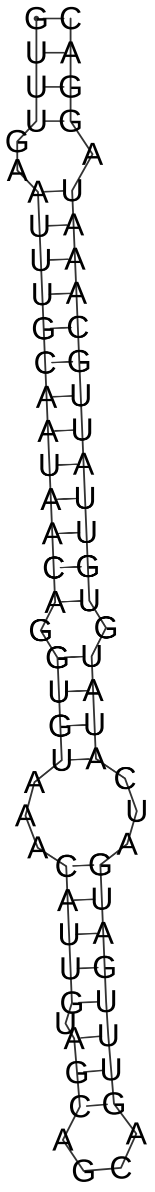

1_110690638-11069071_mir-6516_low

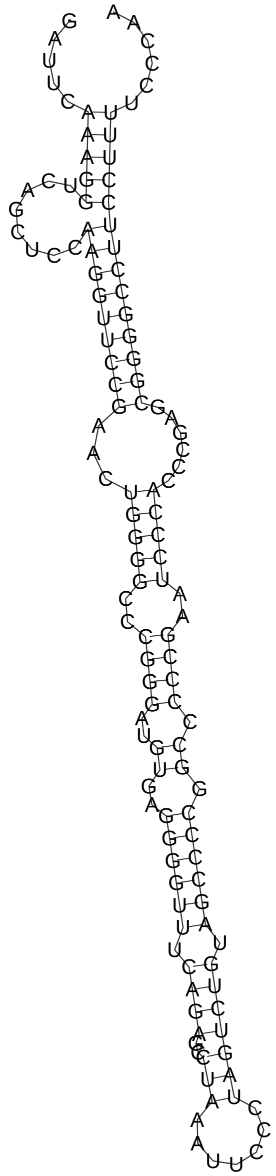

1_113031313-11303143_cfa-mir-8892_low

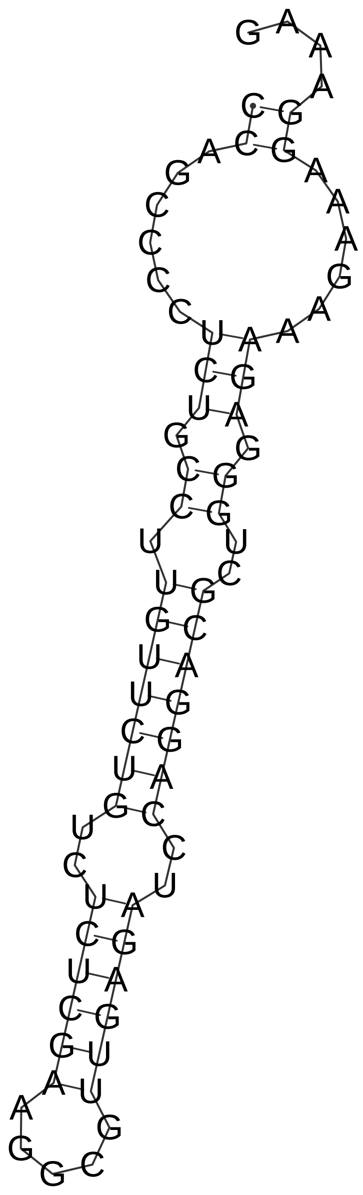

1_114501780-11450184_mir-9087_low

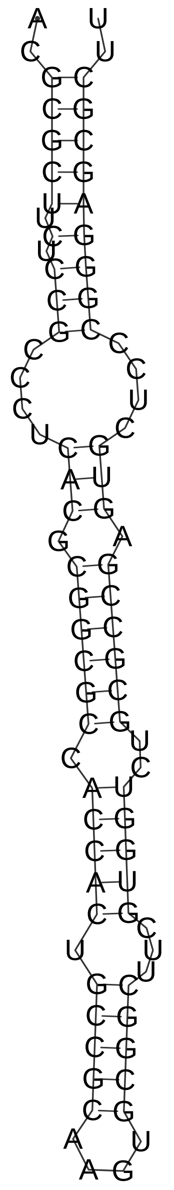

1_121781461-12178154_mir-2901_low

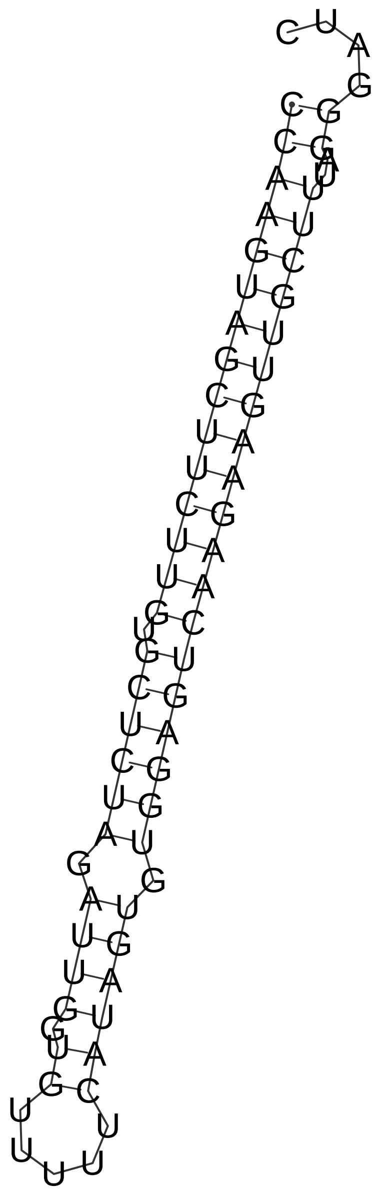

1_122413078-12241314_cfa-mir-578_low

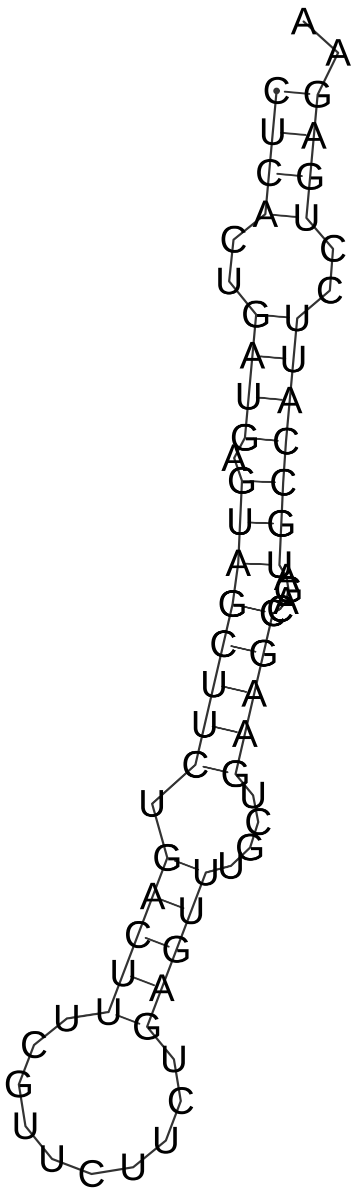

10_874423-87449_mir-677_high

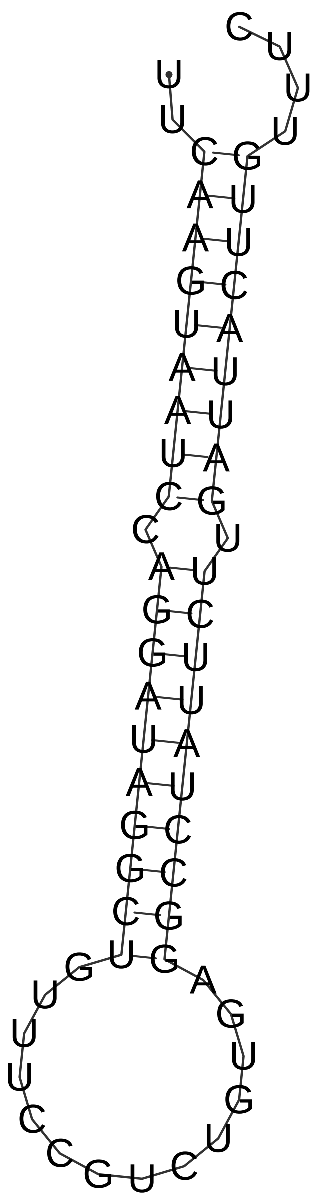

10_1880513-188057_cfa-mir-26a-2_high

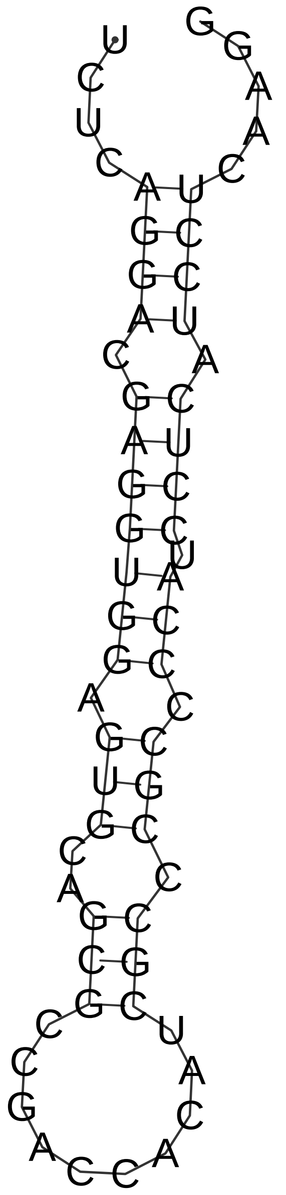

10_6071841-607190_mir-8104_low

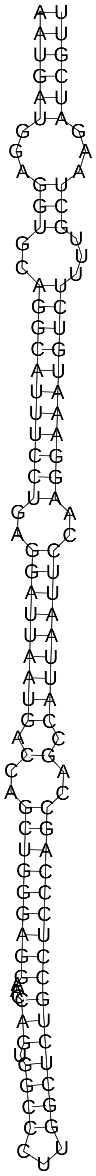

10_8382355-838247_mir-763_low

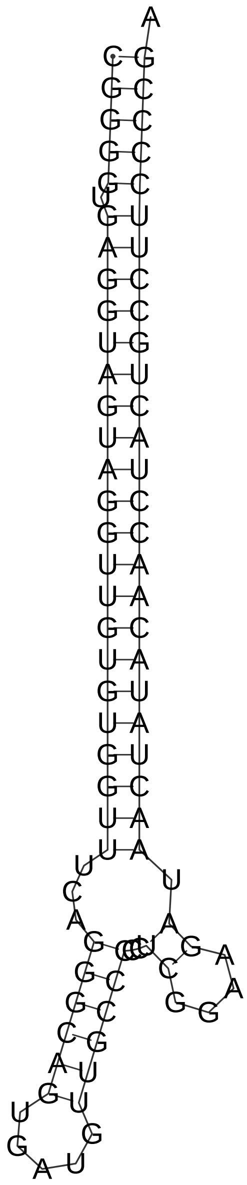

10_20033388-2003347_cfa-let-7b_high

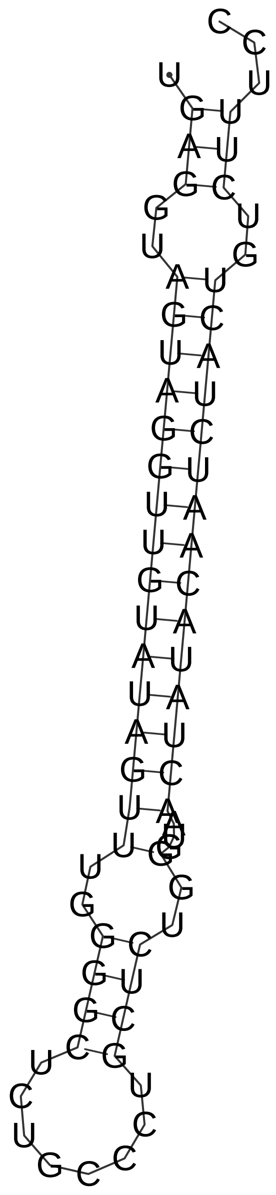

10_20034319-2003438_cfa-let-7a-1_high

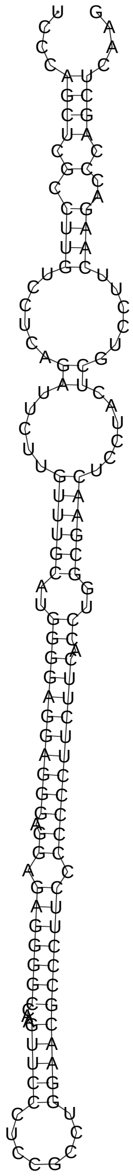

10_20818801-2081893_cfa-mir-1249_high

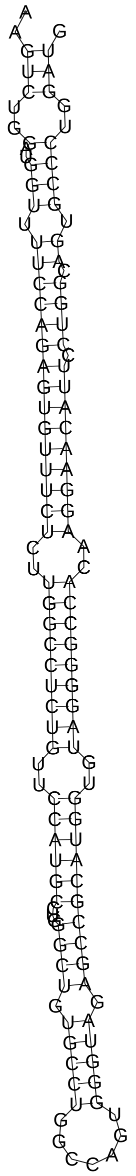

10_23246268-2324639_cfa-mir-8829_high

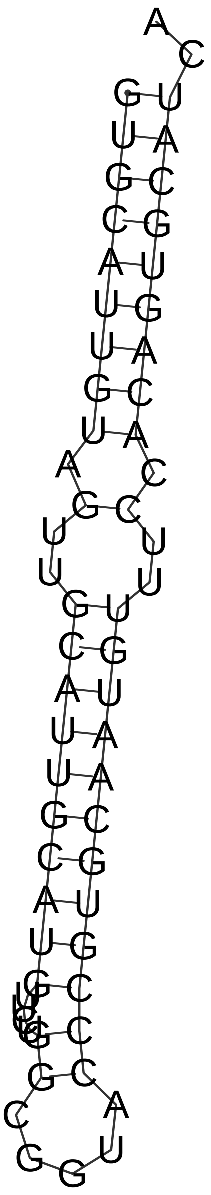

10_23456613-2345667_cfa-mir-33a_high

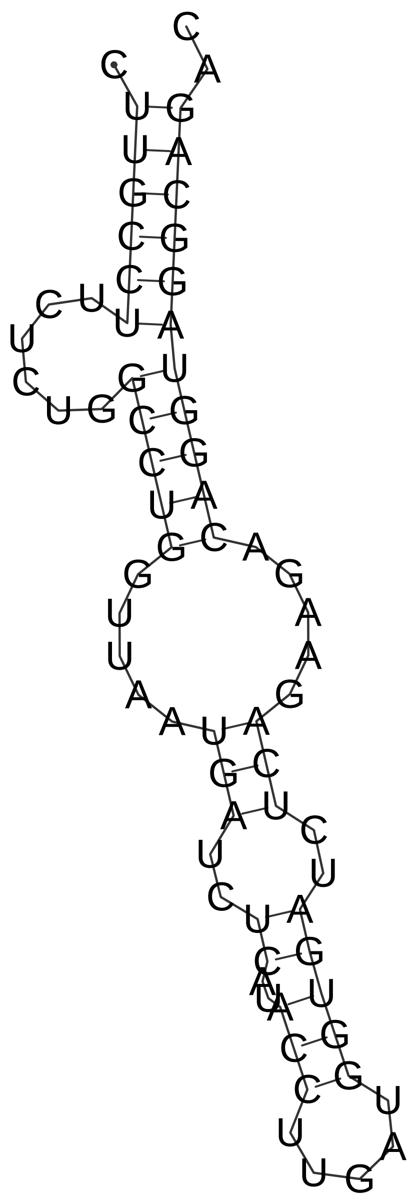

10_24665092-2466515_mir-2439_low

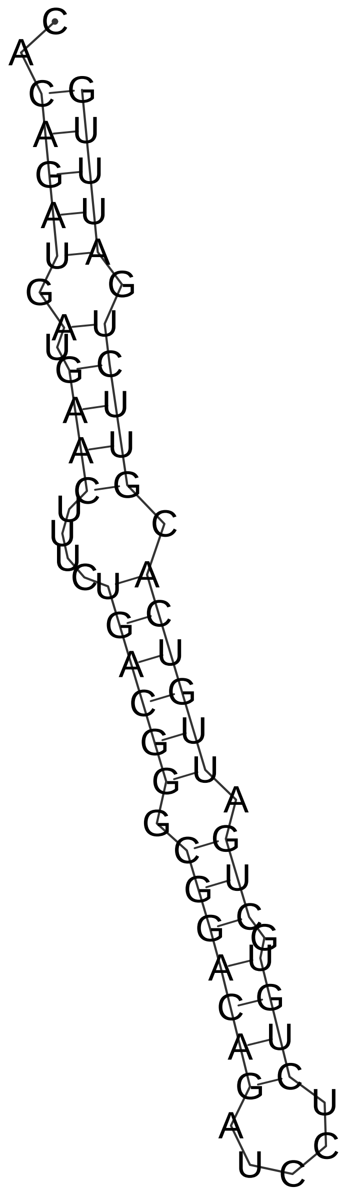

10_25739564-2573962_mir-9298_high

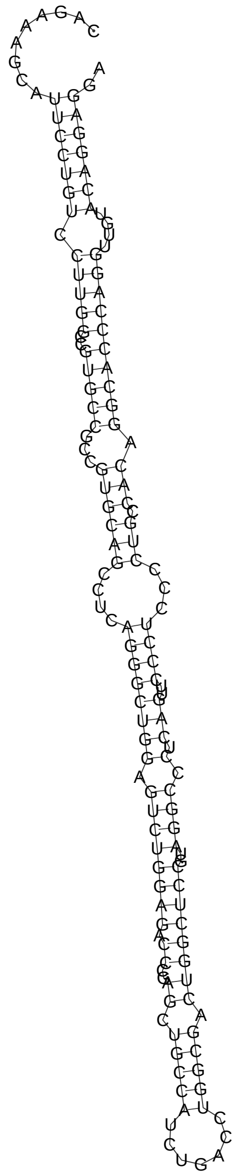

10_27182717-2718286_cfa-mir-8828_low

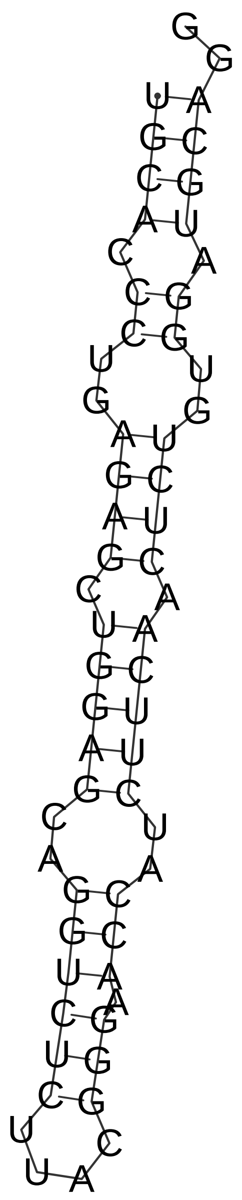

10_27296054-2729611_cfa-mir-1835_high

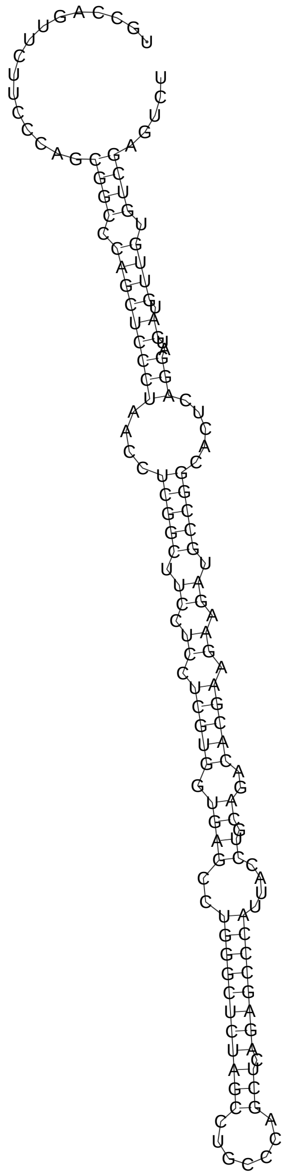

10_27860823-2786096_cfa-mir-8827_low

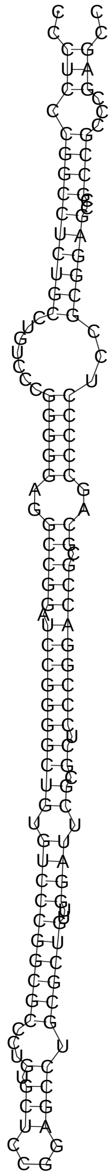

10_37131530-3713166_cfa-mir-8799c_low

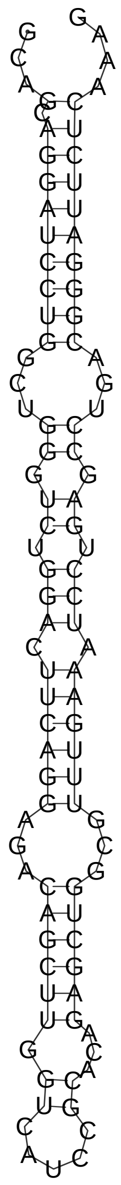

10_40365571-4036566_cfa-mir-8826_high

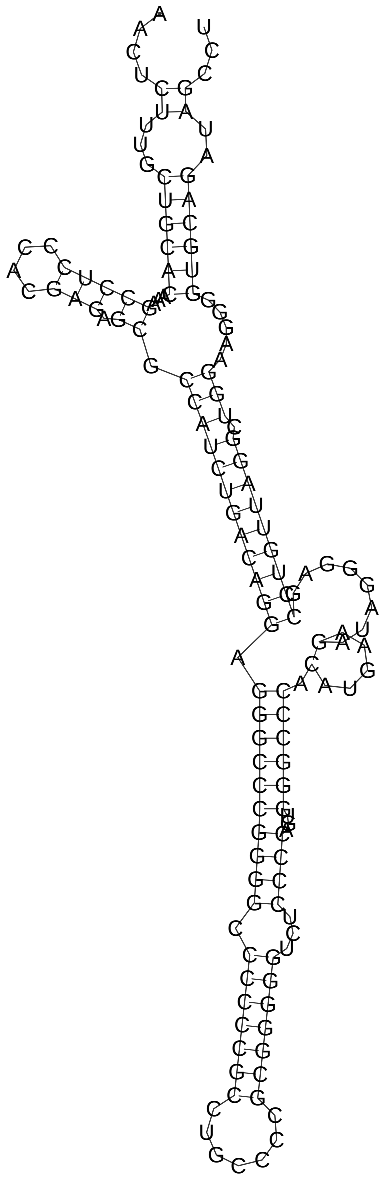

10_45159756-4515989_mir-9144_low

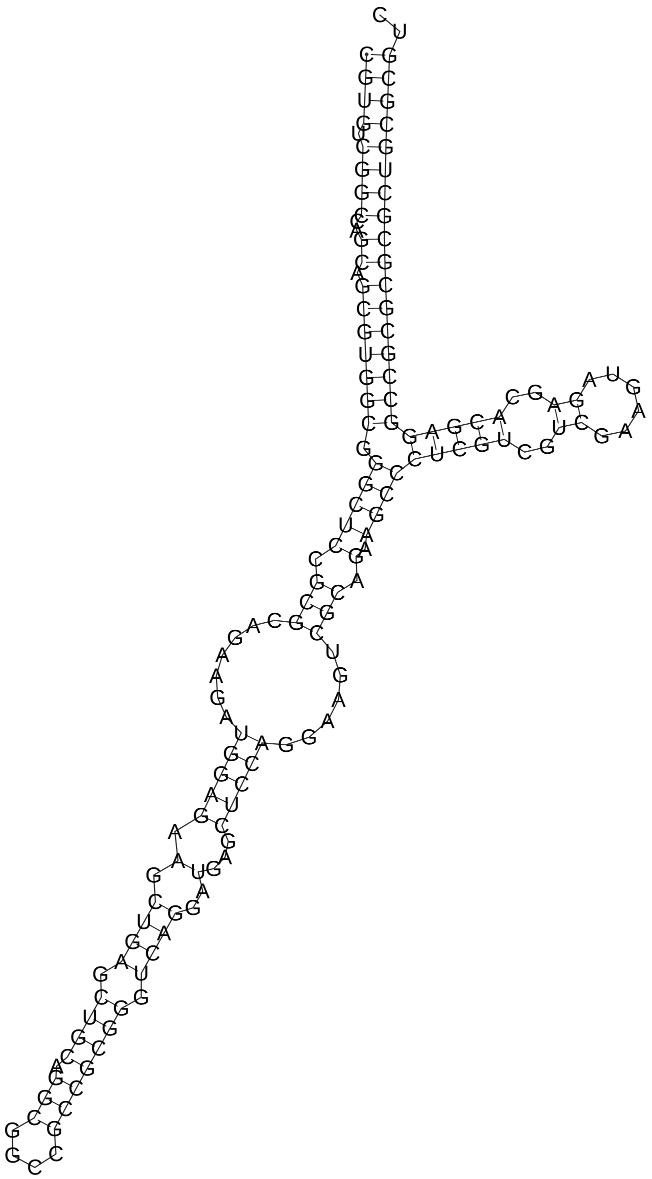

10_52666322-5266646_mir-9153_low

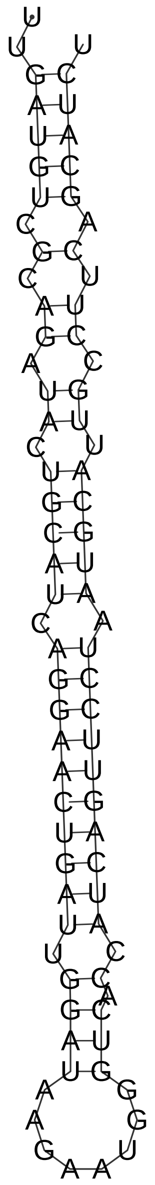

10_56754566-5675464_cfa-mir-217_high

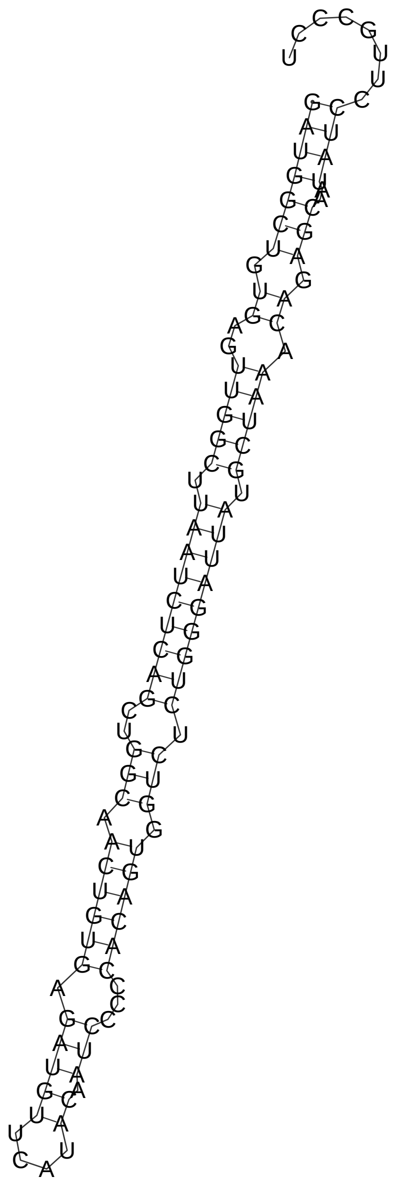

10_56761430-5676153_cfa-mir-216a_high

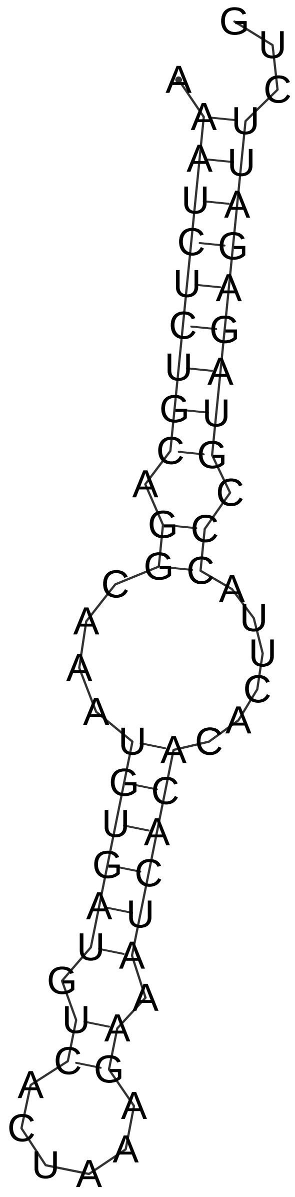

10_56772853-5677291_cfa-mir-216b_high

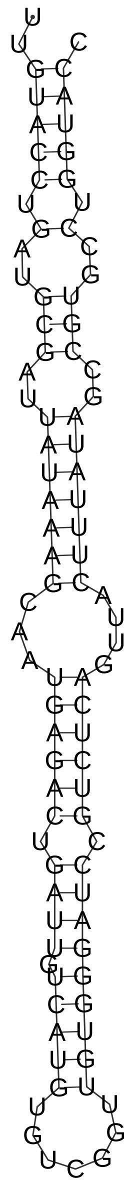

11_1704582-170467_cfa-mir-340_high

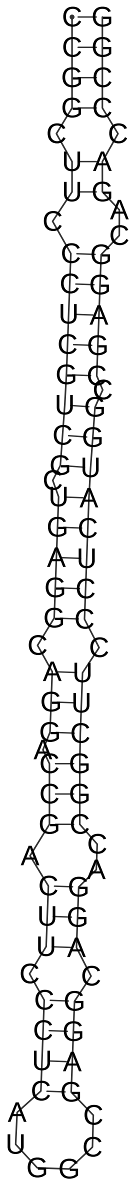

11_14017075-1401715_cfa-mir-8830-2_low

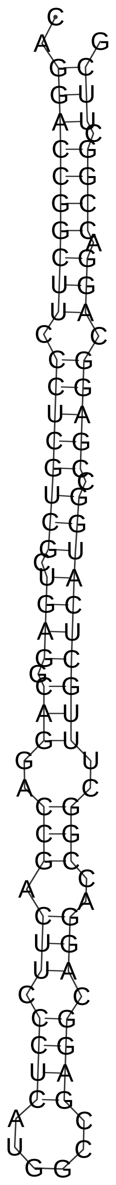

11_14017421-1401751_cfa-mir-8830-1_low

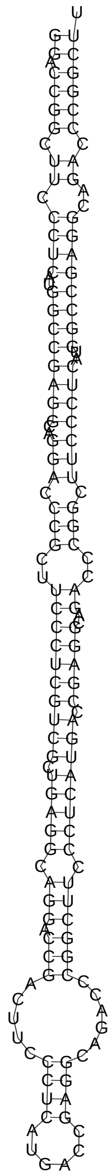

11_14017612-1401775_cfa-mir-8830-3_low

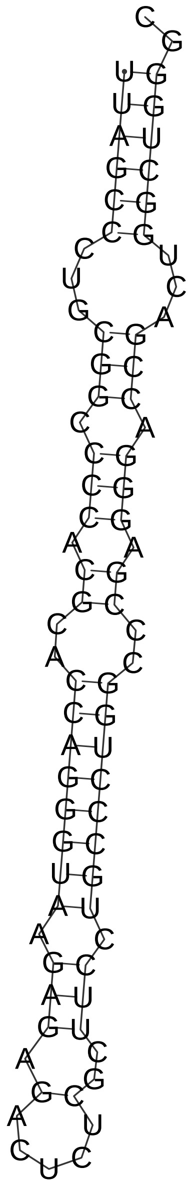

11_25355355-2535543_cfa-mir-874_high

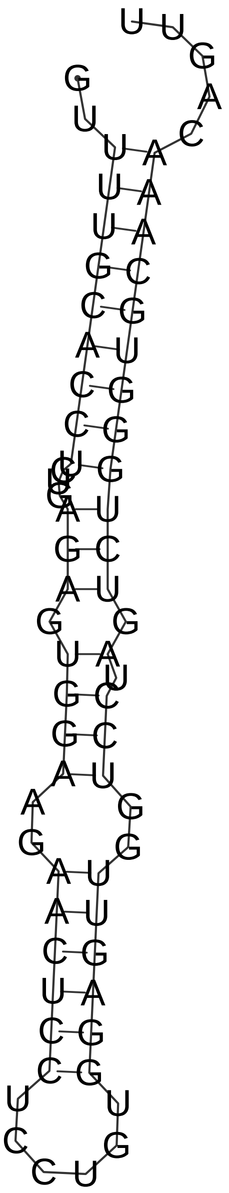

11_26741534-2674160_miR-1543_high

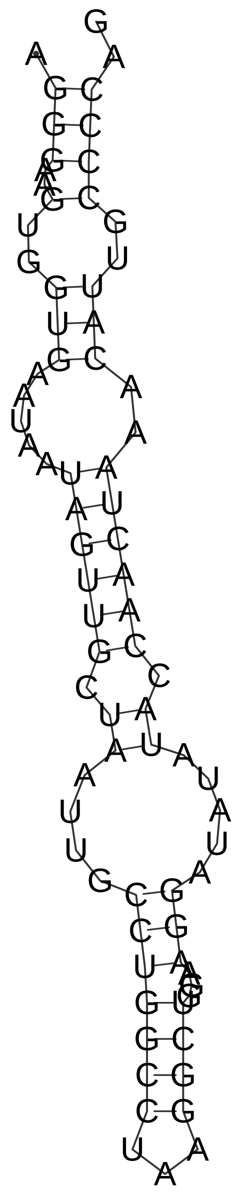

11_28565345-2856542_mir-8953_low

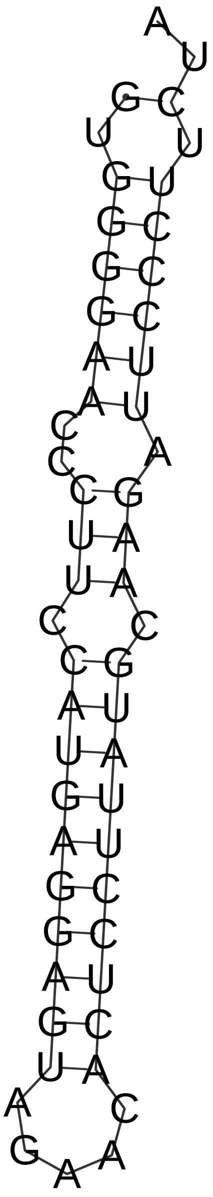

11_40300967-4030102_cfa-mir-491_high

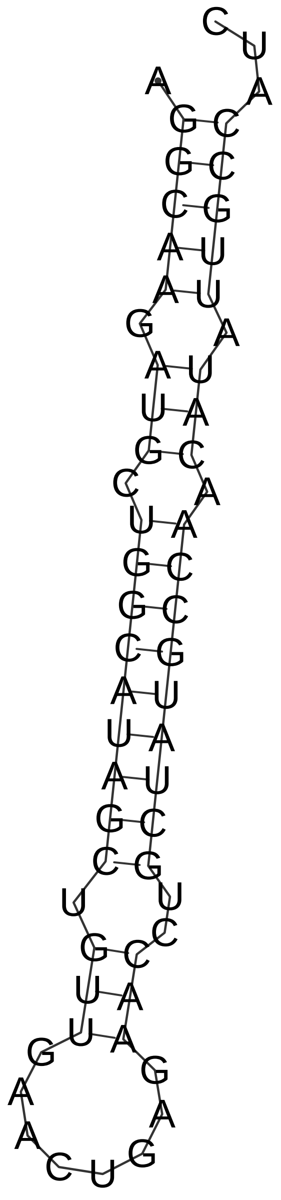

11_40904438-4090449_cfa-mir-31_high

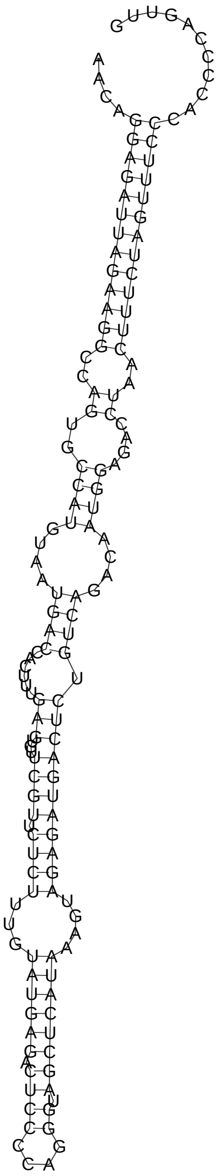

11_44895300-4489544_cfa-mir-8831_low

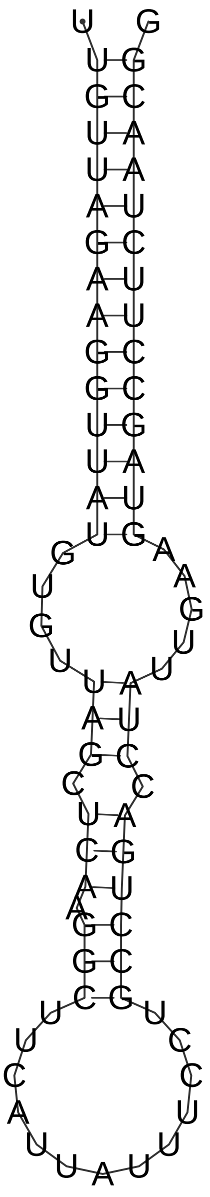

11_45208764-4520883_cfa-mir-872_low

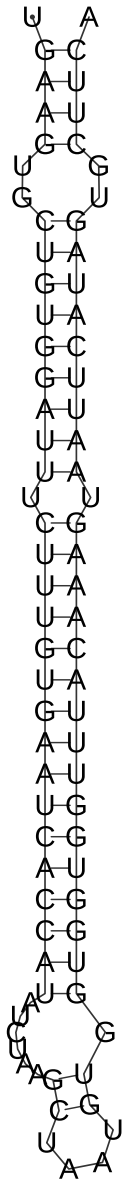

11_46819464-4681954_cfa-mir-876_high

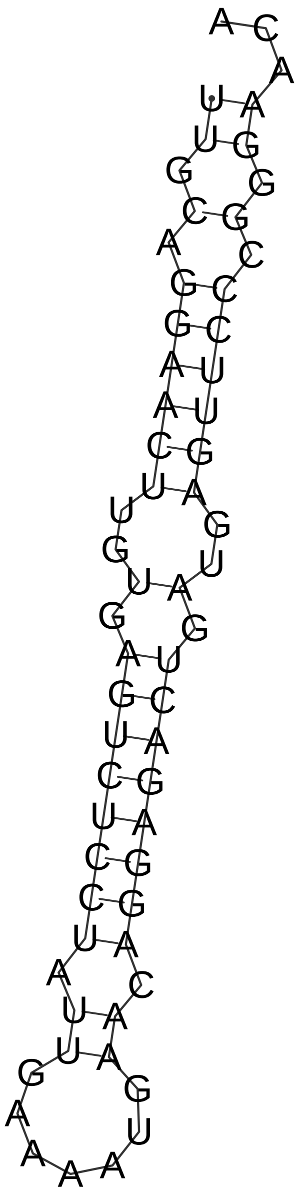

11_46838185-4683824_mir-873_high

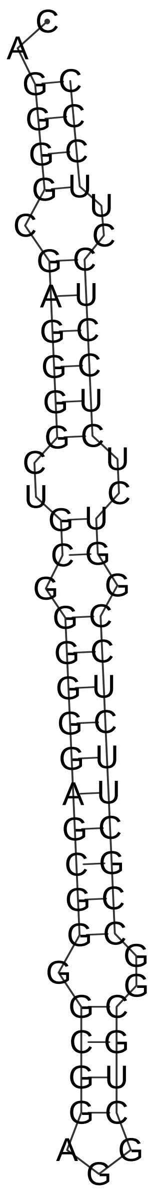

11_50156577-5015664_cfa-mir-207_low

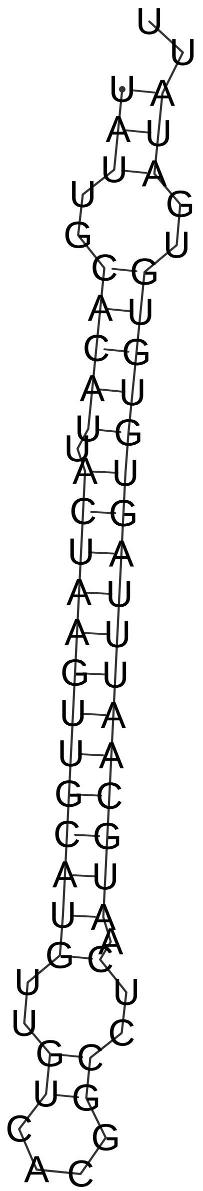

11_64197130-6419719_cfa-mir-32_high

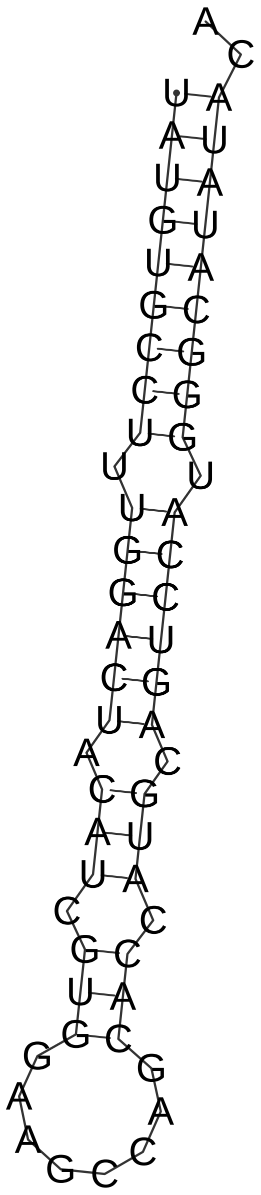

11_68461911-6846196_cfa-mir-455_high

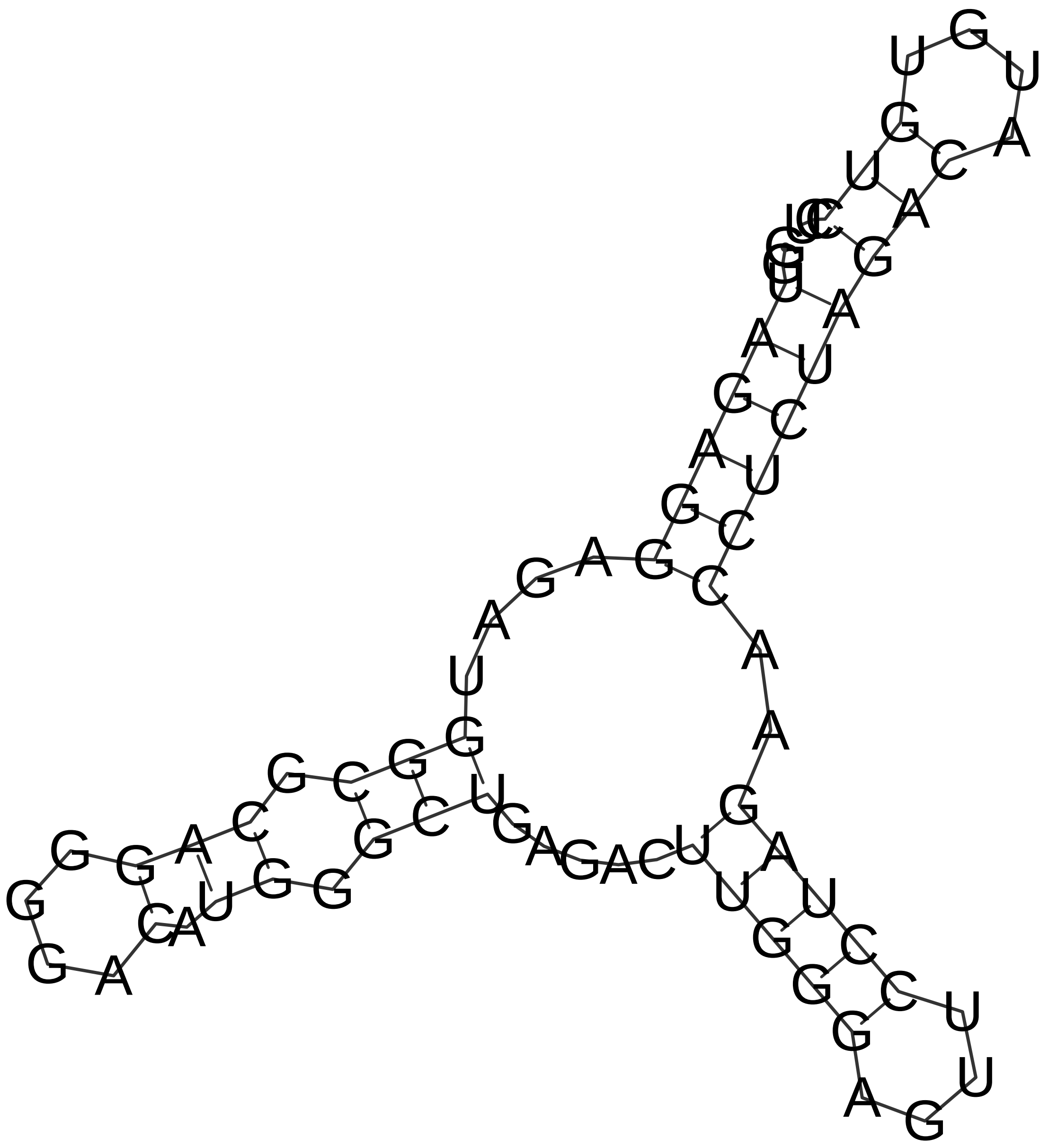

12_406899-40696_mir-877_high

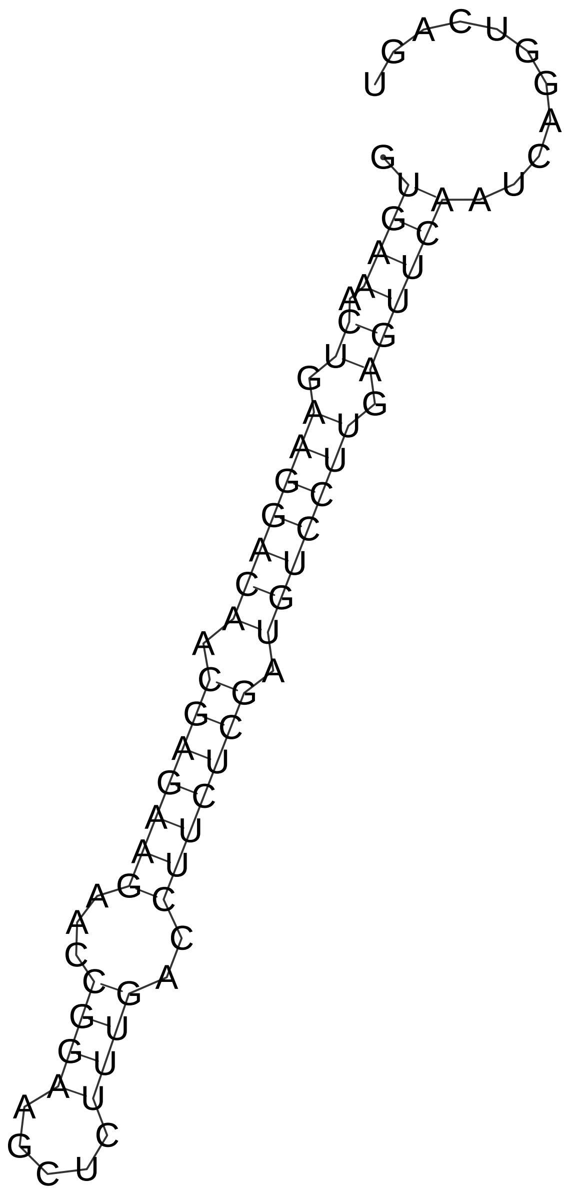

12_1034339-103441_mir-8094_low

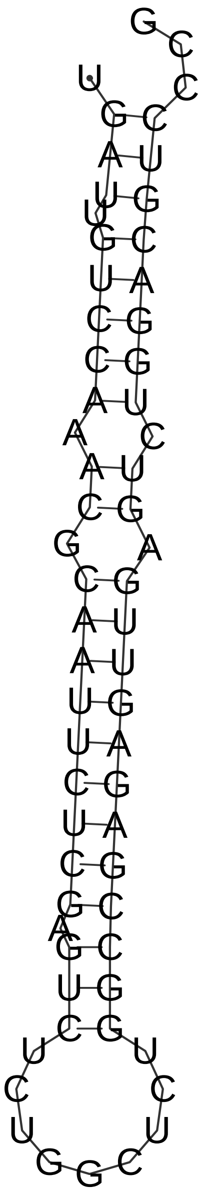

12_2670928-267099_cfa-mir-219-1_high

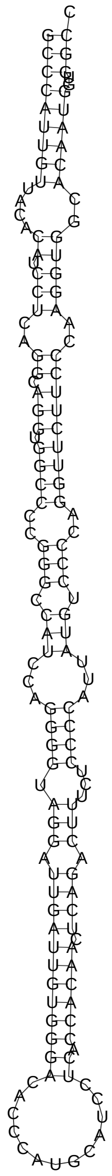

12_3200669-320081_cfa-mir-8832_low

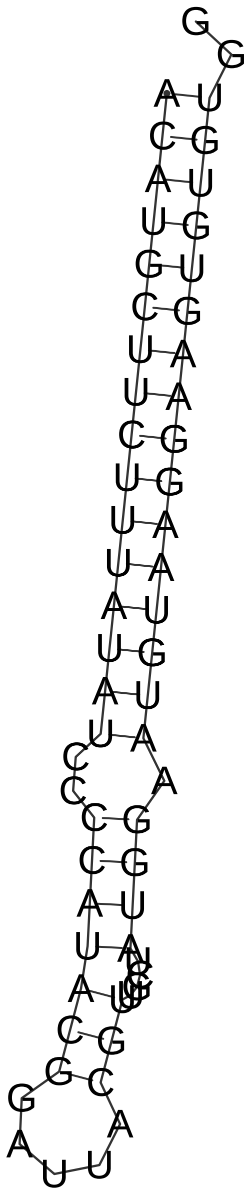

12_19805523-1980558_cfa-mir-206_high

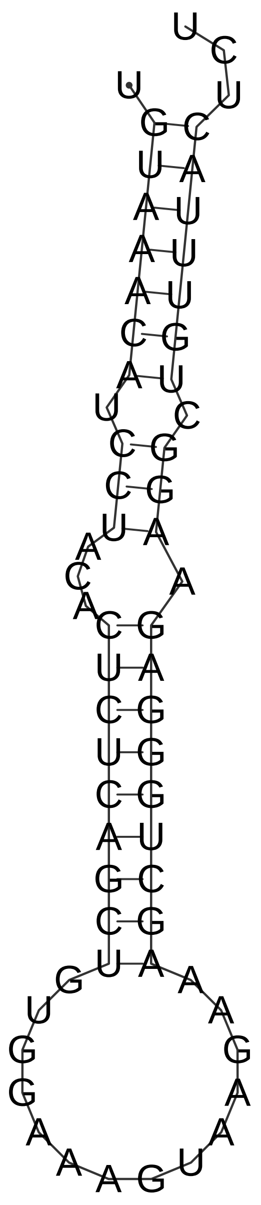

12_33757899-3375796_cfa-mir-30c-2_high

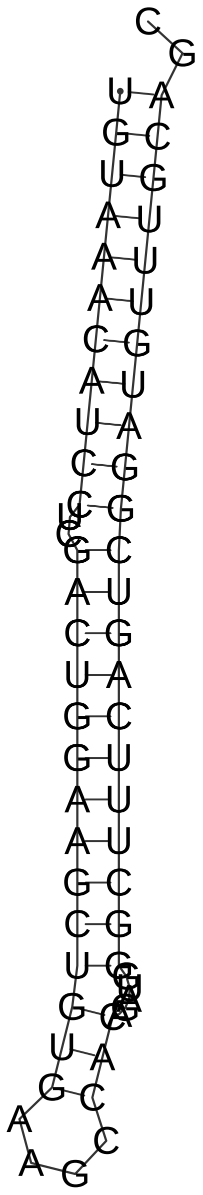

12_33783894-3378395_cfa-mir-30a_high

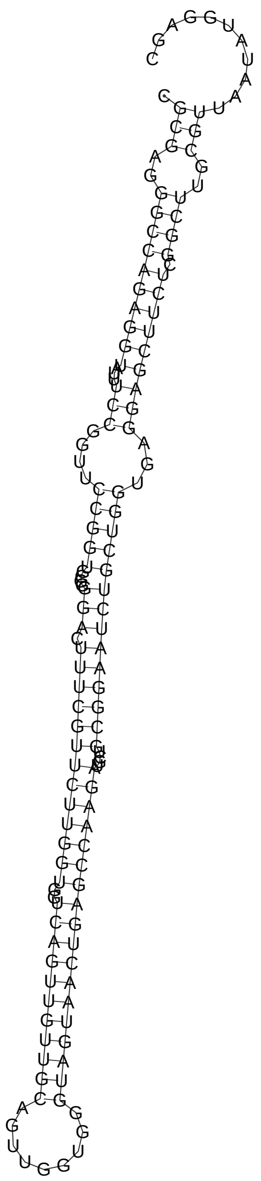

12_36887516-3688765_cfa-mir-8835_low

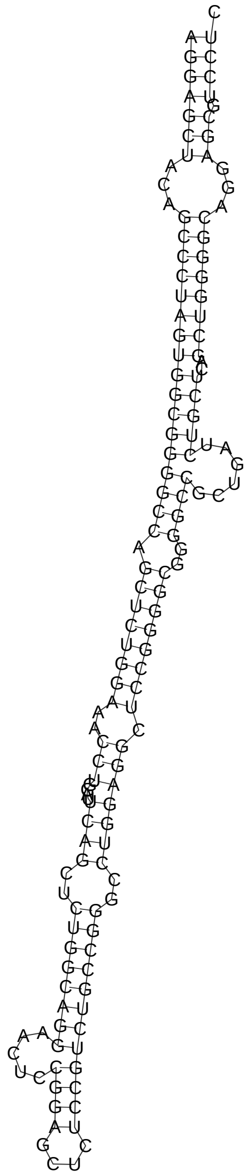

12_40720025-4072016_cfa-mir-8834a-1_low

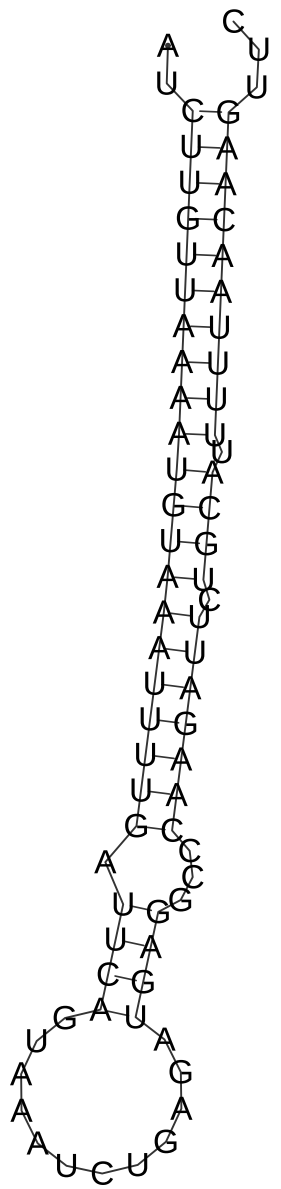

12_48210886-4821095_mir-544_low

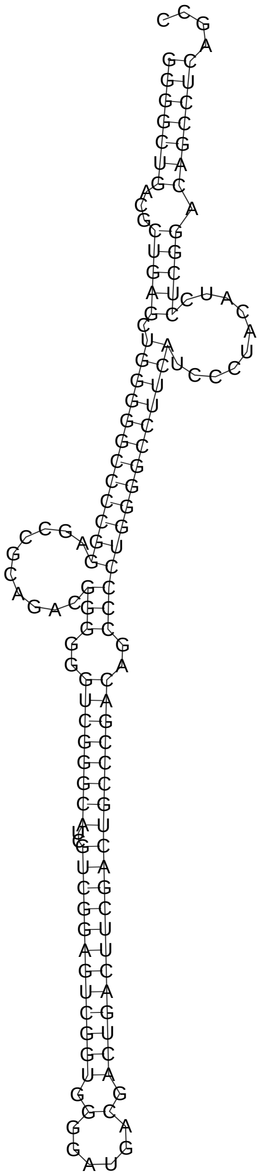

12_48374871-4837500_cfa-mir-8833_low

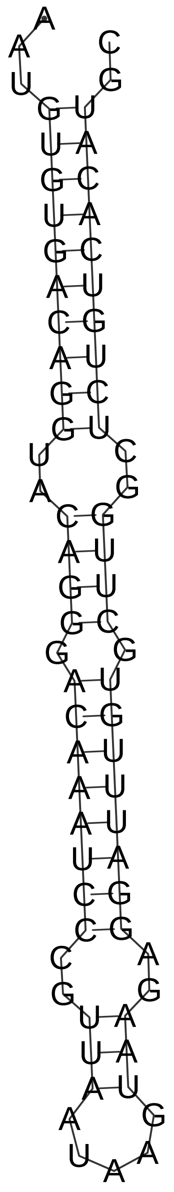

12_56421084-5642115_mir-2113_high

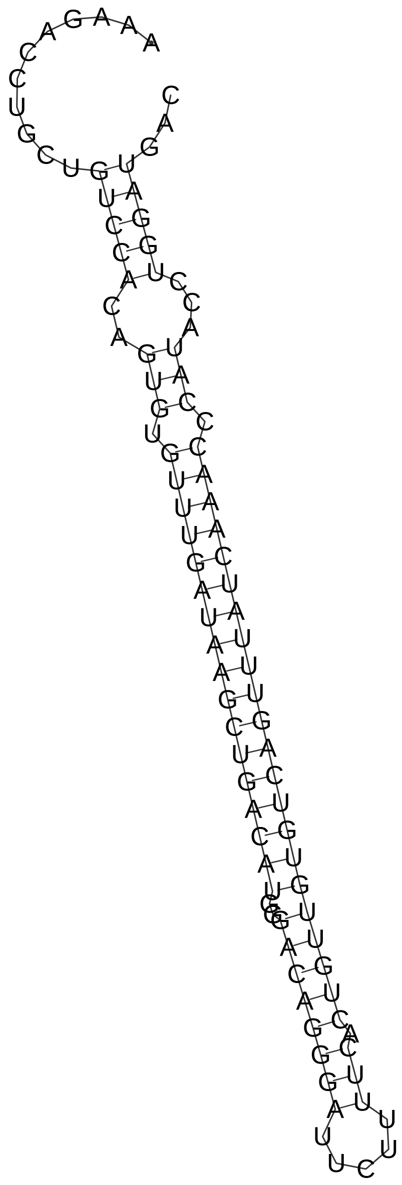

13_1538928-153902_cfa-mir-599_high

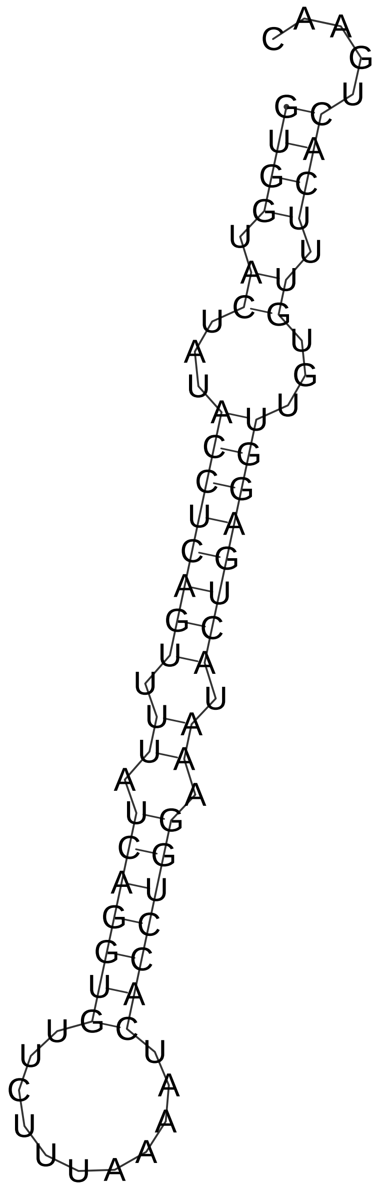

13_1539078-153915_cfa-mir-875_low

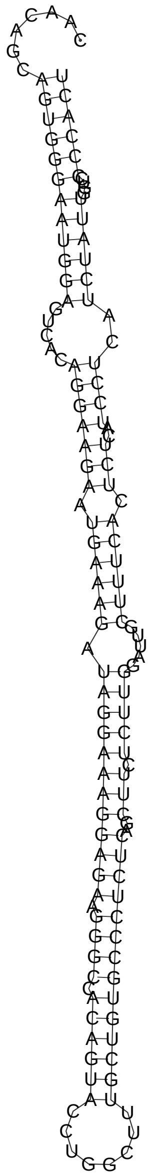

13_9751650-975178_cfa-mir-8836_low

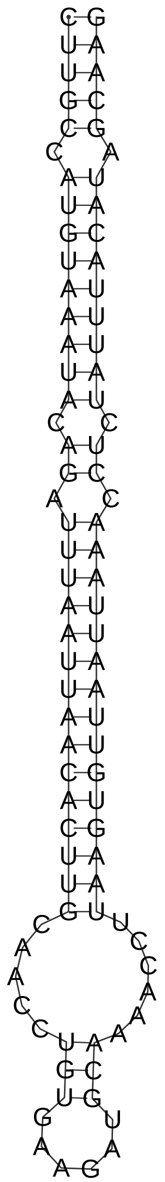

13_12774532-1277462_mir-2053_low

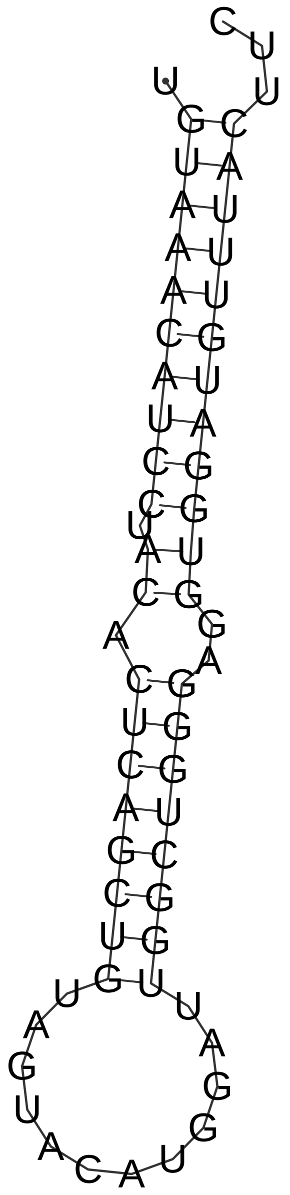

13_30906038-3090609_cfa-mir-30b_high

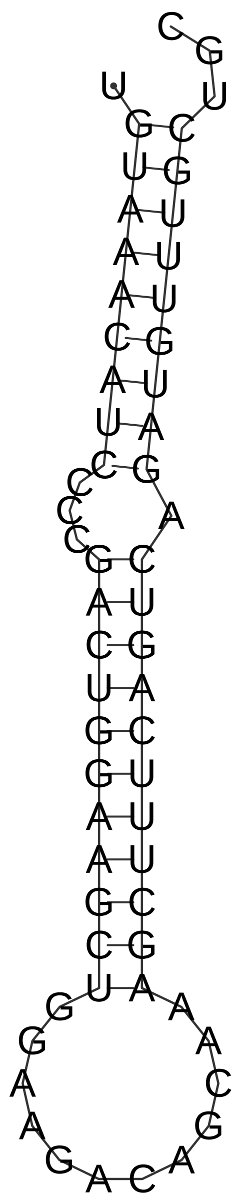

13_30910306-3091036_cfa-mir-30d_high

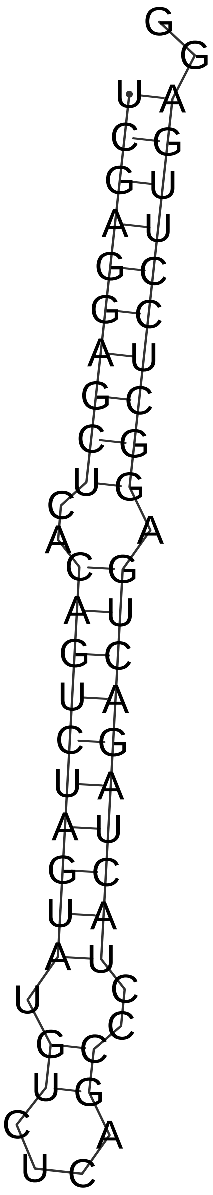

13_35353689-3535374_cfa-mir-151_high

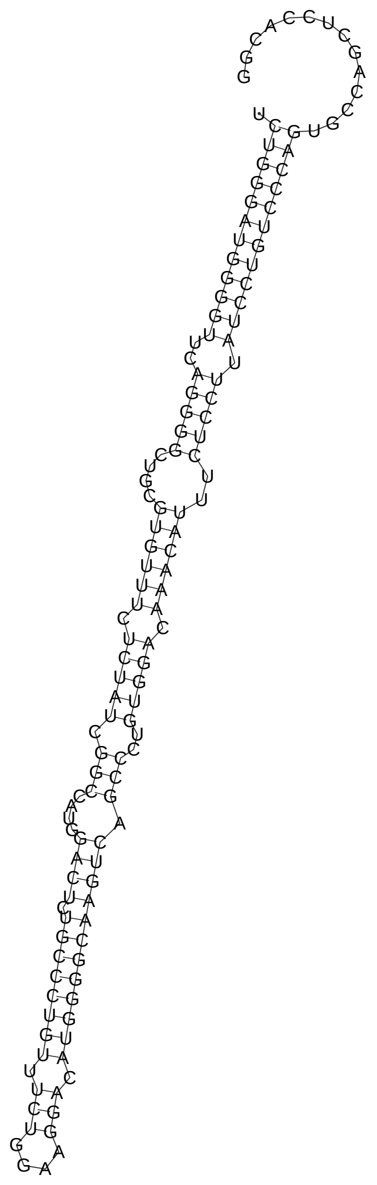

13_40256908-4025704_cfa-mir-8880_low

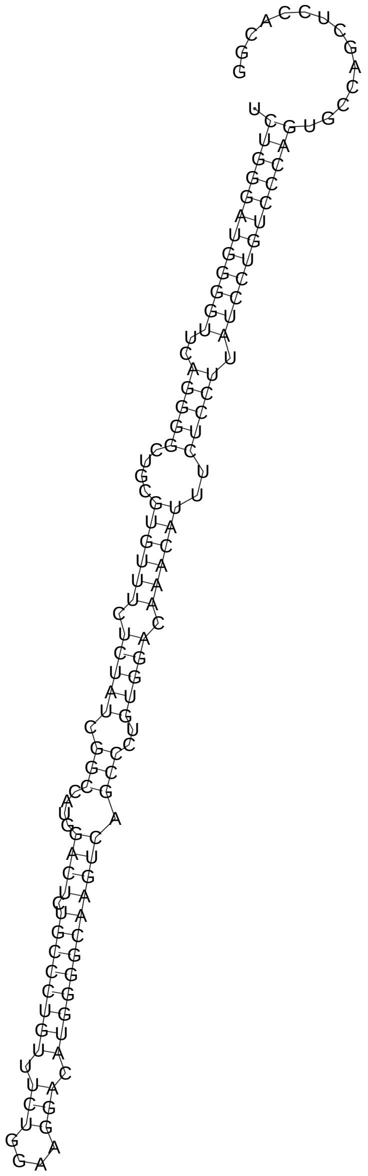

13_40692846-4069297_cfa-mir-8880_low

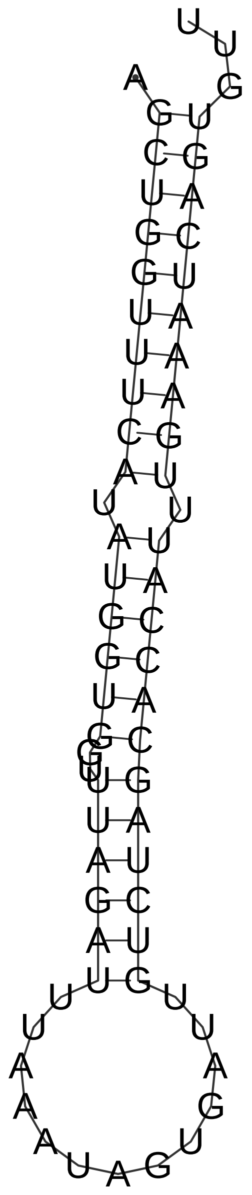

14_6184457-618452_cfa-mir-29b-1_high

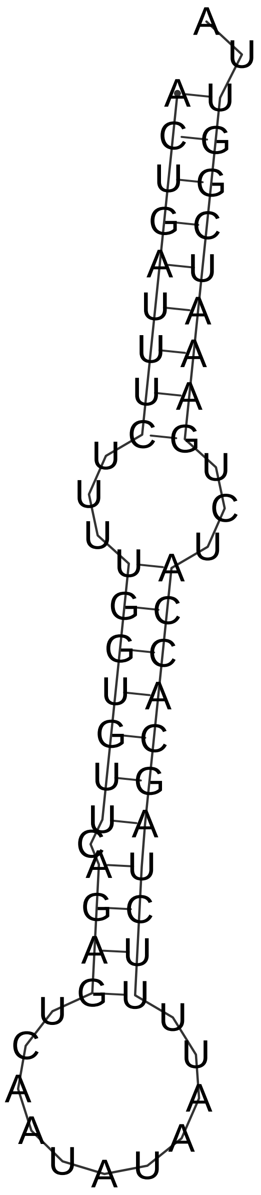

14_6184844-618490_cfa-mir-29a_high

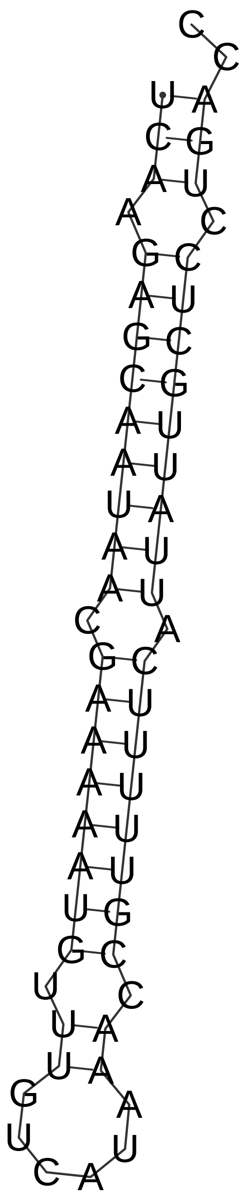

14_6444861-644491_cfa-mir-335_high

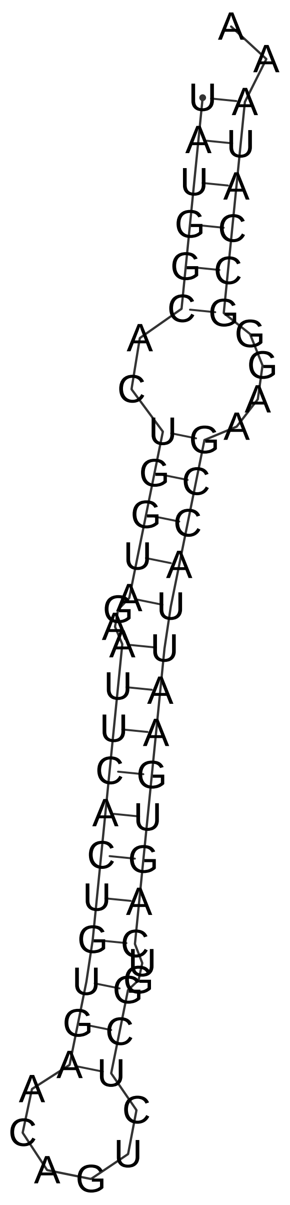

14_7068542-706860_cfa-mir-183_high

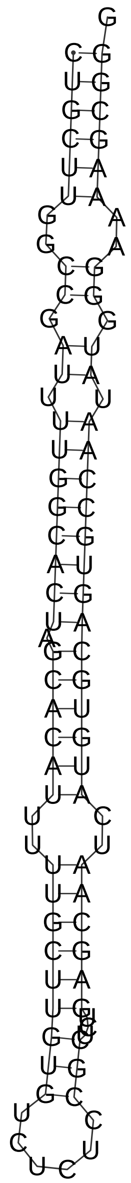

14_7068754-706884_cfa-mir-96_high

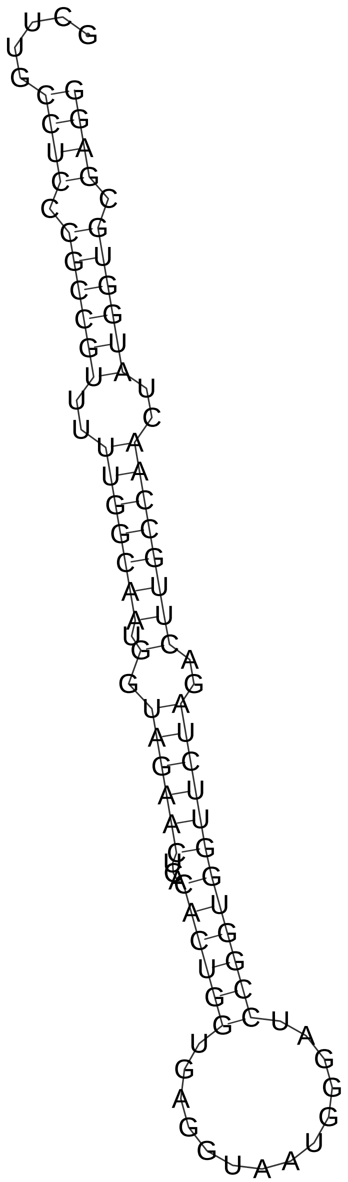

14_7072705-707279_cfa-mir-182_high

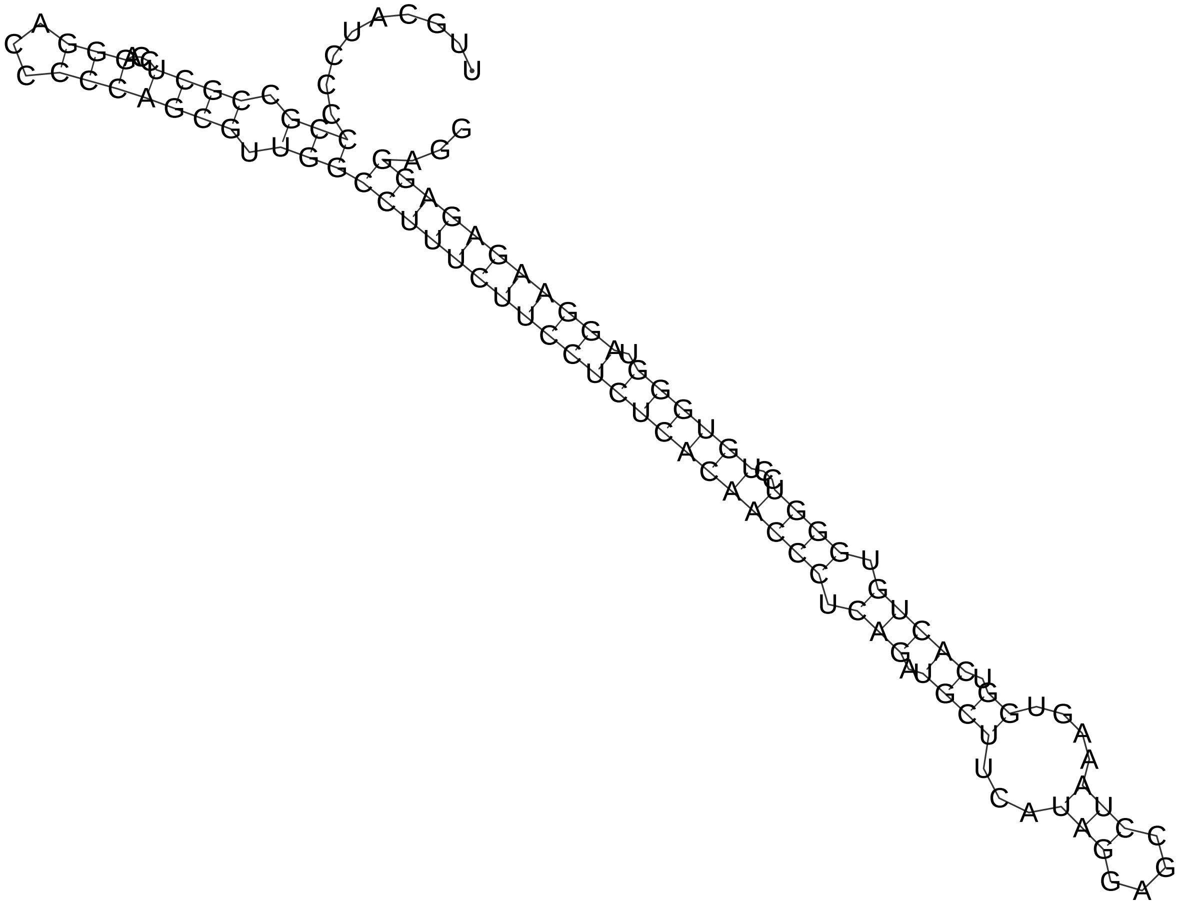

14_7850266-785038_mir-9082_low

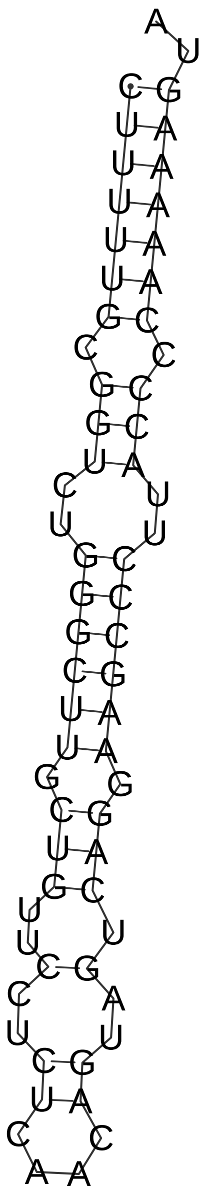

14_8165110-816517_cfa-mir-129-1_high

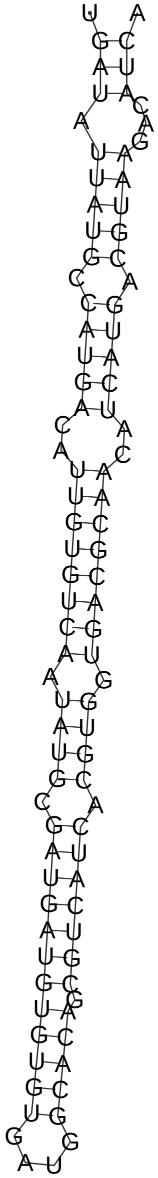

14_9182237-918233_cfa-mir-592_high

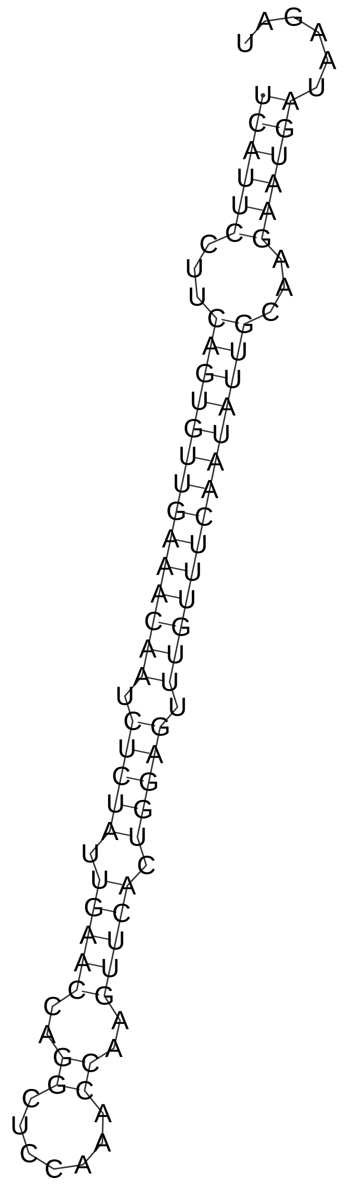

14_18979467-1897955_cfa-mir-653_high

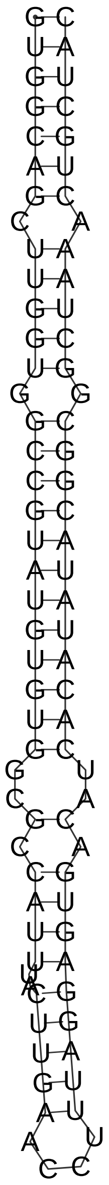

14_18980180-1898026_cfa-mir-489_high

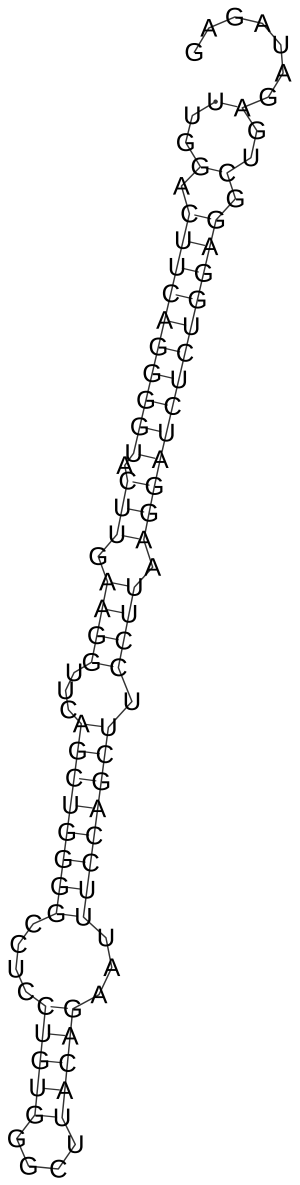

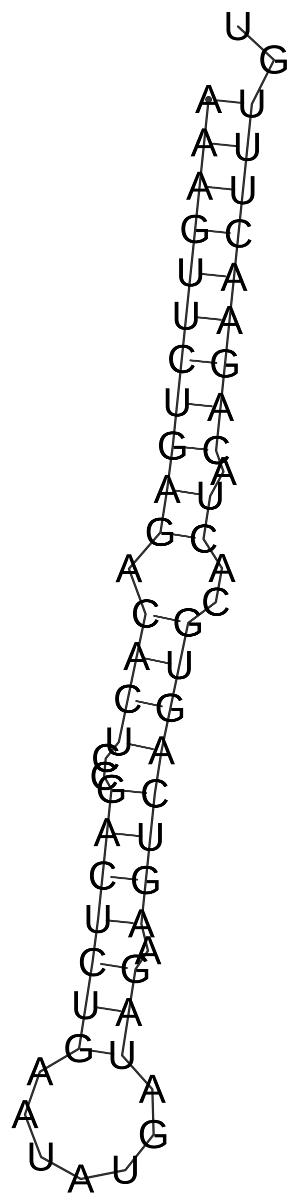

14_39301847-3930190_cfa-mir-148a_high

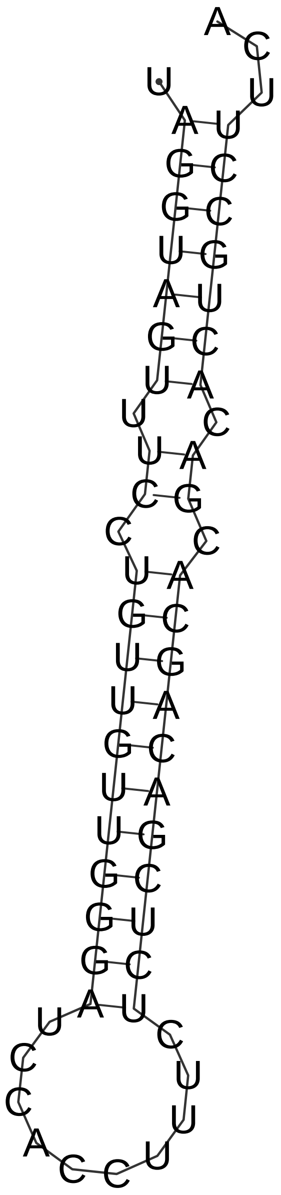

14_40344005-4034406_cfa-mir-196b_high

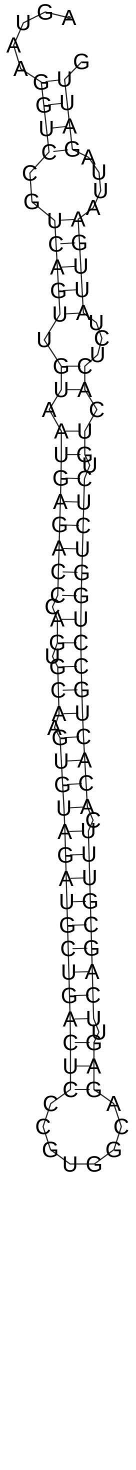

14_53825737-5382584_mir-3666_low

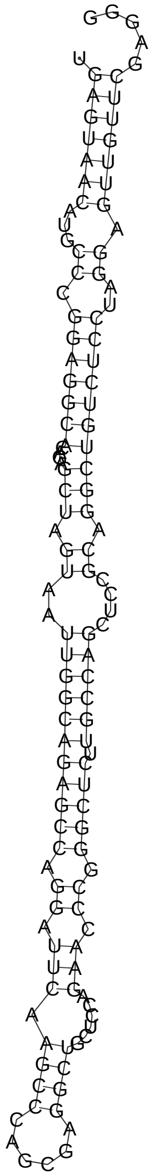

15_1285317-128544_cfa-mir-8839_low

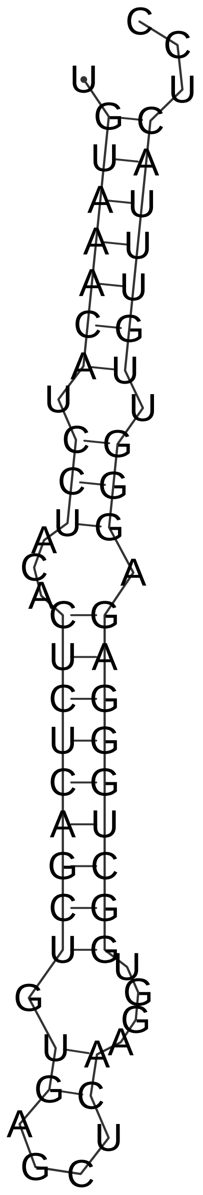

15_2280050-228011_cfa-mir-30c-1_high

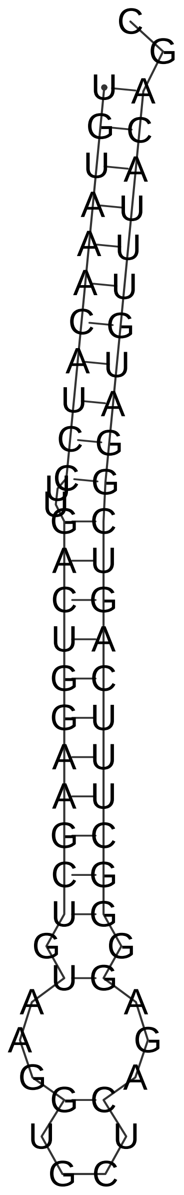

15_2283306-228336_cfa-mir-30e_high

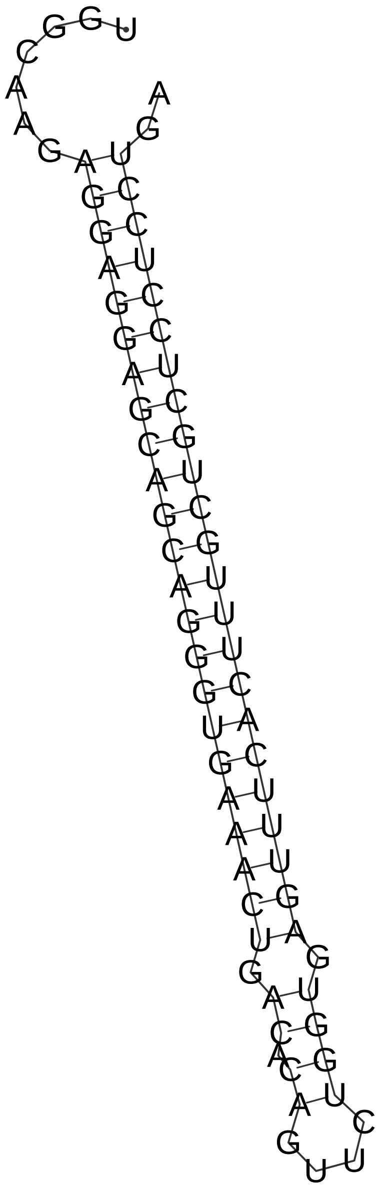

15_9455388-945545_cfa-mir-761_low

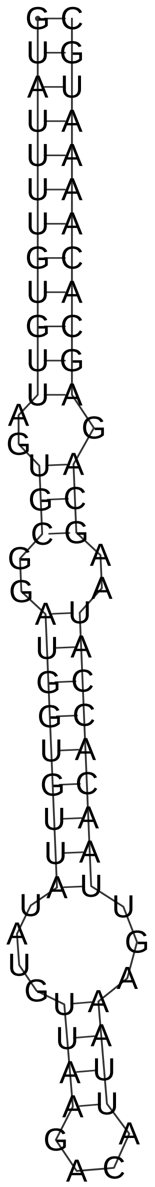

15_15969633-1596970_mir-2414_low

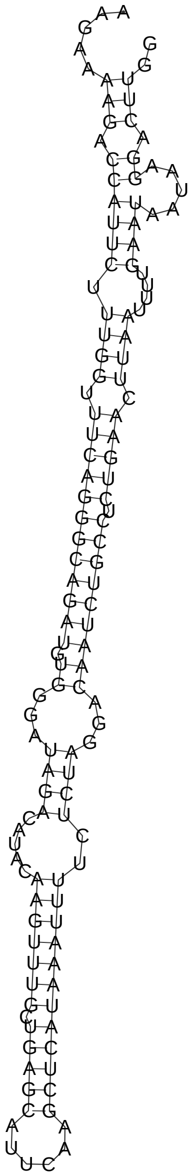

15_26986548-2698667_cfa-mir-8838_low

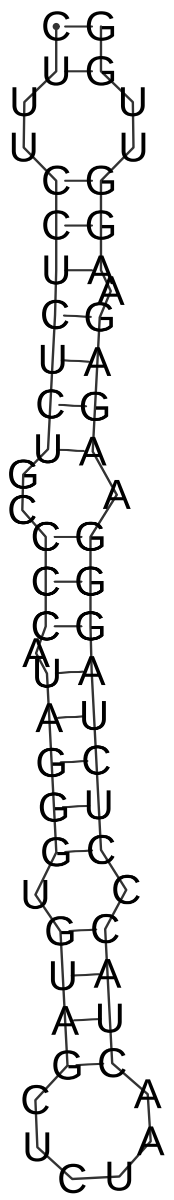

15_28107144-2810720_mir-3059_high

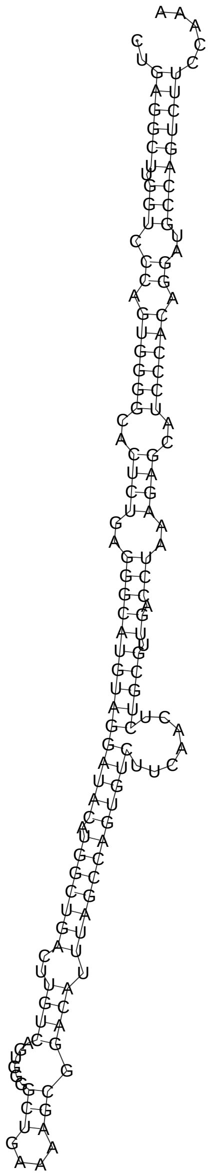

15_32468976-3246912_cfa-mir-8840_low

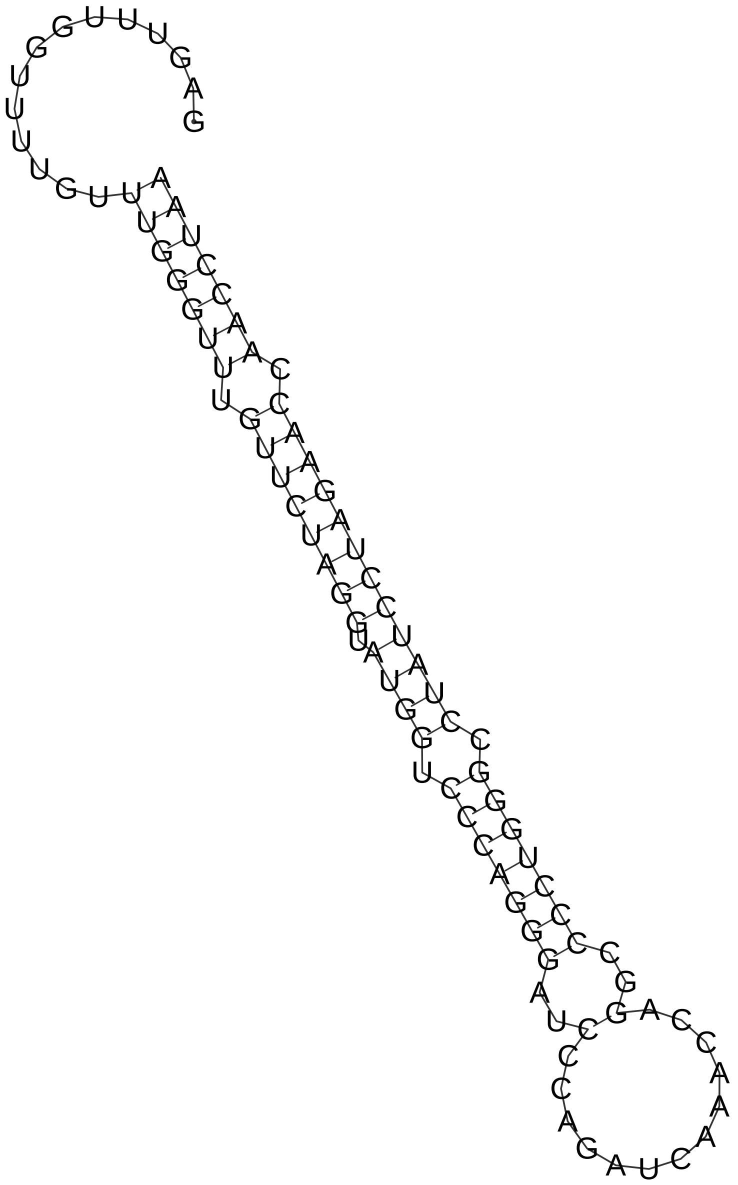

15_35234750-3523483_cfa-mir-331_high

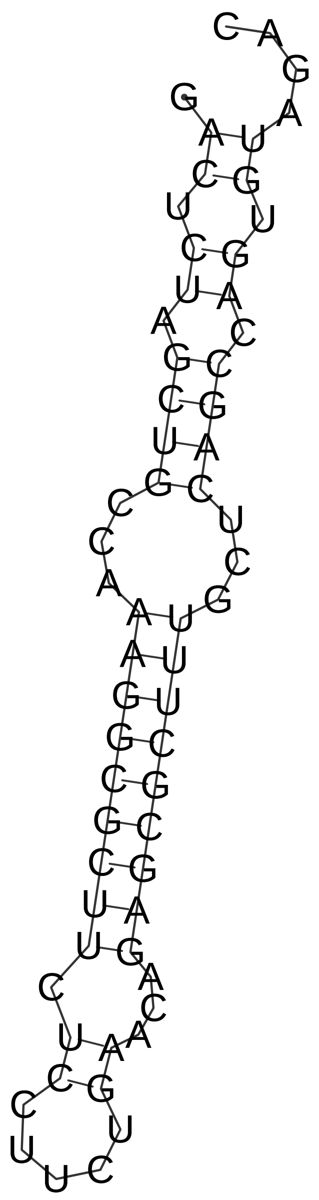

15_37111041-3711110_mir-1251_high

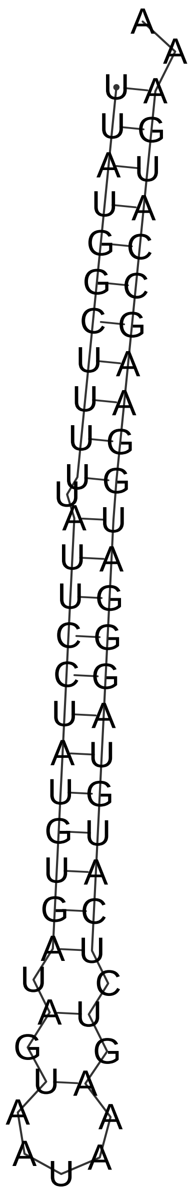

15_37181419-3718148_cfa-mir-135a-2_high

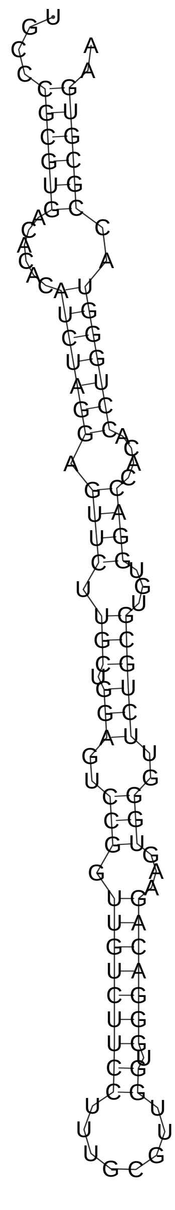

15_39178848-3917895_cfa-mir-8837_low

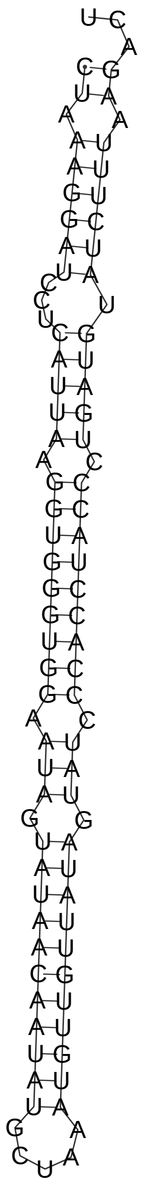

15_54704749-5470484_mir-2985_low

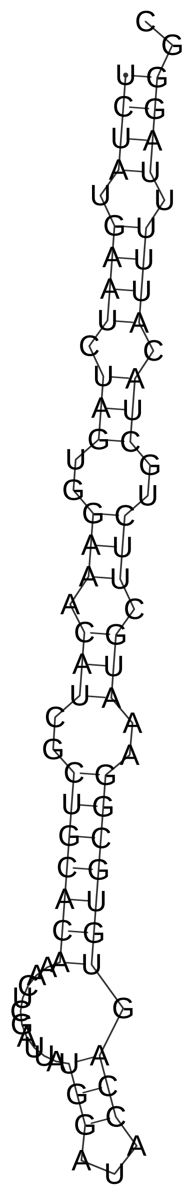

15_58855306-5885538_mir-147_high

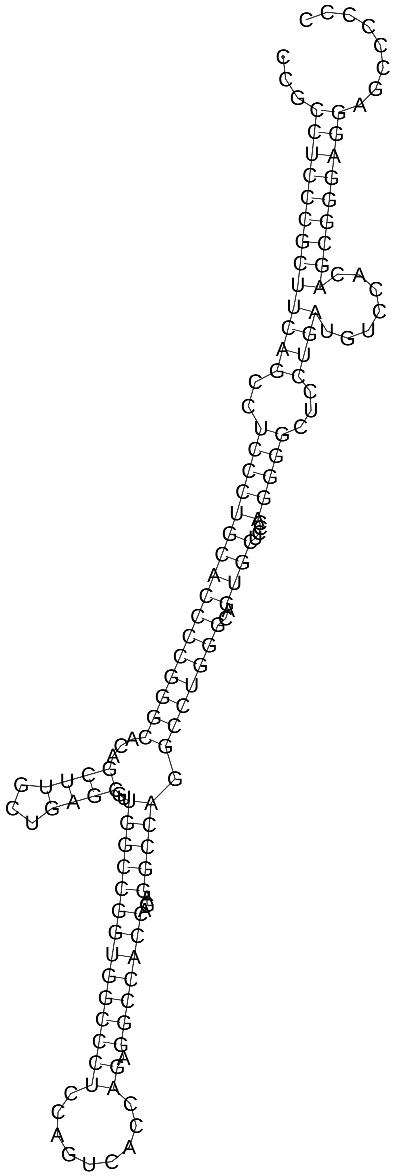

16_1101349-110149_cfa-mir-8862_low

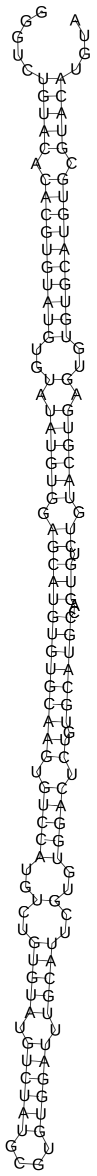

16_1148170-114831_cfa-mir-8852_low

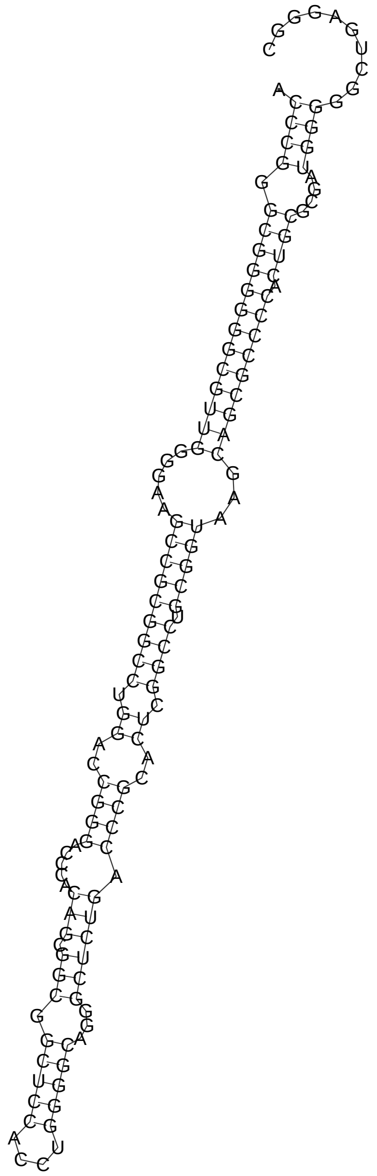

16_1275336-127546_cfa-mir-8853_low

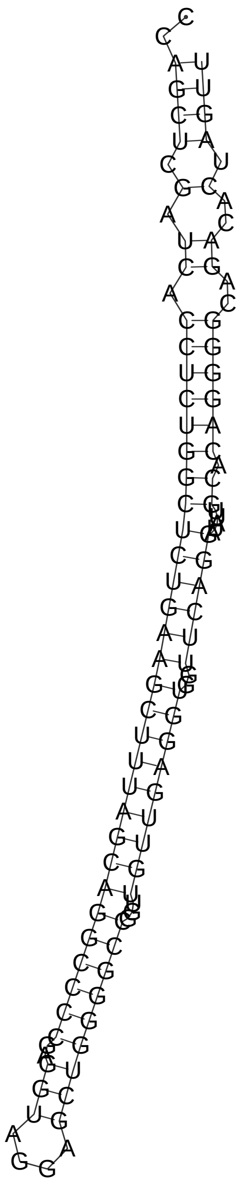

16_1707011-170711_cfa-mir-8793-1_low

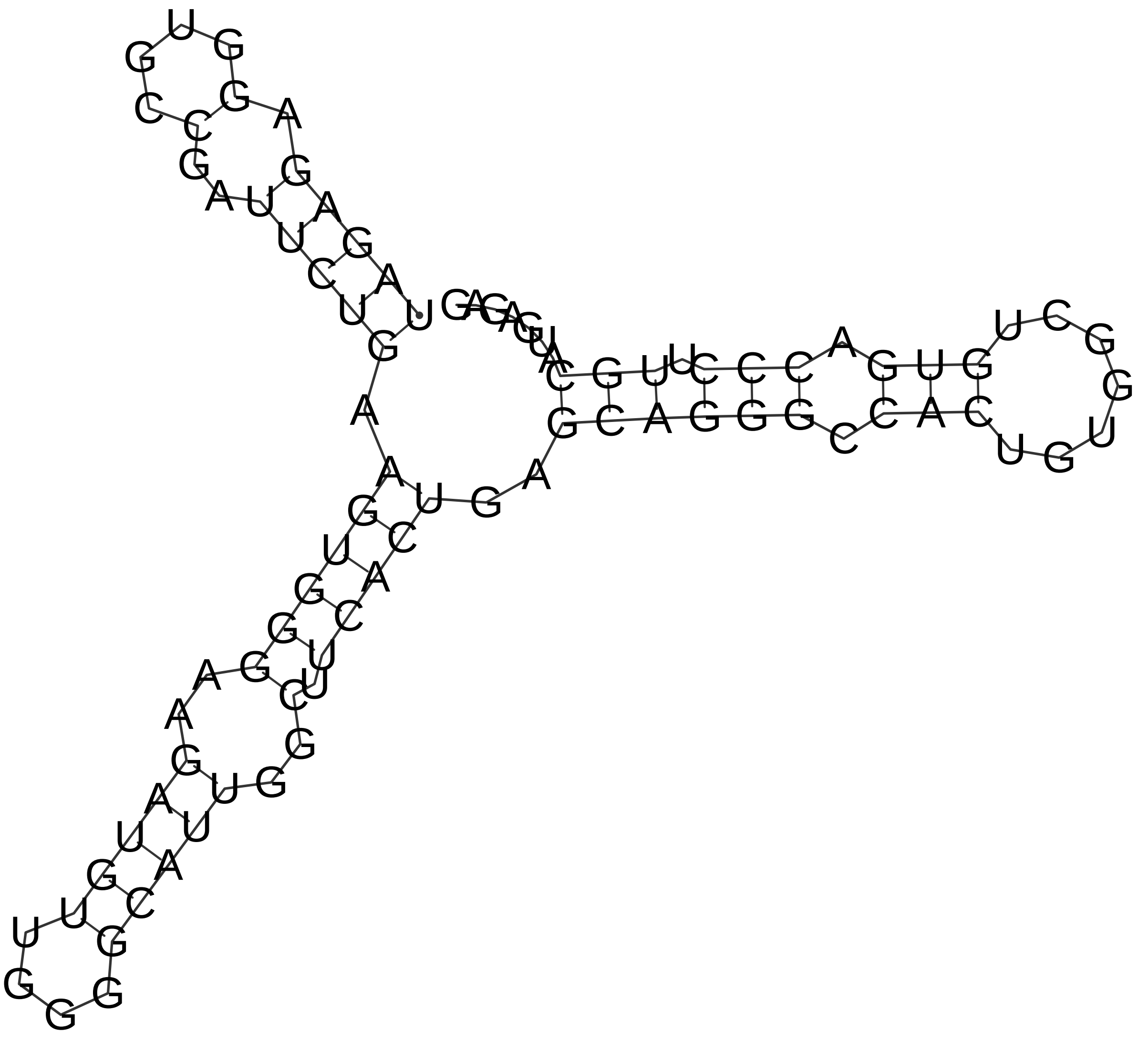

16_1725693-172578_mir-8907_low

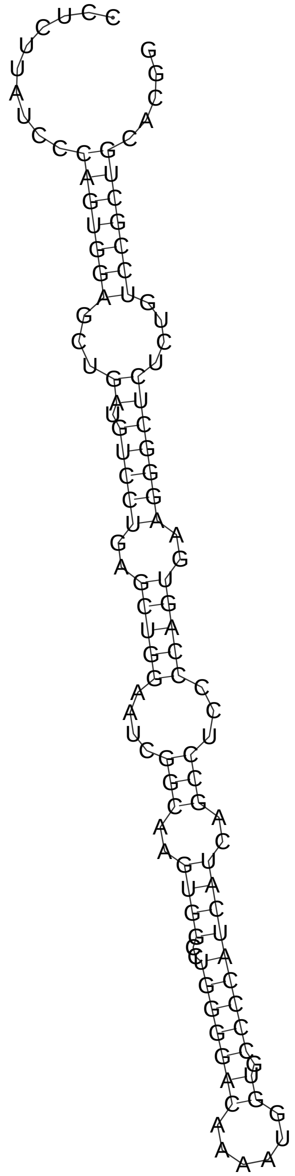

16_1728901-172901_mir-8794_low

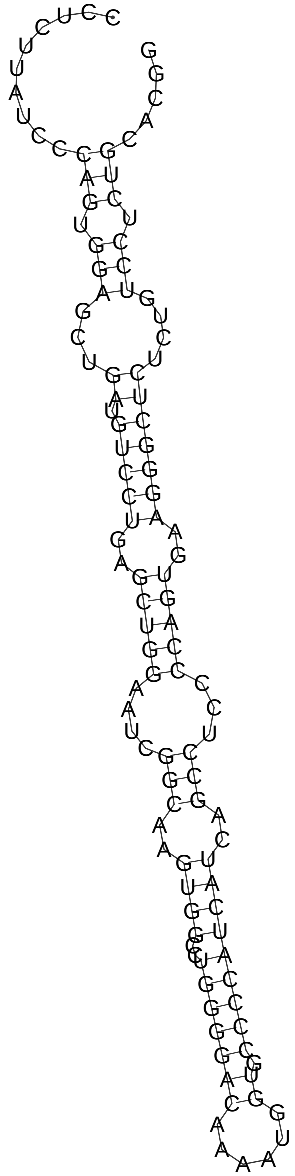

16_10434119-1043423_cfa-mir-8794-1_low

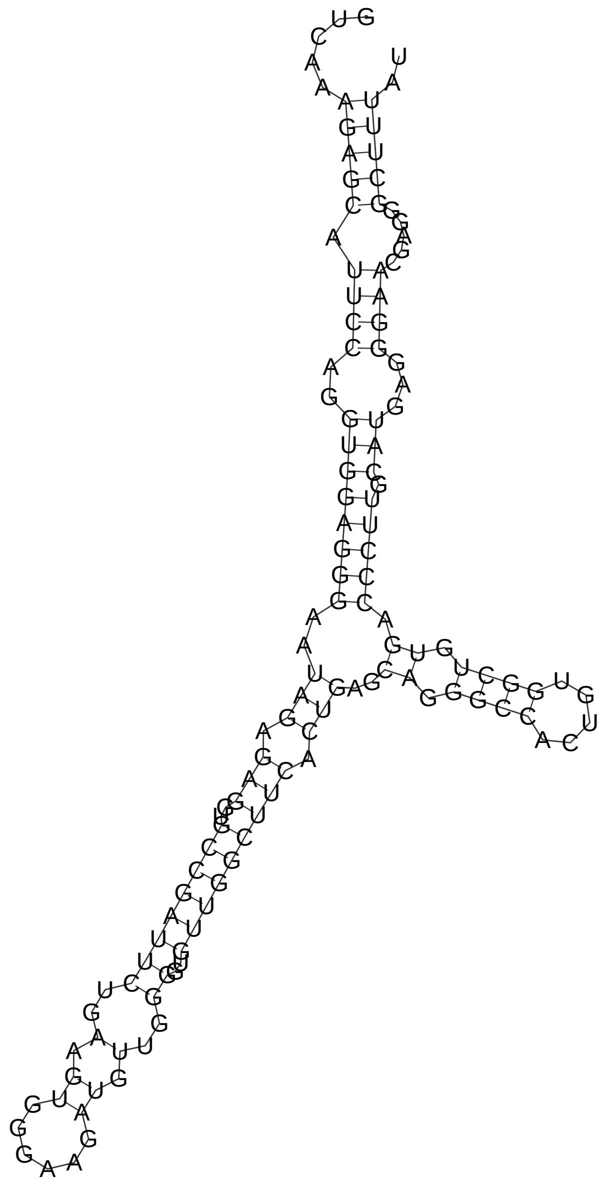

16_10437283-1043741_cfa-mir-8907_low

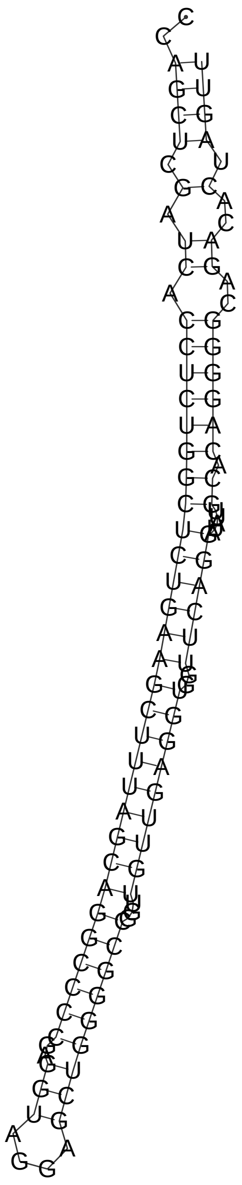

16_10452169-1045227_cfa-mir-8793-2_low

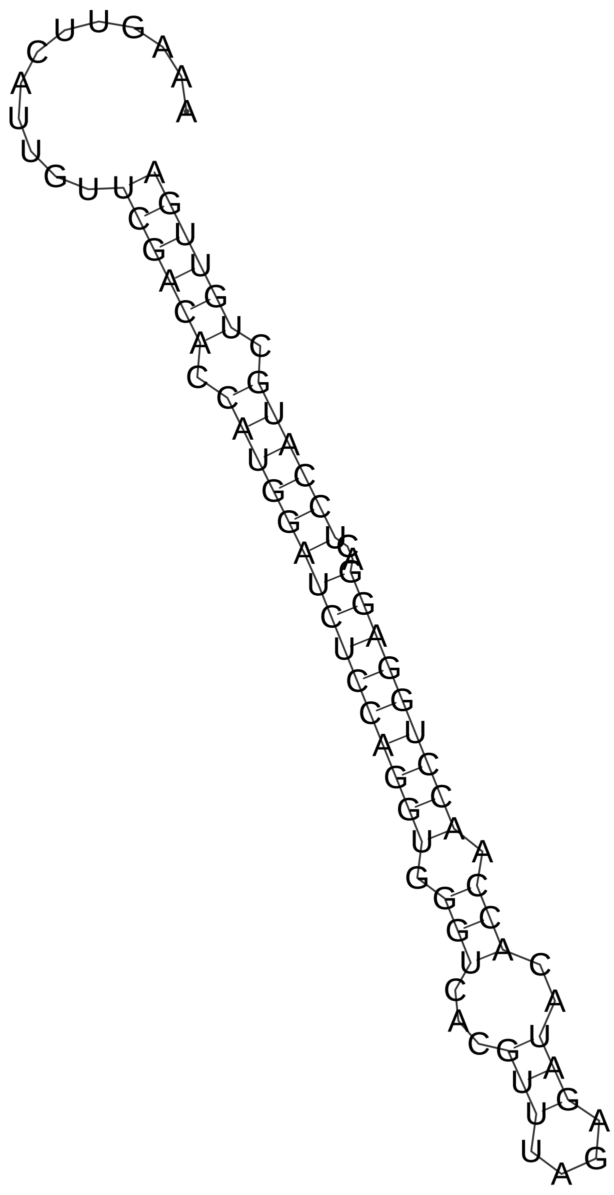

16_11628312-1162839_cfa-mir-490_high

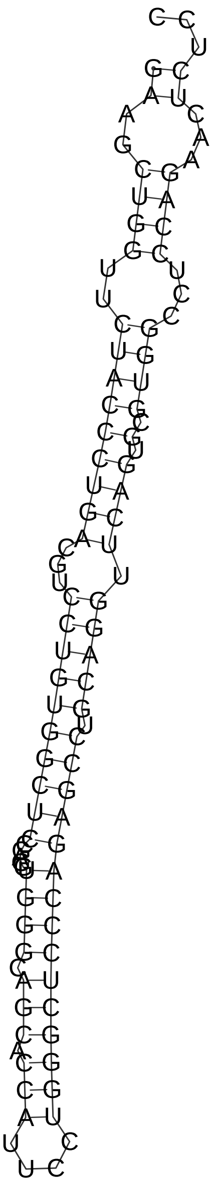

16_12928966-1292906_cfa-mir-8847_low

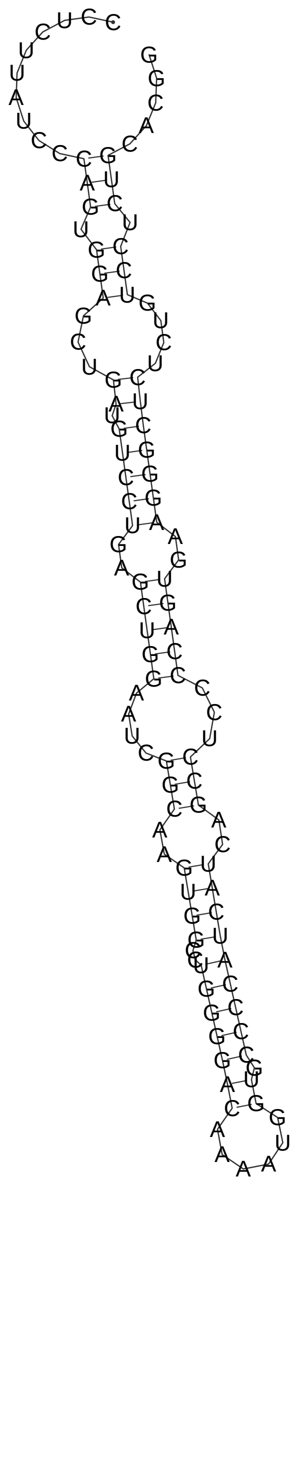

16_13901238-1390135_cfa-mir-8794-2_low

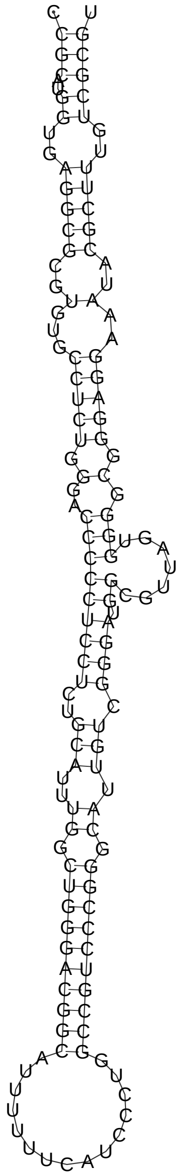

16_13916150-1391628_cfa-mir-8906_low

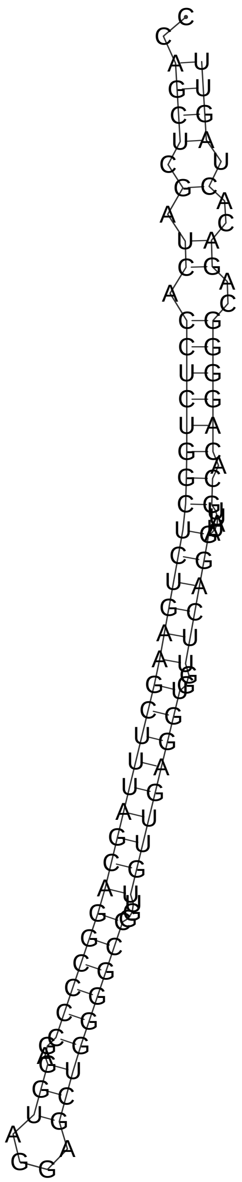

16_13946165-1394626_cfa-mir-8793-3_low

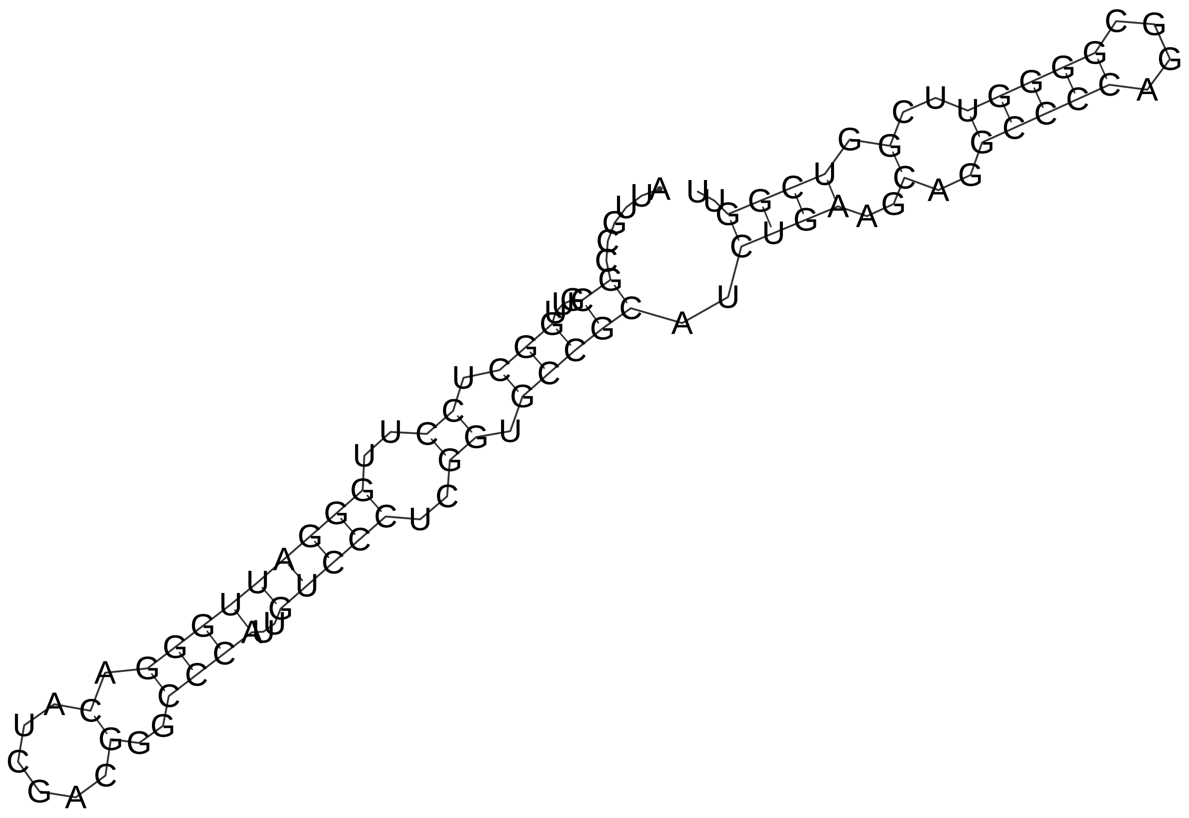

16_13954683-1395477_cfa-mir-8849_low

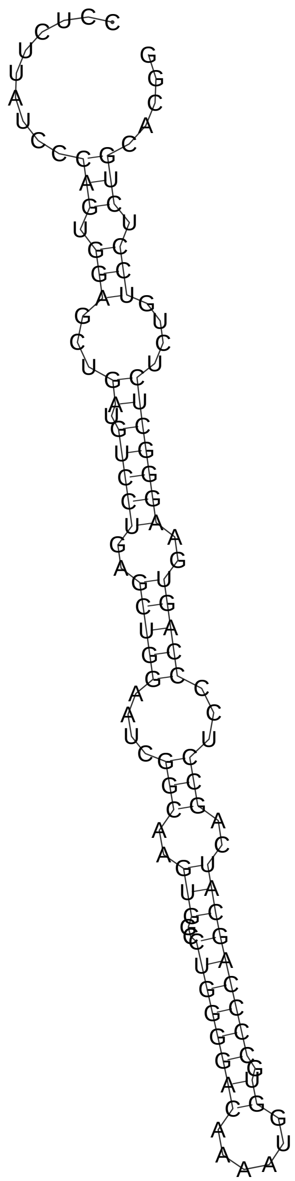

16_13963440-1396355_mir-8794_low

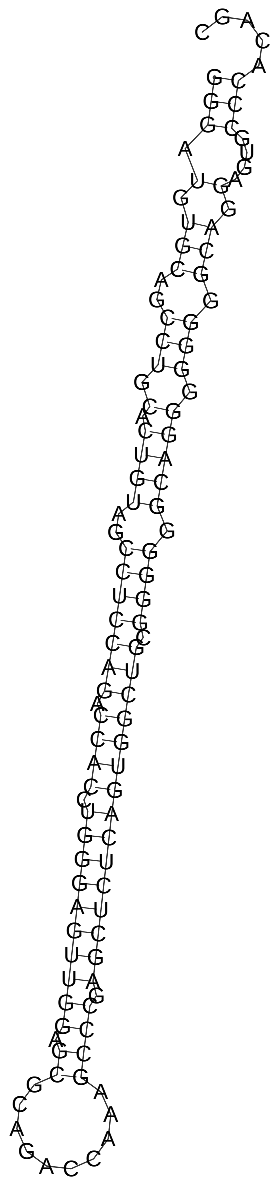

16_13973805-1397391_cfa-mir-8863_low

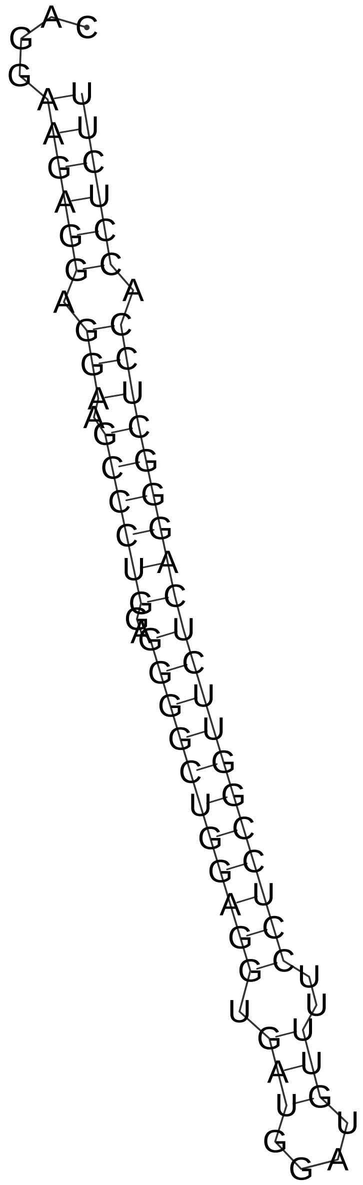

16_15278782-1527885_cfa-mir-671_high

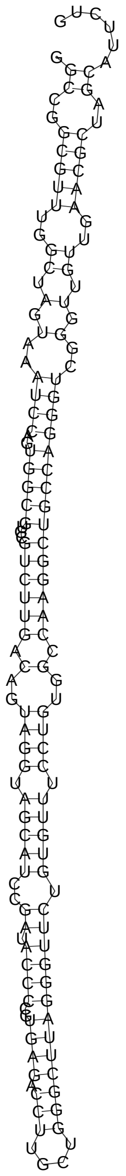

16_15641487-1564163_cfa-mir-8855_low

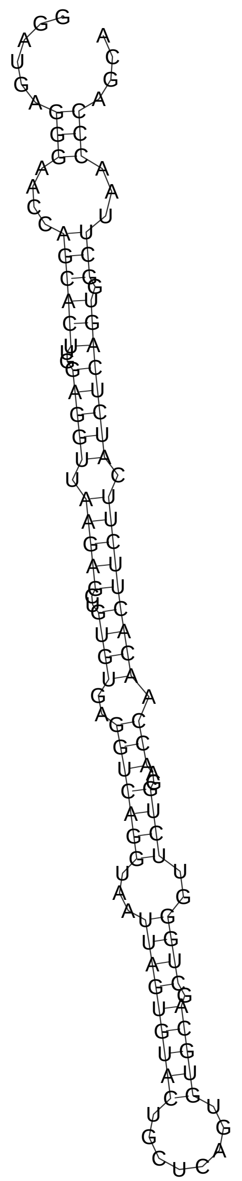

16_17313541-1731366_cfa-mir-8850_low

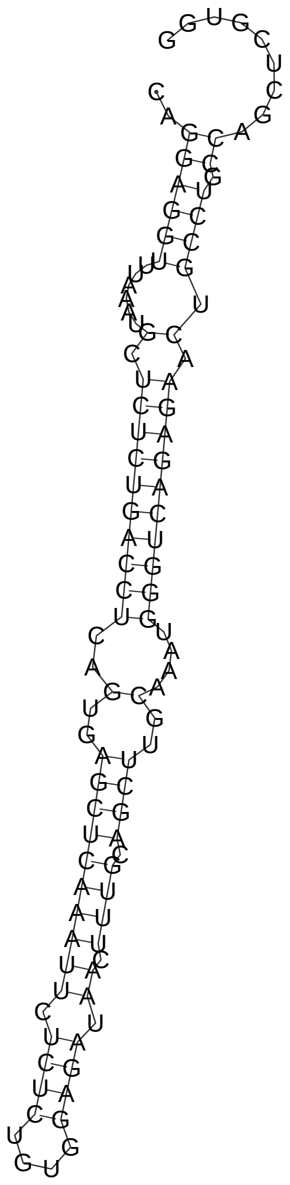

16_17324652-1732475_cfa-mir-8851_low

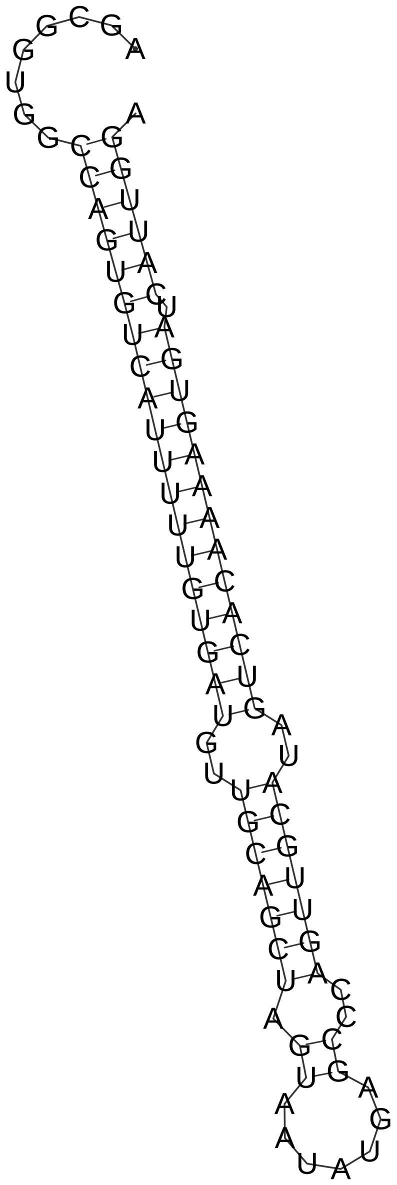

16_19932378-1993245_cfa-mir-153_high

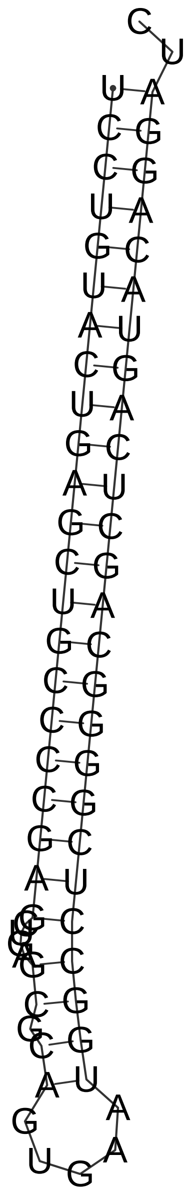

16_23958963-2395902_cfa-mir-486_low

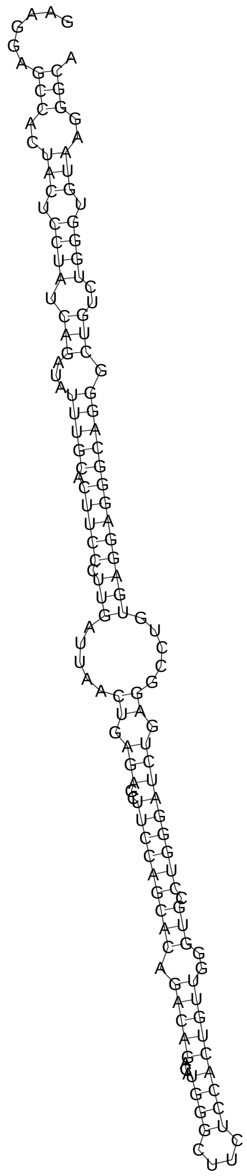

16_23989851-2398999_cfa-mir-8854_low

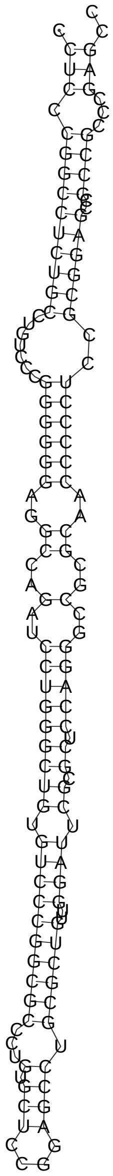

16_26379772-2637990_cfa-mir-8799d_low

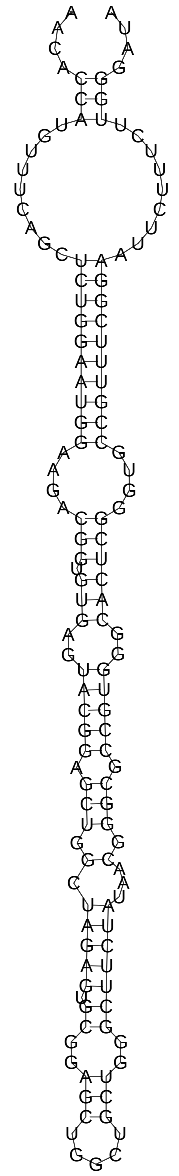

16_34814768-3481490_cfa-mir-8856_low

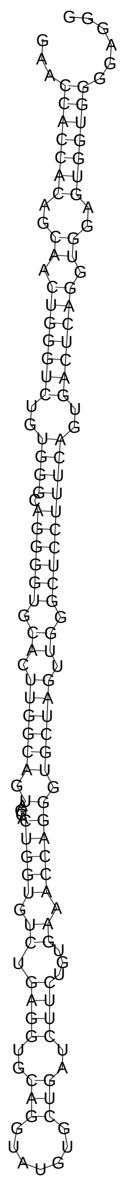

16_35465007-3546515_cfa-mir-8848_low

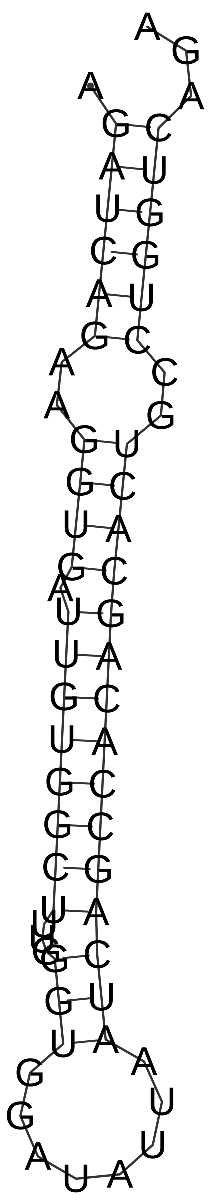

16_38173722-3817378_cfa-mir-383_high

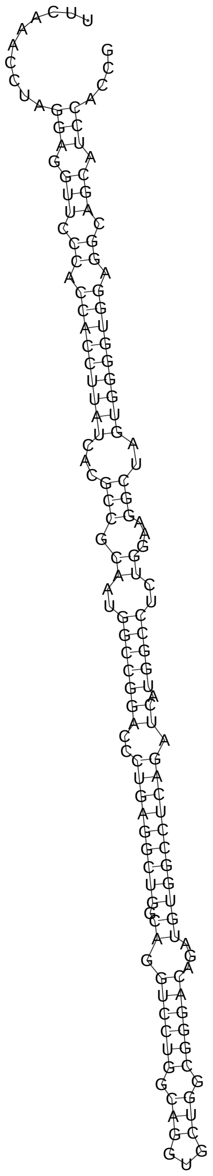

17_7152907-715305_cfa-mir-8846_low

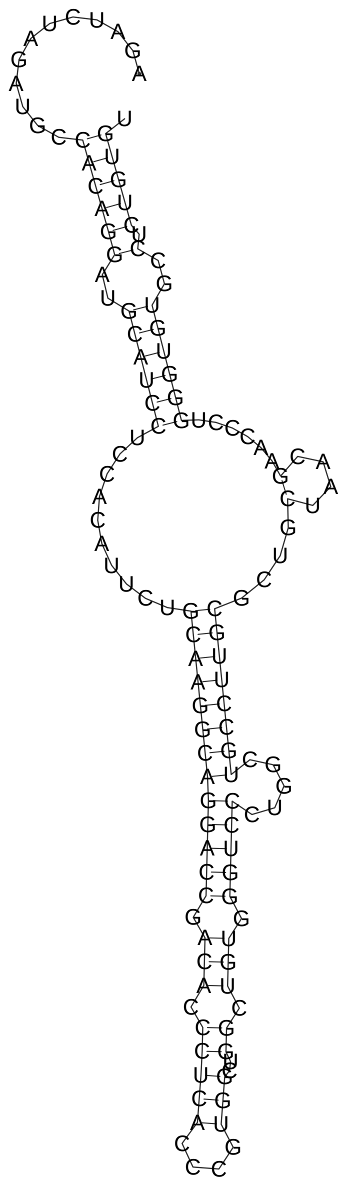

17_13107265-1310738_cfa-mir-8845_low

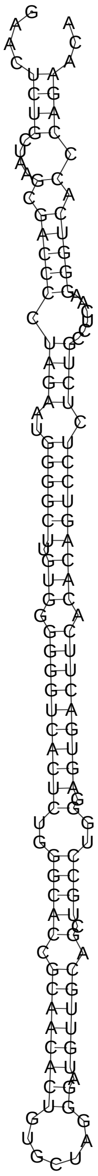

17_19575798-1957593_cfa-mir-1301_high

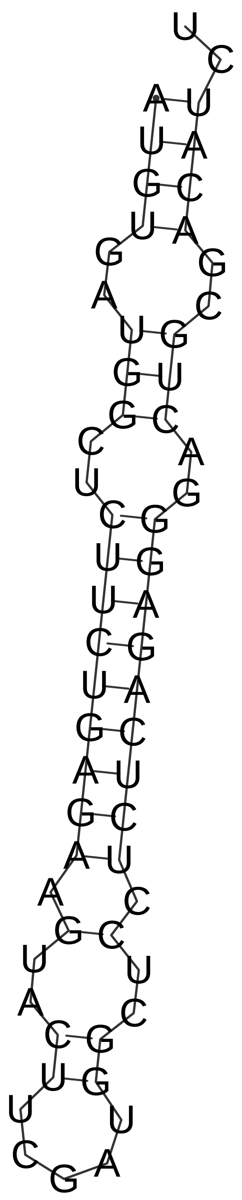

17_21491603-2149166_cfa-mir-1837-1_high

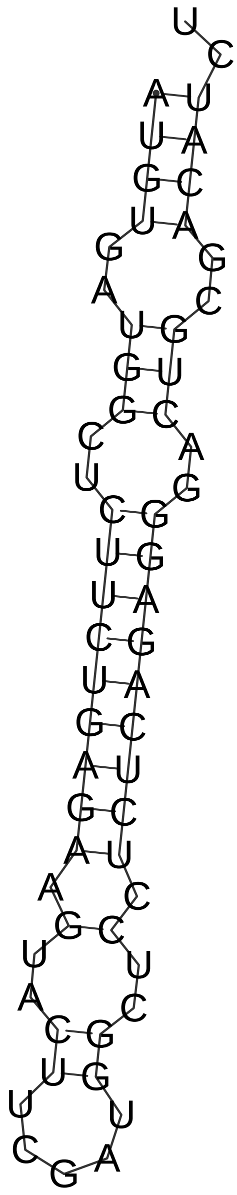

17_21620453-2162051_cfa-mir-1837-2_high

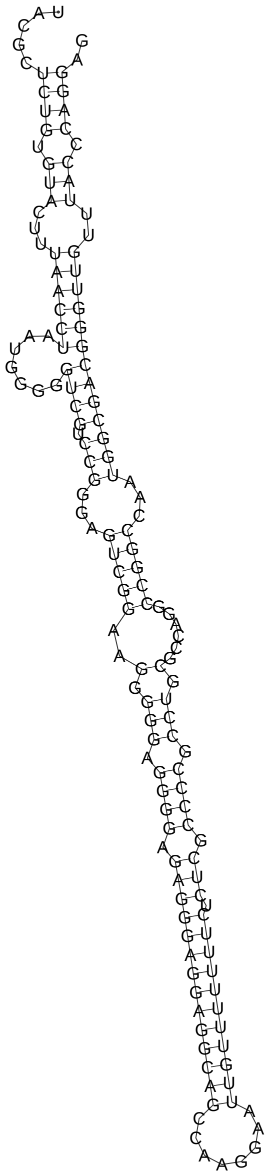

17_38744548-3874469_cfa-mir-8843_low

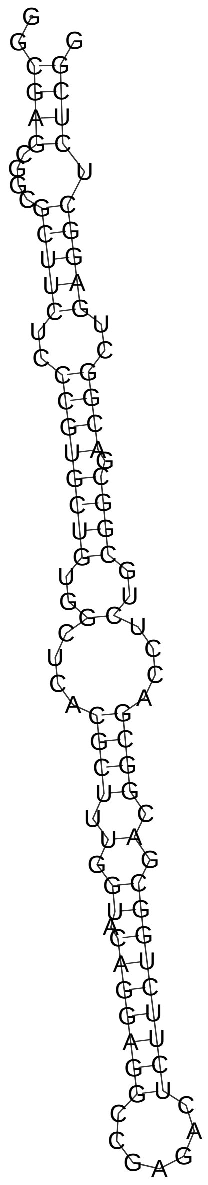

17_51412614-5141271_cfa-mir-8842_low

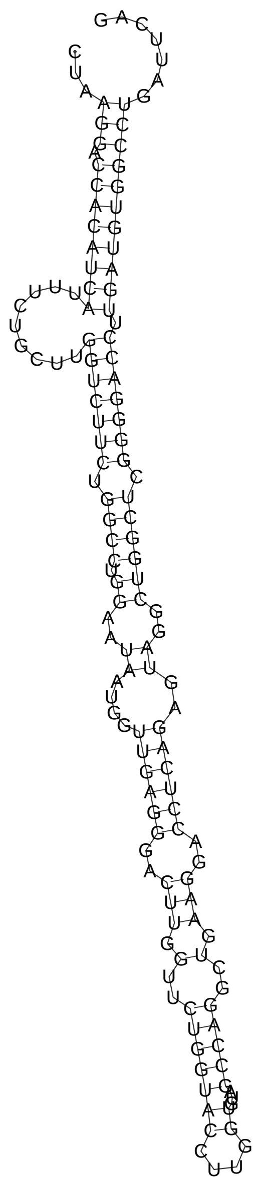

17_61805689-6180582_cfa-mir-8844_low

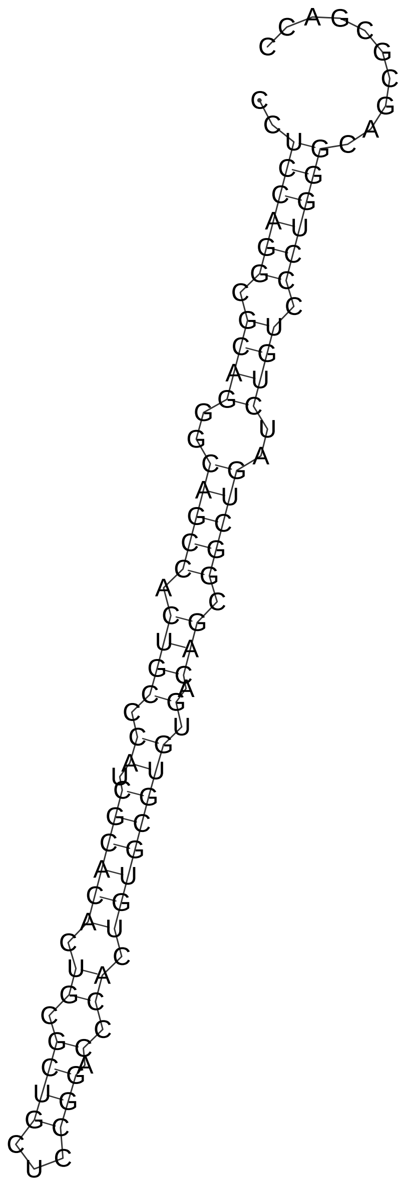

18_25675449-2567554_cfa-mir-210_high

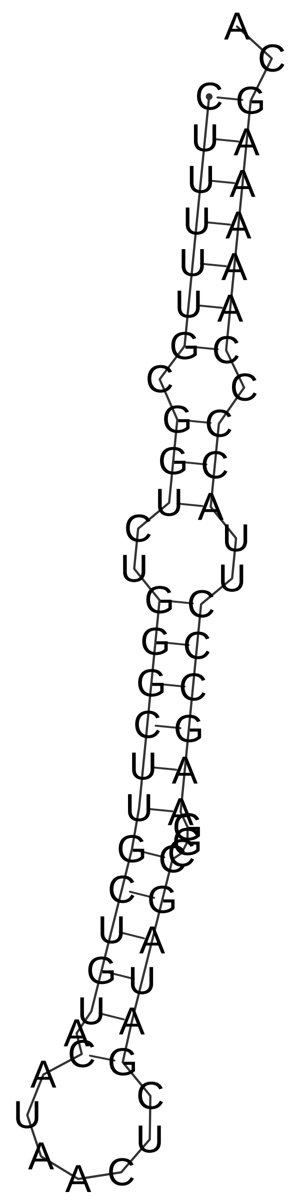

18_26154534-2615459_cfa-mir-129-2_high

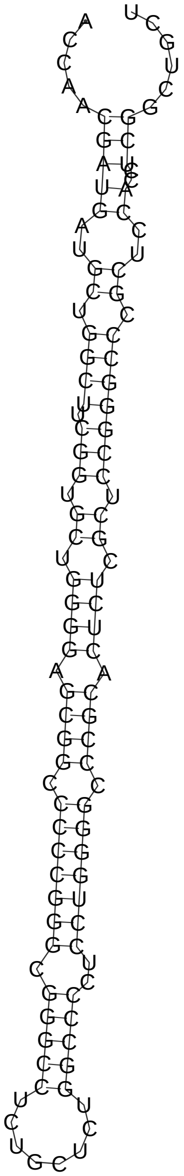

18_32990094-3299020_cfa-mir-1343_high

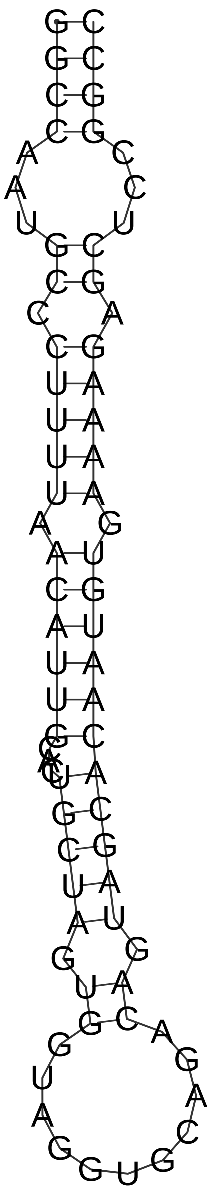

18_38543613-3854368_mir-3590_high

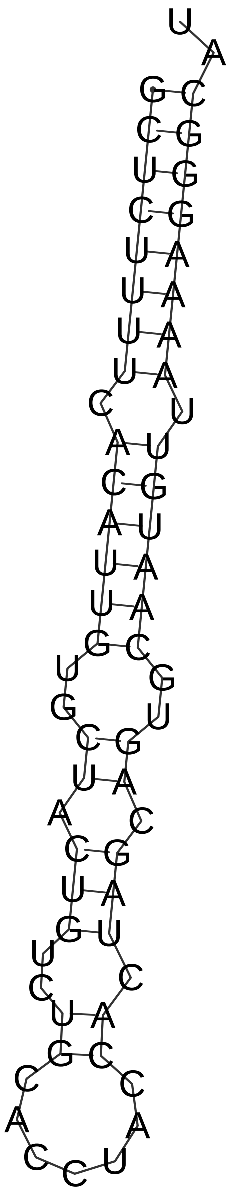

18_38543618-3854367_cfa-mir-130a_high

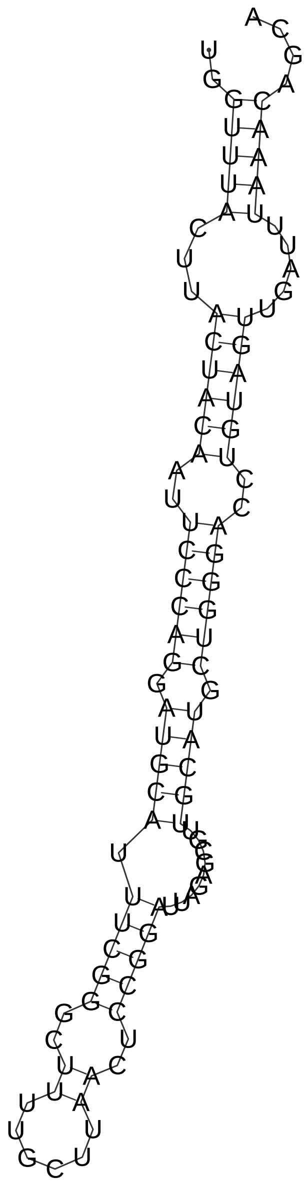

18_43307787-4330788_mir-1955_low

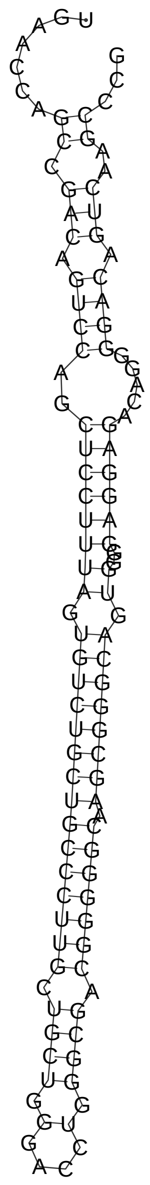

18_43668954-4366906_cfa-mir-8861_low

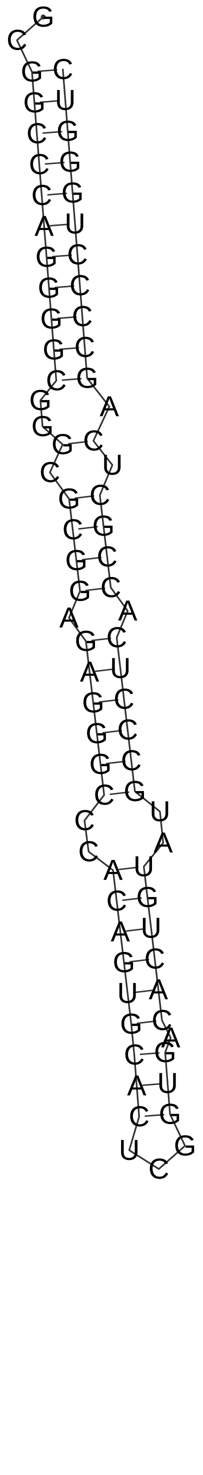

18_46206396-4620647_mir-675_high

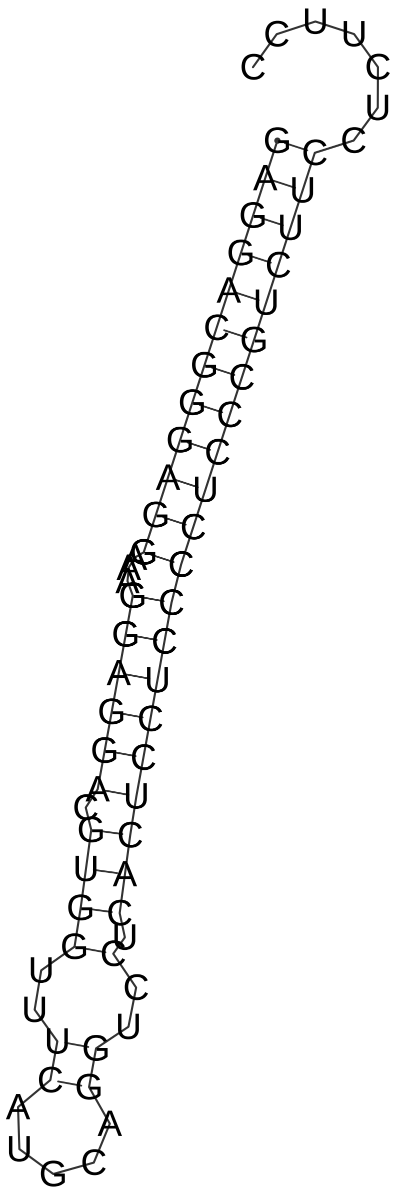

18_46298392-4629846_cfa-mir-483_high

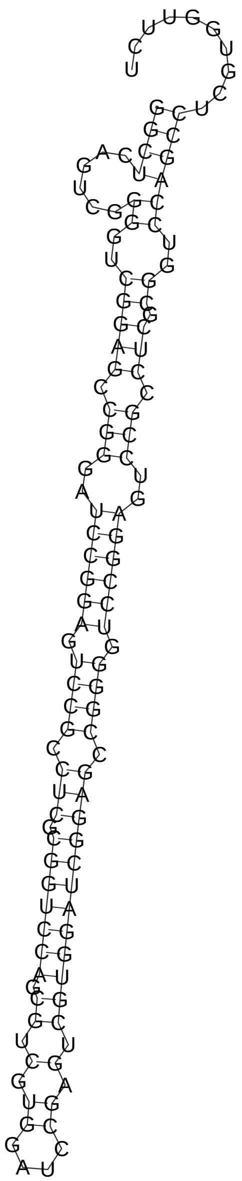

18_46690380-4669049_cfa-mir-8859a_high

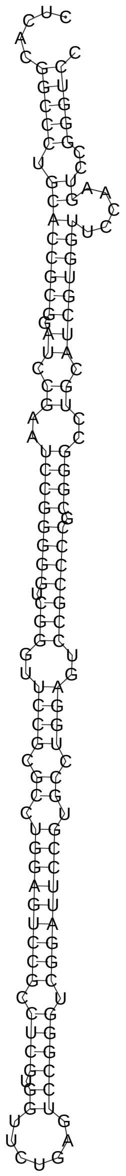

18_46690541-4669067_cfa-mir-8859b_high

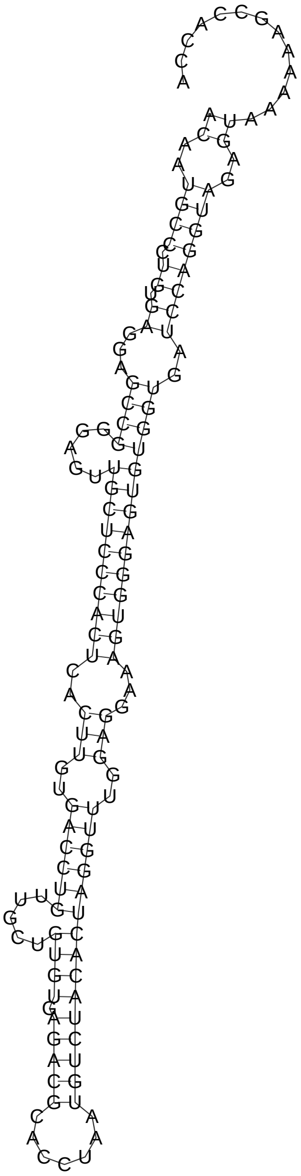

18_47168318-4716845_cfa-mir-8860_low

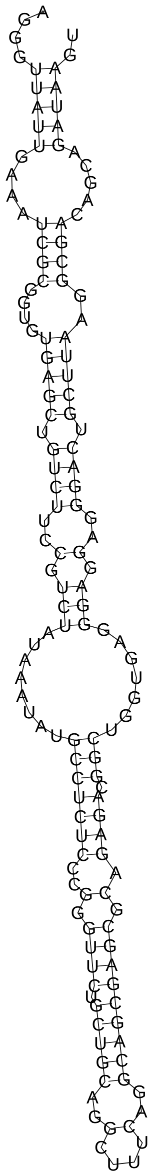

18_51707336-5170747_cfa-mir-8858_low

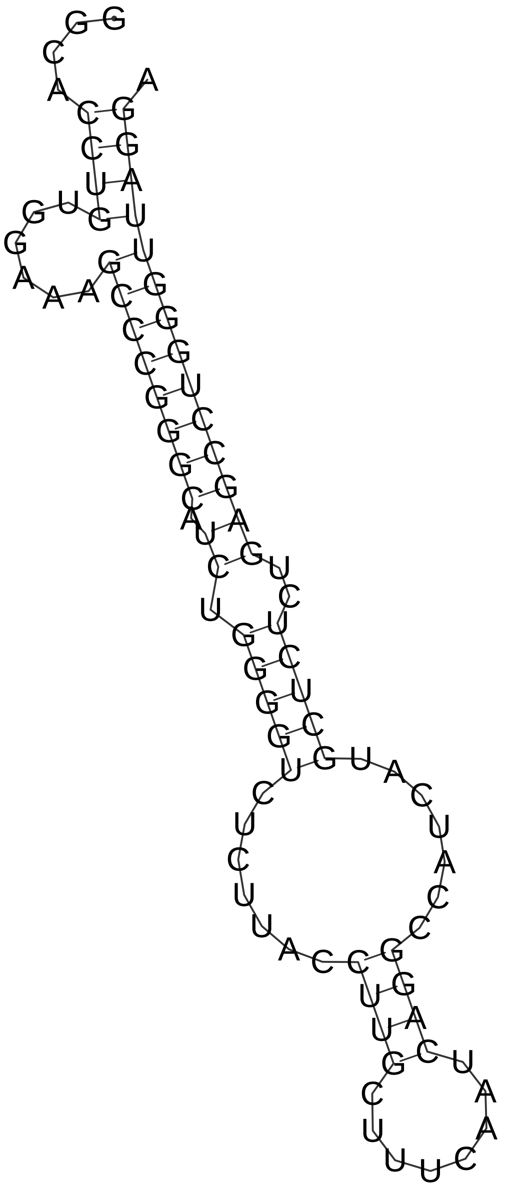

18_52251213-5225129_mir-9112_low

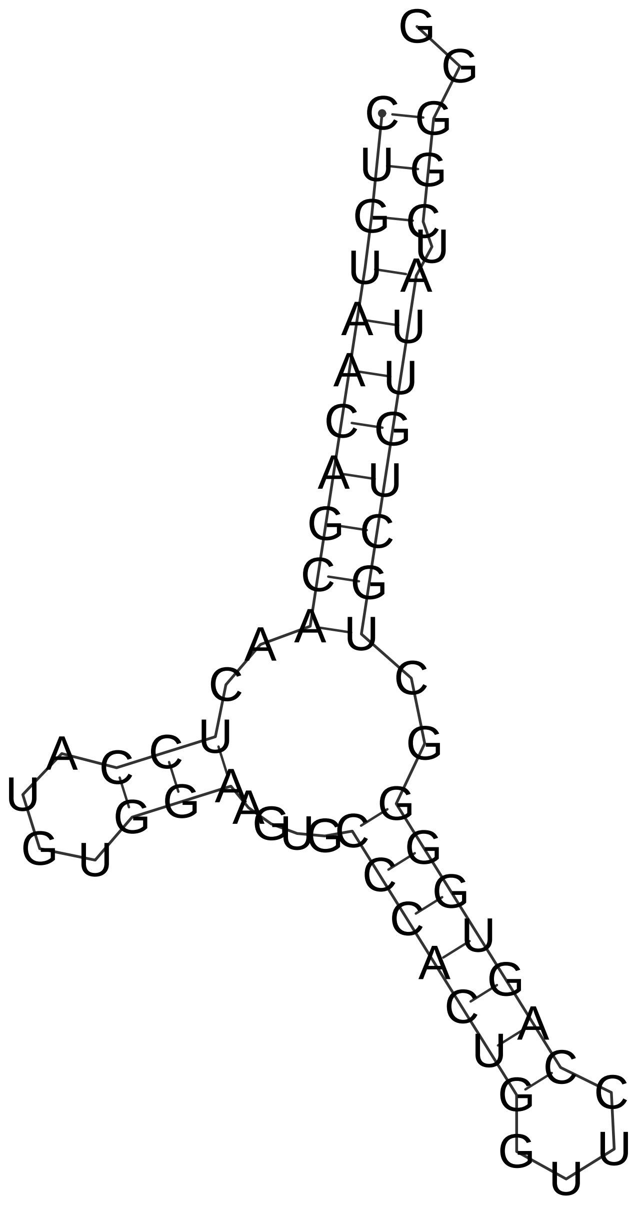

18_52280928-5228098_mir-194_high

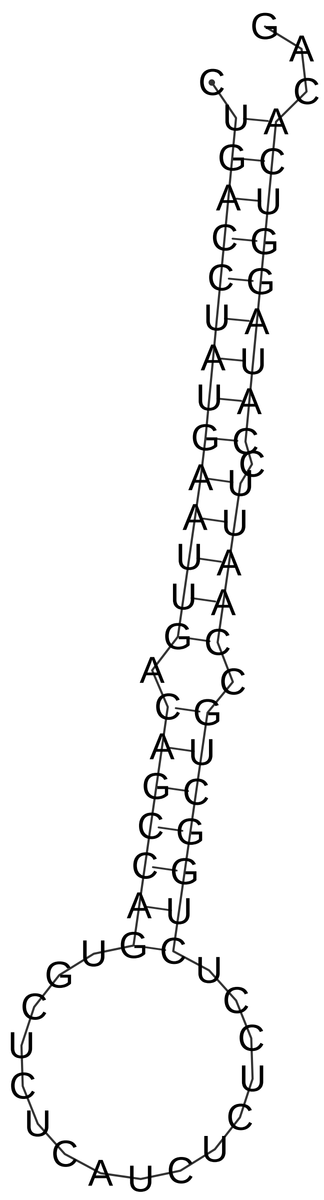

18_52281129-5228119_cfa-mir-192_high

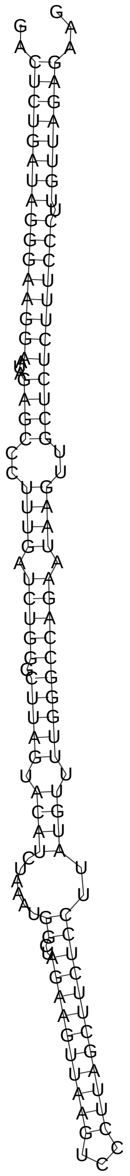

19_13642448-1364257_cfa-mir-8857_low

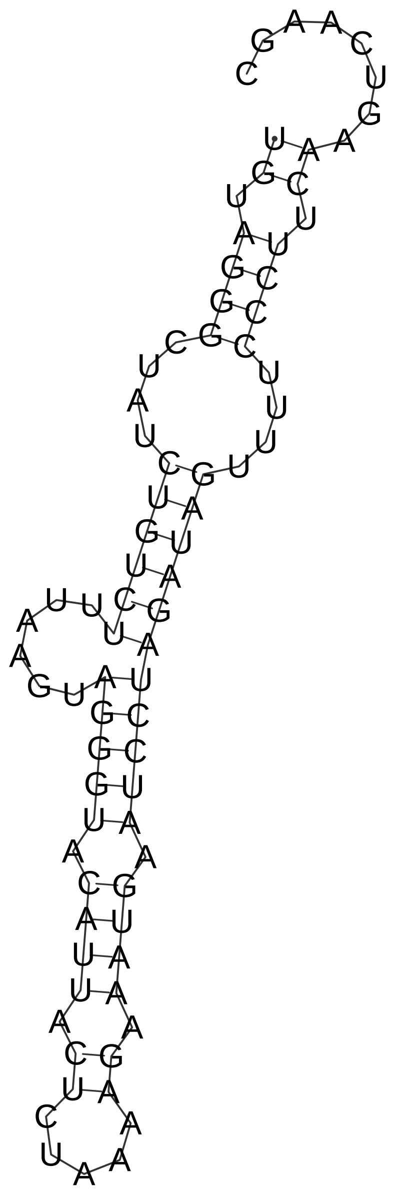

19_16390623-1639070_mir-684_low

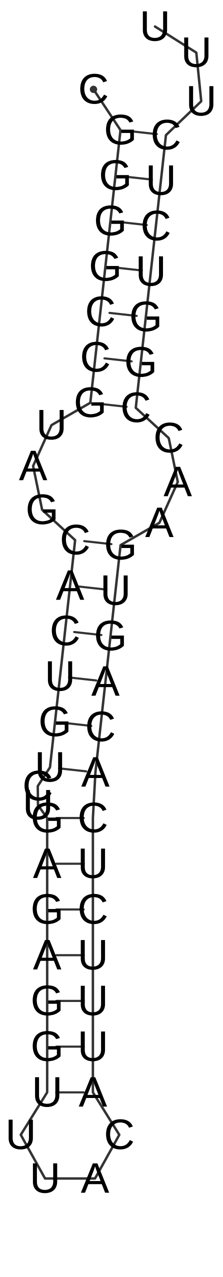

19_38435956-3843601_cfa-mir-128-1_high

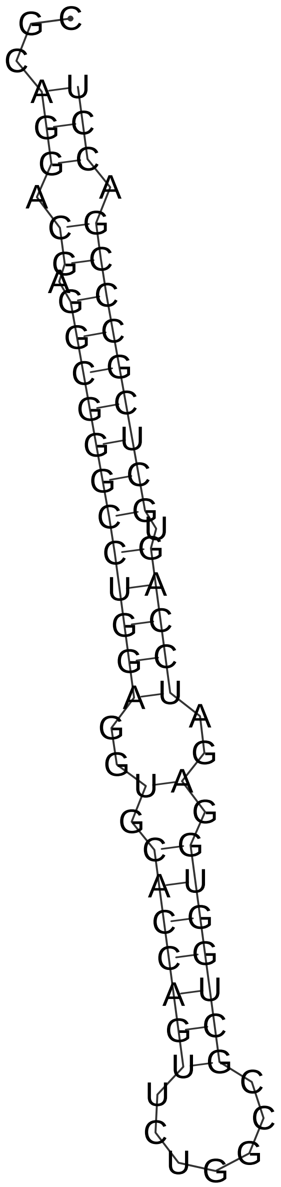

2_1319449-131951_mir-4683_high

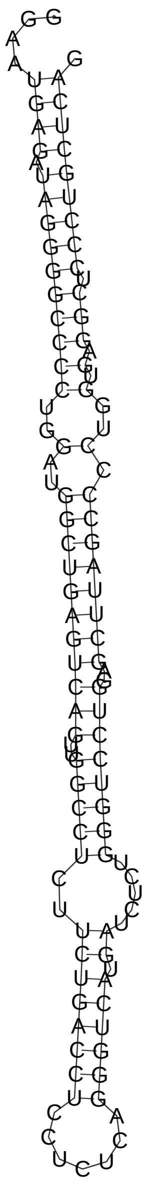

2_4216693-421680_cfa-mir-8864-1_low

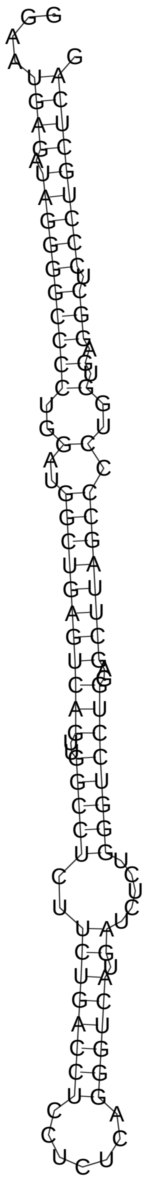

2_4601780-460189_cfa-mir-8864-4_low

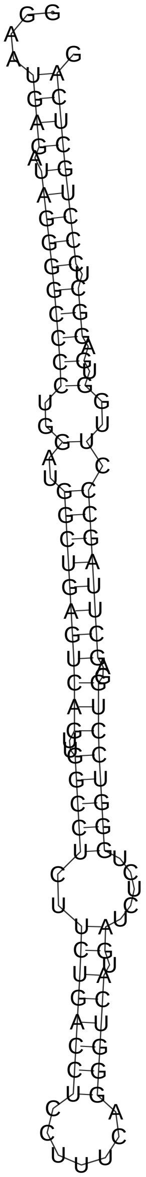

2_5658031-565814_cfa-mir-8864-3_low

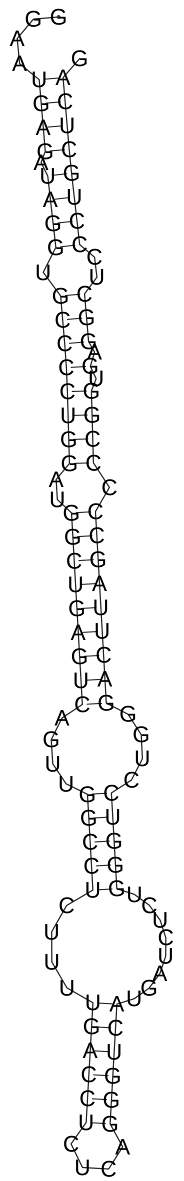

2_6435916-643602_mir-8864_low

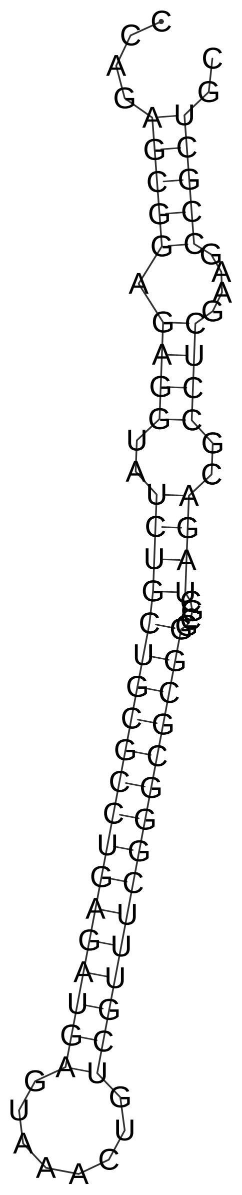

2_14591216-1459129_mir-1893_low

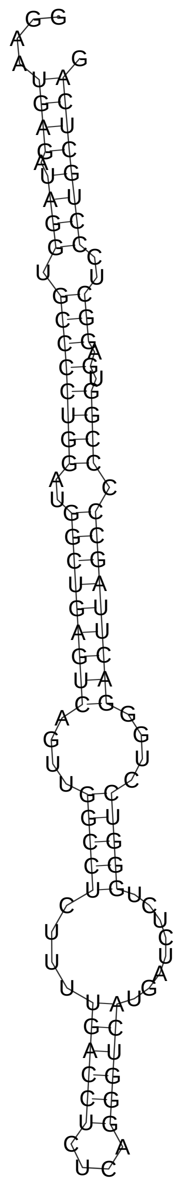

2_18383163-1838327_mir-8864_low

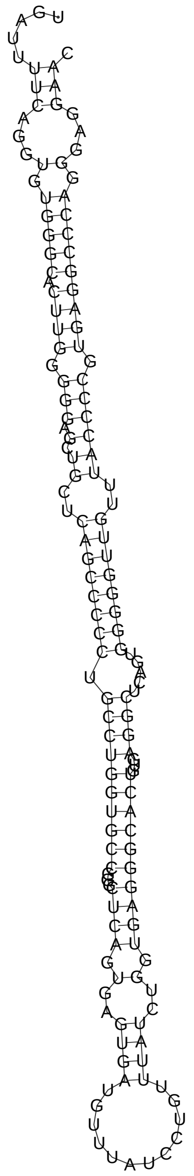

2_18385514-1838565_cfa-mir-8895_low

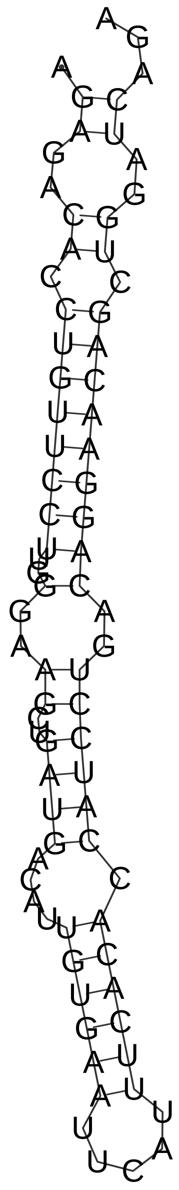

2_22199888-2219996_mir-4293_low

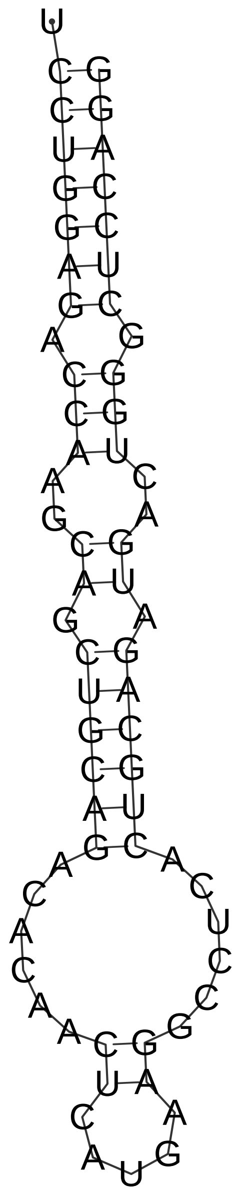

2_37910194-3791025_mir-9162_low

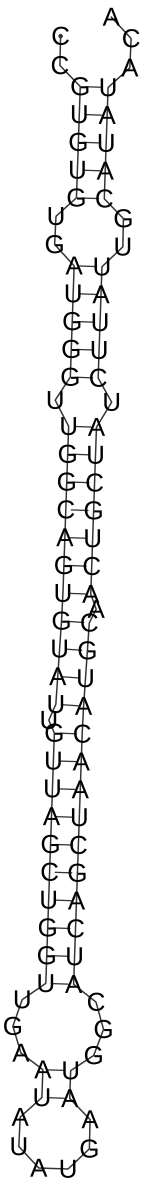

2_42541949-4254203_cfa-mir-449a_high

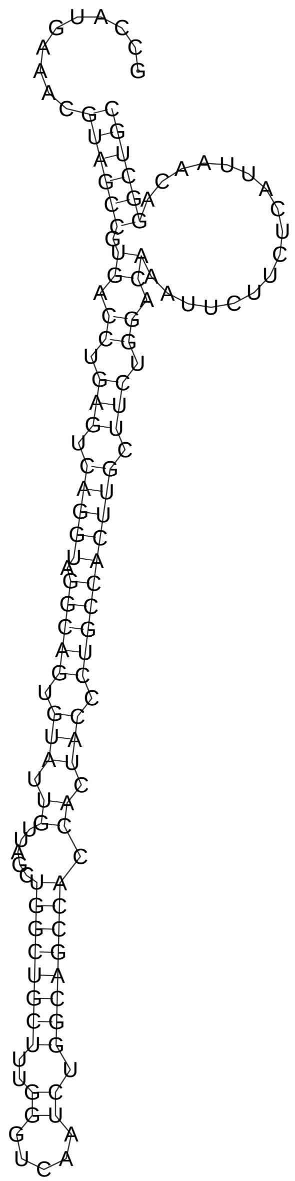

2_42542049-4254217_cfa-mir-449b_high

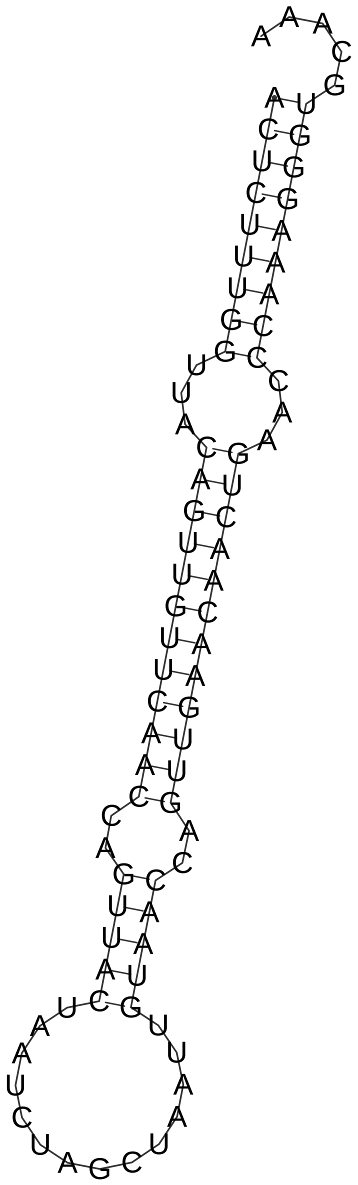

2_46497270-4649735_cfa-mir-582_high

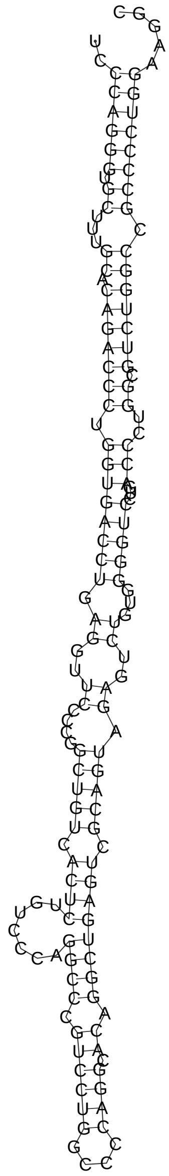

2_57407598-5740774_cfa-mir-8813-2_low

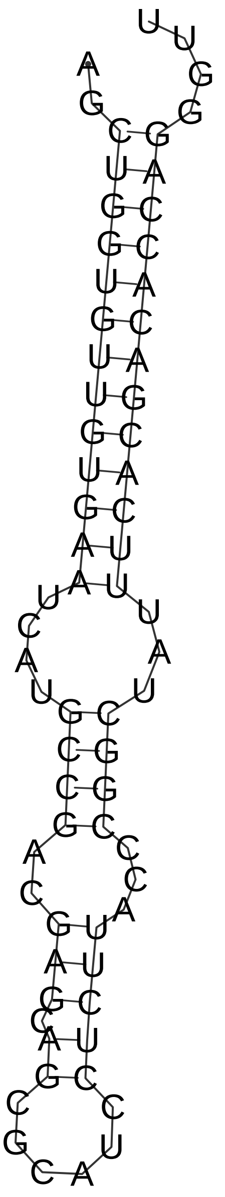

2_59454083-5945415_cfa-mir-138b_high

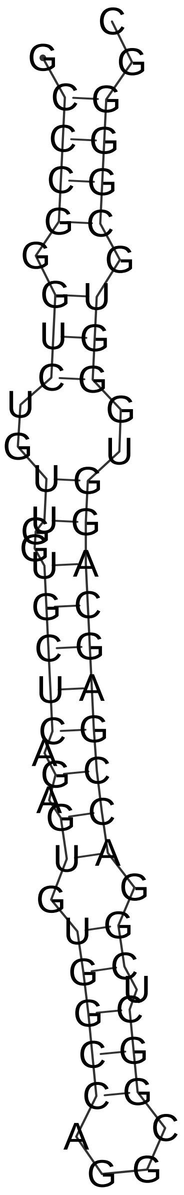

2_74037172-7403723_mir-3917_low

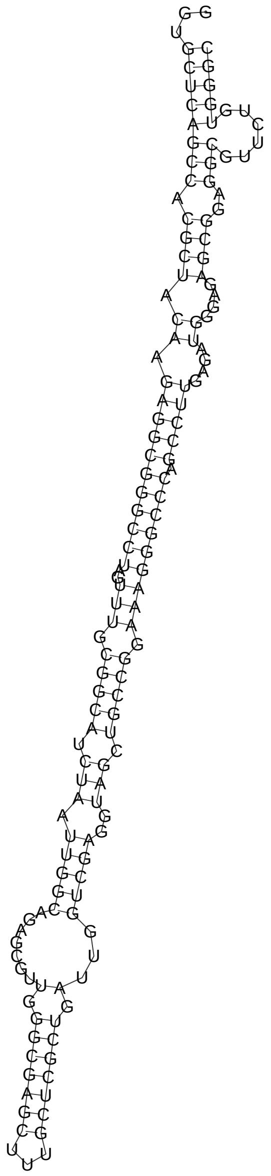

2_76050573-7605071_cfa-mir-8897_low

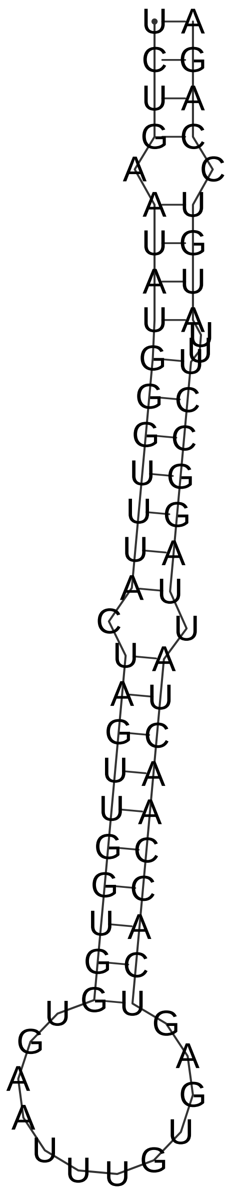

2_76351689-7635175_mir-3115_low

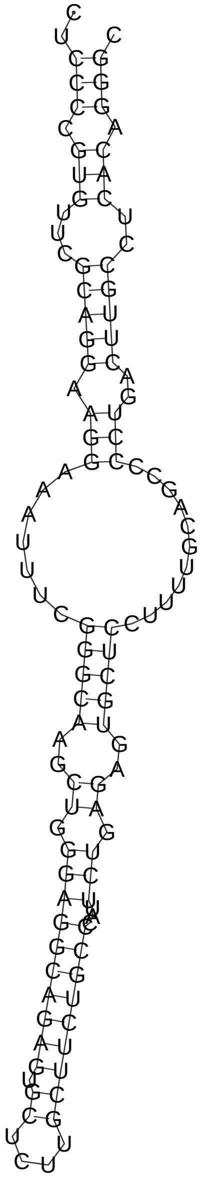

2_83927298-8392740_cfa-mir-8896_low

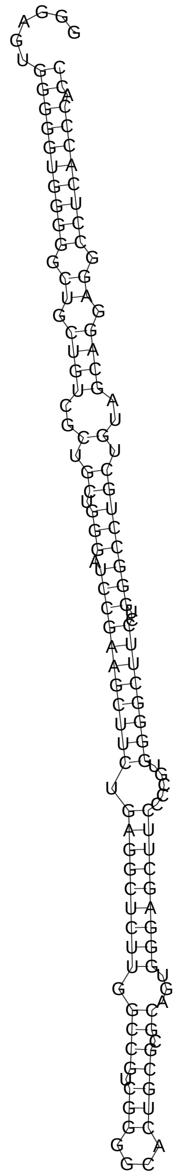

2_84472247-8447238_cfa-mir-8894_low

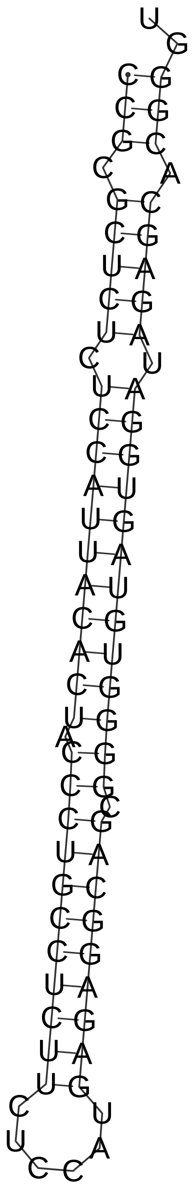

20_7968529-796860_cfa-mir-885_high

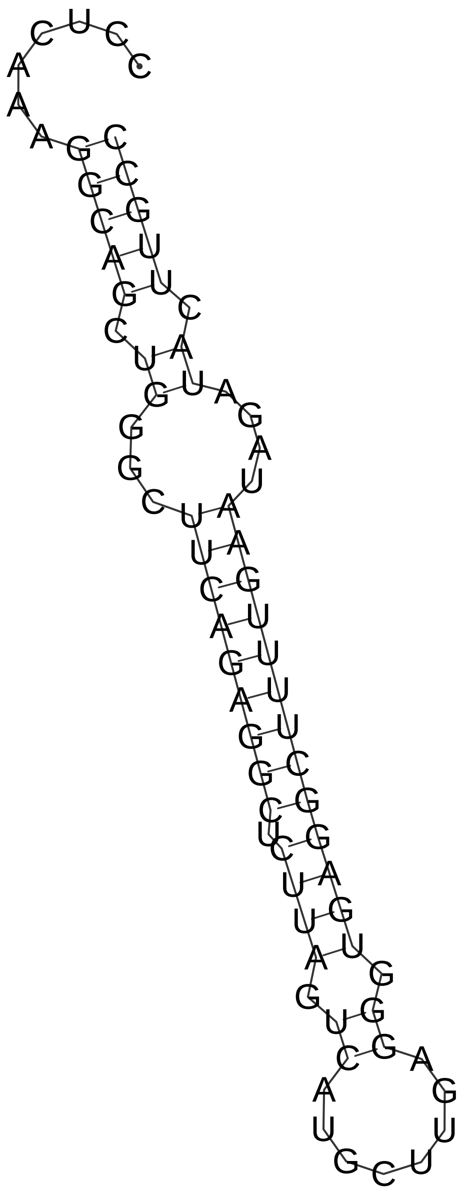

20_19236073-1923614_mir-9260_low

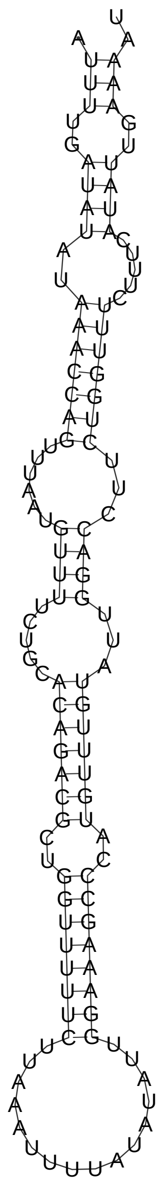

20_20479023-2047913_mir-1284_low

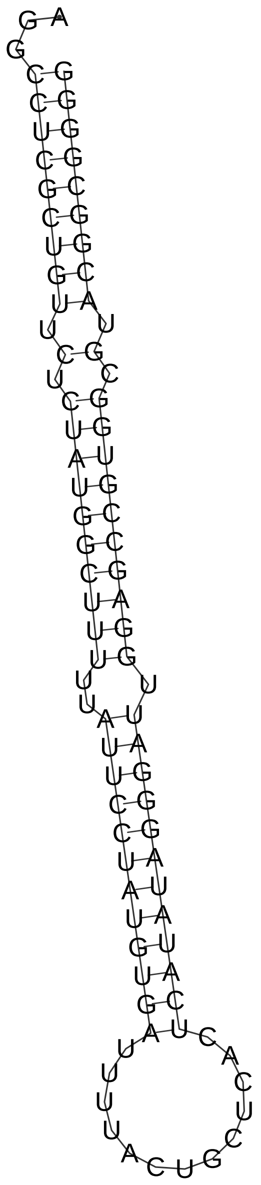

20_37461817-3746190_cfa-mir-135a-1_high

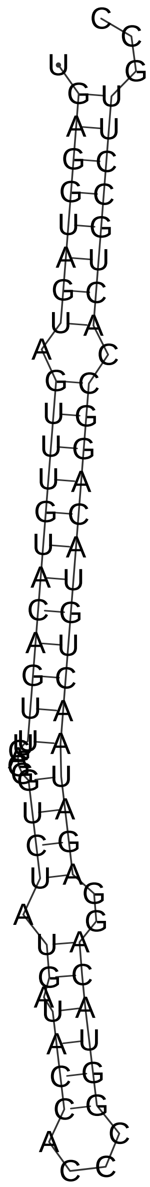

20_37501074-3750115_cfa-let-7g_high

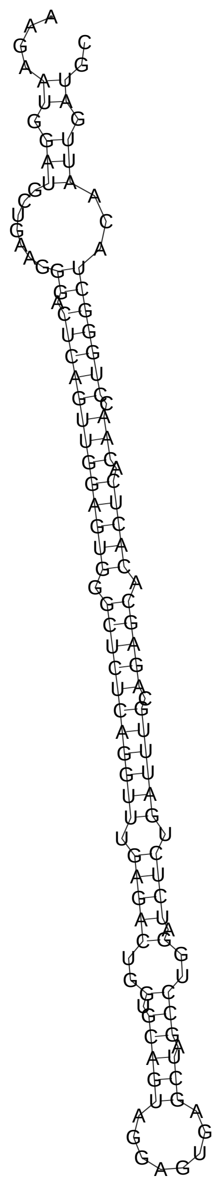

20_37964390-3796451_cfa-mir-8800_low

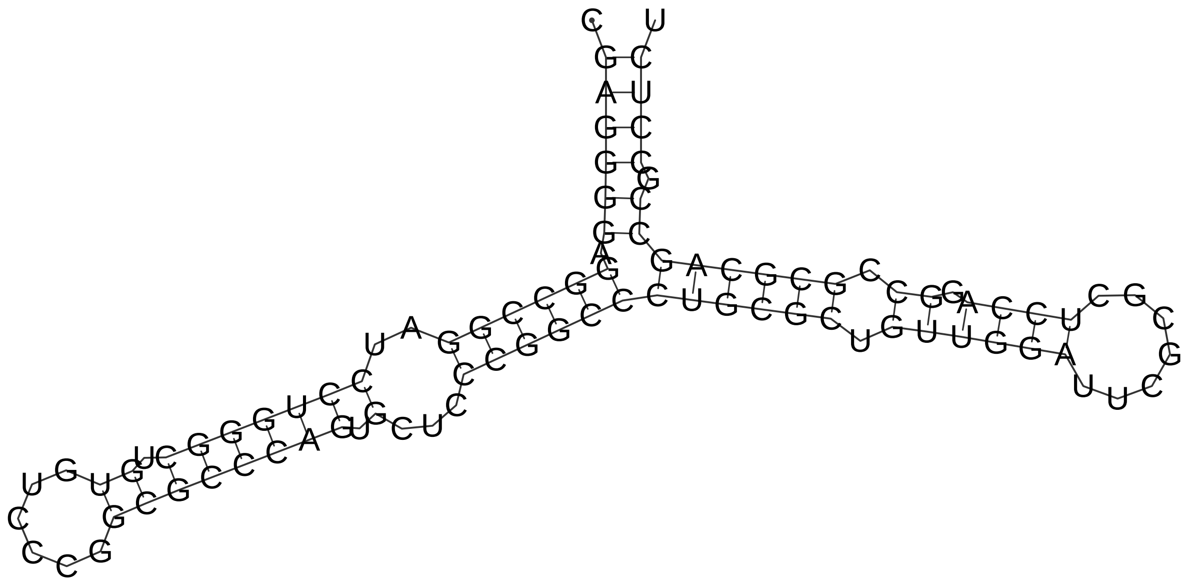

20_38527785-3852787_cfa-mir-8799a_low

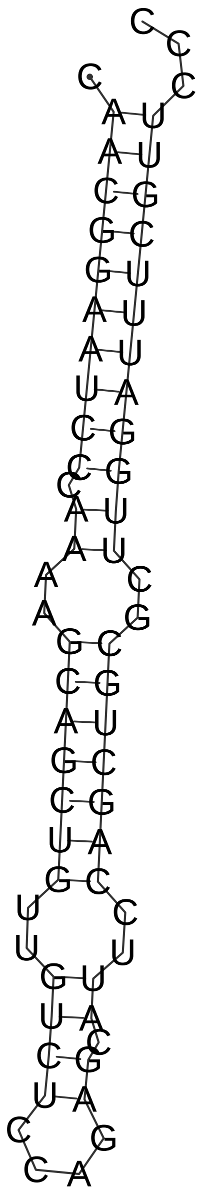

20_40144135-4014419_cfa-mir-191_high

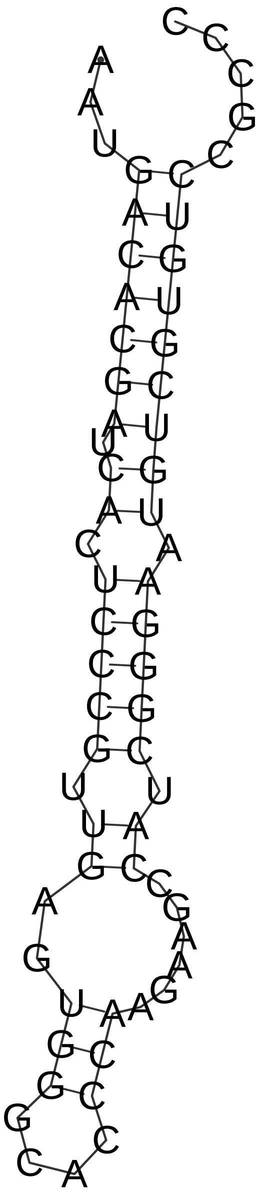

20_40144613-4014467_cfa-mir-425_high

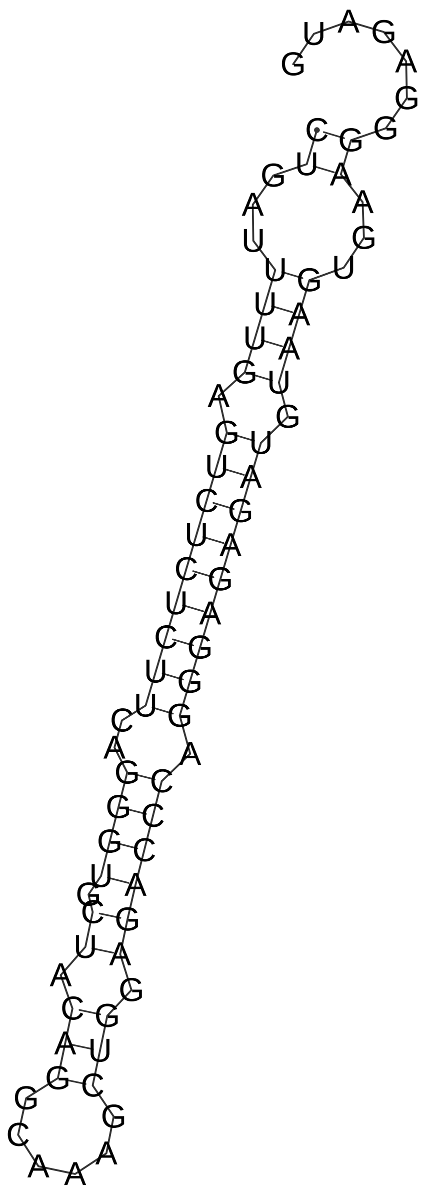

20_40535521-4053559_mir-711_low

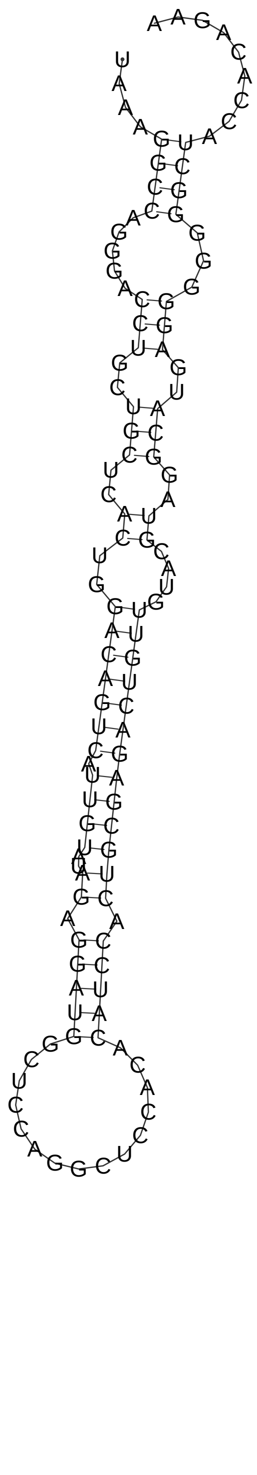

20_43603630-4360374_cfa-mir-8801_low

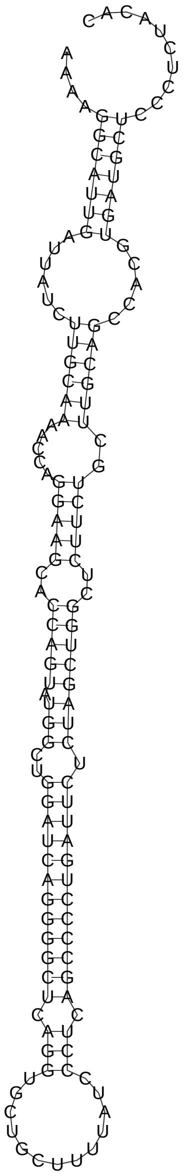

20_44332243-4433237_cfa-mir-8803_high

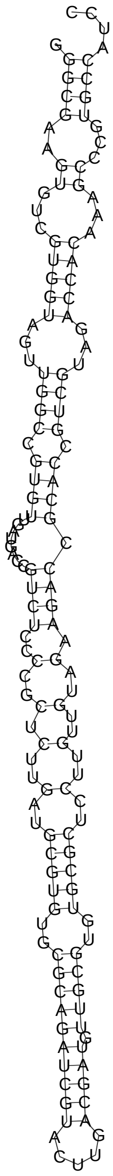

20_48369940-4837008_mir-8994_low

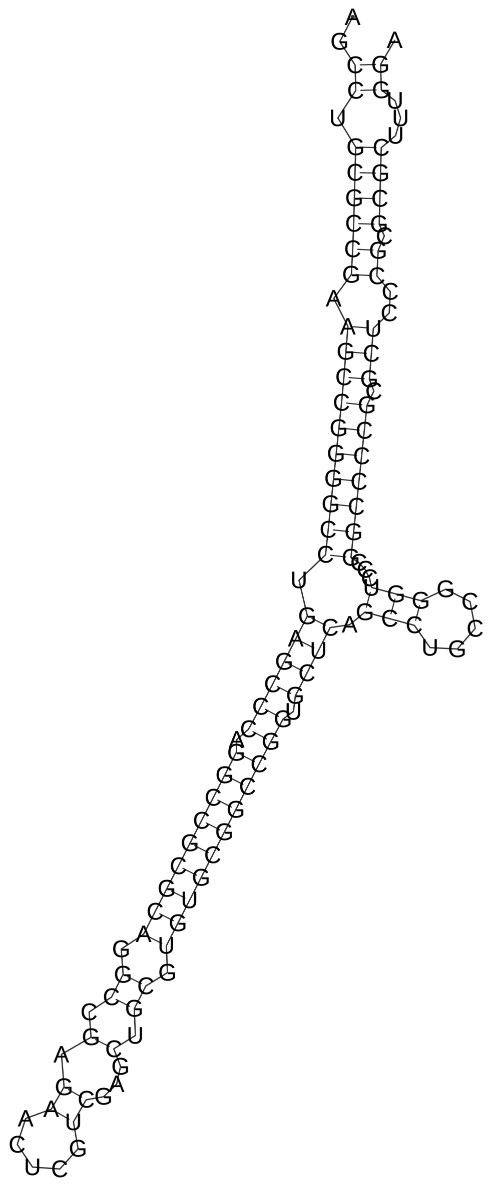

20_48441016-4844113_cfa-mir-1199_low

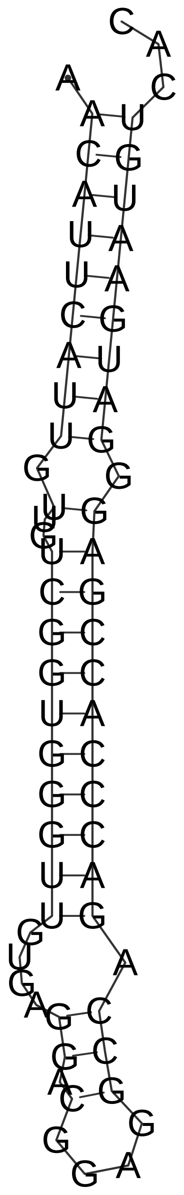

20_48593158-4859322_cfa-mir-181d_high

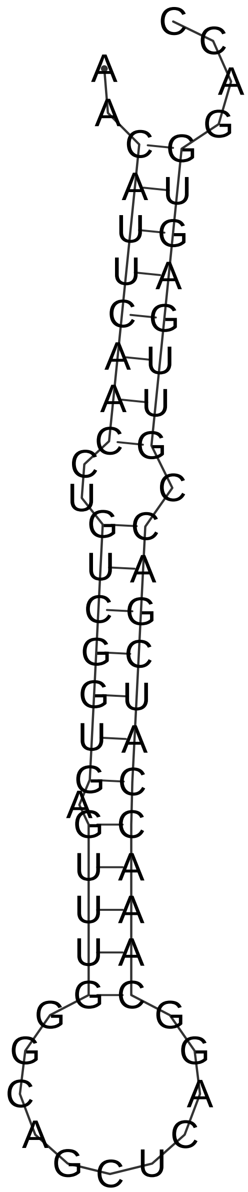

20_48593343-4859340_cfa-mir-181c_high

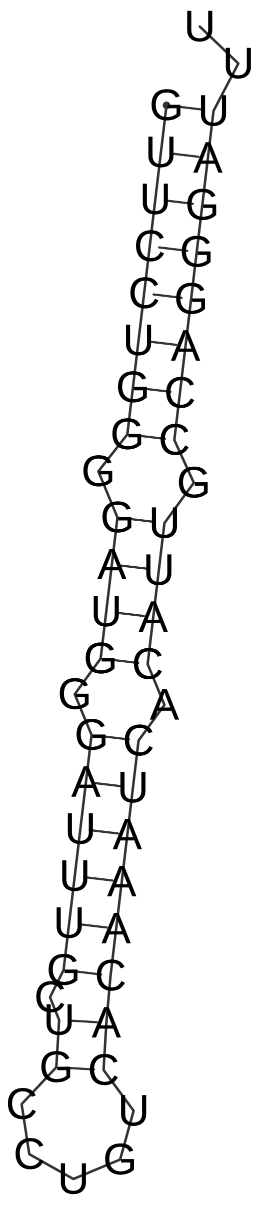

20_48620335-4862038_cfa-mir-23a_high

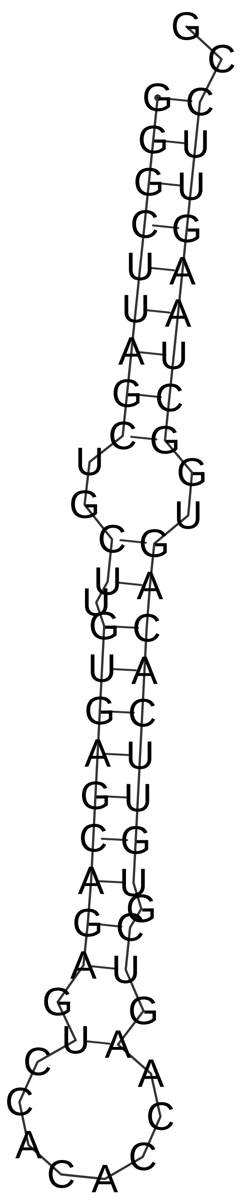

20_48620556-4862061_cfa-mir-27a_high

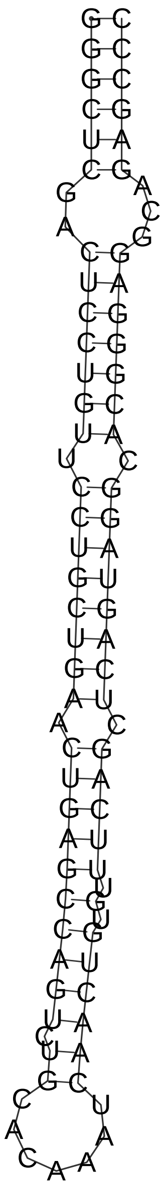

20_48620671-4862075_mir-3074_high

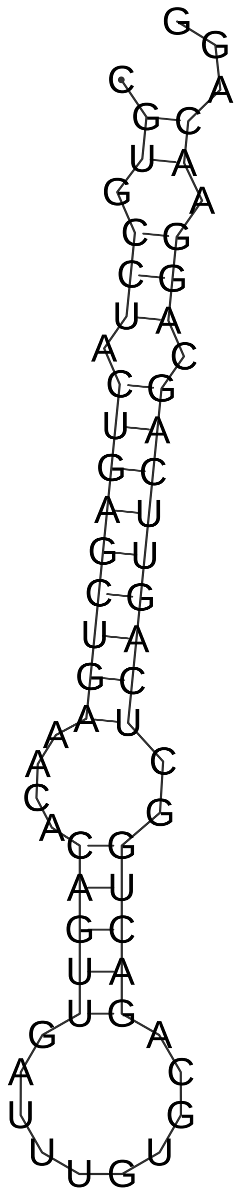

20_48620684-4862074_cfa-mir-24-2_high

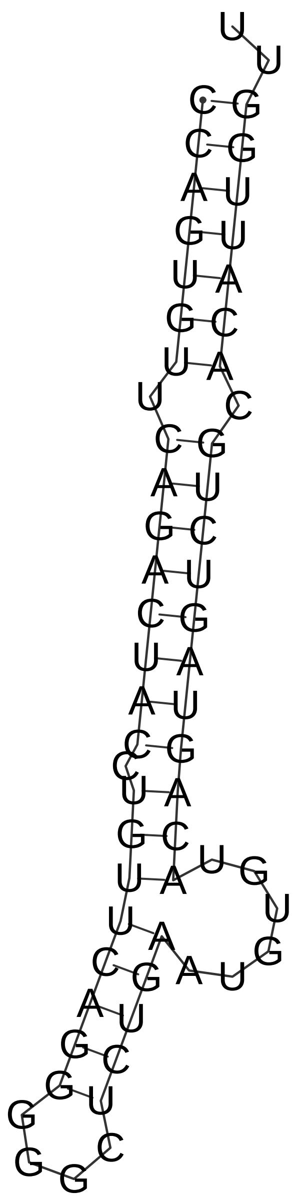

20_50410924-5041098_cfa-mir-199-1_high

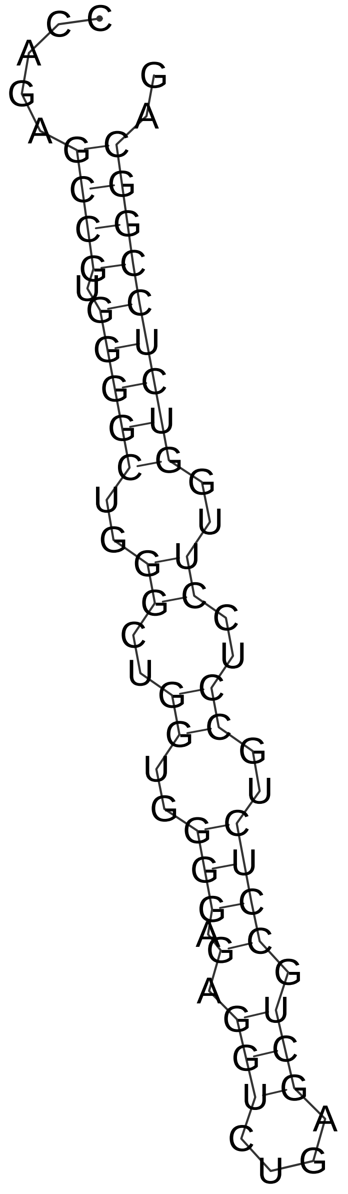

20_53555280-5355534_mir-6791_low

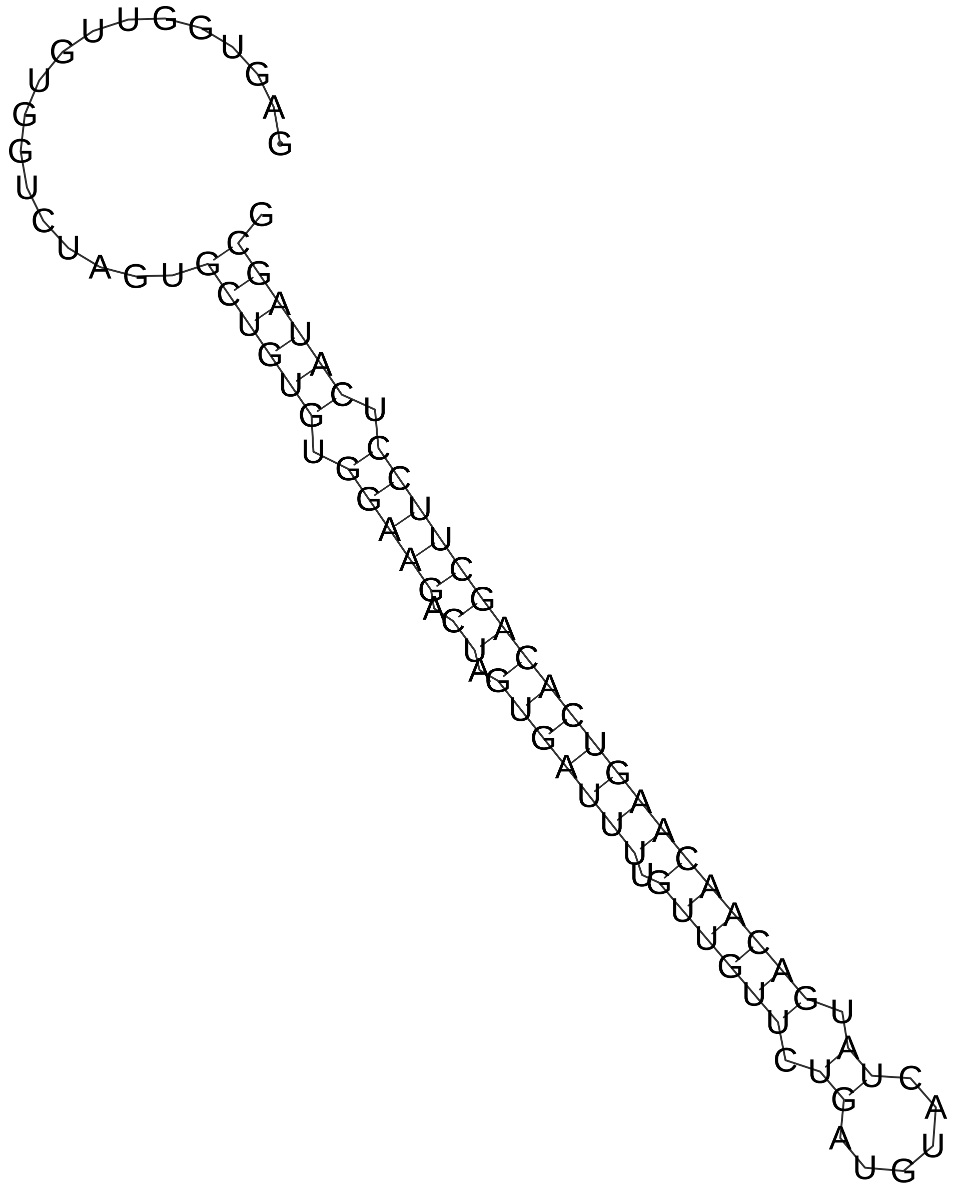

20_54993200-5499328_cfa-mir-7-3_high

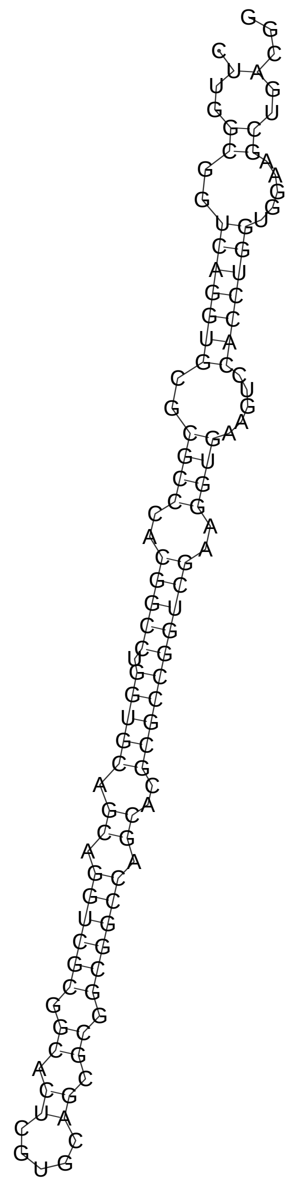

20_55100020-5510013_cfa-mir-8804_low

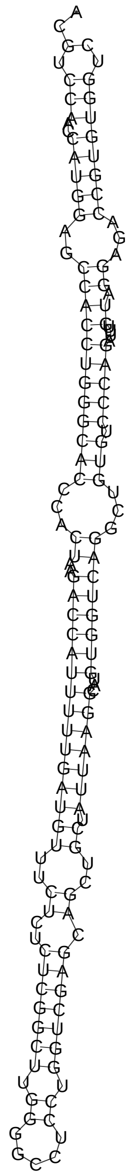

20_55613223-5561336_cfa-mir-8802_low

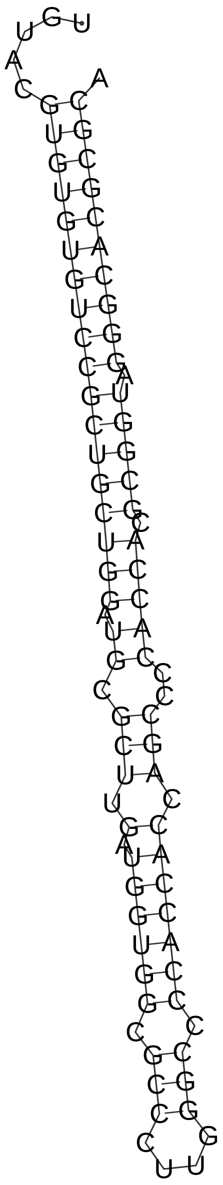

20_57321911-5732199_mir-1905_low

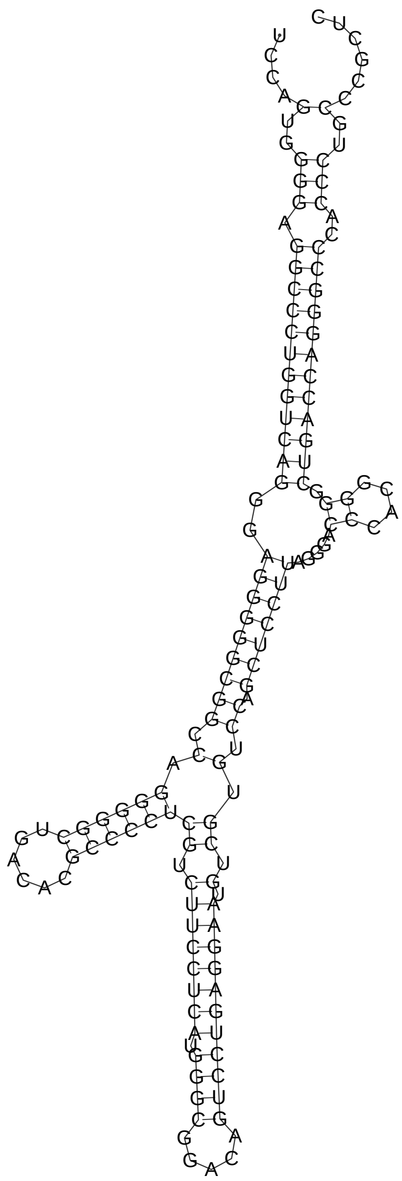

20_57346963-5734710_cfa-mir-8805_low

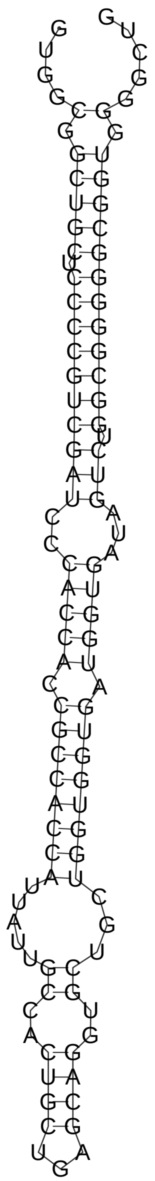

21_4824763-482486_mir-1260b_low

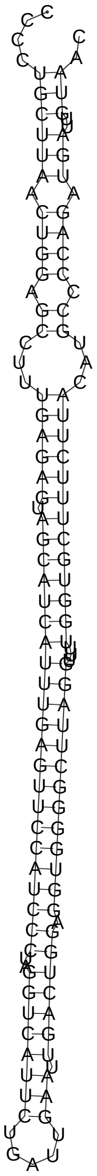

21_6284255-628438_cfa-mir-8812_low

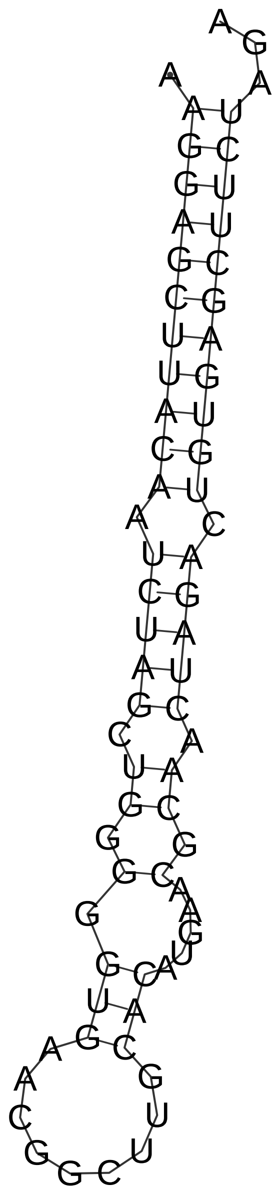

21_19542577-1954264_cfa-mir-708_high

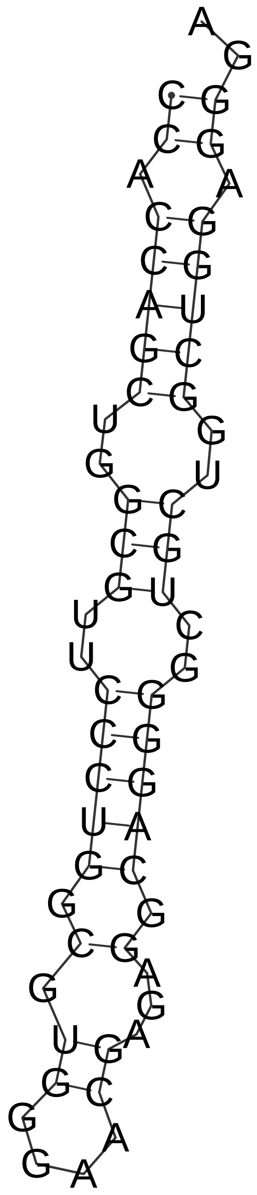

21_20592019-2059207_cfa-mir-1838_high

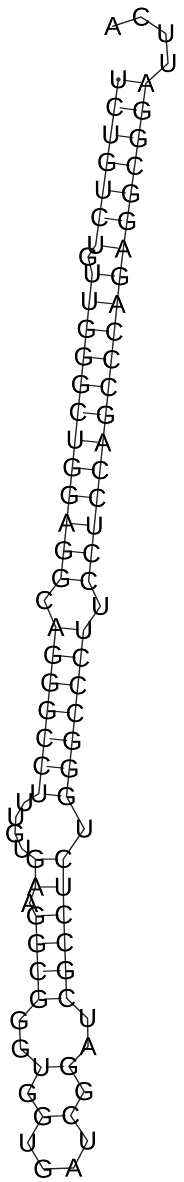

21_23240463-2324055_cfa-mir-326_high

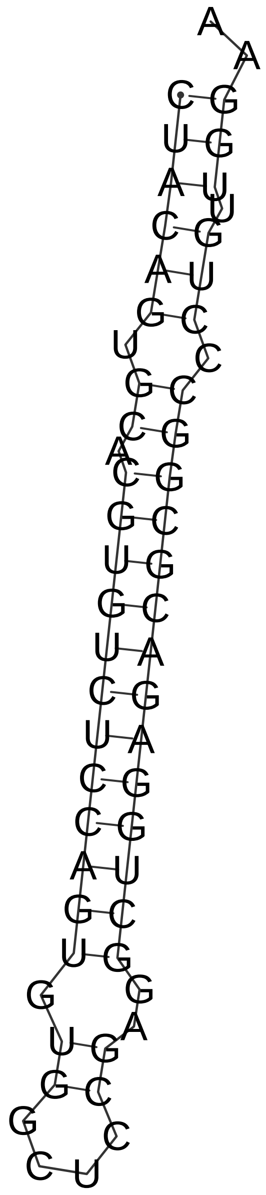

21_25597025-2559708_cfa-mir-139_high

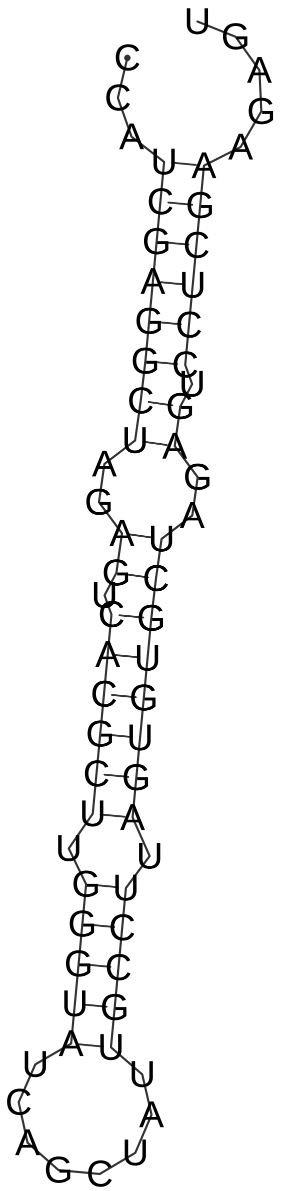

21_32100953-3210101_mir-8986a_high

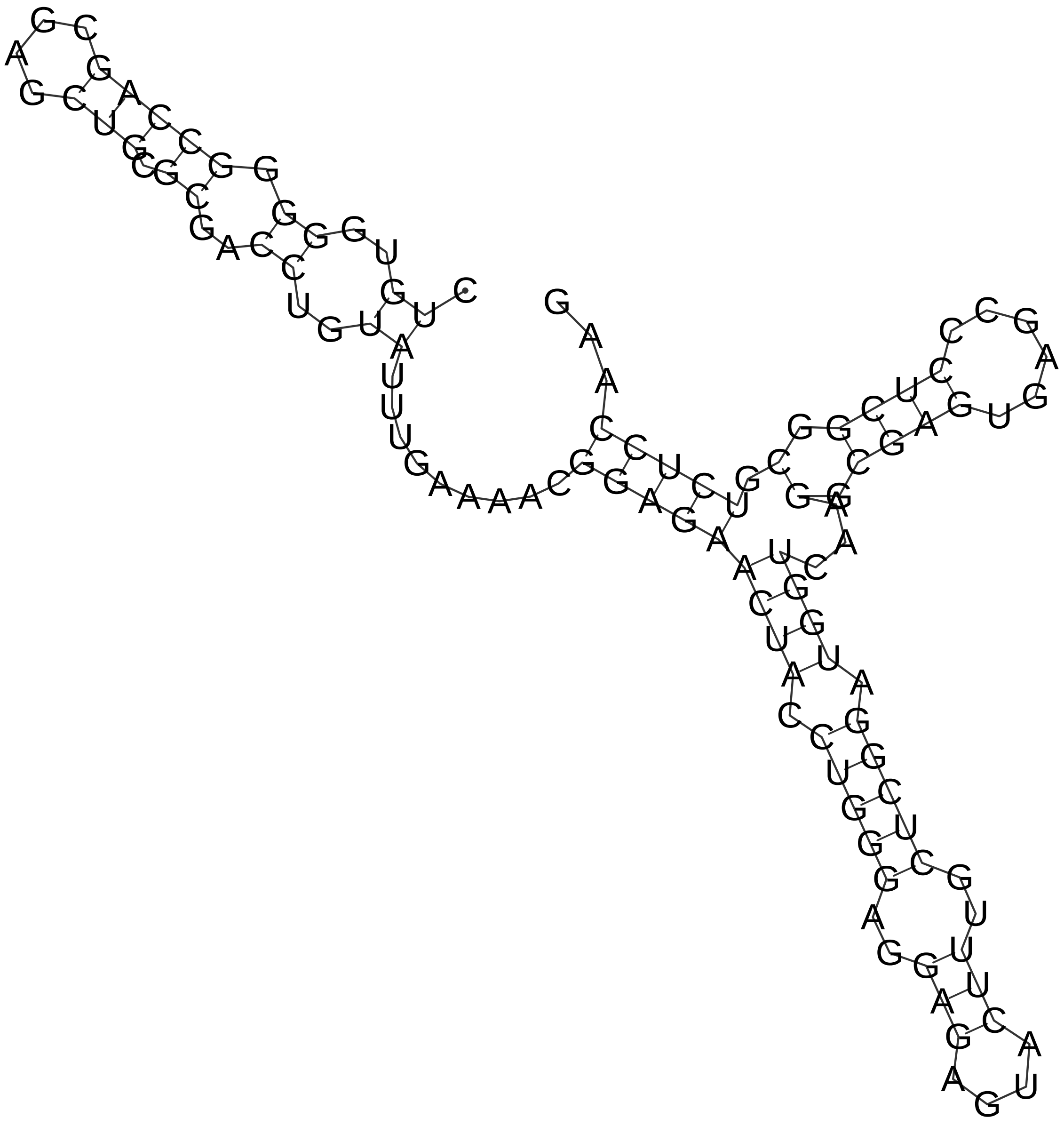

21_33645436-3364554_cfa-mir-8810_low

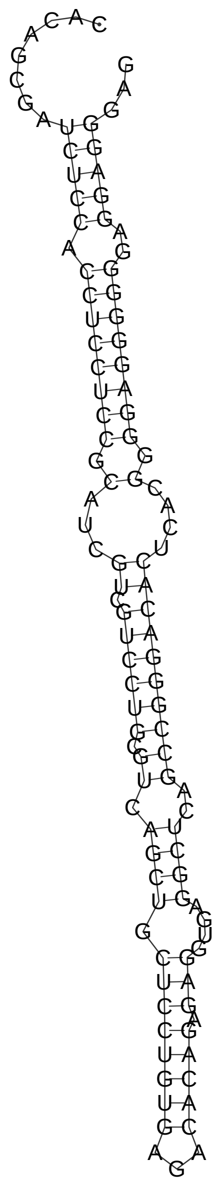

21_50309884-5030999_cfa-mir-8811_low

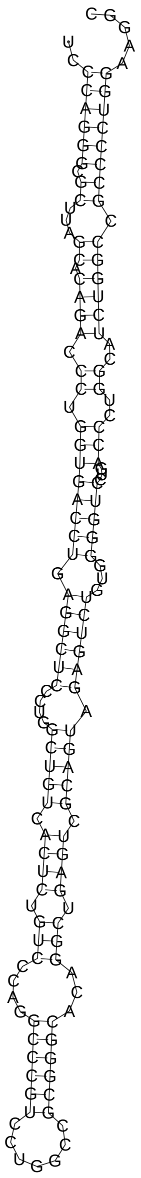

21_50751931-5075207_cfa-mir-8813-1_low

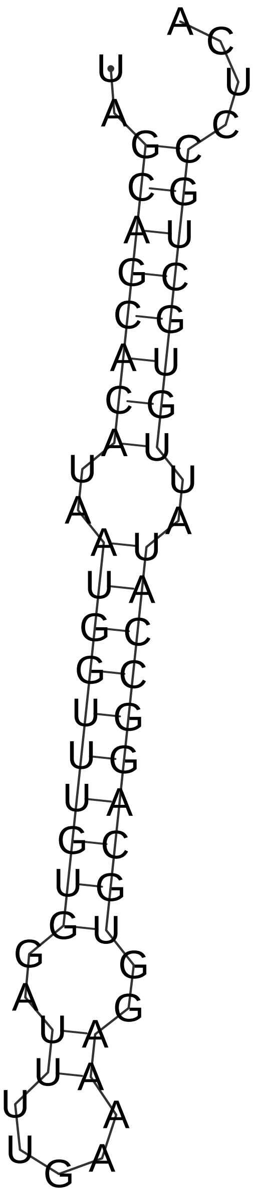

22_1846880-184693_cfa-mir-15a_high

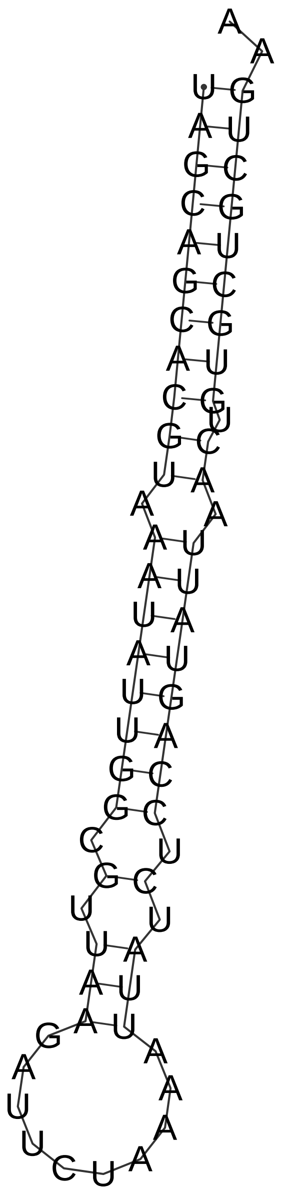

22_1847020-184708_cfa-mir-16-1_high

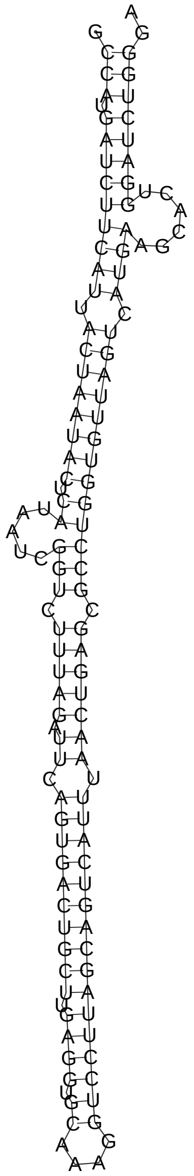

22_6109249-610937_cfa-mir-8814_low

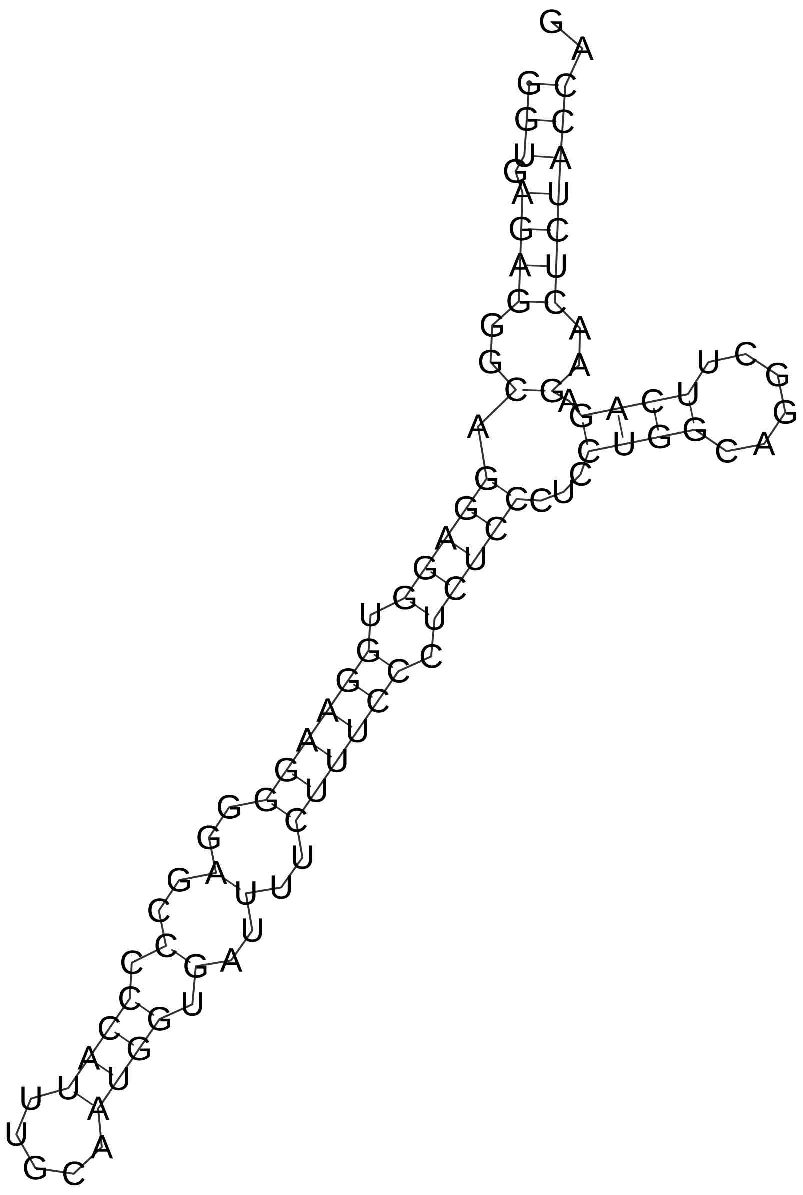

22_9147830-914792_mir-5006_low

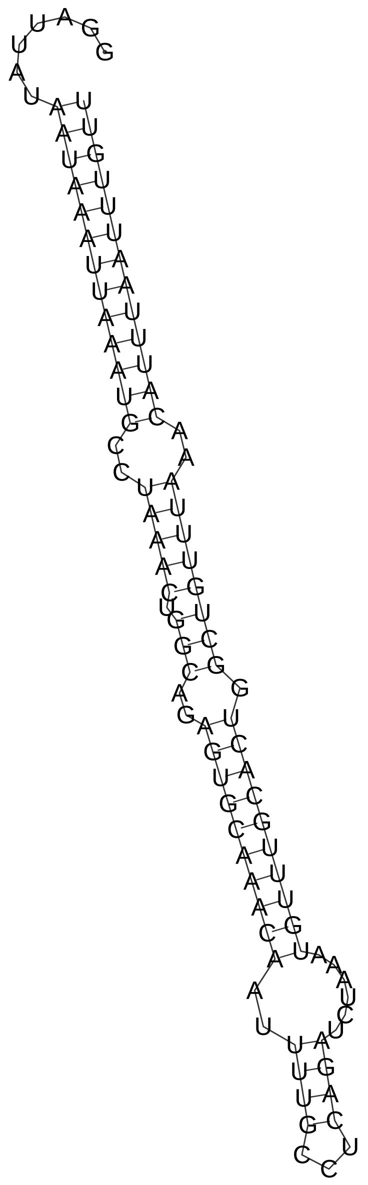

22_9834874-983496_cfa-mir-759_low

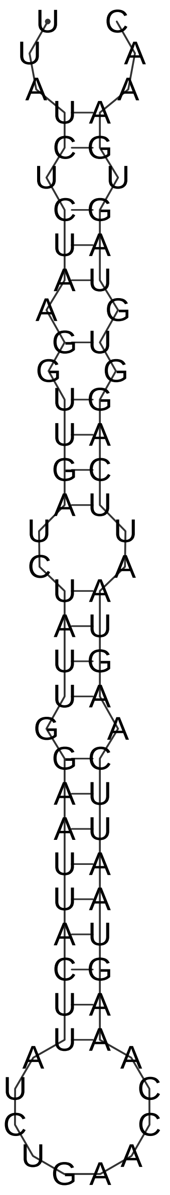

22_11262816-1126288_mir-1297_low

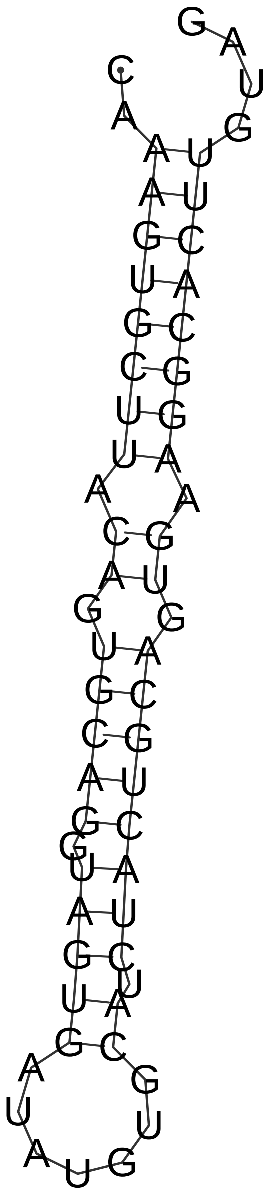

22_42478013-4247807_cfa-mir-17_high

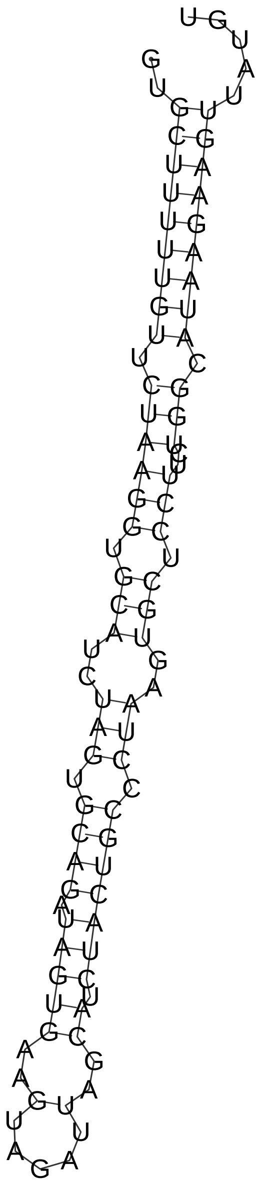

22_42478138-4247822_cfa-mir-18a_high

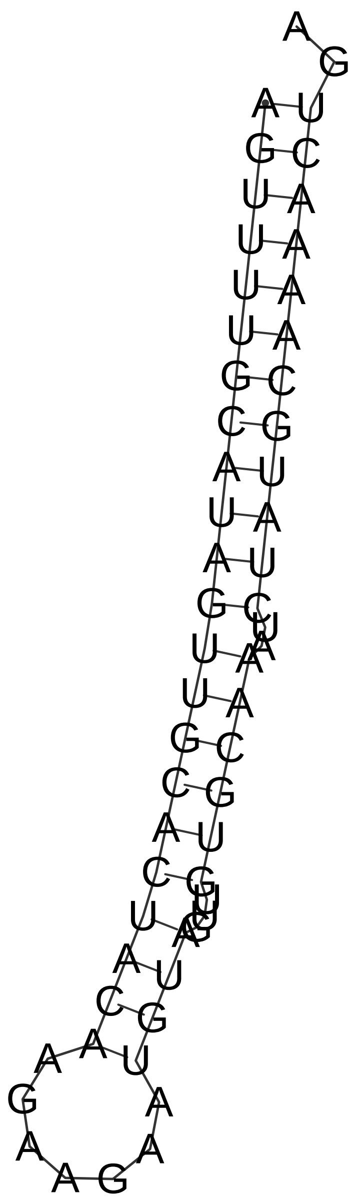

22_42478298-4247835_cfa-mir-19a_high

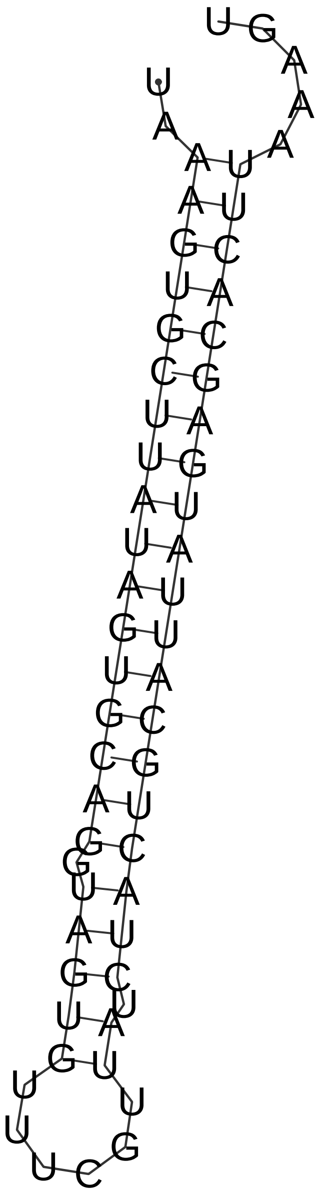

22_42478462-4247852_cfa-mir-20a_high

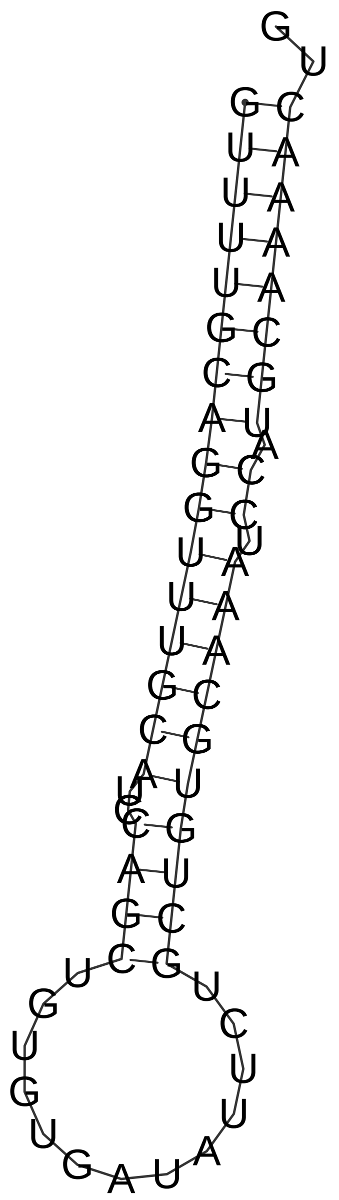

22_42478598-4247865_cfa-mir-19b-1_high

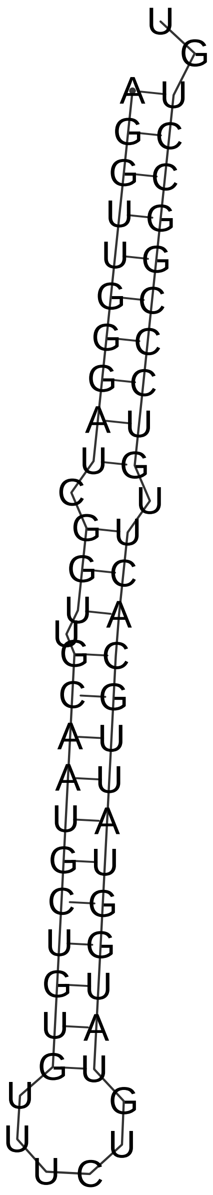

22_42478714-4247877_cfa-mir-92a-1_high

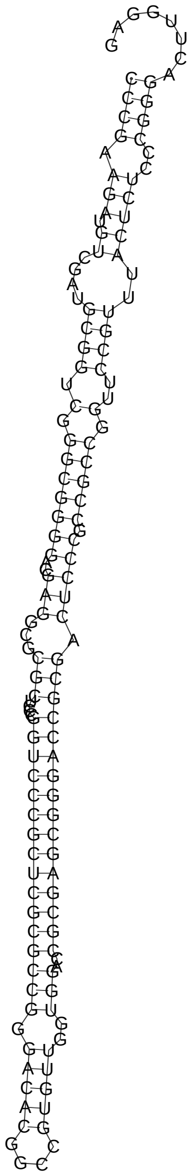

22_55639667-5563980_mir-9334_low

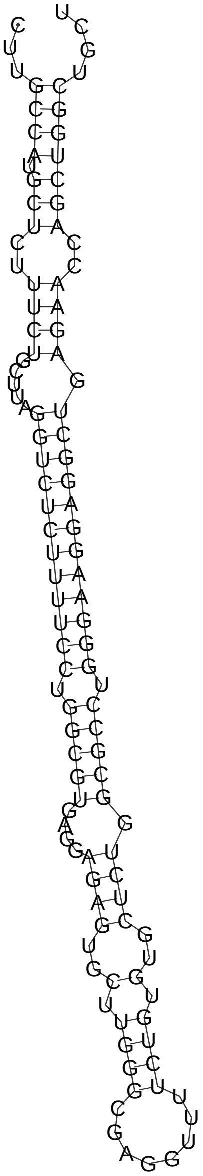

23_1777021-177713_cfa-mir-8807_low

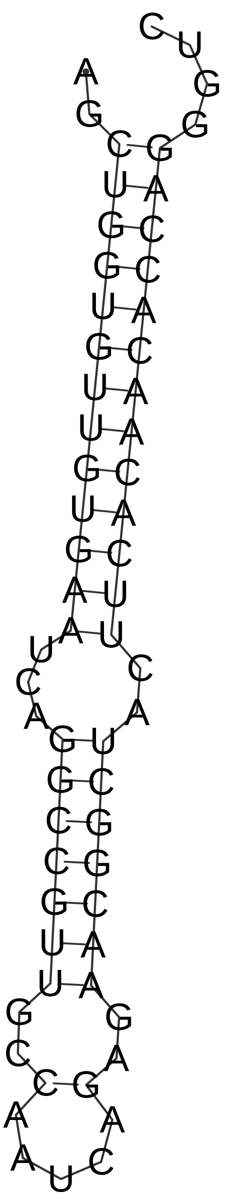

23_2223136-222319_cfa-mir-138a_high

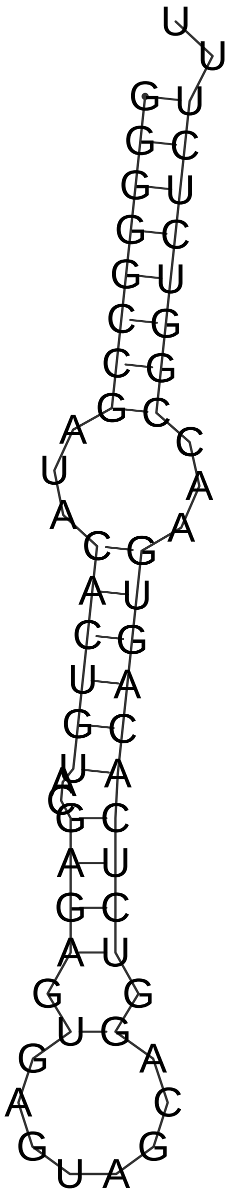

23_5826463-582652_cfa-mir-128-2_high

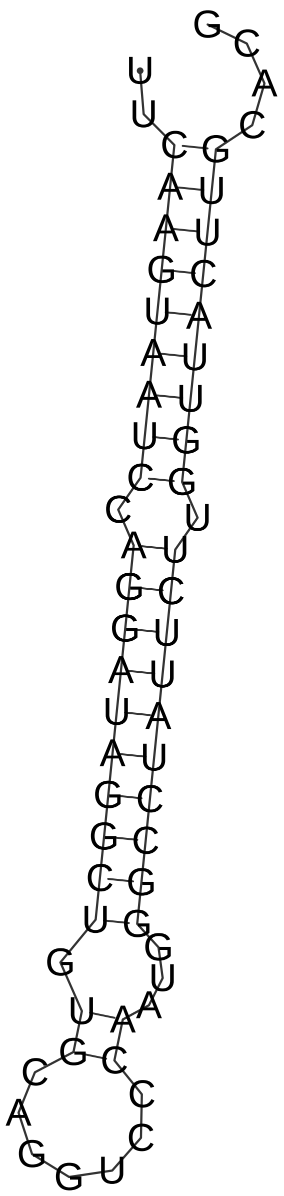

23_7747073-774713_cfa-mir-26a-1_high

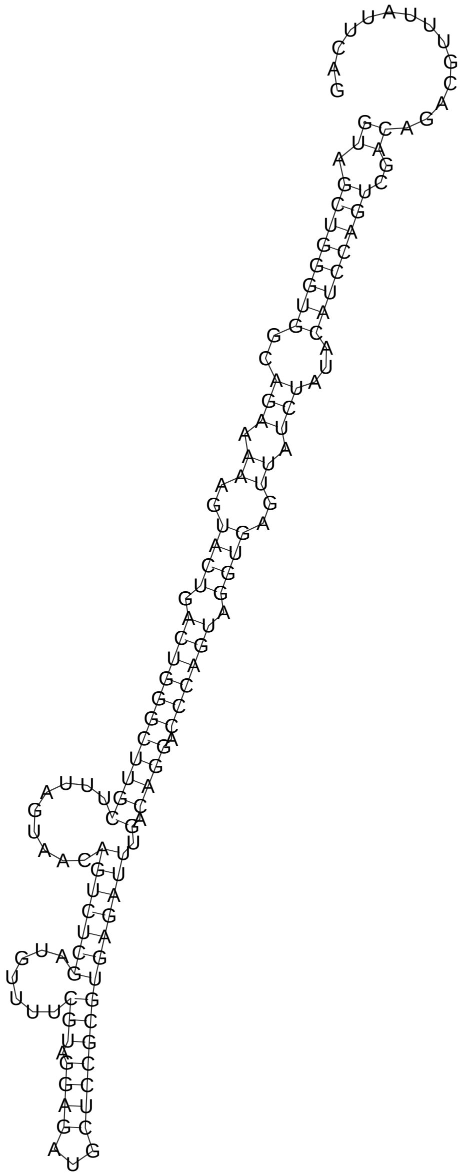

23_12234924-1223506_cfa-mir-8806_low

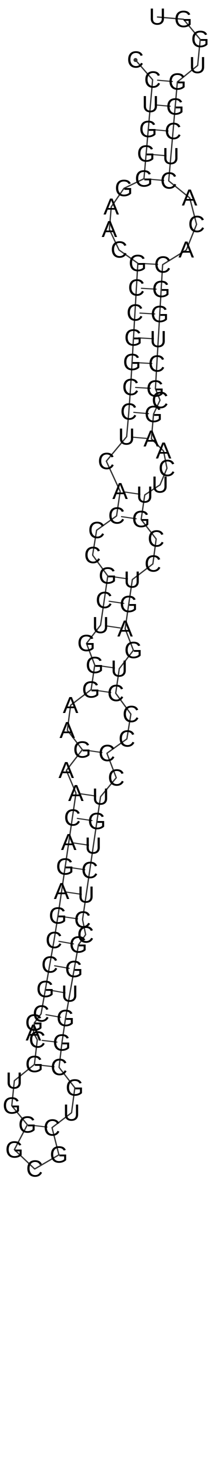

23_49185460-4918556_cfa-mir-8808_low

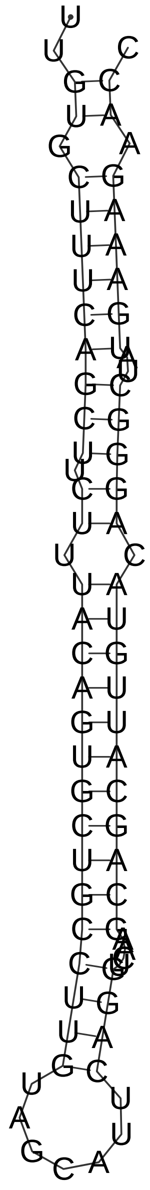

24_17468354-1746843_cfa-mir-103-2_high

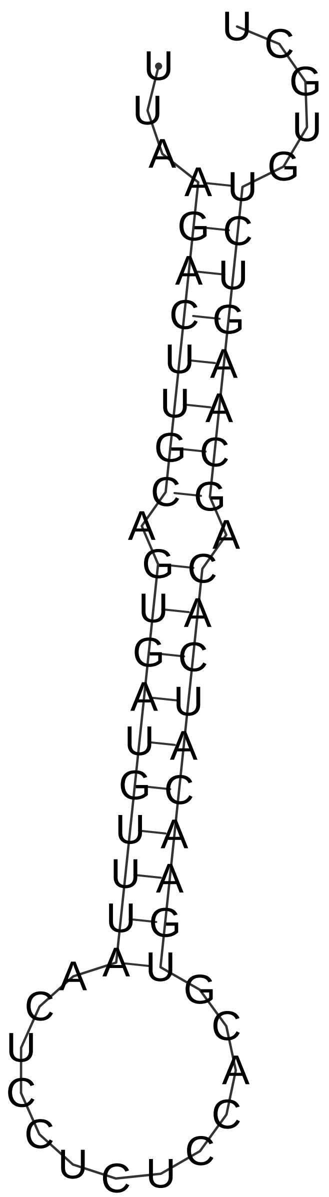

24_24029549-2402960_cfa-mir-499_high

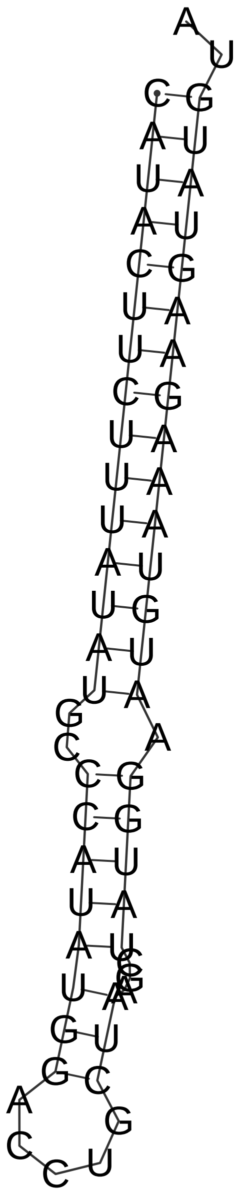

24_46481078-4648113_cfa-mir-1-1_high

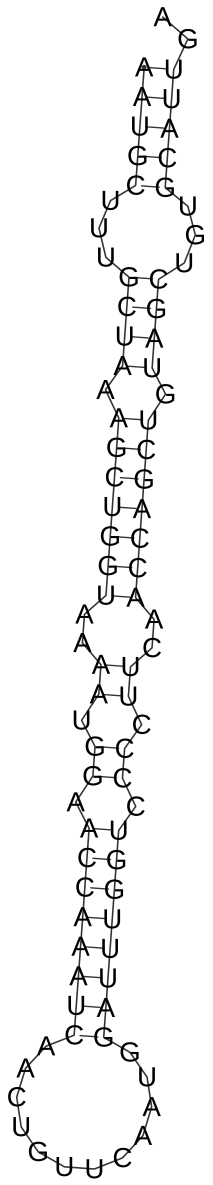

24_46490501-4649058_cfa-mir-133c_high

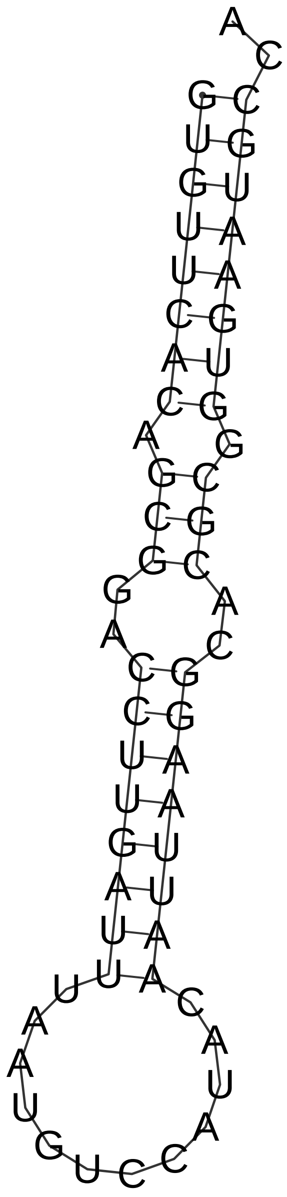

24_46916732-4691678_cfa-mir-124-3_high

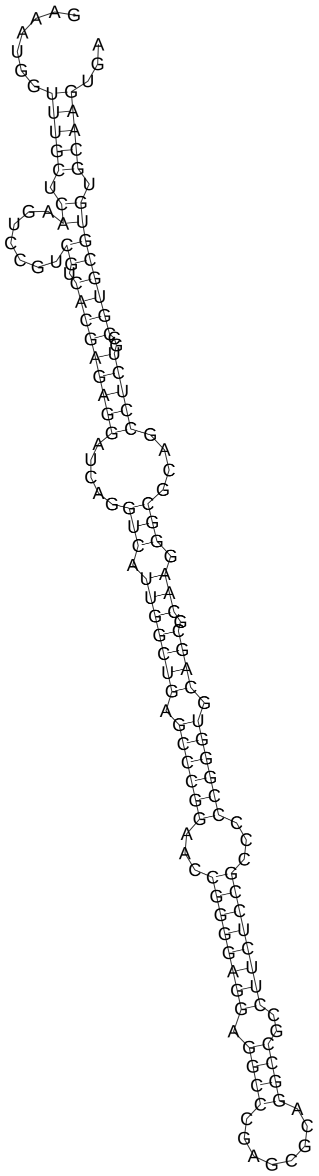

24_47407915-4740805_cfa-mir-8809_high

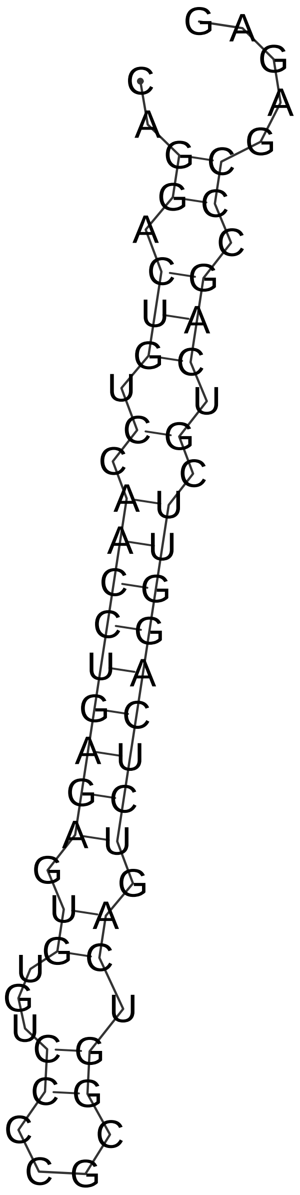

24_47411216-4741127_mir-1388_high

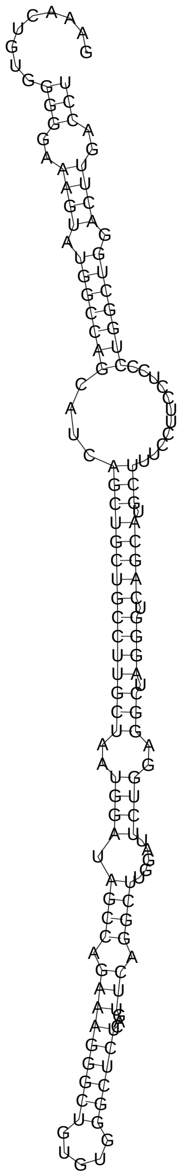

25_9793701-979384_cfa-mir-8825_low

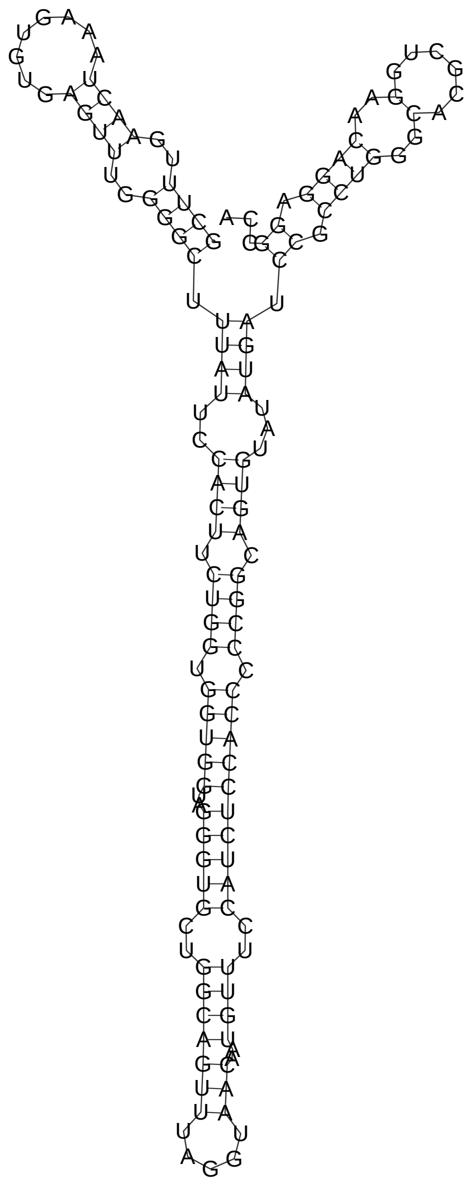

25_19022858-1902299_cfa-mir-8824_low

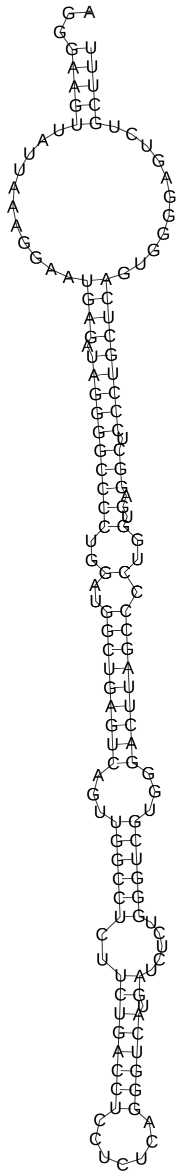

25_19949304-1994944_cfa-mir-8864-2_low

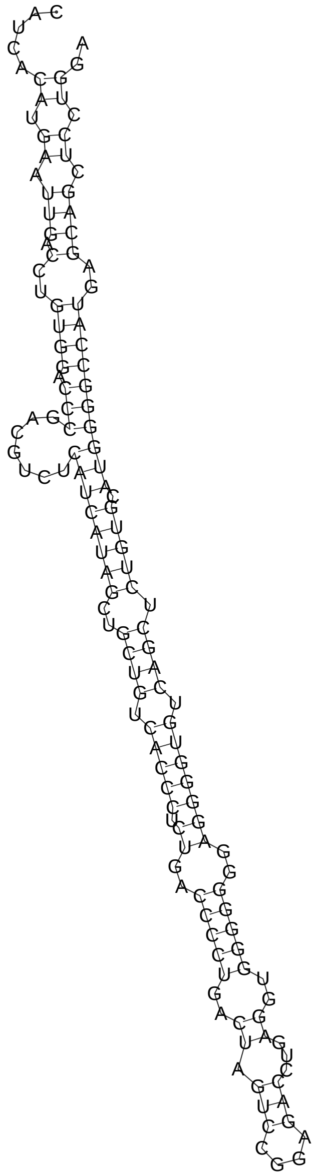

25_27536348-2753648_cfa-mir-8820_low

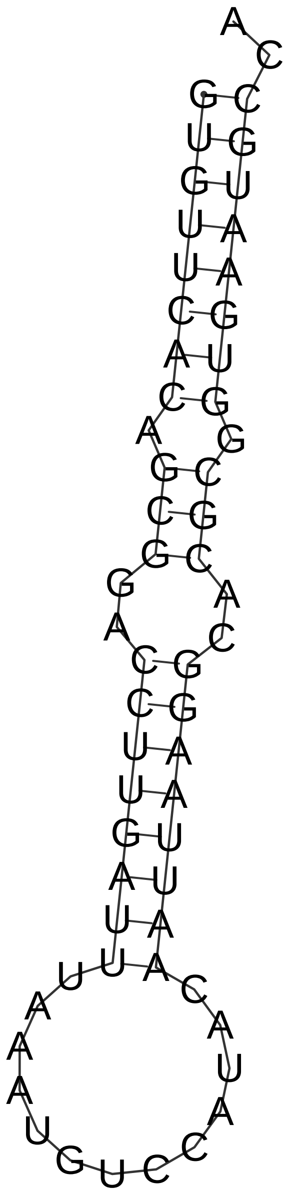

25_28188264-2818832_cfa-mir-124-1_high

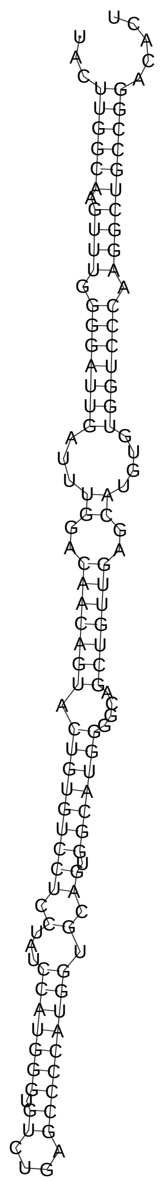

25_28497353-2849748_cfa-mir-8821_low

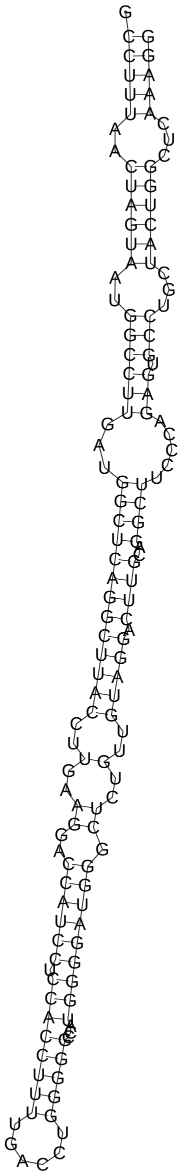

25_34860432-3486056_mir-9064_low

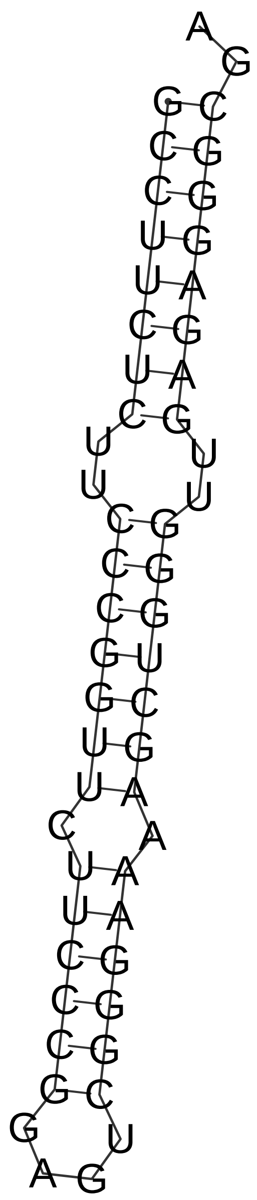

25_35016676-3501672_cfa-mir-320_high

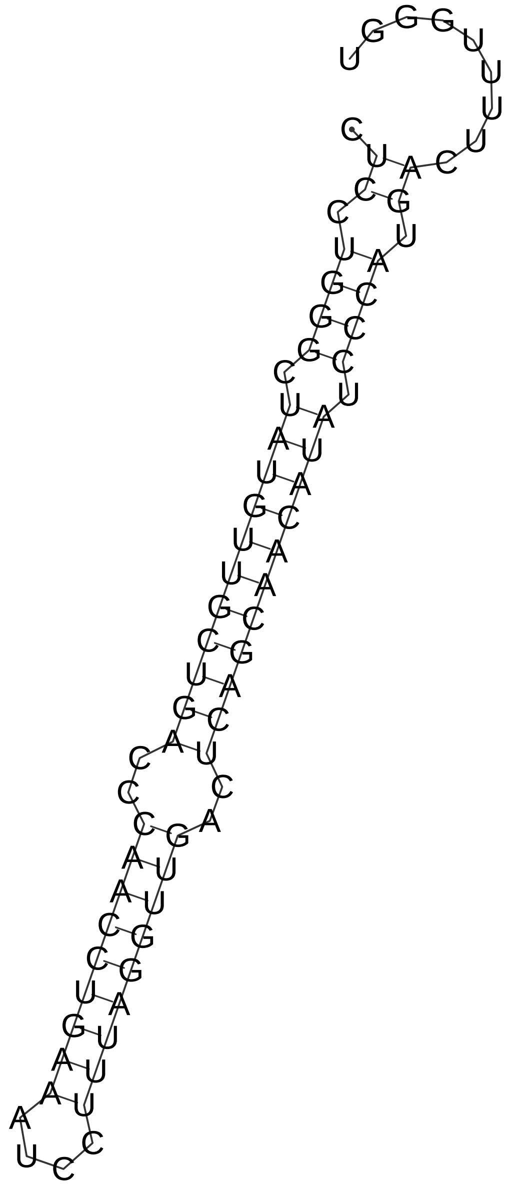

25_39560796-3956086_mir-5702_low

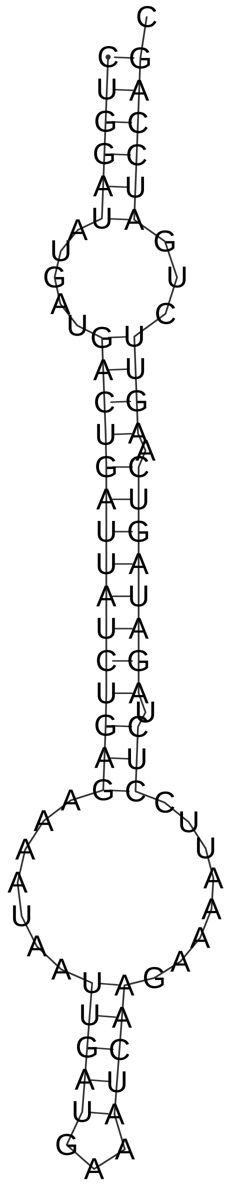

25_43332306-4333238_mir-3535_high

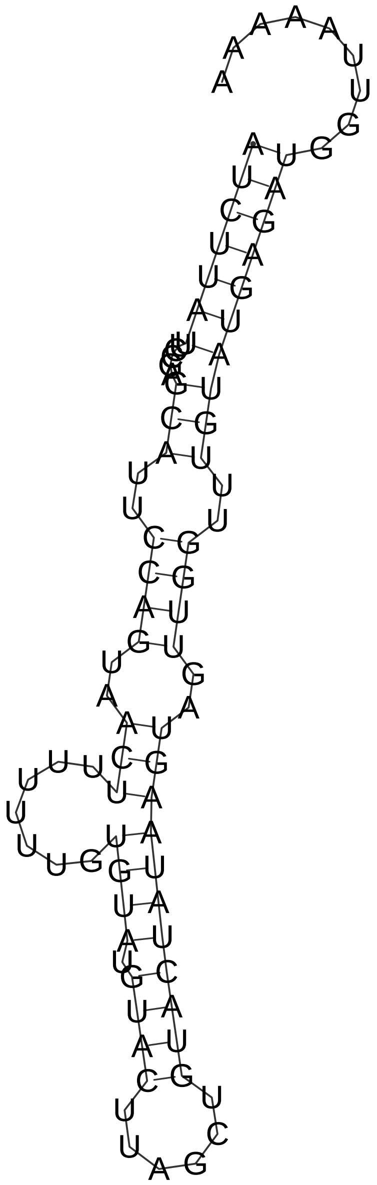

25_43567761-4356784_mir-1244_low

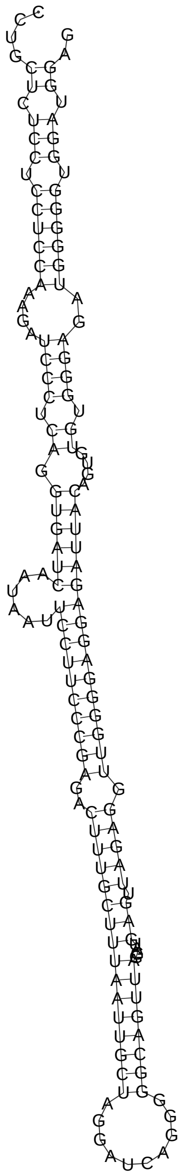

25_45164925-4516506_cfa-mir-8823_low

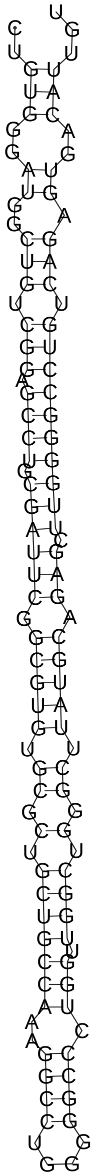

25_48871813-4887192_cfa-mir-8822_low

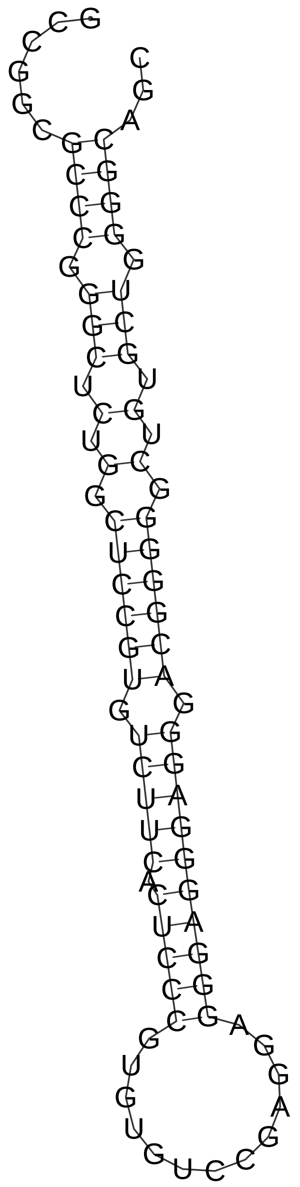

25_50483520-5048360_cfa-mir-149_high

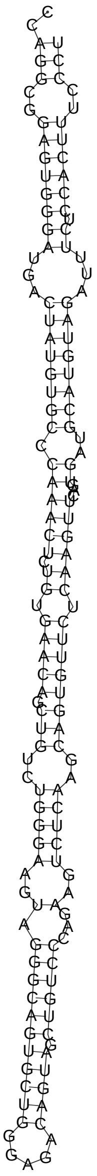

26_14526313-1452645_cfa-mir-8819_low

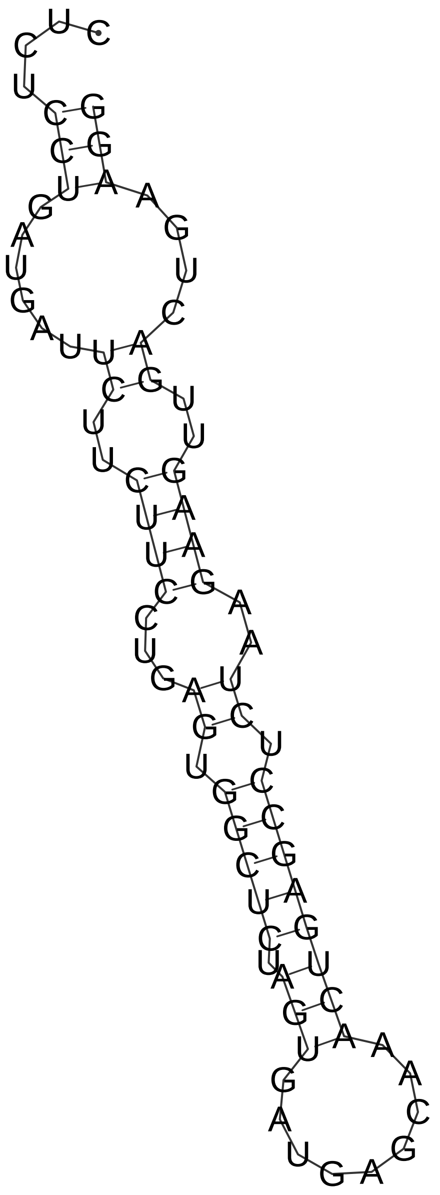

26_22638642-2263871_mir-3653_low

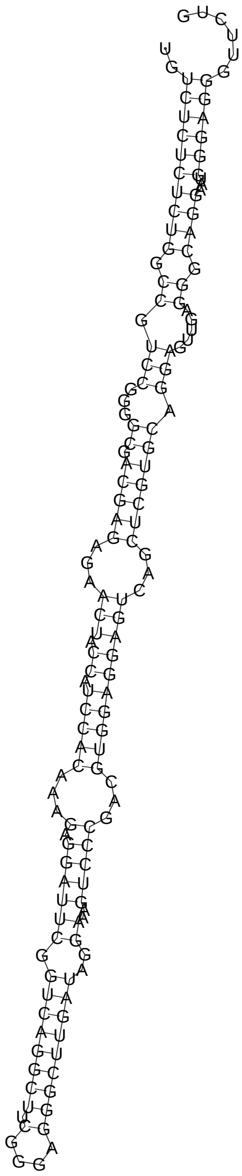

26_28860341-2886048_cfa-mir-8905_low

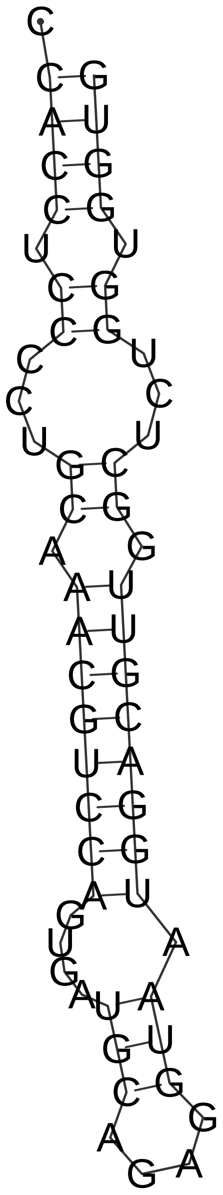

26_29255671-2925572_cfa-mir-1306_high

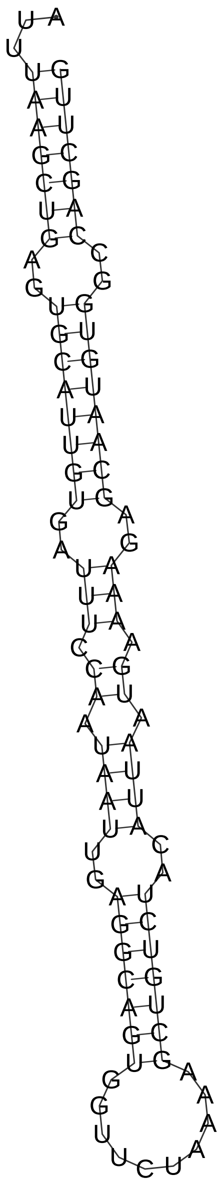

26_29255971-2925606_mir-3618_low

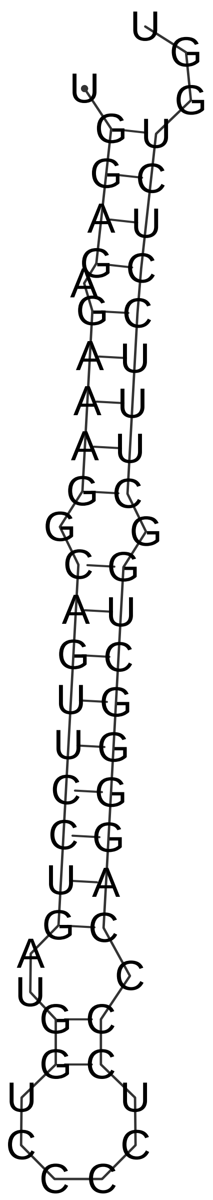

26_29296014-2929606_cfa-mir-185_high

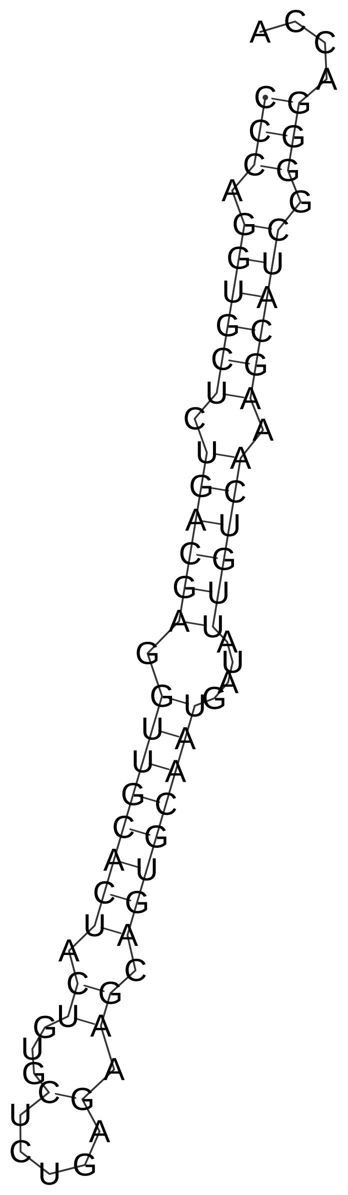

26_30924103-3092417_cfa-mir-301b_high

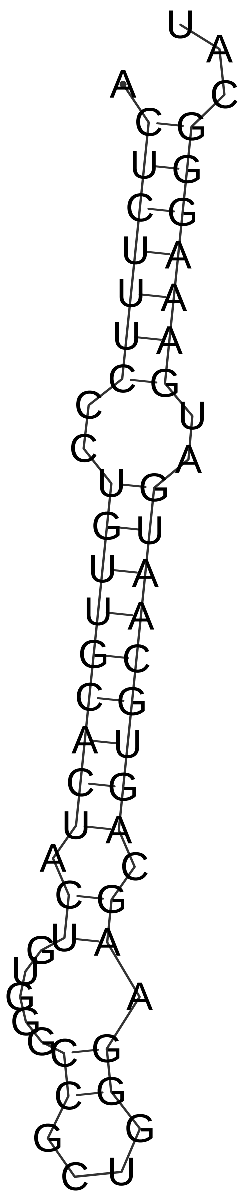

26_30924432-3092449_cfa-mir-130b_high

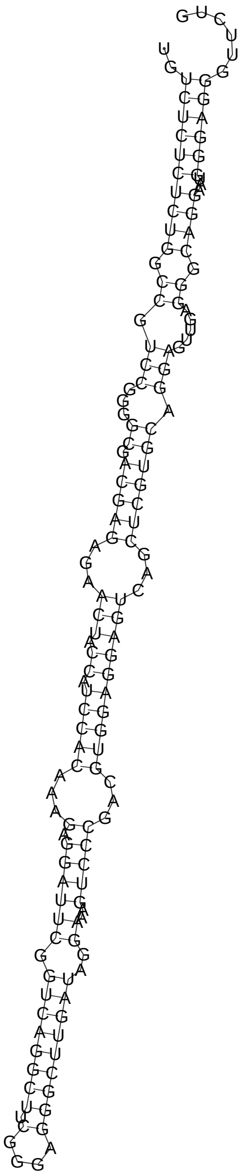

26_31301037-3130118_cfa-mir-8905_low

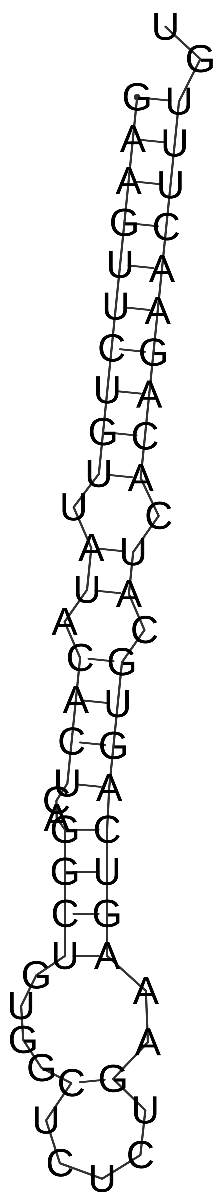

27_975146-97520_cfa-mir-148b_high

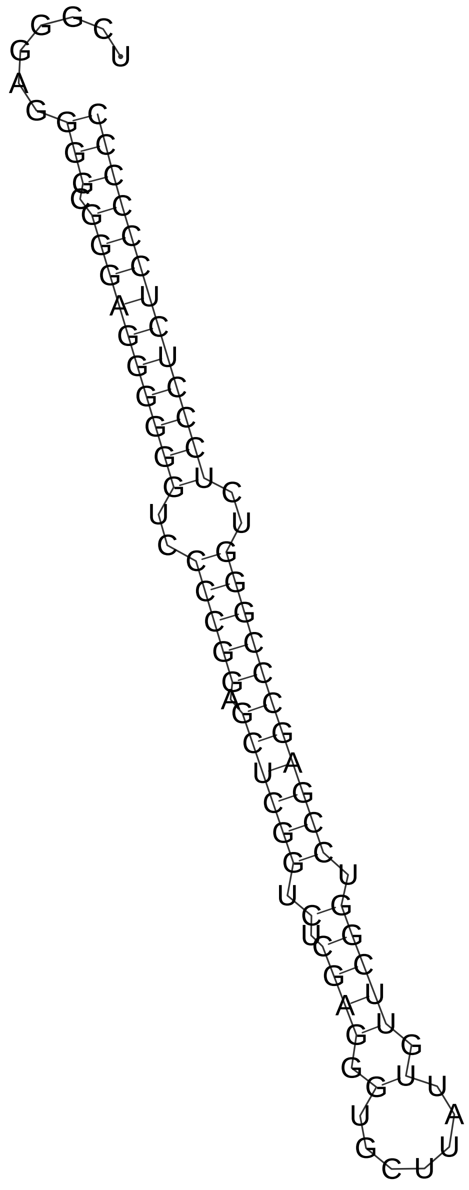

27_1230447-123053_cfa-mir-615_high

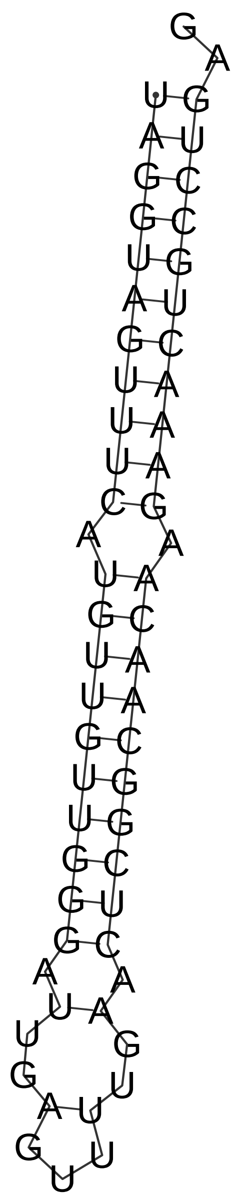

27_1270718-127077_cfa-mir-196a-2_high

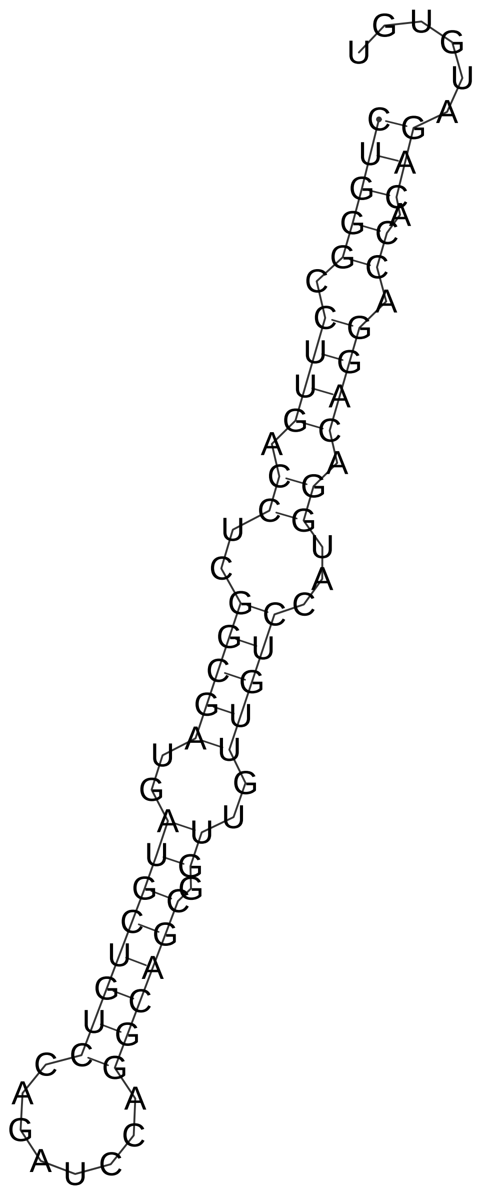

27_2342884-234296_mir-9321_low

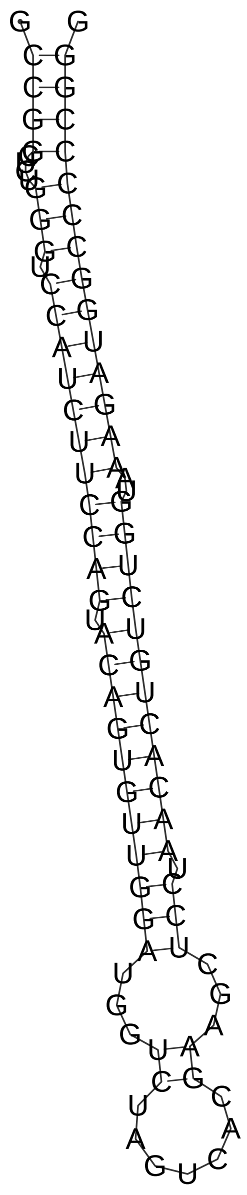

27_38081668-3808175_cfa-mir-141_high

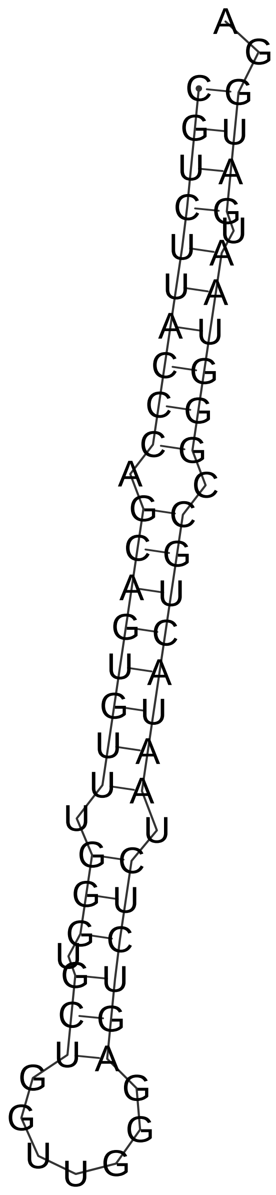

27_38082097-3808215_cfa-mir-200c_high

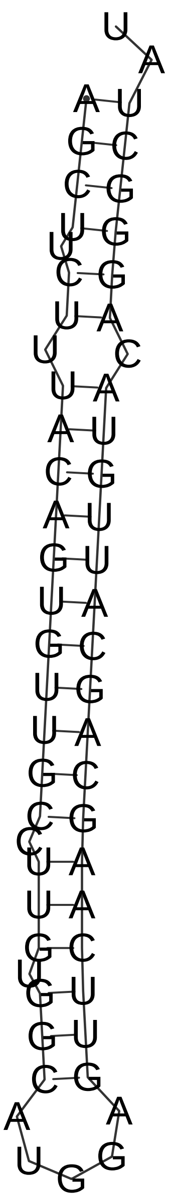

28_4484653-448471_cfa-mir-107_high

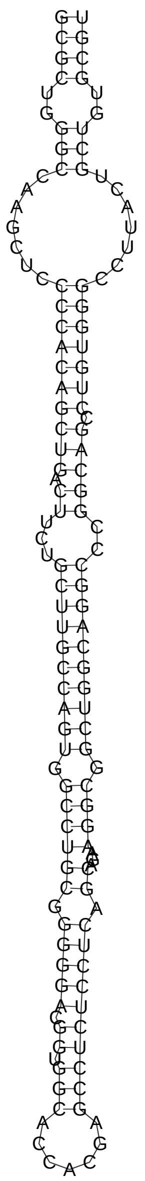

28_4705462-470559_cfa-mir-8815-1_low

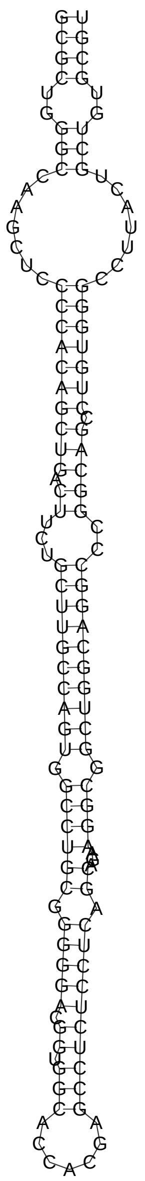

28_6819312-681944_cfa-mir-8815-2_low

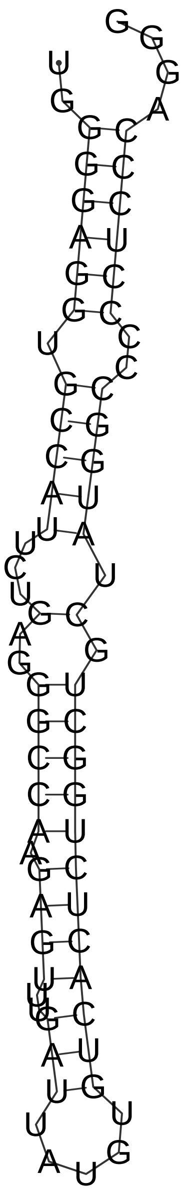

28_11089077-1108914_mir-3085_high

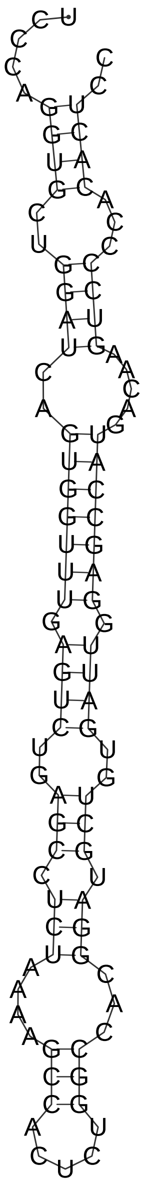

28_11528123-1152821_mir-1287_low

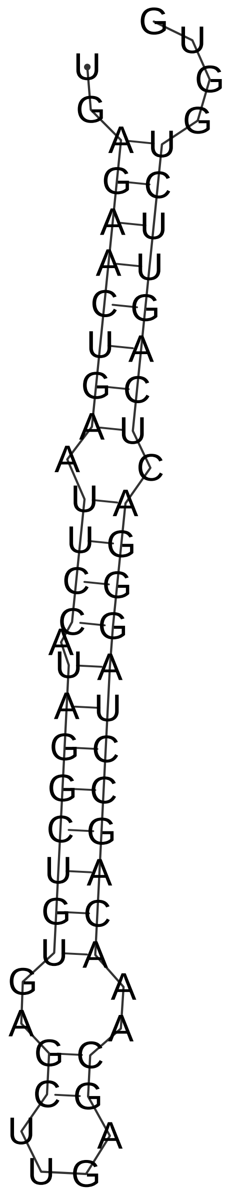

28_14940550-1494061_cfa-mir-146b_high

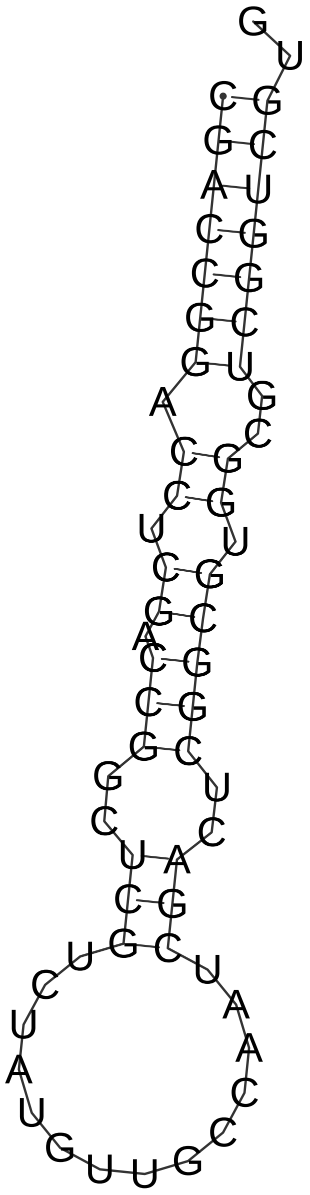

28_15765927-1576598_cfa-mir-1307_high

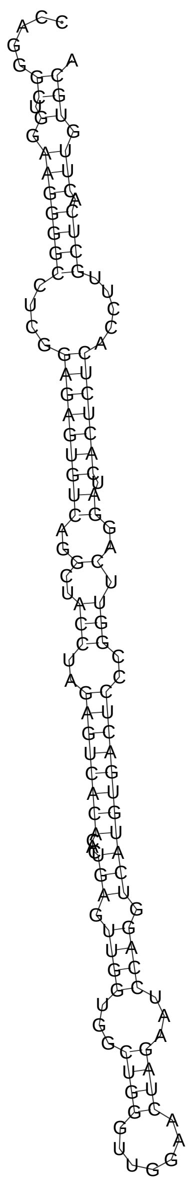

28_15916916-1591704_cfa-mir-8816_low

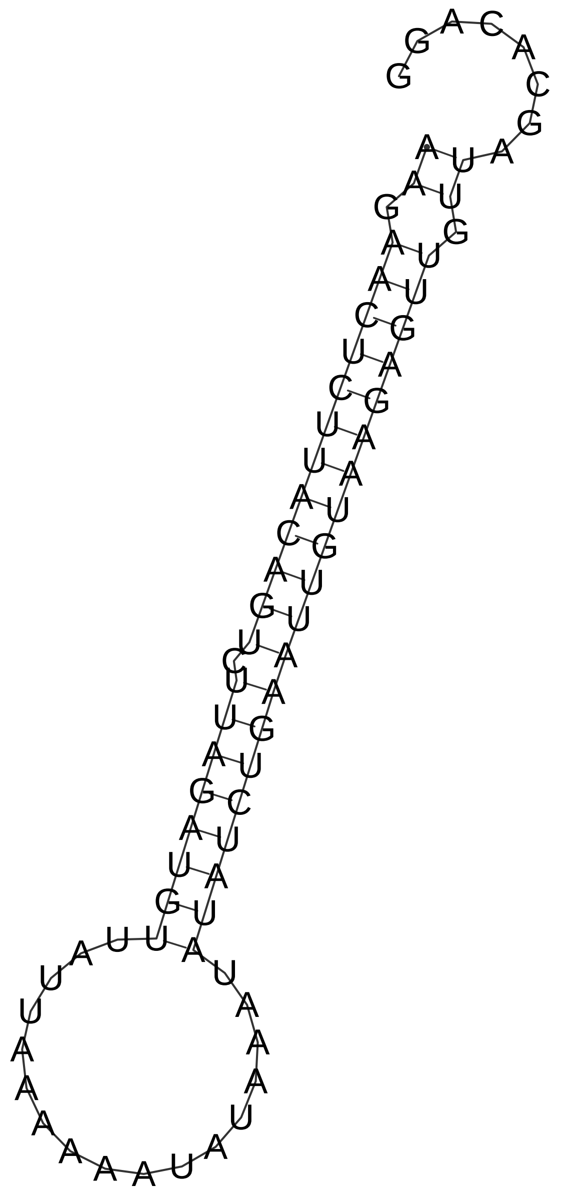

28_22226258-2222632_mir-4680_low

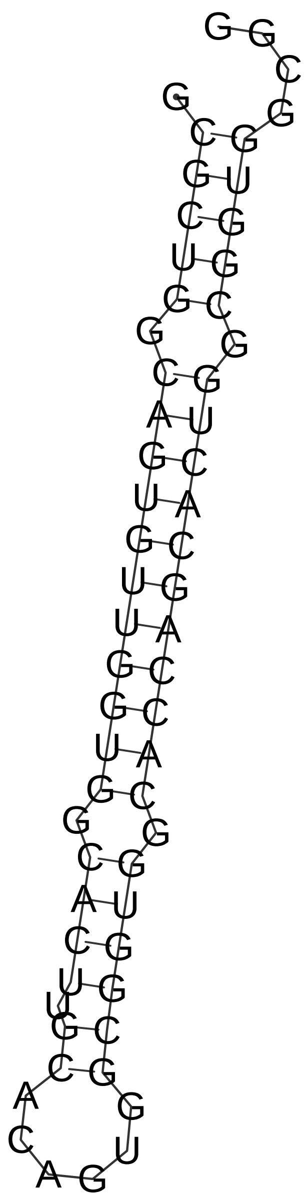

28_27372107-2737216_mir-9851_high

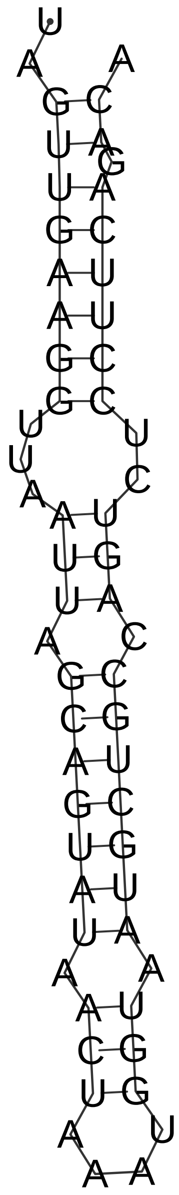

28_29309122-2930918_mir-3084_high

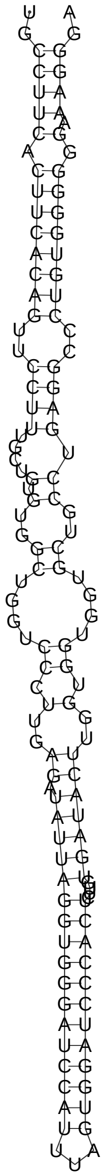

28_37593890-3759401_cfa-mir-8817_low

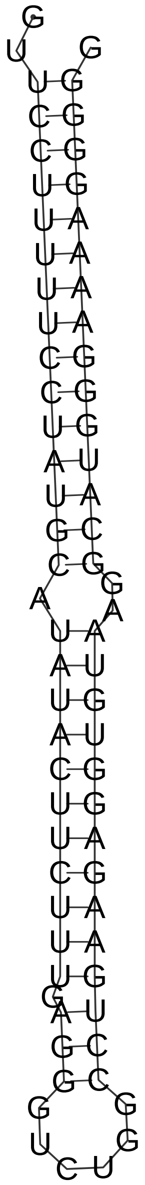

28_40842076-4084214_cfa-mir-202_high

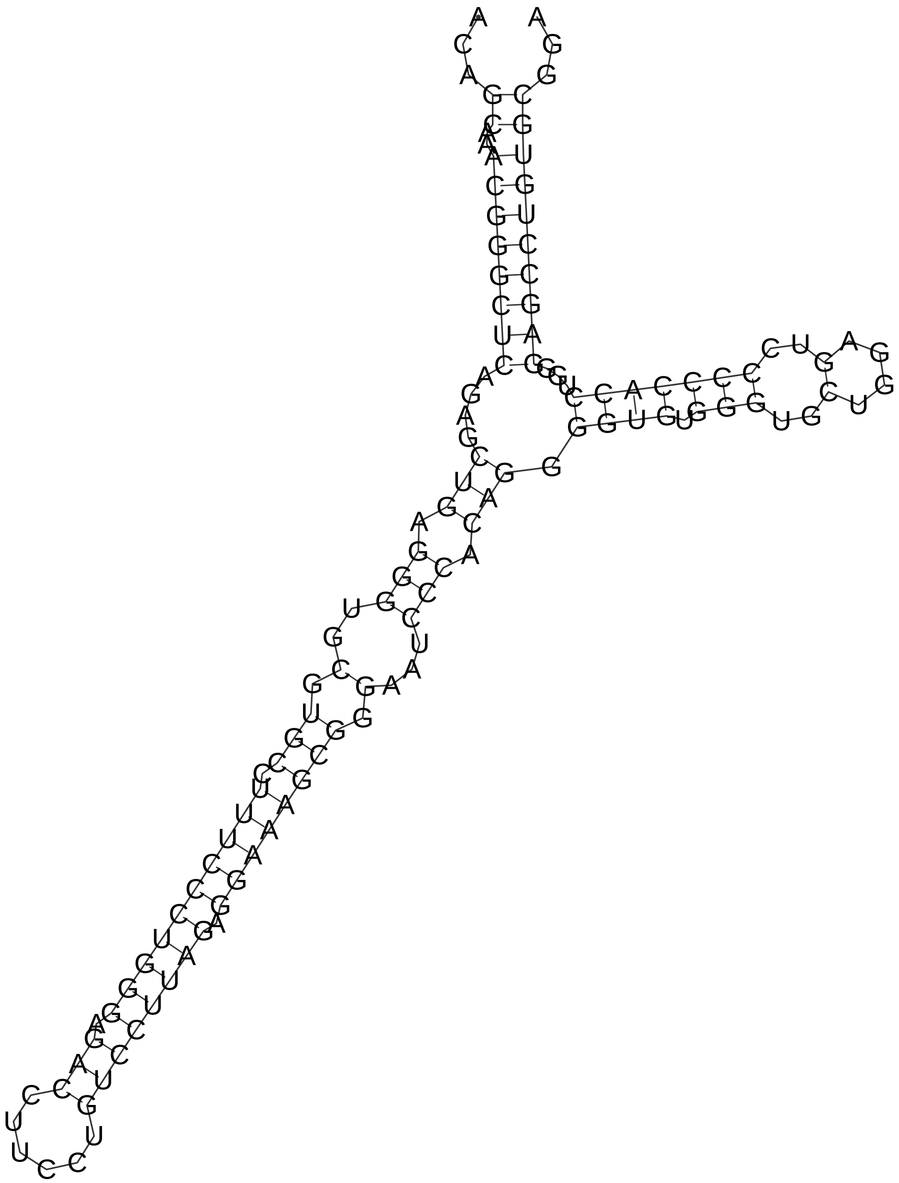

28_41168746-4116887_cfa-mir-8818_low

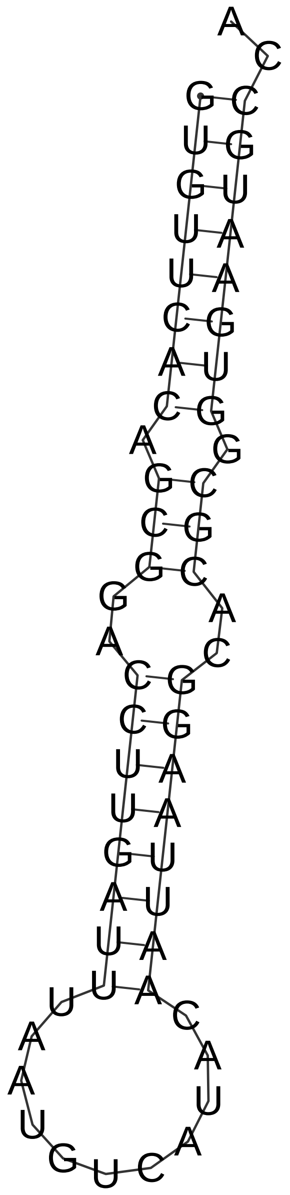

29_14264389-1426444_cfa-mir-124-2_high

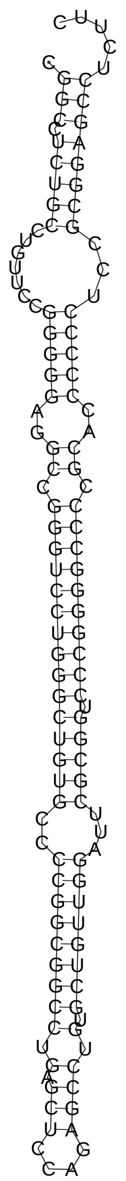

29_29264004-2926412_cfa-mir-8799b_low

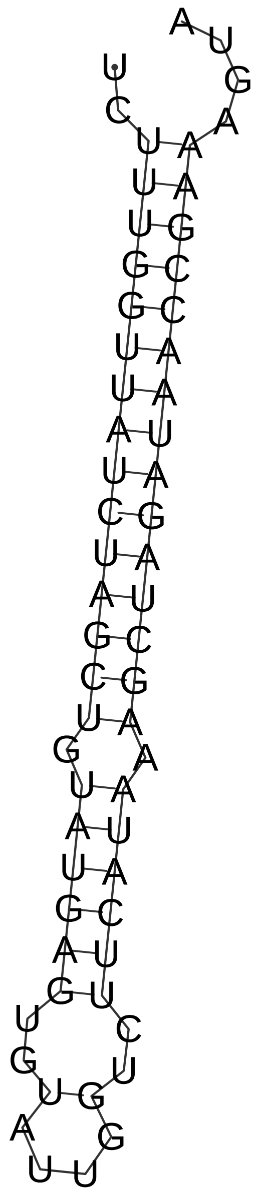

3_19997697-1999775_cfa-mir-9-2_high

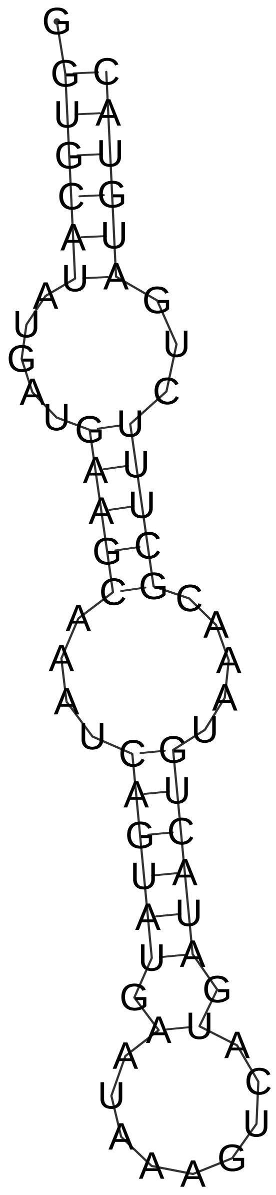

3_21703284-2170334_mir-3607_high

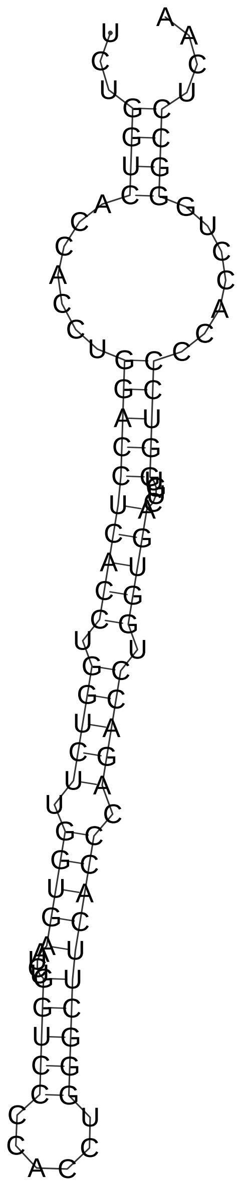

3_31262394-3126249_cfa-mir-8901_low

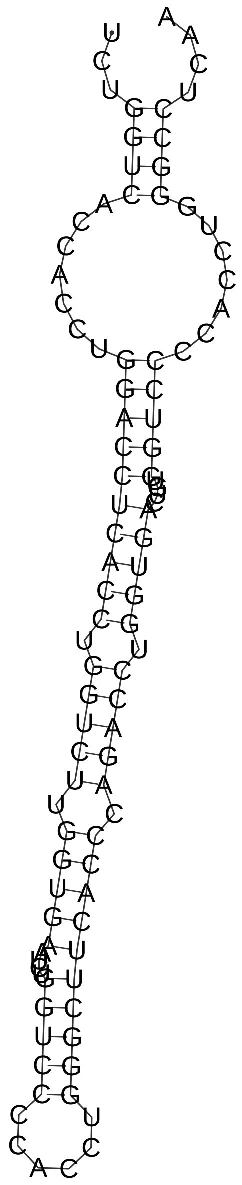

3_31617282-3161737_cfa-mir-8901_low

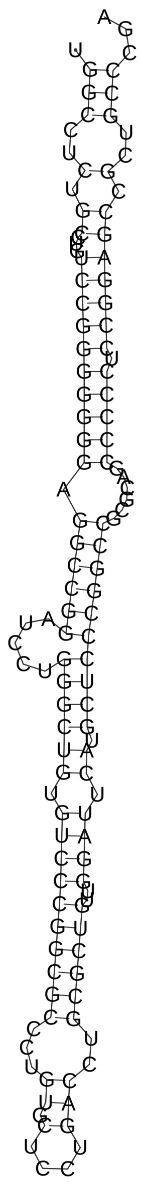

3_32684275-3268439_cfa-mir-8799g_low

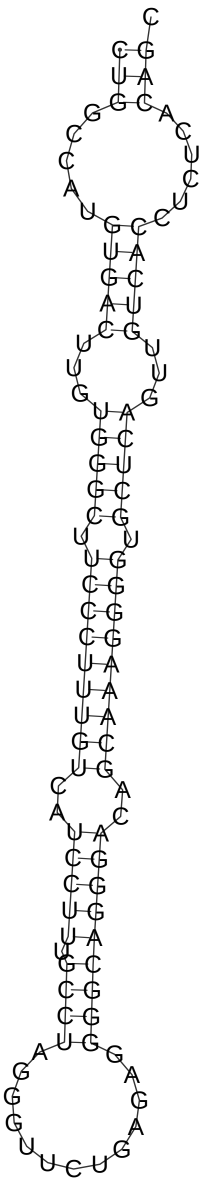

3_37882660-3788275_cfa-mir-211_high

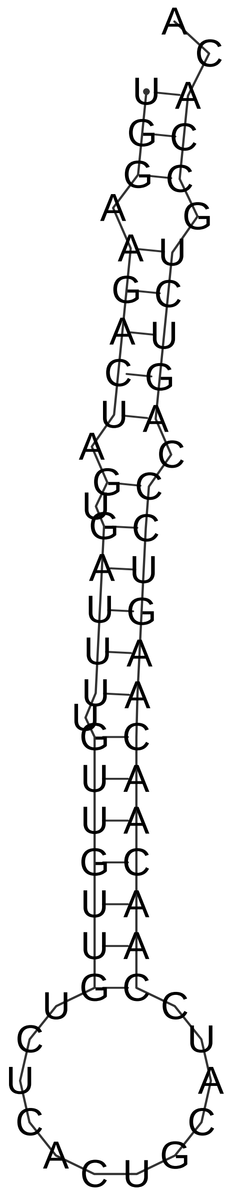

3_51802454-5180251_cfa-mir-7-2_high

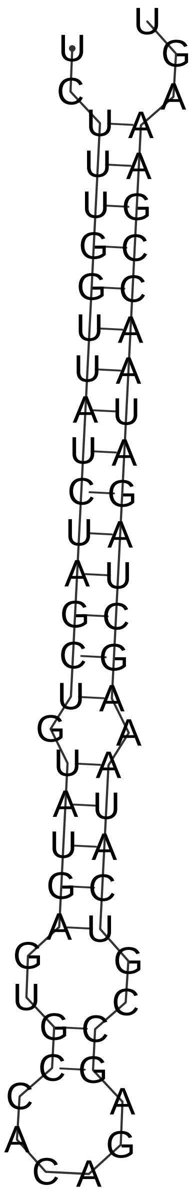

3_52410840-5241090_cfa-mir-9-3_high

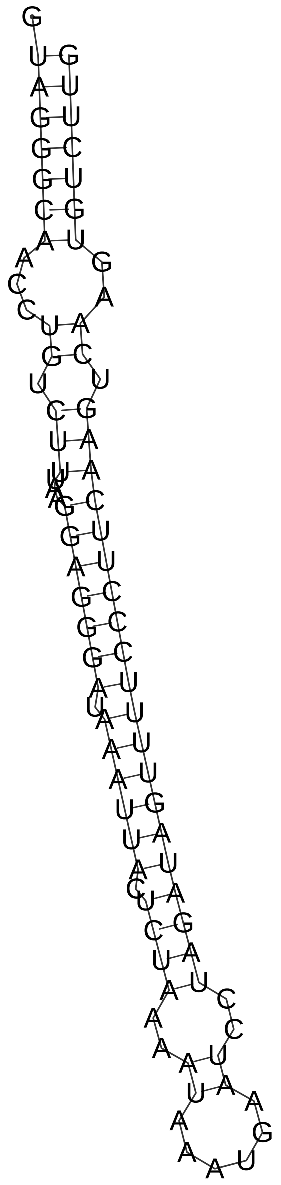

3_54138599-5413868_mir-684_low

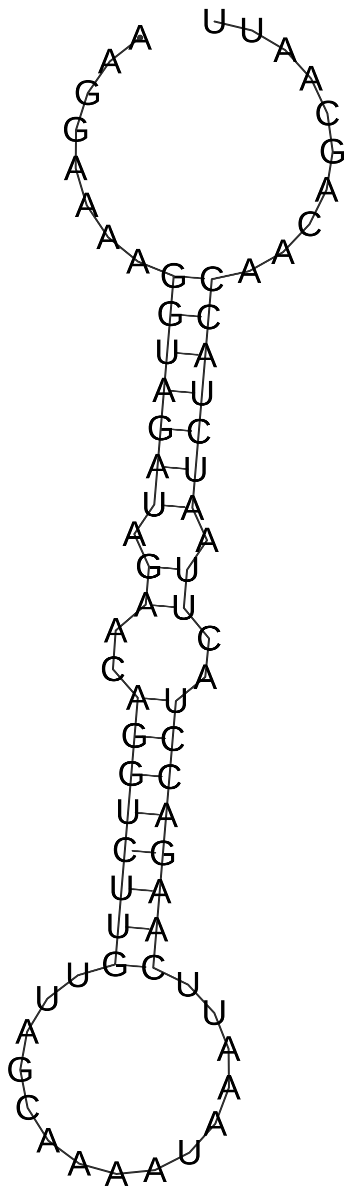

3_54579692-5457976_cfa-mir-1839_high

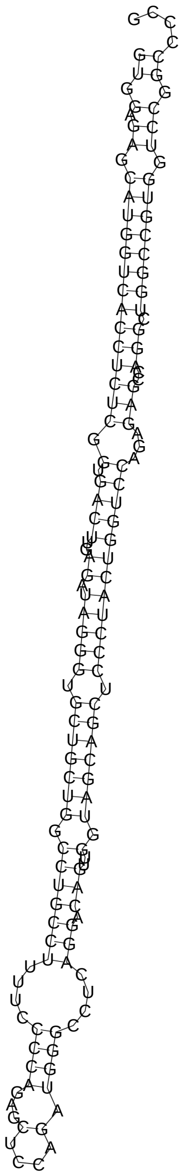

3_54657737-5465787_cfa-mir-8898_low

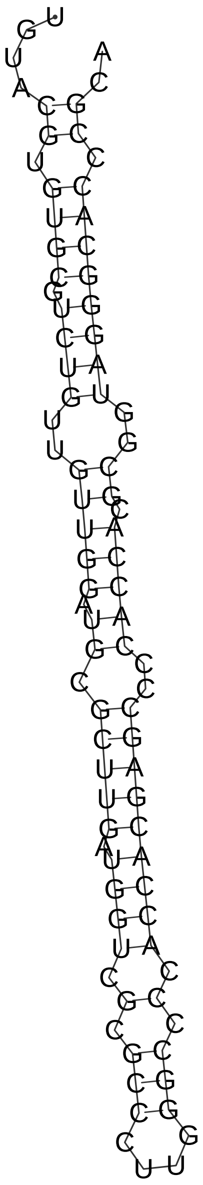

3_55912906-5591299_mir-1905_low

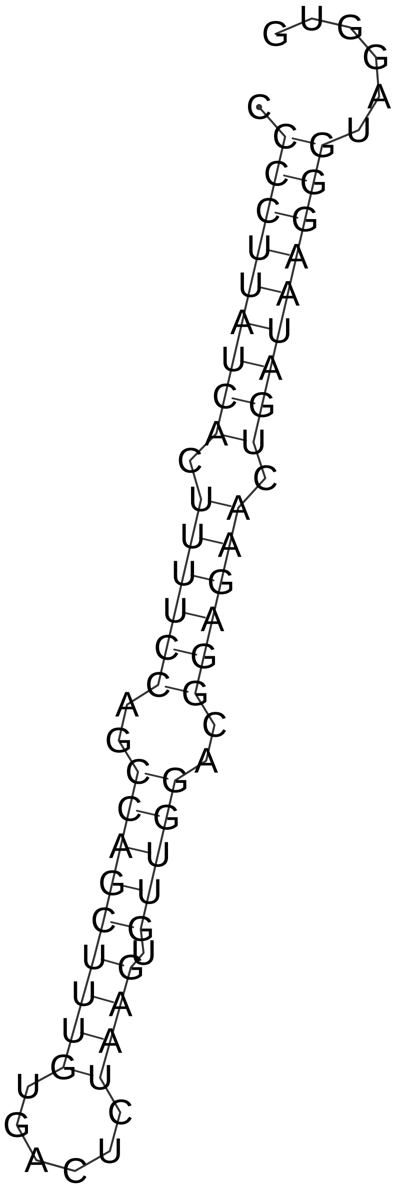

3_57914444-5791451_cfa-mir-184_high

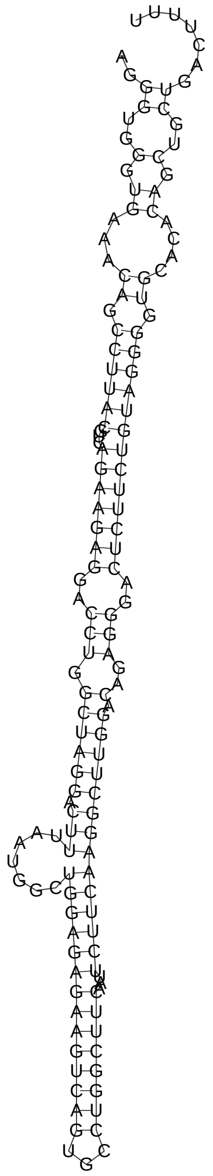

3_58385089-5838522_cfa-mir-8900_low

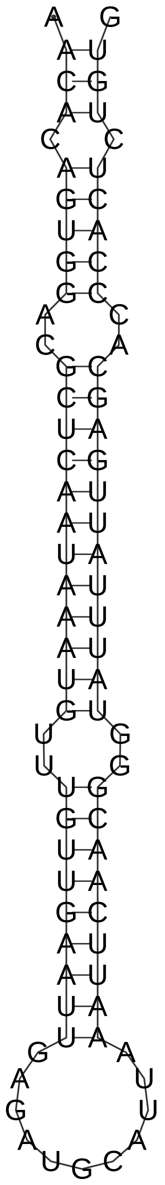

3_59867521-5986760_cfa-mir-95_high

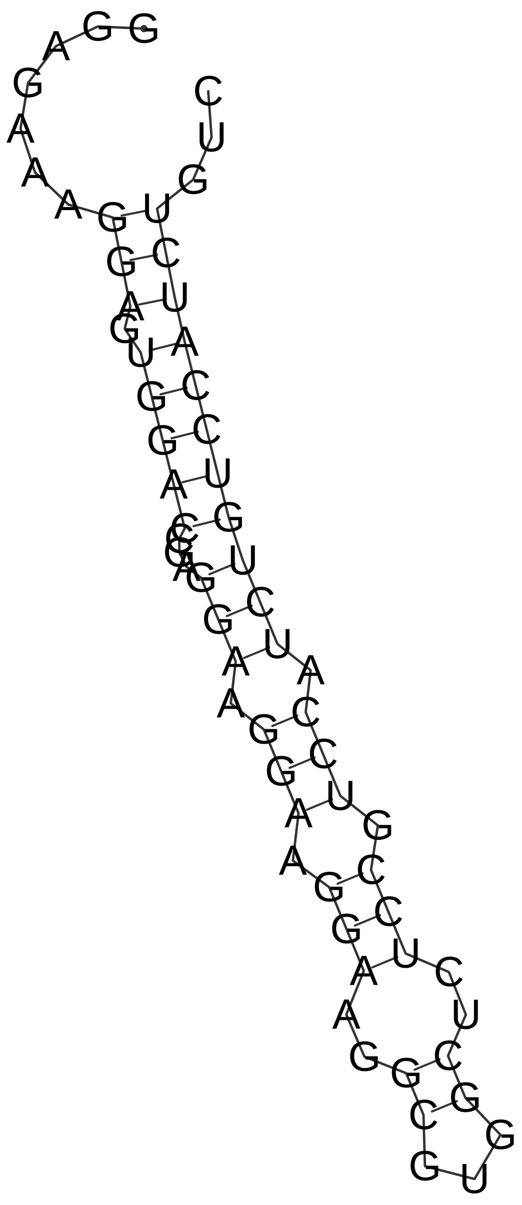

3_61939007-6193906_mir-4800_low

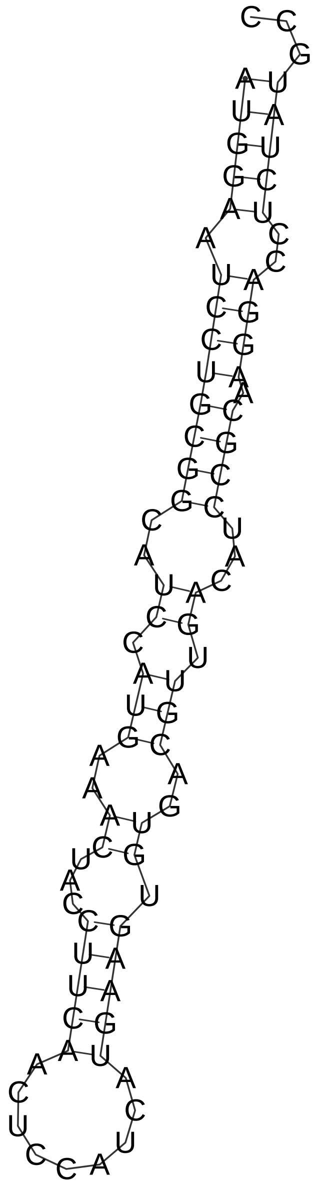

3_63027413-6302749_mir-3533_low

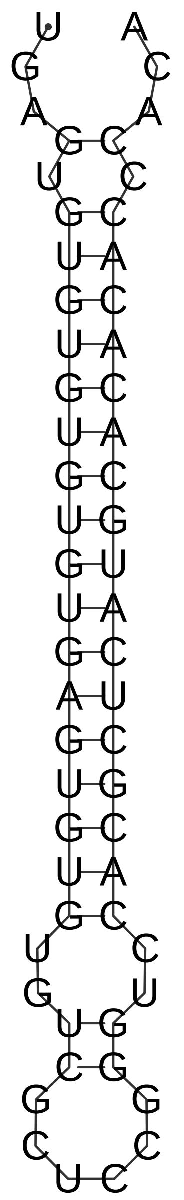

3_73494062-7349411_cfa-mir-574_high

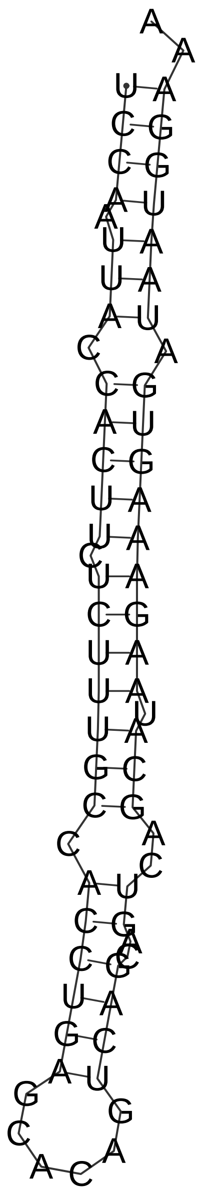

3_82049047-8204911_mir-4275_low

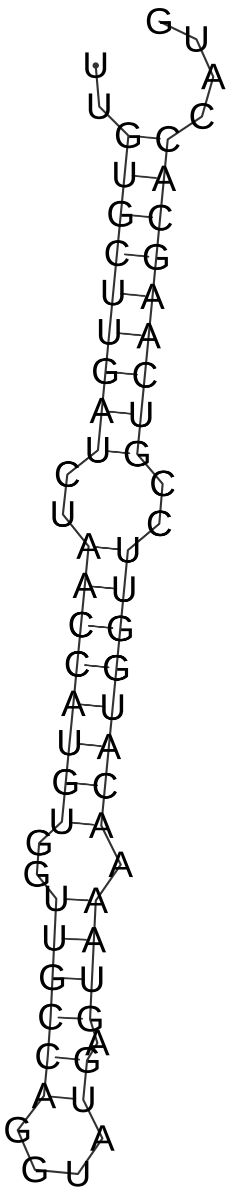

3_89044709-8904477_cfa-mir-218-1_high

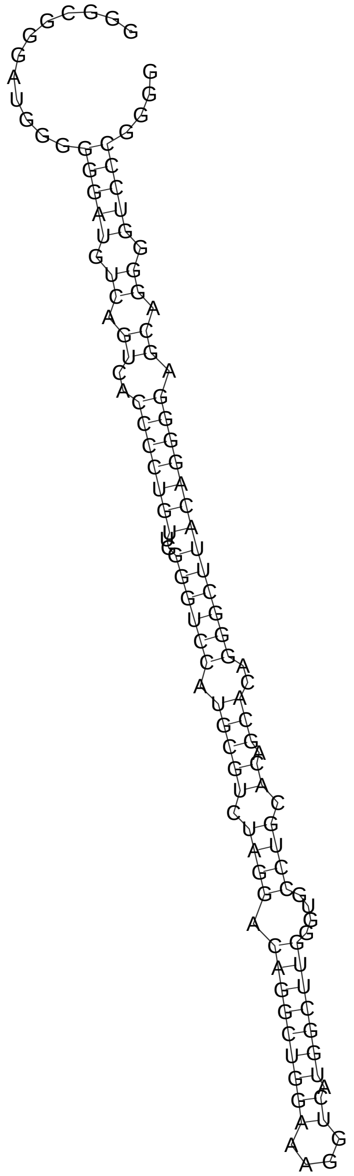

3_91886956-9188707_cfa-mir-8899_low

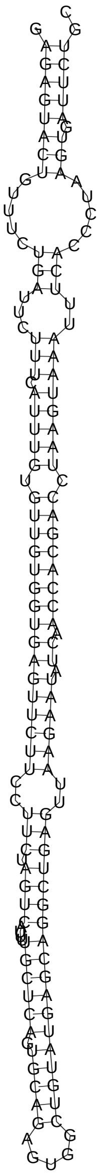

30_3219341-321948_cfa-mir-8792_low

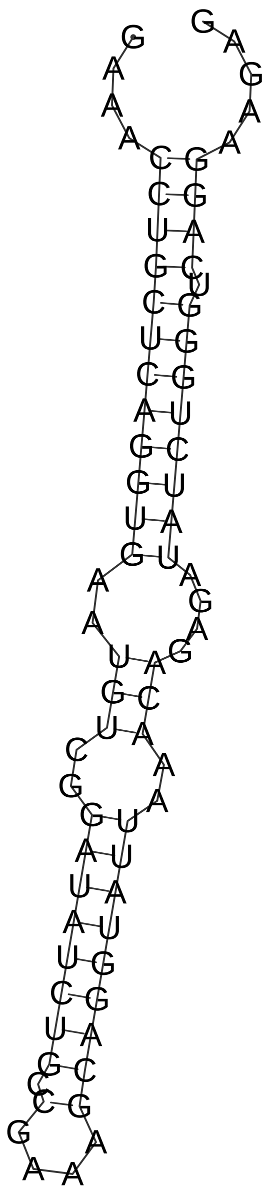

30_10022251-1002232_mir-9838_low

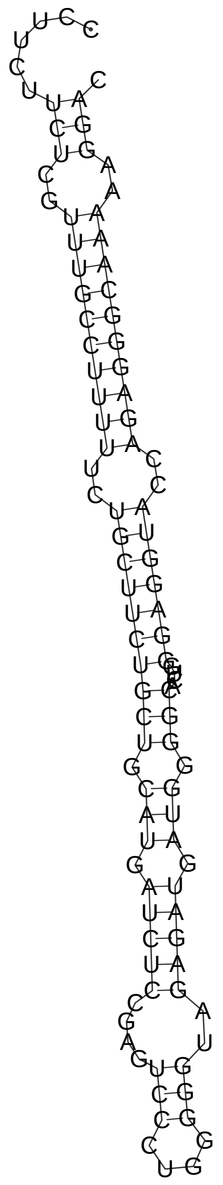

30_10536862-1053696_mir-1282_low

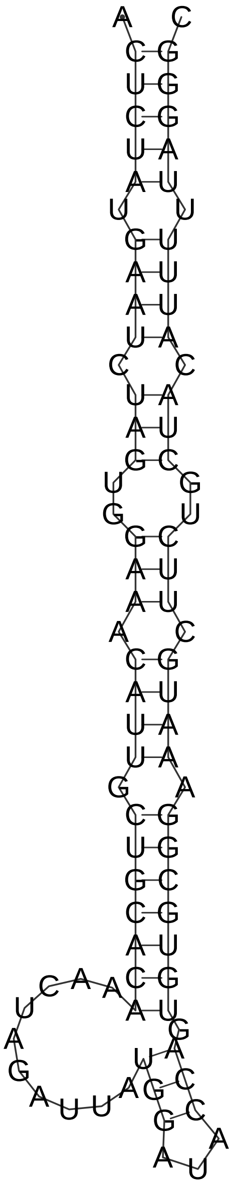

30_11800621-1180070_cfa-mir-147_high

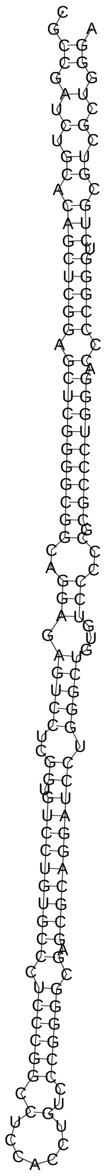

30_16018399-1601854_cfa-mir-8791a_low

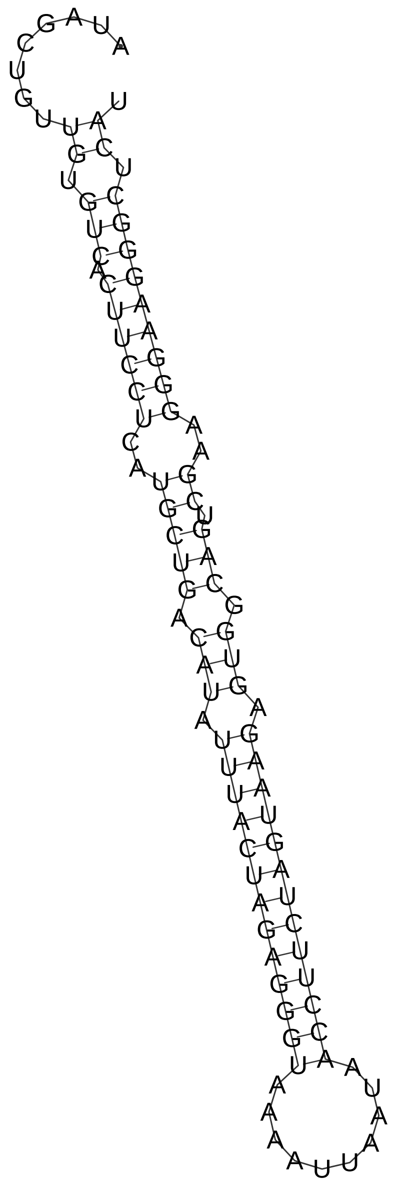

30_20769709-2076980_cfa-mir-628_high

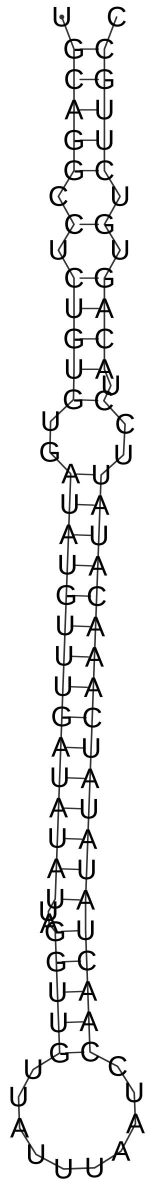

30_27433122-2743320_cfa-mir-190a_high

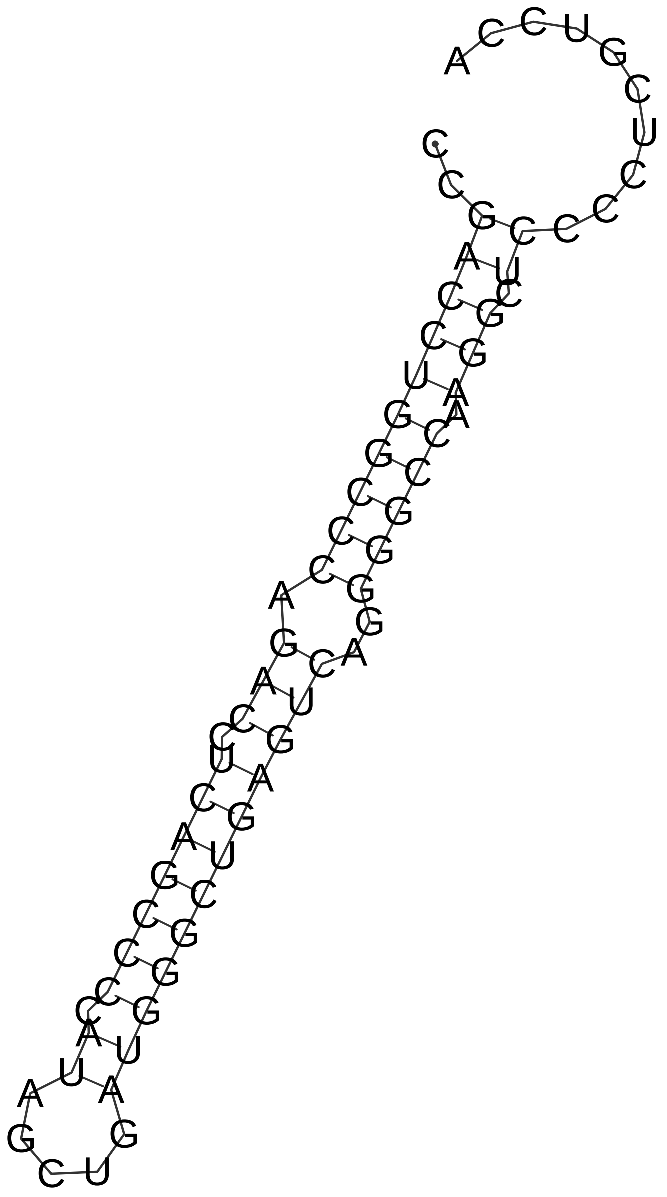

30_38216543-3821661_cfa-mir-631_low

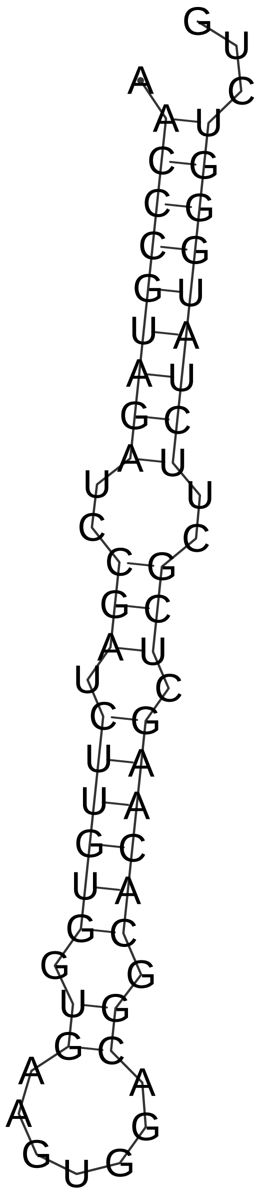

31_13258782-1325884_cfa-mir-99a-1_high

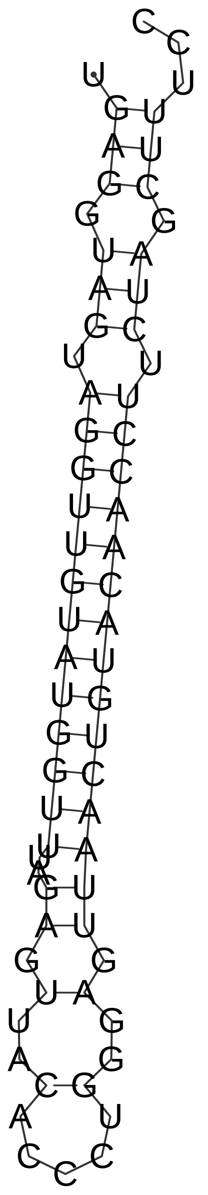

31_13259522-1325958_cfa-let-7c_high

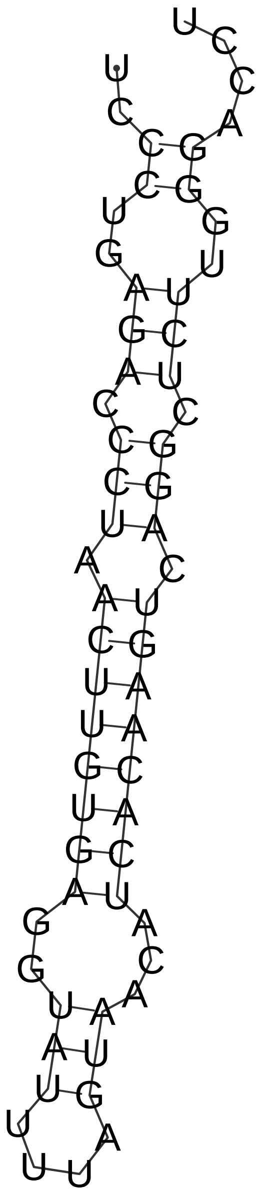

31_13304980-1330504_cfa-mir-125b-2_high

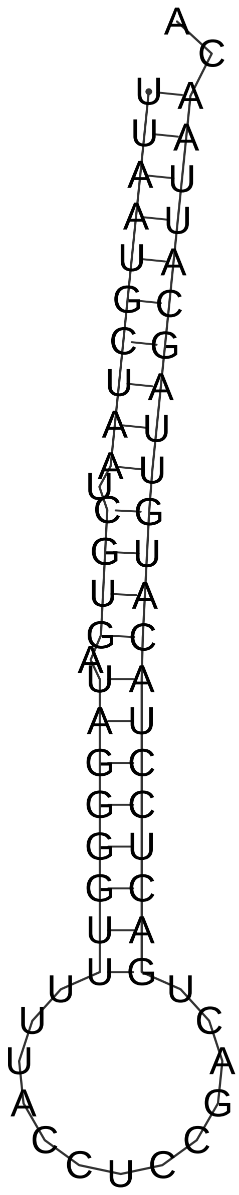

31_21078768-2107882_cfa-mir-155_high

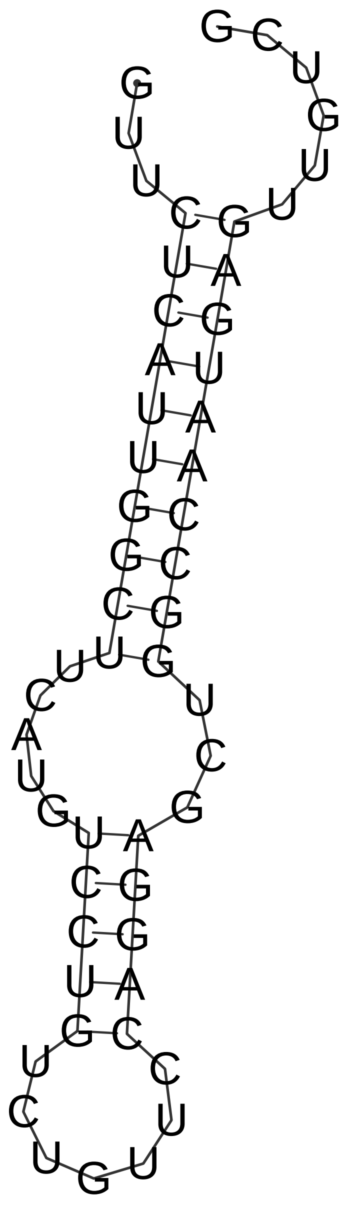

31_24034697-2403475_mir-9027_low

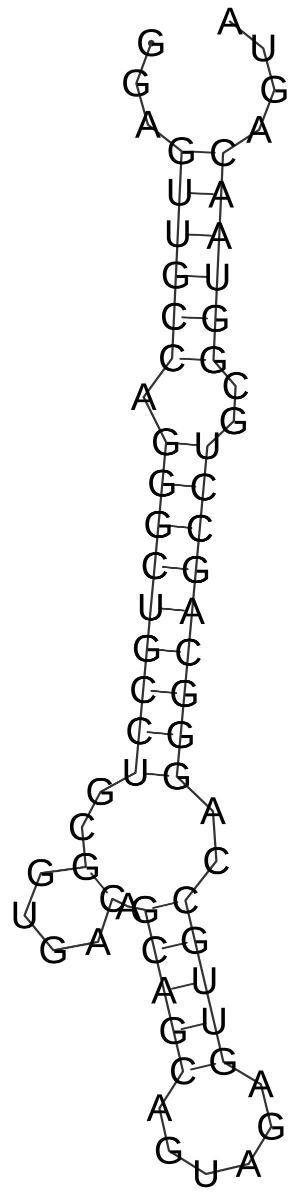

31_28172857-2817292_mir-6501_low

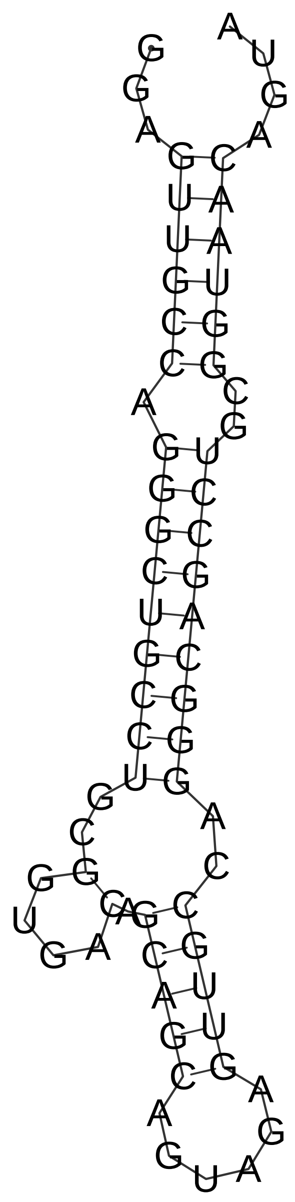

31_29071706-2907177_mir-6501_low

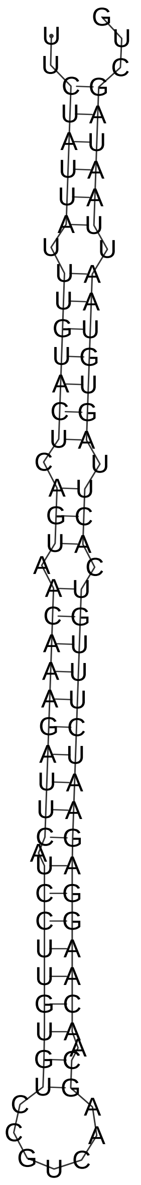

31_31005200-3100528_cfa-mir-802_high

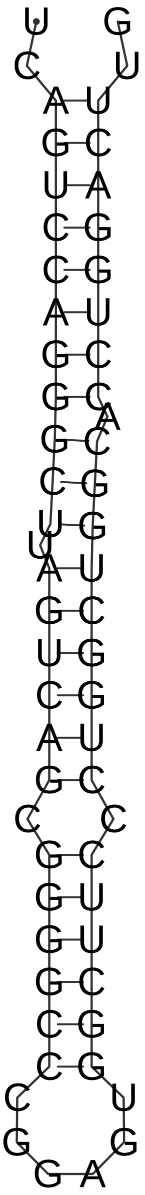

32_21108324-2110838_mir-1956_low

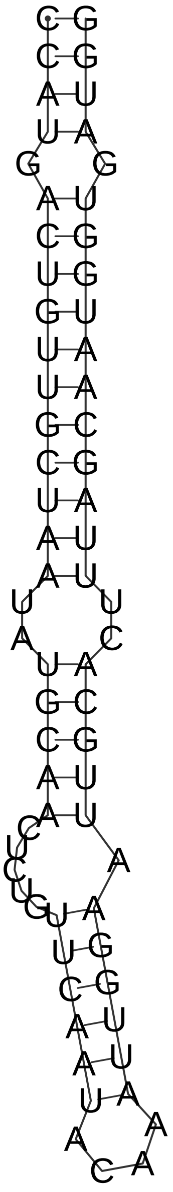

32_32428905-3242897_cfa-mir-367_low

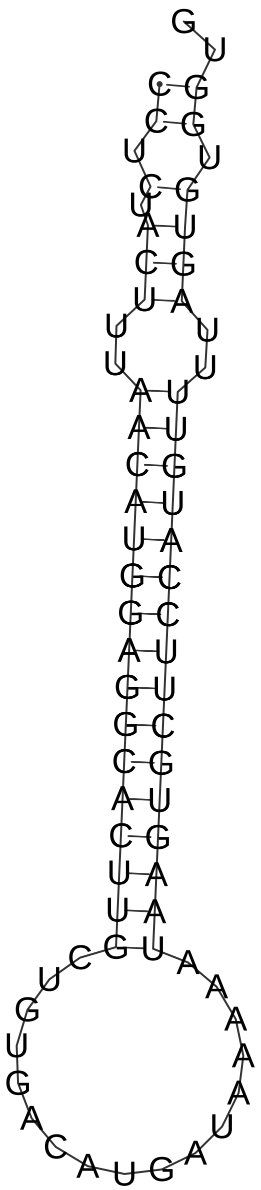

32_32429032-3242910_cfa-mir-302d_low

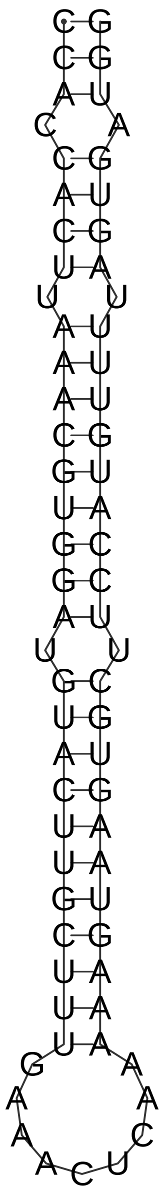

32_32429209-3242927_cfa-mir-302a_high

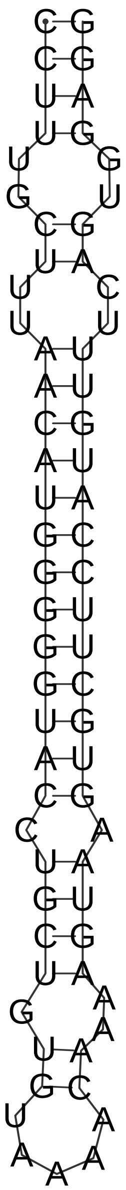

32_32429366-3242943_cfa-mir-302c_low

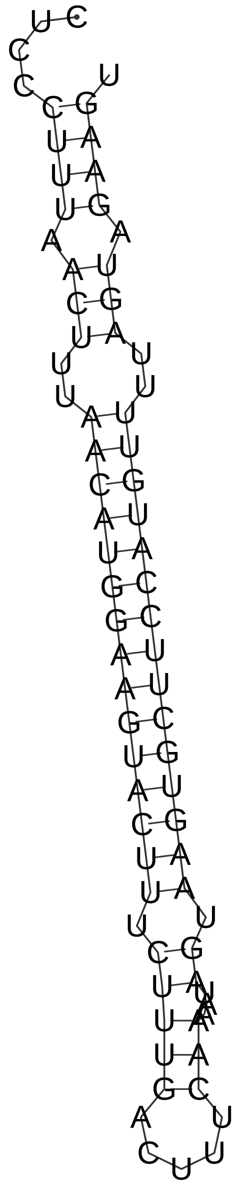

32_32429500-3242957_cfa-mir-302b_low

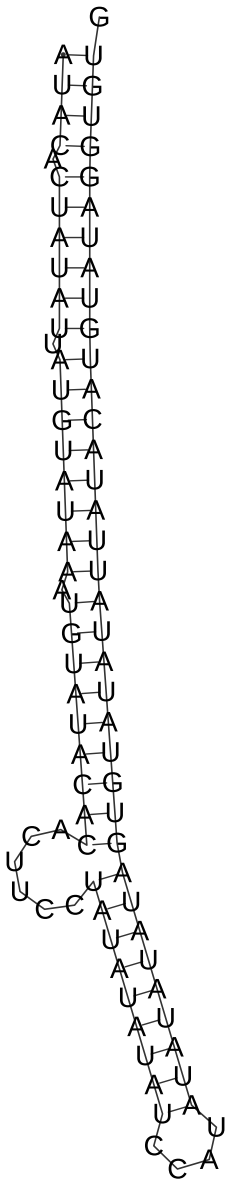

33_18506878-1850696_cfa-mir-568_low

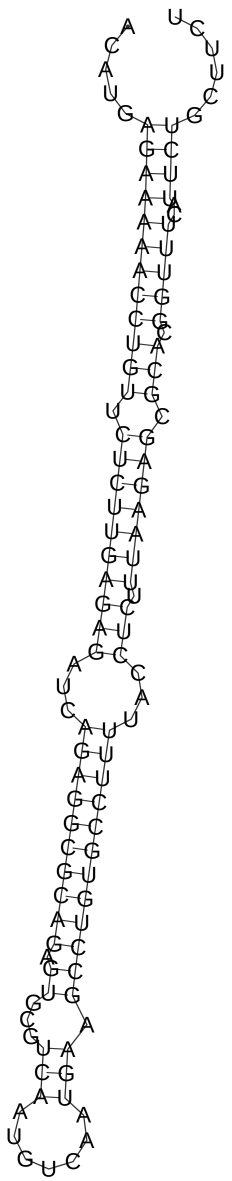

33_23649303-2364941_cfa-mir-6529_high

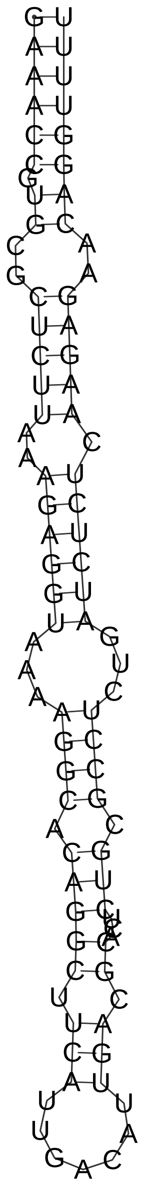

33_23649314-2364940_mir-6529_low

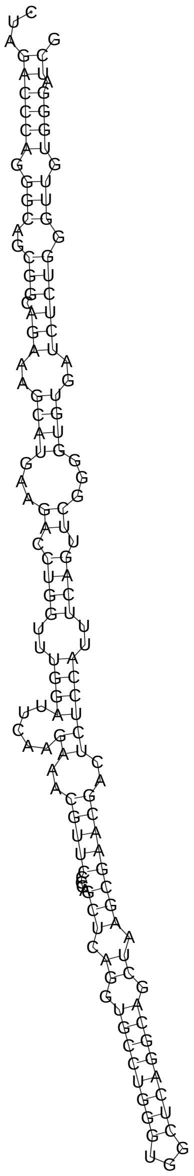

33_29672097-2967224_cfa-mir-8789_low

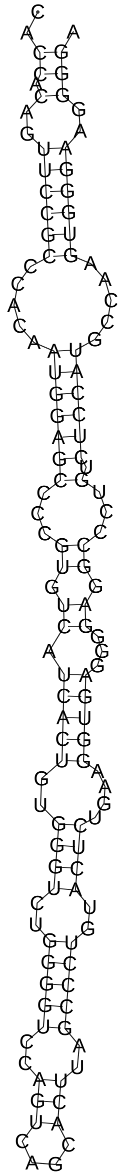

34_12141935-1214205_mir-8790_low

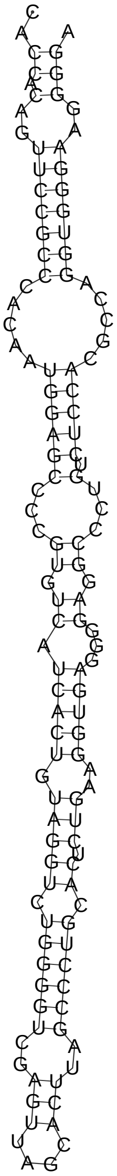

34_12372846-1237296_cfa-mir-8790_low

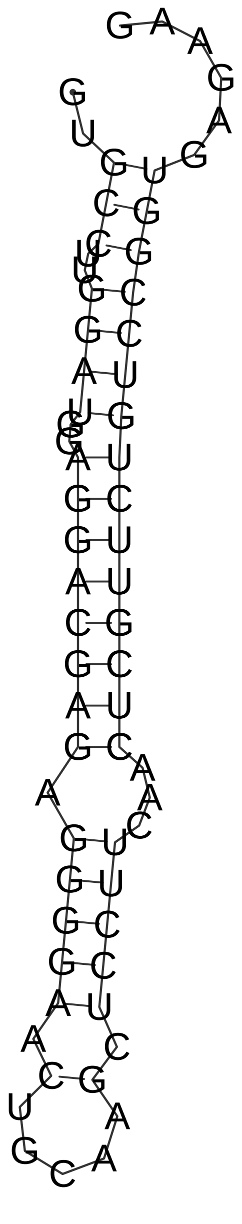

34_14840248-1484031_mir-1897_low

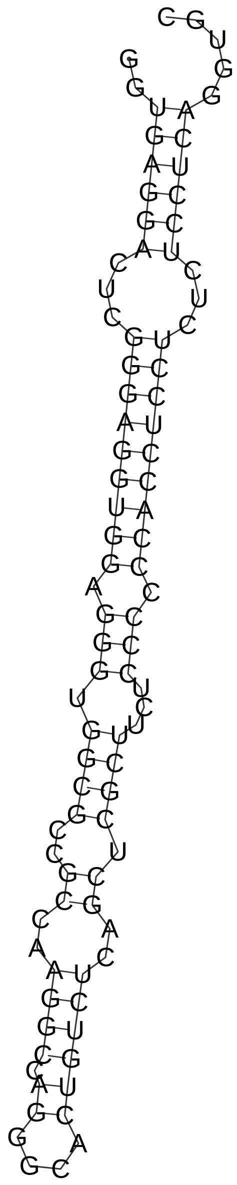

34_17134367-1713445_mir-1224_high

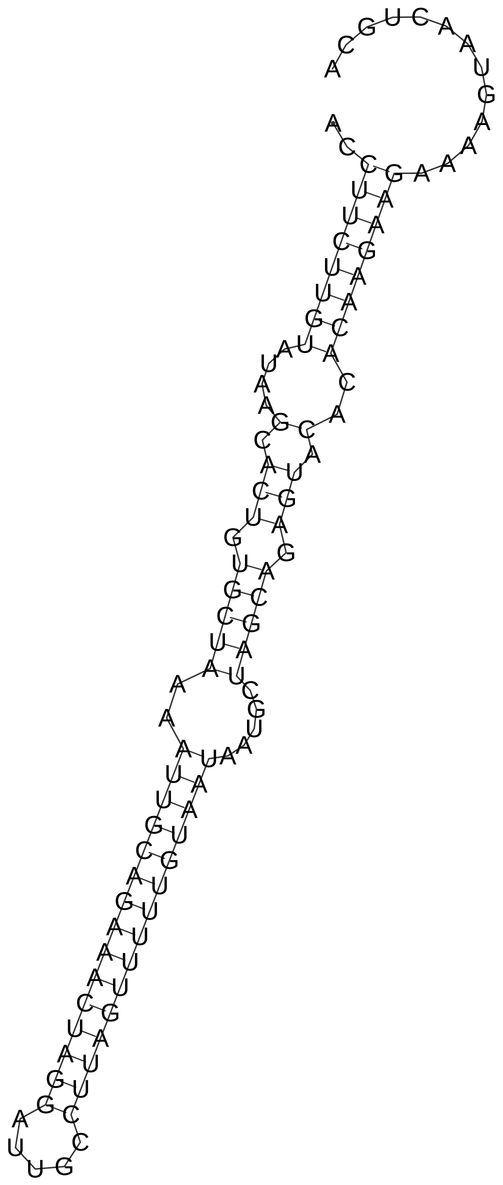

34_19357972-1935807_miR-1248_high

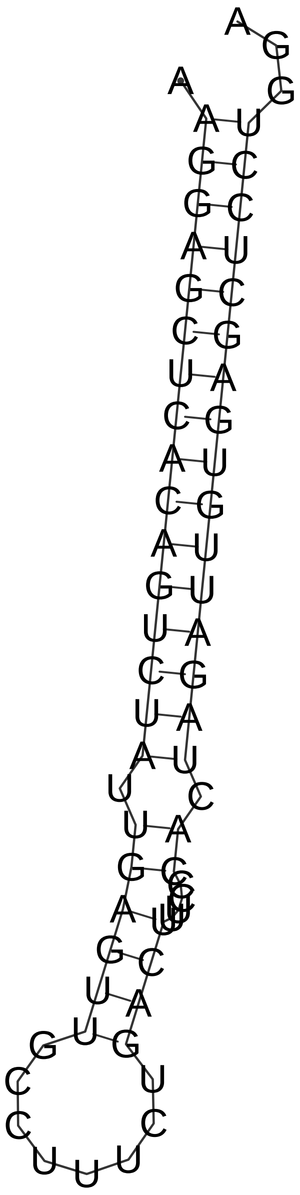

34_20971517-2097157_cfa-mir-28_high

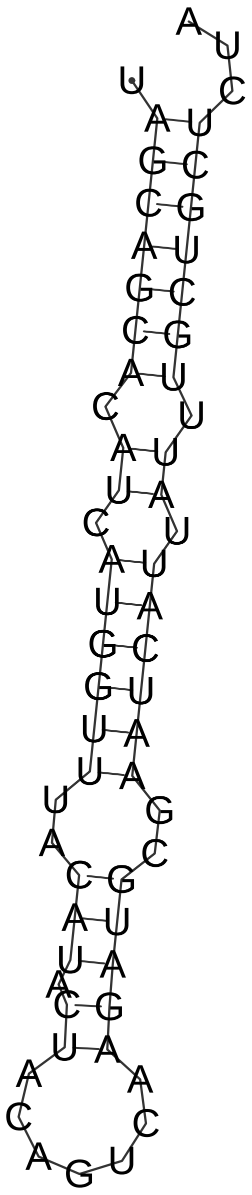

34_26460663-2646072_cfa-mir-15b_high

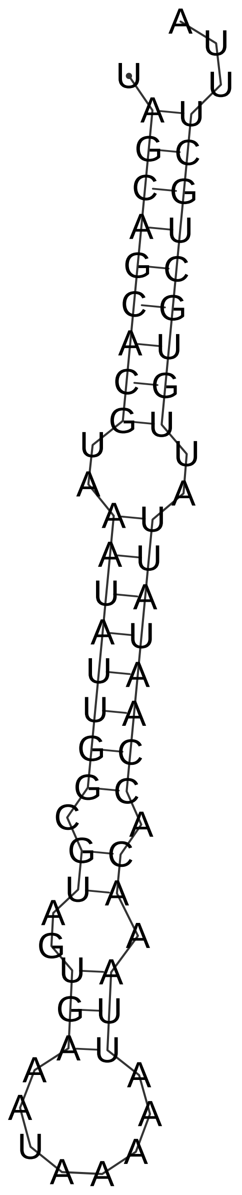

34_26460807-2646087_cfa-mir-16-2_high

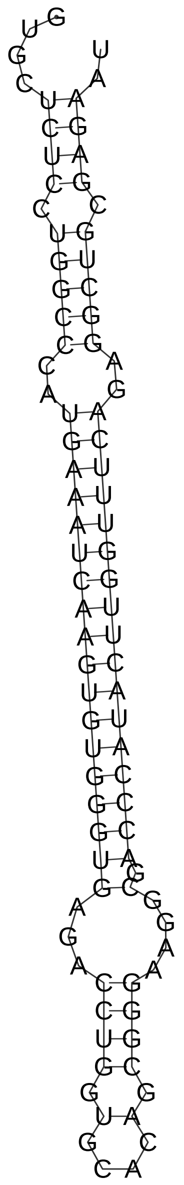

34_33292499-3329258_cfa-mir-551b_high

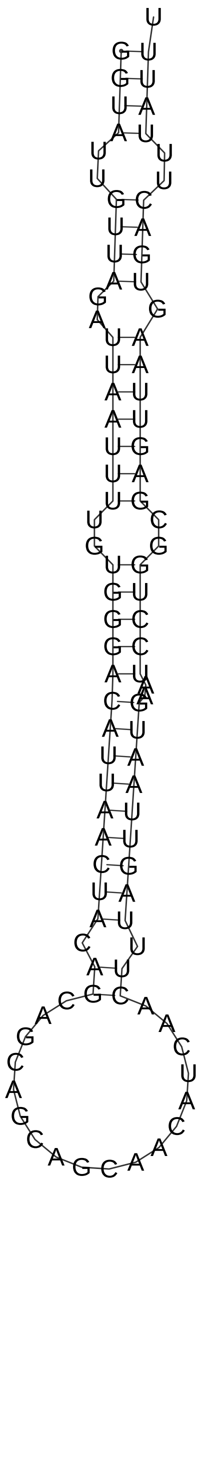

34_35465927-3546602_mir-569_low

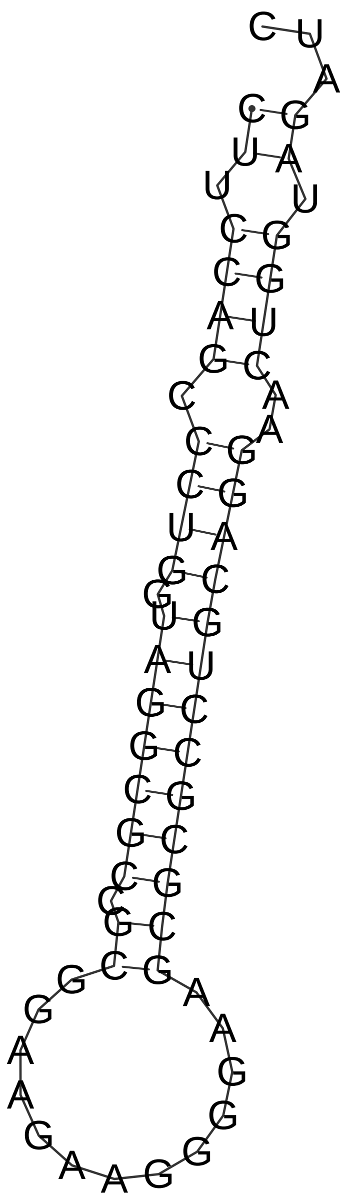

35_1841482-184154_mir-6720_high

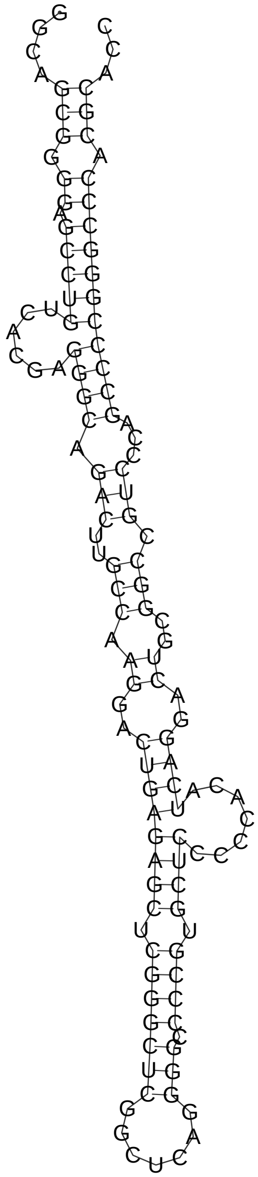

36_1546410-154653_cfa-mir-8797_low

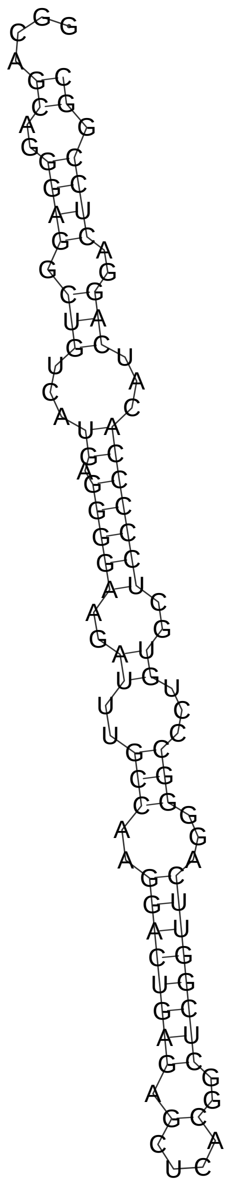

36_10420663-1042076_mir-8797_low

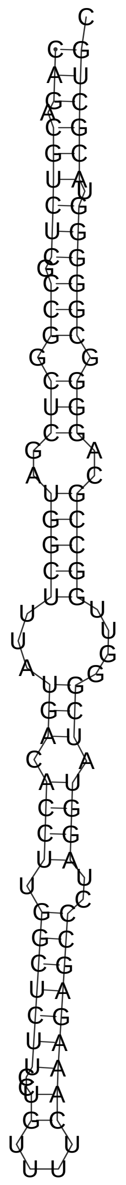

36_19933715-1993381_mir-9183_low

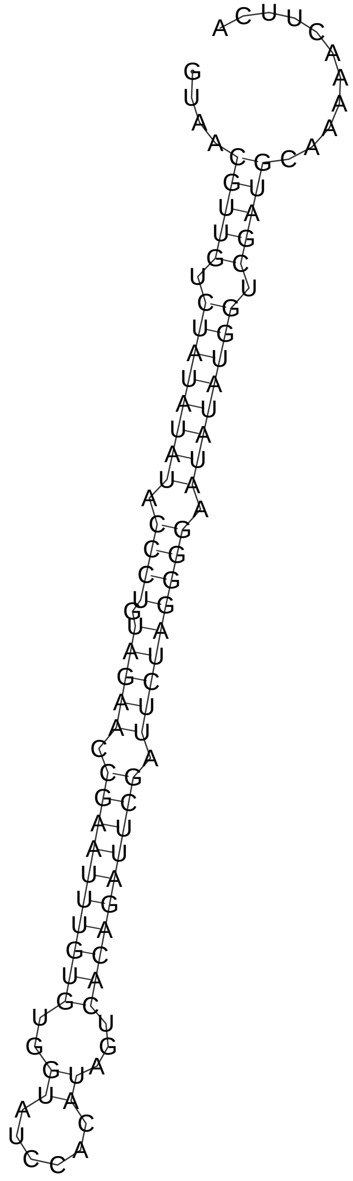

36_19958913-1995901_cfa-mir-10b_high

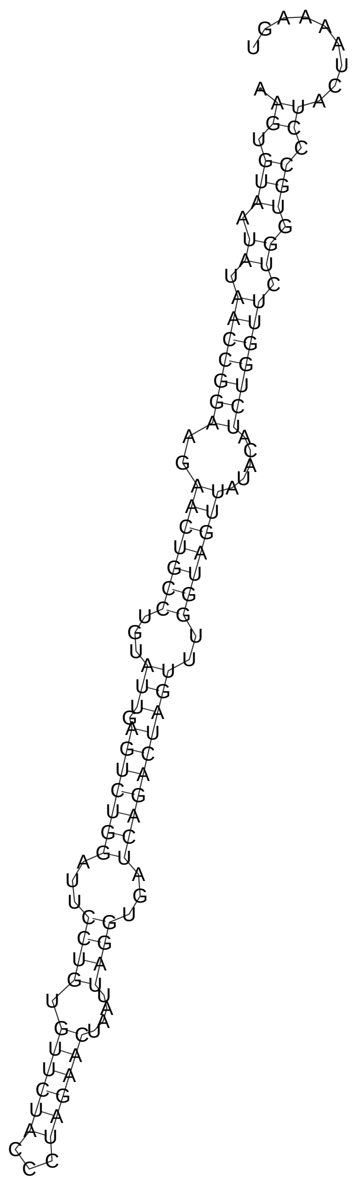

36_21115493-2111561_cfa-mir-8796_low

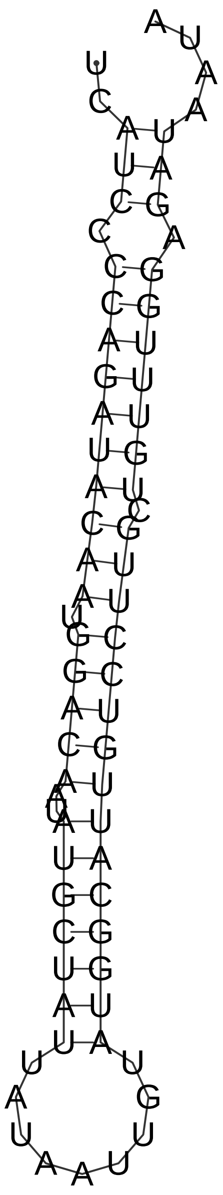

37_15549130-1554920_mir-2355_high

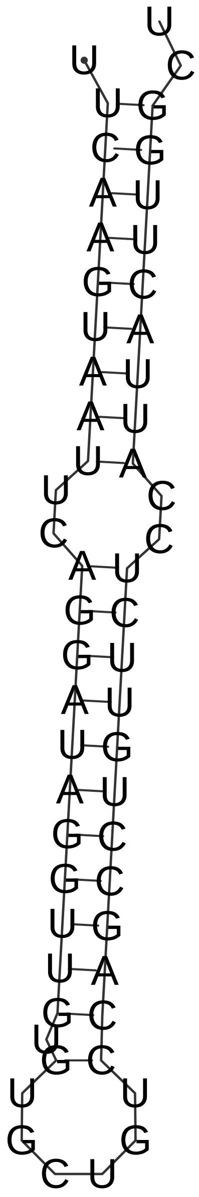

37_25054488-2505454_cfa-mir-26b_high

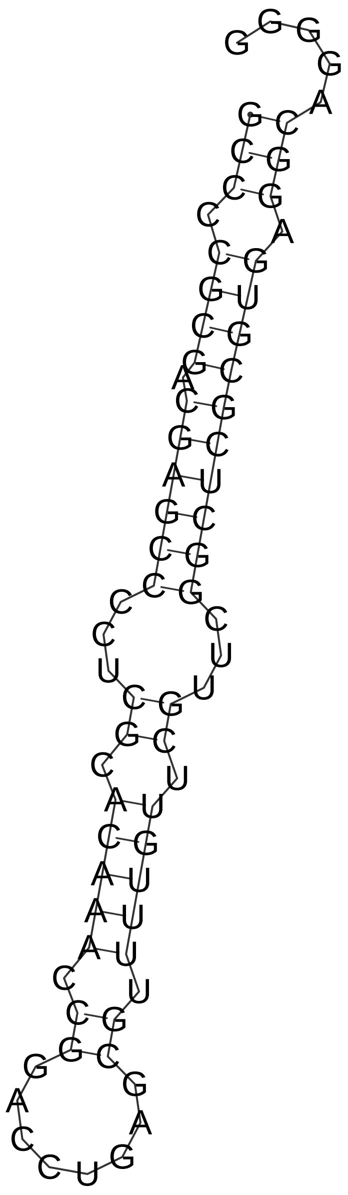

37_25568052-2556812_cfa-mir-375_high

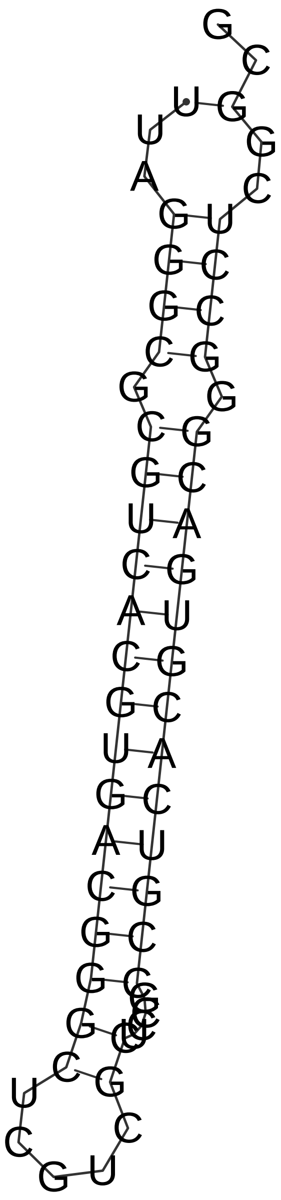

37_25734793-2573484_cfa-mir-1840_high

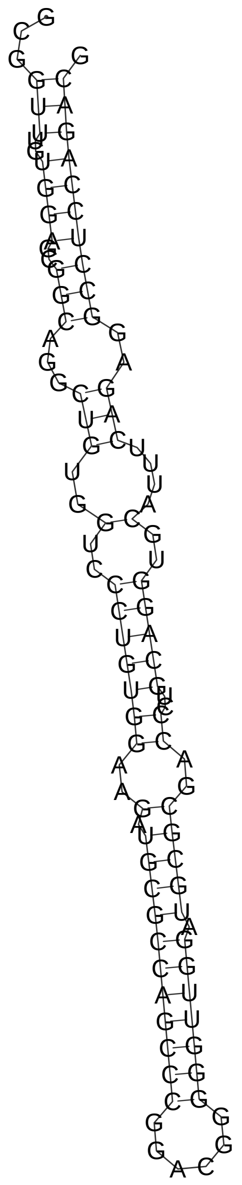

37_30320456-3032055_cfa-mir-8795_low

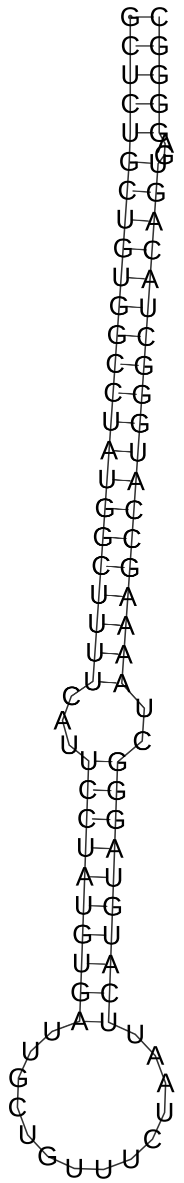

38_1926558-192664_cfa-mir-135b_high

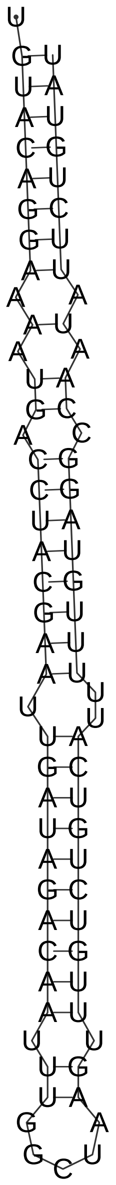

38_14895108-1489518_cfa-mir-215_high

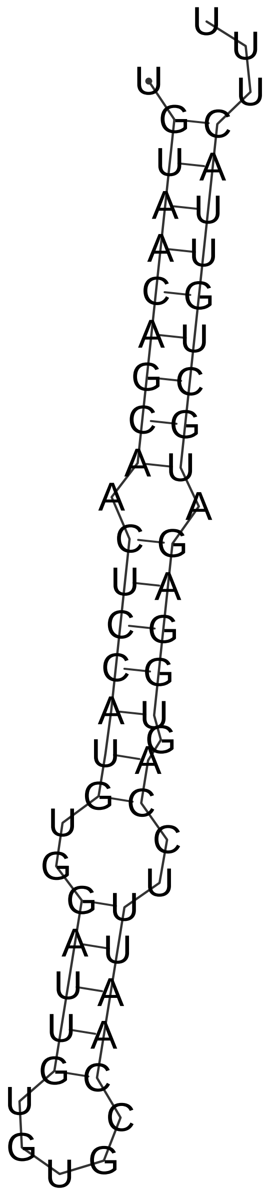

38_14895401-1489545_cfa-mir-194_high

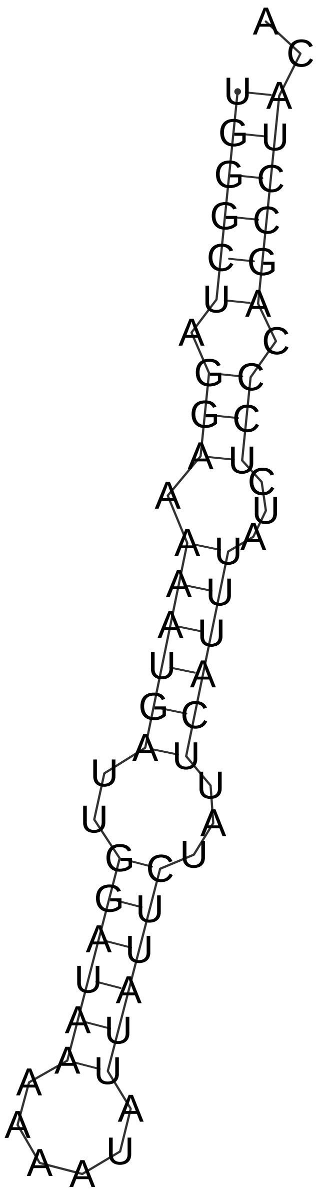

38_14977030-1497709_cfa-mir-664_high

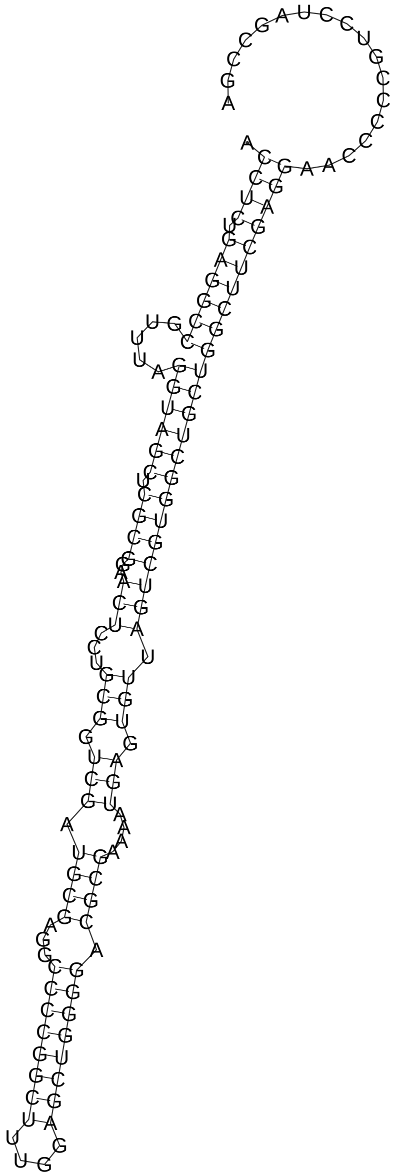

38_17579162-1757929_cfa-mir-8798_low

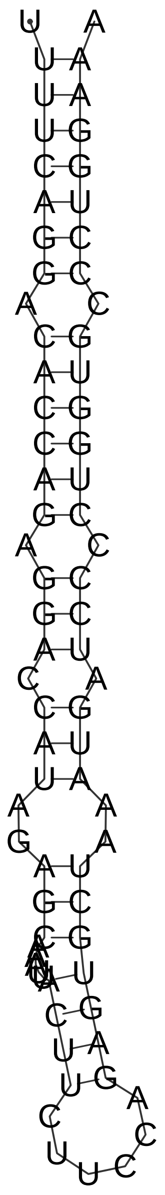

38_18675020-1867509_mir-7177_high

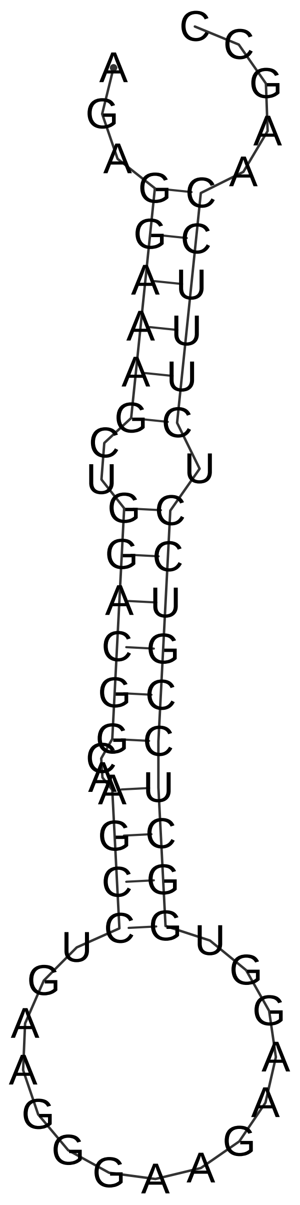

4_8702838-870289_cfa-mir-1841_high

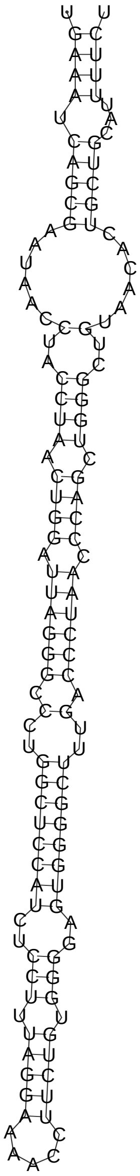

4_15180088-1518021_cfa-mir-1296_high

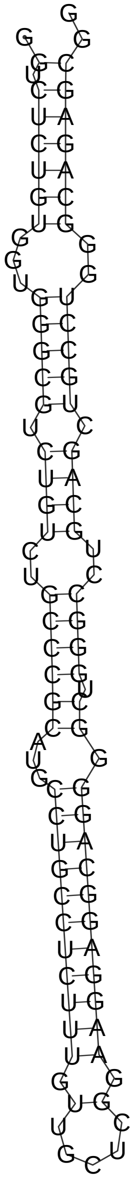

4_34073335-3407342_cfa-mir-346_high

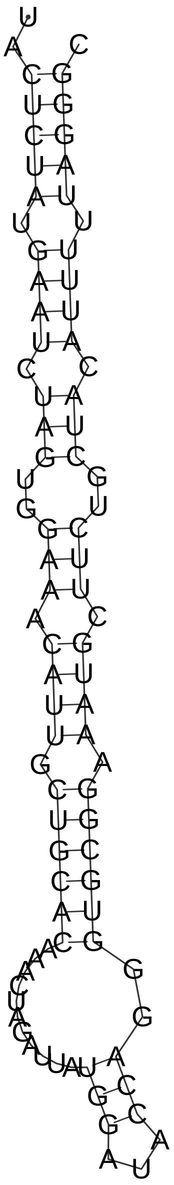

4_36279254-3627934_mir-147_high

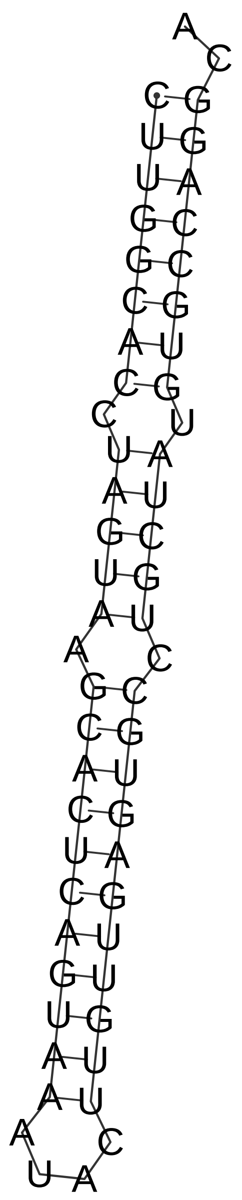

4_36951021-3695107_cfa-mir-1271_high

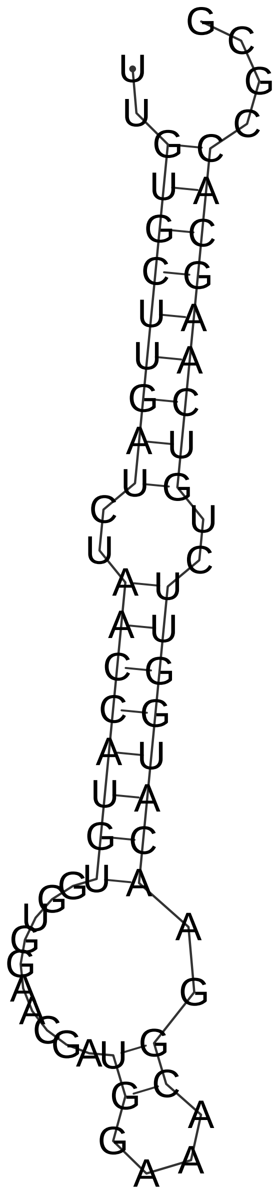

4_43010775-4301083_cfa-mir-218-2_high

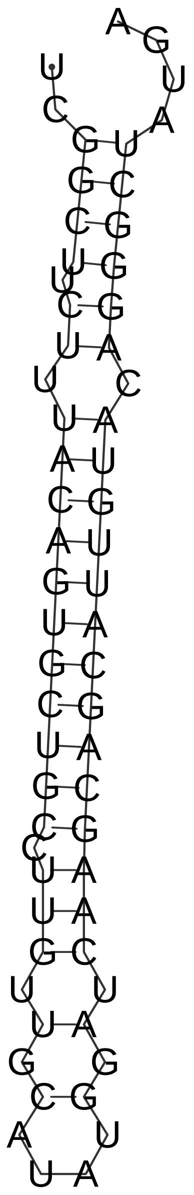

4_43182876-4318293_cfa-mir-103-1_high

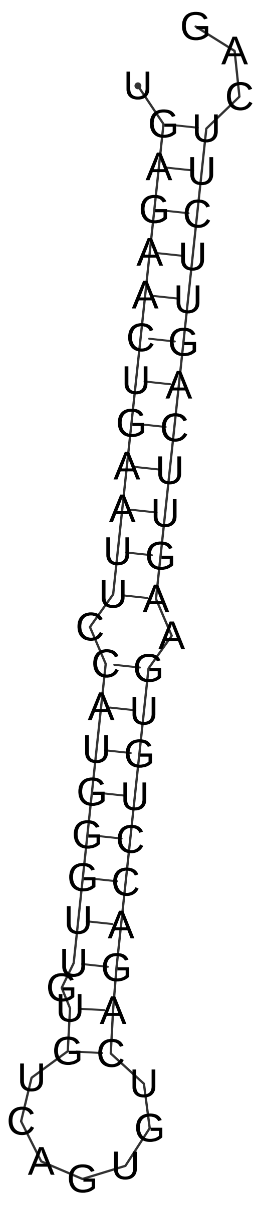

4_50248597-5024865_cfa-mir-146a_high

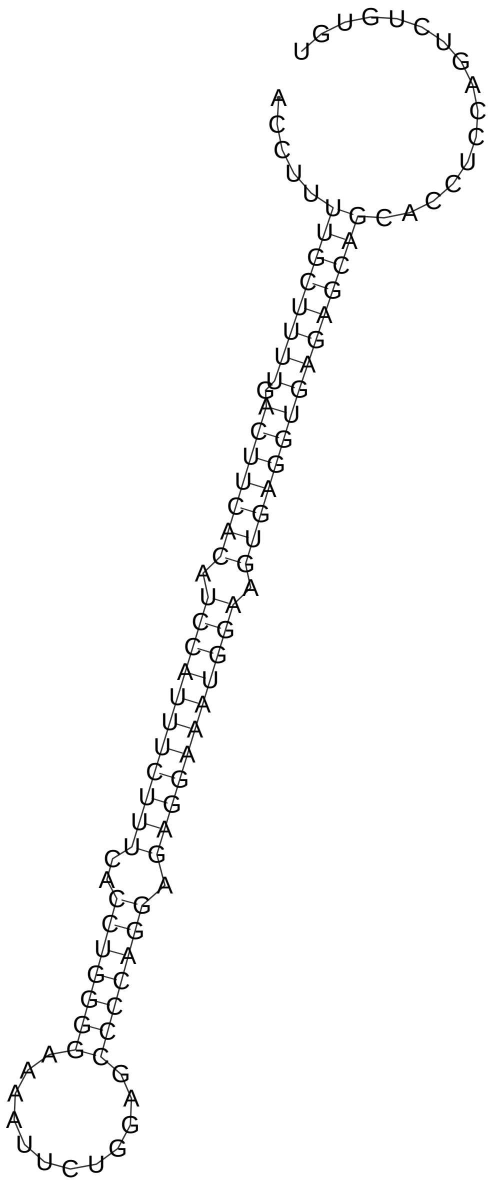

4_50474008-5047411_cfa-mir-8902_high

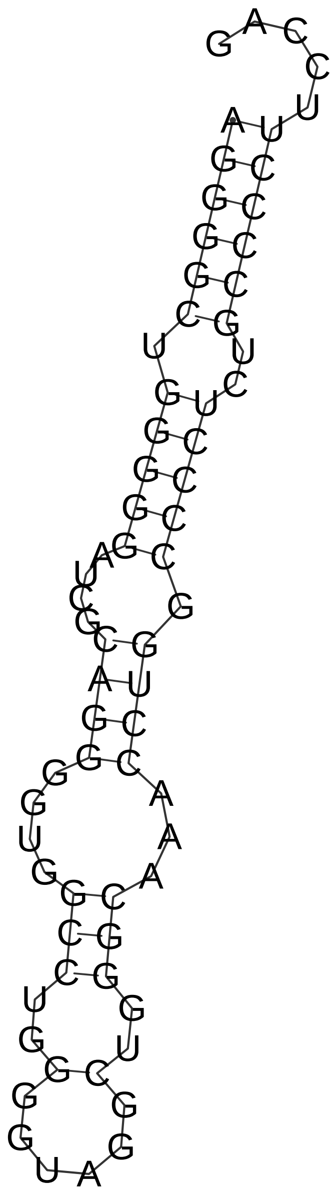

4_58838021-5883808_mir-6982_low

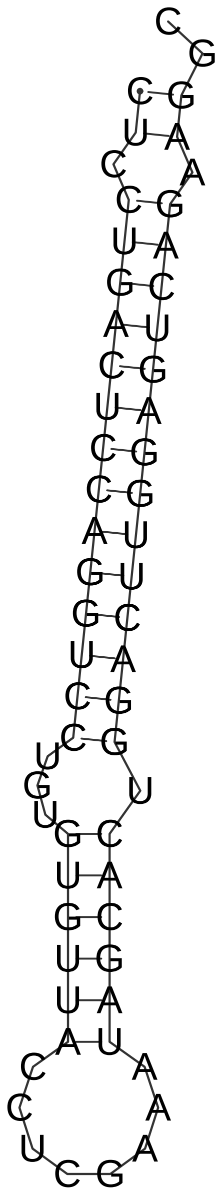

4_59276347-5927640_cfa-mir-378_high

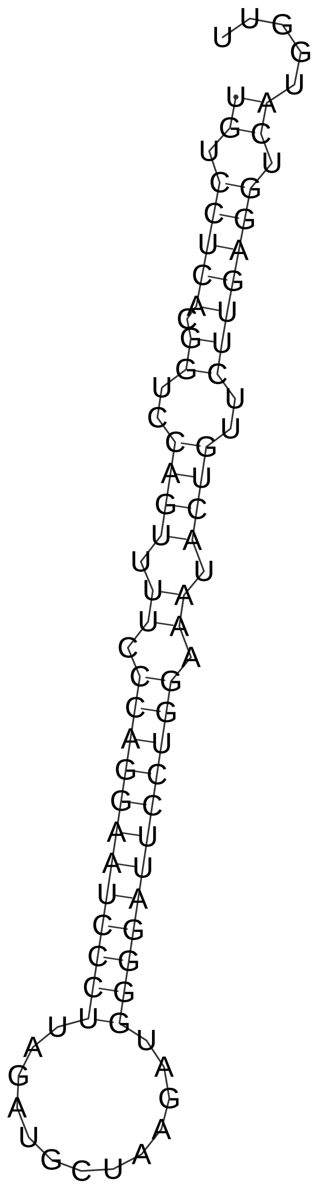

4_59533128-5953321_cfa-mir-145_high

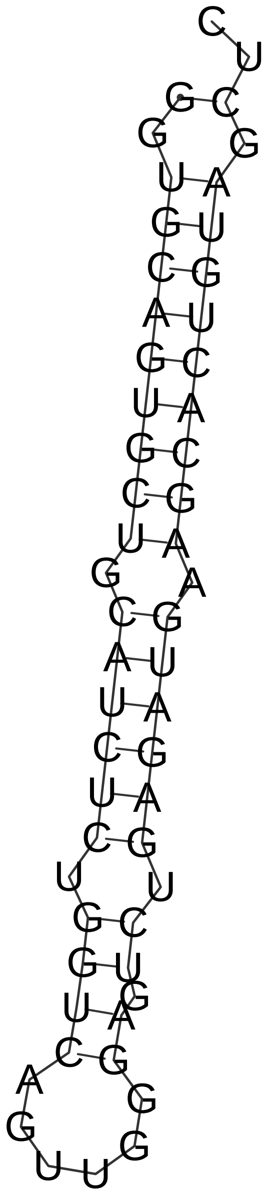

4_59534535-5953458_cfa-mir-143_high

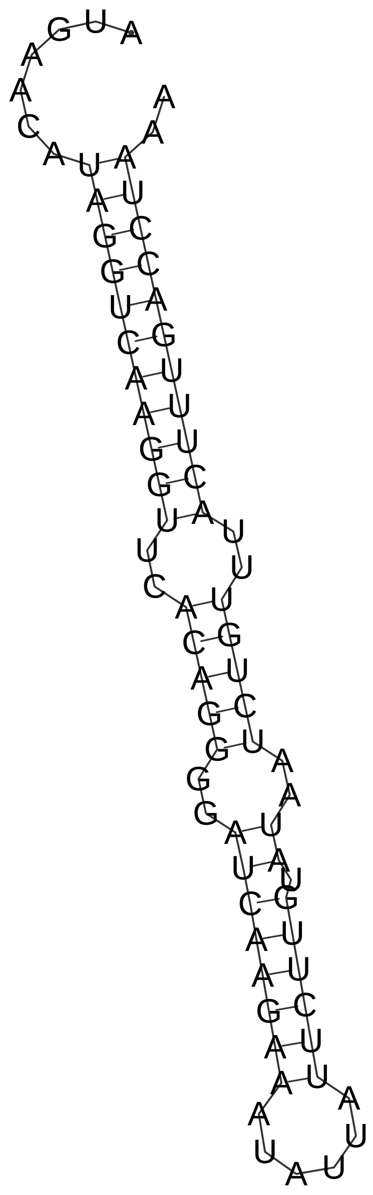

4_75097832-7509790_mir-1898_low

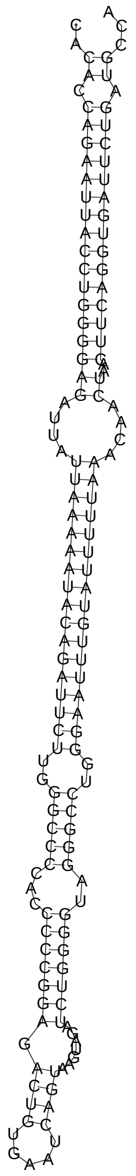

4_75786287-7578642_cfa-mir-8903_high

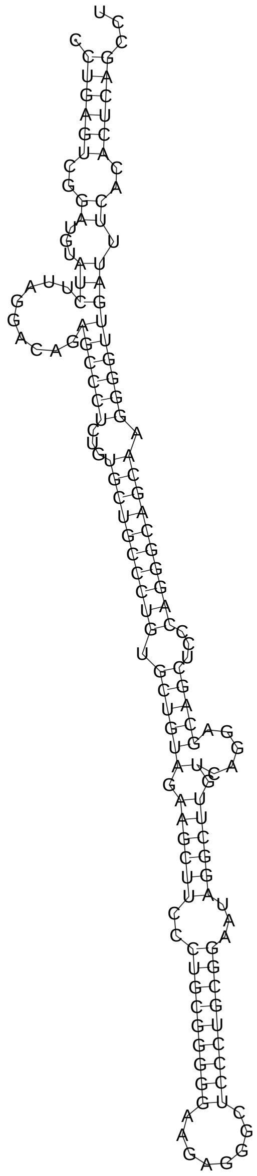

5_1012814-101295_cfa-mir-8868_low

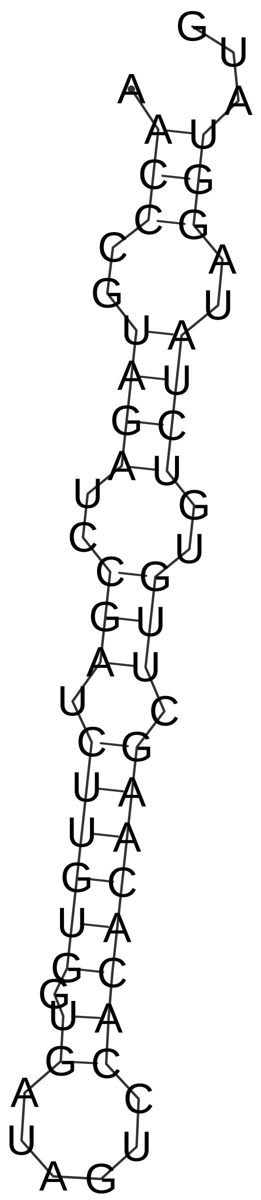

5_12047369-1204742_cfa-mir-99a-2_high

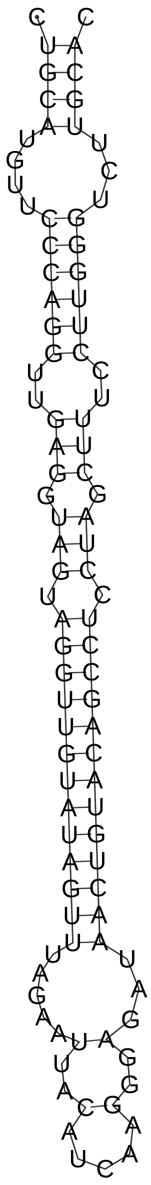

5_12052786-1205288_cfa-let-7a-2_high

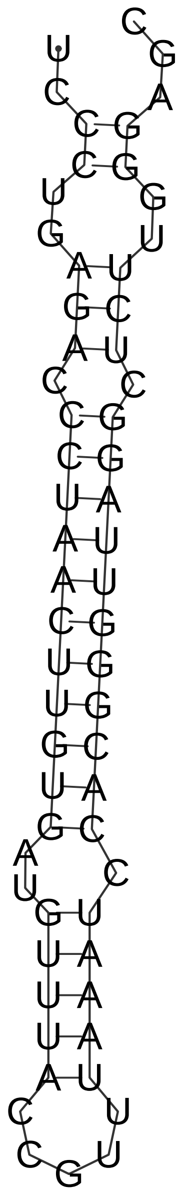

5_12097672-1209773_cfa-mir-125b-1_high

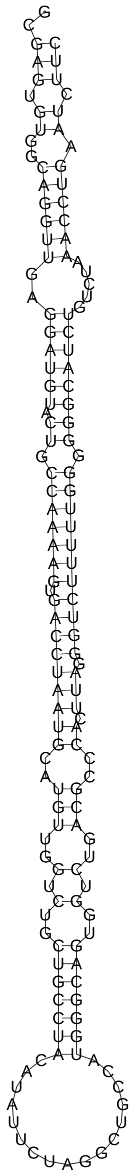

5_16420822-1642096_cfa-mir-8871_low

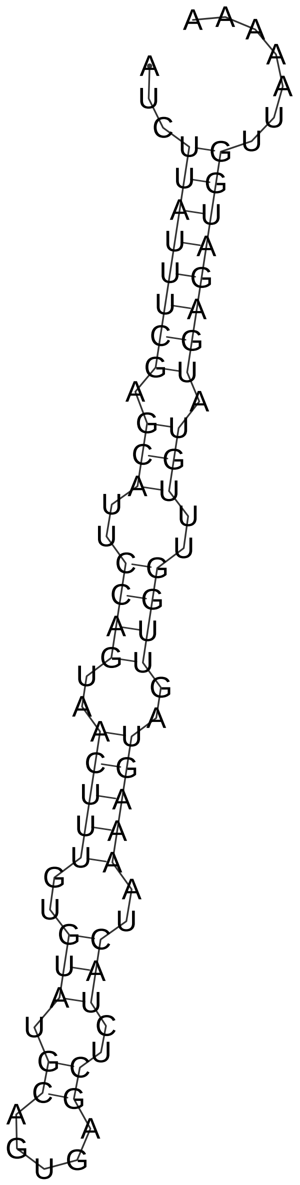

5_18630015-1863009_mir-1244_low

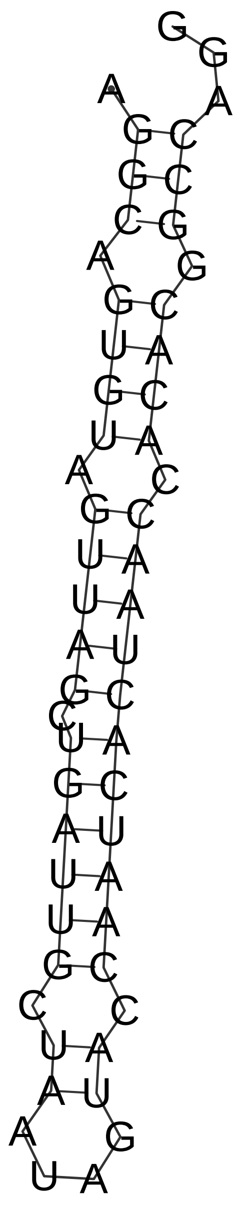

5_21558106-2155816_cfa-mir-34c_high

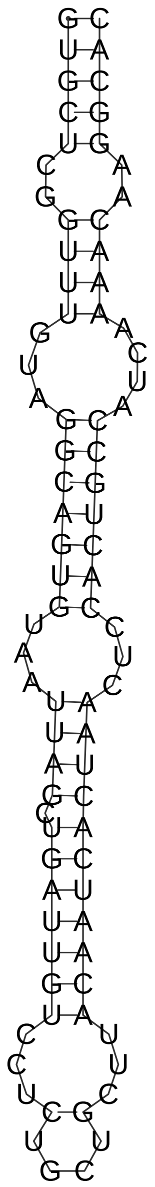

5_21558685-2155876_cfa-mir-34b_high

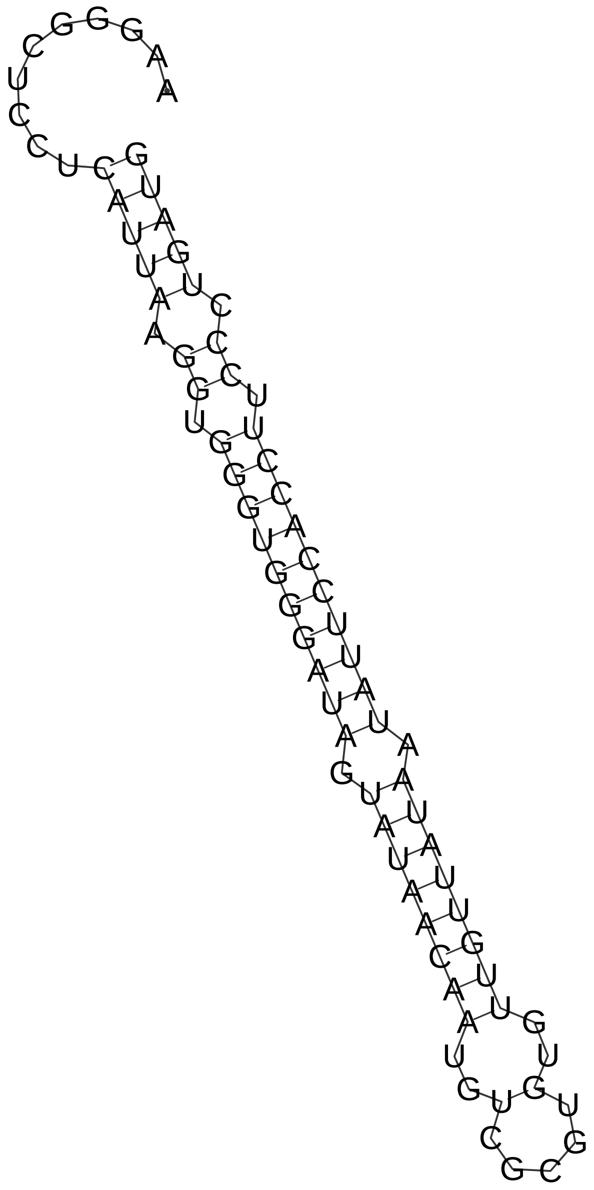

5_26311212-2631128_mir-2985_low

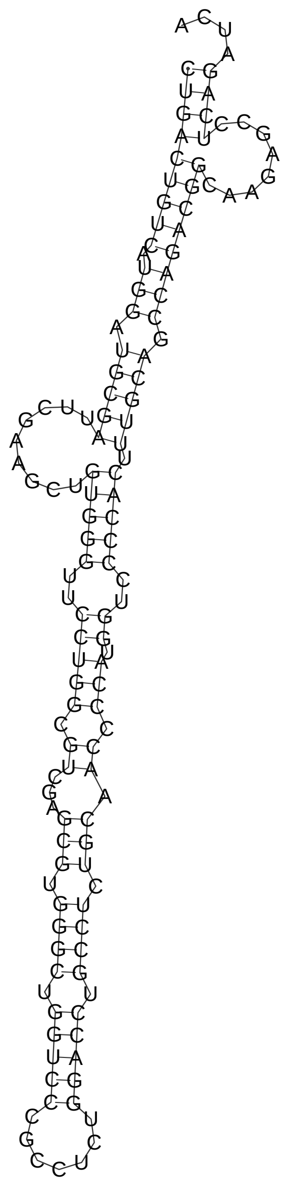

5_31002729-3100285_cfa-mir-8870_low

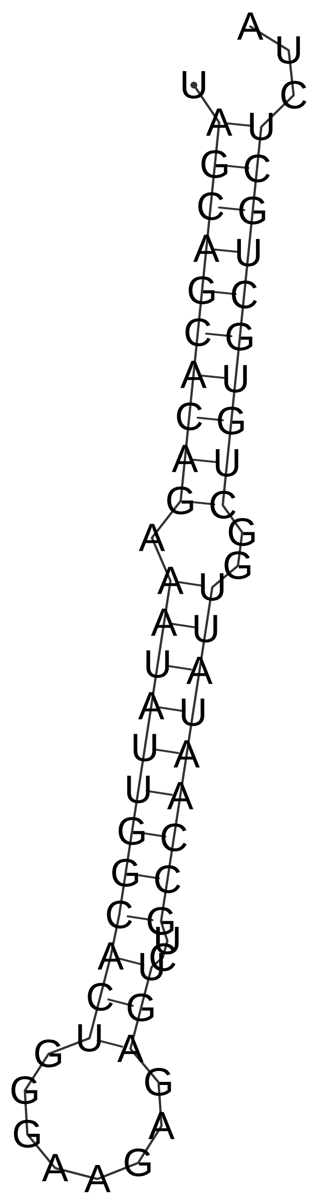

5_32045450-3204551_cfa-mir-195_high

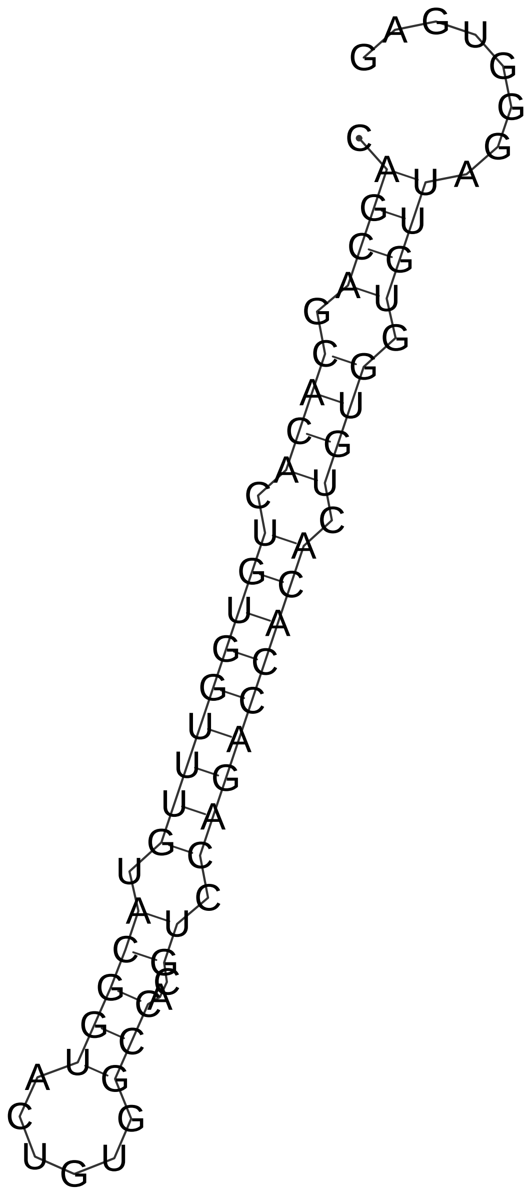

5_32045759-3204582_cfa-mir-497_high

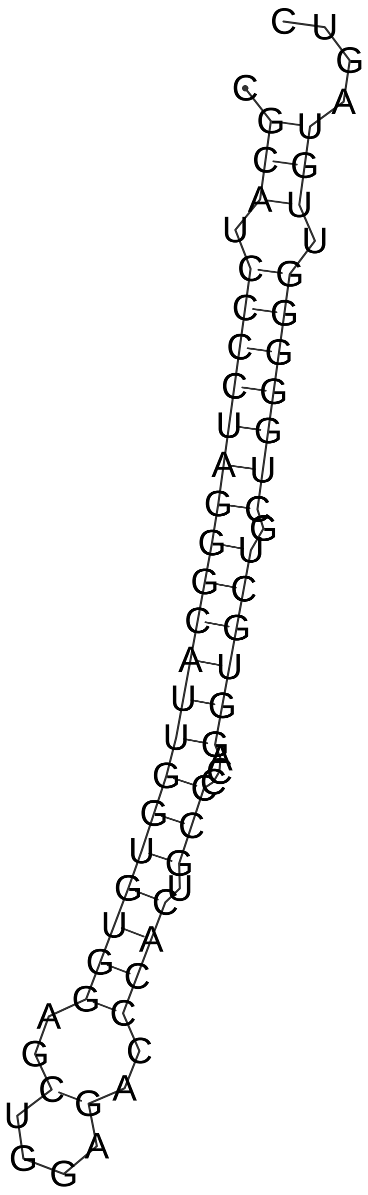

5_32192273-3219234_cfa-mir-324_high

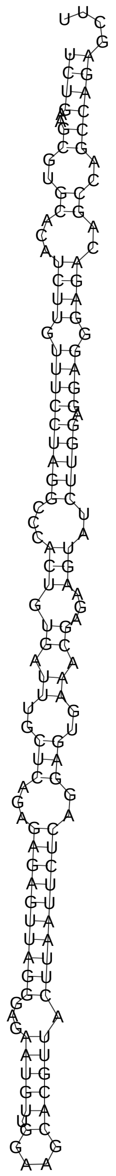

5_32715835-3271597_cfa-mir-8867_low

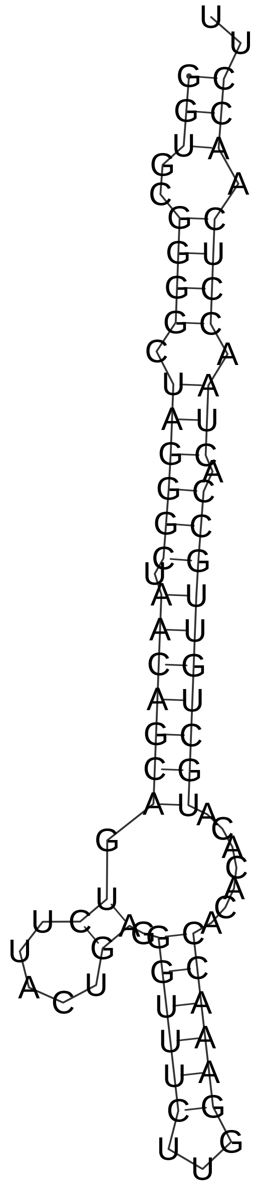

5_36178243-3617832_mir-744_high

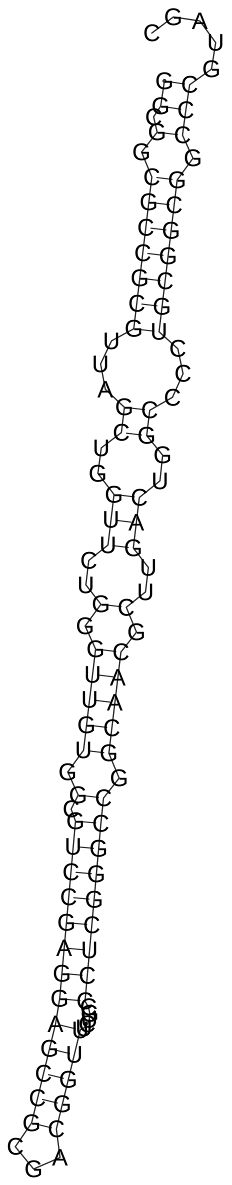

5_41262556-4126266_cfa-mir-8872_low

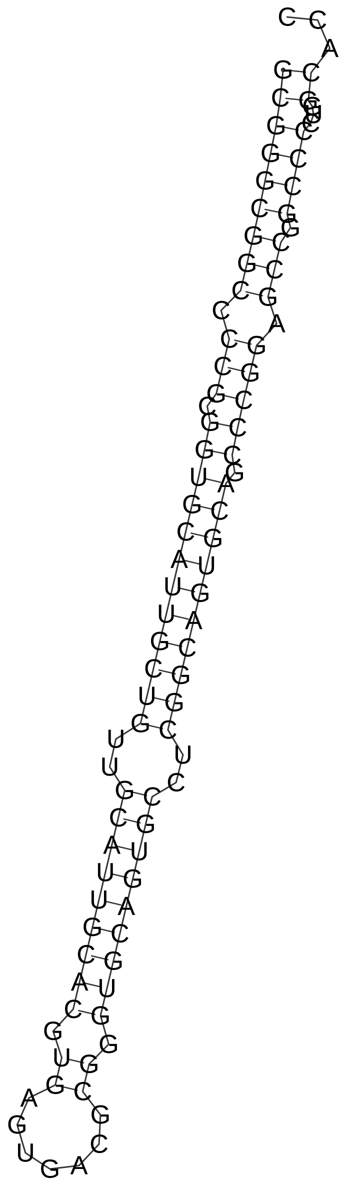

5_41685165-4168525_cfa-mir-33b_high

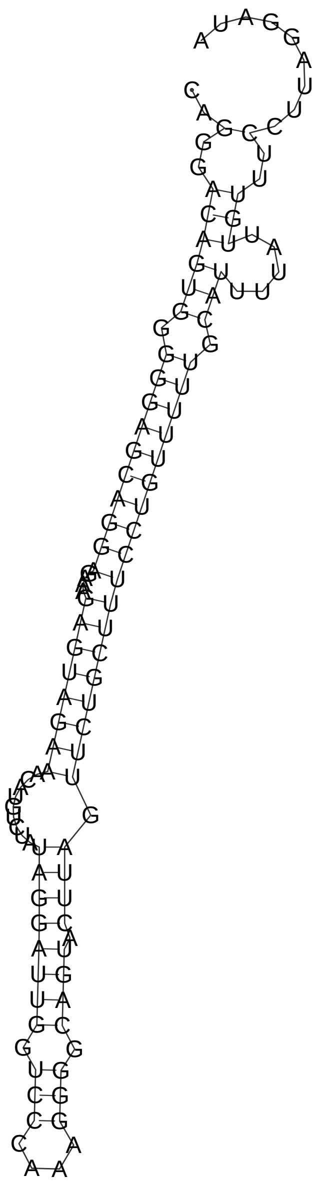

5_42890895-4289100_cfa-mir-8869_low

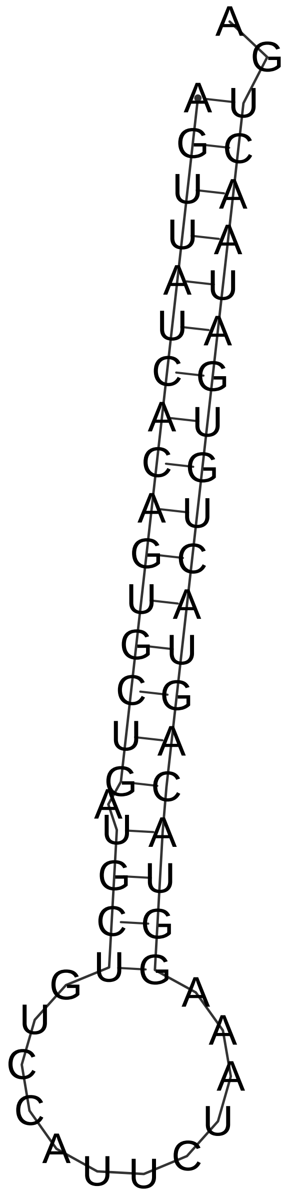

5_45184774-4518482_cfa-mir-101-1_high

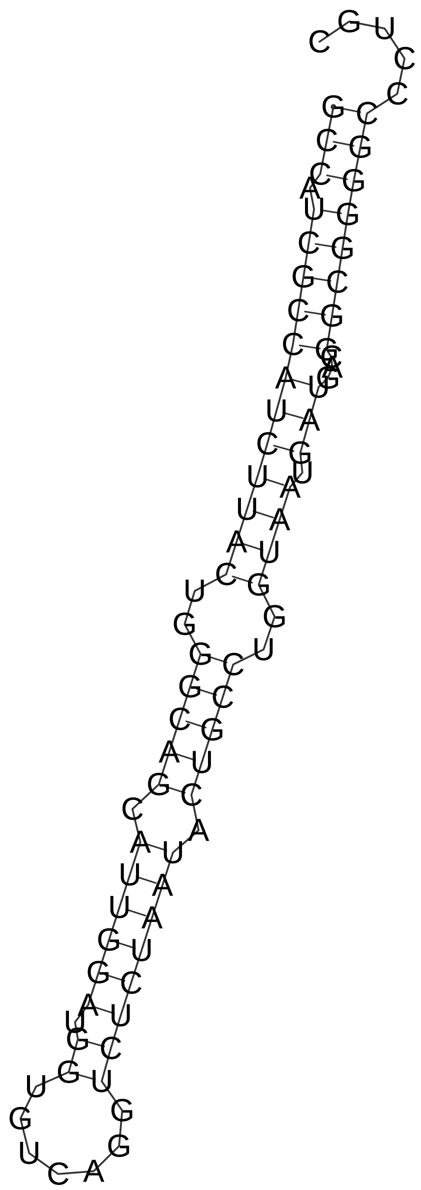

5_56368400-5636847_cfa-mir-200b_high

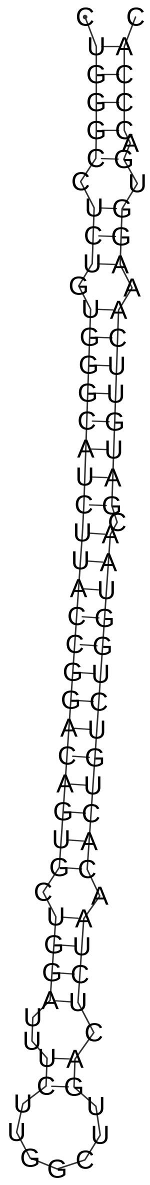

5_56369063-5636915_cfa-mir-200a_high

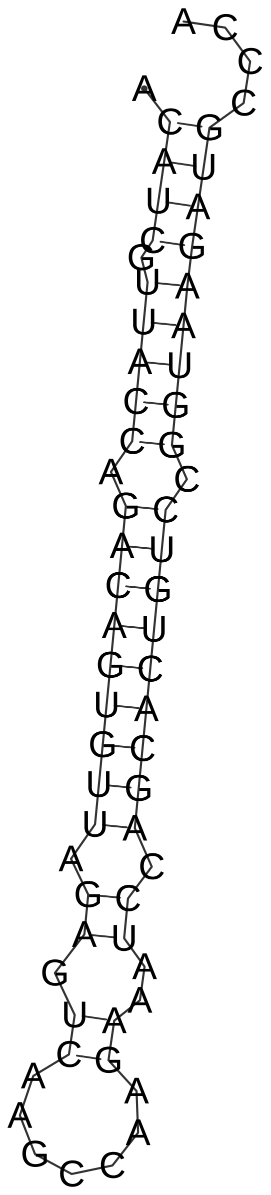

5_56369074-5636913_mir-3548_high

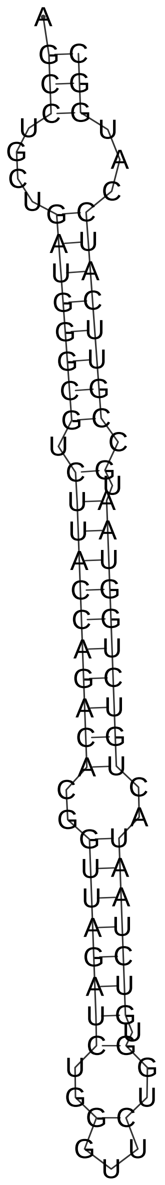

5_56370509-5637059_cfa-mir-429_high

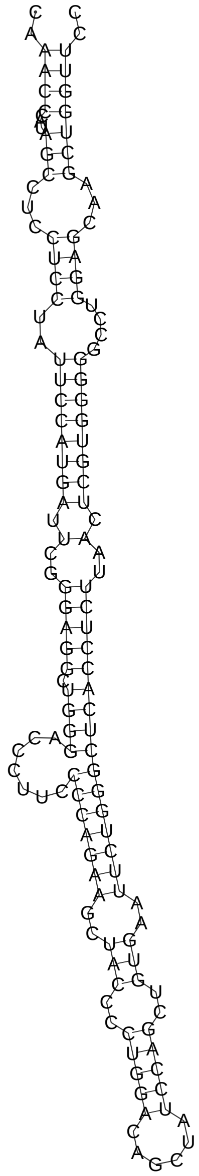

5_57395454-5739558_cfa-mir-8866_low

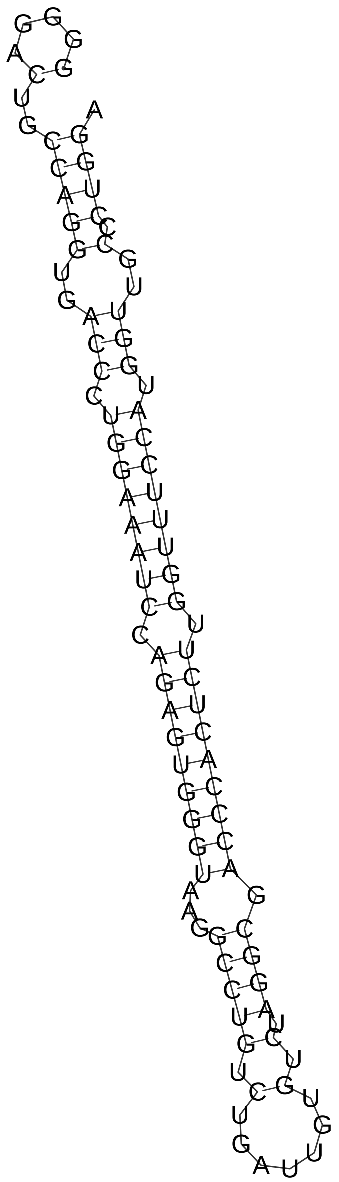

5_58112708-5811280_cfa-mir-551a_high

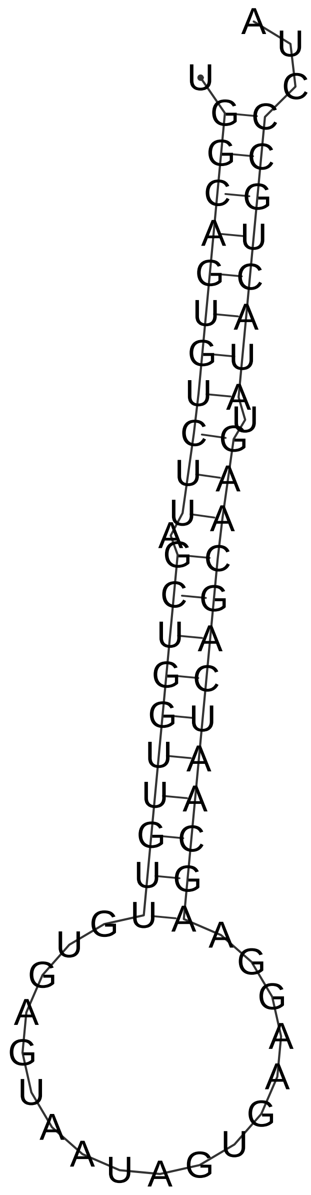

5_62485832-6248589_cfa-mir-34a_high

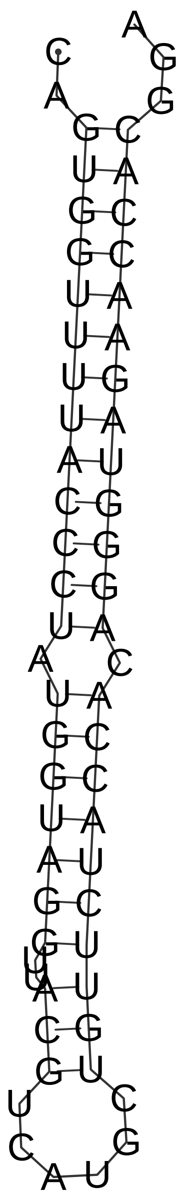

5_79814256-7981431_cfa-mir-140_high

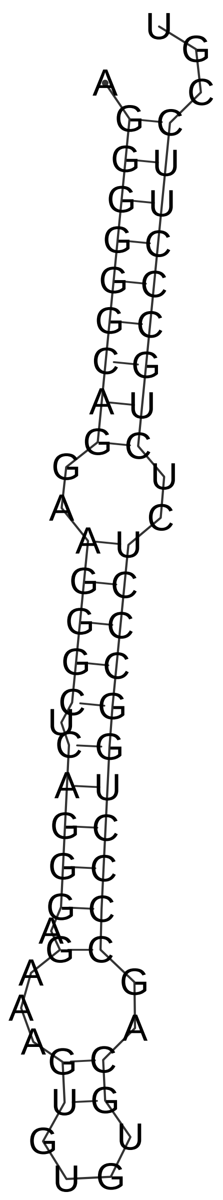

5_82166208-8216627_cfa-mir-328_high

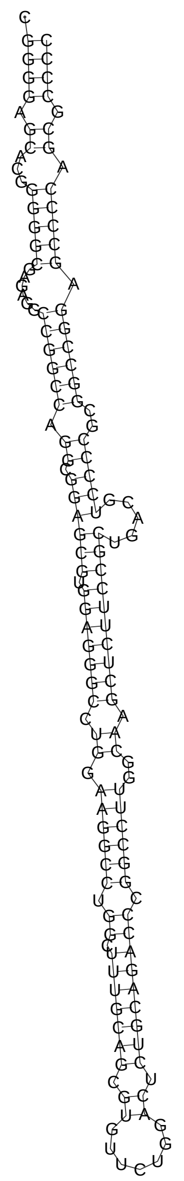

6_5910293-591043_cfa-mir-2387_high

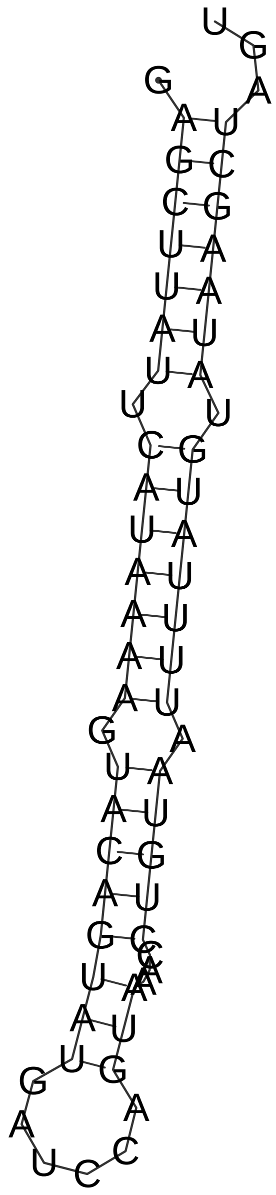

6_6186538-618659_cfa-mir-590_high

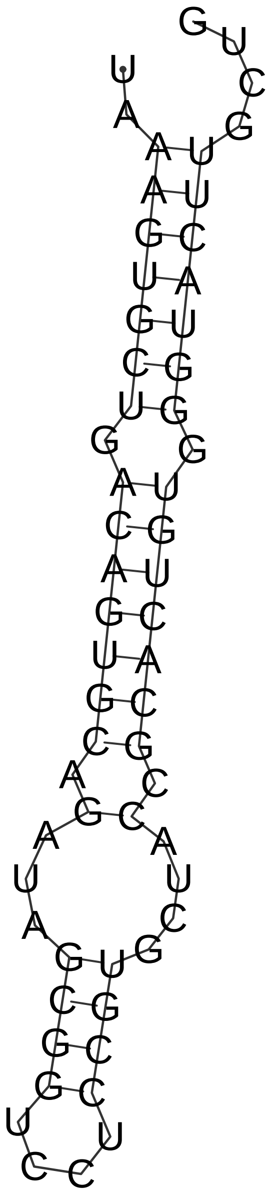

6_9498558-949861_cfa-mir-106b_high

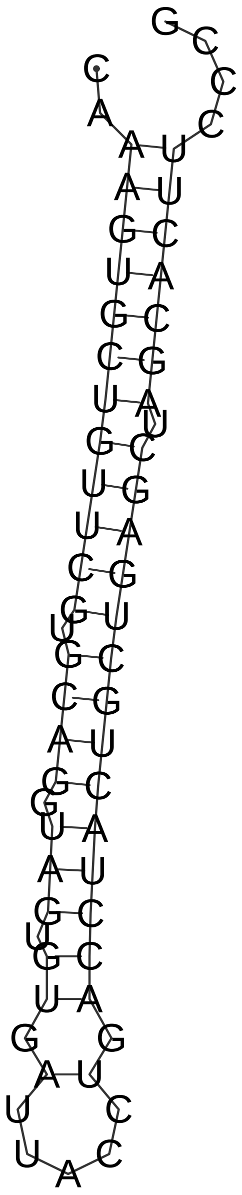

6_9498780-949884_cfa-mir-93_high

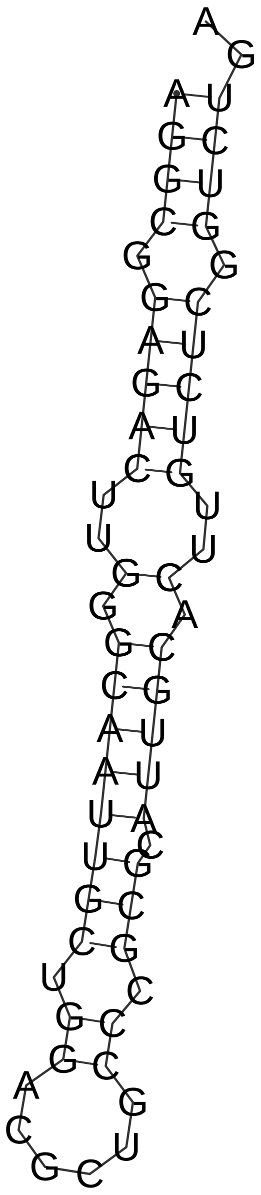

6_9498993-949905_cfa-mir-25_high

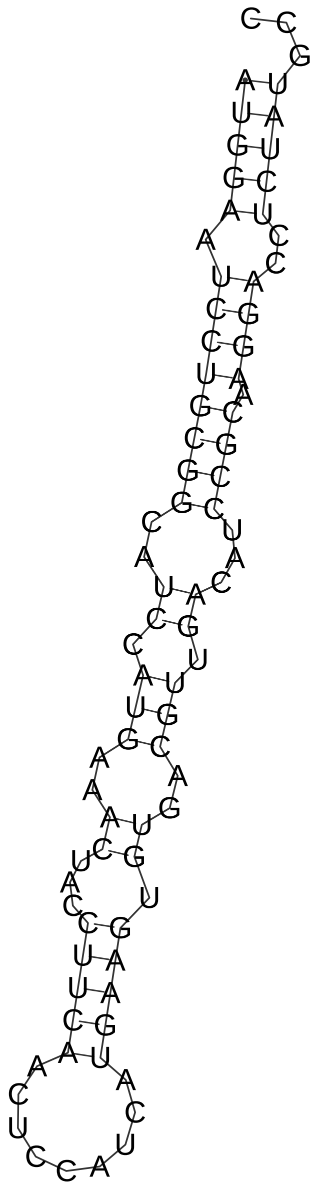

6_12423353-1242343_mir-3533_low

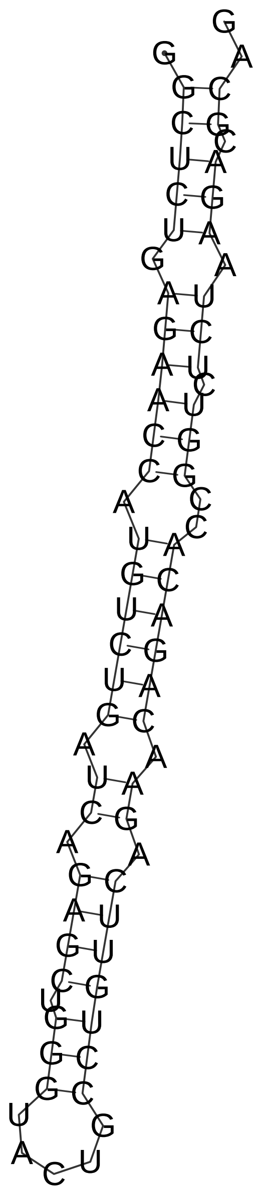

6_12453870-1245394_cfa-mir-589_low

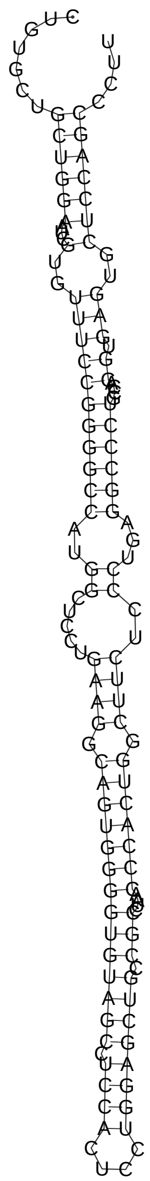

6_13982905-1398303_cfa-mir-8873a_low

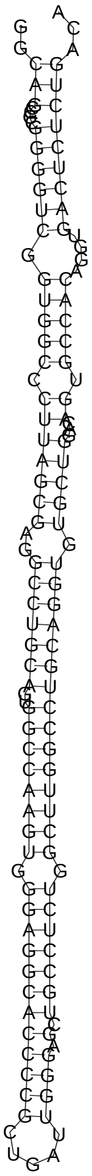

6_14518647-1451877_cfa-mir-8876_high

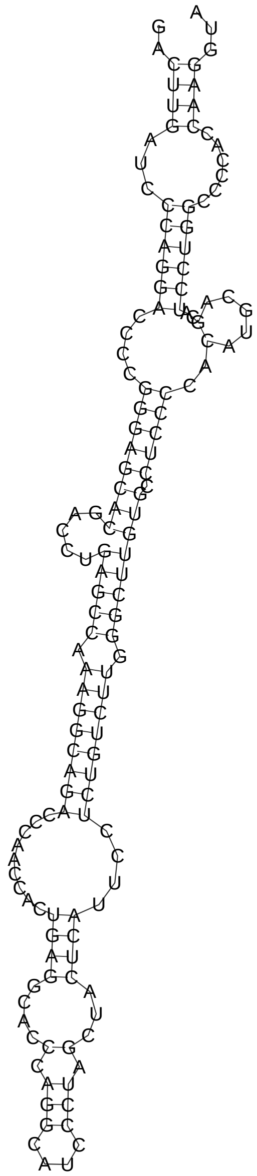

6_15817410-1581755_cfa-mir-8879_low

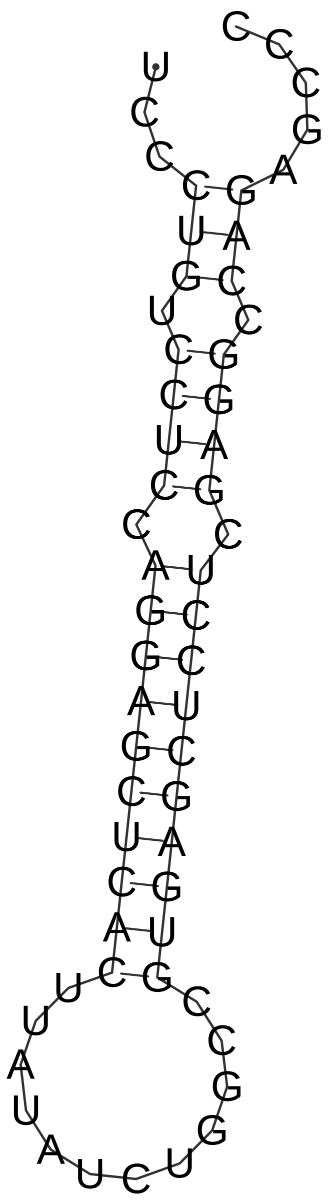

6_15853520-1585357_cfa-mir-339-1_high

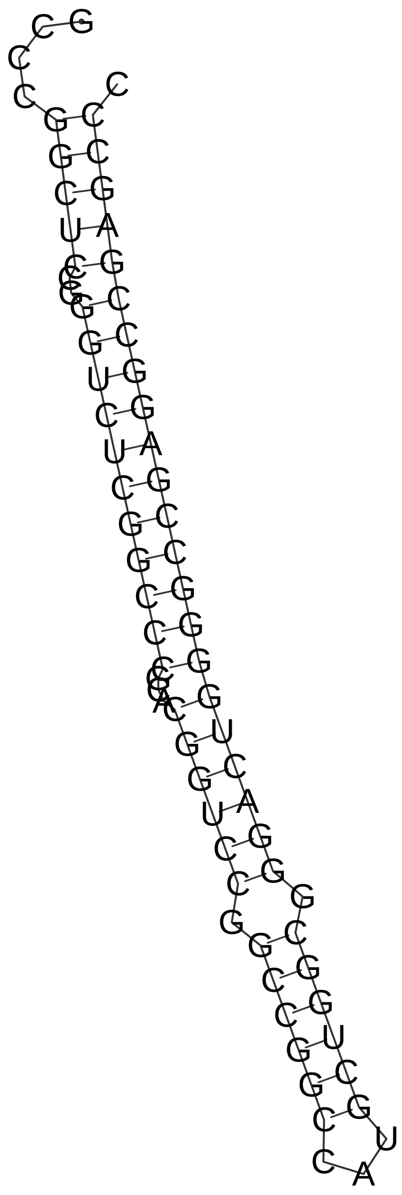

6_17305798-1730586_mir-762_low

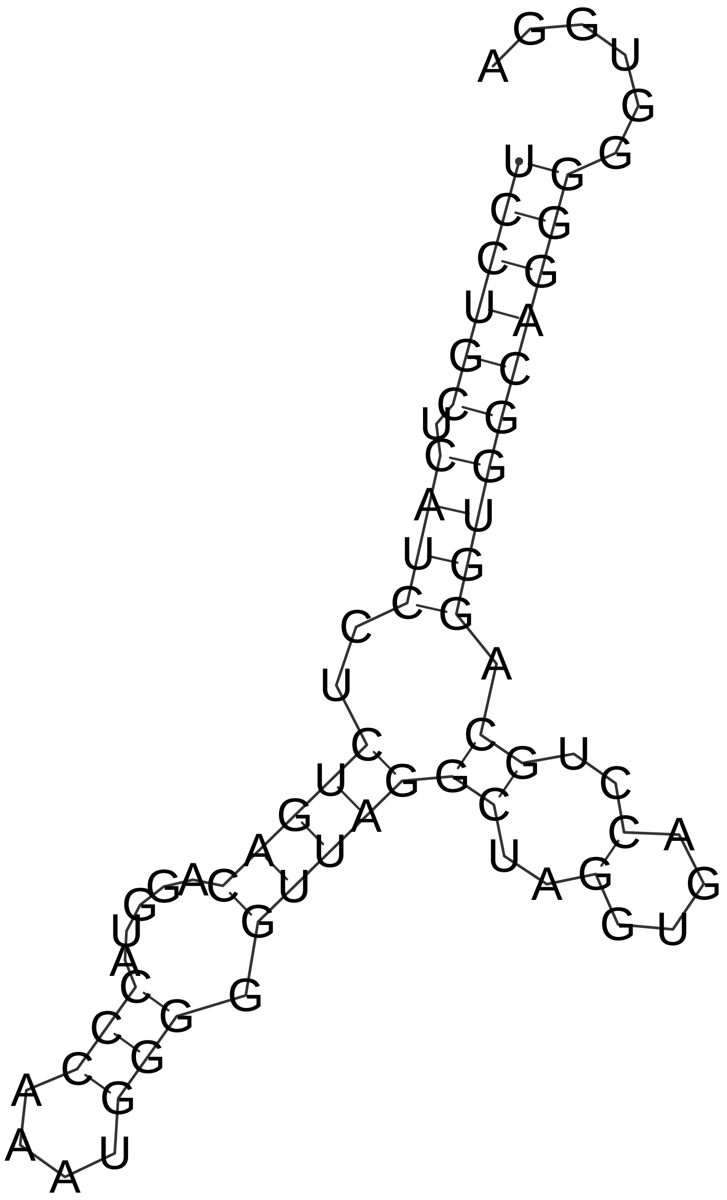

6_18455993-1845606_mir-9130_low

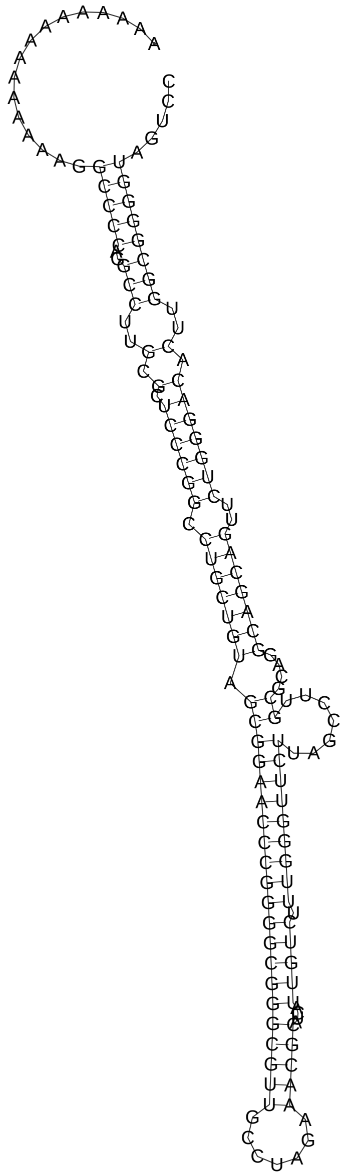

6_21081056-2108120_cfa-mir-8875_low

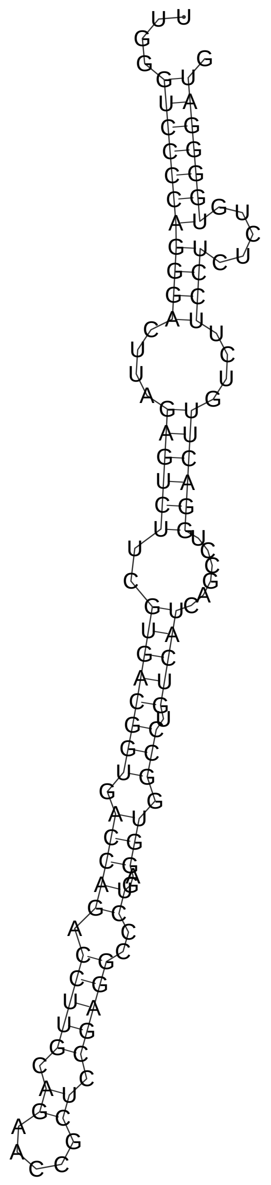

6_21119782-2111989_cfa-mir-8878_low

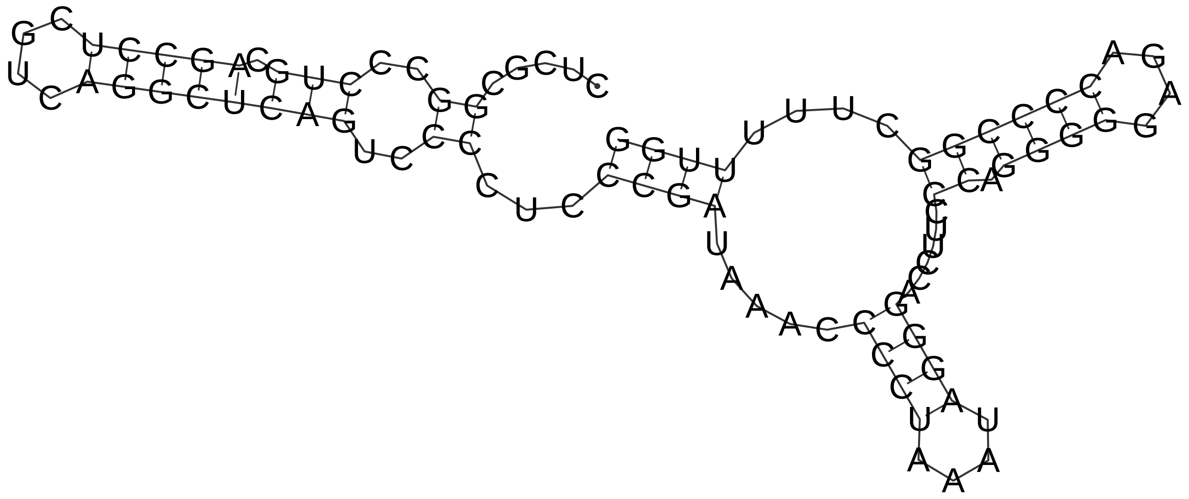

6_28190589-2819067_mir-484_high

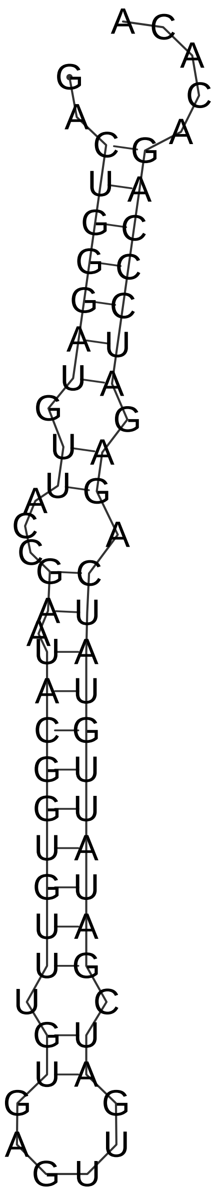

6_28215949-2821601_mir-6506_low

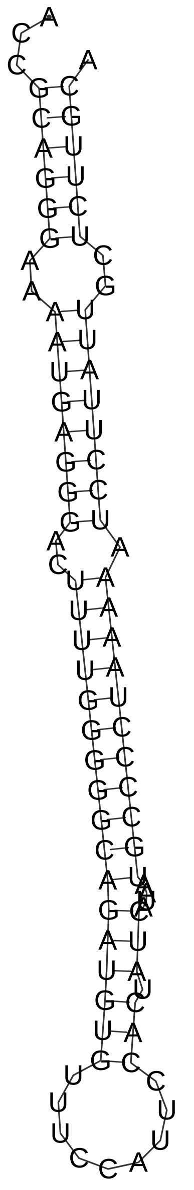

6_28873135-2887322_cfa-mir-365-1_high

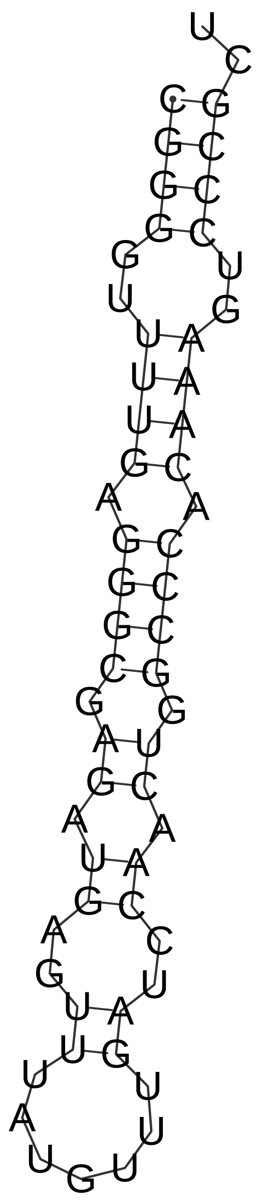

6_28878050-2887810_cfa-mir-193b_high

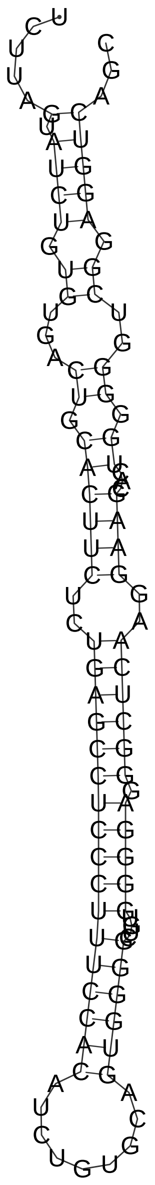

6_31385077-3138517_cfa-mir-8874_low

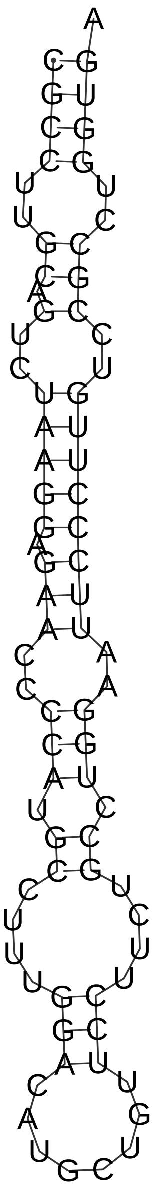

6_38309139-3830921_mir-5125_low

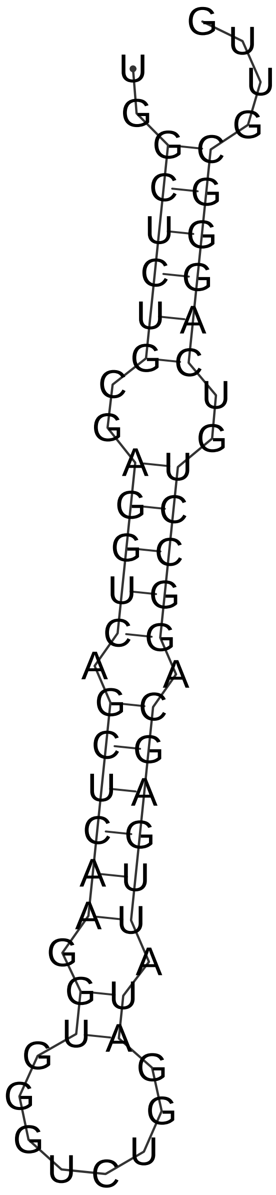

6_38759035-3875909_cfa-mir-1842_high

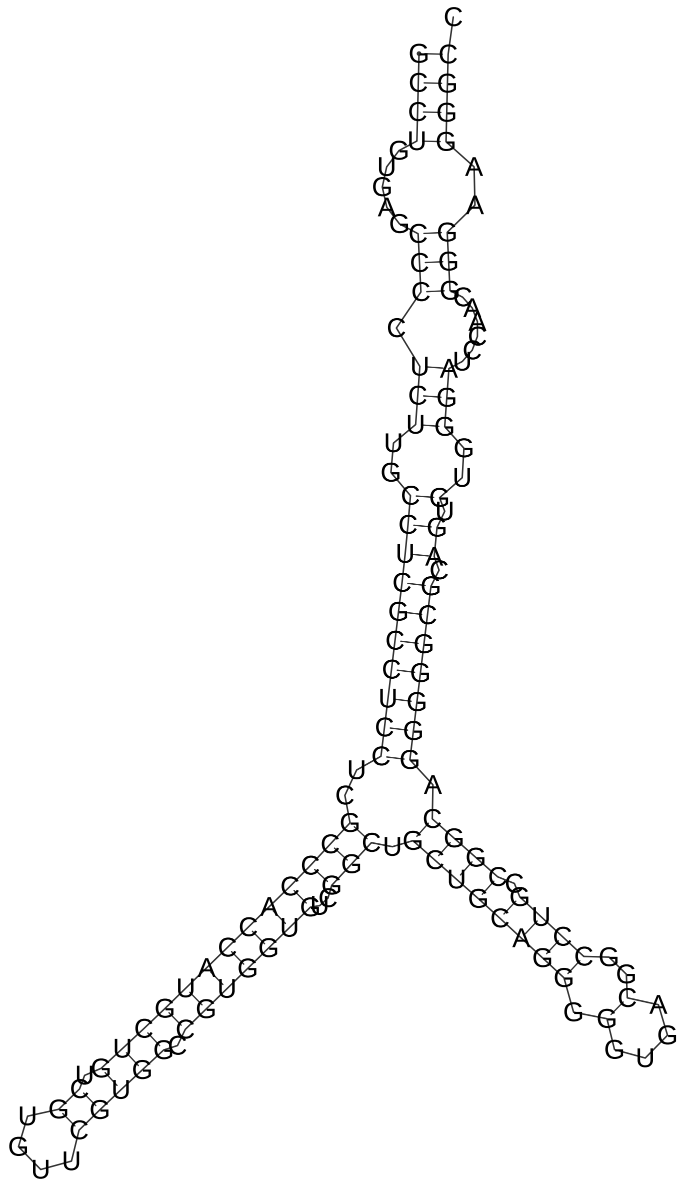

6_39752034-3975216_cfa-mir-8877_low

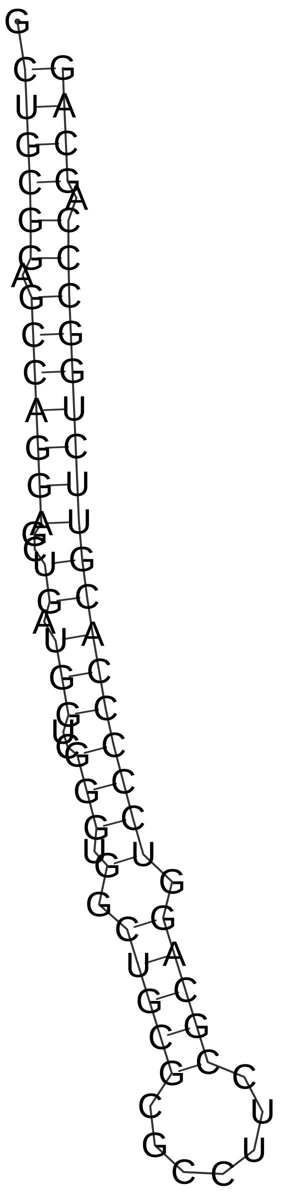

6_39826605-3982667_mir-662_low

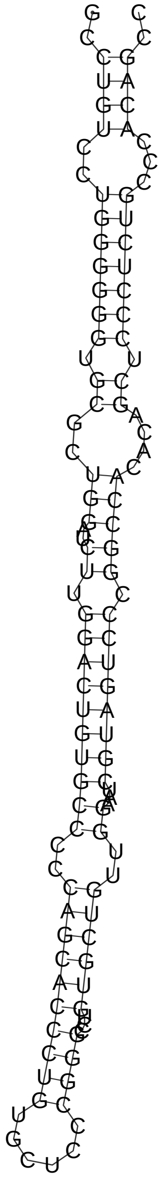

6_40559217-4055932_cfa-mir-8799f_low

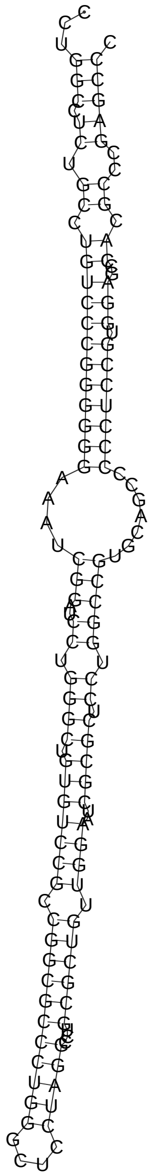

6_41017022-4101715_cfa-mir-8799e-1_low

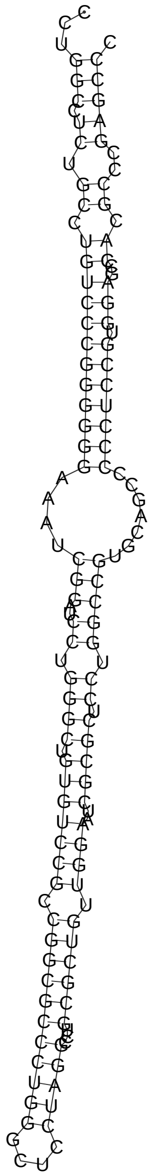

6_41185994-4118612_cfa-mir-8799e-2_low

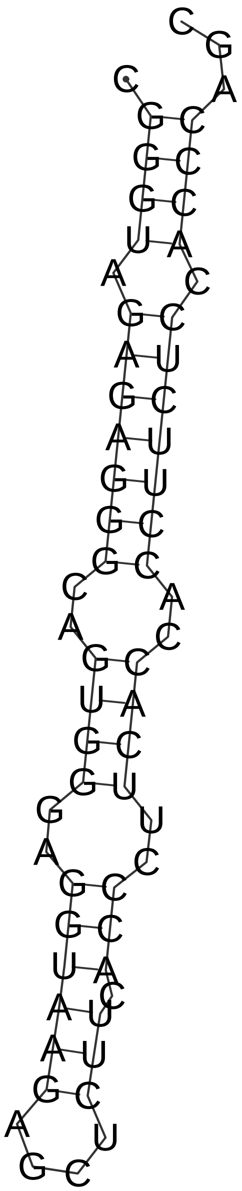

6_42271719-4227177_cfa-mir-197_high

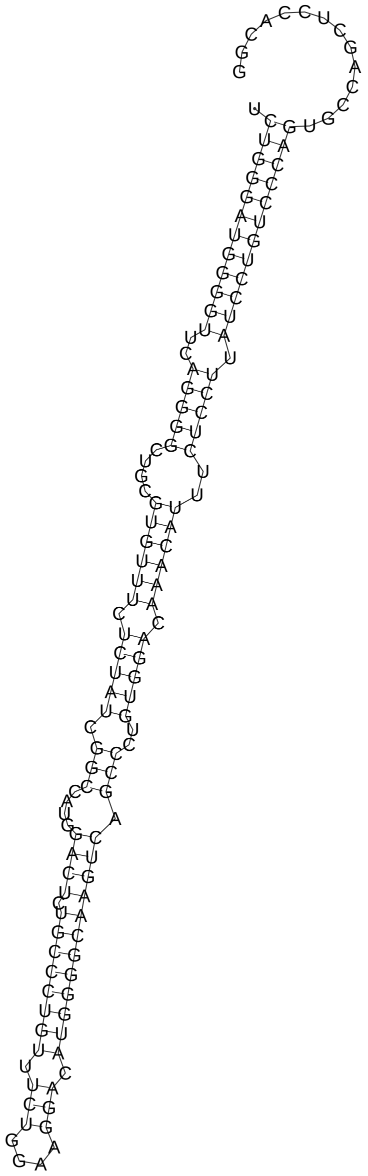

6_43075720-4307585_cfa-mir-8880_low

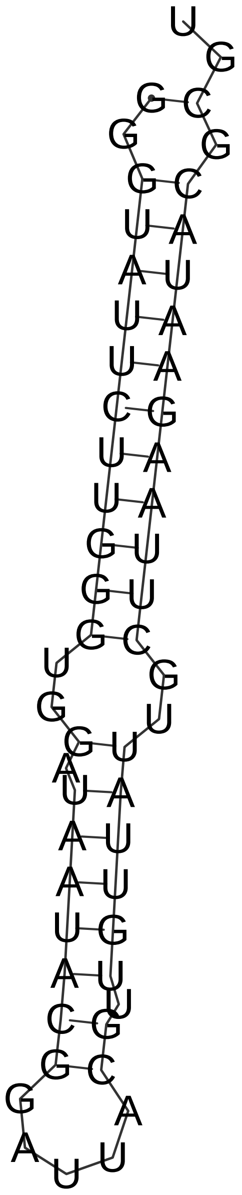

6_51640476-5164053_cfa-mir-137_high

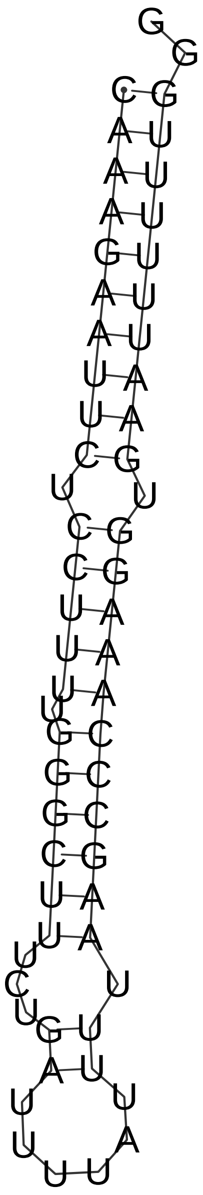

6_74881615-7488167_cfa-mir-186_high

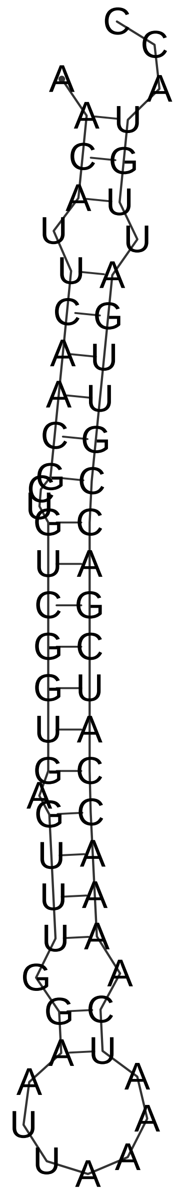

7_4055465-405552_cfa-mir-181a-1_high

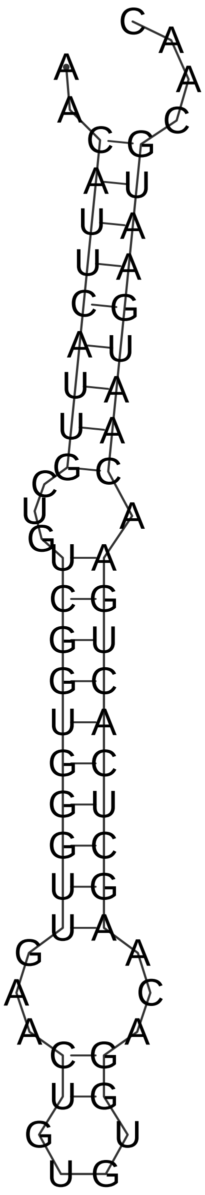

7_4055651-405571_cfa-mir-181b-1_high

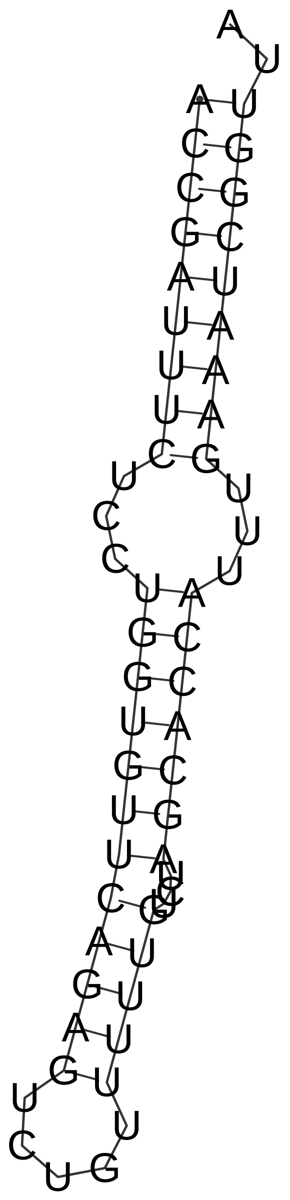

7_6588010-658806_cfa-mir-29c-1_high

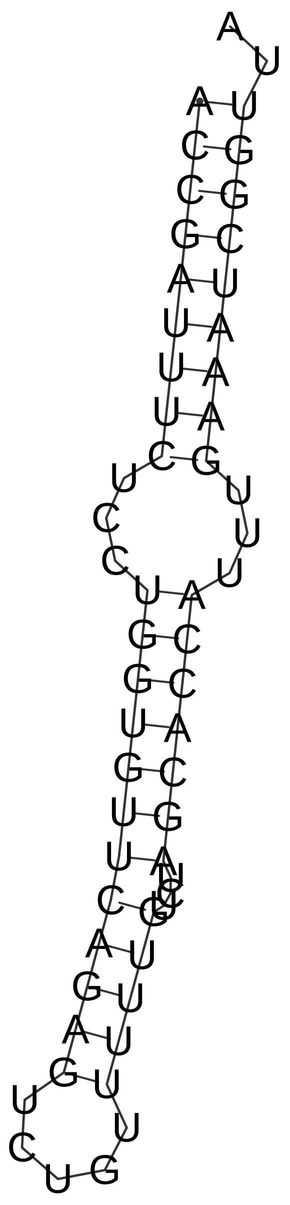

7_6651339-665139_cfa-mir-29c-2_high

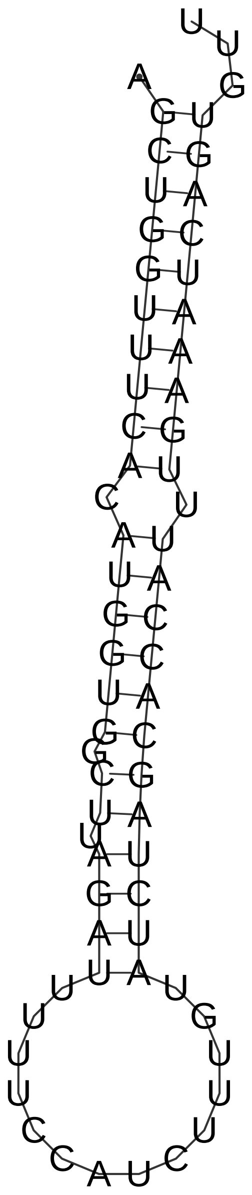

7_6651878-665194_cfa-mir-29b-2_high

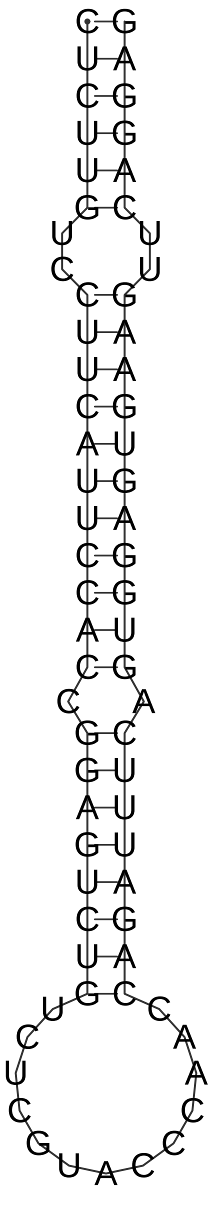

7_8090511-809057_cfa-mir-205_high

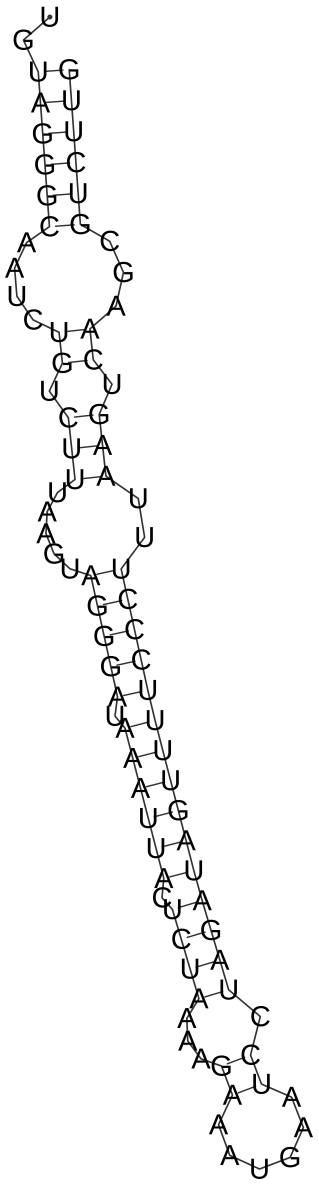

7_13400475-1340056_mir-684_low

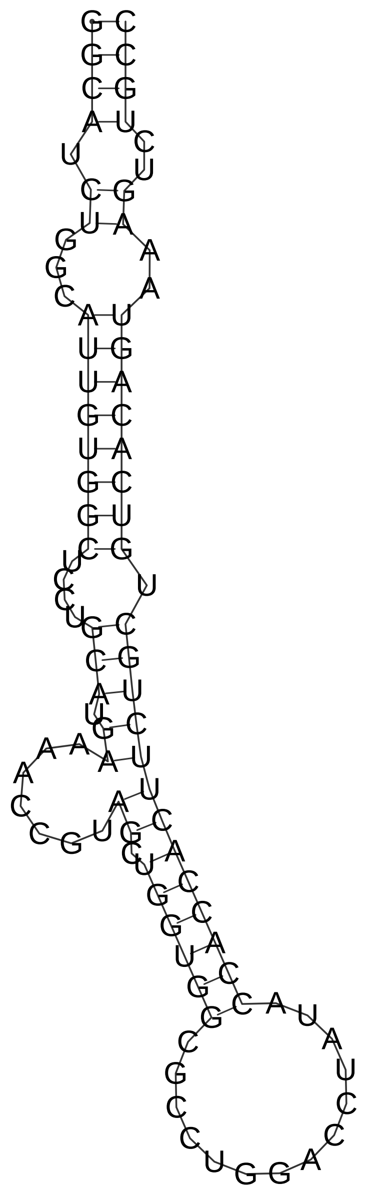

7_14180244-1418033_mir-682_low

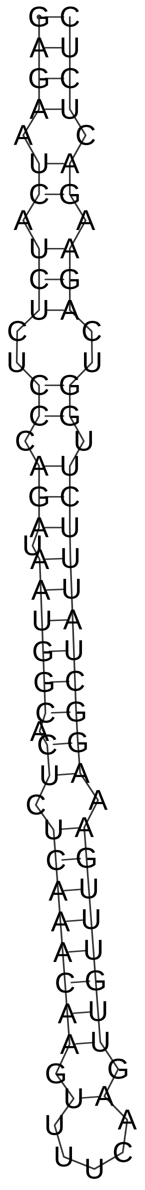

7_22597953-2259803_cfa-mir-488_high

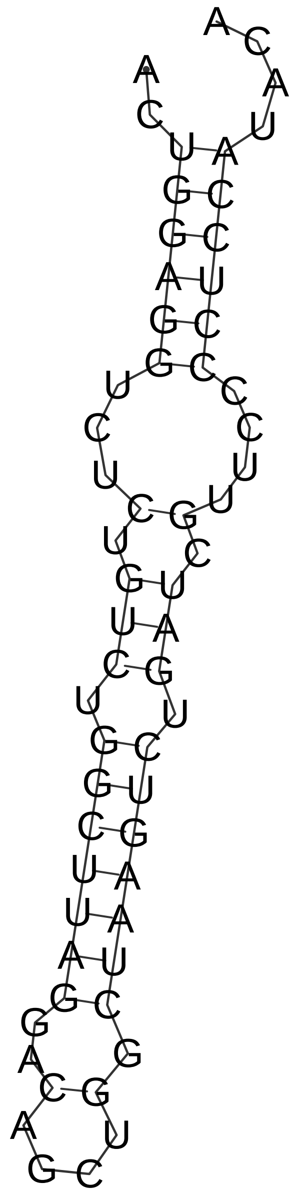

7_23539367-2353942_cfa-mir-1843_high

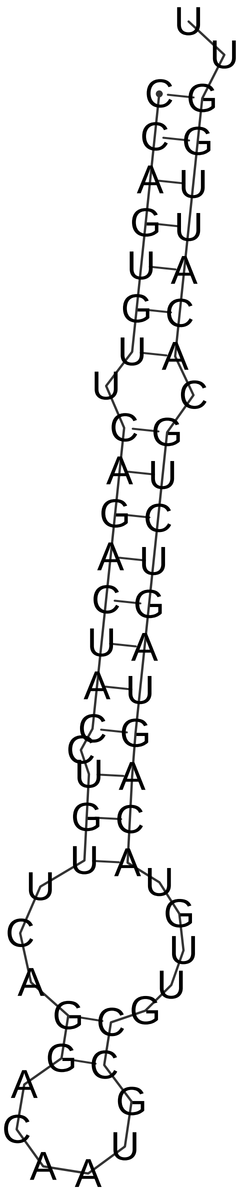

7_26812766-2681282_cfa-mir-199-2_high

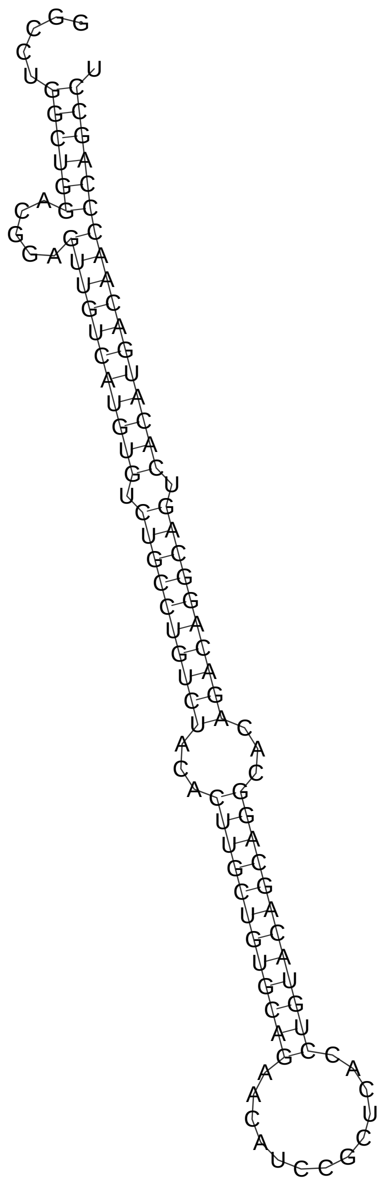

7_26818559-2681866_cfa-mir-214_high

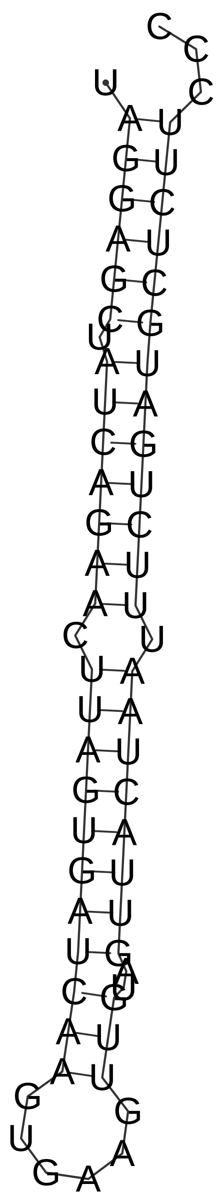

7_27375123-2737518_mir-7178_high

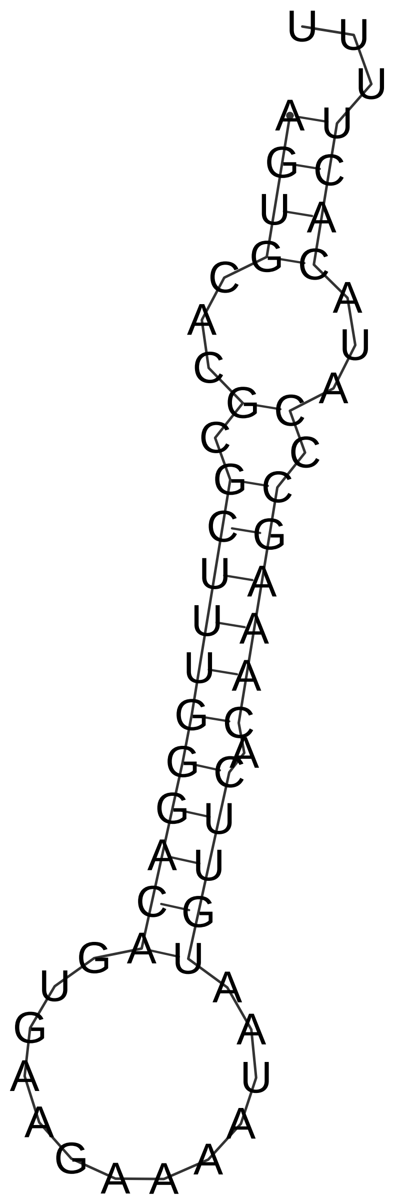

7_34314838-3431489_cfa-mir-350_high

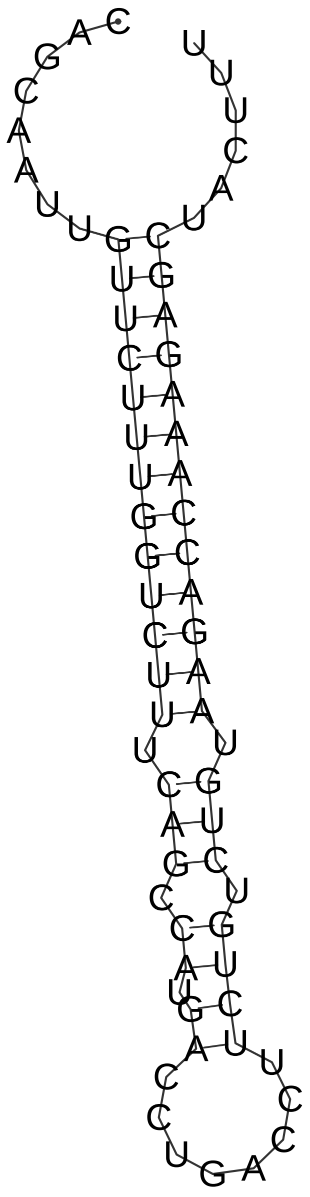

7_34480300-3448036_mir-4677_high

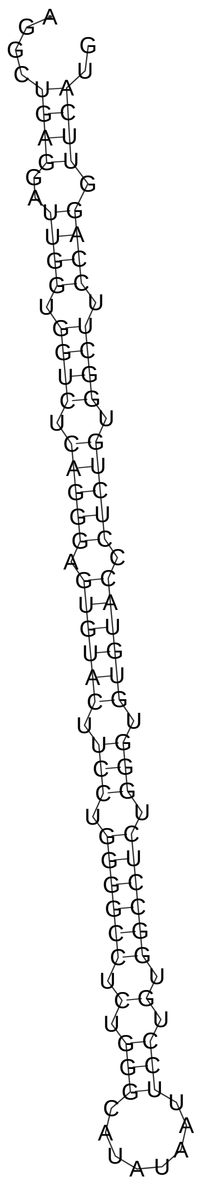

7_35996308-3599641_cfa-mir-7180_high

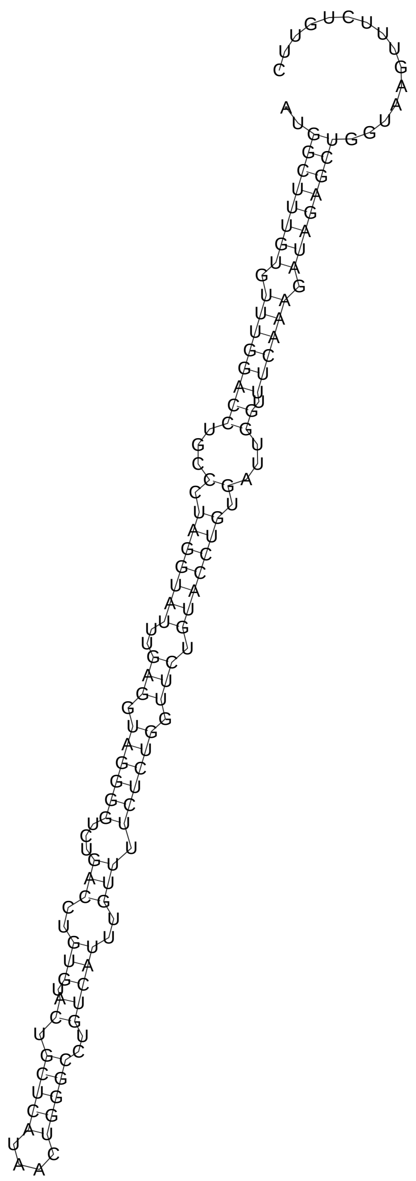

7_37488050-3748819_cfa-mir-8881_low

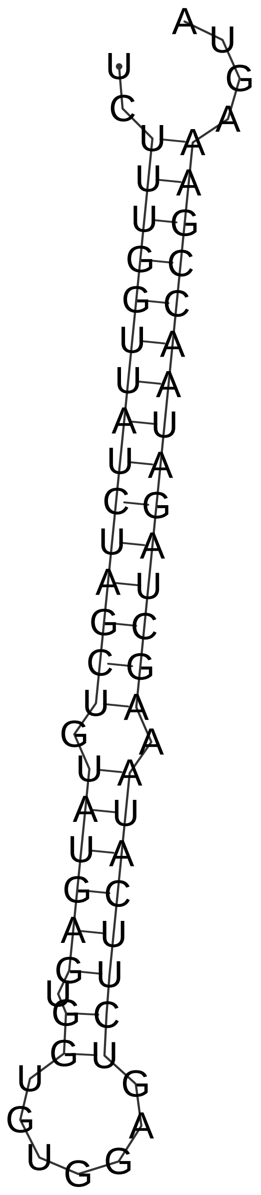

7_41509404-4150946_cfa-mir-9-1_high

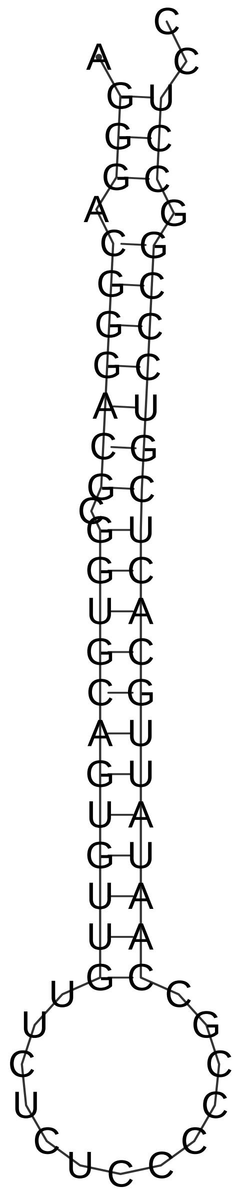

7_42338480-4233854_cfa-mir-92b_high

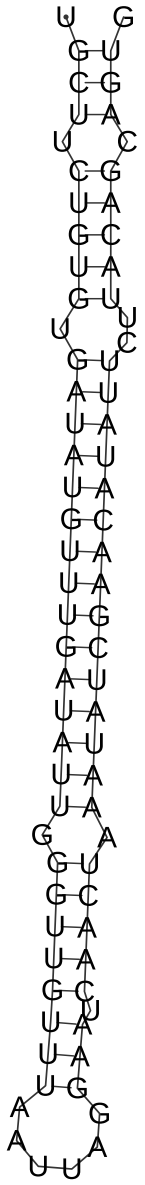

7_43037530-4303760_cfa-mir-190b_high

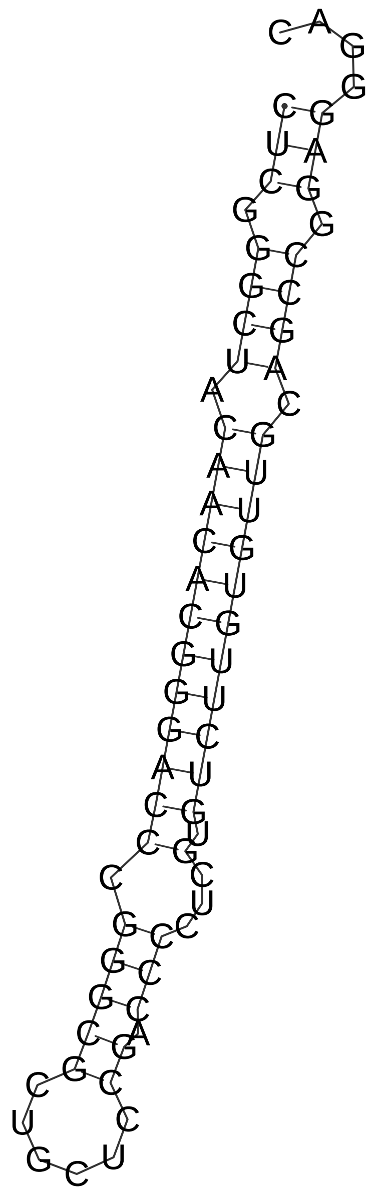

7_54261943-5426201_cfa-mir-187_high

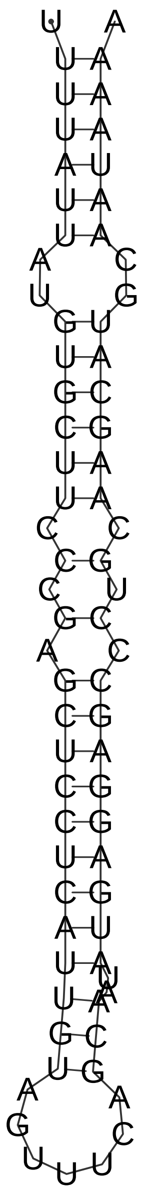

7_63206303-6320637_mir-1597_low

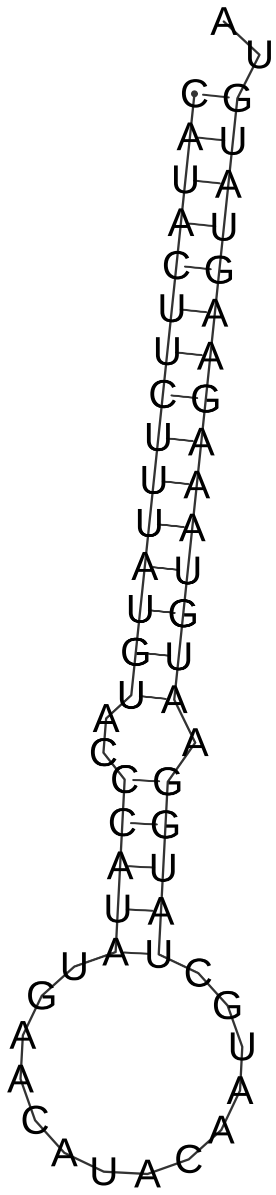

7_66214380-6621443_cfa-mir-1-2_high

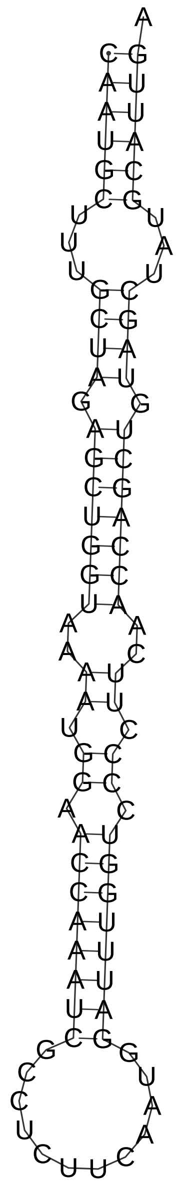

7_66217486-6621757_cfa-mir-133a_high

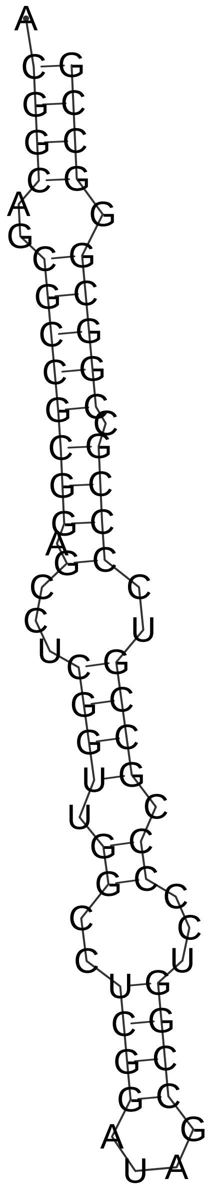

7_80965885-8096595_mir-2904-1_low

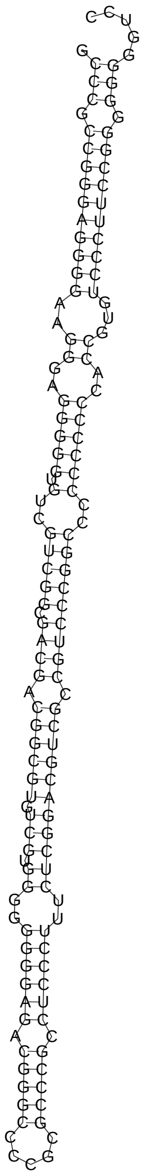

7_80973870-8097401_cfa-mir-8865_low

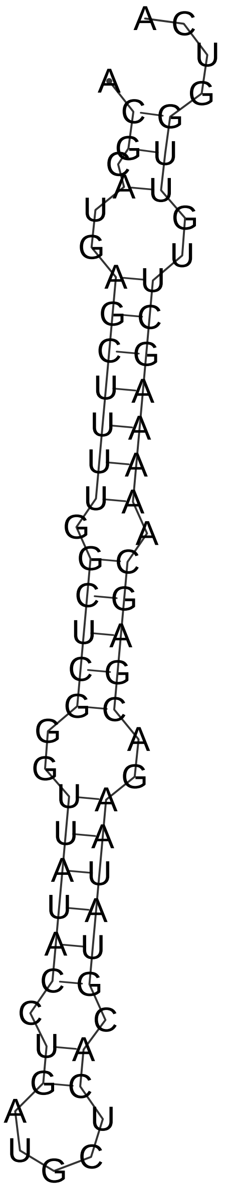

8_3636919-363698_cfa-mir-208a_high

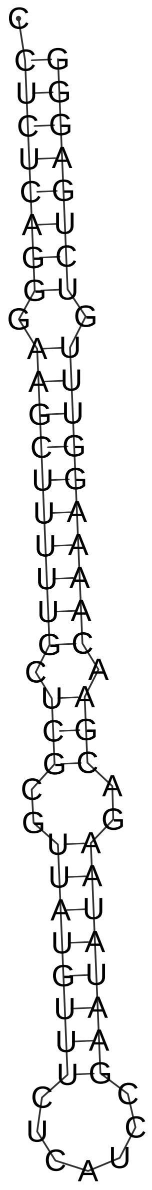

8_3665163-366523_cfa-mir-208b_high

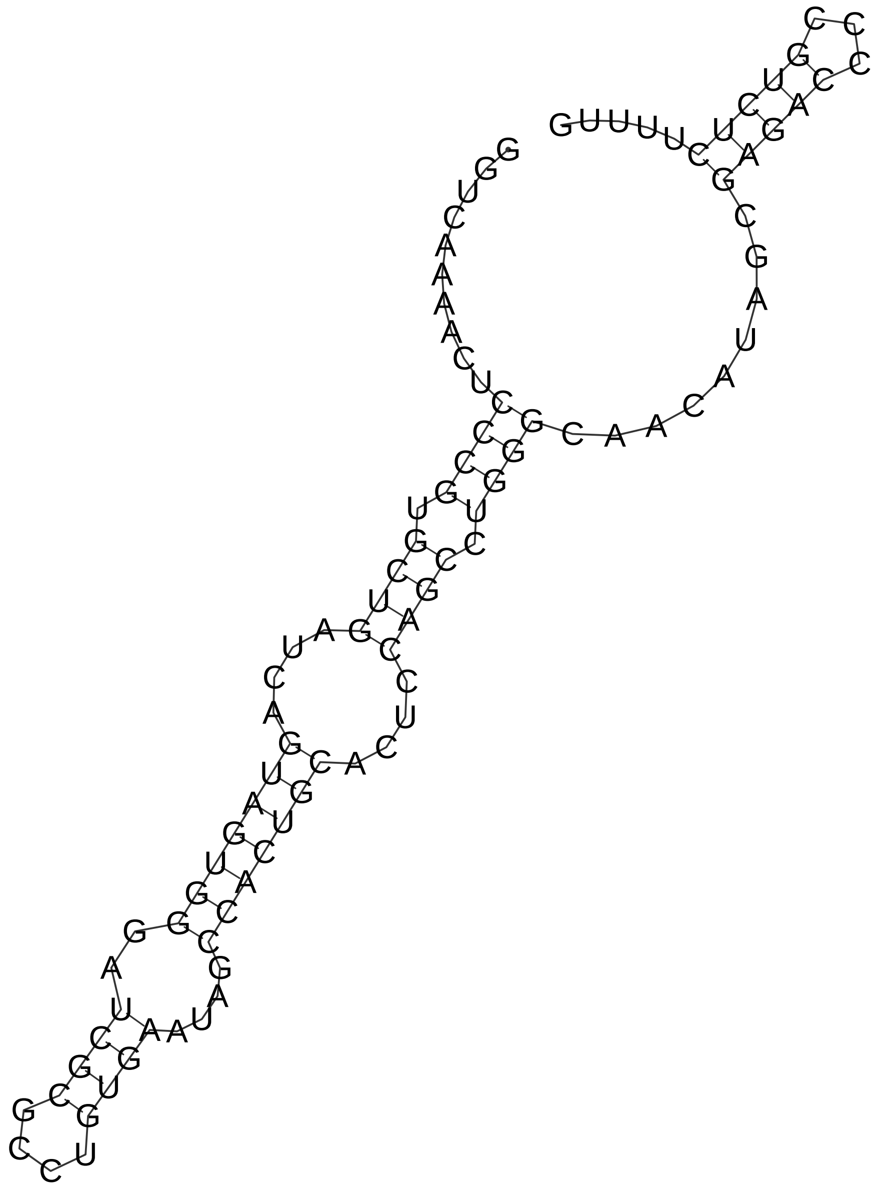

8_26223920-2622401_mir-1285_low

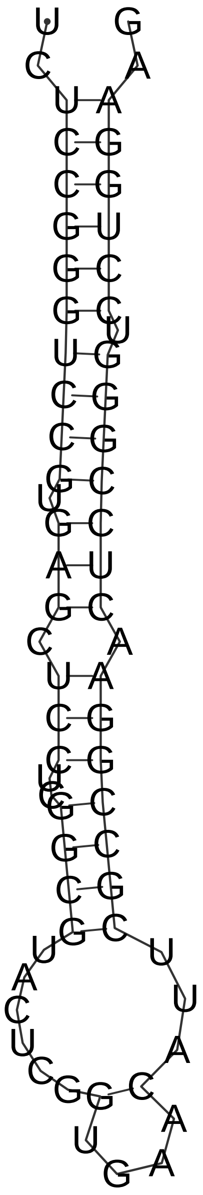

8_26256213-2625627_mir-6517_high

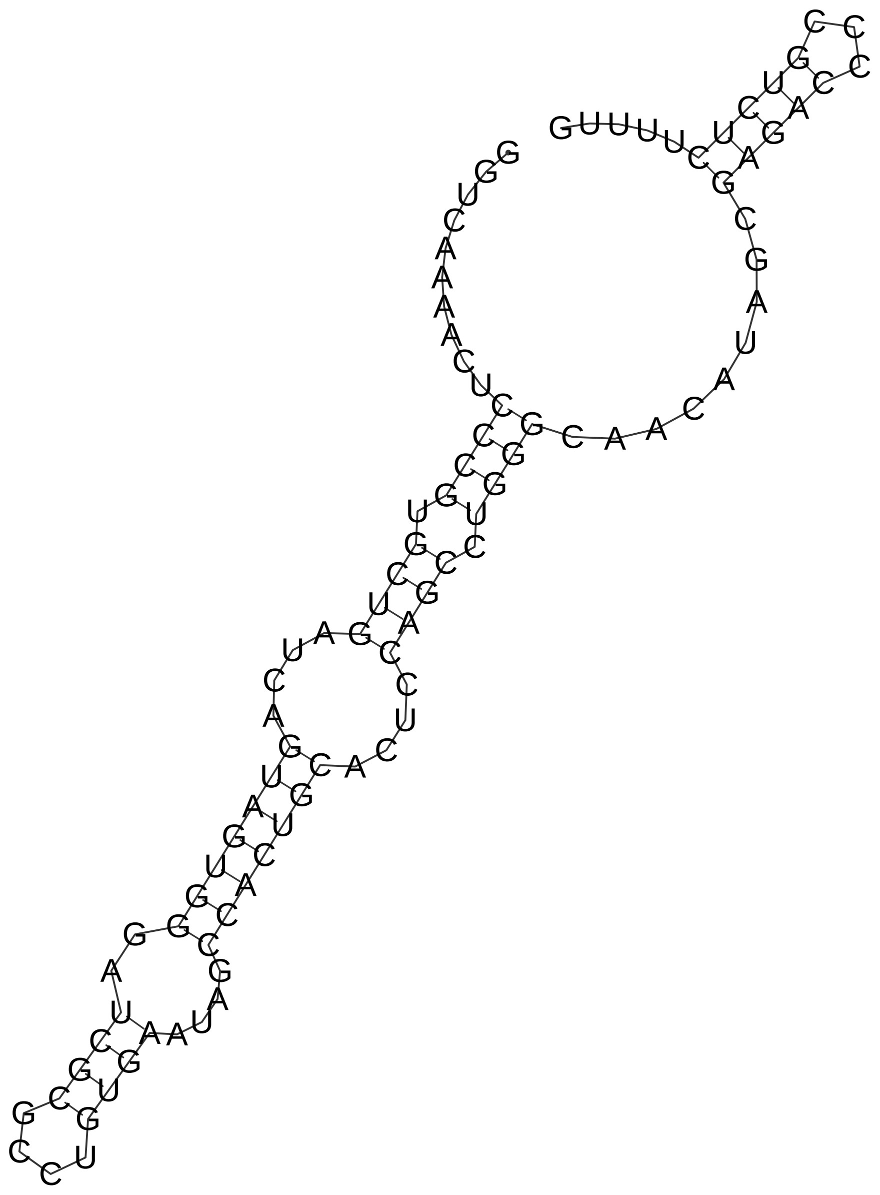

8_26412225-2641231_mir-1285_low

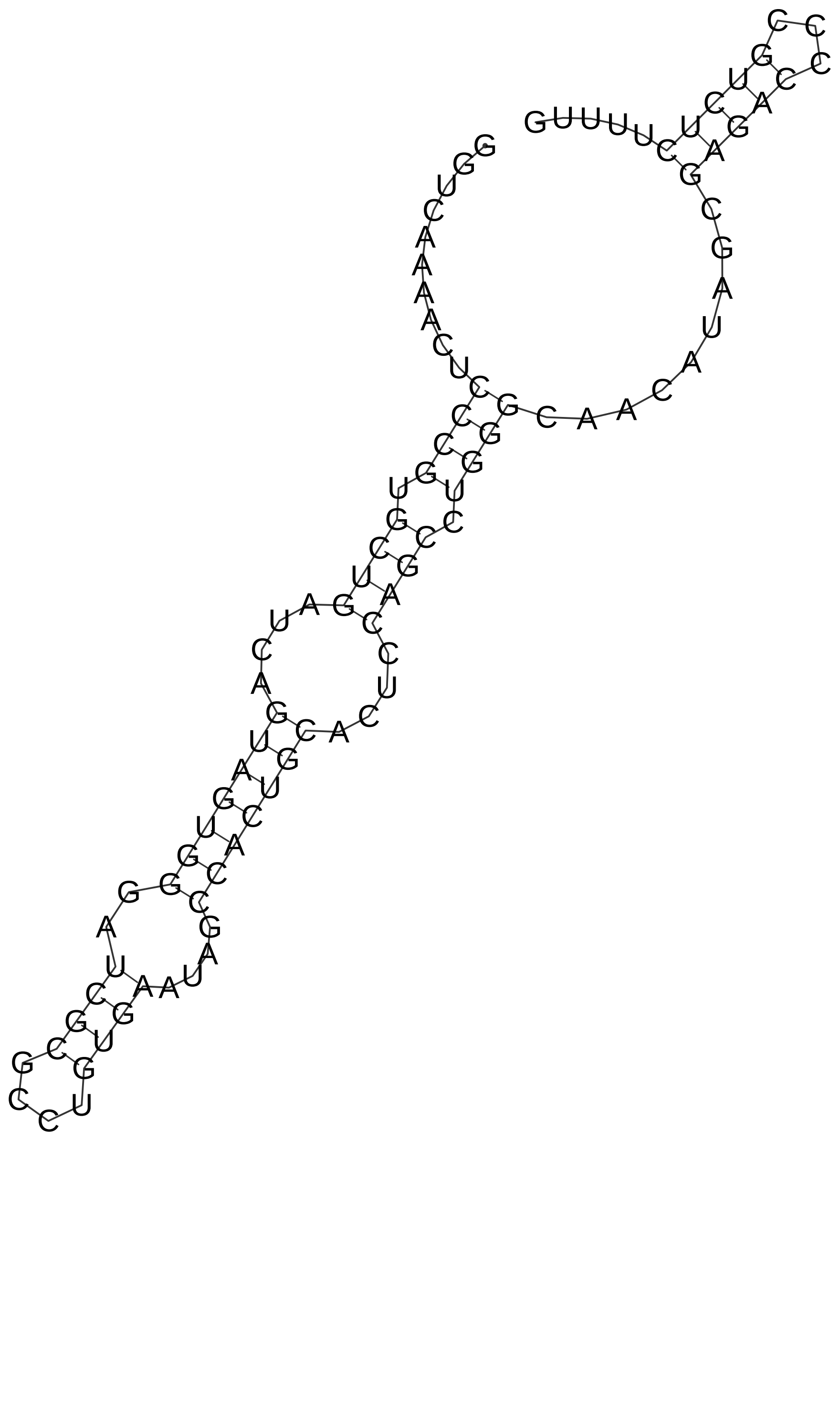

8_26422918-2642301_mir-1285_low

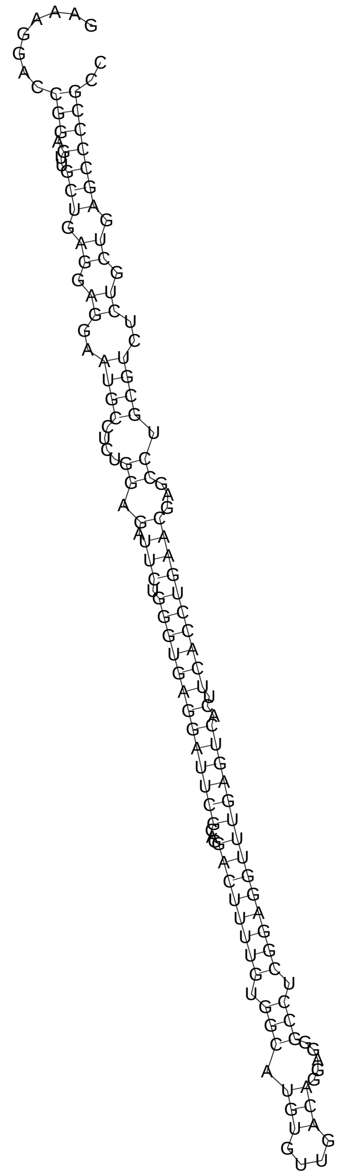

8_32848208-3284835_cfa-mir-8883_low

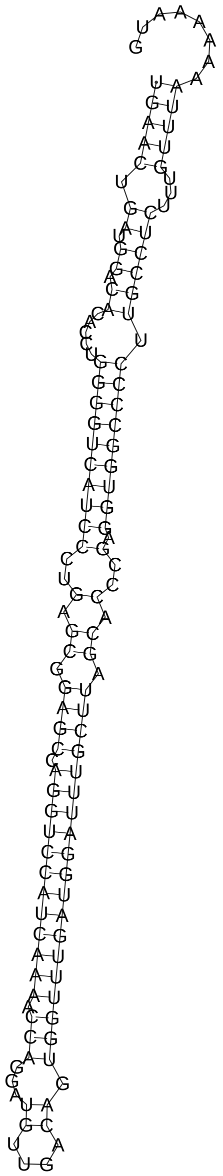

8_53823682-5382381_cfa-mir-8884_high

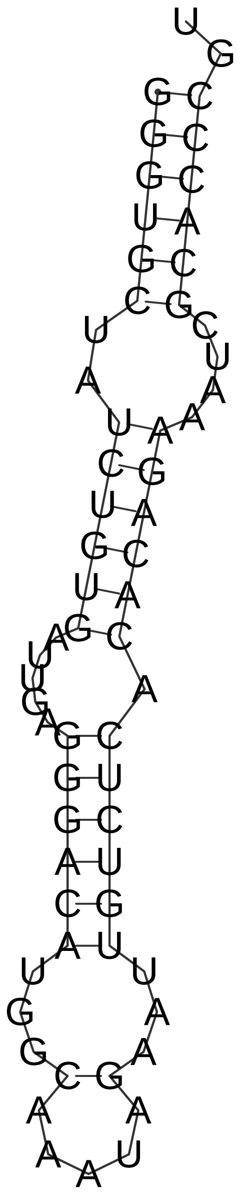

8_68424656-6842471_cfa-mir-342_high

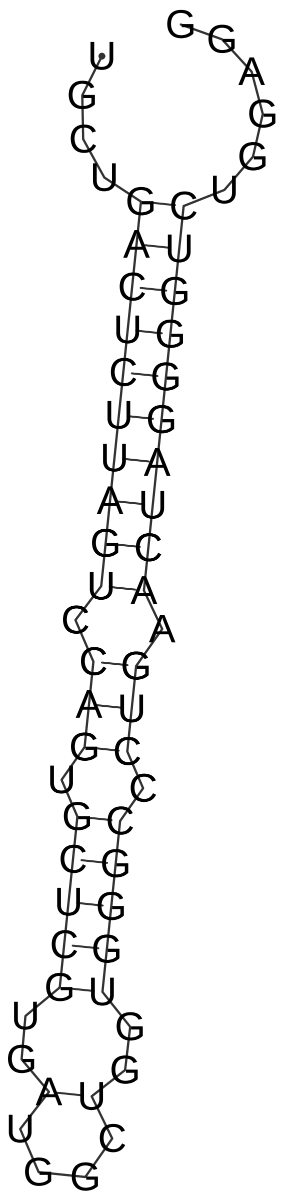

8_68592447-6859250_cfa-mir-345_high

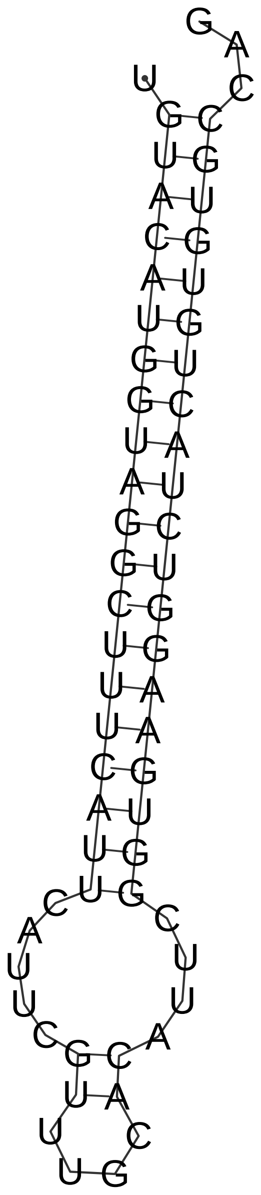

8_69094352-6909441_cfa-mir-493_high

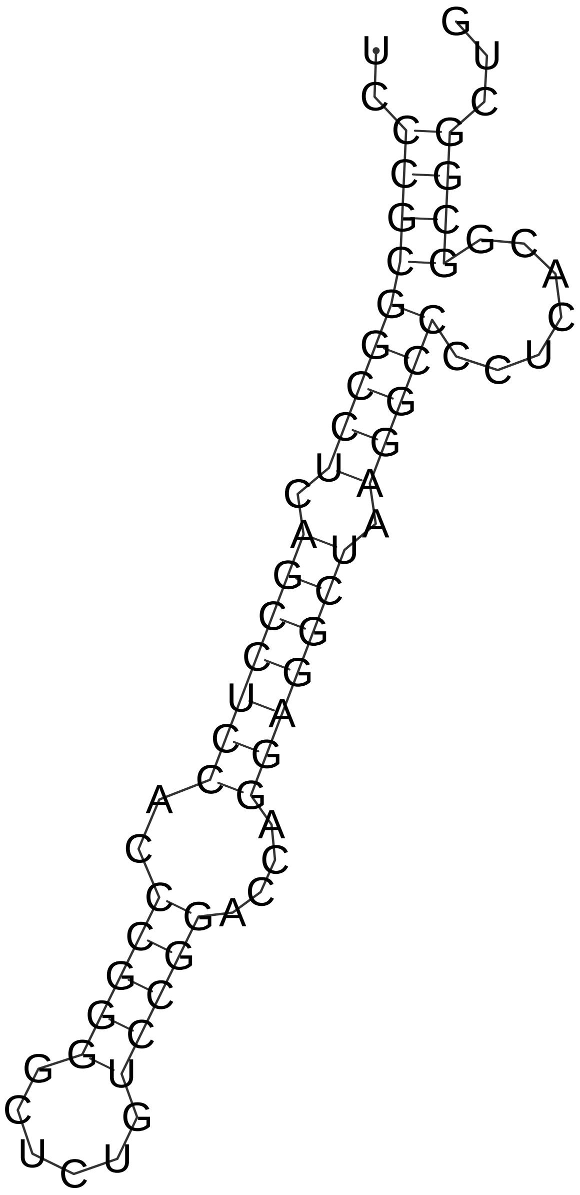

8_69097904-6909797_cfa-mir-665_low

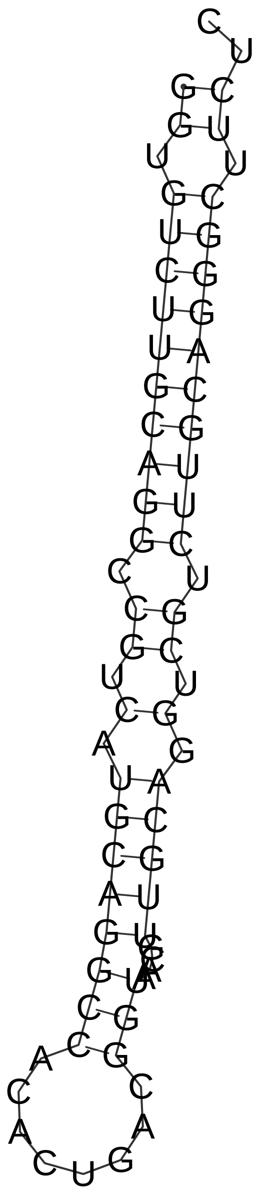

8_69102956-6910302_mir-431_high

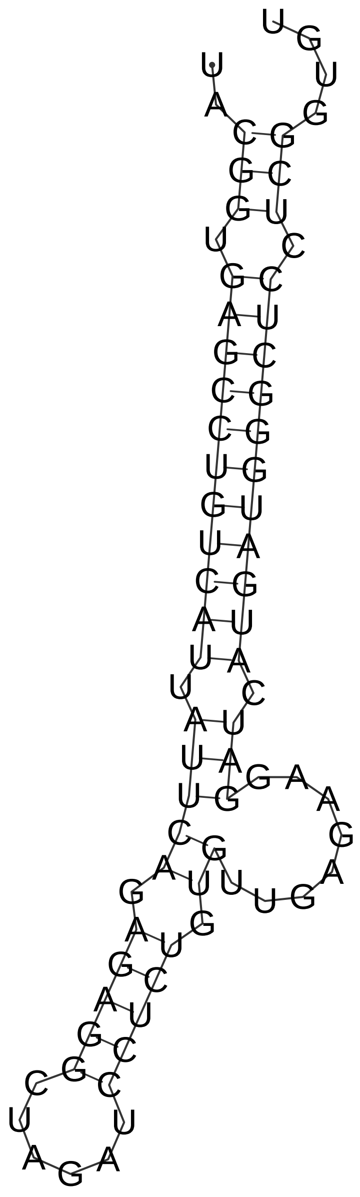

8_69103838-6910391_cfa-mir-433_high

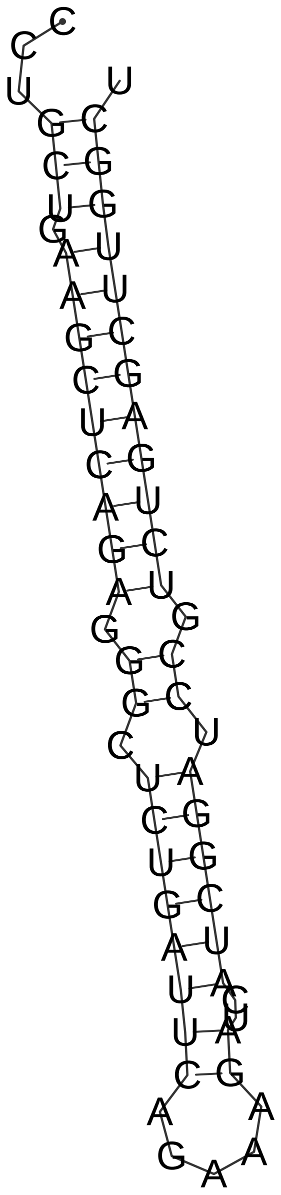

8_69104938-6910499_cfa-mir-127_high

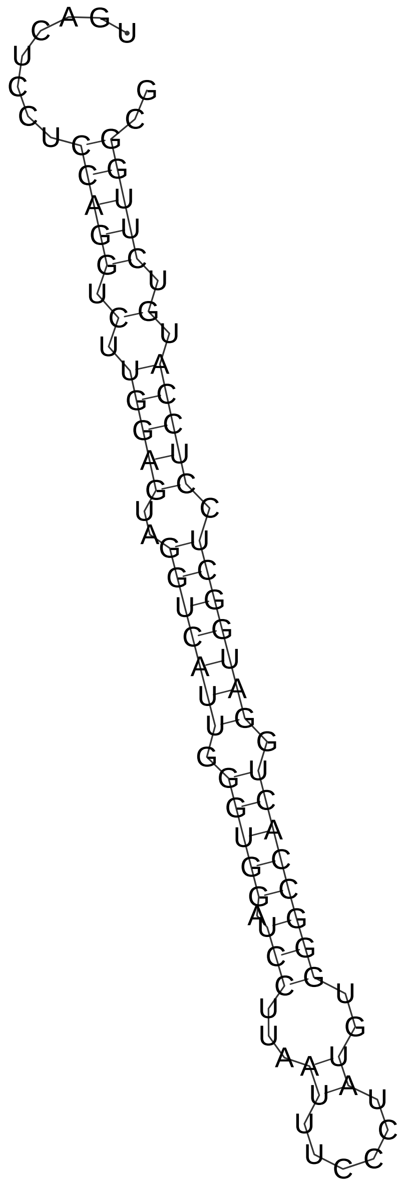

8_69106415-6910650_cfa-mir-432_high

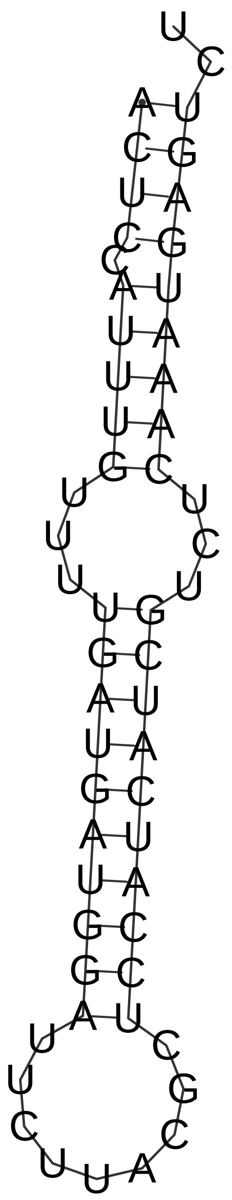

8_69106615-6910667_cfa-mir-136_high

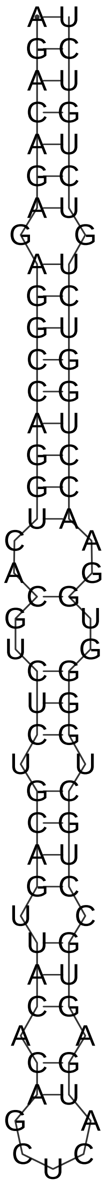

8_69132786-6913286_cfa-mir-370_high

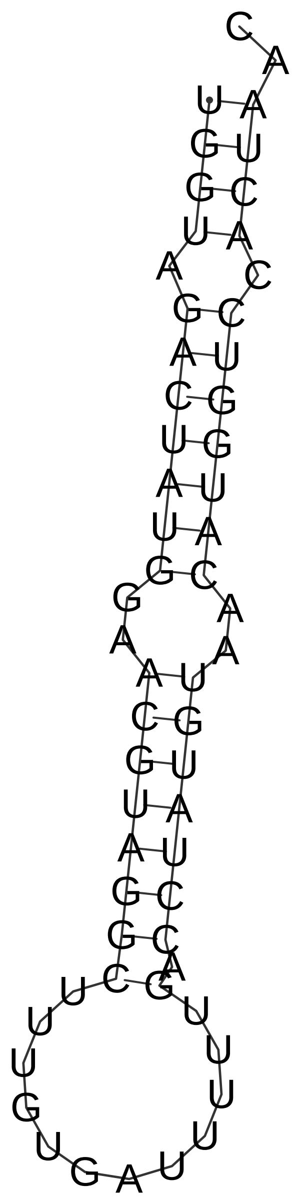

8_69253808-6925386_cfa-mir-379_high

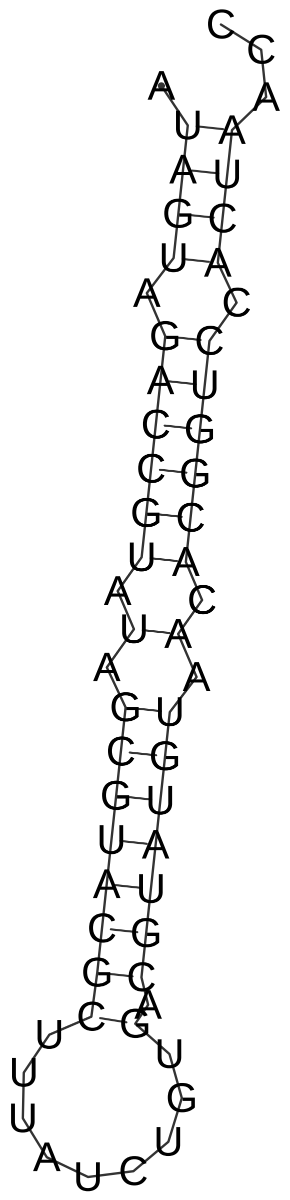

8_69255094-6925515_cfa-mir-411_high

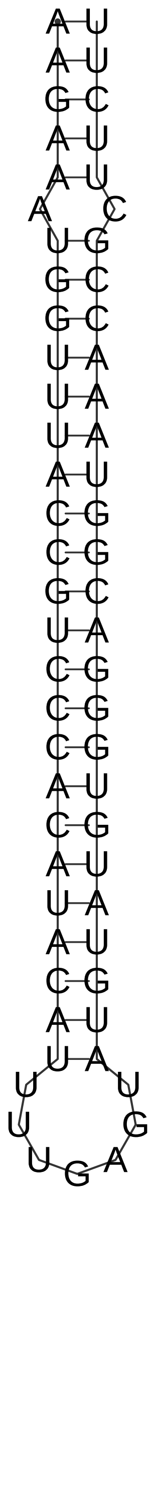

8_69255548-6925561_cfa-mir-299_high

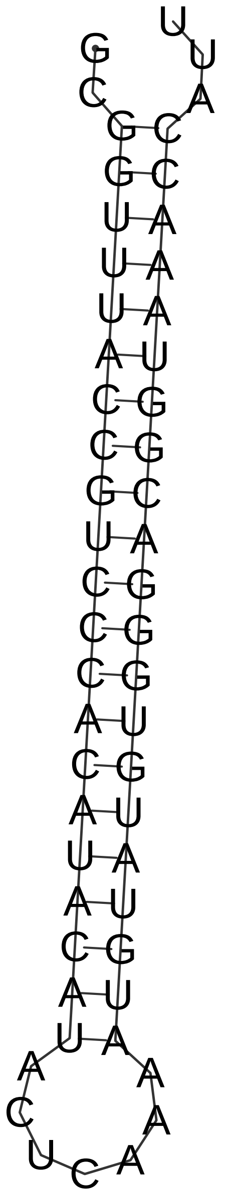

8_69255552-6925560_mir-299b_low

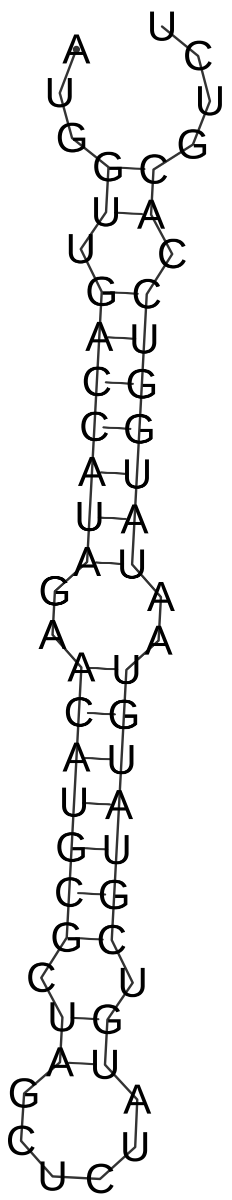

8_69256764-6925682_cfa-mir-380_high

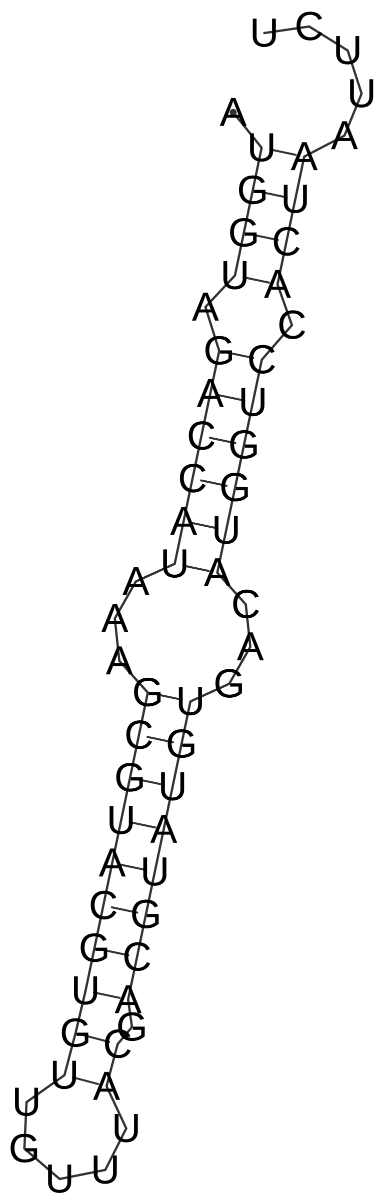

8_69256924-6925698_mir-411b_low

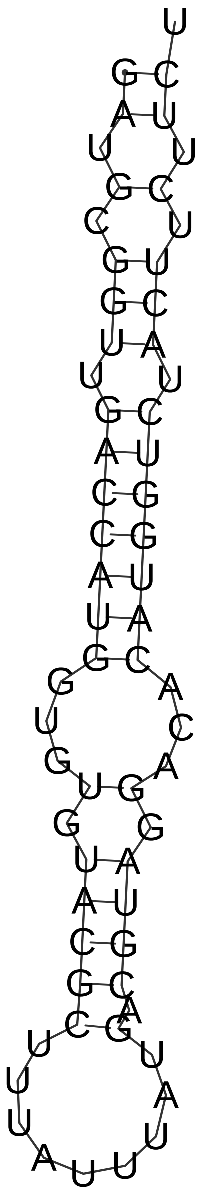

8_69257328-6925739_mir-1197_high

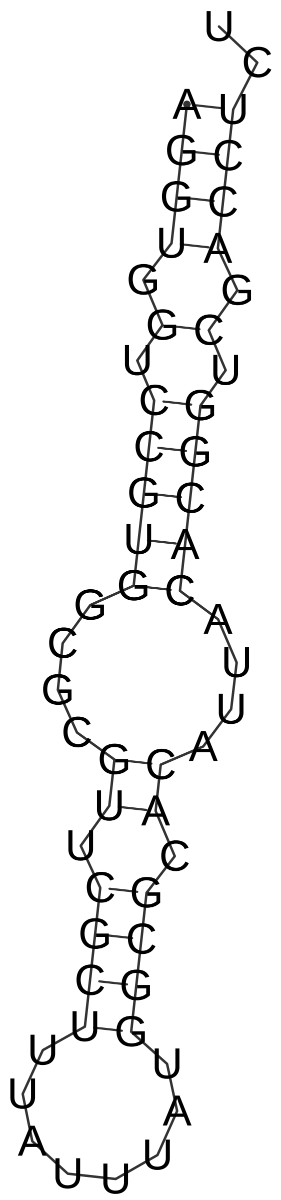

8_69257494-6925754_cfa-mir-323_high

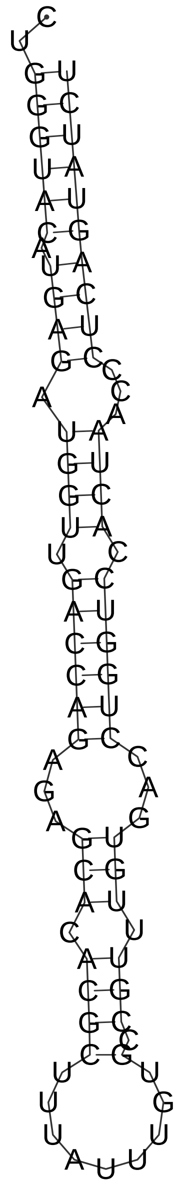

8_69257782-6925786_cfa-mir-758_high

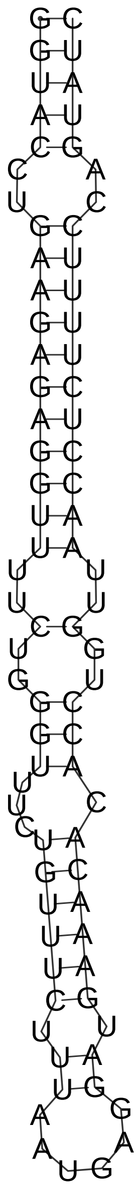

8_69258528-6925860_cfa-mir-329b_high

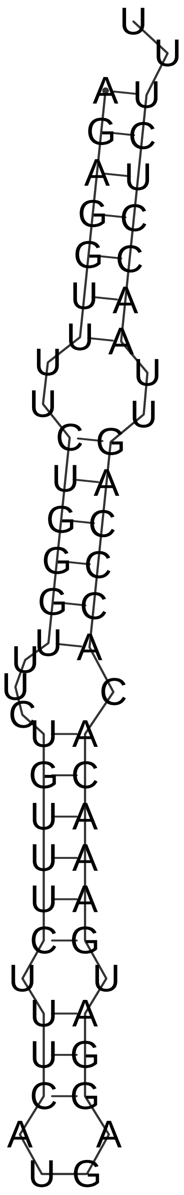

8_69258855-6925891_cfa-mir-329a_high

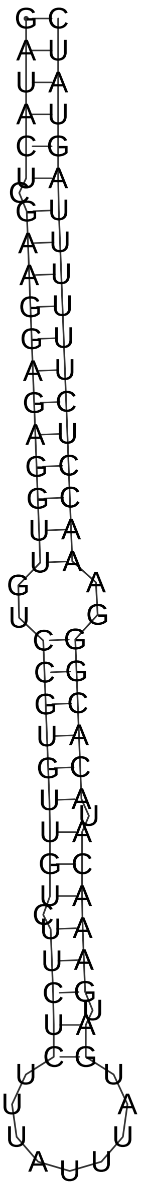

8_69261385-6926146_cfa-mir-494_high

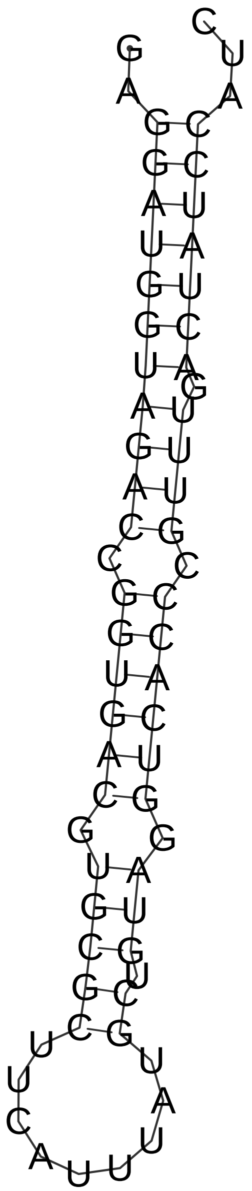

8_69261833-6926189_mir-1193_high

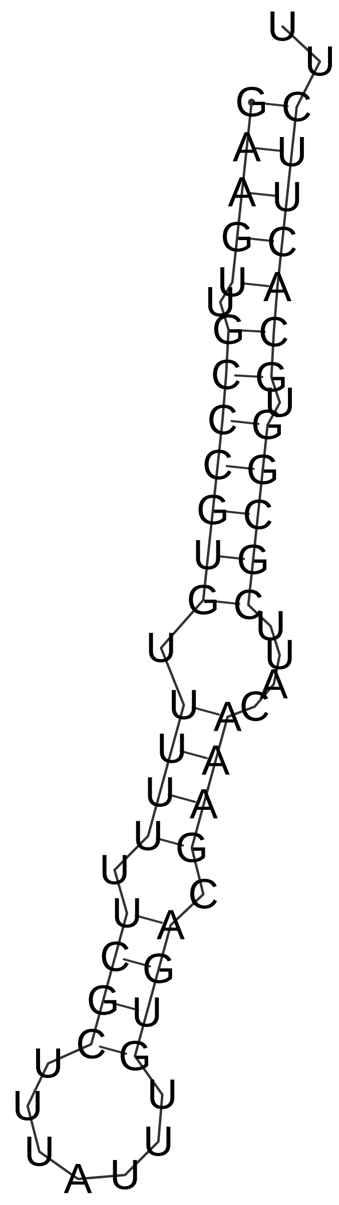

8_69263406-6926346_cfa-mir-543_high

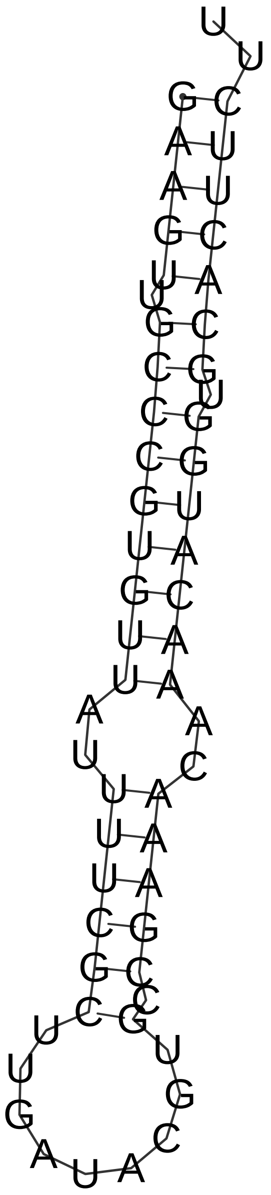

8_69265169-6926522_cfa-mir-495_high

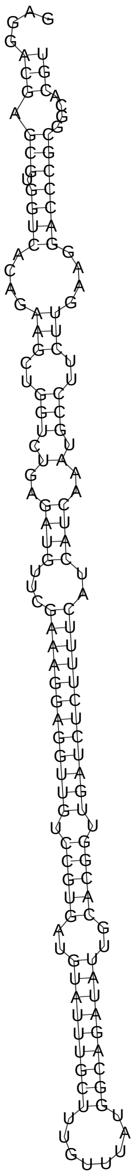

8_69267134-6926727_cfa-mir-3958_high

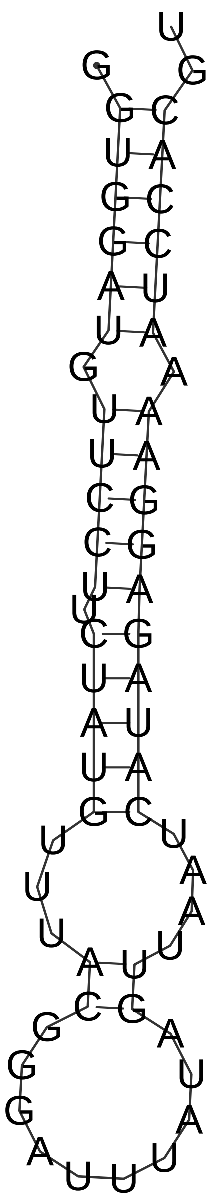

8_69269752-6926981_cfa-mir-376a-3_high

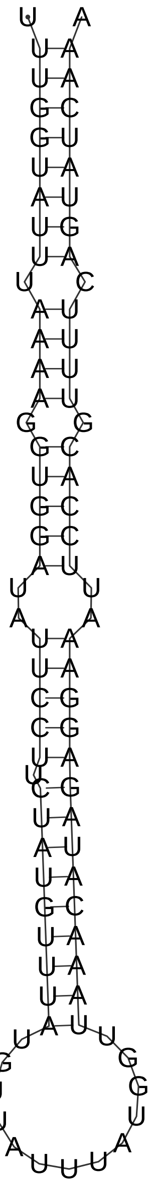

8_69270100-6927018_cfa-mir-376c_high

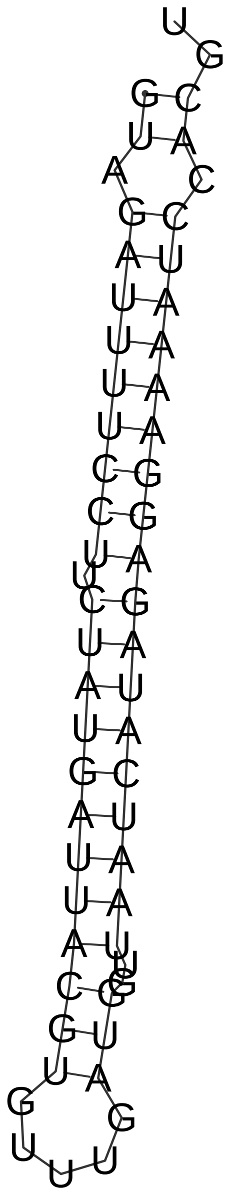

8_69270499-6927055_cfa-mir-376a-2_high

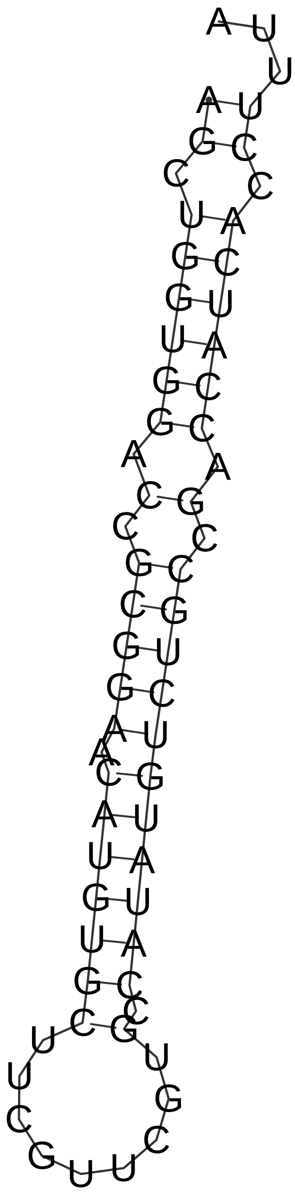

8_69270651-6927071_mir-654_high

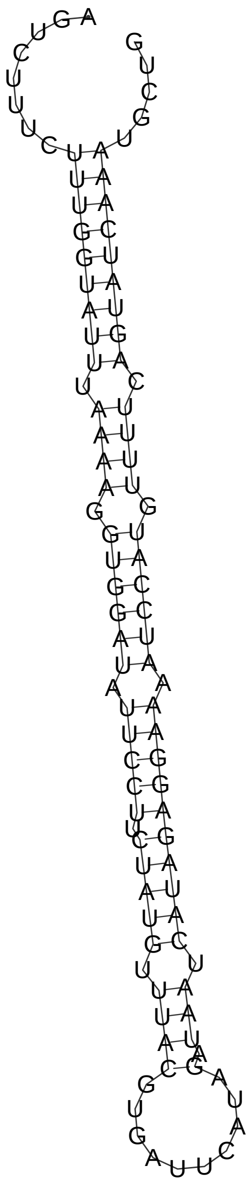

8_69270858-6927095_cfa-mir-376b_high

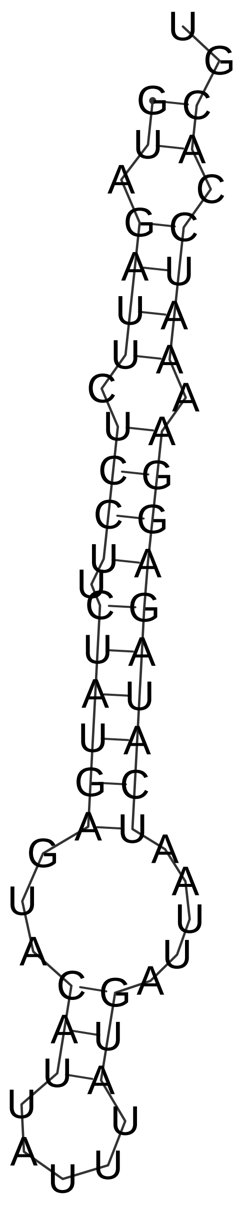

8_69271259-6927131_cfa-mir-376a-1_high

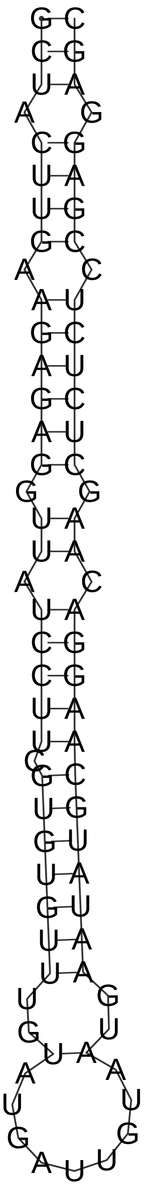

8_69271815-6927189_cfa-mir-300_high

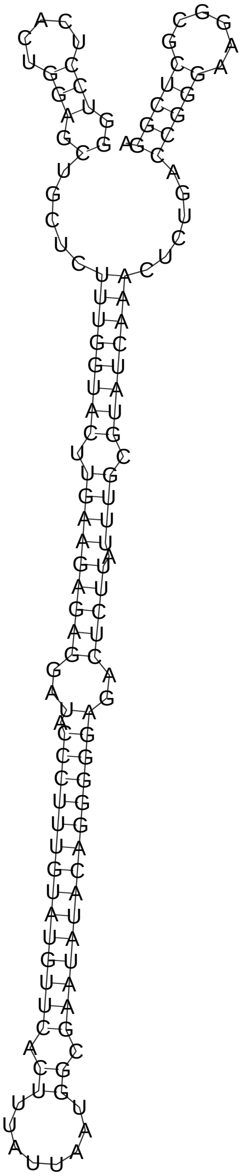

8_69273204-6927333_cfa-mir-1185_high

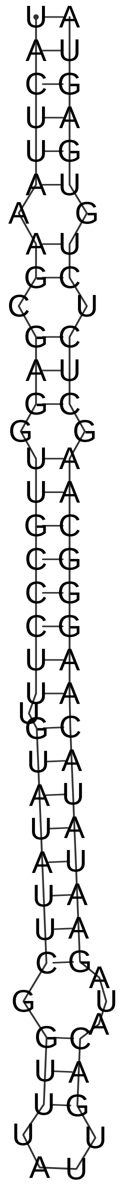

8_69274983-6927505_cfa-mir-381_high

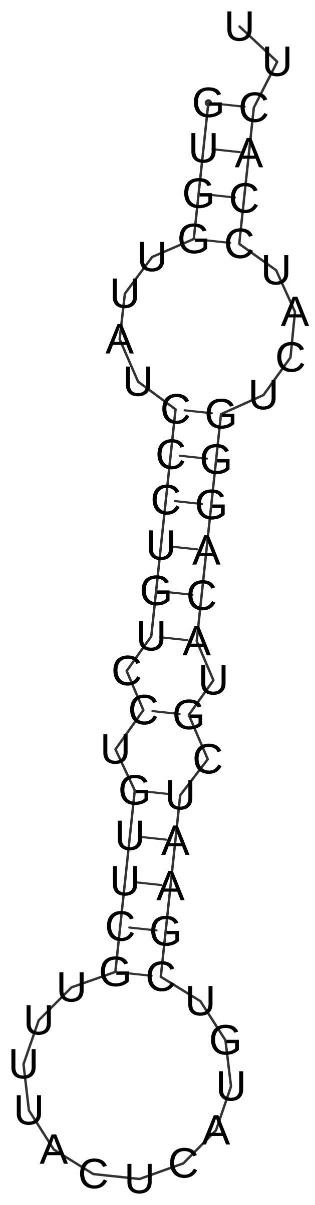

8_69275496-6927555_cfa-mir-487b_high

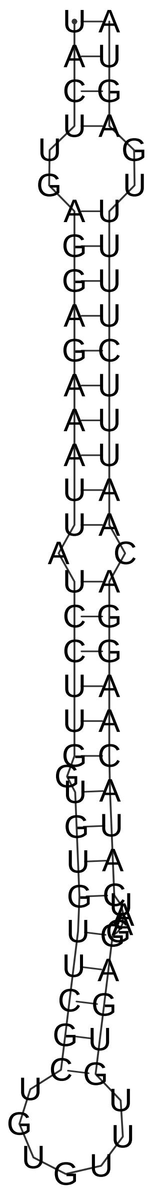

8_69276311-6927638_cfa-mir-539_high

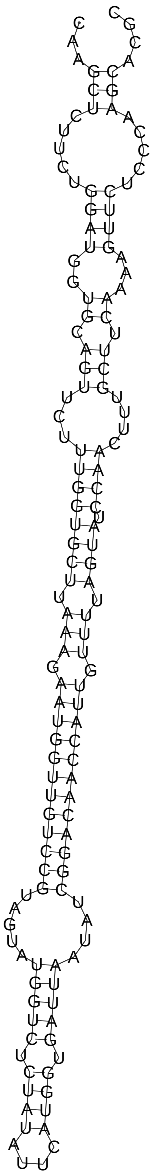

8_69276842-6927698_cfa-mir-889_high

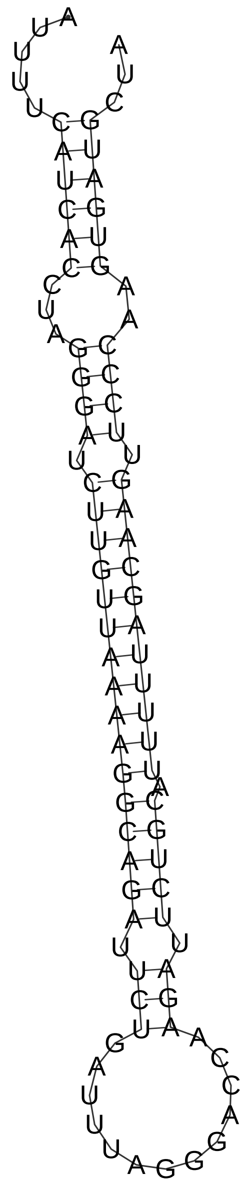

8_69277656-6927774_cfa-mir-544_high

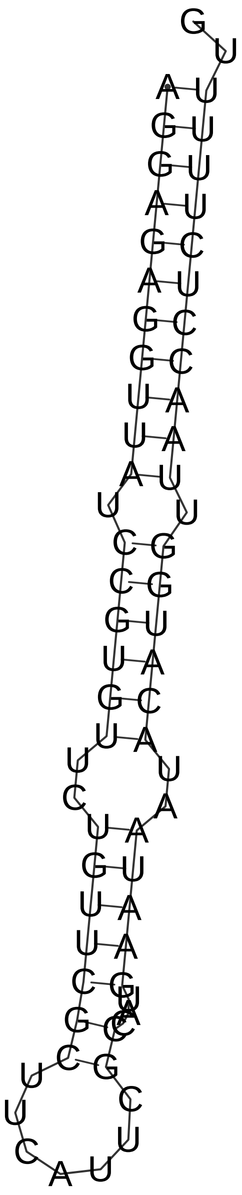

8_69278521-6927858_mir-655_high

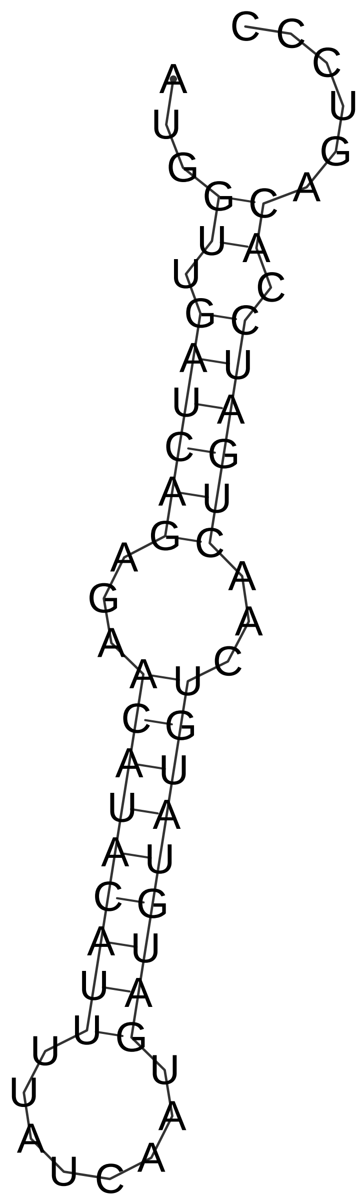

8_69280666-6928072_mir-3959_high

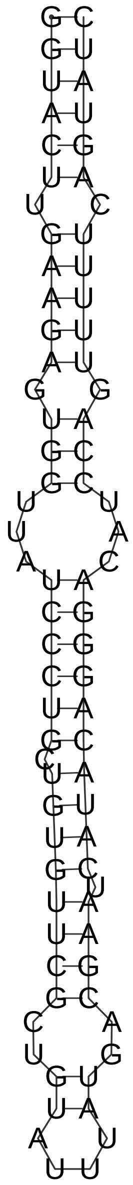

8_69281078-6928115_cfa-mir-487a_high

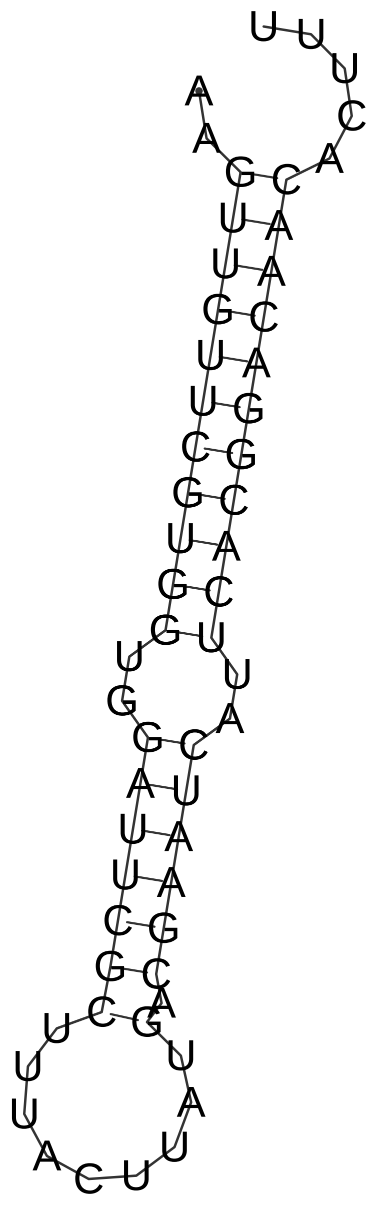

8_69283123-6928317_cfa-mir-382_high

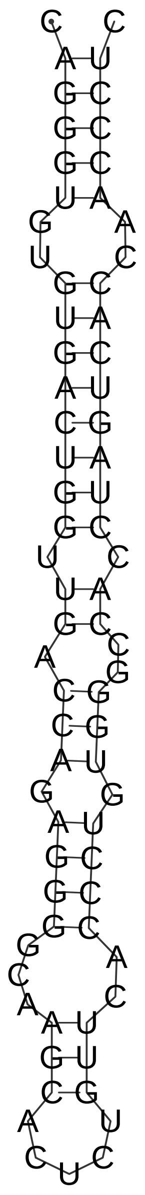

8_69283496-6928356_cfa-mir-134_high

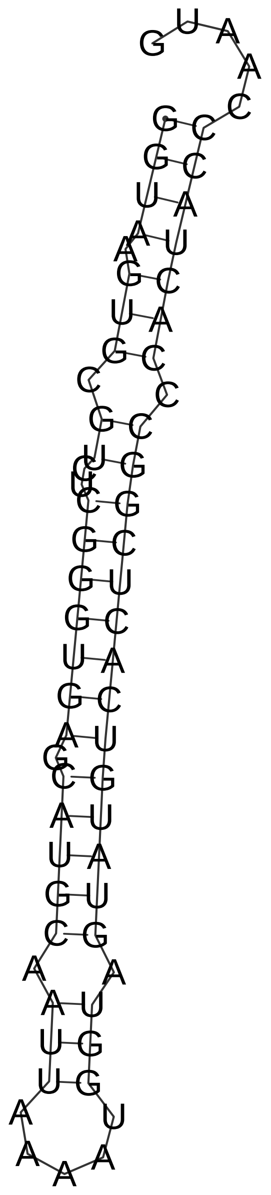

8_69284077-6928414_mir-668_high

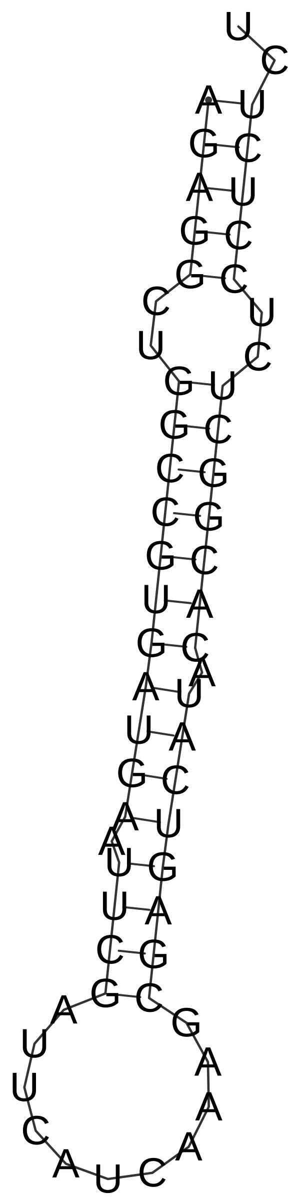

8_69284239-6928429_cfa-mir-485_high

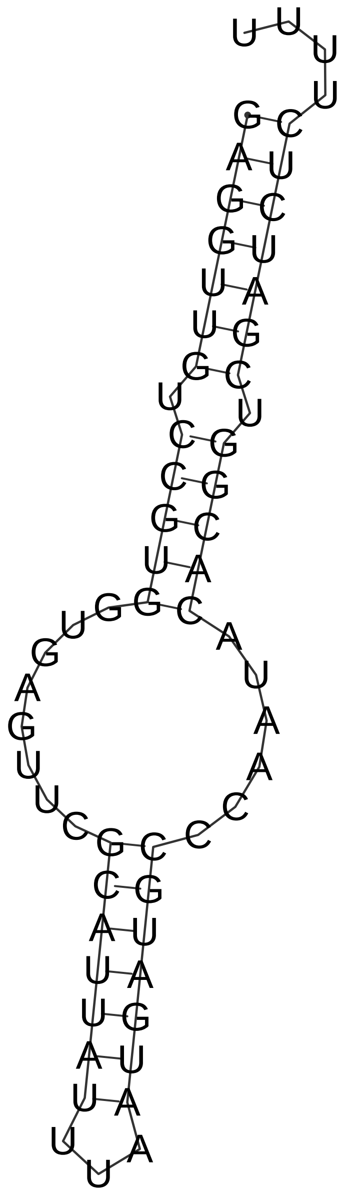

8_69285041-6928510_mir-323b_high

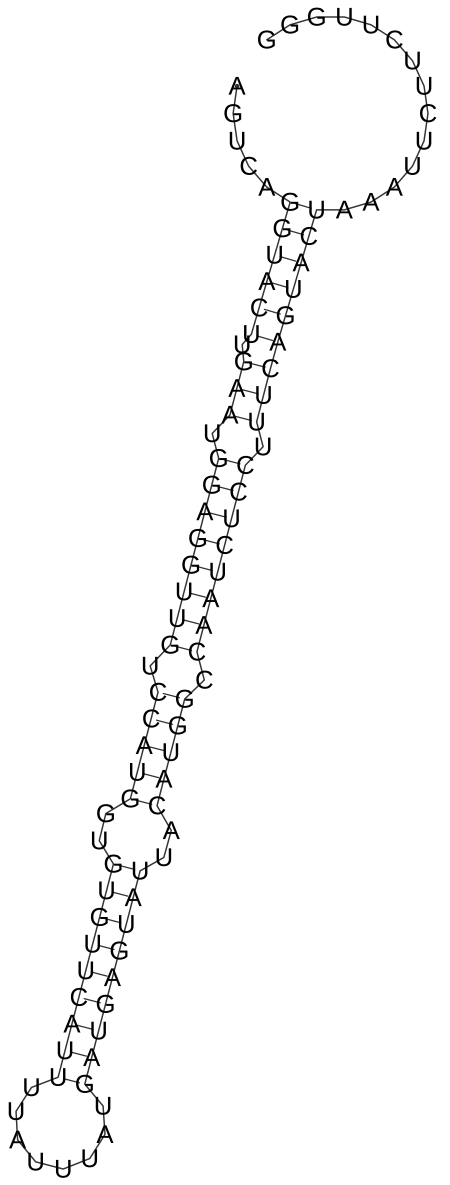

8_69289410-6928950_cfa-mir-496_high

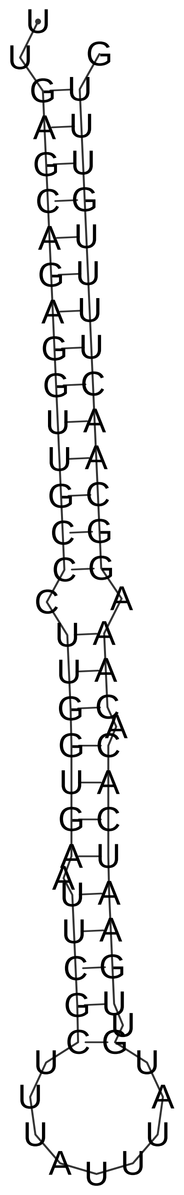

8_69290937-6929100_cfa-mir-377_high

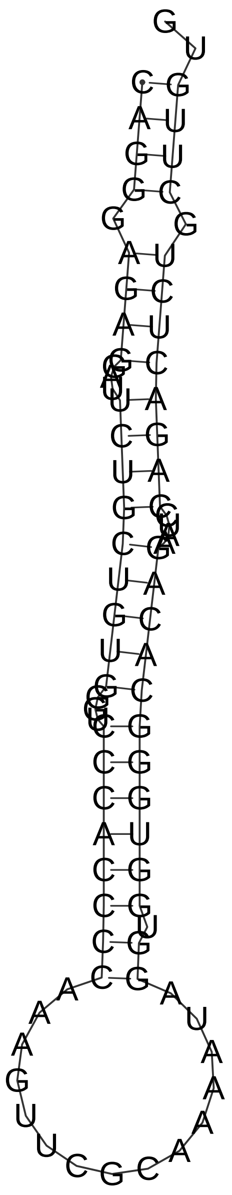

8_69292811-6929289_mir-541_low

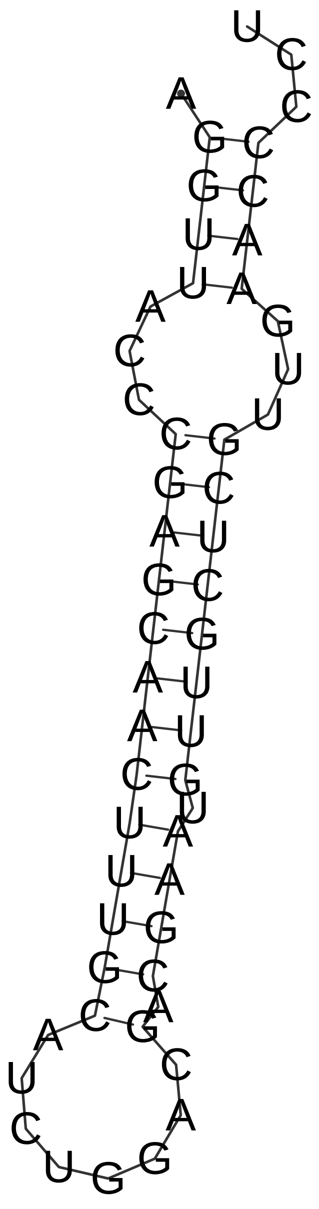

8_69293782-6929383_cfa-mir-409_high

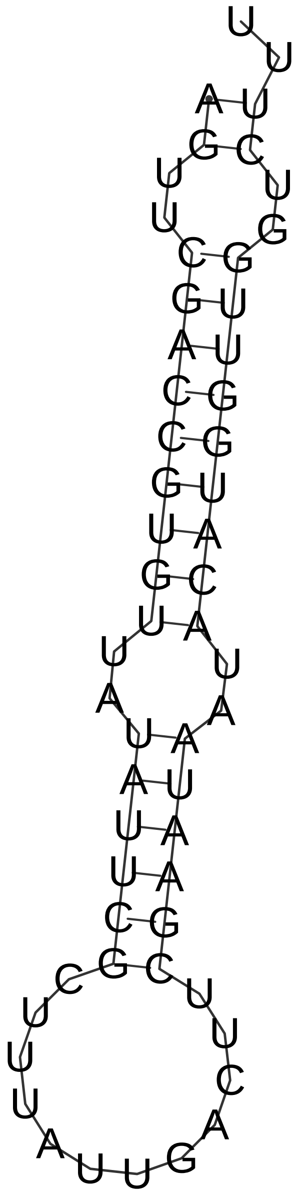

8_69294073-6929412_cfa-mir-369_high

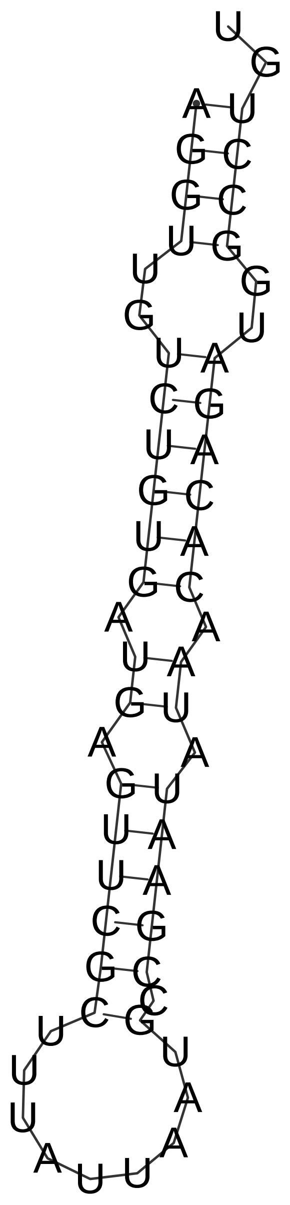

8_69294377-6929443_cfa-mir-410_high

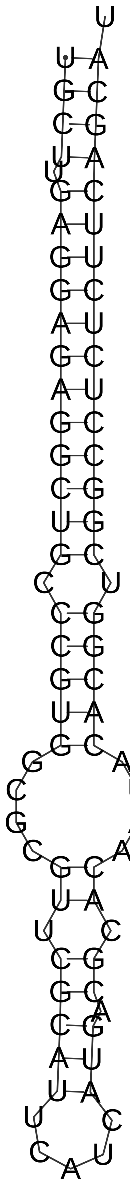

8_69294874-6929494_mir-541_low

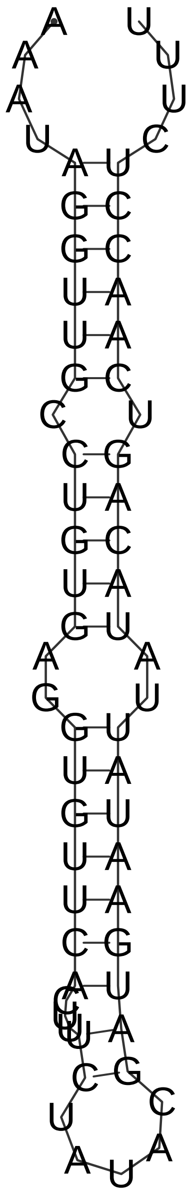

8_69295119-6929518_mir-656_high

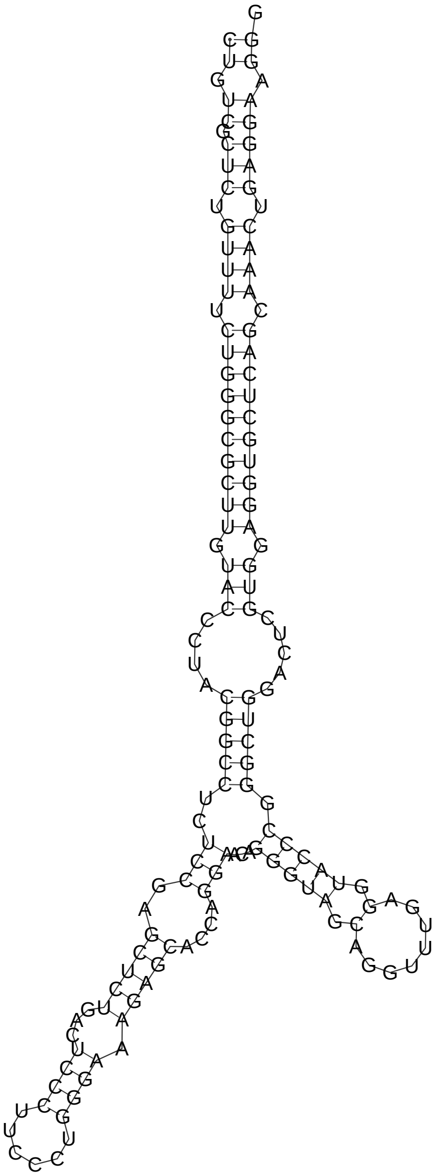

8_70657032-7065717_cfa-mir-8882_low

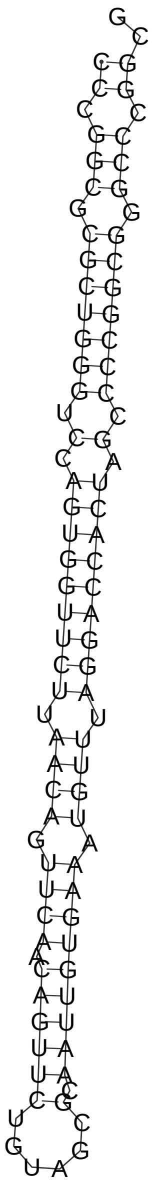

8_71774247-7177434_cfa-mir-203_high

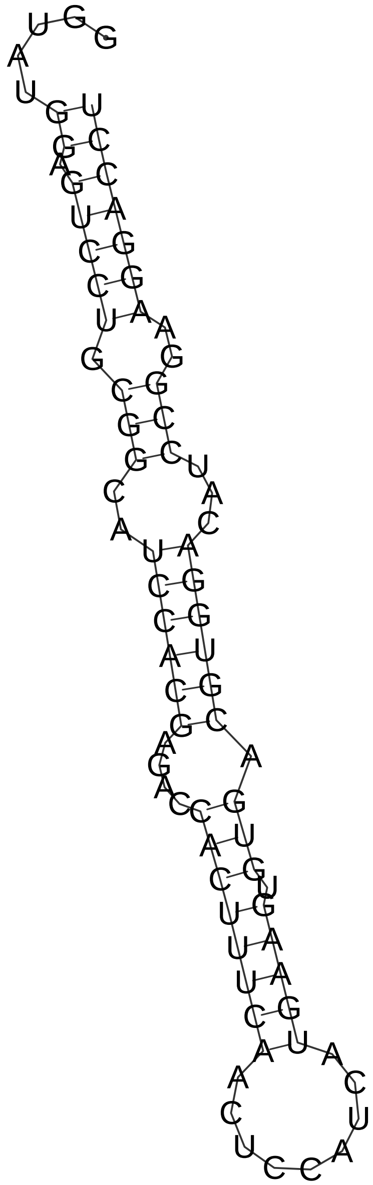

9_637175-63725_mir-3533_low

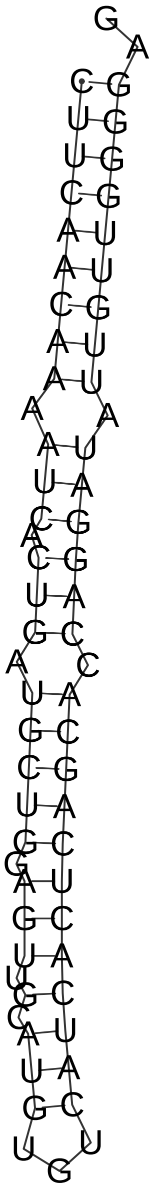

9_918646-91871_mir-3065_high

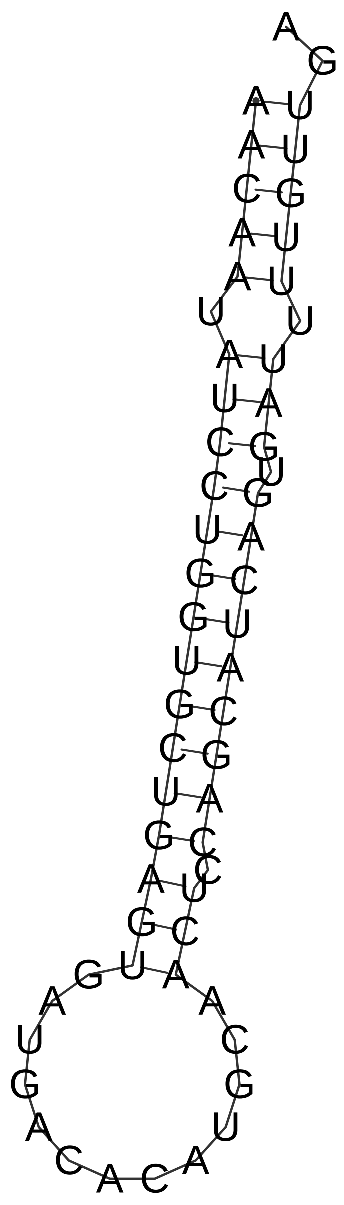

9_918652-91871_cfa-mir-338_high

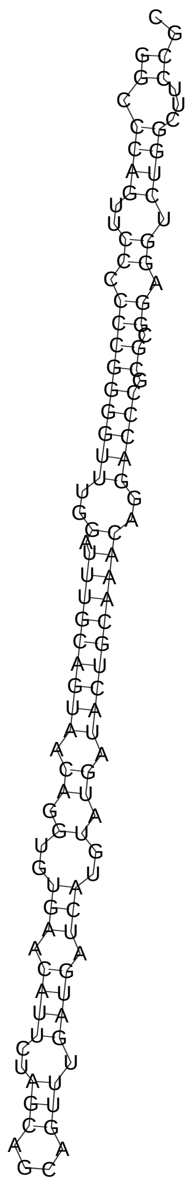

9_3776763-377687_cfa-mir-6516_high

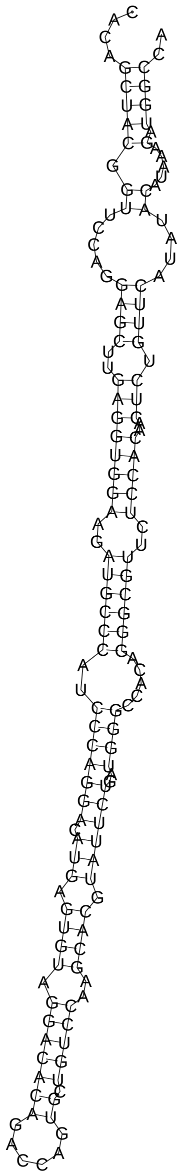

9_4378704-437884_cfa-mir-8886_low

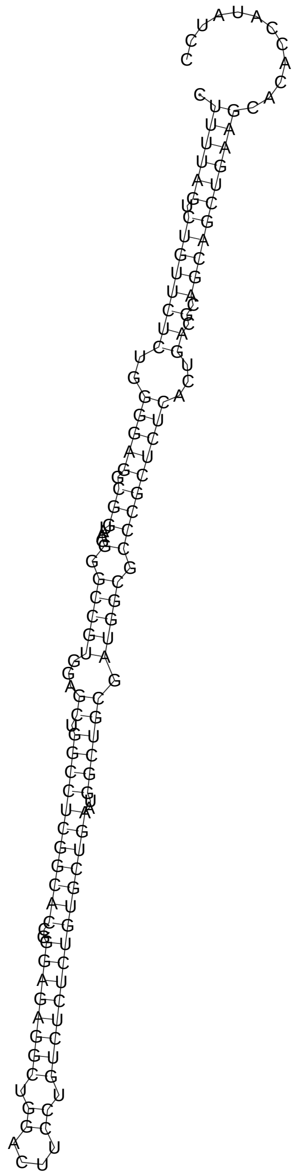

9_9429575-942971_cfa-mir-8885_low

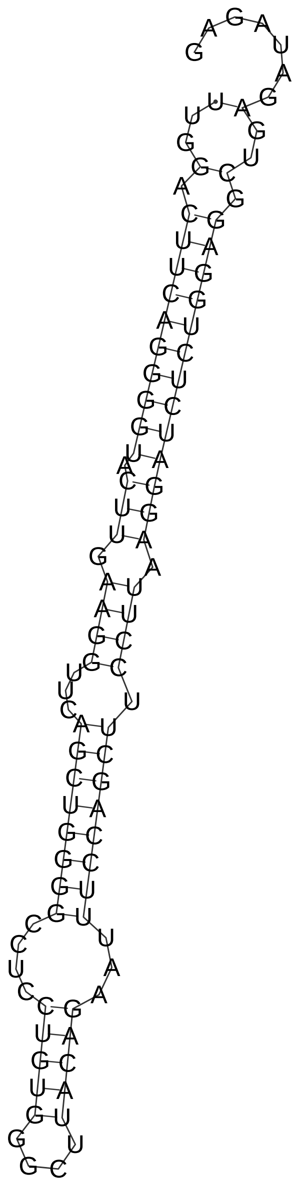

9_10288946-1028904_cfa-mir-8841-1_low

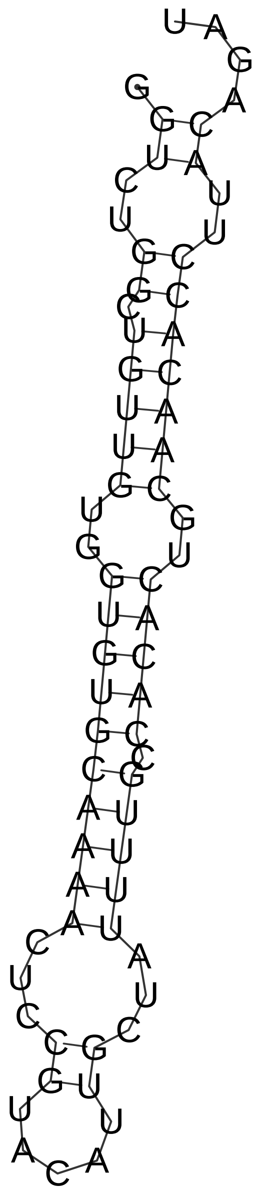

9_12301854-1230192_mir-3064_high

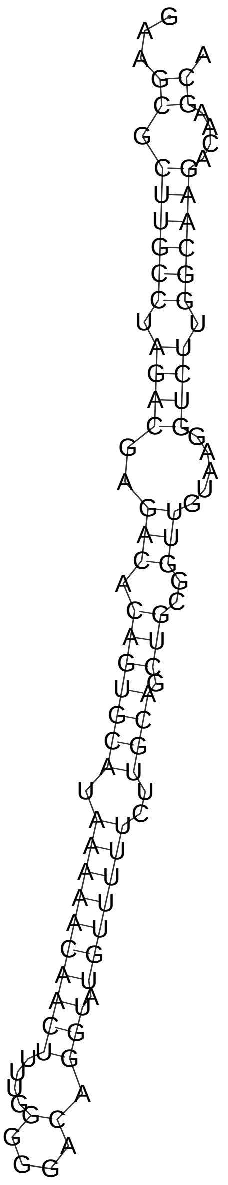

9_12302297-1230239_mir-5047_low

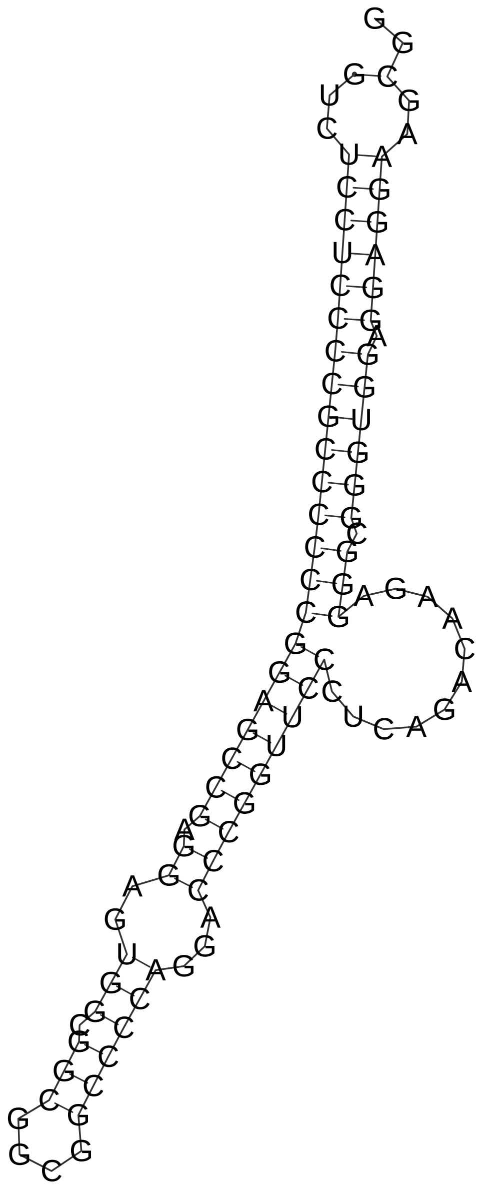

9_12471995-1247208_mir-3141_low

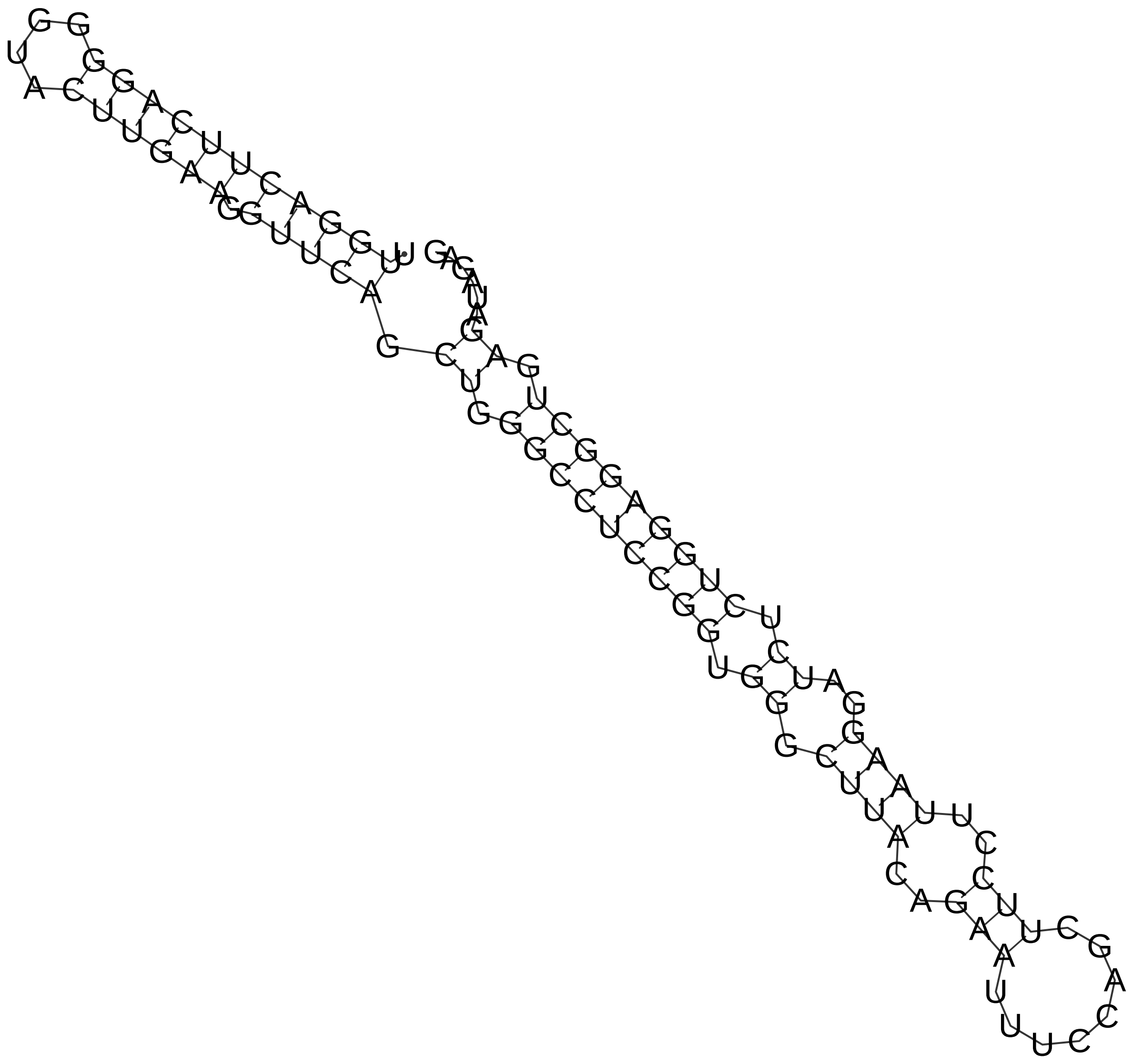

9_17998769-1799886_mir-8841_low

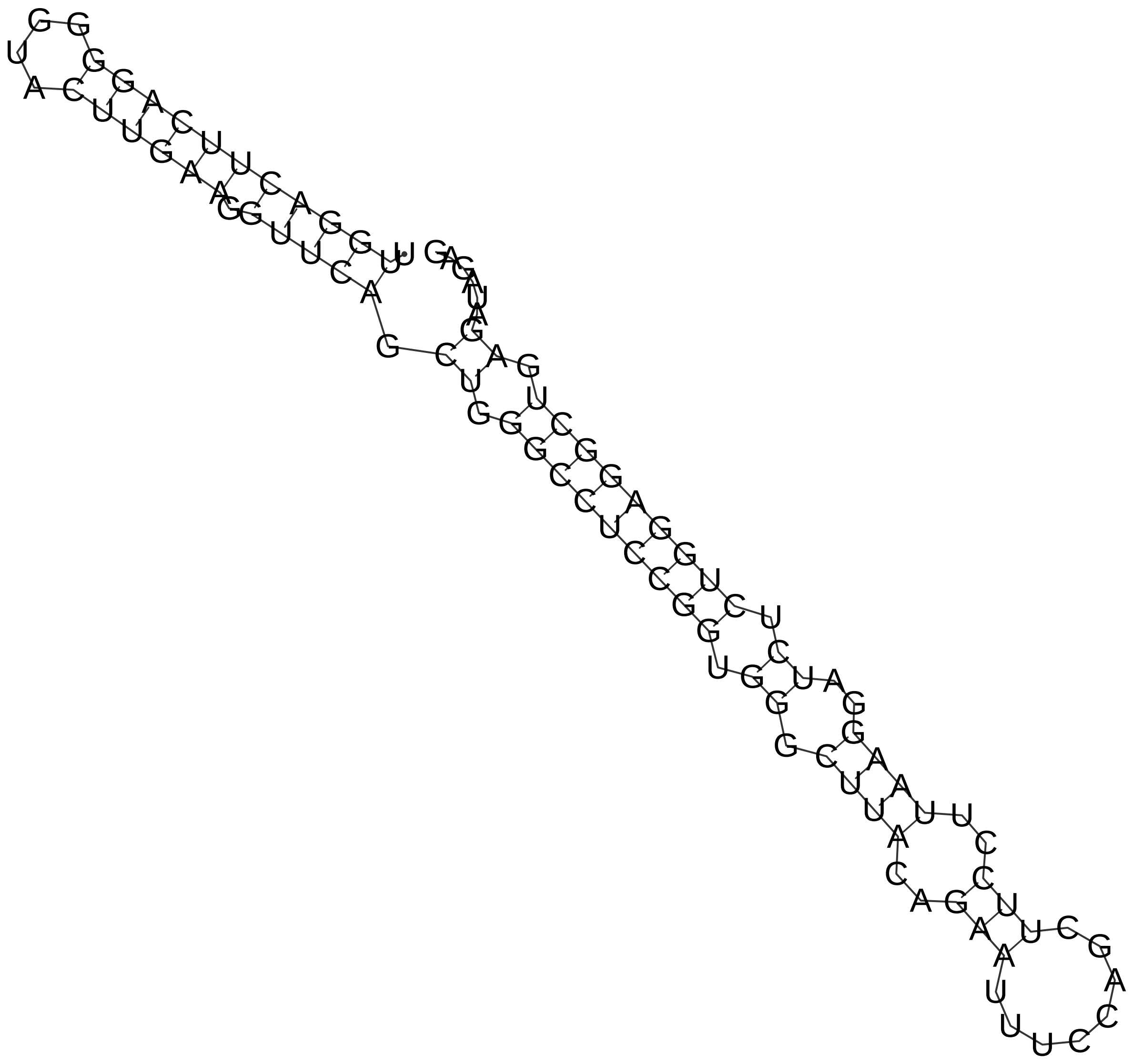

9_18025439-1802553_mir-8841_low

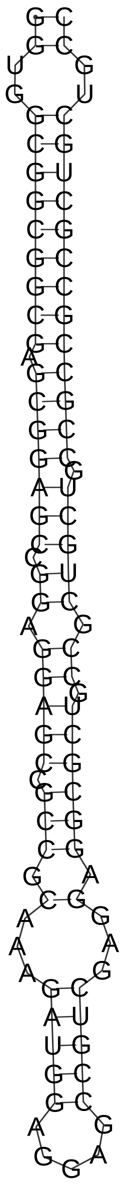

9_19261101-1926119_mir-8101_low

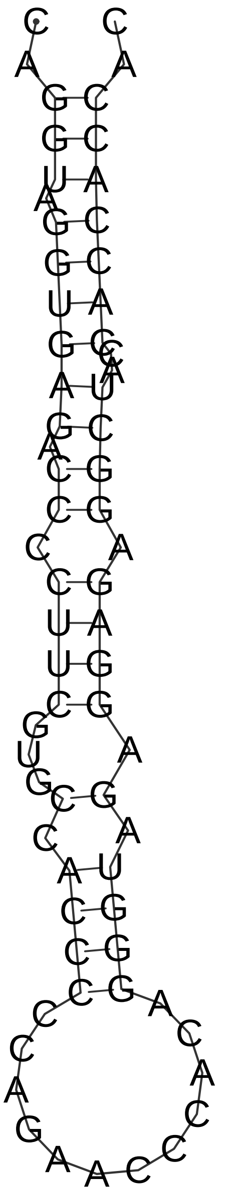

9_20322763-2032283_mir-9788_high

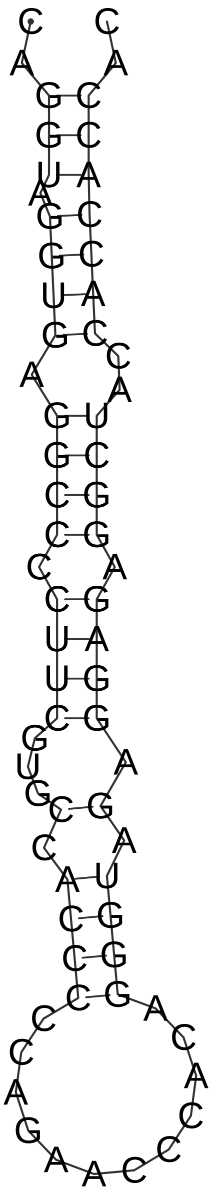

9_20359448-2035951_mir-9788_high

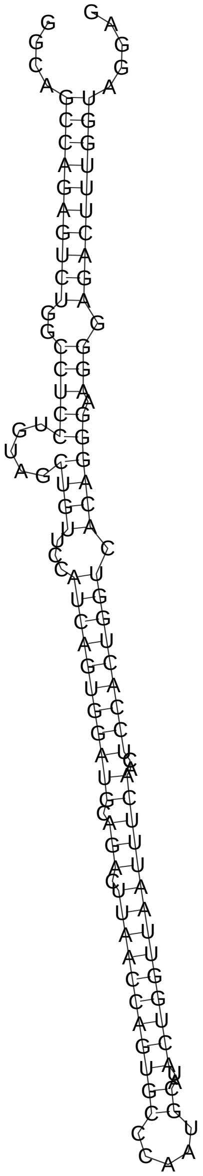

9_22156111-2215623_cfa-mir-8888_low

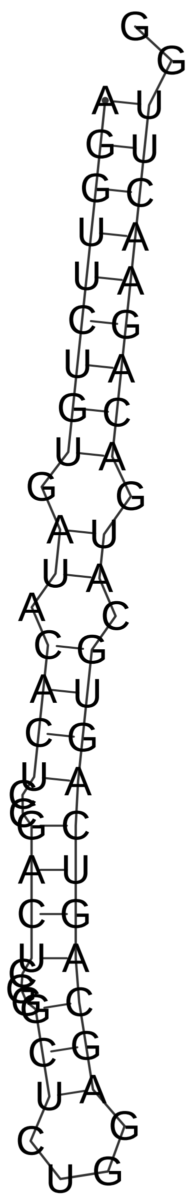

9_24335161-2433521_cfa-mir-152_high

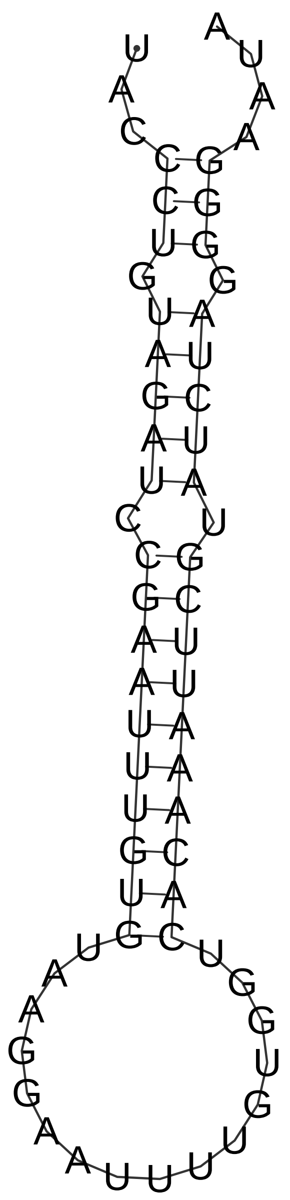

9_24842171-2484223_cfa-mir-10a_high

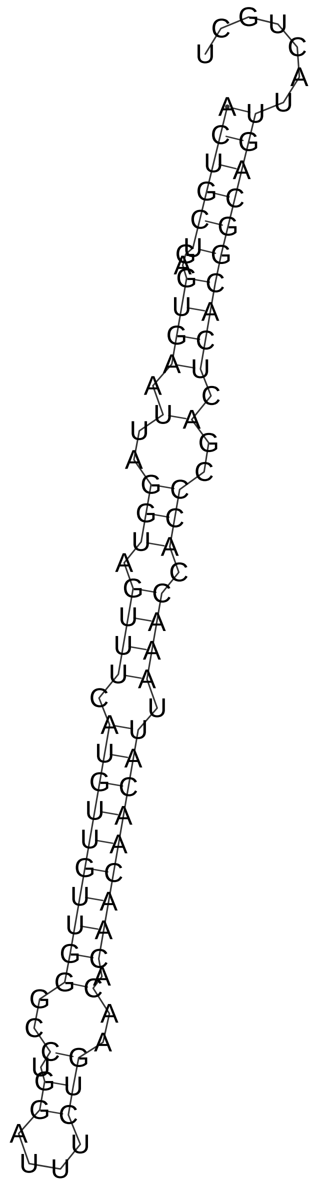

9_24895666-2489575_cfa-mir-196a-1_high

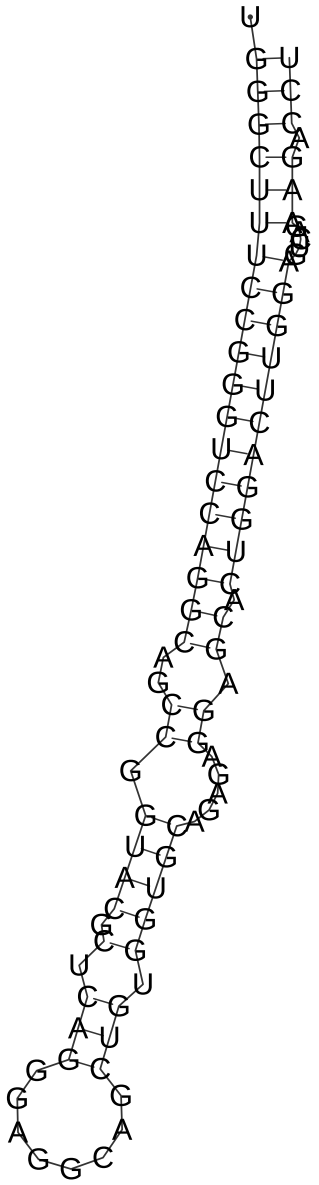

9_32395941-3239602_mir-378_high

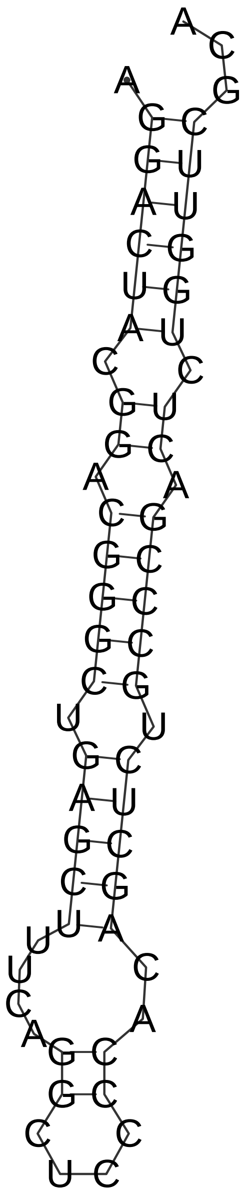

9_32760904-3276096_cfa-mir-1844_high

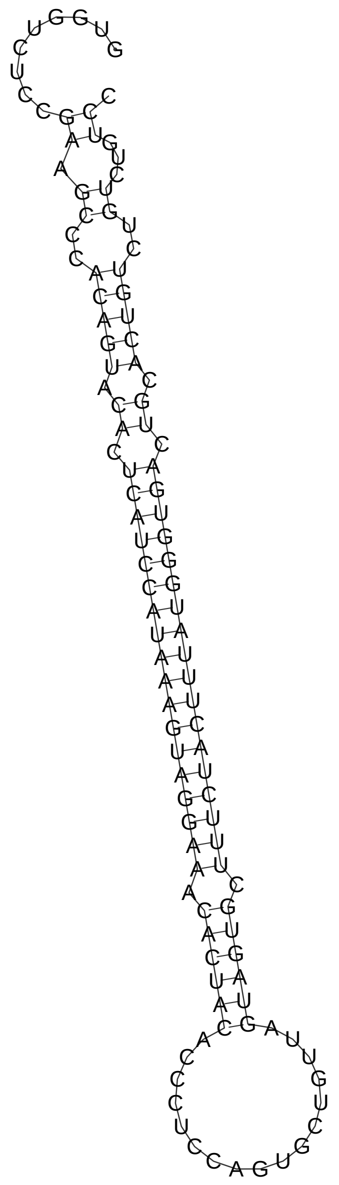

9_32977367-3297747_mir-142_low

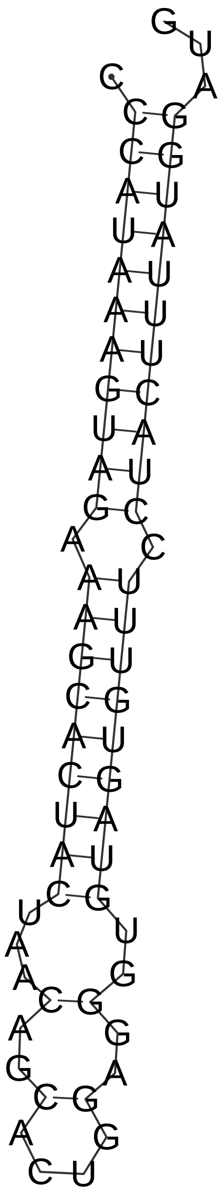

9_32977393-3297745_cfa-mir-142_high

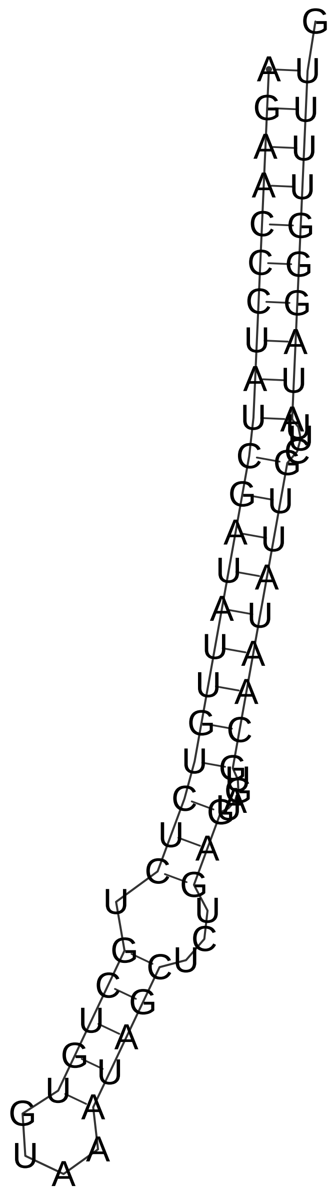

9_33714192-3371426_cfa-mir-454_high

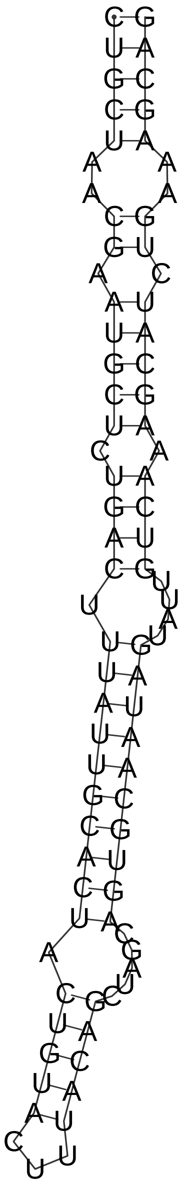

9_33724572-3372465_cfa-mir-301a_high

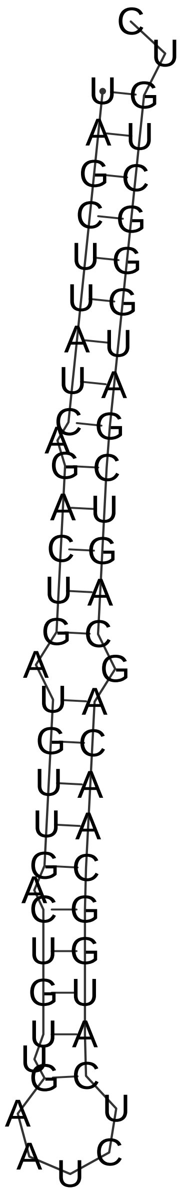

9_34340550-3434060_cfa-mir-21_high

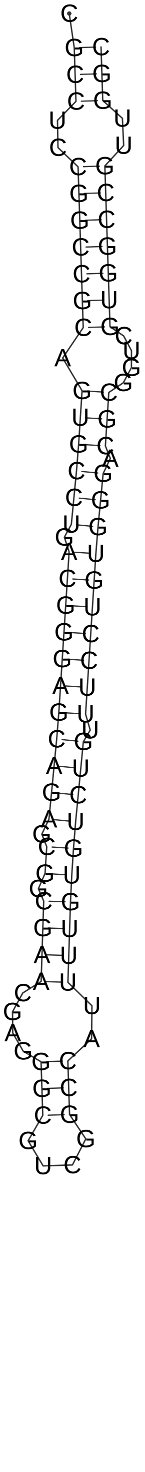

9_40612586-4061268_cfa-mir-632_low

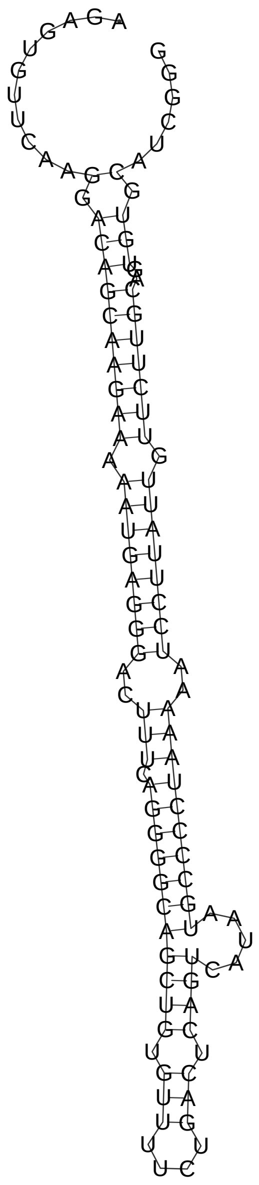

9_41303092-4130320_cfa-mir-365-2_high

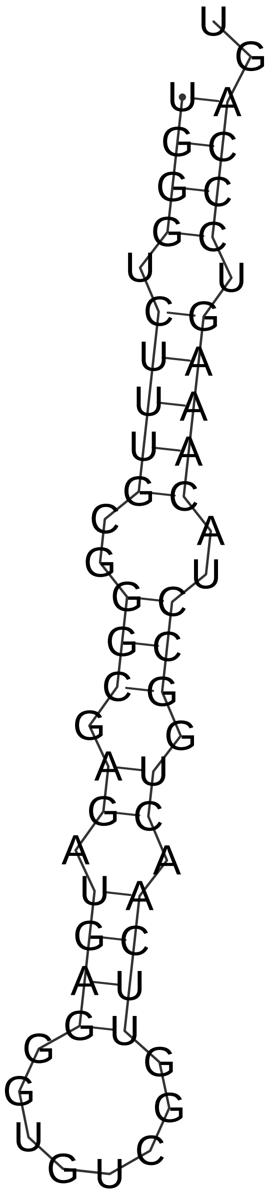

9_41316481-4131653_cfa-mir-193a_high

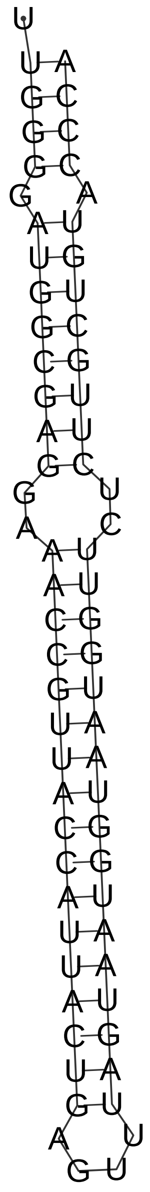

9_43030863-4303093_cfa-mir-451_high

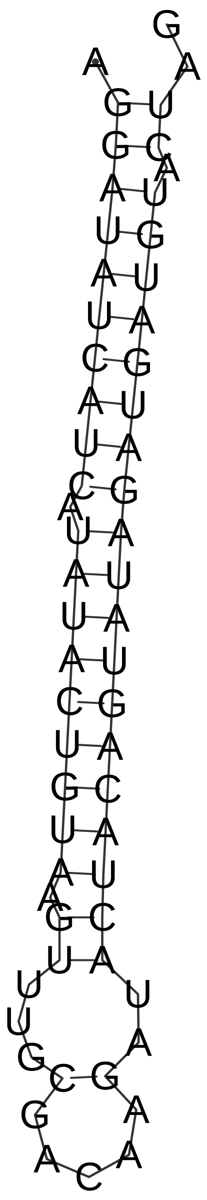

9_43031035-4303109_cfa-mir-144_high

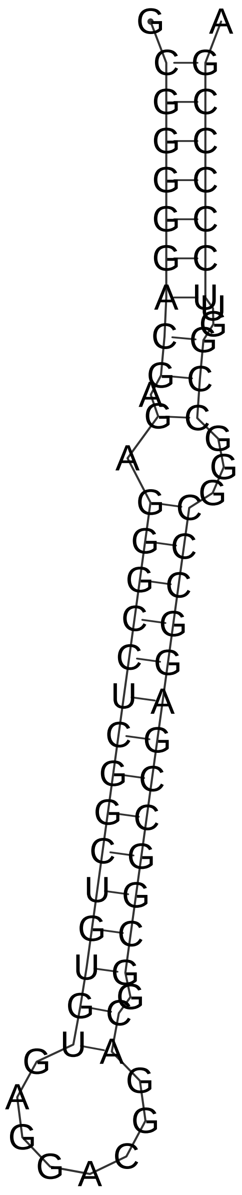

9_43501537-4350160_mir-4523_low

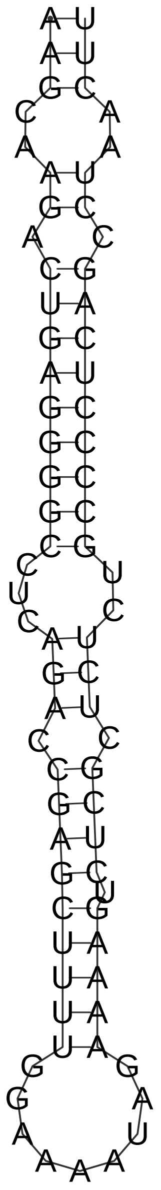

9_44155904-4415597_mir-423_low

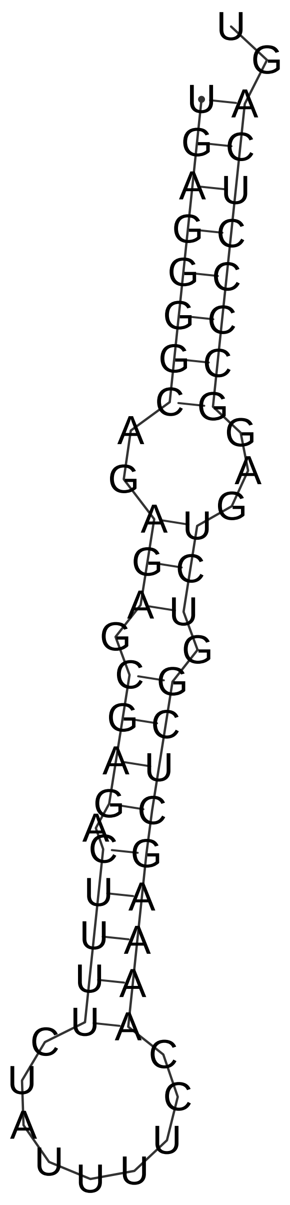

9_44155913-4415597_cfa-mir-423a_high

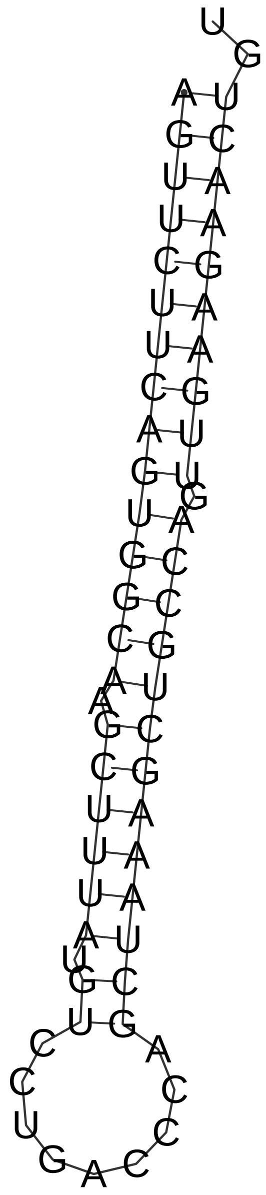

9_45852534-4585259_cfa-mir-22_high

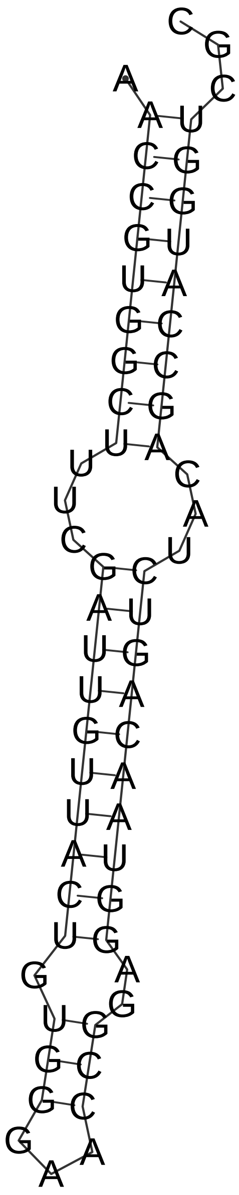

9_46153531-4615359_cfa-mir-132_high

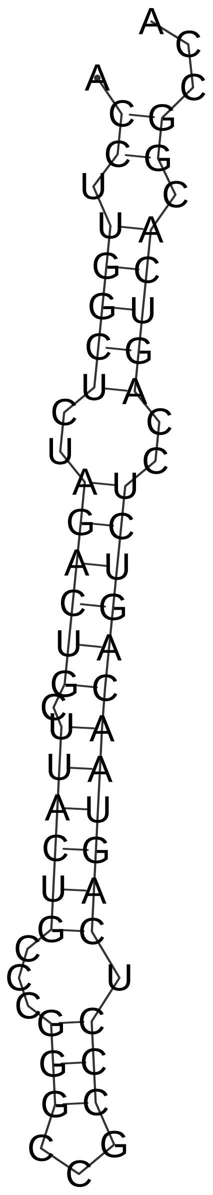

9_46153913-4615397_cfa-mir-212_high

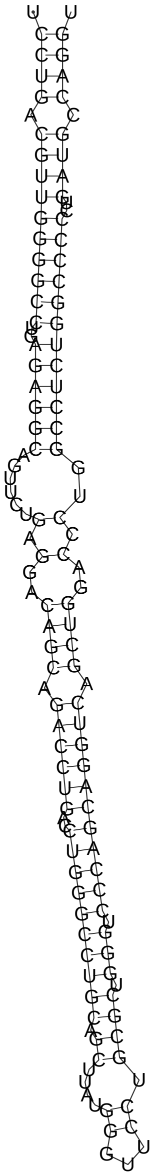

9_47791955-4779208_cfa-mir-8889_low

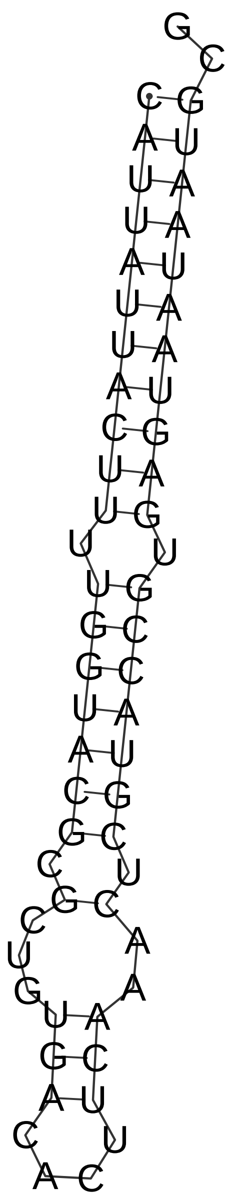

9_48879438-4887949_cfa-mir-126_high

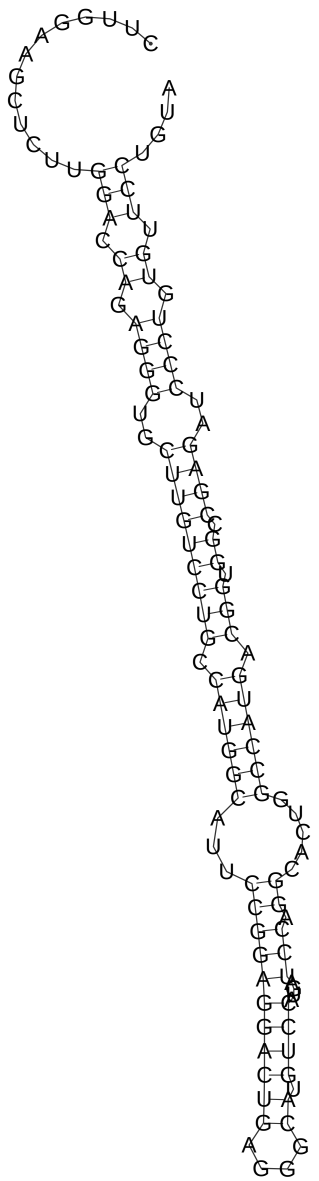

9_54627793-5462790_cfa-mir-8887_low

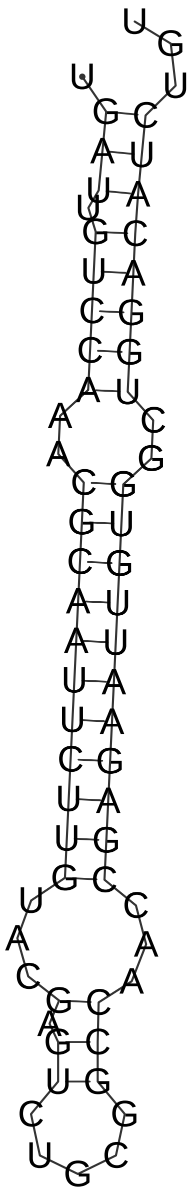

9_55139107-5513917_cfa-mir-219-2_high

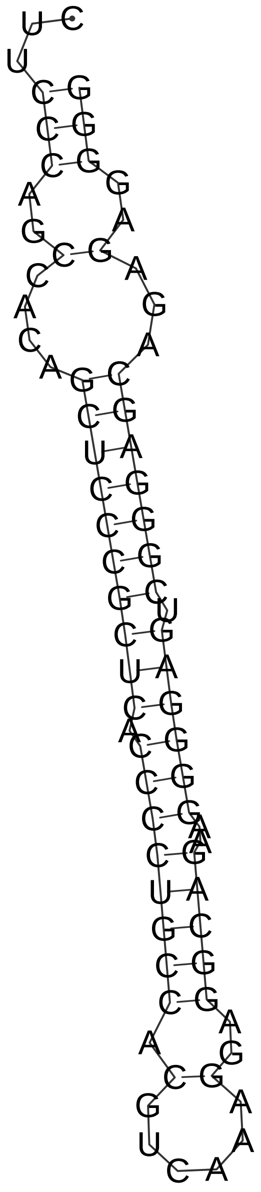

9_55262159-5526223_mir-3154_high

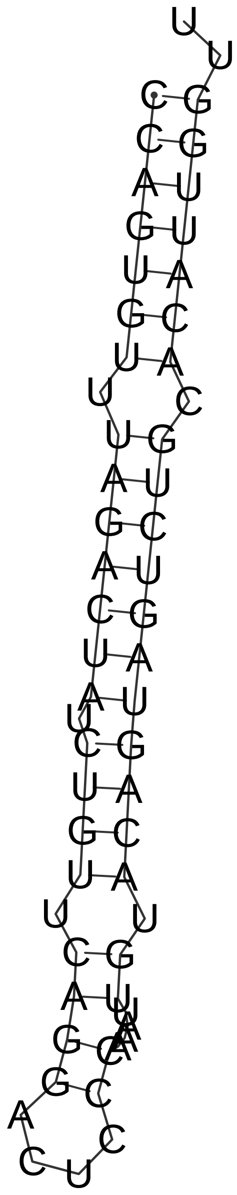

9_55262354-5526241_cfa-mir-199-3_high

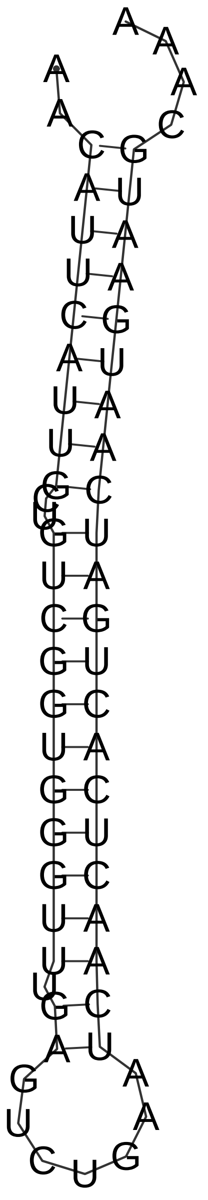

9_58301314-5830137_cfa-mir-181b-2_high

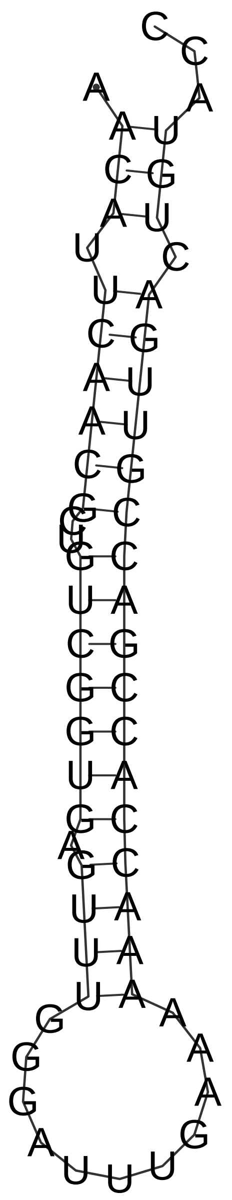

9_58302547-5830260_cfa-mir-181a-2_high

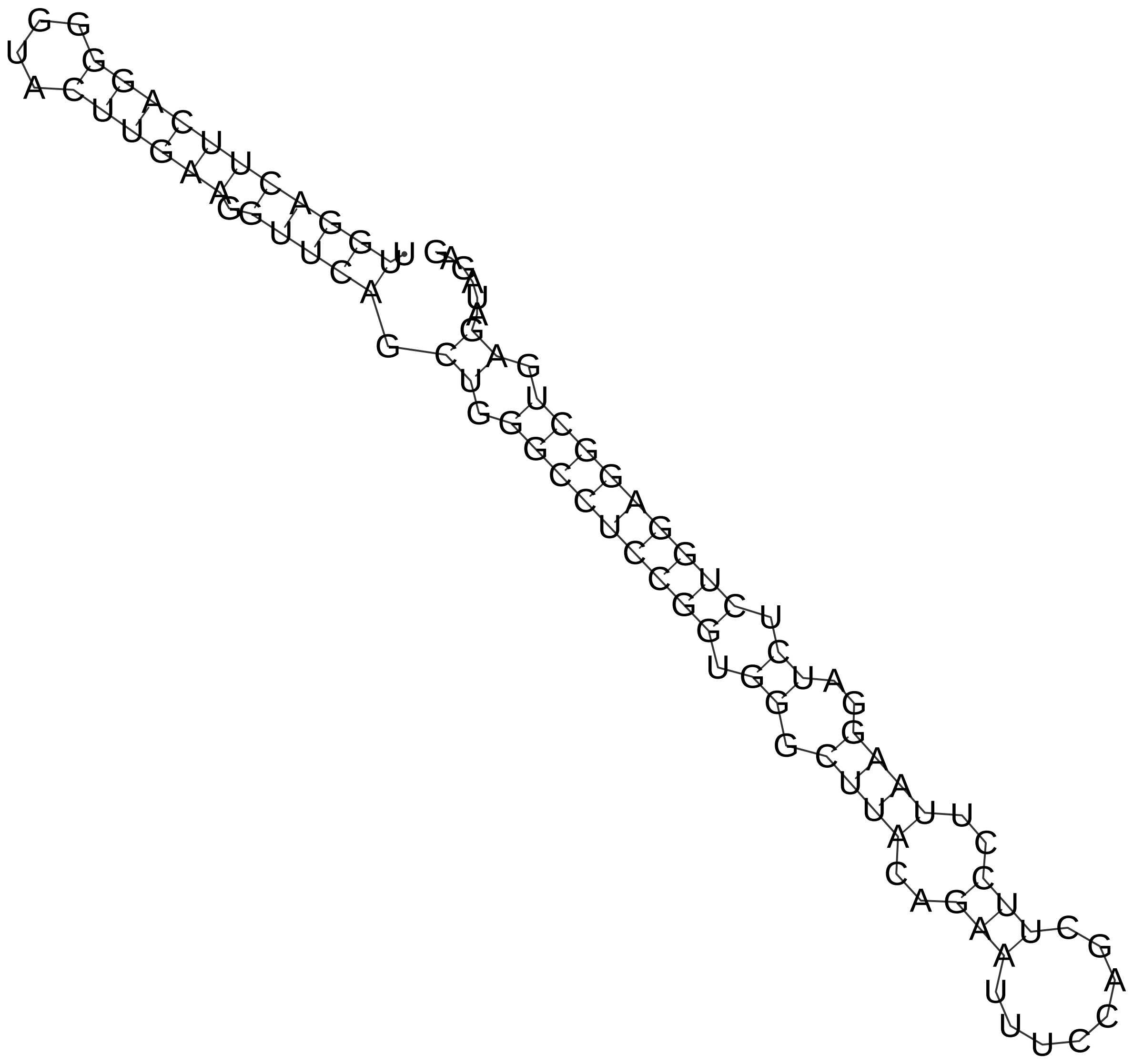

AAEX03022212_16938-1703_mir-8841_low

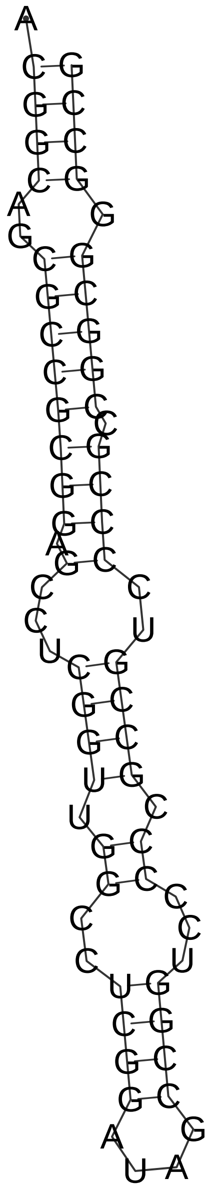

AAEX03024183_8568-863_mir-2904_low

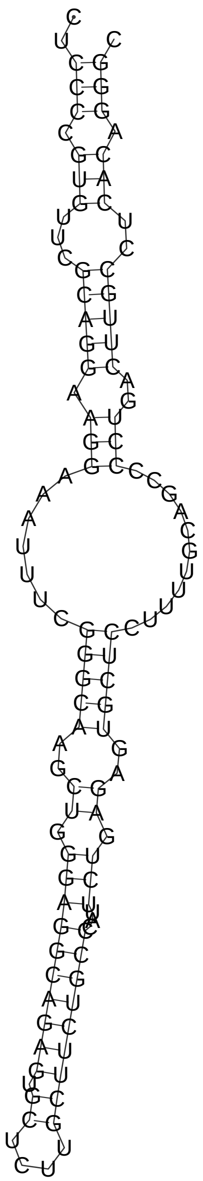

AAEX03024228_2238-234_cfa-mir-8896_low

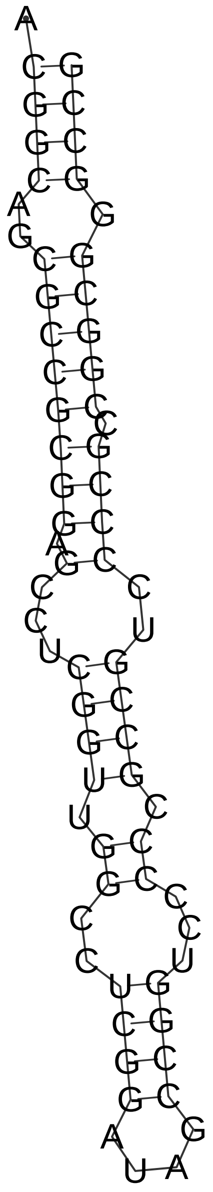

AAEX03024828_6548-661_mir-2904_low

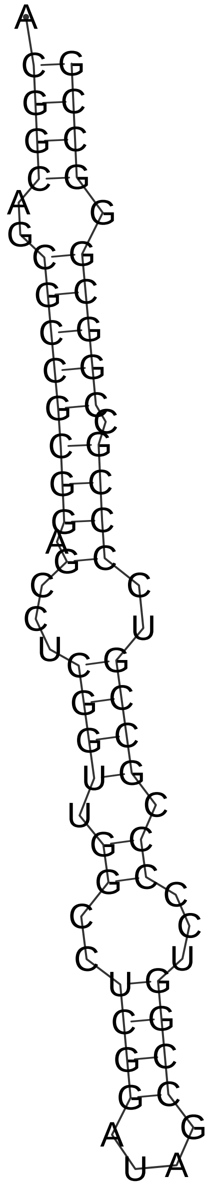

AAEX03025000_461-52_mir-2904_low

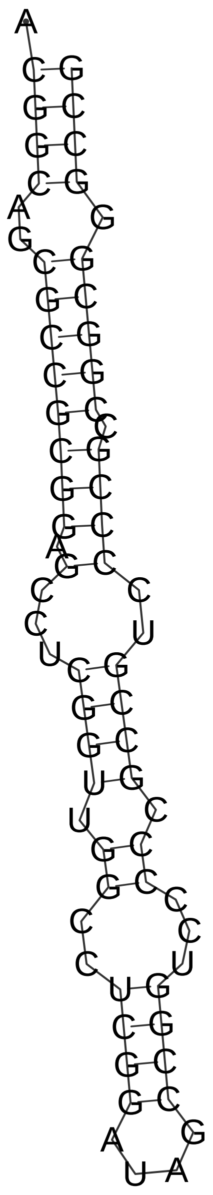

AAEX03025102_5518-558_mir-2904_low

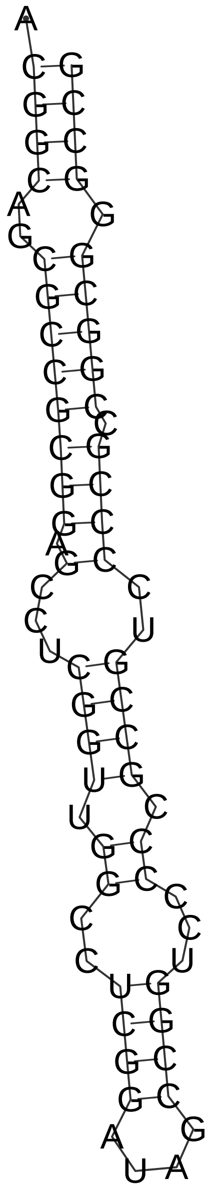

AAEX03025132_5588-565_mir-2904_low

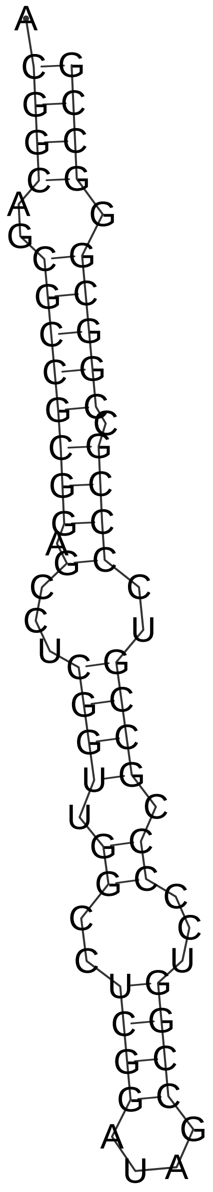

AAEX03025416_5934-600_mir-2904_low

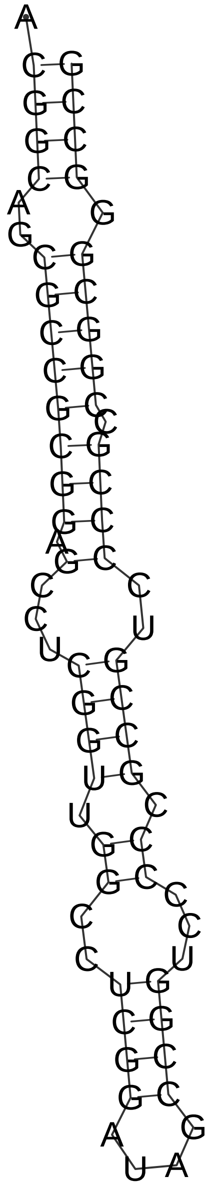

AAEX03025524_4458-452_mir-2904_low

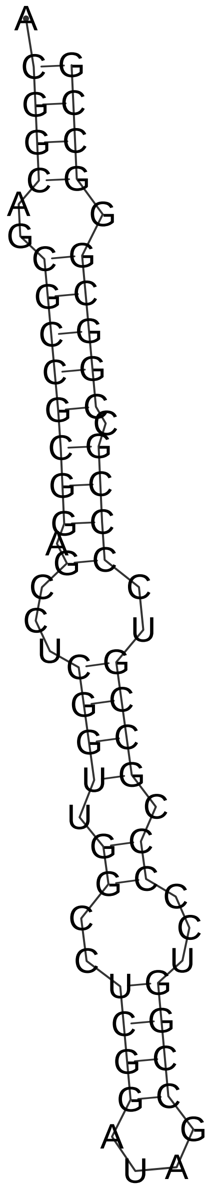

AAEX03025644_1229-129_mir-2904_low

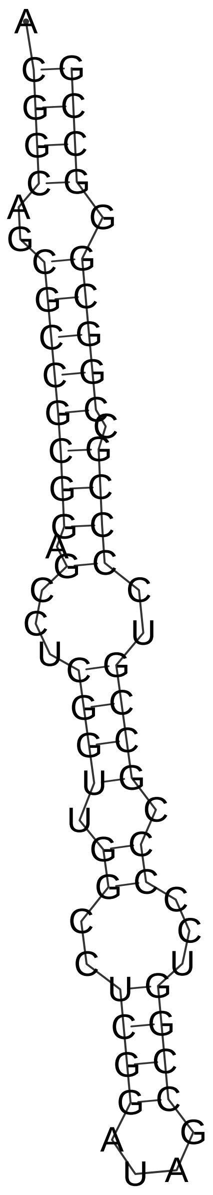

AAEX03025665_4046-411_mir-2904_low

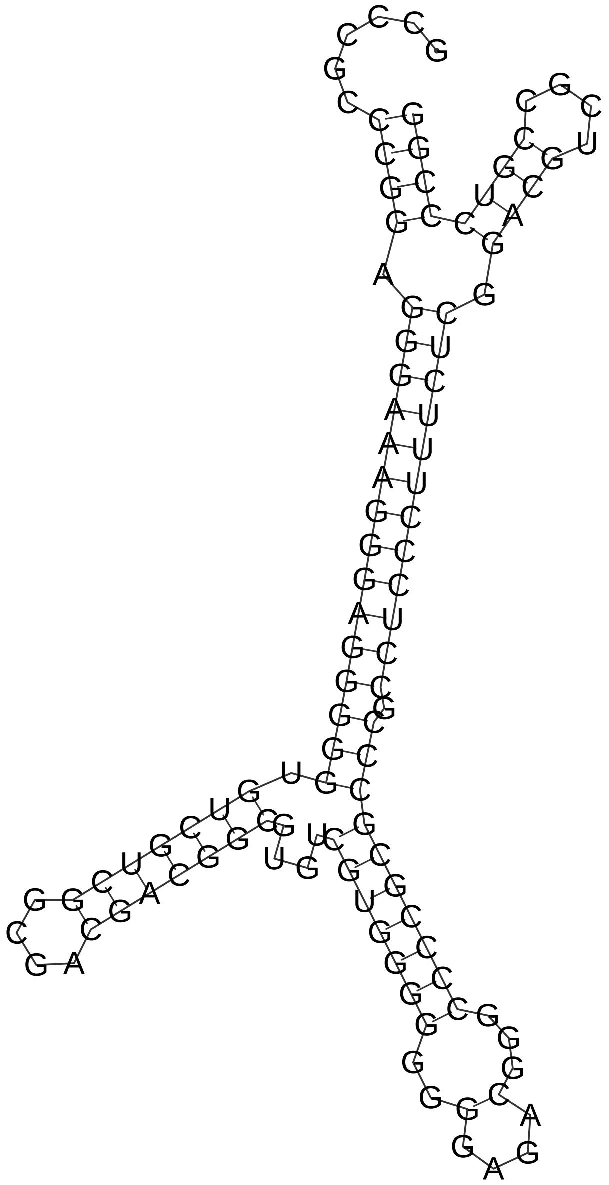

AAEX03025866_3500-360_mir-8865_low

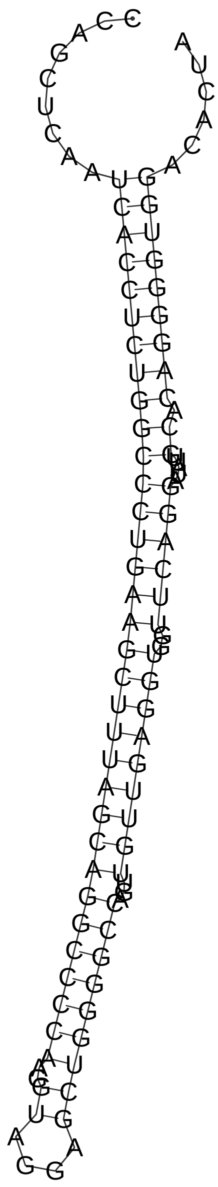

JH373239_456681-45678_mir-8793_low

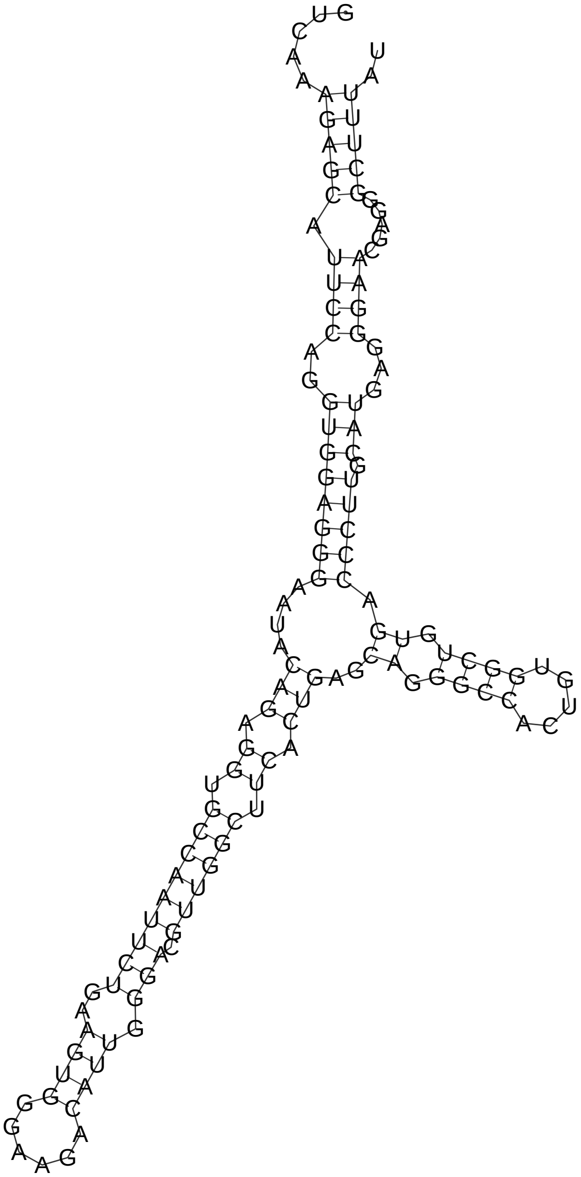

JH373239_484473-48460_mir-8907_low

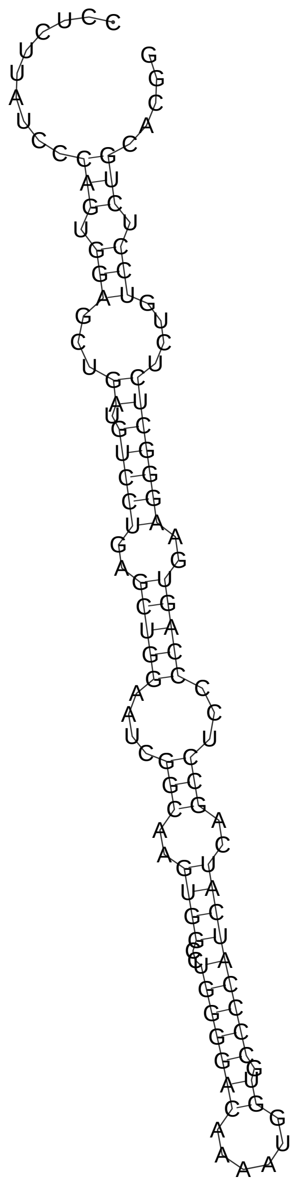

JH373319_81142-8125_mir-8794_low

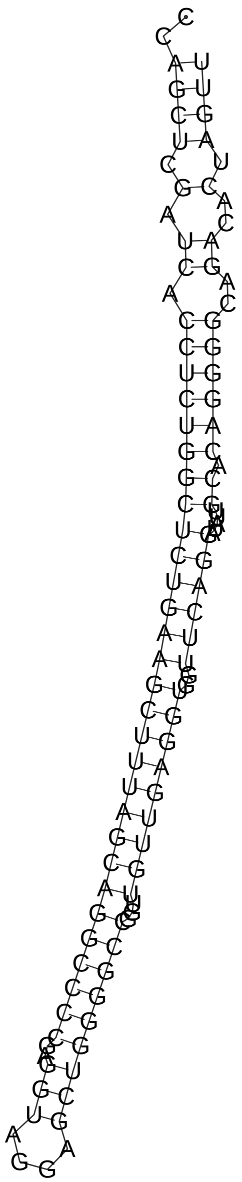

JH373319_98211-9831_mir-8793_low

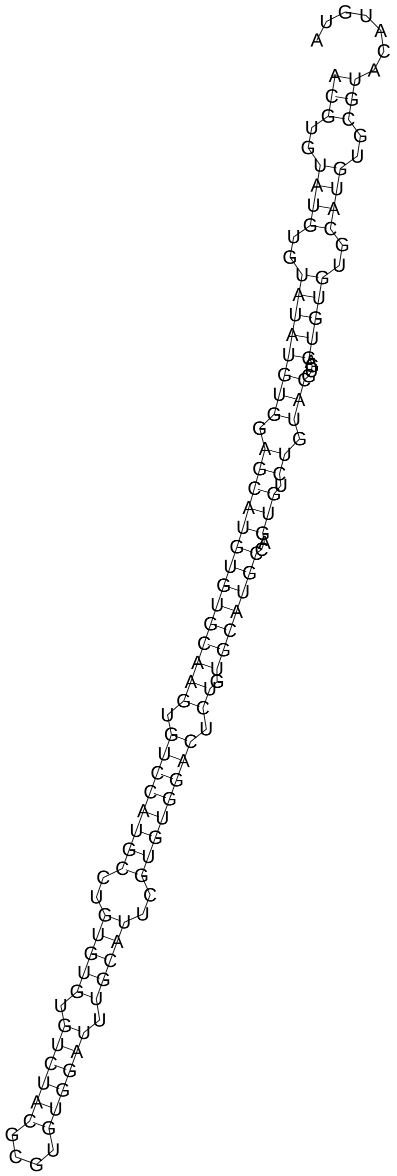

JH373319_109588-10972_mir-8852_low

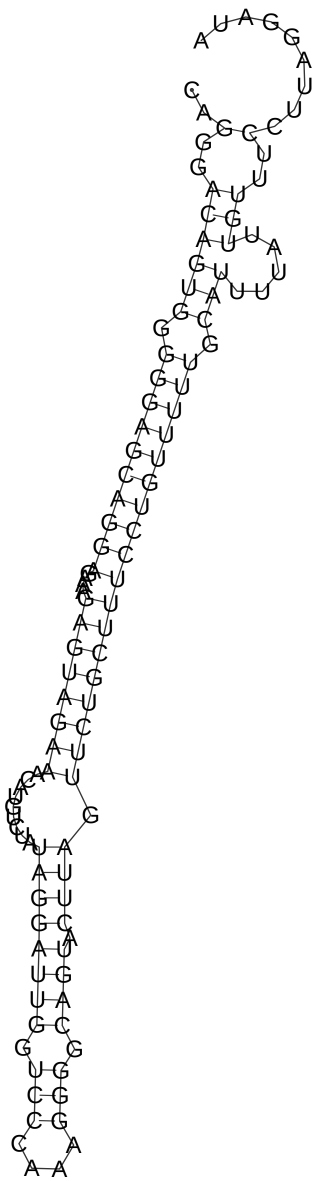

JH373377_11739-1185_cfa-mir-8869_low

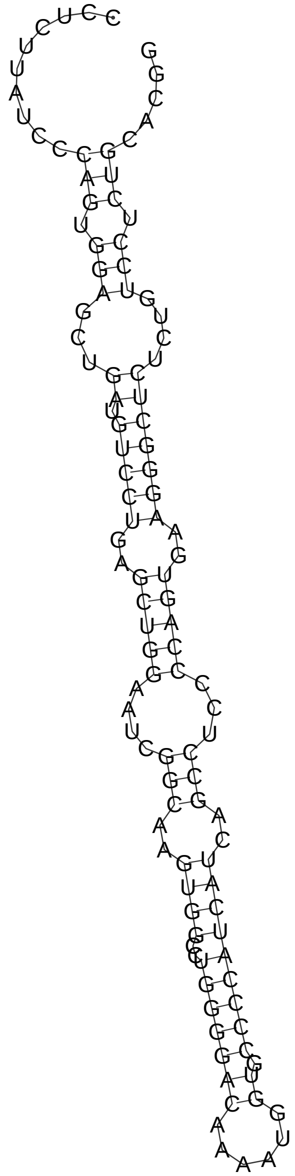

JH373389_12198-1231_mir-8794_low

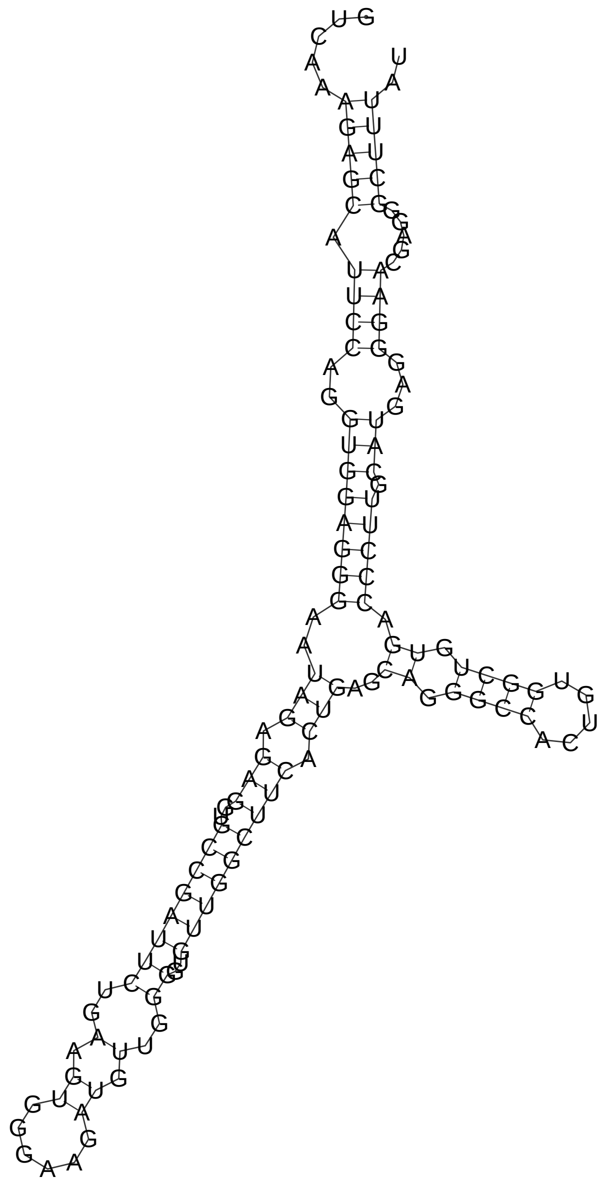

JH373389_15358-1548_cfa-mir-8907_low

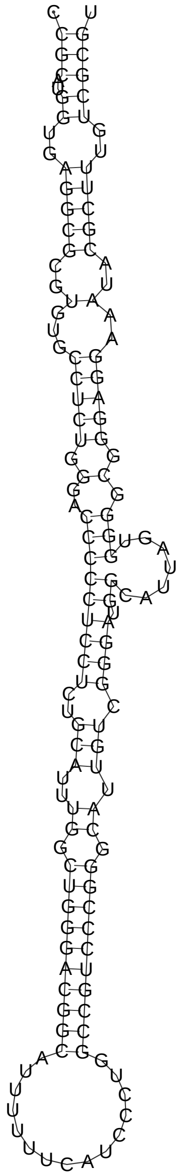

JH373389_61631-6176_mir-8906_low

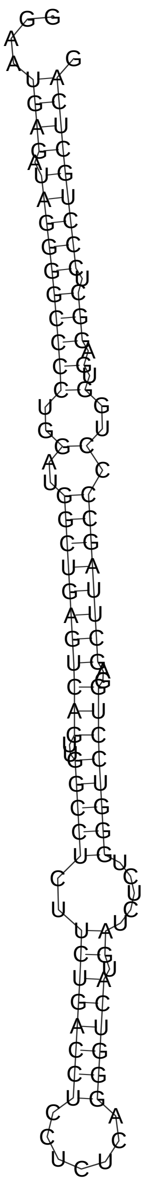

JH373412_34331-3444_mir-8864_low

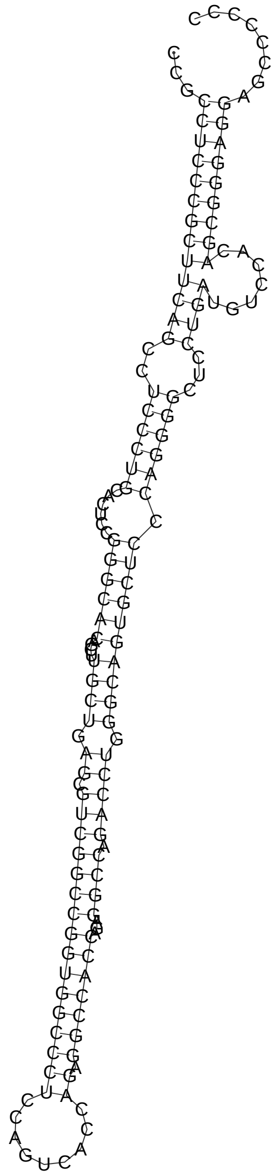

JH373426_11694-1183_mir-8862_low

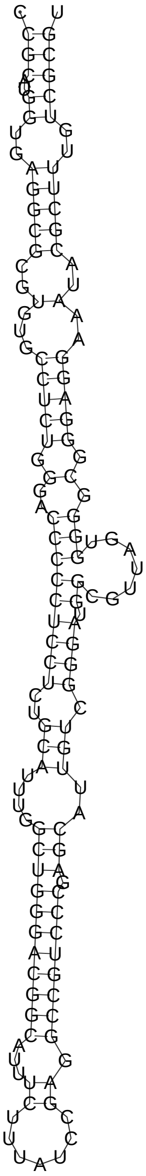

JH373426_19686-1982_mir-8906_low

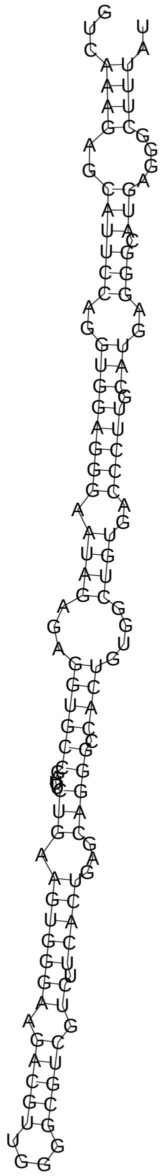

JH373426_37343-3747_mir-8907_low

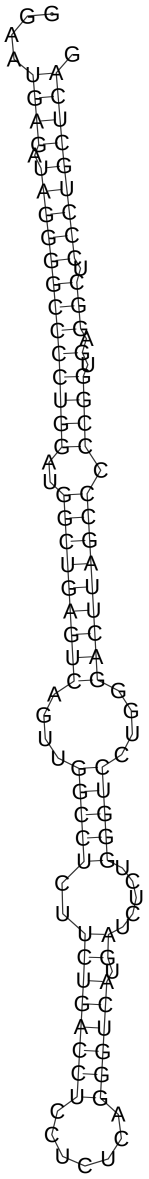

JH373432_27872-2798_mir-8864_low

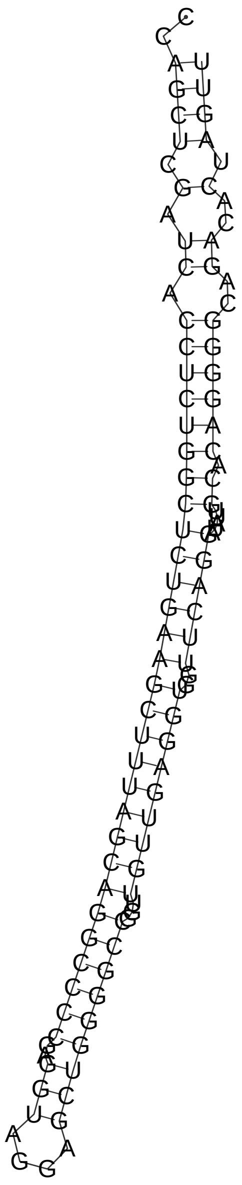

JH373475_25818-2592_mir-8793_low

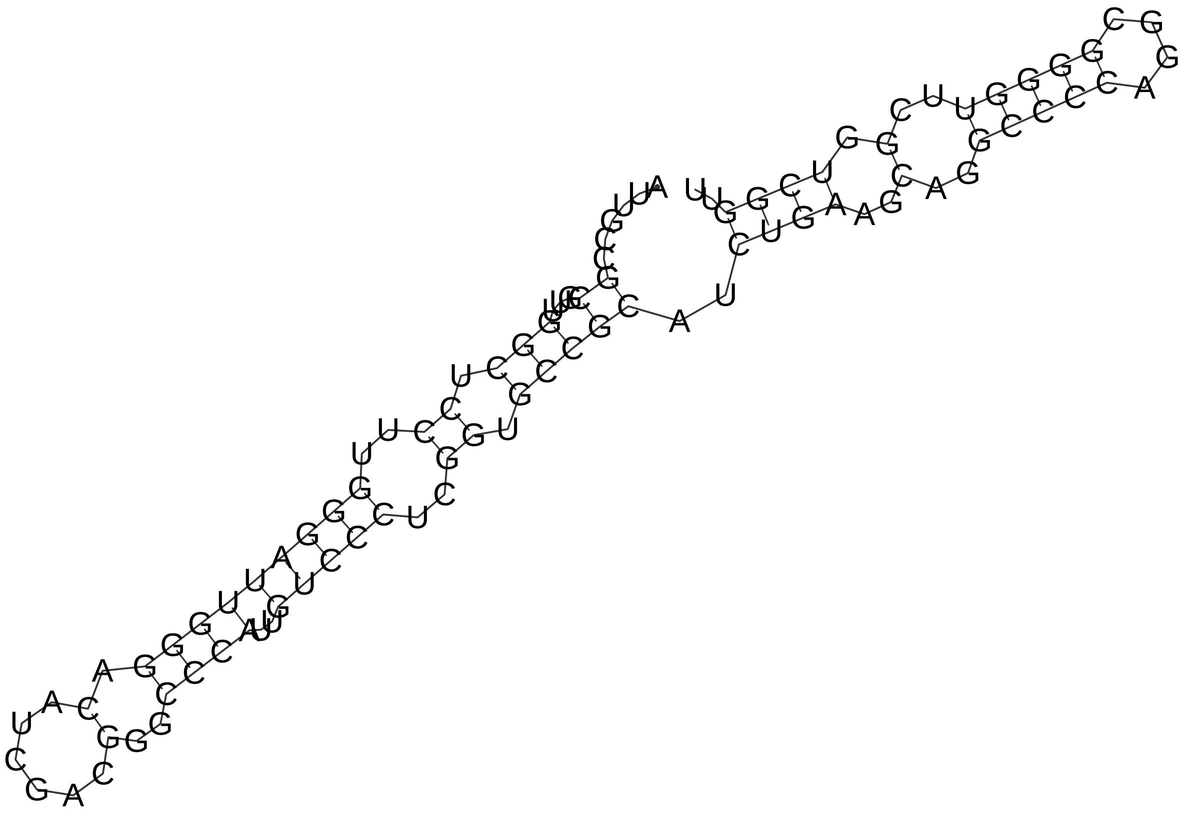

JH373475_34171-3426_cfa-mir-8849_low

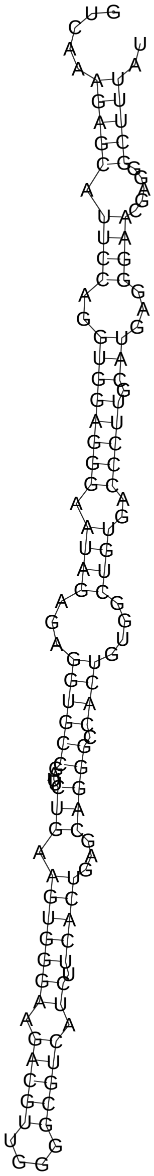

JH373475_42635-4276_mir-8907_low

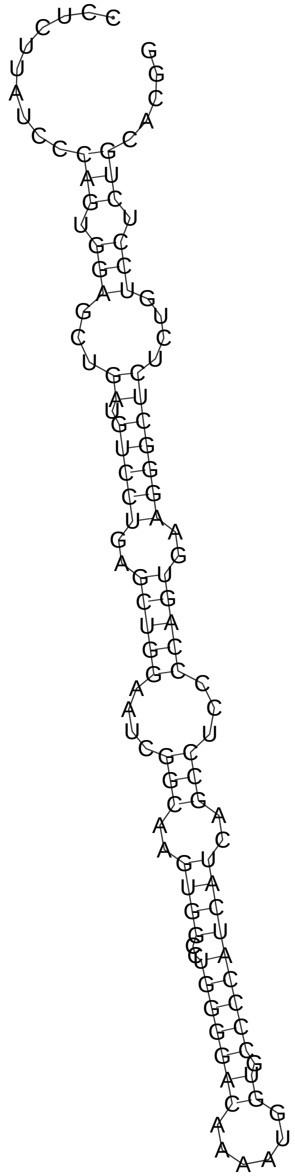

JH373475_45815-4592_mir-8794_low

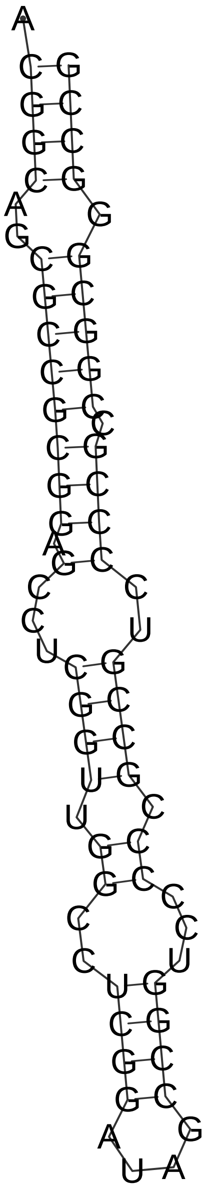

JH373485_14855-1492_mir-2904_low

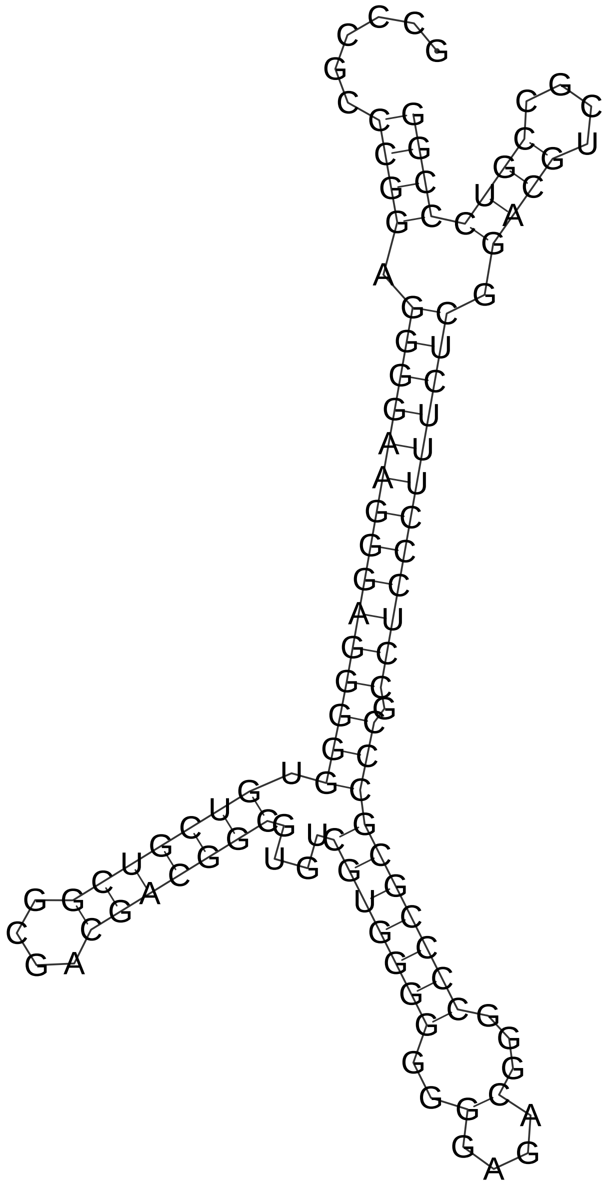

JH373485_22229-2233_mir-8865_low

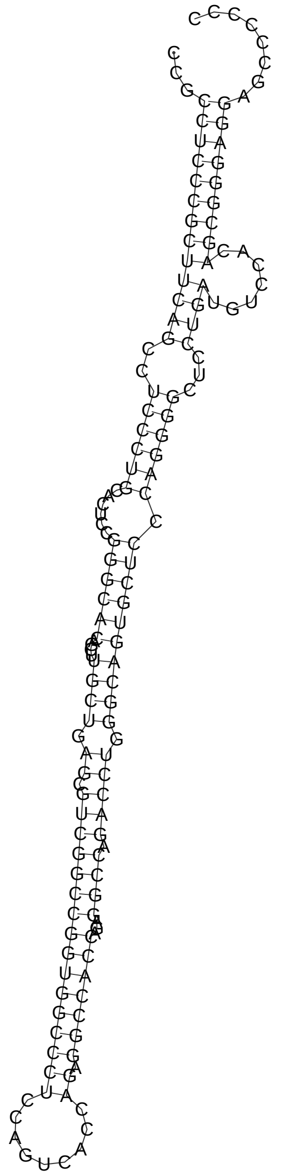

JH373579_13947-1409_mir-8862_low

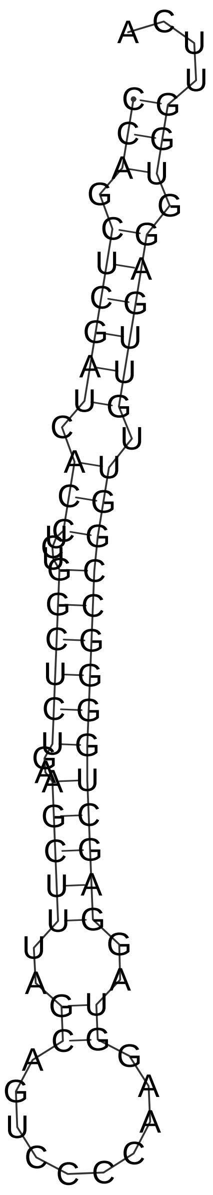

JH373600_19752-1982_mir-8793_low

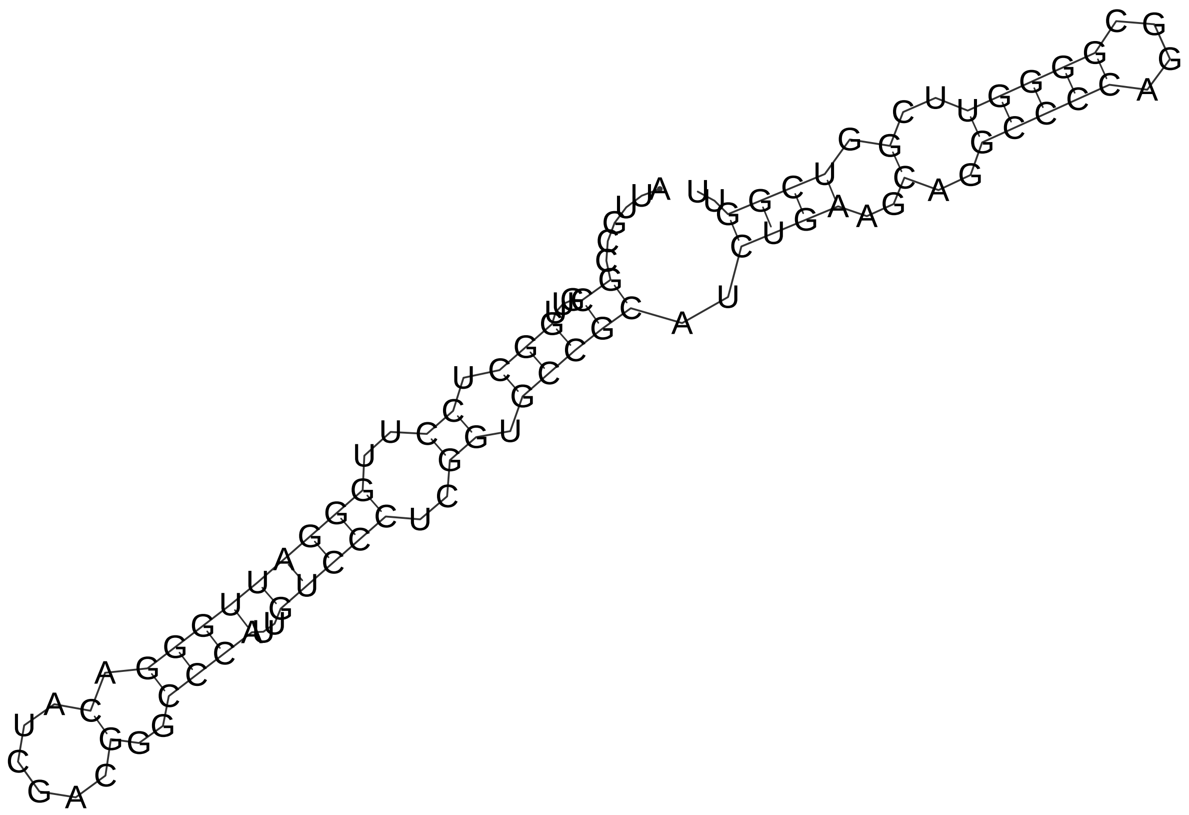

JH373600_27983-2807_cfa-mir-8849_low

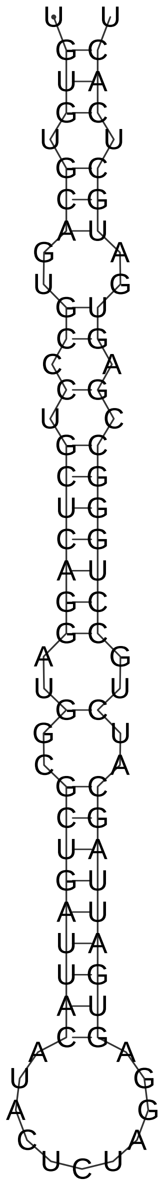

JH373668_949-103_cfa-mir-8908c_high

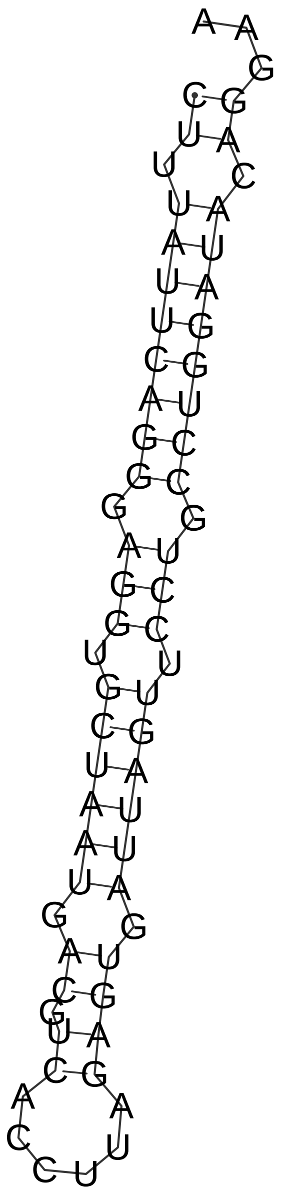

JH373668_7412-747_mir-8908b_high

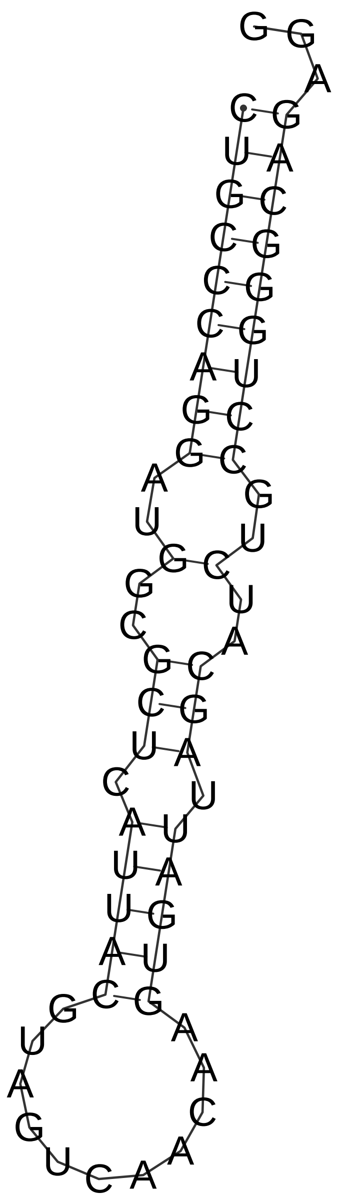

JH373668_10365-1042_mir-8908_high

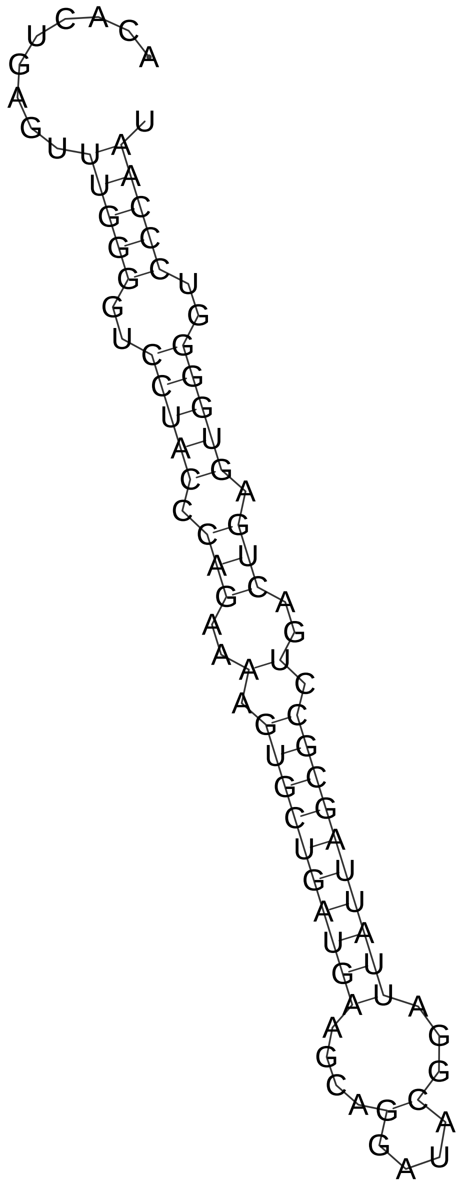

JH373668_23671-2375_mir-8908_high

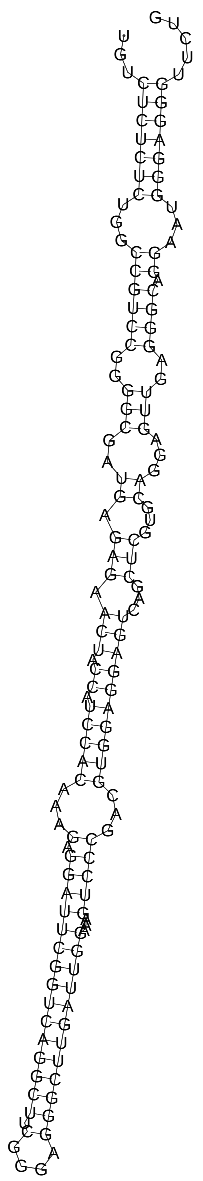

JH373721_20094-2023_mir-8905_low

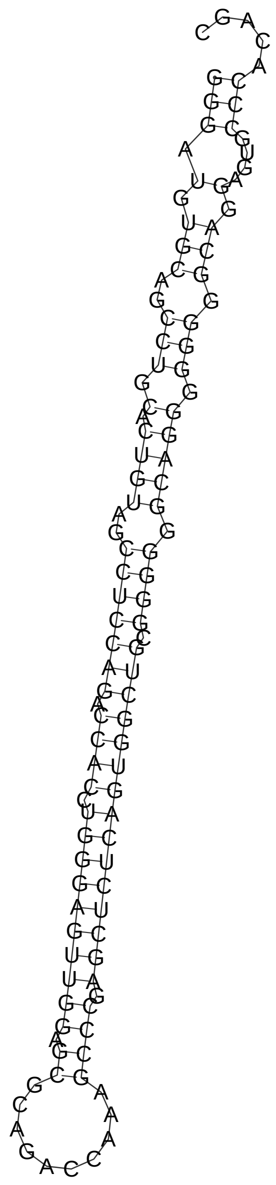

JH373733_3510-362_cfa-mir-8863_low

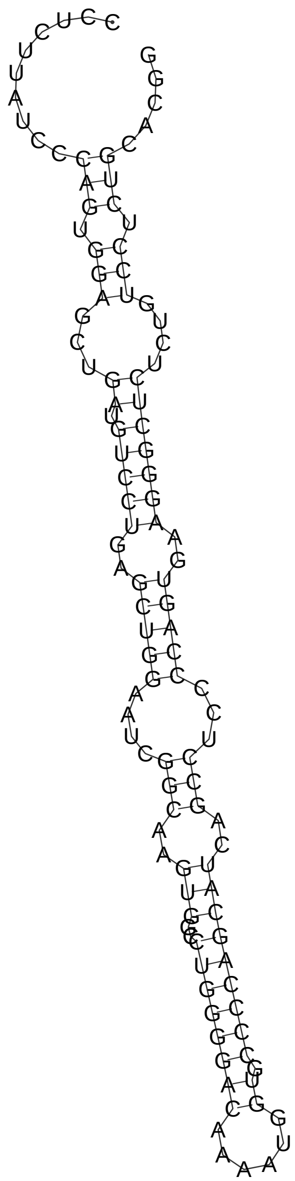

JH373733_12166-1227_mir-8794_low

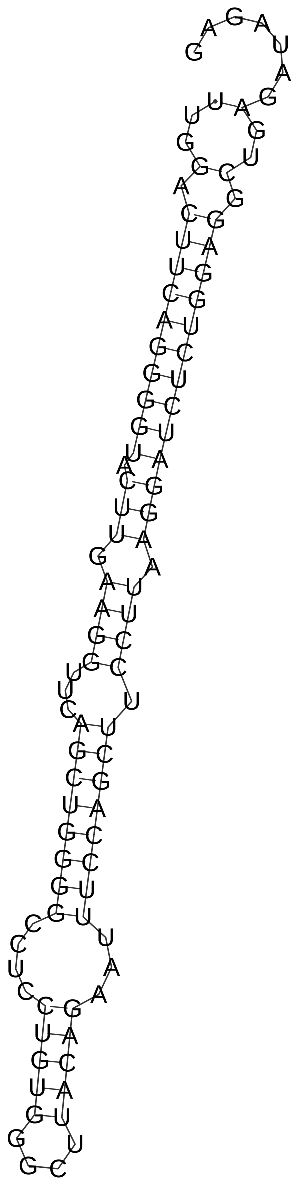

JH373758_16181-1627_mir-8841_low

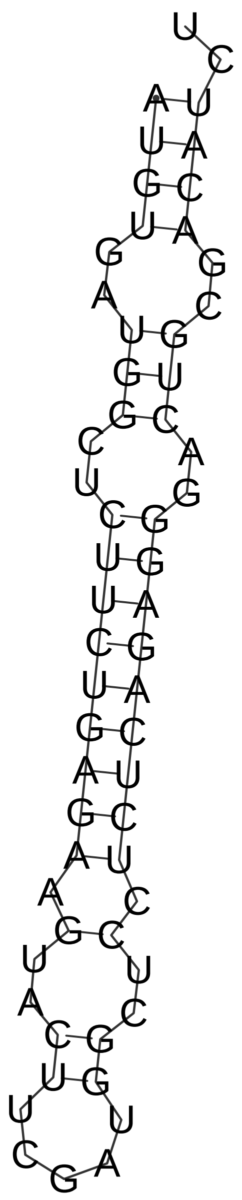

JH373785_7319-737_cfa-mir-1837-3_high

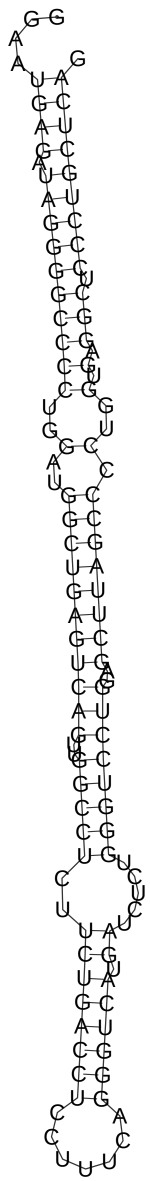

JH373864_5187-529_mir-8864_low

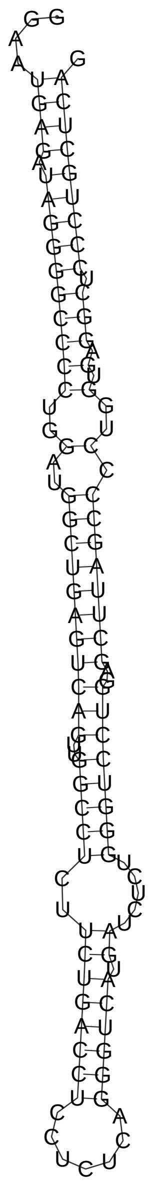

JH374064_3469-358_mir-8864_low

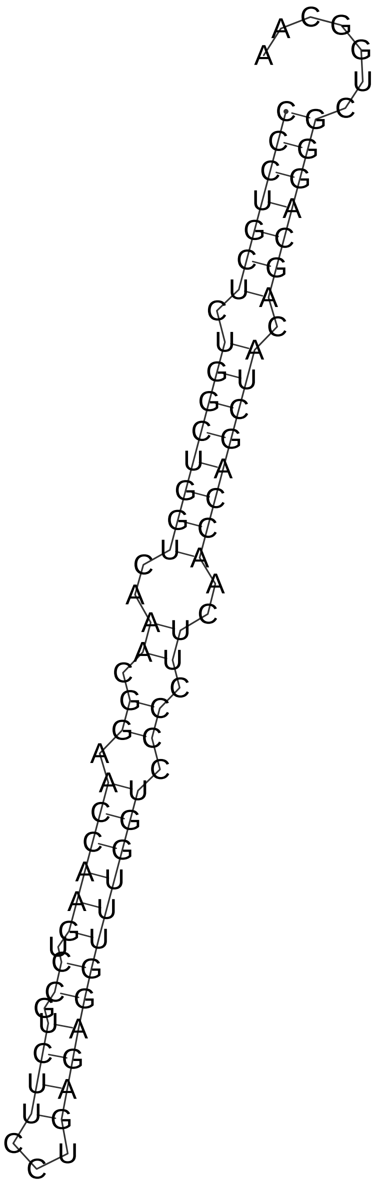

JH374091_8441-852_cfa-mir-133b_high

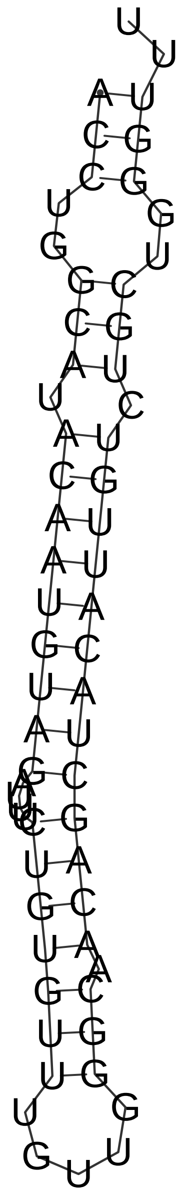

X_39523890-3952395_cfa-mir-221_high

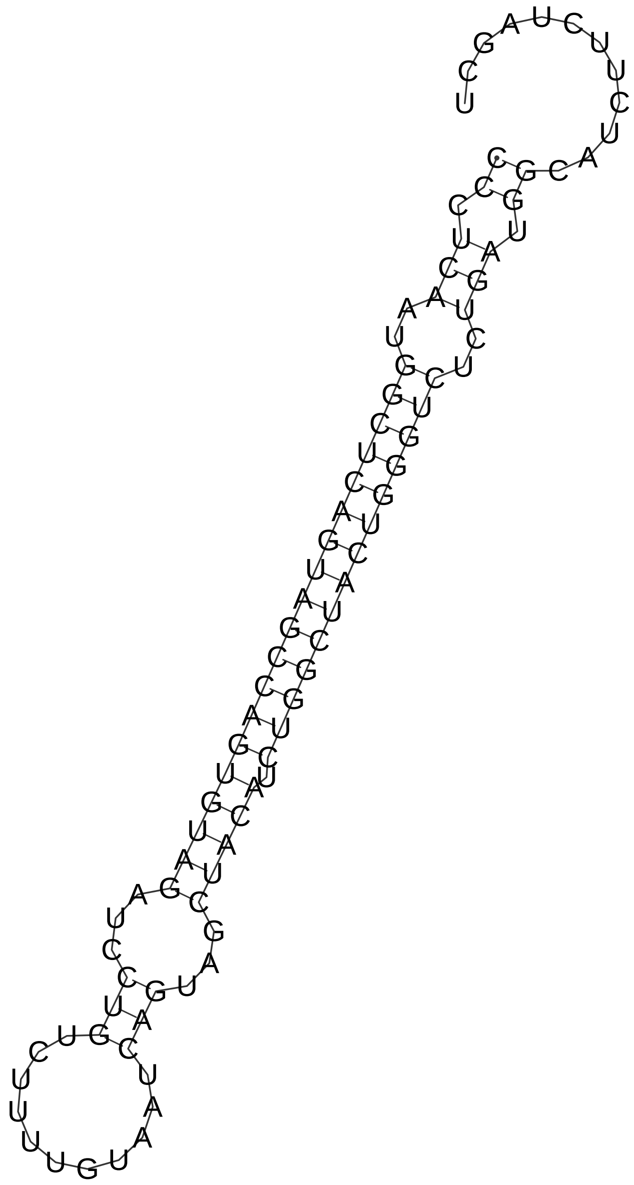

X_39524519-3952460_cfa-mir-222_high

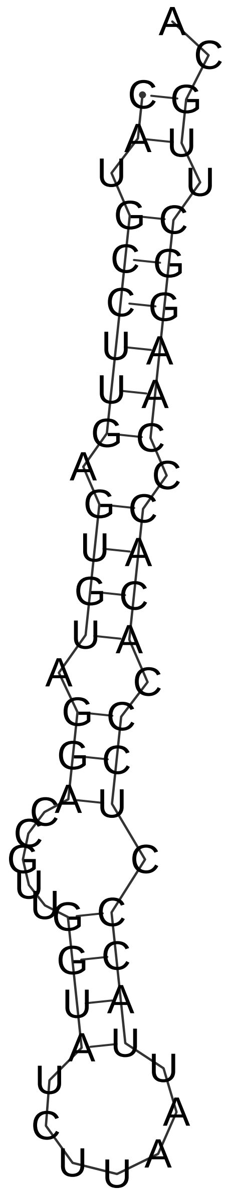

X_42774680-4277473_cfa-mir-532_high

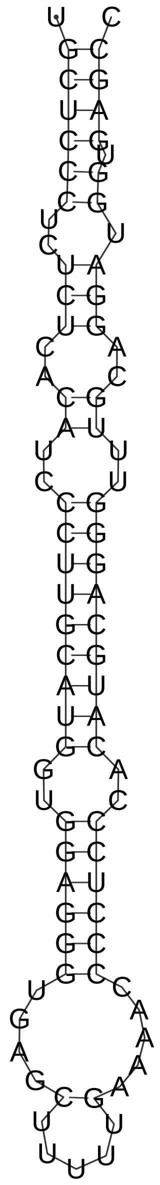

X_42775058-4277514_cfa-mir-188_high

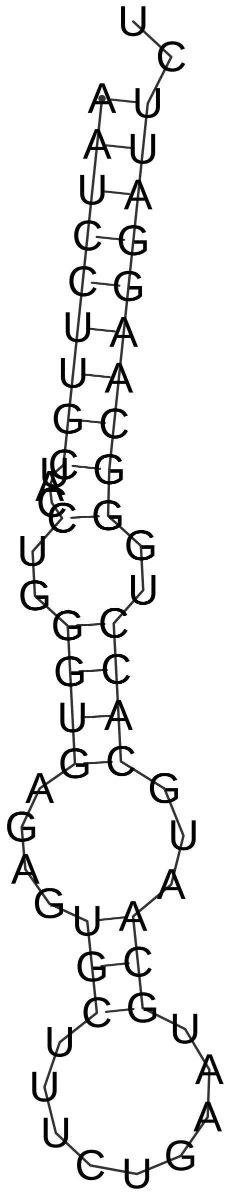

X_42779872-4277993_cfa-mir-500_high

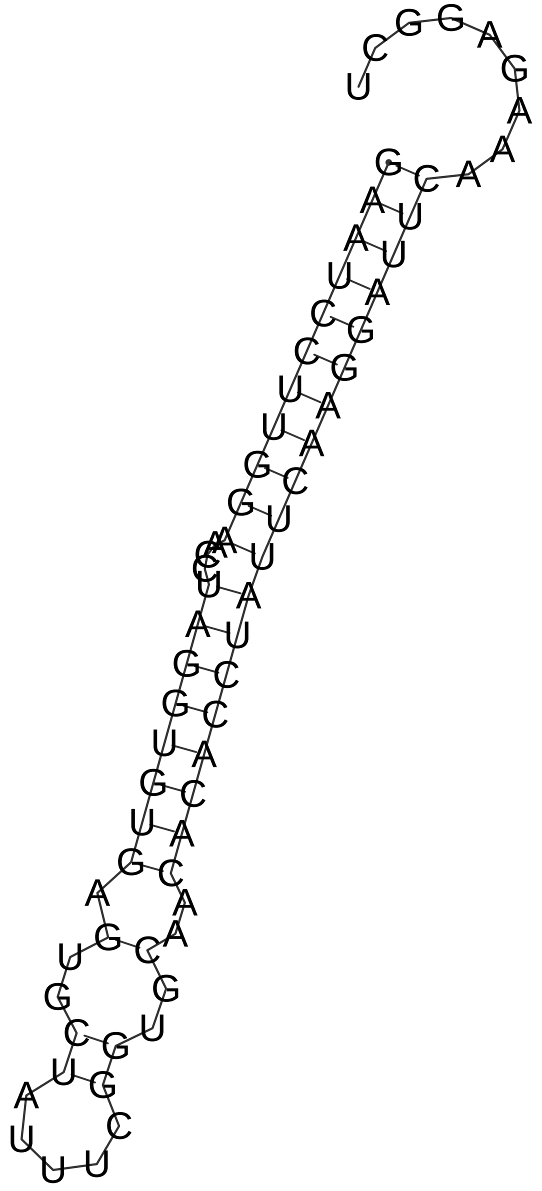

X_42780368-4278043_cfa-mir-362_high

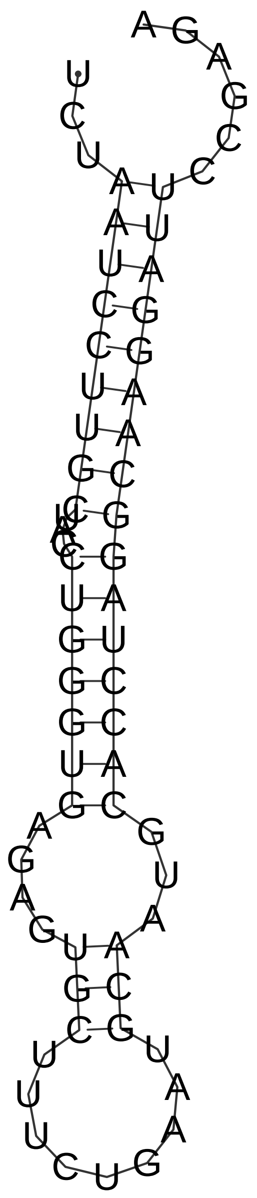

X_42781867-4278193_mir-500_high

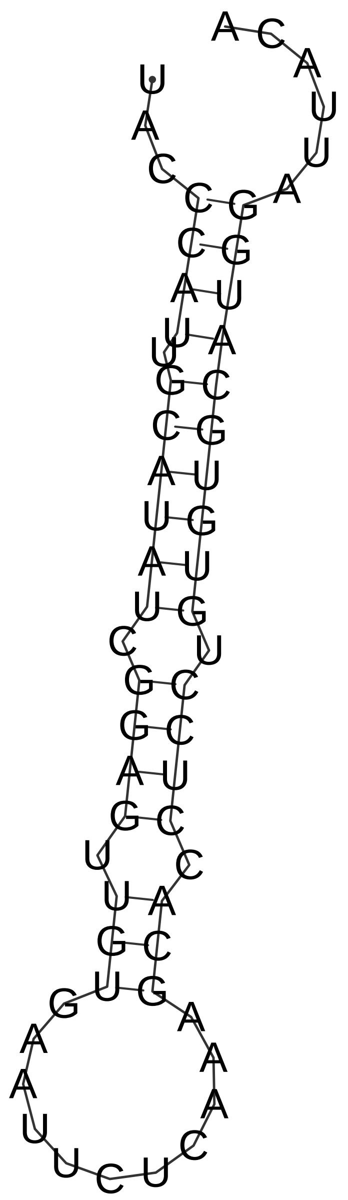

X_42783965-4278402_cfa-mir-660_high

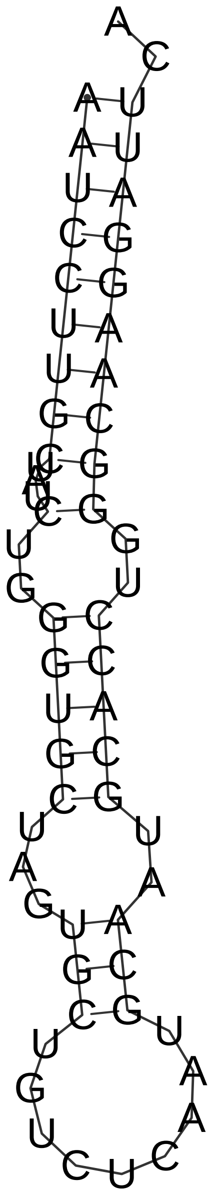

X_42785601-4278565_cfa-mir-502_high

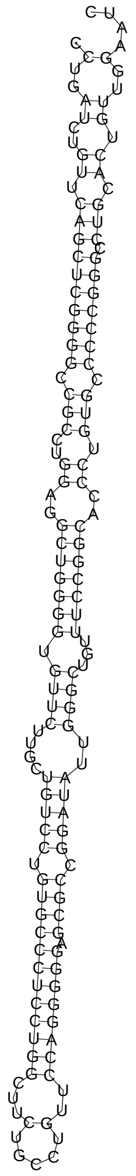

X_43758547-4375869_cfa-mir-8791b_low

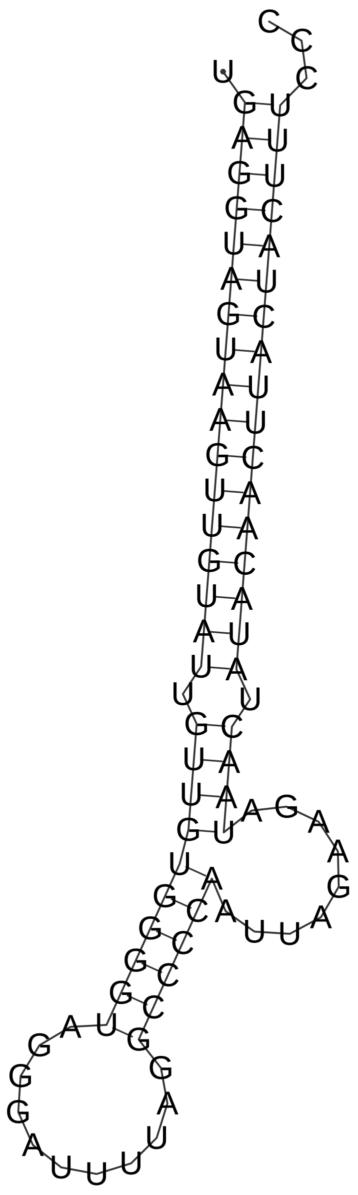

X_45253693-4525377_cfa-mir-98_high

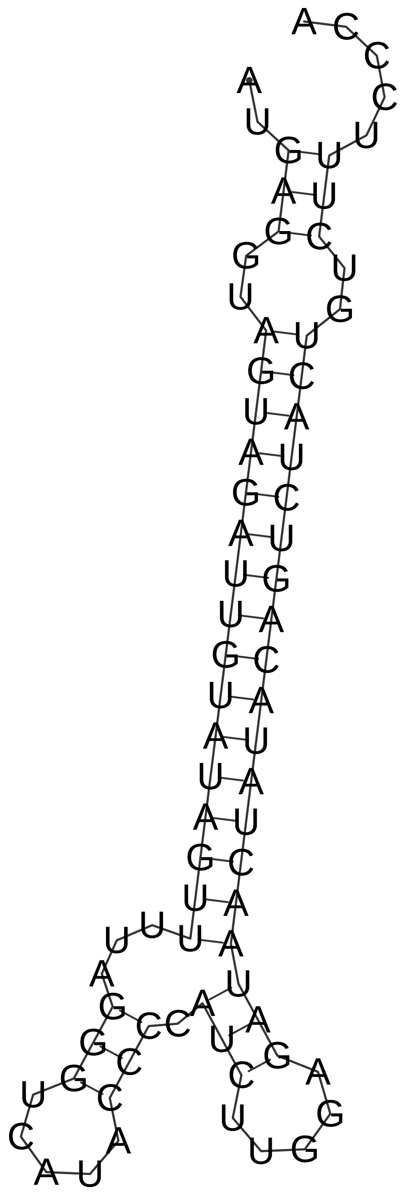

X_45254534-4525460_let-7f_high

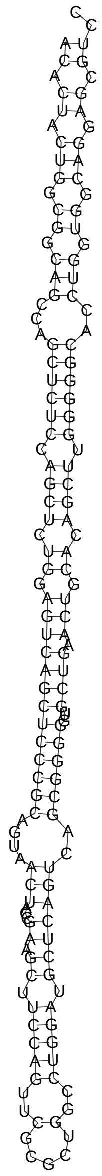

X_47487713-4748785_cfa-mir-8834b_low

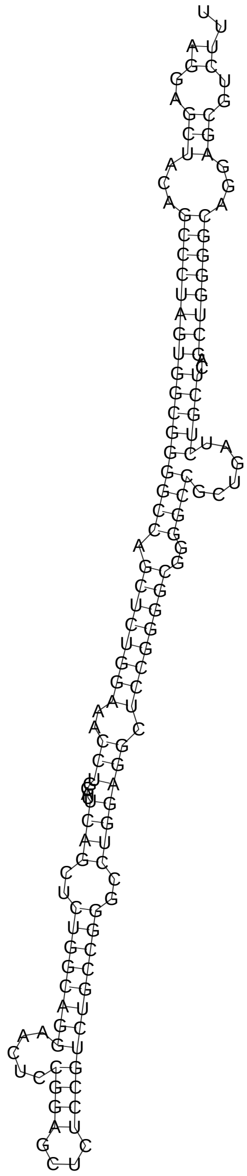

X_48070440-4807058_cfa-mir-8834a-2_low

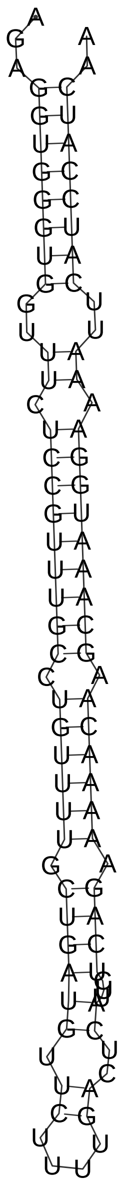

X_49313381-4931347_cfa-mir-1468_high

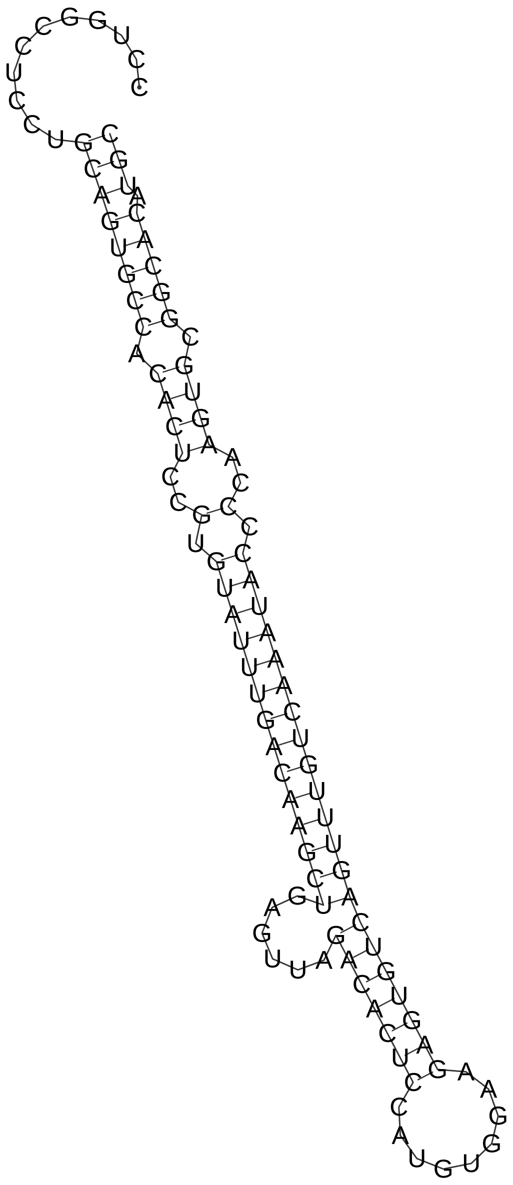

X_50838131-5083823_cfa-mir-223_high

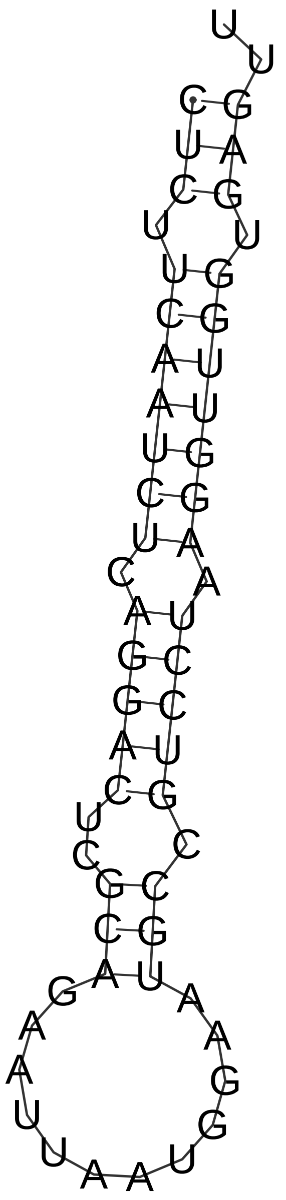

X_54495387-5449544_cfa-mir-676_high

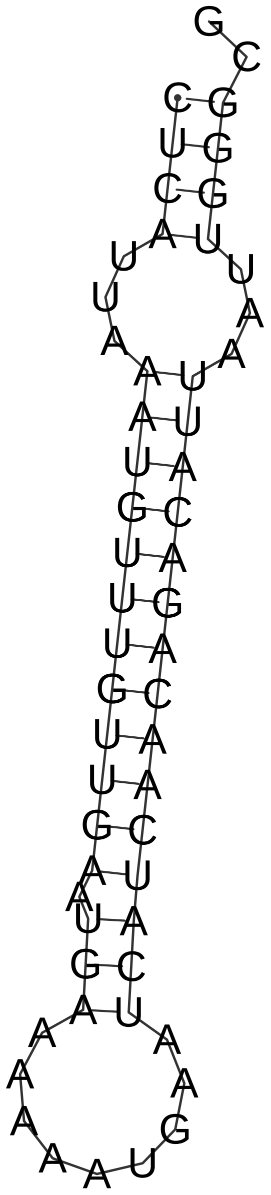

X_57550055-5755011_cfa-mir-421_high

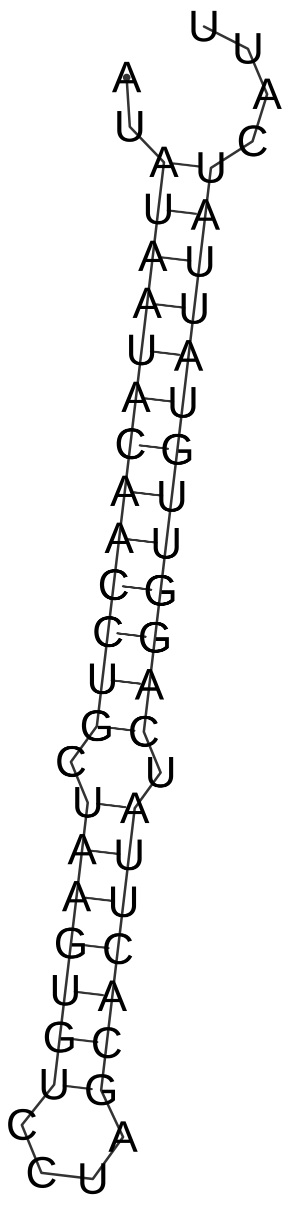

X_57550217-5755026_cfa-mir-374b_high

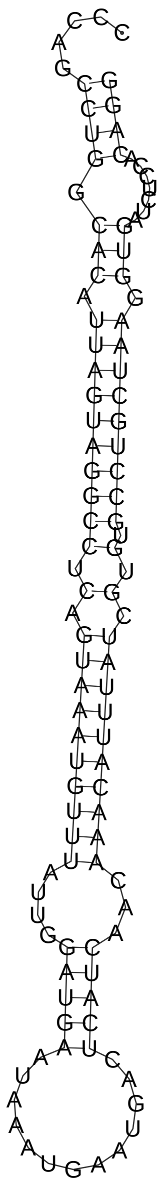

X_57590201-5759030_cfa-mir-545_high

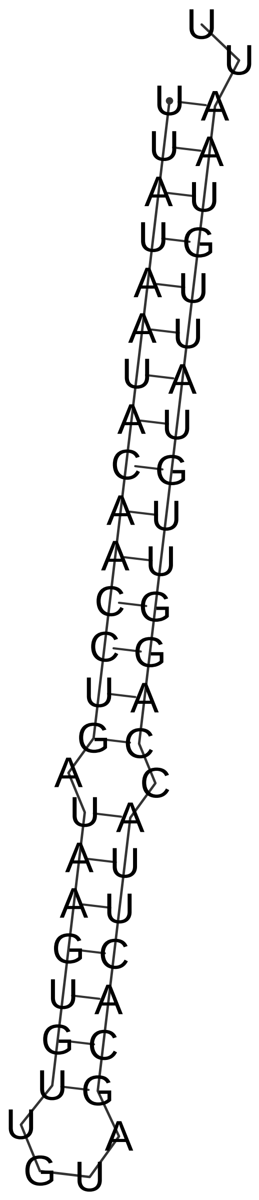

X_57590394-5759044_cfa-mir-374a_high

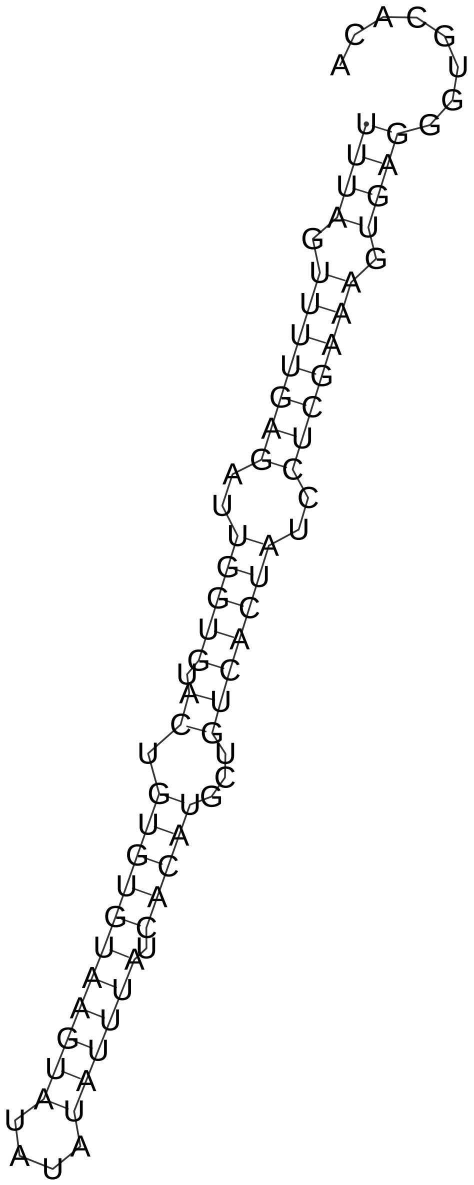

X_58042211-5804229_mir-672_low

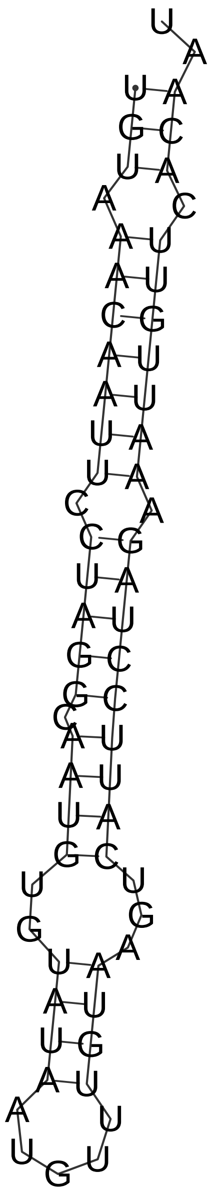

X_59390415-5939047_cfa-mir-384_high

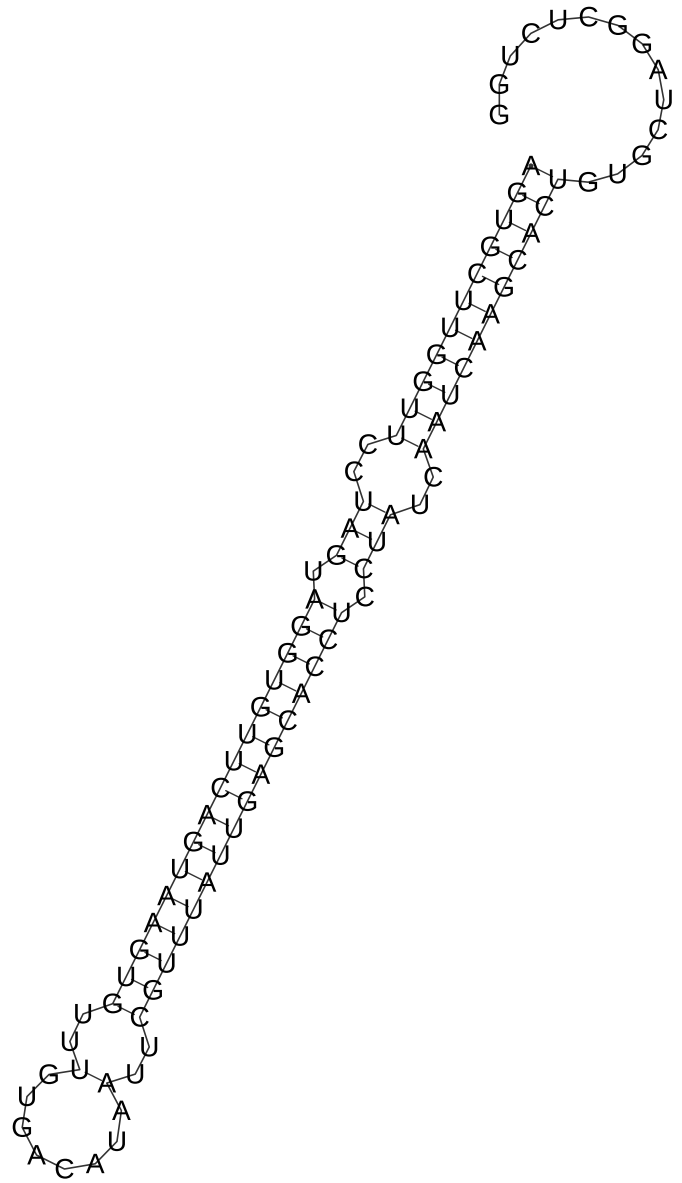

X_59443927-5944402_cfa-mir-325_high

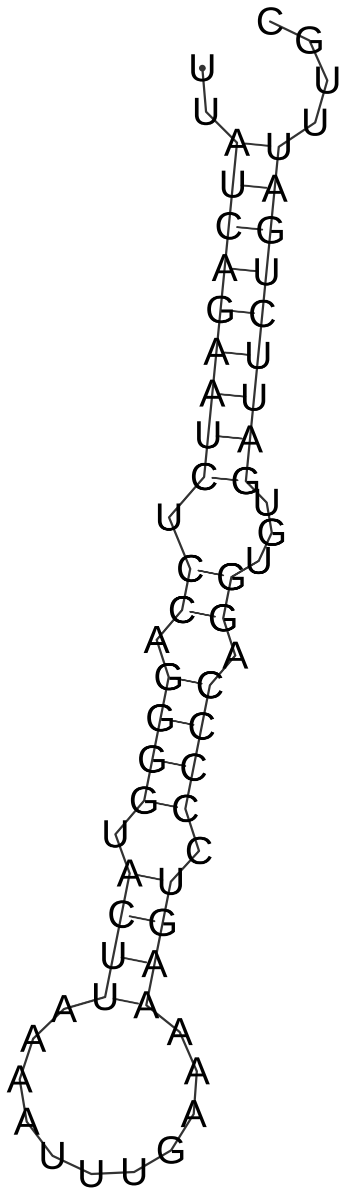

X_66698698-6669876_cfa-mir-361_high

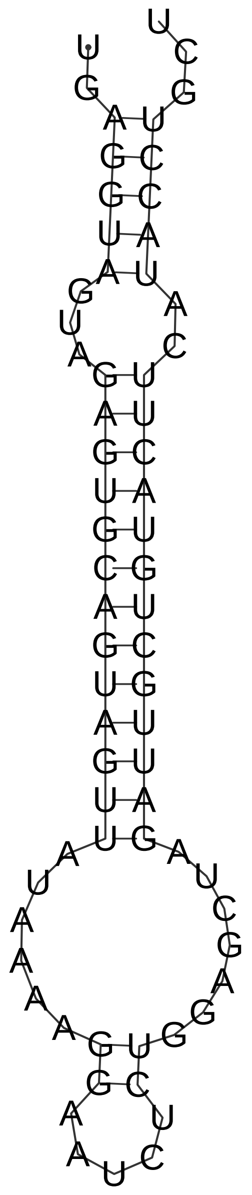

X_71568579-7156864_cfa-let-7j_low

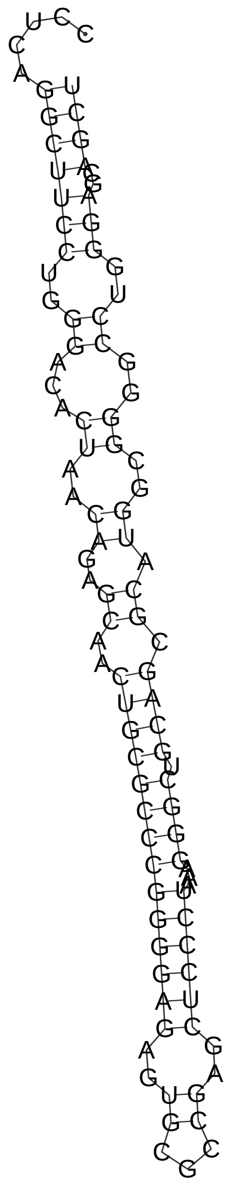

X_76666587-7666668_cfa-mir-8904a_low

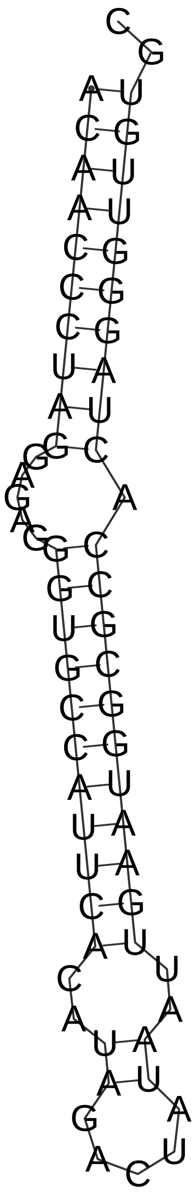

X_83271857-8327191_cfa-mir-652_high

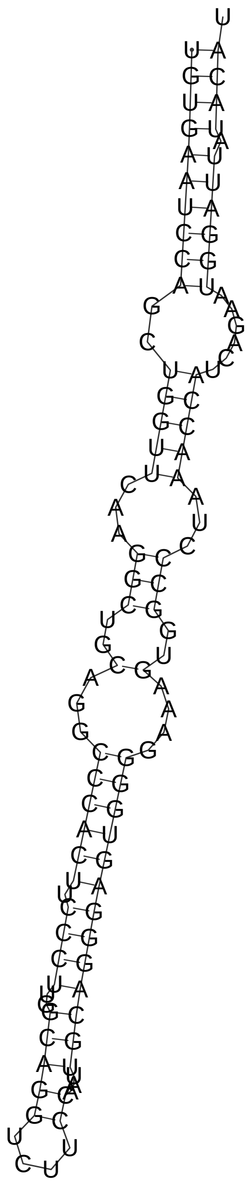

X_87387040-8738714_mir-3552_high

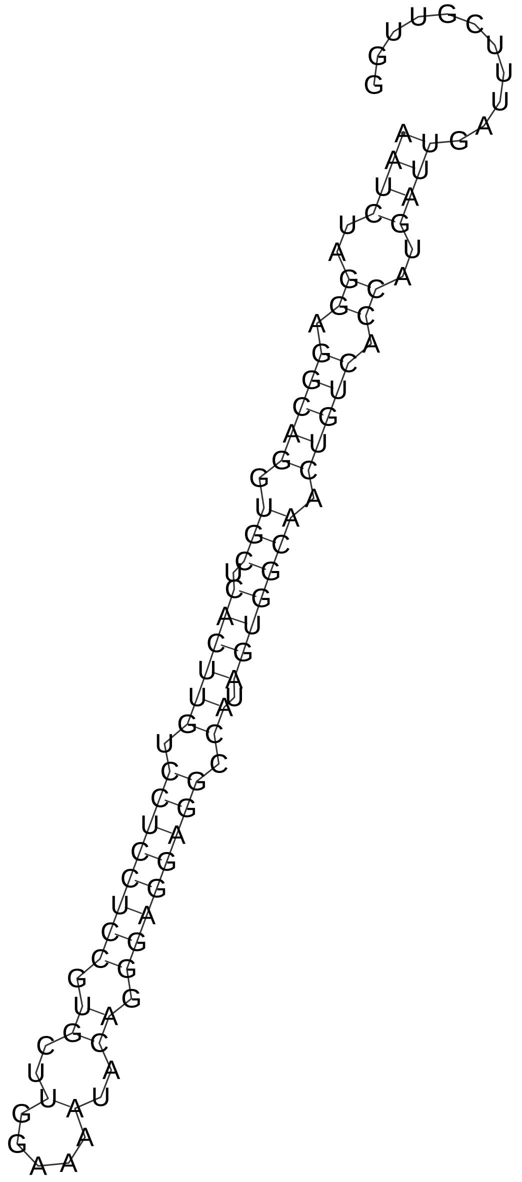

X_87389790-8738988_cfa-mir-764_high

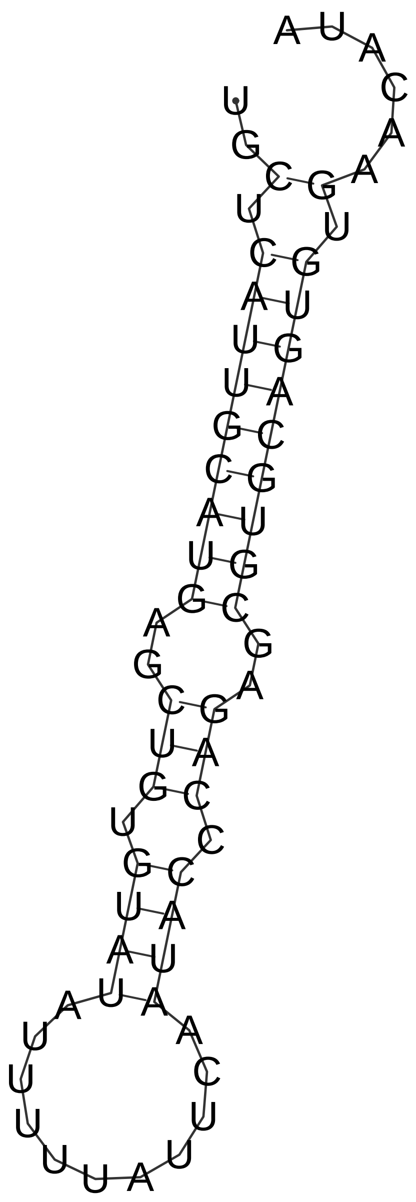

X_87400541-8740060_mir-1912_high

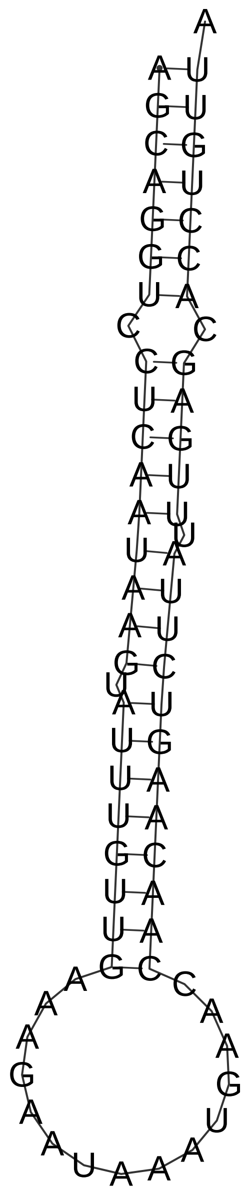

X_87401649-8740171_mir-1264_high

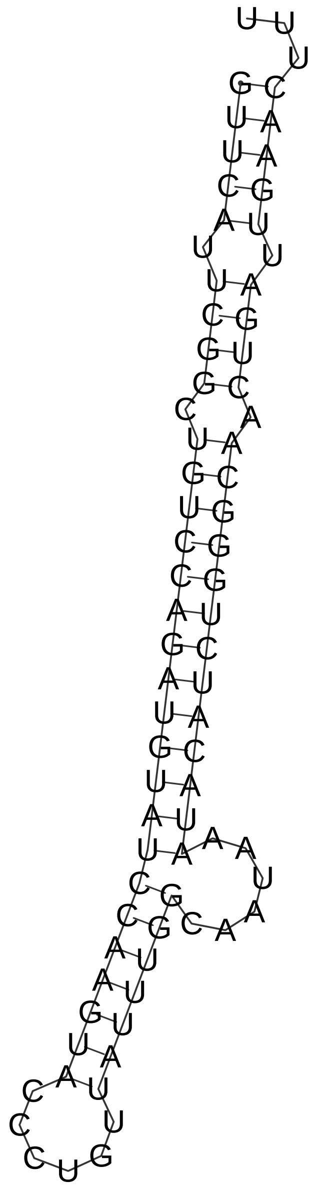

X_87468911-8746898_mir-1298_high

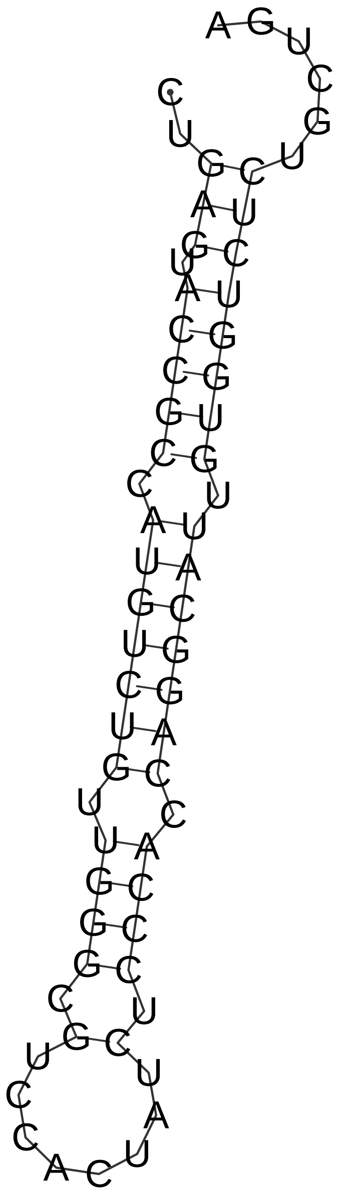

X_87505481-8750554_mir-1911_high

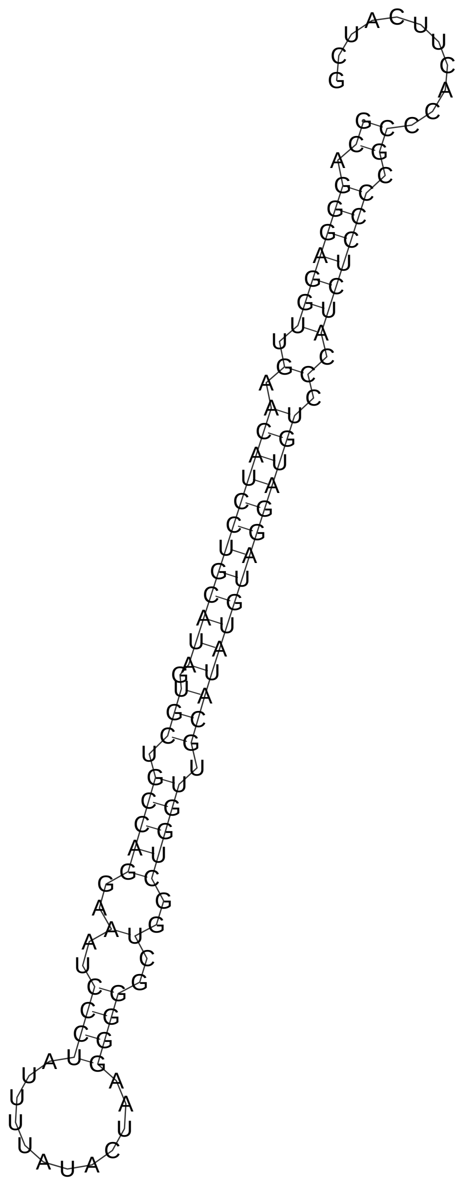

X_87558063-8755817_cfa-mir-448_high

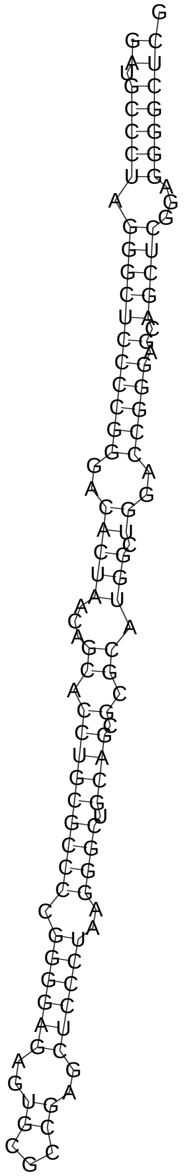

X_93909532-9390964_cfa-mir-8904b_low

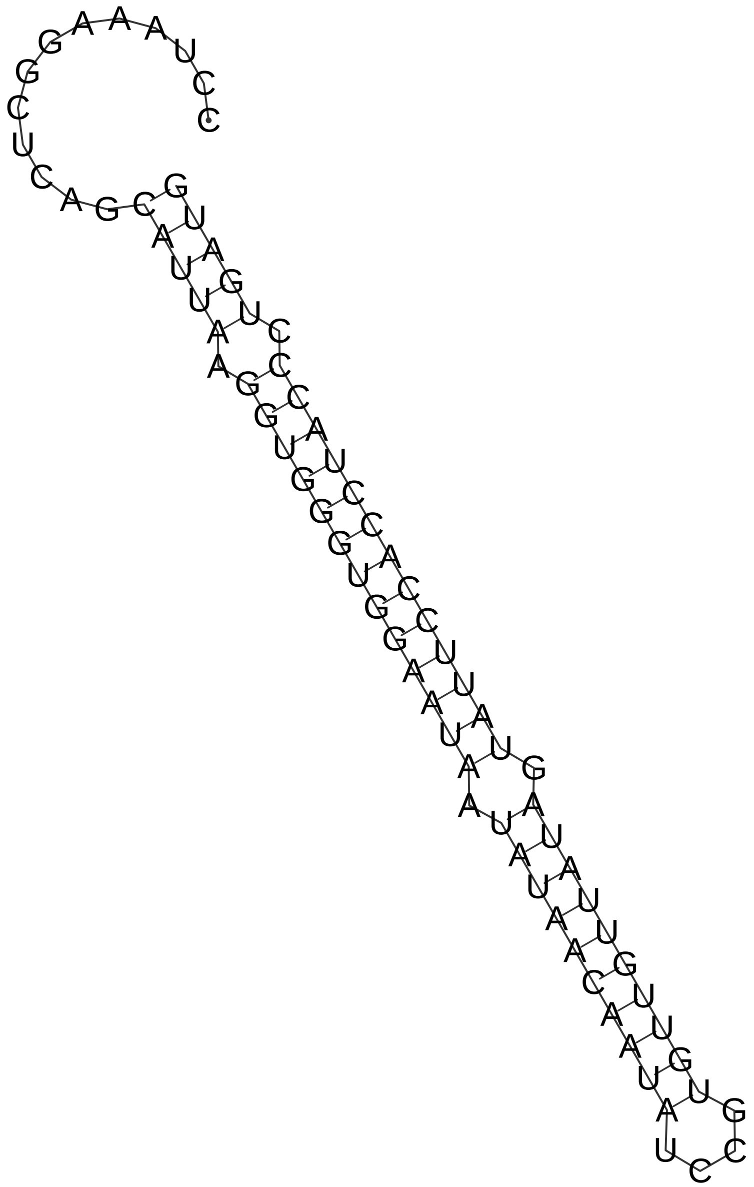

X_94981706-9498178_mir-2985_low

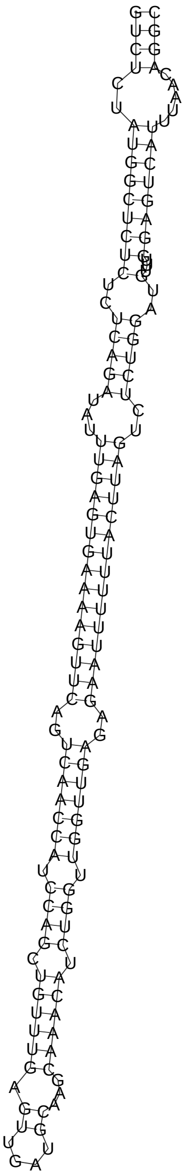

X_96560867-9656100_cfa-mir-2483_high

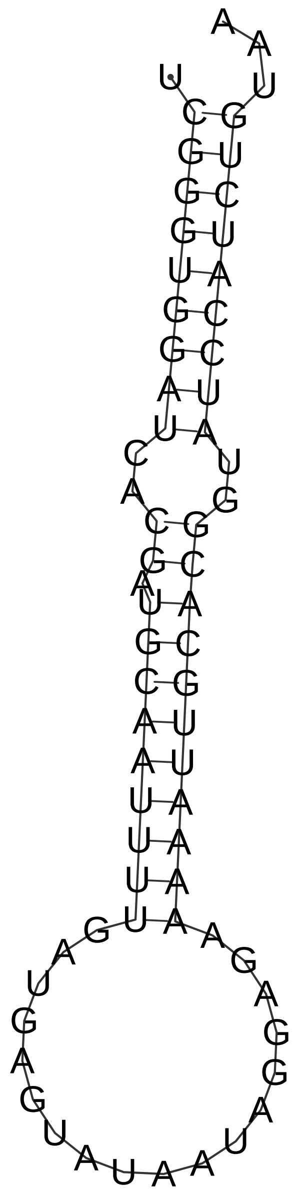

X_104895657-10489572_cfa-mir-363_high

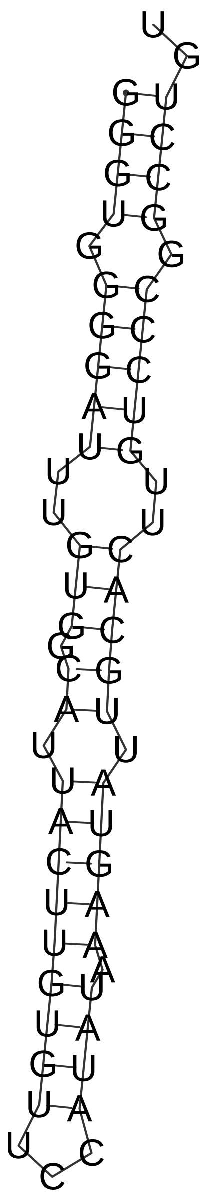

X_104895826-10489588_cfa-mir-92a-2_high

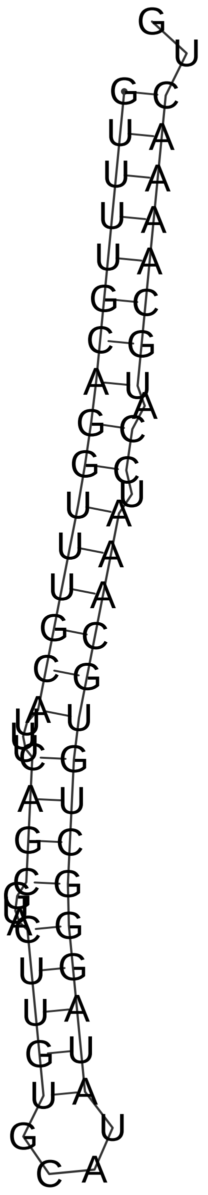

X_104895962-10489602_cfa-mir-19b-2_high

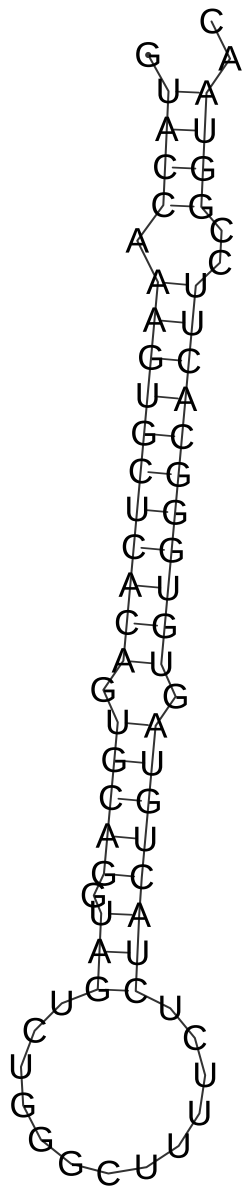

X_104896091-10489615_cfa-mir-20b_high

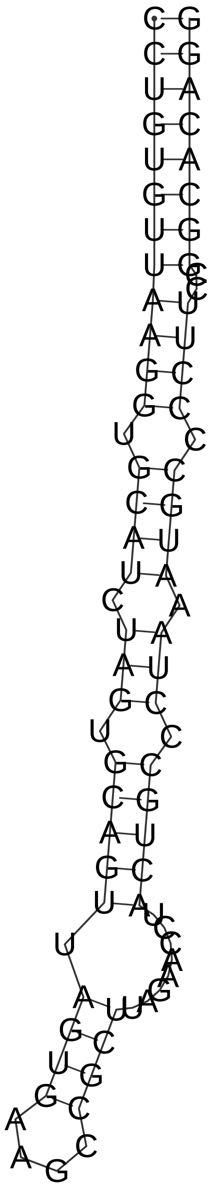

X_104896339-10489641_cfa-mir-18b_high

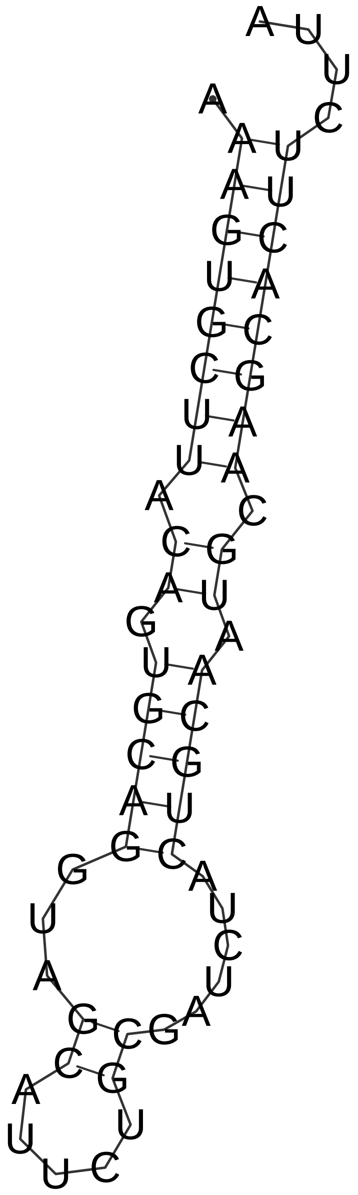

X_104896518-10489657_cfa-mir-106a_high

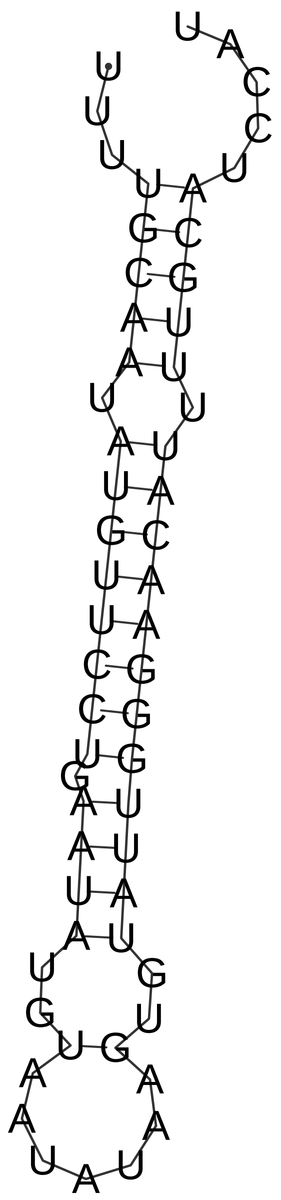

X_105177287-10517734_cfa-mir-450b_high

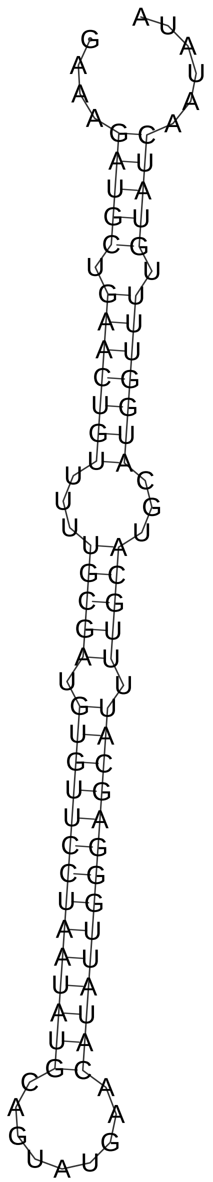

X_105177439-10517752_cfa-mir-450a_high

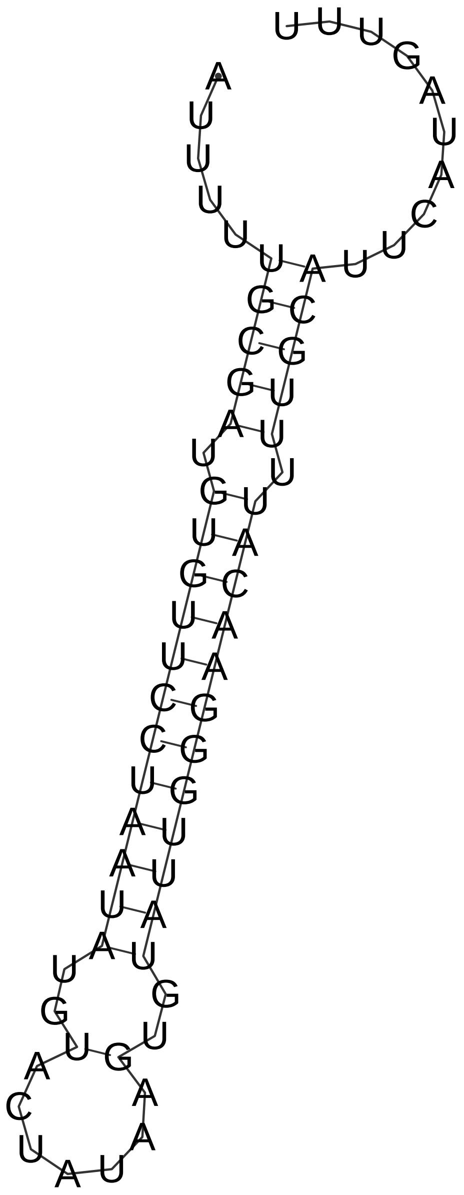

X_105177585-10517764_mir-450_high

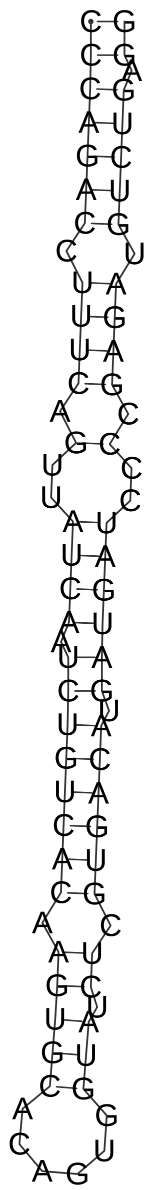

X_105178351-10517842_mir-542_low

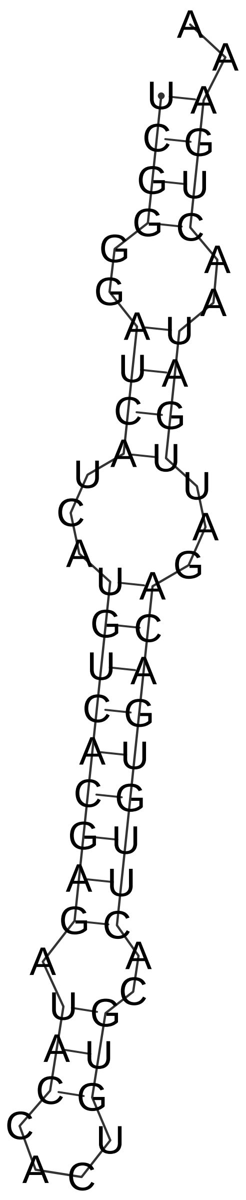

X_105178359-10517841_cfa-mir-542_high

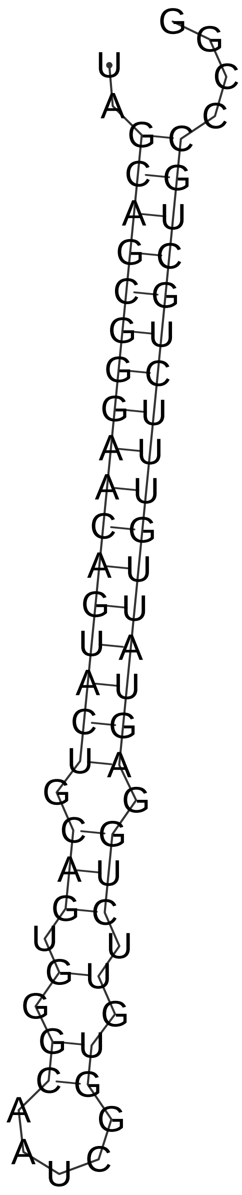

X_105183139-10518320_cfa-mir-503_high

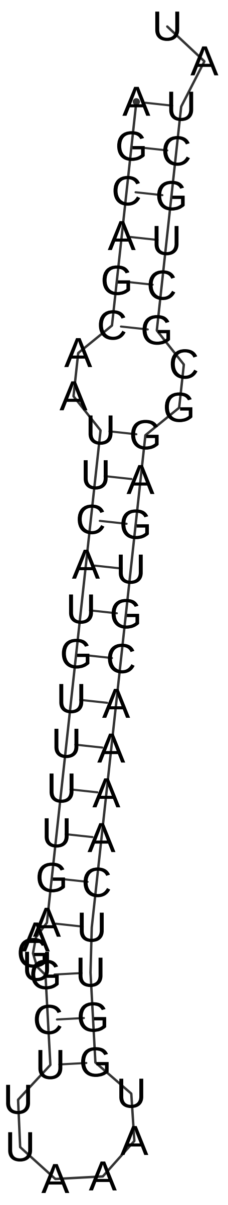

X_105183437-10518349_cfa-mir-424_high

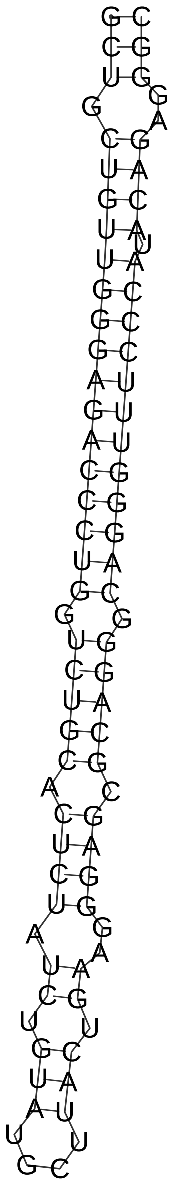

X_108693063-10869314_cfa-mir-504_high

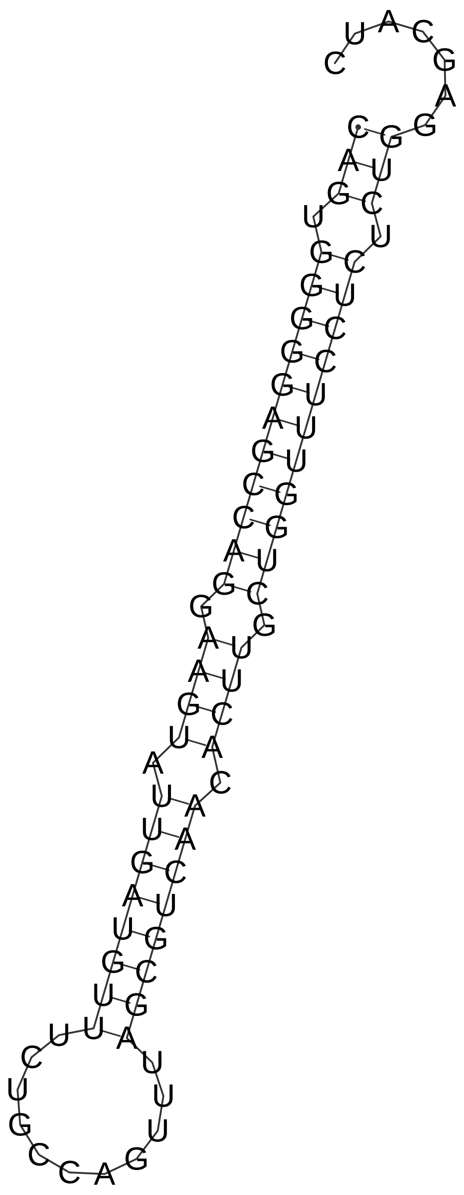

X_109866448-10986652_cfa-mir-505_high

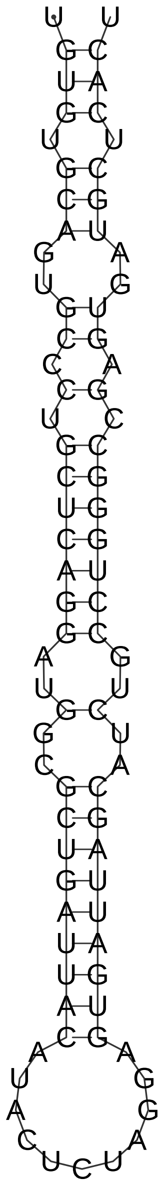

X_114675824-11467590_cfa-mir-8908c_high

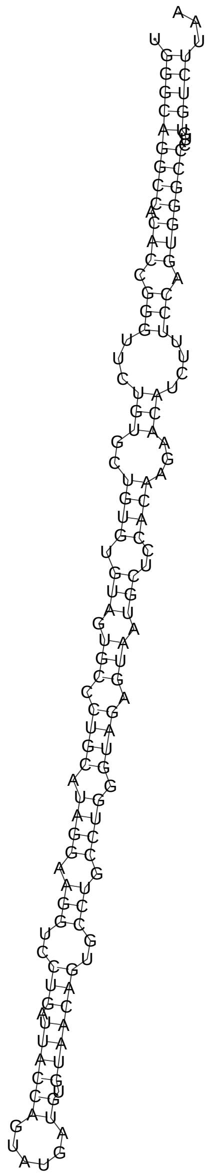

X_114676803-11467694_cfa-mir-8908e_high

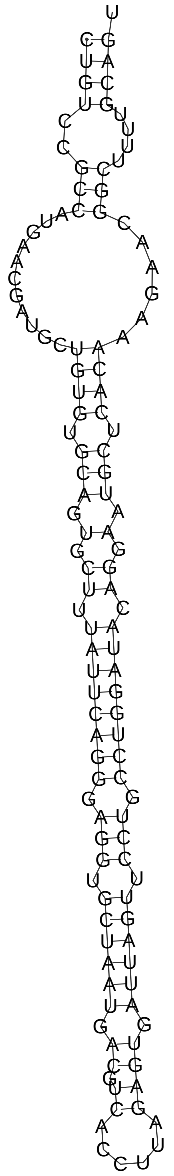

X_114678475-11467859_cfa-mir-8908b_high

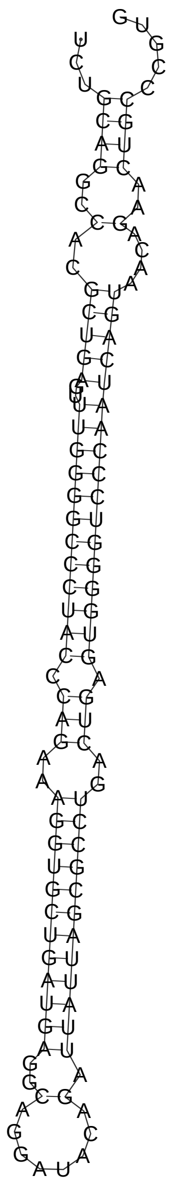

X_114679949-11468006_cfa-mir-8908d-2_high

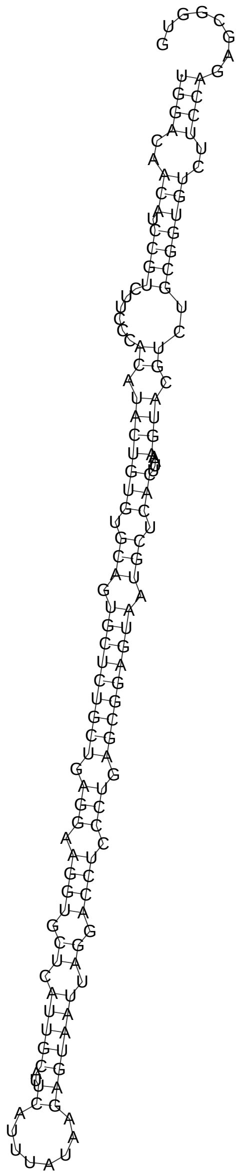

X_114680866-11468101_cfa-mir-8908a-4_high

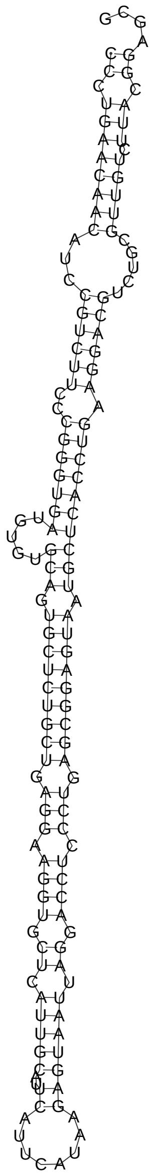

X_114684058-11468420_cfa-mir-8908a-1_high

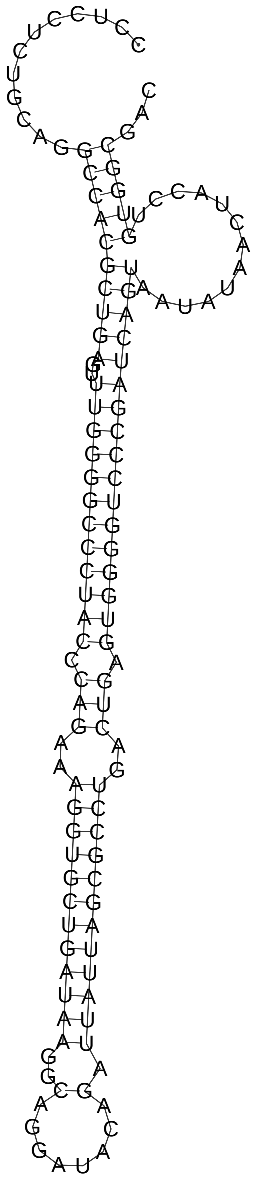

X_114686137-11468625_cfa-mir-8908d-3_high

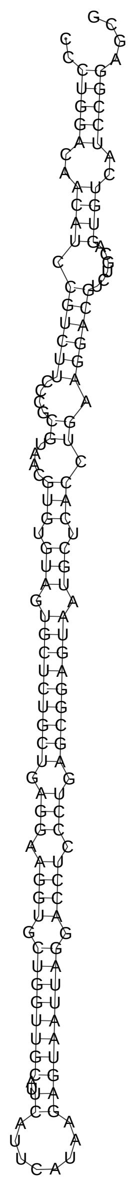

X_114686988-11468713_cfa-mir-8908a-2_high

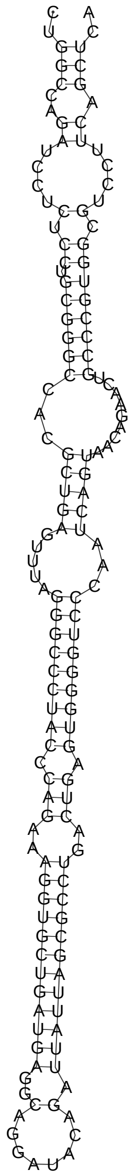

X_114688835-11468897_cfa-mir-8908d-1_high

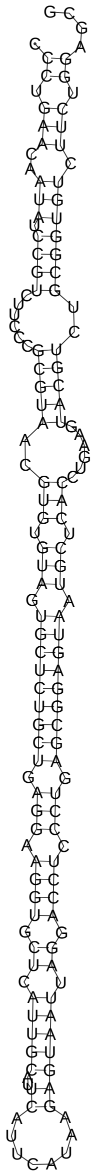

X_114689771-11468991_cfa-mir-8908a-3_high

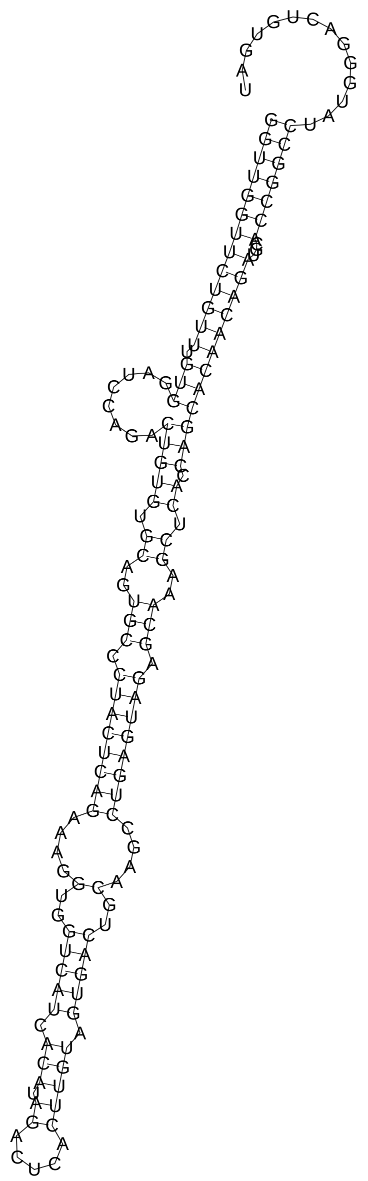

X_114717056-11471720_cfa-mir-8908f_high

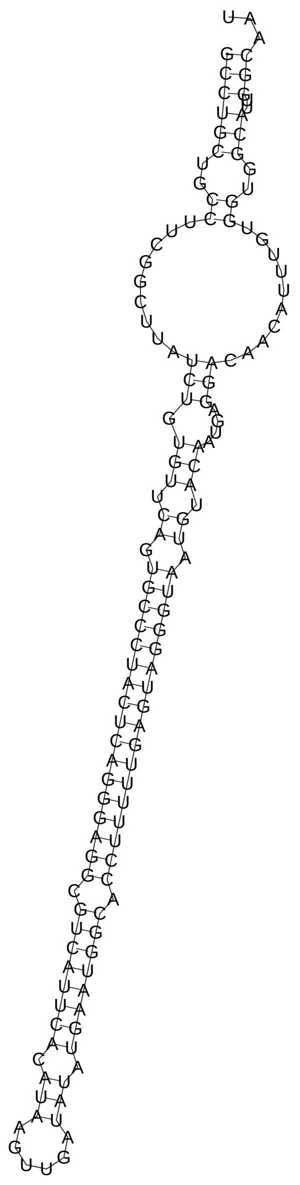

X_115652383-11565251_cfa-mir-506_high

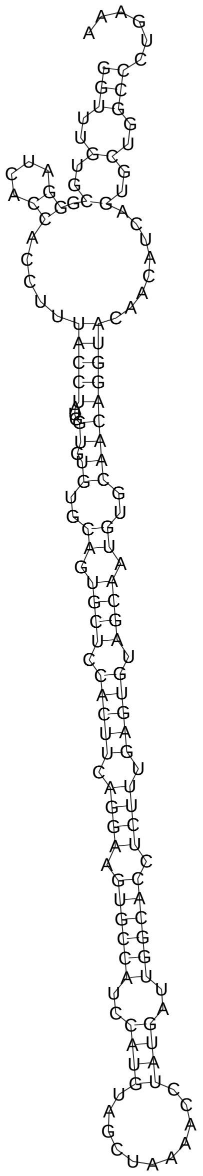

X_115652681-11565282_cfa-mir-507a_high

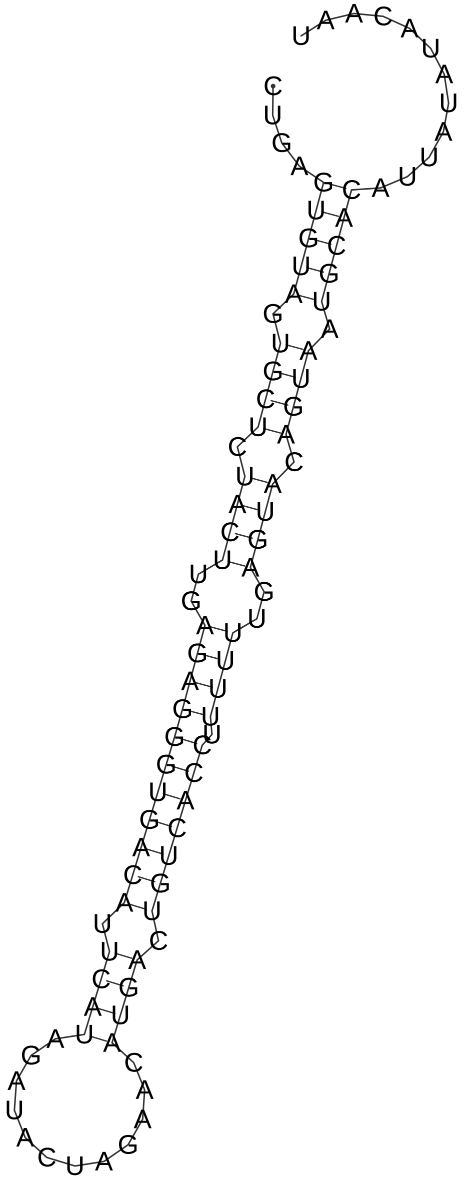

X_115658377-11565847_cfa-mir-508a_high

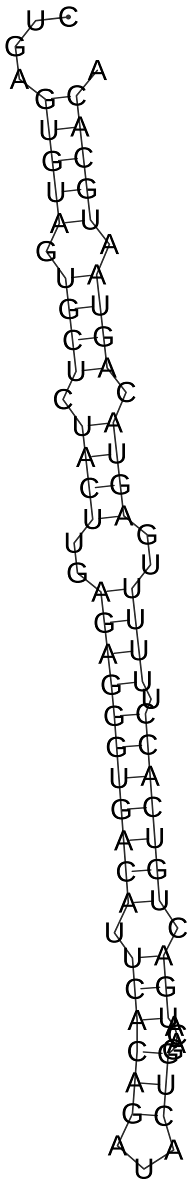

X_115658599-11565868_cfa-mir-508b_high

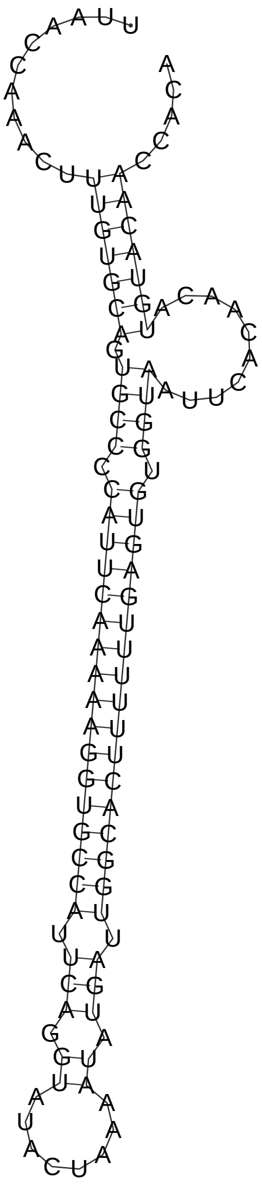

X_115665368-11566547_cfa-mir-507b_high

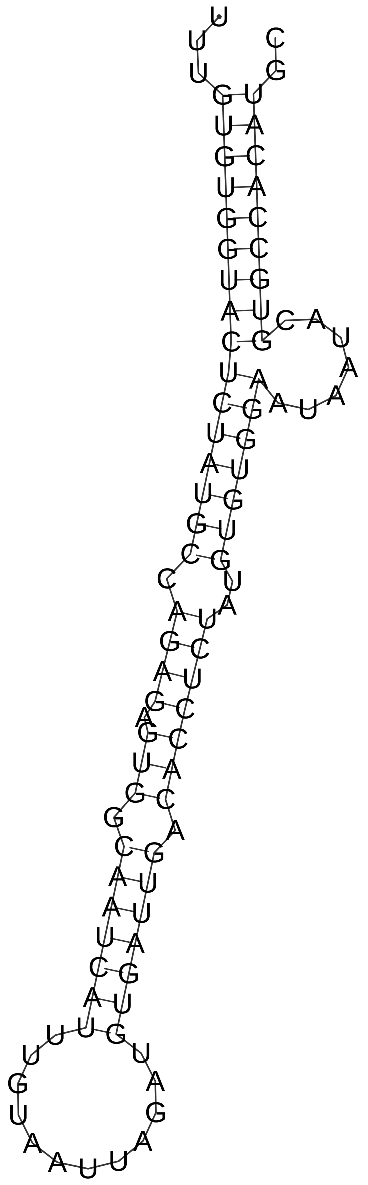

X_115674734-11567482_mir-514_low

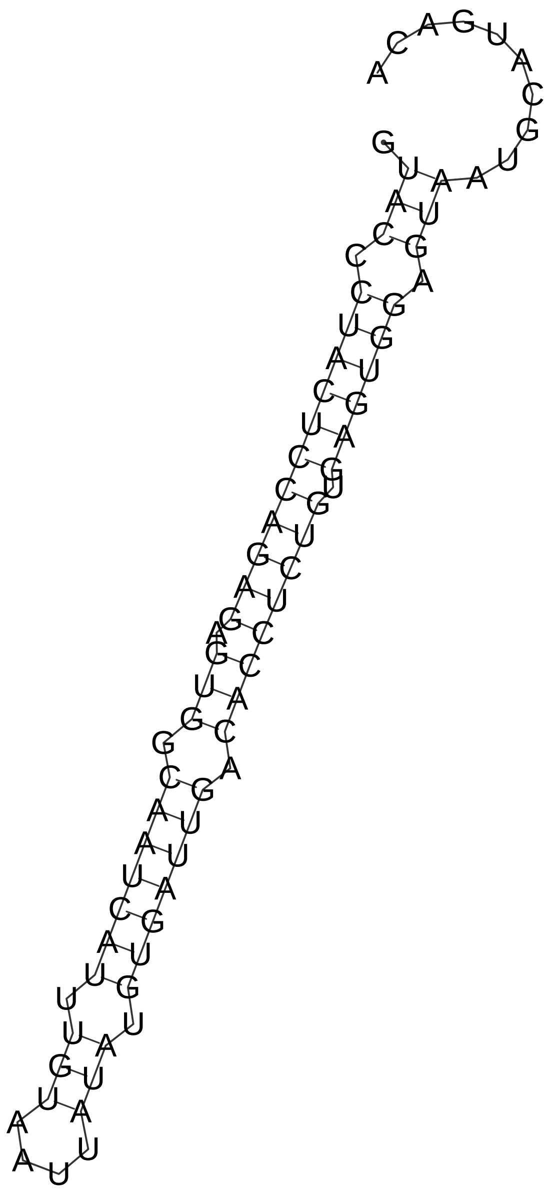

X_115688006-11568808_cfa-mir-514_high

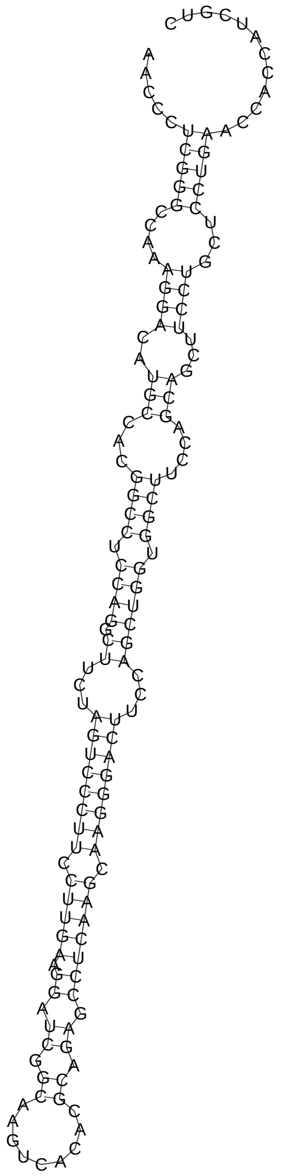

X_118541319-11854146_cfa-mir-2114_high

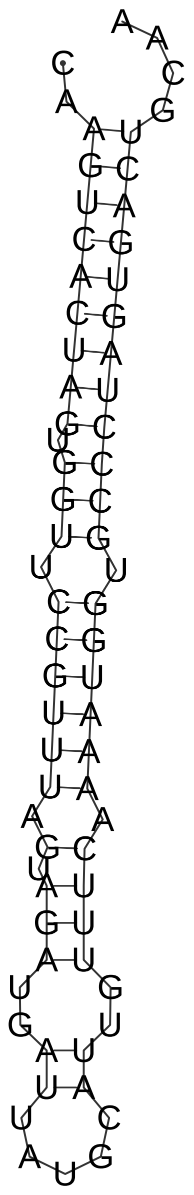

X_119925345-11992541_cfa-mir-224_high

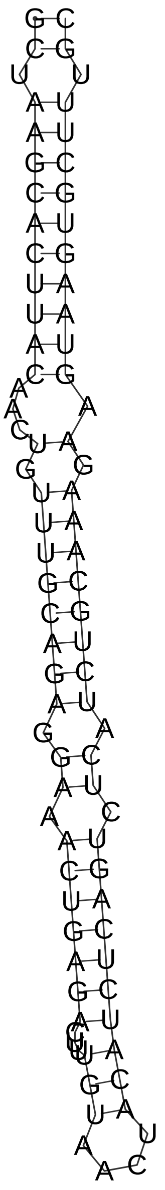

X_119926379-11992646_cfa-mir-452_high

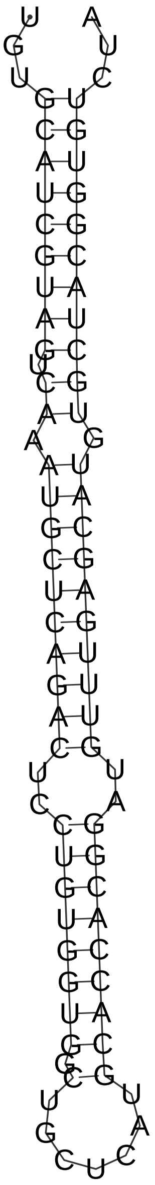

X_120403195-12040327_cfa-mir-105a_high

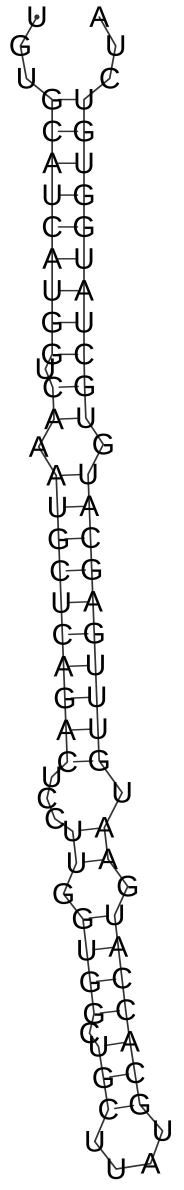

X_120405349-12040542_cfa-mir-105b_high

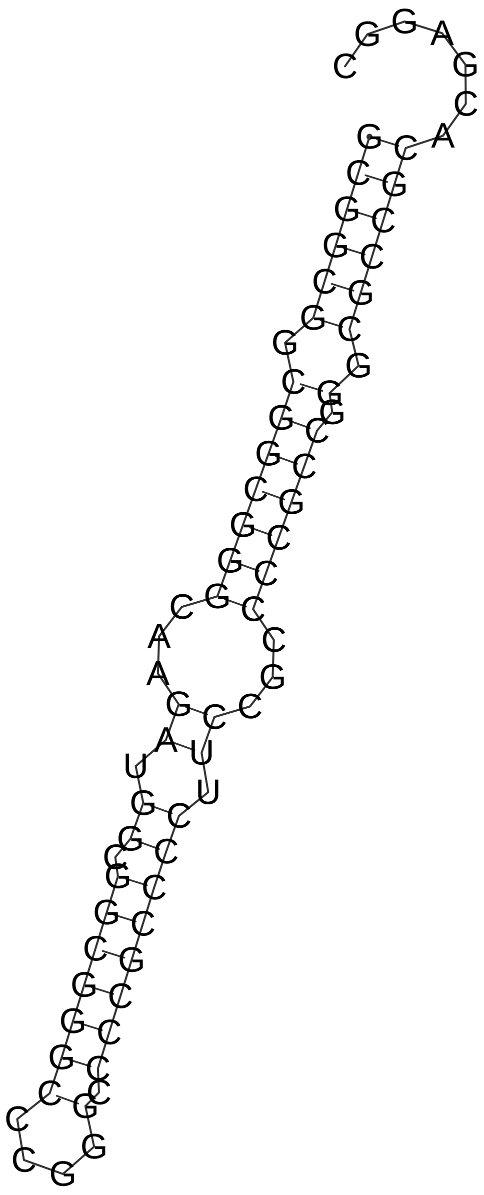

X_121862617-12186268_cfa-mir-718_low

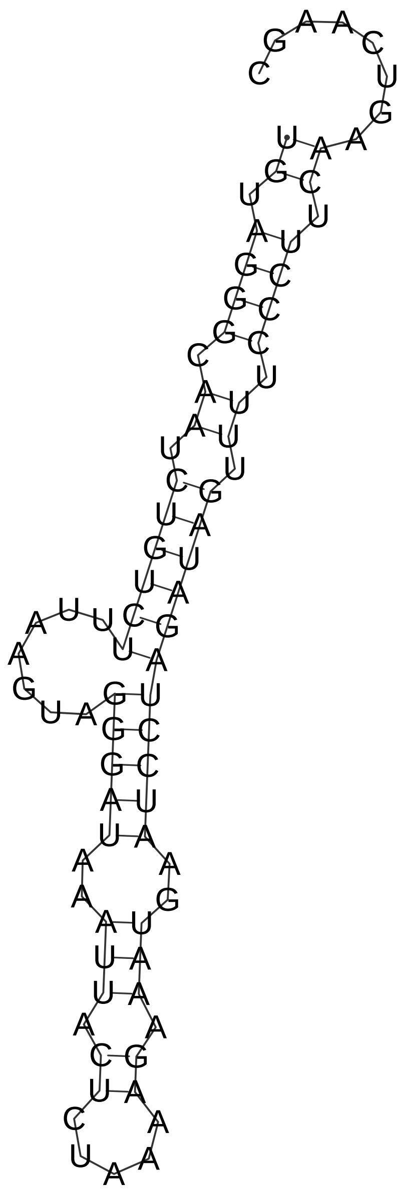

12_33290936-3329101_mir-684_low

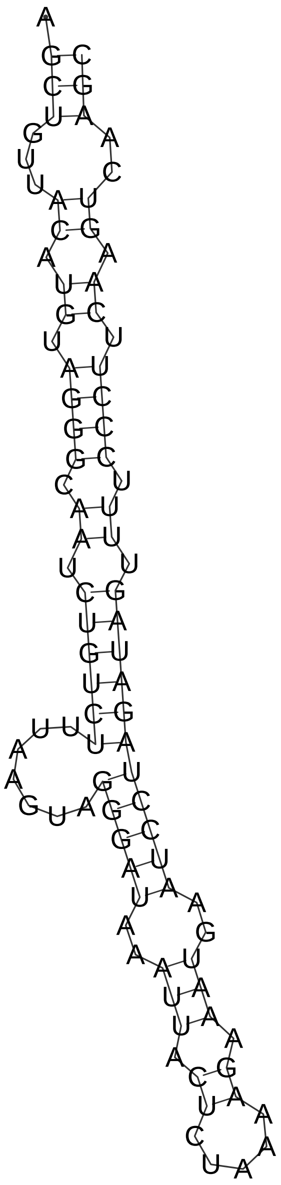

16_4113941-411403_mir-684_low

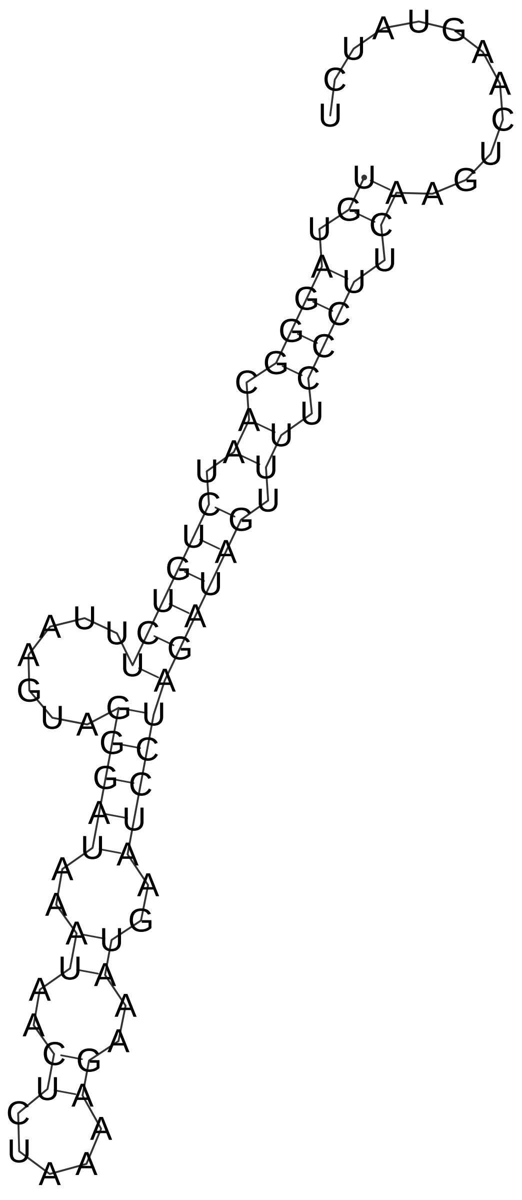

31_28082610-2808269_mir-684_low

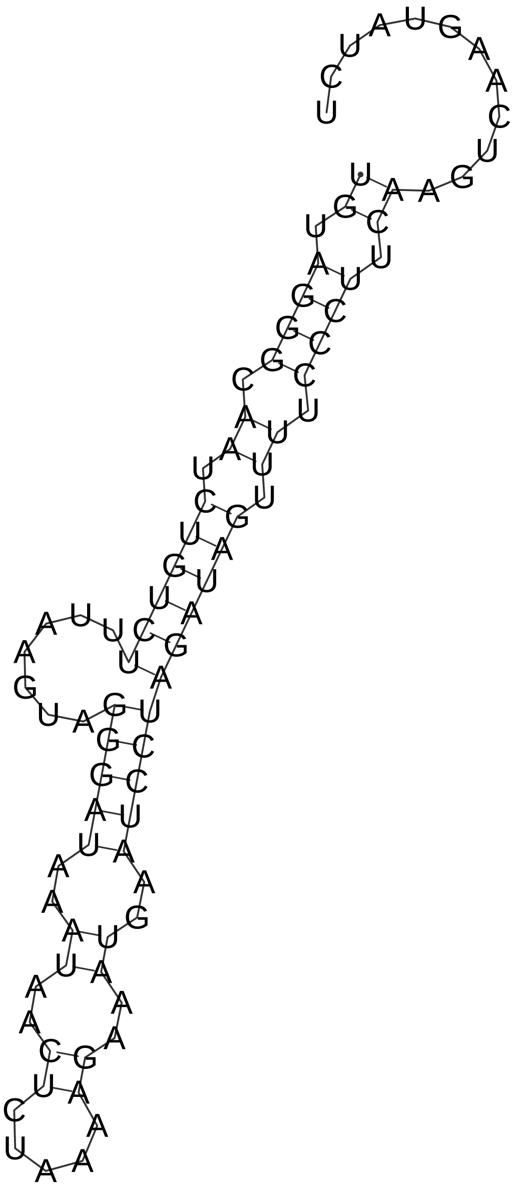

31_28981557-2898164_mir-684_low

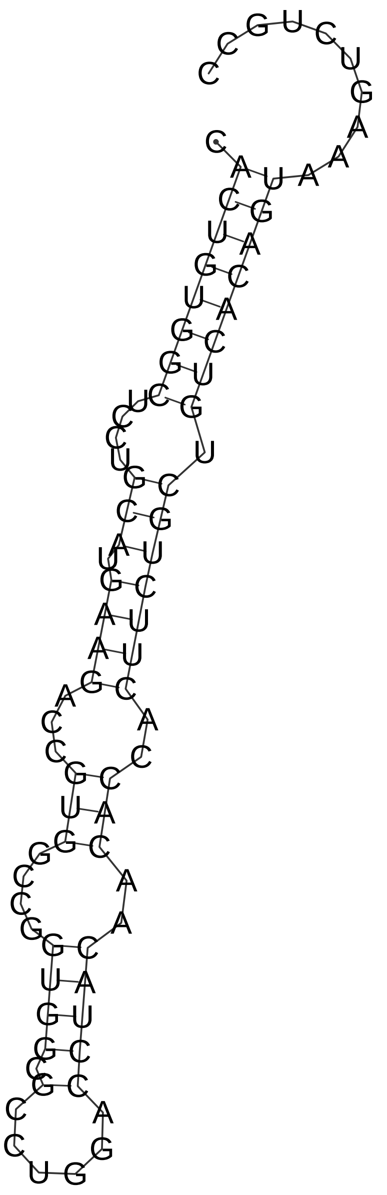

32_5648665-564874_mir-682_low

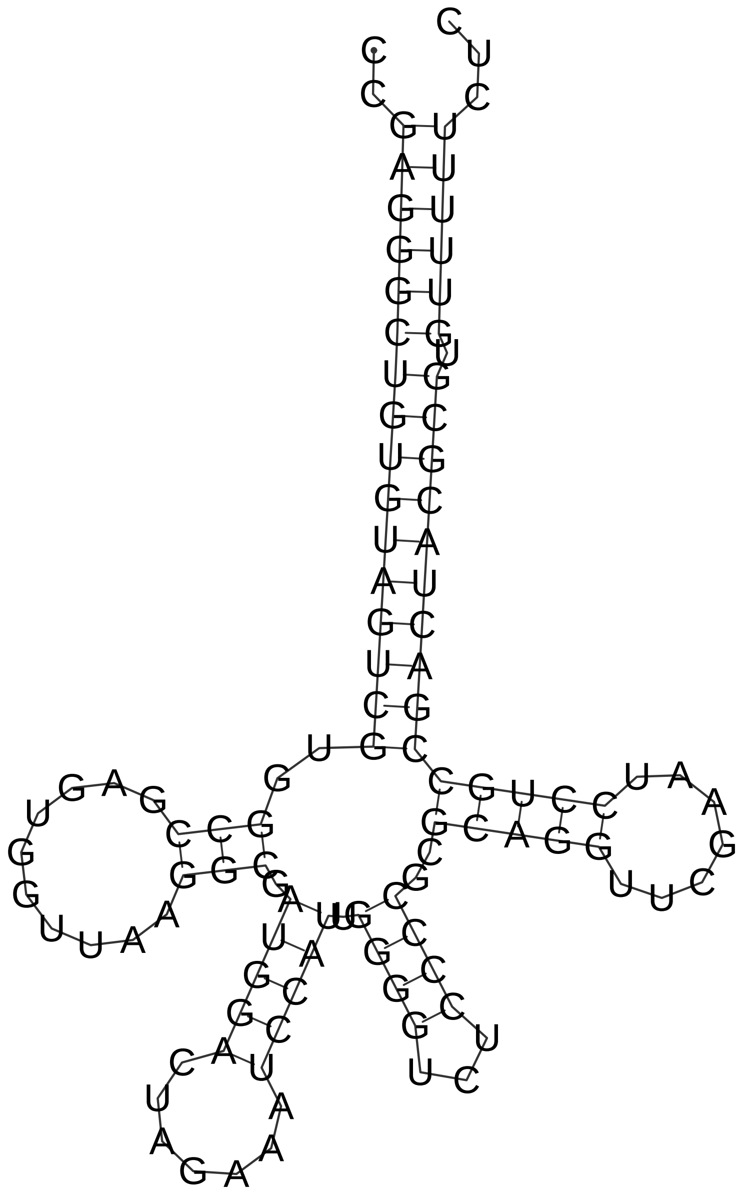

35_24800423-2480052_mir-9277_low

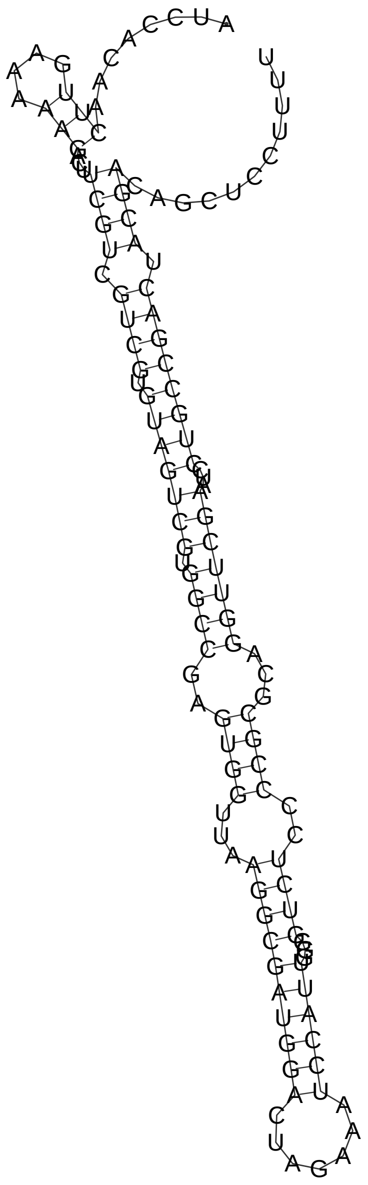

35_24816215-2481633_mir-9277_low

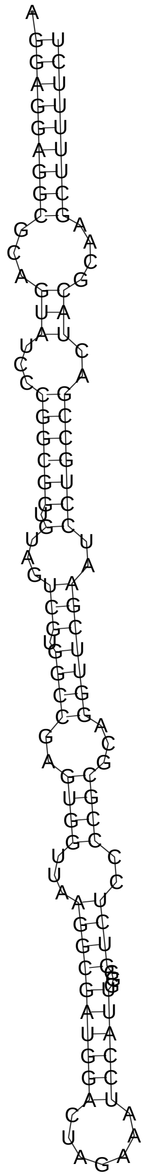

5_33032200-3303231_mir-9277_low

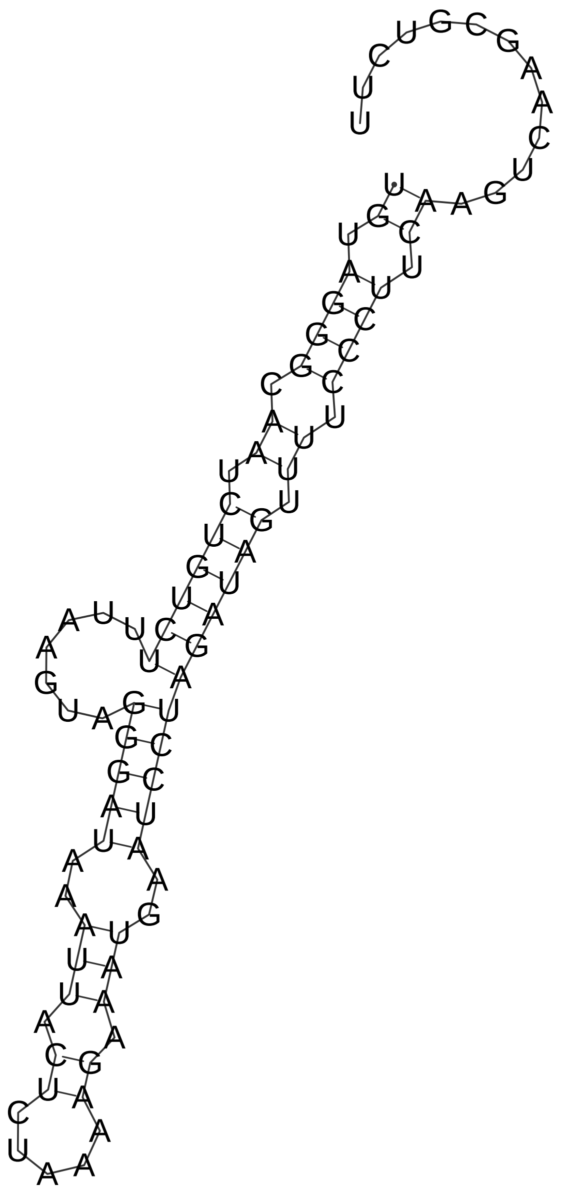

9_5311077-531116_mir-684_low
